# Prevalence, years lived with disability, and trends in anaemia burden by severity and cause, 1990–2021: findings from the Global Burden of Disease Study 2021

**DOI:** 10.1016/S2352-3026(23)00160-6

**Published:** 2023-07-31

**Authors:** GBD 2021 Anaemia Collaborators

## Abstract

**Background:**

Anaemia is a major health problem worldwide. Global estimates of anaemia burden are crucial for developing appropriate interventions to meet current international targets for disease mitigation. We describe the prevalence, years lived with disability, and trends of anaemia and its underlying causes in 204 countries and territories.

**Methods:**

We estimated population-level distributions of haemoglobin concentration by age and sex for each location from 1990 to 2021. We then calculated anaemia burden by severity and associated years lived with disability (YLDs). With data on prevalence of the causes of anaemia and associated cause-specific shifts in haemoglobin concentrations, we modelled the proportion of anaemia attributed to 37 underlying causes for all locations, years, and demographics in the Global Burden of Disease Study 2021.

**Findings:**

In 2021, the global prevalence of anaemia across all ages was 24·3% (95% uncertainty interval [UI] 23·9–24·7), corresponding to 1·92 billion (1·89–1·95) prevalent cases, compared with a prevalence of 28·2% (27·8–28·5) and 1·50 billion (1·48–1·52) prevalent cases in 1990. Large variations were observed in anaemia burden by age, sex, and geography, with children younger than 5 years, women, and countries in sub-Saharan Africa and south Asia being particularly affected. Anaemia caused 52·0 million (35·1–75·1) YLDs in 2021, and the YLD rate due to anaemia declined with increasing Socio-demographic Index. The most common causes of anaemia YLDs in 2021 were dietary iron deficiency (cause-specific anaemia YLD rate per 100 000 population: 422·4 [95% UI 286·1–612·9]), haemoglobinopathies and haemolytic anaemias (89·0 [58·2–123·7]), and other neglected tropical diseases (36·3 [24·4–52·8]), collectively accounting for 84·7% (84·1–85·2) of anaemia YLDs.

**Interpretation:**

Anaemia remains a substantial global health challenge, with persistent disparities according to age, sex, and geography. Estimates of cause-specific anaemia burden can be used to design locally relevant health interventions aimed at improving anaemia management and prevention.

**Funding:**

Bill & Melinda Gates Foundation.

## Introduction

Anaemia is a widespread global health problem associated with poor health outcomes, increased morbidity and mortality, and substantial health and economic costs.^1^ Anaemia in pregnancy is associated with increased rates of preterm labour, postpartum haemorrhage, low birthweight, short gestation, stillbirth, and infections for both child and mother.^2^ Anaemia in children is linked with impaired cognitive and motor development and susceptibility to infections, potentially increasing the risk of mortality during childhood from severe infections, such as those due to malaria.^3^, ^4^ Symptoms of anaemia in adults include weakness, fatigue, difficulty concentrating, and challenges with work and activities of daily life.^5^ Anaemia in older adults (>65 years) is an identified risk factor for hospitalisation, poorer surgical outcomes,^6^ and increased all-cause mortality.^7^

Anaemia can be caused by numerous conditions that result in blood loss, reduce the lifespan of red blood cells, or trigger reductions in the synthesis of haemoglobin or red blood cells. Chronic inflammation can lead to hepcidin-mediated inhibition of iron absorption that renders oral iron administration ineffective,^8^, ^9^ and some data suggest that iron supplementation could increase susceptibility to malaria^8^ and potentially to other acute inflammatory conditions. Despite the complexity of underlying causes of anaemia, most anaemia reduction strategies have focused only on iron delivery, probably because iron deficiency is a common manifestation of anaemia in most countries and dietary interventions are comparatively simple.^10^, ^11^ However, iron administration alone is likely to be insufficient because inadequate iron intake is only one of numerous underlying reasons for an individual to be iron-deficient or anaemic.^12^ Fewer than half of individuals with anaemia will respond to iron interventions if the causes of anaemia that are unrelated to inadequate iron intake are not addressed,^13^ and it should therefore not be surprising that progress in reducing the burden of anaemia has been slow and uneven in regions around the world.^14^, ^15^

**Research in context**

**Evidence before this study**

The Global Burden of Disease, Injuries, and Risk Factors Study (GBD) is a comprehensive effort to systematically measure the causes and risk factors of death and disability. Anaemia-specific manuscripts relating to the 2010 and 2013 GBDs have previously been published. Other studies examining trends in anaemia burden at the global level have often been limited to particular demographic groups (eg, children or women of reproductive age), geographies (eg, low-income settings), or specific underlying causes (eg, iron deficiency), and therefore do not capture the full extent of the anaemia burden. For example, 2022 WHO estimates present anaemia burden among children aged 6–59 months and women aged 15–49 years from 2000 to 2019. In this context, the GBD provides an ideal framework to comprehensively quantify the prevalence, years lived with disability, and trends of anaemia burden across all geographies, demographics, and causes. To estimate total anaemia prevalence, we used individual-level and tabulated survey and report data from the Global Health Data Exchange, identified using the keywords “anemia” and “hemoglobin”, supplemented with sources from the WHO Vitamin and Mineral Nutrition Information System, a comprehensive database including measurements of haemoglobin concentration and anaemia prevalence collected from systematic reviews of scientific literature databases; WHO regional and country offices; other research, governmental and non-governmental organisations; and manual searches of non-indexed journals. Inclusion criteria were quantitative measurement of haemoglobin concentrations in either a population-based sample or a group judged to adequately represent the sex, age groups, and location of the study.

**Added value of this study**

To our knowledge, this study presents the most up-to-date and complete estimates of global anaemia burden, covering 204 countries and territories, 25 age groups, and male and female sexes from 1990 to 2021. We provide a comprehensive account of anaemia prevalence, associated years lived with disability, and the trends in these values, including an examination of underlying causes of anaemia and associations with Socio-demographic Index. We have improved on previous estimates through the addition of numerous data sources, enhanced data processing algorithms for pregnancy adjustment, and revised modelling techniques to strengthen estimates in locations and populations in which data are sparse. Our causal attribution models included additional causes of anaemia, amended methods for estimating cause-specific anaemia burden, and optimised redistribution algorithms to account for the varied effect of different diseases on haemoglobin concentrations.

**Implications of all the available evidence**

We show that progress towards alleviating the anaemia burden across age, sex, and geography is varied and often slow. Our analysis of the underlying causes of anaemia provides insights for the design of effective, contextualised disease surveillance programmes and public health interventions. Although iron deficiency is often the most frequent cause of anaemia, much of the iron-related anaemia burden could be unresponsive to iron treatments if the underlying issues relating to iron deficiency are not addressed. Interventions to deal with major causes of anaemia—such as chronic kidney disease, gastrointestinal disorders, malaria, and, where prevalent, neglected tropical diseases such as schistosomiasis and hookworm disease—are necessary to substantially reduce anaemia burden. Important gaps in data and knowledge remain, necessitating the development of evidence-based definitions of anaemia centred on actual health loss, quantification of morbidity and mortality associated with low haemoglobin concentrations as a risk factor for other illnesses, and analysis of the effect of comorbid diseases on the incidence and severity of anaemia. Investments in closing these gaps will improve future estimates of the burden of anaemia and will provide policy makers, stakeholders, and health practitioners with the comprehensive data needed to reduce persistently high anaemia prevalence and associated health loss.

WHO's Global Nutrition Target calls for a 50% reduction in anaemia prevalence among women of reproductive age (15–49 years) by 2030^16^ to meet the targets of Sustainable Development Goals 2 and 3, which relate to improved nutrition, good health, and wellbeing.^17^ Accordingly, many organisations—including WHO, UNICEF, the United States Agency for International Development (USAID), and the Biomarkers Reflecting Inflammation and Nutritional Determinants of Anemia (BRINDA) project—have called for a comprehensive anaemia monitoring framework.^18^ Estimates from WHO of the overall anaemia prevalence in women of reproductive age and children were published in 2022,^15^ and other studies highlight anaemia burden in low-income and middle-income countries.^19^

High-quality, internally consistent, and comprehensive estimates of the distribution of anaemia and its underlying causes are essential for policy makers and health practitioners to develop interventions that are contextually appropriate and likely to reduce anaemia-associated morbidity and mortality.^13^ This study aims to update and expand on our previous estimates of global anaemia burden.^20^, ^21^ This manuscript was produced as part of the Global Burden of Disease, Injuries, and Risk Factors Study (GBD) Collaborator Network and in accordance with the GBD Protocol.^22^

## Methods

### Overview and definitions

Anaemia is defined by decreased blood concentration of haemoglobin.^23^ We estimated unique, continuous distributions of elevation-adjusted haemoglobin concentrations (g/L), anaemia prevalence, and years lived with disability (YLDs) by severity and 37 underlying causes of anaemia annually from 1990 to 2021 for 204 countries and territories, 21 GBD regions, male and female sexes, and 25 age groups (0–6 days, 7–27 days, 1–5 months, 6–11 months, 12–23 months, 2–4 years, 5–94 years in five-year age bins, and ≥95 years). Anaemia severity levels (mild, moderate, and severe) were defined using specific haemoglobin concentration thresholds that vary by age, sex, and pregnancy status (table). This study complies with the Guidelines for Accurate and Transparent Health Estimates Reporting (appendix pp 3–4).^24^

**Table:** Haemoglobin concentration thresholds (g/L) for classification of anaemia severity by age, sex, and pregnancy status

	**Mild anaemia**	**Moderate anaemia**	**Severe anaemia**
**0–6 days**			
Males	145–159	100–144	<100
Females	145–159	100–144	<100
**7–27 days**			
Males	120–134	85–119	<85
Females	120–134	85–119	<85
**1 month–4 years**			
Males	100–109	70–99	<70
Females	100–109	70–99	<70
**5 – 14 years**			
Males	110–114	80–109	<80
Females	110–114	80–109	<80
**≥15 years**			
Males	110–129	80–109	<80
Females, non–pregnant	110–119	80–109	<80
Females, pregnant	100–109	70–99	<70

Published WHO thresholds^23^ were used for males and females aged 6 months and older; thresholds for those younger than 6 months were imputed as described in the appendix (p 6).

### Anaemia prevalence estimation

#### Input data and data processing

We used representative data on mean haemoglobin concentrations and severity-specific anaemia prevalence from population surveys, published studies, and government reports. All input sources are listed in the appendix (pp 888–923) and at the Global Health Data Exchange. Each input datum was assigned to a specific GBD location, age, sex, and pregnancy status. Elevation-adjusted or elevation-adjusted and smoking-adjusted data were used in the model directly, raw data were adjusted using the WHO elevation-adjustment formula (appendix p 8), and no additional adjustments were made for smoking status, method of haemoglobin sampling (eg, whole blood *vs* capillary), or analysis method (eg, Coulter counter *vs* point-of-care testing).

#### Spatiotemporal Gaussian process regression

We estimated log-transformed mean haemoglobin concentration and logit-transformed prevalence of severe, moderate plus severe, and total anaemia using spatiotemporal Gaussian process regression models.^25^ The first-stage prediction was an ensemble of linear mixed-effects models (for a list of all covariates considered in submodels, see appendix pp 10–11), for which we retained only those submodels with significant covariate coefficients in the expected direction and each retained submodel was weighted inversely proportionally to its out-of-sample root mean squared error.^26^ In stage two, we calculated the residuals between our ensemble model and our input data and smoothed these residuals over space, age, and time, producing a revised estimate for every location, year, age, and sex. The final step was a Gaussian process regression that further smoothed the residuals between our data and step two estimates, from which we quantified uncertainty in our final model estimates by taking 1000 draws from the posterior Gaussian process. More detailed information on the spatiotemporal Gaussian process regression modelling process can be found in the appendix (pp 10–11, 32–36).

#### Ensemble distribution modelling and calculation of prevalence

We estimated final distributions of haemoglobin concentration using ensemble modelling in three phases (appendix pp 11–13). First, to identify which distribution families to use, we tested both single candidate two-parameter distributions (gamma, mirrored gamma, Weibull, mirrored lognormal, and mirrored Gumbel) and weighted ensembles of those same candidate distributions, fitting to individual-level data on haemoglobin concentration from population surveys, and evaluating the fit using a loss function of severity-specific prediction error weighted by the severity-specific GBD disability weights for anaemia. We selected the combination that minimised test set error, defined as the absolute difference between the observed severity-specific prevalence in a given survey and the prevalence predicted by a given combination of distributions, with the severity-specific errors summed and weighted proportionally to the disability weights for mild, moderate, and severe anaemia (appendix pp 11–12).

Second, we estimated the variance in haemoglobin concentration distributions for each GBD demographic by using mean haemoglobin concentration and anaemia prevalence estimates from spatiotemporal Gaussian process regression modelling and pairing with the selected distributions from phase one. To calculate variance, we anchored each distribution at the modelled mean estimate of haemoglobin concentration, then used an optimisation algorithm to find the variance value that minimised the distance between our modelled anaemia prevalence estimates and those implied by the given mean haemoglobin and variance combination. We again weighted severity-specific errors proportionally to severity-specific disability weights, such that errors for more severe anaemia were more heavily penalised.

Third, we derived haemoglobin distributions for every location, year, age group, and sex using mean haemoglobin concentrations from spatiotemporal Gaussian process regression modelling, optimised variance, and ensemble distribution weights, with separate distributions by pregnancy status for females aged 10–54 years. Final anaemia prevalence was calculated by finding the area under the probability density curve between the corresponding haemoglobin concentration thresholds for each severity by age, sex, and pregnancy status. Separate prevalence estimates by pregnancy status were aggregated into final combined estimates for all females aged 10–54 years.

### Causal attribution of anaemia

Each case of anaemia was assigned to a single cause in a mutually exclusive, collectively exhaustive manner for each of 37 underlying GBD causes considered in this analysis (appendix pp 13–16). We used four inputs for this process: haemoglobin concentration distributions including mean and variance; overall anaemia prevalence by severity; prevalence or incidence of each disease from the GBD 2021 study; and cause-specific haemoglobin shifts. Haemoglobin shifts represent mean difference in haemoglobin concentration associated with a given cause. These values were derived from published cohort studies, case-control studies, and intervention trials and have been described previously.^20^ A minimum of 10% of prevalent anaemia cases were reserved for five residual causes for which haemoglobin shift information, estimates of cause-specific prevalence, or both were absent. Anaemia cases due to these causes were assigned using fixed-proportion redistribution, with most assigned to dietary iron deficiency (appendix p 15).

We multiplied each cause-specific haemoglobin shift by the prevalence of that cause, giving a prevalence-weighted haemoglobin shift for every location, year, age, sex, and cause. We added this shift to our estimate of mean haemoglobin concentration and recalculated a counterfactual haemoglobin concentration distribution (assuming unchanged variance) that represents a theoretical population distribution that would exist in the absence of each underlying cause. We then calculated the difference in anaemia prevalence by severity between the counterfactual and original haemoglobin distributions and assigned the difference to a specific cause and severity combination (eg, moderate anaemia due to clinical malaria). We scaled the results to ensure that cause-specific prevalence of anaemia could not exceed the total prevalence of a given cause and that all estimated cases of anaemia were attributed to an underlying cause.

### YLDs

The YLD metric allows for standardised comparisons of non-fatal health burden between diseases.^27^ We calculated YLDs by multiplying our estimates of severity-specific anaemia cases by associated severity-specific disability weights, which represent the level of health loss associated with a given disease state, where 0 is no health loss and 1 is death. The disability weights were 0·004 (95% uncertainty interval [UI] 0·001–0·008) for mild anaemia, 0.052 (0·034–0·076) for moderate anaemia, and 0·149 (0·101–0·209) for severe anaemia (for more information on this calculation see appendix pp 16–17).

### Epidemiological transition and annualised rates of change

As secondary analyses, we explored the association between anaemia and socioeconomic development by analysing the relationship between anaemia burden (prevalence and YLDs) and the Socio-demographic Index (SDI) for each location in the analysis. SDI is a composite indicator based on estimates of total fertility rate in those younger than 25 years, mean years of education in individuals older than 15 years, and lag-distributed income per capita.^28^ We conducted a meta-analysis of the relationship between country-level logit-transformed anaemia prevalence and log-transformed YLDs and SDI using meta-regression—Bayesian, regularised, trimmed models,^29^ fitting restricted cubic splines that allowed for estimation of non-linear associations between anaemia burden and SDI. The splines contained two internal knots: one placed at the 33·33rd percentile and one at the 66·66th percentile of observed SDI values. From these models, we predicted expected values of anaemia prevalence and YLDs for each SDI value and calculated observed-to-expected ratios of anaemia burden for each location to identify countries with an anaemia burden substantially larger or smaller than expected on the basis of each country's level of socioeconomic development. Results stratified by SDI are based on each country's 2021 SDI value. To supplement trend assessments, we calculated annualised rate of change between two years as log-transformed difference in anaemia prevalence or YLD rate divided by the total number of years between the two values (for more information on this analysis see appendix p 17).

### Uncertainty

We propagated uncertainty through each step of the estimation process by sampling draws from the posterior distribution of each estimated quantity. Uncertainty was not captured for population size, covariates used in spatiotemporal Gaussian process regression models, pregnancy prevalence, or haemoglobin shifts. Aggregations by geography, age, sex, and cause were made at the draw level, assuming uncorrelated uncertainty. The 95% UIs for each quantity correspond to the 2·5th and 97·5th percentiles of the draws.

### Role of the funding source

The funder had no role in study design, data collection, analysis, interpretation, or manuscript preparation.

## Results

### Overview

**Figure 1:**
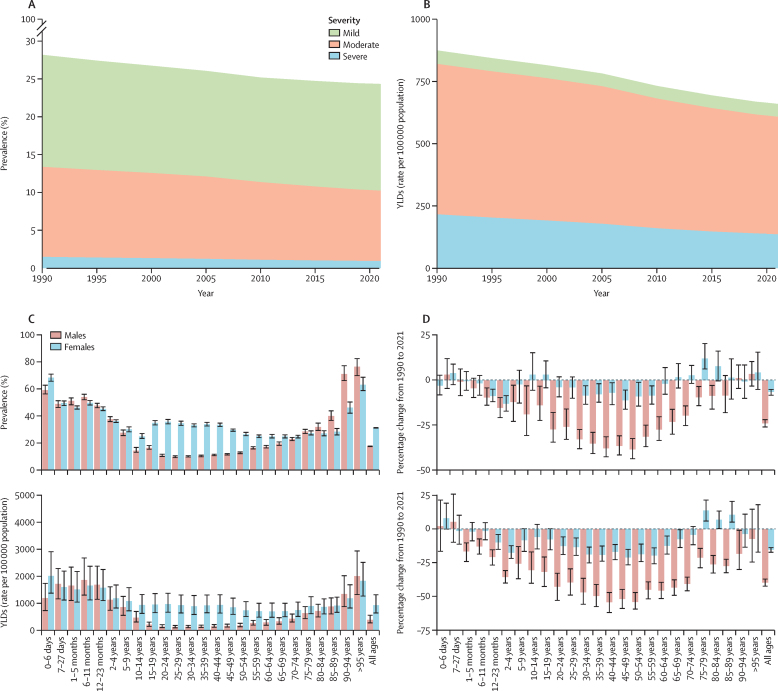
Anaemia prevalence and YLDs (rate per 100 000 population) (A) Global anaemia prevalence by severity for all ages and male and female sexes, 1990–2021. (B) YLDs (rate per 100 000 population) by severity for all ages and male and female sexes, 1990–2021. (C) Global anaemia prevalence (top) and YLDs (rate per 100 000 population; bottom) for all ages and male and female sexes in 2021. Error bars are 95% CI. (D) Percentage change between 1990 and 2021 in anaemia prevalence (top) and YLDs (rate per 100 000 population; bottom) for all ages and male and female sexes. Error bars are 95% CI. YLDs=years lived with disability.

In 2021, the global prevalence of anaemia across all ages was 24·3% (95% UI 23·9–24·7), a decrease from 28·2% (27·8–28·5) in 1990 (figure 1A). The prevalence of severe anaemia was 0·9% (0·9–1·0), moderate anaemia was 9·3% (9·1–9·4), and mild anaemia was 14·1% (13·8–14·5; appendix pp 38–43). Despite a decrease in prevalence, the total number of people with anaemia increased from 1·50 billion (95% UI 1·48–1·52) in 1990 to 1·92 billion (1·89–1·95) in 2021, a difference attributable primarily to population growth. Total anaemia YLDs increased from 46·6 million (31·6–65·7) in 1990 to 52·0 million (35·1–75·1) in 2021, while YLD rate per 100 000 population decreased from 874·2 (591·7–1232·6) in 1990 to 659·2 (444·9–952·3) in 2021 (figure 1B; appendix pp 38–43). Country profiles and additional results can be found in the appendix (p 48–887).

### Age and sex trends

**Figure 2:**
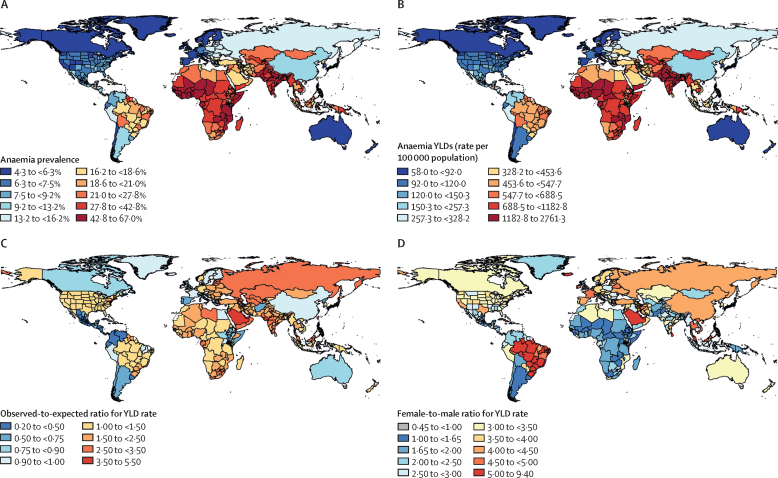
All-ages anaemia burden, 2021 (A) Anaemia prevalence for all ages and male and female sexes, 2021. (B) Anaemia YLDs (rate per 100 000 population) for all ages and male and female sexes, 2021. (C) Observed-to-expected ratio for YLD rate per 100 000 population for all ages and male and female sexes, 2021. Expected values were based on Socio-demographic Index. (D) Male-to-female ratio for YLD rate per 100,000 population, 2021. YLDs=years lived with disability.

Across all ages, males had a lower prevalence of anaemia than females (figure 1C). The 2021 all-ages prevalence was 17·5% (95% UI 17·0–18·0) in males and 31·2% (30·7–31·7) in females. Differences by sex were particularly large among adolescents and adults (aged 10–64 years); whereas children younger than 5 years had a comparatively high anaemia prevalence of 41·4% (40·7–42·2), the differences between males and females were smaller for that age group. Prevalence and YLDs in males and females began to increasingly diverge after age 5 years and did not begin to reconverge until age 80 years (figure 1C). For females aged 15–49 years the anaemia prevalence in 2021 was 33·7% (33·0–34·4), compared with 11·3% (10·9–11·8) for males. The corresponding YLD rates per 100 000 population were 926·8 (628·3–1328·7) for females and 151·8 (97·5–230·2) for males. In every region, females had a higher anaemia YLD rate than males. Regions with the largest female-to-male YLD ratio in 2021 were tropical Latin America (6·0 [4·5–7·6]), east Asia (4·2 [3·7–4·5]), and eastern Europe (4·2 [3·5–5·2]; figure 2D). The decrease in anaemia prevalence over time was also larger for males (reduction of 24·1% [21·8–26·3] from 1990 to 2021) than for females (reduction of 6·6% [4·9–8·6]; figure 1D). This disparity was similar in the YLD results, with males having a reduction of 40·2% (37·2–43·1) and females having a reduction of 15·4% (13·6–17·7) between 1990 and 2021. The reduction in anaemia burden over this period was greatest in adults, particularly those aged 20–74 years, for both males and females.

### Geographical trends

We found large geographical disparities in anaemia burden (figure 2). Total anaemia prevalence in 2021 was greatest in western sub-Saharan Africa (47·4% [95% UI 45·1–49·5]), south Asia (43·0% [41·9–44·0), and central sub-Saharan Africa (35·7% [33·1–39·3]; figure 2A). These regions had the largest YLD rates per 100 000 population in 2021 (western sub-Saharan Africa 1540·8 [1042·1–2202·4], south Asia 1264·5 [856·5–1821·7], and central sub-Saharan Africa 962·6 [635·6–1429·6]; figure 2B). These regions also had the greatest anaemia burden in 1990 (appendix pp 38–43). By contrast, the regions with the lowest prevalence in 2021 were Australasia (5·7% [5·1–6·9]), western Europe (6·0% [5·7–6·4]), and high-income North America (6·8% [6·2–7·6]; figure 2A). Disparities in anaemia prevalence were more pronounced at the country level. In 2021, the three countries with the highest burden (Mali, Zambia, and Togo) all had an anaemia prevalence of greater than 50%, whereas the three countries with the lowest burden (Iceland, Norway, and Monaco) all had an anaemia prevalence of less than 5%. Whereas 22 countries had an anaemia prevalence of greater than 50% in 1990, only four countries (Mali, Zambia, Togo, and Senegal) had such a high prevalence in 2021.

### Epidemiological transition

We observed a large negative association between SDI and anaemia burden (appendix p 17); countries at higher SDI levels tended to have lower anaemia prevalence and YLD rates. Expected anaemia prevalence based on SDI varied from 52·9% at the lowest observed SDI value to 3·5% at the highest observed SDI value; the equivalent range for anaemia YLD rates per 100 000 population was 1735·2 to 42·1. Substantial variation in anaemia YLD rates were also observed within SDI levels. Countries with the highest observed-to-expected ratios for YLDs based on SDI in 2021 were the United Arab Emirates (ratio 5·3), Saudi Arabia (4·1), and the Bahamas (4·1), and those with the lowest ratios were El Salvador (0·3), Ecuador (0·4), and Nicaragua (0·4; figure 2C).

### Trends in causes of anaemia

**Figure 3:**
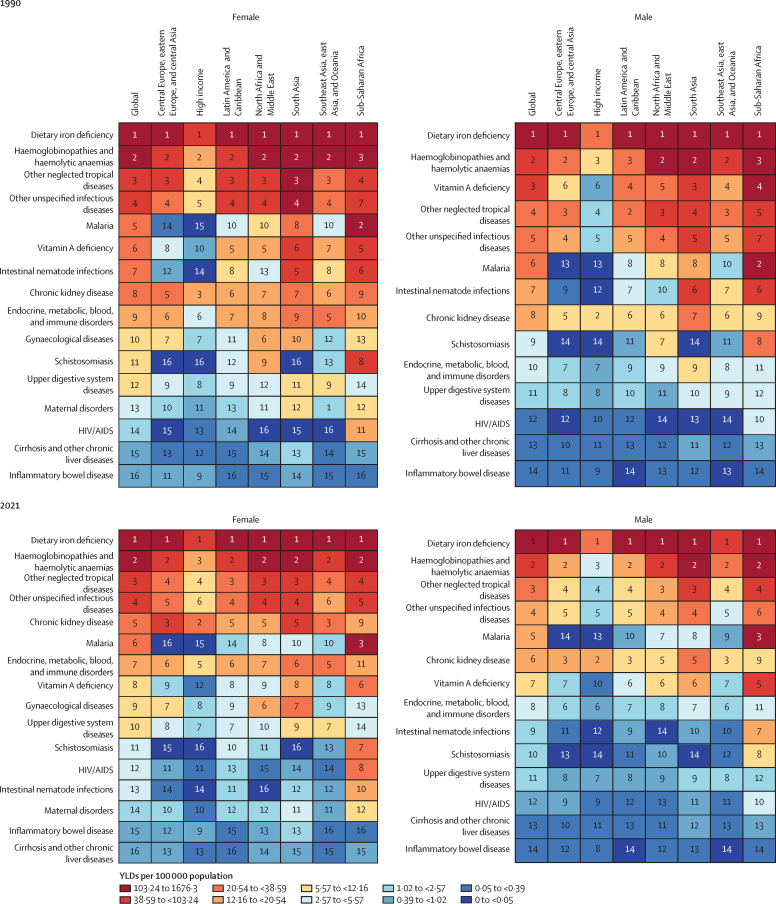
Causes of anaemia ranked by all-ages YLD rate, globally and by super-region, in males and females in 1990 and 2021 YLDs=years lived with disability.

Across all ages and male and female sexes, the leading causes of anaemia YLDs globally in 2021 were dietary iron deficiency (cause-specific anaemia YLD rate per 100 000 population: 422·4 [95% UI 286·1–612·9]), haemoglobinopathies and haemolytic anaemias (89·0 [58·2–123·7]), and other neglected tropical diseases (36·3 [24·4–52·8]; figure 3), collectively accounting for 84·7% (84·1–85·2) of anaemia YLDs. The burden due to dietary iron deficiency anaemia was particularly large, comprising 66·2% (65·5–66·8) of total anaemia cases with 444 million (433–455) cases among males and 825 million (811–839) cases among females globally in 2021. Dietary iron deficiency was the leading cause of all-ages anaemia YLDs in every GBD super-region (figure 3). Compared with prevalence, dietary iron deficiency was responsible for a smaller share of anaemia YLDs (64·0% [63·3–64·9]), reflective of dietary iron deficiency being associated with comparatively less severe forms of anaemia.

**Figure 4:**
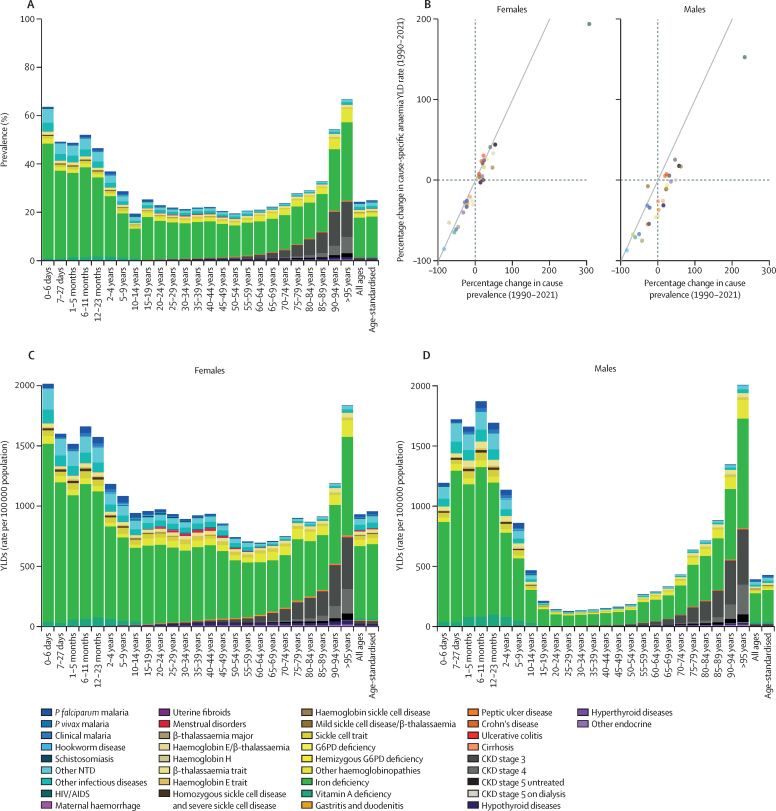
Global distribution of anaemia causes (A) Cause-specific anaemia prevalence by age, for male and female sexes, 2021. (B) Percentage change in YLDs versus the percentage change in cause prevalence, all ages, for male and female sexes, 1990–2021. (C) Cause-specific anaemia YLD rate per 100 000 population by age, for males, 2021. (D) Cause-specific anaemia YLD rate per 100 000 population by age, for females, 2021. CKD=chronic kidney disease. Endocrine=endocrine, metabolic, blood, and immune disorders. G6PD=glucose-6-phosphate dehydrogenase. NTD=neglected tropical diseases. *P falciparum*=*Plasmodium falciparum*. *P vivax*=*Plasmodium vivax*. YLDs=years lived with disability.

We observed variation in the relative contribution of causes of anaemia by age and sex that mirrored the underlying epidemiology of the causes. For example, in the oldest age groups, anaemia due to chronic kidney disease increased considerably, accounting for the second-largest share of anaemia YLDs globally for people older than 80 years (figure 4). Among children younger than 5 years, the most frequent cause of anaemia was dietary iron deficiency, but haemoglobinopathies, other infectious diseases, and malaria were also important contributors in locations where these diseases were prevalent. Gynaecological disorders and maternal haemorrhage were important contributors to anaemia burden among women of reproductive age, although dietary iron deficiency was the largest contributor for females in this age group and accounted for a substantial portion of the difference in anaemia burden between males and females (figure 4C, D). In general, changes in the epidemiology and prevalence of underlying causes over time were similarly reflected in changes in anaemia burden (figure 4B).

Regional variation in disease distribution was also reflected in cause-specific anaemia burden. For example, HIV/AIDS was the second largest contributor to anaemia YLDs in southern sub-Saharan Africa. Anaemia due to malaria was most prominent in the central, eastern, and western sub-Saharan Africa regions, and this cause ranked second or third in YLD burden in each of these regions. Chronic illnesses—particularly chronic kidney disease—contributed the second-largest share of anaemia YLDs across all ages in western Europe, high-income North America, and high-income Asia Pacific, whereas infections caused less anaemia in these than in other regions (figure 3).

## Discussion

Anaemia affected more than 1·9 billion people and caused 52·0 million YLDs in 2021. This massive burden represented 5·7% of all YLDs in 2021, with only two level 3 GBD causes (low back pain and depressive disorders) responsible for more disability. Reductions in anaemia YLDs outpaced prevalence changes between 1990 and 2021, reflecting a global shift towards less severe anaemia,^30^ but progress was variable and comparatively slow. The largest decreases were among males and adults aged 20–74 years and not the young children (<5 years) and women of reproductive age that are the focus of international targets on anaemia and nutrition. All but one region saw decreases at the aggregate level, but considerable geographical disparities within regions remain and the range in prevalence between countries with the highest and lowest burdens is more than 50 percentage points.

Preventing diseases and injuries that cause anaemia is a crucial component of any public health strategy for anaemia. For example, antiretroviral therapy can help to reverse anaemia and improve survival in people living with HIV.^31^ Malaria control methods (eg, mosquito vector management and treatment of bed nets with insecticide), malaria vaccination,^32^ and increased access to antimalarial medications^47^ can reduce the incidence of malaria and subsequently reduce anaemia.^39^ Curing parasitic infections with antihelminthics can reverse chronic inflammation and blood loss due to conditions such as hookworm,^33^ schistosomiasis,^34^ and other neglected tropical diseases. Chronic kidney disease and other diseases that lead to chronic inflammation, decreased erythropoietin production, or both are an important cause of anaemia among older populations;^35^ monitoring and preventing the onset and progression of these diseases can substantially reduce the anaemia burden in these groups.^36^ Because of the importance of considering underlying causes, cause-specific anaemia YLD estimates are the most informative view of underlying epidemiology, especially with our approach, which assigns each prevalent case of anaemia to a single underlying cause.

A substantial component of the higher prevalence of anaemia in women of reproductive age is probably related to unmet needs for family planning services. Hormonal contraception has shown to be effective^37^ as both prevention and treatment for anaemia caused by abnormal uterine bleeding,^38^, ^39^ as its use has been associated with lower anaemia rates in the community.^40^ Gender inequalities related to household food consumption and division of labour probably exacerbate disparities in conditions such as anaemia, because women might be the most likely among household members to be affected by food insecurity^41^, ^42^ and to lack access to sufficient quantities of iron-rich foods,^43^ and be less likely to receive health screening and care, whether due to domestic work demands, lack of autonomy, or prioritisation of other family members' care.^44^, ^45^ Social interventions, including education for girls and women^46^ and expanded agricultural empowerment (eg, access to productive resources, self-managed time, decision-making power, and financial control) of women,^47^ could help to reduce these disparities. Further research into gender inequalities is needed to evaluate the relative contributions of each of these factors in different contexts and to target interventions aimed at reducing the enormous anaemia burden among women of reproductive age.

Dietary iron deficiency was the leading cause of anaemia in most demographics, including children and women of reproductive age, and iron delivery interventions remain a mainstay of public health programmes. Iron delivery approaches include large-scale food fortification,^48^ oral iron supplements,^49^ and intravenous iron (depending on context and severity).^50^ In addition to reducing iron-deficiency anaemia directly, iron delivery can have other positive effects. As part of a multiple micronutrient supplementation regimen, iron administration during the antenatal period is associated with improved birth outcomes and improved maternal and infant health.^51^ Iron repletion might improve cognition,^52^ although benefits are not uniform across age groups and have not been shown in children younger than 5 years.^53^, ^54^ Delayed cord clamping, breastfeeding, and vitamin A supplementation for severe vitamin A deficiency^55^ could also reduce the anaemia burden in newborn babies, especially those born preterm who might also benefit from early erythropoietin administration.^56^ However, iron delivery alone is unlikely to prevent or treat all iron deficiency, and a growing body of evidence suggests that iron supplementation could be harmful to some children with acute or chronic infections.^38^ Population-level interventions for anaemia in children should therefore consider the infectious status of the community and the individual.^57^, ^58^

Several fundamental unknowns remain in our collective understanding of anaemia; many researchers worldwide are working on these problems and that focus must be sustained. First and foremost, we do not truly know what the ideal definition of anaemia should be. Although many health conditions are defined on the basis of empirical assessments of increased rates of poor health outcomes (eg, diabetes, hypertension, obesity, and chronic kidney disease) or statistical deviations in representative populations (eg, childhood growth standards), WHO anaemia definitions do not have a similar rigorous clinical basis.^18^ Evidence-based definitions of anaemia according to health loss associated with low haemoglobin concentrations are urgently needed to guide updated burden assessments, clinical standards,^59^ and subsequent prioritisation of this public health problem.^60^ Work from the BRINDA team suggests that existing formulae for altitude adjustment could lead to underestimation of anaemia at lower altitudes (<2000 m) and overestimation at higher altitudes (>3000 m), with differences in total anaemia prevalence ranging from three to 22 percentage points in the locations studied.^61^ Second, methods of haemoglobin sampling (eg, whole *vs* capillary blood) and analysis (eg, laboratory *vs* point-of-care testing) vary considerably; the effect of this heterogeneity on estimates of anaemia burden and trends requires further exploration, as some evaluations have suggested up to a 28% variation between sampling methods.^62^ Third, although we assess the direct disability burden of anaemia in the form of YLDs, this measure probably represents only a small part of the full health effects of low haemoglobin concentrations. In addition to anaemia-related risks of preterm labour, low birthweight, short gestation, stillbirth, and impaired motor and cognitive development, anaemia has been associated with increased risk of several conditions including stroke,^63^ cardiovascular disease,^64^ dementia,^65^ vision problems,^66^ low bone mineral density,^67^ and increased all-cause mortality after surgery^68^ and in older adults.^7^ A rigorous assessment of the evidence for low haemoglobin concentrations as an upstream risk factor for morbidity and mortality is necessary to close this knowledge gap. Similarly, although acute and chronic anaemia are common comorbidities in the hours and days preceding death, and blood transfusion is universally regarded as a life-saving intervention, there has been no comprehensive accounting for anaemia-associated mortality during this period.^69^ Furthermore, disability weights and YLDs do not account for changes in health loss associated with adaptation and chronicity of illness. As such, these and similar analyses capture disease burden before longer-term compensatory changes.

This study has a number of limitations. First, data availability varied considerably by age and sex. Although comparatively many haemoglobin surveys exist for children older than 6 months and women of reproductive age, data are much sparser for younger children, adult males, and males and females older than 60 years. In addition, high-quality data at more granular levels to inform subnational estimation, including those subnational locations included in this analysis, are comparatively sparse. Second, WHO anaemia definitions are not available for children younger than 6 months, so we imputed thresholds based on median haemoglobin concentrations for older children; this probably imparted additional uncertainty in burden estimates beyond what is presented. Third, in our causal analysis, we assumed a linear cause-specific haemoglobin shift for any starting haemoglobin value, and we assumed that the shape of the population haemoglobin distribution was constant, which might not capture the true variation in cause-specific effects on haemoglobin distributions across geography and cause. Fourth, because the amount of data available to inform haemoglobin shifts varied substantially by cause (appendix pp 18–25) we were not able to capture uncertainty in the shifts themselves. Fifth, the assumption that each anaemia case has only a single underlying cause, although reflecting a sparsity of data to inform the interplay between diseases, nonetheless is a limitation in that many people with anaemia are likely to have multiple comorbid conditions contributing to their anaemia. Additional data on the combined effects of comorbid conditions on haemoglobin concentrations are needed to fully account for the multiple causes underlying many cases of anaemia. Finally, we were unable to capture all potential causes of anaemia in our causal analysis, owing to an absence of either estimates of disease prevalence or associated haemoglobin shifts. These potential causes include cancers, injuries, some micronutrient deficiencies (eg, folate and cobalamin), causes of inflammation not already captured in this analysis, and drug reactions. In addition, we had to estimate some causes as residual causes, again because either cause prevalence estimates or haemoglobin shifts were not available; this includes dietary iron deficiency, the largest cause of anaemia in our analyses.

Anaemia remains a major public health issue across the life course. The persistently high anaemia burden—particularly in women of reproductive age and young children—underscores the need for renewed attention on accurately measuring the prevalence of anaemia and its underlying causes, and using these data to design comprehensive policies and interventions that reflect the context-specific epidemiology of the disease and its determinants. Analyses of population-level anaemia burden can provide the insights required to appropriately tailor interventions at the country and subnational levels in an effort to reduce the prevalence of anaemia across all ages and sexes. Although probably not obtainable for most countries, multifaceted and contextual approaches will be necessary to ensure substantive progress towards Sustainable Development Goals 2 and 3 and WHO Global Nutrition Targets.

Correspondence to: Prof Nicholas J Kassebaum, Institute for Health Metrics and Evaluation, Seattle, WA 98195, USA **nickjk@uw.edu**

## Data sharing

To download the data used in these analyses and corresponding results, please visit the Global Health Data Exchange at http://ghdx.healthdata.org.

## Declaration of interests

S Afzal reports participation on a data safety monitoring board or advisory board with the National Bioethics Committee of Pakistan, King Edward Medical University Institutional Review Board, and the Ethics Review Board in Board of Studies; a leadership or fiduciary role with the Pakistan Association of Medical Editors, as a Fellow of Faculty of Public Health (FFPH) Royal Colleges UK, and an advocacy role in the Society of Prevention and Advocacy Research with King Edward Medical University, Lahore, Pakistan, all outside the submitted work. R Agustina reports leadership or fiduciary roles with the Multiple Micronutrient Supplementation Technical Advisory Group of the New York Academy of Sciences and with the Indonesia Technical Advisory Board for Multimicronutrient Supplemenation, outside the submitted work. R Ancuceanu reports payment or honoraria for lectures, presentations, speakers bureaus, manuscript writing or educational events from AbbVie, Sandoz, B Braun, and Laropharm, outside the submitted work. S Das is a member of the Personalized Medicine Division of the American Association of Clinical Chemistry and a member of the Royal College of Biology; and reports other financial interests through a research grant of 1·6 million INR from the Department of Science and Technology, Government of India; all outside the submitted work. C Hennessy reports support for the present work from the Institute for Health Metrics and Evaluation and the Center for Health System Effectiveness. N E Ismail is a council member of the Malaysian Academy of Pharmacy, outside the submitted work. J J Jozwiak reports payment or honoraria for lectures, presentations, speakers bureaus, manuscript writing or educational events from Novartis and Adamed as personal payments, outside the submitted work. N J Kassebaum reports support for this manuscript from the Bill & Melinda Gates Foundation as grant payments to their institution (Institute of Health Metrics and Evaluation, University of Washington, Seattle, WA, USA). K Krishan reports non-financial support from UGC Centre of Advanced Study, CAS II, Department of Anthropology, Panjab University, Chandigarh, India, outside the submitted work. W Mendoza is a staff member at the UNFPA Peru Country Office, which does not necessarily endorse these results. A-F A Mentis reports grants or contracts from MilkSafe: a novel pipeline to enrich formula milk using omics technologies, a project co-financed by the European Regional Development Fund of the European Union and Greek national funds through the Operational Program Competitiveness, Entrepreneurship and Innovation, under the call RESEARCH – CREATE – INNOVATE (project code T2EDK-02222) and from ELIDEK (Hellenic Foundation for Research and Innovation, MIMS-860); has received payment for expert testimony as a peer-reviewer for Fondazione Cariplo, Italy; serves as an editorial board member for the journals *Systematic Reviews* and *Annals of Epidemiology*, and as an Associate Editor for *Translational Psychiatry*; and is a scientific officer with the BGI Group; all outside the submitted work. N Moka is Treasurer of the Kentucky Society of Clinical Oncology, outside the submitted work. A Ortiz reports grants or contracts from Sanofi as payments to their institution (IIS-Fundacion Jiménez Díaz UAM, Madrid, Spain); consulting fees from Advicienne, Astellas Pharma, AstraZeneca, Amicus Therapeutics, Amgen, Boehringer Ingelheim, Fresenius Medical Care, GSK, Bayer, Sanofi-Genzyme, Menarini, Mundipharma, Kyowa Kirin, Lilly, Alexion Pharmaceuticals, Freeline Therapeutics, Idorsia, Chiesi, Otsuka Pharmaceutical, Novo Nordisk, Sysmex, and Vifor Fresenius Medical Care Renal Pharma; support for travel from Advicienne, Astellas Pharma, AstraZeneca, Amicus Therapeutics, Amgen, Boehringer Ingelheim, Fresenius Medical Care, GSK, Bayer, Sanofi-Genzyme, Menarini, Mundipharma, Kyowa Kirin, Lilly, Alexion Pharmaceuticals, Freeline Therapeutics, Idorsia, Chiesi, Otsuka Pharmaceutical, Novo Nordisk, Sysmex, and Vifor Fresenius Medical Care Renal Pharma; a leadership or fiduciary role with the European Renal Association, a role as Director of the Catedra Mundipharma-UAM of diabetic kidney disease and the Catedra AstraZeneca-UAM of chronic kidney disease and electrolytes; and stock or stock options in Telara Pharma; all outside the submitted work. Z Quazi Syed reports support for this manuscript from the South Asia Infant Feeding Research Network and Datta Meghe Institute of Higher Education and Research, Wardha, India; grants or contracts from the Global Consortium for Public Health and Research and Datta Meghe Institute of Higher Education and Research; support for attending meetings and/or travel from the Division of Evidence Synthesis, Jawaharlal Nehru Medical College, Wardha, India, and Datta Meghe Institute of Medical Sciences, Wardha, India, outside the submitted work. T Richards reports grants or contracts from Vifor Pharma for PREVENT laboratory analysis; consulting fees from BioAge Labs for a clinical trial design; payment or honoraria for lectures, presentations, speakers bureaus, manuscript writing or educational events from Pharmacosmos through the CAVIAR education grant and from Pfizer for the IRONWOMAN trial; support for attending meetings from Pfizer, Pharmacosmos, and Vifor Pharma; a role as treasurer with NATA; and a role as Director of The Iron Clinic; all outside the submitted work. V Shivarov is an employee of ICON and reports stock or stock options in the company, all outside the submitted work. J A Singh reports consulting fees from Crealta/Horizon, MediSys, Fidia Farmaceutici, PK Med, Two Labs, Adept Field Solutions, Clinical Care Options, Clearview Healthcare Partners, Putnam Associates, Focus Forward, Navigant Consulting, Spherix Global Insights, Mediq, Jupiter Life Science, UBM, Trio Health, Medscape, WebMD, and Practice Point Communications, the National Institutes of Health, and the American College of Rheumatology; payment or honoraria for speakers bureaus from Simply Speaking; support for attending meetings or travel from the steering committee of OMERACT; participation on a data safety monitoring board or advisory board with the US Food and Drug Administration Arthritis Advisory Committee; membership of the steering committee of OMERACT, a role as Chair (unpaid) of the Veterans Affairs Rheumatology Field Advisory Committee, and roles as Editor and Director (unpaid) with the UAB Cochrane Musculoskeletal Group Satellite Center on Network Meta-analysis; stock or stock options in TPT Global Tech, Vaxart Pharmaceuticals, Aytu BioPharma, Adaptimmune Therapeutics, GeoVax Labs, Pieris Pharmaceuticals, Enzolytics, Seres Therapeutics, Tonix Pharmaceuticals, and Charlotte's Web Holdings, and previous stock options in Amarin, Viking Therapeutics, and Moderna Pharmaceuticals; all outside the submitted work. J D Stanaway reports support for this manuscript from the Bill & Melinda Gates Foundation as grants to their institution (Institute of Health Metrics and Evaluation, University of Washington, Seattle, WA, USA). P S Suchdev reports grants or contracts from the Centers for Disease Control and Prevention (CDC) and the Bill & Melinda Gates Foundation, all outside the submitted work. M F Young reports grants from the Bill & Melinda Gates Foundation and the CDC for a project titled Biomarkers reflecting inflammation and nutritional determinants of anemia (BRINDA) and from the National Institutes of Health for a project titled Mother–child hemoglobin at preconception and first 1000 days and child development at 6 years; roles in the *Lancet Haematology* Commission on anaemia, the Anaemia Evidence Gap Map Advisory Group, as a subject matter expert on the advisory committee related to the Improving Estimates of Anemia in Global Burden of Disease research project, as a member of the technical advisory group for the Redefining Maternal Anaemia in Pregnancy and Postpartum project and a member of the WHO Guideline Development Group for Anemia; all outside the submitted work. All other authors declare no competing interests.
