# Correction to *Lancet Haematol* 2023; 10: e713–34

**DOI:** 10.1016/S2352-3026(23)00283-1

**Published:** 2023-10-02

*GBD 2021 Anaemia Collaborators. Prevalence, years lived with disability, and trends in anaemia burden by severity and cause, 1990–2021: findings from the Global Burden of Disease Study 2021.* Lancet Haematol *2023; **10:** e713–34*—In this Article, Hossein Akbarialiabad's affiliation should have been “St George and Sutherland Clinical School, UNSW Medicine, University of New South Wales, Sydney, NSW, Australia”. This correction has been made as of Oct 2, 2023.

