# Correction to *Lancet Haematol* 2023; 10: e713–34

**DOI:** 10.1016/S2352-3026(23)00373-3

**Published:** 2023-12-20

*GBD 2021 Anaemia Collaborators. Prevalence, years lived with disability, and trends in anaemia burden by severity and cause, 1990–2021: findings from the Global Burden of Disease Study 2021.* Lancet Haematol *2023; **10:** e713–34*—The author list of this Article has been supplied for indexing on PubMed. This correction was made to the online version on Oct 31, 2023.

