# Supplementary material for: Prevalence, years lived with disability, and trends in anaemia burden by severity and cause, 1990–2021: findings from the Global Burden of Disease Study 2021
Source: Lancet Haematol. 2023 Jul 31;10(9):e713–34. doi: 10.1016/S2352-3026(23)00160-6 (PMC10465717; doi:10.1016/S2352-3026(23)00160-6)
Supplement: Supplementary appendix [file mmc1.pdf]

THE LANCET
Haematology

Supplementary appendix

This appendix formed part of the original submission and has been peer reviewed. We post it as supplied by the authors.

Supplement to: GBD 2021 Anaemia Collaborators. Prevalence, years lived with disability, and trends in anaemia burden by severity and cause, 1990–2021: findings from the Global Burden of Disease Study 2021. *Lancet Haematol* 2023; published online July 31. https://doi.org/10.1016/S2352-3026(23)00160-6.

Appendix to “Levels and trends in anaemia burden by severity and cause from 1990–2021: findings from the Global Burden of Disease Study 2021”

Contents

Section 1: GATHER Checklist 3

Section 2: Anaemia Estimation Methods 5

Figure S0. GBD Estimation flowchart for anaemia and causes of anaemia 5

Anaemia envelope: input data and methodological summary 6

Case definition 6

Table S1. Definitions of mild, moderate, and severe anaemia based on blood haemoglobin concentration 6

Input data 6

Figure S1. Anaemia 2021 – Unique source-years of data by country and sub-national unit 7

Data processing 7

Method of blood sampling and method of testing 7

Elevation adjustment and smoking adjustment 8

Figure S2. Haemoglobin adjustment for elevation. 8

Age-sex splitting and crosswalking data by pregnancy status 8

Table S2. MR-BRT mean haemoglobin crosswalk values 9

Figure S3. MR-BRT mean haemoglobin crosswalk funnel plot. 9

Modelling strategy 9

ST-GPR models of mean haemoglobin and anaemia prevalence 10

Table S3. ST-GPR ensemble stage 1 covariates and expected directions 10

Ensemble distribution modeling 11

Generation of ensemble weights 11

Haemoglobin variance optimisation 12

Fitting ensemble distributions with method of moments 13

Finding the area under the curve to calculate anaemia prevalence 13

Anaemia causal attribution: input data and methodological summary 13

Causes and inputs 13

Table S4. Causes included in GBD Anaemia Causal Attribution. 14

Modelling strategy 15

Table S5. GBD 2021 residual aetiology distribution proportions 15

Years lived with disability (YLDs) calculation 16

Table S6. Anaemia disability weights and health states 16

Epidemiological transition analyses 17
Section 3: Cause-Specific Haemoglobin Shifts 18
Overview 18
Table S7. GBD 2021 cause-specific haemoglobin shifts 18
Malaria: PfPR, PvPR, and clinical malaria 19
Hookworm disease 20
Schistosomiasis 21
HIV/AIDS 21
Other neglected tropical diseases and other infectious diseases 21
Maternal haemorrhage 21
Uterine fibroids 22
Menstrual disorders 22
Haemoglobinopathies: thalassaemias, thalassaemia trait, sickle cell disorders, sickle cell trait, glucose-6-phosphate dehydrogenase (G6PD) deficiency, G6PD trait, and other haemoglobinopathies and haemolytic anaemias 22
Nutritional deficiencies 23
Vitamin A deficiency 23
Iron deficiency 23
Gastrointestinal 23
Upper GI 23
Gastritis, duodenitis, and peptic ulcer disease 23
Inflammatory bowel disease 24
Crohn's disease and ulcerative colitis 24
Decompensated Cirrhosis 24
Chronic kidney disease 24
Thyroid disorders 24
Other endocrine, nutrition, blood, and immune disorders 25
References 25
Section 4: Spatiotemporal Gaussian process regression methods 32
Section 5: Supplemental Tables and Figures 37
Section 6: Anaemia burden location profiles 48
Section 7: Anaemia prevalence input data sources 888
Authors' Contributions 924

Section 1: GATHER Checklist

#	GATHER checklist item	Description of compliance	Reference
**Objectives and funding**			
**1**	Define the indicator(s), populations (including age, sex, and geographic entities), and time period(s) for which estimates were made.	Narrative provided in paper and appendix describing indicators, definitions, populations, and time periods	Main text (Methods) and Appendix (Section 2)
**2**	List the funding sources for the work.	Funding sources listed in paper	Summary (Funding)
**Data Inputs**			
For all data inputs from multiple sources that are synthesized as part of the study:			
**3**	Describe how the data were identified and how the data were accessed.	Narrative description of data seeking methods provided	Main text (Methods) and Appendix (Section 2)
**4**	Specify the inclusion and exclusion criteria. Identify all ad-hoc exclusions.	Narrative about inclusion and exclusion criteria provided; ad hoc exclusions in appendix supplementary methods	Main text (Methods) and Appendix (Section 2)
**5**	Provide information on all included data sources and their main characteristics. For each data source used, report reference information or contact name/institution, population represented, data collection method, year(s) of data collection, sex and age range, diagnostic criteria or measurement method, and sample size, as relevant.	An interactive, online data source tool that provides metadata for data sources by component, geography, cause, risk, or impairment has been developed	Appendix (Section 7) and Global Health Data Exchange
**6**	Identify and describe any categories of input data that have potentially important biases (e.g., based on characteristics listed in item 5).	Summary of known biases included in appendix supplementary methods	Appendix (Section 2)
For data inputs that contribute to the analysis but were not synthesized as part of the study:			
**7**	Describe and give sources for any other data inputs.	Included in online data source tool	Global Health Data Exchange
For all data inputs:			
**8**	Provide all data inputs in a file format from which data can be efficiently extracted (e.g., a spreadsheet rather than a PDF), including all relevant meta-data listed in item 5. For any data inputs that cannot be shared because of ethical or legal reasons, such as third-party ownership, provide a contact	Downloads of input data available through online data tools, including data visualisation tools and data query tools; input data not available in tools will be made available upon request	Online data visualisation tools, data query tools, and the Global Health Data Exchange

	name or the name of the institution that retains the right to the data.		
**Data analysis**			
**9**	Provide a conceptual overview of the data analysis method. A diagram may be helpful.	Flow diagram of methodological process provided, as well as narrative descriptions of modelling process	Main text (Methods) and Appendix (Section 2)
**10**	Provide a detailed description of all steps of the analysis, including mathematical formulae. This description should cover, as relevant, data cleaning, data pre-processing, data adjustments and weighting of data sources, and mathematical or statistical model(s).	Flow diagram and detailed methods write-up covering all data extraction, processing, and modelling processes provided	Main text (Methods) and Appendix (Section 2)
**11**	Describe how candidate models were evaluated and how the final model(s) were selected.	Provided in methodological write-up	Appendix (Section 2)
**12**	Provide the results of an evaluation of model performance, if done, as well as the results of any relevant sensitivity analysis.	Provided in methodological write-up	Appendix (Section 2)
**13**	Describe methods for calculating uncertainty of the estimates. State which sources of uncertainty were, and were not, accounted for in the uncertainty analysis.	Provided in main text methods narrative description and appendix methodological write-up	Main text (Methods) and Appendix (Section 2)
**14**	State how analytic or statistical source code used to generate estimates can be accessed.	Remote code repository for access to analytic code provided	Remote code repository
**Results and Discussion**			
**15**	Provide published estimates in a file format from which data can be efficiently extracted.	GBD 2021 results available through online data and visualisation tools, the Global Health Data Exchange, and the online data query tool	Main text, appendix, online data and visualisation tools
**16**	Report a quantitative measure of the uncertainty of the estimates (e.g. uncertainty intervals).	Uncertainty provided with all results	Main text, appendix, online data and visualisation tools
**17**	Interpret results in light of existing evidence. If updating a previous set of estimates, describe the reasons for changes in estimates.	Discussion of results and methodological changes between GBD rounds provided in manuscript narrative and appendix	Main text (Methods and Discussion) and Appendix (Section 2)
**18**	Discuss limitations of the estimates. Include a discussion of any modelling assumptions or data limitations that affect interpretation of the estimates.	Discussion of limitations, including modelling assumptions and data limitations, included in manuscript narrative and appendix	Main text (Methods and Discussion) and Appendix (Section 2)

Section 2: Anaemia Estimation Methods

Figure S0. GBD Estimation flowchart for anaemia and causes of anaemia

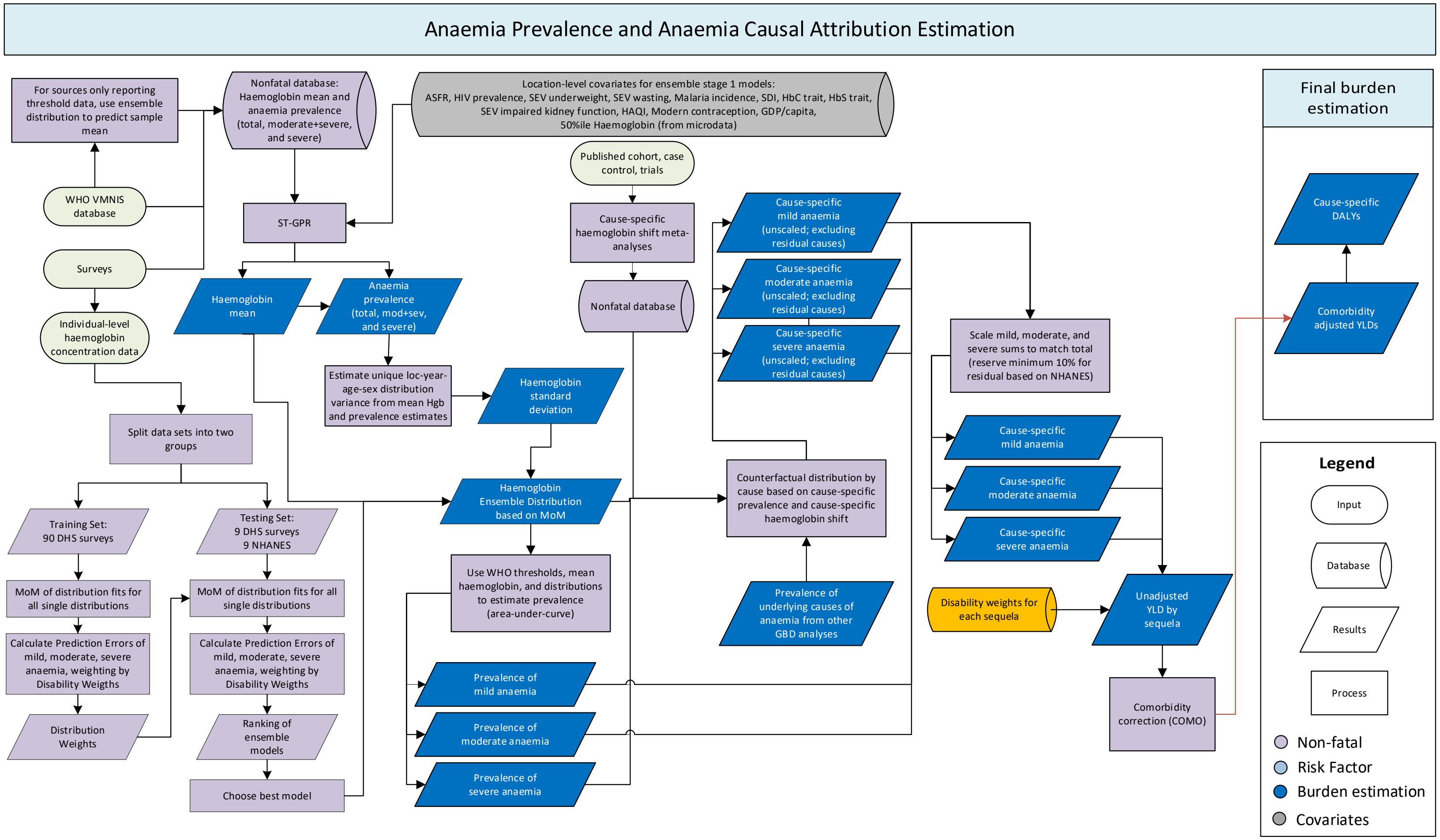

Anaemia envelope: input data and methodological summary

Case definition

Anaemia is defined as decreased blood concentration of haemoglobin, irrespective of underlying cause, red blood cell morphology, or red blood cell function. Thresholds for defining individuals as being anaemic, as well as thresholds for anaemia severity, are based on WHO thresholds for haemoglobin in g/L.[1] There are no international guidelines on appropriate thresholds for diagnosing anaemia in children younger than 6 months of age. To estimate thresholds for these age groups, we calculated the ratio of "normal" haemoglobin (defined as the 50th percentile of the global haemoglobin distribution) for the periods 0–6 days, 7–27 days, and 1–5 months over the 6–11 months age group by severity, and then multiplied the WHO thresholds for 6–59 months by these ratios.

Table S1. Definitions of mild, moderate, and severe anaemia based on blood haemoglobin concentration.

Sex	Age	Mild	Moderate	Severe
Both	0 - 6 days	145 - 159 g/L	100 - 144 g/L	< 100 g/L
Both	7 - 27 days	120 - 134 g/L	85 - 119 g/L	< 85 g/L
Both	1 month - 4 years	100 - 109 g/L	70 - 99 g/L	< 70 g/L
Both	5 - 14 years	110 - 114 g/L	80 - 109 g/L	< 80 g/L
Male	15+ years	110 - 129 g/L	80 - 109 g/L	< 80 g/L
Female, non-pregnant	15+ years	110 - 119 g/L	80 - 109 g/L	< 80 g/L
Female, pregnant	15+ years	100 - 109 g/L	70 - 99 g/L	< 70 g/L

Input data

To estimate total anaemia prevalence (called the "envelope"), we utilised data from a variety of sources. Inclusion criteria comprised quantitative measurement of haemoglobin in either a population-based sample or group judged to adequately represent the sex, age groups, and location of the study.

We searched the Global Health Data Exchange (GHDx) using the keywords "anemia" and "hemoglobin" to identify potential data sources for inclusion. Population-based surveys including the Demographic and Health Survey (DHS), Multiple Indicator Cluster Survey (MICS) series, national micronutrient surveys, and other national and subnational nutrition surveys comprised the bulk of input data. We extracted all sources with individual-level data into GBD age groups by sex and pregnancy status in six different formats: mean haemoglobin concentration, severe anaemia prevalence, moderate anaemia prevalence, moderate+severe anaemia prevalence, mild anaemia prevalence, and total anaemia prevalence. We supplemented these data with additional sources of mean haemoglobin concentration and anaemia prevalence from the WHO Vitamin and Mineral Nutrition Information System (available at https://www.who.int/teams/nutrition-and-food-safety/databases/vitamin-and-mineral-nutrition-information-system/data) and other literature sources, where available.

All input data are available for download through the Global Health Data Exchange (GHDx), and a list of references is included in Section 7 of this appendix.

Figure S1. Anaemia 2021 – Unique source-years of data by country and sub-national unit

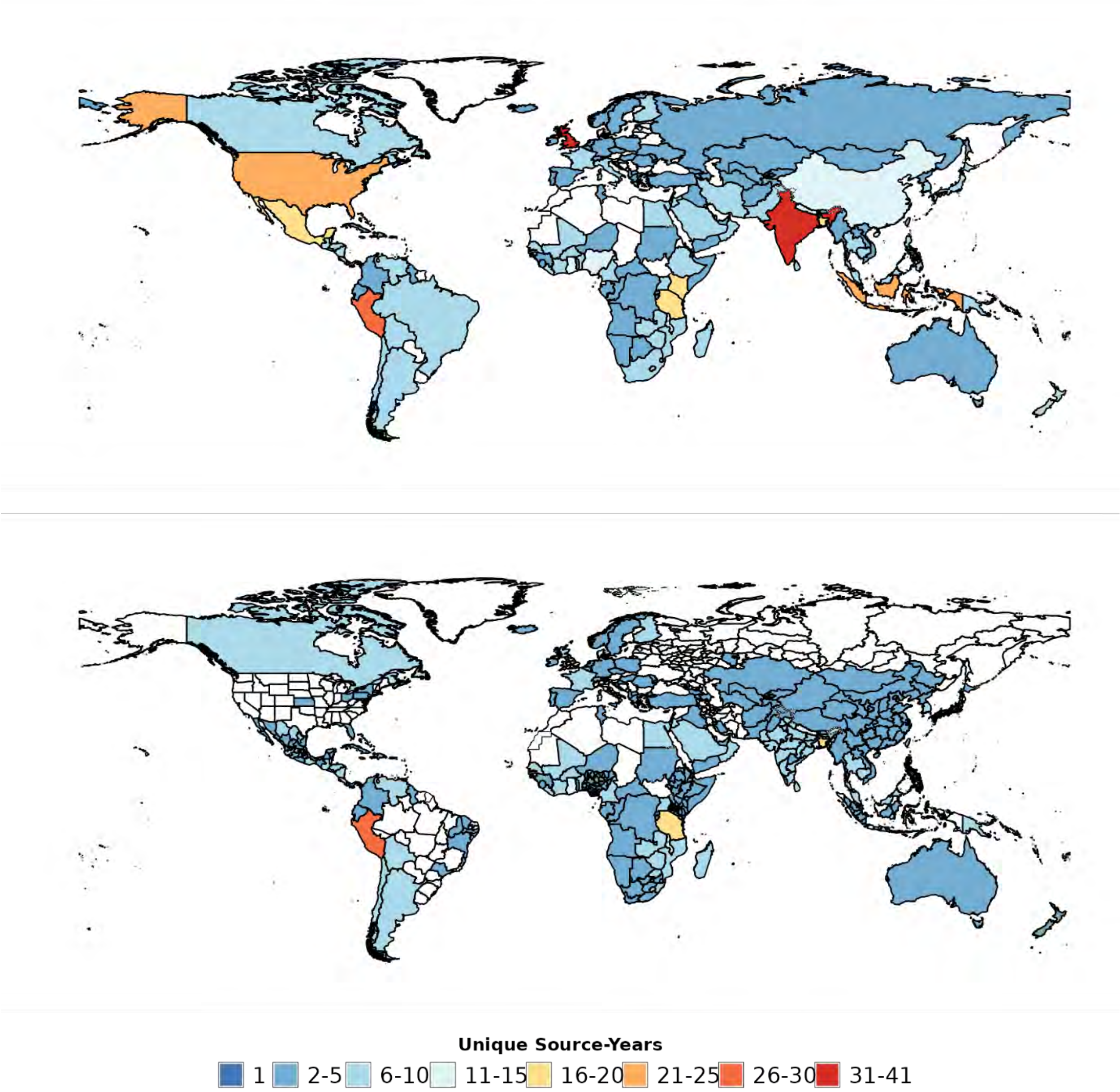

Data processing

Method of blood sampling and method of testing

Most surveys used a HemoCue test, adjusted for elevation, and excluded those with terminal or acute medical conditions. Published scientific literature studies and those from higher income locations typically measured haemoglobin with a Coulter counter. These methods, which both operate by reacting haemoglobin with a specific reagent (Drabkin's solution) and measuring absorbance wavelengths, were treated as equivalent for this analysis. We also did not make any formal distinction in data processing between studies that drew whole blood from participants and those that used capillary venous sampling. Further investigation is needed to determine if formal data adjustment for HemoCue and

capillary venous sampling is needed or if their additional variability, which leads to higher uncertainty in input data sources (and intrinsically lower influence on the model), is sufficient.

Elevation adjustment and smoking adjustment

Haemoglobin concentration increases with increasing elevation, a physiologic response to lower ambient oxygen levels that aims to maintain oxygen delivery throughout the body. Under 1000 meters, there appears to be little effect on haemoglobin, but previous studies have suggested an exponentially-increasing effect of elevation, as illustrated by Figure S2, which is the WHO recommended formula for haemoglobin adjustment.[1]

We used all survey-reported, elevation-adjusted, or elevation- and smoking-adjusted haemoglobin data directly without further adjustment. Using the equation above, we adjusted individual-level data that did not present elevation-adjusted haemoglobin values but did include elevation levels. Testing alternative approaches for elevation adjustment is an area for further investigation. Where smoking-adjusted data were unavailable, no additional adjustments were made in the GBD 2021 analysis.

Figure S2. Haemoglobin adjustment for elevation.

WHO haemoglobin adjustment factor (difference in haemoglobin) by elevation in meters. Adjustments are based on the formula: $\Delta Hb = -0{\cdot}32 \times (\text{elevation in meters} \times 0{\cdot}0033) + 0{\cdot}22 \times (\text{elevation in meters} \times 0{\cdot}0033)^2$

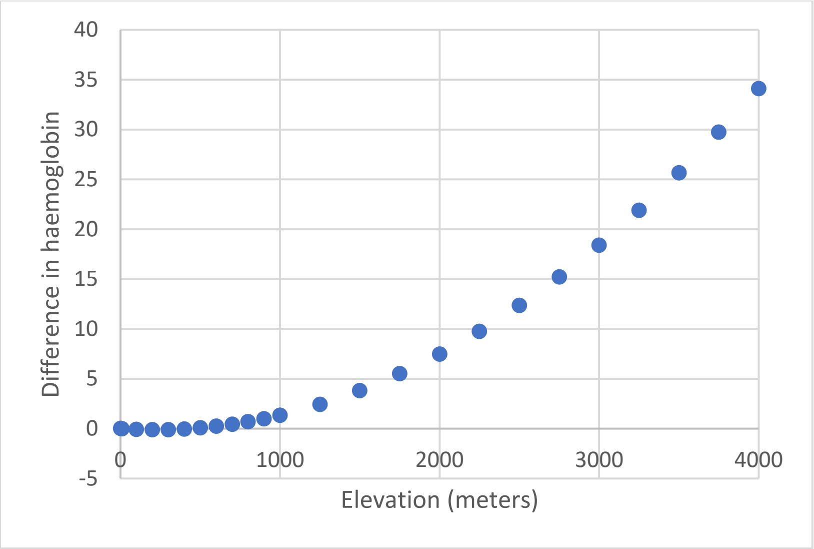

Age-sex splitting and crosswalking data by pregnancy status

We pooled all data that fit entirely within single GBD age-sex groups to calculate a global age and sex pattern of mean haemoglobin concentration and anaemia prevalence by severity. We then used these global age and sex patterns to proportionally split any datum that spanned more than a single GBD age-sex group into the age- and sex-specific GBD groups.

Haemoglobin concentration varies systematically based on pregnancy status. Our models of mean haemoglobin concentration and anaemia prevalence are based on these values among non-pregnant females, and these estimates are later adjusted to account for pregnancy prevalence for each location/year/age/sex as described in the "Fitting ensemble distributions with method of moments" methods section below. Studies that only reported mean haemoglobin among pregnant women were crosswalked to fit our model case definition of non-pregnant women. To estimate this crosswalk, we

matched data sources where we had age-specific estimates of mean haemoglobin separately by pregnancy status and calculated the ratio of these values among pregnant females versus non-pregnant females within a single study. We log-transformed these ratios and calculated standard errors of the ratios using the delta method. We then meta-analysed these ratios using the Meta-Regression – Bayesian, Regularized, Trimmed (MR-BRT) meta-analytic tool[2] with a 10% trim setting. Although age was tested as a potential predictor in the model, we did not observe a significant age dependence of the ratios. The crosswalk effects are illustrated below.

Table S2. MR-BRT mean haemoglobin crosswalk values.

Model	Data input	Reference or alternative case definition	Gamma	Beta Coefficient, Log (Mean [Hb]) (95% UI)*	Adjustment factor**
**Mean Haemoglobin**	Non-Pregnant Women	Ref	0·033	---	---
	Pregnant Women	Alt		-0·09<br>(-0·15 – -0·02)	0·92<br>(0·86 – 0·98)

**MR-BRT crosswalk adjustments can be interpreted as the factor the alternative case definition is adjusted by to reflect what it would have been had it been measured using the reference case definition. If the log/logit beta coefficient is negative, then the alternative is adjusted up to the reference. If the log/logit beta coefficient is positive, then the alternative is adjusted down to the reference.*
***The adjustment factor column is the exponentiated beta coefficient. For log beta coefficients, this is the relative rate between the two case definitions. For logit beta coefficients, this is the relative odds between the two case definitions.*

Figure S3. MR-BRT mean haemoglobin crosswalk funnel plot.
Funnel plot for MR-BRT meta-analysis of mean haemoglobin by pregnancy status, standard error versus effect size. Each data point represents a within-study, age-specific ratio of haemoglobin between pregnant and non-pregnant women (log-transformed).

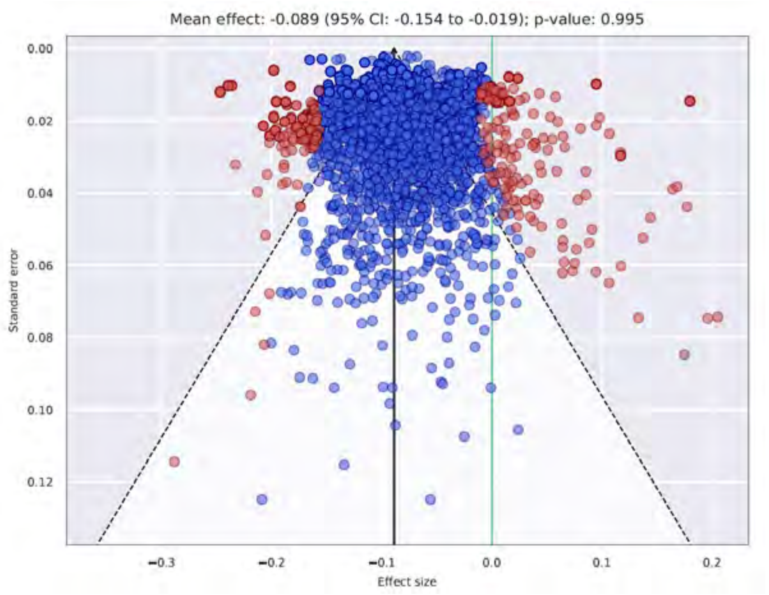

Modelling strategy

Estimation of overall anaemia prevalence occurred in four steps: 1) spatiotemporal Gaussian process regression (ST-GPR) models of mean haemoglobin and prevalence of total, moderate+severe, and

severe anaemia, 2) calculation of ensemble weights, 3) generation of ensemble distributions, and 4) calculation of anaemia prevalence based on the ensemble distributions.

ST-GPR models of mean haemoglobin and anaemia prevalence

In previous GBD iterations, we modelled the mean and standard deviation of haemoglobin and used these as inputs to our ensemble model of haemoglobin concentration. To utilise additional data sources that report prevalence of anaemia directly, in GBD 2021 we amended our modelling process to include models of anaemia prevalence (total, moderate+severe, and severe) instead of modelling the standard deviation of haemoglobin directly.

We modelled 1) mean haemoglobin, 2) total anaemia prevalence, 3) moderate+severe anaemia prevalence, and 4) severe anaemia prevalence using ST-GPR, a three-step modeling process for generating estimates for every location, year, age, and sex in the GBD Study.[3] In all models, mean haemoglobin data were log-transformed and anaemia prevalence data were logit-transformed. The first step of the ST-GPR process is an ensemble linear mixed-effects regression of our data on a set of potentially predictive covariates taken from the GBD Study covariates database, using the lme4 package[4] in R.[5] We tested every combination of these covariates in individual linear mixed-effects regressions with nested random intercepts at the super-region, region, and location levels, where the random intercepts are assumed to be sampled from a Gaussian distribution with mean 0 and an estimated variance, Σ.[4] The random intercepts were used only in fitting the models, and were not used when producing predictions. We then evaluated and ranked each of the sub-models by their out-of-sample root-mean-squared error (RMSE). Finally, to produce initial estimates for every location, year, age, and sex in the analysis, we averaged the 50 top-performing models where the estimated coefficients were 1) statistically significant at $p < 0{\cdot}05$ and 2) in the expected direction, weighting each sub-model's contribution to the final ensemble inverse-proportionally to its RMSE. Table S3 lists each covariate tested in the ensemble regression and the expected sign of the regression coefficient for mean haemoglobin and each anaemia prevalence model. Diagnostics showing the frequency of covariate selection and standardized coefficients can be found in Section 8 of this appendix.

Table S3. ST-GPR ensemble stage 1 covariates and expected directions

Covariate	Mean haemoglobin	Anaemia Prevalence
Age-specific fertility rate	–	+
HIV prevalence	–	+
Summary exposure value (SEV[3]) for child underweight	–	+
SEV for child wasting	–	+
Malaria incidence	–	+
Haemoglobin C (sickle type C) trait (all ages)	–	+
Haemoglobin S (sickle type S) trait (all ages)	–	+
Sociodemographic index[4] (SDI)	+	–
SEV for impaired kidney function	–	+

Healthcare access and quality index[5] (HAQI)	+	–
Modern contraception prevalence	+	–
50th percentile of haemoglobin	+	–

The second spatiotemporal smoothing step of ST-GPR calculates the residual between the stage 1 regression estimate and each of the observed data points and then smooths this residual, drawing strength over space, age, and time and producing a revised stage 2 estimate for every location, year, age, and sex. The third step of ST-GPR is a Gaussian process regression, using the stage 2 estimates as a prior and the observed data points and their variance to 1) further smooth the residual between the stage 2 predictions and observed data and produce a final mean estimate for each location, year, age, and sex and 2) estimate uncertainty around this mean estimate, quantified by taking 1,000 draws from the posterior Gaussian process. More detailed information on the ST-GPR modelling process can be found in Section 4.

Ensemble distribution modeling

We modelled the full distribution of haemoglobin for each population (location/age/year/sex), from which we applied the WHO thresholds to calculate prevalence of anaemia by severity. In GBD 2015, a Weibull distribution was fitted using shape and scale parameters estimated from mean haemoglobin. Since GBD 2016, we now combine multiple two-parameter distributions to create a more precise and unbiased ensemble distribution.

Generation of ensemble weights

First, we created a training and testing set of individual-level haemoglobin measurements. The training set consisted of 90 DHS surveys, providing 290 group-specific samples of microdata from children younger than 5 years, males aged 15–45 years, pregnant females aged 15–45 years, and non-pregnant females aged 15–45 years (not all groups were sampled in each DHS). A set of two-parameter distributions (gamma, mirrored gamma, Weibull, mirrored lognormal, and mirrored Gumbel) were fit to the sample's haemoglobin mean and variance. These distributions were combined using weights optimised by a loss function of severity-specific prediction error weighted by the ratio of the severity's disability weight (DW) to the mild anaemia DW. Weights were constrained to be positive and sum to 1, so that the resultant ensemble distribution is a proper probability density function. All permutations of the five distributions were tested (i.e., we optimised weights for both a mix of all five distributions as well as a gamma-Weibull two-way combination).

The loss function is

$$\sum_{i=1}^{n_i}\sum_{j=1}^{n_j}\sum_{k=1}^{n_k} r_j|p_{ijk} - \hat{p}_{ijk}|$$

Where

$$\hat{p}_{ijk} = \sum_{z=1}^{n_z} w_z \int_{t1_{jk}}^{t2_{jk}} PDF_{ijz}$$

n_i is a list of surveys (in either the training or testing set)

n_j is the list of groups: children <5, males 15–45, pregnant females 15–45, non-pregnant females 15–45, males >45, and females >45

n_k is the list of severities (mild, moderate, severe)

n_z is the list of distributions (gamma, mirrored gamma, Weibull, mirrored lognormal, and mirrored Gumbel)

r is the ratio of the severity j disability weight to that of mild anaemia

$r_k = 13$ for moderate and $r_k = 40$ for severe

PDF is a probability density function fit to the sample mean and variance

t1 and t2 are the lower and upper bounds to the WHO anaemia definition for the group

w is the set of distribution weights (each constrained to be positive) such that

$\sum_{z=1}^{n_z} w_z = 1$ and all $w_z > 0$

Therefore $\sum_{z=1}^{n_z} w_z * PDF_z$ describes the ensemble probability density function that can be integrated to calculate prevalence for any severity.

The testing set consisted of nine National Health and Nutrition Examination Surveys (NHANES) and nine Demographic and Health Surveys (DHS) not included in the training data. Inclusion of NHANES as half the testing set ensured out of sample predictive validity by challenging the global weights, as it provided the ensemble distribution with high-income data (while DHS data is from low- and middle-income countries) and data from adults older than 45 years (DHS did not take blood tests from older populations). We selected the combination of distributions (including all individual component distributions) that minimised the loss function. For GBD 2021, this resulted in an ensemble distribution that was 40% gamma and 60% mirrored Gumbel.

Haemoglobin variance optimisation

In order to generate an ensemble distribution of haemoglobin concentration, we needed an estimate of the variance of the haemoglobin distribution. In previous GBD cycles, we directly modeled the standard deviation of haemoglobin concentration, but, as previously described, in GBD 2021 we transitioned to modeling anaemia prevalence instead of standard deviation of haemoglobin. To generate an estimate of distribution variance, we used our ST-GPR estimates of mean haemoglobin and prevalence of total, moderate+severe, and severe anaemia prevalence in a variance-optimisation algorithm. For every location, year, age, and sex, we anchored the distributions at the estimated mean haemoglobin concentration value and found the variance value that minimized the error between our estimates of severe, moderate+severe, and total anaemia and the corresponding values implied by a given haemoglobin mean and variance combination. We weighted the errors in the variance optimisation algorithm based on the severity-specific disability weights, such that the severe anaemia error was weighted the most and total anaemia was weighted the least.

Fitting ensemble distributions with method of moments

Using our global model weights and our estimates of mean haemoglobin and variance of haemoglobin, we modelled the full distribution of haemoglobin for each location, year, age, and sex by fitting each component distribution on the modelled mean and variance and weighting each individual distribution to create the ensemble distribution: $\sum_{z=1}^{n_z} w_z * PDF_z$.

Because anaemia thresholds depend on pregnancy, we separately modelled the distribution of haemoglobin among pregnant and non-pregnant women. Our modelling process directly provided estimates of mean haemoglobin concentration and variance of haemoglobin among non-pregnant women; to estimate the mean haemoglobin among pregnant women, we shifted the non-pregnant mean haemoglobin estimate for each location, year, age, and sex by the crosswalk adjustment factor calculated as described in the data processing section. We assumed that the distribution variance was the same among pregnant and non-pregnant women for this analysis.

We used these estimates of mean haemoglobin concentration and haemoglobin variance by pregnancy status to produce separate weighted ensemble distributions. To combine these distributions to represent the entire population of females in a given location, year, and age, we estimated the proportion of pregnant women for each location, year, and age using the formula:

$$pregnancy\_prevalence = (ASFR + (ASFR * SBR)) * 46/52$$

where *ASFR* is the location- and age-specific fertility rate and *SBR* is the location-specific stillbirth rate. We then weighted each of the haemoglobin distributions among pregnant and non-pregnant women accordingly to produce a final general population distribution.

Finding the area under the curve to calculate anaemia prevalence

Using the WHO anaemia thresholds shown in Table S1, we calculated the prevalence of mild, moderate, and severe anaemia for each location, year, age group, and sex as the area under the haemoglobin concentration curve between the thresholds for each severity.

Anaemia causal attribution: input data and methodological summary

Causes and inputs

Anaemia can arise as a result of many different diseases. Each round of GBD, as evidence is identified and synthesised, additional GBD causes are added to the GBD Anaemia Causal Attribution analysis. Additional changes for specific causes are incorporated to either improve efficiency or reflect changes to modelling approaches for underlying causes. For GBD 2021, these cause changes included adding explicit estimates for anaemia attributable to hypothyroid disease and hyperthyroid disease (previously included in the "Other endocrine, nutrition, blood, and immune disorders" category).

For each cause included in GBD Anaemia Causal Attribution, two inputs are required. The first required input is cause-level prevalence generated from other cause-specific GBD estimates. For estimation details of each cause, see the corresponding section of the GBD Diseases and Injuries Capstone methods appendix corresponding to that cause.[6] The second required input is a cause-specific haemoglobin shift. These shifts were derived from published cohort studies, case control studies, or treatment trials, depending on the cause. Data on haemoglobin concentration for diseased versus non-diseased persons in each of the studies were meta-analysed. A majority of the haemoglobin shifts have not changed since GBD 2010 and are described in detail in Section 3 of the appendix. There were a few causes shown in

Table S4 below that were granted exceptions to the above requirements of needing cause-level prevalence and haemoglobin shift. Exceptions were made because these cause groups—mostly "other" causes in the GBD cause hierarchy—are known or suspected to lead to anaemia. Anaemia burden for each of these causes was assigned from the residual anaemia envelope in a manner analogous to fixed proportion redistribution used in the GBD cause-specific mortality analysis.[7] Our goal is to systematically add all causes of anaemia as specific inputs to GBD Anaemia Causal Attribution and eliminate the need for residual attribution.

Table S4. Causes included in GBD Anaemia Causal Attribution.

**Causes with prevalence and haemoglobin shift inputs**
Malaria<br>*Plasmodium falciparum* parasitaemia without clinical malaria<br>*P. vivax* parasitaemia without clinical malaria<br>Clinical malaria
Hookworm Disease
Schistosomiasis
HIV/AIDS
Maternal hemorrhage
Gynaecological disorders<br>Uterine fibroids<br>Menstrual disorders
Thalassaemias<br>Beta-thalassaemia major<br>Haemoglobin E/beta-thalassaemia<br>Haemoglobin H disease
Thalasasemias trait<br>Beta-thalassaemia trait<br>Haemoglobin E trait
Sickle cell disorders<br>Homozygous sickle cell and severe sickle cell/beta-thalassaemia parent<br>Haemoglobin SC disease<br>Mild sickle cell/beta-thalassaemia
Sickle cell trait
G6PD deficiency
Hemizygous G6PD deficiency
Vitamin A deficiency (under 15 years only)
Gastrointestinal disorders<br>Gastritis & Duodenitis<br>Peptic ulcer disease<br>Crohn's disease<br>Ulcerative colitis<br>Cirrhosis and other chronic liver diseases, decompensated
Chronic kidney disease<br>Stage III chronic kidney disease<br>Stave IV chronic kidney disease<br>Stage V chronic kidney disease<br>End stage renal disease
Thyroid diseases

Hyperthyroid disease
Hypothyroid disease
Other endocrine, nutrition, blood, and immune disorders
**Estimated via Fixed Proportion Redistribution Methods* in GBD Anaemia Causal Attribution**
Other neglected tropical diseases
Other infectious diseases
Dietary iron deficiency
Other haemoglobinopathies and haemolytic anaemias

* A minimum of 10% of all anaemia was assigned to residual categories based on analysis of NHANES-III data from the United States.

Modelling strategy

To estimate anaemia prevalence attributable to each cause, we used the modelled full distribution of haemoglobin for each location, year, age, and sex combined with the cause-specific prevalence and haemoglobin shift associated with each cause. We multiplied the haemoglobin shift by the estimated cause prevalence, returning a prevalence-weighted shift in the haemoglobin distribution specific to each cause, location, year, age, and sex. We then shifted our mean haemoglobin estimate by this cause prevalence-weighted haemoglobin shift and re-estimated the haemoglobin distribution, using the same distribution variance value. We calculated the difference in anaemia prevalence between our original distribution and this shifted counterfactual distribution for each anaemia severity, and this difference was the estimated prevalence of anaemia attributable to a given cause. Whenever the sum of the cause-specific anaemia prevalences was greater than the initial estimated anaemia prevalence, we proportionally squeezed the estimated cause-specific anaemia prevalences to sum to 90% of the total anaemia prevalence envelope. For all causes with population-specific prevalence estimates, we enforced a similar condition where the sum of mild, moderate, and severe cause-specific anaemia would not exceed the total cause prevalence within each population. We thus scaled the results to ensure the sum across anaemia severity matched the total prevalence for each cause.

As noted in Table S4, we reserved a minimum of 10% of all anaemia for each location, year, age, and sex to be assigned to residual causes based on a review of findings from NHANES in the United States.[8] The residual envelope was distributed among the four residual aetiologies in an age- and sex-specific manner according to the proportions shown in Table S5.

Table S5. GBD 2021 residual aetiology distribution proportions

Cause	Age (years)					
	Under-15		15-59		60+	
	Males	Females	Males	Females	Males	Females
Dietary iron deficiency	80·50%	80·50%	80·50%	80·50%	80·50%	80·50%
Other neglected tropical diseases	9·63%	9·63%	4·875%	6·00%	2·785%	2·785%
Other infectious diseases	6·26%	6·26%	4·875%	6·00%	2·785%	2·785%
Other haemoglobinopathies and haemolytic anaemias	3·61%	3·61%	9·75%	7·50%	13·93%	13·93%

It is important to take note of the difference between “dietary iron deficiency” as a GBD cause and “iron deficiency” as a GBD risk. Many GBD causes lead to anaemia that clinically manifests as iron deficiency (or microcytosis), but where inadequate intake is not the underlying problem. Examples include neglected tropical diseases such as malaria, hookworm, and schistosomiasis, gastrointestinal disorders,

cirrhosis, maternal haemorrhage, menstrual disorders, uterine fibroids, and vitamin A deficiency. The name "dietary iron deficiency" is intended to differentiate, therefore, between inadequate intake and haemorrhagic conditions or disorders of iron metabolism. Additionally, because we have yet to include 100% of anaemia causes, estimates should be interpreted to also include some acute and chronic haemorrhagic states for which supplementation may be helpful, but poor nutritional intake is not the only underlying problem. Examples include malabsorption syndromes, other micronutrient deficiencies besides vitamin A deficiency, and injuries with associated acute blood loss anaemia. "Iron deficiency" exposure as estimated for the GBD risk factors analysis, in contrast, includes a combined assessment of the magnitude of haemotologic insult from all causes that manifest as iron deficiency. As mentioned above, our goal is to systematically add all causes of anaemia as specific inputs to GBD Anaemia Causal Attribution, including inadequate iron intake, and eliminate the need for residual attribution.

Years lived with disability (YLDs) calculation

Total years lived with disability (YLDs) for a given disease were calculated as the prevalence of the disease multiplied by the disability weight (DW) associated with that disease. The disability weight captures the level of health loss due to that illness and ranges between zero (no health loss) and one (death). Table S6 details the disability weights and health state descriptions associated with each severity of anaemia. We calculated YLDs by anaemia severity and cause for every location, year, age, and sex in the analysis.

Table S6. Anaemia disability weights and health states

Anaemia Severity	Health State Description	Disability Weight
Mild anaemia	feels slightly tired and weak at times, but this does not interfere with normal daily activities.	0.004 (0.001 – 0.008)
Moderate anaemia	feels moderate fatigue, weakness, and shortness of breath after exercise, making daily activities more difficult.	0.052 (0.034 – 0.076)
Severe anaemia	feels very weak, tired and short of breath, and has problems with activities that require physical effort or deep concentration.	0.149 (0.101 – 0.209)

To account for co-occurrence of different diseases, we adjusted YLDs in a micro-simulation process that ensures the implied disability weight for someone with comorbid illnesses does not exceed one. A simulated dataset of 40,000 individuals for every location-age-sex-year estimated in the GBD was run through a four-step process:

1) Each individual was estimated to have zero to multiple disease sequelae.

2) Each individual's assigned disease sequelae disability weights (DW) were estimated (one minus the multiplicative sum of one minus each DW present).

3) The DW attributable to each sequela for the individual was calculated (DW of the disease sequela divided by the sum of the disease sequelae across all individuals, multiplied by the

individual's total attributed DW).

4) YLDs per capita for every location-age-sex-year were calculated by summing the attributable DWs for a disease across individuals, and then multiplying this rate by the location-age-sex-year population.

We propagated uncertainty by repeating this process 500 times for each location, year, age, and sex, and we calculated 95% uncertainty intervals as the 2.5th and 97.5th percentiles of these iterations.

Epidemiological transition analyses

We analysed the relationship between anaemia burden (prevalence and YLDs) and the Socio-demographic Index (SDI) using meta-regression—Bayesian, regularised, trimmed (MR-BRT) models.[2] For each age and sex combination, we fit models on national-level estimates of anaemia burden from 1990-2021, including an intercept and cubic spline on SDI to predict the average relationship between socioeconomic development and anaemia burden over time. The splines contained two internal knots placed at the 33.33rd and 66.66th percentiles of the observed SDI values. We constrained both tails of the spline to be linear.

For each measure of burden, we aggregated the age- and sex-specific expected values predicted from the spline models to create aggregated expected values at the location, region, super-region, and global levels. We calculated observed-to-expected (OE) ratios for each aggregate and calculated the annualised rate of change in the OE ratio between years *y1* and *y2* with the formula:

$$Annualized\ Rate\ of\ Change_{OE,y1,y2} = \frac{\log\left(\frac{OE_{y2}}{OE_{y1}}\right)}{y2 - y1}$$

Figure S4. Epidemiological transition of all-ages anaemia burden on the basis of SDI.

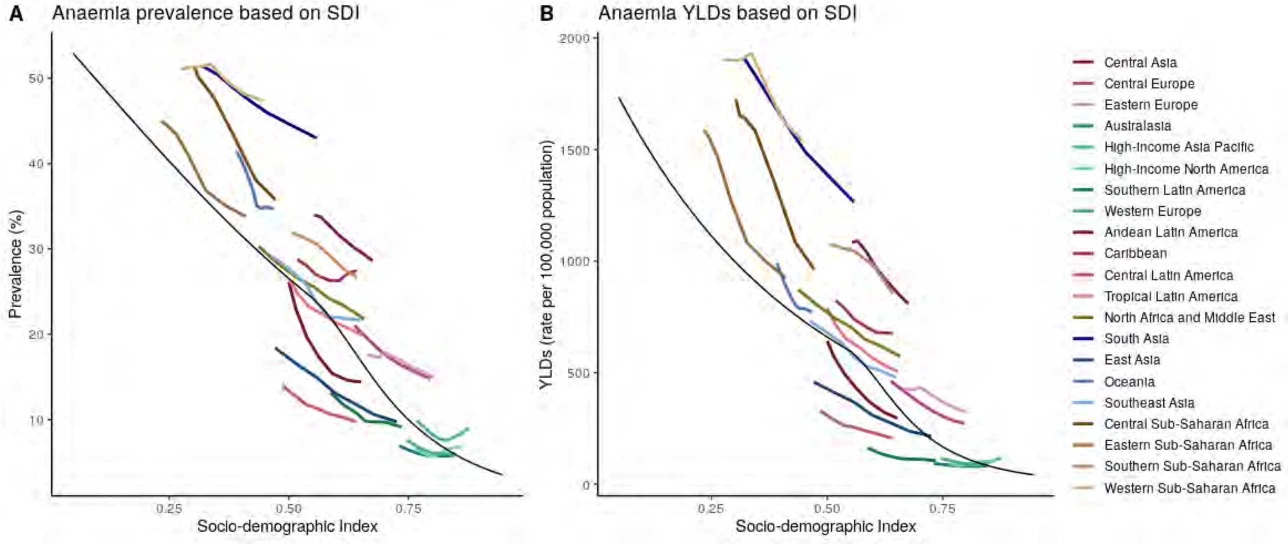

(A) Anaemia prevalence for all ages and both sexes vs. SDI by region, 1990–2021. (B) Anaemia years lived with disability (YLDs) rate per 100,000 population for all ages and both sexes vs. SDI by region, 1990–2021. Fit lines are based on relationship between SDI and anemia burden at the national level; regional aggregates are displayed.

Section 3: Cause-Specific Haemoglobin Shifts

Overview

Haemoglobin shifts represent either the differences in haematologic parameters in those with versus without disease (most non-communicable diseases) or the haematologic changes observed when the condition was effectively cured (many infections). Subgroup data on haemoglobin concentration is rarely reported as a primary, or even secondary, outcome of clinical research studies. As such, there is comparative data sparsity on this component of the GBD analysis of cause-specific anaemia burden. Cause-specific haemoglobin shifts were determined primarily via review of published studies identified during data-seeking efforts for each GBD analysis. The table below details the haemoglobin shifts used in GBD 2021 for each of the GBD causes analysed as leading to anaemia. This is followed by a text description of the source of information for each GBD cause. As described in the text, uncertainty of haemoglobin shifts was calculated whenever input data were sufficient to support meta-analysis, but uncertainty was not included in the estimation of cause-specific anaemia.

Table S7. GBD 2021 cause-specific haemoglobin shifts

**Cause**	**Sub-group of cause**	**Haemoglobin shift (g/L)**	
		**Males**	**Females**
Malaria	*Plasmodium falciparum* parasite rate (*Pf*PR)	8·37	8·37
	Plasmodium vivax parasite rate (*Pf*PR)	11·3	11·3
	Clinical malaria	20	20
Hookworm disease		2·08	2·08
Schistosomiasis		2·5	2·5
HIV/AIDS		9·7	7
Other neglected tropical diseases	Residual cause	--	--
Other infectious diseases	Residual cause	--	--
Maternal haemorrhage	Incident cases of maternal haemorrhage	--	6·8
Gynaecological disorders	Uterine fibroids (symptomatic only)	--	13·7
	Menstrual disorders	--	3
Thalassaemias	Beta Thalassaemia Major (Hbβ-Hbβ)	70	70
	Compound heterozygous “E-β” Thalassaemia (HbE-Hbβ)	64	64
	Haemoglobin H disease (- - / - α)	56	56
Thalassaemia trait	Haemoglobin E trait	6	6
	Beta Thalassaemia trait	19	19
Sickle cell disorders	Homozygous Sickle Cell Disease (HbS-HbS)	54	54
	Haemoglobin SC Disease	36	36
	Compound heterozygous Sickle-Thalassaemia (HbS-Hbβ)	52	52
Sickle cell trait	Haemoglobin S trait	5	5
G6PD deficiency		0·152	0·074
G6PD trait	Heterozygous (female only)	--	0·016
Other haemoglobinopathies and haemolytic anaemias	Residual cause	--	--
Vitamin A deficiency		3·6	3·6
Dietary iron deficiency	Residual cause	*4·01*	*4·01*
Gastrointestinal disorders	Gastritis and Duodenitis	15·11	15·11
	Peptic Ulcer Disease	15·11	15·11
	Crohn’s disease	23	23
	Ulcerative colitis	20	20
	Cirrhosis (decompensated only)	18	18
Chronic kidney disease	Stage III	16·34	11·84
	Stage IV	27·82	21·83
	Stage V, untreated	37·62	32·43

	End Stage Renal Disease, on dialysis	30·7	30·7
Thyroid diseases	Hyperthyroid disease	4·2	4·2
	Hypothyroid disease	2·8	2·8
Other endocrine, nutrition, blood, and immune disorders		15	15

Malaria: PfPR, PvPR, and clinical malaria

For GBD 2017, the Malaria Atlas Project team in Oxford, United Kingdom updated the GBD 2010 analysis on the relationship between *Plasmodium falciparum* Parasite Rate (PfPR) and hemoglobin concentration to facilitate inclusion of *Plasmodium vivax* (Pv) and clinical malaria in the cause-specific anaemia analysis. The aim was to use household survey data to estimate the haemoglobin shift associated with malaria parasite infection. Data on rapid diagnostic test (RDT) status, blood haemoglobin concentration, fever (as an indicator of symptomatic illness), location and household were collected from 38 national surveys from the Demographic and Health Surveys (DHS) Program. Information for a total of 142,408 children younger than 5 years of age across 10,932 clusters was included. Households with two or more children of differing RDT status were also included, of which there were 20,633 households. Figure S5 shows the prevalence of anaemia (no anemia; mild, moderate, or severe anemia) with *Pf*PR in all children (left plot), in children with a positive RDT at interview (centre plot), and interview RDT negatives (right plot). The proportion of children with mild or no anaemia decreased with increasing PR. RDT-negative children were approximately twice as likely to have no anaemia and were also slightly more commonly found to have mild anaemia than RDT-positive children. Moderate and severe anaemia both increased with increasing PR and both were much more common in RDT-positive children.

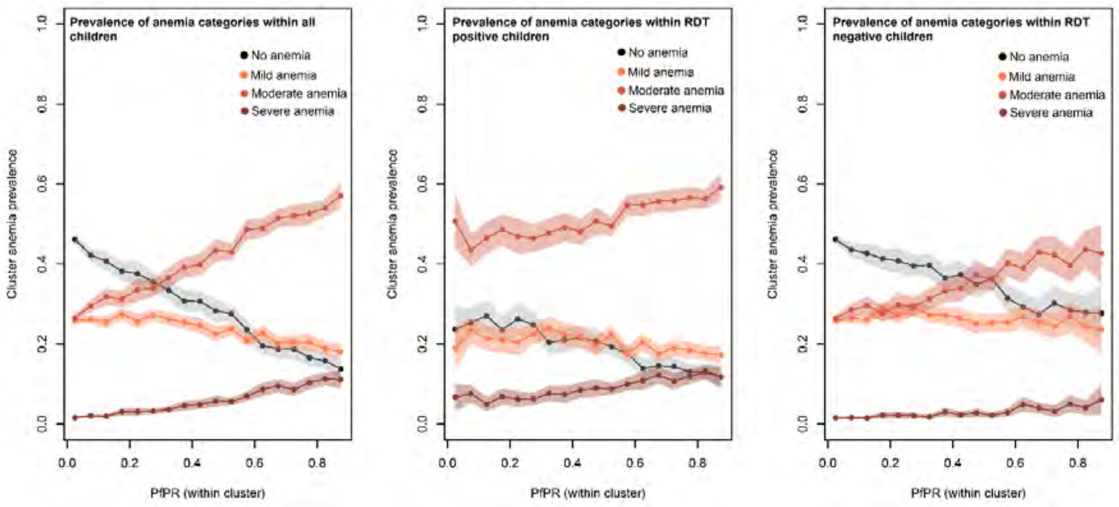

Figure S5. Summary of anaemia status with increasing *Pf*PR for children within household surveys.

To analyse quantitative differences, children from household surveys were aggregated by cluster and separated by RDT and fever status (Figure S6). For each cluster, the mean haemoglobin of all RDT-positive individuals was subtracted from that of RDT-negative individuals within the same cluster. For each of the three sub-categories (febrile, afebrile, and all RDT-positive), the resulting distribution of differences was plotted as a kernel density function with bandwidth equal to 3. Average haemoglobin in RDT-positive individuals was reduced compared to average haemoglobin in RDT-negative individuals,

with a larger reduction in haemoglobin in children with symptomatic illness. Final haemoglobin shifts for each of PvPR and PfPR were approximated as the differences in the modes of the distribution of the kernel density functions. This resulted in an estimated shift of 11·3 g/L for *Plasmodium vivax* PR and 8·37 g/L for *Plasmodium falciparum* PR. The analysis was repeated within three PR bins to represent low, medium, and high transmission, and we found little difference in haemoglobin shift by PR.

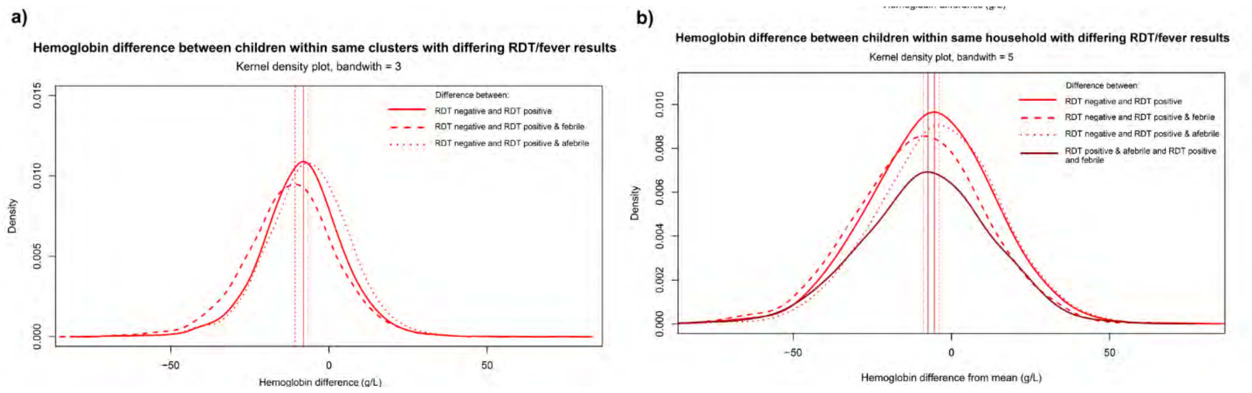

Figure S6. Haemoglobin difference within clusters and households

a) clusters, displaying the difference in mean haemoglobin density between all RDT positives, febrile RDT positives, and afebrile RDT positives from RDT negatives as a kernel density function with bandwidth equal to 3, and b) households, displaying the difference in mean haemoglobin density between all RDT positives, febrile RDT positives, and afebrile RDT positives from RDT negatives, and the difference between febrile RDT positives and afebrile RDT positives, as a kernel density function with bandwidth equal to 5. A negative difference indicates that the population displayed has less haemoglobin, on average, than the reference population.

In addition to comparing aggregate differences between RDT-positive and RDT-negative children, we also evaluated by differing fever status within households as a potential proxy for clinical malaria. The aim of splitting by household was to control for individual-level factors that influence malaria positivity, anaemia, and fever that would differ within households in a cluster, but not within individuals in a household (such as wealth, housing and sanitation quality, or maternal education). Households were removed from the dataset if all children had the same RDT status, and we increased the kernel density function bandwidth to 5 to account for the increased noise. Not surprisingly, we found a larger reduction in haemoglobin in children with symptomatic illness and the resultant estimated shift of 17·1 g/L for *Plasmodium vivax* was comparable to what we found for a series of specific studies on prevalence and incidence of clinical malaria.[9–36] Due to the higher specificity of these studies as compared to the DHS analysis, we used the finding of a haemoglobin shift of 20 g/L for clinical malaria.

Hookworm disease

We identified two studies to inform the haemoglobin shift due to hookworm infection.[37,38] In each systematic review, the authors evaluated studies published between 1980 and 2009 for inclusion. Studies prior to 1980 were uniformly found to use different diagnostic tests and intensity thresholds which prevented comparability, so these were excluded. The authors performed random effects meta-analyses to compare haemoglobin levels in infected versus non-infected individuals across a variety of demographic groups.

We judged the pooled effect of albendazole treatment to likely be that most-closely correlated with the haematologic effects of hookworm infection and used this for determining the haemoglobin shift for

hookworm disease. The haemoglobin shift due to hookworm infection was 2·08 (95% UI 0·17 – 4·00) g/L. We did not find sufficient evidence to support different shifts by age or sex. There are not separate hookworm prevalence estimates for pregnant versus non-pregnant females in the 2021 GBD Study so shifts were assigned to the general population.

Schistosomiasis

Haemoglobin shifts due to schistosomiasis infection were derived from a systematic review by King and colleagues.[39] This study included a comprehensive literature review for studies published between 1921 and 2002 that reported on disabling sequelae of schistosomiasis infection by subtype. There were a total of five studies that reported on differences in mean haemoglobin concentrations between those with infection prior to, as compared to after, antihelminthic treatment. A DerSimonian-Laird (i.e., random effects) meta-analysis was performed on each subtype and across all subtypes. Because of the comparative data sparsity, we used only the pooled haemoglobin shift for all schistosomiasis infections in the GBD analysis of anemia causes. The pooled value for the haemoglobin shift due to schistosomiasis was 2·5 (95% UI 1·5-3·6) g/L.

HIV/AIDS

We derived data on haematologic status in males and females with HIV/AIDS from a published study by Adamu and colleagues.[40] The authors pooled data on mean and standard deviation of haemoglobin for both males and females (non-pregnant and pregnant) for those that were HIV-positive and compared these with a matched control population that was HIV-negative.

Although the GBD modelling process for HIV/AIDS considers antiretroviral therapy (ART) coverage, there are not currently separate distinct estimates of prevalence by receipt of ART status. The same is true for pregnancy status. We therefore pooled all those with HIV together for males and females. The haemoglobin shift for HIV/AIDS was 7 (95% UI 6·28–7·72) g/L for females and 9·7 (8·7–10·7) g/L for males.

Other neglected tropical diseases and other infectious diseases

There are a number of infectious diseases that are not modelled directly in GBD, including those in the GBD cause categories of “other neglected tropical diseases” and “other infectious diseases.” To account for the contribution each of these groups likely make to anaemia burden, a fixed proportion of the residual anaemia burden are assigned to these causes. There are a number of additional infectious conditions where prevalence and incidence estimates are produced, but for which haemoglobin shift data has not been identified to date. Future systematic reviews and data identification on haemoglobin shifts could facilitate the inclusion of conditions such as tuberculosis, typhoid, paratyphoid, other invasive non-typhoidal salmonellae disease, and diarrhea, particularly in children.

Maternal haemorrhage

A systematic review of studies published between 1990 and 2009 by Wagner and colleagues[41] served as the basis of our assessment of the haematologic consequences of maternal haemorrhage. Although the acute duration of a maternal haemorrhage event is short, the resultant haemoglobin decrement can be comparatively long-lasting. The time after delivery at which comparisons were made varied amongst the component studies with an average of approximately 6 months. The pooled haemoglobin shift assigned to *incident* (rather than prevalent) cases of maternal haemorrhage was 6·8 (5·3-8·3) g/L and all cases of anaemia were correspondingly assigned to be of one year in duration.

Uterine fibroids

The link between uterine fibroids and anaemia has been described to be dependent on the size and severity of the leiomyomas present in the uterus. Isolated submucosal lesions, likely the most common type of fibroids, are not significantly associated with decreased haemoglobin,[42] but the degree of haemoglobin decrement can be quite substantial for the subset that are severe and requires intervention as demonstrated in a study by Volkers and colleagues of 88 women undergoing uterine artery embolisation for fibroids.[43] We paired the haemoglobin shift of 13·7 (9·03 - 18·4) g/L from this study with the subset of fibroids estimated in the GBD to require hospitalization. Fibroids that do not require hospitalization were not assumed to experience any anaemia, but this could be amended if further data is identified to support an effect.

Menstrual disorders

Menstrual disorders is estimated in the GBD as a component of "other gynaecological disorders." We identified only a small number of sources that reported on either haemoglobin variation or typical blood loss during normal menstrual cycles.[44–46] Due to heterogeneity in the study design and outcomes, quantitative haemoglobin difference in the general population of those with menstrual disorders was not readily discernible. We therefore approximated with a conservative haemoglobin shift of 3 g/L and plan to continue seeking additional primary data to inform this assessment.

Haemoglobinopathies: thalassaemias, thalassaemia trait, sickle cell disorders, sickle cell trait, glucose-6-phosphate dehydrogenase (G6PD) deficiency, G6PD trait, and other haemoglobinopathies and haemolytic anaemias

We identified a number of small published studies reporting data concurrently on haemoglobin genotype and overall haemoglobin concentration in each of three subtypes of sickle cell disease and sickle cell trait[47–55] (HbS only), thalassaemias and thalassaemia trait[56–59] (HbE and Hb-beta), G6PD deficiency, and G6PD trait.[60–64] The genetics of each of the sickle cell and thalassaemia variants are reasonably straightforward and facilitated simple comparative meta-analyses. The haemoglobin shifts for three types of sickle cell disorders were 54 g/L, 36 g/L, and 52 g/L for homozygous sickle cell disease (SS and Sβ°), haemoglobin SC Disease (SC), and mild sickle-cell/beta-thalassaemia (Sβ+), respectively. Thalassaemia shifts were 70 g/L, 64 g/L, and 56 g/L for beta-thalassaemia major (β°β° or β° β+), compound heterozygous "E-β" thalassaemia (Eβ), and haemoglobin H disease (- - / - α), respectively. Sickle cell trait was estimated to have a haemoglobin shift of 5 g/L, haemoglobin E trait was 6 g/L, and beta-thalaessemia trait was 19 g/L.

G6PD deficiency was more complex. Class I through Class V severities of G6PD deficiency correspond to different strata of enzyme activity. The proportion of the general population with each severity class is not readily known, but we found several descriptions that the prevalence of chronic haemolysis in G6PD deficiency is roughly approximated at 1 per 1,000,000 persons with G6PD deficiency. Systematic surveys of prevalence of Class II G6PD deficiency have not been completed, but even many of these individuals are asymptomatic most of the time. They experience crises when exposed to triggers such as fava beans, some pharmaceuticals, or severe infection. Based on these qualitative descriptions, we settled on a value of 1% of the prevalent population as a reasonable approximation of those who are experiencing a haemoglobin shift from their G6PD deficiency. Another way we thought about this issue was to consider those with G6PD deficiency to be completely asymptomatic except for during episodes of haemolytic crisis. If every person had an average of six weeks of severe anaemia at some point during their lifetime,

the amount of disability would still be nearly identical to the 1% figure in the discussion above. For this reason, and in recognition that the available studies were performed on populations in Sardinia and Calabria known to have very high rates of G6PD-Mediterranean (a particularly severe mutation), we elected to apply the shifts from Piomelli and colleagues,[61] down-weighted to 1% based on the conservative assessment of the likely proportion who have Class II disease. G6PD trait was subsequently reduced for the GBD 2021 analysis to be half of the reported shift based on an assumption of 50% X-inactivation in adult mammals.

We assigned a fixed proportion of the residual anaemia envelope to the GBD cause of "other haemoglobinopathies and haemolytic anaemias" in recognition that this category includes known anaemia-inducing conditions such as alpha thalassaemia and aplastic anaemia. The addition of separate prevalence estimates for these conditions in the future would facilitate their direct inclusion in the GBD estimation of cause-specific anaemia.

Nutritional deficiencies

Vitamin A deficiency

We identified five studies in preschool and school-aged children that reported changes in haemoglobin levels following vitamin A supplementation.[65–69] Each trial reported haemoglobin levels pre- and post-supplementation for children receiving the intervention and at equivalent time points for control groups. We used the difference in the observed haemoglobin changes between intervention and control groups as the basis of our haemoglobin shift due to vitamin A. We pooled the reported results and estimated a pooled vitamin A haemoglobin shift of 3·6 g/L. We restricted estimates of anaemia due to vitamin A deficiency to children and adolescents younger than 15 years, as these were the age groups for which we found clear evidence of an effect of vitamin A on haemoglobin levels.

Iron deficiency

We estimated the iron deficiency haemoglobin shift by pooling the results of multiple iron fortification trials.[70–78] We extracted the haemoglobin differences pre- and post-iron intervention and weighted these by the sample size of the study population to produce a pooled estimate of the haemoglobin shift associated with iron deficiency. This shift was 4·01 g/L.

However, because estimates of dietary iron deficiency prevalence do not currently exist in the GBD Study, we were unable to use this haemoglobin shift to produce cause-specific anaemia prevalence estimates directly. Instead, anaemia due to dietary iron deficiency was estimated via fixed proportion redistribution in our analysis, accounting for 80·5% of all residual anaemia cases. Estimation of underlying prevalence of dietary iron deficiency in future GBD iterations will allow for direct estimation of dietary iron deficiency anaemia using the haemoglobin shift derived above.

Gastrointestinal

Upper GI

Gastritis, duodenitis, and peptic ulcer disease

To estimate the haemoglobin shift attributable to gastritis and duodenitis and peptic ulcer disease, we used results from a meta-analyses by Huang and colleagues.[79] The authors identified randomised controlled trials of *Helicobacter pylori* eradication treatment that reported haemoglobin concentrations pre- and post-treatment. They evaluated the difference between the pre- and post-treatment haemoglobin concentrations in the treatment group and equivalent values in the control group, and the

difference between these was the basis of the haemoglobin shift attributable to *H. pylori* eradication. We used a haemoglobin shift of 15·11 g/L as reported in this meta-analysis for both gastritis and duodenitis and peptic ulcer disease.

Inflammatory bowel disease

Crohn's disease and ulcerative colitis

The haemoglobin shifts for Crohn's disease and ulcerative colitis were derived from two studies examining changes in haemoglobin concentration among participants with inflammatory bowel disease and iron deficiency anaemia who were supplemented with iron.[80,81] Differences in mean haemoglobin concentration at baseline and follow-up were pooled across studies and analyzed to produce haemoglobin shifts for both Crohn's disease and ulcerative colitis. We estimated haemoglobin shifts of 23 g/L for Crohn's disease and 20 g/L for ulcerative colitis. We used the same values for males and females, as we did not find data to suggest a significant difference by sex.

Decompensated Cirrhosis

Anaemia is a common consequence of liver disease and can arise from a variety of causes, most significantly hemolysis and acute and chronic blood loss. We used data from a study comparing participants with decompensated cirrhosis to healthy controls with reported haemoglobin concentrations for each group.[82] This difference, the haemoglobin shift used in this analysis, was reported as 18 g/L.

Chronic kidney disease

We identified six sources to inform the haemoglobin shift due to chronic kidney diseases (CKD).[83–88] Together these sources informed differential severity of haemotologic decrement by severity of CKD, but did not differentiate by subcause of CKD (hypertension, diabetes mellitus, congenital, hypertension, or other). We extracted the mean haemoglobin concentration, standard error, and sample size for each CKD stage based on glomerular filtration rate ranges. We applied sex ratios from Hsu and colleagues[89] and the comparative differences were pooled together to calculate stage-specific haemoglobin shifts using fixed effects meta-analysis. End stage renal disease (ESRD) on dialysis was added to the cause-specific anaemia analysis in GBD 2019 following identification of corresponding shift data comparing to other stages of CKD.[90] No data stratified by sex were identified for ESRD on dialysis. The final shifts used in the GBD 2021 analysis were Stage III (16·34 g/L for males; 11·84 g/L for females); Stage IV (27·82 g/L for males; 21·83 g/L for females), Stage V, untreated (37·62 g/L for males; 32·43 g/L for females), and ESRD, on dialysis (30·7 for both sexes).

Thyroid disorders

To quantify the effects of hypothyroidism and hyperthyroidism on haemoglobin, we included data from a prospective observational study that examined differences in haemoglobin levels by thyroid stimulating hormone (TSH) levels.[91] Participants were categorized as having hypothyroidism, euthyroidism, or hyperthyroidism based on TSH levels, with further differentiation into subclinical or overt disease based on free thyroxine (fT4) levels. The authors assessed differences in haemoglobin levels within these categories using multiple linear regression with adjustment for a variety of potential confounding variables. We used the results for overt hypothyroidism and overt hyperthyroidism compared to normal thyroid function as the bases of our haemoglobin shifts. The resulting shifts used in this analysis were 2·8 g/L for hypothyroidism and 4·2 g/L for hyperthyroidism.

Other endocrine, nutrition, blood, and immune disorders

There are a number of additional endocrine, nutrition, blood, and immune disorders which are known to contribute to anaemia but for which direct prevalence estimates do not exist in the GBD. Conditions in this category include, for example, cystic fibrosis,[92] hypothalamic-pituitary-adrenal (HPA) axis disorders,[93,94] metabolic disease,[95] haemophilias, and splenic disorders.[96] To account for these causes in our analysis, we estimated a haemoglobin shift of 15 g/L based on published shifts for similar conditions to those included in this category. Estimation of the prevalences of these underlying causes and evaluation of associated haemoglobin shifts will allow for the direct estimation of anaemia due to these causes in the future.

References

1 World Health Organization. Haemoglobin concentrations for the diagnosis of anaemia and assessment of severity. 2011. https://apps.who.int/iris/bitstream/handle/10665/85839/WHO_NMH_NHD_MNM_11.1_eng.pdf.

2 Zheng P, Barber R, Sorensen RJD, Murray CJL, Aravkin AY. Trimmed Constrained Mixed Effects Models: Formulations and Algorithms. *Journal of Computational and Graphical Statistics* 2021; **30**: 544–56.

3 Murray CJL, Aravkin AY, Zheng P, *et al.* Global burden of 87 risk factors in 204 countries and territories, 1990–2019: a systematic analysis for the Global Burden of Disease Study 2019. *The Lancet* 2020; **396**: 1223–49.

4 Bates D, Mächler M, Bolker B, Walker S. Fitting Linear Mixed-Effects Models Using lme4. *Journal of Statistical Software* 2015; **67**: 1–48.

5 R Core Team. R: A language and environment for statistical computing. 2022. https://www.R-project.org/.

6 GBD 2021 DALYs Collaborators. Global years lived with disability (YLDs), disability-adjusted life-years (DALYs), and healthy life expectancy (HALE) for 370 diseases and injuries in 204 countries and territories, 1990–2020: a systematic analysis for the Global Burden of Disease Study 2020. **in draft**.

7 GBD 2019 Diseases and Injuries Collaborators. Global burden of 369 diseases and injuries, 1990–2019: a systematic analysis for the Global Burden of Disease Study 2019. *The Lancet* Forthcoming.

8 Looker AC, Dallman PR, Carroll MD, Gunter EW, Johnson CL. Prevalence of iron deficiency in the United States. *JAMA* 1997; **277**: 973–6.

9 Cameron E, Battle KE, Bhatt S, *et al.* Defining the relationship between infection prevalence and clinical incidence of Plasmodium falciparum malaria. *Nat Commun* 2015; **6**: 8170.

10 Ba F. THÈSE DE TROISIÈME CYCLE DE BIOLOGIE ANIMALE FATOU BA épouse FALL. *Université Cheikh Anta Diop de Dakar* 2000.

11 Bloland PB, Boriga DA, Ruebush TK, *et al.* Longitudinal cohort study of the epidemiology of malaria infections in an area of intense malaria transmission II. Descriptive epidemiology of malaria infection and disease among children. *Am J Trop Med Hyg* 1999; **60**: 641–8.

12 Bonnet S, Paul RE l, Gouagna C, *et al.* Level and dynamics of malaria transmission and morbidity in an equatorial area of South Cameroon. *Trop Med Int Health* 2002; **7**: 249–56.

13 Bougouma EC, Tiono AB, Ouédraogo A, *et al.* Haemoglobin variants and Plasmodium falciparum malaria in children under five years of age living in a high and seasonal malaria transmission area of Burkina Faso. *Malar J* 2012; **11**: 154.

14 Ouédraogo A, Tiono AB, Diarra A, *et al.* Malaria Morbidity in High and Seasonal Malaria Transmission Area of Burkina Faso. *PLOS ONE* 2013; **8**: e50036.

15 Coulibaly D, Diallo DA, Thera MA, *et al.* Impact of preseason treatment on incidence of falciparum malaria and parasite density at a site for testing malaria vaccines in Bandiagara, Mali. *Am J Trop Med Hyg* 2002; **67**: 604–10.

16 Diallo S, Konate L, Ndir O, *et al.* [Malaria in the central health district of Dakar (Senegal). Entomological, parasitological and clinical data]. *Sante* 2000; **10**: 221–9.

17 Diallo S, Ndir O, Faye O, *et al.* [Malaria in the southern sanitary district of Dakar (Senegal). 1. Parasitemia and malarial attacks]. *Bull Soc Pathol Exot* 1998; **91**: 208–13.

18 Dicko A, Sagara I, Diemert D, *et al.* Year-to-year variation in the age-specific incidence of clinical malaria in two potential vaccine testing sites in Mali with different levels of malaria transmission intensity. *Am J Trop Med Hyg* 2007; **77**: 1028–33.

19 Fillol F, Cournil A, Boulanger D, *et al.* Influence of wasting and stunting at the onset of the rainy season on subsequent malaria morbidity among rural preschool children in Senegal. *Am J Trop Med Hyg* 2009; **80**: 202–8.

20 Greenwood BM, Bradley AK, Greenwood AM, *et al.* Mortality and morbidity from malaria among children in a rural area of The Gambia, West Africa. *Transactions of the Royal Society of Tropical Medicine and Hygiene* 1987; **81**: 478–86.

21 Henry M-C, Rogier C, Nzeyimana I, *et al.* Inland valley rice production systems and malaria infection and disease in the savannah of Côte d'Ivoire. *Trop Med Int Health* 2003; **8**: 449–58.

22 Guinovart C, Bassat Q, Sigaúque B, *et al.* Malaria in rural Mozambique. Part I: Children attending the outpatient clinic. *Malaria Journal* 2008; **7**: 36.

23 Loha E, Lunde TM, Lindtjørn B. Effect of Bednets and Indoor Residual Spraying on Spatio-Temporal Clustering of Malaria in a Village in South Ethiopia: A Longitudinal Study. *PLOS ONE* 2012; **7**: e47354.

24 Lusingu JP, Vestergaard LS, Mmbando BP, *et al.* Malaria morbidity and immunity among residents of villages with different Plasmodium falciparum transmission intensity in North-Eastern Tanzania. *Malar J* 2004; **3**: 26.

25 Molez JF, Diop A, Gaye O, Lemasson JJ, Fontenille D. [Malaria morbidity in Barkedji, village of Ferlo, in Senegal Sahelian area]. *Bull Soc Pathol Exot* 2006; **99**: 187–90.

26 Mwangi TW, Ross A, Marsh K, Snow RW. The effects of untreated bednets on malaria infection and morbidity on the Kenyan coast. *Trans R Soc Trop Med Hyg* 2003; **97**: 369–72.

27 Mwangi TW, Ross A, Snow RW, Marsh K. Case definitions of clinical malaria under different transmission conditions in Kilifi District, Kenya. *J Infect Dis* 2005; **191**: 1932–9.

28 Nebie I, Diarra A, Ouedraogo A, *et al.* Humoral responses to Plasmodium falciparum blood-stage antigens and association with incidence of clinical malaria in children living in an area of seasonal malaria transmission in Burkina Faso, West Africa. *Infect Immun* 2008; **76**: 759–66.

29 Owusu-Agyei S, Asante KP, Adjuik M, *et al.* Epidemiology of malaria in the forest-savanna transitional zone of Ghana. *Malaria Journal* 2009; **8**: 220.

30 Rogier C, Trape JF. Malaria attacks in children exposed to high transmission: who is protected? *Trans R Soc Trop Med Hyg* 1993; **87**: 245–6.

31 Saúte F, Aponte J, Ahmeda J, *et al.* Malaria in southern Mozambique: Incidence of clinical malaria in children living in a rural community in Manhiça district. *Transactions of The Royal Society of Tropical Medicine and Hygiene* 2003; **97**: 655–60.

32 Thompson R, Begtrup K, Cuamba N, *et al.* The Matola malaria project: a temporal and spatial study of malaria transmission and disease in a suburban area of Maputo, Mozambique. *Am J Trop Med Hyg* 1997; **57**: 550–9.

33 Trape JF, Zoulani A, Quinet MC. Assessment of the incidence and prevalence of clinical malaria in semi-immune children exposed to intense and perennial transmission. *Am J Epidemiol* 1987; **126**: 193–201.

34 Velema JP, Alihonou EM, Chippaux JP, van Boxel Y, Gbedji E, Adegbini R. Malaria morbidity and mortality in children under three years of age on the coast of Benin, West Africa. *Trans R Soc Trop Med Hyg* 1991; **85**: 430–5.

35 Schellenberg DM, Aponte JJ, Kahigwa EA, *et al.* The incidence of clinical malaria detected by active case detection in children in Ifakara, southern Tanzania. *Trans R Soc Trop Med Hyg* 2003; **97**: 647–54.

36 Trape JF, Rogier C. Combating malaria morbidity and mortality by reducing transmission. *Parasitol Today* 1996; **12**: 236–40.

37 Brooker S, Hotez PJ, Bundy DAP. Hookworm-Related Anaemia among Pregnant Women: A Systematic Review. *PLoS Negl Trop Dis* 2008; **2**: e291.

38 Smith JL, Brooker S. Impact of hookworm infection and deworming on anaemia in non-pregnant populations: a systematic review. *Trop Med Int Health* 2010; **15**: 776–95.

39 King CH, Dickman K, Tisch DJ. Reassessment of the cost of chronic helmintic infection: a meta-analysis of disability-related outcomes in endemic schistosomiasis. *The Lancet* 2005; **365**: 1561–9.

40 Adamu AL, Crampin A, Kayuni N, *et al.* Prevalence and risk factors for anemia severity and type in Malawian men and women: urban and rural differences. *Popul Health Metr* 2017; **15**: 12.

41 Wagner KS, Ronsmans C, Thomas SL, *et al.* Women who experience obstetric haemorrhage are at higher risk of anaemia, in both rich and poor countries. *Trop Med Int Health* 2012; **17**: 9–22.

42 Puri K, Famuyide AO, Erwin PJ, Stewart EA, Laughlin-Tommaso SK. Submucosal fibroids and the relation to heavy menstrual bleeding and anemia. *American Journal of Obstetrics & Gynecology* 2014; **210**: 38.e1-38.e7.

43 Volkers NA, Hehenkamp WJK, Birnie E, Ankum WM, Reekers JA. Uterine artery embolization versus hysterectomy in the treatment of symptomatic uterine fibroids: 2 years' outcome from the randomized EMMY trial. *American Journal of Obstetrics & Gynecology* 2007; **196**: 519.e1-519.e11.

44 Ofojekwu M-JN, Nnanna OU, Okolie CE, Odewumi LA, Isiguzoro IOU, Lugos MosesD. Hemoglobin and Serum Iron Concentrations in Menstruating Nulliparous Women in Jos, Nigeria. *Laboratory Medicine* 2013; **44**: 121–4.

45 Andrade ATL, Souza JP, Shaw ST, Belsey EM, Rowe PJ. Menstrual blood loss and body iron stores in Brazilian women. *Contraception* 1991; **43**: 241–9.

46 Peuranpää P, Heliövaara-Peippo S, Fraser I, Paavonen J, Hurskainen R. Effects of anemia and iron deficiency on quality of life in women with heavy menstrual bleeding. *Acta Obstetricia et Gynecologica Scandinavica* 2014; **93**: 654–60.

47 Steinberg MH, Ohene-Frempong K. Clinical Aspects of Sickle Cell Anemia in Adults and Children. In: Disorders of Hemoglobin: Genetics, Pathophysiology, and Clinical Management. Cambridge University Press, 2001: 611–70.

48 Deyde VM, Lo BB, Khalifa IO, Ly B, Ball A, Fattoum S. Epidemiological profile of hemoglobinopathies in the Mauritanian population. *Ann Hematol* 2002; **81**: 320–1.

49 Zago MA, Costa FF. Hereditary haemoglobin disorders in Brazil. *Trans R Soc Trop Med Hyg* 1985; **79**: 385–8.

50 Bayoumi RA, Abu Zeid YA, Abdul Sadig A, Awad Elkarim O. Sickle cell disease in Sudan. *Trans R Soc Trop Med Hyg* 1988; **82**: 164–8.

51 Bashir N, Barkawi M, Sharif L. Sickle cell/beta-thalassemia in North Jordan. *J Trop Pediatr* 1992; **38**: 196–8.

52 Van-Dunem JCC, Alves JGB, Bernardino L, *et al.* Factors associated with sickle cell disease mortality among hospitalized Angolan children and adolescents. *West Afr J Med* 2007; **26**: 269–73.

53 Steinberg M. Sickle Cell Trait. In: Disorders of Hemoglobin: Genetics, Pathophysiology, and Clinical Management. Cambridge University Press, 2001: 811–30.

54 Steinberg M, Nagel R. Hemoglobin SC Disease and HbC Disorders. In: Disorders of Hemoglobin: Genetics, Pathophysiology, and Clinical Management. Cambridge University Press, 2001: 756–85.

55 Al-Riyami AA, Suleiman AJ, Afifi M, Al-Lamki ZM, Daar S. A community-based study of common hereditary blood disorders in Oman. *East Mediterr Health J* 2001; **7**: 1004–11.

56 Olivieri NF, Weatherall DJ. Clinical Aspects of Beta Thalassemia. In: Disorders of Hemoglobin: Genetics, Pathophysiology, and Clinical Management. Cambridge University Press, 2001: 277–341.

57 Steinberg MH. Sickle Cell Trait. In: Disorders of Hemoglobin: Genetics, Pathophysiology, and Clinical Management. Cambridge University Press, 2001: 811–30.

58 Venugopal S, Dhuri S, Al Jabal KB, Shaju A. Hemoglobin H disease in Muscat, Oman - A 5 year study. *Oman Med J* 2008; **23**: 82–5.

59 Ankra-Badu GA, Al-Jama A, Al Kadim Y. Hemoglobin H disease in the Al-Qatif Region of Saudi Arabia. *Ann Saudi Med* 2001; **21**: 308–11.

60 Tagarelli A, Piro A, Bastone L, Tagarelli G. Identification of glucose 6-phosphate dehydrogenase deficiency in a population with a high frequency of thalassemia. *FEBS Lett* 2000; **466**: 139–42.

61 Piomelli S, Siniscalco M. The haematological effects of glucose-6-phosphate dehydrogenase deficiency and thalassaemia trait: interaction between the two genes at the phenotype level. *Br J Haematol* 1969; **16**: 537–49.

62 Mason PJ, Bautista JM, Gilsanz F. G6PD deficiency: the genotype-phenotype association. *Blood Rev* 2007; **21**: 267–83.

63 Zipursky A, Rowland M, Peters JC, Israels LG. Congenital non-spherocytic hemolytic anemia. *Can Med Assoc J* 1965; **93**: 1141–6.

64 Gupte SC, Shaw AN, Shah KC. Hematological findings and severity of G6PD deficiency in Vataliya Prajapati subjects. *J Assoc Physicians India* 2005; **53**: 1027–30.

65 Muhilal null, Permeisih D, Idjradinata YR, Muherdiyantiningsih null, Karyadi D. Vitamin A-fortified monosodium glutamate and health, growth, and survival of children: a controlled field trial. *Am J Clin Nutr* 1988; **48**: 1271–6.

66 Mejía LA, Chew F. Hematological effect of supplementing anemic children with vitamin A alone and in combination with iron. *Am J Clin Nutr* 1988; **48**: 595–600.

67 Bloem MW, Wedel M, van Agtmaal EJ, Speek AJ, Saowakontha S, Schreurs WH. Vitamin A intervention: short-term effects of a single, oral, massive dose on iron metabolism. *Am J Clin Nutr* 1990; **51**: 76–9.

68 Semba RD, Muhilal MPH, West KP, *et al.* Impact of vitamin a supplementation on hematological indicators of iron metabolism and protein status in children. *Nutrition Research* 1992; **12**: 469–78.

69 Mwanri L, Worsley A, Ryan P, Masika J. Supplemental vitamin A improves anemia and growth in anemic school children in Tanzania. *J Nutr* 2000; **130**: 2691–6.

70 de Almeida CAN, Dutra-De-Oliveira JE, Crott GC, *et al.* Effect of fortification of drinking water with iron plus ascorbic acid or with ascorbic acid alone on hemoglobin values and anthropometric indicators in preschool children in day-care centers in Southeast Brazil. *Food Nutr Bull* 2005; **26**: 259–65.

71 Ballot DE, MacPhail AP, Bothwell TH, Gillooly M, Mayet FG. Fortification of curry powder with NaFe(111)EDTA in an iron-deficient population: report of a controlled iron-fortification trial. *Am J Clin Nutr* 1989; **49**: 162–9.

72 Abrams SA, Mushi A, Hilmers DC, Griffin IJ, Davila P, Allen L. A multinutrient-fortified beverage enhances the nutritional status of children in Botswana. *J Nutr* 2003; **133**: 1834–40.

73 Faber M, Kvalsvig JD, Lombard CJ, Benadé AJS. Effect of a fortified maize-meal porridge on anemia, micronutrient status, and motor development of infants. *Am J Clin Nutr* 2005; **82**: 1032–9.

74 Thuy PV, Berger J, Davidsson L, *et al.* Regular consumption of NaFeEDTA-fortified fish sauce improves iron status and reduces the prevalence of anemia in anemic Vietnamese women. *Am J Clin Nutr* 2003; **78**: 284–90.

75 Sari M, Bloem MW, de Pee S, Schultink WJ, Sastroamidjojo S. Effect of iron-fortified candies on the iron status of children aged 4–6 y in East Jakarta, Indonesia. *The American Journal of Clinical Nutrition* 2001; **73**: 1034–9.

76 Nadiger HA, Krishnamachari KA, Naidu AN, Rao BS, Srikantia SG. The use of common salt (sodium chloride) fortified with iron to control anaemia: results of a preliminary study. *Br J Nutr* 1980; **43**: 45–51.

77 van Stuijvenberg ME, Smuts CM, Wolmarans P, Lombard CJ, Dhansay MA. The efficacy of ferrous bisglycinate and electrolytic iron as fortificants in bread in iron-deficient school children. *Br J Nutr* 2006; **95**: 532–8.

78 Viteri FE, Alvarez E, Batres R, *et al.* Fortification of sugar with iron sodium ethylenediaminotetraacetate (FeNaEDTA) improves iron status in semirural Guatemalan populations. *Am J Clin Nutr* 1995; **61**: 1153–63.

79 Huang X, Qu X, Yan W, *et al.* Iron deficiency anaemia can be improved after eradication of Helicobacter pylori. *Postgraduate Medical Journal* 2010; **86**: 272–8.

80 Wells CW, Lewis S, Barton RJ, Corbett S. Effects of Changes in Hemoglobin Level on Quality of Life and Cognitive Function in Inflammatory Bowel Disease Patients. *Inflammatory Bowel Diseases* 2006; **12**: 123–30.

81 Evstatiev R, Marteau P, Iqbal T, *et al.* FERGIcor, a Randomized Controlled Trial on Ferric Carboxymaltose for Iron Deficiency Anemia in Inflammatory Bowel Disease. *Gastroenterology* 2011; **141**: 846-853.e2.

82 Yang Y-Y, Lin H-C, Lee W-C, *et al.* Plasma erythropoietin level in patients with cirrhosis and its relationship to the severity of cirrhosis and renal function. *Journal of Gastroenterology and Hepatology* 2003; **18**: 1156–61.

83 Finkelstein FO, Story K, Firanek C, *et al.* Health-related quality of life and hemoglobin levels in chronic kidney disease patients. *Clin J Am Soc Nephrol* 2009; **4**: 33–8.

84 Levin A, Djurdjev O, Beaulieu M, Er L. Variability and risk factors for kidney disease progression and death following attainment of stage 4 CKD in a referred cohort. *Am J Kidney Dis* 2008; **52**: 661–71.

85 McClellan W, Aronoff SL, Bolton WK, *et al.* The prevalence of anemia in patients with chronic kidney disease. *Curr Med Res Opin* 2004; **20**: 1501–10.

86 Li Y, Shi H, Wang W-M, *et al.* Prevalence, awareness, and treatment of anemia in Chinese patients with nondialysis chronic kidney disease: First multicenter, cross-sectional study. *Medicine (Baltimore)* 2016; **95**: e3872.

87 Poudel B, Yadav DB, Jha B, Raut K, Pandeya D. Prevalence and association of anemia with CKD: A hospital based crosssectional study from Nepal. *Biomedical Research* 2013; **24**: 99–103.

88 Astor BC, Muntner P, Levin A, Eustace JA, Coresh J. Association of kidney function with anemia: the Third National Health and Nutrition Examination Survey (1988-1994). *Arch Intern Med* 2002; **162**: 1401–8.

89 Hsu C, McCulloch CE, Curhan GC. Epidemiology of anemia associated with chronic renal insufficiency among adults in the United States: results from the Third National Health and Nutrition Examination Survey. *J Am Soc Nephrol* 2002; **13**: 504–10.

90 Assiri AMA, Kamel HFM, ALrefai AA. Critical Appraisal of Advanced Glycation End Products (AGEs) and Circulating Soluble Receptors for Advanced Glycation End Products (sRAGE) as a Predictive Biomarkers for Cardiovascular Disease in Hemodialysis Patients. *Med Sci (Basel)* 2018; **6**: E38.

91 Floriani C, Feller M, Aubert CE, *et al.* Thyroid Dysfunction and Anemia: A Prospective Cohort Study and a Systematic Review. *Thyroid* 2018; **28**: 575–82.

92 von Drygalski A, Biller J. Anemia in Cystic Fibrosis (CF): Prevalence, Mechanisms and Correlation with Pulmonary Function. *Blood* 2006; **108**: 1286.

93 Giraldi FP, Ascoli P, De MM, Polli N, Cavagnini F. Anemia in male patients with cushing. *Endocrine Abstracts* 2009; **20**. https://www.endocrine-abstracts.org/ea/0020/ea0020p39 (accessed July 25, 2022).

94 Ambrogio AG, Martin MD, Ascoli P, Cavagnini F, Giraldi FP. Gender-dependent changes in haematological parameters in patients with Cushing's disease before and after remission. *European Journal of Endocrinology* 2014; **170**: 393–400.

95 Finsterer J, Frank M. Haematological abnormalities in mitochondrial disorders. *Singapore Med J* 2015; **56**: 412–9.

96 Kirkineska L, Perifanis V, Vasiliadis T. Functional hyposplenism. *Hippokratia* 2014; **18**: 7–11.

Section 4: Spatiotemporal Gaussian process regression methods

The following is adapted from the GBD 2019 Risk Factors Capstone paper supplementary methods appendix[1], with permission.

This type of regression is used when data density is sufficient to estimate a very flexible time trend. The approach is a stochastic modelling technique that is designed to detect signals amidst noisy data. It also serves as a powerful tool for interpolating non-linear trends. Unlike classical linear models that assume that the trend underlying data follows a definitive functional form, GPR assumes that the specific trend of interest follows a Gaussian process, which is defined by a mean function $m(\cdot)$ and a covariance function $Cov(\cdot)$. For example, let $p_{c,a,s,t}$ be the data, in normal, log, or logit space, observed in country c, for age group a, and sex s at time t:

$$\left(p_{c,a,s,t}\right) = g_{c,a,s}(t) + \epsilon_{c,a,s,t}$$

where

$$\epsilon_{c,a,s,t} \sim Normal\left(0, \sigma_p^2\right),$$

$$g_{c,a,s}(t) \sim GP\left(m_{c,a,s}(t), Cov\left(g_{c,a,s}(t)\right)\right).$$

The derivation of the mean and covariance functions, $m_{c,a,s}(t)$ and $Cov\left(g_{c,a,s}(t)\right)$, along with a more detailed description of the error variance (σ_p^2), is described below.

Estimating mean functions

We estimated mean functions by using a two-step approach. $m_{c,a,s}(t)$ can be expressed, depending on the exposure transformation, as:

$$log\left(p_{c,a,s}(t)\right) = X_{c,a,s}\beta + h(r_{c,a,s,t})$$

$$logit\left(p_{c,a,s}(t)\right) = X_{c,a,s}\beta + h(r_{c,a,s,t})$$

$$p_{c,a,s}(t) = X_{c,a,s}\beta + h(r_{c,a,s,t})$$

where $X\beta$ is the summation of the components of a hierarchical linear mixed-effects regression, including the intercept and the product of covariates with their corresponding fixed-effect coefficients. Models were run as hierarchical linear mixed-effects regressions with random intercepts on the levels of the geographic hierarchy. For these mixed-effects models, random intercepts were only used in the fit, not in the prediction. We combined these linear mixed-effects regressions in an ensemble model as described in Section 1 of this appendix. The second part of the equation, $h(r_{c,a,s,t})$, is a smoothing function for the residuals, $r_{c,a,s,t}$, derived from the initial ensemble linear model.

Although the linear component captures the general trend in exposures over time, much of the data variability may still not be adequately accounted for. To address this, we fit a locally weighted polynomial regression (locally estimated scatterplot smoothing, or LOESS) function $h(r_{c,a,s,t})$ to

[1] *GBD 2019 Risk Factors Collaborators. Global burden of 87 risk factors in 204 countries and territories, 1990–2019: a systematic analysis for the Global Burden of Disease Study 2019. The Lancet. 2020 Oct 17;396(10258):1223–49.*

systematically estimate this residual variability by borrowing strength across time, age, and space patterns (the spatiotemporal component of ST-GPR). The time adjustment parameter, defined by λ , aims to borrow strength from neighboring time points (i.e., the exposure in this year is highly correlated with exposure in the previous year but less so further back in time). The age-adjustment parameter, defined by ω, borrows strength from data in neighboring age groups. The space-adjustment parameter, defined by ξ, aims to borrow strength across the hierarchy of geographic locations. The spatial and temporal weights are combined into a single space-time weight to allow the amount of spatial weight given to a particular point $r_{c,a,s,t}$ to fluctuate given the data availability at each time *t* and location-level *l* in the location hierarchy.

Let $w_{c,a,s,t}$ be the final weight assigned to observation $r_{c,a,s,t}$ with reference to a focal observation r_{c_0,a_0,s_0,t_0}. We first generated a temporal weight $t.w_{c,a,s,t}$ for smoothing over time, which was based on the scaled distance along the time dimension of the two observations:

$$t.w_{c,a,s,t} = \frac{1}{e^{\lambda|t-t_0|}}$$

Next, we generated a spatial weight to smooth over geography. Specifically, we defined a geospatial relationship by categorizing data based on the GBD location hierarchy. ζ acts as a scalar on a given datapoint given its proximity to the target location:

$$t.w_{c,a,s,t} = \zeta^{|c-c_0|}$$

For example, estimating a country would use the following weighting scheme:

- Country data: ζ^0 = 1
- Regional data not from the country being estimated: ζ^1
- Data from other regions in the same super region: ξ^2
- Global data from other super regions: ζ^3

Under the spatial weighting specification, typical values of ζ range from [0.001, 0.2], where ζ can be interpreted as the amount to downweight regional datapoints compared to country datapoints for a given estimating country. For example, for a given datapoint $r_{c,a,s,t}$ and ζ = 0.01, a datapoint not within country *c* but within the same region *r* as $r_{c,a,s,t}$ would be assigned $\frac{1}{100}$ the weight of a datapoint within the country.

The spatial and temporal weights were then multiplied and summed across each level of the location hierarchy and normalised for each time period *t* . This procedure allowed the space-time weight to implicitly take into account the amount of data available at the country vs. region vs. super-region level and attribute spatial weight accordingly.

Given a normalisation constant,

$$K_i = \sum_{c \epsilon C} s.w_{c,t} * t.w_{c.t} + \sum_{c \epsilon R} s.w_{c,t} * t.w_{c.t} + \sum_{c \epsilon SR} s.w_{c,t} * t.w_{c.t}$$

the final space-time weight would then equal

$$w'_{c,a,s,t} = \frac{s.w_{c,t} * t.w_{c,t}}{K_i}$$

Finally, we calculated the weight $w''_{c,a,s,t}$ to smooth over age, which is based on a distance along the age dimension of two observations. For a point between the age a of the observation $r_{c,a,s,t}$ and a focal observation r_{c_0,a_0,s_0,t_0}, the weight is defined as follows:

$$w''_{c,a,s,t} = \frac{1}{e^{\omega|a-a_0|}}$$

The final weights would then be computed by simply multiplying the space-time weights and age weights and normalising so all weights for a given time period *t* sum to 1. A full derivation of weights for each category, assuming the location being estimated was a country, follows:

1) If the observation $r_{c,t}$ belongs to the same country c_0 of the focal observation r_{c_0,t_0}:

$$w_{c,a,s,t} = \frac{(w'_{c,a,s,t}w''_{c,a,s,t})}{\sum_{c=c_0}(w'_{c,a,s,t}w''_{c,a,s,t})} \qquad \forall c = c_0$$

2) If the observation $r_{c,t}$ belongs to a different country than the focal observation r_{c_0,t_0}, but both belong to the same region *R*:

$$w_{c,a,s,t} = \frac{(w'_{c,a,s,t}w''_{c,a,s,t})}{\sum_{c\neq c_0}(w'_{c,a,s,t}w''_{c,a,s,t})} \qquad \forall c \neq c_0 \cap R[c] = R[c_0]$$

3) If the observation $r_{c,t}$ belongs to the same super region *SR* but to both a different country c_0 and a different region $R[c_0]$ than the focal observation r_{c_0,t_0}:

$$w_{c,a,s,t} = \frac{(w'_{c,a,s,t}w''_{c,a,s,t})}{\sum_{c\neq c_0}(w'_{c,a,s,t}w''_{c,a,s,t})} \qquad \forall c \neq c_0 \cap R[c] \neq R[c_0] \cap SR[c] = SR[c_0]$$

4) If the observation $r_{c,t}$ is from a different super region than the focal observation r_{c_0,t_0}(Ie, all other data currently not receiving a weight):

$$w_{c,a,s,t} = \frac{(w'_{c,a,s,t}w''_{c,a,s,t})}{\sum_{c\neq c_0}(w'_{c,a,s,t}w''_{c,a,s,t})} \qquad \forall c \neq c_0 \cap R[c] \neq R[c_0] \cap SR[c] \neq SR[c_0]$$

The final weights were then normalised such that the sum of weights across age, time, and geographic hierarchy for a reference group was 1.

Estimating error variance

σ_p^2 represents the error variance in normal or transformed space including the sampling variance of the estimates and prediction error from any crosswalks performed. First, variance was systematically imputed if the data extraction did not include any measure of uncertainty. When some sample sizes for data were available, missing sample sizes were imputed as the 5th percentile of available sample sizes. Missing variances were then calculated as $\sigma_p^2 = \frac{p*(1-p)}{n}$ for proportions or were predicted from the

mean by using a regression for continuous values. When sample sizes were entirely missing and could not be imputed, the 95th percentile of available variances at the most granular geographic level (i.e.,, first country, then region, etc.) were used to impute missing variances. For proportions where *p*n* or *(1-p)*n* is <20, variance was replaced by using the Wilson Interval Score method.

Next, if the exposure was modelled as a log transformation, the error variance was transformed into log-space by using the delta method approximation as follows:

$$\sigma_p^2 \cong \frac{\sigma_{p\prime}^2}{p_{c,a,s,t}^2}$$

where $\sigma_{p\prime}^2$ represents the error variance in normal space. If the exposure was modelled as a logit transformation, the error variance was transformed into logit-space by using the delta method approximation as follows:

$$\sigma_p^2 \cong \frac{\sigma_{p\prime}^2}{(p_{c,a,s,t} * (1 - p_{c,a,s,t}))^2}$$

Finally, prior to GPR, an approximation of non-sampling variance was added to the error variance. Calculations of non-sampling variance were done on normal-space variances. Non-sampling variance was calculated as the variance of inverse-variance weighted residuals from the space-time estimate at a given location-level hierarchy. If there were <10 data points at a given level of the location hierarchy, the non-sampling variance was replaced with that of the next highest geography level with >10 data points.

Estimating the covariance function

The final input into GPR is the covariance function, which defines the shape and distribution of the trends. Here, we have chosen the Matern-Euclidian covariance function, which offers the flexibility to model a wide spectrum of trends with varying degrees of smoothness. The function is defined as follows:

$$M(t,t') = \sigma^2 \frac{2^{1-v}}{\Gamma(v)} \left(\frac{d(t,t')\sqrt{2v}}{l}\right)^v K_v\left(\frac{d(t,t')\sqrt{2v}}{l}\right)$$

where $d(\cdot)$is a distance function; σ^2, v, l, and K_v are hyperparameters of the covariance function—specifically σ^2 is the marginal variance, v is the smoothness parameter that defines the differentiability of the function, l is the length scale, which roughly defines the distance between which two points become uncorrelated, and K_v is the Bessel function. We approximated σ^2 by taking the normalised median absolute deviation $MADN(r_c')$ of the difference, which is the normalised absolute deviation of the difference of the first-stage linear regression estimate from the second-stage spatiotemporal smoothing step for each country. We then took the mean of these country-level MADN estimates for all countries with 10+ country-years of data to ensure that differences between first- and second-stage estimates had sufficient data to truly convey meaningful information on model uncertainty. We used the parameter specification $v = 2$ for all models.

Prediction using GPR

We integrated over $g_{c,t}(t_*)$ to predict a full time series for country c, age a, sex s, and prediction time t_*as follows:

$$p_{c,a,s}(t_*) \sim N\left(m_{c,a,s,t}(t_*), \sigma_p^2 I + Cov\left(g_{c,a,s,t}(t_*)\right)\right)$$

Random draws of 1000 samples were obtained from the distributions above for every country for a given indicator. The final estimated mean for each country was the mean of the draws. In addition, 95% UIs were calculated by taking the 2.5th and 97.5th percentiles of the sample distribution.

Subnational Scaling and Aggregation

To ensure internal consistency of the estimates between countries and their respective subnational locations, national estimates were either created by population-weighted aggregation or subnational estimates were adjusted by population-weighted scaling to the national estimates, depending on the data coverage of a given country compared to that of its subnational locations. For example, if data coverage was better at the national level than at its corresponding subnational locations for a given country and risk across age, sex, and time, estimates were rescaled to be consistent with the national level. Conversely, if data coverage was better at the subnational level, estimates for its parent country were generated through population-weighted aggregation of subnational estimates.

Estimates can also be scaled within logit space. Scaling in logit space ensures that subnational estimates of proportion models do not exceed one after being rescaled to the national estimate.

Section 5: Supplemental Tables and Figures

Anaemia prevalence (total, mild, moderate, and severe) and YLD rate (total), 1990 and 2021

	Prevalence (%)								YLDs (rate per 100,000 population)	
	Total		Mild		Moderate		Severe		Total	
	1990	2021	1990	2021	1990	2021	1990	2021	1990	2021
Global	28·2 (27·8–28·5)	24·3 (23·9–24·7)	14·8 (14·6–15·1)	14·1 (13·8–14·5)	11·9 (11·7–12·0)	9·3 (9·1–9·4)	1·5 (1·4–1·5)	0·9 (0·9–1·0)	874·8 (592·1–1233·4)	659·6 (445·2–952·9)
Central Europe, Eastern Europe, and Central Asia	21·3 (20·5–22·0)	18·0 (17·4–18·7)	13·0 (12·4–13·6)	11·6 (11·1–12·2)	7·6 (7·3–7·9)	5·9 (5·6–6·2)	0·8 (0·7–0·8)	0·5 (0·5–0·6)	545·3 (365·1–806·3)	419·7 (285·8–605·8)
Central Asia	34·0 (32·4–36·1)	28·6 (27·3–30·3)	17·1 (15·7–18·6)	16·0 (14·8–17·7)	15·2 (14·6–16·1)	11·5 (11·0–12·2)	1·7 (1·4–1·9)	1·1 (0·9–1·3)	1083·7 (712·8–1528·6)	808·8 (563·5–1141·3)
Armenia	21·5 (19·8–22·9)	18·1 (16·7–19·5)	11·4 (10·4–12·6)	11·3 (10·1–12·5)	8·9 (7·9–9·7)	6·2 (5·6–7·0)	1·2 (0·9–1·5)	0·5 (0·4–0·7)	669·6 (422·3–923·7)	435·9 (284·1–615·3)
Azerbaijan	30·3 (28·2–32·7)	24·1 (22·4–26·1)	15·3 (13·6–17·6)	14·4 (13·1–15·9)	13·4 (12·1–14·5)	8·9 (8·1–9·8)	1·7 (1·2–2·4)	0·8 (0·5–1·1)	985·2 (645·4–1412·5)	622·8 (376·9–861·1)
Georgia	22·9 (21·0–24·9)	20·7 (18·8–22·0)	13·3 (11·9–15·3)	12·4 (11·0–13·9)	8·8 (7·8–9·8)	7·6 (6·8–8·3)	0·8 (0·6–1·1)	0·7 (0·5–1·0)	615·6 (401·3–941·8)	536·5 (348·6–800·7)
Kazakhstan	34·8 (31·5–38·6)	26·7 (23·6–30·4)	18·6 (15·7–22·1)	16·7 (13·8–20·4)	14·8 (13·2–16·5)	9·3 (8·3–11·4)	1·5 (1·0–2·1)	0·6 (0·4–0·9)	1040·5 (635·9–1496·4)	630·9 (416·2–943·8)
Kyrgyzstan	31·2 (28·7–35·2)	25·4 (22·8–28·4)	16·3 (13·8–20·4)	14·5 (12·2–17·5)	13·5 (12·2–15·5)	10·1 (9·1–11·2)	1·3 (1·0–1·8)	0·8 (0·6–1·1)	948·2 (641·1–1316·9)	683·4 (438·2–994·6)
Mongolia	31·2 (29·4–33·3)	22·4 (21·0–24·0)	12·5 (11·4–14·6)	10·6 (9·7–12·2)	15·4 (14·1–16·6)	10·1 (9·2–10·9)	3·4 (2·5–4·2)	1·8 (1·4–2·2)	1323·8 (914·1–1835·5)	816·0 (523·8–1198·1)
Tajikistan	31·1 (29·0–33·9)	28·0 (26·3–30·2)	14·2 (12·2–16·6)	14·3 (12·9–16·3)	14·7 (13·3–16·1)	12·2 (11·3–13·2)	2·3 (1·6–2·9)	1·5 (1·2–1·9)	1139·7 (763·2–1678·9)	902·2 (606·9–1271·1)
Turkmenistan	30·0 (27·8–33·2)	23·9 (21·8–26·4)	14·1 (12·1–17·8)	13·2 (11·7–15·7)	13·9 (12·7–15·0)	9·6 (8·8–10·6)	2·0 (1·5–2·6)	1·0 (0·7–1·3)	1065·7 (700·9–1476·5)	690·2 (456·8–1004·0)
Uzbekistan	41·8 (37·7–46·2)	35·0 (31·7–39·3)	20·4 (17·4–24·9)	18·6 (15·8–22·6)	19·6 (18·0–21·8)	15·0 (13·6–16·8)	1·7 (1·3–2·3)	1·4 (1·0–1·9)	1335·9 (858·0–1882·0)	1042·0 (712·6–1466·3)
Central Europe	21·0 (20·2–22·2)	14·8 (14·2–15·7)	14·1 (13·3–15·2)	10·7 (10·1–11·5)	6·4 (6·1–6·8)	3·8 (3·6–4·0)	0·6 (0·5–0·6)	0·3 (0·2–0·3)	462·2 (304·4–681·6)	273·0 (180·6–390·3)
Albania	31·3 (27·4–38·1)	17·7 (16·3–19·5)	22·4 (18·2–28·7)	13·4 (12·1–15·2)	8·5 (7·1–10·4)	4·1 (3·8–4·5)	0·4 (0·2–0·7)	0·2 (0·1–0·2)	580·8 (356·7–844·5)	284·3 (186·3–425·3)
Bosnia and Herzegovina	22·0 (20·0–25·1)	16·1 (14·4–17·8)	14·5 (12·7–17·0)	11·5 (10·1–13·2)	6·9 (6·1–7·9)	4·3 (3·8–4·8)	0·6 (0·4–0·8)	0·3 (0·2–0·4)	494·4 (331·9–698·0)	303·0 (194·8–452·4)
Bulgaria	20·0 (18·4–22·0)	17·4 (16·2–19·1)	13·3 (11·7–15·1)	12·3 (11·2–14·0)	6·2 (5·6–6·9)	4·7 (4·2–5·3)	0·5 (0·4–0·7)	0·3 (0·2–0·5)	442·6 (287·2–635·4)	334·9 (213·4–467·8)
Croatia	15·8 (14·5–17·6)	12·3 (11·2–13·5)	10·7 (9·5–12·6)	8·9 (7·9–10·1)	4·8 (4·3–5·2)	3·3 (2·8–3·7)	0·4 (0·3–0·5)	0·2 (0·2–0·3)	339·9 (229·3–500·9)	230·9 (147·0–342·8)
Czechia	17·0 (15·5–19·6)	12·2 (10·7–13·4)	11·6 (10·2–14·0)	8·8 (7·7–9·9)	5·1 (4·4–5·7)	3·1 (2·8–3·6)	0·4 (0·3–0·5)	0·2 (0·1–0·3)	359·5 (232·8–529·1)	223·0 (145·7–340·0)
Hungary	17·5 (16·1–19·1)	12·7 (11·3–14·5)	11·3 (9·9–12·9)	8·8 (7·6–10·7)	5·7 (5·1–6·5)	3·6 (3·2–4·1)	0·5 (0·4–0·7)	0·3 (0·2–0·4)	411·4 (255·5–608·0)	259·1 (168·3–388·3)
Montenegro	16·3 (14·9–18·6)	14·4 (12·8–15·9)	11·1 (9·9–13·0)	10·3 (9·0–12·0)	4·8 (4·4–5·4)	3·8 (3·5–4·2)	0·3 (0·3–0·5)	0·2 (0·2–0·3)	339·5 (220·2–499·7)	268·8 (177·6–381·9)
North Macedonia	20·8 (18·9–24·0)	15·1 (14·0–16·6)	12·8 (11·3–15·0)	10·2 (9·0–11·7)	7·2 (6·4–8·3)	4·5 (4·0–5·0)	0·8 (0·6–1·0)	0·4 (0·3–0·5)	535·4 (351·7–763·4)	325·9 (207·1–471·4)
Poland	23·1 (20·8–25·8)	15·5 (14·0–17·9)	15·6 (13·5–18·4)	11·4 (10·2–13·6)	6·8 (6·1–7·7)	3·8 (3·4–4·2)	0·6 (0·5–0·9)	0·3 (0·2–0·4)	498·4 (326·6–731·7)	274·4 (180·4–398·8)
Romania	22·3 (20·0–25·0)	15·6 (14·2–17·2)	14·5 (12·7–17·3)	11·2 (9·8–12·7)	7·1 (6·4–7·9)	4·1 (3·6–4·6)	0·6 (0·5–0·8)	0·3 (0·2–0·4)	509·7 (327·1–737·0)	291·6 (185·3–432·2)
Serbia	20·6 (18·8–23·1)	14·7 (13·5–16·0)	13·8 (12·3–16·3)	10·6 (9·5–12·1)	6·3 (5·6–7·1)	3·9 (3·4–4·4)	0·5 (0·4–0·7)	0·3 (0·2–0·4)	447·0 (284·3–668·1)	275·7 (175·0–412·6)
Slovakia	18·6 (17·0–20·7)	14·0 (12·6–15·9)	12·5 (11·0–14·2)	10·1 (8·8–11·8)	5·7 (5·0–6·4)	3·6 (3·1–4·1)	0·4 (0·3–0·6)	0·2 (0·2–0·3)	402·1 (262·6–584·6)	255·5 (160·6–381·2)
Slovenia	15·3 (14·0–17·3)	10·8 (9·7–12·0)	10·5 (9·5–12·2)	7·8 (6·9–8·9)	4·5 (4·0–5·0)	2·8 (2·4–3·2)	0·3 (0·2–0·4)	0·2 (0·1–0·3)	317·0 (203·5–458·7)	196·9 (120·7–302·1)
Eastern Europe	17·6 (16·4–18·4)	14·9 (13·8–15·9)	11·1 (10·2–12·0)	10·1 (9·2–11·0)	5·9 (5·5–6·3)	4·5 (4·1–5·0)	0·6 (0·5–0·7)	0·4 (0·3–0·5)	426·5 (279·3–646·5)	321·8 (209·9–476·1)
Belarus	17·4 (16·1–19·2)	13·6 (12·7–15·0)	11·1 (9·8–12·8)	9·2 (8·3–10·7)	5·8 (5·3–6·5)	4·1 (3·7–4·8)	0·5 (0·3–0·7)	0·3 (0·2–0·4)	408·7 (262·3–592·9)	286·4 (188·7–418·5)
Estonia	17·1 (15·9–18·5)	13·1 (12·1–14·4)	10·9 (10·0–12·2)	9·1 (8·1–10·3)	5·7 (5·1–6·4)	3·7 (3·3–4·2)	0·5 (0·3–0·6)	0·2 (0·2–0·3)	398·2 (264·1–589·3)	257·6 (165·1–380·7)
Latvia	17·4 (16·1–19·1)	14·7 (13·5–16·2)	11·2 (10·1–12·6)	10·2 (9·3–11·5)	5·8 (5·2–6·4)	4·3 (3·8–4·9)	0·5 (0·3–0·6)	0·3 (0·2–0·4)	402·6 (264·5–571·5)	296·7 (181·1–440·0)
Lithuania	16·9 (15·5–18·5)	14·7 (13·5–15·8)	10·9 (9·6–12·2)	10·2 (9·2–11·3)	5·6 (5·1–6·3)	4·2 (3·8–4·8)	0·4 (0·3–0·6)	0·3 (0·2–0·4)	391·5 (253·6–579·4)	293·1 (181·6–447·5)
Republic of Moldova	22·4 (20·2–25·3)	17·9 (16·6–19·6)	14·5 (12·7–17·7)	12·0 (10·9–13·3)	7·4 (6·5–8·3)	5·5 (4·9–6·2)	0·5 (0·3–0·6)	0·3 (0·2–0·5)	495·4 (323·1–689·2)	376·4 (235·7–536·7)
Russian Federation	18·3 (16·5–19·7)	15·1 (13·7–16·6)	11·7 (10·4–13·1)	10·4 (9·2–11·9)	6·0 (5·4–6·6)	4·4 (3·9–5·0)	0·6 (0·4–0·7)	0·3 (0·3–0·5)	430·7 (277·3–666·7)	310·4 (202·4–468·0)
Ukraine	15·3 (14·4–16·5)	14·4 (13·4–15·9)	9·1 (8·4–9·9)	9·0 (8·2–9·7)	5·5 (5·0–6·1)	4·9 (4·4–5·5)	0·7 (0·5–0·9)	0·6 (0·4–0·8)	416·5 (279·4–604·8)	368·0 (241·4–536·5)
High-income	8·0 (7·6–8·5)	7·0 (6·6–7·4)	6·5 (6·1–6·9)	5·6 (5·2–5·9)	1·4 (1·4–1·5)	1·3 (1·3–1·4)	0·1 (0·1–0·1)	0·1 (0·1–0·1)	106·8 (68·2–163·7)	99·6 (64·9–149·9)
Australasia	6·9 (6·0–8·6)	5·7 (5·1–6·9)	5·6 (4·7–7·1)	4·6 (4·0–5·7)	1·3 (1·1–1·4)	1·1 (1·0–1·3)	0·1 (0·0–0·1)	0·0 (0·0–0·1)	93·2 (56·1–133·9)	81·3 (49·5–122·2)
Australia	6·7 (5·7–8·9)	5·7 (5·0–7·1)	5·5 (4·4–7·5)	4·5 (3·9–5·8)	1·2 (1·0–1·5)	1·1 (0·9–1·3)	0·0 (0·0–0·1)	0·0 (0·0–0·1)	89·0 (53·2–130·2)	79·5 (48·0–124·8)
New Zealand	7·8 (6·8–10·1)	6·1 (5·1–8·0)	6·2 (5·3–8·2)	4·8 (4·0–6·4)	1·5 (1·3–2·0)	1·2 (1·0–1·6)	0·1 (0·1–0·2)	0·1 (0·0–0·1)	114·1 (65·6–170·3)	90·1 (54·7–136·6)
High-income Asia Pacific	9·9 (8·8–11·3)	9·0 (8·0–10·5)	8·5 (7·4–9·9)	7·4 (6·5–8·7)	1·3 (1·2–1·6)	1·5 (1·3–1·7)	0·0 (0·0–0·0)	0·1 (0·1–0·2)	104·1 (65·2–167·4)	118·1 (71·2–179·7)

Brunei Darussalam	10·4 (9·2–12·3)	8·3 (7·3–10·1)	8·5 (7·4–10·3)	6·6 (5·8–8·3)	1·8 (1·4–2·2)	1·6 (1·2–1·9)	0·1 (0·0–0·1)	0·1 (0·0–0·1)	131·3 (80·1–212·9)	114·3 (68·9–189·5)
Japan	9·9 (8·5–11·7)	10·6 (9·1–12·8)	8·5 (7·1–10·3)	8·7 (7·3–10·7)	1·3 (1·0–1·6)	1·7 (1·5–2·1)	0·0 (0·0–0·1)	0·2 (0·1–0·2)	102·9 (64·9–164·0)	141·2 (82·7–214·2)
Republic of Korea	9·5 (7·9–11·3)	5·2 (4·6–5·7)	8·1 (6·8–9·8)	4·2 (3·7–4·7)	1·4 (1·0–1·8)	0·9 (0·7–1·1)	0·0 (0·0–0·0)	0·0 (0·0–0·0)	104·8 (58·5–187·7)	66·5 (40·9–103·0)
Singapore	16·0 (10·9–21·0)	7·7 (5·2–12·6)	14·3 (9·7–18·9)	6·9 (4·6–11·5)	1·7 (1·0–2·4)	0·8 (0·5–1·3)	0·0 (0·0–0·0)	0·0 (0·0–0·0)	142·6 (72·8–252·9)	68·5 (32·2–134·7)
High-income North America	6·5 (6·1–7·0)	6·8 (6·2–7·6)	5·3 (4·9–5·7)	5·3 (4·8–6·0)	1·2 (1·1–1·3)	1·4 (1·3–1·6)	0·0 (0·0–0·1)	0·1 (0·1–0·1)	88·1 (57·2–133·4)	103·4 (65·9–158·5)
Canada	6·5 (4·3–10·3)	5·1 (4·0–8·0)	5·7 (3·8–8·9)	4·3 (3·3–7·1)	0·7 (0·4–1·2)	0·8 (0·5–1·1)	0·0 (0·0–0·0)	0·0 (0·0–0·0)	61·0 (31·4–104·3)	58·9 (33·3–96·6)
Greenland	8·9 (7·2–11·4)	6·3 (5·7–7·0)	7·2 (5·9–9·6)	5·0 (4·5–5·6)	1·6 (1·3–2·0)	1·2 (1·0–1·5)	0·1 (0·0–0·1)	0·1 (0·0–0·1)	117·1 (67·9–191·2)	89·1 (53·3–144·7)
United States of America	6·5 (6·2–6·9)	7·0 (6·4–7·7)	5·2 (4·9–5·5)	5·4 (4·9–6·0)	1·3 (1·2–1·4)	1·5 (1·4–1·7)	0·0 (0·0–0·1)	0·1 (0·1–0·1)	91·0 (58·6–138·9)	108·4 (69·4–166·5)
Southern Latin America	13·2 (10·7–16·2)	9·1 (6·6–12·4)	10·9 (8·5–14·0)	7·7 (5·5–10·7)	2·1 (1·6–3·0)	1·3 (1·0–1·8)	0·1 (0·1–0·2)	0·1 (0·0–0·1)	162·6 (92·7–288·9)	105·5 (57·9–183·1)
Argentina	14·9 (11·3–19·6)	10·6 (7·1–15·6)	12·6 (9·0–17·1)	9·1 (6·0–13·6)	2·2 (1·4–3·6)	1·4 (0·9–2·1)	0·1 (0·0–0·2)	0·1 (0·0–0·1)	174·4 (93·5–310·1)	114·5 (57·1–212·4)
Chile	8·9 (7·9–10·4)	5·6 (5·1–6·1)	6·9 (6·1–8·2)	4·4 (4·0–5·0)	1·9 (1·5–2·4)	1·2 (1·0–1·3)	0·1 (0·1–0·1)	0·1 (0·0–0·1)	136·6 (85·4–213·5)	84·0 (53·1–123·8)
Uruguay	13·0 (9·2–17·9)	8·6 (7·0–11·5)	11·0 (7·7–15·8)	7·2 (5·7–10·1)	1·9 (1·4–2·8)	1·3 (1·0–1·7)	0·1 (0·0–0·1)	0·1 (0·0–0·1)	148·6 (88·1–255·7)	103·4 (60·8–185·9)
Western Europe	7·6 (7·0–8·2)	6·0 (5·7–6·4)	5·9 (5·4–6·5)	4·7 (4·4–5·1)	1·6 (1·5–1·7)	1·2 (1·2–1·3)	0·1 (0·1–0·1)	0·1 (0·1–0·1)	115·2 (73·7–172·5)	89·0 (57·3–131·7)
Andorra	5·6 (5·0–6·3)	5·2 (4·7–5·8)	4·4 (3·9–5·1)	4·1 (3·7–4·7)	1·1 (0·9–1·3)	1·0 (0·9–1·2)	0·0 (0·0–0·1)	0·0 (0·0–0·1)	79·7 (45·0–121·0)	72·0 (43·8–111·5)
Austria	7·0 (6·2–8·3)	5·7 (5·3–6·3)	5·5 (4·8–6·5)	4·5 (4·1–5·0)	1·5 (1·3–1·8)	1·2 (1·0–1·4)	0·1 (0·0–0·1)	0·0 (0·0–0·1)	107·7 (68·5–164·9)	82·8 (53·5–122·4)
Belgium	6·7 (6·1–7·3)	5·7 (5·1–6·3)	5·0 (4·5–5·5)	4·3 (3·7–4·8)	1·7 (1·5–1·9)	1·3 (1·1–1·5)	0·1 (0·1–0·1)	0·1 (0·0–0·1)	115·0 (76·4–175·3)	92·2 (57·9–139·5)
Cyprus	8·6 (7·5–10·7)	5·6 (4·9–6·2)	6·6 (5·5–8·5)	4·4 (3·8–4·9)	1·9 (1·5–2·2)	1·2 (1·0–1·4)	0·1 (0·0–0·1)	0·0 (0·0–0·1)	134·2 (80·6–196·7)	82·1 (52·2–129·4)
Denmark	7·5 (6·7–8·4)	5·8 (5·2–6·8)	5·9 (5·3–6·7)	4·6 (4·0–5·5)	1·5 (1·3–1·8)	1·1 (1·0–1·3)	0·1 (0·0–0·1)	0·0 (0·0–0·1)	107·7 (67·8–165·3)	81·8 (49·8–128·4)
Finland	7·0 (6·3–7·8)	5·3 (4·8–6·0)	5·3 (4·8–5·9)	4·1 (3·7–4·6)	1·6 (1·4–1·9)	1·2 (1·0–1·3)	0·1 (0·0–0·1)	0·0 (0·0–0·1)	112·0 (70·1–180·7)	81·6 (50·3–123·9)
France	6·8 (6·3–7·6)	5·5 (4·9–6·1)	4·8 (4·5–5·5)	4·1 (3·6–4·5)	1·8 (1·6–2·2)	1·3 (1·2–1·5)	0·1 (0·1–0·1)	0·1 (0·1–0·1)	127·7 (78·9–194·6)	92·9 (60·3–138·0)
Germany	9·1 (6·9–11·9)	6·6 (5·4–8·1)	7·6 (5·6–10·2)	5·4 (4·4–6·9)	1·4 (1·1–1·8)	1·1 (0·9–1·3)	0·1 (0·0–0·1)	0·0 (0·0–0·1)	107·4 (65·3–177·6)	80·6 (48·8–123·4)
Greece	7·5 (6·7–9·1)	7·6 (6·8–8·4)	5·7 (4·9–7·2)	5·9 (5·1–6·6)	1·7 (1·4–2·0)	1·6 (1·4–1·9)	0·1 (0·1–0·1)	0·1 (0·0–0·1)	119·3 (74·4–180·7)	116·8 (72·2–172·3)
Iceland	5·7 (5·2–6·3)	4·5 (4·0–5·0)	4·3 (3·8–4·7)	3·4 (3·0–3·9)	1·4 (1·1–1·5)	1·0 (0·9–1·1)	0·1 (0·0–0·1)	0·0 (0·0–0·1)	93·5 (58·3–139·5)	69·8 (42·4–103·8)
Ireland	7·3 (6·6–8·3)	4·9 (4·5–5·4)	5·5 (4·9–6·5)	3·9 (3·5–4·4)	1·7 (1·4–1·9)	1·0 (0·8–1·2)	0·1 (0·1–0·1)	0·0 (0·0–0·1)	118·6 (75·6–175·9)	71·0 (46·6–107·2)
Israel	9·0 (7·8–10·6)	6·6 (6·0–7·6)	6·7 (5·7–8·3)	5·1 (4·6–5·9)	2·2 (1·8–2·5)	1·5 (1·2–1·7)	0·1 (0·1–0·2)	0·1 (0·0–0·1)	150·9 (97·0–241·1)	102·3 (62·7–153·7)
Italy	7·0 (6·0–8·2)	5·8 (5·2–6·6)	5·5 (4·7–6·7)	4·5 (4·0–5·2)	1·4 (1·2–1·6)	1·2 (1·1–1·4)	0·1 (0·1–0·1)	0·1 (0·0–0·1)	103·0 (69·3–155·5)	85·7 (53·2–126·1)
Luxembourg	7·2 (6·5–8·5)	5·1 (4·6–5·7)	5·7 (5·0–7·0)	4·1 (3·7–4·6)	1·5 (1·2–1·7)	1·0 (0·9–1·2)	0·1 (0·0–0·1)	0·0 (0·0–0·1)	105·0 (63·7–154·6)	69·9 (43·9–105·8)
Malta	8·4 (7·4–10·3)	6·5 (5·9–7·3)	6·4 (5·5–8·1)	5·1 (4·5–5·9)	2·0 (1·6–2·3)	1·4 (1·2–1·6)	0·1 (0·1–0·1)	0·1 (0·0–0·1)	136·9 (88·4–208·0)	96·5 (62·4–141·4)
Monaco	5·3 (4·8–6·0)	4·8 (4·3–5·4)	4·3 (3·8–5·0)	4·0 (3·5–4·6)	0·9 (0·8–1·0)	0·8 (0·7–1·0)	0·0 (0·0–0·0)	0·0 (0·0–0·0)	66·7 (44·0–98·0)	59·0 (37·5–90·4)
Netherlands	6·5 (5·8–7·2)	5·4 (4·8–6·1)	5·0 (4·4–5·6)	4·2 (3·8–4·8)	1·4 (1·3–1·6)	1·1 (1·0–1·3)	0·1 (0·0–0·1)	0·0 (0·0–0·1)	101·8 (62·3–152·4)	80·0 (48·2–119·8)
Norway	6·5 (5·8–7·3)	4·7 (4·3–5·2)	5·1 (4·5–5·8)	3·7 (3·3–4·2)	1·4 (1·2–1·6)	0·9 (0·8–1·1)	0·1 (0·0–0·1)	0·0 (0·0–0·1)	98·4 (59·0–144·9)	67·5 (41·1–102·1)
Portugal	9·4 (8·3–10·9)	6·8 (6·1–7·7)	7·1 (6·1–8·6)	5·3 (4·7–6·1)	2·1 (1·7–2·5)	1·5 (1·2–1·7)	0·1 (0·1–0·2)	0·1 (0·0–0·1)	149·2 (88·9–226·4)	103·2 (64·8–162·9)
San Marino	5·5 (5·0–6·2)	5·8 (5·1–6·6)	4·3 (3·9–5·0)	4·6 (4·0–5·2)	1·2 (1·0–1·3)	1·2 (1·0–1·3)	0·0 (0·0–0·1)	0·0 (0·0–0·1)	81·9 (51·6–129·4)	82·2 (51·3–125·5)
Spain	7·3 (6·5–8·7)	5·8 (5·1–6·5)	5·7 (5·0–7·0)	4·5 (4·0–5·2)	1·5 (1·3–1·8)	1·2 (1·0–1·4)	0·1 (0·0–0·1)	0·0 (0·0–0·1)	108·5 (68·6–171·2)	82·7 (48·7–128·7)
Sweden	6·7 (5·9–7·7)	5·4 (4·9–6·1)	5·3 (4·6–6·2)	4·3 (3·9–4·9)	1·4 (1·2–1·6)	1·1 (0·9–1·3)	0·1 (0·0–0·1)	0·0 (0·0–0·1)	101·4 (64·4–153·3)	77·2 (47·3–118·1)
Switzerland	7·8 (5·8–11·1)	6·6 (5·2–9·3)	6·7 (4·9–9·7)	5·6 (4·2–8·1)	1·1 (0·9–1·5)	1·0 (0·8–1·3)	0·0 (0·0–0·1)	0·0 (0·0–0·0)	87·1 (50·3–149·2)	75·0 (44·3–129·1)
United Kingdom	7·5 (6·8–8·5)	6·5 (5·8–7·4)	5·6 (5·0–6·4)	5·0 (4·4–5·7)	1·8 (1·6–1·9)	1·4 (1·2–1·6)	0·1 (0·1–0·2)	0·1 (0·1–0·1)	132·0 (85·6–195·3)	105·5 (65·6–174·9)
Latin America and Caribbean	21·4 (20·4–22·6)	15·6 (14·9–16·3)	12·5 (11·7–13·3)	9·9 (9·3–10·5)	8·1 (7·7–8·6)	5·3 (5·0–5·5)	0·8 (0·7–1·0)	0·4 (0·4–0·5)	584·2 (380·6–839·5)	367·9 (239·3–535·0)
Andean Latin America	26·1 (23·6–29·6)	14·4 (13·1–16·0)	16·0 (13·7–19·8)	9·8 (8·5–11·3)	9·3 (8·5–10·8)	4·3 (4·0–4·7)	0·7 (0·5–0·9)	0·3 (0·2–0·3)	641·9 (413·1–897·9)	295·5 (194·2–436·3)
Bolivia (Plurinational State of)	37·0 (32·3–42·8)	23·5 (18·4–30·1)	20·0 (15·8–24·5)	14·4 (9·4–20·5)	15·1 (13·6–16·7)	8·4 (7·5–9·5)	1·9 (1·3–2·6)	0·7 (0·5–1·1)	1119·3 (763·9–1697·5)	587·9 (408·3–864·8)
Ecuador	13·3 (10·9–18·1)	6·8 (6·2–7·4)	9·8 (7·8–14·6)	4·9 (4·5–5·6)	3·4 (2·8–4·5)	1·7 (1·6–1·9)	0·2 (0·1–0·3)	0·1 (0·1–0·1)	234·0 (139·1–406·5)	119·3 (75·2–187·8)
Peru	28·8 (24·9–33·3)	15·2 (13·8–17·6)	17·8 (14·2–21·7)	10·7 (9·4–13·1)	10·4 (9·0–12·4)	4·3 (3·7–5·0)	0·6 (0·4–1·0)	0·2 (0·1–0·3)	689·3 (429·5–963·0)	288·1 (181·0–433·9)
Caribbean	28·8 (27·6–30·3)	27·4 (25·9–28·8)	16·1 (14·9–17·7)	16·7 (15·3–18·5)	11·5 (11·0–12·1)	10·1 (9·5–10·8)	1·2 (1·0–1·4)	0·7 (0·5–0·9)	824·5 (550·9–1157·0)	675·1 (442·8–973·6)

Antigua and Barbuda	28·1 (24·9–32·6)	21·3 (18·7–24·4)	18·8 (15·7–23·1)	14·9 (12·3–18·3)	8·9 (7·7–10·8)	6·1 (5·2–7·0)	0·5 (0·3–0·8)	0·3 (0·1–0·5)	595·9 (363·1–876·7)	408·9 (242·6–631·1)
Bahamas	26·1 (23·1–30·0)	22·0 (19·3–26·3)	16·7 (13·5–20·9)	14·9 (12·4–18·8)	8·9 (8·0–10·1)	6·8 (5·9–7·8)	0·5 (0·3–0·8)	0·3 (0·2–0·5)	594·1 (386·9–872·7)	451·3 (286·5–661·7)
Barbados	22·9 (21·0–25·6)	18·6 (16·9–20·7)	14·3 (12·4–16·8)	12·3 (10·8–14·2)	8·0 (7·2–8·8)	5·8 (5·1–6·5)	0·6 (0·4–0·9)	0·4 (0·3–0·6)	546·6 (351·0–837·6)	407·0 (247·8–625·2)
Belize	31·1 (27·5–37·4)	26·3 (24·1–30·6)	17·9 (14·6–22·7)	16·5 (14·0–19·8)	12·2 (10·7–13·9)	9·2 (8·0–10·5)	1·0 (0·7–1·4)	0·6 (0·3–0·9)	836·2 (553·5–1176·6)	622·3 (405·3–912·7)
Bermuda	18·4 (16·5–20·7)	11·8 (10·5–13·4)	12·7 (10·9–14·6)	8·8 (7·7–10·3)	5·4 (4·8–6·3)	2·9 (2·5–3·2)	0·3 (0·2–0·4)	0·1 (0·1–0·2)	361·7 (236·0–560·5)	195·9 (120·2–293·8)
Cuba	21·1 (18·6–24·4)	16·9 (15·1–19·3)	13·5 (11·5–17·0)	11·6 (10·0–14·0)	7·1 (6·2–8·0)	5·1 (4·4–5·7)	0·5 (0·3–0·7)	0·3 (0·2–0·4)	484·2 (304·6–723·6)	343·8 (215·9–484·4)
Dominica	26·1 (23·8–28·5)	22·3 (20·0–24·0)	15·1 (13·0–18·0)	14·1 (12·2–15·6)	10·1 (9·1–11·0)	7·6 (6·5–8·4)	0·9 (0·6–1·2)	0·6 (0·3–0·8)	700·7 (459·3–1009·7)	525·5 (346·3–757·5)
Dominican Republic	28·8 (26·1–32·1)	21·9 (20·3–23·9)	15·8 (13·6–18·8)	13·5 (12·1–15·4)	11·8 (10·5–12·9)	7·8 (7·2–8·7)	1·2 (0·8–1·8)	0·6 (0·4–0·8)	842·2 (529·0–1180·8)	537·1 (353·1–809·6)
Grenada	28·0 (26·4–30·0)	22·0 (19·3–24·9)	15·4 (14·0–17·1)	14·0 (11·8–16·5)	11·5 (10·5–12·4)	7·4 (6·6–8·3)	1·1 (0·7–1·4)	0·6 (0·4–0·9)	803·5 (532·0–1131·1)	515·7 (324·5–759·2)
Guyana	40·8 (35·7–46·1)	29·9 (27·1–34·5)	26·3 (21·3–31·2)	19·4 (16·8–23·6)	13·8 (12·1–15·7)	10·0 (9·1–11·3)	0·8 (0·5–1·1)	0·6 (0·3–0·8)	915·2 (586·2–1381·9)	669·4 (425·7–1018·2)
Haiti	46·0 (42·2–50·9)	46·8 (41·8–51·3)	20·1 (16·9–24·7)	25·9 (20·9–30·5)	22·4 (20·1–25·1)	19·6 (17·5–21·7)	3·5 (2·8–4·1)	1·3 (0·8–1·9)	1730·1 (1173·6–2523·5)	1289·9 (833·3–1895·2)
Jamaica	27·5 (24·2–30·8)	22·9 (20·6–25·4)	17·3 (14·7–20·9)	15·4 (13·0–18·2)	9·6 (8·6–10·8)	7·1 (6·2–7·9)	0·6 (0·3–0·9)	0·4 (0·2–0·6)	638·9 (419·9–952·2)	479·7 (286·9–717·7)
Puerto Rico	19·9 (18·0–23·0)	14·6 (12·7–16·9)	13·4 (11·4–16·1)	10·7 (8·9–12·6)	6·2 (5·5–6·9)	3·7 (3·3–4·3)	0·3 (0·2–0·5)	0·2 (0·1–0·3)	413·3 (256·8–634·8)	256·9 (156·7–396·3)
Saint Kitts and Nevis	31·6 (27·9–36·2)	22·4 (19·5–26·2)	19·2 (15·9–23·9)	15·5 (12·7–19·3)	11·5 (10·2–13·1)	6·5 (5·7–7·4)	0·9 (0·5–1·2)	0·3 (0·2–0·6)	785·1 (501·5–1214·7)	442·5 (245·6–644·5)
Saint Lucia	30·4 (27·1–35·9)	23·1 (20·6–26·6)	18·0 (15·0–23·6)	15·4 (13·3–18·9)	11·5 (10·2–13·0)	7·3 (6·4–8·0)	0·9 (0·5–1·4)	0·5 (0·3–0·7)	788·7 (494·4–1125·0)	494·4 (306·5–749·5)
Saint Vincent and the Grenadines	29·0 (25·3–34·4)	24·6 (21·7–28·3)	17·2 (13·6–22·1)	16·2 (13·6–20·2)	11·0 (9·6–12·8)	7·9 (7·1–8·6)	0·8 (0·5–1·3)	0·5 (0·3–0·7)	748·1 (480·2–1095·7)	533·6 (358·1–765·3)
Suriname	32·3 (28·1–37·0)	27·5 (25·0–31·0)	19·2 (15·9–23·5)	17·9 (14·9–21·3)	12·1 (10·6–14·0)	9·0 (8·2–10·2)	1·0 (0·6–1·4)	0·6 (0·4–0·8)	832·8 (534·3–1172·4)	609·5 (394·8–875·8)
Trinidad and Tobago	28·7 (26·1–32·5)	22·0 (19·3–24·7)	17·4 (14·5–21·7)	15·2 (12·9–17·7)	10·5 (9·4–11·7)	6·5 (5·8–7·5)	0·8 (0·4–1·1)	0·3 (0·2–0·5)	713·8 (452·2–1046·3)	440·4 (277·3–664·9)
United States Virgin Islands	23·1 (20·4–27·7)	18·0 (15·6–21·0)	15·4 (13·0–19·1)	13·2 (11·0–16·0)	7·2 (6·4–8·1)	4·6 (4·1–5·3)	0·4 (0·2–0·6)	0·2 (0·1–0·3)	483·4 (323·7–747·6)	313·2 (195·2–489·6)
Central Latin America	14·0 (13·6–14·6)	9·7 (9·5–10·0)	8·9 (8·5–9·5)	6·5 (6·3–6·8)	4·8 (4·6–5·0)	3·0 (2·9–3·1)	0·4 (0·3–0·4)	0·2 (0·2–0·2)	328·3 (216·6–483·3)	206·1 (135·2–307·1)
Colombia	15·0 (13·6–17·1)	7·9 (7·2–8·6)	10·2 (8·8–12·2)	5·3 (4·8–5·9)	4·6 (3·9–5·2)	2·4 (2·1–2·6)	0·3 (0·2–0·4)	0·2 (0·1–0·2)	310·3 (181·4–459·8)	164·4 (102·9–244·5)
Costa Rica	13·6 (11·7–15·9)	9·9 (8·3–12·2)	10·3 (8·6–12·3)	7·7 (6·4–9·9)	3·2 (2·6–3·9)	2·1 (1·8–2·5)	0·1 (0·1–0·2)	0·1 (0·1–0·1)	223·8 (134·4–334·6)	151·2 (94·9–235·9)
El Salvador	14·1 (12·7–16·4)	9·3 (8·5–10·3)	8·9 (7·8–10·8)	6·6 (5·9–7·5)	4·9 (4·3–5·5)	2·5 (2·2–2·8)	0·4 (0·2–0·5)	0·1 (0·1–0·2)	335·9 (207·7–535·6)	174·6 (113·1–270·7)
Guatemala	26·2 (24·0–29·3)	19·7 (18·2–21·4)	13·9 (12·2–17·1)	11·6 (10·1–13·4)	11·2 (10·0–12·5)	7·5 (6·7–8·4)	1·1 (0·8–1·5)	0·6 (0·4–0·8)	781·6 (510·5–1079·4)	512·9 (337·1–769·3)
Honduras	20·5 (18·7–22·5)	14·5 (13·0–16·4)	12·3 (11·2–13·5)	9·6 (8·4–11·2)	7·7 (6·7–8·7)	4·6 (4·2–5·2)	0·5 (0·4–0·7)	0·3 (0·2–0·3)	512·4 (334·7–790·5)	311·9 (198·2–454·5)
Mexico	11·1 (10·9–11·5)	8·2 (8·0–8·3)	6·9 (6·7–7·1)	5·5 (5·4–5·7)	4·0 (3·8–4·1)	2·5 (2·4–2·5)	0·3 (0·3–0·3)	0·2 (0·2–0·2)	274·5 (187·8–405·8)	170·8 (112·3–252·1)
Nicaragua	17·5 (15·8–19·3)	11·6 (10·6–12·7)	10·7 (9·3–12·2)	7·4 (6·7–8·3)	6·4 (5·2–7·4)	3·9 (3·5–4·4)	0·5 (0·3–0·7)	0·3 (0·2–0·4)	432·8 (268·1–653·3)	270·2 (172·2–382·5)
Panama	17·7 (15·4–20·1)	12·6 (10·8–15·8)	13·1 (11·3–15·2)	9·7 (8·1–12·4)	4·4 (3·8–5·1)	2·8 (2·3–3·3)	0·2 (0·1–0·3)	0·1 (0·1–0·1)	302·7 (194·2–438·8)	195·3 (122·4–304·5)
Venezuela (Bolivarian Republic of)	17·4 (14·3–21·2)	12·2 (10·8–15·1)	12·0 (9·6–15·3)	8·7 (7·5–11·2)	5·0 (4·2–5·9)	3·4 (2·9–3·9)	0·3 (0·2–0·5)	0·2 (0·2–0·3)	351·4 (220·4–523·9)	235·5 (147·6–373·3)
Tropical Latin America	26·5 (24·0–29·0)	19·9 (18·4–21·6)	14·5 (12·6–16·6)	12·2 (10·7–13·6)	10·7 (9·4–11·9)	7·1 (6·4–7·6)	1·3 (1·0–1·7)	0·7 (0·5–0·9)	790·2 (500·3–1123·2)	504·8 (311·7–717·6)
Brazil	26·6 (24·1–29·2)	20·0 (18·4–21·8)	14·6 (12·6–16·8)	12·2 (10·8–13·7)	10·7 (9·4–12·0)	7·1 (6·4–7·7)	1·3 (1·0–1·7)	0·7 (0·5–0·9)	796·2 (505·1–1131·9)	509·1 (313·9–721·0)
Paraguay	21·8 (19·5–24·6)	16·3 (14·8–18·6)	12·8 (11·0–15·0)	10·4 (9·2–12·3)	8·3 (7·2–9·7)	5·6 (4·9–6·6)	0·6 (0·4–1·0)	0·3 (0·2–0·5)	567·2 (333·5–835·2)	373·1 (238·7–557·3)
North Africa and Middle East	30·3 (29·0–31·8)	21·7 (21·1–22·4)	17·0 (16·0–18·2)	13·1 (12·5–13·6)	11·9 (11·4–12·3)	7·8 (7·6–8·1)	1·4 (1·2–1·5)	0·9 (0·8–0·9)	871·7 (580·3–1241·0)	572·2 (383·4–812·0)
North Africa and Middle East	30·3 (29·0–31·8)	21·7 (21·1–22·4)	17·0 (16·0–18·2)	13·1 (12·5–13·6)	11·9 (11·4–12·3)	7·8 (7·6–8·1)	1·4 (1·2–1·5)	0·9 (0·8–0·9)	871·7 (580·3–1241·0)	572·2 (383·4–812·0)
Afghanistan	29·0 (27·1–32·0)	21·0 (19·3–23·5)	14·6 (13·2–17·1)	11·7 (10·6–14·0)	12·7 (11·4–14·0)	8·5 (7·6–9·5)	1·7 (1·3–2·1)	0·8 (0·6–1·1)	946·5 (661·2–1308·9)	598·0 (374·6–874·8)
Algeria	30·5 (27·6–35·5)	22·4 (20·1–25·6)	18·2 (15·4–22·6)	14·4 (12·3–17·6)	11·4 (10·0–12·9)	7·5 (6·5–8·4)	0·9 (0·6–1·2)	0·5 (0·3–0·7)	779·1 (496·6–1121·3)	507·3 (321·0–740·7)
Bahrain	29·9 (26·3–33·3)	18·6 (16·6–21·4)	19·9 (16·7–23·0)	13·3 (11·3–16·2)	9·4 (8·3–11·0)	5·1 (4·3–5·9)	0·5 (0·4–0·7)	0·2 (0·2–0·4)	632·8 (402·4–924·7)	343·7 (213·7–533·3)
Egypt	31·3 (28·6–35·8)	21·1 (19·7–23·1)	19·4 (16·9–23·6)	14·7 (13·4–16·4)	11·2 (10·0–12·5)	6·2 (5·6–6·8)	0·7 (0·5–1·0)	0·3 (0·2–0·4)	750·5 (458·3–1091·6)	411·2 (268·5–620·0)
Iran (Islamic Republic of)	27·0 (24·4–29·3)	18·0 (16·6–19·7)	15·5 (13·2–17·6)	11·1 (9·8–12·8)	10·4 (9·1–11·4)	6·3 (5·6–6·8)	1·1 (0·8–1·4)	0·6 (0·4–0·8)	744·6 (488·3–1077·1)	453·6 (316·4–672·6)
Iraq	28·5 (25·3–32·6)	19·8 (17·6–22·6)	16·1 (13·7–19·7)	12·5 (10·5–15·5)	11·4 (9·9–12·8)	6·9 (5·9–7·6)	1·1 (0·7–1·5)	0·5 (0·3–0·7)	795·6 (484·5–1177·3)	470·1 (289·3–682·5)
Jordan	28·3 (25·6–31·7)	21·7 (20·3–23·3)	15·6 (13·3–18·2)	12·9 (11·6–14·3)	11·7 (10·3–13·0)	8·2 (7·5–9·0)	1·0 (0·7–1·3)	0·6 (0·4–0·7)	804·6 (516·5–1209·8)	548·6 (357·9–805·2)
Kuwait	21·0 (19·2–23·1)	14·9 (13·3–17·3)	14·7 (13·2–16·6)	10·0 (8·6–12·6)	6·1 (5·3–7·0)	4·6 (3·9–5·2)	0·3 (0·2–0·4)	0·3 (0·2–0·4)	403·7 (279·5–606·1)	311·5 (202·3–475·6)
Lebanon	25·1 (23·5–28·3)	16·5 (15·2–17·8)	14·1 (12·7–16·9)	9·8 (8·8–10·9)	10·1 (9·1–11·2)	6·1 (5·6–6·7)	0·9 (0·7–1·2)	0·6 (0·5–0·8)	700·3 (456·7–995·1)	440·4 (296·2–625·9)

Libya	26·8 (23·7–30·8)	20·4 (18·0–23·3)	16·6 (13·7–20·2)	13·0 (10·7–15·8)	9·5 (8·5–10·8)	6·9 (6·1–7·7)	0·6 (0·4–0·8)	0·5 (0·3–0·7)	643·2 (398·5–909·7)	473·4 (307·0–699·6)
Morocco	31·7 (28·2–35·5)	22·9 (20·7–25·8)	18·3 (15·0–23·2)	14·3 (12·3–17·2)	12·4 (10·7–13·8)	8·0 (7·2–9·1)	1·1 (0·7–1·5)	0·6 (0·4–0·9)	862·6 (562·2–1244·4)	552·1 (373·2–804·0)
Oman	36·7 (32·6–41·1)	19·2 (16·8–22·8)	26·2 (22·3–30·5)	14·6 (12·1–18·1)	10·1 (8·5–11·8)	4·4 (3·7–5·0)	0·3 (0·2–0·5)	0·1 (0·1–0·2)	666·5 (395·3–1057·6)	301·8 (179·6–453·6)
Palestine	26·0 (23·1–31·8)	17·5 (15·5–19·8)	16·3 (13·8–21·4)	12·0 (10·4–14·4)	9·1 (8·0–10·3)	5·2 (4·6–5·9)	0·6 (0·4–0·9)	0·2 (0·2–0·3)	614·9 (394·7–973·4)	345·6 (224·7–518·1)
Qatar	21·5 (19·0–24·0)	12·5 (10·8–15·1)	14·6 (12·2–17·2)	9·2 (7·6–11·8)	6·5 (5·8–7·3)	3·1 (2·7–3·6)	0·4 (0·3–0·6)	0·2 (0·1–0·3)	442·0 (286·4–637·2)	217·5 (128·5–321·8)
Saudi Arabia	25·1 (23·3–26·7)	16·8 (15·0–19·2)	14·1 (12·8–15·9)	11·3 (9·5–13·7)	10·0 (9·1–11·2)	5·2 (4·5–5·8)	1·0 (0·7–1·3)	0·3 (0·2–0·5)	713·5 (482·2–961·8)	352·7 (228·5–507·7)
Sudan	41·2 (37·5–45·1)	31·5 (28·7–37·0)	19·7 (16·6–23·7)	17·6 (14·5–22·7)	19·1 (16·8–21·7)	12·9 (11·7–14·5)	2·4 (1·7–3·2)	1·1 (0·8–1·6)	1397·9 (906·4–1972·4)	881·3 (554·1–1296·4)
Syrian Arab Republic	31·7 (28·2–35·8)	22·6 (20·4–25·5)	18·0 (14·9–22·0)	14·1 (12·2–16·5)	12·7 (11·3–14·5)	8·0 (7·0–8·9)	1·0 (0·6–1·4)	0·6 (0·4–0·8)	866·6 (544·4–1232·5)	542·9 (339·4–776·0)
Tunisia	21·8 (20·4–23·4)	17·1 (16·0–18·7)	11·6 (10·6–12·8)	10·0 (8·9–11·5)	9·1 (8·2–10·0)	6·5 (5·8–7·2)	1·1 (0·8–1·4)	0·7 (0·5–0·9)	667·7 (458·3–927·6)	469·1 (314·6–682·1)
Türkiye	29·0 (26·5–33·7)	18·0 (16·2–20·4)	17·3 (14·6–21·0)	11·7 (10·2–13·8)	10·9 (9·6–11·9)	5·9 (5·2–6·7)	0·9 (0·7–1·3)	0·4 (0·3–0·6)	752·8 (489·7–1098·3)	403·6 (264·9–570·0)
United Arab Emirates	28·6 (25·7–32·4)	18·2 (15·6–21·0)	21·5 (18·7–25·4)	13·8 (11·3–16·6)	6·8 (5·9–7·9)	4·2 (3·5–4·9)	0·3 (0·2–0·5)	0·2 (0·1–0·3)	475·2 (293·7–715·2)	291·1 (176·0–434·5)
Yemen	43·3 (41·0–46·8)	42·5 (40·5–44·3)	11·9 (10·5–13·4)	13·1 (12·0–14·2)	22·4 (20·2–25·6)	22·7 (21·0–24·6)	9·1 (7·3–10·7)	6·7 (5·9–7·6)	2507·6 (1717·3–3390·3)	2183·2 (1471·5–2949·3)
South Asia	51·4 (50·4–52·2)	43·0 (41·9–44·0)	22·3 (21·5–23·1)	23·4 (22·4–24·4)	25·4 (24·8–26·0)	17·6 (17·1–18·1)	3·7 (3·5–3·9)	2·0 (1·9–2·1)	1911·5 (1301·1–2636·8)	1264·5 (856·5–1821·7)
South Asia	51·4 (50·4–52·2)	43·0 (41·9–44·0)	22·3 (21·5–23·1)	23·4 (22·4–24·4)	25·4 (24·8–26·0)	17·6 (17·1–18·1)	3·7 (3·5–3·9)	2·0 (1·9–2·1)	1911·5 (1301·1–2636·8)	1264·5 (856·5–1821·7)
Bangladesh	48·2 (43·1–53·5)	32·2 (29·1–36·8)	25·5 (20·6–31·0)	20·2 (17·1–23·9)	20·5 (18·6–23·7)	11·1 (10·0–12·2)	2·2 (1·7–2·8)	1·0 (0·7–1·3)	1459·1 (969·7–2154·5)	777·0 (525·0–1118·1)
Bhutan	50·2 (39·6–60·3)	43·5 (39·4–47·2)	19·3 (16·3–23·6)	23·5 (19·3–27·7)	26·6 (18·8–32·6)	17·8 (15·5–20·1)	4·4 (2·5–6·6)	2·2 (1·4–3·2)	2053·6 (1259·0–3135·3)	1321·4 (768·5–1941·7)
India	51·6 (50·7–52·4)	43·7 (42·7–44·6)	22·0 (21·2–22·7)	24·0 (23·0–24·9)	25·9 (25·2–26·5)	17·8 (17·2–18·5)	3·8 (3·6–4·0)	2·0 (1·8–2·1)	1943·7 (1325·2–2678·8)	1272·2 (856·4–1811·5)
Nepal	52·7 (48·5–57·5)	40·0 (35·9–45·1)	26·8 (21·5–32·4)	25·5 (21·4–31·6)	23·4 (20·4–25·9)	13·5 (12·3–15·4)	2·5 (1·9–3·2)	0·9 (0·7–1·2)	1651·9 (1118·2–2326·0)	916·2 (604·6–1315·4)
Pakistan	52·6 (47·2–56·3)	46·6 (43·1–50·8)	20·7 (17·8–23·9)	22·3 (19·1–26·4)	27·1 (24·2–29·4)	21·3 (19·8–23·2)	4·8 (3·8–5·9)	3·0 (2·4–3·7)	2153·0 (1472·3–3003·1)	1604·3 (1054·8–2362·2)
Southeast Asia, East Asia, and Oceania	21·5 (20·8–22·0)	13·7 (13·3–14·2)	13·3 (12·9–13·7)	9·1 (8·8–9·4)	7·5 (7·3–7·7)	4·3 (4·1–4·5)	0·7 (0·7–0·8)	0·3 (0·3–0·4)	535·4 (359·8–767·8)	302·6 (201·9–438·6)
East Asia	18·4 (17·9–18·9)	9·7 (9·4–10·1)	11·5 (11·1–11·8)	6·5 (6·2–6·7)	6·3 (6·1–6·5)	3·0 (2·9–3·1)	0·6 (0·6–0·7)	0·3 (0·2–0·3)	458·4 (312·7–657·9)	214·8 (144·9–309·0)
China	18·4 (17·8–18·9)	9·6 (9·2–9·9)	11·4 (11·0–11·8)	6·3 (6·1–6·6)	6·3 (6·1–6·6)	3·0 (2·8–3·1)	0·6 (0·6–0·7)	0·3 (0·2–0·3)	460·0 (313·9–658·4)	211·9 (142·8–304·4)
Democratic People's Republic of Korea	25·5 (23·1–27·9)	17·6 (16·2–19·2)	16·9 (14·9–19·1)	11·9 (10·8–13·4)	8·1 (7·1–9·4)	5·3 (4·7–5·9)	0·5 (0·3–0·7)	0·4 (0·3–0·5)	553·4 (341·1–801·0)	371·0 (239·8–545·4)
Taiwan (Province of China)	13·0 (11·7–14·7)	10·8 (9·8–11·8)	8·9 (7·9–10·4)	7·6 (6·8–8·5)	3·9 (3·4–4·5)	3·0 (2·7–3·5)	0·3 (0·2–0·3)	0·2 (0·1–0·3)	271·5 (175·4–431·6)	211·6 (130·5–328·9)
Oceania	41·5 (37·0–50·6)	34·7 (29·9–40·7)	25·6 (21·3–31·0)	22·5 (17·9–27·5)	15·0 (12·5–19·5)	11·6 (10·2–13·5)	0·9 (0·6–1·3)	0·6 (0·5–0·9)	990·3 (602·6–1492·5)	769·8 (470·6–1155·3)
American Samoa	24·5 (22·1–28·0)	23·4 (21·0–26·7)	15·3 (12·6–18·3)	15·5 (13·1–18·4)	8·6 (7·6–9·6)	7·4 (6·7–8·2)	0·6 (0·4–0·8)	0·5 (0·3–0·7)	583·6 (372·5–855·0)	510·2 (321·7–723·0)
Cook Islands	24·5 (21·8–27·4)	20·1 (17·7–23·4)	16·6 (14·3–19·5)	14·6 (12·4–17·7)	7·5 (6·5–8·7)	5·2 (4·7–6·0)	0·3 (0·2–0·5)	0·2 (0·2–0·4)	497·9 (294·3–739·3)	355·6 (222·7–540·5)
Fiji	27·0 (25·4–29·7)	29·9 (26·1–34·9)	16·3 (14·8–18·6)	20·4 (16·9–24·9)	10·1 (9·1–11·1)	9·0 (8·1–10·2)	0·6 (0·4–1·0)	0·5 (0·3–0·6)	668·2 (425·9–921·3)	603·1 (366·5–901·2)
Guam	21·1 (18·3–24·4)	21·4 (18·9–24·4)	15·2 (12·5–18·9)	15·5 (12·8–18·2)	5·7 (4·9–6·5)	5·6 (4·9–6·4)	0·2 (0·1–0·3)	0·3 (0·2–0·4)	380·1 (242·2–551·3)	383·6 (250·0–573·0)
Kiribati	37·7 (34·1–42·7)	33·6 (29·7–38·0)	21·0 (17·8–26·0)	20·0 (16·8–23·6)	15·3 (13·8–16·9)	12·6 (11·4–14·2)	1·4 (0·9–1·8)	1·0 (0·6–1·5)	1057·1 (725·1–1471·1)	869·4 (566·6–1282·3)
Marshall Islands	32·2 (29·4–35·6)	28·4 (25·5–31·9)	18·1 (15·9–21·5)	17·3 (15·0–20·9)	13·1 (11·3–15·1)	10·3 (9·1–11·7)	1·0 (0·7–1·4)	0·8 (0·6–1·1)	884·1 (549·5–1236·2)	707·4 (421·7–996·1)
Micronesia (Federated States of)	35·8 (32·3–41·0)	28·9 (26·1–32·3)	21·3 (18·3–26·5)	18·5 (15·8–21·7)	13·6 (11·8–15·4)	9·8 (8·8–10·9)	0·9 (0·5–1·3)	0·6 (0·4–0·9)	902·9 (558·7–1280·6)	659·4 (411·3–984·5)
Nauru	33·2 (29·4–38·5)	29·0 (25·5–33·7)	20·9 (17·4–25·6)	18·8 (15·2–22·8)	11·6 (10·2–12·9)	9·7 (8·6–10·9)	0·7 (0·4–1·0)	0·5 (0·3–0·8)	770·6 (487·7–1108·2)	642·8 (420·2–969·4)
Niue	28·8 (26·0–33·3)	25·4 (22·2–28·6)	18·6 (15·2–22·7)	17·6 (14·7–20·5)	9·6 (8·6–10·7)	7·4 (6·6–8·2)	0·6 (0·4–0·8)	0·4 (0·3–0·5)	640·4 (386·1–931·1)	500·6 (320·7–736·9)
Northern Mariana Islands	22·3 (19·2–25·5)	20·9 (18·2–23·6)	16·1 (13·0–19·0)	15·2 (12·8–17·8)	6·1 (5·2–6·8)	5·5 (4·9–6·3)	0·2 (0·1–0·3)	0·2 (0·2–0·4)	402·3 (224·8–614·9)	372·5 (236·7–551·9)
Palau	27·1 (24·4–30·6)	24·3 (21·5–28·1)	18·0 (15·4–21·2)	17·4 (14·4–20·6)	8·6 (7·7–9·5)	6·6 (5·8–7·6)	0·4 (0·2–0·6)	0·3 (0·2–0·5)	571·8 (351·4–819·6)	451·4 (300·3–688·0)
Papua New Guinea	47·3 (40·8–60·1)	36·2 (30·0–43·7)	29·5 (22·9–37·8)	23·5 (17·8–29·9)	16·8 (13·3–23·6)	12·1 (10·3–14·6)	1·0 (0·5–1·5)	0·6 (0·4–1·0)	1108·6 (640·2–1681·2)	797·9 (475·6–1237·9)
Samoa	25·2 (22·9–29·2)	22·4 (20·1–25·6)	14·2 (11·9–18·4)	13·5 (11·2–16·2)	10·0 (8·9–11·2)	8·2 (7·4–9·3)	1·0 (0·7–1·3)	0·7 (0·5–1·0)	705·5 (436·5–1067·8)	575·1 (388·8–818·2)
Solomon Islands	39·6 (33·0–52·1)	33·6 (29·8–39·0)	22·6 (18·0–30·4)	20·5 (17·2–24·7)	15·8 (12·8–21·4)	12·2 (10·5–14·2)	1·2 (0·8–1·7)	0·8 (0·5–1·3)	1064·3 (684·1–1805·8)	824·5 (556·7–1248·8)
Tokelau	31·5 (28·4–35·8)	25·0 (21·9–28·3)	19·6 (16·8–23·4)	16·8 (13·7–19·9)	11·2 (10·1–12·8)	7·8 (6·8–8·6)	0·7 (0·5–1·0)	0·4 (0·3–0·6)	747·2 (498·4–1085·6)	519·1 (325·8–749·6)
Tonga	26·4 (23·6–29·5)	24·1 (21·5–28·0)	16·0 (13·4–19·0)	15·3 (13·1–19·3)	9·7 (8·7–10·8)	8·3 (7·3–9·2)	0·7 (0·5–1·0)	0·6 (0·4–0·8)	656·9 (426·6–959·2)	560·5 (351·2–813·9)
Tuvalu	37·7 (33·5–41·2)	28·6 (25·8–32·4)	22·4 (18·4–26·2)	18·7 (15·8–22·4)	14·2 (12·7–16·1)	9·3 (8·3–10·5)	1·1 (0·6–1·5)	0·5 (0·3–0·8)	964·1 (632·1–1442·4)	624·6 (418·4–911·2)
Vanuatu	31·2 (28·6–34·7)	31·4 (28·3–34·8)	18·4 (15·7–21·3)	19·3 (16·3–22·5)	11·8 (10·2–13·5)	11·2 (10·1–12·4)	1·0 (0·6–1·3)	0·9 (0·6–1·2)	813·4 (505·0–1207·1)	770·1 (505·4–1104·7)

Southeast Asia	29·2 (28·1–30·6)	21·7 (20·8–22·7)	17·8 (16·7–19·2)	14·3 (13·6–15·2)	10·5 (9·9–11·0)	6·8 (6·5–7·2)	0·9 (0·8–1·0)	0·5 (0·5–0·6)	730·4 (479·0–1038·5)	478·7 (315·5–698·9)
Cambodia	46·0 (41·4–51·5)	31·0 (27·5–36·0)	22·3 (18·7–28·9)	18·5 (15·3–23·9)	21·8 (19·2–24·2)	11·8 (10·4–13·4)	1·9 (1·3–2·8)	0·7 (0·5–1·1)	1479·9 (967·1–2202·1)	772·3 (518·1–1122·1)
Indonesia	31·3 (28·8–34·5)	21·2 (19·7–22·9)	21·5 (19·2–24·3)	15·5 (14·1–17·0)	9·3 (8·1–10·4)	5·4 (5·0–6·0)	0·6 (0·4–0·8)	0·3 (0·3–0·4)	636·0 (407·1–958·6)	380·9 (237·9–568·0)
Lao People's Democratic Republic	38·2 (34·5–42·9)	29·2 (26·6–32·4)	20·8 (17·3–24·9)	19·1 (16·8–21·8)	16·0 (14·1–17·9)	9·7 (8·7–10·6)	1·4 (1·0–1·9)	0·5 (0·3–0·7)	1106·1 (717·9–1644·8)	631·9 (409·8–943·8)
Malaysia	24·1 (22·5–25·3)	27·2 (23·6–31·3)	13·9 (12·7–15·0)	20·3 (16·1–24·8)	9·5 (8·4–10·3)	6·6 (5·5–7·8)	0·7 (0·5–1·0)	0·3 (0·2–0·4)	644·0 (430·7–956·6)	449·7 (268·4–666·0)
Maldives	48·8 (45·3–53·4)	26·2 (23·2–30·2)	20·0 (16·2–25·6)	14·6 (12·3–18·1)	26·0 (23·2–29·2)	10·9 (9·7–12·2)	2·8 (1·8–3·9)	0·8 (0·5–1·0)	1805·0 (1108·2–2518·3)	717·5 (465·5–1030·9)
Mauritius	28·7 (25·2–33·2)	24·5 (21·6–29·2)	19·1 (15·7–24·0)	17·2 (14·4–22·2)	9·1 (7·9–11·5)	7·0 (6·1–7·8)	0·5 (0·3–0·8)	0·4 (0·2–0·6)	610·7 (368·2–901·2)	469·3 (299·7–685·3)
Myanmar	40·1 (36·8–44·4)	36·3 (33·6–40·1)	20·4 (17·1–23·9)	22·6 (19·2–26·7)	18·0 (16·0–19·9)	13·0 (11·6–14·6)	1·7 (1·2–2·4)	0·7 (0·4–1·0)	1244·3 (772·1–1803·3)	840·3 (539·7–1198·7)
Philippines	25·2 (23·1–27·7)	18·3 (16·9–19·6)	14·5 (12·6–16·6)	9·9 (8·9–10·8)	9·7 (8·7–10·7)	7·4 (6·7–8·1)	1·1 (0·8–1·4)	1·0 (0·8–1·3)	706·5 (469·3–1034·9)	566·1 (371·9–779·5)
Seychelles	25·6 (22·7–29·7)	20·6 (18·6–24·3)	16·0 (13·6–20·1)	13·9 (11·9–17·4)	9·0 (8·0–10·0)	6·3 (5·8–7·1)	0·6 (0·4–0·8)	0·4 (0·2–0·5)	604·8 (418·0–882·9)	425·1 (278·6–606·2)
Sri Lanka	34·1 (30·5–38·2)	21·3 (19·3–24·3)	22·0 (17·7–26·0)	13·5 (11·7–16·7)	11·4 (10·0–13·1)	7·3 (6·5–8·1)	0·7 (0·4–0·9)	0·5 (0·3–0·7)	761·2 (487·6–1136·1)	490·8 (328·5–686·8)
Thailand	21·2 (19·6–22·5)	19·3 (16·9–21·6)	12·4 (11·3–13·4)	12·8 (11·0–15·4)	8·1 (7·0–8·9)	5·9 (5·2–6·5)	0·8 (0·5–1·1)	0·5 (0·4–0·7)	569·0 (380·0–817·1)	425·2 (271·5–632·5)
Timor-Leste	33·8 (25·7–42·3)	26·8 (23·5–30·5)	21·8 (16·0–30·0)	19·5 (16·7–23·0)	11·4 (8·4–14·3)	7·1 (6·3–7·8)	0·7 (0·4–1·1)	0·2 (0·2–0·3)	753·8 (450·3–1102·1)	467·8 (290·5–699·9)
Viet Nam	23·9 (22·5–25·6)	16·0 (14·8–17·1)	12·8 (11·6–14·0)	9·4 (8·4–10·3)	10·1 (9·2–11·0)	6·1 (5·5–6·7)	1·1 (0·8–1·4)	0·6 (0·4–0·8)	714·8 (455·1–978·0)	428·3 (289·5–591·2)
Sub-Saharan Africa	46·7 (45·4–47·9)	39·4 (38·0–40·6)	20·9 (20·0–21·8)	20·6 (19·7–21·6)	22·7 (22·0–23·4)	17·1 (16·4–17·9)	3·0 (2·8–3·2)	1·7 (1·5–1·8)	1671·2 (1144·2–2343·8)	1191·1 (799·6–1716·4)
Central Sub-Saharan Africa	51·3 (47·6–55·9)	35·7 (33·1–39·3)	24·1 (20·6–28·1)	20·3 (17·9–23·6)	24·4 (22·3–26·8)	14·4 (13·0–16·0)	2·7 (2·2–3·5)	1·0 (0·8–1·3)	1725·1 (1138·3–2432·5)	962·6 (635·6–1429·6)
Angola	35·9 (31·5–40·0)	31·4 (27·7–36·4)	18·6 (15·5–22·0)	19·1 (16·2–23·5)	15·2 (13·3–17·3)	11·6 (9·9–13·3)	2·1 (1·5–2·7)	0·8 (0·5–1·1)	1147·9 (749·7–1686·7)	772·3 (495·9–1127·8)
Central African Republic	45·6 (39·3–51·8)	41·7 (35·6–48·8)	22·1 (18·1–27·1)	21·6 (17·6–27·4)	21·6 (17·5–25·8)	18·5 (15·0–22·5)	1·9 (1·2–2·8)	1·5 (1·0–2·2)	1453·4 (923·5–2003·1)	1245·0 (781·2–1872·2)
Congo	42·3 (37·6–49·0)	37·1 (31·4–44·8)	20·5 (17·4–26·2)	20·4 (16·3–26·6)	19·8 (16·8–22·5)	15·5 (12·9–19·2)	2·1 (1·5–2·7)	1·2 (0·8–1·7)	1388·6 (923·8–2016·6)	1037·8 (610·5–1630·6)
Democratic Republic of the Congo	56·4 (51·1–62·5)	36·7 (32·5–42·0)	26·1 (21·4–31·5)	20·6 (17·5–25·3)	27·4 (24·7–30·8)	15·0 (12·9–17·3)	3·0 (2·2–4·1)	1·1 (0·7–1·5)	1918·6 (1261·7–2774·8)	1005·6 (673·8–1540·7)
Equatorial Guinea	49·0 (42·9–56·5)	34·1 (28·3–40·3)	22·1 (18·3–28·1)	20·7 (16·1–25·6)	24·2 (20·7–27·8)	12·6 (10·0–16·0)	2·6 (1·7–3·5)	0·8 (0·5–1·3)	1699·1 (1138·0–2477·1)	835·0 (553·1–1240·9)
Gabon	51·2 (45·0–59·9)	43·7 (37·3–51·4)	22·6 (18·7–29·9)	23·2 (19·1–29·6)	25·4 (21·3–30·0)	18·9 (15·7–22·0)	3·2 (2·3–4·0)	1·6 (1·1–2·3)	1834·1 (1200·8–2695·4)	1291·6 (839·8–1887·9)
Eastern Sub-Saharan Africa	45·0 (43·5–46·5)	33·8 (32·5–35·3)	20·8 (19·6–22·3)	19·4 (18·3–20·9)	21·3 (20·4–22·2)	13·1 (12·5–13·7)	2·9 (2·7–3·3)	1·3 (1·1–1·4)	1586·2 (1074·1–2246·4)	925·5 (624·5–1352·7)
Burundi	41·6 (34·3–48·4)	34·0 (28·6–40·3)	20·9 (16·2–28·3)	19·0 (15·0–24·9)	18·5 (14·8–23·0)	13·9 (10·9–18·1)	2·1 (1·3–3·3)	1·1 (0·6–1·9)	1331·3 (802·1–2027·3)	938·0 (498·3–1508·8)
Comoros	43·0 (26·2–60·1)	30·2 (21·9–35·4)	26·0 (16·7–36·3)	19·7 (14·9–24·0)	15·9 (8·2–24·5)	9·9 (6·3–12·0)	1·0 (0·5–1·9)	0·6 (0·3–0·9)	1059·7 (503·0–1744·6)	664·9 (341·3–969·2)
Djibouti	41·0 (35·4–46·2)	30·3 (25·2–36·2)	26·3 (21·3–31·8)	20·2 (16·5–25·4)	13·8 (11·6–17·1)	9·6 (7·7–11·8)	0·8 (0·4–1·2)	0·5 (0·3–0·8)	919·0 (587·2–1397·1)	638·7 (368·4–974·5)
Eritrea	48·0 (42·2–54·2)	35·9 (30·7–41·7)	27·8 (23·1–33·2)	22·4 (18·0–27·7)	18·9 (15·6–22·1)	12·7 (11·3–15·6)	1·4 (0·8–2·2)	0·8 (0·5–1·2)	1263·1 (802·9–1956·3)	841·0 (572·6–1271·2)
Ethiopia	37·5 (35·7–39·8)	26·0 (24·3–28·3)	17·1 (15·4–18·8)	14·2 (12·8–16·4)	18·1 (17·0–19·5)	10·8 (10·1–11·9)	2·3 (2·0–2·9)	1·0 (0·8–1·2)	1324·9 (907·9–1889·8)	750·1 (490·6–1078·1)
Kenya	28·0 (26·8–29·3)	20·3 (19·2–21·3)	16·2 (15·3–17·2)	12·8 (11·9–13·5)	10·5 (9·8–11·0)	6·7 (6·3–7·1)	1·4 (1·3–1·5)	0·8 (0·7–0·8)	792·6 (539·3–1134·8)	502·9 (339·9–743·8)
Madagascar	43·5 (39·4–51·4)	33·6 (30·3–39·2)	21·9 (18·1–29·4)	20·0 (17·3–25·7)	19·6 (17·2–22·1)	12·7 (11·2–15·1)	2·0 (1·5–2·7)	0·9 (0·6–1·3)	1376·7 (867·9–2020·7)	853·8 (561·8–1230·8)
Malawi	58·5 (53·7–64·8)	46·0 (38·3–55·0)	22·5 (18·9–27·6)	28·2 (22·2–38·2)	30·6 (27·1–34·0)	16·9 (13·7–20·9)	5·3 (3·7–7·0)	1·0 (0·5–1·9)	2401·2 (1621·7–3420·8)	1107·7 (643·8–1668·8)
Mozambique	48·7 (45·0–54·2)	44·0 (39·7–50·6)	18·8 (15·6–23·6)	18·1 (14·5–23·4)	24·8 (22·6–27·5)	20·4 (18·0–23·3)	5·2 (4·3–6·1)	5·5 (4·9–6·6)	2085·5 (1429·4–2899·7)	1904·6 (1305·9–2622·0)
Rwanda	32·5 (28·7–37·8)	20·1 (17·9–22·7)	17·0 (14·7–21·3)	12·9 (11·2–15·4)	14·0 (12·1–16·4)	6·8 (5·9–7·8)	1·5 (1·1–2·1)	0·4 (0·3–0·6)	1000·8 (602·8–1463·7)	458·7 (292·5–643·6)
Somalia	53·3 (47·1–59·3)	44·4 (38·1–52·0)	28·1 (21·8–34·7)	26·1 (20·5–33·6)	23·1 (19·8–27·4)	17·0 (14·5–19·6)	2·1 (1·3–3·2)	1·2 (0·7–1·8)	1593·8 (1035·7–2452·9)	1142·8 (709·9–1729·9)
South Sudan	48·7 (41·2–57·9)	38·2 (31·5–46·6)	28·5 (22·6–36·0)	23·2 (18·4–31·0)	18·8 (15·6–23·9)	14·2 (11·1–17·7)	1·4 (0·8–2·1)	0·9 (0·5–1·4)	1263·5 (807·5–2019·4)	938·4 (561·8–1510·1)
Uganda	45·3 (39·4–51·3)	30·0 (26·7–35·1)	20·9 (16·8–27·7)	17·8 (14·4–22·7)	21·7 (18·6–25·1)	11·5 (9·7–13·7)	2·7 (1·8–3·6)	0·7 (0·5–1·1)	1574·2 (1046·0–2343·4)	761·4 (468·2–1182·4)
United Republic of Tanzania	66·6 (61·7–73·0)	44·6 (37·8–53·6)	28·0 (22·0–34·4)	27·2 (21·7–35·0)	33·5 (30·4–37·0)	16·4 (14·3–18·9)	5·1 (3·9–6·3)	0·9 (0·5–1·6)	2540·9 (1697·4–3651·3)	1071·0 (696·6–1572·8)
Zambia	52·2 (46·6–57·2)	54·6 (48·7–61·1)	20·7 (17·4–25·8)	32·3 (25·7–39·8)	27·2 (23·7–30·0)	20·8 (17·8–23·9)	4·4 (3·2–5·4)	1·5 (0·9–2·7)	2095·2 (1372·2–3023·9)	1401·1 (850·6–2101·9)
Southern Sub-Saharan Africa	31·9 (30·1–33·7)	26·7 (25·4–28·1)	16·1 (14·5–17·6)	14·0 (12·9–15·2)	13·5 (12·6–14·3)	11·1 (10·6–11·8)	2·2 (1·9–2·7)	1·6 (1·4–1·9)	1075·7 (711·1–1510·5)	855·1 (566·7–1178·2)
Botswana	35·6 (31·1–41·7)	27·0 (23·5–31·1)	21·3 (17·1–27·5)	17·3 (14·4–21·3)	13·5 (11·7–16·2)	9·1 (8·0–10·9)	0·8 (0·5–1·4)	0·5 (0·3–0·8)	891·6 (557·5–1337·3)	606·3 (370·9–975·3)
Eswatini	29·9 (26·1–36·5)	24·7 (21·8–29·4)	17·3 (14·4–23·8)	14·8 (12·3–19·4)	11·8 (10·2–14·0)	9·3 (7·8–11·6)	0·8 (0·5–1·2)	0·6 (0·5–1·0)	787·1 (520·9–1259·6)	623·3 (381·9–1005·4)
Lesotho	28·8 (26·1–33·2)	25·0 (22·9–27·8)	15·7 (13·4–19·9)	14·1 (12·4–17·2)	12·1 (10·7–14·0)	10·1 (9·0–11·6)	1·0 (0·7–1·4)	0·8 (0·6–1·1)	825·8 (515·2–1200·8)	688·0 (453·0–1005·5)
Namibia	44·4 (34·3–52·3)	24·6 (21·7–28·5)	28·6 (22·8–34·6)	16·1 (13·1–19·4)	14·8 (9·6–19·2)	8·2 (7·1–9·4)	1·0 (0·6–1·6)	0·4 (0·3–0·6)	1012·5 (527·1–1574·6)	536·3 (354·2–779·5)

South Africa	30·6 (28·3–33·3)	25·7 (24·4–27·2)	14·6 (12·5–16·7)	13·1 (12·0–14·2)	13·3 (12·3–14·3)	10·7 (10·0–11·5)	2·7 (2·2–3·2)	1·9 (1·6–2·2)	1127·7 (758·2–1581·0)	872·5 (571·1–1195·7)
Zimbabwe	34·8 (31·1–39·0)	30·9 (27·7–34·8)	19·3 (16·0–23·6)	16·4 (14·0–20·6)	14·3 (13·1–16·6)	13·4 (11·7–15·8)	1·2 (0·8–1·8)	1·2 (0·9–1·6)	980·8 (635·5–1424·6)	916·8 (609·2–1295·4)
Western Sub-Saharan Africa	51·0 (48·9–53·2)	47·4 (45·1–49·5)	21·5 (20·1–22·8)	22·8 (21·5–24·2)	26·2 (24·8–27·7)	22·3 (21·0–23·8)	3·4 (3·1–3·7)	2·2 (2·0–2·5)	1901·5 (1303·0–2663·6)	1540·8 (1042·1–2202·4)
Benin	43·5 (40·1–47·2)	47·4 (41·9–54·6)	16·3 (14·3–20·2)	24·2 (20·2–29·8)	23·2 (21·0–25·9)	21·5 (18·0–24·8)	4·0 (3·1–5·0)	1·7 (1·0–2·4)	1830·6 (1245·0–2624·0)	1425·9 (943·8–2101·8)
Burkina Faso	55·1 (49·9–60·5)	48·8 (41·6–55·4)	20·0 (17·6–23·5)	19·9 (17·1–24·3)	31·5 (27·4–36·4)	25·5 (21·5–29·4)	3·7 (2·7–4·9)	3·4 (2·3–4·4)	2214·4 (1444·4–3278·0)	1867·6 (1226·8–2670·1)
Cabo Verde	38·2 (33·5–43·9)	27·8 (24·6–33·6)	20·8 (16·3–26·8)	17·7 (14·7–22·5)	16·2 (14·0–18·8)	9·6 (8·4–10·9)	1·3 (0·8–1·8)	0·6 (0·4–0·9)	1091·6 (723·8–1549·7)	635·8 (417·0–919·0)
Cameroon	34·7 (31·6–38·8)	32·6 (29·3–37·6)	15·6 (13·4–19·4)	17·4 (14·9–21·2)	16·9 (15·3–18·9)	14·1 (12·2–16·4)	2·1 (1·6–2·9)	1·1 (0·8–1·5)	1231·0 (836·8–1716·7)	952·0 (637·4–1433·7)
Chad	50·9 (44·6–56·9)	49·3 (43·2–59·2)	21·7 (17·8–26·7)	23·3 (18·3–31·5)	26·4 (22·4–30·4)	23·8 (20·5–27·9)	2·9 (2·0–4·1)	2·2 (1·4–3·2)	1844·8 (1171·1–2728·2)	1623·8 (1017·1–2375·7)
Côte d'Ivoire	51·4 (46·3–56·6)	43·6 (37·1–50·0)	24·1 (19·6–29·2)	21·7 (18·0–26·2)	25·0 (22·3–28·0)	20·1 (16·7–23·3)	2·3 (1·5–3·1)	1·8 (1·2–2·6)	1699·5 (1126·3–2499·0)	1367·2 (860·2–1972·4)
Gambia	50·8 (47·0–55·3)	46·1 (41·5–51·8)	20·2 (16·9–24·4)	21·2 (16·8–27·4)	27·2 (23·8–30·0)	22·7 (19·3–25·2)	3·4 (2·4–4·7)	2·2 (1·5–3·1)	1961·5 (1261·7–2838·0)	1561·1 (978·5–2298·7)
Ghana	52·9 (47·5–58·8)	44·4 (39·0–51·0)	24·3 (20·1–29·8)	24·6 (20·2–31·6)	25·9 (22·5–30·3)	18·5 (16·4–21·1)	2·7 (2·0–3·8)	1·3 (0·6–2·0)	1801·2 (1167·5–2515·4)	1218·6 (813·8–1819·3)
Guinea	46·6 (41·0–52·8)	45·9 (40·2–54·2)	20·7 (17·4–26·4)	21·7 (18·3–29·6)	23·4 (20·2–27·5)	22·1 (19·2–25·5)	2·4 (1·7–3·3)	2·0 (1·4–2·7)	1622·8 (1065·8–2376·3)	1496·7 (1002·8–2118·5)
Guinea-Bissau	53·0 (46·6–60·5)	44·8 (39·5–50·5)	21·8 (17·8–28·2)	22·6 (18·6–29·2)	28·1 (24·1–32·9)	20·3 (17·4–23·9)	3·1 (2·3–4·6)	1·8 (1·2–2·6)	1963·4 (1386·4–2871·1)	1390·8 (871·9–2072·6)
Liberia	54·3 (46·6–62·8)	48·4 (41·5–57·5)	20·2 (16·8–25·1)	19·5 (15·4–25·8)	29·5 (24·3–35·1)	25·2 (21·6–30·8)	4·6 (3·3–6·2)	3·8 (2·7–5·0)	2248·5 (1443·6–3340·8)	1897·6 (1219·6–2613·5)
Mali	55·6 (50·9–60·7)	57·6 (51·8–63·3)	21·1 (17·9–26·1)	22·4 (19·0–27·4)	30·2 (27·1–33·9)	31·2 (28·0–34·6)	4·2 (2·9–5·5)	4·0 (3·0–5·2)	2228·7 (1478·1–3101·8)	2254·3 (1462·6–3188·9)
Mauritania	47·6 (43·8–52·9)	38·2 (33·2–44·1)	22·3 (19·3–27·8)	22·1 (17·7–28·5)	23·0 (20·4–26·4)	15·1 (12·9–17·8)	2·3 (1·6–2·9)	1·0 (0·6–1·6)	1588·8 (1072·3–2281·0)	1005·0 (628·7–1594·9)
Niger	55·3 (48·1–63·2)	48·4 (42·1–54·3)	23·2 (18·1–28·1)	22·7 (18·0–27·8)	29·0 (23·6–35·0)	23·8 (20·1–27·6)	3·1 (1·9–4·5)	1·9 (1·2–2·8)	2007·5 (1363·4–2977·4)	1576·5 (1007·0–2388·7)
Nigeria	51·5 (47·6–55·4)	48·7 (45·1–52·2)	21·7 (19·6–23·9)	23·6 (21·0–26·3)	26·1 (23·7–29·0)	22·8 (20·3–25·4)	3·7 (3·0–4·4)	2·3 (1·9–2·8)	1937·1 (1336·4–2699·9)	1580·3 (1045·2–2249·5)
Sao Tome and Principe	43·9 (30·5–57·3)	36·5 (31·9–41·9)	22·5 (16·6–30·8)	21·8 (17·2–27·8)	19·7 (12·4–25·4)	13·8 (12·3–16·0)	1·6 (0·8–2·6)	0·8 (0·5–1·4)	1330·1 (676·3–2141·8)	911·0 (576·8–1435·9)
Senegal	57·1 (52·3–63·0)	51·9 (46·6–57·4)	20·1 (17·2–24·7)	25·9 (21·2–32·6)	32·0 (28·4–35·9)	24·1 (21·4–26·4)	5·1 (3·8–6·6)	1·9 (1·2–2·8)	2441·0 (1553·1–3548·7)	1601·1 (1055·2–2277·0)
Sierra Leone	48·9 (41·2–56·7)	44·7 (38·2–51·1)	22·8 (18·8–28·8)	22·1 (18·3–26·7)	23·6 (19·0–29·0)	20·6 (17·4–24·9)	2·5 (1·7–3·5)	2·0 (1·4–2·9)	1651·7 (1004·2–2483·2)	1431·7 (890·7–2261·4)
Togo	55·9 (50·6–63·0)	53·4 (49·1–60·3)	23·5 (19·4–28·6)	25·6 (21·3–31·9)	29·6 (25·4–33·1)	25·3 (22·0–27·4)	2·9 (1·8–3·9)	2·5 (1·4–3·8)	2007·2 (1305·6–2830·1)	1739·7 (1154·6–2536·6)

ST-GPR Ensemble Stage 1 Covariate Coefficients

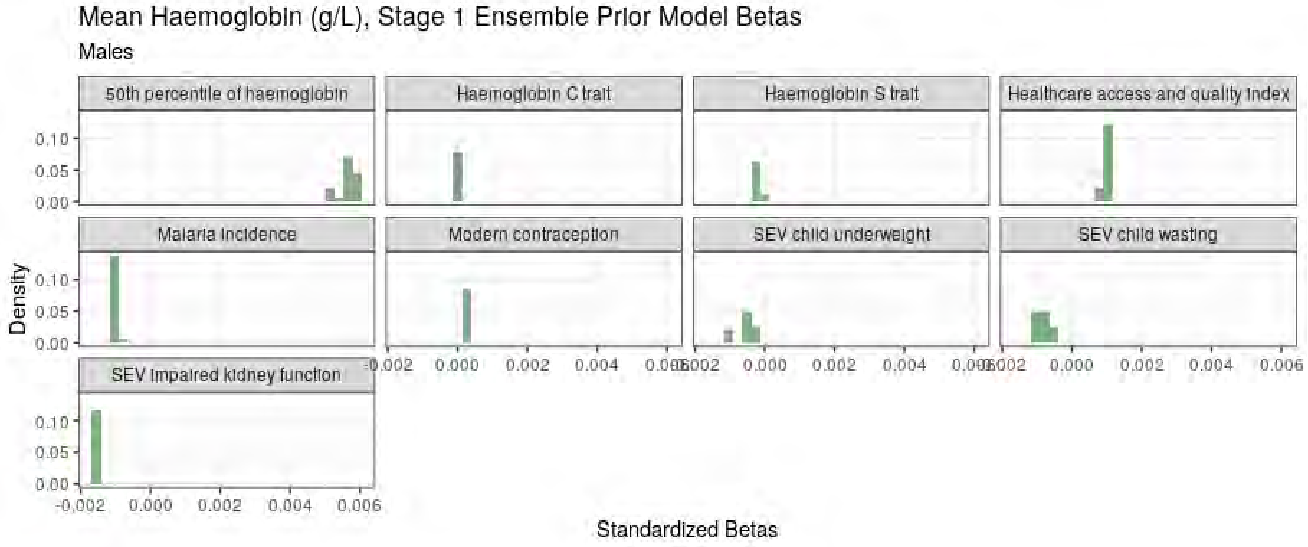

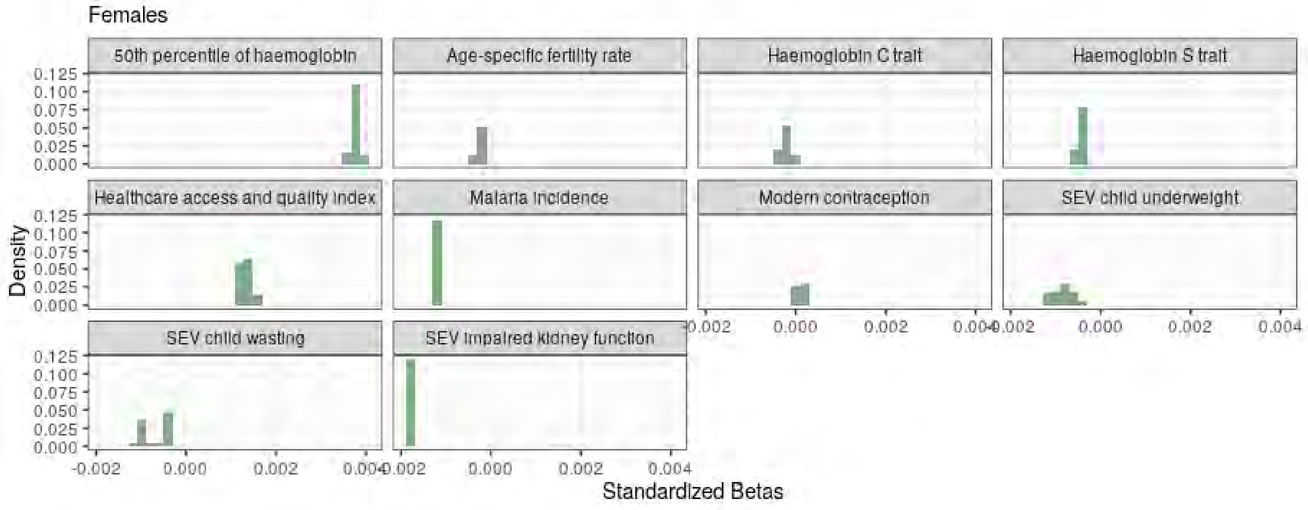

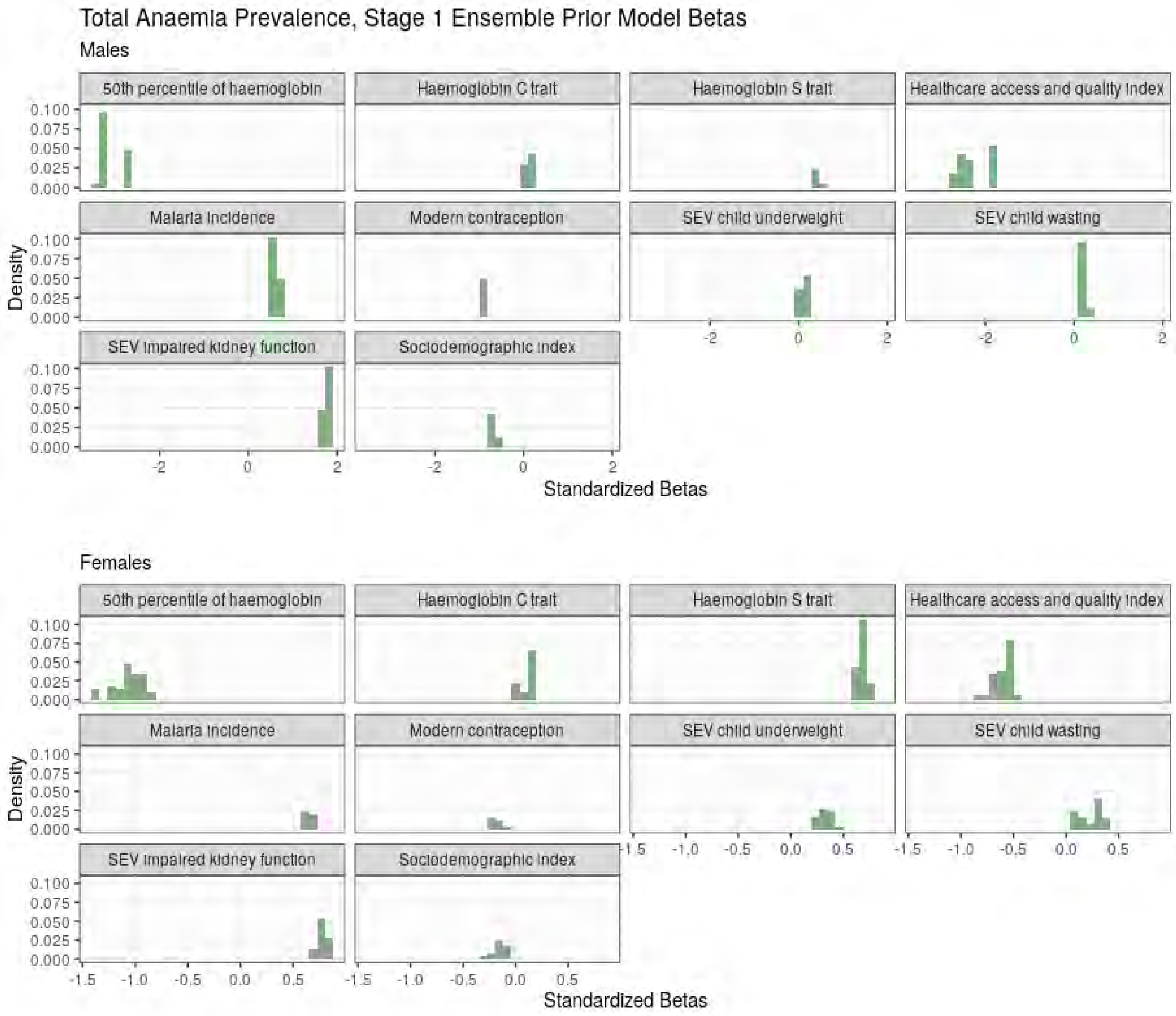
Total Anaemia Prevalence, Stage 1 Ensemble Prior Model Betas
Males
50th percentile of haemoglobin
Haemoglobin C trait
Haemoglobin S trait
Healthcare access and quality index
Malaria incidence
Modern contraception
SEV child underweight
SEV child wasting
SEV impaired kidney function
Sociodemographic index
Density
Standardized Betas
Females
50th percentile of haemoglobin
Haemoglobin C trait
Haemoglobin S trait
Healthcare access and quality index
Malaria incidence
Modern contraception
SEV child underweight
SEV child wasting
SEV impaired kidney function
Sociodemographic index
Density
Standardized Betas

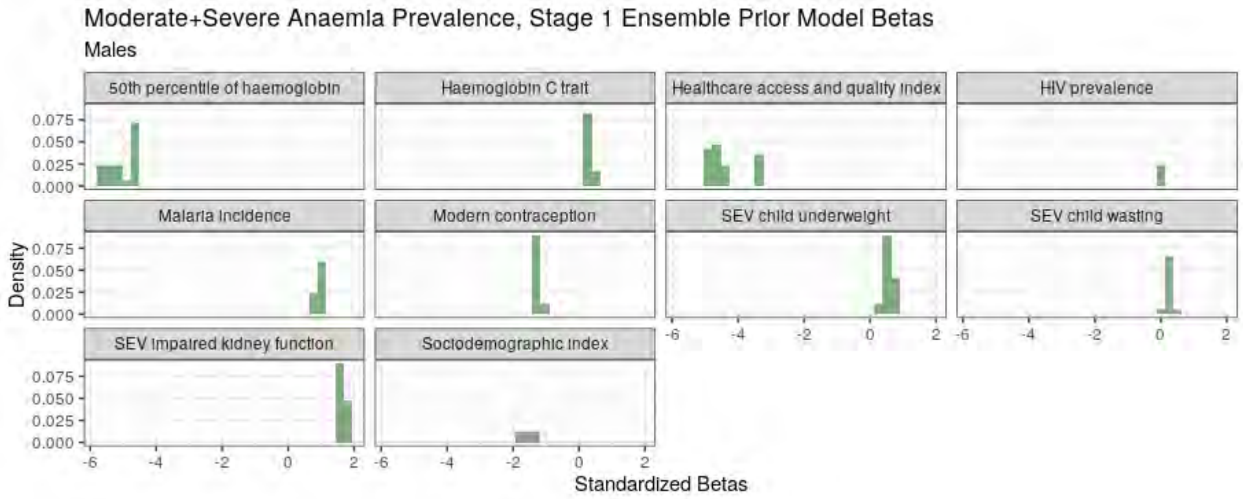
Moderate+Severe Anaemia Prevalence, Stage 1 Ensemble Prior Model Betas
Males
50th percentile of haemoglobin
Haemoglobin C trait
Healthcare access and quality index
HIV prevalence
Malaria incidence
Modern contraception
SEV child underweight
SEV child wasting
SEV impaired kidney function
Sociodemographic index
Density
Standardized Betas

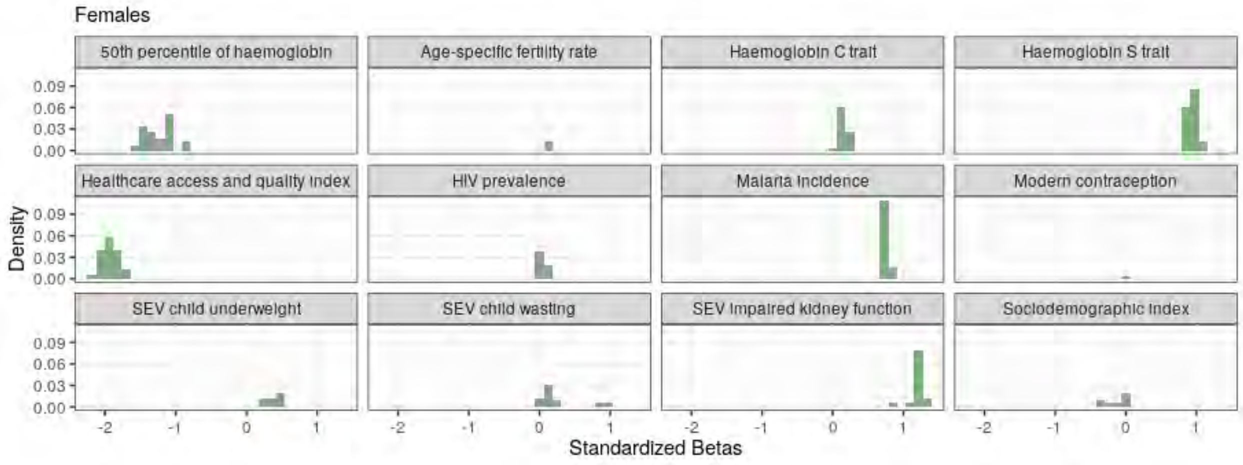
Females
50th percentile of haemoglobin
Age-specific fertility rate
Haemoglobin C trait
Haemoglobin S trait
Healthcare access and quality index
HIV prevalence
Malaria incidence
Modern contraception
SEV child underweight
SEV child wasting
SEV impaired kidney function
Sociodemographic index
Density
Standardized Betas

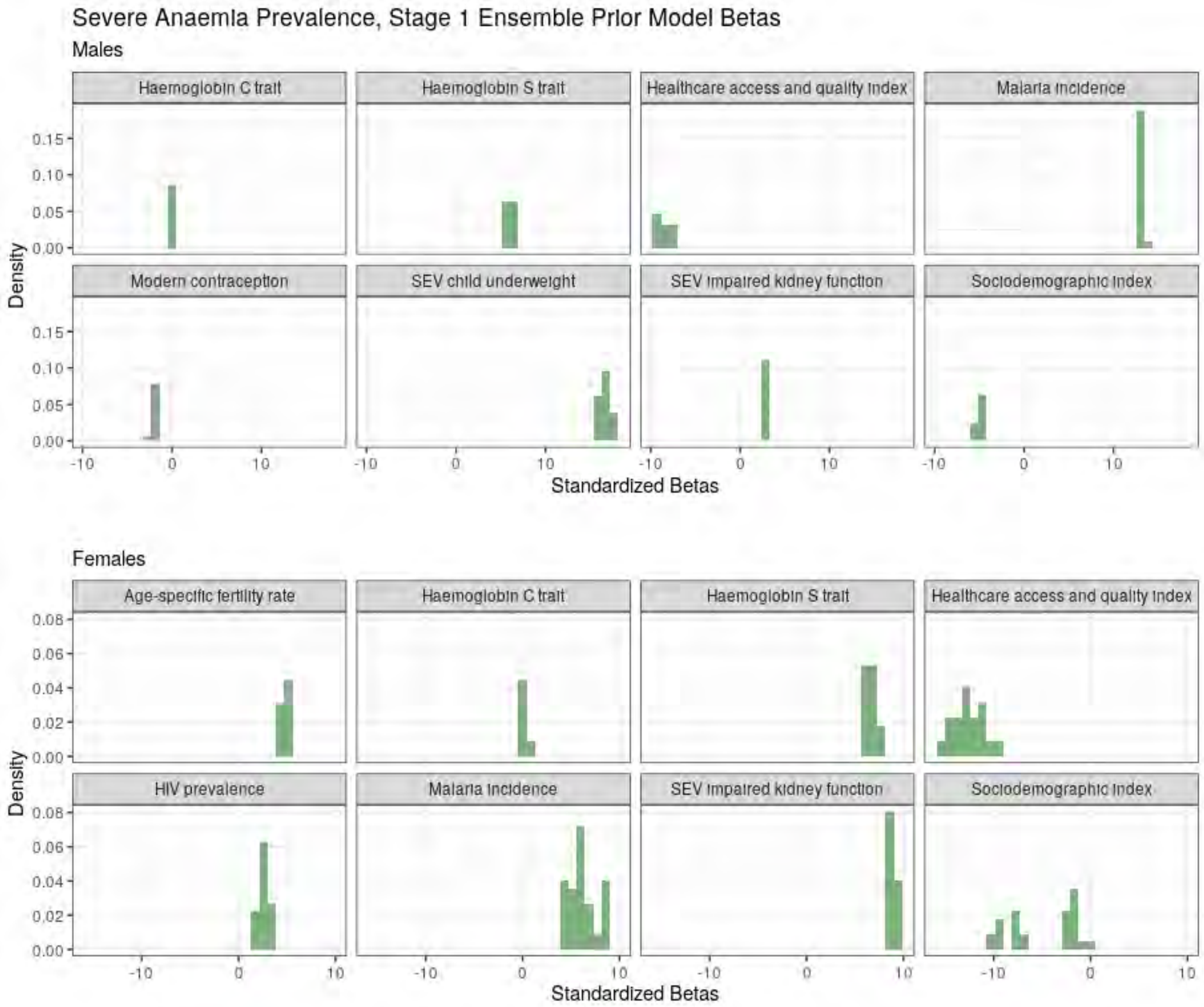
Severe Anaemia Prevalence, Stage 1 Ensemble Prior Model Betas
Males
Haemoglobin C trait
Haemoglobin S trait
Healthcare access and quality index
Malaria incidence
Modern contraception
SEV child underweight
SEV impaired kidney function
Sociodemographic index
Density
Standardized Betas
Females
Age-specific fertility rate
Haemoglobin C trait
Haemoglobin S trait
Healthcare access and quality index
HIV prevalence
Malaria incidence
SEV impaired kidney function
Sociodemographic index
Density
Standardized Betas

Section 6: Anaemia burden location profiles

Location profiles show final anaemia prevalence results for total, moderate + severe, and severe anaemia by GBD age group for both sexes combined, 1990 – 2021 (left); anaemia prevalence results by most-detailed underlying cause of anaemia stratified by age and sex in 2021 (top right); and rankings of GBD "Level 3" causes of anaemia by YLD rate for males and females, all ages combined, 1990 and 2021 (bottom right).

Results are grouped according to the GBD location hierarchy, beginning at the global level and descending through GBD super-regions, regions, countries, and subnational geographies:

Central Europe, Eastern Europe, and Central Asia

Central Asia: Armenia, Azerbaijan, Georgia, Kazakhstan, Kyrgyzstan, Mongolia, Tajikistan, Turkmenistan, Uzbekistan
Central Europe: Albania, Bosnia and Herzegovina, Bulgaria, Croatia, Czechia, Hungary, Montenegro, North Macedonia, Poland, Romania, Serbia, Slovakia, Slovenia,
Eastern Europe: Belarus, Estonia, Latvia, Lithuania, Republic of Moldova, Russian Federation, Ukraine

High Income

Australasia: Australia, New Zealand
High-income Asia Pacific: Brunei Darussalam, Japan, Republic of Korea, Singapore
High-income North America: Canada, Georgia, United States of America
Southern Latin America: Argentina, Chile, Uruguay
Western Europe: Andorra, Austria, Belgium, Cyprus, Denmark, Finland, France, Germany, Greece, Iceland, Ireland, Israel, Italy, Luxembourg, Malta, Monaco, Netherlands, Norway, Portugal, San Marino, Spain, Sweden, Switzerland, United Kingdom

Latin America and the Caribbean

Andean Latin America: Bolivia (Plurinational State of), Ecuador, Peru
Caribbean: Antigua and Barbuda, Bahamas, Barbados, Belize, Cuba, Dominica, Dominican Republic, Grenada, Guyana, Haiti, Jamaica, Saint Kitts and Nevis, Saint Lucia, Saint Vincent and the Grenadines, Suriname, Trinidad and Tobago
Central Latin America: Colombia, Costa Rica, El Salvador, Guatemala, Honduras, Mexico, Nicaragua, Panama, Venezuela (Bolivarian Republic of)
Tropical Latin America: Brazil, Paraguay

North Africa and the Middle East

Algeria, Bahrain, Egypt, Iran (Islamic Republic of), Iraq, Jordan, Kuwait, Lebanon, Libya, Morocco, Oman, Palestine, Qatar, Saudi Arabia, Syrian Arab Republic, Tunisia, Turkey, United Arab Emirates, Yemen, Afghanistan, Sudan

South Asia

Bangladesh, Bhutan, India, Nepal, Pakistan

Southeast Asia, East Asia, and Oceania

East Asia: China, Democratic People's Republic of Korea, Taiwan (Province of China)
Oceania: Fiji, Kiribati, Marshall Islands, Micronesia (Federated States of), Nauru, Palau, Papua New Guinea, Samoa, Solomon Islands, Tonga, Tuvalu, Vanuatu
Southeast Asia: Cambodia, Indonesia, Lao People's Democratic Republic, Malaysia, Maldives, Mauritius, Myanmar, Philippines, Seychelles, Sri Lanka, Thailand, Timor-Leste, Viet Nam

Sub-Saharan Africa

Central sub-Saharan Africa: Angola, Central African Republic, Congo, Democratic Republic of the Congo, Equatorial Guinea, Gabon
Eastern Sub-Saharan Africa: Burundi, Comoros, Djibouti, Eritrea, Ethiopia, Kenya, Madagascar, Malawi, Mozambique, Rwanda, Somalia, South Sudan, Uganda, United Republic of Tanzania, Zambia
Southern Sub-Saharan Africa: Botswana, Eswatini, Lesotho, Namibia, South Africa, Zimbabwe
Western Sub-Saharan Africa: Benin, Burkina Faso, Cabo Verde, Cameroon, Chad, Côte d'Ivoire, Gambia, Ghana, Guinea, Guinea-Bissau, Liberia, Mali, Mauritania, Niger, Nigeria, Sao Tome and Principe, Senegal, Sierra Leone, Togo

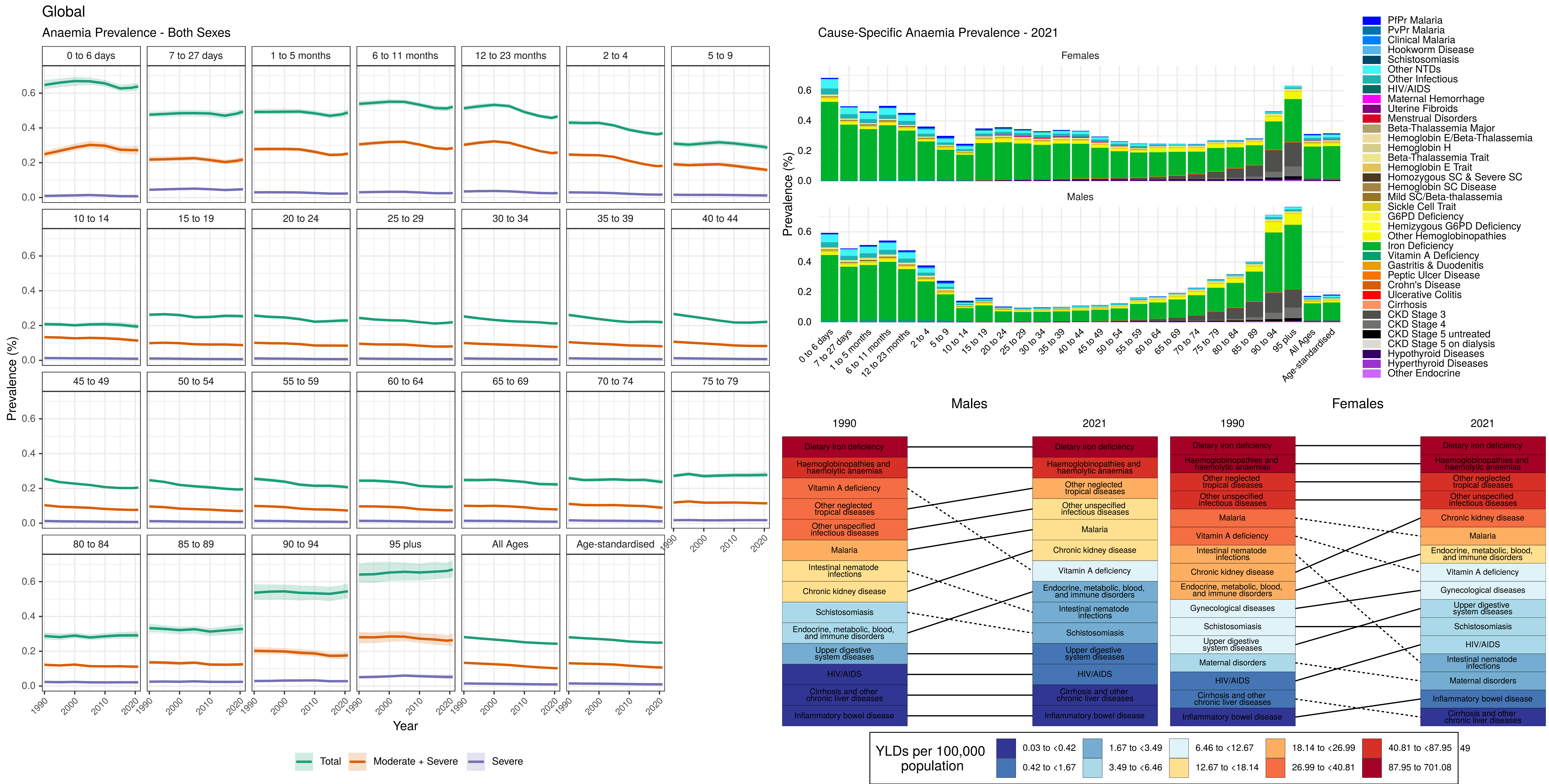
Global
Anaemia Prevalence - Both Sexes
0 to 6 days
7 to 27 days
1 to 5 months
6 to 11 months
12 to 23 months
2 to 4
5 to 9
10 to 14
15 to 19
20 to 24
25 to 29
30 to 34
35 to 39
40 to 44
45 to 49
50 to 54
55 to 59
60 to 64
65 to 69
70 to 74
75 to 79
80 to 84
85 to 89
90 to 94
95 plus
All Ages
Age-standardised
Prevalence (%)
Year
1990
2000
2010
2020
Total
Moderate + Severe
Severe
Cause-Specific Anaemia Prevalence - 2021
Females
Males
PfPr Malaria
PvPr Malaria
Clinical Malaria
Hookworm Disease
Schistosomiasis
Other NTDs
Other Infectious
HIV/AIDS
Maternal Hemorrhage
Uterine Fibroids
Menstrual Disorders
Beta-Thalassemia Major
Hemoglobin E/Beta-Thalassemia
Hemoglobin H
Beta-Thalassemia Trait
Hemoglobin E Trait
Homozygous SC & Severe SC
Hemoglobin SC Disease
Mild SC/Beta-thalassemia
Sickle Cell Trait
G6PD Deficiency
Hemizygous G6PD Deficiency
Other Hemoglobinopathies
Iron Deficiency
Vitamin A Deficiency
Gastritis & Duodenitis
Peptic Ulcer Disease
Crohn's Disease
Ulcerative Colitis
Cirrhosis
CKD Stage 3
CKD Stage 4
CKD Stage 5 untreated
CKD Stage 5 on dialysis
Hypothyroid Diseases
Hyperthyroid Diseases
Other Endocrine
Males
Females
1990
2021
Dietary iron deficiency
Haemoglobinopathies and haemolytic anaemias
Vitamin A deficiency
Other neglected tropical diseases
Other unspecified infectious diseases
Malaria
Intestinal nematode infections
Chronic kidney disease
Schistosomiasis
Endocrine, metabolic, blood, and immune disorders
Upper digestive system diseases
HIV/AIDS
Cirrhosis and other chronic liver diseases
Inflammatory bowel disease
Gynecological diseases
Maternal disorders
YLDs per 100,000 population
0.03 to <0.42
0.42 to <1.67
1.67 to <3.49
3.49 to <6.46
6.46 to <12.67
12.67 to <18.14
18.14 to <26.99
26.99 to <40.81
40.81 to <87.95
87.95 to 701.08

Central Europe, eastern Europe, and central Asia

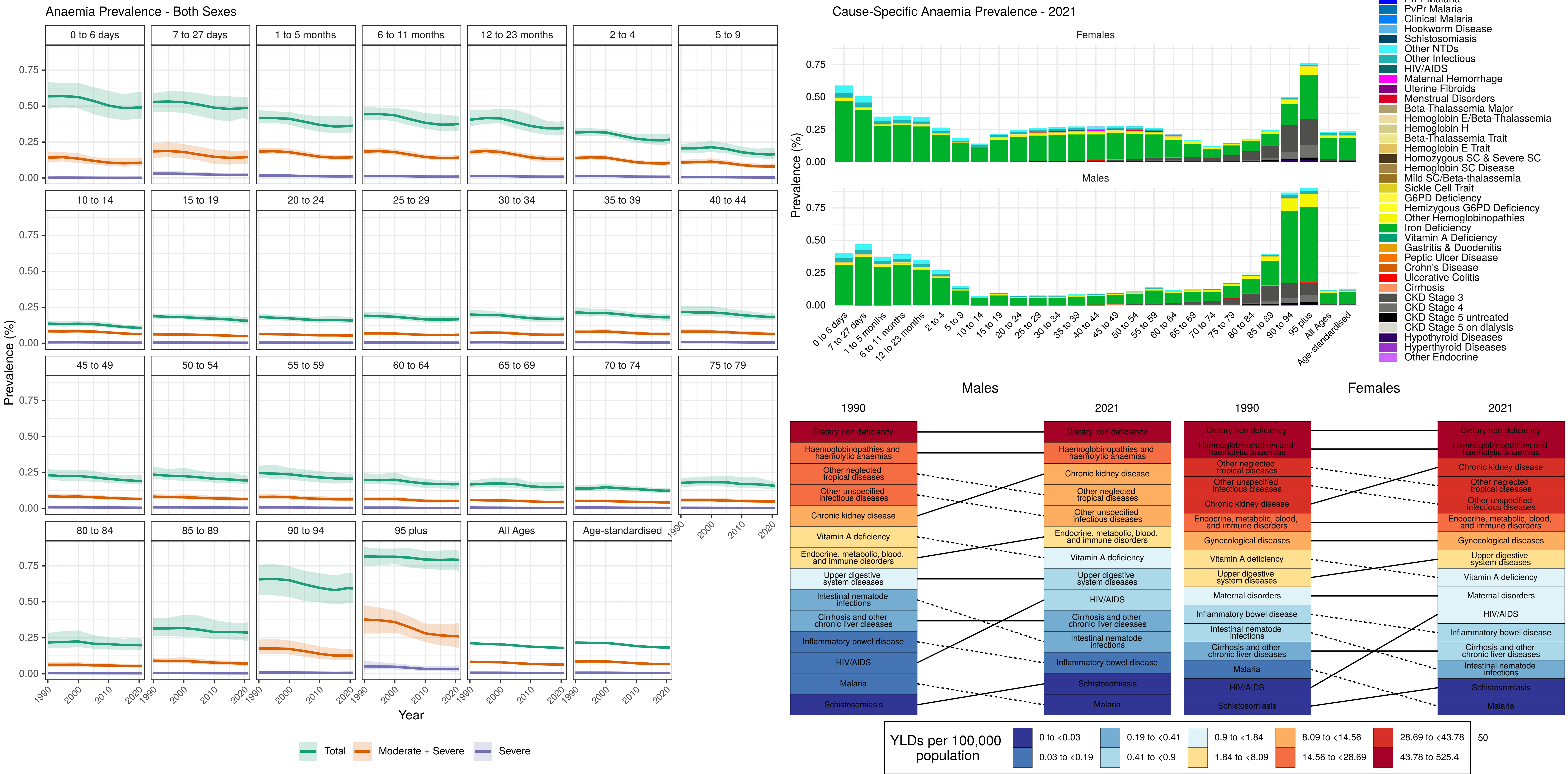

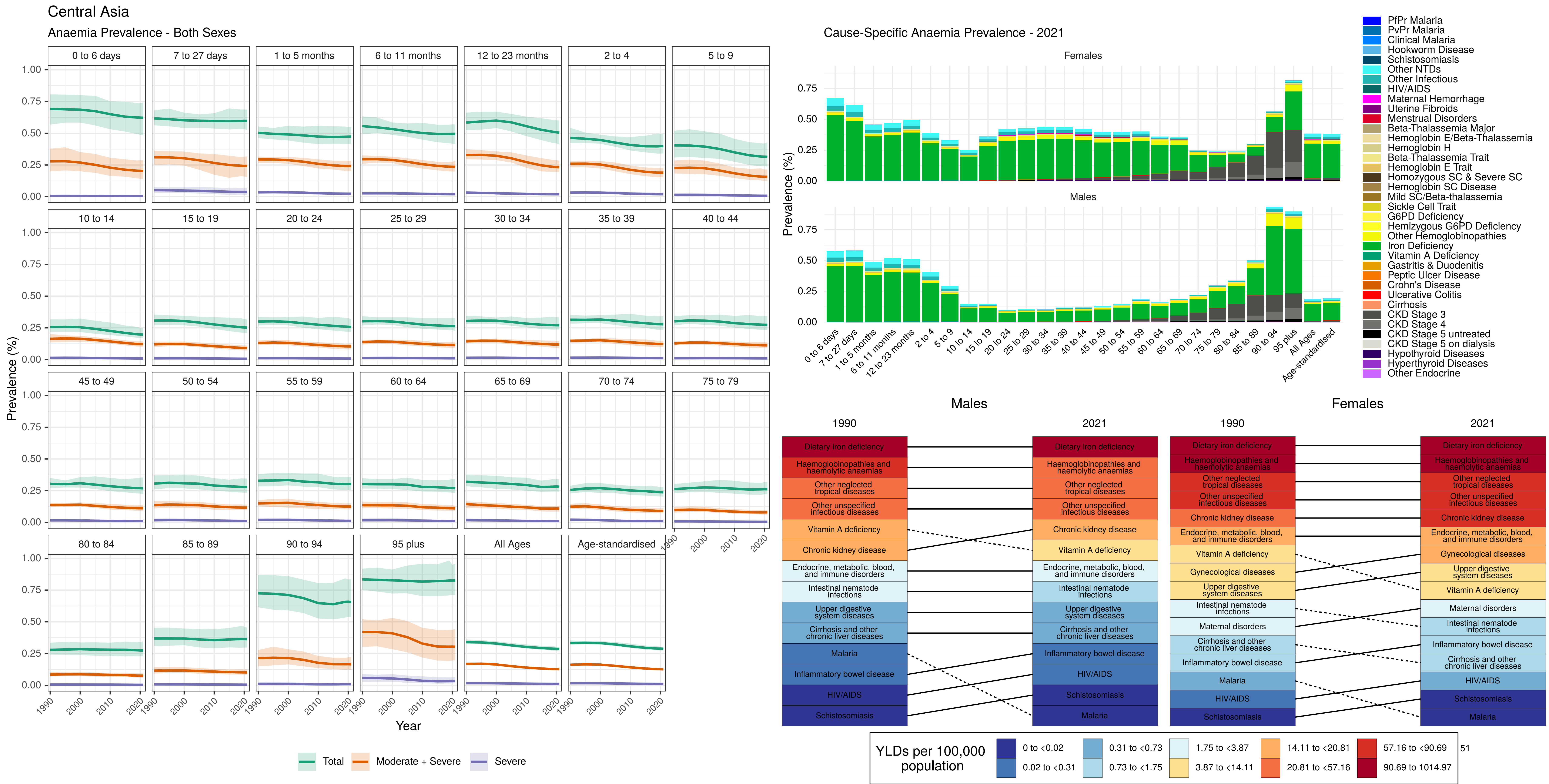

Central Asia
Anaemia Prevalence - Both Sexes
0 to 6 days
7 to 27 days
1 to 5 months
6 to 11 months
12 to 23 months
2 to 4
5 to 9
10 to 14
15 to 19
20 to 24
25 to 29
30 to 34
35 to 39
40 to 44
45 to 49
50 to 54
55 to 59
60 to 64
65 to 69
70 to 74
75 to 79
80 to 84
85 to 89
90 to 94
95 plus
All Ages
Age-standardised
Prevalence (%)
Year
Total
Moderate + Severe
Severe
Cause-Specific Anaemia Prevalence - 2021
Females
Males
Prevalence (%)
PfPr Malaria
PvPr Malaria
Clinical Malaria
Hookworm Disease
Schistosomiasis
Other NTDs
Other Infectious
HIV/AIDS
Maternal Hemorrhage
Uterine Fibroids
Menstrual Disorders
Beta-Thalassemia Major
Hemoglobin E/Beta-Thalassemia
Hemoglobin H
Beta-Thalassemia Trait
Hemoglobin E Trait
Homozygous SC & Severe SC
Hemoglobin SC Disease
Mild SC/Beta-thalassemia
Sickle Cell Trait
G6PD Deficiency
Hemizygous G6PD Deficiency
Other Hemoglobinopathies
Iron Deficiency
Vitamin A Deficiency
Gastritis & Duodenitis
Peptic Ulcer Disease
Crohn's Disease
Ulcerative Colitis
Cirrhosis
CKD Stage 3
CKD Stage 4
CKD Stage 5 untreated
CKD Stage 5 on dialysis
Hypothyroid Diseases
Hyperthyroid Diseases
Other Endocrine
Males
1990
Dietary iron deficiency
Haemoglobinopathies and haemolytic anaemias
Other neglected tropical diseases
Other unspecified infectious diseases
Vitamin A deficiency
Chronic kidney disease
Endocrine, metabolic, blood, and immune disorders
Intestinal nematode infections
Upper digestive system diseases
Cirrhosis and other chronic liver diseases
Malaria
Inflammatory bowel disease
HIV/AIDS
Schistosomiasis
2021
Dietary iron deficiency
Haemoglobinopathies and haemolytic anaemias
Other neglected tropical diseases
Other unspecified infectious diseases
Chronic kidney disease
Vitamin A deficiency
Endocrine, metabolic, blood, and immune disorders
Intestinal nematode infections
Upper digestive system diseases
Cirrhosis and other chronic liver diseases
Inflammatory bowel disease
HIV/AIDS
Schistosomiasis
Malaria
Females
1990
Dietary iron deficiency
Haemoglobinopathies and haemolytic anaemias
Other neglected tropical diseases
Other unspecified infectious diseases
Chronic kidney disease
Endocrine, metabolic, blood, and immune disorders
Vitamin A deficiency
Gynecological diseases
Upper digestive system diseases
Intestinal nematode infections
Maternal disorders
Cirrhosis and other chronic liver diseases
Inflammatory bowel disease
Malaria
HIV/AIDS
Schistosomiasis
2021
Dietary iron deficiency
Haemoglobinopathies and haemolytic anaemias
Other neglected tropical diseases
Other unspecified infectious diseases
Chronic kidney disease
Endocrine, metabolic, blood, and immune disorders
Gynecological diseases
Upper digestive system diseases
Vitamin A deficiency
Maternal disorders
Intestinal nematode infections
Inflammatory bowel disease
Cirrhosis and other chronic liver diseases
HIV/AIDS
Schistosomiasis
Malaria
YLDs per 100,000 population
0 to <0.02
0.02 to <0.31
0.31 to <0.73
0.73 to <1.75
1.75 to <3.87
3.87 to <14.11
14.11 to <20.81
20.81 to <57.16
57.16 to <90.69
90.69 to 1014.97

Armenia

Anaemia Prevalence - Both Sexes

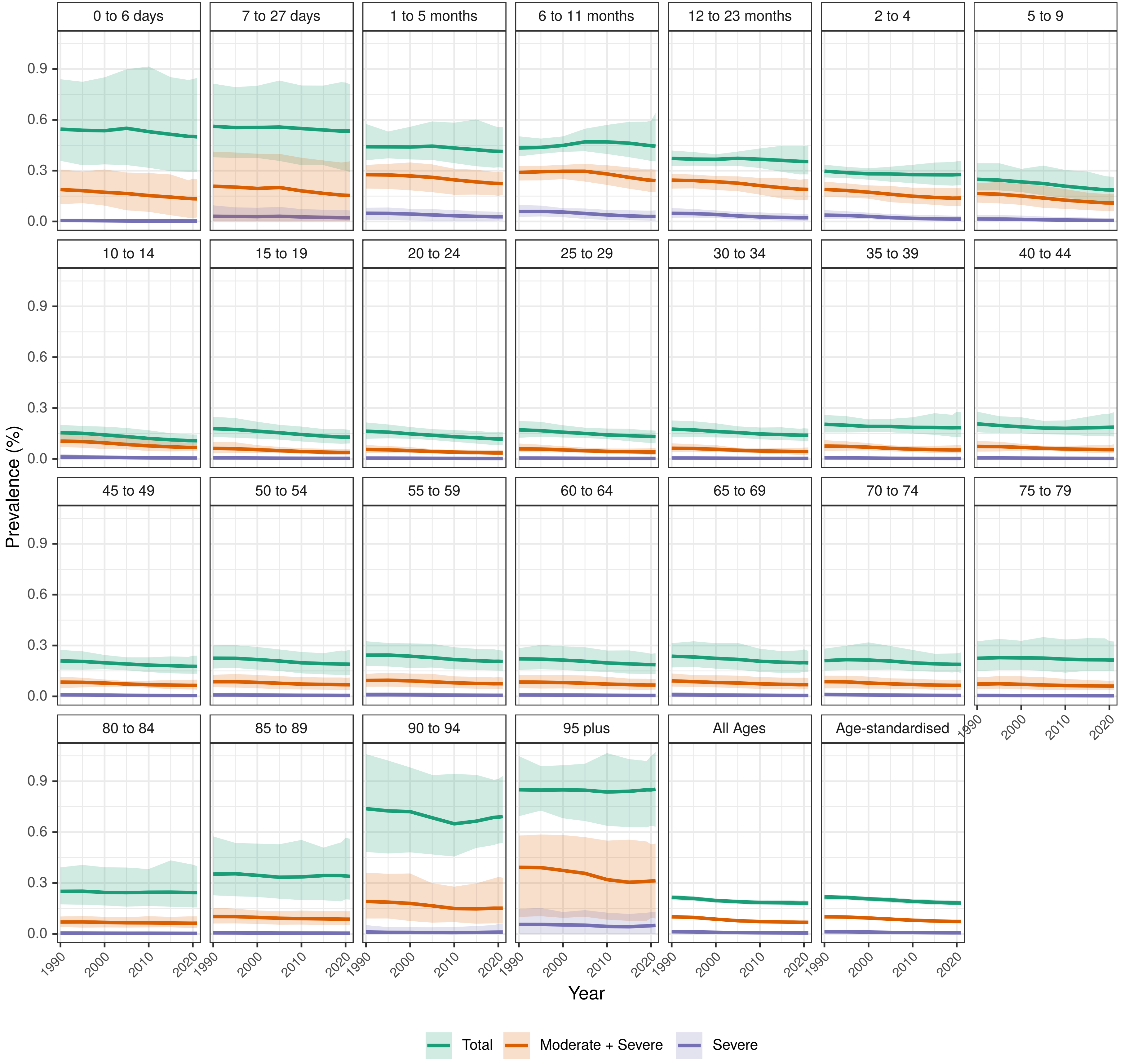

Cause-Specific Anaemia Prevalence - 2021

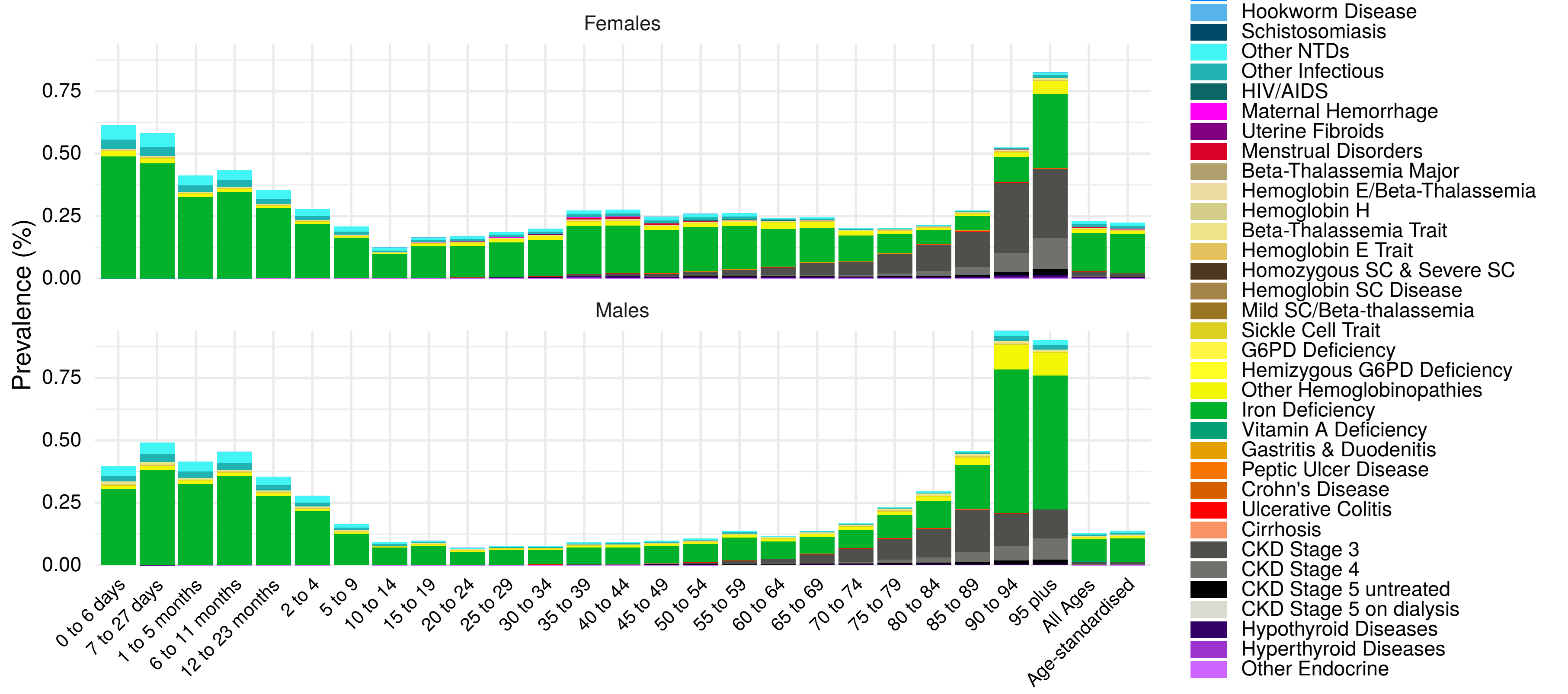

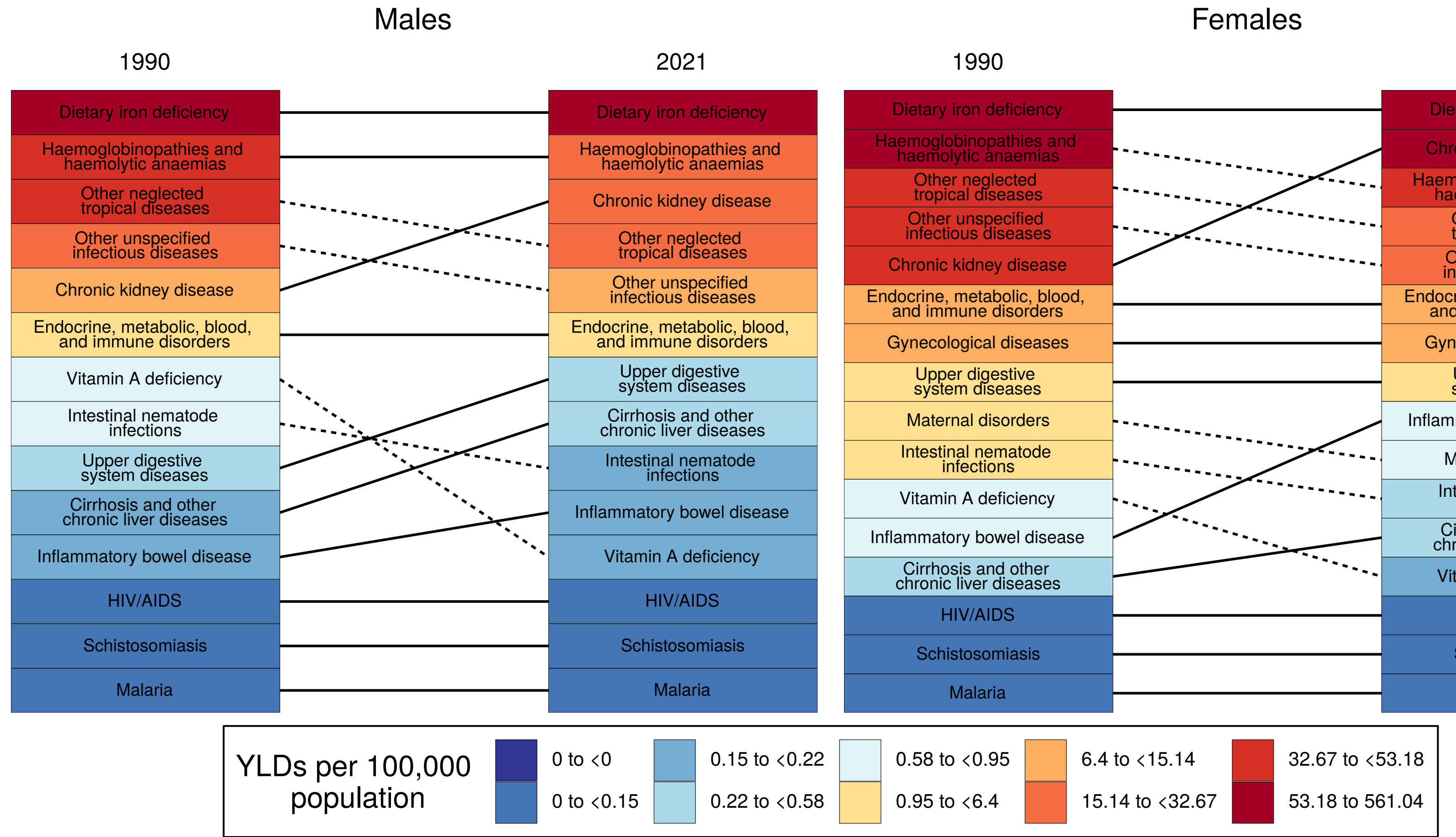

Azerbaijan

Anaemia Prevalence - Both Sexes

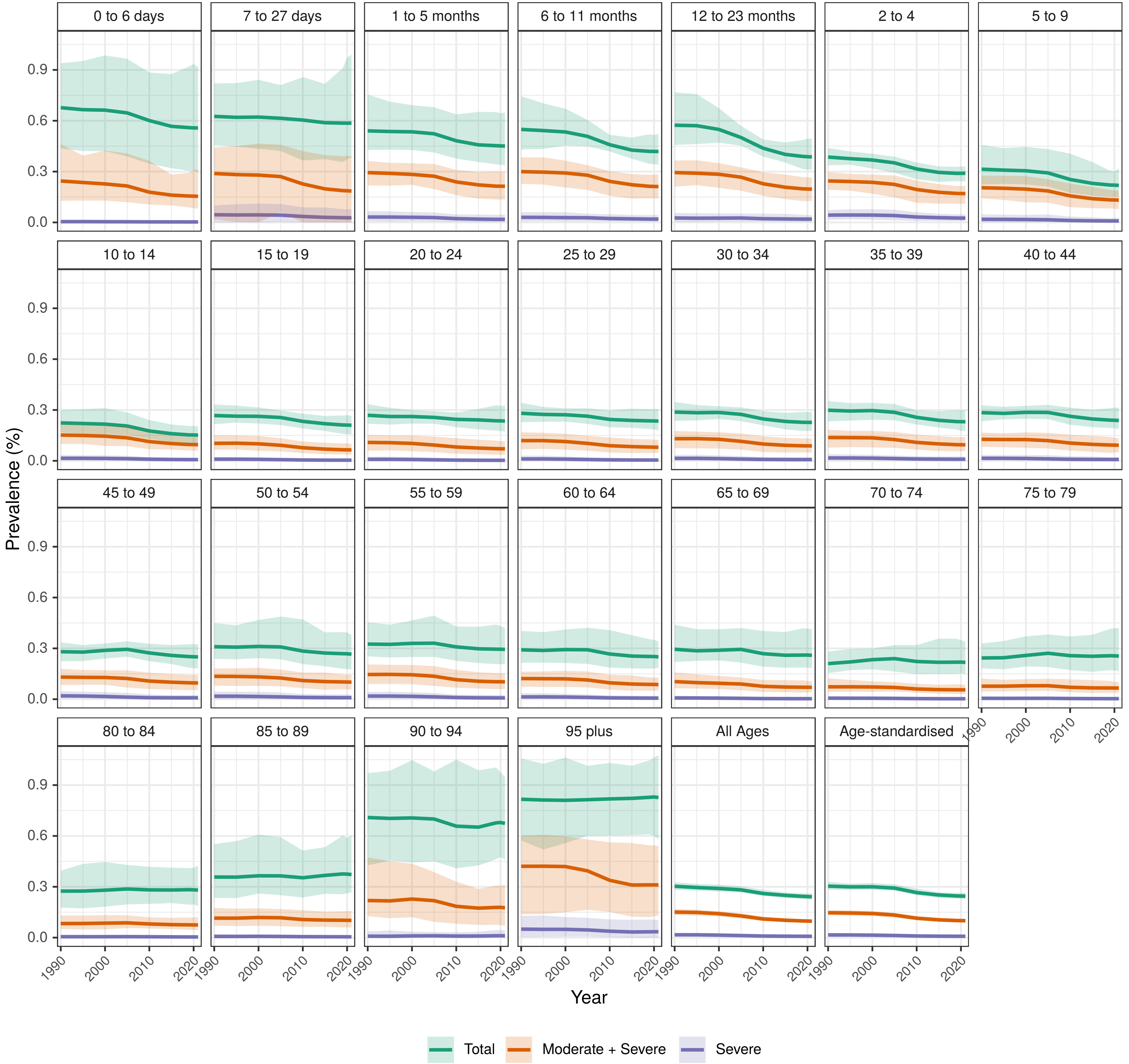

Cause-Specific Anaemia Prevalence - 2021

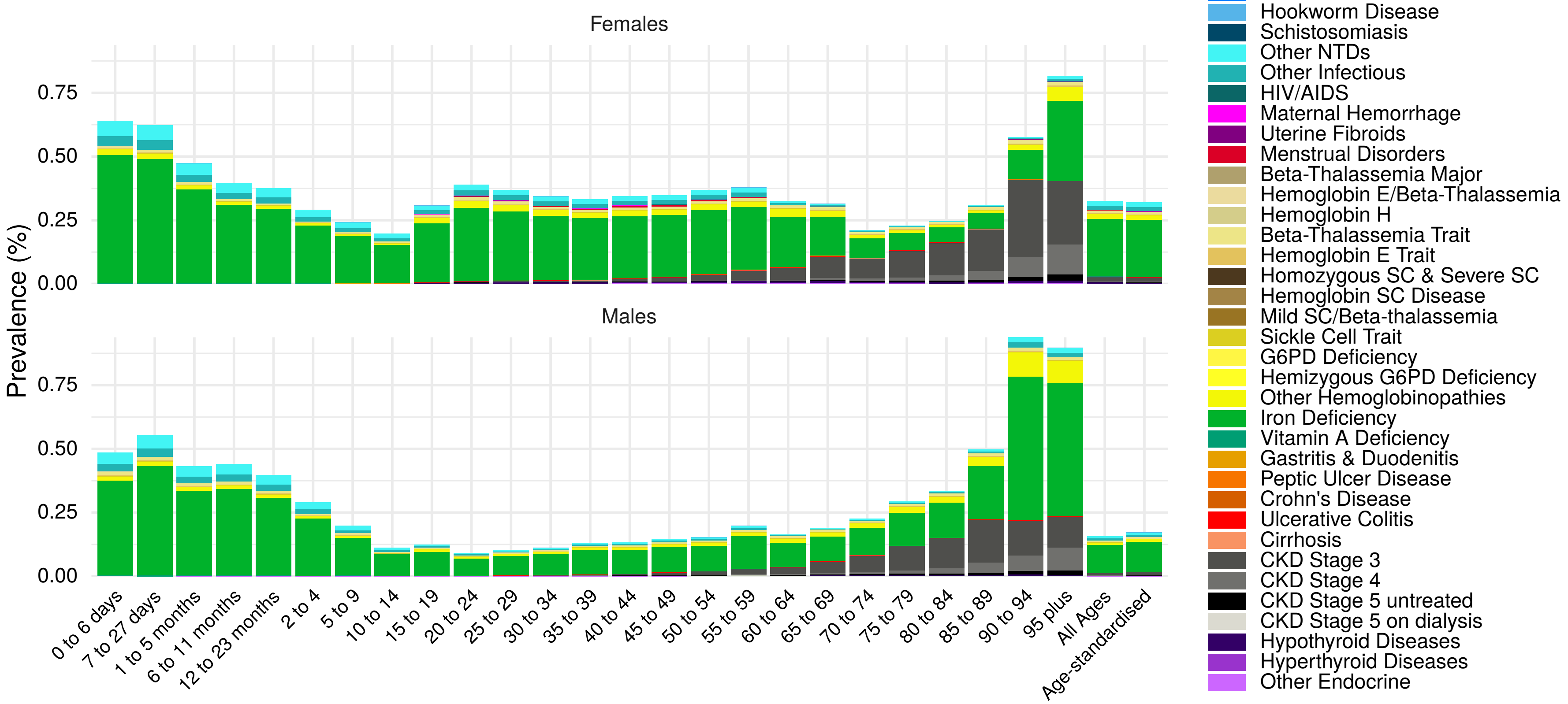

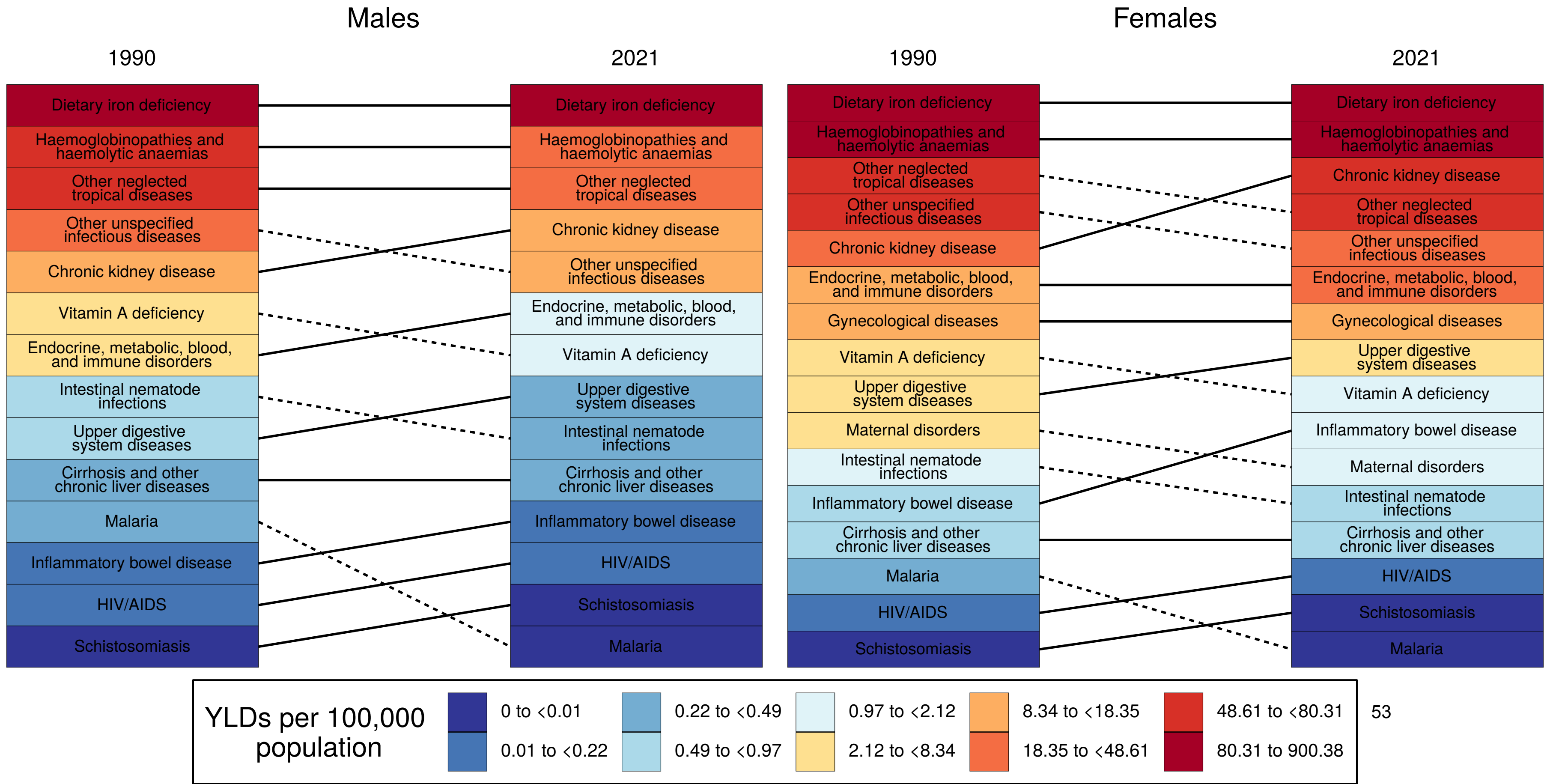

Georgia

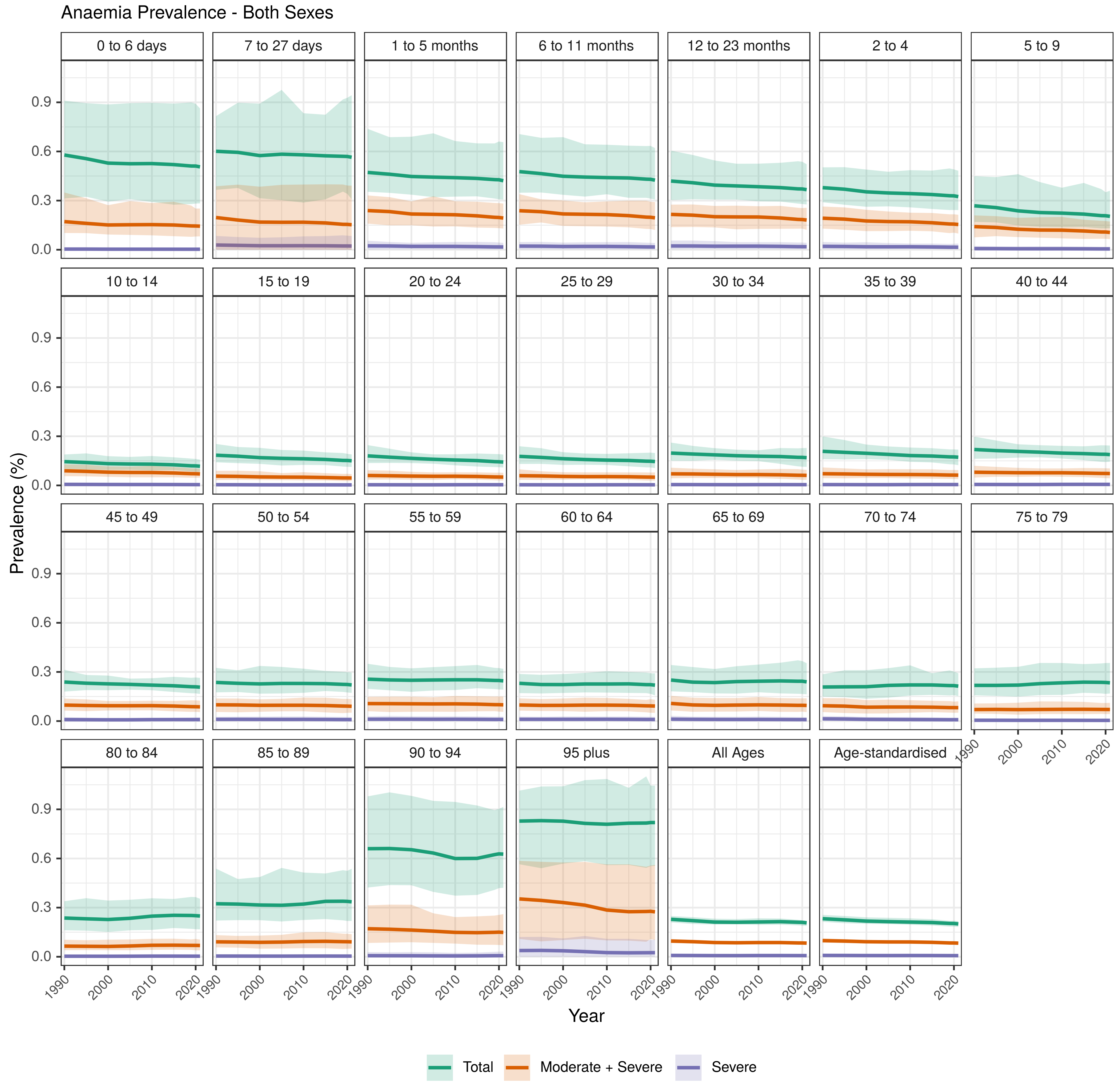
Anaemia Prevalence - Both Sexes
0 to 6 days
7 to 27 days
1 to 5 months
6 to 11 months
12 to 23 months
2 to 4
5 to 9
10 to 14
15 to 19
20 to 24
25 to 29
30 to 34
35 to 39
40 to 44
45 to 49
50 to 54
55 to 59
60 to 64
65 to 69
70 to 74
75 to 79
80 to 84
85 to 89
90 to 94
95 plus
All Ages
Age-standardised
Prevalence (%)
Year
Total
Moderate + Severe
Severe

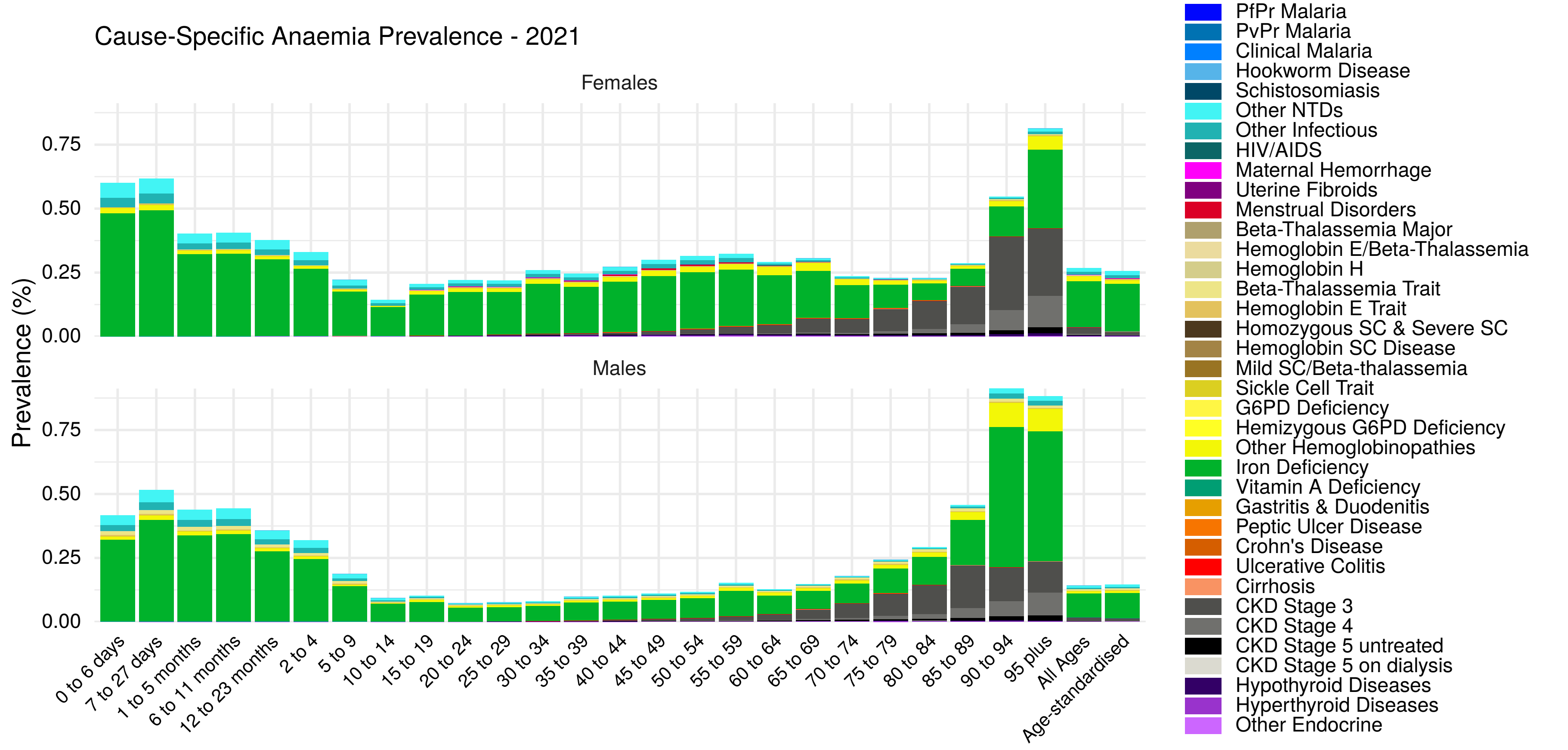
Cause-Specific Anaemia Prevalence - 2021
Females
Males
Prevalence (%)
PfPr Malaria
PvPr Malaria
Clinical Malaria
Hookworm Disease
Schistosomiasis
Other NTDs
Other Infectious
HIV/AIDS
Maternal Hemorrhage
Uterine Fibroids
Menstrual Disorders
Beta-Thalassemia Major
Hemoglobin E/Beta-Thalassemia
Hemoglobin H
Beta-Thalassemia Trait
Hemoglobin E Trait
Homozygous SC & Severe SC
Hemoglobin SC Disease
Mild SC/Beta-thalassemia
Sickle Cell Trait
G6PD Deficiency
Hemizygous G6PD Deficiency
Other Hemoglobinopathies
Iron Deficiency
Vitamin A Deficiency
Gastritis & Duodenitis
Peptic Ulcer Disease
Crohn's Disease
Ulcerative Colitis
Cirrhosis
CKD Stage 3
CKD Stage 4
CKD Stage 5 untreated
CKD Stage 5 on dialysis
Hypothyroid Diseases
Hyperthyroid Diseases
Other Endocrine

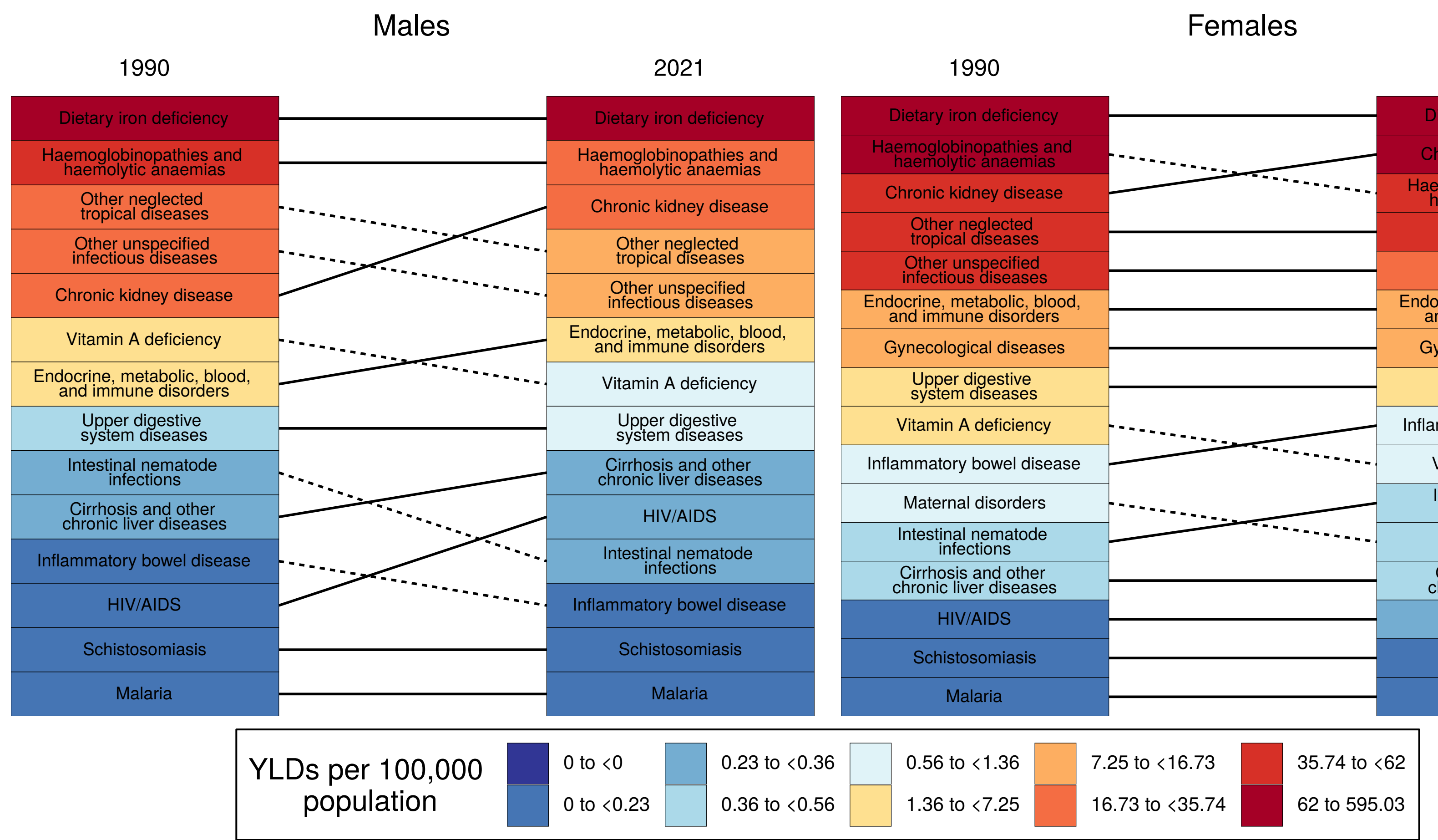
Males
Females
1990
2021
Dietary iron deficiency
Haemoglobinopathies and haemolytic anaemias
Other neglected tropical diseases
Other unspecified infectious diseases
Chronic kidney disease
Vitamin A deficiency
Endocrine, metabolic, blood, and immune disorders
Upper digestive system diseases
Intestinal nematode infections
Cirrhosis and other chronic liver diseases
Inflammatory bowel disease
HIV/AIDS
Schistosomiasis
Malaria
Gynecological diseases
Maternal disorders
YLDs per 100,000 population
0 to <0
0 to <0.23
0.23 to <0.36
0.36 to <0.56
0.56 to <1.36
1.36 to <7.25
7.25 to <16.73
16.73 to <35.74
35.74 to <62
62 to 595.03

Kazakhstan

Anaemia Prevalence - Both Sexes

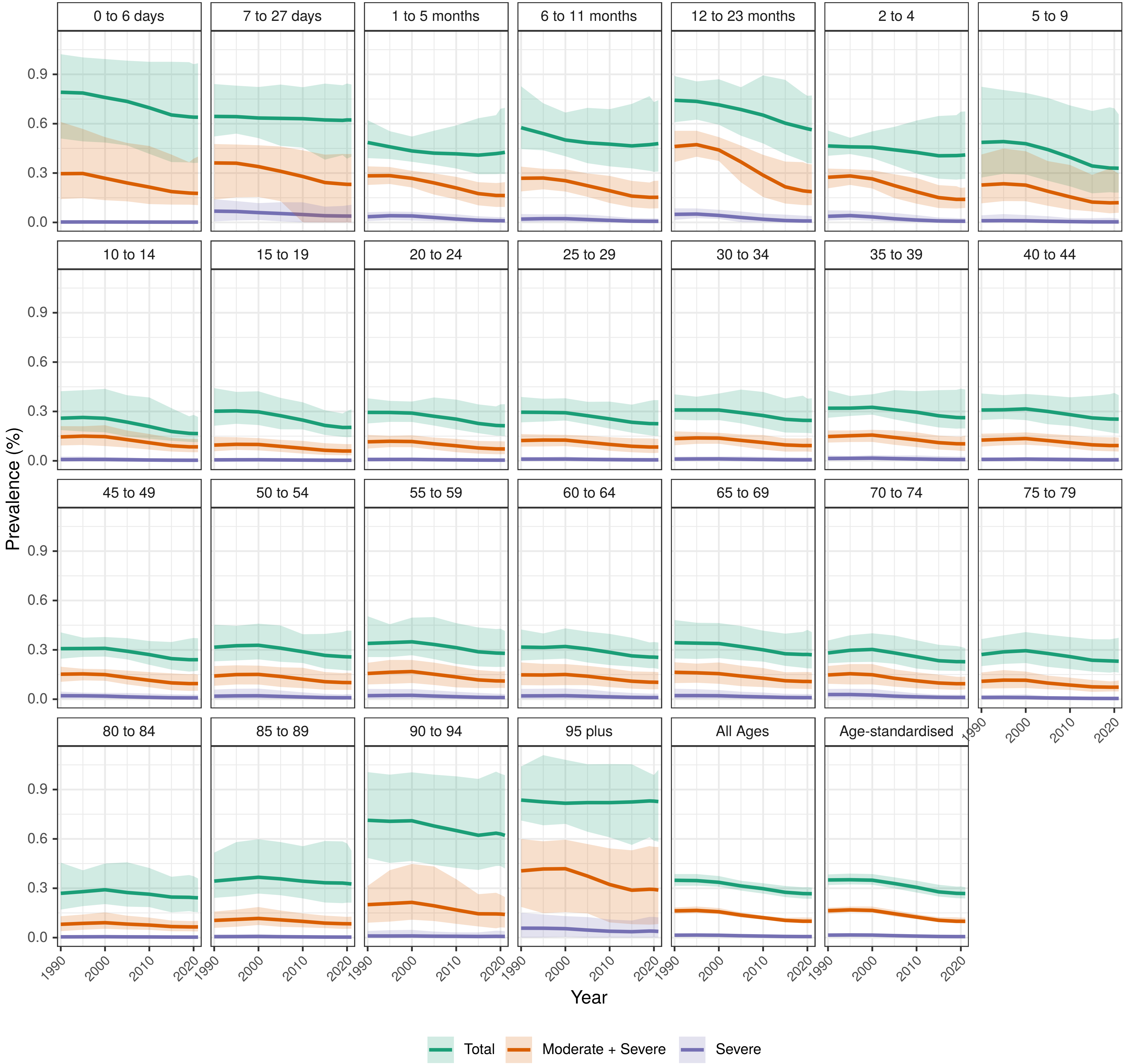

Cause-Specific Anaemia Prevalence - 2021

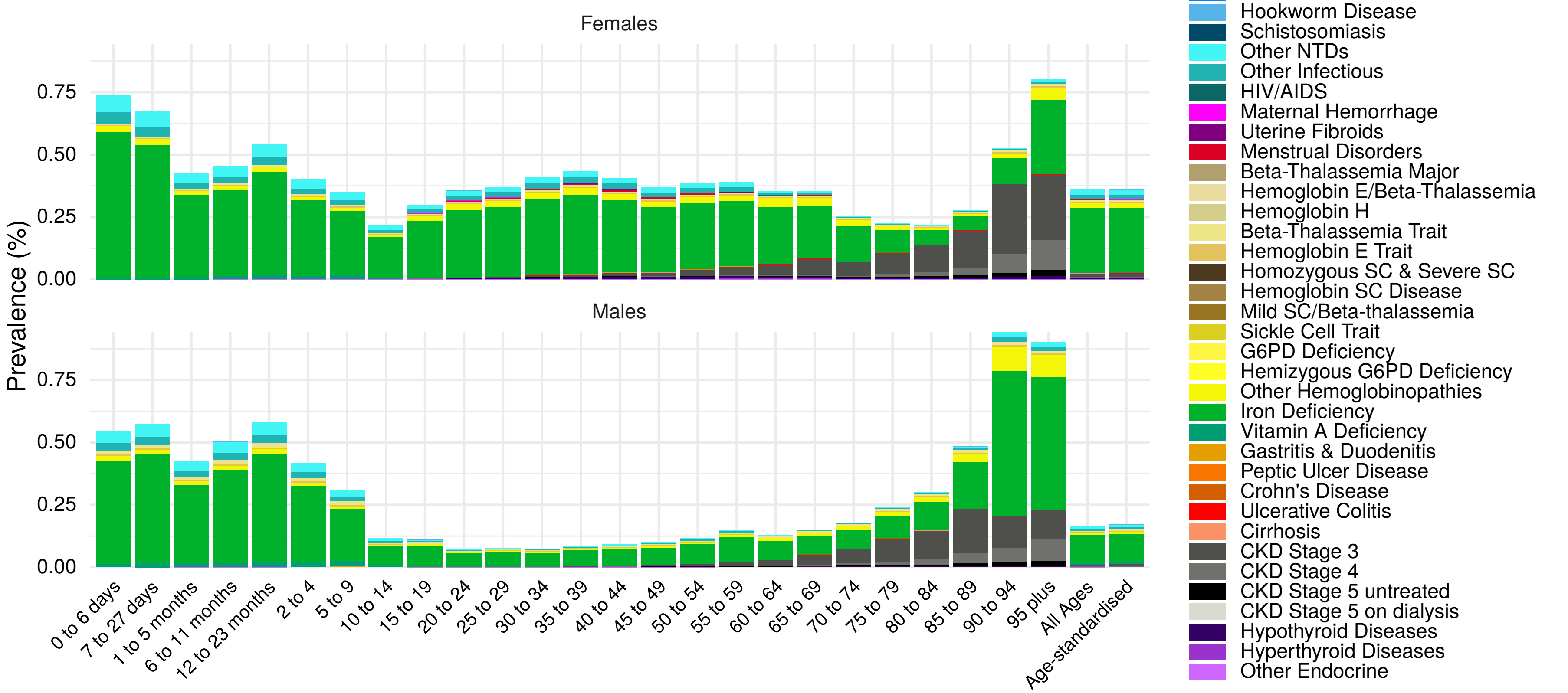

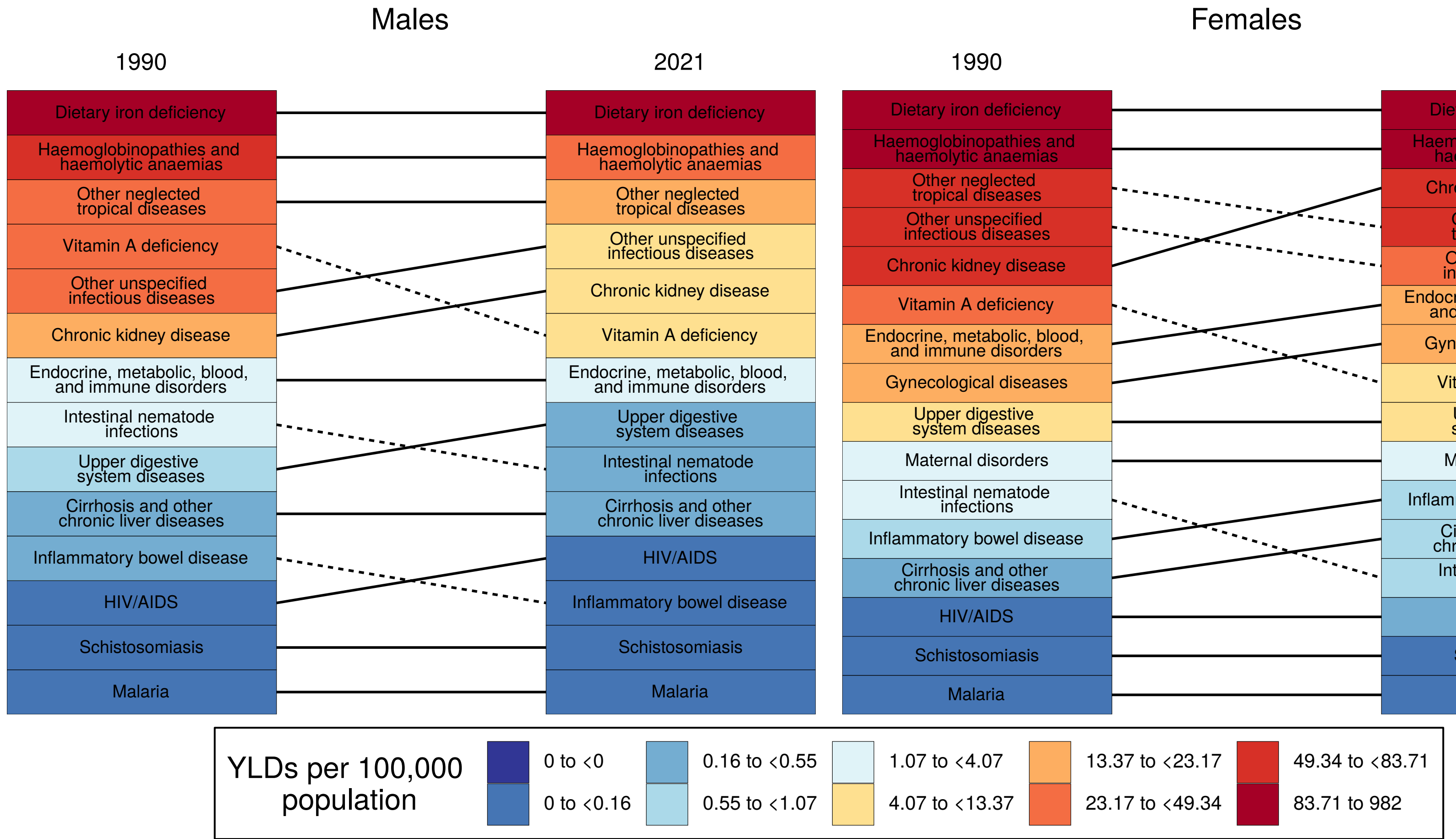

Kyrgyzstan

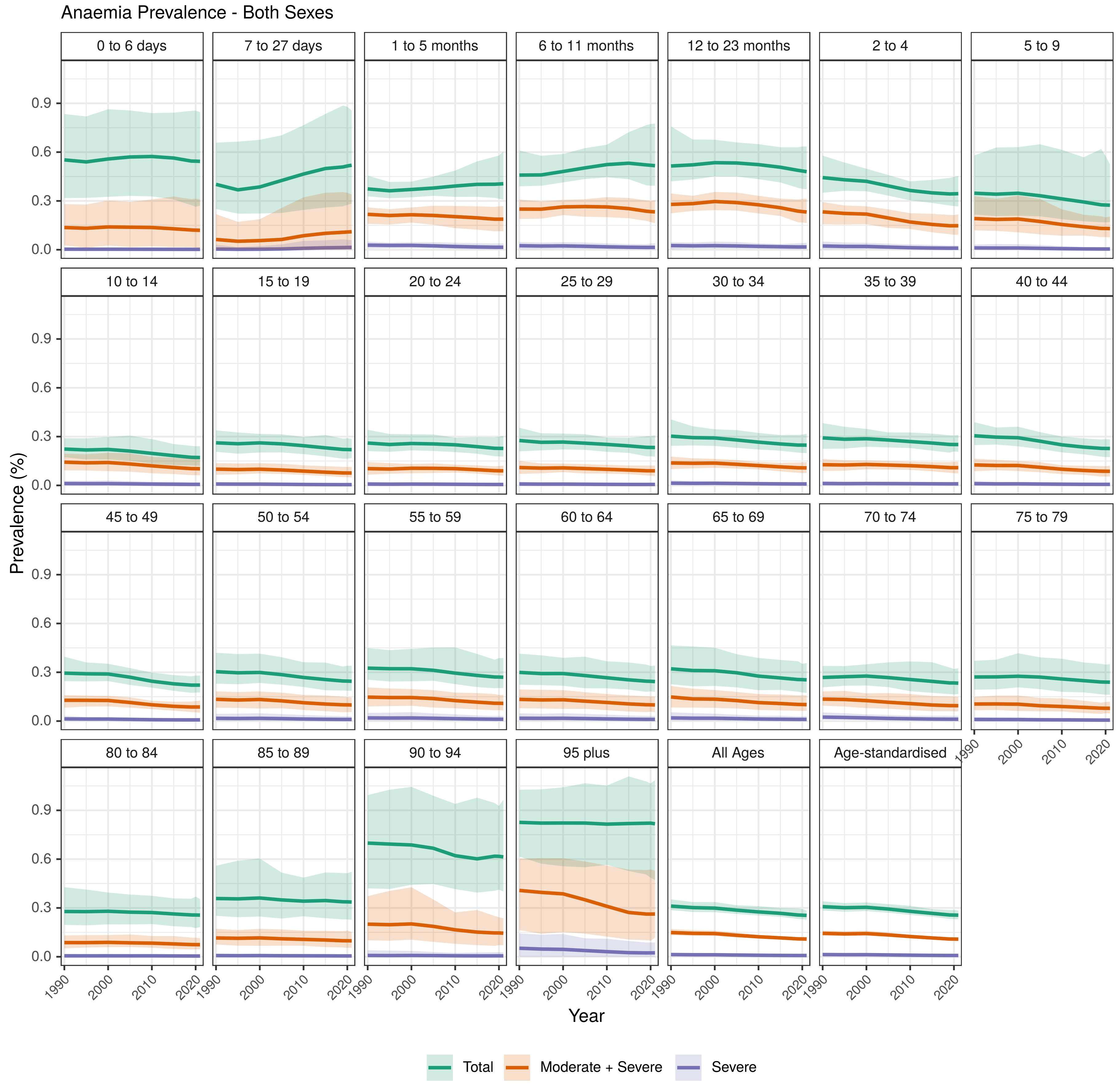
Anaemia Prevalence - Both Sexes
0 to 6 days
7 to 27 days
1 to 5 months
6 to 11 months
12 to 23 months
2 to 4
5 to 9
10 to 14
15 to 19
20 to 24
25 to 29
30 to 34
35 to 39
40 to 44
45 to 49
50 to 54
55 to 59
60 to 64
65 to 69
70 to 74
75 to 79
80 to 84
85 to 89
90 to 94
95 plus
All Ages
Age-standardised
Prevalence (%)
Year
0.0
0.3
0.6
0.9
1990
2000
2010
2020
Total
Moderate + Severe
Severe

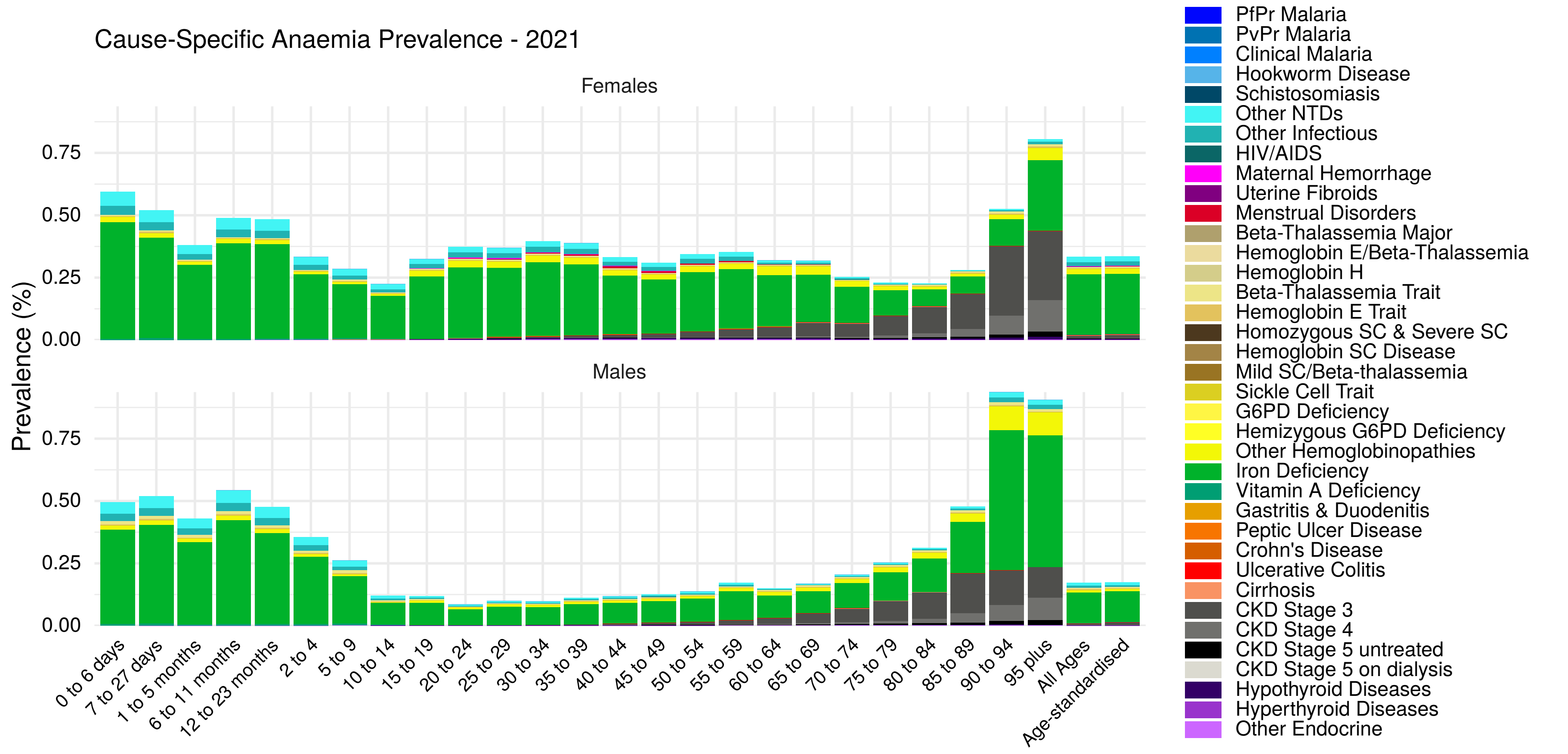
Cause-Specific Anaemia Prevalence - 2021
Females
Males
Prevalence (%)
0.00
0.25
0.50
0.75
0 to 6 days
7 to 27 days
1 to 5 months
6 to 11 months
12 to 23 months
2 to 4
5 to 9
10 to 14
15 to 19
20 to 24
25 to 29
30 to 34
35 to 39
40 to 44
45 to 49
50 to 54
55 to 59
60 to 64
65 to 69
70 to 74
75 to 79
80 to 84
85 to 89
90 to 94
95 plus
All Ages
Age-standardised
PfPr Malaria
PvPr Malaria
Clinical Malaria
Hookworm Disease
Schistosomiasis
Other NTDs
Other Infectious
HIV/AIDS
Maternal Hemorrhage
Uterine Fibroids
Menstrual Disorders
Beta-Thalassemia Major
Hemoglobin E/Beta-Thalassemia
Hemoglobin H
Beta-Thalassemia Trait
Hemoglobin E Trait
Homozygous SC & Severe SC
Hemoglobin SC Disease
Mild SC/Beta-thalassemia
Sickle Cell Trait
G6PD Deficiency
Hemizygous G6PD Deficiency
Other Hemoglobinopathies
Iron Deficiency
Vitamin A Deficiency
Gastritis & Duodenitis
Peptic Ulcer Disease
Crohn's Disease
Ulcerative Colitis
Cirrhosis
CKD Stage 3
CKD Stage 4
CKD Stage 5 untreated
CKD Stage 5 on dialysis
Hypothyroid Diseases
Hyperthyroid Diseases
Other Endocrine

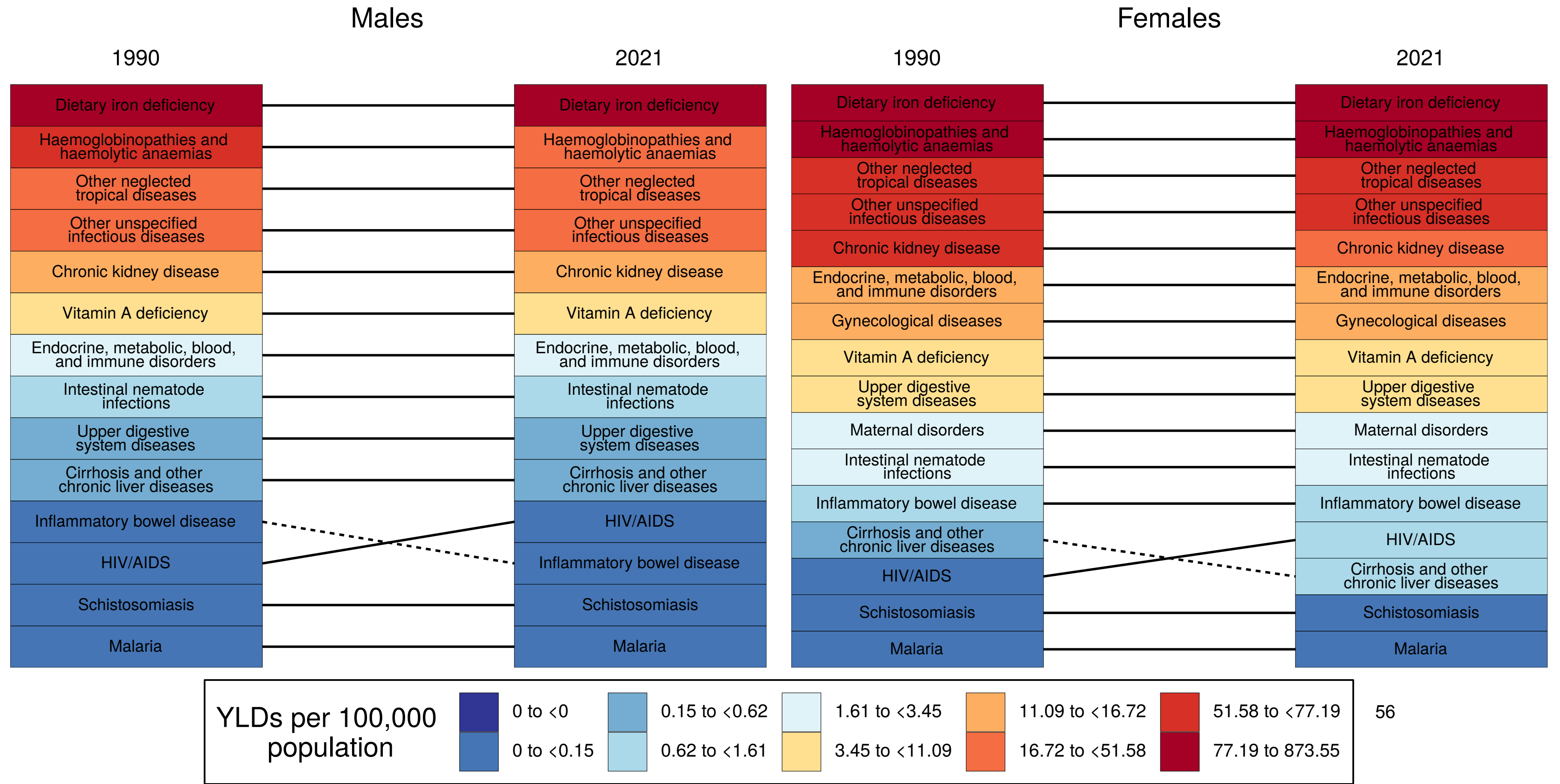
Males
Females
1990
2021
Dietary iron deficiency
Haemoglobinopathies and haemolytic anaemias
Other neglected tropical diseases
Other unspecified infectious diseases
Chronic kidney disease
Vitamin A deficiency
Endocrine, metabolic, blood, and immune disorders
Intestinal nematode infections
Upper digestive system diseases
Cirrhosis and other chronic liver diseases
Inflammatory bowel disease
HIV/AIDS
Schistosomiasis
Malaria
Gynecological diseases
Maternal disorders
YLDs per 100,000 population
0 to <0
0 to <0.15
0.15 to <0.62
0.62 to <1.61
1.61 to <3.45
3.45 to <11.09
11.09 to <16.72
16.72 to <51.58
51.58 to <77.19
77.19 to 873.55

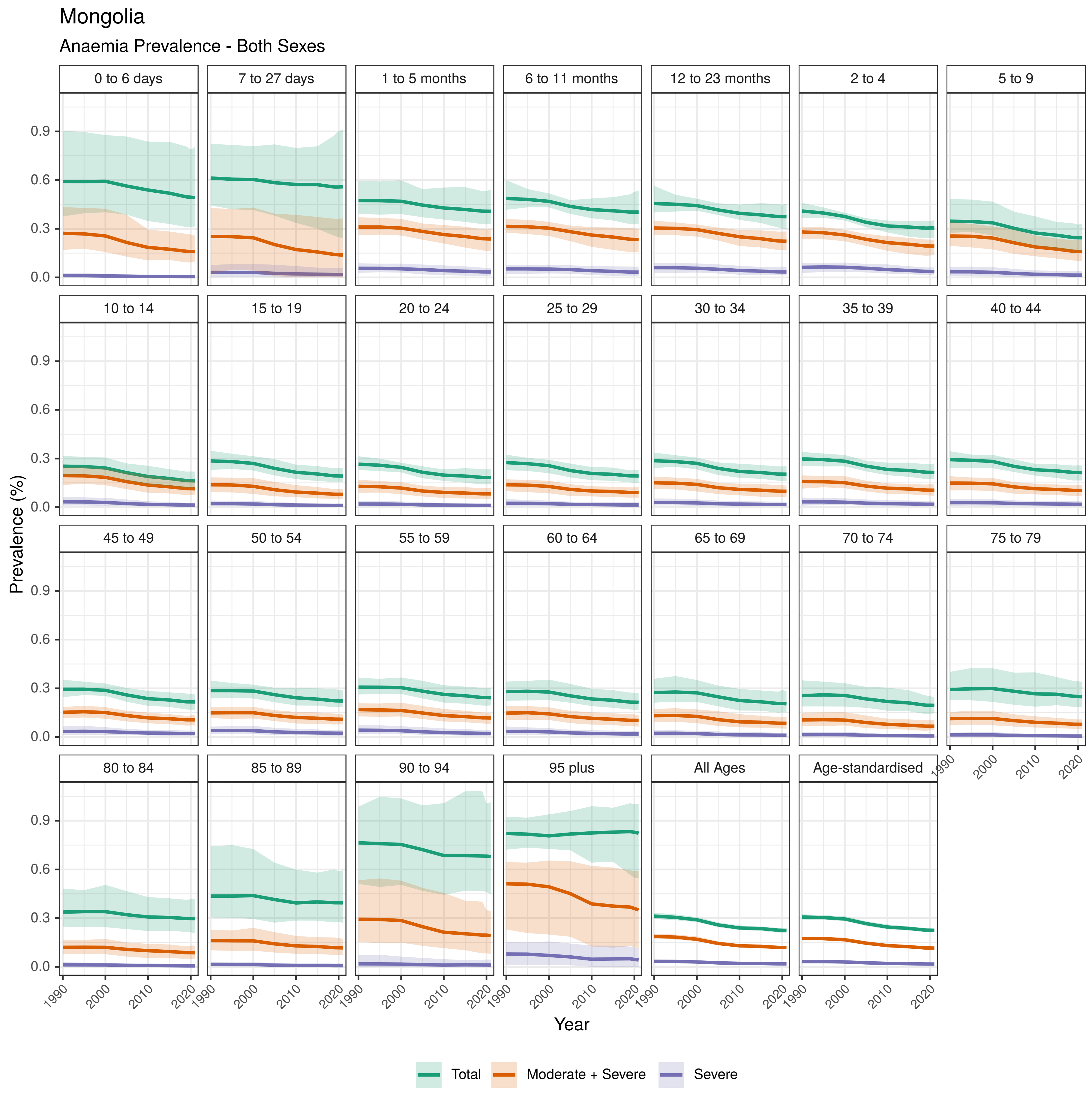
Mongolia
Anaemia Prevalence - Both Sexes
0 to 6 days
7 to 27 days
1 to 5 months
6 to 11 months
12 to 23 months
2 to 4
5 to 9
10 to 14
15 to 19
20 to 24
25 to 29
30 to 34
35 to 39
40 to 44
45 to 49
50 to 54
55 to 59
60 to 64
65 to 69
70 to 74
75 to 79
80 to 84
85 to 89
90 to 94
95 plus
All Ages
Age-standardised
Prevalence (%)
0.0
0.3
0.6
0.9
1990
2000
2010
2020
Year
Total
Moderate + Severe
Severe

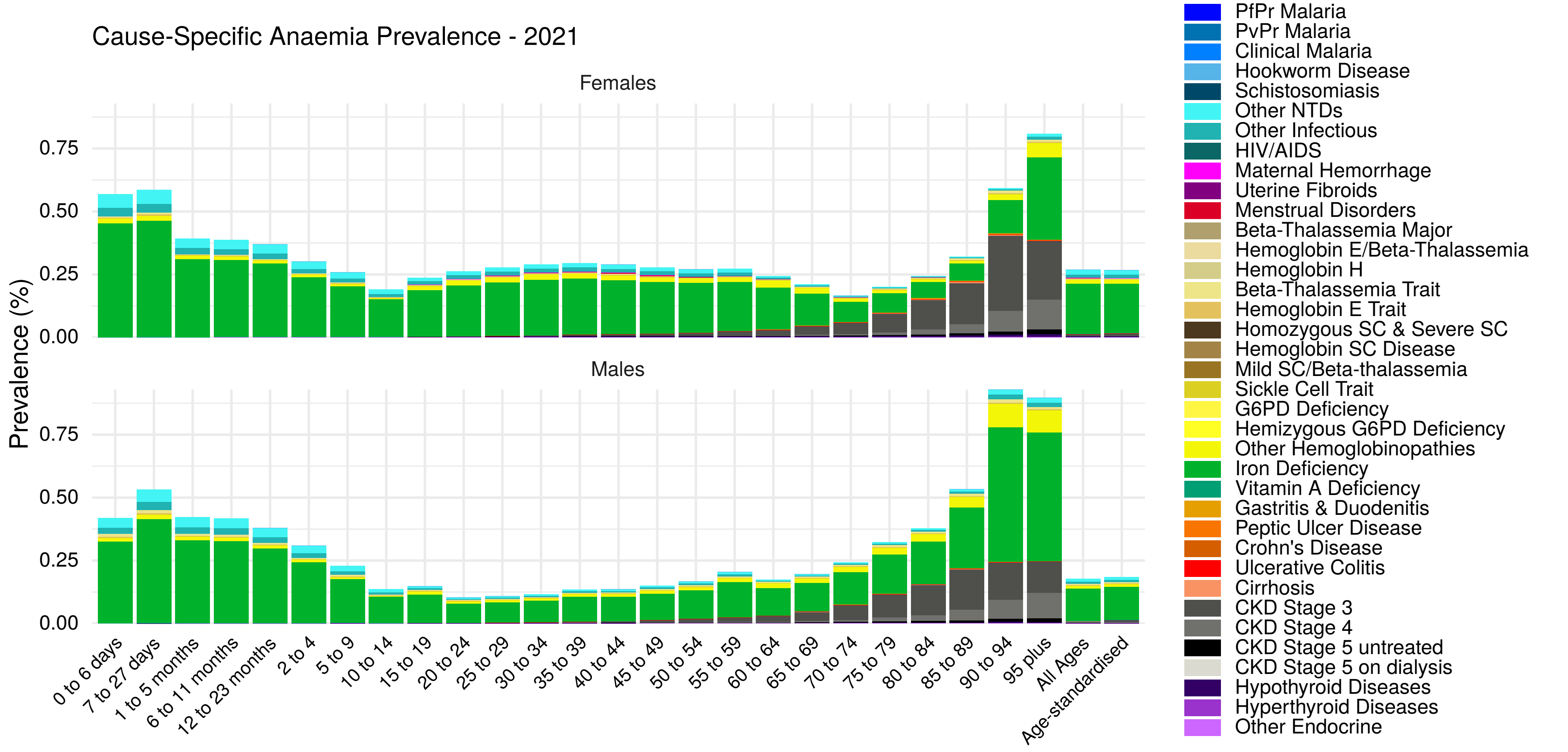
Cause-Specific Anaemia Prevalence - 2021
Females
Males
Prevalence (%)
0.00
0.25
0.50
0.75
0 to 6 days
7 to 27 days
1 to 5 months
6 to 11 months
12 to 23 months
2 to 4
5 to 9
10 to 14
15 to 19
20 to 24
25 to 29
30 to 34
35 to 39
40 to 44
45 to 49
50 to 54
55 to 59
60 to 64
65 to 69
70 to 74
75 to 79
80 to 84
85 to 89
90 to 94
95 plus
All Ages
Age-standardised
PfPr Malaria
PvPr Malaria
Clinical Malaria
Hookworm Disease
Schistosomiasis
Other NTDs
Other Infectious
HIV/AIDS
Maternal Hemorrhage
Uterine Fibroids
Menstrual Disorders
Beta-Thalassemia Major
Hemoglobin E/Beta-Thalassemia
Hemoglobin H
Beta-Thalassemia Trait
Hemoglobin E Trait
Homozygous SC & Severe SC
Hemoglobin SC Disease
Mild SC/Beta-thalassemia
Sickle Cell Trait
G6PD Deficiency
Hemizygous G6PD Deficiency
Other Hemoglobinopathies
Iron Deficiency
Vitamin A Deficiency
Gastritis & Duodenitis
Peptic Ulcer Disease
Crohn's Disease
Ulcerative Colitis
Cirrhosis
CKD Stage 3
CKD Stage 4
CKD Stage 5 untreated
CKD Stage 5 on dialysis
Hypothyroid Diseases
Hyperthyroid Diseases
Other Endocrine

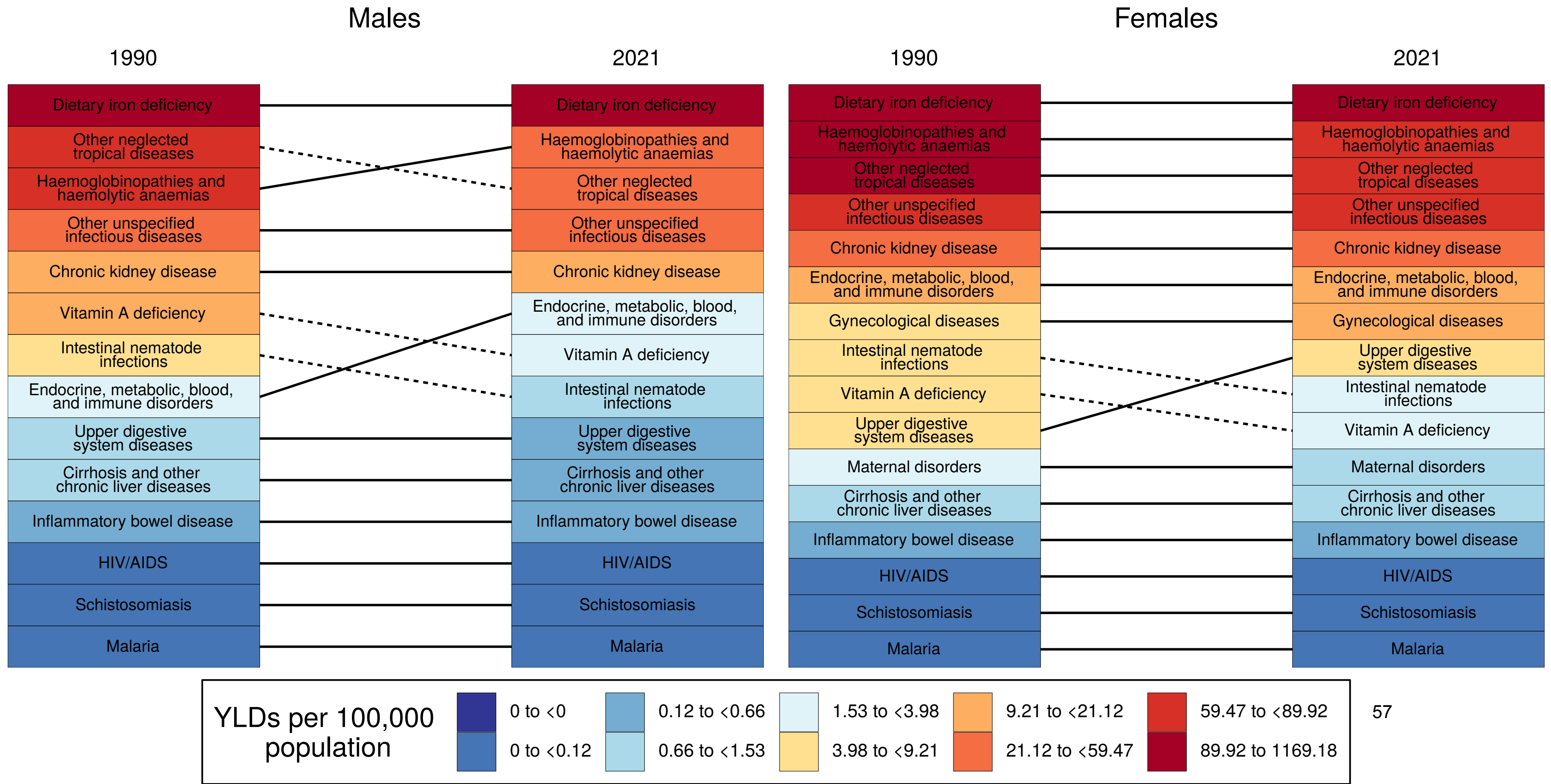
Males
Females
1990
2021
Dietary iron deficiency
Other neglected tropical diseases
Haemoglobinopathies and haemolytic anaemias
Other unspecified infectious diseases
Chronic kidney disease
Vitamin A deficiency
Intestinal nematode infections
Endocrine, metabolic, blood, and immune disorders
Upper digestive system diseases
Cirrhosis and other chronic liver diseases
Inflammatory bowel disease
HIV/AIDS
Schistosomiasis
Malaria
Gynecological diseases
Maternal disorders
YLDs per 100,000 population
0 to <0
0 to <0.12
0.12 to <0.66
0.66 to <1.53
1.53 to <3.98
3.98 to <9.21
9.21 to <21.12
21.12 to <59.47
59.47 to <89.92
89.92 to 1169.18

Tajikistan

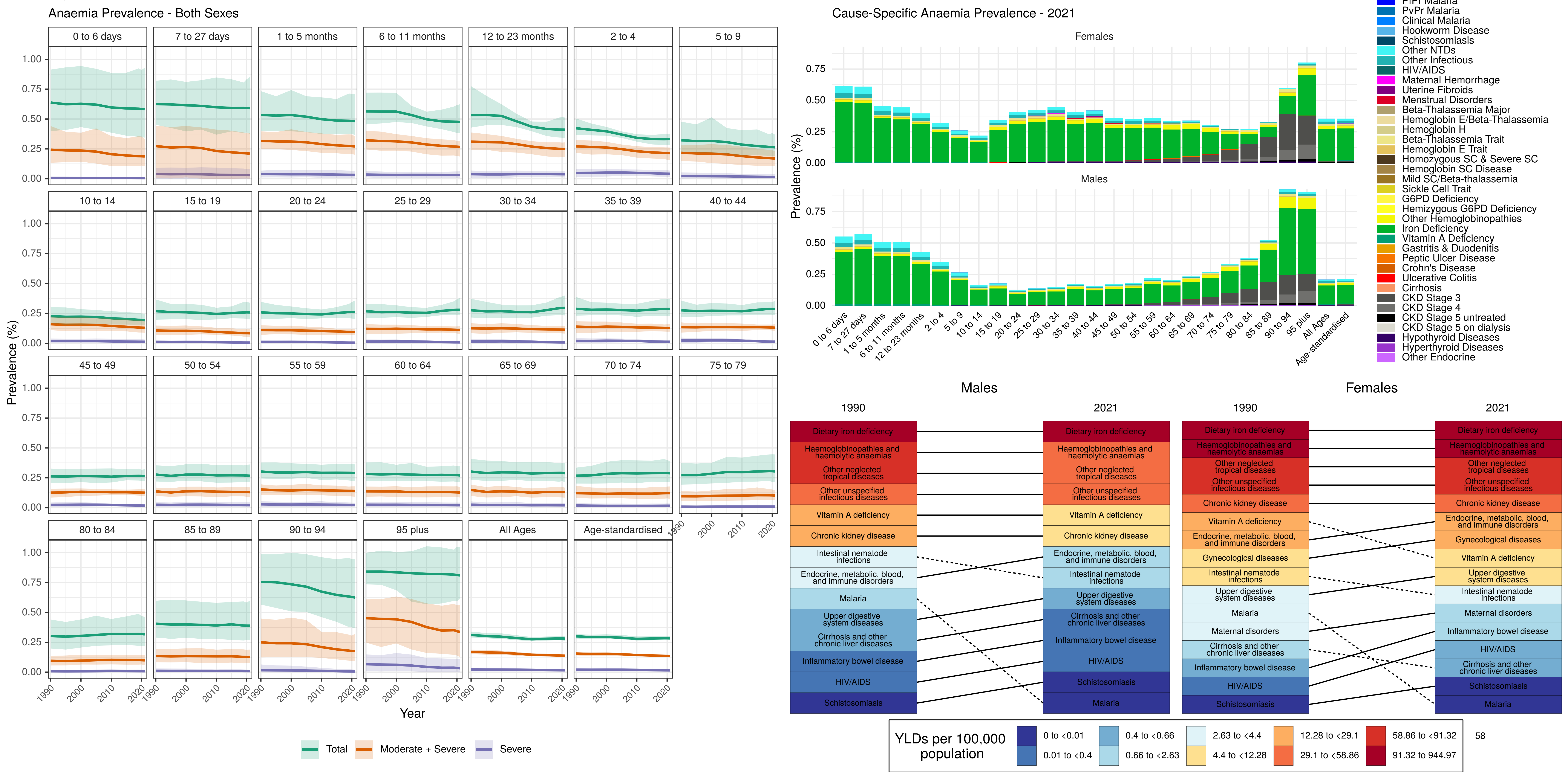

Anaemia Prevalence - Both Sexes
0 to 6 days
7 to 27 days
1 to 5 months
6 to 11 months
12 to 23 months
2 to 4
5 to 9
10 to 14
15 to 19
20 to 24
25 to 29
30 to 34
35 to 39
40 to 44
45 to 49
50 to 54
55 to 59
60 to 64
65 to 69
70 to 74
75 to 79
80 to 84
85 to 89
90 to 94
95 plus
All Ages
Age-standardised
Prevalence (%)
Year
Total
Moderate + Severe
Severe
Cause-Specific Anaemia Prevalence - 2021
Females
Males
PfPr Malaria
PvPr Malaria
Clinical Malaria
Hookworm Disease
Schistosomiasis
Other NTDs
Other Infectious
HIV/AIDS
Maternal Hemorrhage
Uterine Fibroids
Menstrual Disorders
Beta-Thalassemia Major
Hemoglobin E/Beta-Thalassemia
Hemoglobin H
Beta-Thalassemia Trait
Hemoglobin E Trait
Homozygous SC & Severe SC
Hemoglobin SC Disease
Mild SC/Beta-thalassemia
Sickle Cell Trait
G6PD Deficiency
Hemizygous G6PD Deficiency
Other Hemoglobinopathies
Iron Deficiency
Vitamin A Deficiency
Gastritis & Duodenitis
Peptic Ulcer Disease
Crohn's Disease
Ulcerative Colitis
Cirrhosis
CKD Stage 3
CKD Stage 4
CKD Stage 5 untreated
CKD Stage 5 on dialysis
Hypothyroid Diseases
Hyperthyroid Diseases
Other Endocrine
Males
Females
1990
2021
Males 1990: Dietary iron deficiency; Haemoglobinopathies and haemolytic anaemias; Other neglected tropical diseases; Other unspecified infectious diseases; Vitamin A deficiency; Chronic kidney disease; Intestinal nematode infections; Endocrine, metabolic, blood, and immune disorders; Malaria; Upper digestive system diseases; Cirrhosis and other chronic liver diseases; Inflammatory bowel disease; HIV/AIDS; Schistosomiasis
Males 2021: Dietary iron deficiency; Haemoglobinopathies and haemolytic anaemias; Other neglected tropical diseases; Other unspecified infectious diseases; Vitamin A deficiency; Chronic kidney disease; Endocrine, metabolic, blood, and immune disorders; Intestinal nematode infections; Upper digestive system diseases; Cirrhosis and other chronic liver diseases; Inflammatory bowel disease; HIV/AIDS; Schistosomiasis; Malaria
Females 1990: Dietary iron deficiency; Haemoglobinopathies and haemolytic anaemias; Other neglected tropical diseases; Other unspecified infectious diseases; Chronic kidney disease; Vitamin A deficiency; Endocrine, metabolic, blood, and immune disorders; Gynecological diseases; Intestinal nematode infections; Upper digestive system diseases; Malaria; Maternal disorders; Cirrhosis and other chronic liver diseases; Inflammatory bowel disease; HIV/AIDS; Schistosomiasis
Females 2021: Dietary iron deficiency; Haemoglobinopathies and haemolytic anaemias; Other neglected tropical diseases; Other unspecified infectious diseases; Chronic kidney disease; Endocrine, metabolic, blood, and immune disorders; Gynecological diseases; Vitamin A deficiency; Upper digestive system diseases; Intestinal nematode infections; Maternal disorders; Inflammatory bowel disease; HIV/AIDS; Cirrhosis and other chronic liver diseases; Schistosomiasis; Malaria
YLDs per 100,000 population
0 to <0.01
0.01 to <0.4
0.4 to <0.66
0.66 to <2.63
2.63 to <4.4
4.4 to <12.28
12.28 to <29.1
29.1 to <58.86
58.86 to <91.32
91.32 to 944.97

Turkmenistan

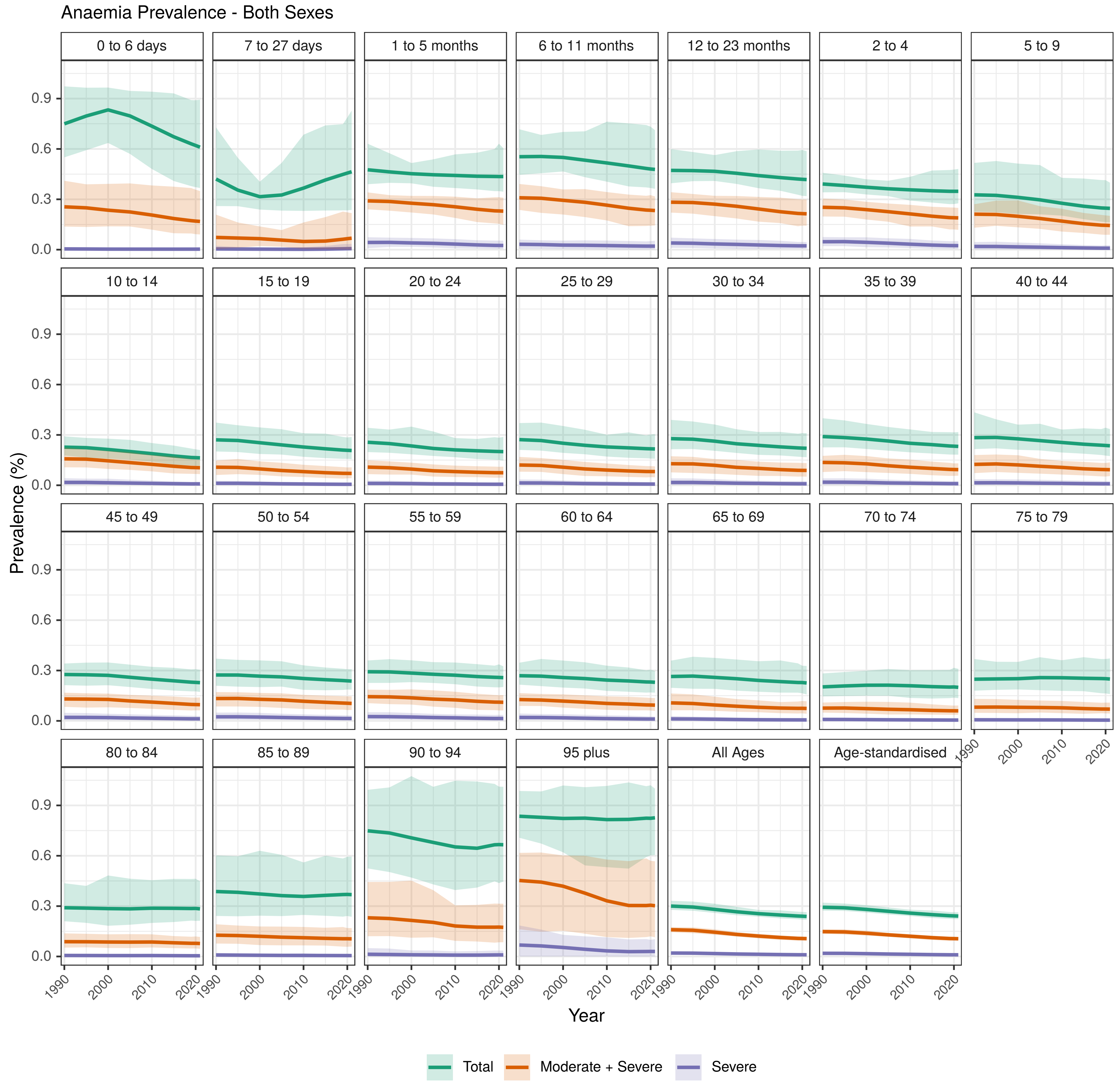

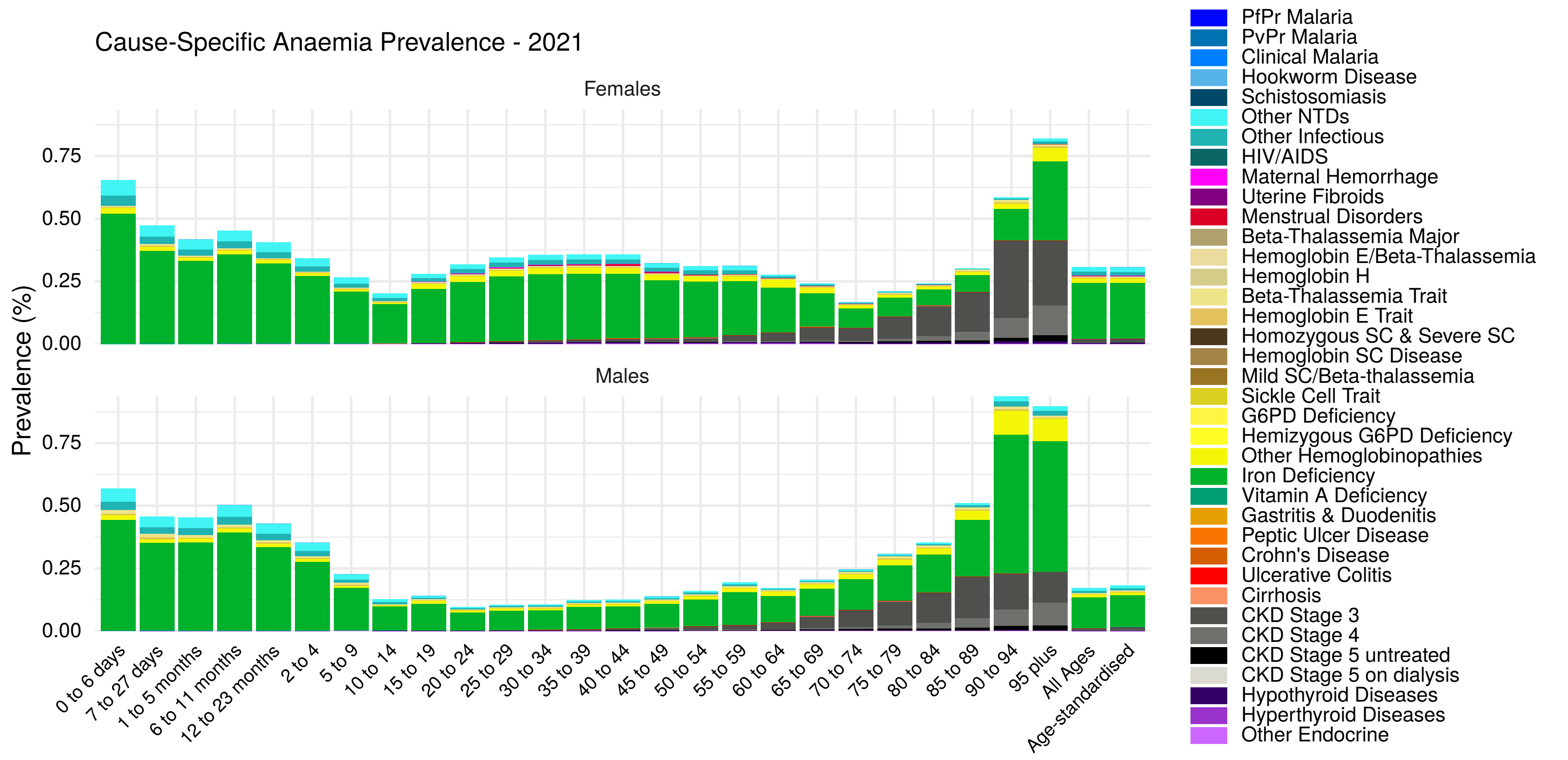

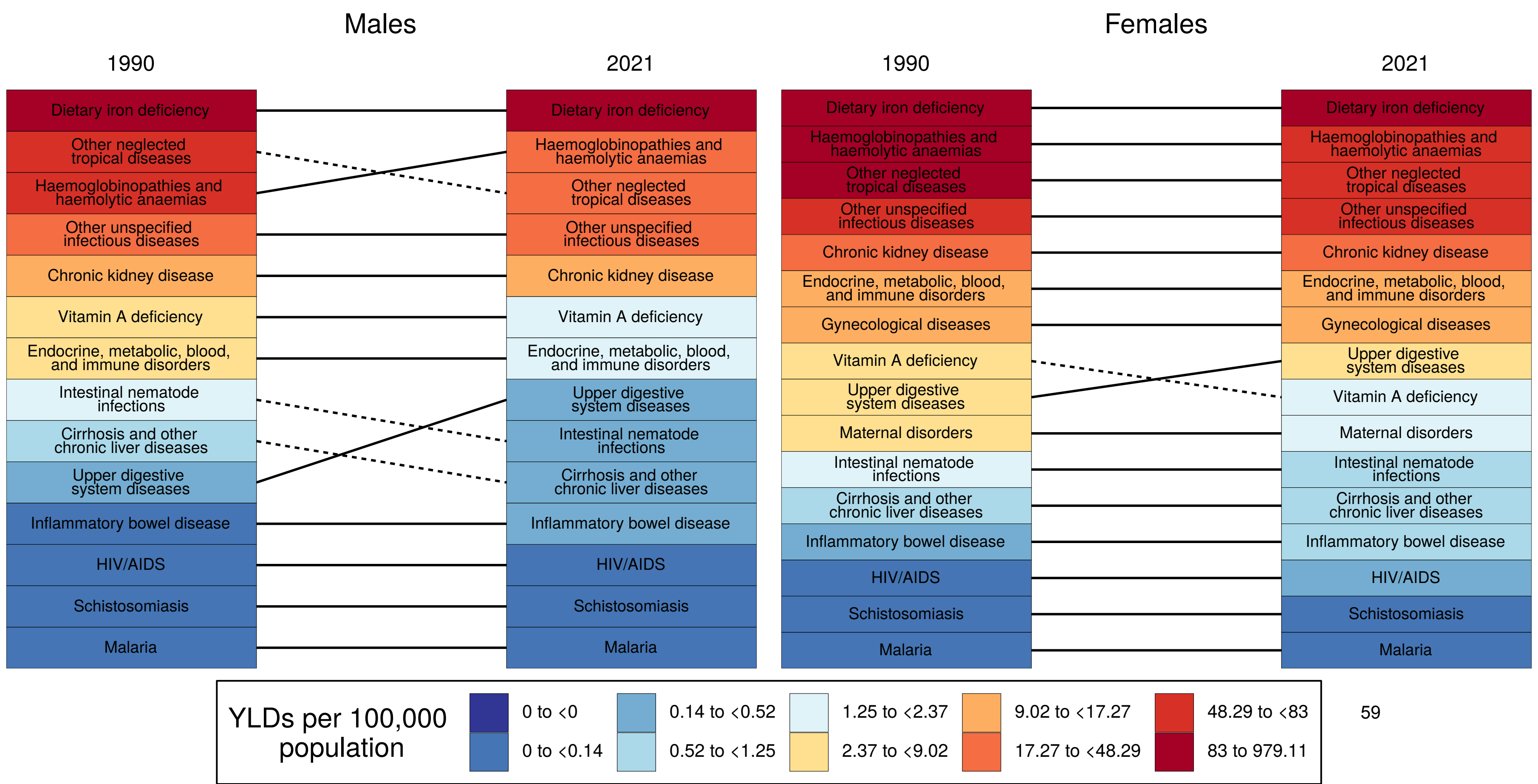

Uzbekistan

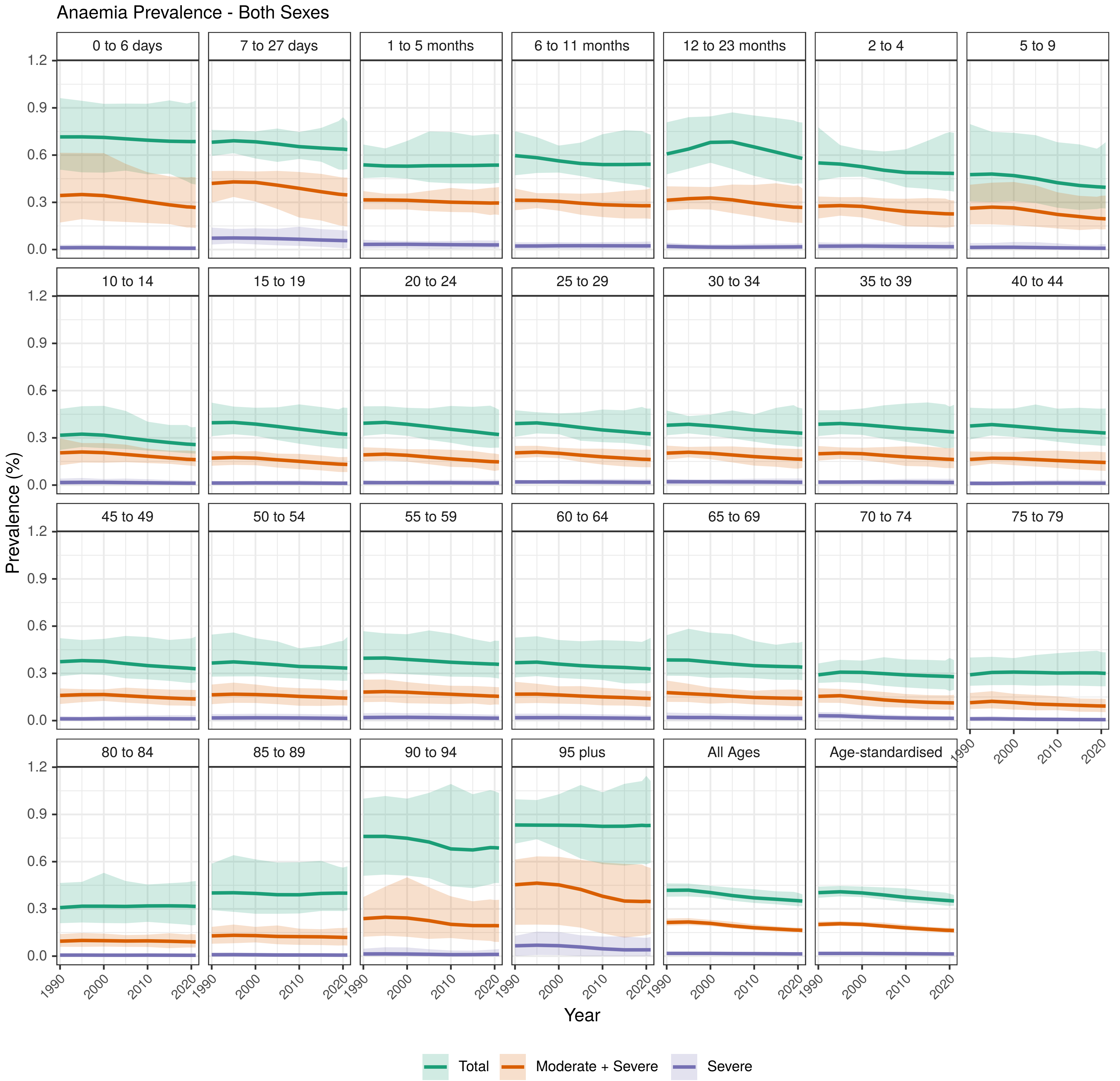
Anaemia Prevalence - Both Sexes
0 to 6 days
7 to 27 days
1 to 5 months
6 to 11 months
12 to 23 months
2 to 4
5 to 9
10 to 14
15 to 19
20 to 24
25 to 29
30 to 34
35 to 39
40 to 44
45 to 49
50 to 54
55 to 59
60 to 64
65 to 69
70 to 74
75 to 79
80 to 84
85 to 89
90 to 94
95 plus
All Ages
Age-standardised
Prevalence (%)
Year
1990
2000
2010
2020
0.0
0.3
0.6
0.9
1.2
Total
Moderate + Severe
Severe

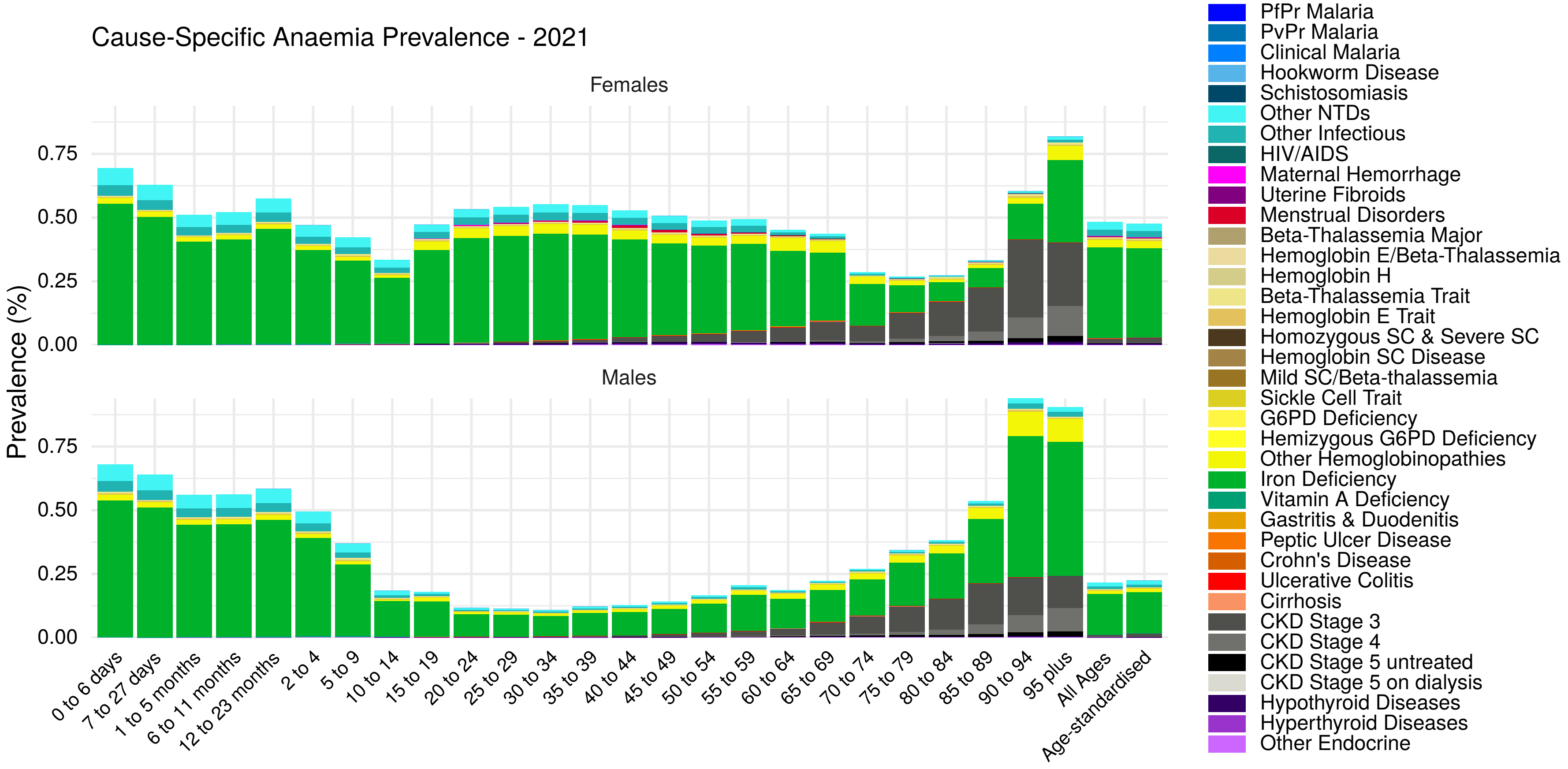
Cause-Specific Anaemia Prevalence - 2021
Females
Males
Prevalence (%)
0.00
0.25
0.50
0.75
0 to 6 days
7 to 27 days
1 to 5 months
6 to 11 months
12 to 23 months
2 to 4
5 to 9
10 to 14
15 to 19
20 to 24
25 to 29
30 to 34
35 to 39
40 to 44
45 to 49
50 to 54
55 to 59
60 to 64
65 to 69
70 to 74
75 to 79
80 to 84
85 to 89
90 to 94
95 plus
All Ages
Age-standardised
PfPr Malaria
PvPr Malaria
Clinical Malaria
Hookworm Disease
Schistosomiasis
Other NTDs
Other Infectious
HIV/AIDS
Maternal Hemorrhage
Uterine Fibroids
Menstrual Disorders
Beta-Thalassemia Major
Hemoglobin E/Beta-Thalassemia
Hemoglobin H
Beta-Thalassemia Trait
Hemoglobin E Trait
Homozygous SC & Severe SC
Hemoglobin SC Disease
Mild SC/Beta-thalassemia
Sickle Cell Trait
G6PD Deficiency
Hemizygous G6PD Deficiency
Other Hemoglobinopathies
Iron Deficiency
Vitamin A Deficiency
Gastritis & Duodenitis
Peptic Ulcer Disease
Crohn's Disease
Ulcerative Colitis
Cirrhosis
CKD Stage 3
CKD Stage 4
CKD Stage 5 untreated
CKD Stage 5 on dialysis
Hypothyroid Diseases
Hyperthyroid Diseases
Other Endocrine

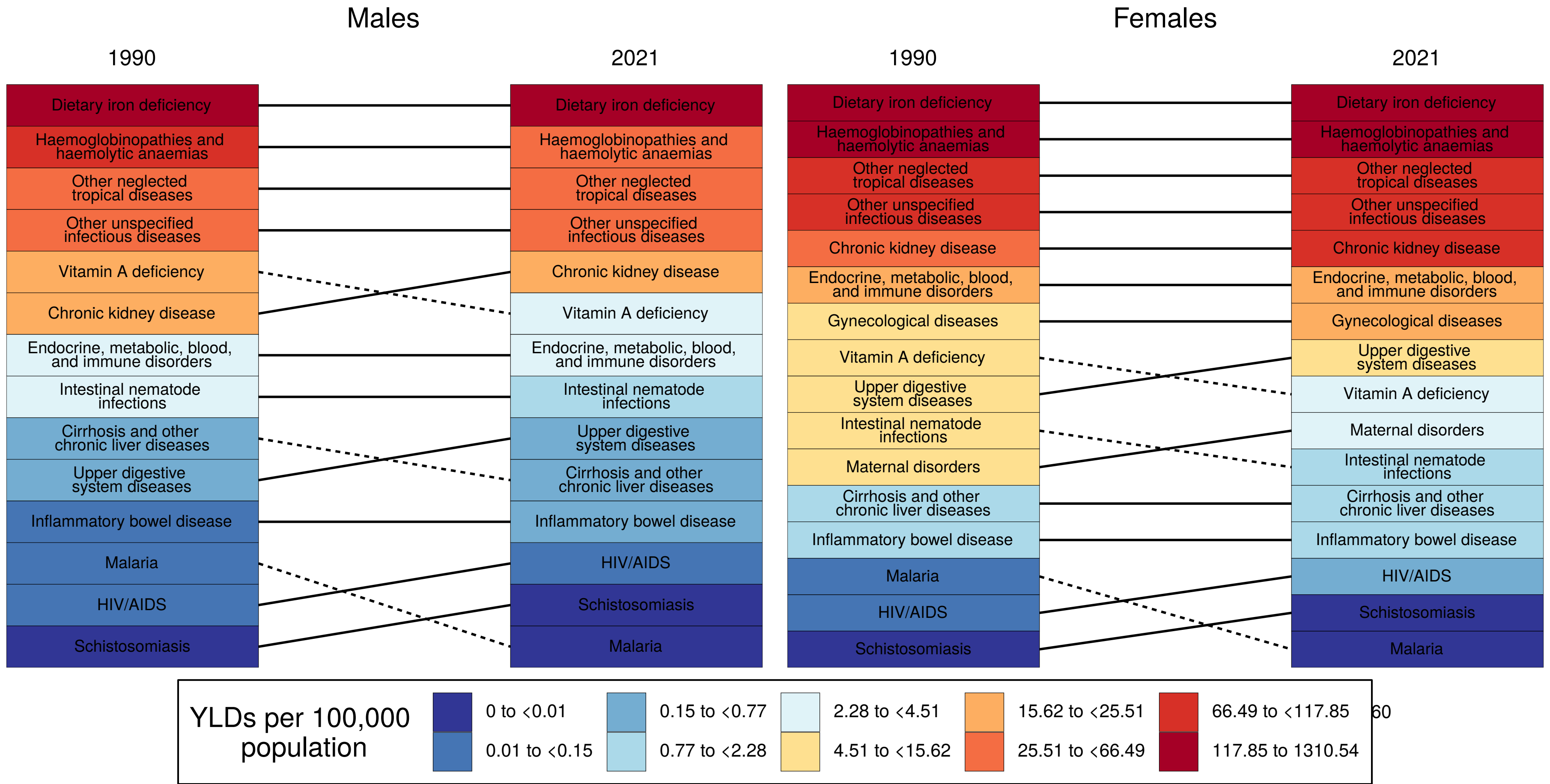
Males
Females
1990
2021
Dietary iron deficiency
Haemoglobinopathies and haemolytic anaemias
Other neglected tropical diseases
Other unspecified infectious diseases
Vitamin A deficiency
Chronic kidney disease
Endocrine, metabolic, blood, and immune disorders
Intestinal nematode infections
Cirrhosis and other chronic liver diseases
Upper digestive system diseases
Inflammatory bowel disease
Malaria
HIV/AIDS
Schistosomiasis
Gynecological diseases
Maternal disorders
YLDs per 100,000 population
0 to <0.01
0.01 to <0.15
0.15 to <0.77
0.77 to <2.28
2.28 to <4.51
4.51 to <15.62
15.62 to <25.51
25.51 to <66.49
66.49 to <117.85
117.85 to 1310.54

Central Europe

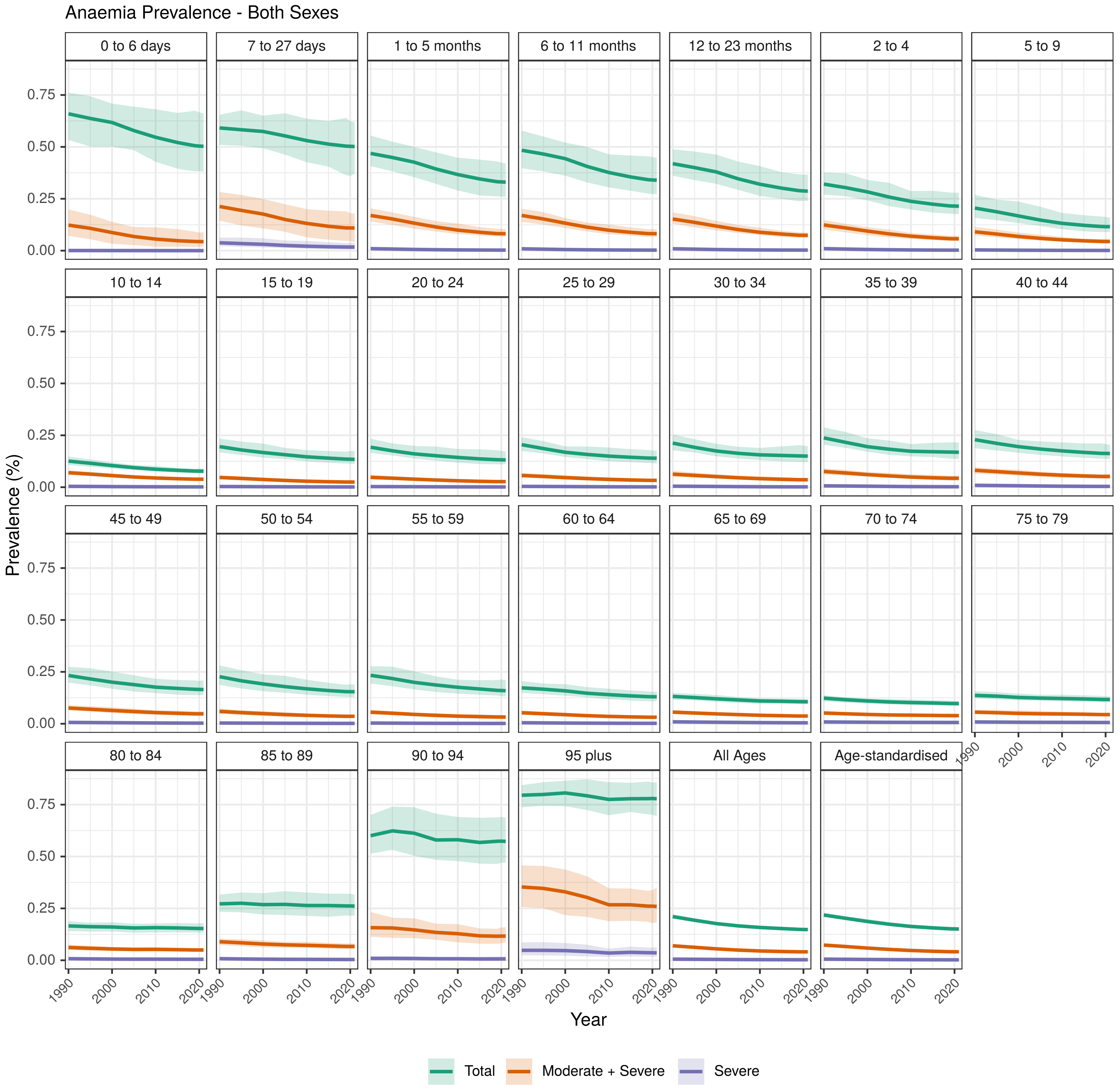

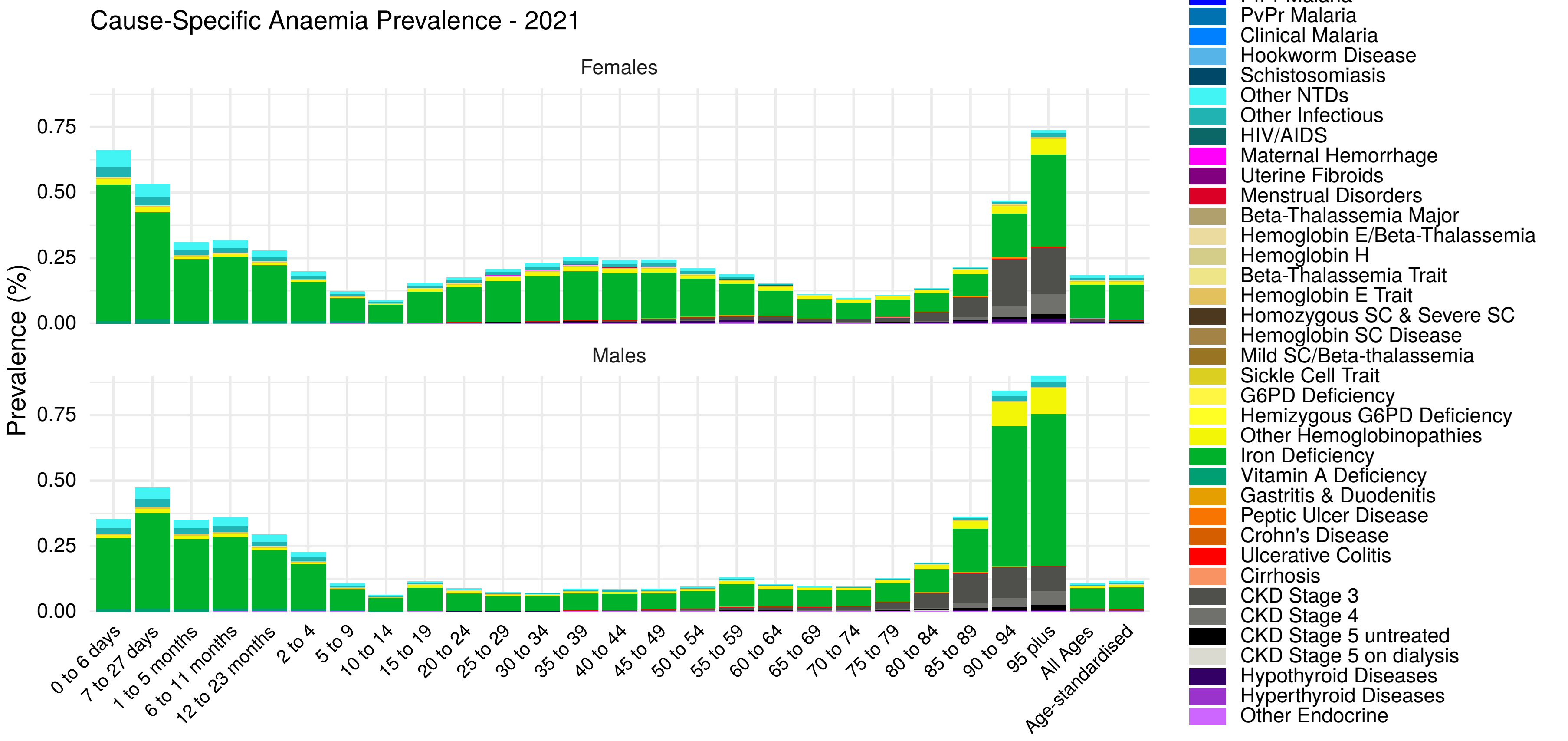

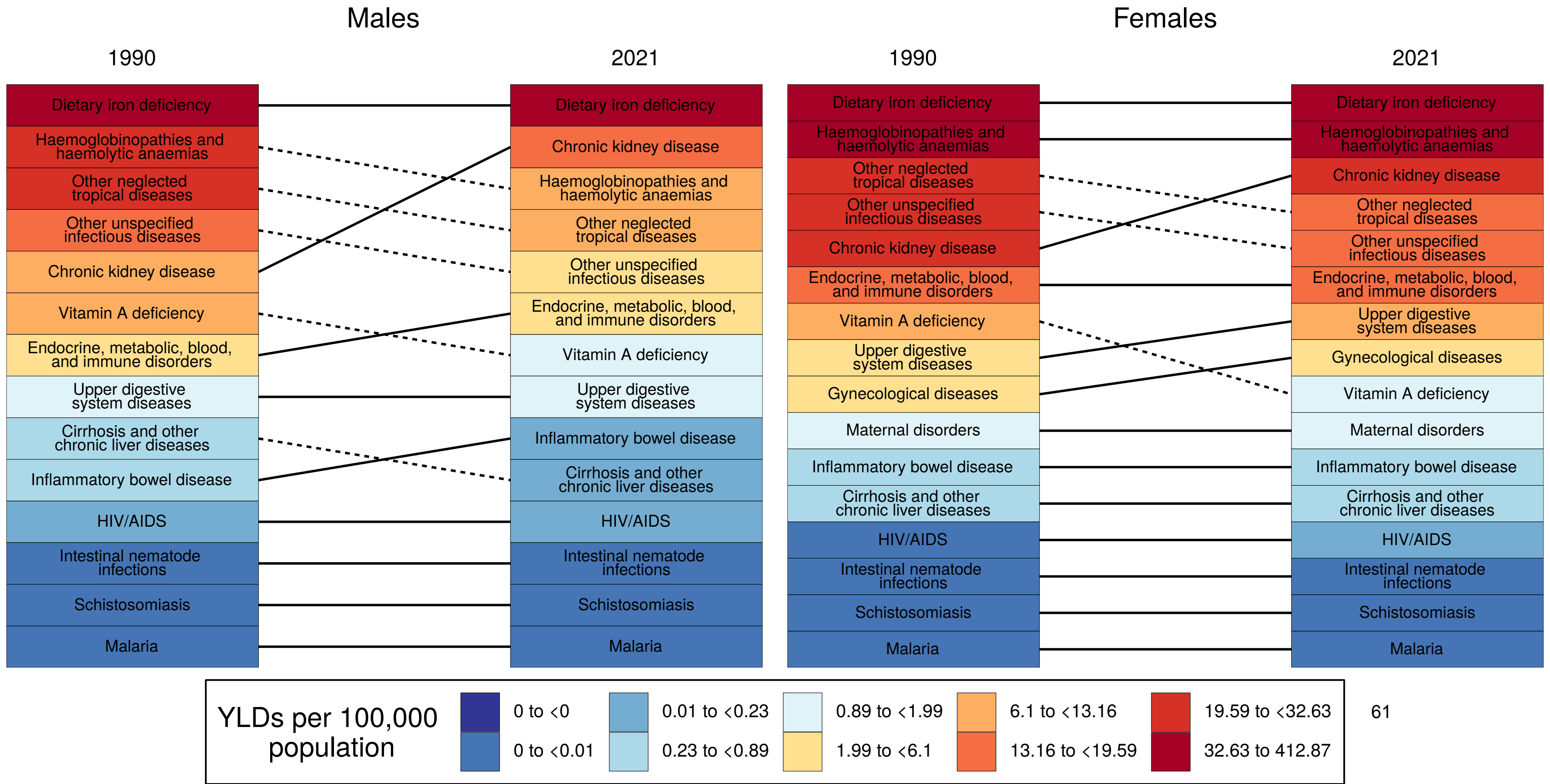

61

Albania

Anaemia Prevalence - Both Sexes

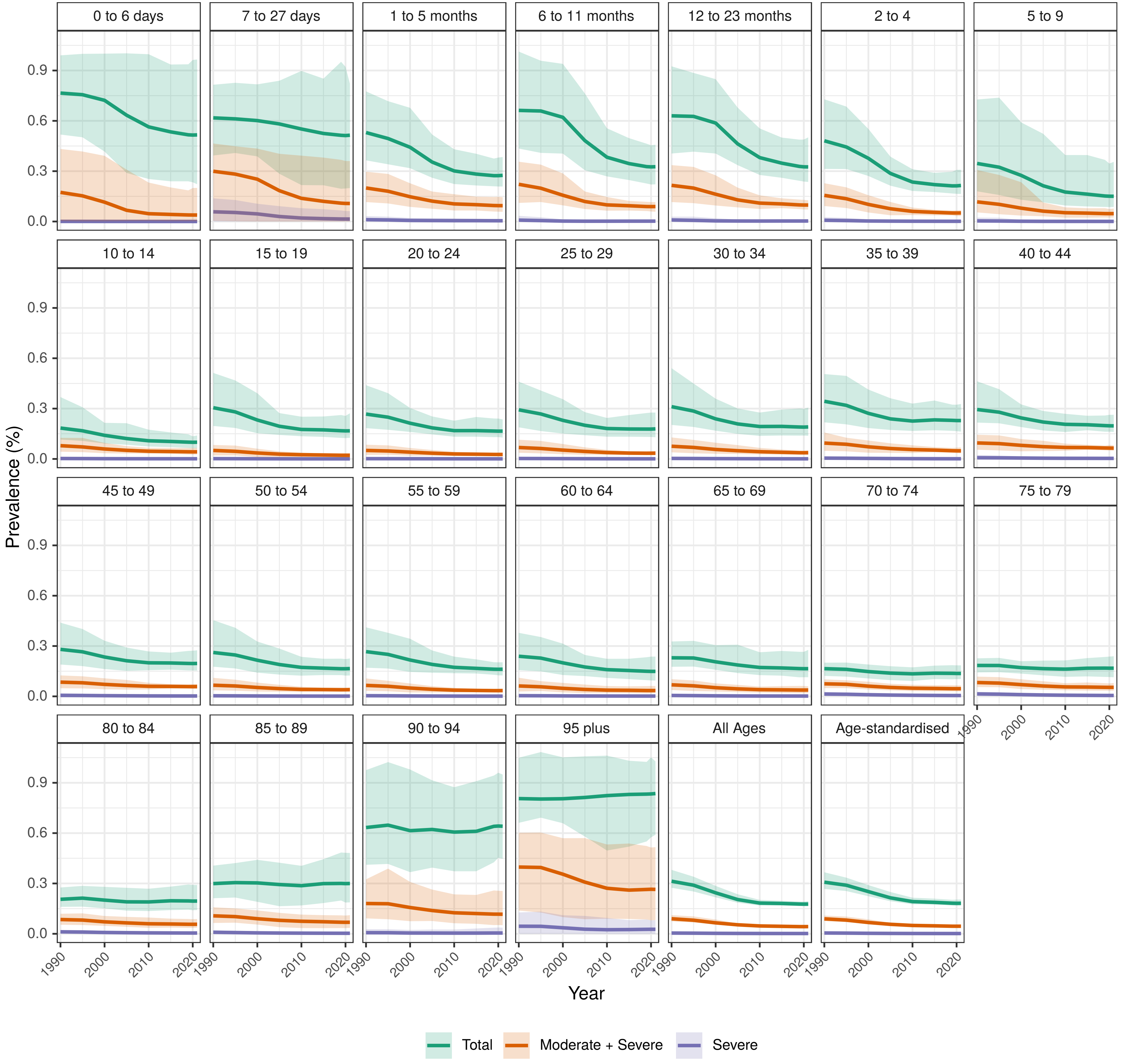

Cause-Specific Anaemia Prevalence - 2021

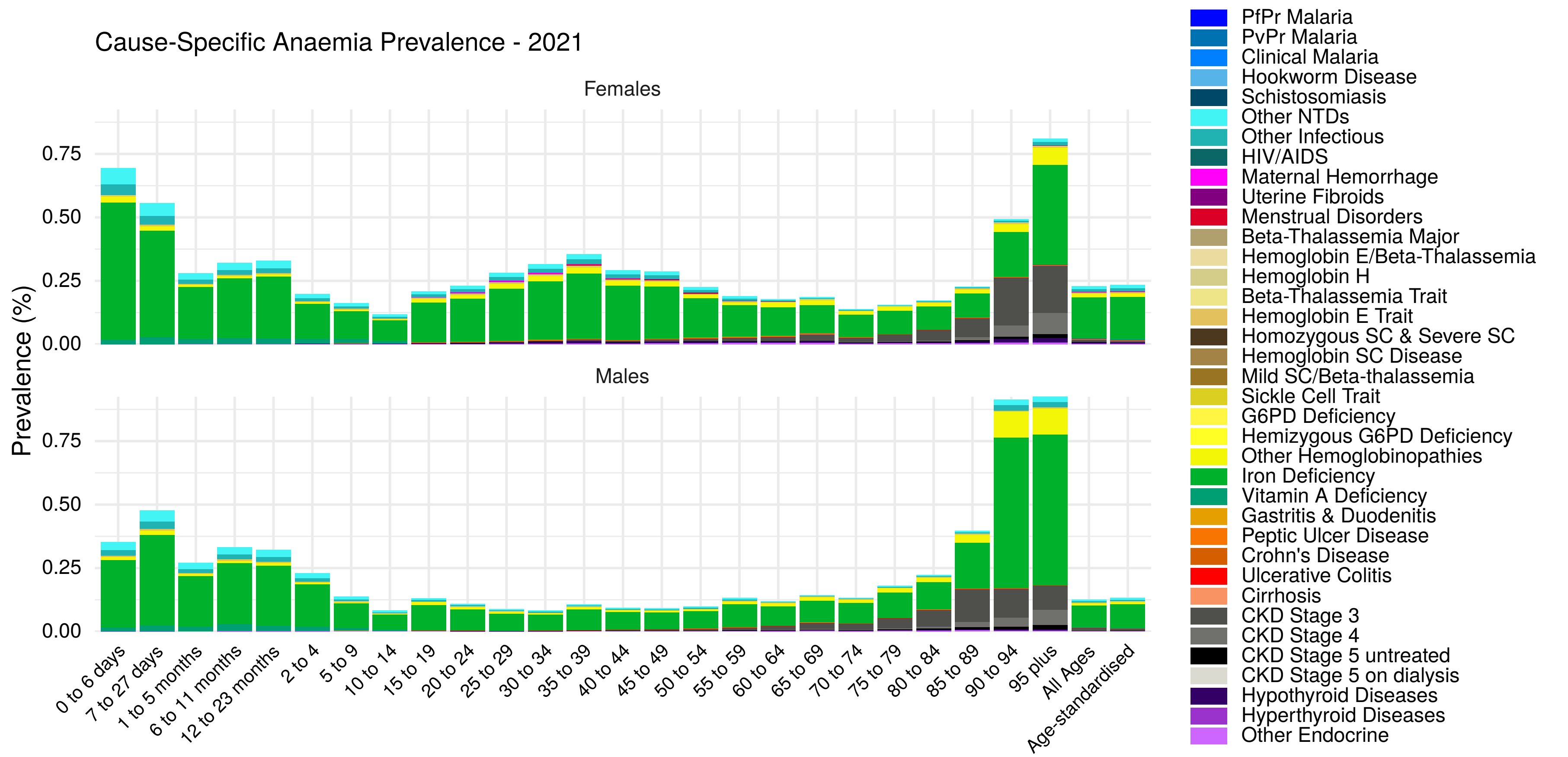

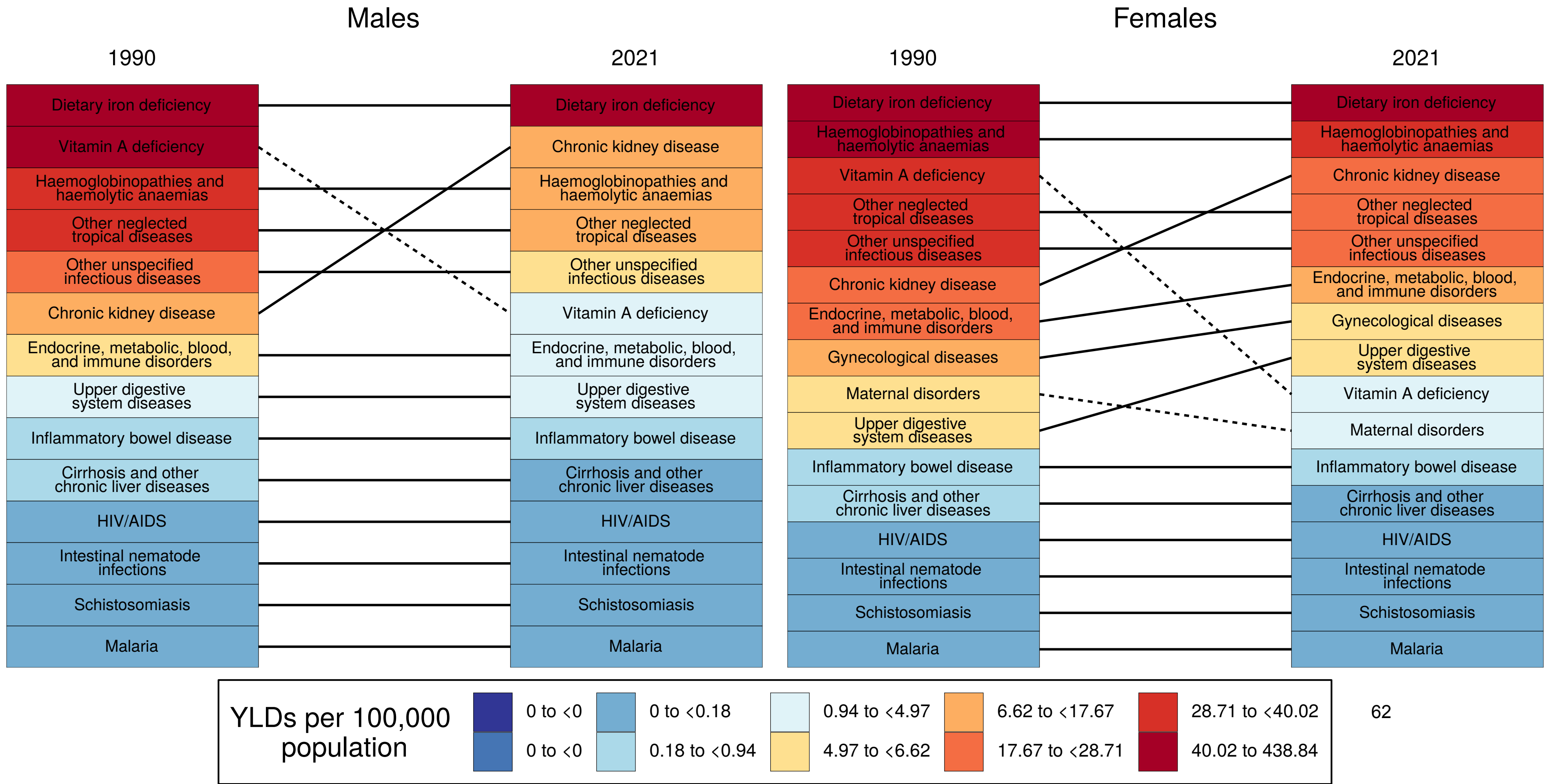

Bosnia and Herzegovina

Anaemia Prevalence - Both Sexes

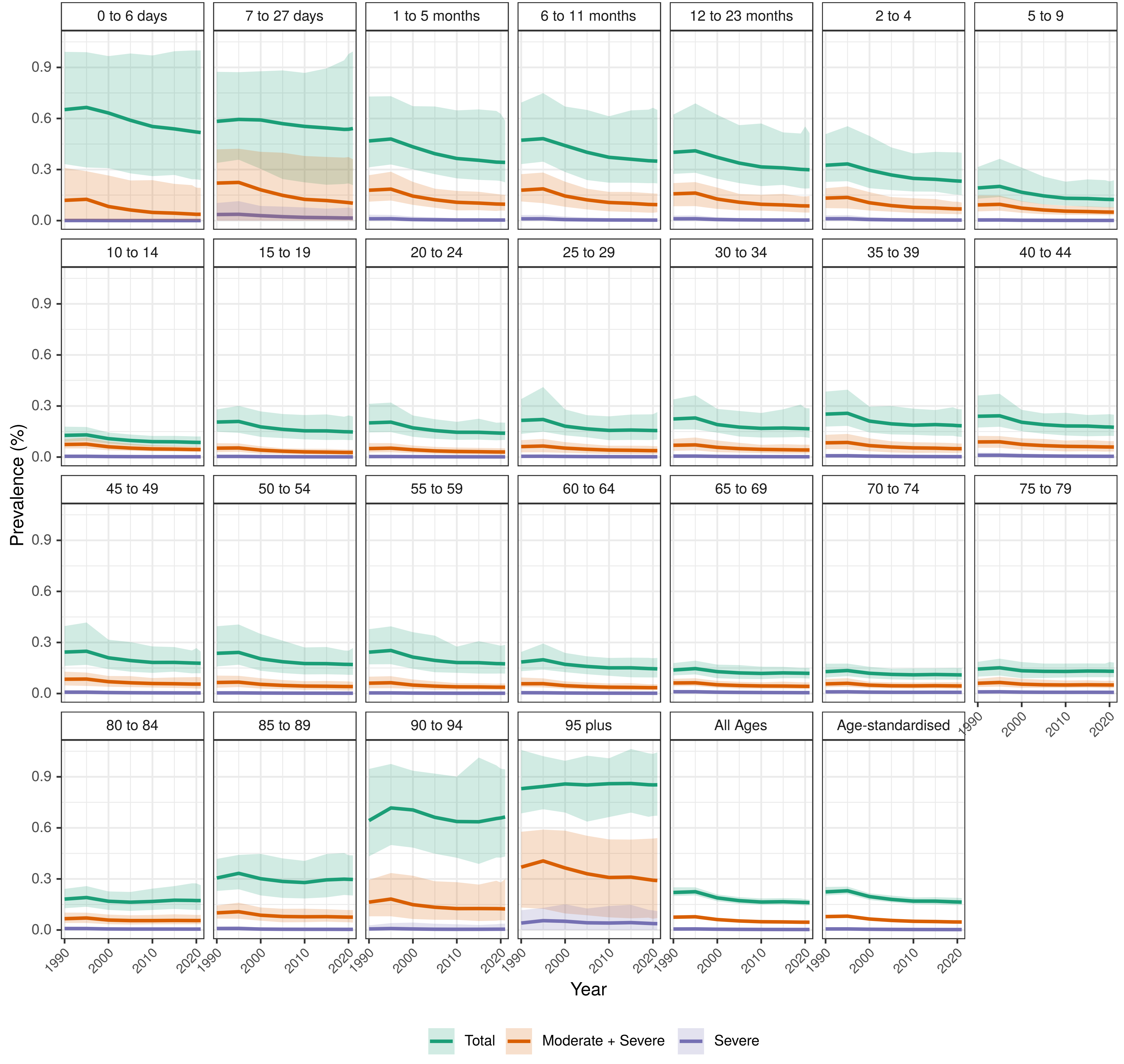

Cause-Specific Anaemia Prevalence - 2021

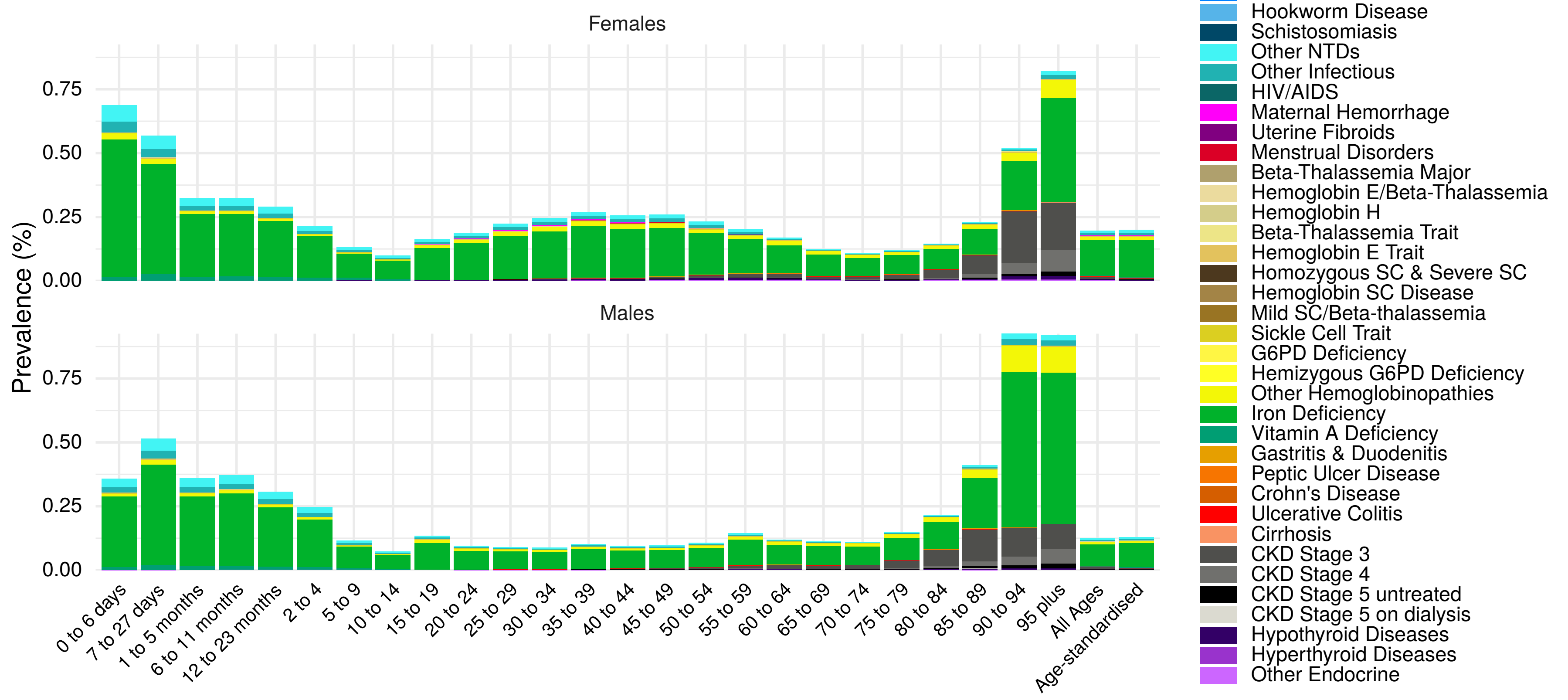

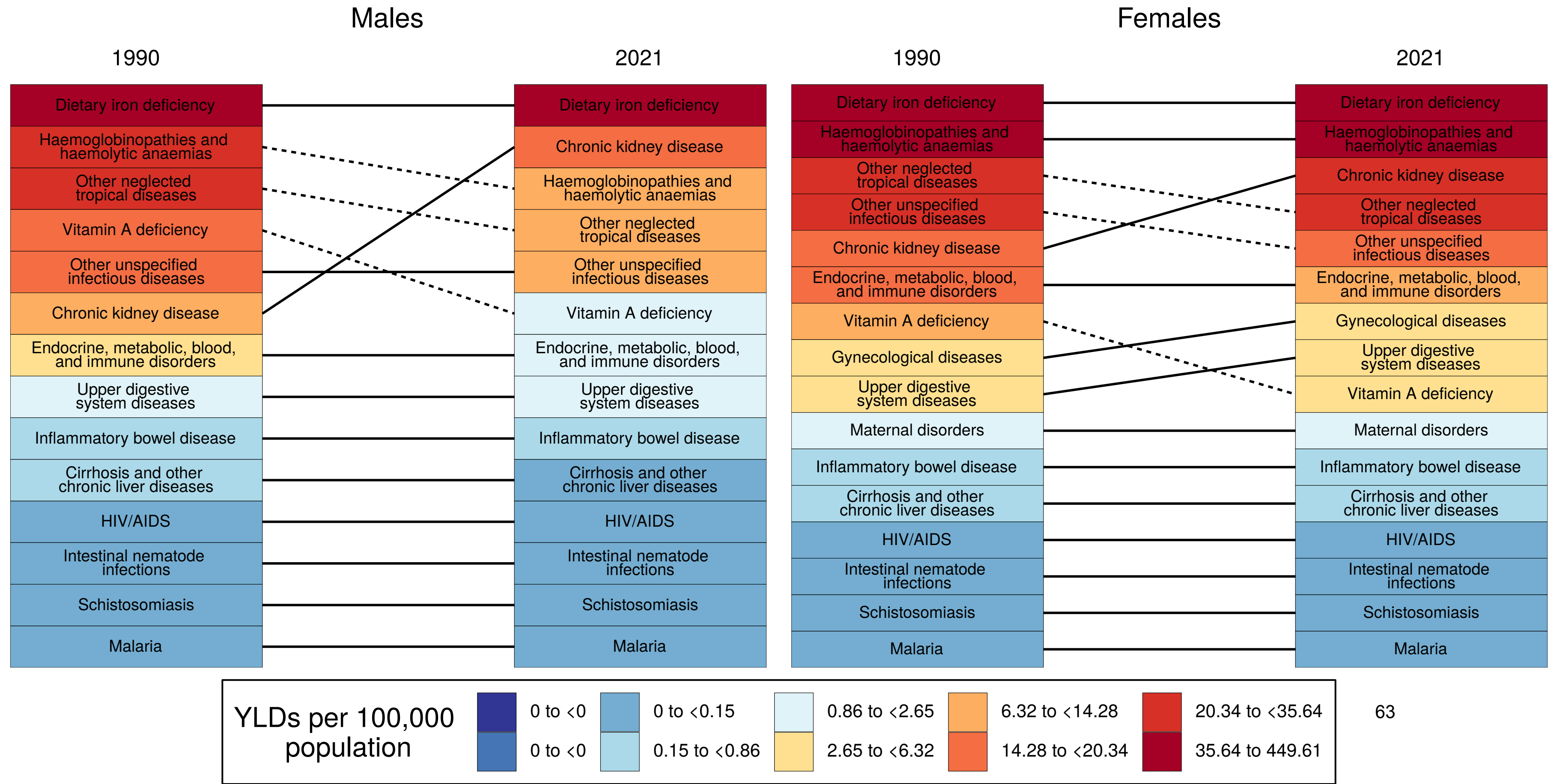

Bulgaria

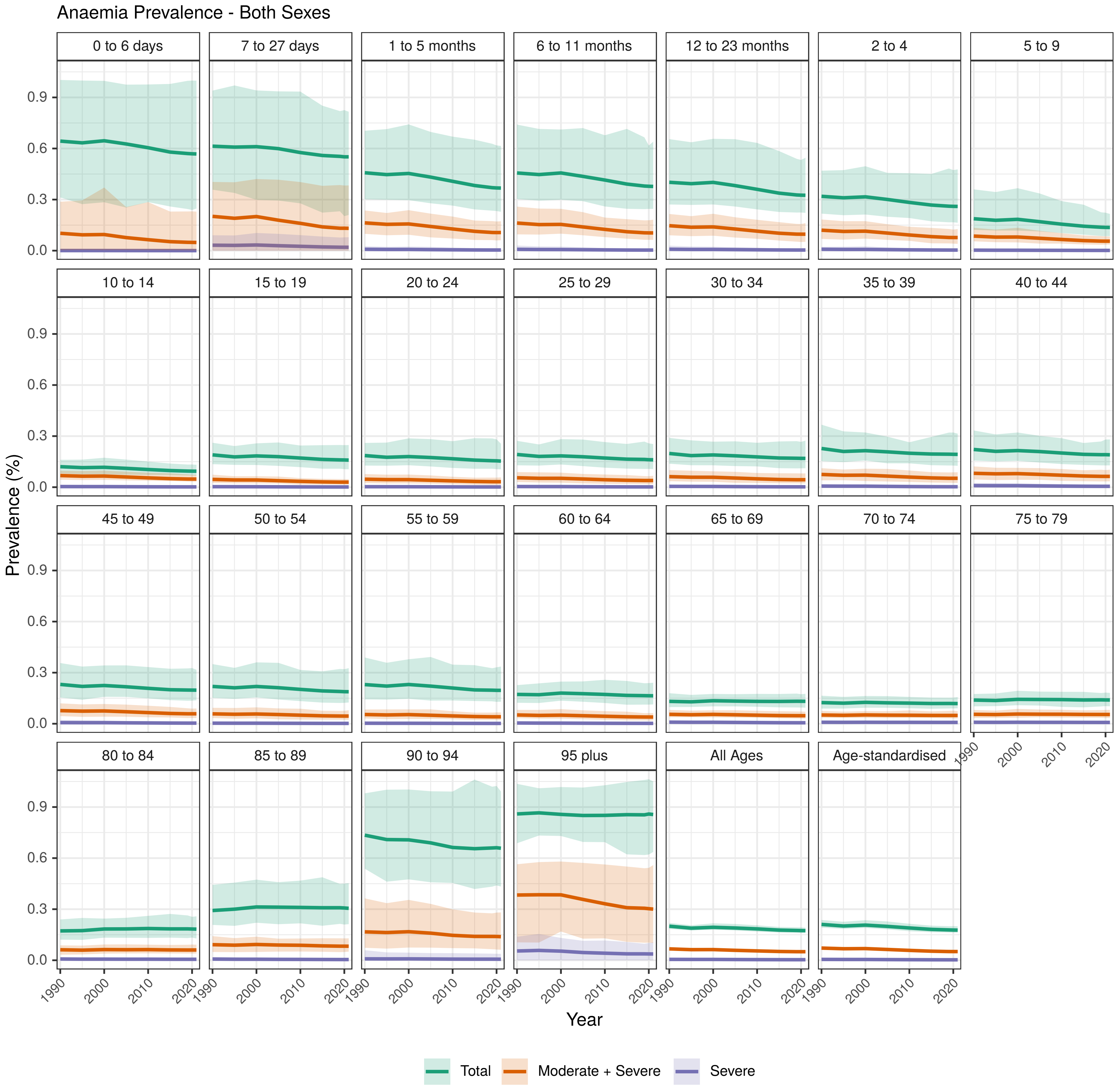

Anaemia Prevalence - Both Sexes
0 to 6 days
7 to 27 days
1 to 5 months
6 to 11 months
12 to 23 months
2 to 4
5 to 9
10 to 14
15 to 19
20 to 24
25 to 29
30 to 34
35 to 39
40 to 44
45 to 49
50 to 54
55 to 59
60 to 64
65 to 69
70 to 74
75 to 79
80 to 84
85 to 89
90 to 94
95 plus
All Ages
Age-standardised
Prevalence (%)
Year
Total
Moderate + Severe
Severe

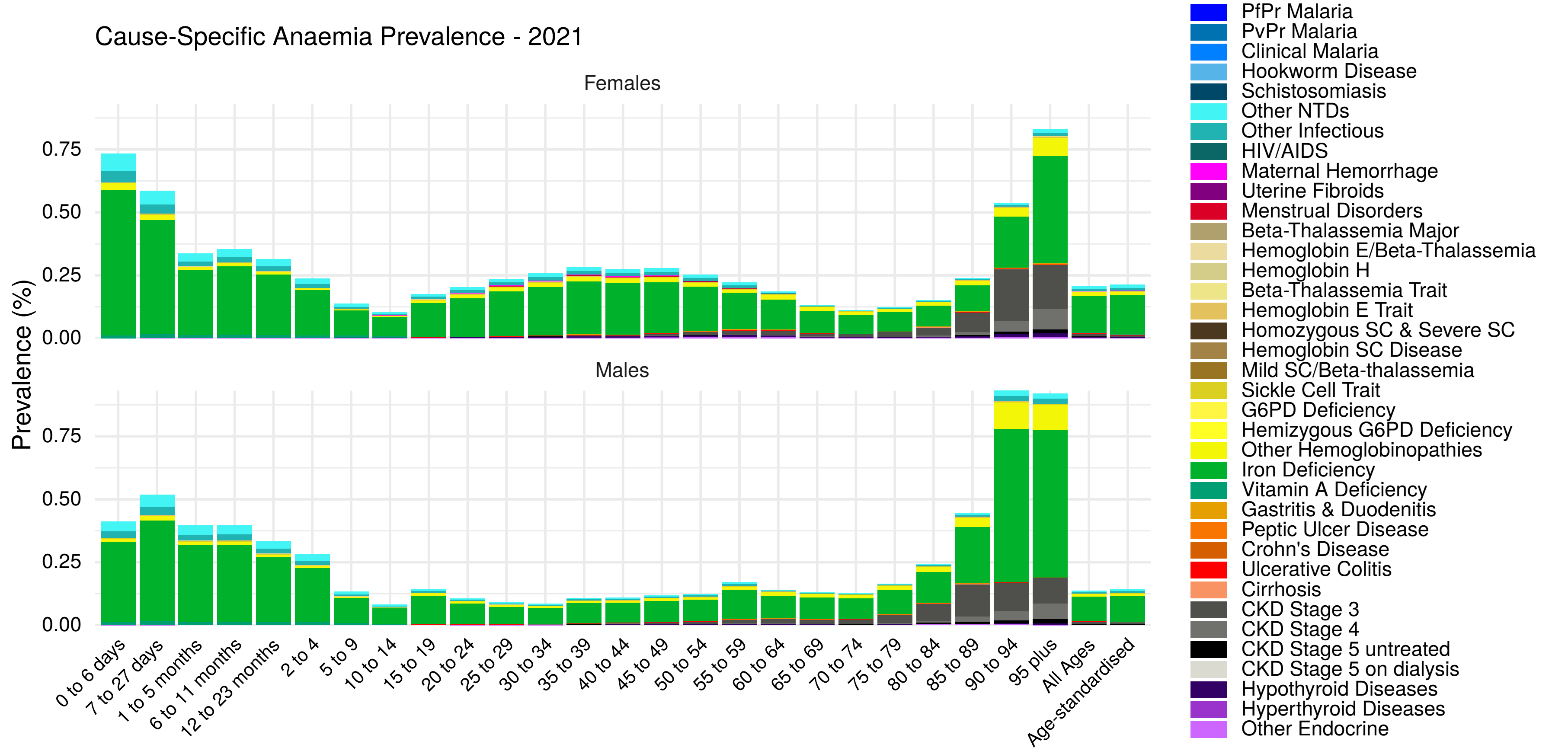

Cause-Specific Anaemia Prevalence - 2021
Females
Males
Prevalence (%)
PfPr Malaria
PvPr Malaria
Clinical Malaria
Hookworm Disease
Schistosomiasis
Other NTDs
Other Infectious
HIV/AIDS
Maternal Hemorrhage
Uterine Fibroids
Menstrual Disorders
Beta-Thalassemia Major
Hemoglobin E/Beta-Thalassemia
Hemoglobin H
Beta-Thalassemia Trait
Hemoglobin E Trait
Homozygous SC & Severe SC
Hemoglobin SC Disease
Mild SC/Beta-thalassemia
Sickle Cell Trait
G6PD Deficiency
Hemizygous G6PD Deficiency
Other Hemoglobinopathies
Iron Deficiency
Vitamin A Deficiency
Gastritis & Duodenitis
Peptic Ulcer Disease
Crohn's Disease
Ulcerative Colitis
Cirrhosis
CKD Stage 3
CKD Stage 4
CKD Stage 5 untreated
CKD Stage 5 on dialysis
Hypothyroid Diseases
Hyperthyroid Diseases
Other Endocrine

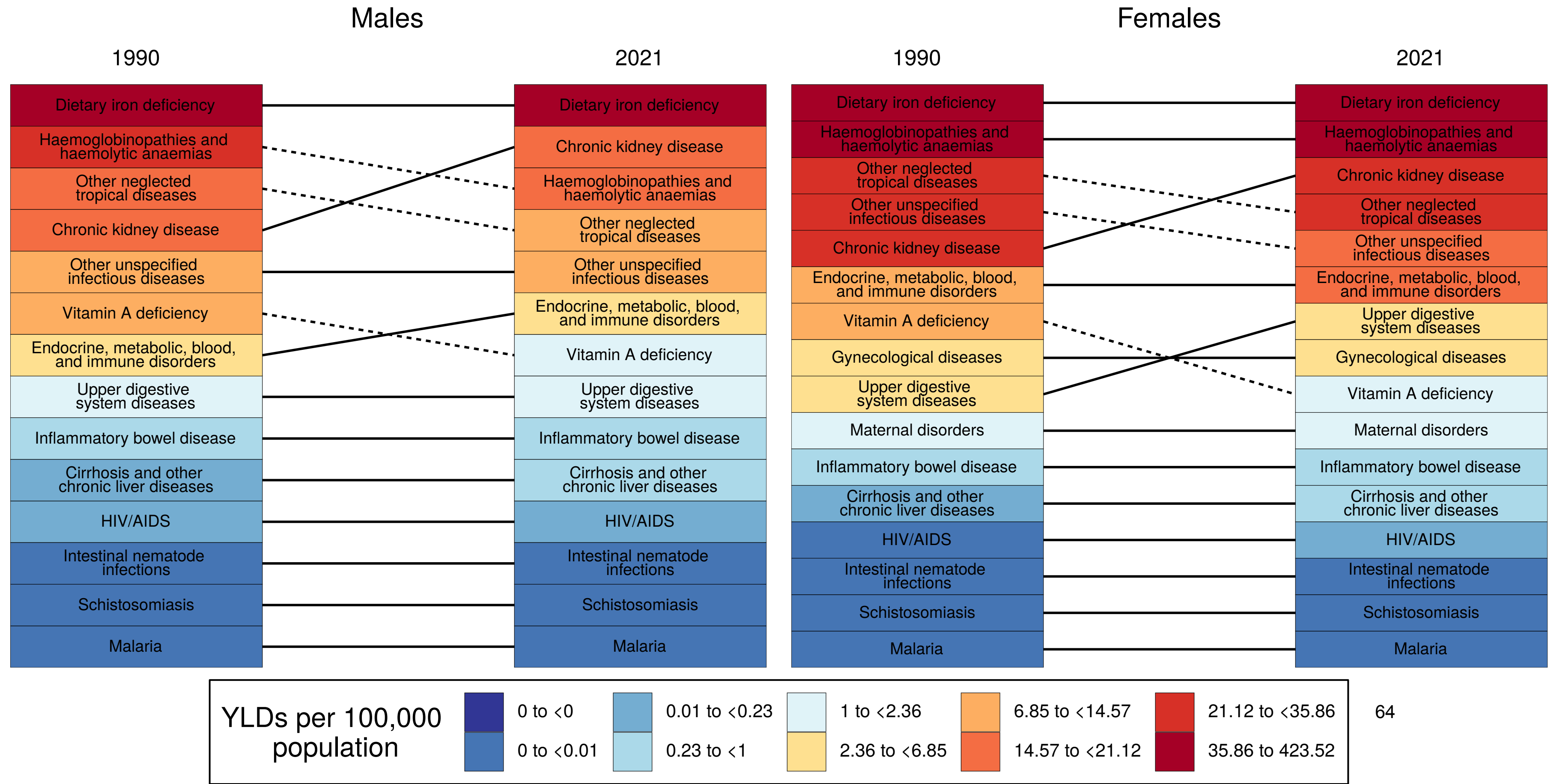

Males
Females
1990
2021
Dietary iron deficiency
Haemoglobinopathies and haemolytic anaemias
Other neglected tropical diseases
Chronic kidney disease
Other unspecified infectious diseases
Vitamin A deficiency
Endocrine, metabolic, blood, and immune disorders
Upper digestive system diseases
Gynecological diseases
Maternal disorders
Inflammatory bowel disease
Cirrhosis and other chronic liver diseases
HIV/AIDS
Intestinal nematode infections
Schistosomiasis
Malaria
YLDs per 100,000 population
0 to <0
0 to <0.01
0.01 to <0.23
0.23 to <1
1 to <2.36
2.36 to <6.85
6.85 to <14.57
14.57 to <21.12
21.12 to <35.86
35.86 to 423.52

Croatia

Anaemia Prevalence - Both Sexes

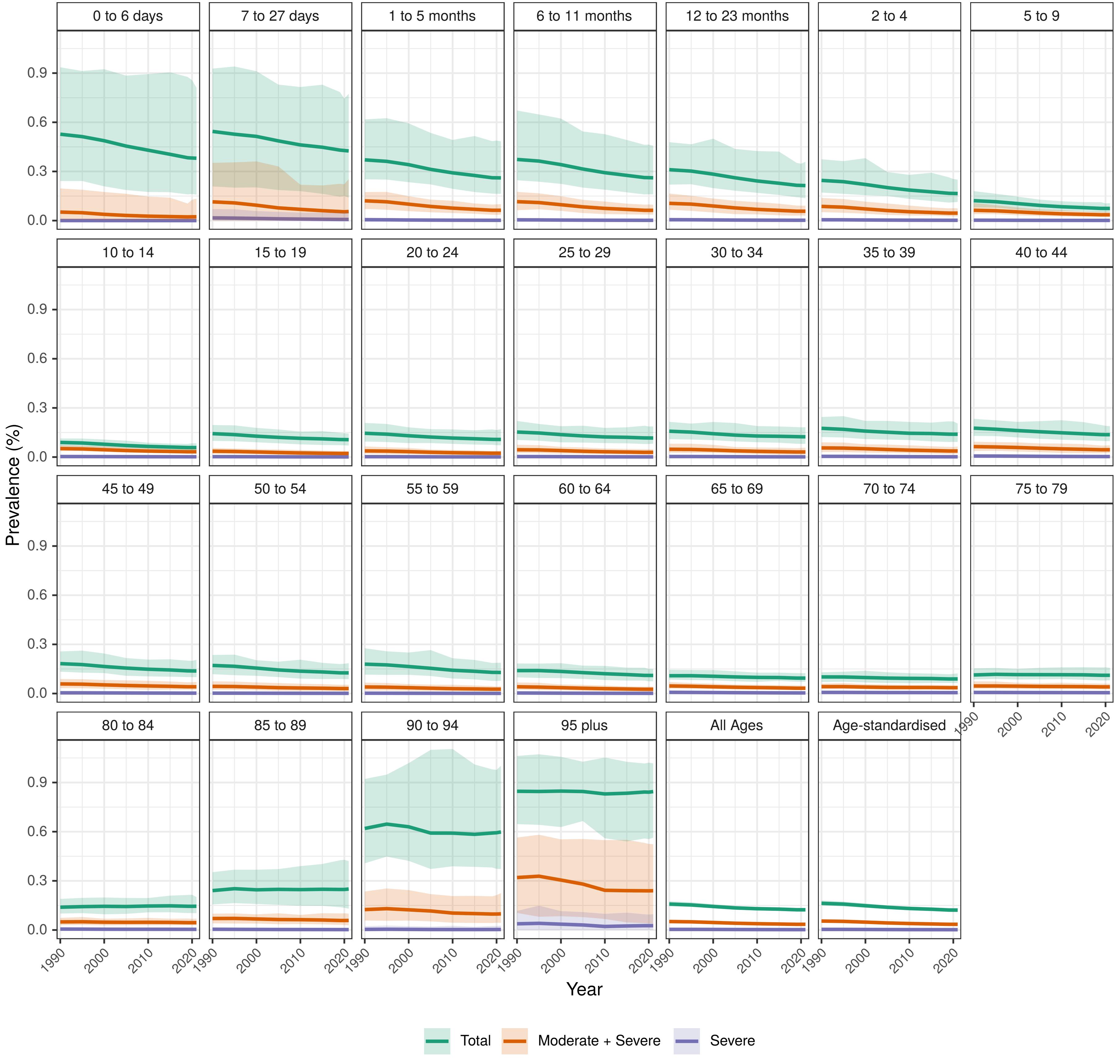

Cause-Specific Anaemia Prevalence - 2021

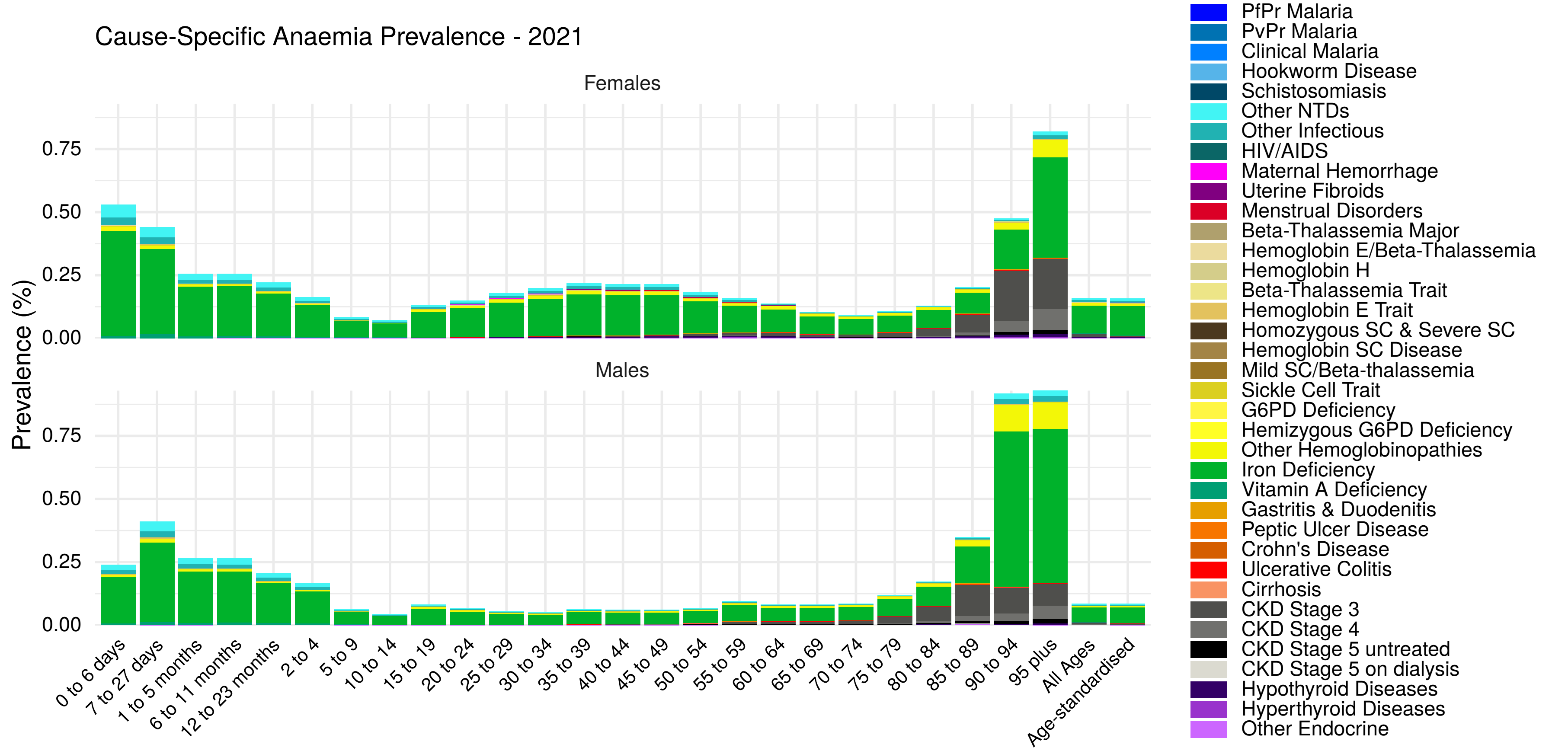

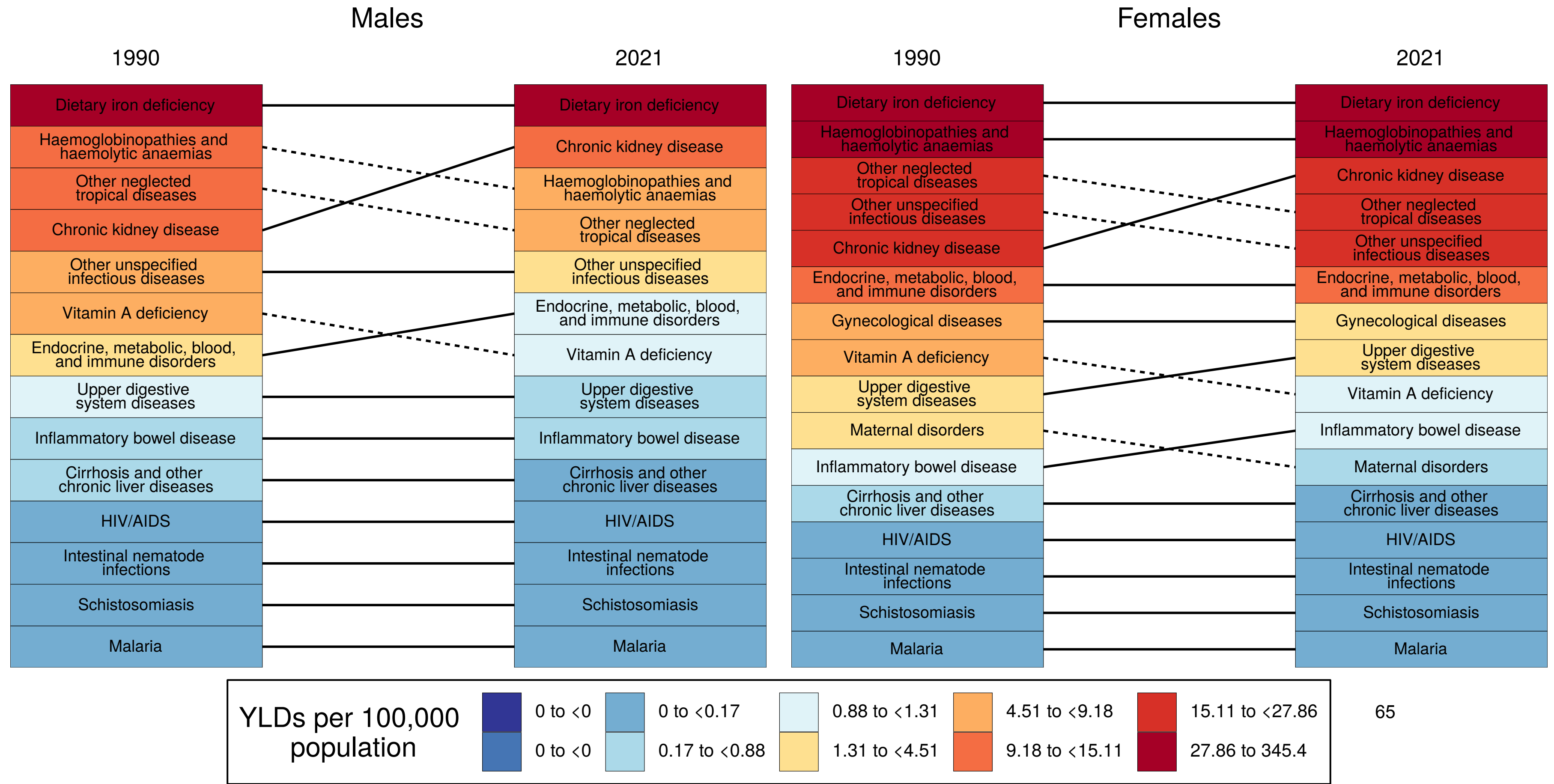

Czechia

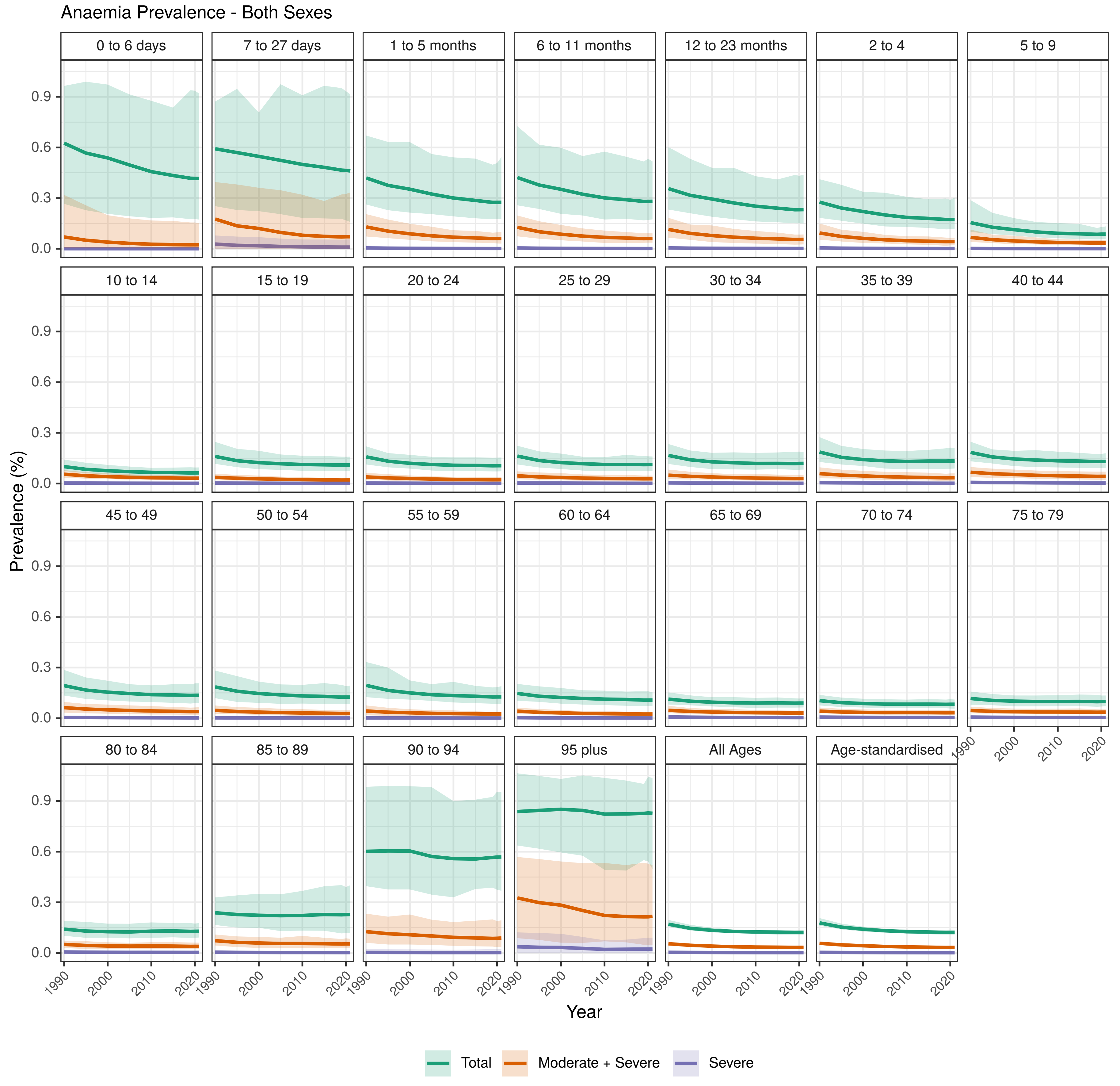

Anaemia Prevalence - Both Sexes
0 to 6 days
7 to 27 days
1 to 5 months
6 to 11 months
12 to 23 months
2 to 4
5 to 9
10 to 14
15 to 19
20 to 24
25 to 29
30 to 34
35 to 39
40 to 44
45 to 49
50 to 54
55 to 59
60 to 64
65 to 69
70 to 74
75 to 79
80 to 84
85 to 89
90 to 94
95 plus
All Ages
Age-standardised
Prevalence (%)
Year
Total
Moderate + Severe
Severe

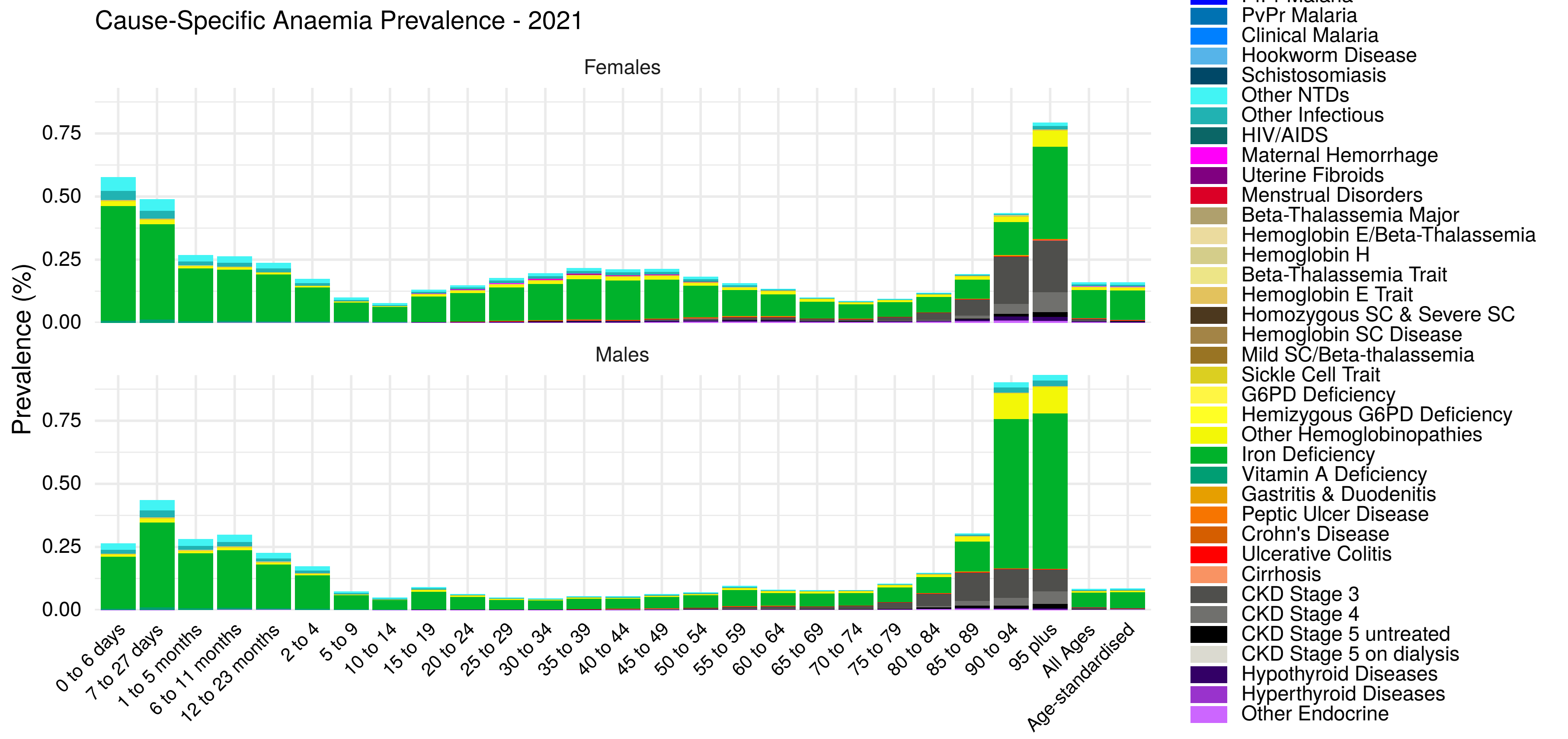

Cause-Specific Anaemia Prevalence - 2021
Females
Males
Prevalence (%)
PfPr Malaria
PvPr Malaria
Clinical Malaria
Hookworm Disease
Schistosomiasis
Other NTDs
Other Infectious
HIV/AIDS
Maternal Hemorrhage
Uterine Fibroids
Menstrual Disorders
Beta-Thalassemia Major
Hemoglobin E/Beta-Thalassemia
Hemoglobin H
Beta-Thalassemia Trait
Hemoglobin E Trait
Homozygous SC & Severe SC
Hemoglobin SC Disease
Mild SC/Beta-thalassemia
Sickle Cell Trait
G6PD Deficiency
Hemizygous G6PD Deficiency
Other Hemoglobinopathies
Iron Deficiency
Vitamin A Deficiency
Gastritis & Duodenitis
Peptic Ulcer Disease
Crohn's Disease
Ulcerative Colitis
Cirrhosis
CKD Stage 3
CKD Stage 4
CKD Stage 5 untreated
CKD Stage 5 on dialysis
Hypothyroid Diseases
Hyperthyroid Diseases
Other Endocrine

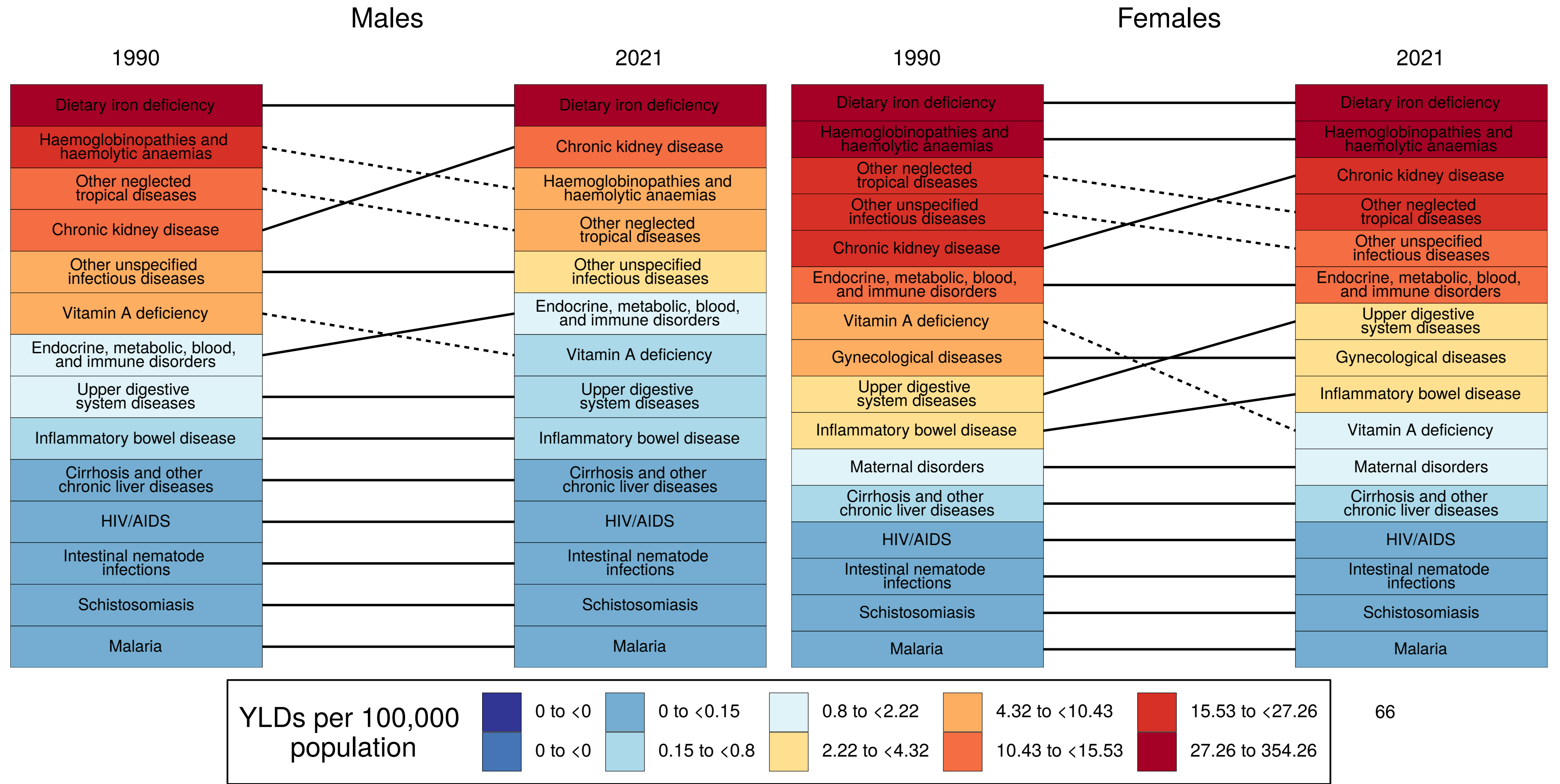

Males
Females
1990
2021
Dietary iron deficiency
Haemoglobinopathies and haemolytic anaemias
Other neglected tropical diseases
Chronic kidney disease
Other unspecified infectious diseases
Vitamin A deficiency
Endocrine, metabolic, blood, and immune disorders
Upper digestive system diseases
Inflammatory bowel disease
Cirrhosis and other chronic liver diseases
HIV/AIDS
Intestinal nematode infections
Schistosomiasis
Malaria
Gynecological diseases
Maternal disorders
YLDs per 100,000 population
0 to <0
0 to <0.15
0.15 to <0.8
0.8 to <2.22
2.22 to <4.32
4.32 to <10.43
10.43 to <15.53
15.53 to <27.26
27.26 to 354.26

Hungary

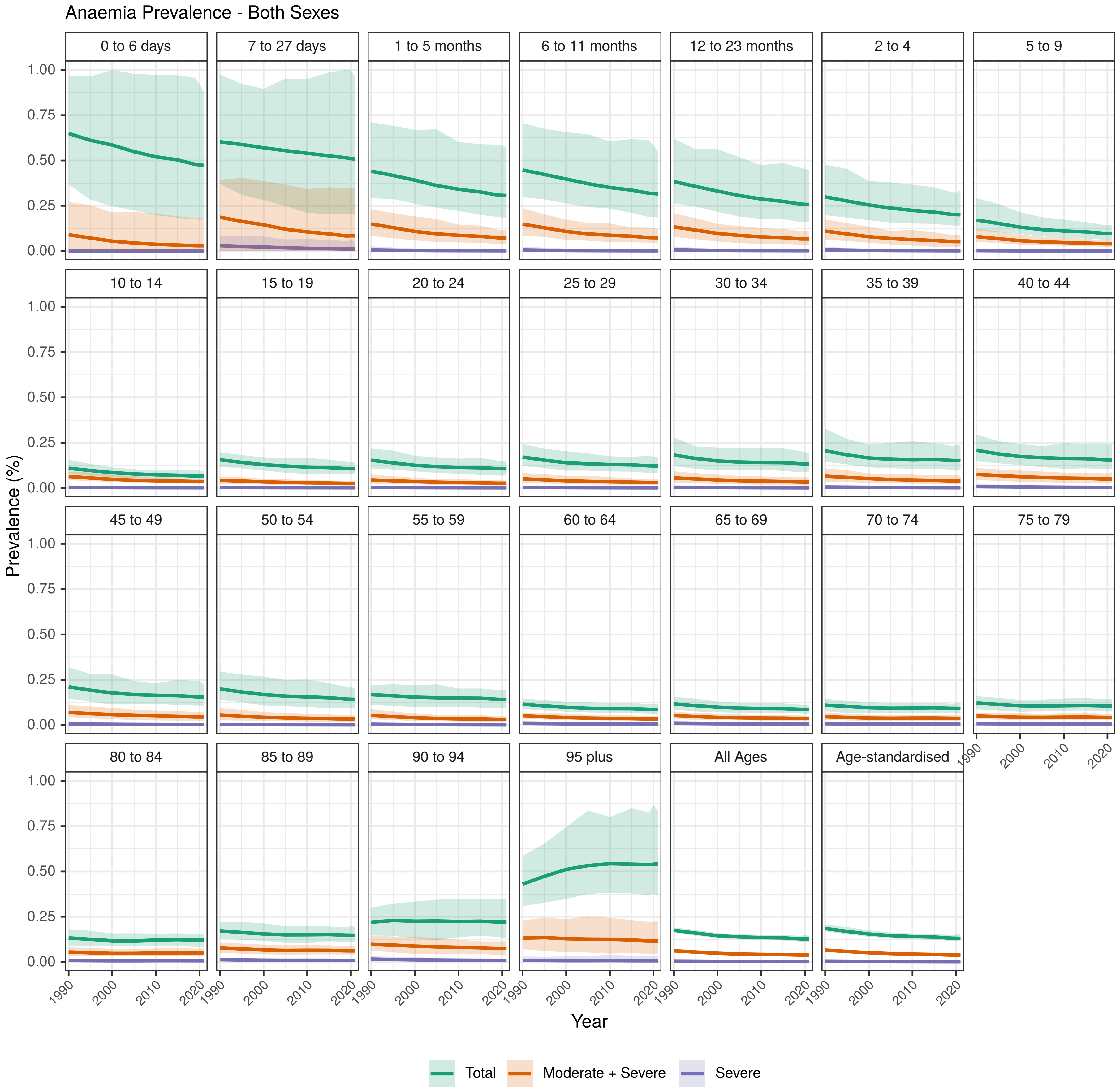

Anaemia Prevalence - Both Sexes
0 to 6 days
7 to 27 days
1 to 5 months
6 to 11 months
12 to 23 months
2 to 4
5 to 9
10 to 14
15 to 19
20 to 24
25 to 29
30 to 34
35 to 39
40 to 44
45 to 49
50 to 54
55 to 59
60 to 64
65 to 69
70 to 74
75 to 79
80 to 84
85 to 89
90 to 94
95 plus
All Ages
Age-standardised
Prevalence (%)
Year
Total
Moderate + Severe
Severe

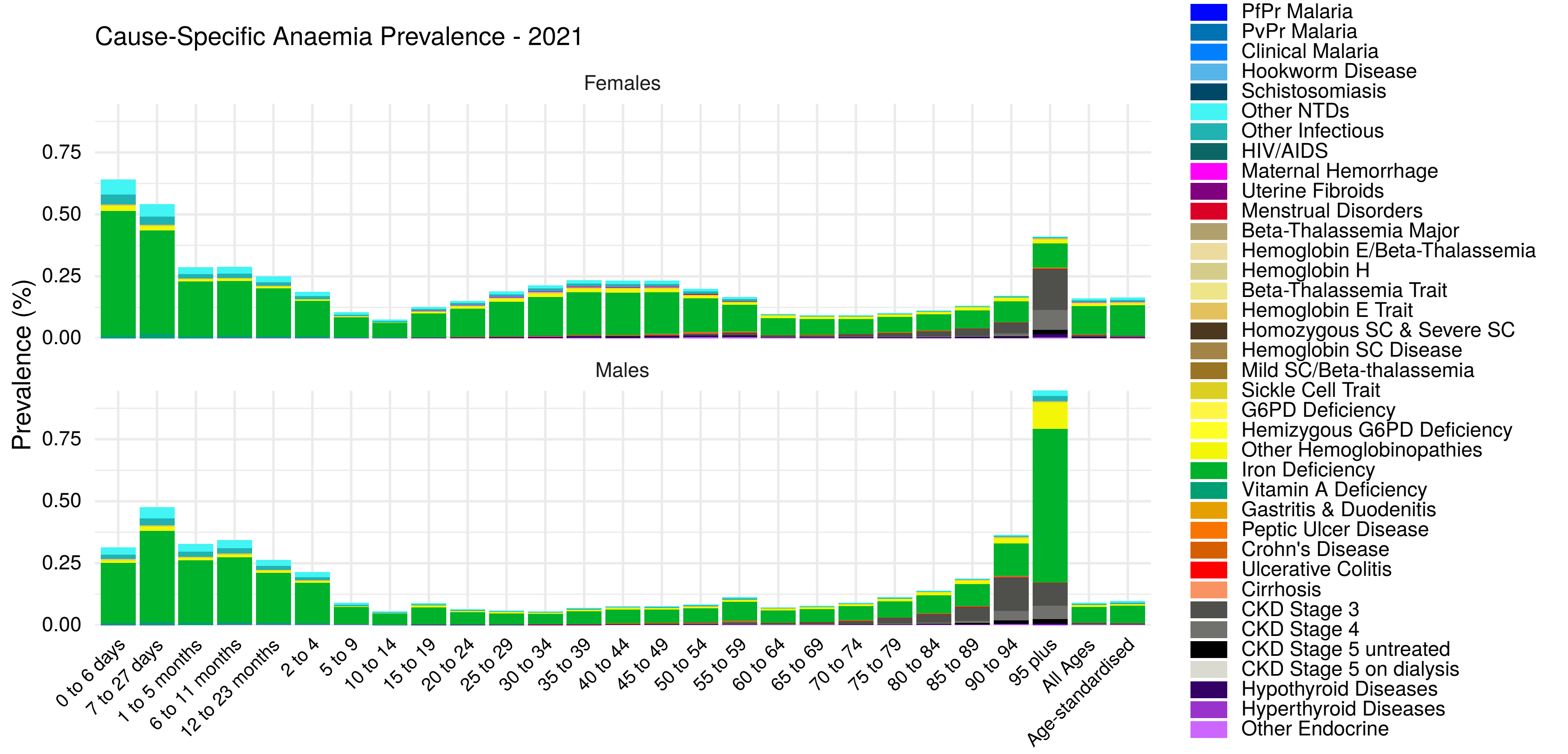

Cause-Specific Anaemia Prevalence - 2021
Females
Males
Prevalence (%)
PfPr Malaria
PvPr Malaria
Clinical Malaria
Hookworm Disease
Schistosomiasis
Other NTDs
Other Infectious
HIV/AIDS
Maternal Hemorrhage
Uterine Fibroids
Menstrual Disorders
Beta-Thalassemia Major
Hemoglobin E/Beta-Thalassemia
Hemoglobin H
Beta-Thalassemia Trait
Hemoglobin E Trait
Homozygous SC & Severe SC
Hemoglobin SC Disease
Mild SC/Beta-thalassemia
Sickle Cell Trait
G6PD Deficiency
Hemizygous G6PD Deficiency
Other Hemoglobinopathies
Iron Deficiency
Vitamin A Deficiency
Gastritis & Duodenitis
Peptic Ulcer Disease
Crohn's Disease
Ulcerative Colitis
Cirrhosis
CKD Stage 3
CKD Stage 4
CKD Stage 5 untreated
CKD Stage 5 on dialysis
Hypothyroid Diseases
Hyperthyroid Diseases
Other Endocrine

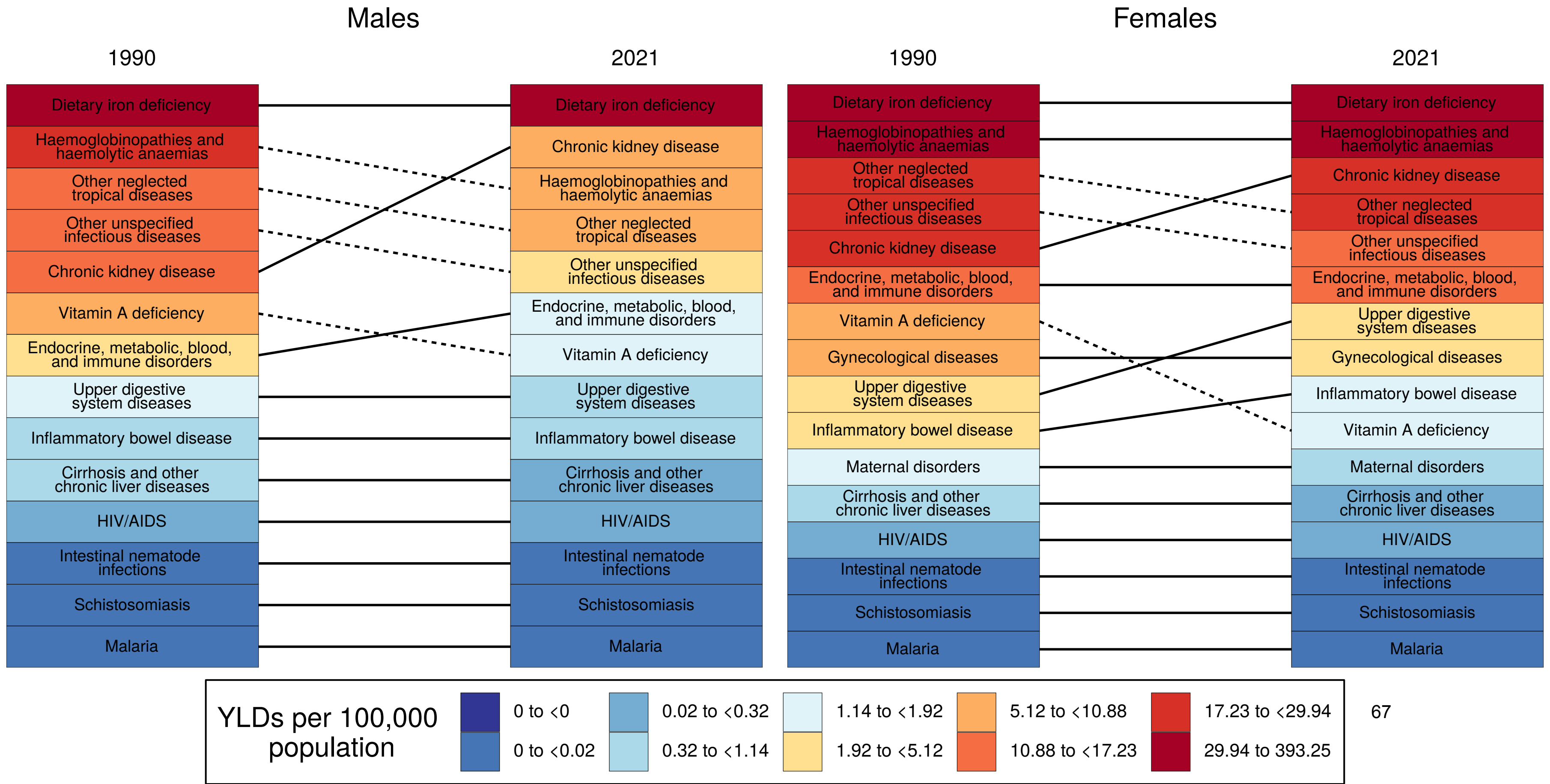

Males
Females
1990
2021
Dietary iron deficiency
Haemoglobinopathies and haemolytic anaemias
Other neglected tropical diseases
Other unspecified infectious diseases
Chronic kidney disease
Vitamin A deficiency
Endocrine, metabolic, blood, and immune disorders
Upper digestive system diseases
Inflammatory bowel disease
Cirrhosis and other chronic liver diseases
HIV/AIDS
Intestinal nematode infections
Schistosomiasis
Malaria
Gynecological diseases
Maternal disorders
YLDs per 100,000 population
0 to <0
0 to <0.02
0.02 to <0.32
0.32 to <1.14
1.14 to <1.92
1.92 to <5.12
5.12 to <10.88
10.88 to <17.23
17.23 to <29.94
29.94 to 393.25

Montenegro

Anaemia Prevalence - Both Sexes

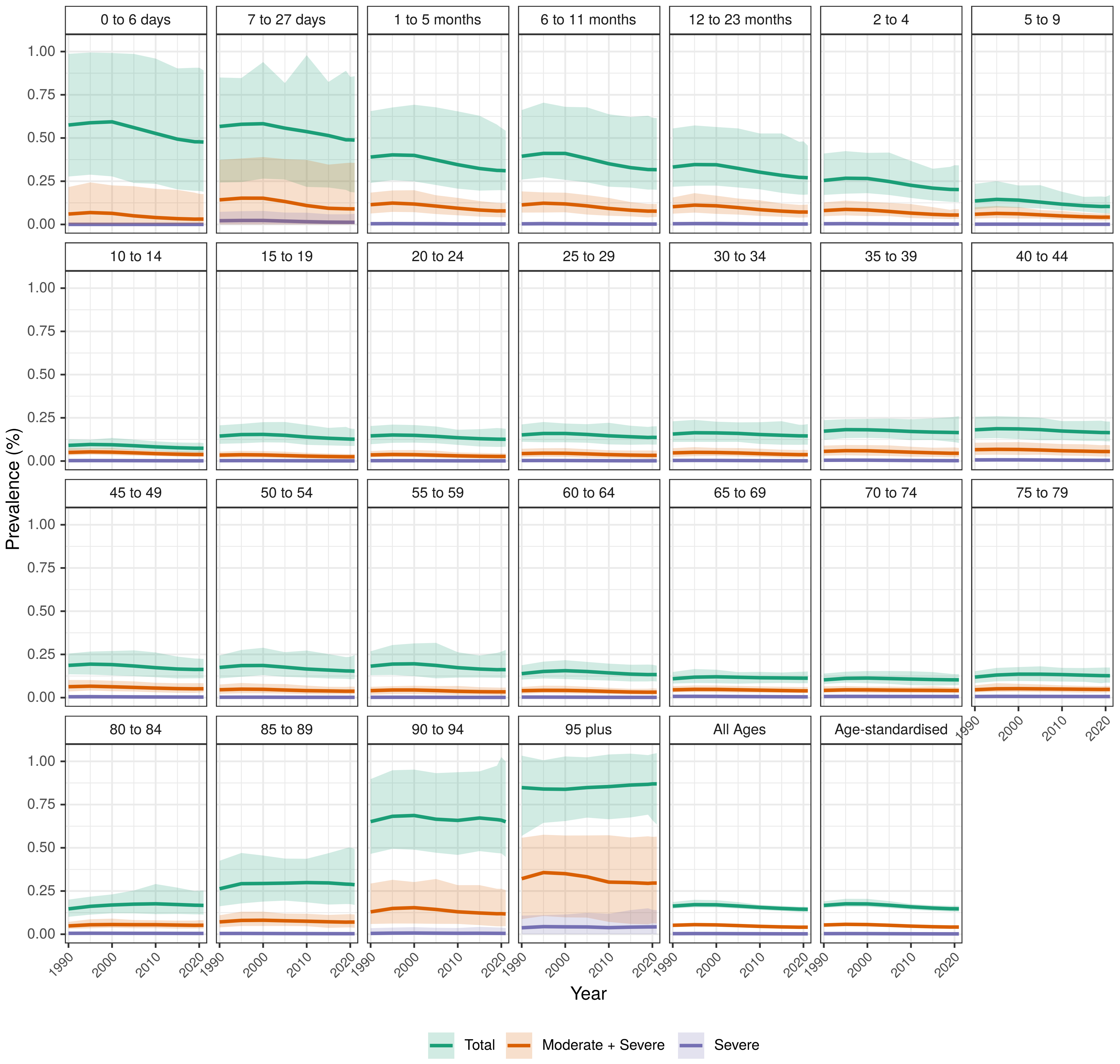

Cause-Specific Anaemia Prevalence - 2021

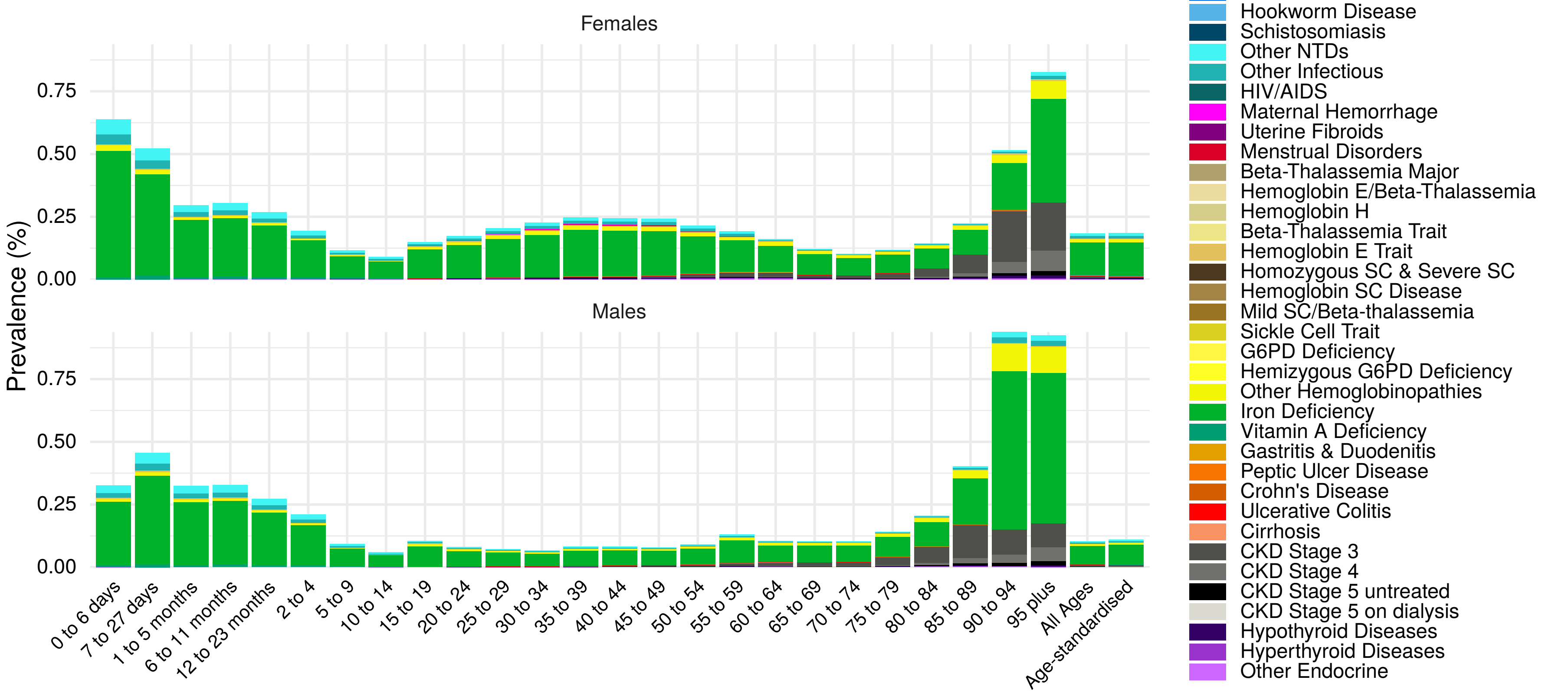

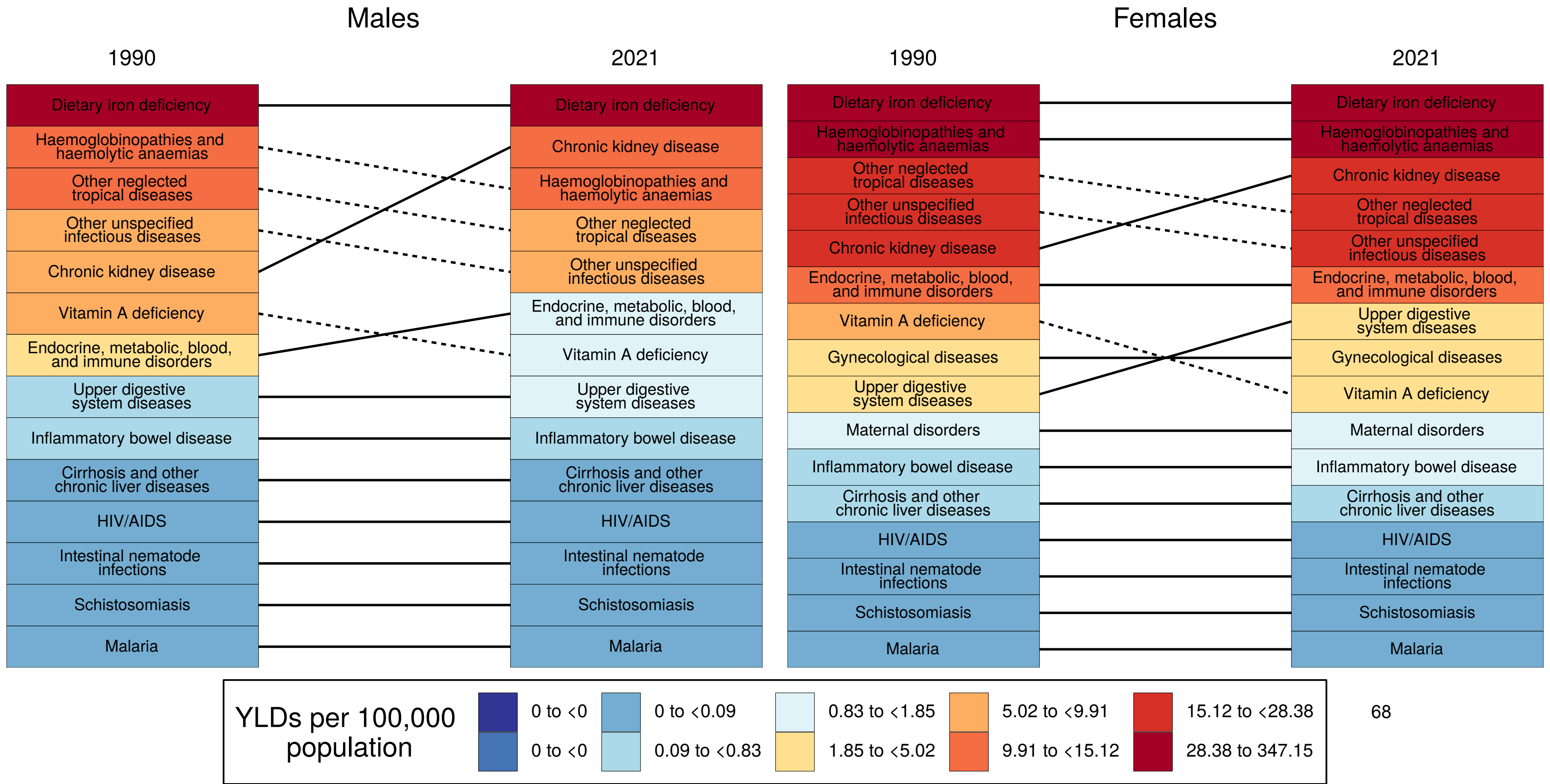

North Macedonia

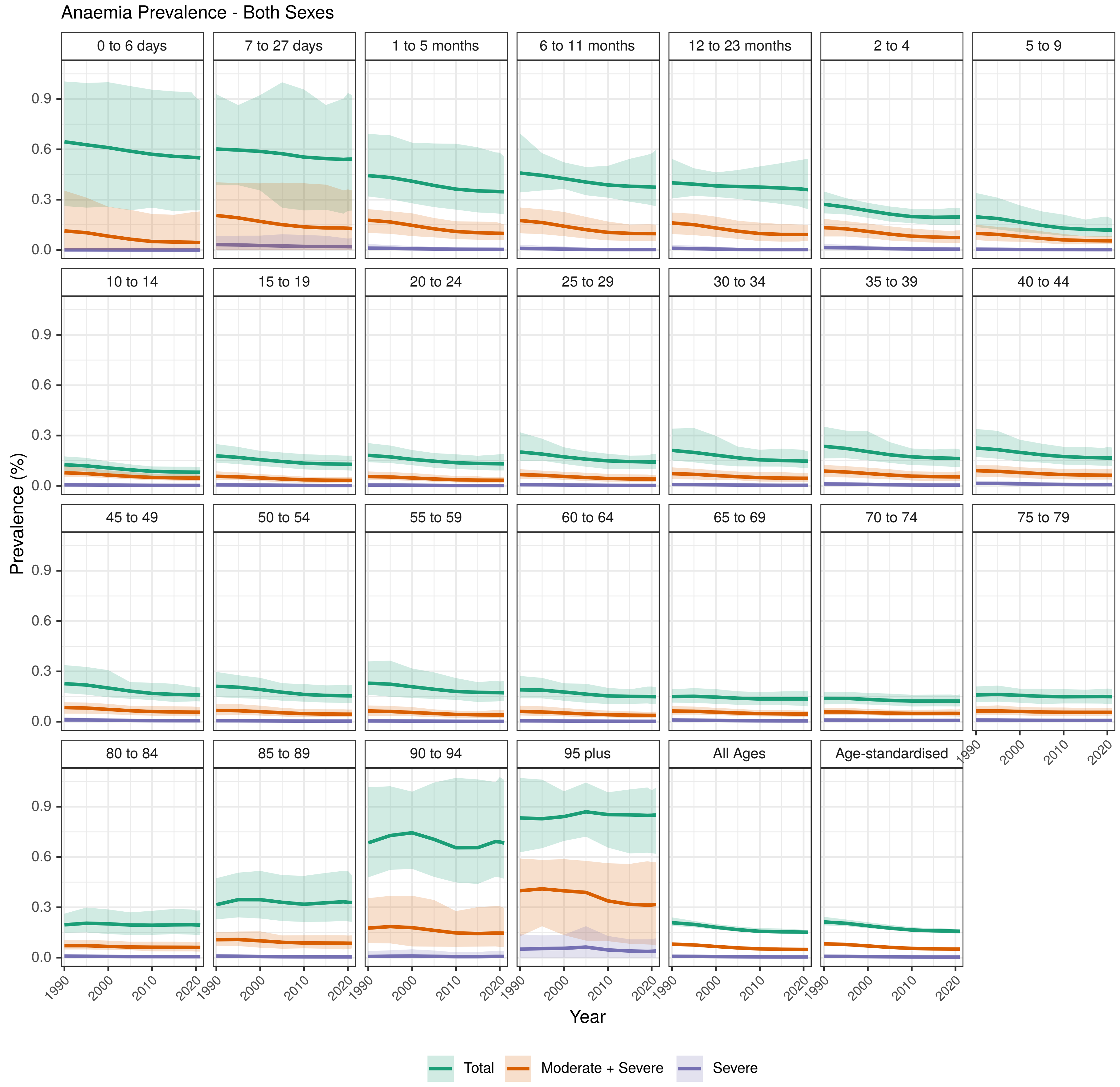

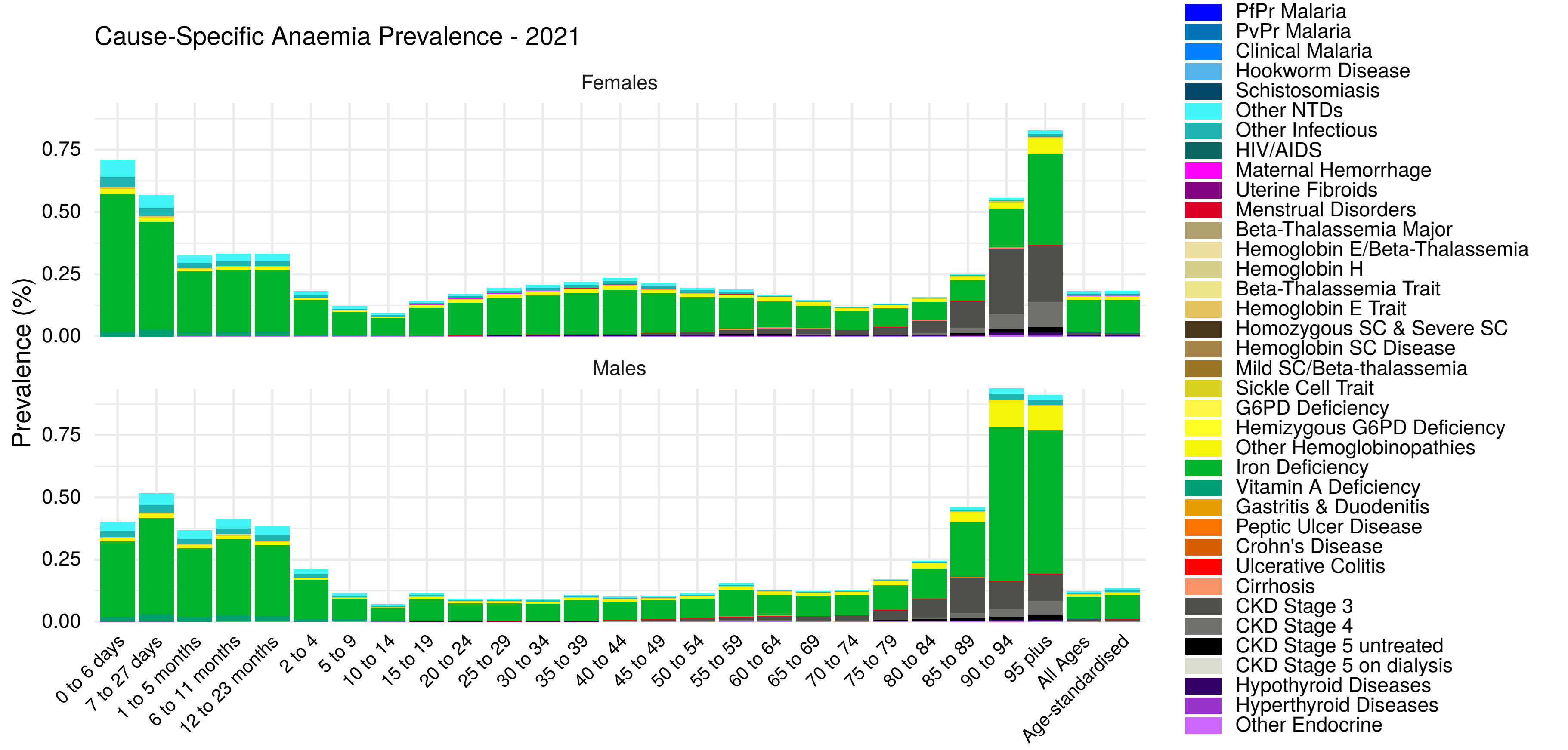

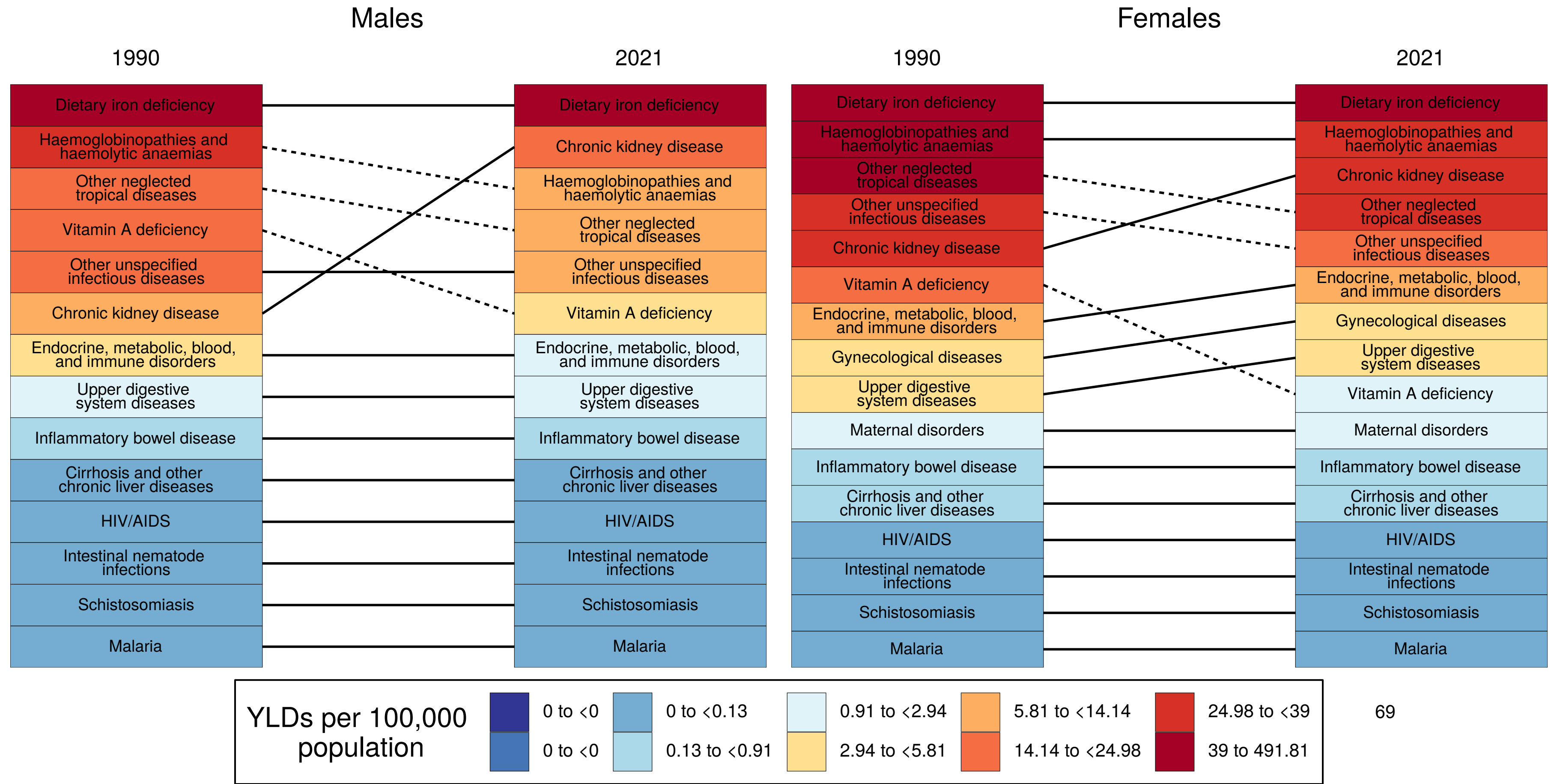

Poland

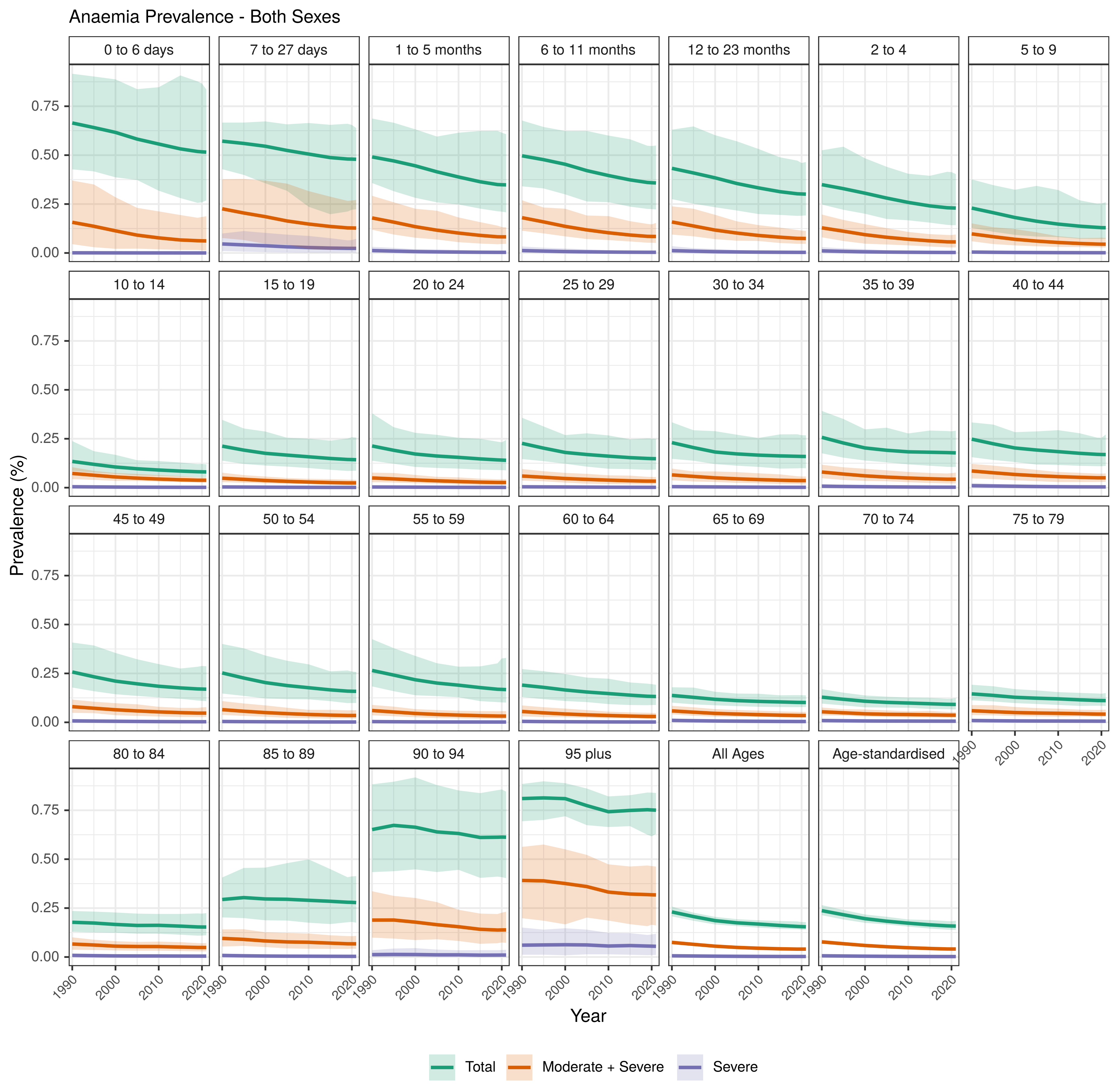

Anaemia Prevalence - Both Sexes
0 to 6 days
7 to 27 days
1 to 5 months
6 to 11 months
12 to 23 months
2 to 4
5 to 9
10 to 14
15 to 19
20 to 24
25 to 29
30 to 34
35 to 39
40 to 44
45 to 49
50 to 54
55 to 59
60 to 64
65 to 69
70 to 74
75 to 79
80 to 84
85 to 89
90 to 94
95 plus
All Ages
Age-standardised
Prevalence (%)
Year
Total
Moderate + Severe
Severe

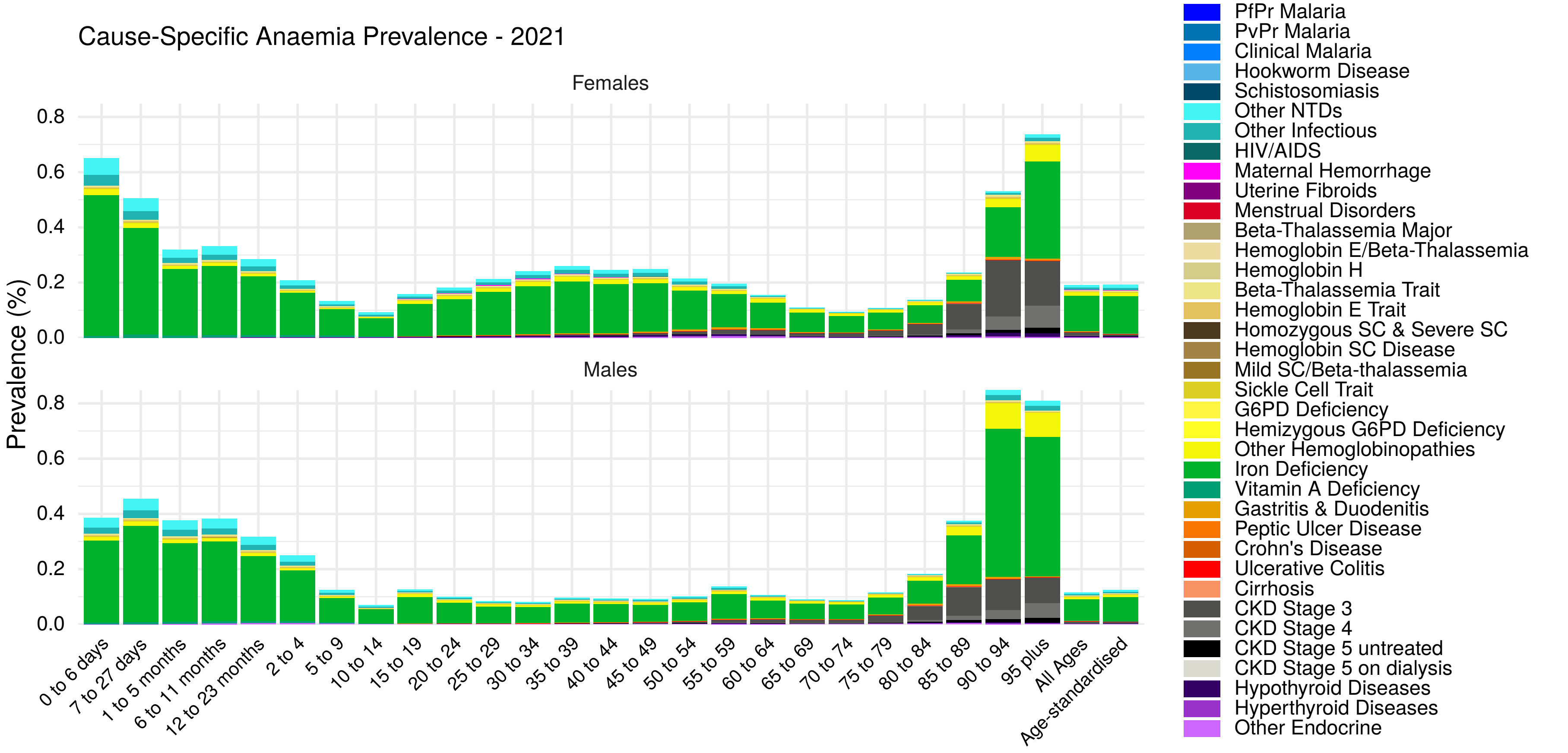

Cause-Specific Anaemia Prevalence - 2021
Females
Males
Prevalence (%)
PfPr Malaria
PvPr Malaria
Clinical Malaria
Hookworm Disease
Schistosomiasis
Other NTDs
Other Infectious
HIV/AIDS
Maternal Hemorrhage
Uterine Fibroids
Menstrual Disorders
Beta-Thalassemia Major
Hemoglobin E/Beta-Thalassemia
Hemoglobin H
Beta-Thalassemia Trait
Hemoglobin E Trait
Homozygous SC & Severe SC
Hemoglobin SC Disease
Mild SC/Beta-thalassemia
Sickle Cell Trait
G6PD Deficiency
Hemizygous G6PD Deficiency
Other Hemoglobinopathies
Iron Deficiency
Vitamin A Deficiency
Gastritis & Duodenitis
Peptic Ulcer Disease
Crohn's Disease
Ulcerative Colitis
Cirrhosis
CKD Stage 3
CKD Stage 4
CKD Stage 5 untreated
CKD Stage 5 on dialysis
Hypothyroid Diseases
Hyperthyroid Diseases
Other Endocrine

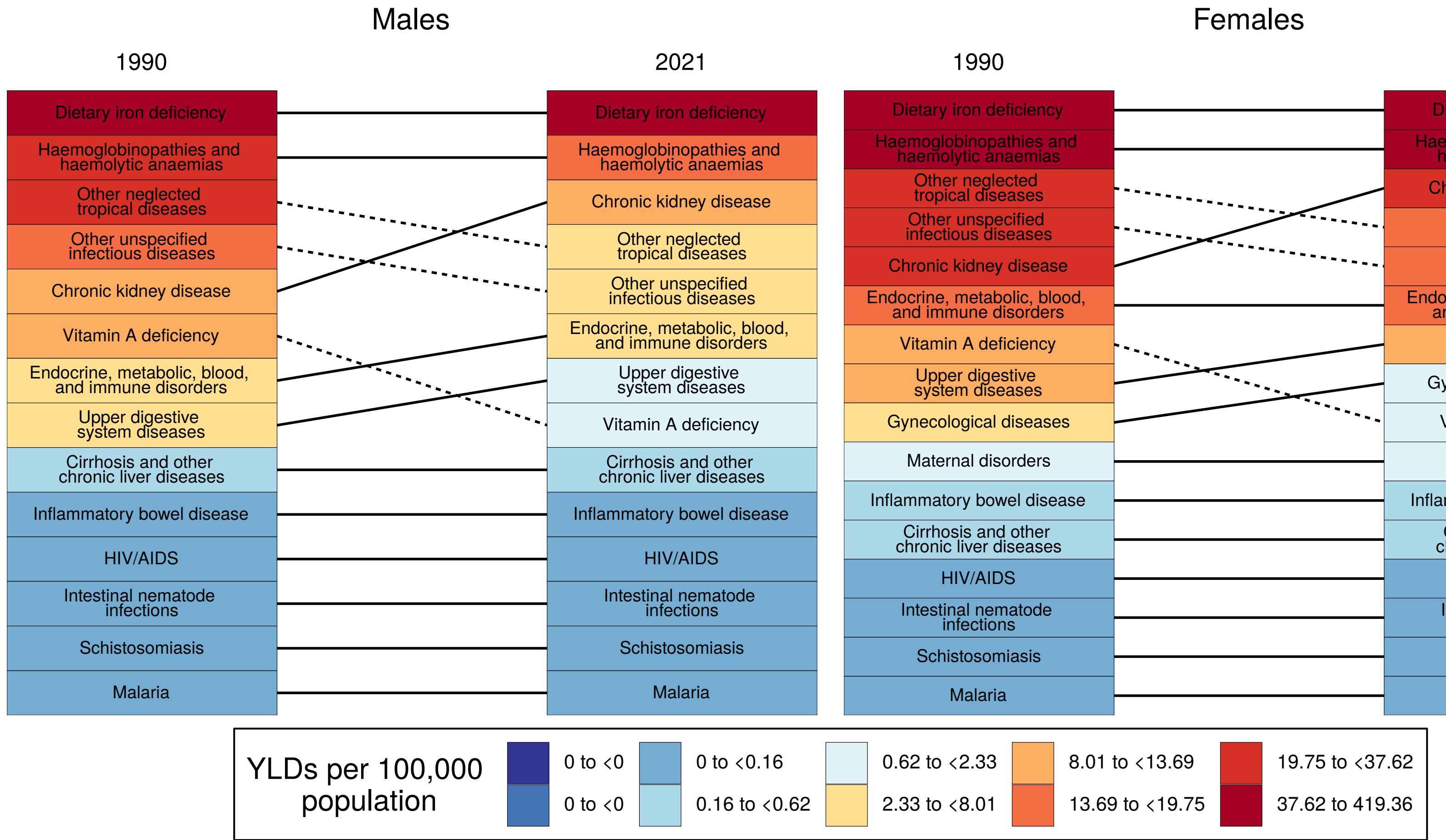

Males
Females
1990
2021
Dietary iron deficiency
Haemoglobinopathies and haemolytic anaemias
Other neglected tropical diseases
Other unspecified infectious diseases
Chronic kidney disease
Vitamin A deficiency
Endocrine, metabolic, blood, and immune disorders
Upper digestive system diseases
Cirrhosis and other chronic liver diseases
Inflammatory bowel disease
HIV/AIDS
Intestinal nematode infections
Schistosomiasis
Malaria
Gynecological diseases
Maternal disorders
YLDs per 100,000 population
0 to <0
0 to <0.16
0.16 to <0.62
0.62 to <2.33
2.33 to <8.01
8.01 to <13.69
13.69 to <19.75
19.75 to <37.62
37.62 to 419.36

Romania

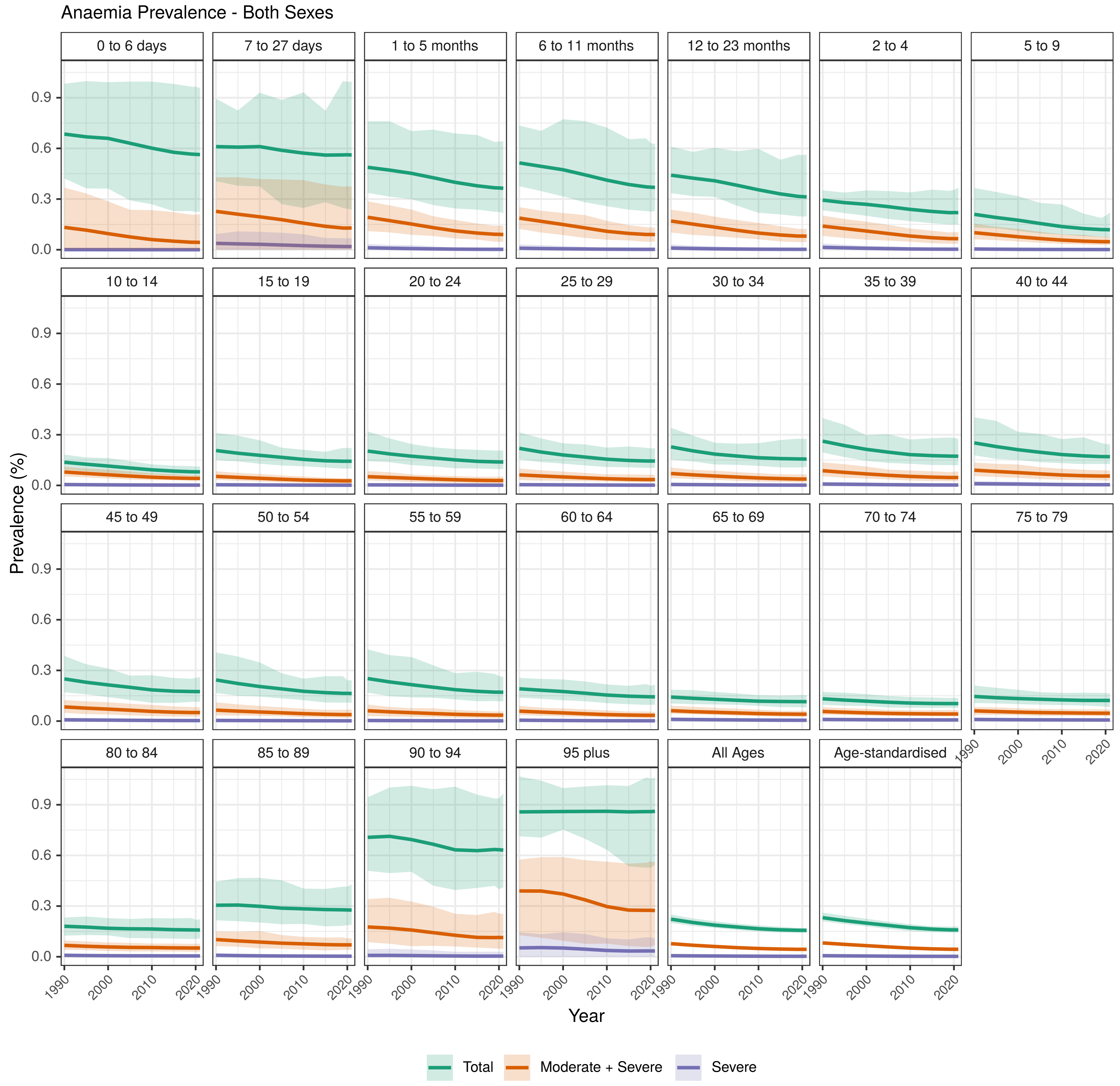
Anaemia Prevalence - Both Sexes
0 to 6 days
7 to 27 days
1 to 5 months
6 to 11 months
12 to 23 months
2 to 4
5 to 9
10 to 14
15 to 19
20 to 24
25 to 29
30 to 34
35 to 39
40 to 44
45 to 49
50 to 54
55 to 59
60 to 64
65 to 69
70 to 74
75 to 79
80 to 84
85 to 89
90 to 94
95 plus
All Ages
Age-standardised
Prevalence (%)
Year
Total
Moderate + Severe
Severe

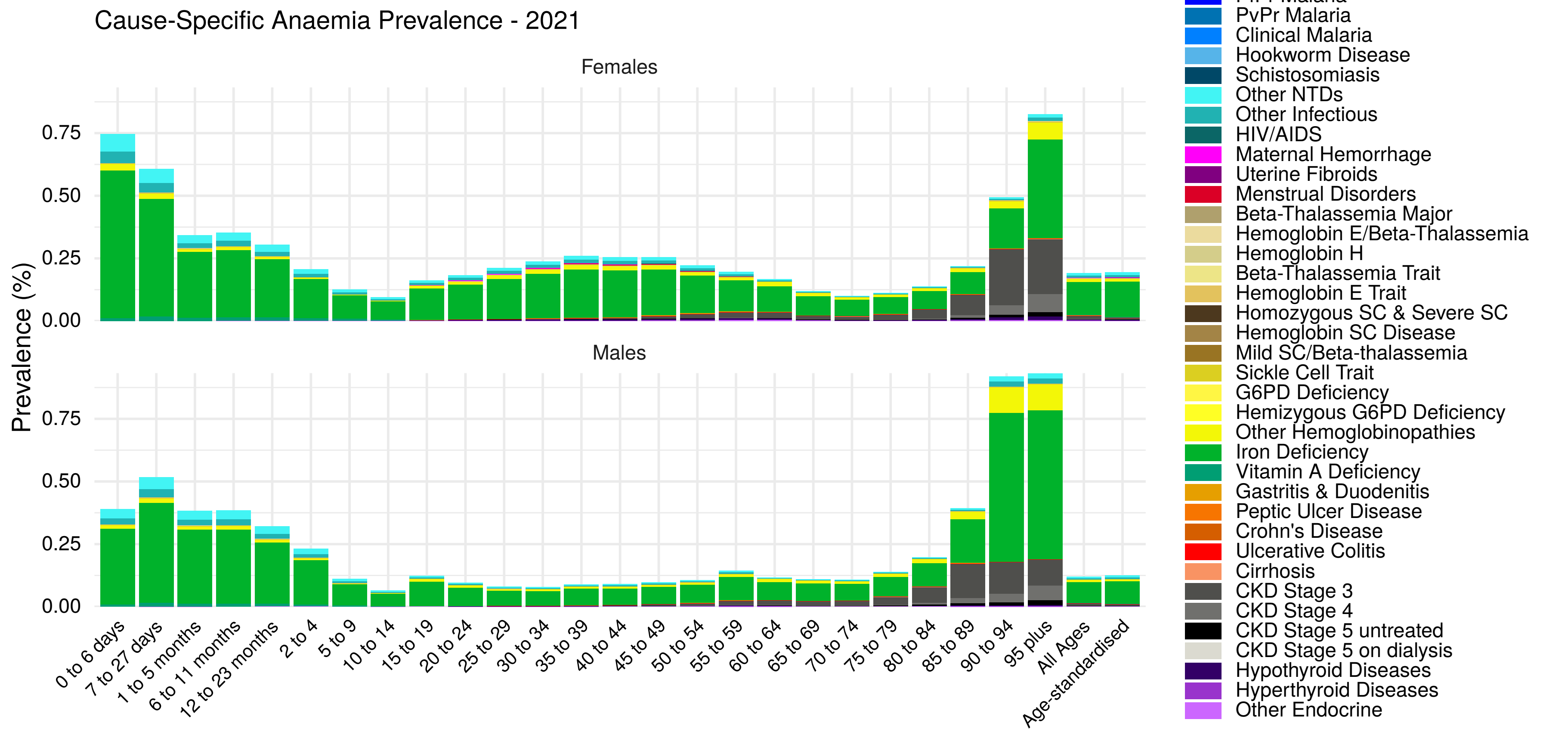
Cause-Specific Anaemia Prevalence - 2021
Females
Males
Prevalence (%)
PfPr Malaria
PvPr Malaria
Clinical Malaria
Hookworm Disease
Schistosomiasis
Other NTDs
Other Infectious
HIV/AIDS
Maternal Hemorrhage
Uterine Fibroids
Menstrual Disorders
Beta-Thalassemia Major
Hemoglobin E/Beta-Thalassemia
Hemoglobin H
Beta-Thalassemia Trait
Hemoglobin E Trait
Homozygous SC & Severe SC
Hemoglobin SC Disease
Mild SC/Beta-thalassemia
Sickle Cell Trait
G6PD Deficiency
Hemizygous G6PD Deficiency
Other Hemoglobinopathies
Iron Deficiency
Vitamin A Deficiency
Gastritis & Duodenitis
Peptic Ulcer Disease
Crohn's Disease
Ulcerative Colitis
Cirrhosis
CKD Stage 3
CKD Stage 4
CKD Stage 5 untreated
CKD Stage 5 on dialysis
Hypothyroid Diseases
Hyperthyroid Diseases
Other Endocrine

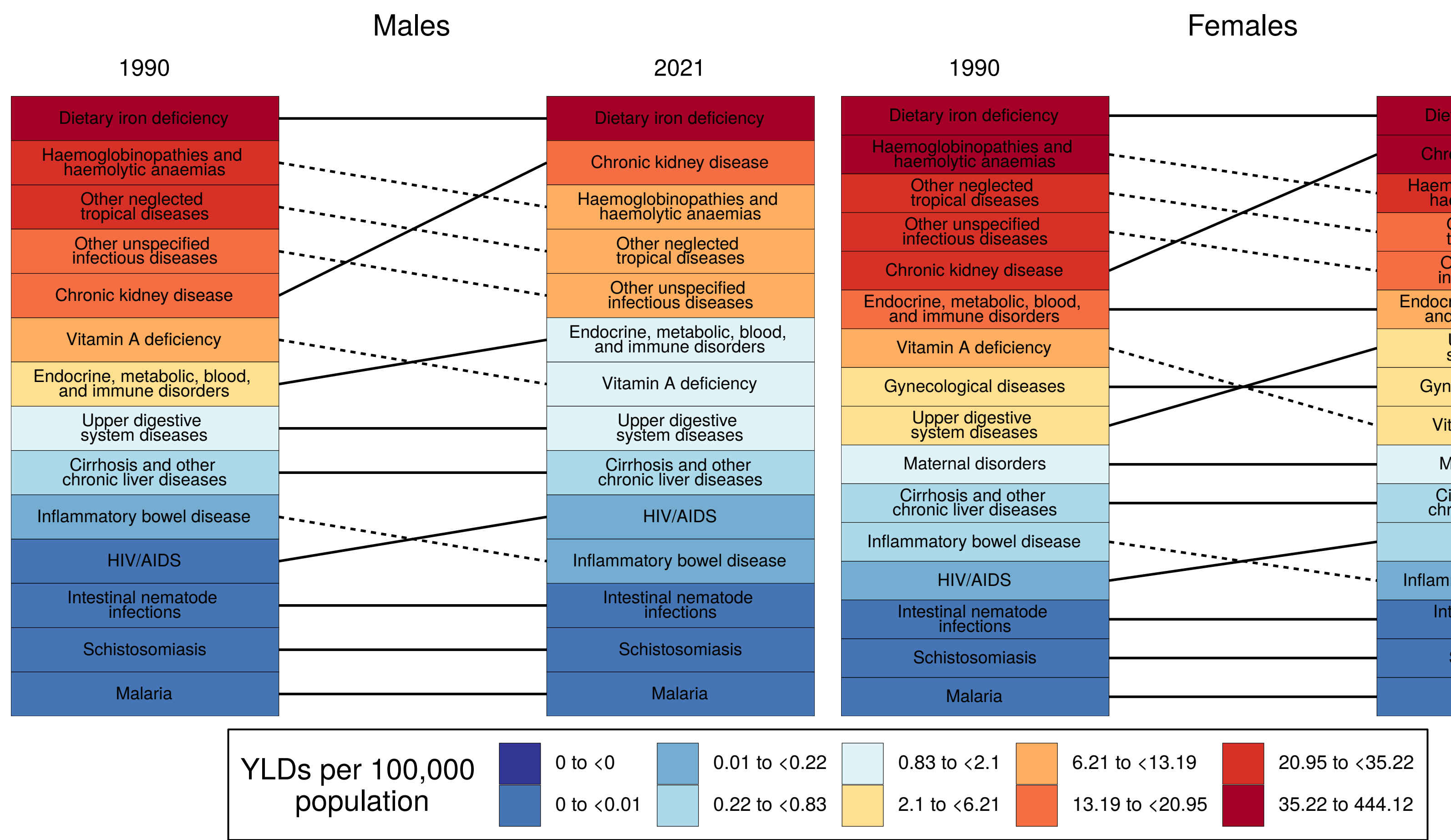
Males
Females
1990
2021
Dietary iron deficiency
Haemoglobinopathies and haemolytic anaemias
Other neglected tropical diseases
Other unspecified infectious diseases
Chronic kidney disease
Vitamin A deficiency
Endocrine, metabolic, blood, and immune disorders
Upper digestive system diseases
Cirrhosis and other chronic liver diseases
Inflammatory bowel disease
HIV/AIDS
Intestinal nematode infections
Schistosomiasis
Malaria
Gynecological diseases
Maternal disorders
YLDs per 100,000 population
0 to <0
0 to <0.01
0.01 to <0.22
0.22 to <0.83
0.83 to <2.1
2.1 to <6.21
6.21 to <13.19
13.19 to <20.95
20.95 to <35.22
35.22 to 444.12

Serbia

Anaemia Prevalence - Both Sexes

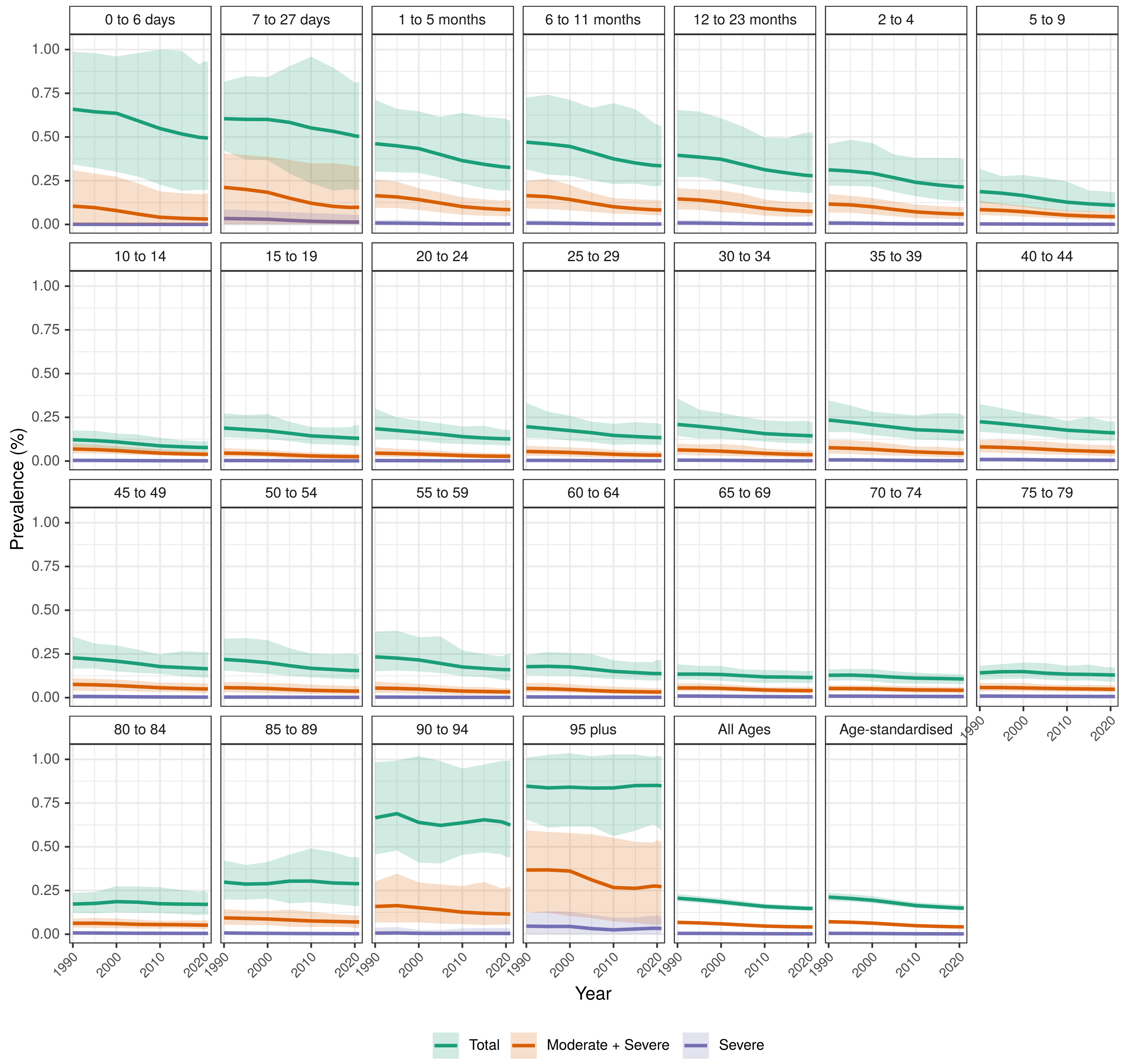

Cause-Specific Anaemia Prevalence - 2021

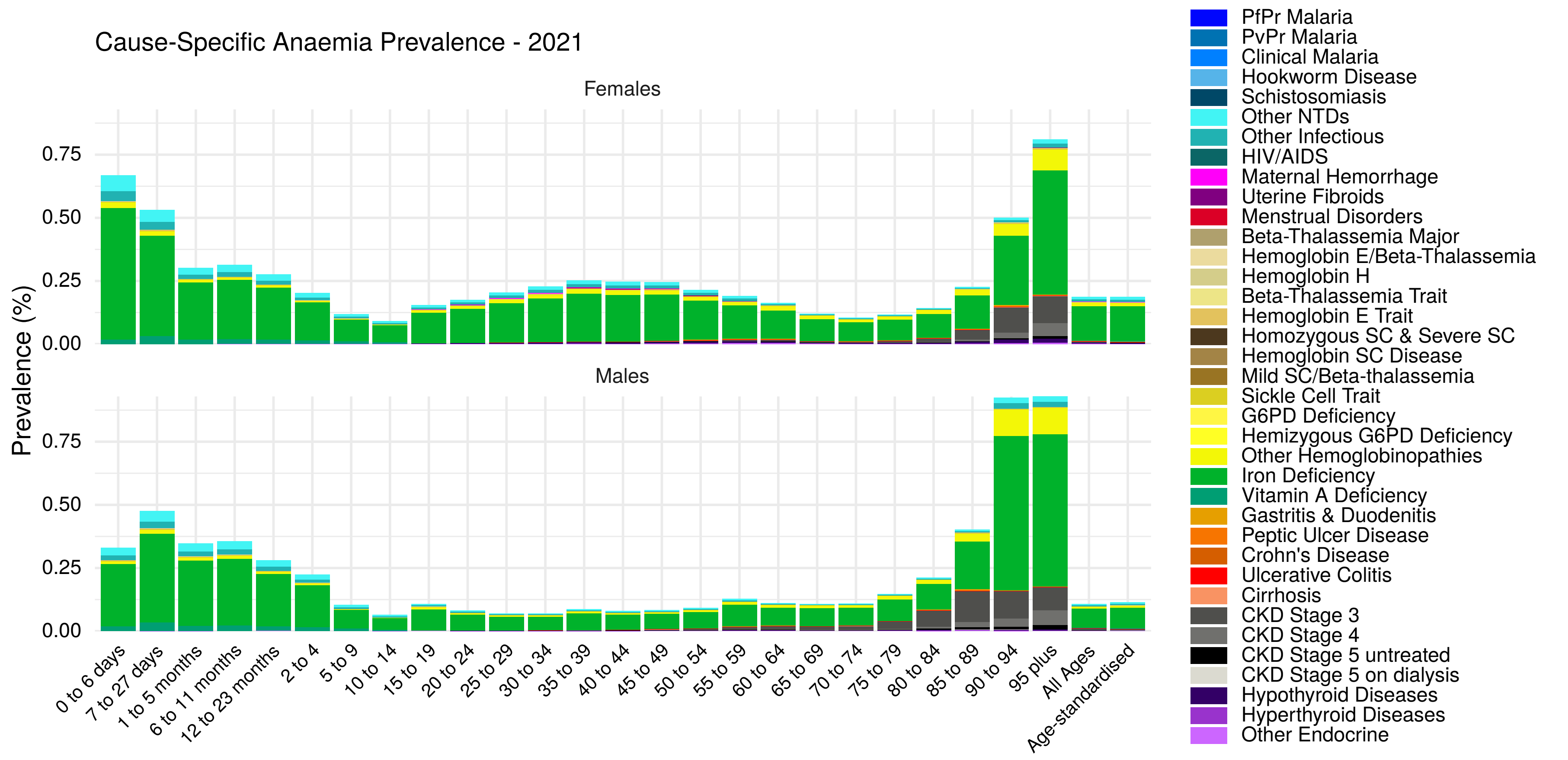

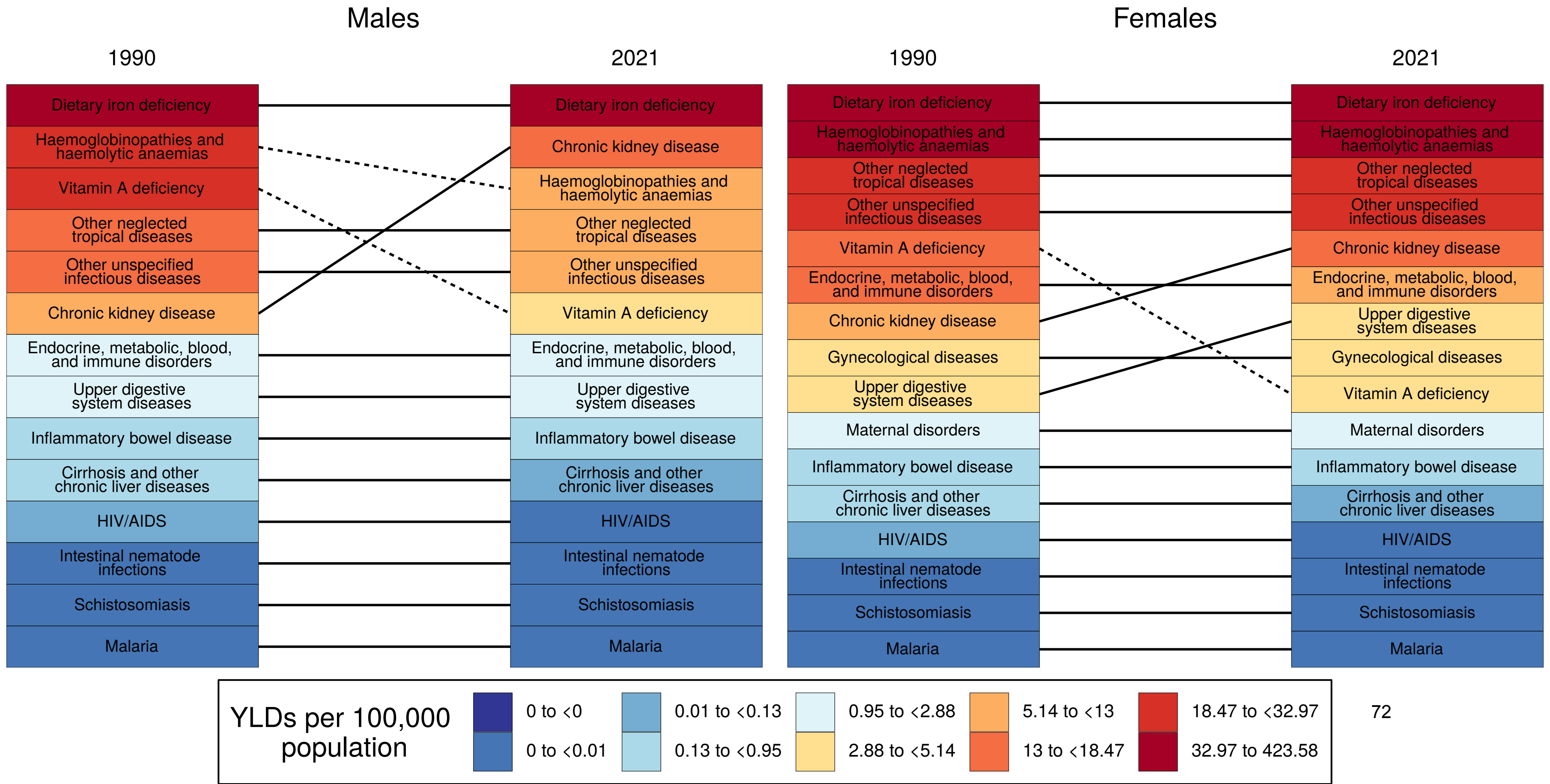

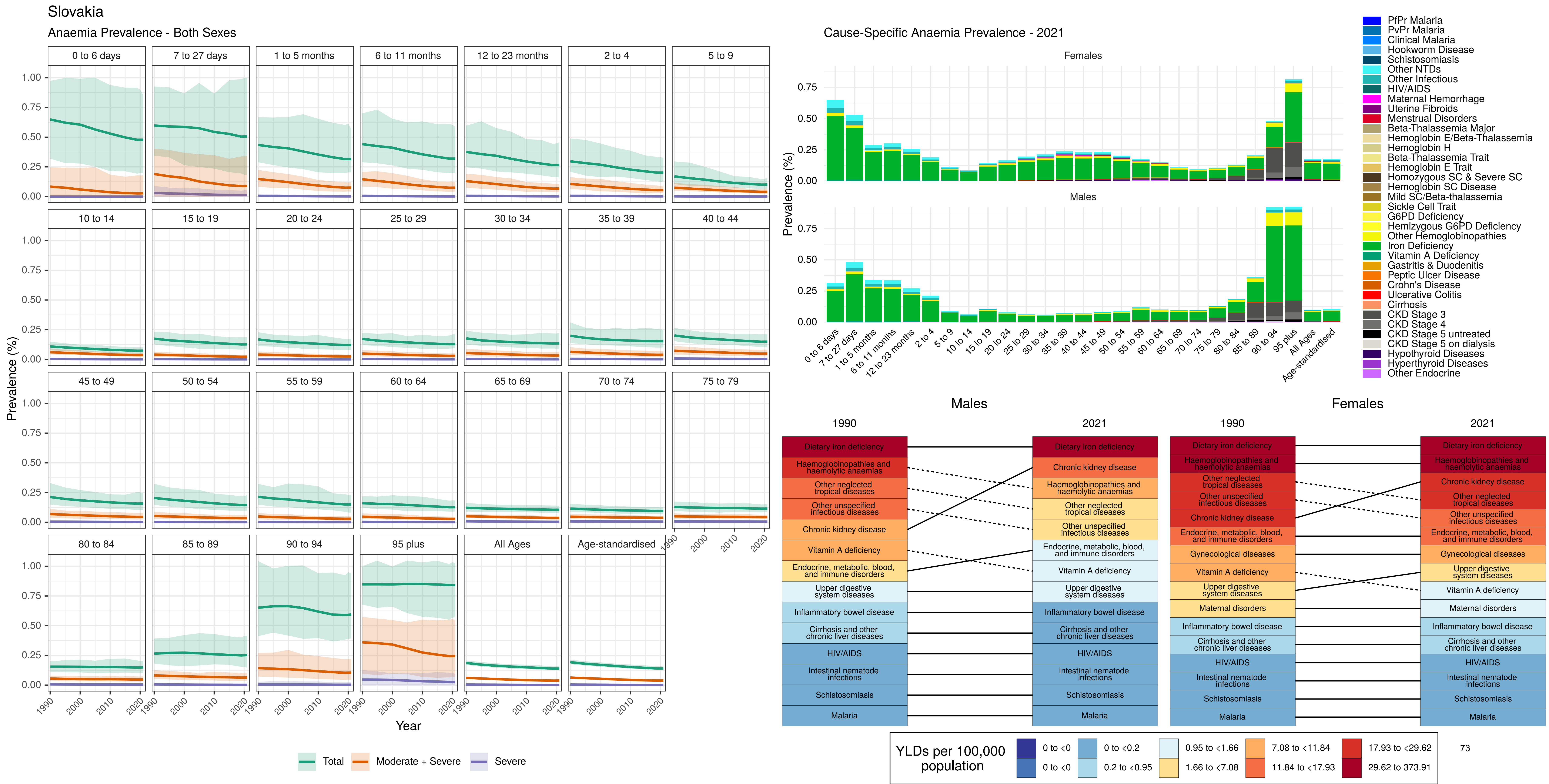
Slovakia
Anaemia Prevalence - Both Sexes
0 to 6 days
7 to 27 days
1 to 5 months
6 to 11 months
12 to 23 months
2 to 4
5 to 9
10 to 14
15 to 19
20 to 24
25 to 29
30 to 34
35 to 39
40 to 44
45 to 49
50 to 54
55 to 59
60 to 64
65 to 69
70 to 74
75 to 79
80 to 84
85 to 89
90 to 94
95 plus
All Ages
Age-standardised
Prevalence (%)
Year
Total
Moderate + Severe
Severe
Cause-Specific Anaemia Prevalence - 2021
Females
Males
Prevalence (%)
PfPr Malaria
PvPr Malaria
Clinical Malaria
Hookworm Disease
Schistosomiasis
Other NTDs
Other Infectious
HIV/AIDS
Maternal Hemorrhage
Uterine Fibroids
Menstrual Disorders
Beta-Thalassemia Major
Hemoglobin E/Beta-Thalassemia
Hemoglobin H
Beta-Thalassemia Trait
Hemoglobin E Trait
Homozygous SC & Severe SC
Hemoglobin SC Disease
Mild SC/Beta-thalassemia
Sickle Cell Trait
G6PD Deficiency
Hemizygous G6PD Deficiency
Other Hemoglobinopathies
Iron Deficiency
Vitamin A Deficiency
Gastritis & Duodenitis
Peptic Ulcer Disease
Crohn's Disease
Ulcerative Colitis
Cirrhosis
CKD Stage 3
CKD Stage 4
CKD Stage 5 untreated
CKD Stage 5 on dialysis
Hypothyroid Diseases
Hyperthyroid Diseases
Other Endocrine
Males
Females
1990
2021
Dietary iron deficiency
Haemoglobinopathies and haemolytic anaemias
Other neglected tropical diseases
Other unspecified infectious diseases
Chronic kidney disease
Vitamin A deficiency
Endocrine, metabolic, blood, and immune disorders
Upper digestive system diseases
Inflammatory bowel disease
Cirrhosis and other chronic liver diseases
HIV/AIDS
Intestinal nematode infections
Schistosomiasis
Malaria
Gynecological diseases
Maternal disorders
YLDs per 100,000 population
0 to <0
0 to <0
0 to <0.2
0.2 to <0.95
0.95 to <1.66
1.66 to <7.08
7.08 to <11.84
11.84 to <17.93
17.93 to <29.62
29.62 to 373.91

Slovenia

Anaemia Prevalence - Both Sexes

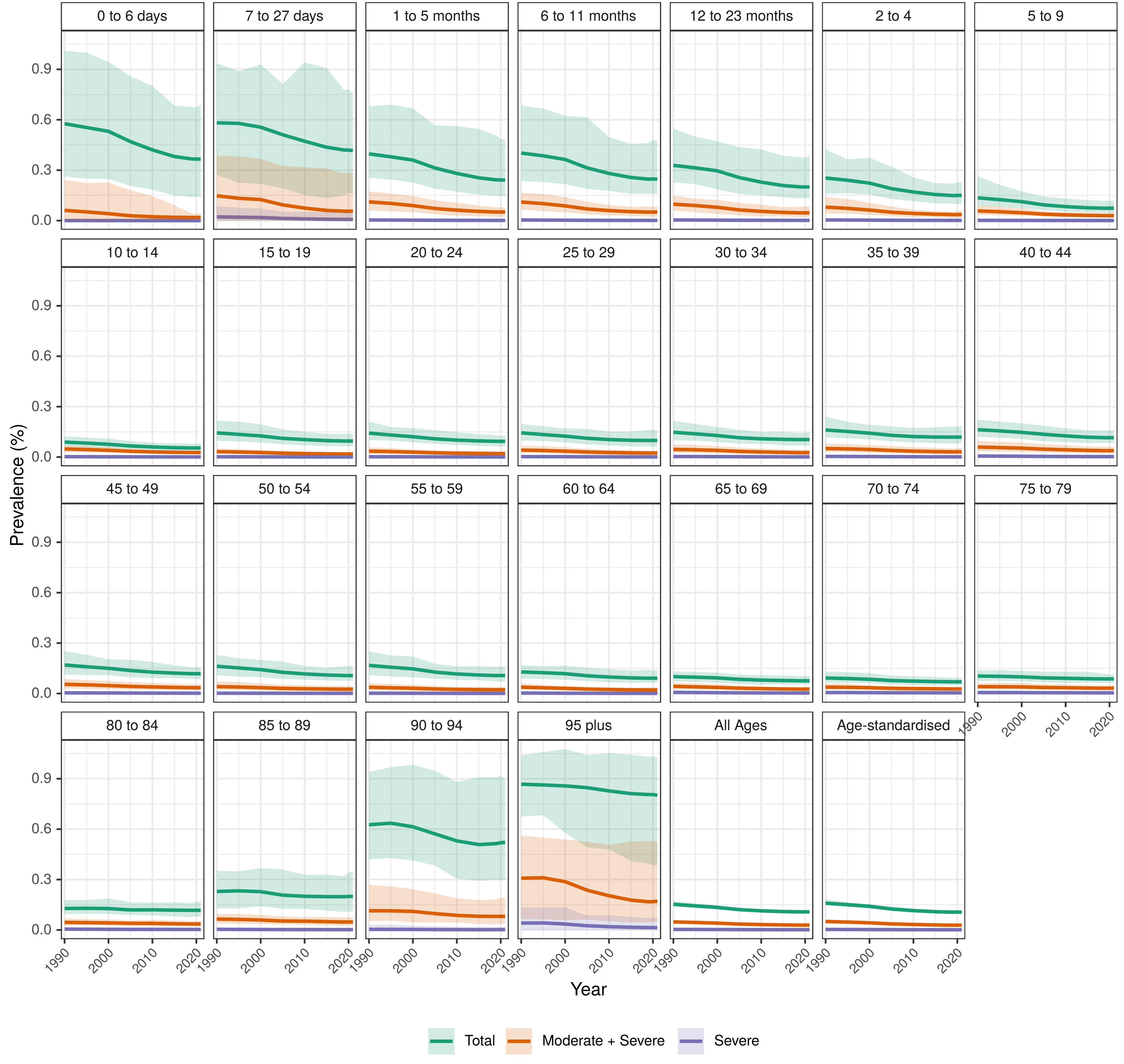

Cause-Specific Anaemia Prevalence - 2021

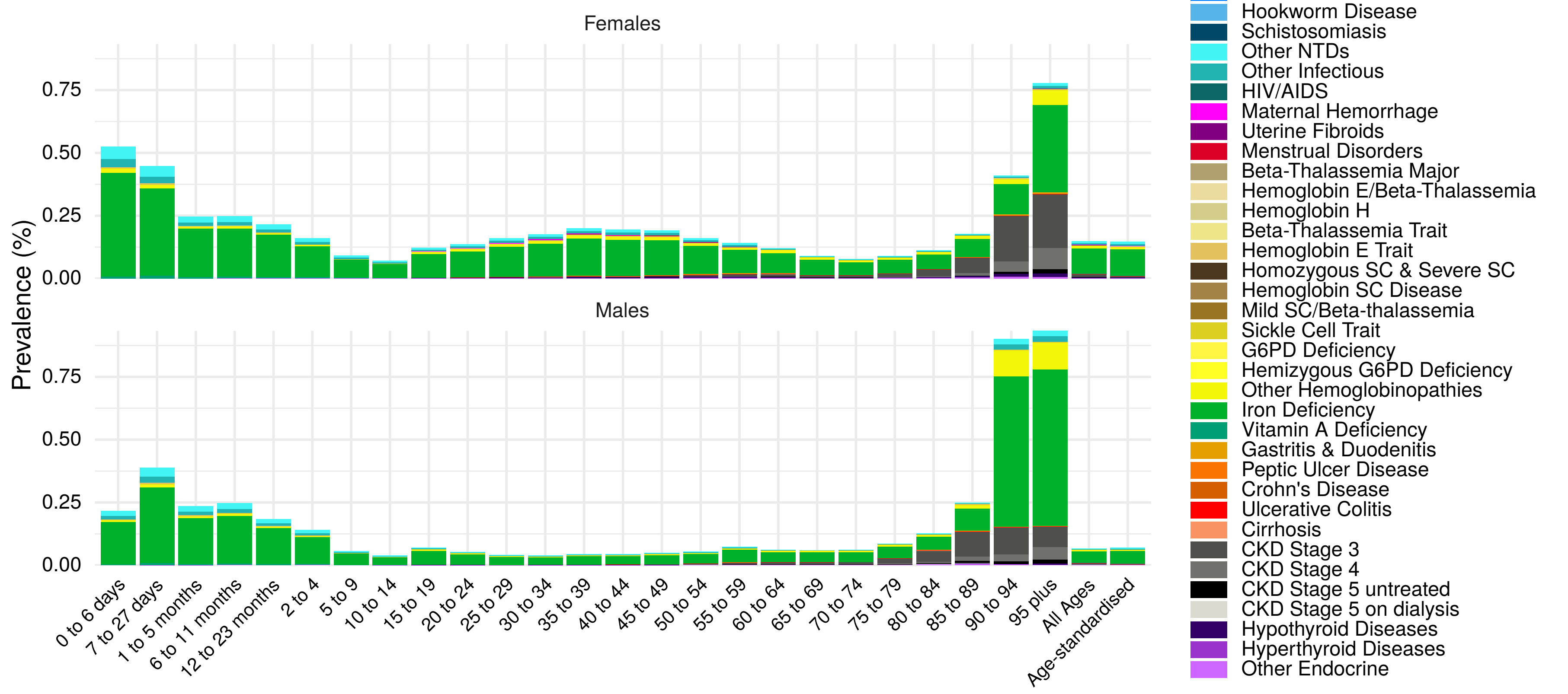

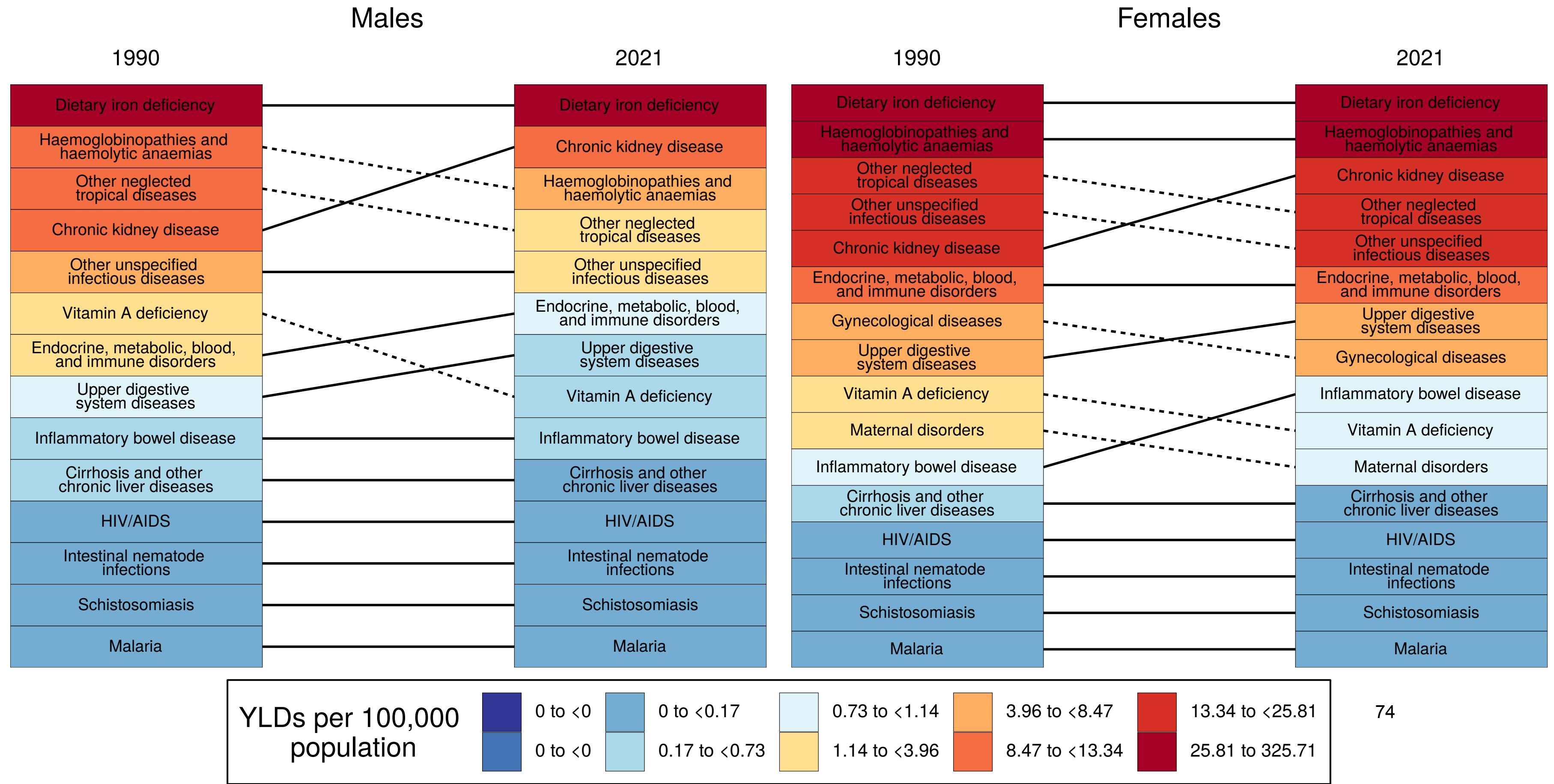

Eastern Europe

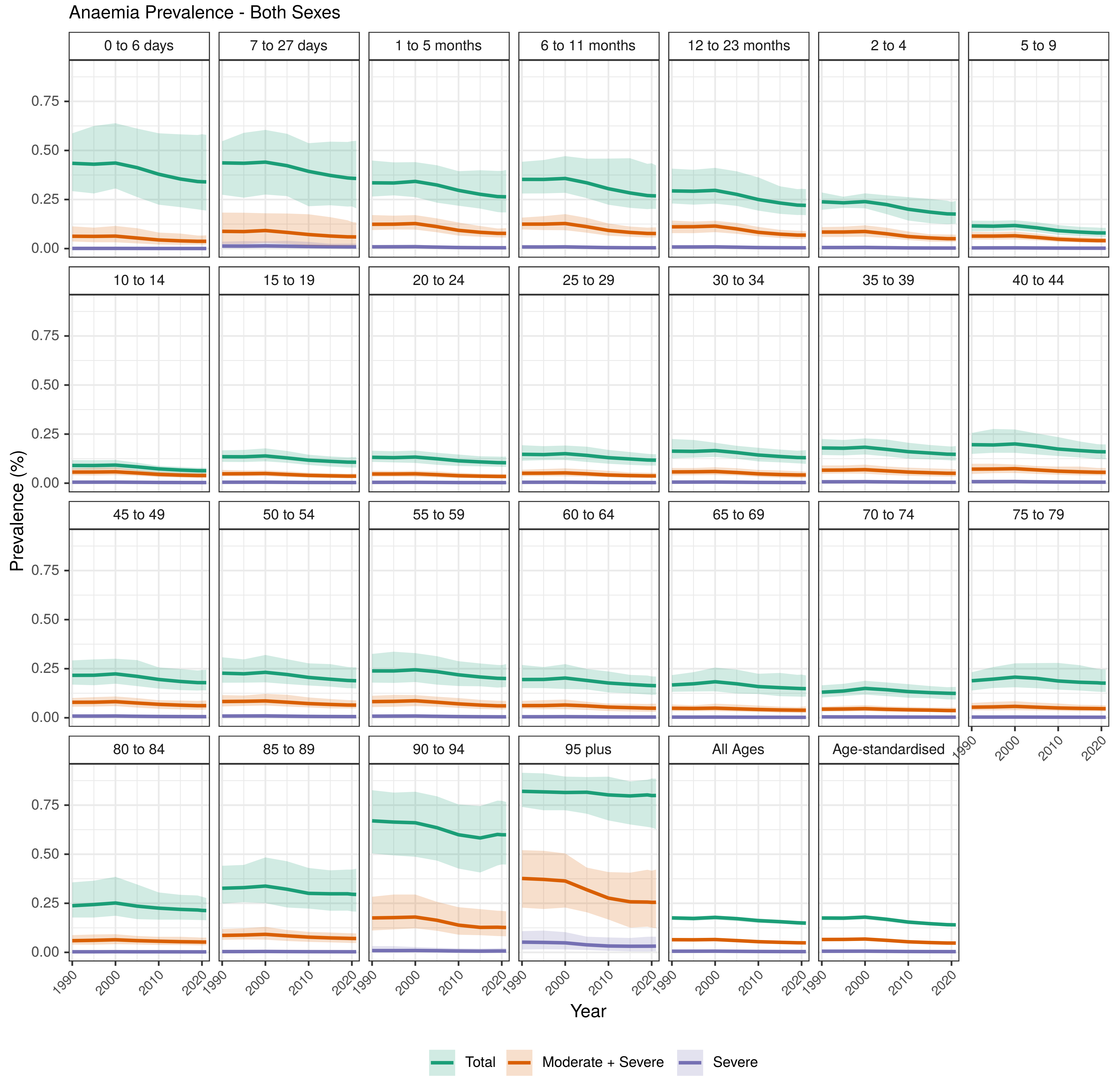

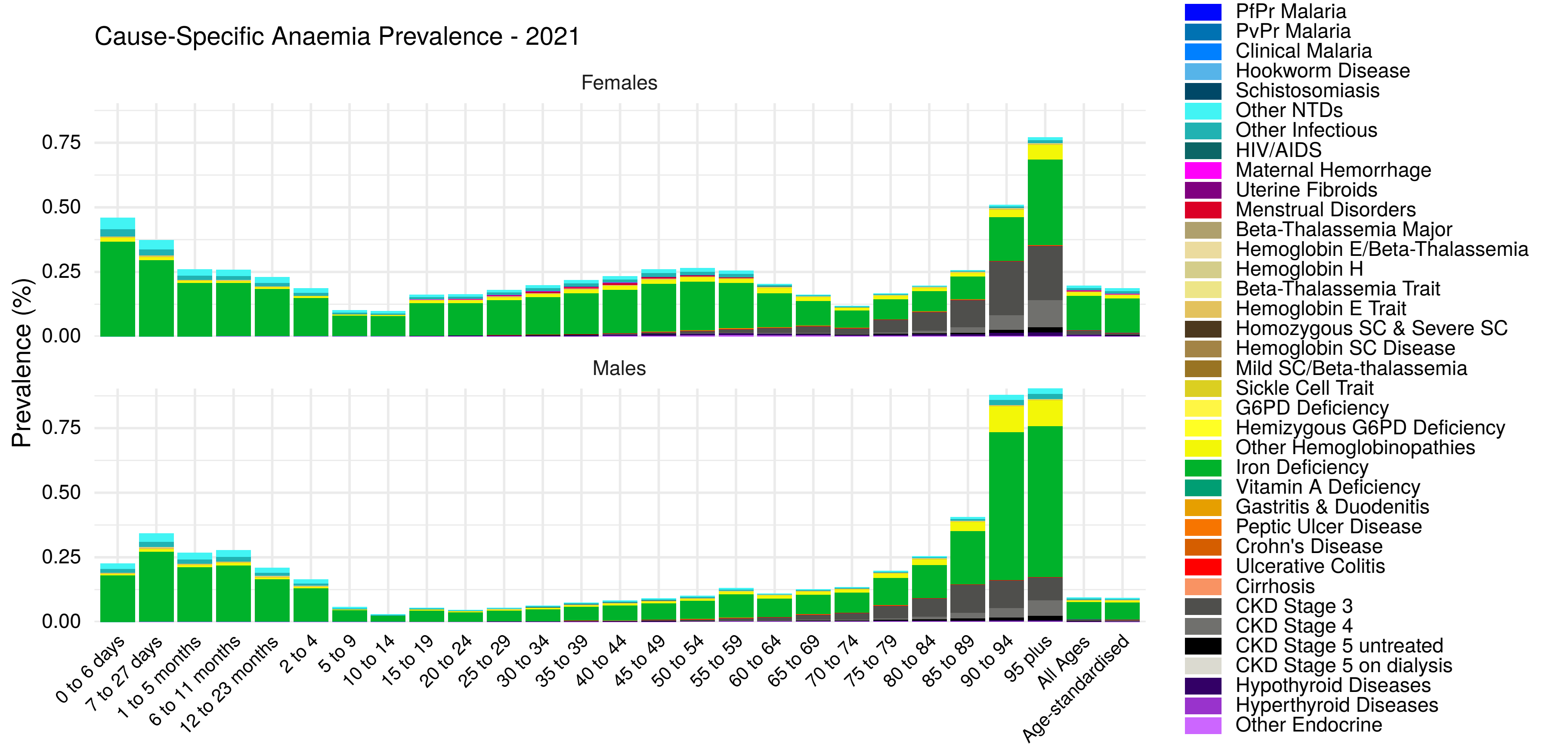

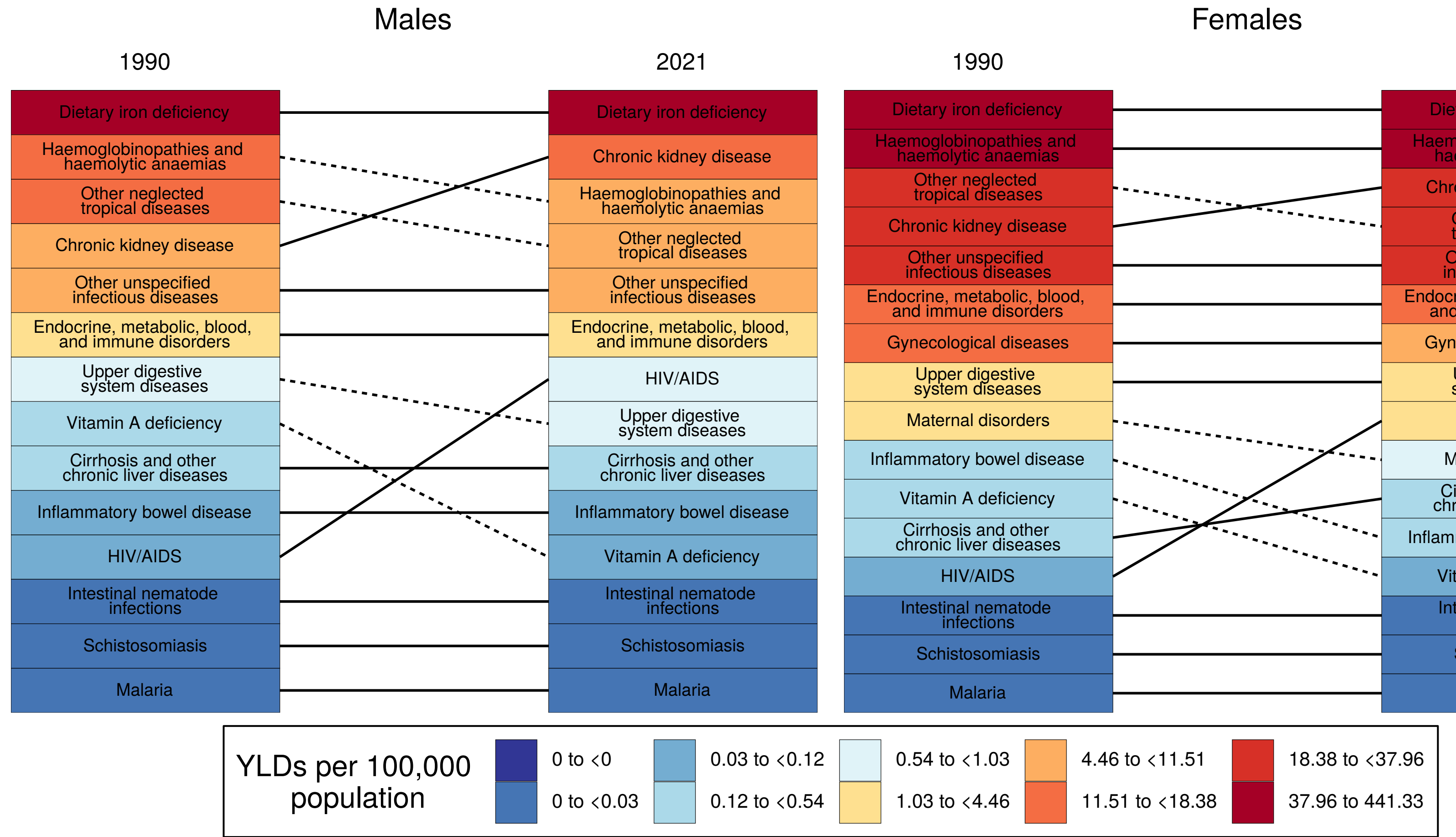

Belarus

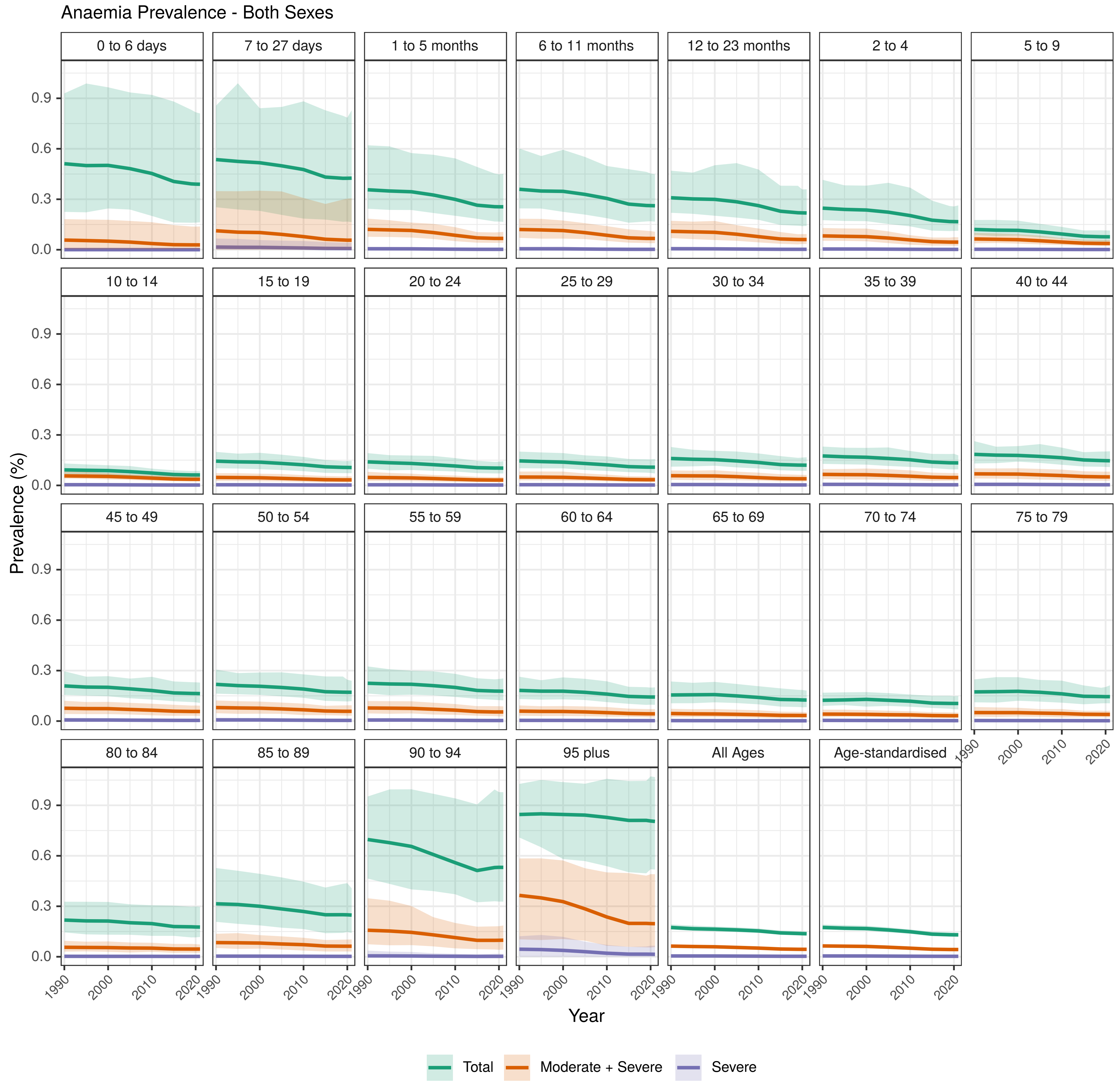

Anaemia Prevalence - Both Sexes
0 to 6 days
7 to 27 days
1 to 5 months
6 to 11 months
12 to 23 months
2 to 4
5 to 9
10 to 14
15 to 19
20 to 24
25 to 29
30 to 34
35 to 39
40 to 44
45 to 49
50 to 54
55 to 59
60 to 64
65 to 69
70 to 74
75 to 79
80 to 84
85 to 89
90 to 94
95 plus
All Ages
Age-standardised
Prevalence (%)
Year
Total
Moderate + Severe
Severe

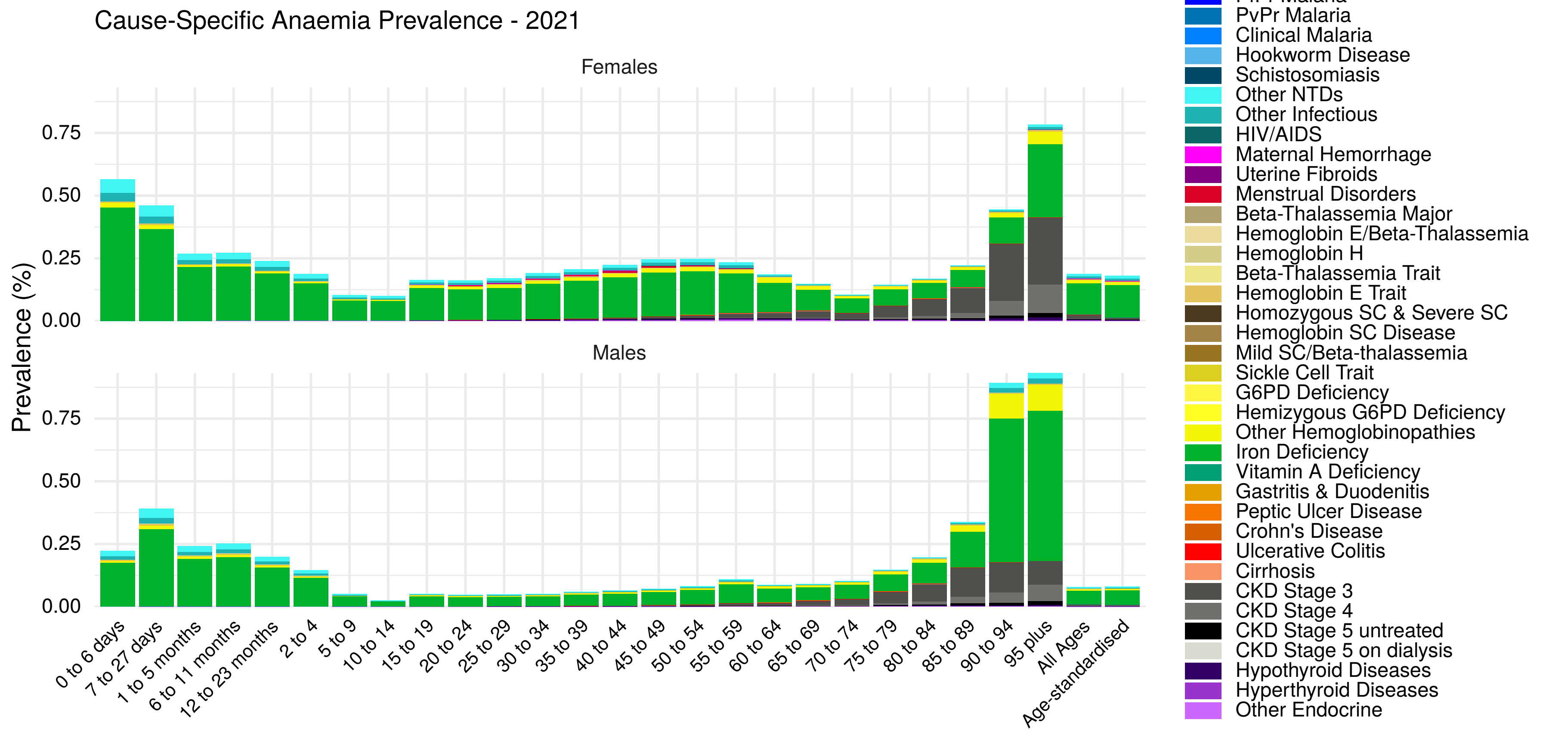

Cause-Specific Anaemia Prevalence - 2021
Females
Males
Prevalence (%)
PfPr Malaria
PvPr Malaria
Clinical Malaria
Hookworm Disease
Schistosomiasis
Other NTDs
Other Infectious
HIV/AIDS
Maternal Hemorrhage
Uterine Fibroids
Menstrual Disorders
Beta-Thalassemia Major
Hemoglobin E/Beta-Thalassemia
Hemoglobin H
Beta-Thalassemia Trait
Hemoglobin E Trait
Homozygous SC & Severe SC
Hemoglobin SC Disease
Mild SC/Beta-thalassemia
Sickle Cell Trait
G6PD Deficiency
Hemizygous G6PD Deficiency
Other Hemoglobinopathies
Iron Deficiency
Vitamin A Deficiency
Gastritis & Duodenitis
Peptic Ulcer Disease
Crohn's Disease
Ulcerative Colitis
Cirrhosis
CKD Stage 3
CKD Stage 4
CKD Stage 5 untreated
CKD Stage 5 on dialysis
Hypothyroid Diseases
Hyperthyroid Diseases
Other Endocrine

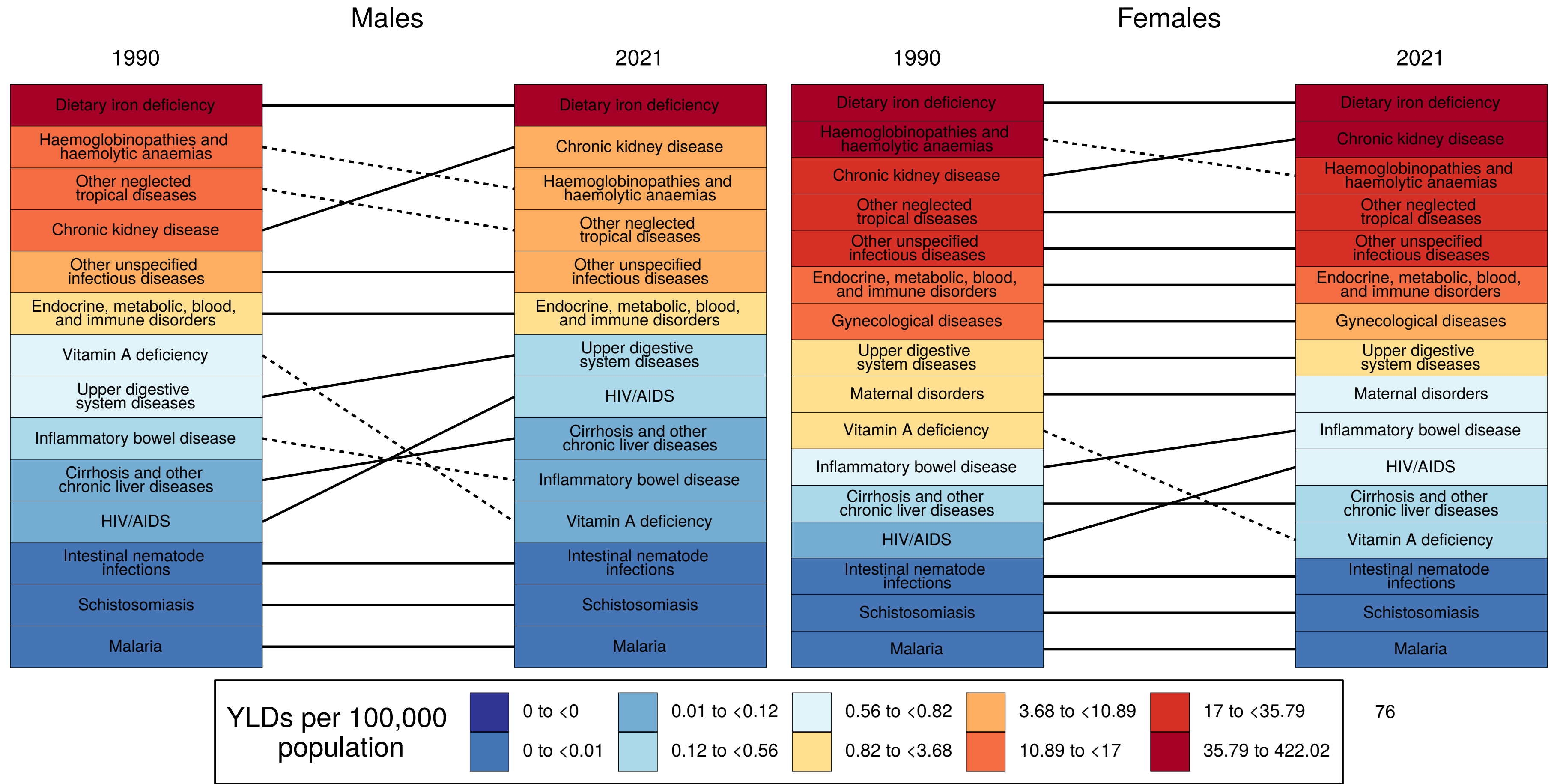

Males
Females
1990
2021
Dietary iron deficiency
Haemoglobinopathies and haemolytic anaemias
Other neglected tropical diseases
Chronic kidney disease
Other unspecified infectious diseases
Endocrine, metabolic, blood, and immune disorders
Vitamin A deficiency
Upper digestive system diseases
Inflammatory bowel disease
Cirrhosis and other chronic liver diseases
HIV/AIDS
Intestinal nematode infections
Schistosomiasis
Malaria
Gynecological diseases
Maternal disorders
YLDs per 100,000 population
0 to <0
0 to <0.01
0.01 to <0.12
0.12 to <0.56
0.56 to <0.82
0.82 to <3.68
3.68 to <10.89
10.89 to <17
17 to <35.79
35.79 to 422.02

Estonia

Anaemia Prevalence - Both Sexes

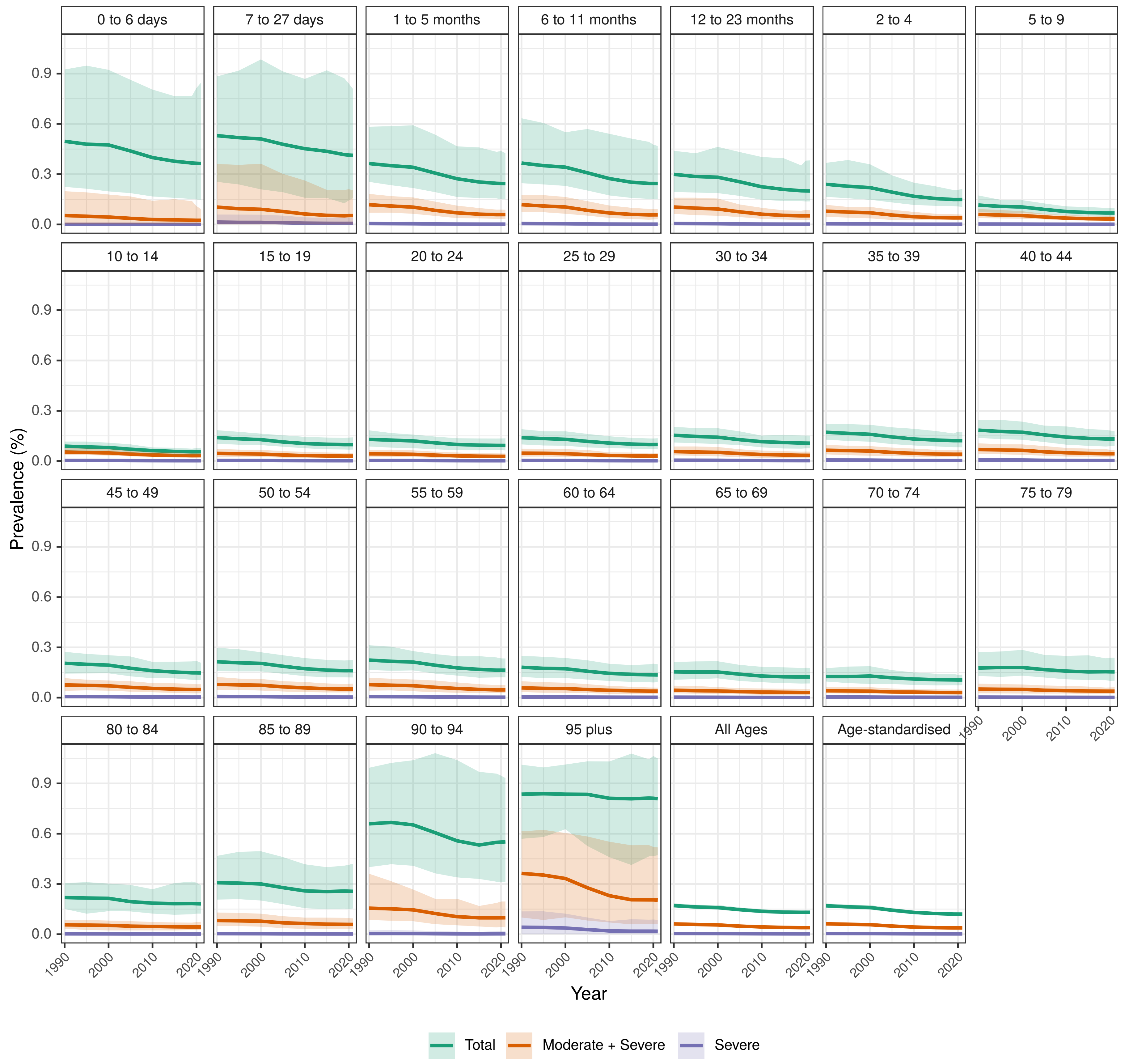

Cause-Specific Anaemia Prevalence - 2021

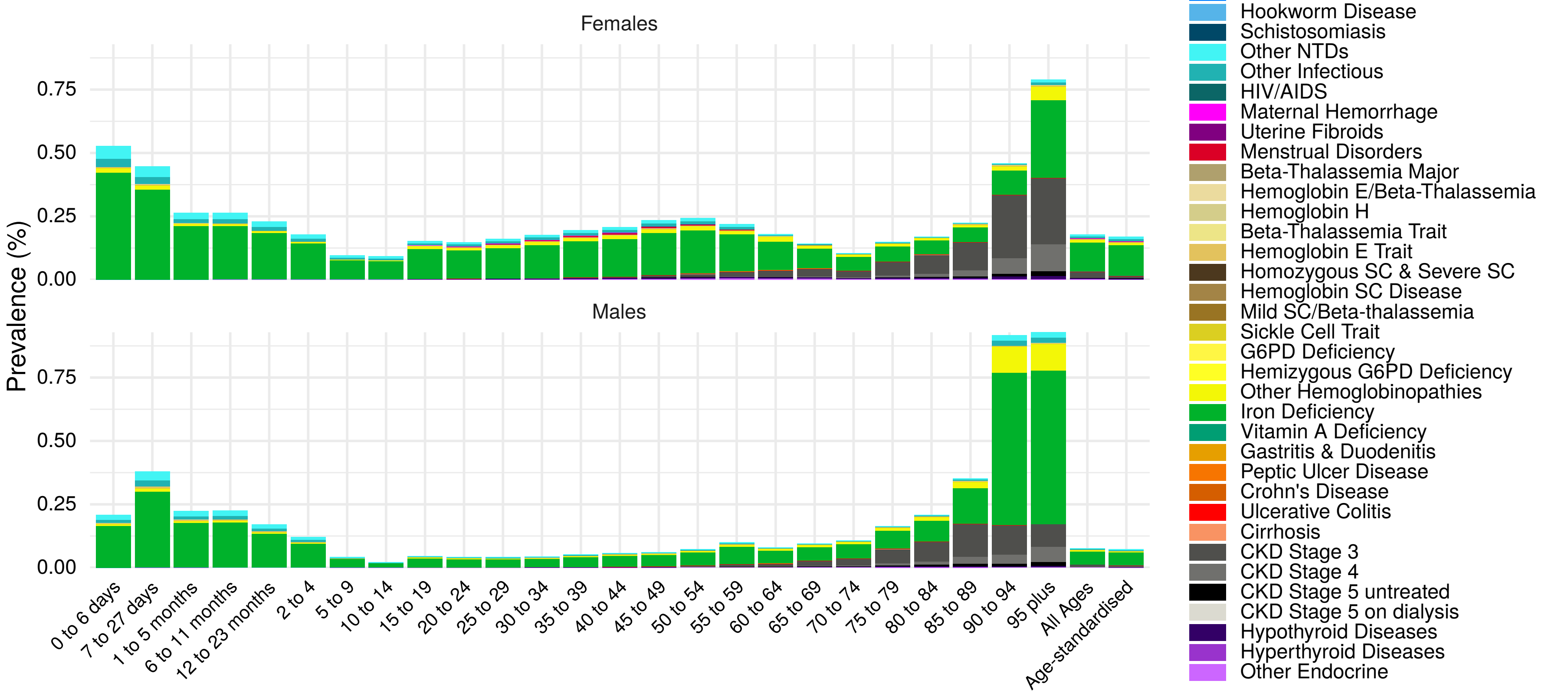

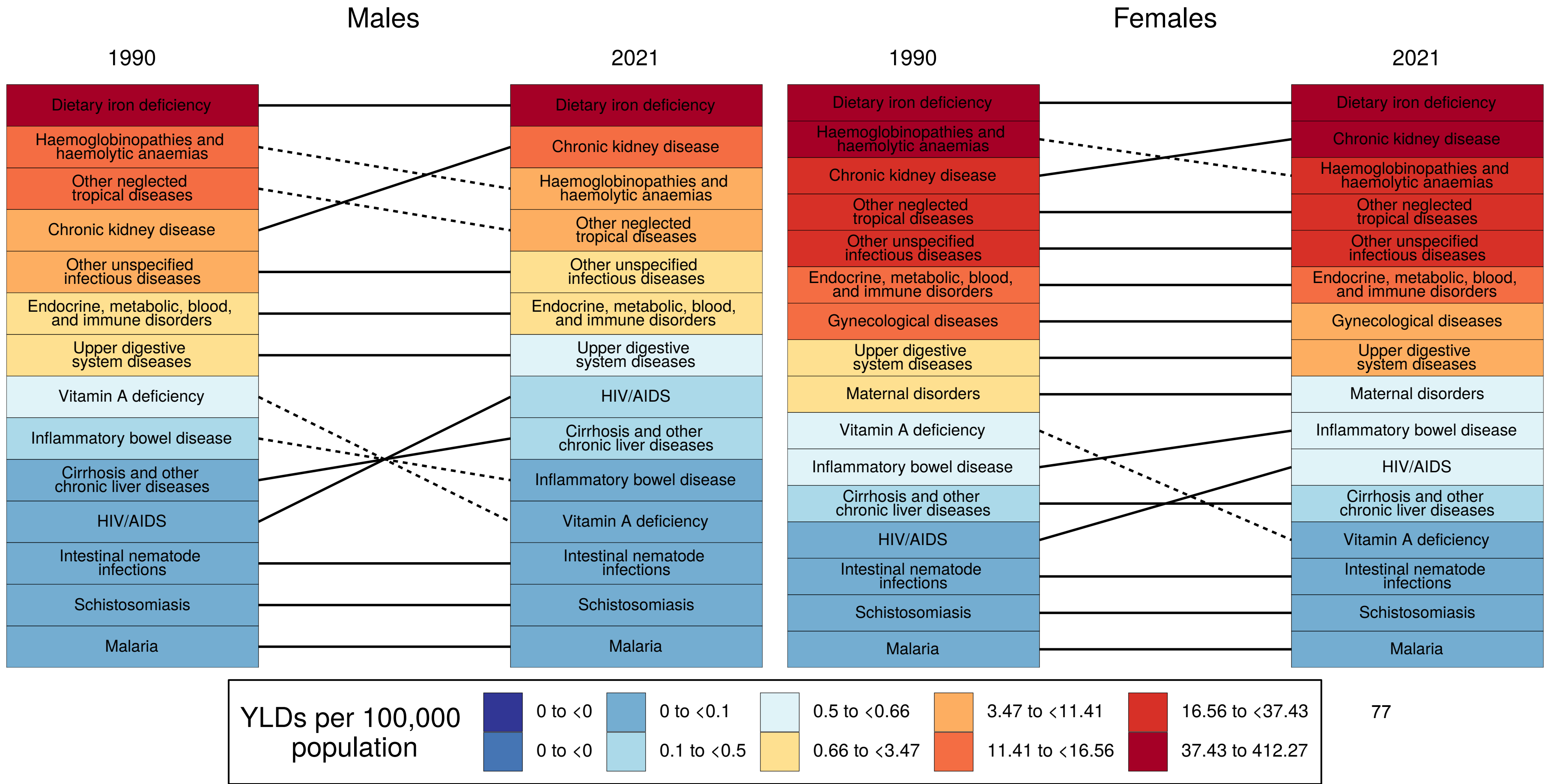

Latvia

Anaemia Prevalence - Both Sexes

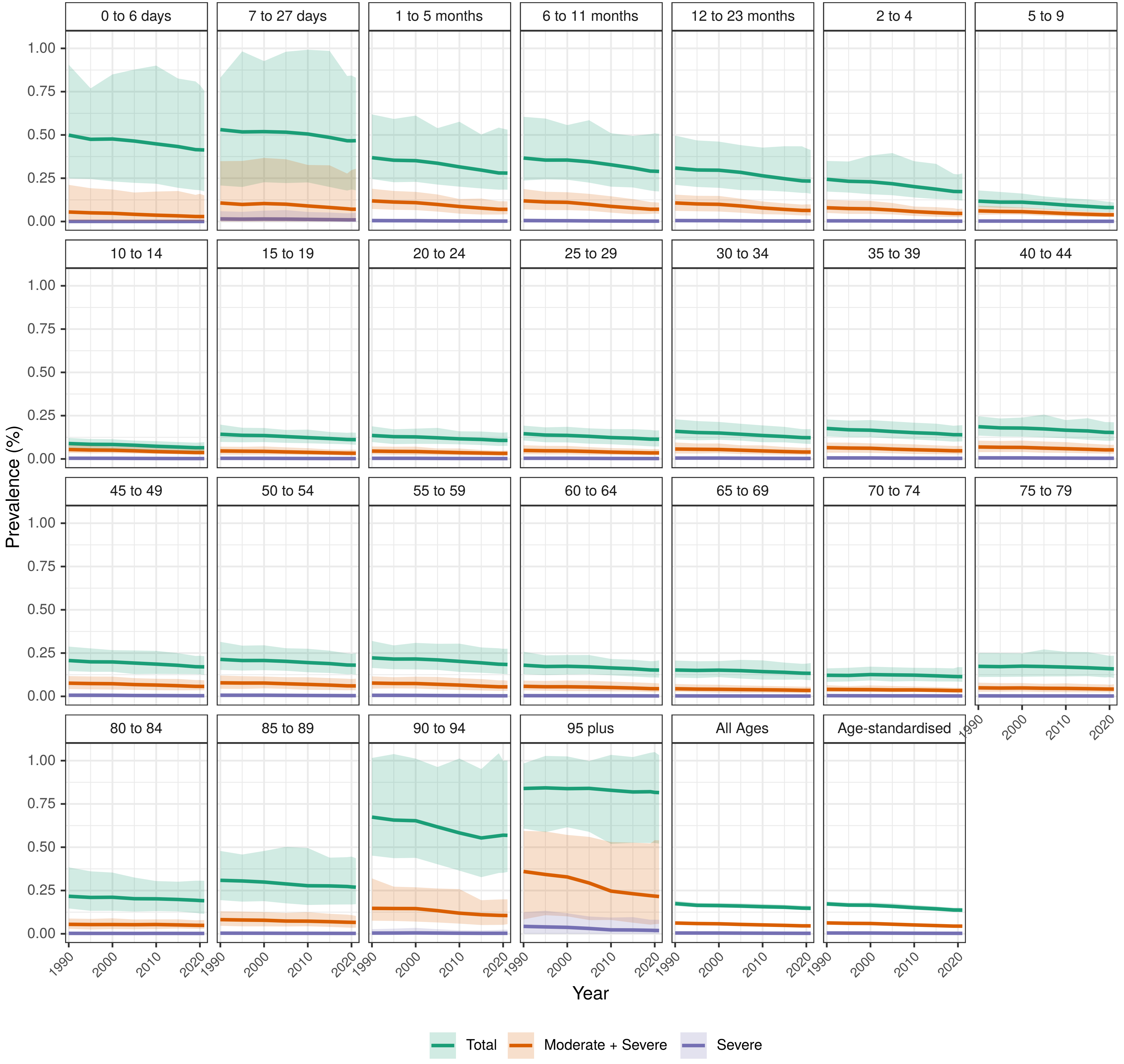

Cause-Specific Anaemia Prevalence - 2021

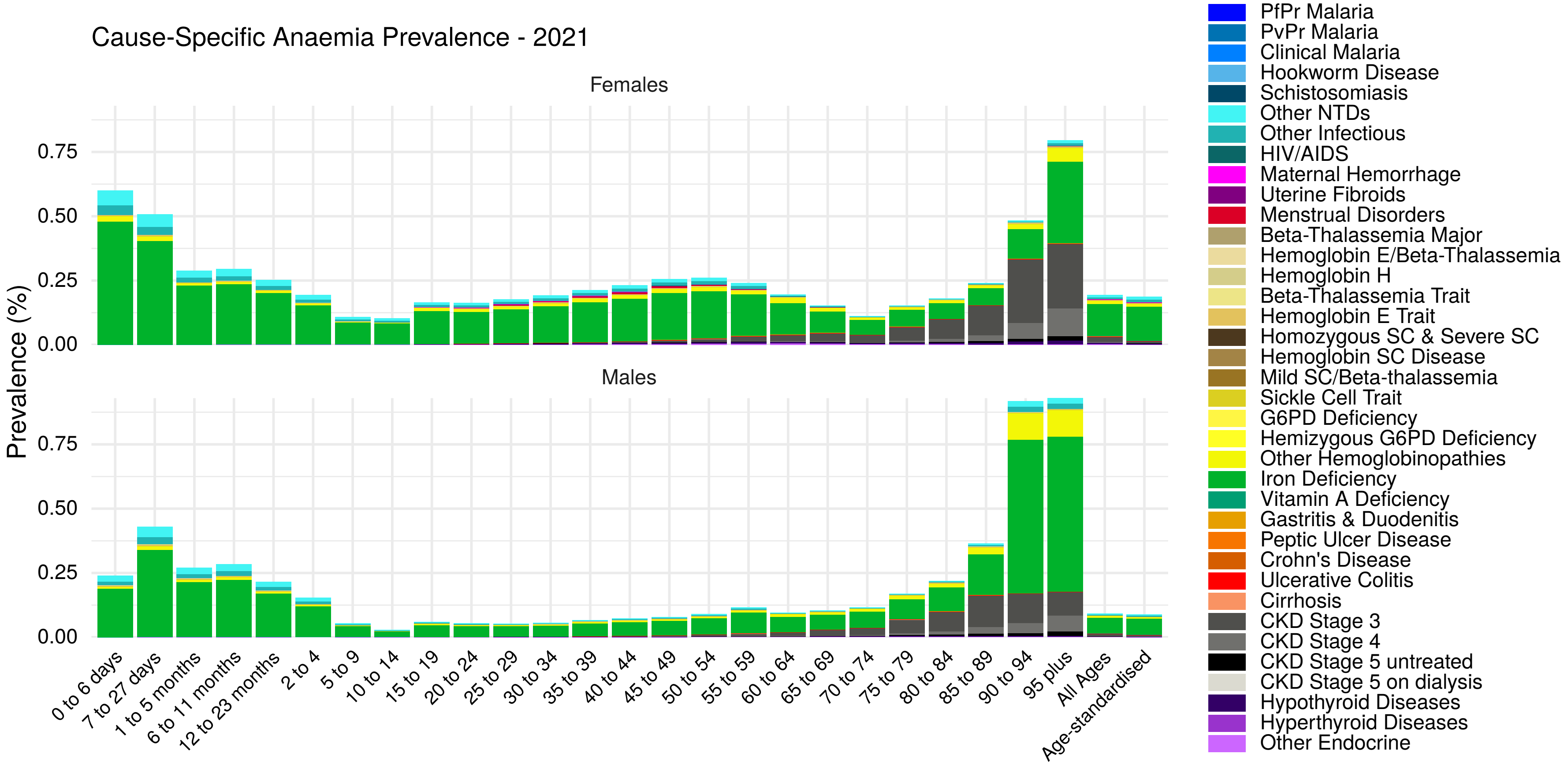

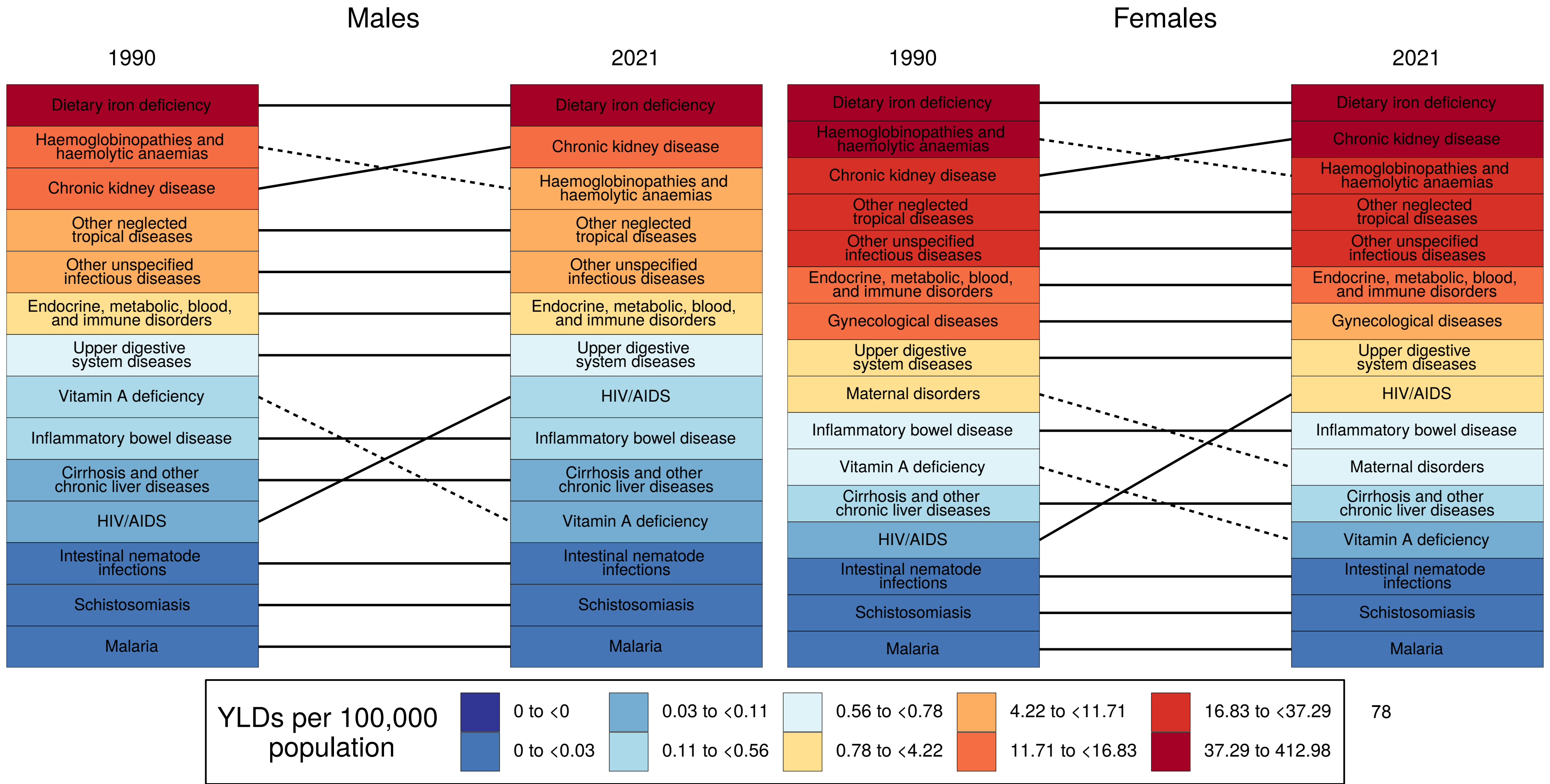

Lithuania

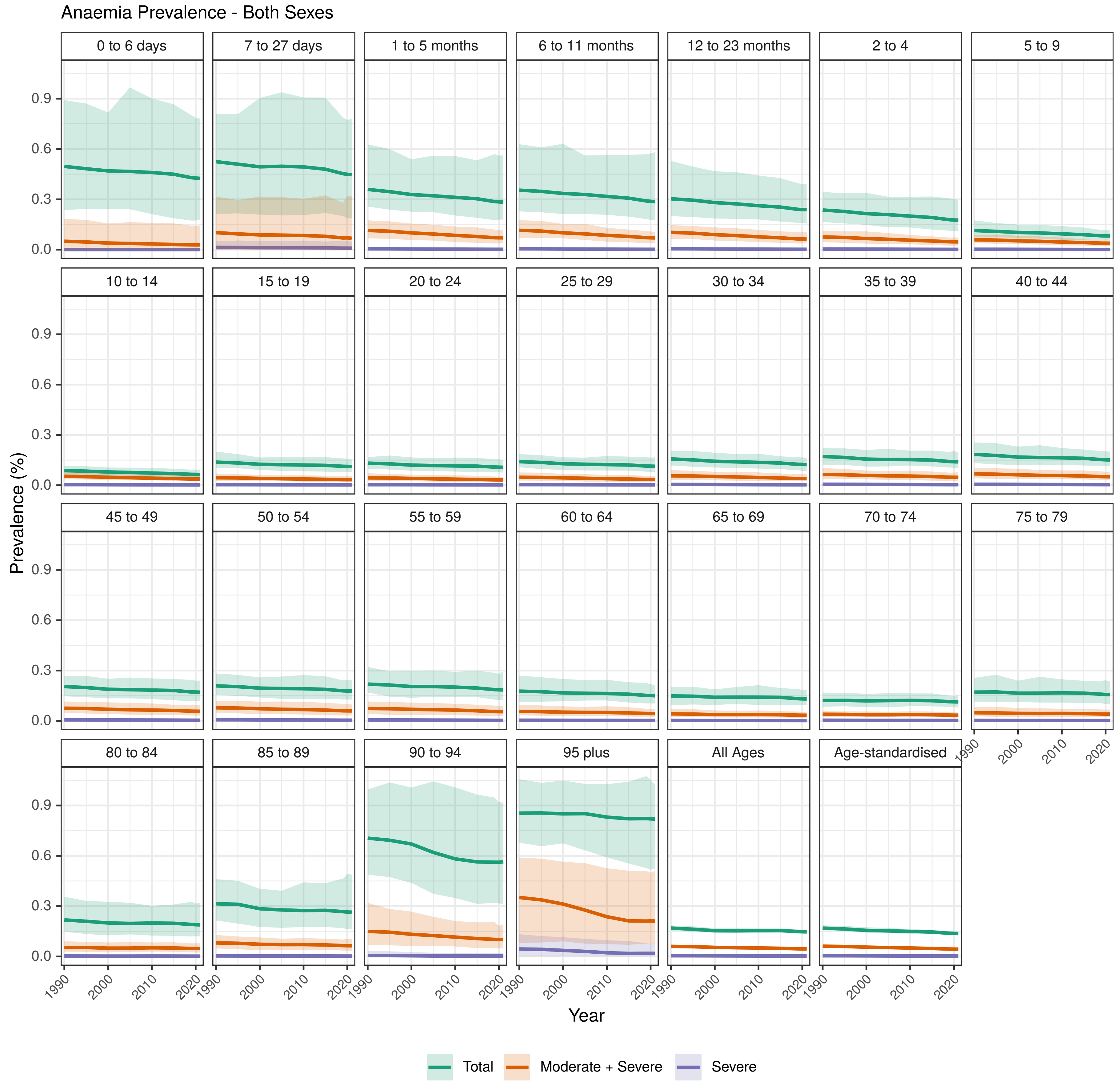
Anaemia Prevalence - Both Sexes
0 to 6 days
7 to 27 days
1 to 5 months
6 to 11 months
12 to 23 months
2 to 4
5 to 9
10 to 14
15 to 19
20 to 24
25 to 29
30 to 34
35 to 39
40 to 44
45 to 49
50 to 54
55 to 59
60 to 64
65 to 69
70 to 74
75 to 79
80 to 84
85 to 89
90 to 94
95 plus
All Ages
Age-standardised
Prevalence (%)
Year
Total
Moderate + Severe
Severe

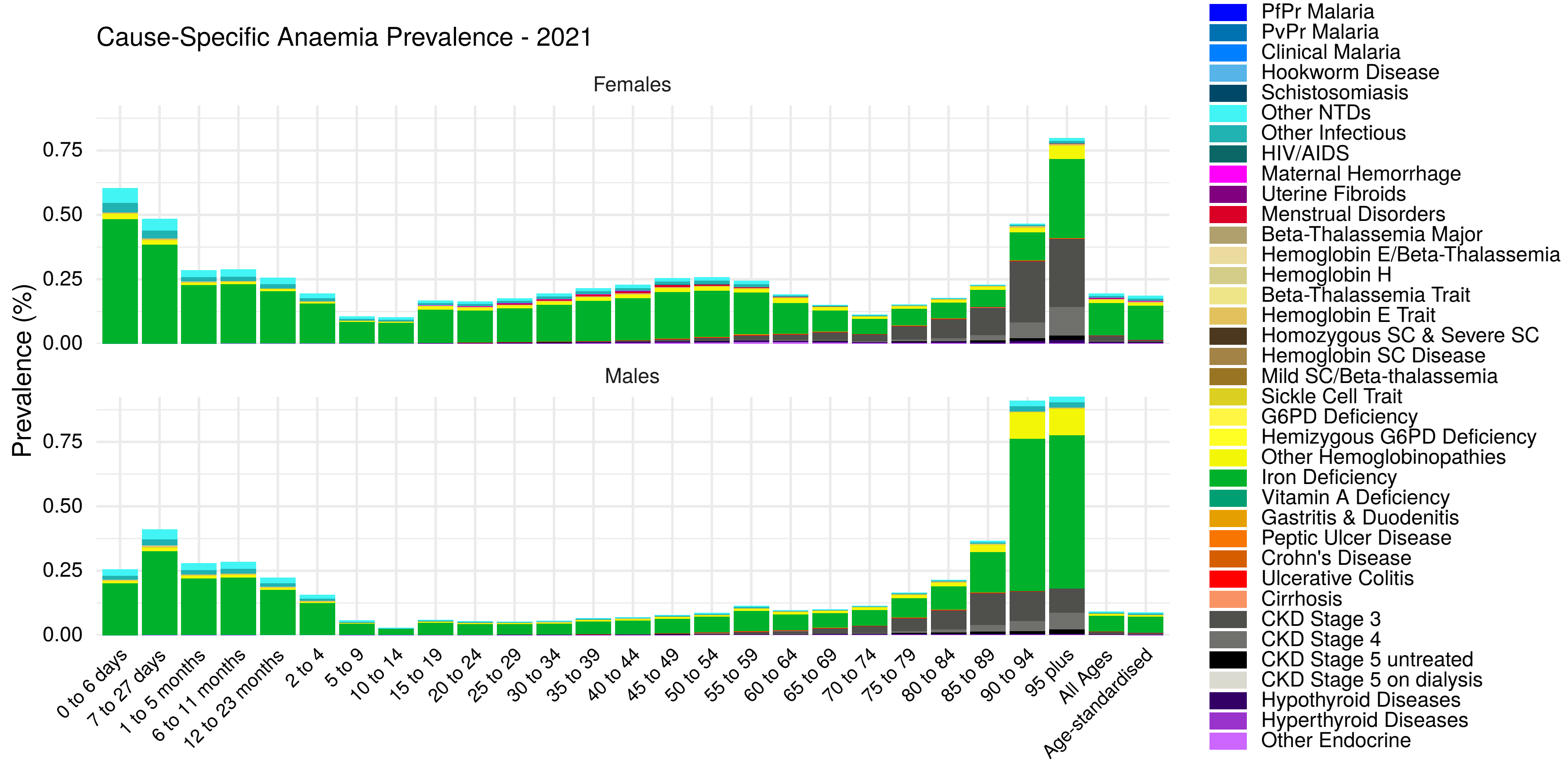
Cause-Specific Anaemia Prevalence - 2021
Females
Males
Prevalence (%)
PfPr Malaria
PvPr Malaria
Clinical Malaria
Hookworm Disease
Schistosomiasis
Other NTDs
Other Infectious
HIV/AIDS
Maternal Hemorrhage
Uterine Fibroids
Menstrual Disorders
Beta-Thalassemia Major
Hemoglobin E/Beta-Thalassemia
Hemoglobin H
Beta-Thalassemia Trait
Hemoglobin E Trait
Homozygous SC & Severe SC
Hemoglobin SC Disease
Mild SC/Beta-thalassemia
Sickle Cell Trait
G6PD Deficiency
Hemizygous G6PD Deficiency
Other Hemoglobinopathies
Iron Deficiency
Vitamin A Deficiency
Gastritis & Duodenitis
Peptic Ulcer Disease
Crohn's Disease
Ulcerative Colitis
Cirrhosis
CKD Stage 3
CKD Stage 4
CKD Stage 5 untreated
CKD Stage 5 on dialysis
Hypothyroid Diseases
Hyperthyroid Diseases
Other Endocrine

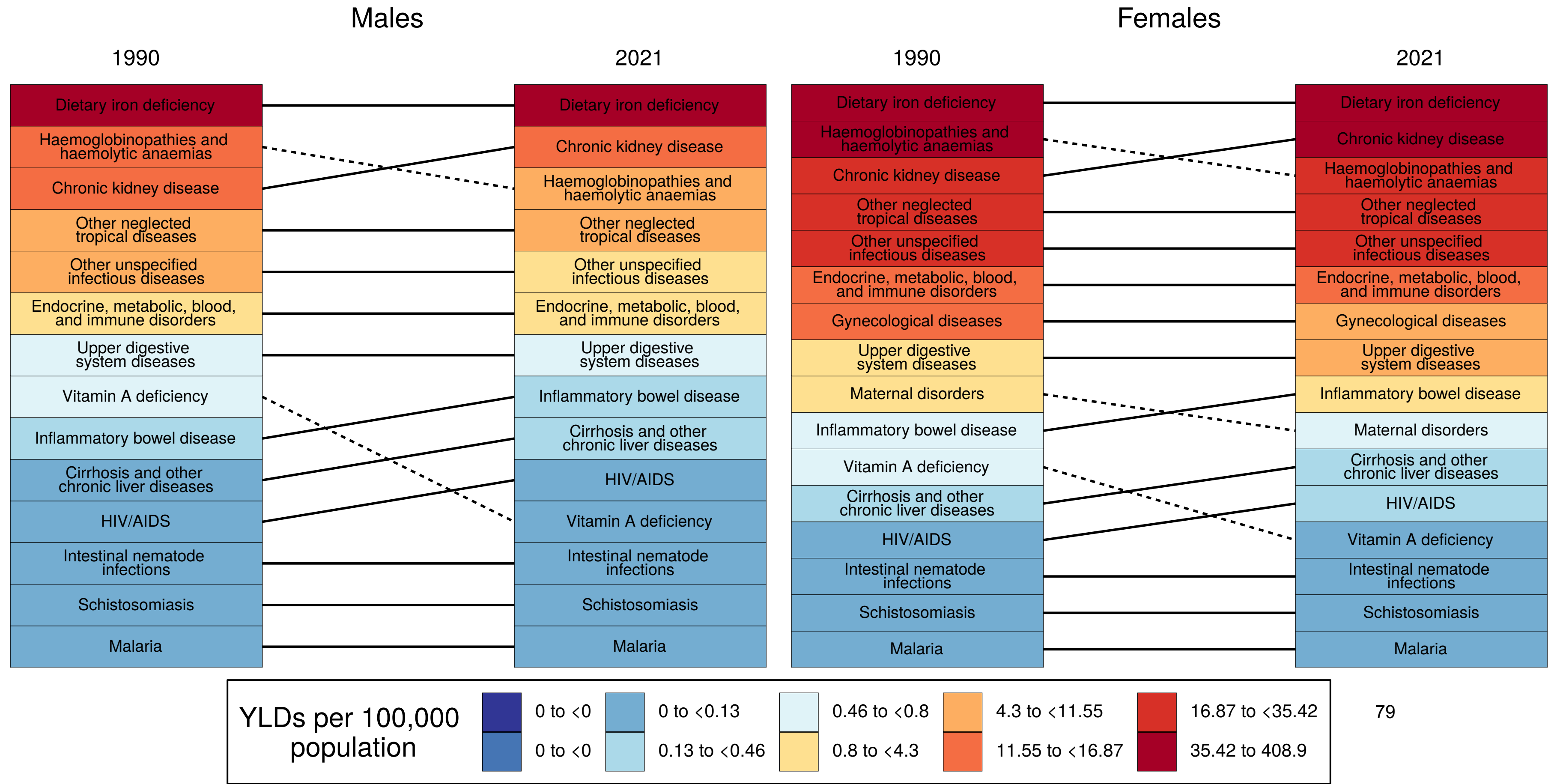
Males
Females
1990
2021
Dietary iron deficiency
Haemoglobinopathies and haemolytic anaemias
Chronic kidney disease
Other neglected tropical diseases
Other unspecified infectious diseases
Endocrine, metabolic, blood, and immune disorders
Gynecological diseases
Upper digestive system diseases
Maternal disorders
Vitamin A deficiency
Inflammatory bowel disease
Cirrhosis and other chronic liver diseases
HIV/AIDS
Intestinal nematode infections
Schistosomiasis
Malaria
YLDs per 100,000 population
0 to <0
0 to <0
0 to <0.13
0.13 to <0.46
0.46 to <0.8
0.8 to <4.3
4.3 to <11.55
11.55 to <16.87
16.87 to <35.42
35.42 to 408.9

Moldova

Anaemia Prevalence - Both Sexes

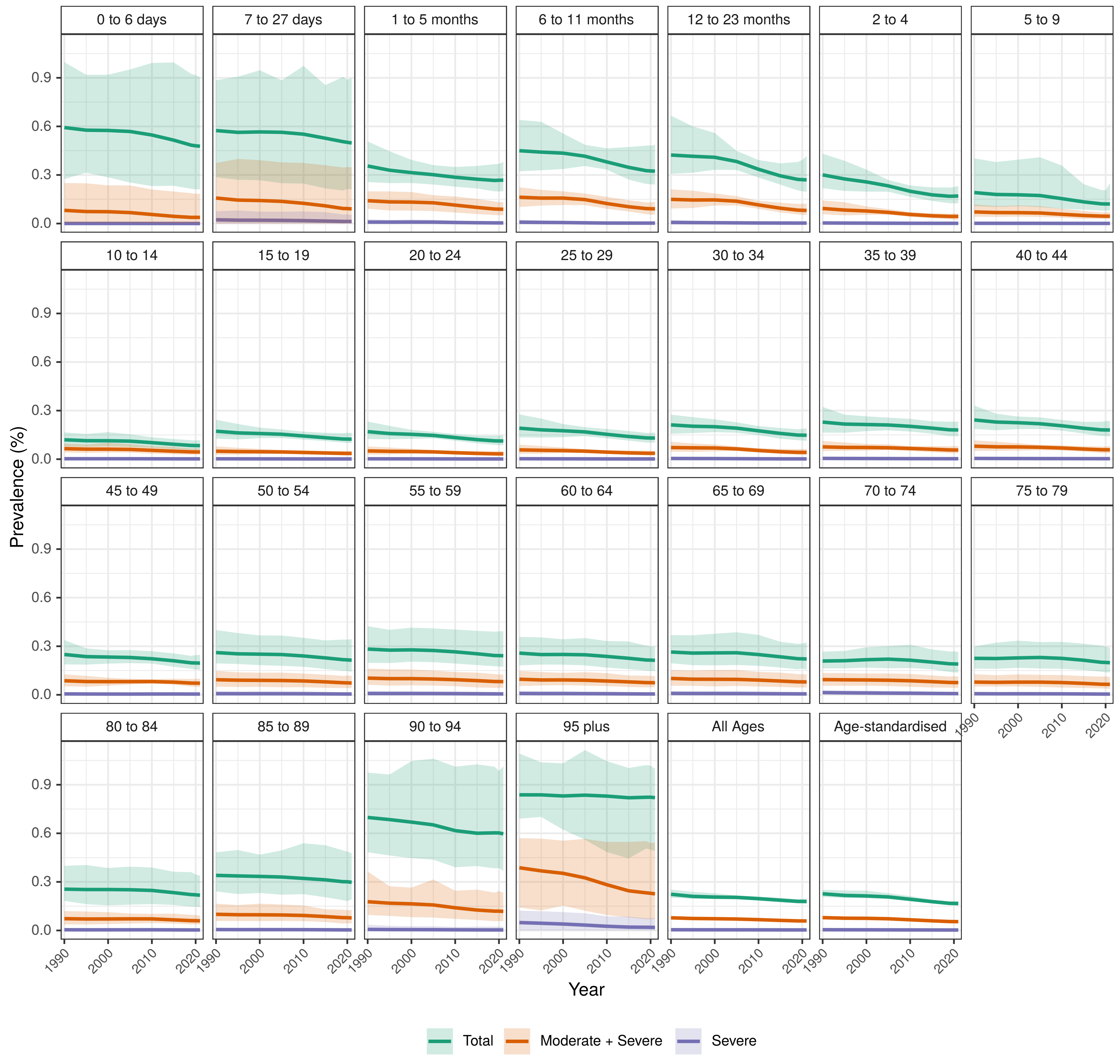

Cause-Specific Anaemia Prevalence - 2021

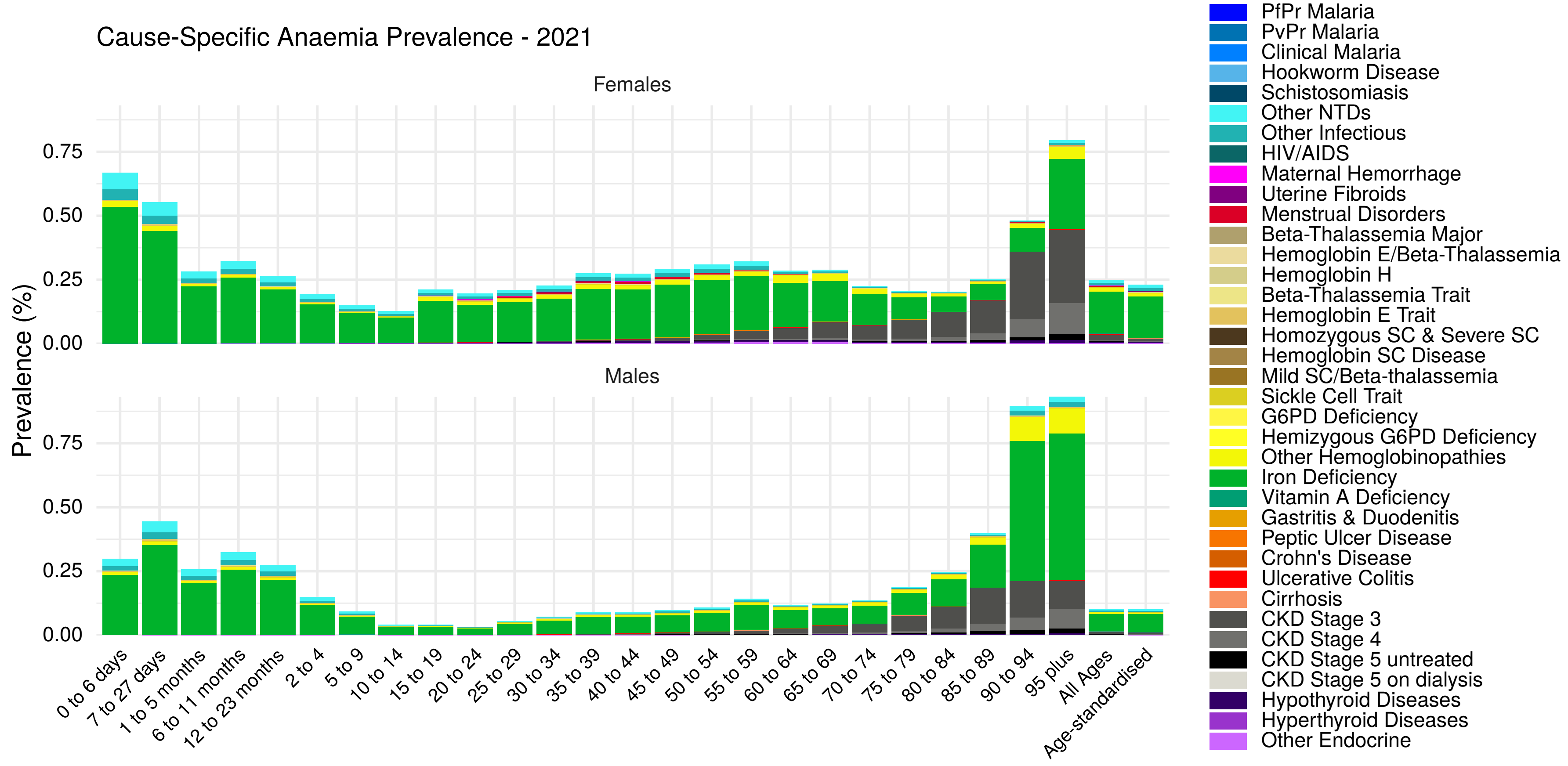

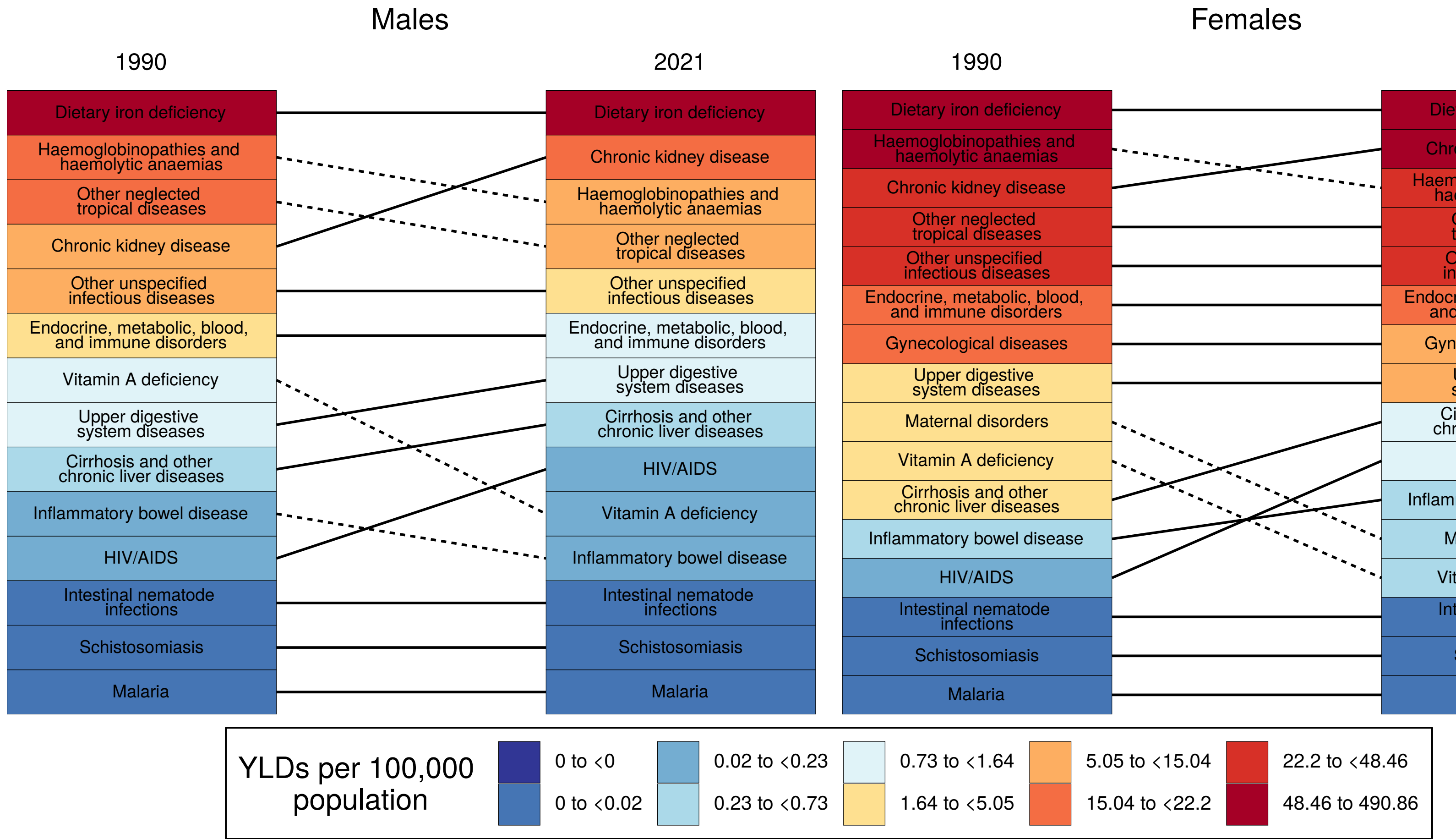

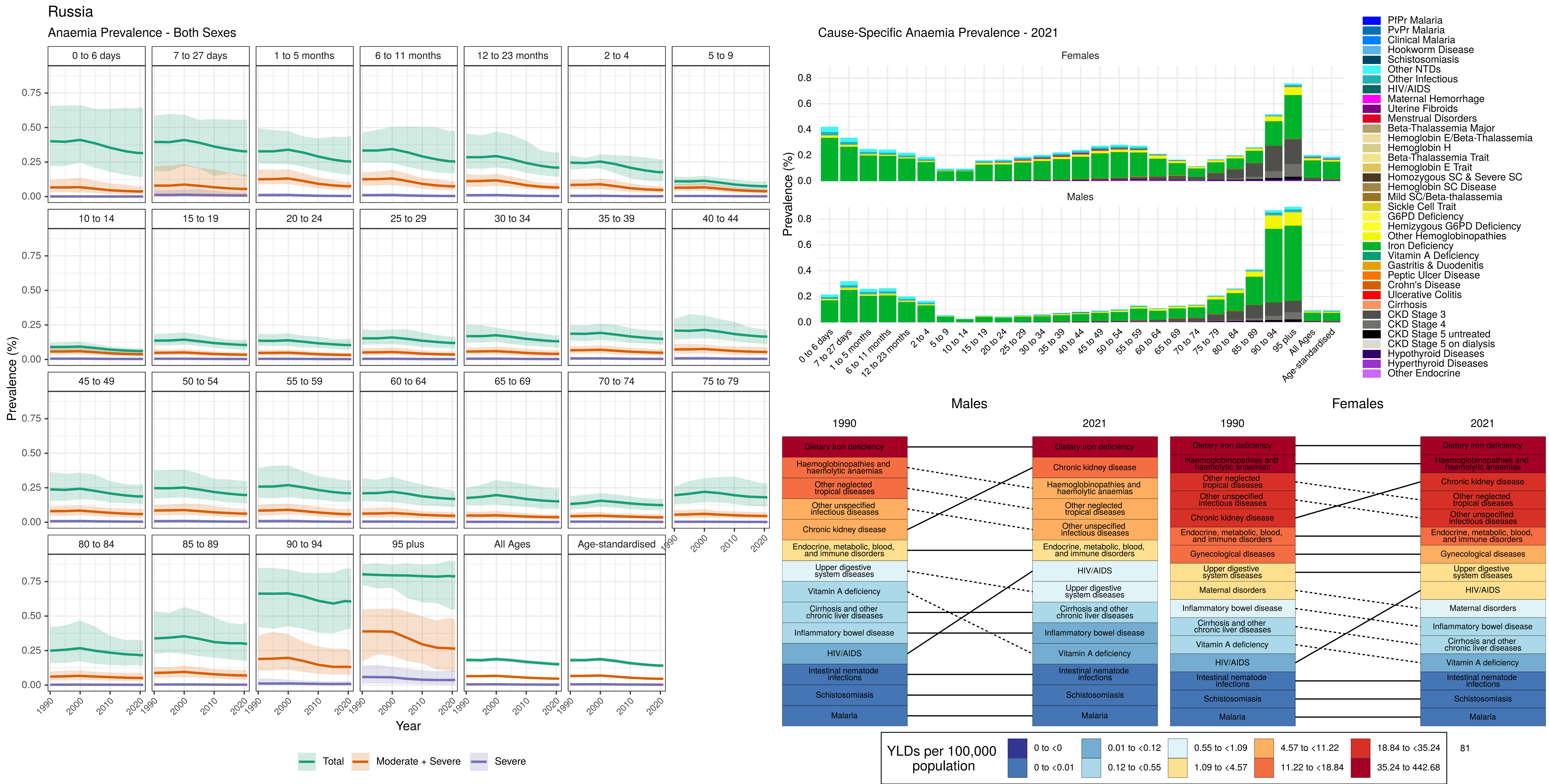

Russia
Anaemia Prevalence - Both Sexes
0 to 6 days
7 to 27 days
1 to 5 months
6 to 11 months
12 to 23 months
2 to 4
5 to 9
10 to 14
15 to 19
20 to 24
25 to 29
30 to 34
35 to 39
40 to 44
45 to 49
50 to 54
55 to 59
60 to 64
65 to 69
70 to 74
75 to 79
80 to 84
85 to 89
90 to 94
95 plus
All Ages
Age-standardised
Prevalence (%)
Year
Total
Moderate + Severe
Severe
Cause-Specific Anaemia Prevalence - 2021
Females
Males
Prevalence (%)
PfPr Malaria
PvPr Malaria
Clinical Malaria
Hookworm Disease
Schistosomiasis
Other NTDs
Other Infectious
HIV/AIDS
Maternal Hemorrhage
Uterine Fibroids
Menstrual Disorders
Beta-Thalassemia Major
Hemoglobin E/Beta-Thalassemia
Hemoglobin H
Beta-Thalassemia Trait
Hemoglobin E Trait
Homozygous SC & Severe SC
Hemoglobin SC Disease
Mild SC/Beta-thalassemia
Sickle Cell Trait
G6PD Deficiency
Hemizygous G6PD Deficiency
Other Hemoglobinopathies
Iron Deficiency
Vitamin A Deficiency
Gastritis & Duodenitis
Peptic Ulcer Disease
Crohn's Disease
Ulcerative Colitis
Cirrhosis
CKD Stage 3
CKD Stage 4
CKD Stage 5 untreated
CKD Stage 5 on dialysis
Hypothyroid Diseases
Hyperthyroid Diseases
Other Endocrine
Males
Females
1990
2021
Males 1990: Dietary iron deficiency; Haemoglobinopathies and haemolytic anaemias; Other neglected tropical diseases; Other unspecified infectious diseases; Chronic kidney disease; Endocrine, metabolic, blood, and immune disorders; Upper digestive system diseases; Vitamin A deficiency; Cirrhosis and other chronic liver diseases; Inflammatory bowel disease; HIV/AIDS; Intestinal nematode infections; Schistosomiasis; Malaria
Males 2021: Dietary iron deficiency; Chronic kidney disease; Haemoglobinopathies and haemolytic anaemias; Other neglected tropical diseases; Other unspecified infectious diseases; Endocrine, metabolic, blood, and immune disorders; HIV/AIDS; Upper digestive system diseases; Cirrhosis and other chronic liver diseases; Inflammatory bowel disease; Vitamin A deficiency; Intestinal nematode infections; Schistosomiasis; Malaria
Females 1990: Dietary iron deficiency; Haemoglobinopathies and haemolytic anaemias; Other neglected tropical diseases; Other unspecified infectious diseases; Chronic kidney disease; Endocrine, metabolic, blood, and immune disorders; Gynecological diseases; Upper digestive system diseases; Maternal disorders; Inflammatory bowel disease; Cirrhosis and other chronic liver diseases; Vitamin A deficiency; HIV/AIDS; Intestinal nematode infections; Schistosomiasis; Malaria
Females 2021: Dietary iron deficiency; Haemoglobinopathies and haemolytic anaemias; Chronic kidney disease; Other neglected tropical diseases; Other unspecified infectious diseases; Endocrine, metabolic, blood, and immune disorders; Gynecological diseases; Upper digestive system diseases; HIV/AIDS; Maternal disorders; Inflammatory bowel disease; Cirrhosis and other chronic liver diseases; Vitamin A deficiency; Intestinal nematode infections; Schistosomiasis; Malaria
YLDs per 100,000 population
0 to <0
0 to <0.01
0.01 to <0.12
0.12 to <0.55
0.55 to <1.09
1.09 to <4.57
4.57 to <11.22
11.22 to <18.84
18.84 to <35.24
35.24 to 442.68

Ukraine

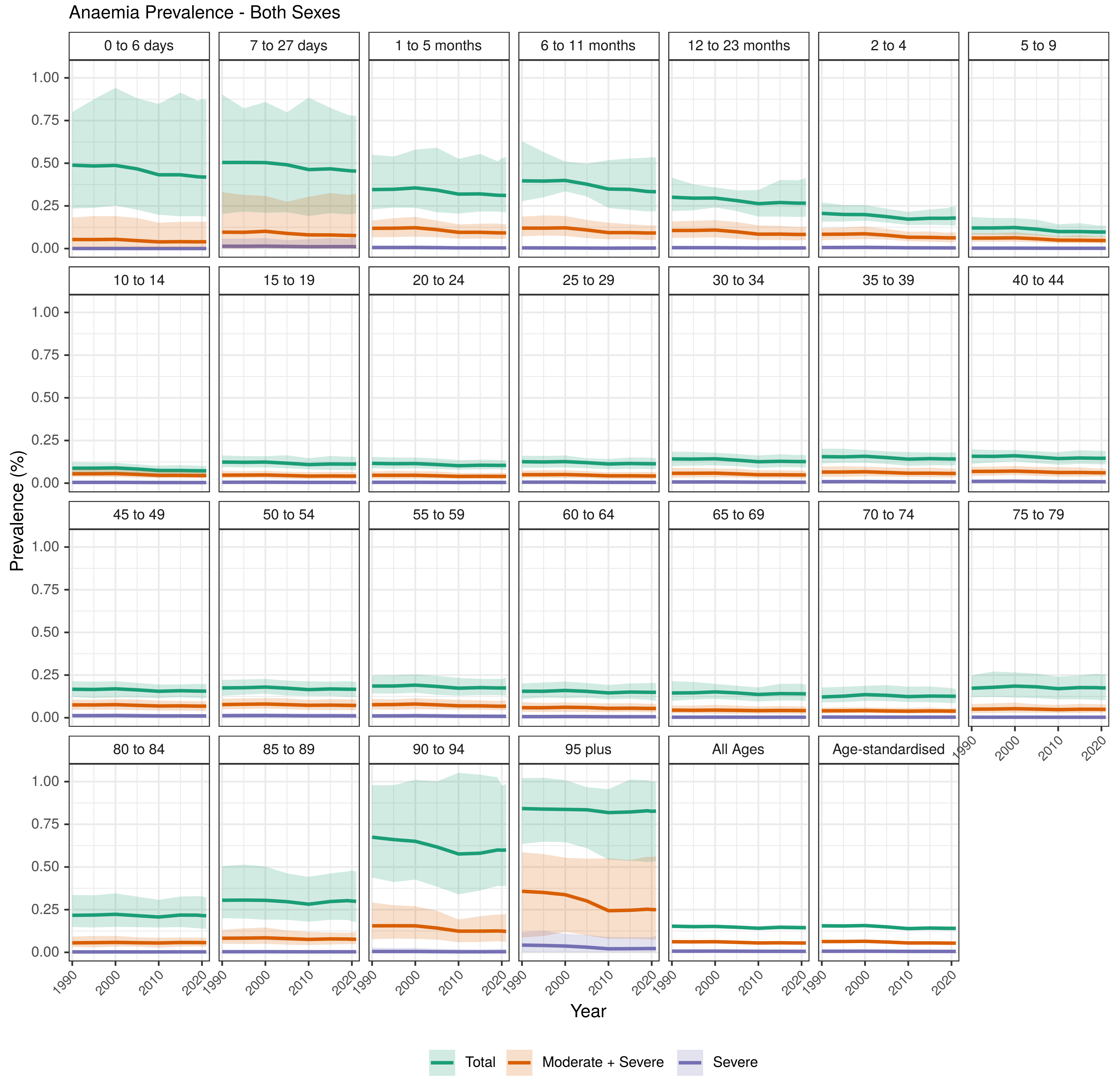

Anaemia Prevalence - Both Sexes
0 to 6 days
7 to 27 days
1 to 5 months
6 to 11 months
12 to 23 months
2 to 4
5 to 9
10 to 14
15 to 19
20 to 24
25 to 29
30 to 34
35 to 39
40 to 44
45 to 49
50 to 54
55 to 59
60 to 64
65 to 69
70 to 74
75 to 79
80 to 84
85 to 89
90 to 94
95 plus
All Ages
Age-standardised
Prevalence (%)
Year
Total
Moderate + Severe
Severe

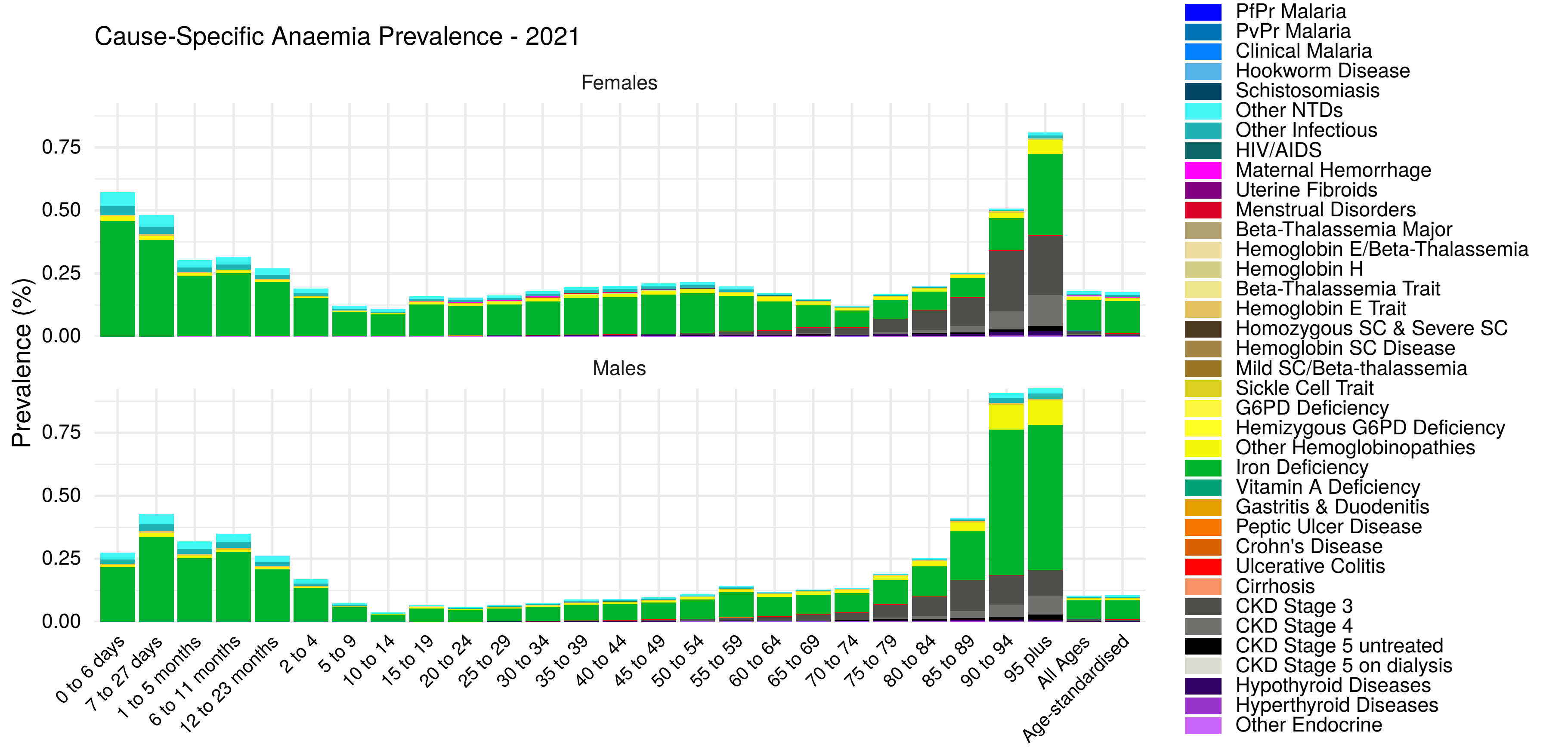

Cause-Specific Anaemia Prevalence - 2021
Females
Males
Prevalence (%)
PfPr Malaria
PvPr Malaria
Clinical Malaria
Hookworm Disease
Schistosomiasis
Other NTDs
Other Infectious
HIV/AIDS
Maternal Hemorrhage
Uterine Fibroids
Menstrual Disorders
Beta-Thalassemia Major
Hemoglobin E/Beta-Thalassemia
Hemoglobin H
Beta-Thalassemia Trait
Hemoglobin E Trait
Homozygous SC & Severe SC
Hemoglobin SC Disease
Mild SC/Beta-thalassemia
Sickle Cell Trait
G6PD Deficiency
Hemizygous G6PD Deficiency
Other Hemoglobinopathies
Iron Deficiency
Vitamin A Deficiency
Gastritis & Duodenitis
Peptic Ulcer Disease
Crohn's Disease
Ulcerative Colitis
Cirrhosis
CKD Stage 3
CKD Stage 4
CKD Stage 5 untreated
CKD Stage 5 on dialysis
Hypothyroid Diseases
Hyperthyroid Diseases
Other Endocrine

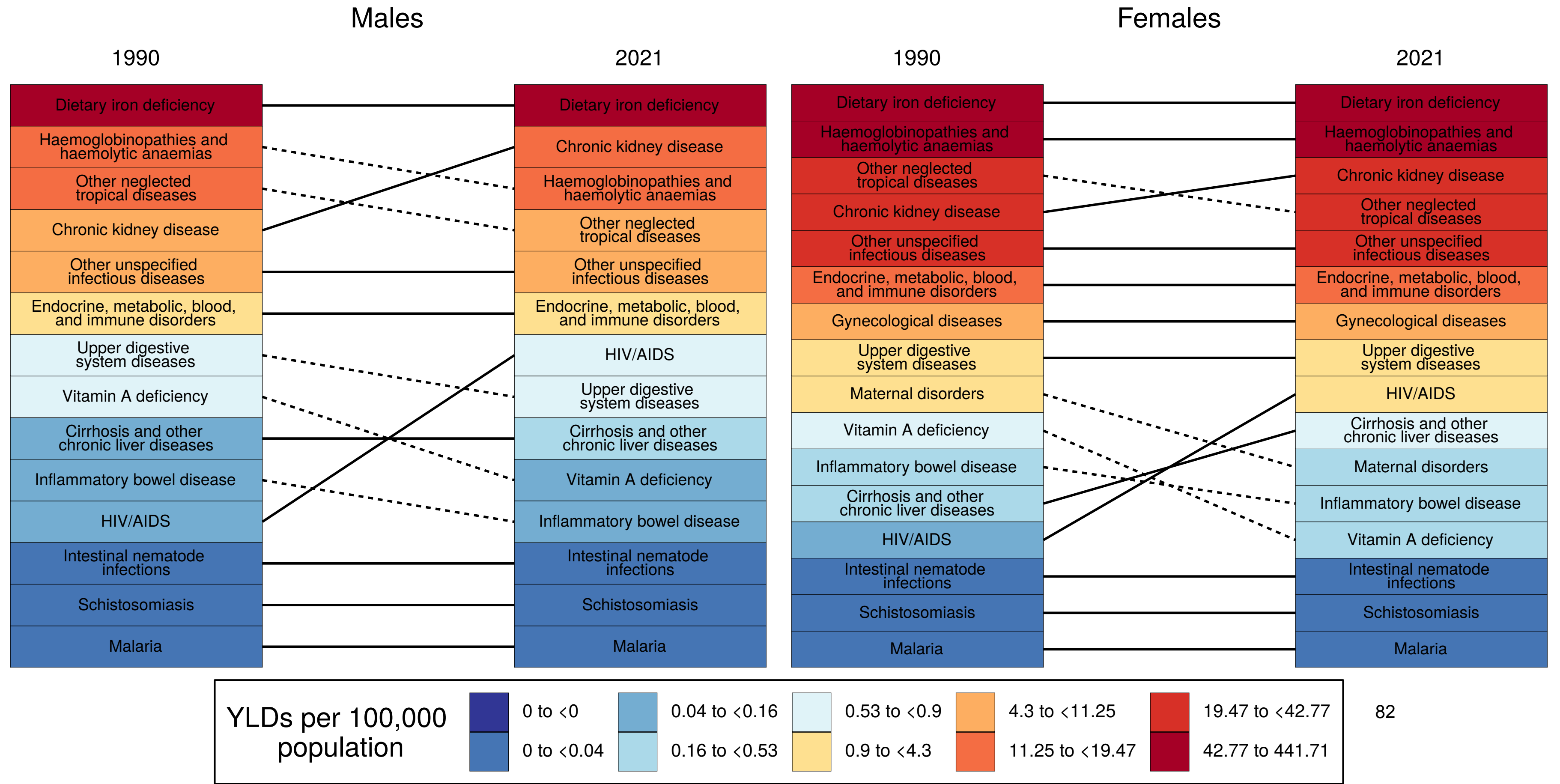

Males
1990
2021
Dietary iron deficiency
Haemoglobinopathies and haemolytic anaemias
Other neglected tropical diseases
Chronic kidney disease
Other unspecified infectious diseases
Endocrine, metabolic, blood, and immune disorders
Upper digestive system diseases
Vitamin A deficiency
Cirrhosis and other chronic liver diseases
Inflammatory bowel disease
HIV/AIDS
Intestinal nematode infections
Schistosomiasis
Malaria
Females
Gynecological diseases
Maternal disorders
YLDs per 100,000 population
0 to <0
0 to <0.04
0.04 to <0.16
0.16 to <0.53
0.53 to <0.9
0.9 to <4.3
4.3 to <11.25
11.25 to <19.47
19.47 to <42.77
42.77 to 441.71

High income

Anaemia Prevalence - Both Sexes

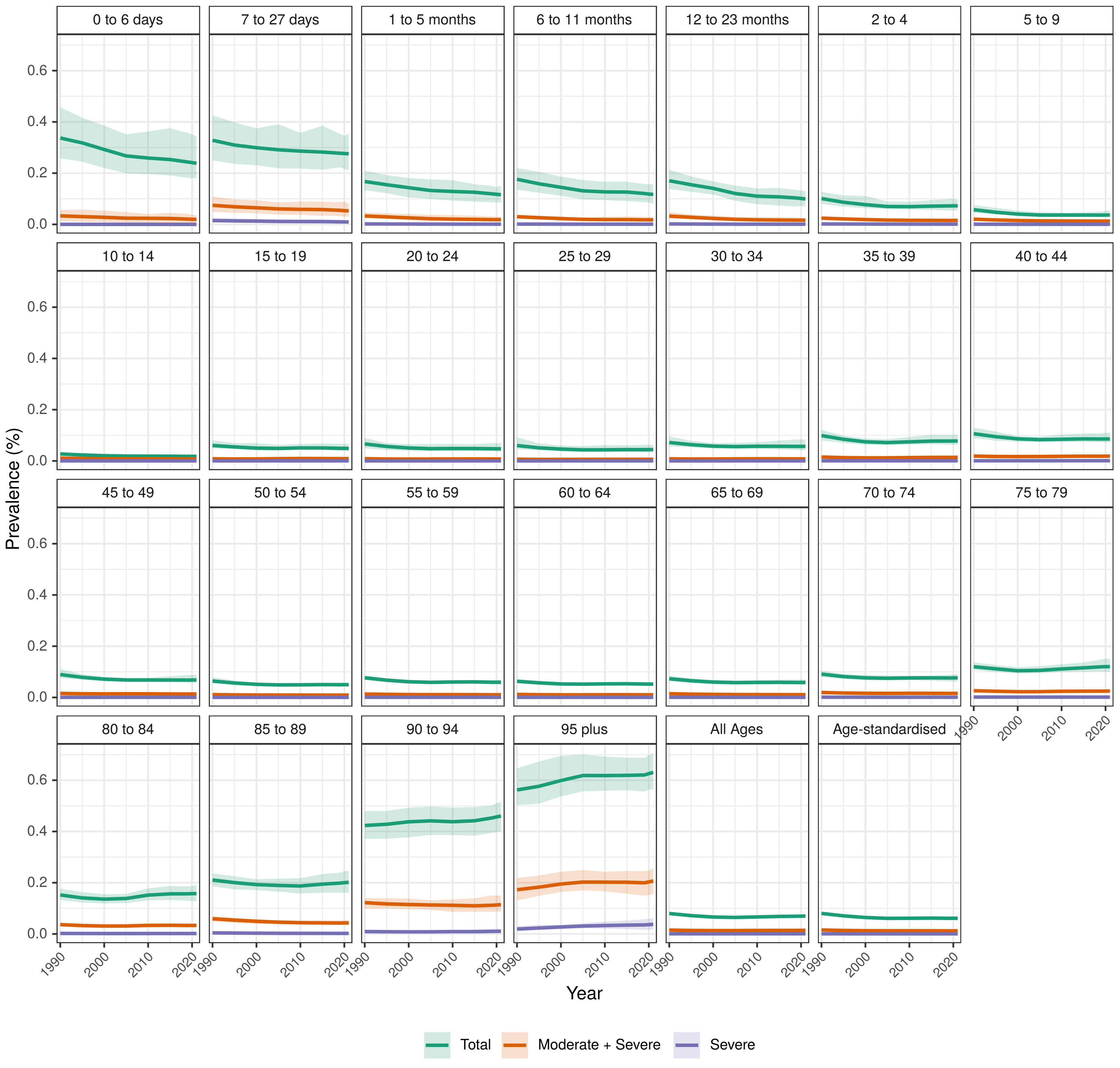

Cause-Specific Anaemia Prevalence - 2021

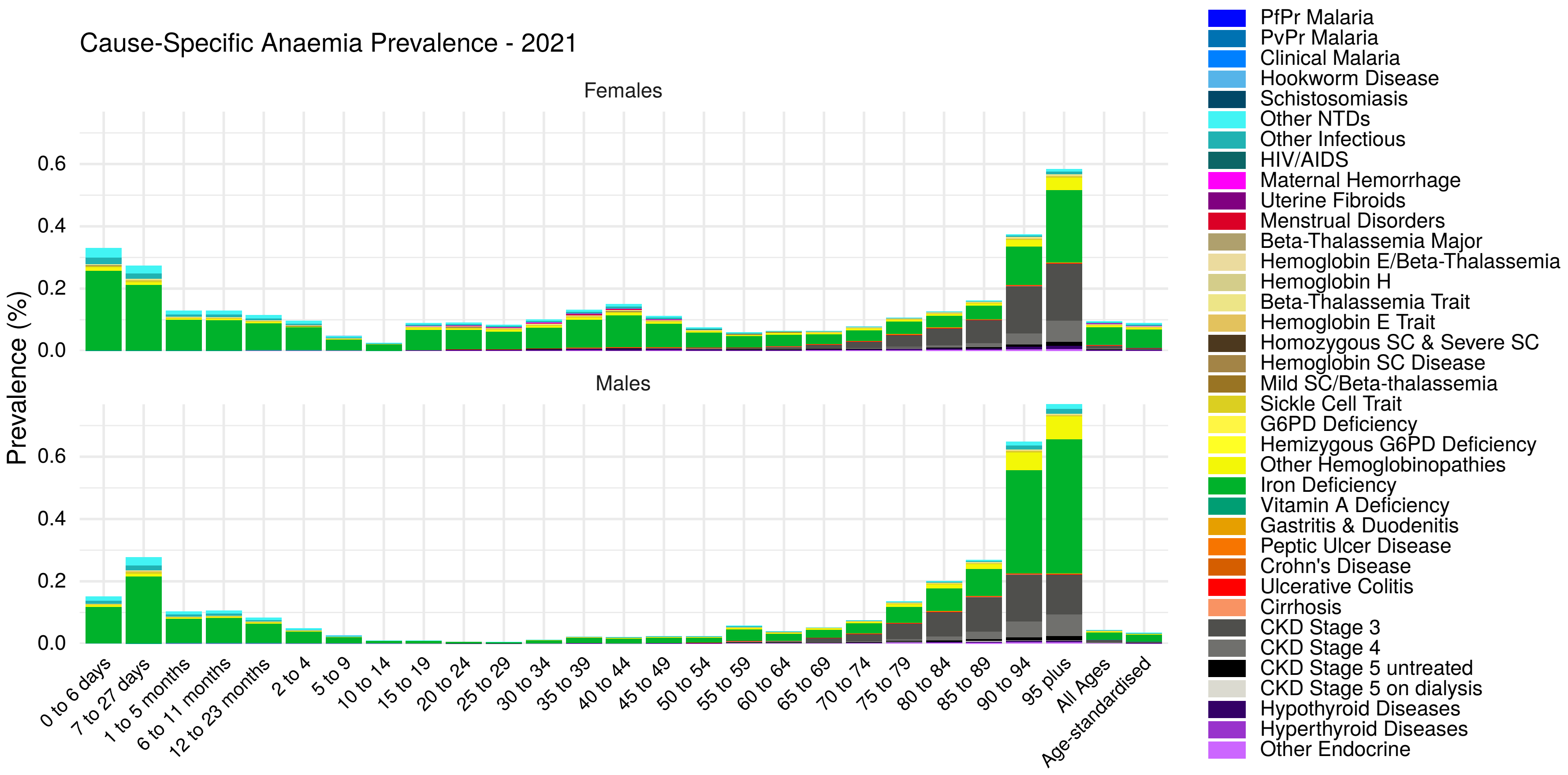

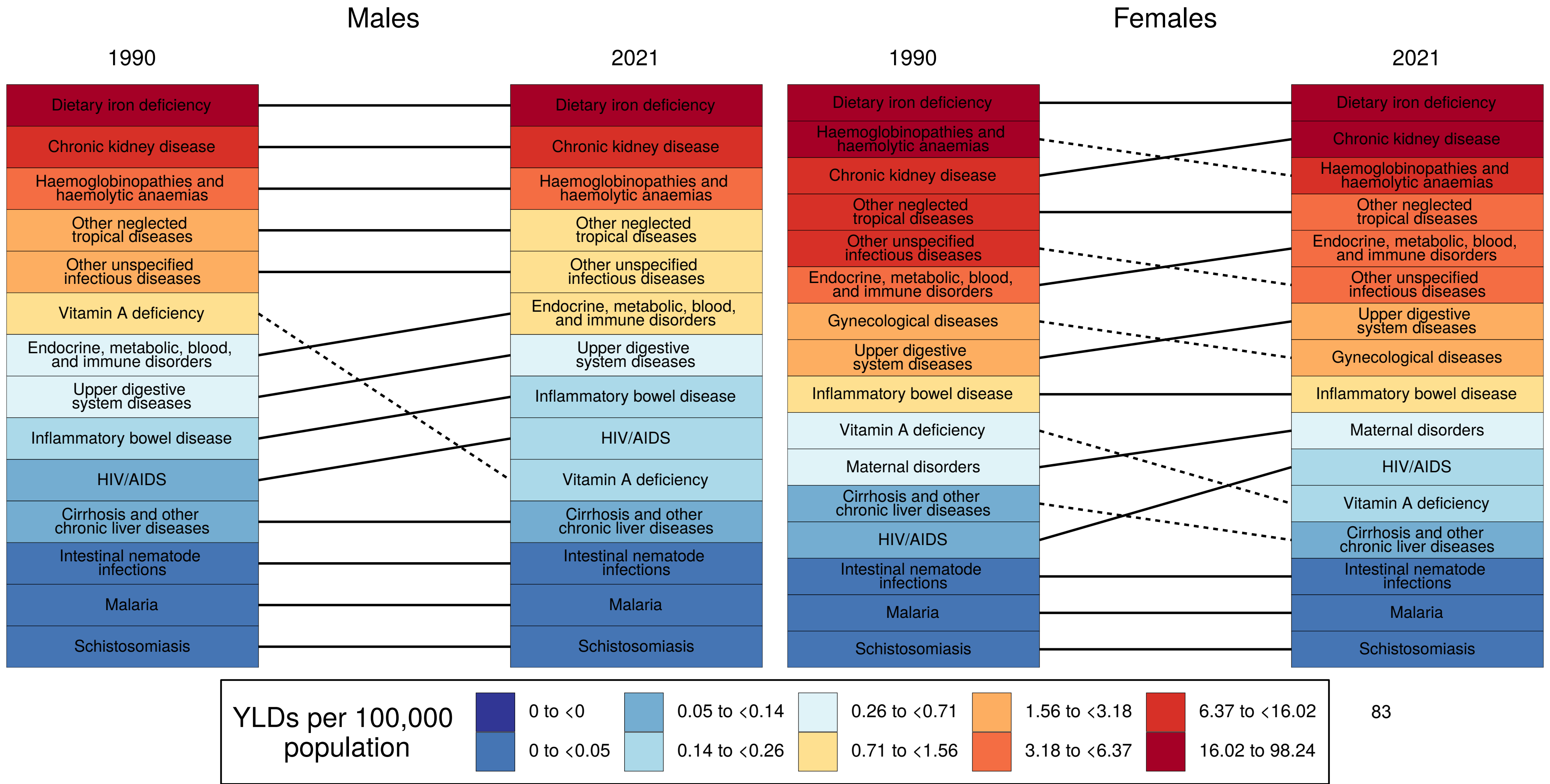

Australasia

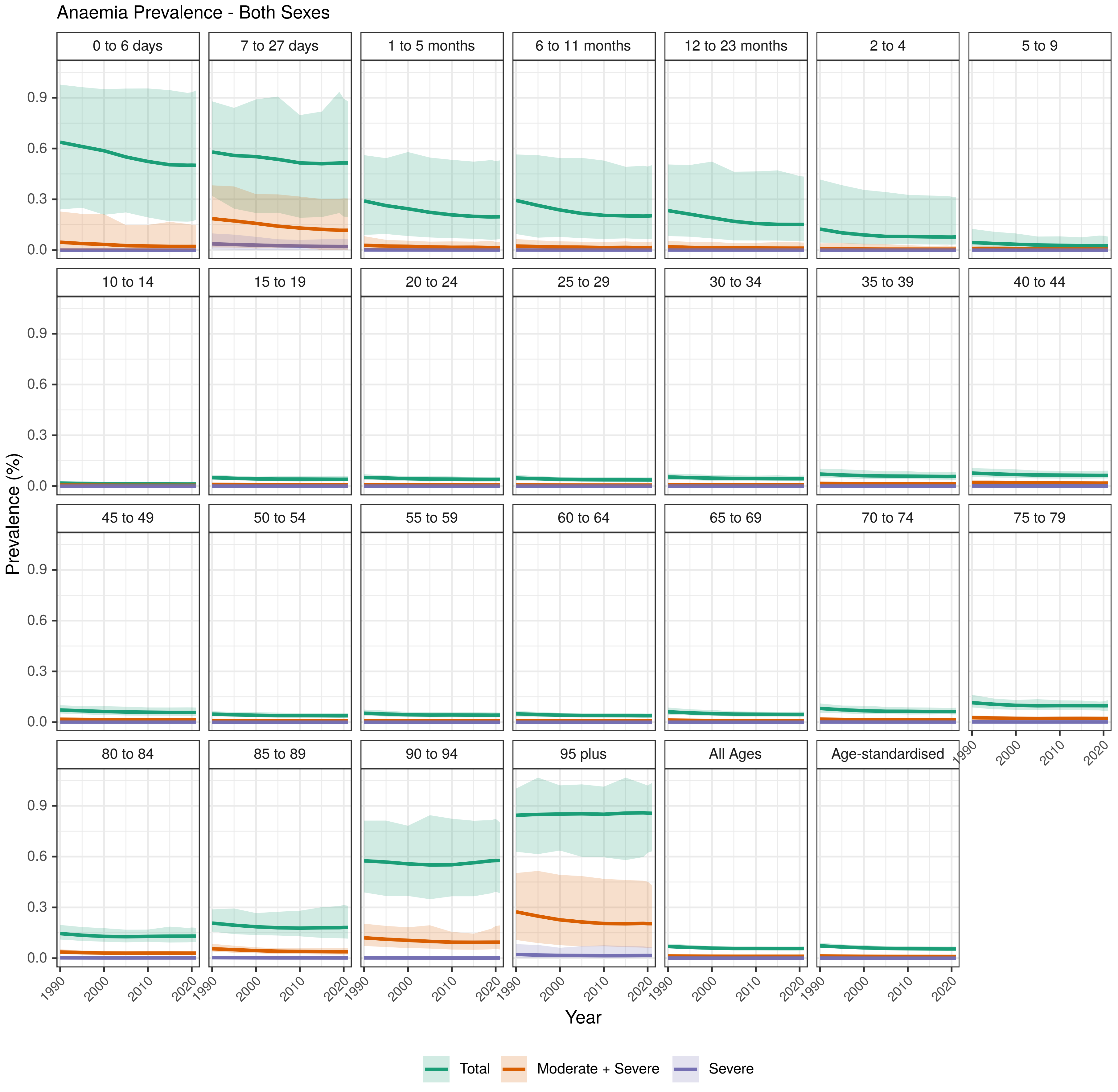

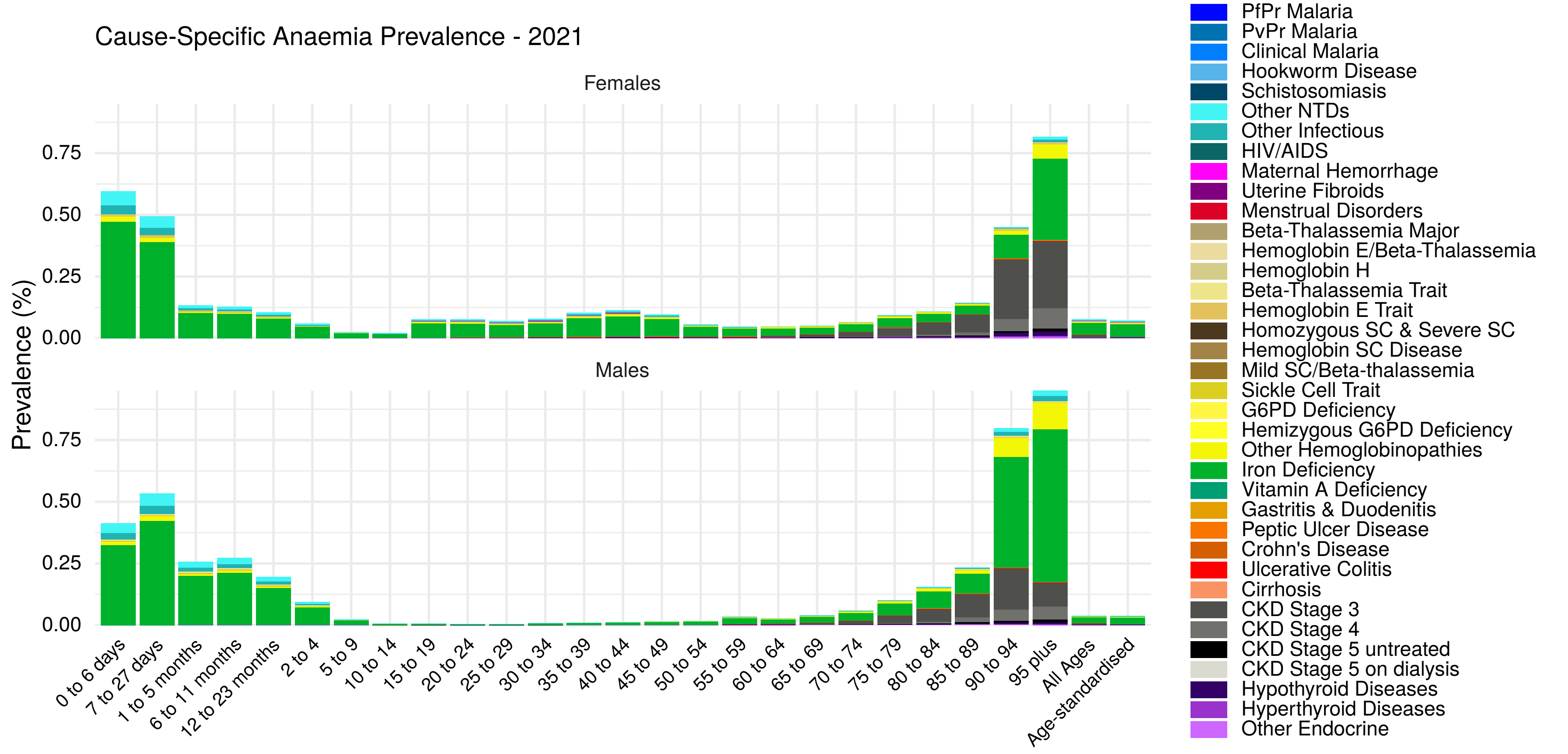

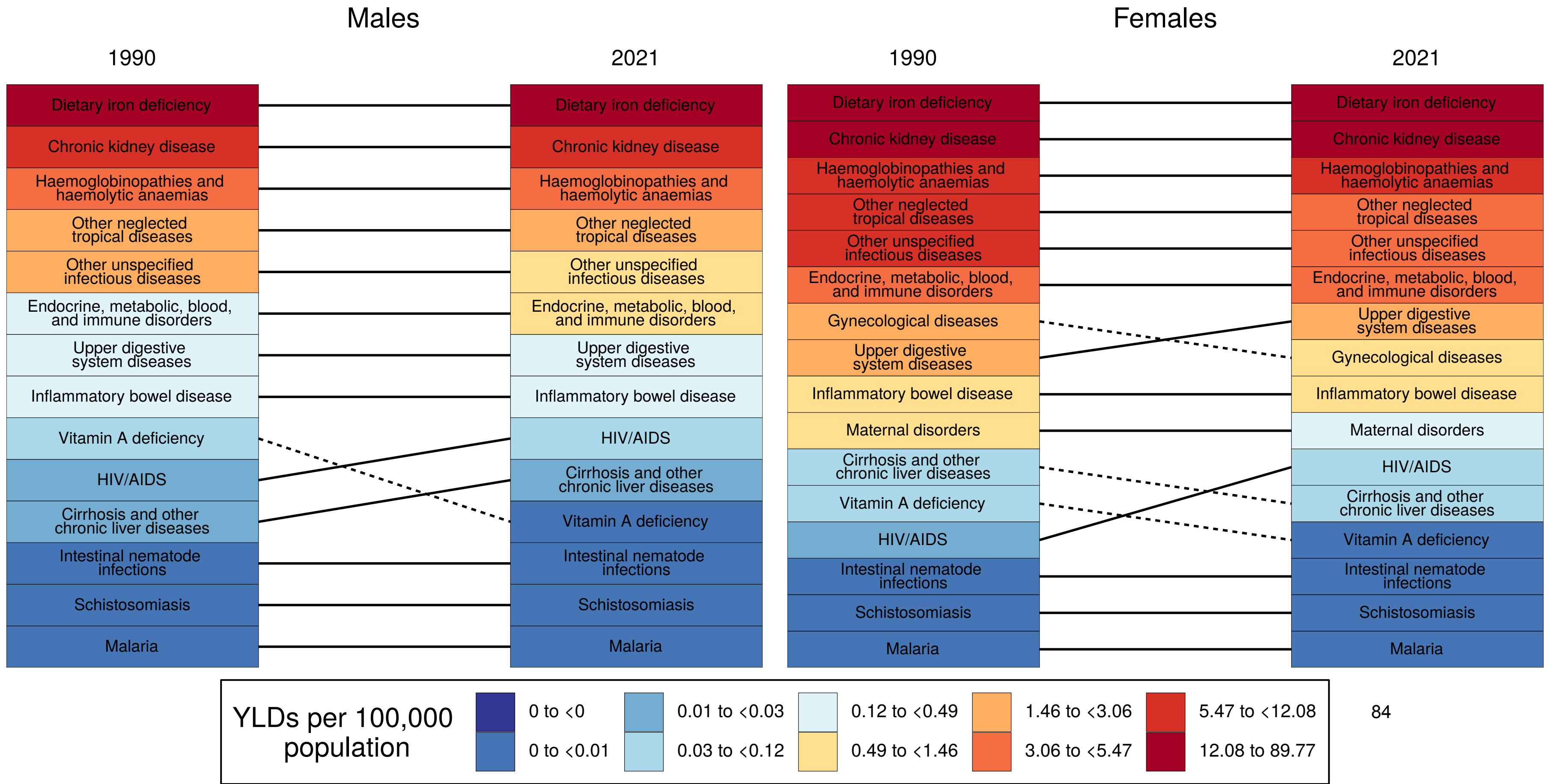

Australia

Anaemia Prevalence - Both Sexes

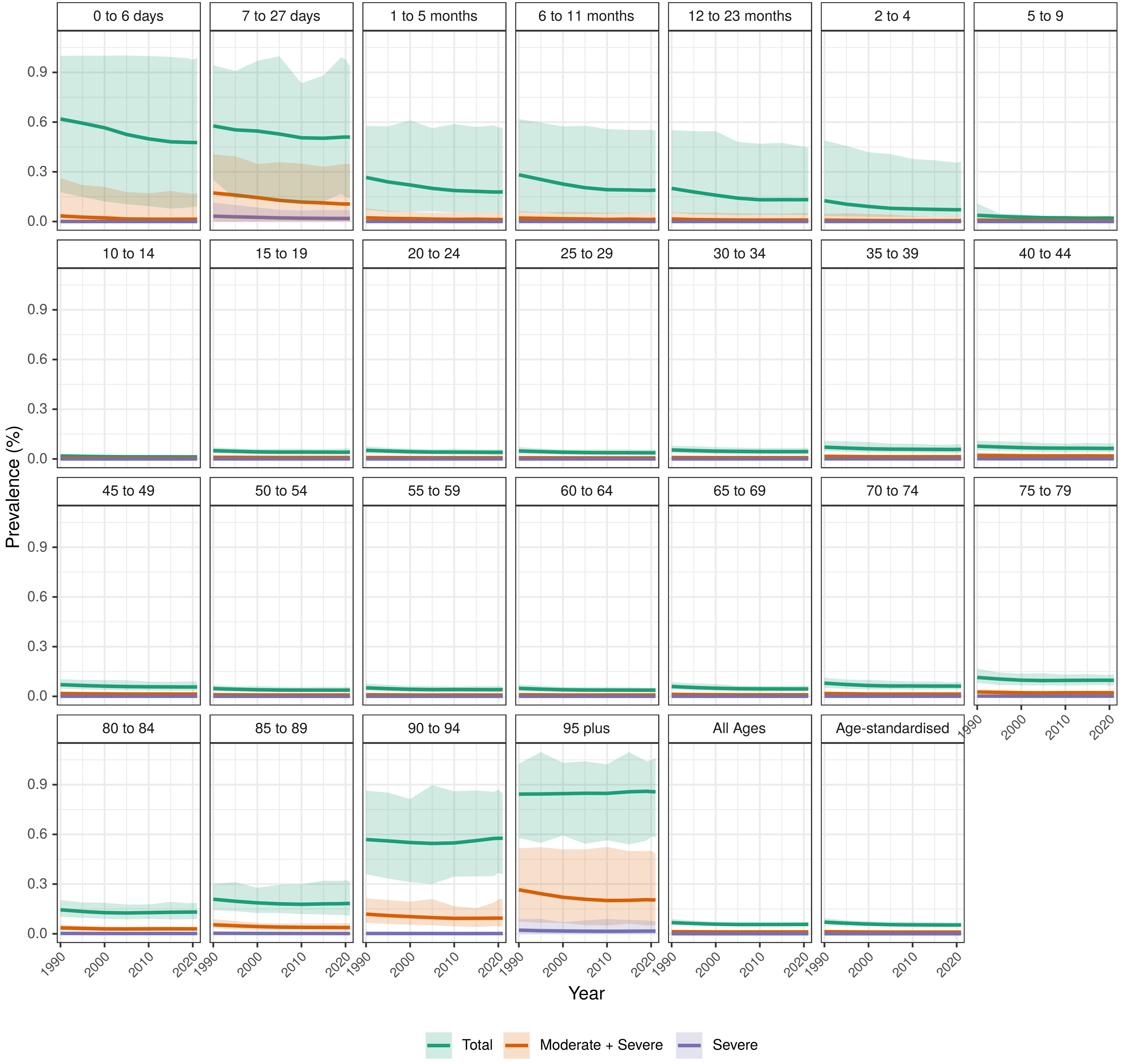

Cause-Specific Anaemia Prevalence - 2021

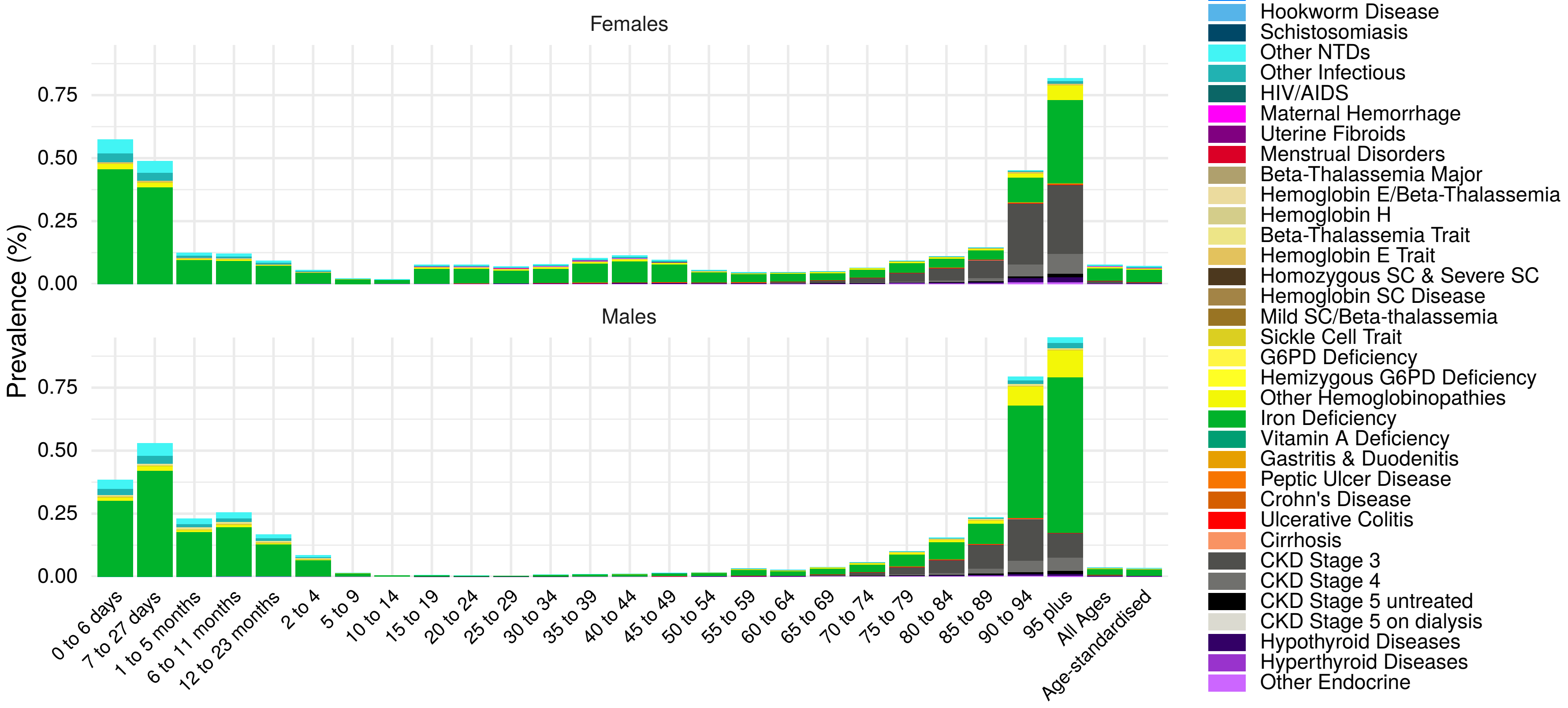

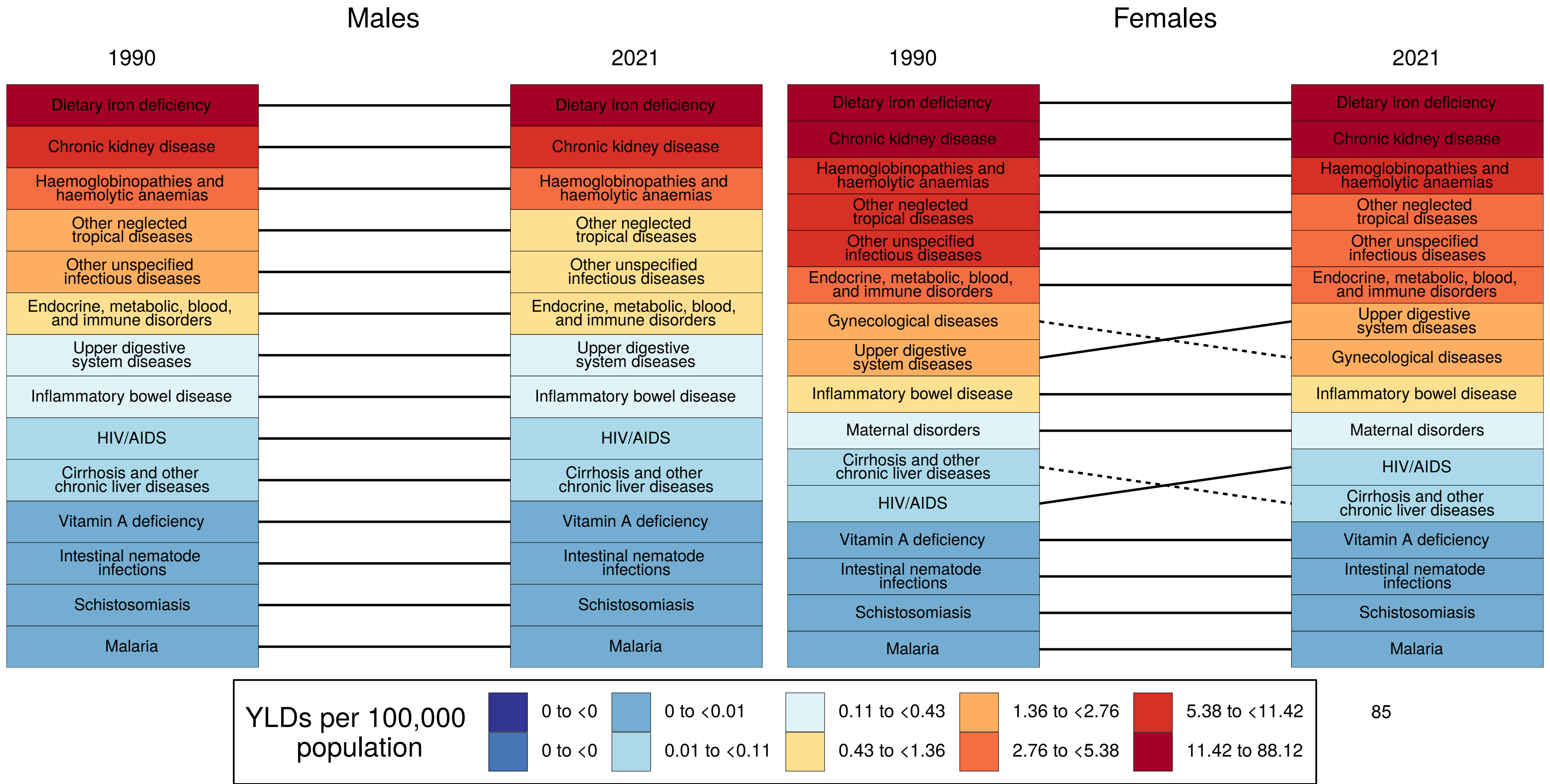

New Zealand

Anaemia Prevalence - Both Sexes

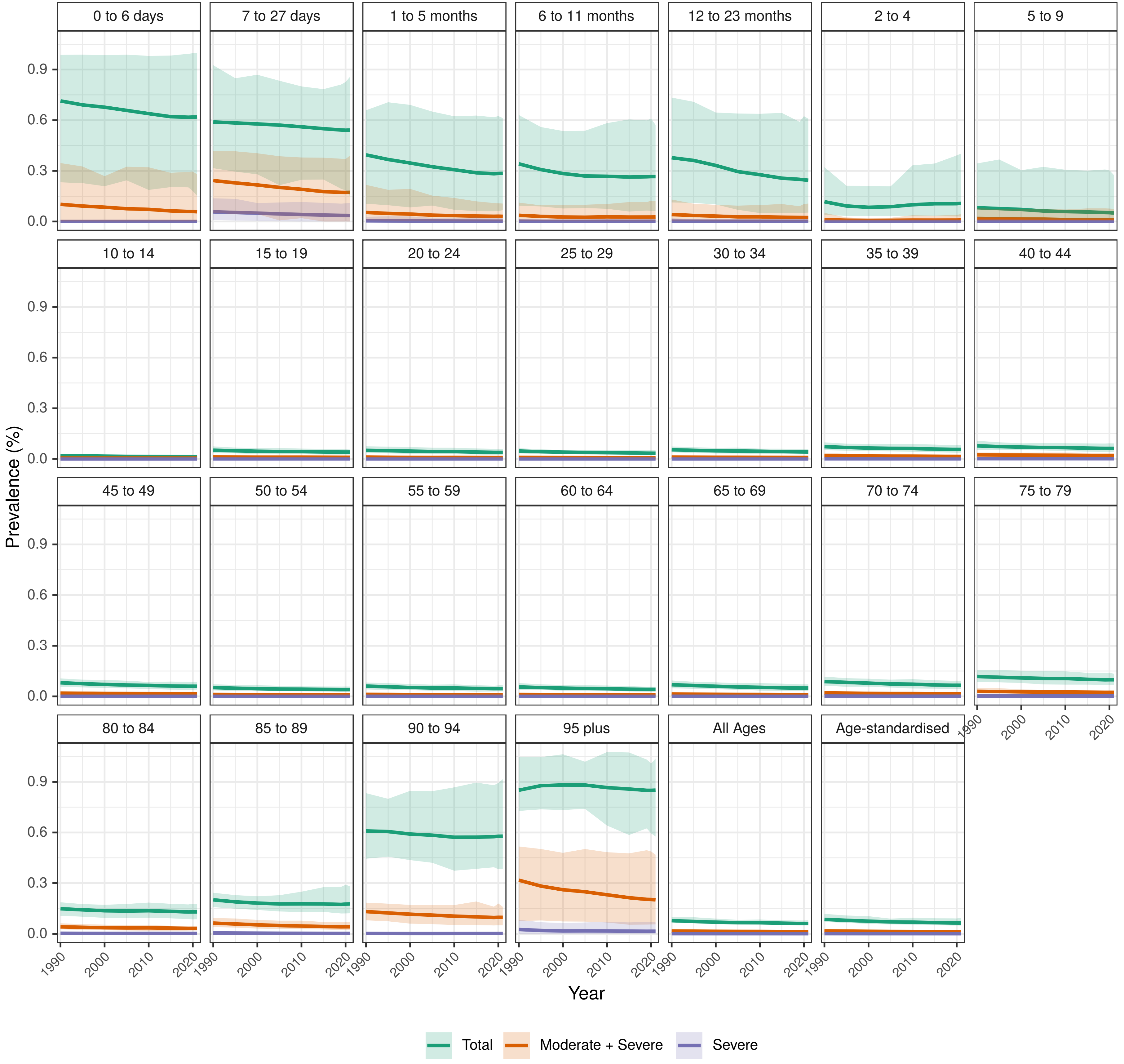

Cause-Specific Anaemia Prevalence - 2021

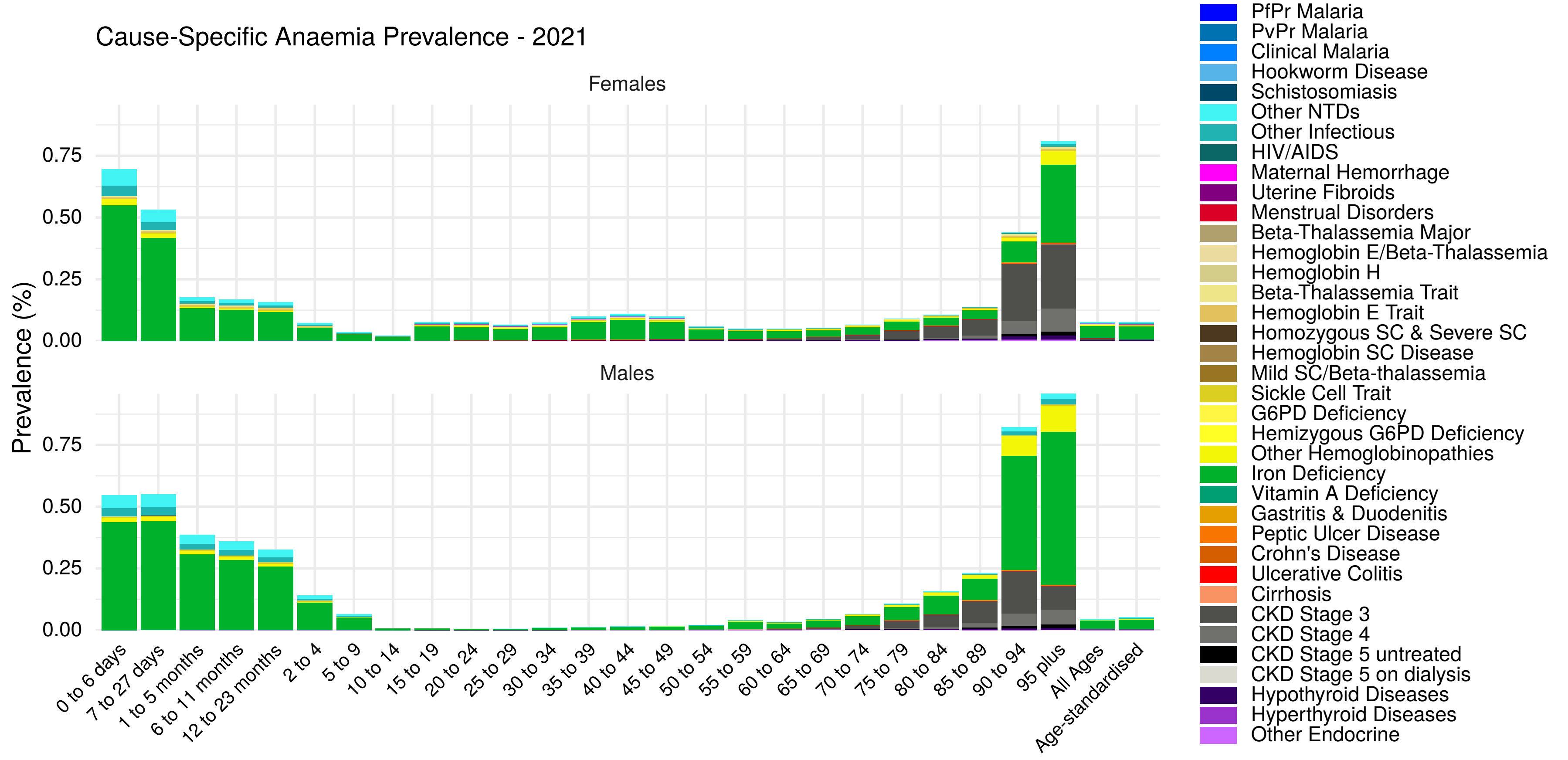

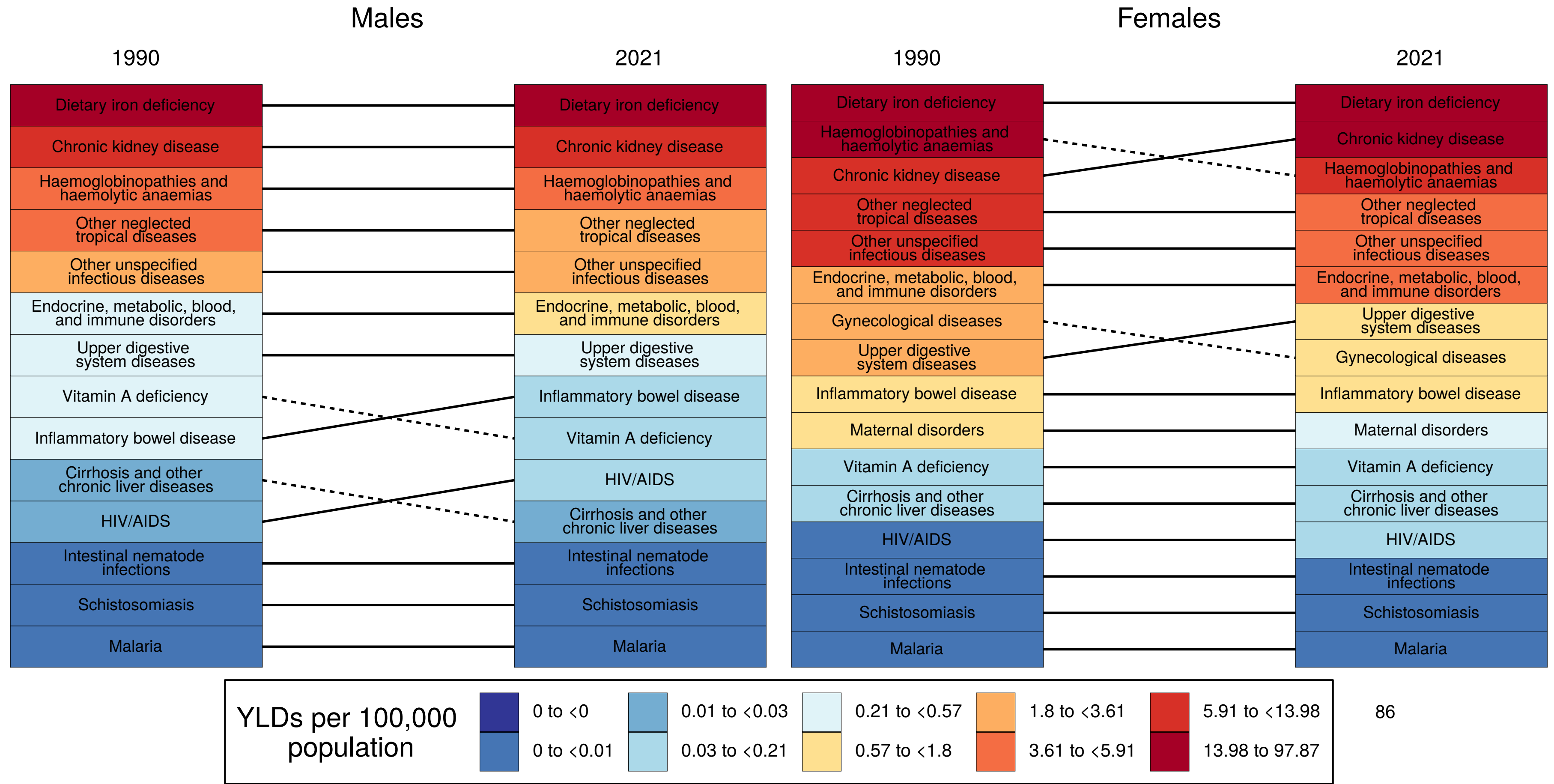

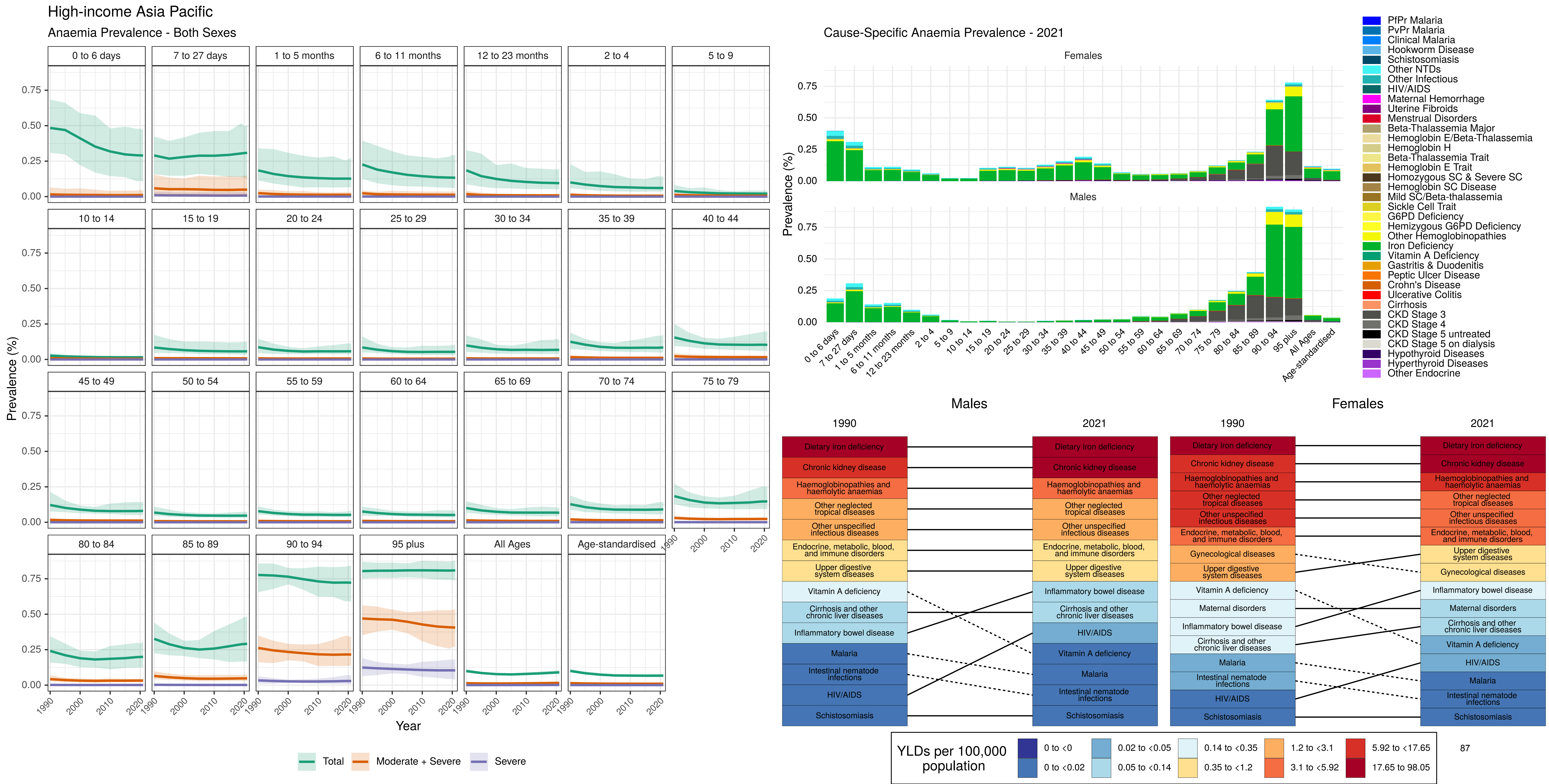
High-income Asia Pacific
Anaemia Prevalence - Both Sexes
0 to 6 days
7 to 27 days
1 to 5 months
6 to 11 months
12 to 23 months
2 to 4
5 to 9
10 to 14
15 to 19
20 to 24
25 to 29
30 to 34
35 to 39
40 to 44
45 to 49
50 to 54
55 to 59
60 to 64
65 to 69
70 to 74
75 to 79
80 to 84
85 to 89
90 to 94
95 plus
All Ages
Age-standardised
Prevalence (%)
0.00
0.25
0.50
0.75
1990
2000
2010
2020
Year
Total
Moderate + Severe
Severe
Cause-Specific Anaemia Prevalence - 2021
Females
Males
Prevalence (%)
PfPr Malaria
PvPr Malaria
Clinical Malaria
Hookworm Disease
Schistosomiasis
Other NTDs
Other Infectious
HIV/AIDS
Maternal Hemorrhage
Uterine Fibroids
Menstrual Disorders
Beta-Thalassemia Major
Hemoglobin E/Beta-Thalassemia
Hemoglobin H
Beta-Thalassemia Trait
Hemoglobin E Trait
Homozygous SC & Severe SC
Hemoglobin SC Disease
Mild SC/Beta-thalassemia
Sickle Cell Trait
G6PD Deficiency
Hemizygous G6PD Deficiency
Other Hemoglobinopathies
Iron Deficiency
Vitamin A Deficiency
Gastritis & Duodenitis
Peptic Ulcer Disease
Crohn's Disease
Ulcerative Colitis
Cirrhosis
CKD Stage 3
CKD Stage 4
CKD Stage 5 untreated
CKD Stage 5 on dialysis
Hypothyroid Diseases
Hyperthyroid Diseases
Other Endocrine
Males
Females
1990
2021
Dietary iron deficiency
Chronic kidney disease
Haemoglobinopathies and haemolytic anaemias
Other neglected tropical diseases
Other unspecified infectious diseases
Endocrine, metabolic, blood, and immune disorders
Upper digestive system diseases
Vitamin A deficiency
Cirrhosis and other chronic liver diseases
Inflammatory bowel disease
Malaria
Intestinal nematode infections
HIV/AIDS
Schistosomiasis
Gynecological diseases
Maternal disorders
YLDs per 100,000 population
0 to <0
0 to <0.02
0.02 to <0.05
0.05 to <0.14
0.14 to <0.35
0.35 to <1.2
1.2 to <3.1
3.1 to <5.92
5.92 to <17.65
17.65 to 98.05

Brunei

Anaemia Prevalence - Both Sexes

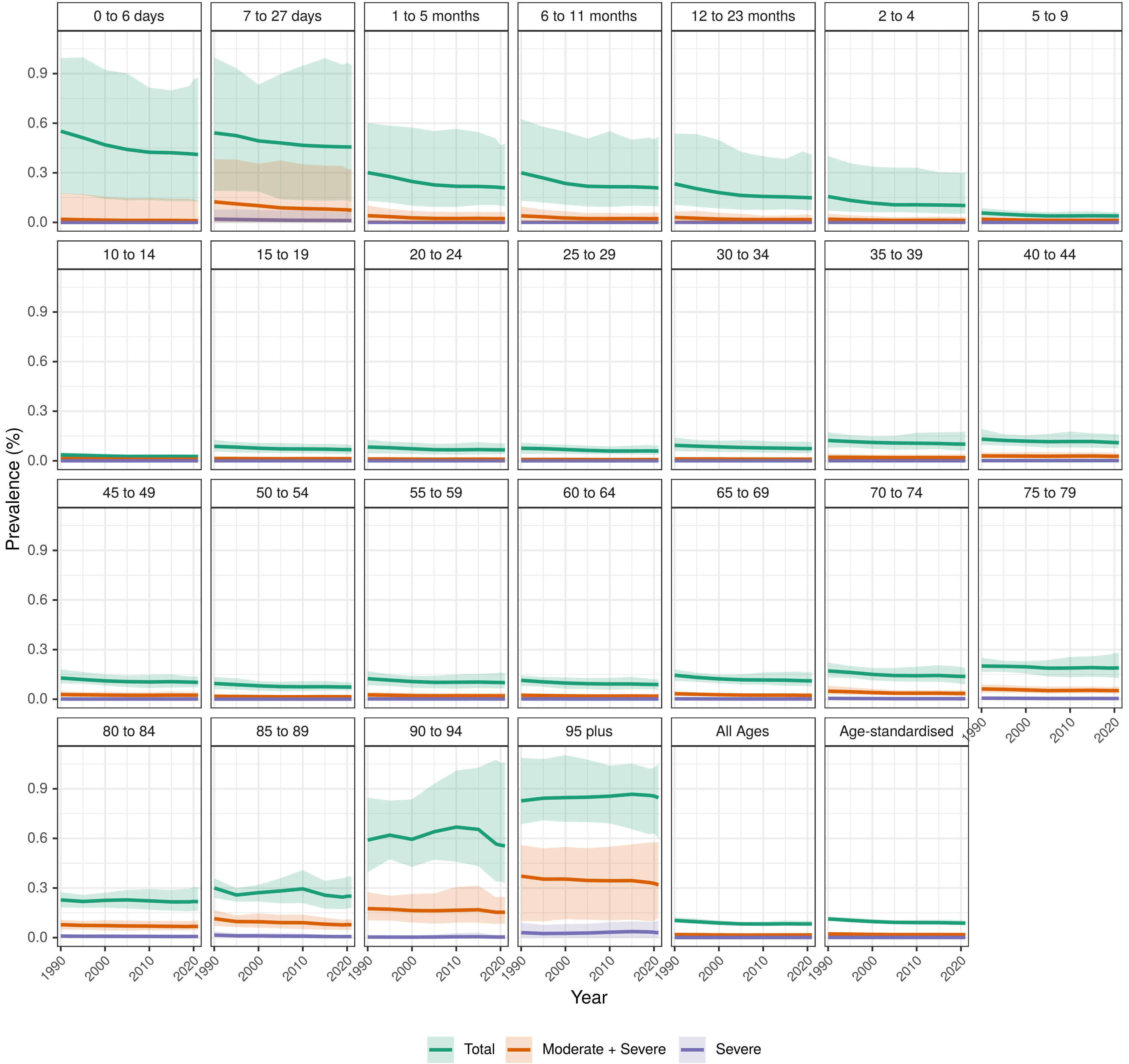

Cause-Specific Anaemia Prevalence - 2021

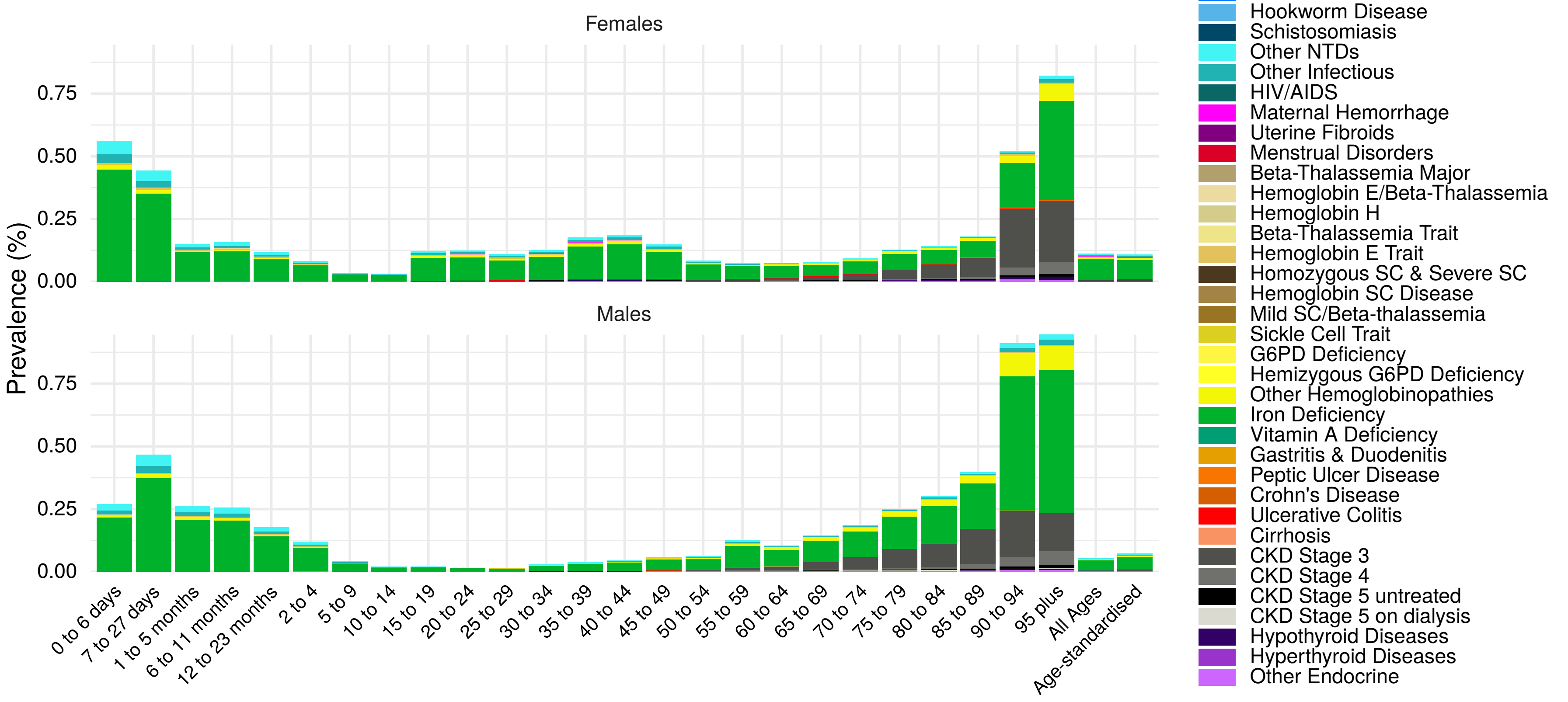

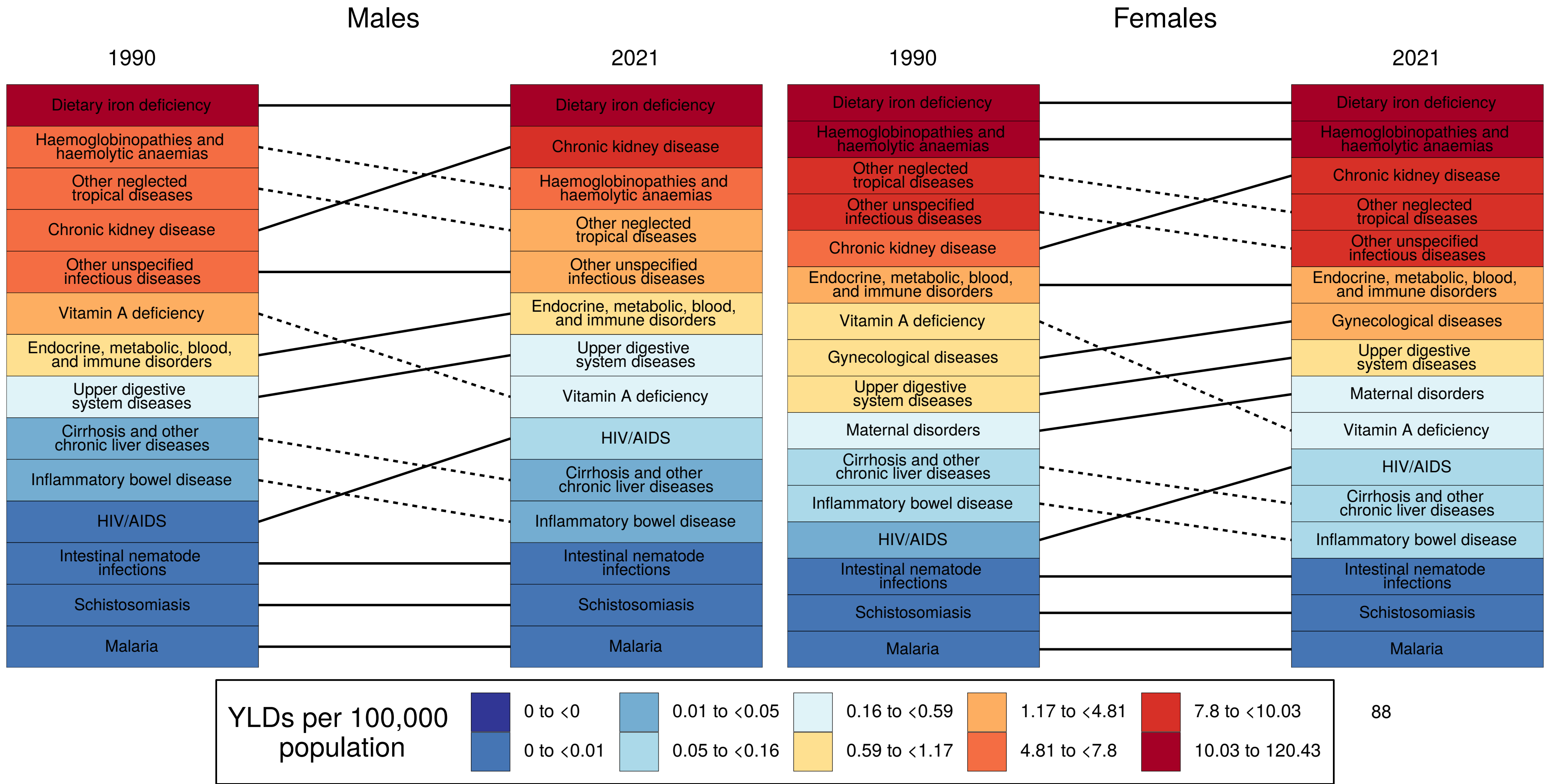

Japan

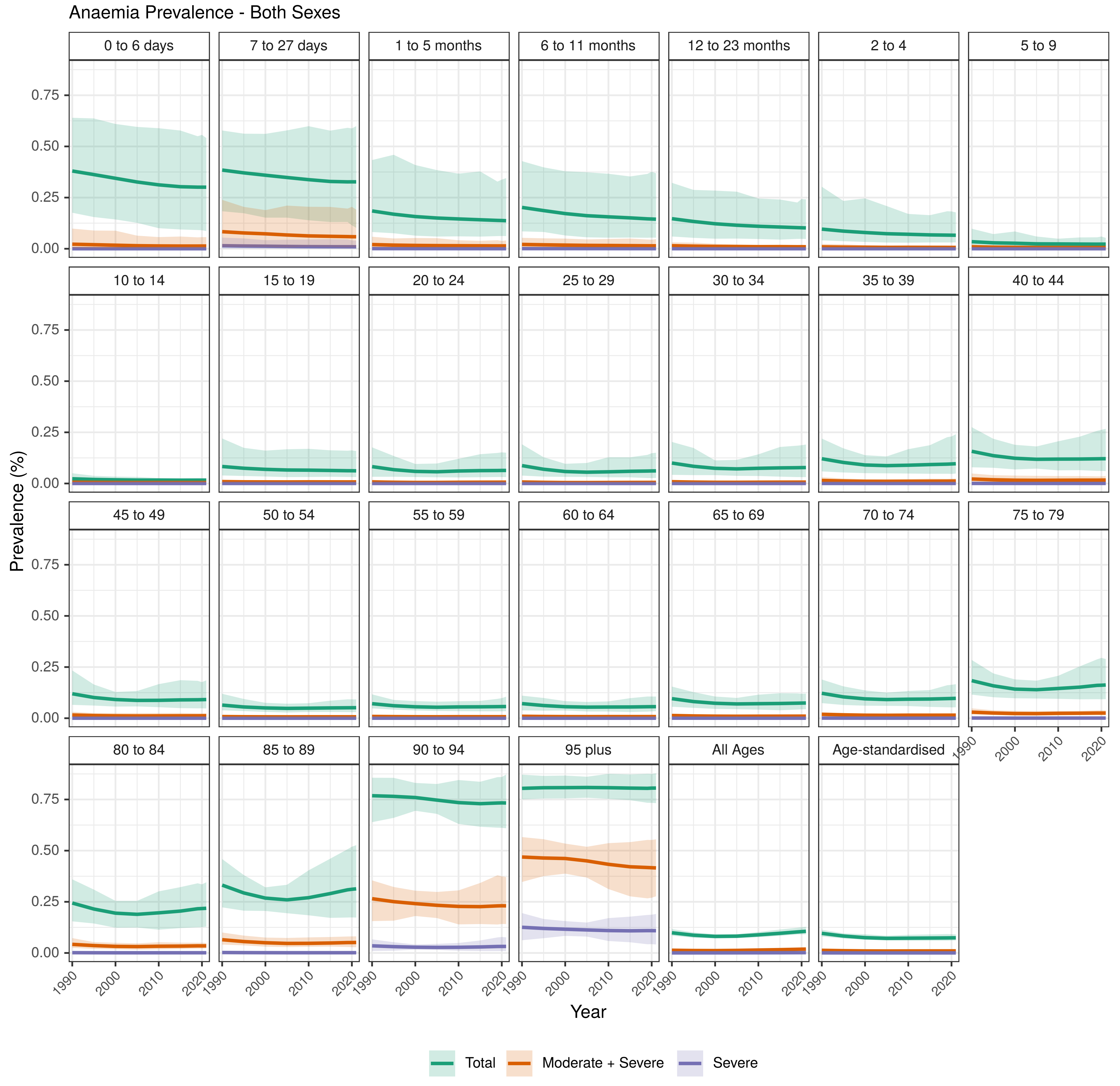

Anaemia Prevalence - Both Sexes
0 to 6 days
7 to 27 days
1 to 5 months
6 to 11 months
12 to 23 months
2 to 4
5 to 9
10 to 14
15 to 19
20 to 24
25 to 29
30 to 34
35 to 39
40 to 44
45 to 49
50 to 54
55 to 59
60 to 64
65 to 69
70 to 74
75 to 79
80 to 84
85 to 89
90 to 94
95 plus
All Ages
Age-standardised
Prevalence (%)
0.00
0.25
0.50
0.75
1990
2000
2010
2020
Year
Total
Moderate + Severe
Severe

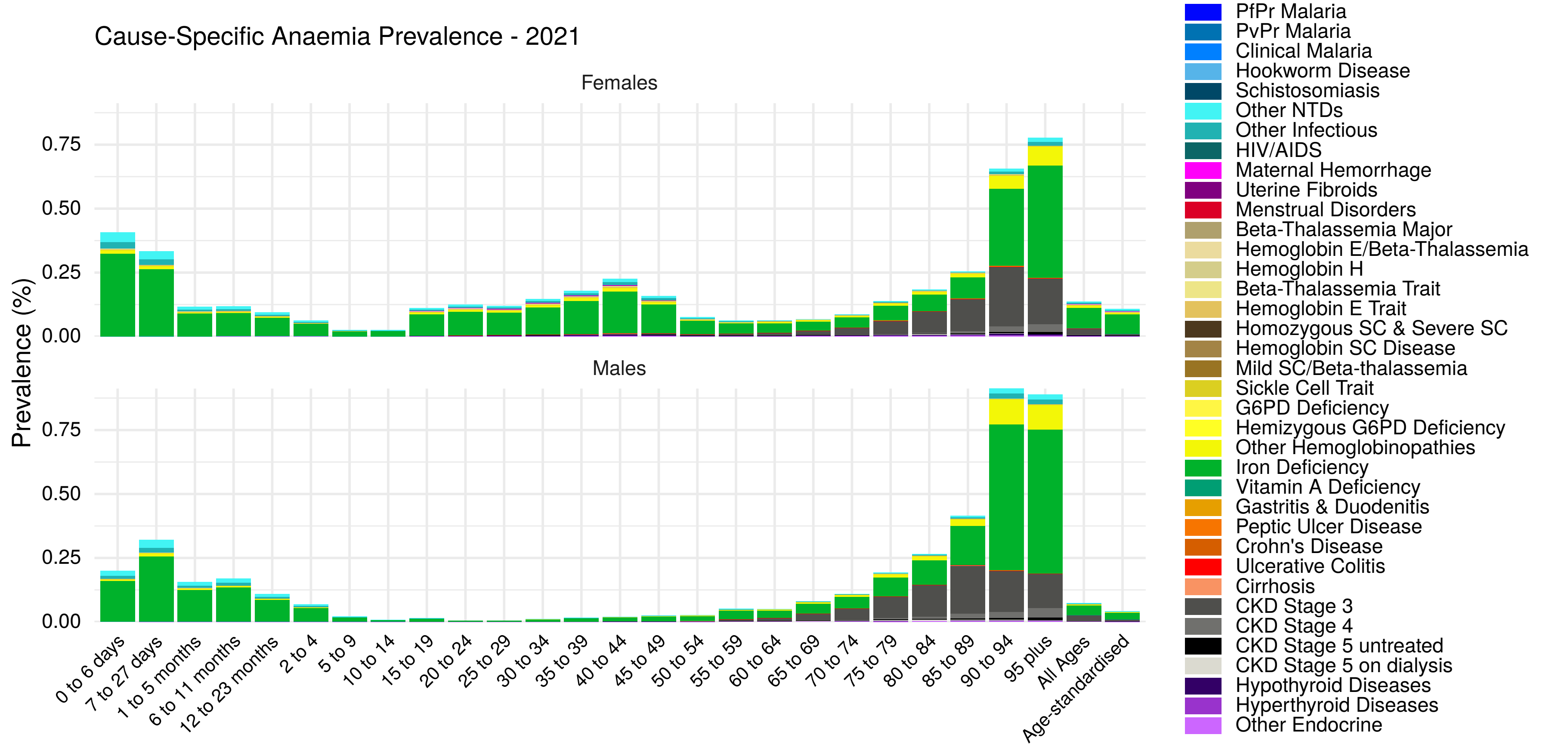

Cause-Specific Anaemia Prevalence - 2021
Females
Males
Prevalence (%)
0.00
0.25
0.50
0.75
0 to 6 days
7 to 27 days
1 to 5 months
6 to 11 months
12 to 23 months
2 to 4
5 to 9
10 to 14
15 to 19
20 to 24
25 to 29
30 to 34
35 to 39
40 to 44
45 to 49
50 to 54
55 to 59
60 to 64
65 to 69
70 to 74
75 to 79
80 to 84
85 to 89
90 to 94
95 plus
All Ages
Age-standardised
PfPr Malaria
PvPr Malaria
Clinical Malaria
Hookworm Disease
Schistosomiasis
Other NTDs
Other Infectious
HIV/AIDS
Maternal Hemorrhage
Uterine Fibroids
Menstrual Disorders
Beta-Thalassemia Major
Hemoglobin E/Beta-Thalassemia
Hemoglobin H
Beta-Thalassemia Trait
Hemoglobin E Trait
Homozygous SC & Severe SC
Hemoglobin SC Disease
Mild SC/Beta-thalassemia
Sickle Cell Trait
G6PD Deficiency
Hemizygous G6PD Deficiency
Other Hemoglobinopathies
Iron Deficiency
Vitamin A Deficiency
Gastritis & Duodenitis
Peptic Ulcer Disease
Crohn's Disease
Ulcerative Colitis
Cirrhosis
CKD Stage 3
CKD Stage 4
CKD Stage 5 untreated
CKD Stage 5 on dialysis
Hypothyroid Diseases
Hyperthyroid Diseases
Other Endocrine

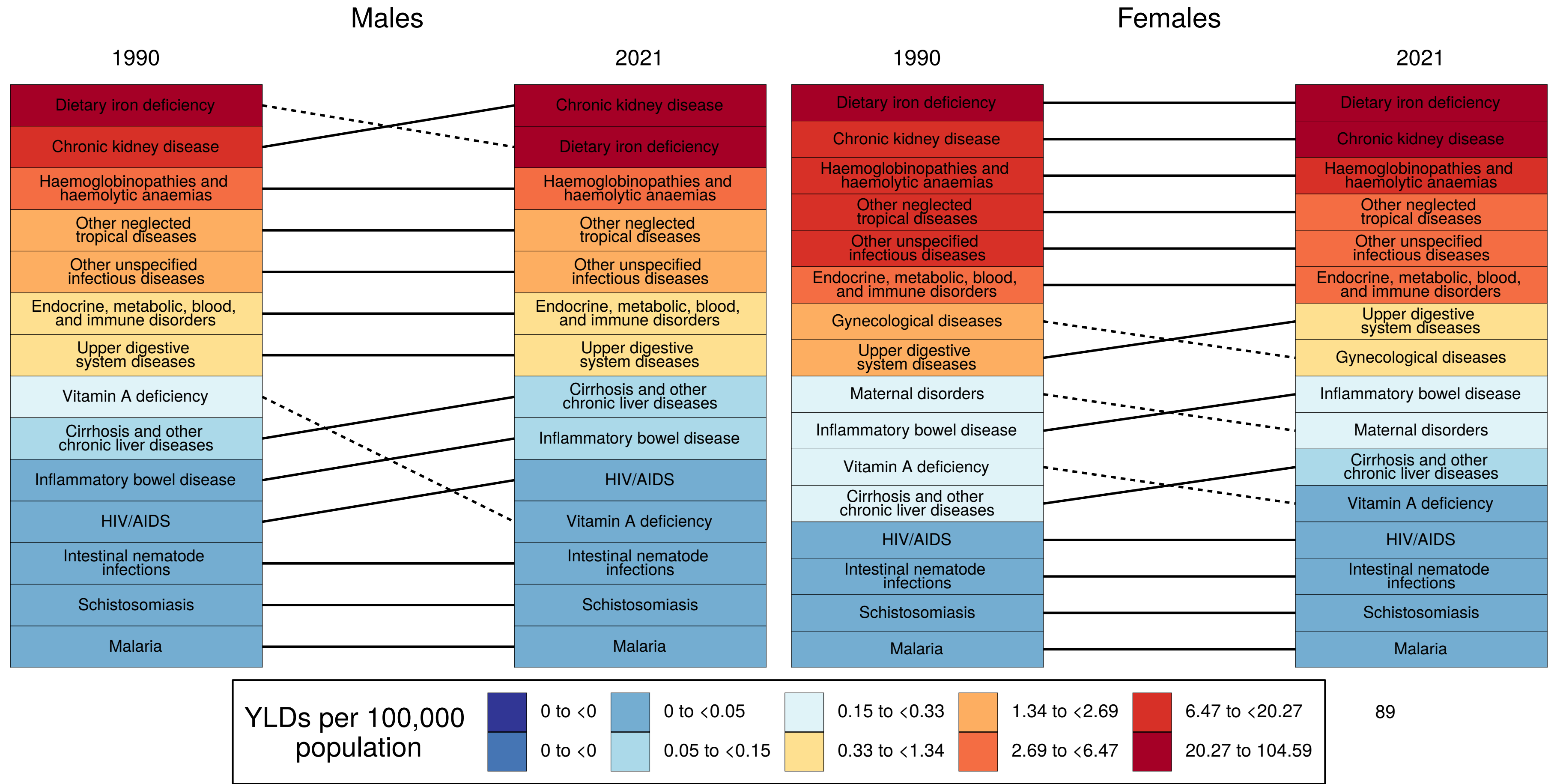

Males
Females
1990
2021
Dietary iron deficiency
Chronic kidney disease
Haemoglobinopathies and haemolytic anaemias
Other neglected tropical diseases
Other unspecified infectious diseases
Endocrine, metabolic, blood, and immune disorders
Upper digestive system diseases
Vitamin A deficiency
Cirrhosis and other chronic liver diseases
Inflammatory bowel disease
HIV/AIDS
Intestinal nematode infections
Schistosomiasis
Malaria
Gynecological diseases
Maternal disorders
YLDs per 100,000 population
0 to <0
0 to <0
0 to <0.05
0.05 to <0.15
0.15 to <0.33
0.33 to <1.34
1.34 to <2.69
2.69 to <6.47
6.47 to <20.27
20.27 to 104.59

Aichi

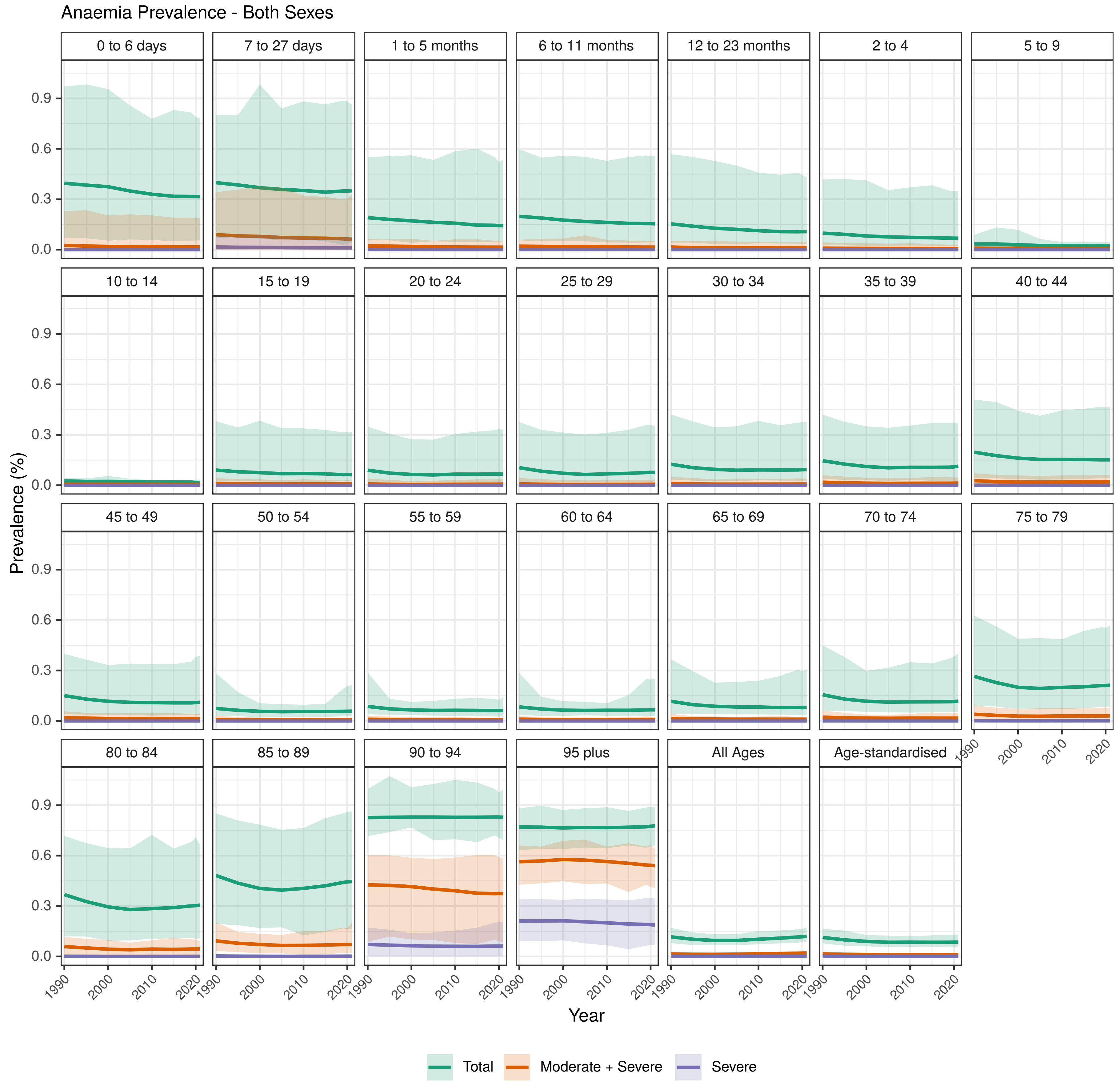
Anaemia Prevalence - Both Sexes
0 to 6 days
7 to 27 days
1 to 5 months
6 to 11 months
12 to 23 months
2 to 4
5 to 9
10 to 14
15 to 19
20 to 24
25 to 29
30 to 34
35 to 39
40 to 44
45 to 49
50 to 54
55 to 59
60 to 64
65 to 69
70 to 74
75 to 79
80 to 84
85 to 89
90 to 94
95 plus
All Ages
Age-standardised
Prevalence (%)
Year
Total
Moderate + Severe
Severe

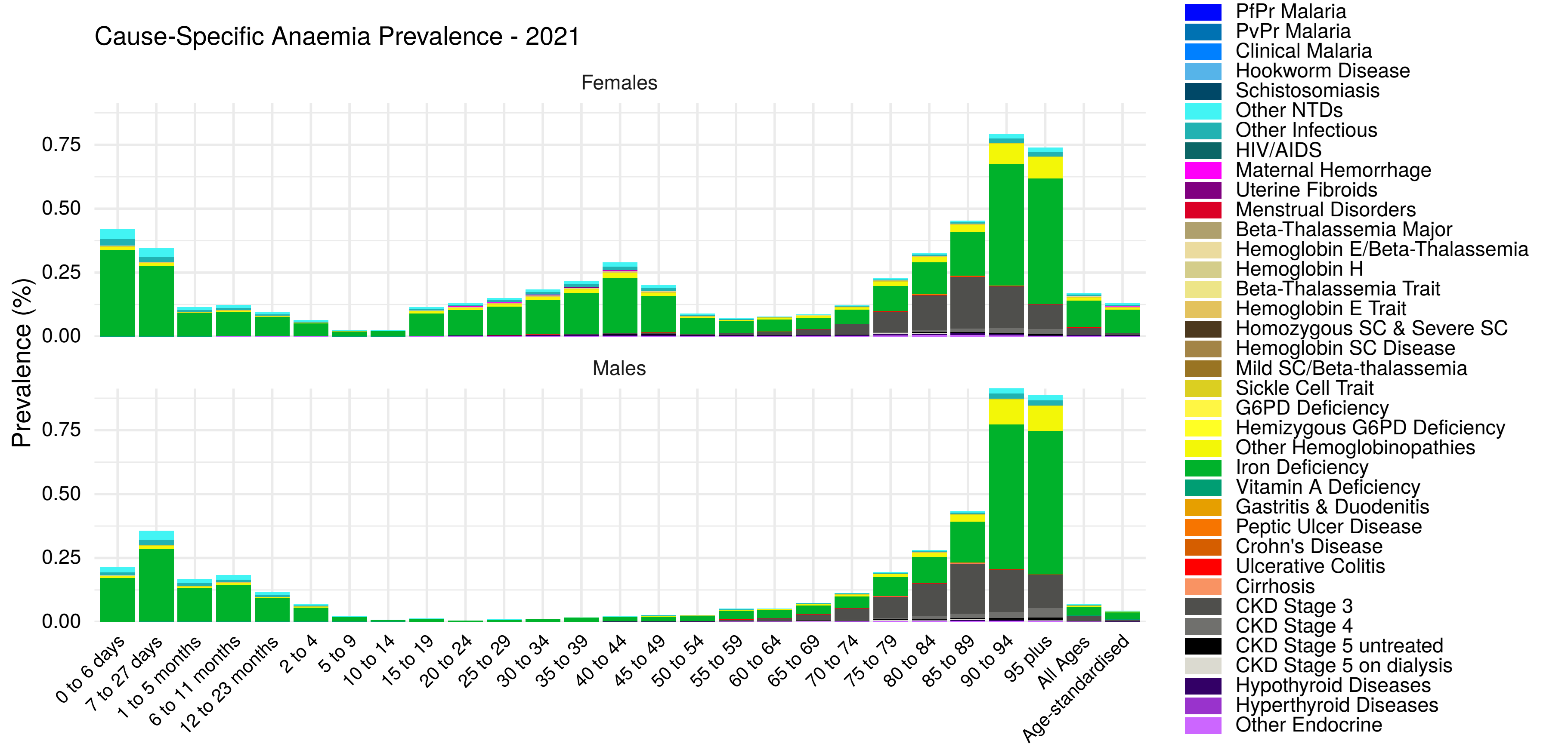
Cause-Specific Anaemia Prevalence - 2021
Females
Males
Prevalence (%)
0 to 6 days
7 to 27 days
1 to 5 months
6 to 11 months
12 to 23 months
2 to 4
5 to 9
10 to 14
15 to 19
20 to 24
25 to 29
30 to 34
35 to 39
40 to 44
45 to 49
50 to 54
55 to 59
60 to 64
65 to 69
70 to 74
75 to 79
80 to 84
85 to 89
90 to 94
95 plus
All Ages
Age-standardised
PfPr Malaria
PvPr Malaria
Clinical Malaria
Hookworm Disease
Schistosomiasis
Other NTDs
Other Infectious
HIV/AIDS
Maternal Hemorrhage
Uterine Fibroids
Menstrual Disorders
Beta-Thalassemia Major
Hemoglobin E/Beta-Thalassemia
Hemoglobin H
Beta-Thalassemia Trait
Hemoglobin E Trait
Homozygous SC & Severe SC
Hemoglobin SC Disease
Mild SC/Beta-thalassemia
Sickle Cell Trait
G6PD Deficiency
Hemizygous G6PD Deficiency
Other Hemoglobinopathies
Iron Deficiency
Vitamin A Deficiency
Gastritis & Duodenitis
Peptic Ulcer Disease
Crohn's Disease
Ulcerative Colitis
Cirrhosis
CKD Stage 3
CKD Stage 4
CKD Stage 5 untreated
CKD Stage 5 on dialysis
Hypothyroid Diseases
Hyperthyroid Diseases
Other Endocrine

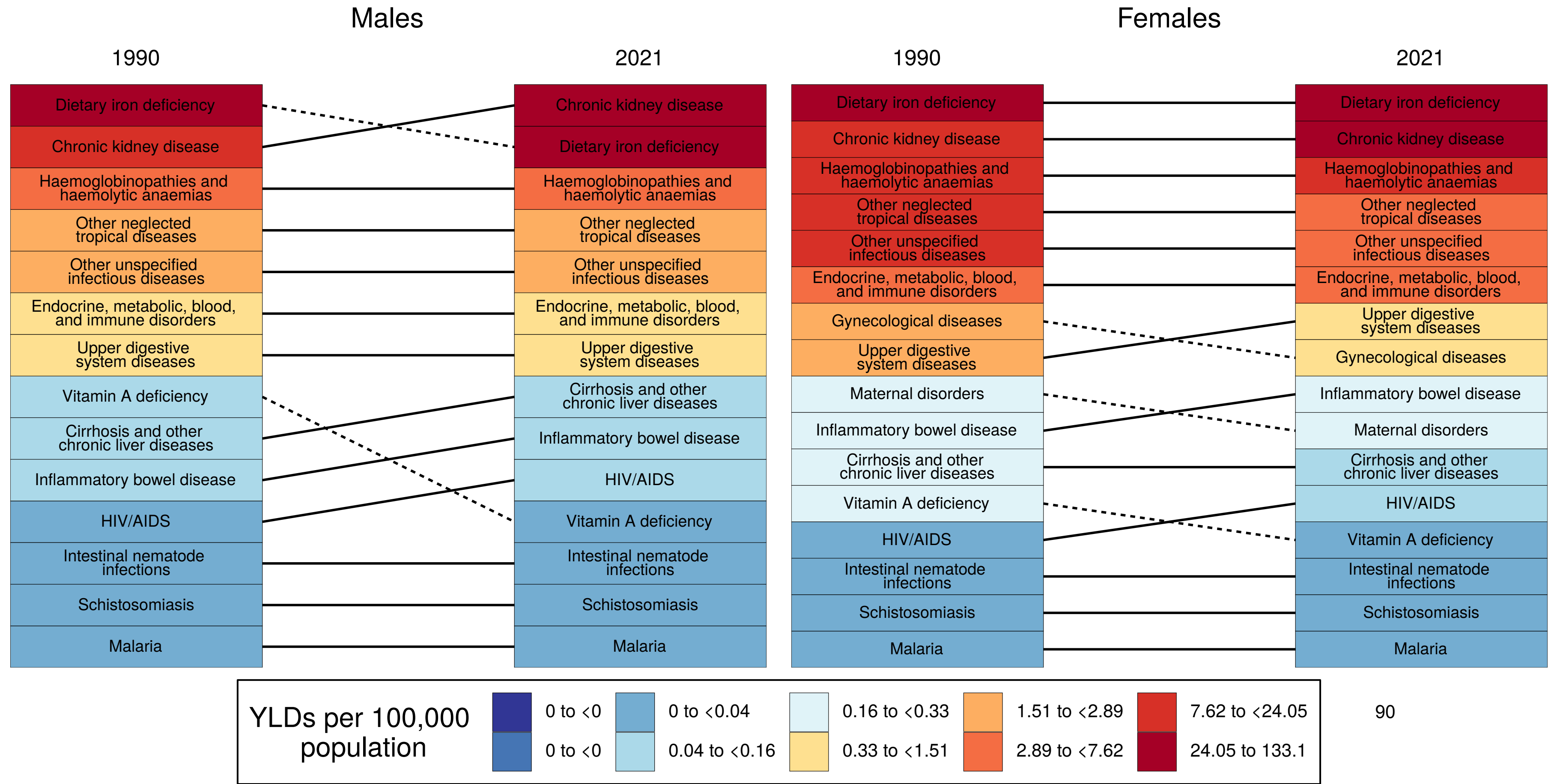
Males
Females
1990
2021
Dietary iron deficiency
Chronic kidney disease
Haemoglobinopathies and haemolytic anaemias
Other neglected tropical diseases
Other unspecified infectious diseases
Endocrine, metabolic, blood, and immune disorders
Upper digestive system diseases
Vitamin A deficiency
Cirrhosis and other chronic liver diseases
Inflammatory bowel disease
HIV/AIDS
Intestinal nematode infections
Schistosomiasis
Malaria
Gynecological diseases
Maternal disorders
YLDs per 100,000 population
0 to <0
0 to <0
0 to <0.04
0.04 to <0.16
0.16 to <0.33
0.33 to <1.51
1.51 to <2.89
2.89 to <7.62
7.62 to <24.05
24.05 to 133.1

Akita

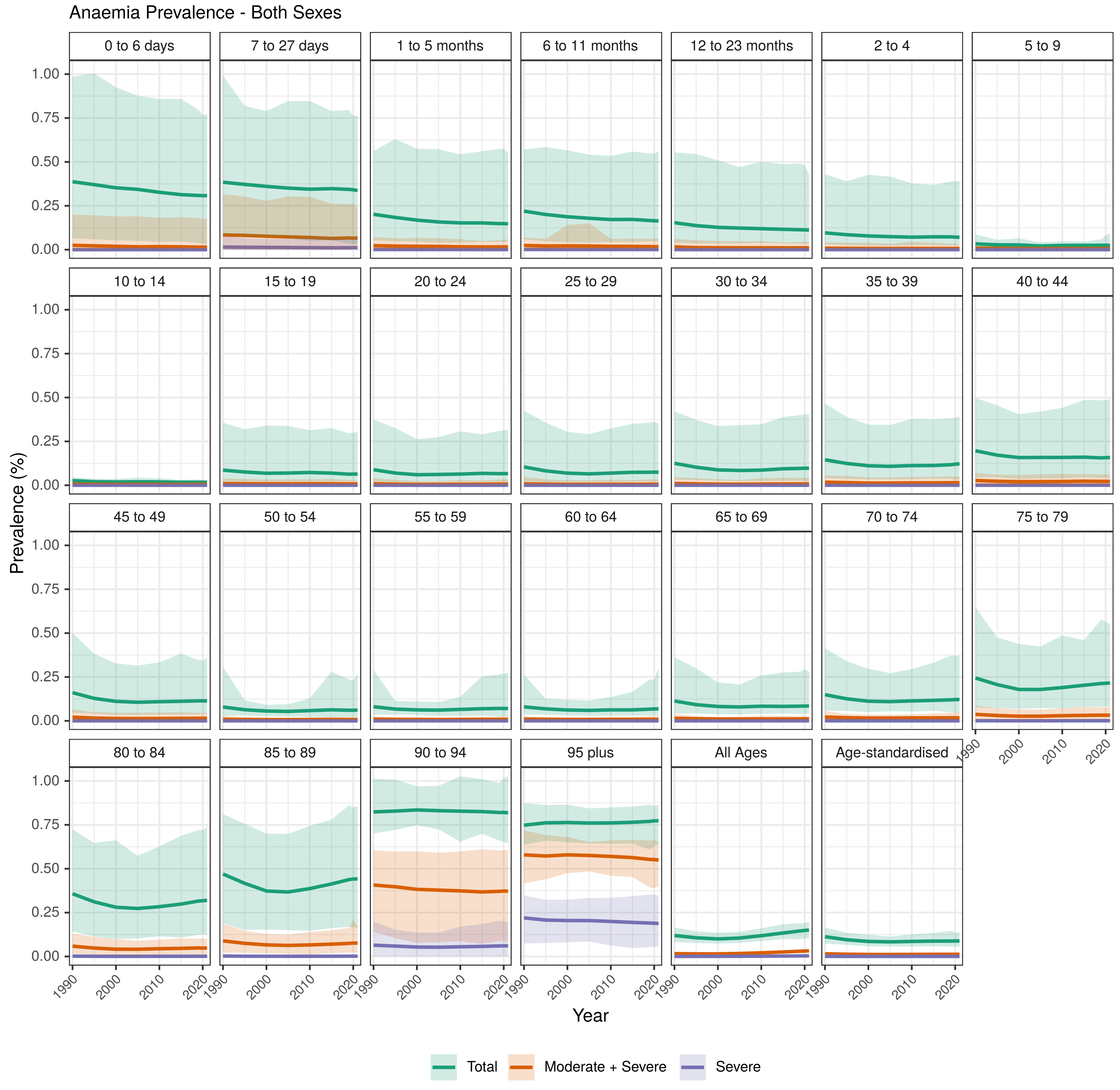

Anaemia Prevalence - Both Sexes
0 to 6 days
7 to 27 days
1 to 5 months
6 to 11 months
12 to 23 months
2 to 4
5 to 9
10 to 14
15 to 19
20 to 24
25 to 29
30 to 34
35 to 39
40 to 44
45 to 49
50 to 54
55 to 59
60 to 64
65 to 69
70 to 74
75 to 79
80 to 84
85 to 89
90 to 94
95 plus
All Ages
Age-standardised
Prevalence (%)
Year
Total
Moderate + Severe
Severe

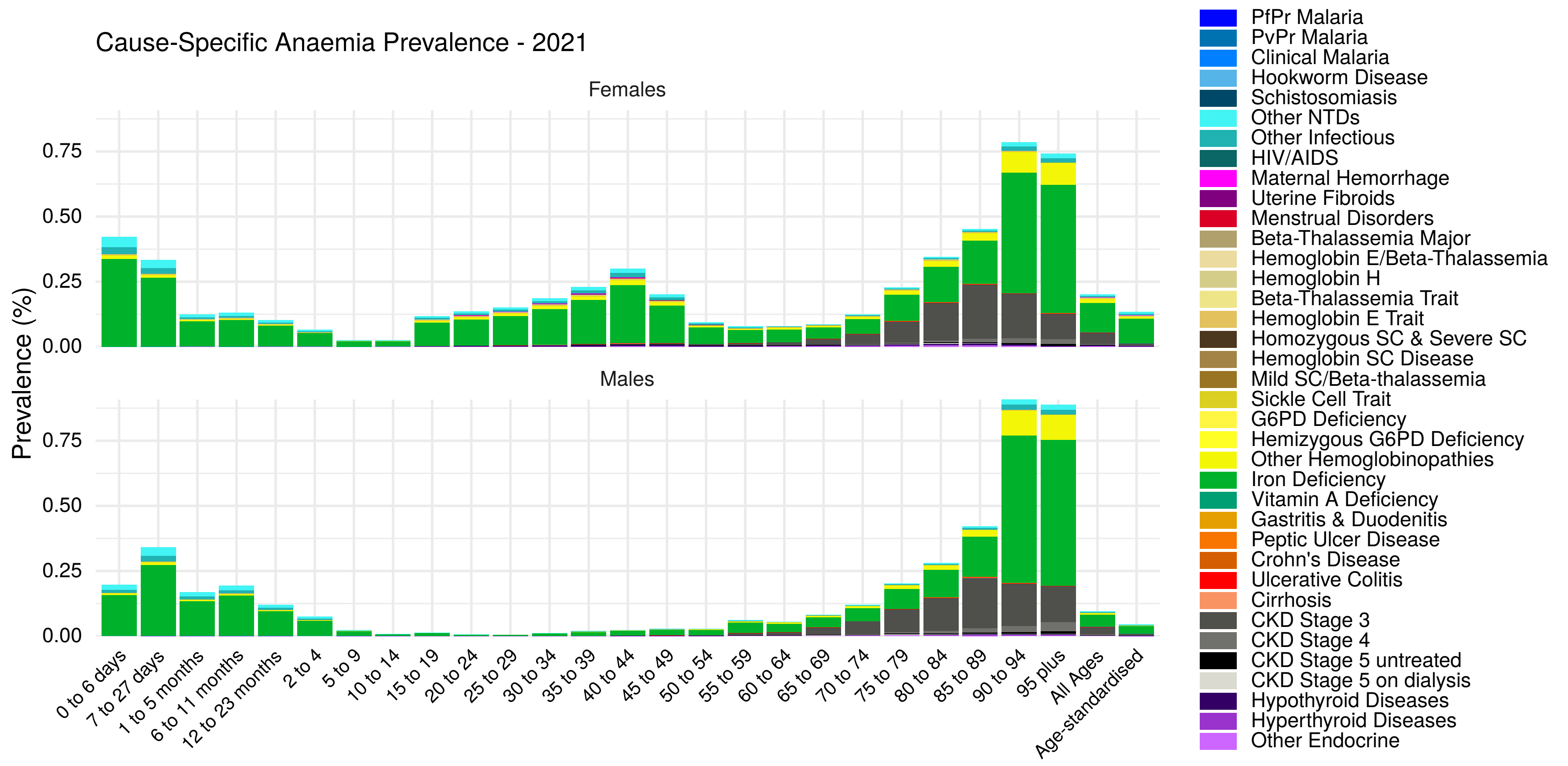

Cause-Specific Anaemia Prevalence - 2021
Females
Males
Prevalence (%)
PfPr Malaria
PvPr Malaria
Clinical Malaria
Hookworm Disease
Schistosomiasis
Other NTDs
Other Infectious
HIV/AIDS
Maternal Hemorrhage
Uterine Fibroids
Menstrual Disorders
Beta-Thalassemia Major
Hemoglobin E/Beta-Thalassemia
Hemoglobin H
Beta-Thalassemia Trait
Hemoglobin E Trait
Homozygous SC & Severe SC
Hemoglobin SC Disease
Mild SC/Beta-thalassemia
Sickle Cell Trait
G6PD Deficiency
Hemizygous G6PD Deficiency
Other Hemoglobinopathies
Iron Deficiency
Vitamin A Deficiency
Gastritis & Duodenitis
Peptic Ulcer Disease
Crohn's Disease
Ulcerative Colitis
Cirrhosis
CKD Stage 3
CKD Stage 4
CKD Stage 5 untreated
CKD Stage 5 on dialysis
Hypothyroid Diseases
Hyperthyroid Diseases
Other Endocrine

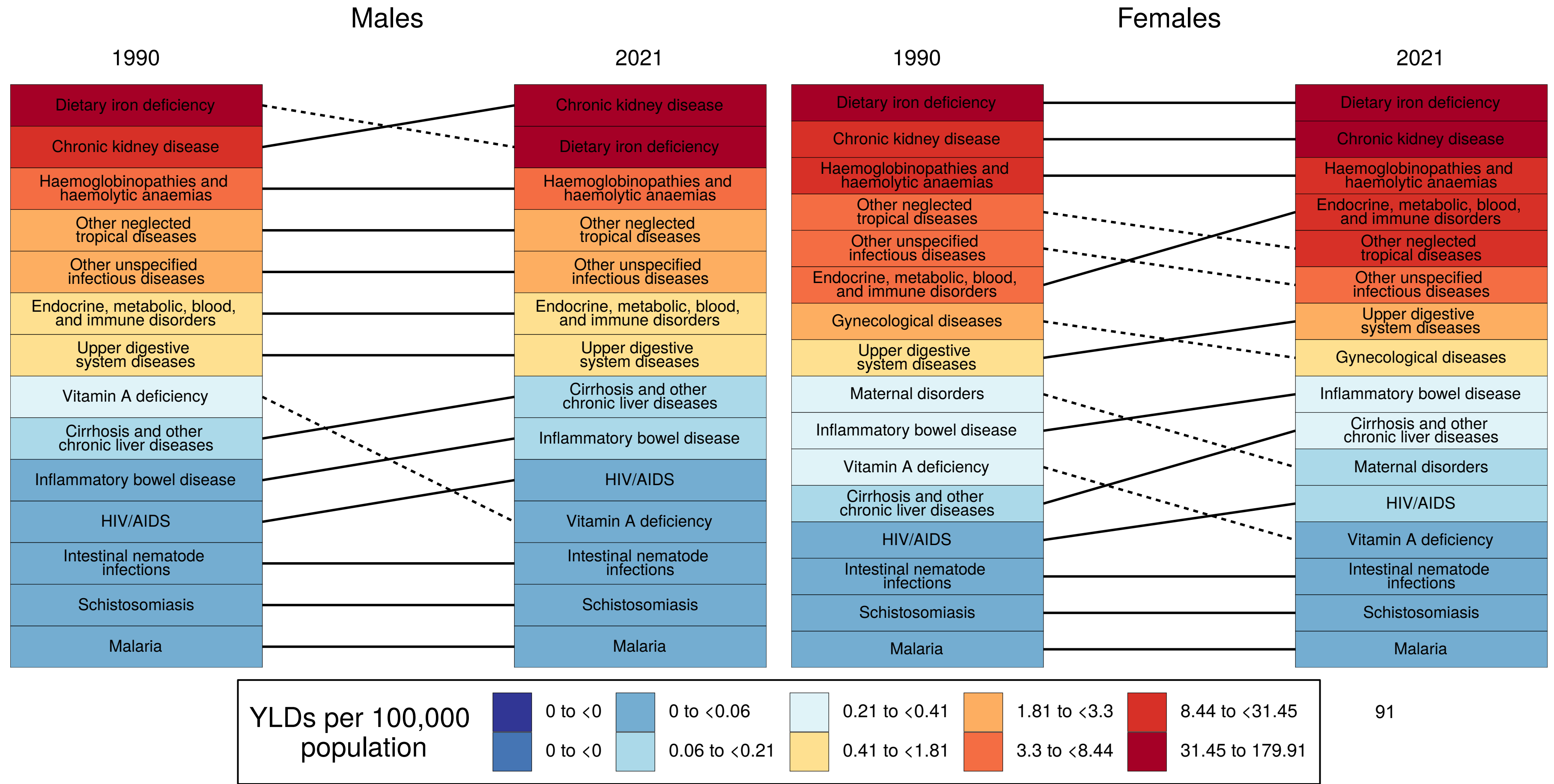

Males
Females
1990
2021
Dietary iron deficiency
Chronic kidney disease
Haemoglobinopathies and haemolytic anaemias
Other neglected tropical diseases
Other unspecified infectious diseases
Endocrine, metabolic, blood, and immune disorders
Upper digestive system diseases
Vitamin A deficiency
Cirrhosis and other chronic liver diseases
Inflammatory bowel disease
HIV/AIDS
Intestinal nematode infections
Schistosomiasis
Malaria
Gynecological diseases
Maternal disorders
YLDs per 100,000 population
0 to <0
0 to <0.06
0.06 to <0.21
0.21 to <0.41
0.41 to <1.81
1.81 to <3.3
3.3 to <8.44
8.44 to <31.45
31.45 to 179.91

Aomori

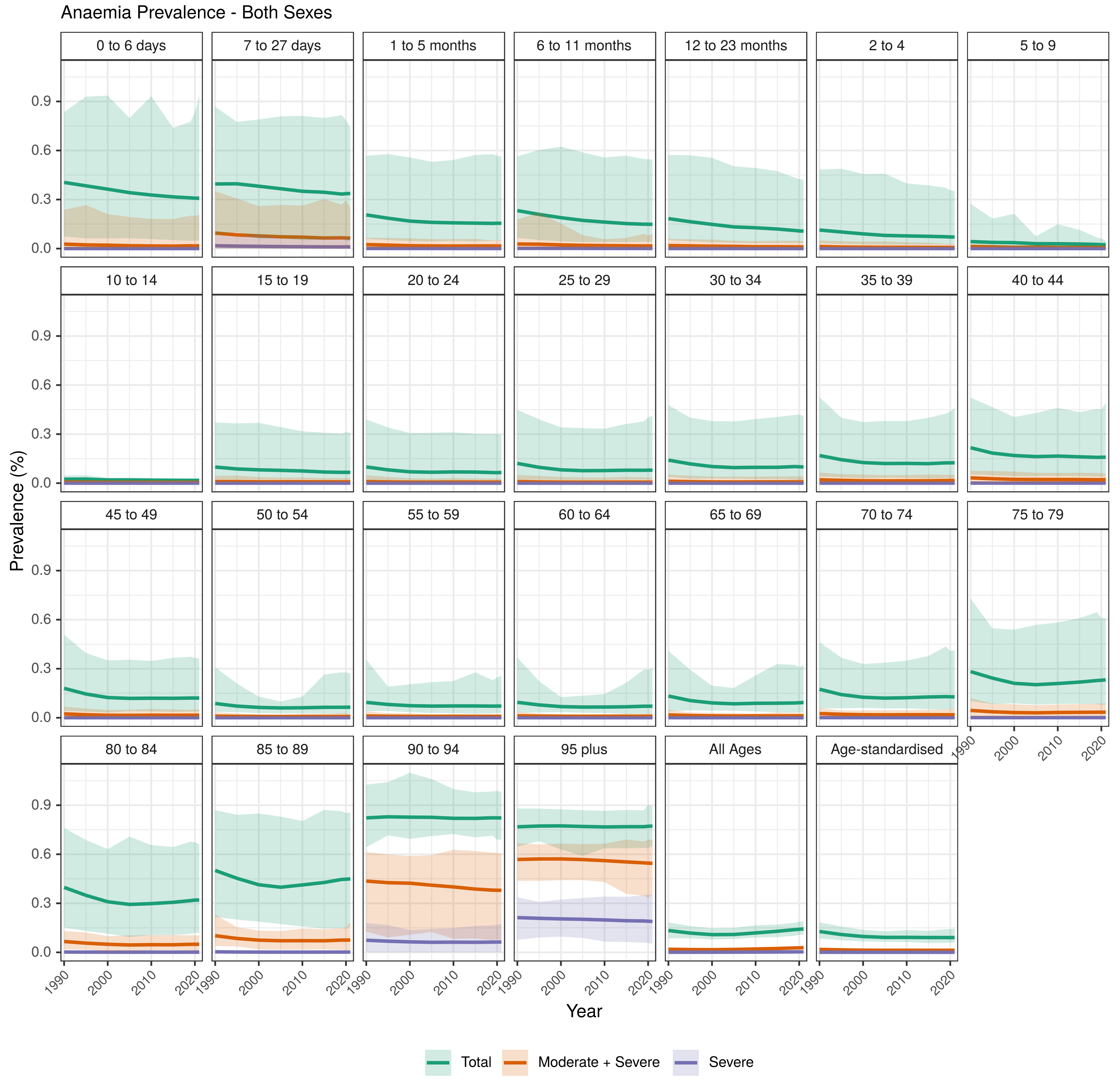

Anaemia Prevalence - Both Sexes
0 to 6 days
7 to 27 days
1 to 5 months
6 to 11 months
12 to 23 months
2 to 4
5 to 9
10 to 14
15 to 19
20 to 24
25 to 29
30 to 34
35 to 39
40 to 44
45 to 49
50 to 54
55 to 59
60 to 64
65 to 69
70 to 74
75 to 79
80 to 84
85 to 89
90 to 94
95 plus
All Ages
Age-standardised
Prevalence (%)
Year
Total
Moderate + Severe
Severe

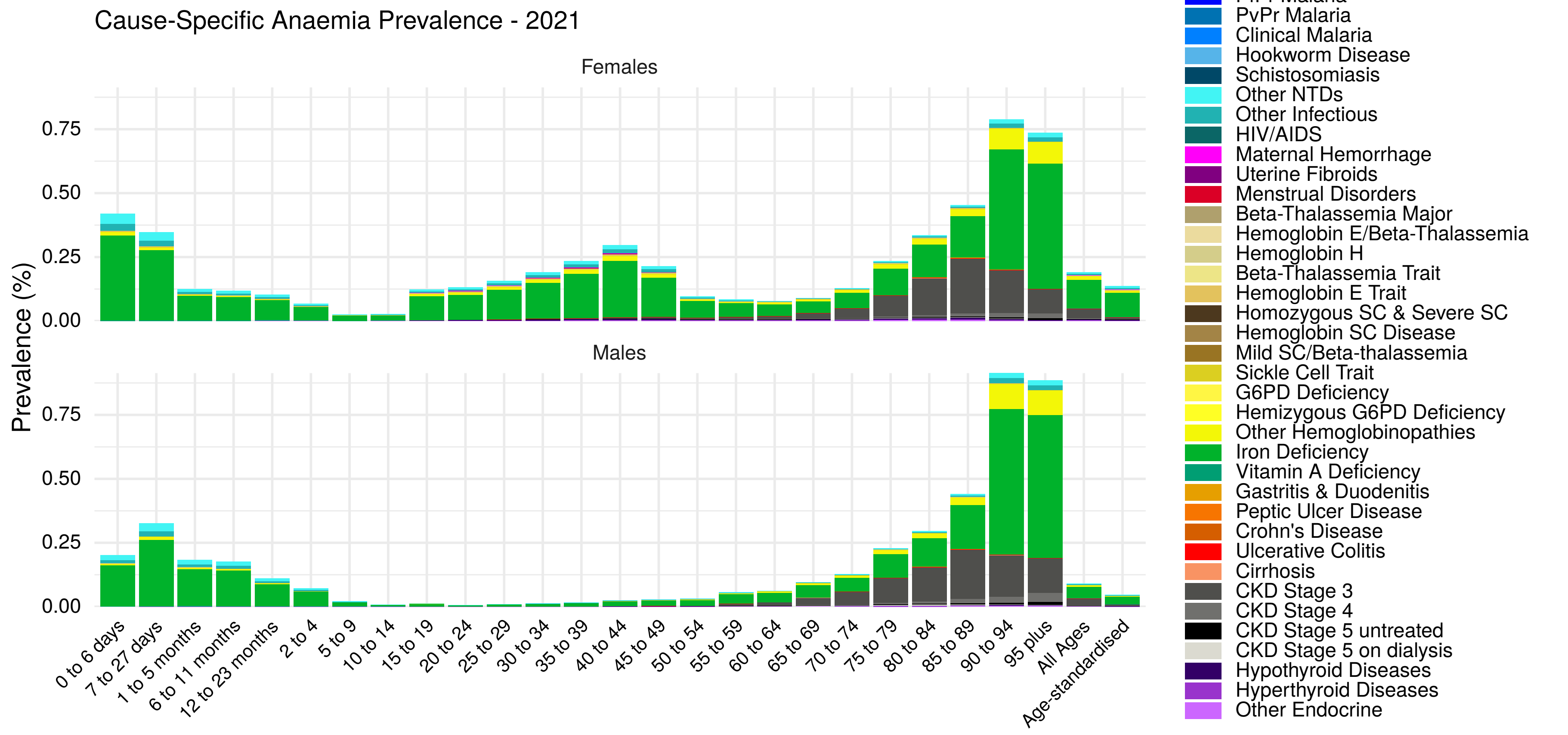

Cause-Specific Anaemia Prevalence - 2021
Females
Males
Prevalence (%)
PfPr Malaria
PvPr Malaria
Clinical Malaria
Hookworm Disease
Schistosomiasis
Other NTDs
Other Infectious
HIV/AIDS
Maternal Hemorrhage
Uterine Fibroids
Menstrual Disorders
Beta-Thalassemia Major
Hemoglobin E/Beta-Thalassemia
Hemoglobin H
Beta-Thalassemia Trait
Hemoglobin E Trait
Homozygous SC & Severe SC
Hemoglobin SC Disease
Mild SC/Beta-thalassemia
Sickle Cell Trait
G6PD Deficiency
Hemizygous G6PD Deficiency
Other Hemoglobinopathies
Iron Deficiency
Vitamin A Deficiency
Gastritis & Duodenitis
Peptic Ulcer Disease
Crohn's Disease
Ulcerative Colitis
Cirrhosis
CKD Stage 3
CKD Stage 4
CKD Stage 5 untreated
CKD Stage 5 on dialysis
Hypothyroid Diseases
Hyperthyroid Diseases
Other Endocrine

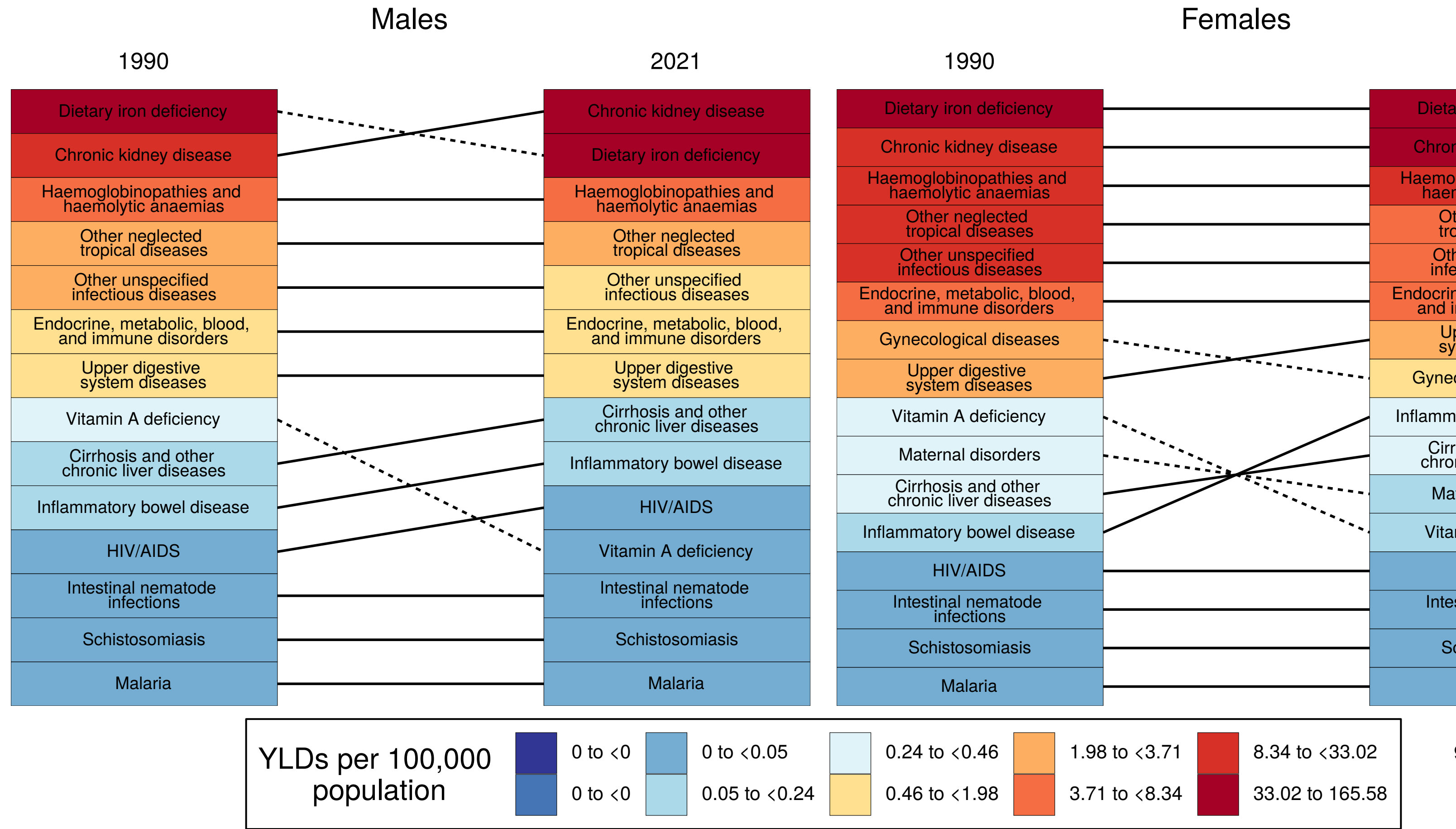

Males
Females
1990
2021
Dietary iron deficiency
Chronic kidney disease
Haemoglobinopathies and haemolytic anaemias
Other neglected tropical diseases
Other unspecified infectious diseases
Endocrine, metabolic, blood, and immune disorders
Upper digestive system diseases
Vitamin A deficiency
Cirrhosis and other chronic liver diseases
Inflammatory bowel disease
HIV/AIDS
Intestinal nematode infections
Schistosomiasis
Malaria
Gynecological diseases
Maternal disorders
YLDs per 100,000 population
0 to <0
0 to <0.05
0.05 to <0.24
0.24 to <0.46
0.46 to <1.98
1.98 to <3.71
3.71 to <8.34
8.34 to <33.02
33.02 to 165.58

Chiba

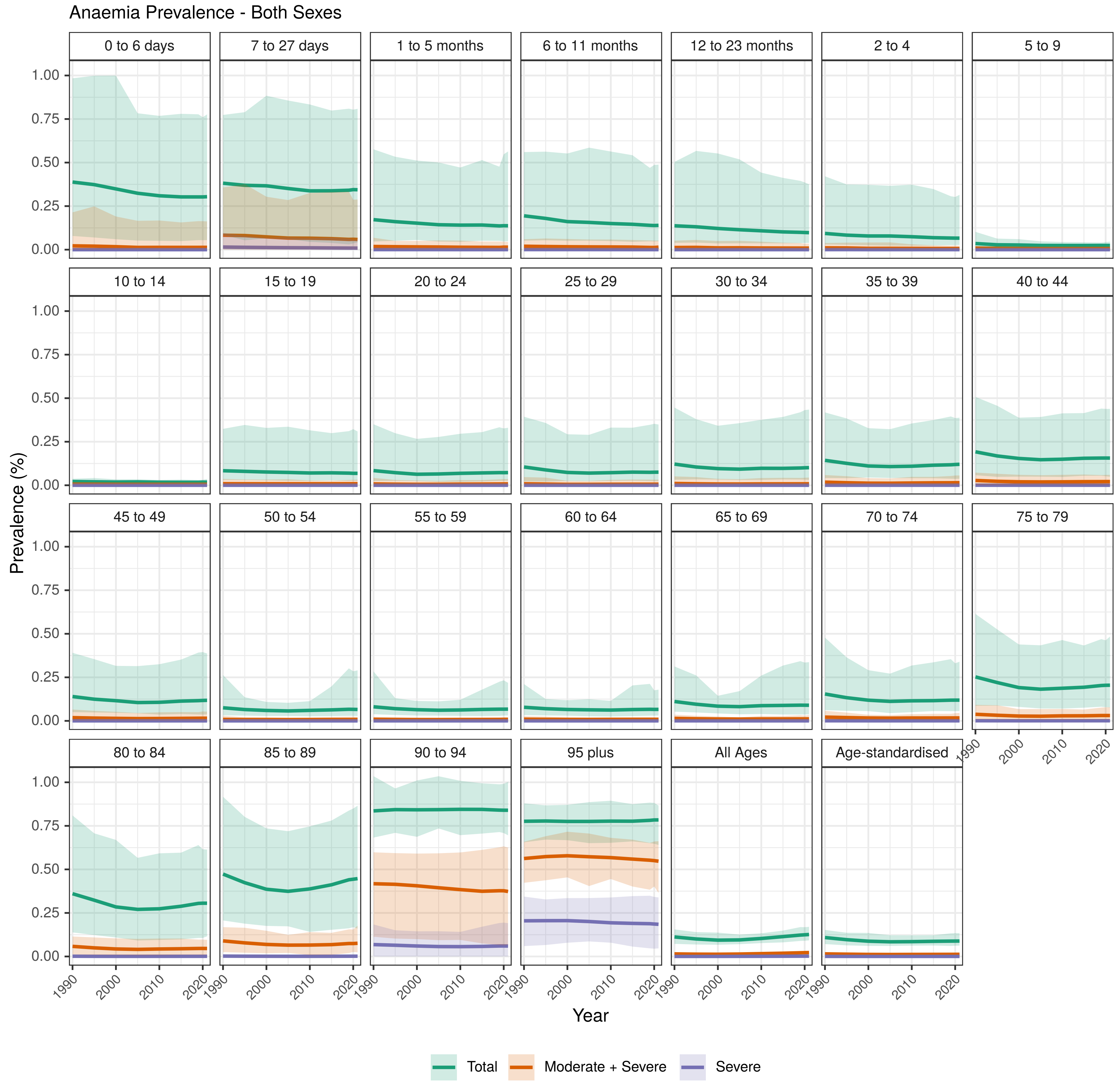

Anaemia Prevalence - Both Sexes
0 to 6 days
7 to 27 days
1 to 5 months
6 to 11 months
12 to 23 months
2 to 4
5 to 9
10 to 14
15 to 19
20 to 24
25 to 29
30 to 34
35 to 39
40 to 44
45 to 49
50 to 54
55 to 59
60 to 64
65 to 69
70 to 74
75 to 79
80 to 84
85 to 89
90 to 94
95 plus
All Ages
Age-standardised
Prevalence (%)
1.00
0.75
0.50
0.25
0.00
1990
2000
2010
2020
Year
Total
Moderate + Severe
Severe

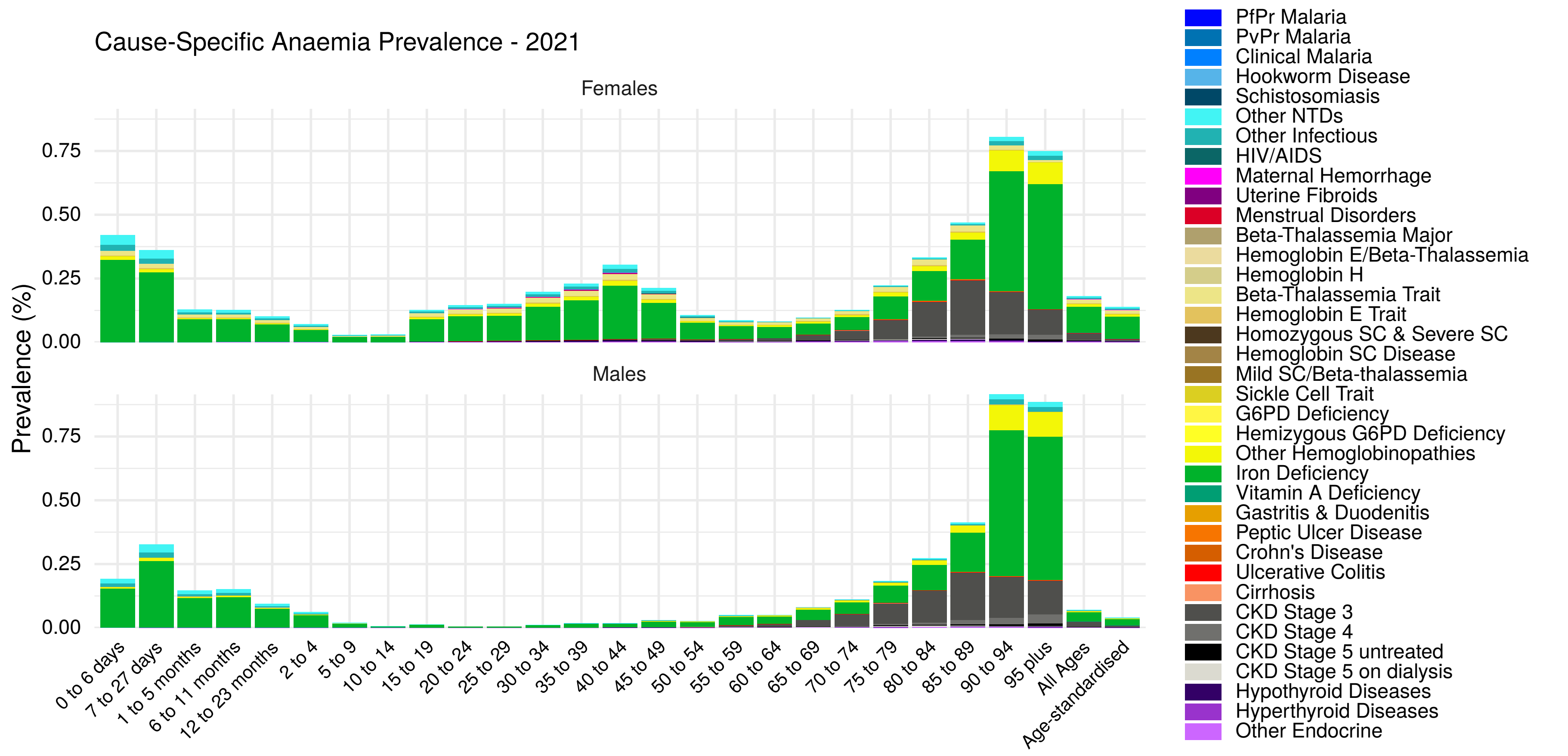

Cause-Specific Anaemia Prevalence - 2021
Females
Males
Prevalence (%)
0.75
0.50
0.25
0.00
0 to 6 days
7 to 27 days
1 to 5 months
6 to 11 months
12 to 23 months
2 to 4
5 to 9
10 to 14
15 to 19
20 to 24
25 to 29
30 to 34
35 to 39
40 to 44
45 to 49
50 to 54
55 to 59
60 to 64
65 to 69
70 to 74
75 to 79
80 to 84
85 to 89
90 to 94
95 plus
All Ages
Age-standardised
PfPr Malaria
PvPr Malaria
Clinical Malaria
Hookworm Disease
Schistosomiasis
Other NTDs
Other Infectious
HIV/AIDS
Maternal Hemorrhage
Uterine Fibroids
Menstrual Disorders
Beta-Thalassemia Major
Hemoglobin E/Beta-Thalassemia
Hemoglobin H
Beta-Thalassemia Trait
Hemoglobin E Trait
Homozygous SC & Severe SC
Hemoglobin SC Disease
Mild SC/Beta-thalassemia
Sickle Cell Trait
G6PD Deficiency
Hemizygous G6PD Deficiency
Other Hemoglobinopathies
Iron Deficiency
Vitamin A Deficiency
Gastritis & Duodenitis
Peptic Ulcer Disease
Crohn's Disease
Ulcerative Colitis
Cirrhosis
CKD Stage 3
CKD Stage 4
CKD Stage 5 untreated
CKD Stage 5 on dialysis
Hypothyroid Diseases
Hyperthyroid Diseases
Other Endocrine

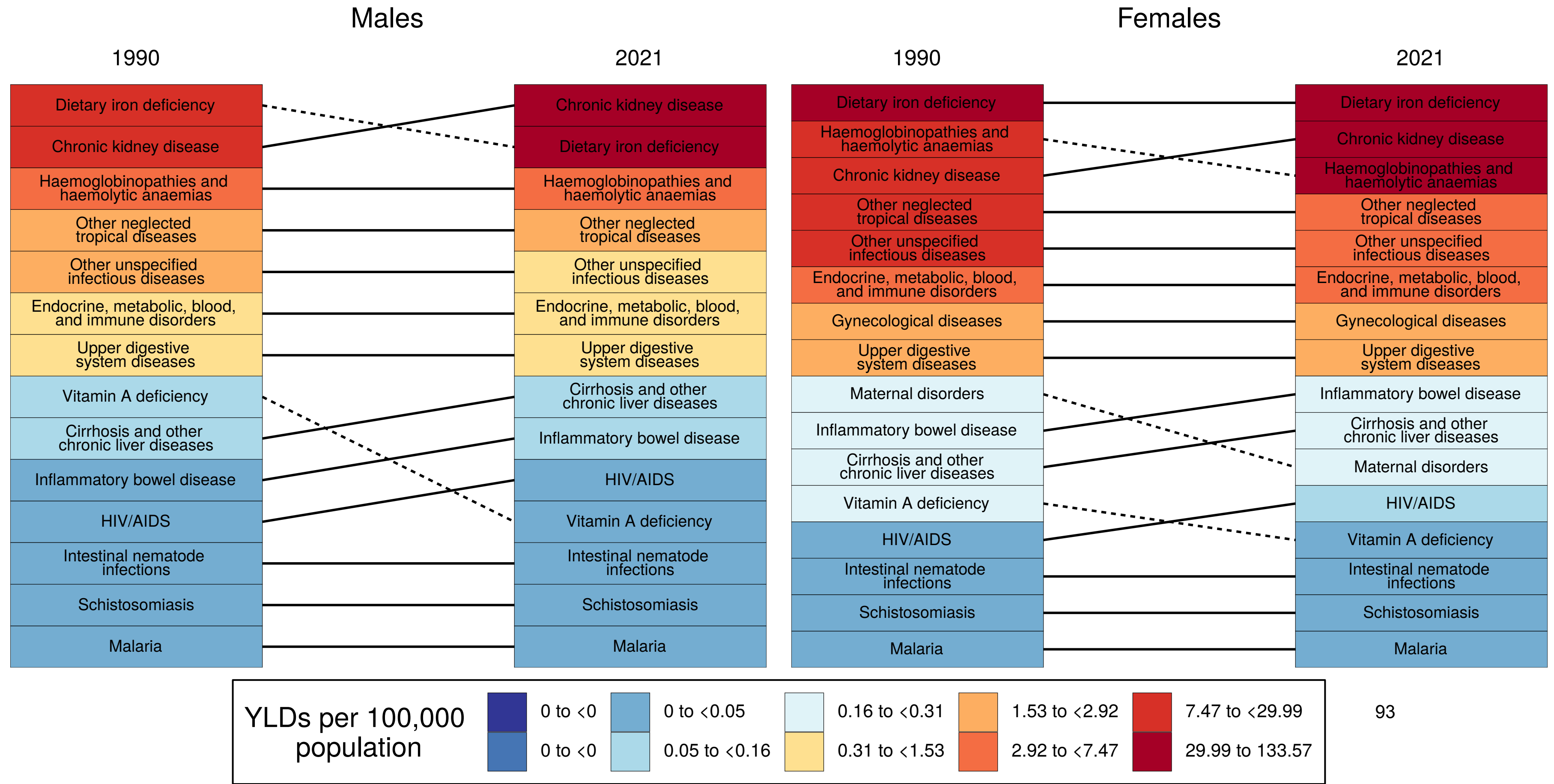

Males
Females
1990
2021
Dietary iron deficiency
Chronic kidney disease
Haemoglobinopathies and haemolytic anaemias
Other neglected tropical diseases
Other unspecified infectious diseases
Endocrine, metabolic, blood, and immune disorders
Upper digestive system diseases
Vitamin A deficiency
Cirrhosis and other chronic liver diseases
Inflammatory bowel disease
HIV/AIDS
Intestinal nematode infections
Schistosomiasis
Malaria
Chronic kidney disease
Dietary iron deficiency
Haemoglobinopathies and haemolytic anaemias
Other neglected tropical diseases
Other unspecified infectious diseases
Endocrine, metabolic, blood, and immune disorders
Upper digestive system diseases
Cirrhosis and other chronic liver diseases
Inflammatory bowel disease
HIV/AIDS
Vitamin A deficiency
Intestinal nematode infections
Schistosomiasis
Malaria
Dietary iron deficiency
Haemoglobinopathies and haemolytic anaemias
Chronic kidney disease
Other neglected tropical diseases
Other unspecified infectious diseases
Endocrine, metabolic, blood, and immune disorders
Gynecological diseases
Upper digestive system diseases
Maternal disorders
Inflammatory bowel disease
Cirrhosis and other chronic liver diseases
Vitamin A deficiency
HIV/AIDS
Intestinal nematode infections
Schistosomiasis
Malaria
Dietary iron deficiency
Chronic kidney disease
Haemoglobinopathies and haemolytic anaemias
Other neglected tropical diseases
Other unspecified infectious diseases
Endocrine, metabolic, blood, and immune disorders
Gynecological diseases
Upper digestive system diseases
Inflammatory bowel disease
Cirrhosis and other chronic liver diseases
Maternal disorders
HIV/AIDS
Vitamin A deficiency
Intestinal nematode infections
Schistosomiasis
Malaria
YLDs per 100,000 population
0 to <0
0 to <0
0 to <0.05
0.05 to <0.16
0.16 to <0.31
0.31 to <1.53
1.53 to <2.92
2.92 to <7.47
7.47 to <29.99
29.99 to 133.57

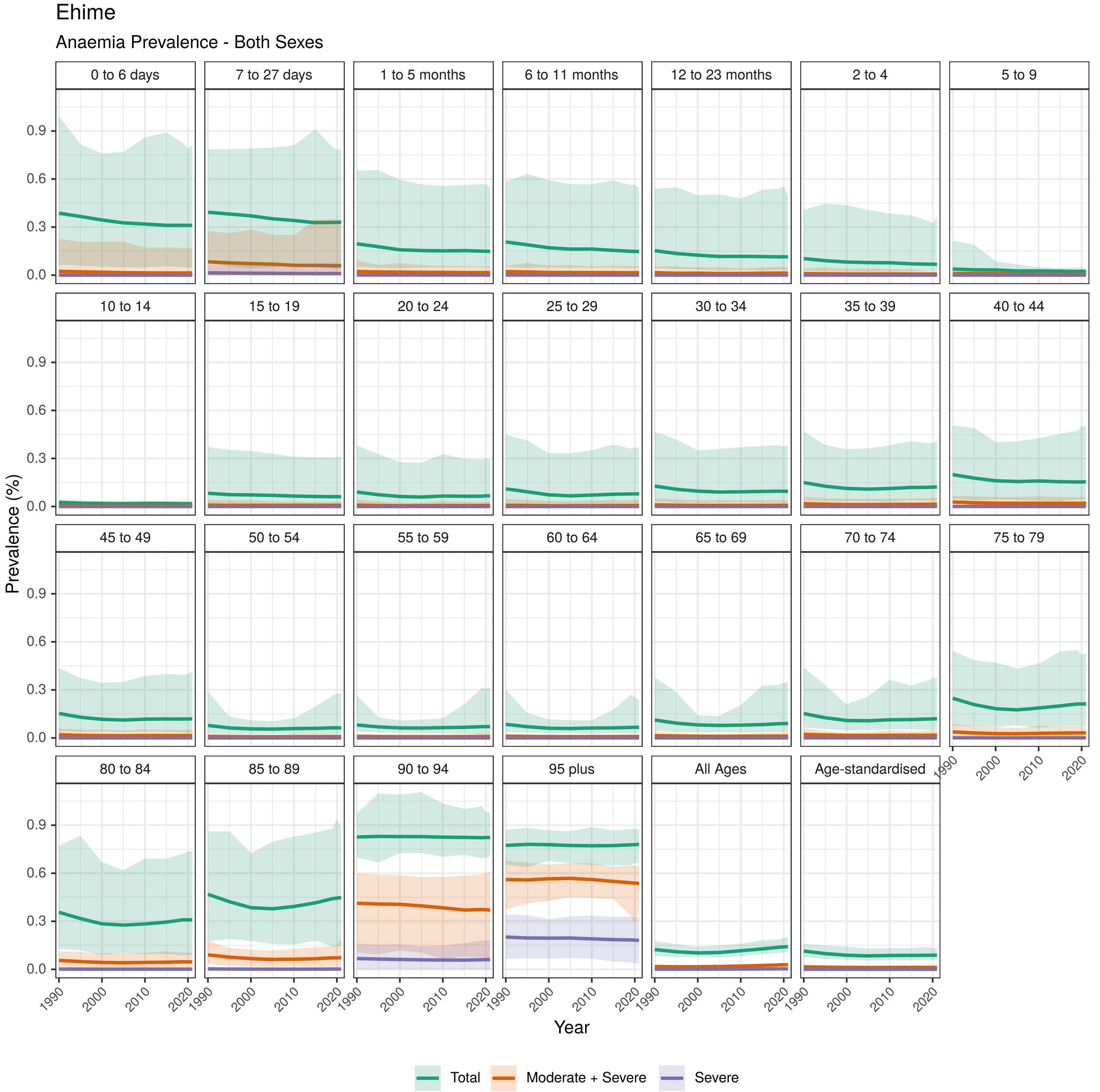
Ehime
Anaemia Prevalence - Both Sexes
0 to 6 days
7 to 27 days
1 to 5 months
6 to 11 months
12 to 23 months
2 to 4
5 to 9
10 to 14
15 to 19
20 to 24
25 to 29
30 to 34
35 to 39
40 to 44
45 to 49
50 to 54
55 to 59
60 to 64
65 to 69
70 to 74
75 to 79
80 to 84
85 to 89
90 to 94
95 plus
All Ages
Age-standardised
Prevalence (%)
Year
Total
Moderate + Severe
Severe

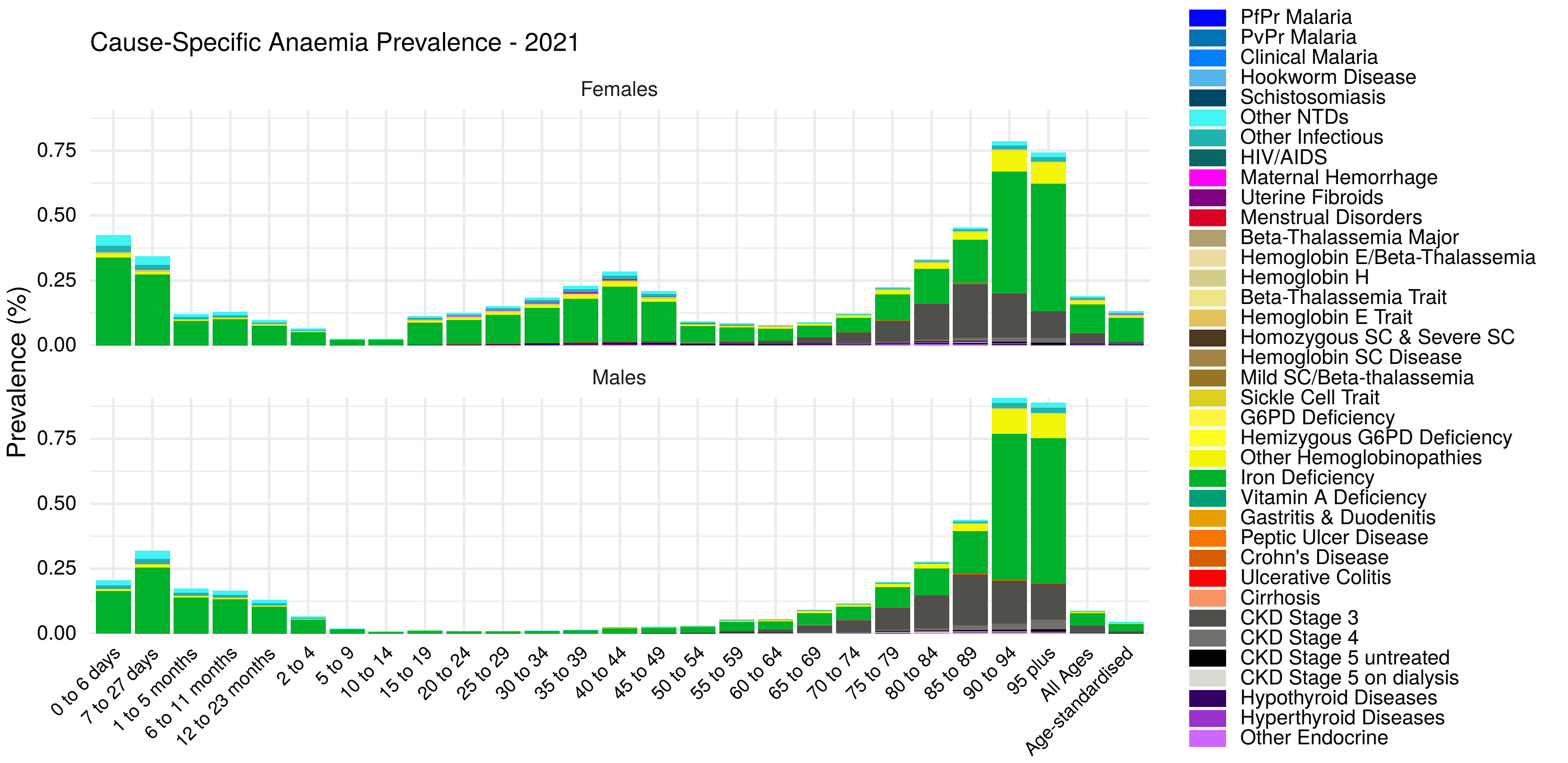
Cause-Specific Anaemia Prevalence - 2021
Females
Males
Prevalence (%)
PfPr Malaria
PvPr Malaria
Clinical Malaria
Hookworm Disease
Schistosomiasis
Other NTDs
Other Infectious
HIV/AIDS
Maternal Hemorrhage
Uterine Fibroids
Menstrual Disorders
Beta-Thalassemia Major
Hemoglobin E/Beta-Thalassemia
Hemoglobin H
Beta-Thalassemia Trait
Hemoglobin E Trait
Homozygous SC & Severe SC
Hemoglobin SC Disease
Mild SC/Beta-thalassemia
Sickle Cell Trait
G6PD Deficiency
Hemizygous G6PD Deficiency
Other Hemoglobinopathies
Iron Deficiency
Vitamin A Deficiency
Gastritis & Duodenitis
Peptic Ulcer Disease
Crohn's Disease
Ulcerative Colitis
Cirrhosis
CKD Stage 3
CKD Stage 4
CKD Stage 5 untreated
CKD Stage 5 on dialysis
Hypothyroid Diseases
Hyperthyroid Diseases
Other Endocrine

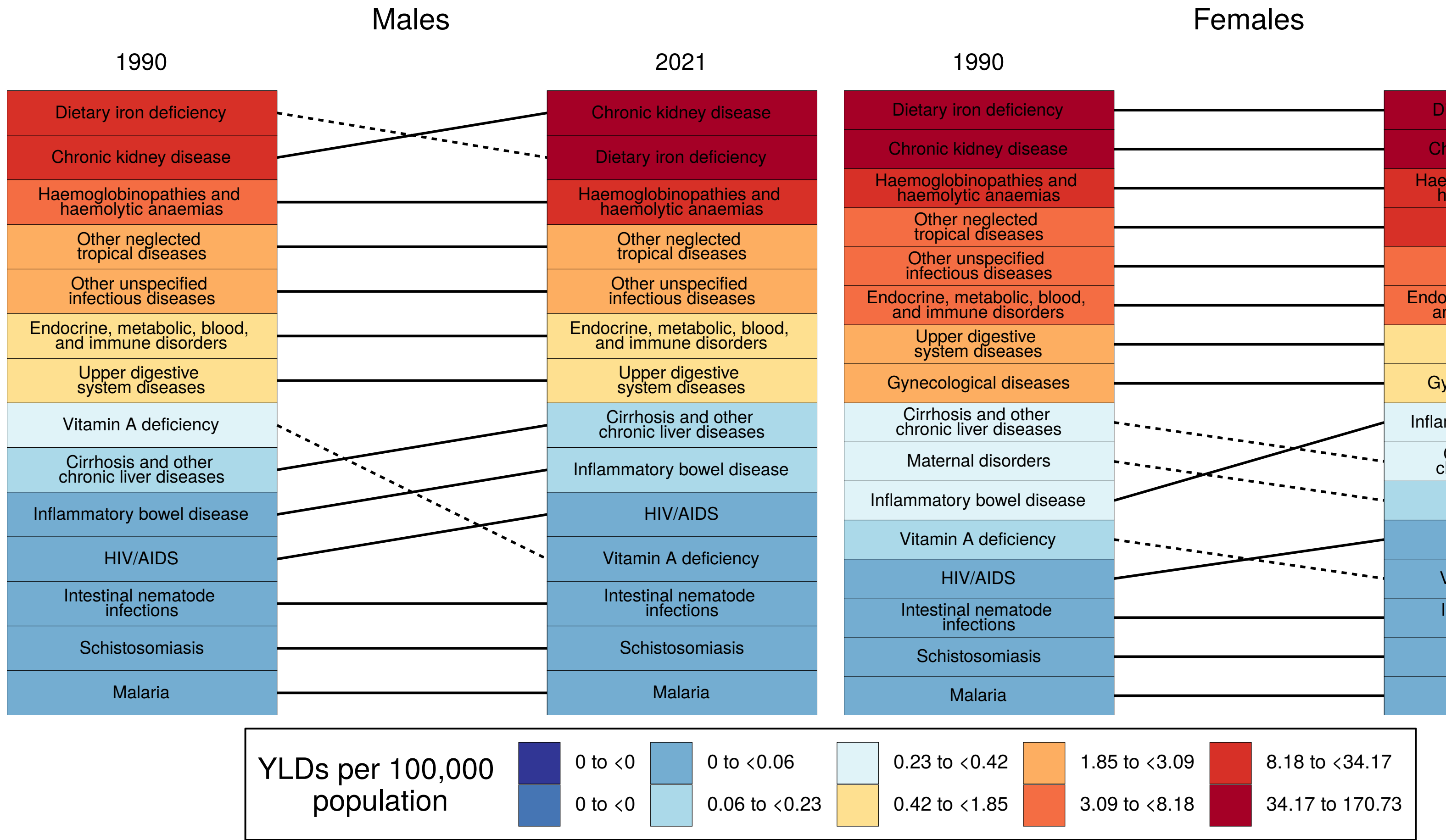
Males
Females
1990
2021
Dietary iron deficiency
Chronic kidney disease
Haemoglobinopathies and haemolytic anaemias
Other neglected tropical diseases
Other unspecified infectious diseases
Endocrine, metabolic, blood, and immune disorders
Upper digestive system diseases
Vitamin A deficiency
Cirrhosis and other chronic liver diseases
Inflammatory bowel disease
HIV/AIDS
Intestinal nematode infections
Schistosomiasis
Malaria
Gynecological diseases
Maternal disorders
YLDs per 100,000 population
0 to <0
0 to <0.06
0.06 to <0.23
0.23 to <0.42
0.42 to <1.85
1.85 to <3.09
3.09 to <8.18
8.18 to <34.17
34.17 to 170.73

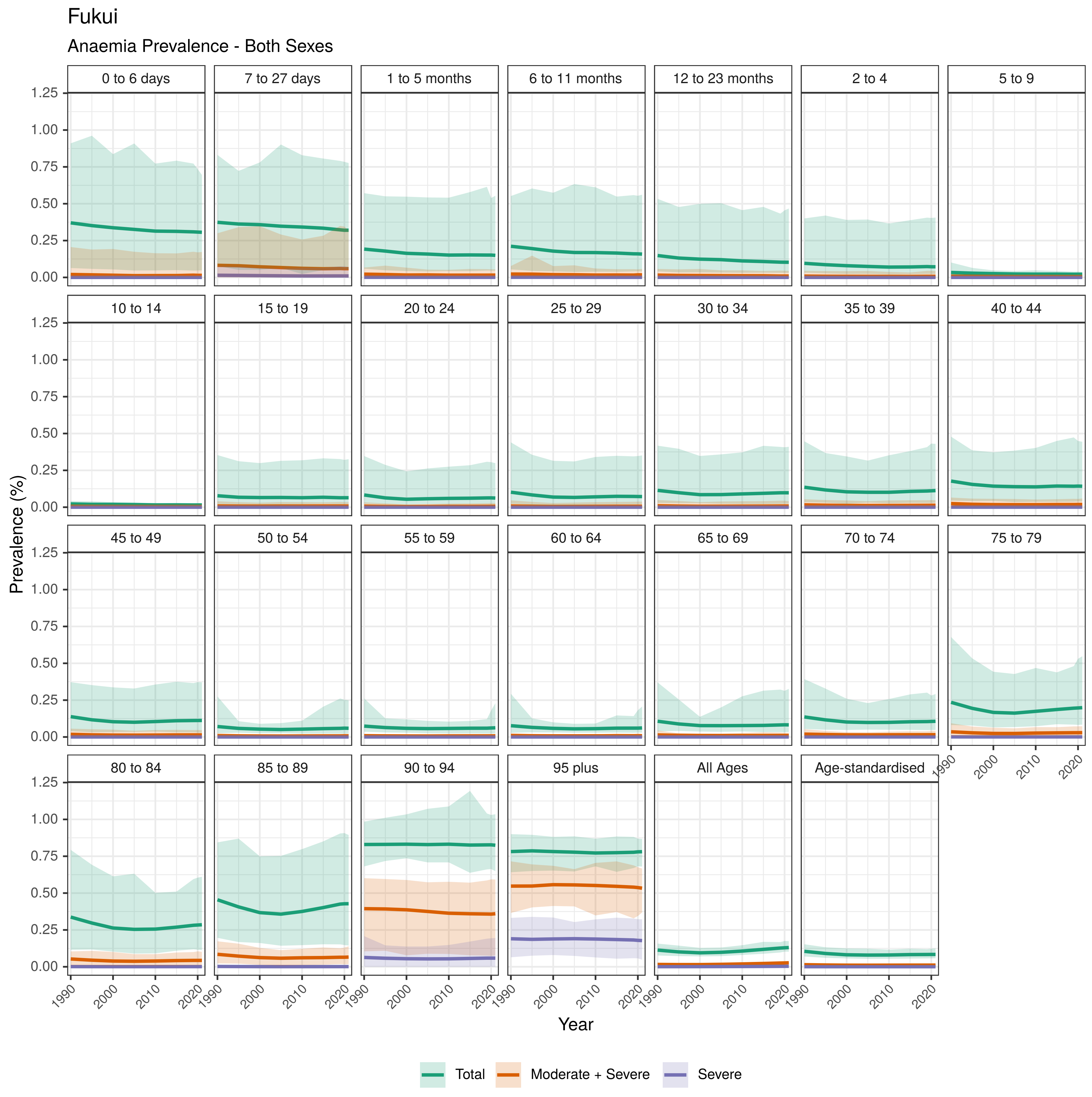
Fukui
Anaemia Prevalence - Both Sexes
0 to 6 days
7 to 27 days
1 to 5 months
6 to 11 months
12 to 23 months
2 to 4
5 to 9
10 to 14
15 to 19
20 to 24
25 to 29
30 to 34
35 to 39
40 to 44
45 to 49
50 to 54
55 to 59
60 to 64
65 to 69
70 to 74
75 to 79
80 to 84
85 to 89
90 to 94
95 plus
All Ages
Age-standardised
Prevalence (%)
Year
Total
Moderate + Severe
Severe

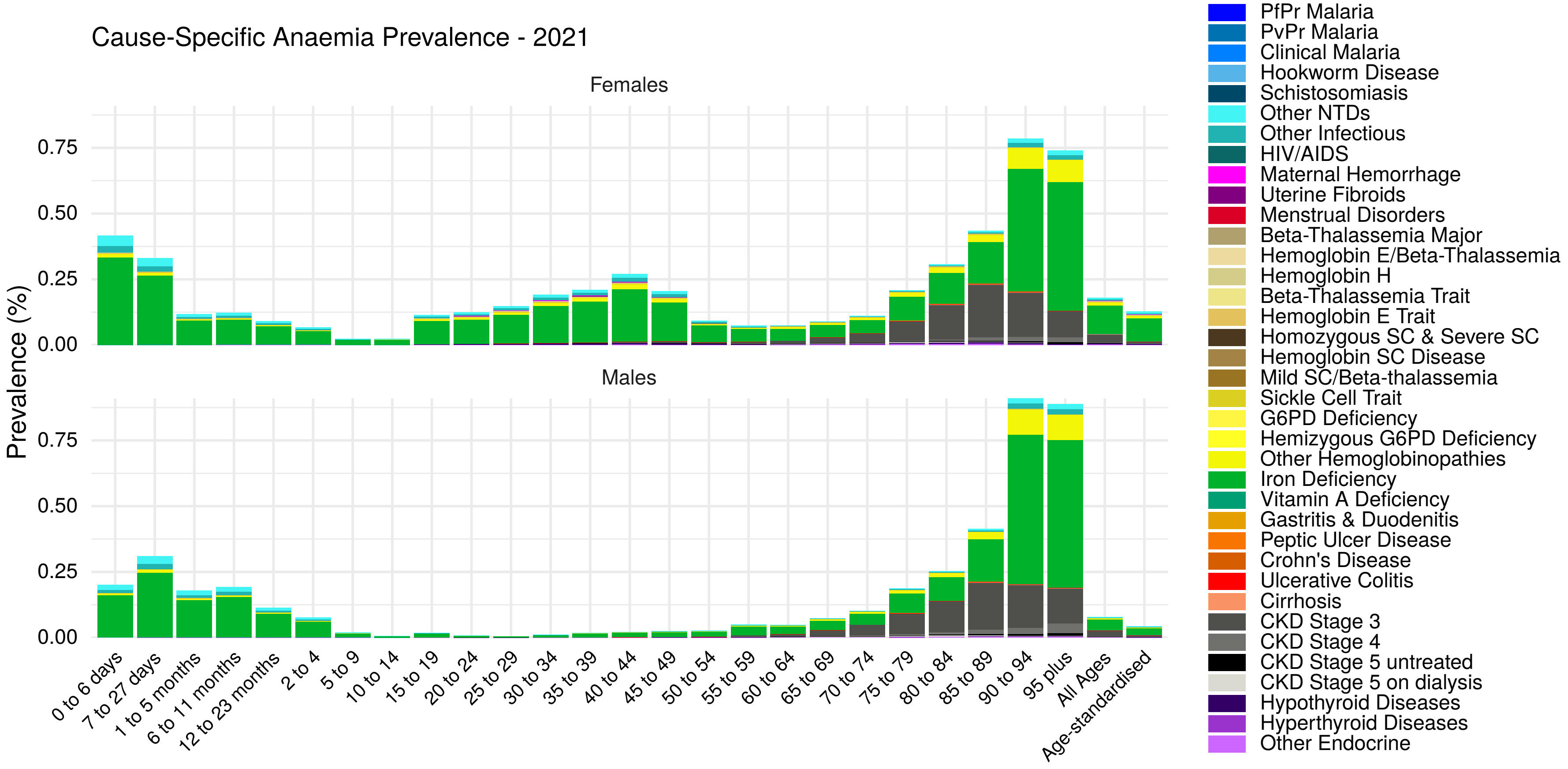
Cause-Specific Anaemia Prevalence - 2021
Females
Males
Prevalence (%)
PfPr Malaria
PvPr Malaria
Clinical Malaria
Hookworm Disease
Schistosomiasis
Other NTDs
Other Infectious
HIV/AIDS
Maternal Hemorrhage
Uterine Fibroids
Menstrual Disorders
Beta-Thalassemia Major
Hemoglobin E/Beta-Thalassemia
Hemoglobin H
Beta-Thalassemia Trait
Hemoglobin E Trait
Homozygous SC & Severe SC
Hemoglobin SC Disease
Mild SC/Beta-thalassemia
Sickle Cell Trait
G6PD Deficiency
Hemizygous G6PD Deficiency
Other Hemoglobinopathies
Iron Deficiency
Vitamin A Deficiency
Gastritis & Duodenitis
Peptic Ulcer Disease
Crohn's Disease
Ulcerative Colitis
Cirrhosis
CKD Stage 3
CKD Stage 4
CKD Stage 5 untreated
CKD Stage 5 on dialysis
Hypothyroid Diseases
Hyperthyroid Diseases
Other Endocrine

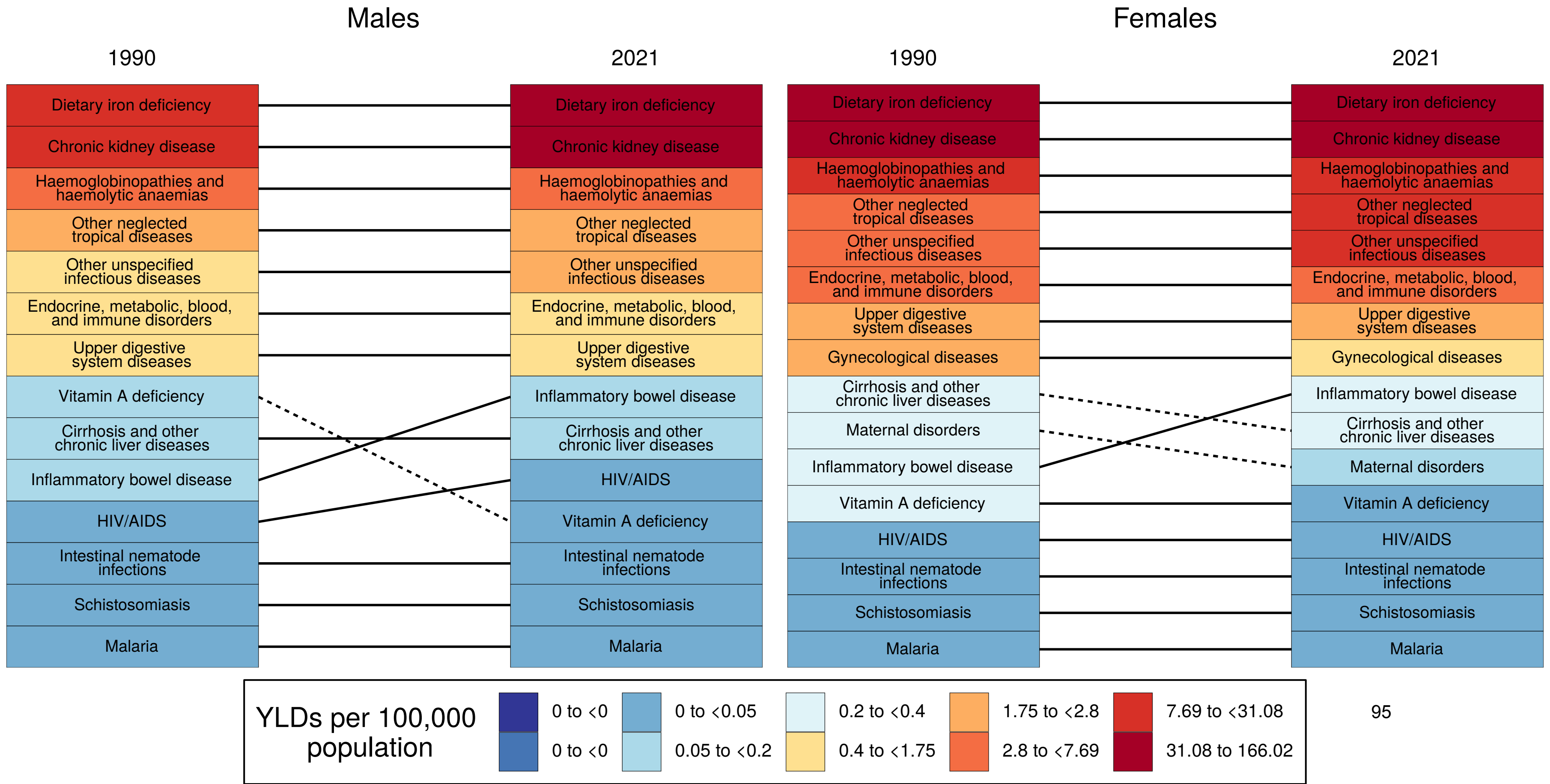
Males
Females
1990
2021
Dietary iron deficiency
Chronic kidney disease
Haemoglobinopathies and haemolytic anaemias
Other neglected tropical diseases
Other unspecified infectious diseases
Endocrine, metabolic, blood, and immune disorders
Upper digestive system diseases
Gynecological diseases
Vitamin A deficiency
Cirrhosis and other chronic liver diseases
Maternal disorders
Inflammatory bowel disease
HIV/AIDS
Intestinal nematode infections
Schistosomiasis
Malaria
YLDs per 100,000 population
0 to <0
0 to <0.05
0.05 to <0.2
0.2 to <0.4
0.4 to <1.75
1.75 to <2.8
2.8 to <7.69
7.69 to <31.08
31.08 to 166.02

Fukuoka

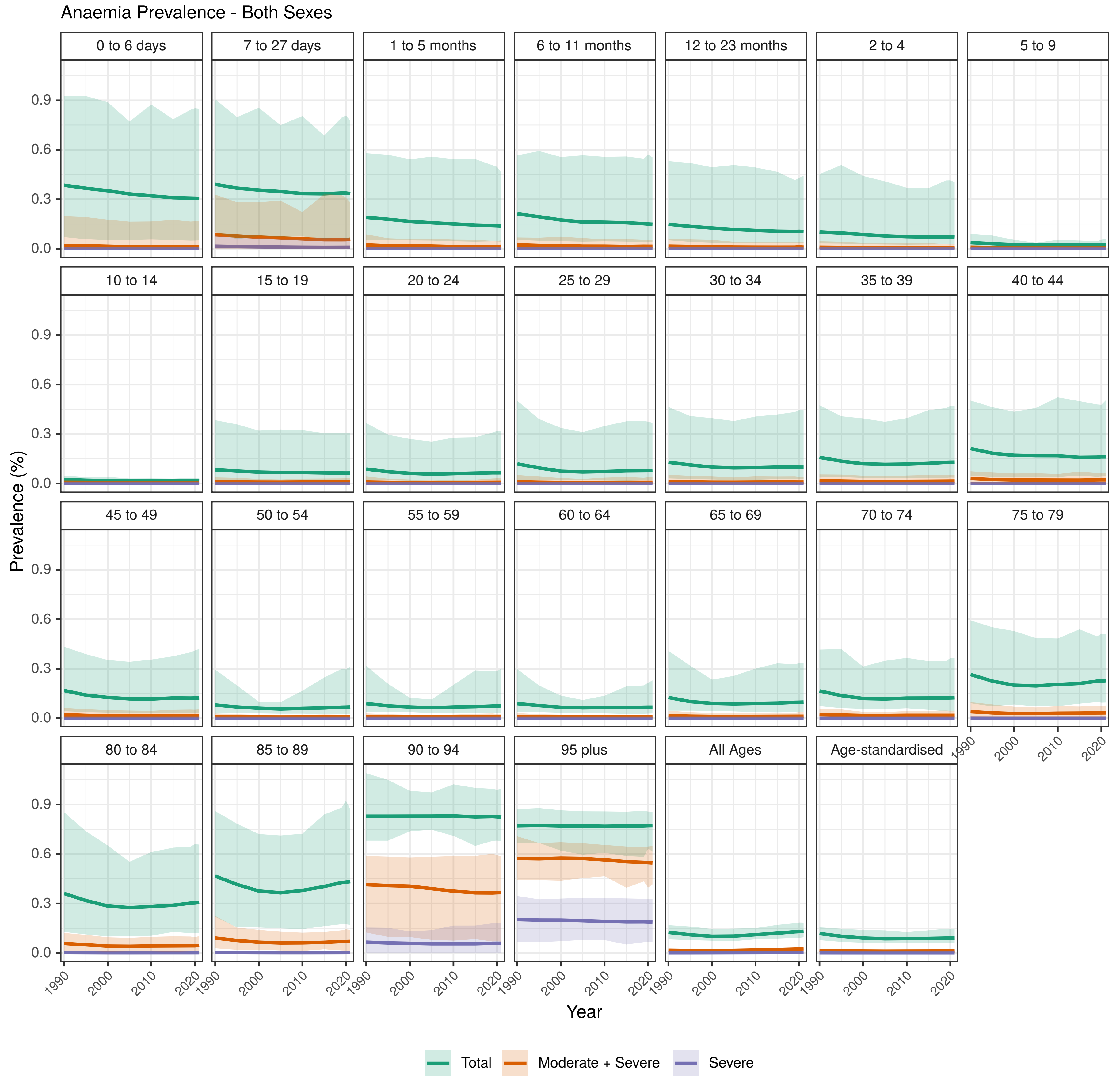

Anaemia Prevalence - Both Sexes
0 to 6 days
7 to 27 days
1 to 5 months
6 to 11 months
12 to 23 months
2 to 4
5 to 9
10 to 14
15 to 19
20 to 24
25 to 29
30 to 34
35 to 39
40 to 44
45 to 49
50 to 54
55 to 59
60 to 64
65 to 69
70 to 74
75 to 79
80 to 84
85 to 89
90 to 94
95 plus
All Ages
Age-standardised
Prevalence (%)
Year
Total
Moderate + Severe
Severe

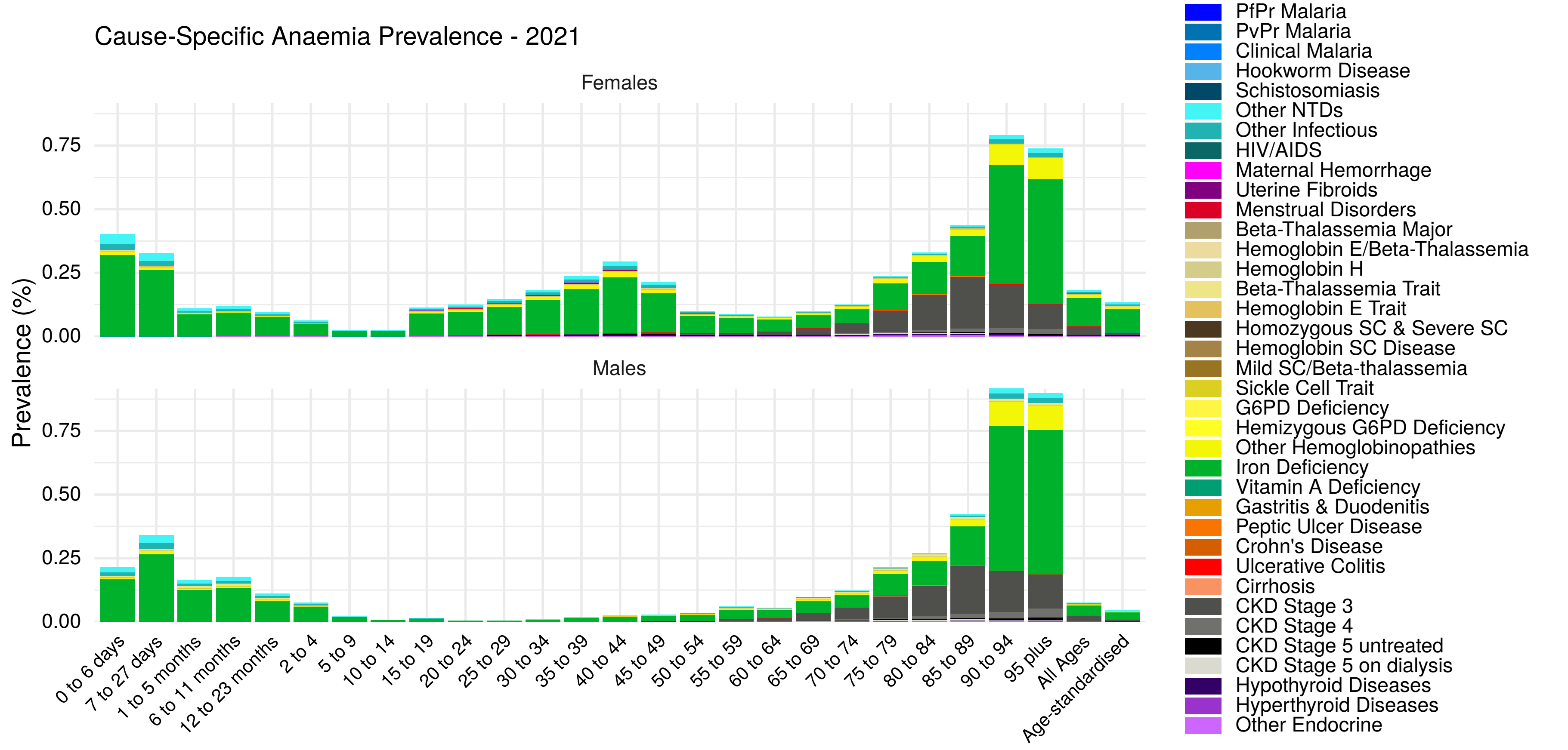

Cause-Specific Anaemia Prevalence - 2021
Females
Males
Prevalence (%)
PfPr Malaria
PvPr Malaria
Clinical Malaria
Hookworm Disease
Schistosomiasis
Other NTDs
Other Infectious
HIV/AIDS
Maternal Hemorrhage
Uterine Fibroids
Menstrual Disorders
Beta-Thalassemia Major
Hemoglobin E/Beta-Thalassemia
Hemoglobin H
Beta-Thalassemia Trait
Hemoglobin E Trait
Homozygous SC & Severe SC
Hemoglobin SC Disease
Mild SC/Beta-thalassemia
Sickle Cell Trait
G6PD Deficiency
Hemizygous G6PD Deficiency
Other Hemoglobinopathies
Iron Deficiency
Vitamin A Deficiency
Gastritis & Duodenitis
Peptic Ulcer Disease
Crohn's Disease
Ulcerative Colitis
Cirrhosis
CKD Stage 3
CKD Stage 4
CKD Stage 5 untreated
CKD Stage 5 on dialysis
Hypothyroid Diseases
Hyperthyroid Diseases
Other Endocrine

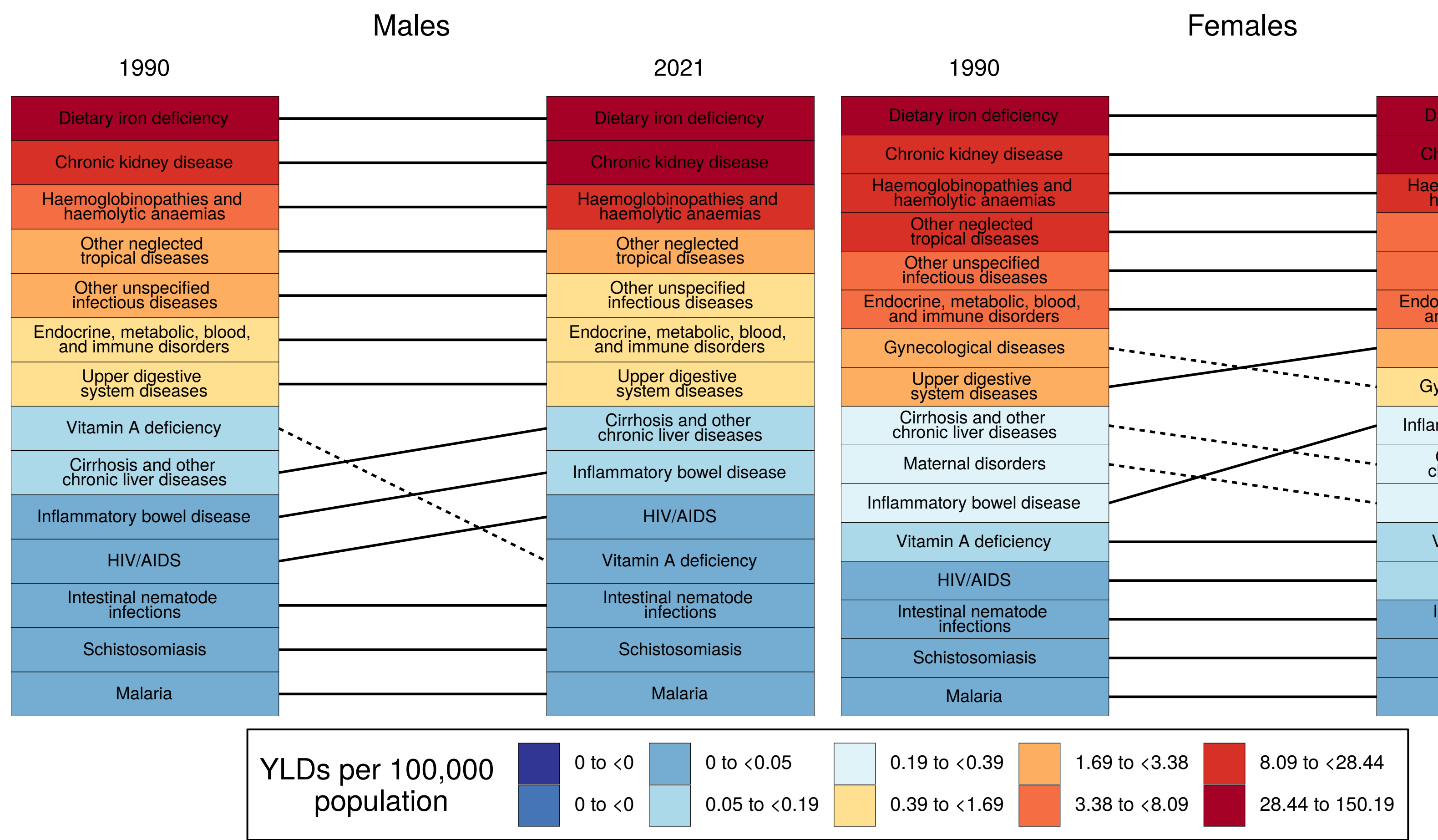

Males
Females
1990
2021
Dietary iron deficiency
Chronic kidney disease
Haemoglobinopathies and haemolytic anaemias
Other neglected tropical diseases
Other unspecified infectious diseases
Endocrine, metabolic, blood, and immune disorders
Upper digestive system diseases
Vitamin A deficiency
Cirrhosis and other chronic liver diseases
Inflammatory bowel disease
HIV/AIDS
Intestinal nematode infections
Schistosomiasis
Malaria
Gynecological diseases
Maternal disorders
YLDs per 100,000 population
0 to <0
0 to <0.05
0.05 to <0.19
0.19 to <0.39
0.39 to <1.69
1.69 to <3.38
3.38 to <8.09
8.09 to <28.44
28.44 to 150.19

Fukushima

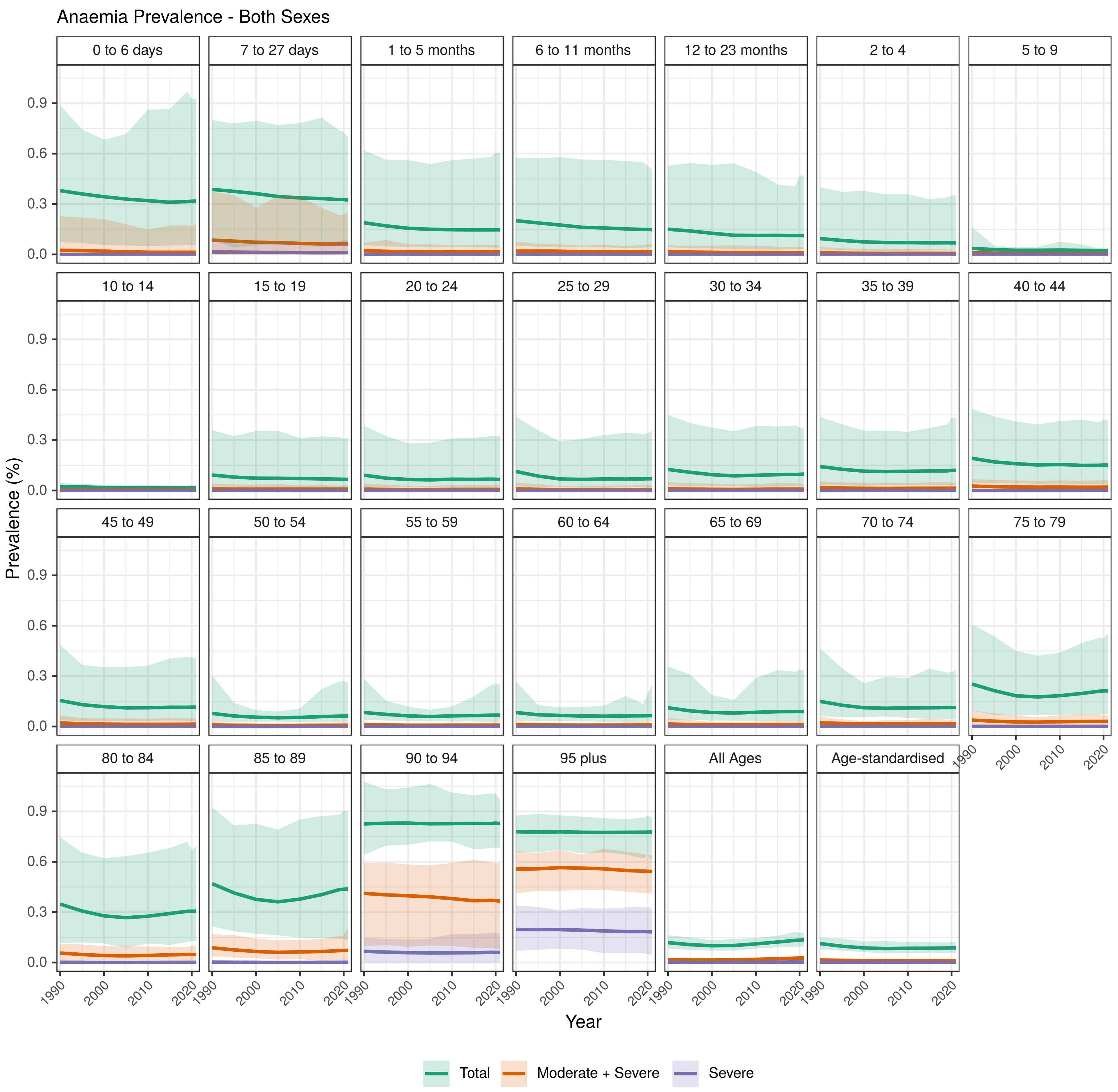
Anaemia Prevalence - Both Sexes
0 to 6 days
7 to 27 days
1 to 5 months
6 to 11 months
12 to 23 months
2 to 4
5 to 9
10 to 14
15 to 19
20 to 24
25 to 29
30 to 34
35 to 39
40 to 44
45 to 49
50 to 54
55 to 59
60 to 64
65 to 69
70 to 74
75 to 79
80 to 84
85 to 89
90 to 94
95 plus
All Ages
Age-standardised
Prevalence (%)
Year
Total
Moderate + Severe
Severe

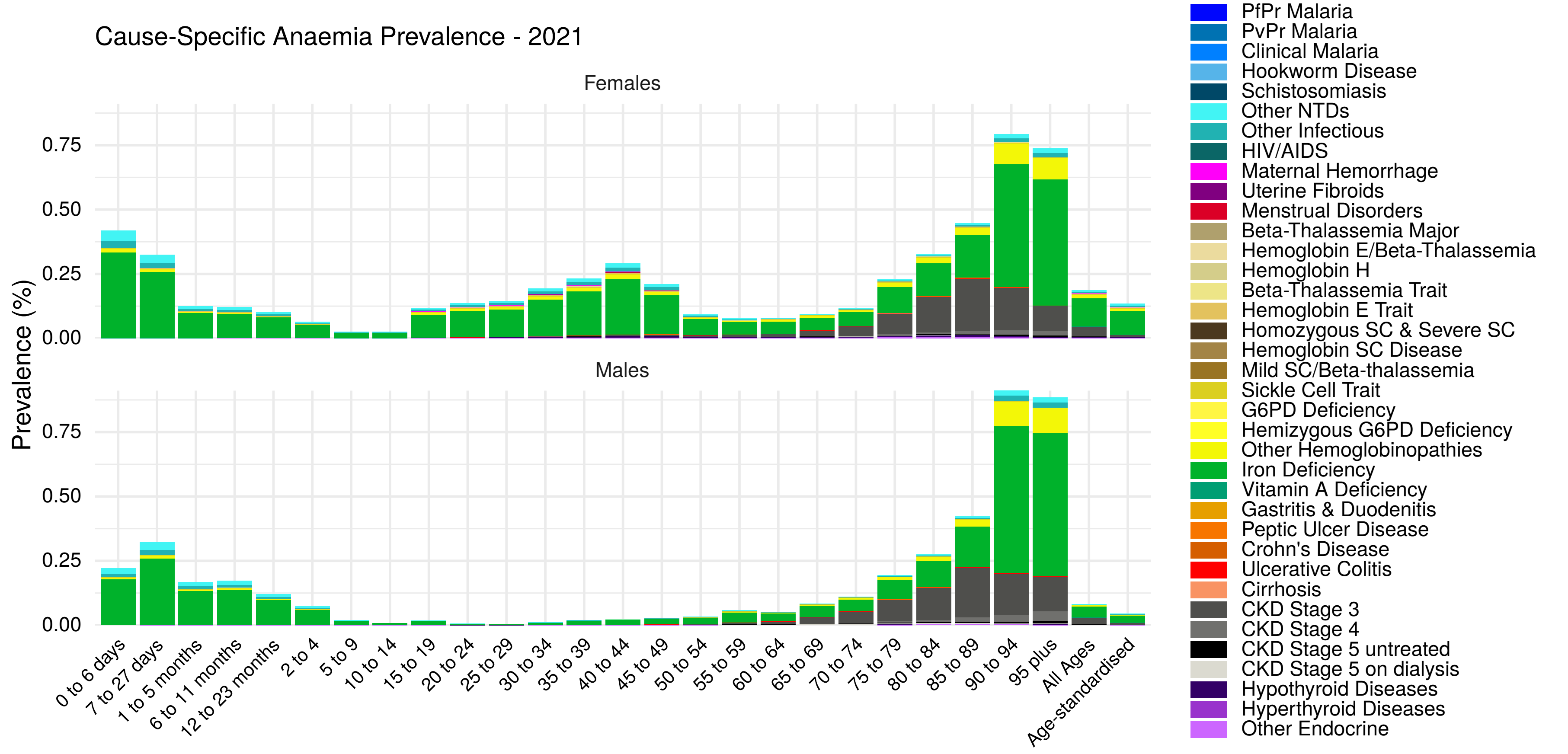
Cause-Specific Anaemia Prevalence - 2021
Females
Males
Prevalence (%)
PfPr Malaria
PvPr Malaria
Clinical Malaria
Hookworm Disease
Schistosomiasis
Other NTDs
Other Infectious
HIV/AIDS
Maternal Hemorrhage
Uterine Fibroids
Menstrual Disorders
Beta-Thalassemia Major
Hemoglobin E/Beta-Thalassemia
Hemoglobin H
Beta-Thalassemia Trait
Hemoglobin E Trait
Homozygous SC & Severe SC
Hemoglobin SC Disease
Mild SC/Beta-thalassemia
Sickle Cell Trait
G6PD Deficiency
Hemizygous G6PD Deficiency
Other Hemoglobinopathies
Iron Deficiency
Vitamin A Deficiency
Gastritis & Duodenitis
Peptic Ulcer Disease
Crohn's Disease
Ulcerative Colitis
Cirrhosis
CKD Stage 3
CKD Stage 4
CKD Stage 5 untreated
CKD Stage 5 on dialysis
Hypothyroid Diseases
Hyperthyroid Diseases
Other Endocrine

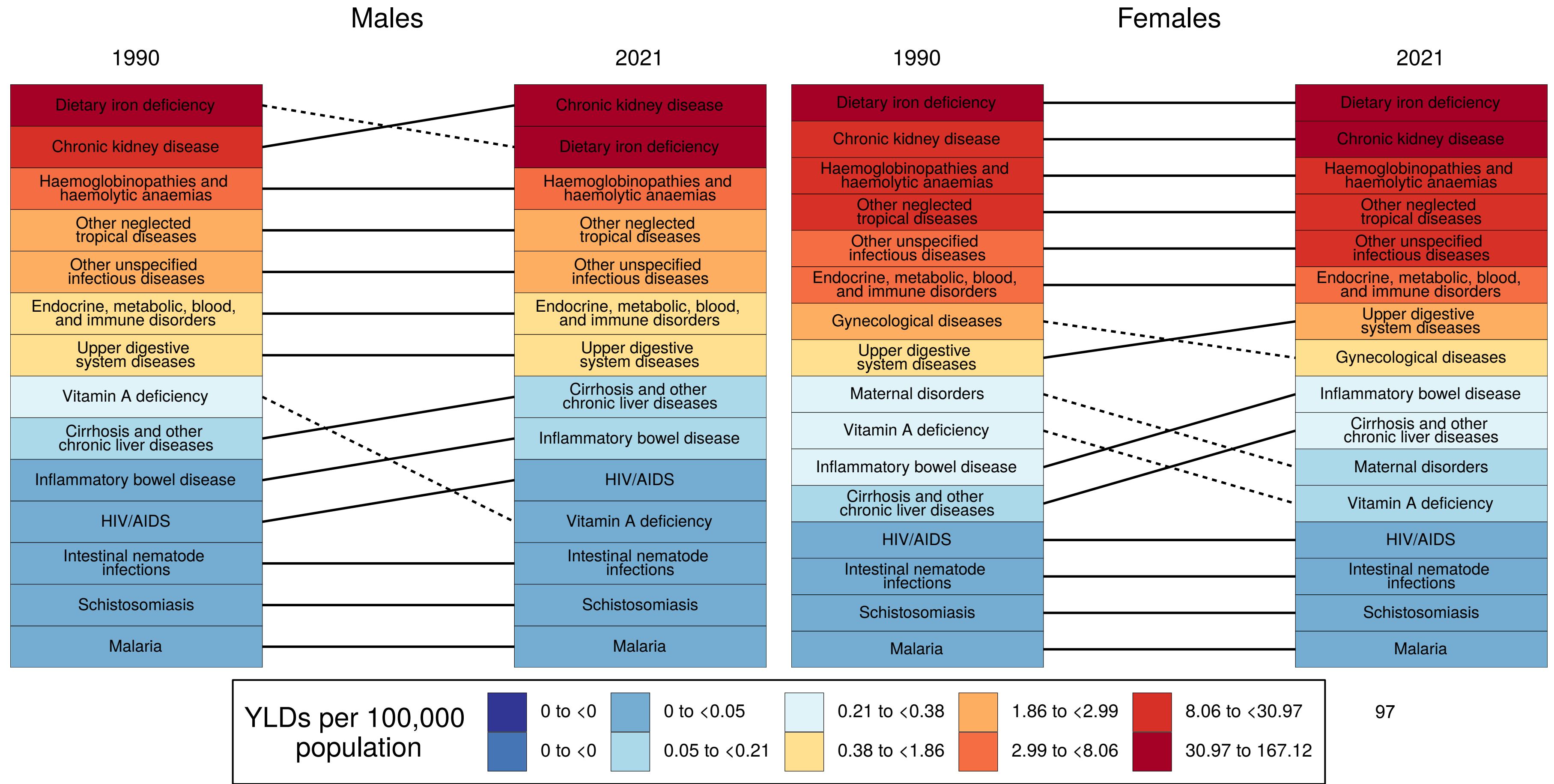
Males
Females
1990
2021
Dietary iron deficiency
Chronic kidney disease
Haemoglobinopathies and haemolytic anaemias
Other neglected tropical diseases
Other unspecified infectious diseases
Endocrine, metabolic, blood, and immune disorders
Upper digestive system diseases
Vitamin A deficiency
Cirrhosis and other chronic liver diseases
Inflammatory bowel disease
HIV/AIDS
Intestinal nematode infections
Schistosomiasis
Malaria
Gynecological diseases
Maternal disorders
YLDs per 100,000 population
0 to <0
0 to <0.05
0.05 to <0.21
0.21 to <0.38
0.38 to <1.86
1.86 to <2.99
2.99 to <8.06
8.06 to <30.97
30.97 to 167.12

Gifu

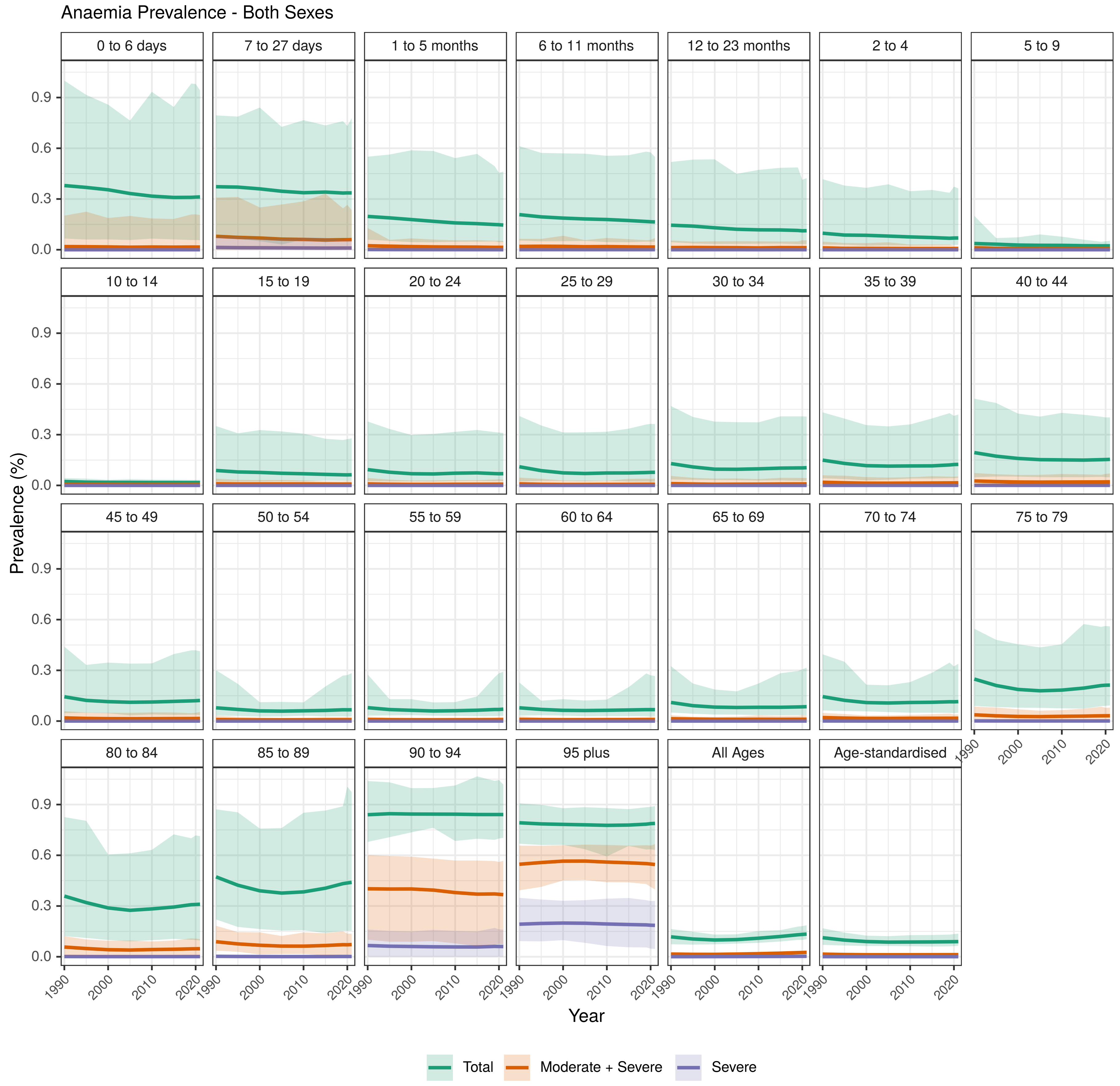
Anaemia Prevalence - Both Sexes
0 to 6 days
7 to 27 days
1 to 5 months
6 to 11 months
12 to 23 months
2 to 4
5 to 9
10 to 14
15 to 19
20 to 24
25 to 29
30 to 34
35 to 39
40 to 44
45 to 49
50 to 54
55 to 59
60 to 64
65 to 69
70 to 74
75 to 79
80 to 84
85 to 89
90 to 94
95 plus
All Ages
Age-standardised
Prevalence (%)
0.0
0.3
0.6
0.9
1990
2000
2010
2020
Year
Total
Moderate + Severe
Severe

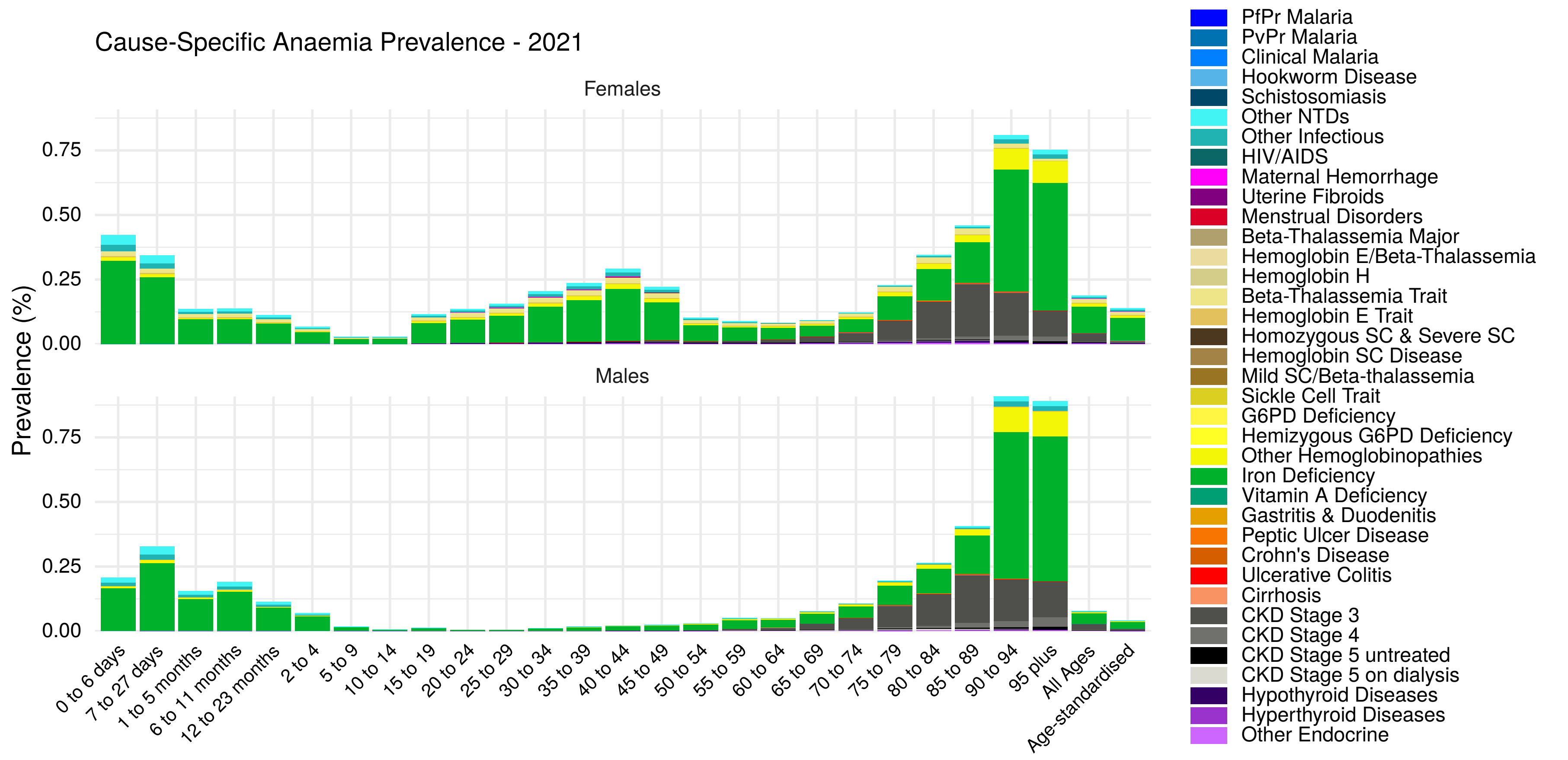
Cause-Specific Anaemia Prevalence - 2021
Females
Males
Prevalence (%)
0.00
0.25
0.50
0.75
0 to 6 days
7 to 27 days
1 to 5 months
6 to 11 months
12 to 23 months
2 to 4
5 to 9
10 to 14
15 to 19
20 to 24
25 to 29
30 to 34
35 to 39
40 to 44
45 to 49
50 to 54
55 to 59
60 to 64
65 to 69
70 to 74
75 to 79
80 to 84
85 to 89
90 to 94
95 plus
All Ages
Age-standardised
PfPr Malaria
PvPr Malaria
Clinical Malaria
Hookworm Disease
Schistosomiasis
Other NTDs
Other Infectious
HIV/AIDS
Maternal Hemorrhage
Uterine Fibroids
Menstrual Disorders
Beta-Thalassemia Major
Hemoglobin E/Beta-Thalassemia
Hemoglobin H
Beta-Thalassemia Trait
Hemoglobin E Trait
Homozygous SC & Severe SC
Hemoglobin SC Disease
Mild SC/Beta-thalassemia
Sickle Cell Trait
G6PD Deficiency
Hemizygous G6PD Deficiency
Other Hemoglobinopathies
Iron Deficiency
Vitamin A Deficiency
Gastritis & Duodenitis
Peptic Ulcer Disease
Crohn's Disease
Ulcerative Colitis
Cirrhosis
CKD Stage 3
CKD Stage 4
CKD Stage 5 untreated
CKD Stage 5 on dialysis
Hypothyroid Diseases
Hyperthyroid Diseases
Other Endocrine

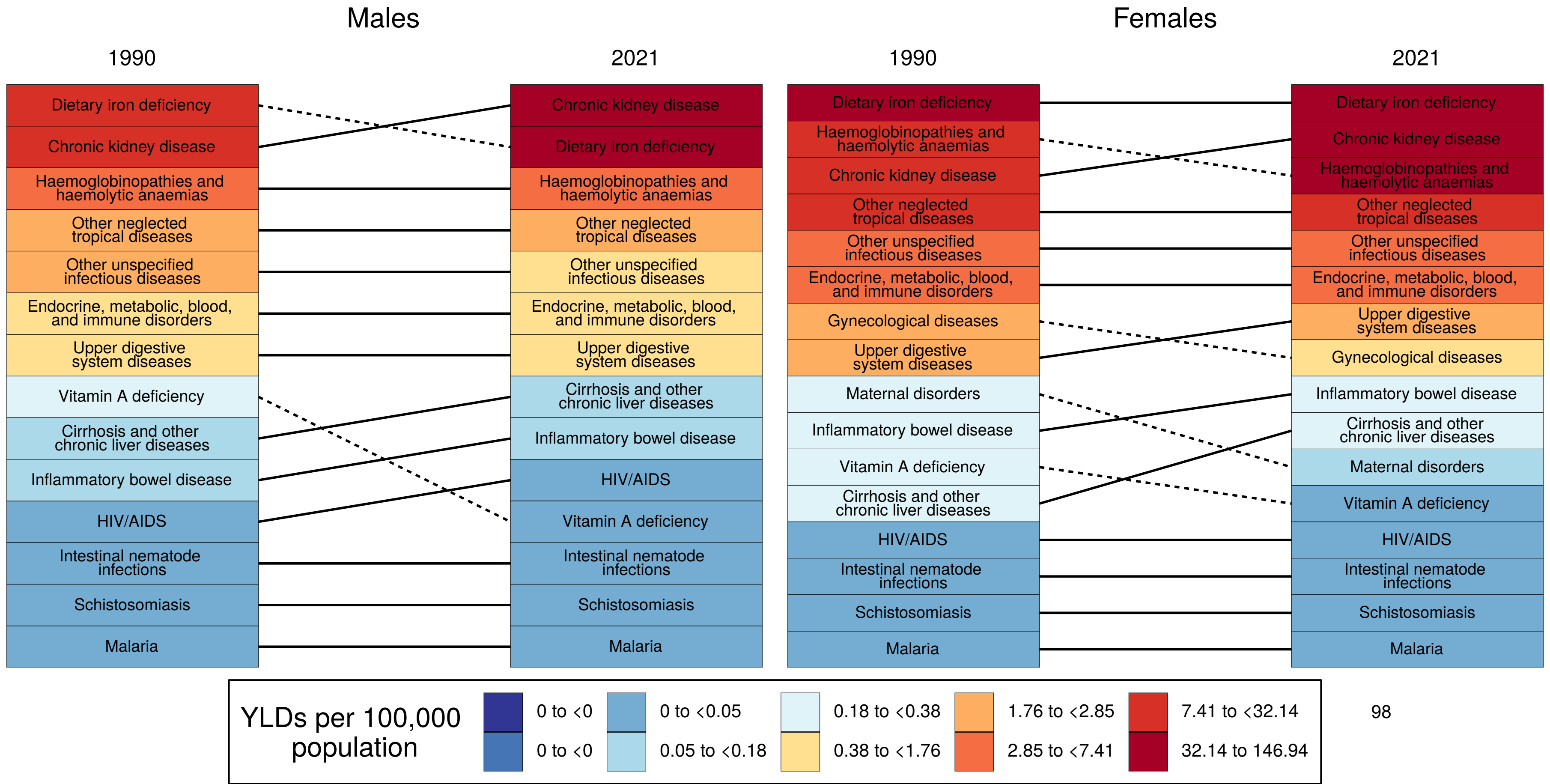
Males
Females
1990
2021
Dietary iron deficiency
Chronic kidney disease
Haemoglobinopathies and haemolytic anaemias
Other neglected tropical diseases
Other unspecified infectious diseases
Endocrine, metabolic, blood, and immune disorders
Upper digestive system diseases
Vitamin A deficiency
Cirrhosis and other chronic liver diseases
Inflammatory bowel disease
HIV/AIDS
Intestinal nematode infections
Schistosomiasis
Malaria
Gynecological diseases
Maternal disorders
YLDs per 100,000 population
0 to <0
0 to <0.05
0.05 to <0.18
0.18 to <0.38
0.38 to <1.76
1.76 to <2.85
2.85 to <7.41
7.41 to <32.14
32.14 to 146.94

Gunma

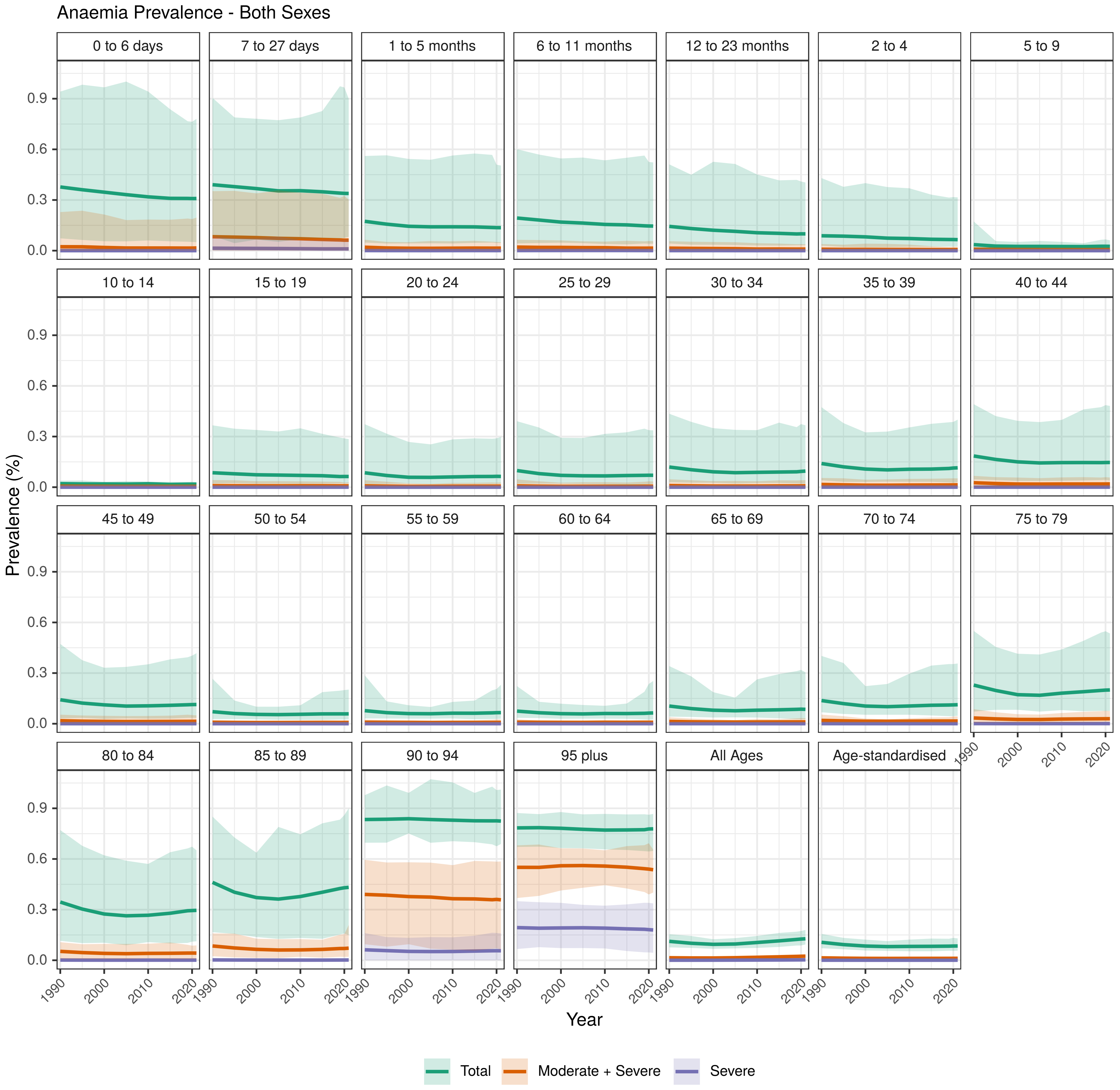
Anaemia Prevalence - Both Sexes
0 to 6 days
7 to 27 days
1 to 5 months
6 to 11 months
12 to 23 months
2 to 4
5 to 9
10 to 14
15 to 19
20 to 24
25 to 29
30 to 34
35 to 39
40 to 44
45 to 49
50 to 54
55 to 59
60 to 64
65 to 69
70 to 74
75 to 79
80 to 84
85 to 89
90 to 94
95 plus
All Ages
Age-standardised
Prevalence (%)
0.0
0.3
0.6
0.9
1990
2000
2010
2020
Year
Total
Moderate + Severe
Severe

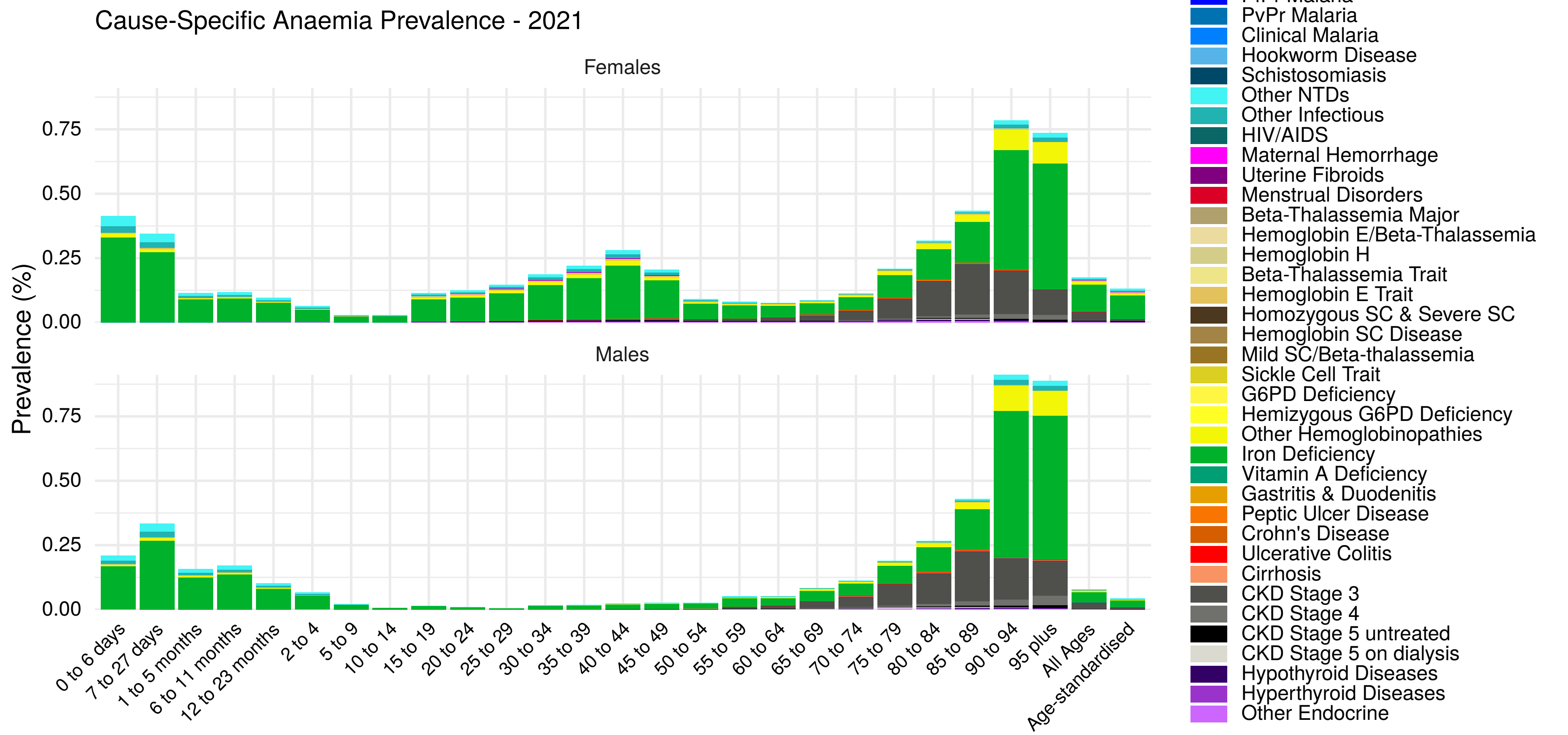
Cause-Specific Anaemia Prevalence - 2021
Females
Males
Prevalence (%)
0.00
0.25
0.50
0.75
0 to 6 days
7 to 27 days
1 to 5 months
6 to 11 months
12 to 23 months
2 to 4
5 to 9
10 to 14
15 to 19
20 to 24
25 to 29
30 to 34
35 to 39
40 to 44
45 to 49
50 to 54
55 to 59
60 to 64
65 to 69
70 to 74
75 to 79
80 to 84
85 to 89
90 to 94
95 plus
All Ages
Age-standardised
PfPr Malaria
PvPr Malaria
Clinical Malaria
Hookworm Disease
Schistosomiasis
Other NTDs
Other Infectious
HIV/AIDS
Maternal Hemorrhage
Uterine Fibroids
Menstrual Disorders
Beta-Thalassemia Major
Hemoglobin E/Beta-Thalassemia
Hemoglobin H
Beta-Thalassemia Trait
Hemoglobin E Trait
Homozygous SC & Severe SC
Hemoglobin SC Disease
Mild SC/Beta-thalassemia
Sickle Cell Trait
G6PD Deficiency
Hemizygous G6PD Deficiency
Other Hemoglobinopathies
Iron Deficiency
Vitamin A Deficiency
Gastritis & Duodenitis
Peptic Ulcer Disease
Crohn's Disease
Ulcerative Colitis
Cirrhosis
CKD Stage 3
CKD Stage 4
CKD Stage 5 untreated
CKD Stage 5 on dialysis
Hypothyroid Diseases
Hyperthyroid Diseases
Other Endocrine

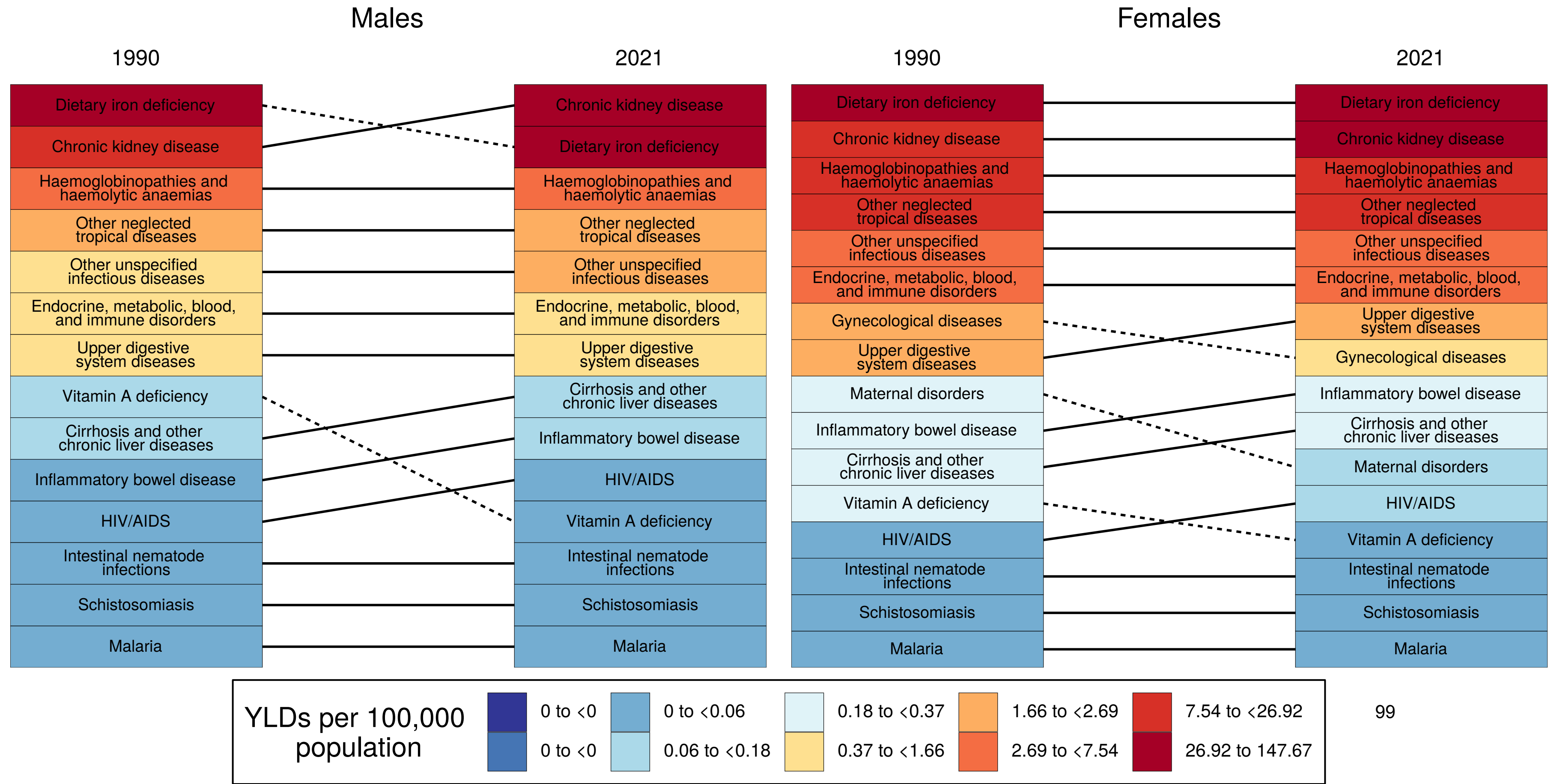
Males
Females
1990
2021
Dietary iron deficiency
Chronic kidney disease
Haemoglobinopathies and haemolytic anaemias
Other neglected tropical diseases
Other unspecified infectious diseases
Endocrine, metabolic, blood, and immune disorders
Upper digestive system diseases
Vitamin A deficiency
Cirrhosis and other chronic liver diseases
Inflammatory bowel disease
HIV/AIDS
Intestinal nematode infections
Schistosomiasis
Malaria
Gynecological diseases
Maternal disorders
YLDs per 100,000 population
0 to <0
0 to <0
0 to <0.06
0.06 to <0.18
0.18 to <0.37
0.37 to <1.66
1.66 to <2.69
2.69 to <7.54
7.54 to <26.92
26.92 to 147.67

Hiroshima

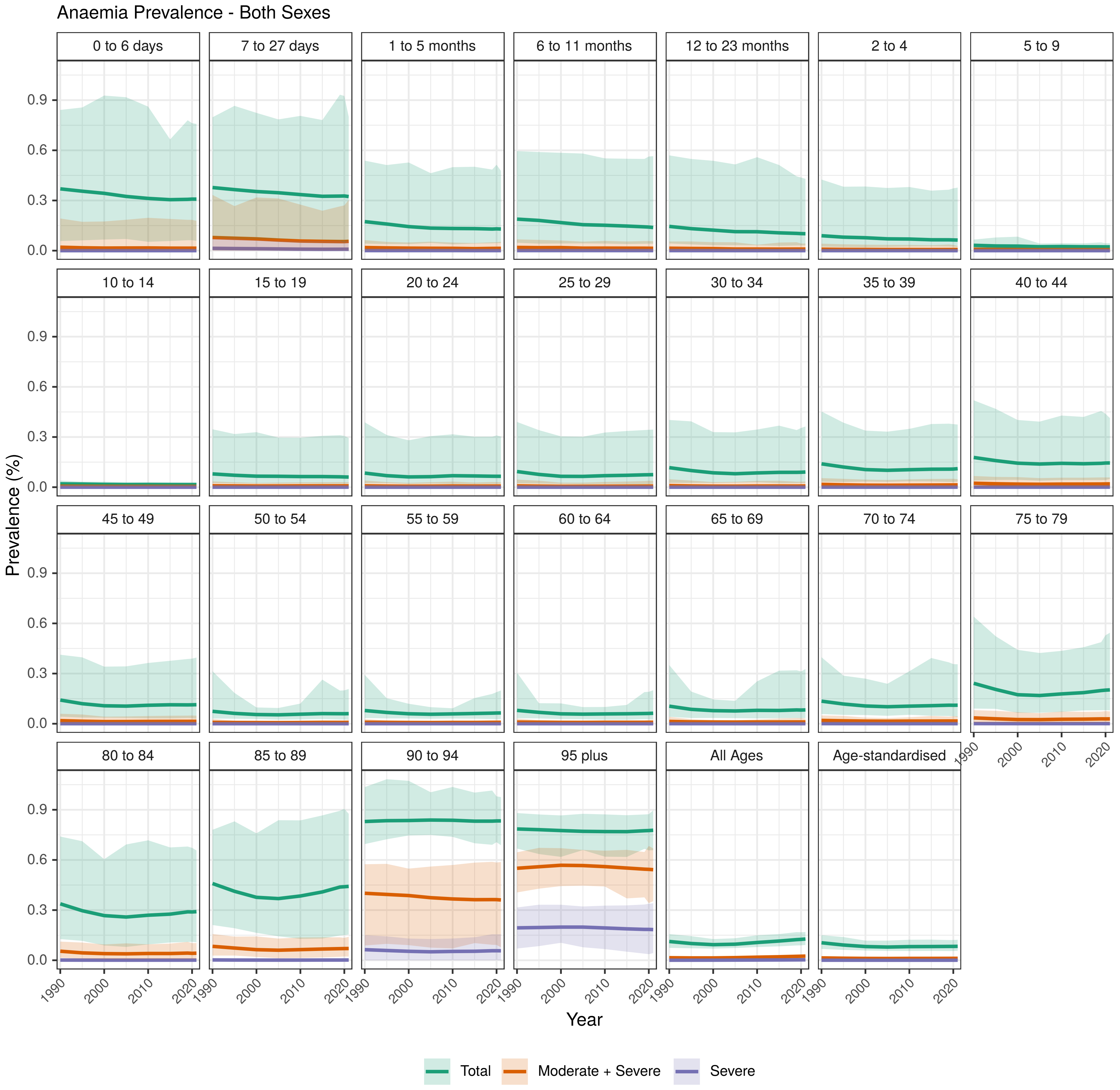

Anaemia Prevalence - Both Sexes
0 to 6 days
7 to 27 days
1 to 5 months
6 to 11 months
12 to 23 months
2 to 4
5 to 9
10 to 14
15 to 19
20 to 24
25 to 29
30 to 34
35 to 39
40 to 44
45 to 49
50 to 54
55 to 59
60 to 64
65 to 69
70 to 74
75 to 79
80 to 84
85 to 89
90 to 94
95 plus
All Ages
Age-standardised
Prevalence (%)
0.0
0.3
0.6
0.9
1990
2000
2010
2020
Year
Total
Moderate + Severe
Severe

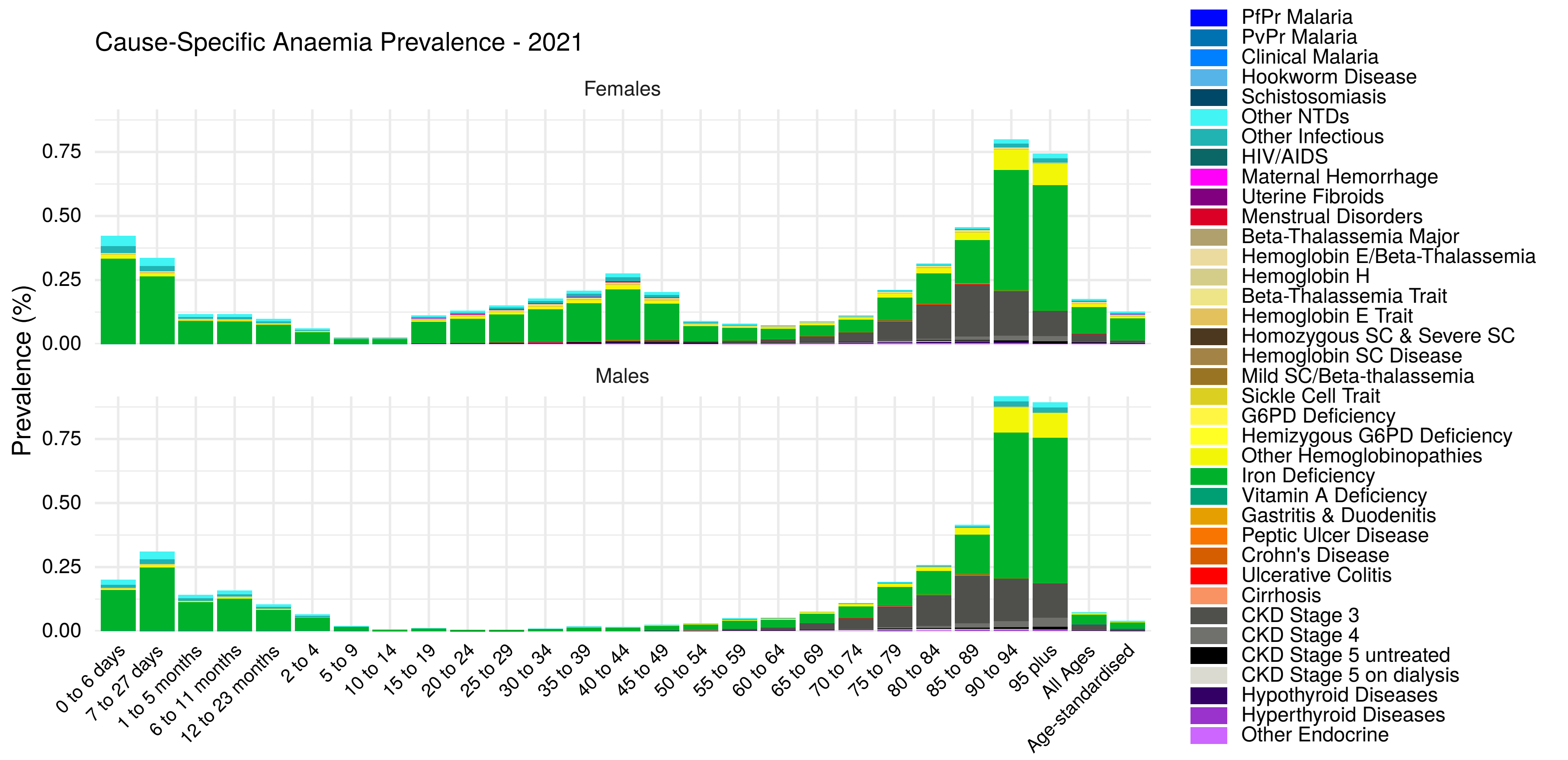

Cause-Specific Anaemia Prevalence - 2021
Females
Males
Prevalence (%)
0.00
0.25
0.50
0.75
0 to 6 days
7 to 27 days
1 to 5 months
6 to 11 months
12 to 23 months
2 to 4
5 to 9
10 to 14
15 to 19
20 to 24
25 to 29
30 to 34
35 to 39
40 to 44
45 to 49
50 to 54
55 to 59
60 to 64
65 to 69
70 to 74
75 to 79
80 to 84
85 to 89
90 to 94
95 plus
All Ages
Age-standardised
PfPr Malaria
PvPr Malaria
Clinical Malaria
Hookworm Disease
Schistosomiasis
Other NTDs
Other Infectious
HIV/AIDS
Maternal Hemorrhage
Uterine Fibroids
Menstrual Disorders
Beta-Thalassemia Major
Hemoglobin E/Beta-Thalassemia
Hemoglobin H
Beta-Thalassemia Trait
Hemoglobin E Trait
Homozygous SC & Severe SC
Hemoglobin SC Disease
Mild SC/Beta-thalassemia
Sickle Cell Trait
G6PD Deficiency
Hemizygous G6PD Deficiency
Other Hemoglobinopathies
Iron Deficiency
Vitamin A Deficiency
Gastritis & Duodenitis
Peptic Ulcer Disease
Crohn's Disease
Ulcerative Colitis
Cirrhosis
CKD Stage 3
CKD Stage 4
CKD Stage 5 untreated
CKD Stage 5 on dialysis
Hypothyroid Diseases
Hyperthyroid Diseases
Other Endocrine

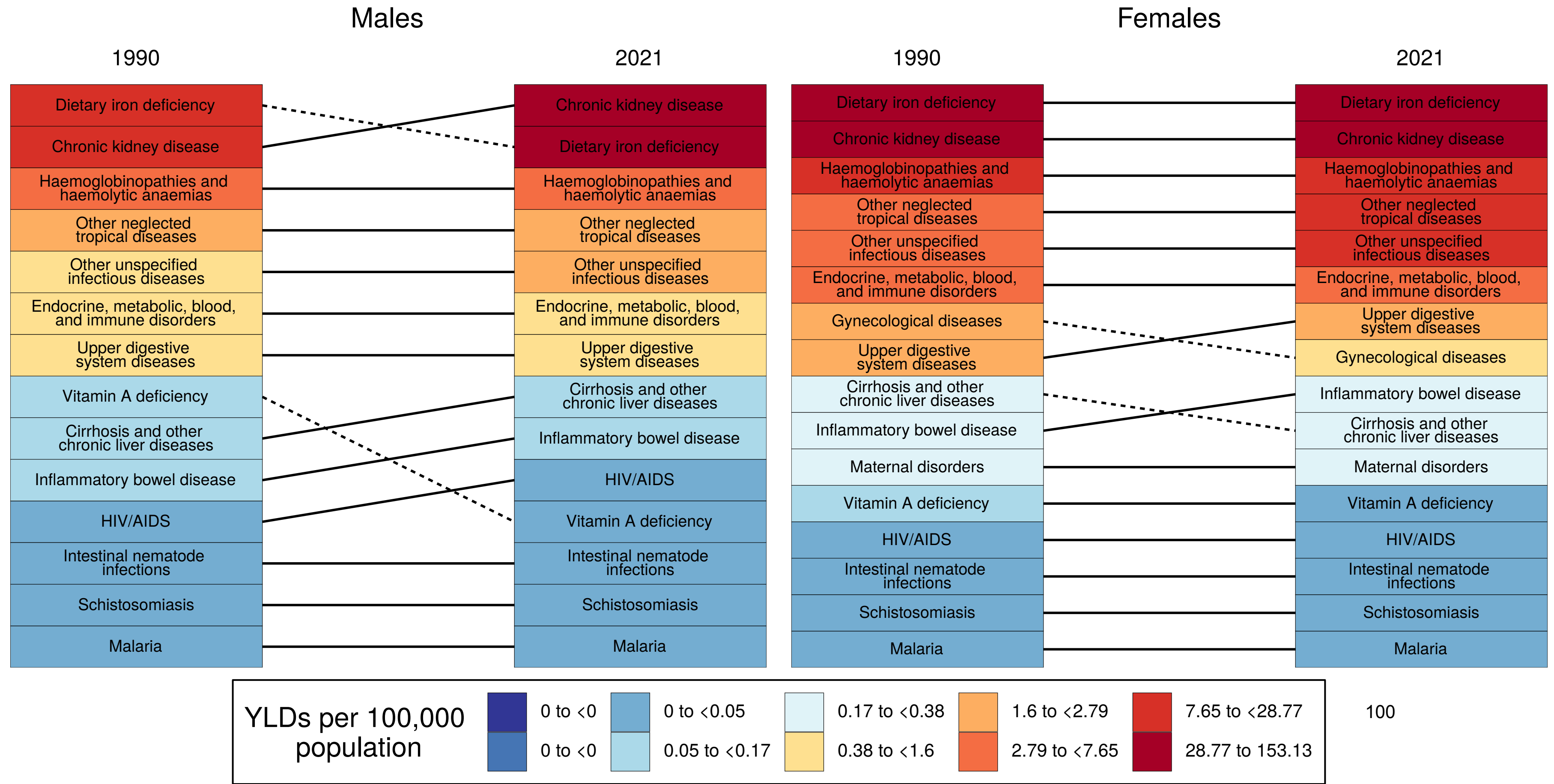

Males
Females
1990
2021
Dietary iron deficiency
Chronic kidney disease
Haemoglobinopathies and haemolytic anaemias
Other neglected tropical diseases
Other unspecified infectious diseases
Endocrine, metabolic, blood, and immune disorders
Upper digestive system diseases
Vitamin A deficiency
Cirrhosis and other chronic liver diseases
Inflammatory bowel disease
HIV/AIDS
Intestinal nematode infections
Schistosomiasis
Malaria
Gynecological diseases
Maternal disorders
YLDs per 100,000 population
0 to <0
0 to <0
0 to <0.05
0.05 to <0.17
0.17 to <0.38
0.38 to <1.6
1.6 to <2.79
2.79 to <7.65
7.65 to <28.77
28.77 to 153.13
100

Hokkaidō

Anaemia Prevalence - Both Sexes

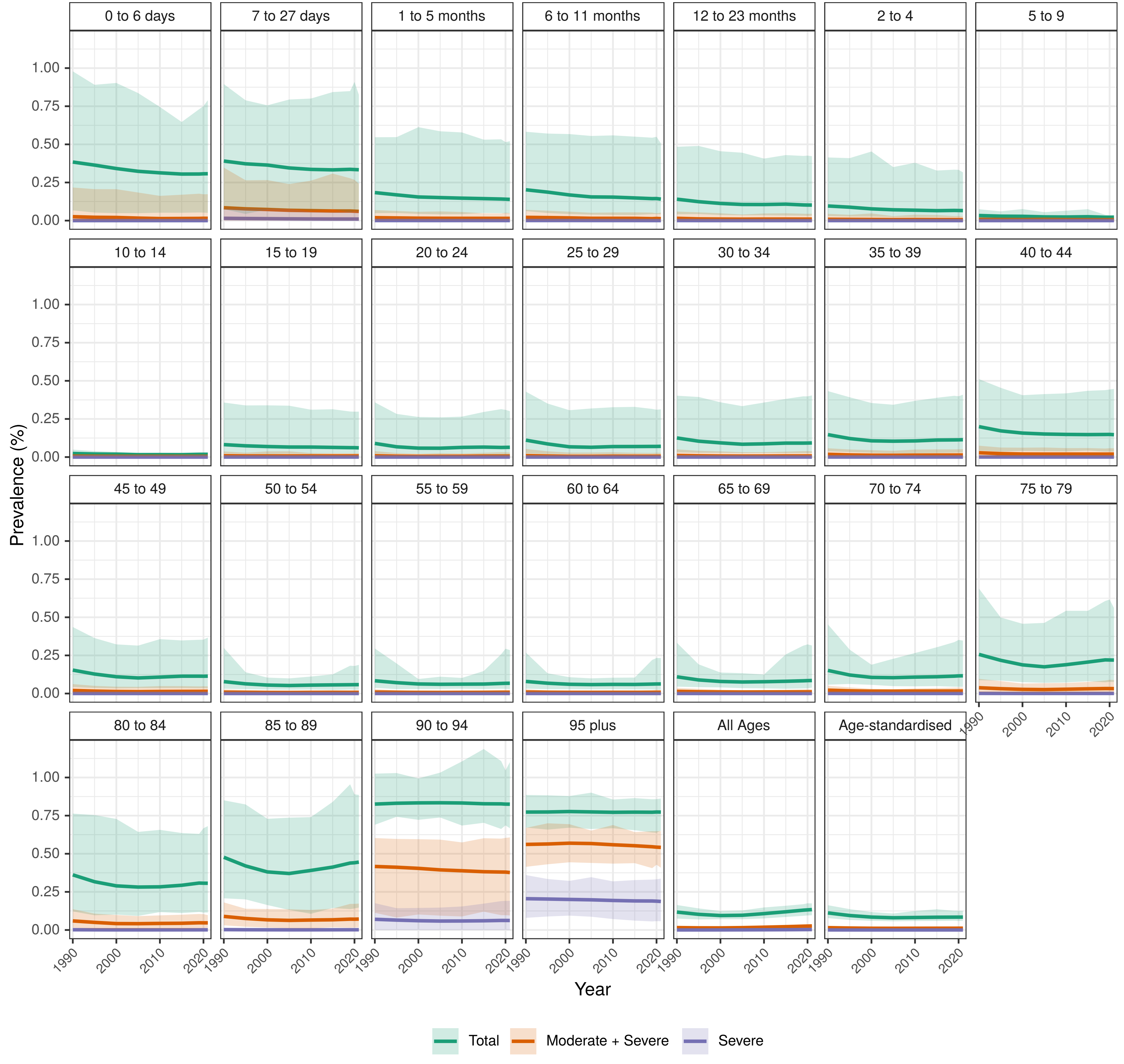

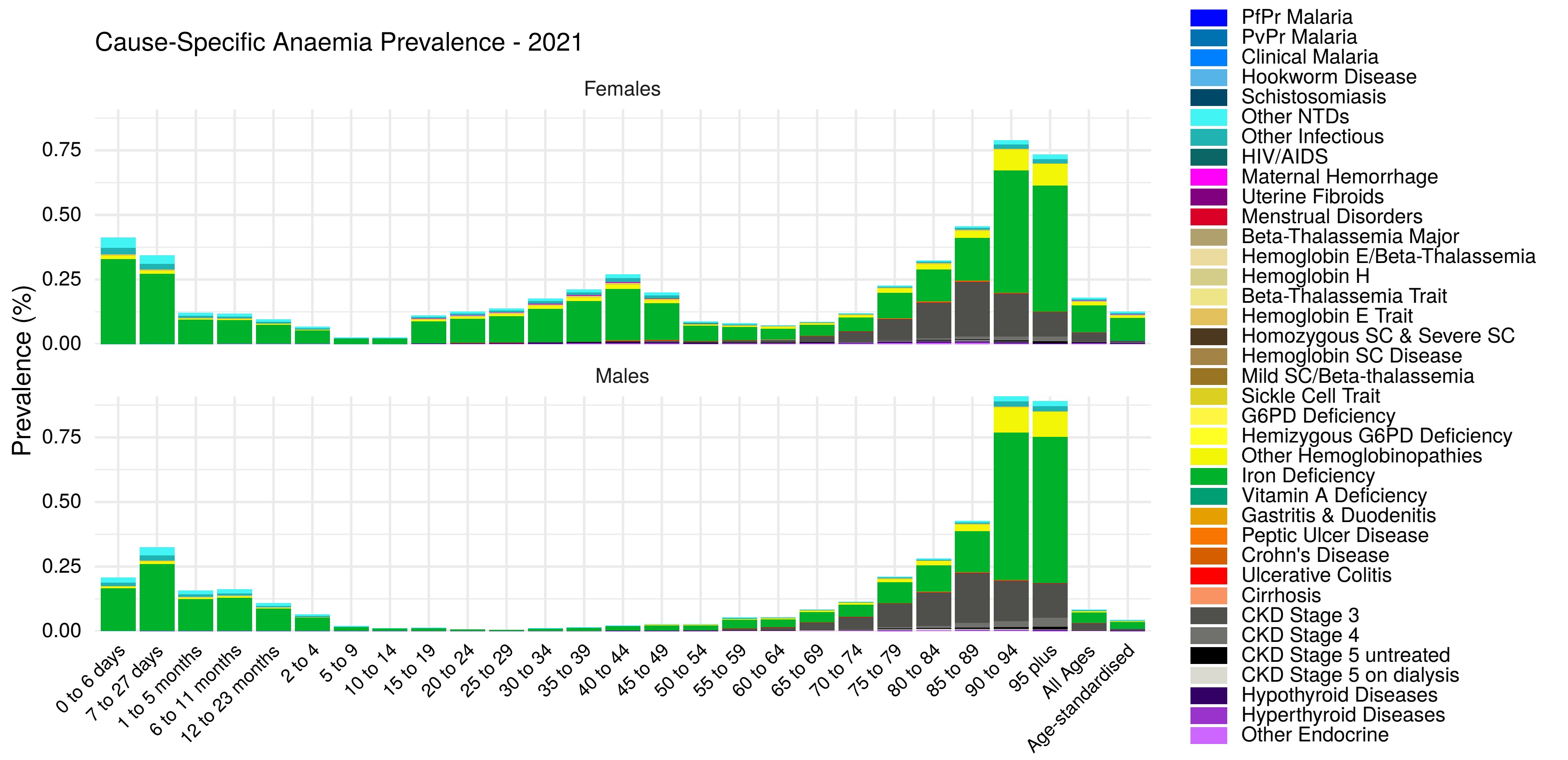

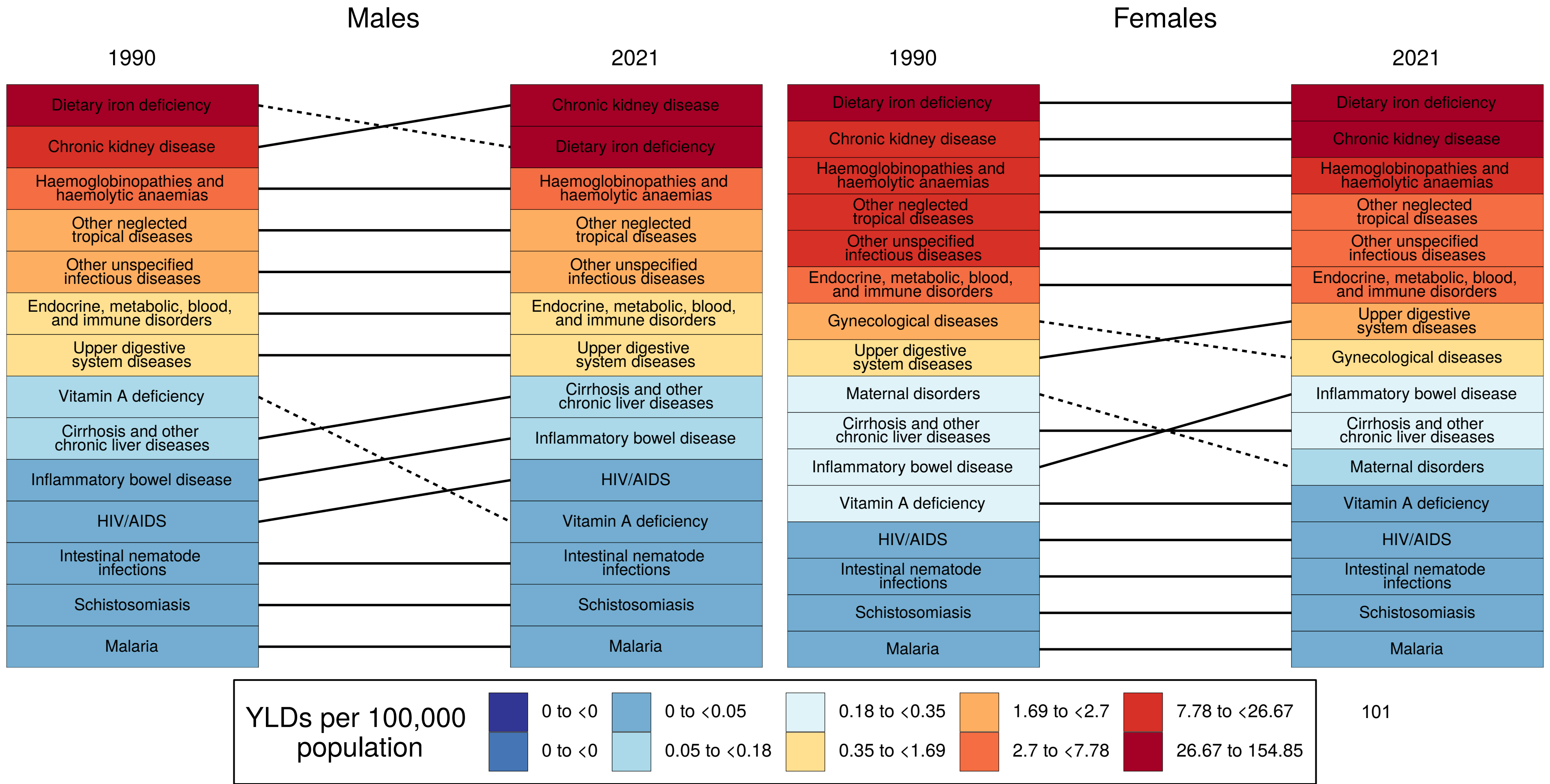

Hyōgo

Anaemia Prevalence - Both Sexes

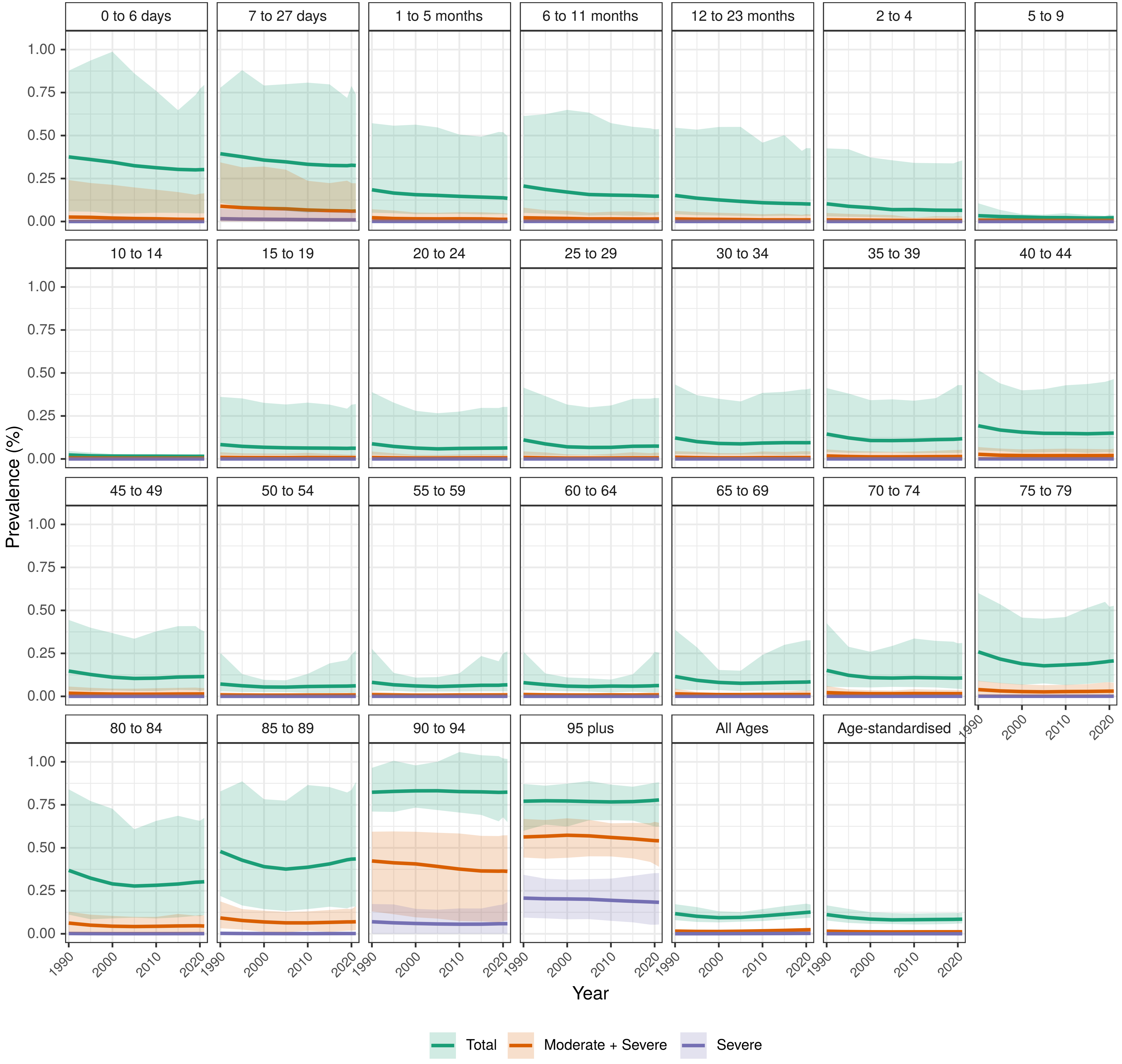

Cause-Specific Anaemia Prevalence - 2021

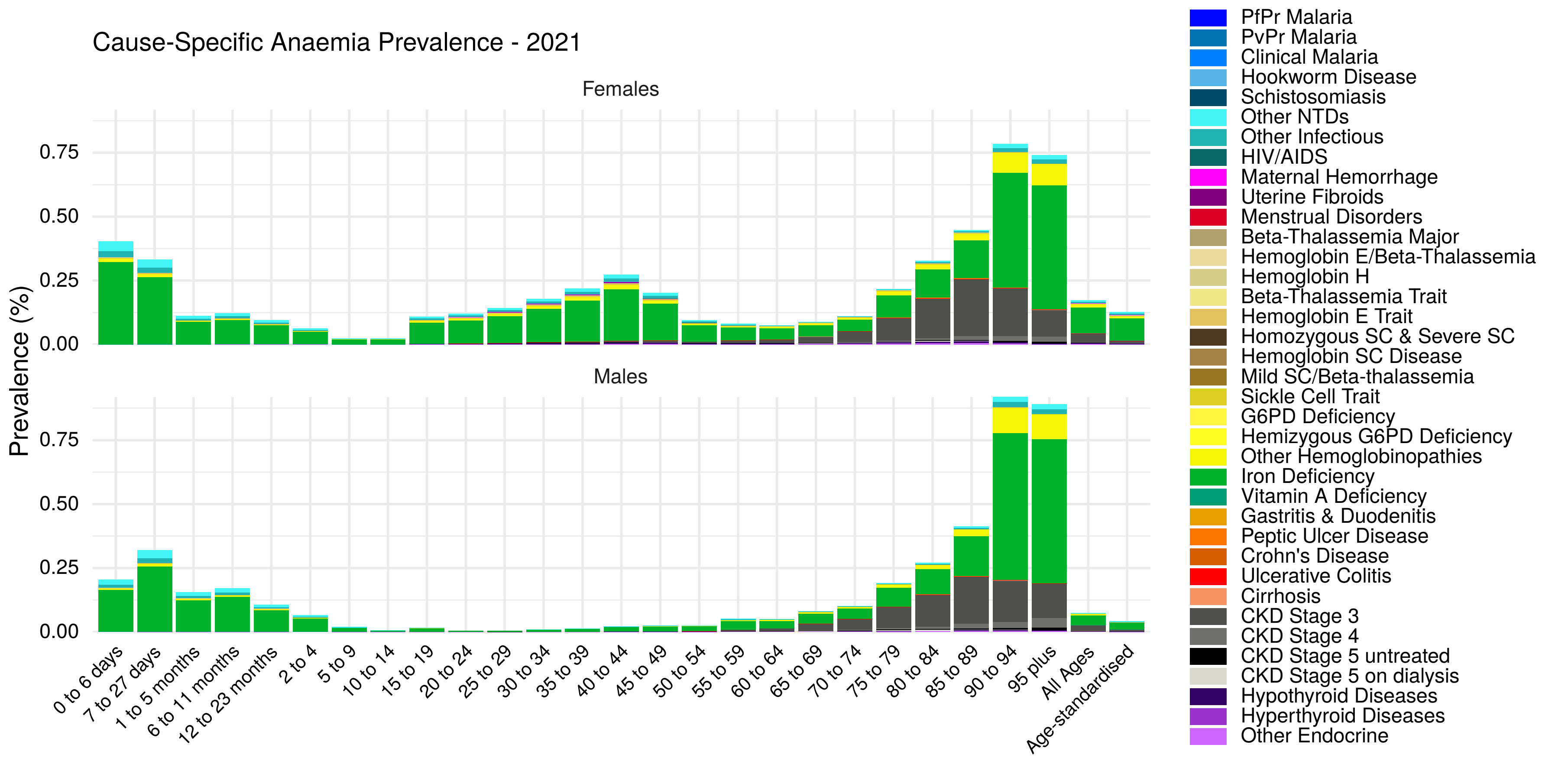

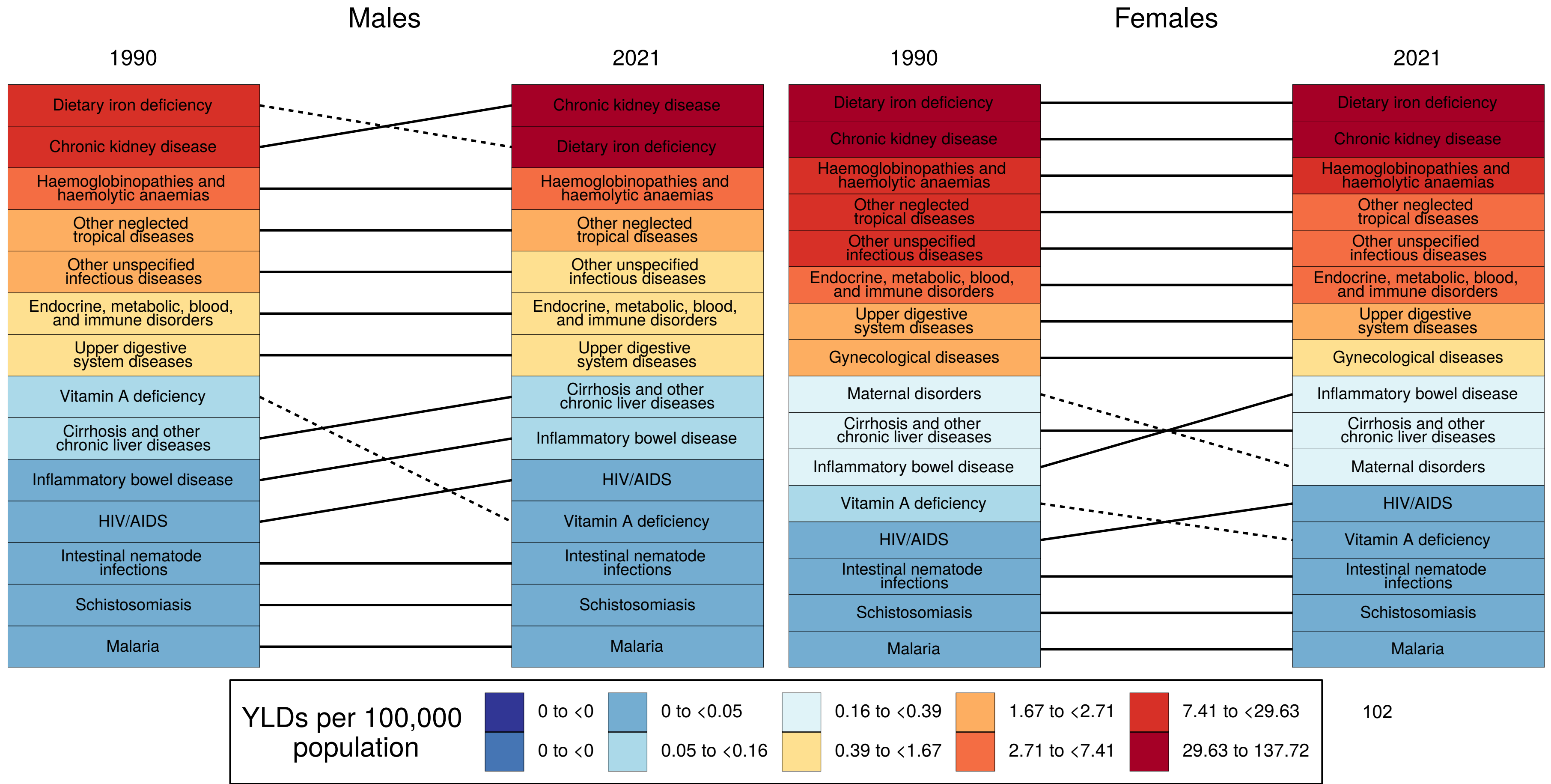

Ibaraki

Anaemia Prevalence - Both Sexes

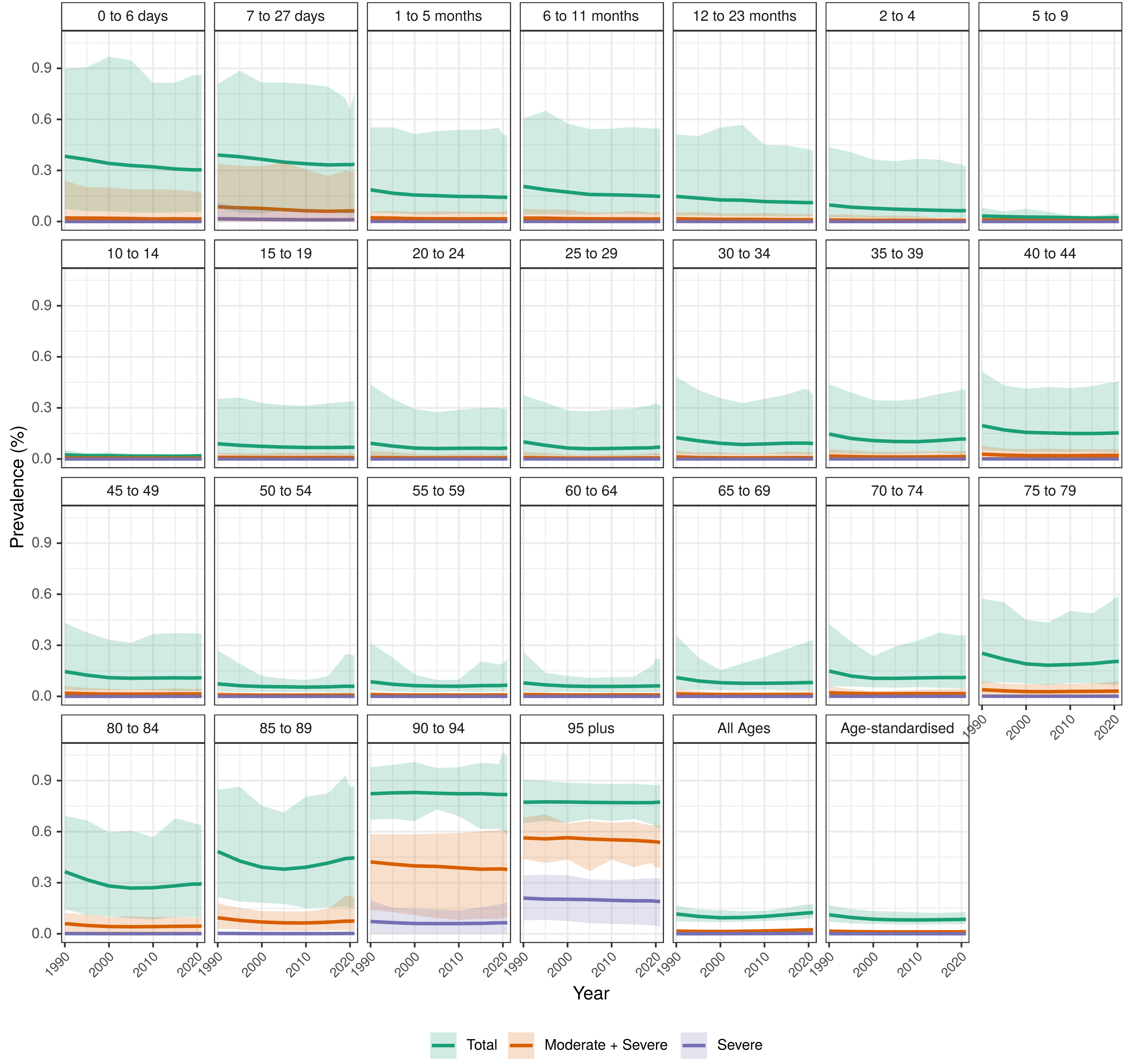

Cause-Specific Anaemia Prevalence - 2021

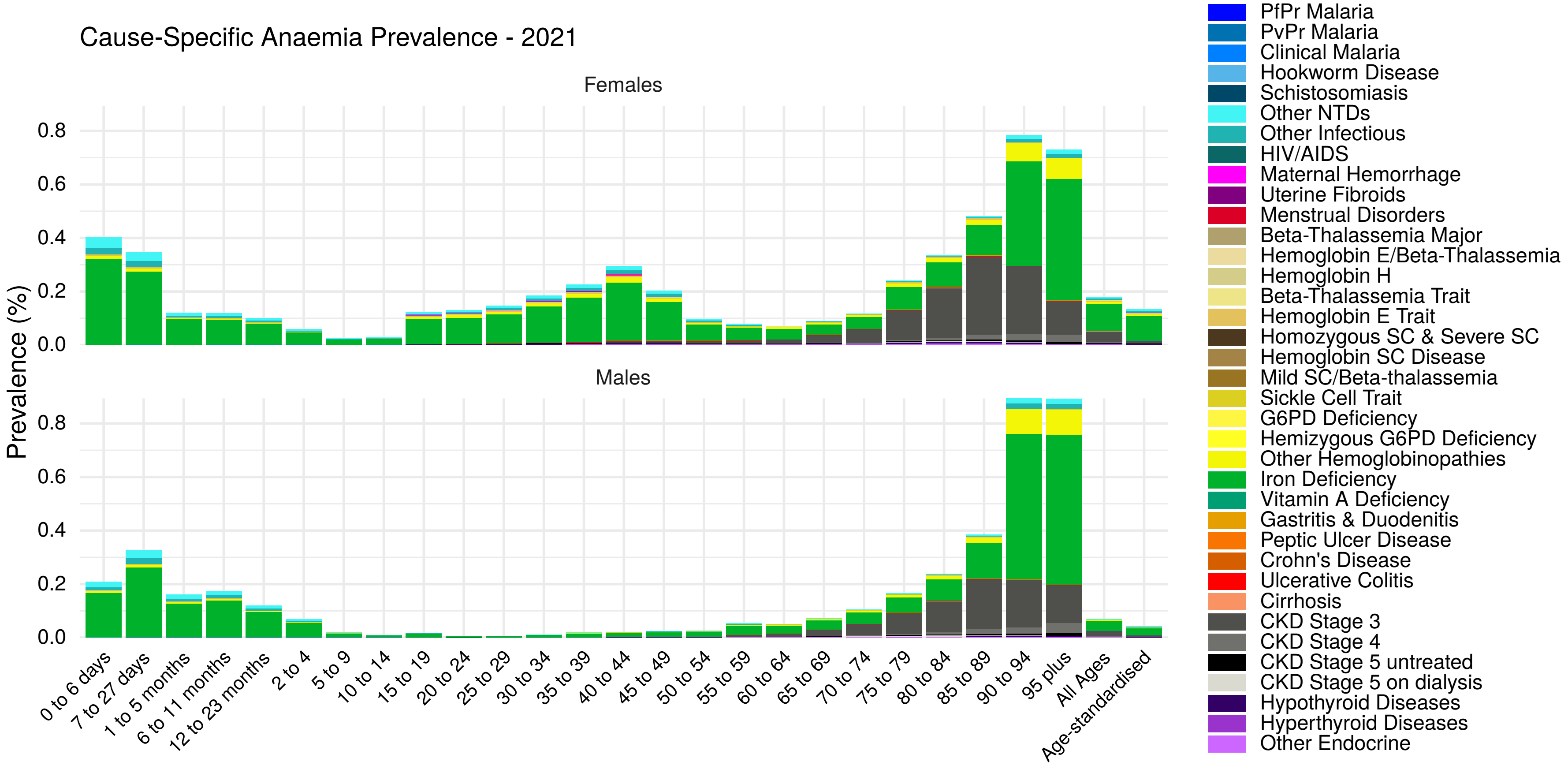

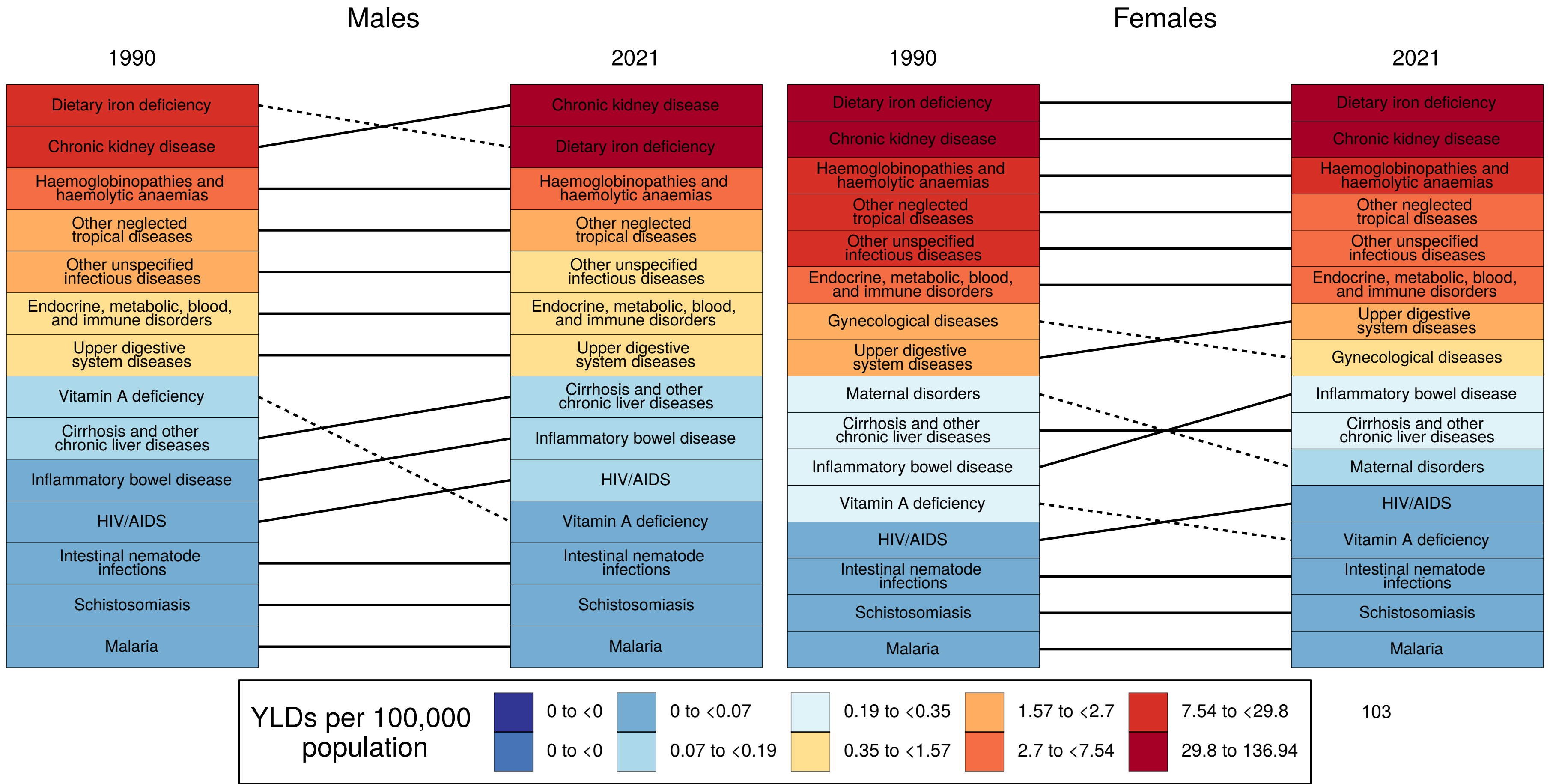

Ishikawa

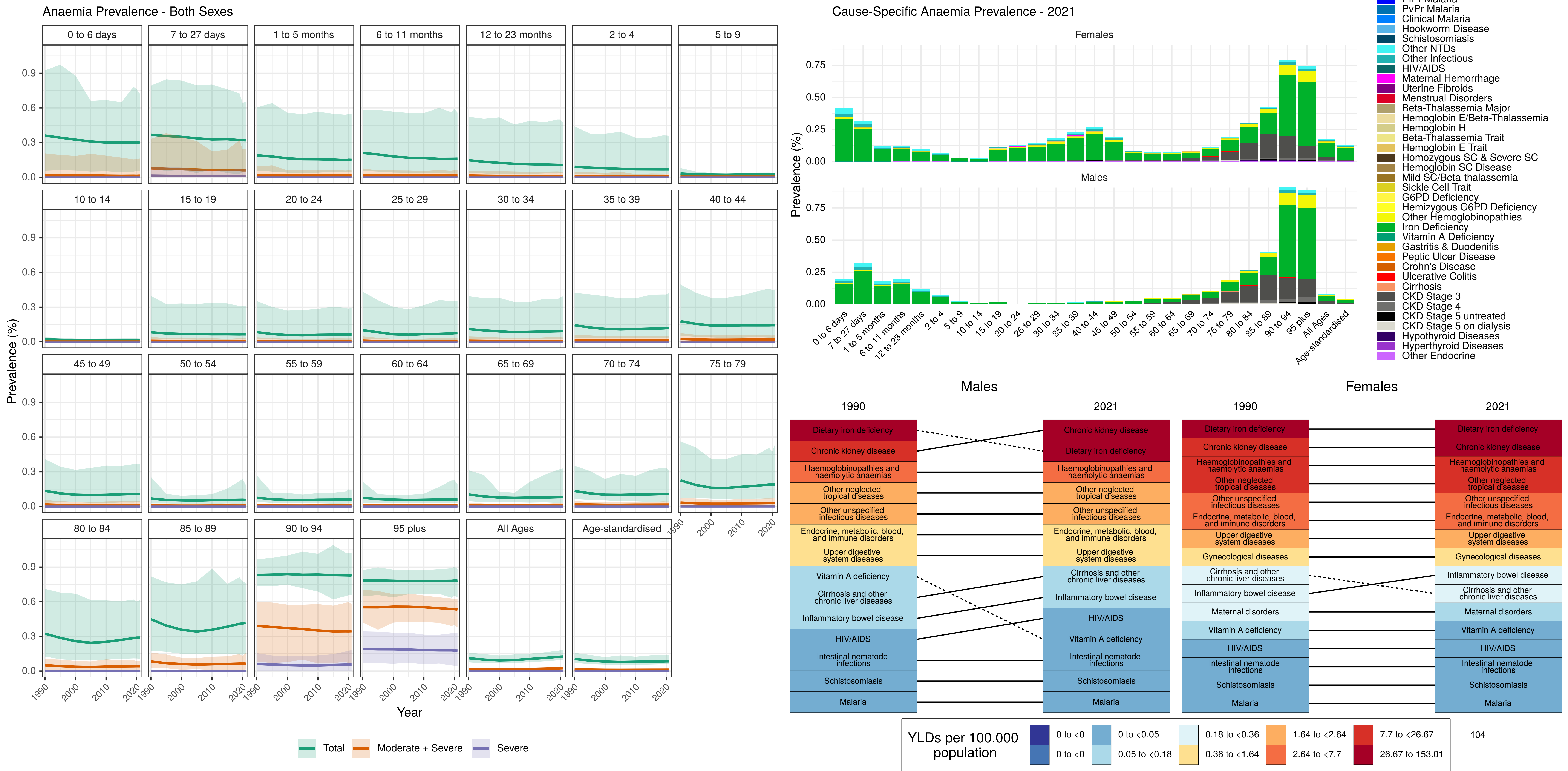

Anaemia Prevalence - Both Sexes
0 to 6 days
7 to 27 days
1 to 5 months
6 to 11 months
12 to 23 months
2 to 4
5 to 9
10 to 14
15 to 19
20 to 24
25 to 29
30 to 34
35 to 39
40 to 44
45 to 49
50 to 54
55 to 59
60 to 64
65 to 69
70 to 74
75 to 79
80 to 84
85 to 89
90 to 94
95 plus
All Ages
Age-standardised
Prevalence (%)
Year
Total
Moderate + Severe
Severe
Cause-Specific Anaemia Prevalence - 2021
Females
Males
PfPr Malaria
PvPr Malaria
Clinical Malaria
Hookworm Disease
Schistosomiasis
Other NTDs
Other Infectious
HIV/AIDS
Maternal Hemorrhage
Uterine Fibroids
Menstrual Disorders
Beta-Thalassemia Major
Hemoglobin E/Beta-Thalassemia
Hemoglobin H
Beta-Thalassemia Trait
Hemoglobin E Trait
Homozygous SC & Severe SC
Hemoglobin SC Disease
Mild SC/Beta-thalassemia
Sickle Cell Trait
G6PD Deficiency
Hemizygous G6PD Deficiency
Other Hemoglobinopathies
Iron Deficiency
Vitamin A Deficiency
Gastritis & Duodenitis
Peptic Ulcer Disease
Crohn's Disease
Ulcerative Colitis
Cirrhosis
CKD Stage 3
CKD Stage 4
CKD Stage 5 untreated
CKD Stage 5 on dialysis
Hypothyroid Diseases
Hyperthyroid Diseases
Other Endocrine
Males
Females
1990
2021
Dietary iron deficiency
Chronic kidney disease
Haemoglobinopathies and haemolytic anaemias
Other neglected tropical diseases
Other unspecified infectious diseases
Endocrine, metabolic, blood, and immune disorders
Upper digestive system diseases
Vitamin A deficiency
Cirrhosis and other chronic liver diseases
Inflammatory bowel disease
HIV/AIDS
Intestinal nematode infections
Schistosomiasis
Malaria
Gynecological diseases
Maternal disorders
YLDs per 100,000 population
0 to <0
0 to <0
0 to <0.05
0.05 to <0.18
0.18 to <0.36
0.36 to <1.64
1.64 to <2.64
2.64 to <7.7
7.7 to <26.67
26.67 to 153.01

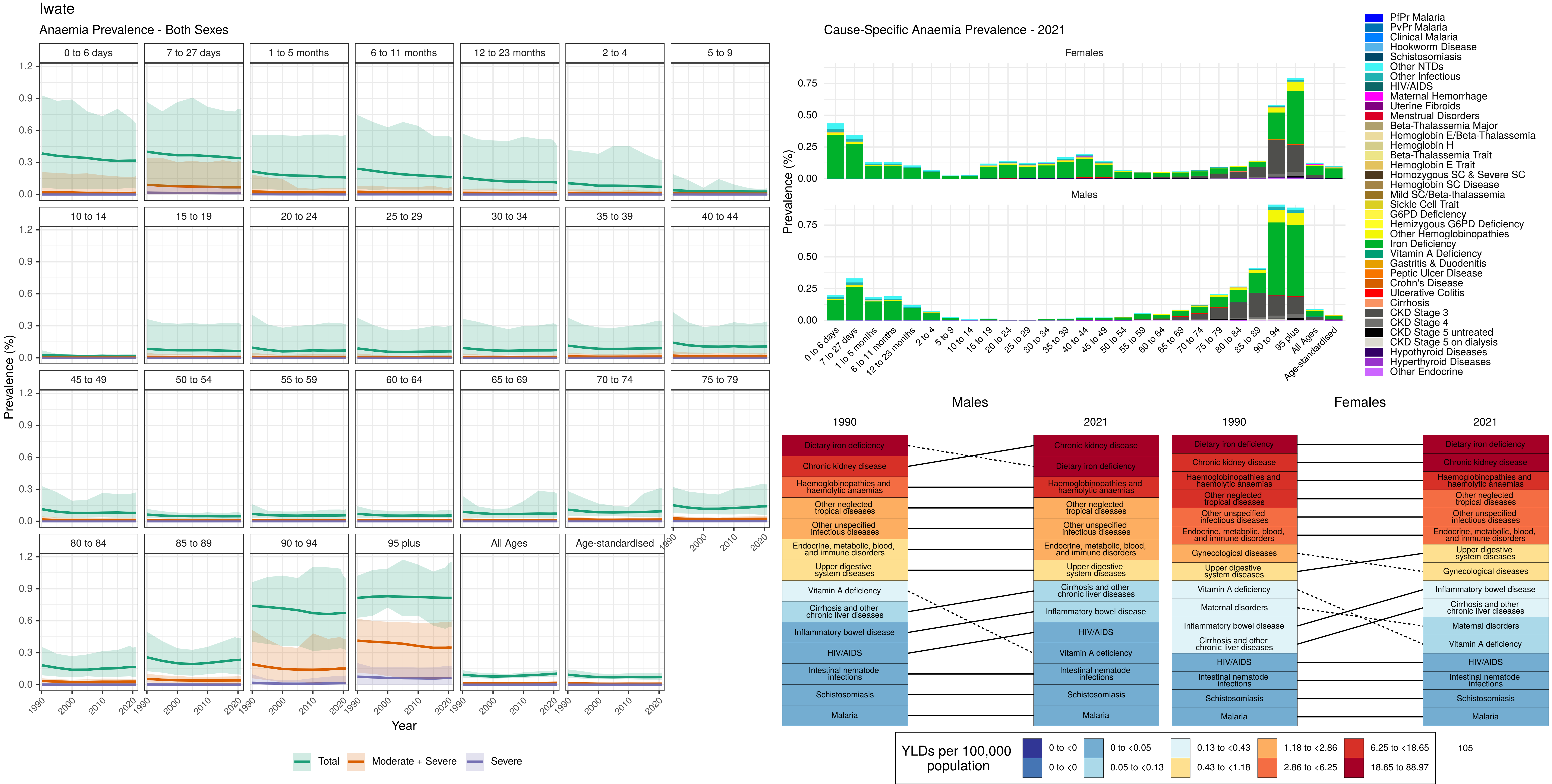
Iwate
Anaemia Prevalence - Both Sexes
0 to 6 days
7 to 27 days
1 to 5 months
6 to 11 months
12 to 23 months
2 to 4
5 to 9
10 to 14
15 to 19
20 to 24
25 to 29
30 to 34
35 to 39
40 to 44
45 to 49
50 to 54
55 to 59
60 to 64
65 to 69
70 to 74
75 to 79
80 to 84
85 to 89
90 to 94
95 plus
All Ages
Age-standardised
Prevalence (%)
Year
Total
Moderate + Severe
Severe
Cause-Specific Anaemia Prevalence - 2021
Females
Males
Prevalence (%)
PfPr Malaria
PvPr Malaria
Clinical Malaria
Hookworm Disease
Schistosomiasis
Other NTDs
Other Infectious
HIV/AIDS
Maternal Hemorrhage
Uterine Fibroids
Menstrual Disorders
Beta-Thalassemia Major
Hemoglobin E/Beta-Thalassemia
Hemoglobin H
Beta-Thalassemia Trait
Hemoglobin E Trait
Homozygous SC & Severe SC
Hemoglobin SC Disease
Mild SC/Beta-thalassemia
Sickle Cell Trait
G6PD Deficiency
Hemizygous G6PD Deficiency
Other Hemoglobinopathies
Iron Deficiency
Vitamin A Deficiency
Gastritis & Duodenitis
Peptic Ulcer Disease
Crohn's Disease
Ulcerative Colitis
Cirrhosis
CKD Stage 3
CKD Stage 4
CKD Stage 5 untreated
CKD Stage 5 on dialysis
Hypothyroid Diseases
Hyperthyroid Diseases
Other Endocrine
Males
Females
1990
2021
Dietary iron deficiency
Chronic kidney disease
Haemoglobinopathies and haemolytic anaemias
Other neglected tropical diseases
Other unspecified infectious diseases
Endocrine, metabolic, blood, and immune disorders
Upper digestive system diseases
Vitamin A deficiency
Cirrhosis and other chronic liver diseases
Inflammatory bowel disease
HIV/AIDS
Intestinal nematode infections
Schistosomiasis
Malaria
Gynecological diseases
Maternal disorders
YLDs per 100,000 population
0 to <0
0 to <0
0 to <0.05
0.05 to <0.13
0.13 to <0.43
0.43 to <1.18
1.18 to <2.86
2.86 to <6.25
6.25 to <18.65
18.65 to 88.97

Kagawa

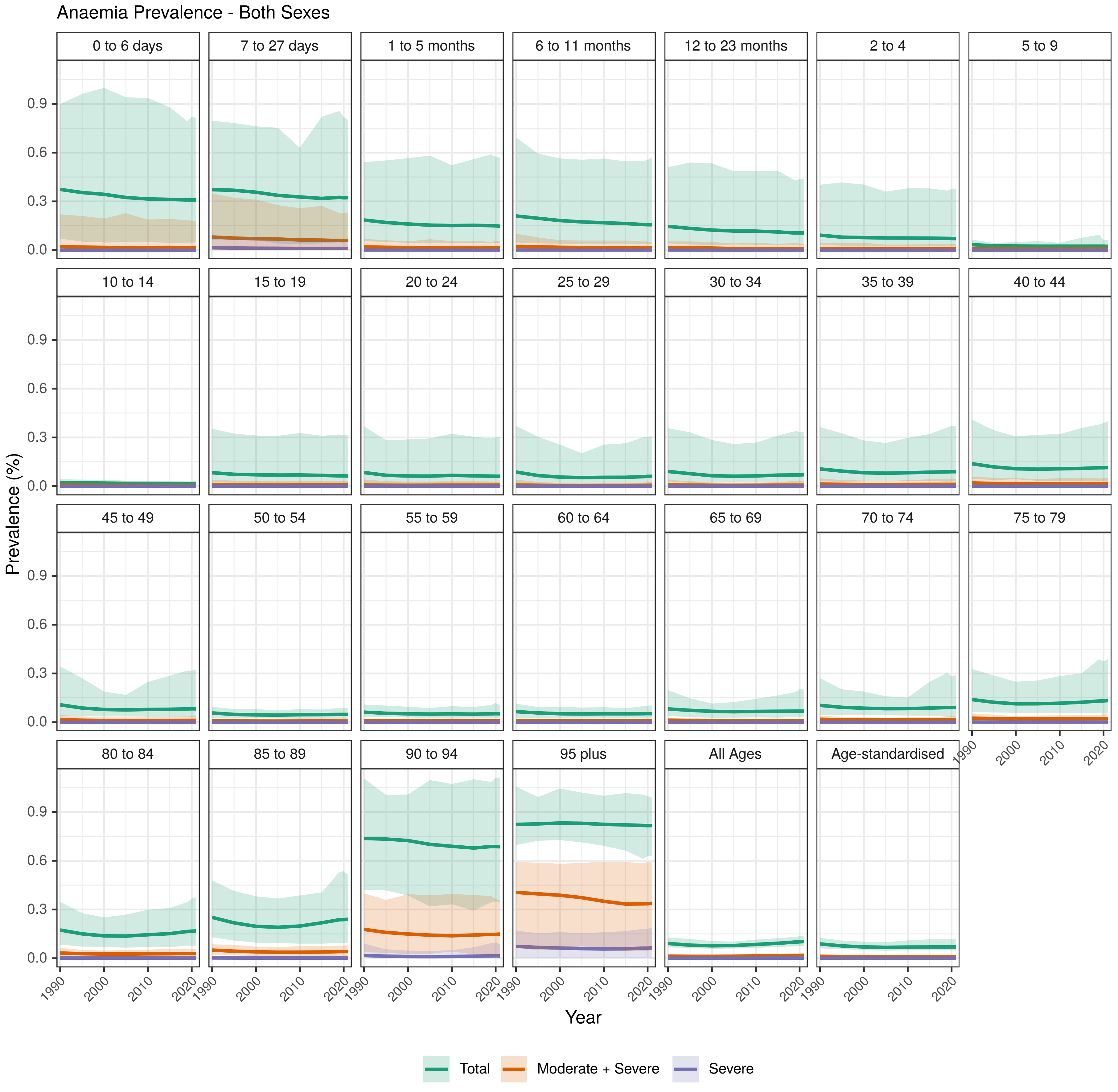

Anaemia Prevalence - Both Sexes
0 to 6 days
7 to 27 days
1 to 5 months
6 to 11 months
12 to 23 months
2 to 4
5 to 9
10 to 14
15 to 19
20 to 24
25 to 29
30 to 34
35 to 39
40 to 44
45 to 49
50 to 54
55 to 59
60 to 64
65 to 69
70 to 74
75 to 79
80 to 84
85 to 89
90 to 94
95 plus
All Ages
Age-standardised
0.0
0.3
0.6
0.9
1990
2000
2010
2020
Prevalence (%)
Year
Total
Moderate + Severe
Severe

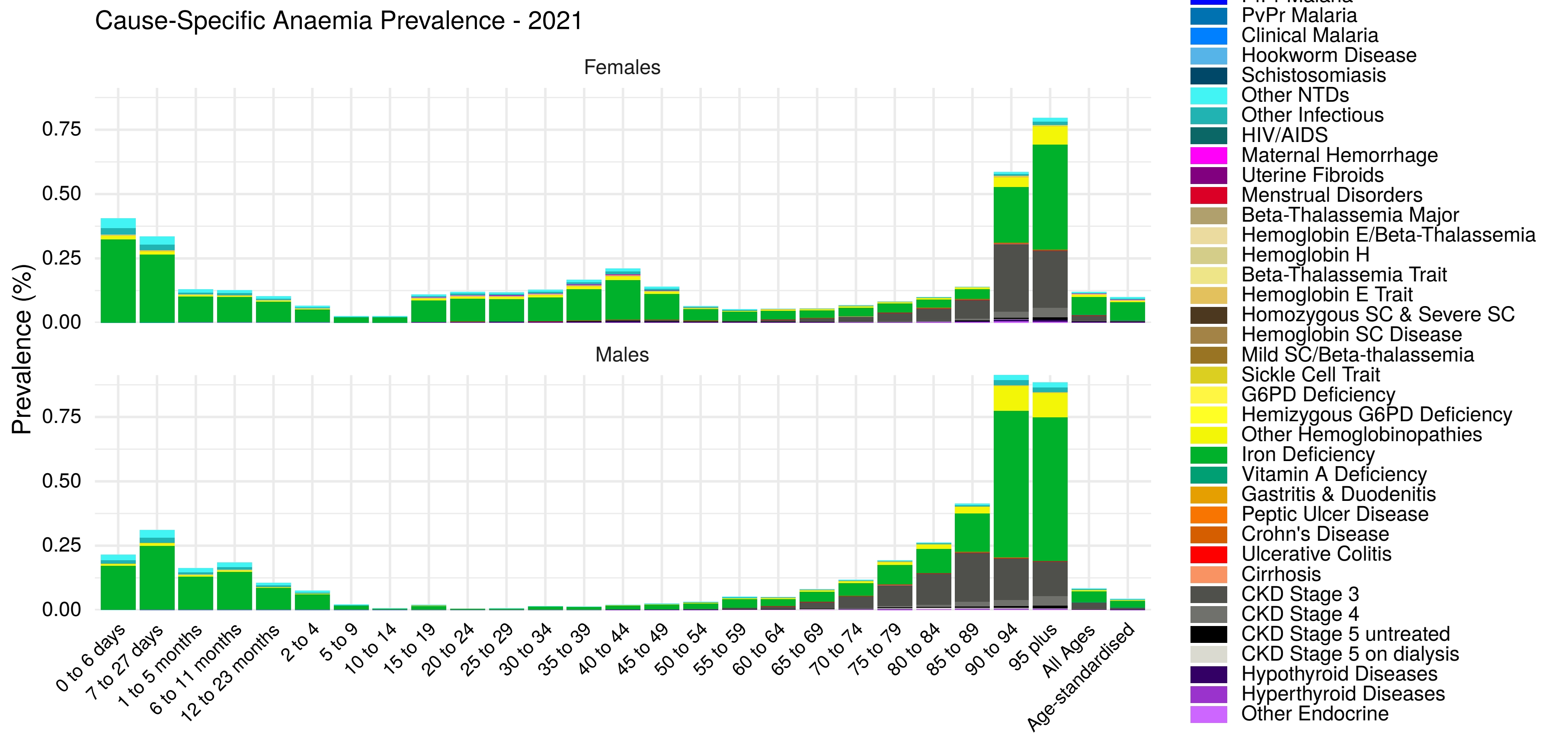

Cause-Specific Anaemia Prevalence - 2021
Females
Males
Prevalence (%)
0.00
0.25
0.50
0.75
0 to 6 days
7 to 27 days
1 to 5 months
6 to 11 months
12 to 23 months
2 to 4
5 to 9
10 to 14
15 to 19
20 to 24
25 to 29
30 to 34
35 to 39
40 to 44
45 to 49
50 to 54
55 to 59
60 to 64
65 to 69
70 to 74
75 to 79
80 to 84
85 to 89
90 to 94
95 plus
All Ages
Age-standardised
PfPr Malaria
PvPr Malaria
Clinical Malaria
Hookworm Disease
Schistosomiasis
Other NTDs
Other Infectious
HIV/AIDS
Maternal Hemorrhage
Uterine Fibroids
Menstrual Disorders
Beta-Thalassemia Major
Hemoglobin E/Beta-Thalassemia
Hemoglobin H
Beta-Thalassemia Trait
Hemoglobin E Trait
Homozygous SC & Severe SC
Hemoglobin SC Disease
Mild SC/Beta-thalassemia
Sickle Cell Trait
G6PD Deficiency
Hemizygous G6PD Deficiency
Other Hemoglobinopathies
Iron Deficiency
Vitamin A Deficiency
Gastritis & Duodenitis
Peptic Ulcer Disease
Crohn's Disease
Ulcerative Colitis
Cirrhosis
CKD Stage 3
CKD Stage 4
CKD Stage 5 untreated
CKD Stage 5 on dialysis
Hypothyroid Diseases
Hyperthyroid Diseases
Other Endocrine

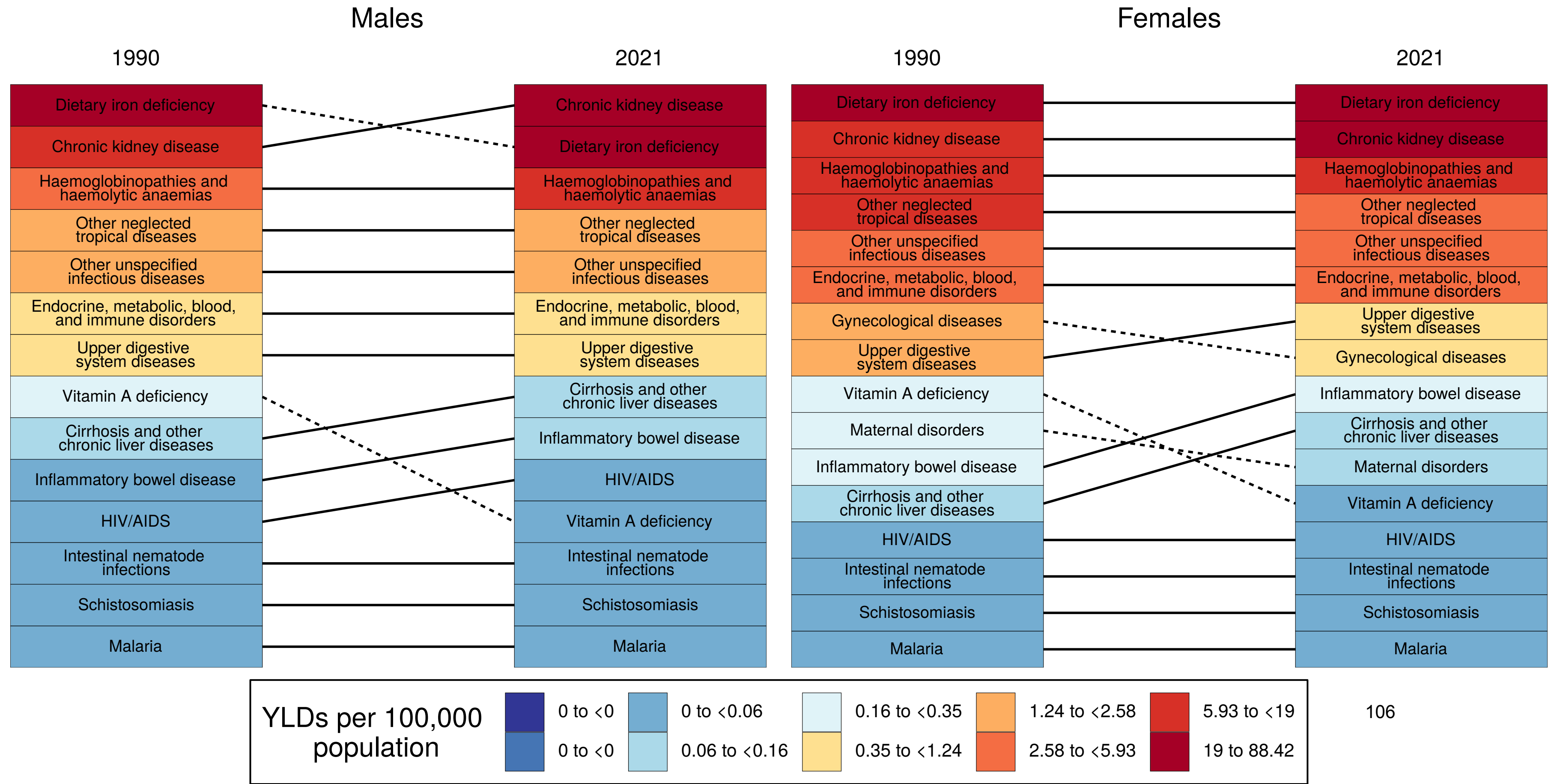

Males
Females
1990
2021
Dietary iron deficiency
Chronic kidney disease
Haemoglobinopathies and haemolytic anaemias
Other neglected tropical diseases
Other unspecified infectious diseases
Endocrine, metabolic, blood, and immune disorders
Upper digestive system diseases
Vitamin A deficiency
Cirrhosis and other chronic liver diseases
Inflammatory bowel disease
HIV/AIDS
Intestinal nematode infections
Schistosomiasis
Malaria
Chronic kidney disease
Dietary iron deficiency
Haemoglobinopathies and haemolytic anaemias
Other neglected tropical diseases
Other unspecified infectious diseases
Endocrine, metabolic, blood, and immune disorders
Upper digestive system diseases
Cirrhosis and other chronic liver diseases
Inflammatory bowel disease
HIV/AIDS
Vitamin A deficiency
Intestinal nematode infections
Schistosomiasis
Malaria
Dietary iron deficiency
Chronic kidney disease
Haemoglobinopathies and haemolytic anaemias
Other neglected tropical diseases
Other unspecified infectious diseases
Endocrine, metabolic, blood, and immune disorders
Gynecological diseases
Upper digestive system diseases
Vitamin A deficiency
Maternal disorders
Inflammatory bowel disease
Cirrhosis and other chronic liver diseases
HIV/AIDS
Intestinal nematode infections
Schistosomiasis
Malaria
Dietary iron deficiency
Chronic kidney disease
Haemoglobinopathies and haemolytic anaemias
Other neglected tropical diseases
Other unspecified infectious diseases
Endocrine, metabolic, blood, and immune disorders
Upper digestive system diseases
Gynecological diseases
Inflammatory bowel disease
Cirrhosis and other chronic liver diseases
Maternal disorders
Vitamin A deficiency
HIV/AIDS
Intestinal nematode infections
Schistosomiasis
Malaria
YLDs per 100,000 population
0 to <0
0 to <0
0 to <0.06
0.06 to <0.16
0.16 to <0.35
0.35 to <1.24
1.24 to <2.58
2.58 to <5.93
5.93 to <19
19 to 88.42

Kagoshima

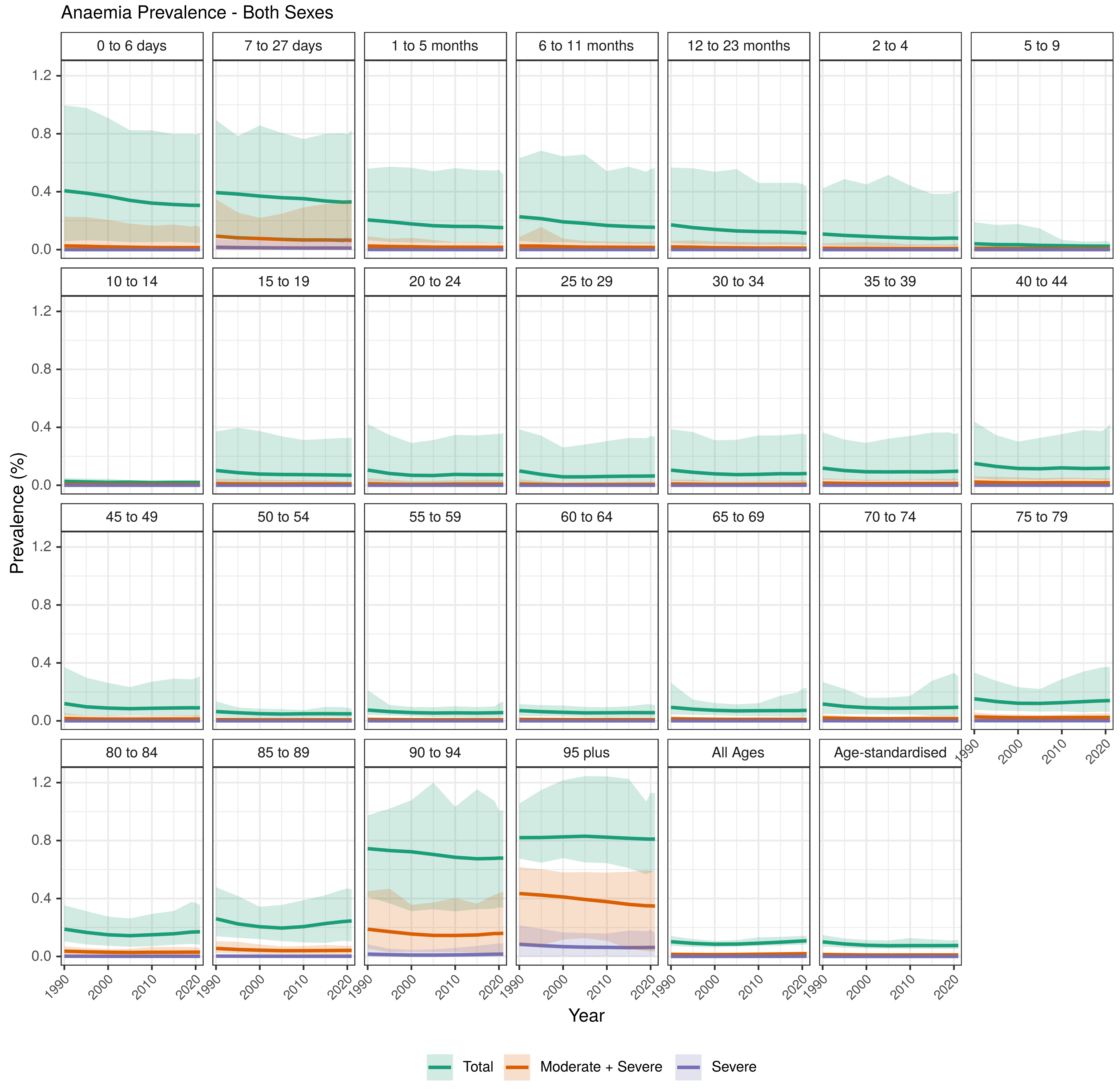
Anaemia Prevalence - Both Sexes
0 to 6 days
7 to 27 days
1 to 5 months
6 to 11 months
12 to 23 months
2 to 4
5 to 9
10 to 14
15 to 19
20 to 24
25 to 29
30 to 34
35 to 39
40 to 44
45 to 49
50 to 54
55 to 59
60 to 64
65 to 69
70 to 74
75 to 79
80 to 84
85 to 89
90 to 94
95 plus
All Ages
Age-standardised
Prevalence (%)
0.0
0.4
0.8
1.2
1990
2000
2010
2020
Year
Total
Moderate + Severe
Severe

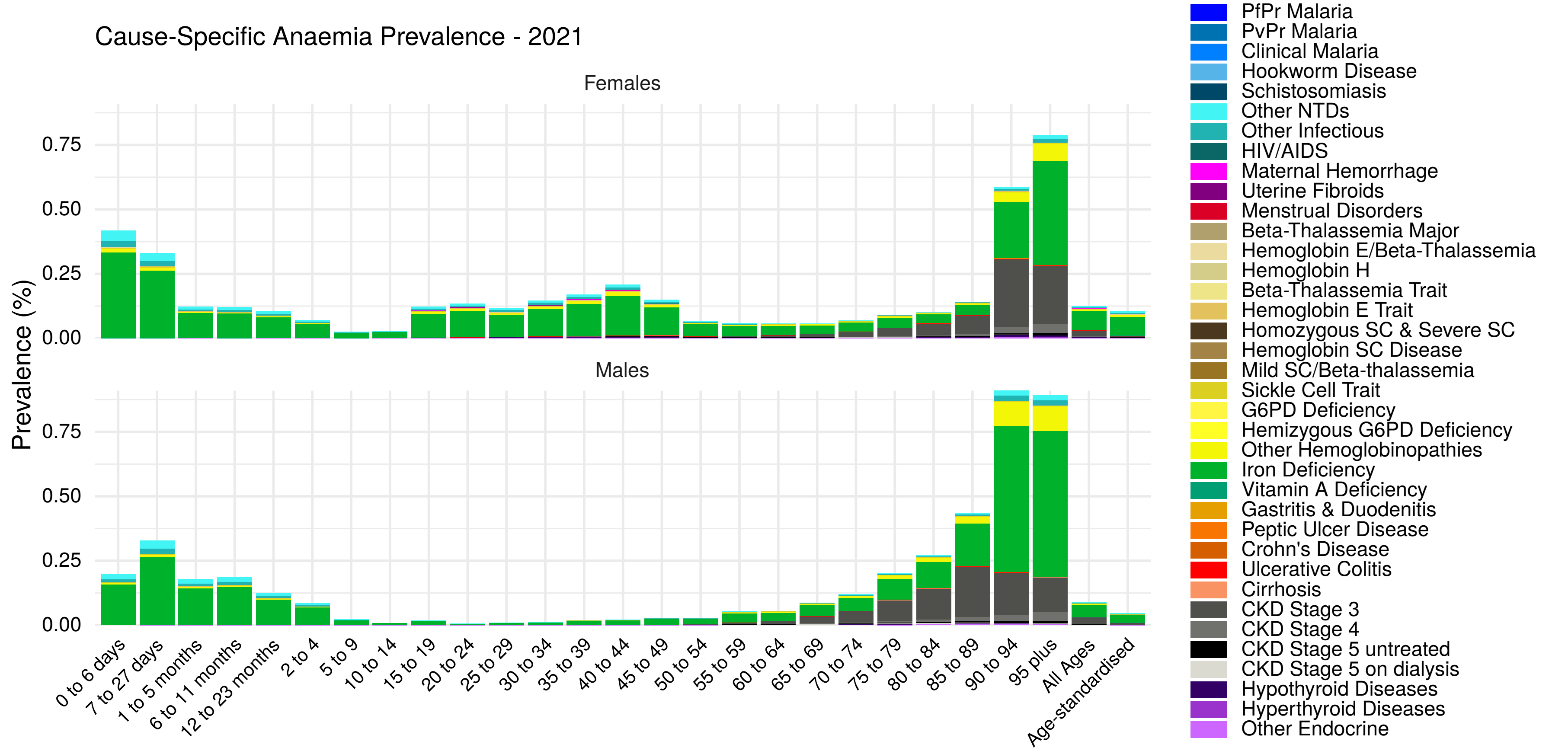
Cause-Specific Anaemia Prevalence - 2021
Females
Males
Prevalence (%)
0.00
0.25
0.50
0.75
0 to 6 days
7 to 27 days
1 to 5 months
6 to 11 months
12 to 23 months
2 to 4
5 to 9
10 to 14
15 to 19
20 to 24
25 to 29
30 to 34
35 to 39
40 to 44
45 to 49
50 to 54
55 to 59
60 to 64
65 to 69
70 to 74
75 to 79
80 to 84
85 to 89
90 to 94
95 plus
All Ages
Age-standardised
PfPr Malaria
PvPr Malaria
Clinical Malaria
Hookworm Disease
Schistosomiasis
Other NTDs
Other Infectious
HIV/AIDS
Maternal Hemorrhage
Uterine Fibroids
Menstrual Disorders
Beta-Thalassemia Major
Hemoglobin E/Beta-Thalassemia
Hemoglobin H
Beta-Thalassemia Trait
Hemoglobin E Trait
Homozygous SC & Severe SC
Hemoglobin SC Disease
Mild SC/Beta-thalassemia
Sickle Cell Trait
G6PD Deficiency
Hemizygous G6PD Deficiency
Other Hemoglobinopathies
Iron Deficiency
Vitamin A Deficiency
Gastritis & Duodenitis
Peptic Ulcer Disease
Crohn's Disease
Ulcerative Colitis
Cirrhosis
CKD Stage 3
CKD Stage 4
CKD Stage 5 untreated
CKD Stage 5 on dialysis
Hypothyroid Diseases
Hyperthyroid Diseases
Other Endocrine

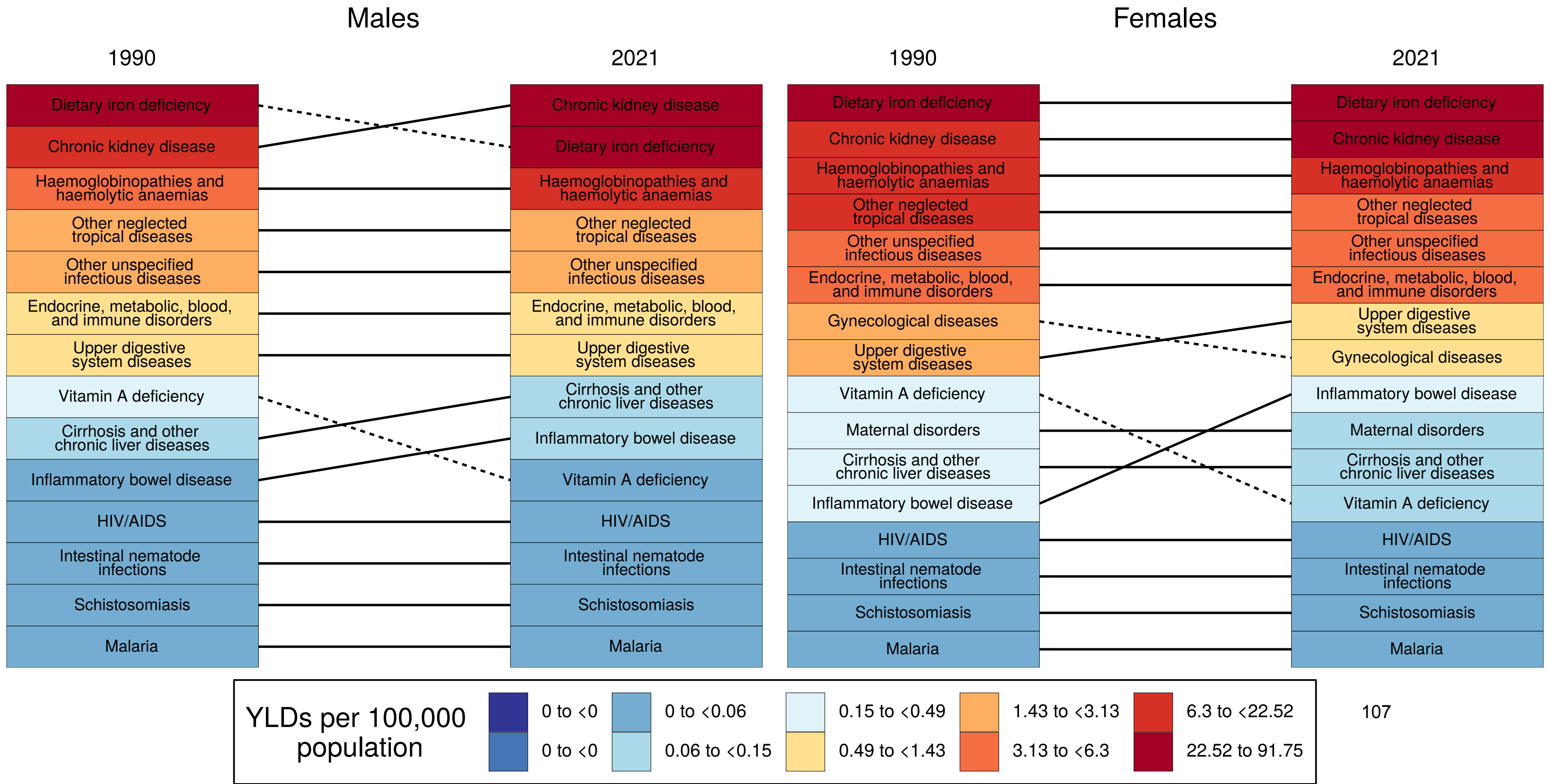
Males
Females
1990
2021
Dietary iron deficiency
Chronic kidney disease
Haemoglobinopathies and haemolytic anaemias
Other neglected tropical diseases
Other unspecified infectious diseases
Endocrine, metabolic, blood, and immune disorders
Upper digestive system diseases
Vitamin A deficiency
Cirrhosis and other chronic liver diseases
Inflammatory bowel disease
HIV/AIDS
Intestinal nematode infections
Schistosomiasis
Malaria
Gynecological diseases
Maternal disorders
YLDs per 100,000 population
0 to <0
0 to <0.06
0.06 to <0.15
0.15 to <0.49
0.49 to <1.43
1.43 to <3.13
3.13 to <6.3
6.3 to <22.52
22.52 to 91.75

Kanagawa

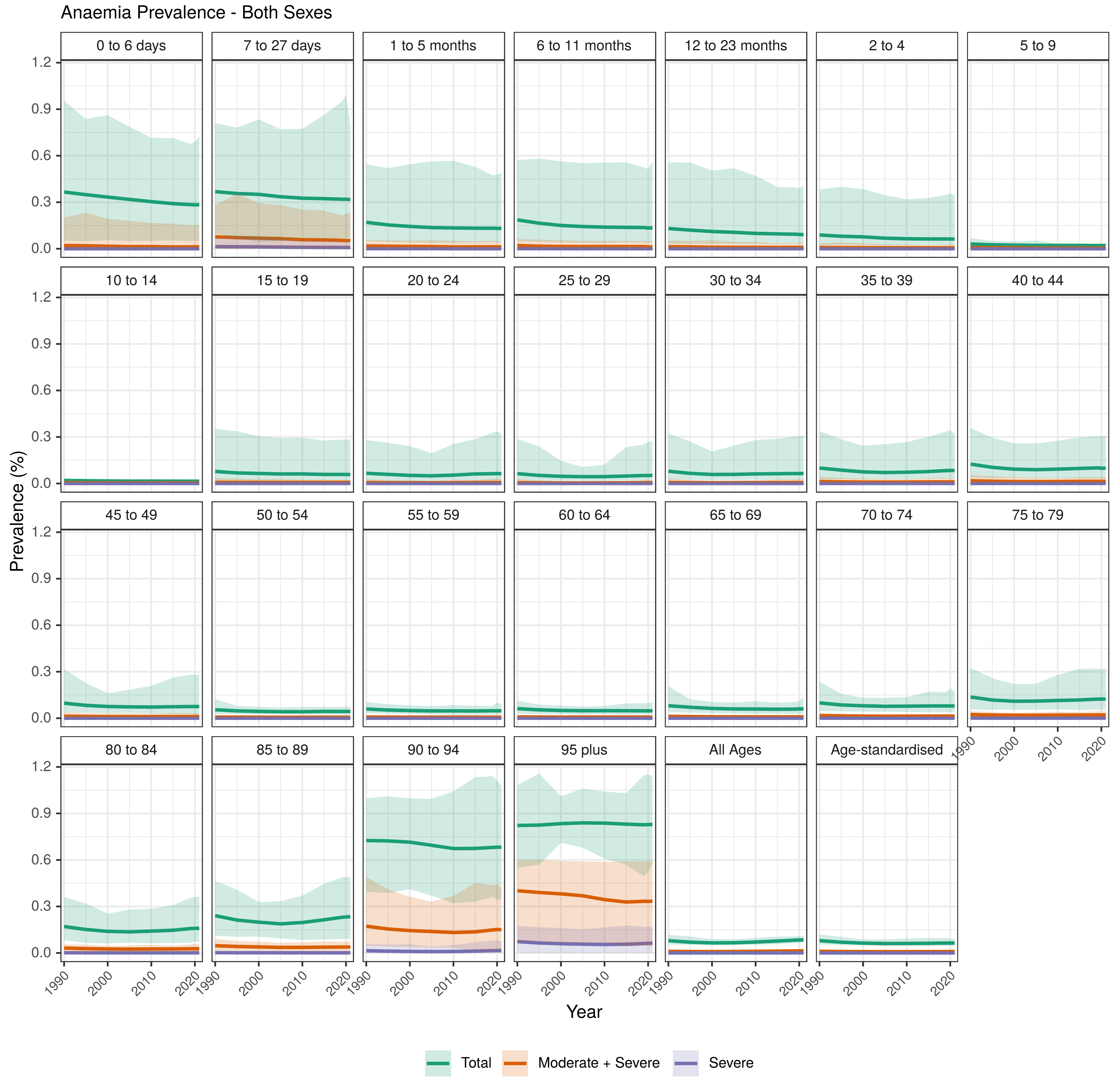

Anaemia Prevalence - Both Sexes
0 to 6 days
7 to 27 days
1 to 5 months
6 to 11 months
12 to 23 months
2 to 4
5 to 9
10 to 14
15 to 19
20 to 24
25 to 29
30 to 34
35 to 39
40 to 44
45 to 49
50 to 54
55 to 59
60 to 64
65 to 69
70 to 74
75 to 79
80 to 84
85 to 89
90 to 94
95 plus
All Ages
Age-standardised
Prevalence (%)
Year
Total
Moderate + Severe
Severe

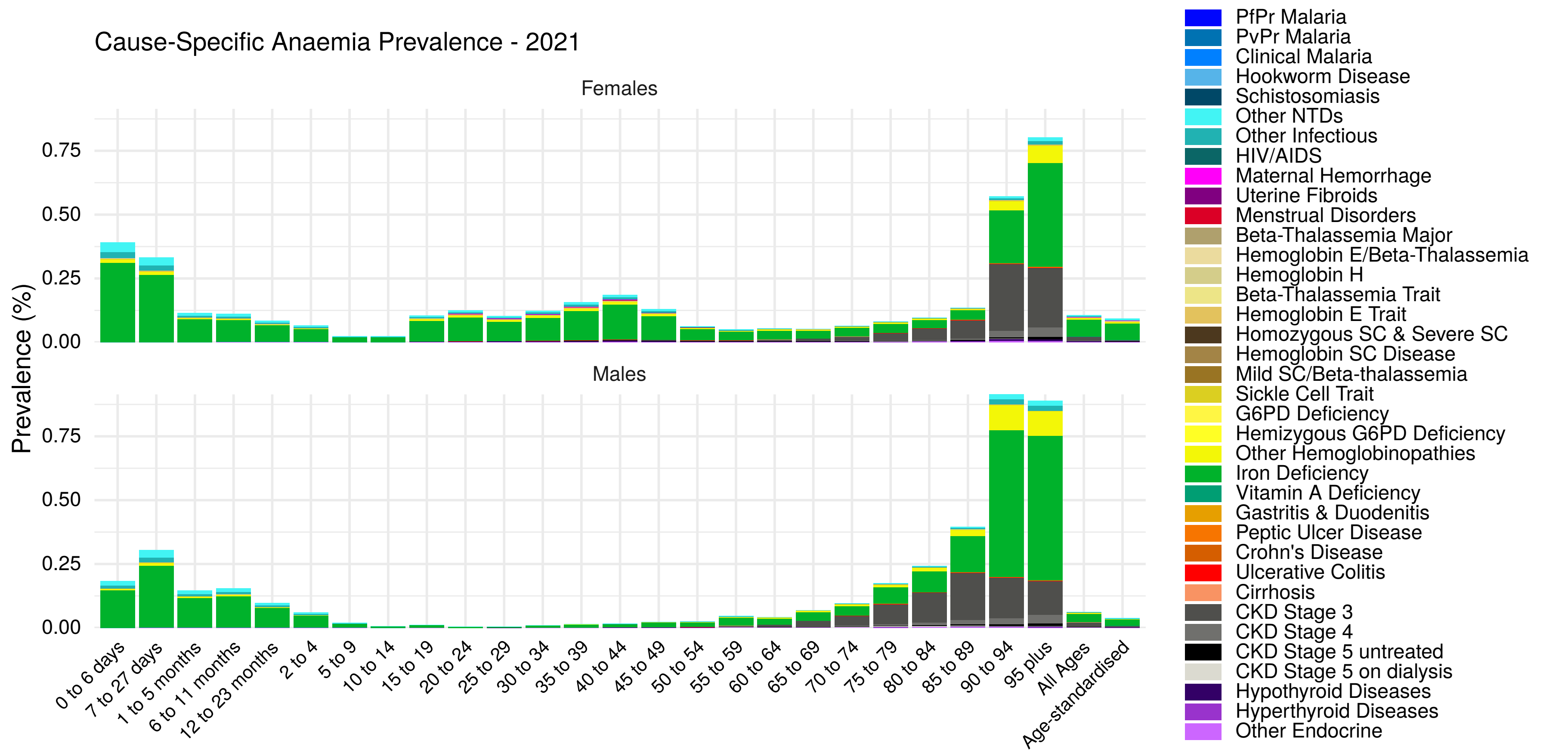

Cause-Specific Anaemia Prevalence - 2021
Females
Males
Prevalence (%)
PfPr Malaria
PvPr Malaria
Clinical Malaria
Hookworm Disease
Schistosomiasis
Other NTDs
Other Infectious
HIV/AIDS
Maternal Hemorrhage
Uterine Fibroids
Menstrual Disorders
Beta-Thalassemia Major
Hemoglobin E/Beta-Thalassemia
Hemoglobin H
Beta-Thalassemia Trait
Hemoglobin E Trait
Homozygous SC & Severe SC
Hemoglobin SC Disease
Mild SC/Beta-thalassemia
Sickle Cell Trait
G6PD Deficiency
Hemizygous G6PD Deficiency
Other Hemoglobinopathies
Iron Deficiency
Vitamin A Deficiency
Gastritis & Duodenitis
Peptic Ulcer Disease
Crohn's Disease
Ulcerative Colitis
Cirrhosis
CKD Stage 3
CKD Stage 4
CKD Stage 5 untreated
CKD Stage 5 on dialysis
Hypothyroid Diseases
Hyperthyroid Diseases
Other Endocrine

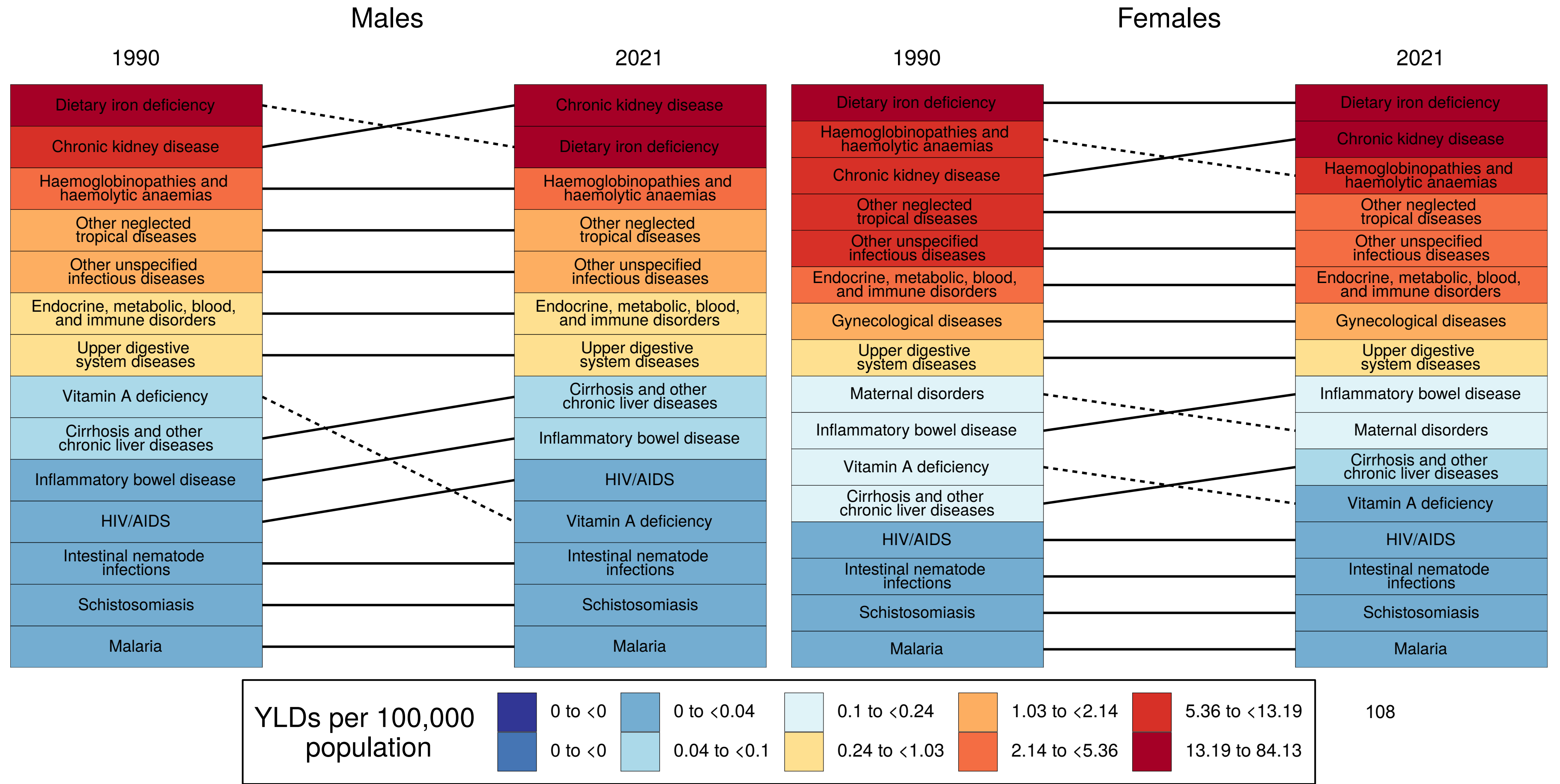

Males
Females
1990
2021
Dietary iron deficiency
Chronic kidney disease
Haemoglobinopathies and haemolytic anaemias
Other neglected tropical diseases
Other unspecified infectious diseases
Endocrine, metabolic, blood, and immune disorders
Upper digestive system diseases
Vitamin A deficiency
Cirrhosis and other chronic liver diseases
Inflammatory bowel disease
HIV/AIDS
Intestinal nematode infections
Schistosomiasis
Malaria
Gynecological diseases
Maternal disorders
YLDs per 100,000 population
0 to <0
0 to <0.04
0.04 to <0.1
0.1 to <0.24
0.24 to <1.03
1.03 to <2.14
2.14 to <5.36
5.36 to <13.19
13.19 to 84.13

Kōchi

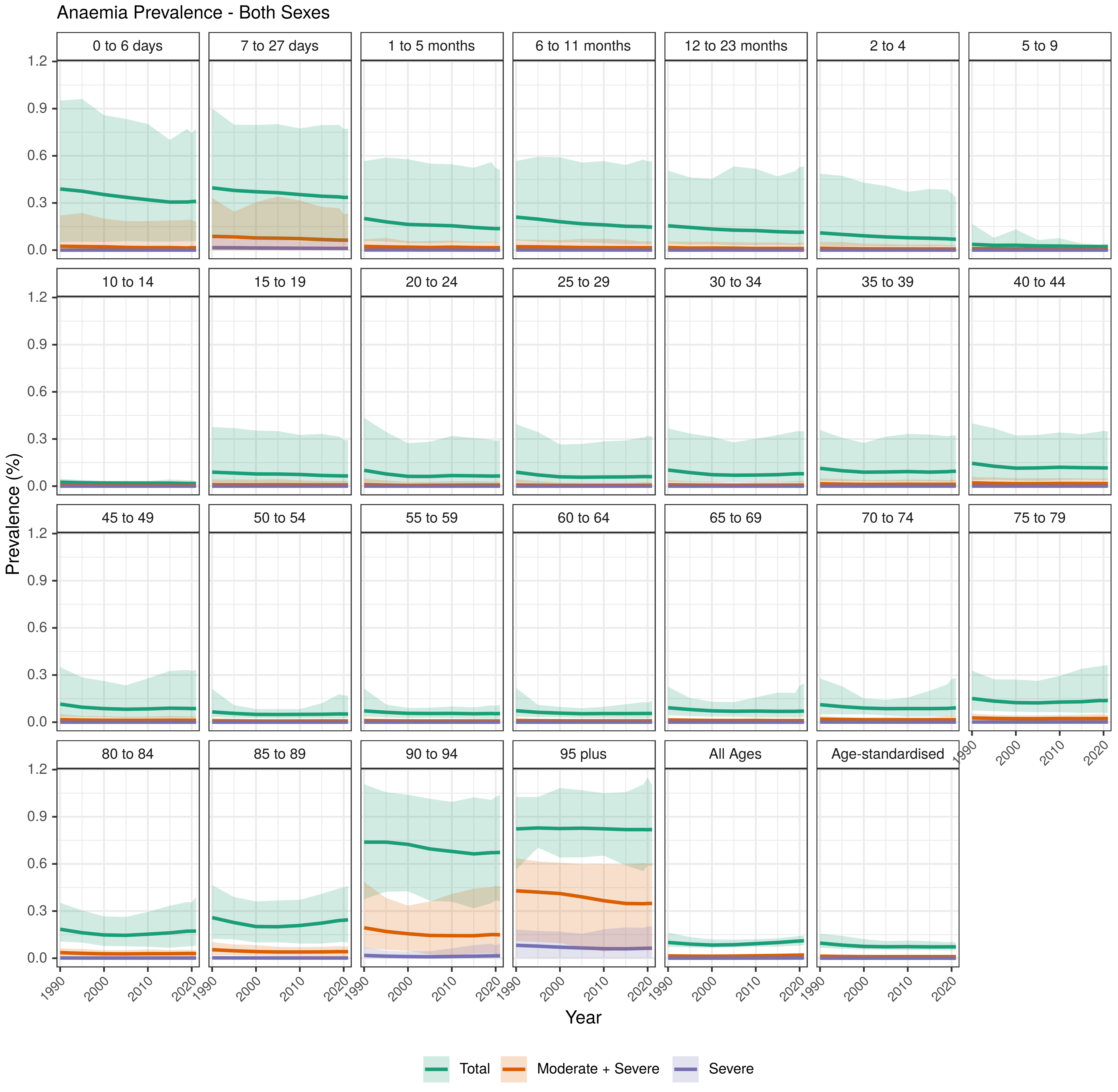
Anaemia Prevalence - Both Sexes
0 to 6 days
7 to 27 days
1 to 5 months
6 to 11 months
12 to 23 months
2 to 4
5 to 9
10 to 14
15 to 19
20 to 24
25 to 29
30 to 34
35 to 39
40 to 44
45 to 49
50 to 54
55 to 59
60 to 64
65 to 69
70 to 74
75 to 79
80 to 84
85 to 89
90 to 94
95 plus
All Ages
Age-standardised
Prevalence (%)
Year
Total
Moderate + Severe
Severe

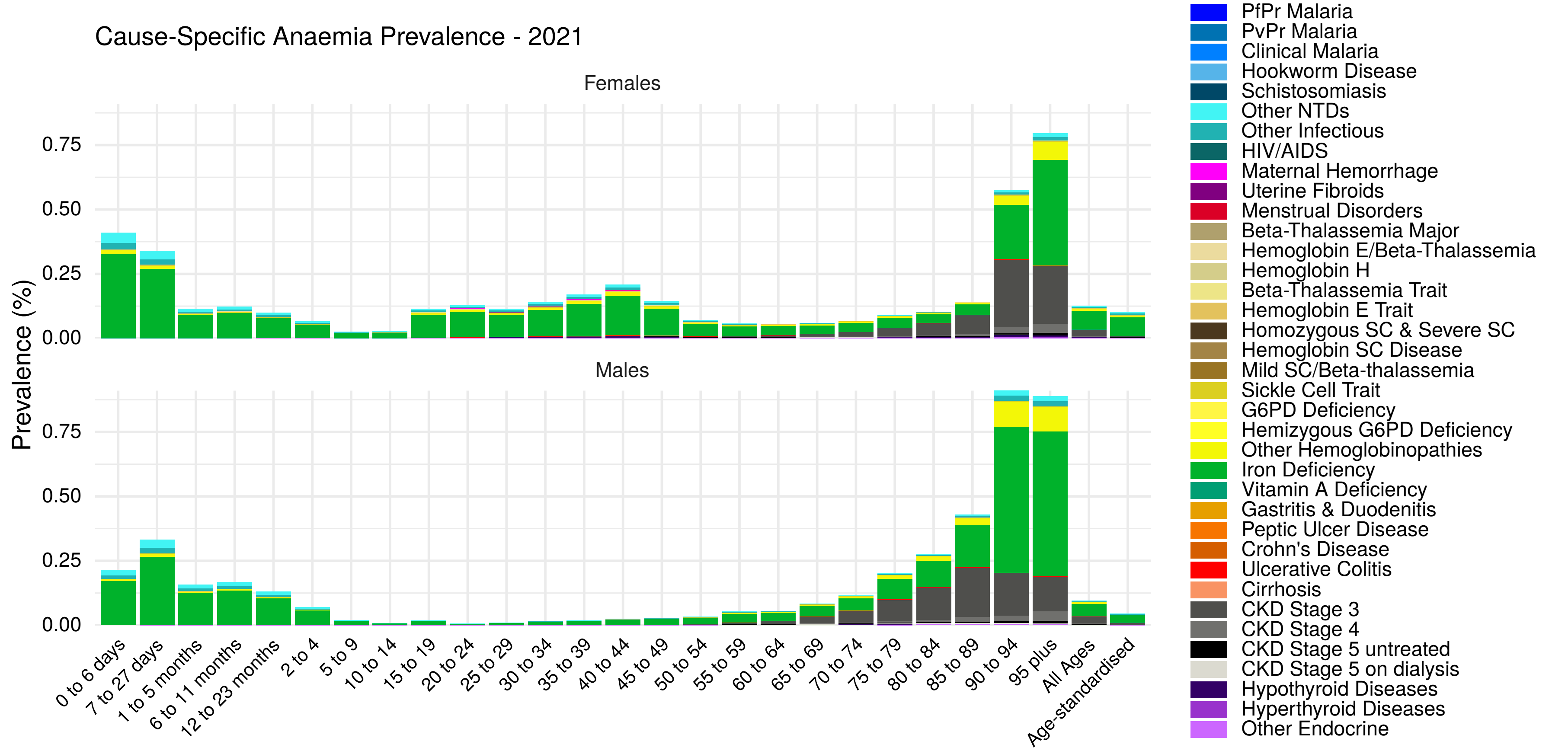
Cause-Specific Anaemia Prevalence - 2021
Females
Males
Prevalence (%)
PfPr Malaria
PvPr Malaria
Clinical Malaria
Hookworm Disease
Schistosomiasis
Other NTDs
Other Infectious
HIV/AIDS
Maternal Hemorrhage
Uterine Fibroids
Menstrual Disorders
Beta-Thalassemia Major
Hemoglobin E/Beta-Thalassemia
Hemoglobin H
Beta-Thalassemia Trait
Hemoglobin E Trait
Homozygous SC & Severe SC
Hemoglobin SC Disease
Mild SC/Beta-thalassemia
Sickle Cell Trait
G6PD Deficiency
Hemizygous G6PD Deficiency
Other Hemoglobinopathies
Iron Deficiency
Vitamin A Deficiency
Gastritis & Duodenitis
Peptic Ulcer Disease
Crohn's Disease
Ulcerative Colitis
Cirrhosis
CKD Stage 3
CKD Stage 4
CKD Stage 5 untreated
CKD Stage 5 on dialysis
Hypothyroid Diseases
Hyperthyroid Diseases
Other Endocrine

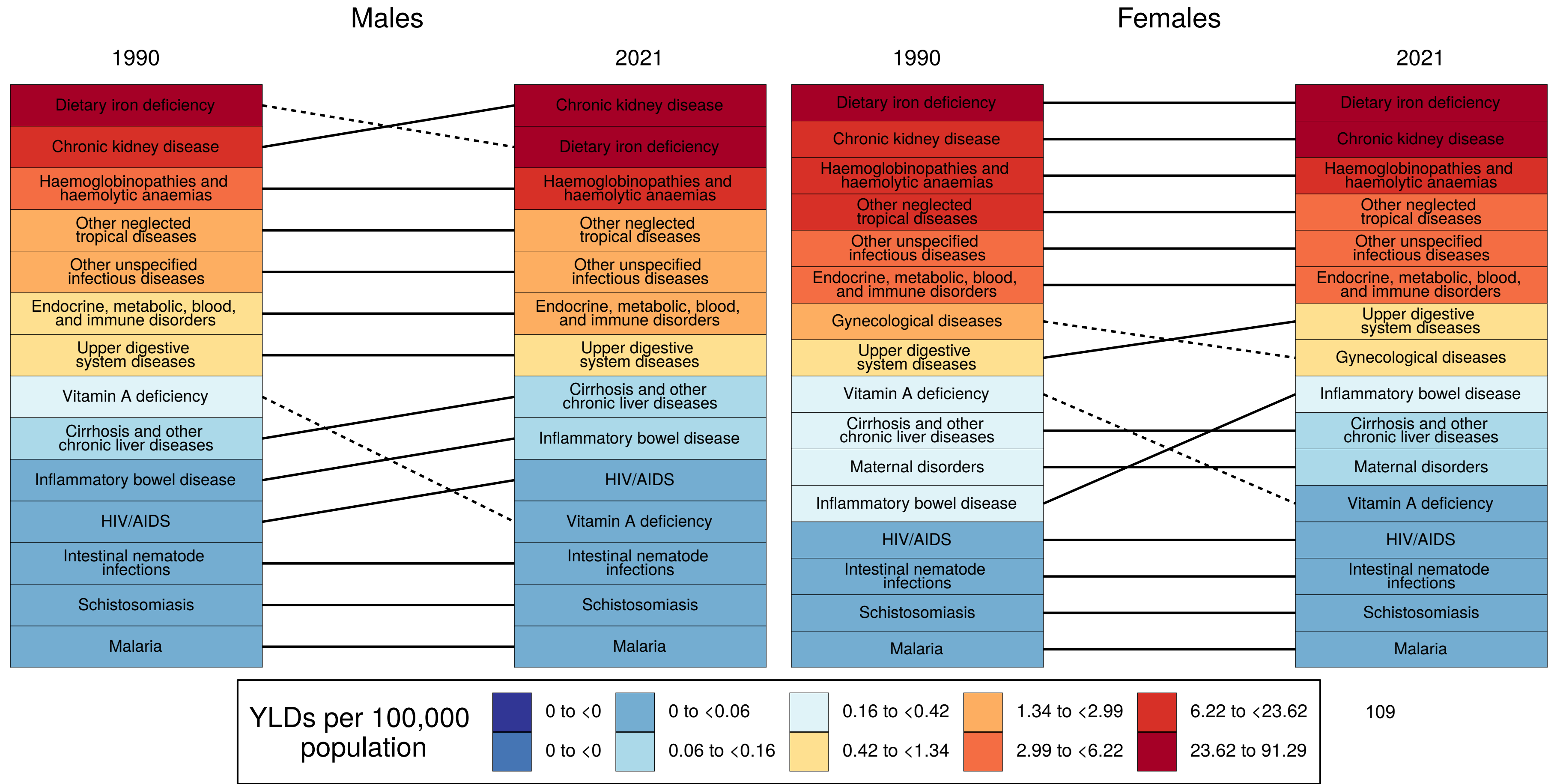
Males
Females
1990
2021
Dietary iron deficiency
Chronic kidney disease
Haemoglobinopathies and haemolytic anaemias
Other neglected tropical diseases
Other unspecified infectious diseases
Endocrine, metabolic, blood, and immune disorders
Upper digestive system diseases
Vitamin A deficiency
Cirrhosis and other chronic liver diseases
Inflammatory bowel disease
HIV/AIDS
Intestinal nematode infections
Schistosomiasis
Malaria
Gynecological diseases
Maternal disorders
YLDs per 100,000 population
0 to <0
0 to <0.06
0.06 to <0.16
0.16 to <0.42
0.42 to <1.34
1.34 to <2.99
2.99 to <6.22
6.22 to <23.62
23.62 to 91.29

Kumamoto

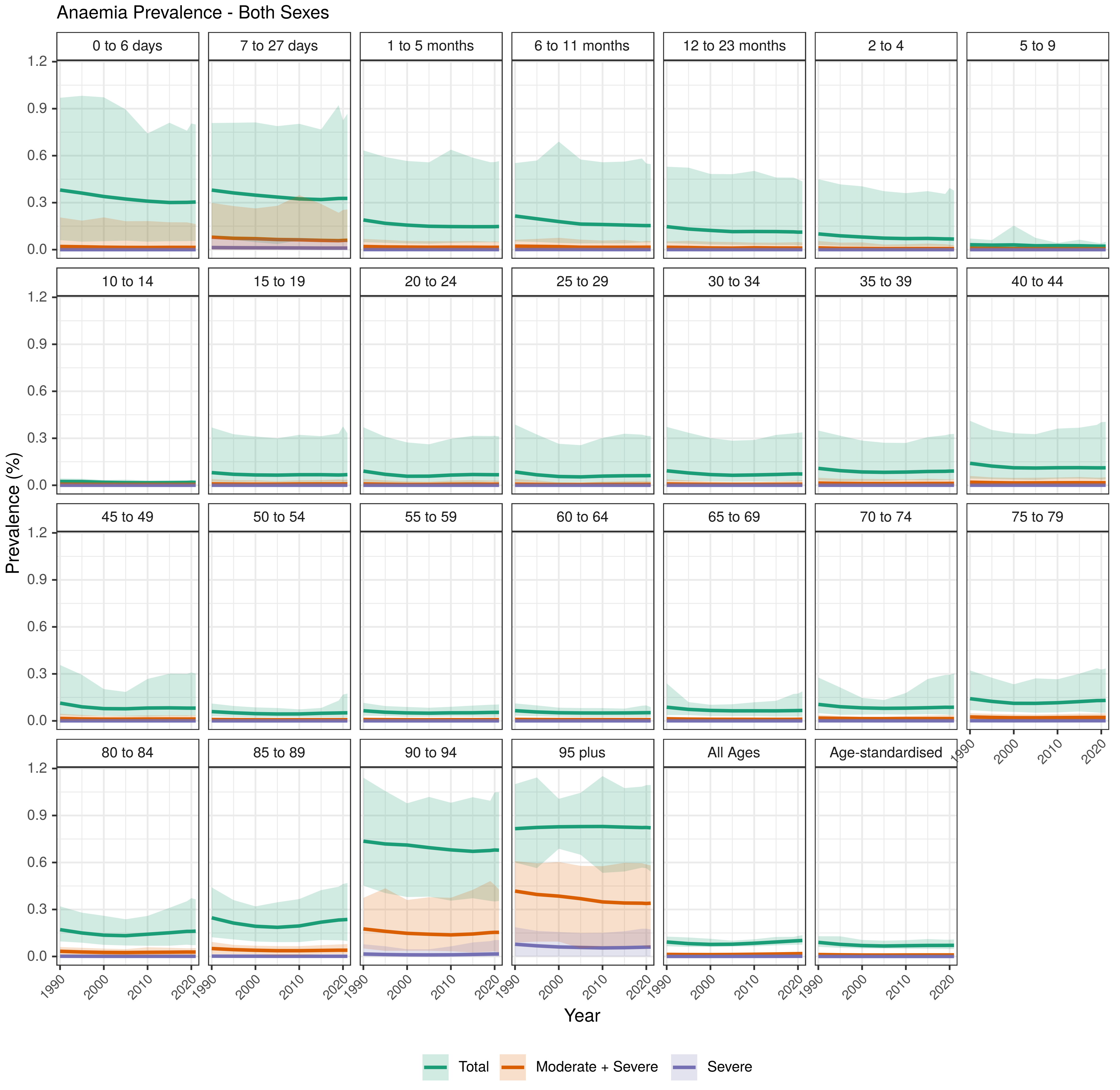

Anaemia Prevalence - Both Sexes
0 to 6 days
7 to 27 days
1 to 5 months
6 to 11 months
12 to 23 months
2 to 4
5 to 9
10 to 14
15 to 19
20 to 24
25 to 29
30 to 34
35 to 39
40 to 44
45 to 49
50 to 54
55 to 59
60 to 64
65 to 69
70 to 74
75 to 79
80 to 84
85 to 89
90 to 94
95 plus
All Ages
Age-standardised
Prevalence (%)
0.0
0.3
0.6
0.9
1.2
1990
2000
2010
2020
Year
Total
Moderate + Severe
Severe

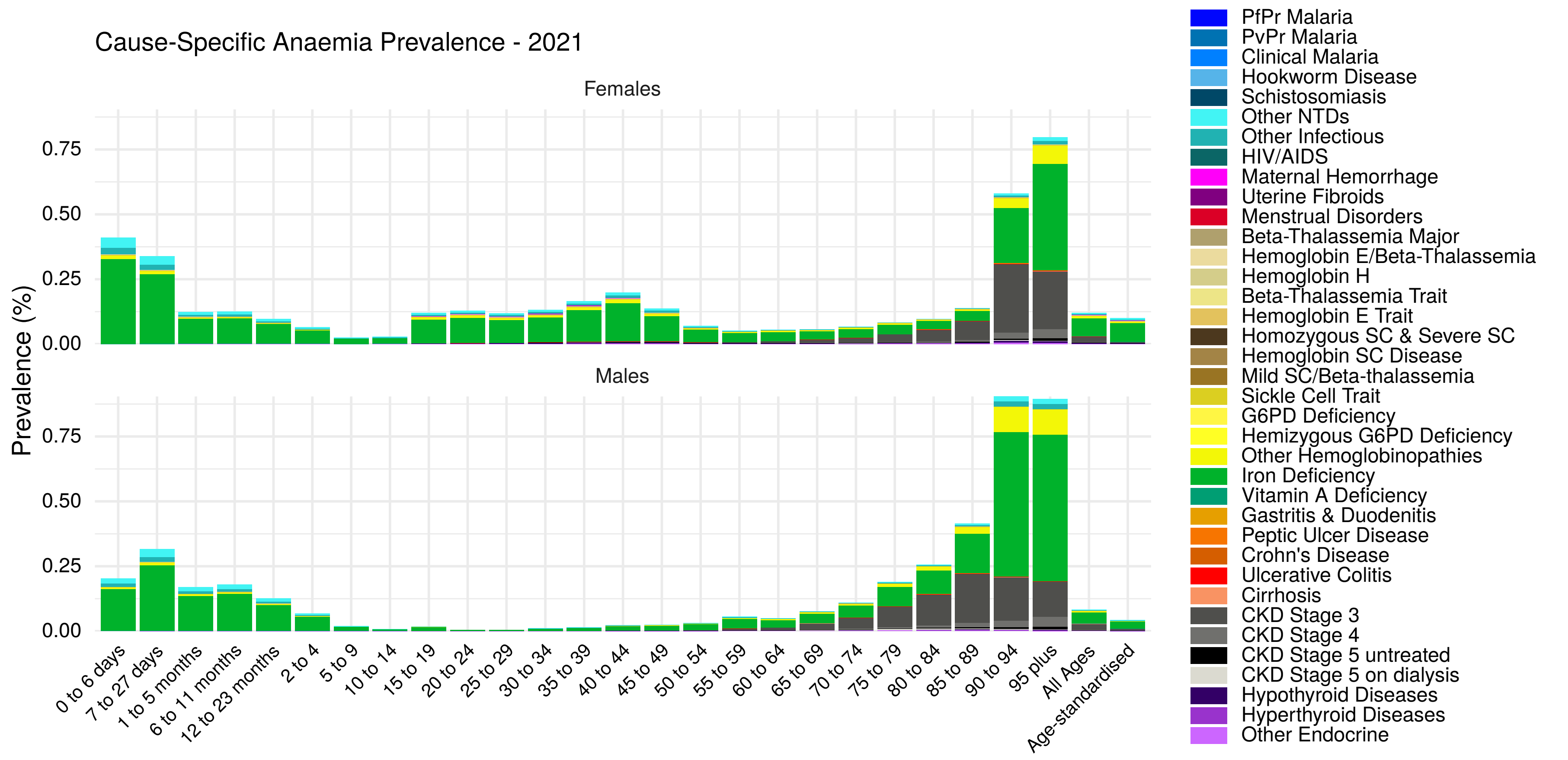

Cause-Specific Anaemia Prevalence - 2021
Females
Males
Prevalence (%)
0.00
0.25
0.50
0.75
0 to 6 days
7 to 27 days
1 to 5 months
6 to 11 months
12 to 23 months
2 to 4
5 to 9
10 to 14
15 to 19
20 to 24
25 to 29
30 to 34
35 to 39
40 to 44
45 to 49
50 to 54
55 to 59
60 to 64
65 to 69
70 to 74
75 to 79
80 to 84
85 to 89
90 to 94
95 plus
All Ages
Age-standardised
PfPr Malaria
PvPr Malaria
Clinical Malaria
Hookworm Disease
Schistosomiasis
Other NTDs
Other Infectious
HIV/AIDS
Maternal Hemorrhage
Uterine Fibroids
Menstrual Disorders
Beta-Thalassemia Major
Hemoglobin E/Beta-Thalassemia
Hemoglobin H
Beta-Thalassemia Trait
Hemoglobin E Trait
Homozygous SC & Severe SC
Hemoglobin SC Disease
Mild SC/Beta-thalassemia
Sickle Cell Trait
G6PD Deficiency
Hemizygous G6PD Deficiency
Other Hemoglobinopathies
Iron Deficiency
Vitamin A Deficiency
Gastritis & Duodenitis
Peptic Ulcer Disease
Crohn's Disease
Ulcerative Colitis
Cirrhosis
CKD Stage 3
CKD Stage 4
CKD Stage 5 untreated
CKD Stage 5 on dialysis
Hypothyroid Diseases
Hyperthyroid Diseases
Other Endocrine

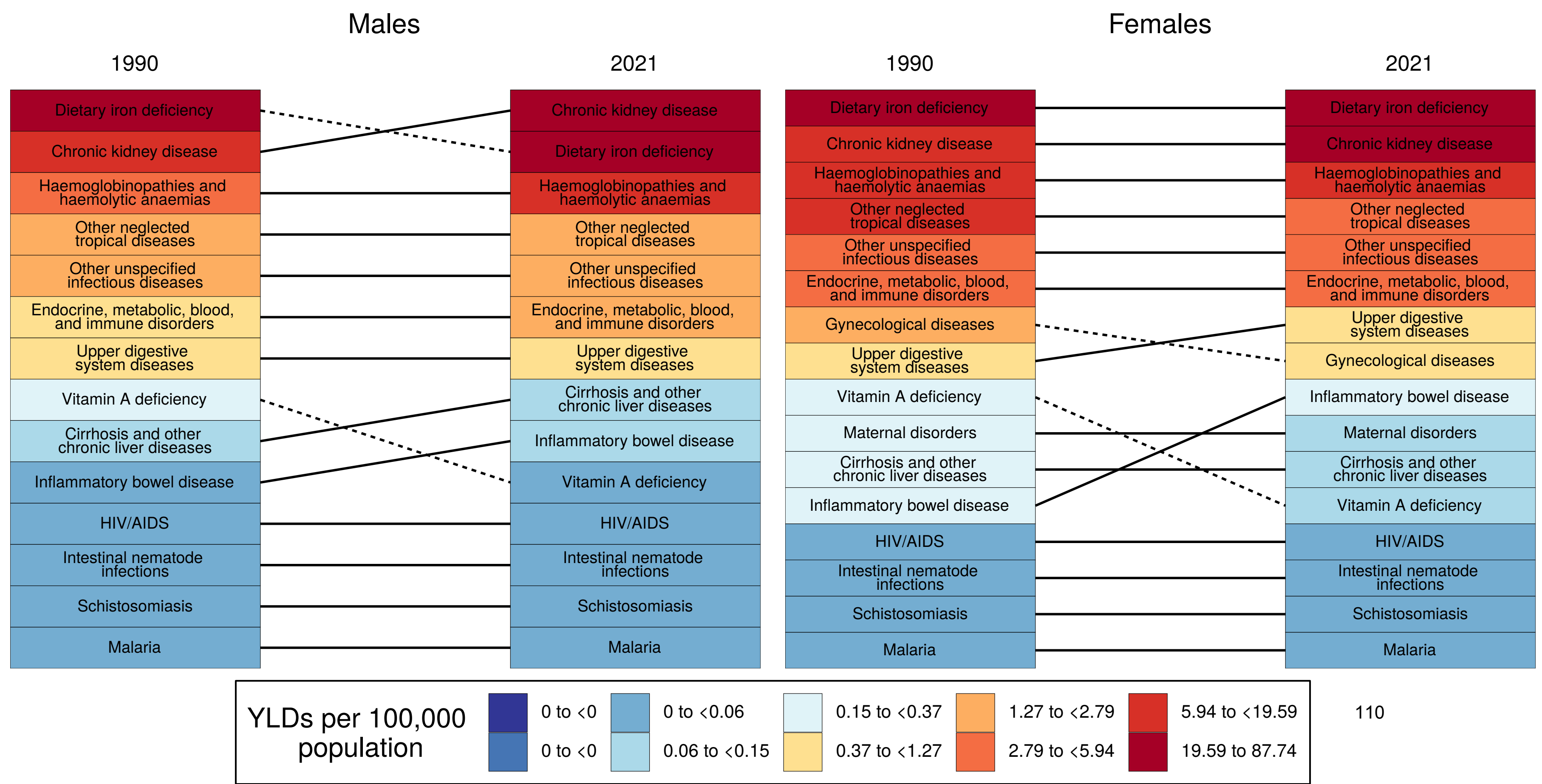

Males
Females
1990
2021
1990
2021
Dietary iron deficiency
Chronic kidney disease
Haemoglobinopathies and haemolytic anaemias
Other neglected tropical diseases
Other unspecified infectious diseases
Endocrine, metabolic, blood, and immune disorders
Upper digestive system diseases
Vitamin A deficiency
Cirrhosis and other chronic liver diseases
Inflammatory bowel disease
HIV/AIDS
Intestinal nematode infections
Schistosomiasis
Malaria
Chronic kidney disease
Dietary iron deficiency
Haemoglobinopathies and haemolytic anaemias
Other neglected tropical diseases
Other unspecified infectious diseases
Endocrine, metabolic, blood, and immune disorders
Upper digestive system diseases
Cirrhosis and other chronic liver diseases
Inflammatory bowel disease
Vitamin A deficiency
HIV/AIDS
Intestinal nematode infections
Schistosomiasis
Malaria
Dietary iron deficiency
Chronic kidney disease
Haemoglobinopathies and haemolytic anaemias
Other neglected tropical diseases
Other unspecified infectious diseases
Endocrine, metabolic, blood, and immune disorders
Gynecological diseases
Upper digestive system diseases
Vitamin A deficiency
Maternal disorders
Cirrhosis and other chronic liver diseases
Inflammatory bowel disease
HIV/AIDS
Intestinal nematode infections
Schistosomiasis
Malaria
Dietary iron deficiency
Chronic kidney disease
Haemoglobinopathies and haemolytic anaemias
Other neglected tropical diseases
Other unspecified infectious diseases
Endocrine, metabolic, blood, and immune disorders
Upper digestive system diseases
Gynecological diseases
Inflammatory bowel disease
Maternal disorders
Cirrhosis and other chronic liver diseases
Vitamin A deficiency
HIV/AIDS
Intestinal nematode infections
Schistosomiasis
Malaria
YLDs per 100,000 population
0 to <0
0 to <0
0 to <0.06
0.06 to <0.15
0.15 to <0.37
0.37 to <1.27
1.27 to <2.79
2.79 to <5.94
5.94 to <19.59
19.59 to 87.74

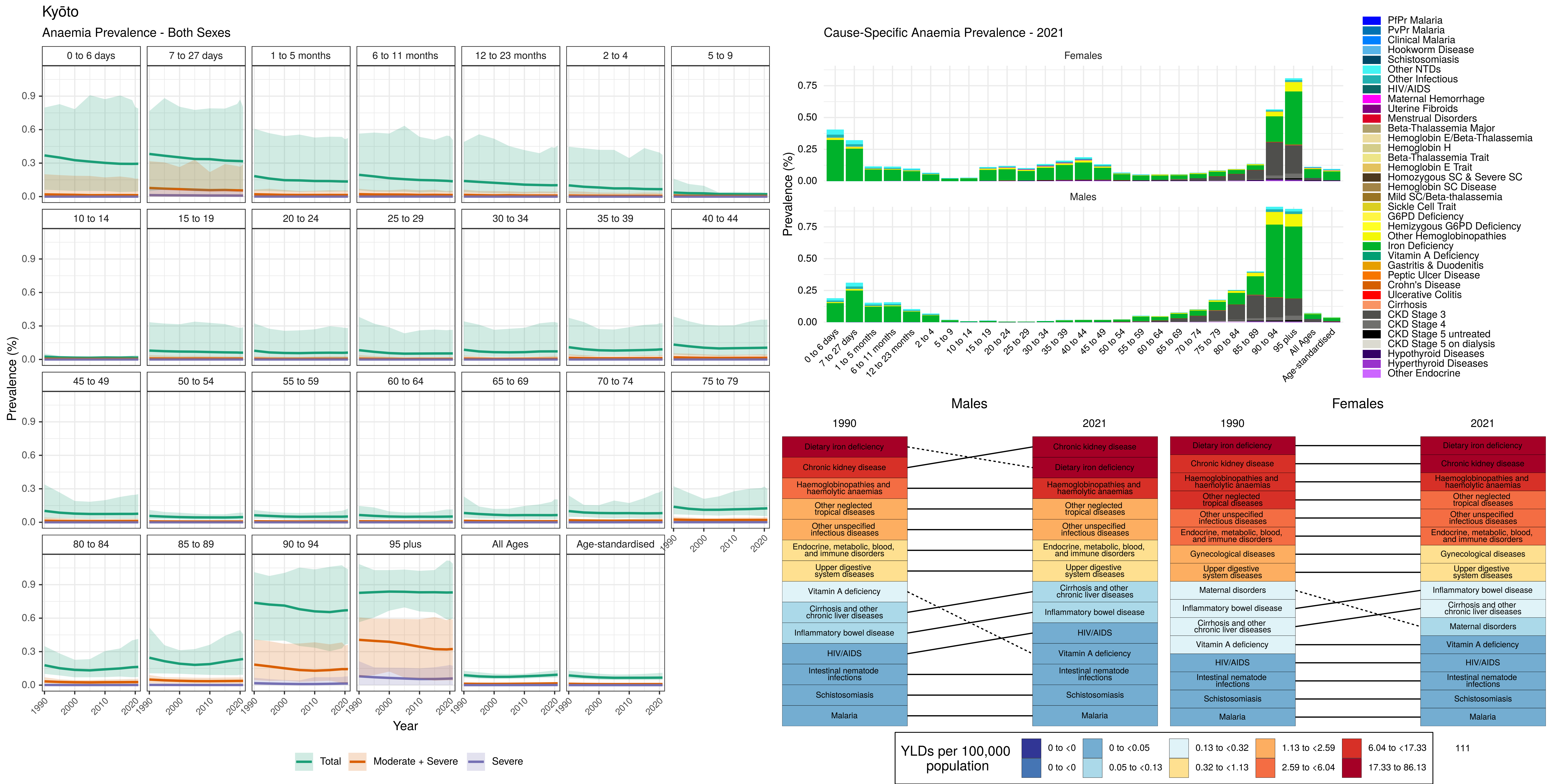
Kyōto
Anaemia Prevalence - Both Sexes
0 to 6 days
7 to 27 days
1 to 5 months
6 to 11 months
12 to 23 months
2 to 4
5 to 9
10 to 14
15 to 19
20 to 24
25 to 29
30 to 34
35 to 39
40 to 44
45 to 49
50 to 54
55 to 59
60 to 64
65 to 69
70 to 74
75 to 79
80 to 84
85 to 89
90 to 94
95 plus
All Ages
Age-standardised
Prevalence (%)
Year
Total
Moderate + Severe
Severe
Cause-Specific Anaemia Prevalence - 2021
Females
Males
PfPr Malaria
PvPr Malaria
Clinical Malaria
Hookworm Disease
Schistosomiasis
Other NTDs
Other Infectious
HIV/AIDS
Maternal Hemorrhage
Uterine Fibroids
Menstrual Disorders
Beta-Thalassemia Major
Hemoglobin E/Beta-Thalassemia
Hemoglobin H
Beta-Thalassemia Trait
Hemoglobin E Trait
Homozygous SC & Severe SC
Hemoglobin SC Disease
Mild SC/Beta-thalassemia
Sickle Cell Trait
G6PD Deficiency
Hemizygous G6PD Deficiency
Other Hemoglobinopathies
Iron Deficiency
Vitamin A Deficiency
Gastritis & Duodenitis
Peptic Ulcer Disease
Crohn's Disease
Ulcerative Colitis
Cirrhosis
CKD Stage 3
CKD Stage 4
CKD Stage 5 untreated
CKD Stage 5 on dialysis
Hypothyroid Diseases
Hyperthyroid Diseases
Other Endocrine
Males
Females
1990
2021
Dietary iron deficiency
Chronic kidney disease
Haemoglobinopathies and haemolytic anaemias
Other neglected tropical diseases
Other unspecified infectious diseases
Endocrine, metabolic, blood, and immune disorders
Upper digestive system diseases
Vitamin A deficiency
Cirrhosis and other chronic liver diseases
Inflammatory bowel disease
HIV/AIDS
Intestinal nematode infections
Schistosomiasis
Malaria
Gynecological diseases
Maternal disorders
YLDs per 100,000 population
0 to <0
0 to <0
0 to <0.05
0.05 to <0.13
0.13 to <0.32
0.32 to <1.13
1.13 to <2.59
2.59 to <6.04
6.04 to <17.33
17.33 to 86.13

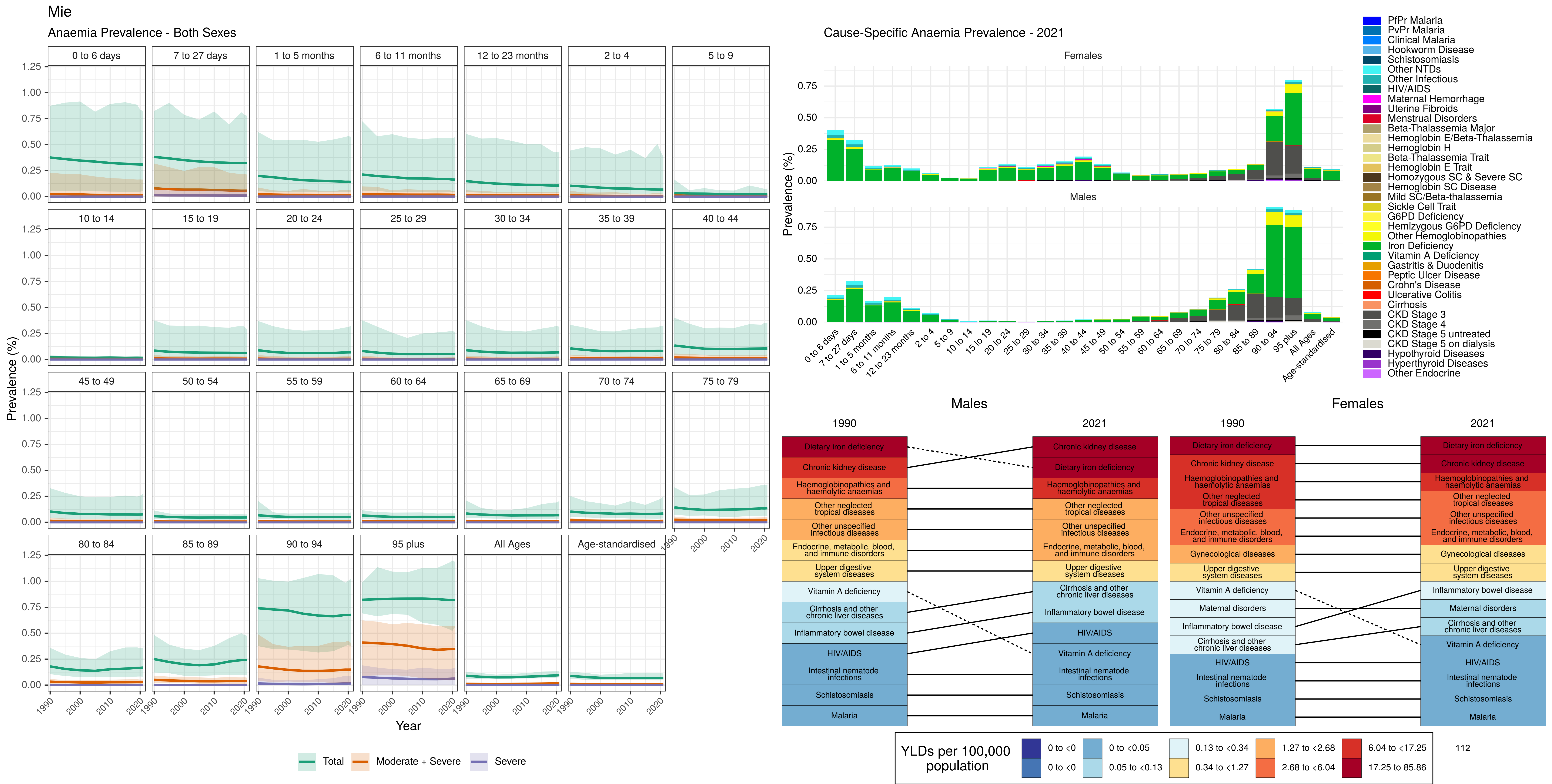
Mie
Anaemia Prevalence - Both Sexes
0 to 6 days
7 to 27 days
1 to 5 months
6 to 11 months
12 to 23 months
2 to 4
5 to 9
10 to 14
15 to 19
20 to 24
25 to 29
30 to 34
35 to 39
40 to 44
45 to 49
50 to 54
55 to 59
60 to 64
65 to 69
70 to 74
75 to 79
80 to 84
85 to 89
90 to 94
95 plus
All Ages
Age-standardised
Prevalence (%)
Year
Total
Moderate + Severe
Severe
Cause-Specific Anaemia Prevalence - 2021
Females
Males
PfPr Malaria
PvPr Malaria
Clinical Malaria
Hookworm Disease
Schistosomiasis
Other NTDs
Other Infectious
HIV/AIDS
Maternal Hemorrhage
Uterine Fibroids
Menstrual Disorders
Beta-Thalassemia Major
Hemoglobin E/Beta-Thalassemia
Hemoglobin H
Beta-Thalassemia Trait
Hemoglobin E Trait
Homozygous SC & Severe SC
Hemoglobin SC Disease
Mild SC/Beta-thalassemia
Sickle Cell Trait
G6PD Deficiency
Hemizygous G6PD Deficiency
Other Hemoglobinopathies
Iron Deficiency
Vitamin A Deficiency
Gastritis & Duodenitis
Peptic Ulcer Disease
Crohn's Disease
Ulcerative Colitis
Cirrhosis
CKD Stage 3
CKD Stage 4
CKD Stage 5 untreated
CKD Stage 5 on dialysis
Hypothyroid Diseases
Hyperthyroid Diseases
Other Endocrine
Males
Females
1990
2021
Dietary iron deficiency
Chronic kidney disease
Haemoglobinopathies and haemolytic anaemias
Other neglected tropical diseases
Other unspecified infectious diseases
Endocrine, metabolic, blood, and immune disorders
Upper digestive system diseases
Vitamin A deficiency
Cirrhosis and other chronic liver diseases
Inflammatory bowel disease
HIV/AIDS
Intestinal nematode infections
Schistosomiasis
Malaria
Gynecological diseases
Maternal disorders
YLDs per 100,000 population
0 to <0
0 to <0.05
0.05 to <0.13
0.13 to <0.34
0.34 to <1.27
1.27 to <2.68
2.68 to <6.04
6.04 to <17.25
17.25 to 85.86

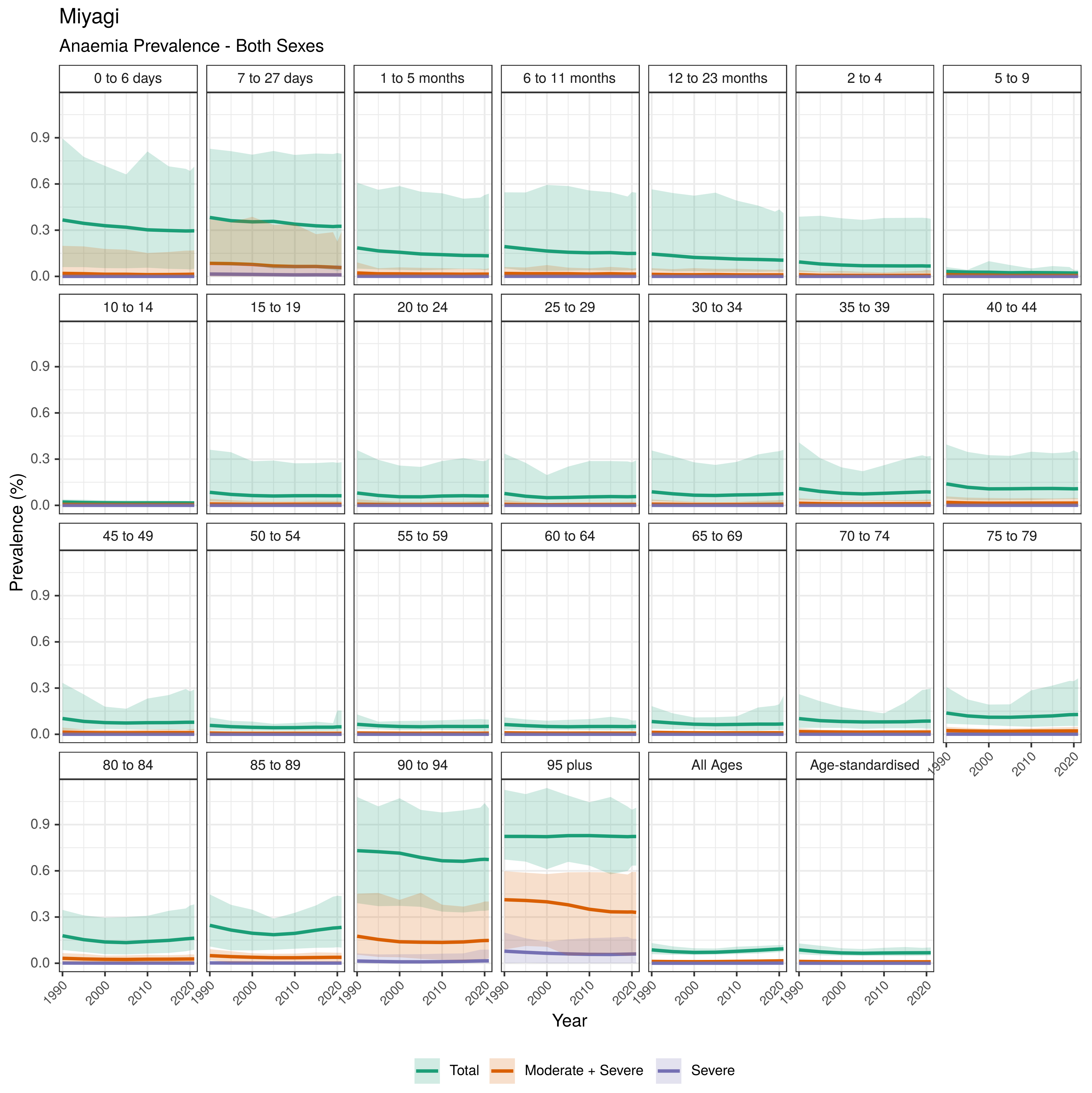
Miyagi
Anaemia Prevalence - Both Sexes
0 to 6 days
7 to 27 days
1 to 5 months
6 to 11 months
12 to 23 months
2 to 4
5 to 9
10 to 14
15 to 19
20 to 24
25 to 29
30 to 34
35 to 39
40 to 44
45 to 49
50 to 54
55 to 59
60 to 64
65 to 69
70 to 74
75 to 79
80 to 84
85 to 89
90 to 94
95 plus
All Ages
Age-standardised
Prevalence (%)
Year
Total
Moderate + Severe
Severe

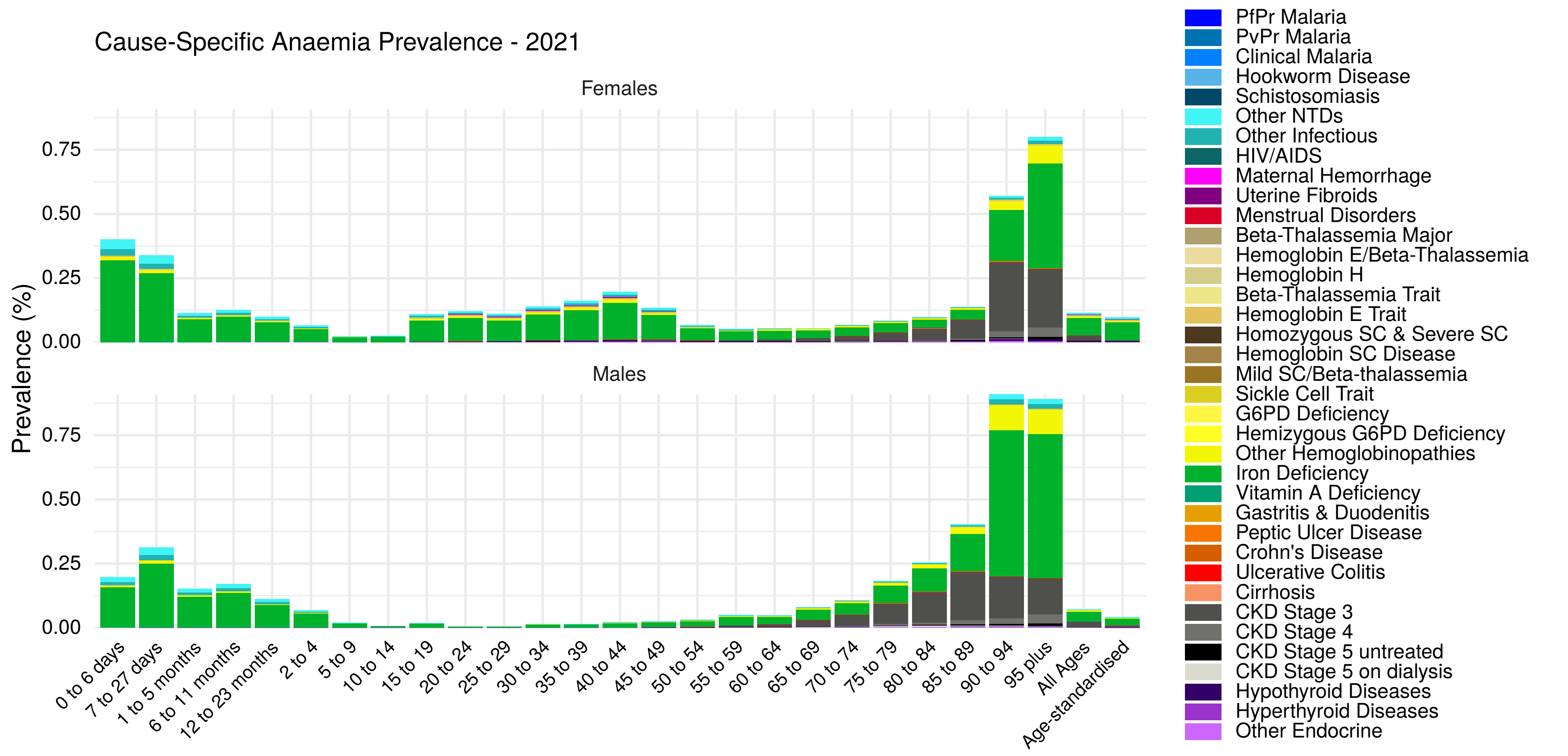
Cause-Specific Anaemia Prevalence - 2021
Females
Males
Prevalence (%)
PfPr Malaria
PvPr Malaria
Clinical Malaria
Hookworm Disease
Schistosomiasis
Other NTDs
Other Infectious
HIV/AIDS
Maternal Hemorrhage
Uterine Fibroids
Menstrual Disorders
Beta-Thalassemia Major
Hemoglobin E/Beta-Thalassemia
Hemoglobin H
Beta-Thalassemia Trait
Hemoglobin E Trait
Homozygous SC & Severe SC
Hemoglobin SC Disease
Mild SC/Beta-thalassemia
Sickle Cell Trait
G6PD Deficiency
Hemizygous G6PD Deficiency
Other Hemoglobinopathies
Iron Deficiency
Vitamin A Deficiency
Gastritis & Duodenitis
Peptic Ulcer Disease
Crohn's Disease
Ulcerative Colitis
Cirrhosis
CKD Stage 3
CKD Stage 4
CKD Stage 5 untreated
CKD Stage 5 on dialysis
Hypothyroid Diseases
Hyperthyroid Diseases
Other Endocrine

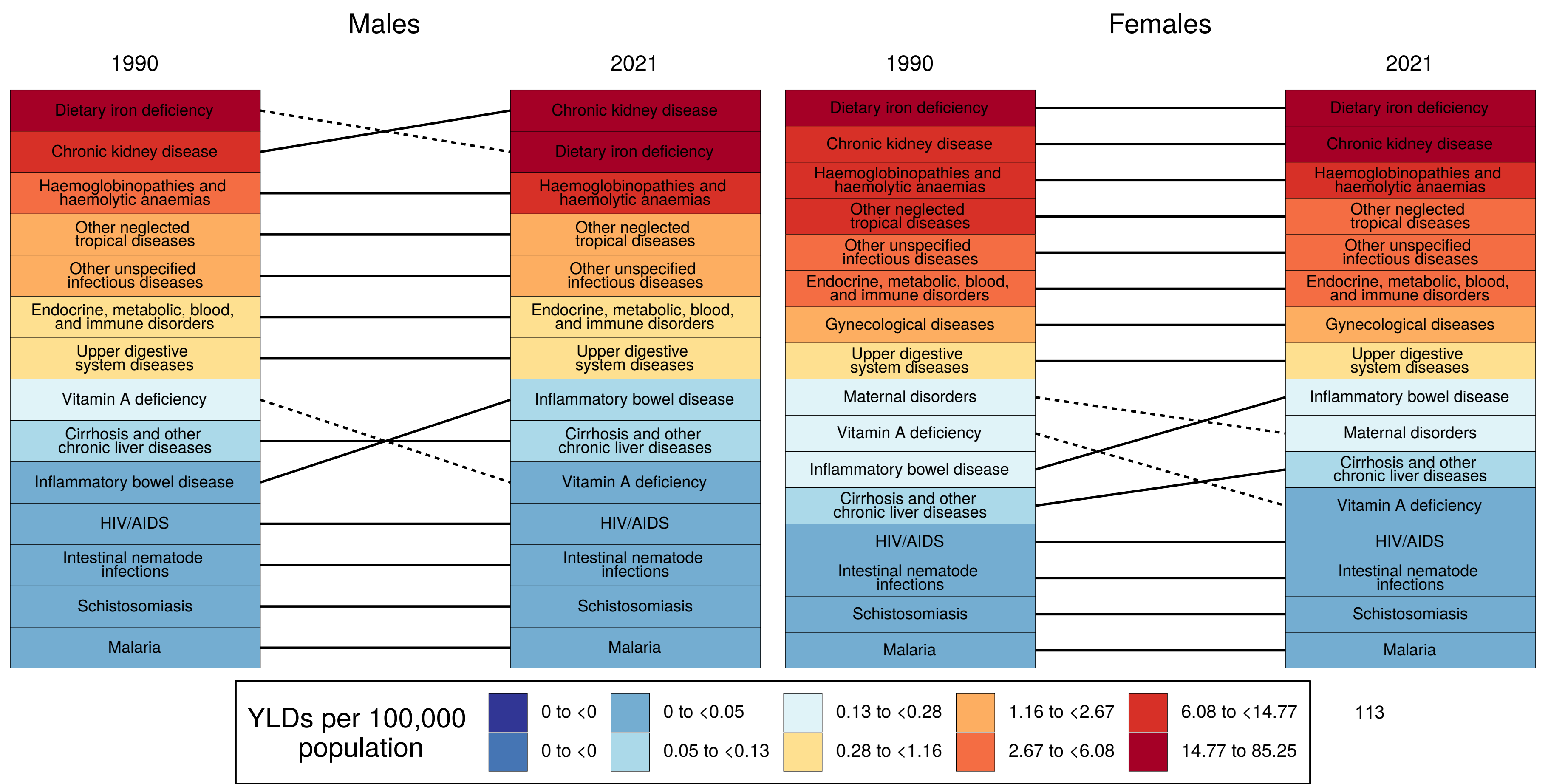
Males
Females
1990
2021
Dietary iron deficiency
Chronic kidney disease
Haemoglobinopathies and haemolytic anaemias
Other neglected tropical diseases
Other unspecified infectious diseases
Endocrine, metabolic, blood, and immune disorders
Upper digestive system diseases
Vitamin A deficiency
Cirrhosis and other chronic liver diseases
Inflammatory bowel disease
HIV/AIDS
Intestinal nematode infections
Schistosomiasis
Malaria
Gynecological diseases
Maternal disorders
YLDs per 100,000 population
0 to <0
0 to <0.05
0.05 to <0.13
0.13 to <0.28
0.28 to <1.16
1.16 to <2.67
2.67 to <6.08
6.08 to <14.77
14.77 to 85.25

Miyazaki

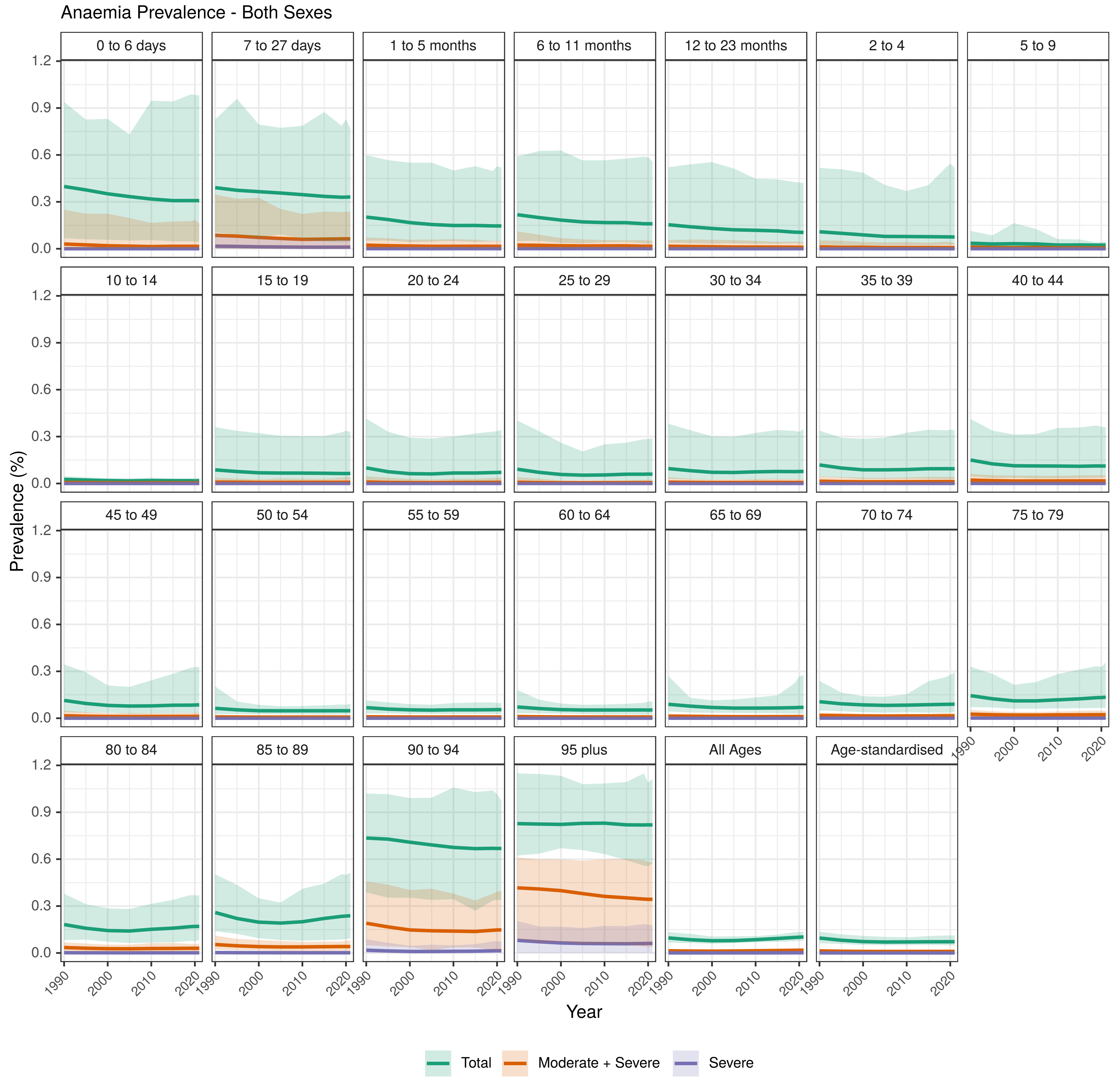

Anaemia Prevalence - Both Sexes
0 to 6 days
7 to 27 days
1 to 5 months
6 to 11 months
12 to 23 months
2 to 4
5 to 9
10 to 14
15 to 19
20 to 24
25 to 29
30 to 34
35 to 39
40 to 44
45 to 49
50 to 54
55 to 59
60 to 64
65 to 69
70 to 74
75 to 79
80 to 84
85 to 89
90 to 94
95 plus
All Ages
Age-standardised
Prevalence (%)
Year
Total
Moderate + Severe
Severe

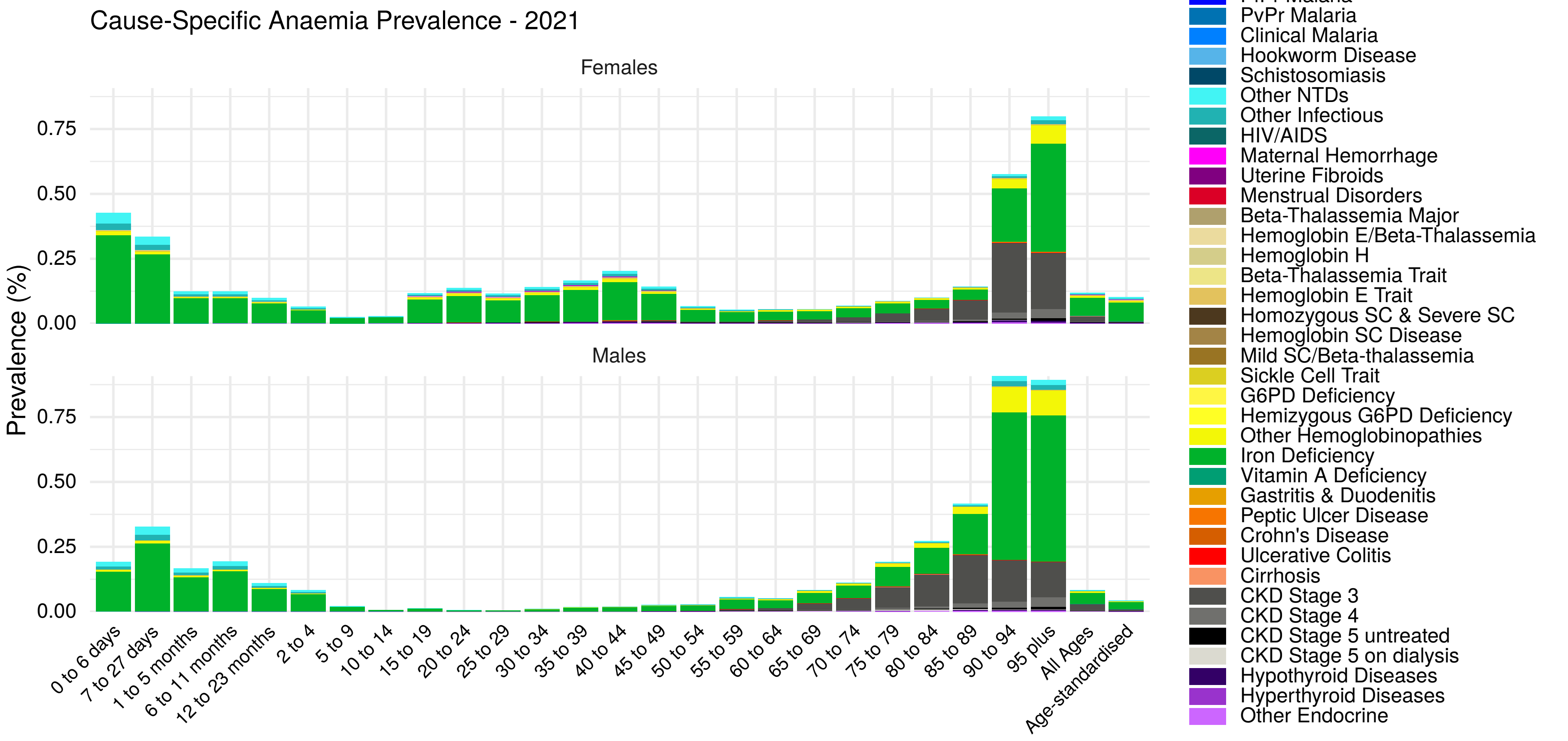

Cause-Specific Anaemia Prevalence - 2021
Females
Males
Prevalence (%)
PfPr Malaria
PvPr Malaria
Clinical Malaria
Hookworm Disease
Schistosomiasis
Other NTDs
Other Infectious
HIV/AIDS
Maternal Hemorrhage
Uterine Fibroids
Menstrual Disorders
Beta-Thalassemia Major
Hemoglobin E/Beta-Thalassemia
Hemoglobin H
Beta-Thalassemia Trait
Hemoglobin E Trait
Homozygous SC & Severe SC
Hemoglobin SC Disease
Mild SC/Beta-thalassemia
Sickle Cell Trait
G6PD Deficiency
Hemizygous G6PD Deficiency
Other Hemoglobinopathies
Iron Deficiency
Vitamin A Deficiency
Gastritis & Duodenitis
Peptic Ulcer Disease
Crohn's Disease
Ulcerative Colitis
Cirrhosis
CKD Stage 3
CKD Stage 4
CKD Stage 5 untreated
CKD Stage 5 on dialysis
Hypothyroid Diseases
Hyperthyroid Diseases
Other Endocrine

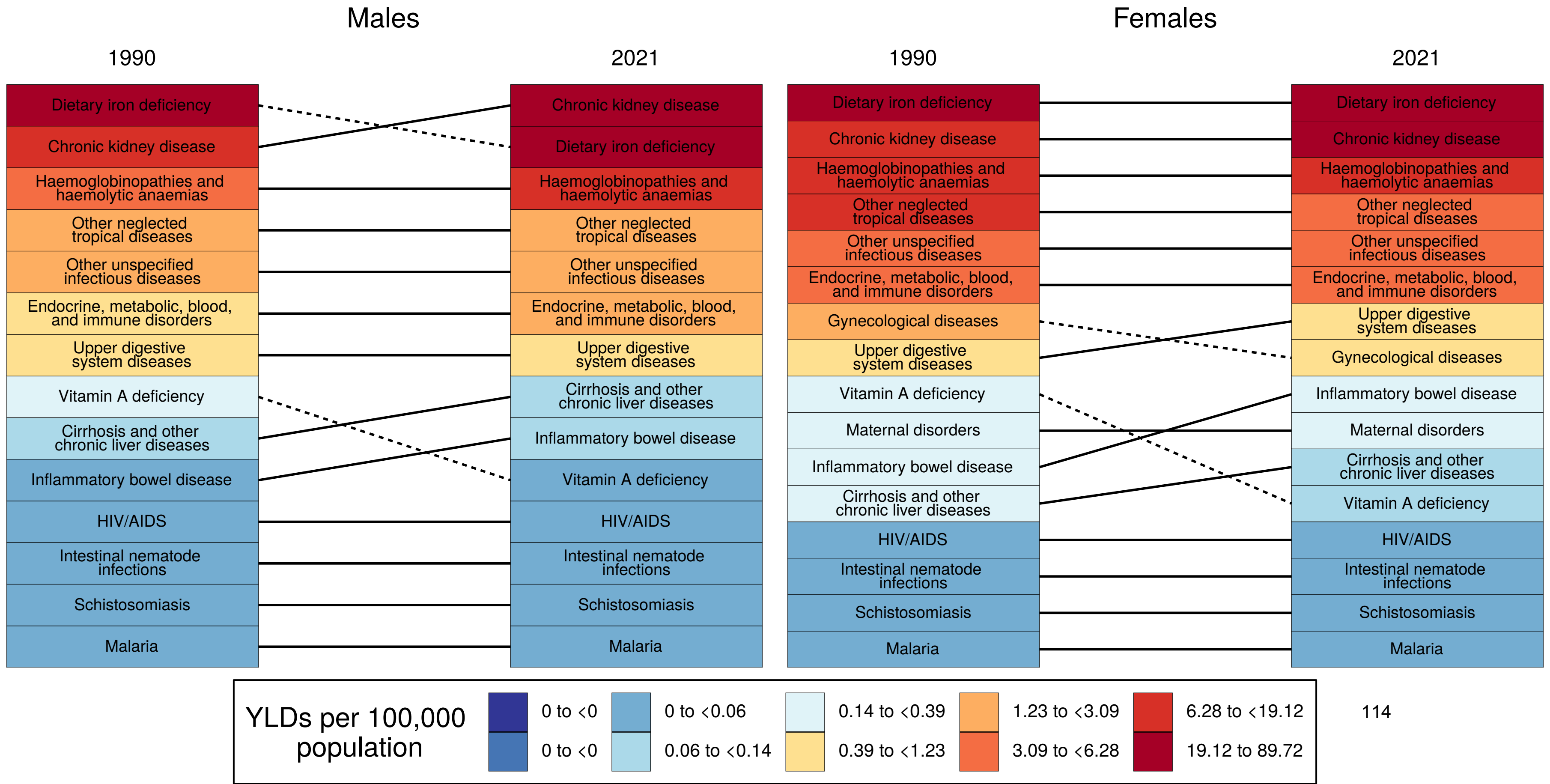

Males
Females
1990
2021
Dietary iron deficiency
Chronic kidney disease
Haemoglobinopathies and haemolytic anaemias
Other neglected tropical diseases
Other unspecified infectious diseases
Endocrine, metabolic, blood, and immune disorders
Upper digestive system diseases
Vitamin A deficiency
Cirrhosis and other chronic liver diseases
Inflammatory bowel disease
HIV/AIDS
Intestinal nematode infections
Schistosomiasis
Malaria
Gynecological diseases
Maternal disorders
YLDs per 100,000 population
0 to <0
0 to <0.06
0.06 to <0.14
0.14 to <0.39
0.39 to <1.23
1.23 to <3.09
3.09 to <6.28
6.28 to <19.12
19.12 to 89.72

Nagano

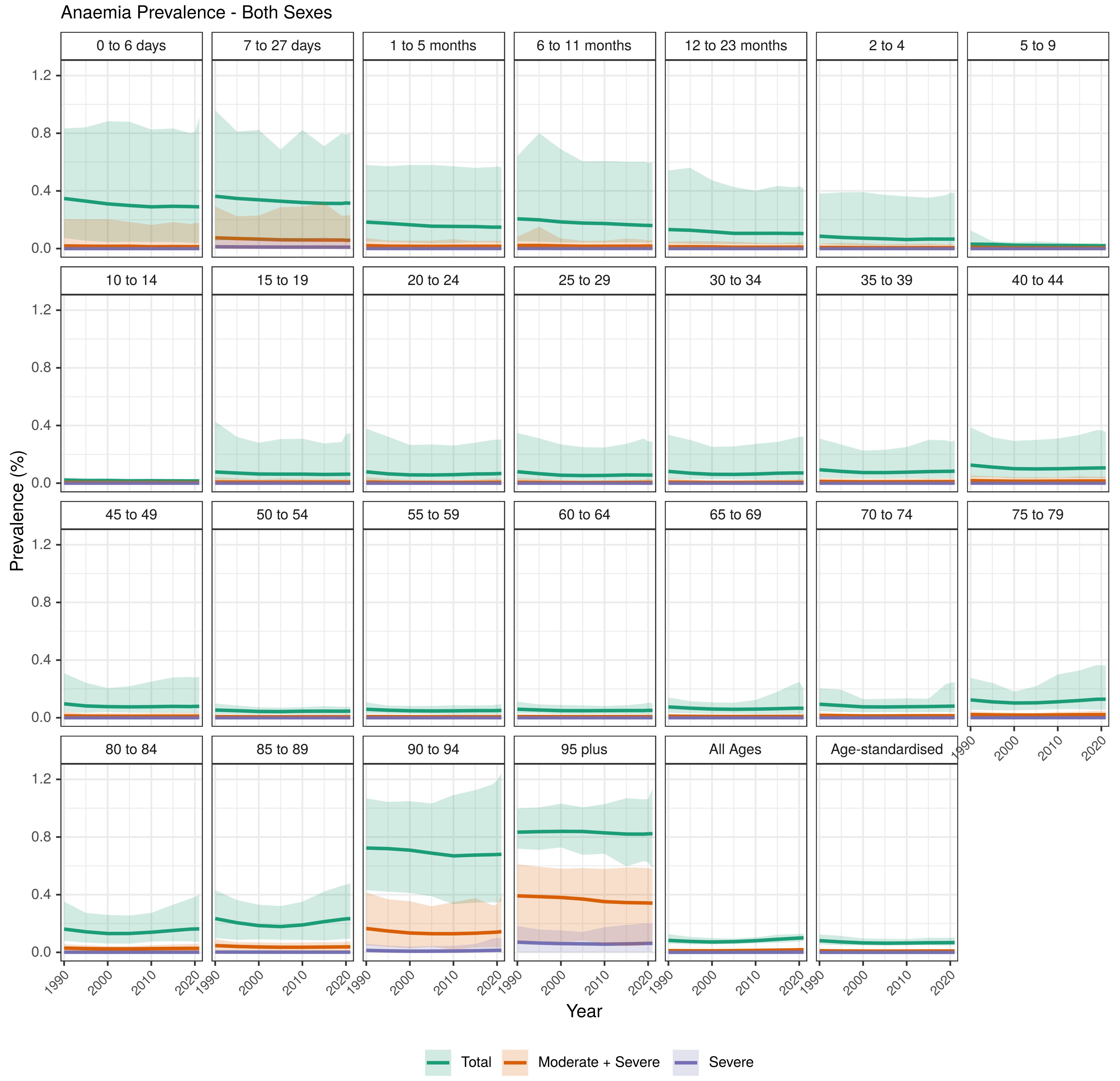

Anaemia Prevalence - Both Sexes
0 to 6 days
7 to 27 days
1 to 5 months
6 to 11 months
12 to 23 months
2 to 4
5 to 9
10 to 14
15 to 19
20 to 24
25 to 29
30 to 34
35 to 39
40 to 44
45 to 49
50 to 54
55 to 59
60 to 64
65 to 69
70 to 74
75 to 79
80 to 84
85 to 89
90 to 94
95 plus
All Ages
Age-standardised
Prevalence (%)
Year
Total
Moderate + Severe
Severe

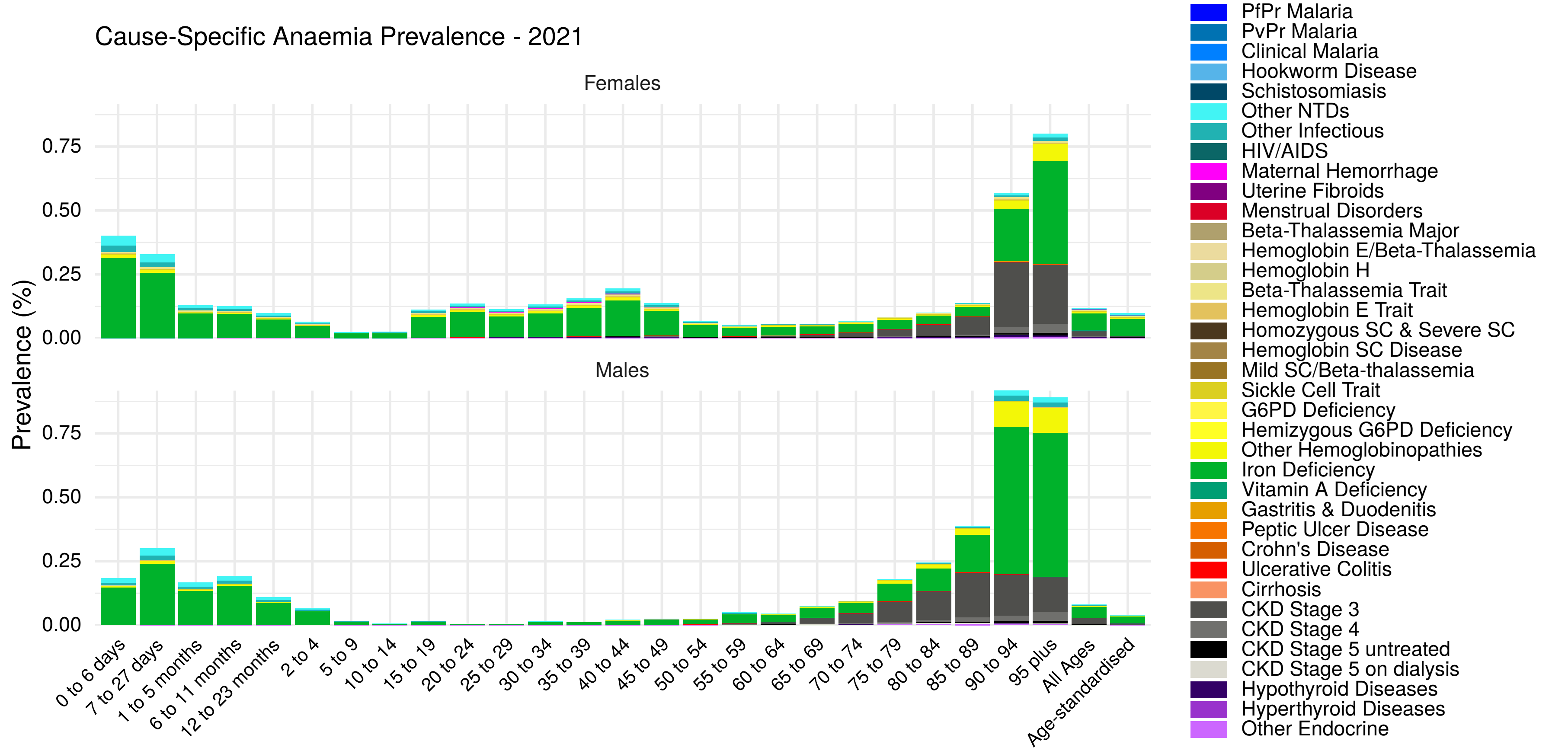

Cause-Specific Anaemia Prevalence - 2021
Females
Males
Prevalence (%)
PfPr Malaria
PvPr Malaria
Clinical Malaria
Hookworm Disease
Schistosomiasis
Other NTDs
Other Infectious
HIV/AIDS
Maternal Hemorrhage
Uterine Fibroids
Menstrual Disorders
Beta-Thalassemia Major
Hemoglobin E/Beta-Thalassemia
Hemoglobin H
Beta-Thalassemia Trait
Hemoglobin E Trait
Homozygous SC & Severe SC
Hemoglobin SC Disease
Mild SC/Beta-thalassemia
Sickle Cell Trait
G6PD Deficiency
Hemizygous G6PD Deficiency
Other Hemoglobinopathies
Iron Deficiency
Vitamin A Deficiency
Gastritis & Duodenitis
Peptic Ulcer Disease
Crohn's Disease
Ulcerative Colitis
Cirrhosis
CKD Stage 3
CKD Stage 4
CKD Stage 5 untreated
CKD Stage 5 on dialysis
Hypothyroid Diseases
Hyperthyroid Diseases
Other Endocrine

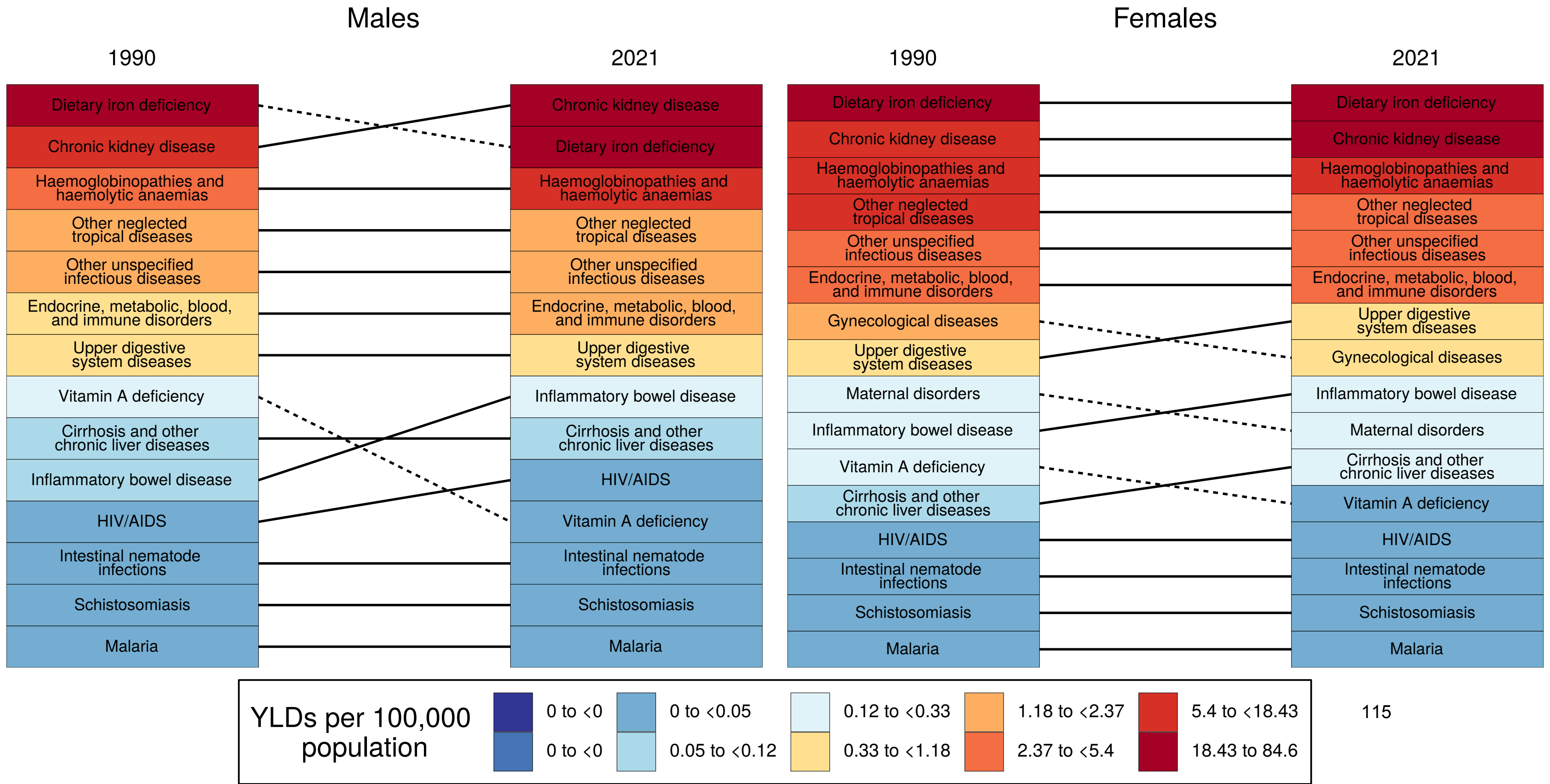

Males
Females
1990
2021
Dietary iron deficiency
Chronic kidney disease
Haemoglobinopathies and haemolytic anaemias
Other neglected tropical diseases
Other unspecified infectious diseases
Endocrine, metabolic, blood, and immune disorders
Upper digestive system diseases
Vitamin A deficiency
Cirrhosis and other chronic liver diseases
Inflammatory bowel disease
HIV/AIDS
Intestinal nematode infections
Schistosomiasis
Malaria
Gynecological diseases
Maternal disorders
YLDs per 100,000 population
0 to <0
0 to <0.05
0.05 to <0.12
0.12 to <0.33
0.33 to <1.18
1.18 to <2.37
2.37 to <5.4
5.4 to <18.43
18.43 to 84.6

Nagasaki

Anaemia Prevalence - Both Sexes

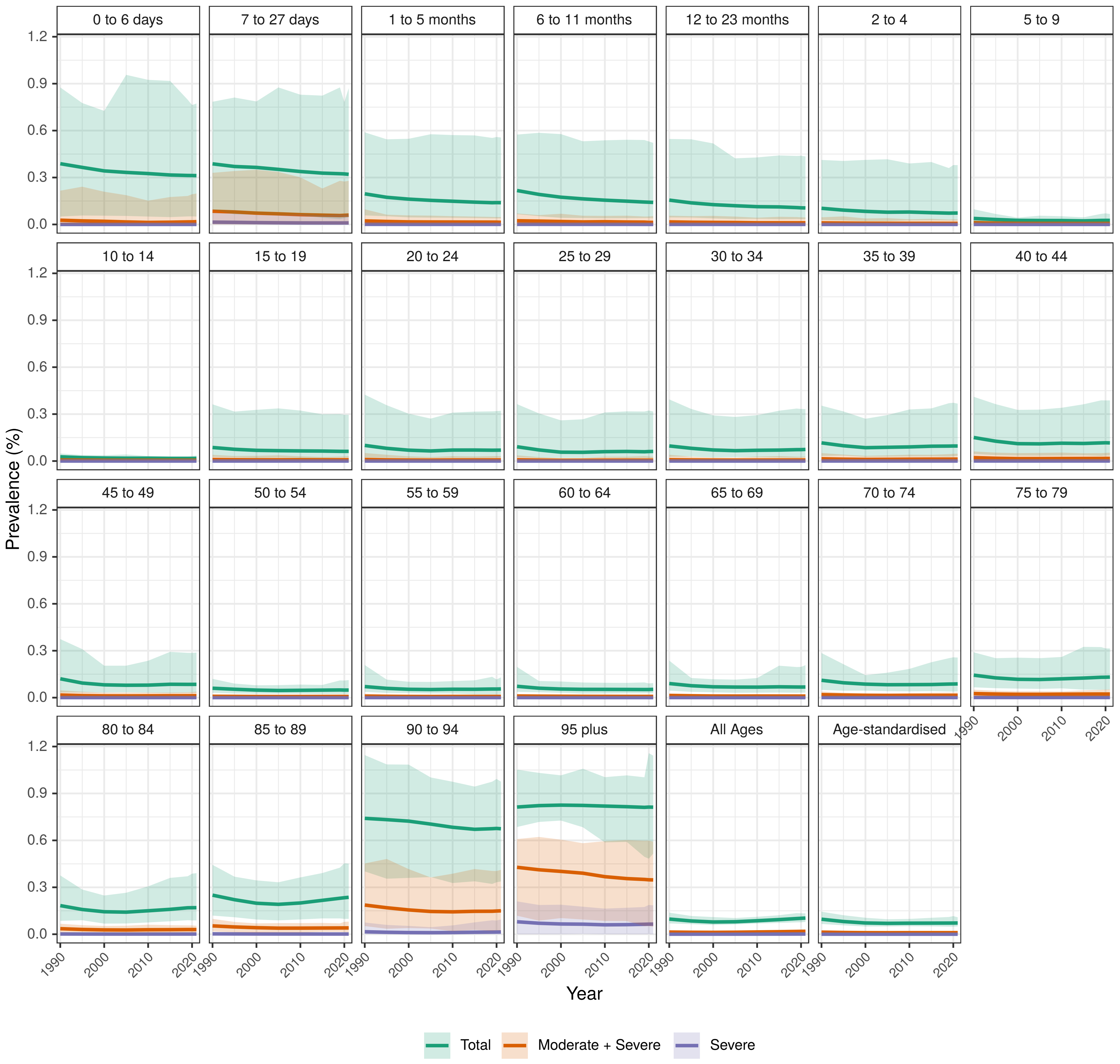

Cause-Specific Anaemia Prevalence - 2021

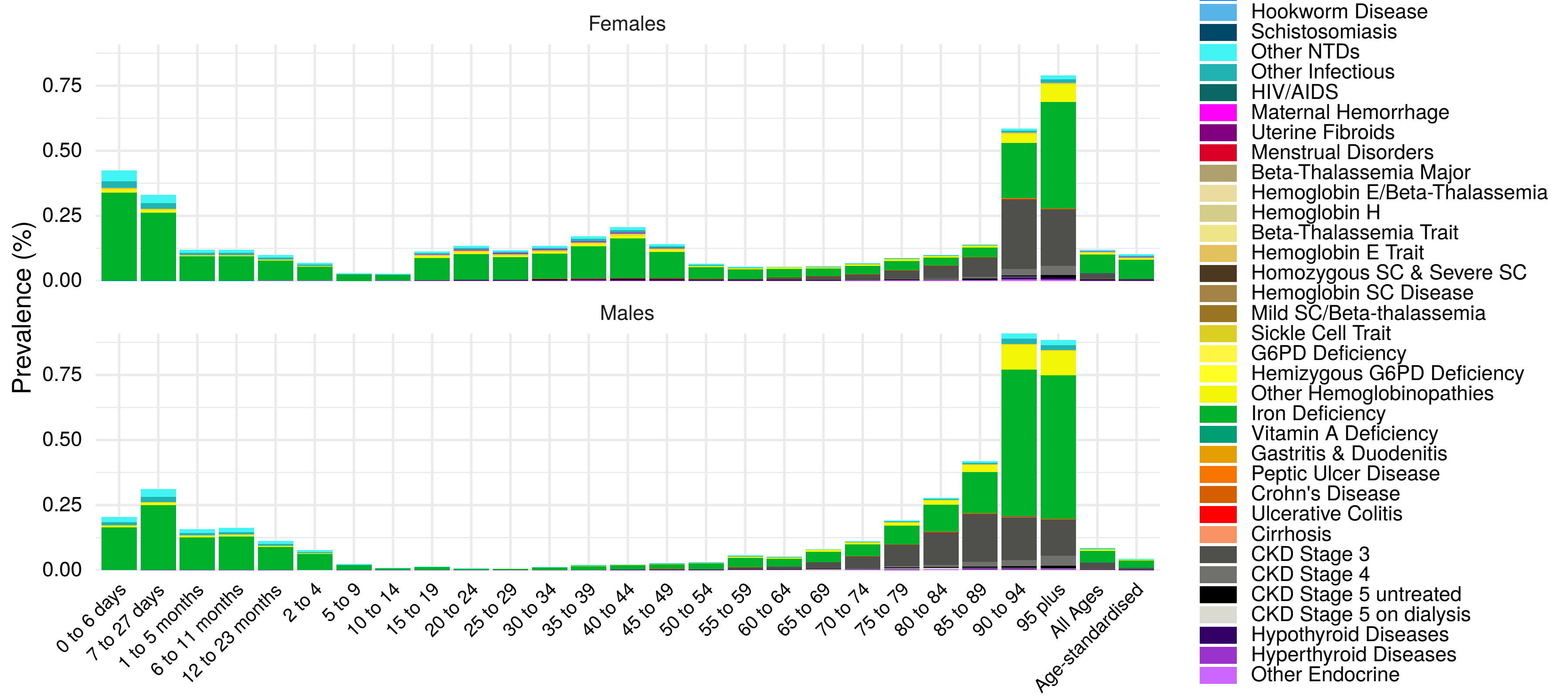

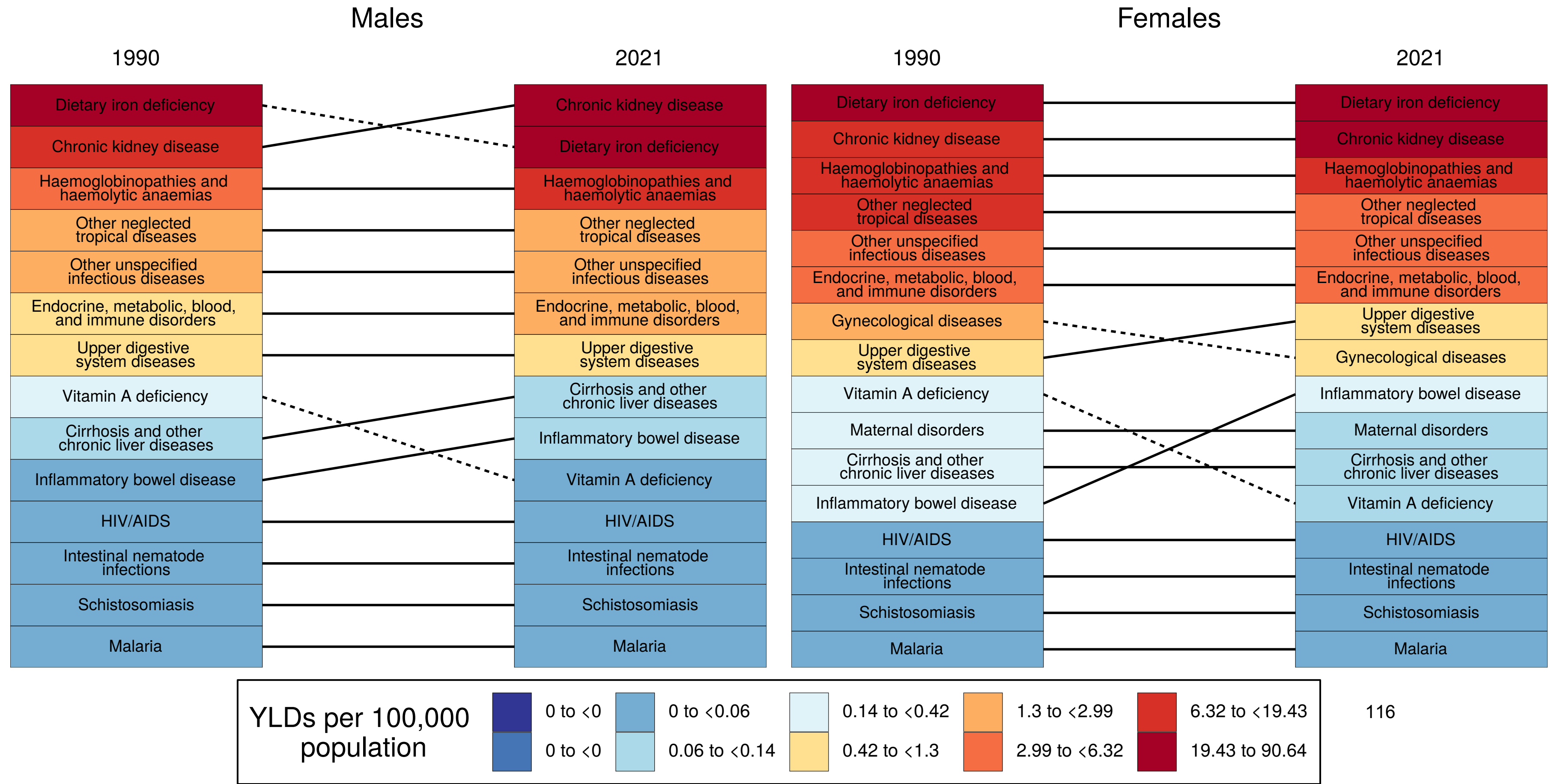

Nara

Anaemia Prevalence - Both Sexes

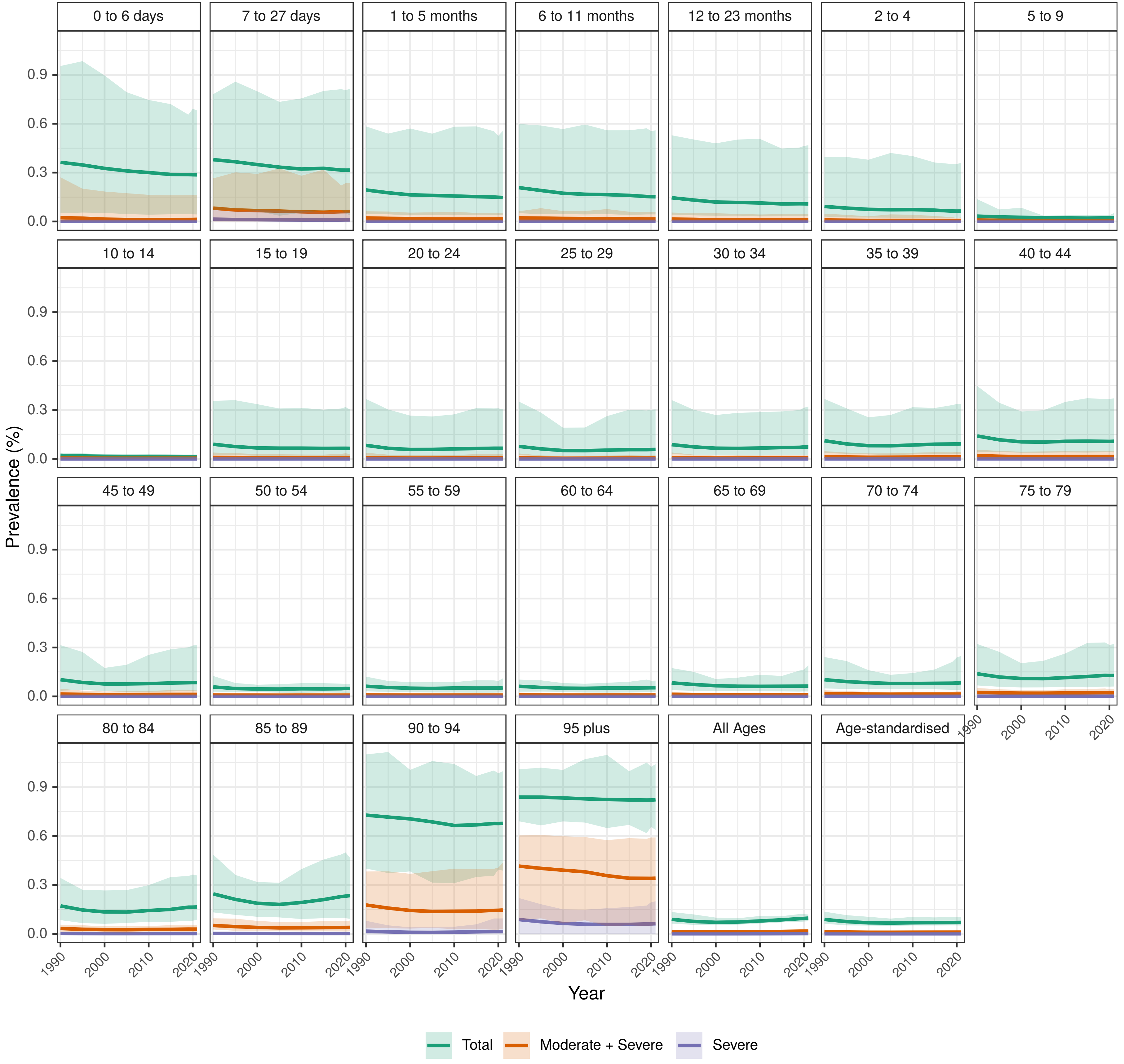

Cause-Specific Anaemia Prevalence - 2021

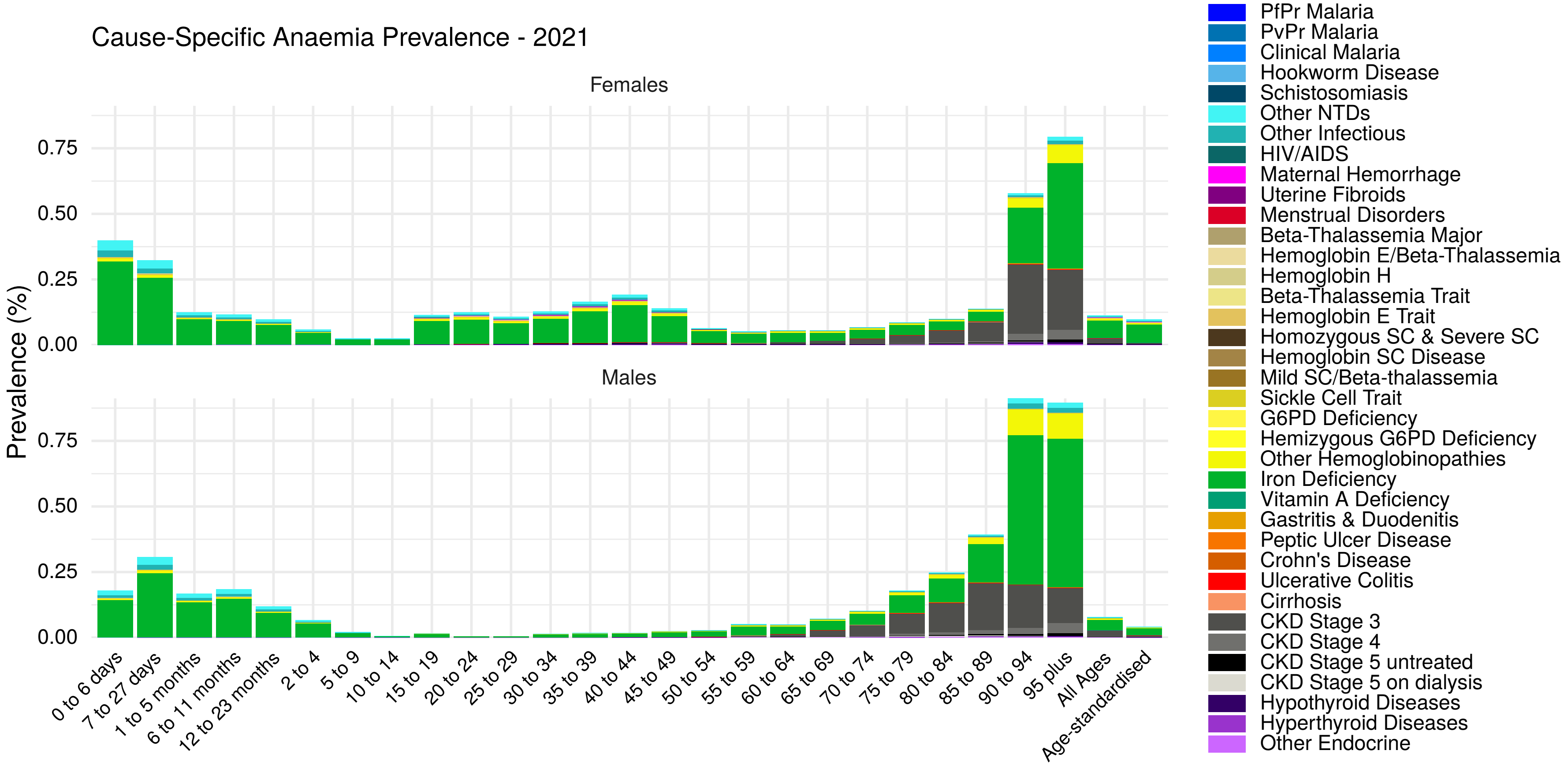

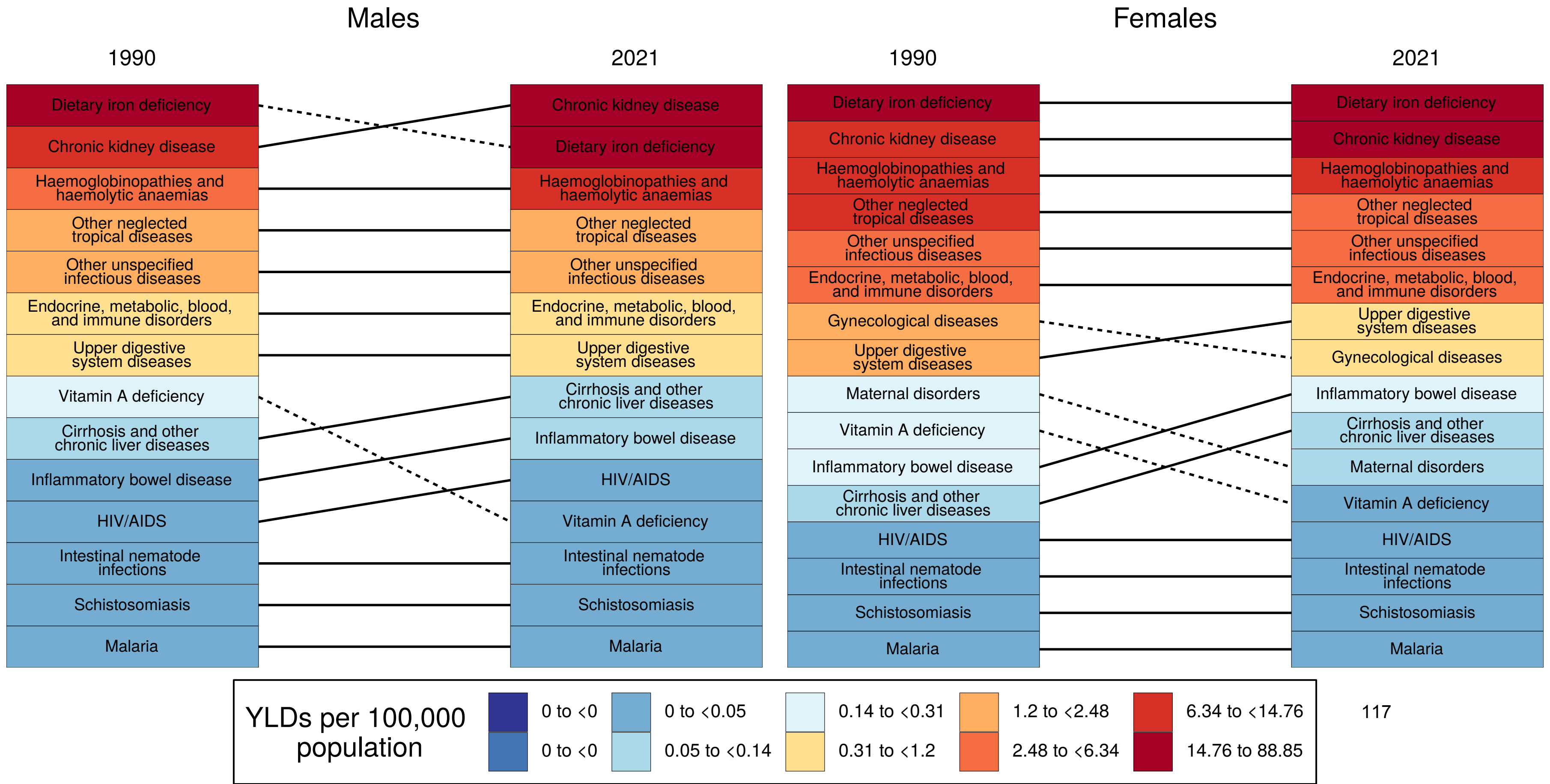

Niigata

Anaemia Prevalence - Both Sexes

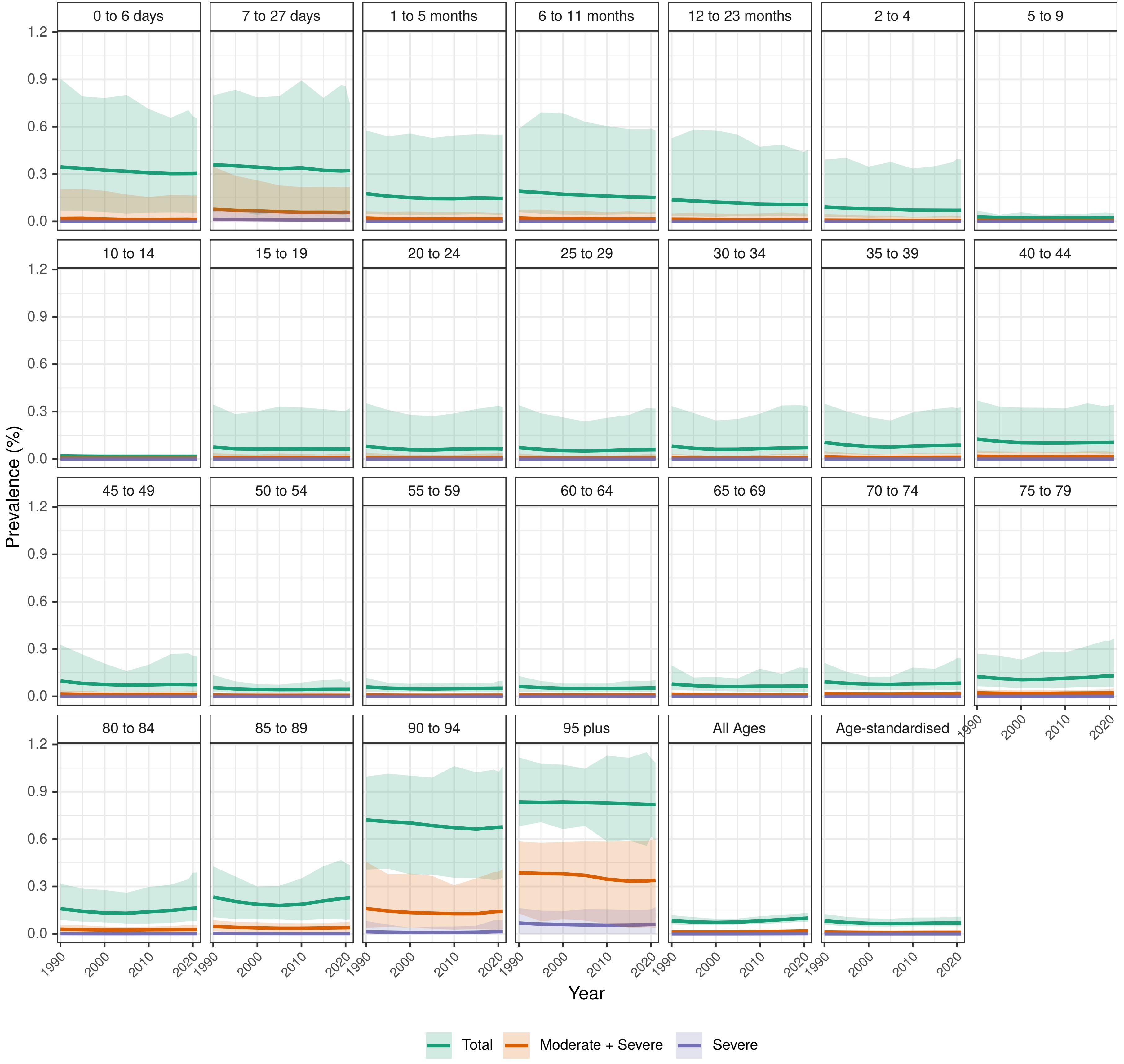

Cause-Specific Anaemia Prevalence - 2021

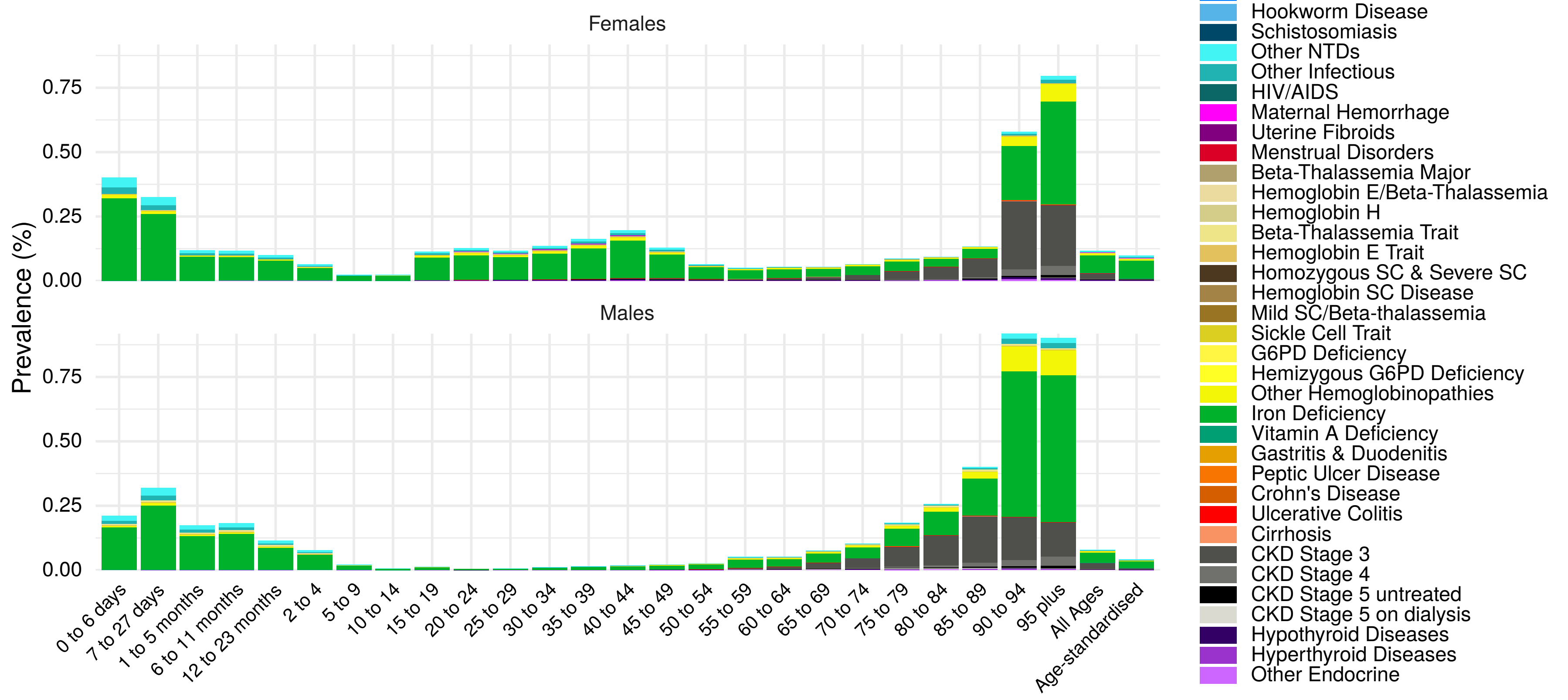

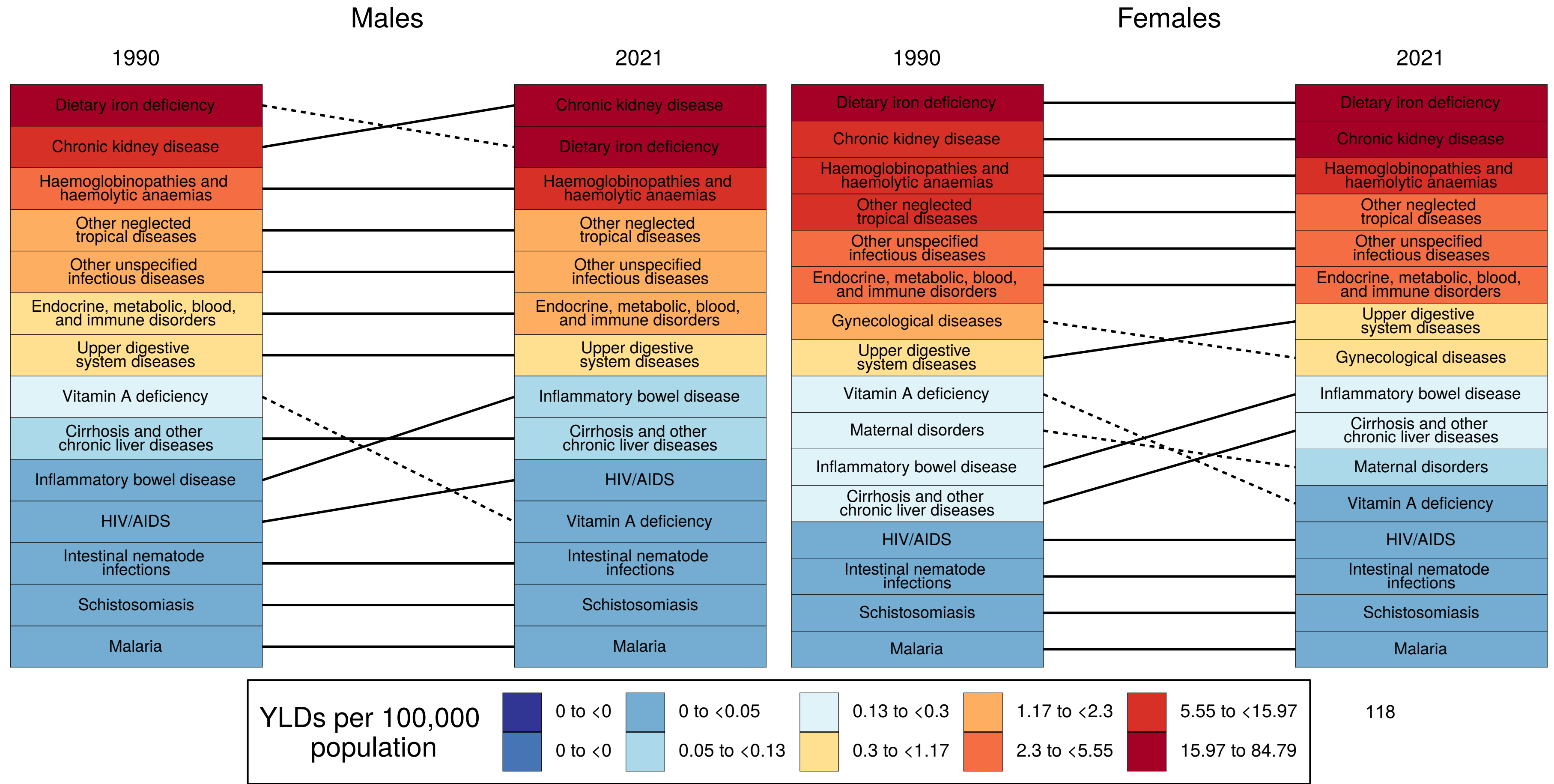

Ōita

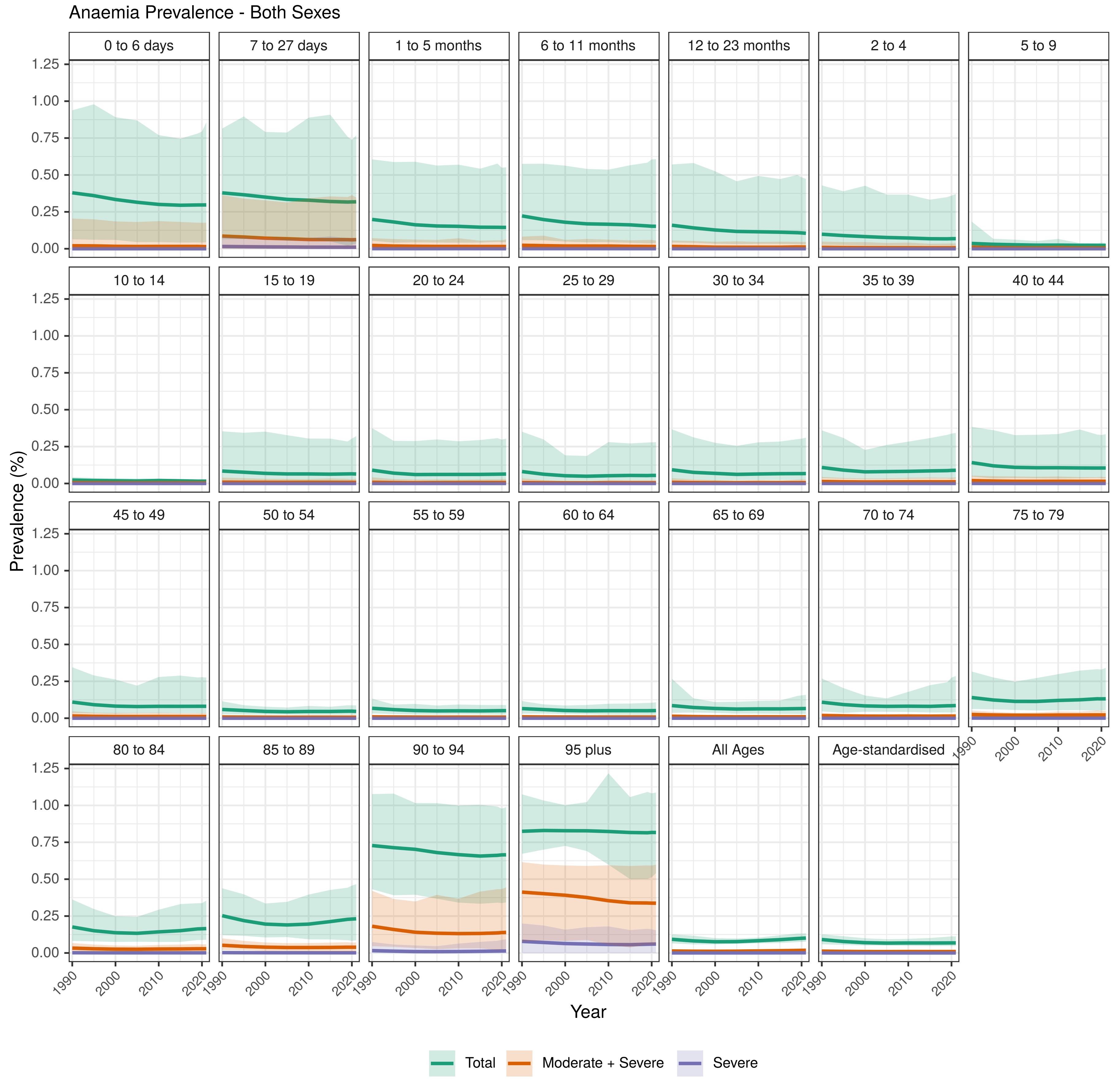

Anaemia Prevalence - Both Sexes
0 to 6 days
7 to 27 days
1 to 5 months
6 to 11 months
12 to 23 months
2 to 4
5 to 9
10 to 14
15 to 19
20 to 24
25 to 29
30 to 34
35 to 39
40 to 44
45 to 49
50 to 54
55 to 59
60 to 64
65 to 69
70 to 74
75 to 79
80 to 84
85 to 89
90 to 94
95 plus
All Ages
Age-standardised
Prevalence (%)
Year
Total
Moderate + Severe
Severe

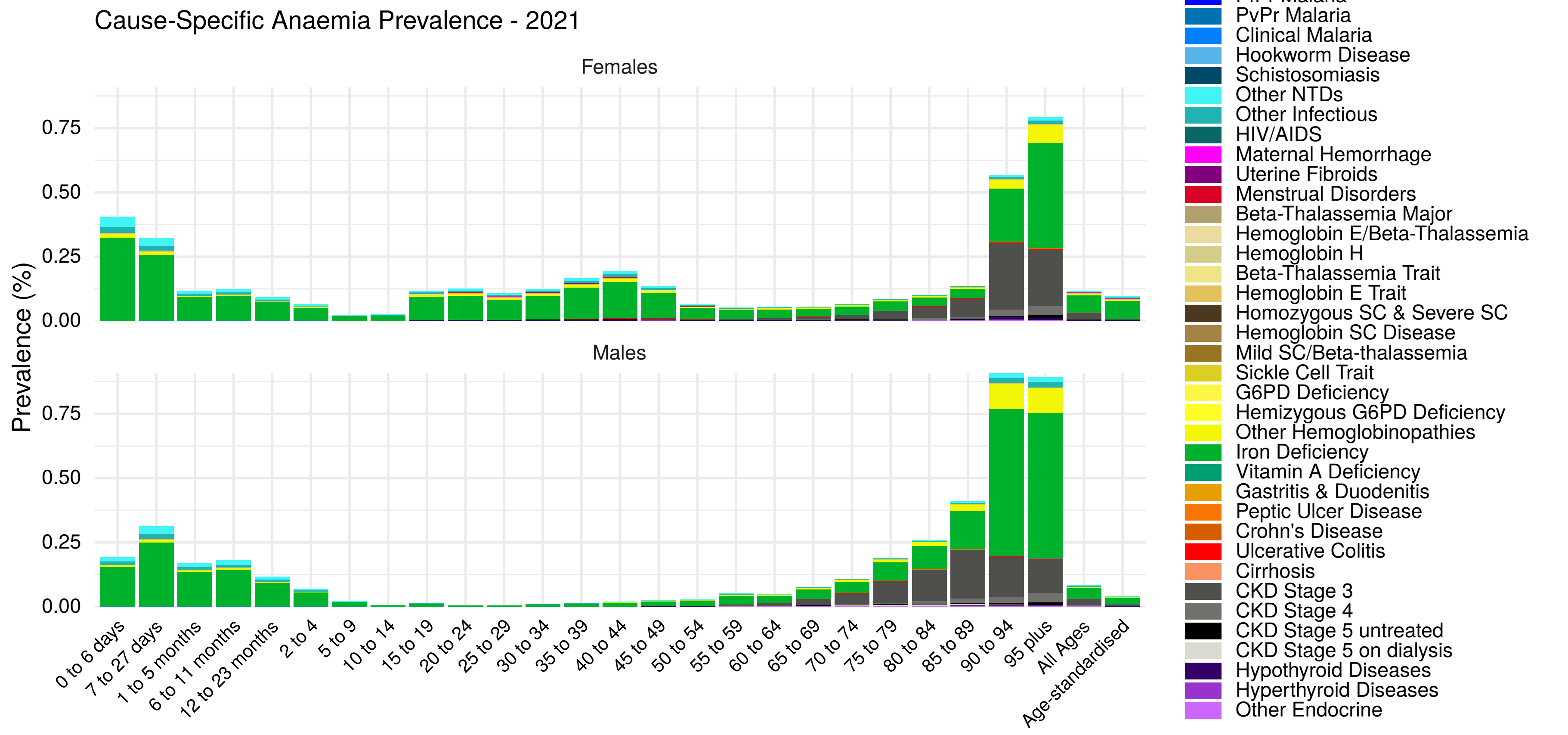

Cause-Specific Anaemia Prevalence - 2021
Females
Males
Prevalence (%)
PfPr Malaria
PvPr Malaria
Clinical Malaria
Hookworm Disease
Schistosomiasis
Other NTDs
Other Infectious
HIV/AIDS
Maternal Hemorrhage
Uterine Fibroids
Menstrual Disorders
Beta-Thalassemia Major
Hemoglobin E/Beta-Thalassemia
Hemoglobin H
Beta-Thalassemia Trait
Hemoglobin E Trait
Homozygous SC & Severe SC
Hemoglobin SC Disease
Mild SC/Beta-thalassemia
Sickle Cell Trait
G6PD Deficiency
Hemizygous G6PD Deficiency
Other Hemoglobinopathies
Iron Deficiency
Vitamin A Deficiency
Gastritis & Duodenitis
Peptic Ulcer Disease
Crohn's Disease
Ulcerative Colitis
Cirrhosis
CKD Stage 3
CKD Stage 4
CKD Stage 5 untreated
CKD Stage 5 on dialysis
Hypothyroid Diseases
Hyperthyroid Diseases
Other Endocrine

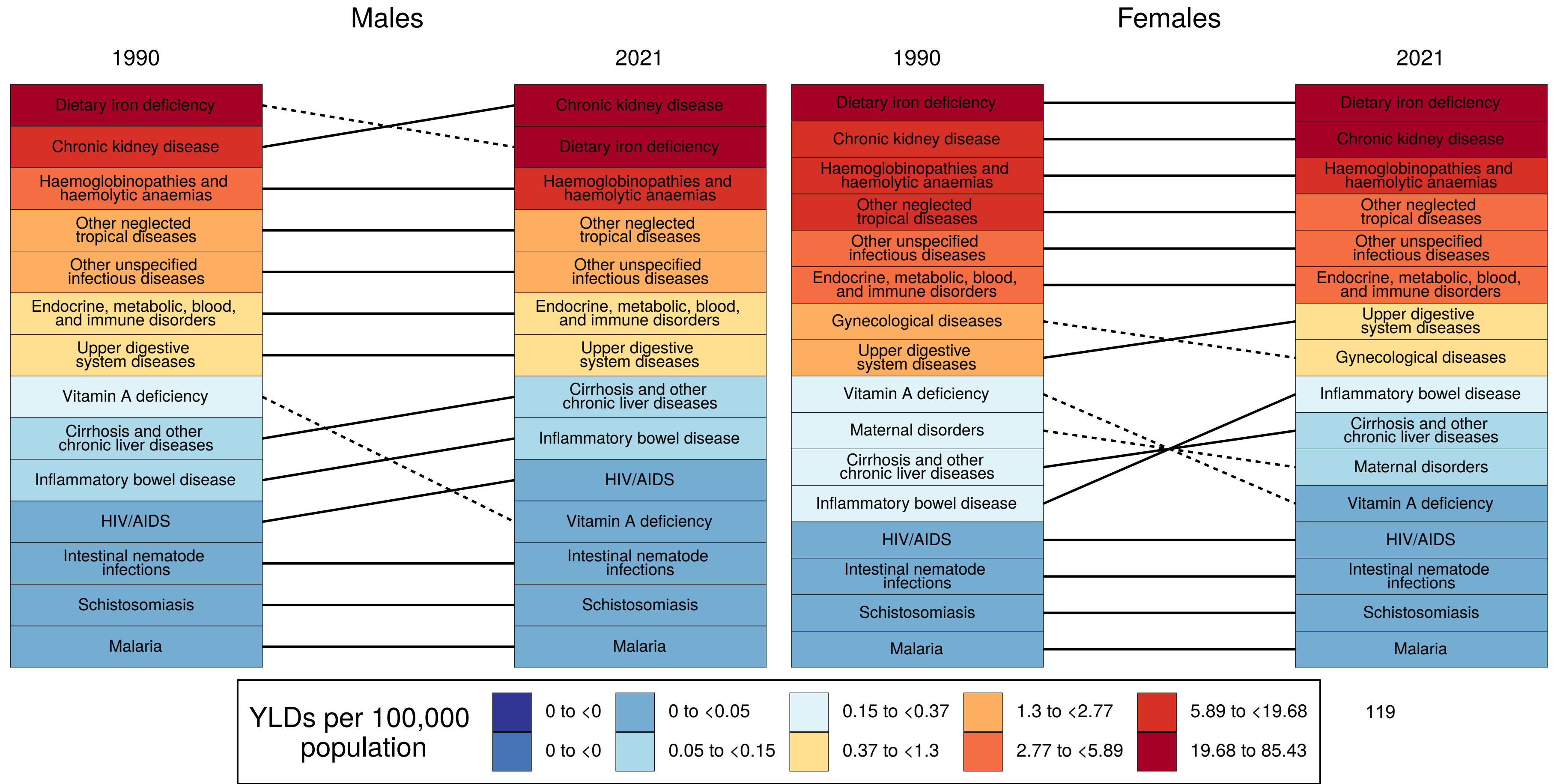

Males
Females
1990
2021
Dietary iron deficiency
Chronic kidney disease
Haemoglobinopathies and haemolytic anaemias
Other neglected tropical diseases
Other unspecified infectious diseases
Endocrine, metabolic, blood, and immune disorders
Upper digestive system diseases
Vitamin A deficiency
Cirrhosis and other chronic liver diseases
Inflammatory bowel disease
HIV/AIDS
Intestinal nematode infections
Schistosomiasis
Malaria
Gynecological diseases
Maternal disorders
YLDs per 100,000 population
0 to <0
0 to <0.05
0.05 to <0.15
0.15 to <0.37
0.37 to <1.3
1.3 to <2.77
2.77 to <5.89
5.89 to <19.68
19.68 to 85.43

Okayama

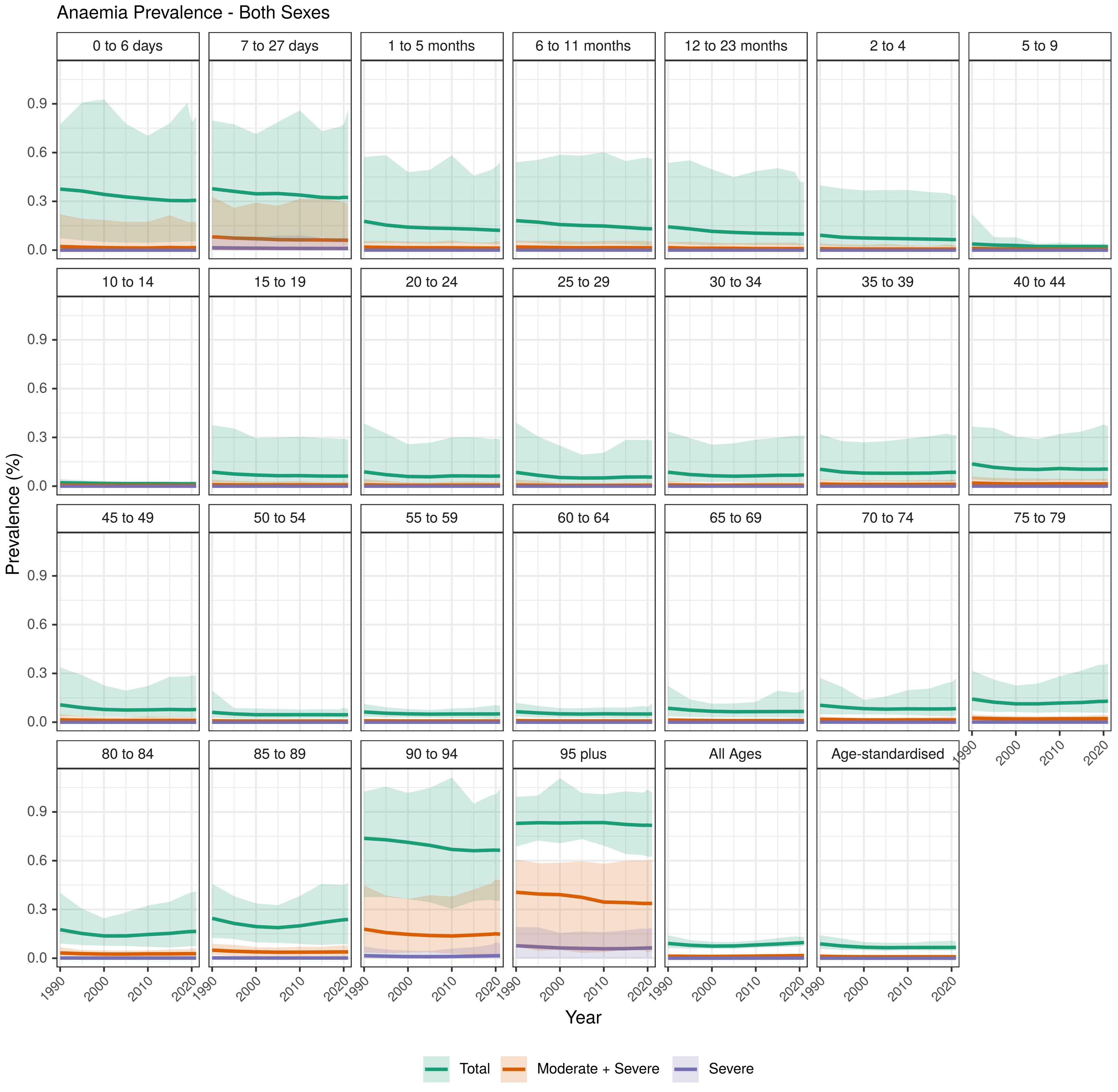

Anaemia Prevalence - Both Sexes
0 to 6 days
7 to 27 days
1 to 5 months
6 to 11 months
12 to 23 months
2 to 4
5 to 9
10 to 14
15 to 19
20 to 24
25 to 29
30 to 34
35 to 39
40 to 44
45 to 49
50 to 54
55 to 59
60 to 64
65 to 69
70 to 74
75 to 79
80 to 84
85 to 89
90 to 94
95 plus
All Ages
Age-standardised
Prevalence (%)
0.0
0.3
0.6
0.9
1990
2000
2010
2020
Year
Total
Moderate + Severe
Severe

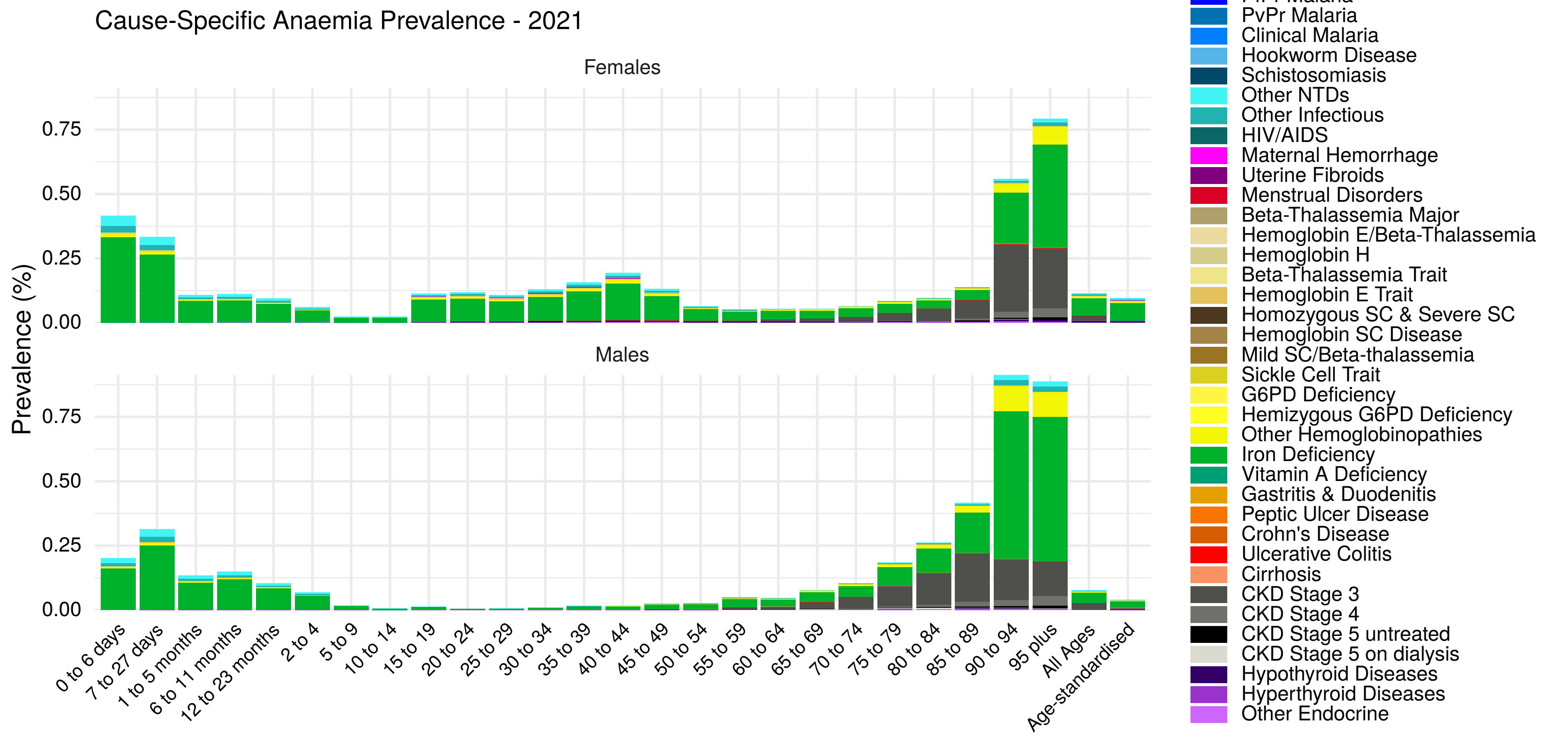

Cause-Specific Anaemia Prevalence - 2021
Females
Males
Prevalence (%)
0.00
0.25
0.50
0.75
0 to 6 days
7 to 27 days
1 to 5 months
6 to 11 months
12 to 23 months
2 to 4
5 to 9
10 to 14
15 to 19
20 to 24
25 to 29
30 to 34
35 to 39
40 to 44
45 to 49
50 to 54
55 to 59
60 to 64
65 to 69
70 to 74
75 to 79
80 to 84
85 to 89
90 to 94
95 plus
All Ages
Age-standardised
PfPr Malaria
PvPr Malaria
Clinical Malaria
Hookworm Disease
Schistosomiasis
Other NTDs
Other Infectious
HIV/AIDS
Maternal Hemorrhage
Uterine Fibroids
Menstrual Disorders
Beta-Thalassemia Major
Hemoglobin E/Beta-Thalassemia
Hemoglobin H
Beta-Thalassemia Trait
Hemoglobin E Trait
Homozygous SC & Severe SC
Hemoglobin SC Disease
Mild SC/Beta-thalassemia
Sickle Cell Trait
G6PD Deficiency
Hemizygous G6PD Deficiency
Other Hemoglobinopathies
Iron Deficiency
Vitamin A Deficiency
Gastritis & Duodenitis
Peptic Ulcer Disease
Crohn's Disease
Ulcerative Colitis
Cirrhosis
CKD Stage 3
CKD Stage 4
CKD Stage 5 untreated
CKD Stage 5 on dialysis
Hypothyroid Diseases
Hyperthyroid Diseases
Other Endocrine

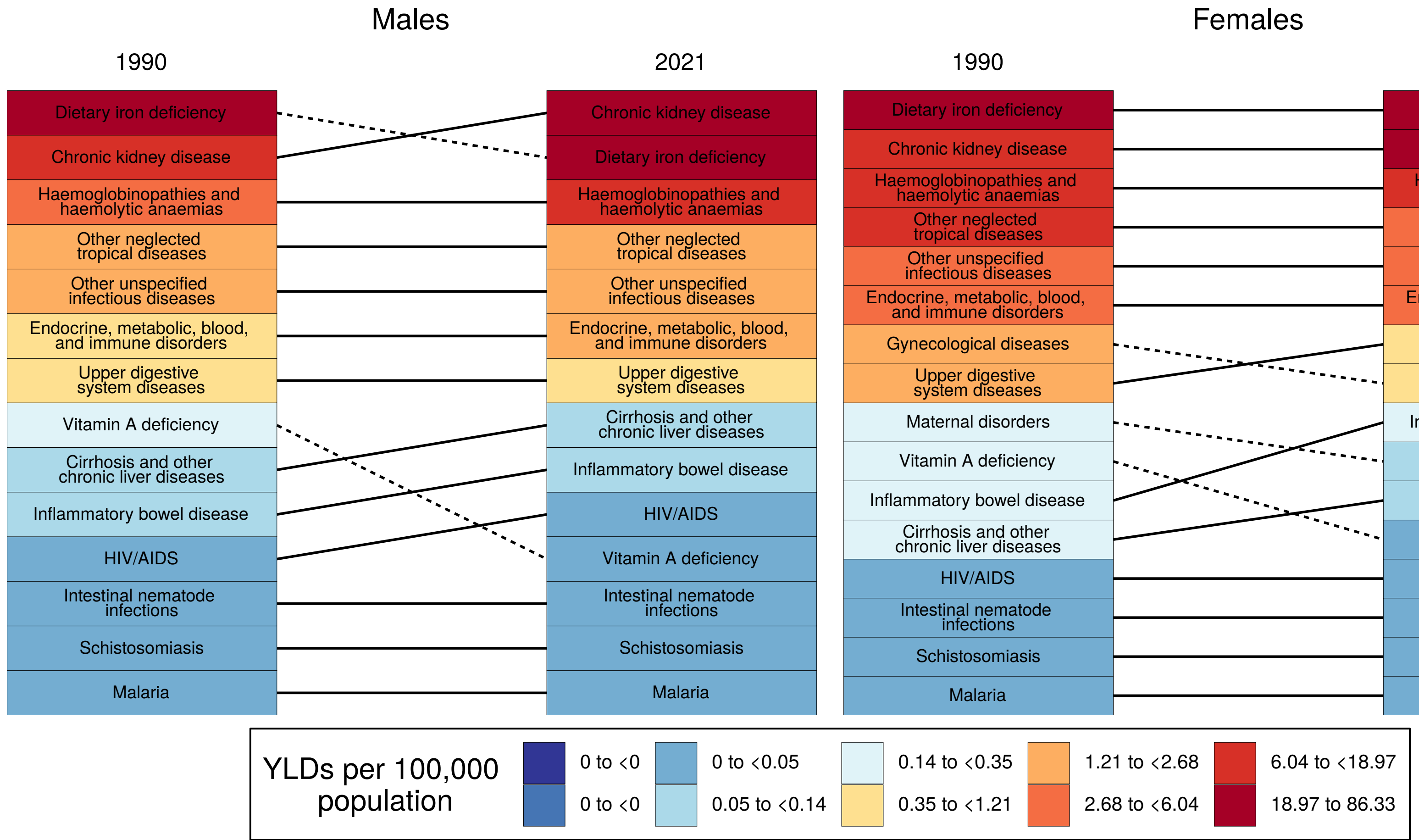

Males
Females
1990
2021
Dietary iron deficiency
Chronic kidney disease
Haemoglobinopathies and haemolytic anaemias
Other neglected tropical diseases
Other unspecified infectious diseases
Endocrine, metabolic, blood, and immune disorders
Upper digestive system diseases
Vitamin A deficiency
Cirrhosis and other chronic liver diseases
Inflammatory bowel disease
HIV/AIDS
Intestinal nematode infections
Schistosomiasis
Malaria
Gynecological diseases
Maternal disorders
YLDs per 100,000 population
0 to <0
0 to <0.05
0.05 to <0.14
0.14 to <0.35
0.35 to <1.21
1.21 to <2.68
2.68 to <6.04
6.04 to <18.97
18.97 to 86.33

120

Okinawa

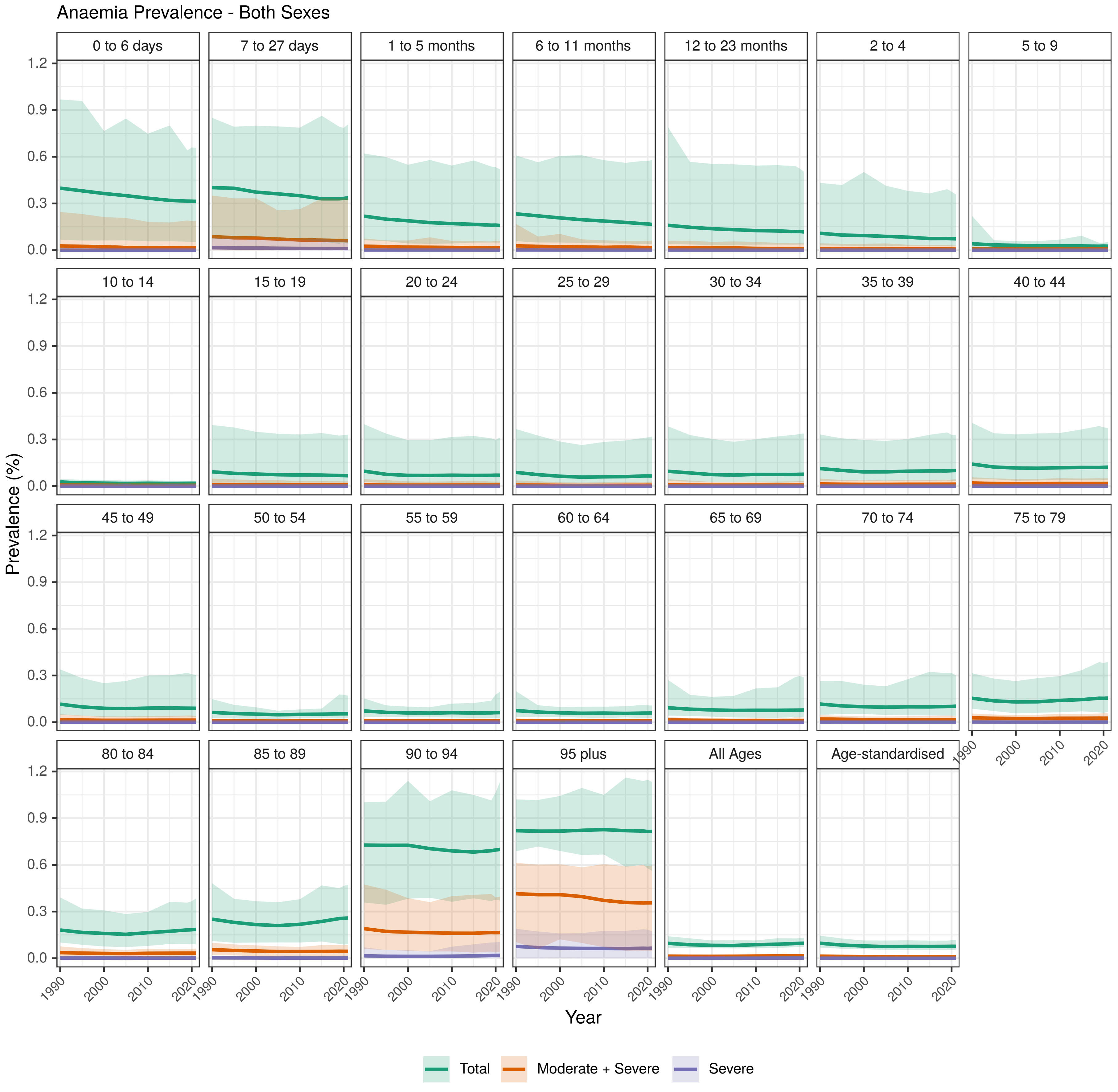

Anaemia Prevalence - Both Sexes
0 to 6 days
7 to 27 days
1 to 5 months
6 to 11 months
12 to 23 months
2 to 4
5 to 9
10 to 14
15 to 19
20 to 24
25 to 29
30 to 34
35 to 39
40 to 44
45 to 49
50 to 54
55 to 59
60 to 64
65 to 69
70 to 74
75 to 79
80 to 84
85 to 89
90 to 94
95 plus
All Ages
Age-standardised
Prevalence (%)
Year
Total
Moderate + Severe
Severe

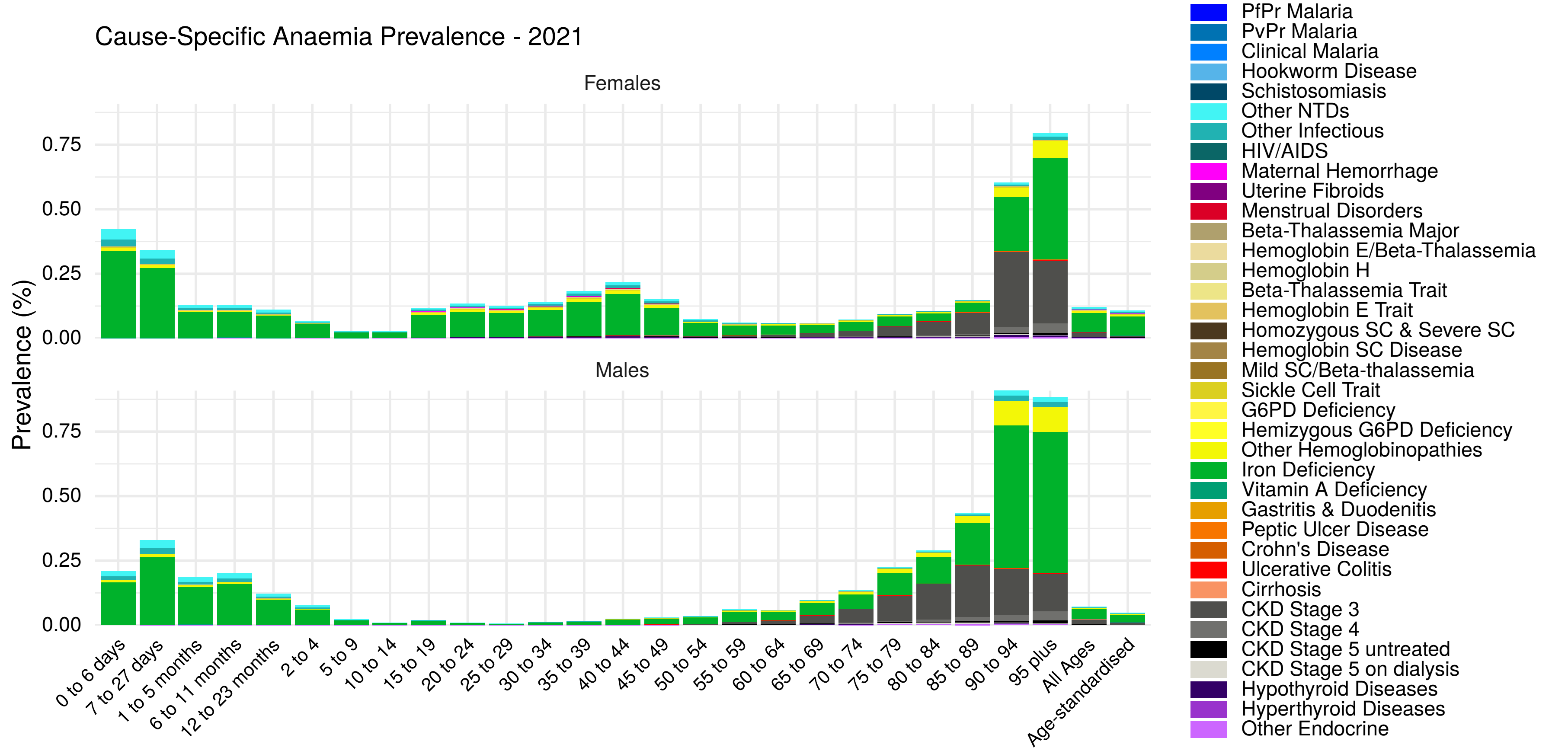

Cause-Specific Anaemia Prevalence - 2021
Females
Males
Prevalence (%)
PfPr Malaria
PvPr Malaria
Clinical Malaria
Hookworm Disease
Schistosomiasis
Other NTDs
Other Infectious
HIV/AIDS
Maternal Hemorrhage
Uterine Fibroids
Menstrual Disorders
Beta-Thalassemia Major
Hemoglobin E/Beta-Thalassemia
Hemoglobin H
Beta-Thalassemia Trait
Hemoglobin E Trait
Homozygous SC & Severe SC
Hemoglobin SC Disease
Mild SC/Beta-thalassemia
Sickle Cell Trait
G6PD Deficiency
Hemizygous G6PD Deficiency
Other Hemoglobinopathies
Iron Deficiency
Vitamin A Deficiency
Gastritis & Duodenitis
Peptic Ulcer Disease
Crohn's Disease
Ulcerative Colitis
Cirrhosis
CKD Stage 3
CKD Stage 4
CKD Stage 5 untreated
CKD Stage 5 on dialysis
Hypothyroid Diseases
Hyperthyroid Diseases
Other Endocrine

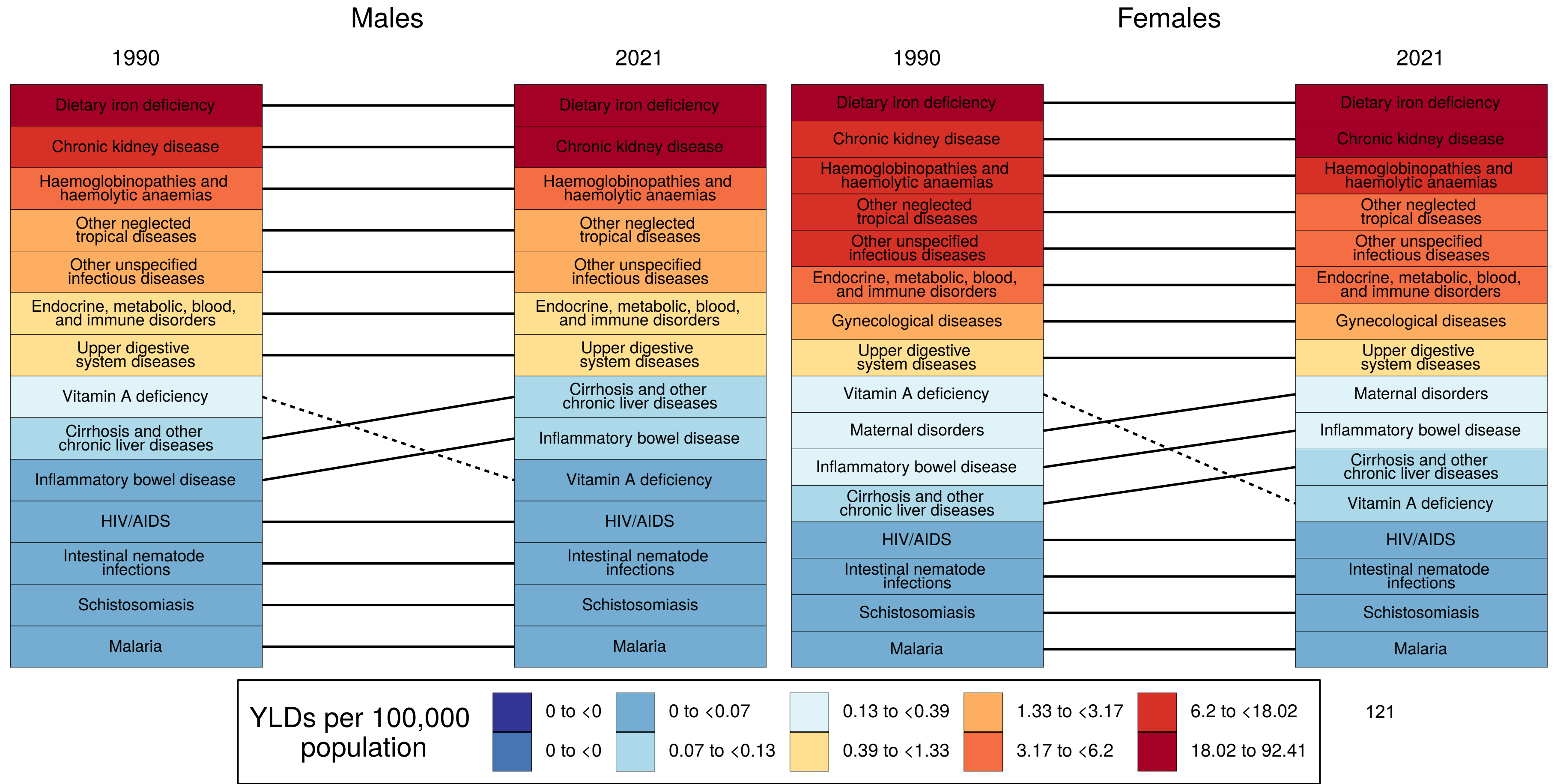

Males
Females
1990
2021
Dietary iron deficiency
Chronic kidney disease
Haemoglobinopathies and haemolytic anaemias
Other neglected tropical diseases
Other unspecified infectious diseases
Endocrine, metabolic, blood, and immune disorders
Upper digestive system diseases
Vitamin A deficiency
Cirrhosis and other chronic liver diseases
Inflammatory bowel disease
HIV/AIDS
Intestinal nematode infections
Schistosomiasis
Malaria
Gynecological diseases
Maternal disorders
YLDs per 100,000 population
0 to <0
0 to <0.07
0.07 to <0.13
0.13 to <0.39
0.39 to <1.33
1.33 to <3.17
3.17 to <6.2
6.2 to <18.02
18.02 to 92.41

Ōsaka

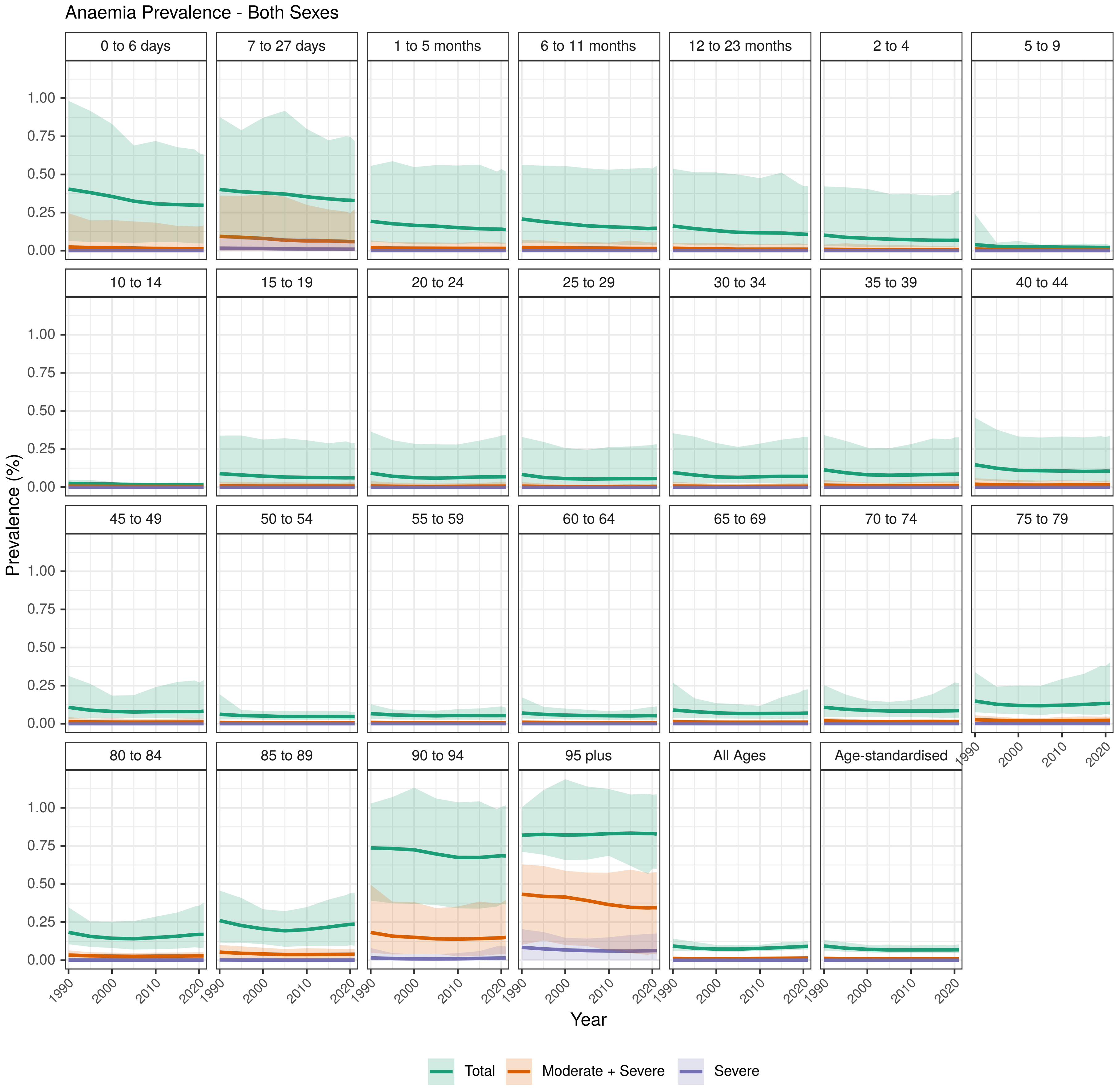

Anaemia Prevalence - Both Sexes
0 to 6 days
7 to 27 days
1 to 5 months
6 to 11 months
12 to 23 months
2 to 4
5 to 9
10 to 14
15 to 19
20 to 24
25 to 29
30 to 34
35 to 39
40 to 44
45 to 49
50 to 54
55 to 59
60 to 64
65 to 69
70 to 74
75 to 79
80 to 84
85 to 89
90 to 94
95 plus
All Ages
Age-standardised
Prevalence (%)
0.00
0.25
0.50
0.75
1.00
1990
2000
2010
2020
Year
Total
Moderate + Severe
Severe

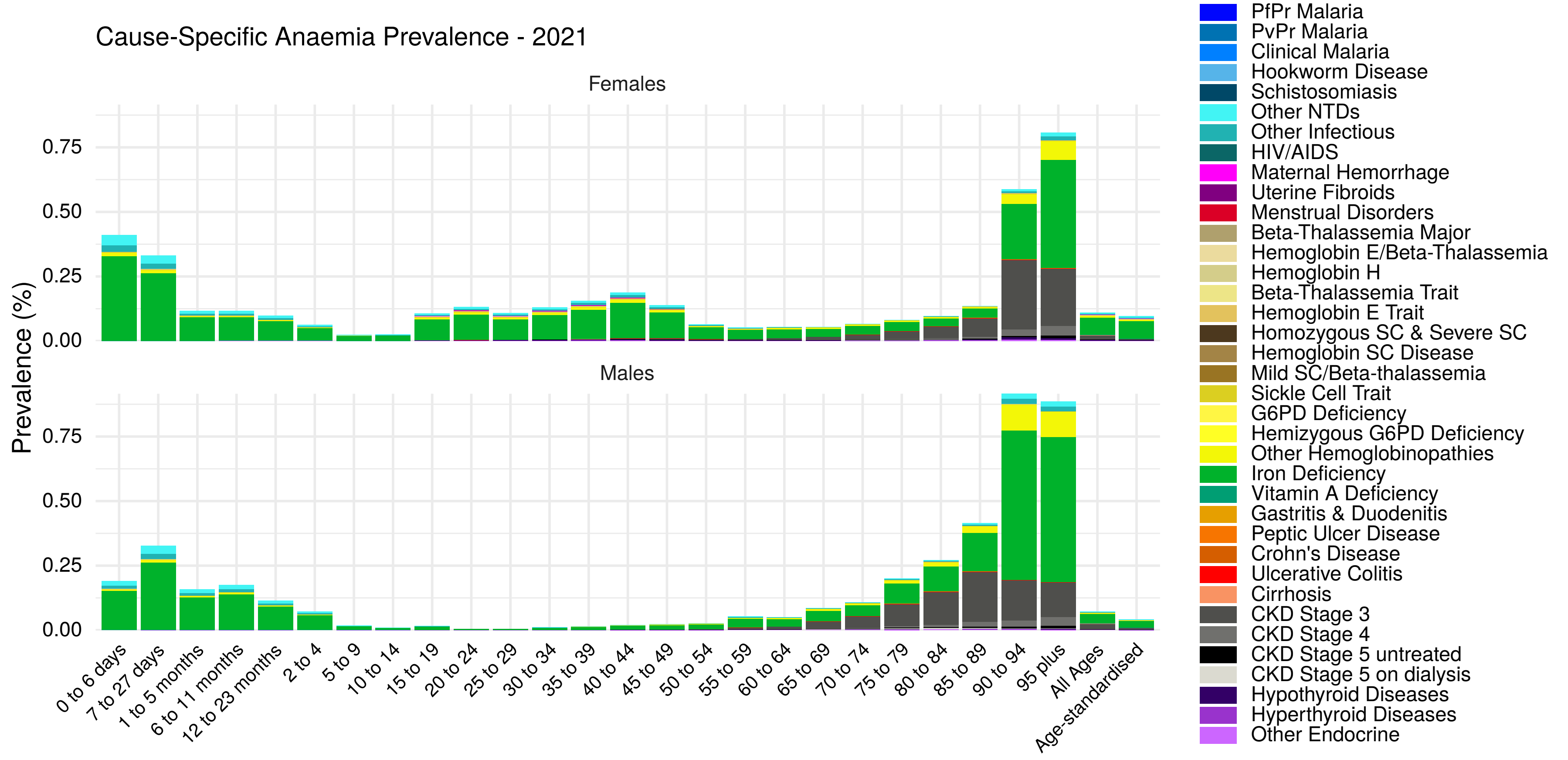

Cause-Specific Anaemia Prevalence - 2021
Females
Males
Prevalence (%)
0.00
0.25
0.50
0.75
0 to 6 days
7 to 27 days
1 to 5 months
6 to 11 months
12 to 23 months
2 to 4
5 to 9
10 to 14
15 to 19
20 to 24
25 to 29
30 to 34
35 to 39
40 to 44
45 to 49
50 to 54
55 to 59
60 to 64
65 to 69
70 to 74
75 to 79
80 to 84
85 to 89
90 to 94
95 plus
All Ages
Age-standardised
PfPr Malaria
PvPr Malaria
Clinical Malaria
Hookworm Disease
Schistosomiasis
Other NTDs
Other Infectious
HIV/AIDS
Maternal Hemorrhage
Uterine Fibroids
Menstrual Disorders
Beta-Thalassemia Major
Hemoglobin E/Beta-Thalassemia
Hemoglobin H
Beta-Thalassemia Trait
Hemoglobin E Trait
Homozygous SC & Severe SC
Hemoglobin SC Disease
Mild SC/Beta-thalassemia
Sickle Cell Trait
G6PD Deficiency
Hemizygous G6PD Deficiency
Other Hemoglobinopathies
Iron Deficiency
Vitamin A Deficiency
Gastritis & Duodenitis
Peptic Ulcer Disease
Crohn's Disease
Ulcerative Colitis
Cirrhosis
CKD Stage 3
CKD Stage 4
CKD Stage 5 untreated
CKD Stage 5 on dialysis
Hypothyroid Diseases
Hyperthyroid Diseases
Other Endocrine

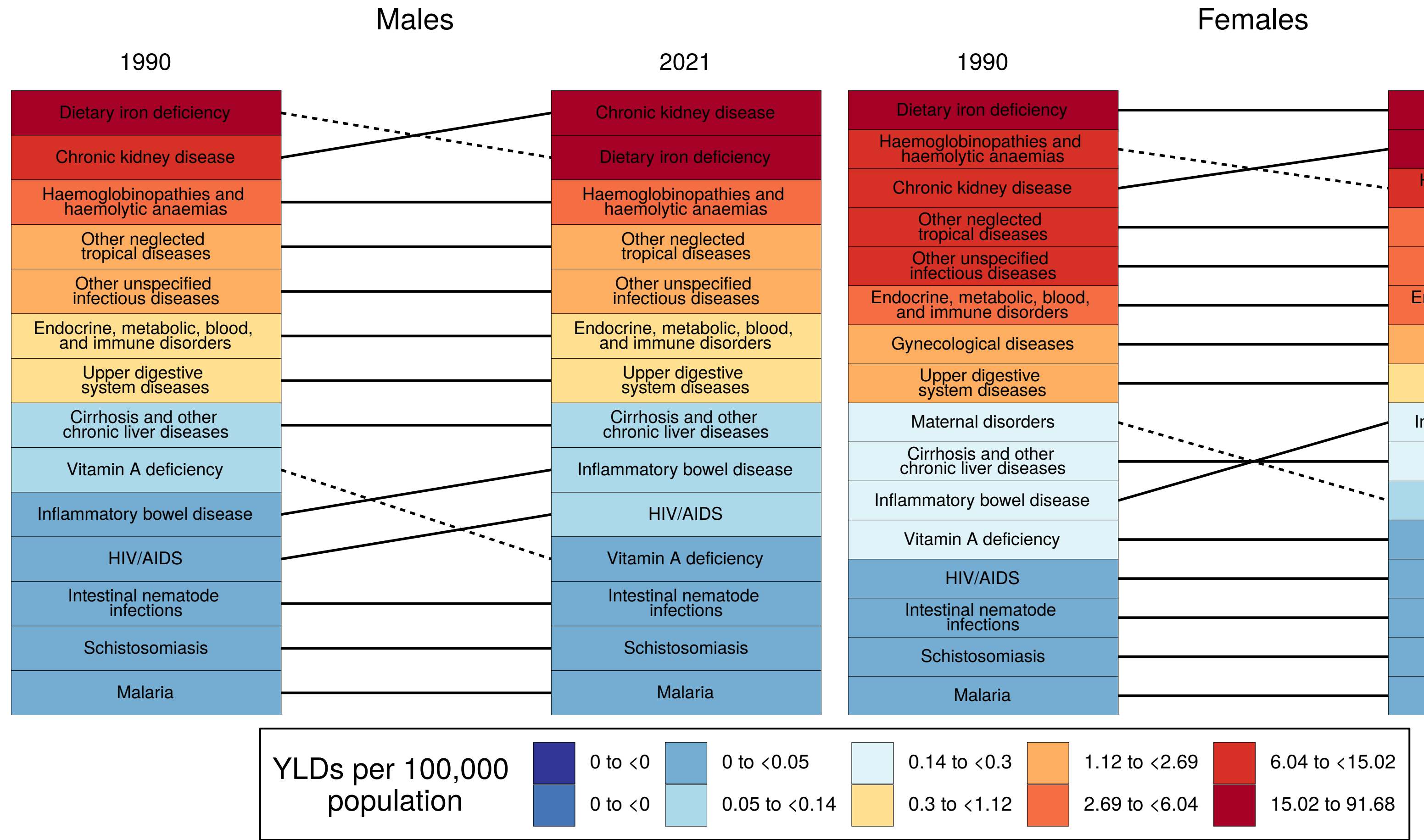

Males
1990
Dietary iron deficiency
Chronic kidney disease
Haemoglobinopathies and haemolytic anaemias
Other neglected tropical diseases
Other unspecified infectious diseases
Endocrine, metabolic, blood, and immune disorders
Upper digestive system diseases
Cirrhosis and other chronic liver diseases
Vitamin A deficiency
Inflammatory bowel disease
HIV/AIDS
Intestinal nematode infections
Schistosomiasis
Malaria
2021
Chronic kidney disease
Dietary iron deficiency
Haemoglobinopathies and haemolytic anaemias
Other neglected tropical diseases
Other unspecified infectious diseases
Endocrine, metabolic, blood, and immune disorders
Upper digestive system diseases
Cirrhosis and other chronic liver diseases
Inflammatory bowel disease
HIV/AIDS
Vitamin A deficiency
Intestinal nematode infections
Schistosomiasis
Malaria
Females
1990
Dietary iron deficiency
Haemoglobinopathies and haemolytic anaemias
Chronic kidney disease
Other neglected tropical diseases
Other unspecified infectious diseases
Endocrine, metabolic, blood, and immune disorders
Gynecological diseases
Upper digestive system diseases
Maternal disorders
Cirrhosis and other chronic liver diseases
Inflammatory bowel disease
Vitamin A deficiency
HIV/AIDS
Intestinal nematode infections
Schistosomiasis
Malaria
2021
Dietary iron deficiency
Chronic kidney disease
Haemoglobinopathies and haemolytic anaemias
Other neglected tropical diseases
Other unspecified infectious diseases
Endocrine, metabolic, blood, and immune disorders
Gynecological diseases
Upper digestive system diseases
Inflammatory bowel disease
Cirrhosis and other chronic liver diseases
Maternal disorders
Vitamin A deficiency
HIV/AIDS
Intestinal nematode infections
Schistosomiasis
Malaria
YLDs per 100,000 population
0 to <0
0 to <0
0 to <0.05
0.05 to <0.14
0.14 to <0.3
0.3 to <1.12
1.12 to <2.69
2.69 to <6.04
6.04 to <15.02
15.02 to 91.68

Saga

Anaemia Prevalence - Both Sexes

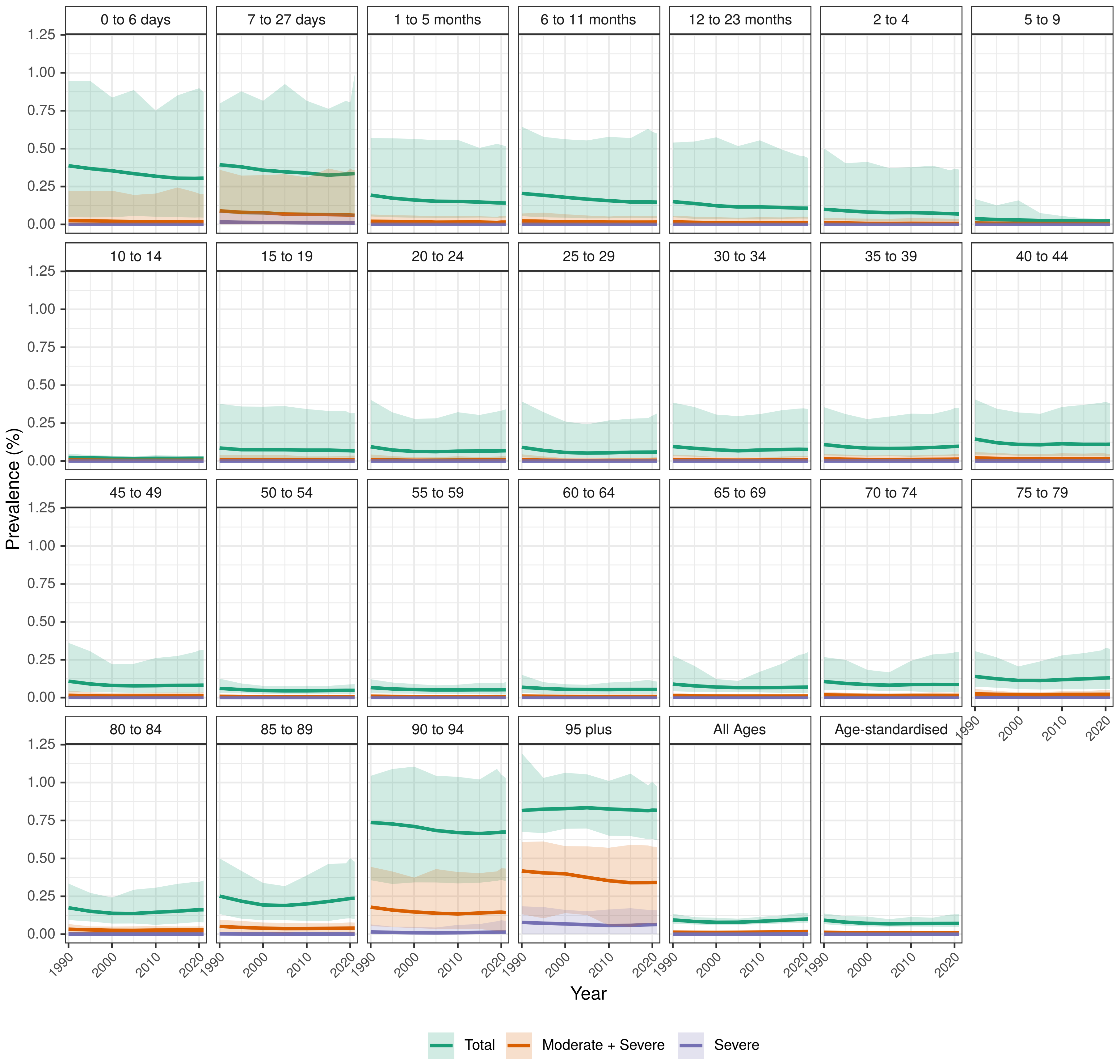

Cause-Specific Anaemia Prevalence - 2021

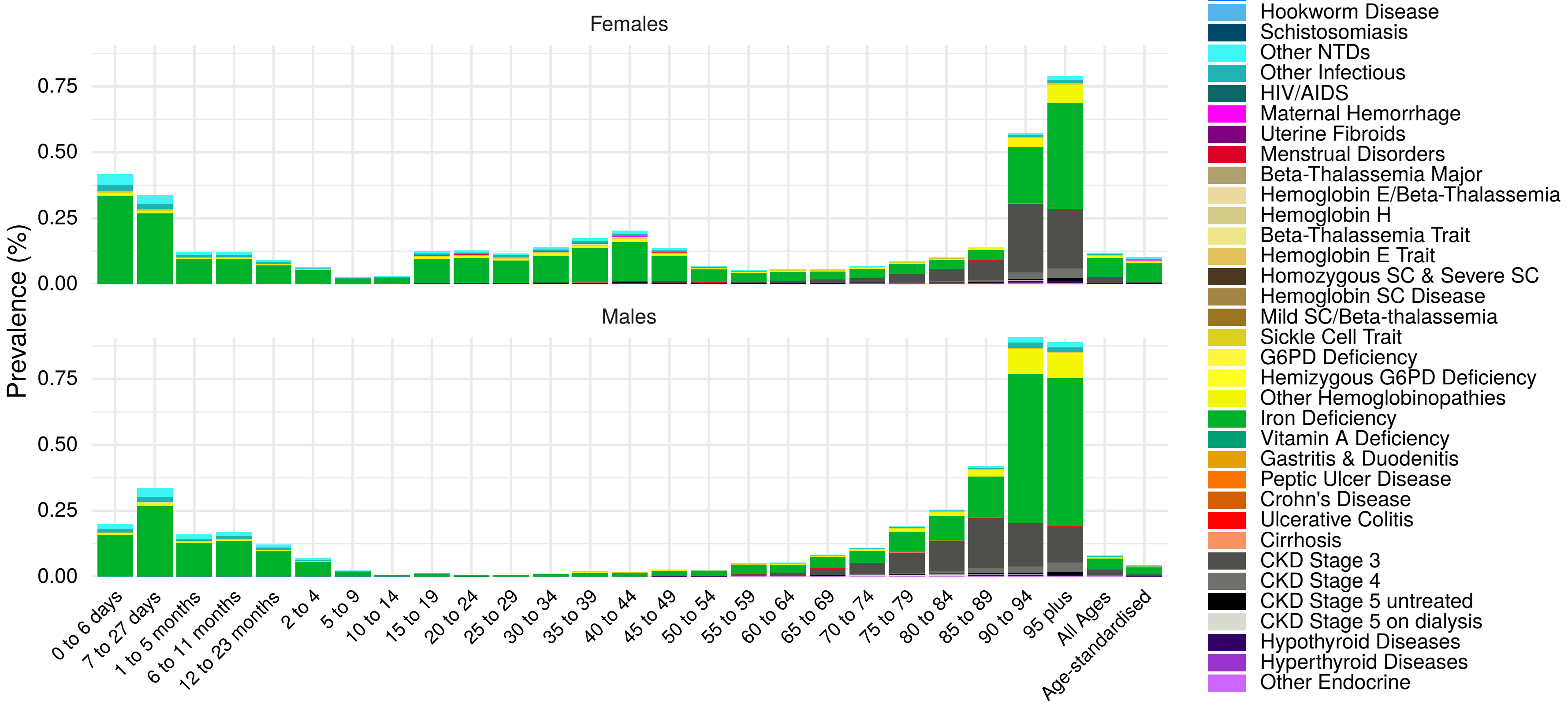

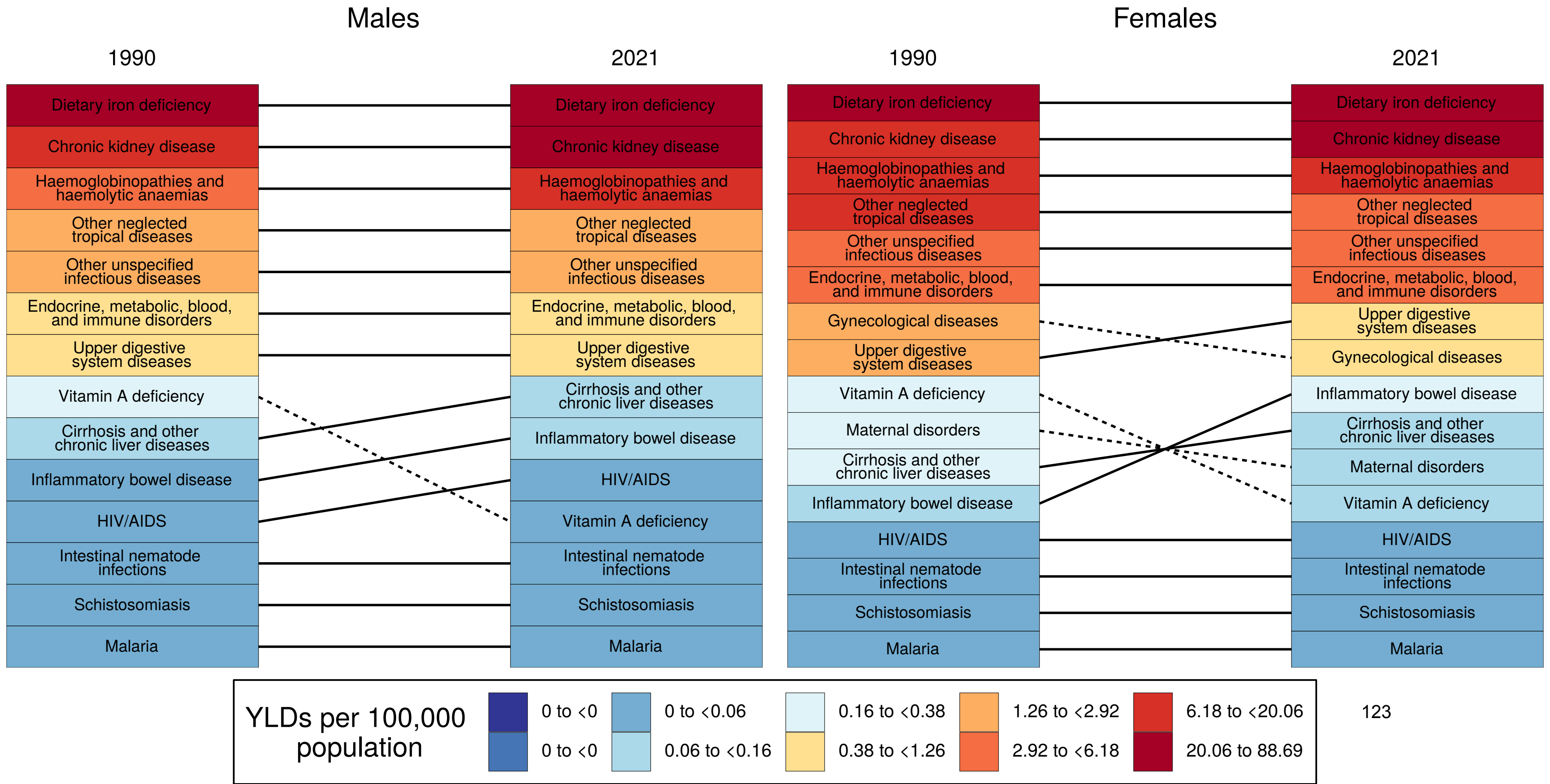

Saitama

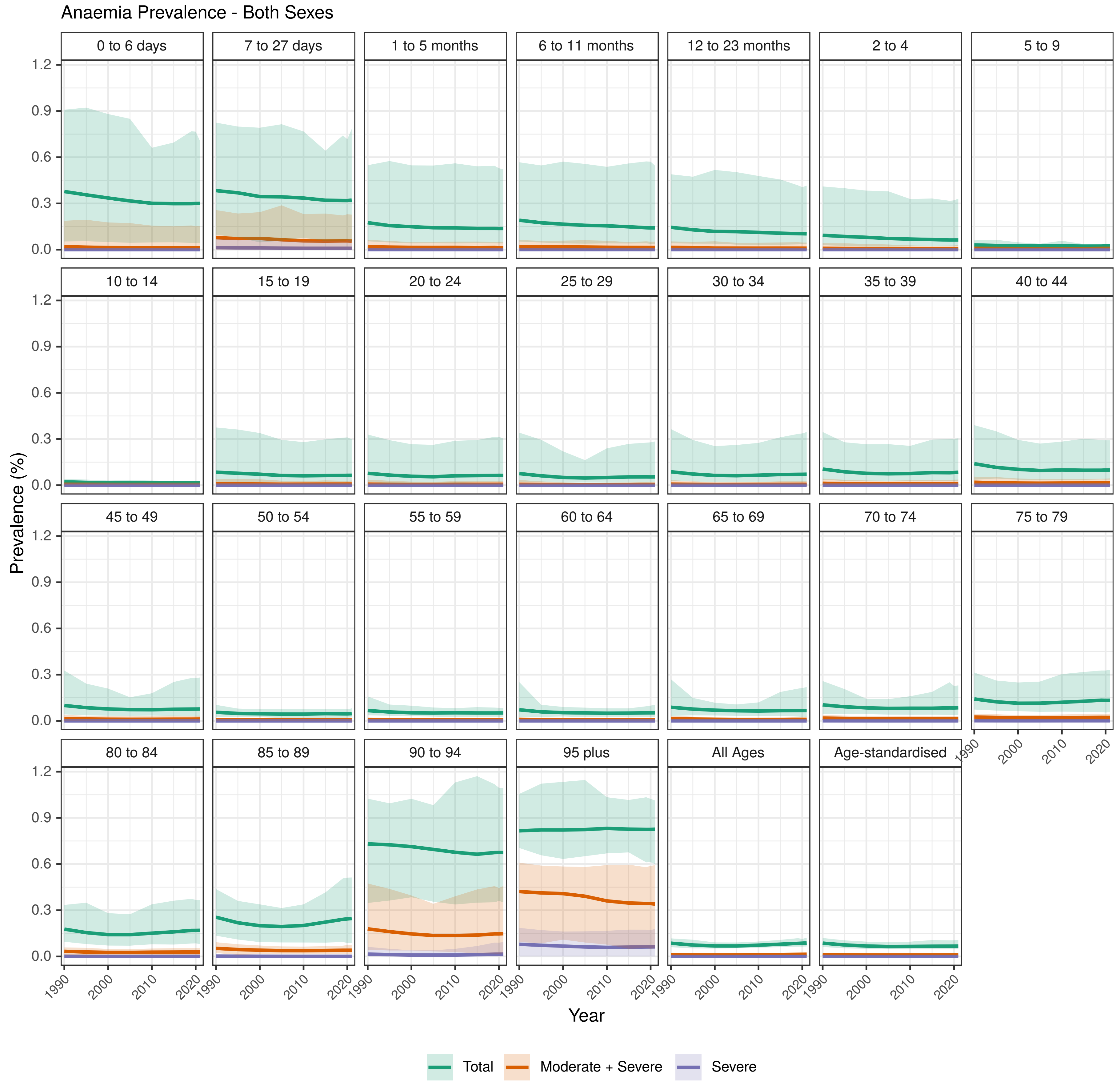

Anaemia Prevalence - Both Sexes
0 to 6 days
7 to 27 days
1 to 5 months
6 to 11 months
12 to 23 months
2 to 4
5 to 9
10 to 14
15 to 19
20 to 24
25 to 29
30 to 34
35 to 39
40 to 44
45 to 49
50 to 54
55 to 59
60 to 64
65 to 69
70 to 74
75 to 79
80 to 84
85 to 89
90 to 94
95 plus
All Ages
Age-standardised
Prevalence (%)
Year
Total
Moderate + Severe
Severe

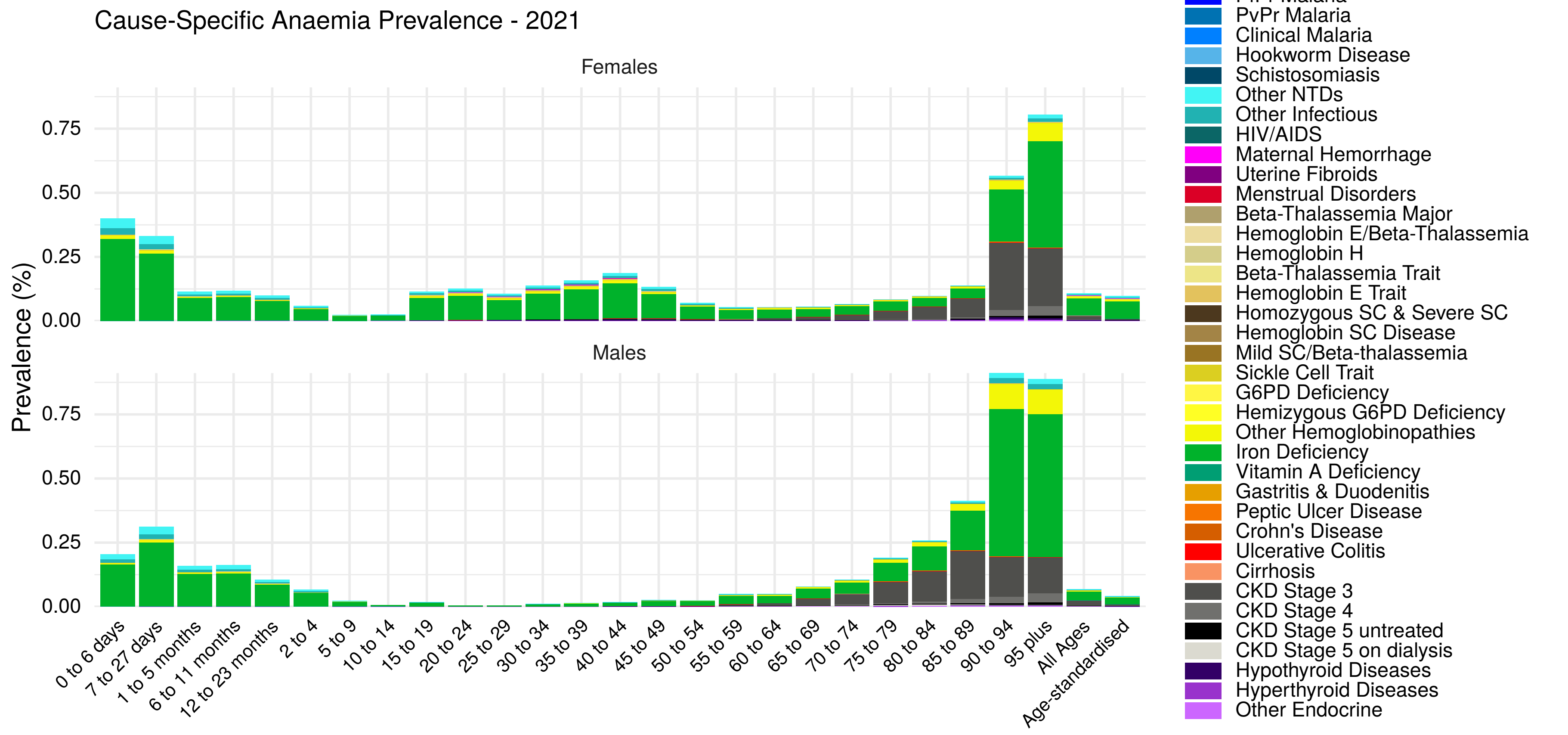

Cause-Specific Anaemia Prevalence - 2021
Females
Males
Prevalence (%)
PfPr Malaria
PvPr Malaria
Clinical Malaria
Hookworm Disease
Schistosomiasis
Other NTDs
Other Infectious
HIV/AIDS
Maternal Hemorrhage
Uterine Fibroids
Menstrual Disorders
Beta-Thalassemia Major
Hemoglobin E/Beta-Thalassemia
Hemoglobin H
Beta-Thalassemia Trait
Hemoglobin E Trait
Homozygous SC & Severe SC
Hemoglobin SC Disease
Mild SC/Beta-thalassemia
Sickle Cell Trait
G6PD Deficiency
Hemizygous G6PD Deficiency
Other Hemoglobinopathies
Iron Deficiency
Vitamin A Deficiency
Gastritis & Duodenitis
Peptic Ulcer Disease
Crohn's Disease
Ulcerative Colitis
Cirrhosis
CKD Stage 3
CKD Stage 4
CKD Stage 5 untreated
CKD Stage 5 on dialysis
Hypothyroid Diseases
Hyperthyroid Diseases
Other Endocrine

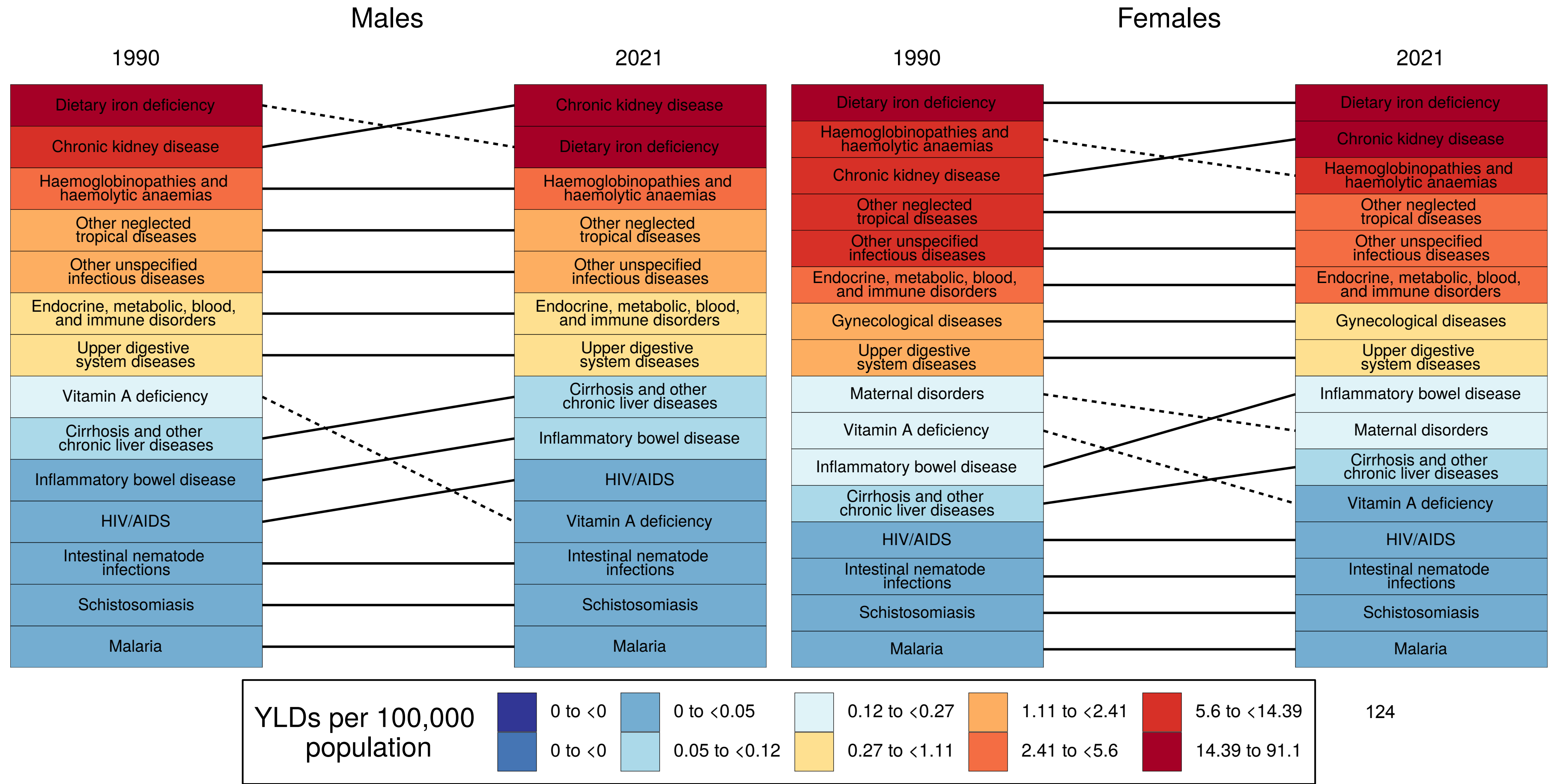

Males
Females
1990
2021
Dietary iron deficiency
Chronic kidney disease
Haemoglobinopathies and haemolytic anaemias
Other neglected tropical diseases
Other unspecified infectious diseases
Endocrine, metabolic, blood, and immune disorders
Upper digestive system diseases
Vitamin A deficiency
Cirrhosis and other chronic liver diseases
Inflammatory bowel disease
HIV/AIDS
Intestinal nematode infections
Schistosomiasis
Malaria
Gynecological diseases
Maternal disorders
YLDs per 100,000 population
0 to <0
0 to <0.05
0.05 to <0.12
0.12 to <0.27
0.27 to <1.11
1.11 to <2.41
2.41 to <5.6
5.6 to <14.39
14.39 to 91.1

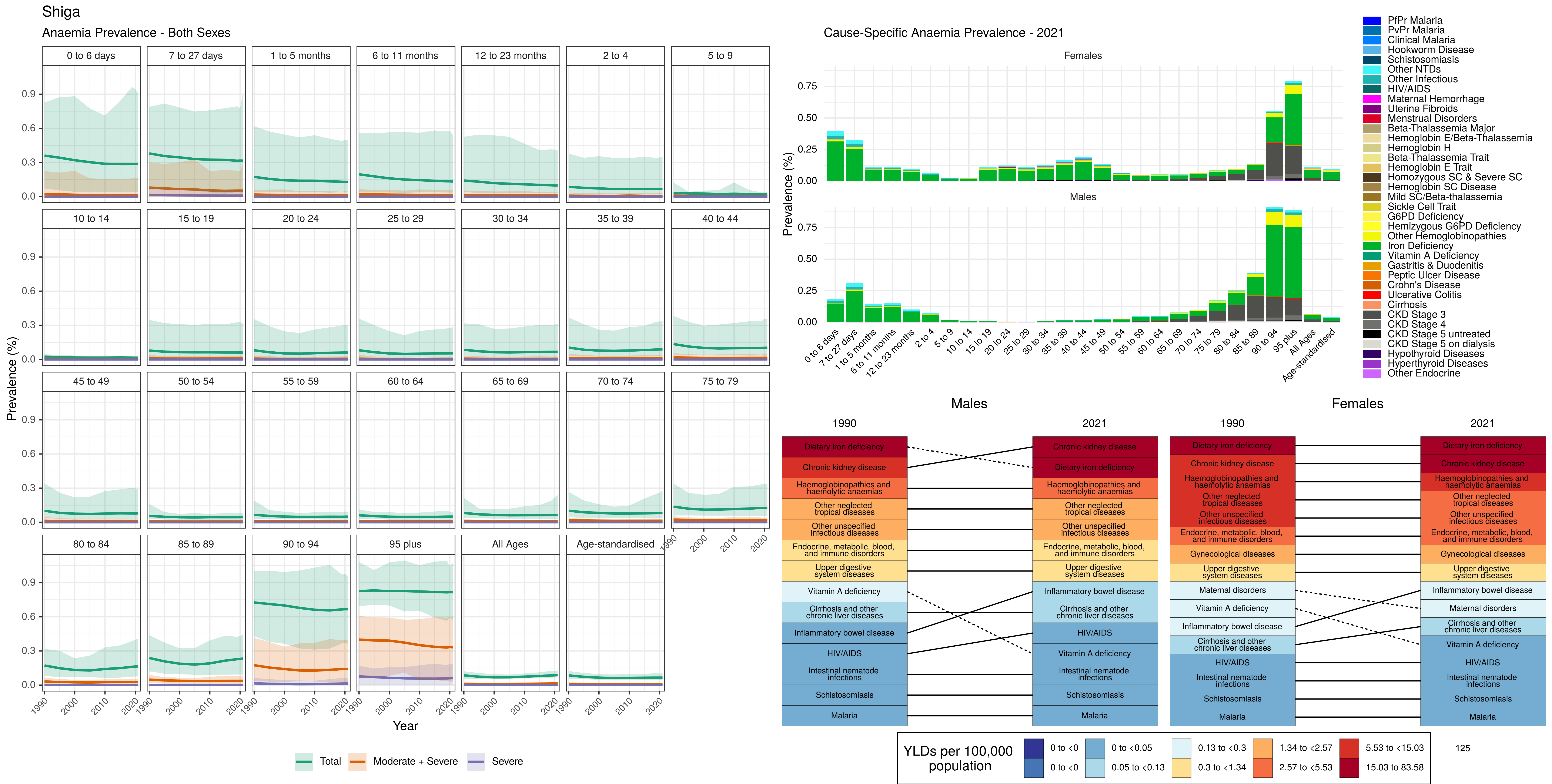

Shiga
Anaemia Prevalence - Both Sexes
0 to 6 days
7 to 27 days
1 to 5 months
6 to 11 months
12 to 23 months
2 to 4
5 to 9
10 to 14
15 to 19
20 to 24
25 to 29
30 to 34
35 to 39
40 to 44
45 to 49
50 to 54
55 to 59
60 to 64
65 to 69
70 to 74
75 to 79
80 to 84
85 to 89
90 to 94
95 plus
All Ages
Age-standardised
Prevalence (%)
Year
Total
Moderate + Severe
Severe
Cause-Specific Anaemia Prevalence - 2021
Females
Males
PfPr Malaria
PvPr Malaria
Clinical Malaria
Hookworm Disease
Schistosomiasis
Other NTDs
Other Infectious
HIV/AIDS
Maternal Hemorrhage
Uterine Fibroids
Menstrual Disorders
Beta-Thalassemia Major
Hemoglobin E/Beta-Thalassemia
Hemoglobin H
Beta-Thalassemia Trait
Hemoglobin E Trait
Homozygous SC & Severe SC
Hemoglobin SC Disease
Mild SC/Beta-thalassemia
Sickle Cell Trait
G6PD Deficiency
Hemizygous G6PD Deficiency
Other Hemoglobinopathies
Iron Deficiency
Vitamin A Deficiency
Gastritis & Duodenitis
Peptic Ulcer Disease
Crohn's Disease
Ulcerative Colitis
Cirrhosis
CKD Stage 3
CKD Stage 4
CKD Stage 5 untreated
CKD Stage 5 on dialysis
Hypothyroid Diseases
Hyperthyroid Diseases
Other Endocrine
Males
Females
1990
2021
Dietary iron deficiency
Chronic kidney disease
Haemoglobinopathies and haemolytic anaemias
Other neglected tropical diseases
Other unspecified infectious diseases
Endocrine, metabolic, blood, and immune disorders
Gynecological diseases
Upper digestive system diseases
Maternal disorders
Vitamin A deficiency
Inflammatory bowel disease
Cirrhosis and other chronic liver diseases
HIV/AIDS
Intestinal nematode infections
Schistosomiasis
Malaria
YLDs per 100,000 population
0 to <0
0 to <0
0 to <0.05
0.05 to <0.13
0.13 to <0.3
0.3 to <1.34
1.34 to <2.57
2.57 to <5.53
5.53 to <15.03
15.03 to 83.58

125

Shimane

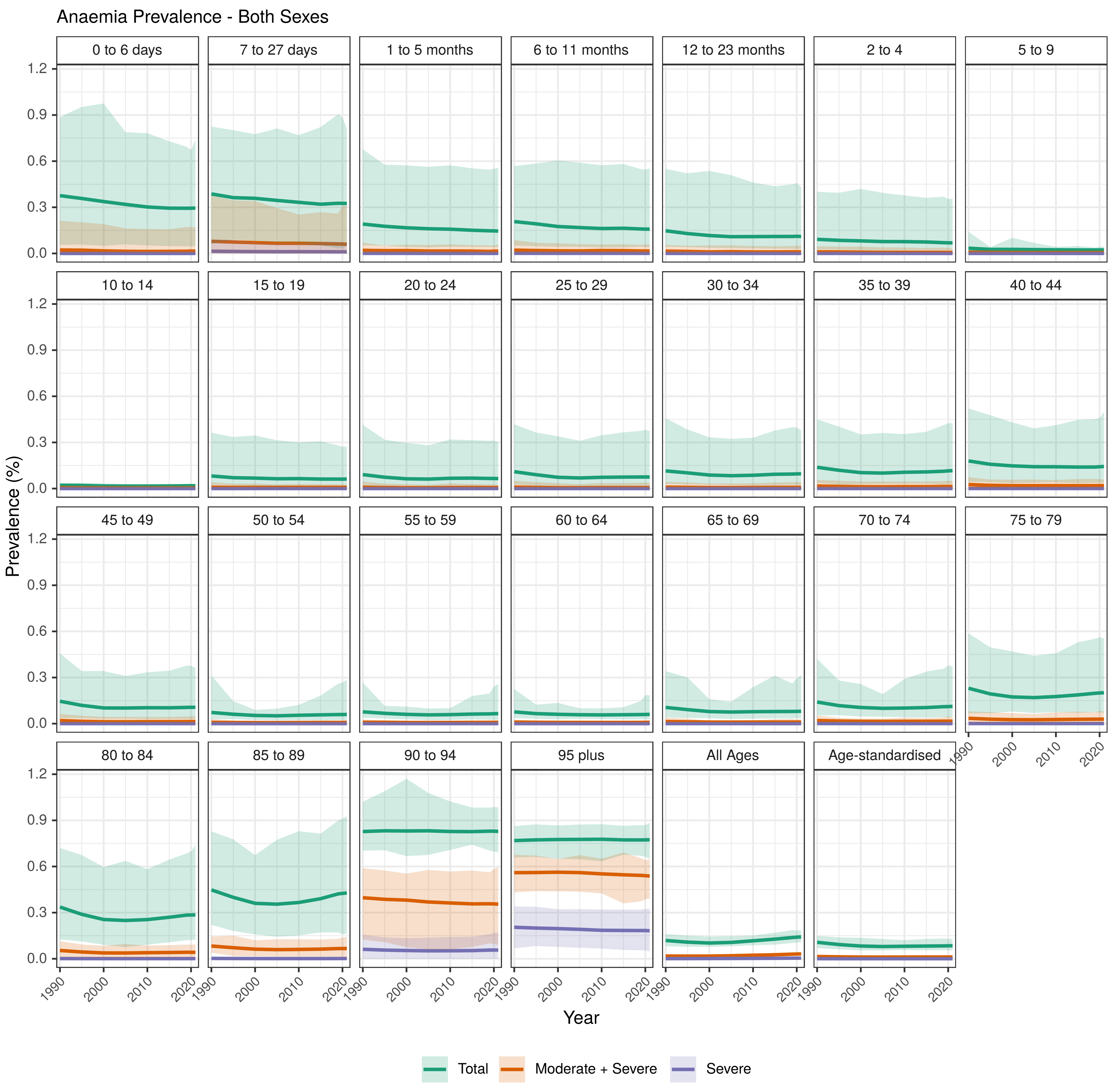

Anaemia Prevalence - Both Sexes
0 to 6 days
7 to 27 days
1 to 5 months
6 to 11 months
12 to 23 months
2 to 4
5 to 9
10 to 14
15 to 19
20 to 24
25 to 29
30 to 34
35 to 39
40 to 44
45 to 49
50 to 54
55 to 59
60 to 64
65 to 69
70 to 74
75 to 79
80 to 84
85 to 89
90 to 94
95 plus
All Ages
Age-standardised
Prevalence (%)
Year
1.2
0.9
0.6
0.3
0.0
1990
2000
2010
2020
Total
Moderate + Severe
Severe

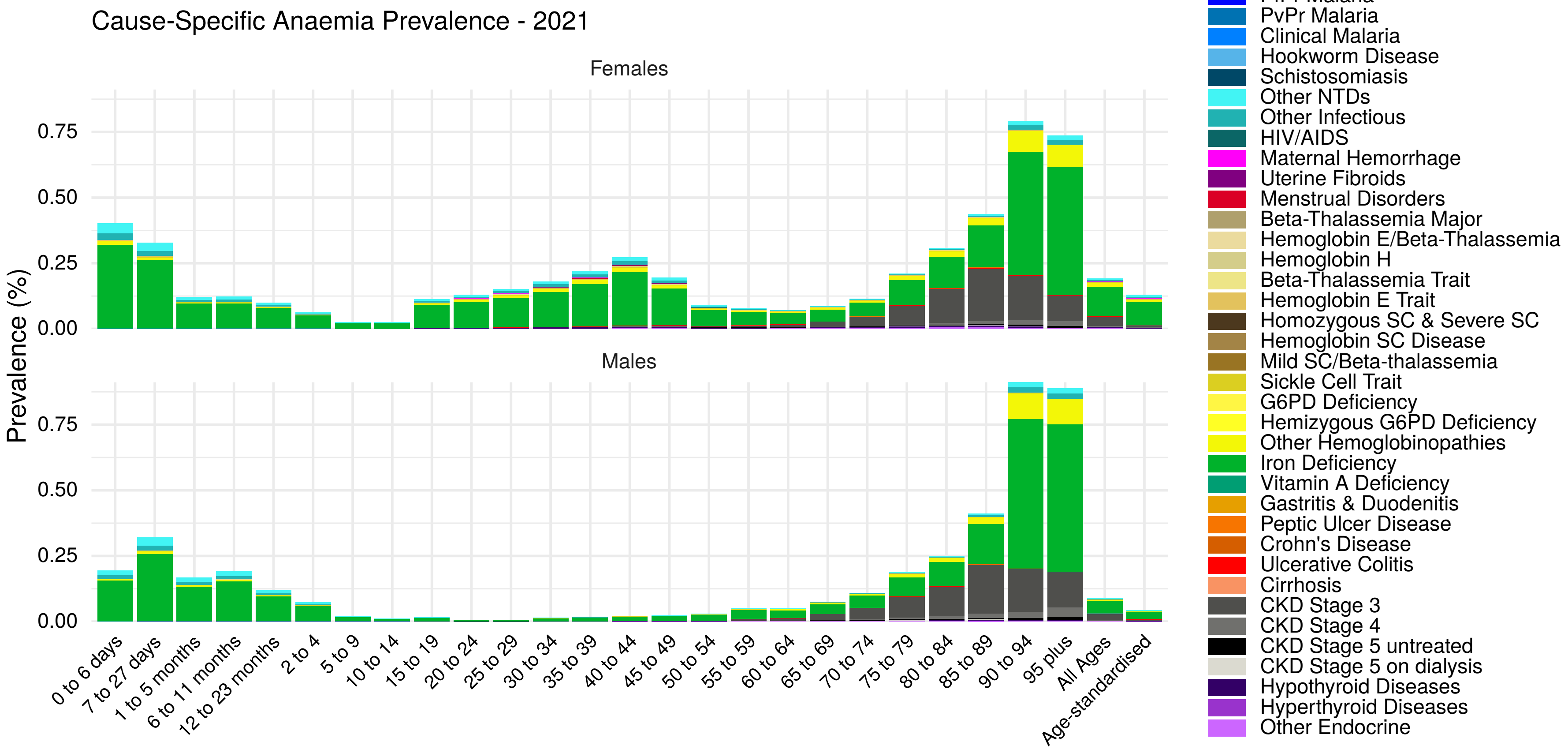

Cause-Specific Anaemia Prevalence - 2021
Females
Males
Prevalence (%)
0.75
0.50
0.25
0.00
0 to 6 days
7 to 27 days
1 to 5 months
6 to 11 months
12 to 23 months
2 to 4
5 to 9
10 to 14
15 to 19
20 to 24
25 to 29
30 to 34
35 to 39
40 to 44
45 to 49
50 to 54
55 to 59
60 to 64
65 to 69
70 to 74
75 to 79
80 to 84
85 to 89
90 to 94
95 plus
All Ages
Age-standardised
PfPr Malaria
PvPr Malaria
Clinical Malaria
Hookworm Disease
Schistosomiasis
Other NTDs
Other Infectious
HIV/AIDS
Maternal Hemorrhage
Uterine Fibroids
Menstrual Disorders
Beta-Thalassemia Major
Hemoglobin E/Beta-Thalassemia
Hemoglobin H
Beta-Thalassemia Trait
Hemoglobin E Trait
Homozygous SC & Severe SC
Hemoglobin SC Disease
Mild SC/Beta-thalassemia
Sickle Cell Trait
G6PD Deficiency
Hemizygous G6PD Deficiency
Other Hemoglobinopathies
Iron Deficiency
Vitamin A Deficiency
Gastritis & Duodenitis
Peptic Ulcer Disease
Crohn's Disease
Ulcerative Colitis
Cirrhosis
CKD Stage 3
CKD Stage 4
CKD Stage 5 untreated
CKD Stage 5 on dialysis
Hypothyroid Diseases
Hyperthyroid Diseases
Other Endocrine

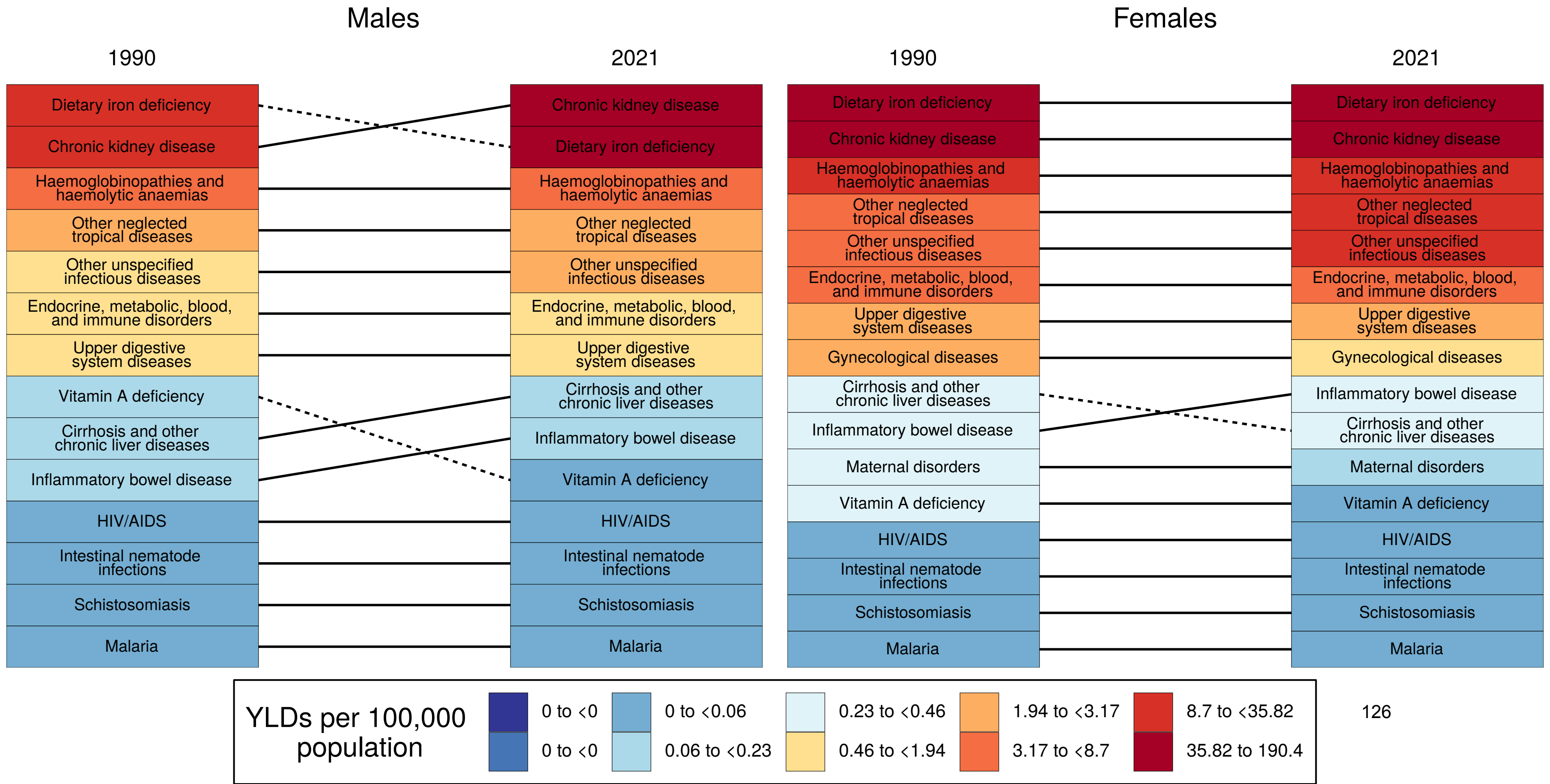

Males
Females
1990
2021
Dietary iron deficiency
Chronic kidney disease
Haemoglobinopathies and haemolytic anaemias
Other neglected tropical diseases
Other unspecified infectious diseases
Endocrine, metabolic, blood, and immune disorders
Upper digestive system diseases
Vitamin A deficiency
Cirrhosis and other chronic liver diseases
Inflammatory bowel disease
HIV/AIDS
Intestinal nematode infections
Schistosomiasis
Malaria
Gynecological diseases
Maternal disorders
YLDs per 100,000 population
0 to <0
0 to <0
0 to <0.06
0.06 to <0.23
0.23 to <0.46
0.46 to <1.94
1.94 to <3.17
3.17 to <8.7
8.7 to <35.82
35.82 to 190.4

Shizuoka

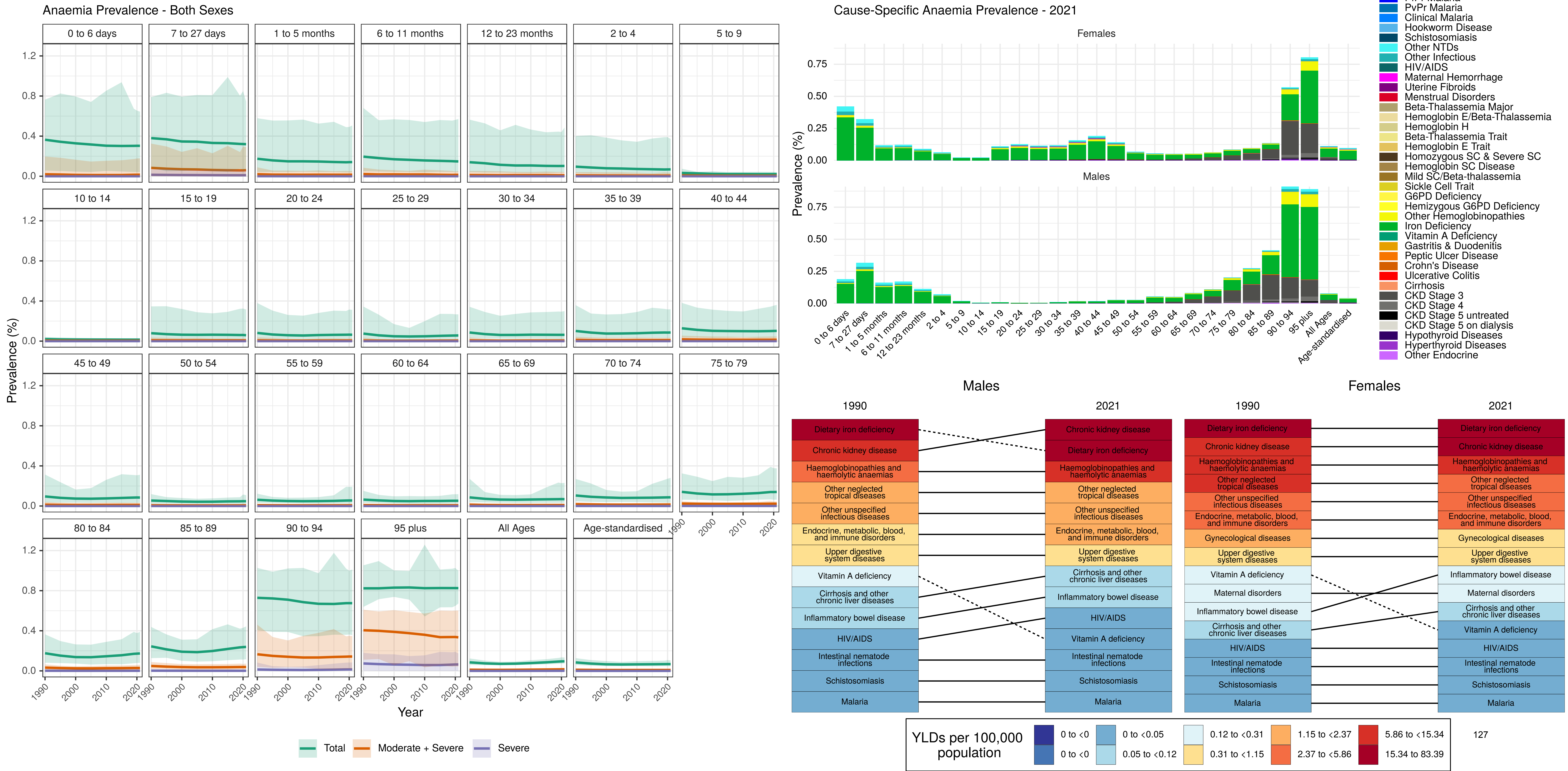

Anaemia Prevalence - Both Sexes
0 to 6 days
7 to 27 days
1 to 5 months
6 to 11 months
12 to 23 months
2 to 4
5 to 9
10 to 14
15 to 19
20 to 24
25 to 29
30 to 34
35 to 39
40 to 44
45 to 49
50 to 54
55 to 59
60 to 64
65 to 69
70 to 74
75 to 79
80 to 84
85 to 89
90 to 94
95 plus
All Ages
Age-standardised
Prevalence (%)
Year
Total
Moderate + Severe
Severe
Cause-Specific Anaemia Prevalence - 2021
Females
Males
PfPr Malaria
PvPr Malaria
Clinical Malaria
Hookworm Disease
Schistosomiasis
Other NTDs
Other Infectious
HIV/AIDS
Maternal Hemorrhage
Uterine Fibroids
Menstrual Disorders
Beta-Thalassemia Major
Hemoglobin E/Beta-Thalassemia
Hemoglobin H
Beta-Thalassemia Trait
Hemoglobin E Trait
Homozygous SC & Severe SC
Hemoglobin SC Disease
Mild SC/Beta-thalassemia
Sickle Cell Trait
G6PD Deficiency
Hemizygous G6PD Deficiency
Other Hemoglobinopathies
Iron Deficiency
Vitamin A Deficiency
Gastritis & Duodenitis
Peptic Ulcer Disease
Crohn's Disease
Ulcerative Colitis
Cirrhosis
CKD Stage 3
CKD Stage 4
CKD Stage 5 untreated
CKD Stage 5 on dialysis
Hypothyroid Diseases
Hyperthyroid Diseases
Other Endocrine
Males
Females
1990
2021
Dietary iron deficiency
Chronic kidney disease
Haemoglobinopathies and haemolytic anaemias
Other neglected tropical diseases
Other unspecified infectious diseases
Endocrine, metabolic, blood, and immune disorders
Gynecological diseases
Upper digestive system diseases
Vitamin A deficiency
Maternal disorders
Cirrhosis and other chronic liver diseases
Inflammatory bowel disease
HIV/AIDS
Intestinal nematode infections
Schistosomiasis
Malaria
YLDs per 100,000 population
0 to <0
0 to <0
0 to <0.05
0.05 to <0.12
0.12 to <0.31
0.31 to <1.15
1.15 to <2.37
2.37 to <5.86
5.86 to <15.34
15.34 to 83.39

Tochigi

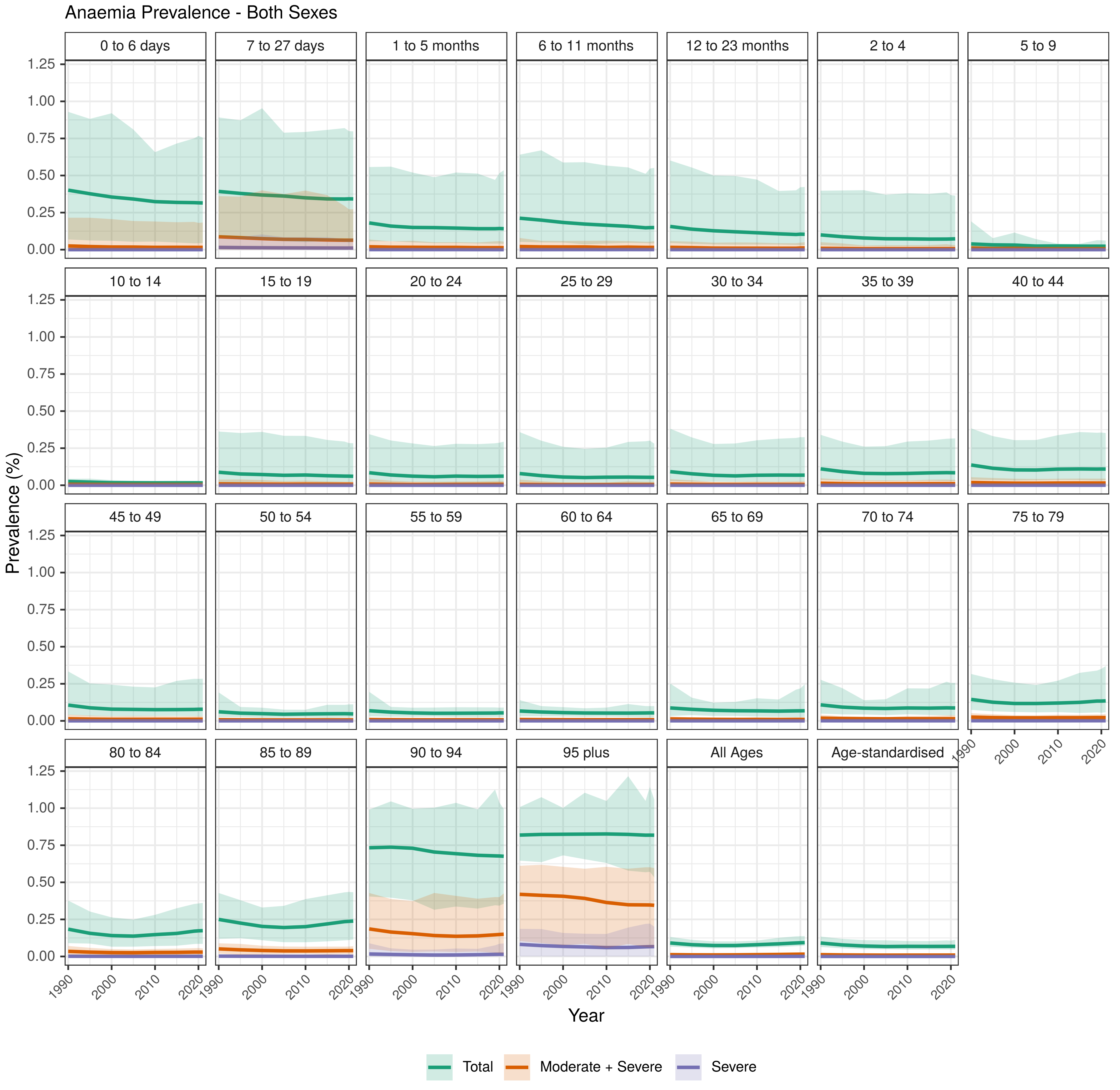

Anaemia Prevalence - Both Sexes
0 to 6 days
7 to 27 days
1 to 5 months
6 to 11 months
12 to 23 months
2 to 4
5 to 9
10 to 14
15 to 19
20 to 24
25 to 29
30 to 34
35 to 39
40 to 44
45 to 49
50 to 54
55 to 59
60 to 64
65 to 69
70 to 74
75 to 79
80 to 84
85 to 89
90 to 94
95 plus
All Ages
Age-standardised
Prevalence (%)
Year
Total
Moderate + Severe
Severe

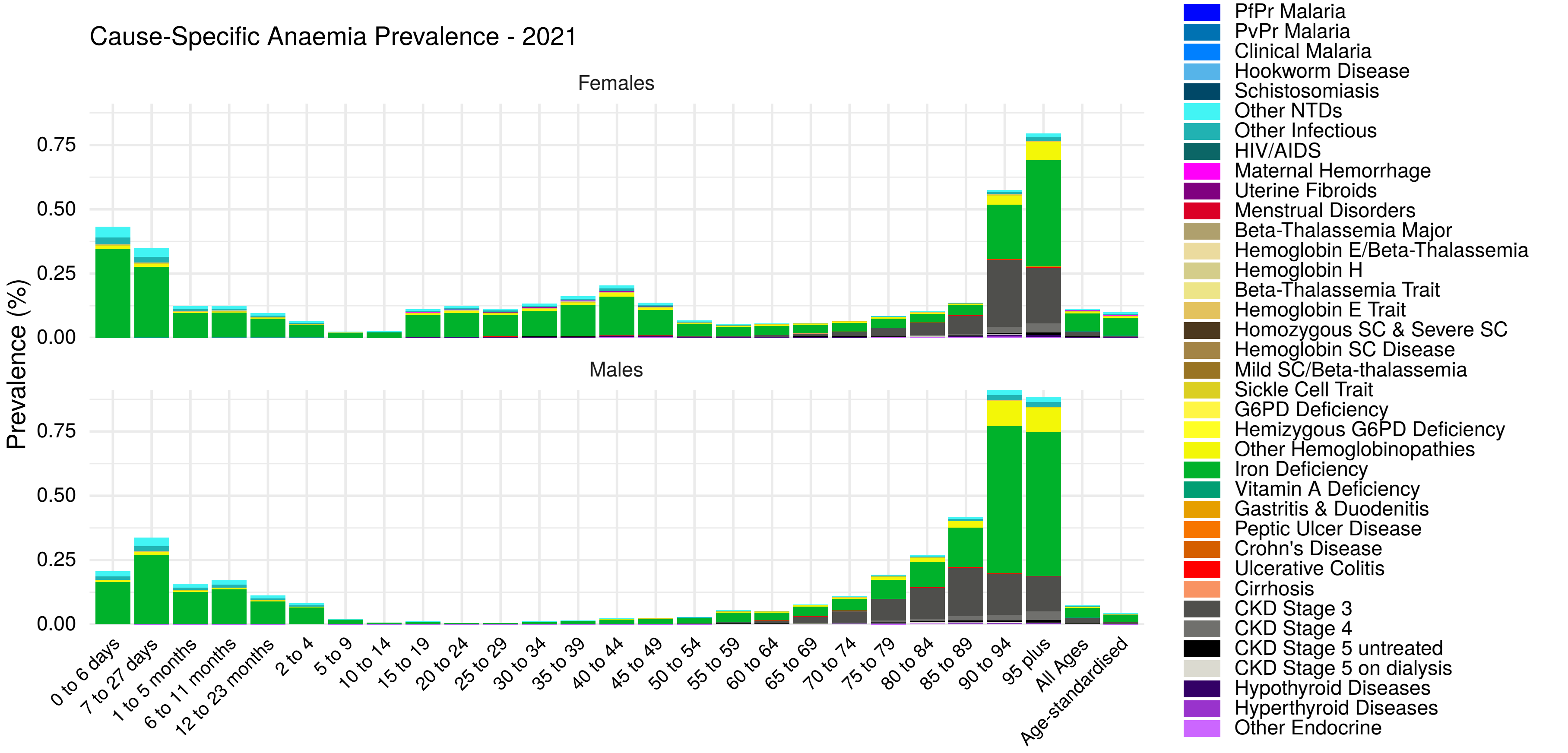

Cause-Specific Anaemia Prevalence - 2021
Females
Males
Prevalence (%)
PfPr Malaria
PvPr Malaria
Clinical Malaria
Hookworm Disease
Schistosomiasis
Other NTDs
Other Infectious
HIV/AIDS
Maternal Hemorrhage
Uterine Fibroids
Menstrual Disorders
Beta-Thalassemia Major
Hemoglobin E/Beta-Thalassemia
Hemoglobin H
Beta-Thalassemia Trait
Hemoglobin E Trait
Homozygous SC & Severe SC
Hemoglobin SC Disease
Mild SC/Beta-thalassemia
Sickle Cell Trait
G6PD Deficiency
Hemizygous G6PD Deficiency
Other Hemoglobinopathies
Iron Deficiency
Vitamin A Deficiency
Gastritis & Duodenitis
Peptic Ulcer Disease
Crohn's Disease
Ulcerative Colitis
Cirrhosis
CKD Stage 3
CKD Stage 4
CKD Stage 5 untreated
CKD Stage 5 on dialysis
Hypothyroid Diseases
Hyperthyroid Diseases
Other Endocrine

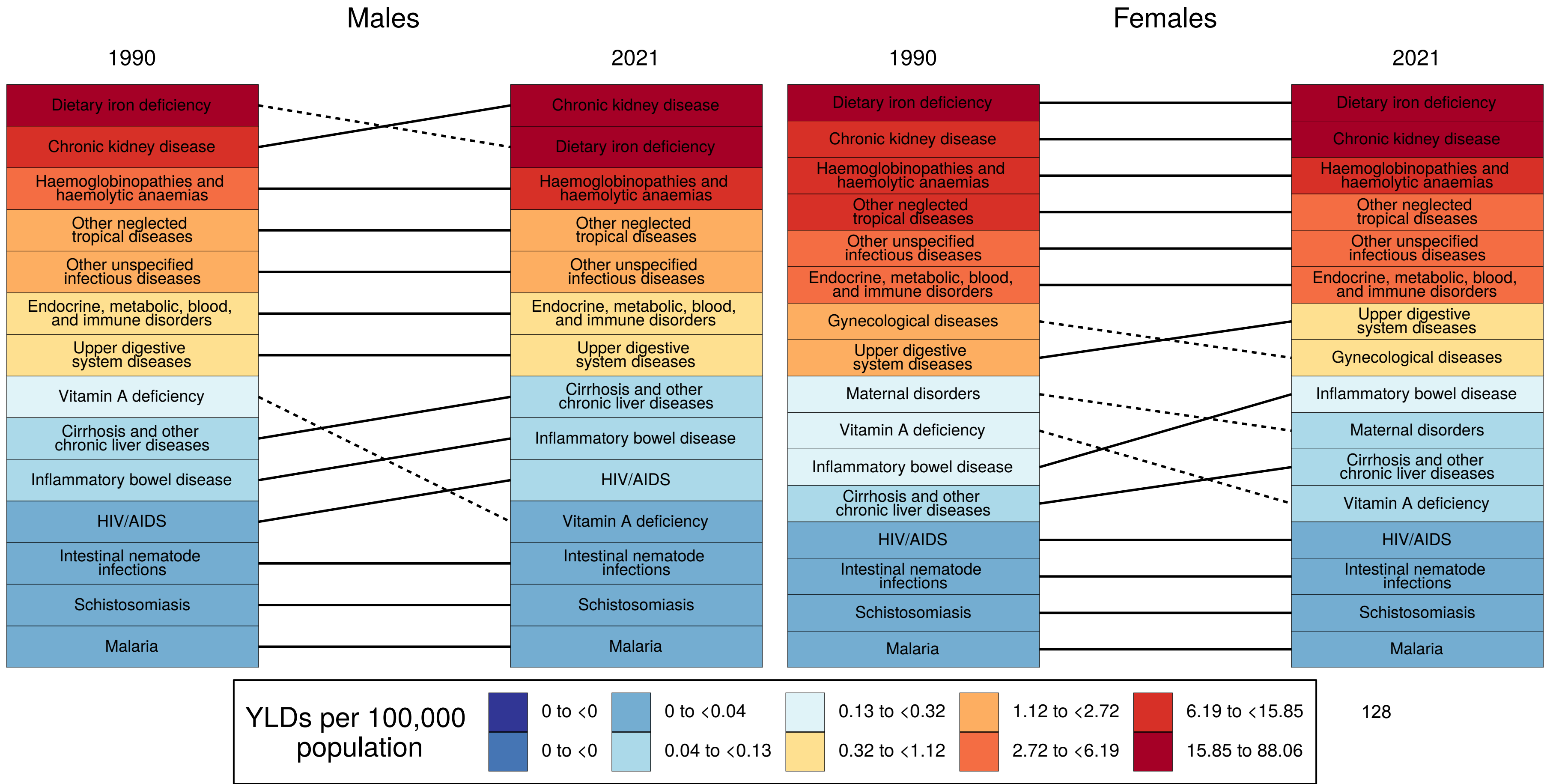

Males
Females
1990
2021
Dietary iron deficiency
Chronic kidney disease
Haemoglobinopathies and haemolytic anaemias
Other neglected tropical diseases
Other unspecified infectious diseases
Endocrine, metabolic, blood, and immune disorders
Upper digestive system diseases
Vitamin A deficiency
Cirrhosis and other chronic liver diseases
Inflammatory bowel disease
HIV/AIDS
Intestinal nematode infections
Schistosomiasis
Malaria
Gynecological diseases
Maternal disorders
YLDs per 100,000 population
0 to <0
0 to <0
0 to <0.04
0.04 to <0.13
0.13 to <0.32
0.32 to <1.12
1.12 to <2.72
2.72 to <6.19
6.19 to <15.85
15.85 to 88.06

Tokushima

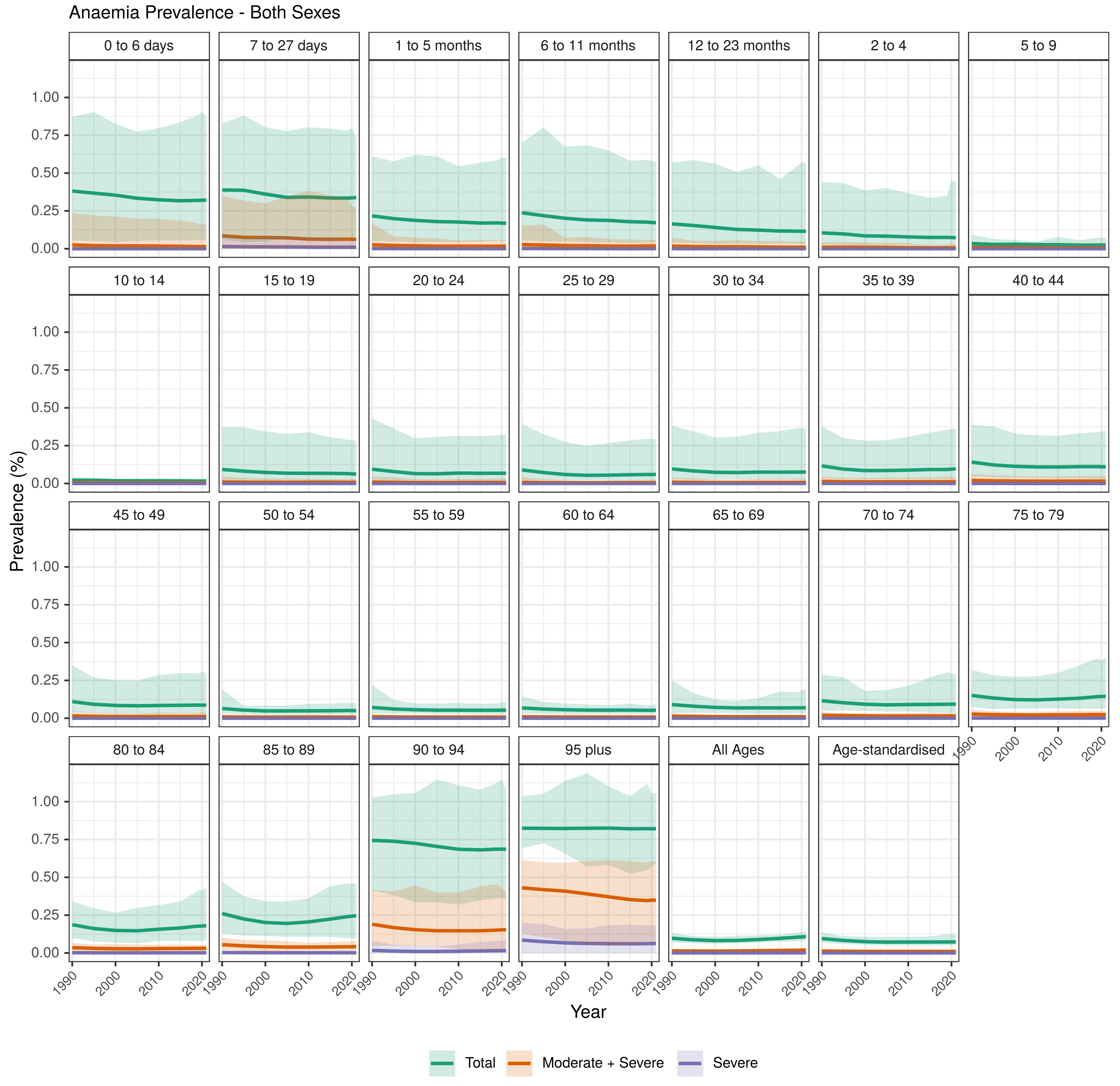

Anaemia Prevalence - Both Sexes
0 to 6 days
7 to 27 days
1 to 5 months
6 to 11 months
12 to 23 months
2 to 4
5 to 9
10 to 14
15 to 19
20 to 24
25 to 29
30 to 34
35 to 39
40 to 44
45 to 49
50 to 54
55 to 59
60 to 64
65 to 69
70 to 74
75 to 79
80 to 84
85 to 89
90 to 94
95 plus
All Ages
Age-standardised
Prevalence (%)
Year
Total
Moderate + Severe
Severe

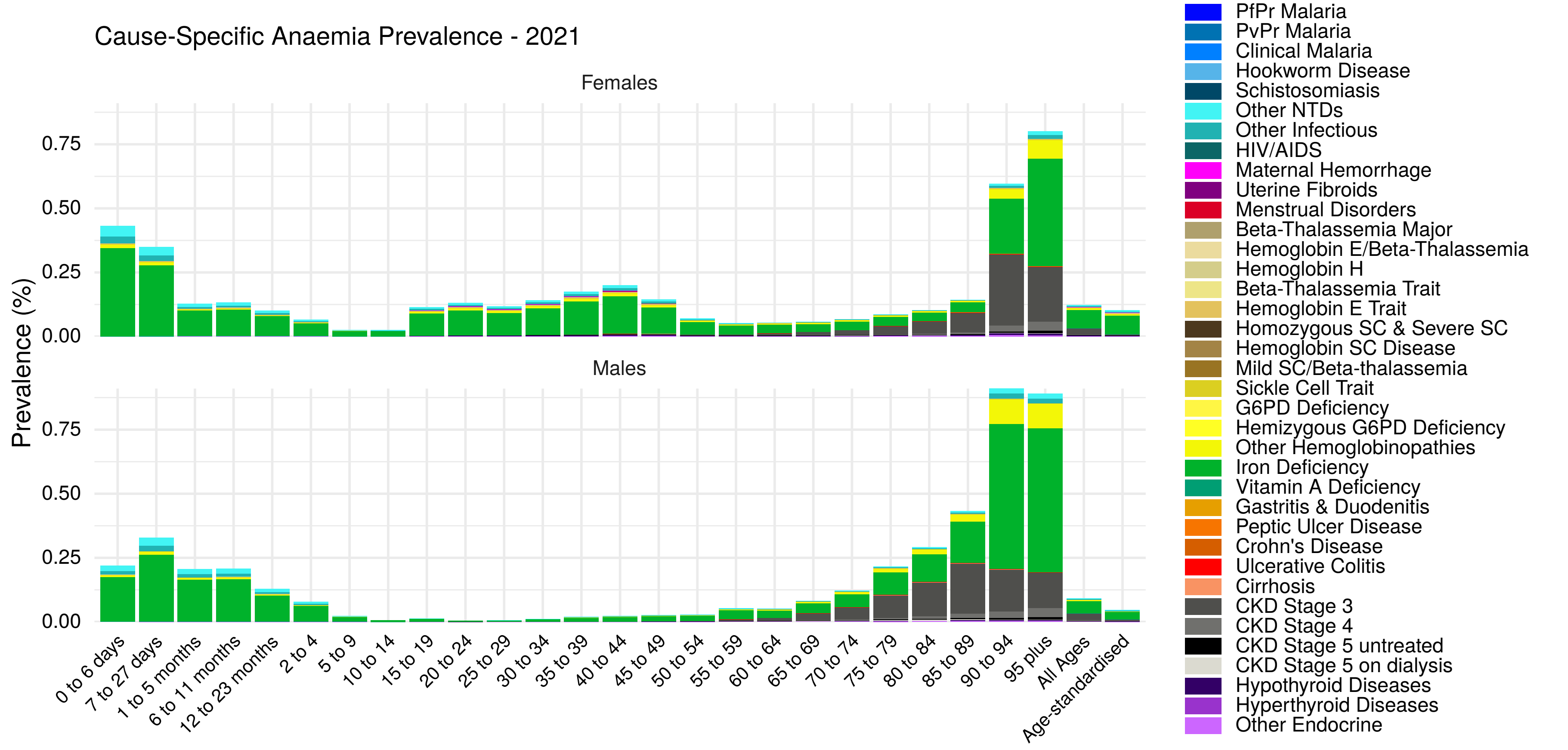

Cause-Specific Anaemia Prevalence - 2021
Females
Males
Prevalence (%)
PfPr Malaria
PvPr Malaria
Clinical Malaria
Hookworm Disease
Schistosomiasis
Other NTDs
Other Infectious
HIV/AIDS
Maternal Hemorrhage
Uterine Fibroids
Menstrual Disorders
Beta-Thalassemia Major
Hemoglobin E/Beta-Thalassemia
Hemoglobin H
Beta-Thalassemia Trait
Hemoglobin E Trait
Homozygous SC & Severe SC
Hemoglobin SC Disease
Mild SC/Beta-thalassemia
Sickle Cell Trait
G6PD Deficiency
Hemizygous G6PD Deficiency
Other Hemoglobinopathies
Iron Deficiency
Vitamin A Deficiency
Gastritis & Duodenitis
Peptic Ulcer Disease
Crohn's Disease
Ulcerative Colitis
Cirrhosis
CKD Stage 3
CKD Stage 4
CKD Stage 5 untreated
CKD Stage 5 on dialysis
Hypothyroid Diseases
Hyperthyroid Diseases
Other Endocrine

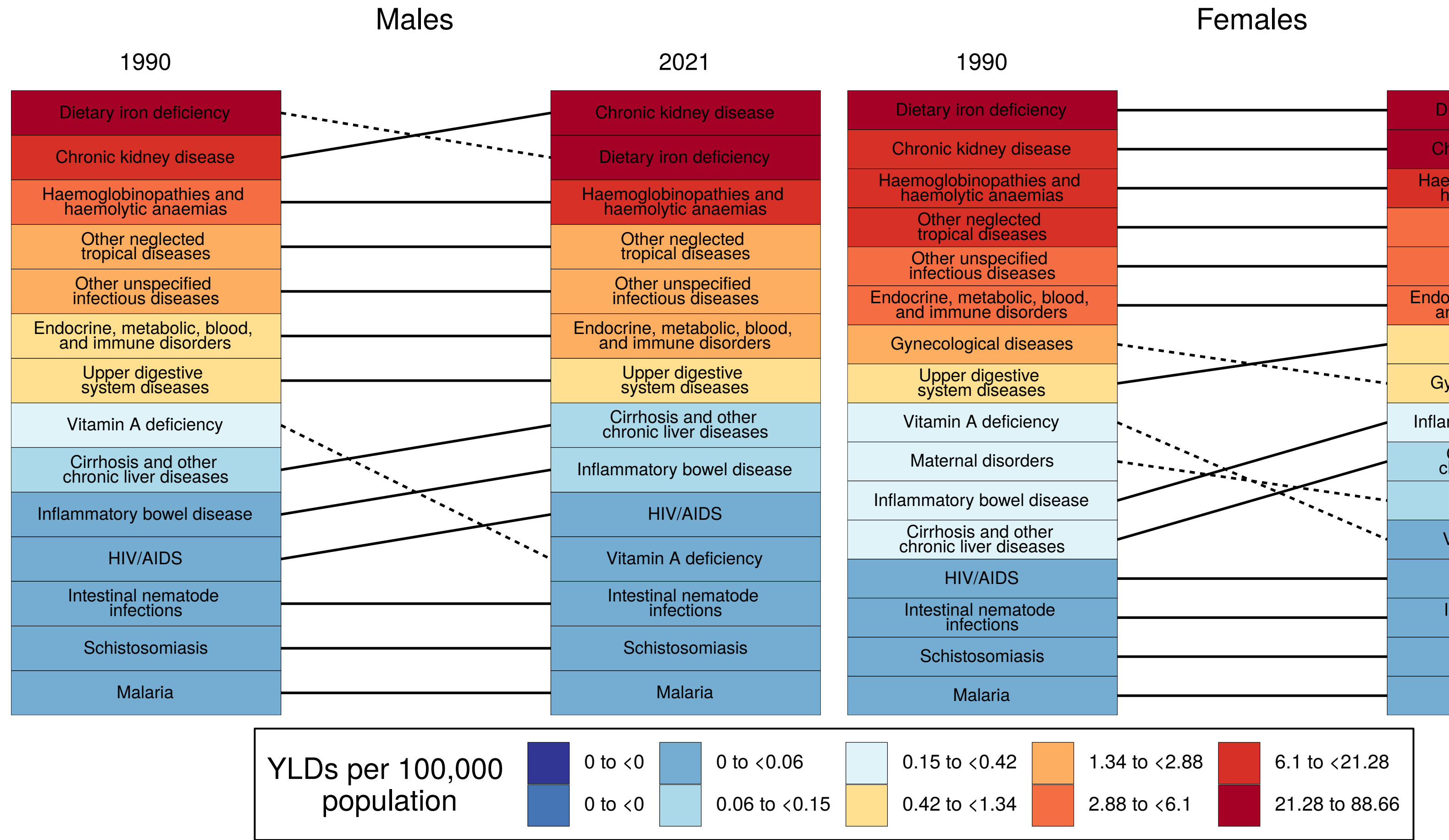

Males
Females
1990
2021
Dietary iron deficiency
Chronic kidney disease
Haemoglobinopathies and haemolytic anaemias
Other neglected tropical diseases
Other unspecified infectious diseases
Endocrine, metabolic, blood, and immune disorders
Upper digestive system diseases
Vitamin A deficiency
Cirrhosis and other chronic liver diseases
Inflammatory bowel disease
HIV/AIDS
Intestinal nematode infections
Schistosomiasis
Malaria
Gynecological diseases
Maternal disorders
YLDs per 100,000 population
0 to <0
0 to <0.06
0.06 to <0.15
0.15 to <0.42
0.42 to <1.34
1.34 to <2.88
2.88 to <6.1
6.1 to <21.28
21.28 to 88.66

Tōkyō

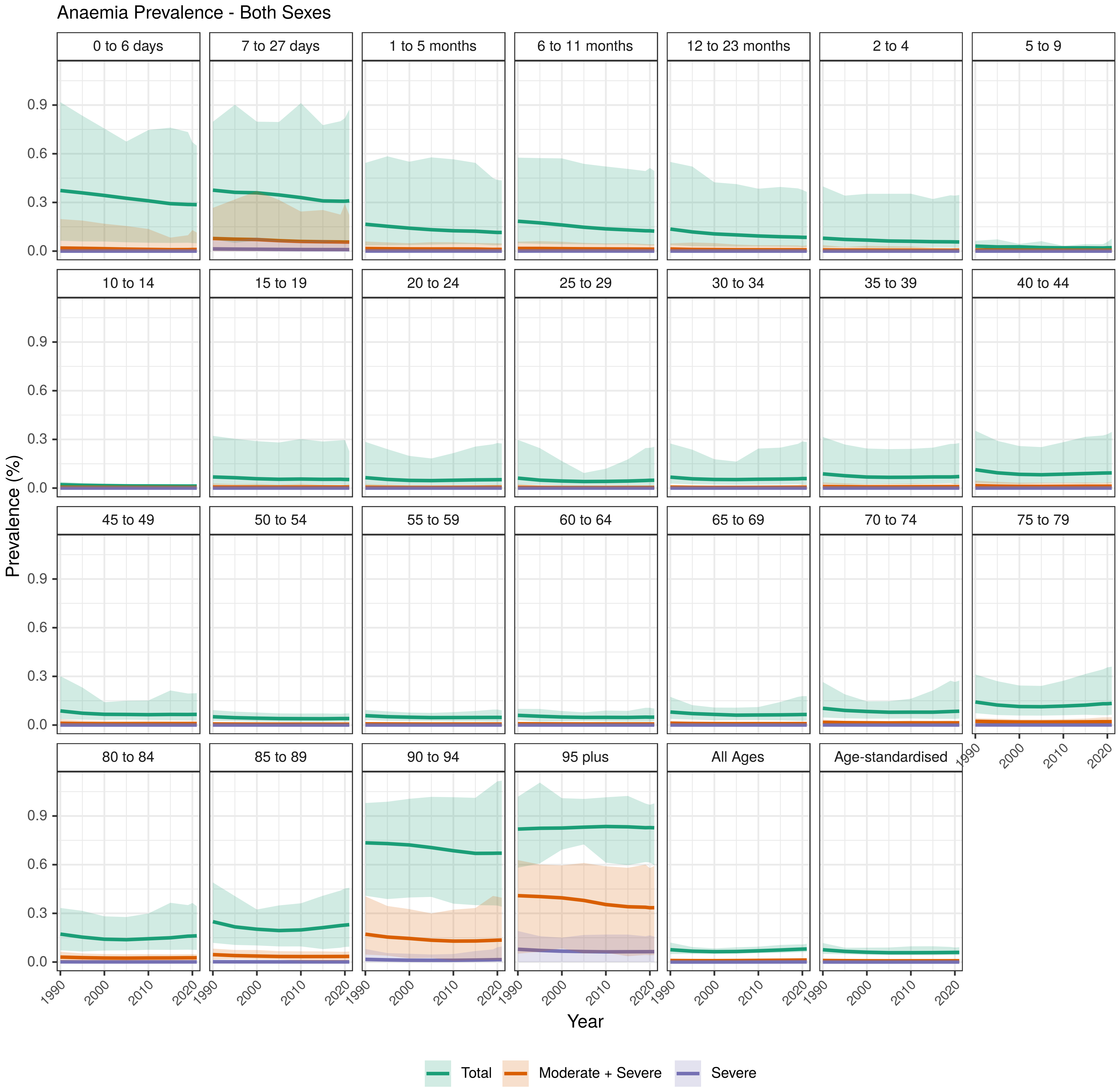
Anaemia Prevalence - Both Sexes
0 to 6 days
7 to 27 days
1 to 5 months
6 to 11 months
12 to 23 months
2 to 4
5 to 9
10 to 14
15 to 19
20 to 24
25 to 29
30 to 34
35 to 39
40 to 44
45 to 49
50 to 54
55 to 59
60 to 64
65 to 69
70 to 74
75 to 79
80 to 84
85 to 89
90 to 94
95 plus
All Ages
Age-standardised
Prevalence (%)
0.0
0.3
0.6
0.9
1990
2000
2010
2020
Year
Total
Moderate + Severe
Severe

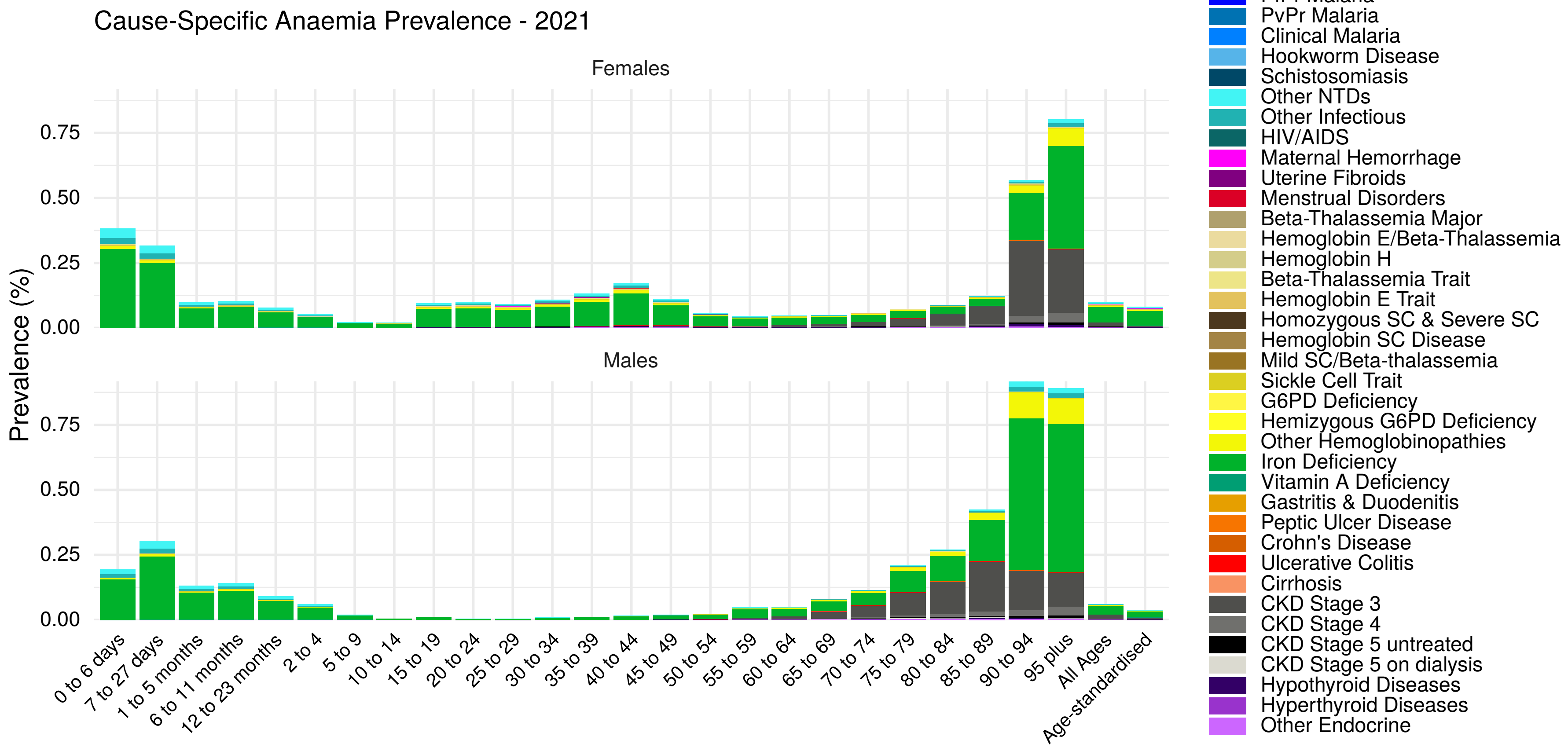
Cause-Specific Anaemia Prevalence - 2021
Females
Males
Prevalence (%)
0.00
0.25
0.50
0.75
0 to 6 days
7 to 27 days
1 to 5 months
6 to 11 months
12 to 23 months
2 to 4
5 to 9
10 to 14
15 to 19
20 to 24
25 to 29
30 to 34
35 to 39
40 to 44
45 to 49
50 to 54
55 to 59
60 to 64
65 to 69
70 to 74
75 to 79
80 to 84
85 to 89
90 to 94
95 plus
All Ages
Age-standardised
PfPr Malaria
PvPr Malaria
Clinical Malaria
Hookworm Disease
Schistosomiasis
Other NTDs
Other Infectious
HIV/AIDS
Maternal Hemorrhage
Uterine Fibroids
Menstrual Disorders
Beta-Thalassemia Major
Hemoglobin E/Beta-Thalassemia
Hemoglobin H
Beta-Thalassemia Trait
Hemoglobin E Trait
Homozygous SC & Severe SC
Hemoglobin SC Disease
Mild SC/Beta-thalassemia
Sickle Cell Trait
G6PD Deficiency
Hemizygous G6PD Deficiency
Other Hemoglobinopathies
Iron Deficiency
Vitamin A Deficiency
Gastritis & Duodenitis
Peptic Ulcer Disease
Crohn's Disease
Ulcerative Colitis
Cirrhosis
CKD Stage 3
CKD Stage 4
CKD Stage 5 untreated
CKD Stage 5 on dialysis
Hypothyroid Diseases
Hyperthyroid Diseases
Other Endocrine

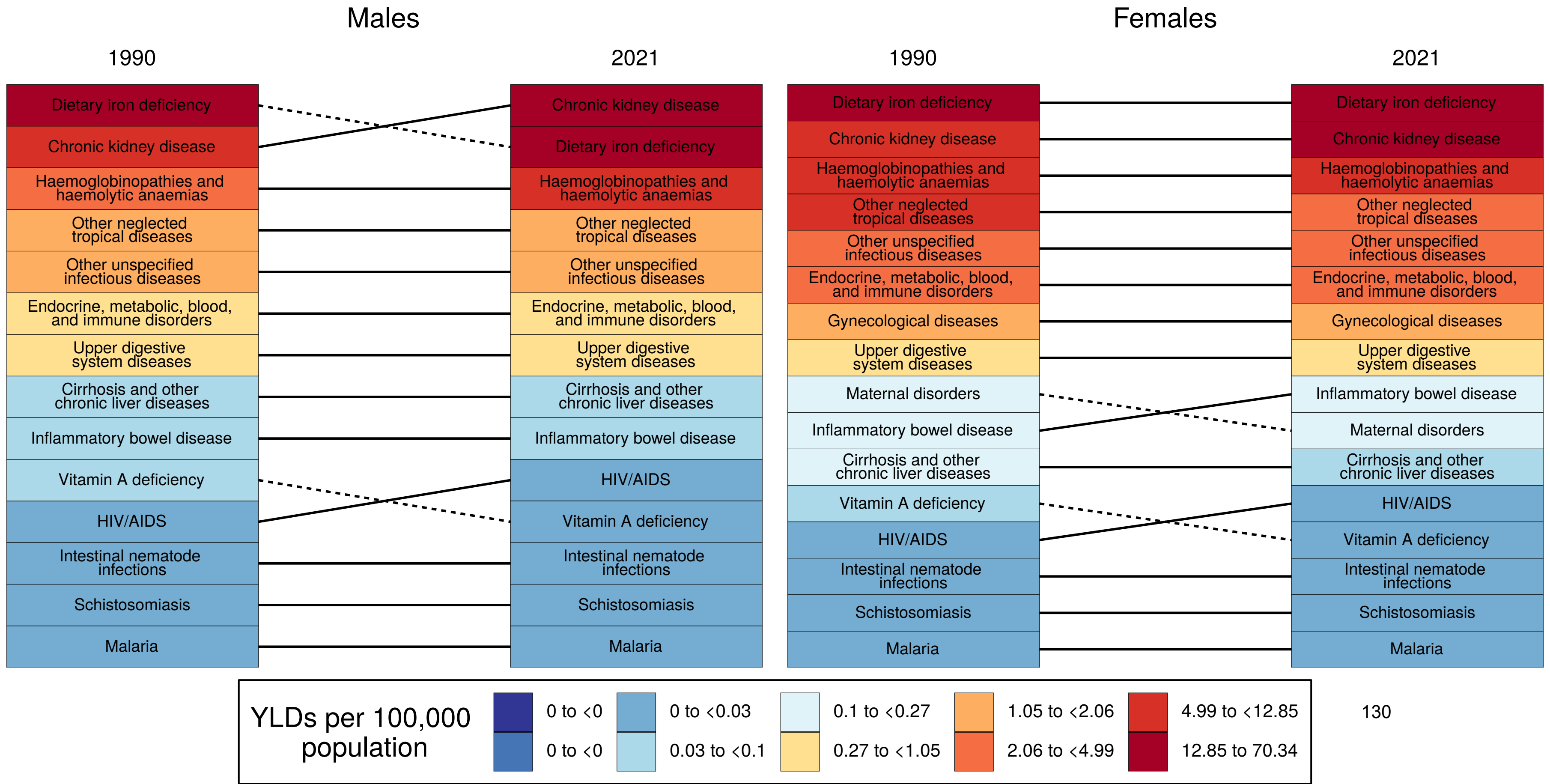
Males
Females
1990
2021
Dietary iron deficiency
Chronic kidney disease
Haemoglobinopathies and haemolytic anaemias
Other neglected tropical diseases
Other unspecified infectious diseases
Endocrine, metabolic, blood, and immune disorders
Upper digestive system diseases
Cirrhosis and other chronic liver diseases
Inflammatory bowel disease
Vitamin A deficiency
HIV/AIDS
Intestinal nematode infections
Schistosomiasis
Malaria
Gynecological diseases
Maternal disorders
YLDs per 100,000 population
0 to <0
0 to <0
0 to <0.03
0.03 to <0.1
0.1 to <0.27
0.27 to <1.05
1.05 to <2.06
2.06 to <4.99
4.99 to <12.85
12.85 to 70.34

Tottori

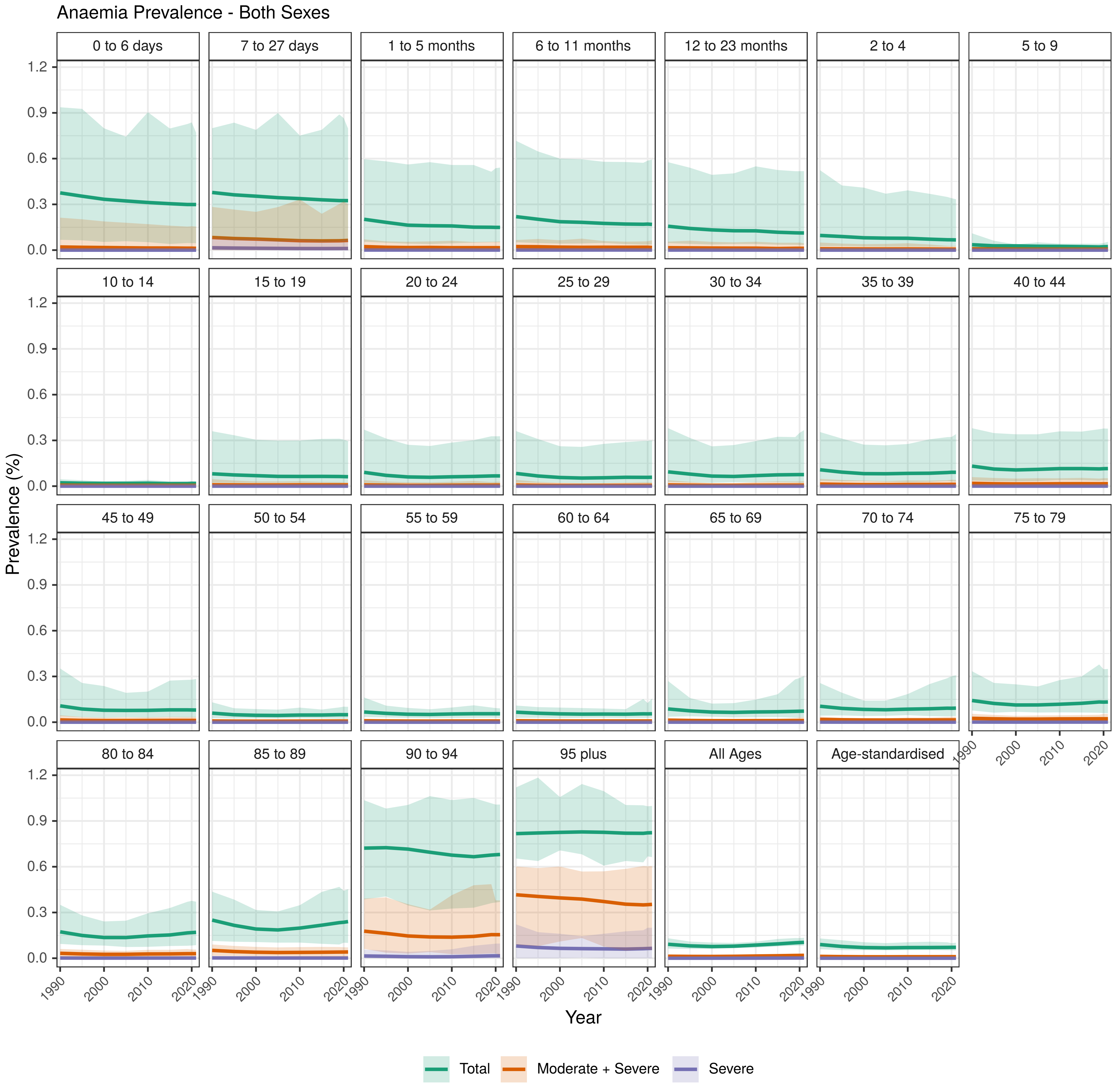
Anaemia Prevalence - Both Sexes
0 to 6 days
7 to 27 days
1 to 5 months
6 to 11 months
12 to 23 months
2 to 4
5 to 9
10 to 14
15 to 19
20 to 24
25 to 29
30 to 34
35 to 39
40 to 44
45 to 49
50 to 54
55 to 59
60 to 64
65 to 69
70 to 74
75 to 79
80 to 84
85 to 89
90 to 94
95 plus
All Ages
Age-standardised
Prevalence (%)
Year
Total
Moderate + Severe
Severe

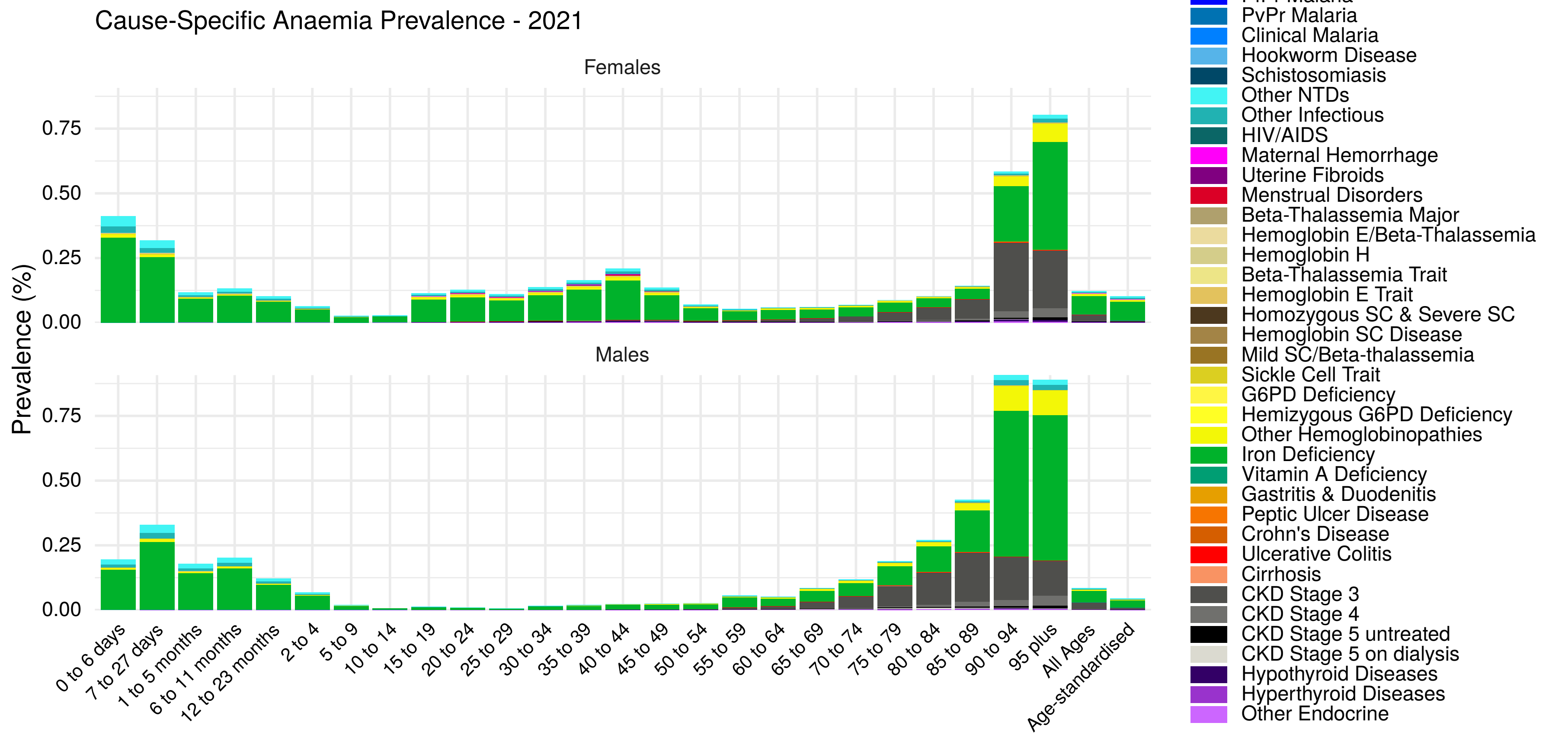
Cause-Specific Anaemia Prevalence - 2021
Females
Males
Prevalence (%)
PfPr Malaria
PvPr Malaria
Clinical Malaria
Hookworm Disease
Schistosomiasis
Other NTDs
Other Infectious
HIV/AIDS
Maternal Hemorrhage
Uterine Fibroids
Menstrual Disorders
Beta-Thalassemia Major
Hemoglobin E/Beta-Thalassemia
Hemoglobin H
Beta-Thalassemia Trait
Hemoglobin E Trait
Homozygous SC & Severe SC
Hemoglobin SC Disease
Mild SC/Beta-thalassemia
Sickle Cell Trait
G6PD Deficiency
Hemizygous G6PD Deficiency
Other Hemoglobinopathies
Iron Deficiency
Vitamin A Deficiency
Gastritis & Duodenitis
Peptic Ulcer Disease
Crohn's Disease
Ulcerative Colitis
Cirrhosis
CKD Stage 3
CKD Stage 4
CKD Stage 5 untreated
CKD Stage 5 on dialysis
Hypothyroid Diseases
Hyperthyroid Diseases
Other Endocrine

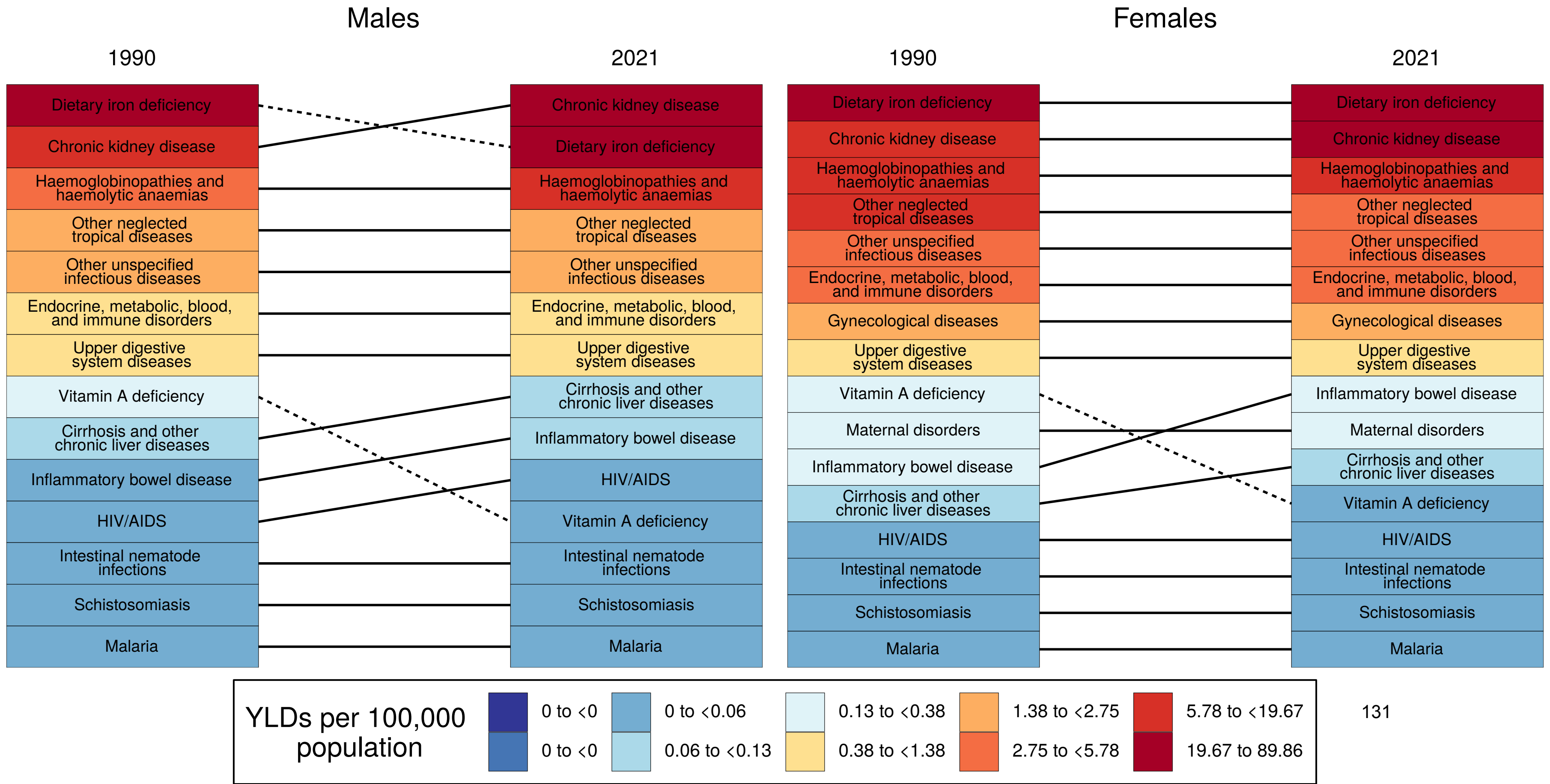
Males
Females
1990
2021
Dietary iron deficiency
Chronic kidney disease
Haemoglobinopathies and haemolytic anaemias
Other neglected tropical diseases
Other unspecified infectious diseases
Endocrine, metabolic, blood, and immune disorders
Upper digestive system diseases
Vitamin A deficiency
Cirrhosis and other chronic liver diseases
Inflammatory bowel disease
HIV/AIDS
Intestinal nematode infections
Schistosomiasis
Malaria
Gynecological diseases
Maternal disorders
YLDs per 100,000 population
0 to <0
0 to <0.06
0.06 to <0.13
0.13 to <0.38
0.38 to <1.38
1.38 to <2.75
2.75 to <5.78
5.78 to <19.67
19.67 to 89.86

Toyama

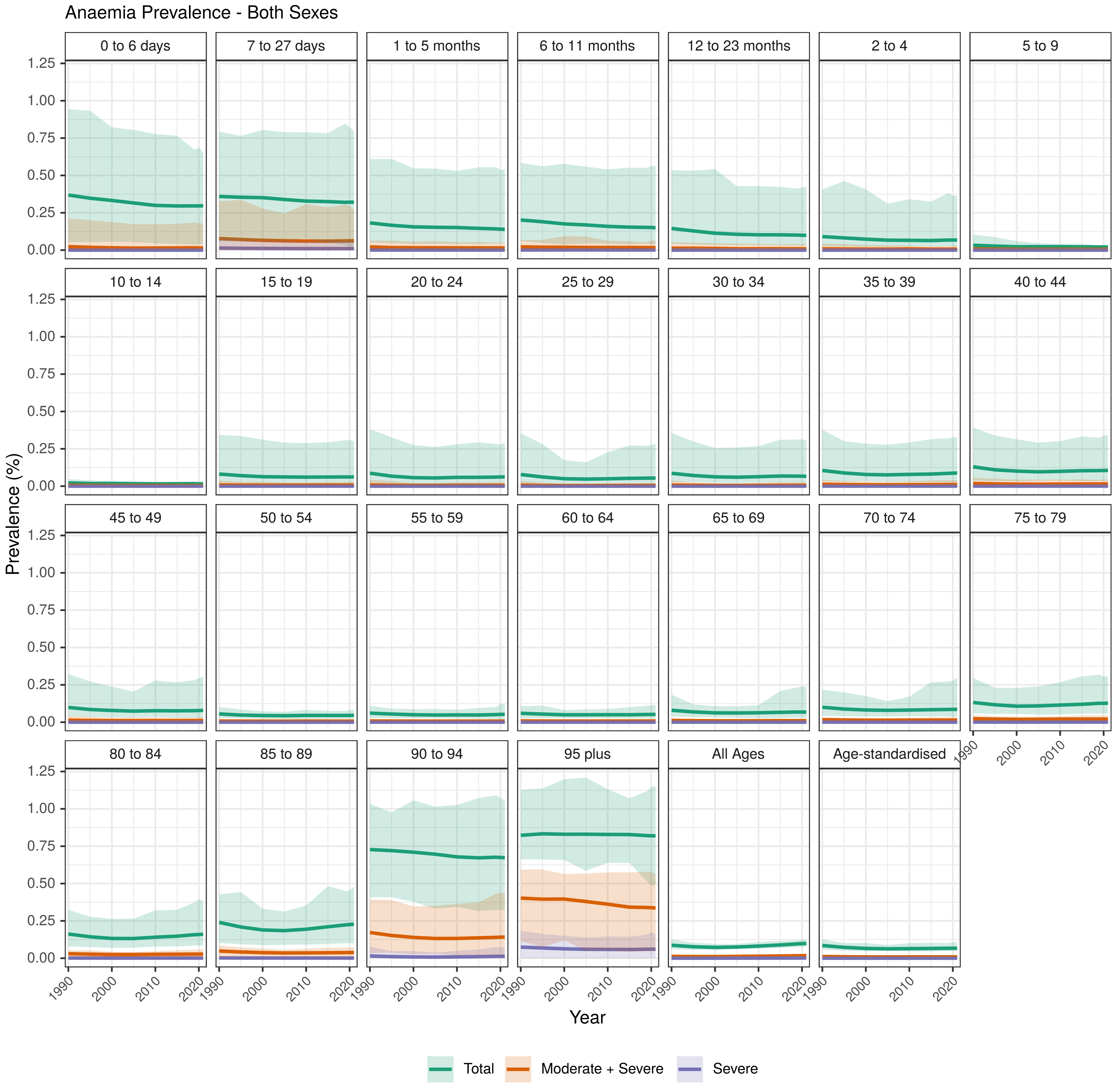

Anaemia Prevalence - Both Sexes
0 to 6 days
7 to 27 days
1 to 5 months
6 to 11 months
12 to 23 months
2 to 4
5 to 9
10 to 14
15 to 19
20 to 24
25 to 29
30 to 34
35 to 39
40 to 44
45 to 49
50 to 54
55 to 59
60 to 64
65 to 69
70 to 74
75 to 79
80 to 84
85 to 89
90 to 94
95 plus
All Ages
Age-standardised
Prevalence (%)
Year
Total
Moderate + Severe
Severe

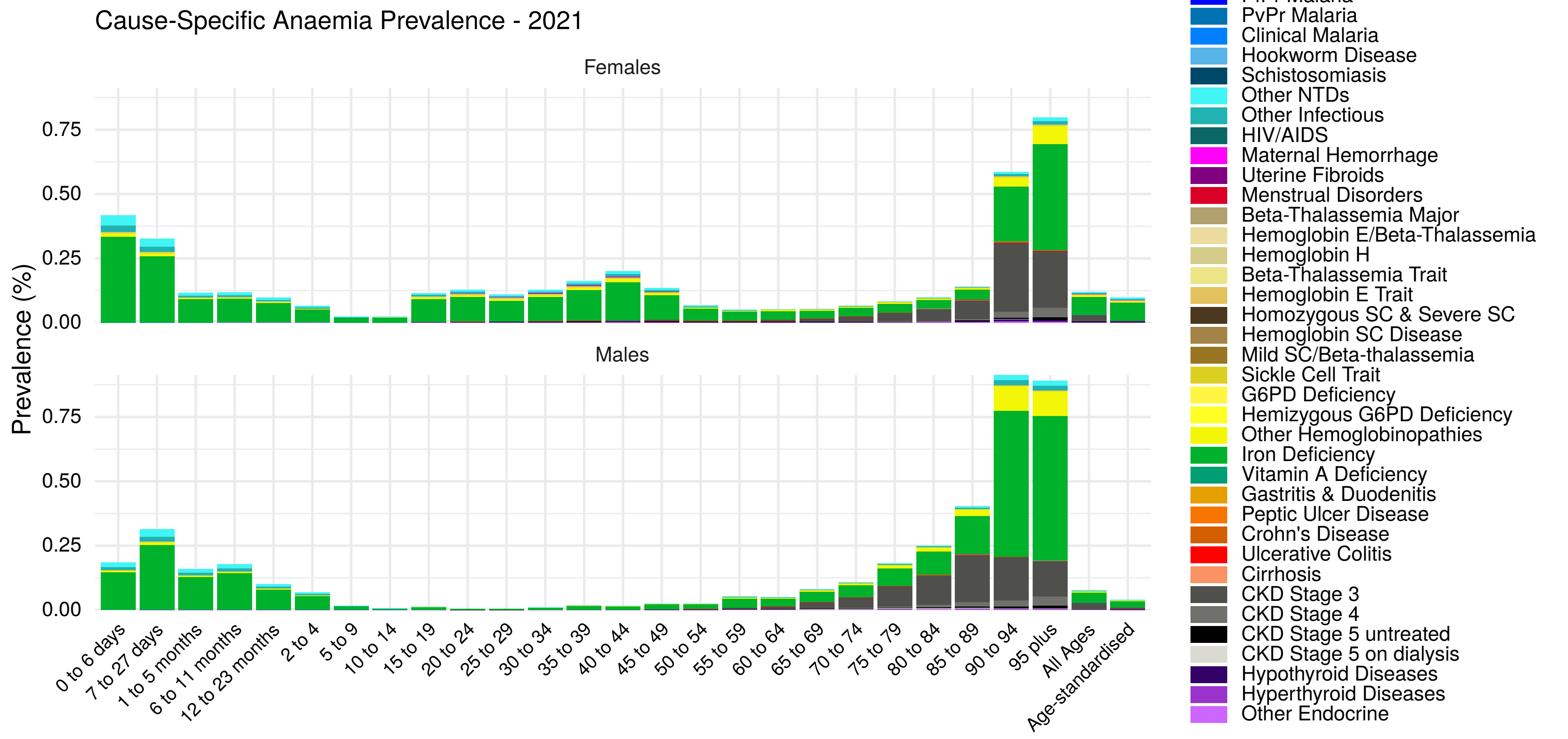

Cause-Specific Anaemia Prevalence - 2021
Females
Males
Prevalence (%)
PfPr Malaria
PvPr Malaria
Clinical Malaria
Hookworm Disease
Schistosomiasis
Other NTDs
Other Infectious
HIV/AIDS
Maternal Hemorrhage
Uterine Fibroids
Menstrual Disorders
Beta-Thalassemia Major
Hemoglobin E/Beta-Thalassemia
Hemoglobin H
Beta-Thalassemia Trait
Hemoglobin E Trait
Homozygous SC & Severe SC
Hemoglobin SC Disease
Mild SC/Beta-thalassemia
Sickle Cell Trait
G6PD Deficiency
Hemizygous G6PD Deficiency
Other Hemoglobinopathies
Iron Deficiency
Vitamin A Deficiency
Gastritis & Duodenitis
Peptic Ulcer Disease
Crohn's Disease
Ulcerative Colitis
Cirrhosis
CKD Stage 3
CKD Stage 4
CKD Stage 5 untreated
CKD Stage 5 on dialysis
Hypothyroid Diseases
Hyperthyroid Diseases
Other Endocrine

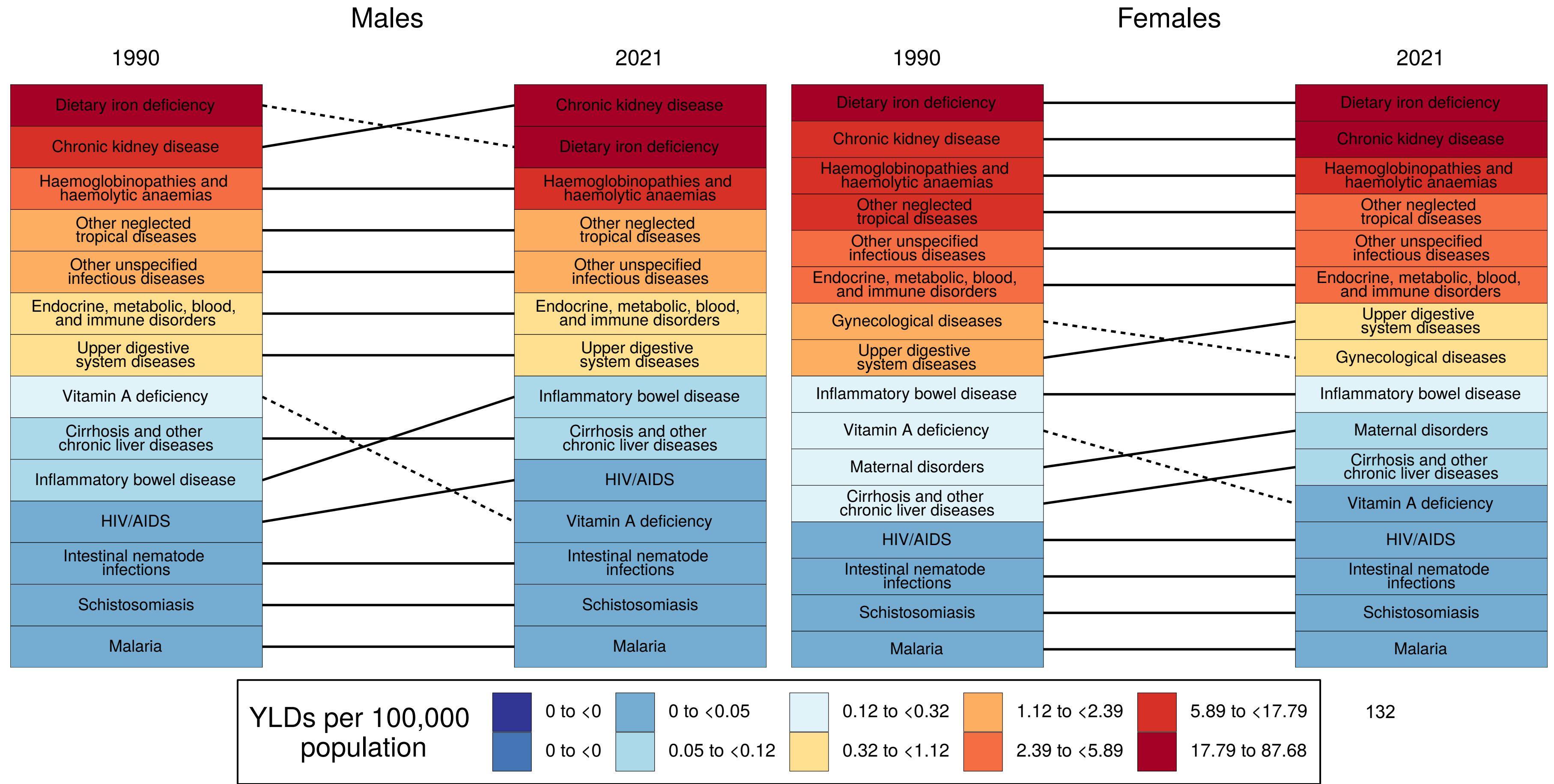

Males
Females
1990
2021
Dietary iron deficiency
Chronic kidney disease
Haemoglobinopathies and haemolytic anaemias
Other neglected tropical diseases
Other unspecified infectious diseases
Endocrine, metabolic, blood, and immune disorders
Upper digestive system diseases
Vitamin A deficiency
Cirrhosis and other chronic liver diseases
Inflammatory bowel disease
HIV/AIDS
Intestinal nematode infections
Schistosomiasis
Malaria
Gynecological diseases
Maternal disorders
YLDs per 100,000 population
0 to <0
0 to <0.05
0.05 to <0.12
0.12 to <0.32
0.32 to <1.12
1.12 to <2.39
2.39 to <5.89
5.89 to <17.79
17.79 to 87.68

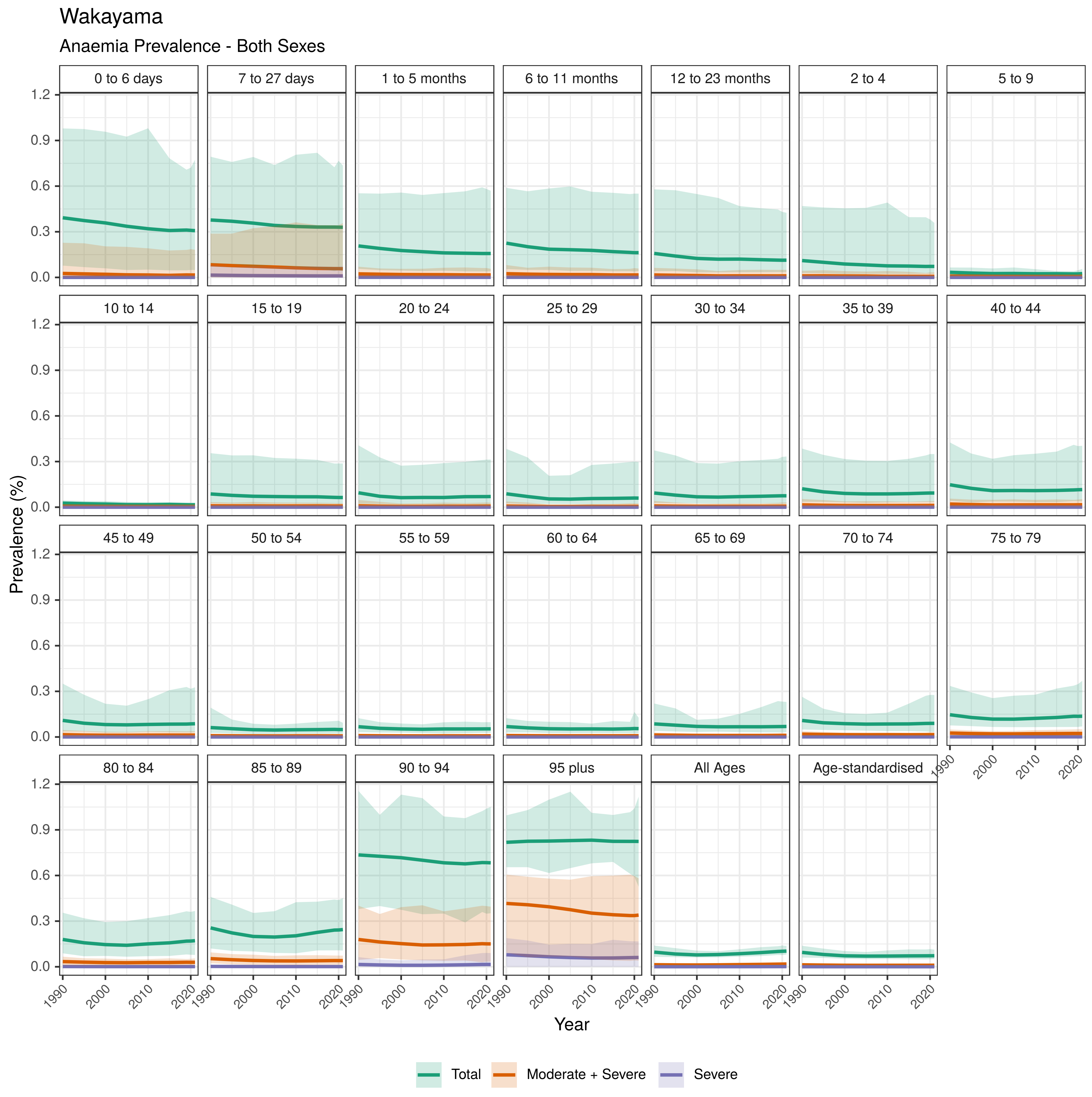
Wakayama
Anaemia Prevalence - Both Sexes
0 to 6 days
7 to 27 days
1 to 5 months
6 to 11 months
12 to 23 months
2 to 4
5 to 9
10 to 14
15 to 19
20 to 24
25 to 29
30 to 34
35 to 39
40 to 44
45 to 49
50 to 54
55 to 59
60 to 64
65 to 69
70 to 74
75 to 79
80 to 84
85 to 89
90 to 94
95 plus
All Ages
Age-standardised
Prevalence (%)
Year
Total
Moderate + Severe
Severe

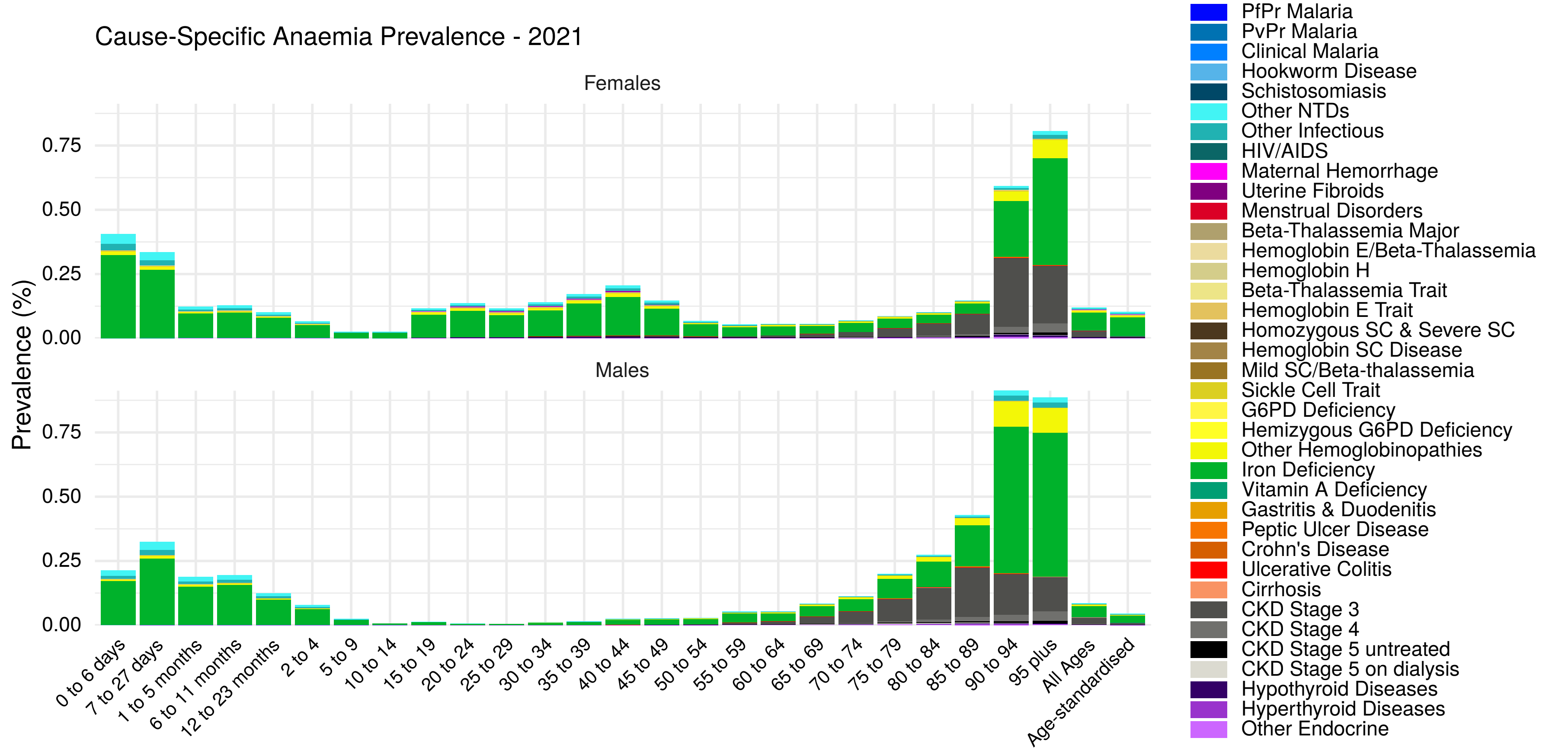
Cause-Specific Anaemia Prevalence - 2021
Females
Males
Prevalence (%)
PfPr Malaria
PvPr Malaria
Clinical Malaria
Hookworm Disease
Schistosomiasis
Other NTDs
Other Infectious
HIV/AIDS
Maternal Hemorrhage
Uterine Fibroids
Menstrual Disorders
Beta-Thalassemia Major
Hemoglobin E/Beta-Thalassemia
Hemoglobin H
Beta-Thalassemia Trait
Hemoglobin E Trait
Homozygous SC & Severe SC
Hemoglobin SC Disease
Mild SC/Beta-thalassemia
Sickle Cell Trait
G6PD Deficiency
Hemizygous G6PD Deficiency
Other Hemoglobinopathies
Iron Deficiency
Vitamin A Deficiency
Gastritis & Duodenitis
Peptic Ulcer Disease
Crohn's Disease
Ulcerative Colitis
Cirrhosis
CKD Stage 3
CKD Stage 4
CKD Stage 5 untreated
CKD Stage 5 on dialysis
Hypothyroid Diseases
Hyperthyroid Diseases
Other Endocrine

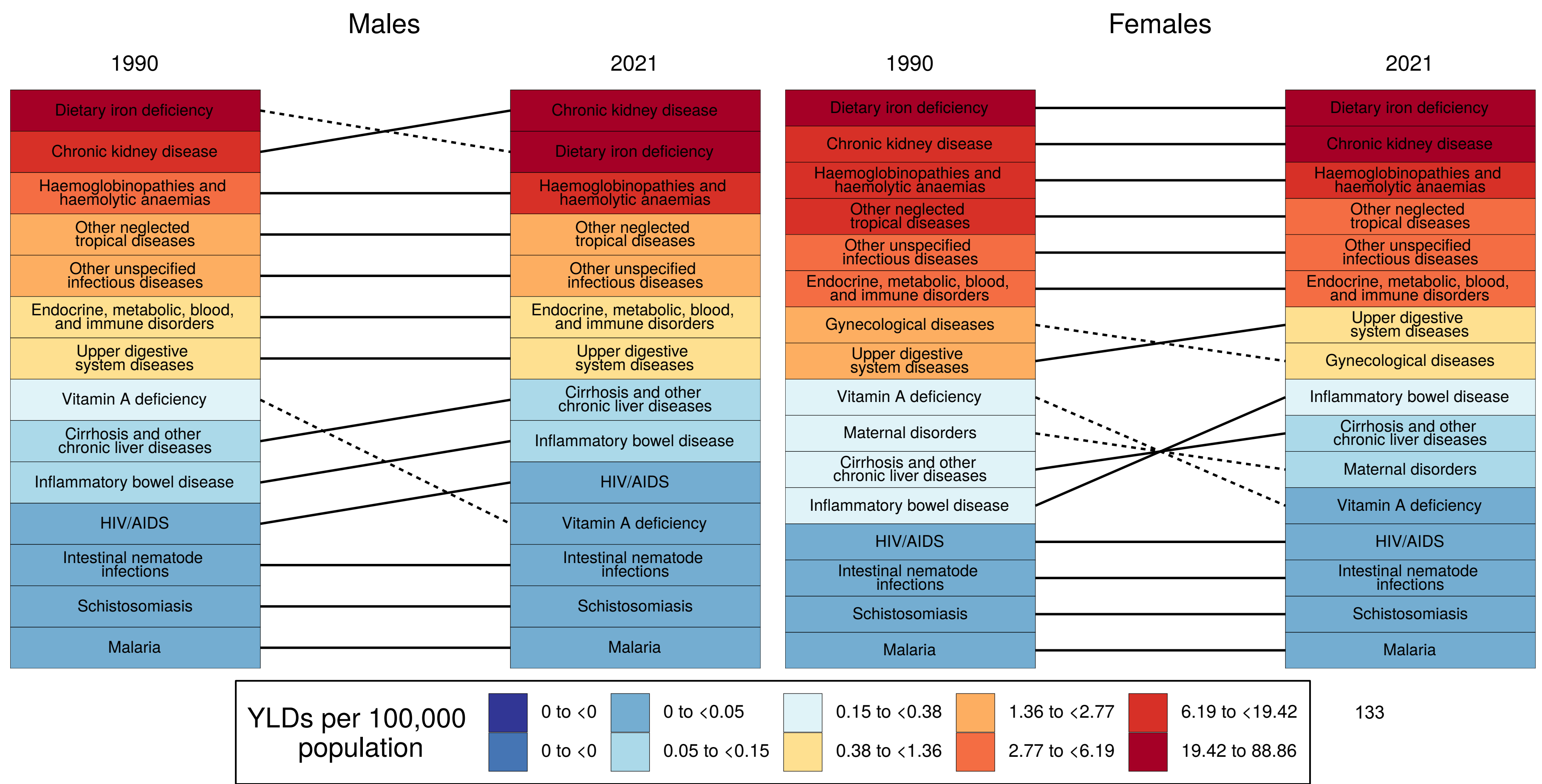
Males
Females
1990
2021
Dietary iron deficiency
Chronic kidney disease
Haemoglobinopathies and haemolytic anaemias
Other neglected tropical diseases
Other unspecified infectious diseases
Endocrine, metabolic, blood, and immune disorders
Upper digestive system diseases
Vitamin A deficiency
Cirrhosis and other chronic liver diseases
Inflammatory bowel disease
HIV/AIDS
Intestinal nematode infections
Schistosomiasis
Malaria
Gynecological diseases
Maternal disorders
YLDs per 100,000 population
0 to <0
0 to <0.05
0.05 to <0.15
0.15 to <0.38
0.38 to <1.36
1.36 to <2.77
2.77 to <6.19
6.19 to <19.42
19.42 to 88.86

Yamagata

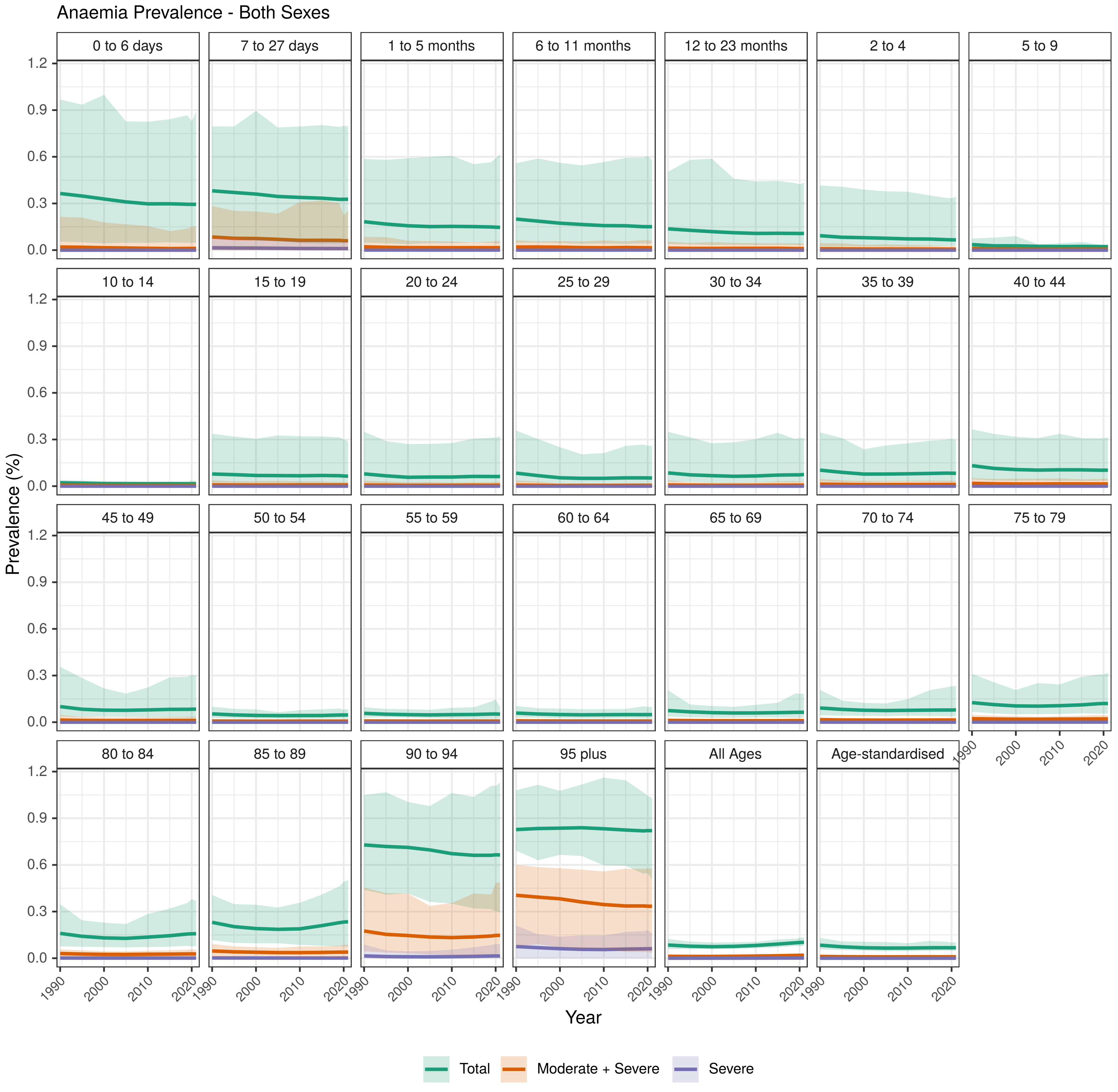

Anaemia Prevalence - Both Sexes
0 to 6 days
7 to 27 days
1 to 5 months
6 to 11 months
12 to 23 months
2 to 4
5 to 9
10 to 14
15 to 19
20 to 24
25 to 29
30 to 34
35 to 39
40 to 44
45 to 49
50 to 54
55 to 59
60 to 64
65 to 69
70 to 74
75 to 79
80 to 84
85 to 89
90 to 94
95 plus
All Ages
Age-standardised
Prevalence (%)
Year
Total
Moderate + Severe
Severe

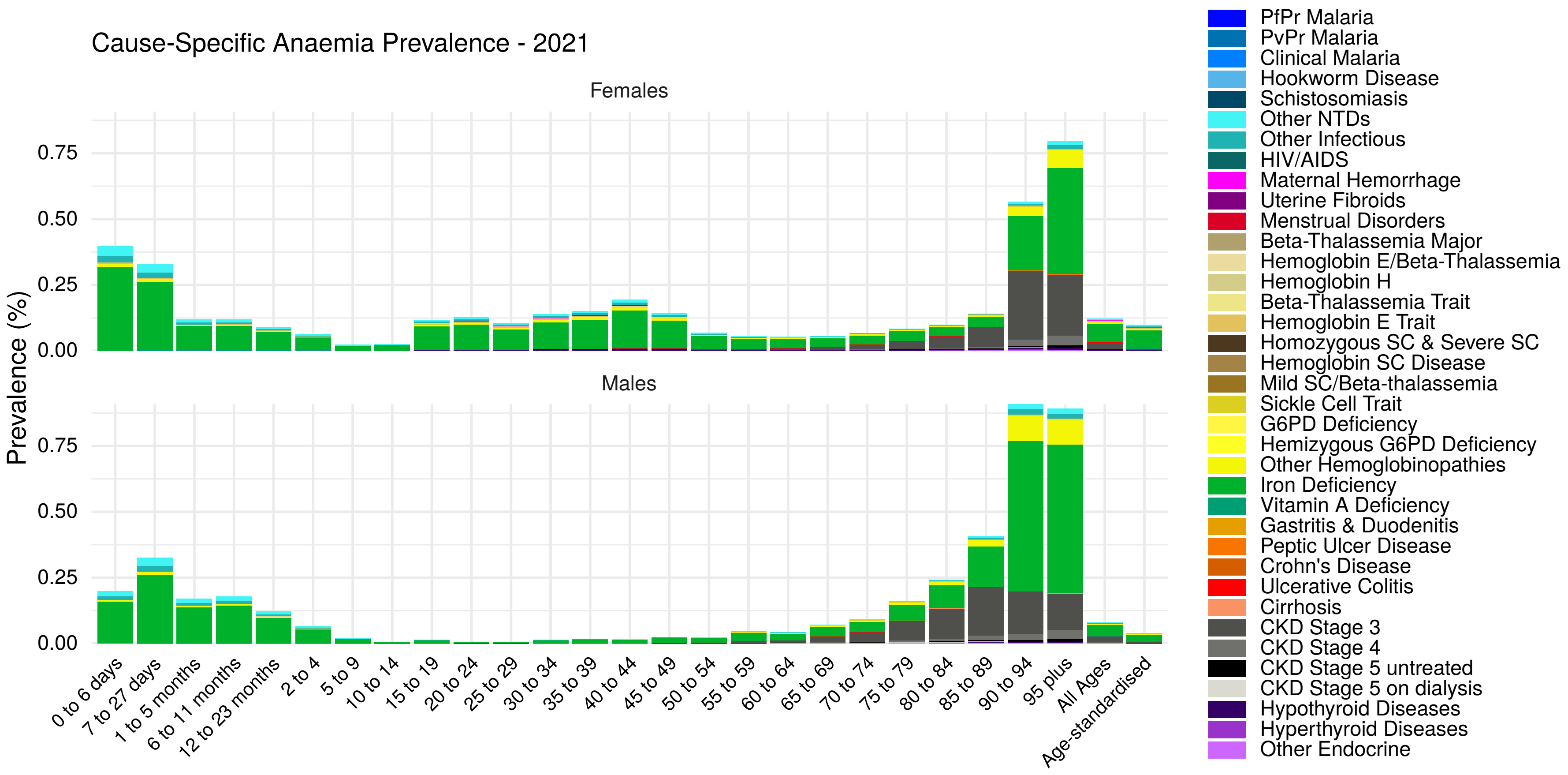

Cause-Specific Anaemia Prevalence - 2021
Females
Males
Prevalence (%)
PfPr Malaria
PvPr Malaria
Clinical Malaria
Hookworm Disease
Schistosomiasis
Other NTDs
Other Infectious
HIV/AIDS
Maternal Hemorrhage
Uterine Fibroids
Menstrual Disorders
Beta-Thalassemia Major
Hemoglobin E/Beta-Thalassemia
Hemoglobin H
Beta-Thalassemia Trait
Hemoglobin E Trait
Homozygous SC & Severe SC
Hemoglobin SC Disease
Mild SC/Beta-thalassemia
Sickle Cell Trait
G6PD Deficiency
Hemizygous G6PD Deficiency
Other Hemoglobinopathies
Iron Deficiency
Vitamin A Deficiency
Gastritis & Duodenitis
Peptic Ulcer Disease
Crohn's Disease
Ulcerative Colitis
Cirrhosis
CKD Stage 3
CKD Stage 4
CKD Stage 5 untreated
CKD Stage 5 on dialysis
Hypothyroid Diseases
Hyperthyroid Diseases
Other Endocrine

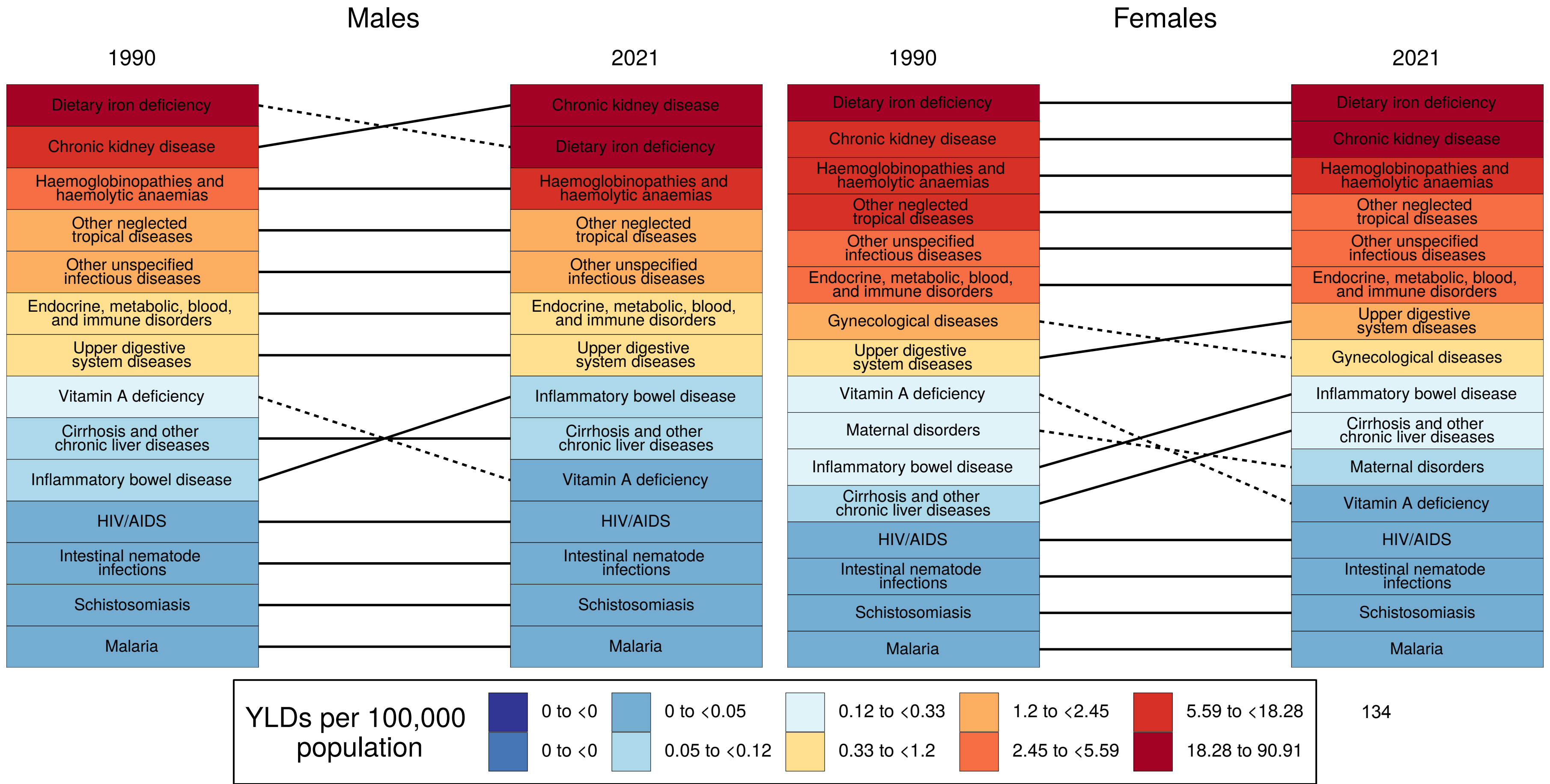

Males
Females
1990
2021
Dietary iron deficiency
Chronic kidney disease
Haemoglobinopathies and haemolytic anaemias
Other neglected tropical diseases
Other unspecified infectious diseases
Endocrine, metabolic, blood, and immune disorders
Upper digestive system diseases
Gynecological diseases
Vitamin A deficiency
Maternal disorders
Cirrhosis and other chronic liver diseases
Inflammatory bowel disease
HIV/AIDS
Intestinal nematode infections
Schistosomiasis
Malaria
YLDs per 100,000 population
0 to <0
0 to <0.05
0.05 to <0.12
0.12 to <0.33
0.33 to <1.2
1.2 to <2.45
2.45 to <5.59
5.59 to <18.28
18.28 to 90.91

Yamaguchi

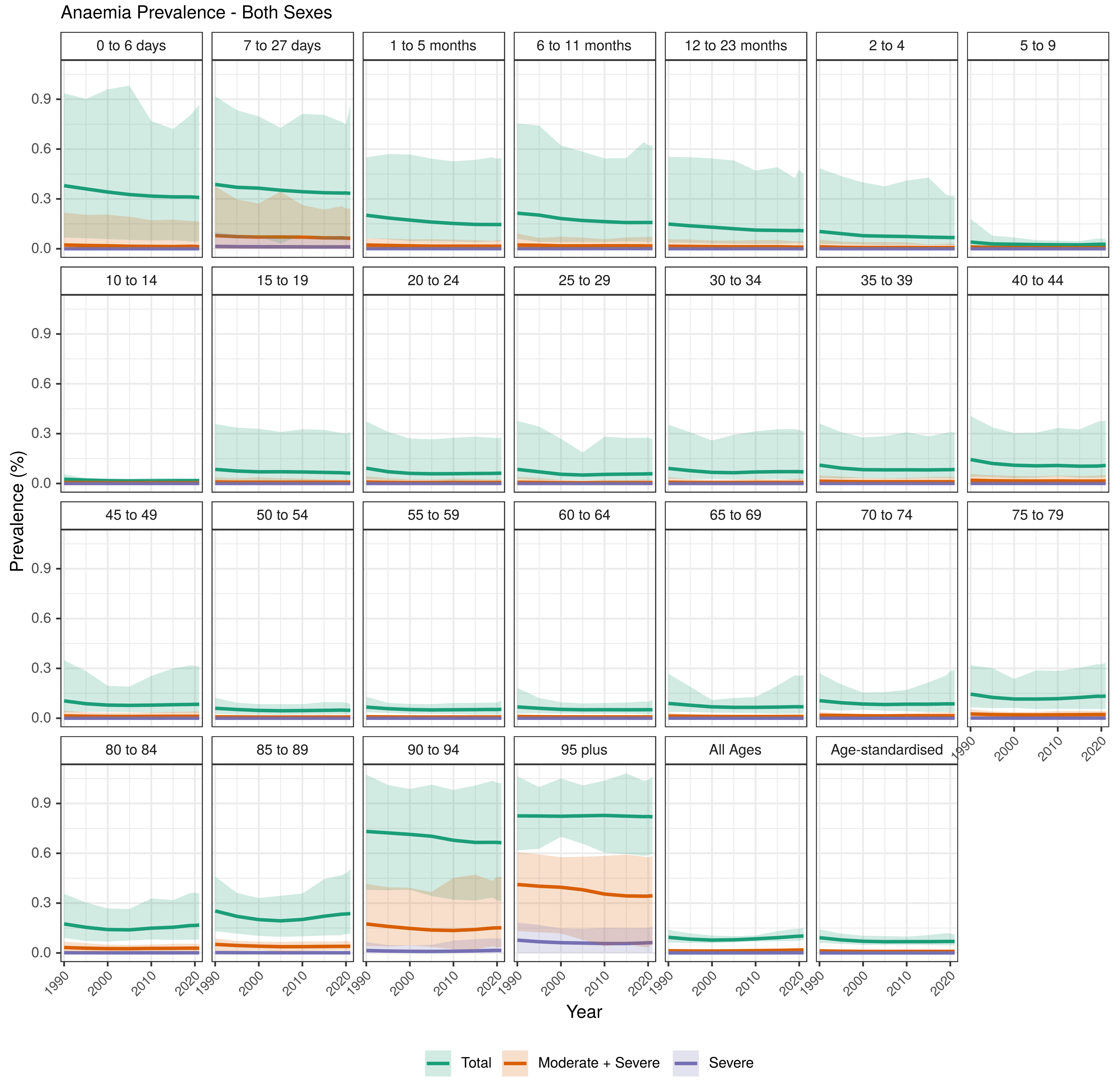
Anaemia Prevalence - Both Sexes
0 to 6 days
7 to 27 days
1 to 5 months
6 to 11 months
12 to 23 months
2 to 4
5 to 9
10 to 14
15 to 19
20 to 24
25 to 29
30 to 34
35 to 39
40 to 44
45 to 49
50 to 54
55 to 59
60 to 64
65 to 69
70 to 74
75 to 79
80 to 84
85 to 89
90 to 94
95 plus
All Ages
Age-standardised
0.0
0.3
0.6
0.9
Prevalence (%)
1990
2000
2010
2020
Year
Total
Moderate + Severe
Severe

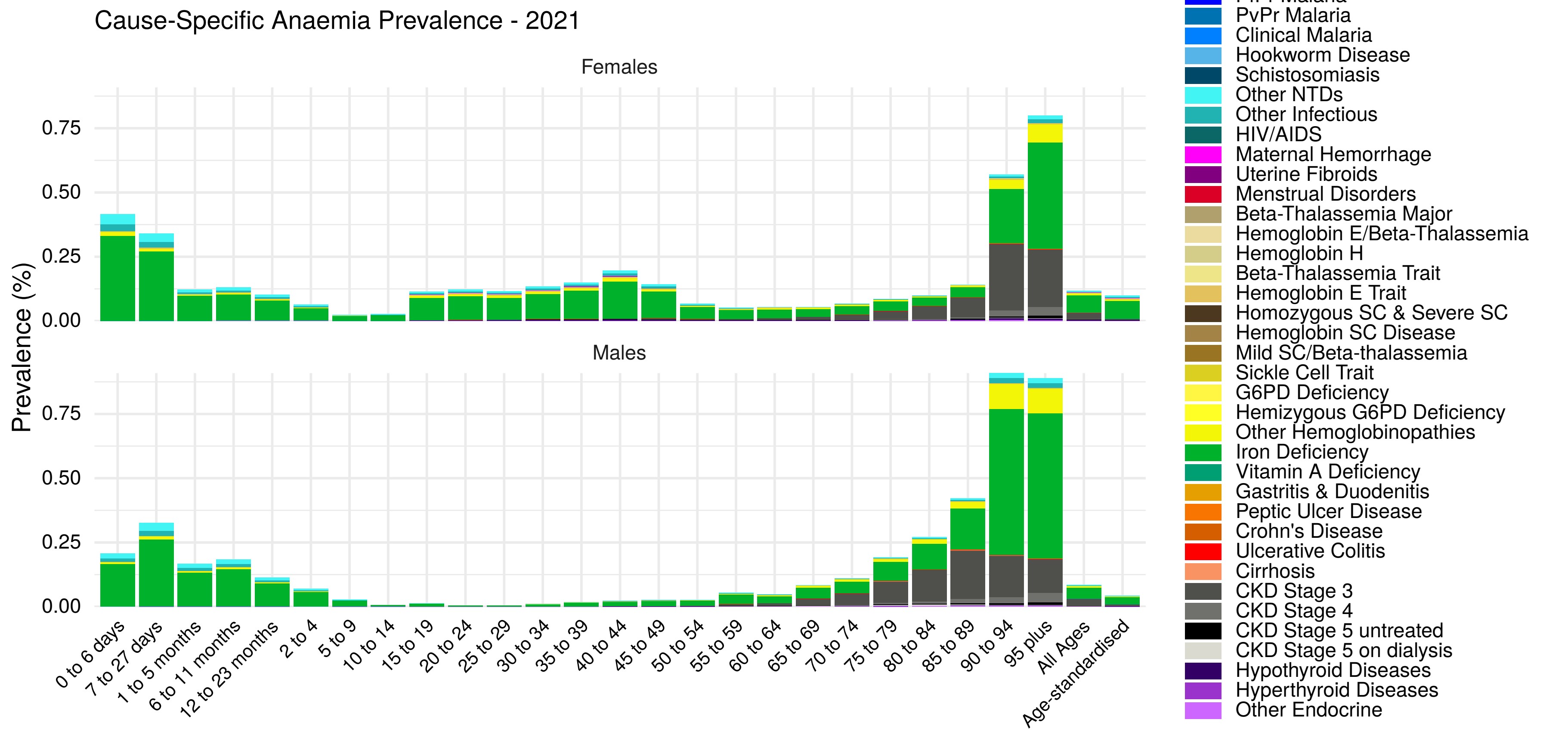
Cause-Specific Anaemia Prevalence - 2021
Females
Males
Prevalence (%)
0.00
0.25
0.50
0.75
0 to 6 days
7 to 27 days
1 to 5 months
6 to 11 months
12 to 23 months
2 to 4
5 to 9
10 to 14
15 to 19
20 to 24
25 to 29
30 to 34
35 to 39
40 to 44
45 to 49
50 to 54
55 to 59
60 to 64
65 to 69
70 to 74
75 to 79
80 to 84
85 to 89
90 to 94
95 plus
All Ages
Age-standardised
PfPr Malaria
PvPr Malaria
Clinical Malaria
Hookworm Disease
Schistosomiasis
Other NTDs
Other Infectious
HIV/AIDS
Maternal Hemorrhage
Uterine Fibroids
Menstrual Disorders
Beta-Thalassemia Major
Hemoglobin E/Beta-Thalassemia
Hemoglobin H
Beta-Thalassemia Trait
Hemoglobin E Trait
Homozygous SC & Severe SC
Hemoglobin SC Disease
Mild SC/Beta-thalassemia
Sickle Cell Trait
G6PD Deficiency
Hemizygous G6PD Deficiency
Other Hemoglobinopathies
Iron Deficiency
Vitamin A Deficiency
Gastritis & Duodenitis
Peptic Ulcer Disease
Crohn's Disease
Ulcerative Colitis
Cirrhosis
CKD Stage 3
CKD Stage 4
CKD Stage 5 untreated
CKD Stage 5 on dialysis
Hypothyroid Diseases
Hyperthyroid Diseases
Other Endocrine

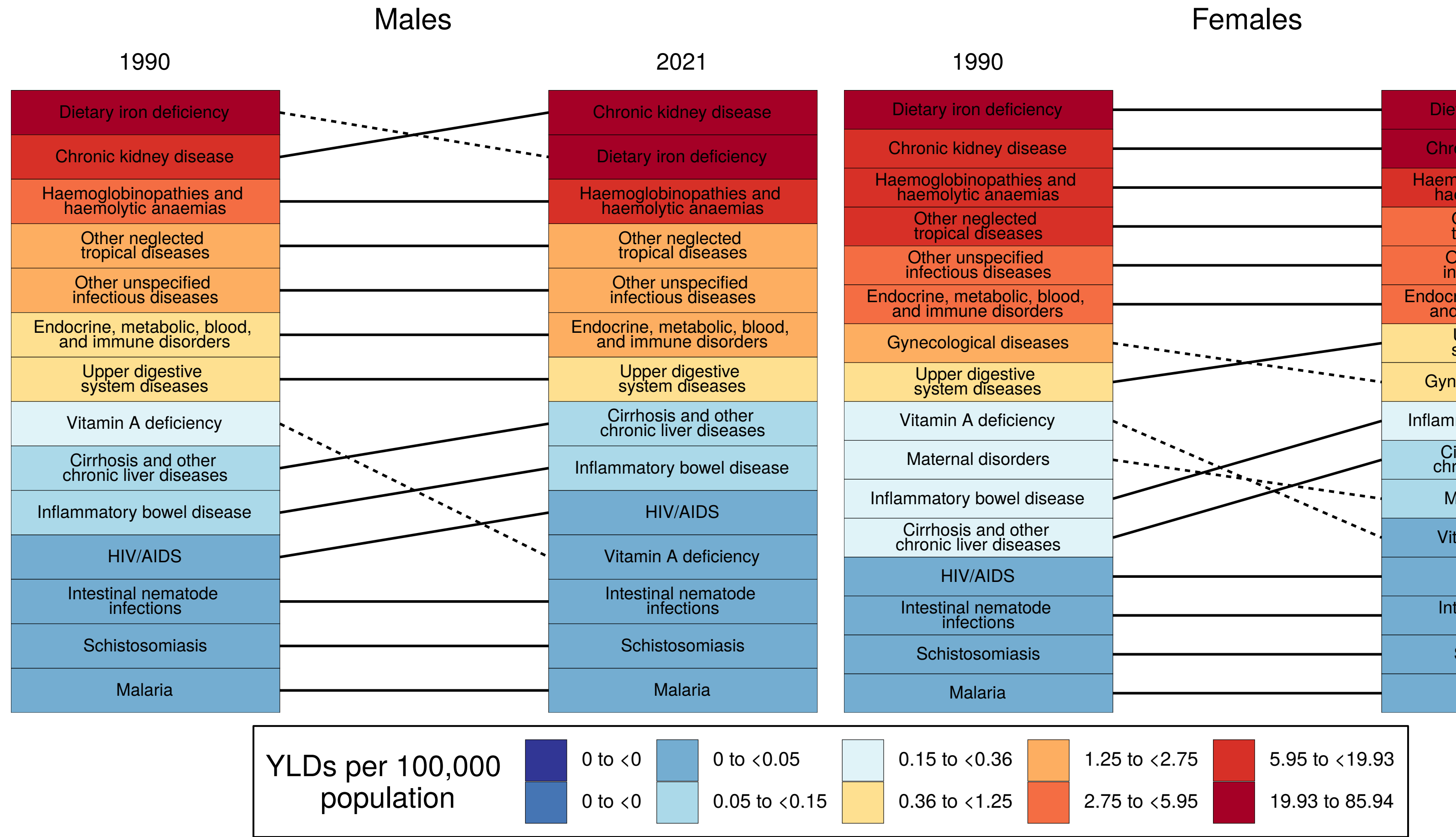
Males
Females
1990
2021
Dietary iron deficiency
Chronic kidney disease
Haemoglobinopathies and haemolytic anaemias
Other neglected tropical diseases
Other unspecified infectious diseases
Endocrine, metabolic, blood, and immune disorders
Upper digestive system diseases
Vitamin A deficiency
Cirrhosis and other chronic liver diseases
Inflammatory bowel disease
HIV/AIDS
Intestinal nematode infections
Schistosomiasis
Malaria
Gynecological diseases
Maternal disorders
YLDs per 100,000 population
0 to <0
0 to <0
0 to <0.05
0.05 to <0.15
0.15 to <0.36
0.36 to <1.25
1.25 to <2.75
2.75 to <5.95
5.95 to <19.93
19.93 to 85.94

Yamanashi

Anaemia Prevalence - Both Sexes

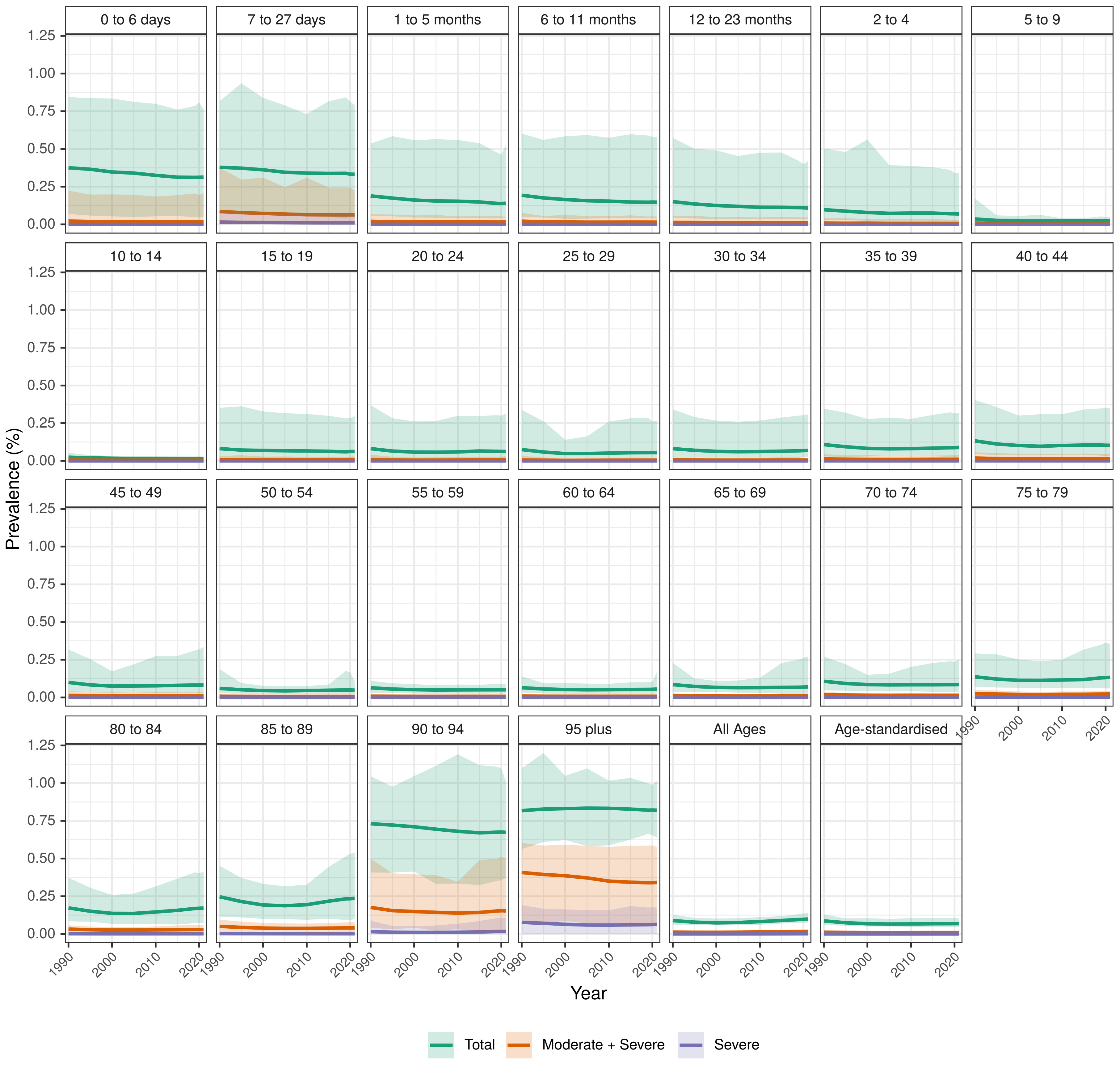

Cause-Specific Anaemia Prevalence - 2021

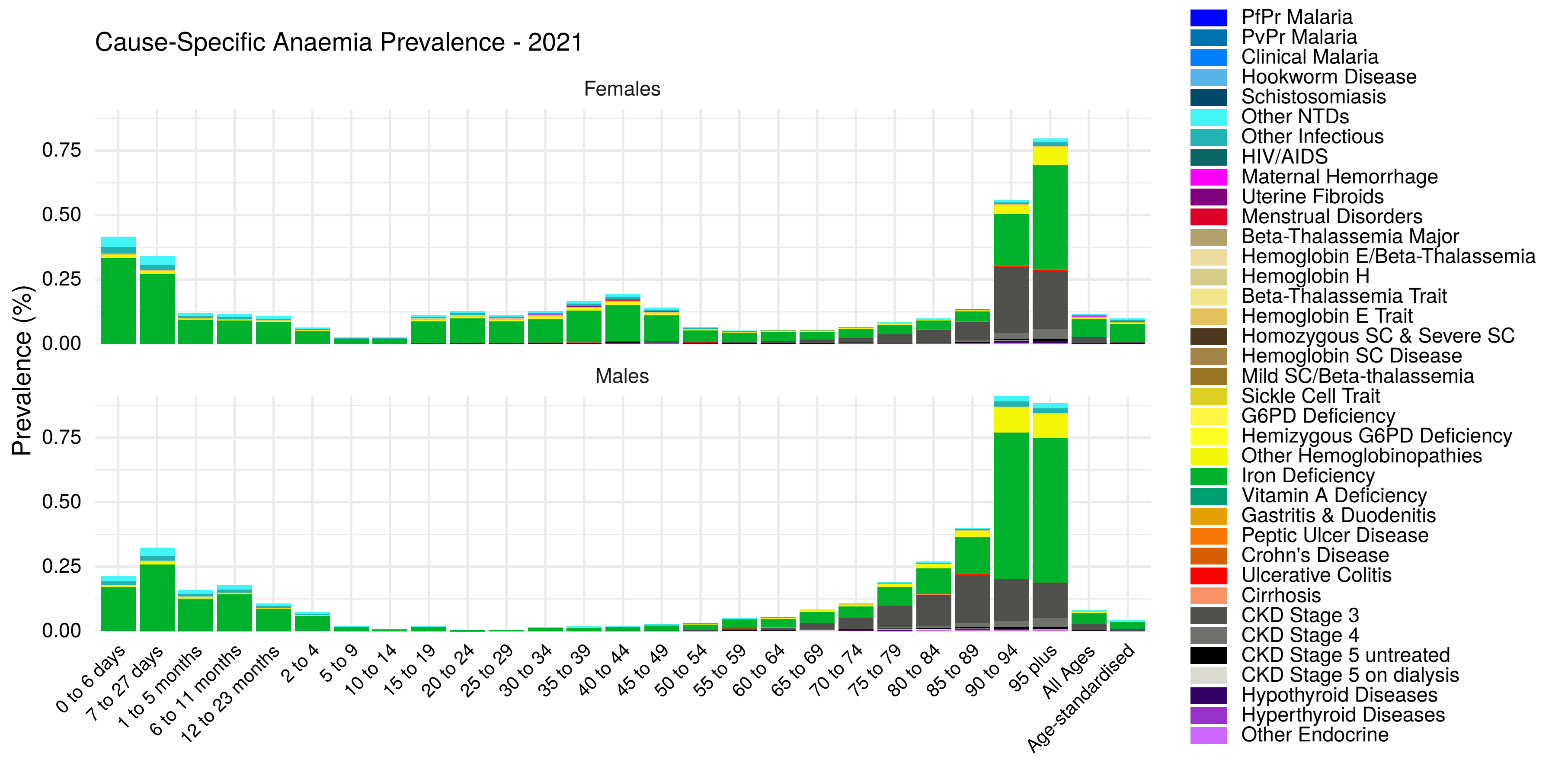

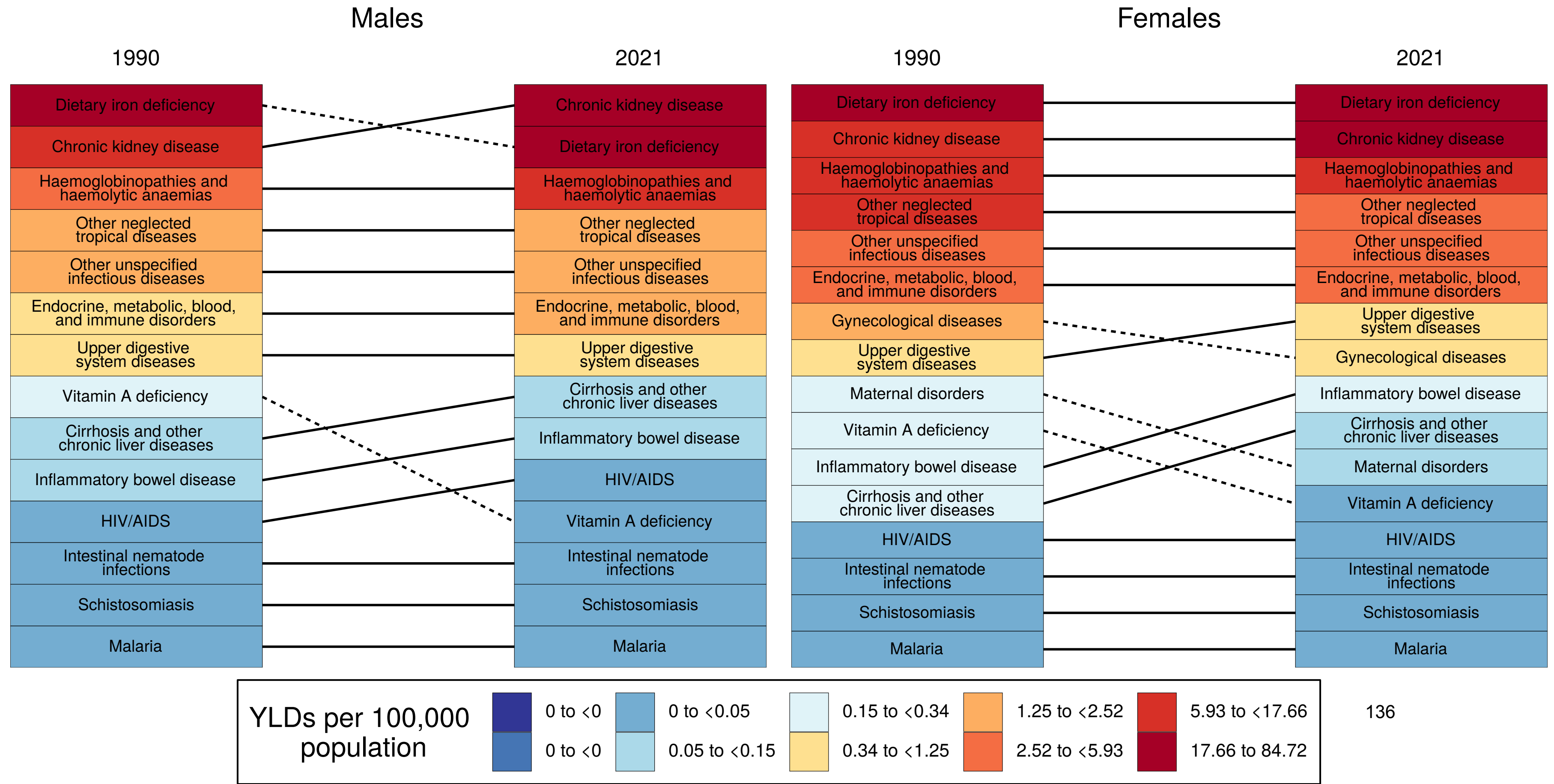

South Korea

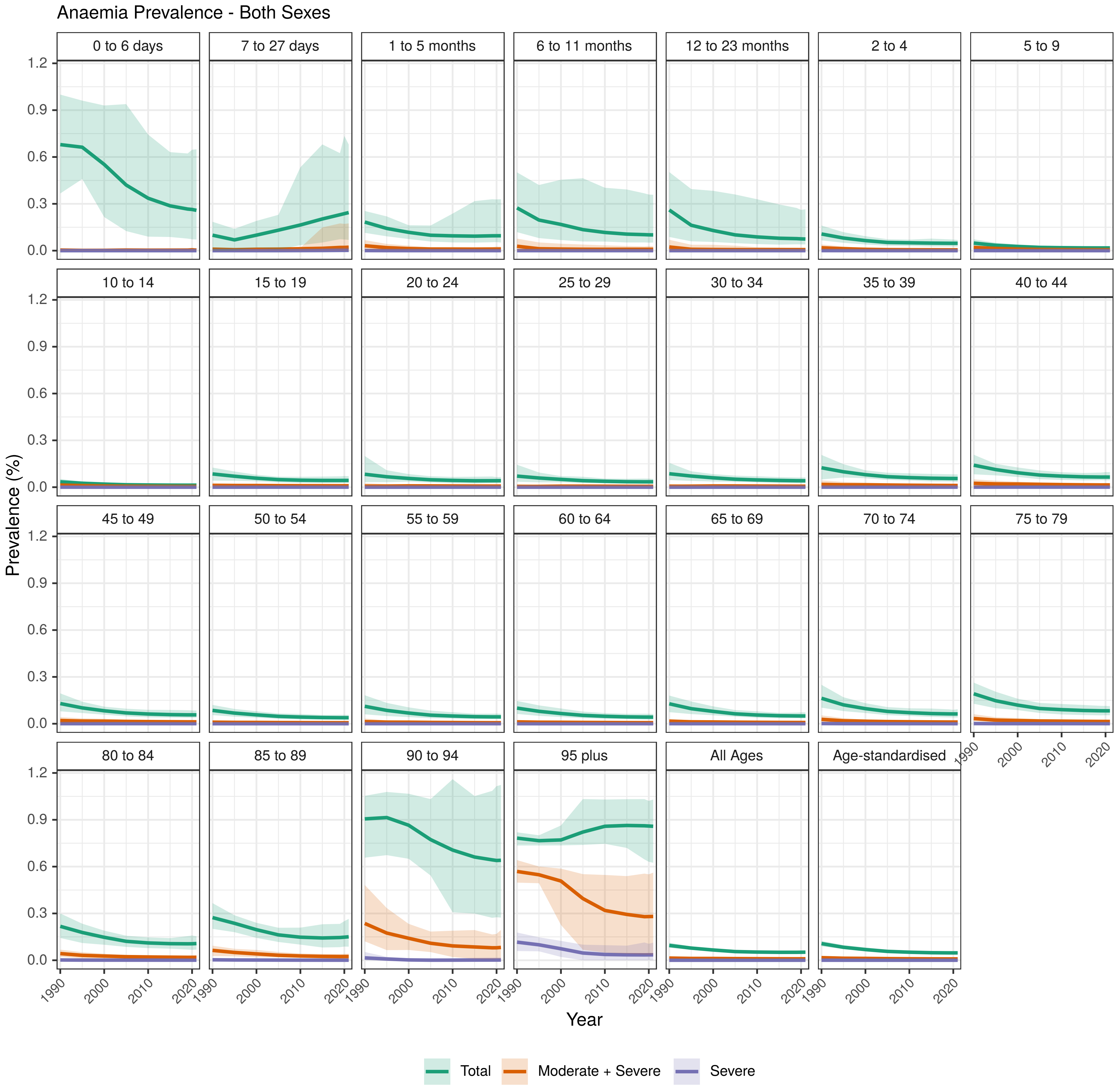

Anaemia Prevalence - Both Sexes
0 to 6 days
7 to 27 days
1 to 5 months
6 to 11 months
12 to 23 months
2 to 4
5 to 9
10 to 14
15 to 19
20 to 24
25 to 29
30 to 34
35 to 39
40 to 44
45 to 49
50 to 54
55 to 59
60 to 64
65 to 69
70 to 74
75 to 79
80 to 84
85 to 89
90 to 94
95 plus
All Ages
Age-standardised
Prevalence (%)
0.0
0.3
0.6
0.9
1.2
1990
2000
2010
2020
Year
Total
Moderate + Severe
Severe

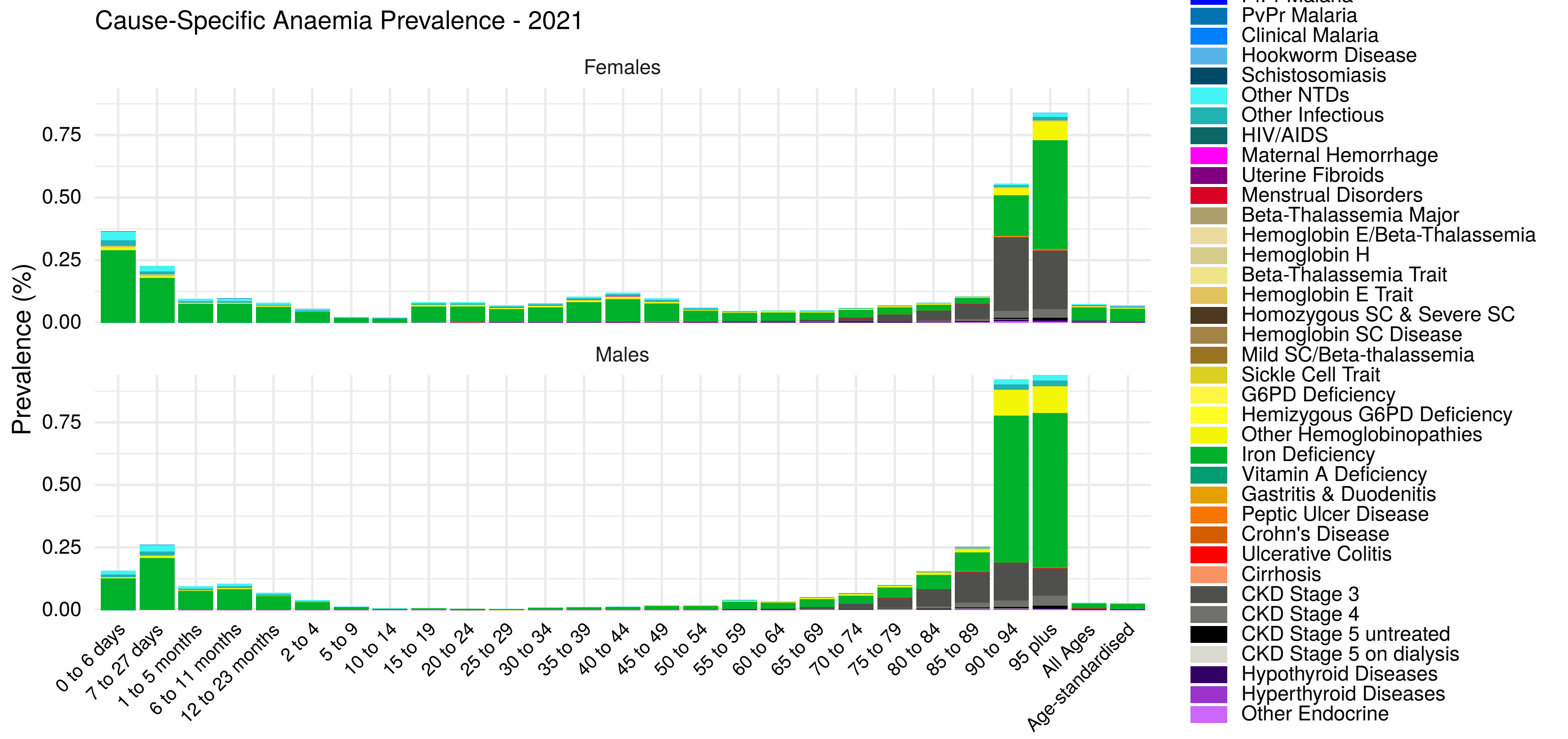

Cause-Specific Anaemia Prevalence - 2021
Females
Males
Prevalence (%)
0.00
0.25
0.50
0.75
0 to 6 days
7 to 27 days
1 to 5 months
6 to 11 months
12 to 23 months
2 to 4
5 to 9
10 to 14
15 to 19
20 to 24
25 to 29
30 to 34
35 to 39
40 to 44
45 to 49
50 to 54
55 to 59
60 to 64
65 to 69
70 to 74
75 to 79
80 to 84
85 to 89
90 to 94
95 plus
All Ages
Age-standardised
PfPr Malaria
PvPr Malaria
Clinical Malaria
Hookworm Disease
Schistosomiasis
Other NTDs
Other Infectious
HIV/AIDS
Maternal Hemorrhage
Uterine Fibroids
Menstrual Disorders
Beta-Thalassemia Major
Hemoglobin E/Beta-Thalassemia
Hemoglobin H
Beta-Thalassemia Trait
Hemoglobin E Trait
Homozygous SC & Severe SC
Hemoglobin SC Disease
Mild SC/Beta-thalassemia
Sickle Cell Trait
G6PD Deficiency
Hemizygous G6PD Deficiency
Other Hemoglobinopathies
Iron Deficiency
Vitamin A Deficiency
Gastritis & Duodenitis
Peptic Ulcer Disease
Crohn's Disease
Ulcerative Colitis
Cirrhosis
CKD Stage 3
CKD Stage 4
CKD Stage 5 untreated
CKD Stage 5 on dialysis
Hypothyroid Diseases
Hyperthyroid Diseases
Other Endocrine

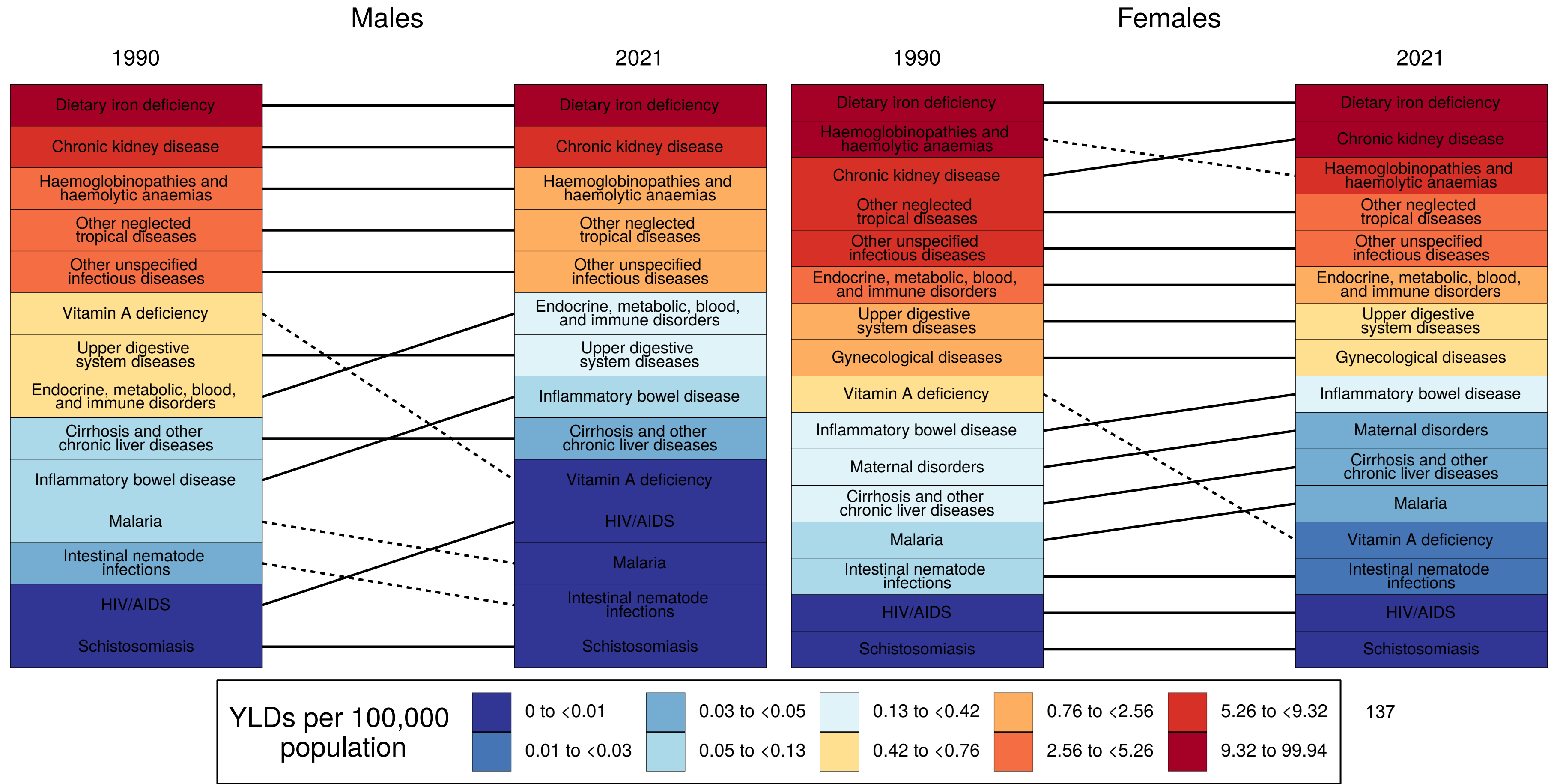

Males
Females
1990
2021
Dietary iron deficiency
Chronic kidney disease
Haemoglobinopathies and haemolytic anaemias
Other neglected tropical diseases
Other unspecified infectious diseases
Vitamin A deficiency
Upper digestive system diseases
Endocrine, metabolic, blood, and immune disorders
Cirrhosis and other chronic liver diseases
Inflammatory bowel disease
Malaria
Intestinal nematode infections
HIV/AIDS
Schistosomiasis
Gynecological diseases
Maternal disorders
YLDs per 100,000 population
0 to <0.01
0.01 to <0.03
0.03 to <0.05
0.05 to <0.13
0.13 to <0.42
0.42 to <0.76
0.76 to <2.56
2.56 to <5.26
5.26 to <9.32
9.32 to 99.94

Singapore

Anaemia Prevalence - Both Sexes

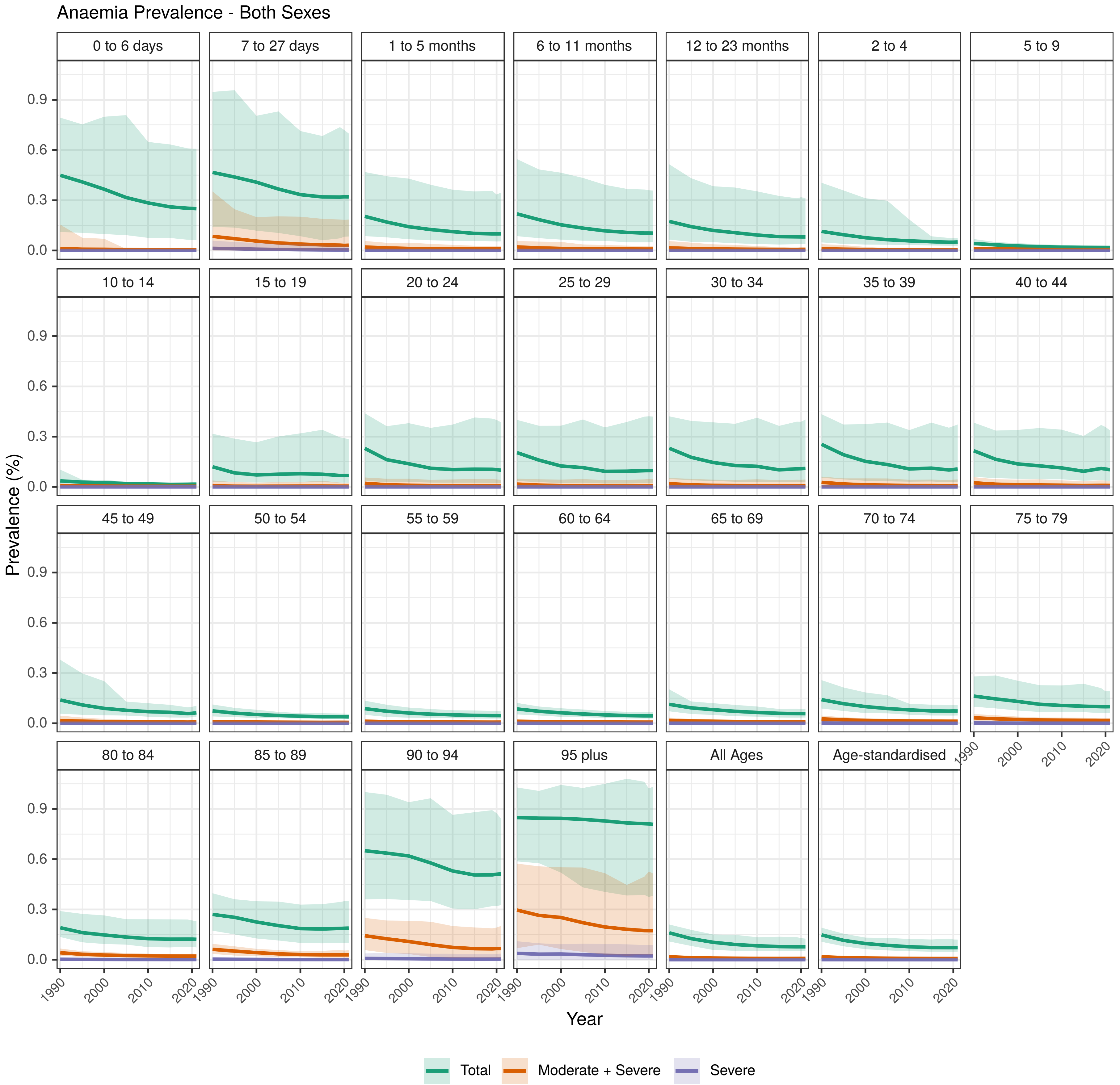

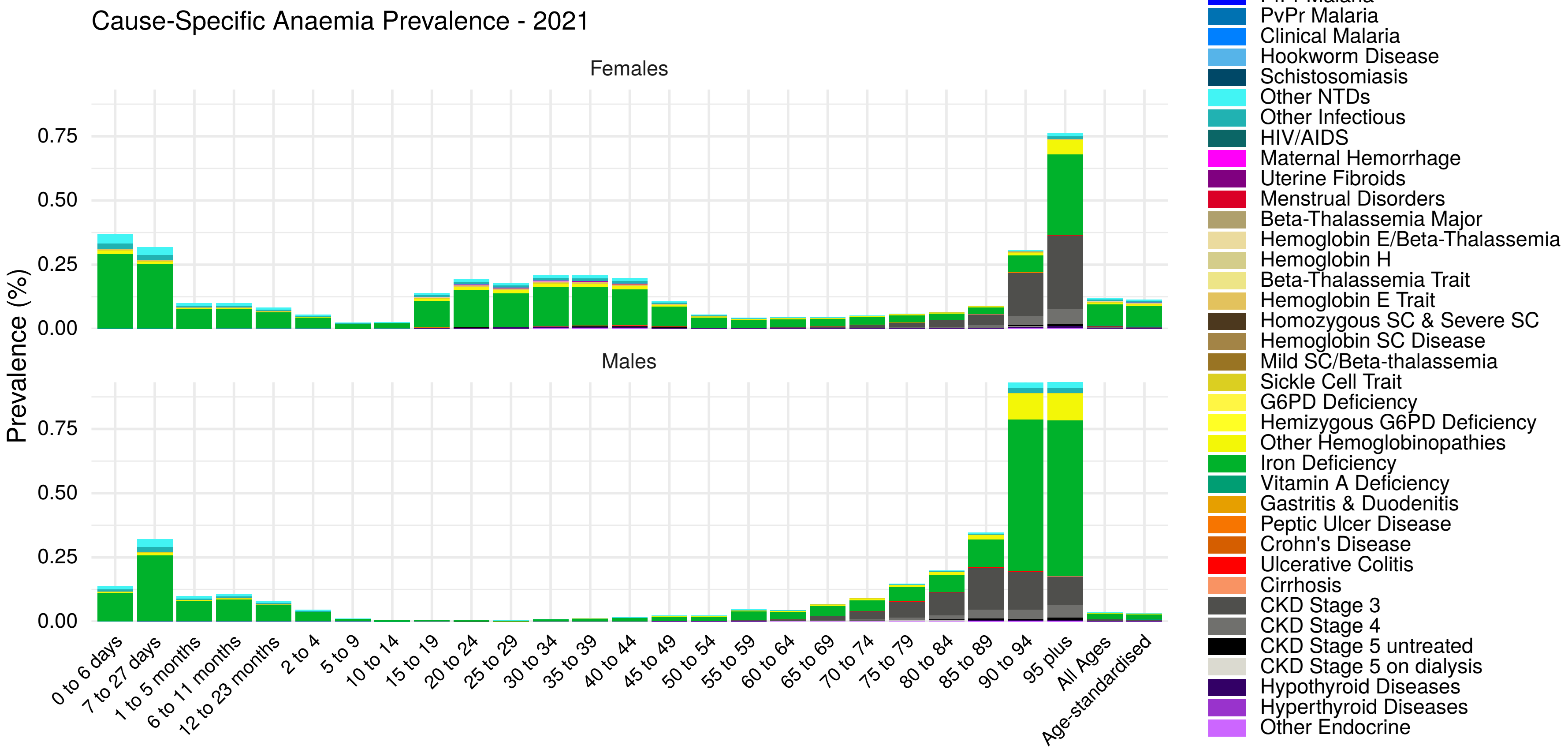

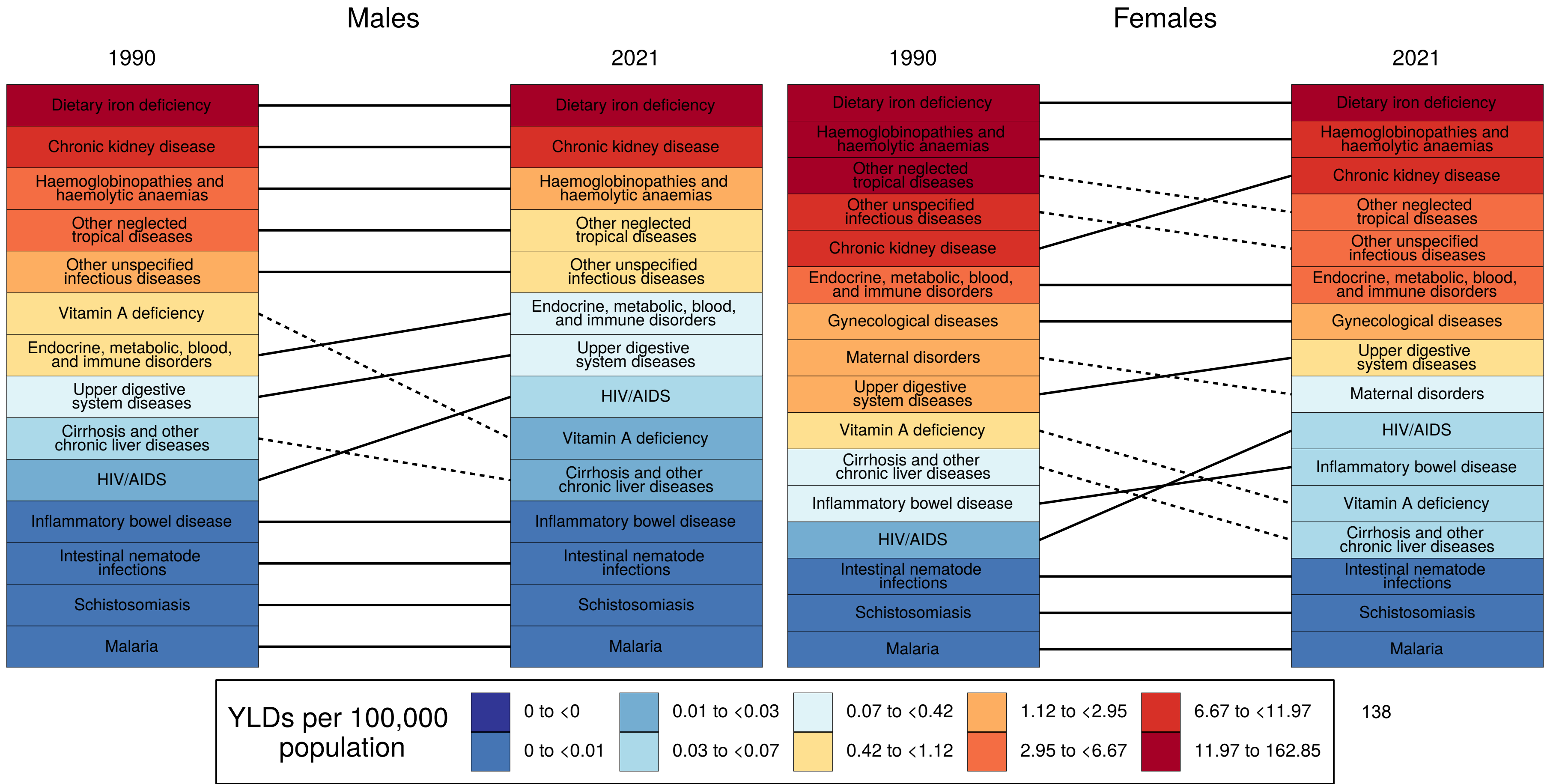

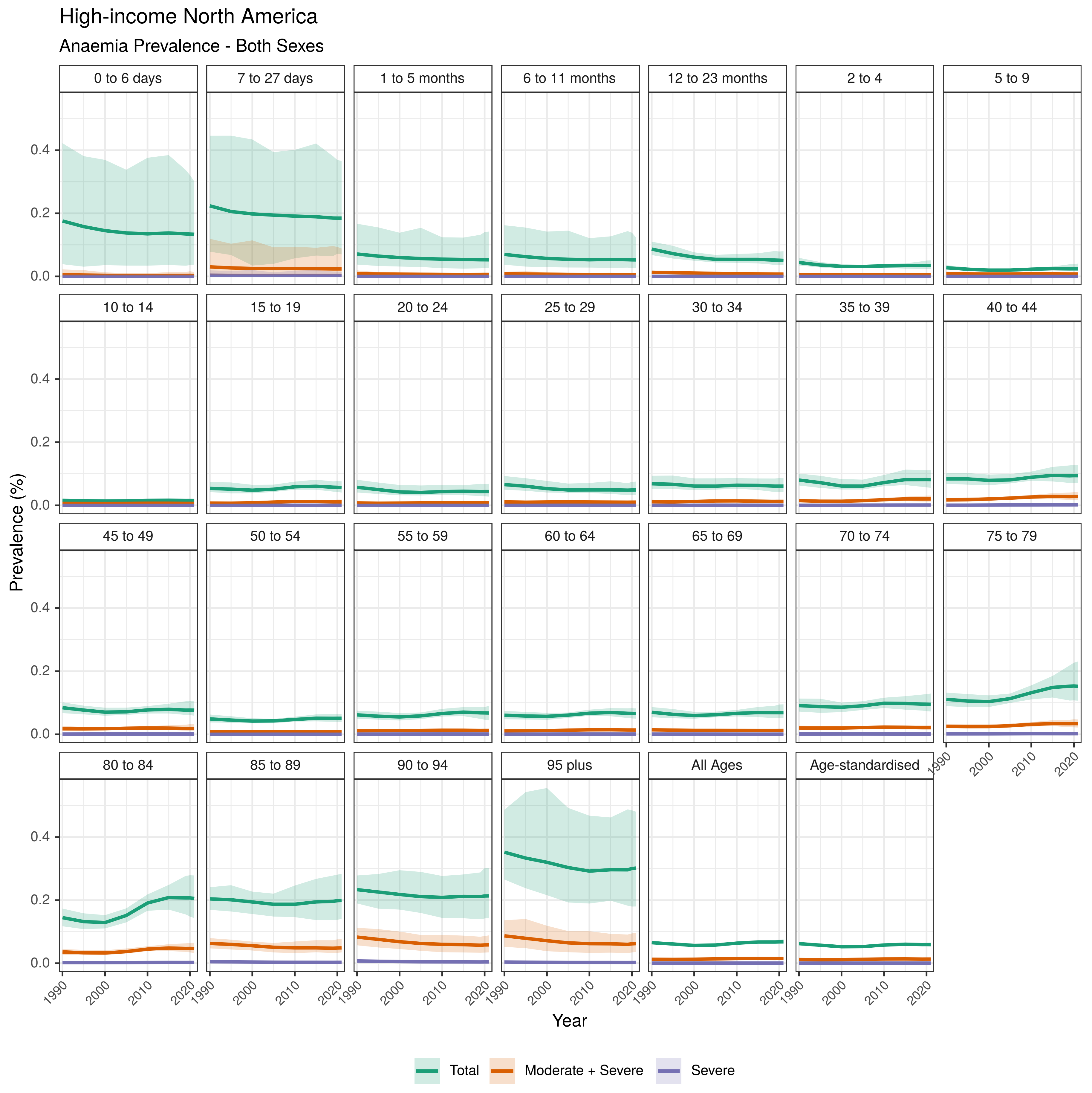

High-income North America
Anaemia Prevalence - Both Sexes
0 to 6 days
7 to 27 days
1 to 5 months
6 to 11 months
12 to 23 months
2 to 4
5 to 9
10 to 14
15 to 19
20 to 24
25 to 29
30 to 34
35 to 39
40 to 44
45 to 49
50 to 54
55 to 59
60 to 64
65 to 69
70 to 74
75 to 79
80 to 84
85 to 89
90 to 94
95 plus
All Ages
Age-standardised
Prevalence (%)
0.0
0.2
0.4
1990
2000
2010
2020
Year
Total
Moderate + Severe
Severe

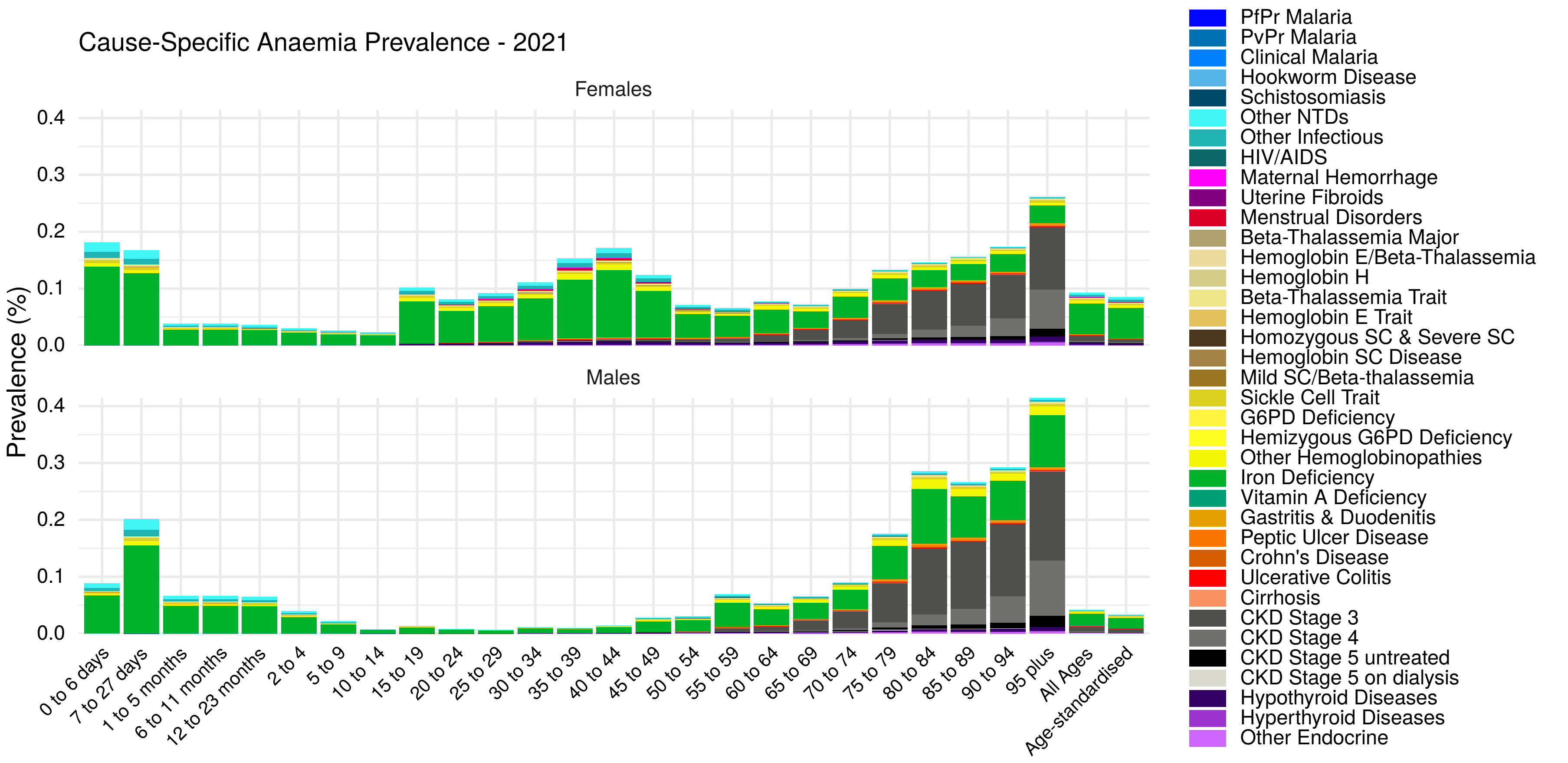

Cause-Specific Anaemia Prevalence - 2021
Females
Males
Prevalence (%)
0.0
0.1
0.2
0.3
0.4
0 to 6 days
7 to 27 days
1 to 5 months
6 to 11 months
12 to 23 months
2 to 4
5 to 9
10 to 14
15 to 19
20 to 24
25 to 29
30 to 34
35 to 39
40 to 44
45 to 49
50 to 54
55 to 59
60 to 64
65 to 69
70 to 74
75 to 79
80 to 84
85 to 89
90 to 94
95 plus
All Ages
Age-standardised
PfPr Malaria
PvPr Malaria
Clinical Malaria
Hookworm Disease
Schistosomiasis
Other NTDs
Other Infectious
HIV/AIDS
Maternal Hemorrhage
Uterine Fibroids
Menstrual Disorders
Beta-Thalassemia Major
Hemoglobin E/Beta-Thalassemia
Hemoglobin H
Beta-Thalassemia Trait
Hemoglobin E Trait
Homozygous SC & Severe SC
Hemoglobin SC Disease
Mild SC/Beta-thalassemia
Sickle Cell Trait
G6PD Deficiency
Hemizygous G6PD Deficiency
Other Hemoglobinopathies
Iron Deficiency
Vitamin A Deficiency
Gastritis & Duodenitis
Peptic Ulcer Disease
Crohn's Disease
Ulcerative Colitis
Cirrhosis
CKD Stage 3
CKD Stage 4
CKD Stage 5 untreated
CKD Stage 5 on dialysis
Hypothyroid Diseases
Hyperthyroid Diseases
Other Endocrine

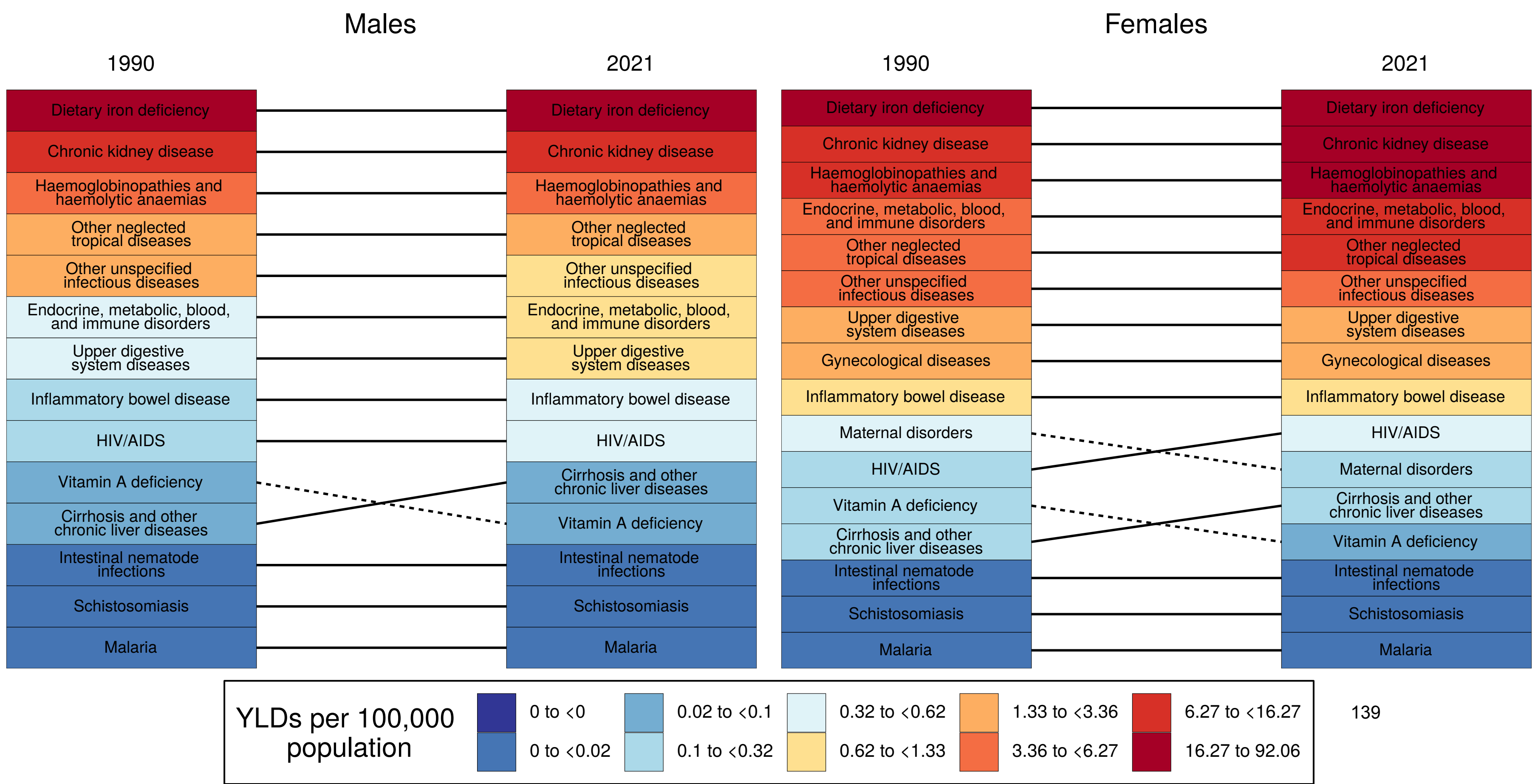

Males
Females
1990
2021
Dietary iron deficiency
Chronic kidney disease
Haemoglobinopathies and haemolytic anaemias
Other neglected tropical diseases
Other unspecified infectious diseases
Endocrine, metabolic, blood, and immune disorders
Upper digestive system diseases
Inflammatory bowel disease
HIV/AIDS
Vitamin A deficiency
Cirrhosis and other chronic liver diseases
Intestinal nematode infections
Schistosomiasis
Malaria
Gynecological diseases
Maternal disorders
YLDs per 100,000 population
0 to <0
0 to <0.02
0.02 to <0.1
0.1 to <0.32
0.32 to <0.62
0.62 to <1.33
1.33 to <3.36
3.36 to <6.27
6.27 to <16.27
16.27 to 92.06

Canada

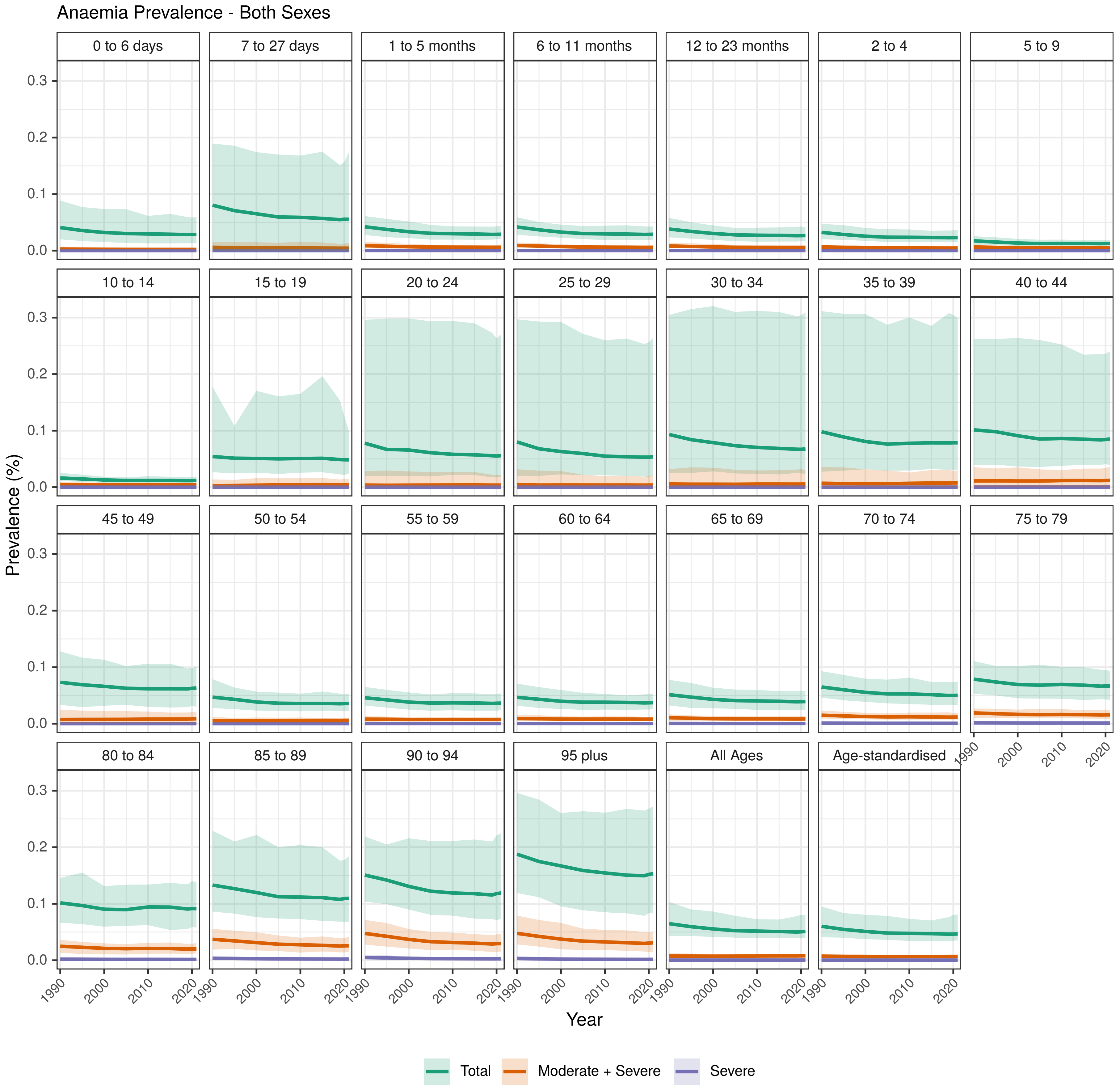

Anaemia Prevalence - Both Sexes
0 to 6 days
7 to 27 days
1 to 5 months
6 to 11 months
12 to 23 months
2 to 4
5 to 9
10 to 14
15 to 19
20 to 24
25 to 29
30 to 34
35 to 39
40 to 44
45 to 49
50 to 54
55 to 59
60 to 64
65 to 69
70 to 74
75 to 79
80 to 84
85 to 89
90 to 94
95 plus
All Ages
Age-standardised
Prevalence (%)
0.0
0.1
0.2
0.3
1990
2000
2010
2020
Year
Total
Moderate + Severe
Severe

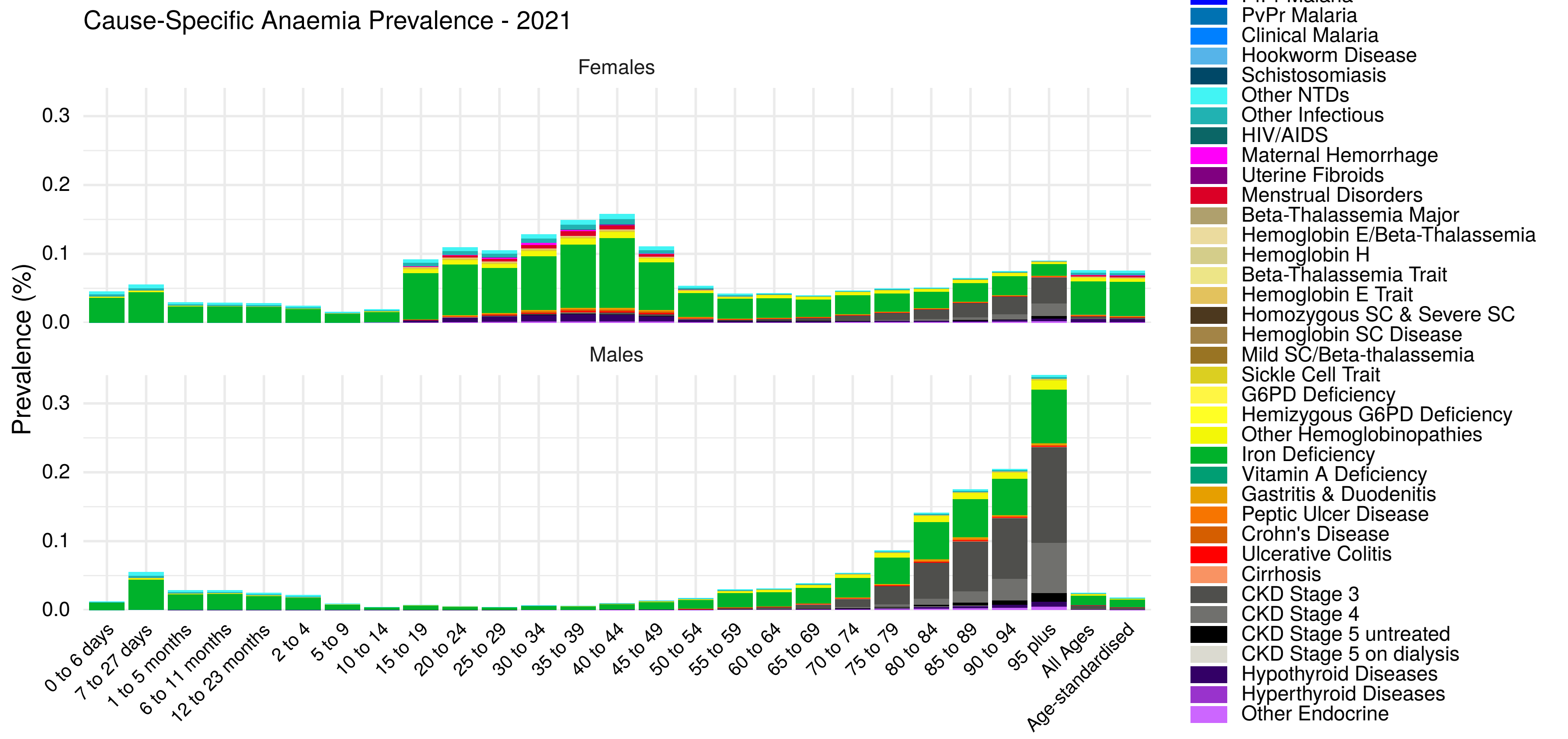

Cause-Specific Anaemia Prevalence - 2021
Females
Males
Prevalence (%)
0.0
0.1
0.2
0.3
0 to 6 days
7 to 27 days
1 to 5 months
6 to 11 months
12 to 23 months
2 to 4
5 to 9
10 to 14
15 to 19
20 to 24
25 to 29
30 to 34
35 to 39
40 to 44
45 to 49
50 to 54
55 to 59
60 to 64
65 to 69
70 to 74
75 to 79
80 to 84
85 to 89
90 to 94
95 plus
All Ages
Age-standardised
PfPr Malaria
PvPr Malaria
Clinical Malaria
Hookworm Disease
Schistosomiasis
Other NTDs
Other Infectious
HIV/AIDS
Maternal Hemorrhage
Uterine Fibroids
Menstrual Disorders
Beta-Thalassemia Major
Hemoglobin E/Beta-Thalassemia
Hemoglobin H
Beta-Thalassemia Trait
Hemoglobin E Trait
Homozygous SC & Severe SC
Hemoglobin SC Disease
Mild SC/Beta-thalassemia
Sickle Cell Trait
G6PD Deficiency
Hemizygous G6PD Deficiency
Other Hemoglobinopathies
Iron Deficiency
Vitamin A Deficiency
Gastritis & Duodenitis
Peptic Ulcer Disease
Crohn's Disease
Ulcerative Colitis
Cirrhosis
CKD Stage 3
CKD Stage 4
CKD Stage 5 untreated
CKD Stage 5 on dialysis
Hypothyroid Diseases
Hyperthyroid Diseases
Other Endocrine

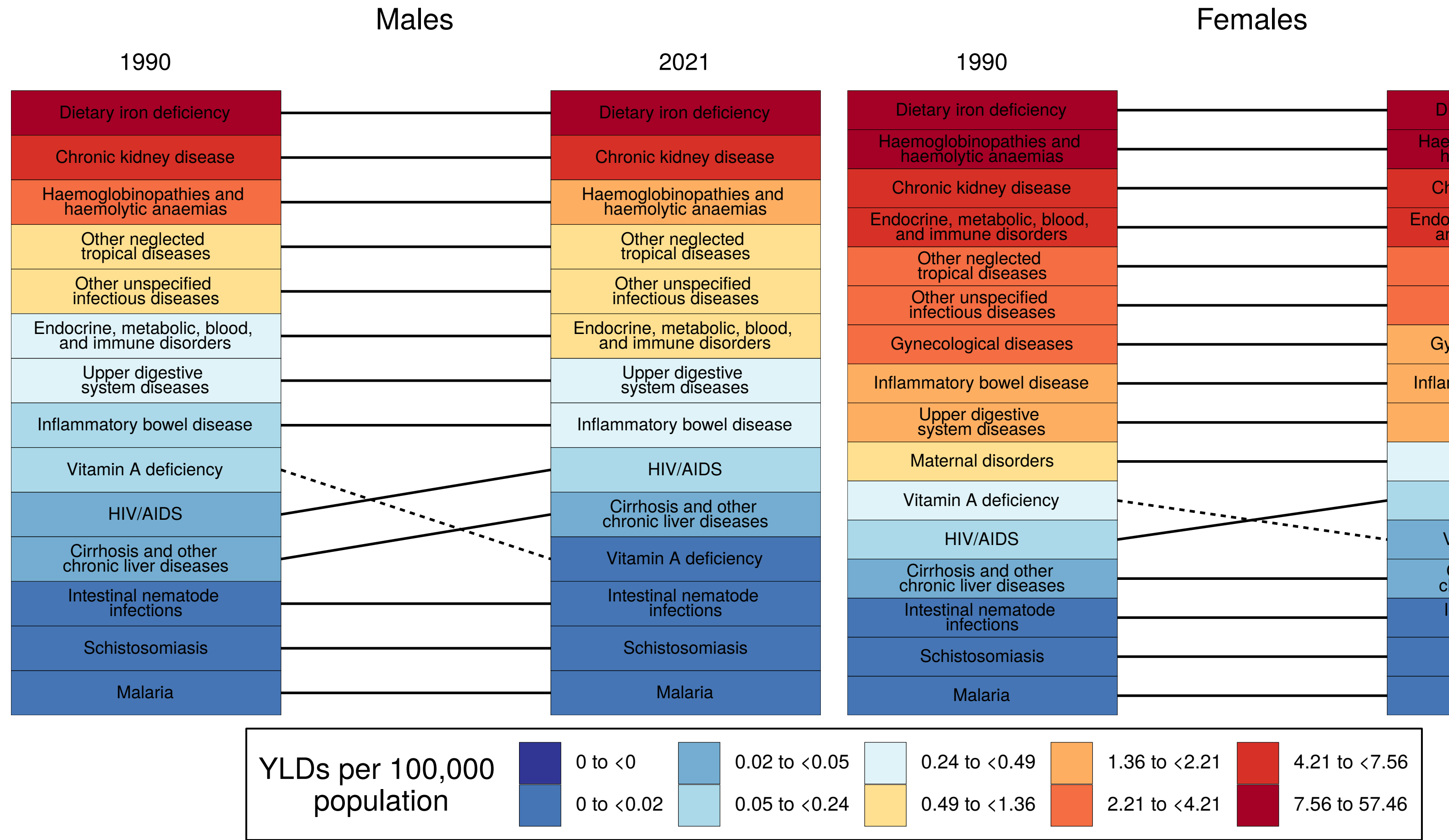

Males
Females
1990
2021
Dietary iron deficiency
Chronic kidney disease
Haemoglobinopathies and haemolytic anaemias
Other neglected tropical diseases
Other unspecified infectious diseases
Endocrine, metabolic, blood, and immune disorders
Upper digestive system diseases
Inflammatory bowel disease
Vitamin A deficiency
HIV/AIDS
Cirrhosis and other chronic liver diseases
Intestinal nematode infections
Schistosomiasis
Malaria
Gynecological diseases
Maternal disorders
YLDs per 100,000 population
0 to <0
0 to <0.02
0.02 to <0.05
0.05 to <0.24
0.24 to <0.49
0.49 to <1.36
1.36 to <2.21
2.21 to <4.21
4.21 to <7.56
7.56 to 57.46

140

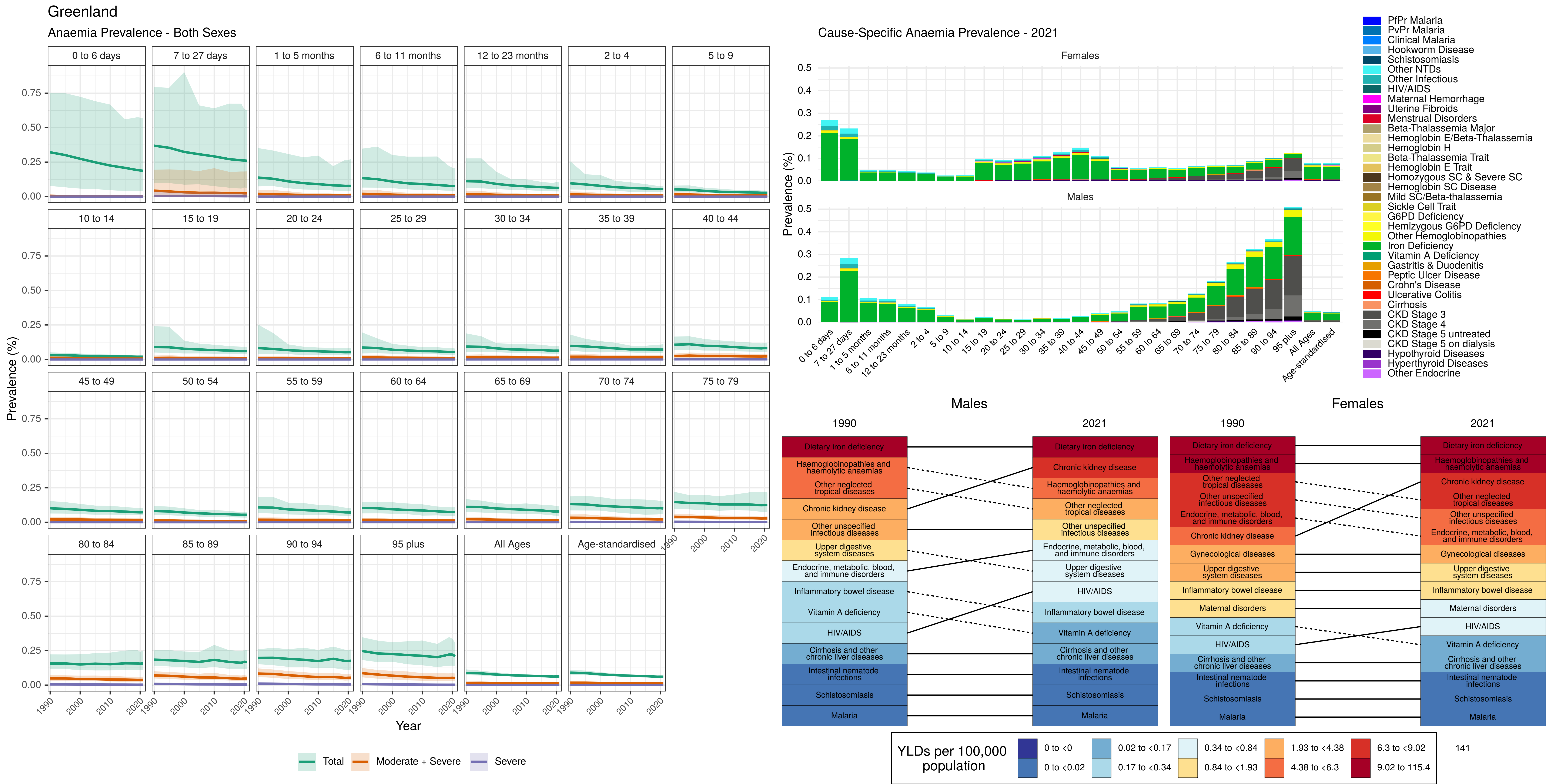

Greenland
Anaemia Prevalence - Both Sexes
0 to 6 days
7 to 27 days
1 to 5 months
6 to 11 months
12 to 23 months
2 to 4
5 to 9
10 to 14
15 to 19
20 to 24
25 to 29
30 to 34
35 to 39
40 to 44
45 to 49
50 to 54
55 to 59
60 to 64
65 to 69
70 to 74
75 to 79
80 to 84
85 to 89
90 to 94
95 plus
All Ages
Age-standardised
Prevalence (%)
Year
Total
Moderate + Severe
Severe
Cause-Specific Anaemia Prevalence - 2021
Females
Males
PfPr Malaria
PvPr Malaria
Clinical Malaria
Hookworm Disease
Schistosomiasis
Other NTDs
Other Infectious
HIV/AIDS
Maternal Hemorrhage
Uterine Fibroids
Menstrual Disorders
Beta-Thalassemia Major
Hemoglobin E/Beta-Thalassemia
Hemoglobin H
Beta-Thalassemia Trait
Hemoglobin E Trait
Homozygous SC & Severe SC
Hemoglobin SC Disease
Mild SC/Beta-thalassemia
Sickle Cell Trait
G6PD Deficiency
Hemizygous G6PD Deficiency
Other Hemoglobinopathies
Iron Deficiency
Vitamin A Deficiency
Gastritis & Duodenitis
Peptic Ulcer Disease
Crohn's Disease
Ulcerative Colitis
Cirrhosis
CKD Stage 3
CKD Stage 4
CKD Stage 5 untreated
CKD Stage 5 on dialysis
Hypothyroid Diseases
Hyperthyroid Diseases
Other Endocrine
Males
Females
1990
2021
Dietary iron deficiency
Haemoglobinopathies and haemolytic anaemias
Other neglected tropical diseases
Chronic kidney disease
Other unspecified infectious diseases
Upper digestive system diseases
Endocrine, metabolic, blood, and immune disorders
Inflammatory bowel disease
Vitamin A deficiency
HIV/AIDS
Cirrhosis and other chronic liver diseases
Intestinal nematode infections
Schistosomiasis
Malaria
Gynecological diseases
Maternal disorders
YLDs per 100,000 population
0 to <0
0 to <0.02
0.02 to <0.17
0.17 to <0.34
0.34 to <0.84
0.84 to <1.93
1.93 to <4.38
4.38 to <6.3
6.3 to <9.02
9.02 to 115.4

USA

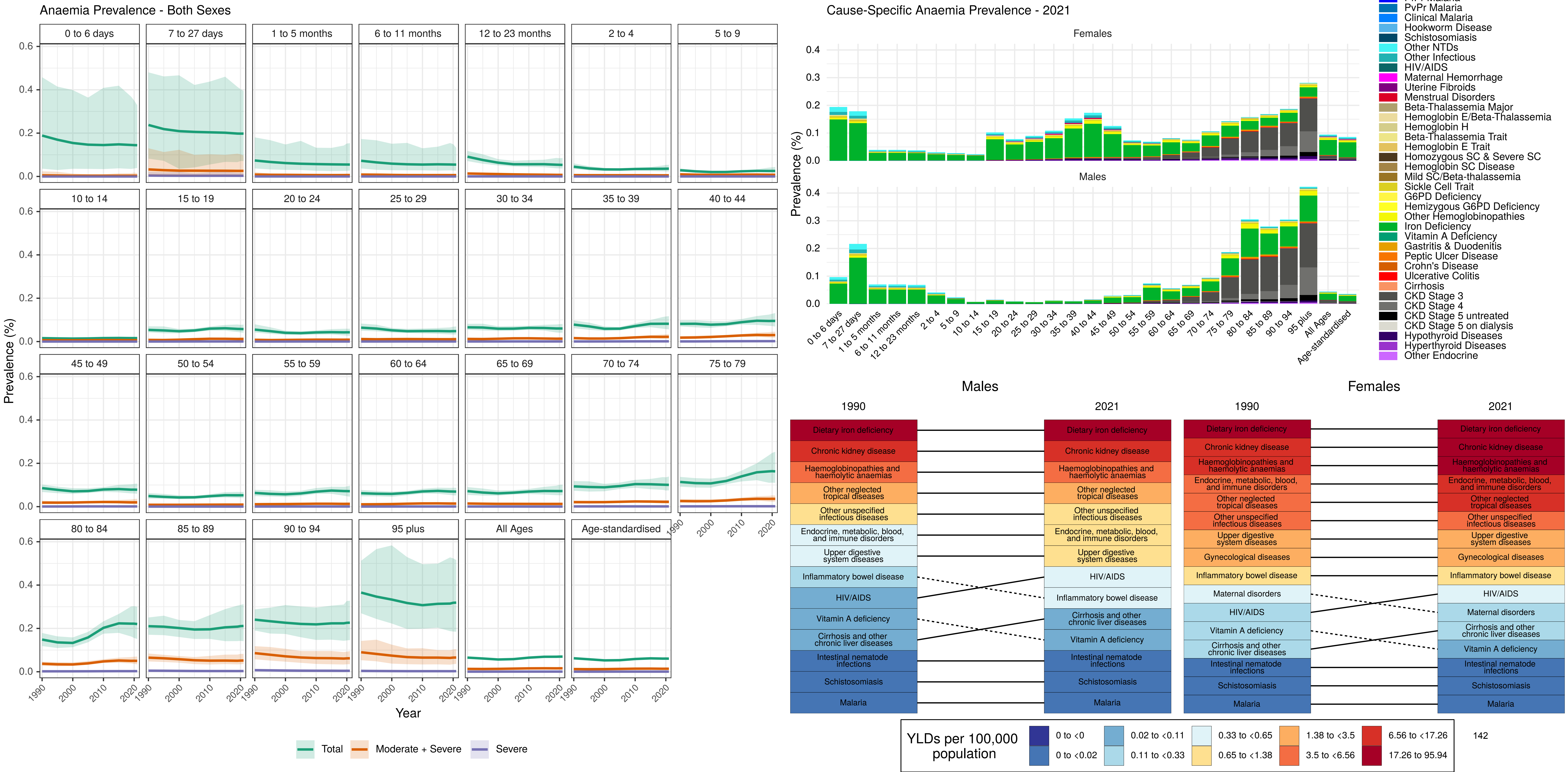

Anaemia Prevalence - Both Sexes
0 to 6 days
7 to 27 days
1 to 5 months
6 to 11 months
12 to 23 months
2 to 4
5 to 9
10 to 14
15 to 19
20 to 24
25 to 29
30 to 34
35 to 39
40 to 44
45 to 49
50 to 54
55 to 59
60 to 64
65 to 69
70 to 74
75 to 79
80 to 84
85 to 89
90 to 94
95 plus
All Ages
Age-standardised
Prevalence (%)
Year
Total
Moderate + Severe
Severe
Cause-Specific Anaemia Prevalence - 2021
Females
Males
PfPr Malaria
PvPr Malaria
Clinical Malaria
Hookworm Disease
Schistosomiasis
Other NTDs
Other Infectious
HIV/AIDS
Maternal Hemorrhage
Uterine Fibroids
Menstrual Disorders
Beta-Thalassemia Major
Hemoglobin E/Beta-Thalassemia
Hemoglobin H
Beta-Thalassemia Trait
Hemoglobin E Trait
Homozygous SC & Severe SC
Hemoglobin SC Disease
Mild SC/Beta-thalassemia
Sickle Cell Trait
G6PD Deficiency
Hemizygous G6PD Deficiency
Other Hemoglobinopathies
Iron Deficiency
Vitamin A Deficiency
Gastritis & Duodenitis
Peptic Ulcer Disease
Crohn's Disease
Ulcerative Colitis
Cirrhosis
CKD Stage 3
CKD Stage 4
CKD Stage 5 untreated
CKD Stage 5 on dialysis
Hypothyroid Diseases
Hyperthyroid Diseases
Other Endocrine
Males
1990
2021
Dietary iron deficiency
Chronic kidney disease
Haemoglobinopathies and haemolytic anaemias
Other neglected tropical diseases
Other unspecified infectious diseases
Endocrine, metabolic, blood, and immune disorders
Upper digestive system diseases
Inflammatory bowel disease
HIV/AIDS
Vitamin A deficiency
Cirrhosis and other chronic liver diseases
Intestinal nematode infections
Schistosomiasis
Malaria
Females
1990
2021
Dietary iron deficiency
Chronic kidney disease
Haemoglobinopathies and haemolytic anaemias
Endocrine, metabolic, blood, and immune disorders
Other neglected tropical diseases
Other unspecified infectious diseases
Upper digestive system diseases
Gynecological diseases
Inflammatory bowel disease
Maternal disorders
HIV/AIDS
Vitamin A deficiency
Cirrhosis and other chronic liver diseases
Intestinal nematode infections
Schistosomiasis
Malaria
YLDs per 100,000 population
0 to <0
0 to <0.02
0.02 to <0.11
0.11 to <0.33
0.33 to <0.65
0.65 to <1.38
1.38 to <3.5
3.5 to <6.56
6.56 to <17.26
17.26 to 95.94

142

Alabama

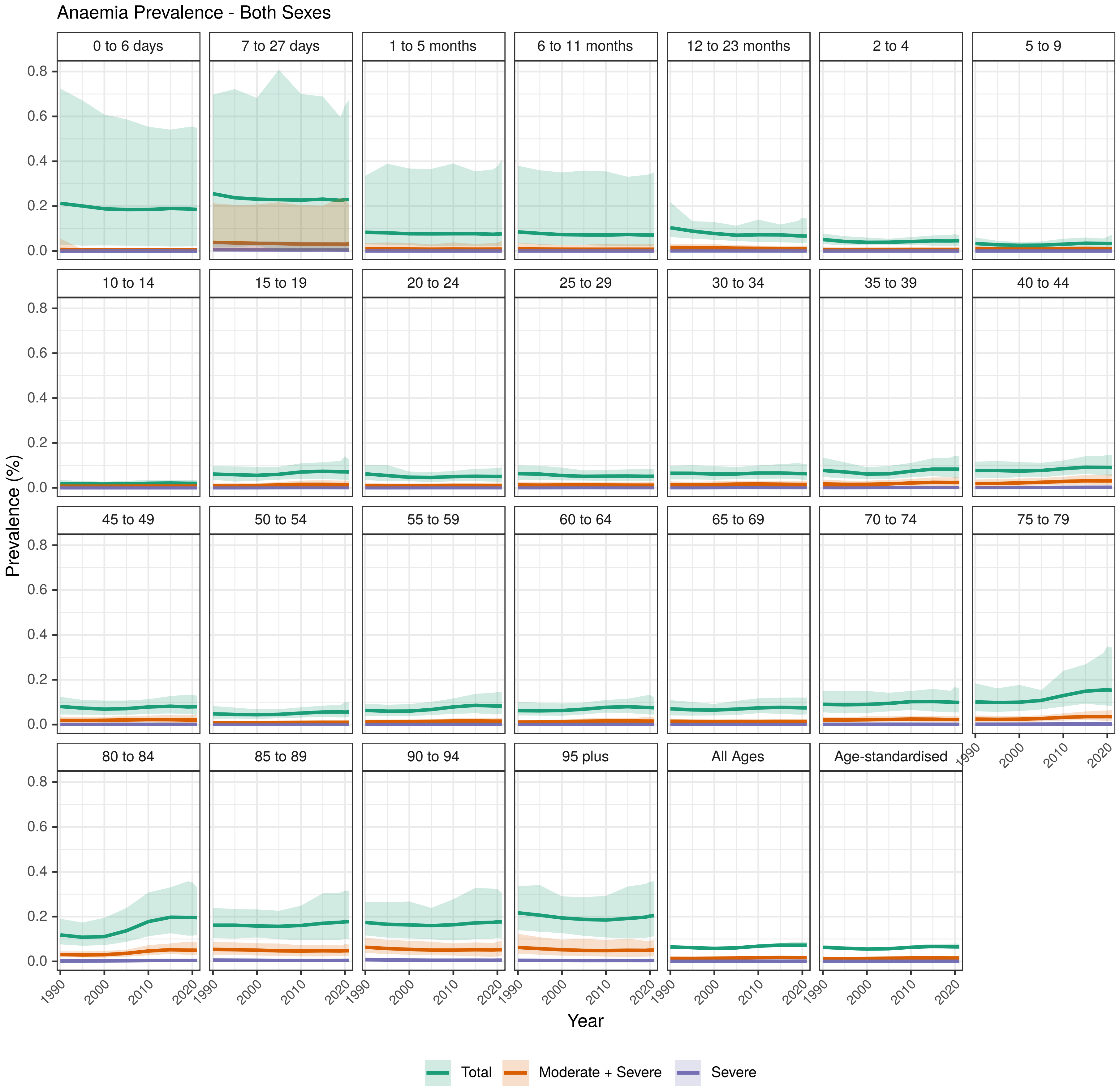

Anaemia Prevalence - Both Sexes
0 to 6 days
7 to 27 days
1 to 5 months
6 to 11 months
12 to 23 months
2 to 4
5 to 9
10 to 14
15 to 19
20 to 24
25 to 29
30 to 34
35 to 39
40 to 44
45 to 49
50 to 54
55 to 59
60 to 64
65 to 69
70 to 74
75 to 79
80 to 84
85 to 89
90 to 94
95 plus
All Ages
Age-standardised
0.0
0.2
0.4
0.6
0.8
Prevalence (%)
1990
2000
2010
2020
Year
Total
Moderate + Severe
Severe

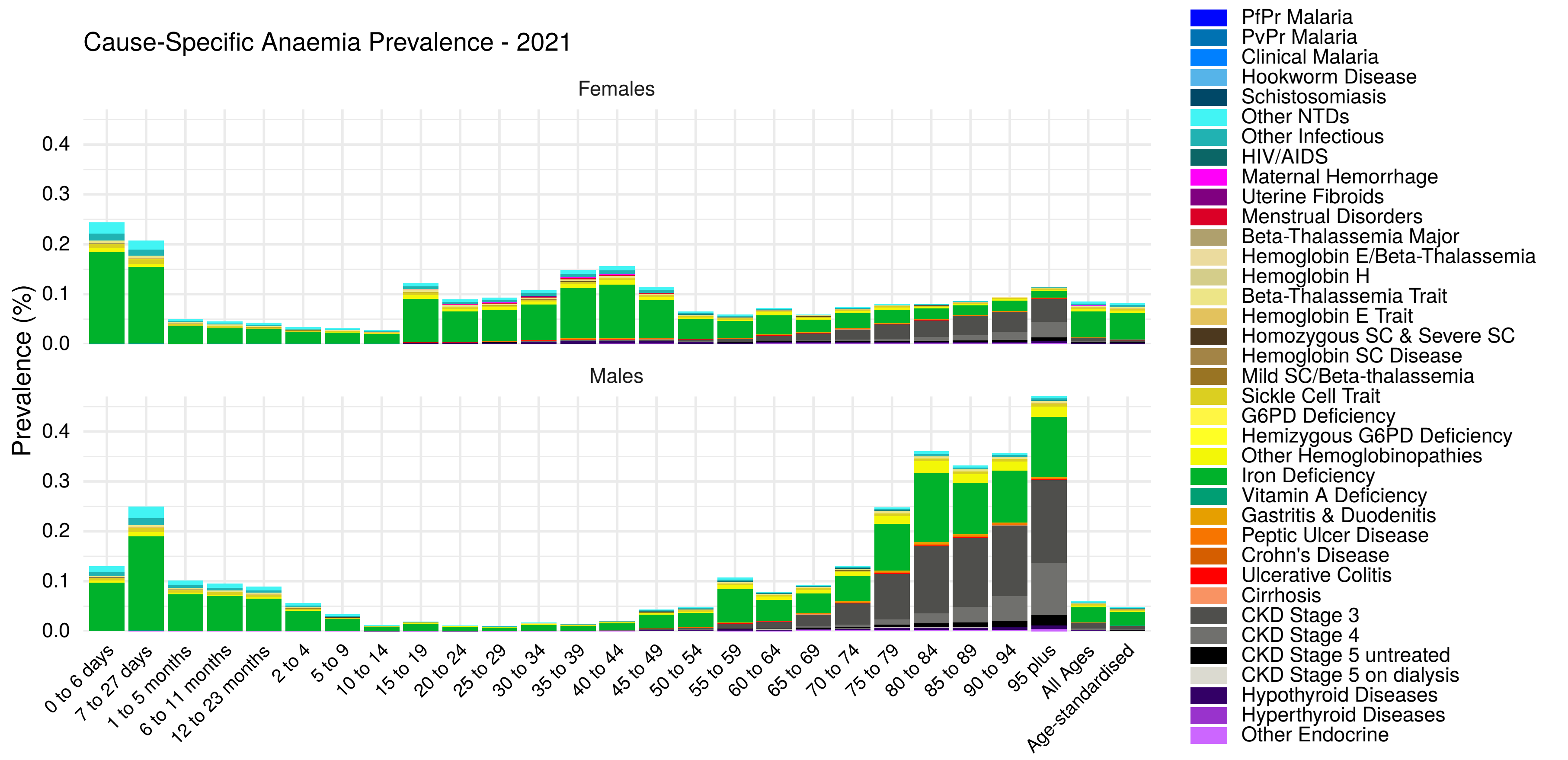

Cause-Specific Anaemia Prevalence - 2021
Females
Males
Prevalence (%)
0.0
0.1
0.2
0.3
0.4
0 to 6 days
7 to 27 days
1 to 5 months
6 to 11 months
12 to 23 months
2 to 4
5 to 9
10 to 14
15 to 19
20 to 24
25 to 29
30 to 34
35 to 39
40 to 44
45 to 49
50 to 54
55 to 59
60 to 64
65 to 69
70 to 74
75 to 79
80 to 84
85 to 89
90 to 94
95 plus
All Ages
Age-standardised
PfPr Malaria
PvPr Malaria
Clinical Malaria
Hookworm Disease
Schistosomiasis
Other NTDs
Other Infectious
HIV/AIDS
Maternal Hemorrhage
Uterine Fibroids
Menstrual Disorders
Beta-Thalassemia Major
Hemoglobin E/Beta-Thalassemia
Hemoglobin H
Beta-Thalassemia Trait
Hemoglobin E Trait
Homozygous SC & Severe SC
Hemoglobin SC Disease
Mild SC/Beta-thalassemia
Sickle Cell Trait
G6PD Deficiency
Hemizygous G6PD Deficiency
Other Hemoglobinopathies
Iron Deficiency
Vitamin A Deficiency
Gastritis & Duodenitis
Peptic Ulcer Disease
Crohn's Disease
Ulcerative Colitis
Cirrhosis
CKD Stage 3
CKD Stage 4
CKD Stage 5 untreated
CKD Stage 5 on dialysis
Hypothyroid Diseases
Hyperthyroid Diseases
Other Endocrine

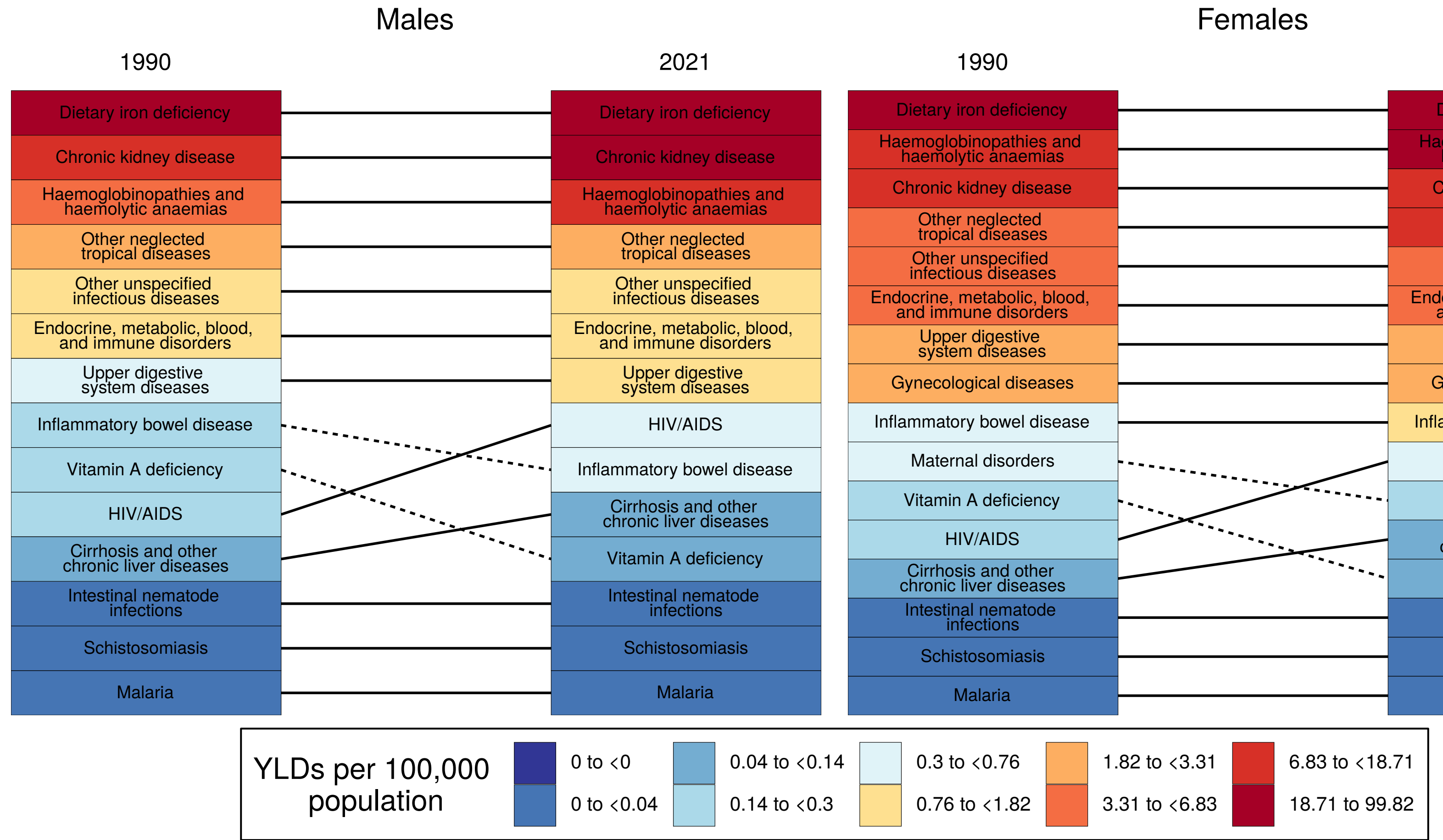

Males
Females
1990
2021
Dietary iron deficiency
Chronic kidney disease
Haemoglobinopathies and haemolytic anaemias
Other neglected tropical diseases
Other unspecified infectious diseases
Endocrine, metabolic, blood, and immune disorders
Upper digestive system diseases
Inflammatory bowel disease
Vitamin A deficiency
HIV/AIDS
Cirrhosis and other chronic liver diseases
Intestinal nematode infections
Schistosomiasis
Malaria
Gynecological diseases
Maternal disorders
YLDs per 100,000 population
0 to <0
0 to <0.04
0.04 to <0.14
0.14 to <0.3
0.3 to <0.76
0.76 to <1.82
1.82 to <3.31
3.31 to <6.83
6.83 to <18.71
18.71 to 99.82

Alaska

Anaemia Prevalence - Both Sexes

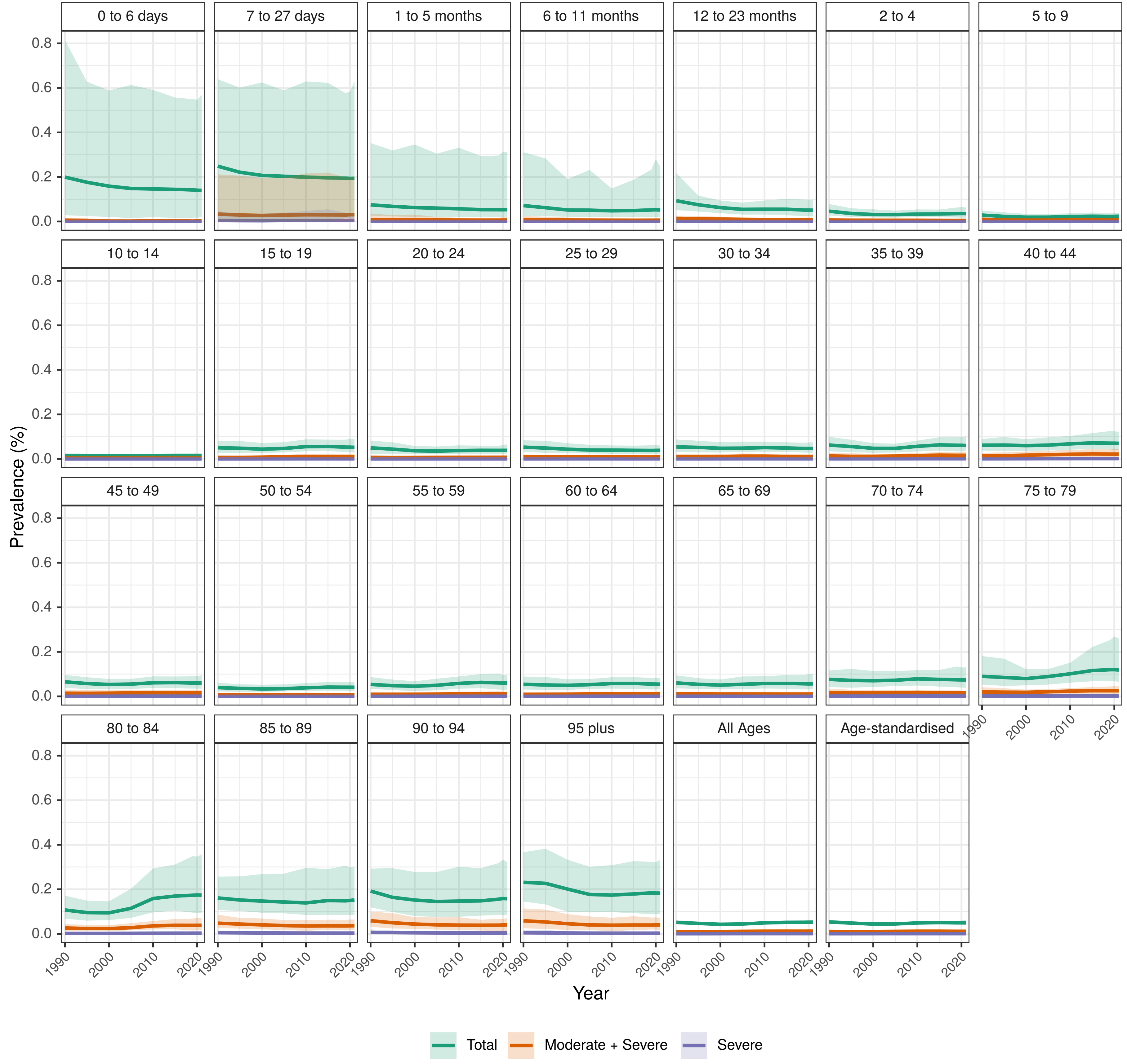

Cause-Specific Anaemia Prevalence - 2021

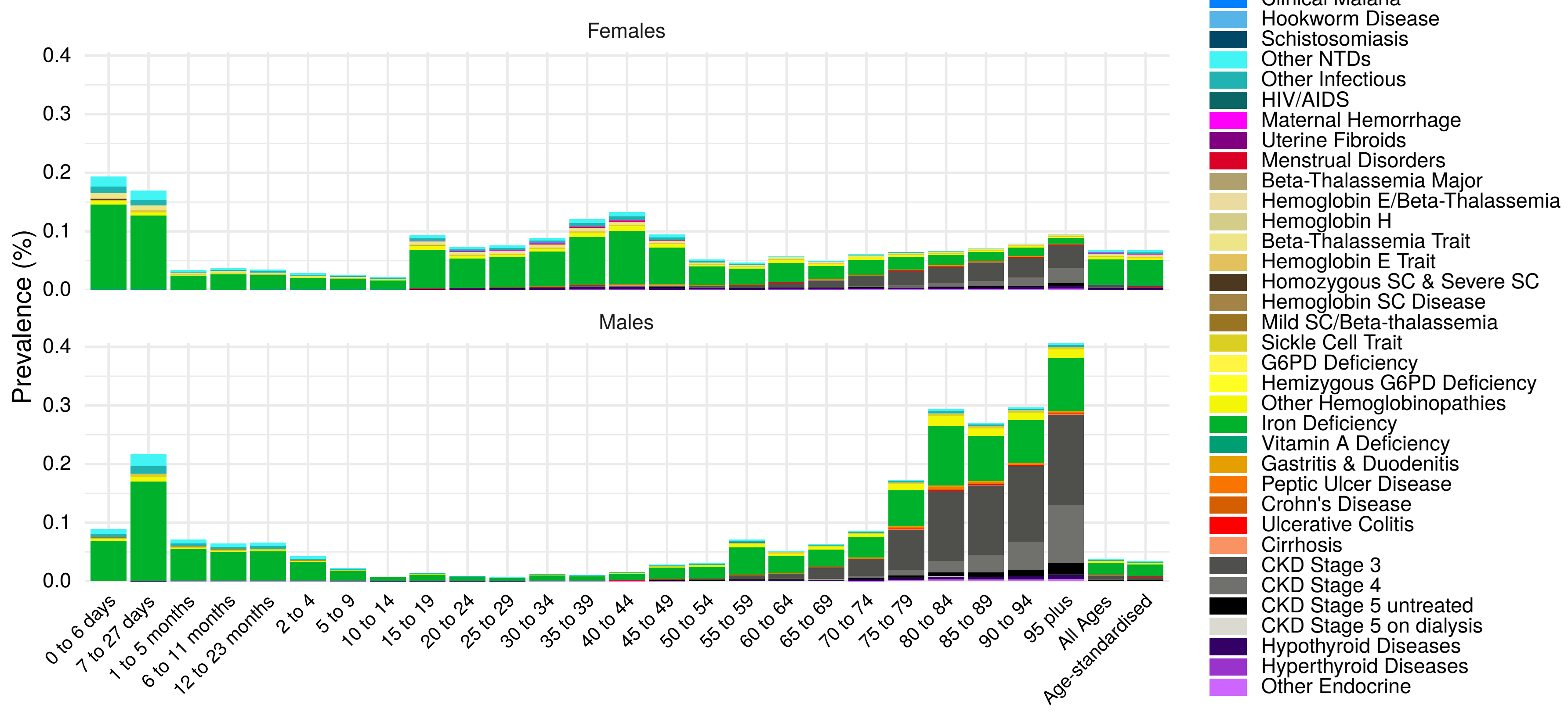

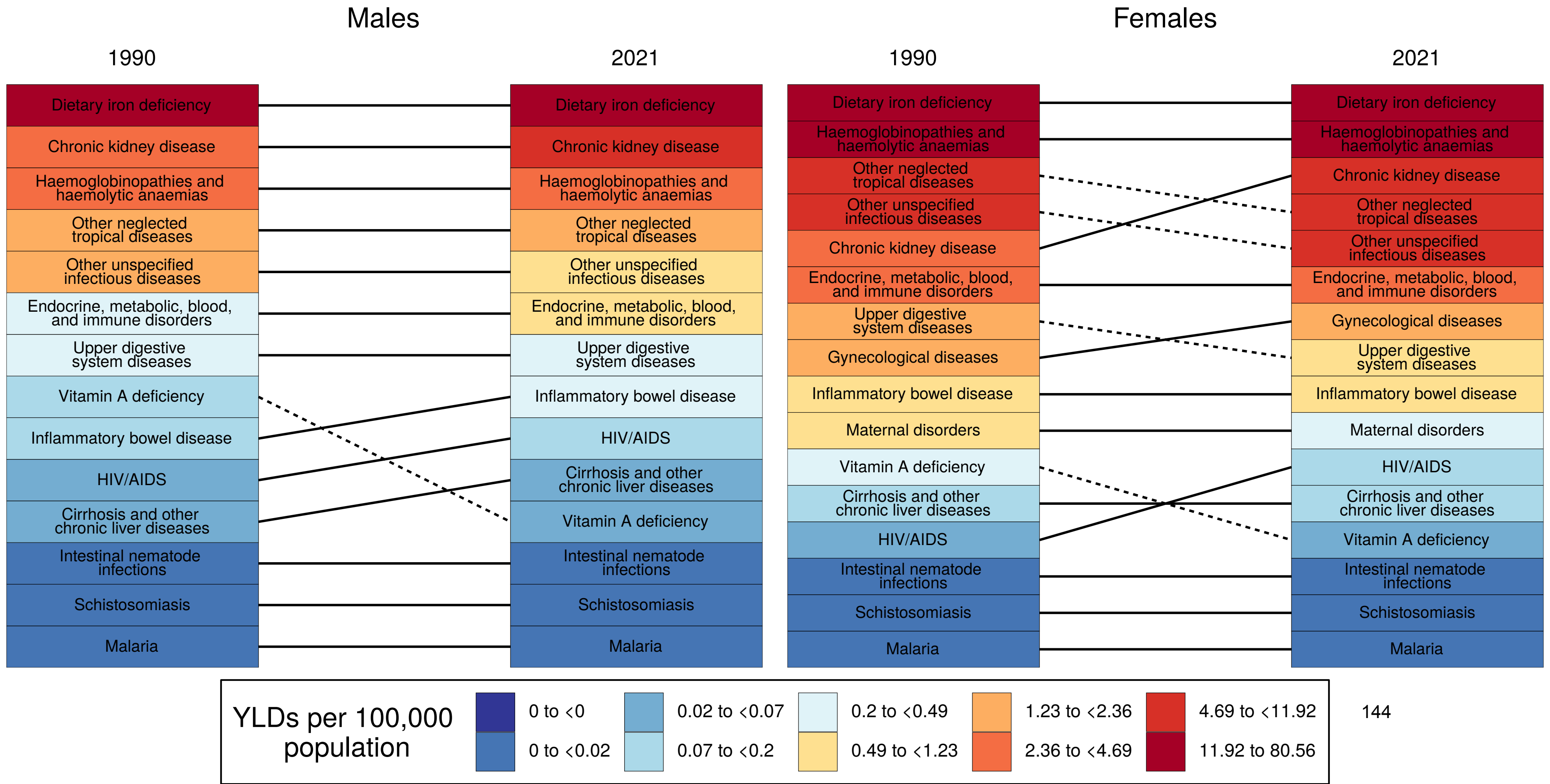

Arizona

Anaemia Prevalence - Both Sexes

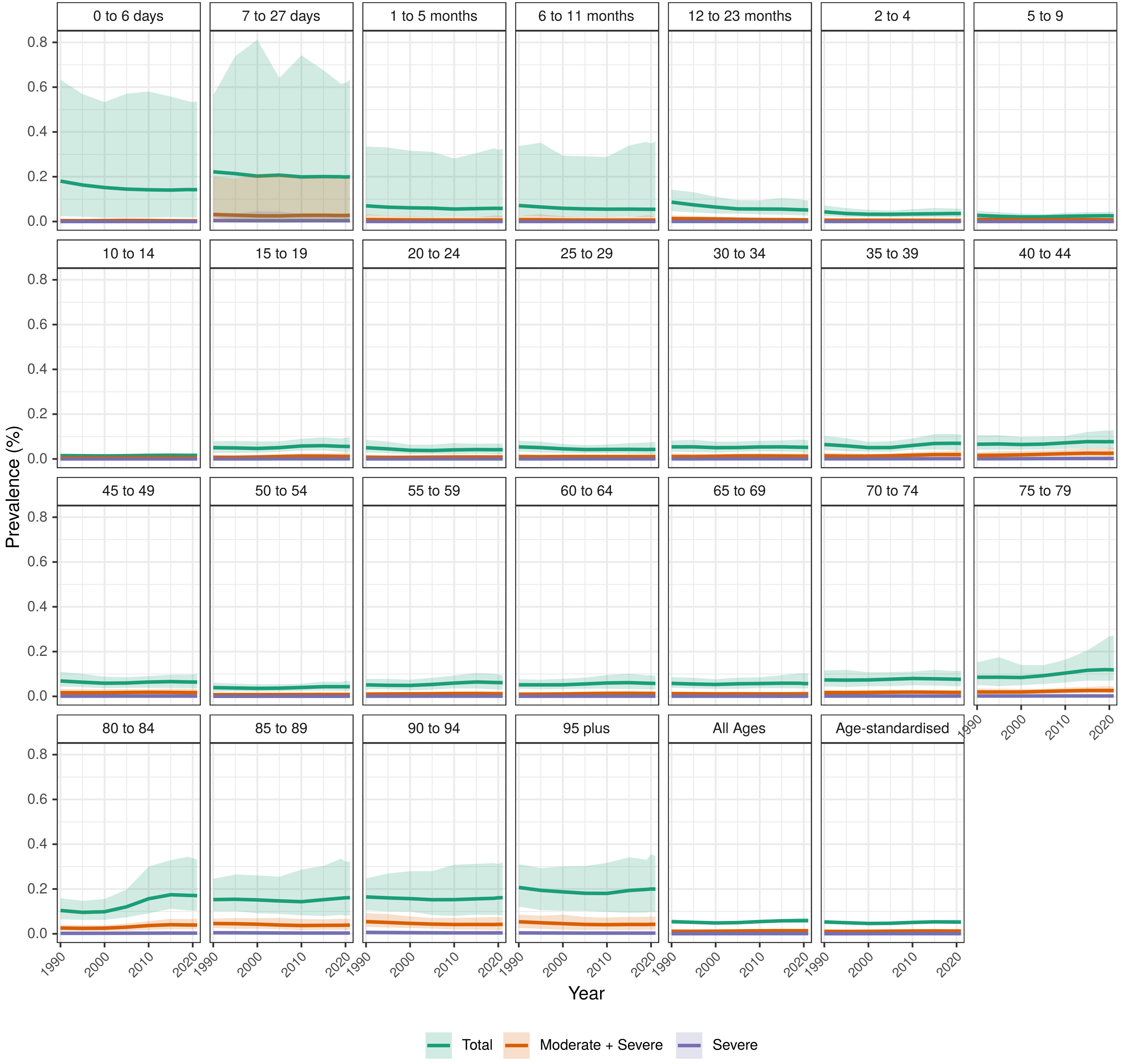

Cause-Specific Anaemia Prevalence - 2021

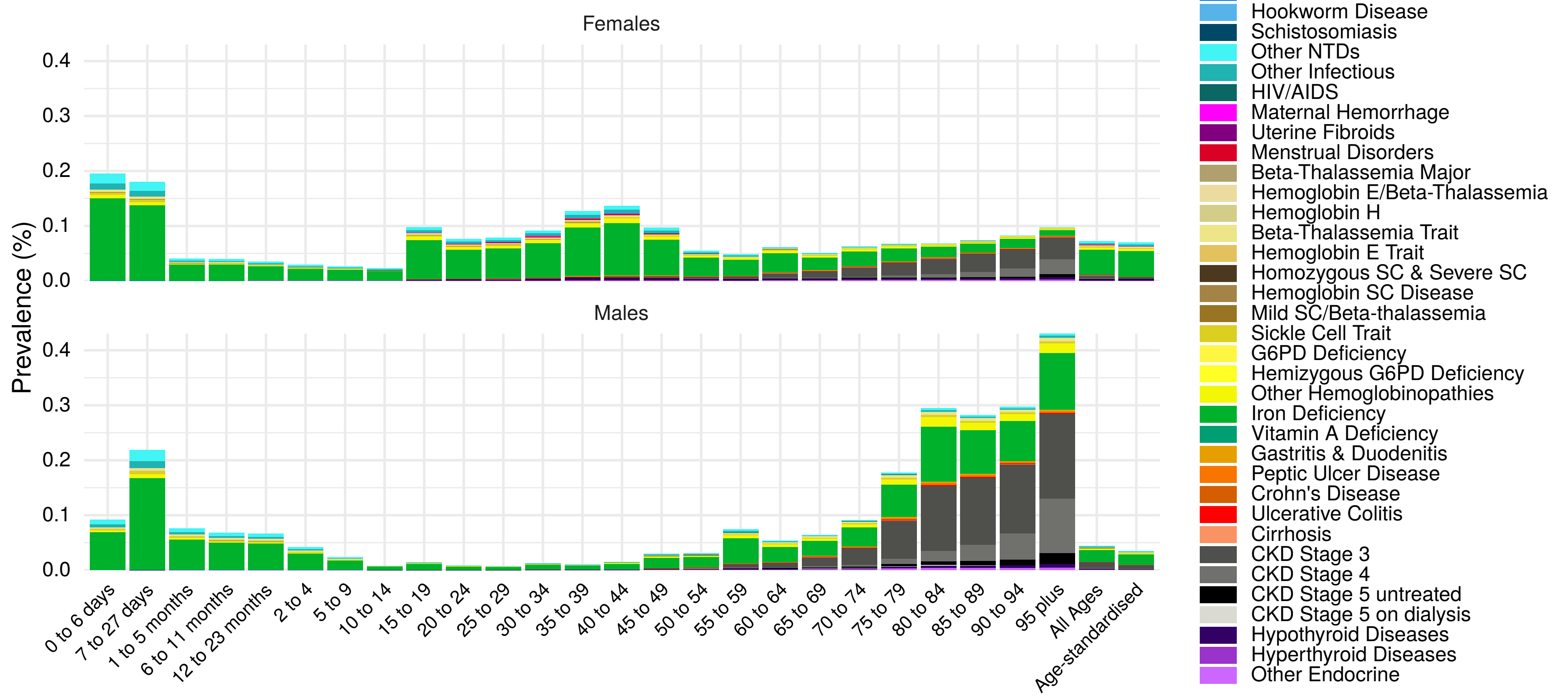

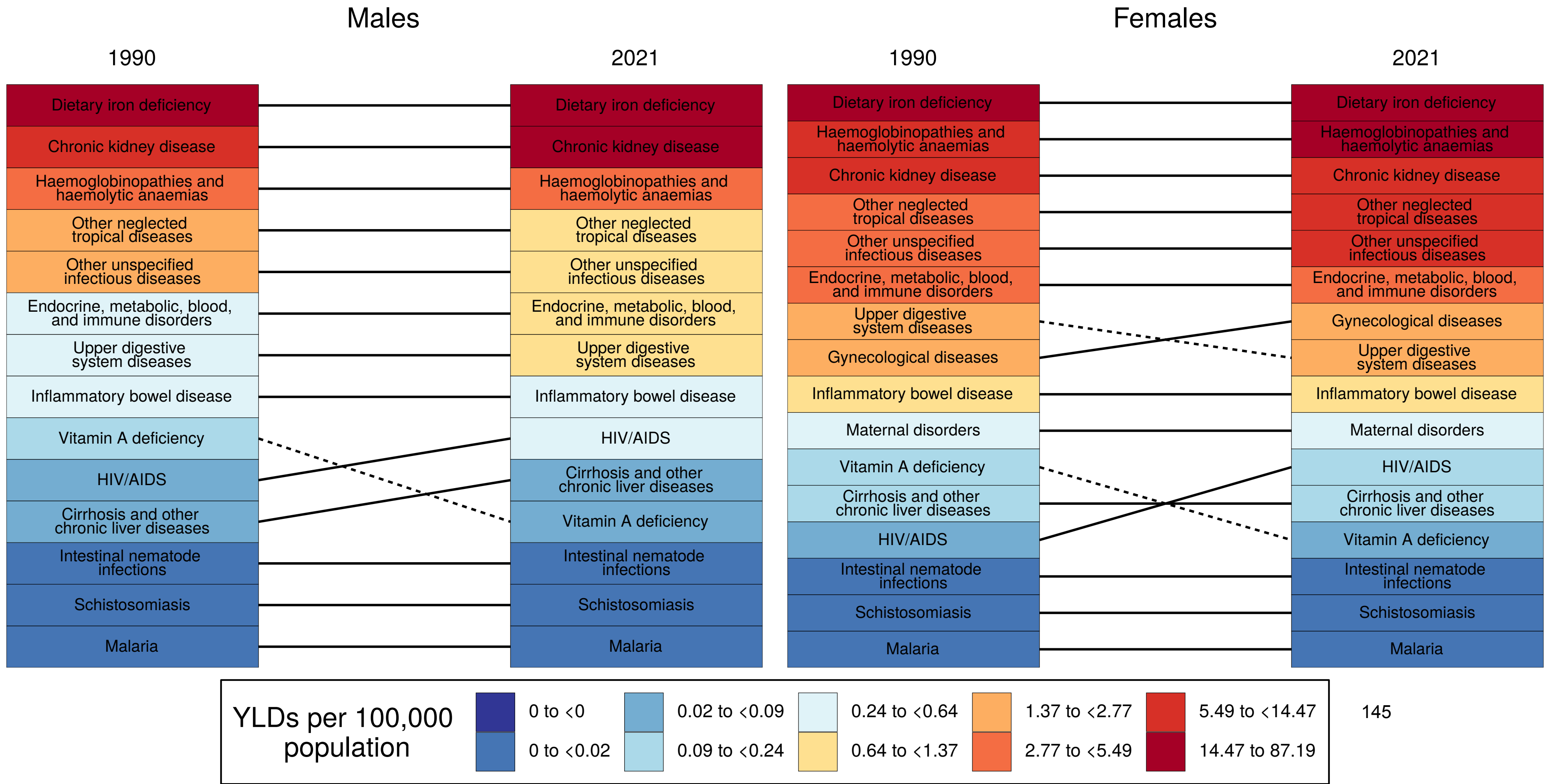

Arkansas

Anaemia Prevalence - Both Sexes

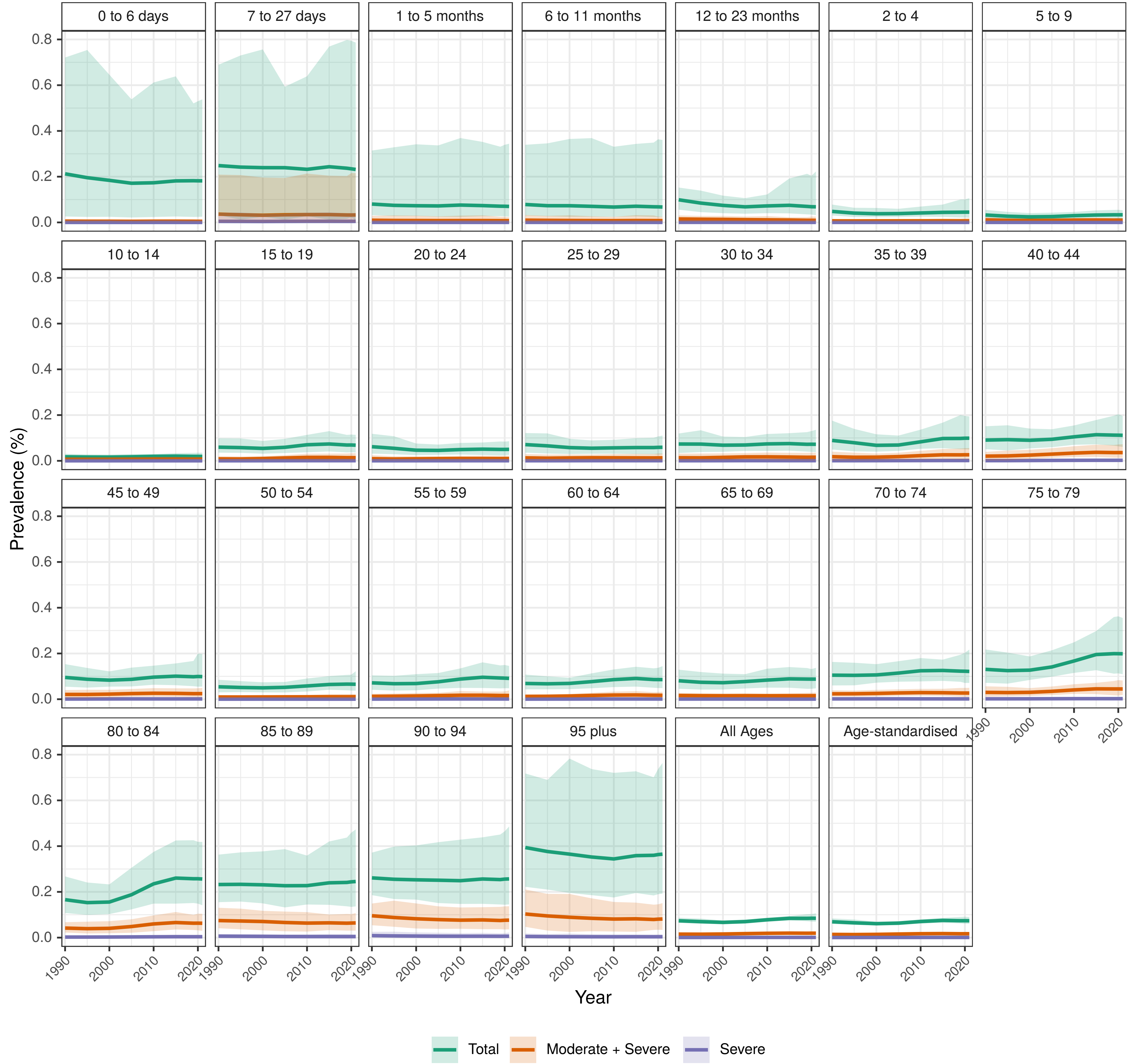

Cause-Specific Anaemia Prevalence - 2021

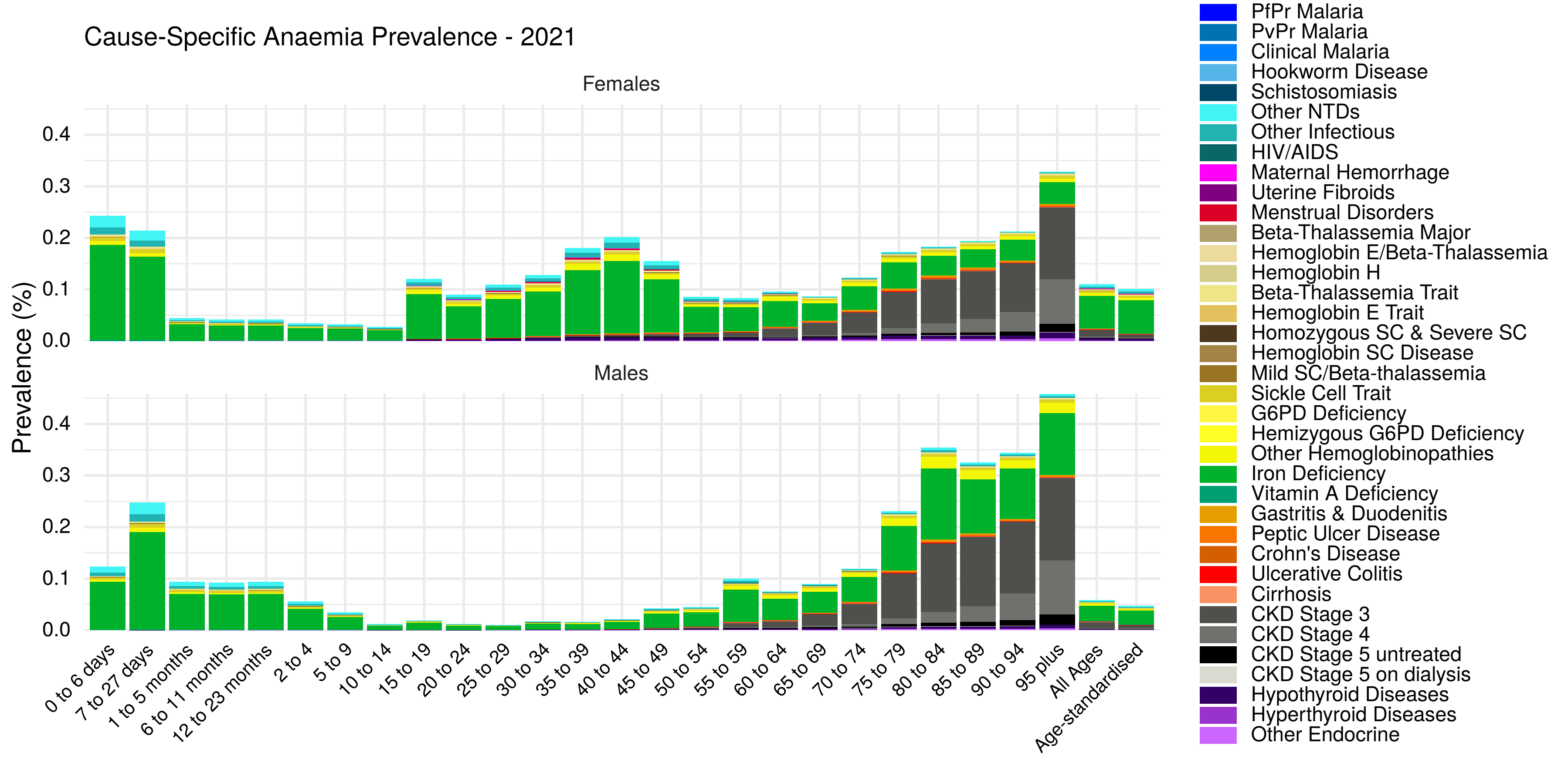

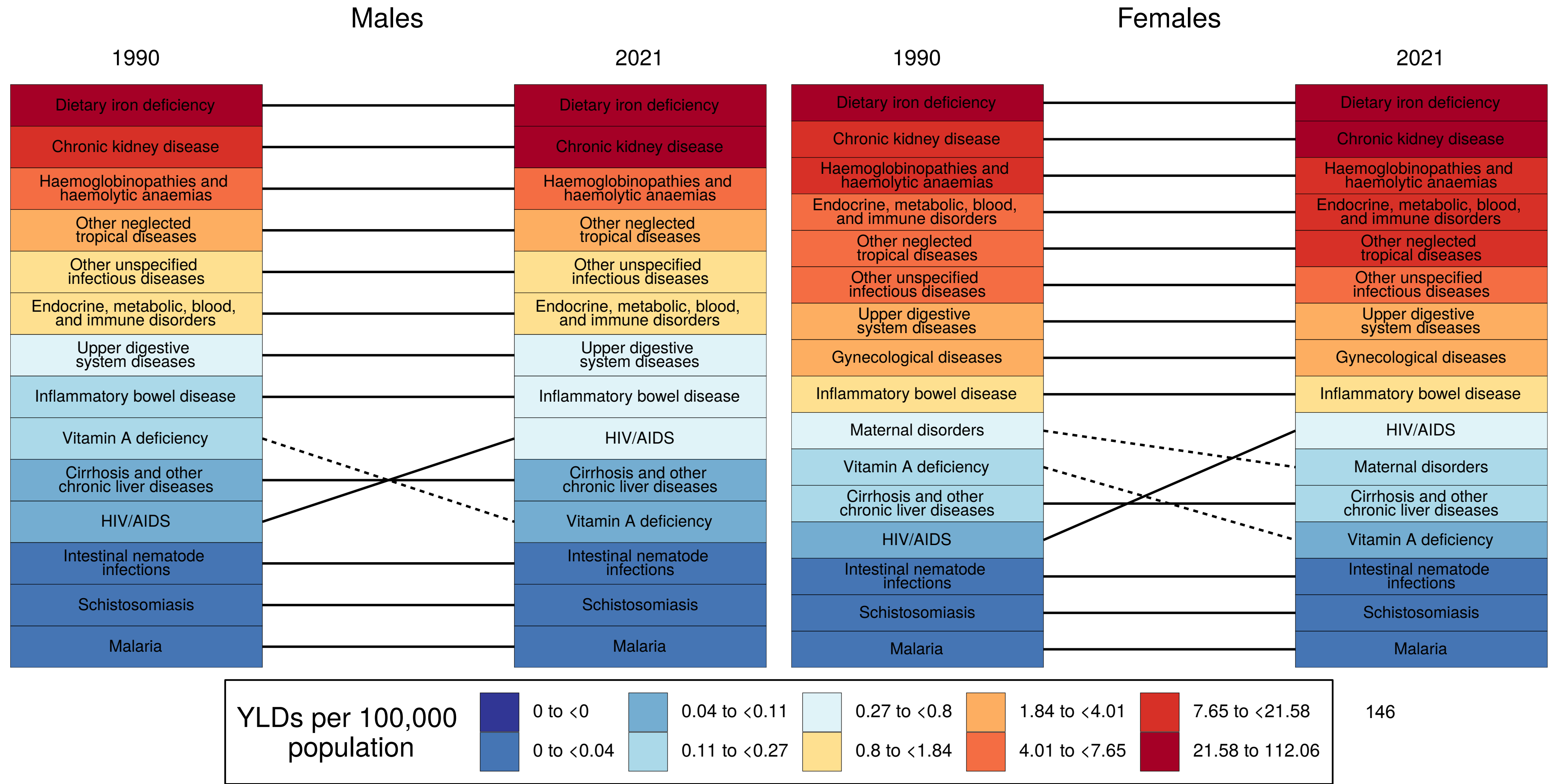

California

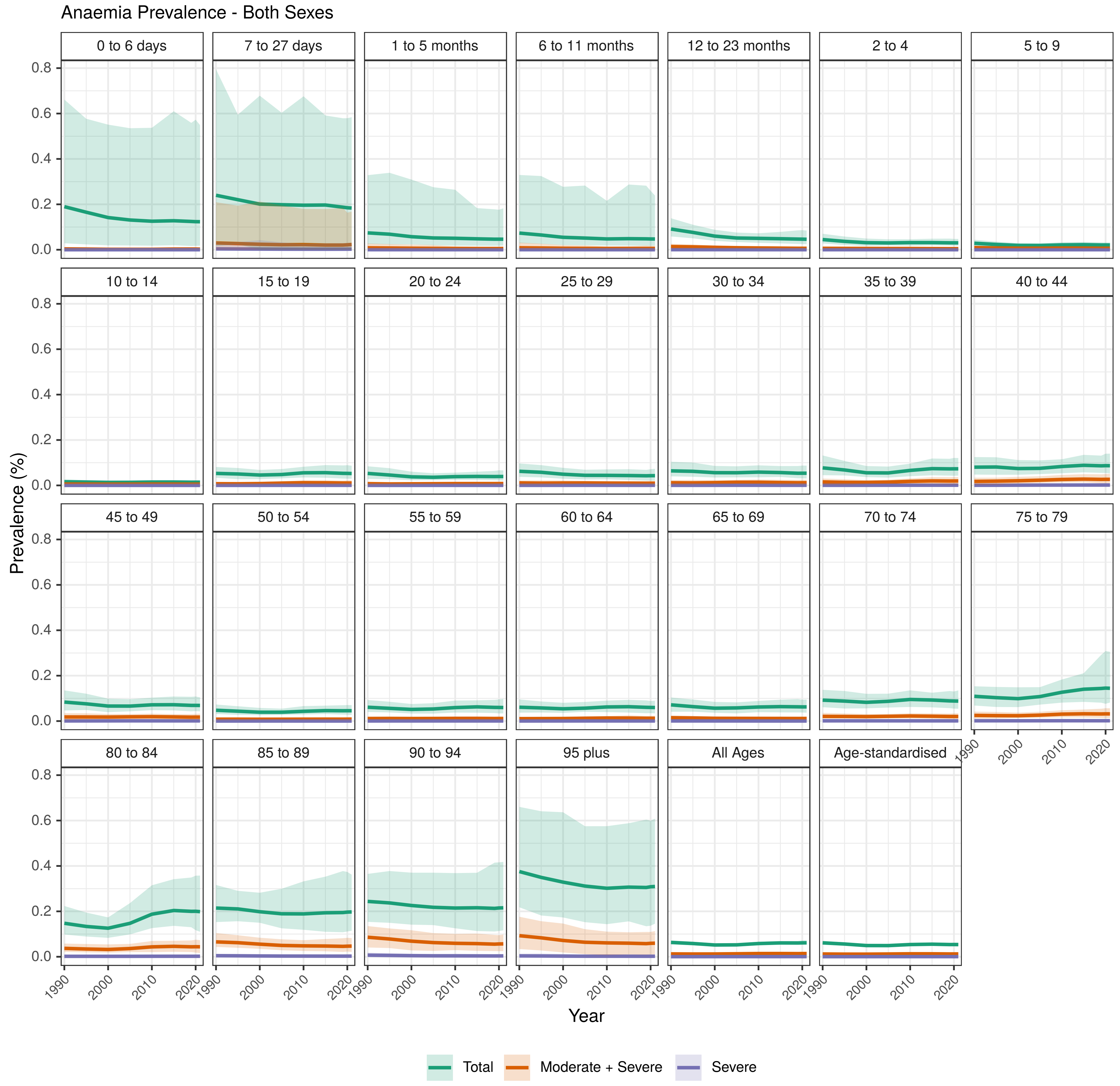

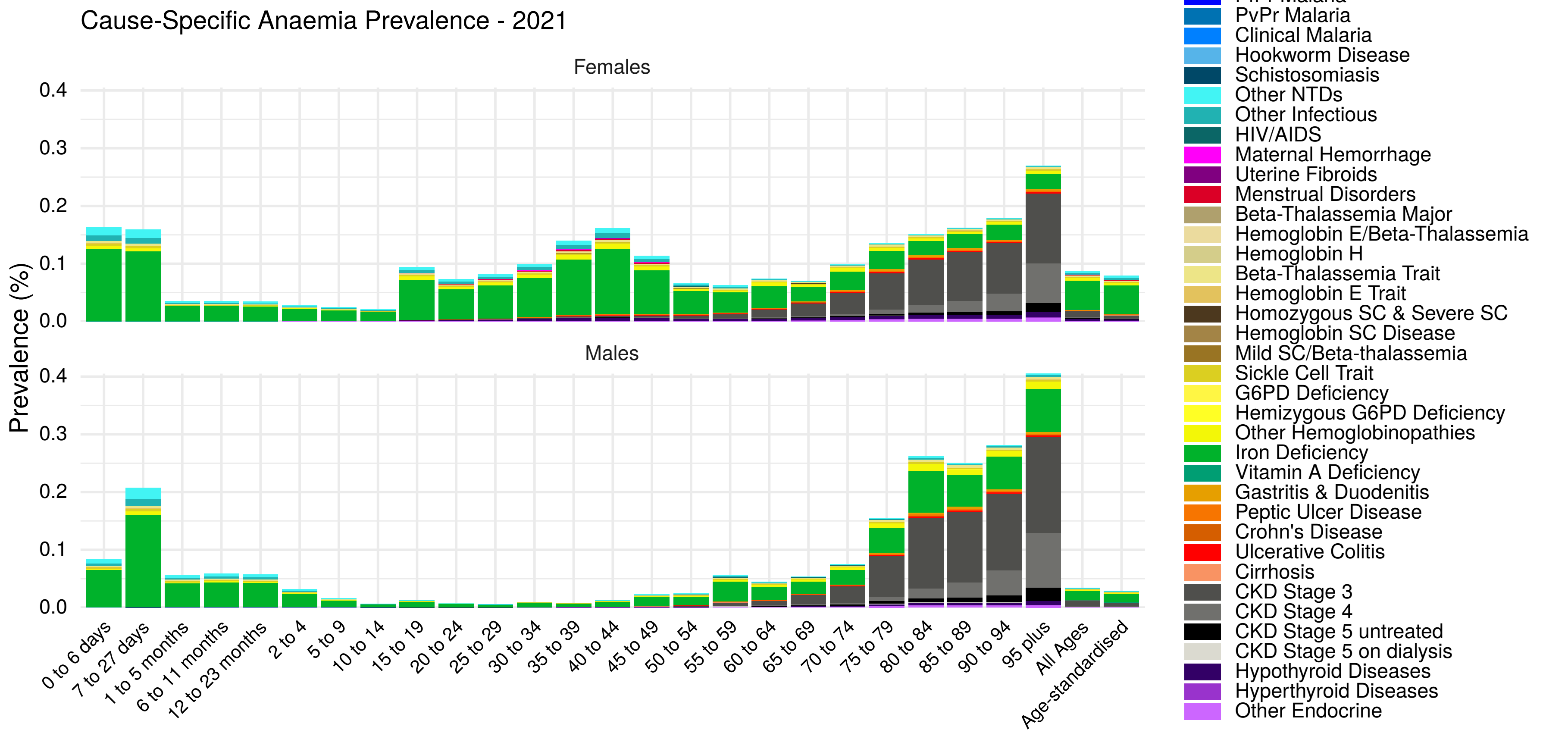

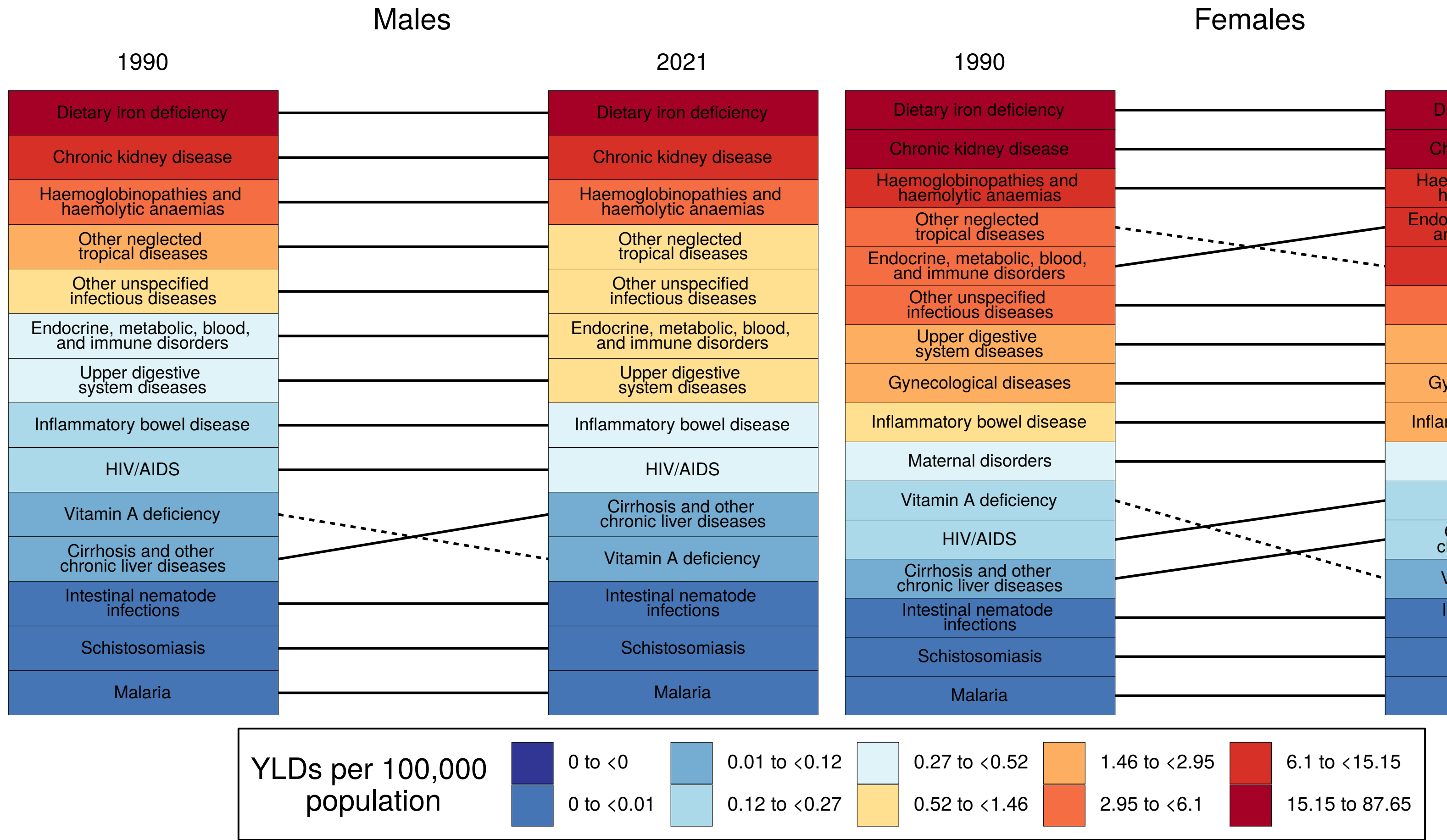

147

Colorado

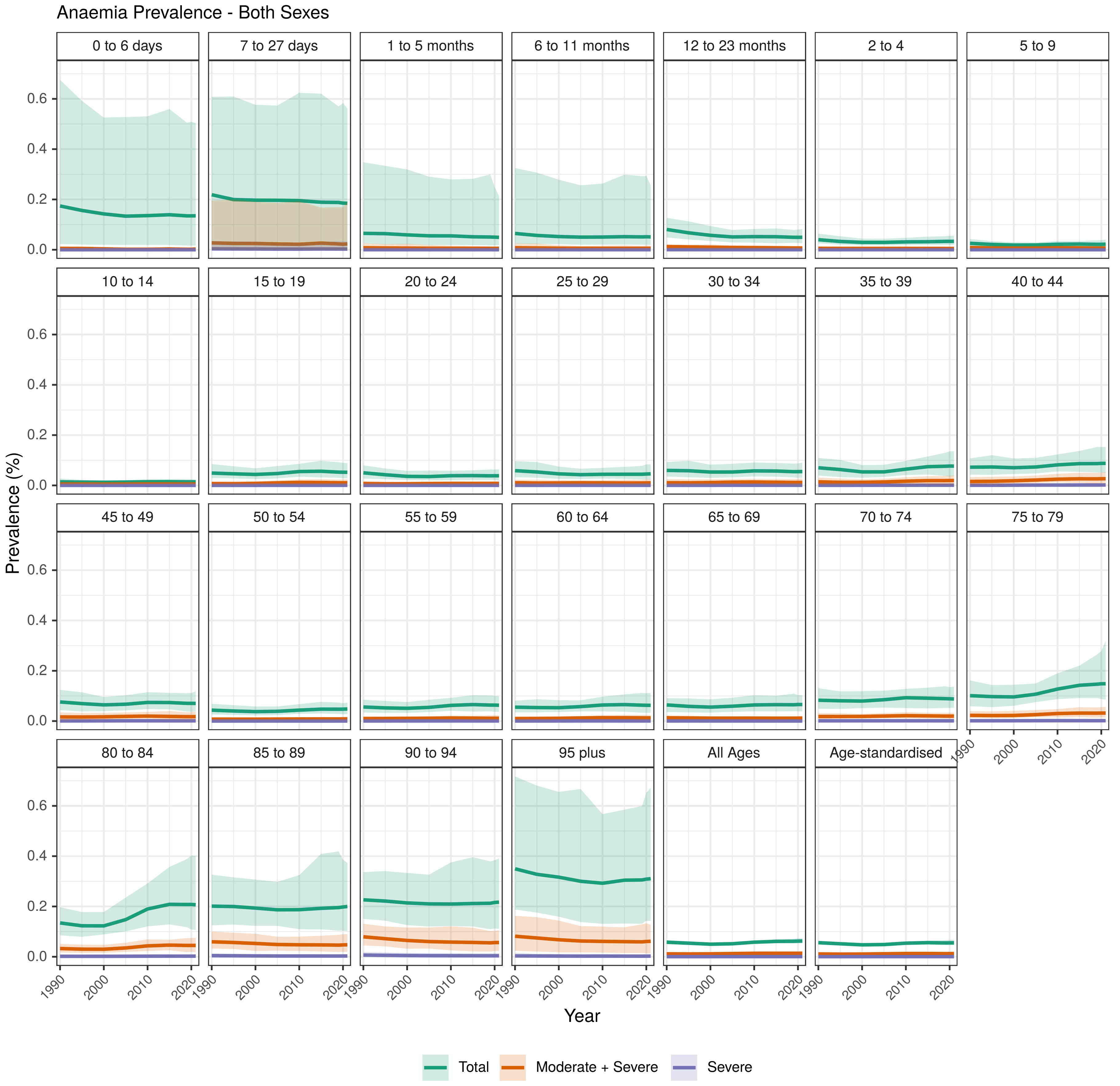
Anaemia Prevalence - Both Sexes
0 to 6 days
7 to 27 days
1 to 5 months
6 to 11 months
12 to 23 months
2 to 4
5 to 9
10 to 14
15 to 19
20 to 24
25 to 29
30 to 34
35 to 39
40 to 44
45 to 49
50 to 54
55 to 59
60 to 64
65 to 69
70 to 74
75 to 79
80 to 84
85 to 89
90 to 94
95 plus
All Ages
Age-standardised
Prevalence (%)
Year
Total
Moderate + Severe
Severe

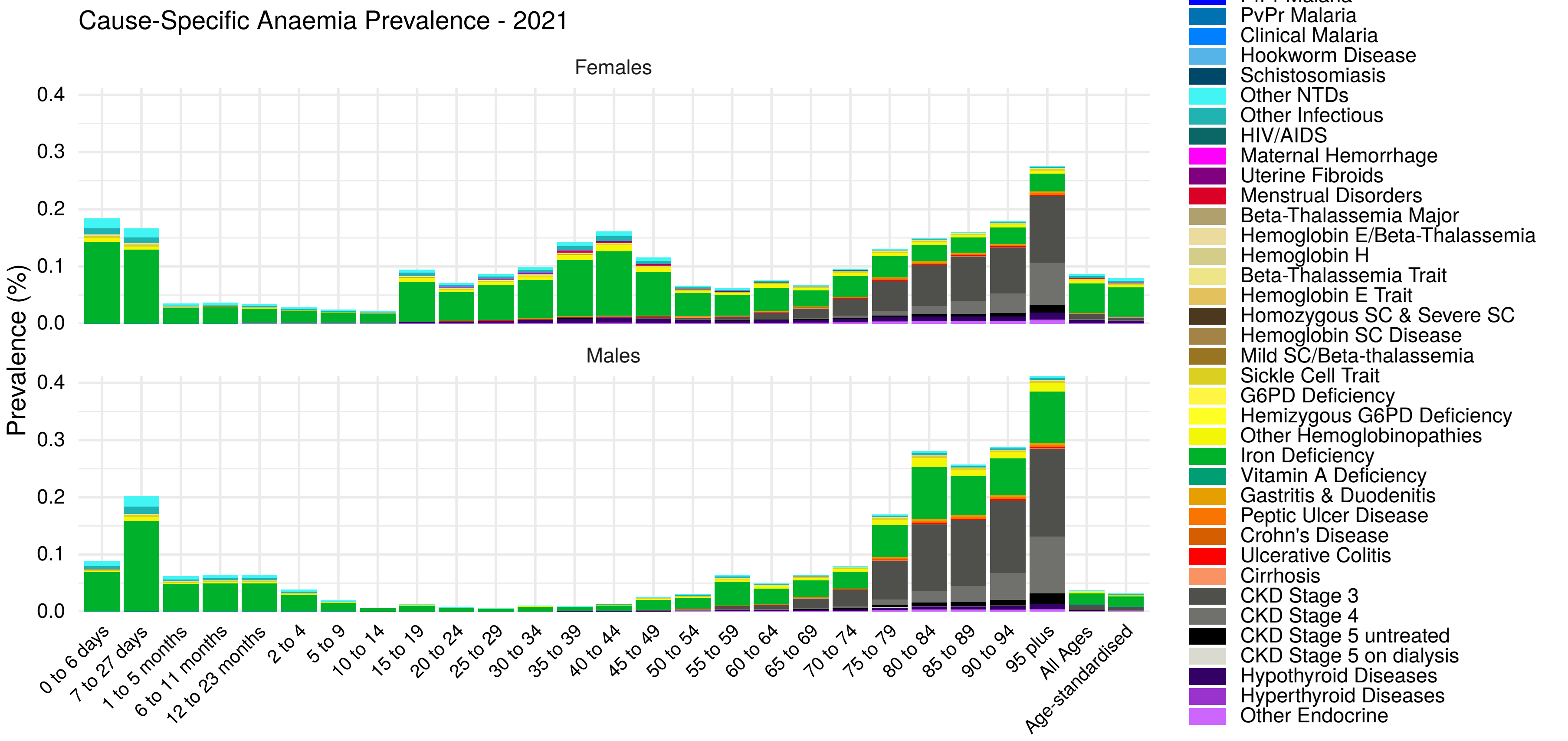
Cause-Specific Anaemia Prevalence - 2021
Females
Males
Prevalence (%)
PfPr Malaria
PvPr Malaria
Clinical Malaria
Hookworm Disease
Schistosomiasis
Other NTDs
Other Infectious
HIV/AIDS
Maternal Hemorrhage
Uterine Fibroids
Menstrual Disorders
Beta-Thalassemia Major
Hemoglobin E/Beta-Thalassemia
Hemoglobin H
Beta-Thalassemia Trait
Hemoglobin E Trait
Homozygous SC & Severe SC
Hemoglobin SC Disease
Mild SC/Beta-thalassemia
Sickle Cell Trait
G6PD Deficiency
Hemizygous G6PD Deficiency
Other Hemoglobinopathies
Iron Deficiency
Vitamin A Deficiency
Gastritis & Duodenitis
Peptic Ulcer Disease
Crohn's Disease
Ulcerative Colitis
Cirrhosis
CKD Stage 3
CKD Stage 4
CKD Stage 5 untreated
CKD Stage 5 on dialysis
Hypothyroid Diseases
Hyperthyroid Diseases
Other Endocrine

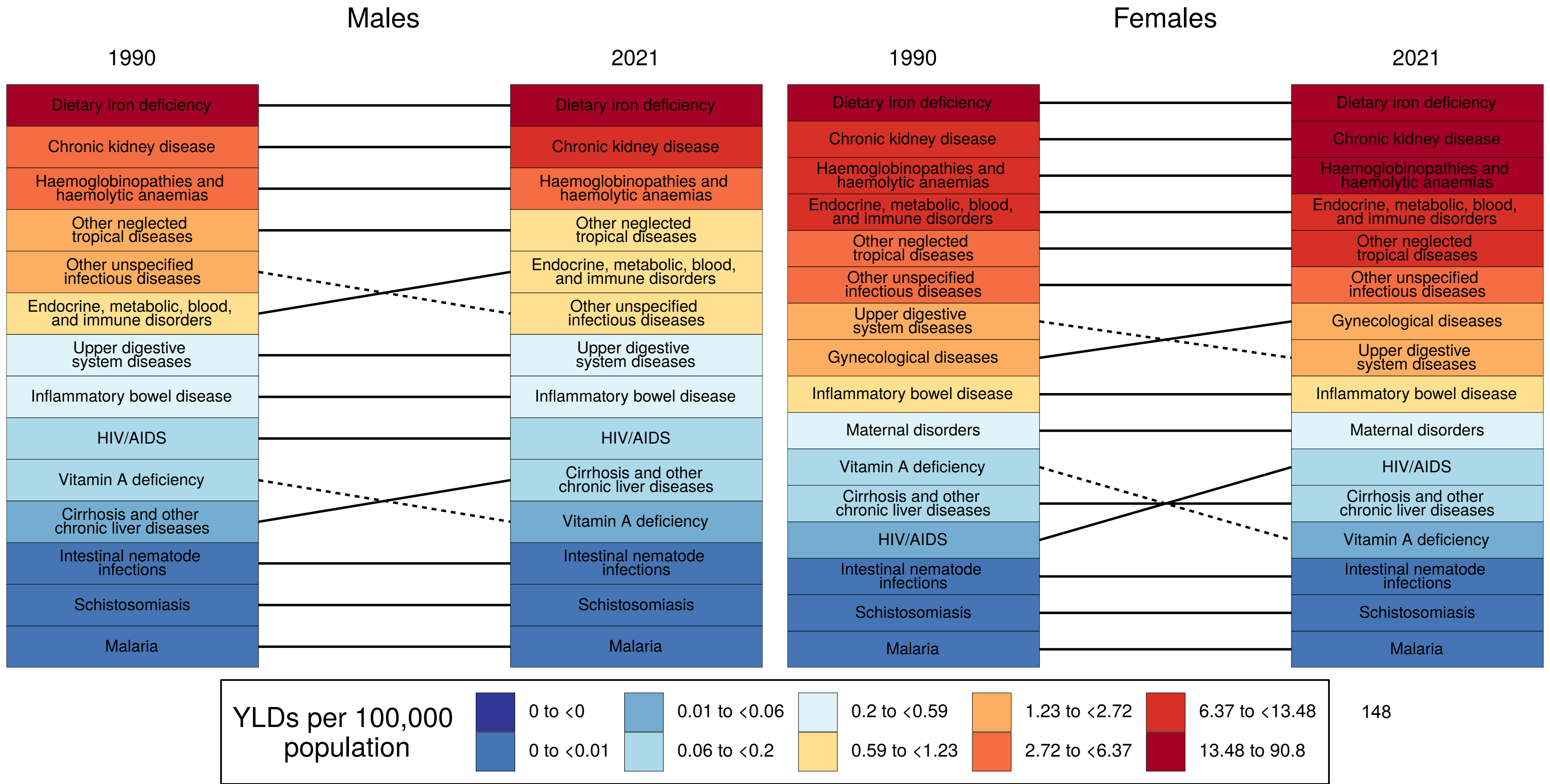
Males
Females
1990
2021
Dietary iron deficiency
Chronic kidney disease
Haemoglobinopathies and haemolytic anaemias
Other neglected tropical diseases
Other unspecified infectious diseases
Endocrine, metabolic, blood, and immune disorders
Upper digestive system diseases
Inflammatory bowel disease
HIV/AIDS
Vitamin A deficiency
Cirrhosis and other chronic liver diseases
Intestinal nematode infections
Schistosomiasis
Malaria
Gynecological diseases
Maternal disorders
YLDs per 100,000 population
0 to <0
0 to <0.01
0.01 to <0.06
0.06 to <0.2
0.2 to <0.59
0.59 to <1.23
1.23 to <2.72
2.72 to <6.37
6.37 to <13.48
13.48 to 90.8

Connecticut

Anaemia Prevalence - Both Sexes

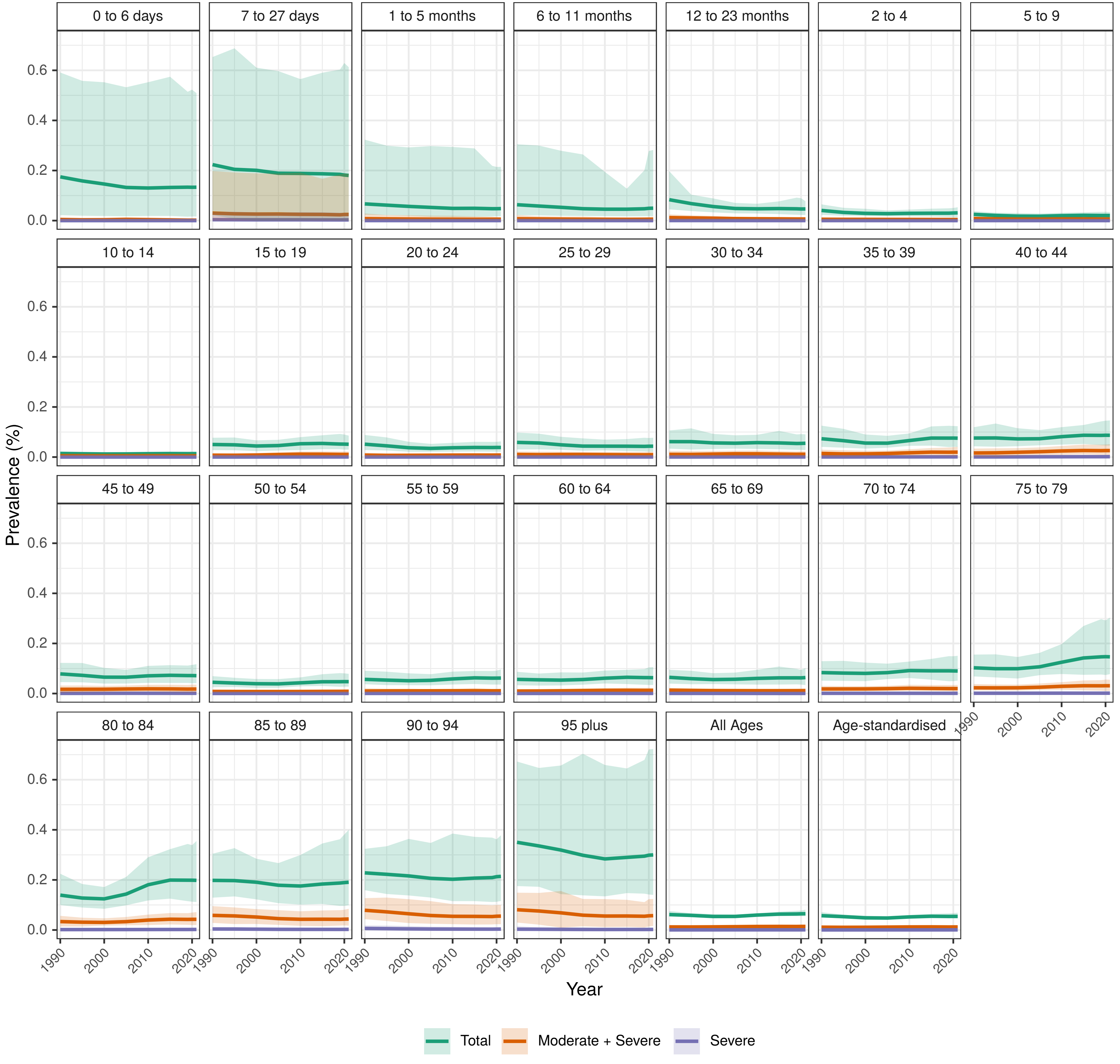

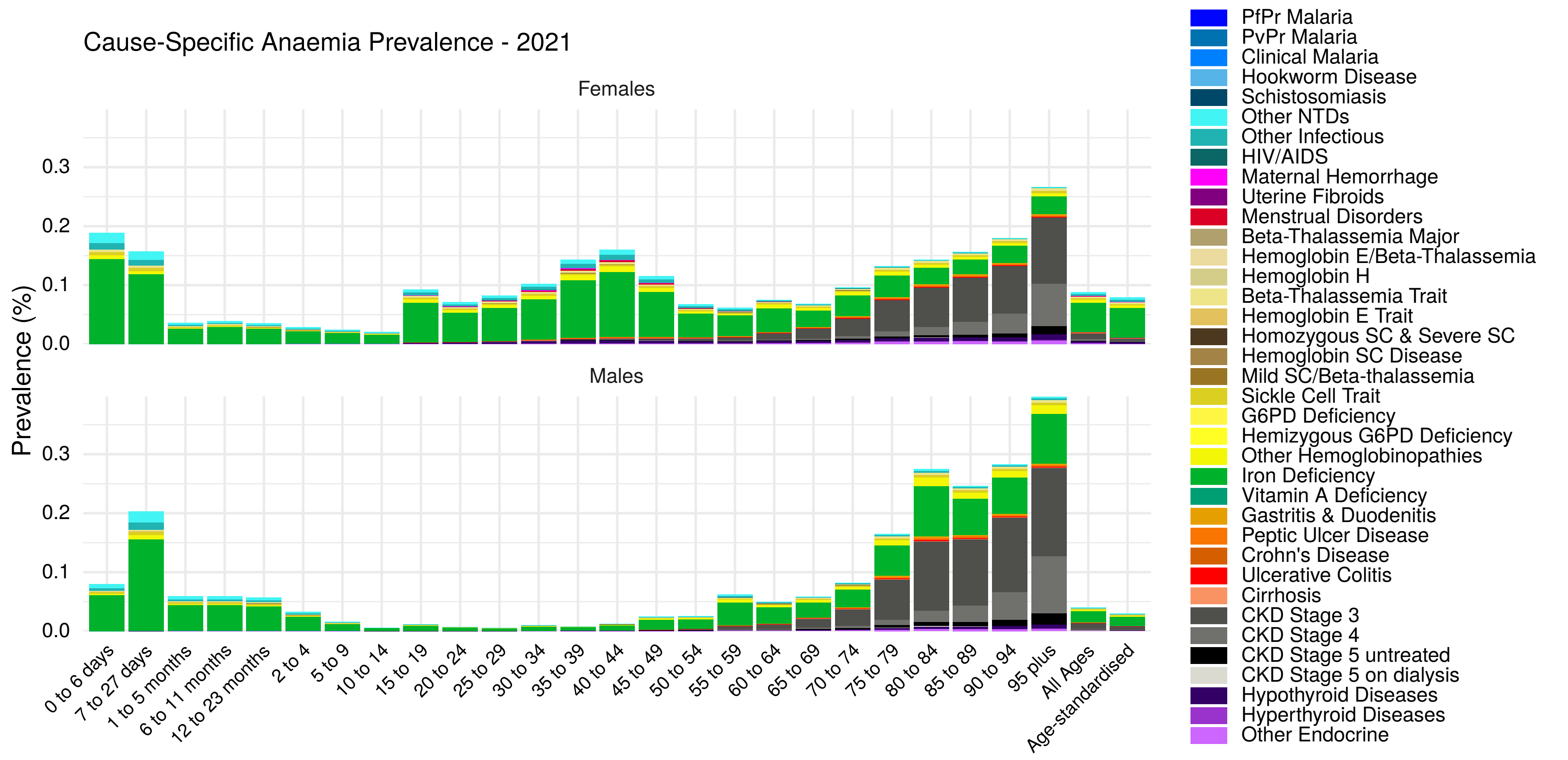

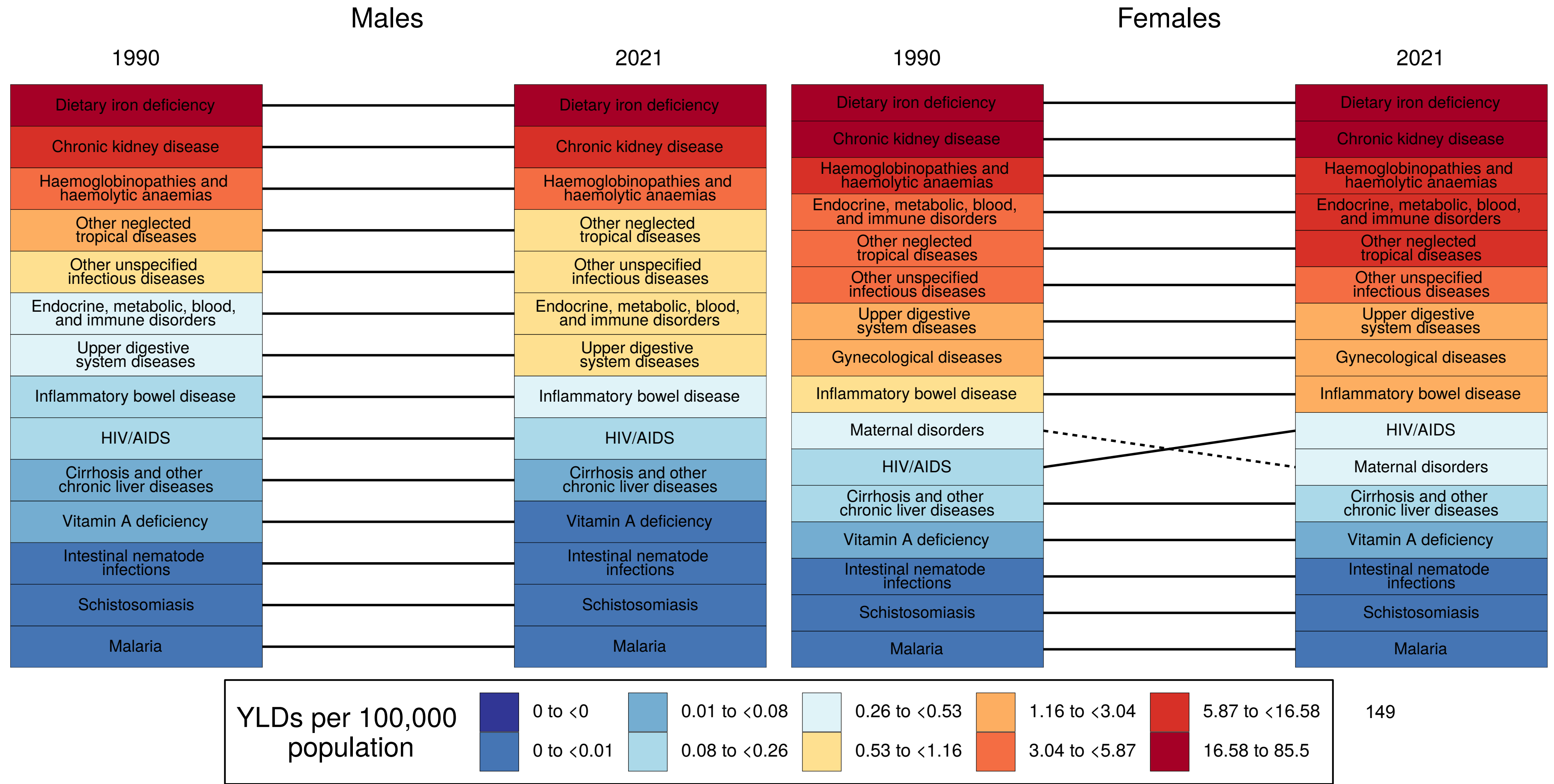

Delaware

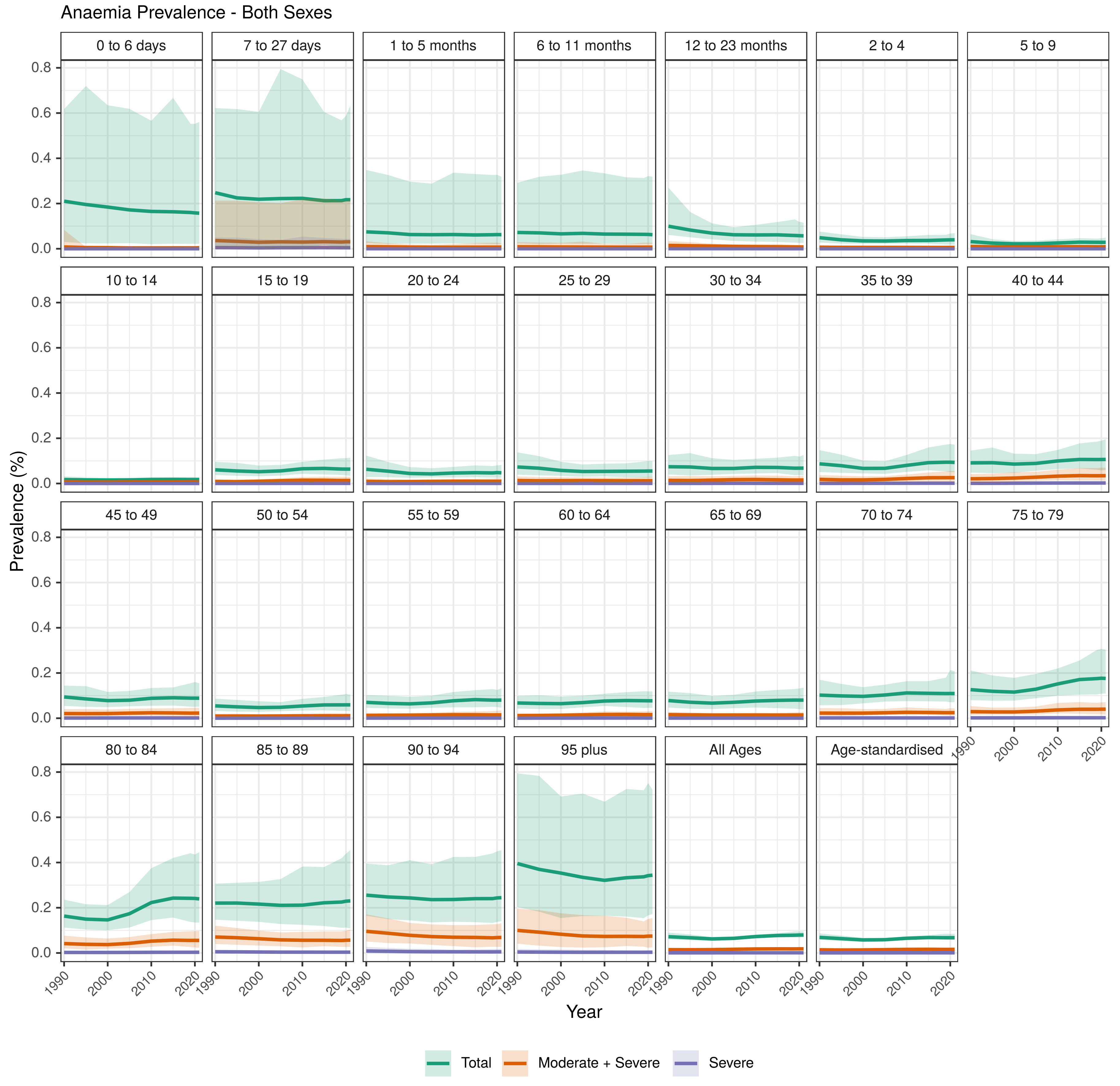

Anaemia Prevalence - Both Sexes
0 to 6 days
7 to 27 days
1 to 5 months
6 to 11 months
12 to 23 months
2 to 4
5 to 9
10 to 14
15 to 19
20 to 24
25 to 29
30 to 34
35 to 39
40 to 44
45 to 49
50 to 54
55 to 59
60 to 64
65 to 69
70 to 74
75 to 79
80 to 84
85 to 89
90 to 94
95 plus
All Ages
Age-standardised
Prevalence (%)
0.0
0.2
0.4
0.6
0.8
1990
2000
2010
2020
Year
Total
Moderate + Severe
Severe

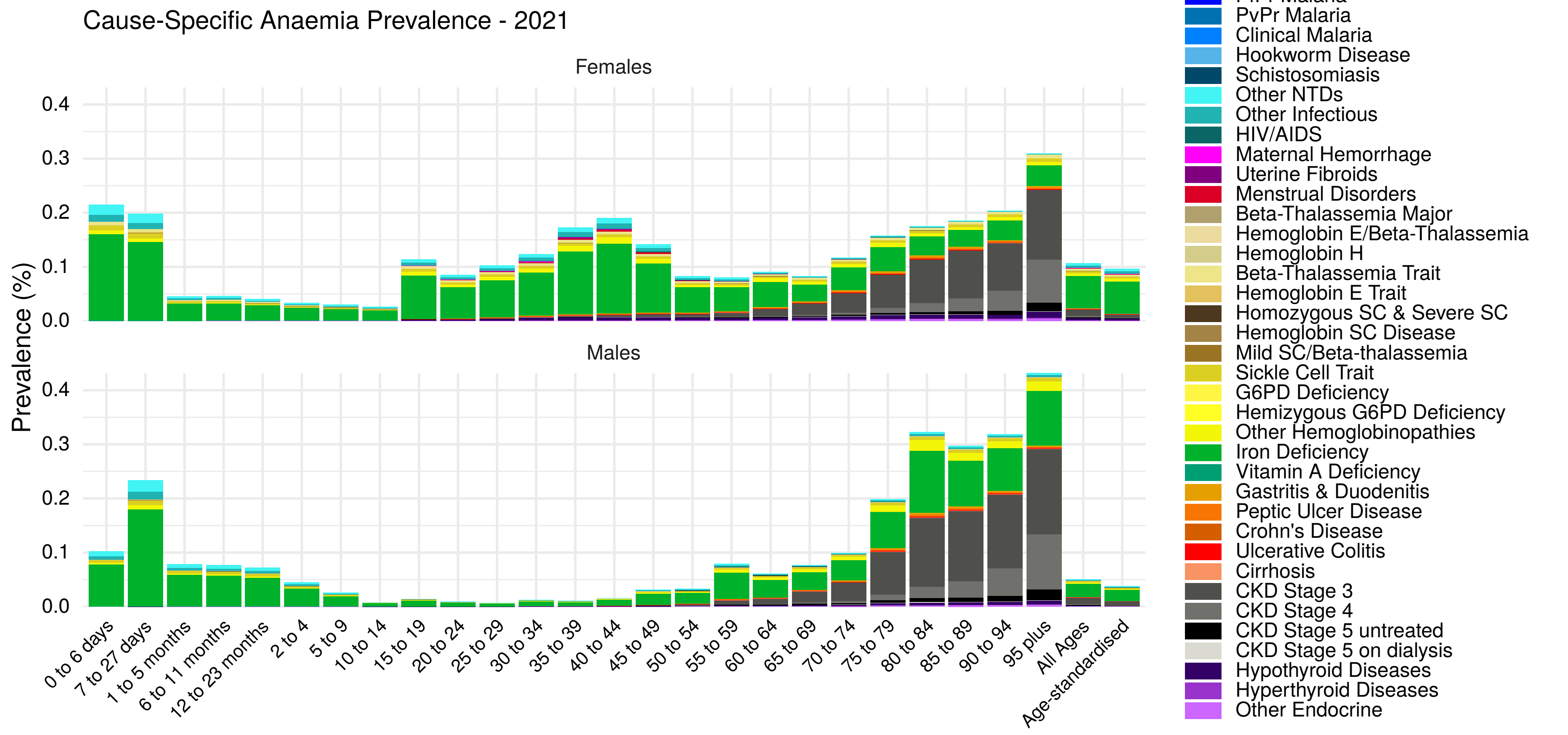

Cause-Specific Anaemia Prevalence - 2021
Females
Males
Prevalence (%)
0.0
0.1
0.2
0.3
0.4
0 to 6 days
7 to 27 days
1 to 5 months
6 to 11 months
12 to 23 months
2 to 4
5 to 9
10 to 14
15 to 19
20 to 24
25 to 29
30 to 34
35 to 39
40 to 44
45 to 49
50 to 54
55 to 59
60 to 64
65 to 69
70 to 74
75 to 79
80 to 84
85 to 89
90 to 94
95 plus
All Ages
Age-standardised
PfPr Malaria
PvPr Malaria
Clinical Malaria
Hookworm Disease
Schistosomiasis
Other NTDs
Other Infectious
HIV/AIDS
Maternal Hemorrhage
Uterine Fibroids
Menstrual Disorders
Beta-Thalassemia Major
Hemoglobin E/Beta-Thalassemia
Hemoglobin H
Beta-Thalassemia Trait
Hemoglobin E Trait
Homozygous SC & Severe SC
Hemoglobin SC Disease
Mild SC/Beta-thalassemia
Sickle Cell Trait
G6PD Deficiency
Hemizygous G6PD Deficiency
Other Hemoglobinopathies
Iron Deficiency
Vitamin A Deficiency
Gastritis & Duodenitis
Peptic Ulcer Disease
Crohn's Disease
Ulcerative Colitis
Cirrhosis
CKD Stage 3
CKD Stage 4
CKD Stage 5 untreated
CKD Stage 5 on dialysis
Hypothyroid Diseases
Hyperthyroid Diseases
Other Endocrine

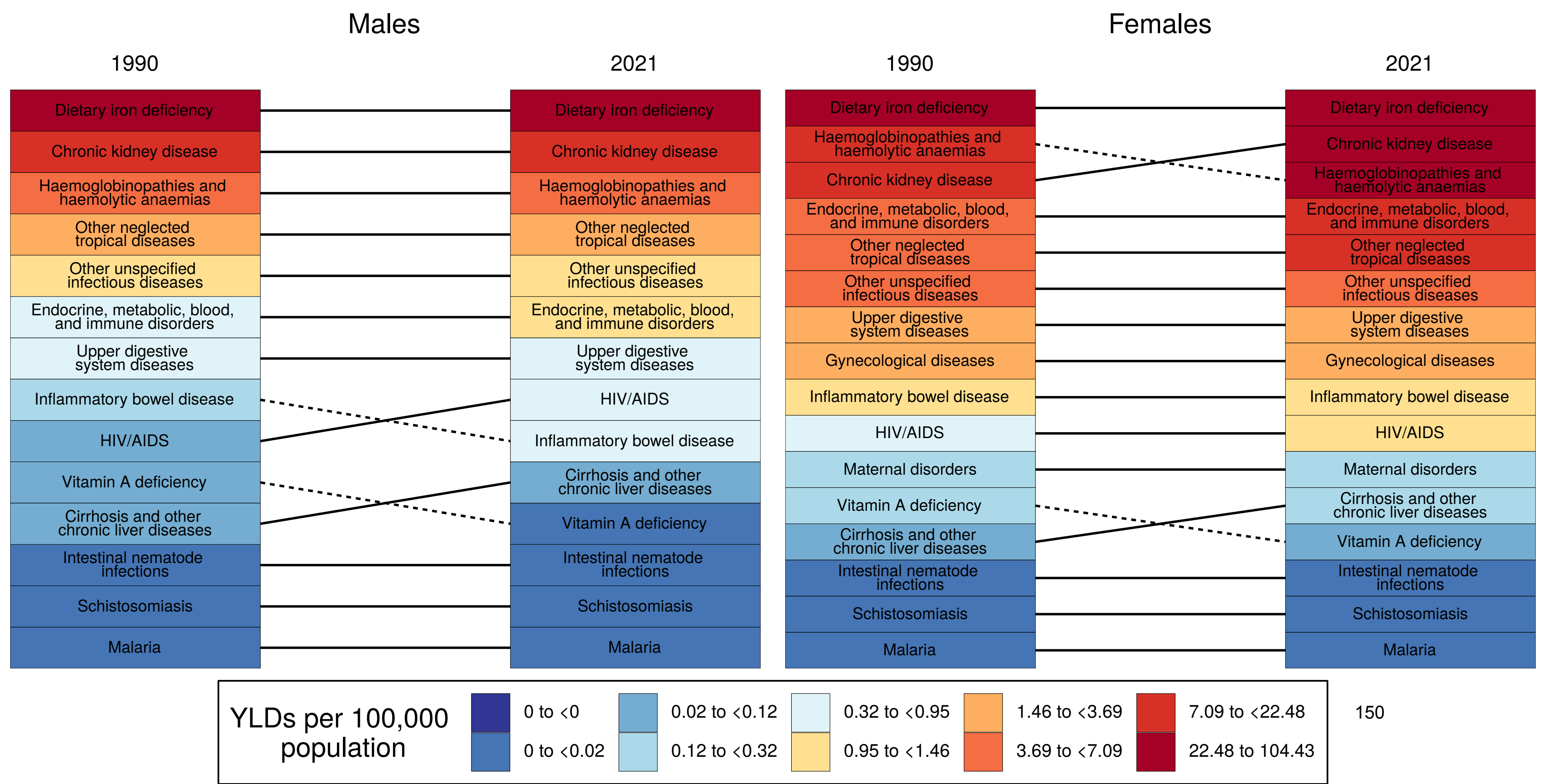

Males
Females
1990
2021
Dietary iron deficiency
Chronic kidney disease
Haemoglobinopathies and haemolytic anaemias
Other neglected tropical diseases
Other unspecified infectious diseases
Endocrine, metabolic, blood, and immune disorders
Upper digestive system diseases
Inflammatory bowel disease
HIV/AIDS
Vitamin A deficiency
Cirrhosis and other chronic liver diseases
Intestinal nematode infections
Schistosomiasis
Malaria
Gynecological diseases
Maternal disorders
YLDs per 100,000 population
0 to <0
0 to <0.02
0.02 to <0.12
0.12 to <0.32
0.32 to <0.95
0.95 to <1.46
1.46 to <3.69
3.69 to <7.09
7.09 to <22.48
22.48 to 104.43
150

Washington, DC

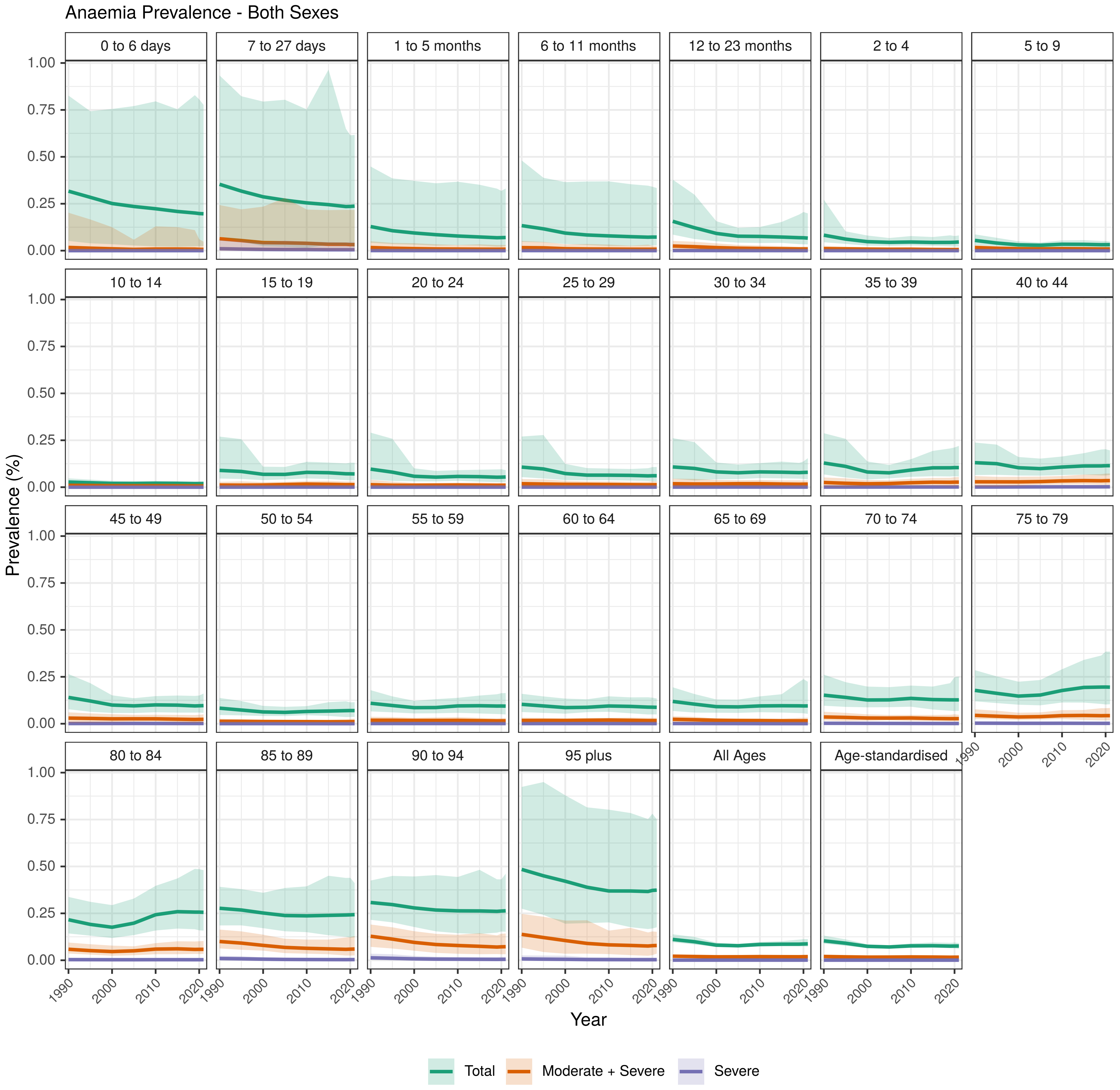

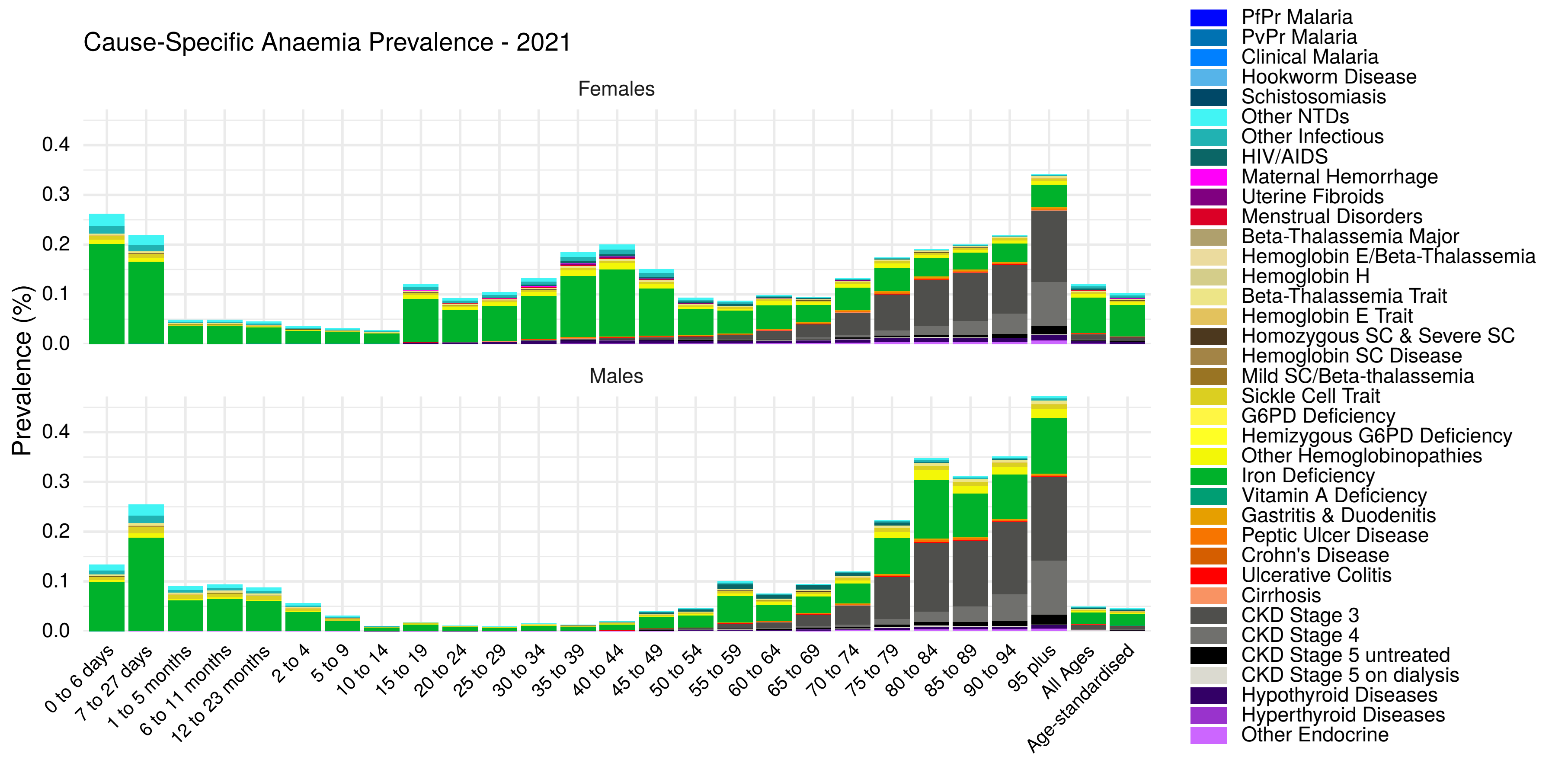

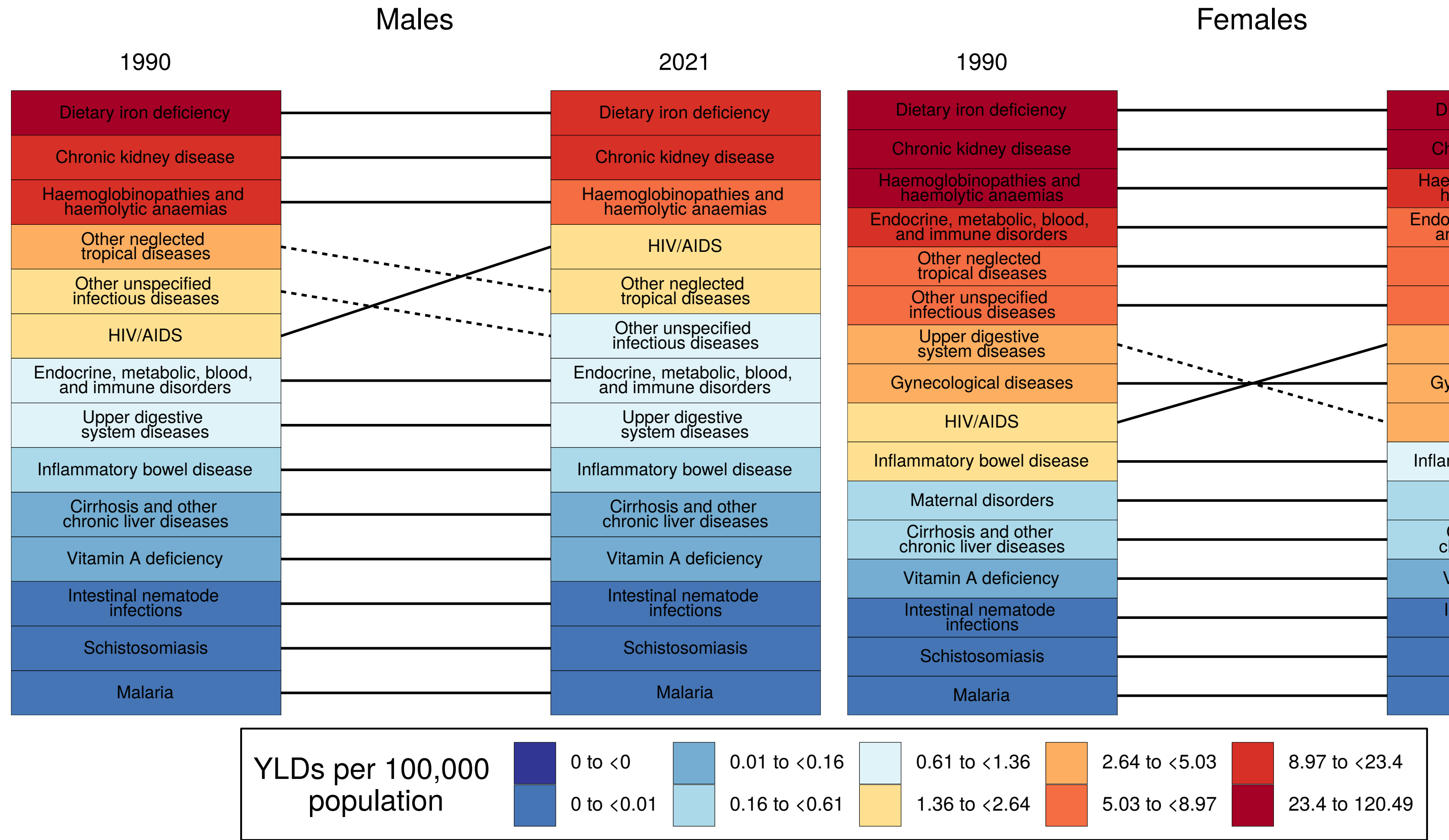

Florida

Anaemia Prevalence - Both Sexes

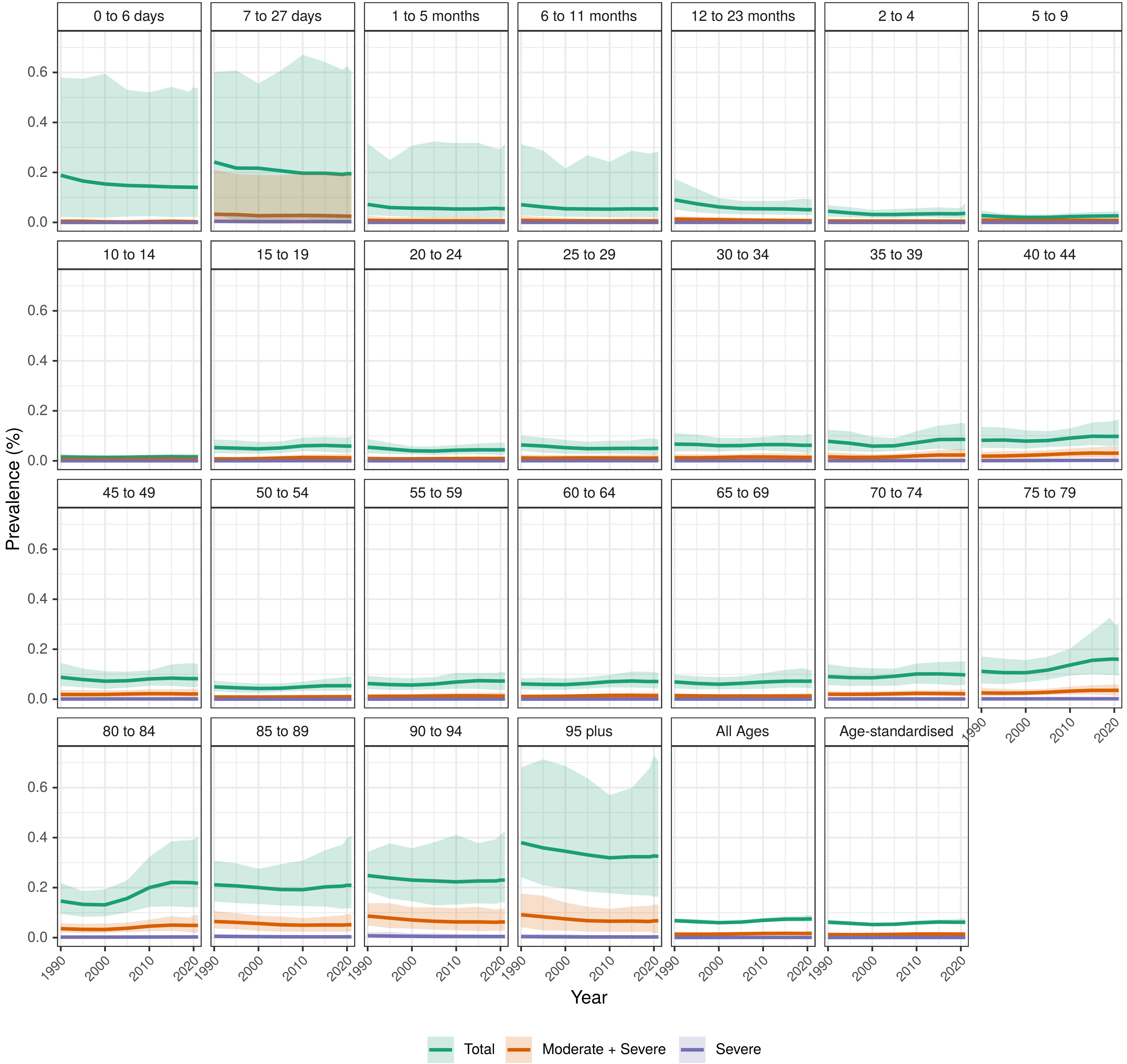

Cause-Specific Anaemia Prevalence - 2021

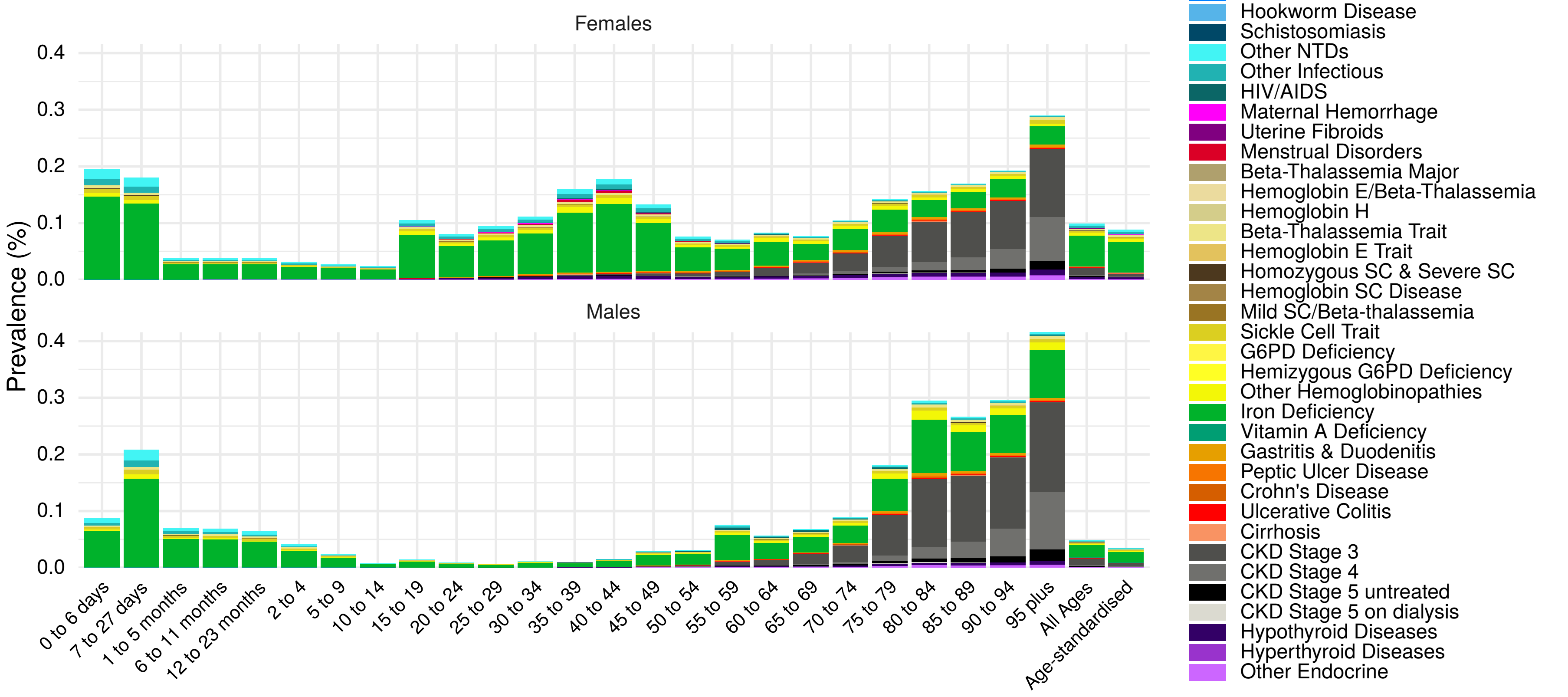

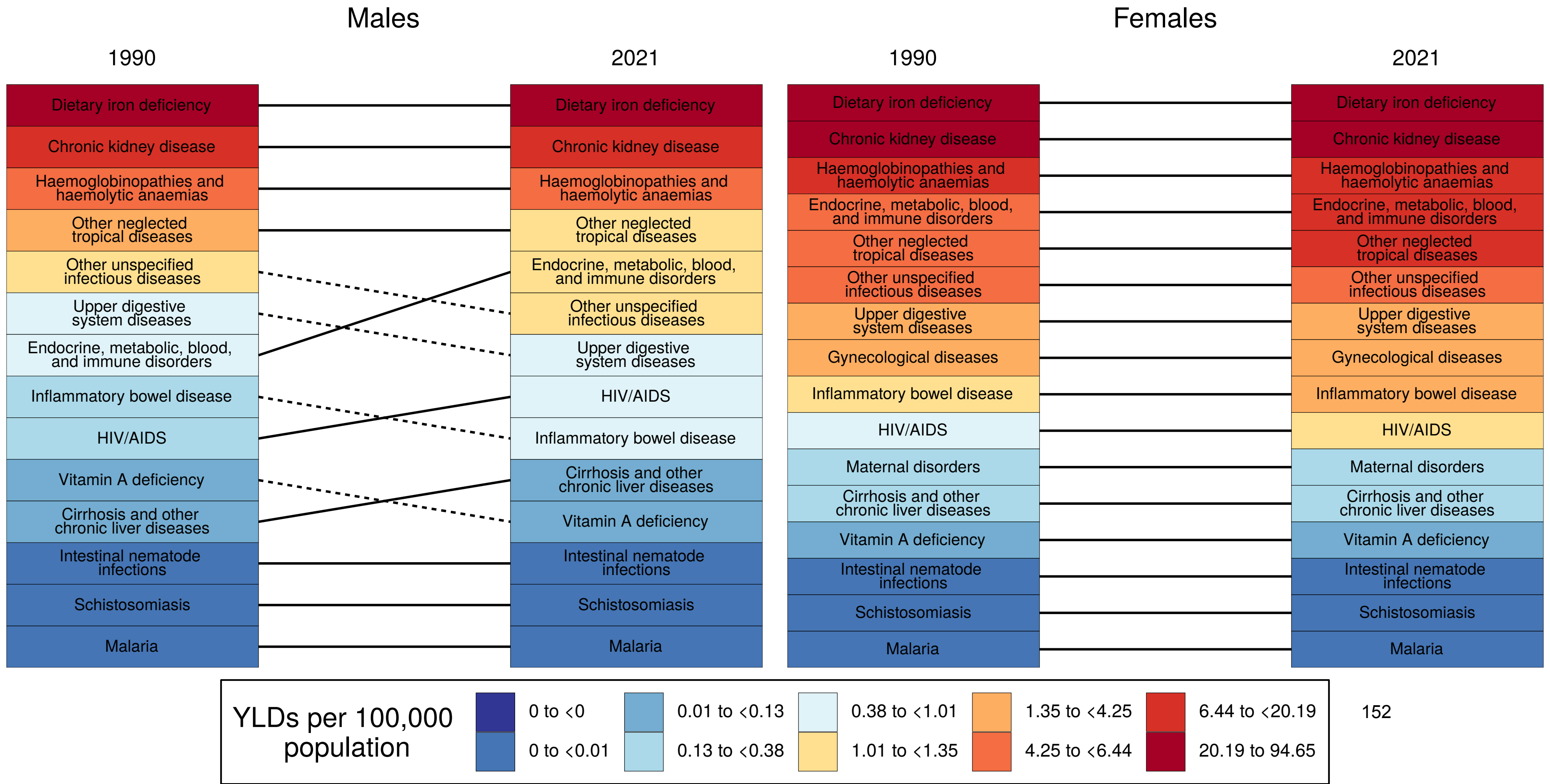

Georgia

Anaemia Prevalence - Both Sexes

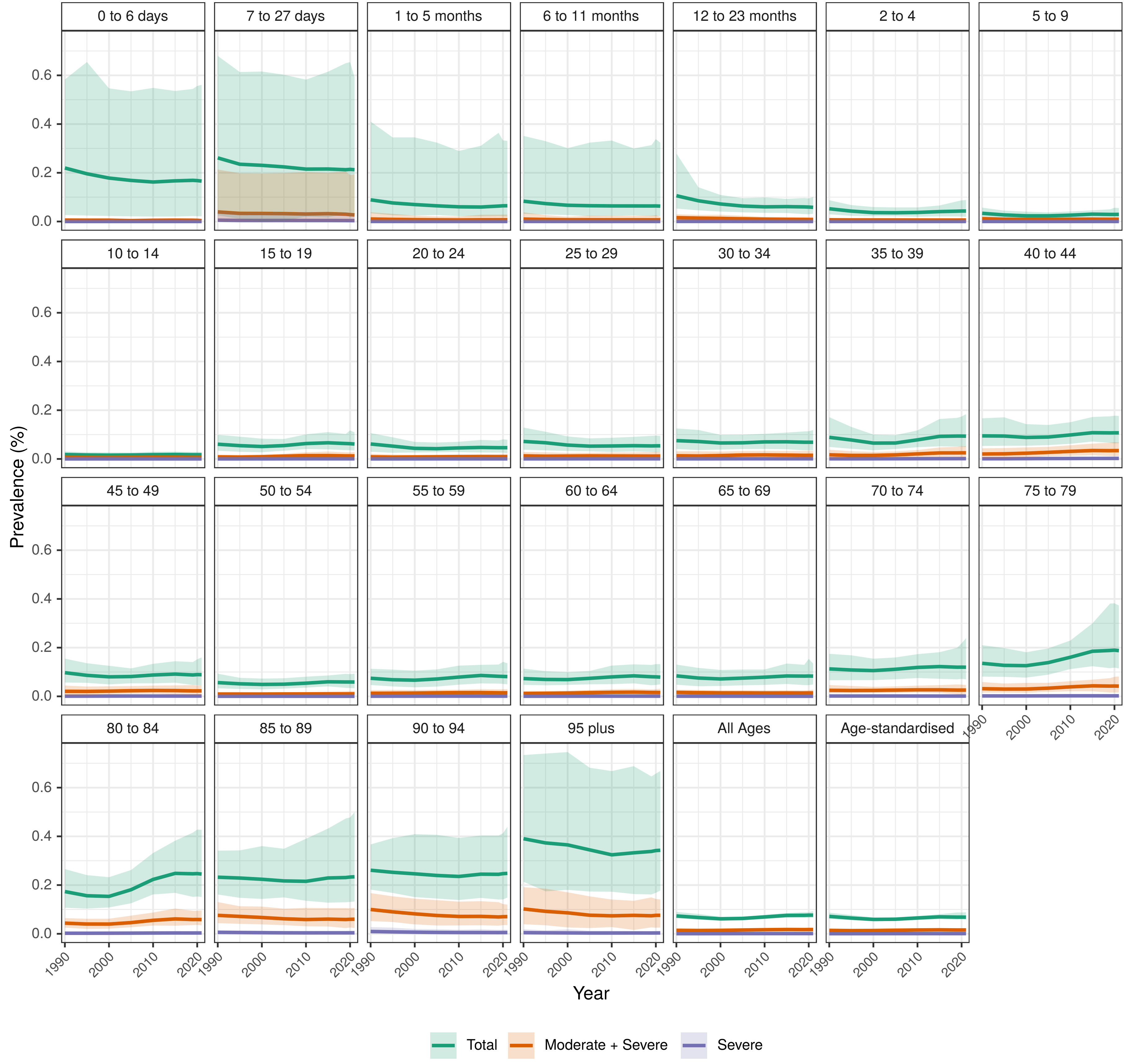

Cause-Specific Anaemia Prevalence - 2021

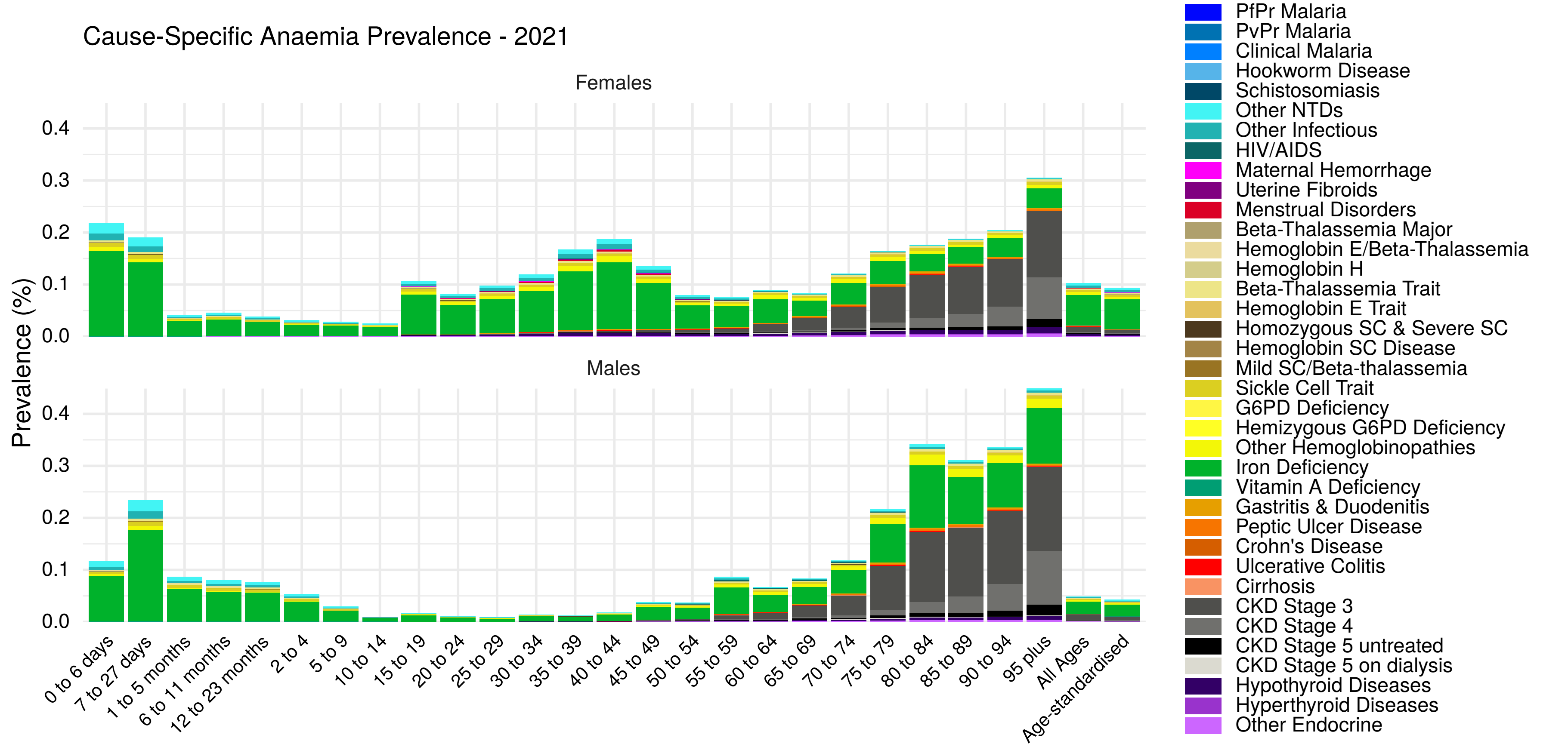

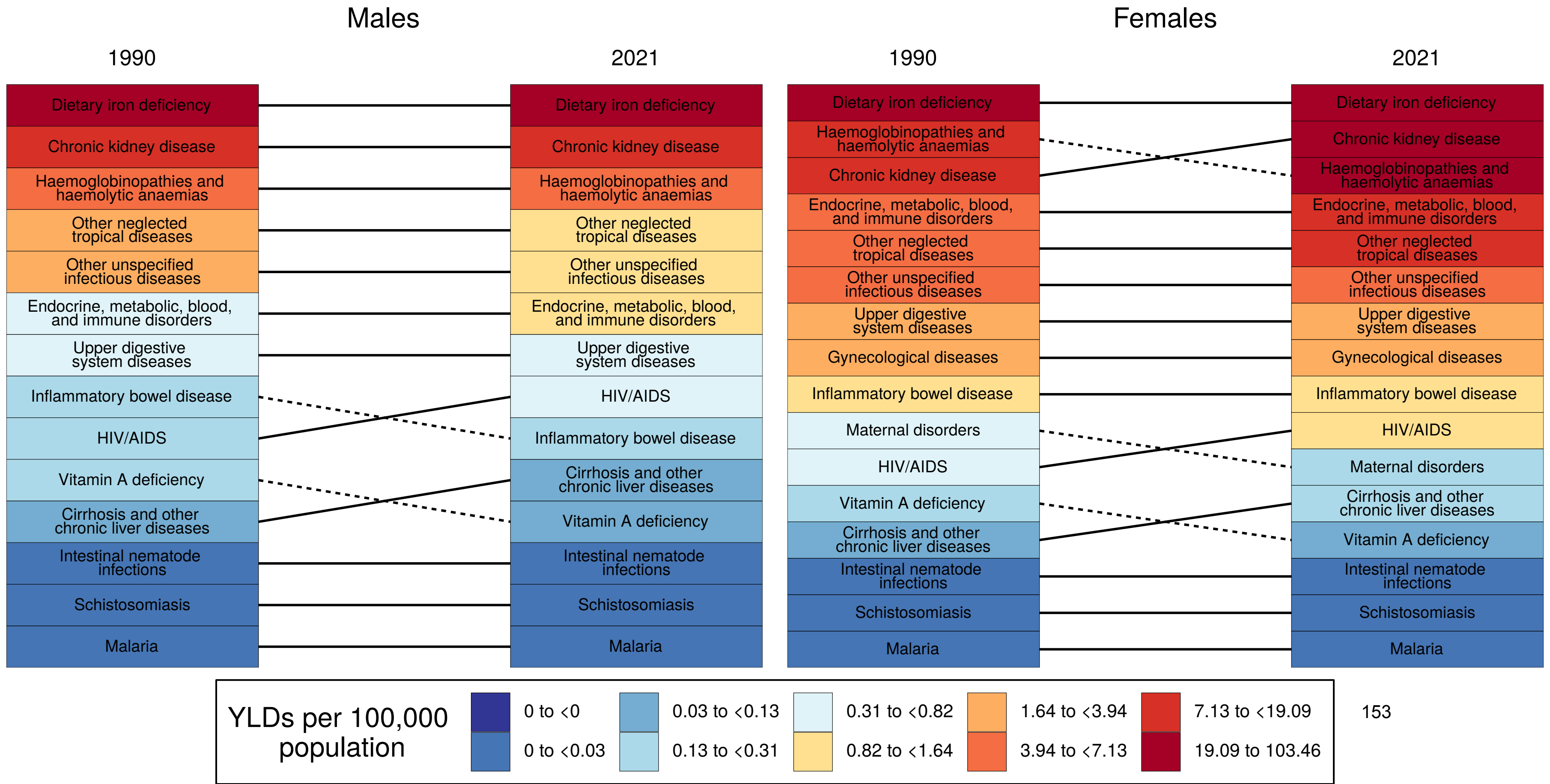

Hawaii

Anaemia Prevalence - Both Sexes

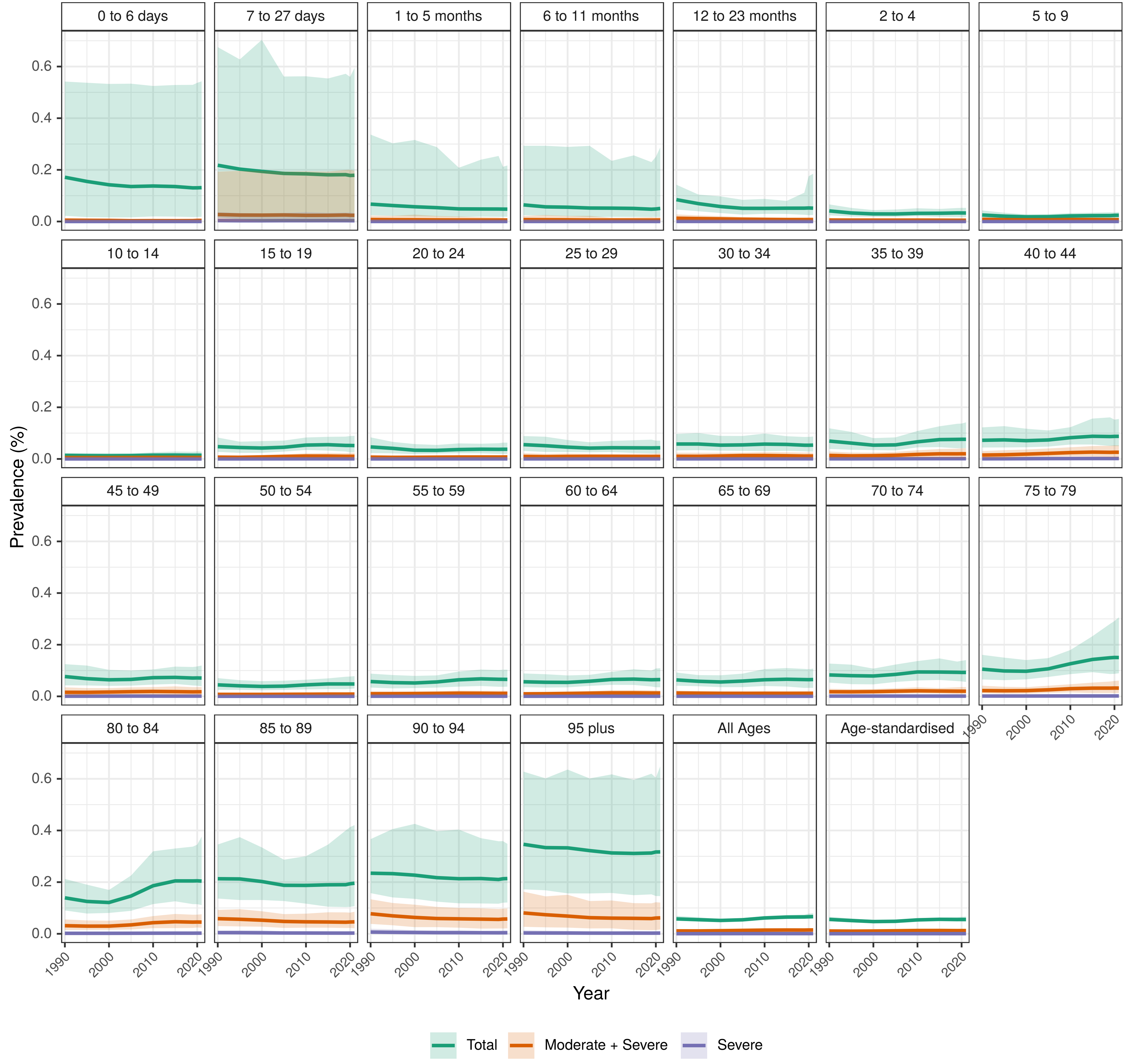

Cause-Specific Anaemia Prevalence - 2021

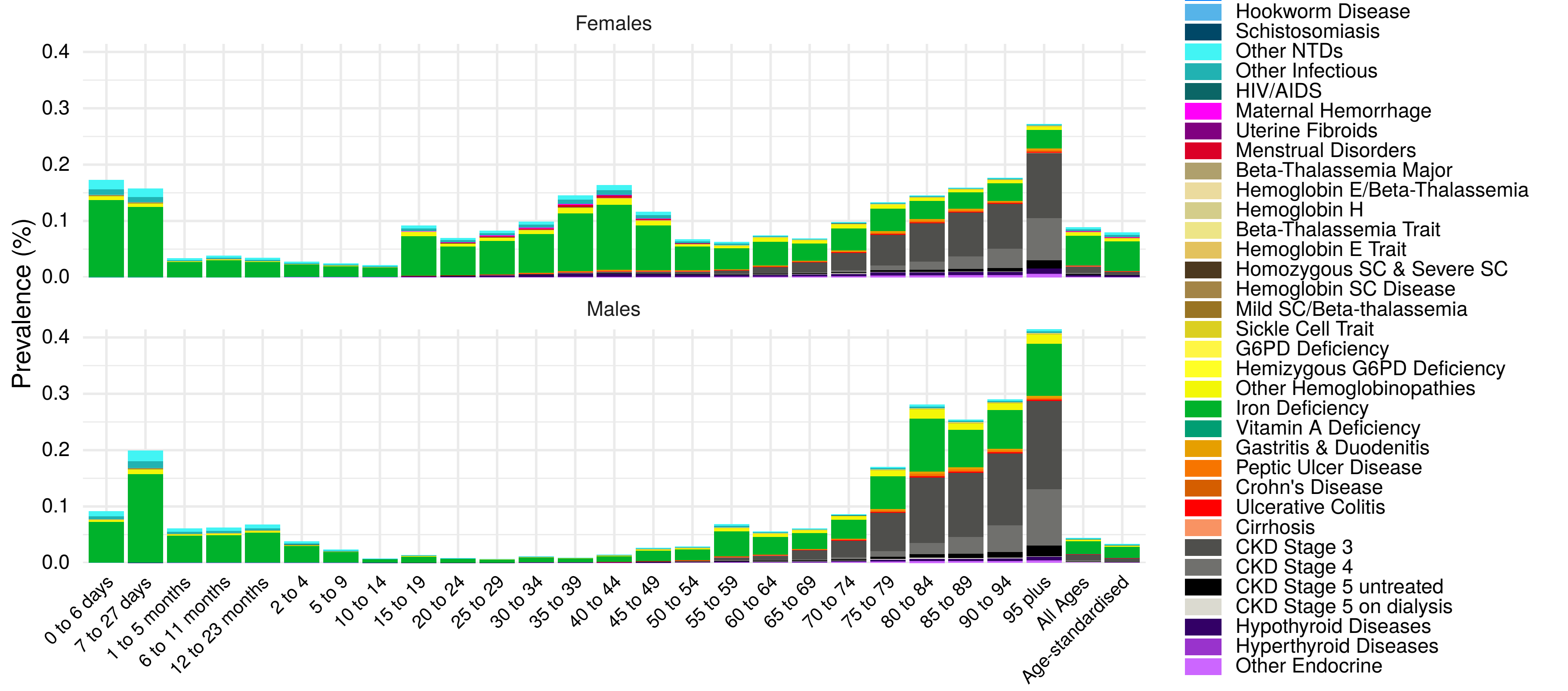

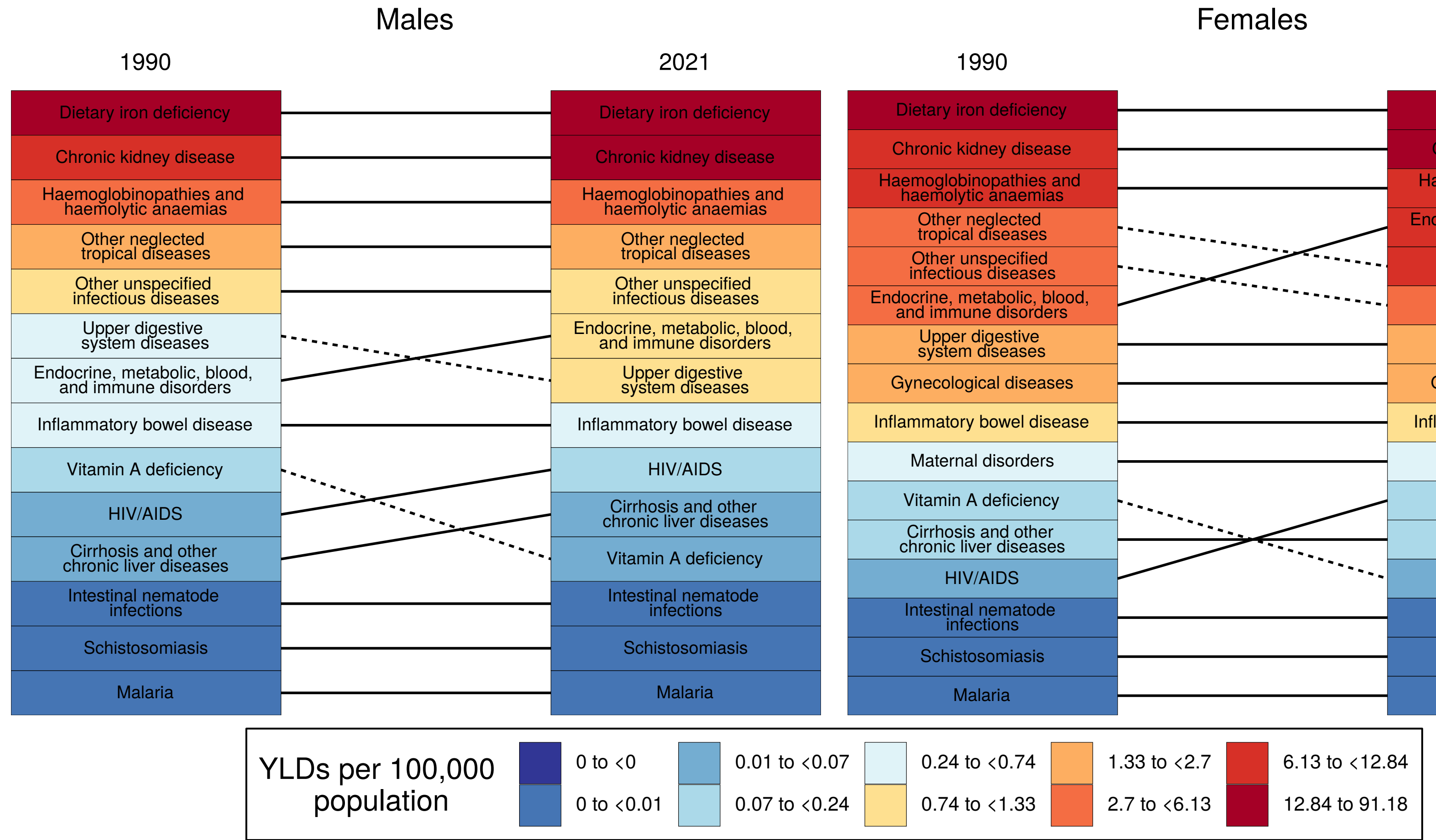

Idaho

Anaemia Prevalence - Both Sexes

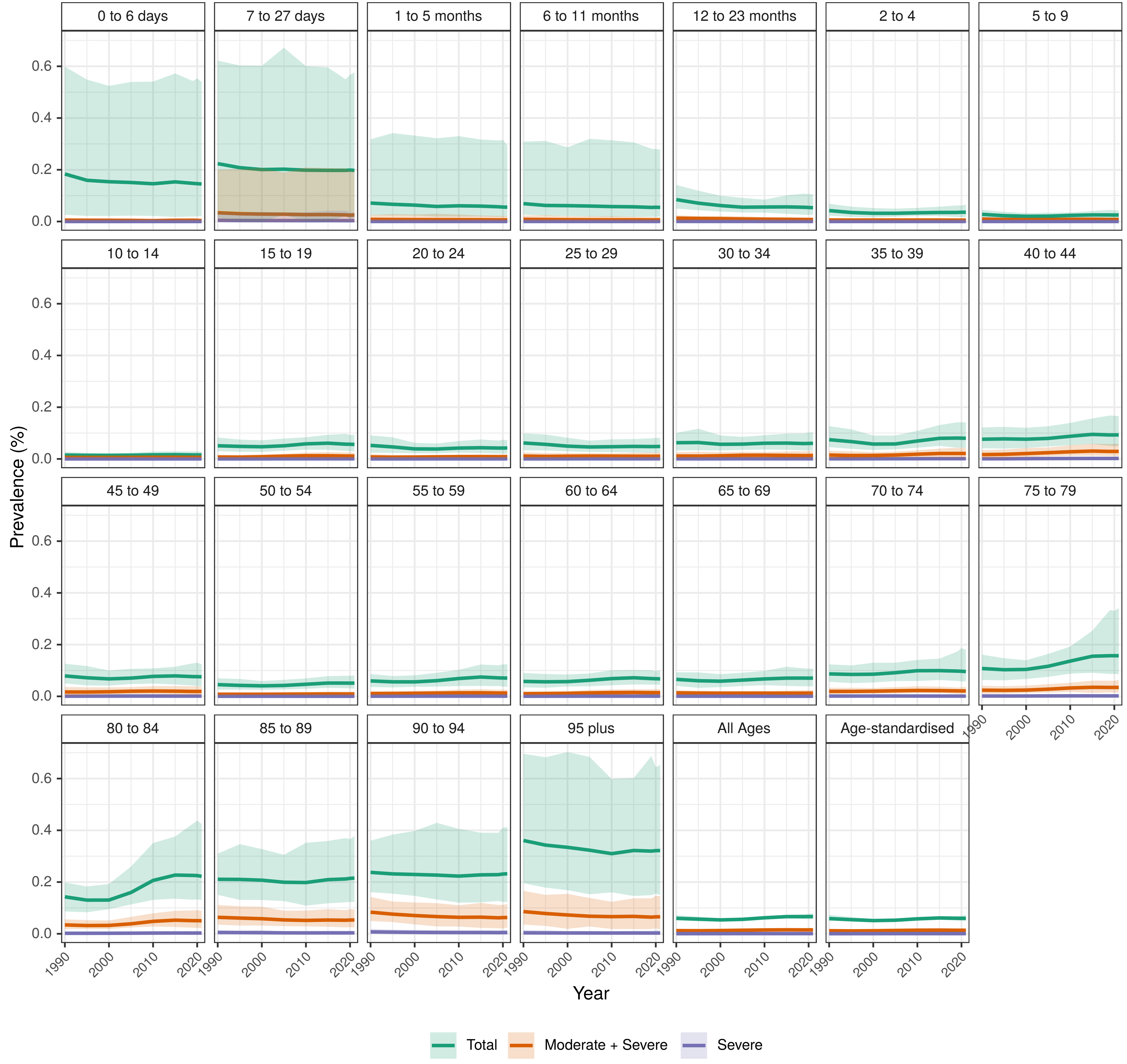

Cause-Specific Anaemia Prevalence - 2021

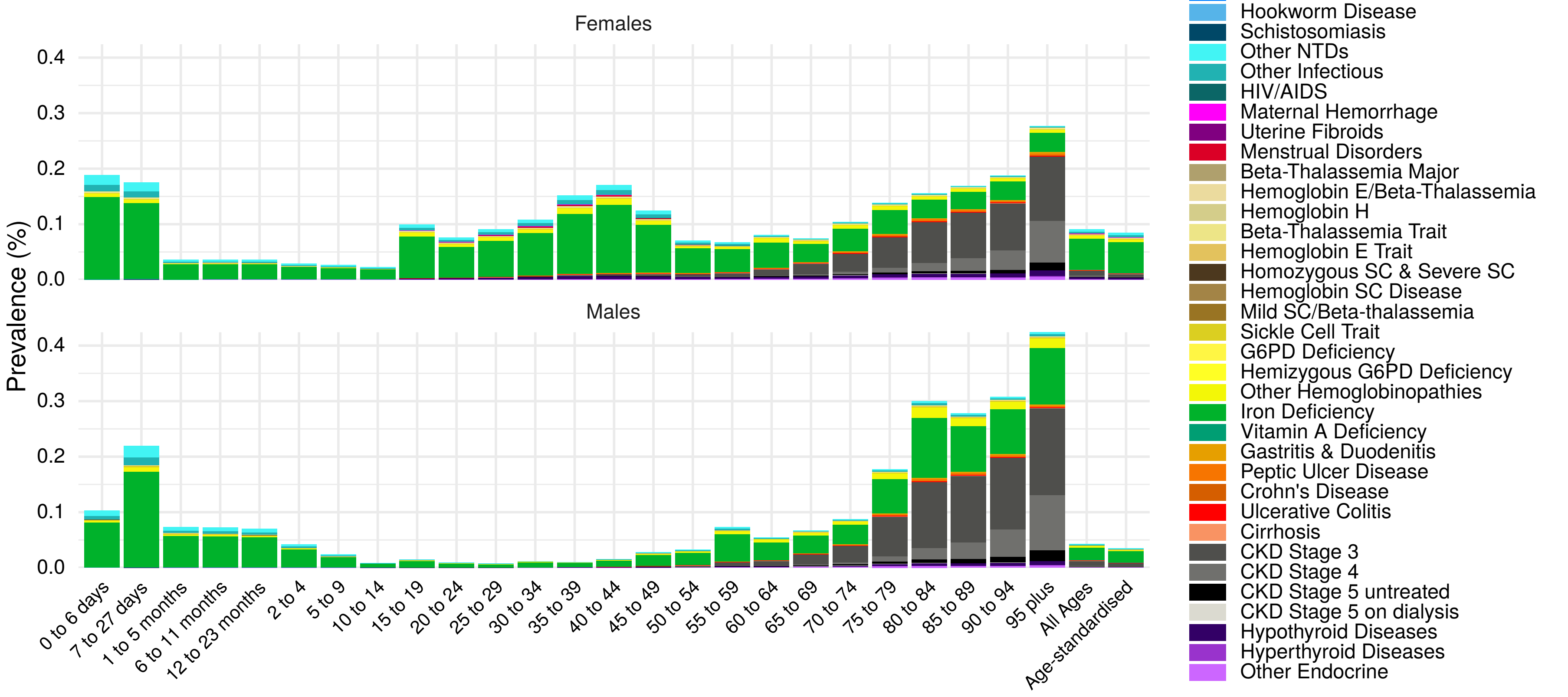

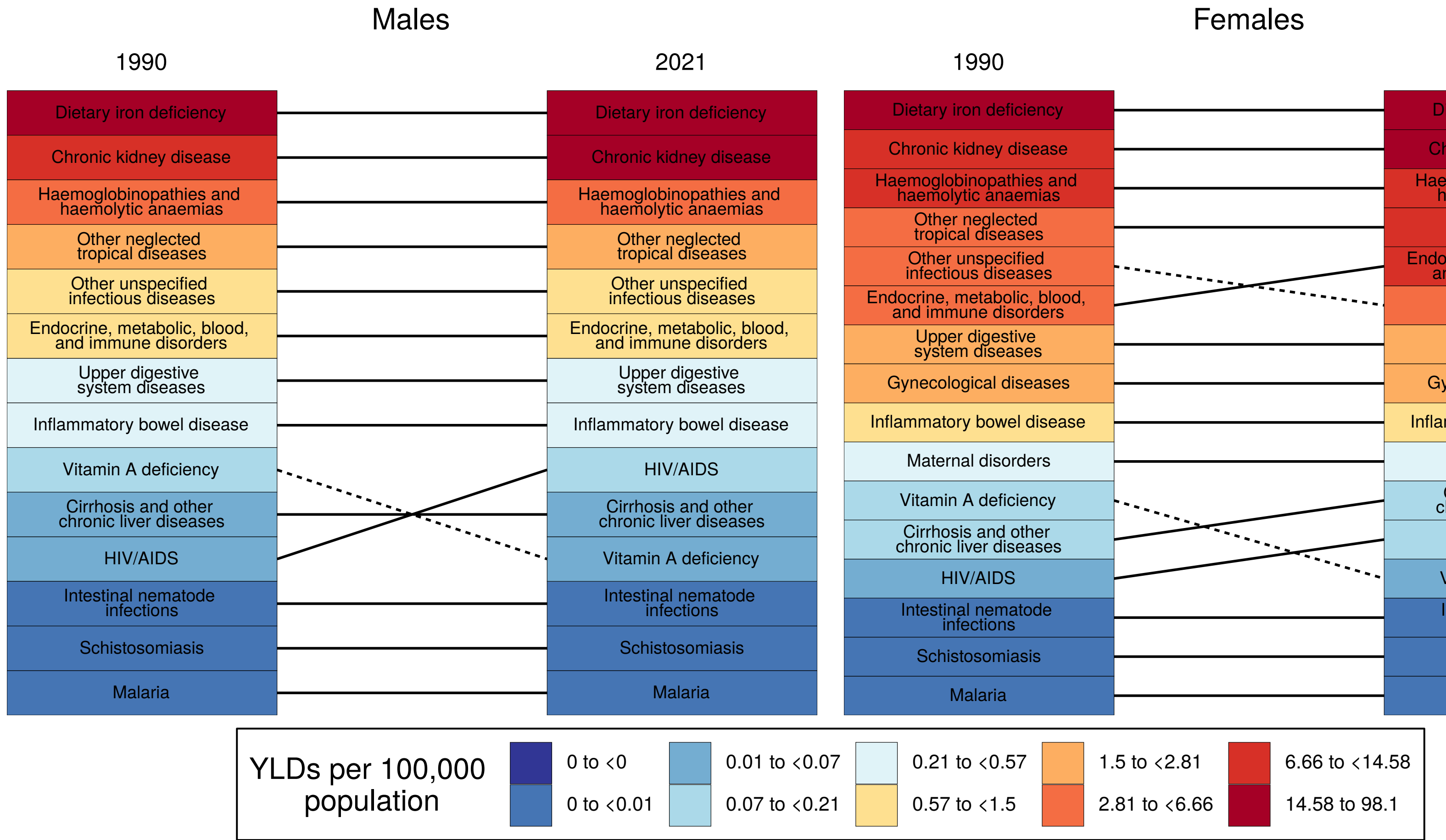

Illinois

Anaemia Prevalence - Both Sexes

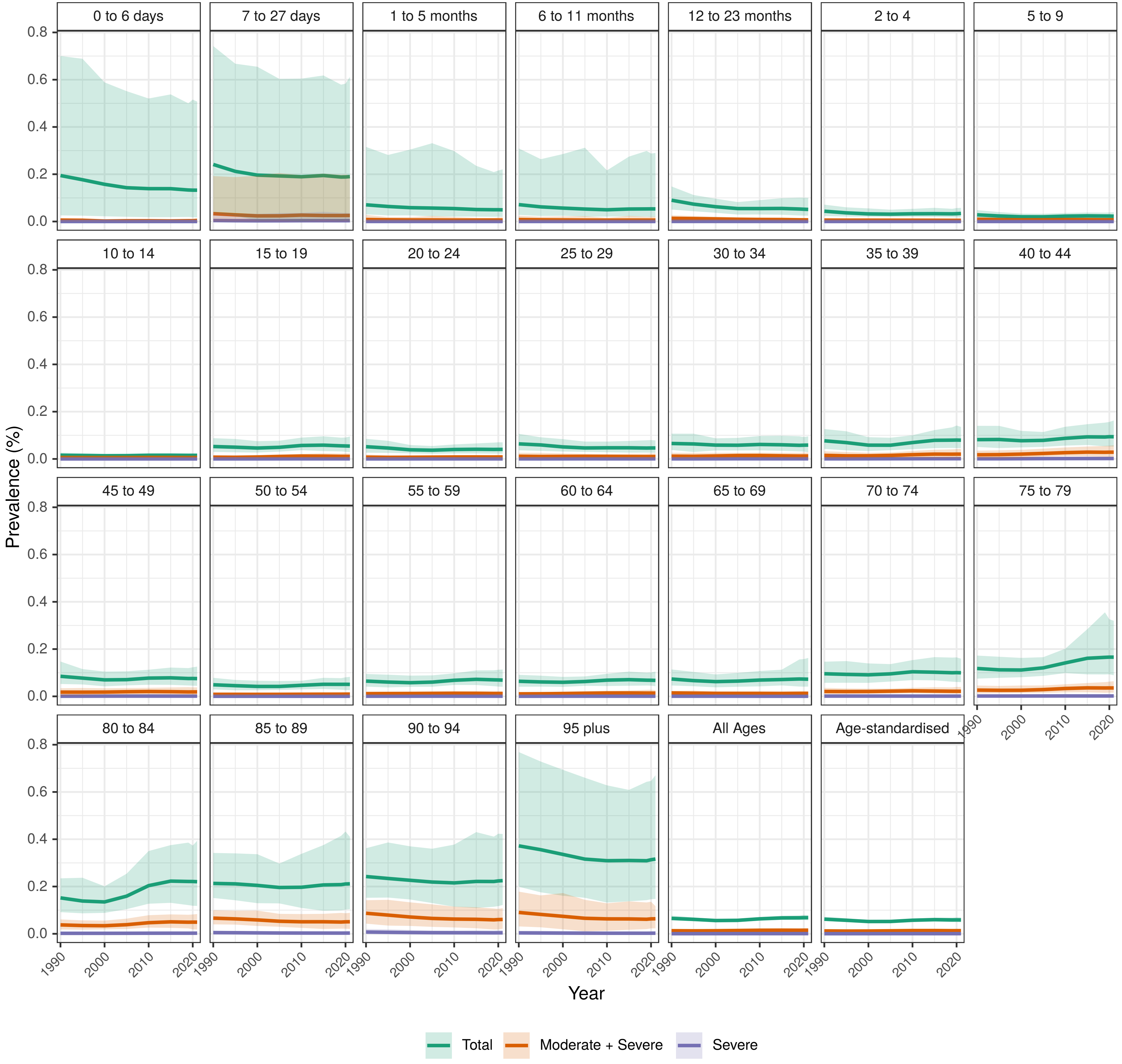

Cause-Specific Anaemia Prevalence - 2021

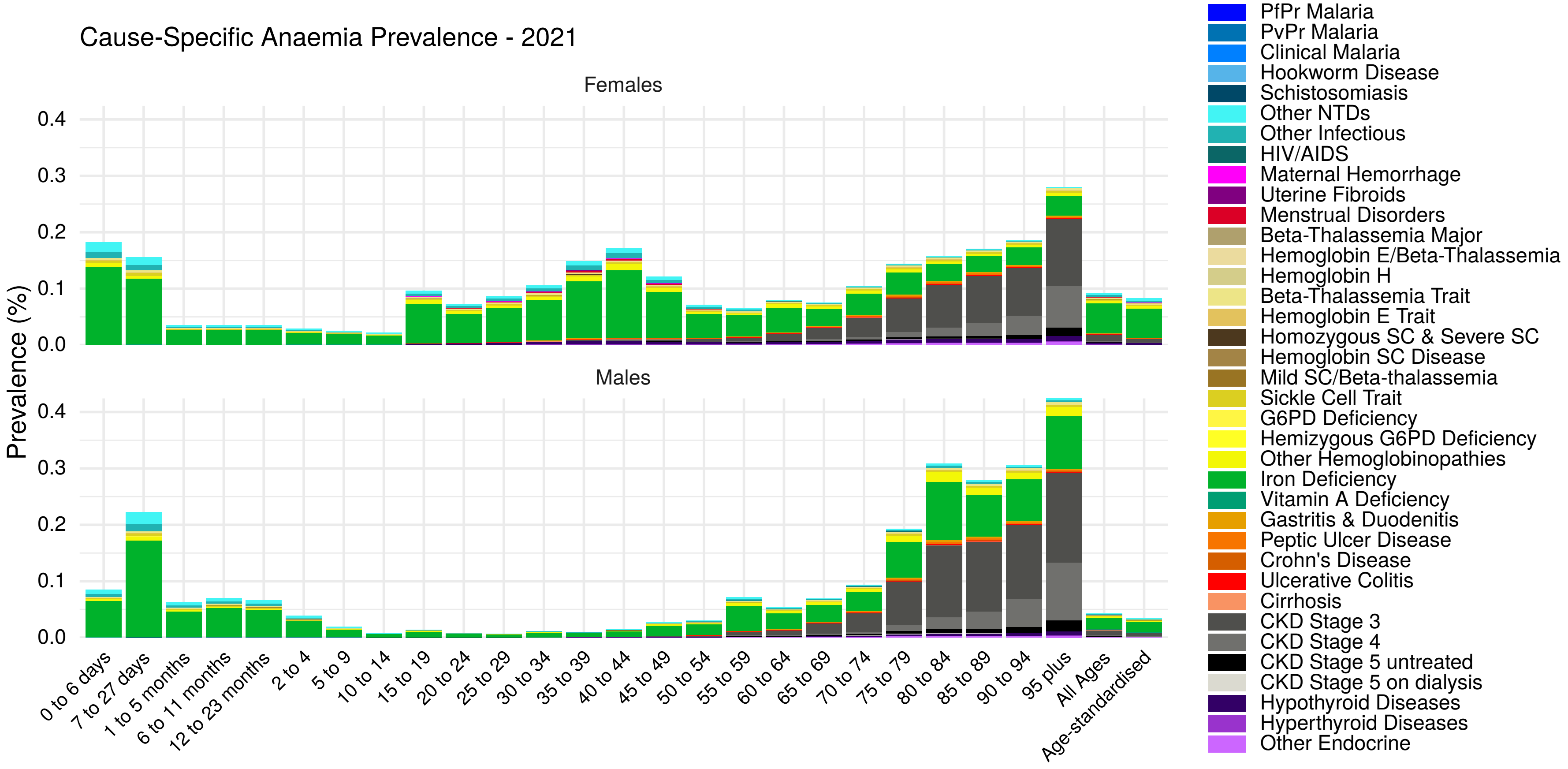

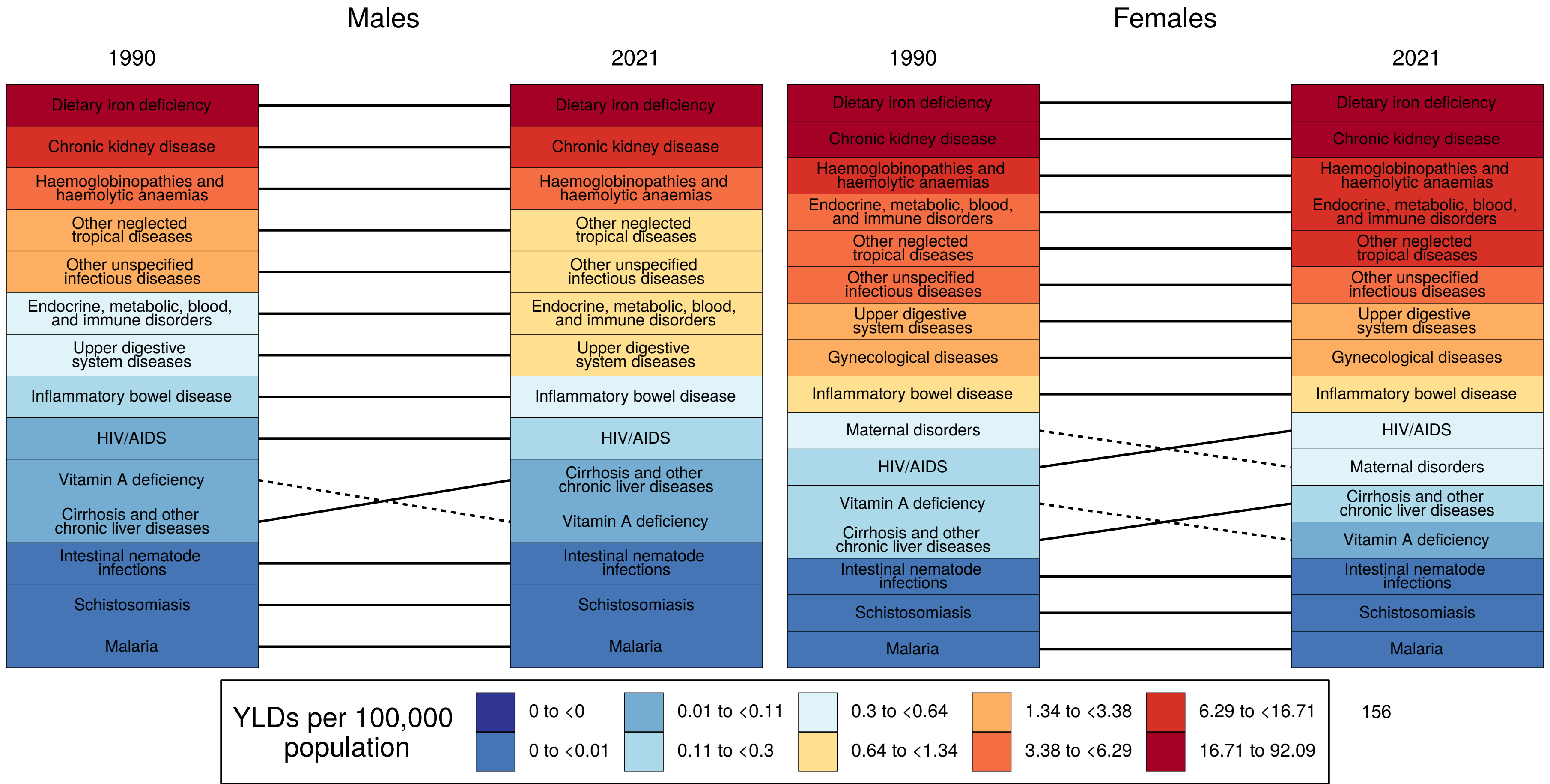

Indiana

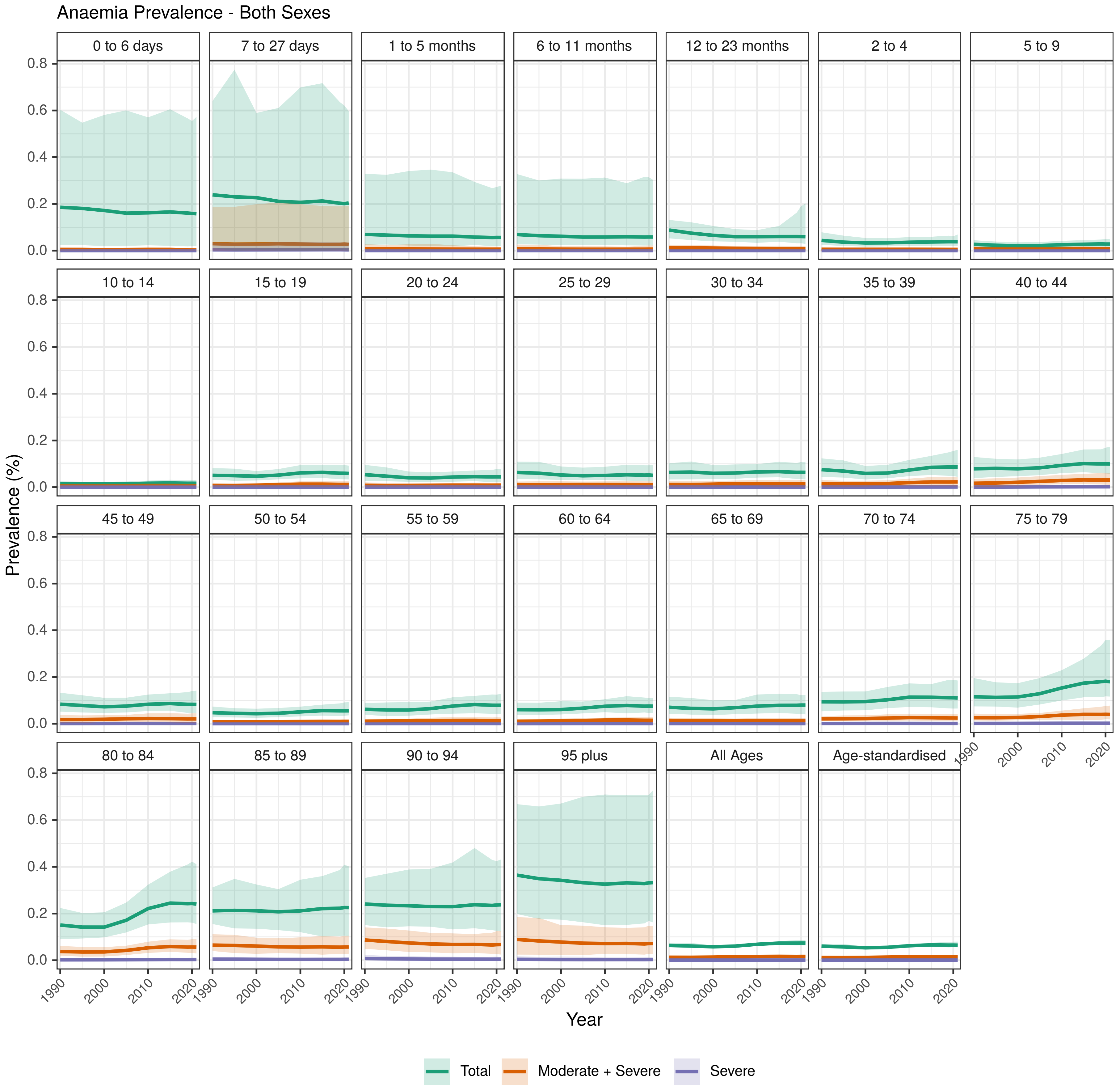

Anaemia Prevalence - Both Sexes
0 to 6 days
7 to 27 days
1 to 5 months
6 to 11 months
12 to 23 months
2 to 4
5 to 9
10 to 14
15 to 19
20 to 24
25 to 29
30 to 34
35 to 39
40 to 44
45 to 49
50 to 54
55 to 59
60 to 64
65 to 69
70 to 74
75 to 79
80 to 84
85 to 89
90 to 94
95 plus
All Ages
Age-standardised
Prevalence (%)
Year
Total
Moderate + Severe
Severe

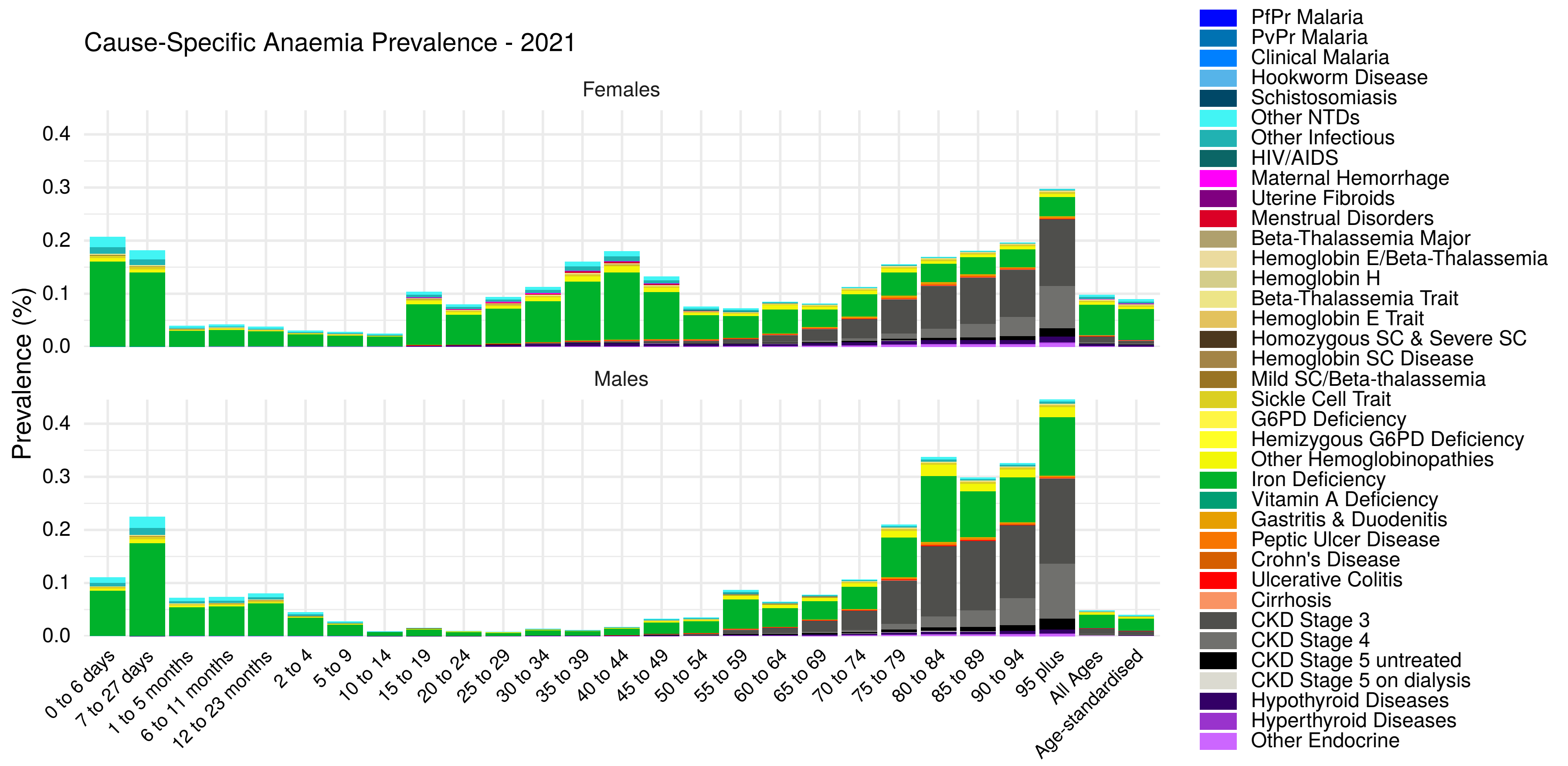

Cause-Specific Anaemia Prevalence - 2021
Females
Males
Prevalence (%)
PfPr Malaria
PvPr Malaria
Clinical Malaria
Hookworm Disease
Schistosomiasis
Other NTDs
Other Infectious
HIV/AIDS
Maternal Hemorrhage
Uterine Fibroids
Menstrual Disorders
Beta-Thalassemia Major
Hemoglobin E/Beta-Thalassemia
Hemoglobin H
Beta-Thalassemia Trait
Hemoglobin E Trait
Homozygous SC & Severe SC
Hemoglobin SC Disease
Mild SC/Beta-thalassemia
Sickle Cell Trait
G6PD Deficiency
Hemizygous G6PD Deficiency
Other Hemoglobinopathies
Iron Deficiency
Vitamin A Deficiency
Gastritis & Duodenitis
Peptic Ulcer Disease
Crohn's Disease
Ulcerative Colitis
Cirrhosis
CKD Stage 3
CKD Stage 4
CKD Stage 5 untreated
CKD Stage 5 on dialysis
Hypothyroid Diseases
Hyperthyroid Diseases
Other Endocrine

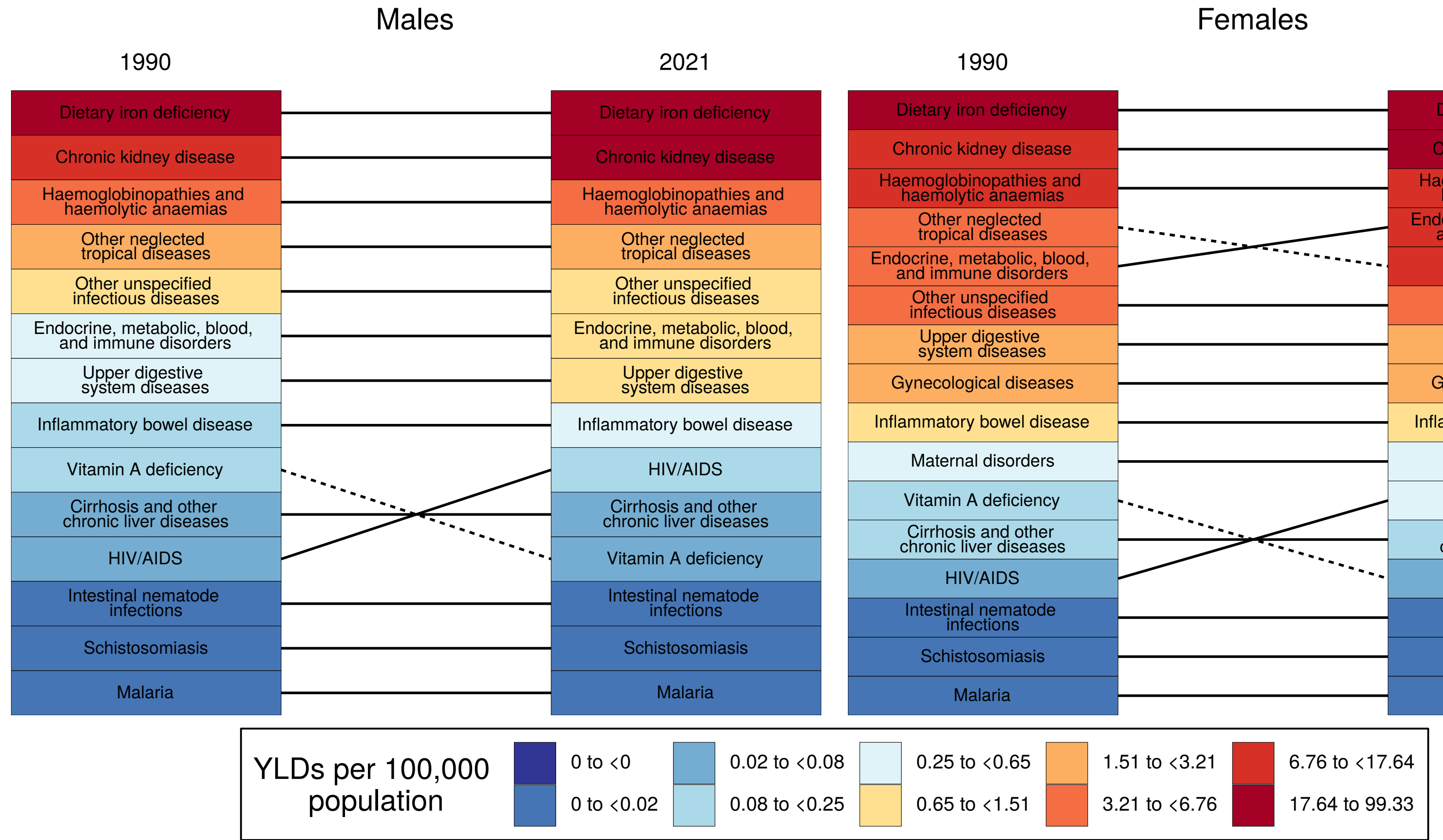

Males
Females
1990
2021
Dietary iron deficiency
Chronic kidney disease
Haemoglobinopathies and haemolytic anaemias
Other neglected tropical diseases
Other unspecified infectious diseases
Endocrine, metabolic, blood, and immune disorders
Upper digestive system diseases
Inflammatory bowel disease
Vitamin A deficiency
Cirrhosis and other chronic liver diseases
HIV/AIDS
Intestinal nematode infections
Schistosomiasis
Malaria
Gynecological diseases
Maternal disorders
YLDs per 100,000 population
0 to <0
0 to <0.02
0.02 to <0.08
0.08 to <0.25
0.25 to <0.65
0.65 to <1.51
1.51 to <3.21
3.21 to <6.76
6.76 to <17.64
17.64 to 99.33

Iowa

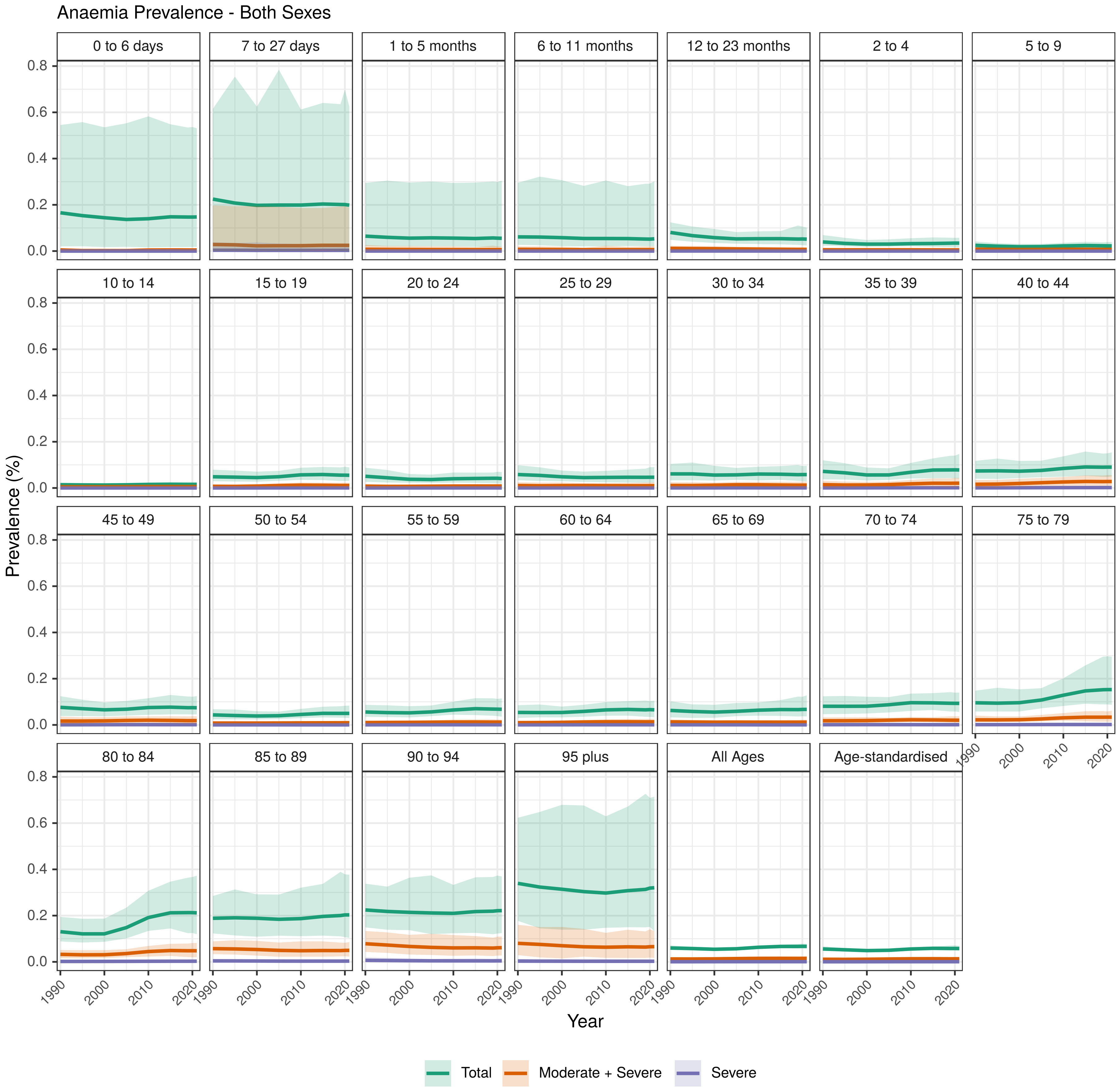

Anaemia Prevalence - Both Sexes
0 to 6 days
7 to 27 days
1 to 5 months
6 to 11 months
12 to 23 months
2 to 4
5 to 9
10 to 14
15 to 19
20 to 24
25 to 29
30 to 34
35 to 39
40 to 44
45 to 49
50 to 54
55 to 59
60 to 64
65 to 69
70 to 74
75 to 79
80 to 84
85 to 89
90 to 94
95 plus
All Ages
Age-standardised
Prevalence (%)
Year
Total
Moderate + Severe
Severe

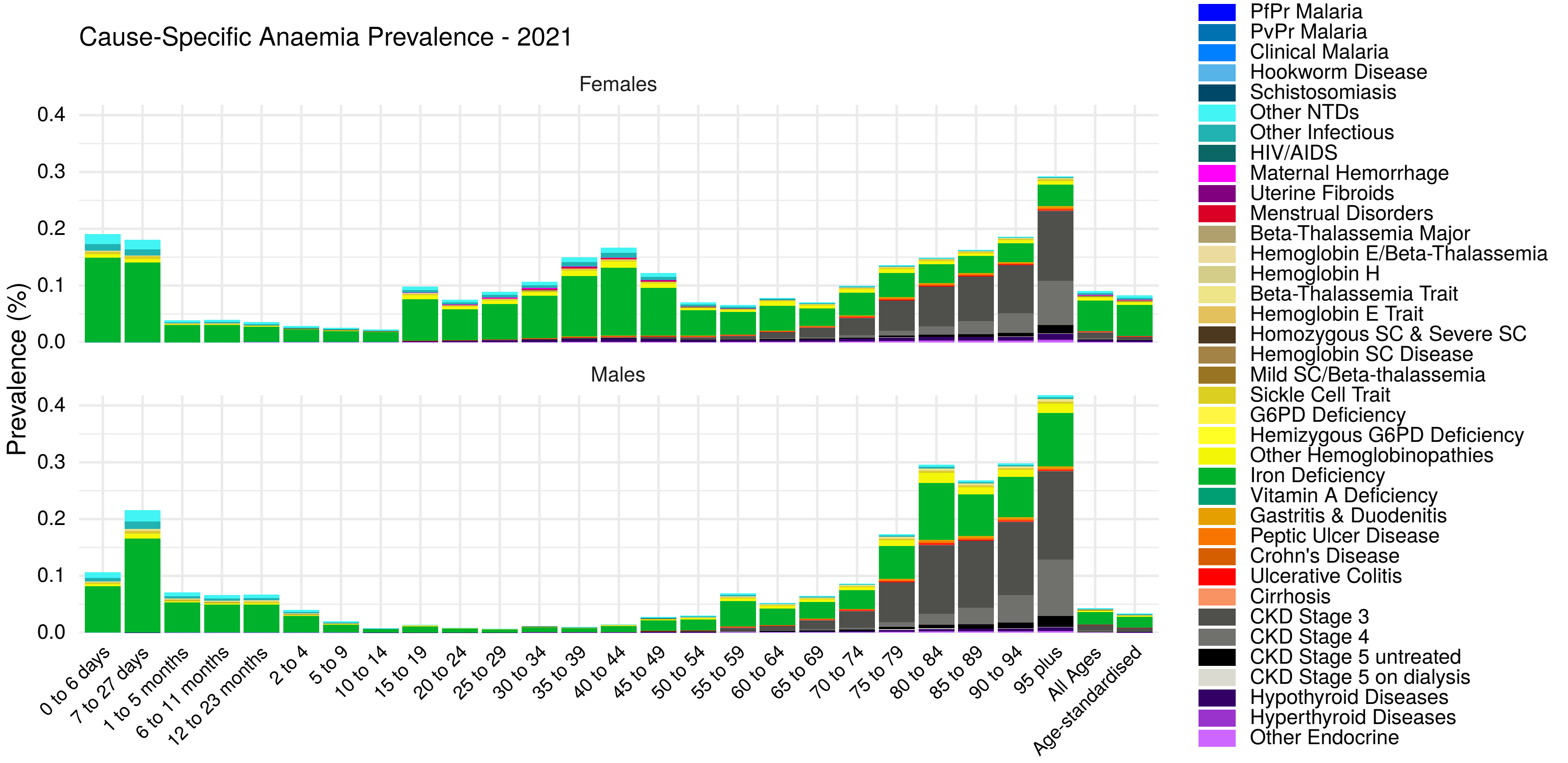

Cause-Specific Anaemia Prevalence - 2021
Females
Males
Prevalence (%)
PfPr Malaria
PvPr Malaria
Clinical Malaria
Hookworm Disease
Schistosomiasis
Other NTDs
Other Infectious
HIV/AIDS
Maternal Hemorrhage
Uterine Fibroids
Menstrual Disorders
Beta-Thalassemia Major
Hemoglobin E/Beta-Thalassemia
Hemoglobin H
Beta-Thalassemia Trait
Hemoglobin E Trait
Homozygous SC & Severe SC
Hemoglobin SC Disease
Mild SC/Beta-thalassemia
Sickle Cell Trait
G6PD Deficiency
Hemizygous G6PD Deficiency
Other Hemoglobinopathies
Iron Deficiency
Vitamin A Deficiency
Gastritis & Duodenitis
Peptic Ulcer Disease
Crohn's Disease
Ulcerative Colitis
Cirrhosis
CKD Stage 3
CKD Stage 4
CKD Stage 5 untreated
CKD Stage 5 on dialysis
Hypothyroid Diseases
Hyperthyroid Diseases
Other Endocrine

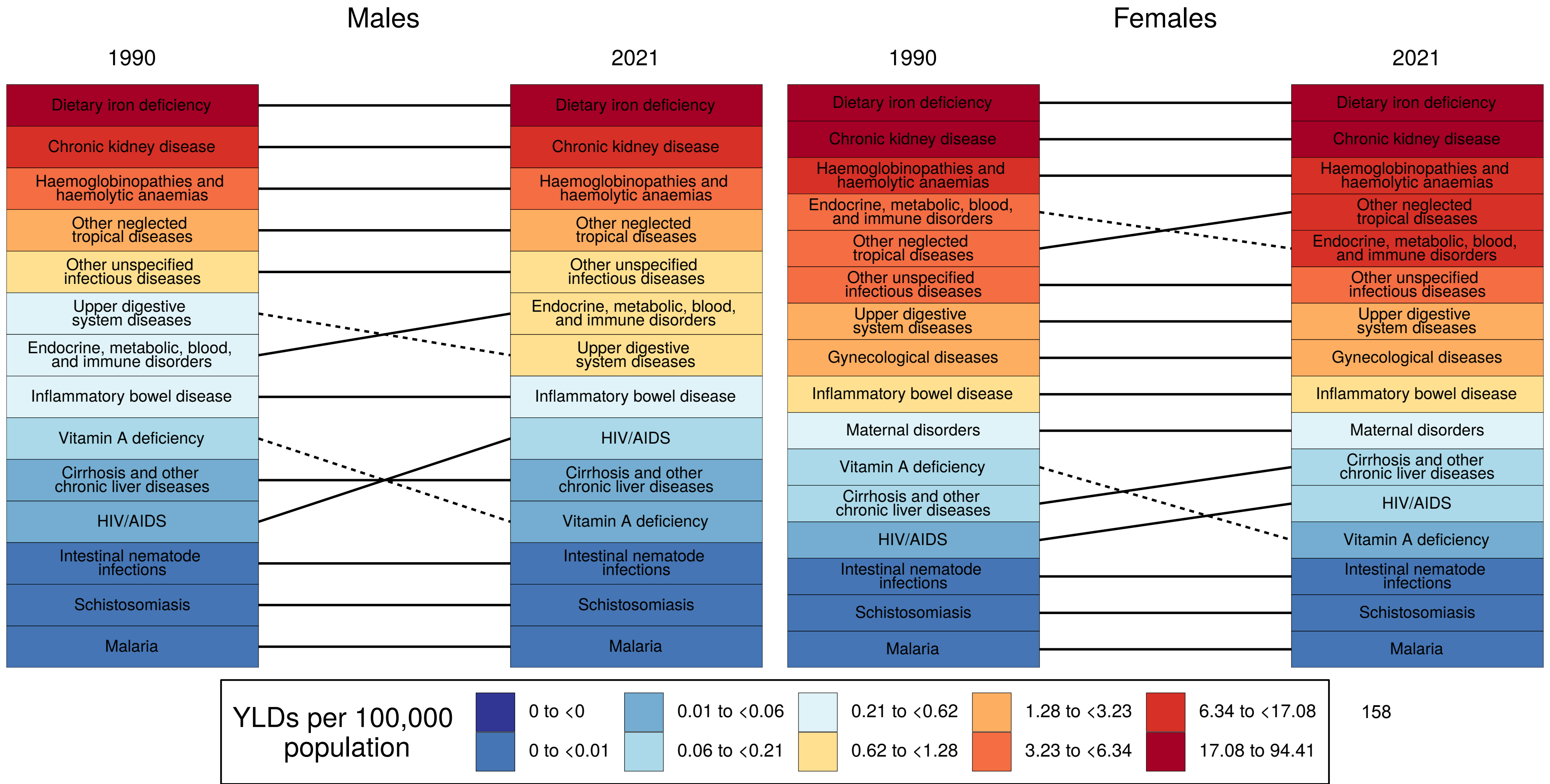

Males
Females
1990
2021
Dietary iron deficiency
Chronic kidney disease
Haemoglobinopathies and haemolytic anaemias
Other neglected tropical diseases
Other unspecified infectious diseases
Upper digestive system diseases
Endocrine, metabolic, blood, and immune disorders
Inflammatory bowel disease
Vitamin A deficiency
Cirrhosis and other chronic liver diseases
HIV/AIDS
Intestinal nematode infections
Schistosomiasis
Malaria
Gynecological diseases
Maternal disorders
YLDs per 100,000 population
0 to <0
0 to <0.01
0.01 to <0.06
0.06 to <0.21
0.21 to <0.62
0.62 to <1.28
1.28 to <3.23
3.23 to <6.34
6.34 to <17.08
17.08 to 94.41

Kansas

Anaemia Prevalence - Both Sexes

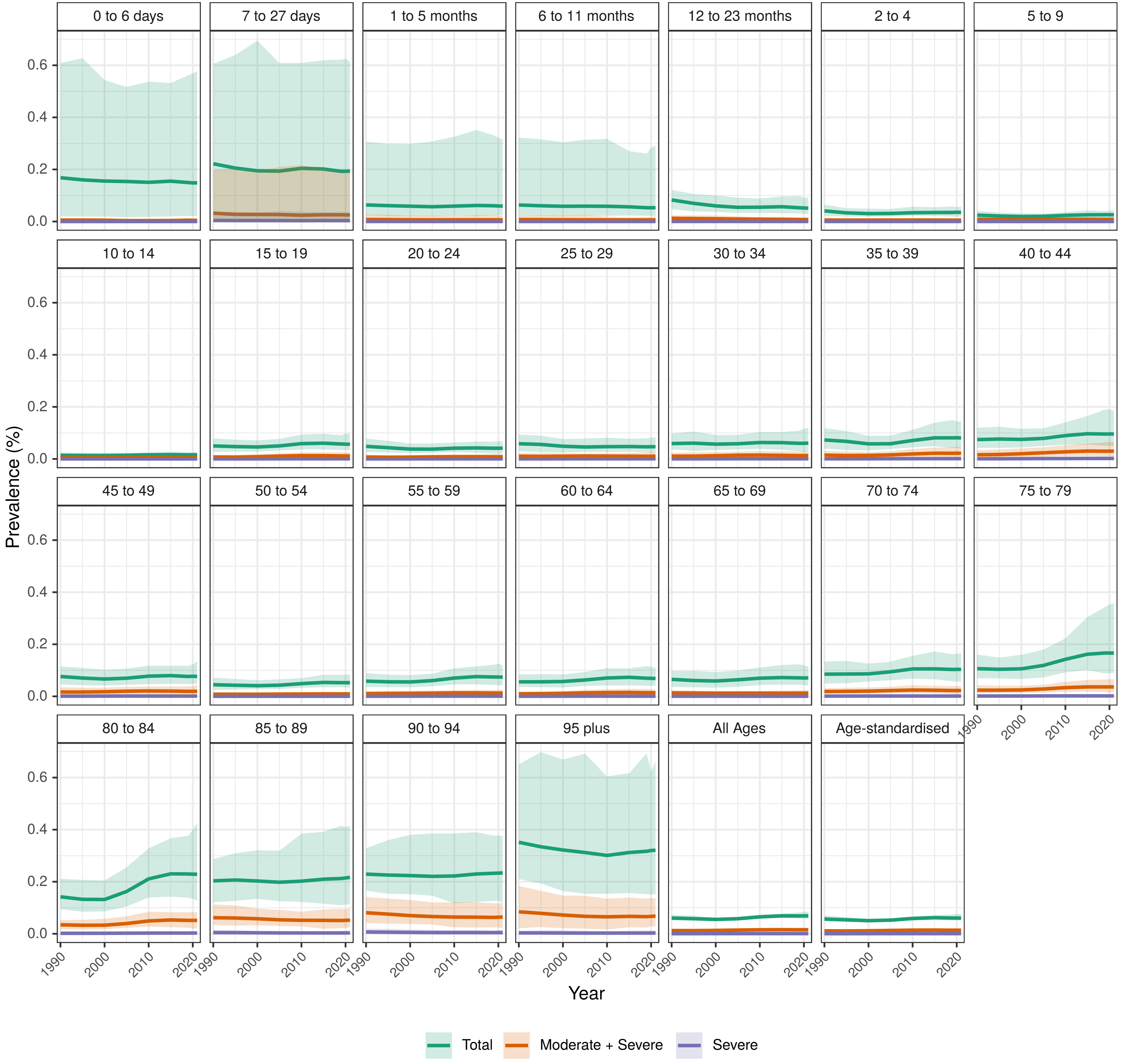

Cause-Specific Anaemia Prevalence - 2021

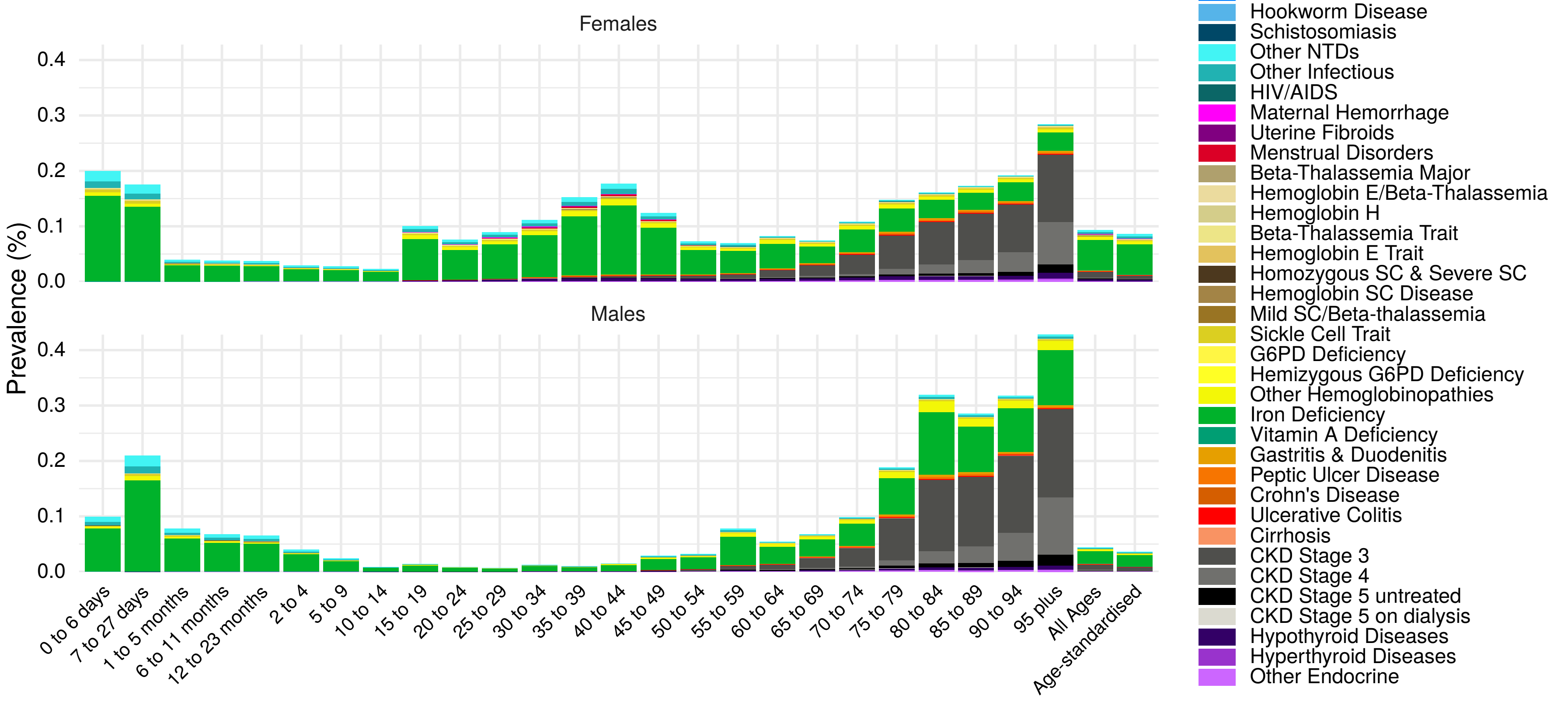

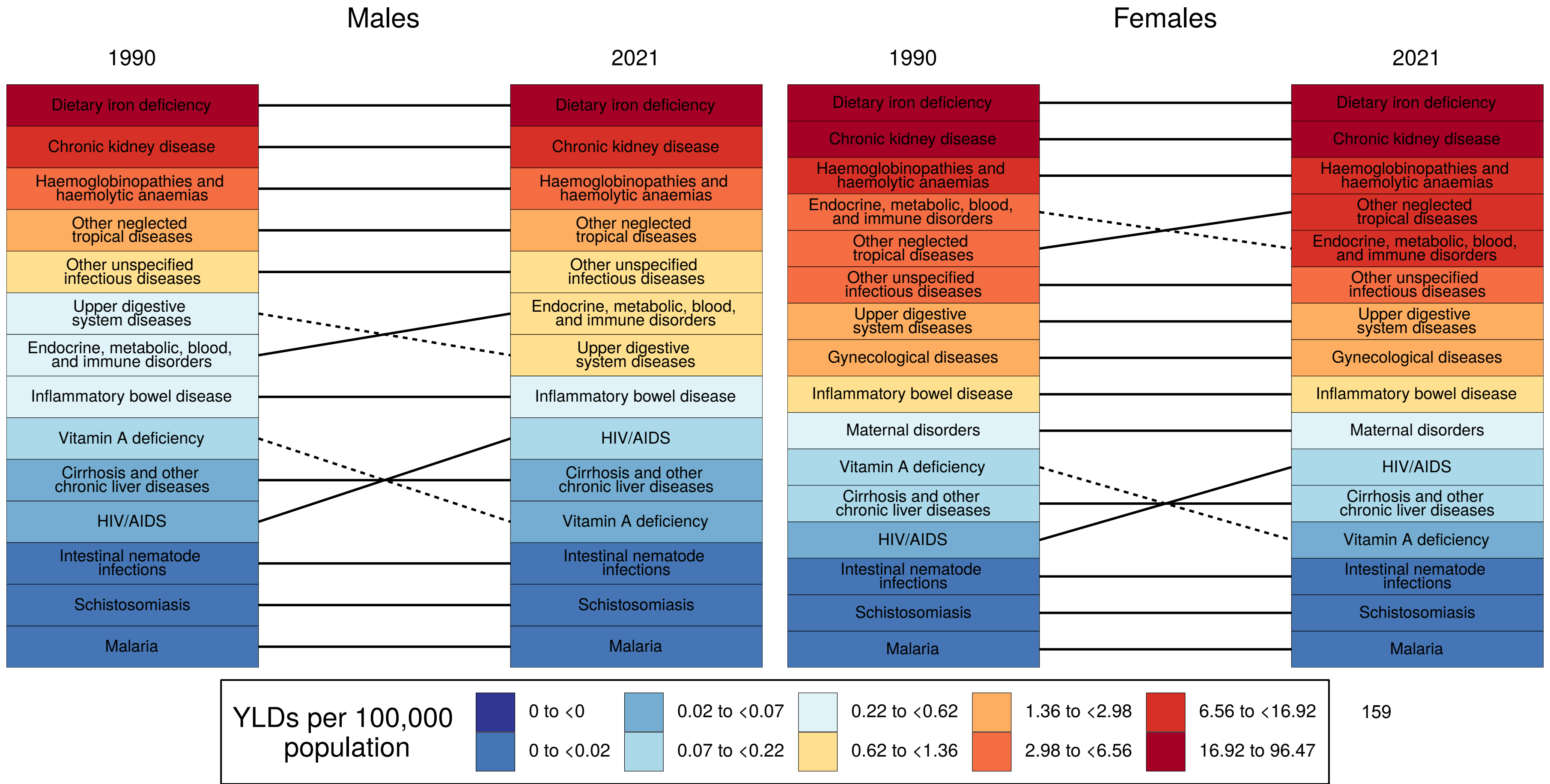

Kentucky

Anaemia Prevalence - Both Sexes

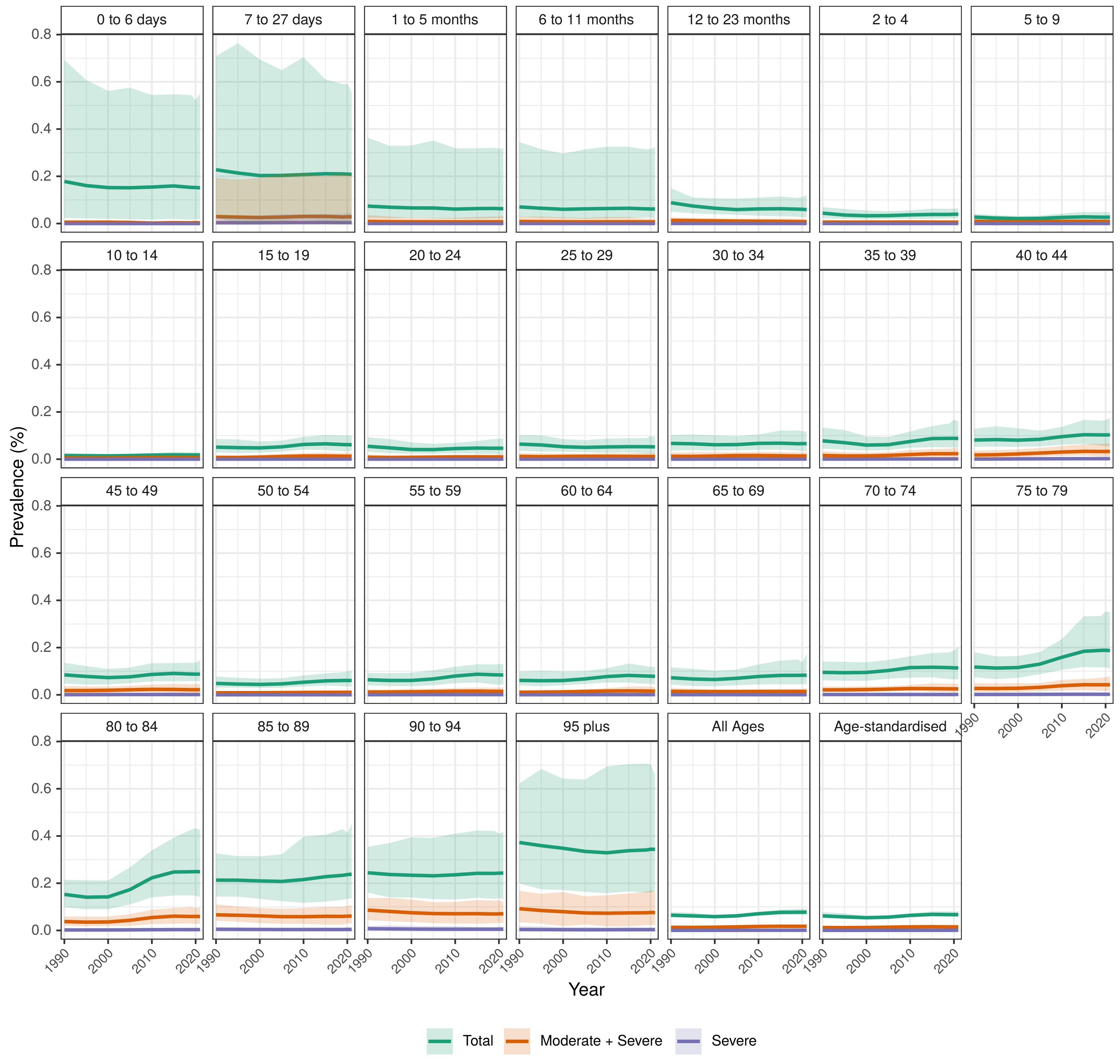

Cause-Specific Anaemia Prevalence - 2021

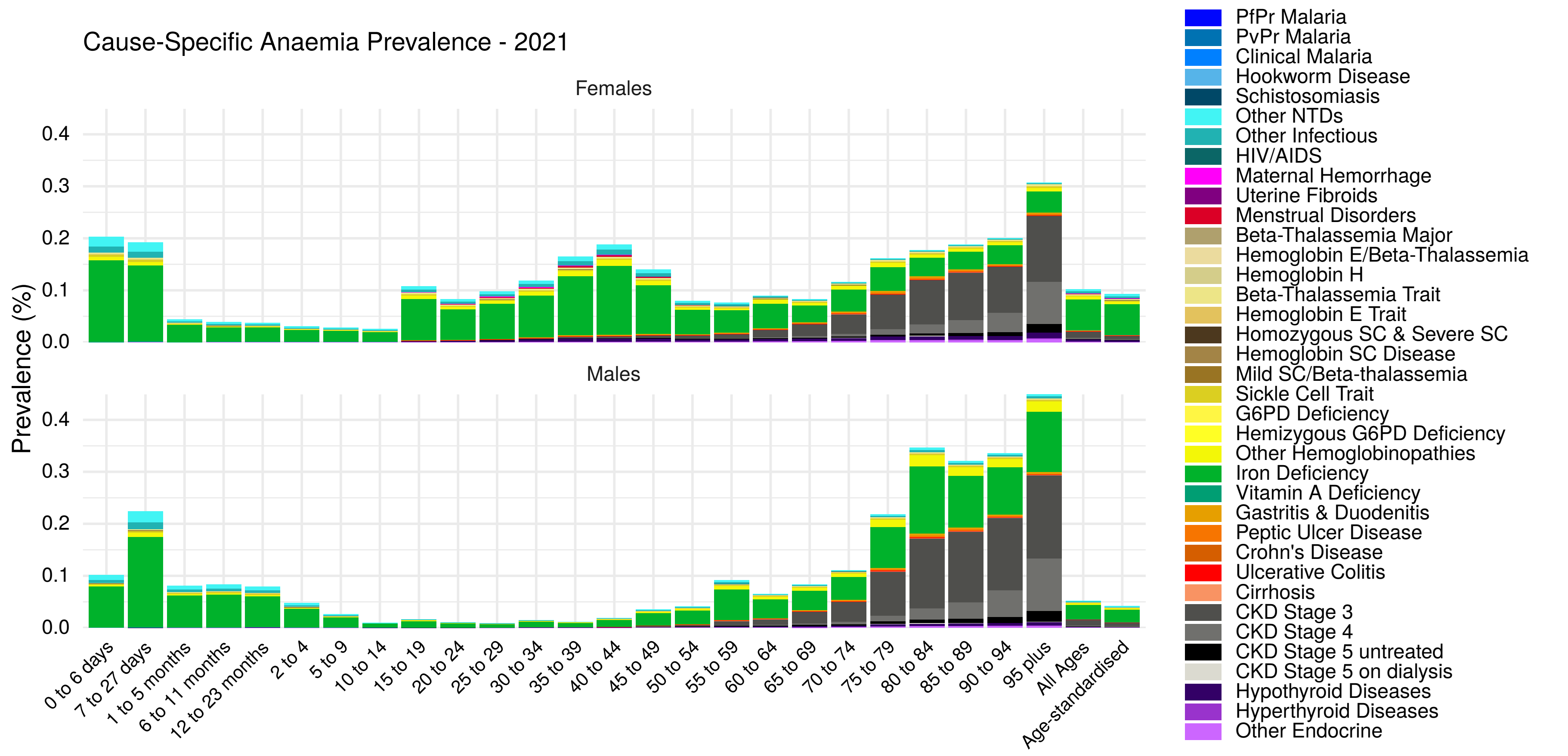

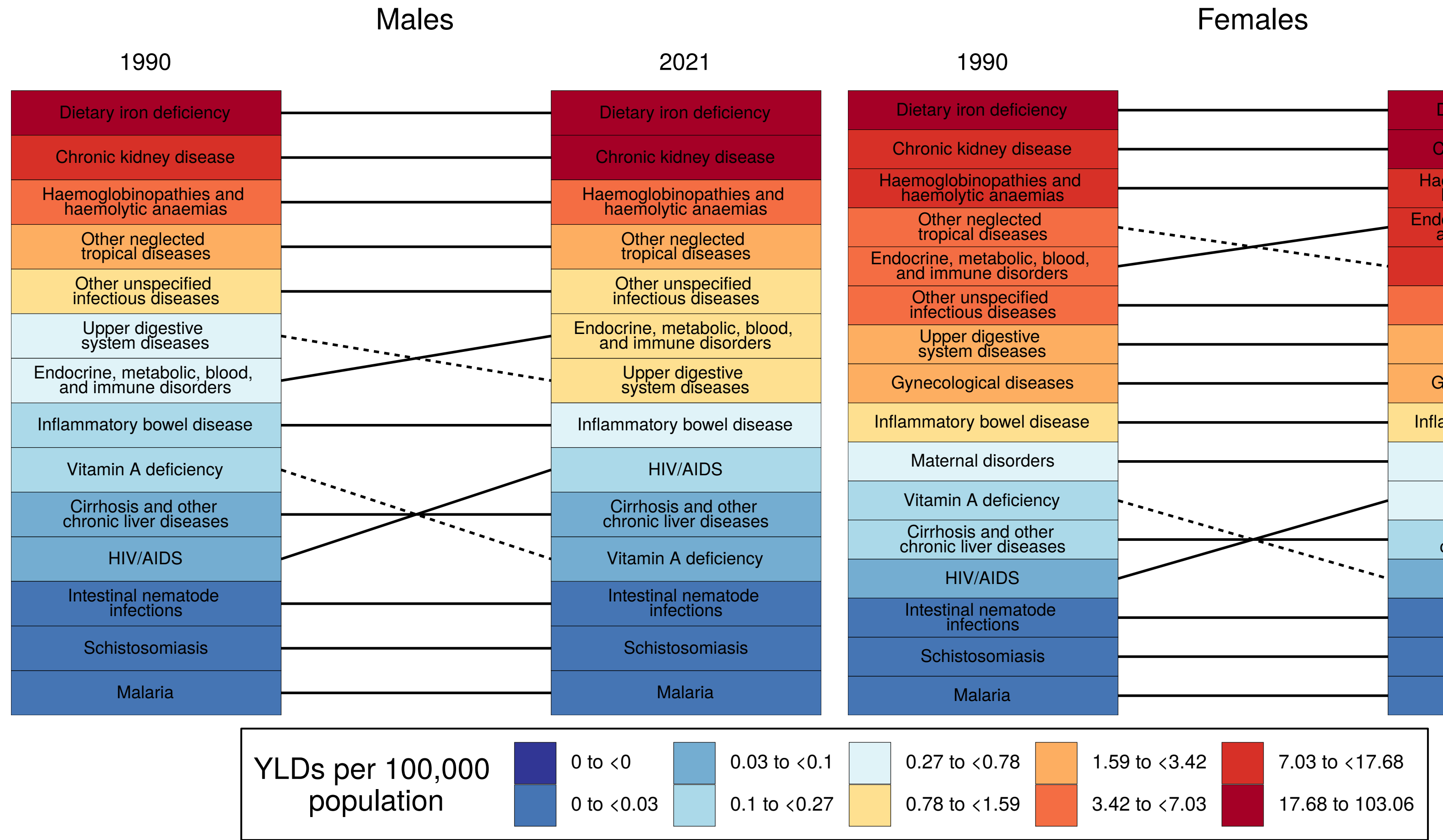

Louisiana

Anaemia Prevalence - Both Sexes

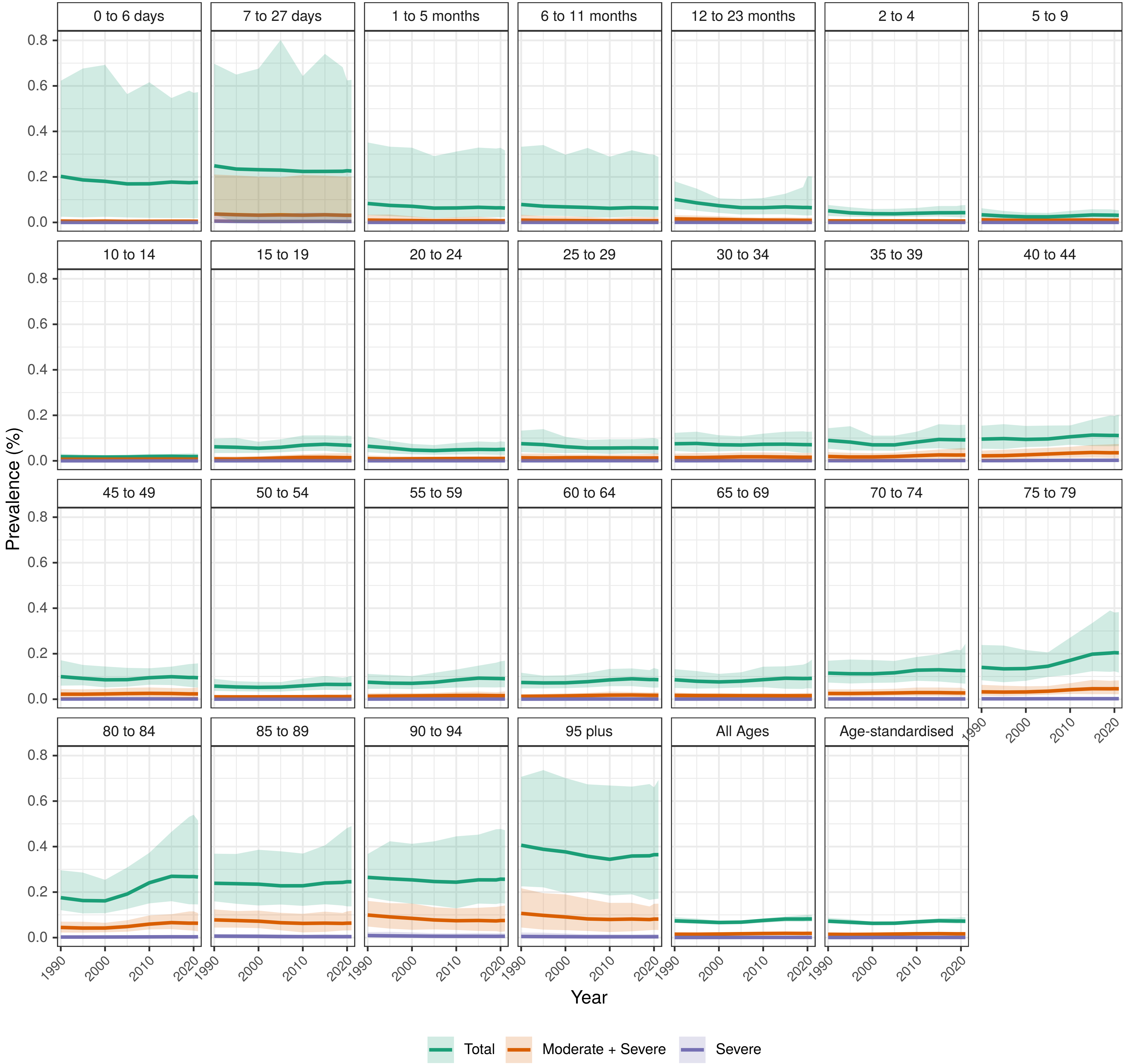

Cause-Specific Anaemia Prevalence - 2021

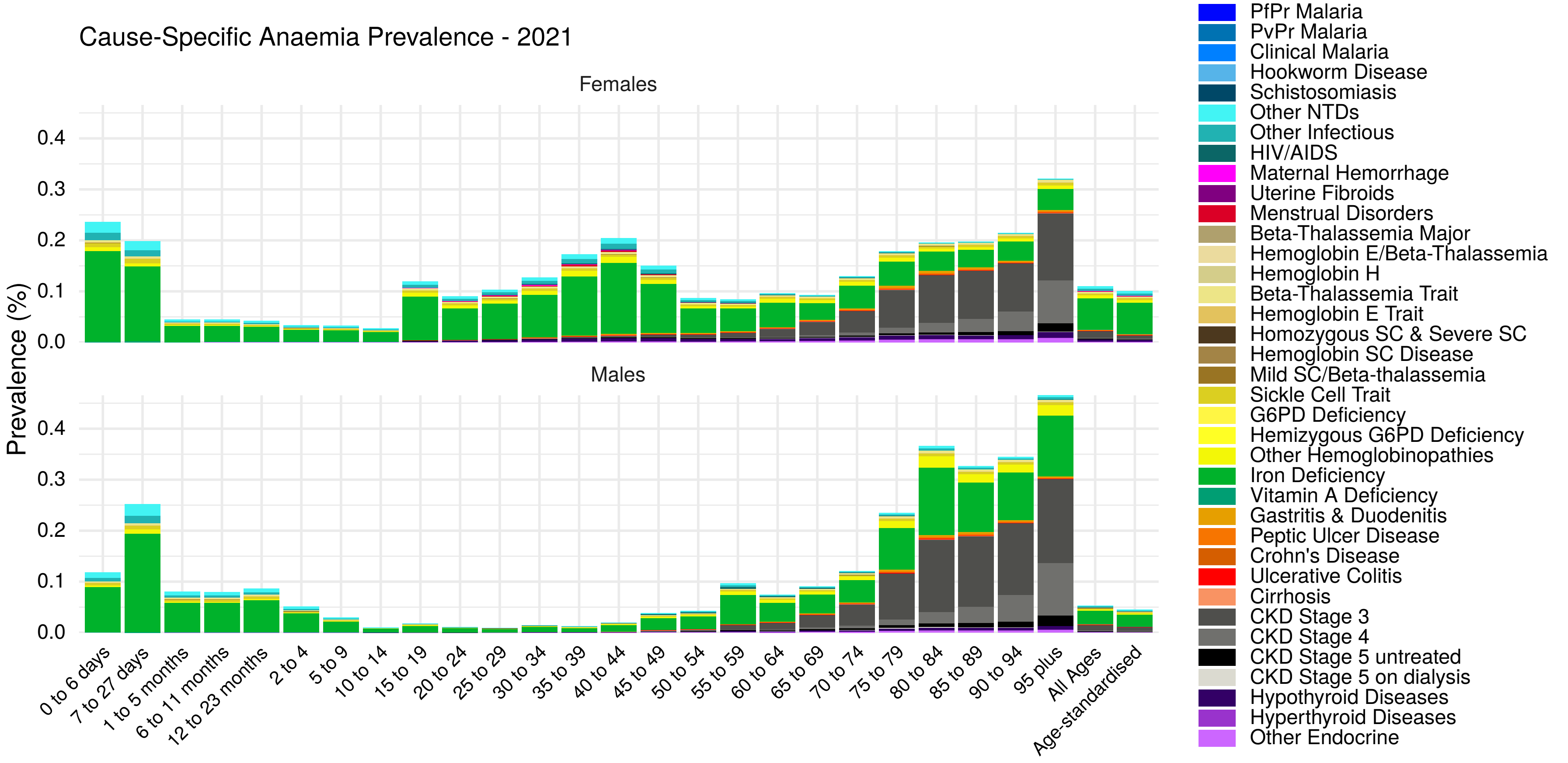

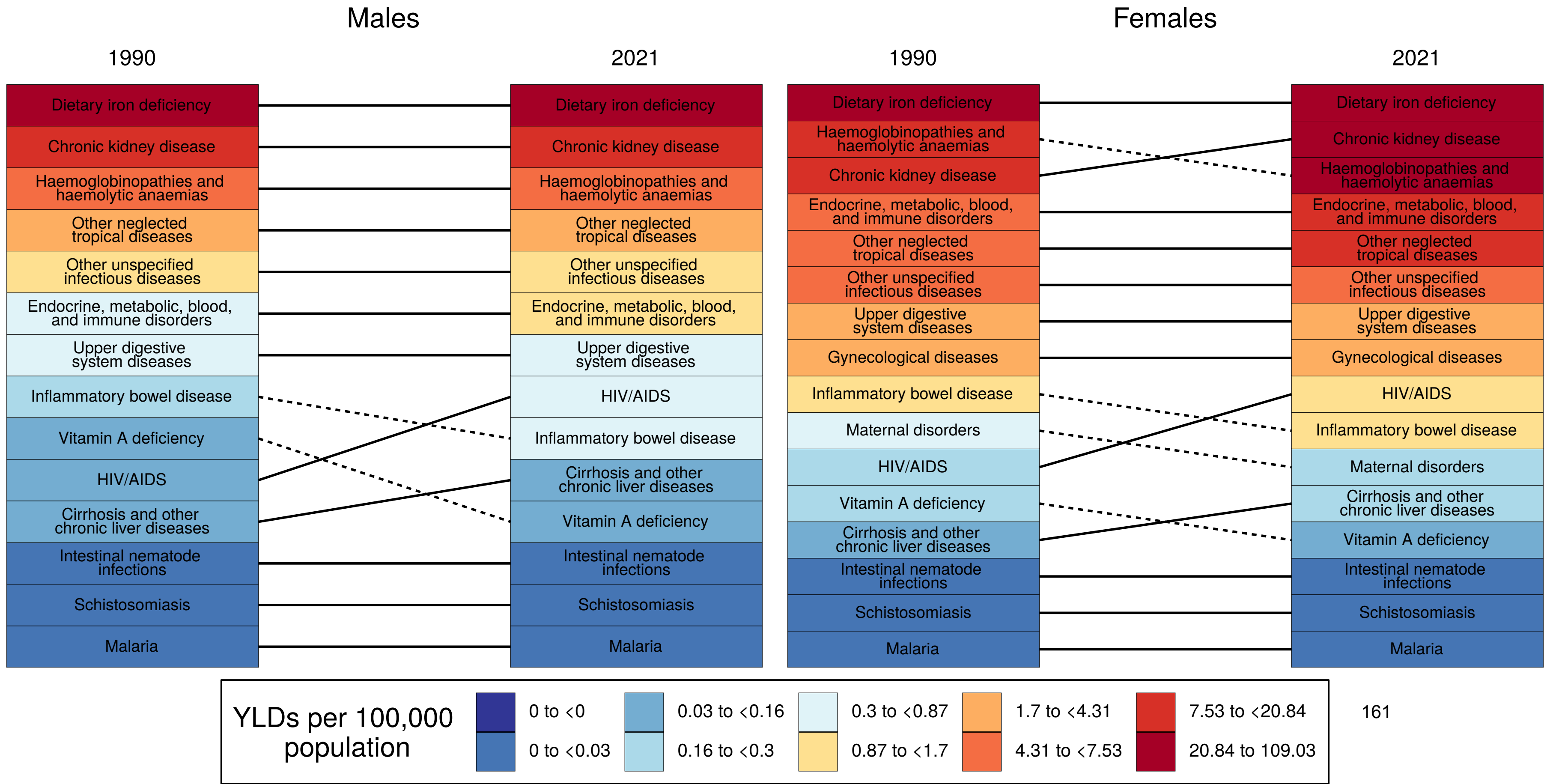

Maine

Anaemia Prevalence - Both Sexes

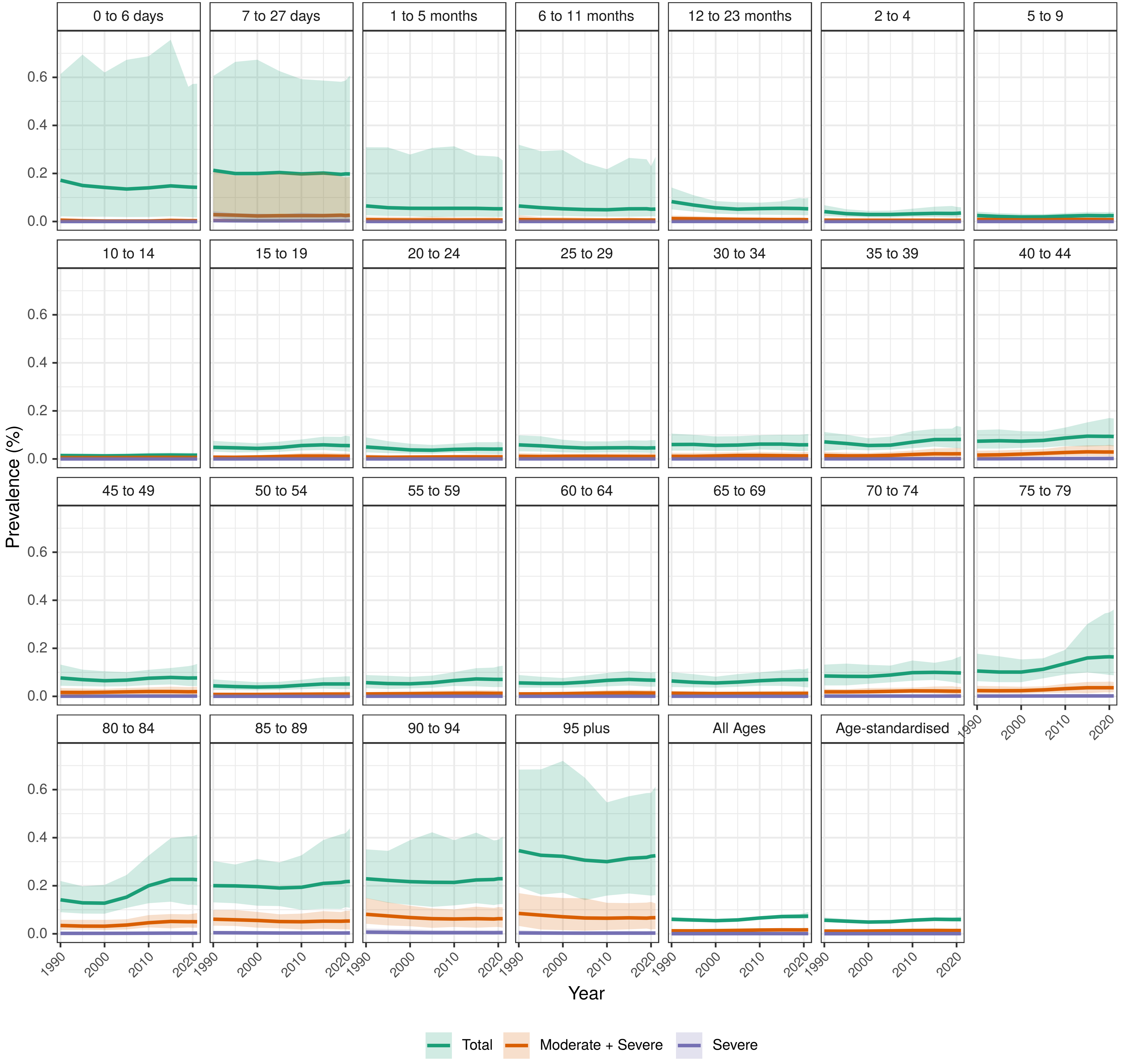

Cause-Specific Anaemia Prevalence - 2021

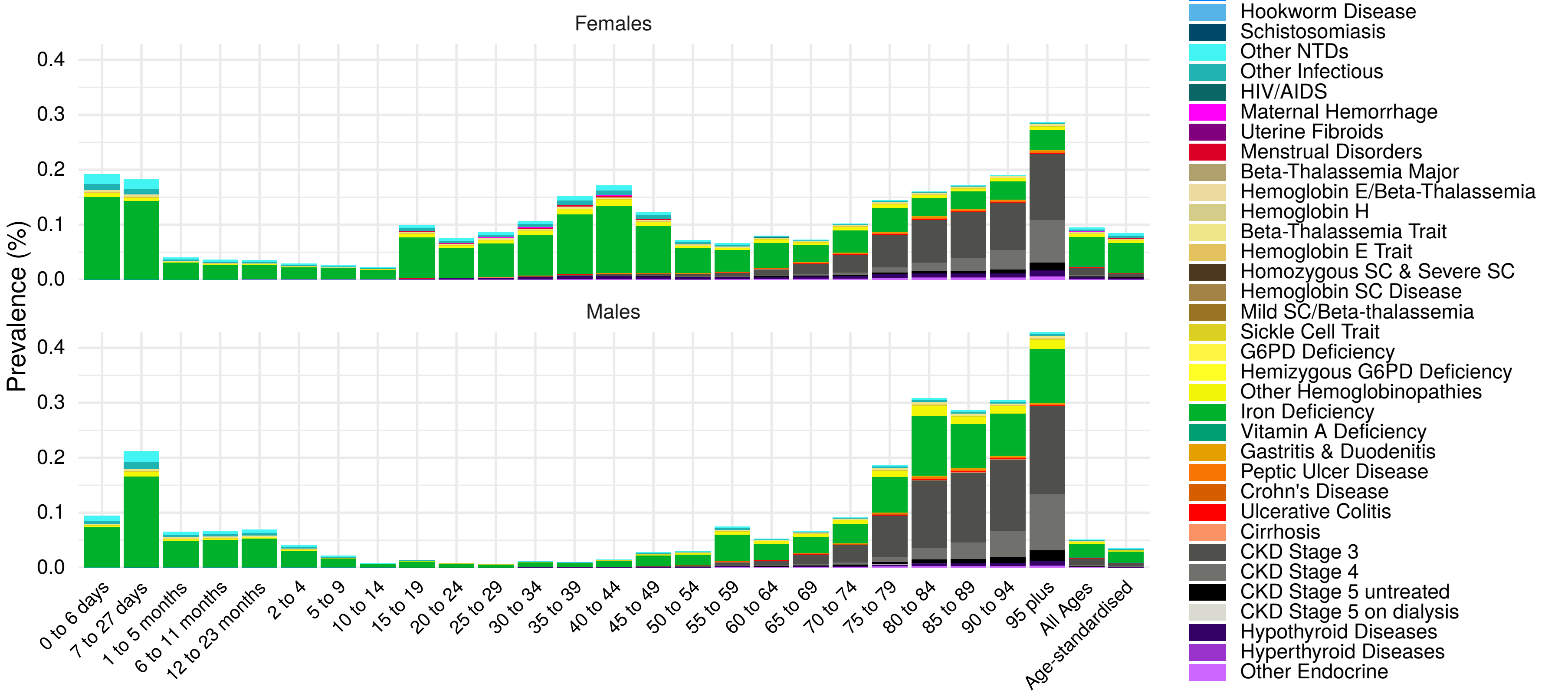

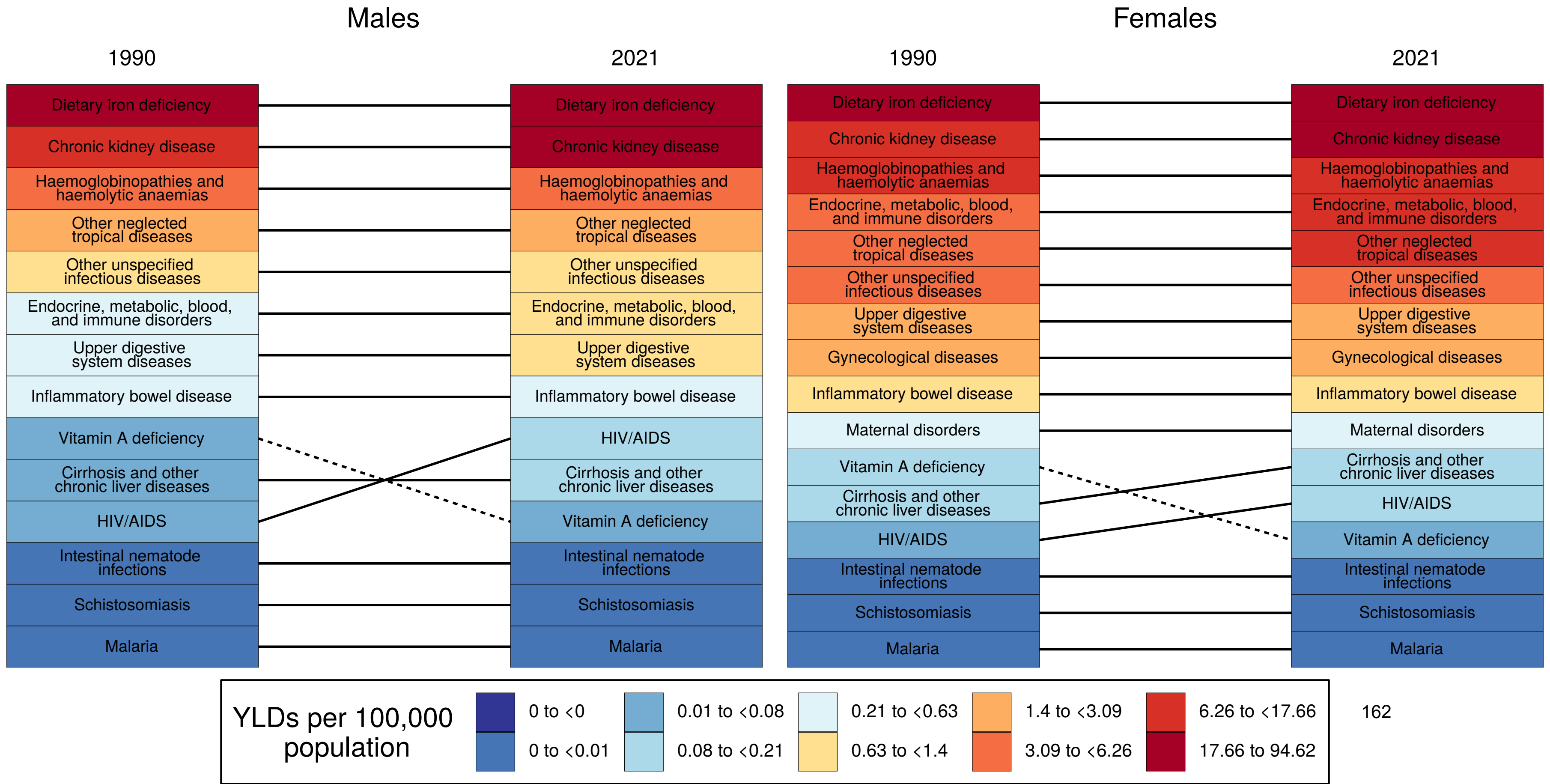

Maryland

Anaemia Prevalence - Both Sexes

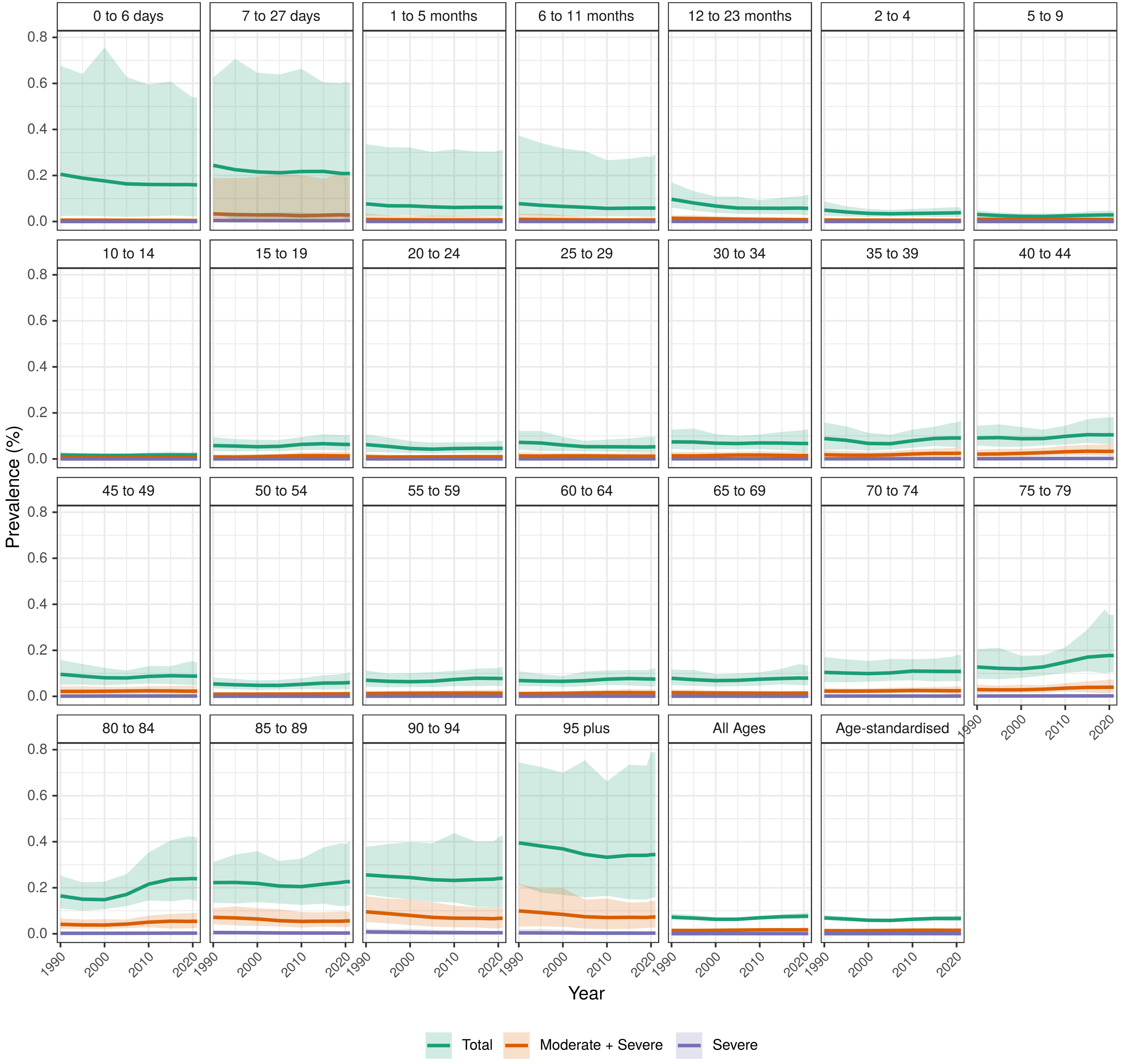

Cause-Specific Anaemia Prevalence - 2021

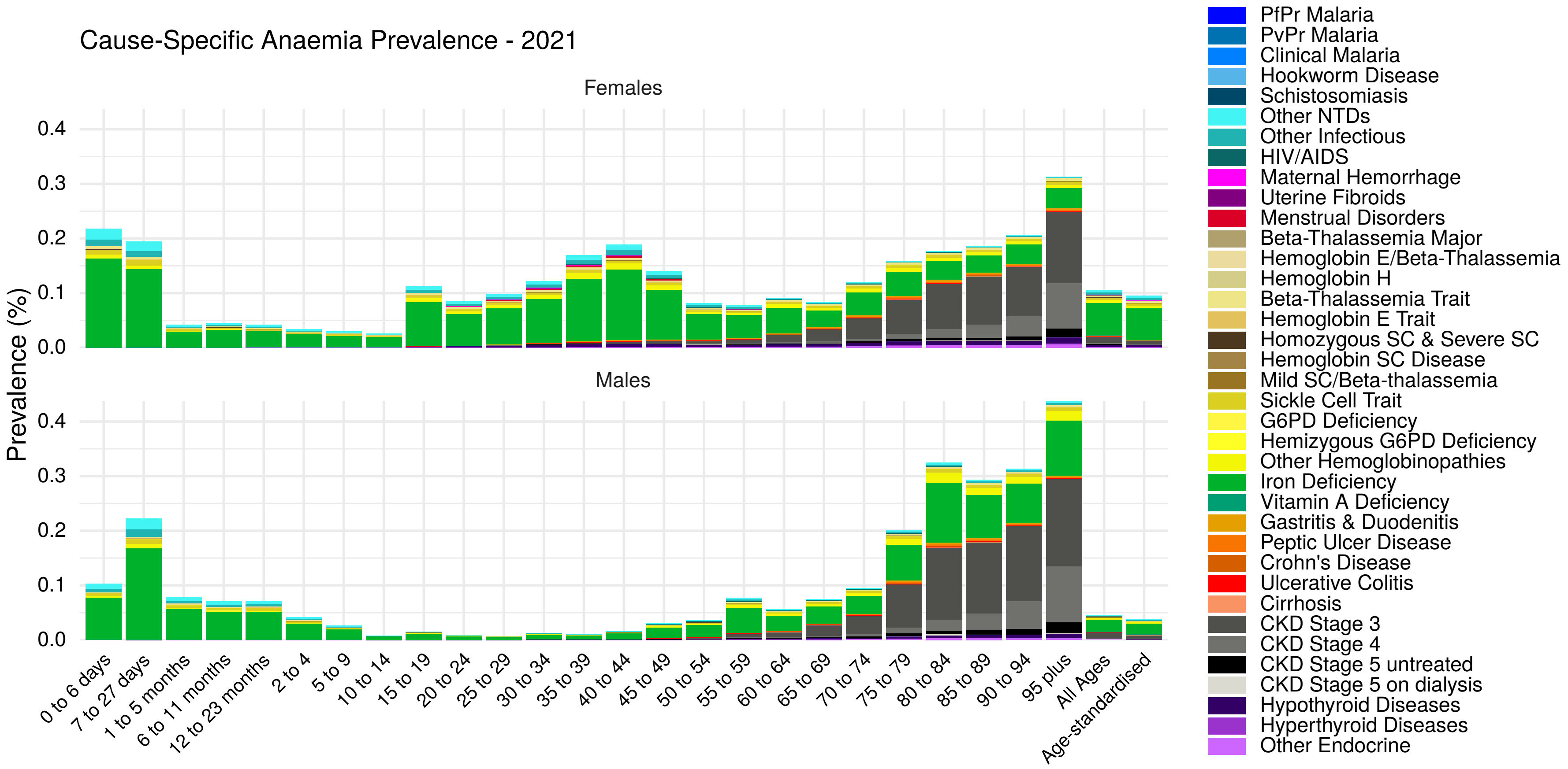

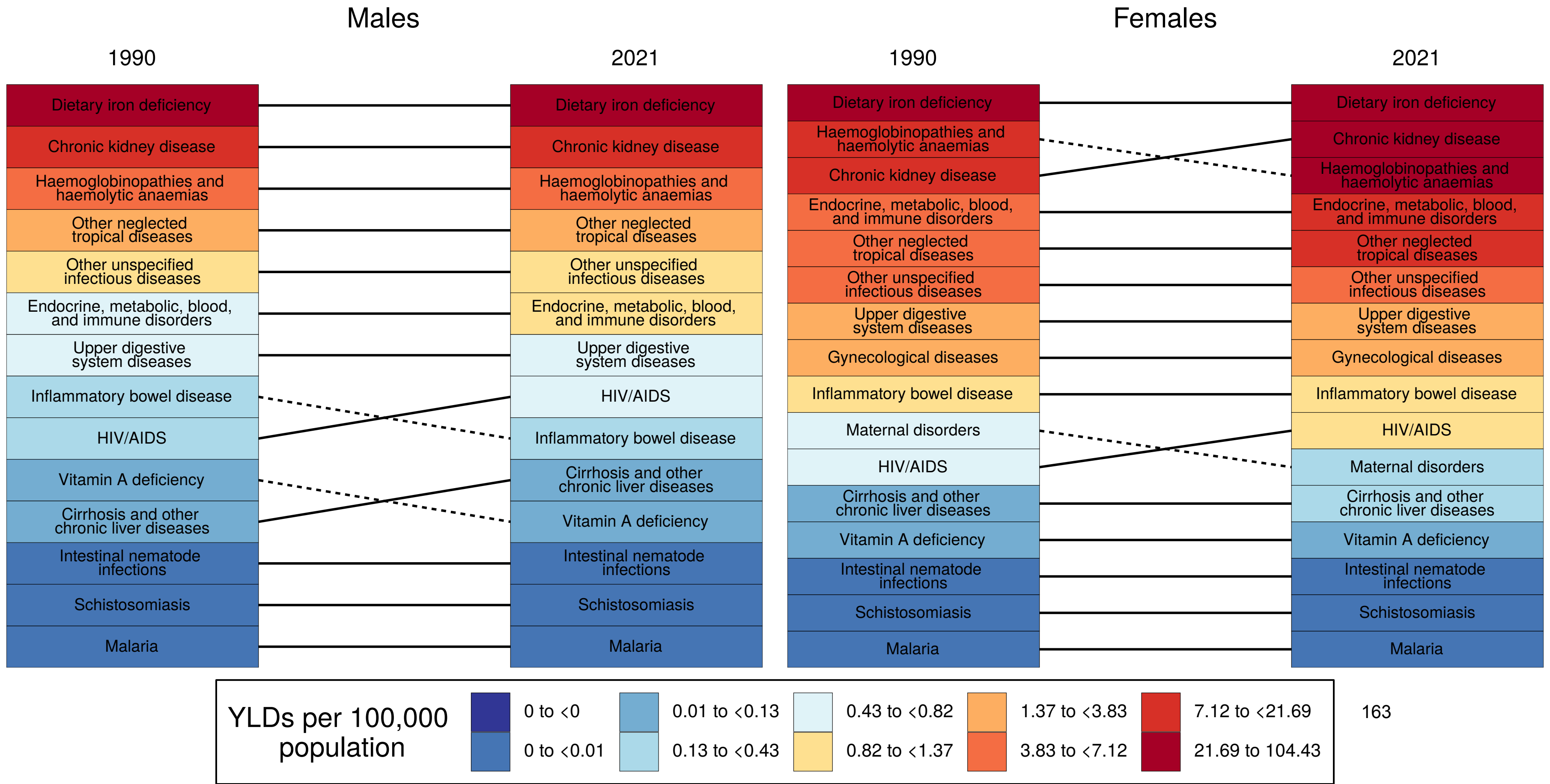

163

Massachusetts

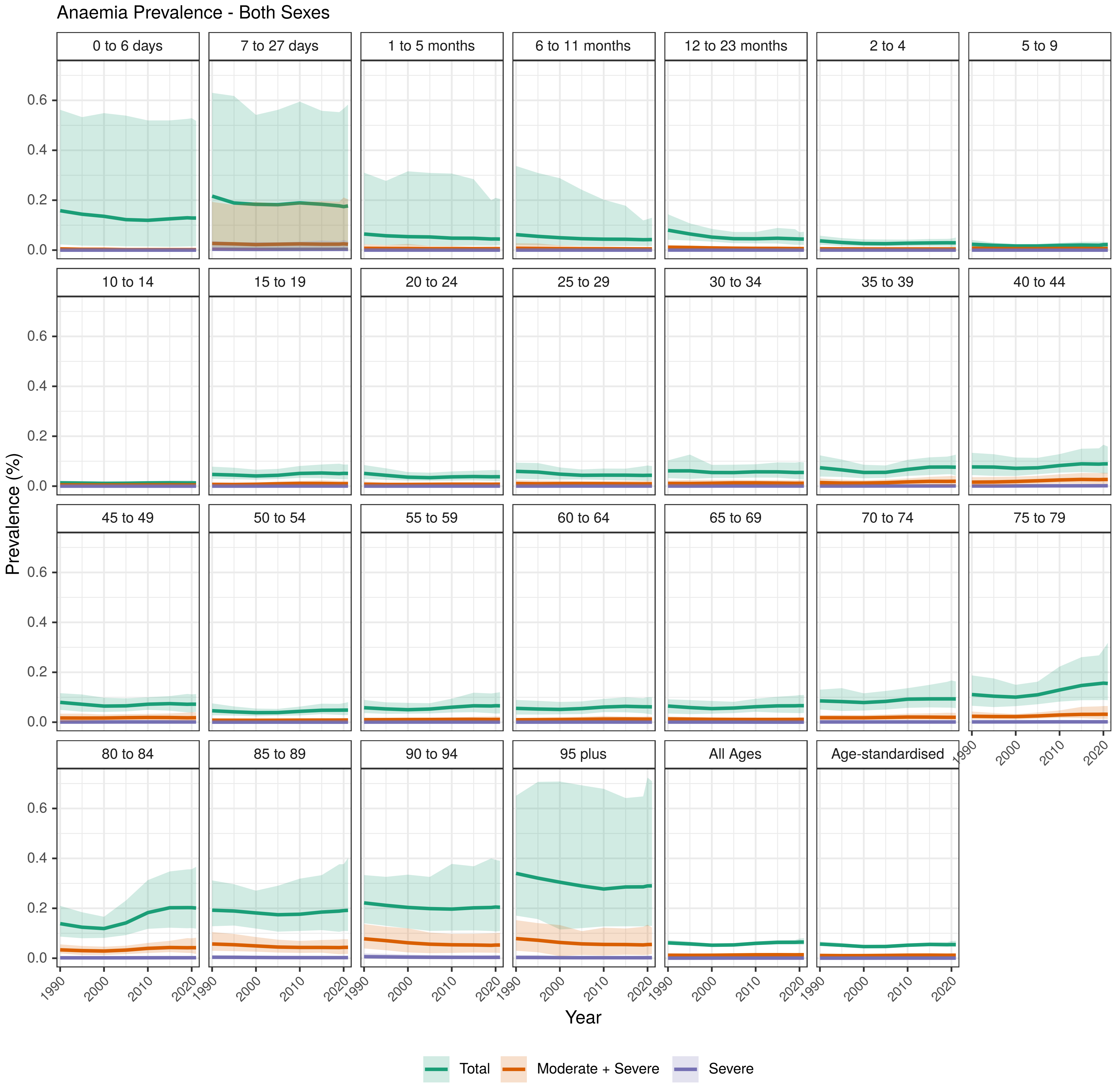

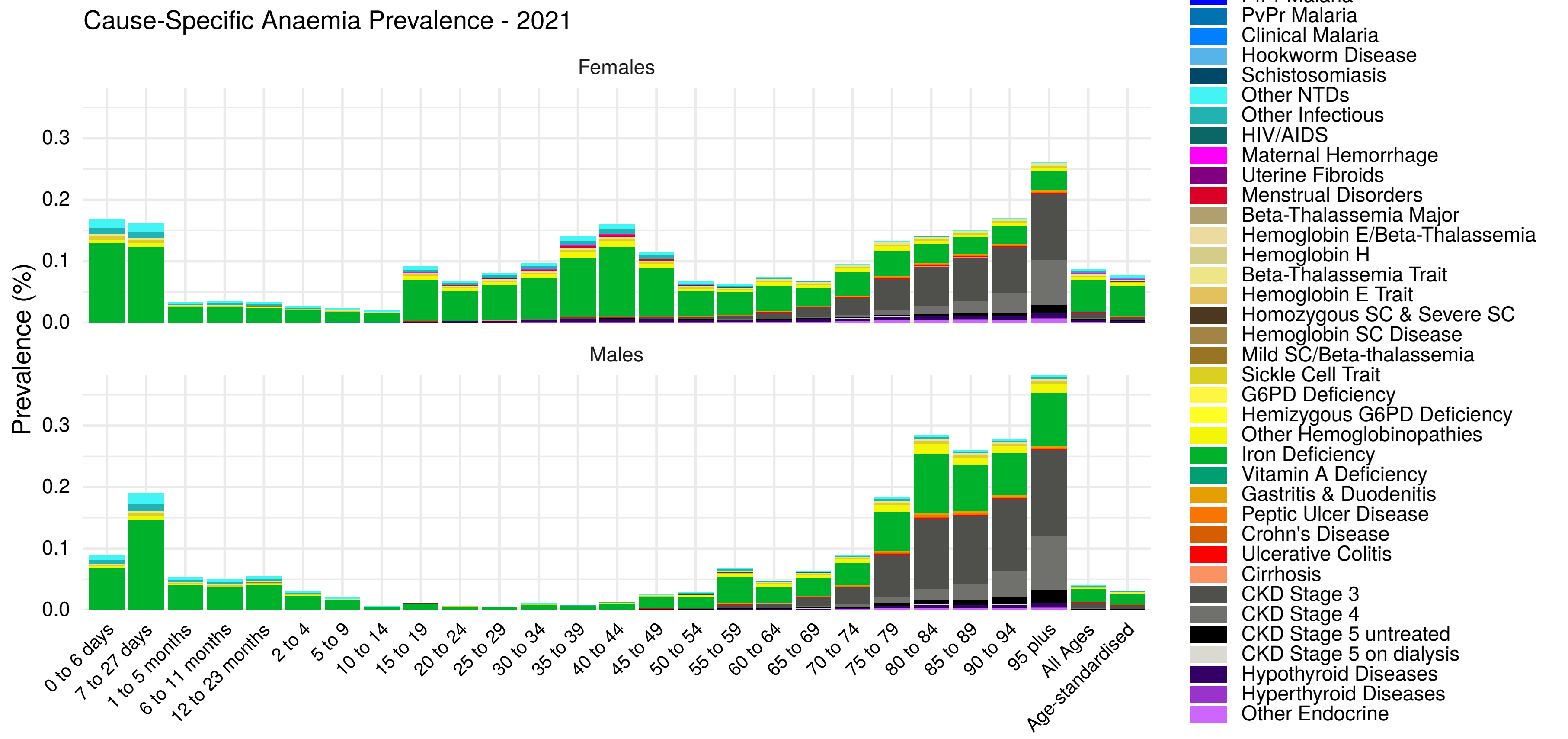

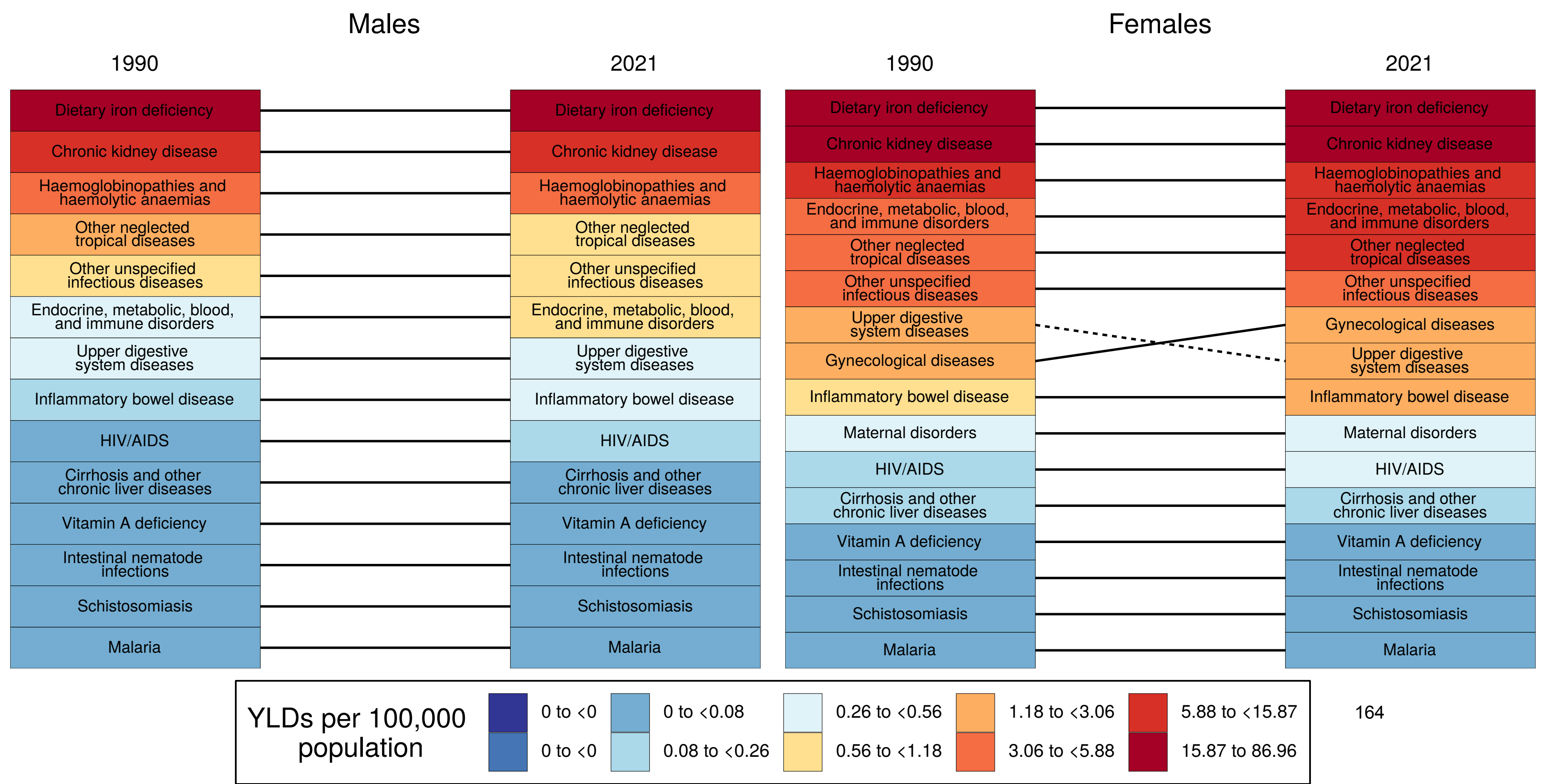

Michigan

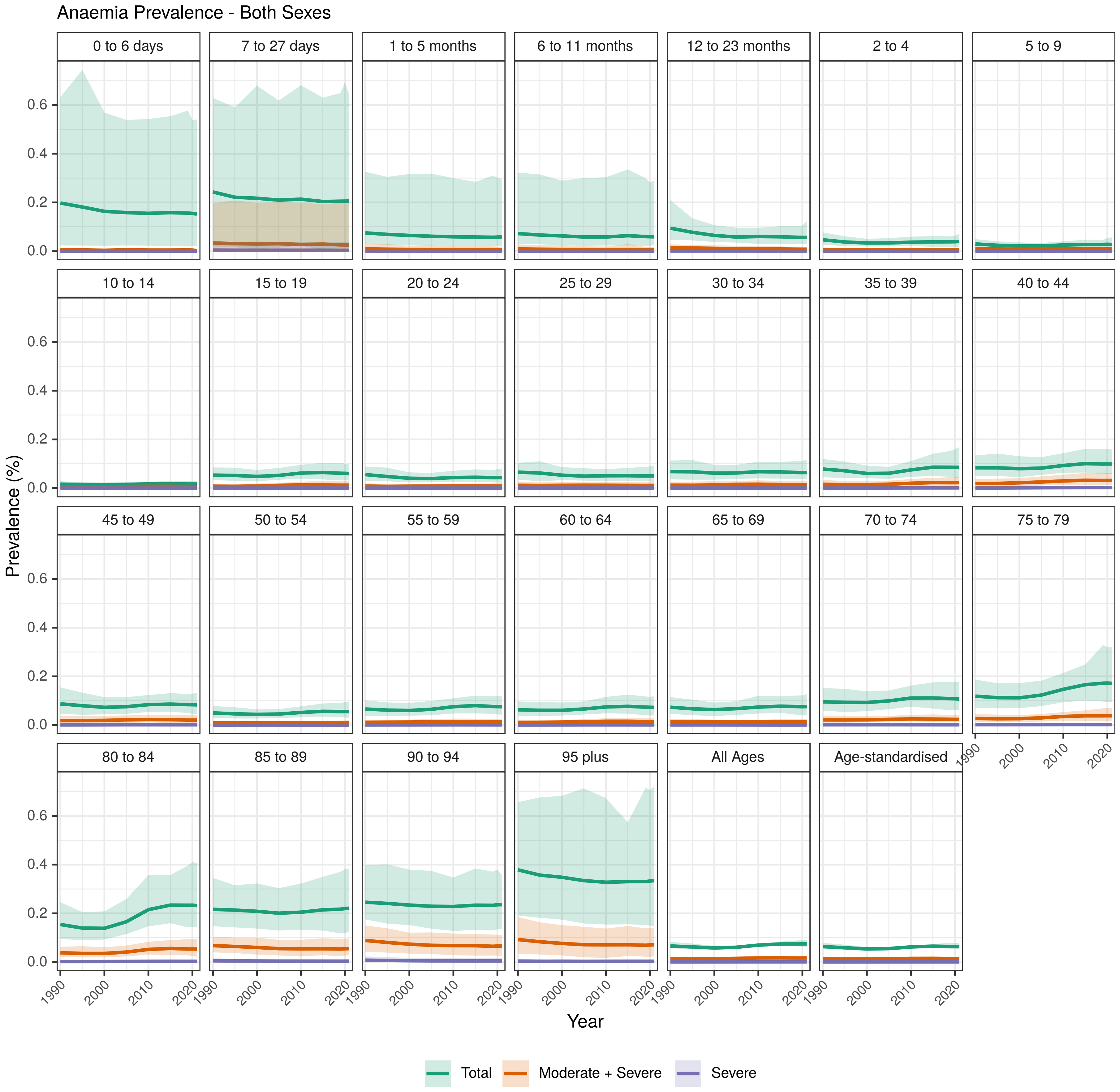

Anaemia Prevalence - Both Sexes
0 to 6 days
7 to 27 days
1 to 5 months
6 to 11 months
12 to 23 months
2 to 4
5 to 9
10 to 14
15 to 19
20 to 24
25 to 29
30 to 34
35 to 39
40 to 44
45 to 49
50 to 54
55 to 59
60 to 64
65 to 69
70 to 74
75 to 79
80 to 84
85 to 89
90 to 94
95 plus
All Ages
Age-standardised
Prevalence (%)
0.0
0.2
0.4
0.6
1990
2000
2010
2020
Year
Total
Moderate + Severe
Severe

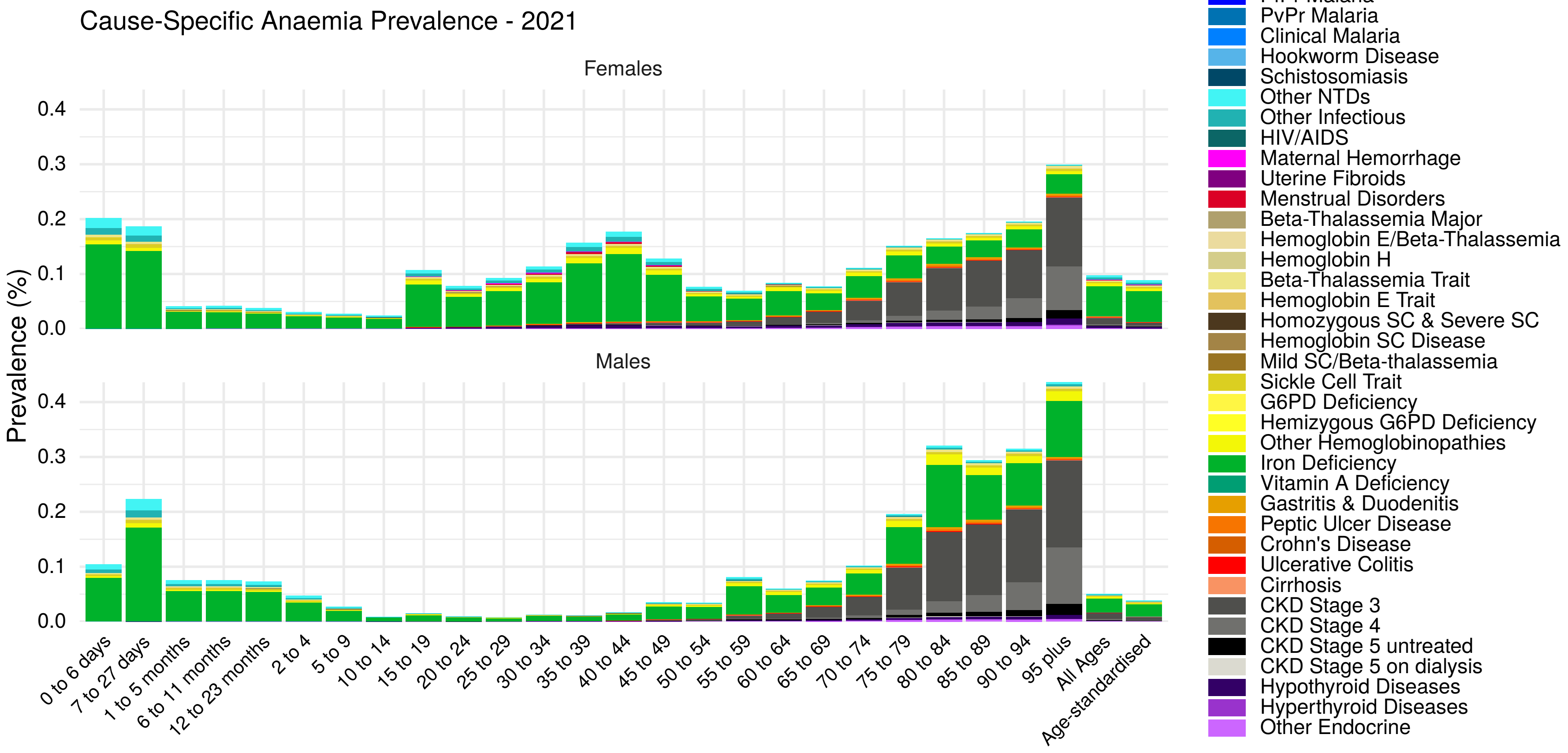

Cause-Specific Anaemia Prevalence - 2021
Females
Males
Prevalence (%)
0.0
0.1
0.2
0.3
0.4
0 to 6 days
7 to 27 days
1 to 5 months
6 to 11 months
12 to 23 months
2 to 4
5 to 9
10 to 14
15 to 19
20 to 24
25 to 29
30 to 34
35 to 39
40 to 44
45 to 49
50 to 54
55 to 59
60 to 64
65 to 69
70 to 74
75 to 79
80 to 84
85 to 89
90 to 94
95 plus
All Ages
Age-standardised
PfPr Malaria
PvPr Malaria
Clinical Malaria
Hookworm Disease
Schistosomiasis
Other NTDs
Other Infectious
HIV/AIDS
Maternal Hemorrhage
Uterine Fibroids
Menstrual Disorders
Beta-Thalassemia Major
Hemoglobin E/Beta-Thalassemia
Hemoglobin H
Beta-Thalassemia Trait
Hemoglobin E Trait
Homozygous SC & Severe SC
Hemoglobin SC Disease
Mild SC/Beta-thalassemia
Sickle Cell Trait
G6PD Deficiency
Hemizygous G6PD Deficiency
Other Hemoglobinopathies
Iron Deficiency
Vitamin A Deficiency
Gastritis & Duodenitis
Peptic Ulcer Disease
Crohn's Disease
Ulcerative Colitis
Cirrhosis
CKD Stage 3
CKD Stage 4
CKD Stage 5 untreated
CKD Stage 5 on dialysis
Hypothyroid Diseases
Hyperthyroid Diseases
Other Endocrine

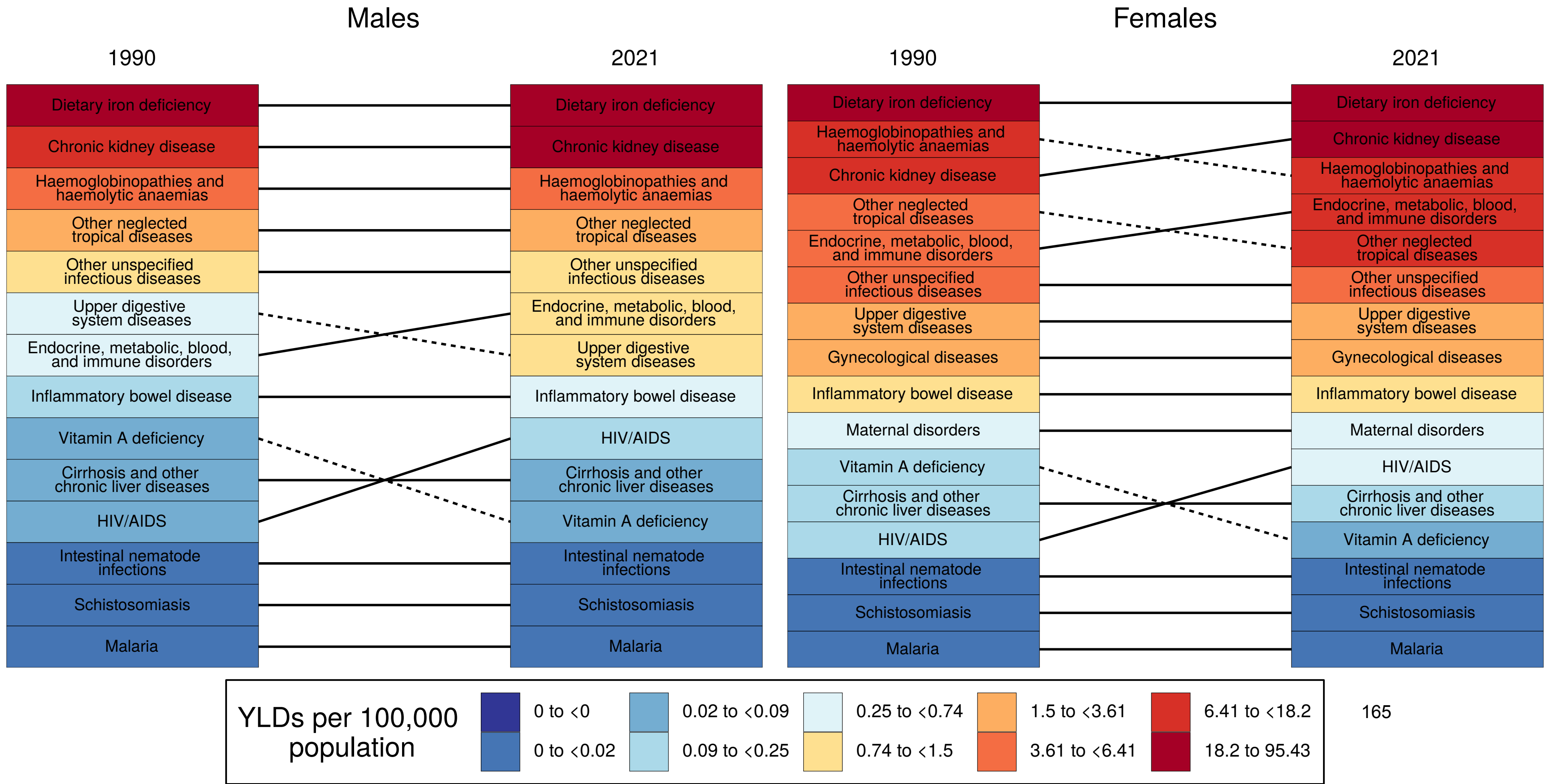

Males
1990
Dietary iron deficiency
Chronic kidney disease
Haemoglobinopathies and haemolytic anaemias
Other neglected tropical diseases
Other unspecified infectious diseases
Upper digestive system diseases
Endocrine, metabolic, blood, and immune disorders
Inflammatory bowel disease
Vitamin A deficiency
Cirrhosis and other chronic liver diseases
HIV/AIDS
Intestinal nematode infections
Schistosomiasis
Malaria
2021
Dietary iron deficiency
Chronic kidney disease
Haemoglobinopathies and haemolytic anaemias
Other neglected tropical diseases
Other unspecified infectious diseases
Endocrine, metabolic, blood, and immune disorders
Upper digestive system diseases
Inflammatory bowel disease
HIV/AIDS
Cirrhosis and other chronic liver diseases
Vitamin A deficiency
Intestinal nematode infections
Schistosomiasis
Malaria
Females
1990
Dietary iron deficiency
Haemoglobinopathies and haemolytic anaemias
Chronic kidney disease
Other neglected tropical diseases
Endocrine, metabolic, blood, and immune disorders
Other unspecified infectious diseases
Upper digestive system diseases
Gynecological diseases
Inflammatory bowel disease
Maternal disorders
Vitamin A deficiency
Cirrhosis and other chronic liver diseases
HIV/AIDS
Intestinal nematode infections
Schistosomiasis
Malaria
2021
Dietary iron deficiency
Chronic kidney disease
Haemoglobinopathies and haemolytic anaemias
Endocrine, metabolic, blood, and immune disorders
Other neglected tropical diseases
Other unspecified infectious diseases
Upper digestive system diseases
Gynecological diseases
Inflammatory bowel disease
Maternal disorders
HIV/AIDS
Cirrhosis and other chronic liver diseases
Vitamin A deficiency
Intestinal nematode infections
Schistosomiasis
Malaria
YLDs per 100,000 population
0 to <0
0 to <0.02
0.02 to <0.09
0.09 to <0.25
0.25 to <0.74
0.74 to <1.5
1.5 to <3.61
3.61 to <6.41
6.41 to <18.2
18.2 to 95.43

Minnesota

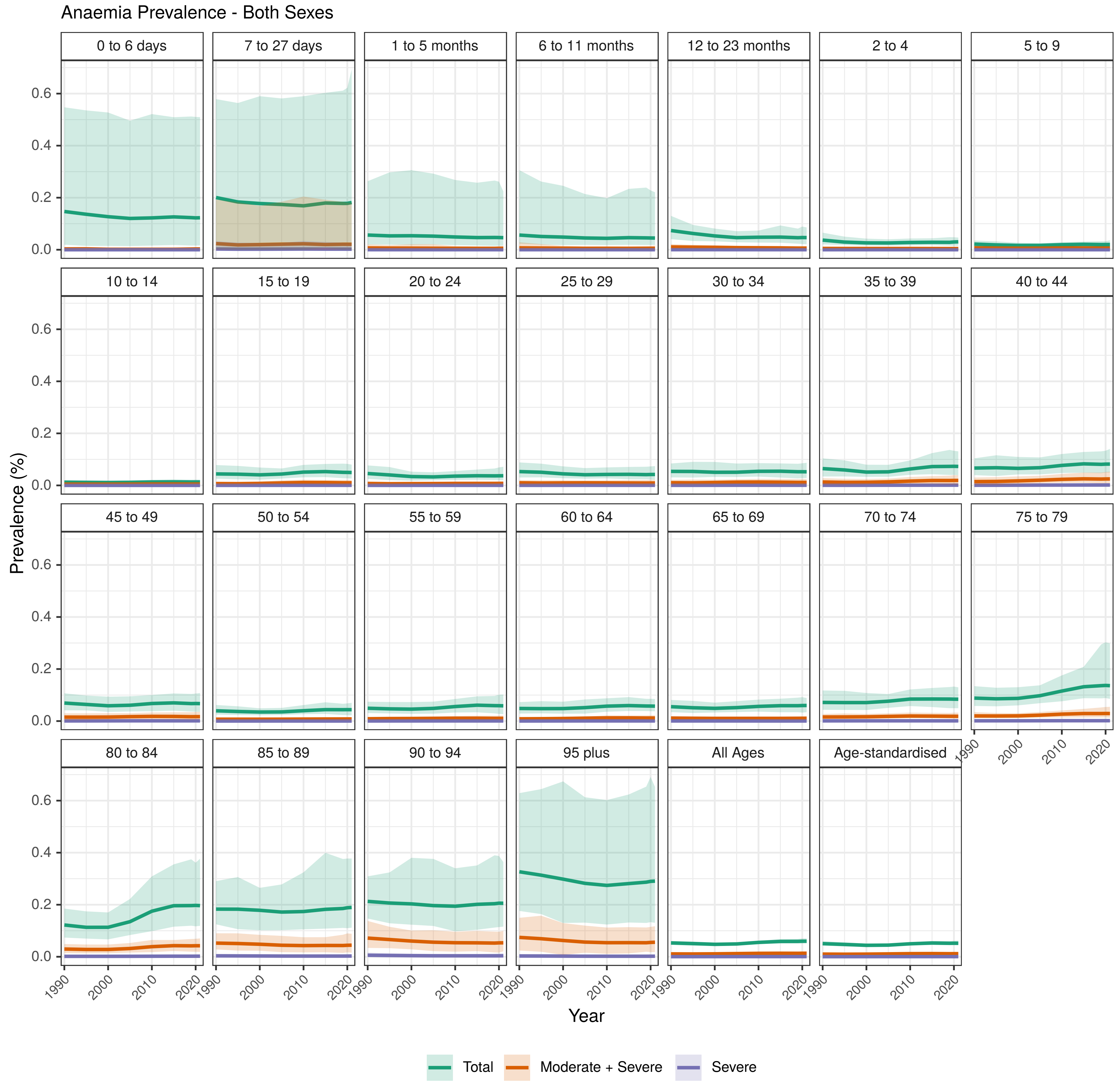

Anaemia Prevalence - Both Sexes
0 to 6 days
7 to 27 days
1 to 5 months
6 to 11 months
12 to 23 months
2 to 4
5 to 9
10 to 14
15 to 19
20 to 24
25 to 29
30 to 34
35 to 39
40 to 44
45 to 49
50 to 54
55 to 59
60 to 64
65 to 69
70 to 74
75 to 79
80 to 84
85 to 89
90 to 94
95 plus
All Ages
Age-standardised
Prevalence (%)
Year
Total
Moderate + Severe
Severe

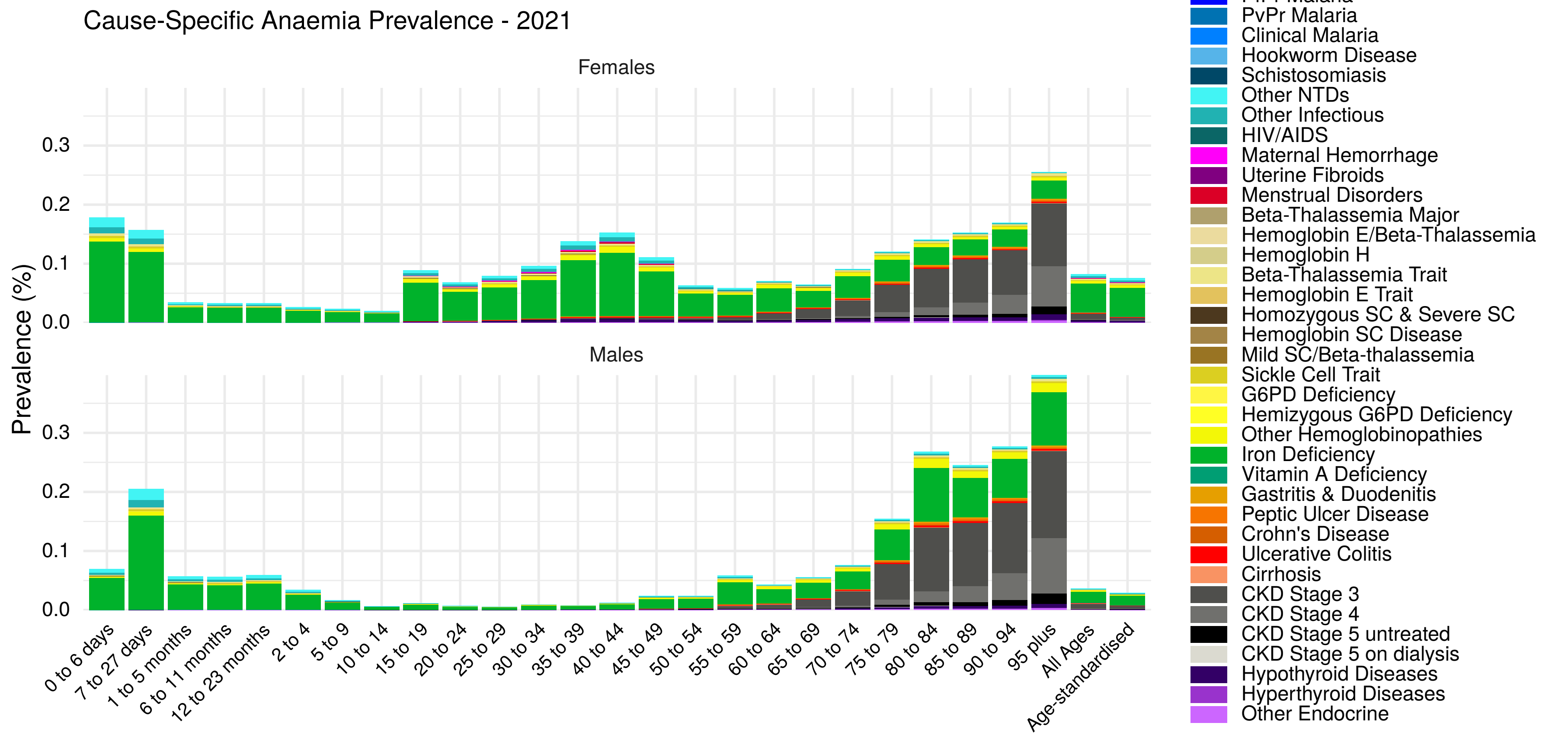

Cause-Specific Anaemia Prevalence - 2021
Females
Males
Prevalence (%)
PfPr Malaria
PvPr Malaria
Clinical Malaria
Hookworm Disease
Schistosomiasis
Other NTDs
Other Infectious
HIV/AIDS
Maternal Hemorrhage
Uterine Fibroids
Menstrual Disorders
Beta-Thalassemia Major
Hemoglobin E/Beta-Thalassemia
Hemoglobin H
Beta-Thalassemia Trait
Hemoglobin E Trait
Homozygous SC & Severe SC
Hemoglobin SC Disease
Mild SC/Beta-thalassemia
Sickle Cell Trait
G6PD Deficiency
Hemizygous G6PD Deficiency
Other Hemoglobinopathies
Iron Deficiency
Vitamin A Deficiency
Gastritis & Duodenitis
Peptic Ulcer Disease
Crohn's Disease
Ulcerative Colitis
Cirrhosis
CKD Stage 3
CKD Stage 4
CKD Stage 5 untreated
CKD Stage 5 on dialysis
Hypothyroid Diseases
Hyperthyroid Diseases
Other Endocrine

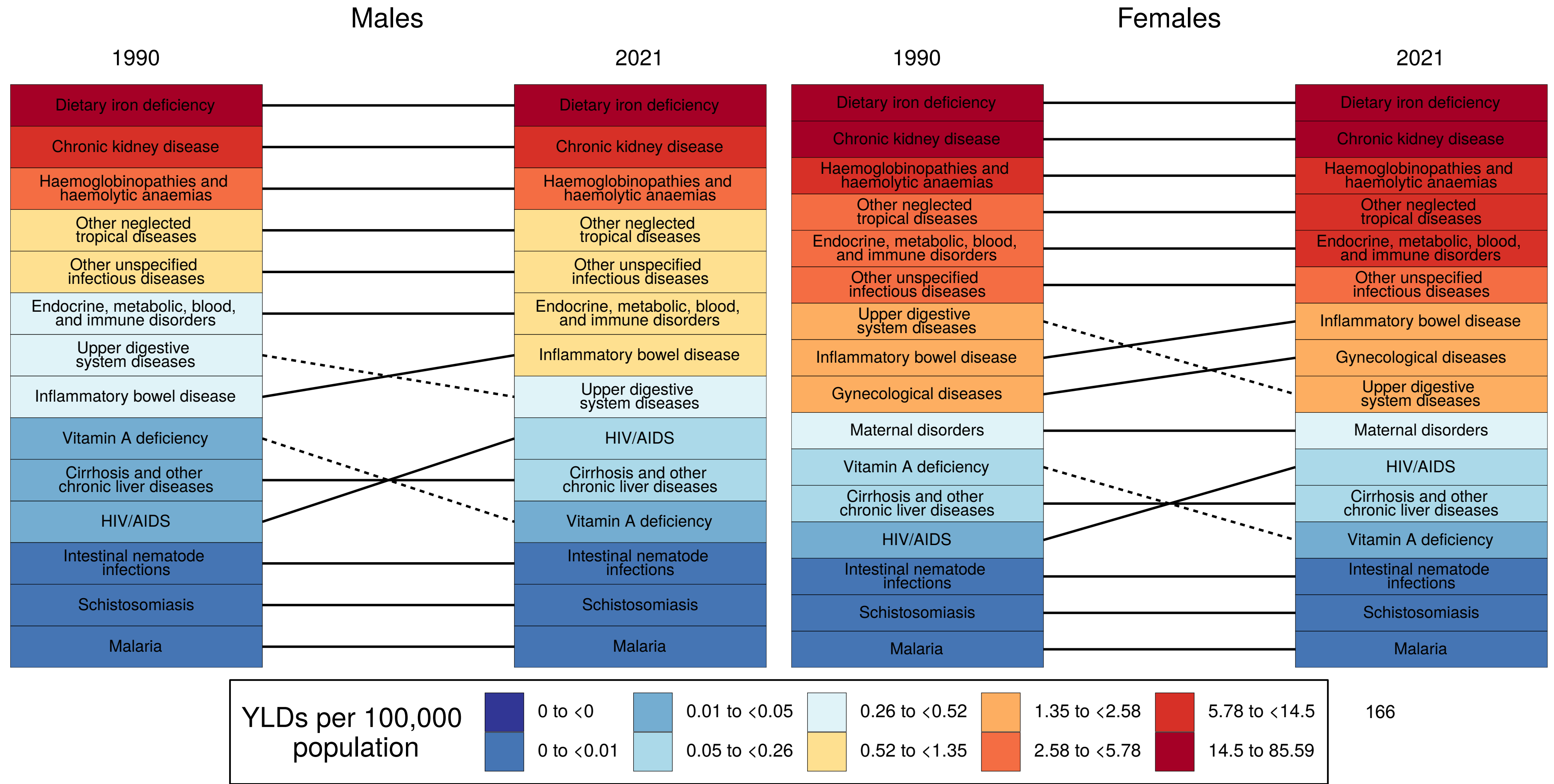

Males
Females
1990
2021
Dietary iron deficiency
Chronic kidney disease
Haemoglobinopathies and haemolytic anaemias
Other neglected tropical diseases
Other unspecified infectious diseases
Endocrine, metabolic, blood, and immune disorders
Upper digestive system diseases
Inflammatory bowel disease
Vitamin A deficiency
Cirrhosis and other chronic liver diseases
HIV/AIDS
Intestinal nematode infections
Schistosomiasis
Malaria
Gynecological diseases
Maternal disorders
YLDs per 100,000 population
0 to <0
0 to <0.01
0.01 to <0.05
0.05 to <0.26
0.26 to <0.52
0.52 to <1.35
1.35 to <2.58
2.58 to <5.78
5.78 to <14.5
14.5 to 85.59

Mississippi

Anaemia Prevalence - Both Sexes

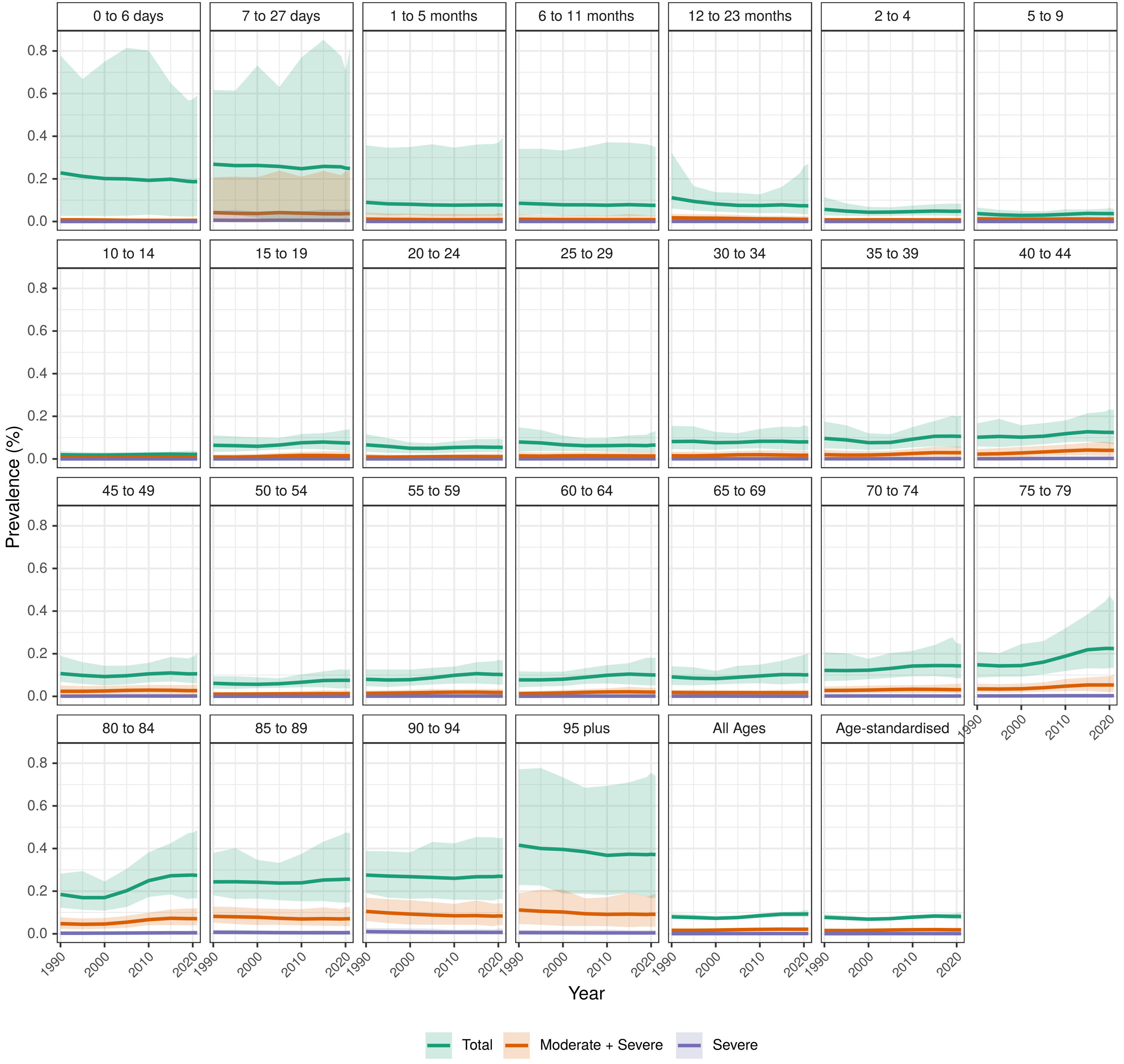

Cause-Specific Anaemia Prevalence - 2021

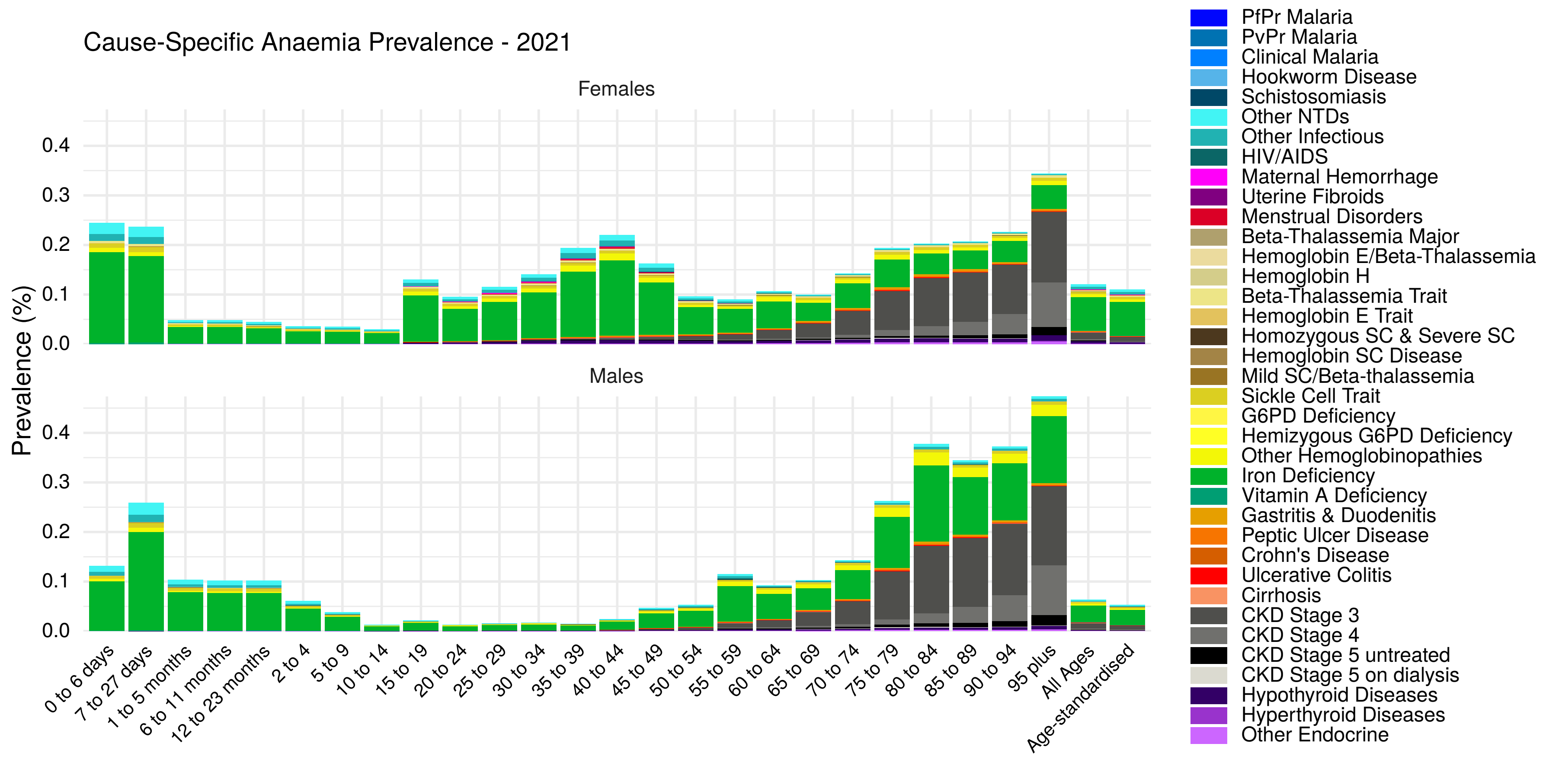

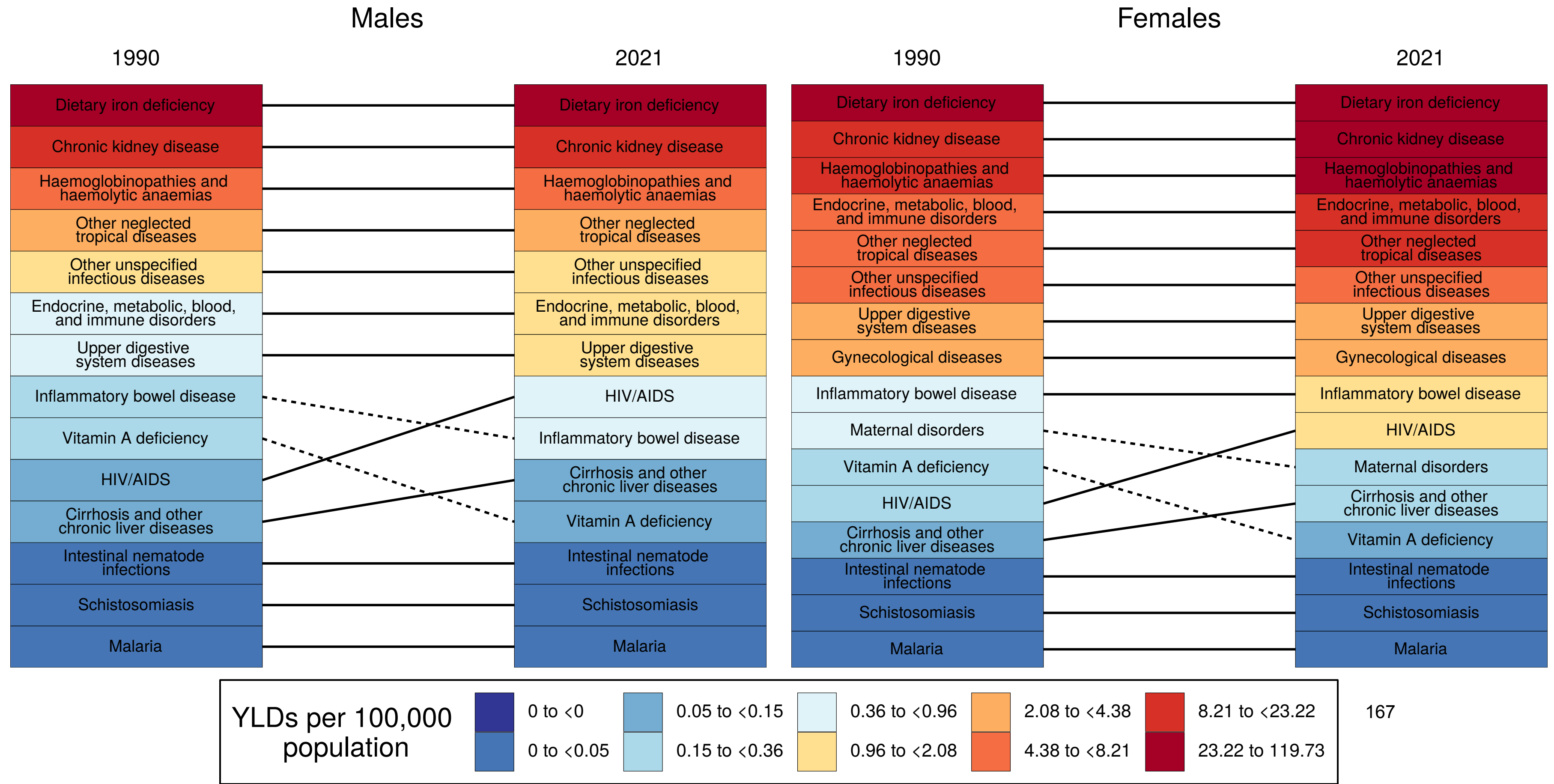

Missouri

Anaemia Prevalence - Both Sexes

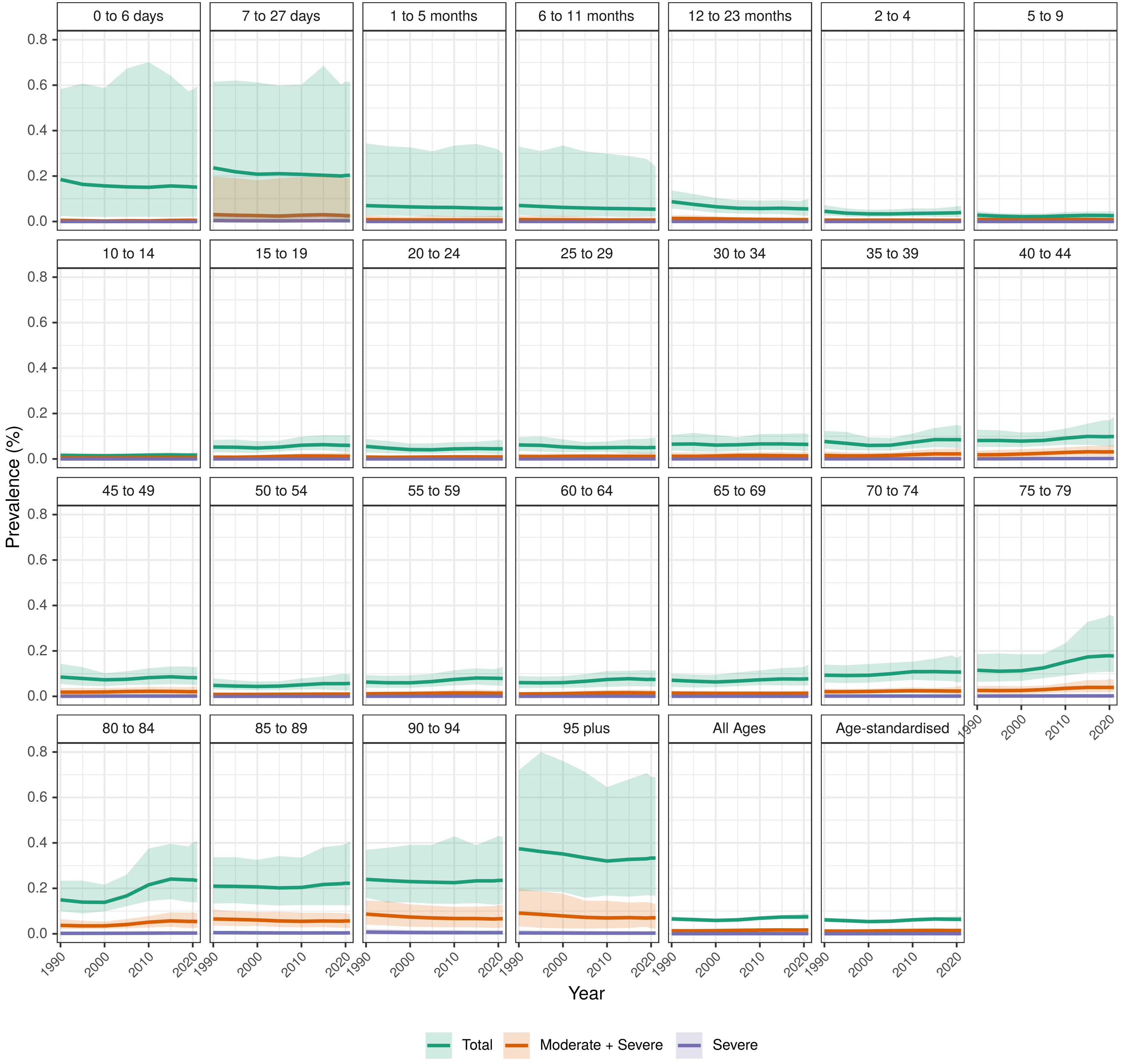

Cause-Specific Anaemia Prevalence - 2021

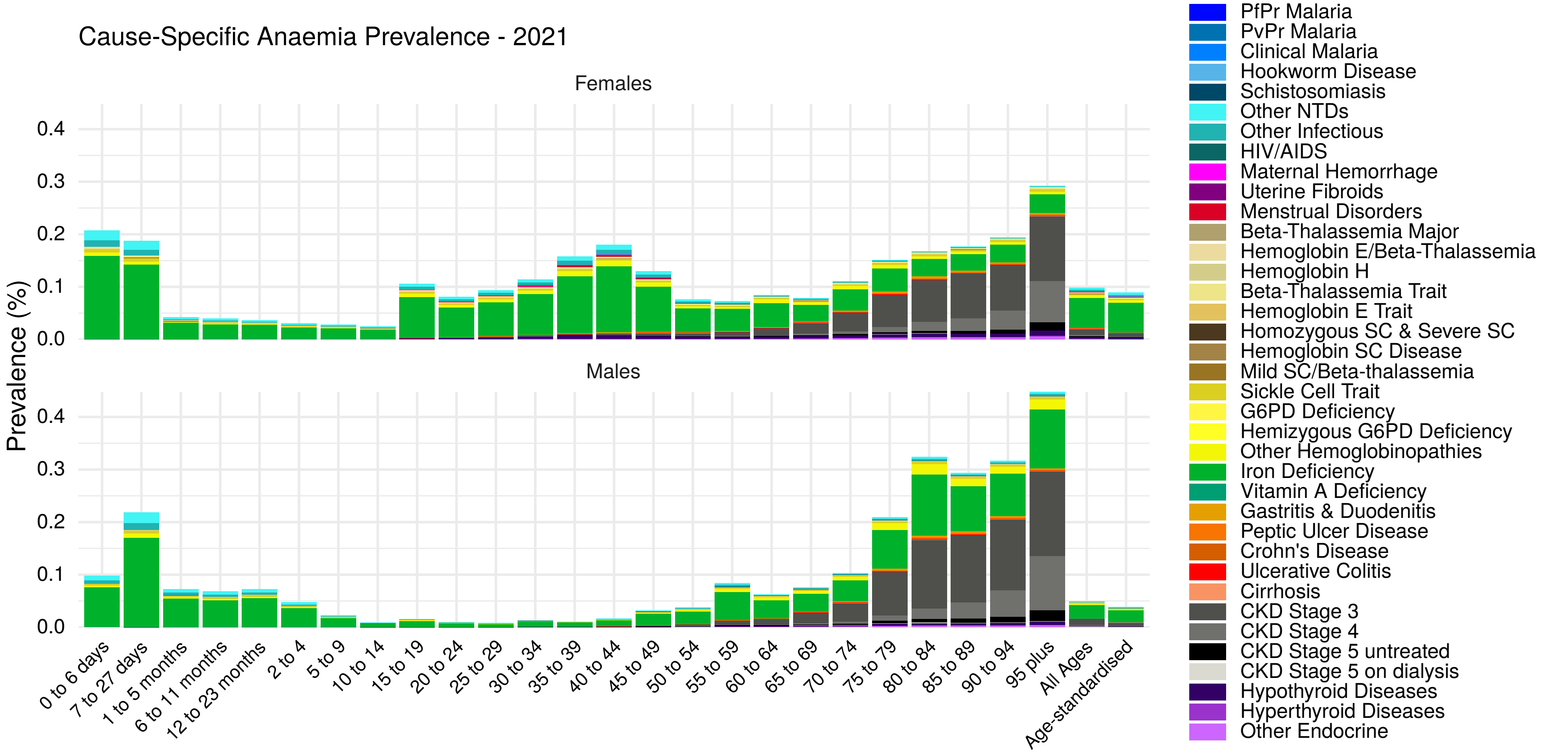

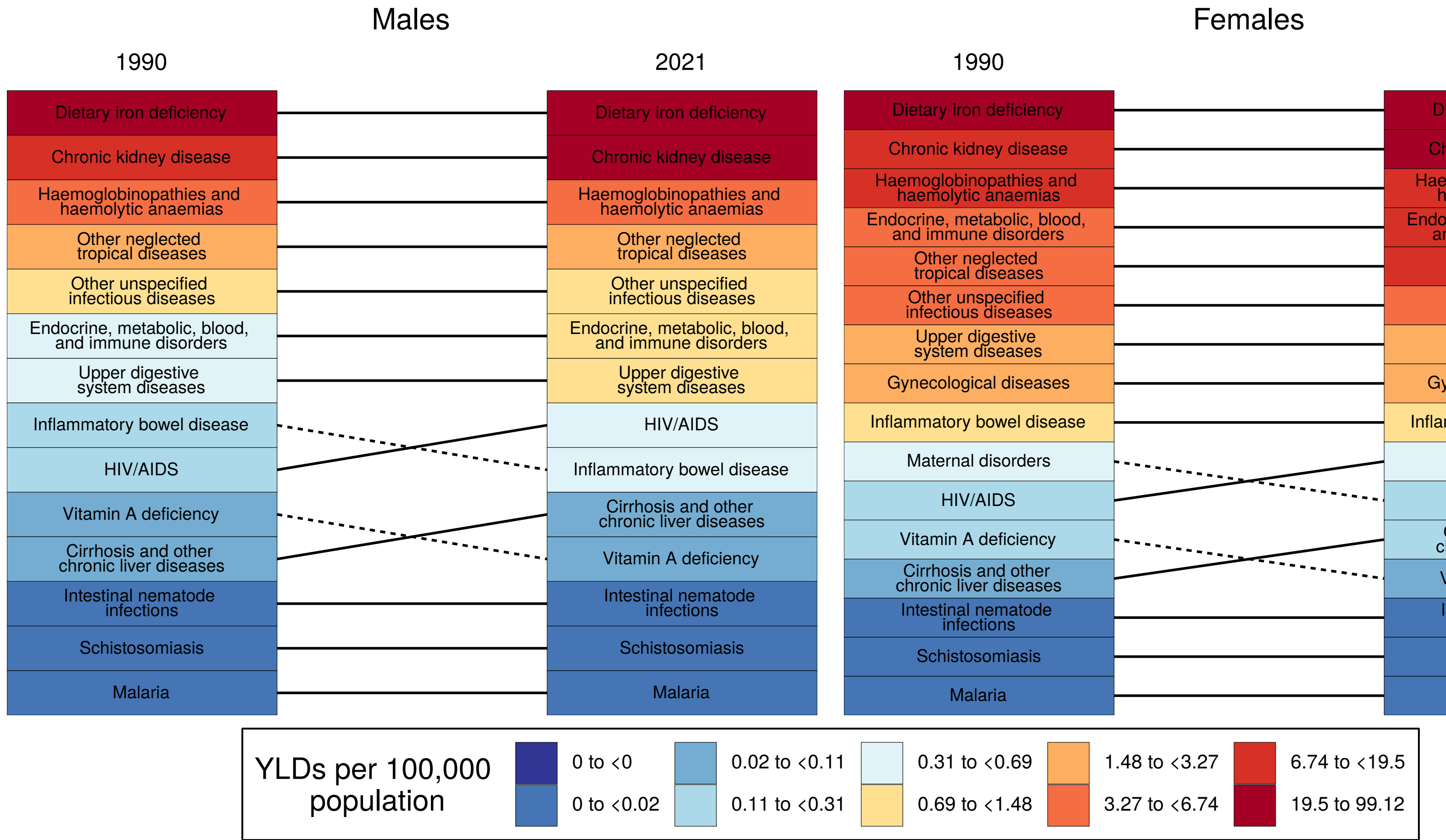

Montana

Anaemia Prevalence - Both Sexes

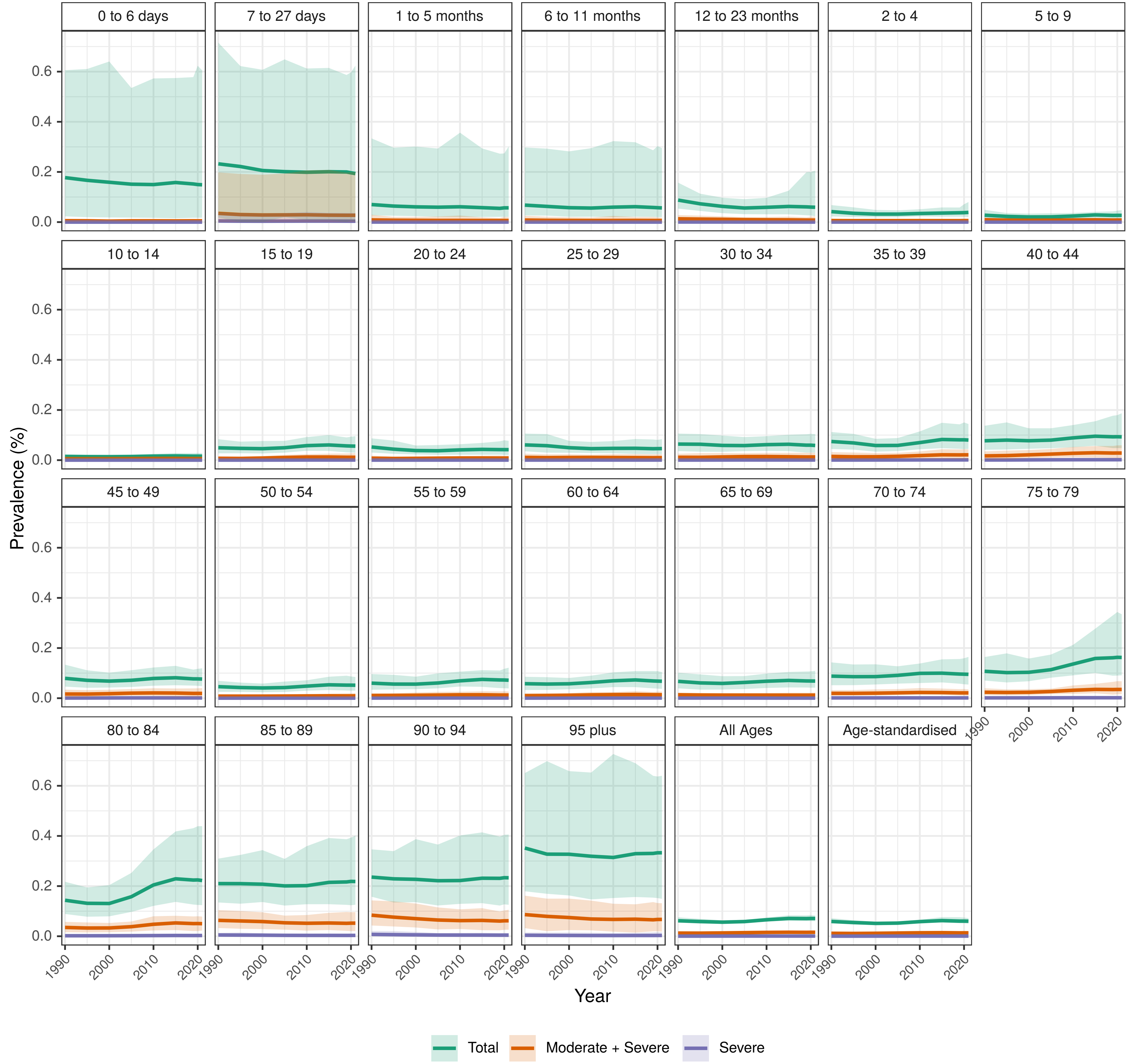

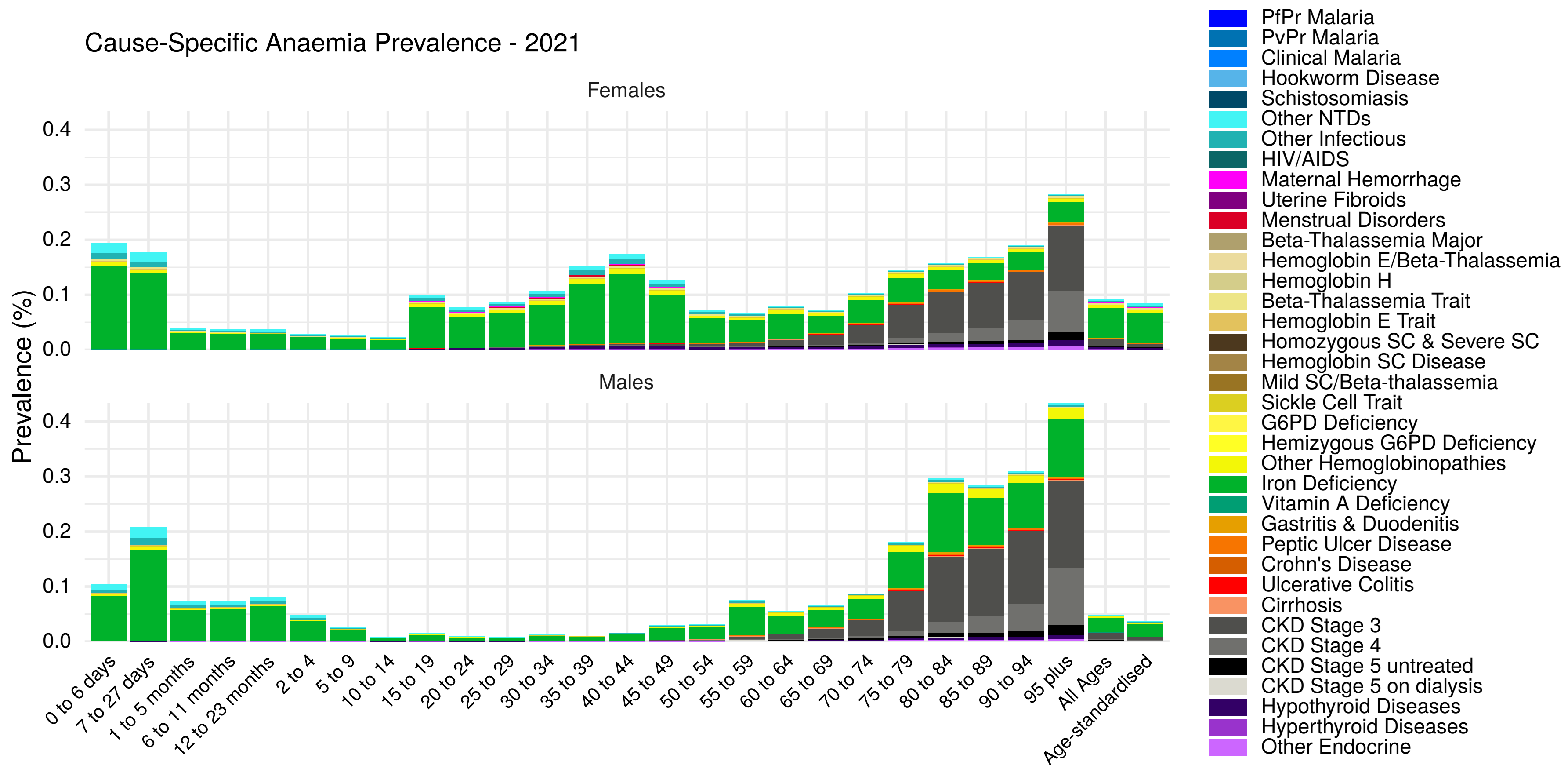

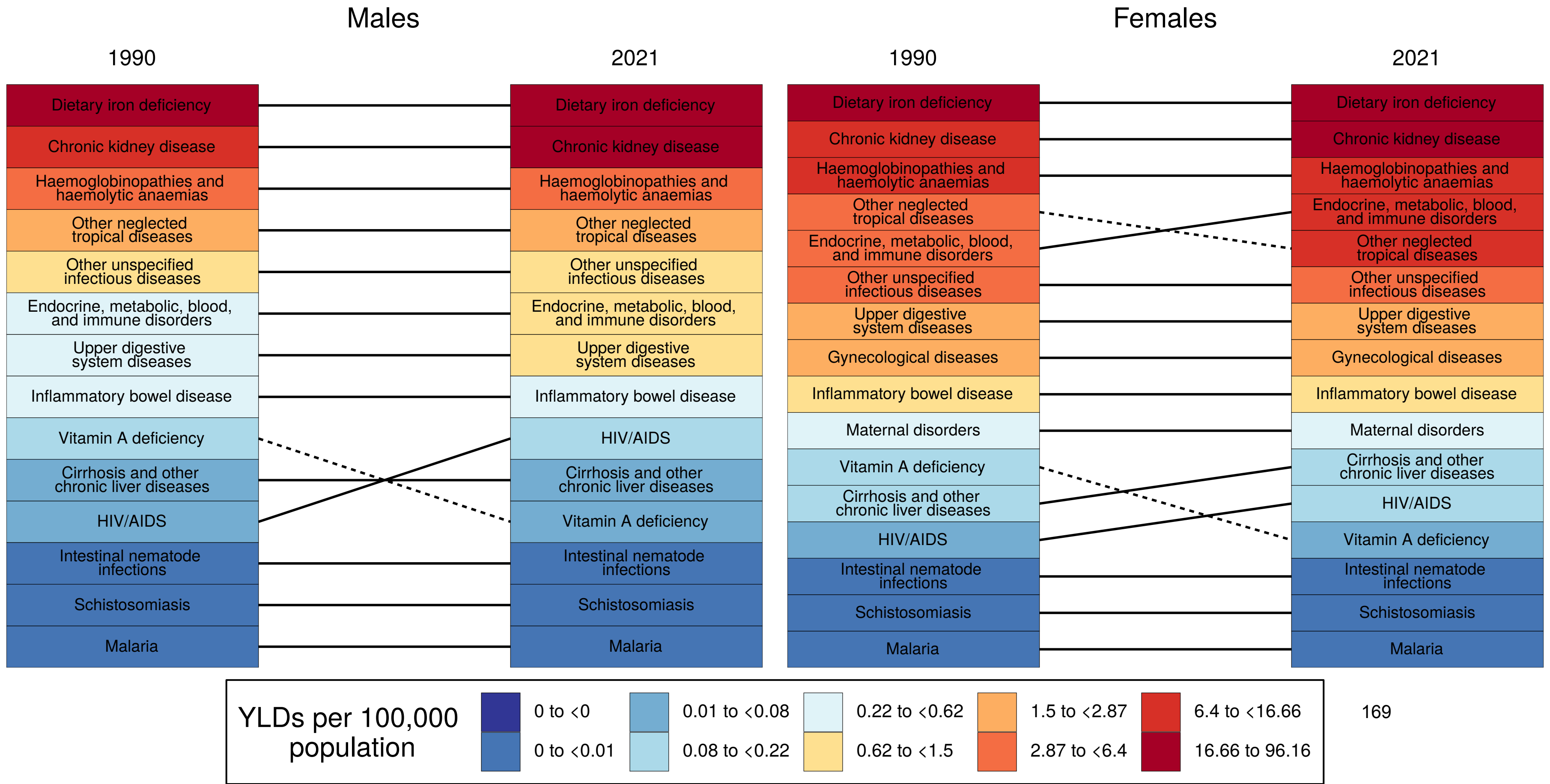

Nebraska

Anaemia Prevalence - Both Sexes

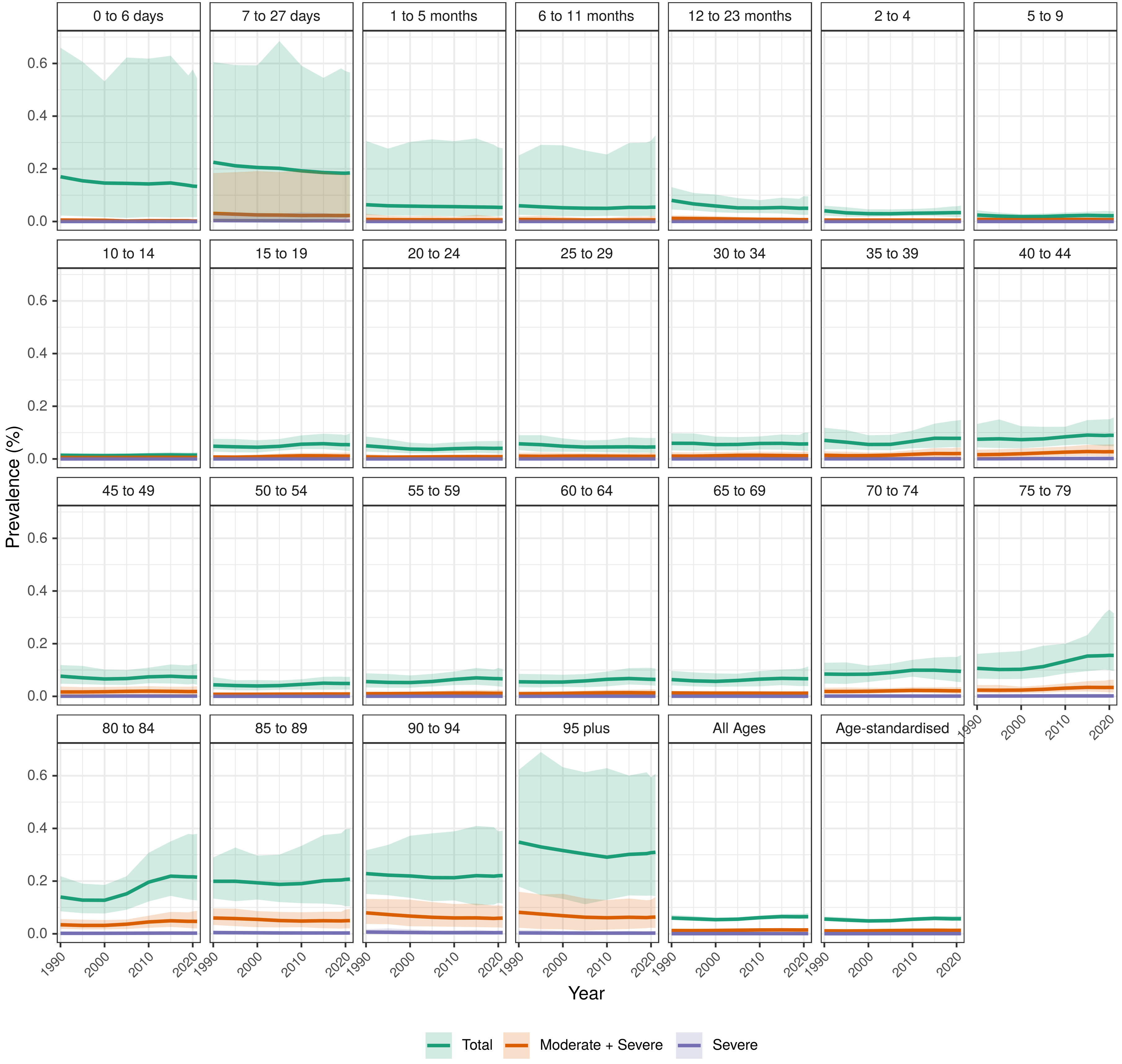

Cause-Specific Anaemia Prevalence - 2021

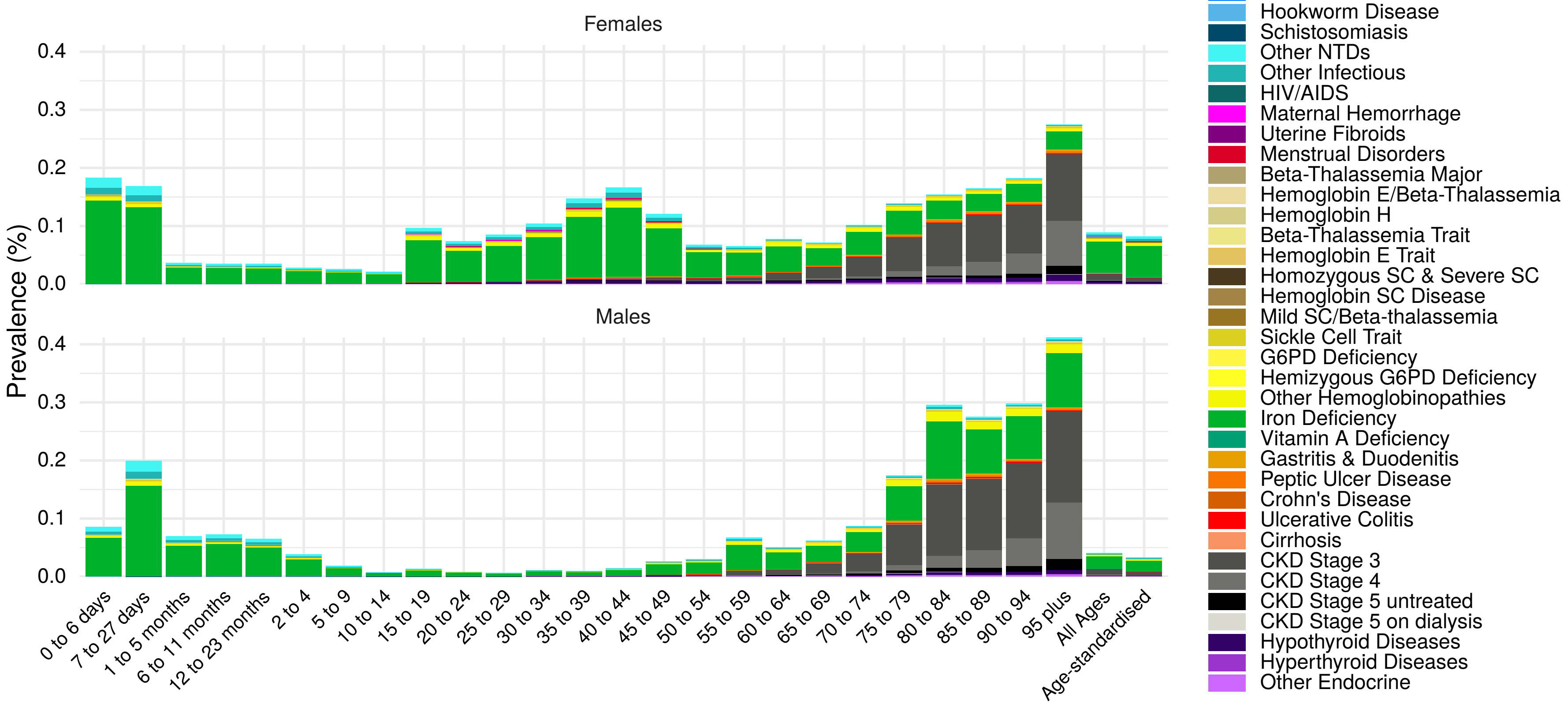

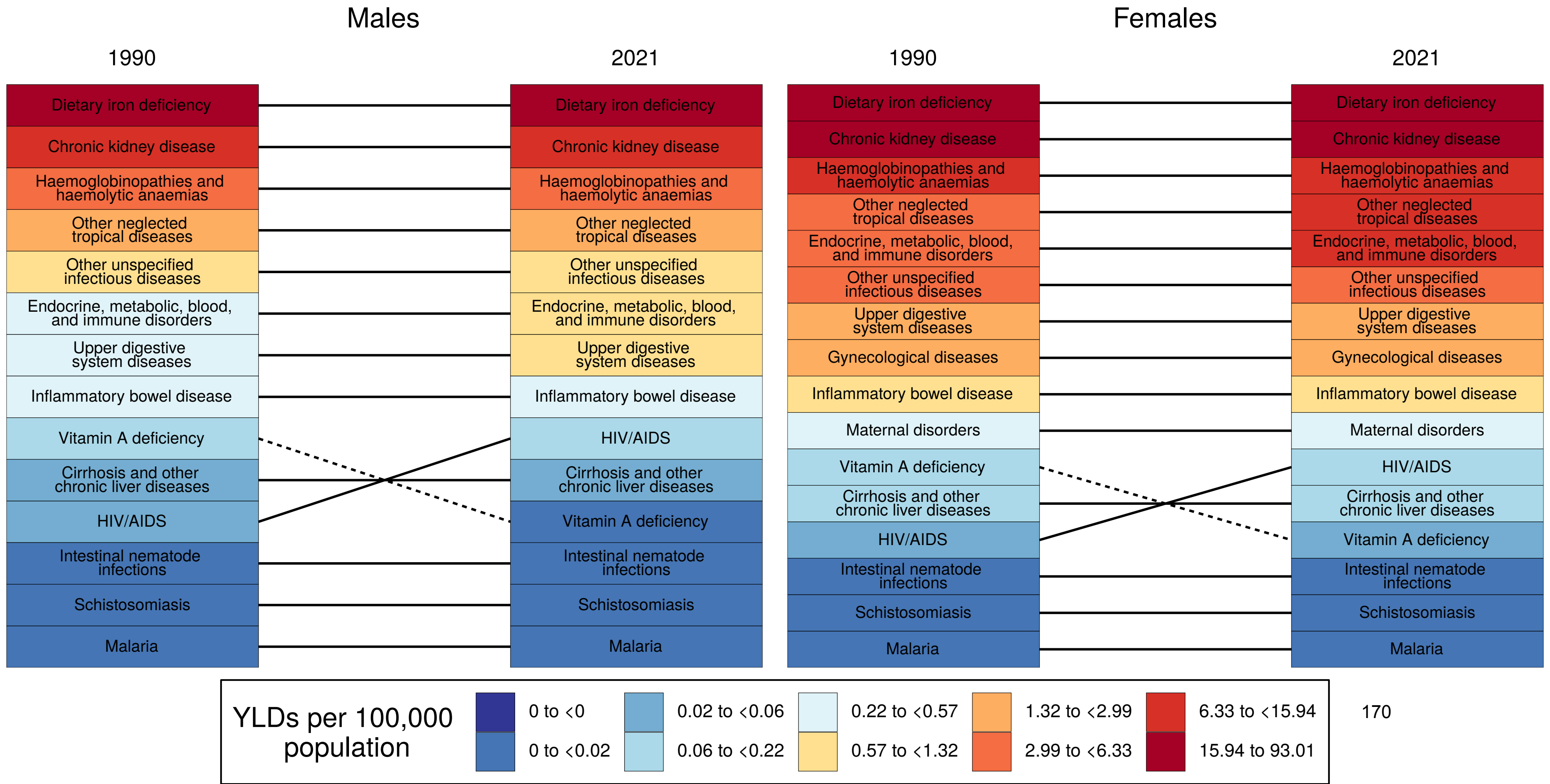

Nevada

Anaemia Prevalence - Both Sexes

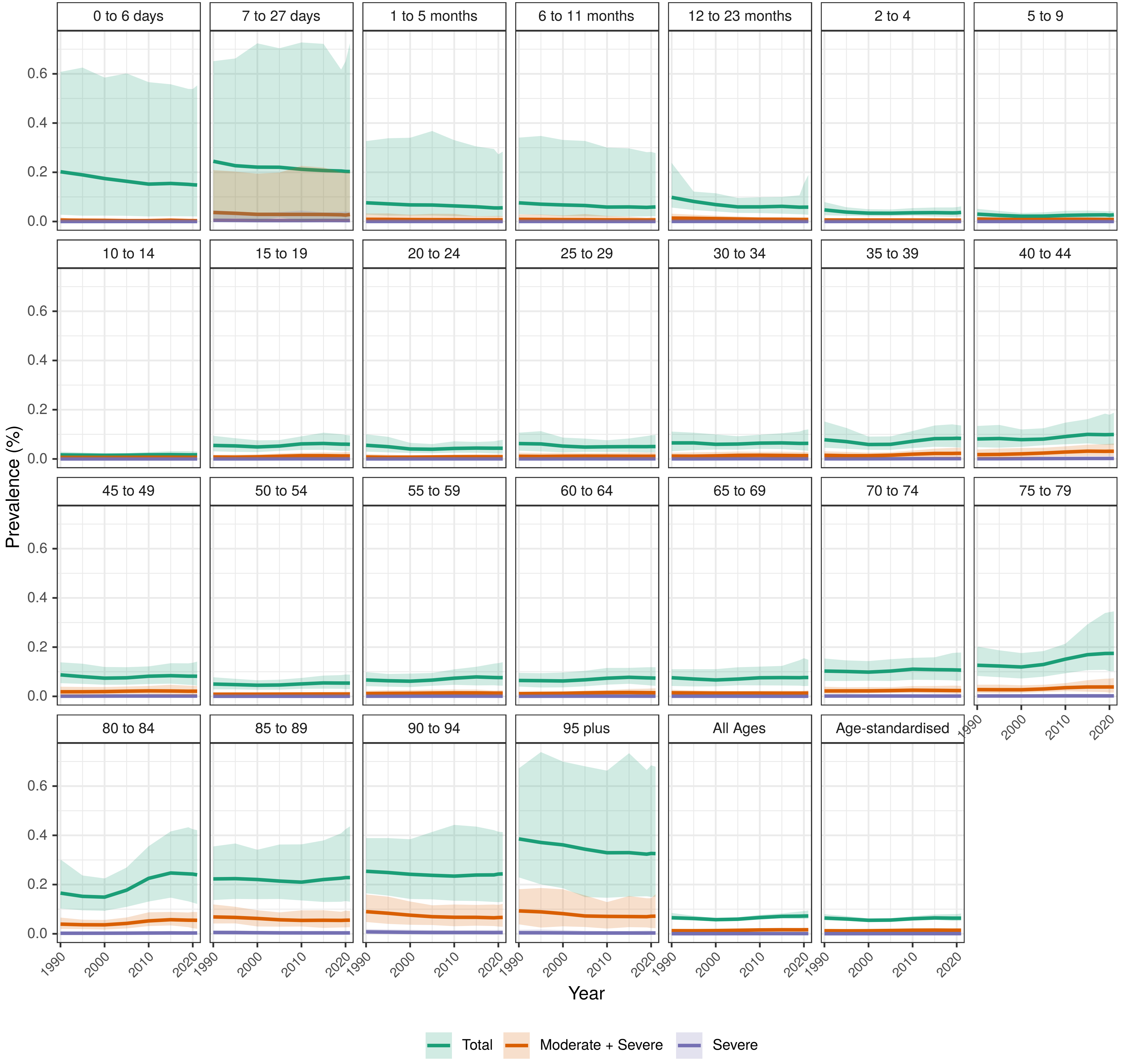

Cause-Specific Anaemia Prevalence - 2021

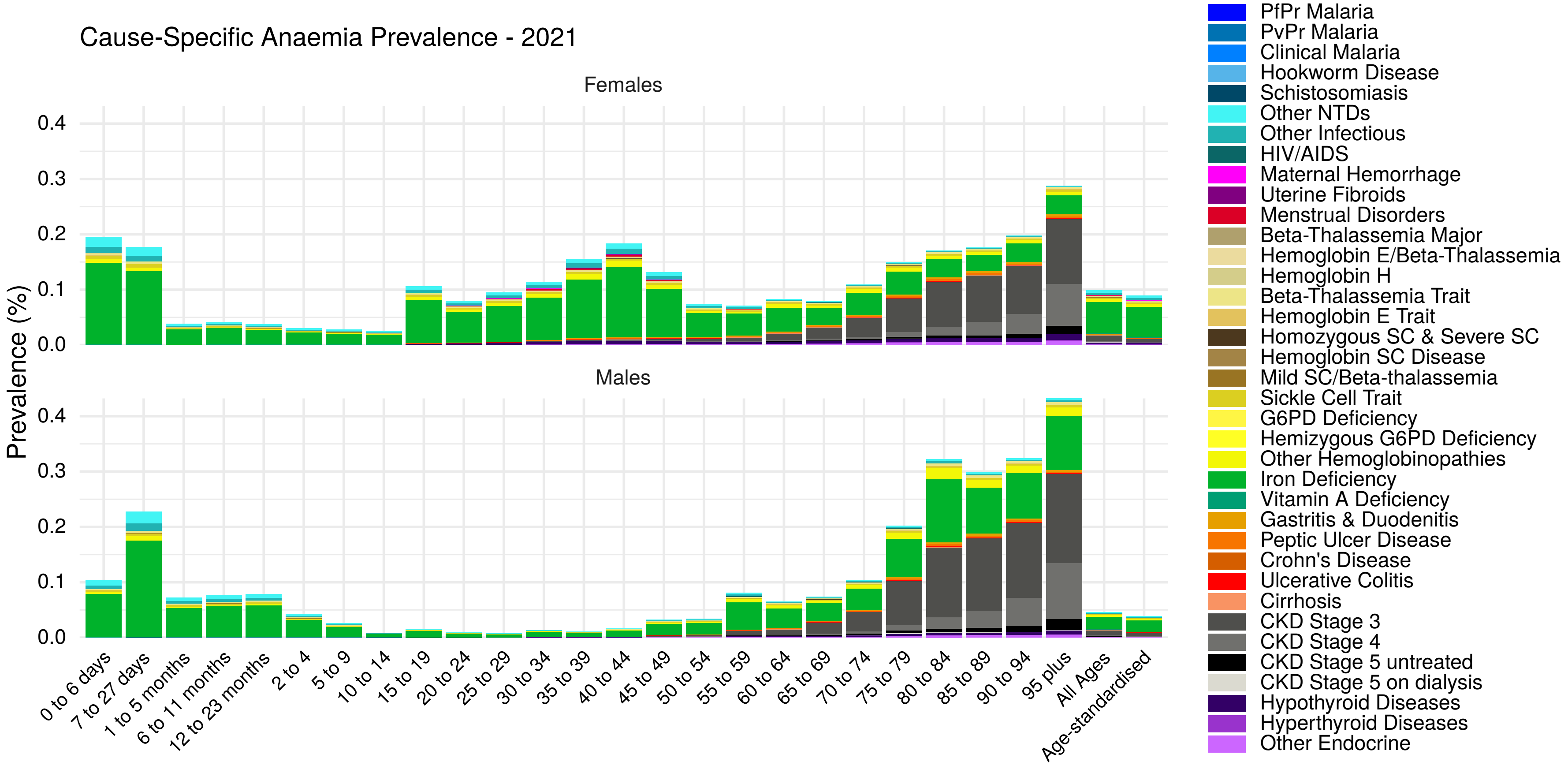

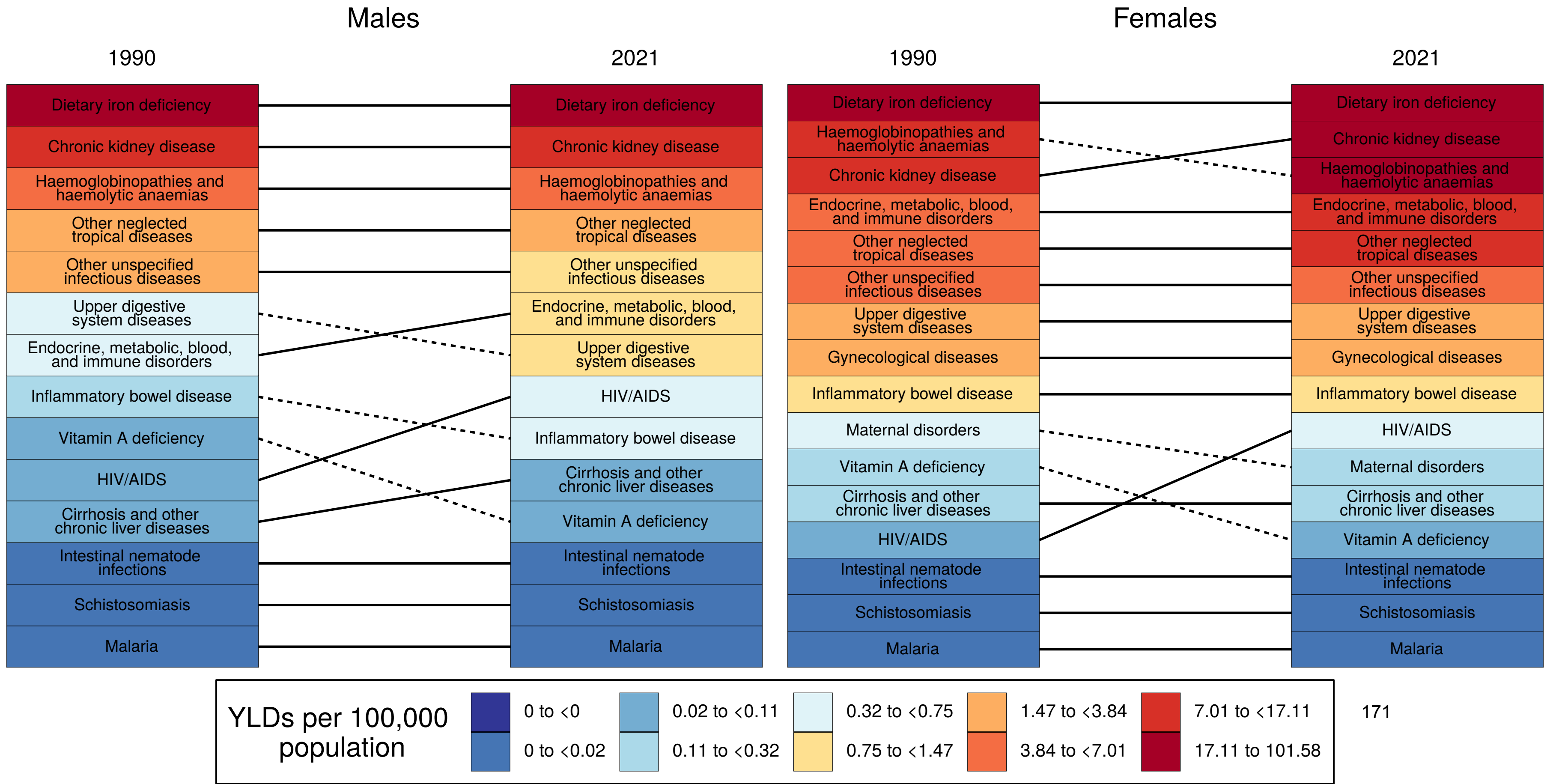

New Hampshire

Anaemia Prevalence - Both Sexes

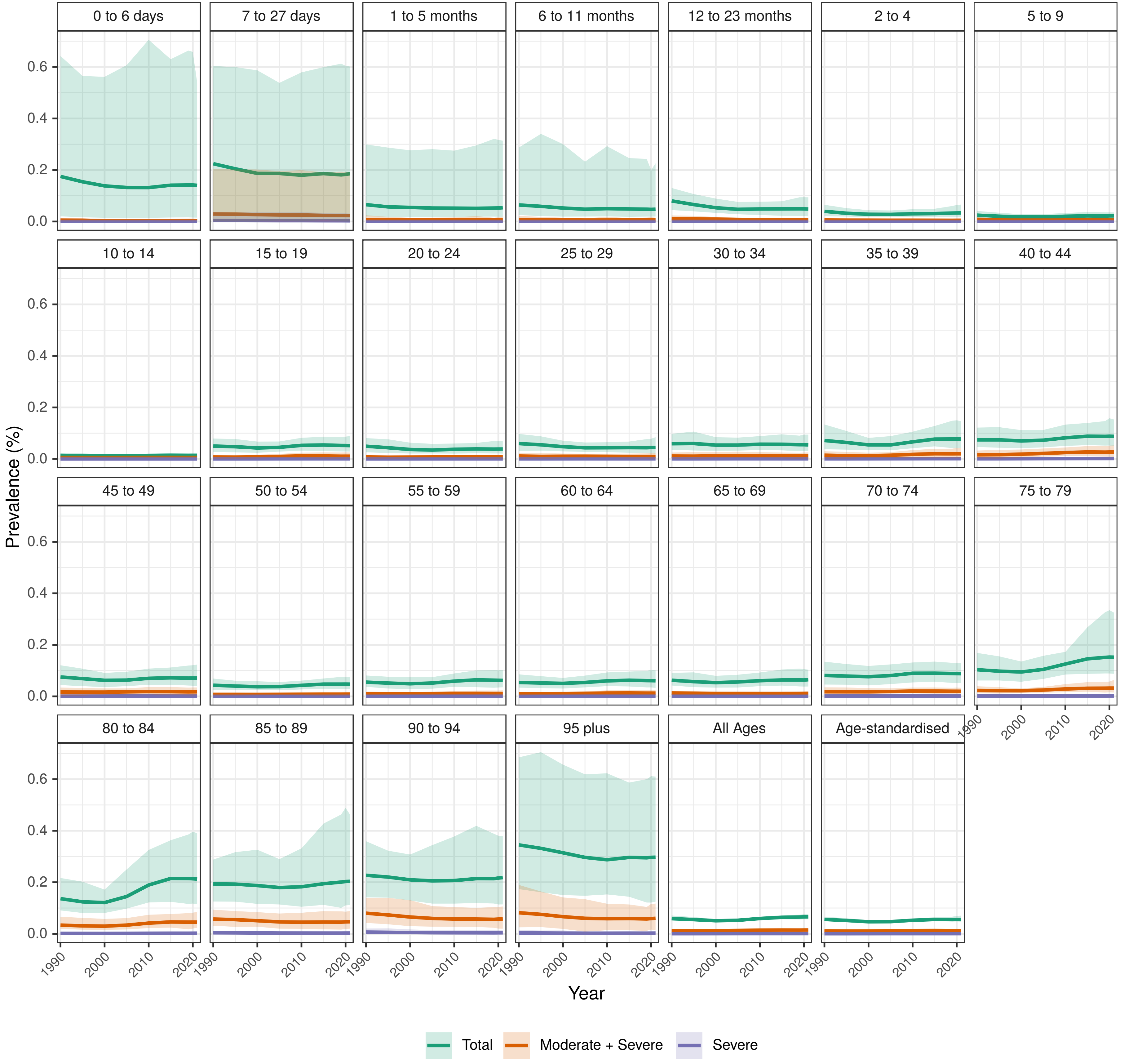

Cause-Specific Anaemia Prevalence - 2021

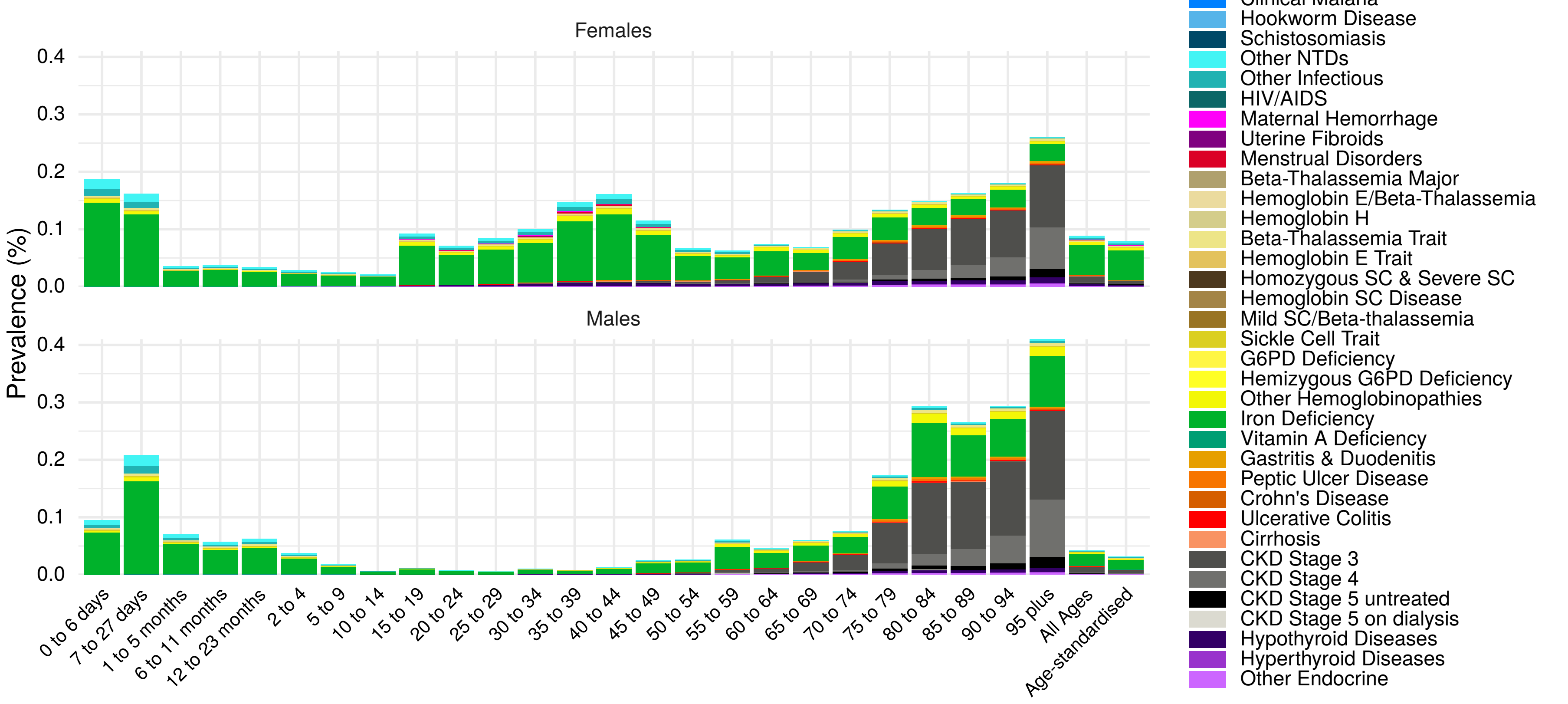

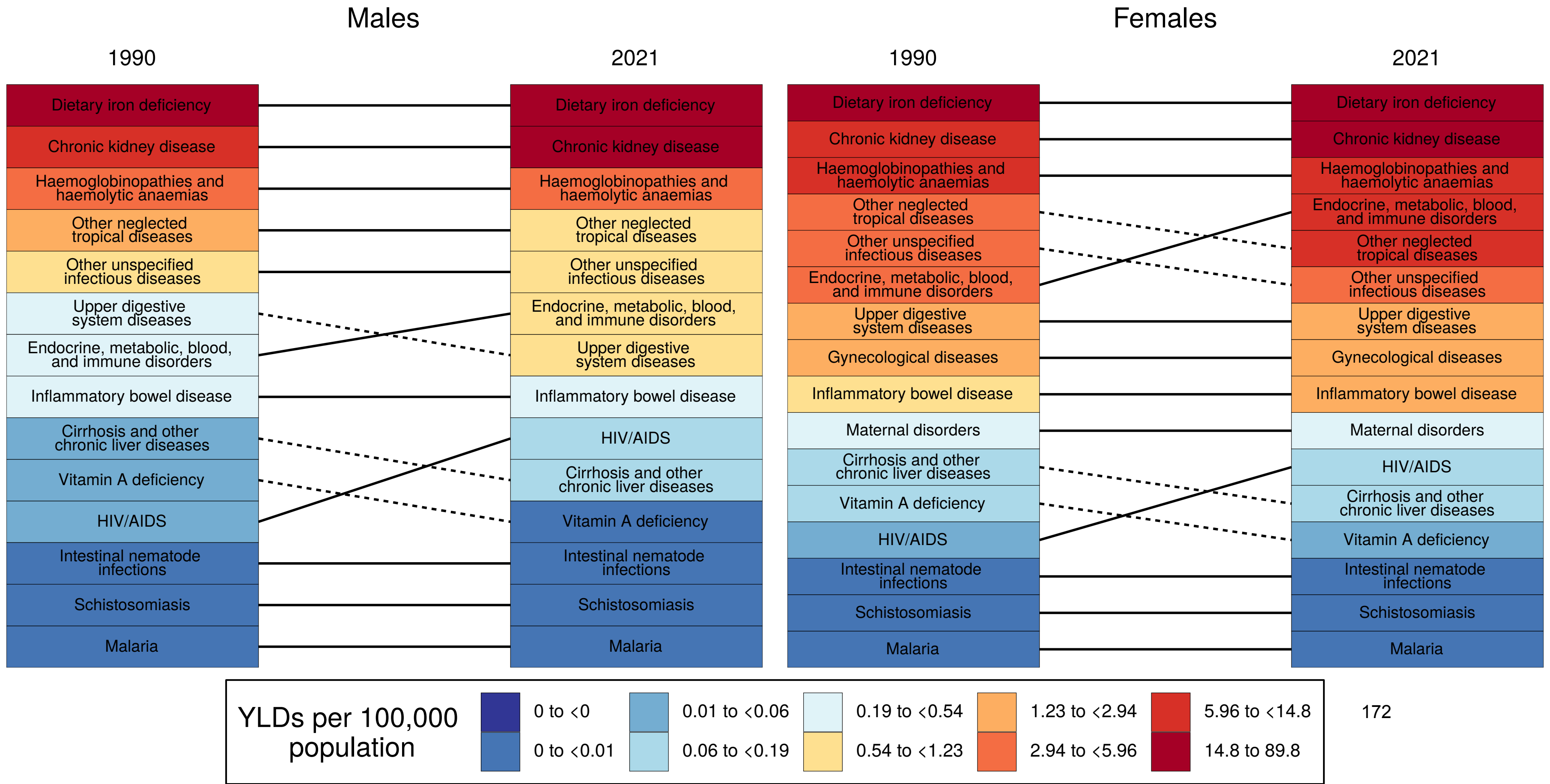

New Jersey

Anaemia Prevalence - Both Sexes

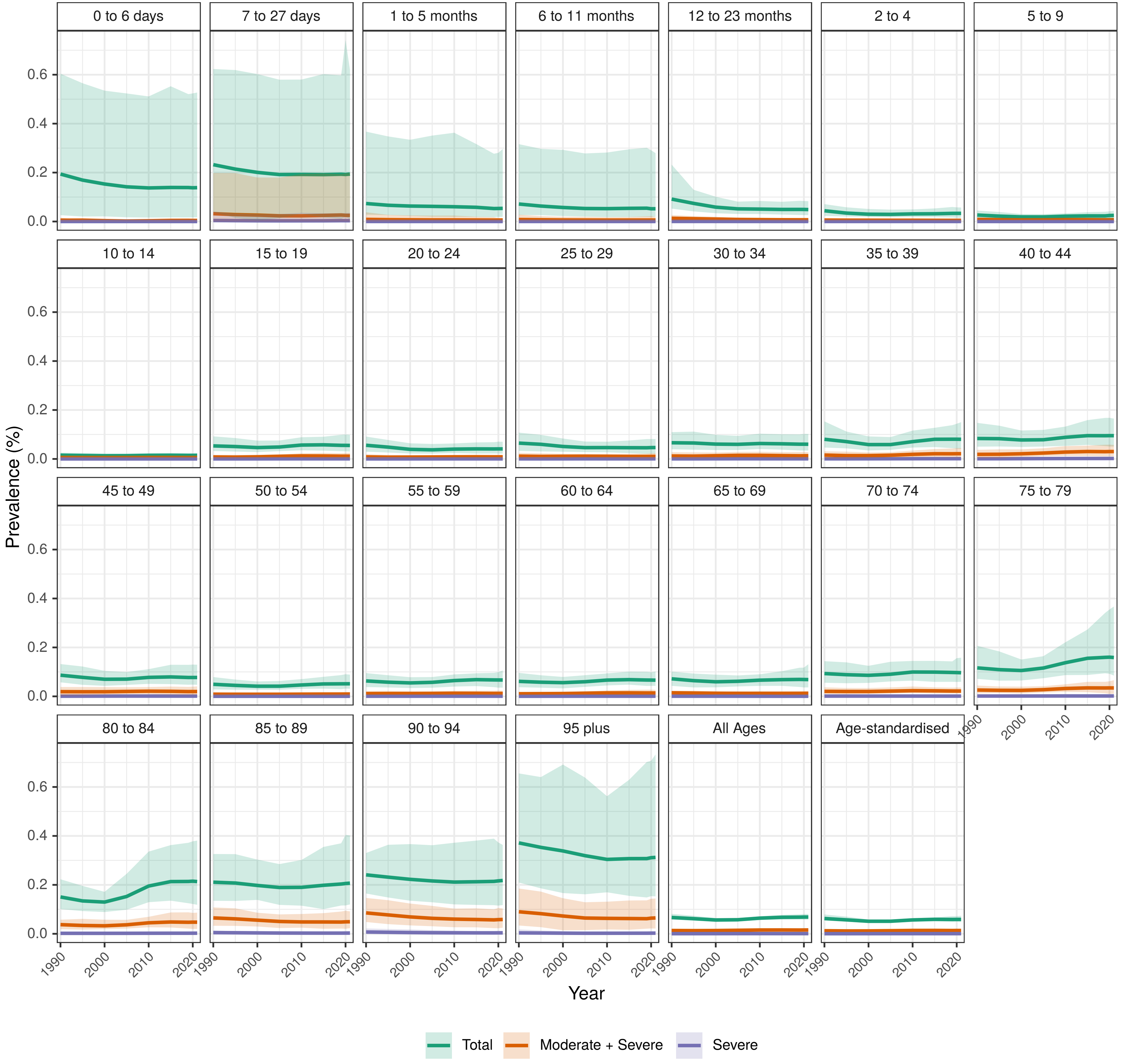

Cause-Specific Anaemia Prevalence - 2021

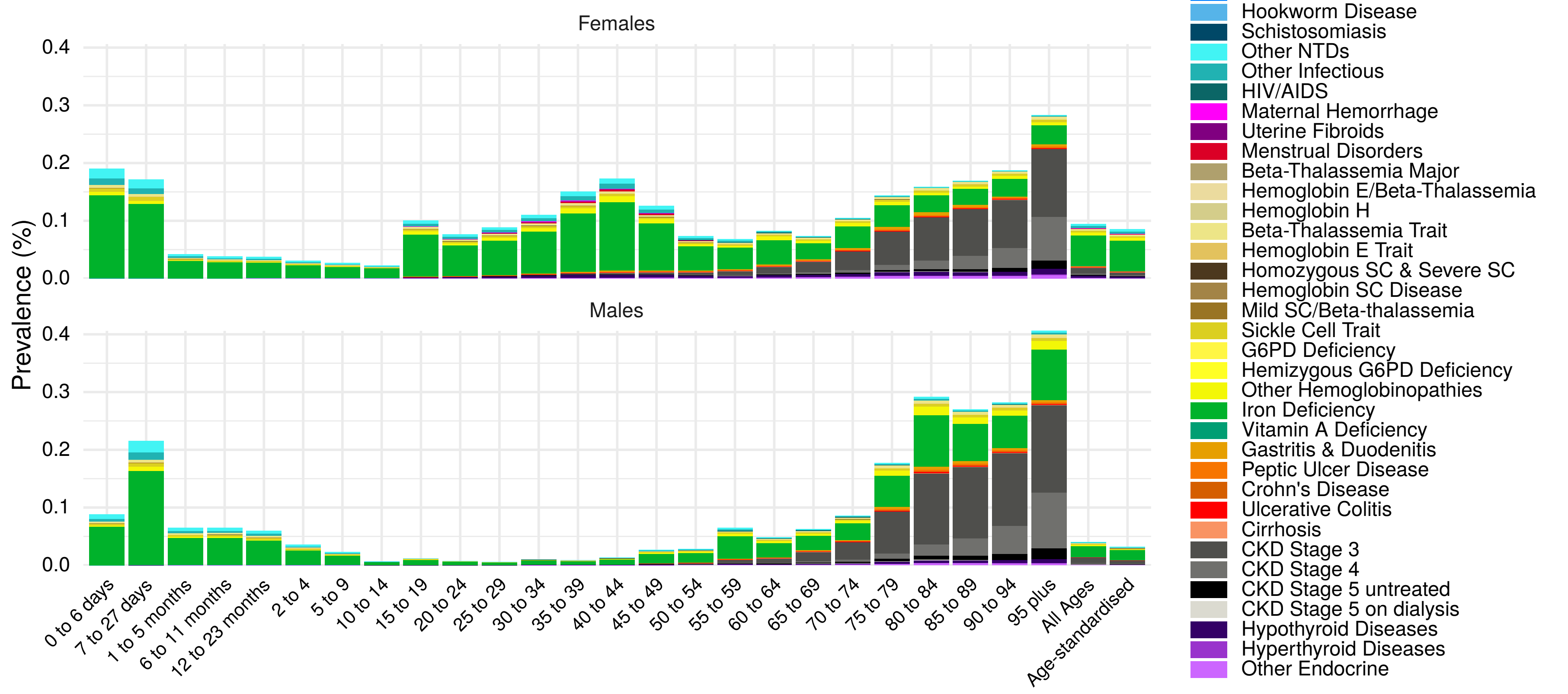

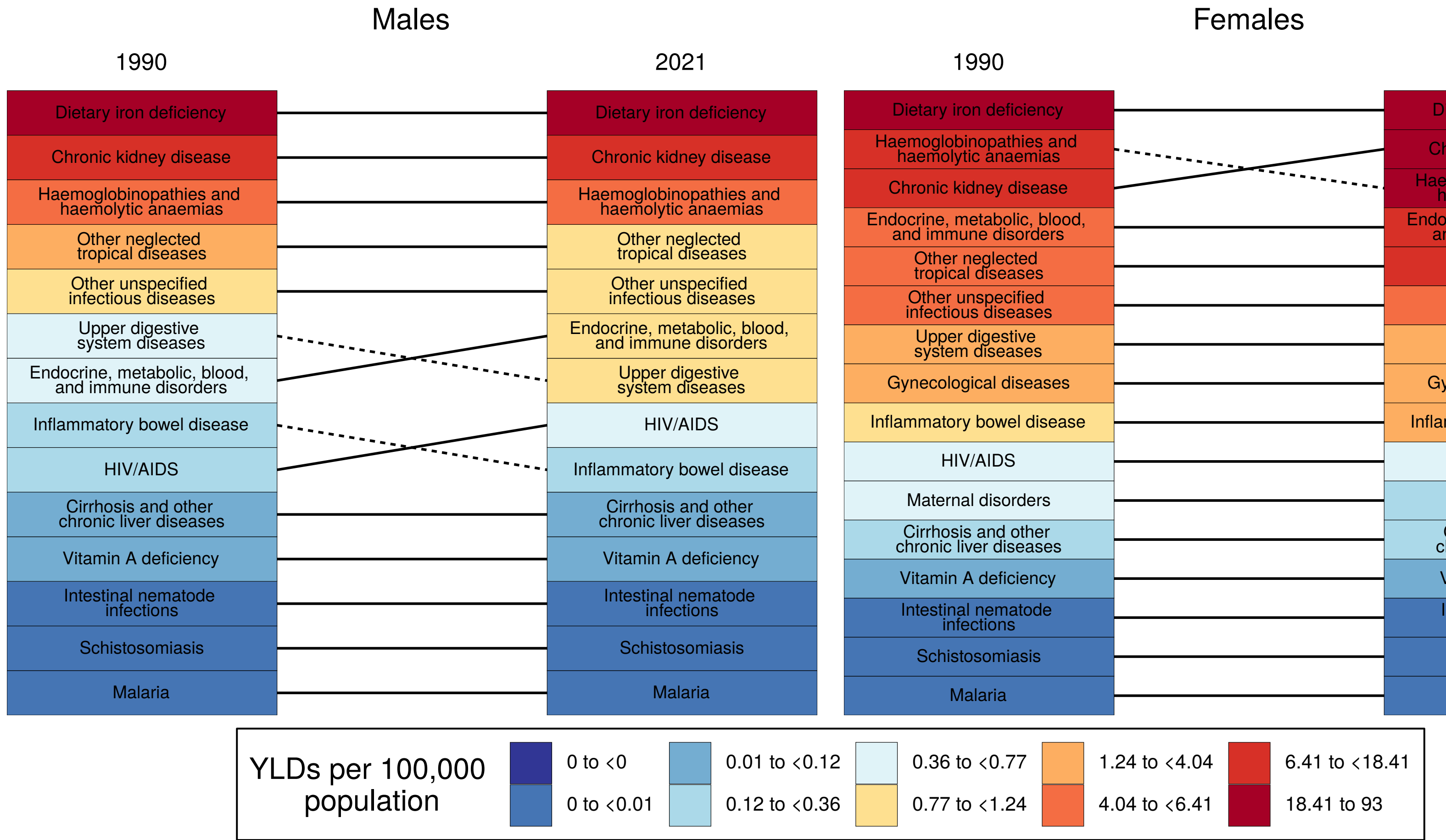

New Mexico

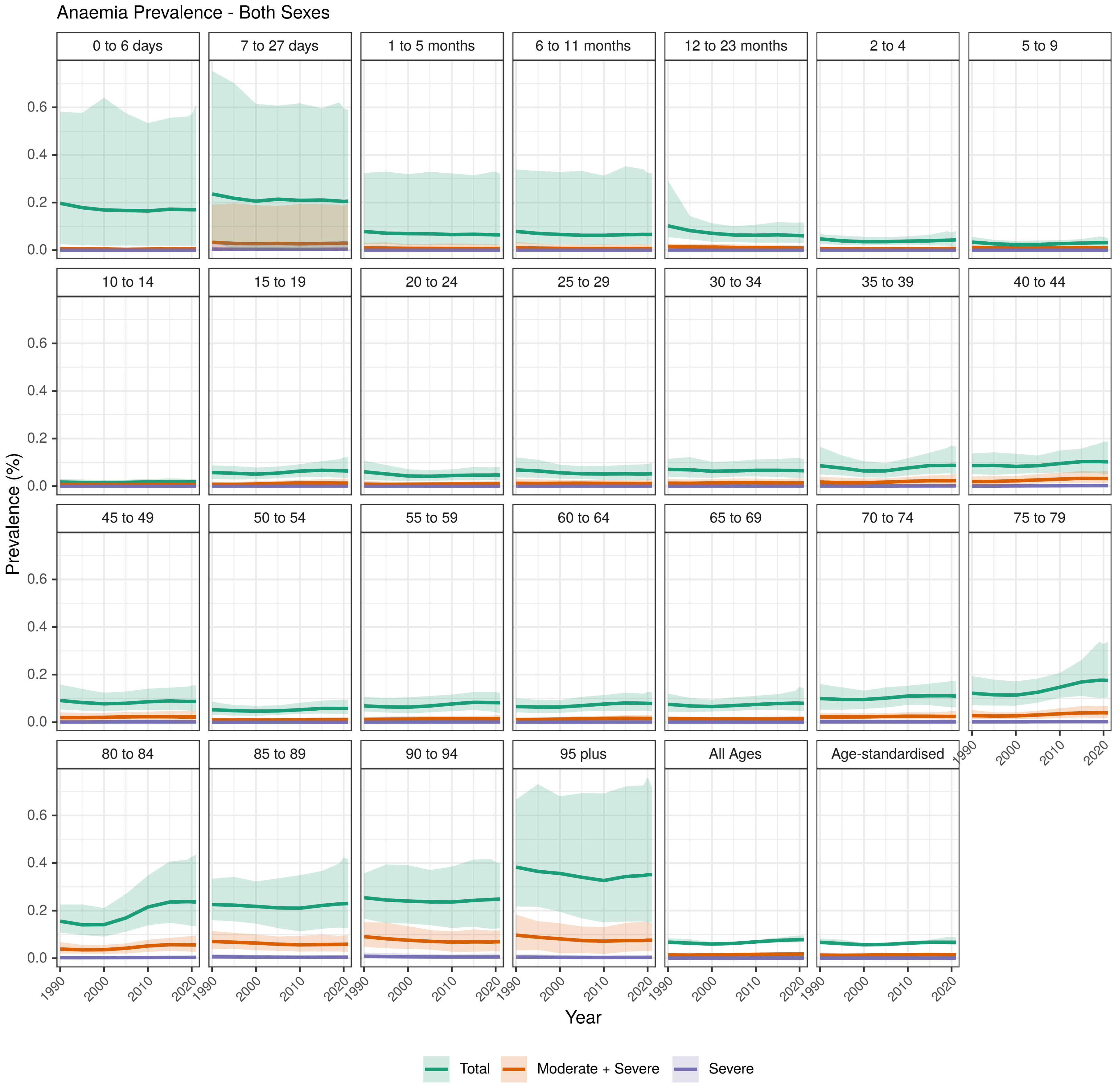
Anaemia Prevalence - Both Sexes
0 to 6 days
7 to 27 days
1 to 5 months
6 to 11 months
12 to 23 months
2 to 4
5 to 9
10 to 14
15 to 19
20 to 24
25 to 29
30 to 34
35 to 39
40 to 44
45 to 49
50 to 54
55 to 59
60 to 64
65 to 69
70 to 74
75 to 79
80 to 84
85 to 89
90 to 94
95 plus
All Ages
Age-standardised
Prevalence (%)
0.0
0.2
0.4
0.6
1990
2000
2010
2020
Year
Total
Moderate + Severe
Severe

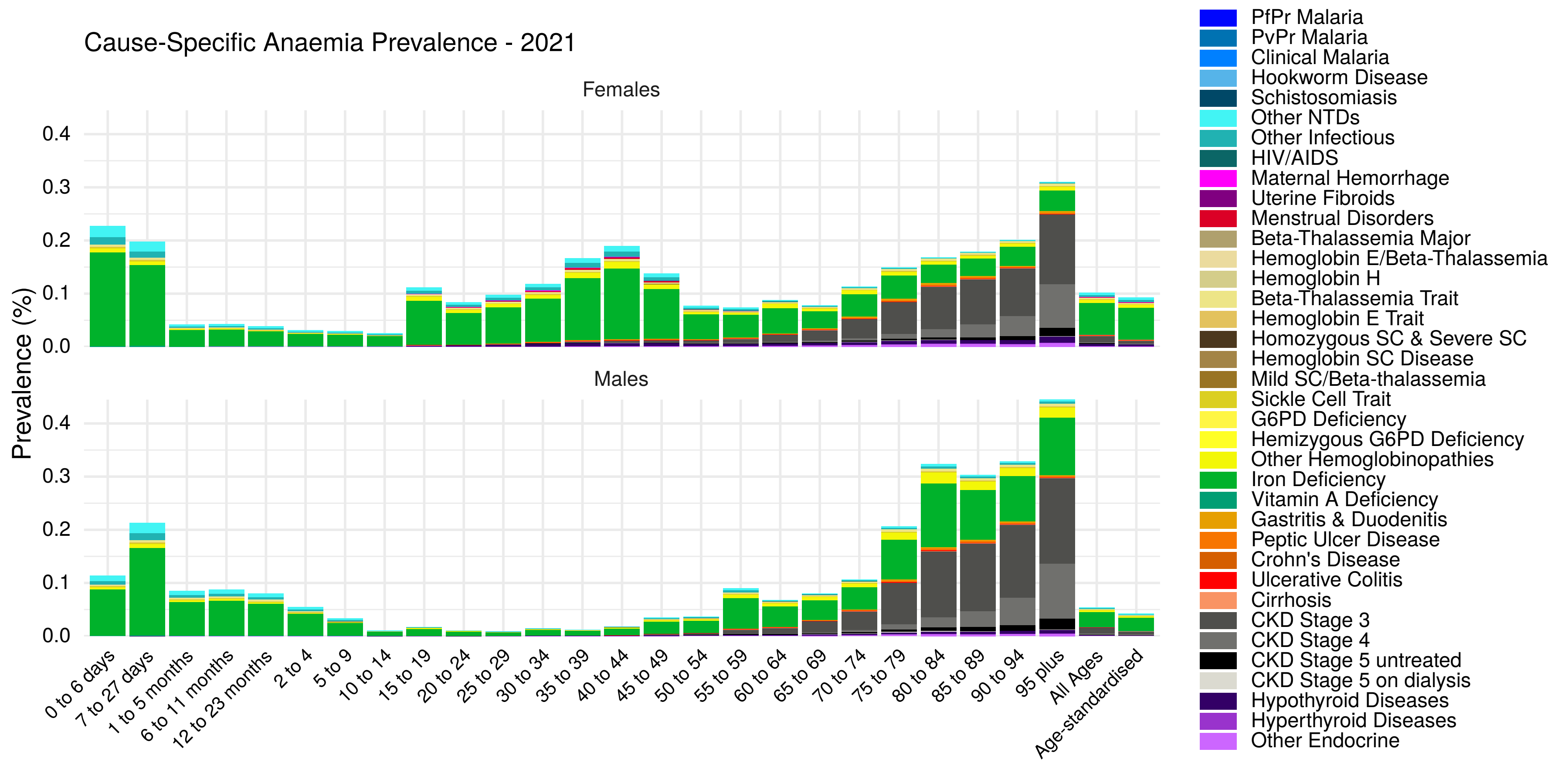
Cause-Specific Anaemia Prevalence - 2021
Females
Males
Prevalence (%)
0.0
0.1
0.2
0.3
0.4
0 to 6 days
7 to 27 days
1 to 5 months
6 to 11 months
12 to 23 months
2 to 4
5 to 9
10 to 14
15 to 19
20 to 24
25 to 29
30 to 34
35 to 39
40 to 44
45 to 49
50 to 54
55 to 59
60 to 64
65 to 69
70 to 74
75 to 79
80 to 84
85 to 89
90 to 94
95 plus
All Ages
Age-standardised
PfPr Malaria
PvPr Malaria
Clinical Malaria
Hookworm Disease
Schistosomiasis
Other NTDs
Other Infectious
HIV/AIDS
Maternal Hemorrhage
Uterine Fibroids
Menstrual Disorders
Beta-Thalassemia Major
Hemoglobin E/Beta-Thalassemia
Hemoglobin H
Beta-Thalassemia Trait
Hemoglobin E Trait
Homozygous SC & Severe SC
Hemoglobin SC Disease
Mild SC/Beta-thalassemia
Sickle Cell Trait
G6PD Deficiency
Hemizygous G6PD Deficiency
Other Hemoglobinopathies
Iron Deficiency
Vitamin A Deficiency
Gastritis & Duodenitis
Peptic Ulcer Disease
Crohn's Disease
Ulcerative Colitis
Cirrhosis
CKD Stage 3
CKD Stage 4
CKD Stage 5 untreated
CKD Stage 5 on dialysis
Hypothyroid Diseases
Hyperthyroid Diseases
Other Endocrine

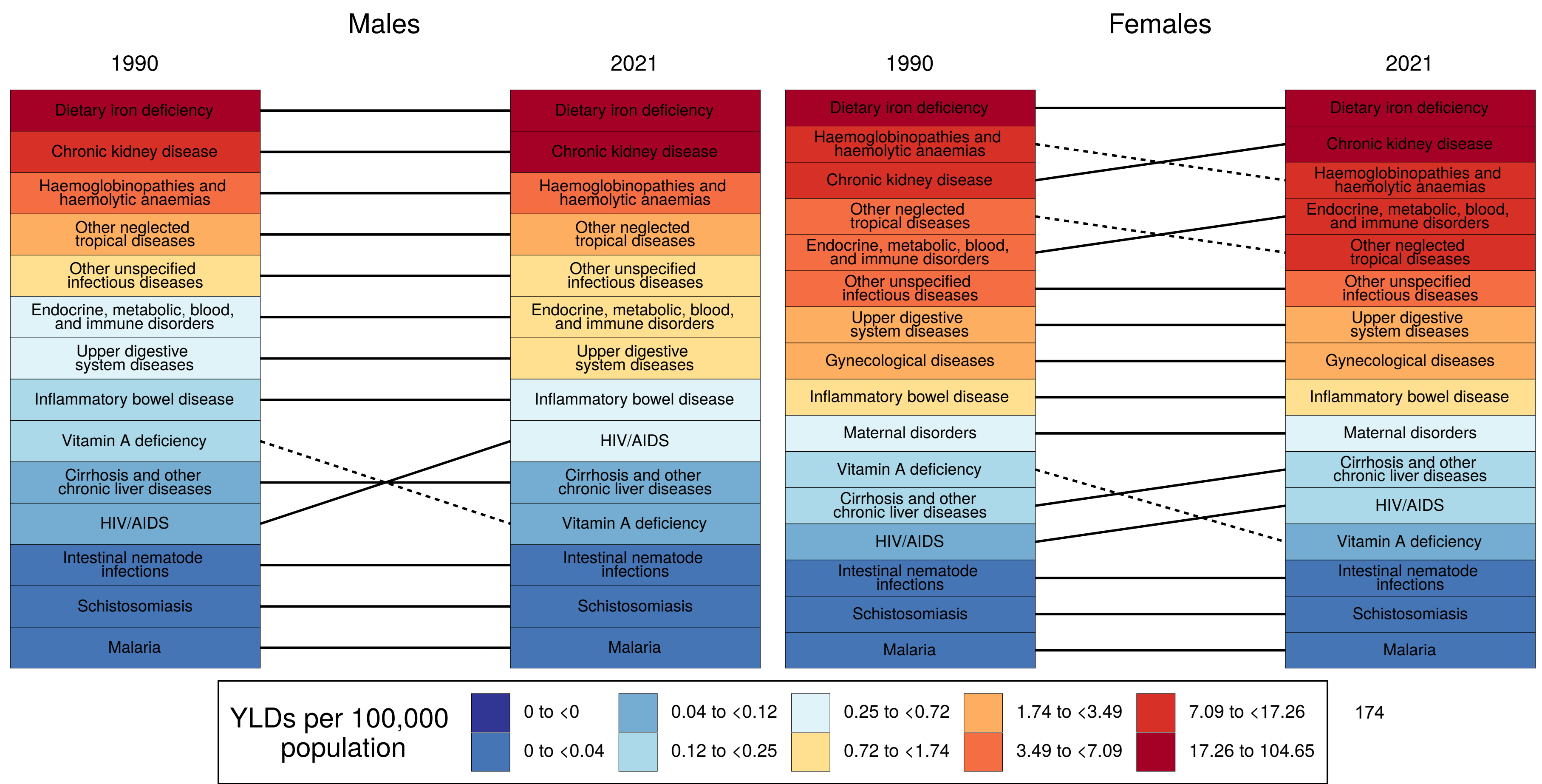
Males
Females
1990
2021
Dietary iron deficiency
Chronic kidney disease
Haemoglobinopathies and haemolytic anaemias
Other neglected tropical diseases
Other unspecified infectious diseases
Endocrine, metabolic, blood, and immune disorders
Upper digestive system diseases
Inflammatory bowel disease
Vitamin A deficiency
Cirrhosis and other chronic liver diseases
HIV/AIDS
Intestinal nematode infections
Schistosomiasis
Malaria
Gynecological diseases
Maternal disorders
YLDs per 100,000 population
0 to <0
0 to <0.04
0.04 to <0.12
0.12 to <0.25
0.25 to <0.72
0.72 to <1.74
1.74 to <3.49
3.49 to <7.09
7.09 to <17.26
17.26 to 104.65

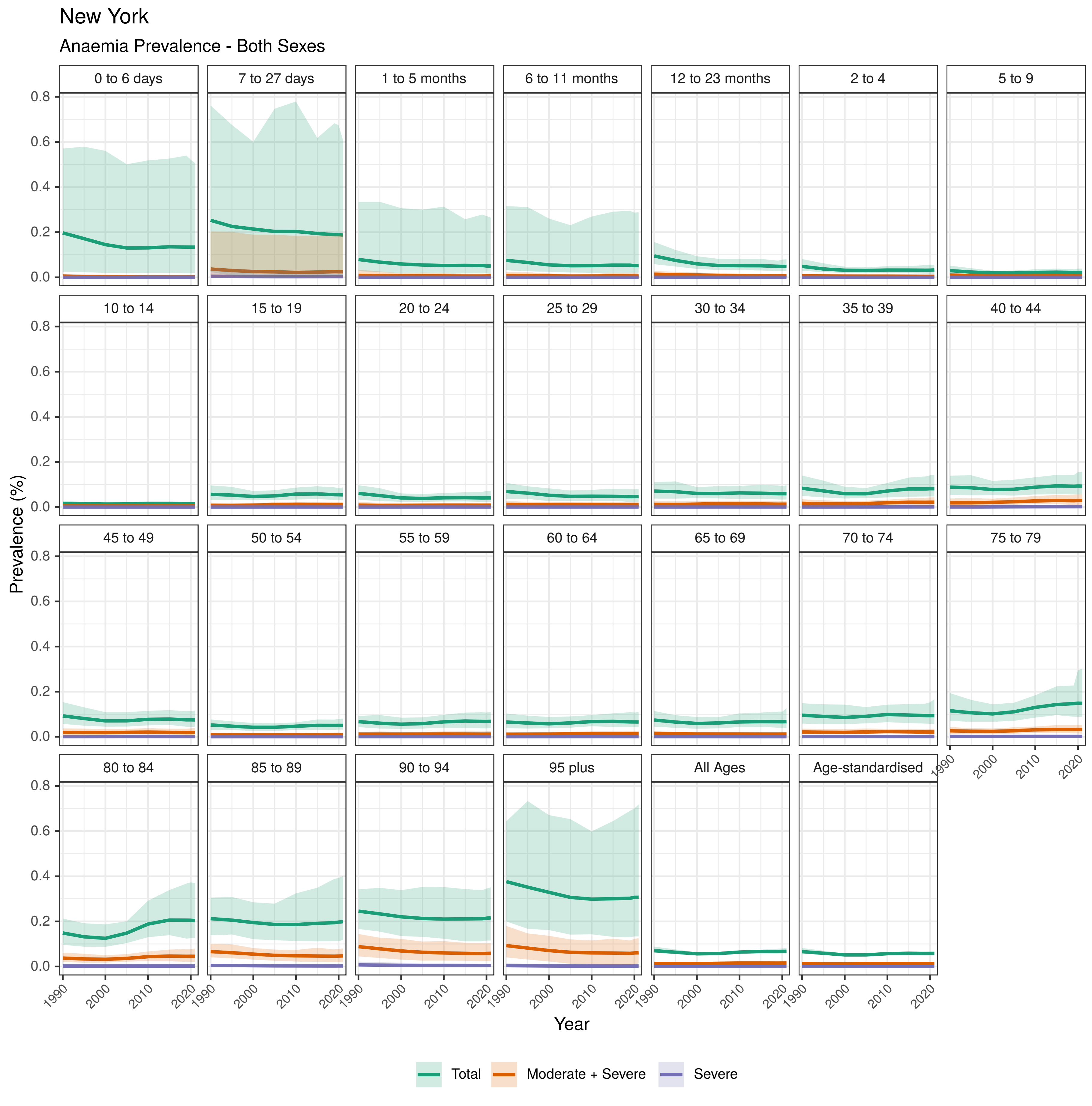

New York
Anaemia Prevalence - Both Sexes
0 to 6 days
7 to 27 days
1 to 5 months
6 to 11 months
12 to 23 months
2 to 4
5 to 9
10 to 14
15 to 19
20 to 24
25 to 29
30 to 34
35 to 39
40 to 44
45 to 49
50 to 54
55 to 59
60 to 64
65 to 69
70 to 74
75 to 79
80 to 84
85 to 89
90 to 94
95 plus
All Ages
Age-standardised
Prevalence (%)
Year
Total
Moderate + Severe
Severe

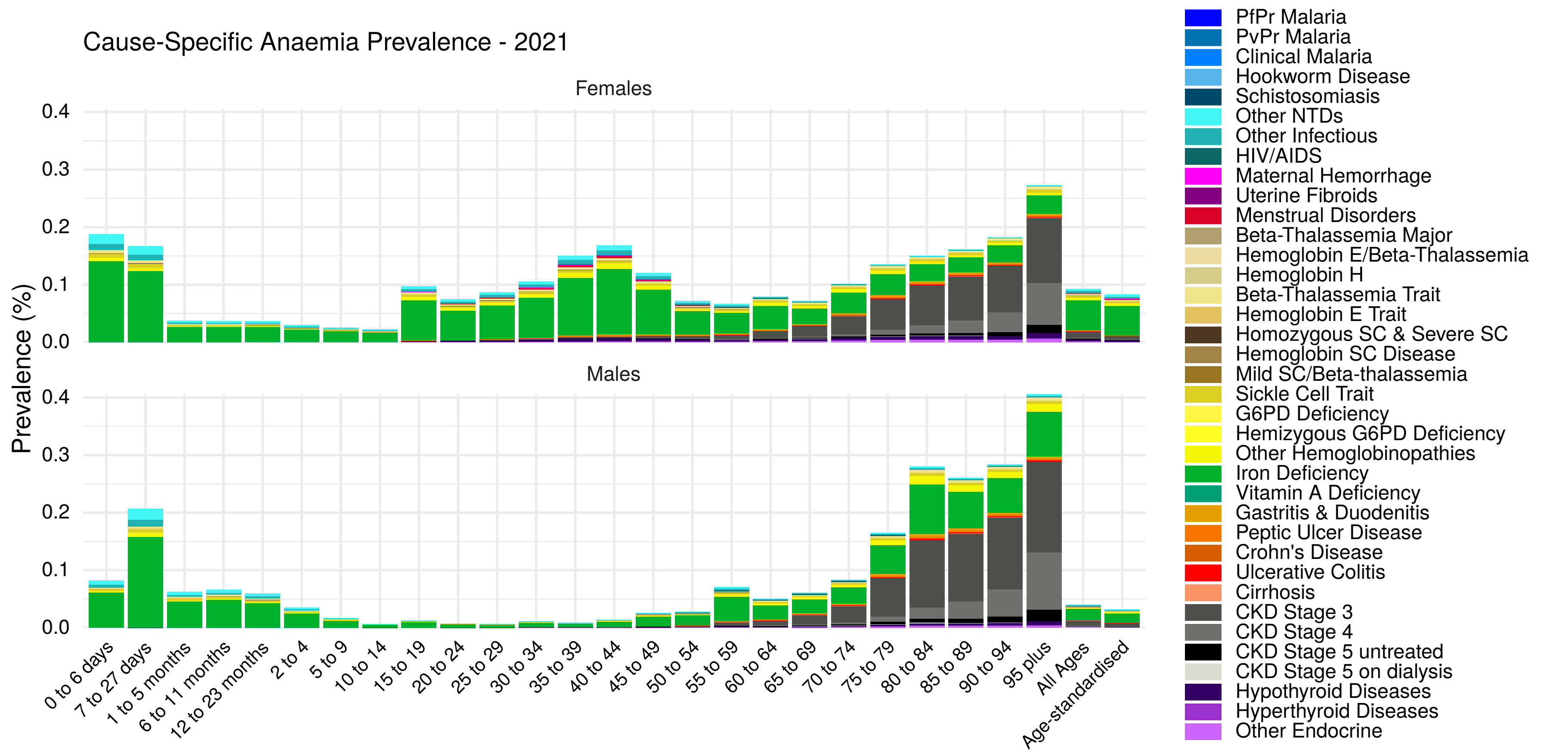

Cause-Specific Anaemia Prevalence - 2021
Females
Males
Prevalence (%)
PfPr Malaria
PvPr Malaria
Clinical Malaria
Hookworm Disease
Schistosomiasis
Other NTDs
Other Infectious
HIV/AIDS
Maternal Hemorrhage
Uterine Fibroids
Menstrual Disorders
Beta-Thalassemia Major
Hemoglobin E/Beta-Thalassemia
Hemoglobin H
Beta-Thalassemia Trait
Hemoglobin E Trait
Homozygous SC & Severe SC
Hemoglobin SC Disease
Mild SC/Beta-thalassemia
Sickle Cell Trait
G6PD Deficiency
Hemizygous G6PD Deficiency
Other Hemoglobinopathies
Iron Deficiency
Vitamin A Deficiency
Gastritis & Duodenitis
Peptic Ulcer Disease
Crohn's Disease
Ulcerative Colitis
Cirrhosis
CKD Stage 3
CKD Stage 4
CKD Stage 5 untreated
CKD Stage 5 on dialysis
Hypothyroid Diseases
Hyperthyroid Diseases
Other Endocrine

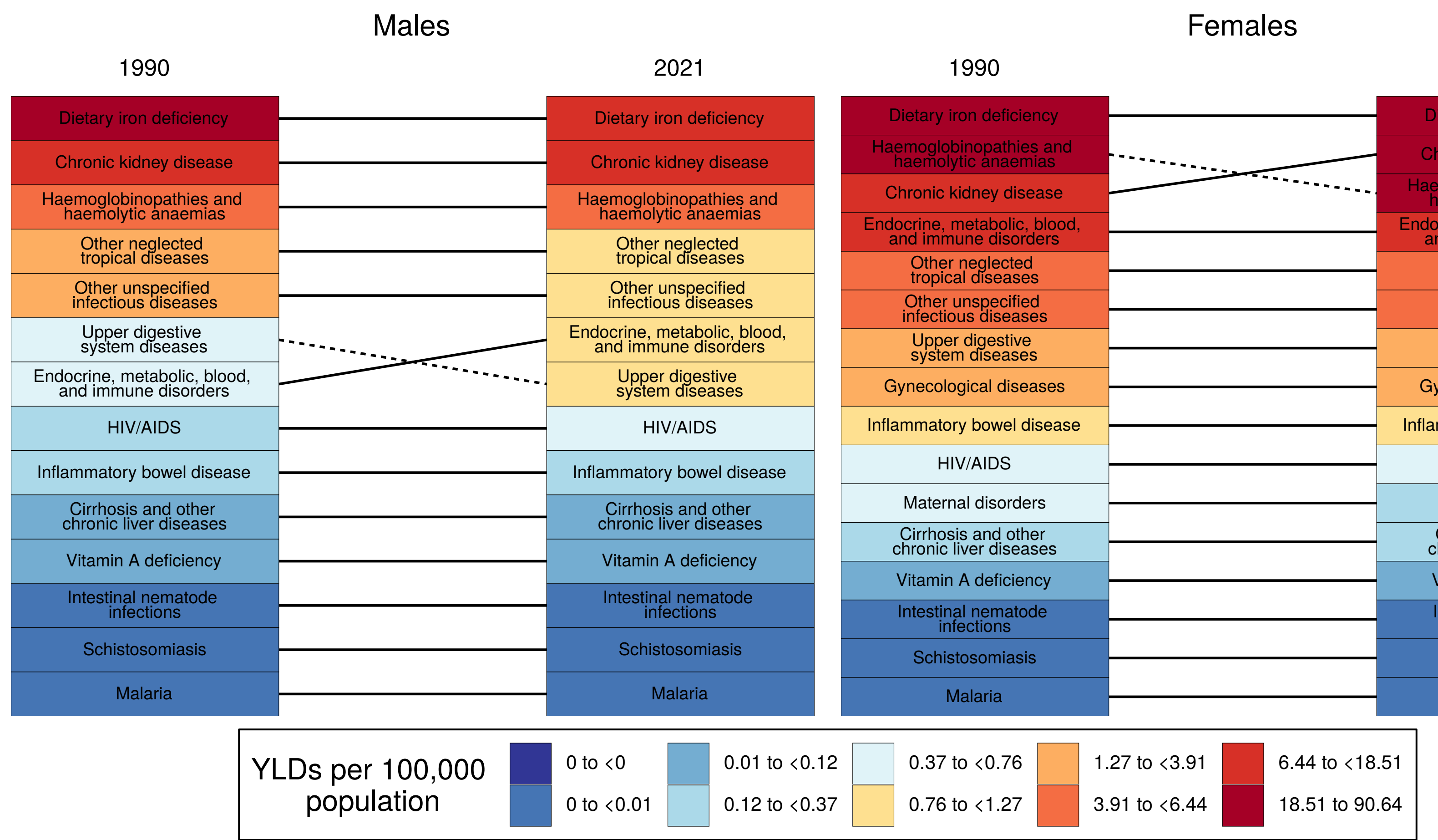

Males
Females
1990
2021
Dietary iron deficiency
Chronic kidney disease
Haemoglobinopathies and haemolytic anaemias
Other neglected tropical diseases
Other unspecified infectious diseases
Upper digestive system diseases
Endocrine, metabolic, blood, and immune disorders
HIV/AIDS
Inflammatory bowel disease
Cirrhosis and other chronic liver diseases
Vitamin A deficiency
Intestinal nematode infections
Schistosomiasis
Malaria
Gynecological diseases
Maternal disorders
YLDs per 100,000 population
0 to <0
0 to <0.01
0.01 to <0.12
0.12 to <0.37
0.37 to <0.76
0.76 to <1.27
1.27 to <3.91
3.91 to <6.44
6.44 to <18.51
18.51 to 90.64

North Carolina

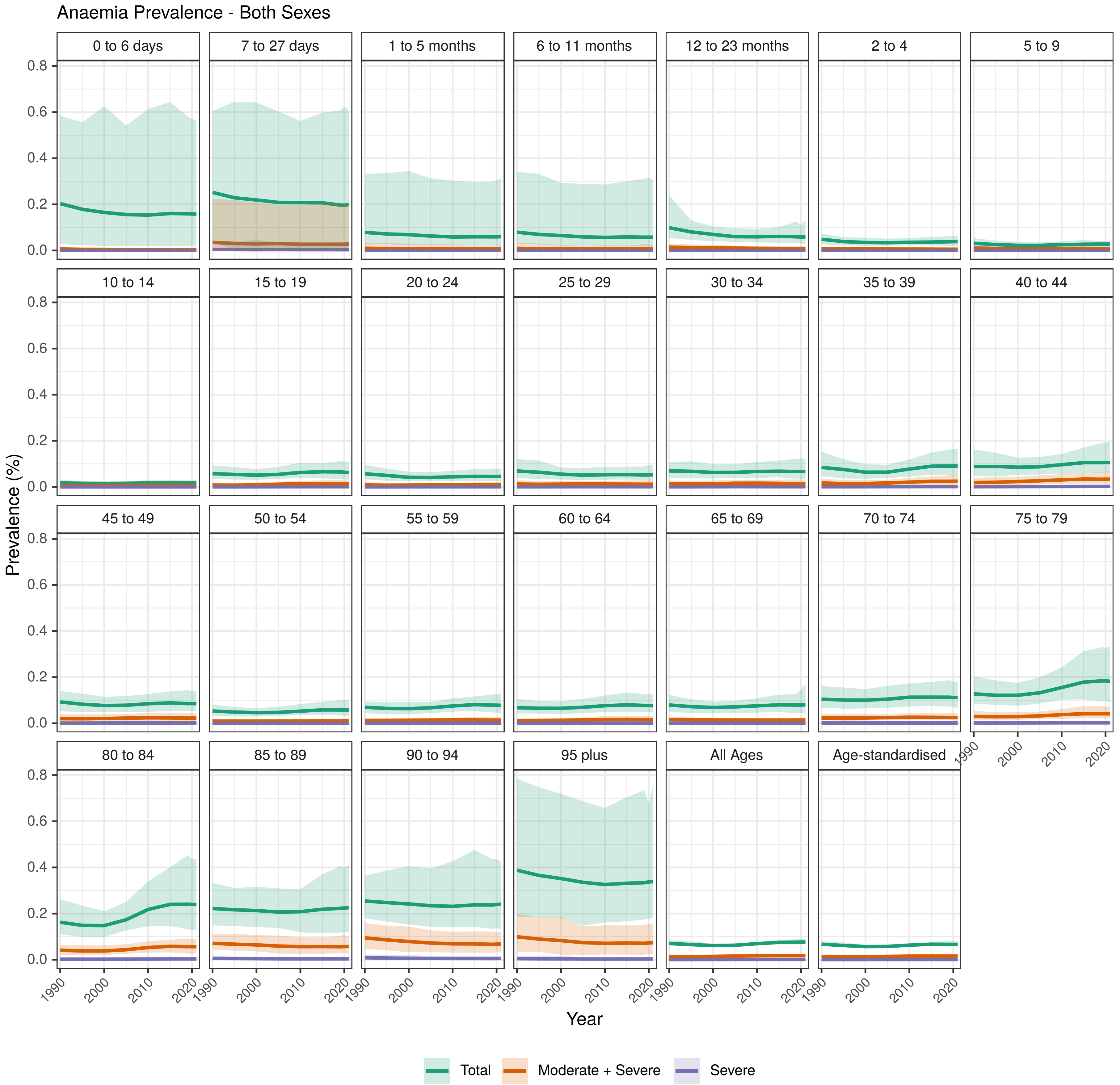

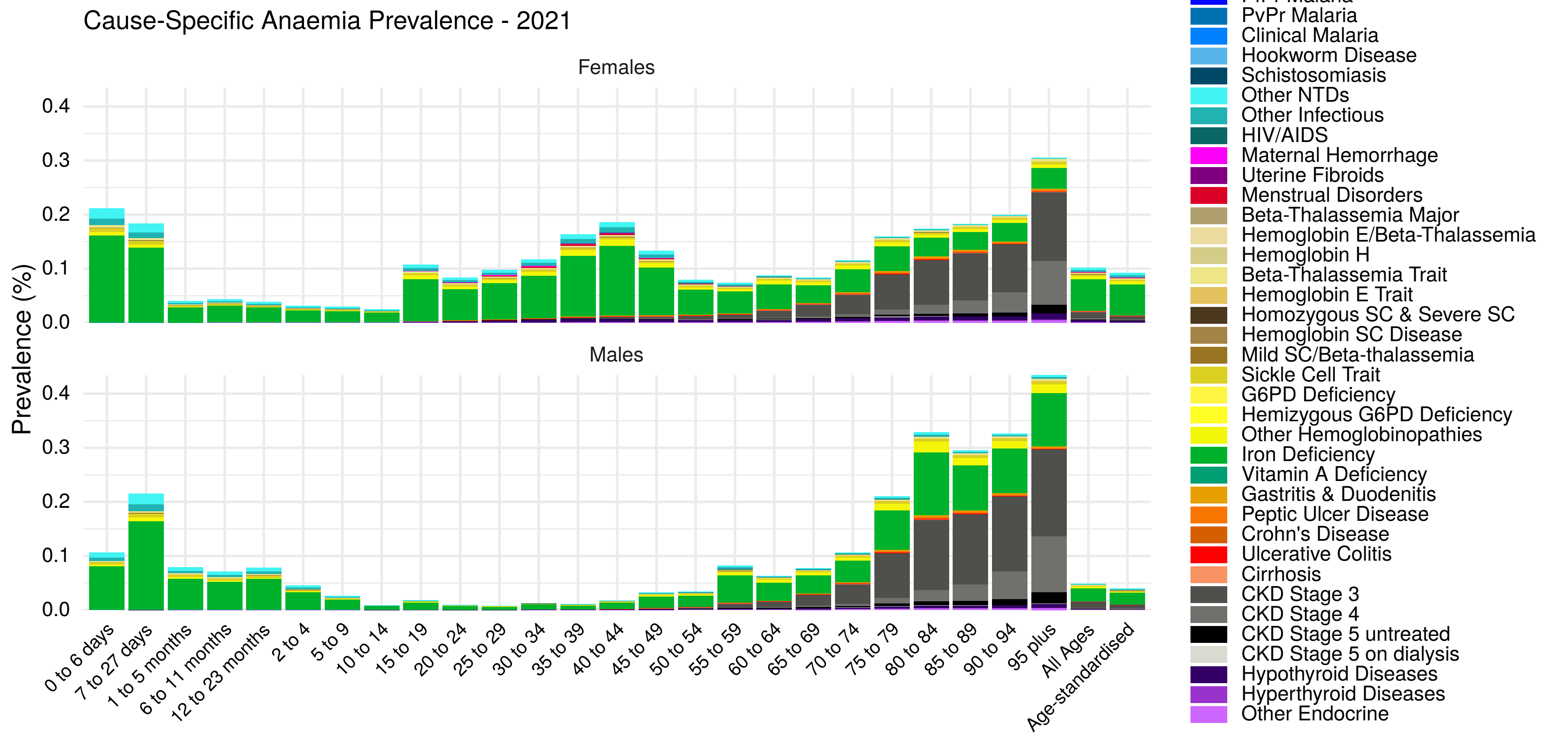

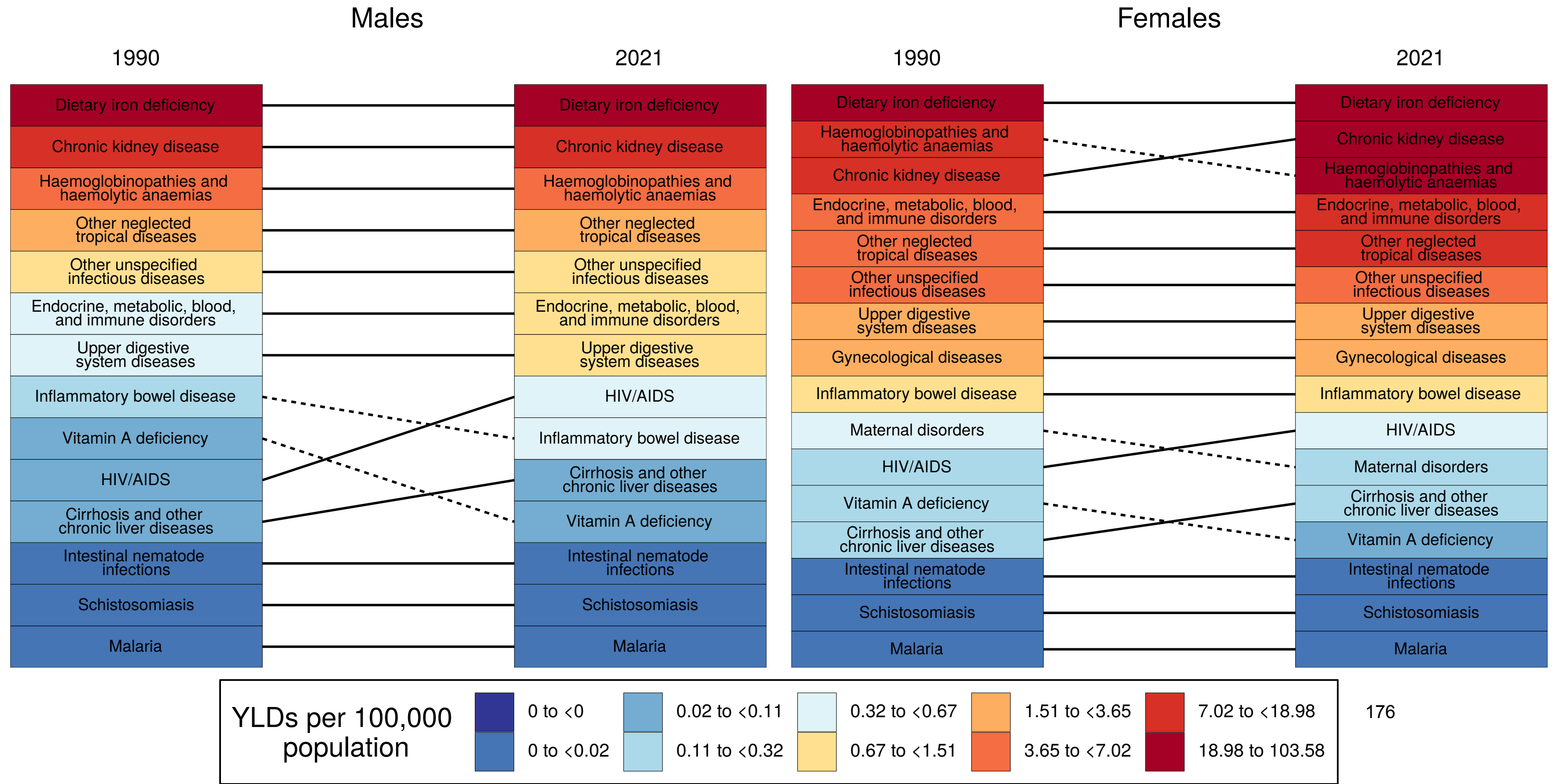

North Dakota

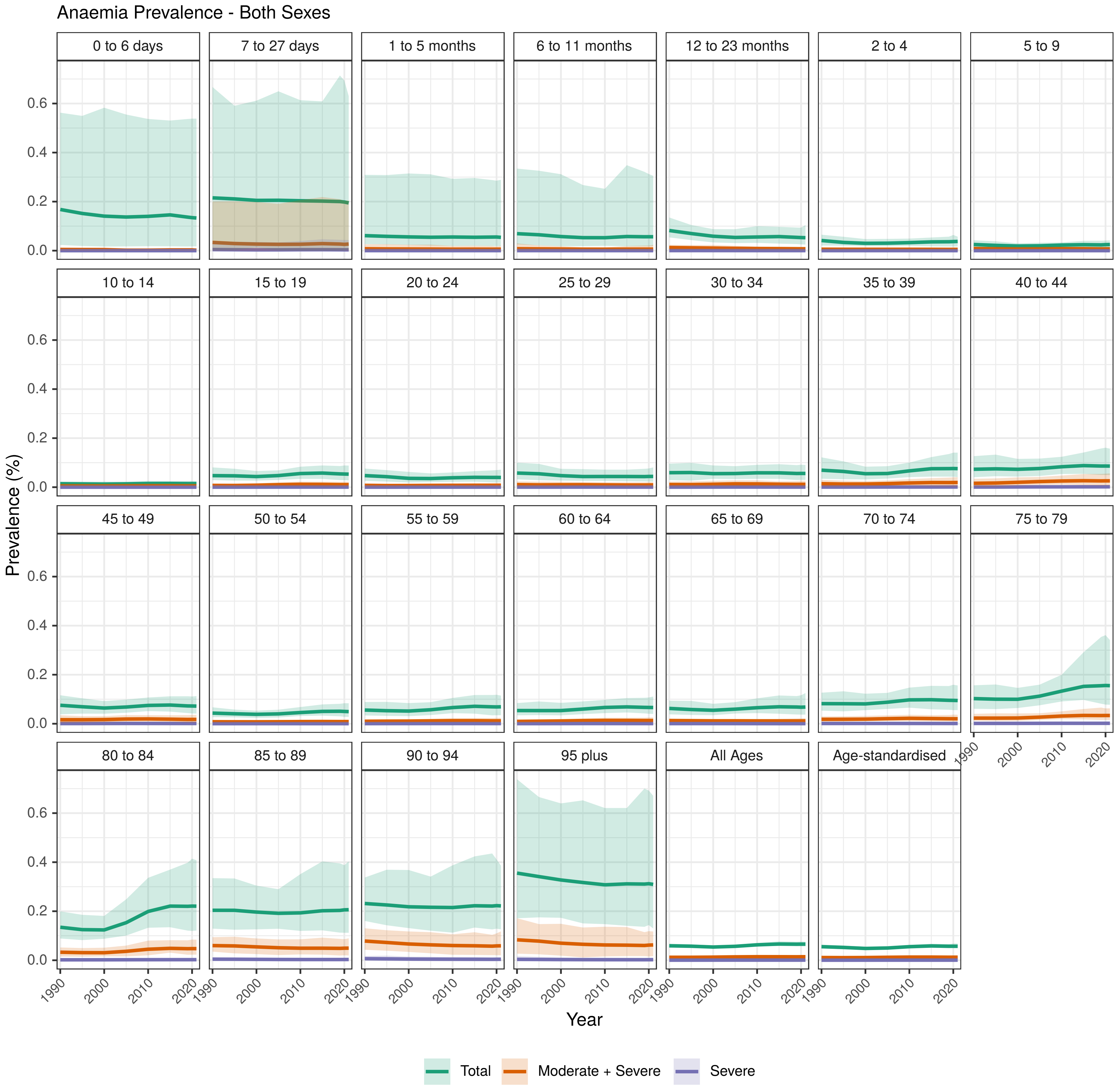

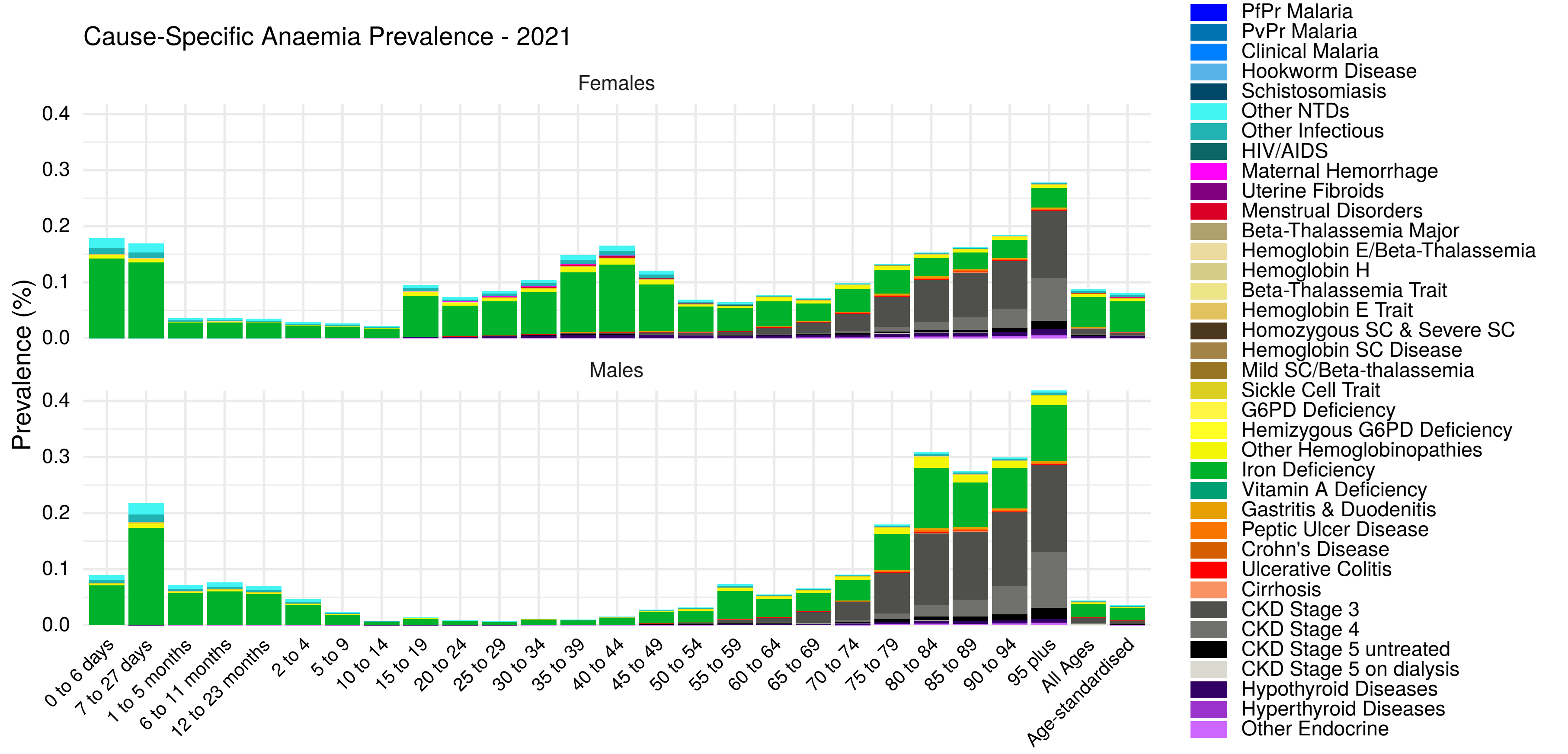

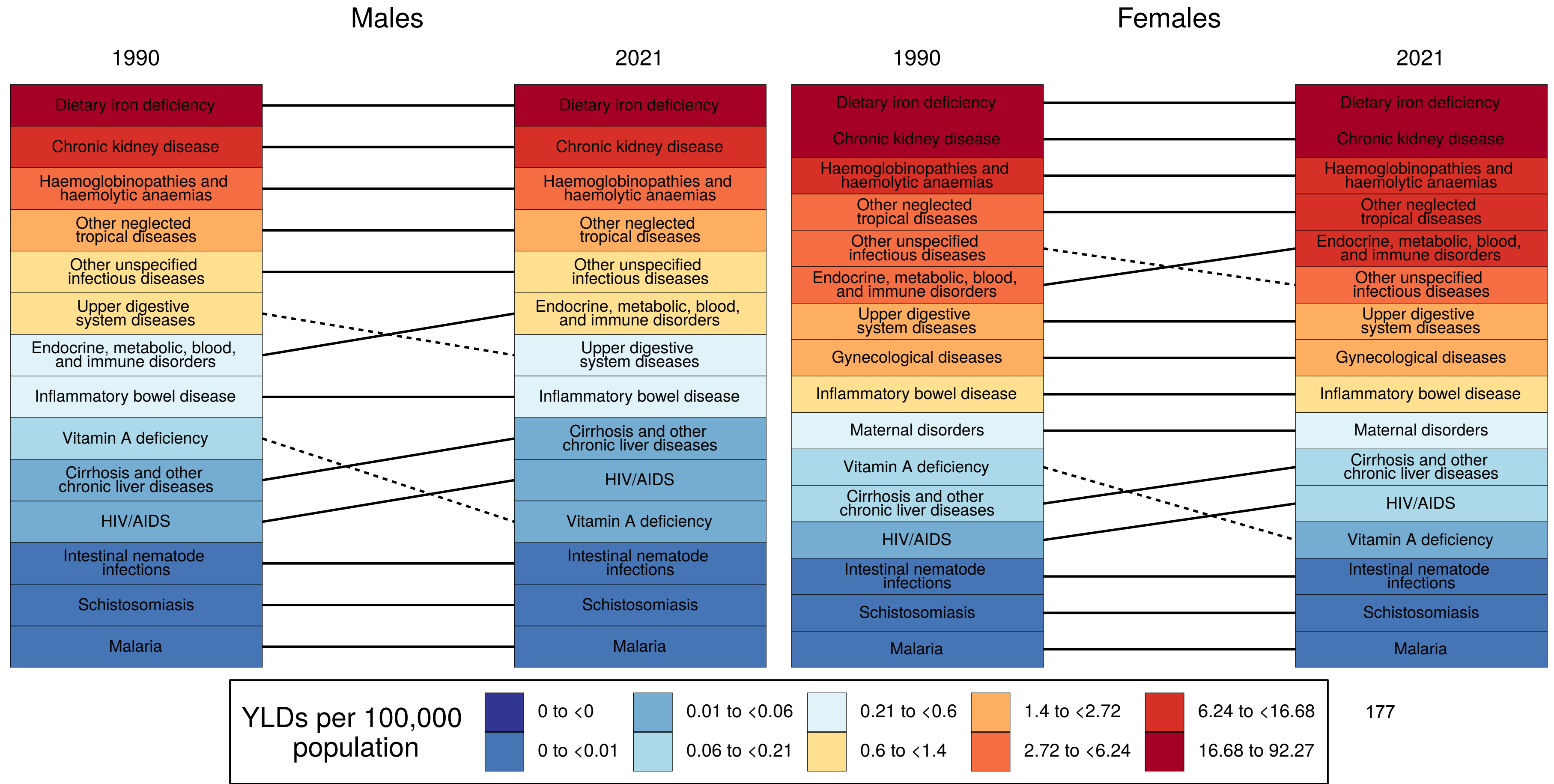

Ohio

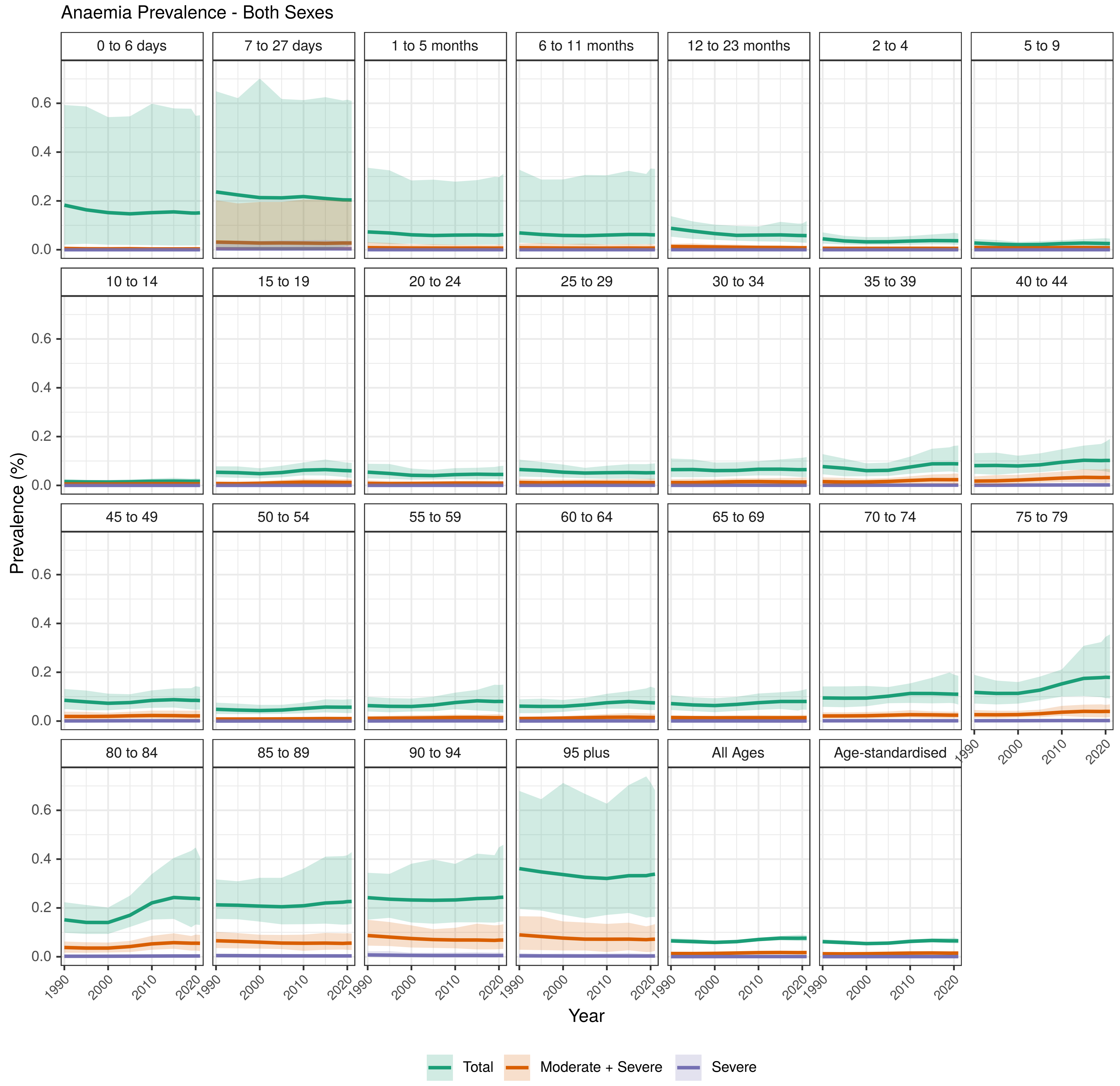

Anaemia Prevalence - Both Sexes
0 to 6 days
7 to 27 days
1 to 5 months
6 to 11 months
12 to 23 months
2 to 4
5 to 9
10 to 14
15 to 19
20 to 24
25 to 29
30 to 34
35 to 39
40 to 44
45 to 49
50 to 54
55 to 59
60 to 64
65 to 69
70 to 74
75 to 79
80 to 84
85 to 89
90 to 94
95 plus
All Ages
Age-standardised
Prevalence (%)
0.0
0.2
0.4
0.6
1990
2000
2010
2020
Year
Total
Moderate + Severe
Severe

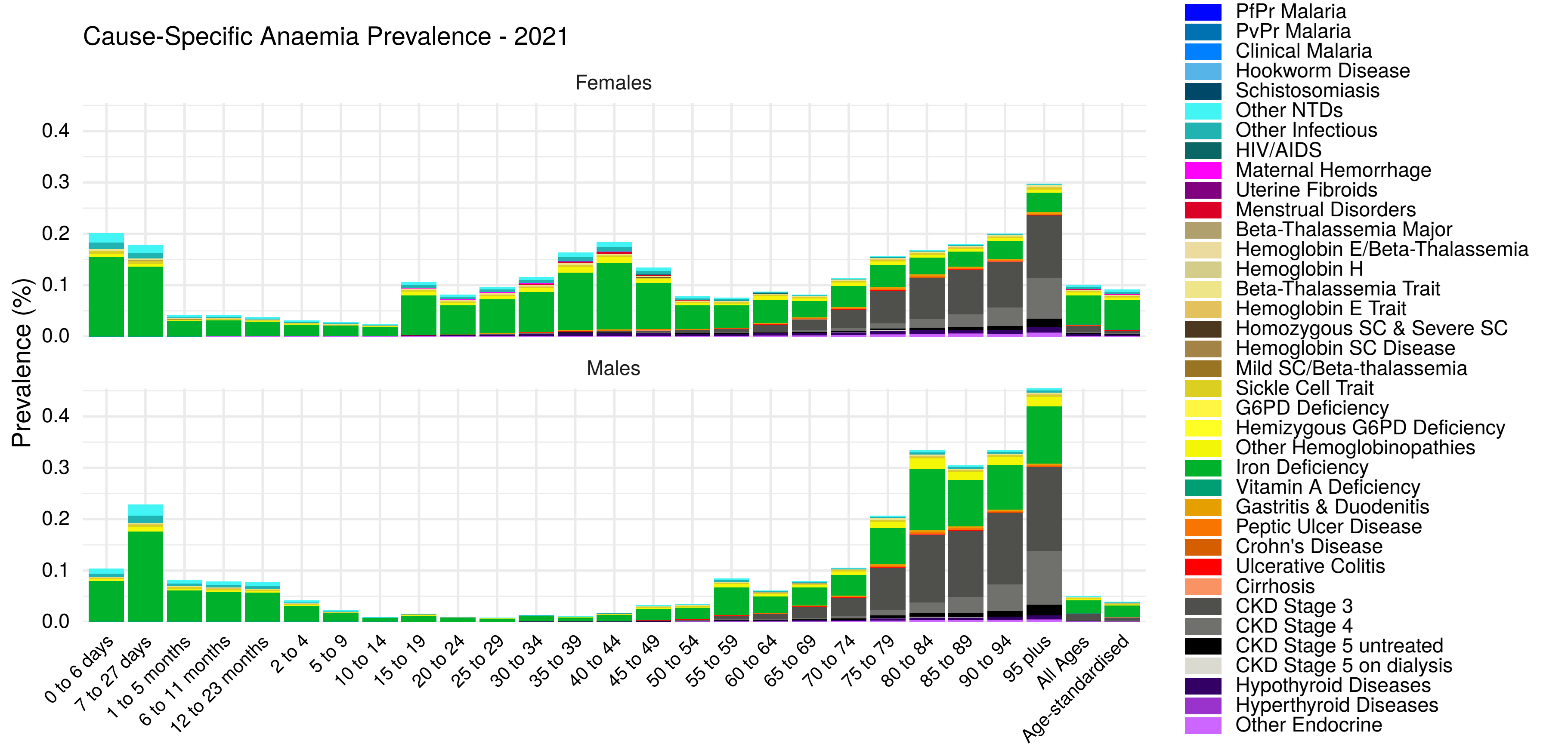

Cause-Specific Anaemia Prevalence - 2021
Females
Males
Prevalence (%)
0.0
0.1
0.2
0.3
0.4
0 to 6 days
7 to 27 days
1 to 5 months
6 to 11 months
12 to 23 months
2 to 4
5 to 9
10 to 14
15 to 19
20 to 24
25 to 29
30 to 34
35 to 39
40 to 44
45 to 49
50 to 54
55 to 59
60 to 64
65 to 69
70 to 74
75 to 79
80 to 84
85 to 89
90 to 94
95 plus
All Ages
Age-standardised
PfPr Malaria
PvPr Malaria
Clinical Malaria
Hookworm Disease
Schistosomiasis
Other NTDs
Other Infectious
HIV/AIDS
Maternal Hemorrhage
Uterine Fibroids
Menstrual Disorders
Beta-Thalassemia Major
Hemoglobin E/Beta-Thalassemia
Hemoglobin H
Beta-Thalassemia Trait
Hemoglobin E Trait
Homozygous SC & Severe SC
Hemoglobin SC Disease
Mild SC/Beta-thalassemia
Sickle Cell Trait
G6PD Deficiency
Hemizygous G6PD Deficiency
Other Hemoglobinopathies
Iron Deficiency
Vitamin A Deficiency
Gastritis & Duodenitis
Peptic Ulcer Disease
Crohn's Disease
Ulcerative Colitis
Cirrhosis
CKD Stage 3
CKD Stage 4
CKD Stage 5 untreated
CKD Stage 5 on dialysis
Hypothyroid Diseases
Hyperthyroid Diseases
Other Endocrine

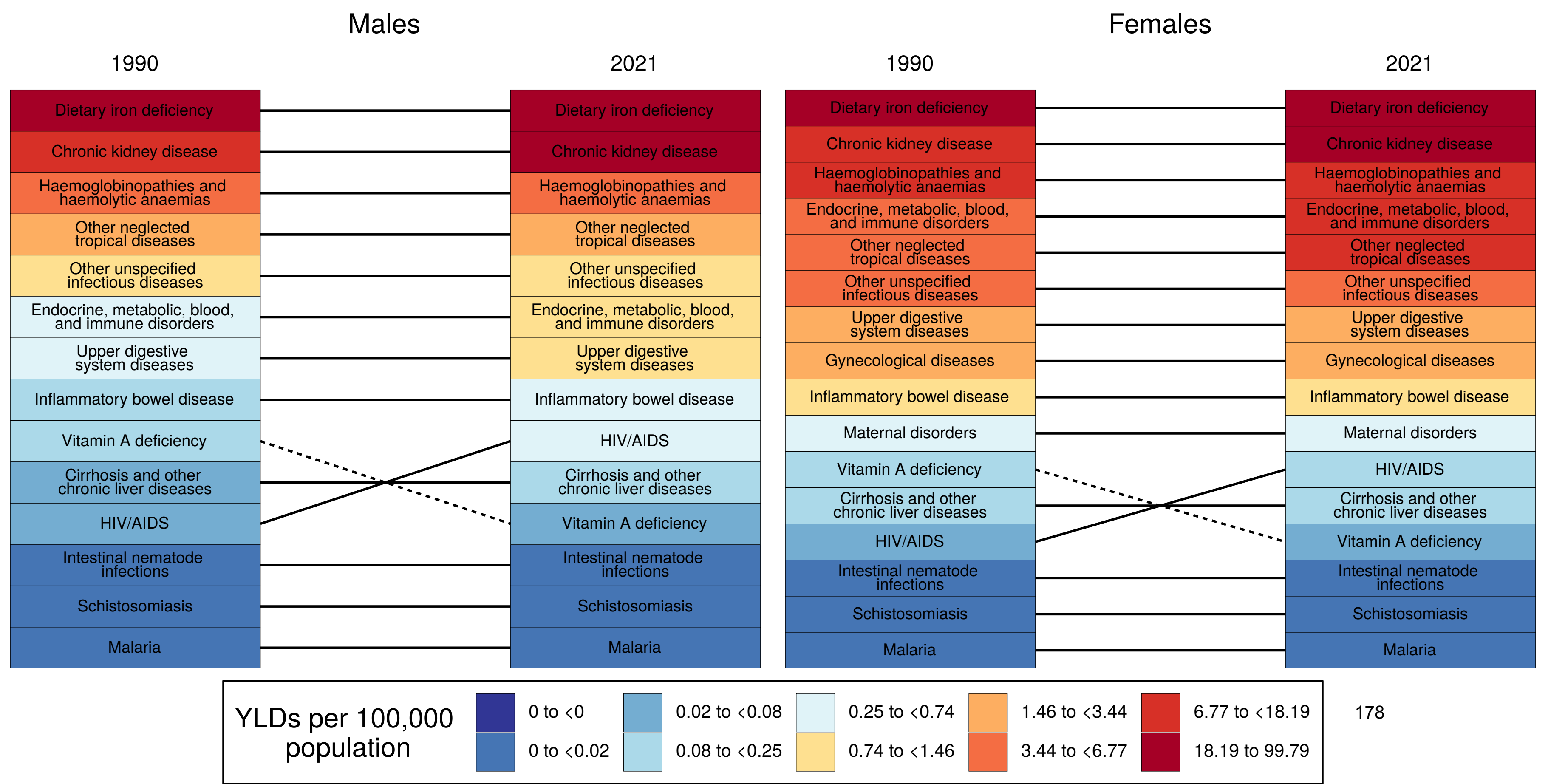

Males
Females
1990
2021
Dietary iron deficiency
Chronic kidney disease
Haemoglobinopathies and haemolytic anaemias
Other neglected tropical diseases
Other unspecified infectious diseases
Endocrine, metabolic, blood, and immune disorders
Upper digestive system diseases
Inflammatory bowel disease
Vitamin A deficiency
Cirrhosis and other chronic liver diseases
HIV/AIDS
Intestinal nematode infections
Schistosomiasis
Malaria
Gynecological diseases
Maternal disorders
YLDs per 100,000 population
0 to <0
0 to <0.02
0.02 to <0.08
0.08 to <0.25
0.25 to <0.74
0.74 to <1.46
1.46 to <3.44
3.44 to <6.77
6.77 to <18.19
18.19 to 99.79

Oklahoma

Anaemia Prevalence - Both Sexes

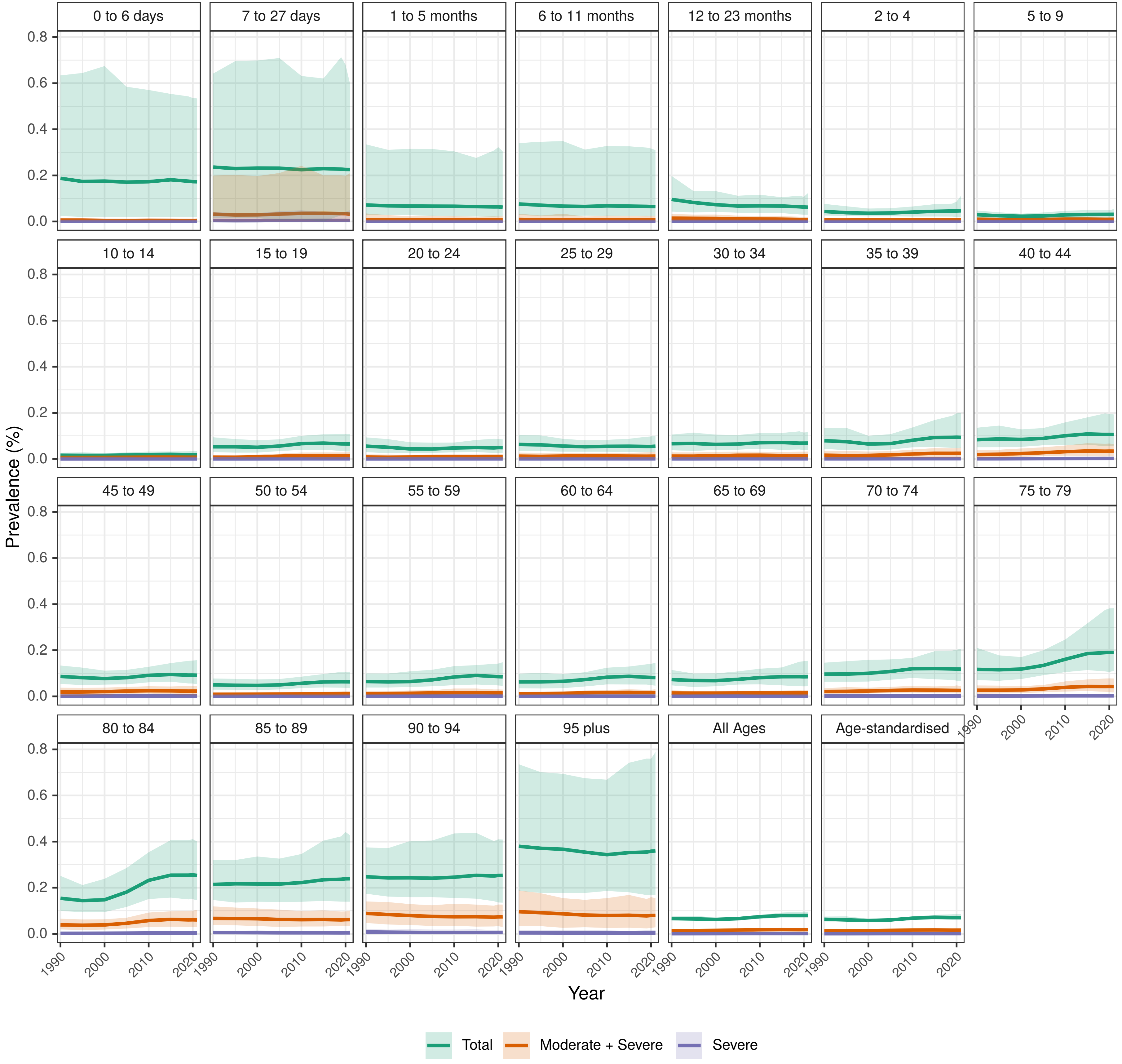

Cause-Specific Anaemia Prevalence - 2021

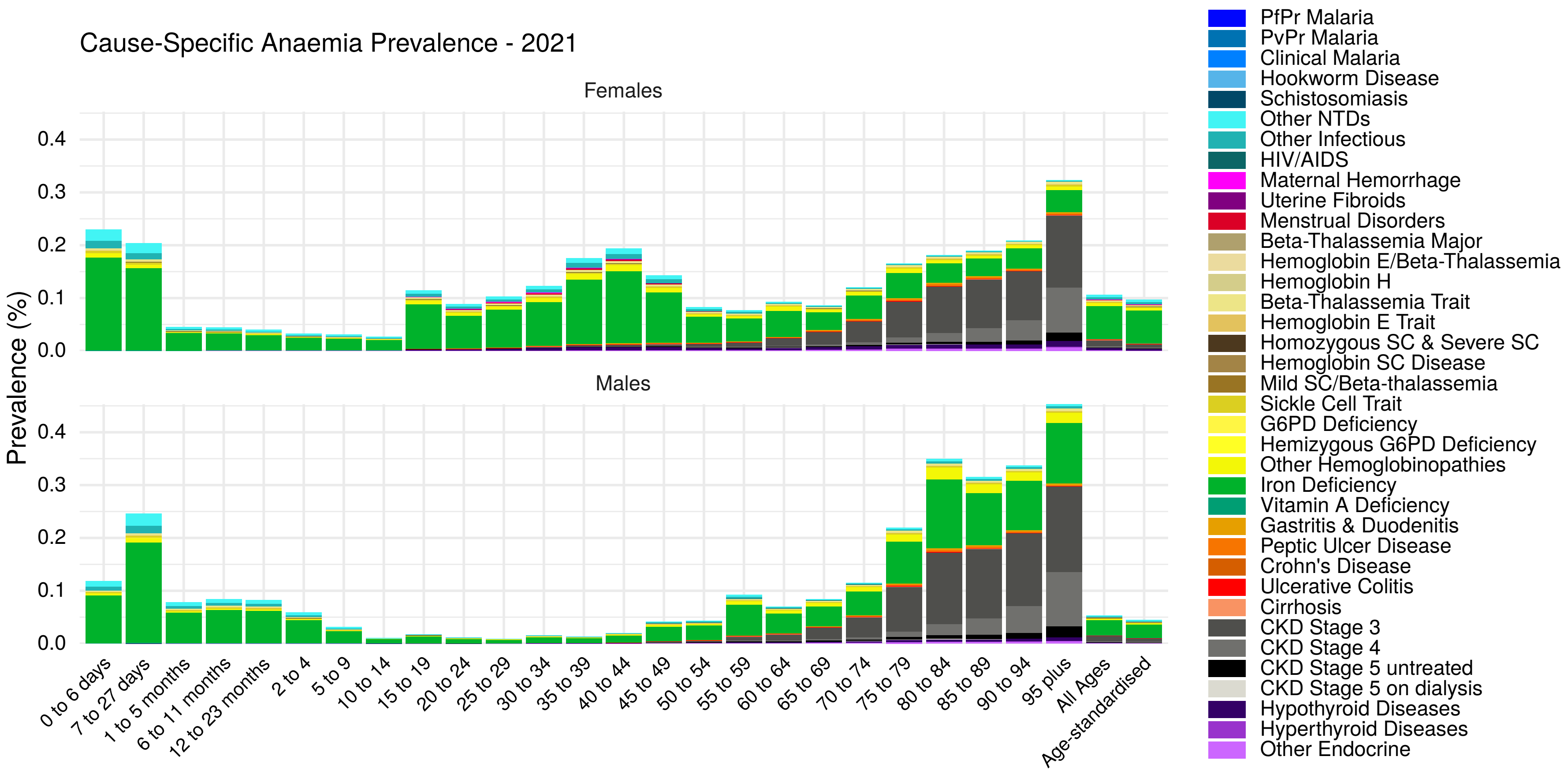

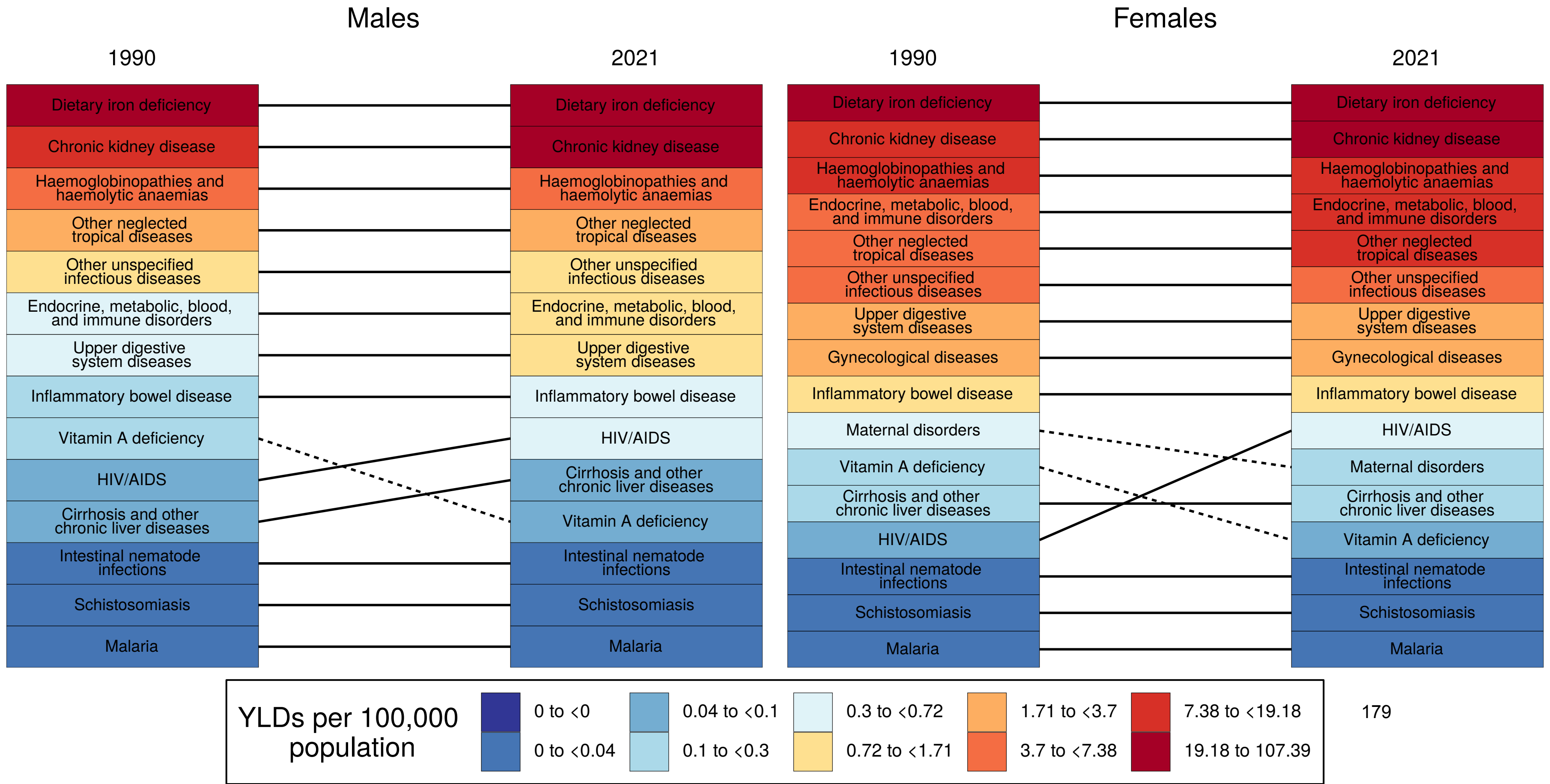

Oregon

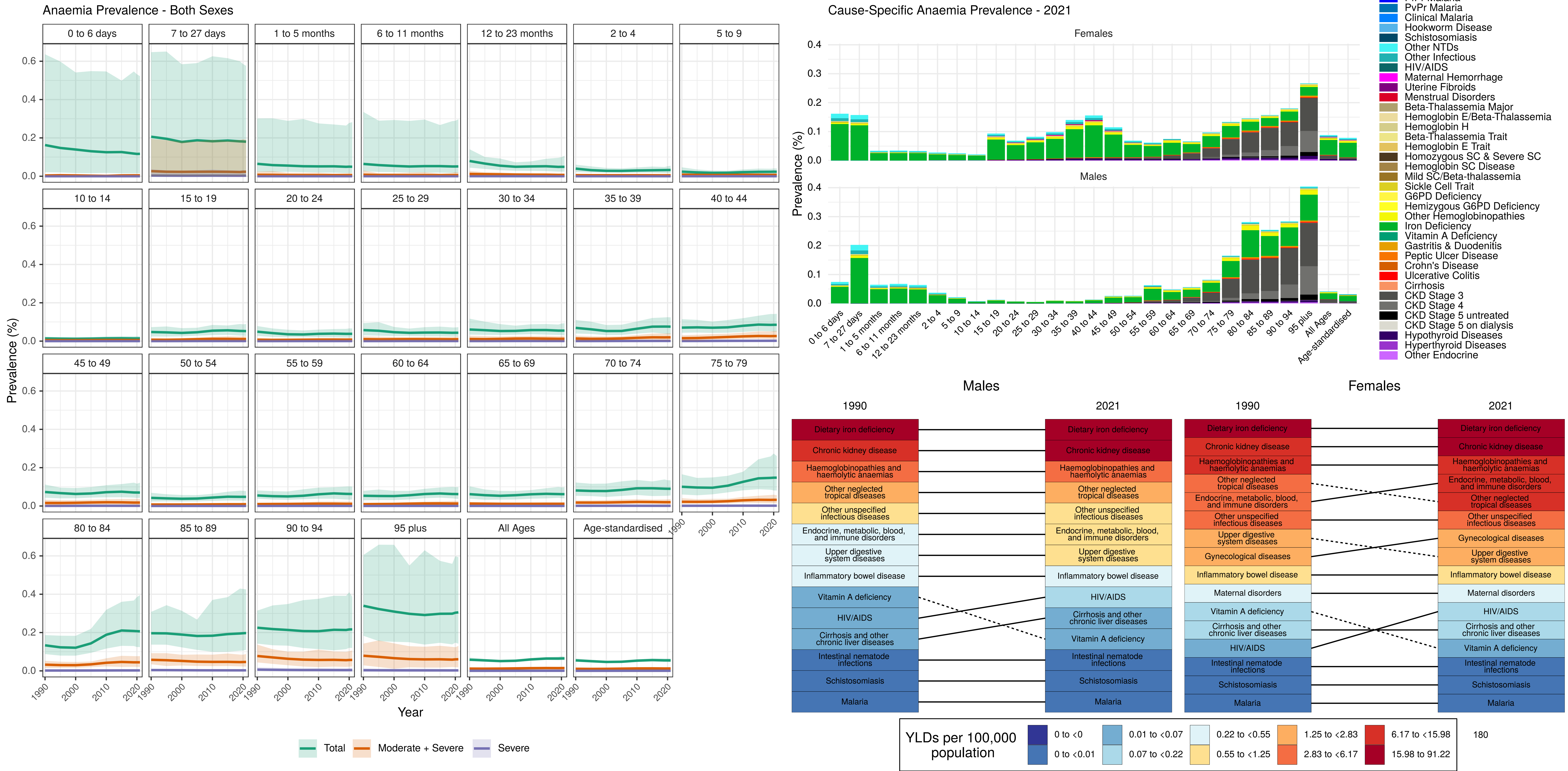

Anaemia Prevalence - Both Sexes
0 to 6 days
7 to 27 days
1 to 5 months
6 to 11 months
12 to 23 months
2 to 4
5 to 9
10 to 14
15 to 19
20 to 24
25 to 29
30 to 34
35 to 39
40 to 44
45 to 49
50 to 54
55 to 59
60 to 64
65 to 69
70 to 74
75 to 79
80 to 84
85 to 89
90 to 94
95 plus
All Ages
Age-standardised
Prevalence (%)
Year
Total
Moderate + Severe
Severe
Cause-Specific Anaemia Prevalence - 2021
Females
Males
PfPr Malaria
PvPr Malaria
Clinical Malaria
Hookworm Disease
Schistosomiasis
Other NTDs
Other Infectious
HIV/AIDS
Maternal Hemorrhage
Uterine Fibroids
Menstrual Disorders
Beta-Thalassemia Major
Hemoglobin E/Beta-Thalassemia
Hemoglobin H
Beta-Thalassemia Trait
Hemoglobin E Trait
Homozygous SC & Severe SC
Hemoglobin SC Disease
Mild SC/Beta-thalassemia
Sickle Cell Trait
G6PD Deficiency
Hemizygous G6PD Deficiency
Other Hemoglobinopathies
Iron Deficiency
Vitamin A Deficiency
Gastritis & Duodenitis
Peptic Ulcer Disease
Crohn's Disease
Ulcerative Colitis
Cirrhosis
CKD Stage 3
CKD Stage 4
CKD Stage 5 untreated
CKD Stage 5 on dialysis
Hypothyroid Diseases
Hyperthyroid Diseases
Other Endocrine
Males
Females
1990
2021
Dietary iron deficiency
Chronic kidney disease
Haemoglobinopathies and haemolytic anaemias
Other neglected tropical diseases
Other unspecified infectious diseases
Endocrine, metabolic, blood, and immune disorders
Upper digestive system diseases
Inflammatory bowel disease
Vitamin A deficiency
HIV/AIDS
Cirrhosis and other chronic liver diseases
Intestinal nematode infections
Schistosomiasis
Malaria
Gynecological diseases
Maternal disorders
YLDs per 100,000 population
0 to <0
0 to <0.01
0.01 to <0.07
0.07 to <0.22
0.22 to <0.55
0.55 to <1.25
1.25 to <2.83
2.83 to <6.17
6.17 to <15.98
15.98 to 91.22

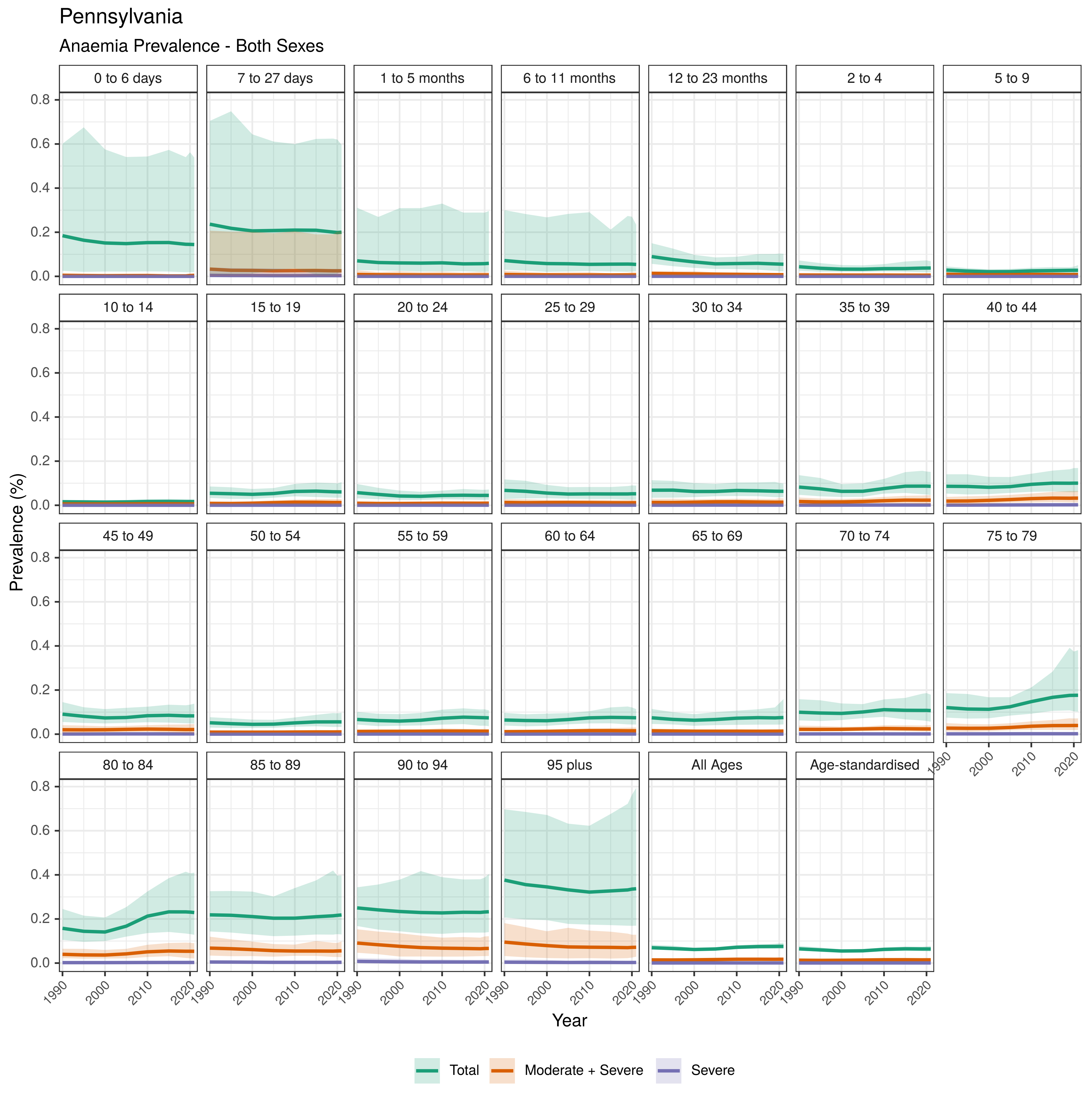

Pennsylvania
Anaemia Prevalence - Both Sexes
0 to 6 days
7 to 27 days
1 to 5 months
6 to 11 months
12 to 23 months
2 to 4
5 to 9
10 to 14
15 to 19
20 to 24
25 to 29
30 to 34
35 to 39
40 to 44
45 to 49
50 to 54
55 to 59
60 to 64
65 to 69
70 to 74
75 to 79
80 to 84
85 to 89
90 to 94
95 plus
All Ages
Age-standardised
Prevalence (%)
Year
Total
Moderate + Severe
Severe

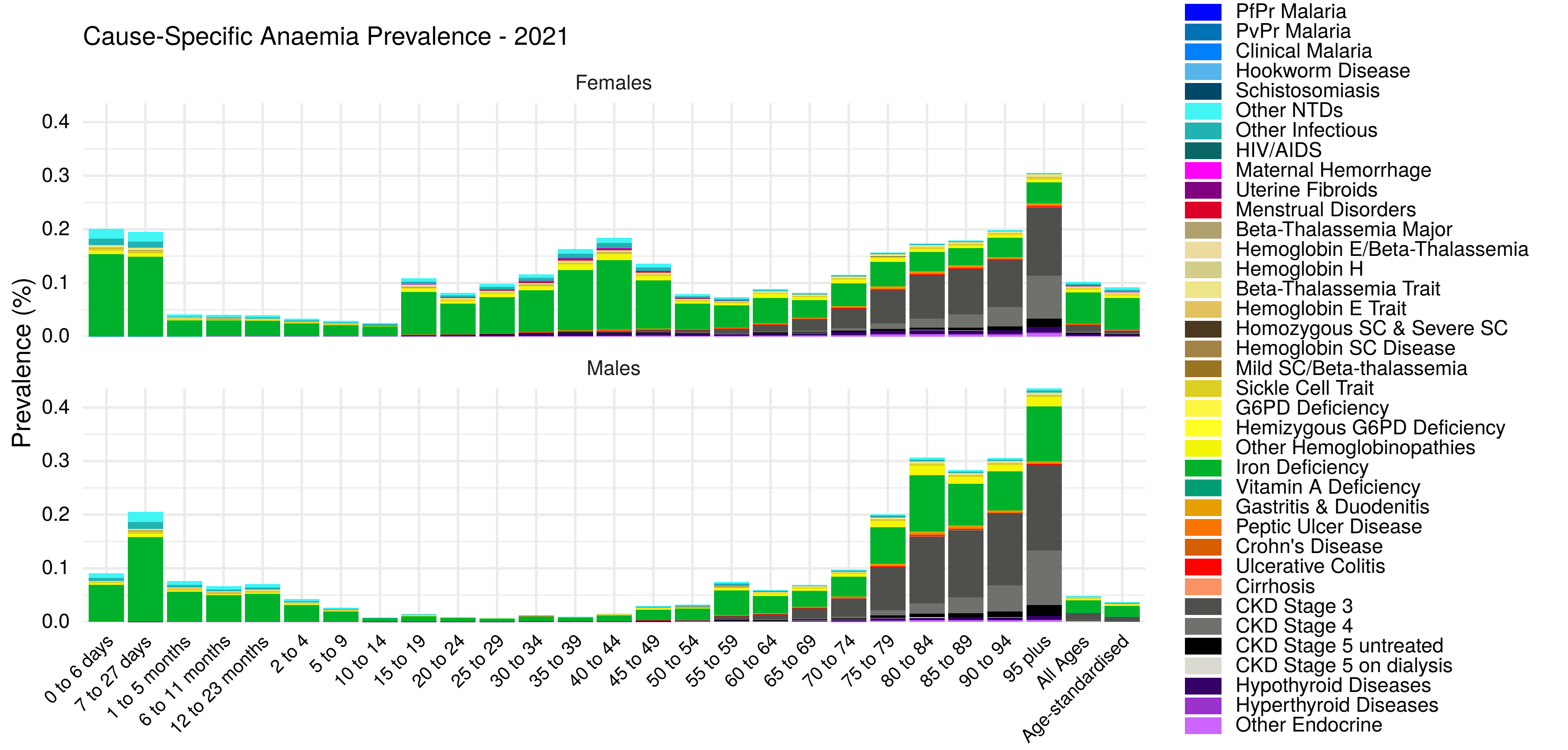

Cause-Specific Anaemia Prevalence - 2021
Females
Males
Prevalence (%)
PfPr Malaria
PvPr Malaria
Clinical Malaria
Hookworm Disease
Schistosomiasis
Other NTDs
Other Infectious
HIV/AIDS
Maternal Hemorrhage
Uterine Fibroids
Menstrual Disorders
Beta-Thalassemia Major
Hemoglobin E/Beta-Thalassemia
Hemoglobin H
Beta-Thalassemia Trait
Hemoglobin E Trait
Homozygous SC & Severe SC
Hemoglobin SC Disease
Mild SC/Beta-thalassemia
Sickle Cell Trait
G6PD Deficiency
Hemizygous G6PD Deficiency
Other Hemoglobinopathies
Iron Deficiency
Vitamin A Deficiency
Gastritis & Duodenitis
Peptic Ulcer Disease
Crohn's Disease
Ulcerative Colitis
Cirrhosis
CKD Stage 3
CKD Stage 4
CKD Stage 5 untreated
CKD Stage 5 on dialysis
Hypothyroid Diseases
Hyperthyroid Diseases
Other Endocrine

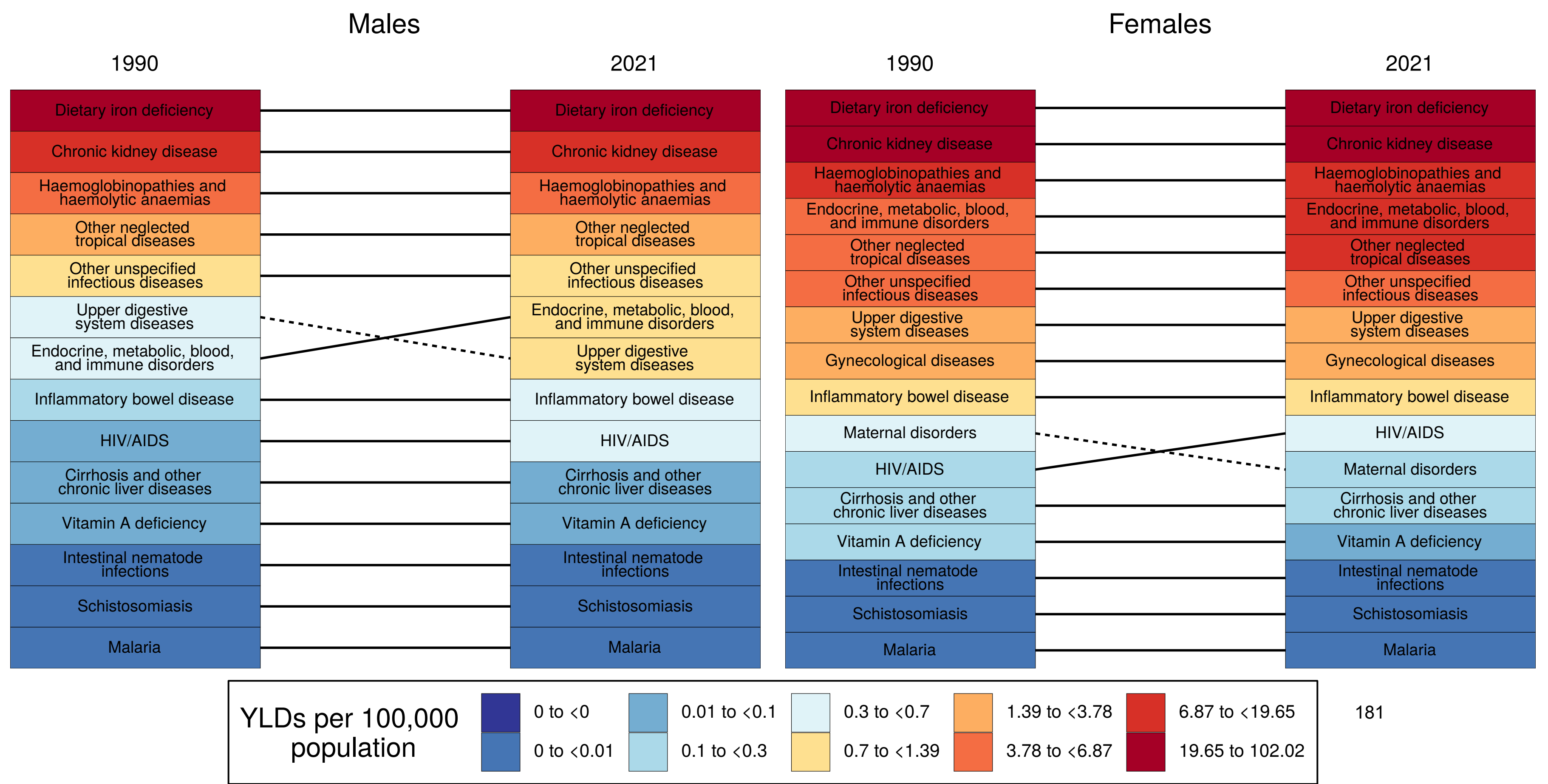

Males
Females
1990
2021
Dietary iron deficiency
Chronic kidney disease
Haemoglobinopathies and haemolytic anaemias
Other neglected tropical diseases
Other unspecified infectious diseases
Upper digestive system diseases
Endocrine, metabolic, blood, and immune disorders
Inflammatory bowel disease
HIV/AIDS
Cirrhosis and other chronic liver diseases
Vitamin A deficiency
Intestinal nematode infections
Schistosomiasis
Malaria
Gynecological diseases
Maternal disorders
YLDs per 100,000 population
0 to <0
0 to <0.01
0.01 to <0.1
0.1 to <0.3
0.3 to <0.7
0.7 to <1.39
1.39 to <3.78
3.78 to <6.87
6.87 to <19.65
19.65 to 102.02

Rhode Island

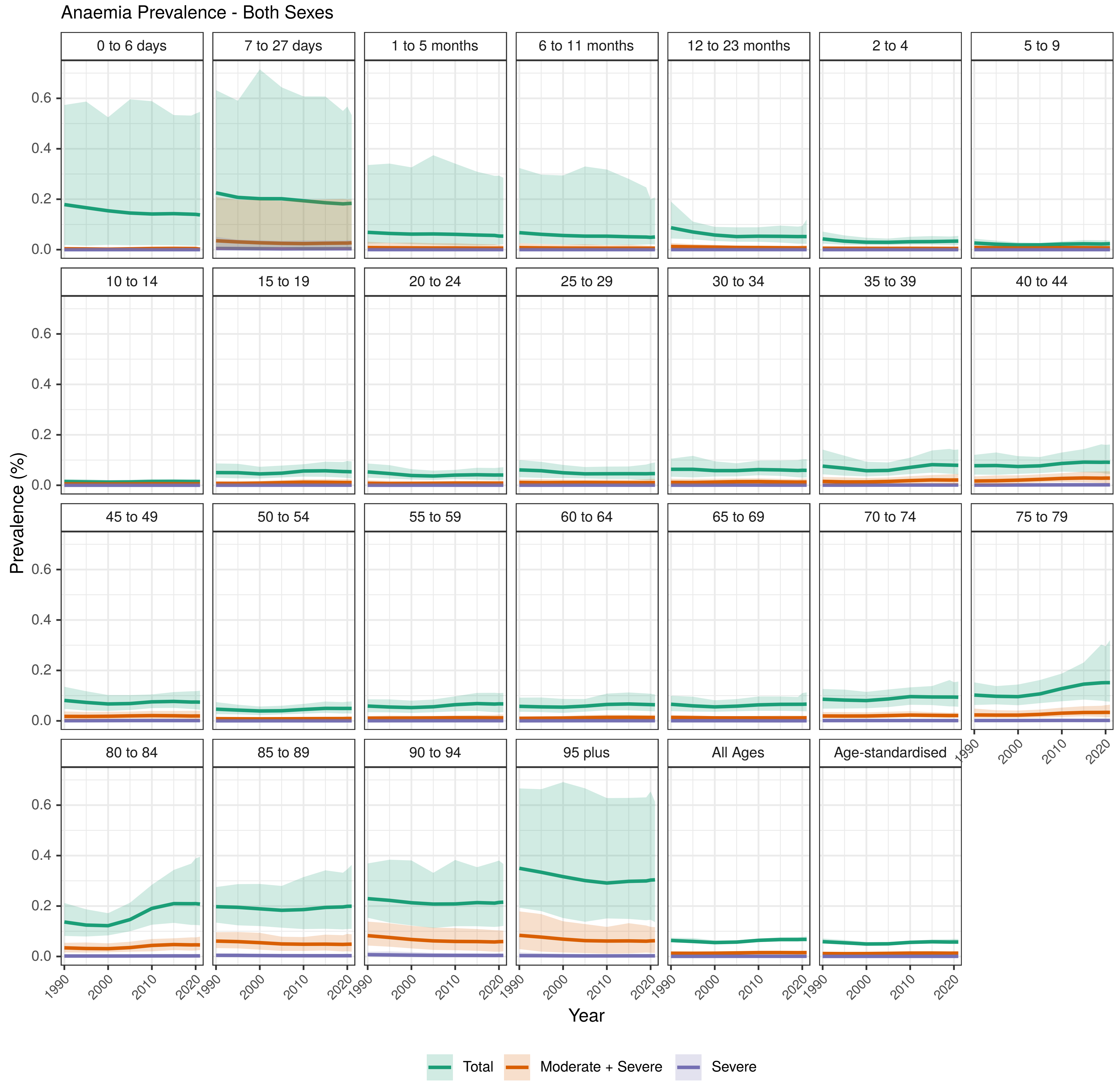

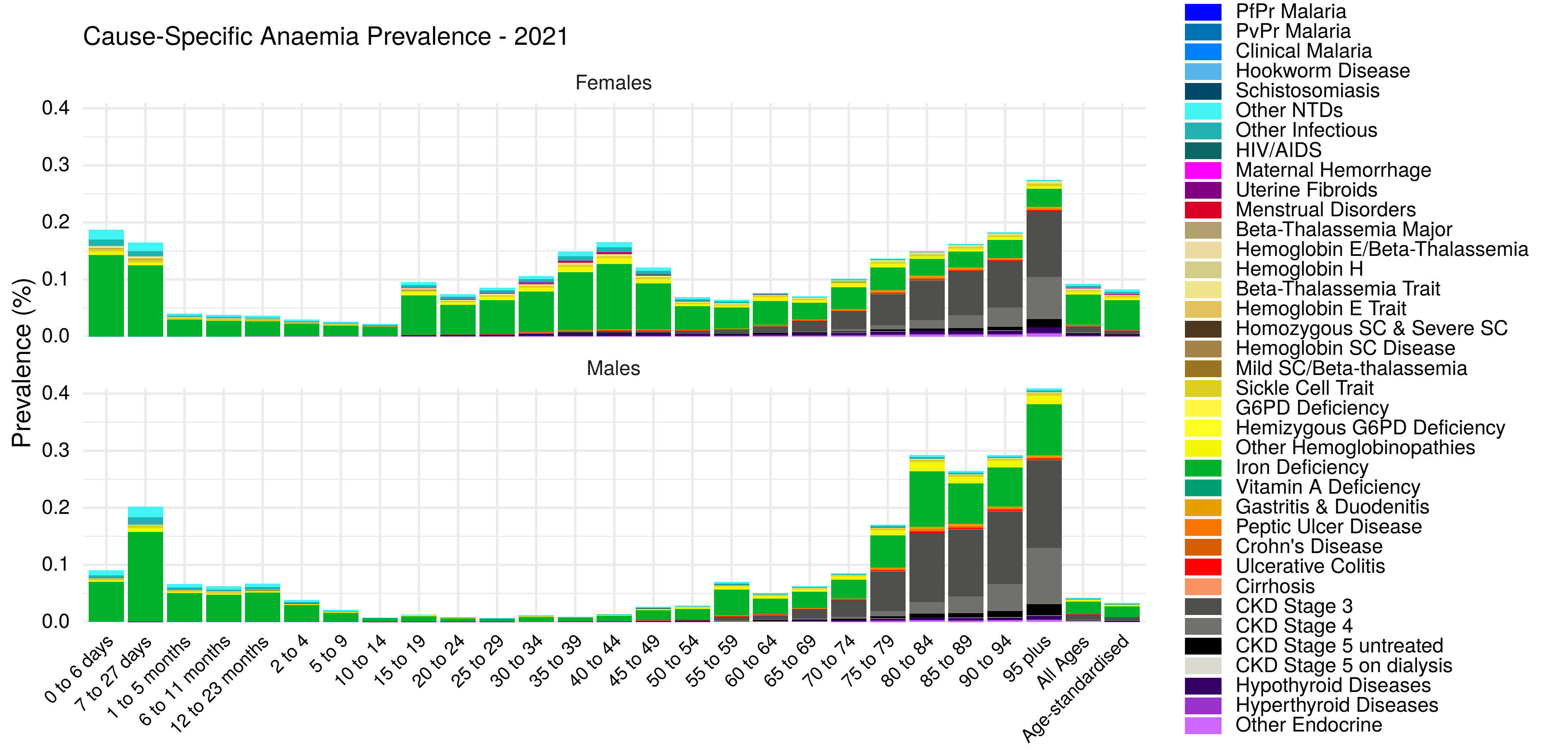

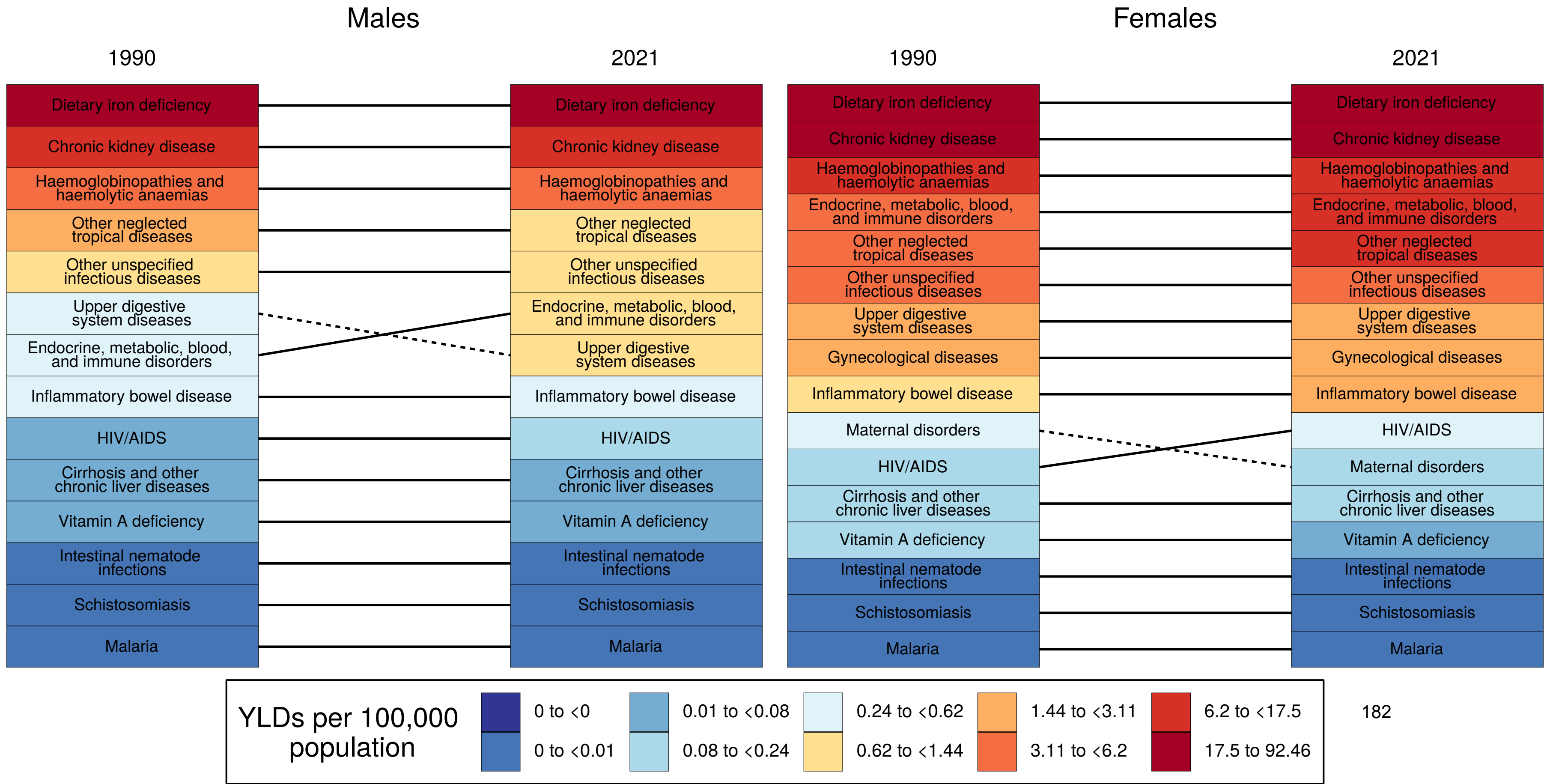

South Carolina

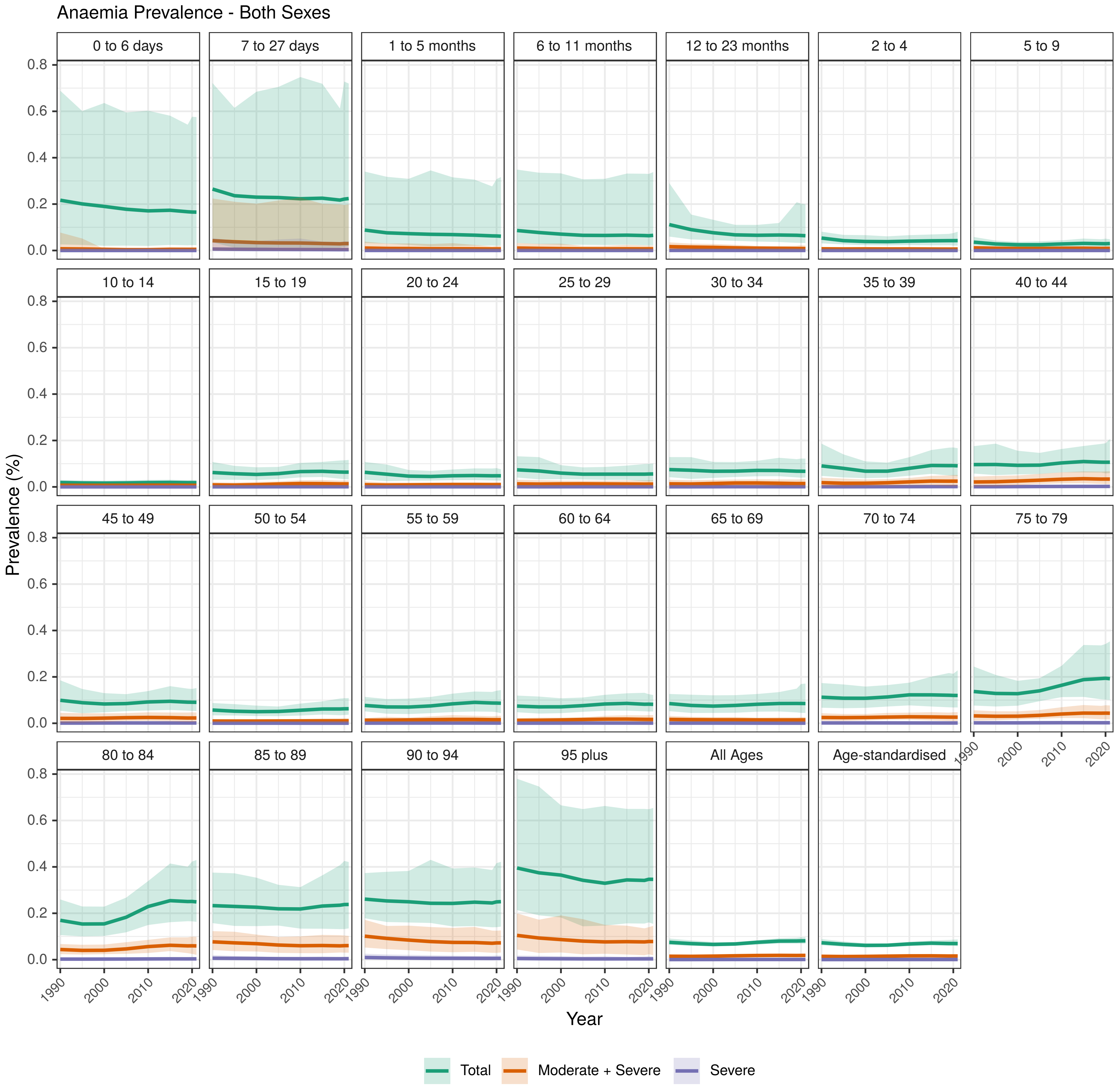

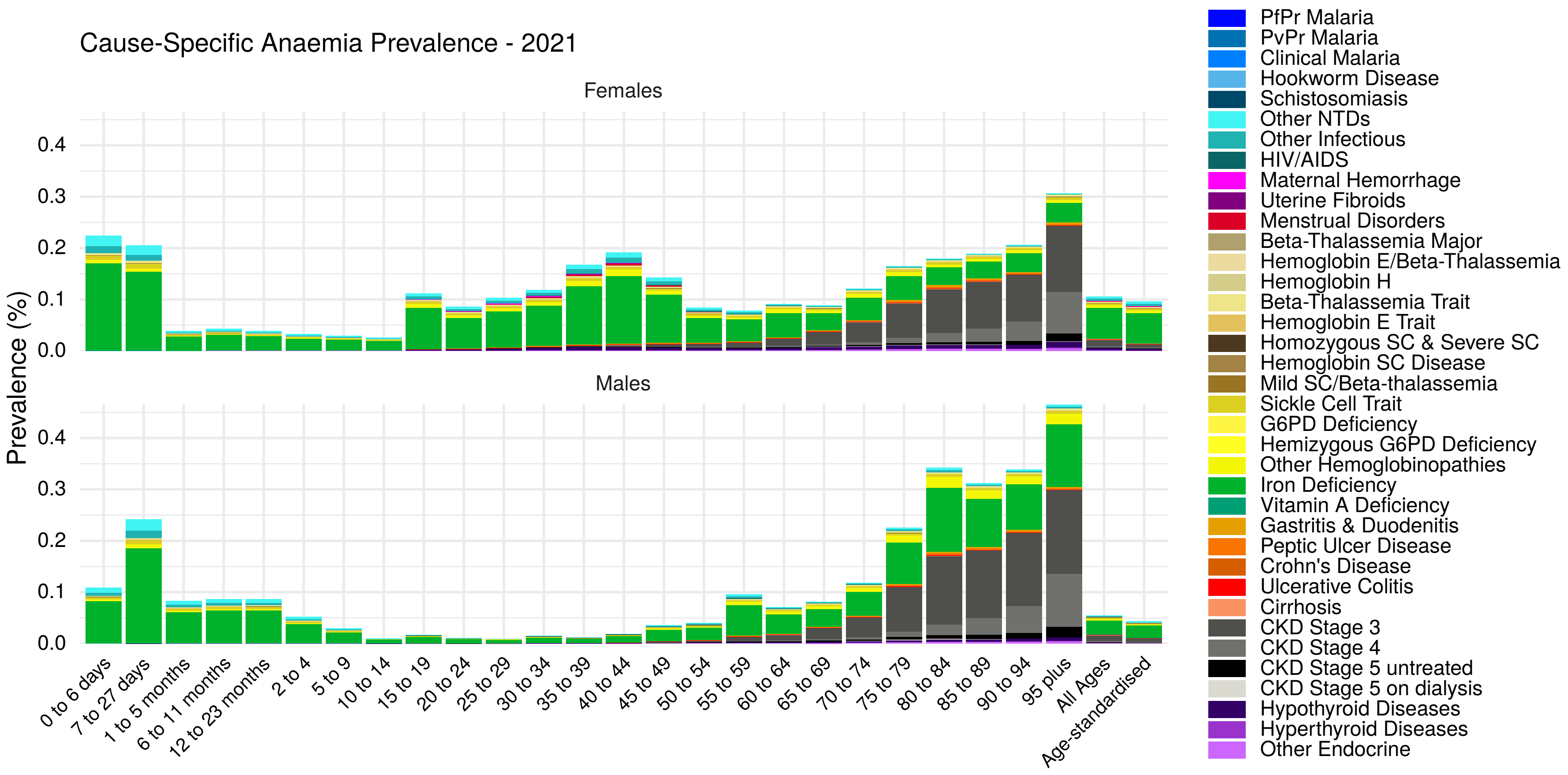

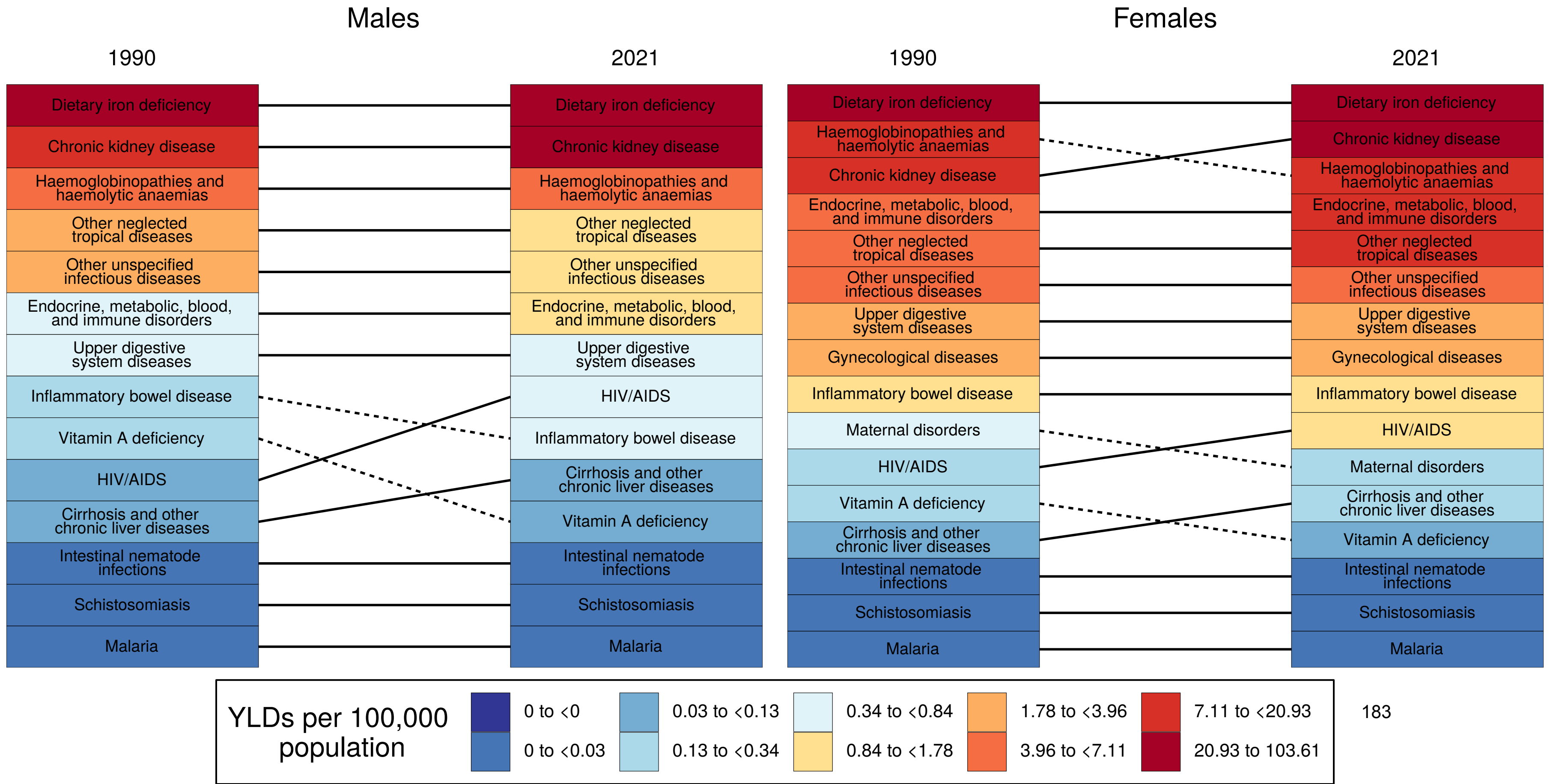

South Dakota

Anaemia Prevalence - Both Sexes

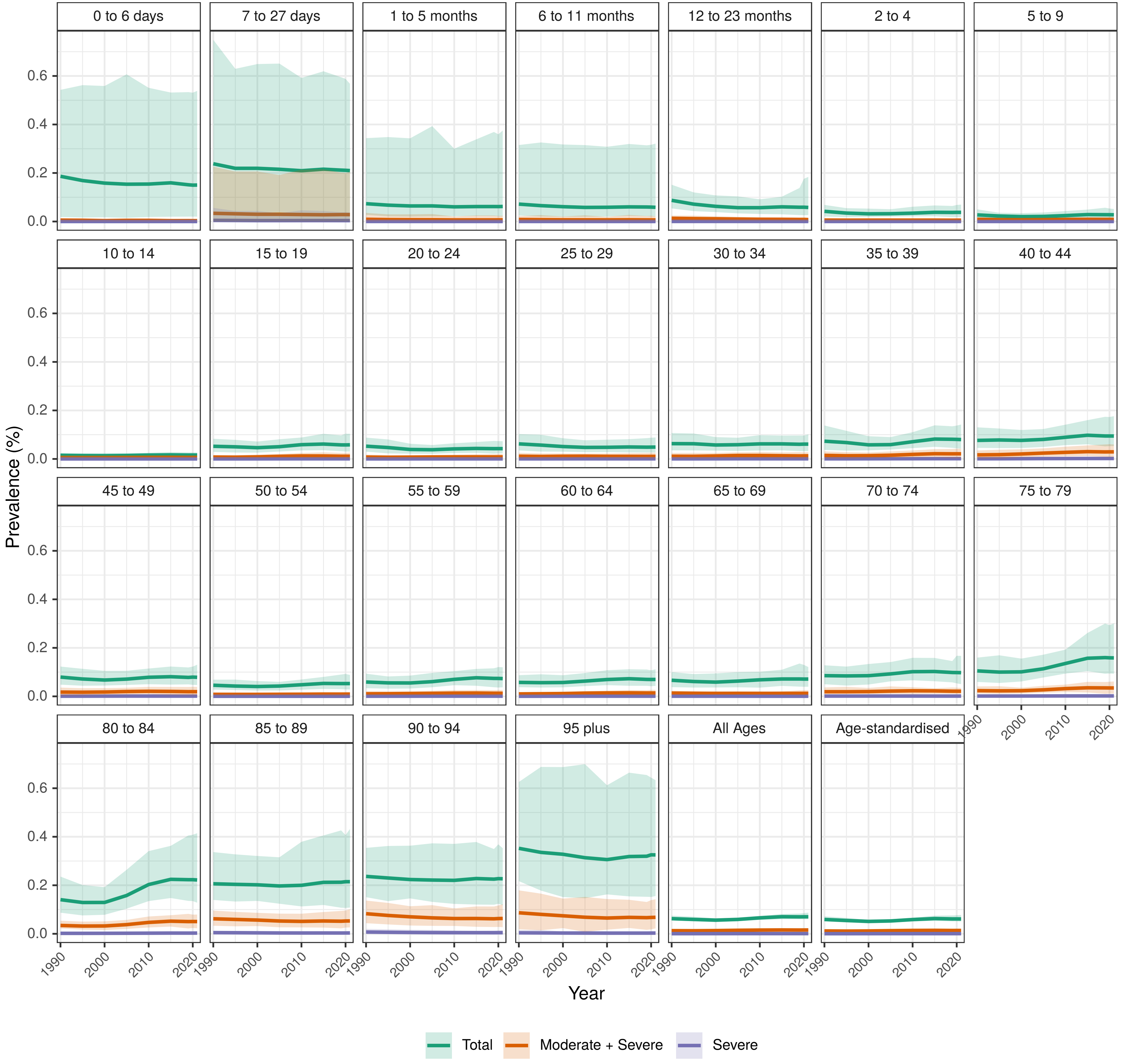

Cause-Specific Anaemia Prevalence - 2021

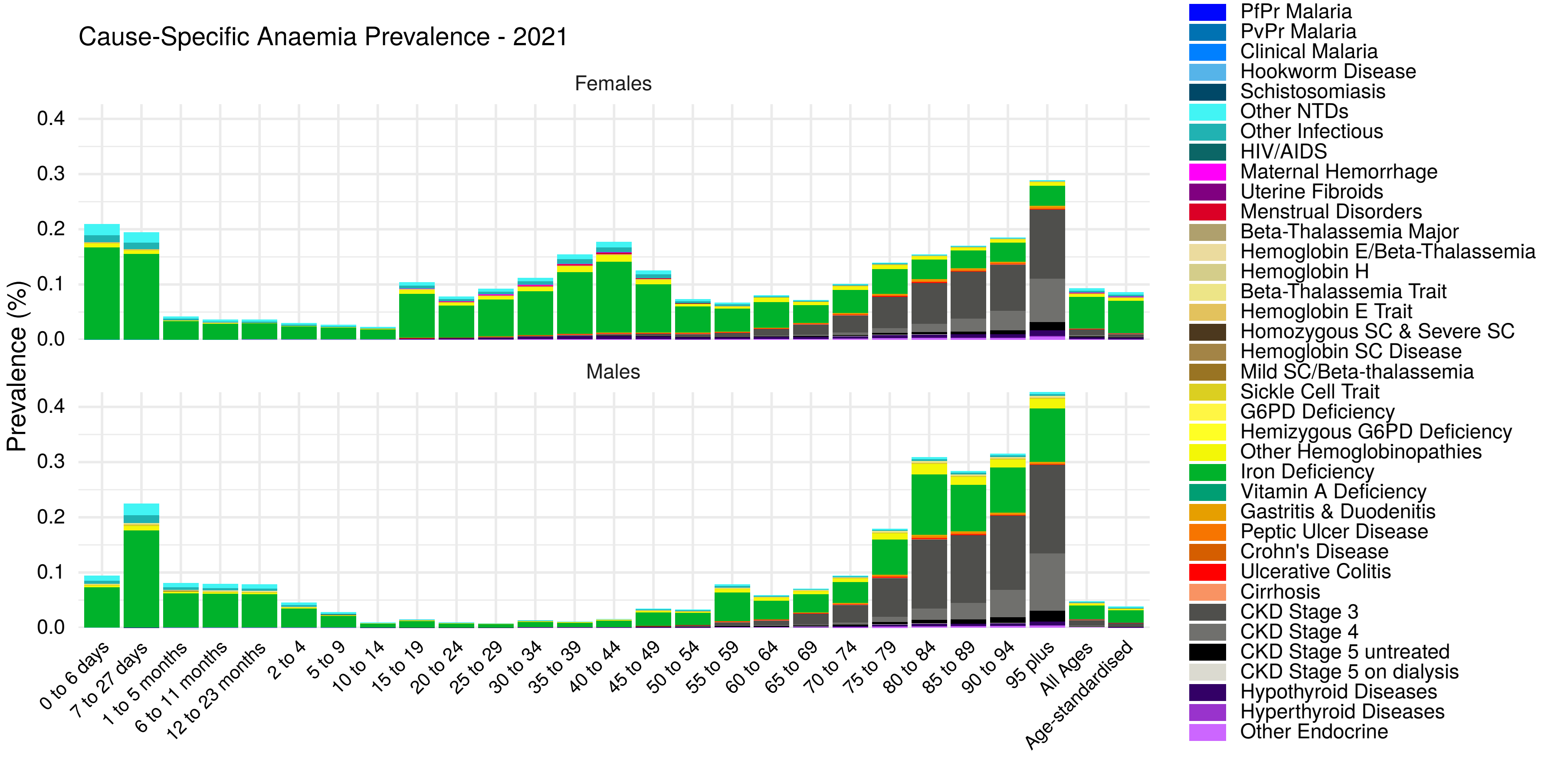

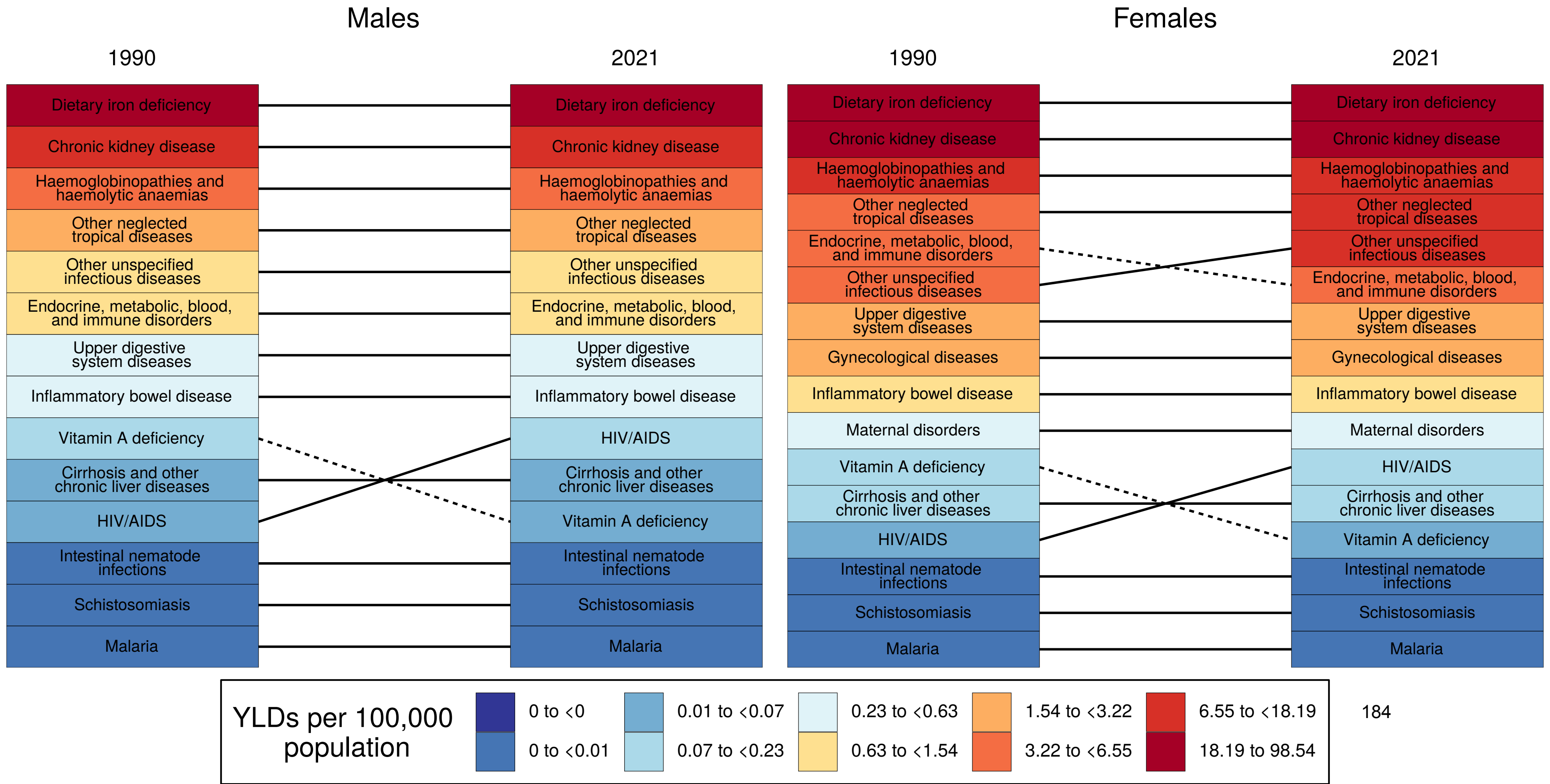

Tennessee

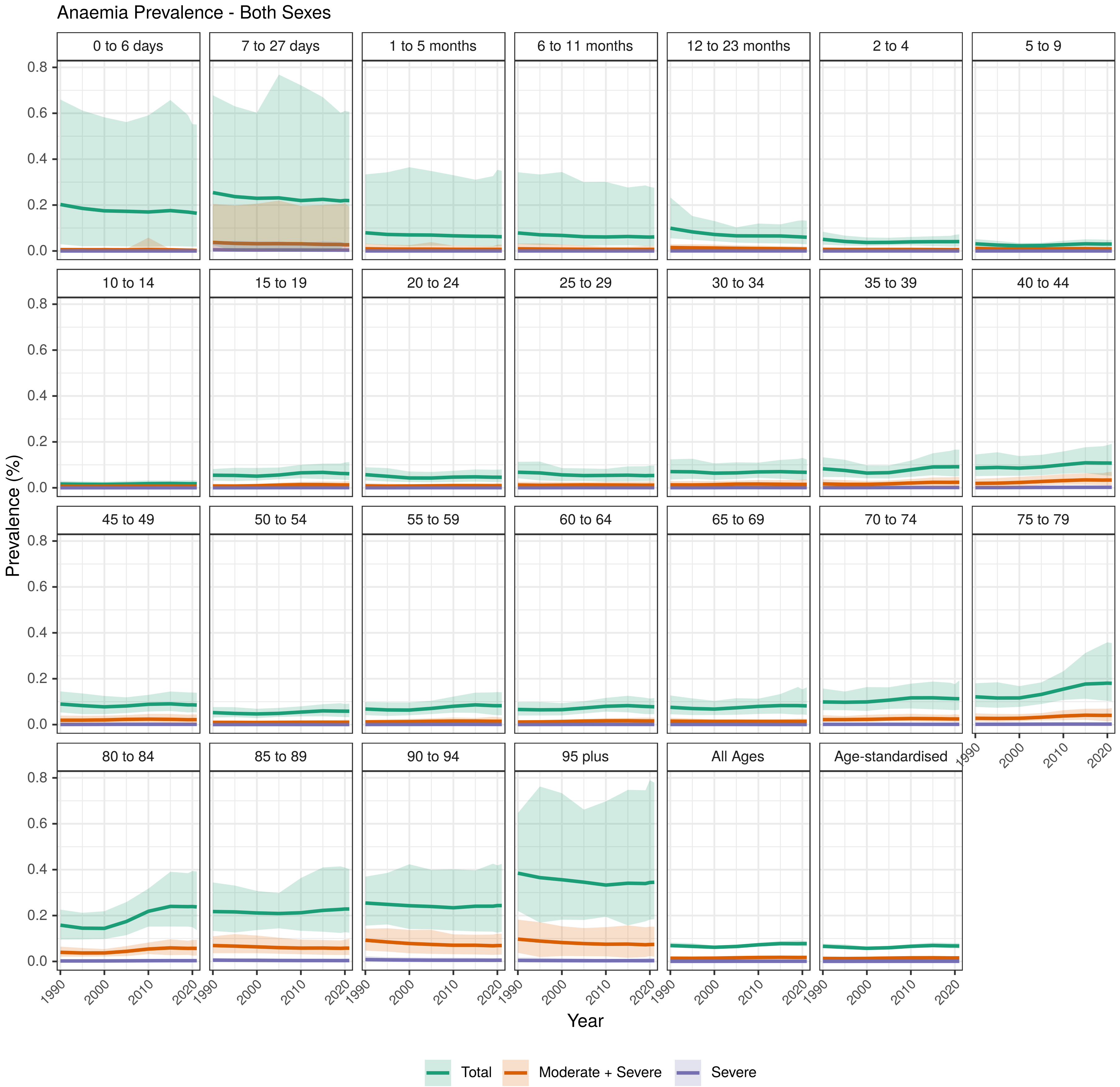

Anaemia Prevalence - Both Sexes
0 to 6 days
7 to 27 days
1 to 5 months
6 to 11 months
12 to 23 months
2 to 4
5 to 9
10 to 14
15 to 19
20 to 24
25 to 29
30 to 34
35 to 39
40 to 44
45 to 49
50 to 54
55 to 59
60 to 64
65 to 69
70 to 74
75 to 79
80 to 84
85 to 89
90 to 94
95 plus
All Ages
Age-standardised
Prevalence (%)
Year
Total
Moderate + Severe
Severe

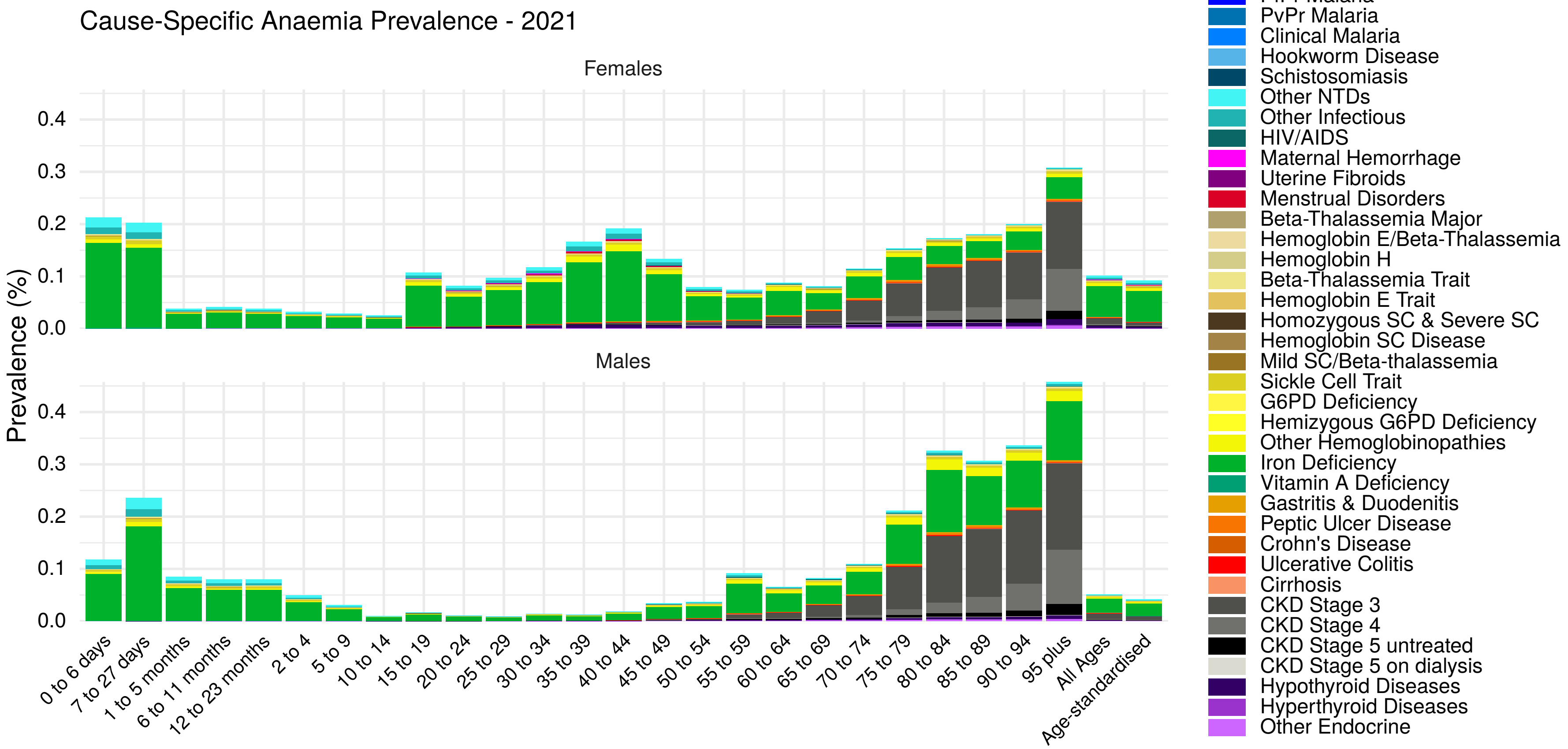

Cause-Specific Anaemia Prevalence - 2021
Females
Males
Prevalence (%)
PfPr Malaria
PvPr Malaria
Clinical Malaria
Hookworm Disease
Schistosomiasis
Other NTDs
Other Infectious
HIV/AIDS
Maternal Hemorrhage
Uterine Fibroids
Menstrual Disorders
Beta-Thalassemia Major
Hemoglobin E/Beta-Thalassemia
Hemoglobin H
Beta-Thalassemia Trait
Hemoglobin E Trait
Homozygous SC & Severe SC
Hemoglobin SC Disease
Mild SC/Beta-thalassemia
Sickle Cell Trait
G6PD Deficiency
Hemizygous G6PD Deficiency
Other Hemoglobinopathies
Iron Deficiency
Vitamin A Deficiency
Gastritis & Duodenitis
Peptic Ulcer Disease
Crohn's Disease
Ulcerative Colitis
Cirrhosis
CKD Stage 3
CKD Stage 4
CKD Stage 5 untreated
CKD Stage 5 on dialysis
Hypothyroid Diseases
Hyperthyroid Diseases
Other Endocrine

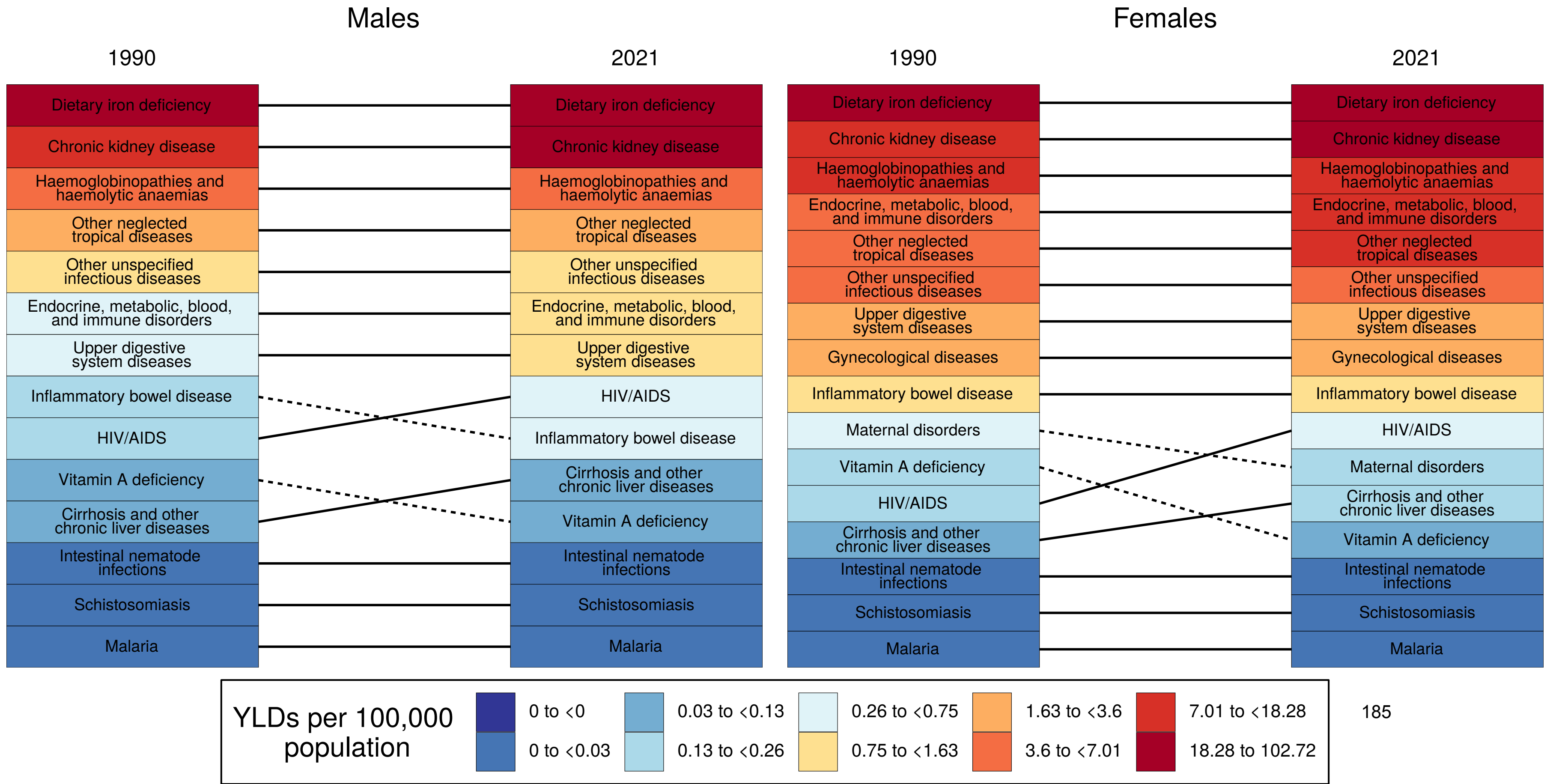

Males
Females
1990
2021
Dietary iron deficiency
Chronic kidney disease
Haemoglobinopathies and haemolytic anaemias
Other neglected tropical diseases
Other unspecified infectious diseases
Endocrine, metabolic, blood, and immune disorders
Upper digestive system diseases
Inflammatory bowel disease
HIV/AIDS
Vitamin A deficiency
Cirrhosis and other chronic liver diseases
Intestinal nematode infections
Schistosomiasis
Malaria
Gynecological diseases
Maternal disorders
YLDs per 100,000 population
0 to <0
0 to <0.03
0.03 to <0.13
0.13 to <0.26
0.26 to <0.75
0.75 to <1.63
1.63 to <3.6
3.6 to <7.01
7.01 to <18.28
18.28 to 102.72

Texas

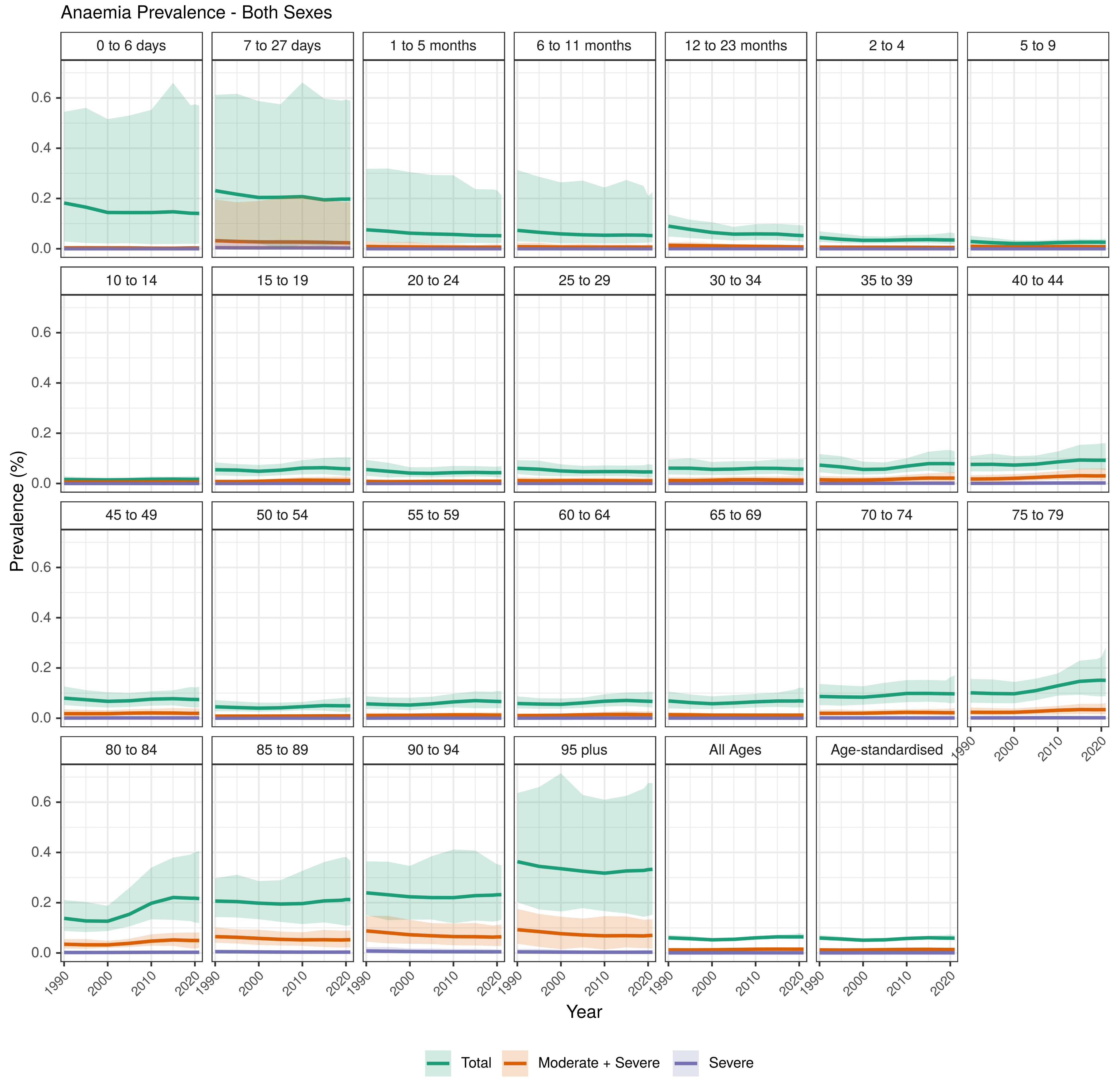
Anaemia Prevalence - Both Sexes
0 to 6 days
7 to 27 days
1 to 5 months
6 to 11 months
12 to 23 months
2 to 4
5 to 9
10 to 14
15 to 19
20 to 24
25 to 29
30 to 34
35 to 39
40 to 44
45 to 49
50 to 54
55 to 59
60 to 64
65 to 69
70 to 74
75 to 79
80 to 84
85 to 89
90 to 94
95 plus
All Ages
Age-standardised
Prevalence (%)
Year
Total
Moderate + Severe
Severe

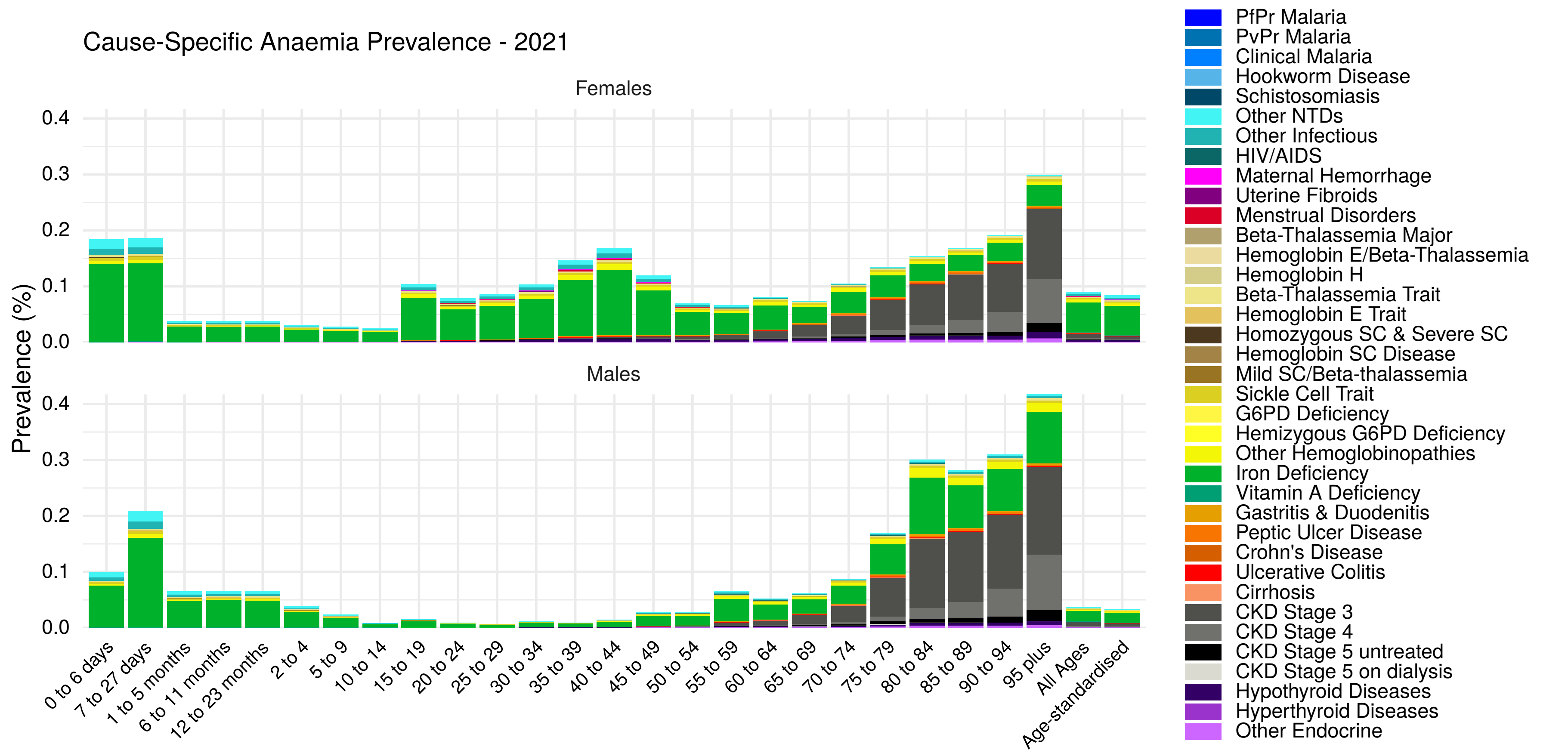
Cause-Specific Anaemia Prevalence - 2021
Females
Males
Prevalence (%)
PfPr Malaria
PvPr Malaria
Clinical Malaria
Hookworm Disease
Schistosomiasis
Other NTDs
Other Infectious
HIV/AIDS
Maternal Hemorrhage
Uterine Fibroids
Menstrual Disorders
Beta-Thalassemia Major
Hemoglobin E/Beta-Thalassemia
Hemoglobin H
Beta-Thalassemia Trait
Hemoglobin E Trait
Homozygous SC & Severe SC
Hemoglobin SC Disease
Mild SC/Beta-thalassemia
Sickle Cell Trait
G6PD Deficiency
Hemizygous G6PD Deficiency
Other Hemoglobinopathies
Iron Deficiency
Vitamin A Deficiency
Gastritis & Duodenitis
Peptic Ulcer Disease
Crohn's Disease
Ulcerative Colitis
Cirrhosis
CKD Stage 3
CKD Stage 4
CKD Stage 5 untreated
CKD Stage 5 on dialysis
Hypothyroid Diseases
Hyperthyroid Diseases
Other Endocrine

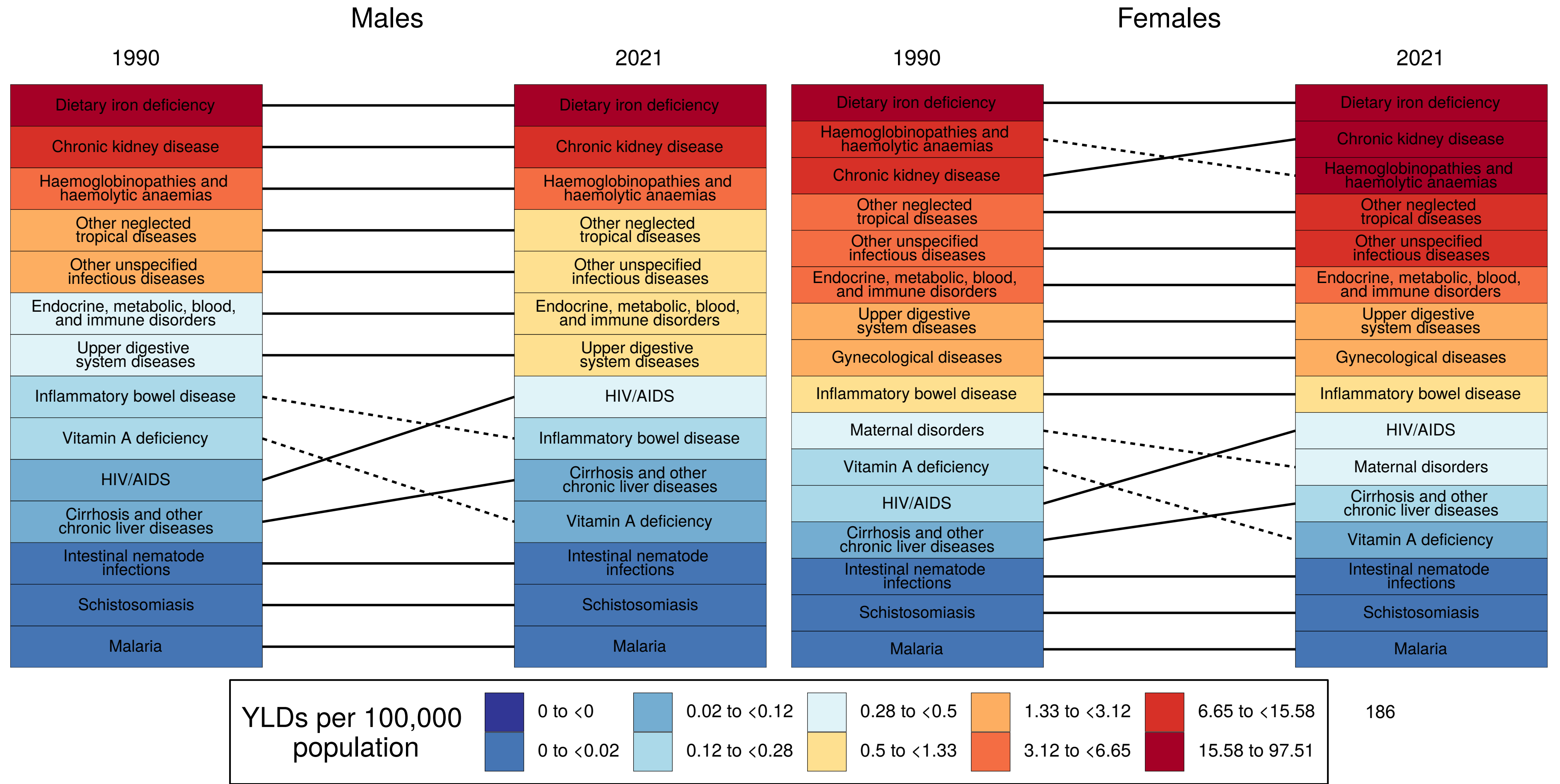
Males
Females
1990
2021
Dietary iron deficiency
Chronic kidney disease
Haemoglobinopathies and haemolytic anaemias
Other neglected tropical diseases
Other unspecified infectious diseases
Endocrine, metabolic, blood, and immune disorders
Upper digestive system diseases
Inflammatory bowel disease
Vitamin A deficiency
HIV/AIDS
Cirrhosis and other chronic liver diseases
Intestinal nematode infections
Schistosomiasis
Malaria
Gynecological diseases
Maternal disorders
YLDs per 100,000 population
0 to <0
0 to <0.02
0.02 to <0.12
0.12 to <0.28
0.28 to <0.5
0.5 to <1.33
1.33 to <3.12
3.12 to <6.65
6.65 to <15.58
15.58 to 97.51

Utah

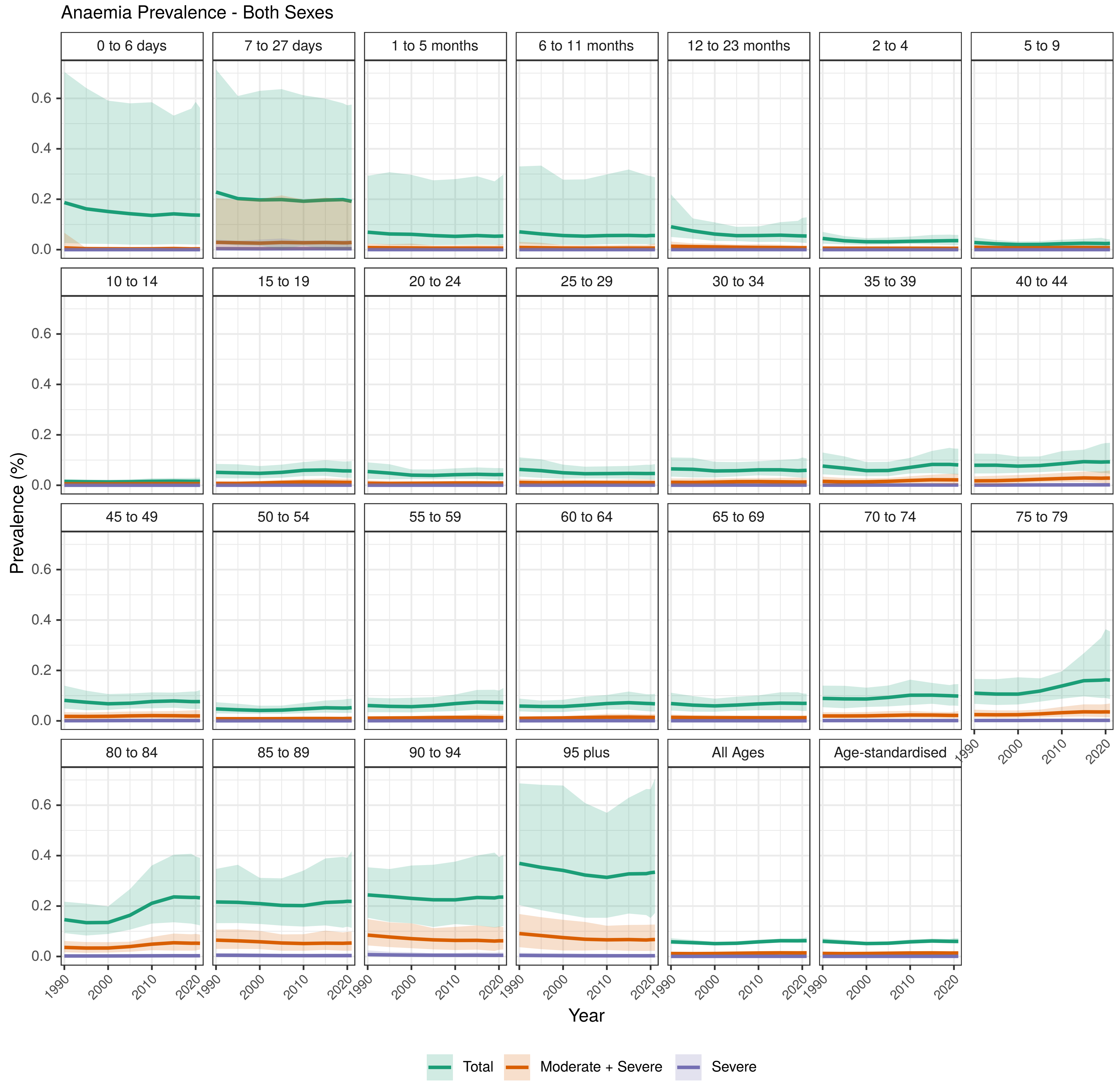
Anaemia Prevalence - Both Sexes
0 to 6 days
7 to 27 days
1 to 5 months
6 to 11 months
12 to 23 months
2 to 4
5 to 9
10 to 14
15 to 19
20 to 24
25 to 29
30 to 34
35 to 39
40 to 44
45 to 49
50 to 54
55 to 59
60 to 64
65 to 69
70 to 74
75 to 79
80 to 84
85 to 89
90 to 94
95 plus
All Ages
Age-standardised
Prevalence (%)
Year
Total
Moderate + Severe
Severe

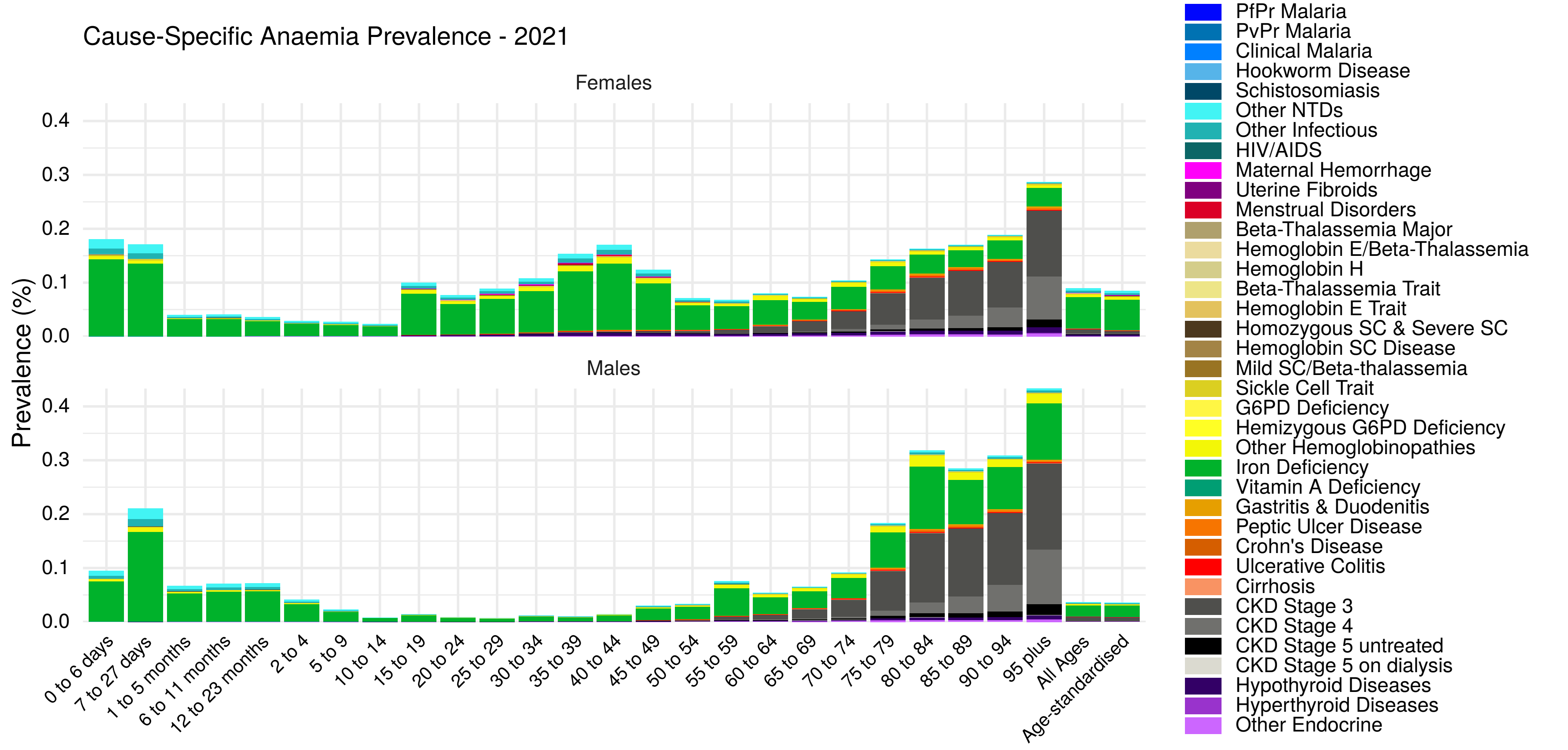
Cause-Specific Anaemia Prevalence - 2021
Females
Males
Prevalence (%)
PfPr Malaria
PvPr Malaria
Clinical Malaria
Hookworm Disease
Schistosomiasis
Other NTDs
Other Infectious
HIV/AIDS
Maternal Hemorrhage
Uterine Fibroids
Menstrual Disorders
Beta-Thalassemia Major
Hemoglobin E/Beta-Thalassemia
Hemoglobin H
Beta-Thalassemia Trait
Hemoglobin E Trait
Homozygous SC & Severe SC
Hemoglobin SC Disease
Mild SC/Beta-thalassemia
Sickle Cell Trait
G6PD Deficiency
Hemizygous G6PD Deficiency
Other Hemoglobinopathies
Iron Deficiency
Vitamin A Deficiency
Gastritis & Duodenitis
Peptic Ulcer Disease
Crohn's Disease
Ulcerative Colitis
Cirrhosis
CKD Stage 3
CKD Stage 4
CKD Stage 5 untreated
CKD Stage 5 on dialysis
Hypothyroid Diseases
Hyperthyroid Diseases
Other Endocrine

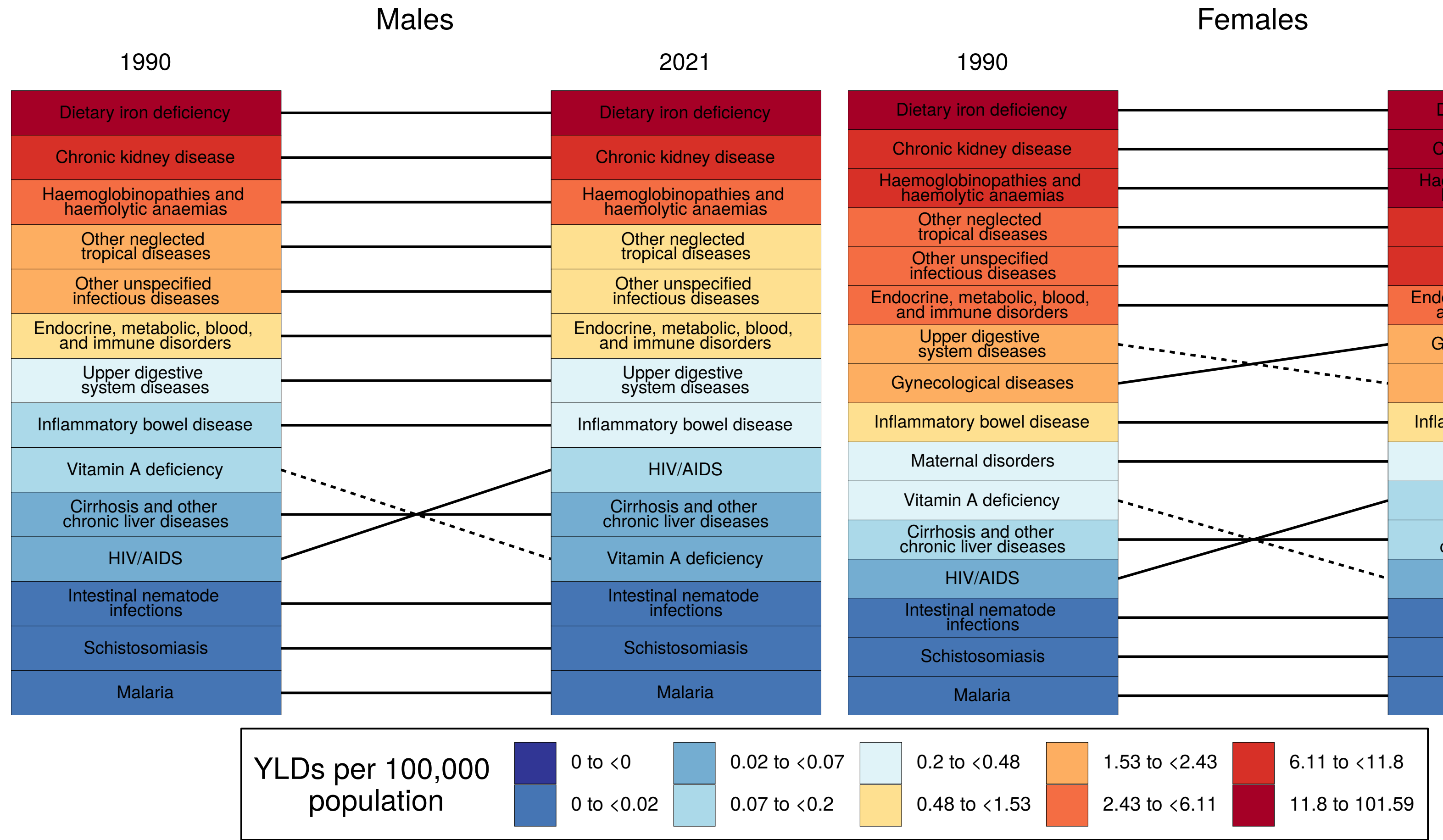
Males
Females
1990
2021
Dietary iron deficiency
Chronic kidney disease
Haemoglobinopathies and haemolytic anaemias
Other neglected tropical diseases
Other unspecified infectious diseases
Endocrine, metabolic, blood, and immune disorders
Upper digestive system diseases
Inflammatory bowel disease
Vitamin A deficiency
Cirrhosis and other chronic liver diseases
HIV/AIDS
Intestinal nematode infections
Schistosomiasis
Malaria
Gynecological diseases
Maternal disorders
YLDs per 100,000 population
0 to <0
0 to <0.02
0.02 to <0.07
0.07 to <0.2
0.2 to <0.48
0.48 to <1.53
1.53 to <2.43
2.43 to <6.11
6.11 to <11.8
11.8 to 101.59

Vermont

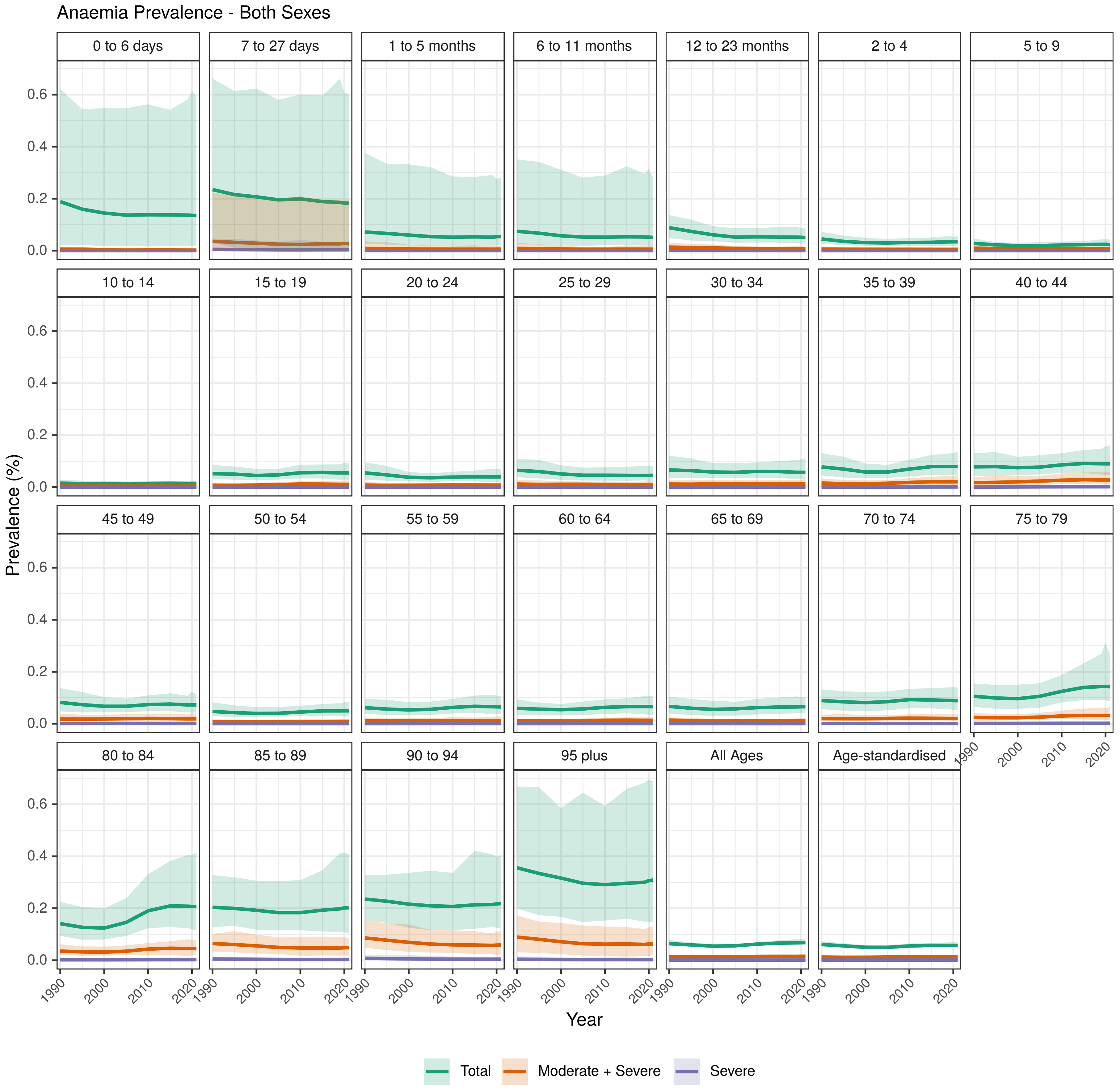
Anaemia Prevalence - Both Sexes
0 to 6 days
7 to 27 days
1 to 5 months
6 to 11 months
12 to 23 months
2 to 4
5 to 9
10 to 14
15 to 19
20 to 24
25 to 29
30 to 34
35 to 39
40 to 44
45 to 49
50 to 54
55 to 59
60 to 64
65 to 69
70 to 74
75 to 79
80 to 84
85 to 89
90 to 94
95 plus
All Ages
Age-standardised
Prevalence (%)
Year
Total
Moderate + Severe
Severe

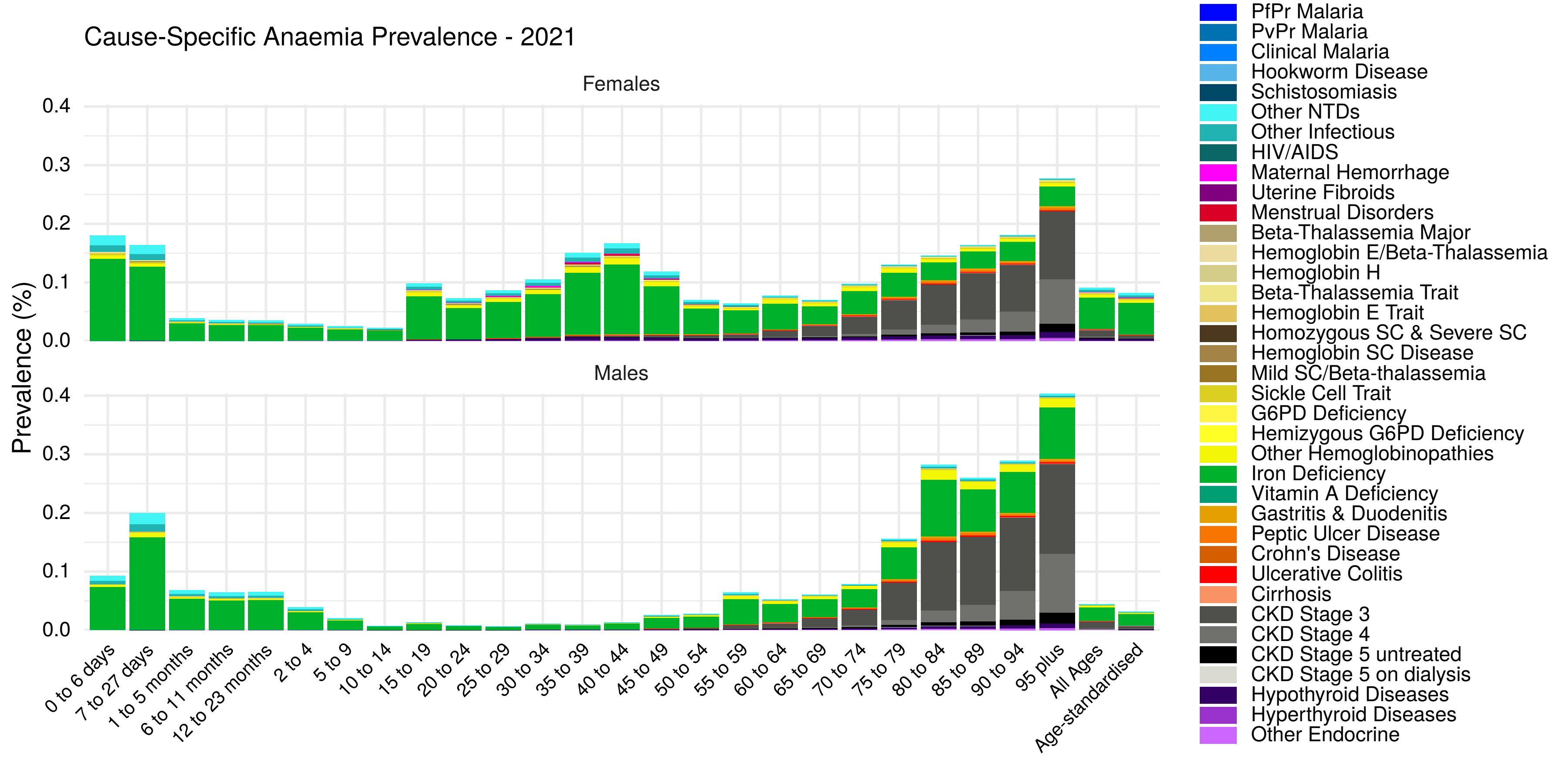
Cause-Specific Anaemia Prevalence - 2021
Females
Males
Prevalence (%)
PfPr Malaria
PvPr Malaria
Clinical Malaria
Hookworm Disease
Schistosomiasis
Other NTDs
Other Infectious
HIV/AIDS
Maternal Hemorrhage
Uterine Fibroids
Menstrual Disorders
Beta-Thalassemia Major
Hemoglobin E/Beta-Thalassemia
Hemoglobin H
Beta-Thalassemia Trait
Hemoglobin E Trait
Homozygous SC & Severe SC
Hemoglobin SC Disease
Mild SC/Beta-thalassemia
Sickle Cell Trait
G6PD Deficiency
Hemizygous G6PD Deficiency
Other Hemoglobinopathies
Iron Deficiency
Vitamin A Deficiency
Gastritis & Duodenitis
Peptic Ulcer Disease
Crohn's Disease
Ulcerative Colitis
Cirrhosis
CKD Stage 3
CKD Stage 4
CKD Stage 5 untreated
CKD Stage 5 on dialysis
Hypothyroid Diseases
Hyperthyroid Diseases
Other Endocrine

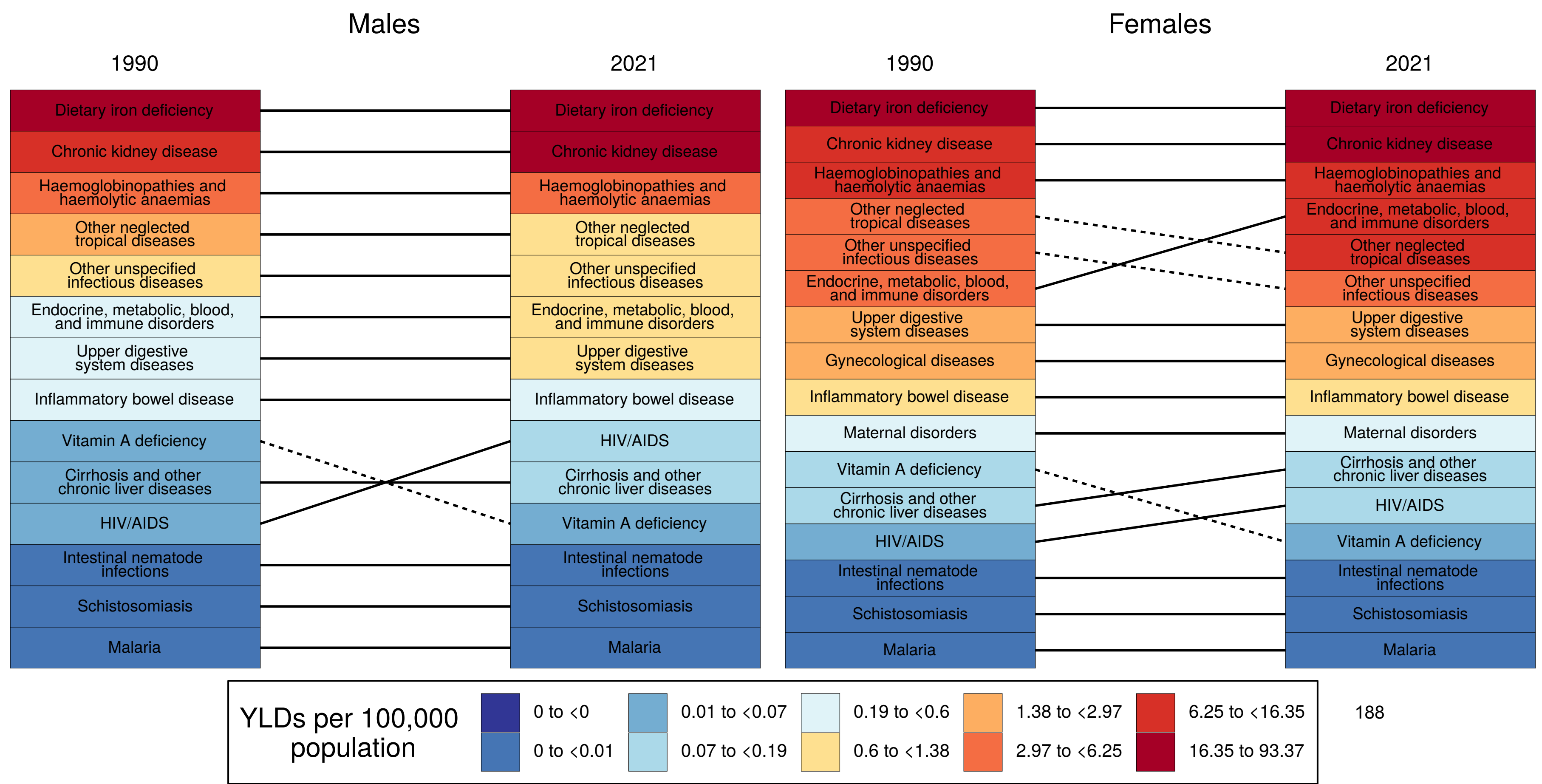
Males
Females
1990
2021
Dietary iron deficiency
Chronic kidney disease
Haemoglobinopathies and haemolytic anaemias
Other neglected tropical diseases
Other unspecified infectious diseases
Endocrine, metabolic, blood, and immune disorders
Upper digestive system diseases
Gynecological diseases
Inflammatory bowel disease
Maternal disorders
Vitamin A deficiency
Cirrhosis and other chronic liver diseases
HIV/AIDS
Intestinal nematode infections
Schistosomiasis
Malaria
YLDs per 100,000 population
0 to <0
0 to <0.01
0.01 to <0.07
0.07 to <0.19
0.19 to <0.6
0.6 to <1.38
1.38 to <2.97
2.97 to <6.25
6.25 to <16.35
16.35 to 93.37

Virginia

Anaemia Prevalence - Both Sexes

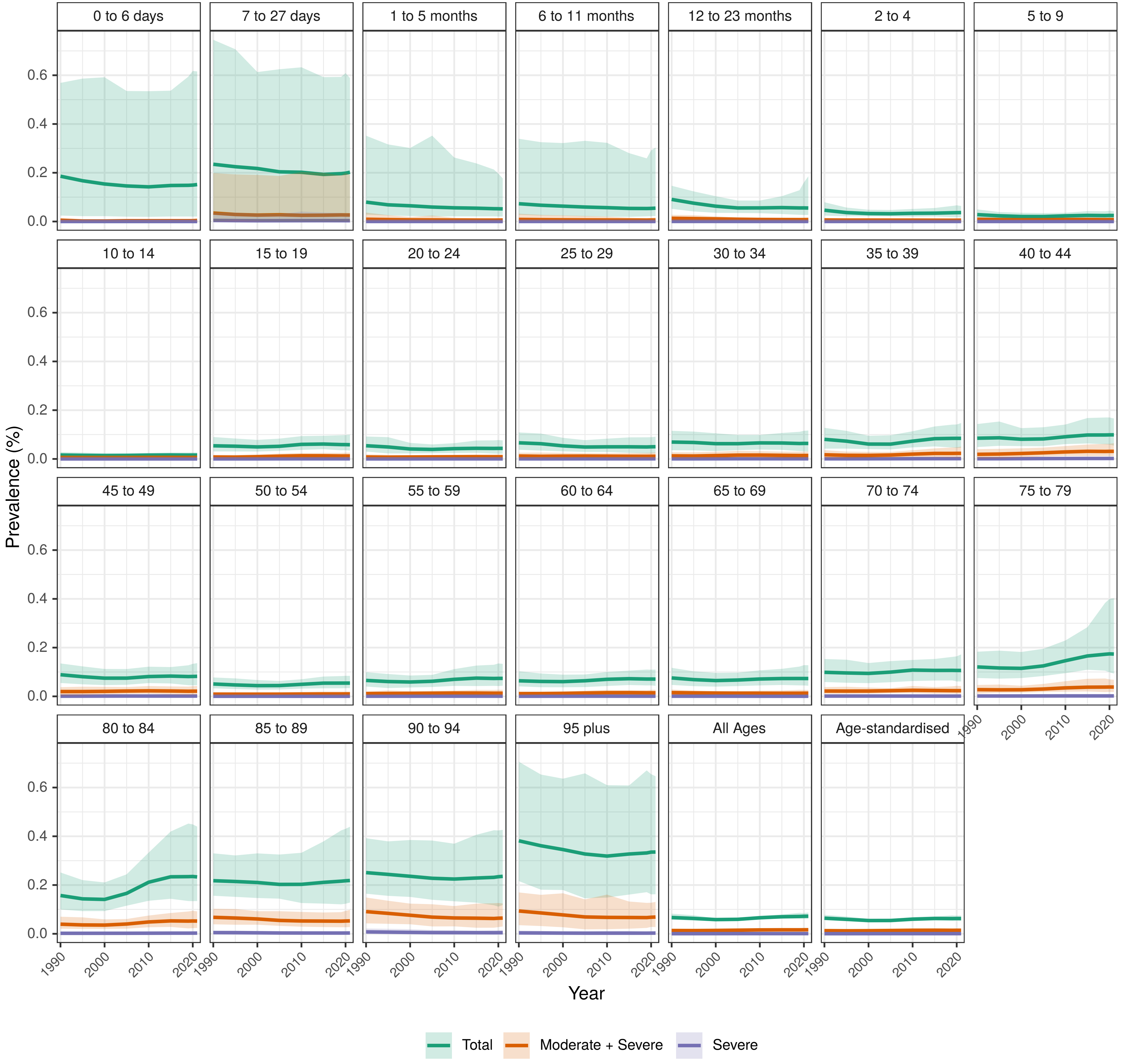

Cause-Specific Anaemia Prevalence - 2021

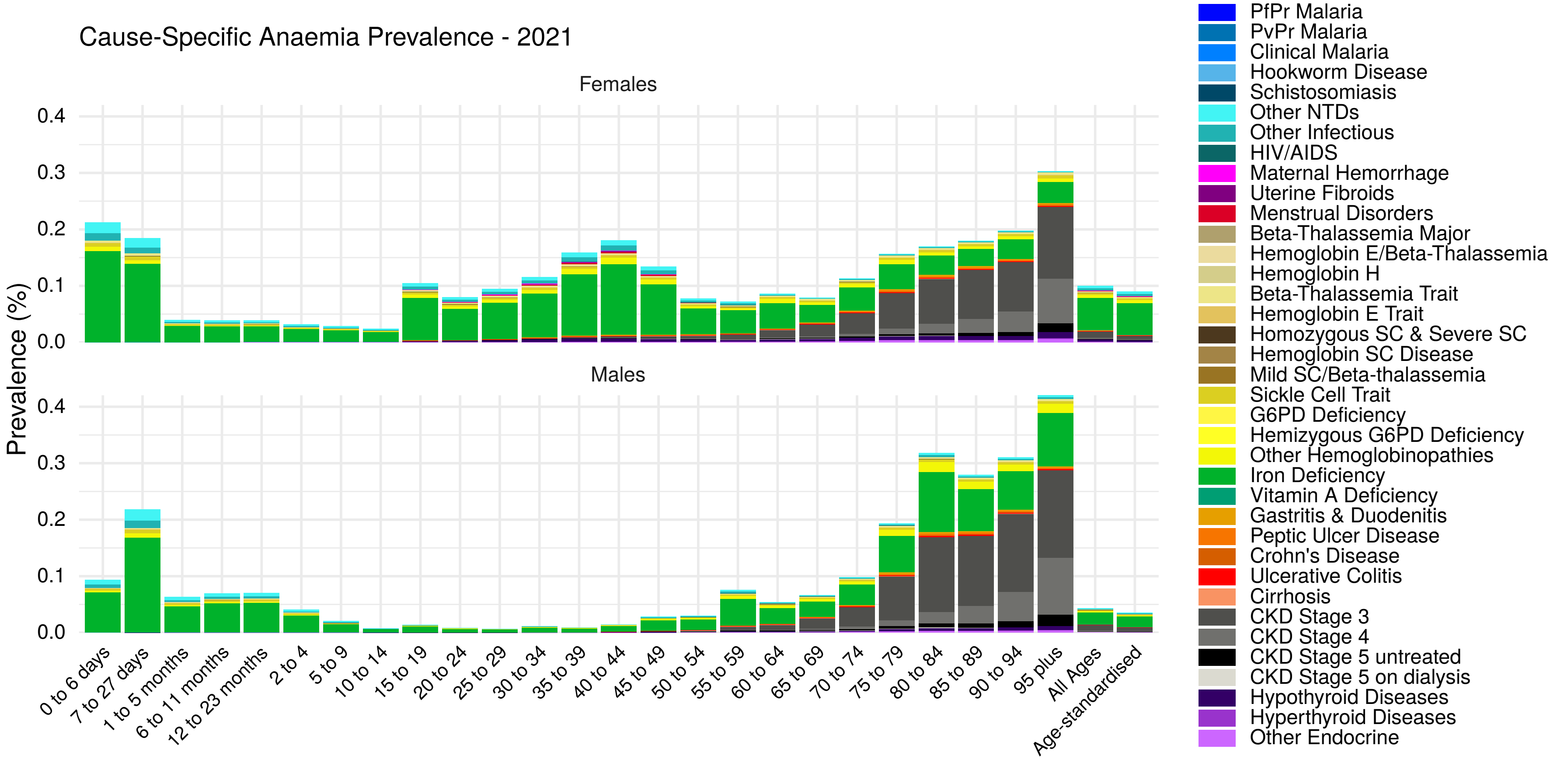

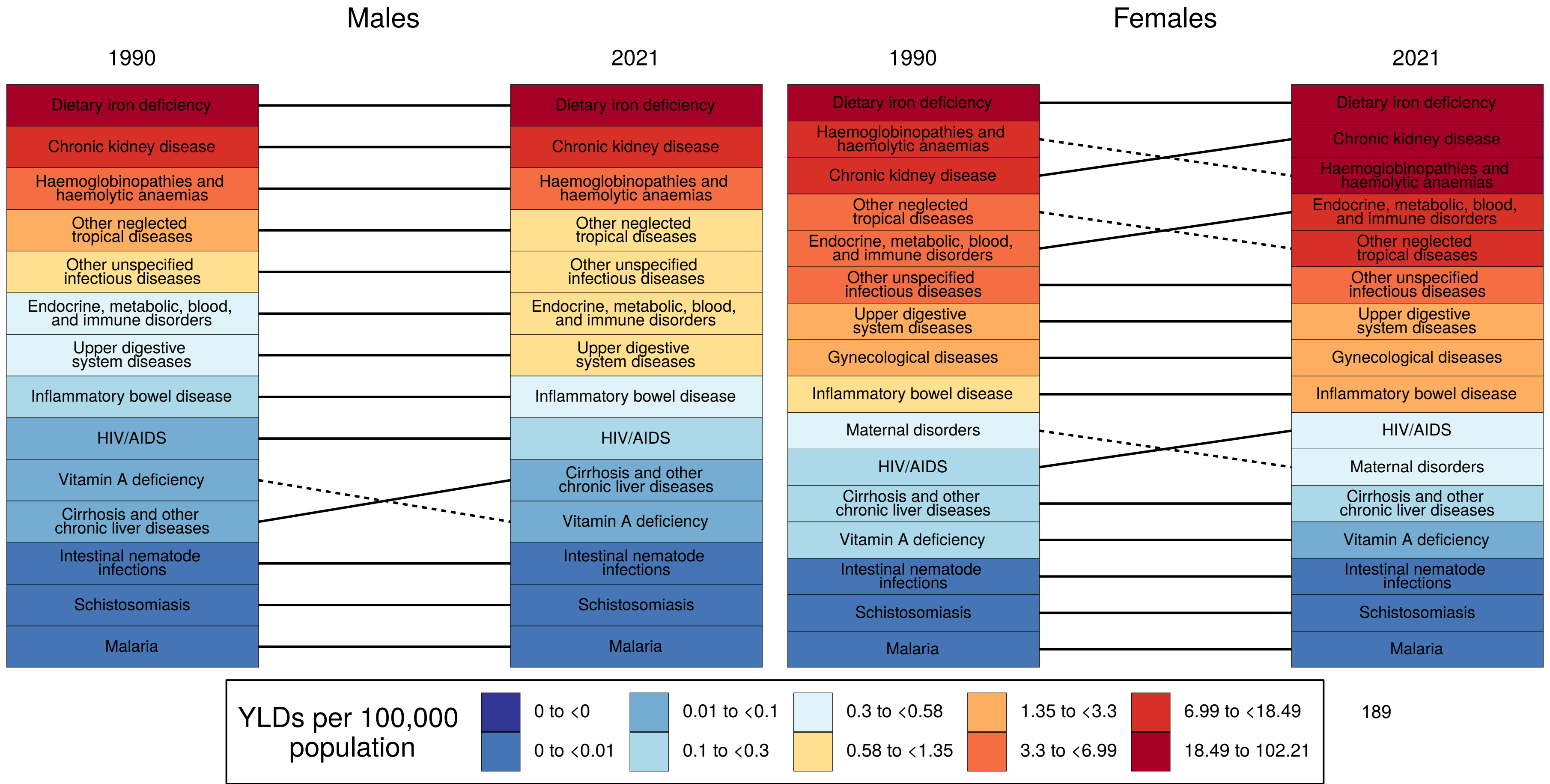

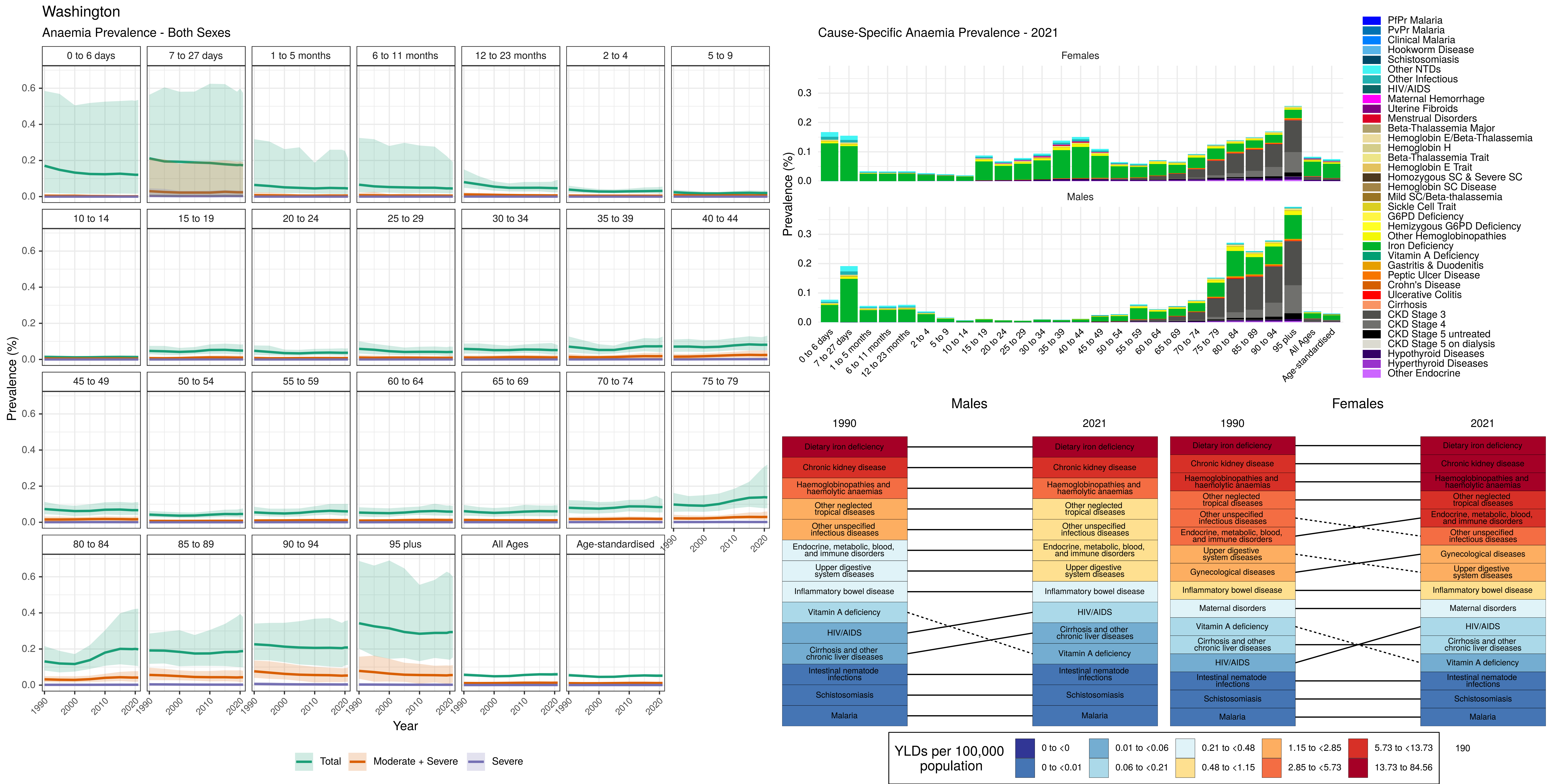

Washington
Anaemia Prevalence - Both Sexes
0 to 6 days
7 to 27 days
1 to 5 months
6 to 11 months
12 to 23 months
2 to 4
5 to 9
10 to 14
15 to 19
20 to 24
25 to 29
30 to 34
35 to 39
40 to 44
45 to 49
50 to 54
55 to 59
60 to 64
65 to 69
70 to 74
75 to 79
80 to 84
85 to 89
90 to 94
95 plus
All Ages
Age-standardised
Prevalence (%)
Year
Total
Moderate + Severe
Severe
Cause-Specific Anaemia Prevalence - 2021
Females
Males
PfPr Malaria
PvPr Malaria
Clinical Malaria
Hookworm Disease
Schistosomiasis
Other NTDs
Other Infectious
HIV/AIDS
Maternal Hemorrhage
Uterine Fibroids
Menstrual Disorders
Beta-Thalassemia Major
Hemoglobin E/Beta-Thalassemia
Hemoglobin H
Beta-Thalassemia Trait
Hemoglobin E Trait
Homozygous SC & Severe SC
Hemoglobin SC Disease
Mild SC/Beta-thalassemia
Sickle Cell Trait
G6PD Deficiency
Hemizygous G6PD Deficiency
Other Hemoglobinopathies
Iron Deficiency
Vitamin A Deficiency
Gastritis & Duodenitis
Peptic Ulcer Disease
Crohn's Disease
Ulcerative Colitis
Cirrhosis
CKD Stage 3
CKD Stage 4
CKD Stage 5 untreated
CKD Stage 5 on dialysis
Hypothyroid Diseases
Hyperthyroid Diseases
Other Endocrine
Males
Females
1990
2021
Dietary iron deficiency
Chronic kidney disease
Haemoglobinopathies and haemolytic anaemias
Other neglected tropical diseases
Other unspecified infectious diseases
Endocrine, metabolic, blood, and immune disorders
Upper digestive system diseases
Gynecological diseases
Inflammatory bowel disease
Maternal disorders
Vitamin A deficiency
HIV/AIDS
Cirrhosis and other chronic liver diseases
Intestinal nematode infections
Schistosomiasis
Malaria
YLDs per 100,000 population
0 to <0
0 to <0.01
0.01 to <0.06
0.06 to <0.21
0.21 to <0.48
0.48 to <1.15
1.15 to <2.85
2.85 to <5.73
5.73 to <13.73
13.73 to 84.56

West Virginia

Anaemia Prevalence - Both Sexes

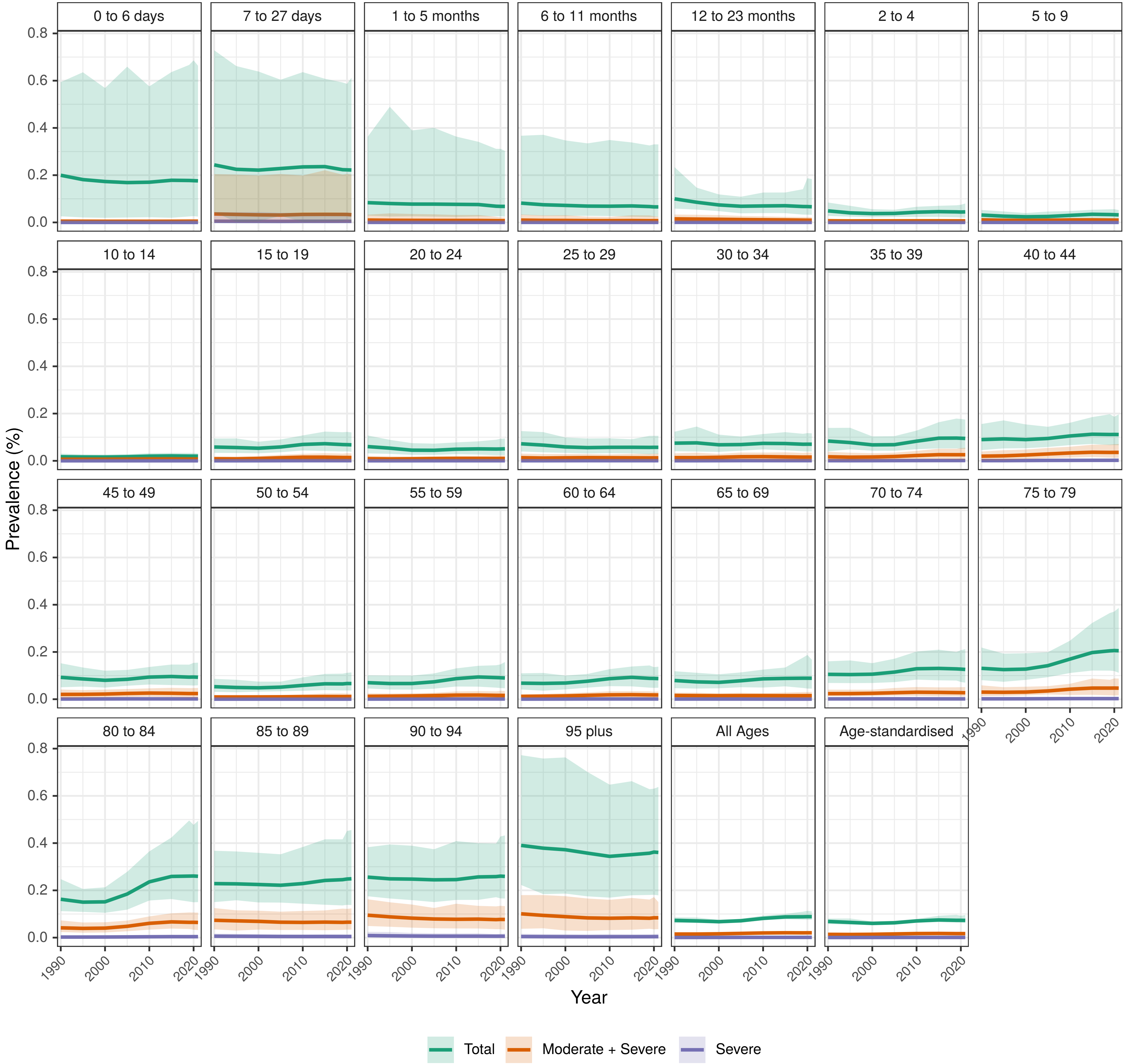

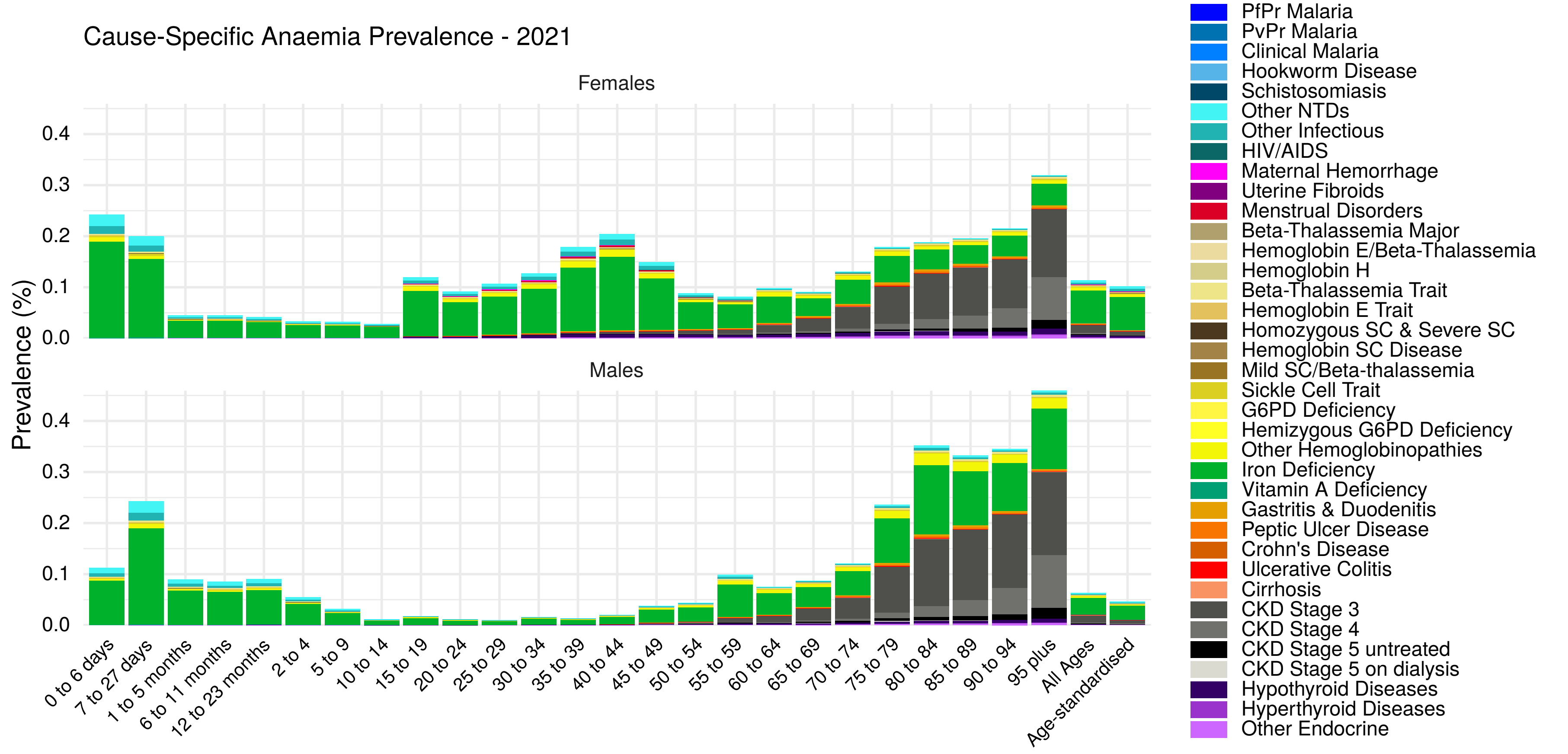

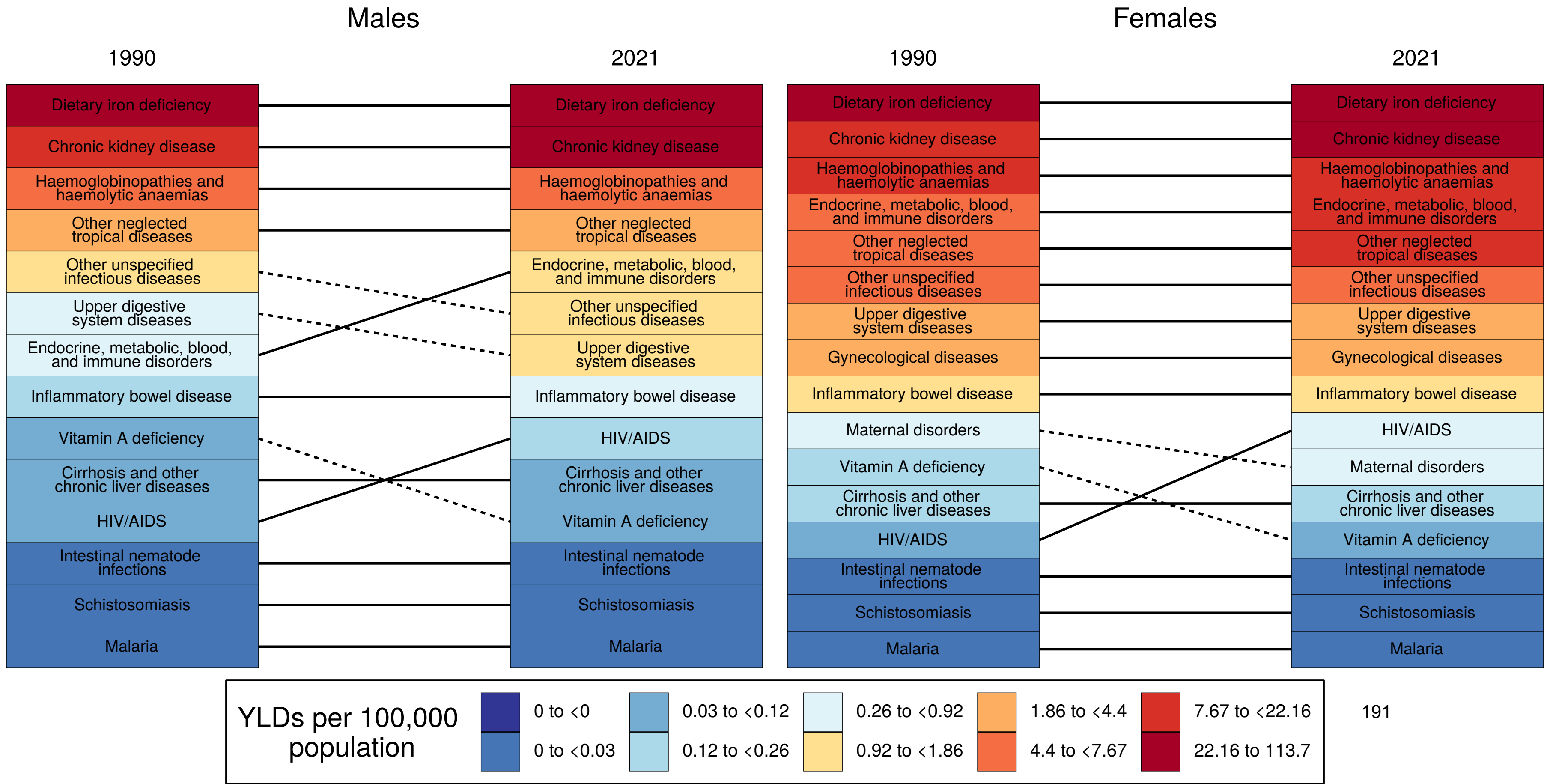

Wisconsin

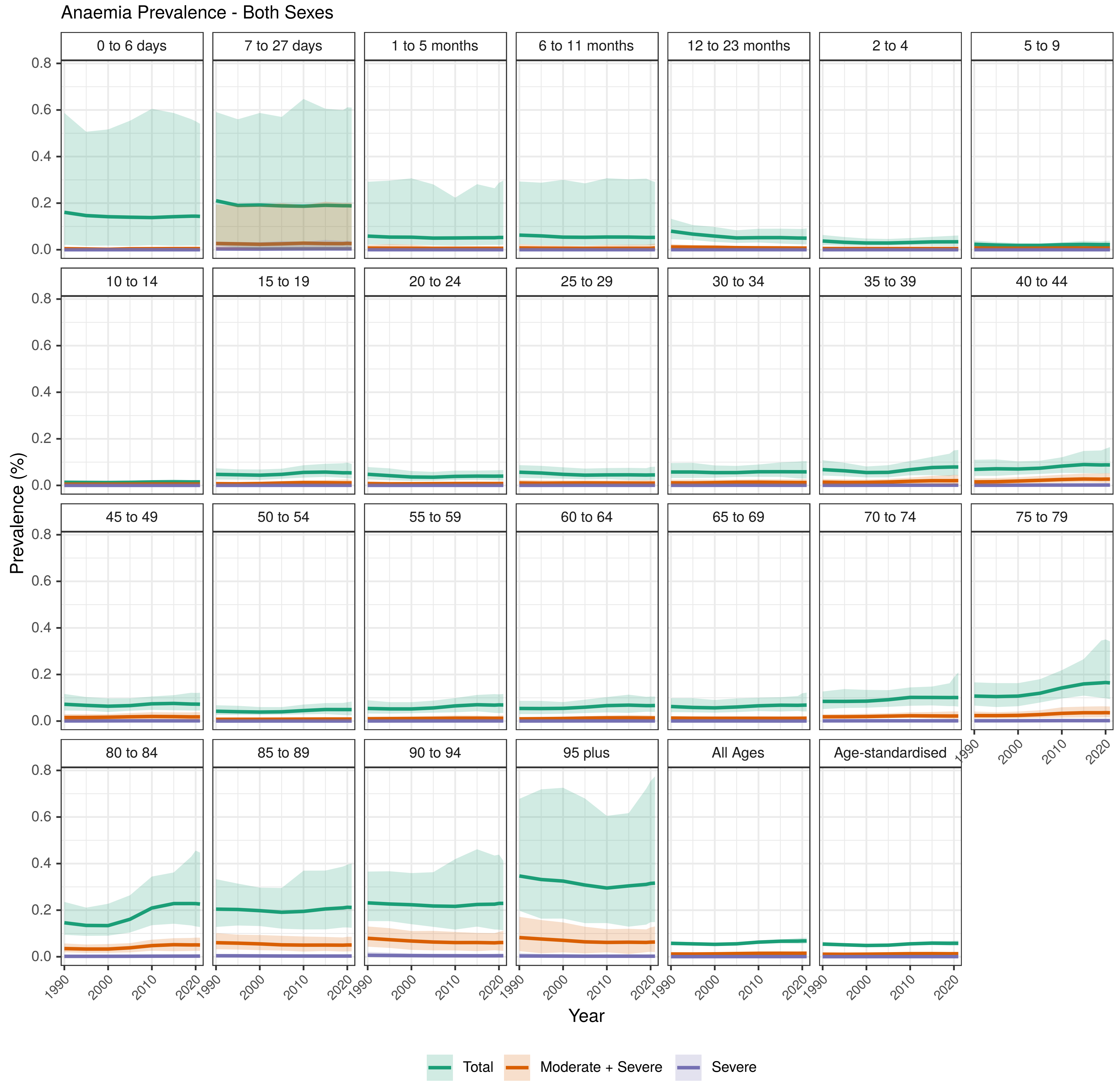

Anaemia Prevalence - Both Sexes
0 to 6 days
7 to 27 days
1 to 5 months
6 to 11 months
12 to 23 months
2 to 4
5 to 9
10 to 14
15 to 19
20 to 24
25 to 29
30 to 34
35 to 39
40 to 44
45 to 49
50 to 54
55 to 59
60 to 64
65 to 69
70 to 74
75 to 79
80 to 84
85 to 89
90 to 94
95 plus
All Ages
Age-standardised
Prevalence (%)
Year
Total
Moderate + Severe
Severe

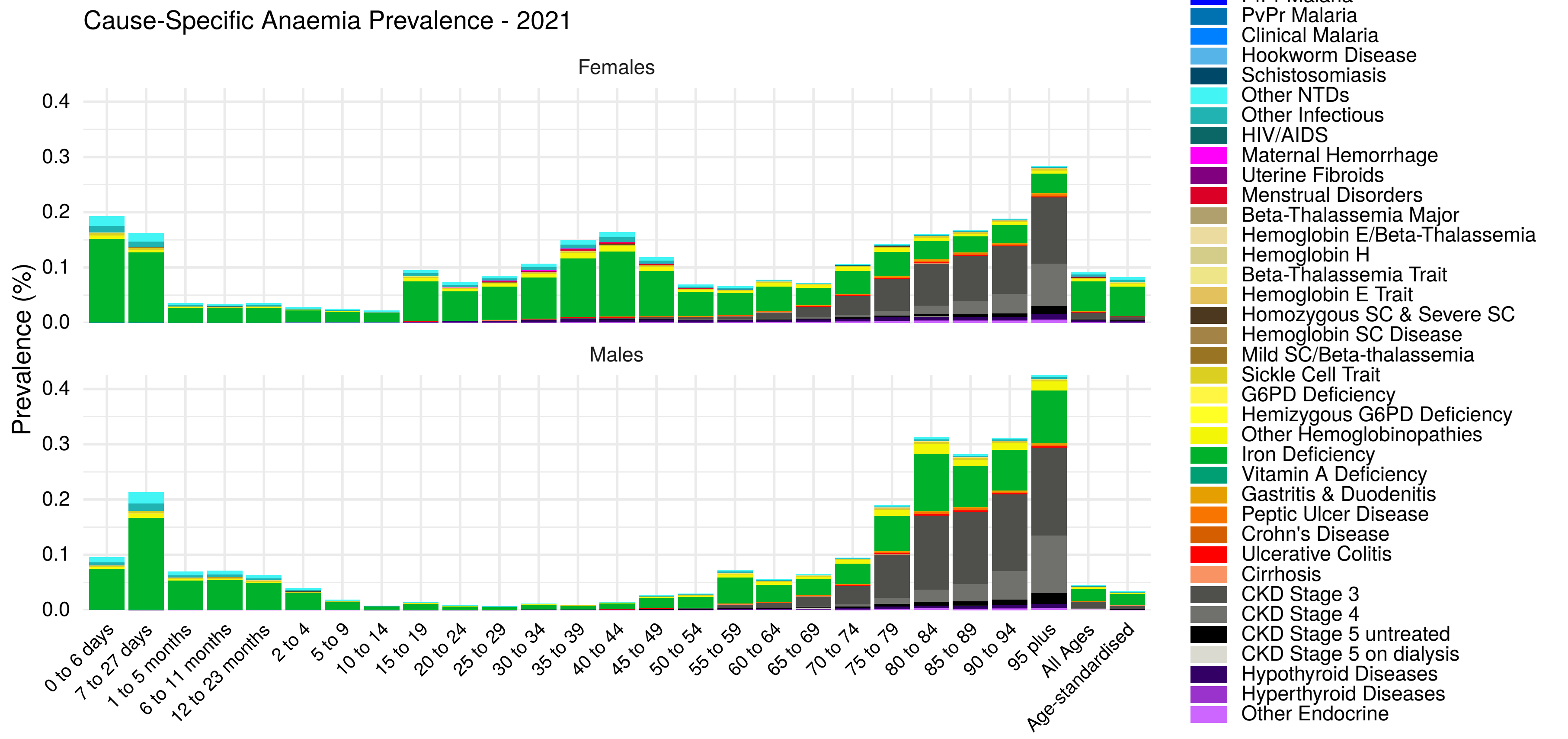

Cause-Specific Anaemia Prevalence - 2021
Females
Males
Prevalence (%)
PfPr Malaria
PvPr Malaria
Clinical Malaria
Hookworm Disease
Schistosomiasis
Other NTDs
Other Infectious
HIV/AIDS
Maternal Hemorrhage
Uterine Fibroids
Menstrual Disorders
Beta-Thalassemia Major
Hemoglobin E/Beta-Thalassemia
Hemoglobin H
Beta-Thalassemia Trait
Hemoglobin E Trait
Homozygous SC & Severe SC
Hemoglobin SC Disease
Mild SC/Beta-thalassemia
Sickle Cell Trait
G6PD Deficiency
Hemizygous G6PD Deficiency
Other Hemoglobinopathies
Iron Deficiency
Vitamin A Deficiency
Gastritis & Duodenitis
Peptic Ulcer Disease
Crohn's Disease
Ulcerative Colitis
Cirrhosis
CKD Stage 3
CKD Stage 4
CKD Stage 5 untreated
CKD Stage 5 on dialysis
Hypothyroid Diseases
Hyperthyroid Diseases
Other Endocrine

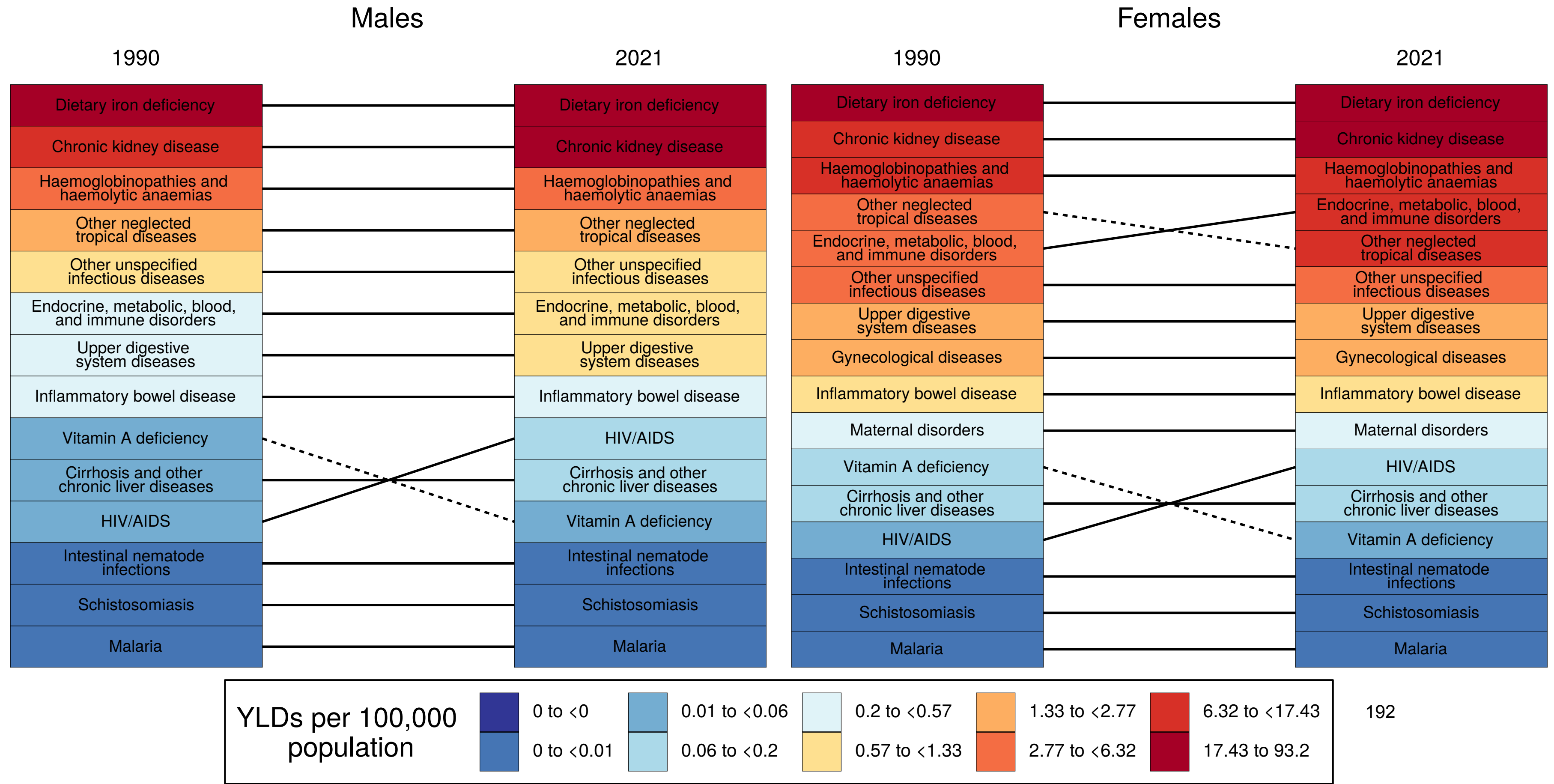

Males
Females
1990
2021
Dietary iron deficiency
Chronic kidney disease
Haemoglobinopathies and haemolytic anaemias
Other neglected tropical diseases
Other unspecified infectious diseases
Endocrine, metabolic, blood, and immune disorders
Upper digestive system diseases
Inflammatory bowel disease
Gynecological diseases
Maternal disorders
Vitamin A deficiency
Cirrhosis and other chronic liver diseases
HIV/AIDS
Intestinal nematode infections
Schistosomiasis
Malaria
YLDs per 100,000 population
0 to <0
0 to <0.01
0.01 to <0.06
0.06 to <0.2
0.2 to <0.57
0.57 to <1.33
1.33 to <2.77
2.77 to <6.32
6.32 to <17.43
17.43 to 93.2

Wyoming

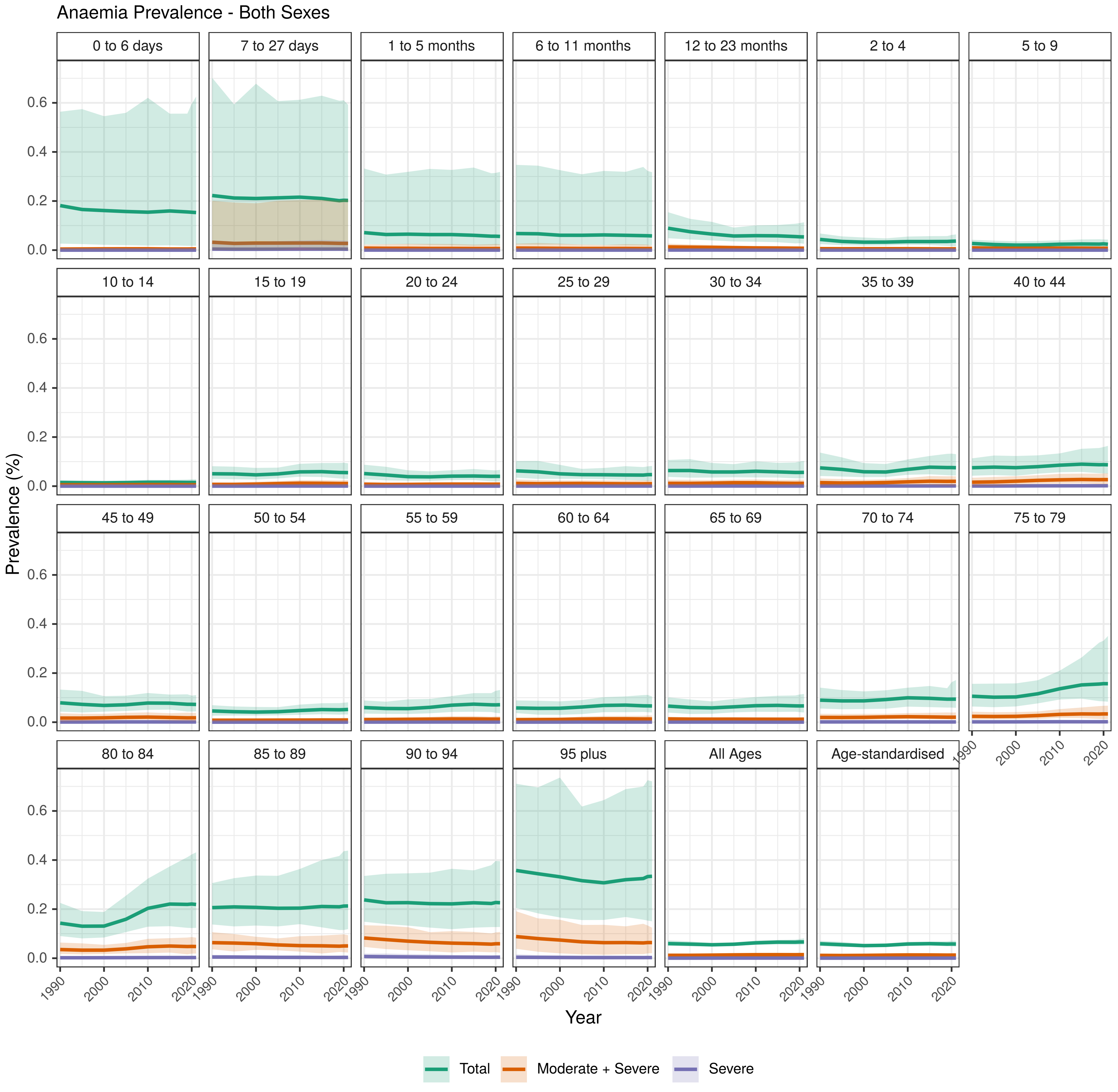

Anaemia Prevalence - Both Sexes
0 to 6 days
7 to 27 days
1 to 5 months
6 to 11 months
12 to 23 months
2 to 4
5 to 9
10 to 14
15 to 19
20 to 24
25 to 29
30 to 34
35 to 39
40 to 44
45 to 49
50 to 54
55 to 59
60 to 64
65 to 69
70 to 74
75 to 79
80 to 84
85 to 89
90 to 94
95 plus
All Ages
Age-standardised
Prevalence (%)
0.0
0.2
0.4
0.6
1990
2000
2010
2020
Year
Total
Moderate + Severe
Severe

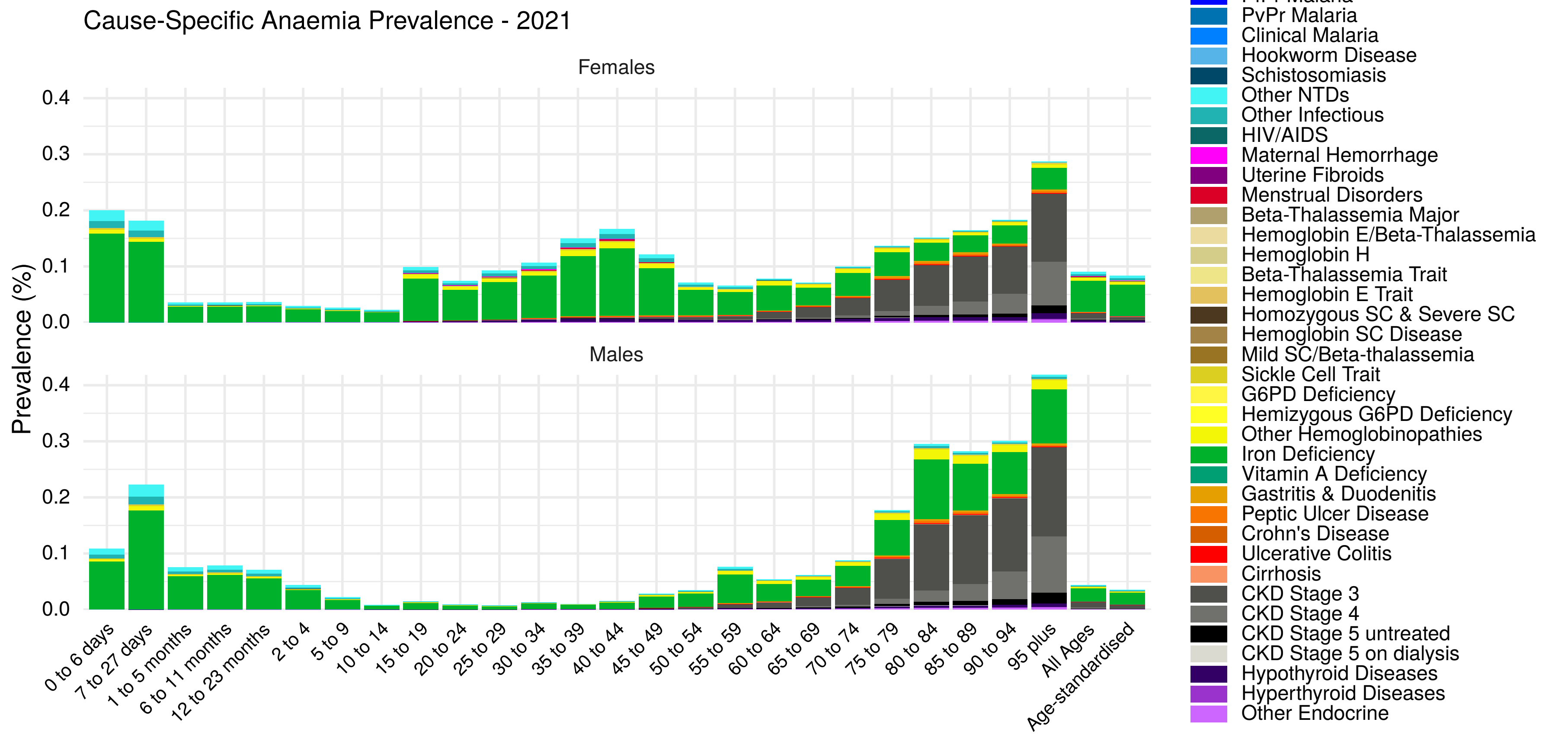

Cause-Specific Anaemia Prevalence - 2021
Females
Males
Prevalence (%)
0.0
0.1
0.2
0.3
0.4
0 to 6 days
7 to 27 days
1 to 5 months
6 to 11 months
12 to 23 months
2 to 4
5 to 9
10 to 14
15 to 19
20 to 24
25 to 29
30 to 34
35 to 39
40 to 44
45 to 49
50 to 54
55 to 59
60 to 64
65 to 69
70 to 74
75 to 79
80 to 84
85 to 89
90 to 94
95 plus
All Ages
Age-standardised
PfPr Malaria
PvPr Malaria
Clinical Malaria
Hookworm Disease
Schistosomiasis
Other NTDs
Other Infectious
HIV/AIDS
Maternal Hemorrhage
Uterine Fibroids
Menstrual Disorders
Beta-Thalassemia Major
Hemoglobin E/Beta-Thalassemia
Hemoglobin H
Beta-Thalasemia Trait
Hemoglobin E Trait
Homozygous SC & Severe SC
Hemoglobin SC Disease
Mild SC/Beta-thalassemia
Sickle Cell Trait
G6PD Deficiency
Hemizygous G6PD Deficiency
Other Hemoglobinopathies
Iron Deficiency
Vitamin A Deficiency
Gastritis & Duodenitis
Peptic Ulcer Disease
Crohn's Disease
Ulcerative Colitis
Cirrhosis
CKD Stage 3
CKD Stage 4
CKD Stage 5 untreated
CKD Stage 5 on dialysis
Hypothyroid Diseases
Hyperthyroid Diseases
Other Endocrine

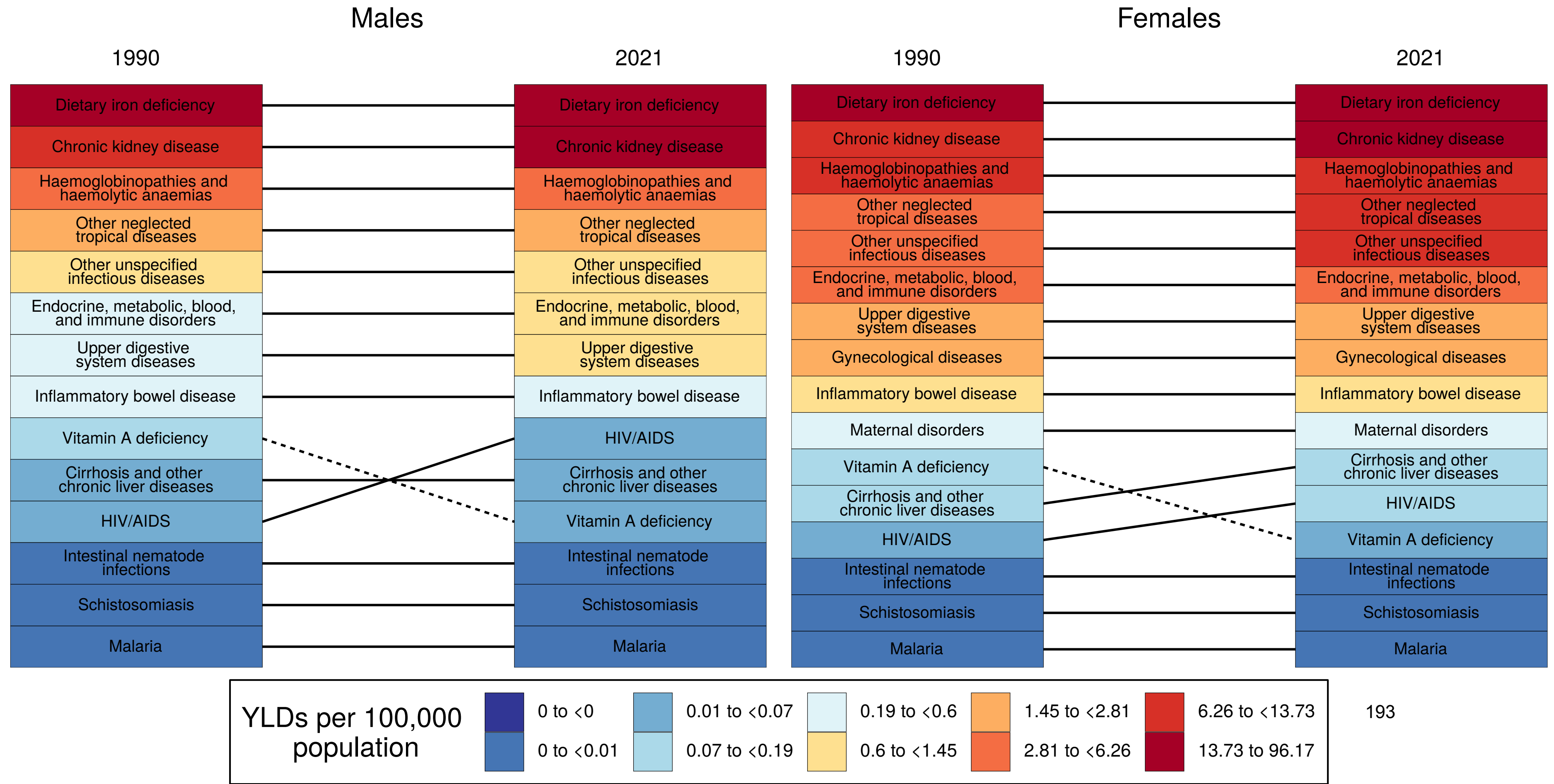

Males
Females
1990
2021
Dietary iron deficiency
Chronic kidney disease
Haemoglobinopathies and haemolytic anaemias
Other neglected tropical diseases
Other unspecified infectious diseases
Endocrine, metabolic, blood, and immune disorders
Upper digestive system diseases
Inflammatory bowel disease
Vitamin A deficiency
Cirrhosis and other chronic liver diseases
HIV/AIDS
Intestinal nematode infections
Schistosomiasis
Malaria
Gynecological diseases
Maternal disorders
YLDs per 100,000 population
0 to <0
0 to <0.01
0.01 to <0.07
0.07 to <0.19
0.19 to <0.6
0.6 to <1.45
1.45 to <2.81
2.81 to <6.26
6.26 to <13.73
13.73 to 96.17

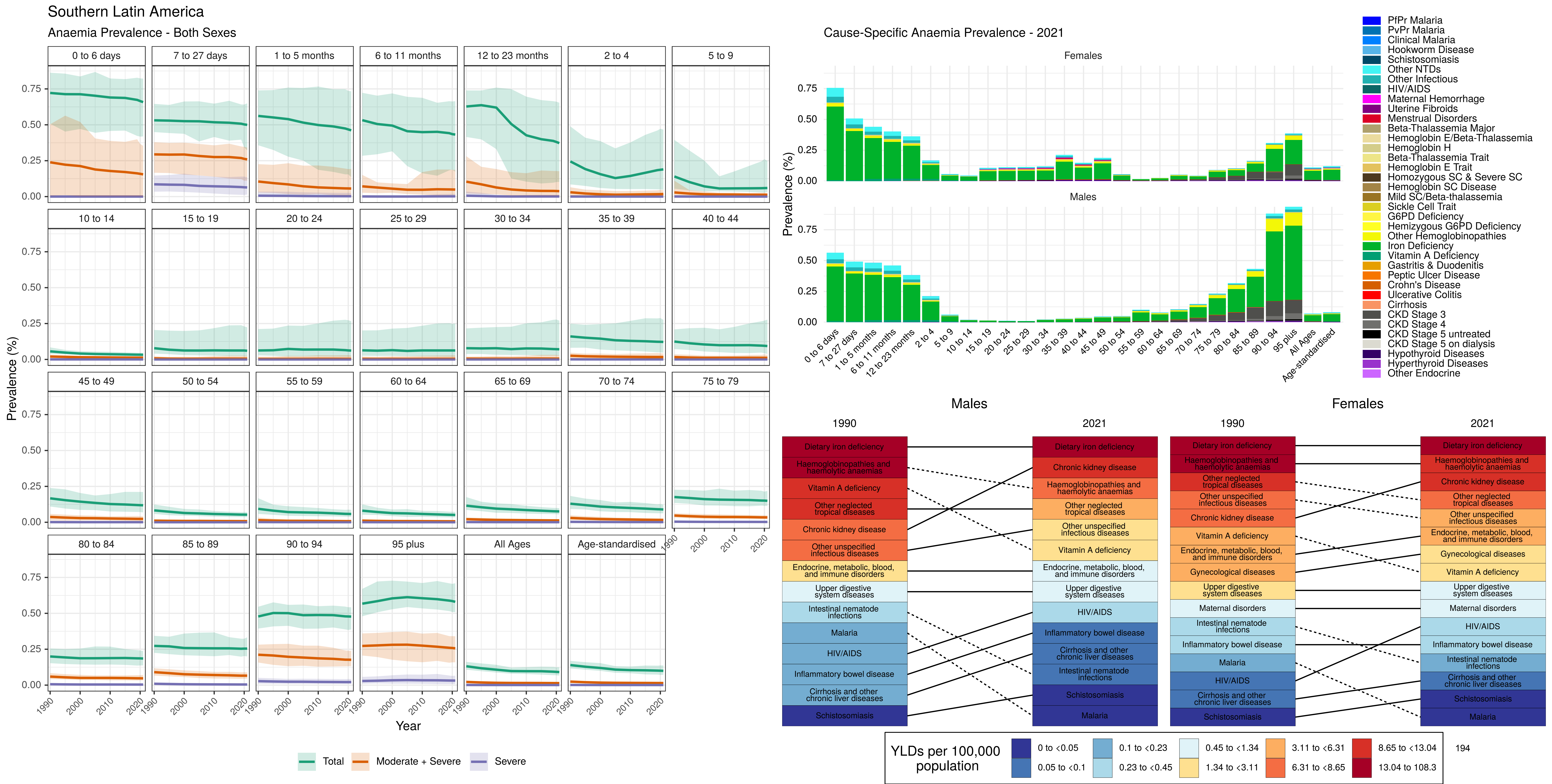

Southern Latin America
Anaemia Prevalence - Both Sexes
0 to 6 days
7 to 27 days
1 to 5 months
6 to 11 months
12 to 23 months
2 to 4
5 to 9
10 to 14
15 to 19
20 to 24
25 to 29
30 to 34
35 to 39
40 to 44
45 to 49
50 to 54
55 to 59
60 to 64
65 to 69
70 to 74
75 to 79
80 to 84
85 to 89
90 to 94
95 plus
All Ages
Age-standardised
Prevalence (%)
Year
Total
Moderate + Severe
Severe
Cause-Specific Anaemia Prevalence - 2021
Females
Males
PfPr Malaria
PvPr Malaria
Clinical Malaria
Hookworm Disease
Schistosomiasis
Other NTDs
Other Infectious
HIV/AIDS
Maternal Hemorrhage
Uterine Fibroids
Menstrual Disorders
Beta-Thalassemia Major
Hemoglobin E/Beta-Thalassemia
Hemoglobin H
Beta-Thalassemia Trait
Hemoglobin E Trait
Homozygous SC & Severe SC
Hemoglobin SC Disease
Mild SC/Beta-thalassemia
Sickle Cell Trait
G6PD Deficiency
Hemizygous G6PD Deficiency
Other Hemoglobinopathies
Iron Deficiency
Vitamin A Deficiency
Gastritis & Duodenitis
Peptic Ulcer Disease
Crohn's Disease
Ulcerative Colitis
Cirrhosis
CKD Stage 3
CKD Stage 4
CKD Stage 5 untreated
CKD Stage 5 on dialysis
Hypothyroid Diseases
Hyperthyroid Diseases
Other Endocrine
Males
Females
1990
2021
Dietary iron deficiency
Haemoglobinopathies and haemolytic anaemias
Vitamin A deficiency
Other neglected tropical diseases
Chronic kidney disease
Other unspecified infectious diseases
Endocrine, metabolic, blood, and immune disorders
Upper digestive system diseases
Intestinal nematode infections
Malaria
HIV/AIDS
Inflammatory bowel disease
Cirrhosis and other chronic liver diseases
Schistosomiasis
Maternal disorders
Gynecological diseases
YLDs per 100,000 population
0 to <0.05
0.05 to <0.1
0.1 to <0.23
0.23 to <0.45
0.45 to <1.34
1.34 to <3.11
3.11 to <6.31
6.31 to <8.65
8.65 to <13.04
13.04 to 108.3

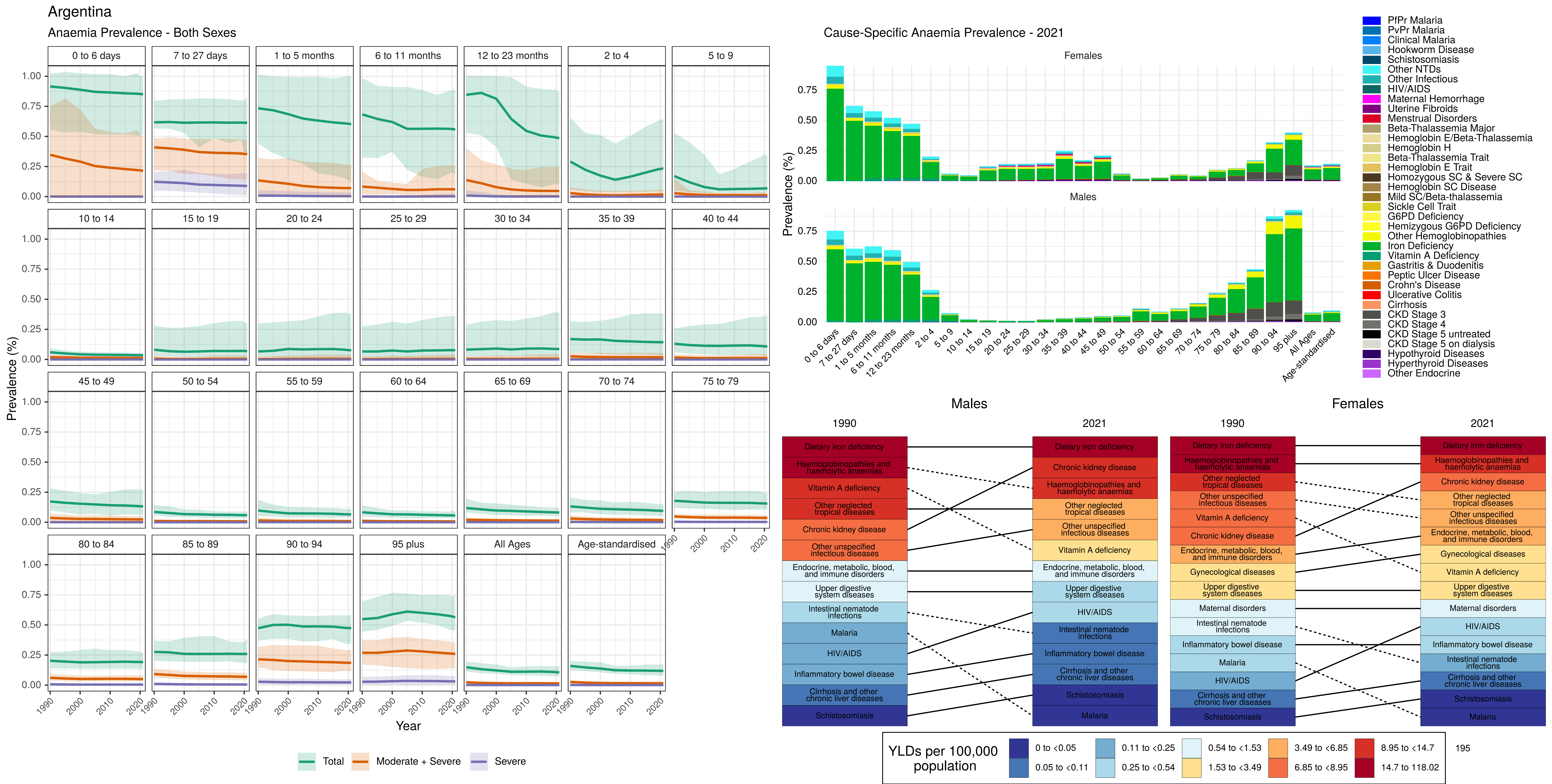
Argentina
Anaemia Prevalence - Both Sexes
0 to 6 days
7 to 27 days
1 to 5 months
6 to 11 months
12 to 23 months
2 to 4
5 to 9
10 to 14
15 to 19
20 to 24
25 to 29
30 to 34
35 to 39
40 to 44
45 to 49
50 to 54
55 to 59
60 to 64
65 to 69
70 to 74
75 to 79
80 to 84
85 to 89
90 to 94
95 plus
All Ages
Age-standardised
Prevalence (%)
Year
Total
Moderate + Severe
Severe
Cause-Specific Anaemia Prevalence - 2021
Females
Males
PfPr Malaria
PvPr Malaria
Clinical Malaria
Hookworm Disease
Schistosomiasis
Other NTDs
Other Infectious
HIV/AIDS
Maternal Hemorrhage
Uterine Fibroids
Menstrual Disorders
Beta-Thalassemia Major
Hemoglobin E/Beta-Thalassemia
Hemoglobin H
Beta-Thalassemia Trait
Hemoglobin E Trait
Homozygous SC & Severe SC
Hemoglobin SC Disease
Mild SC/Beta-thalassemia
Sickle Cell Trait
G6PD Deficiency
Hemizygous G6PD Deficiency
Other Hemoglobinopathies
Iron Deficiency
Vitamin A Deficiency
Gastritis & Duodenitis
Peptic Ulcer Disease
Crohn's Disease
Ulcerative Colitis
Cirrhosis
CKD Stage 3
CKD Stage 4
CKD Stage 5 untreated
CKD Stage 5 on dialysis
Hypothyroid Diseases
Hyperthyroid Diseases
Other Endocrine
Males
1990
Dietary iron deficiency
Haemoglobinopathies and haemolytic anaemias
Vitamin A deficiency
Other neglected tropical diseases
Chronic kidney disease
Other unspecified infectious diseases
Endocrine, metabolic, blood, and immune disorders
Upper digestive system diseases
Intestinal nematode infections
Malaria
HIV/AIDS
Inflammatory bowel disease
Cirrhosis and other chronic liver diseases
Schistosomiasis
2021
Dietary iron deficiency
Chronic kidney disease
Haemoglobinopathies and haemolytic anaemias
Other neglected tropical diseases
Other unspecified infectious diseases
Vitamin A deficiency
Endocrine, metabolic, blood, and immune disorders
Upper digestive system diseases
HIV/AIDS
Intestinal nematode infections
Inflammatory bowel disease
Cirrhosis and other chronic liver diseases
Schistosomiasis
Malaria
Females
1990
Dietary iron deficiency
Haemoglobinopathies and haemolytic anaemias
Other neglected tropical diseases
Other unspecified infectious diseases
Vitamin A deficiency
Chronic kidney disease
Endocrine, metabolic, blood, and immune disorders
Gynecological diseases
Upper digestive system diseases
Maternal disorders
Intestinal nematode infections
Inflammatory bowel disease
Malaria
HIV/AIDS
Cirrhosis and other chronic liver diseases
Schistosomiasis
2021
Dietary iron deficiency
Haemoglobinopathies and haemolytic anaemias
Chronic kidney disease
Other neglected tropical diseases
Other unspecified infectious diseases
Endocrine, metabolic, blood, and immune disorders
Gynecological diseases
Vitamin A deficiency
Upper digestive system diseases
Maternal disorders
HIV/AIDS
Inflammatory bowel disease
Intestinal nematode infections
Cirrhosis and other chronic liver diseases
Schistosomiasis
Malaria
YLDs per 100,000 population
0 to <0.05
0.05 to <0.11
0.11 to <0.25
0.25 to <0.54
0.54 to <1.53
1.53 to <3.49
3.49 to <6.85
6.85 to <8.95
8.95 to <14.7
14.7 to 118.02

Chile

Anaemia Prevalence - Both Sexes

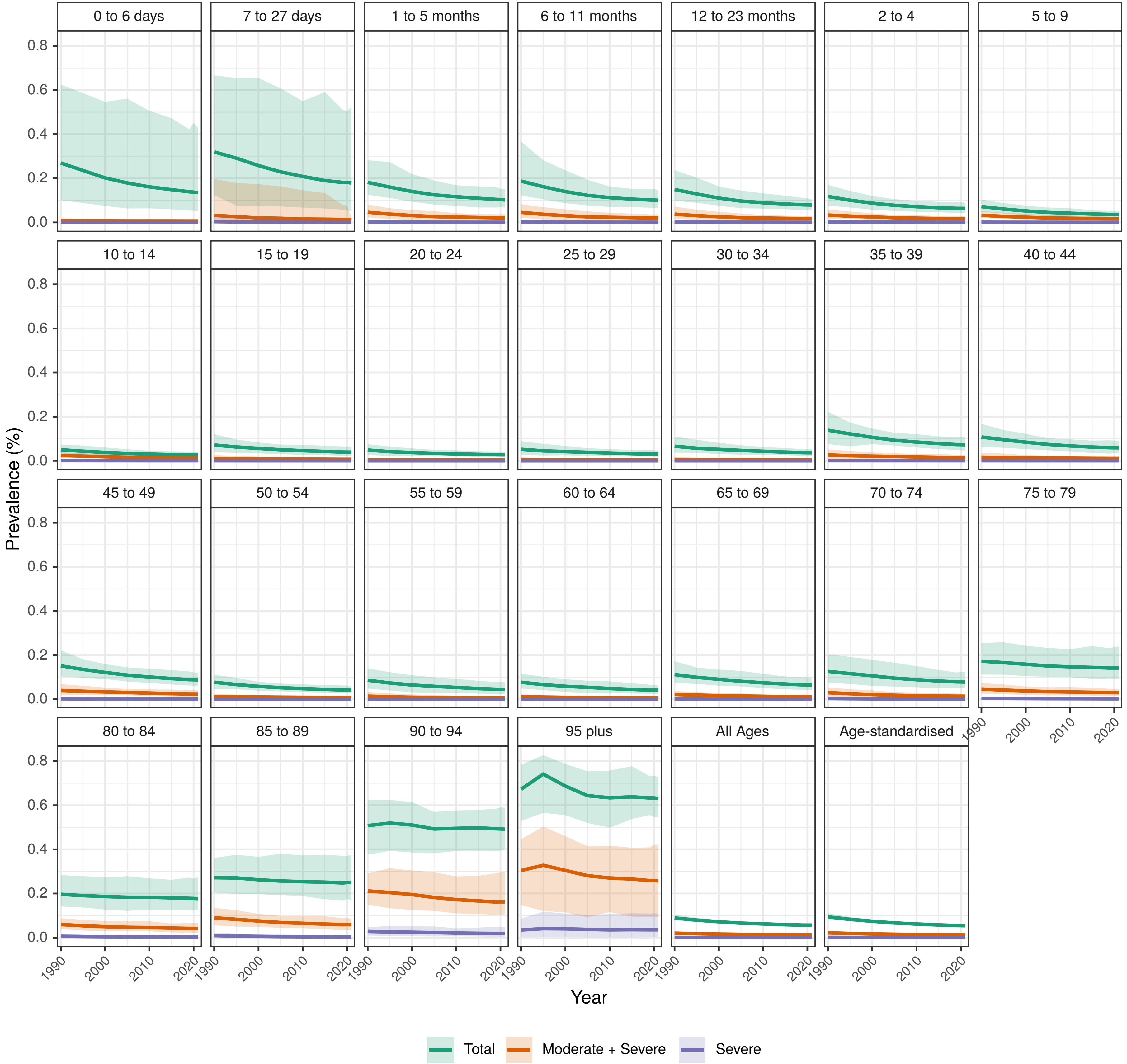

Cause-Specific Anaemia Prevalence - 2021

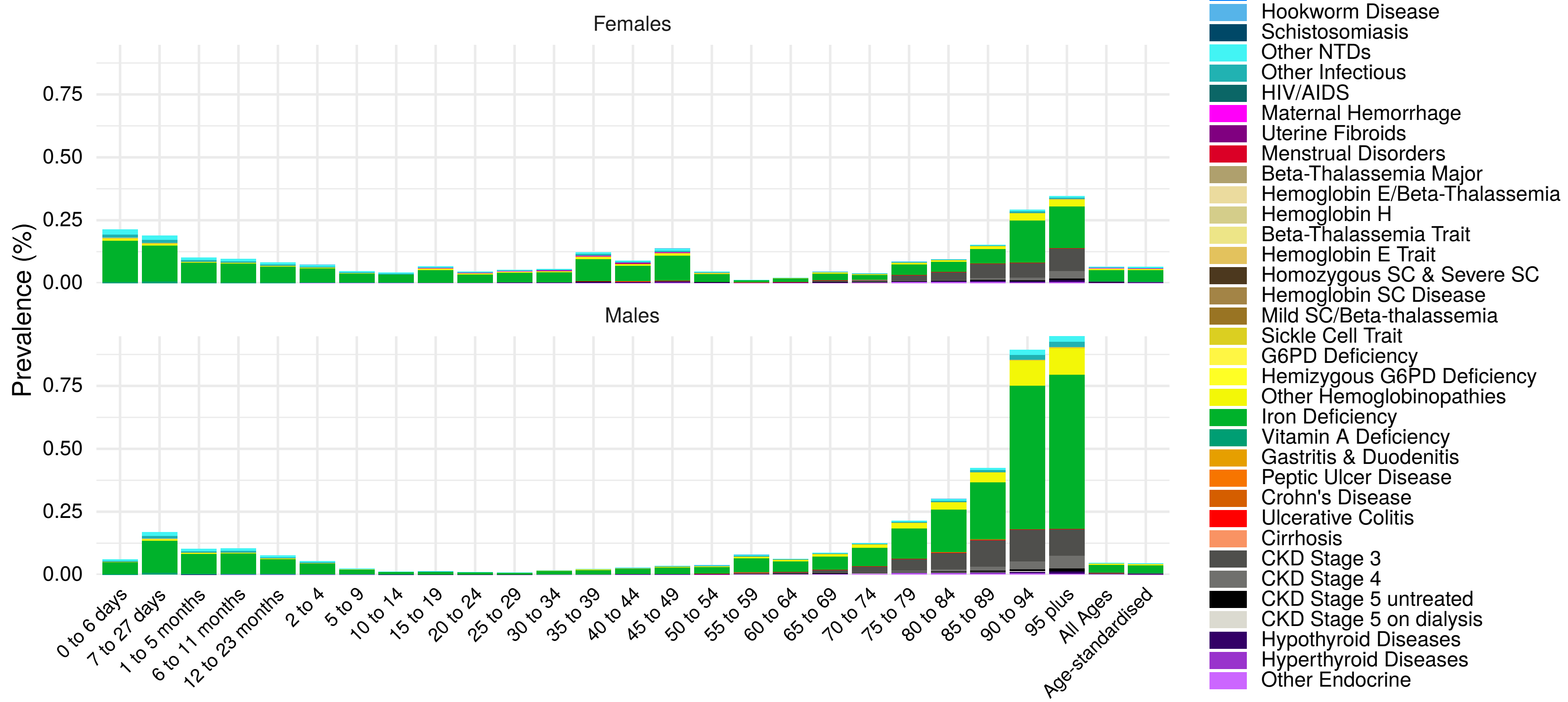

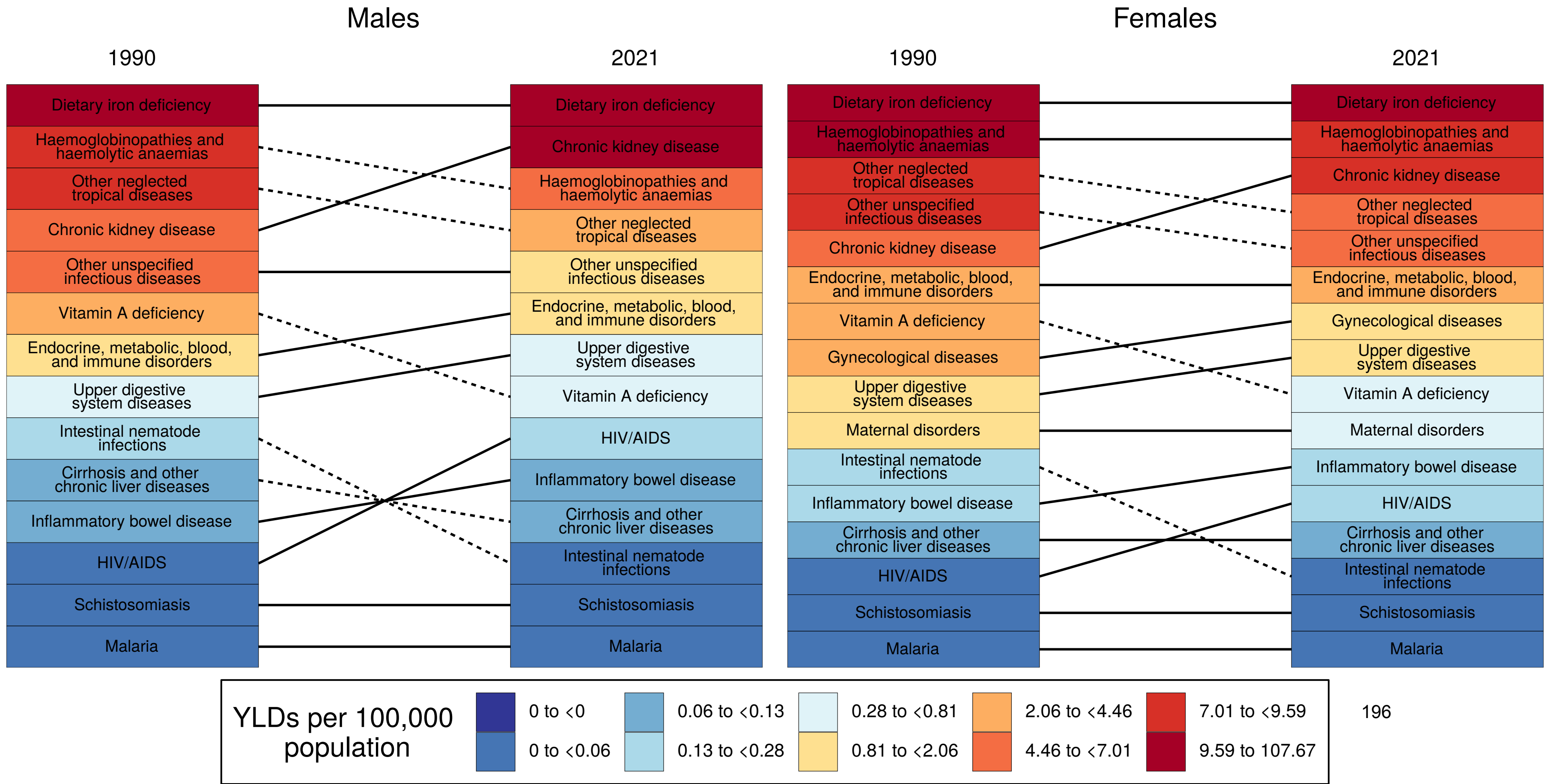

Uruguay

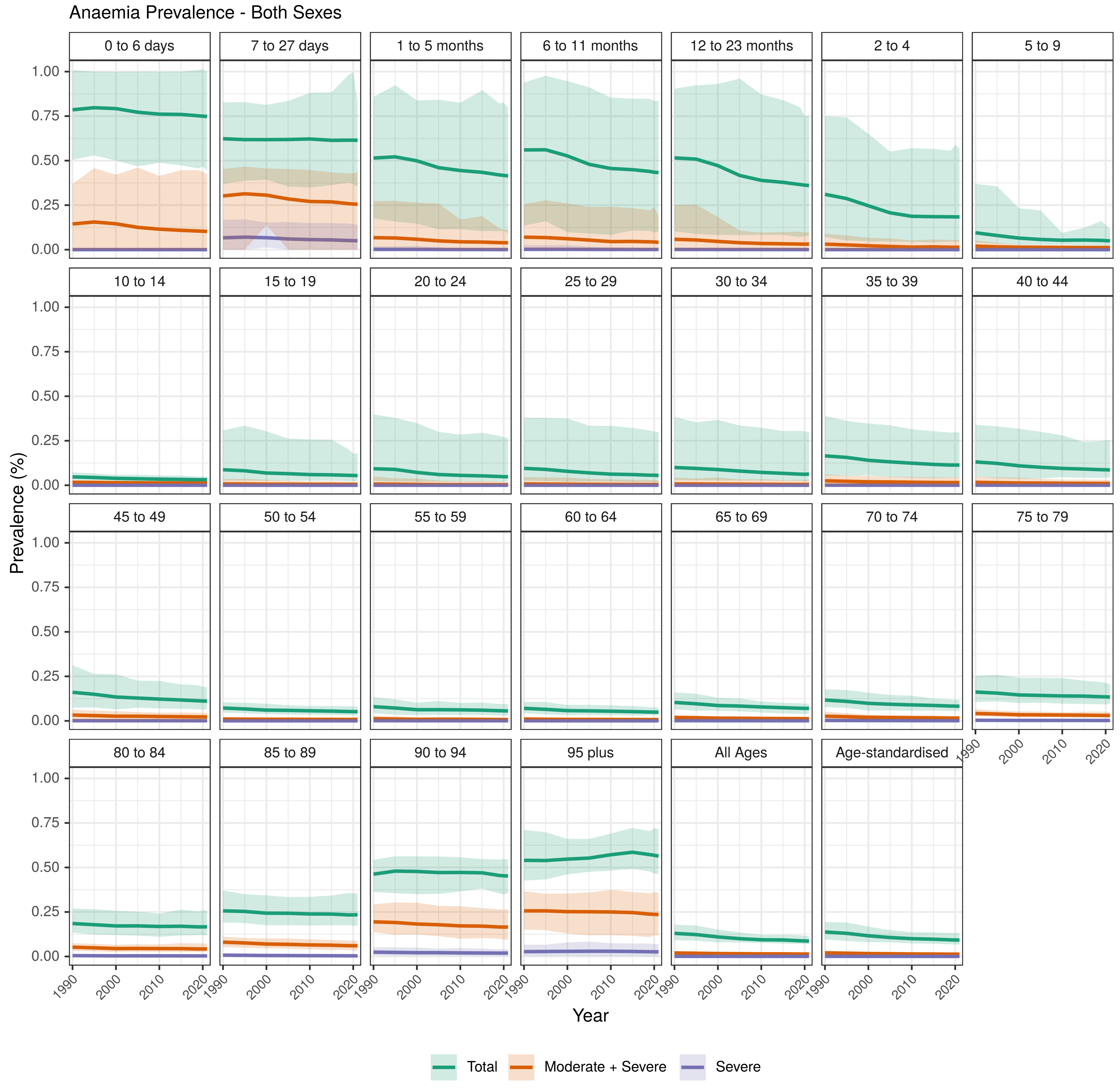

Anaemia Prevalence - Both Sexes
0 to 6 days
7 to 27 days
1 to 5 months
6 to 11 months
12 to 23 months
2 to 4
5 to 9
10 to 14
15 to 19
20 to 24
25 to 29
30 to 34
35 to 39
40 to 44
45 to 49
50 to 54
55 to 59
60 to 64
65 to 69
70 to 74
75 to 79
80 to 84
85 to 89
90 to 94
95 plus
All Ages
Age-standardised
Prevalence (%)
Year
1990 2000 2010 2020
0.00 0.25 0.50 0.75 1.00
Total
Moderate + Severe
Severe

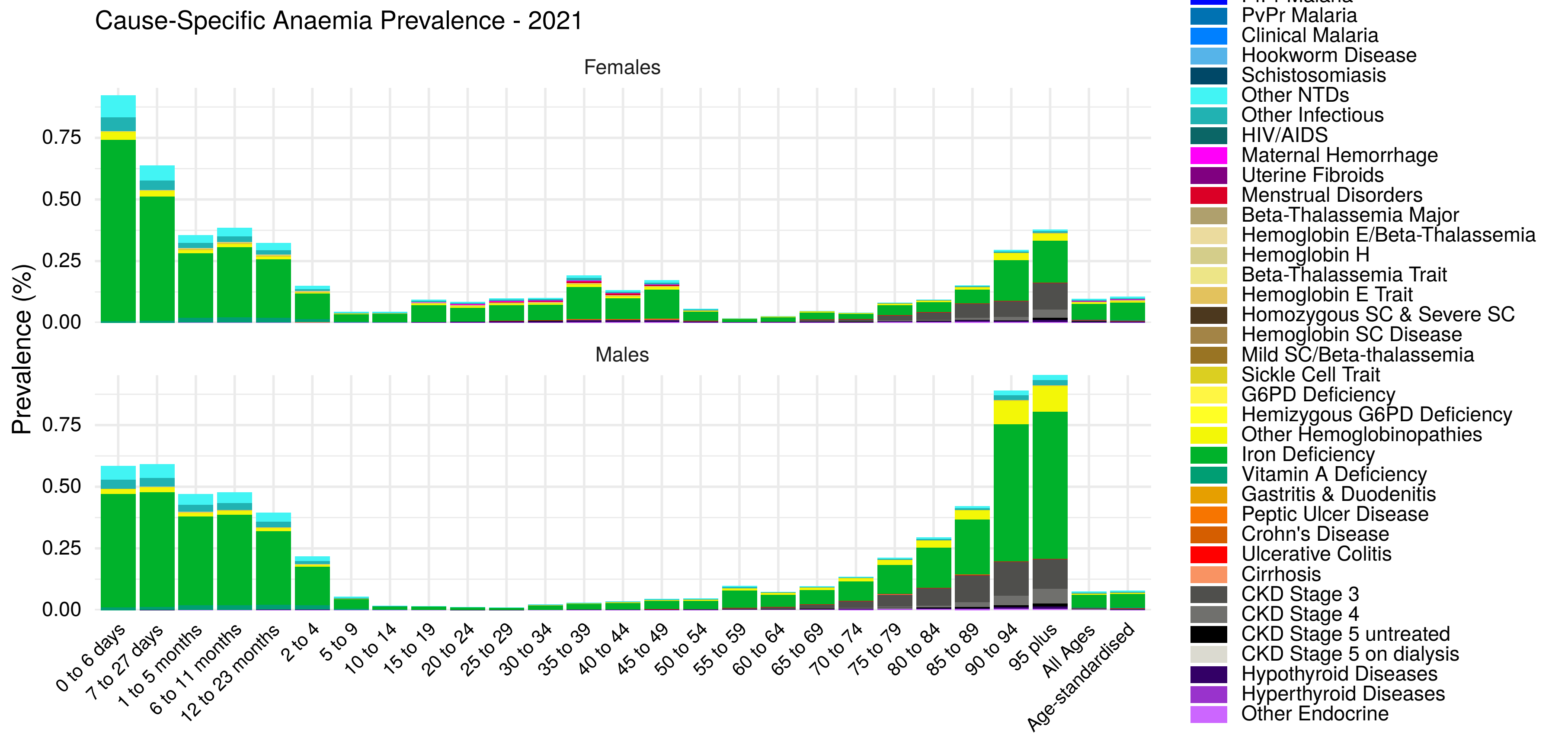

Cause-Specific Anaemia Prevalence - 2021
Females
Males
Prevalence (%)
0.00 0.25 0.50 0.75
0 to 6 days
7 to 27 days
1 to 5 months
6 to 11 months
12 to 23 months
2 to 4
5 to 9
10 to 14
15 to 19
20 to 24
25 to 29
30 to 34
35 to 39
40 to 44
45 to 49
50 to 54
55 to 59
60 to 64
65 to 69
70 to 74
75 to 79
80 to 84
85 to 89
90 to 94
95 plus
All Ages
Age-standardised
PfPr Malaria
PvPr Malaria
Clinical Malaria
Hookworm Disease
Schistosomiasis
Other NTDs
Other Infectious
HIV/AIDS
Maternal Hemorrhage
Uterine Fibroids
Menstrual Disorders
Beta-Thalassemia Major
Hemoglobin E/Beta-Thalassemia
Hemoglobin H
Beta-Thalassemia Trait
Hemoglobin E Trait
Homozygous SC & Severe SC
Hemoglobin SC Disease
Mild SC/Beta-thalassemia
Sickle Cell Trait
G6PD Deficiency
Hemizygous G6PD Deficiency
Other Hemoglobinopathies
Iron Deficiency
Vitamin A Deficiency
Gastritis & Duodenitis
Peptic Ulcer Disease
Crohn's Disease
Ulcerative Colitis
Cirrhosis
CKD Stage 3
CKD Stage 4
CKD Stage 5 untreated
CKD Stage 5 on dialysis
Hypothyroid Diseases
Hyperthyroid Diseases
Other Endocrine

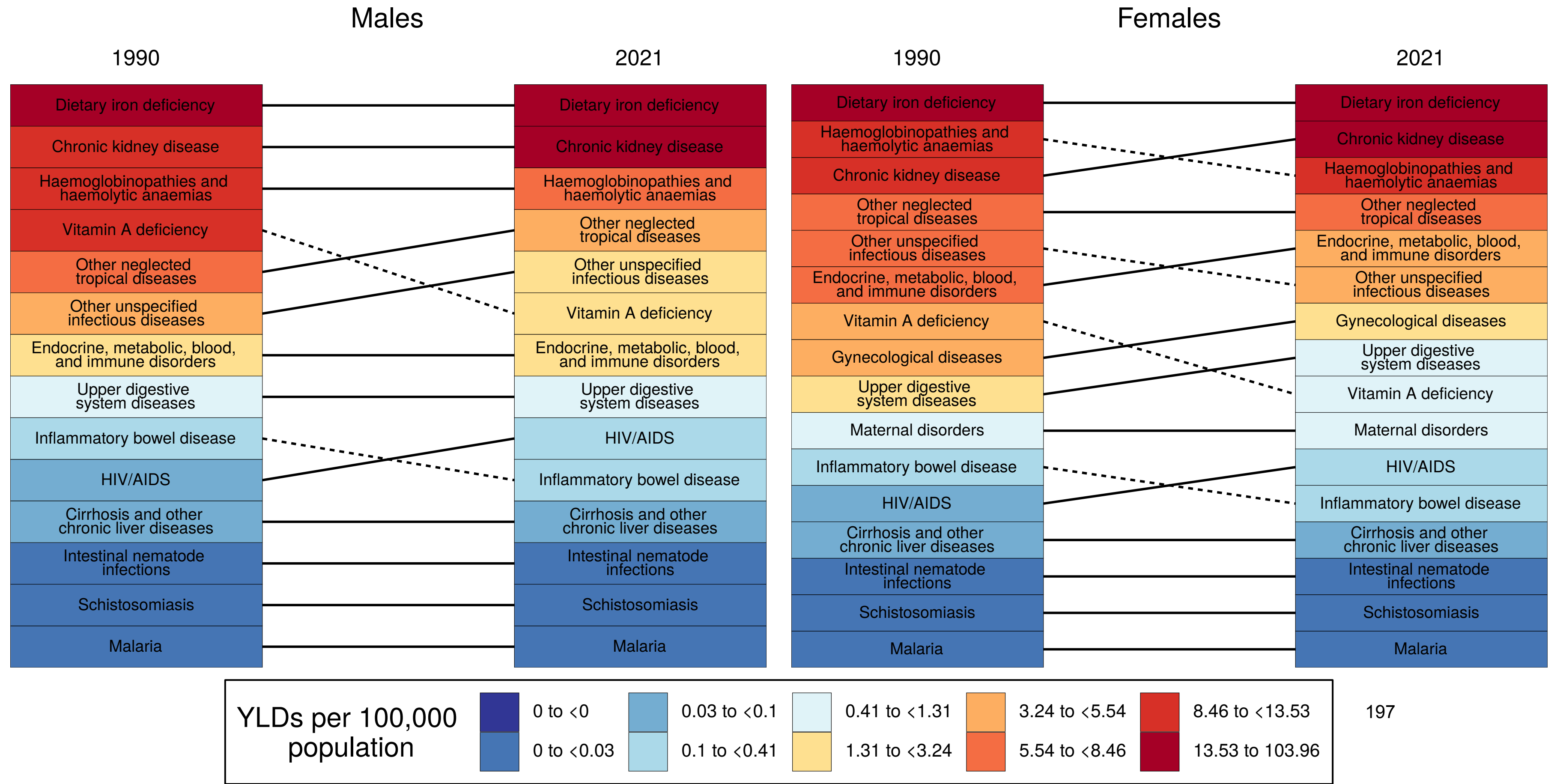

Males
Females
1990
2021
Dietary iron deficiency
Chronic kidney disease
Haemoglobinopathies and haemolytic anaemias
Vitamin A deficiency
Other neglected tropical diseases
Other unspecified infectious diseases
Endocrine, metabolic, blood, and immune disorders
Upper digestive system diseases
Inflammatory bowel disease
HIV/AIDS
Cirrhosis and other chronic liver diseases
Intestinal nematode infections
Schistosomiasis
Malaria
Gynecological diseases
Maternal disorders
YLDs per 100,000 population
0 to <0
0 to <0.03
0.03 to <0.1
0.1 to <0.41
0.41 to <1.31
1.31 to <3.24
3.24 to <5.54
5.54 to <8.46
8.46 to <13.53
13.53 to 103.96

Western Europe

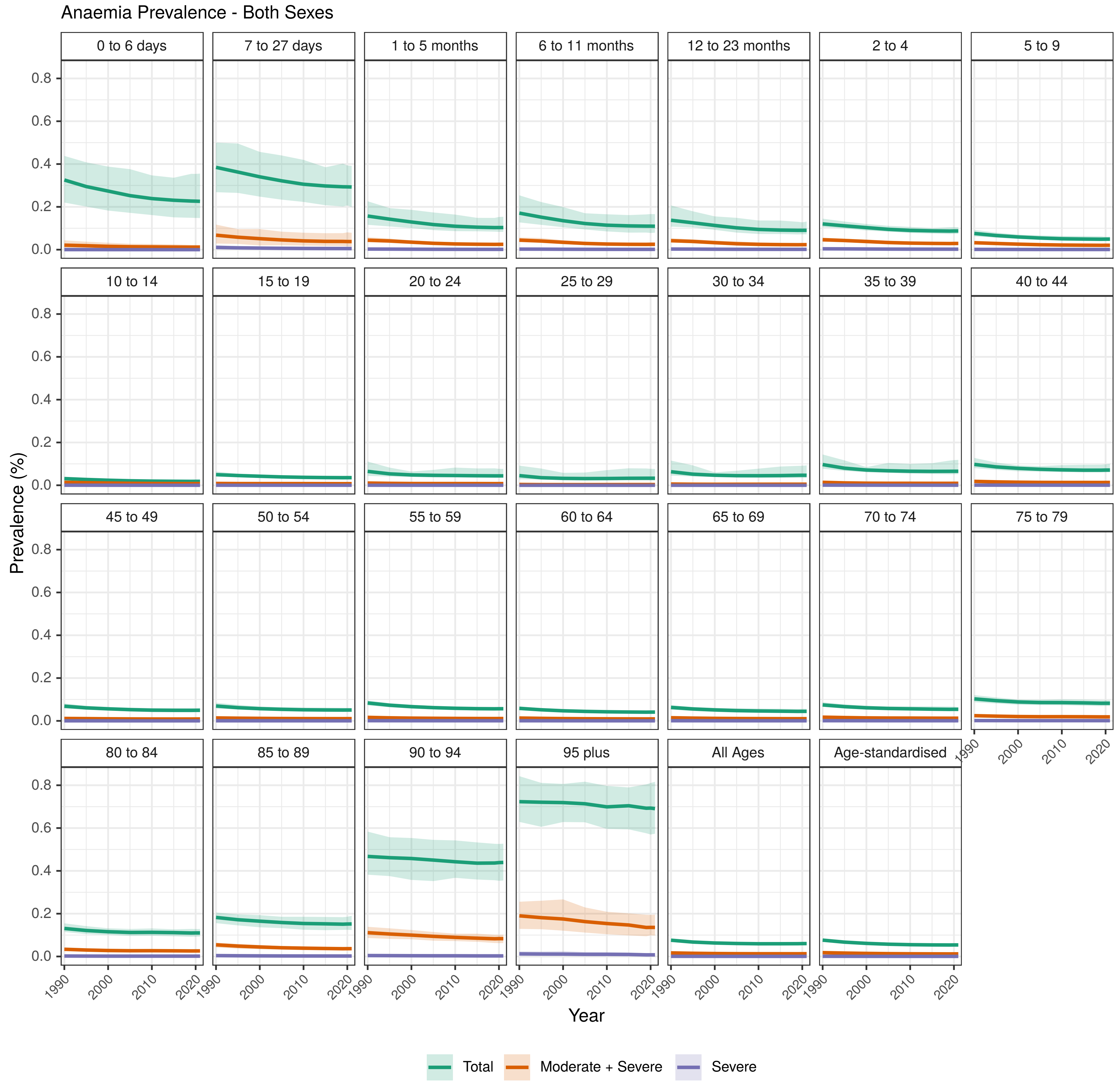

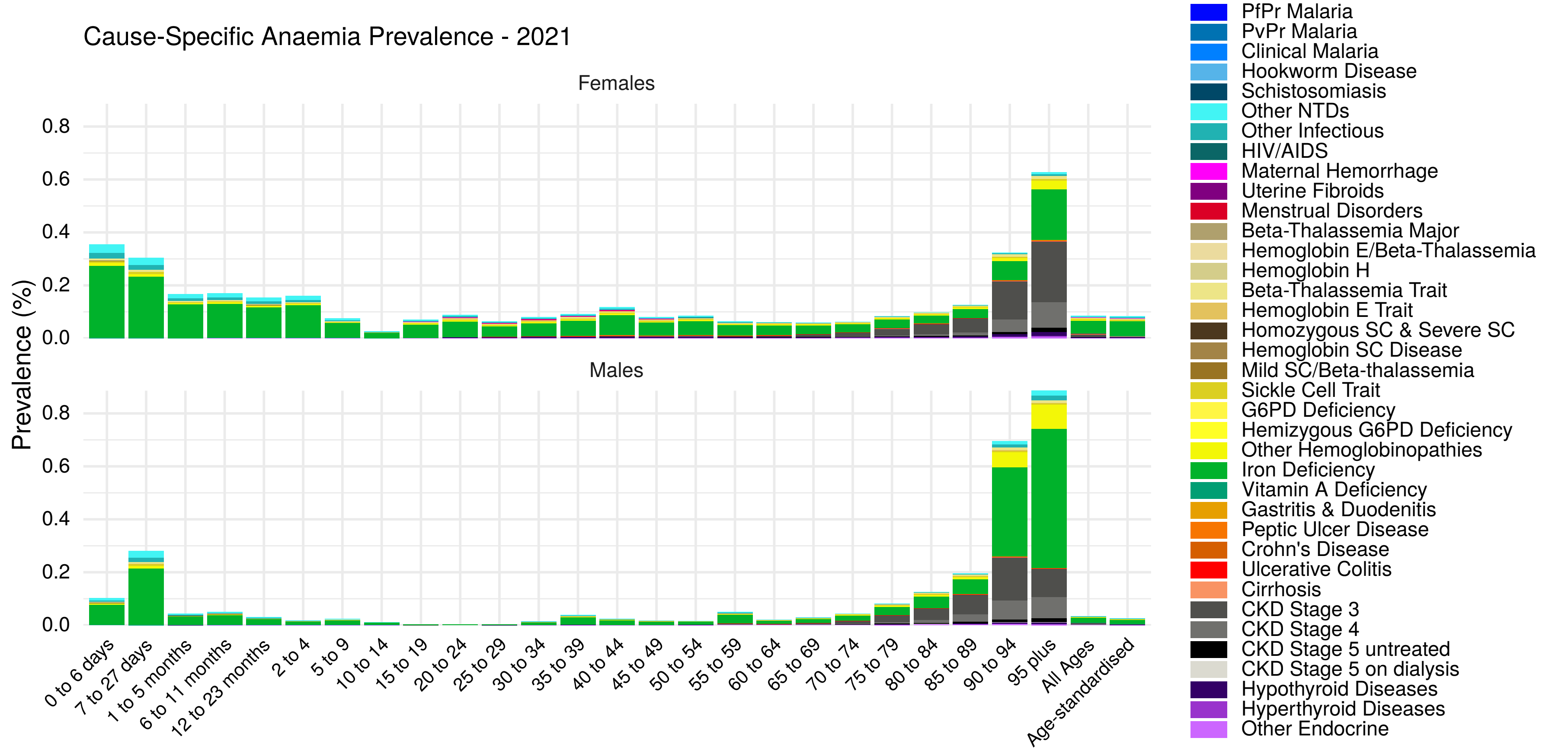

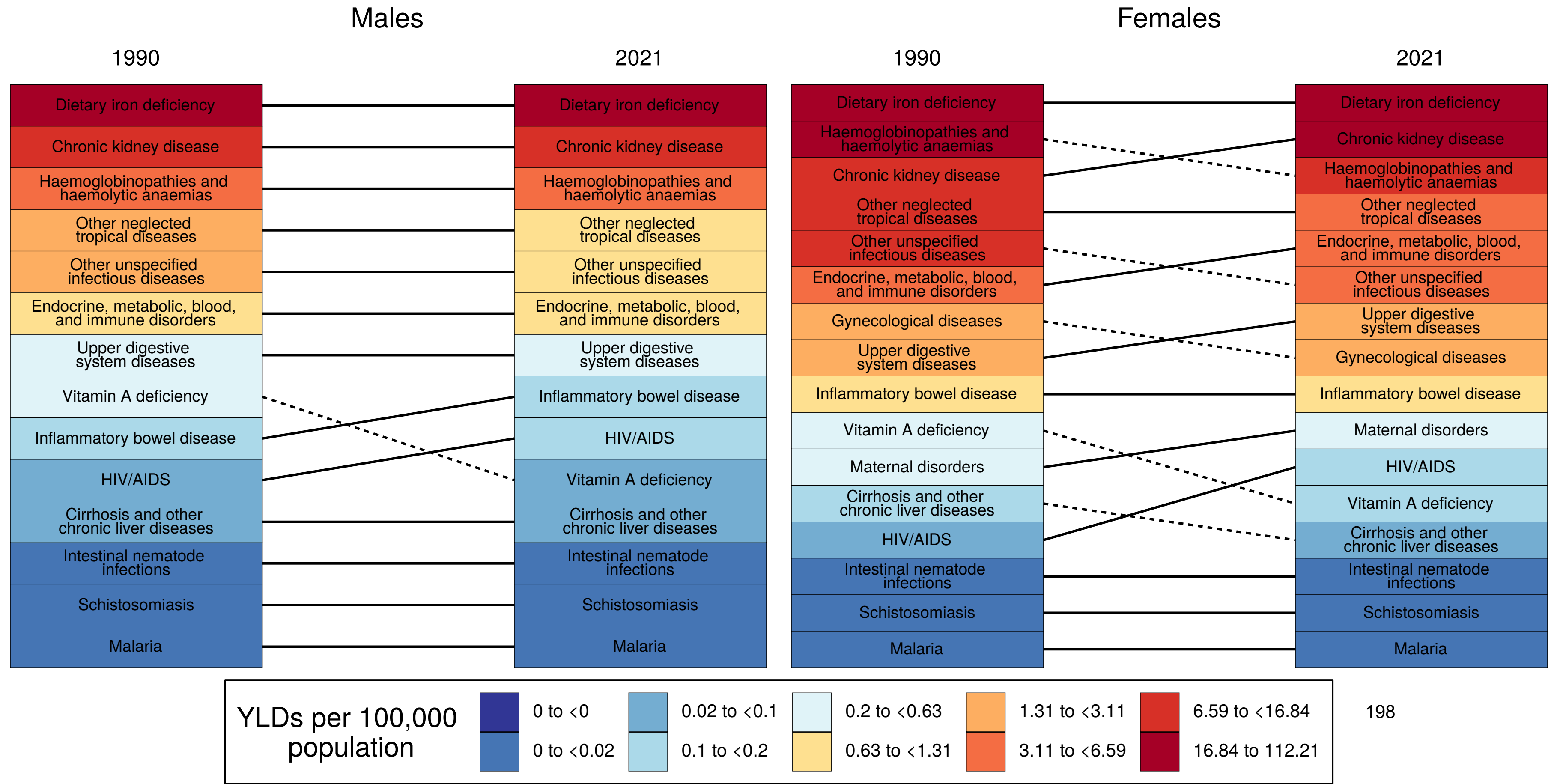

Andorra

Anaemia Prevalence - Both Sexes

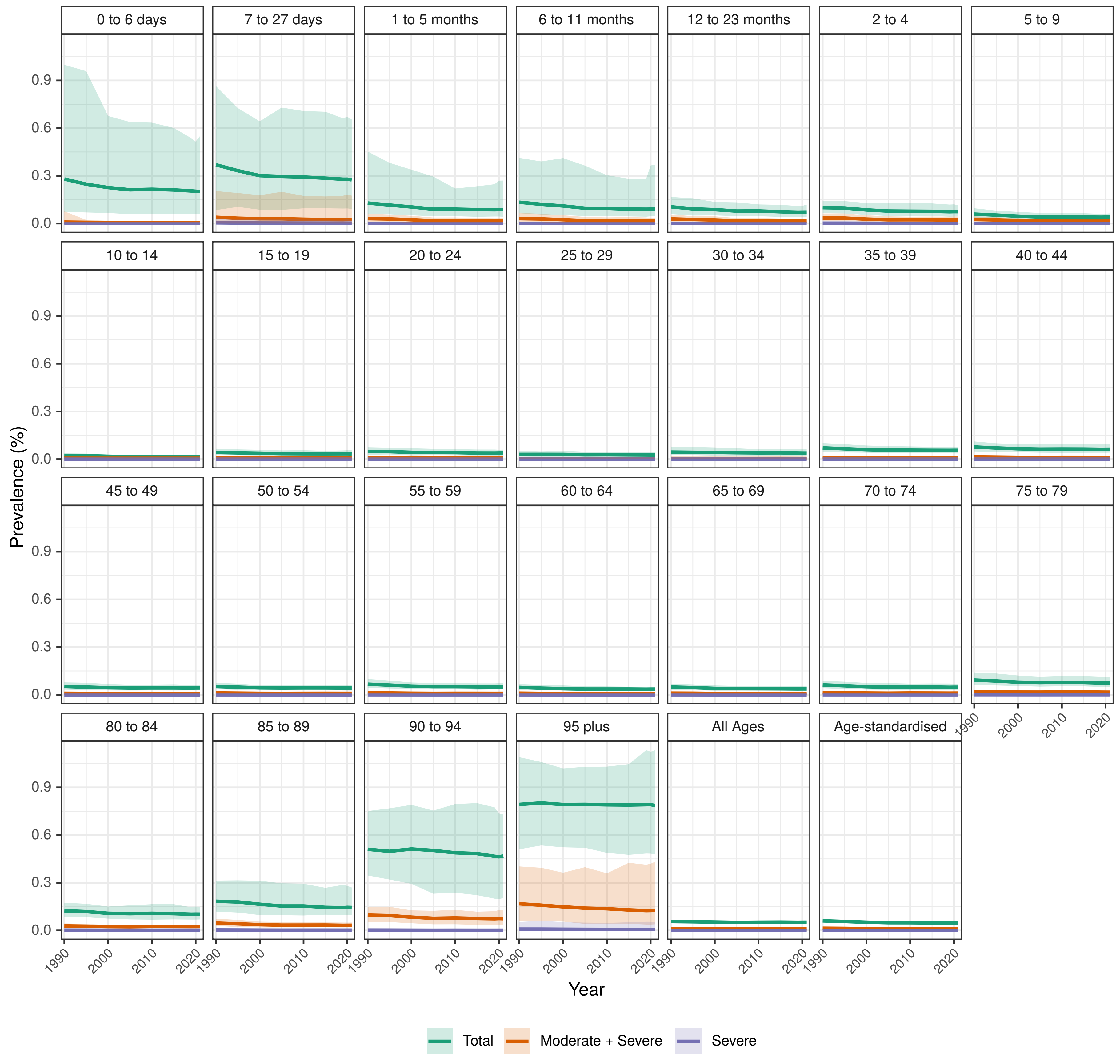

Cause-Specific Anaemia Prevalence - 2021

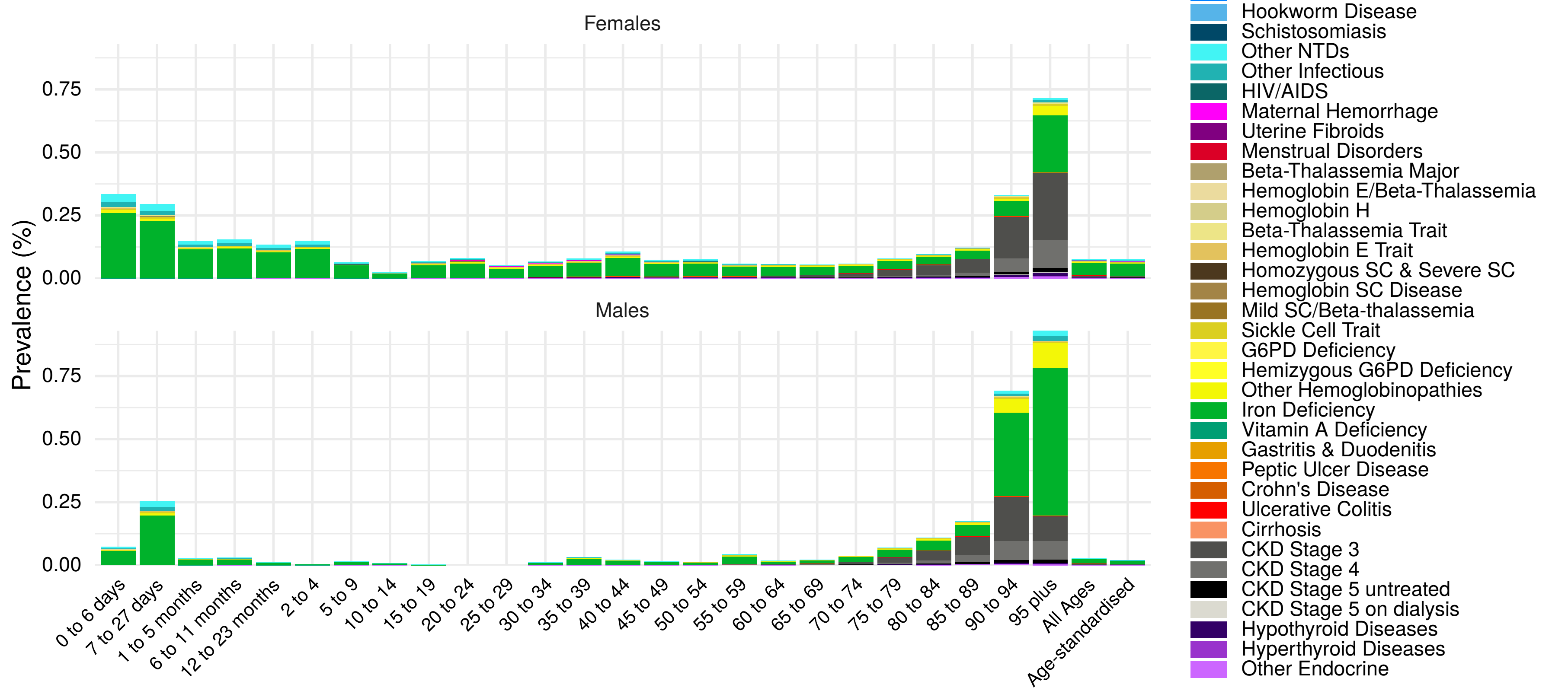

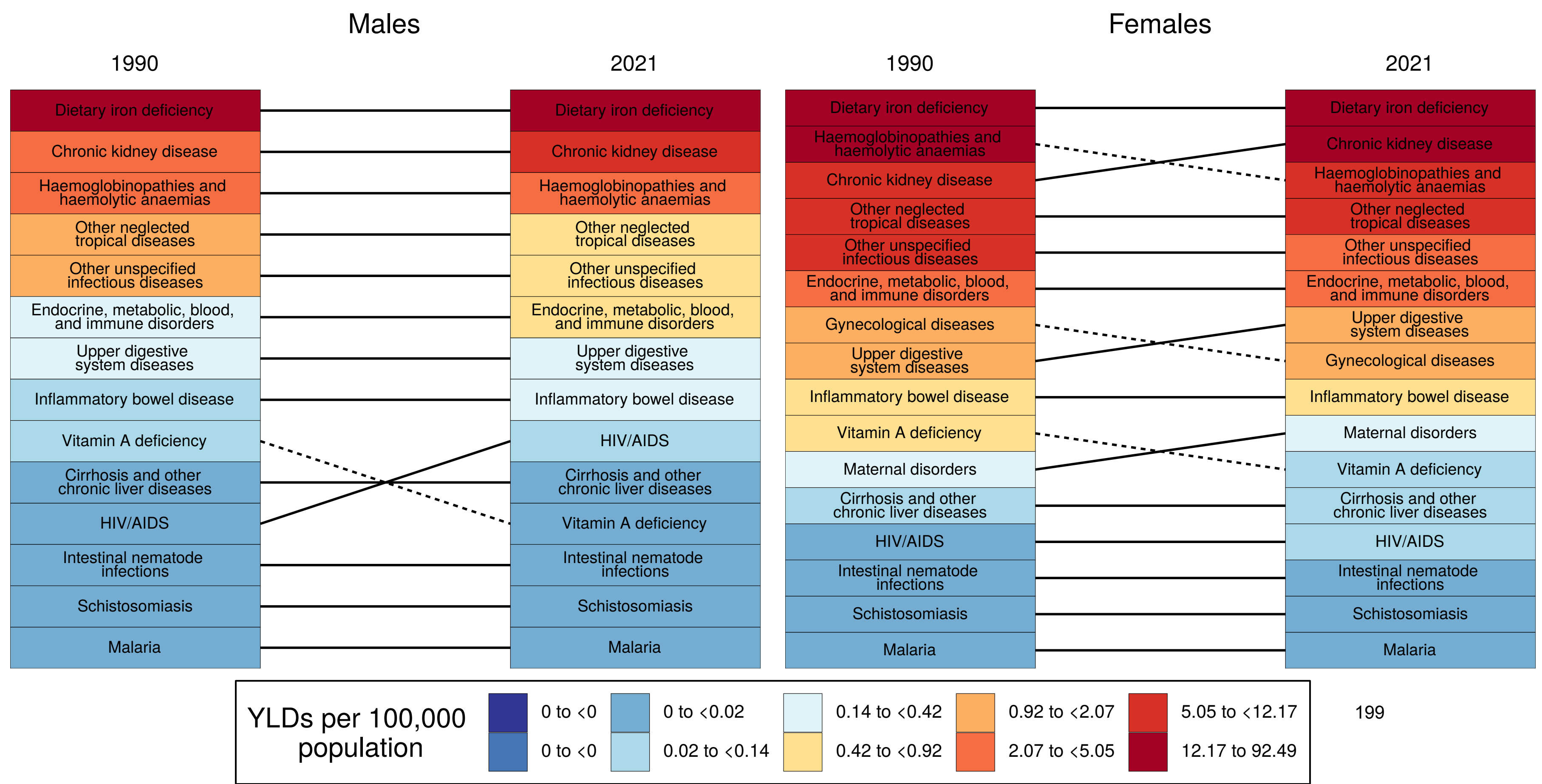

Austria

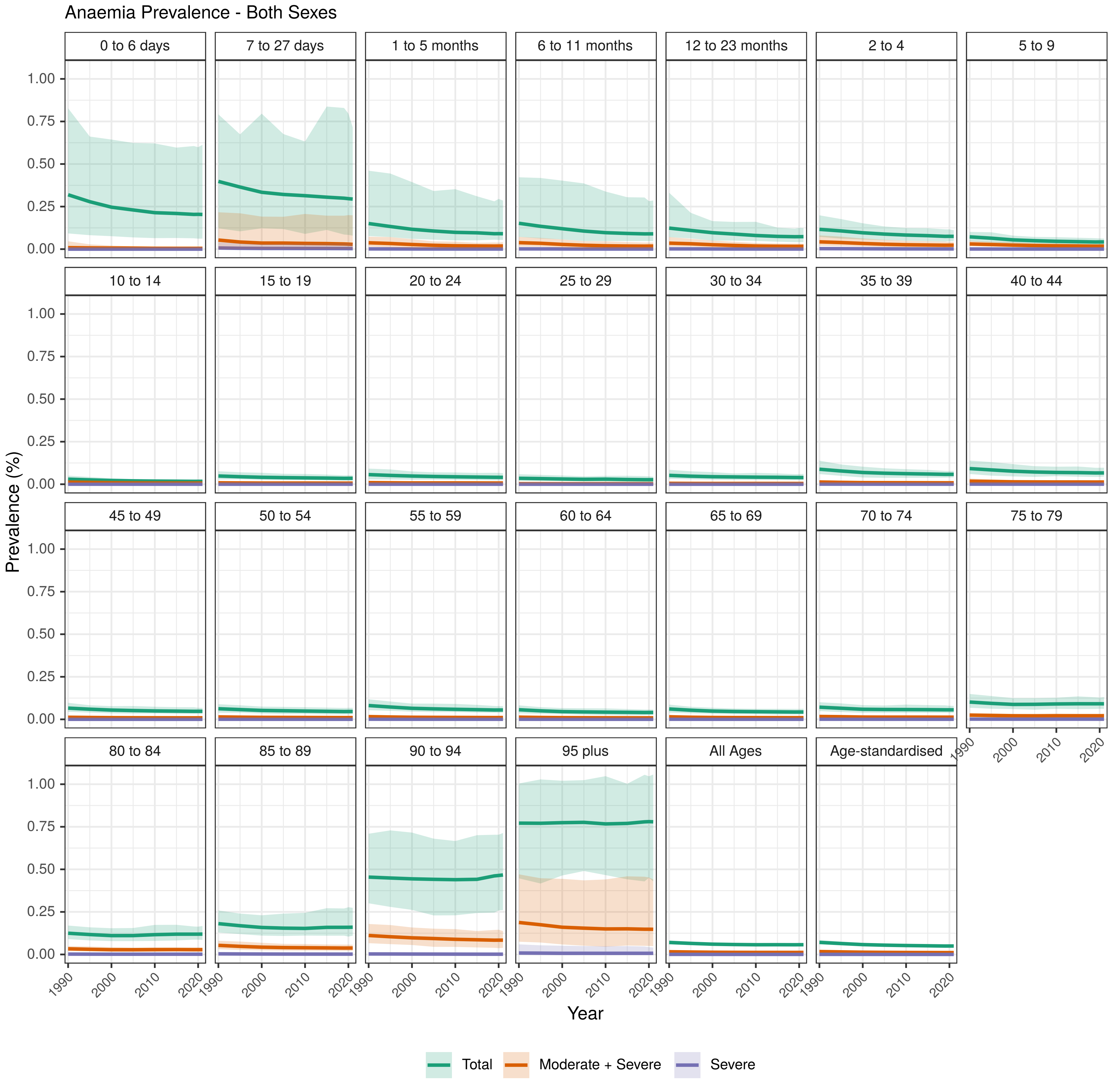

Anaemia Prevalence - Both Sexes
0 to 6 days
7 to 27 days
1 to 5 months
6 to 11 months
12 to 23 months
2 to 4
5 to 9
10 to 14
15 to 19
20 to 24
25 to 29
30 to 34
35 to 39
40 to 44
45 to 49
50 to 54
55 to 59
60 to 64
65 to 69
70 to 74
75 to 79
80 to 84
85 to 89
90 to 94
95 plus
All Ages
Age-standardised
Prevalence (%)
Year
Total
Moderate + Severe
Severe

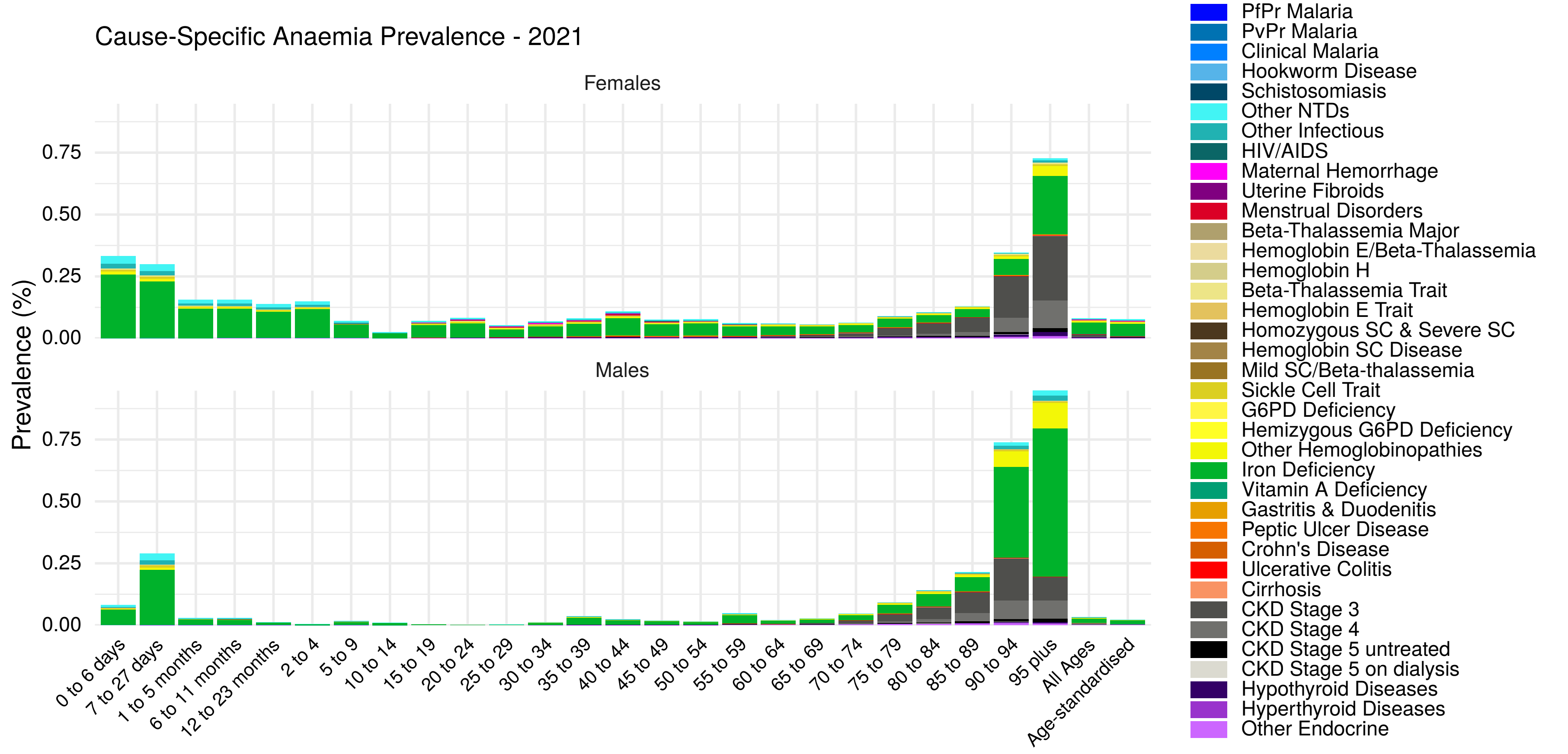

Cause-Specific Anaemia Prevalence - 2021
Females
Males
Prevalence (%)
PfPr Malaria
PvPr Malaria
Clinical Malaria
Hookworm Disease
Schistosomiasis
Other NTDs
Other Infectious
HIV/AIDS
Maternal Hemorrhage
Uterine Fibroids
Menstrual Disorders
Beta-Thalassemia Major
Hemoglobin E/Beta-Thalassemia
Hemoglobin H
Beta-Thalassemia Trait
Hemoglobin E Trait
Homozygous SC & Severe SC
Hemoglobin SC Disease
Mild SC/Beta-thalassemia
Sickle Cell Trait
G6PD Deficiency
Hemizygous G6PD Deficiency
Other Hemoglobinopathies
Iron Deficiency
Vitamin A Deficiency
Gastritis & Duodenitis
Peptic Ulcer Disease
Crohn's Disease
Ulcerative Colitis
Cirrhosis
CKD Stage 3
CKD Stage 4
CKD Stage 5 untreated
CKD Stage 5 on dialysis
Hypothyroid Diseases
Hyperthyroid Diseases
Other Endocrine

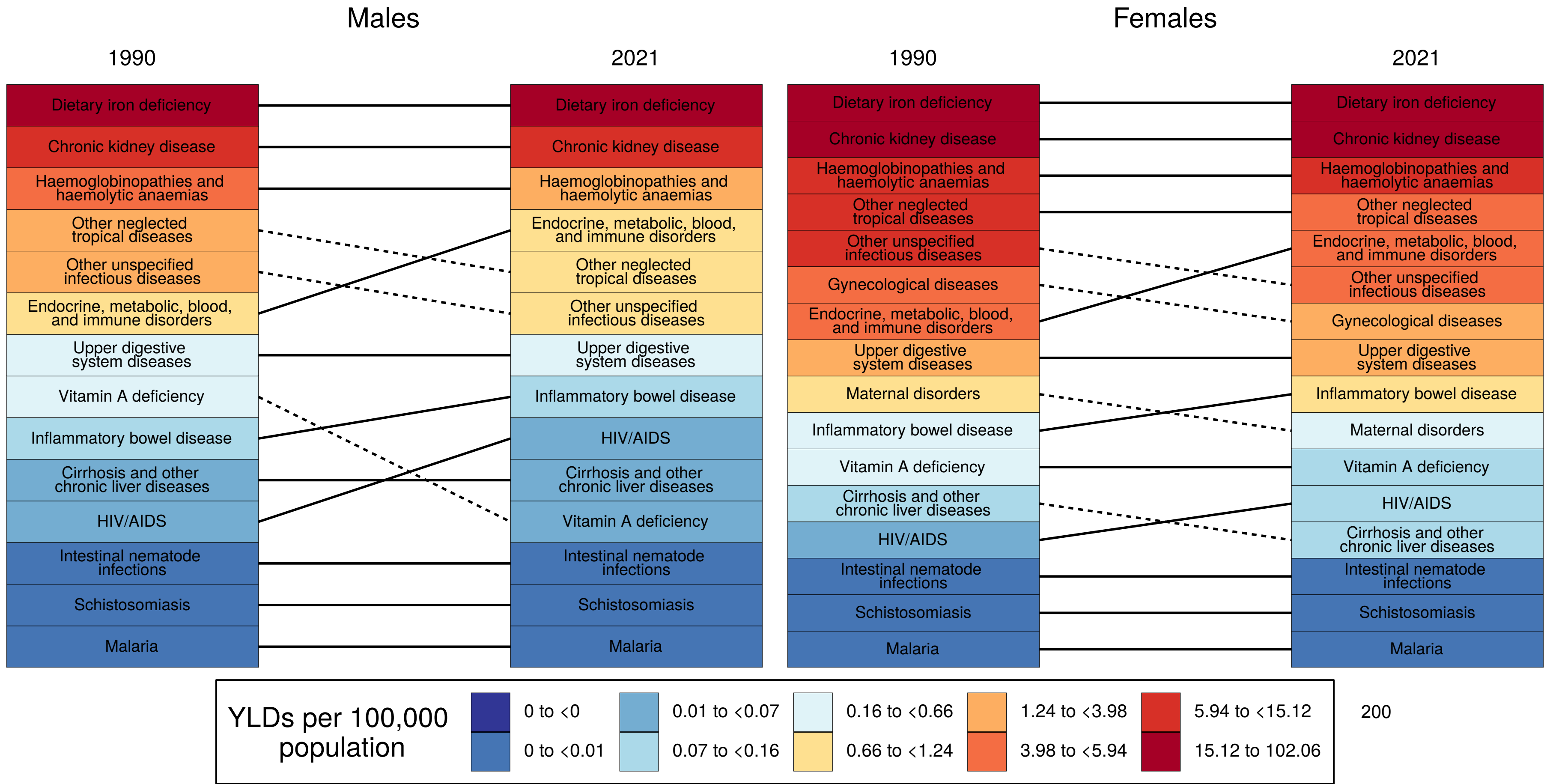

Males
Females
1990
2021
Dietary iron deficiency
Chronic kidney disease
Haemoglobinopathies and haemolytic anaemias
Other neglected tropical diseases
Other unspecified infectious diseases
Endocrine, metabolic, blood, and immune disorders
Upper digestive system diseases
Vitamin A deficiency
Inflammatory bowel disease
Cirrhosis and other chronic liver diseases
HIV/AIDS
Intestinal nematode infections
Schistosomiasis
Malaria
Gynecological diseases
Maternal disorders
YLDs per 100,000 population
0 to <0
0 to <0.01
0.01 to <0.07
0.07 to <0.16
0.16 to <0.66
0.66 to <1.24
1.24 to <3.98
3.98 to <5.94
5.94 to <15.12
15.12 to 102.06

200

Belgium

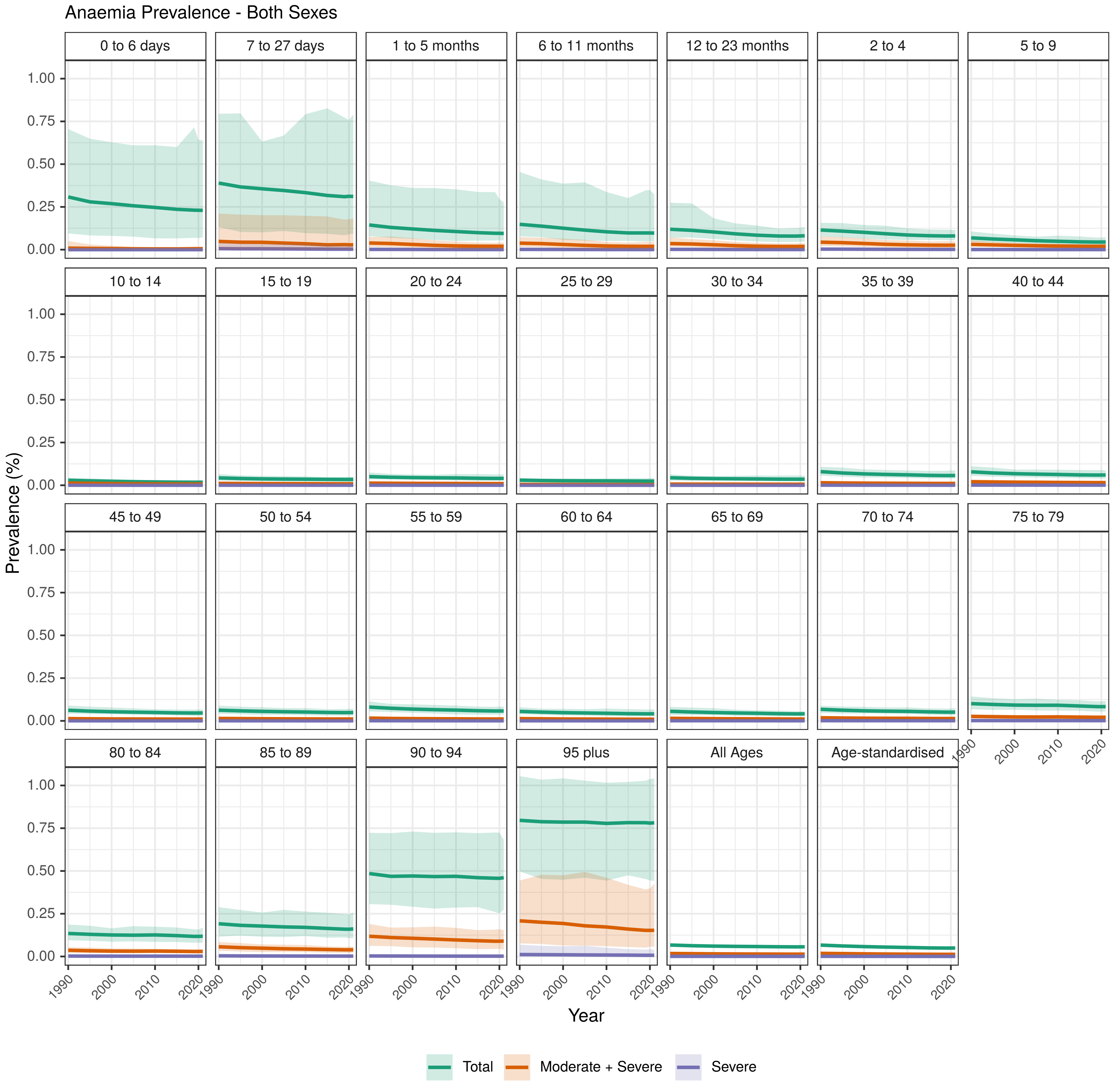

Anaemia Prevalence - Both Sexes
0 to 6 days
7 to 27 days
1 to 5 months
6 to 11 months
12 to 23 months
2 to 4
5 to 9
10 to 14
15 to 19
20 to 24
25 to 29
30 to 34
35 to 39
40 to 44
45 to 49
50 to 54
55 to 59
60 to 64
65 to 69
70 to 74
75 to 79
80 to 84
85 to 89
90 to 94
95 plus
All Ages
Age-standardised
Prevalence (%)
Year
Total
Moderate + Severe
Severe

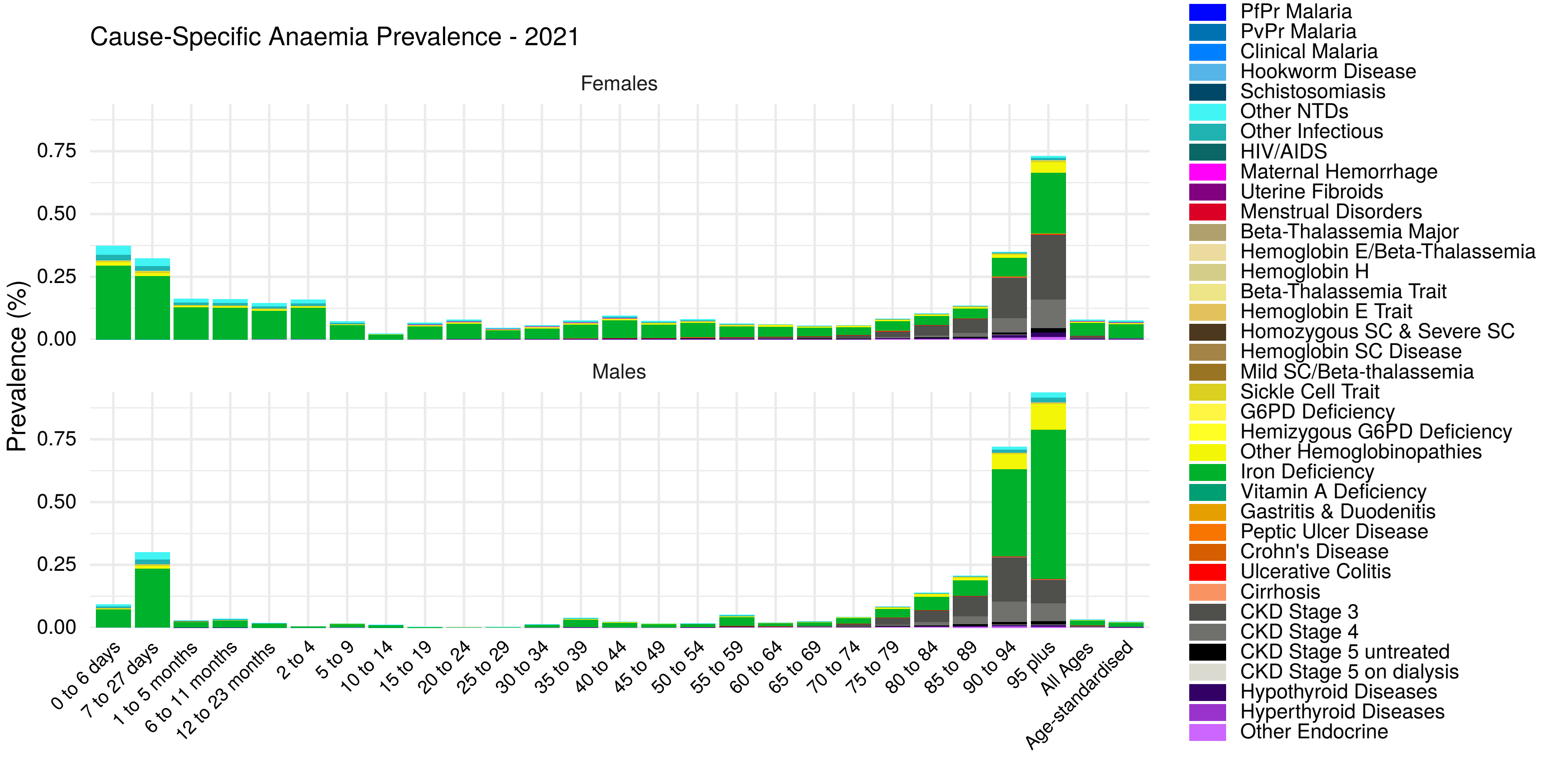

Cause-Specific Anaemia Prevalence - 2021
Females
Males
Prevalence (%)
PfPr Malaria
PvPr Malaria
Clinical Malaria
Hookworm Disease
Schistosomiasis
Other NTDs
Other Infectious
HIV/AIDS
Maternal Hemorrhage
Uterine Fibroids
Menstrual Disorders
Beta-Thalassemia Major
Hemoglobin E/Beta-Thalassemia
Hemoglobin H
Beta-Thalassemia Trait
Hemoglobin E Trait
Homozygous SC & Severe SC
Hemoglobin SC Disease
Mild SC/Beta-thalassemia
Sickle Cell Trait
G6PD Deficiency
Hemizygous G6PD Deficiency
Other Hemoglobinopathies
Iron Deficiency
Vitamin A Deficiency
Gastritis & Duodenitis
Peptic Ulcer Disease
Crohn's Disease
Ulcerative Colitis
Cirrhosis
CKD Stage 3
CKD Stage 4
CKD Stage 5 untreated
CKD Stage 5 on dialysis
Hypothyroid Diseases
Hyperthyroid Diseases
Other Endocrine

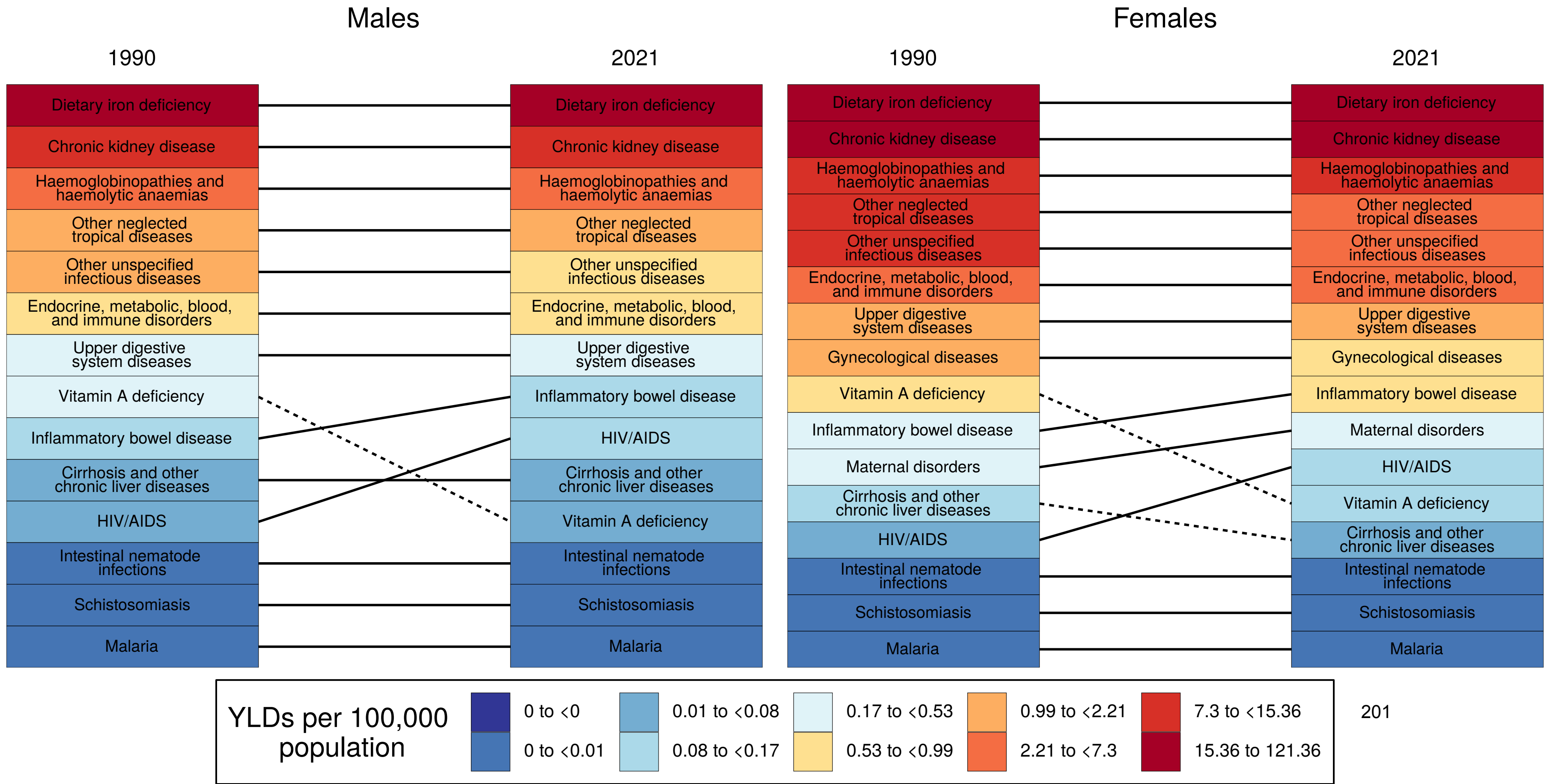

Males
Females
1990
2021
Dietary iron deficiency
Chronic kidney disease
Haemoglobinopathies and haemolytic anaemias
Other neglected tropical diseases
Other unspecified infectious diseases
Endocrine, metabolic, blood, and immune disorders
Upper digestive system diseases
Vitamin A deficiency
Inflammatory bowel disease
Cirrhosis and other chronic liver diseases
HIV/AIDS
Intestinal nematode infections
Schistosomiasis
Malaria
Gynecological diseases
Maternal disorders
YLDs per 100,000 population
0 to <0
0 to <0.01
0.01 to <0.08
0.08 to <0.17
0.17 to <0.53
0.53 to <0.99
0.99 to <2.21
2.21 to <7.3
7.3 to <15.36
15.36 to 121.36

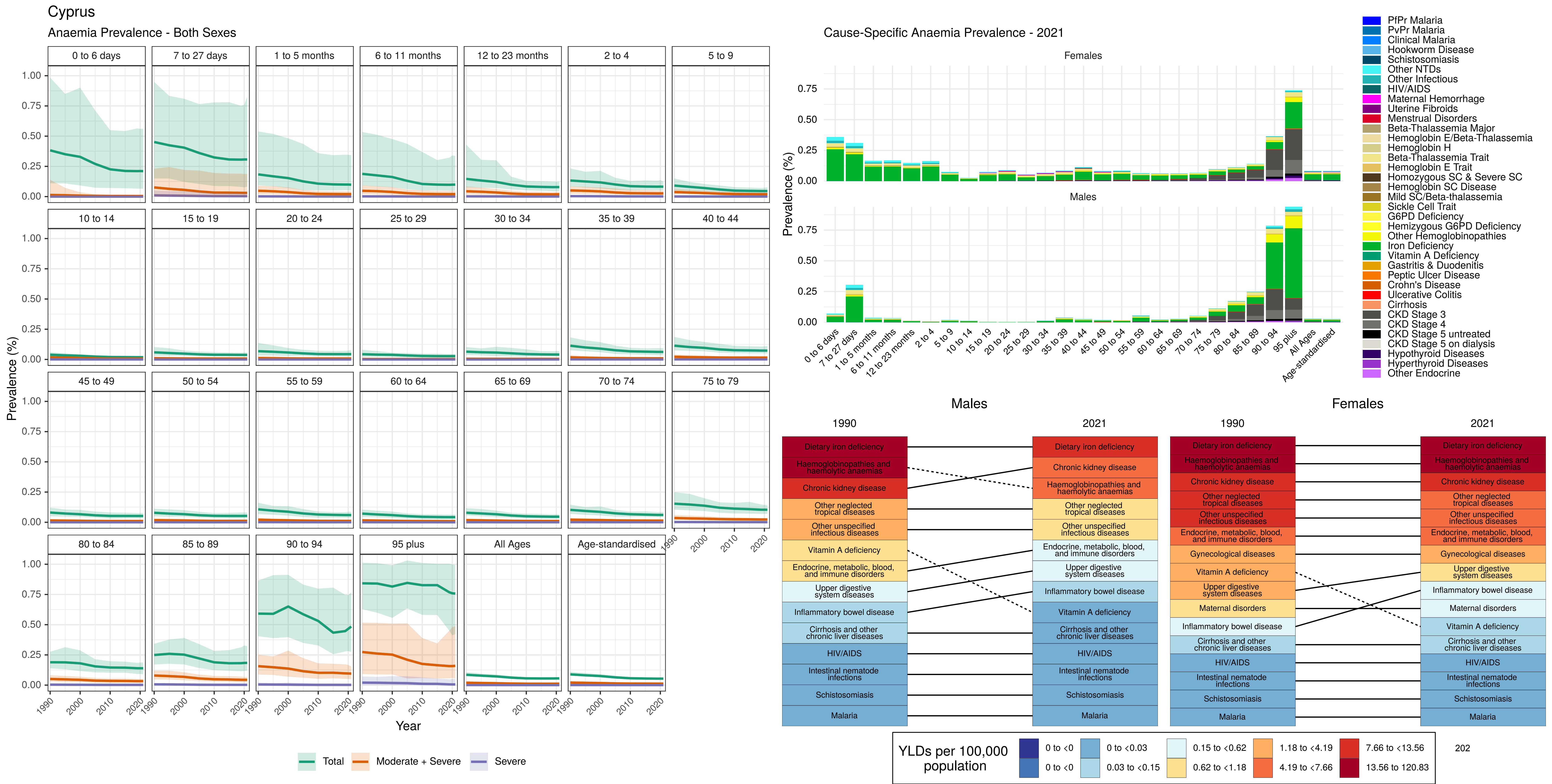

Cyprus
Anaemia Prevalence - Both Sexes
0 to 6 days
7 to 27 days
1 to 5 months
6 to 11 months
12 to 23 months
2 to 4
5 to 9
10 to 14
15 to 19
20 to 24
25 to 29
30 to 34
35 to 39
40 to 44
45 to 49
50 to 54
55 to 59
60 to 64
65 to 69
70 to 74
75 to 79
80 to 84
85 to 89
90 to 94
95 plus
All Ages
Age-standardised
Prevalence (%)
Year
Total
Moderate + Severe
Severe
Cause-Specific Anaemia Prevalence - 2021
Females
Males
Prevalence (%)
PfPr Malaria
PvPr Malaria
Clinical Malaria
Hookworm Disease
Schistosomiasis
Other NTDs
Other Infectious
HIV/AIDS
Maternal Hemorrhage
Uterine Fibroids
Menstrual Disorders
Beta-Thalassemia Major
Hemoglobin E/Beta-Thalassemia
Hemoglobin H
Beta-Thalassemia Trait
Hemoglobin E Trait
Homozygous SC & Severe SC
Hemoglobin SC Disease
Mild SC/Beta-thalassemia
Sickle Cell Trait
G6PD Deficiency
Hemizygous G6PD Deficiency
Other Hemoglobinopathies
Iron Deficiency
Vitamin A Deficiency
Gastritis & Duodenitis
Peptic Ulcer Disease
Crohn's Disease
Ulcerative Colitis
Cirrhosis
CKD Stage 3
CKD Stage 4
CKD Stage 5 untreated
CKD Stage 5 on dialysis
Hypothyroid Diseases
Hyperthyroid Diseases
Other Endocrine
Males
Females
1990
2021
Dietary iron deficiency
Haemoglobinopathies and haemolytic anaemias
Chronic kidney disease
Other neglected tropical diseases
Other unspecified infectious diseases
Vitamin A deficiency
Endocrine, metabolic, blood, and immune disorders
Upper digestive system diseases
Inflammatory bowel disease
Cirrhosis and other chronic liver diseases
HIV/AIDS
Intestinal nematode infections
Schistosomiasis
Malaria
Gynecological diseases
Maternal disorders
YLDs per 100,000 population
0 to <0
0 to <0.03
0.03 to <0.15
0.15 to <0.62
0.62 to <1.18
1.18 to <4.19
4.19 to <7.66
7.66 to <13.56
13.56 to 120.83
202

Denmark

Anaemia Prevalence - Both Sexes

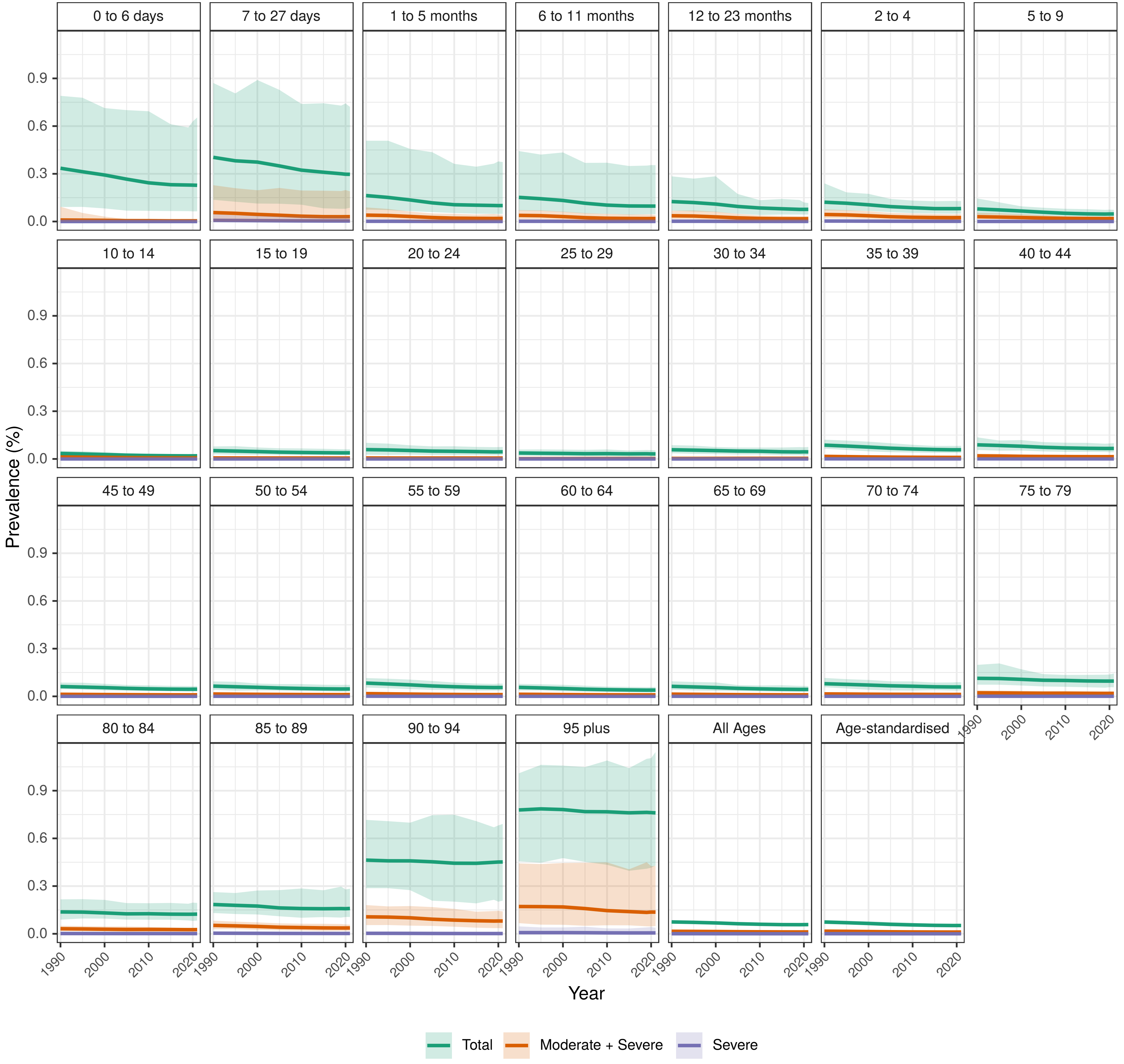

Cause-Specific Anaemia Prevalence - 2021

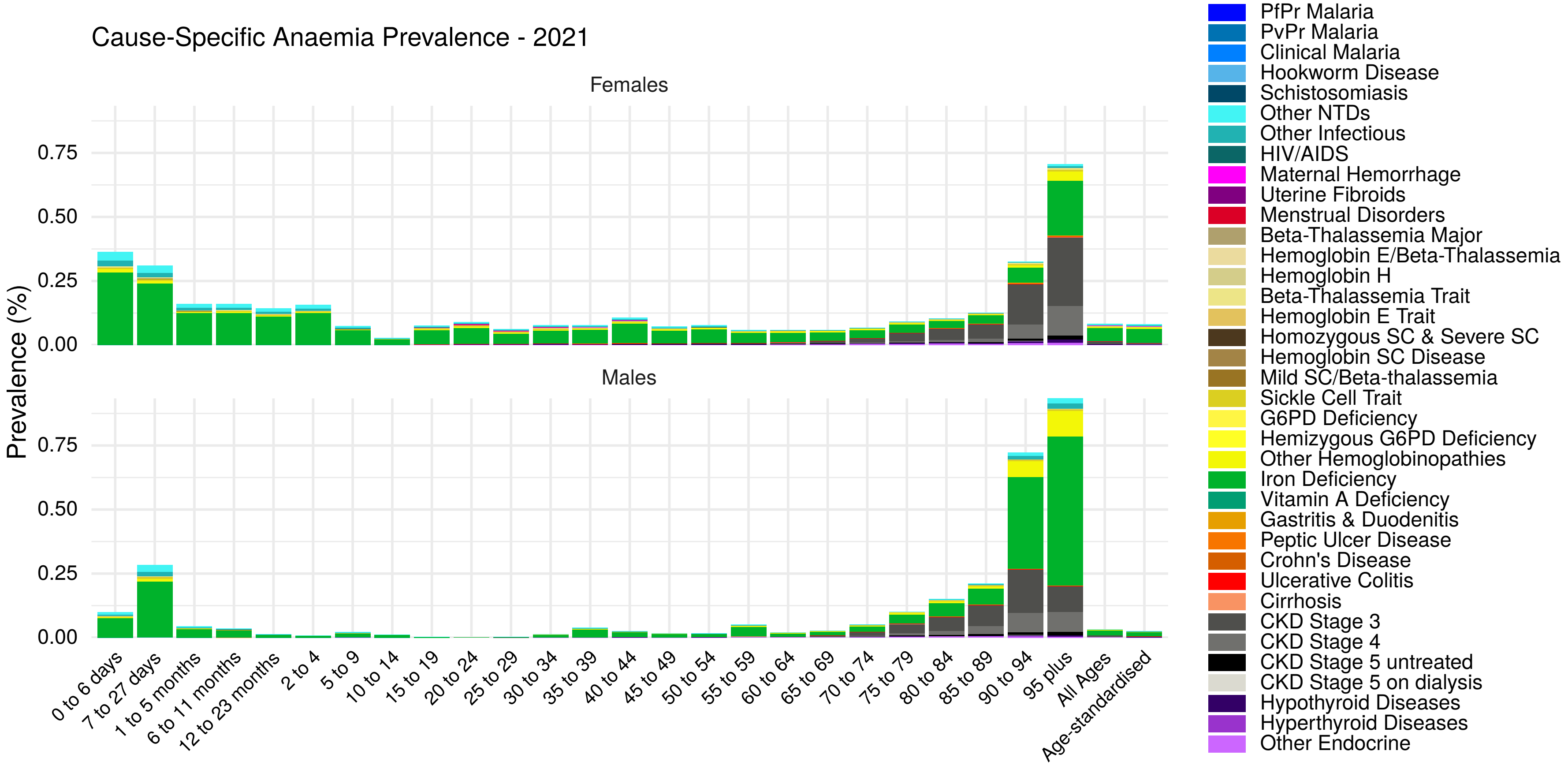

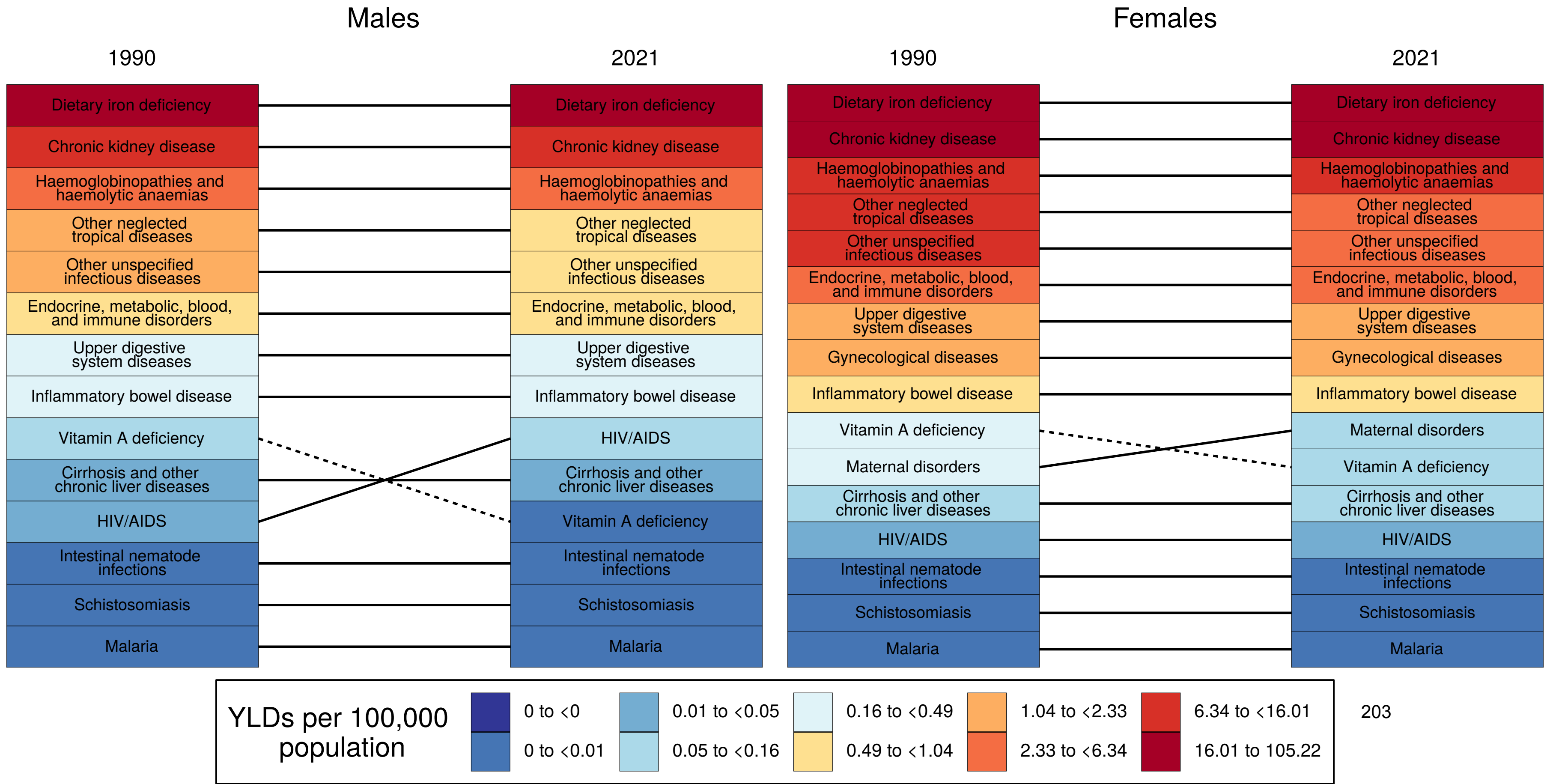

Finland

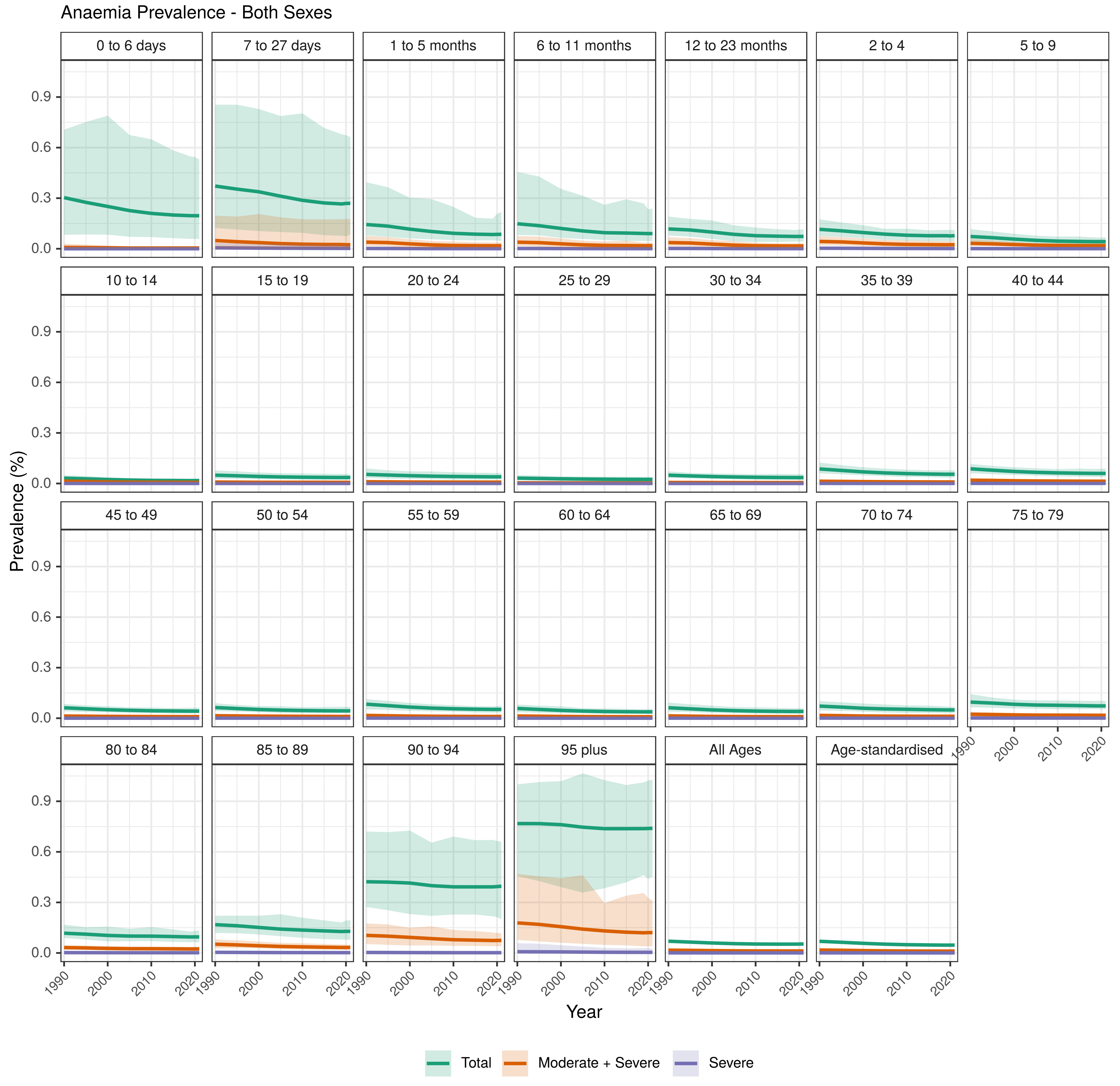
Anaemia Prevalence - Both Sexes
0 to 6 days
7 to 27 days
1 to 5 months
6 to 11 months
12 to 23 months
2 to 4
5 to 9
10 to 14
15 to 19
20 to 24
25 to 29
30 to 34
35 to 39
40 to 44
45 to 49
50 to 54
55 to 59
60 to 64
65 to 69
70 to 74
75 to 79
80 to 84
85 to 89
90 to 94
95 plus
All Ages
Age-standardised
Prevalence (%)
Year
Total
Moderate + Severe
Severe

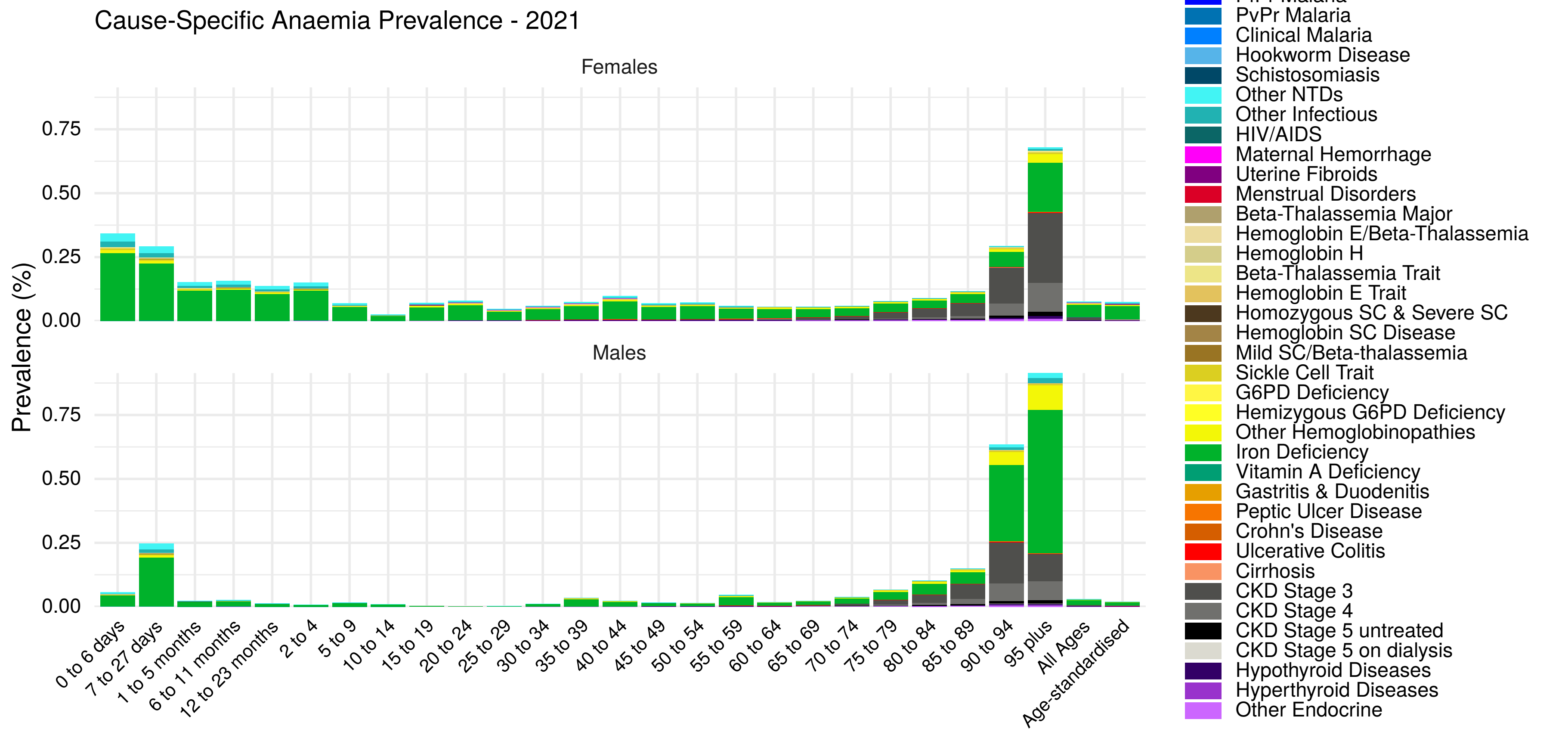
Cause-Specific Anaemia Prevalence - 2021
Females
Males
Prevalence (%)
PfPr Malaria
PvPr Malaria
Clinical Malaria
Hookworm Disease
Schistosomiasis
Other NTDs
Other Infectious
HIV/AIDS
Maternal Hemorrhage
Uterine Fibroids
Menstrual Disorders
Beta-Thalassemia Major
Hemoglobin E/Beta-Thalassemia
Hemoglobin H
Beta-Thalassemia Trait
Hemoglobin E Trait
Homozygous SC & Severe SC
Hemoglobin SC Disease
Mild SC/Beta-thalassemia
Sickle Cell Trait
G6PD Deficiency
Hemizygous G6PD Deficiency
Other Hemoglobinopathies
Iron Deficiency
Vitamin A Deficiency
Gastritis & Duodenitis
Peptic Ulcer Disease
Crohn's Disease
Ulcerative Colitis
Cirrhosis
CKD Stage 3
CKD Stage 4
CKD Stage 5 untreated
CKD Stage 5 on dialysis
Hypothyroid Diseases
Hyperthyroid Diseases
Other Endocrine

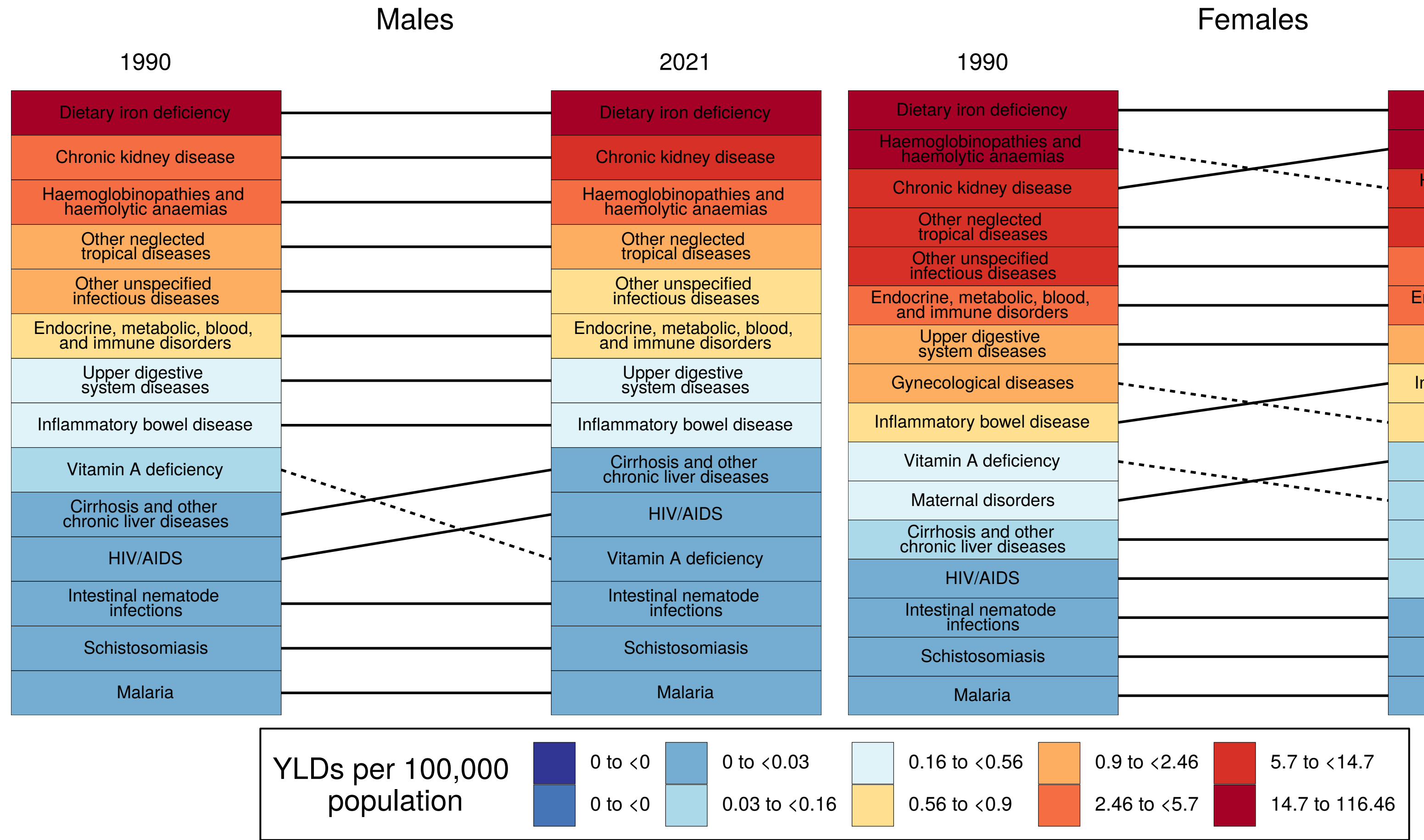
Males
Females
1990
2021
Dietary iron deficiency
Chronic kidney disease
Haemoglobinopathies and haemolytic anaemias
Other neglected tropical diseases
Other unspecified infectious diseases
Endocrine, metabolic, blood, and immune disorders
Upper digestive system diseases
Inflammatory bowel disease
Vitamin A deficiency
Cirrhosis and other chronic liver diseases
HIV/AIDS
Intestinal nematode infections
Schistosomiasis
Malaria
Gynecological diseases
Maternal disorders
YLDs per 100,000 population
0 to <0
0 to <0.03
0.03 to <0.16
0.16 to <0.56
0.56 to <0.9
0.9 to <2.46
2.46 to <5.7
5.7 to <14.7
14.7 to 116.46

France

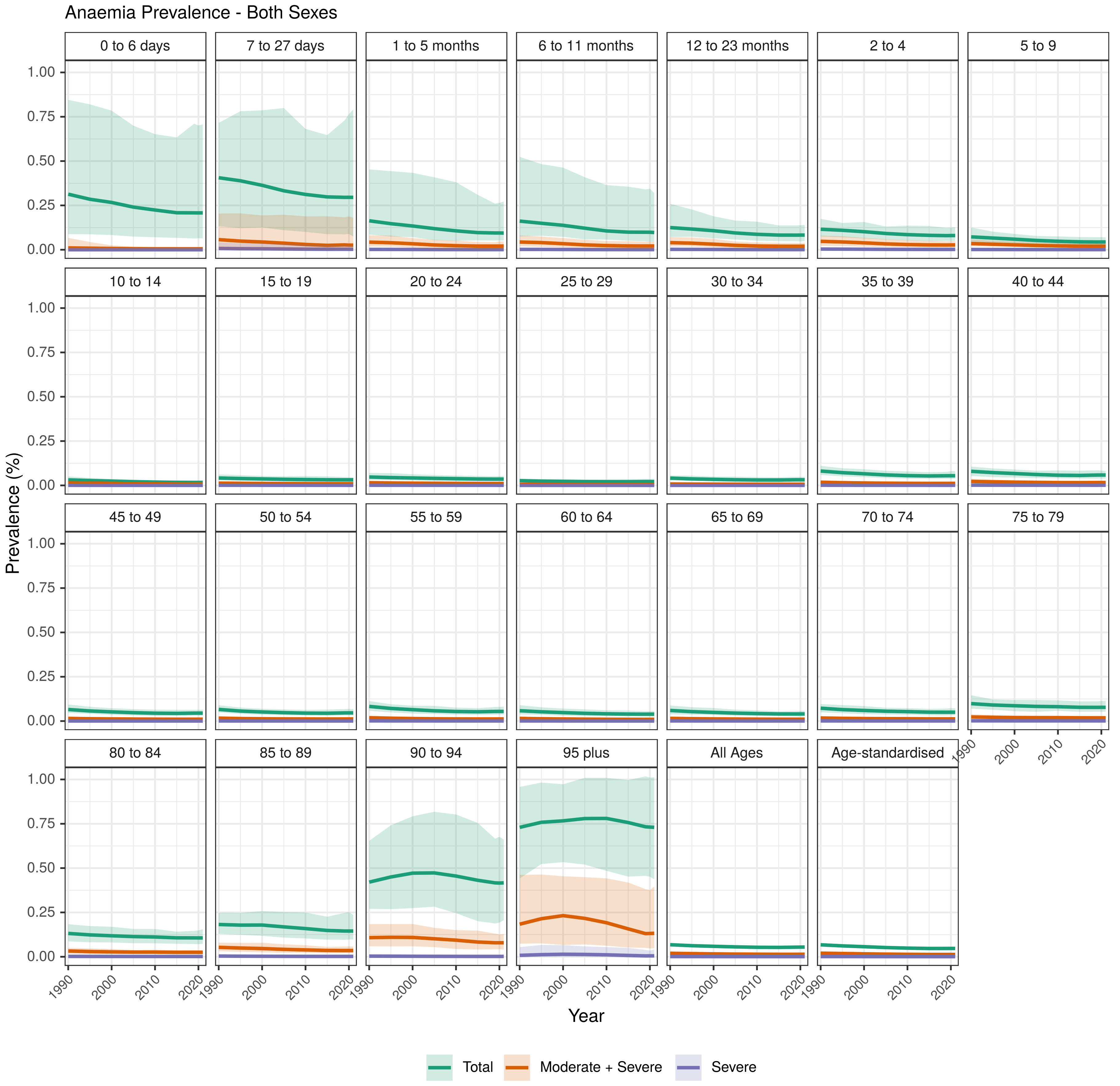
Anaemia Prevalence - Both Sexes
0 to 6 days
7 to 27 days
1 to 5 months
6 to 11 months
12 to 23 months
2 to 4
5 to 9
10 to 14
15 to 19
20 to 24
25 to 29
30 to 34
35 to 39
40 to 44
45 to 49
50 to 54
55 to 59
60 to 64
65 to 69
70 to 74
75 to 79
80 to 84
85 to 89
90 to 94
95 plus
All Ages
Age-standardised
Prevalence (%)
Year
Total
Moderate + Severe
Severe

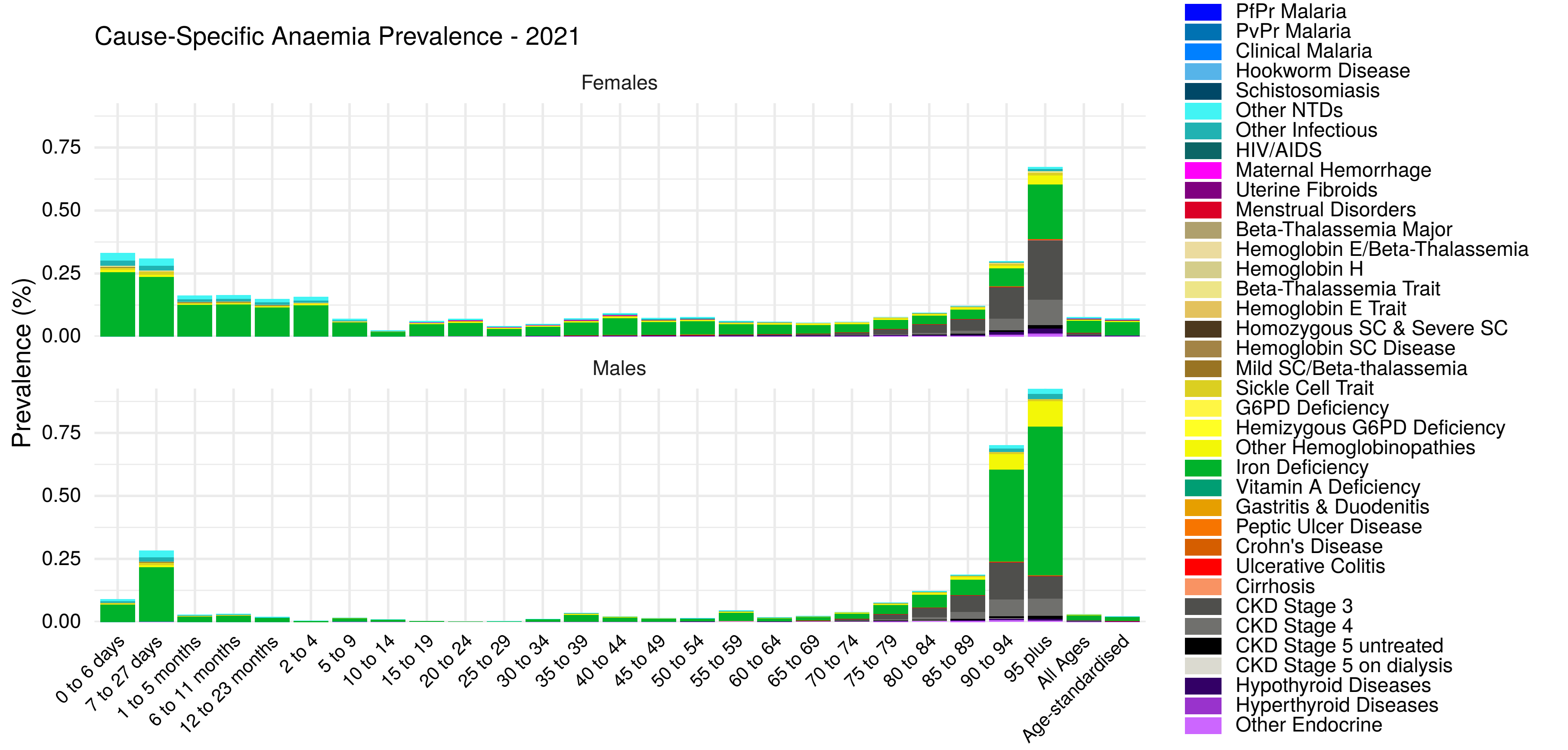
Cause-Specific Anaemia Prevalence - 2021
Females
Males
Prevalence (%)
PfPr Malaria
PvPr Malaria
Clinical Malaria
Hookworm Disease
Schistosomiasis
Other NTDs
Other Infectious
HIV/AIDS
Maternal Hemorrhage
Uterine Fibroids
Menstrual Disorders
Beta-Thalassemia Major
Hemoglobin E/Beta-Thalassemia
Hemoglobin H
Beta-Thalassemia Trait
Hemoglobin E Trait
Homozygous SC & Severe SC
Hemoglobin SC Disease
Mild SC/Beta-thalassemia
Sickle Cell Trait
G6PD Deficiency
Hemizygous G6PD Deficiency
Other Hemoglobinopathies
Iron Deficiency
Vitamin A Deficiency
Gastritis & Duodenitis
Peptic Ulcer Disease
Crohn's Disease
Ulcerative Colitis
Cirrhosis
CKD Stage 3
CKD Stage 4
CKD Stage 5 untreated
CKD Stage 5 on dialysis
Hypothyroid Diseases
Hyperthyroid Diseases
Other Endocrine

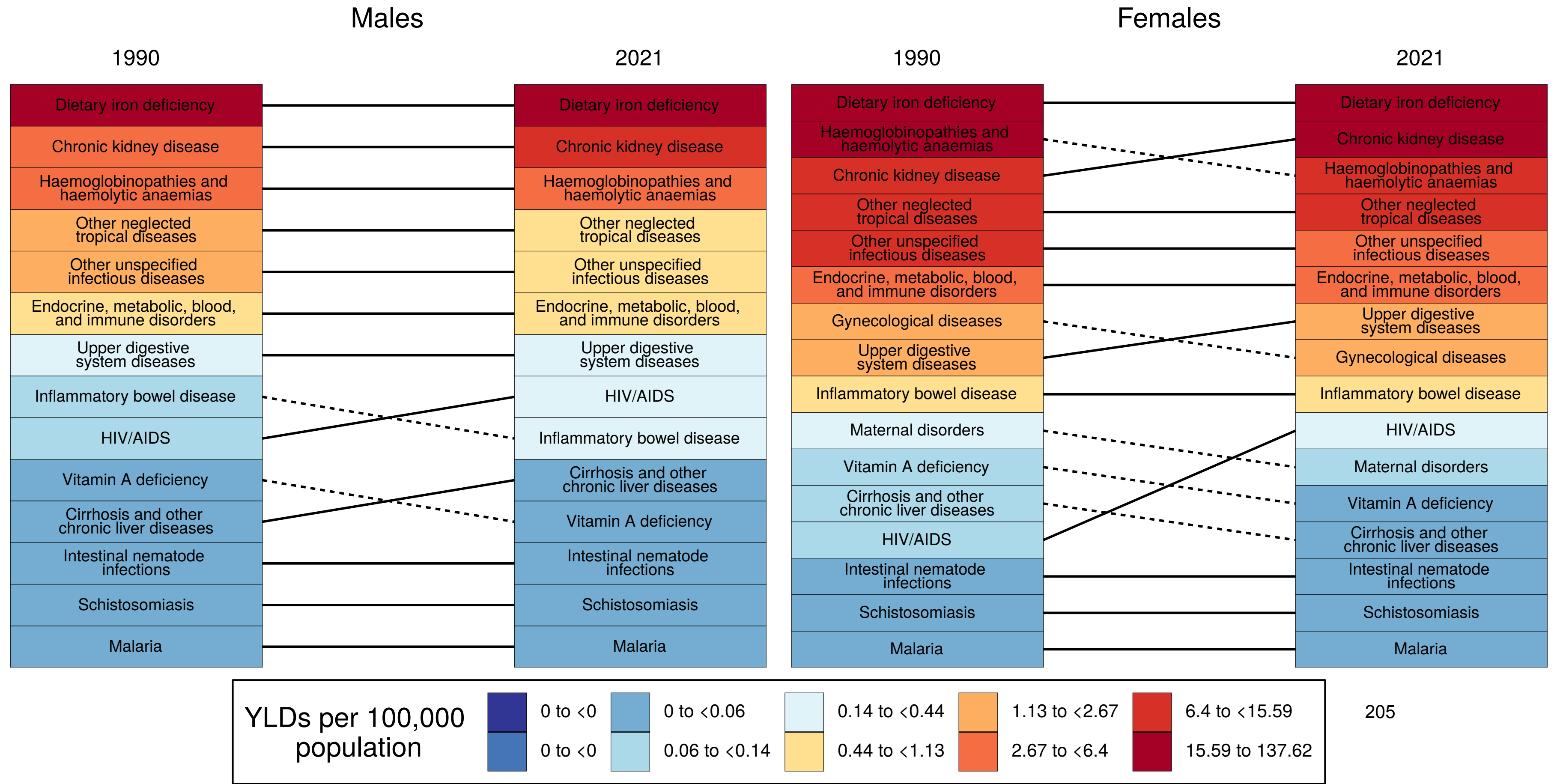
Males
Females
1990
2021
Dietary iron deficiency
Chronic kidney disease
Haemoglobinopathies and haemolytic anaemias
Other neglected tropical diseases
Other unspecified infectious diseases
Endocrine, metabolic, blood, and immune disorders
Upper digestive system diseases
Inflammatory bowel disease
HIV/AIDS
Vitamin A deficiency
Cirrhosis and other chronic liver diseases
Intestinal nematode infections
Schistosomiasis
Malaria
Gynecological diseases
Maternal disorders
YLDs per 100,000 population
0 to <0
0 to <0.06
0.06 to <0.14
0.14 to <0.44
0.44 to <1.13
1.13 to <2.67
2.67 to <6.4
6.4 to <15.59
15.59 to 137.62

Germany

Anaemia Prevalence - Both Sexes

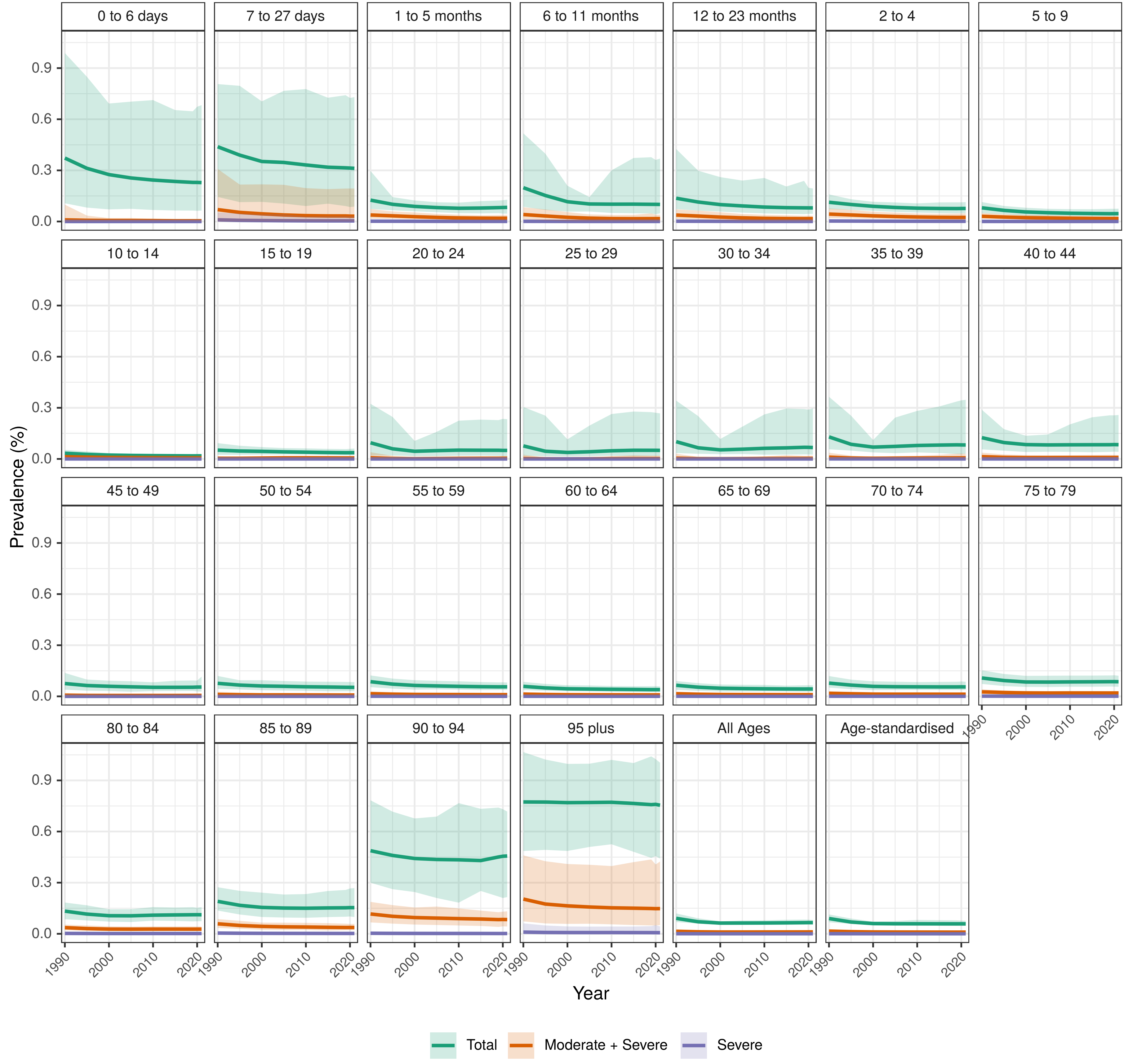

Cause-Specific Anaemia Prevalence - 2021

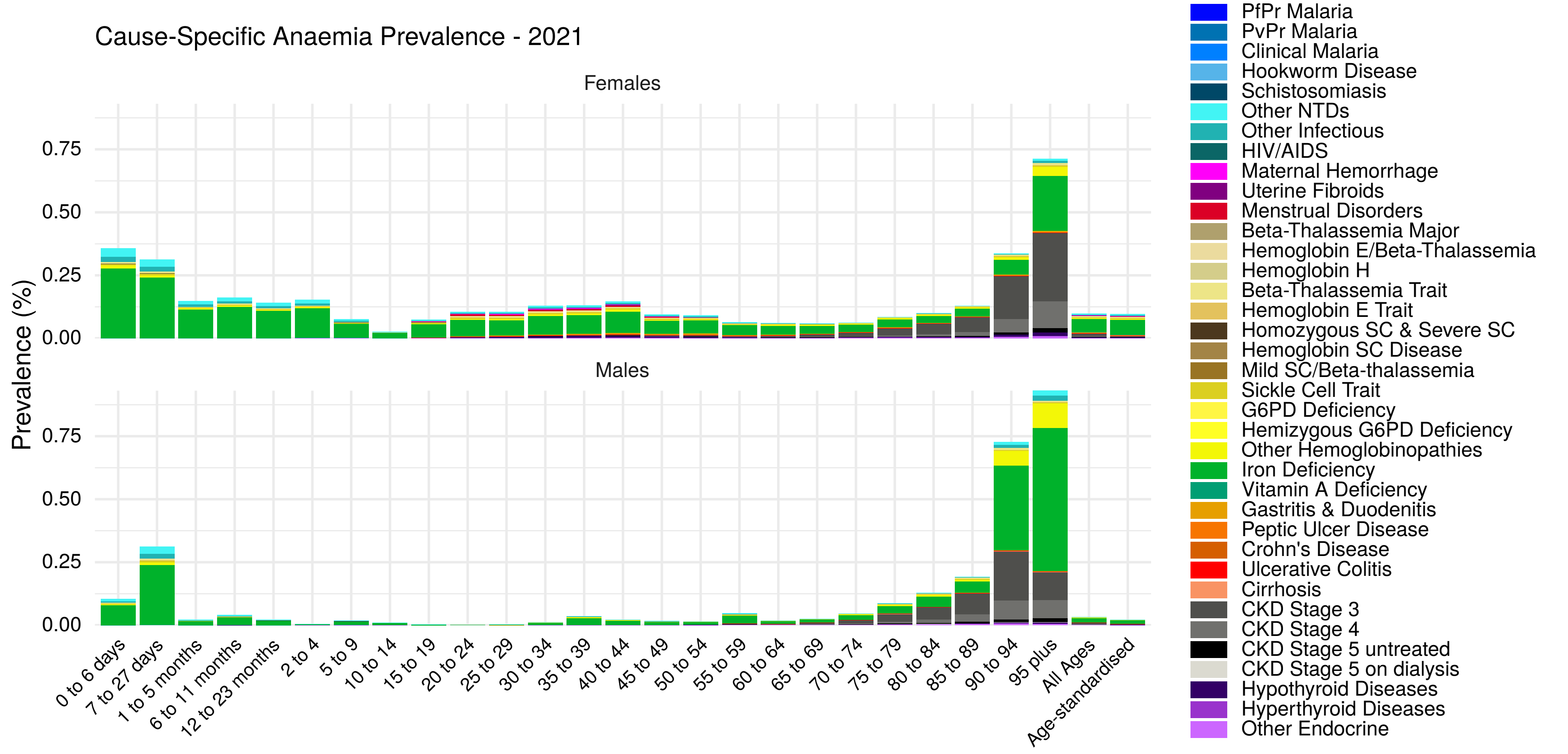

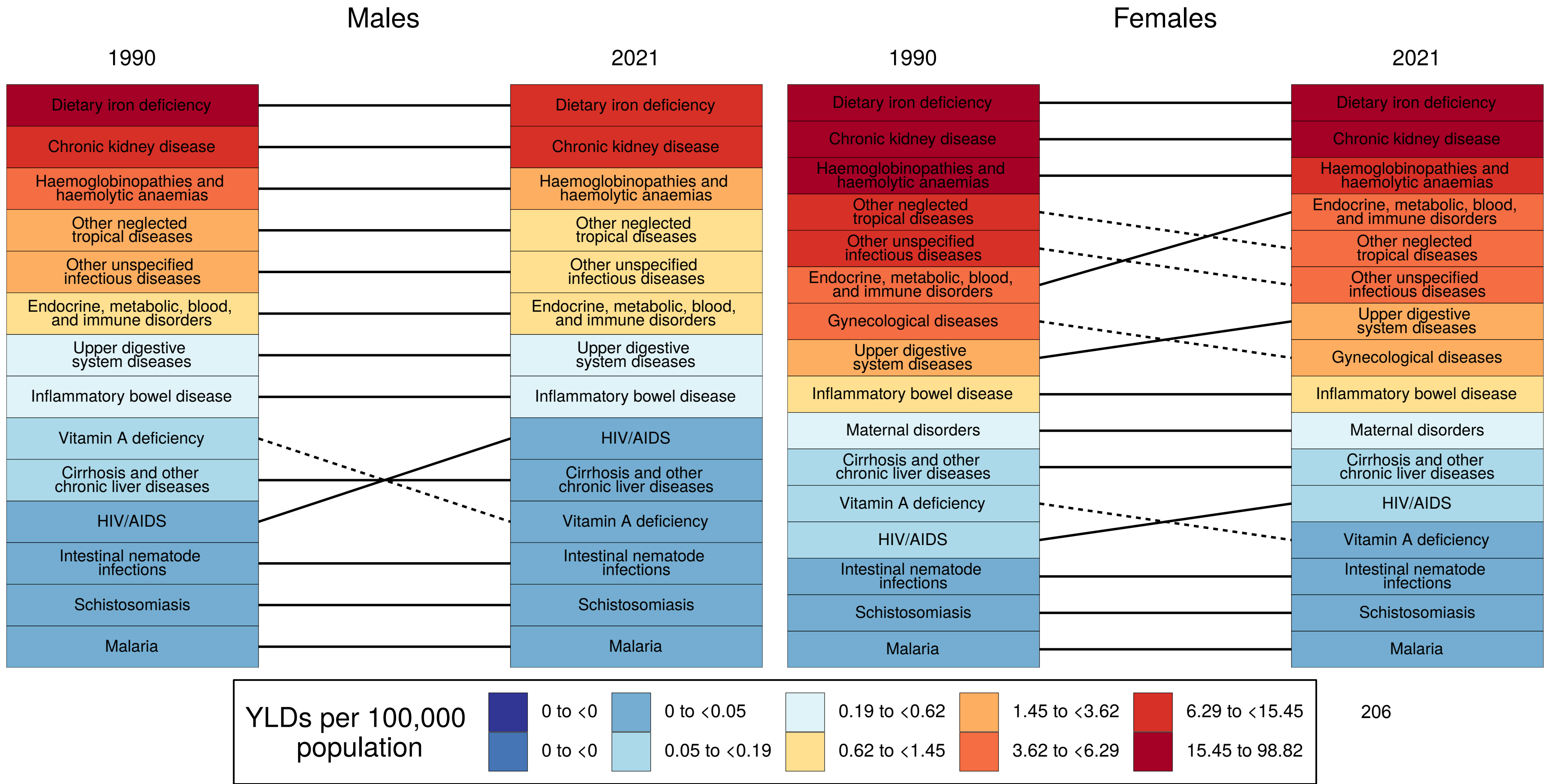

Greece

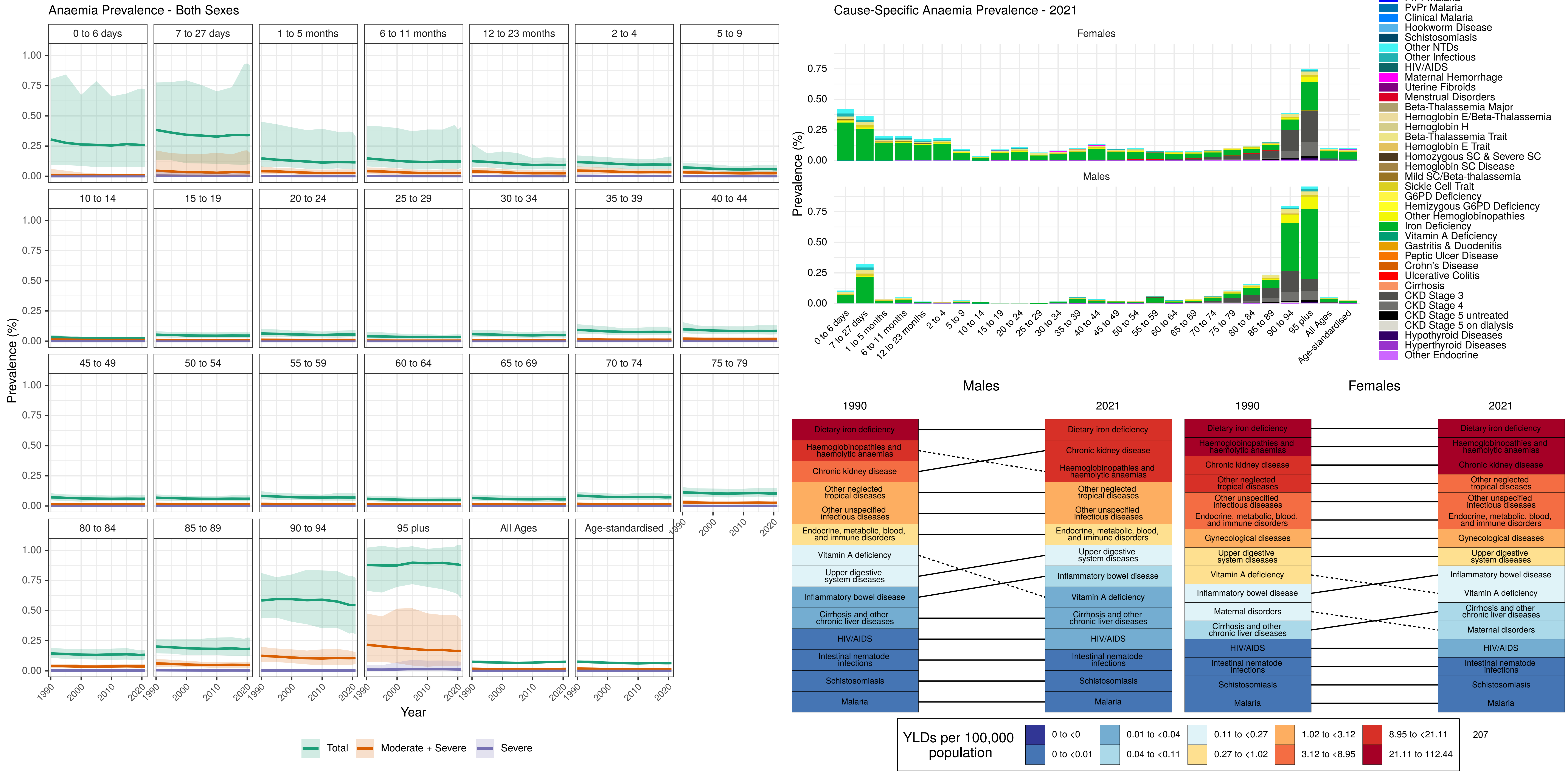

Anaemia Prevalence - Both Sexes
0 to 6 days
7 to 27 days
1 to 5 months
6 to 11 months
12 to 23 months
2 to 4
5 to 9
10 to 14
15 to 19
20 to 24
25 to 29
30 to 34
35 to 39
40 to 44
45 to 49
50 to 54
55 to 59
60 to 64
65 to 69
70 to 74
75 to 79
80 to 84
85 to 89
90 to 94
95 plus
All Ages
Age-standardised
Prevalence (%)
Year
Total
Moderate + Severe
Severe
Cause-Specific Anaemia Prevalence - 2021
Females
Males
Prevalence (%)
PfPr Malaria
PvPr Malaria
Clinical Malaria
Hookworm Disease
Schistosomiasis
Other NTDs
Other Infectious
HIV/AIDS
Maternal Hemorrhage
Uterine Fibroids
Menstrual Disorders
Beta-Thalassemia Major
Hemoglobin E/Beta-Thalassemia
Hemoglobin H
Beta-Thalassemia Trait
Hemoglobin E Trait
Homozygous SC & Severe SC
Hemoglobin SC Disease
Mild SC/Beta-thalassemia
Sickle Cell Trait
G6PD Deficiency
Hemizygous G6PD Deficiency
Other Hemoglobinopathies
Iron Deficiency
Vitamin A Deficiency
Gastritis & Duodenitis
Peptic Ulcer Disease
Crohn's Disease
Ulcerative Colitis
Cirrhosis
CKD Stage 3
CKD Stage 4
CKD Stage 5 untreated
CKD Stage 5 on dialysis
Hypothyroid Diseases
Hyperthyroid Diseases
Other Endocrine
Males
Females
1990
2021
Dietary iron deficiency
Haemoglobinopathies and haemolytic anaemias
Chronic kidney disease
Other neglected tropical diseases
Other unspecified infectious diseases
Endocrine, metabolic, blood, and immune disorders
Vitamin A deficiency
Upper digestive system diseases
Inflammatory bowel disease
Cirrhosis and other chronic liver diseases
HIV/AIDS
Intestinal nematode infections
Schistosomiasis
Malaria
Gynecological diseases
Maternal disorders
YLDs per 100,000 population
0 to <0
0 to <0.01
0.01 to <0.04
0.04 to <0.11
0.11 to <0.27
0.27 to <1.02
1.02 to <3.12
3.12 to <8.95
8.95 to <21.11
21.11 to 112.44

Iceland

Anaemia Prevalence - Both Sexes

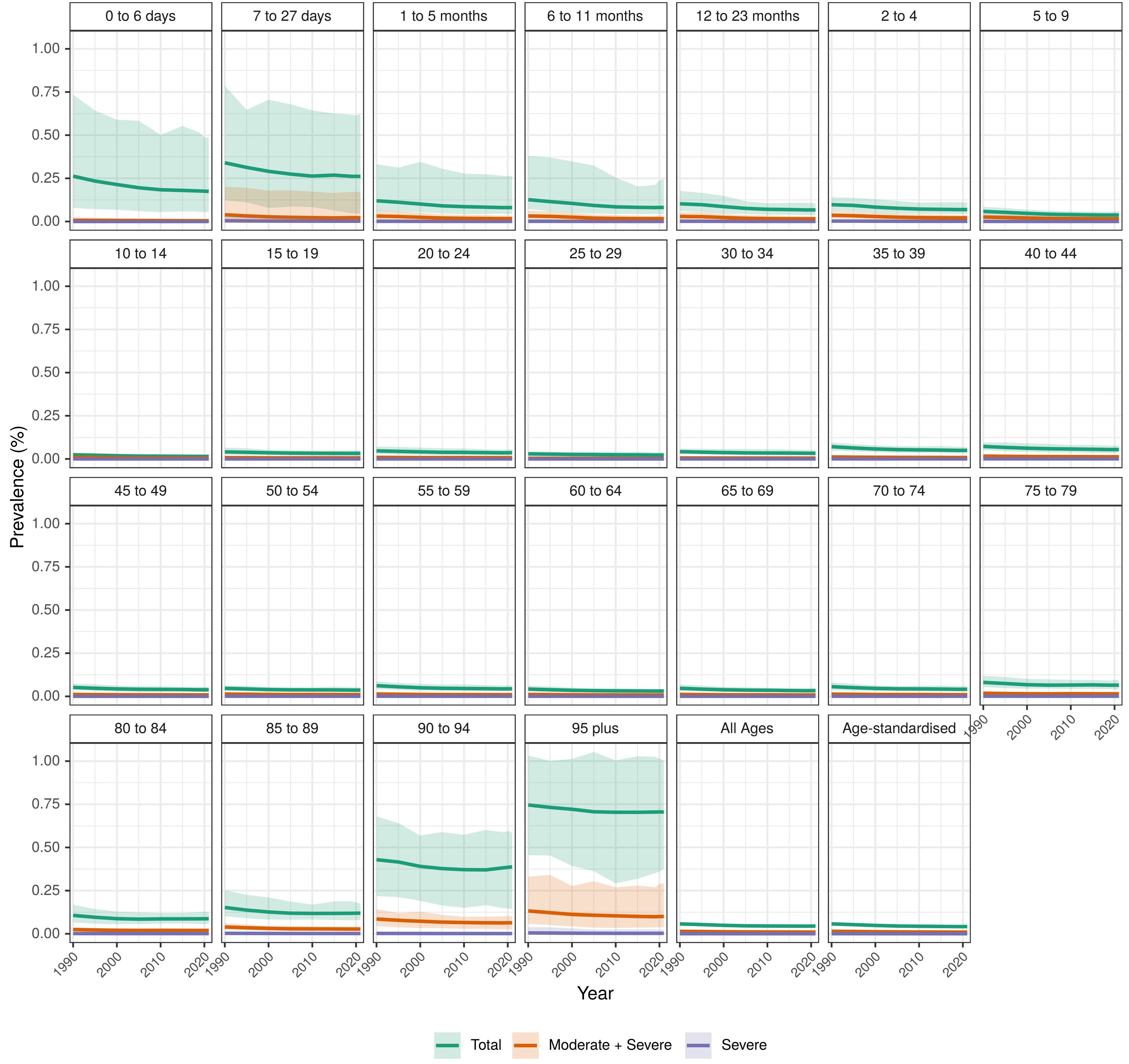

Cause-Specific Anaemia Prevalence - 2021

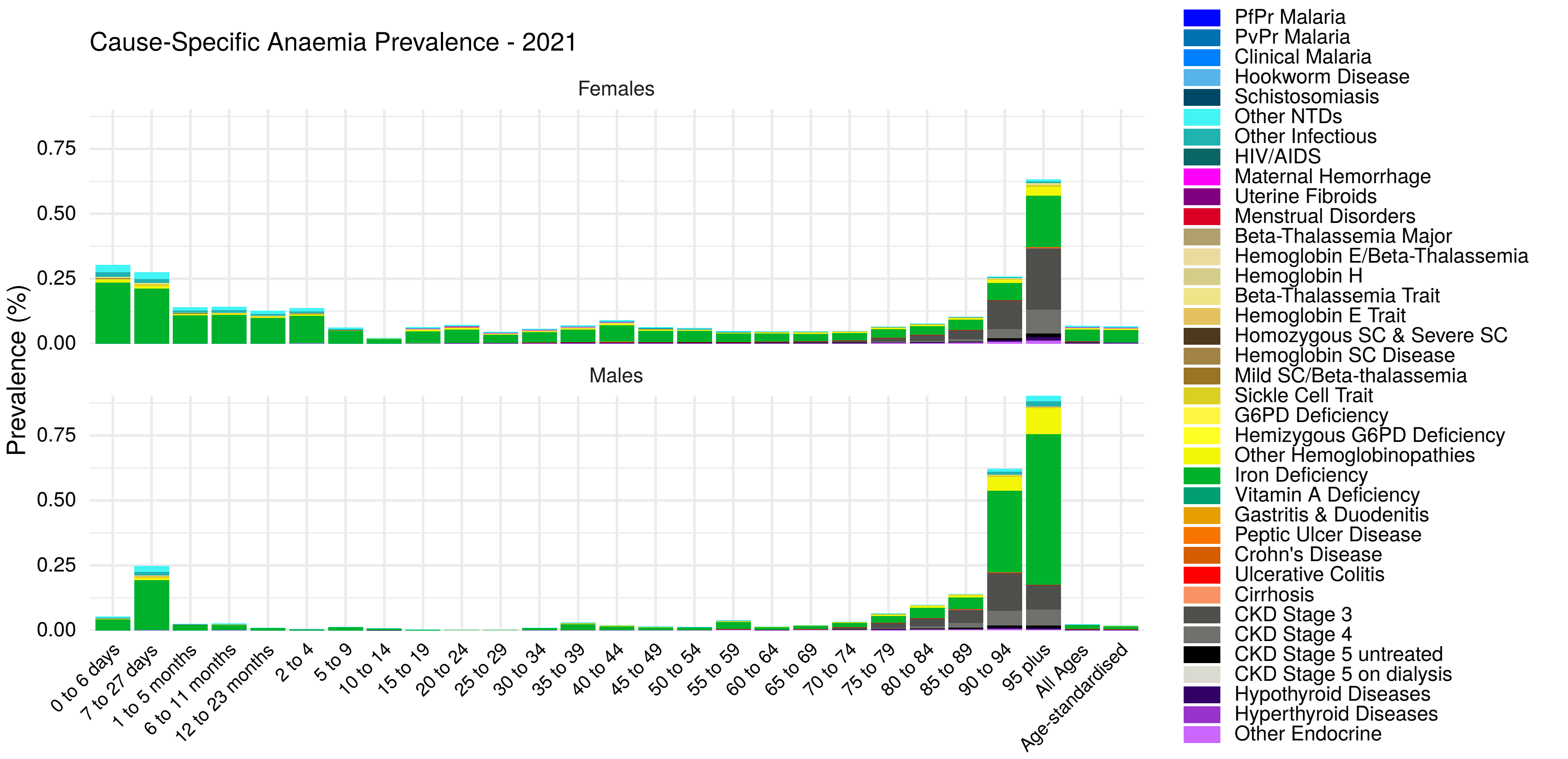

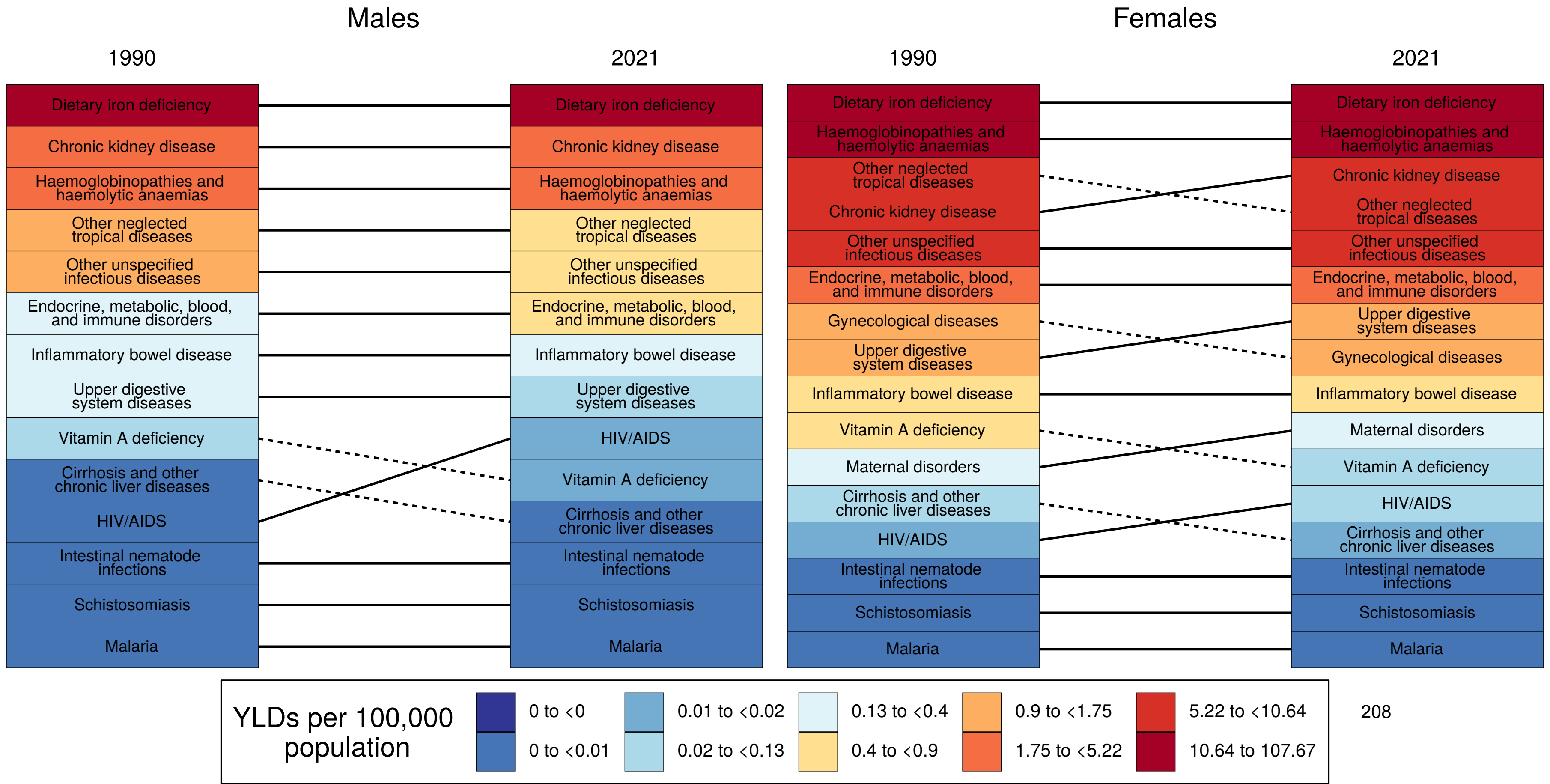

Ireland

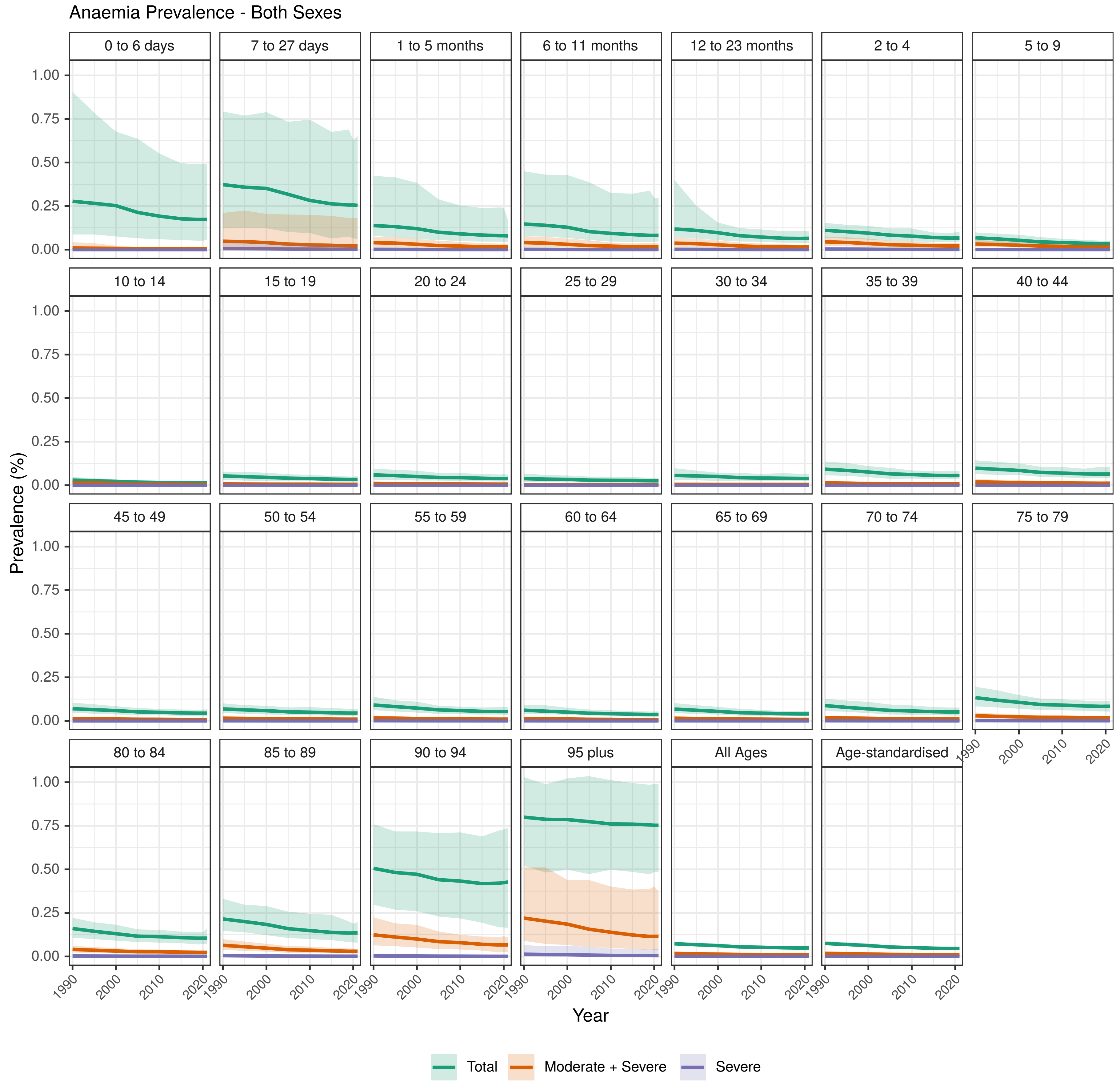

Anaemia Prevalence - Both Sexes
0 to 6 days
7 to 27 days
1 to 5 months
6 to 11 months
12 to 23 months
2 to 4
5 to 9
10 to 14
15 to 19
20 to 24
25 to 29
30 to 34
35 to 39
40 to 44
45 to 49
50 to 54
55 to 59
60 to 64
65 to 69
70 to 74
75 to 79
80 to 84
85 to 89
90 to 94
95 plus
All Ages
Age-standardised
Prevalence (%)
Year
1.00
0.75
0.50
0.25
0.00
1990
2000
2010
2020
Total
Moderate + Severe
Severe

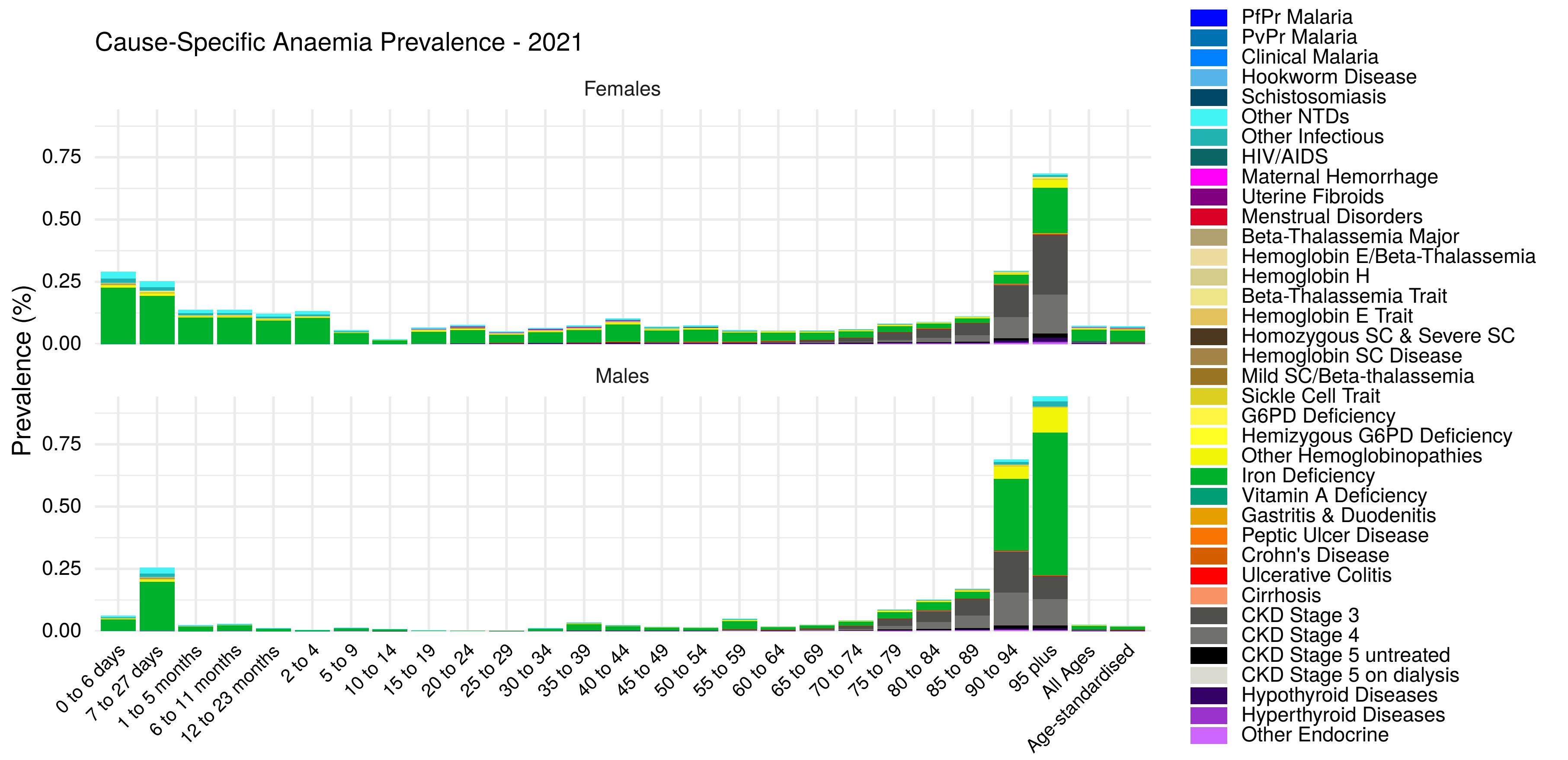

Cause-Specific Anaemia Prevalence - 2021
Females
Males
Prevalence (%)
0.75
0.50
0.25
0.00
0 to 6 days
7 to 27 days
1 to 5 months
6 to 11 months
12 to 23 months
2 to 4
5 to 9
10 to 14
15 to 19
20 to 24
25 to 29
30 to 34
35 to 39
40 to 44
45 to 49
50 to 54
55 to 59
60 to 64
65 to 69
70 to 74
75 to 79
80 to 84
85 to 89
90 to 94
95 plus
All Ages
Age-standardised
PfPr Malaria
PvPr Malaria
Clinical Malaria
Hookworm Disease
Schistosomiasis
Other NTDs
Other Infectious
HIV/AIDS
Maternal Hemorrhage
Uterine Fibroids
Menstrual Disorders
Beta-Thalassemia Major
Hemoglobin E/Beta-Thalassemia
Hemoglobin H
Beta-Thalassemia Trait
Hemoglobin E Trait
Homozygous SC & Severe SC
Hemoglobin SC Disease
Mild SC/Beta-thalassemia
Sickle Cell Trait
G6PD Deficiency
Hemizygous G6PD Deficiency
Other Hemoglobinopathies
Iron Deficiency
Vitamin A Deficiency
Gastritis & Duodenitis
Peptic Ulcer Disease
Crohn's Disease
Ulcerative Colitis
Cirrhosis
CKD Stage 3
CKD Stage 4
CKD Stage 5 untreated
CKD Stage 5 on dialysis
Hypothyroid Diseases
Hyperthyroid Diseases
Other Endocrine

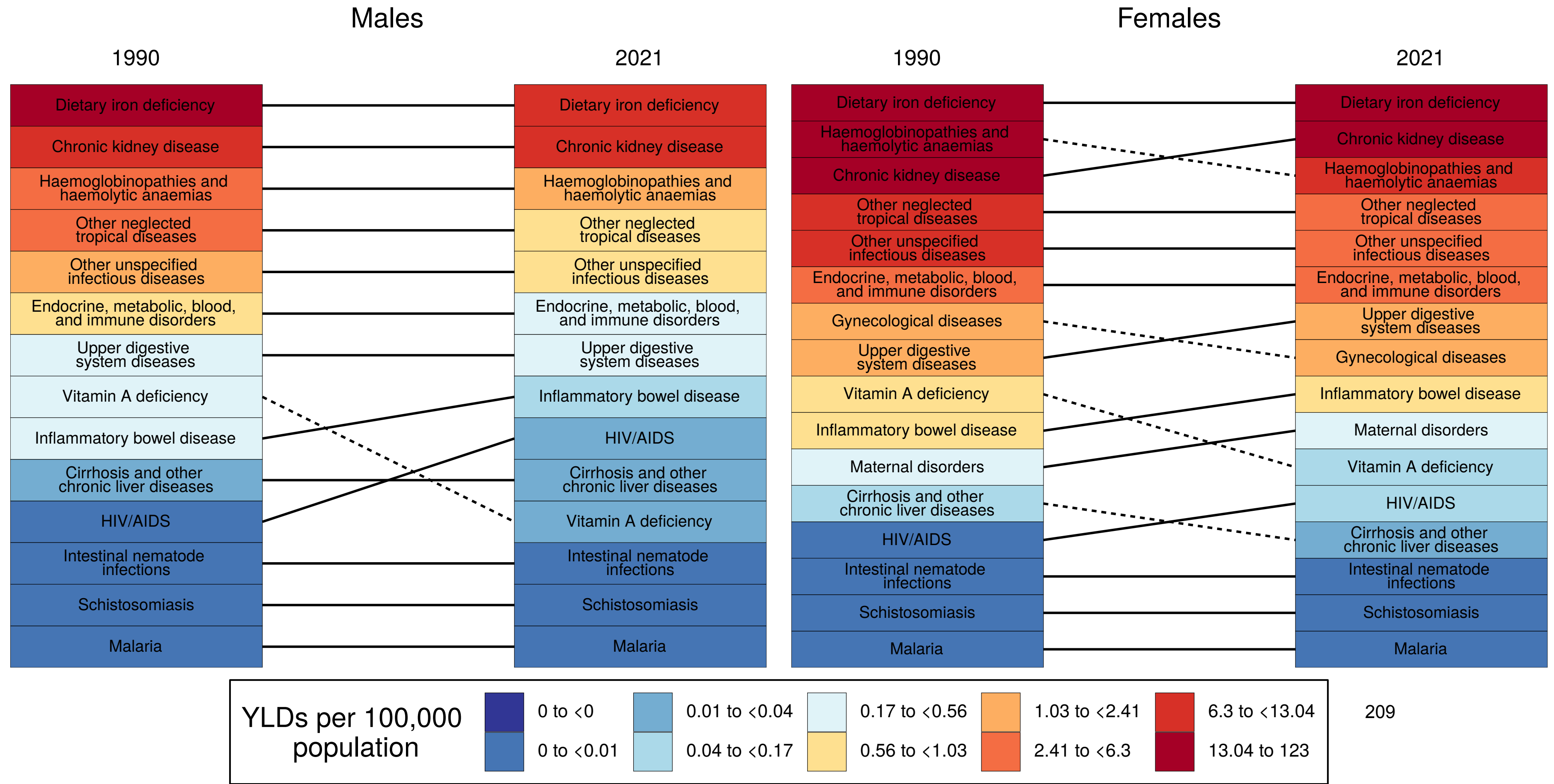

Males
1990
Dietary iron deficiency
Chronic kidney disease
Haemoglobinopathies and haemolytic anaemias
Other neglected tropical diseases
Other unspecified infectious diseases
Endocrine, metabolic, blood, and immune disorders
Upper digestive system diseases
Vitamin A deficiency
Inflammatory bowel disease
Cirrhosis and other chronic liver diseases
HIV/AIDS
Intestinal nematode infections
Schistosomiasis
Malaria
2021
Dietary iron deficiency
Chronic kidney disease
Haemoglobinopathies and haemolytic anaemias
Other neglected tropical diseases
Other unspecified infectious diseases
Endocrine, metabolic, blood, and immune disorders
Upper digestive system diseases
Inflammatory bowel disease
HIV/AIDS
Cirrhosis and other chronic liver diseases
Vitamin A deficiency
Intestinal nematode infections
Schistosomiasis
Malaria
Females
1990
Dietary iron deficiency
Haemoglobinopathies and haemolytic anaemias
Chronic kidney disease
Other neglected tropical diseases
Other unspecified infectious diseases
Endocrine, metabolic, blood, and immune disorders
Gynecological diseases
Upper digestive system diseases
Vitamin A deficiency
Inflammatory bowel disease
Maternal disorders
Cirrhosis and other chronic liver diseases
HIV/AIDS
Intestinal nematode infections
Schistosomiasis
Malaria
2021
Dietary iron deficiency
Chronic kidney disease
Haemoglobinopathies and haemolytic anaemias
Other neglected tropical diseases
Other unspecified infectious diseases
Endocrine, metabolic, blood, and immune disorders
Upper digestive system diseases
Gynecological diseases
Inflammatory bowel disease
Maternal disorders
Vitamin A deficiency
HIV/AIDS
Cirrhosis and other chronic liver diseases
Intestinal nematode infections
Schistosomiasis
Malaria
YLDs per 100,000 population
0 to <0
0 to <0.01
0.01 to <0.04
0.04 to <0.17
0.17 to <0.56
0.56 to <1.03
1.03 to <2.41
2.41 to <6.3
6.3 to <13.04
13.04 to 123

Israel

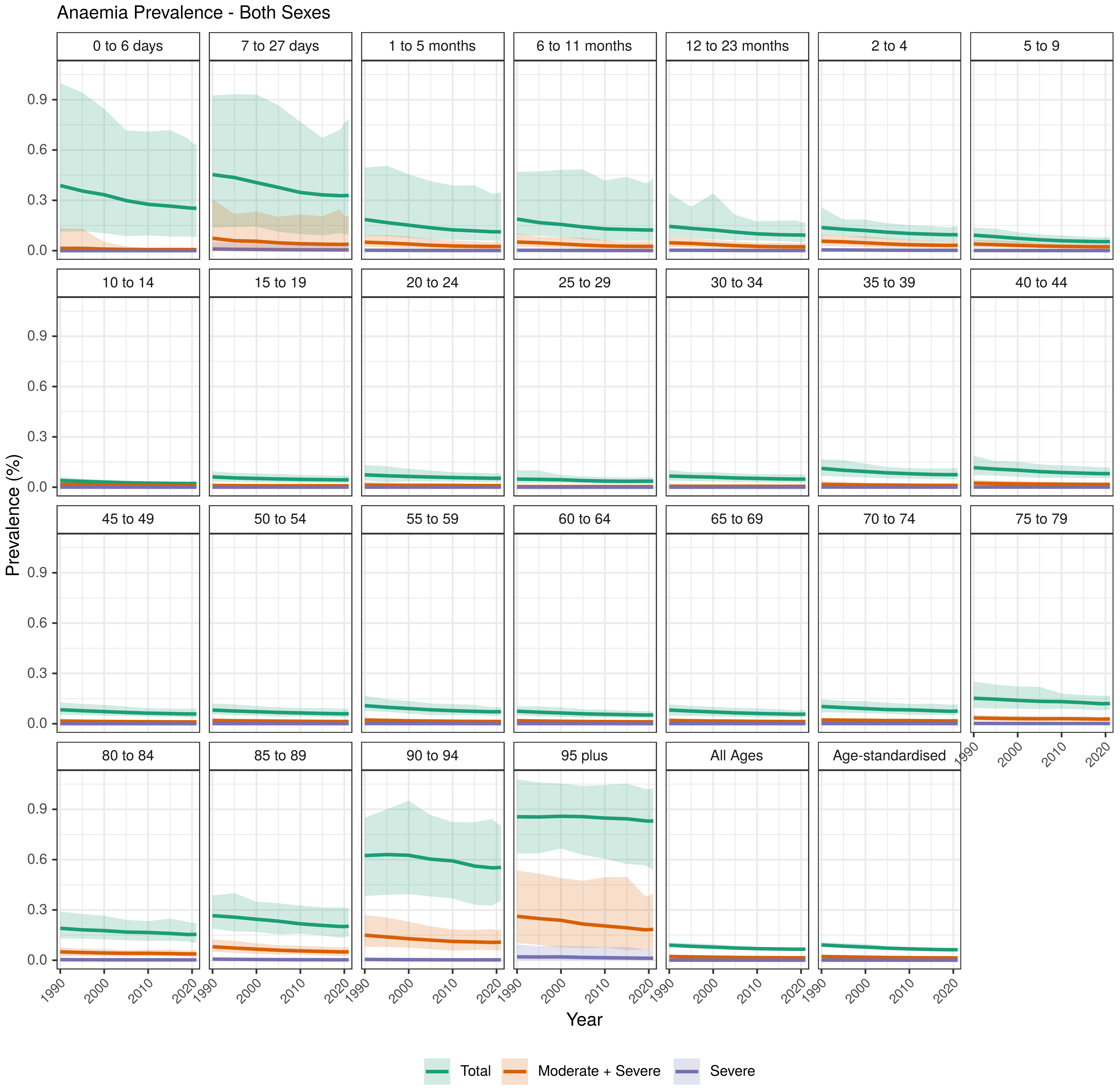

Anaemia Prevalence - Both Sexes
0 to 6 days
7 to 27 days
1 to 5 months
6 to 11 months
12 to 23 months
2 to 4
5 to 9
10 to 14
15 to 19
20 to 24
25 to 29
30 to 34
35 to 39
40 to 44
45 to 49
50 to 54
55 to 59
60 to 64
65 to 69
70 to 74
75 to 79
80 to 84
85 to 89
90 to 94
95 plus
All Ages
Age-standardised
Prevalence (%)
0.0
0.3
0.6
0.9
1990
2000
2010
2020
Year
Total
Moderate + Severe
Severe

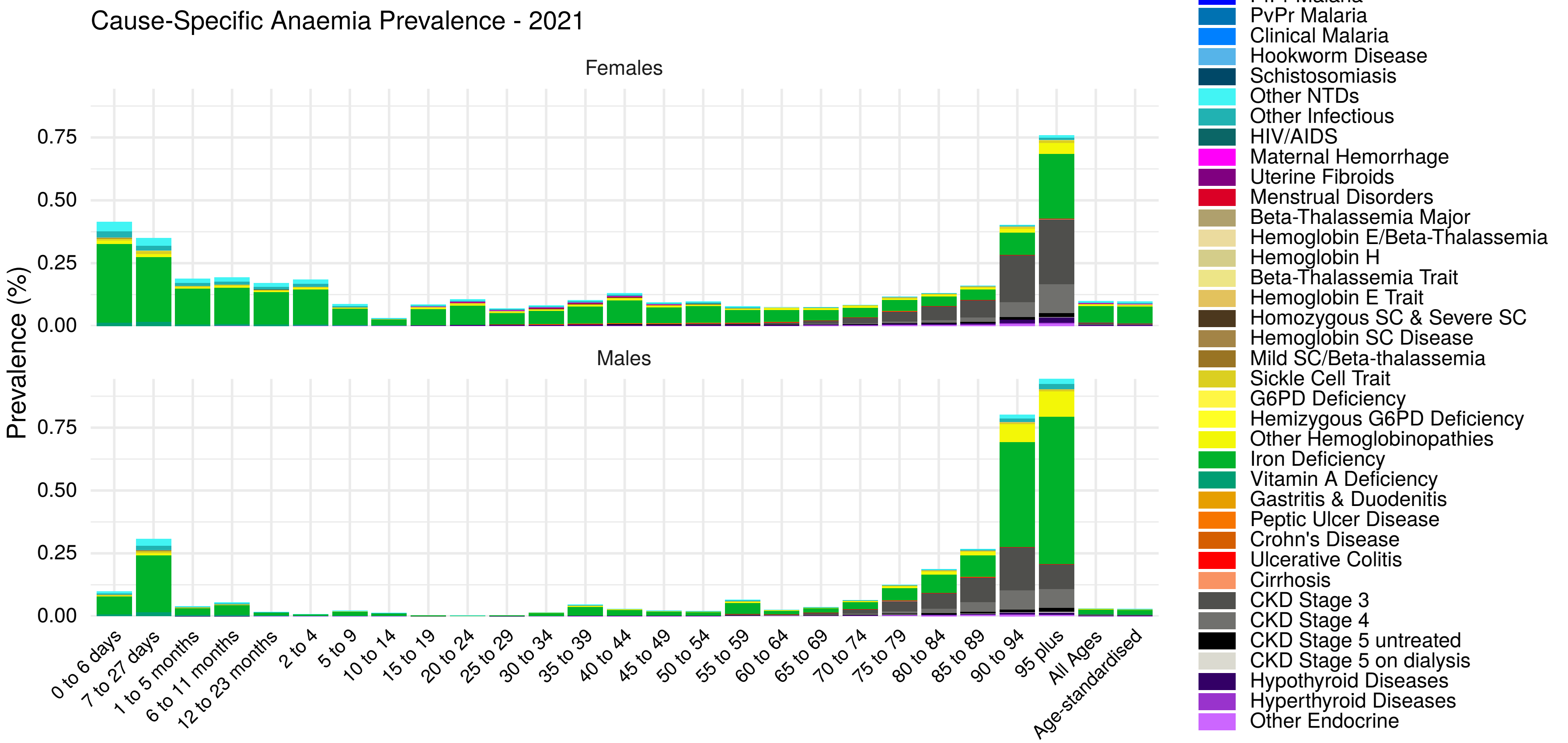

Cause-Specific Anaemia Prevalence - 2021
Females
Males
Prevalence (%)
0.00
0.25
0.50
0.75
0 to 6 days
7 to 27 days
1 to 5 months
6 to 11 months
12 to 23 months
2 to 4
5 to 9
10 to 14
15 to 19
20 to 24
25 to 29
30 to 34
35 to 39
40 to 44
45 to 49
50 to 54
55 to 59
60 to 64
65 to 69
70 to 74
75 to 79
80 to 84
85 to 89
90 to 94
95 plus
All Ages
Age-standardised
PfPr Malaria
PvPr Malaria
Clinical Malaria
Hookworm Disease
Schistosomiasis
Other NTDs
Other Infectious
HIV/AIDS
Maternal Hemorrhage
Uterine Fibroids
Menstrual Disorders
Beta-Thalassemia Major
Hemoglobin E/Beta-Thalassemia
Hemoglobin H
Beta-Thalassemia Trait
Hemoglobin E Trait
Homozygous SC & Severe SC
Hemoglobin SC Disease
Mild SC/Beta-thalassemia
Sickle Cell Trait
G6PD Deficiency
Hemizygous G6PD Deficiency
Other Hemoglobinopathies
Iron Deficiency
Vitamin A Deficiency
Gastritis & Duodenitis
Peptic Ulcer Disease
Crohn's Disease
Ulcerative Colitis
Cirrhosis
CKD Stage 3
CKD Stage 4
CKD Stage 5 untreated
CKD Stage 5 on dialysis
Hypothyroid Diseases
Hyperthyroid Diseases
Other Endocrine

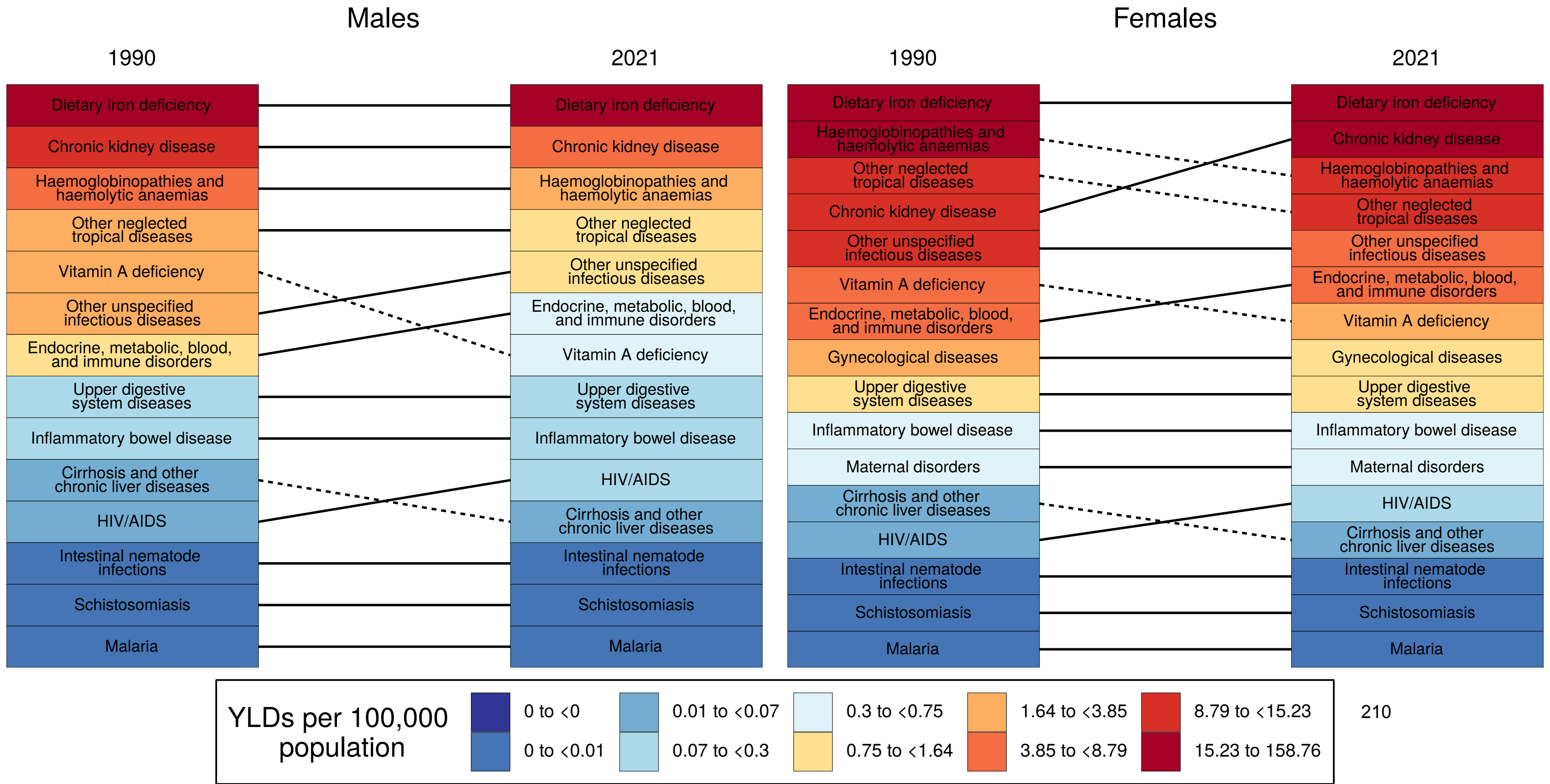

Males
1990
Dietary iron deficiency
Chronic kidney disease
Haemoglobinopathies and haemolytic anaemias
Other neglected tropical diseases
Vitamin A deficiency
Other unspecified infectious diseases
Endocrine, metabolic, blood, and immune disorders
Upper digestive system diseases
Inflammatory bowel disease
Cirrhosis and other chronic liver diseases
HIV/AIDS
Intestinal nematode infections
Schistosomiasis
Malaria
2021
Dietary iron deficiency
Chronic kidney disease
Haemoglobinopathies and haemolytic anaemias
Other neglected tropical diseases
Other unspecified infectious diseases
Endocrine, metabolic, blood, and immune disorders
Vitamin A deficiency
Upper digestive system diseases
Inflammatory bowel disease
HIV/AIDS
Cirrhosis and other chronic liver diseases
Intestinal nematode infections
Schistosomiasis
Malaria
Females
1990
Dietary iron deficiency
Haemoglobinopathies and haemolytic anaemias
Other neglected tropical diseases
Chronic kidney disease
Other unspecified infectious diseases
Vitamin A deficiency
Endocrine, metabolic, blood, and immune disorders
Gynecological diseases
Upper digestive system diseases
Inflammatory bowel disease
Maternal disorders
Cirrhosis and other chronic liver diseases
HIV/AIDS
Intestinal nematode infections
Schistosomiasis
Malaria
2021
Dietary iron deficiency
Chronic kidney disease
Haemoglobinopathies and haemolytic anaemias
Other neglected tropical diseases
Other unspecified infectious diseases
Endocrine, metabolic, blood, and immune disorders
Vitamin A deficiency
Gynecological diseases
Upper digestive system diseases
Inflammatory bowel disease
Maternal disorders
HIV/AIDS
Cirrhosis and other chronic liver diseases
Intestinal nematode infections
Schistosomiasis
Malaria
YLDs per 100,000 population
0 to <0
0 to <0.01
0.01 to <0.07
0.07 to <0.3
0.3 to <0.75
0.75 to <1.64
1.64 to <3.85
3.85 to <8.79
8.79 to <15.23
15.23 to 158.76

Italy

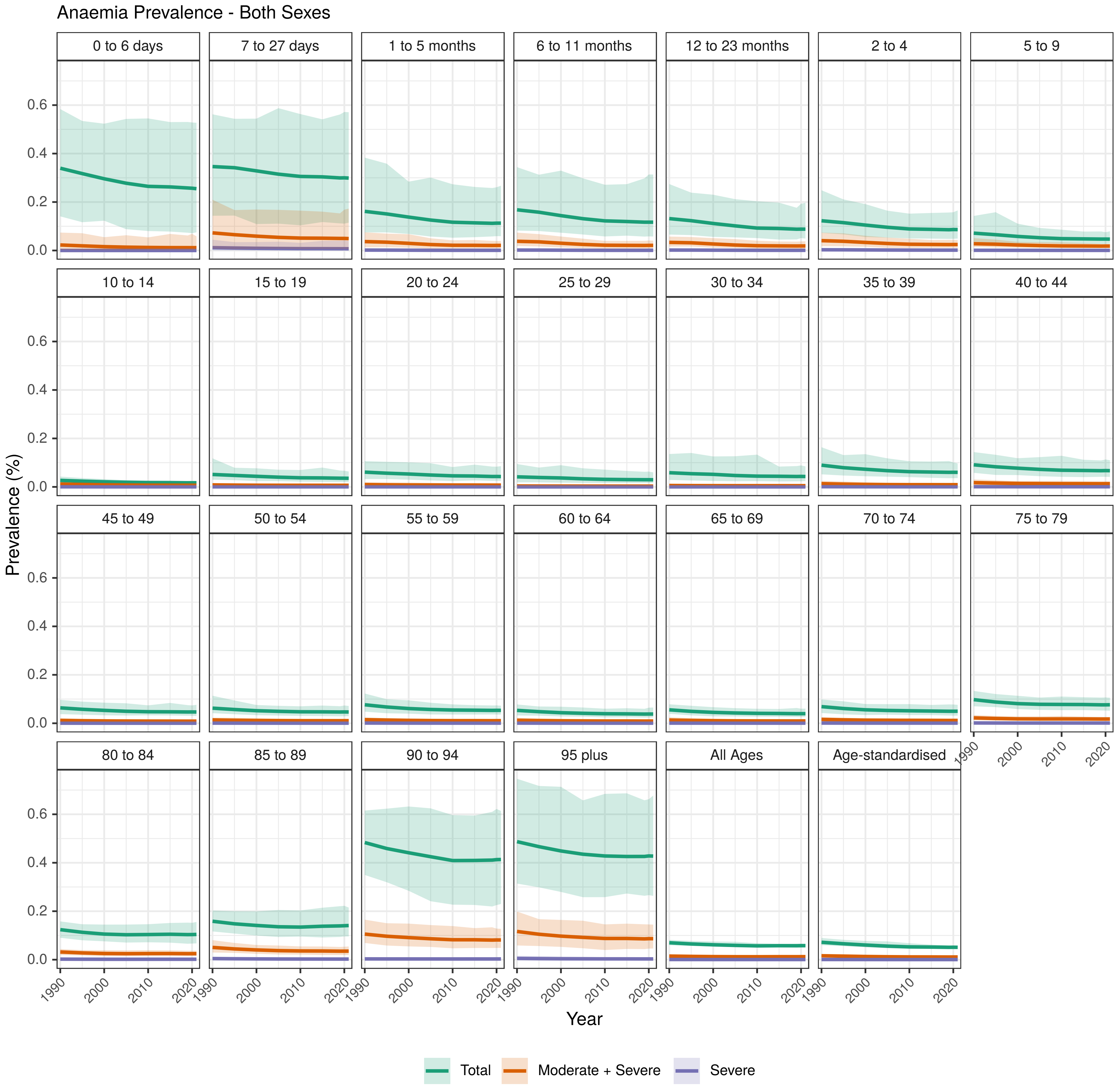

Anaemia Prevalence - Both Sexes
0 to 6 days
7 to 27 days
1 to 5 months
6 to 11 months
12 to 23 months
2 to 4
5 to 9
10 to 14
15 to 19
20 to 24
25 to 29
30 to 34
35 to 39
40 to 44
45 to 49
50 to 54
55 to 59
60 to 64
65 to 69
70 to 74
75 to 79
80 to 84
85 to 89
90 to 94
95 plus
All Ages
Age-standardised
Prevalence (%)
Year
Total
Moderate + Severe
Severe

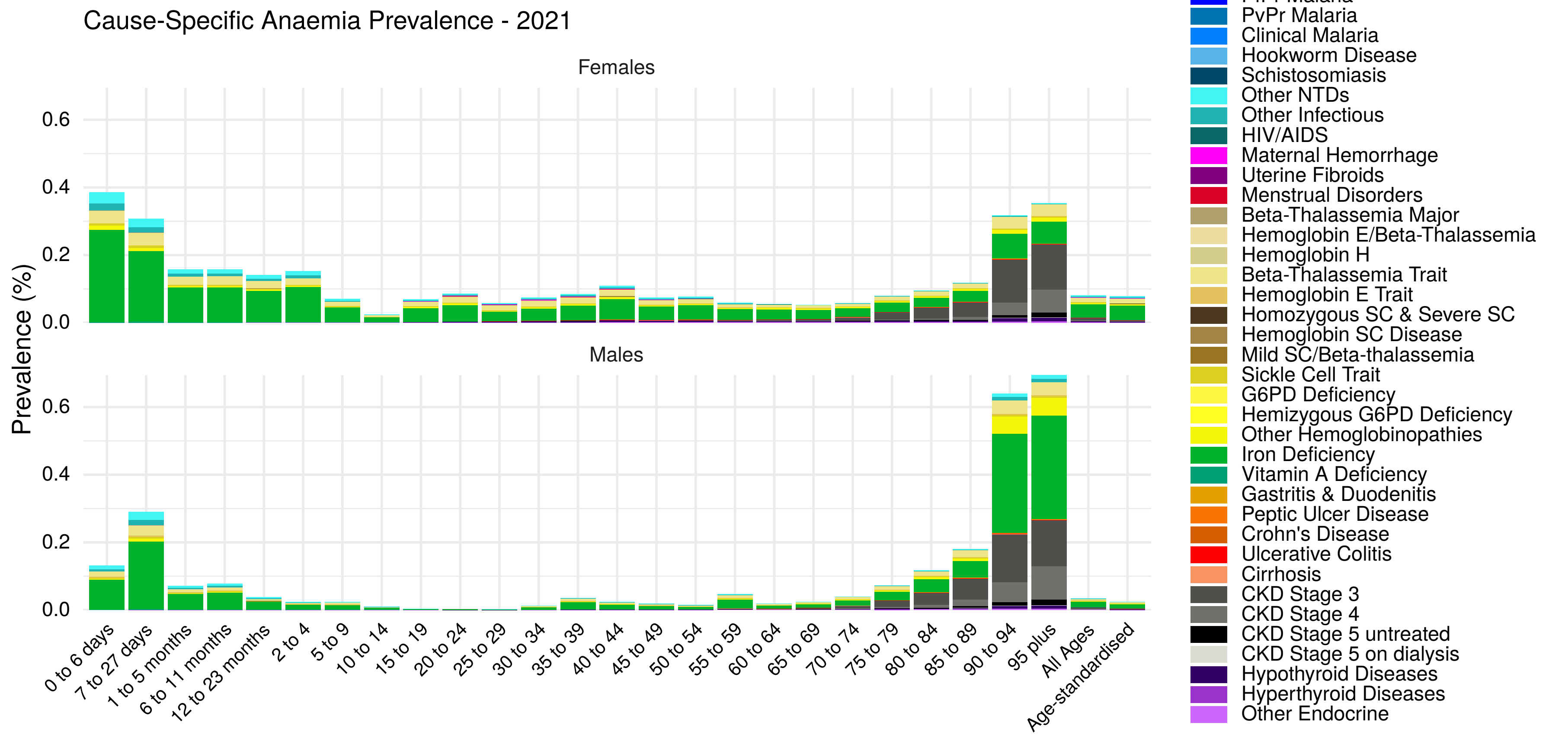

Cause-Specific Anaemia Prevalence - 2021
Females
Males
Prevalence (%)
PfPr Malaria
PvPr Malaria
Clinical Malaria
Hookworm Disease
Schistosomiasis
Other NTDs
Other Infectious
HIV/AIDS
Maternal Hemorrhage
Uterine Fibroids
Menstrual Disorders
Beta-Thalassemia Major
Hemoglobin E/Beta-Thalassemia
Hemoglobin H
Beta-Thalassemia Trait
Hemoglobin E Trait
Homozygous SC & Severe SC
Hemoglobin SC Disease
Mild SC/Beta-thalassemia
Sickle Cell Trait
G6PD Deficiency
Hemizygous G6PD Deficiency
Other Hemoglobinopathies
Iron Deficiency
Vitamin A Deficiency
Gastritis & Duodenitis
Peptic Ulcer Disease
Crohn's Disease
Ulcerative Colitis
Cirrhosis
CKD Stage 3
CKD Stage 4
CKD Stage 5 untreated
CKD Stage 5 on dialysis
Hypothyroid Diseases
Hyperthyroid Diseases
Other Endocrine

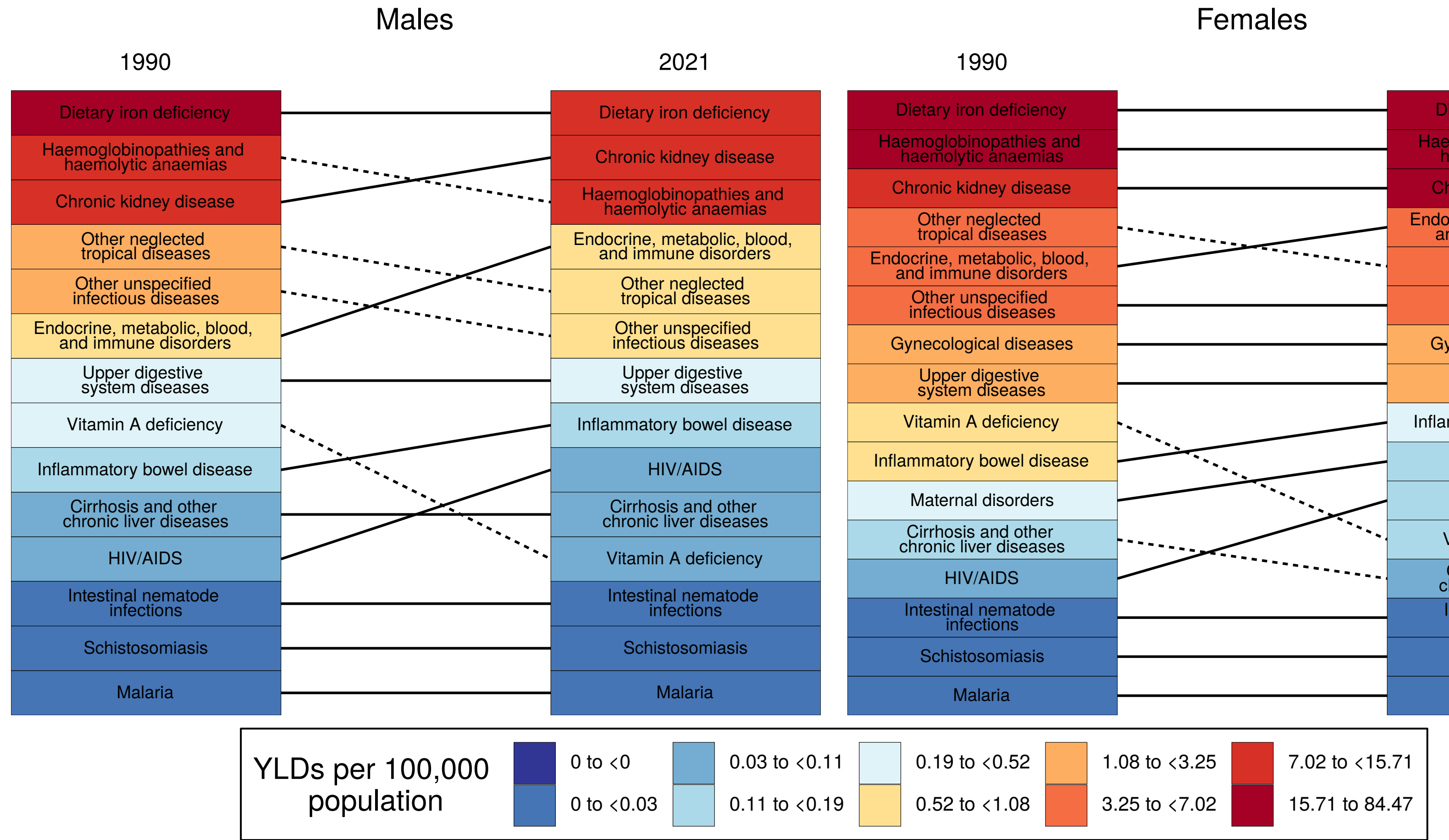

Males
Females
1990
2021
Dietary iron deficiency
Haemoglobinopathies and haemolytic anaemias
Chronic kidney disease
Other neglected tropical diseases
Other unspecified infectious diseases
Endocrine, metabolic, blood, and immune disorders
Upper digestive system diseases
Vitamin A deficiency
Inflammatory bowel disease
Cirrhosis and other chronic liver diseases
HIV/AIDS
Intestinal nematode infections
Schistosomiasis
Malaria
Gynecological diseases
Maternal disorders
YLDs per 100,000 population
0 to <0
0 to <0.03
0.03 to <0.11
0.11 to <0.19
0.19 to <0.52
0.52 to <1.08
1.08 to <3.25
3.25 to <7.02
7.02 to <15.71
15.71 to 84.47

Abruzzo

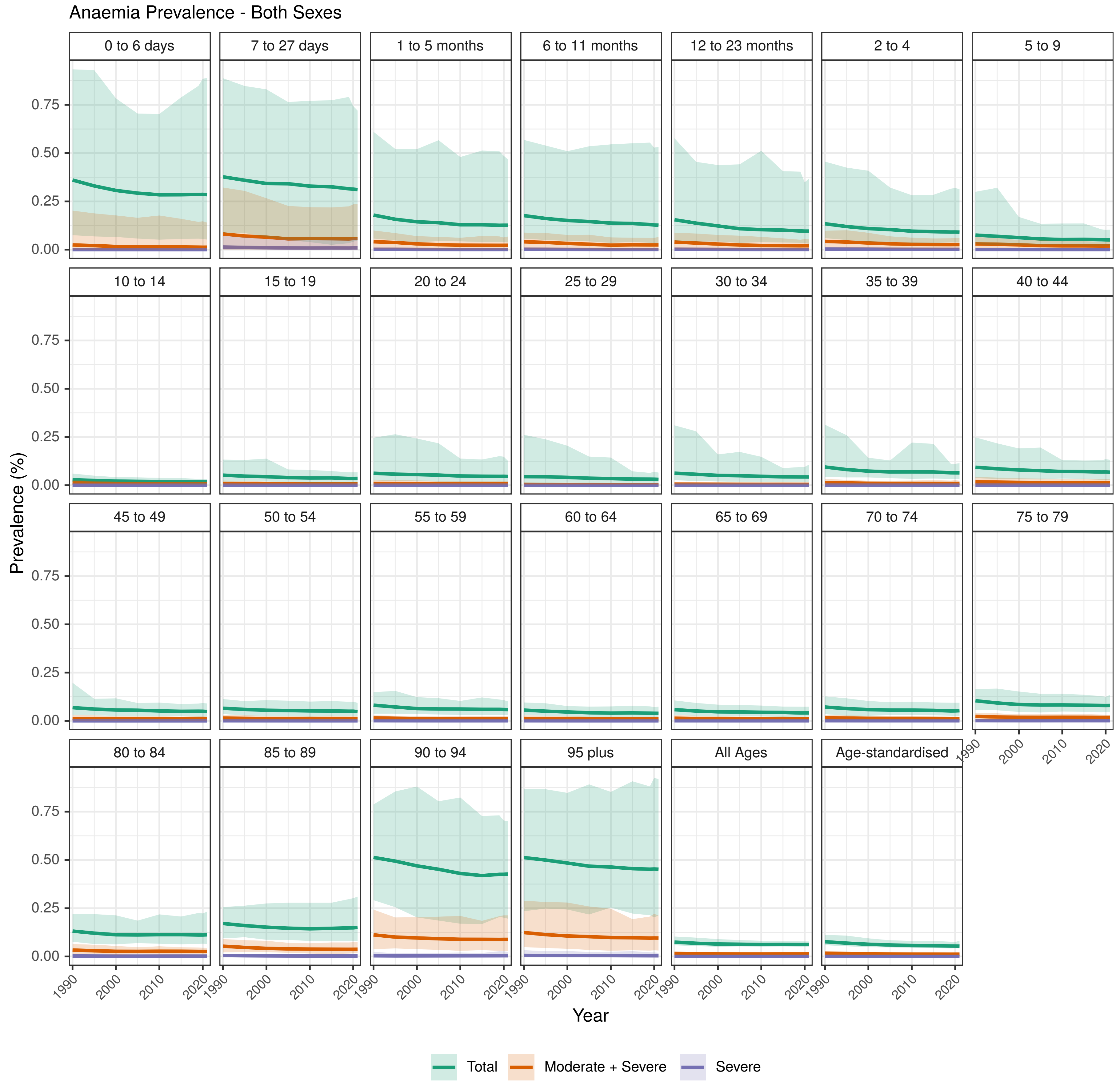
Anaemia Prevalence - Both Sexes
0 to 6 days
7 to 27 days
1 to 5 months
6 to 11 months
12 to 23 months
2 to 4
5 to 9
10 to 14
15 to 19
20 to 24
25 to 29
30 to 34
35 to 39
40 to 44
45 to 49
50 to 54
55 to 59
60 to 64
65 to 69
70 to 74
75 to 79
80 to 84
85 to 89
90 to 94
95 plus
All Ages
Age-standardised
Prevalence (%)
0.00
0.25
0.50
0.75
1990
2000
2010
2020
Year
Total
Moderate + Severe
Severe

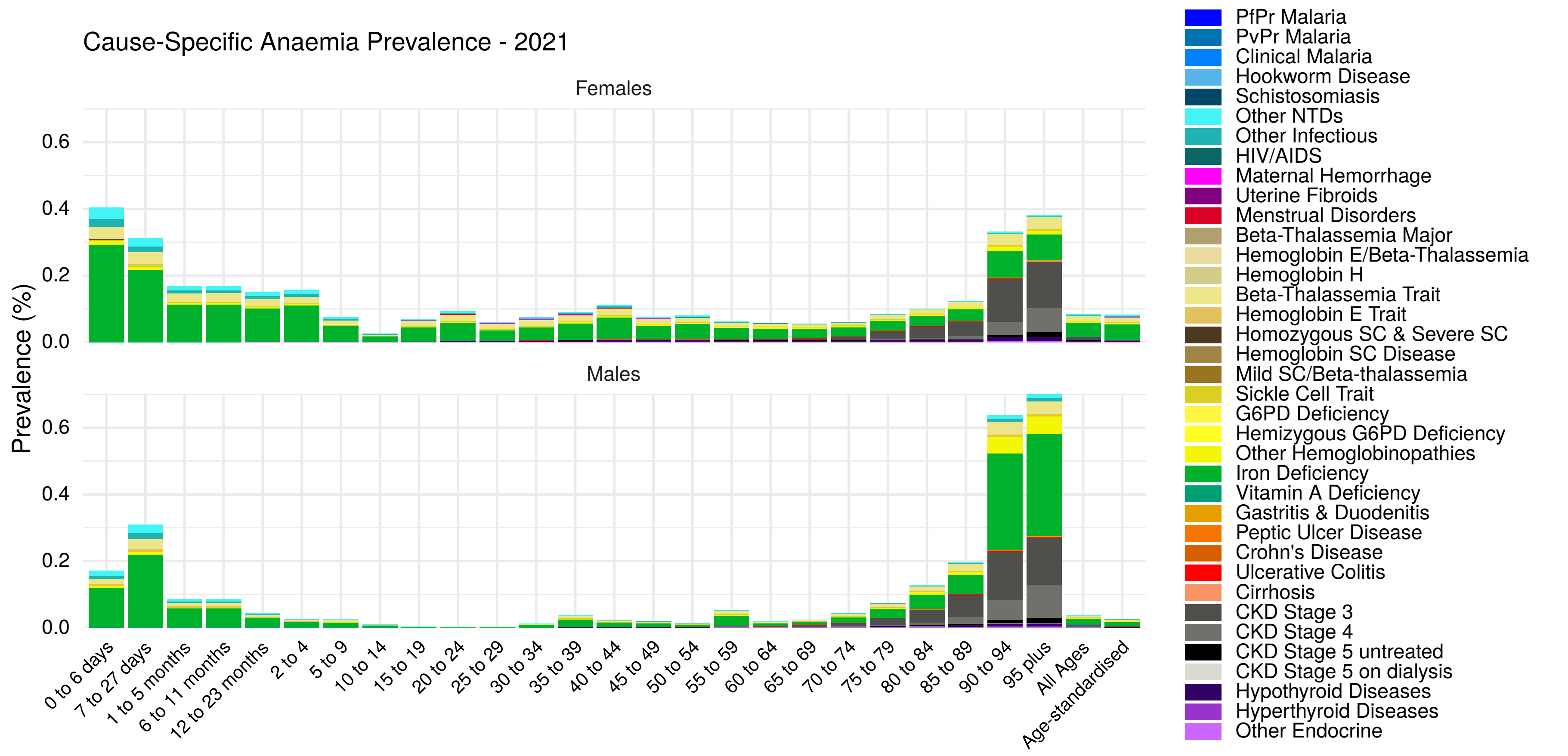
Cause-Specific Anaemia Prevalence - 2021
Females
Males
Prevalence (%)
0.0
0.2
0.4
0.6
0 to 6 days
7 to 27 days
1 to 5 months
6 to 11 months
12 to 23 months
2 to 4
5 to 9
10 to 14
15 to 19
20 to 24
25 to 29
30 to 34
35 to 39
40 to 44
45 to 49
50 to 54
55 to 59
60 to 64
65 to 69
70 to 74
75 to 79
80 to 84
85 to 89
90 to 94
95 plus
All Ages
Age-standardised
PfPr Malaria
PvPr Malaria
Clinical Malaria
Hookworm Disease
Schistosomiasis
Other NTDs
Other Infectious
HIV/AIDS
Maternal Hemorrhage
Uterine Fibroids
Menstrual Disorders
Beta-Thalassemia Major
Hemoglobin E/Beta-Thalassemia
Hemoglobin H
Beta-Thalassemia Trait
Hemoglobin E Trait
Homozygous SC & Severe SC
Hemoglobin SC Disease
Mild SC/Beta-thalassemia
Sickle Cell Trait
G6PD Deficiency
Hemizygous G6PD Deficiency
Other Hemoglobinopathies
Iron Deficiency
Vitamin A Deficiency
Gastritis & Duodenitis
Peptic Ulcer Disease
Crohn's Disease
Ulcerative Colitis
Cirrhosis
CKD Stage 3
CKD Stage 4
CKD Stage 5 untreated
CKD Stage 5 on dialysis
Hypothyroid Diseases
Hyperthyroid Diseases
Other Endocrine

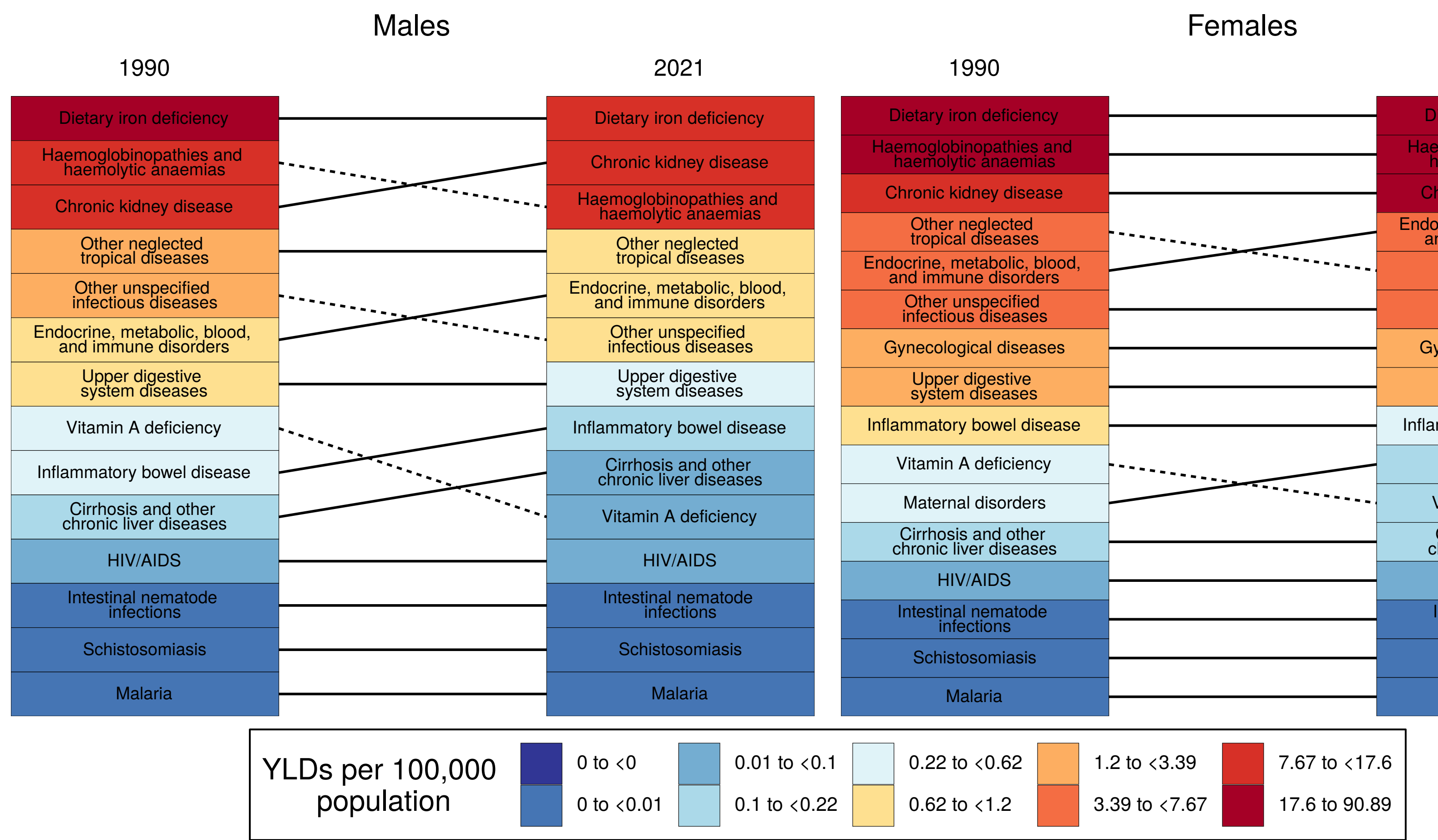
Males
Females
1990
2021
Males 1990:
Dietary iron deficiency
Haemoglobinopathies and haemolytic anaemias
Chronic kidney disease
Other neglected tropical diseases
Other unspecified infectious diseases
Endocrine, metabolic, blood, and immune disorders
Upper digestive system diseases
Vitamin A deficiency
Inflammatory bowel disease
Cirrhosis and other chronic liver diseases
HIV/AIDS
Intestinal nematode infections
Schistosomiasis
Malaria
Males 2021:
Dietary iron deficiency
Chronic kidney disease
Haemoglobinopathies and haemolytic anaemias
Other neglected tropical diseases
Endocrine, metabolic, blood, and immune disorders
Other unspecified infectious diseases
Upper digestive system diseases
Inflammatory bowel disease
Cirrhosis and other chronic liver diseases
Vitamin A deficiency
HIV/AIDS
Intestinal nematode infections
Schistosomiasis
Malaria
Females 1990:
Dietary iron deficiency
Haemoglobinopathies and haemolytic anaemias
Chronic kidney disease
Other neglected tropical diseases
Endocrine, metabolic, blood, and immune disorders
Other unspecified infectious diseases
Gynecological diseases
Upper digestive system diseases
Inflammatory bowel disease
Vitamin A deficiency
Maternal disorders
Cirrhosis and other chronic liver diseases
HIV/AIDS
Intestinal nematode infections
Schistosomiasis
Malaria
Females 2021:
Dietary iron deficiency
Haemoglobinopathies and haemolytic anaemias
Chronic kidney disease
Endocrine, metabolic, blood, and immune disorders
Other neglected tropical diseases
Other unspecified infectious diseases
Gynecological diseases
Upper digestive system diseases
Inflammatory bowel disease
Maternal disorders
Vitamin A deficiency
Cirrhosis and other chronic liver diseases
HIV/AIDS
Intestinal nematode infections
Schistosomiasis
Malaria
YLDs per 100,000 population
0 to <0
0 to <0.01
0.01 to <0.1
0.1 to <0.22
0.22 to <0.62
0.62 to <1.2
1.2 to <3.39
3.39 to <7.67
7.67 to <17.6
17.6 to 90.89

Basilicata

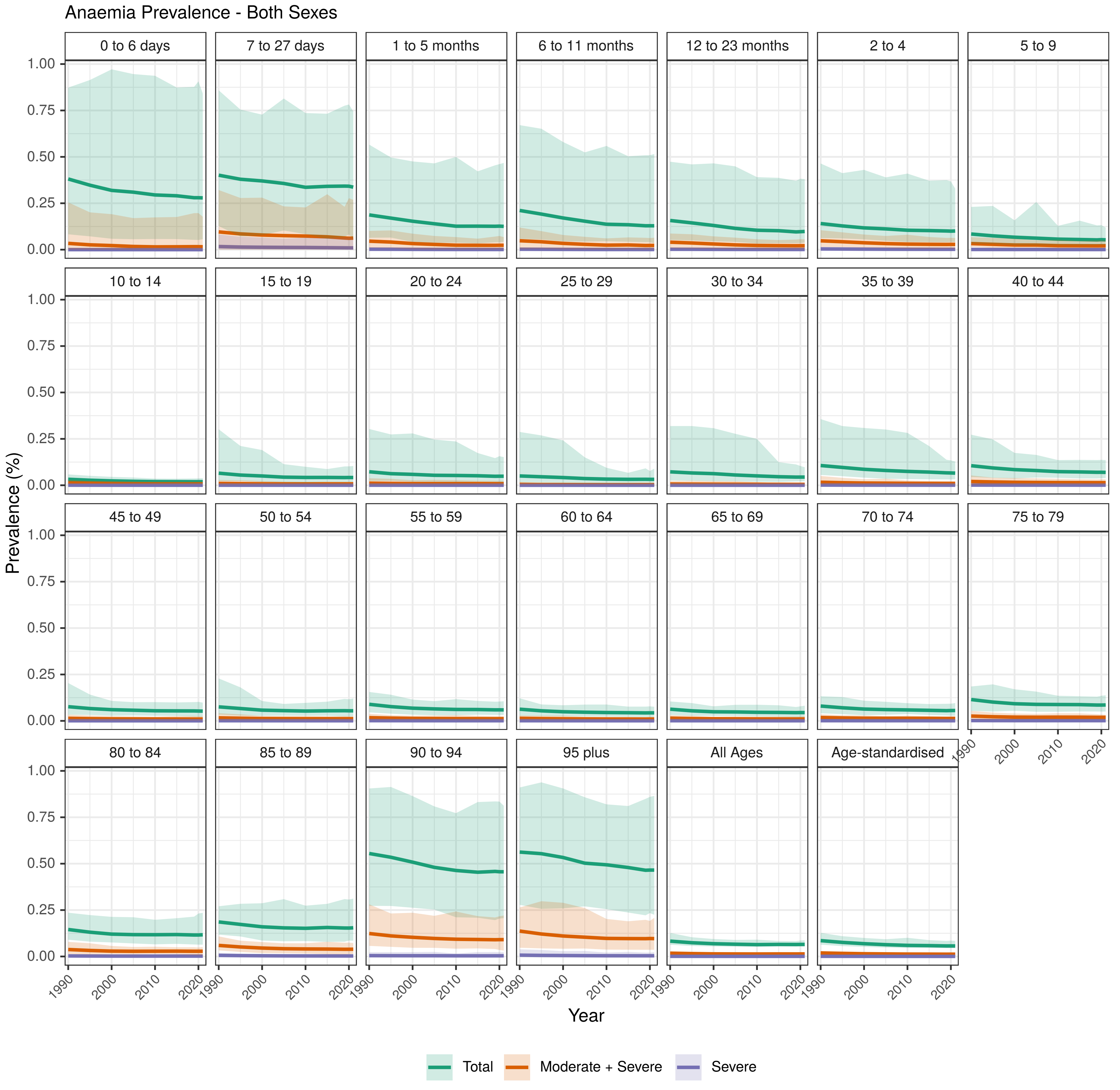

Anaemia Prevalence - Both Sexes
0 to 6 days
7 to 27 days
1 to 5 months
6 to 11 months
12 to 23 months
2 to 4
5 to 9
10 to 14
15 to 19
20 to 24
25 to 29
30 to 34
35 to 39
40 to 44
45 to 49
50 to 54
55 to 59
60 to 64
65 to 69
70 to 74
75 to 79
80 to 84
85 to 89
90 to 94
95 plus
All Ages
Age-standardised
Prevalence (%)
Year
Total
Moderate + Severe
Severe

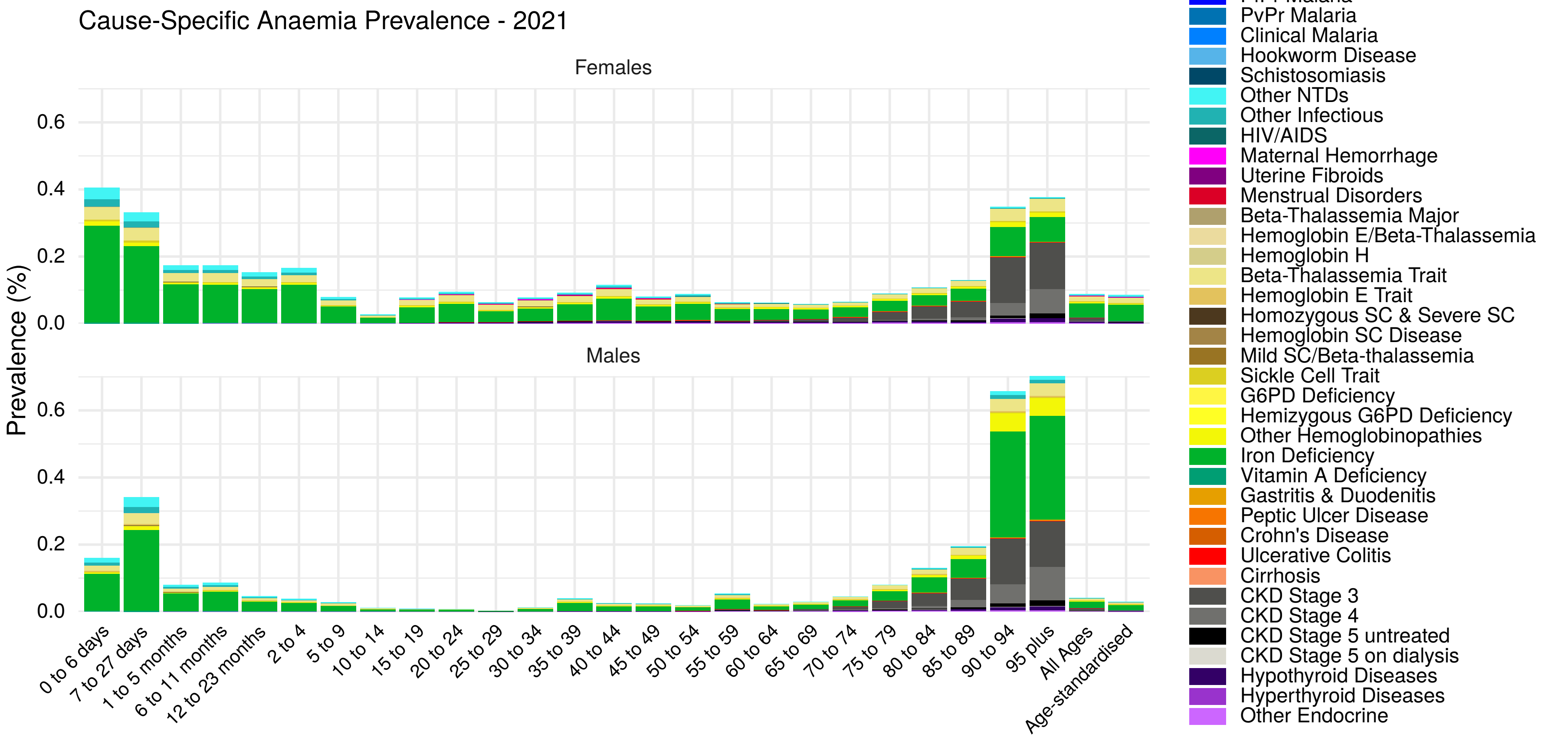

Cause-Specific Anaemia Prevalence - 2021
Females
Males
Prevalence (%)
0 to 6 days
7 to 27 days
1 to 5 months
6 to 11 months
12 to 23 months
2 to 4
5 to 9
10 to 14
15 to 19
20 to 24
25 to 29
30 to 34
35 to 39
40 to 44
45 to 49
50 to 54
55 to 59
60 to 64
65 to 69
70 to 74
75 to 79
80 to 84
85 to 89
90 to 94
95 plus
All Ages
Age-standardised
PfPr Malaria
PvPr Malaria
Clinical Malaria
Hookworm Disease
Schistosomiasis
Other NTDs
Other Infectious
HIV/AIDS
Maternal Hemorrhage
Uterine Fibroids
Menstrual Disorders
Beta-Thalassemia Major
Hemoglobin E/Beta-Thalassemia
Hemoglobin H
Beta-Thalassemia Trait
Hemoglobin E Trait
Homozygous SC & Severe SC
Hemoglobin SC Disease
Mild SC/Beta-thalassemia
Sickle Cell Trait
G6PD Deficiency
Hemizygous G6PD Deficiency
Other Hemoglobinopathies
Iron Deficiency
Vitamin A Deficiency
Gastritis & Duodenitis
Peptic Ulcer Disease
Crohn's Disease
Ulcerative Colitis
Cirrhosis
CKD Stage 3
CKD Stage 4
CKD Stage 5 untreated
CKD Stage 5 on dialysis
Hypothyroid Diseases
Hyperthyroid Diseases
Other Endocrine

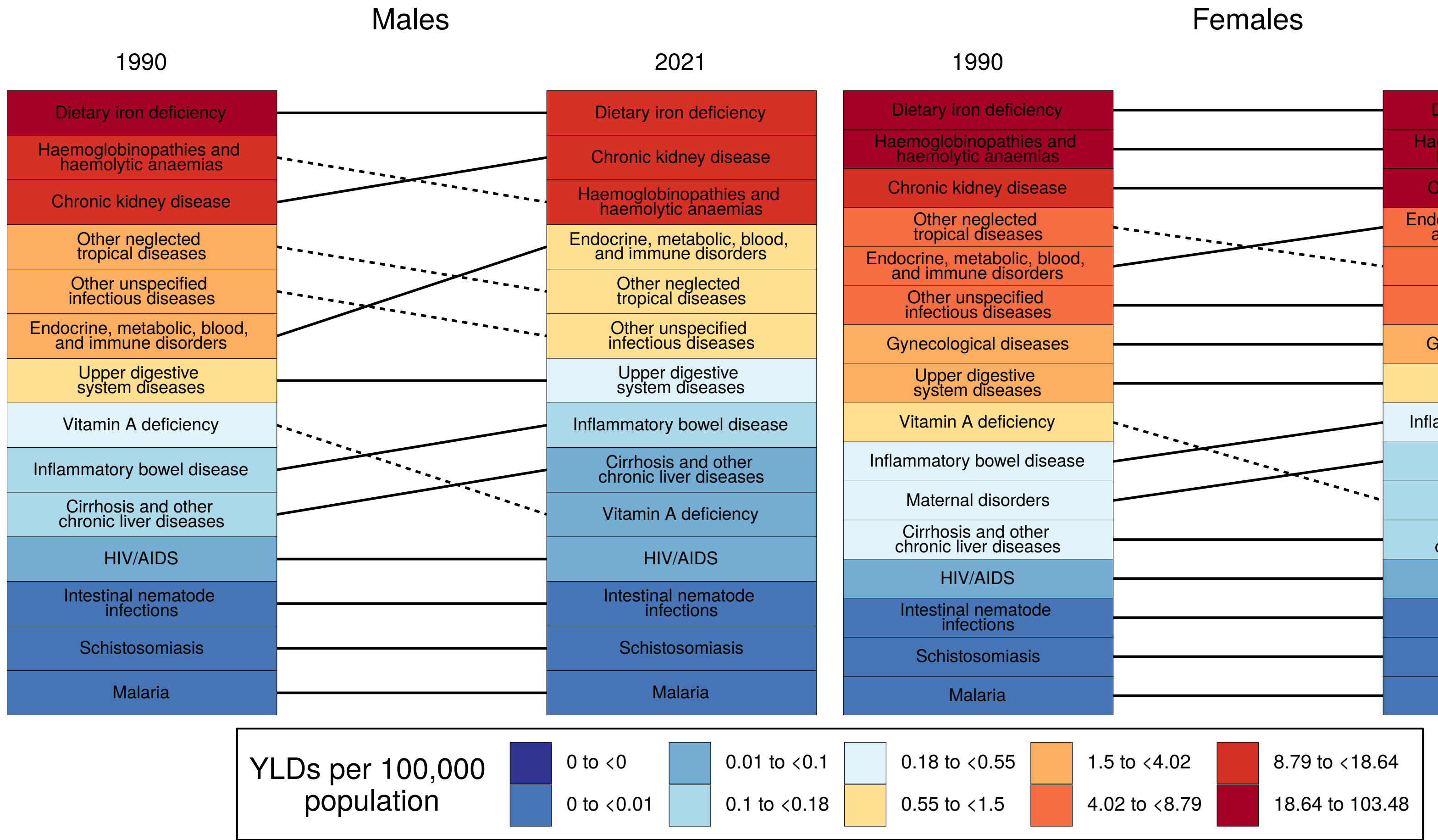

Males
Females
1990
2021
Dietary iron deficiency
Haemoglobinopathies and haemolytic anaemias
Chronic kidney disease
Other neglected tropical diseases
Other unspecified infectious diseases
Endocrine, metabolic, blood, and immune disorders
Upper digestive system diseases
Vitamin A deficiency
Inflammatory bowel disease
Cirrhosis and other chronic liver diseases
HIV/AIDS
Intestinal nematode infections
Schistosomiasis
Malaria
Gynecological diseases
Maternal disorders
YLDs per 100,000 population
0 to <0
0 to <0.01
0.01 to <0.1
0.1 to <0.18
0.18 to <0.55
0.55 to <1.5
1.5 to <4.02
4.02 to <8.79
8.79 to <18.64
18.64 to 103.48

Calabria

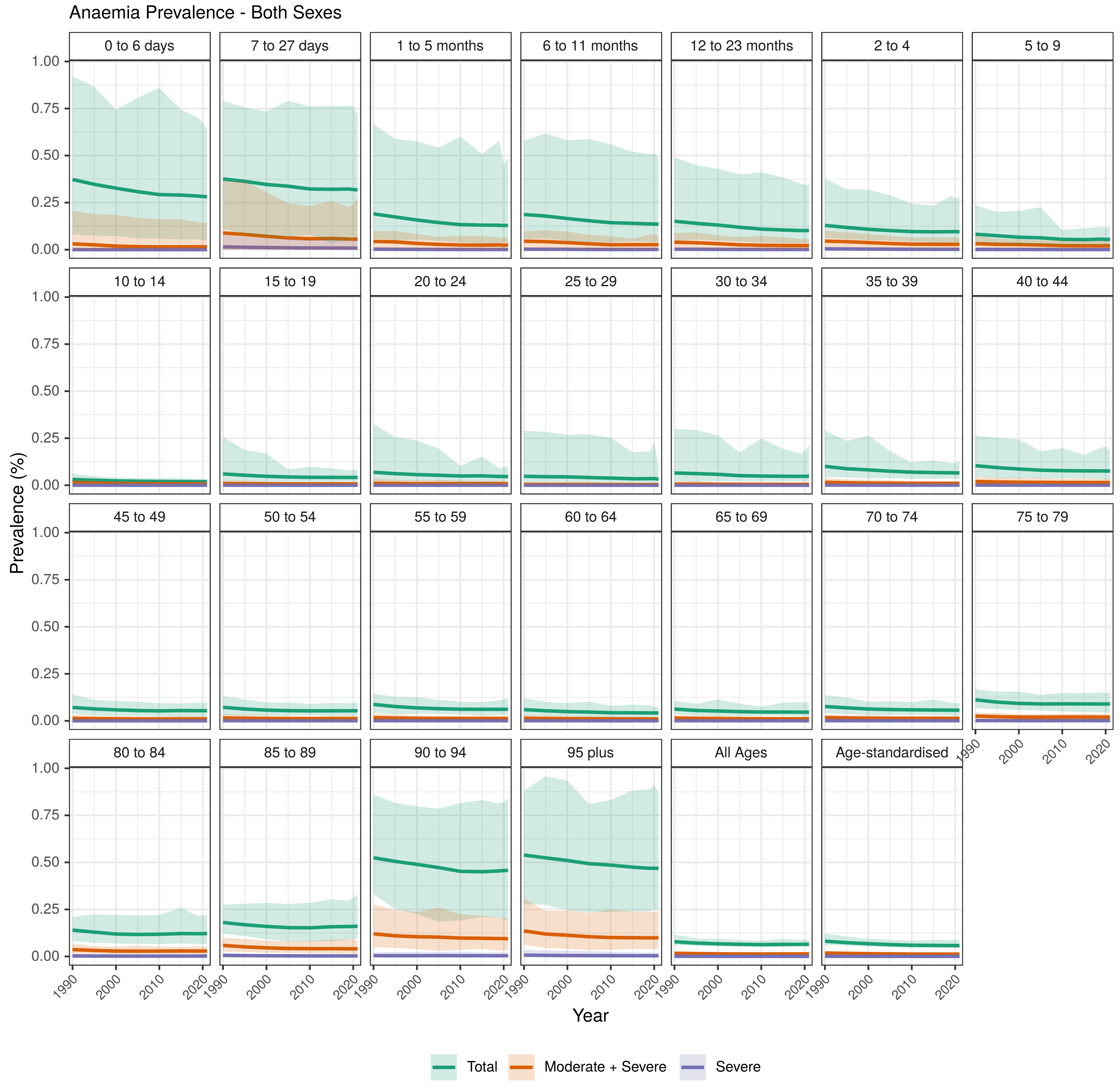

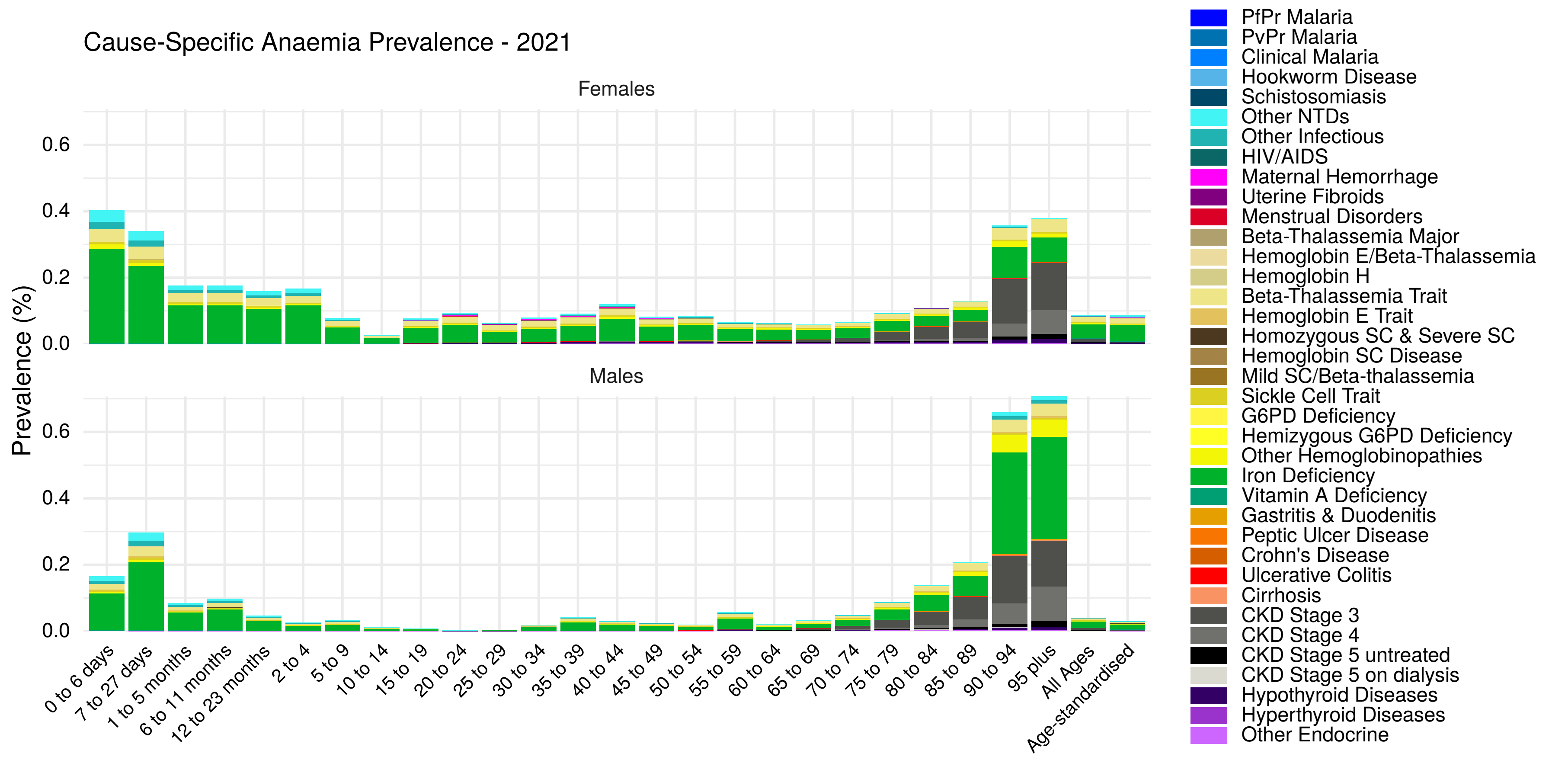

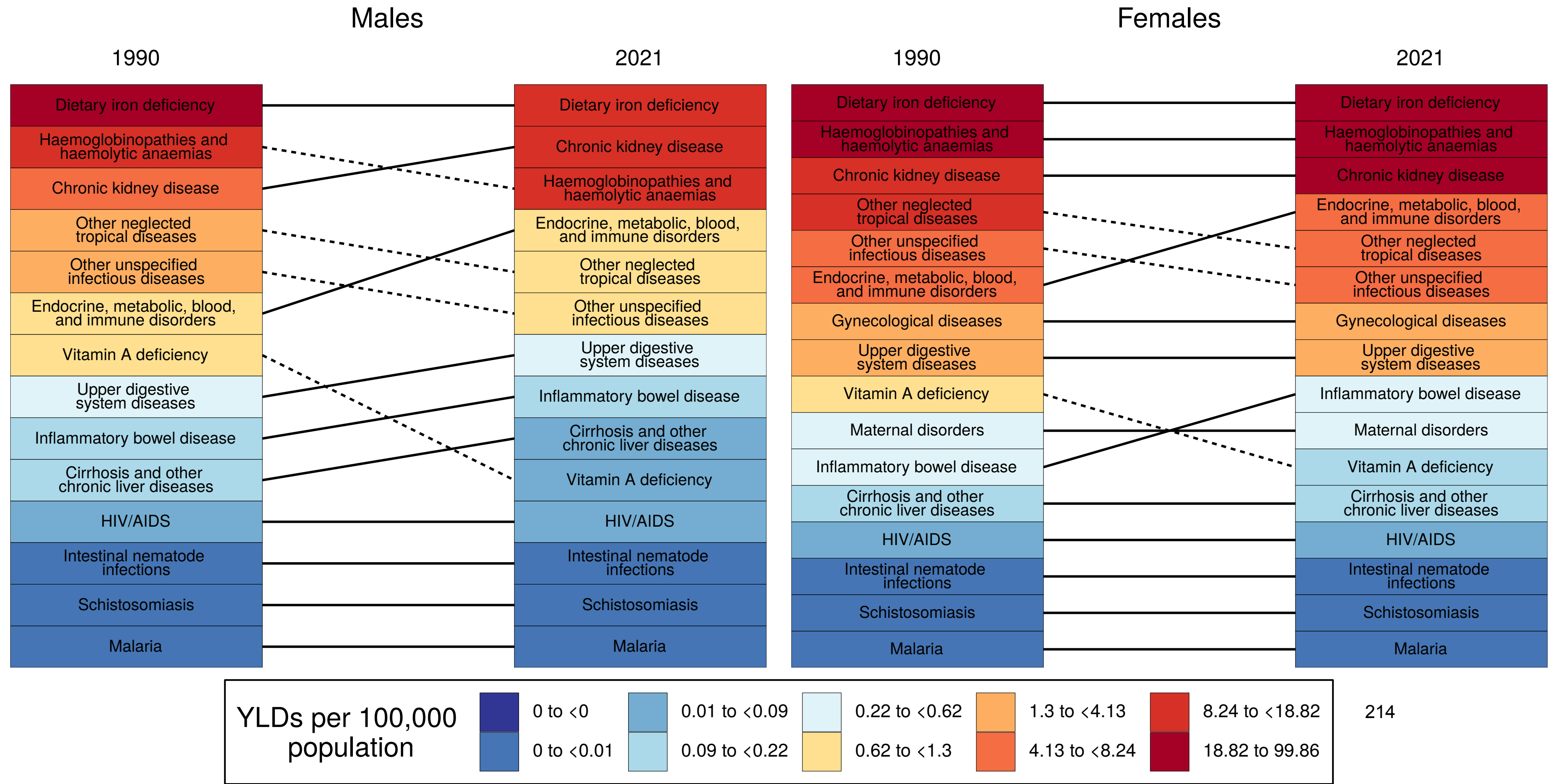

214

Campania

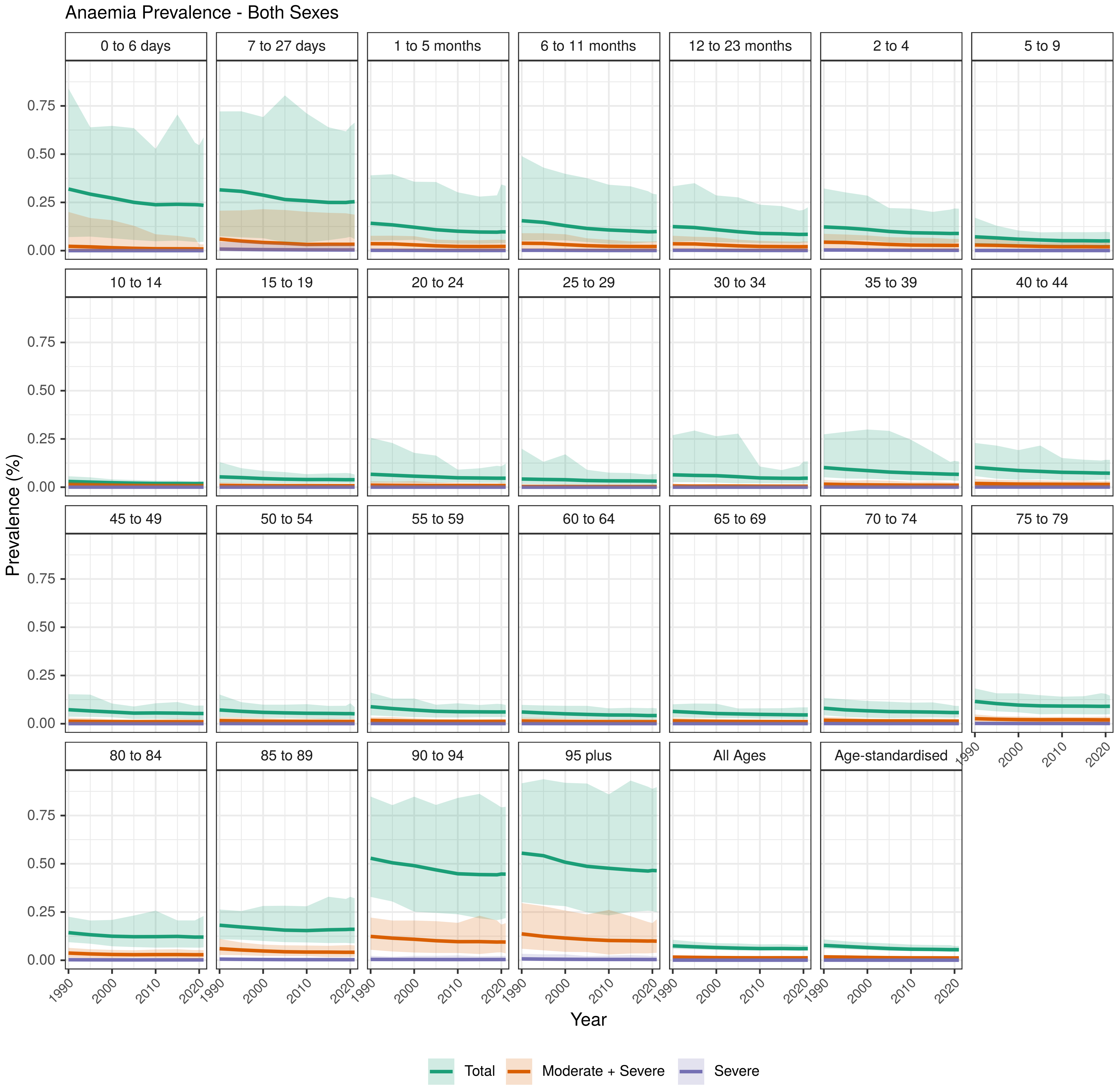

Anaemia Prevalence - Both Sexes
0 to 6 days
7 to 27 days
1 to 5 months
6 to 11 months
12 to 23 months
2 to 4
5 to 9
10 to 14
15 to 19
20 to 24
25 to 29
30 to 34
35 to 39
40 to 44
45 to 49
50 to 54
55 to 59
60 to 64
65 to 69
70 to 74
75 to 79
80 to 84
85 to 89
90 to 94
95 plus
All Ages
Age-standardised
0.00
0.25
0.50
0.75
1990
2000
2010
2020
Prevalence (%)
Year
Total
Moderate + Severe
Severe

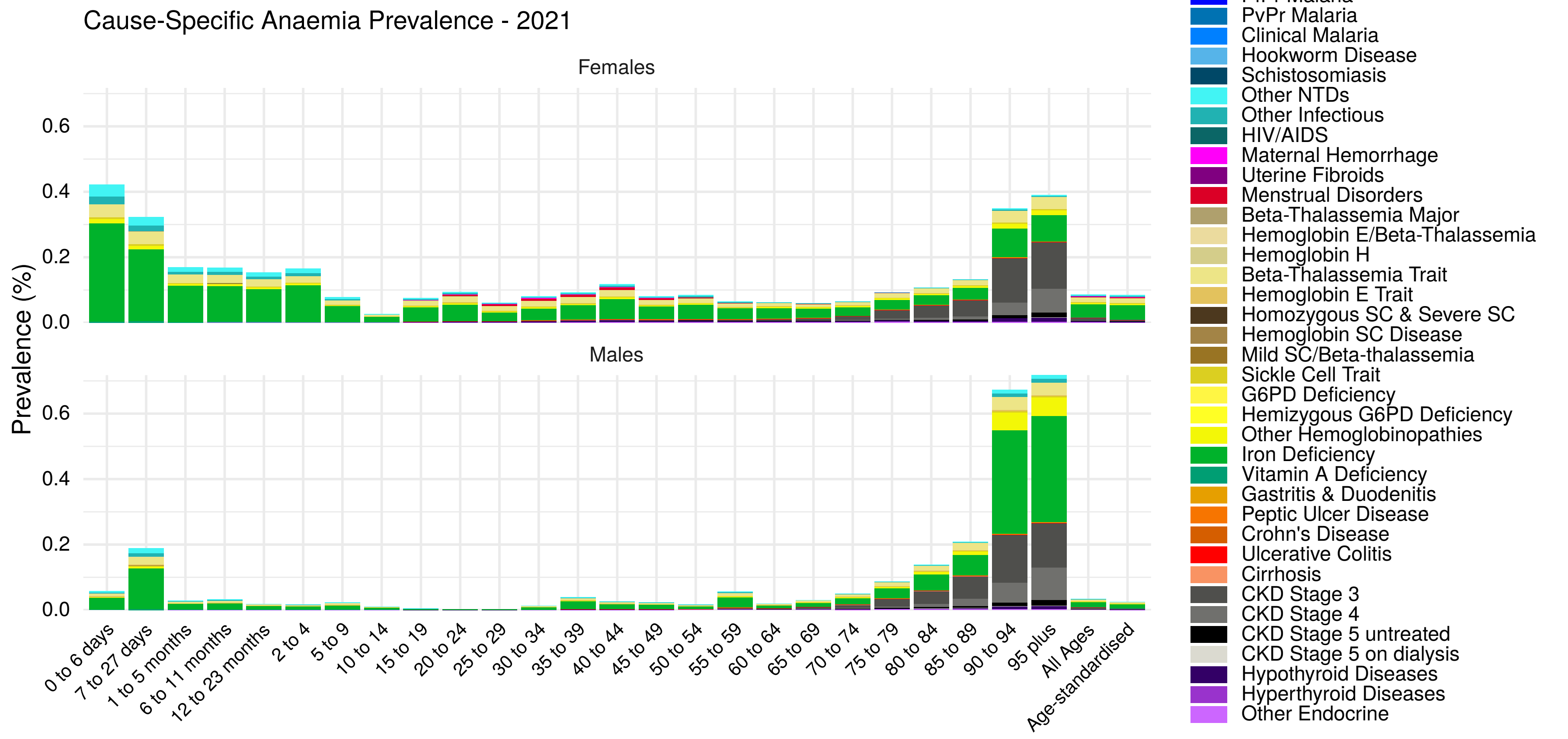

Cause-Specific Anaemia Prevalence - 2021
Females
Males
Prevalence (%)
0.0
0.2
0.4
0.6
0 to 6 days
7 to 27 days
1 to 5 months
6 to 11 months
12 to 23 months
2 to 4
5 to 9
10 to 14
15 to 19
20 to 24
25 to 29
30 to 34
35 to 39
40 to 44
45 to 49
50 to 54
55 to 59
60 to 64
65 to 69
70 to 74
75 to 79
80 to 84
85 to 89
90 to 94
95 plus
All Ages
Age-standardised
PfPr Malaria
PvPr Malaria
Clinical Malaria
Hookworm Disease
Schistosomiasis
Other NTDs
Other Infectious
HIV/AIDS
Maternal Hemorrhage
Uterine Fibroids
Menstrual Disorders
Beta-Thalassemia Major
Hemoglobin E/Beta-Thalassemia
Hemoglobin H
Beta-Thalassemia Trait
Hemoglobin E Trait
Homozygous SC & Severe SC
Hemoglobin SC Disease
Mild SC/Beta-thalassemia
Sickle Cell Trait
G6PD Deficiency
Hemizygous G6PD Deficiency
Other Hemoglobinopathies
Iron Deficiency
Vitamin A Deficiency
Gastritis & Duodenitis
Peptic Ulcer Disease
Crohn's Disease
Ulcerative Colitis
Cirrhosis
CKD Stage 3
CKD Stage 4
CKD Stage 5 untreated
CKD Stage 5 on dialysis
Hypothyroid Diseases
Hyperthyroid Diseases
Other Endocrine

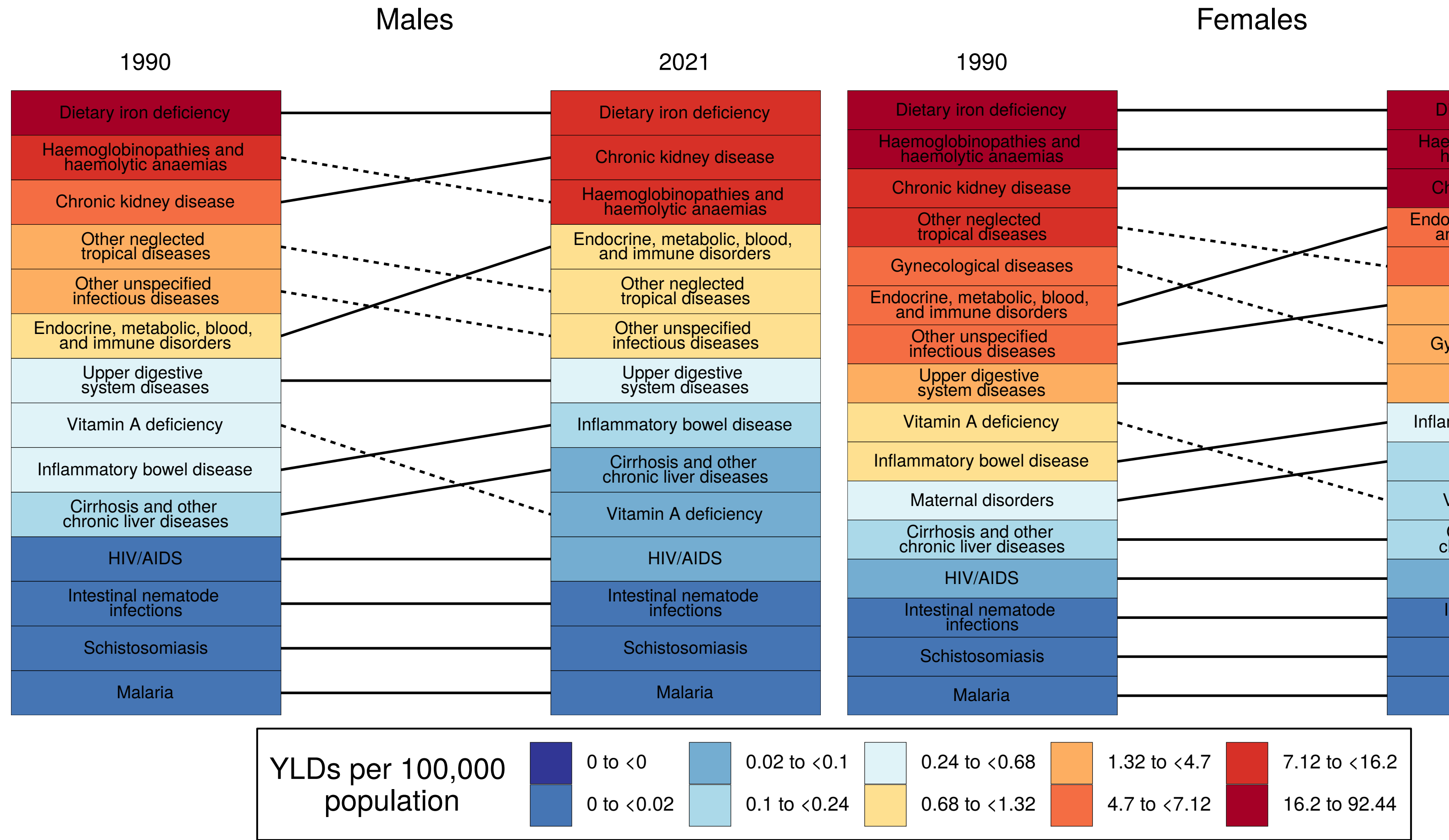

Males
1990
Dietary iron deficiency
Haemoglobinopathies and haemolytic anaemias
Chronic kidney disease
Other neglected tropical diseases
Other unspecified infectious diseases
Endocrine, metabolic, blood, and immune disorders
Upper digestive system diseases
Vitamin A deficiency
Inflammatory bowel disease
Cirrhosis and other chronic liver diseases
HIV/AIDS
Intestinal nematode infections
Schistosomiasis
Malaria
2021
Dietary iron deficiency
Chronic kidney disease
Haemoglobinopathies and haemolytic anaemias
Endocrine, metabolic, blood, and immune disorders
Other neglected tropical diseases
Other unspecified infectious diseases
Upper digestive system diseases
Inflammatory bowel disease
Cirrhosis and other chronic liver diseases
Vitamin A deficiency
HIV/AIDS
Intestinal nematode infections
Schistosomiasis
Malaria
Females
1990
Dietary iron deficiency
Haemoglobinopathies and haemolytic anaemias
Chronic kidney disease
Other neglected tropical diseases
Gynecological diseases
Endocrine, metabolic, blood, and immune disorders
Other unspecified infectious diseases
Upper digestive system diseases
Vitamin A deficiency
Inflammatory bowel disease
Maternal disorders
Cirrhosis and other chronic liver diseases
HIV/AIDS
Intestinal nematode infections
Schistosomiasis
Malaria
2021
Dietary iron deficiency
Haemoglobinopathies and haemolytic anaemias
Chronic kidney disease
Endocrine, metabolic, blood, and immune disorders
Other neglected tropical diseases
Other unspecified infectious diseases
Gynecological diseases
Upper digestive system diseases
Inflammatory bowel disease
Maternal disorders
Vitamin A deficiency
Cirrhosis and other chronic liver diseases
HIV/AIDS
Intestinal nematode infections
Schistosomiasis
Malaria
YLDs per 100,000 population
0 to <0
0 to <0.02
0.02 to <0.1
0.1 to <0.24
0.24 to <0.68
0.68 to <1.32
1.32 to <4.7
4.7 to <7.12
7.12 to <16.2
16.2 to 92.44

Emilia-Romagna

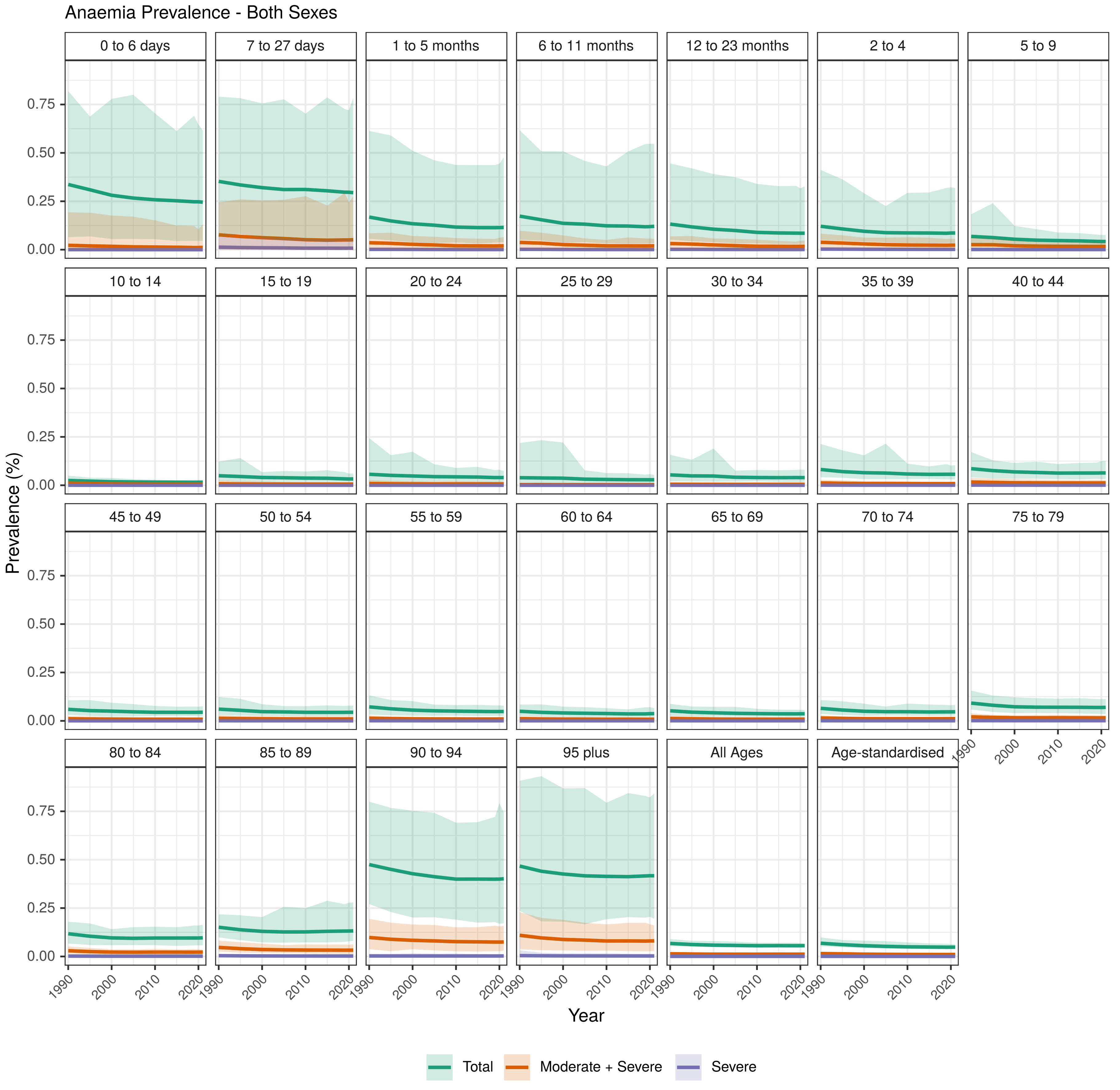

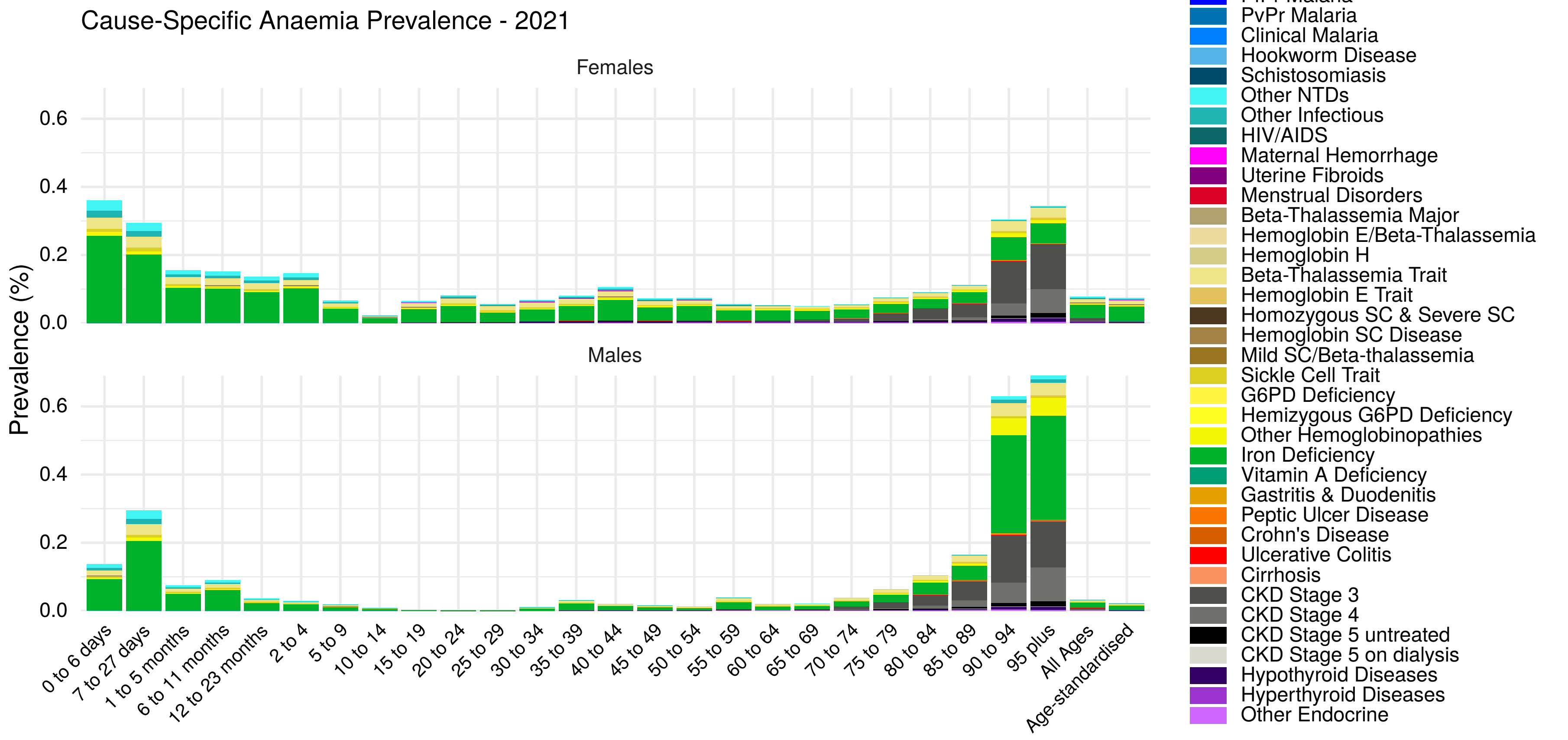

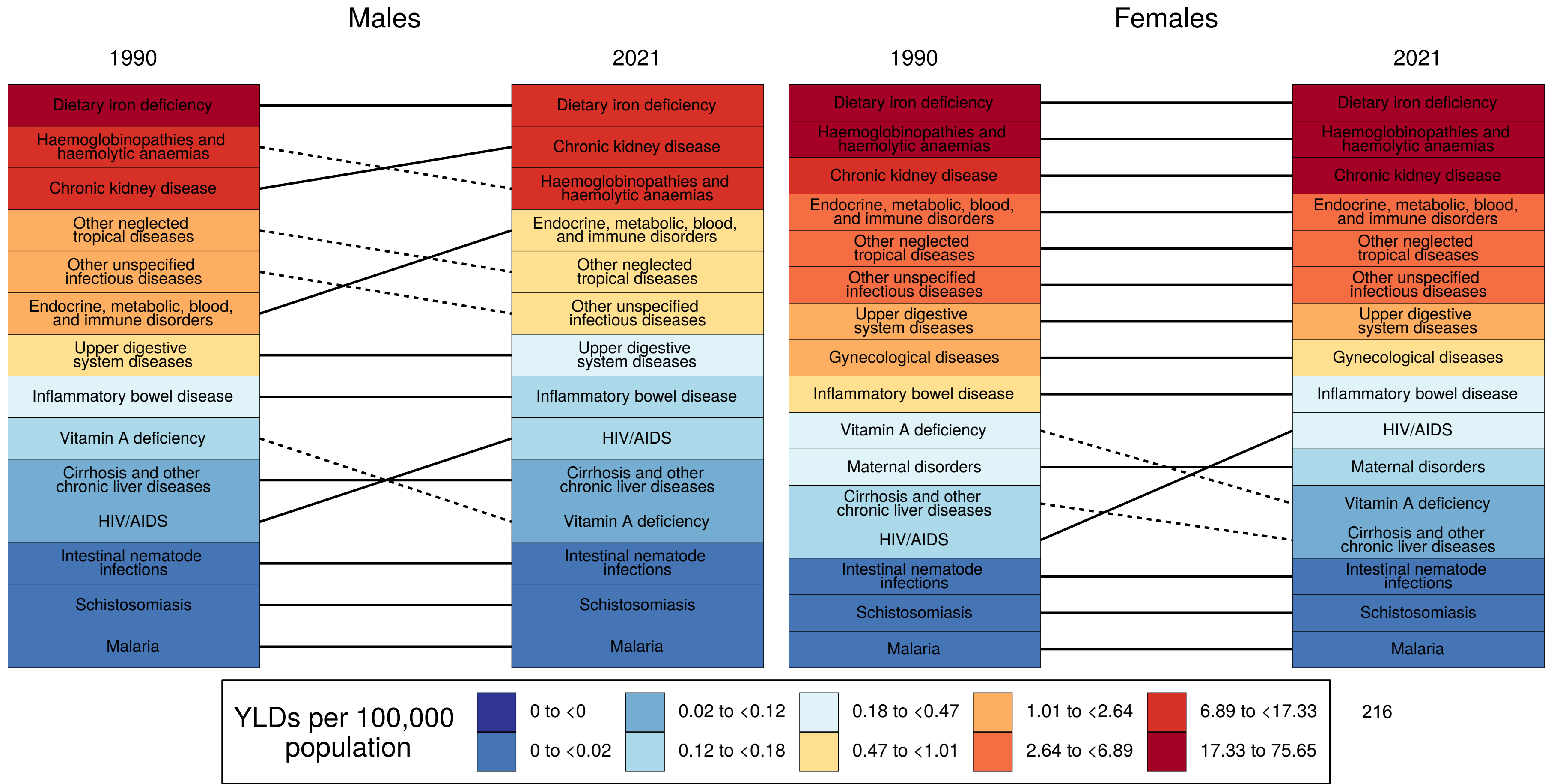

Friuli-Venezia Giulia

Anaemia Prevalence - Both Sexes

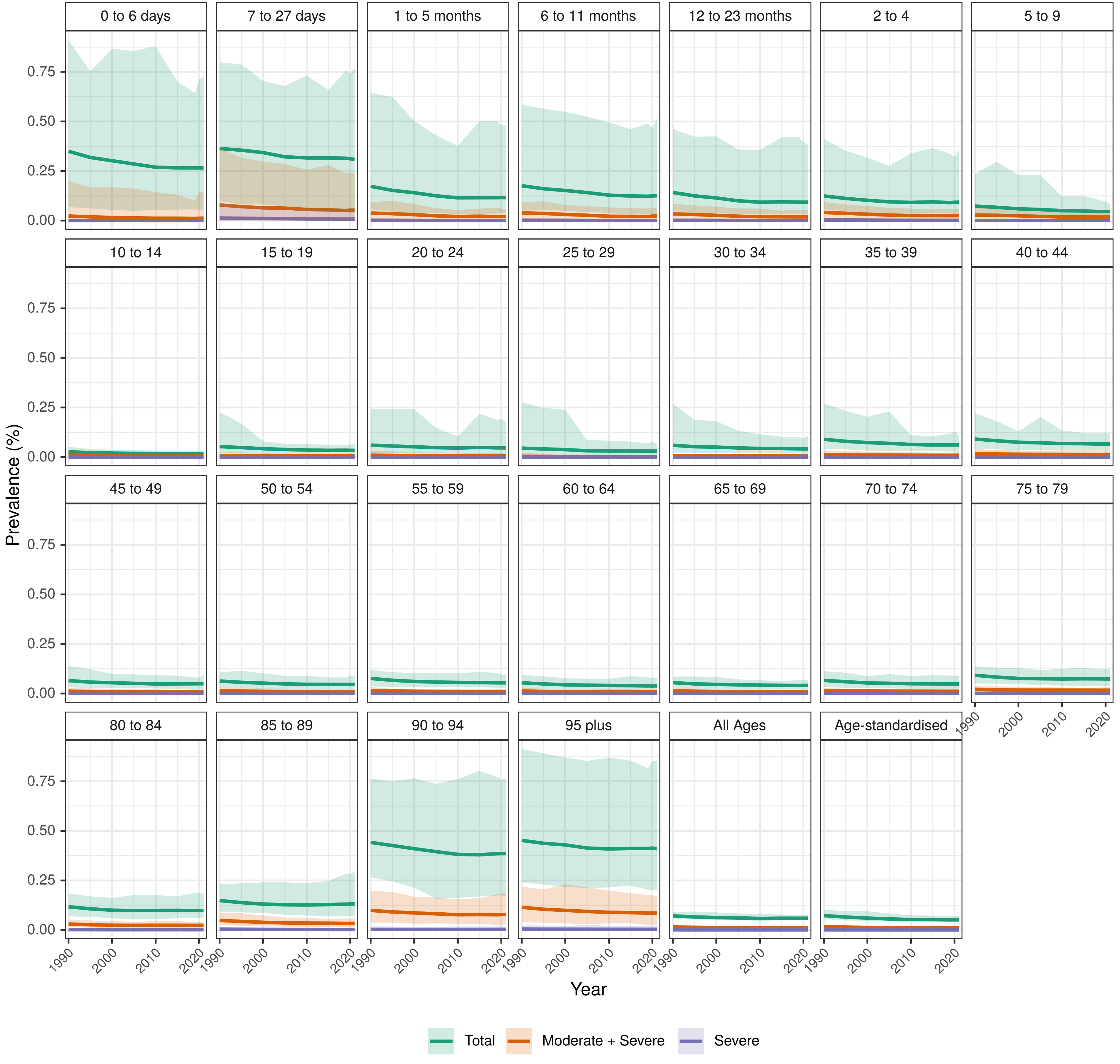

Cause-Specific Anaemia Prevalence - 2021

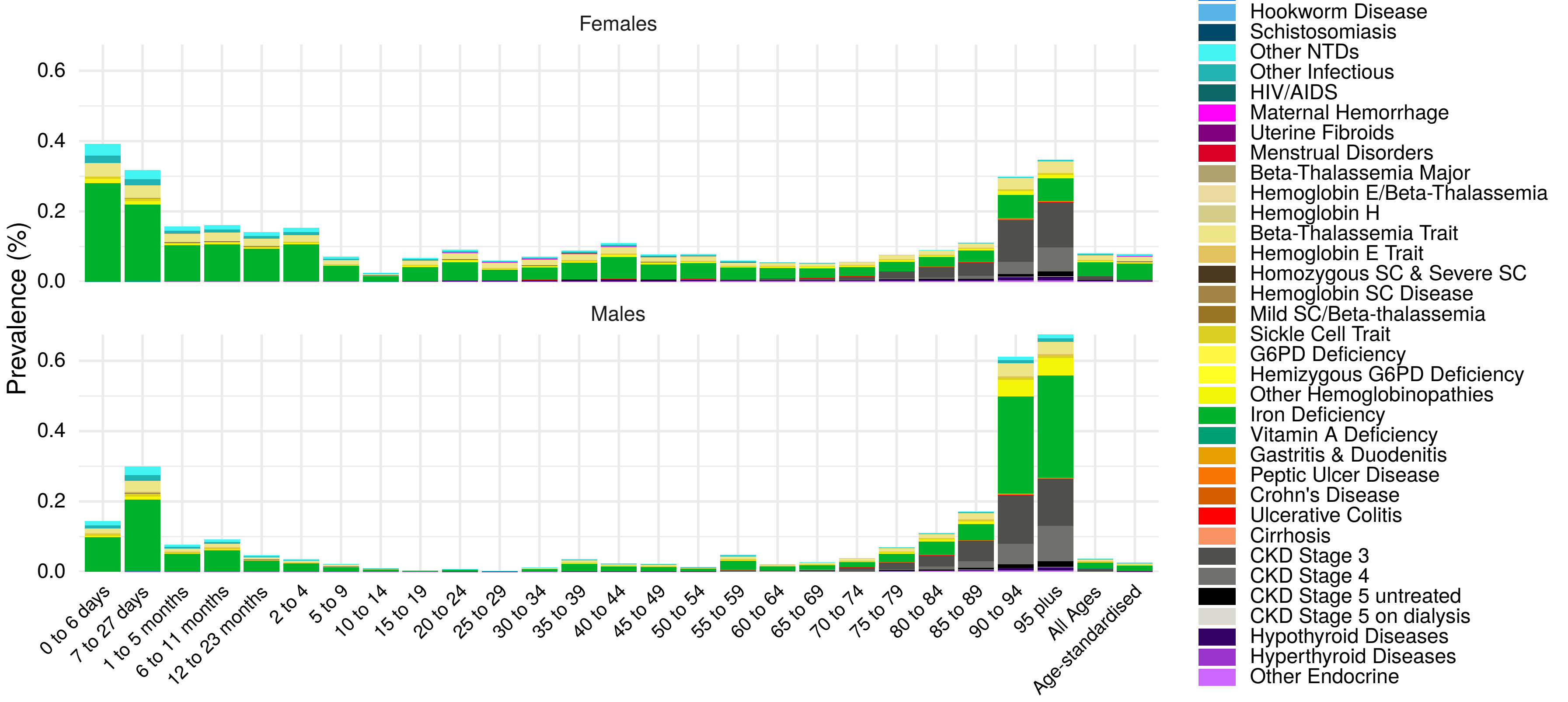

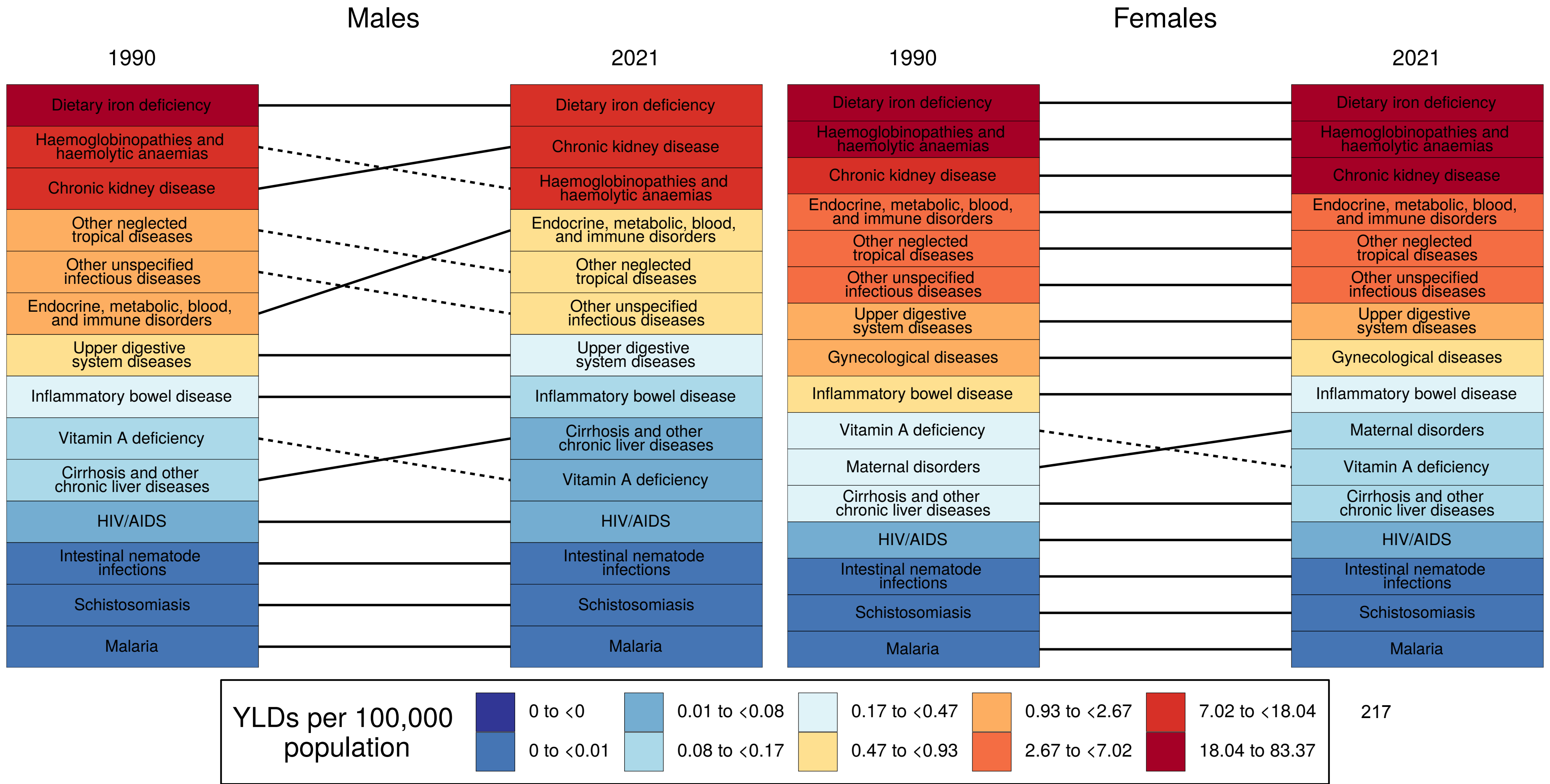

Lazio

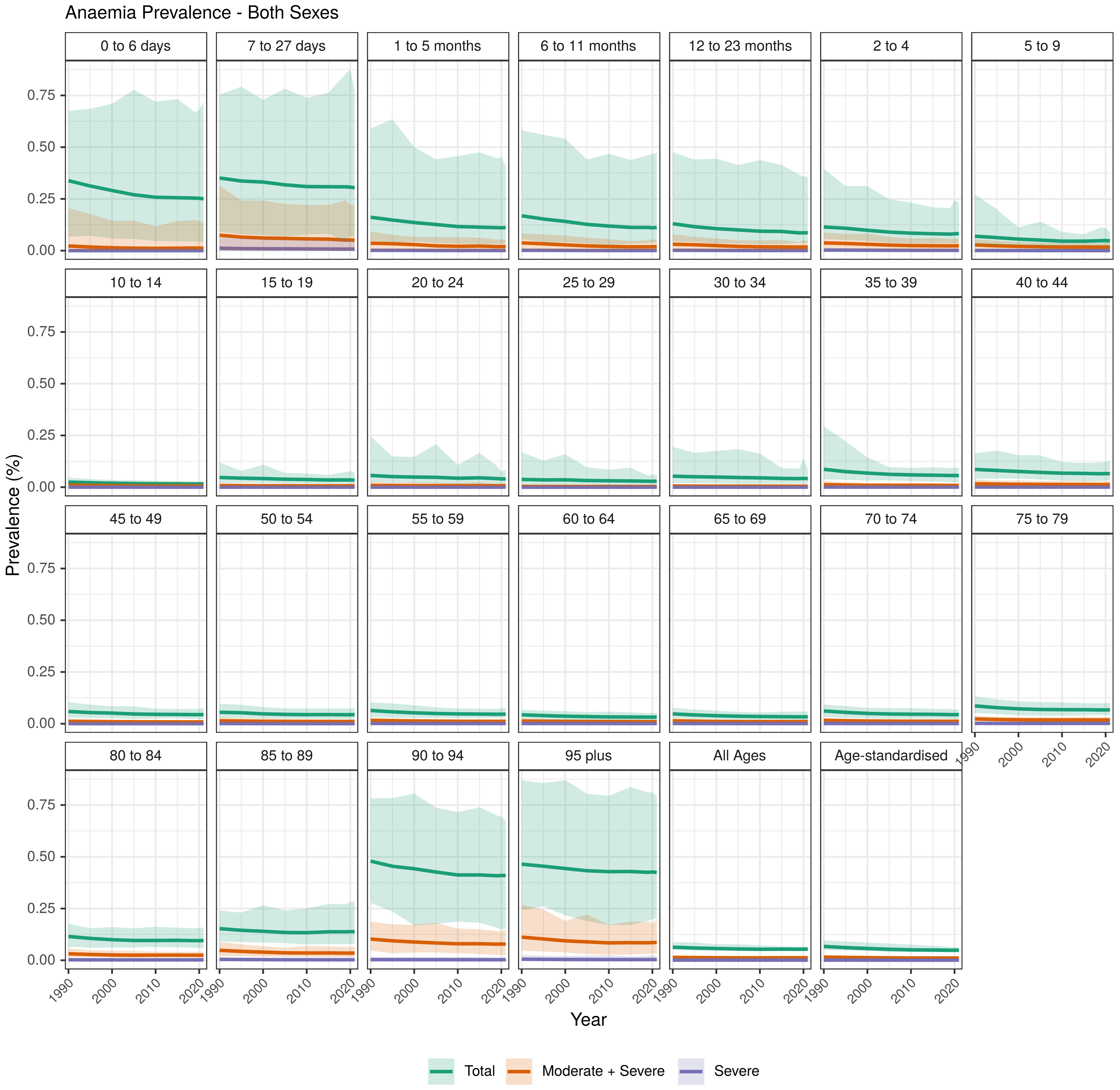

Anaemia Prevalence - Both Sexes
0 to 6 days
7 to 27 days
1 to 5 months
6 to 11 months
12 to 23 months
2 to 4
5 to 9
10 to 14
15 to 19
20 to 24
25 to 29
30 to 34
35 to 39
40 to 44
45 to 49
50 to 54
55 to 59
60 to 64
65 to 69
70 to 74
75 to 79
80 to 84
85 to 89
90 to 94
95 plus
All Ages
Age-standardised
0.00
0.25
0.50
0.75
Prevalence (%)
1990
2000
2010
2020
Year
Total
Moderate + Severe
Severe

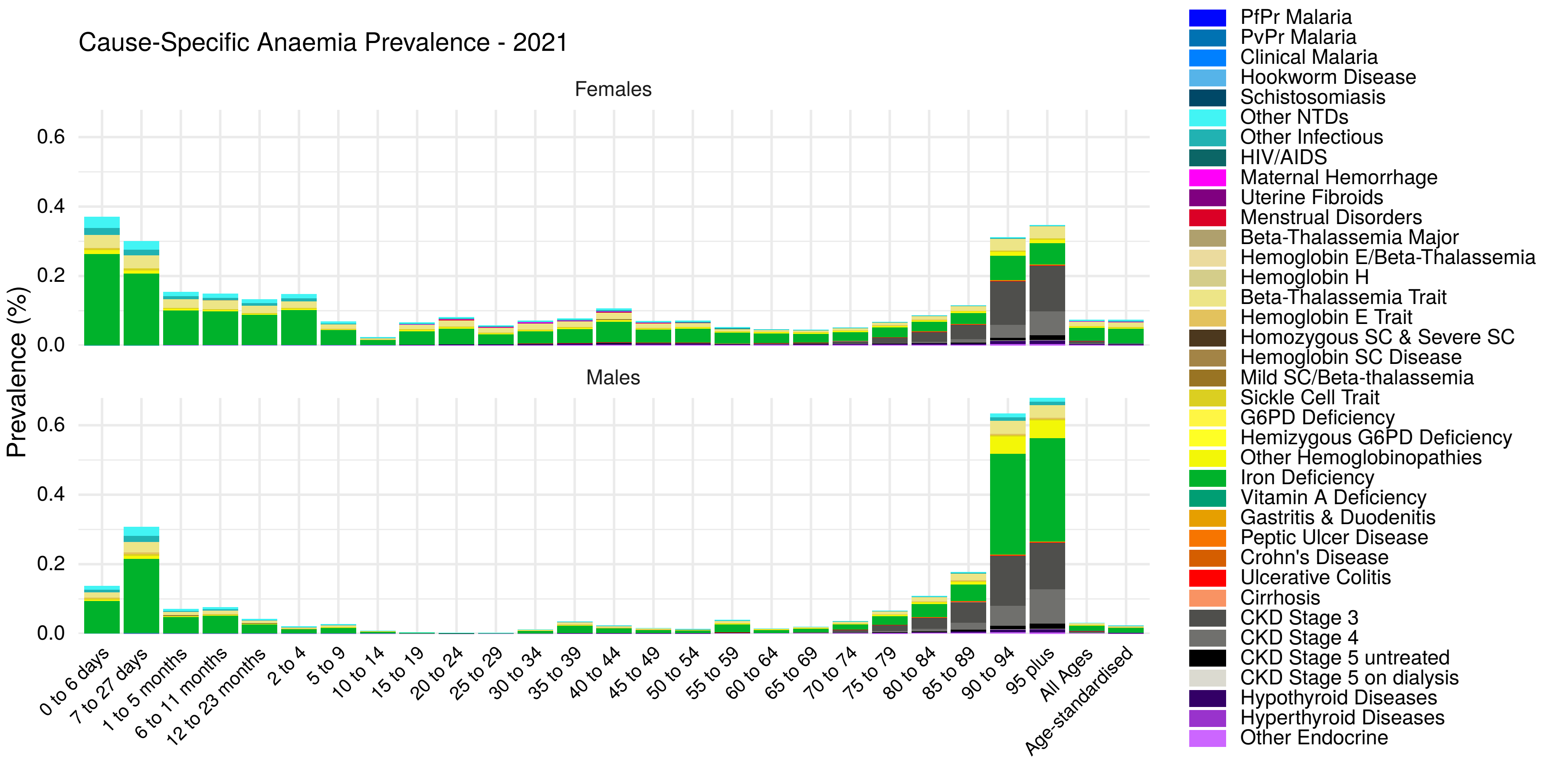

Cause-Specific Anaemia Prevalence - 2021
Females
Males
Prevalence (%)
0.0
0.2
0.4
0.6
0 to 6 days
7 to 27 days
1 to 5 months
6 to 11 months
12 to 23 months
2 to 4
5 to 9
10 to 14
15 to 19
20 to 24
25 to 29
30 to 34
35 to 39
40 to 44
45 to 49
50 to 54
55 to 59
60 to 64
65 to 69
70 to 74
75 to 79
80 to 84
85 to 89
90 to 94
95 plus
All Ages
Age-standardised
PfPr Malaria
PvPr Malaria
Clinical Malaria
Hookworm Disease
Schistosomiasis
Other NTDs
Other Infectious
HIV/AIDS
Maternal Hemorrhage
Uterine Fibroids
Menstrual Disorders
Beta-Thalassemia Major
Hemoglobin E/Beta-Thalassemia
Hemoglobin H
Beta-Thalassemia Trait
Hemoglobin E Trait
Homozygous SC & Severe SC
Hemoglobin SC Disease
Mild SC/Beta-thalassemia
Sickle Cell Trait
G6PD Deficiency
Hemizygous G6PD Deficiency
Other Hemoglobinopathies
Iron Deficiency
Vitamin A Deficiency
Gastritis & Duodenitis
Peptic Ulcer Disease
Crohn's Disease
Ulcerative Colitis
Cirrhosis
CKD Stage 3
CKD Stage 4
CKD Stage 5 untreated
CKD Stage 5 on dialysis
Hypothyroid Diseases
Hyperthyroid Diseases
Other Endocrine

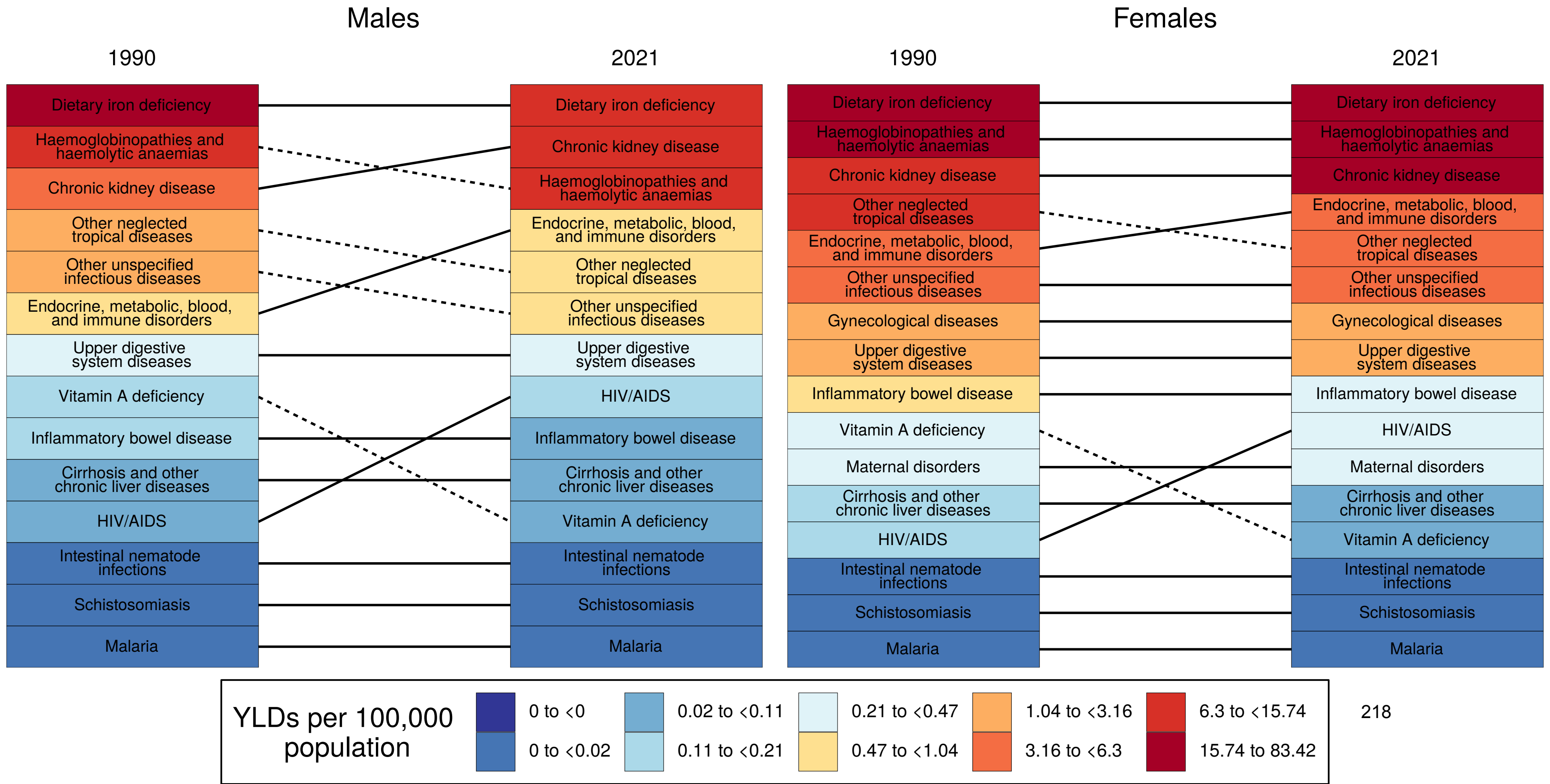

Males
Females
1990
2021
Dietary iron deficiency
Haemoglobinopathies and haemolytic anaemias
Chronic kidney disease
Other neglected tropical diseases
Other unspecified infectious diseases
Endocrine, metabolic, blood, and immune disorders
Upper digestive system diseases
Vitamin A deficiency
Inflammatory bowel disease
Cirrhosis and other chronic liver diseases
HIV/AIDS
Intestinal nematode infections
Schistosomiasis
Malaria
Gynecological diseases
Maternal disorders
YLDs per 100,000 population
0 to <0
0 to <0.02
0.02 to <0.11
0.11 to <0.21
0.21 to <0.47
0.47 to <1.04
1.04 to <3.16
3.16 to <6.3
6.3 to <15.74
15.74 to 83.42

Liguria

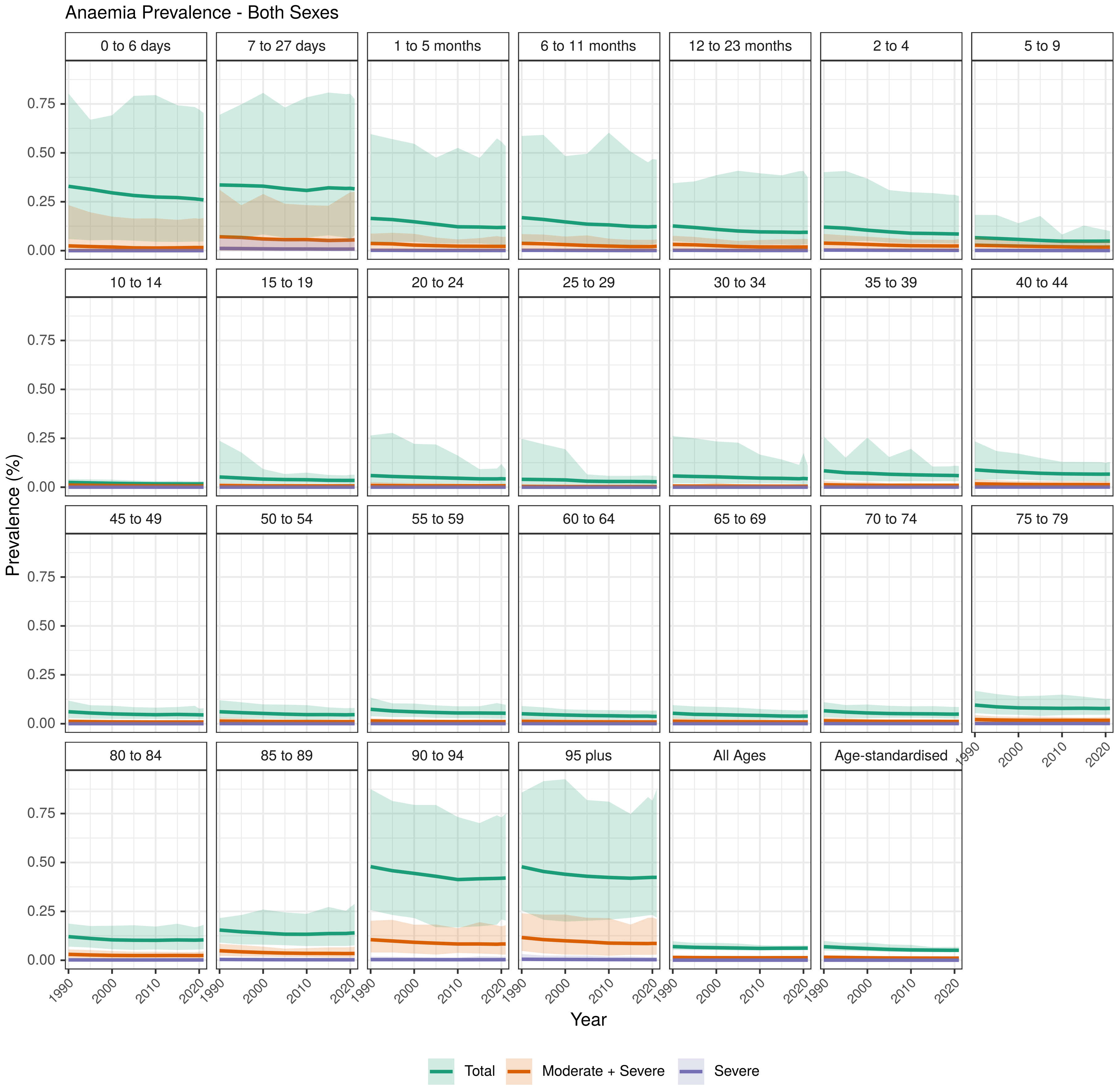

Anaemia Prevalence - Both Sexes
0 to 6 days
7 to 27 days
1 to 5 months
6 to 11 months
12 to 23 months
2 to 4
5 to 9
10 to 14
15 to 19
20 to 24
25 to 29
30 to 34
35 to 39
40 to 44
45 to 49
50 to 54
55 to 59
60 to 64
65 to 69
70 to 74
75 to 79
80 to 84
85 to 89
90 to 94
95 plus
All Ages
Age-standardised
0.00
0.25
0.50
0.75
Prevalence (%)
1990
2000
2010
2020
Year
Total
Moderate + Severe
Severe

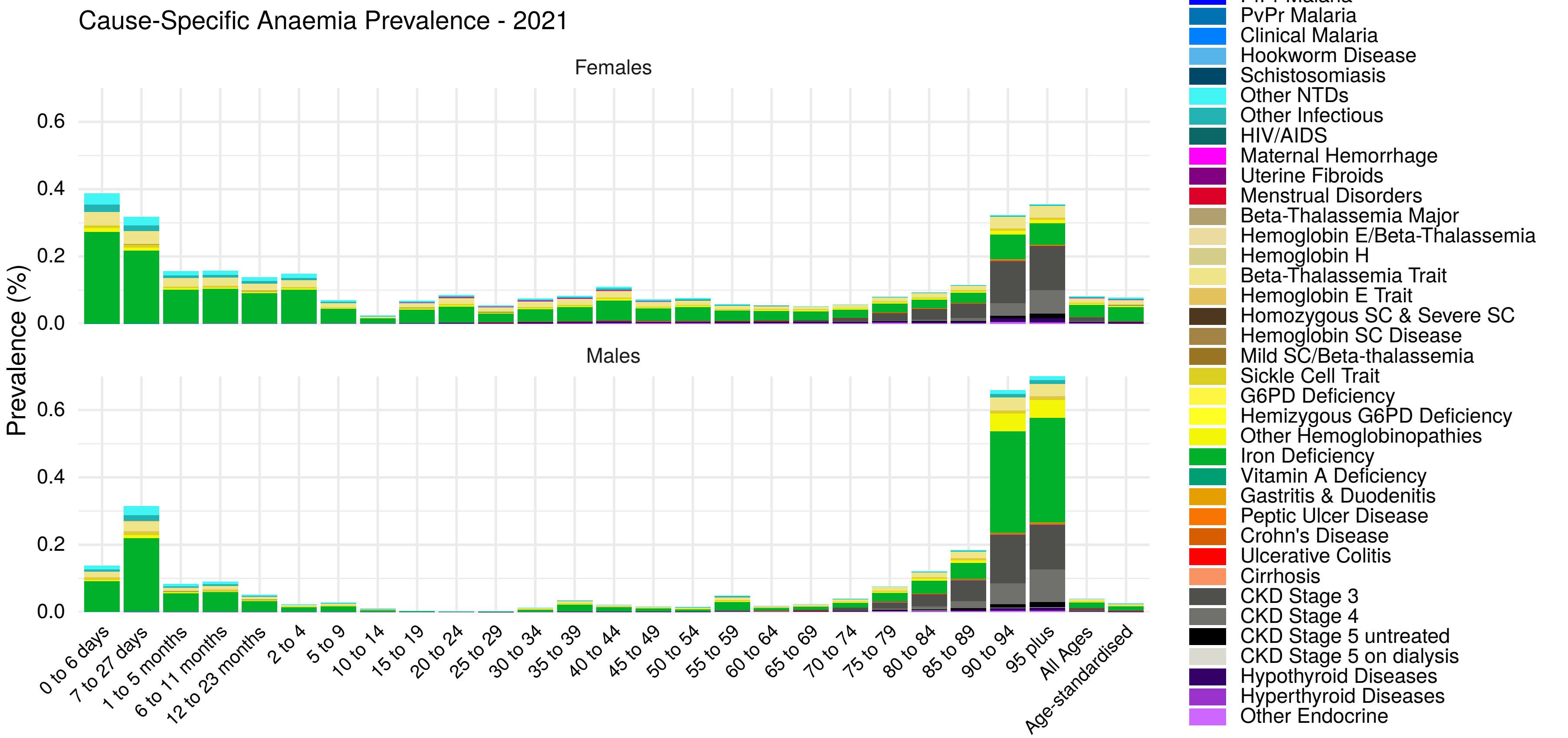

Cause-Specific Anaemia Prevalence - 2021
Females
Males
Prevalence (%)
0.0
0.2
0.4
0.6
0 to 6 days
7 to 27 days
1 to 5 months
6 to 11 months
12 to 23 months
2 to 4
5 to 9
10 to 14
15 to 19
20 to 24
25 to 29
30 to 34
35 to 39
40 to 44
45 to 49
50 to 54
55 to 59
60 to 64
65 to 69
70 to 74
75 to 79
80 to 84
85 to 89
90 to 94
95 plus
All Ages
Age-standardised
PfPr Malaria
PvPr Malaria
Clinical Malaria
Hookworm Disease
Schistosomiasis
Other NTDs
Other Infectious
HIV/AIDS
Maternal Hemorrhage
Uterine Fibroids
Menstrual Disorders
Beta-Thalassemia Major
Hemoglobin E/Beta-Thalassemia
Hemoglobin H
Beta-Thalassemia Trait
Hemoglobin E Trait
Homozygous SC & Severe SC
Hemoglobin SC Disease
Mild SC/Beta-thalassemia
Sickle Cell Trait
G6PD Deficiency
Hemizygous G6PD Deficiency
Other Hemoglobinopathies
Iron Deficiency
Vitamin A Deficiency
Gastritis & Duodenitis
Peptic Ulcer Disease
Crohn's Disease
Ulcerative Colitis
Cirrhosis
CKD Stage 3
CKD Stage 4
CKD Stage 5 untreated
CKD Stage 5 on dialysis
Hypothyroid Diseases
Hyperthyroid Diseases
Other Endocrine

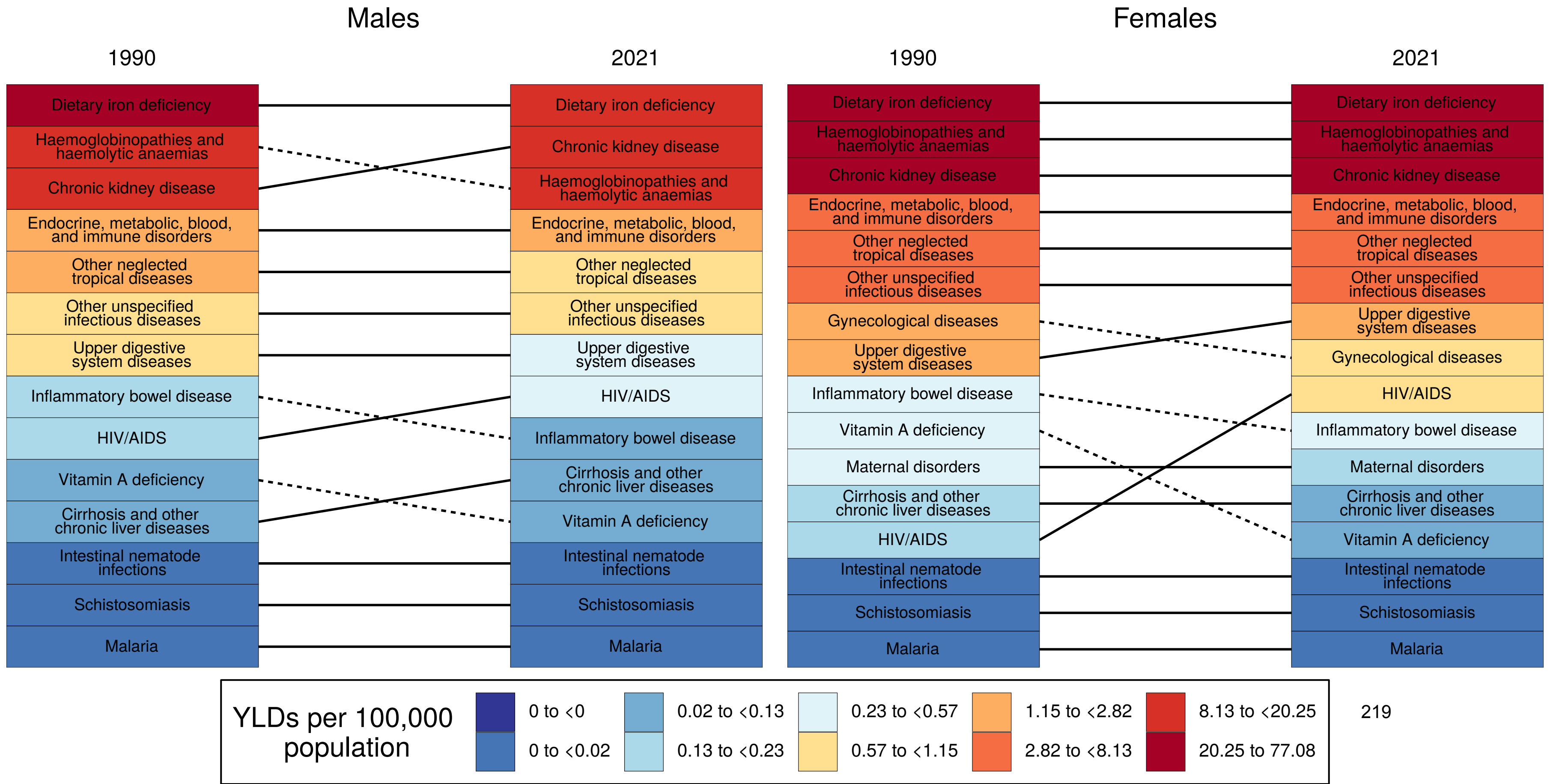

Males
Females
1990
2021
Dietary iron deficiency
Haemoglobinopathies and haemolytic anaemias
Chronic kidney disease
Endocrine, metabolic, blood, and immune disorders
Other neglected tropical diseases
Other unspecified infectious diseases
Upper digestive system diseases
Inflammatory bowel disease
HIV/AIDS
Vitamin A deficiency
Cirrhosis and other chronic liver diseases
Intestinal nematode infections
Schistosomiasis
Malaria
Gynecological diseases
Maternal disorders
YLDs per 100,000 population
0 to <0
0 to <0.02
0.02 to <0.13
0.13 to <0.23
0.23 to <0.57
0.57 to <1.15
1.15 to <2.82
2.82 to <8.13
8.13 to <20.25
20.25 to 77.08

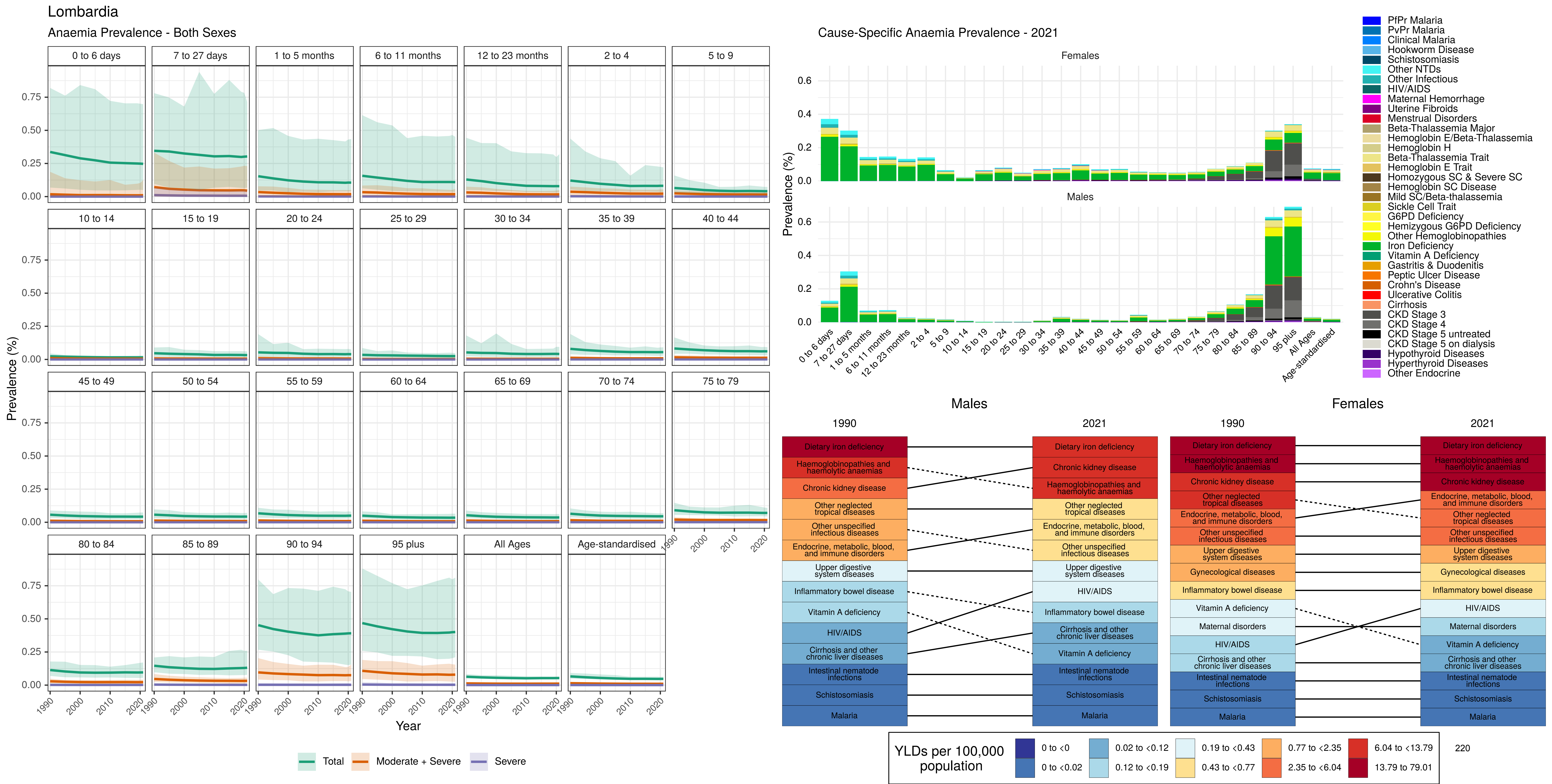
Lombardia
Anaemia Prevalence - Both Sexes
0 to 6 days
7 to 27 days
1 to 5 months
6 to 11 months
12 to 23 months
2 to 4
5 to 9
10 to 14
15 to 19
20 to 24
25 to 29
30 to 34
35 to 39
40 to 44
45 to 49
50 to 54
55 to 59
60 to 64
65 to 69
70 to 74
75 to 79
80 to 84
85 to 89
90 to 94
95 plus
All Ages
Age-standardised
Prevalence (%)
Year
Total
Moderate + Severe
Severe
Cause-Specific Anaemia Prevalence - 2021
Females
Males
PfPr Malaria
PvPr Malaria
Clinical Malaria
Hookworm Disease
Schistosomiasis
Other NTDs
Other Infectious
HIV/AIDS
Maternal Hemorrhage
Uterine Fibroids
Menstrual Disorders
Beta-Thalassemia Major
Hemoglobin E/Beta-Thalassemia
Hemoglobin H
Beta-Thalassemia Trait
Hemoglobin E Trait
Homozygous SC & Severe SC
Hemoglobin SC Disease
Mild SC/Beta-thalassemia
Sickle Cell Trait
G6PD Deficiency
Hemizygous G6PD Deficiency
Other Hemoglobinopathies
Iron Deficiency
Vitamin A Deficiency
Gastritis & Duodenitis
Peptic Ulcer Disease
Crohn's Disease
Ulcerative Colitis
Cirrhosis
CKD Stage 3
CKD Stage 4
CKD Stage 5 untreated
CKD Stage 5 on dialysis
Hypothyroid Diseases
Hyperthyroid Diseases
Other Endocrine
Males
Females
1990
2021
Dietary iron deficiency
Haemoglobinopathies and haemolytic anaemias
Chronic kidney disease
Other neglected tropical diseases
Other unspecified infectious diseases
Endocrine, metabolic, blood, and immune disorders
Upper digestive system diseases
Inflammatory bowel disease
Vitamin A deficiency
HIV/AIDS
Cirrhosis and other chronic liver diseases
Intestinal nematode infections
Schistosomiasis
Malaria
Gynecological diseases
Maternal disorders
YLDs per 100,000 population
0 to <0
0 to <0.02
0.02 to <0.12
0.12 to <0.19
0.19 to <0.43
0.43 to <0.77
0.77 to <2.35
2.35 to <6.04
6.04 to <13.79
13.79 to 79.01

Marche

Anaemia Prevalence - Both Sexes

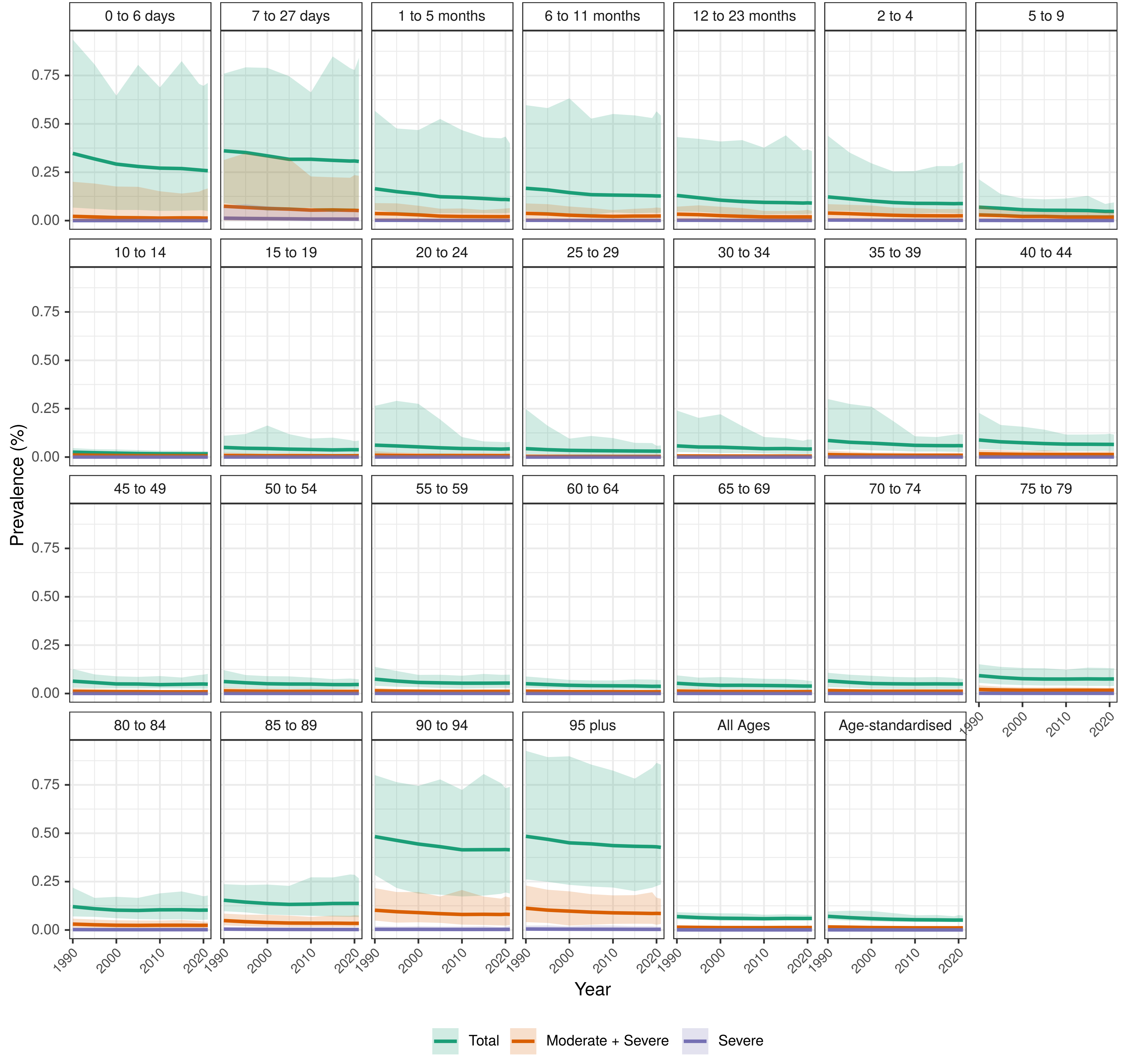

Cause-Specific Anaemia Prevalence - 2021

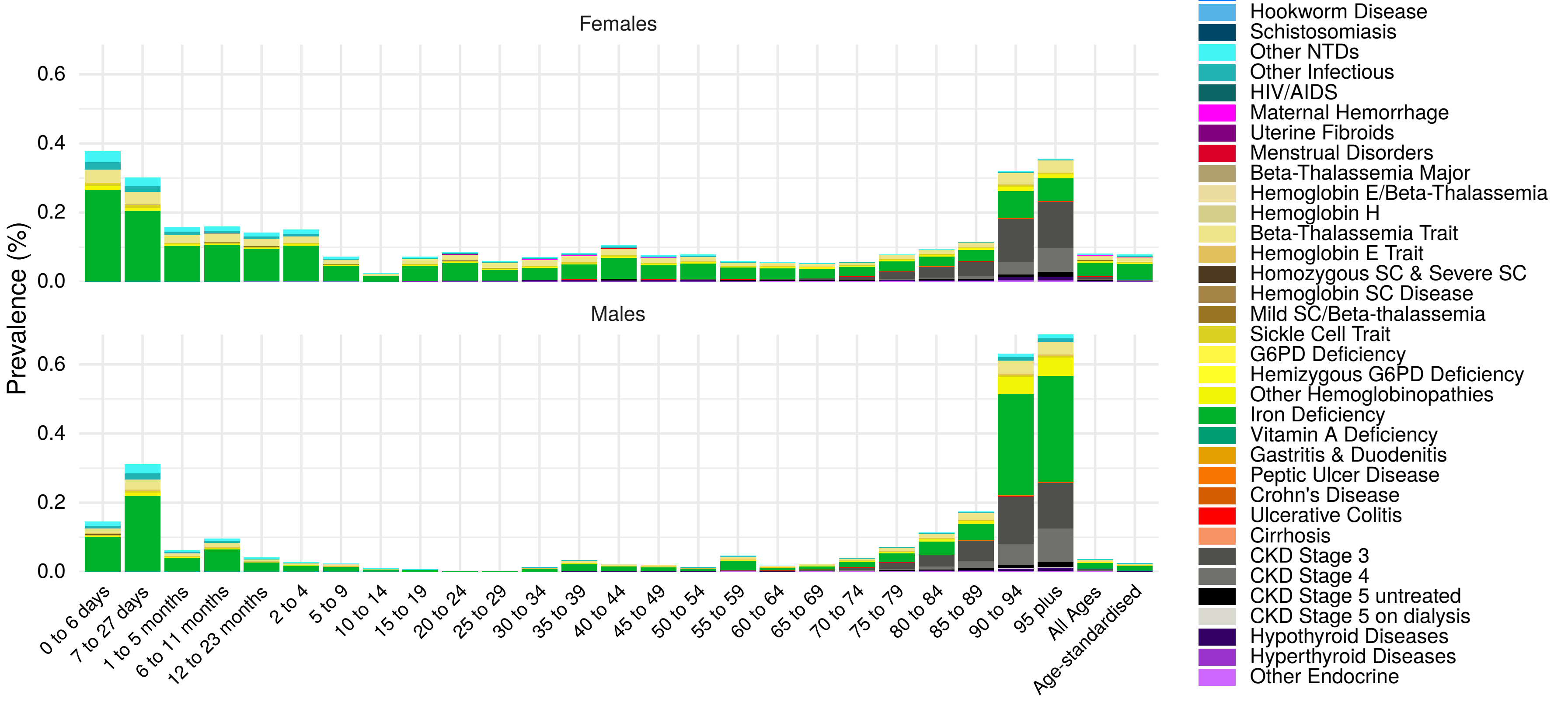

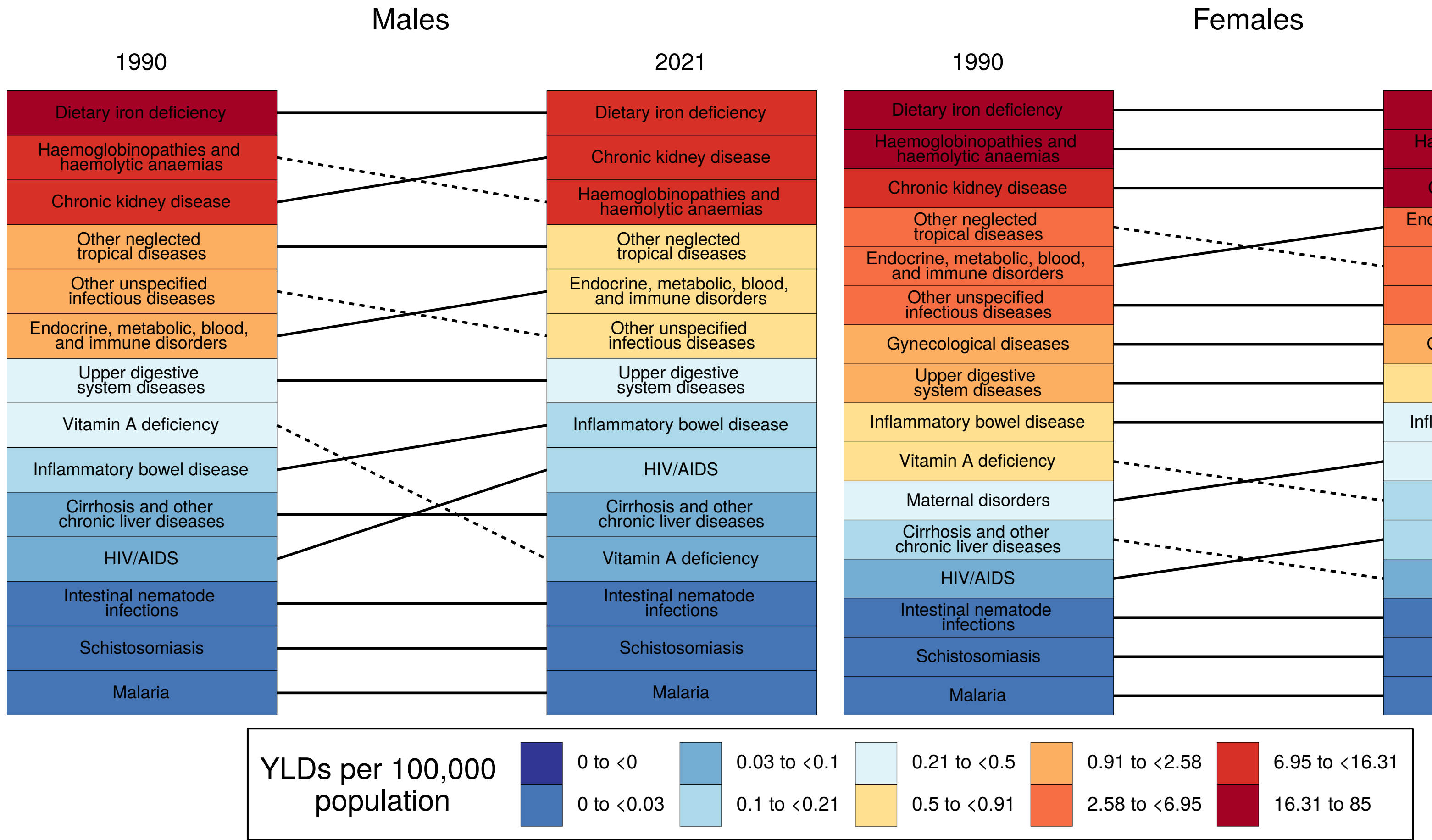

221

Molise

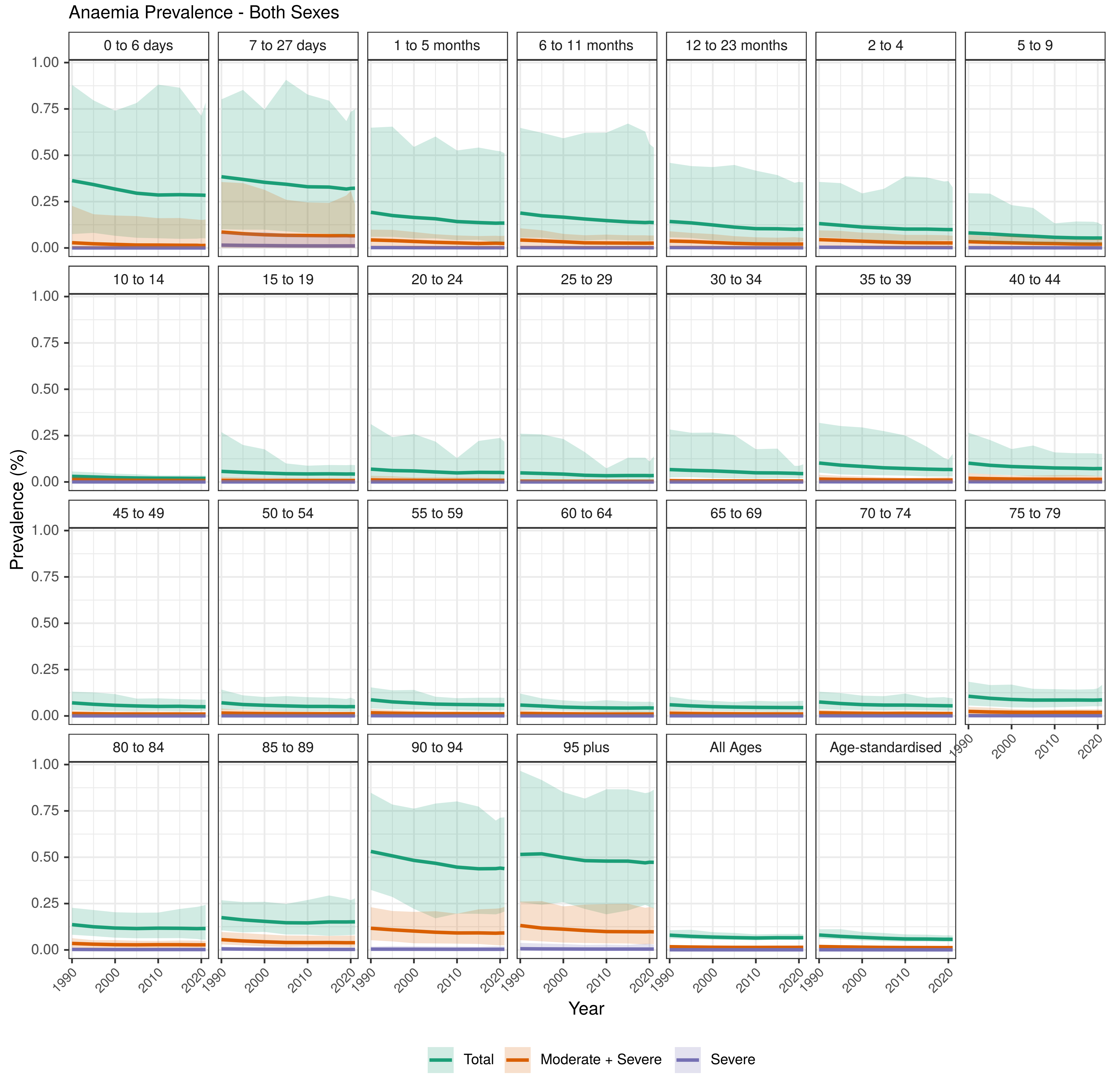

Anaemia Prevalence - Both Sexes
0 to 6 days
7 to 27 days
1 to 5 months
6 to 11 months
12 to 23 months
2 to 4
5 to 9
10 to 14
15 to 19
20 to 24
25 to 29
30 to 34
35 to 39
40 to 44
45 to 49
50 to 54
55 to 59
60 to 64
65 to 69
70 to 74
75 to 79
80 to 84
85 to 89
90 to 94
95 plus
All Ages
Age-standardised
Prevalence (%)
Year
Total
Moderate + Severe
Severe

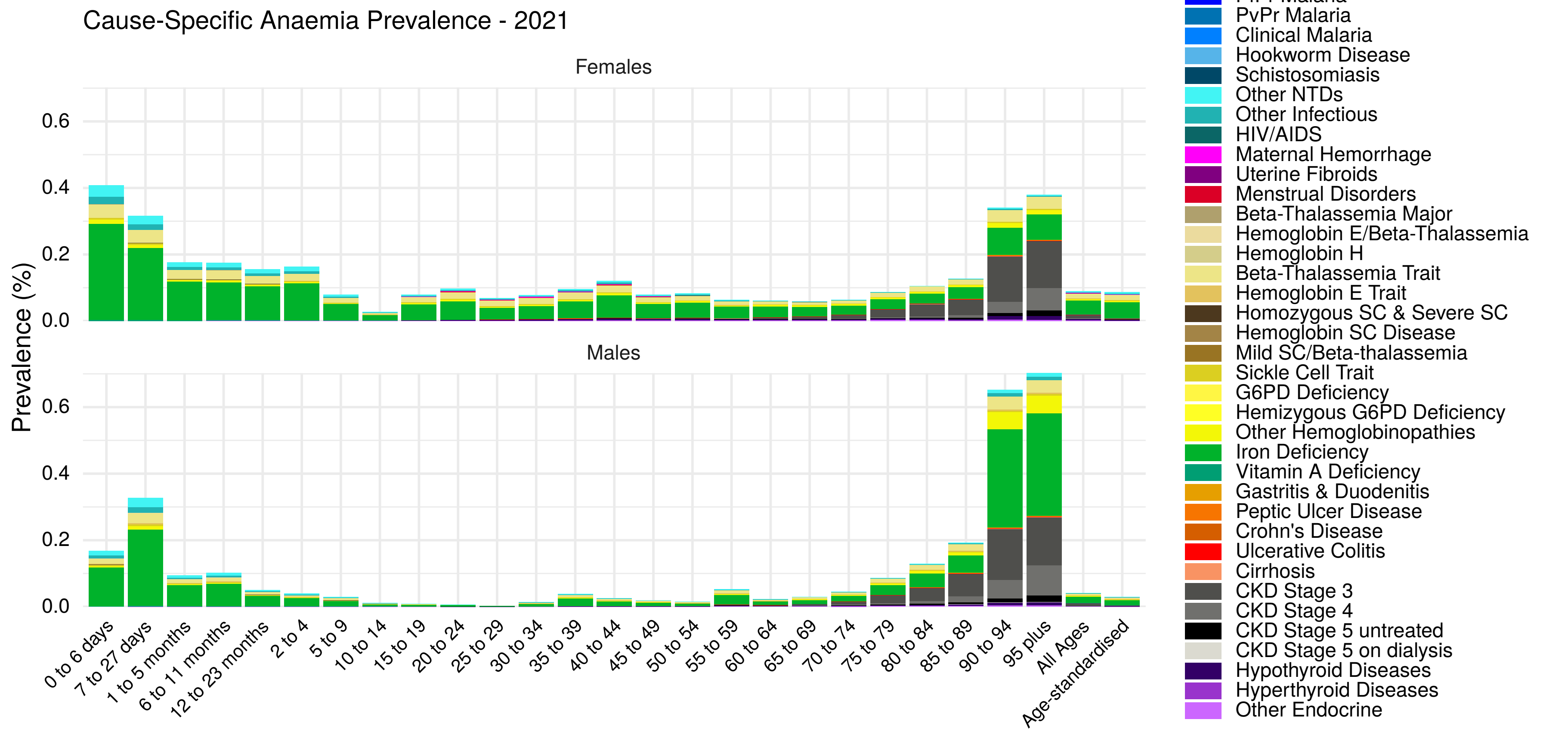

Cause-Specific Anaemia Prevalence - 2021
Females
Males
Prevalence (%)
PfPr Malaria
PvPr Malaria
Clinical Malaria
Hookworm Disease
Schistosomiasis
Other NTDs
Other Infectious
HIV/AIDS
Maternal Hemorrhage
Uterine Fibroids
Menstrual Disorders
Beta-Thalassemia Major
Hemoglobin E/Beta-Thalassemia
Hemoglobin H
Beta-Thalassemia Trait
Hemoglobin E Trait
Homozygous SC & Severe SC
Hemoglobin SC Disease
Mild SC/Beta-thalassemia
Sickle Cell Trait
G6PD Deficiency
Hemizygous G6PD Deficiency
Other Hemoglobinopathies
Iron Deficiency
Vitamin A Deficiency
Gastritis & Duodenitis
Peptic Ulcer Disease
Crohn's Disease
Ulcerative Colitis
Cirrhosis
CKD Stage 3
CKD Stage 4
CKD Stage 5 untreated
CKD Stage 5 on dialysis
Hypothyroid Diseases
Hyperthyroid Diseases
Other Endocrine

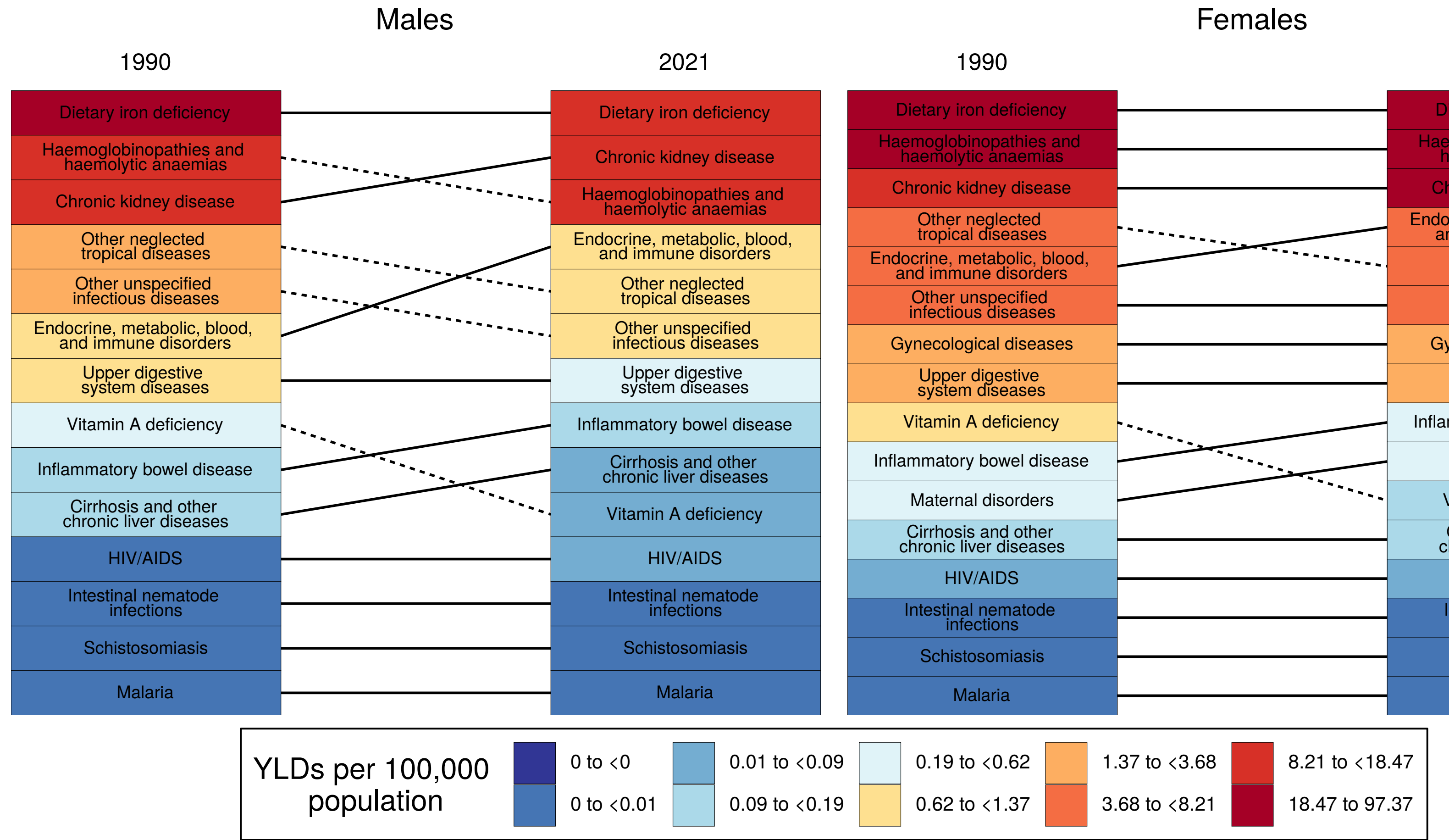

Males
Females
1990
2021
Dietary iron deficiency
Haemoglobinopathies and haemolytic anaemias
Chronic kidney disease
Other neglected tropical diseases
Other unspecified infectious diseases
Endocrine, metabolic, blood, and immune disorders
Upper digestive system diseases
Vitamin A deficiency
Inflammatory bowel disease
Cirrhosis and other chronic liver diseases
HIV/AIDS
Intestinal nematode infections
Schistosomiasis
Malaria
Gynecological diseases
Maternal disorders
YLDs per 100,000 population
0 to <0
0 to <0.01
0.01 to <0.09
0.09 to <0.19
0.19 to <0.62
0.62 to <1.37
1.37 to <3.68
3.68 to <8.21
8.21 to <18.47
18.47 to 97.37

Piemonte

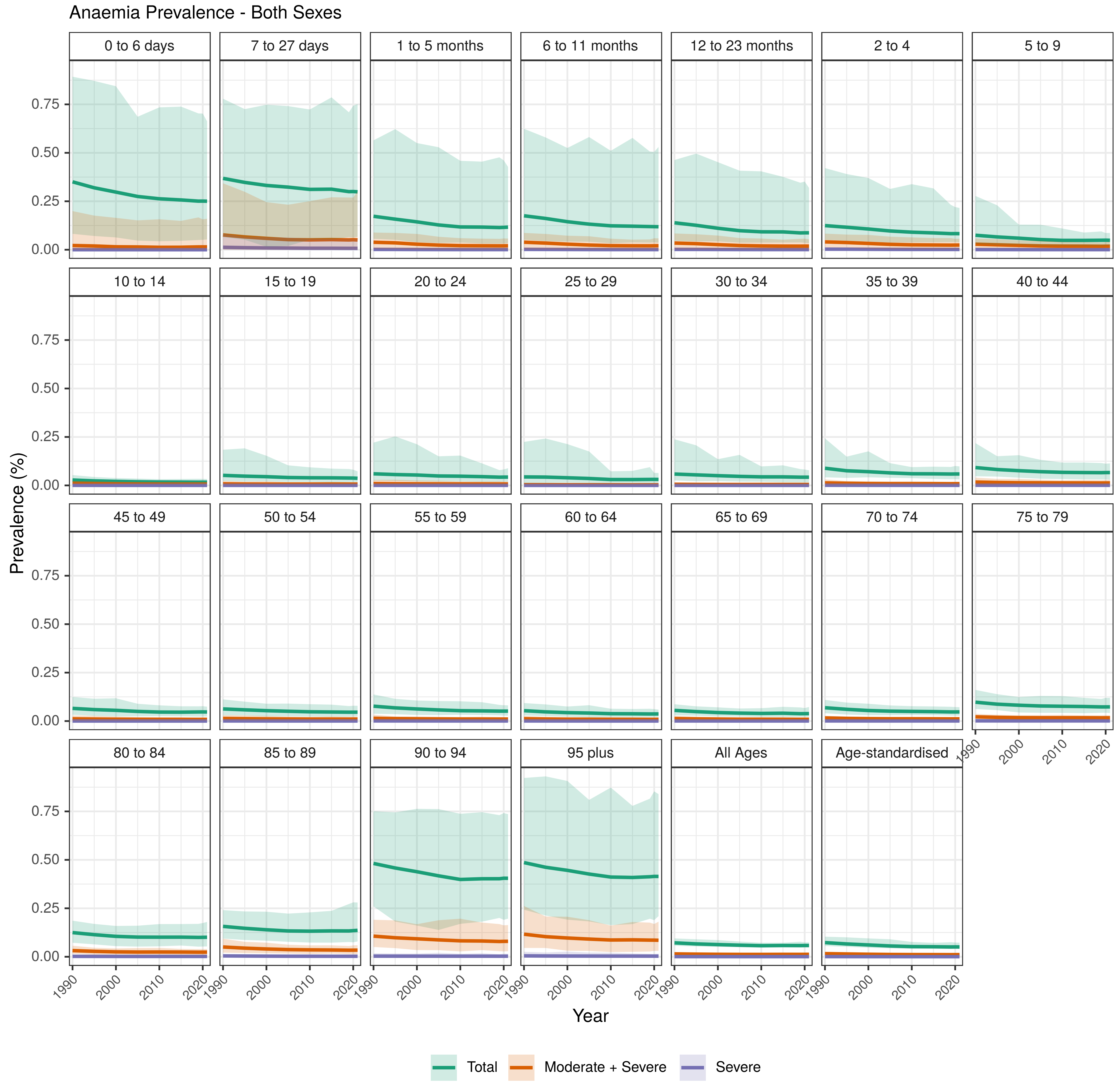
Anaemia Prevalence - Both Sexes
0 to 6 days
7 to 27 days
1 to 5 months
6 to 11 months
12 to 23 months
2 to 4
5 to 9
10 to 14
15 to 19
20 to 24
25 to 29
30 to 34
35 to 39
40 to 44
45 to 49
50 to 54
55 to 59
60 to 64
65 to 69
70 to 74
75 to 79
80 to 84
85 to 89
90 to 94
95 plus
All Ages
Age-standardised
0.00
0.25
0.50
0.75
1990
2000
2010
2020
Prevalence (%)
Year
Total
Moderate + Severe
Severe

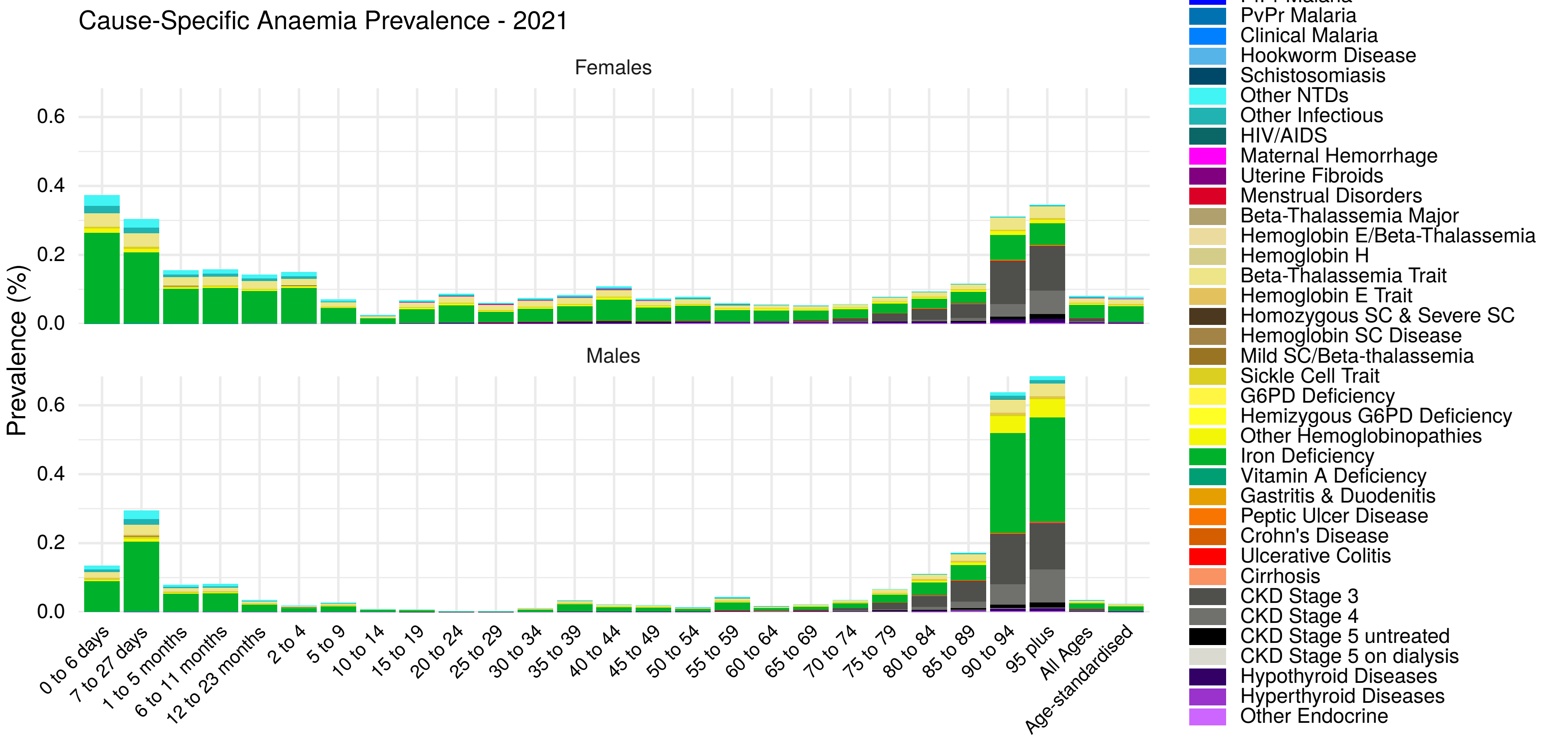
Cause-Specific Anaemia Prevalence - 2021
Females
Males
Prevalence (%)
0.0
0.2
0.4
0.6
0 to 6 days
7 to 27 days
1 to 5 months
6 to 11 months
12 to 23 months
2 to 4
5 to 9
10 to 14
15 to 19
20 to 24
25 to 29
30 to 34
35 to 39
40 to 44
45 to 49
50 to 54
55 to 59
60 to 64
65 to 69
70 to 74
75 to 79
80 to 84
85 to 89
90 to 94
95 plus
All Ages
Age-standardised
PfPr Malaria
PvPr Malaria
Clinical Malaria
Hookworm Disease
Schistosomiasis
Other NTDs
Other Infectious
HIV/AIDS
Maternal Hemorrhage
Uterine Fibroids
Menstrual Disorders
Beta-Thalassemia Major
Hemoglobin E/Beta-Thalassemia
Hemoglobin H
Beta-Thalassemia Trait
Hemoglobin E Trait
Homozygous SC & Severe SC
Hemoglobin SC Disease
Mild SC/Beta-thalassemia
Sickle Cell Trait
G6PD Deficiency
Hemizygous G6PD Deficiency
Other Hemoglobinopathies
Iron Deficiency
Vitamin A Deficiency
Gastritis & Duodenitis
Peptic Ulcer Disease
Crohn's Disease
Ulcerative Colitis
Cirrhosis
CKD Stage 3
CKD Stage 4
CKD Stage 5 untreated
CKD Stage 5 on dialysis
Hypothyroid Diseases
Hyperthyroid Diseases
Other Endocrine

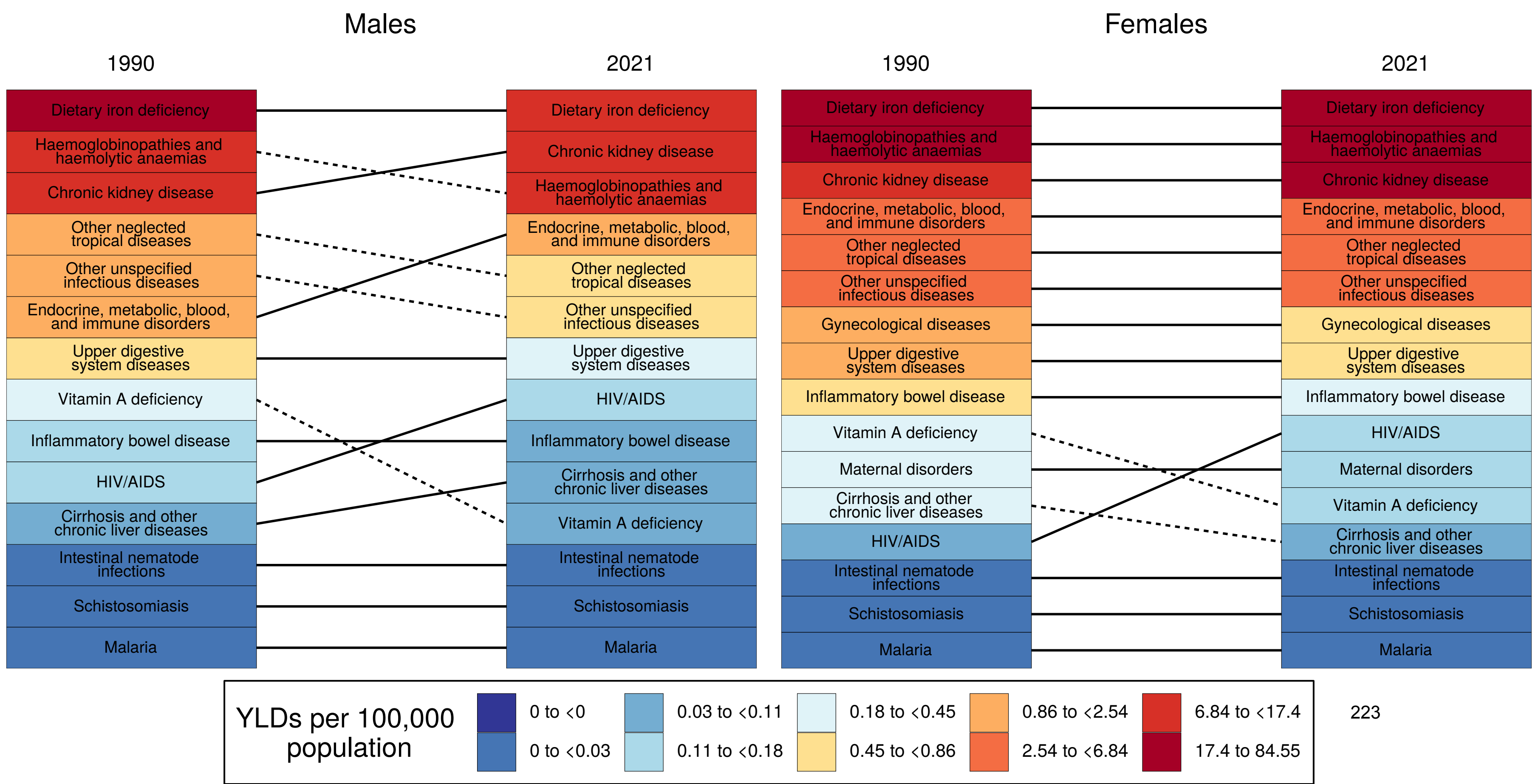
Males
Females
1990
2021
Dietary iron deficiency
Haemoglobinopathies and haemolytic anaemias
Chronic kidney disease
Other neglected tropical diseases
Other unspecified infectious diseases
Endocrine, metabolic, blood, and immune disorders
Upper digestive system diseases
Vitamin A deficiency
Inflammatory bowel disease
HIV/AIDS
Cirrhosis and other chronic liver diseases
Intestinal nematode infections
Schistosomiasis
Malaria
Gynecological diseases
Maternal disorders
YLDs per 100,000 population
0 to <0
0 to <0.03
0.03 to <0.11
0.11 to <0.18
0.18 to <0.45
0.45 to <0.86
0.86 to <2.54
2.54 to <6.84
6.84 to <17.4
17.4 to 84.55

Provincia autonoma di Bolzano

Anaemia Prevalence - Both Sexes

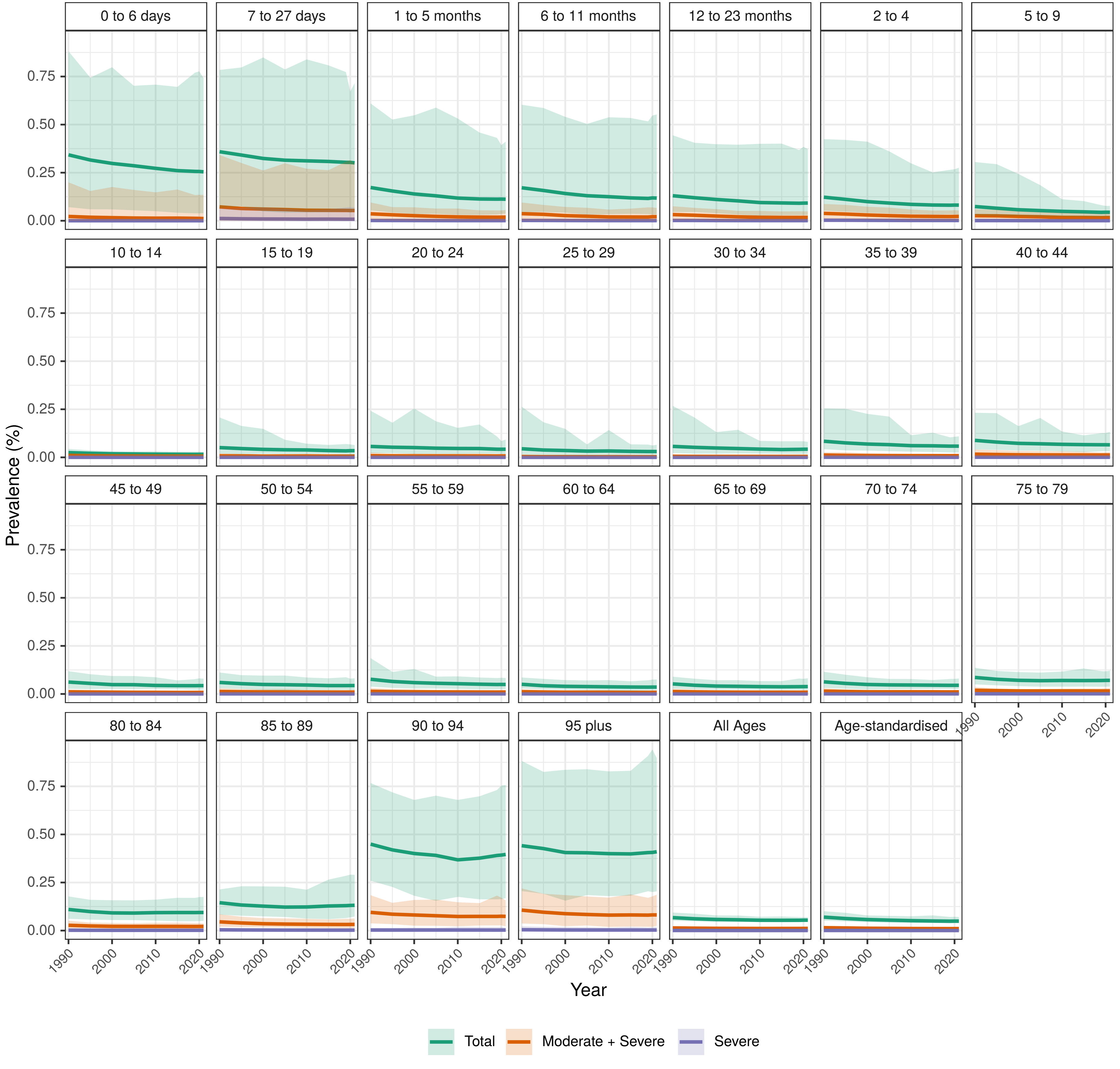

Cause-Specific Anaemia Prevalence - 2021

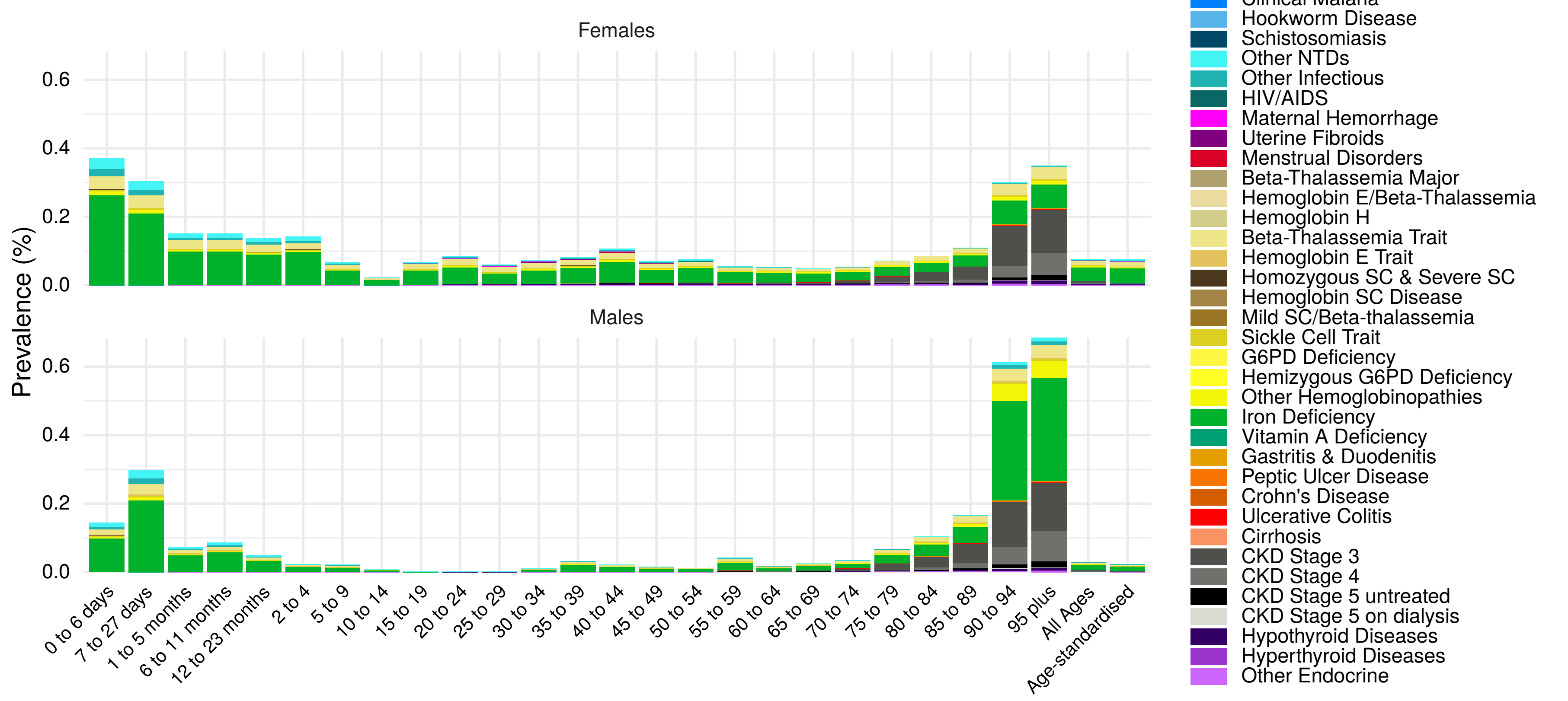

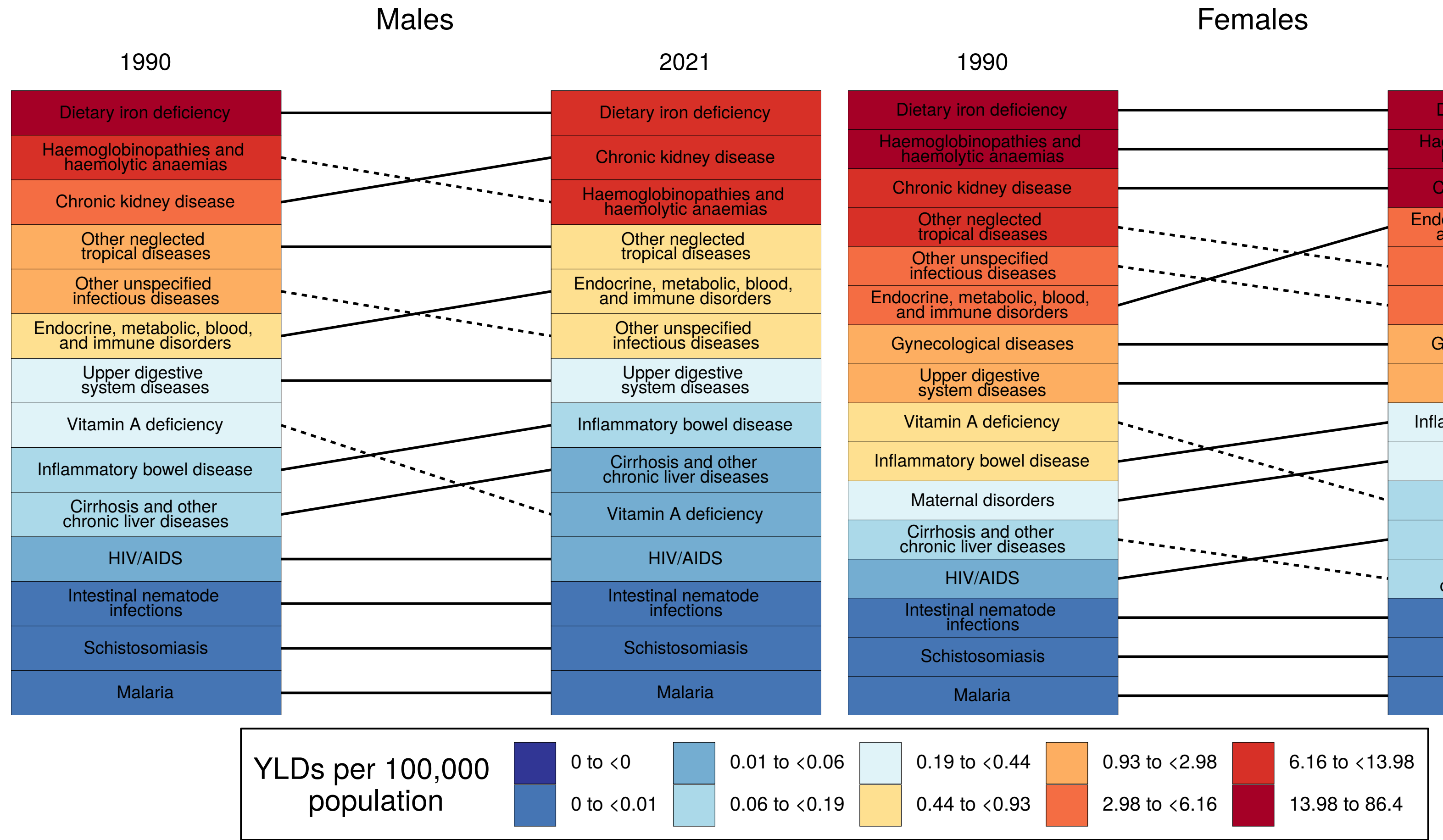

Provincia autonoma di Trento

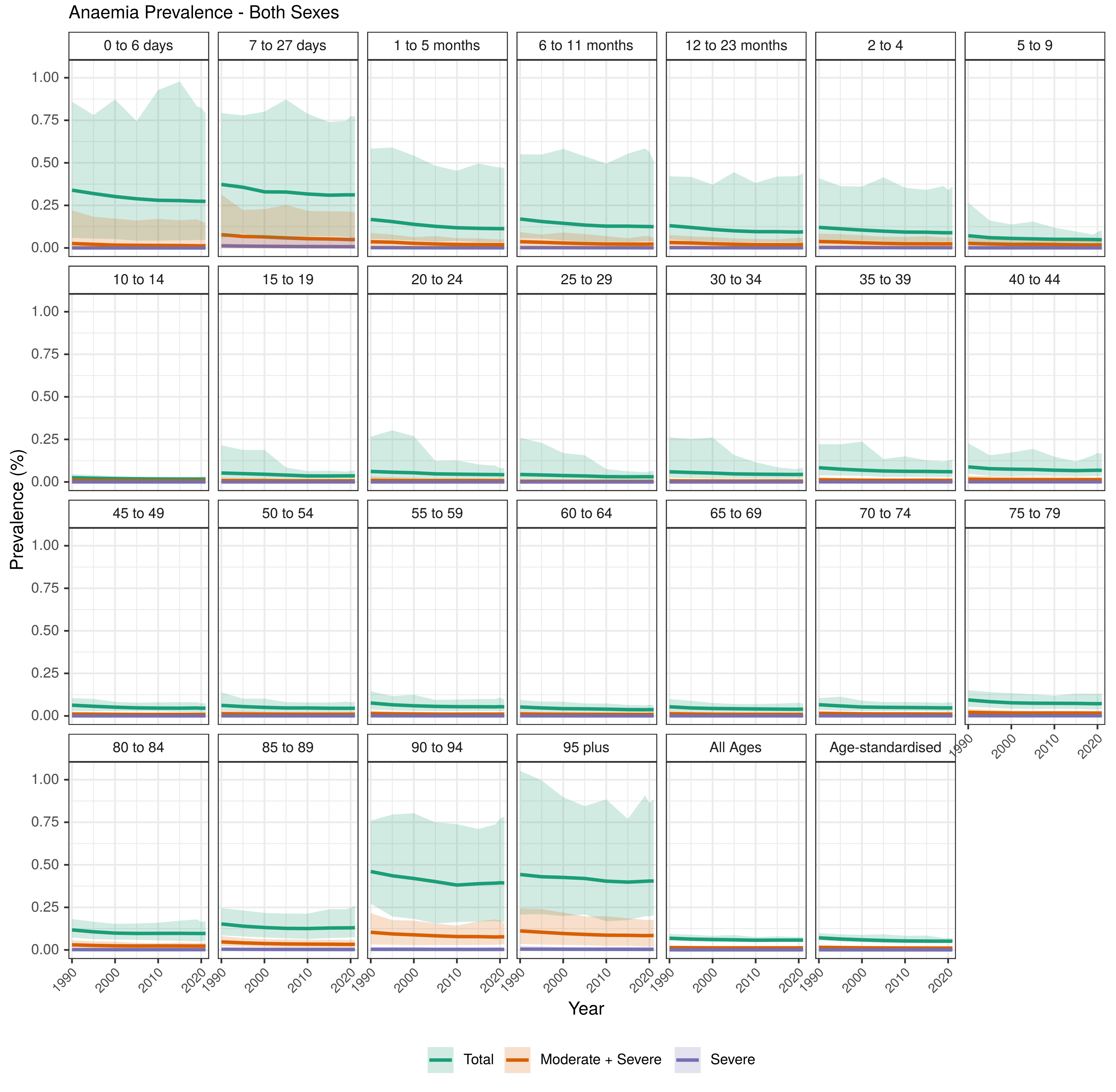

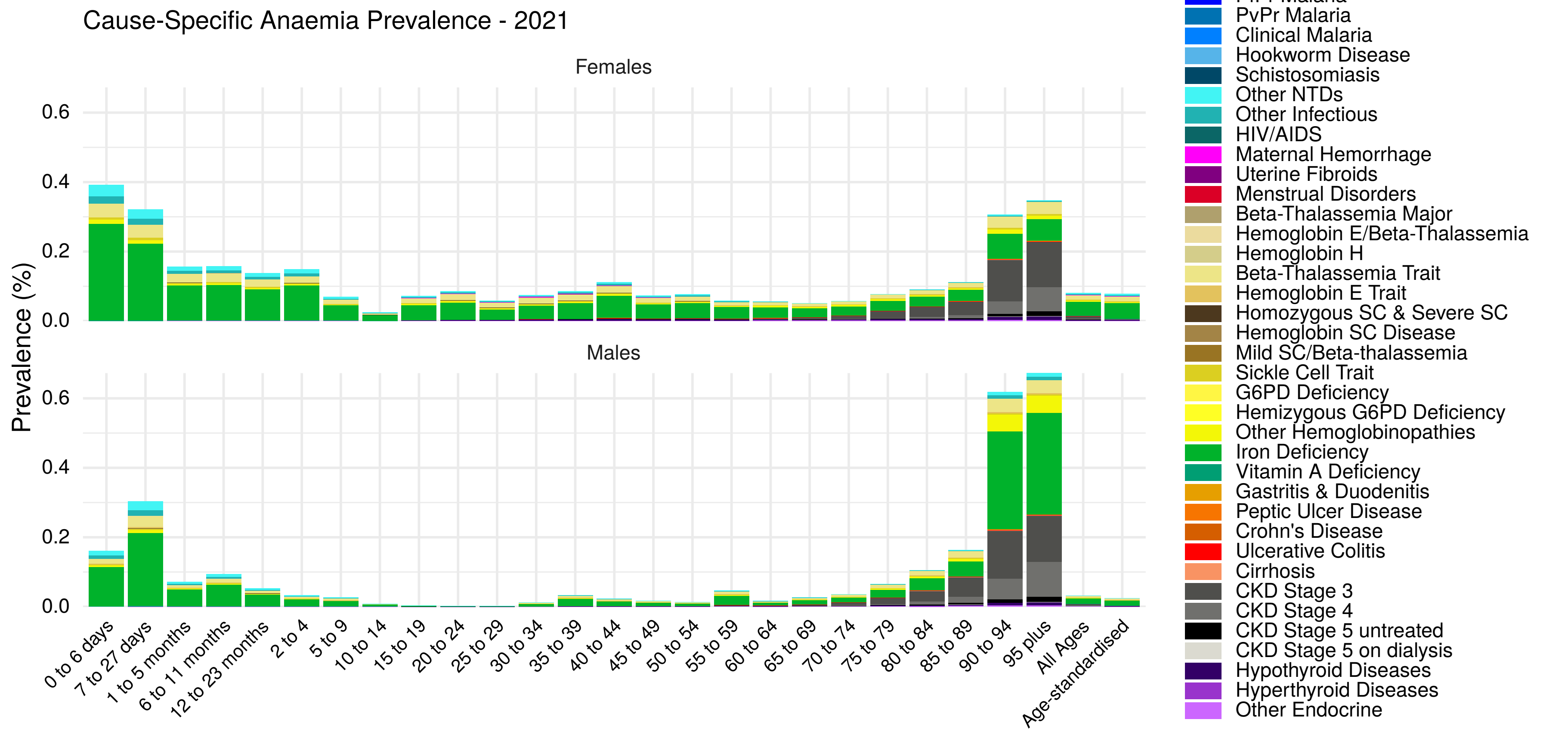

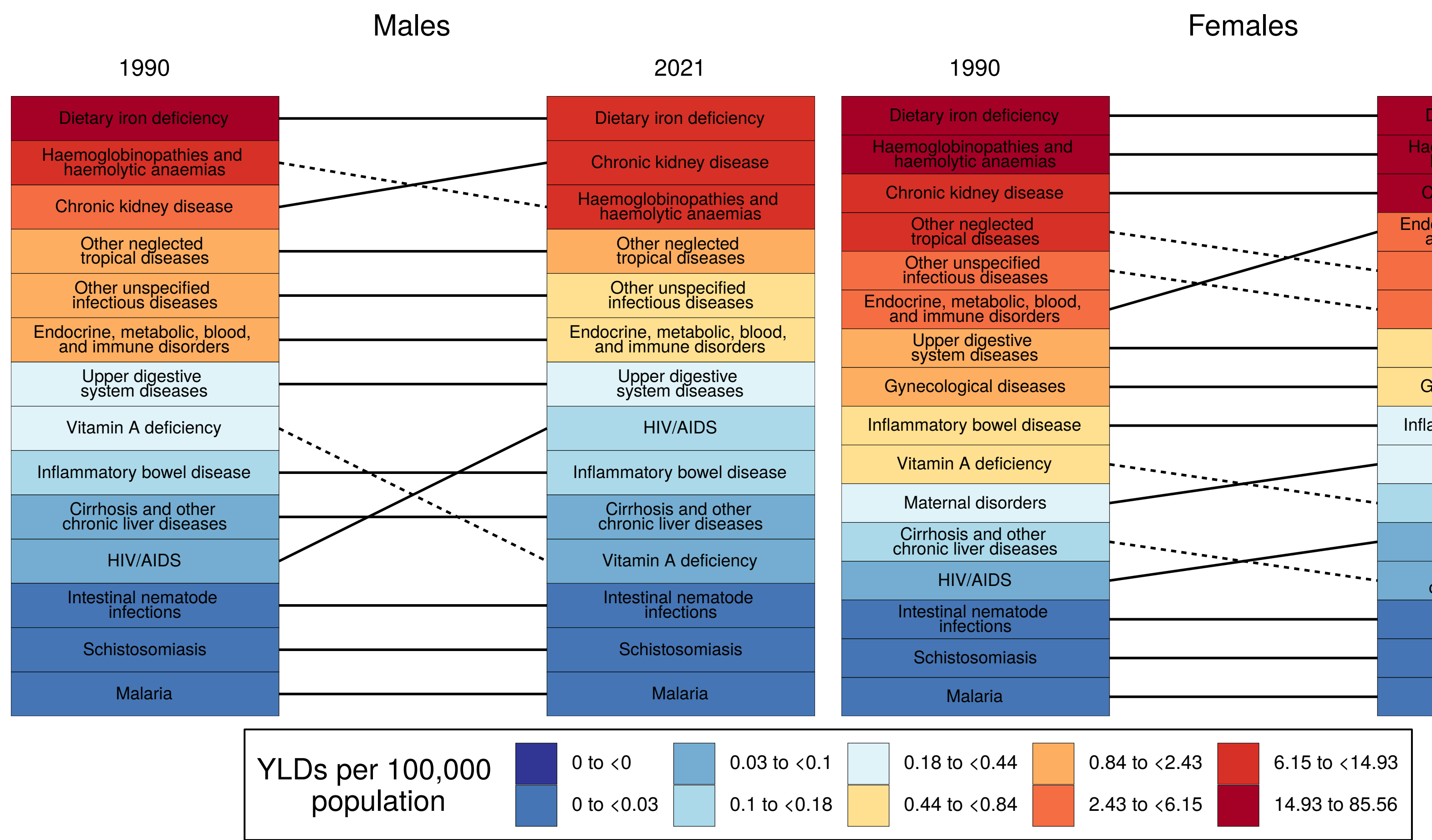

Puglia

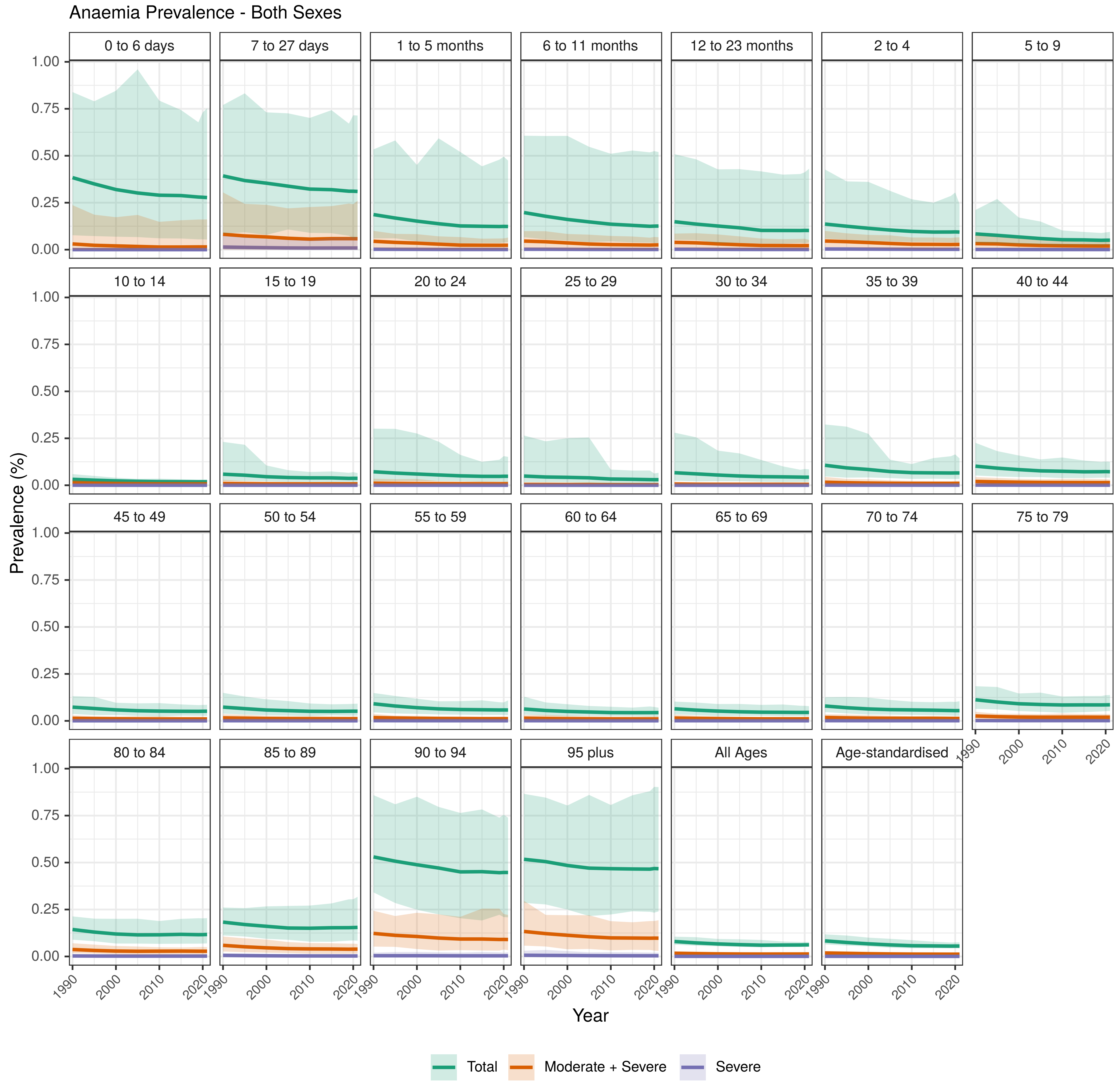

Anaemia Prevalence - Both Sexes
0 to 6 days
7 to 27 days
1 to 5 months
6 to 11 months
12 to 23 months
2 to 4
5 to 9
10 to 14
15 to 19
20 to 24
25 to 29
30 to 34
35 to 39
40 to 44
45 to 49
50 to 54
55 to 59
60 to 64
65 to 69
70 to 74
75 to 79
80 to 84
85 to 89
90 to 94
95 plus
All Ages
Age-standardised
Prevalence (%)
0.00
0.25
0.50
0.75
1.00
1990
2000
2010
2020
Year
Total
Moderate + Severe
Severe

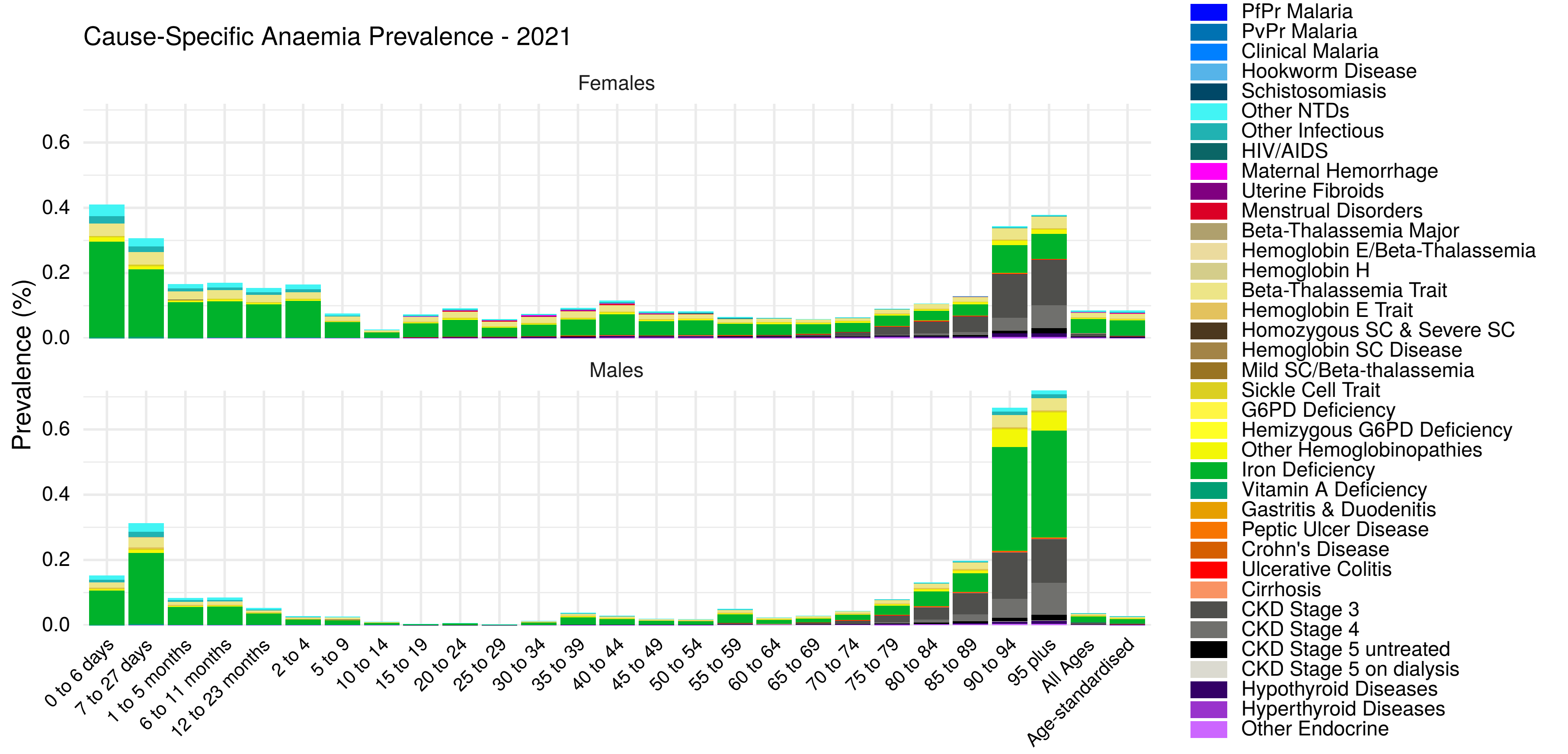

Cause-Specific Anaemia Prevalence - 2021
Females
Males
Prevalence (%)
0.0
0.2
0.4
0.6
0 to 6 days
7 to 27 days
1 to 5 months
6 to 11 months
12 to 23 months
2 to 4
5 to 9
10 to 14
15 to 19
20 to 24
25 to 29
30 to 34
35 to 39
40 to 44
45 to 49
50 to 54
55 to 59
60 to 64
65 to 69
70 to 74
75 to 79
80 to 84
85 to 89
90 to 94
95 plus
All Ages
Age-standardised
PfPr Malaria
PvPr Malaria
Clinical Malaria
Hookworm Disease
Schistosomiasis
Other NTDs
Other Infectious
HIV/AIDS
Maternal Hemorrhage
Uterine Fibroids
Menstrual Disorders
Beta-Thalassemia Major
Hemoglobin E/Beta-Thalassemia
Hemoglobin H
Beta-Thalassemia Trait
Hemoglobin E Trait
Homozygous SC & Severe SC
Hemoglobin SC Disease
Mild SC/Beta-thalassemia
Sickle Cell Trait
G6PD Deficiency
Hemizygous G6PD Deficiency
Other Hemoglobinopathies
Iron Deficiency
Vitamin A Deficiency
Gastritis & Duodenitis
Peptic Ulcer Disease
Crohn's Disease
Ulcerative Colitis
Cirrhosis
CKD Stage 3
CKD Stage 4
CKD Stage 5 untreated
CKD Stage 5 on dialysis
Hypothyroid Diseases
Hyperthyroid Diseases
Other Endocrine

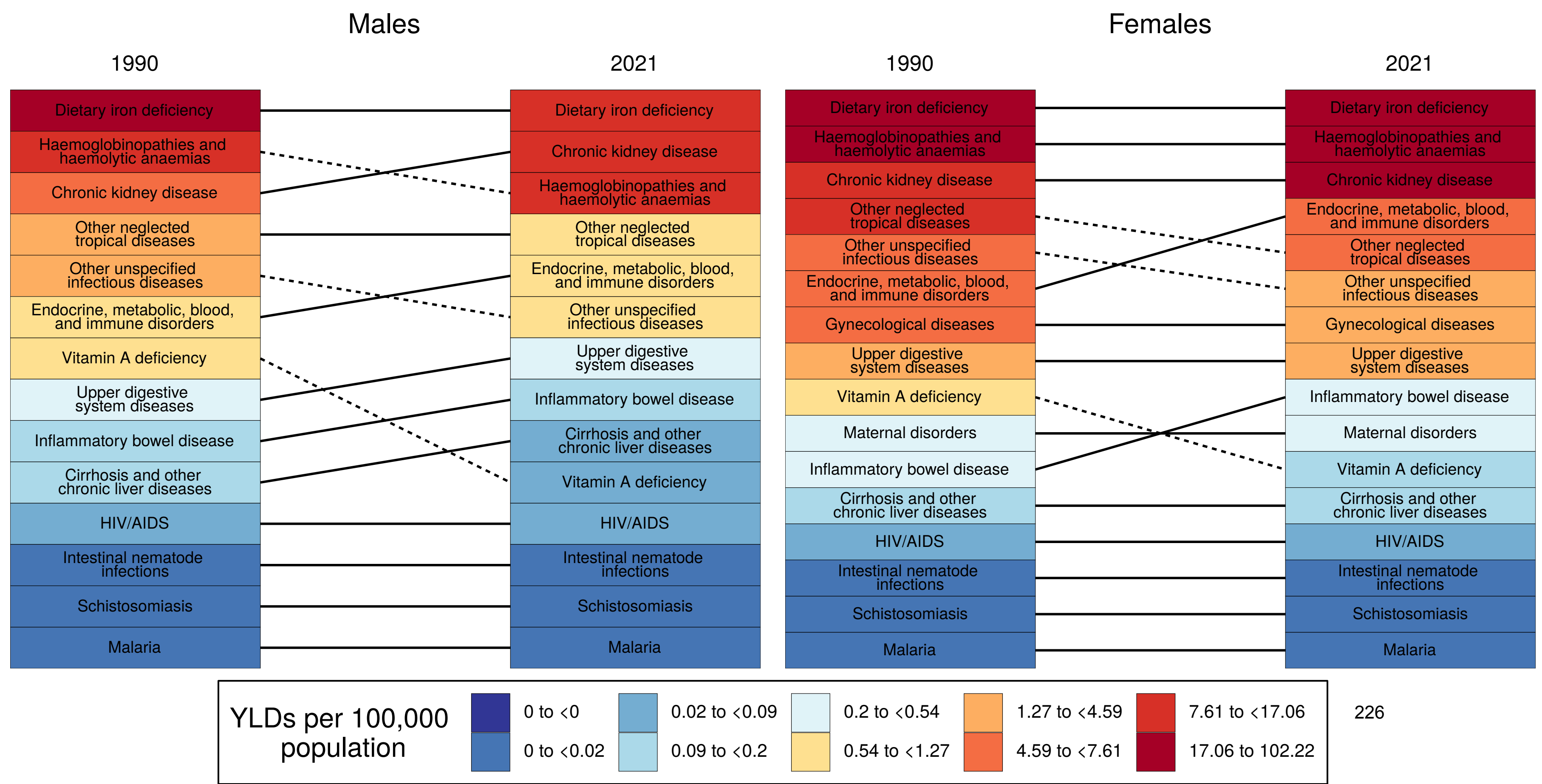

Males
1990
Dietary iron deficiency
Haemoglobinopathies and haemolytic anaemias
Chronic kidney disease
Other neglected tropical diseases
Other unspecified infectious diseases
Endocrine, metabolic, blood, and immune disorders
Vitamin A deficiency
Upper digestive system diseases
Inflammatory bowel disease
Cirrhosis and other chronic liver diseases
HIV/AIDS
Intestinal nematode infections
Schistosomiasis
Malaria
2021
Dietary iron deficiency
Chronic kidney disease
Haemoglobinopathies and haemolytic anaemias
Other neglected tropical diseases
Endocrine, metabolic, blood, and immune disorders
Other unspecified infectious diseases
Upper digestive system diseases
Inflammatory bowel disease
Cirrhosis and other chronic liver diseases
Vitamin A deficiency
HIV/AIDS
Intestinal nematode infections
Schistosomiasis
Malaria
Females
1990
Dietary iron deficiency
Haemoglobinopathies and haemolytic anaemias
Chronic kidney disease
Other neglected tropical diseases
Other unspecified infectious diseases
Endocrine, metabolic, blood, and immune disorders
Gynecological diseases
Upper digestive system diseases
Vitamin A deficiency
Maternal disorders
Inflammatory bowel disease
Cirrhosis and other chronic liver diseases
HIV/AIDS
Intestinal nematode infections
Schistosomiasis
Malaria
2021
Dietary iron deficiency
Haemoglobinopathies and haemolytic anaemias
Chronic kidney disease
Endocrine, metabolic, blood, and immune disorders
Other neglected tropical diseases
Other unspecified infectious diseases
Gynecological diseases
Upper digestive system diseases
Inflammatory bowel disease
Maternal disorders
Vitamin A deficiency
Cirrhosis and other chronic liver diseases
HIV/AIDS
Intestinal nematode infections
Schistosomiasis
Malaria
YLDs per 100,000 population
0 to <0
0 to <0.02
0.02 to <0.09
0.09 to <0.2
0.2 to <0.54
0.54 to <1.27
1.27 to <4.59
4.59 to <7.61
7.61 to <17.06
17.06 to 102.22

Sardegna

Anaemia Prevalence - Both Sexes

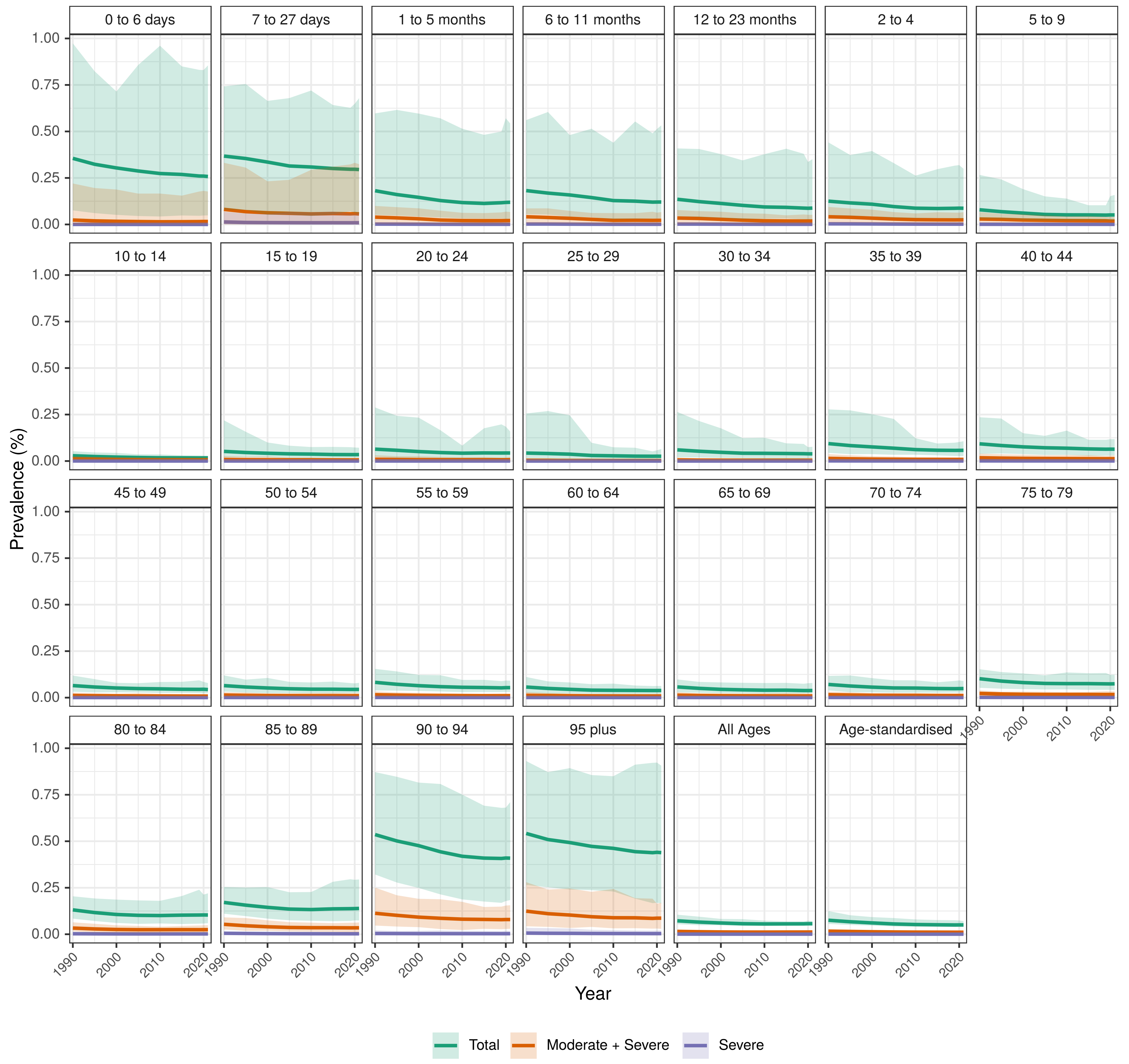

Cause-Specific Anaemia Prevalence - 2021

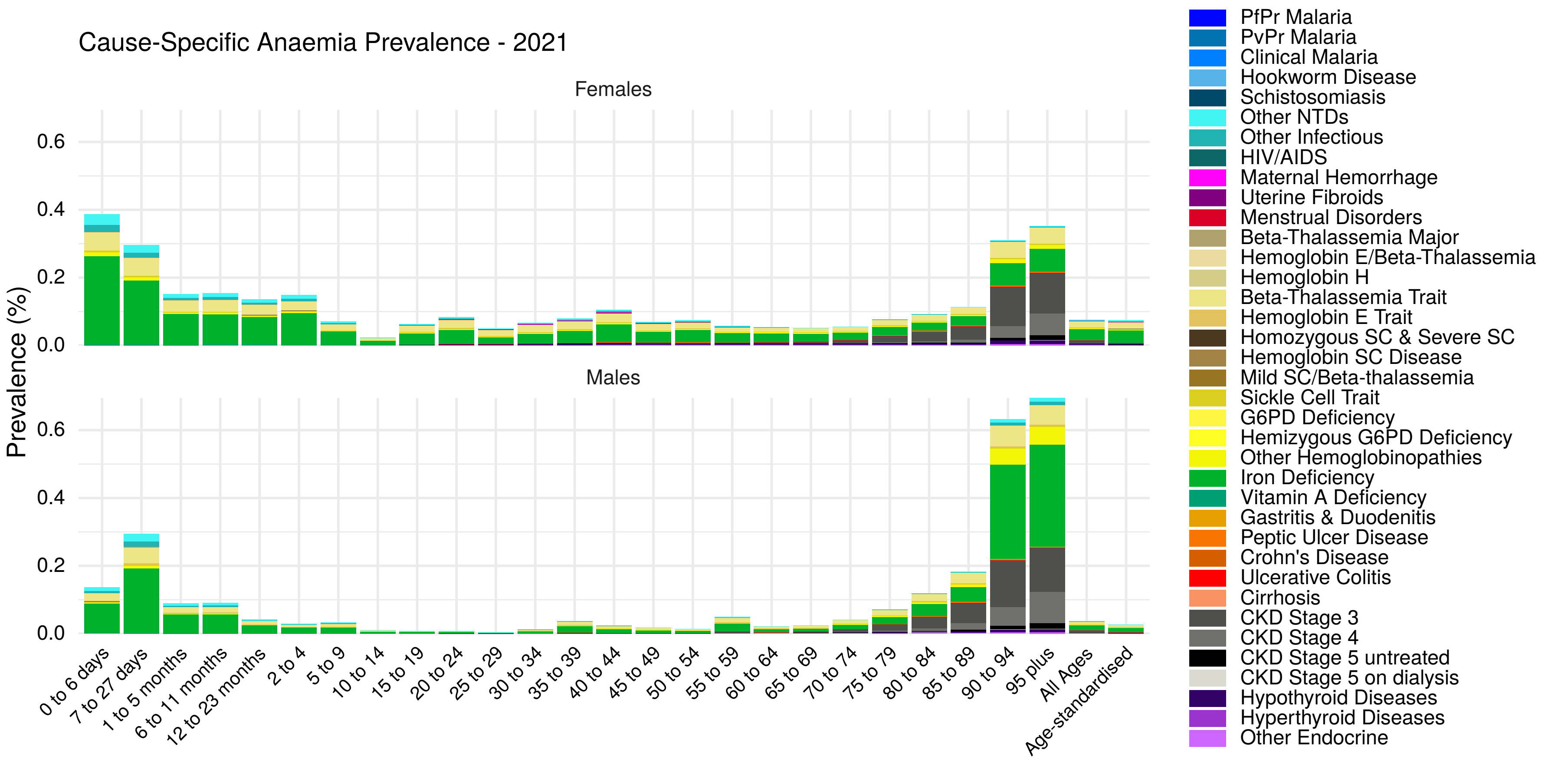

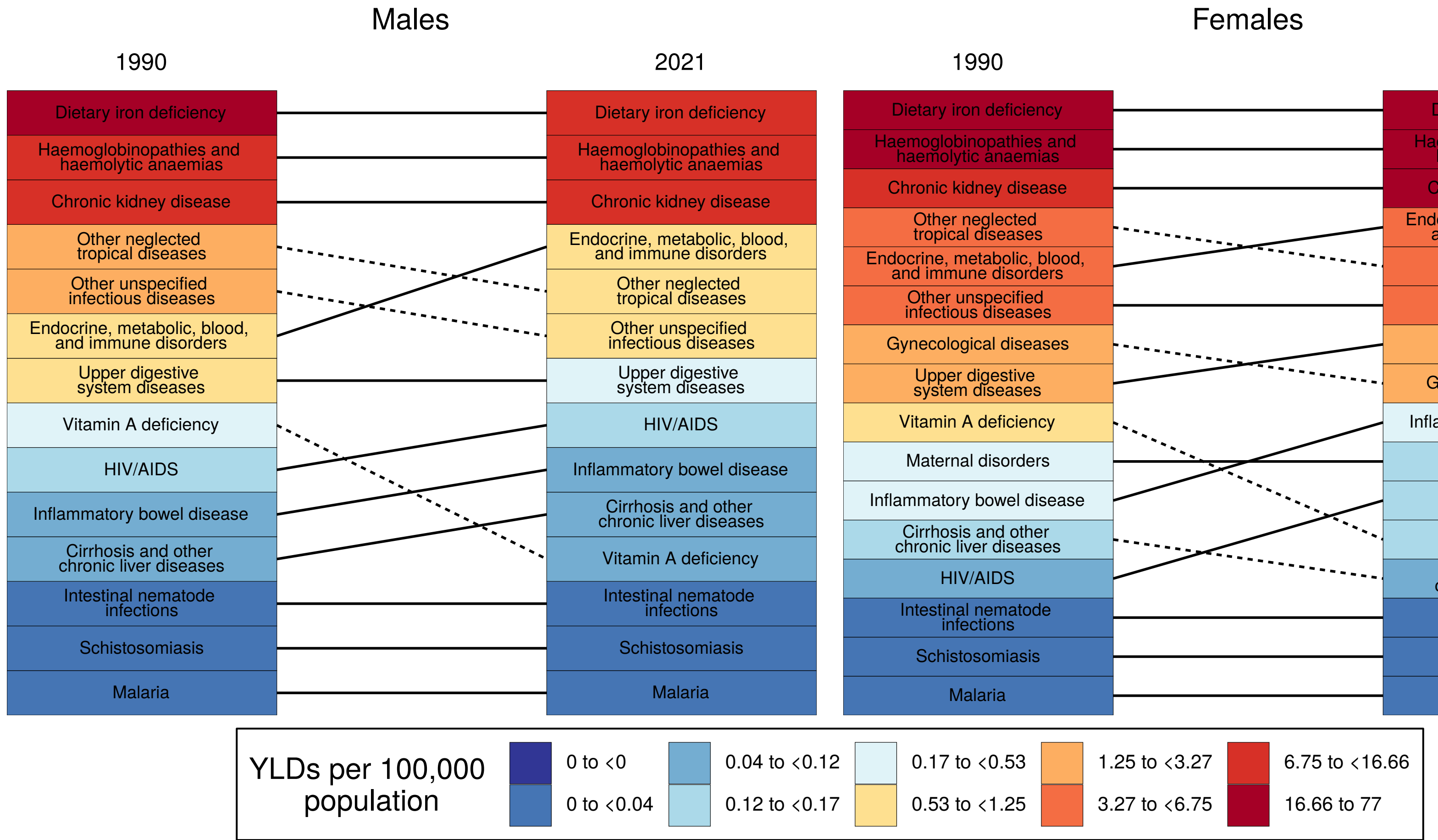

Sicilia

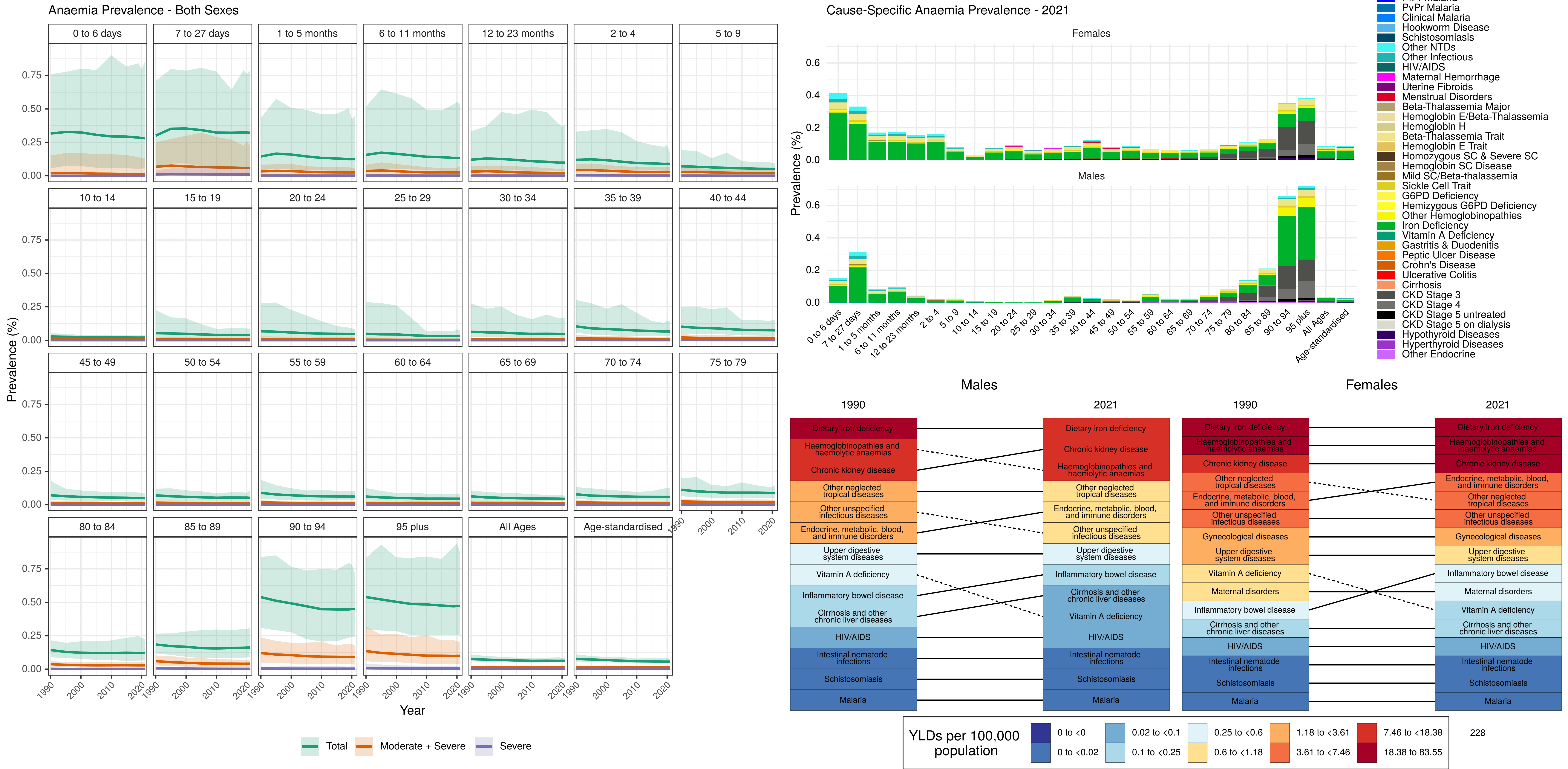

Anaemia Prevalence - Both Sexes
0 to 6 days
7 to 27 days
1 to 5 months
6 to 11 months
12 to 23 months
2 to 4
5 to 9
10 to 14
15 to 19
20 to 24
25 to 29
30 to 34
35 to 39
40 to 44
45 to 49
50 to 54
55 to 59
60 to 64
65 to 69
70 to 74
75 to 79
80 to 84
85 to 89
90 to 94
95 plus
All Ages
Age-standardised
Prevalence (%)
Year
Total
Moderate + Severe
Severe
Cause-Specific Anaemia Prevalence - 2021
Females
Males
PfPr Malaria
PvPr Malaria
Clinical Malaria
Hookworm Disease
Schistosomiasis
Other NTDs
Other Infectious
HIV/AIDS
Maternal Hemorrhage
Uterine Fibroids
Menstrual Disorders
Beta-Thalassemia Major
Hemoglobin E/Beta-Thalassemia
Hemoglobin H
Beta-Thalassemia Trait
Hemoglobin E Trait
Homozygous SC & Severe SC
Hemoglobin SC Disease
Mild SC/Beta-thalassemia
Sickle Cell Trait
G6PD Deficiency
Hemizygous G6PD Deficiency
Other Hemoglobinopathies
Iron Deficiency
Vitamin A Deficiency
Gastritis & Duodenitis
Peptic Ulcer Disease
Crohn's Disease
Ulcerative Colitis
Cirrhosis
CKD Stage 3
CKD Stage 4
CKD Stage 5 untreated
CKD Stage 5 on dialysis
Hypothyroid Diseases
Hyperthyroid Diseases
Other Endocrine
Males
Females
1990
2021
Dietary iron deficiency
Haemoglobinopathies and haemolytic anaemias
Chronic kidney disease
Other neglected tropical diseases
Other unspecified infectious diseases
Endocrine, metabolic, blood, and immune disorders
Upper digestive system diseases
Vitamin A deficiency
Inflammatory bowel disease
Cirrhosis and other chronic liver diseases
HIV/AIDS
Intestinal nematode infections
Schistosomiasis
Malaria
Gynecological diseases
Maternal disorders
YLDs per 100,000 population
0 to <0
0 to <0.02
0.02 to <0.1
0.1 to <0.25
0.25 to <0.6
0.6 to <1.18
1.18 to <3.61
3.61 to <7.46
7.46 to <18.38
18.38 to 83.55

Toscana

Anaemia Prevalence - Both Sexes

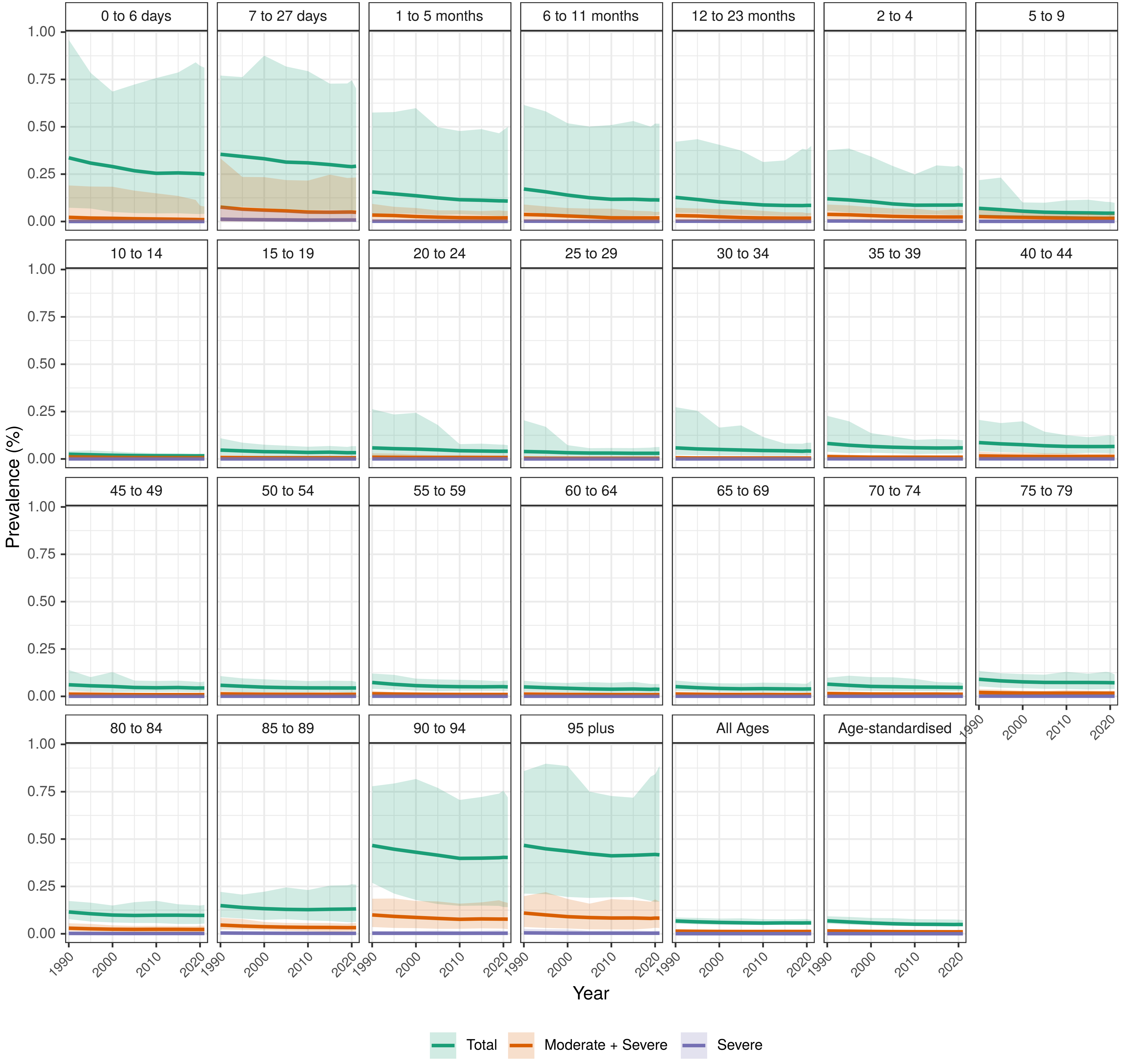

Cause-Specific Anaemia Prevalence - 2021

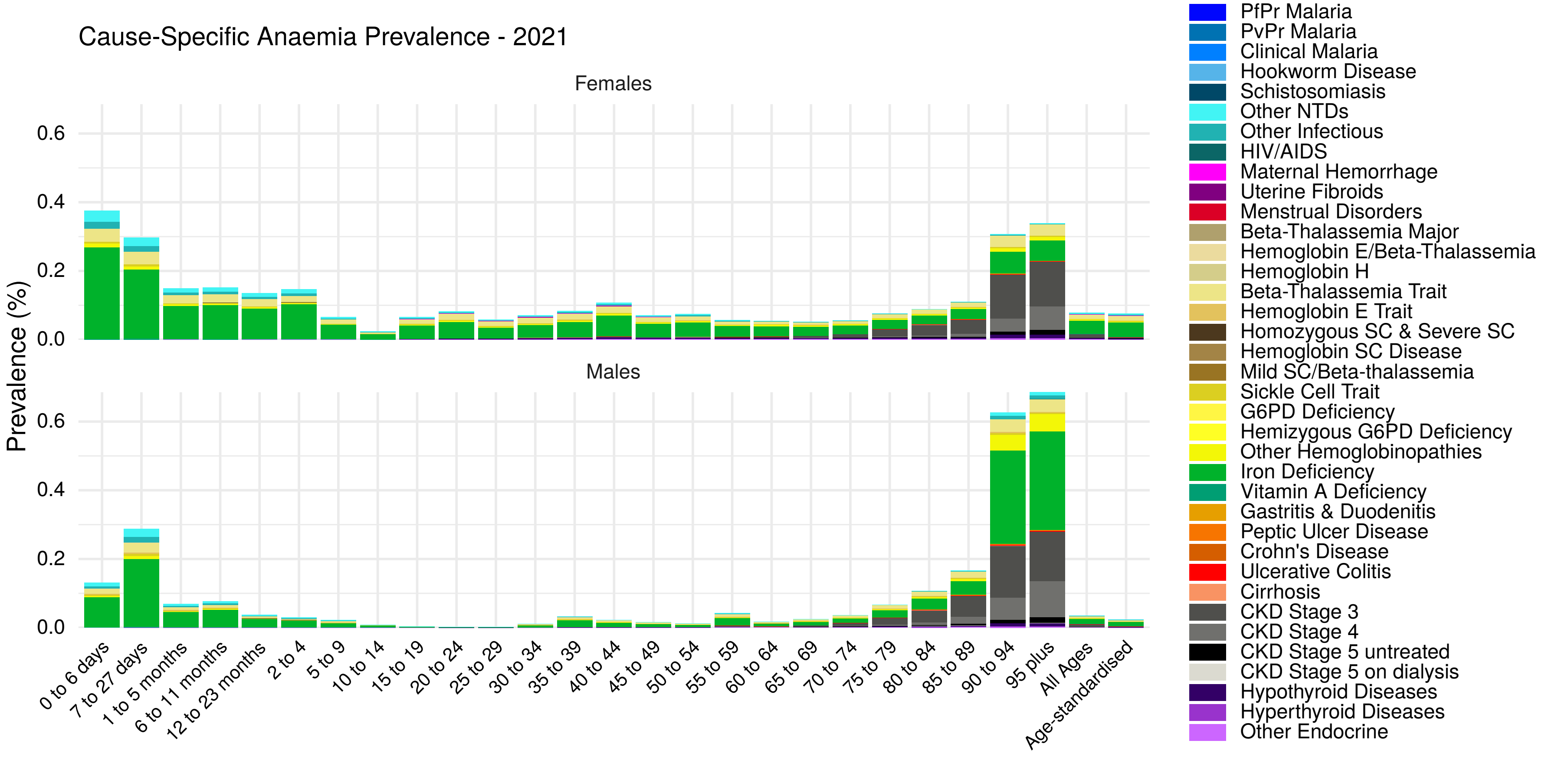

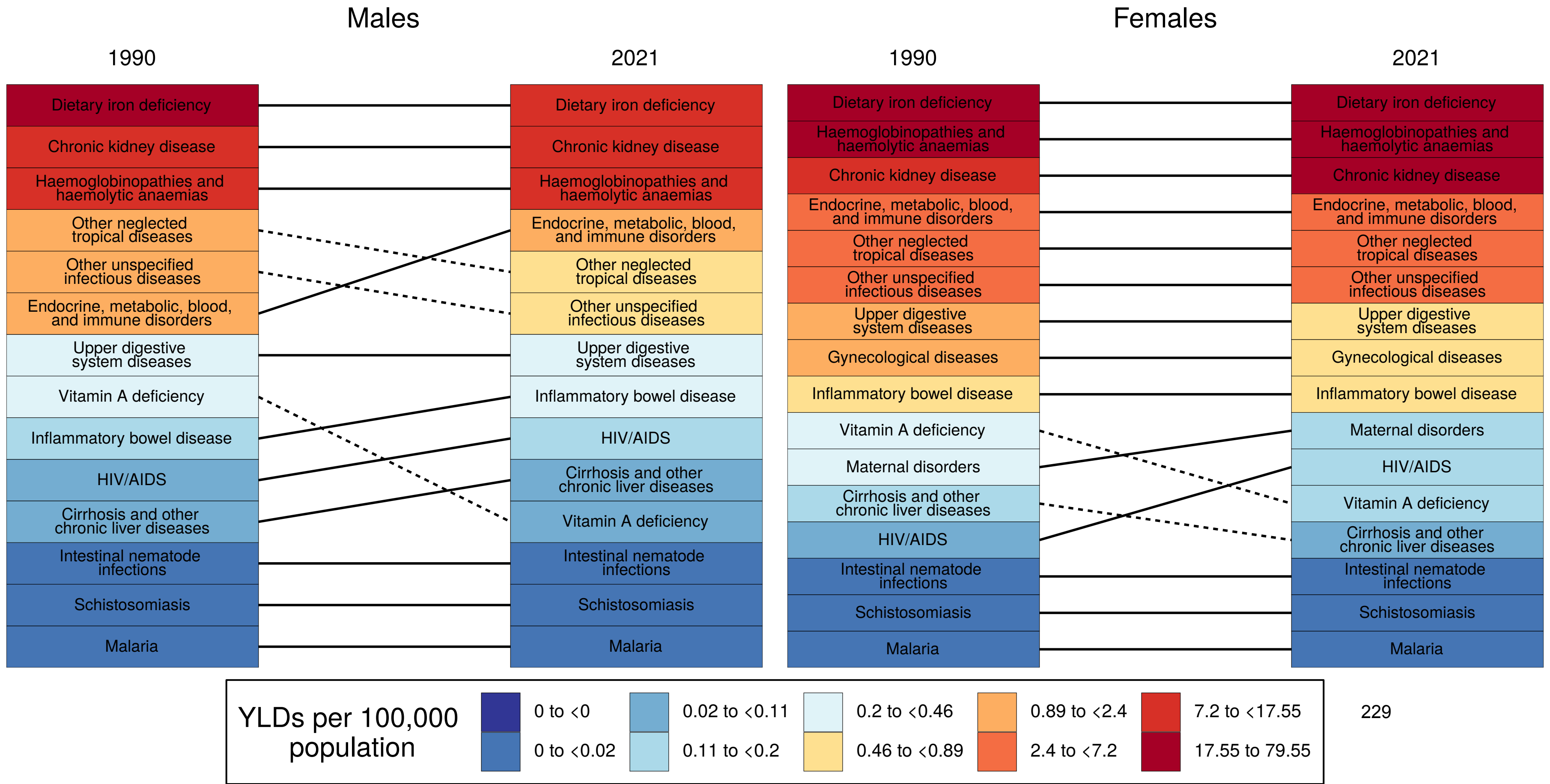

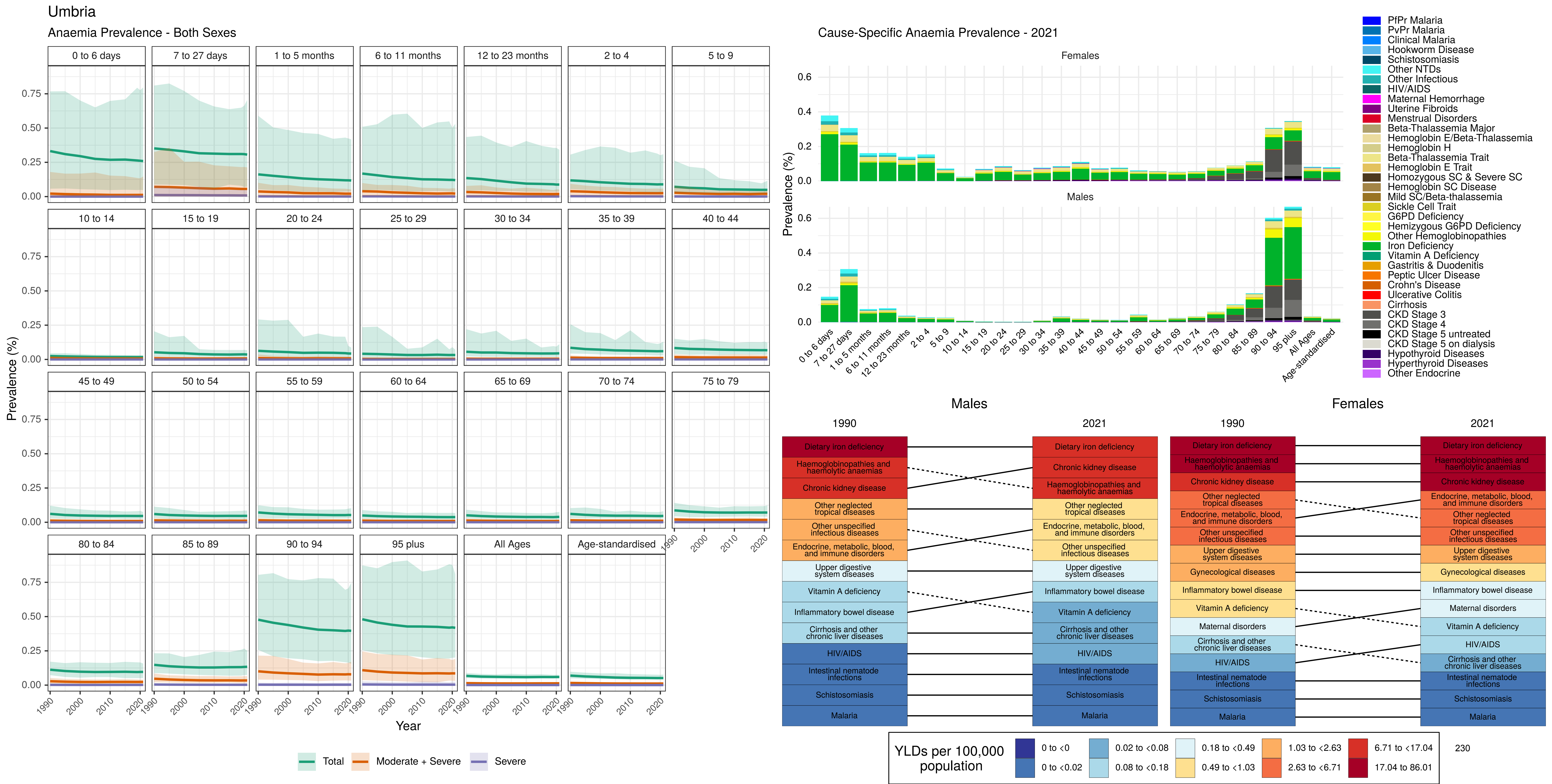
Umbria
Anaemia Prevalence - Both Sexes
0 to 6 days
7 to 27 days
1 to 5 months
6 to 11 months
12 to 23 months
2 to 4
5 to 9
10 to 14
15 to 19
20 to 24
25 to 29
30 to 34
35 to 39
40 to 44
45 to 49
50 to 54
55 to 59
60 to 64
65 to 69
70 to 74
75 to 79
80 to 84
85 to 89
90 to 94
95 plus
All Ages
Age-standardised
Prevalence (%)
0.00
0.25
0.50
0.75
1990
2000
2010
2020
Year
Total
Moderate + Severe
Severe
Cause-Specific Anaemia Prevalence - 2021
Females
Males
Prevalence (%)
0.0
0.2
0.4
0.6
PfPr Malaria
PvPr Malaria
Clinical Malaria
Hookworm Disease
Schistosomiasis
Other NTDs
Other Infectious
HIV/AIDS
Maternal Hemorrhage
Uterine Fibroids
Menstrual Disorders
Beta-Thalassemia Major
Hemoglobin E/Beta-Thalassemia
Hemoglobin H
Beta-Thalassemia Trait
Hemoglobin E Trait
Homozygous SC & Severe SC
Hemoglobin SC Disease
Mild SC/Beta-thalassemia
Sickle Cell Trait
G6PD Deficiency
Hemizygous G6PD Deficiency
Other Hemoglobinopathies
Iron Deficiency
Vitamin A Deficiency
Gastritis & Duodenitis
Peptic Ulcer Disease
Crohn's Disease
Ulcerative Colitis
Cirrhosis
CKD Stage 3
CKD Stage 4
CKD Stage 5 untreated
CKD Stage 5 on dialysis
Hypothyroid Diseases
Hyperthyroid Diseases
Other Endocrine
Males
Females
1990
2021
Dietary iron deficiency
Haemoglobinopathies and haemolytic anaemias
Chronic kidney disease
Other neglected tropical diseases
Other unspecified infectious diseases
Endocrine, metabolic, blood, and immune disorders
Upper digestive system diseases
Vitamin A deficiency
Inflammatory bowel disease
Cirrhosis and other chronic liver diseases
HIV/AIDS
Intestinal nematode infections
Schistosomiasis
Malaria
Gynecological diseases
Maternal disorders
YLDs per 100,000 population
0 to <0
0 to <0.02
0.02 to <0.08
0.08 to <0.18
0.18 to <0.49
0.49 to <1.03
1.03 to <2.63
2.63 to <6.71
6.71 to <17.04
17.04 to 86.01

Valle d'Aosta

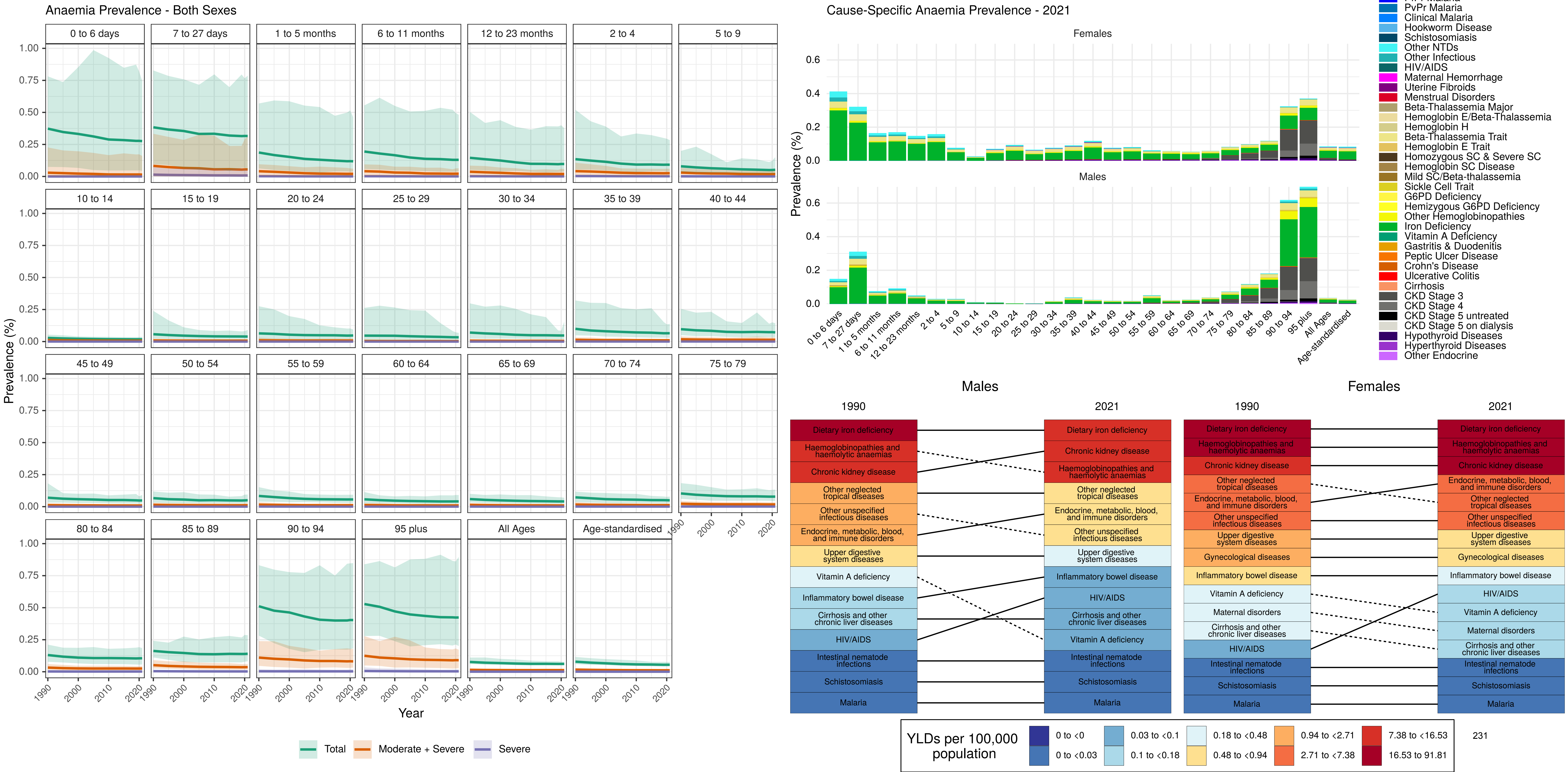

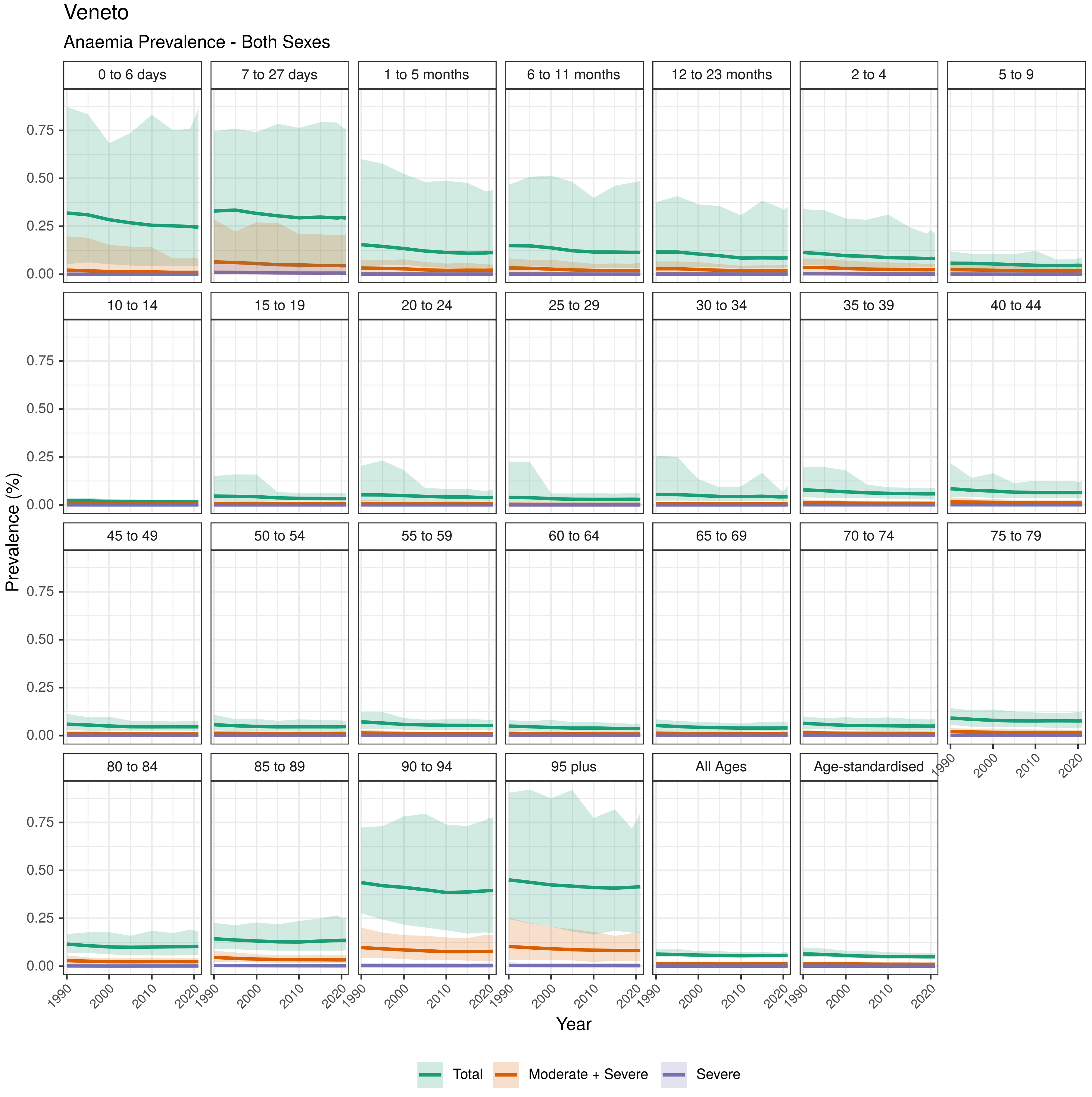
Veneto
Anaemia Prevalence - Both Sexes
0 to 6 days
7 to 27 days
1 to 5 months
6 to 11 months
12 to 23 months
2 to 4
5 to 9
10 to 14
15 to 19
20 to 24
25 to 29
30 to 34
35 to 39
40 to 44
45 to 49
50 to 54
55 to 59
60 to 64
65 to 69
70 to 74
75 to 79
80 to 84
85 to 89
90 to 94
95 plus
All Ages
Age-standardised
Prevalence (%)
Year
Total
Moderate + Severe
Severe

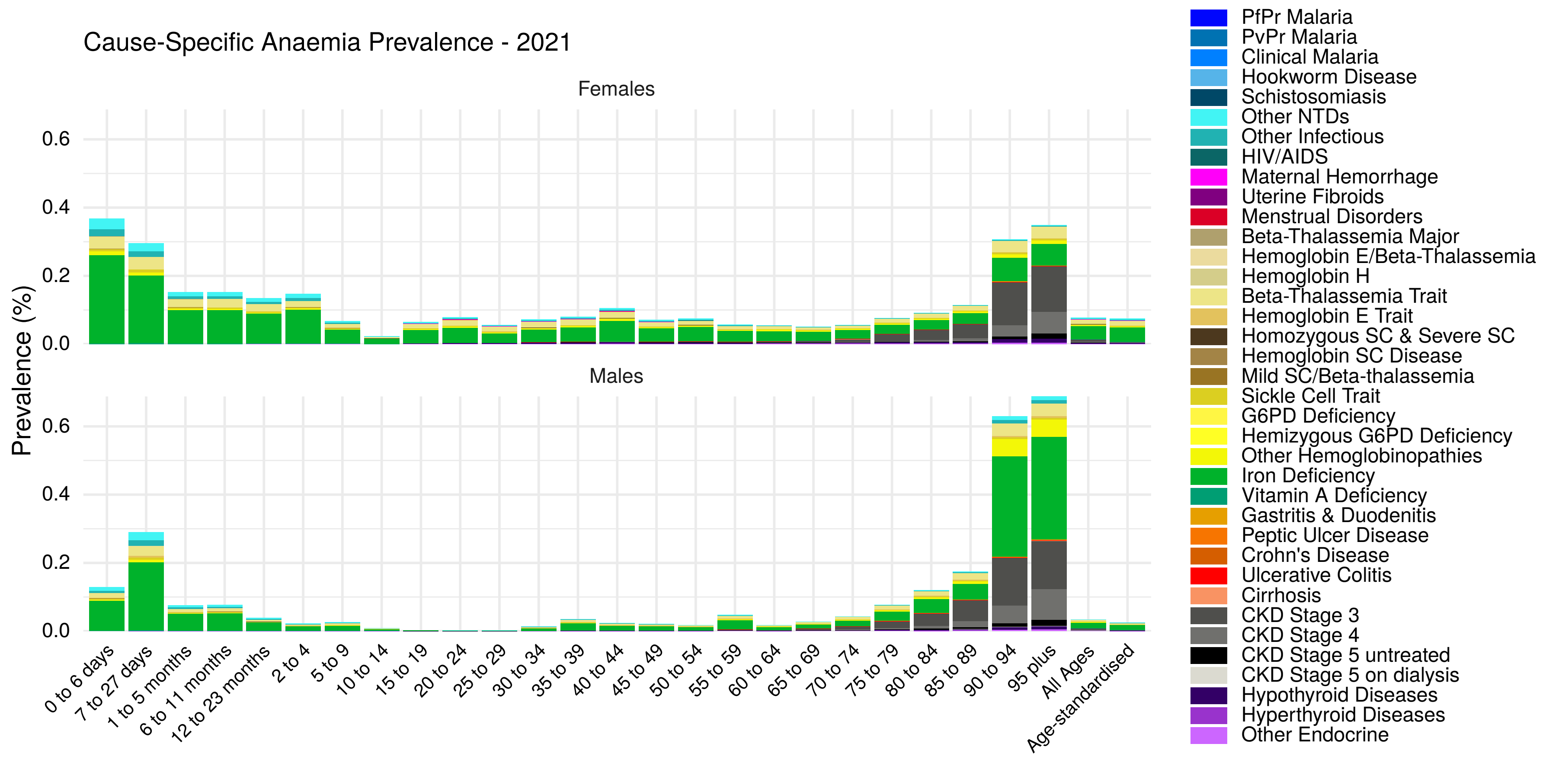
Cause-Specific Anaemia Prevalence - 2021
Females
Males
Prevalence (%)
PfPr Malaria
PvPr Malaria
Clinical Malaria
Hookworm Disease
Schistosomiasis
Other NTDs
Other Infectious
HIV/AIDS
Maternal Hemorrhage
Uterine Fibroids
Menstrual Disorders
Beta-Thalassemia Major
Hemoglobin E/Beta-Thalassemia
Hemoglobin H
Beta-Thalassemia Trait
Hemoglobin E Trait
Homozygous SC & Severe SC
Hemoglobin SC Disease
Mild SC/Beta-thalassemia
Sickle Cell Trait
G6PD Deficiency
Hemizygous G6PD Deficiency
Other Hemoglobinopathies
Iron Deficiency
Vitamin A Deficiency
Gastritis & Duodenitis
Peptic Ulcer Disease
Crohn's Disease
Ulcerative Colitis
Cirrhosis
CKD Stage 3
CKD Stage 4
CKD Stage 5 untreated
CKD Stage 5 on dialysis
Hypothyroid Diseases
Hyperthyroid Diseases
Other Endocrine

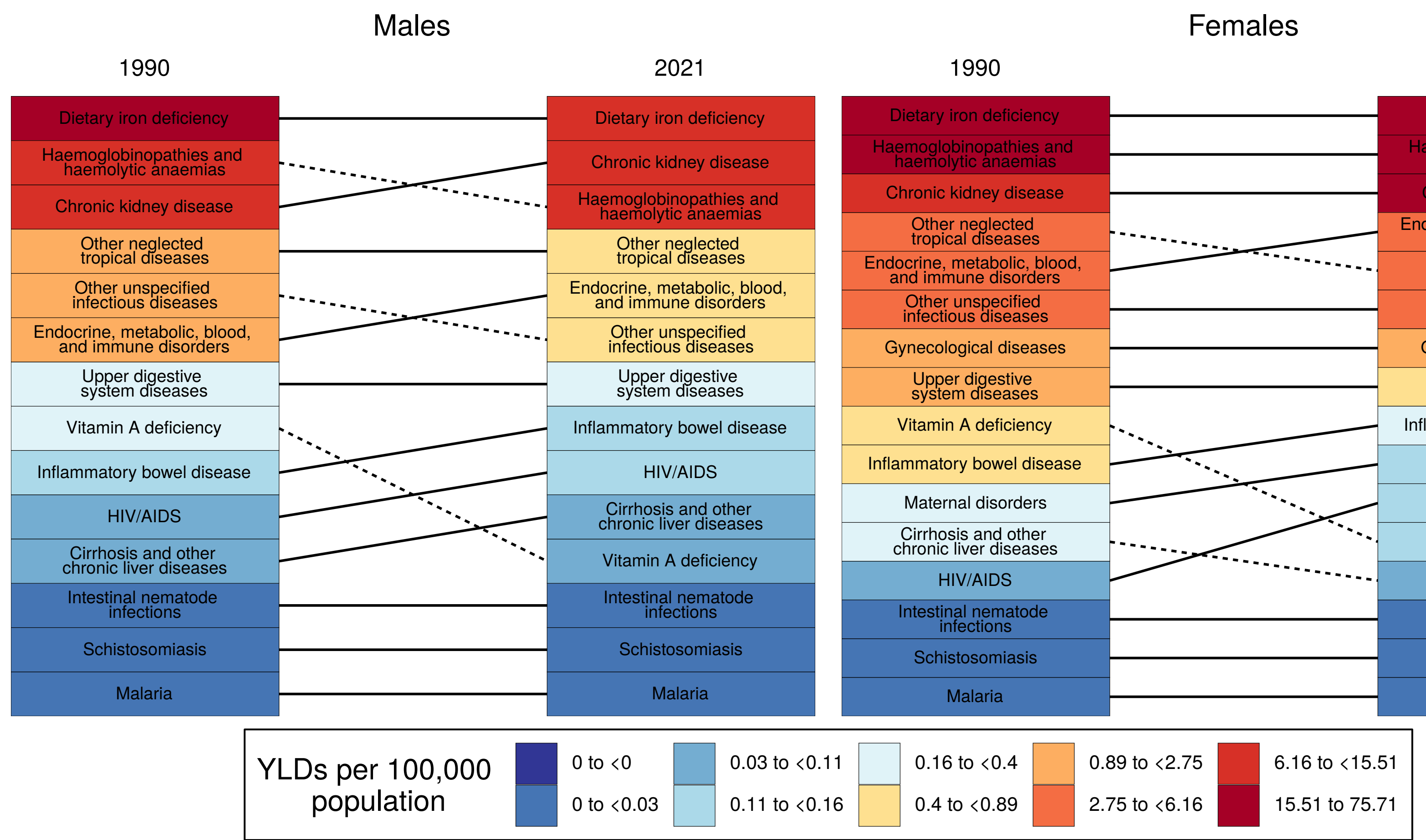
Males
Females
1990
2021
Dietary iron deficiency
Haemoglobinopathies and haemolytic anaemias
Chronic kidney disease
Other neglected tropical diseases
Other unspecified infectious diseases
Endocrine, metabolic, blood, and immune disorders
Upper digestive system diseases
Vitamin A deficiency
Inflammatory bowel disease
HIV/AIDS
Cirrhosis and other chronic liver diseases
Intestinal nematode infections
Schistosomiasis
Malaria
Gynecological diseases
Maternal disorders
YLDs per 100,000 population
0 to <0
0 to <0.03
0.03 to <0.11
0.11 to <0.16
0.16 to <0.4
0.4 to <0.89
0.89 to <2.75
2.75 to <6.16
6.16 to <15.51
15.51 to 75.71

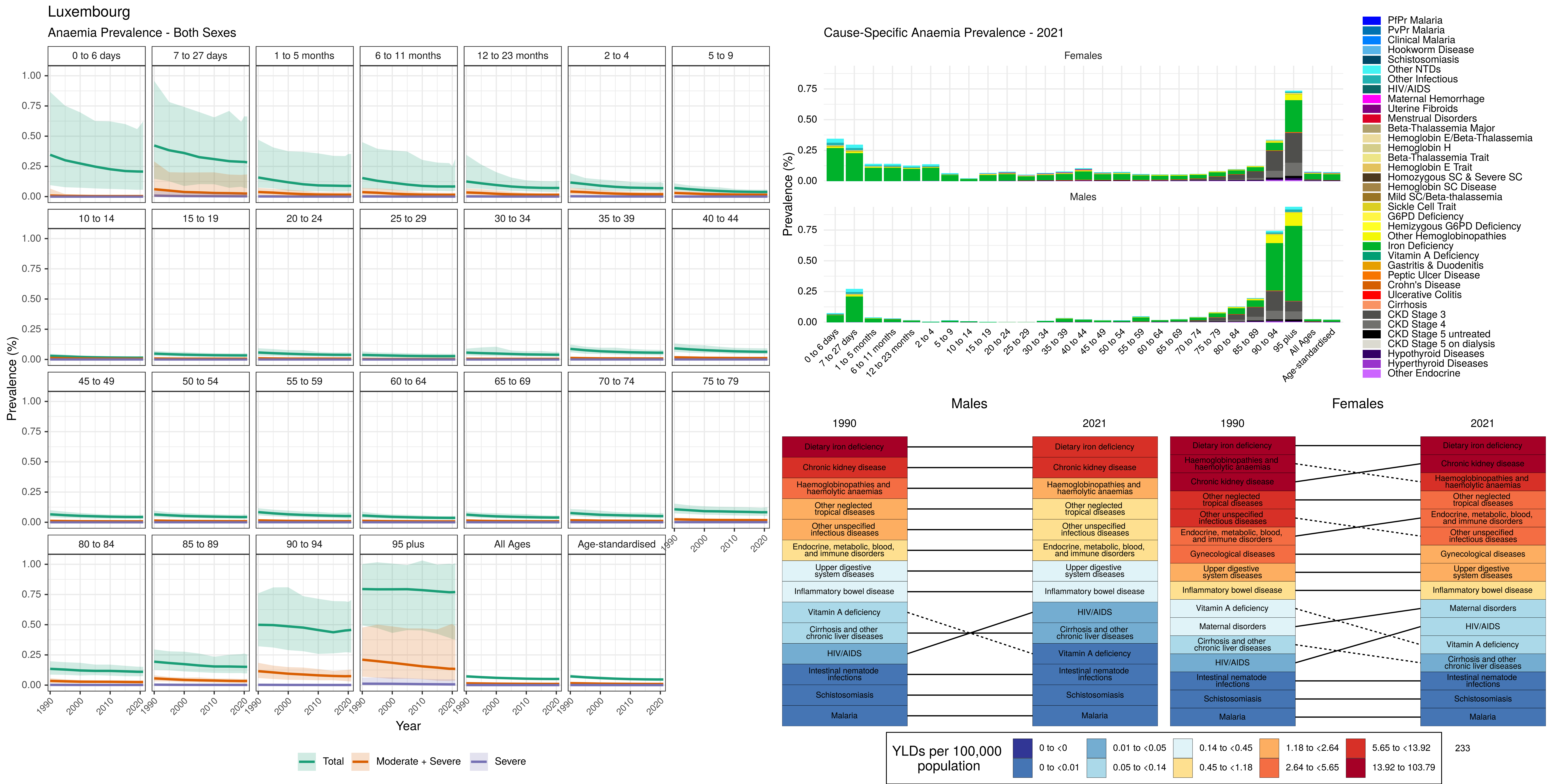
Luxembourg
Anaemia Prevalence - Both Sexes
0 to 6 days
7 to 27 days
1 to 5 months
6 to 11 months
12 to 23 months
2 to 4
5 to 9
10 to 14
15 to 19
20 to 24
25 to 29
30 to 34
35 to 39
40 to 44
45 to 49
50 to 54
55 to 59
60 to 64
65 to 69
70 to 74
75 to 79
80 to 84
85 to 89
90 to 94
95 plus
All Ages
Age-standardised
Prevalence (%)
Year
Total
Moderate + Severe
Severe
Cause-Specific Anaemia Prevalence - 2021
Females
Males
PfPr Malaria
PvPr Malaria
Clinical Malaria
Hookworm Disease
Schistosomiasis
Other NTDs
Other Infectious
HIV/AIDS
Maternal Hemorrhage
Uterine Fibroids
Menstrual Disorders
Beta-Thalassemia Major
Hemoglobin E/Beta-Thalassemia
Hemoglobin H
Beta-Thalassemia Trait
Hemoglobin E Trait
Homozygous SC & Severe SC
Hemoglobin SC Disease
Mild SC/Beta-thalassemia
Sickle Cell Trait
G6PD Deficiency
Hemizygous G6PD Deficiency
Other Hemoglobinopathies
Iron Deficiency
Vitamin A Deficiency
Gastritis & Duodenitis
Peptic Ulcer Disease
Crohn's Disease
Ulcerative Colitis
Cirrhosis
CKD Stage 3
CKD Stage 4
CKD Stage 5 untreated
CKD Stage 5 on dialysis
Hypothyroid Diseases
Hyperthyroid Diseases
Other Endocrine
Males
1990
2021
Dietary iron deficiency
Chronic kidney disease
Haemoglobinopathies and haemolytic anaemias
Other neglected tropical diseases
Other unspecified infectious diseases
Endocrine, metabolic, blood, and immune disorders
Upper digestive system diseases
Inflammatory bowel disease
Vitamin A deficiency
Cirrhosis and other chronic liver diseases
HIV/AIDS
Intestinal nematode infections
Schistosomiasis
Malaria
Females
1990
2021
Dietary iron deficiency
Haemoglobinopathies and haemolytic anaemias
Chronic kidney disease
Other neglected tropical diseases
Other unspecified infectious diseases
Endocrine, metabolic, blood, and immune disorders
Gynecological diseases
Upper digestive system diseases
Inflammatory bowel disease
Vitamin A deficiency
Maternal disorders
Cirrhosis and other chronic liver diseases
HIV/AIDS
Intestinal nematode infections
Schistosomiasis
Malaria
YLDs per 100,000 population
0 to <0
0 to <0.01
0.01 to <0.05
0.05 to <0.14
0.14 to <0.45
0.45 to <1.18
1.18 to <2.64
2.64 to <5.65
5.65 to <13.92
13.92 to 103.79

Malta

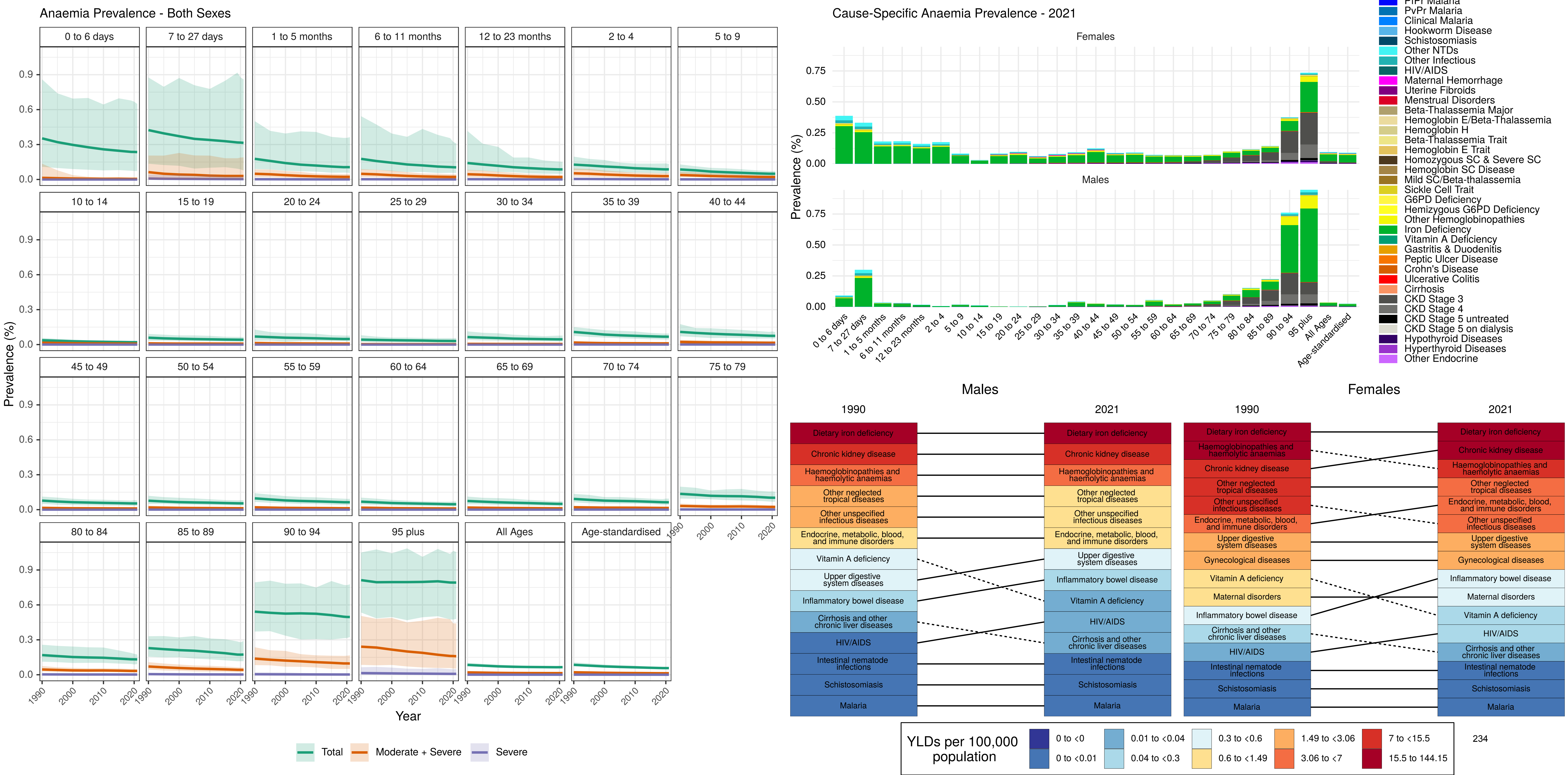

Anaemia Prevalence - Both Sexes
0 to 6 days
7 to 27 days
1 to 5 months
6 to 11 months
12 to 23 months
2 to 4
5 to 9
10 to 14
15 to 19
20 to 24
25 to 29
30 to 34
35 to 39
40 to 44
45 to 49
50 to 54
55 to 59
60 to 64
65 to 69
70 to 74
75 to 79
80 to 84
85 to 89
90 to 94
95 plus
All Ages
Age-standardised
Prevalence (%)
Year
Total
Moderate + Severe
Severe
Cause-Specific Anaemia Prevalence - 2021
Females
Males
PfPr Malaria
PvPr Malaria
Clinical Malaria
Hookworm Disease
Schistosomiasis
Other NTDs
Other Infectious
HIV/AIDS
Maternal Hemorrhage
Uterine Fibroids
Menstrual Disorders
Beta-Thalassemia Major
Hemoglobin E/Beta-Thalassemia
Hemoglobin H
Beta-Thalassemia Trait
Hemoglobin E Trait
Homozygous SC & Severe SC
Hemoglobin SC Disease
Mild SC/Beta-thalassemia
Sickle Cell Trait
G6PD Deficiency
Hemizygous G6PD Deficiency
Other Hemoglobinopathies
Iron Deficiency
Vitamin A Deficiency
Gastritis & Duodenitis
Peptic Ulcer Disease
Crohn's Disease
Ulcerative Colitis
Cirrhosis
CKD Stage 3
CKD Stage 4
CKD Stage 5 untreated
CKD Stage 5 on dialysis
Hypothyroid Diseases
Hyperthyroid Diseases
Other Endocrine
Males
Females
1990
2021
Dietary iron deficiency
Chronic kidney disease
Haemoglobinopathies and haemolytic anaemias
Other neglected tropical diseases
Other unspecified infectious diseases
Endocrine, metabolic, blood, and immune disorders
Vitamin A deficiency
Upper digestive system diseases
Inflammatory bowel disease
Cirrhosis and other chronic liver diseases
HIV/AIDS
Intestinal nematode infections
Schistosomiasis
Malaria
Gynecological diseases
Maternal disorders
YLDs per 100,000 population
0 to <0
0 to <0.01
0.01 to <0.04
0.04 to <0.3
0.3 to <0.6
0.6 to <1.49
1.49 to <3.06
3.06 to <7
7 to <15.5
15.5 to 144.15

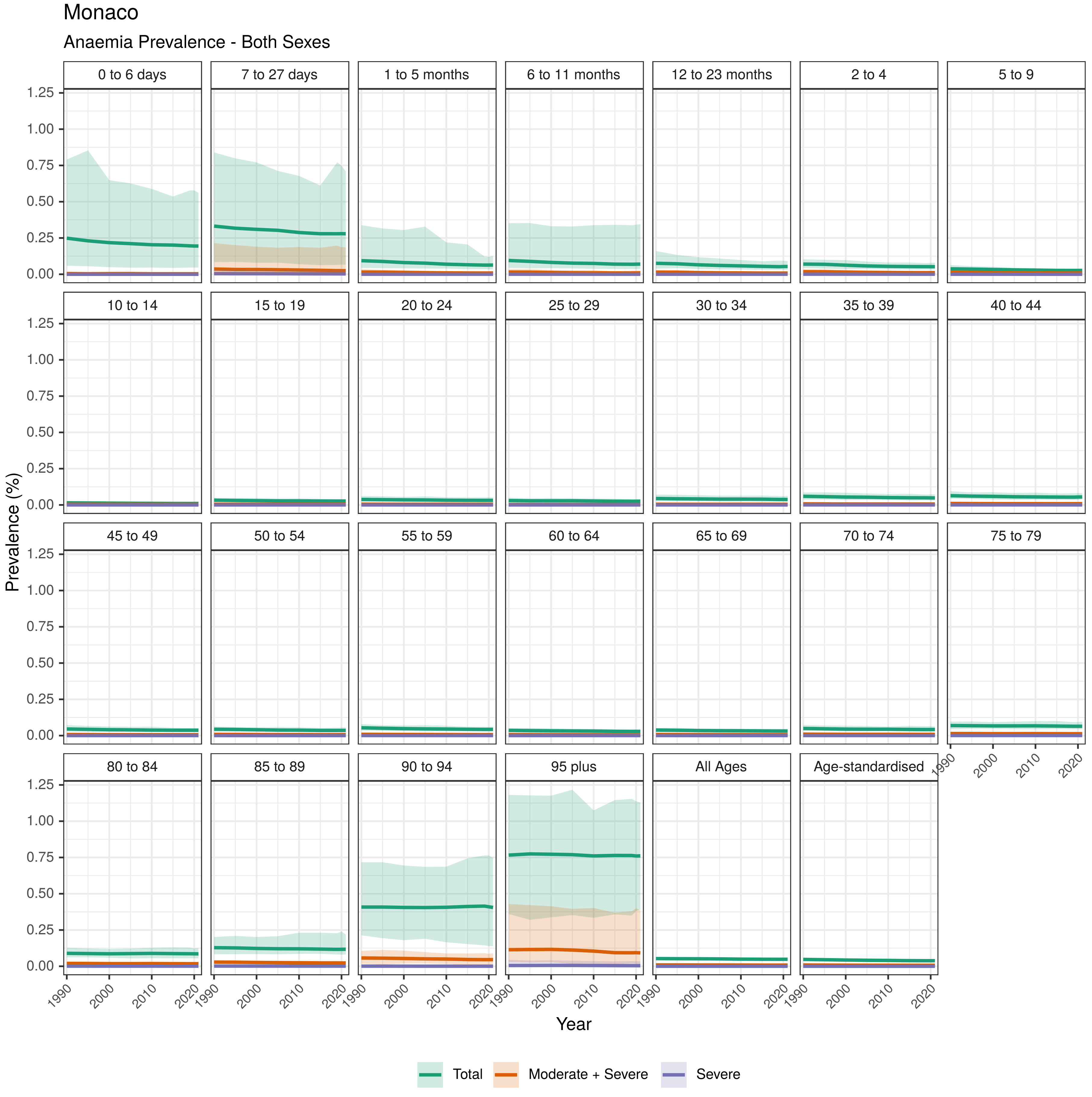
Monaco
Anaemia Prevalence - Both Sexes
0 to 6 days
7 to 27 days
1 to 5 months
6 to 11 months
12 to 23 months
2 to 4
5 to 9
10 to 14
15 to 19
20 to 24
25 to 29
30 to 34
35 to 39
40 to 44
45 to 49
50 to 54
55 to 59
60 to 64
65 to 69
70 to 74
75 to 79
80 to 84
85 to 89
90 to 94
95 plus
All Ages
Age-standardised
Prevalence (%)
0.00
0.25
0.50
0.75
1.00
1.25
1990
2000
2010
2020
Year
Total
Moderate + Severe
Severe

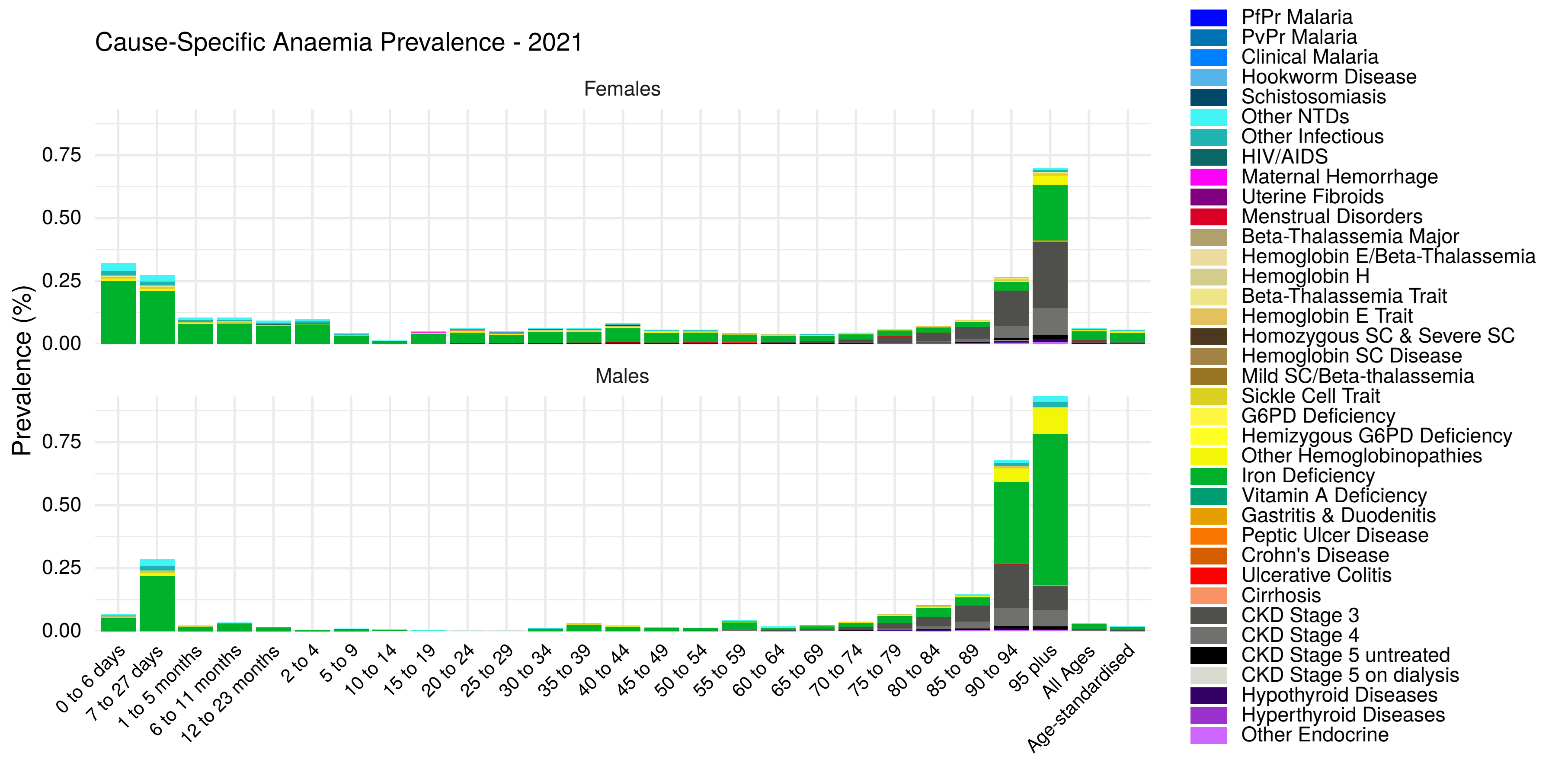
Cause-Specific Anaemia Prevalence - 2021
Females
Males
Prevalence (%)
0.00
0.25
0.50
0.75
0 to 6 days
7 to 27 days
1 to 5 months
6 to 11 months
12 to 23 months
2 to 4
5 to 9
10 to 14
15 to 19
20 to 24
25 to 29
30 to 34
35 to 39
40 to 44
45 to 49
50 to 54
55 to 59
60 to 64
65 to 69
70 to 74
75 to 79
80 to 84
85 to 89
90 to 94
95 plus
All Ages
Age-standardised
PfPr Malaria
PvPr Malaria
Clinical Malaria
Hookworm Disease
Schistosomiasis
Other NTDs
Other Infectious
HIV/AIDS
Maternal Hemorrhage
Uterine Fibroids
Menstrual Disorders
Beta-Thalassemia Major
Hemoglobin E/Beta-Thalassemia
Hemoglobin H
Beta-Thalassemia Trait
Hemoglobin E Trait
Homozygous SC & Severe SC
Hemoglobin SC Disease
Mild SC/Beta-thalassemia
Sickle Cell Trait
G6PD Deficiency
Hemizygous G6PD Deficiency
Other Hemoglobinopathies
Iron Deficiency
Vitamin A Deficiency
Gastritis & Duodenitis
Peptic Ulcer Disease
Crohn's Disease
Ulcerative Colitis
Cirrhosis
CKD Stage 3
CKD Stage 4
CKD Stage 5 untreated
CKD Stage 5 on dialysis
Hypothyroid Diseases
Hyperthyroid Diseases
Other Endocrine

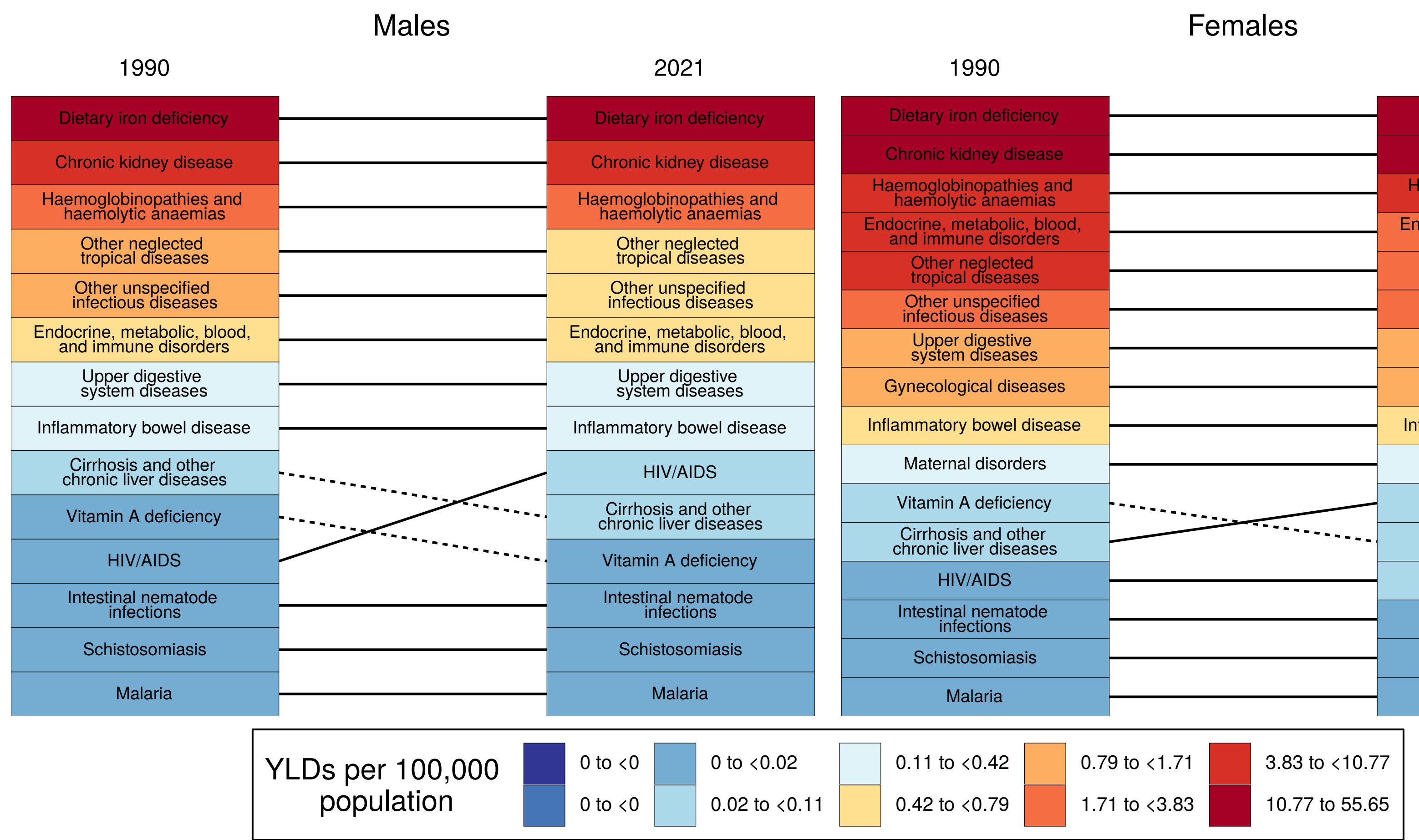
Males
Females
1990
2021
Dietary iron deficiency
Chronic kidney disease
Haemoglobinopathies and haemolytic anaemias
Other neglected tropical diseases
Other unspecified infectious diseases
Endocrine, metabolic, blood, and immune disorders
Upper digestive system diseases
Inflammatory bowel disease
Cirrhosis and other chronic liver diseases
Vitamin A deficiency
HIV/AIDS
Intestinal nematode infections
Schistosomiasis
Malaria
Gynecological diseases
Maternal disorders
YLDs per 100,000 population
0 to <0
0 to <0
0 to <0.02
0.02 to <0.11
0.11 to <0.42
0.42 to <0.79
0.79 to <1.71
1.71 to <3.83
3.83 to <10.77
10.77 to 55.65

Netherlands

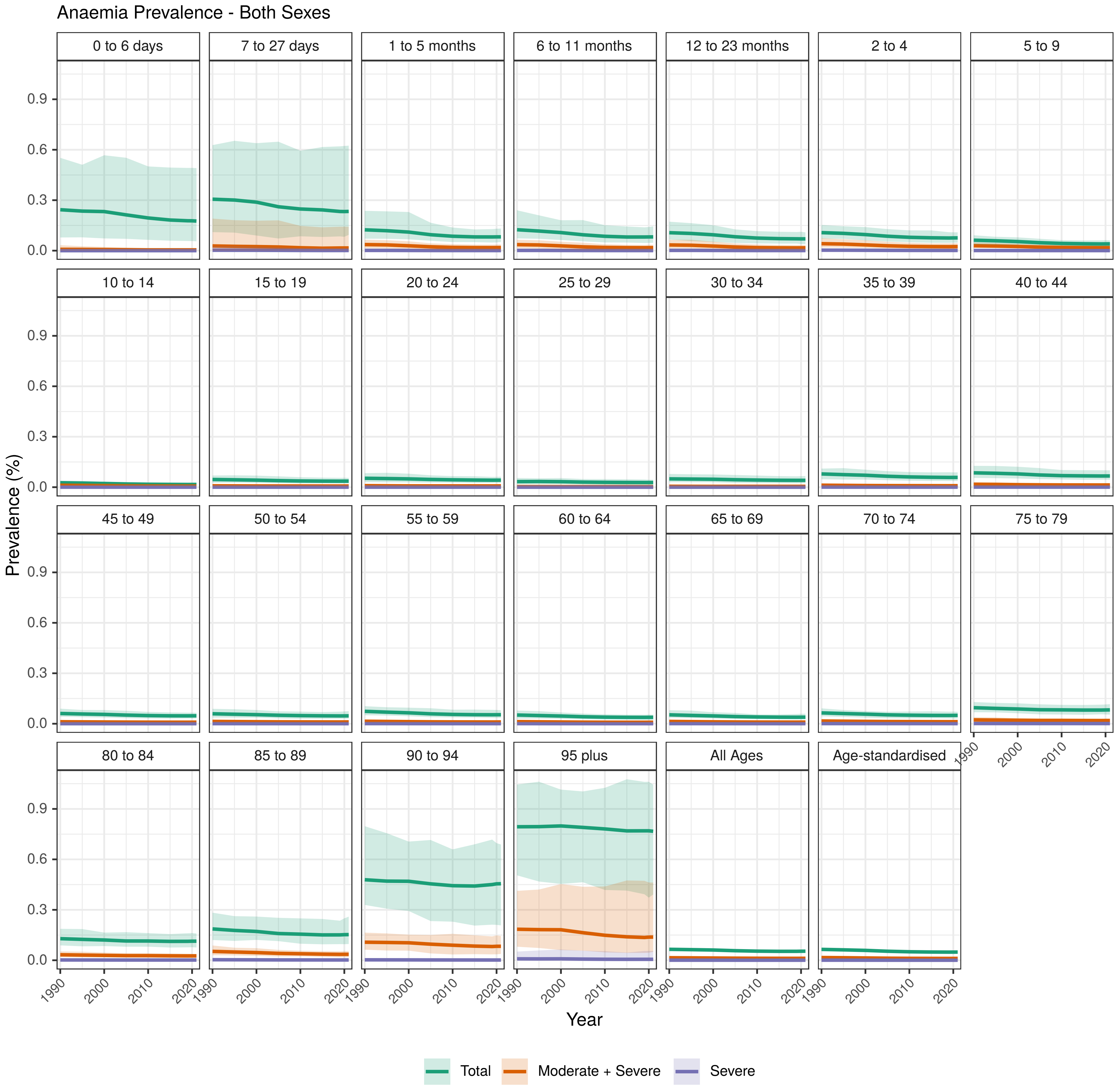

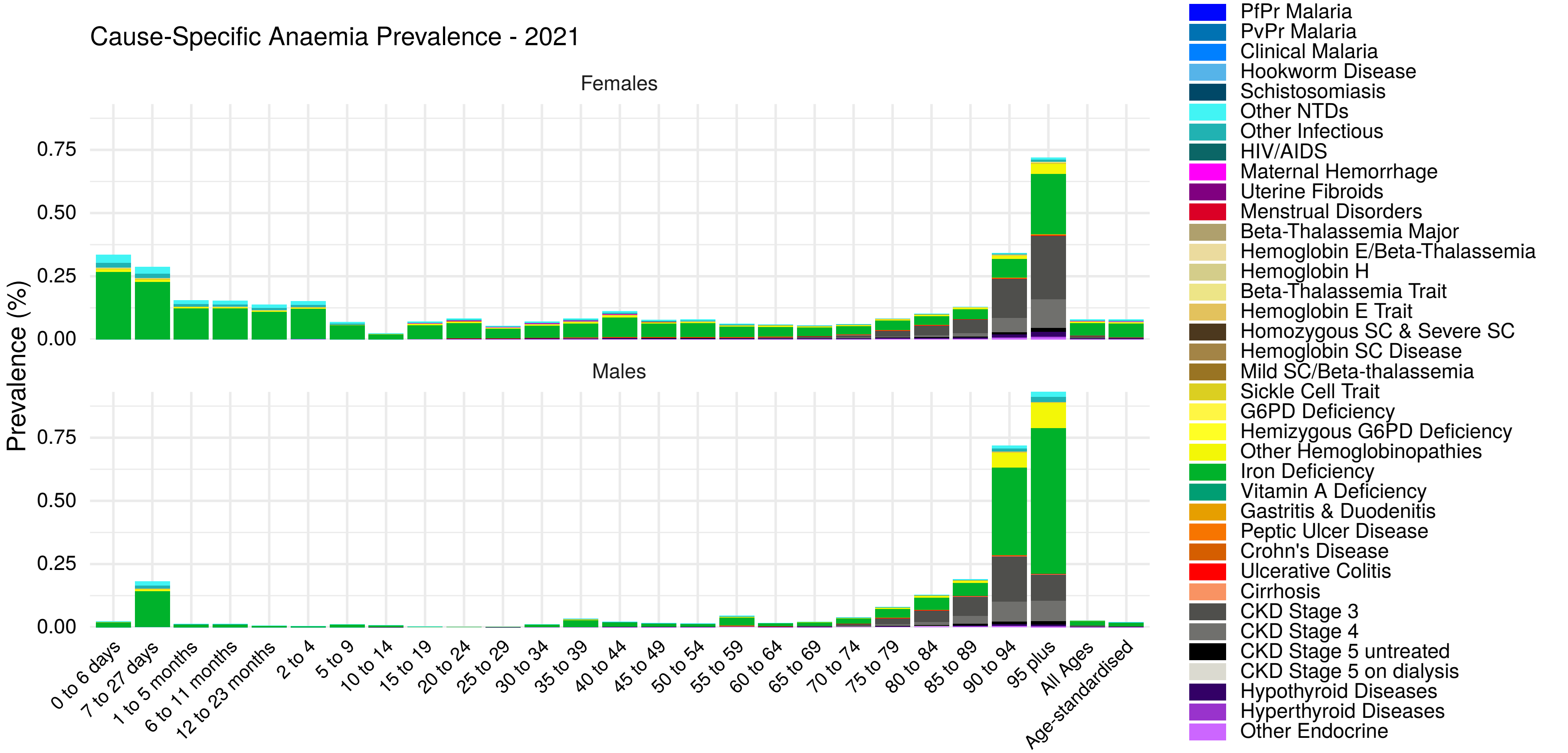

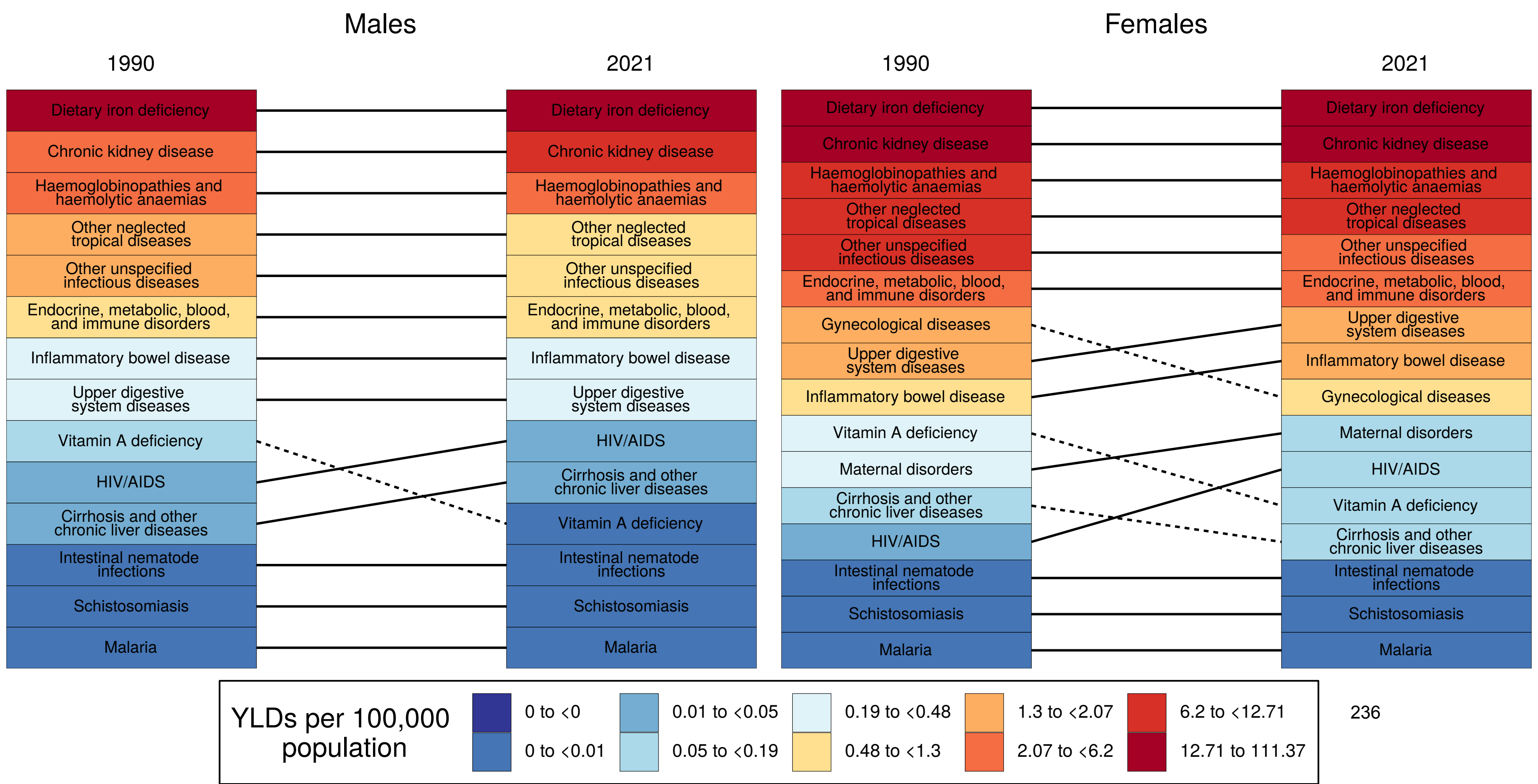

Norway

Anaemia Prevalence - Both Sexes

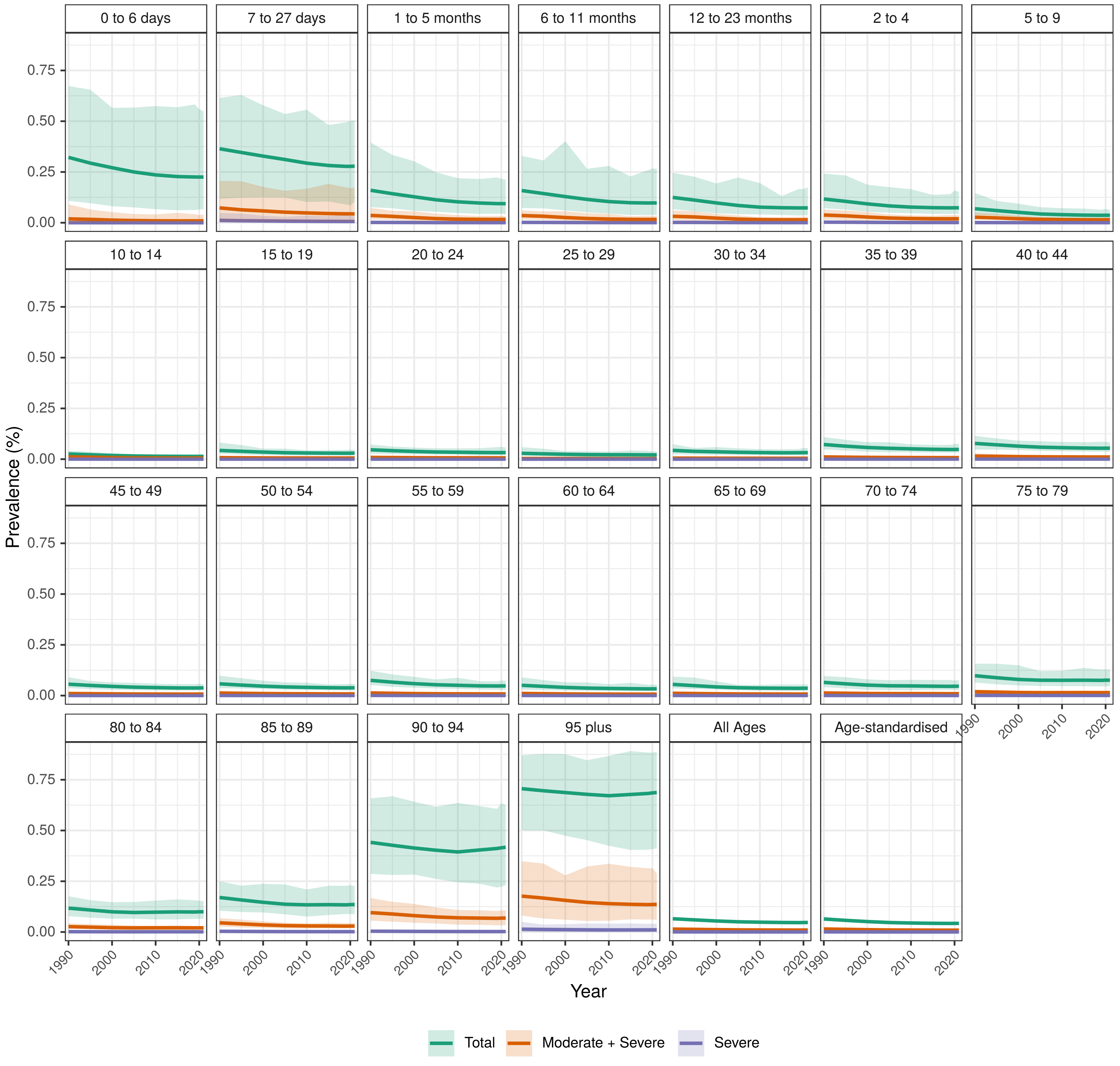

Cause-Specific Anaemia Prevalence - 2021

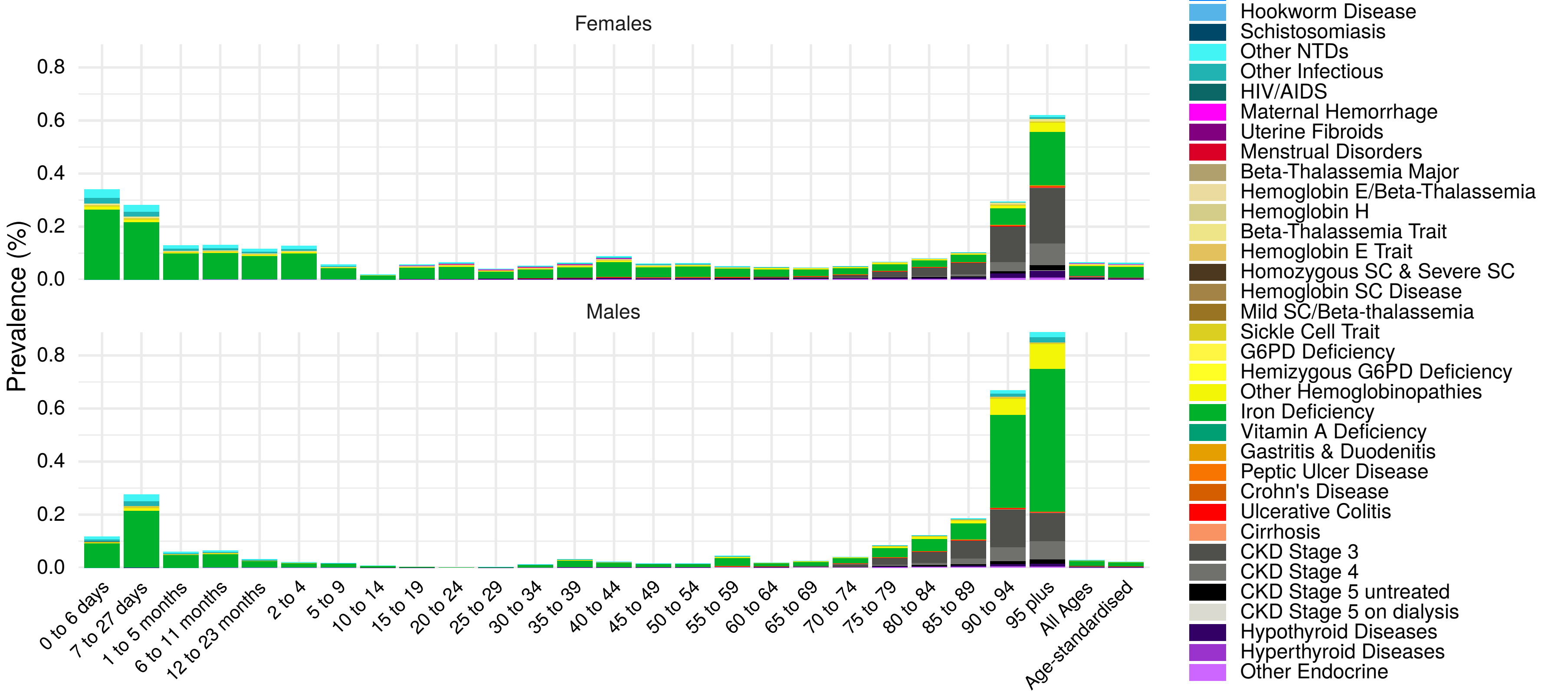

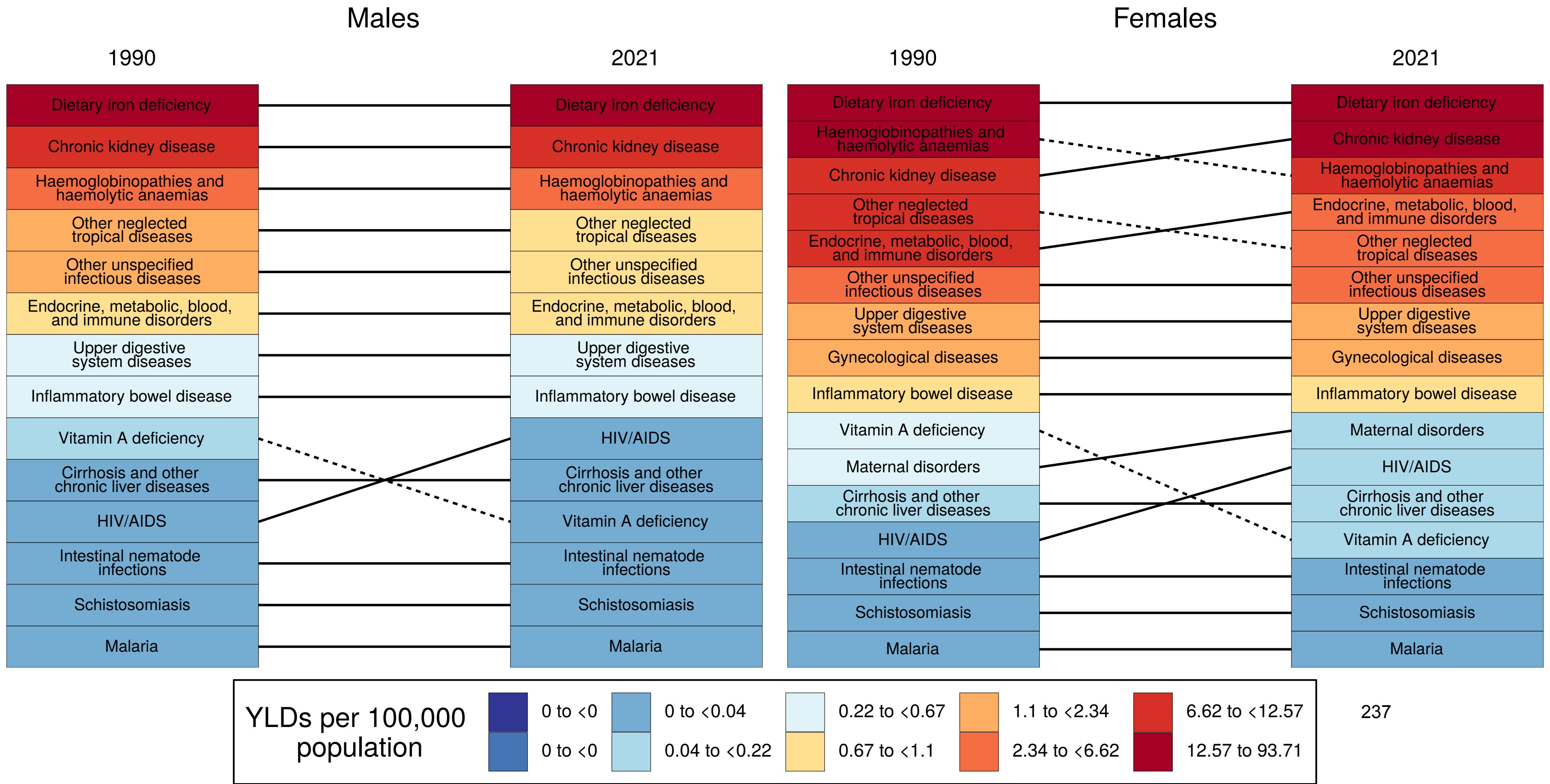

Agder

Anaemia Prevalence - Both Sexes

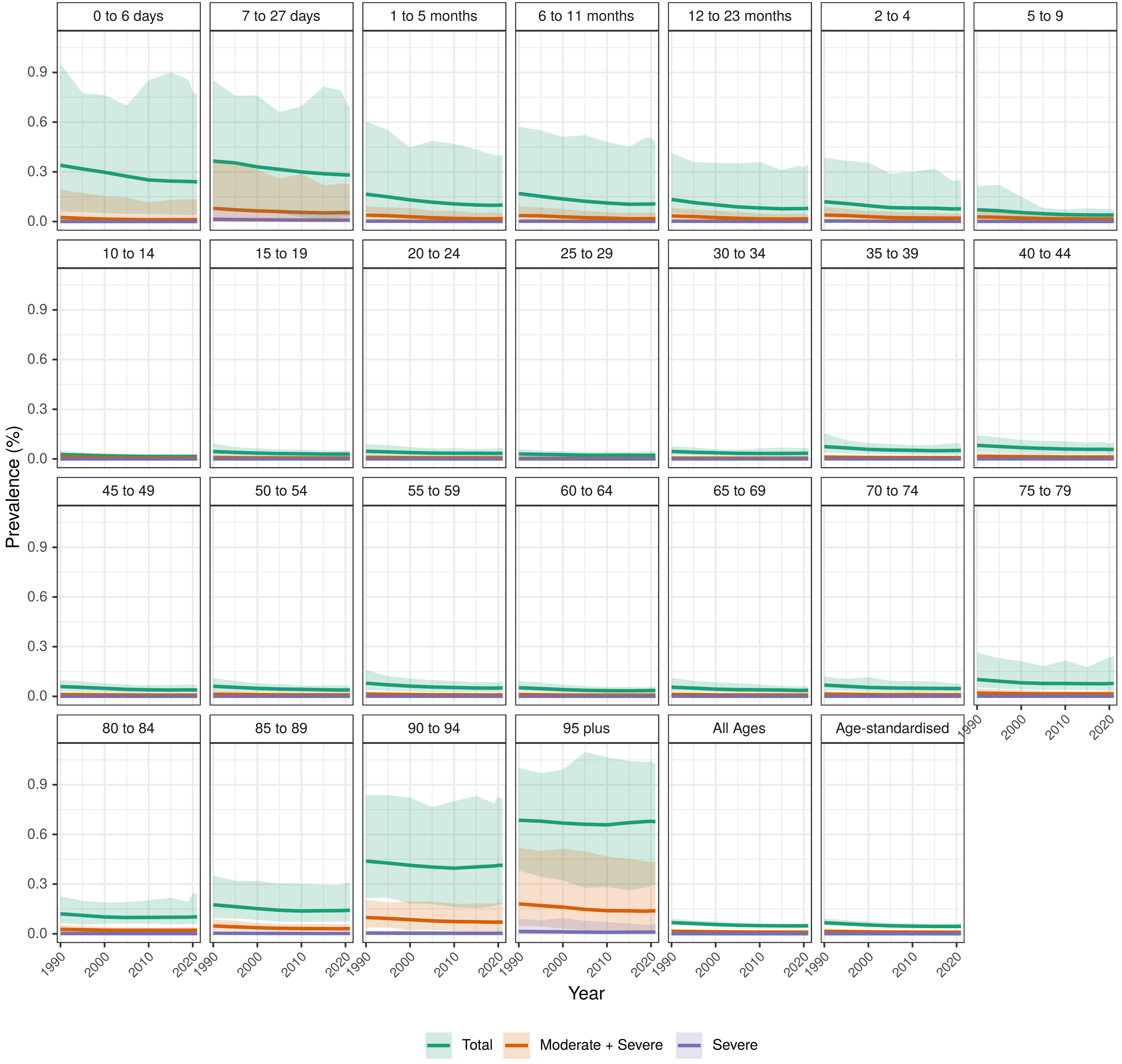

Cause-Specific Anaemia Prevalence - 2021

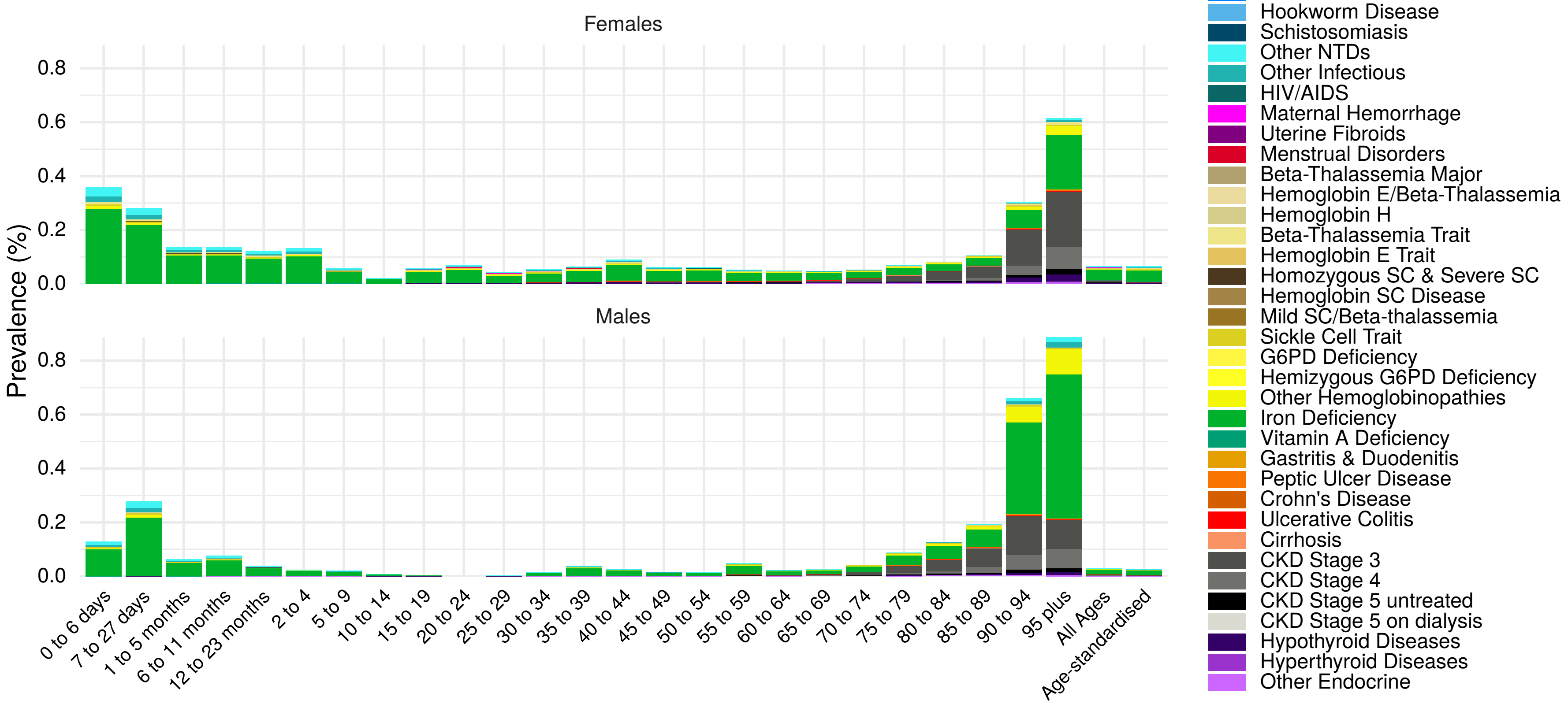

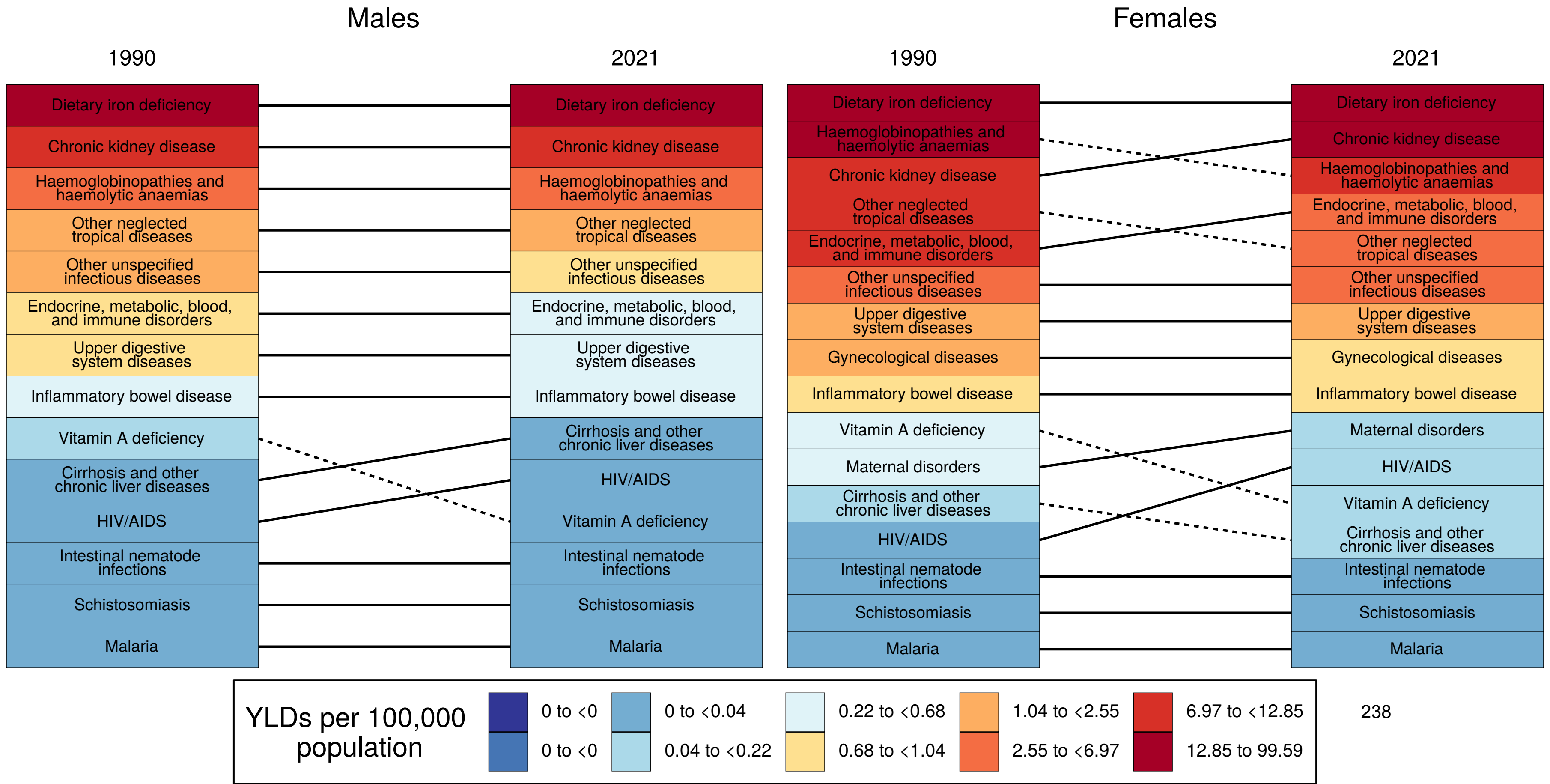

Innlandet

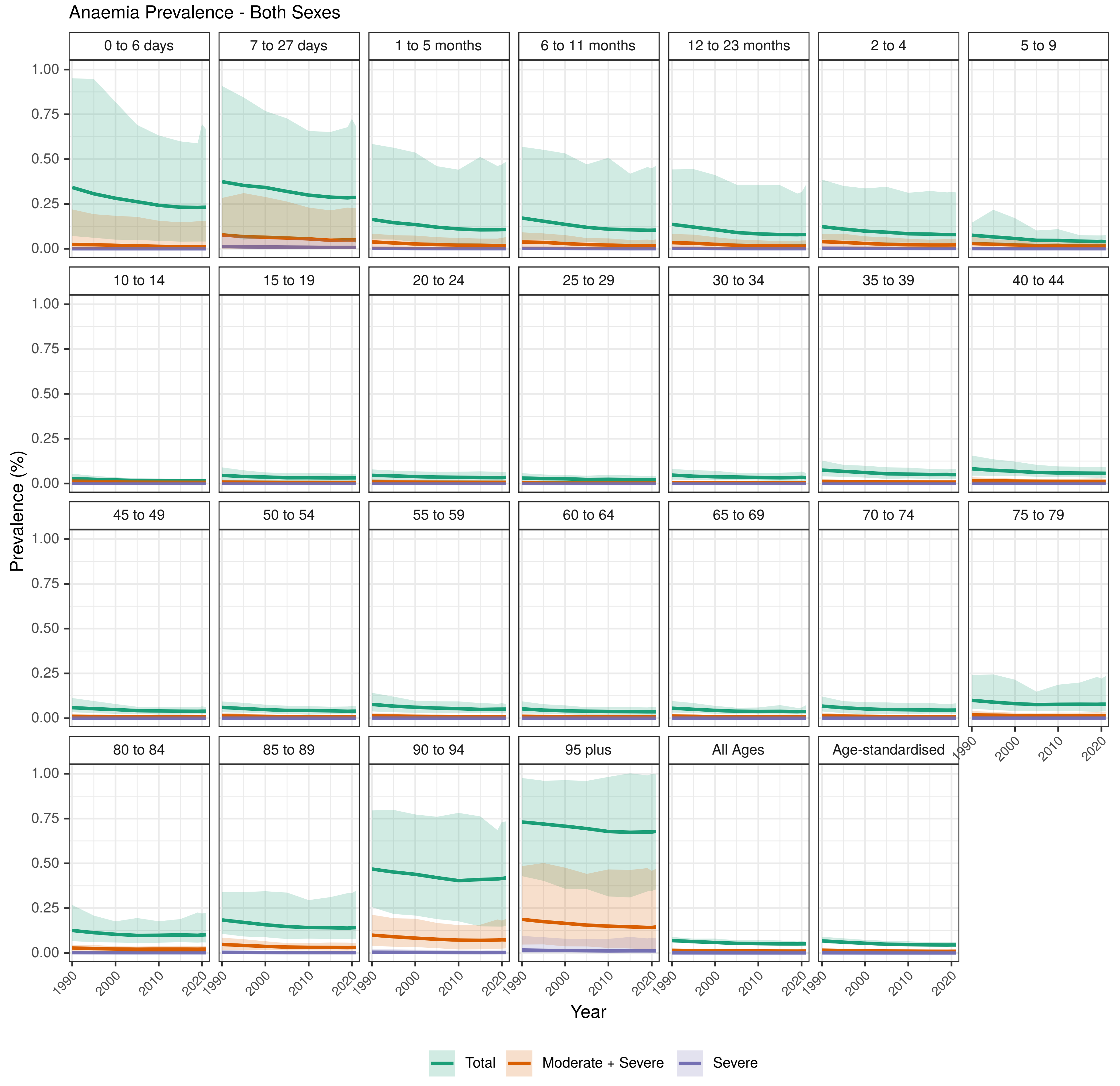
Anaemia Prevalence - Both Sexes
0 to 6 days
7 to 27 days
1 to 5 months
6 to 11 months
12 to 23 months
2 to 4
5 to 9
10 to 14
15 to 19
20 to 24
25 to 29
30 to 34
35 to 39
40 to 44
45 to 49
50 to 54
55 to 59
60 to 64
65 to 69
70 to 74
75 to 79
80 to 84
85 to 89
90 to 94
95 plus
All Ages
Age-standardised
Prevalence (%)
0.00
0.25
0.50
0.75
1.00
1990
2000
2010
2020
Year
Total
Moderate + Severe
Severe

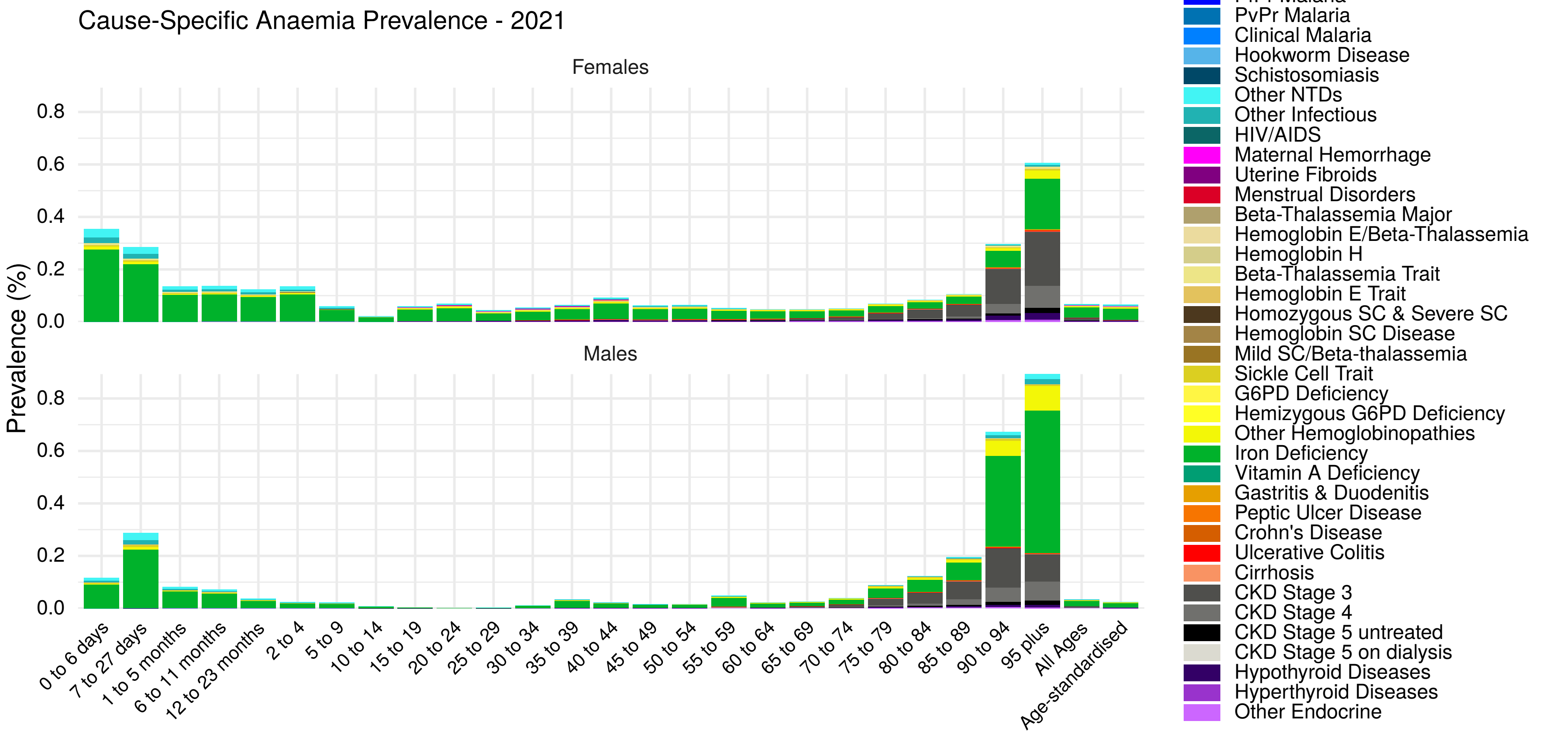
Cause-Specific Anaemia Prevalence - 2021
Females
Males
Prevalence (%)
0.0
0.2
0.4
0.6
0.8
0 to 6 days
7 to 27 days
1 to 5 months
6 to 11 months
12 to 23 months
2 to 4
5 to 9
10 to 14
15 to 19
20 to 24
25 to 29
30 to 34
35 to 39
40 to 44
45 to 49
50 to 54
55 to 59
60 to 64
65 to 69
70 to 74
75 to 79
80 to 84
85 to 89
90 to 94
95 plus
All Ages
Age-standardised
PfPr Malaria
PvPr Malaria
Clinical Malaria
Hookworm Disease
Schistosomiasis
Other NTDs
Other Infectious
HIV/AIDS
Maternal Hemorrhage
Uterine Fibroids
Menstrual Disorders
Beta-Thalassemia Major
Hemoglobin E/Beta-Thalassemia
Hemoglobin H
Beta-Thalassemia Trait
Hemoglobin E Trait
Homozygous SC & Severe SC
Hemoglobin SC Disease
Mild SC/Beta-thalassemia
Sickle Cell Trait
G6PD Deficiency
Hemizygous G6PD Deficiency
Other Hemoglobinopathies
Iron Deficiency
Vitamin A Deficiency
Gastritis & Duodenitis
Peptic Ulcer Disease
Crohn's Disease
Ulcerative Colitis
Cirrhosis
CKD Stage 3
CKD Stage 4
CKD Stage 5 untreated
CKD Stage 5 on dialysis
Hypothyroid Diseases
Hyperthyroid Diseases
Other Endocrine

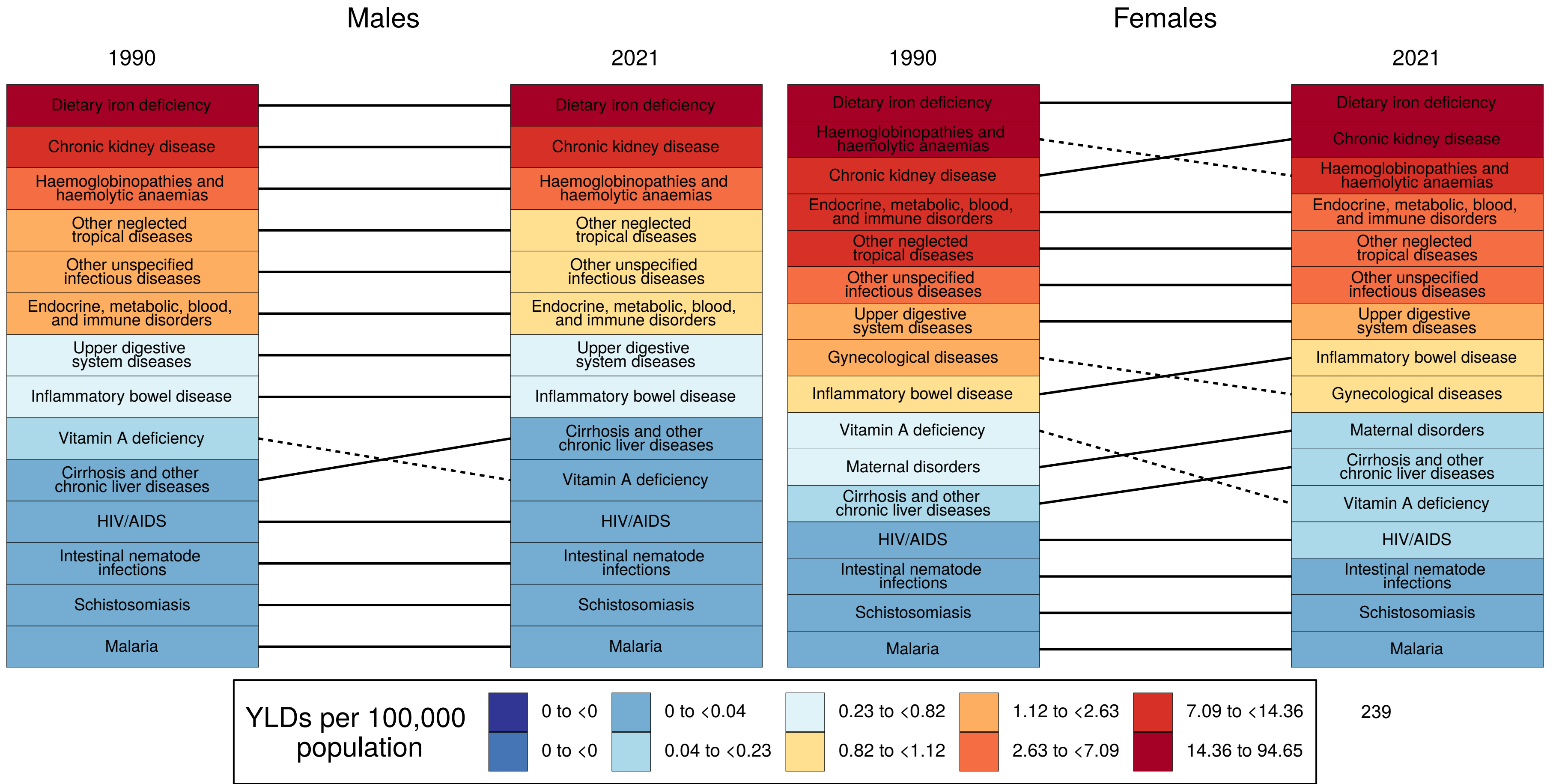
Males
Females
1990
2021
Dietary iron deficiency
Chronic kidney disease
Haemoglobinopathies and haemolytic anaemias
Other neglected tropical diseases
Other unspecified infectious diseases
Endocrine, metabolic, blood, and immune disorders
Upper digestive system diseases
Inflammatory bowel disease
Vitamin A deficiency
Cirrhosis and other chronic liver diseases
HIV/AIDS
Intestinal nematode infections
Schistosomiasis
Malaria
Gynecological diseases
Maternal disorders
YLDs per 100,000 population
0 to <0
0 to <0
0 to <0.04
0.04 to <0.23
0.23 to <0.82
0.82 to <1.12
1.12 to <2.63
2.63 to <7.09
7.09 to <14.36
14.36 to 94.65

Møre og Romsdal

Anaemia Prevalence - Both Sexes

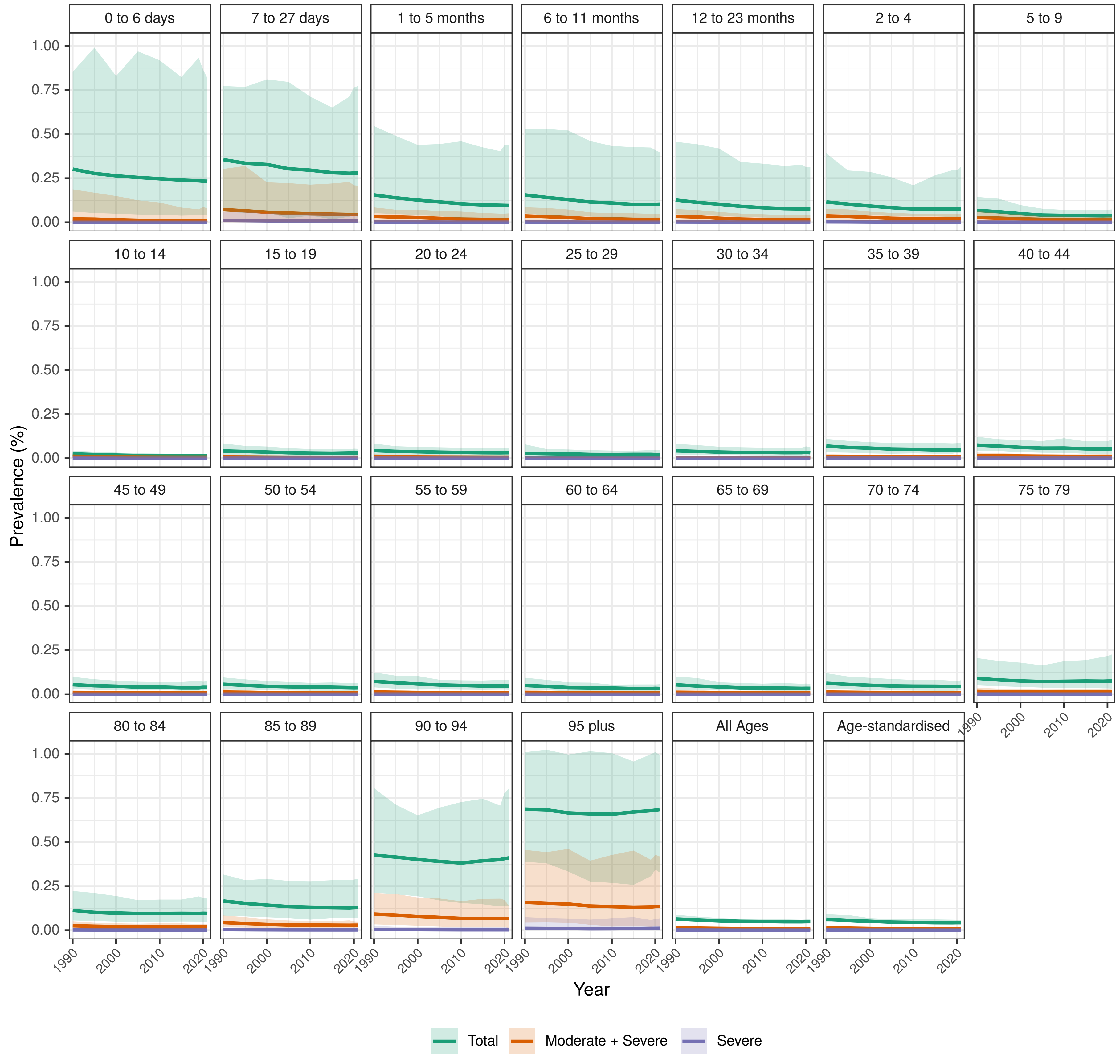

Cause-Specific Anaemia Prevalence - 2021

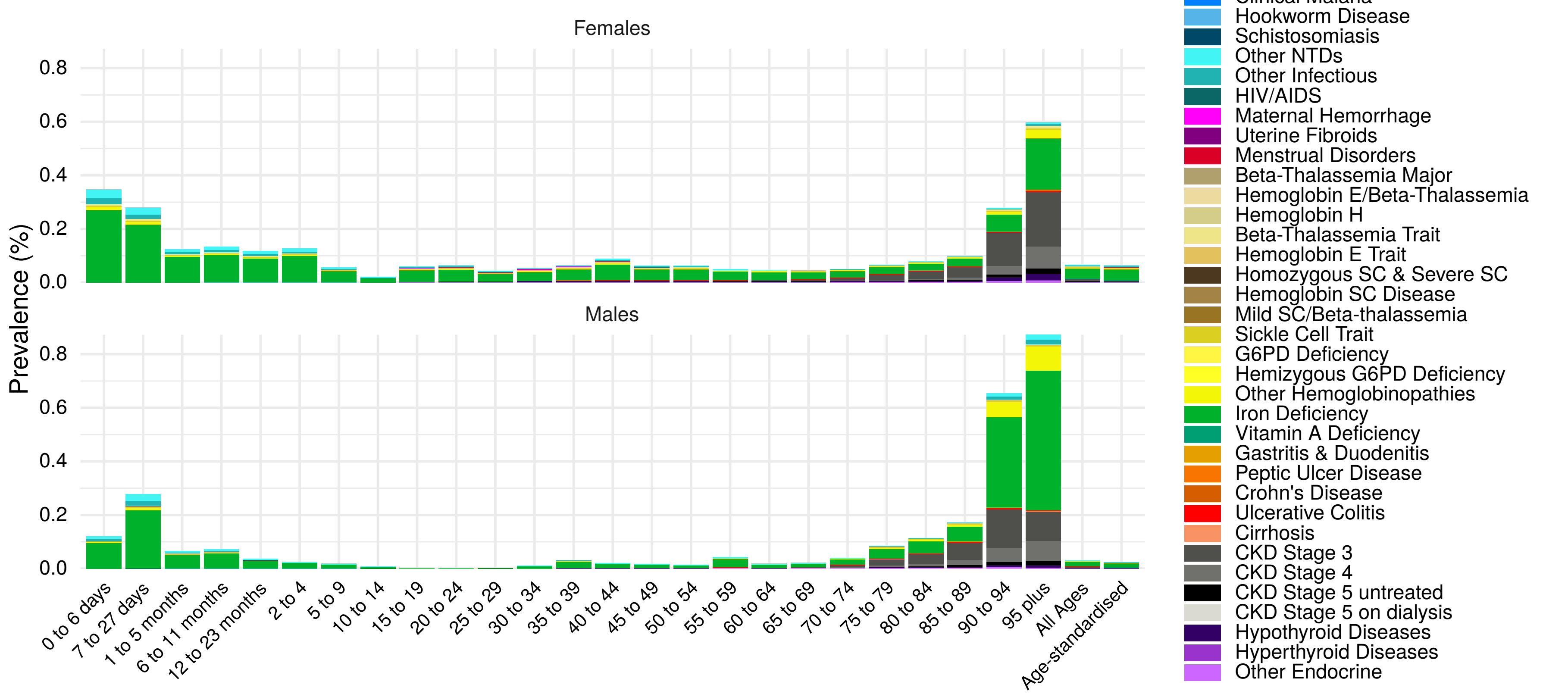

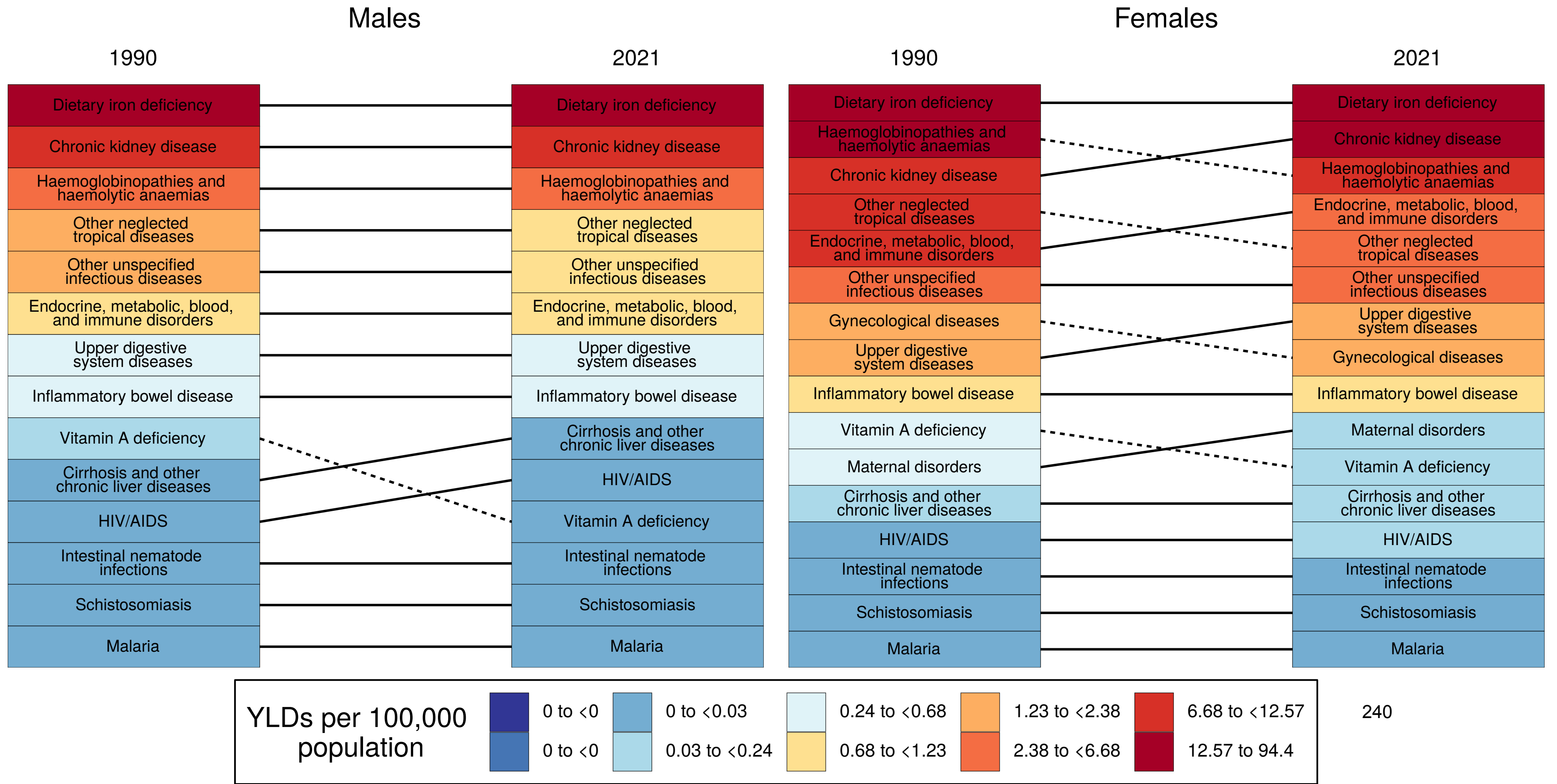

Nordland

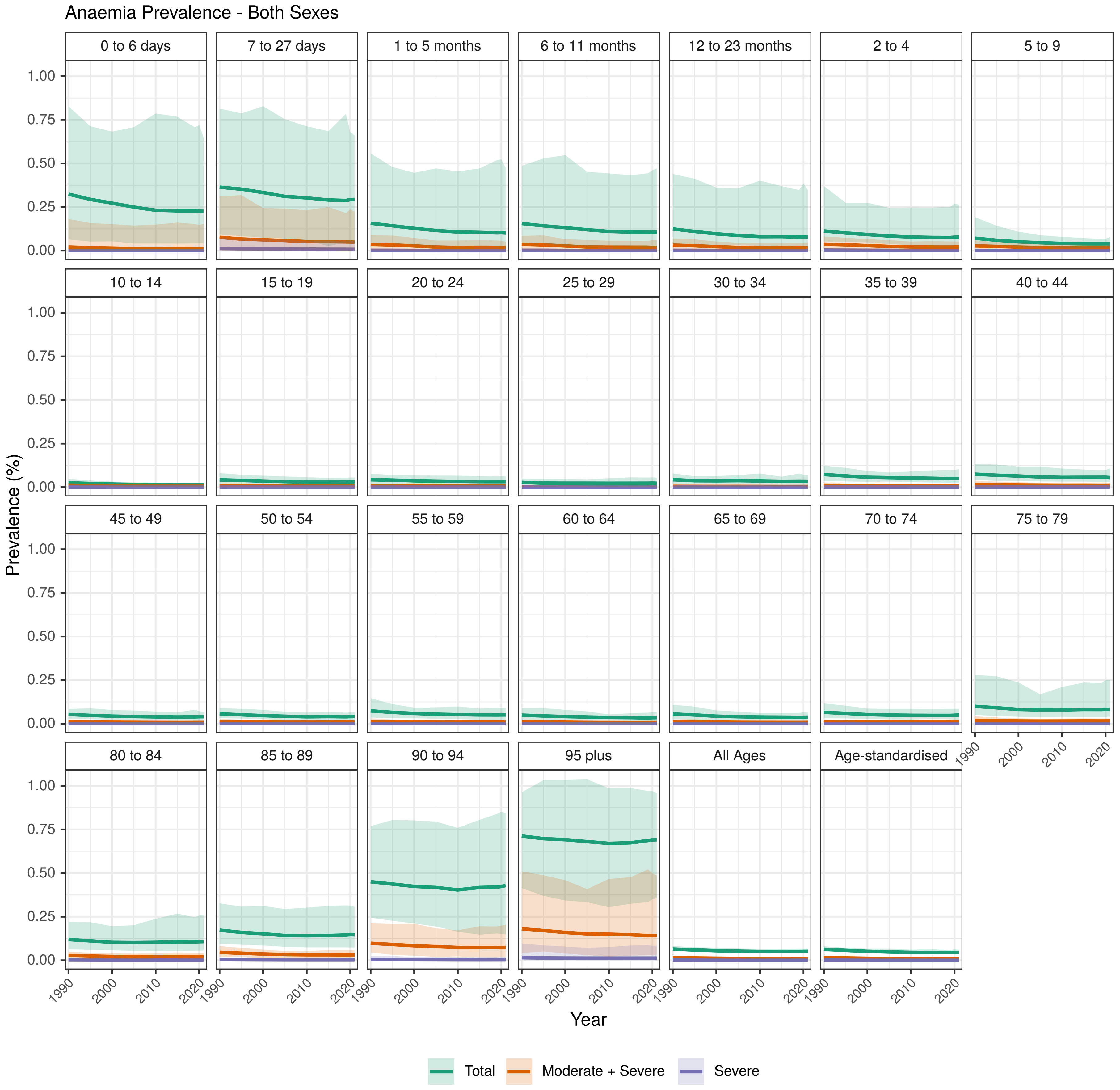

Anaemia Prevalence - Both Sexes
0 to 6 days
7 to 27 days
1 to 5 months
6 to 11 months
12 to 23 months
2 to 4
5 to 9
10 to 14
15 to 19
20 to 24
25 to 29
30 to 34
35 to 39
40 to 44
45 to 49
50 to 54
55 to 59
60 to 64
65 to 69
70 to 74
75 to 79
80 to 84
85 to 89
90 to 94
95 plus
All Ages
Age-standardised
Prevalence (%)
0.00
0.25
0.50
0.75
1.00
1990
2000
2010
2020
Year
Total
Moderate + Severe
Severe

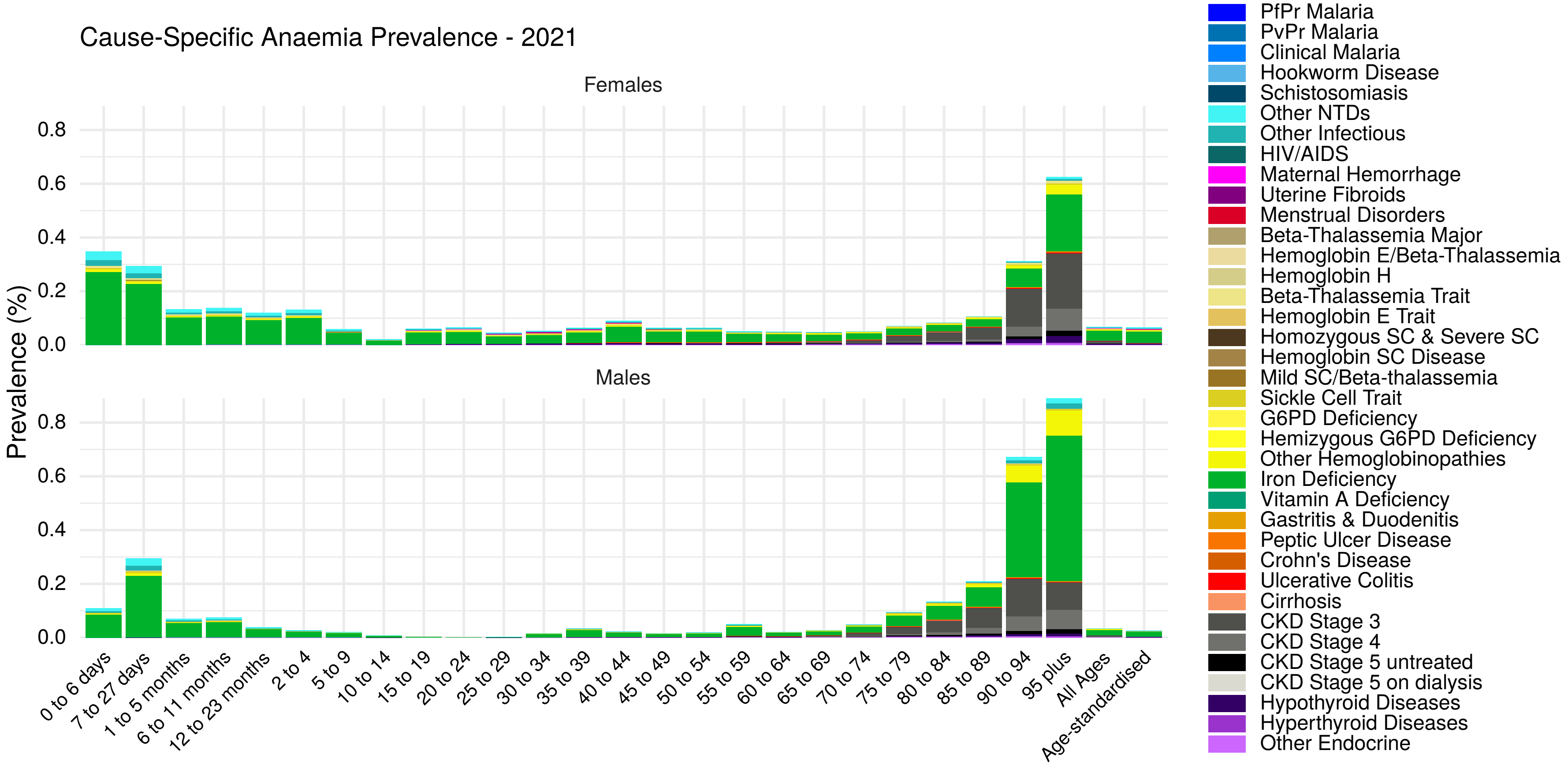

Cause-Specific Anaemia Prevalence - 2021
Females
Males
Prevalence (%)
0.0
0.2
0.4
0.6
0.8
0 to 6 days
7 to 27 days
1 to 5 months
6 to 11 months
12 to 23 months
2 to 4
5 to 9
10 to 14
15 to 19
20 to 24
25 to 29
30 to 34
35 to 39
40 to 44
45 to 49
50 to 54
55 to 59
60 to 64
65 to 69
70 to 74
75 to 79
80 to 84
85 to 89
90 to 94
95 plus
All Ages
Age-standardised
PfPr Malaria
PvPr Malaria
Clinical Malaria
Hookworm Disease
Schistosomiasis
Other NTDs
Other Infectious
HIV/AIDS
Maternal Hemorrhage
Uterine Fibroids
Menstrual Disorders
Beta-Thalassemia Major
Hemoglobin E/Beta-Thalassemia
Hemoglobin H
Beta-Thalassemia Trait
Hemoglobin E Trait
Homozygous SC & Severe SC
Hemoglobin SC Disease
Mild SC/Beta-thalassemia
Sickle Cell Trait
G6PD Deficiency
Hemizygous G6PD Deficiency
Other Hemoglobinopathies
Iron Deficiency
Vitamin A Deficiency
Gastritis & Duodenitis
Peptic Ulcer Disease
Crohn's Disease
Ulcerative Colitis
Cirrhosis
CKD Stage 3
CKD Stage 4
CKD Stage 5 untreated
CKD Stage 5 on dialysis
Hypothyroid Diseases
Hyperthyroid Diseases
Other Endocrine

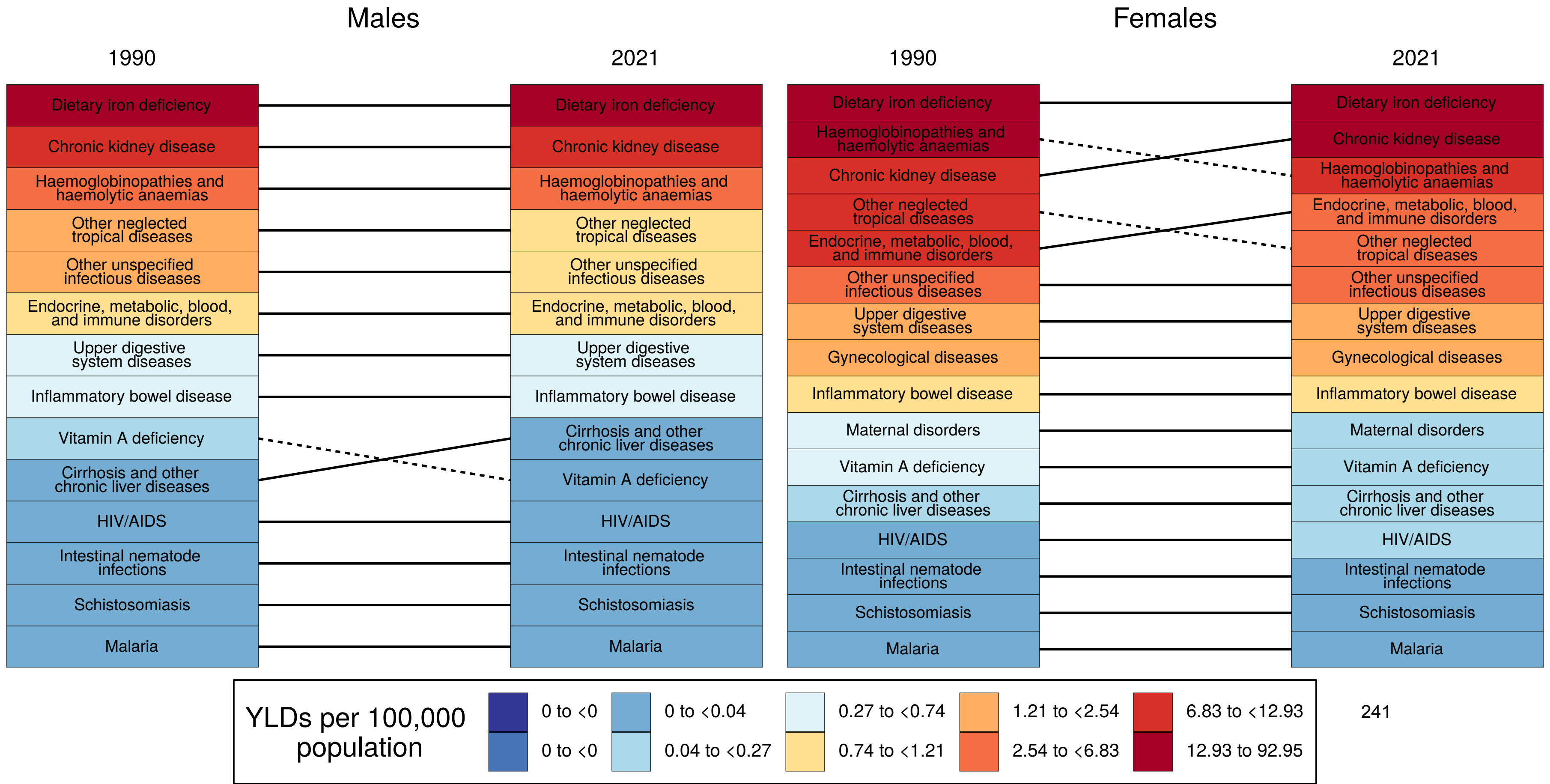

Males
Females
1990
2021
Dietary iron deficiency
Chronic kidney disease
Haemoglobinopathies and haemolytic anaemias
Other neglected tropical diseases
Other unspecified infectious diseases
Endocrine, metabolic, blood, and immune disorders
Upper digestive system diseases
Inflammatory bowel disease
Vitamin A deficiency
Cirrhosis and other chronic liver diseases
HIV/AIDS
Intestinal nematode infections
Schistosomiasis
Malaria
Gynecological diseases
Maternal disorders
YLDs per 100,000 population
0 to <0
0 to <0
0 to <0.04
0.04 to <0.27
0.27 to <0.74
0.74 to <1.21
1.21 to <2.54
2.54 to <6.83
6.83 to <12.93
12.93 to 92.95

Oslo

Anaemia Prevalence - Both Sexes

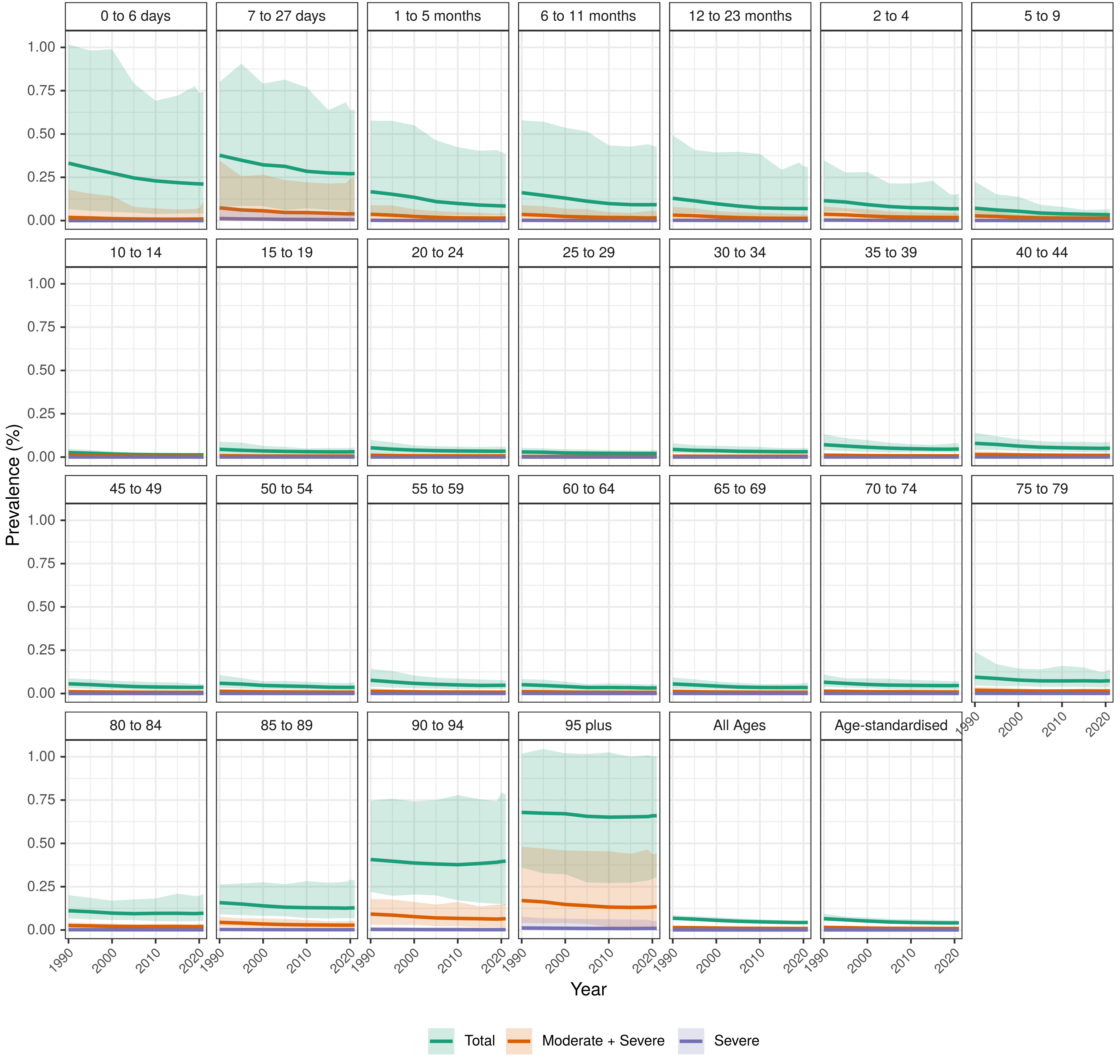

Cause-Specific Anaemia Prevalence - 2021

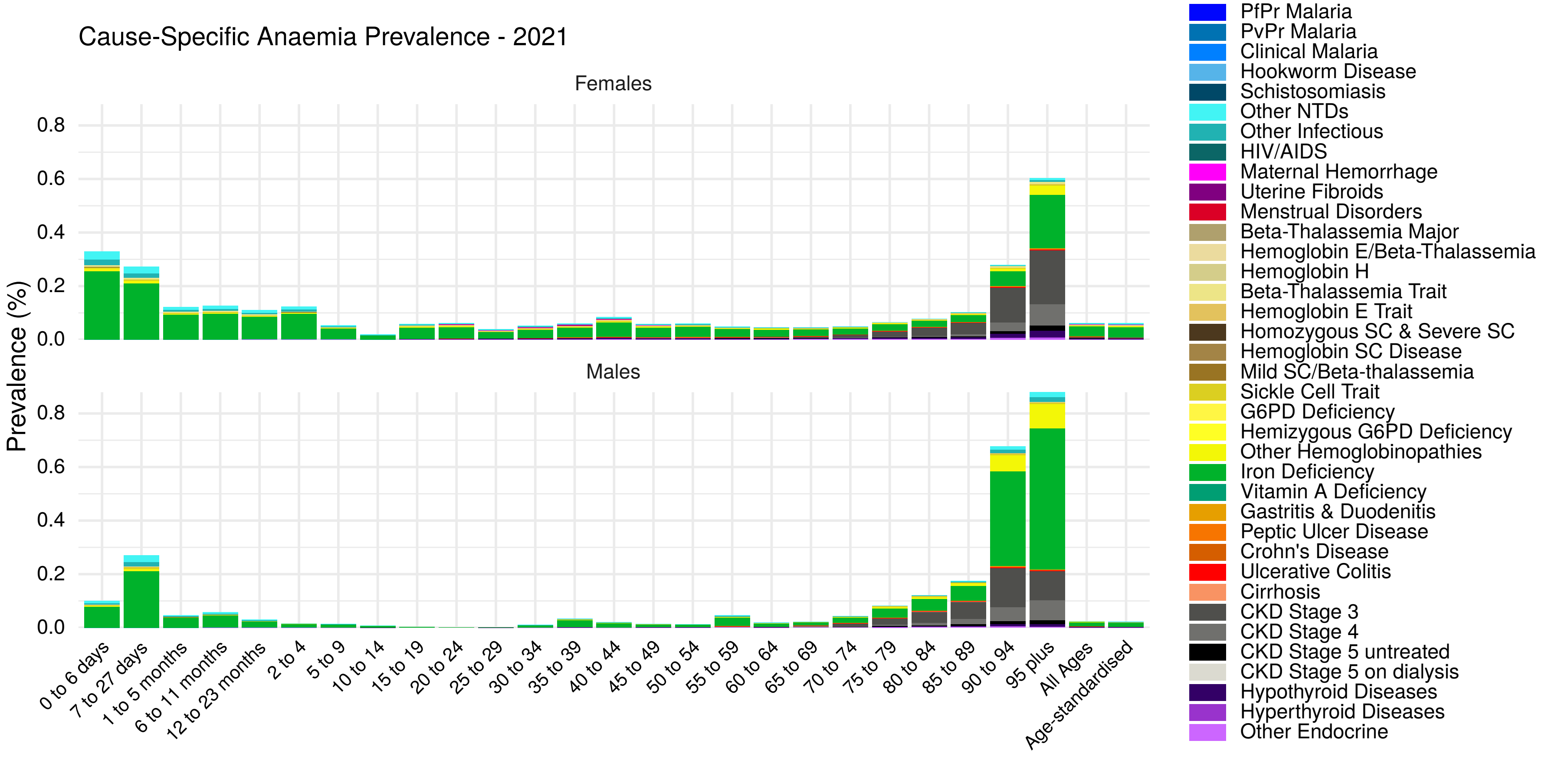

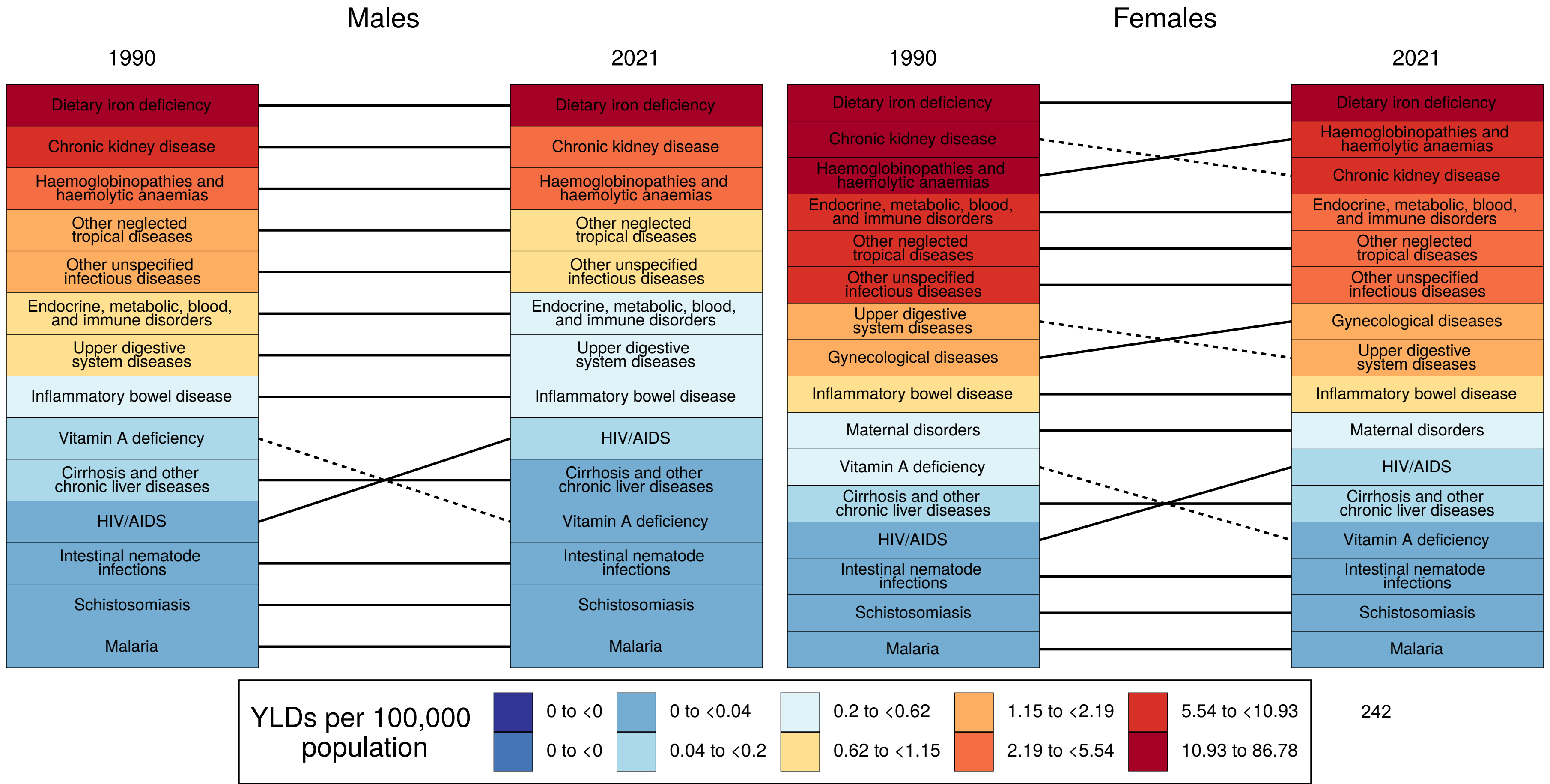

Rogaland

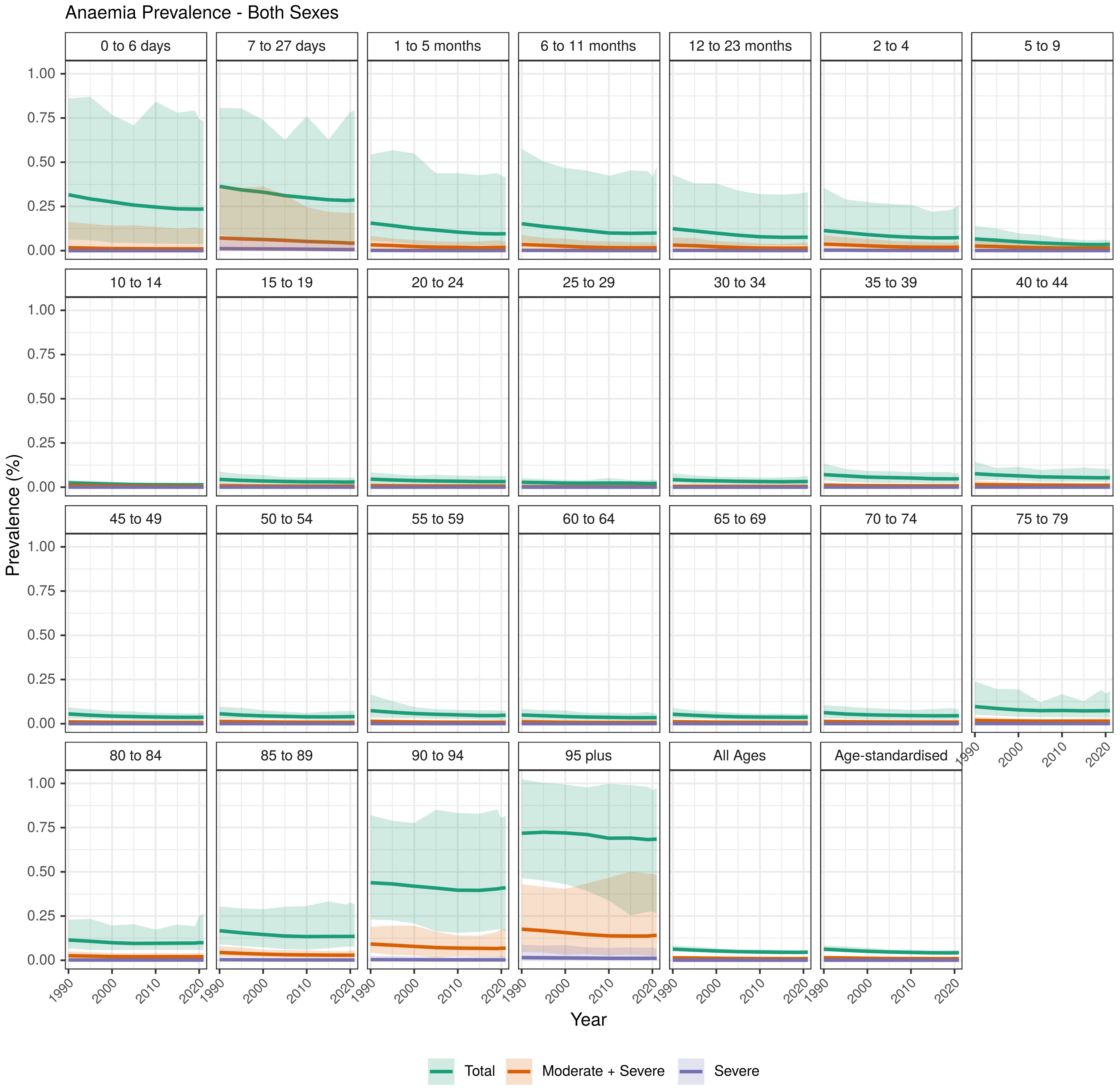

Anaemia Prevalence - Both Sexes
0 to 6 days
7 to 27 days
1 to 5 months
6 to 11 months
12 to 23 months
2 to 4
5 to 9
10 to 14
15 to 19
20 to 24
25 to 29
30 to 34
35 to 39
40 to 44
45 to 49
50 to 54
55 to 59
60 to 64
65 to 69
70 to 74
75 to 79
80 to 84
85 to 89
90 to 94
95 plus
All Ages
Age-standardised
Prevalence (%)
Year
Total
Moderate + Severe
Severe

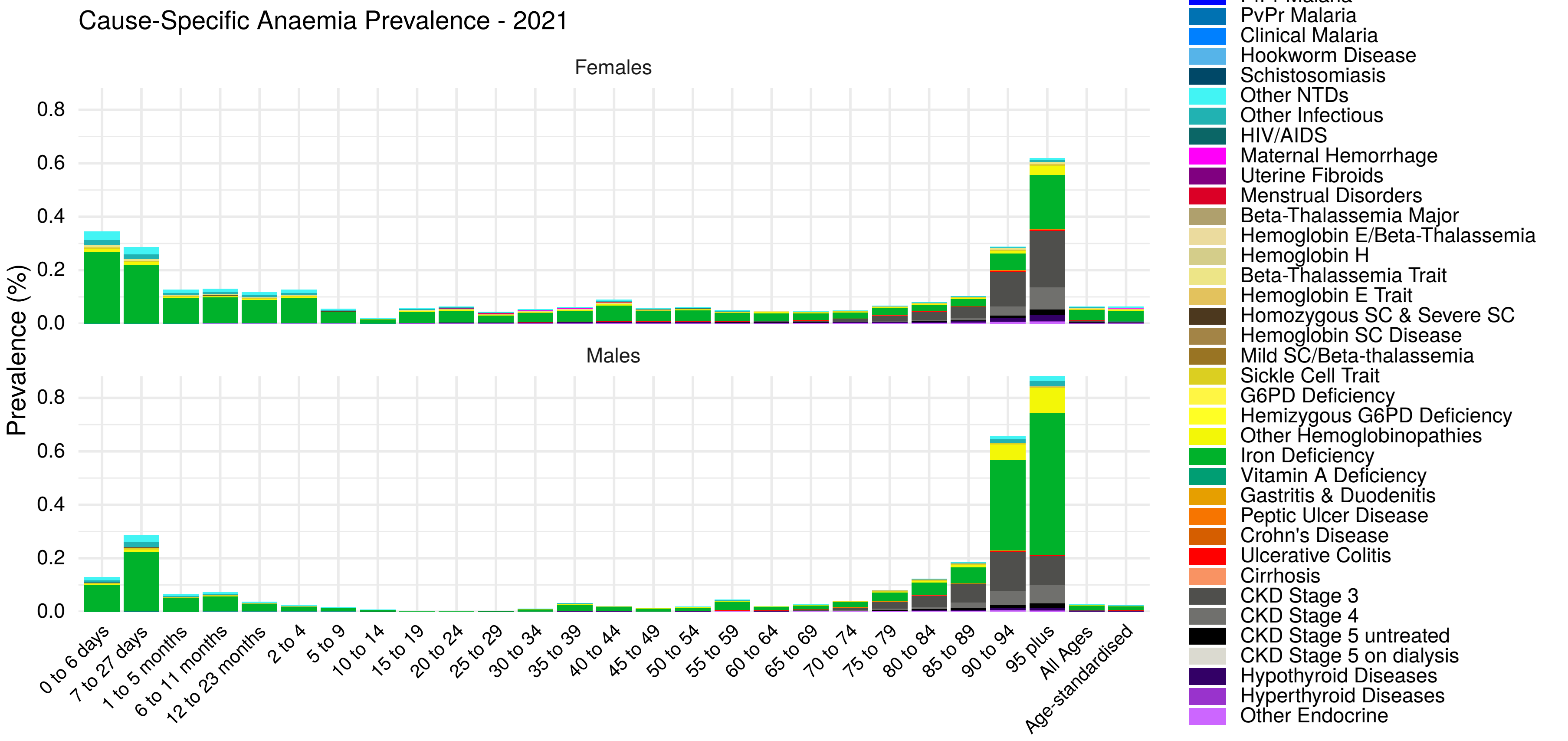

Cause-Specific Anaemia Prevalence - 2021
Females
Males
Prevalence (%)
PfPr Malaria
PvPr Malaria
Clinical Malaria
Hookworm Disease
Schistosomiasis
Other NTDs
Other Infectious
HIV/AIDS
Maternal Hemorrhage
Uterine Fibroids
Menstrual Disorders
Beta-Thalassemia Major
Hemoglobin E/Beta-Thalassemia
Hemoglobin H
Beta-Thalassemia Trait
Hemoglobin E Trait
Homozygous SC & Severe SC
Hemoglobin SC Disease
Mild SC/Beta-thalassemia
Sickle Cell Trait
G6PD Deficiency
Hemizygous G6PD Deficiency
Other Hemoglobinopathies
Iron Deficiency
Vitamin A Deficiency
Gastritis & Duodenitis
Peptic Ulcer Disease
Crohn's Disease
Ulcerative Colitis
Cirrhosis
CKD Stage 3
CKD Stage 4
CKD Stage 5 untreated
CKD Stage 5 on dialysis
Hypothyroid Diseases
Hyperthyroid Diseases
Other Endocrine

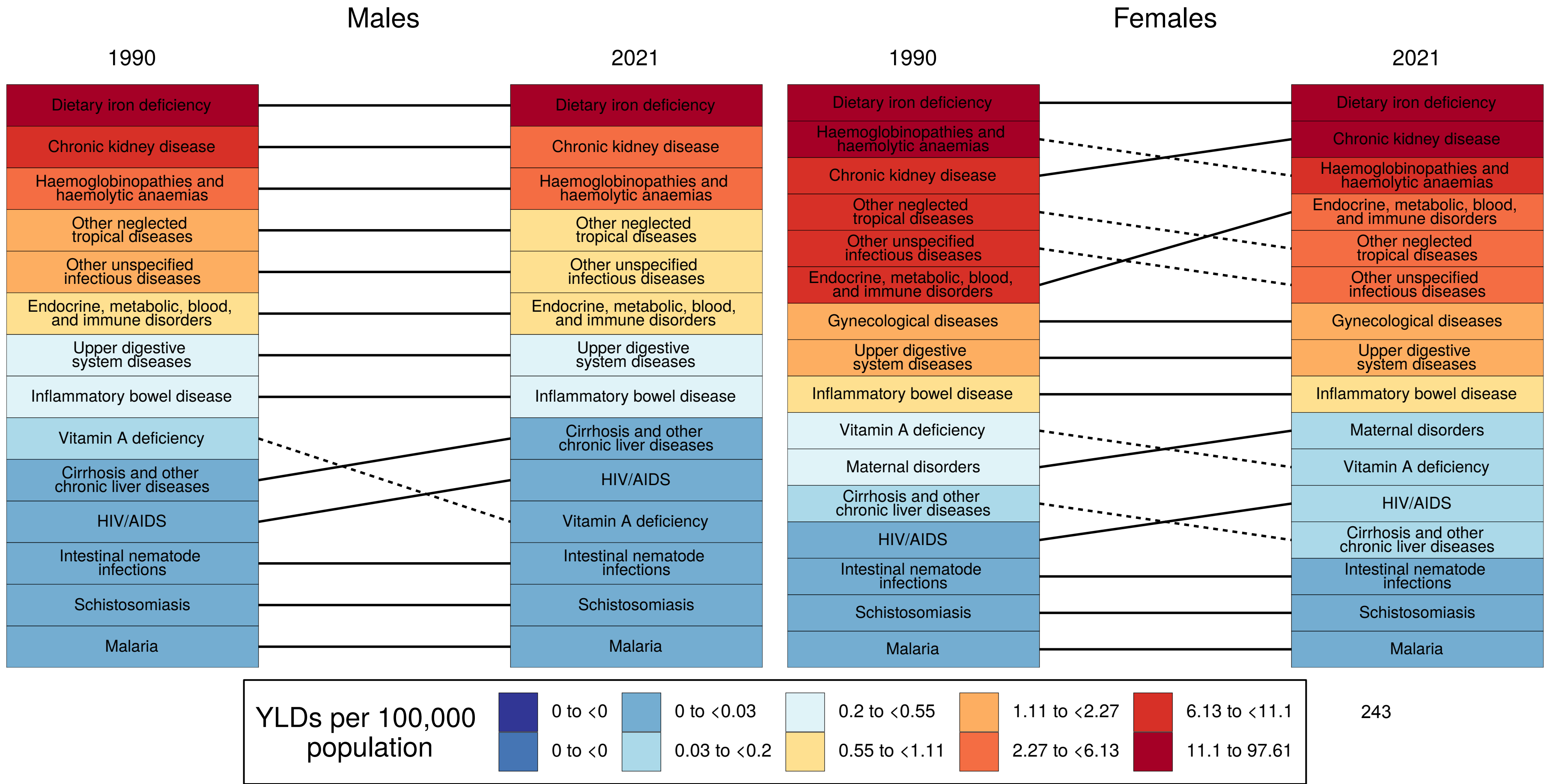

Males
Females
1990
2021
Males 1990: Dietary iron deficiency; Chronic kidney disease; Haemoglobinopathies and haemolytic anaemias; Other neglected tropical diseases; Other unspecified infectious diseases; Endocrine, metabolic, blood, and immune disorders; Upper digestive system diseases; Inflammatory bowel disease; Vitamin A deficiency; Cirrhosis and other chronic liver diseases; HIV/AIDS; Intestinal nematode infections; Schistosomiasis; Malaria
Males 2021: Dietary iron deficiency; Chronic kidney disease; Haemoglobinopathies and haemolytic anaemias; Other neglected tropical diseases; Other unspecified infectious diseases; Endocrine, metabolic, blood, and immune disorders; Upper digestive system diseases; Inflammatory bowel disease; Cirrhosis and other chronic liver diseases; HIV/AIDS; Vitamin A deficiency; Intestinal nematode infections; Schistosomiasis; Malaria
Females 1990: Dietary iron deficiency; Haemoglobinopathies and haemolytic anaemias; Chronic kidney disease; Other neglected tropical diseases; Other unspecified infectious diseases; Endocrine, metabolic, blood, and immune disorders; Gynecological diseases; Upper digestive system diseases; Inflammatory bowel disease; Vitamin A deficiency; Maternal disorders; Cirrhosis and other chronic liver diseases; HIV/AIDS; Intestinal nematode infections; Schistosomiasis; Malaria
Females 2021: Dietary iron deficiency; Chronic kidney disease; Haemoglobinopathies and haemolytic anaemias; Endocrine, metabolic, blood, and immune disorders; Other neglected tropical diseases; Other unspecified infectious diseases; Gynecological diseases; Upper digestive system diseases; Inflammatory bowel disease; Maternal disorders; Vitamin A deficiency; HIV/AIDS; Cirrhosis and other chronic liver diseases; Intestinal nematode infections; Schistosomiasis; Malaria
YLDs per 100,000 population
0 to <0
0 to <0
0 to <0.03
0.03 to <0.2
0.2 to <0.55
0.55 to <1.11
1.11 to <2.27
2.27 to <6.13
6.13 to <11.1
11.1 to 97.61

Troms og Finnmark

Anaemia Prevalence - Both Sexes

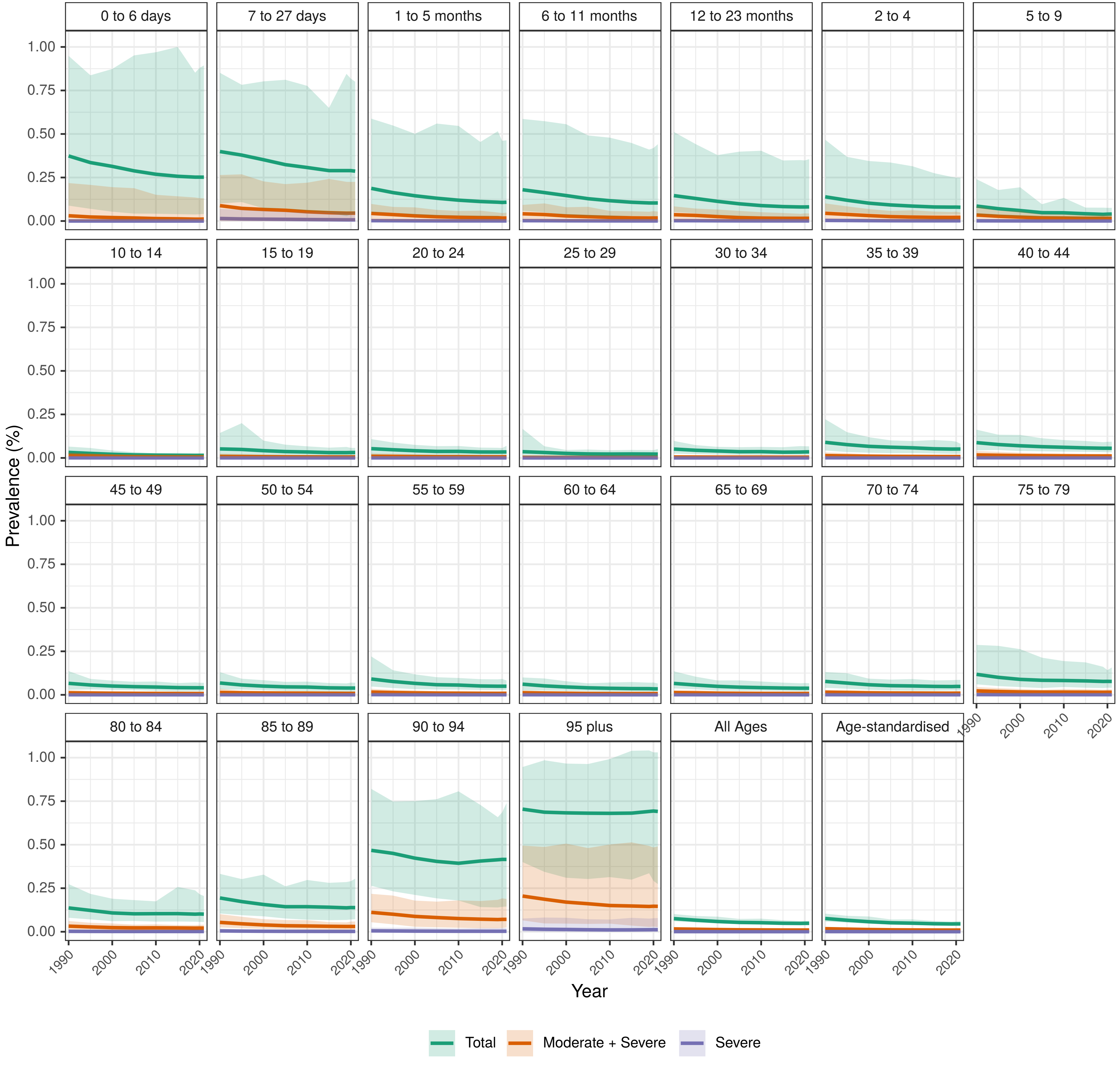

Cause-Specific Anaemia Prevalence - 2021

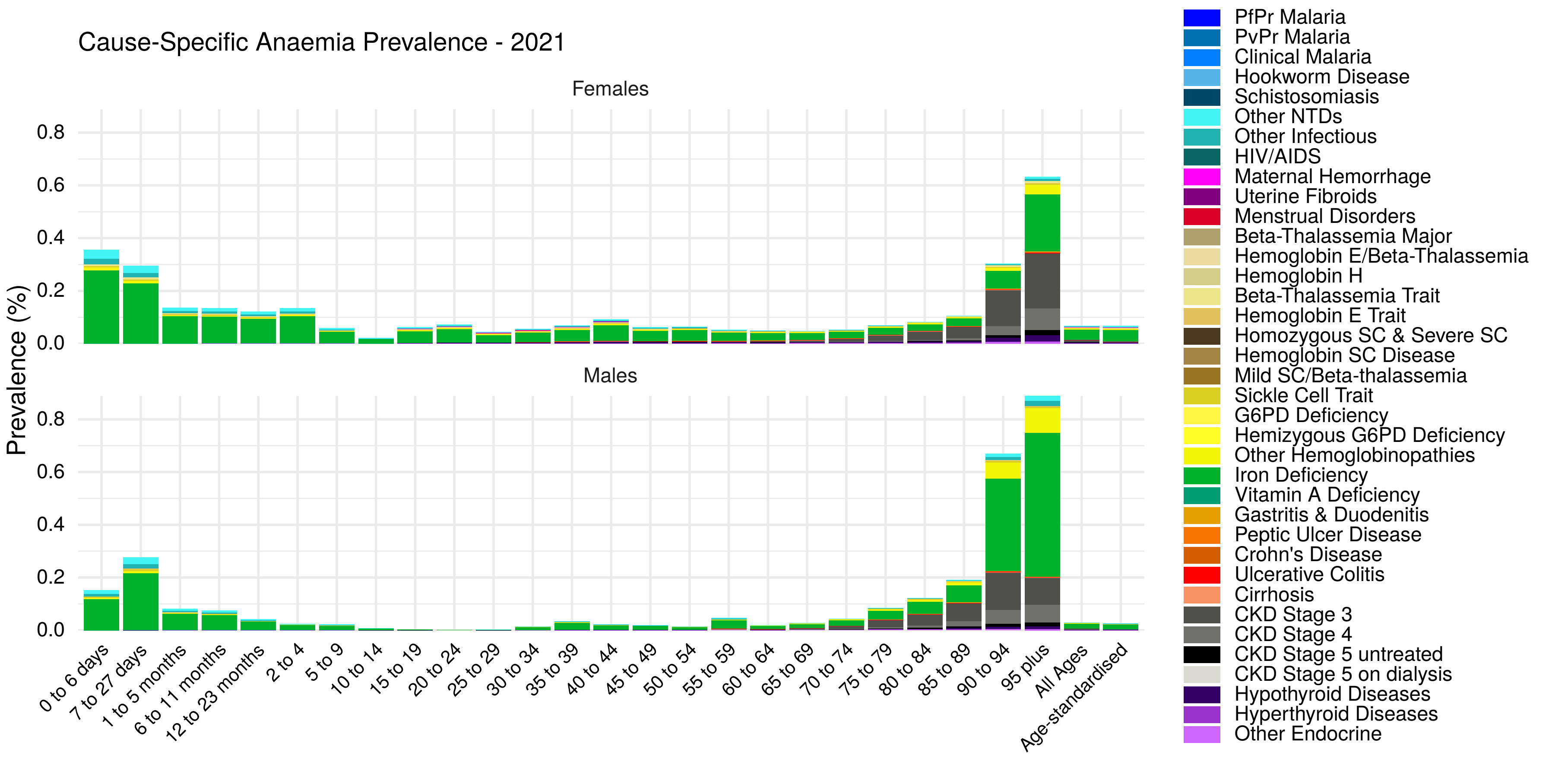

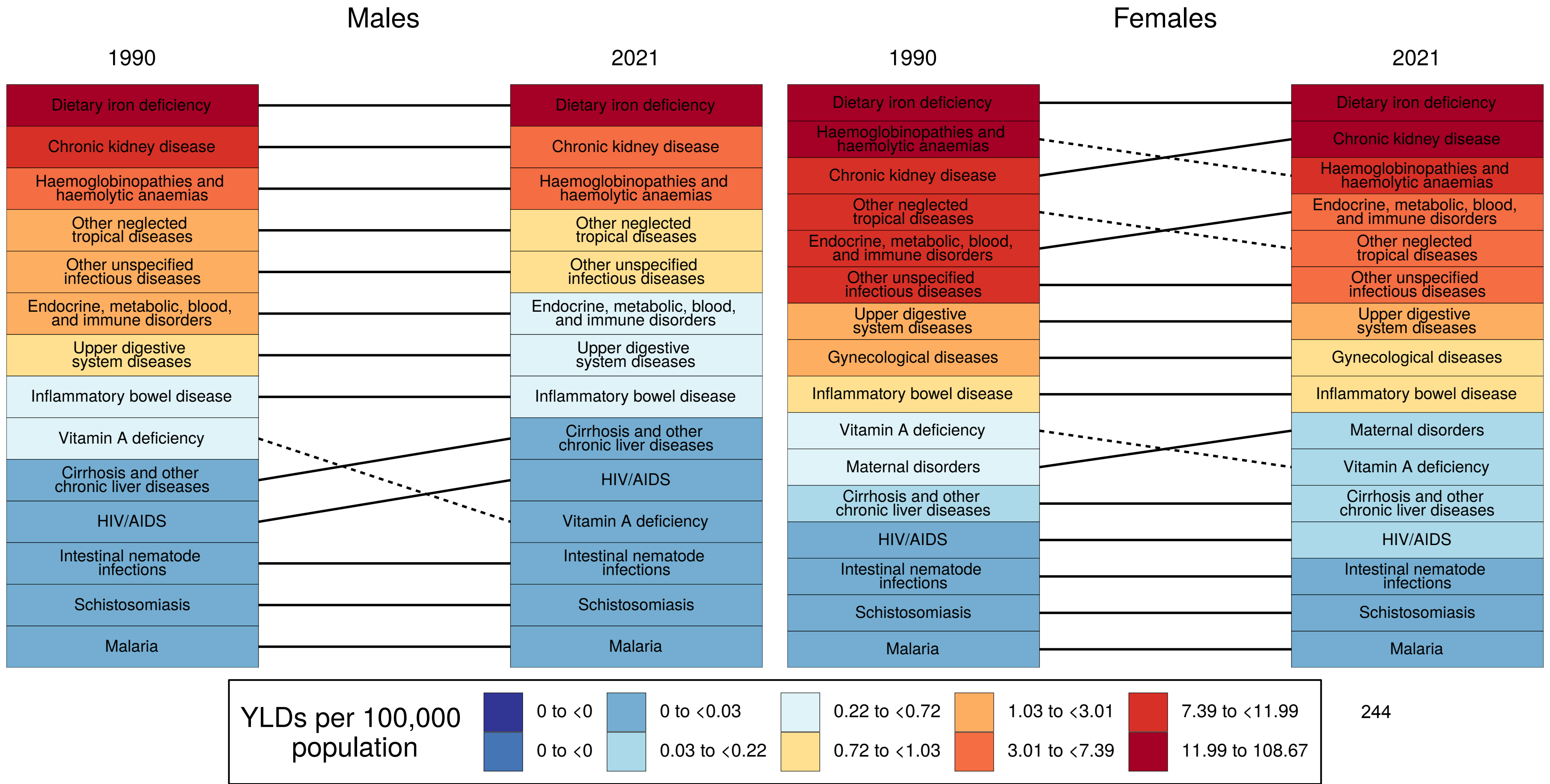

Trøndelag

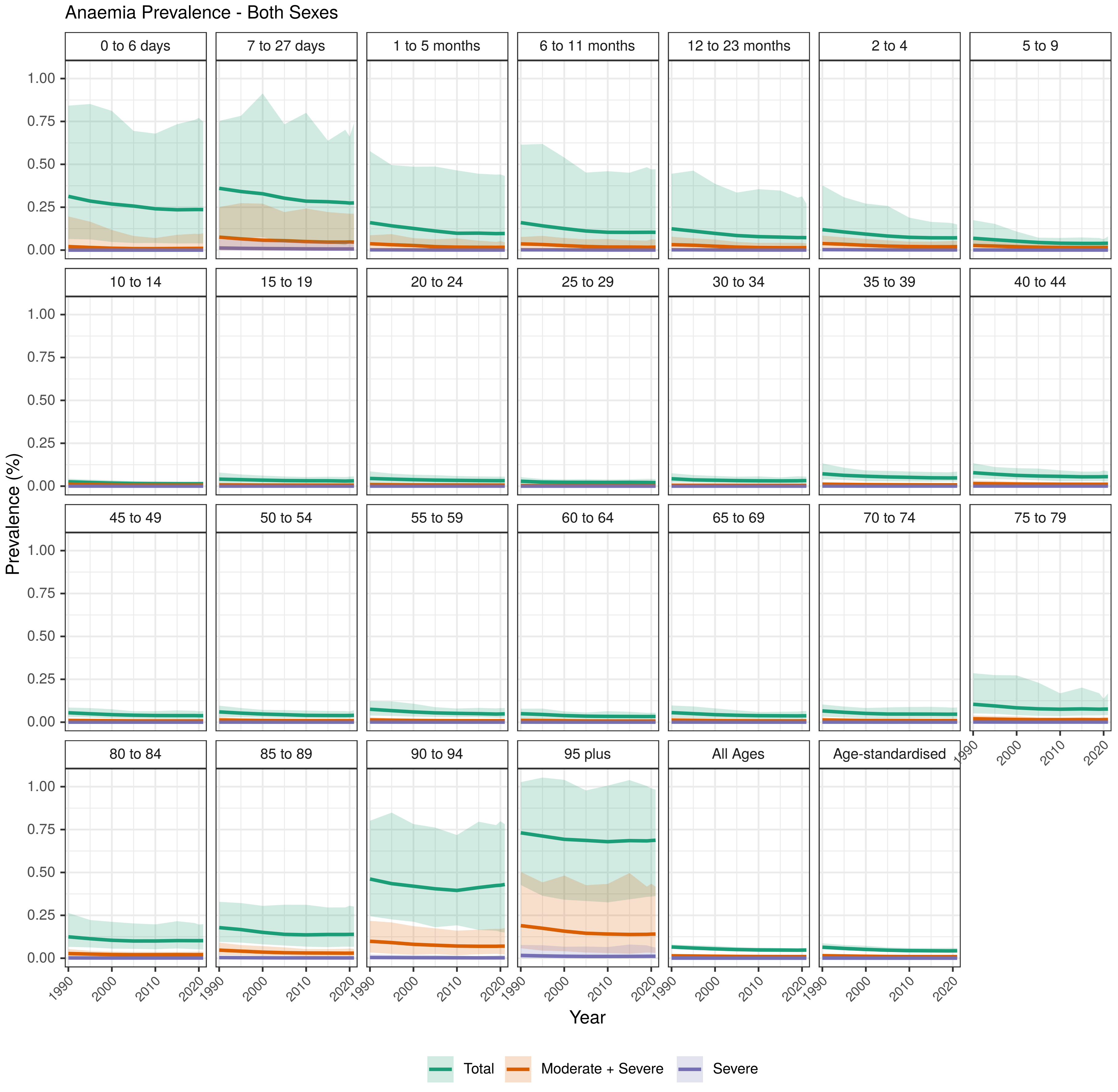

Anaemia Prevalence - Both Sexes
0 to 6 days
7 to 27 days
1 to 5 months
6 to 11 months
12 to 23 months
2 to 4
5 to 9
10 to 14
15 to 19
20 to 24
25 to 29
30 to 34
35 to 39
40 to 44
45 to 49
50 to 54
55 to 59
60 to 64
65 to 69
70 to 74
75 to 79
80 to 84
85 to 89
90 to 94
95 plus
All Ages
Age-standardised
Prevalence (%)
Year
Total
Moderate + Severe
Severe

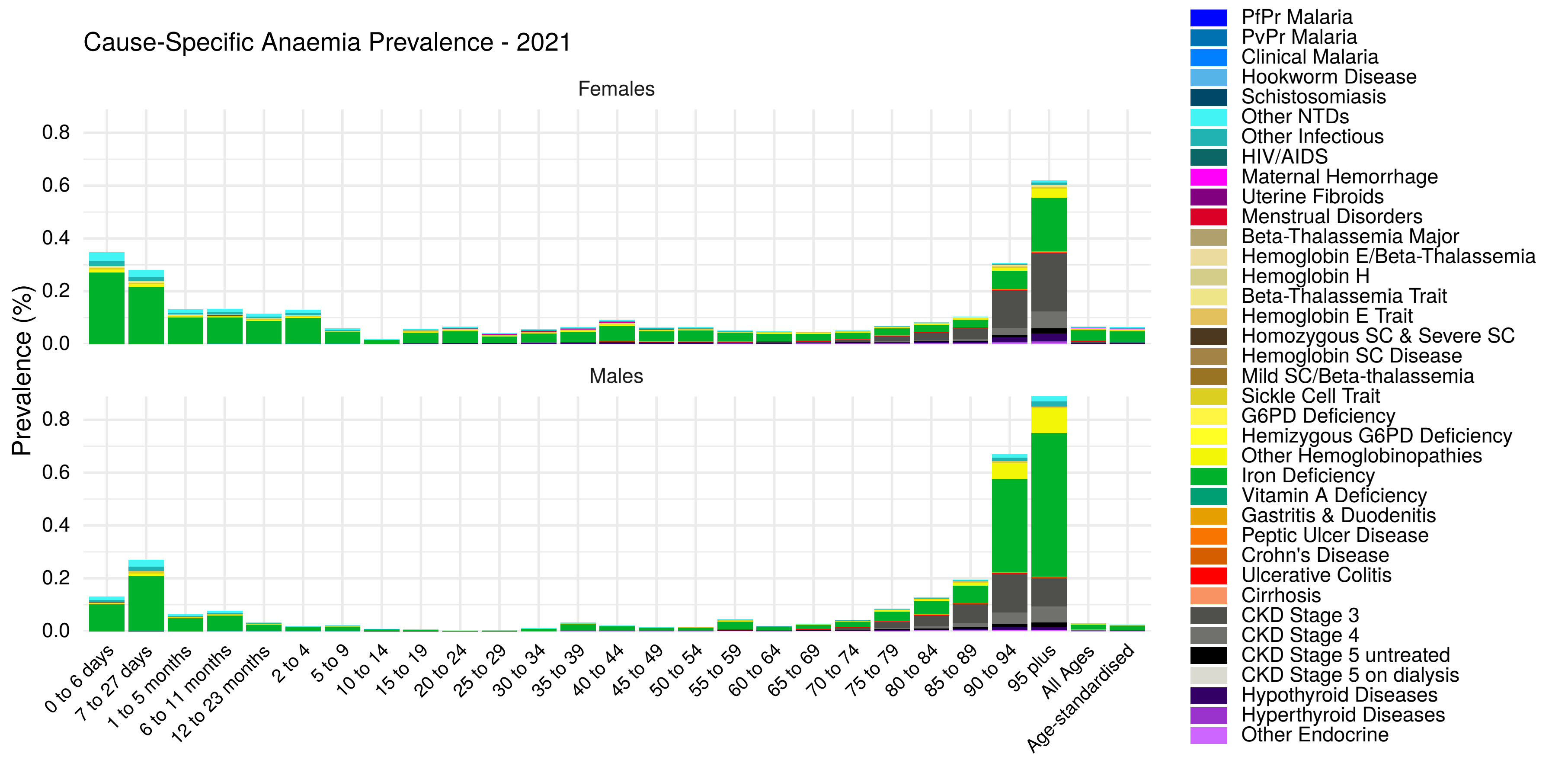

Cause-Specific Anaemia Prevalence - 2021
Females
Males
Prevalence (%)
PfPr Malaria
PvPr Malaria
Clinical Malaria
Hookworm Disease
Schistosomiasis
Other NTDs
Other Infectious
HIV/AIDS
Maternal Hemorrhage
Uterine Fibroids
Menstrual Disorders
Beta-Thalassemia Major
Hemoglobin E/Beta-Thalassemia
Hemoglobin H
Beta-Thalassemia Trait
Hemoglobin E Trait
Homozygous SC & Severe SC
Hemoglobin SC Disease
Mild SC/Beta-thalassemia
Sickle Cell Trait
G6PD Deficiency
Hemizygous G6PD Deficiency
Other Hemoglobinopathies
Iron Deficiency
Vitamin A Deficiency
Gastritis & Duodenitis
Peptic Ulcer Disease
Crohn's Disease
Ulcerative Colitis
Cirrhosis
CKD Stage 3
CKD Stage 4
CKD Stage 5 untreated
CKD Stage 5 on dialysis
Hypothyroid Diseases
Hyperthyroid Diseases
Other Endocrine

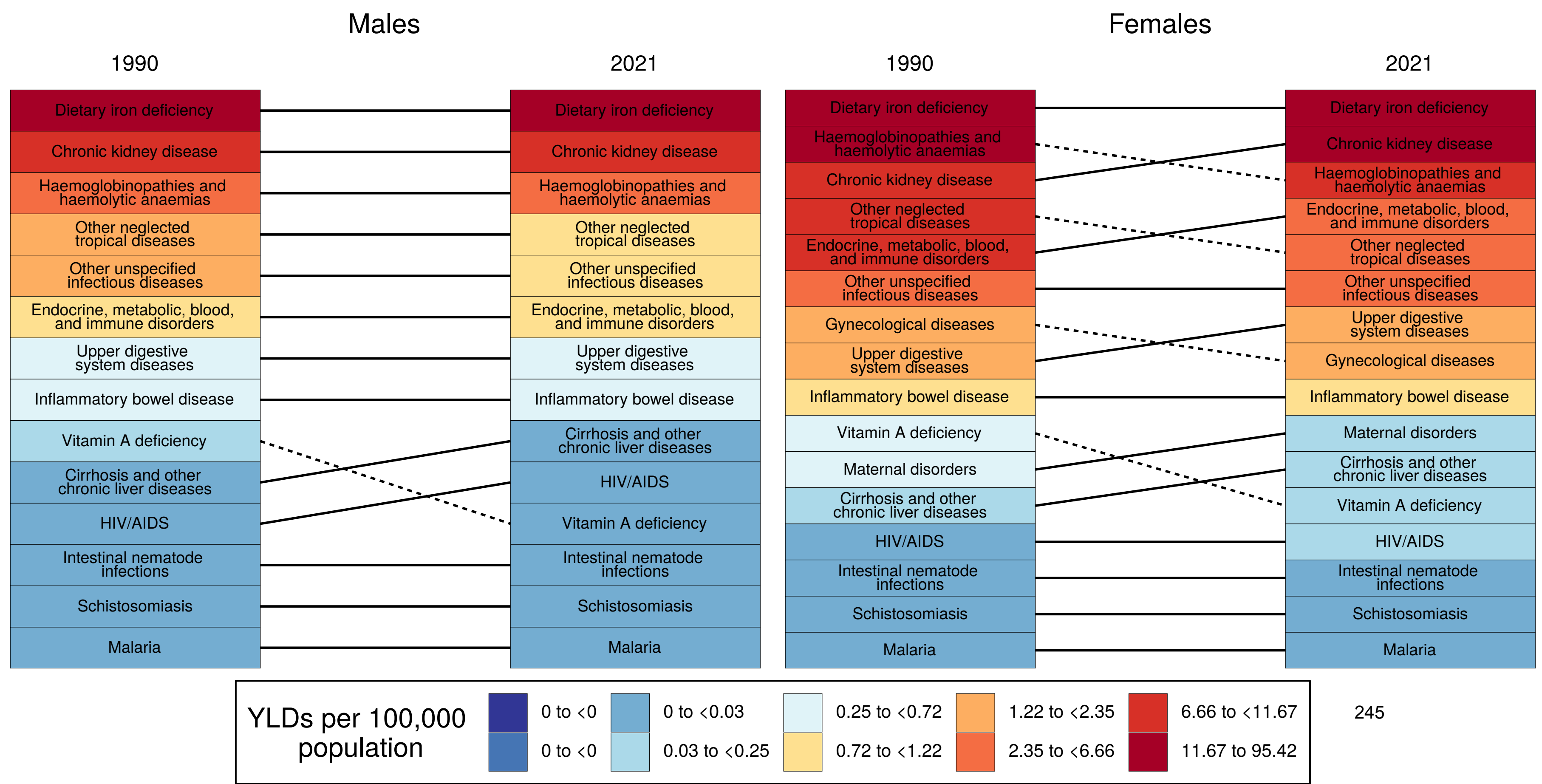

Males
Females
1990
2021
Dietary iron deficiency
Chronic kidney disease
Haemoglobinopathies and haemolytic anaemias
Other neglected tropical diseases
Other unspecified infectious diseases
Endocrine, metabolic, blood, and immune disorders
Upper digestive system diseases
Inflammatory bowel disease
Vitamin A deficiency
Cirrhosis and other chronic liver diseases
HIV/AIDS
Intestinal nematode infections
Schistosomiasis
Malaria
Gynecological diseases
Maternal disorders
YLDs per 100,000 population
0 to <0
0 to <0.03
0.03 to <0.25
0.25 to <0.72
0.72 to <1.22
1.22 to <2.35
2.35 to <6.66
6.66 to <11.67
11.67 to 95.42

Vestfold og Telemark

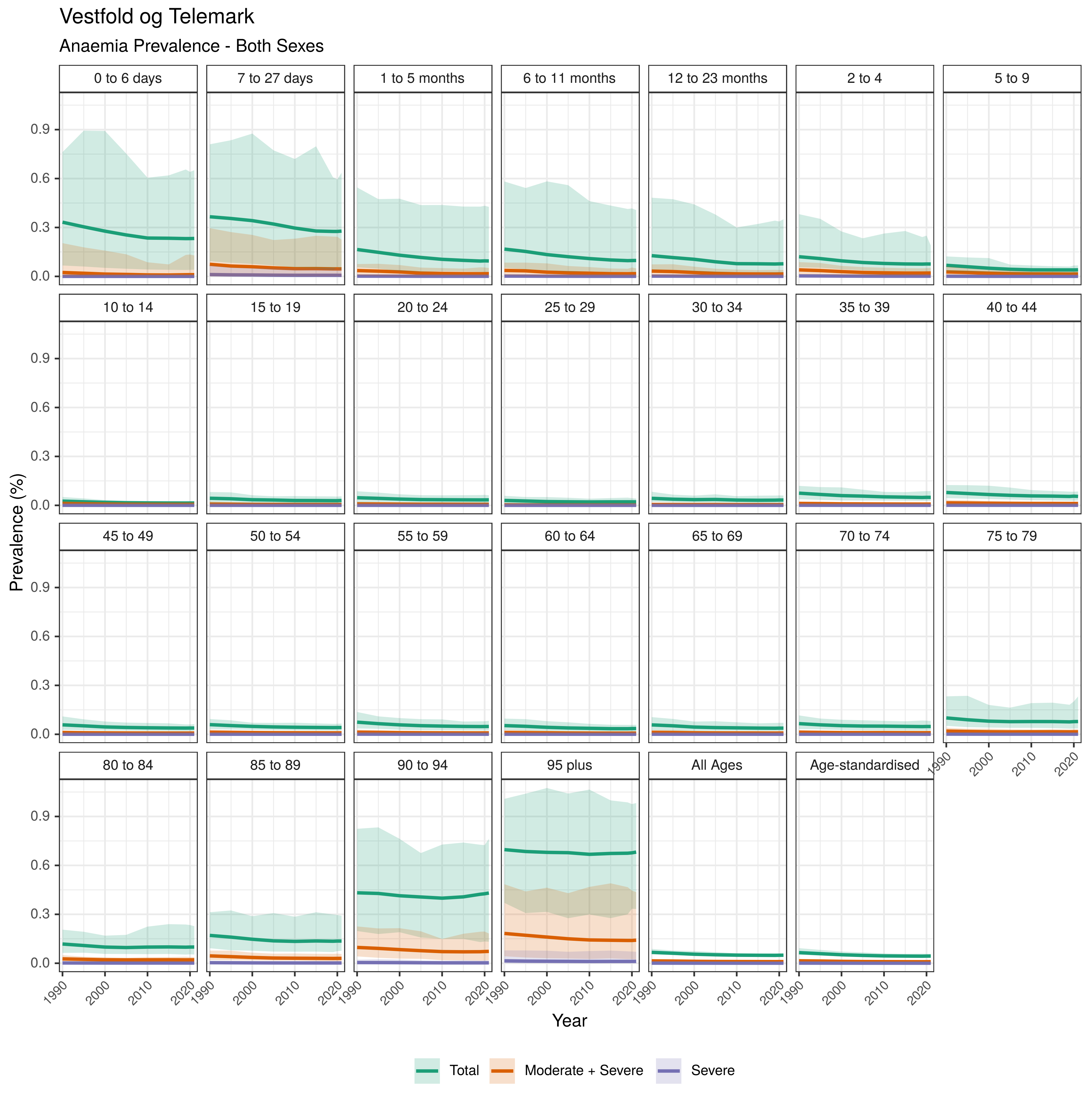

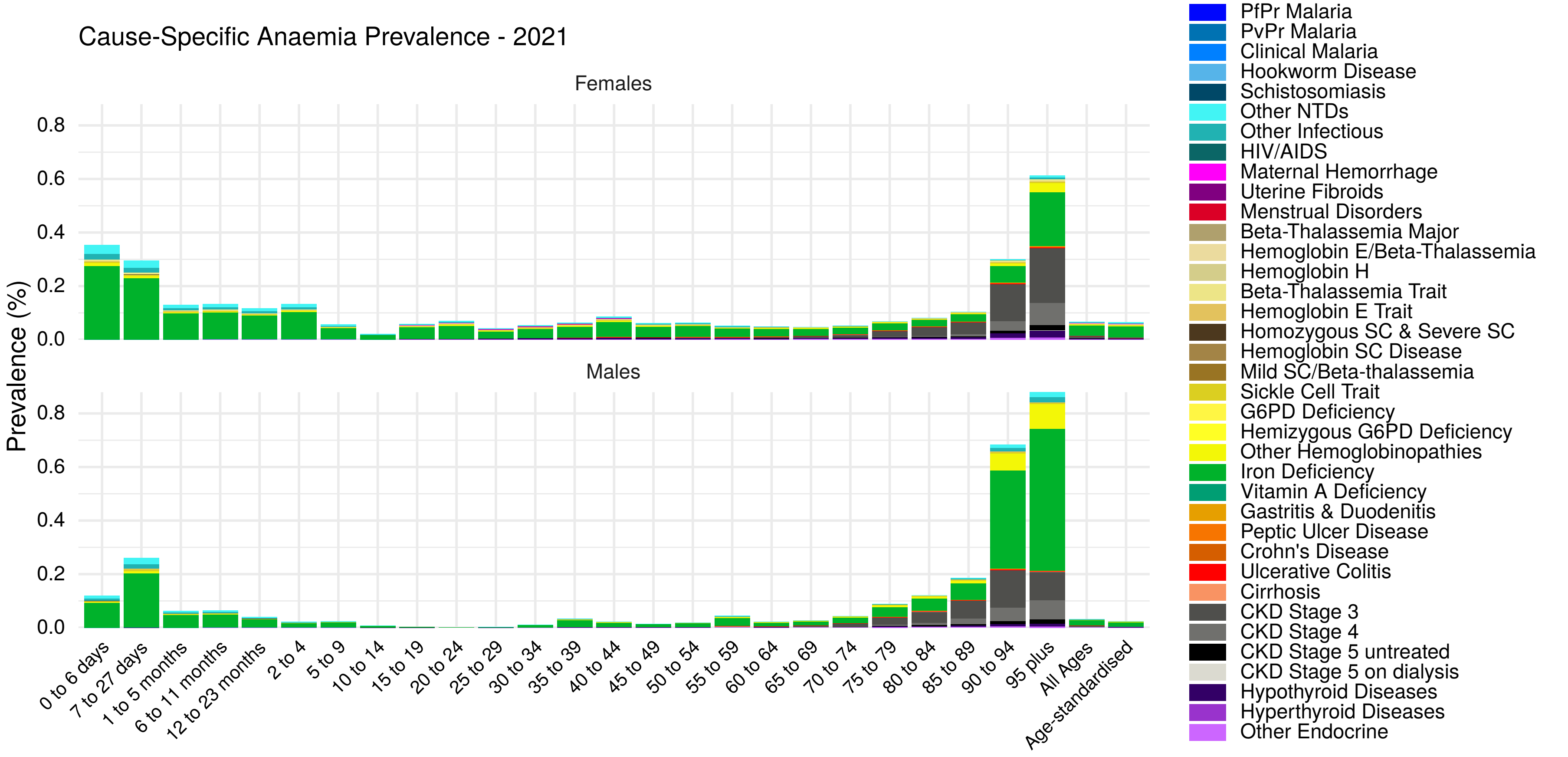

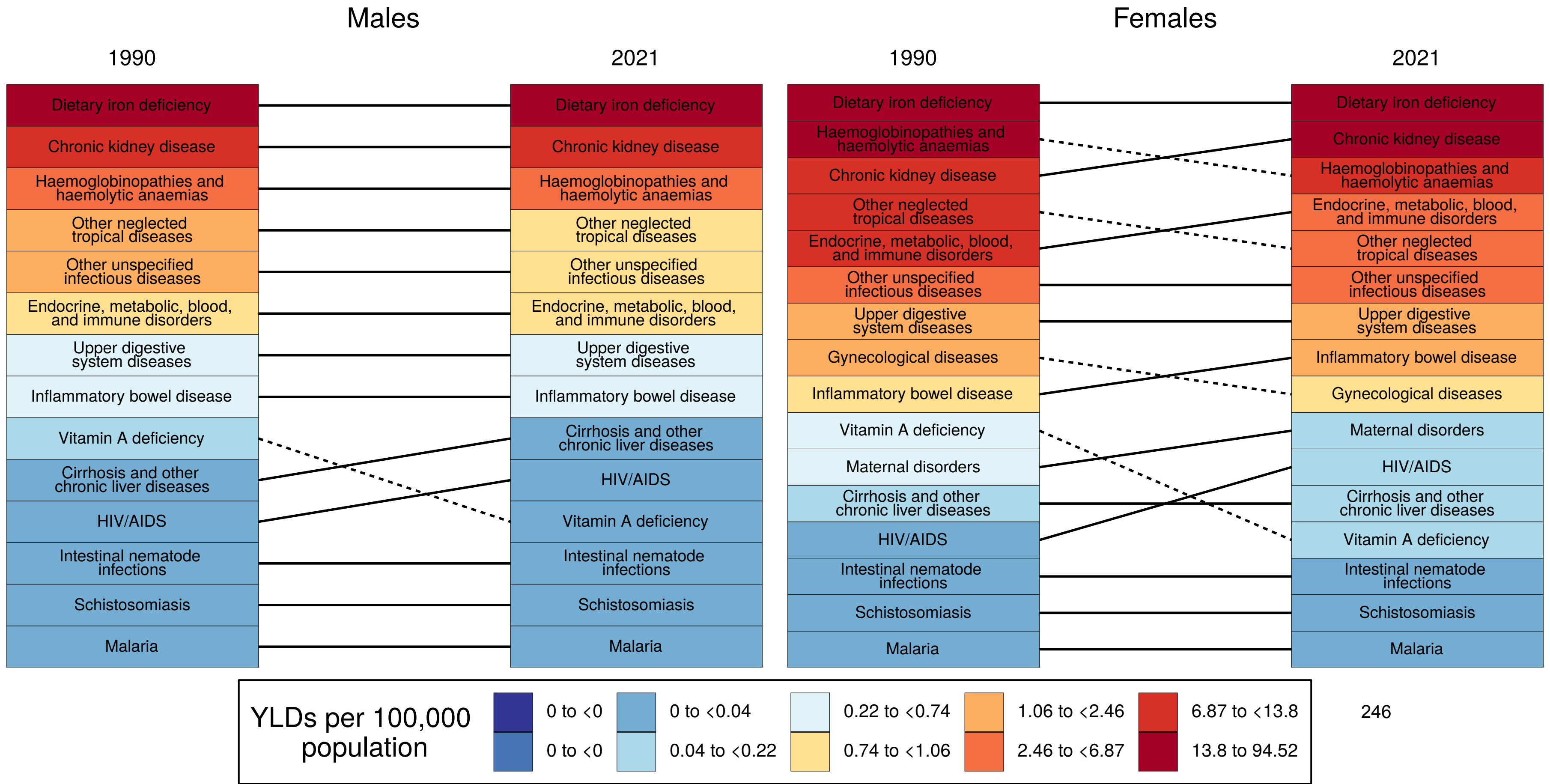

Vestland

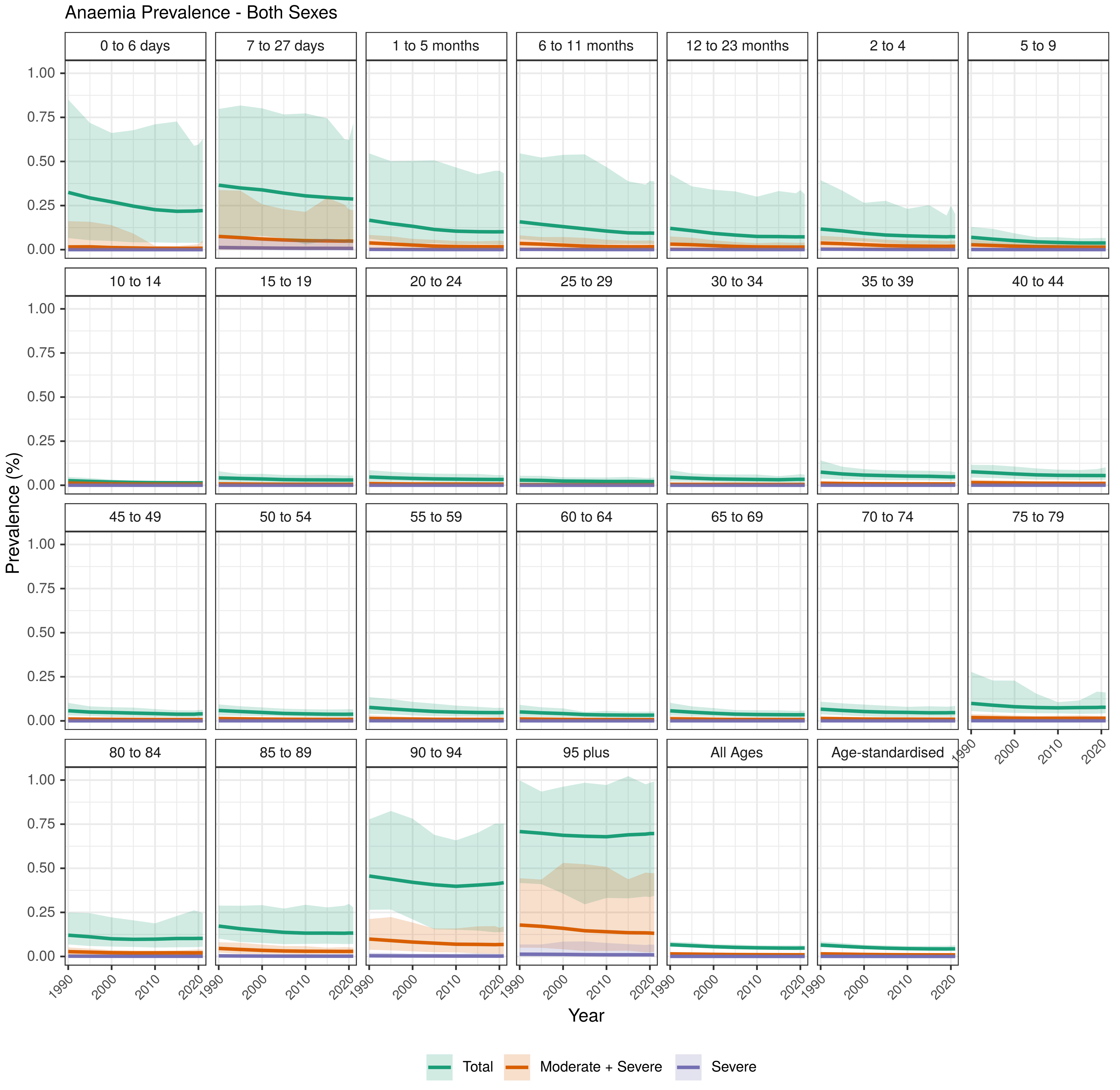

Anaemia Prevalence - Both Sexes
0 to 6 days
7 to 27 days
1 to 5 months
6 to 11 months
12 to 23 months
2 to 4
5 to 9
10 to 14
15 to 19
20 to 24
25 to 29
30 to 34
35 to 39
40 to 44
45 to 49
50 to 54
55 to 59
60 to 64
65 to 69
70 to 74
75 to 79
80 to 84
85 to 89
90 to 94
95 plus
All Ages
Age-standardised
Prevalence (%)
Year
Total
Moderate + Severe
Severe

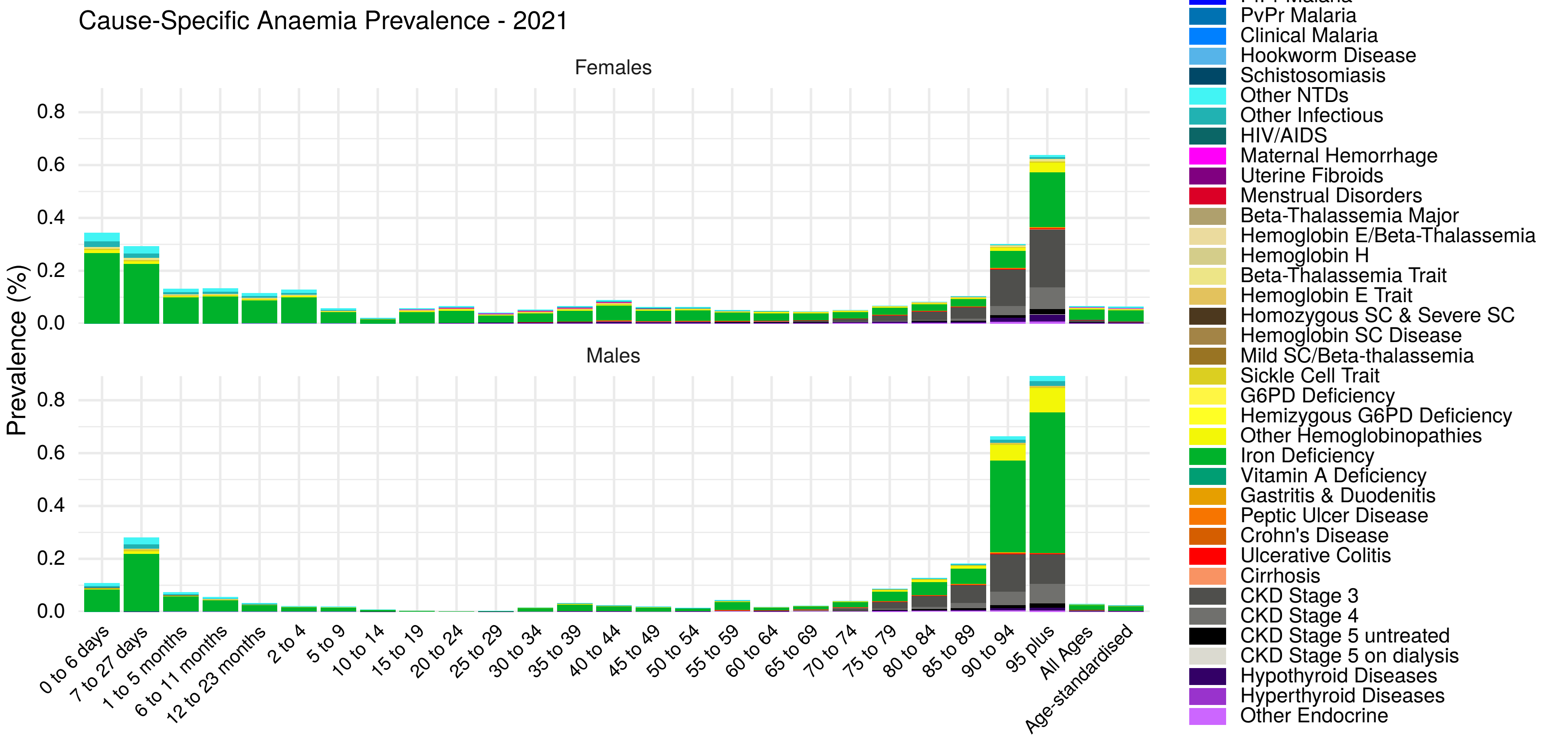

Cause-Specific Anaemia Prevalence - 2021
Females
Males
Prevalence (%)
PfPr Malaria
PvPr Malaria
Clinical Malaria
Hookworm Disease
Schistosomiasis
Other NTDs
Other Infectious
HIV/AIDS
Maternal Hemorrhage
Uterine Fibroids
Menstrual Disorders
Beta-Thalassemia Major
Hemoglobin E/Beta-Thalassemia
Hemoglobin H
Beta-Thalassemia Trait
Hemoglobin E Trait
Homozygous SC & Severe SC
Hemoglobin SC Disease
Mild SC/Beta-thalassemia
Sickle Cell Trait
G6PD Deficiency
Hemizygous G6PD Deficiency
Other Hemoglobinopathies
Iron Deficiency
Vitamin A Deficiency
Gastritis & Duodenitis
Peptic Ulcer Disease
Crohn's Disease
Ulcerative Colitis
Cirrhosis
CKD Stage 3
CKD Stage 4
CKD Stage 5 untreated
CKD Stage 5 on dialysis
Hypothyroid Diseases
Hyperthyroid Diseases
Other Endocrine

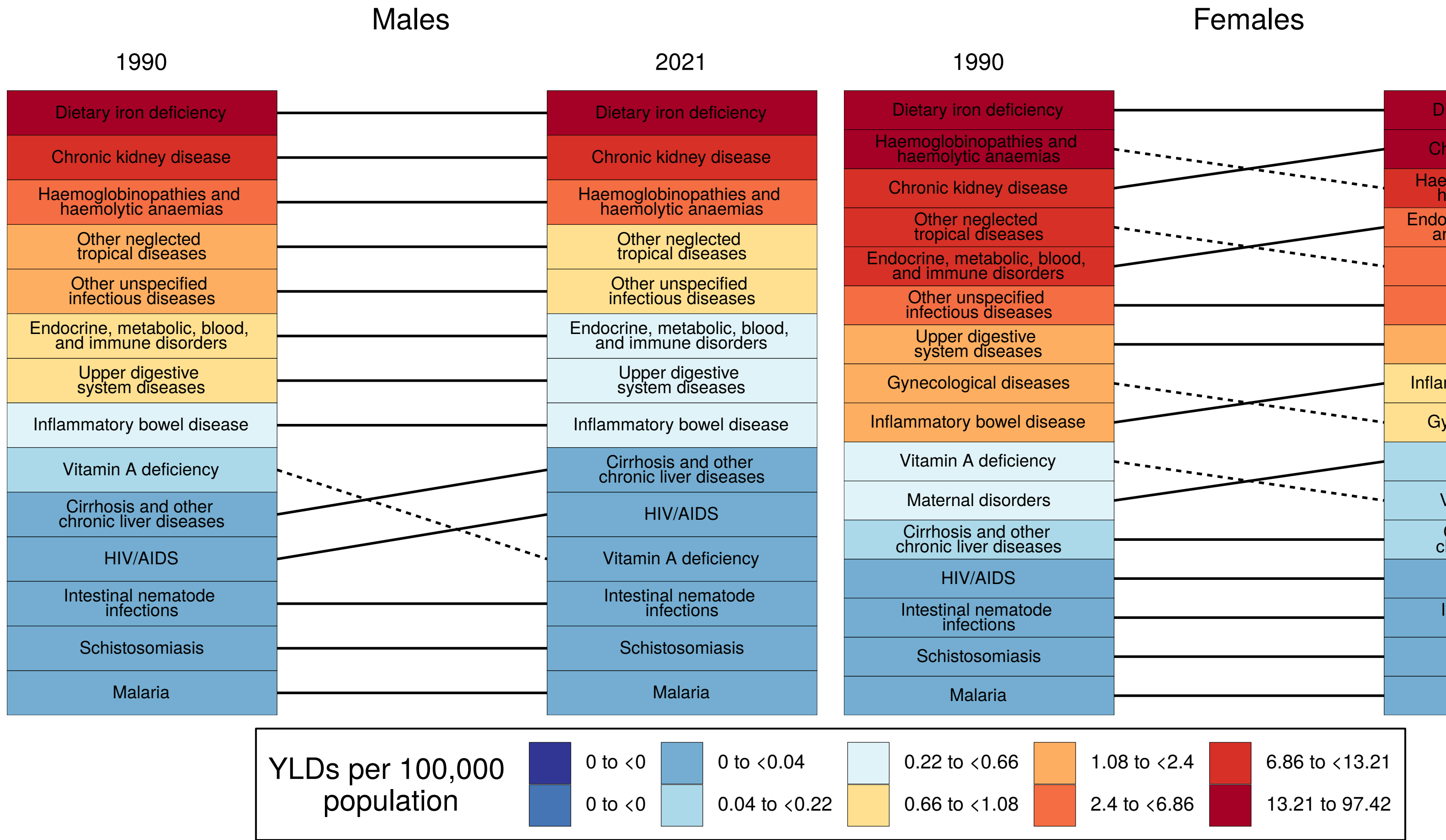

Males
Females
1990
2021
Dietary iron deficiency
Chronic kidney disease
Haemoglobinopathies and haemolytic anaemias
Other neglected tropical diseases
Other unspecified infectious diseases
Endocrine, metabolic, blood, and immune disorders
Upper digestive system diseases
Inflammatory bowel disease
Vitamin A deficiency
Cirrhosis and other chronic liver diseases
HIV/AIDS
Intestinal nematode infections
Schistosomiasis
Malaria
Gynecological diseases
Maternal disorders
YLDs per 100,000 population
0 to <0
0 to <0.04
0.04 to <0.22
0.22 to <0.66
0.66 to <1.08
1.08 to <2.4
2.4 to <6.86
6.86 to <13.21
13.21 to 97.42

Viken

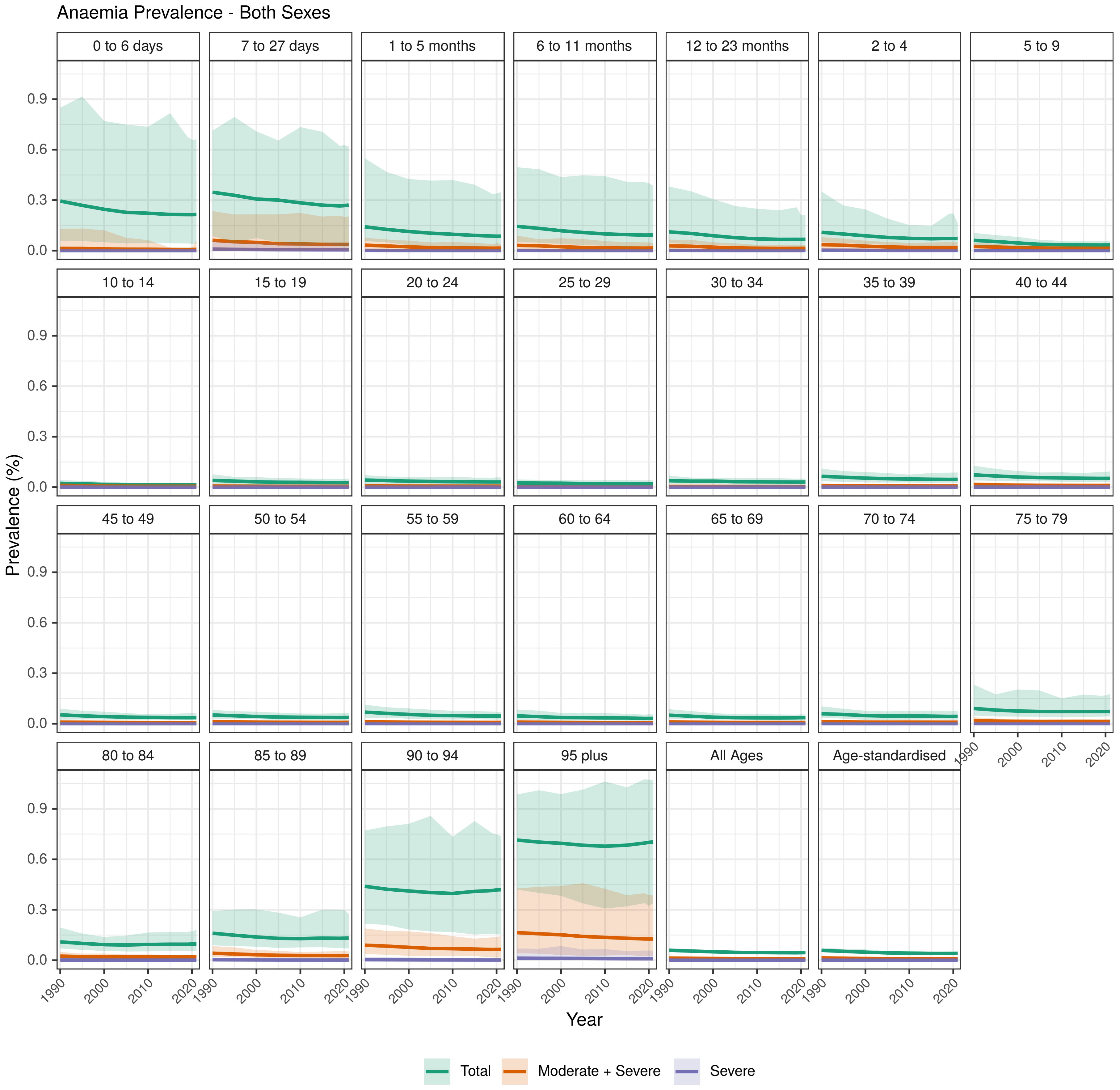

Anaemia Prevalence - Both Sexes
0 to 6 days
7 to 27 days
1 to 5 months
6 to 11 months
12 to 23 months
2 to 4
5 to 9
10 to 14
15 to 19
20 to 24
25 to 29
30 to 34
35 to 39
40 to 44
45 to 49
50 to 54
55 to 59
60 to 64
65 to 69
70 to 74
75 to 79
80 to 84
85 to 89
90 to 94
95 plus
All Ages
Age-standardised
Prevalence (%)
0.0
0.3
0.6
0.9
Year
1990
2000
2010
2020
Total
Moderate + Severe
Severe

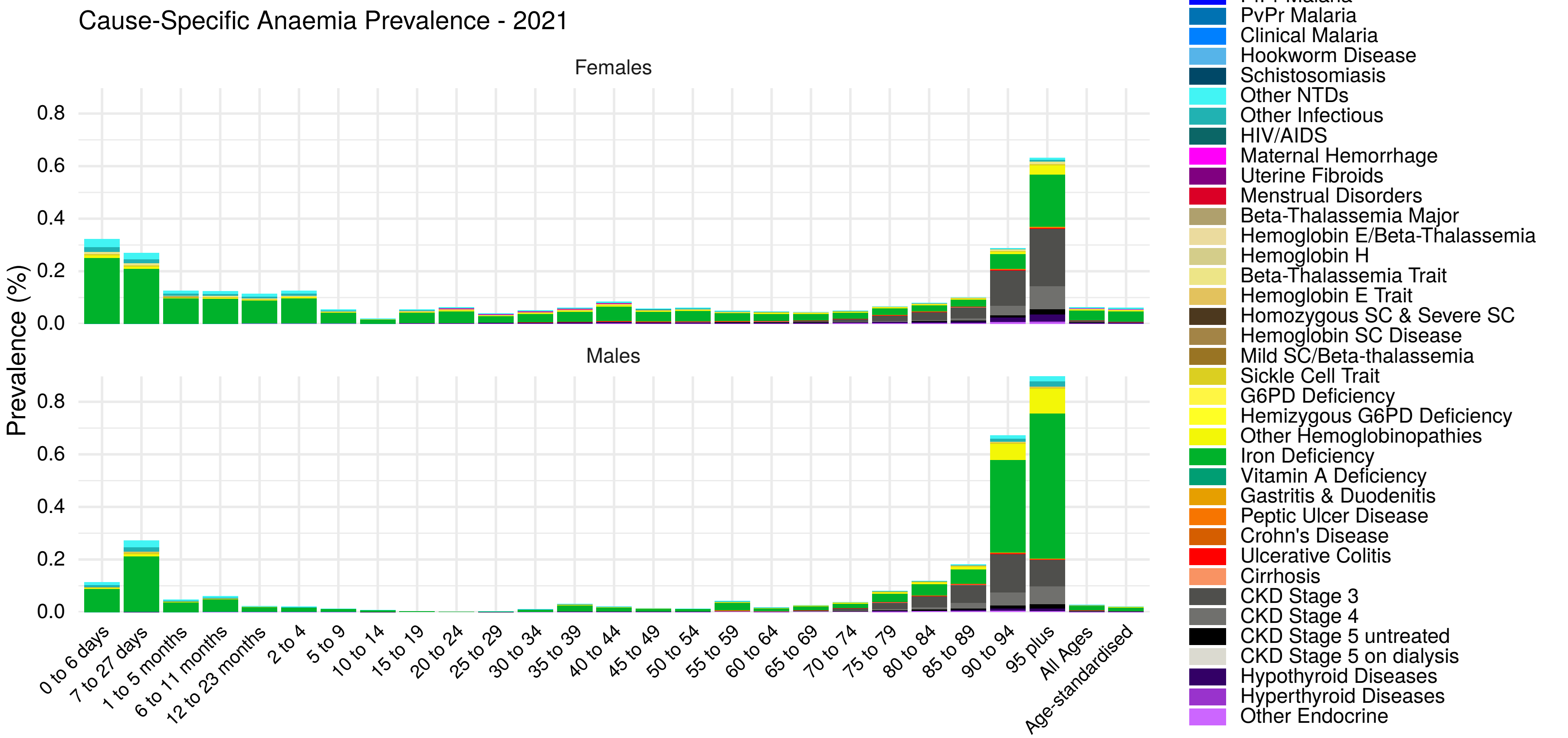

Cause-Specific Anaemia Prevalence - 2021
Females
Males
Prevalence (%)
0.0
0.2
0.4
0.6
0.8
0 to 6 days
7 to 27 days
1 to 5 months
6 to 11 months
12 to 23 months
2 to 4
5 to 9
10 to 14
15 to 19
20 to 24
25 to 29
30 to 34
35 to 39
40 to 44
45 to 49
50 to 54
55 to 59
60 to 64
65 to 69
70 to 74
75 to 79
80 to 84
85 to 89
90 to 94
95 plus
All Ages
Age-standardised
PfPr Malaria
PvPr Malaria
Clinical Malaria
Hookworm Disease
Schistosomiasis
Other NTDs
Other Infectious
HIV/AIDS
Maternal Hemorrhage
Uterine Fibroids
Menstrual Disorders
Beta-Thalassemia Major
Hemoglobin E/Beta-Thalassemia
Hemoglobin H
Beta-Thalassemia Trait
Hemoglobin E Trait
Homozygous SC & Severe SC
Hemoglobin SC Disease
Mild SC/Beta-thalassemia
Sickle Cell Trait
G6PD Deficiency
Hemizygous G6PD Deficiency
Other Hemoglobinopathies
Iron Deficiency
Vitamin A Deficiency
Gastritis & Duodenitis
Peptic Ulcer Disease
Crohn's Disease
Ulcerative Colitis
Cirrhosis
CKD Stage 3
CKD Stage 4
CKD Stage 5 untreated
CKD Stage 5 on dialysis
Hypothyroid Diseases
Hyperthyroid Diseases
Other Endocrine

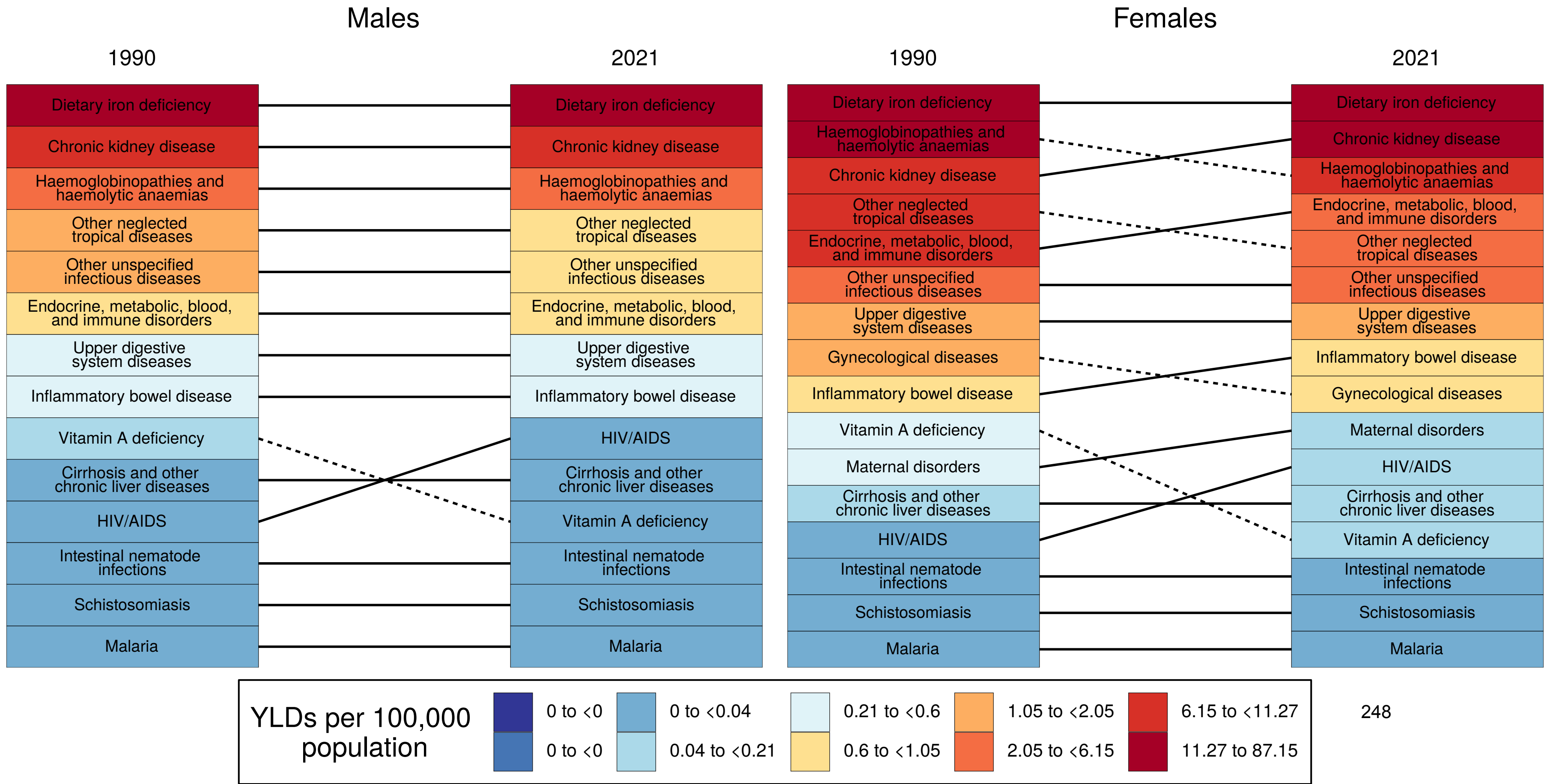

Males
Females
1990
2021
Dietary iron deficiency
Chronic kidney disease
Haemoglobinopathies and haemolytic anaemias
Other neglected tropical diseases
Other unspecified infectious diseases
Endocrine, metabolic, blood, and immune disorders
Upper digestive system diseases
Inflammatory bowel disease
Vitamin A deficiency
Cirrhosis and other chronic liver diseases
HIV/AIDS
Intestinal nematode infections
Schistosomiasis
Malaria
Gynecological diseases
Maternal disorders
YLDs per 100,000 population
0 to <0
0 to <0
0 to <0.04
0.04 to <0.21
0.21 to <0.6
0.6 to <1.05
1.05 to <2.05
2.05 to <6.15
6.15 to <11.27
11.27 to 87.15

Portugal

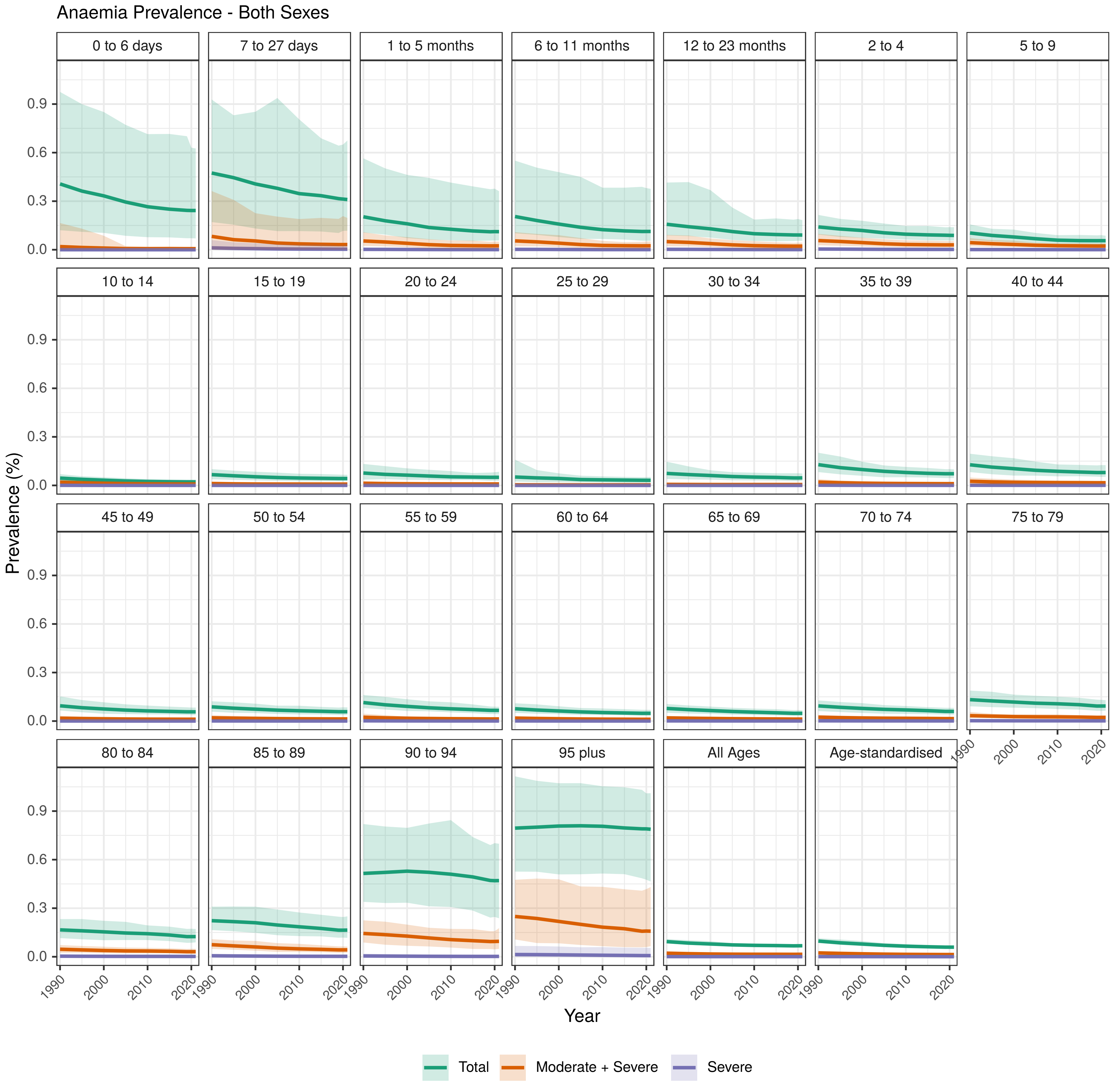

Anaemia Prevalence - Both Sexes
0 to 6 days
7 to 27 days
1 to 5 months
6 to 11 months
12 to 23 months
2 to 4
5 to 9
10 to 14
15 to 19
20 to 24
25 to 29
30 to 34
35 to 39
40 to 44
45 to 49
50 to 54
55 to 59
60 to 64
65 to 69
70 to 74
75 to 79
80 to 84
85 to 89
90 to 94
95 plus
All Ages
Age-standardised
Prevalence (%)
Year
Total
Moderate + Severe
Severe

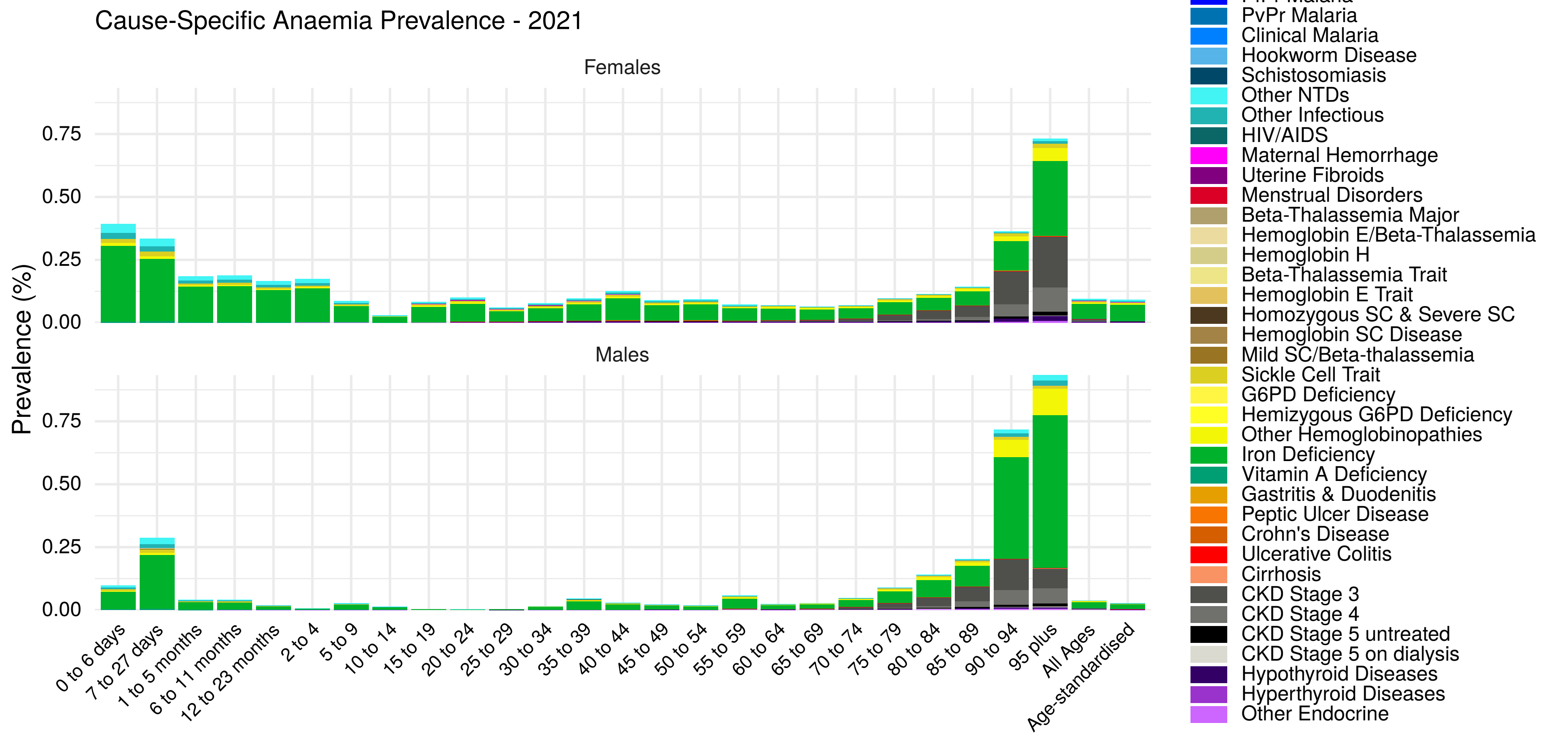

Cause-Specific Anaemia Prevalence - 2021
Females
Males
Prevalence (%)
PfPr Malaria
PvPr Malaria
Clinical Malaria
Hookworm Disease
Schistosomiasis
Other NTDs
Other Infectious
HIV/AIDS
Maternal Hemorrhage
Uterine Fibroids
Menstrual Disorders
Beta-Thalassemia Major
Hemoglobin E/Beta-Thalassemia
Hemoglobin H
Beta-Thalassemia Trait
Hemoglobin E Trait
Homozygous SC & Severe SC
Hemoglobin SC Disease
Mild SC/Beta-thalassemia
Sickle Cell Trait
G6PD Deficiency
Hemizygous G6PD Deficiency
Other Hemoglobinopathies
Iron Deficiency
Vitamin A Deficiency
Gastritis & Duodenitis
Peptic Ulcer Disease
Crohn's Disease
Ulcerative Colitis
Cirrhosis
CKD Stage 3
CKD Stage 4
CKD Stage 5 untreated
CKD Stage 5 on dialysis
Hypothyroid Diseases
Hyperthyroid Diseases
Other Endocrine

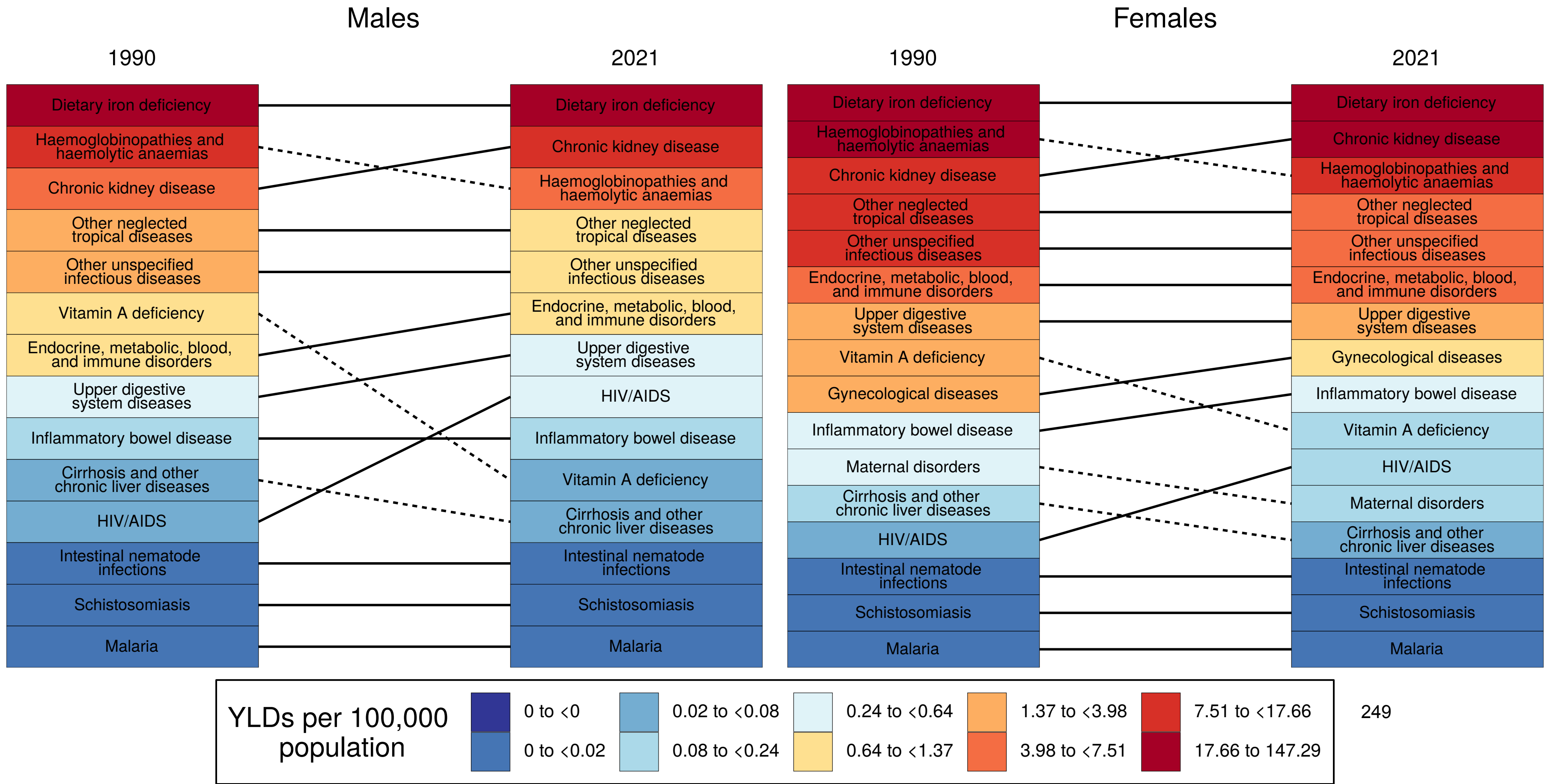

Males
Females
1990
2021
Dietary iron deficiency
Haemoglobinopathies and haemolytic anaemias
Chronic kidney disease
Other neglected tropical diseases
Other unspecified infectious diseases
Vitamin A deficiency
Endocrine, metabolic, blood, and immune disorders
Upper digestive system diseases
Inflammatory bowel disease
Cirrhosis and other chronic liver diseases
HIV/AIDS
Intestinal nematode infections
Schistosomiasis
Malaria
Gynecological diseases
Maternal disorders
YLDs per 100,000 population
0 to <0
0 to <0.02
0.02 to <0.08
0.08 to <0.24
0.24 to <0.64
0.64 to <1.37
1.37 to <3.98
3.98 to <7.51
7.51 to <17.66
17.66 to 147.29

San Marino

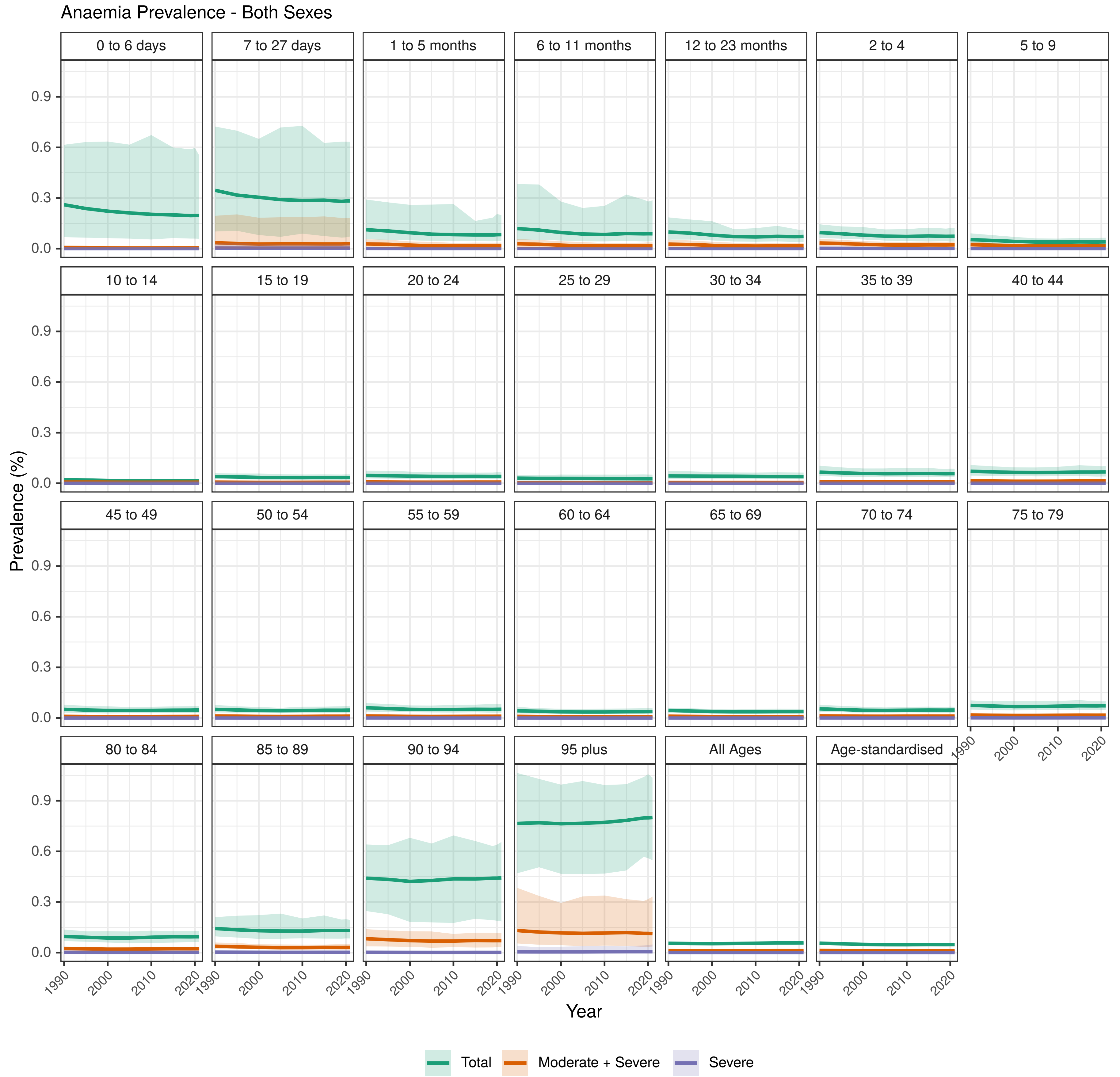
Anaemia Prevalence - Both Sexes
0 to 6 days
7 to 27 days
1 to 5 months
6 to 11 months
12 to 23 months
2 to 4
5 to 9
10 to 14
15 to 19
20 to 24
25 to 29
30 to 34
35 to 39
40 to 44
45 to 49
50 to 54
55 to 59
60 to 64
65 to 69
70 to 74
75 to 79
80 to 84
85 to 89
90 to 94
95 plus
All Ages
Age-standardised
Prevalence (%)
0.0
0.3
0.6
0.9
1990
2000
2010
2020
Year
Total
Moderate + Severe
Severe

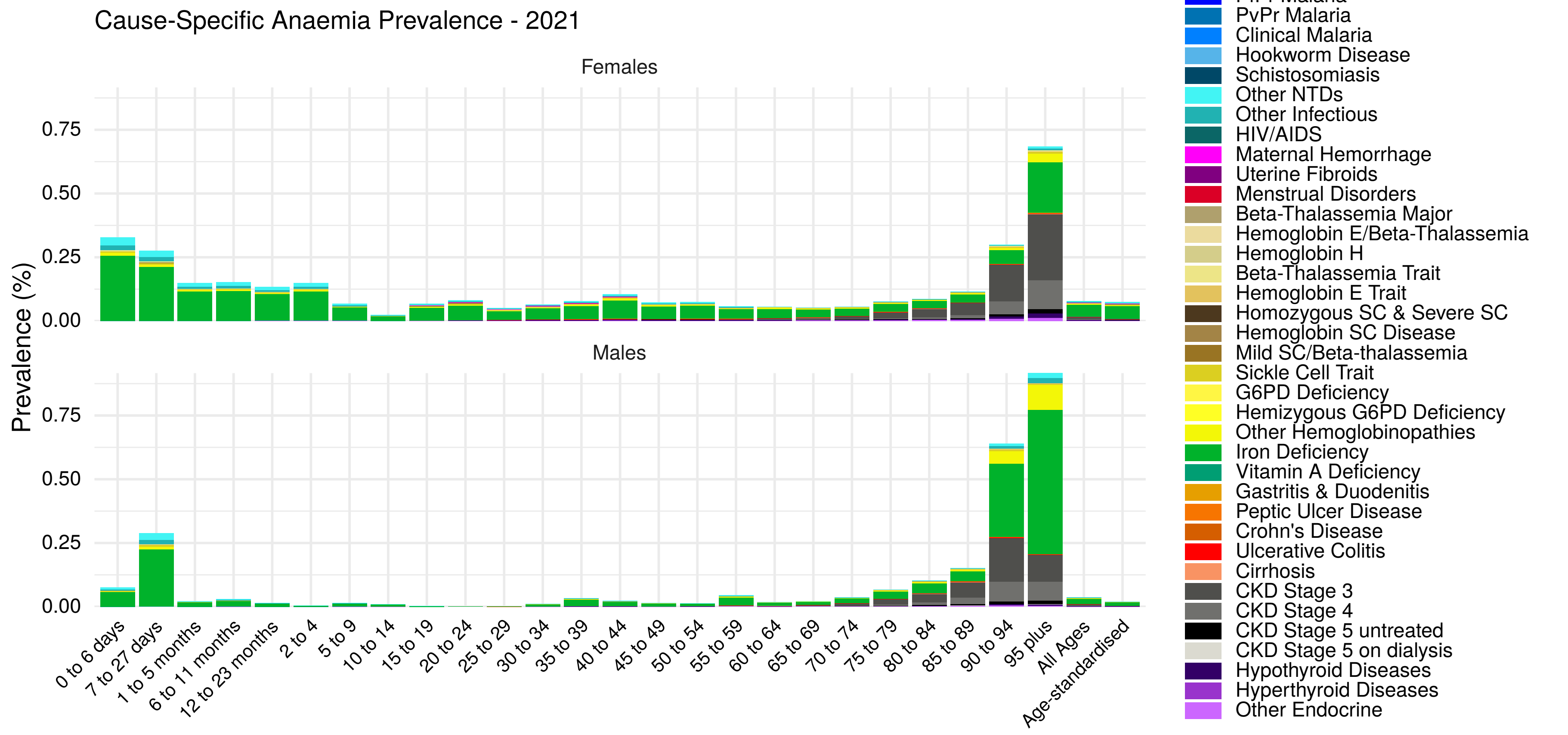
Cause-Specific Anaemia Prevalence - 2021
Females
Males
Prevalence (%)
0.00
0.25
0.50
0.75
0 to 6 days
7 to 27 days
1 to 5 months
6 to 11 months
12 to 23 months
2 to 4
5 to 9
10 to 14
15 to 19
20 to 24
25 to 29
30 to 34
35 to 39
40 to 44
45 to 49
50 to 54
55 to 59
60 to 64
65 to 69
70 to 74
75 to 79
80 to 84
85 to 89
90 to 94
95 plus
All Ages
Age-standardised
PfPr Malaria
PvPr Malaria
Clinical Malaria
Hookworm Disease
Schistosomiasis
Other NTDs
Other Infectious
HIV/AIDS
Maternal Hemorrhage
Uterine Fibroids
Menstrual Disorders
Beta-Thalassemia Major
Hemoglobin E/Beta-Thalassemia
Hemoglobin H
Beta-Thalassemia Trait
Hemoglobin E Trait
Homozygous SC & Severe SC
Hemoglobin SC Disease
Mild SC/Beta-thalassemia
Sickle Cell Trait
G6PD Deficiency
Hemizygous G6PD Deficiency
Other Hemoglobinopathies
Iron Deficiency
Vitamin A Deficiency
Gastritis & Duodenitis
Peptic Ulcer Disease
Crohn's Disease
Ulcerative Colitis
Cirrhosis
CKD Stage 3
CKD Stage 4
CKD Stage 5 untreated
CKD Stage 5 on dialysis
Hypothyroid Diseases
Hyperthyroid Diseases
Other Endocrine

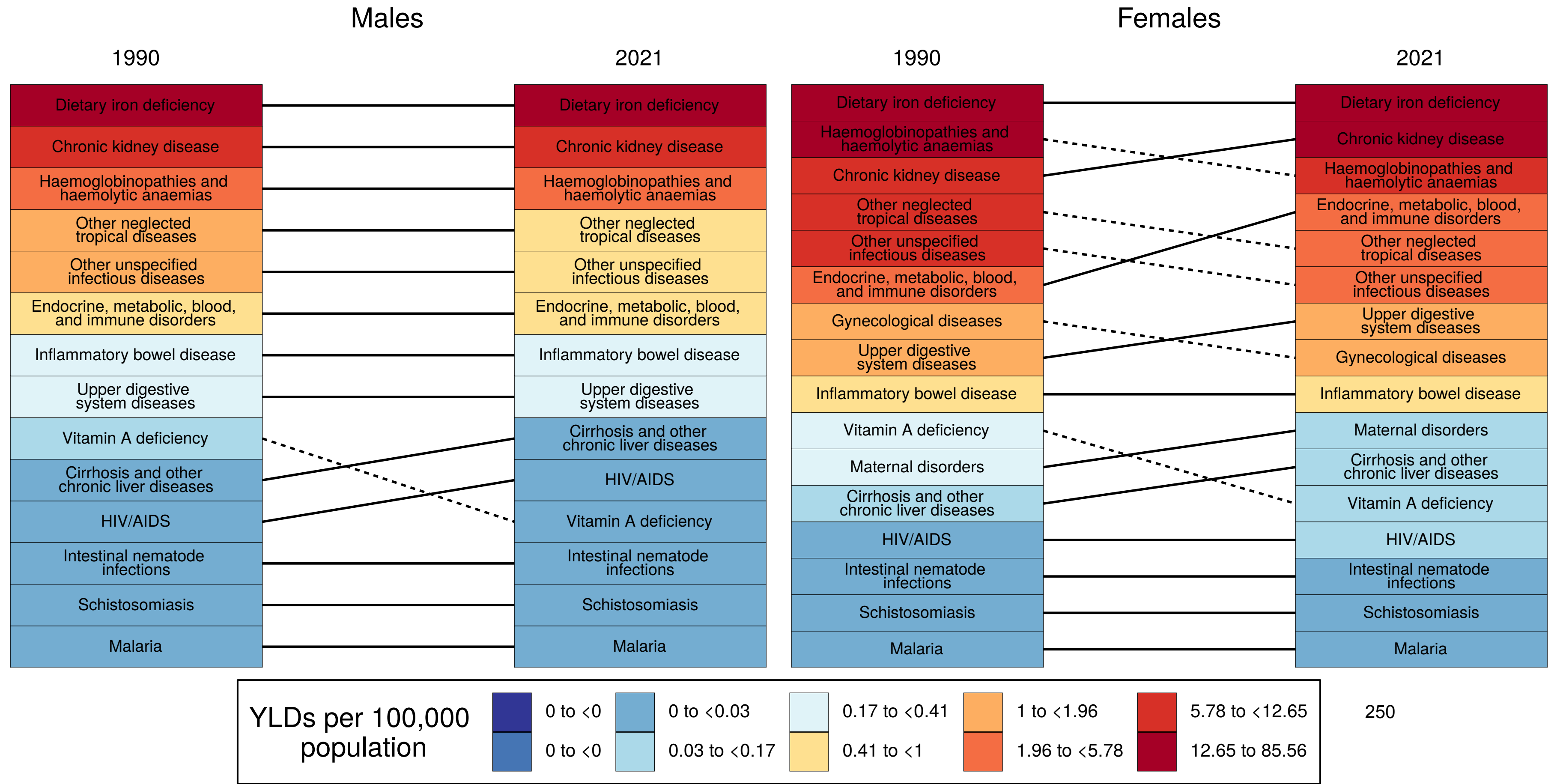
Males
Females
1990
2021
Dietary iron deficiency
Chronic kidney disease
Haemoglobinopathies and haemolytic anaemias
Other neglected tropical diseases
Other unspecified infectious diseases
Endocrine, metabolic, blood, and immune disorders
Inflammatory bowel disease
Upper digestive system diseases
Vitamin A deficiency
Cirrhosis and other chronic liver diseases
HIV/AIDS
Intestinal nematode infections
Schistosomiasis
Malaria
Gynecological diseases
Maternal disorders
YLDs per 100,000 population
0 to <0
0 to <0.03
0.03 to <0.17
0.17 to <0.41
0.41 to <1
1 to <1.96
1.96 to <5.78
5.78 to <12.65
12.65 to 85.56

Spain

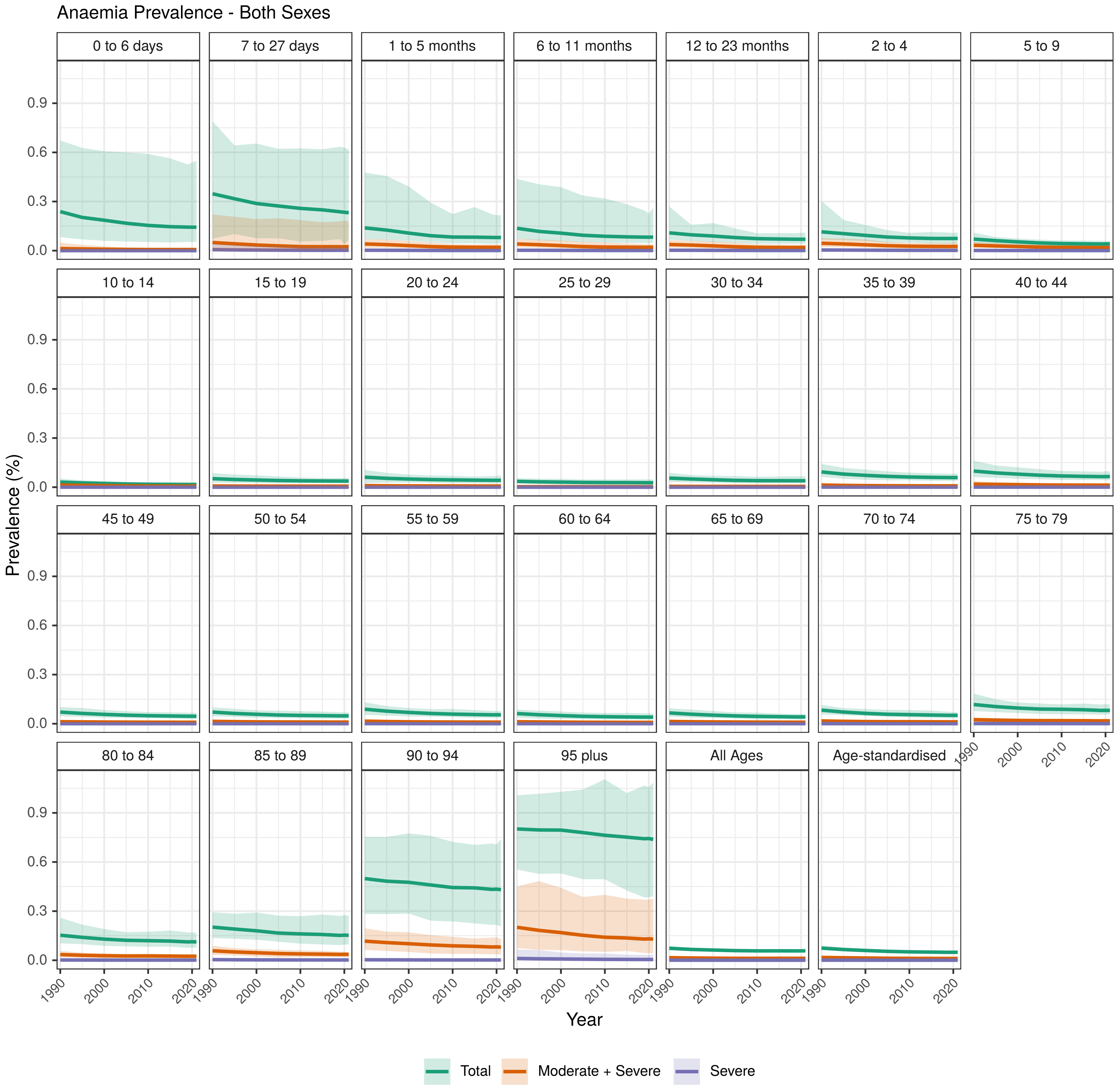
Anaemia Prevalence - Both Sexes
0 to 6 days
7 to 27 days
1 to 5 months
6 to 11 months
12 to 23 months
2 to 4
5 to 9
10 to 14
15 to 19
20 to 24
25 to 29
30 to 34
35 to 39
40 to 44
45 to 49
50 to 54
55 to 59
60 to 64
65 to 69
70 to 74
75 to 79
80 to 84
85 to 89
90 to 94
95 plus
All Ages
Age-standardised
Prevalence (%)
0.0
0.3
0.6
0.9
1990
2000
2010
2020
Year
Total
Moderate + Severe
Severe

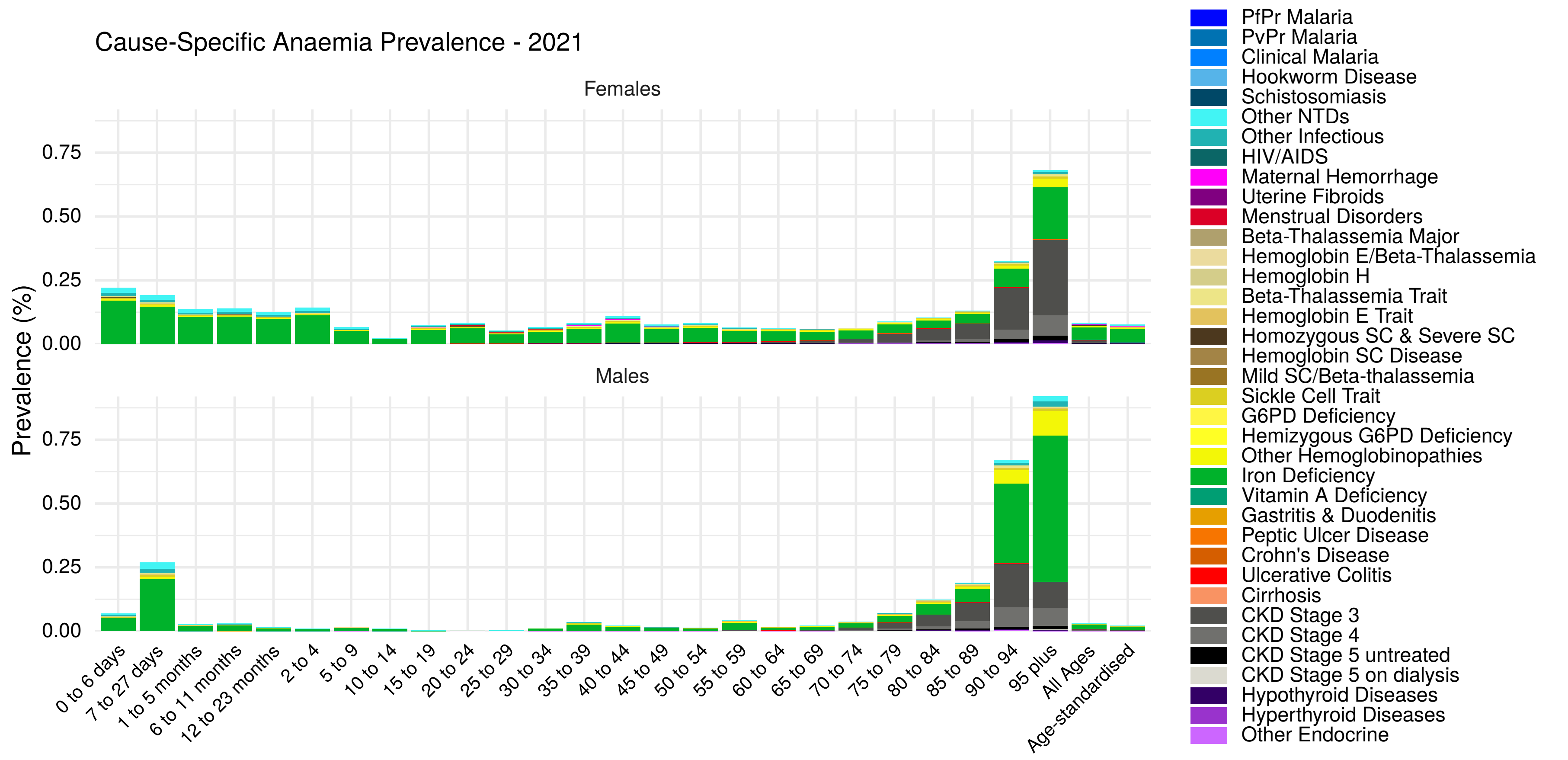
Cause-Specific Anaemia Prevalence - 2021
Females
Males
Prevalence (%)
0.00
0.25
0.50
0.75
0 to 6 days
7 to 27 days
1 to 5 months
6 to 11 months
12 to 23 months
2 to 4
5 to 9
10 to 14
15 to 19
20 to 24
25 to 29
30 to 34
35 to 39
40 to 44
45 to 49
50 to 54
55 to 59
60 to 64
65 to 69
70 to 74
75 to 79
80 to 84
85 to 89
90 to 94
95 plus
All Ages
Age-standardised
PfPr Malaria
PvPr Malaria
Clinical Malaria
Hookworm Disease
Schistosomiasis
Other NTDs
Other Infectious
HIV/AIDS
Maternal Hemorrhage
Uterine Fibroids
Menstrual Disorders
Beta-Thalassemia Major
Hemoglobin E/Beta-Thalassemia
Hemoglobin H
Beta-Thalassemia Trait
Hemoglobin E Trait
Homozygous SC & Severe SC
Hemoglobin SC Disease
Mild SC/Beta-thalassemia
Sickle Cell Trait
G6PD Deficiency
Hemizygous G6PD Deficiency
Other Hemoglobinopathies
Iron Deficiency
Vitamin A Deficiency
Gastritis & Duodenitis
Peptic Ulcer Disease
Crohn's Disease
Ulcerative Colitis
Cirrhosis
CKD Stage 3
CKD Stage 4
CKD Stage 5 untreated
CKD Stage 5 on dialysis
Hypothyroid Diseases
Hyperthyroid Diseases
Other Endocrine

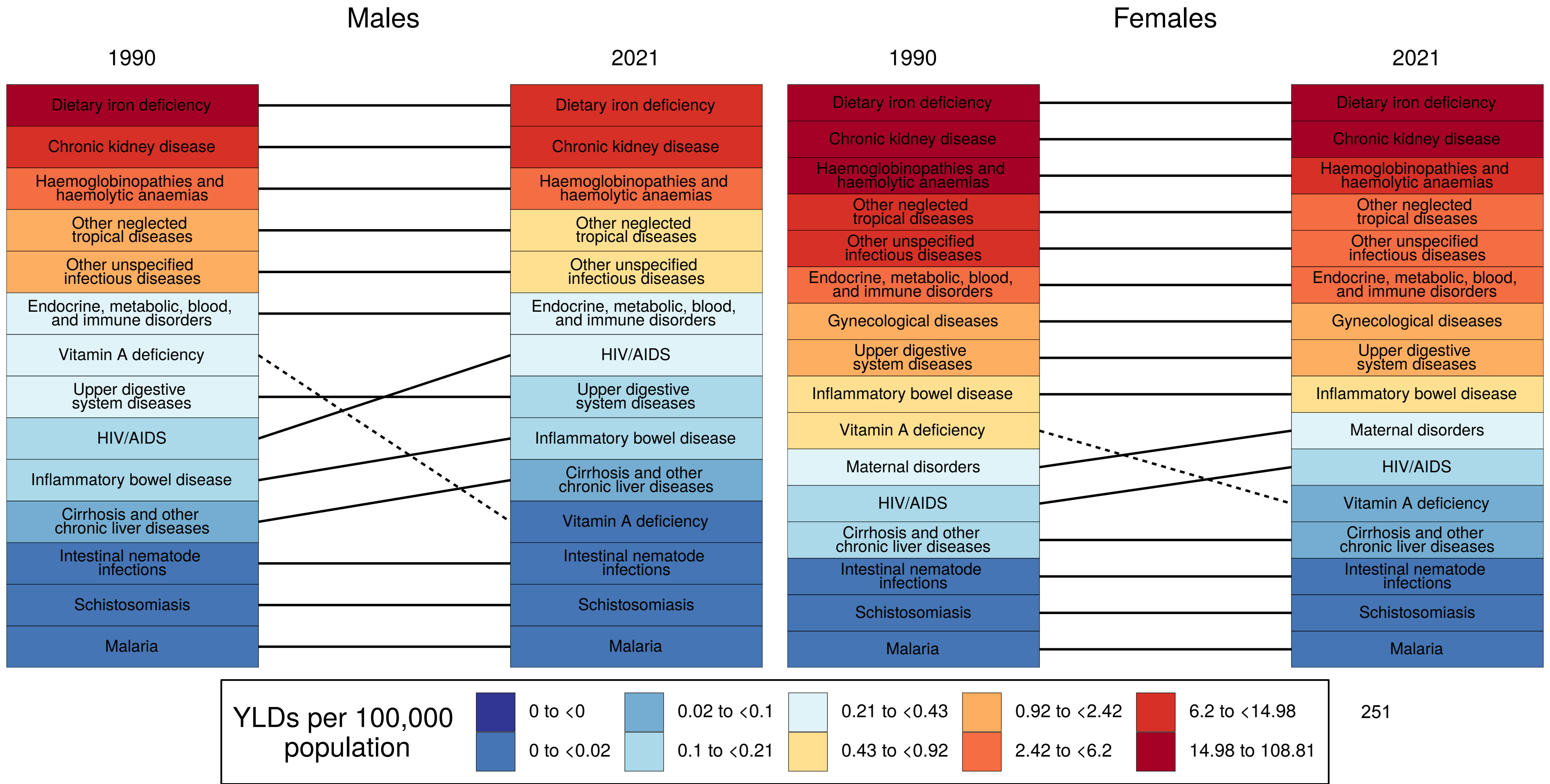
Males
Females
1990
2021
Dietary iron deficiency
Chronic kidney disease
Haemoglobinopathies and haemolytic anaemias
Other neglected tropical diseases
Other unspecified infectious diseases
Endocrine, metabolic, blood, and immune disorders
Vitamin A deficiency
Upper digestive system diseases
HIV/AIDS
Inflammatory bowel disease
Cirrhosis and other chronic liver diseases
Intestinal nematode infections
Schistosomiasis
Malaria
Gynecological diseases
Maternal disorders
YLDs per 100,000 population
0 to <0
0 to <0.02
0.02 to <0.1
0.1 to <0.21
0.21 to <0.43
0.43 to <0.92
0.92 to <2.42
2.42 to <6.2
6.2 to <14.98
14.98 to 108.81

Sweden

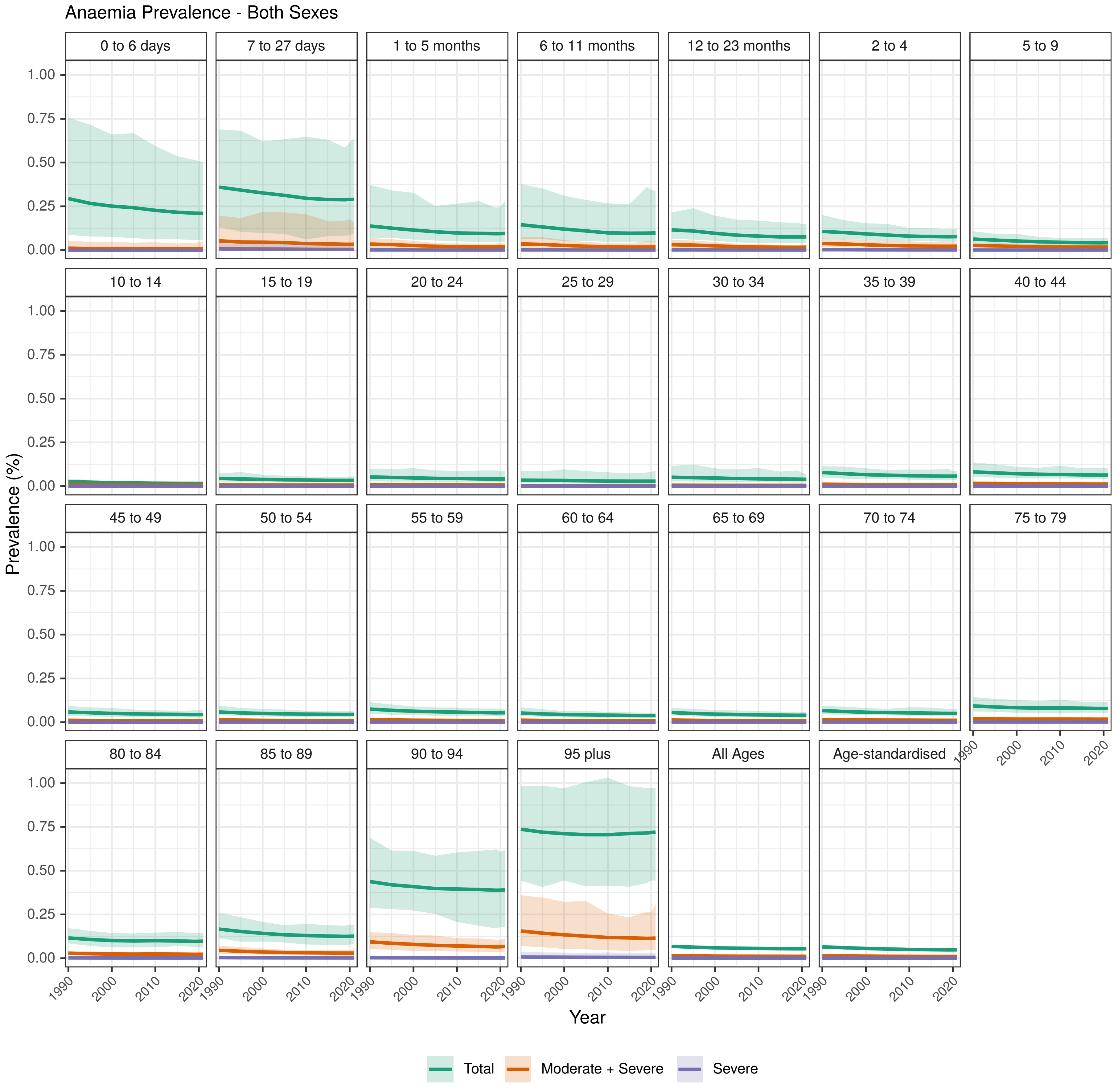
Anaemia Prevalence - Both Sexes
0 to 6 days
7 to 27 days
1 to 5 months
6 to 11 months
12 to 23 months
2 to 4
5 to 9
10 to 14
15 to 19
20 to 24
25 to 29
30 to 34
35 to 39
40 to 44
45 to 49
50 to 54
55 to 59
60 to 64
65 to 69
70 to 74
75 to 79
80 to 84
85 to 89
90 to 94
95 plus
All Ages
Age-standardised
Prevalence (%)
Year
Total
Moderate + Severe
Severe

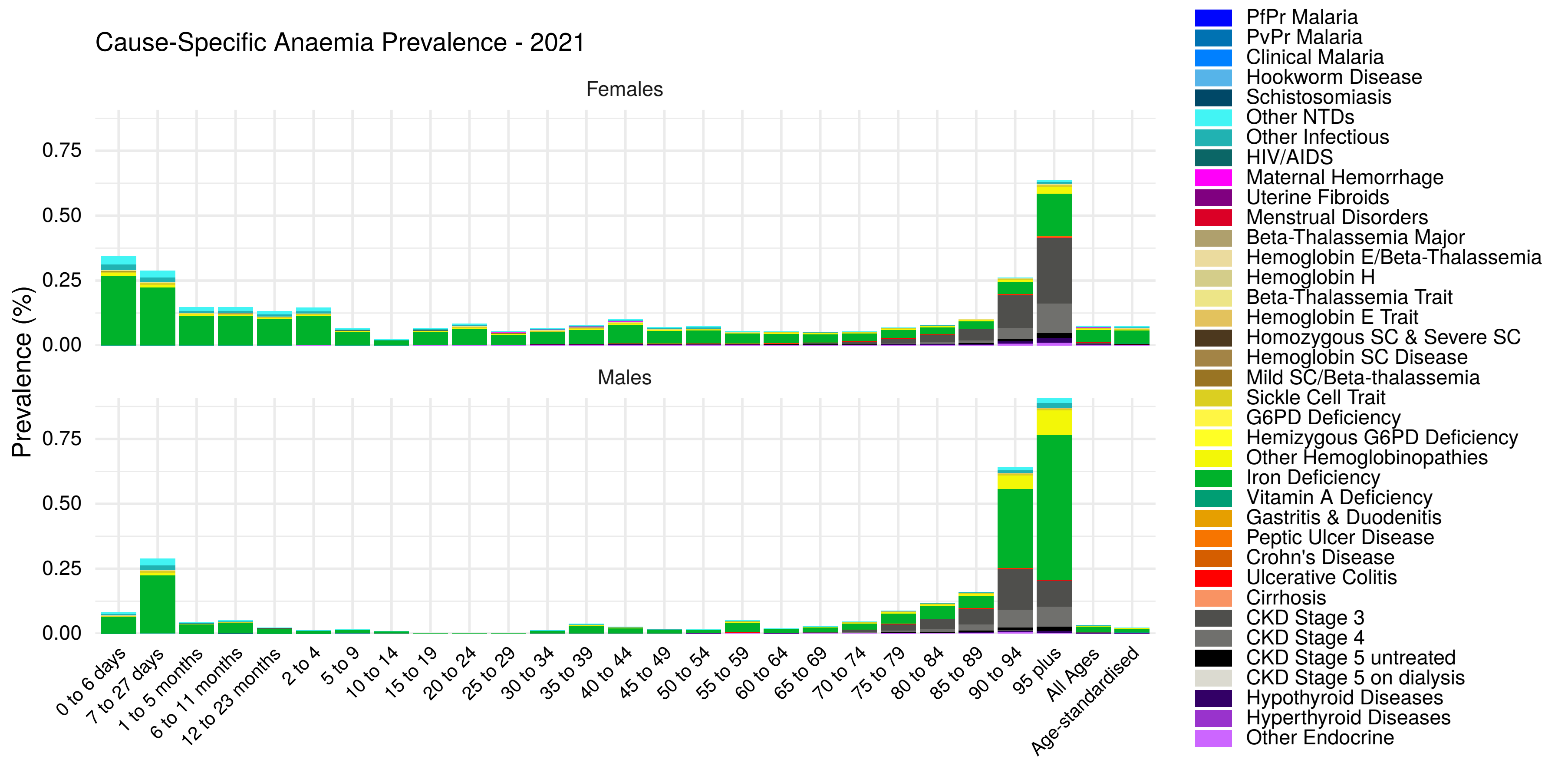
Cause-Specific Anaemia Prevalence - 2021
Females
Males
Prevalence (%)
PfPr Malaria
PvPr Malaria
Clinical Malaria
Hookworm Disease
Schistosomiasis
Other NTDs
Other Infectious
HIV/AIDS
Maternal Hemorrhage
Uterine Fibroids
Menstrual Disorders
Beta-Thalassemia Major
Hemoglobin E/Beta-Thalassemia
Hemoglobin H
Beta-Thalassemia Trait
Hemoglobin E Trait
Homozygous SC & Severe SC
Hemoglobin SC Disease
Mild SC/Beta-thalassemia
Sickle Cell Trait
G6PD Deficiency
Hemizygous G6PD Deficiency
Other Hemoglobinopathies
Iron Deficiency
Vitamin A Deficiency
Gastritis & Duodenitis
Peptic Ulcer Disease
Crohn's Disease
Ulcerative Colitis
Cirrhosis
CKD Stage 3
CKD Stage 4
CKD Stage 5 untreated
CKD Stage 5 on dialysis
Hypothyroid Diseases
Hyperthyroid Diseases
Other Endocrine

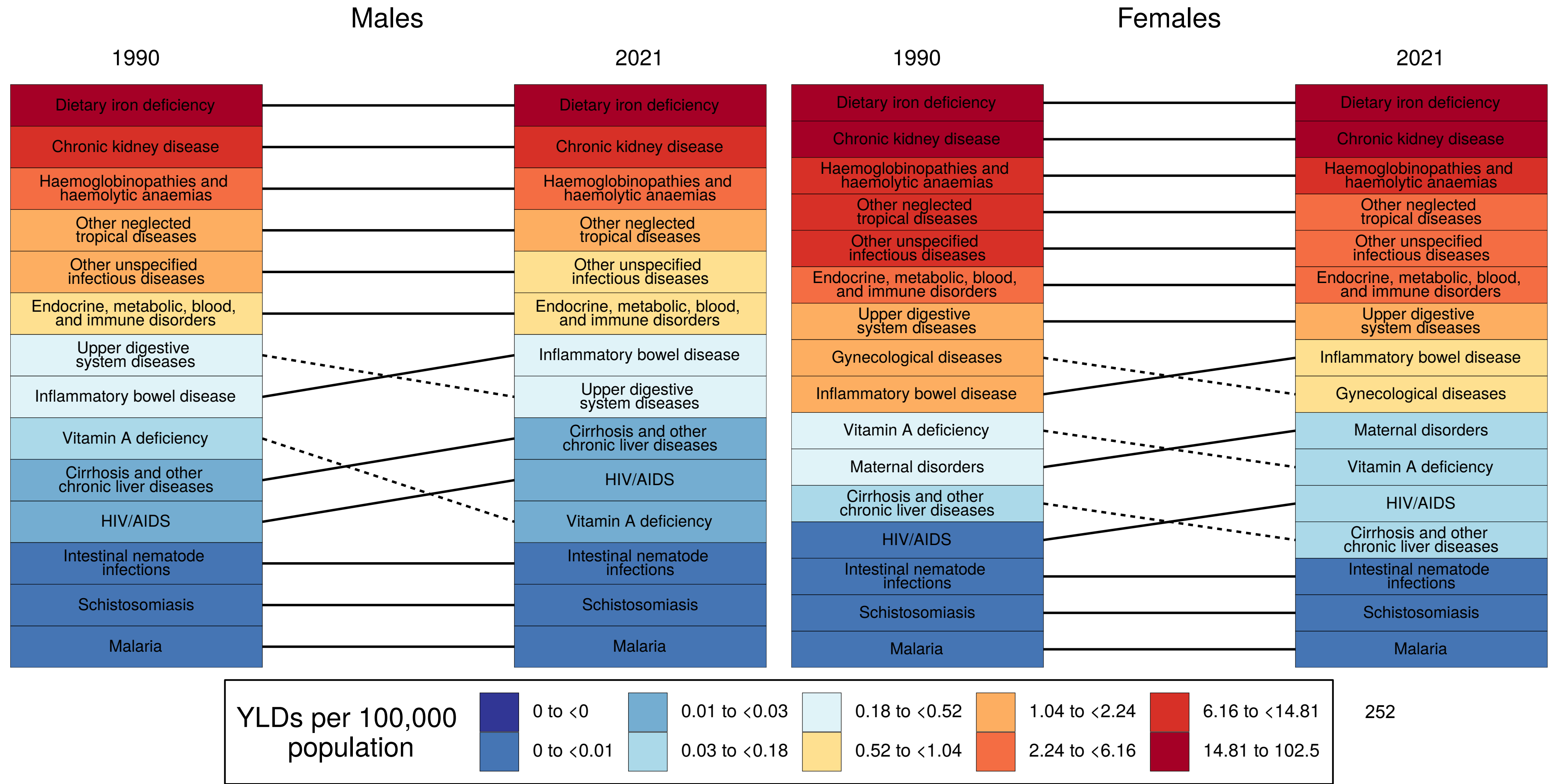
Males
Females
1990
2021
Dietary iron deficiency
Chronic kidney disease
Haemoglobinopathies and haemolytic anaemias
Other neglected tropical diseases
Other unspecified infectious diseases
Endocrine, metabolic, blood, and immune disorders
Upper digestive system diseases
Inflammatory bowel disease
Gynecological diseases
Maternal disorders
Vitamin A deficiency
Cirrhosis and other chronic liver diseases
HIV/AIDS
Intestinal nematode infections
Schistosomiasis
Malaria
YLDs per 100,000 population
0 to <0
0 to <0.01
0.01 to <0.03
0.03 to <0.18
0.18 to <0.52
0.52 to <1.04
1.04 to <2.24
2.24 to <6.16
6.16 to <14.81
14.81 to 102.5

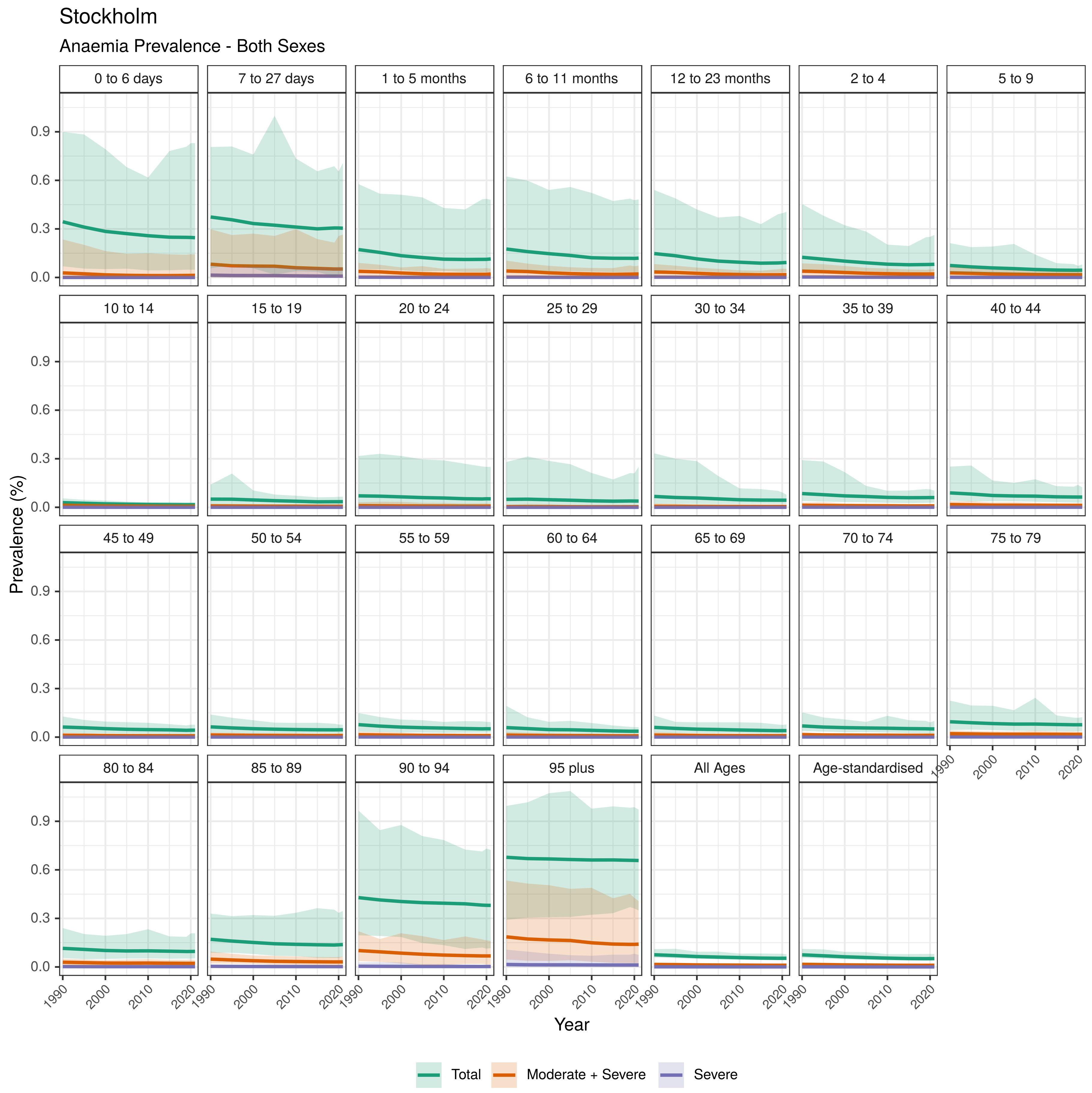
Stockholm
Anaemia Prevalence - Both Sexes
0 to 6 days
7 to 27 days
1 to 5 months
6 to 11 months
12 to 23 months
2 to 4
5 to 9
10 to 14
15 to 19
20 to 24
25 to 29
30 to 34
35 to 39
40 to 44
45 to 49
50 to 54
55 to 59
60 to 64
65 to 69
70 to 74
75 to 79
80 to 84
85 to 89
90 to 94
95 plus
All Ages
Age-standardised
Prevalence (%)
0.0
0.3
0.6
0.9
1990
2000
2010
2020
Year
Total
Moderate + Severe
Severe

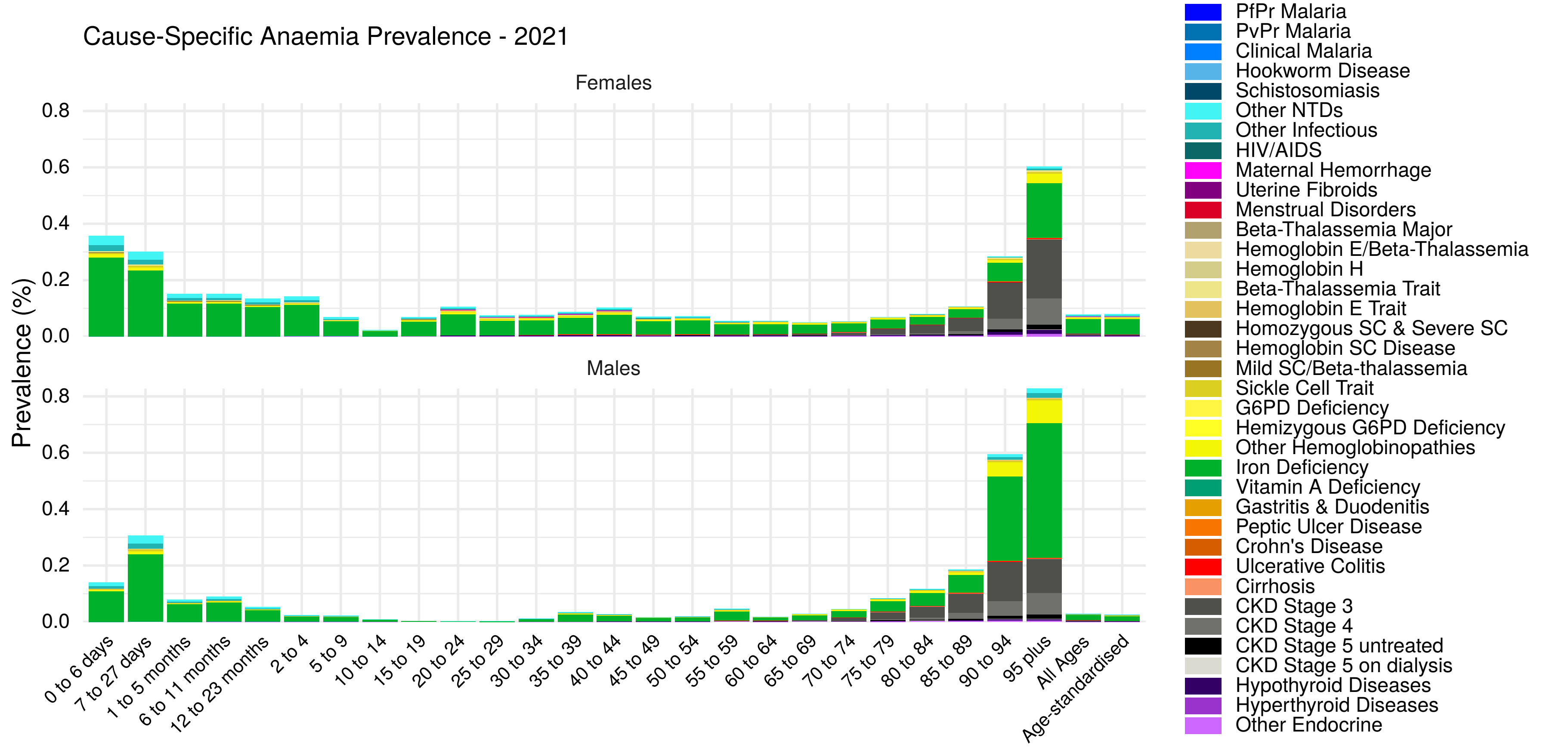
Cause-Specific Anaemia Prevalence - 2021
Females
Males
Prevalence (%)
0.0
0.2
0.4
0.6
0.8
0 to 6 days
7 to 27 days
1 to 5 months
6 to 11 months
12 to 23 months
2 to 4
5 to 9
10 to 14
15 to 19
20 to 24
25 to 29
30 to 34
35 to 39
40 to 44
45 to 49
50 to 54
55 to 59
60 to 64
65 to 69
70 to 74
75 to 79
80 to 84
85 to 89
90 to 94
95 plus
All Ages
Age-standardised
PfPr Malaria
PvPr Malaria
Clinical Malaria
Hookworm Disease
Schistosomiasis
Other NTDs
Other Infectious
HIV/AIDS
Maternal Hemorrhage
Uterine Fibroids
Menstrual Disorders
Beta-Thalassemia Major
Hemoglobin E/Beta-Thalassemia
Hemoglobin H
Beta-Thalassemia Trait
Hemoglobin E Trait
Homozygous SC & Severe SC
Hemoglobin SC Disease
Mild SC/Beta-thalassemia
Sickle Cell Trait
G6PD Deficiency
Hemizygous G6PD Deficiency
Other Hemoglobinopathies
Iron Deficiency
Vitamin A Deficiency
Gastritis & Duodenitis
Peptic Ulcer Disease
Crohn's Disease
Ulcerative Colitis
Cirrhosis
CKD Stage 3
CKD Stage 4
CKD Stage 5 untreated
CKD Stage 5 on dialysis
Hypothyroid Diseases
Hyperthyroid Diseases
Other Endocrine

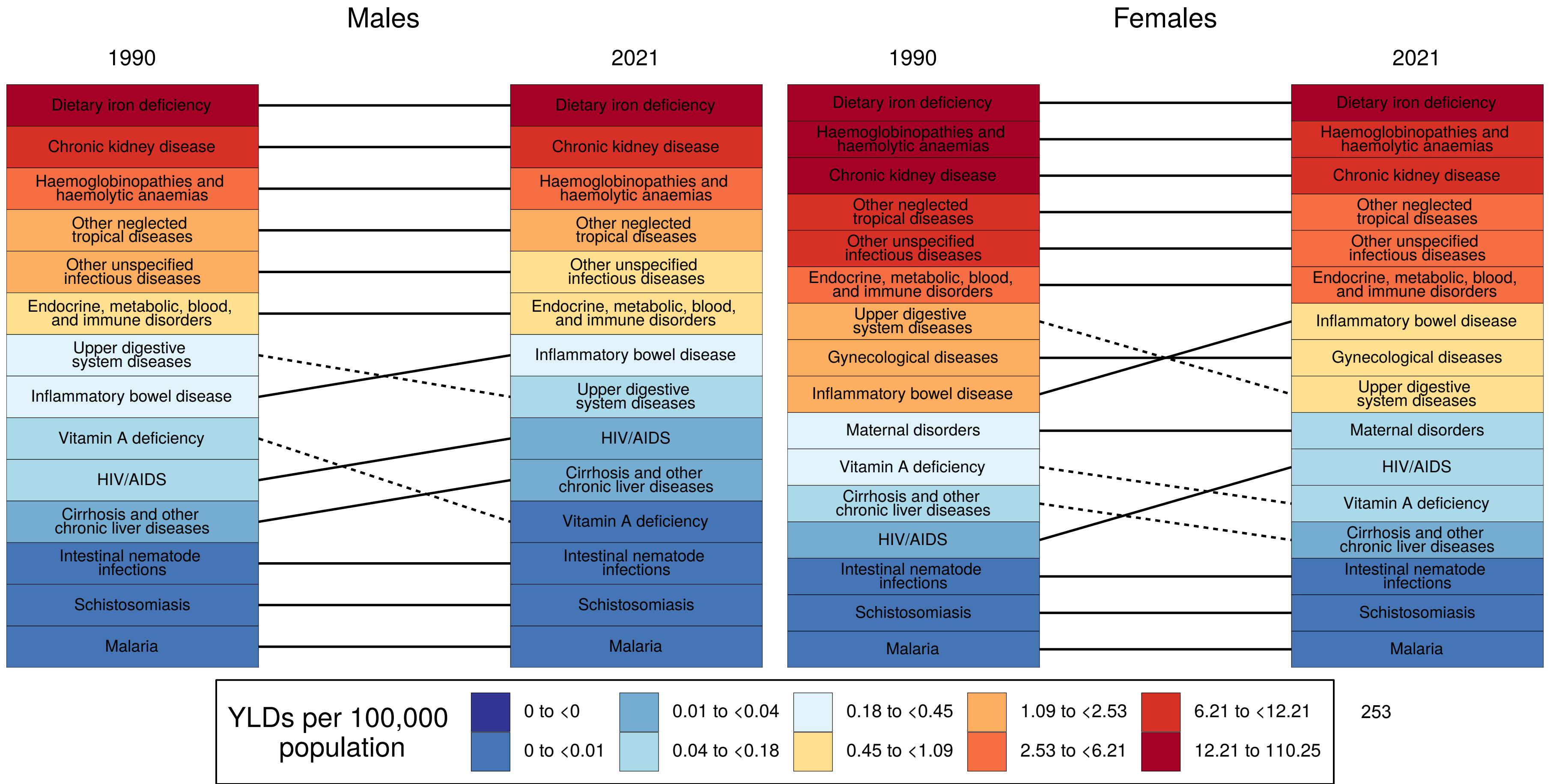
Males
Females
1990
2021
Dietary iron deficiency
Chronic kidney disease
Haemoglobinopathies and haemolytic anaemias
Other neglected tropical diseases
Other unspecified infectious diseases
Endocrine, metabolic, blood, and immune disorders
Upper digestive system diseases
Inflammatory bowel disease
Vitamin A deficiency
HIV/AIDS
Cirrhosis and other chronic liver diseases
Intestinal nematode infections
Schistosomiasis
Malaria
Gynecological diseases
Maternal disorders
YLDs per 100,000 population
0 to <0
0 to <0.01
0.01 to <0.04
0.04 to <0.18
0.18 to <0.45
0.45 to <1.09
1.09 to <2.53
2.53 to <6.21
6.21 to <12.21
12.21 to 110.25

Sweden except Stockholm

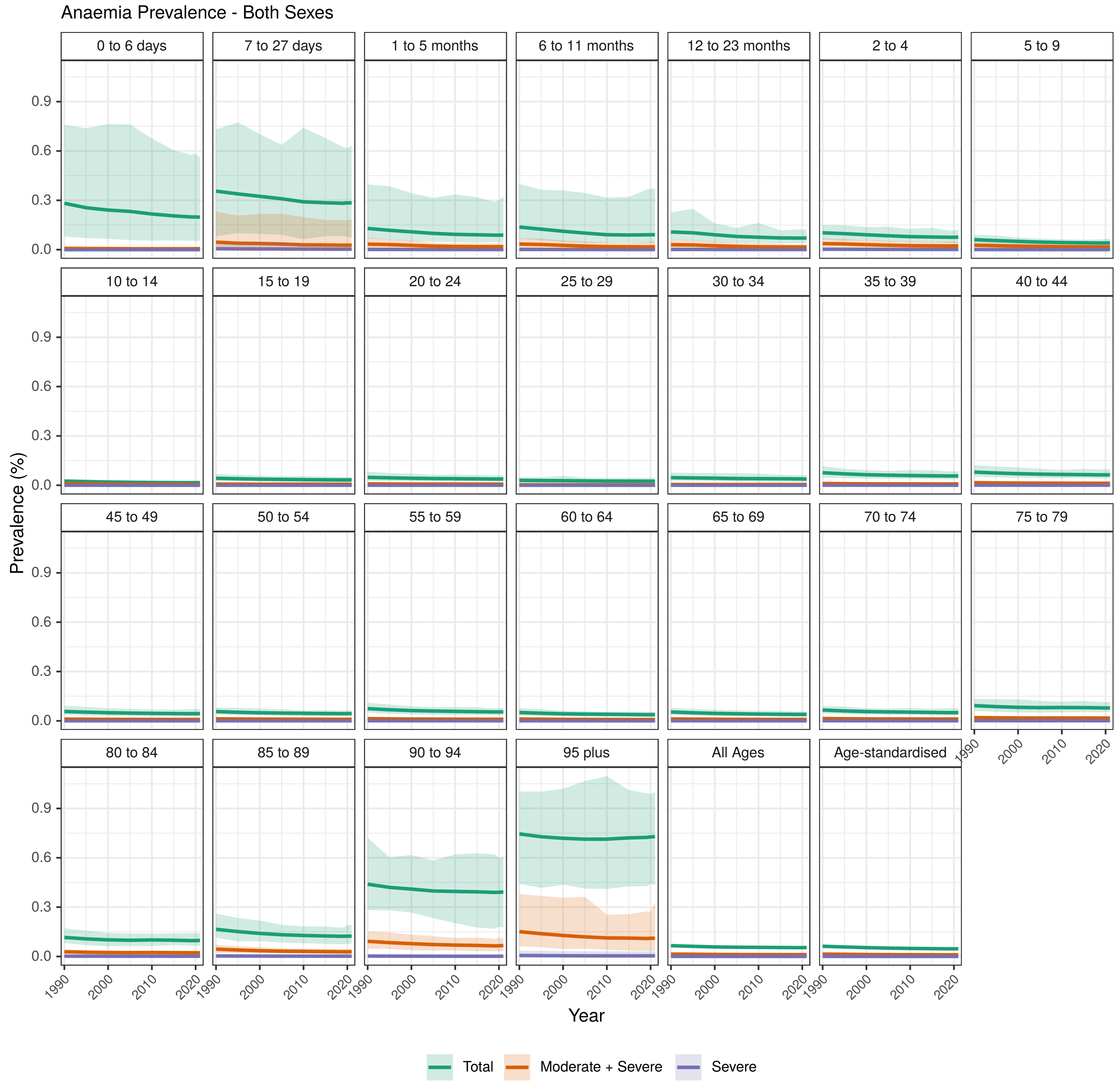

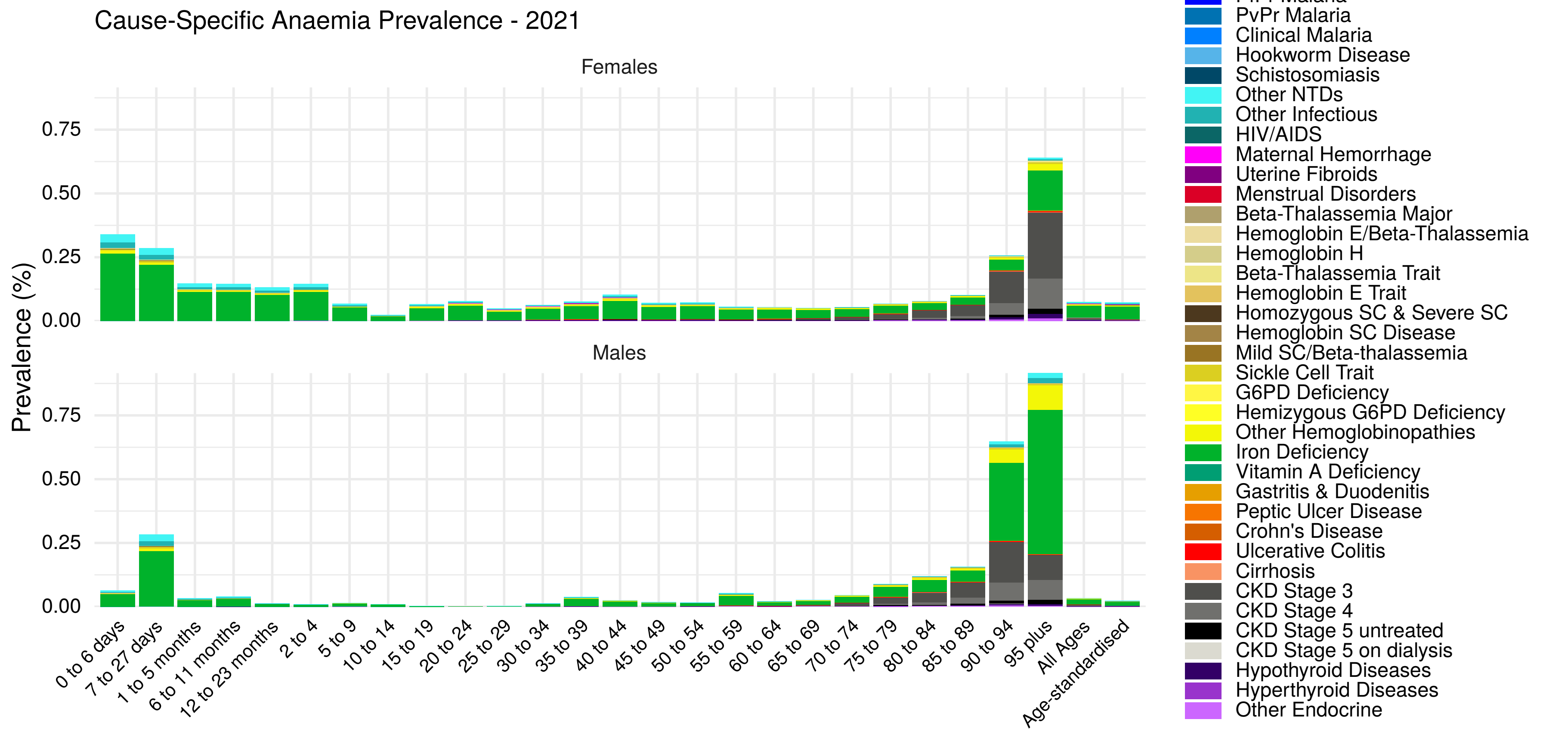

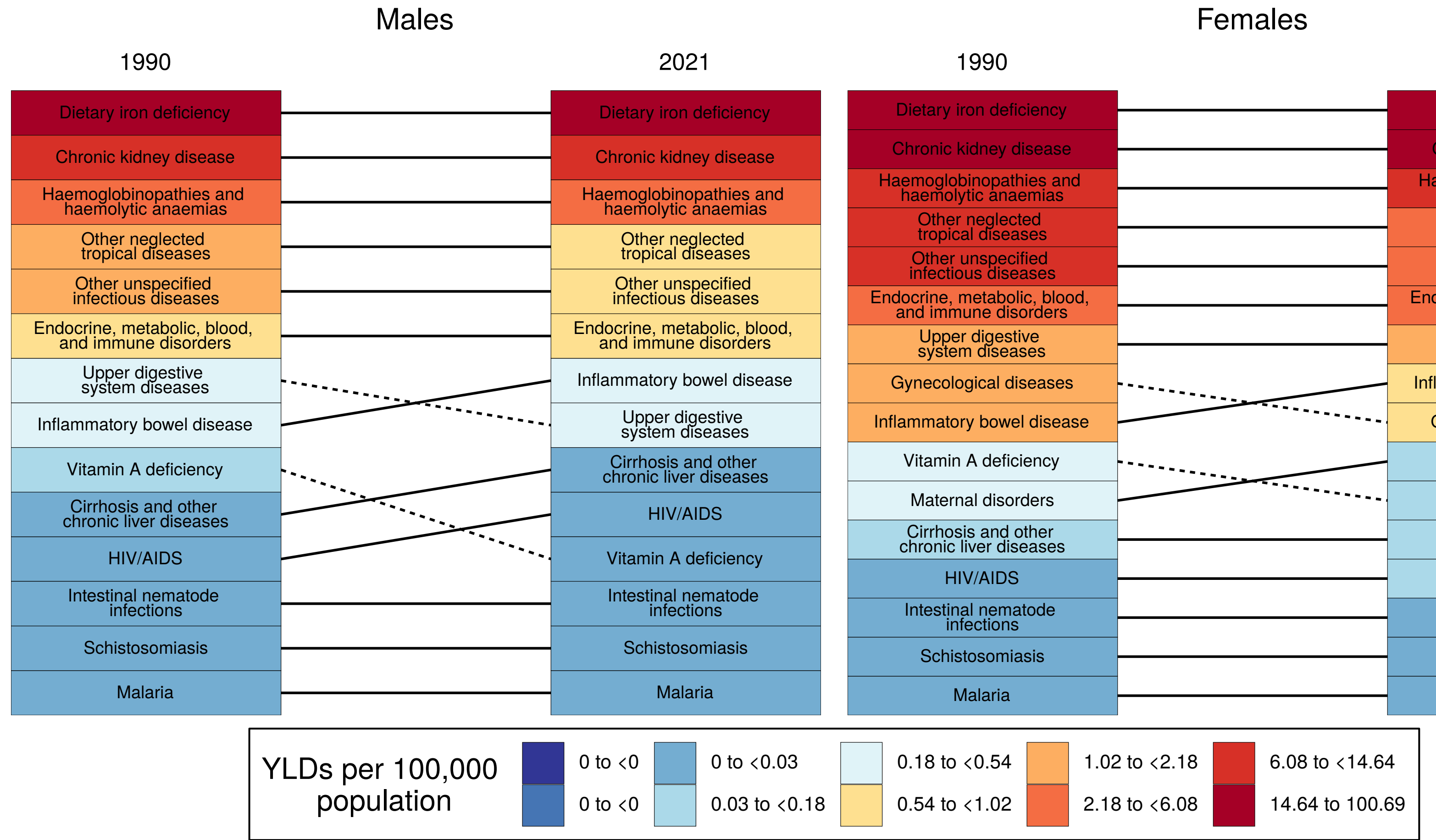

Switzerland

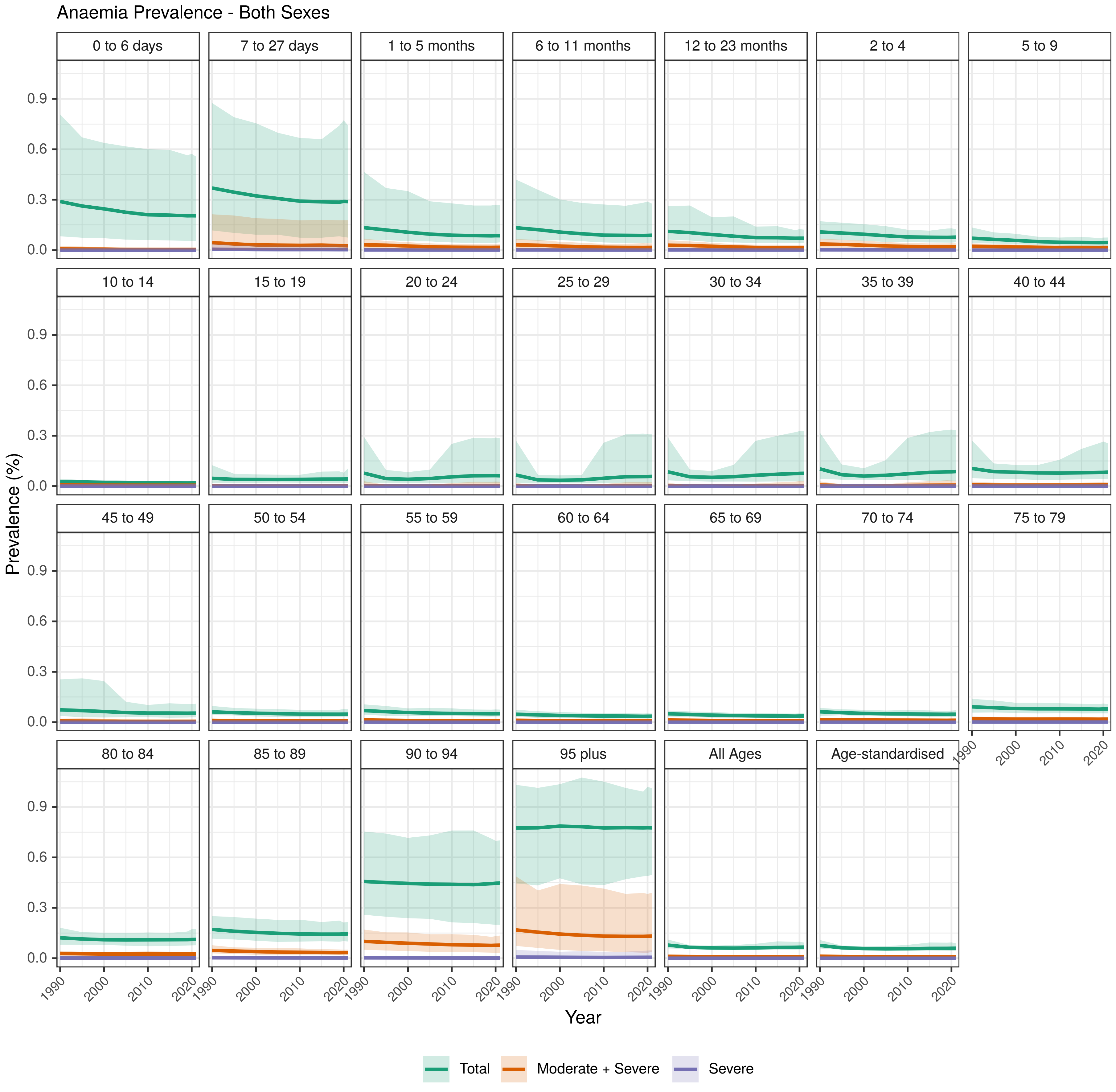

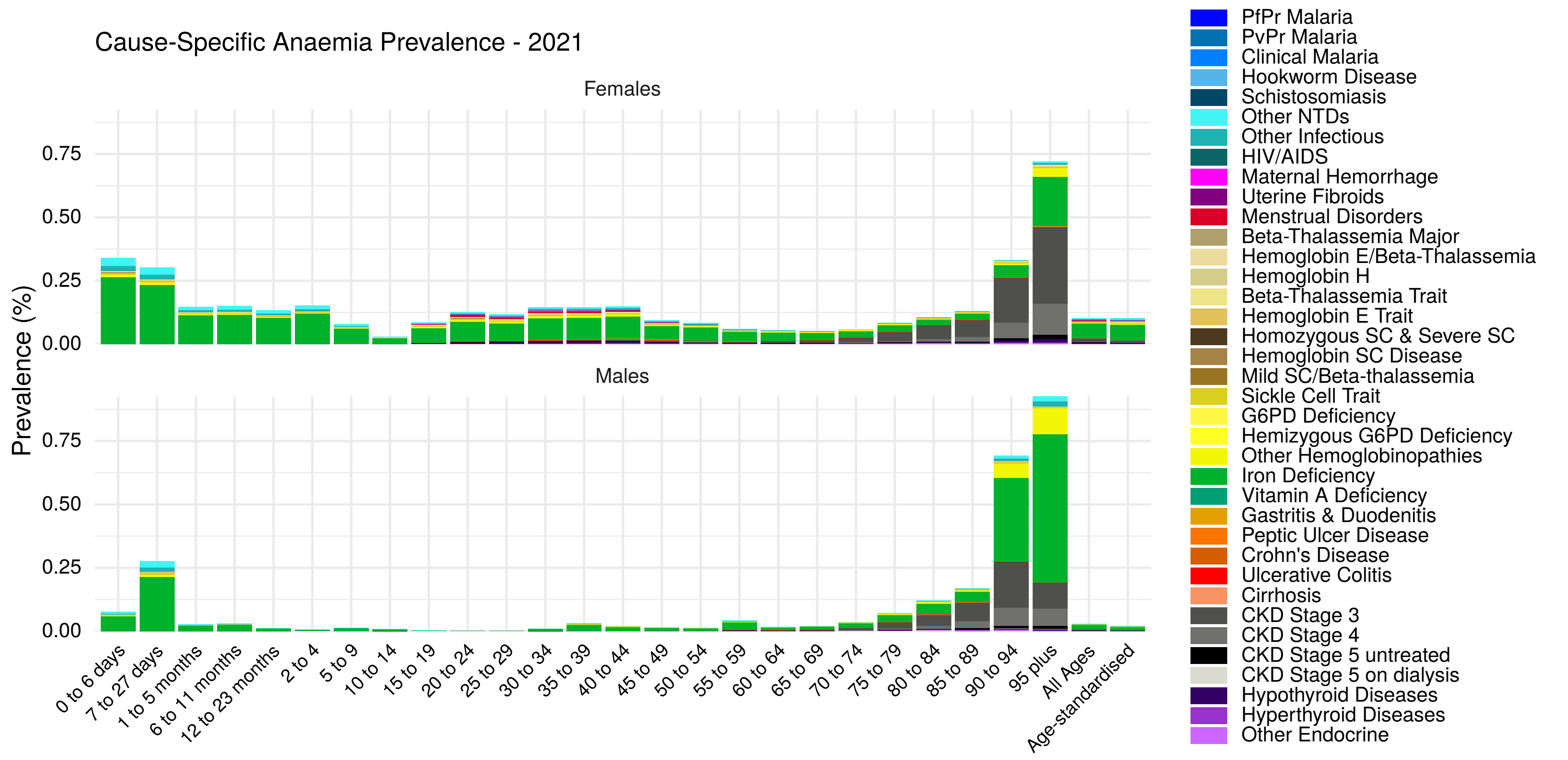

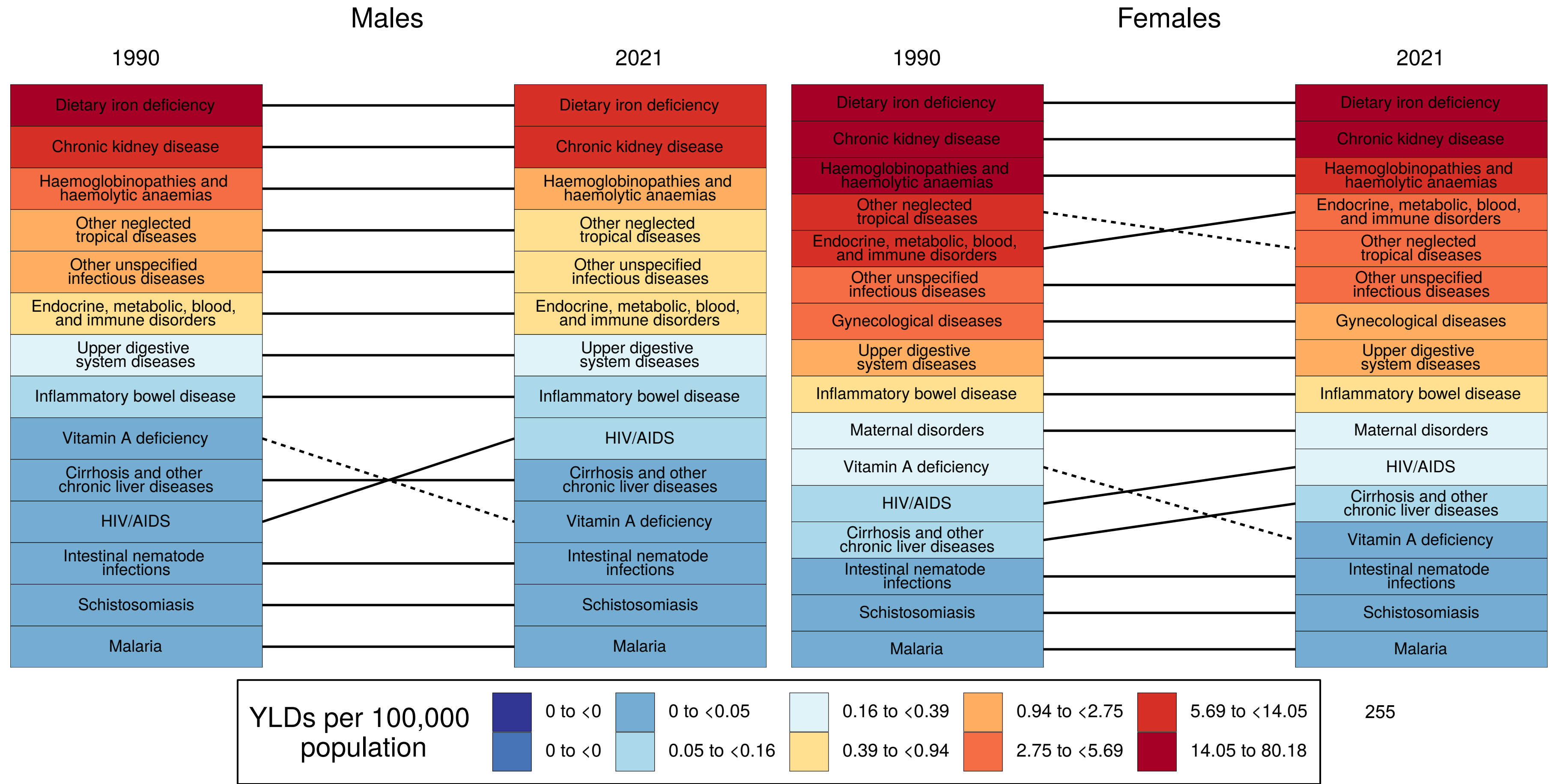

UK

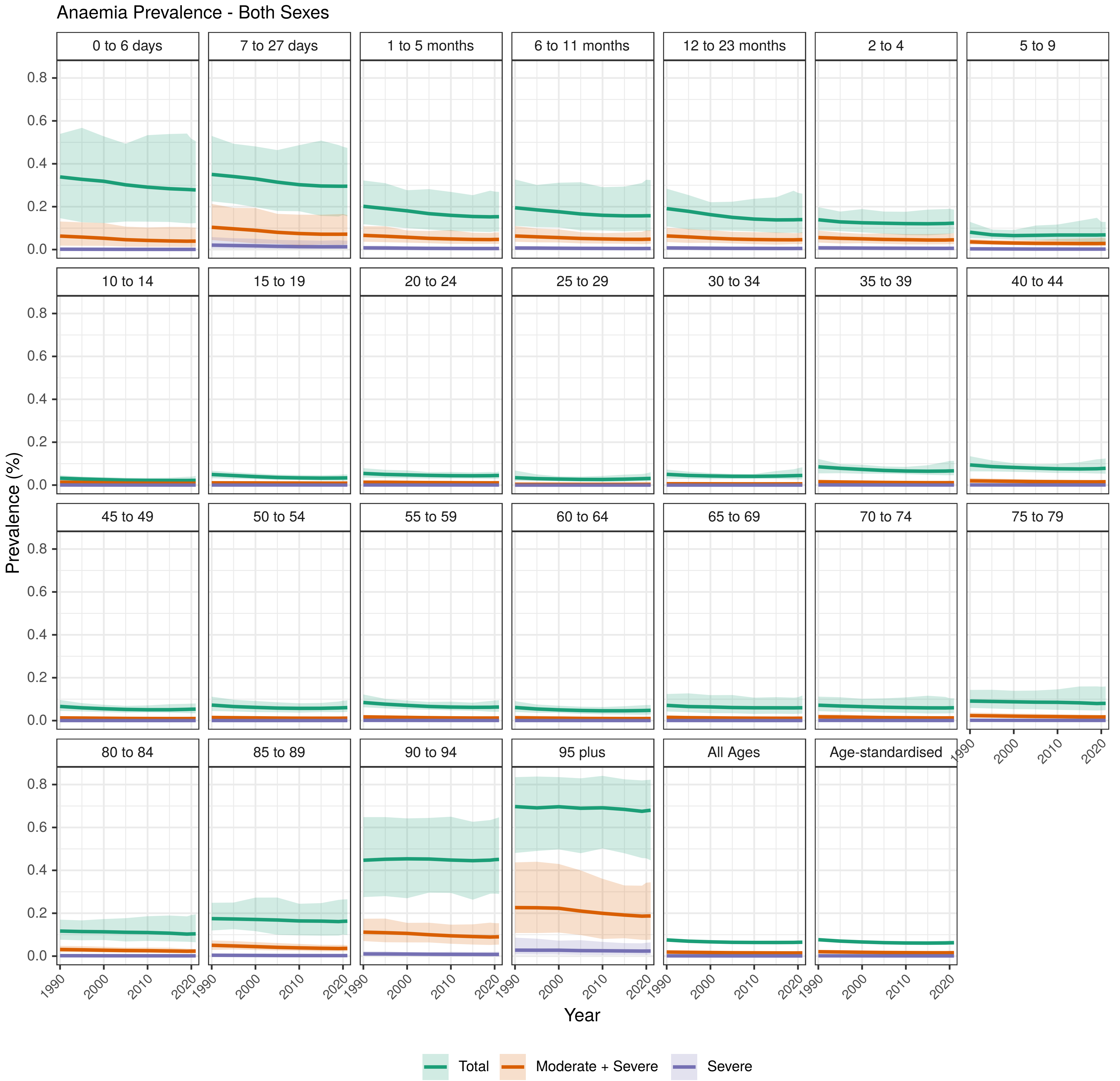
Anaemia Prevalence - Both Sexes
0 to 6 days
7 to 27 days
1 to 5 months
6 to 11 months
12 to 23 months
2 to 4
5 to 9
10 to 14
15 to 19
20 to 24
25 to 29
30 to 34
35 to 39
40 to 44
45 to 49
50 to 54
55 to 59
60 to 64
65 to 69
70 to 74
75 to 79
80 to 84
85 to 89
90 to 94
95 plus
All Ages
Age-standardised
Prevalence (%)
Year
Total
Moderate + Severe
Severe

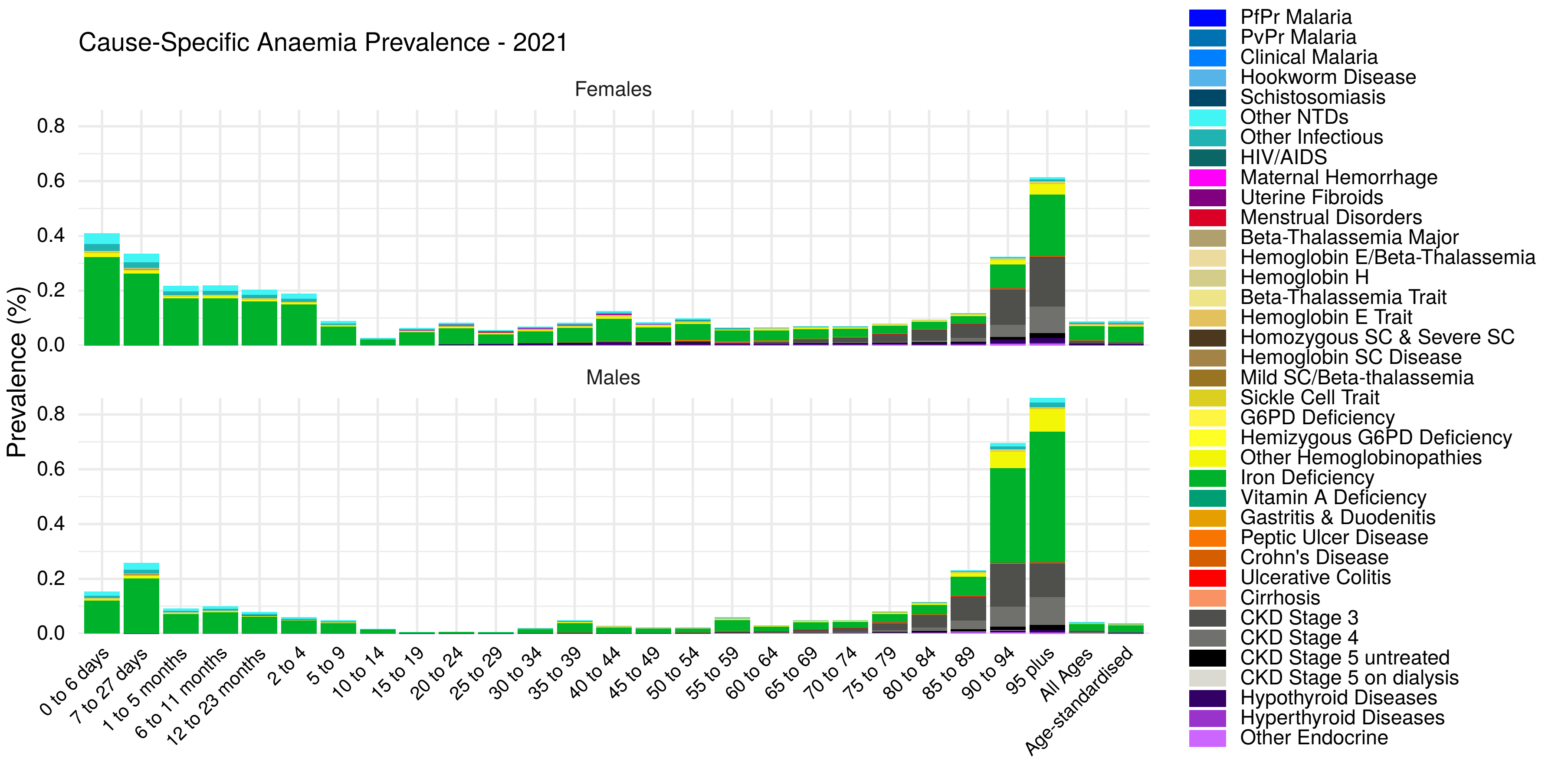
Cause-Specific Anaemia Prevalence - 2021
Females
Males
Prevalence (%)
PfPr Malaria
PvPr Malaria
Clinical Malaria
Hookworm Disease
Schistosomiasis
Other NTDs
Other Infectious
HIV/AIDS
Maternal Hemorrhage
Uterine Fibroids
Menstrual Disorders
Beta-Thalassemia Major
Hemoglobin E/Beta-Thalassemia
Hemoglobin H
Beta-Thalassemia Trait
Hemoglobin E Trait
Homozygous SC & Severe SC
Hemoglobin SC Disease
Mild SC/Beta-thalassemia
Sickle Cell Trait
G6PD Deficiency
Hemizygous G6PD Deficiency
Other Hemoglobinopathies
Iron Deficiency
Vitamin A Deficiency
Gastritis & Duodenitis
Peptic Ulcer Disease
Crohn's Disease
Ulcerative Colitis
Cirrhosis
CKD Stage 3
CKD Stage 4
CKD Stage 5 untreated
CKD Stage 5 on dialysis
Hypothyroid Diseases
Hyperthyroid Diseases
Other Endocrine

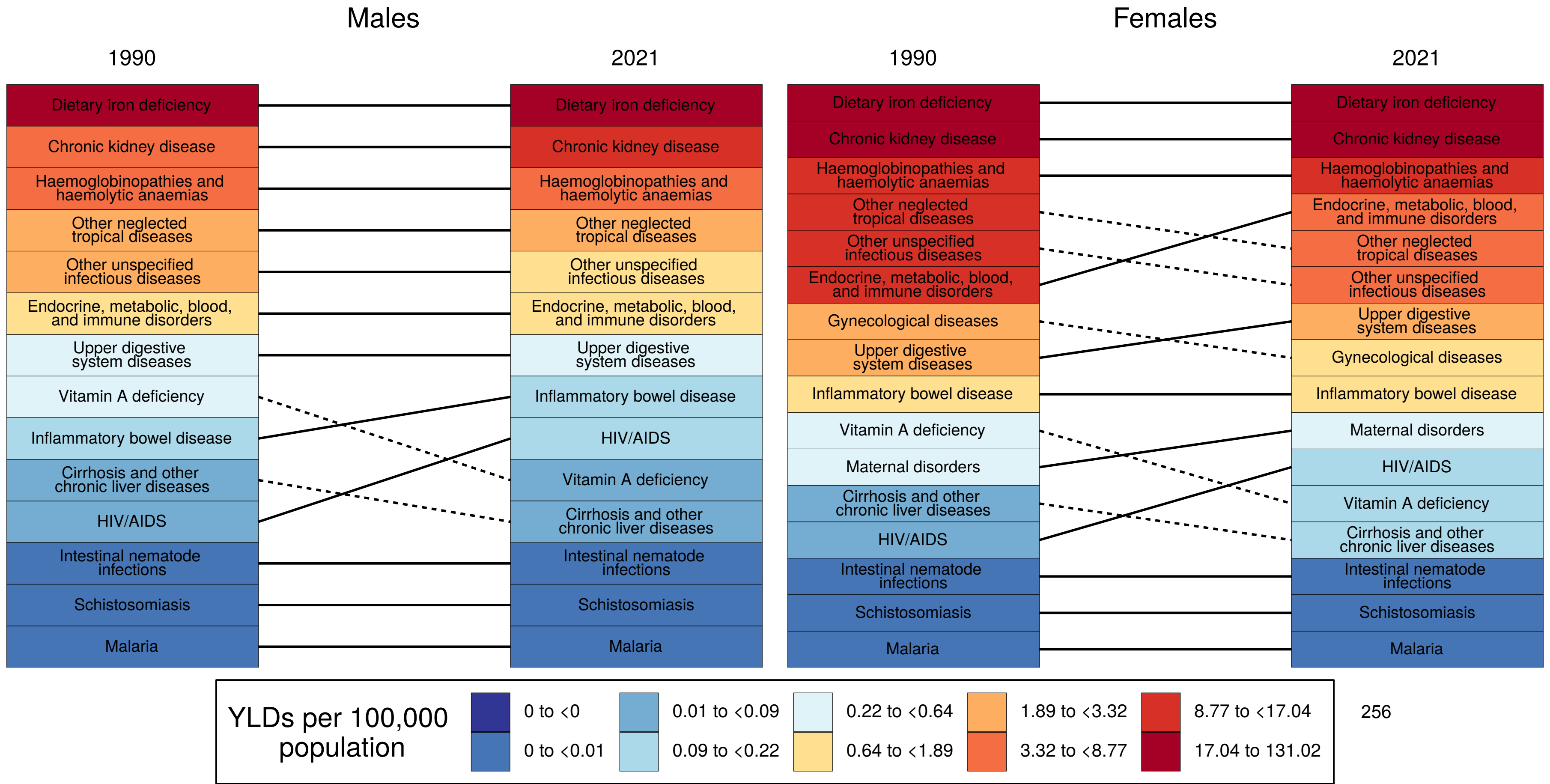
Males
Females
1990
2021
Dietary iron deficiency
Chronic kidney disease
Haemoglobinopathies and haemolytic anaemias
Other neglected tropical diseases
Other unspecified infectious diseases
Endocrine, metabolic, blood, and immune disorders
Upper digestive system diseases
Vitamin A deficiency
Inflammatory bowel disease
Cirrhosis and other chronic liver diseases
HIV/AIDS
Intestinal nematode infections
Schistosomiasis
Malaria
Gynecological diseases
Maternal disorders
YLDs per 100,000 population
0 to <0
0 to <0.01
0.01 to <0.09
0.09 to <0.22
0.22 to <0.64
0.64 to <1.89
1.89 to <3.32
3.32 to <8.77
8.77 to <17.04
17.04 to 131.02

England

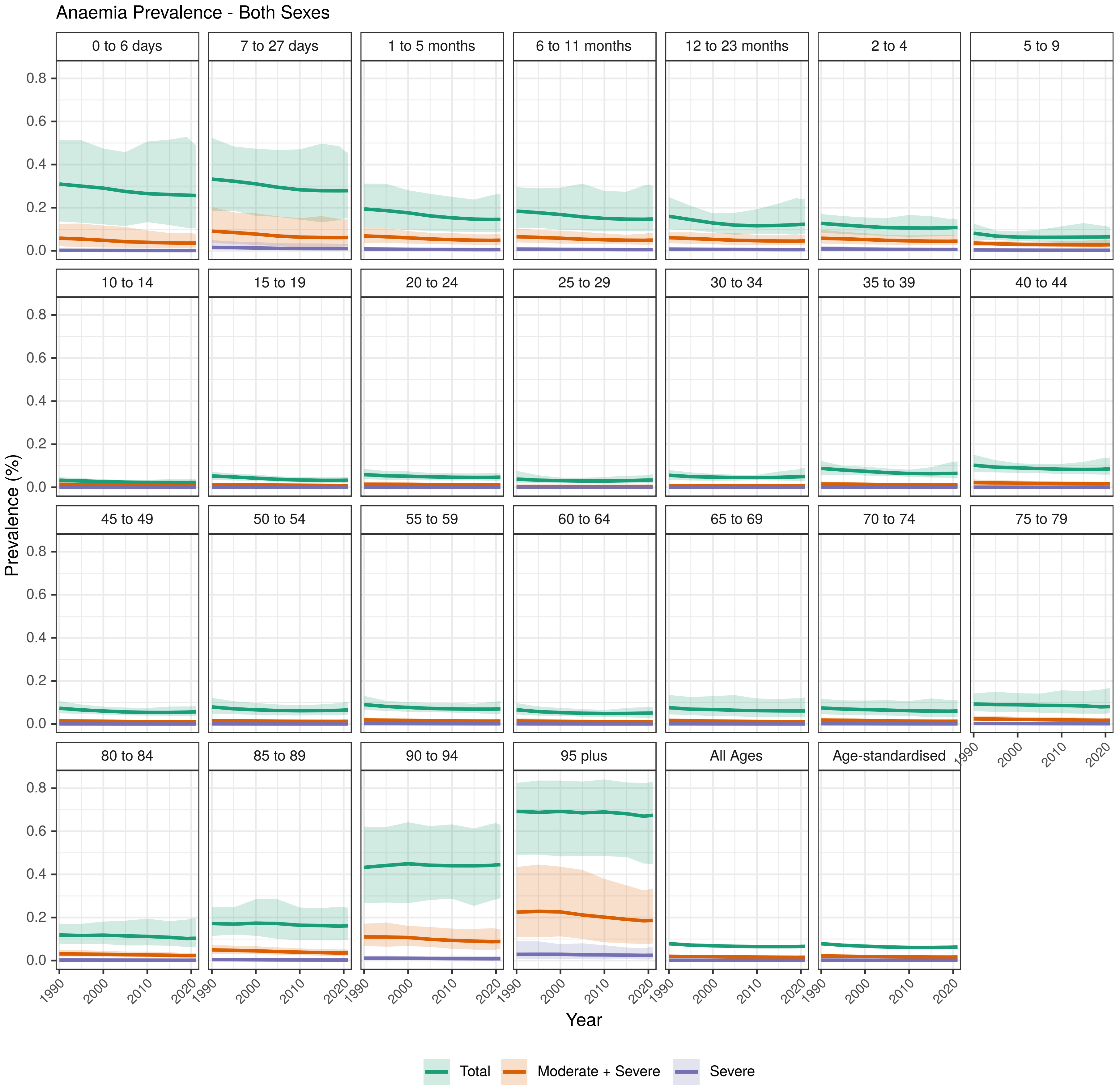

Anaemia Prevalence - Both Sexes
0 to 6 days
7 to 27 days
1 to 5 months
6 to 11 months
12 to 23 months
2 to 4
5 to 9
10 to 14
15 to 19
20 to 24
25 to 29
30 to 34
35 to 39
40 to 44
45 to 49
50 to 54
55 to 59
60 to 64
65 to 69
70 to 74
75 to 79
80 to 84
85 to 89
90 to 94
95 plus
All Ages
Age-standardised
Prevalence (%)
Year
Total
Moderate + Severe
Severe

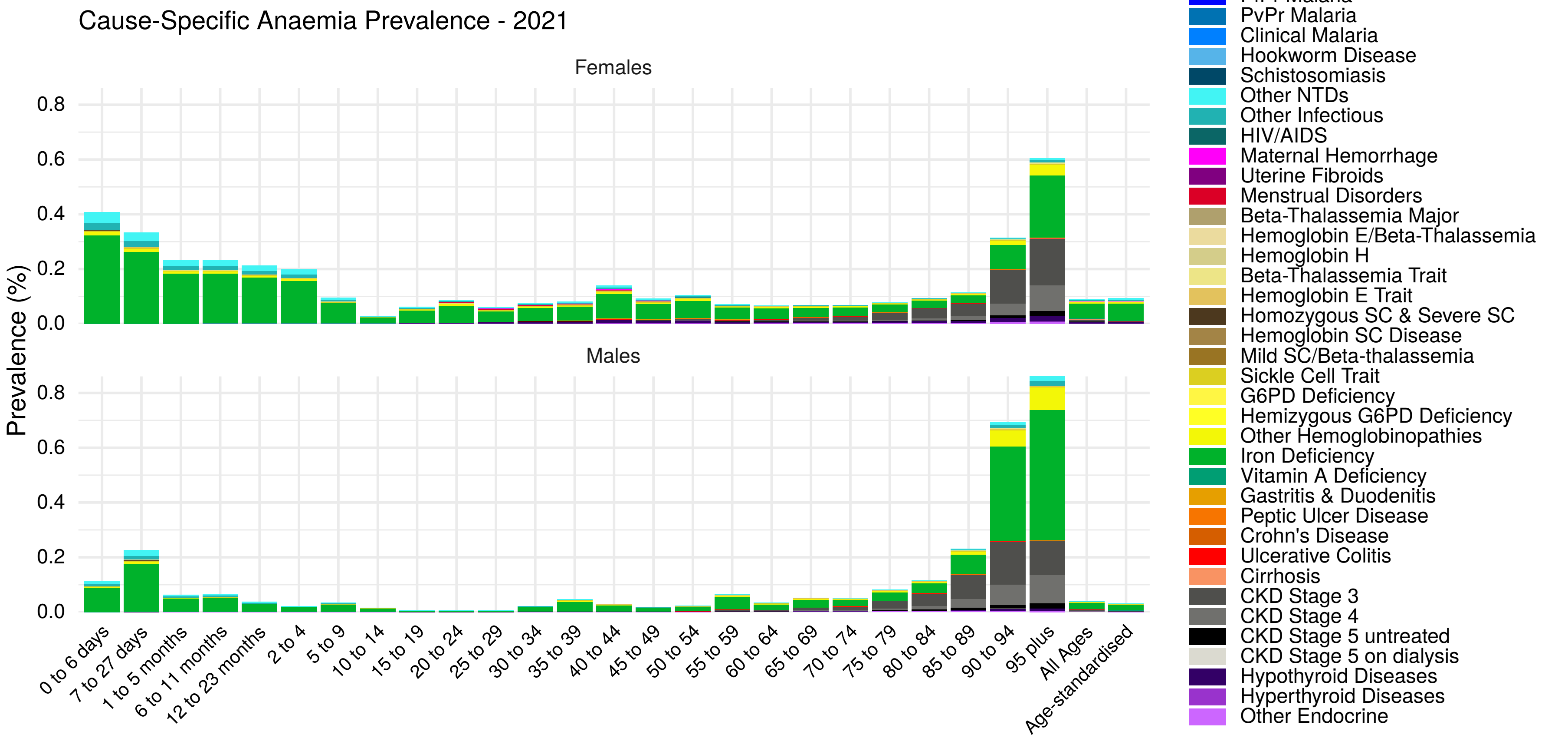

Cause-Specific Anaemia Prevalence - 2021
Females
Males
Prevalence (%)
PfPr Malaria
PvPr Malaria
Clinical Malaria
Hookworm Disease
Schistosomiasis
Other NTDs
Other Infectious
HIV/AIDS
Maternal Hemorrhage
Uterine Fibroids
Menstrual Disorders
Beta-Thalassemia Major
Hemoglobin E/Beta-Thalassemia
Hemoglobin H
Beta-Thalassemia Trait
Hemoglobin E Trait
Homozygous SC & Severe SC
Hemoglobin SC Disease
Mild SC/Beta-thalassemia
Sickle Cell Trait
G6PD Deficiency
Hemizygous G6PD Deficiency
Other Hemoglobinopathies
Iron Deficiency
Vitamin A Deficiency
Gastritis & Duodenitis
Peptic Ulcer Disease
Crohn's Disease
Ulcerative Colitis
Cirrhosis
CKD Stage 3
CKD Stage 4
CKD Stage 5 untreated
CKD Stage 5 on dialysis
Hypothyroid Diseases
Hyperthyroid Diseases
Other Endocrine

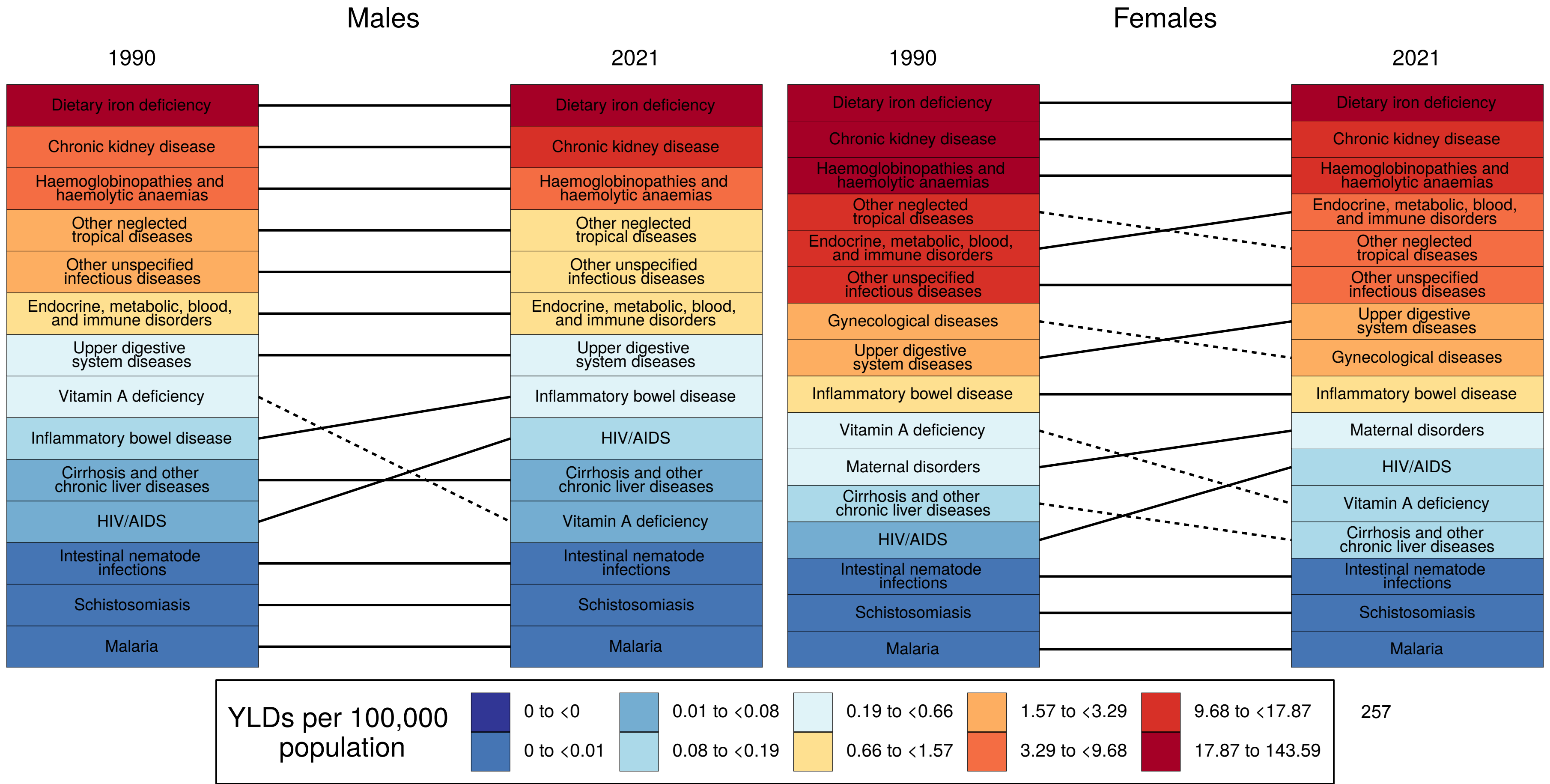

Males
Females
1990
2021
Dietary iron deficiency
Chronic kidney disease
Haemoglobinopathies and haemolytic anaemias
Other neglected tropical diseases
Other unspecified infectious diseases
Endocrine, metabolic, blood, and immune disorders
Upper digestive system diseases
Vitamin A deficiency
Inflammatory bowel disease
Cirrhosis and other chronic liver diseases
HIV/AIDS
Intestinal nematode infections
Schistosomiasis
Malaria
Gynecological diseases
Maternal disorders
YLDs per 100,000 population
0 to <0
0 to <0.01
0.01 to <0.08
0.08 to <0.19
0.19 to <0.66
0.66 to <1.57
1.57 to <3.29
3.29 to <9.68
9.68 to <17.87
17.87 to 143.59

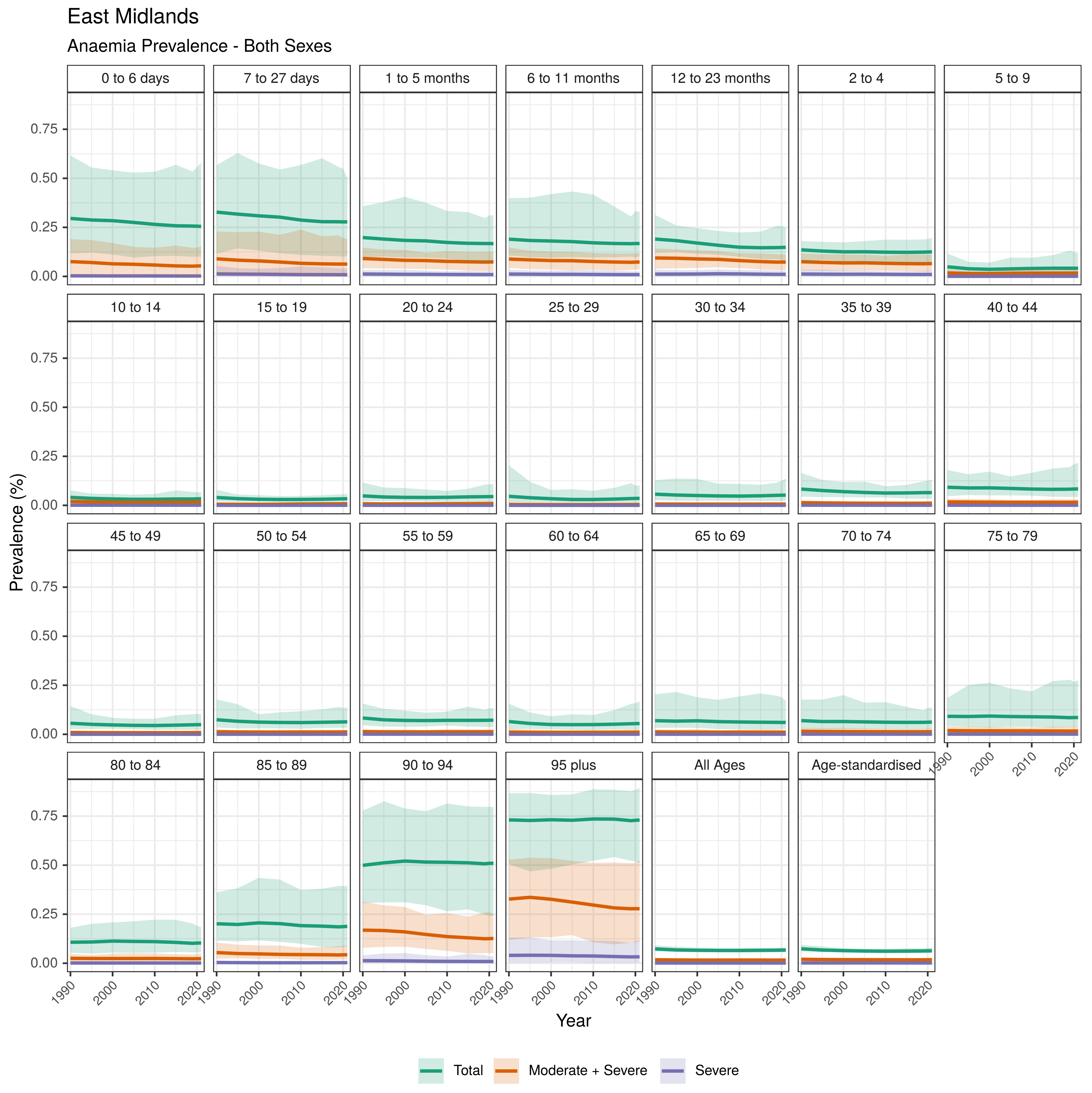
East Midlands
Anaemia Prevalence - Both Sexes
0 to 6 days
7 to 27 days
1 to 5 months
6 to 11 months
12 to 23 months
2 to 4
5 to 9
10 to 14
15 to 19
20 to 24
25 to 29
30 to 34
35 to 39
40 to 44
45 to 49
50 to 54
55 to 59
60 to 64
65 to 69
70 to 74
75 to 79
80 to 84
85 to 89
90 to 94
95 plus
All Ages
Age-standardised
Prevalence (%)
Year
Total
Moderate + Severe
Severe

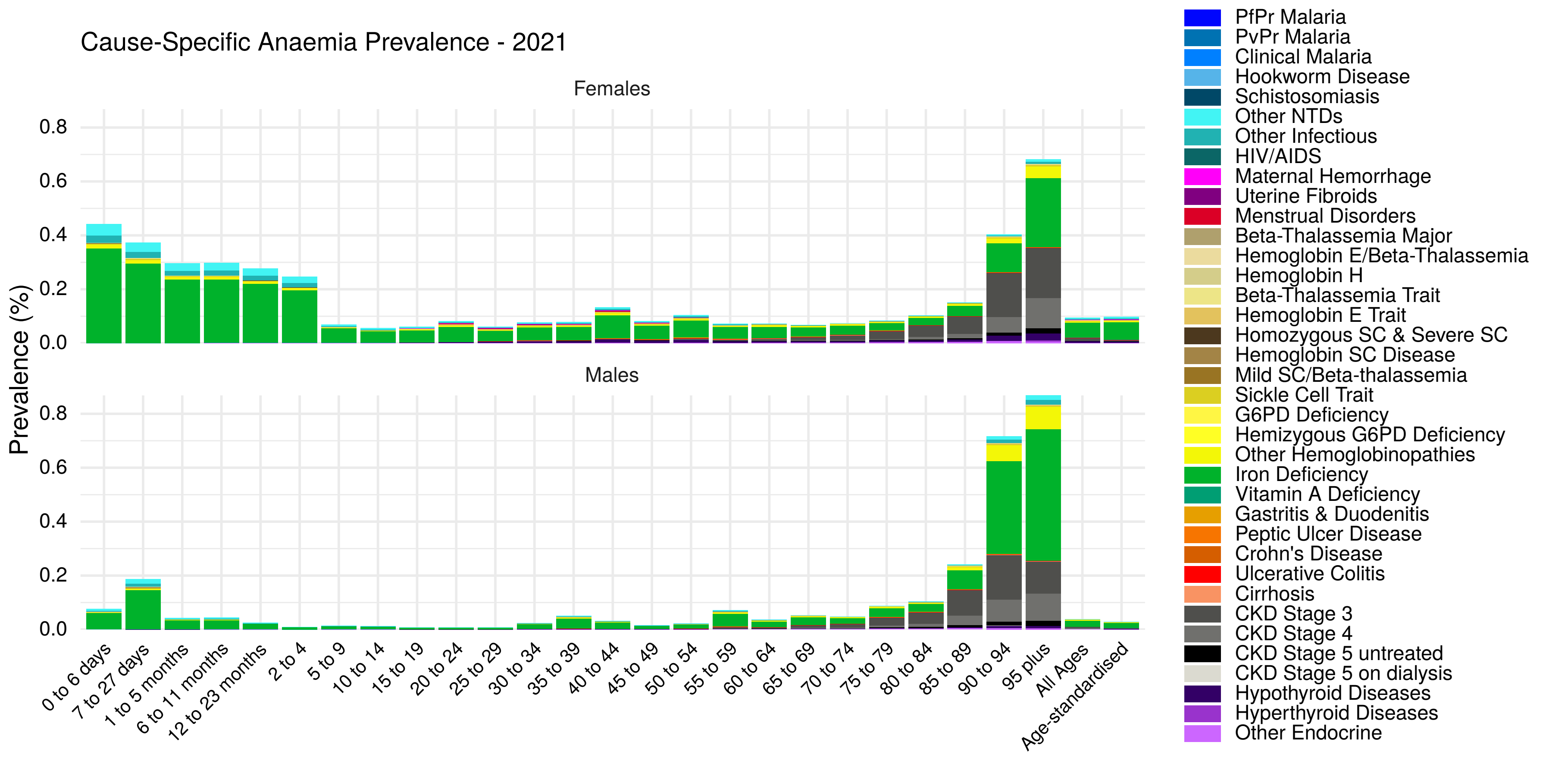
Cause-Specific Anaemia Prevalence - 2021
Females
Males
Prevalence (%)
PfPr Malaria
PvPr Malaria
Clinical Malaria
Hookworm Disease
Schistosomiasis
Other NTDs
Other Infectious
HIV/AIDS
Maternal Hemorrhage
Uterine Fibroids
Menstrual Disorders
Beta-Thalassemia Major
Hemoglobin E/Beta-Thalassemia
Hemoglobin H
Beta-Thalassemia Trait
Hemoglobin E Trait
Homozygous SC & Severe SC
Hemoglobin SC Disease
Mild SC/Beta-thalassemia
Sickle Cell Trait
G6PD Deficiency
Hemizygous G6PD Deficiency
Other Hemoglobinopathies
Iron Deficiency
Vitamin A Deficiency
Gastritis & Duodenitis
Peptic Ulcer Disease
Crohn's Disease
Ulcerative Colitis
Cirrhosis
CKD Stage 3
CKD Stage 4
CKD Stage 5 untreated
CKD Stage 5 on dialysis
Hypothyroid Diseases
Hyperthyroid Diseases
Other Endocrine

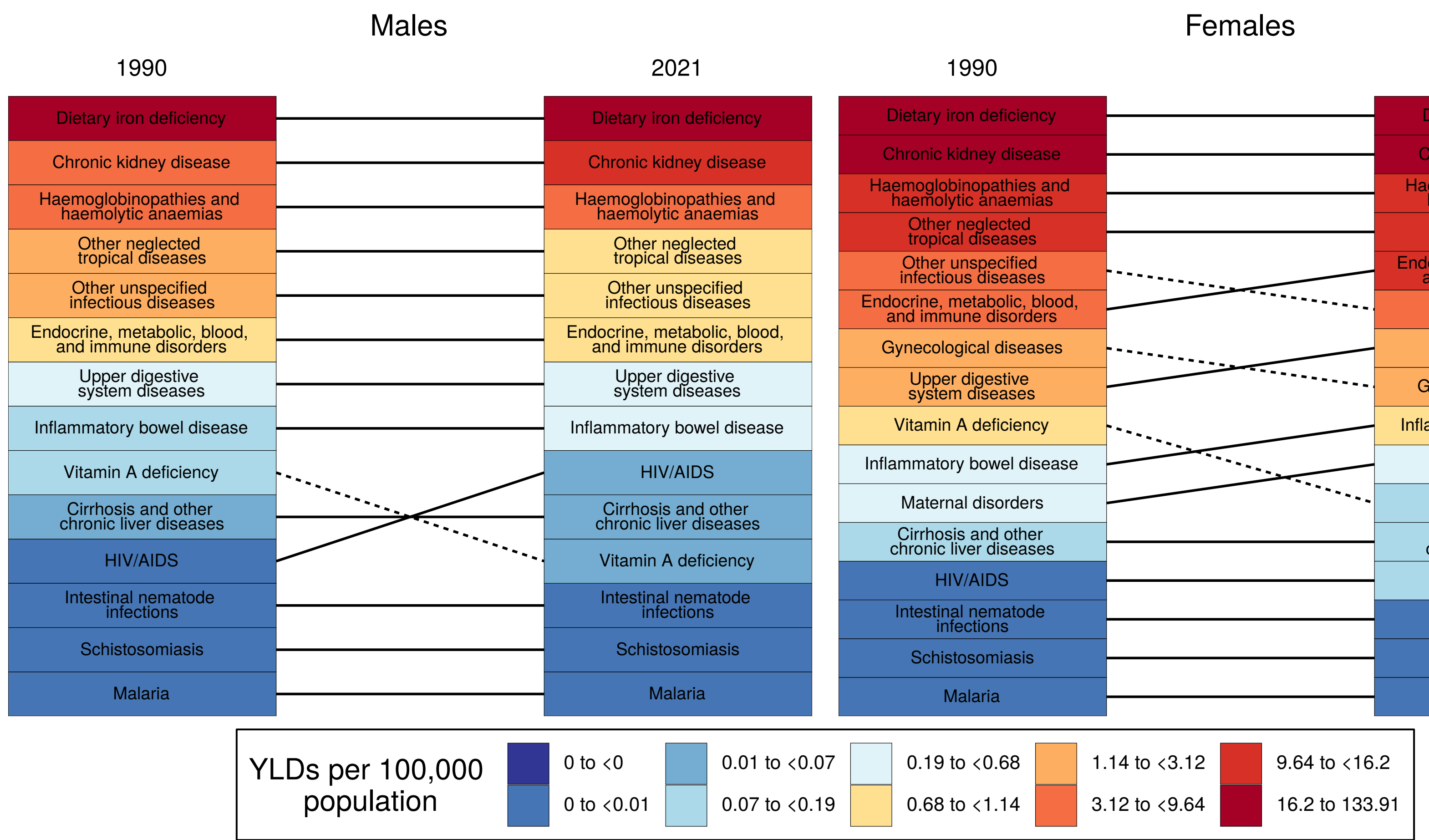
Males
Females
1990
2021
Dietary iron deficiency
Chronic kidney disease
Haemoglobinopathies and haemolytic anaemias
Other neglected tropical diseases
Other unspecified infectious diseases
Endocrine, metabolic, blood, and immune disorders
Upper digestive system diseases
Inflammatory bowel disease
Vitamin A deficiency
Cirrhosis and other chronic liver diseases
HIV/AIDS
Intestinal nematode infections
Schistosomiasis
Malaria
Gynecological diseases
Maternal disorders
YLDs per 100,000 population
0 to <0
0 to <0.01
0.01 to <0.07
0.07 to <0.19
0.19 to <0.68
0.68 to <1.14
1.14 to <3.12
3.12 to <9.64
9.64 to <16.2
16.2 to 133.91

Derby

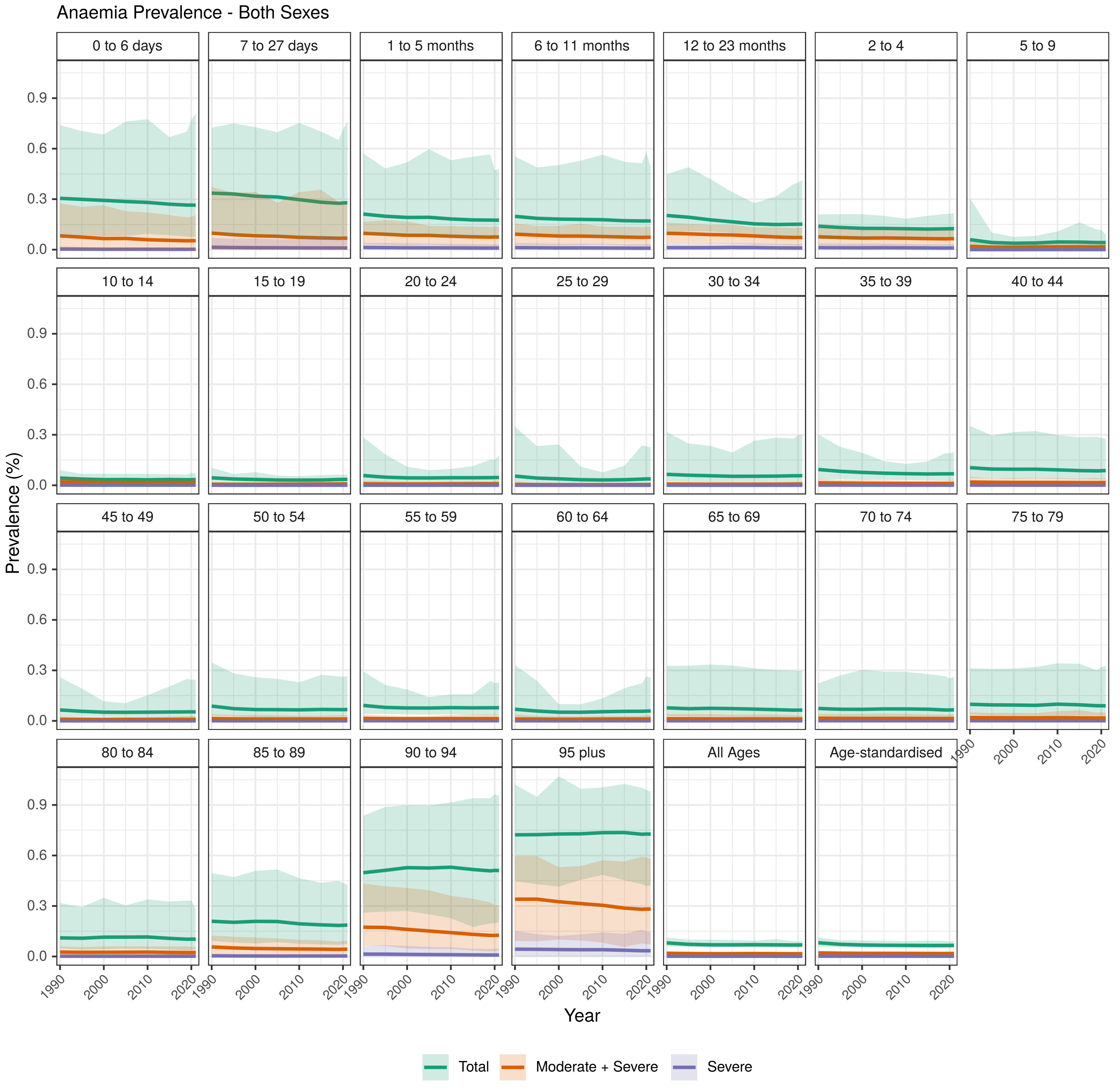

Anaemia Prevalence - Both Sexes
0 to 6 days
7 to 27 days
1 to 5 months
6 to 11 months
12 to 23 months
2 to 4
5 to 9
10 to 14
15 to 19
20 to 24
25 to 29
30 to 34
35 to 39
40 to 44
45 to 49
50 to 54
55 to 59
60 to 64
65 to 69
70 to 74
75 to 79
80 to 84
85 to 89
90 to 94
95 plus
All Ages
Age-standardised
Prevalence (%)
Year
Total
Moderate + Severe
Severe

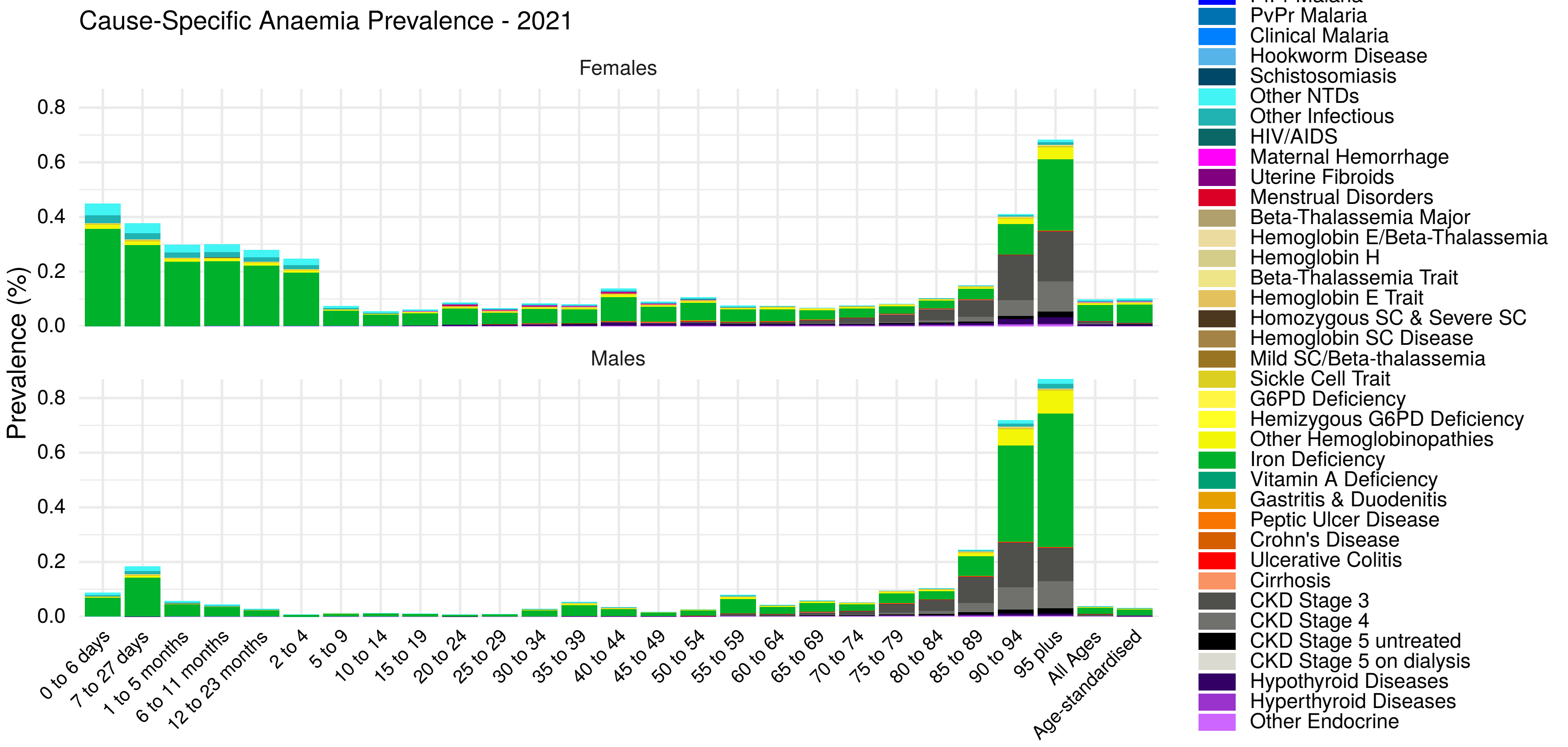

Cause-Specific Anaemia Prevalence - 2021
Females
Males
Prevalence (%)
PfPr Malaria
PvPr Malaria
Clinical Malaria
Hookworm Disease
Schistosomiasis
Other NTDs
Other Infectious
HIV/AIDS
Maternal Hemorrhage
Uterine Fibroids
Menstrual Disorders
Beta-Thalassemia Major
Hemoglobin E/Beta-Thalassemia
Hemoglobin H
Beta-Thalassemia Trait
Hemoglobin E Trait
Homozygous SC & Severe SC
Hemoglobin SC Disease
Mild SC/Beta-thalassemia
Sickle Cell Trait
G6PD Deficiency
Hemizygous G6PD Deficiency
Other Hemoglobinopathies
Iron Deficiency
Vitamin A Deficiency
Gastritis & Duodenitis
Peptic Ulcer Disease
Crohn's Disease
Ulcerative Colitis
Cirrhosis
CKD Stage 3
CKD Stage 4
CKD Stage 5 untreated
CKD Stage 5 on dialysis
Hypothyroid Diseases
Hyperthyroid Diseases
Other Endocrine

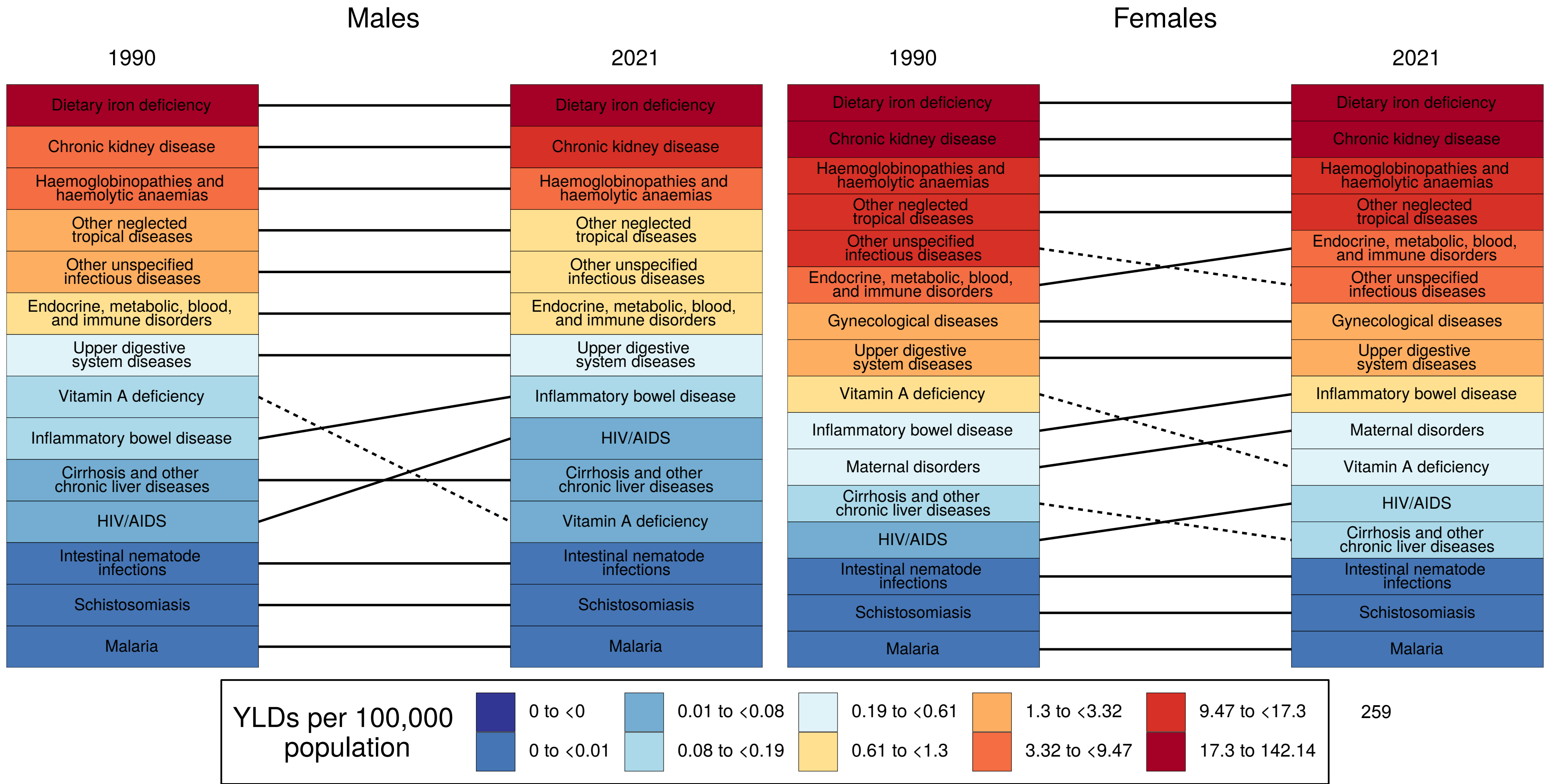

Males
Females
1990
2021
Dietary iron deficiency
Chronic kidney disease
Haemoglobinopathies and haemolytic anaemias
Other neglected tropical diseases
Other unspecified infectious diseases
Endocrine, metabolic, blood, and immune disorders
Upper digestive system diseases
Vitamin A deficiency
Inflammatory bowel disease
Cirrhosis and other chronic liver diseases
HIV/AIDS
Intestinal nematode infections
Schistosomiasis
Malaria
Gynecological diseases
Maternal disorders
YLDs per 100,000 population
0 to <0
0 to <0.01
0.01 to <0.08
0.08 to <0.19
0.19 to <0.61
0.61 to <1.3
1.3 to <3.32
3.32 to <9.47
9.47 to <17.3
17.3 to 142.14

Derbyshire

Anaemia Prevalence - Both Sexes

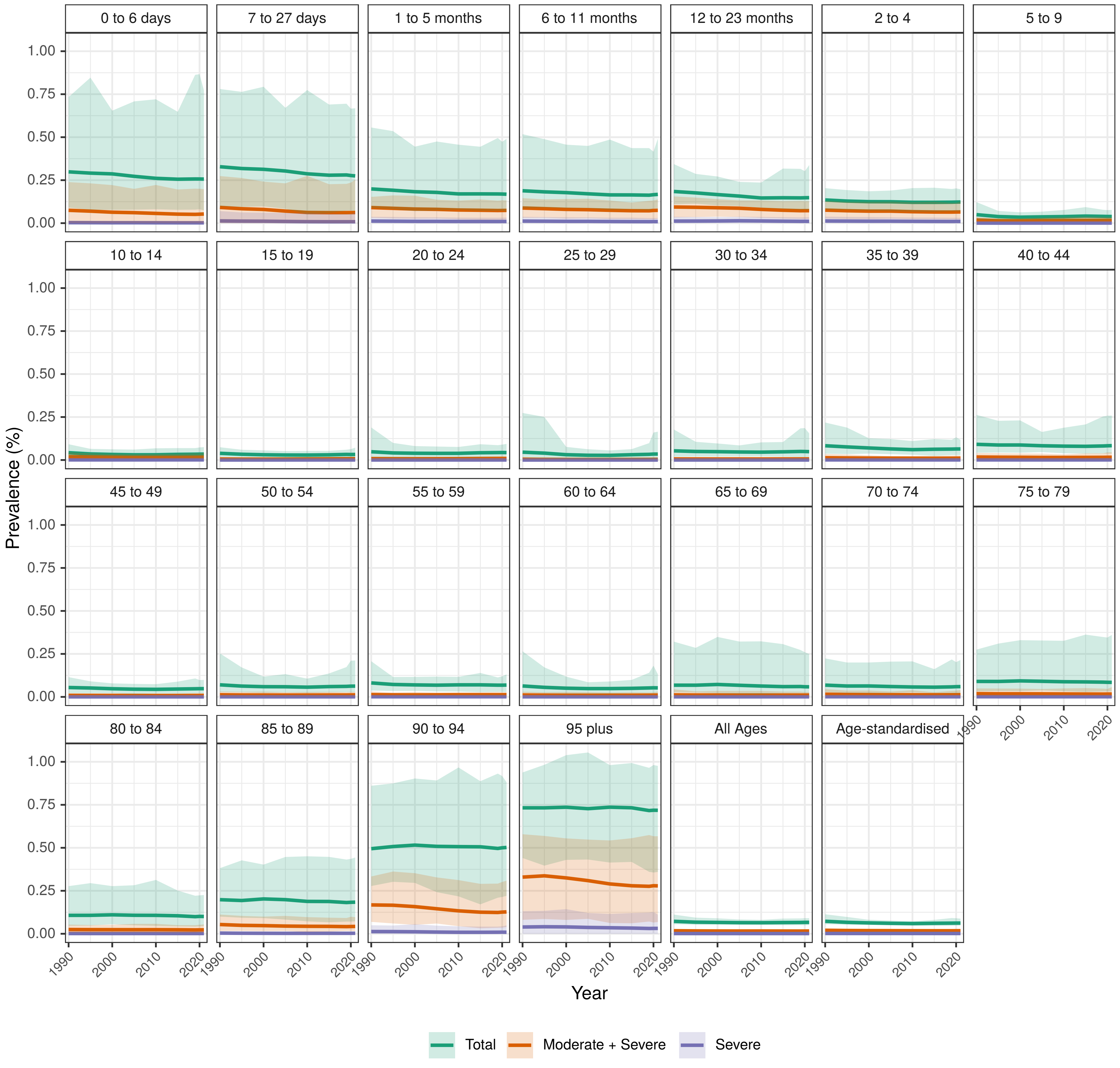

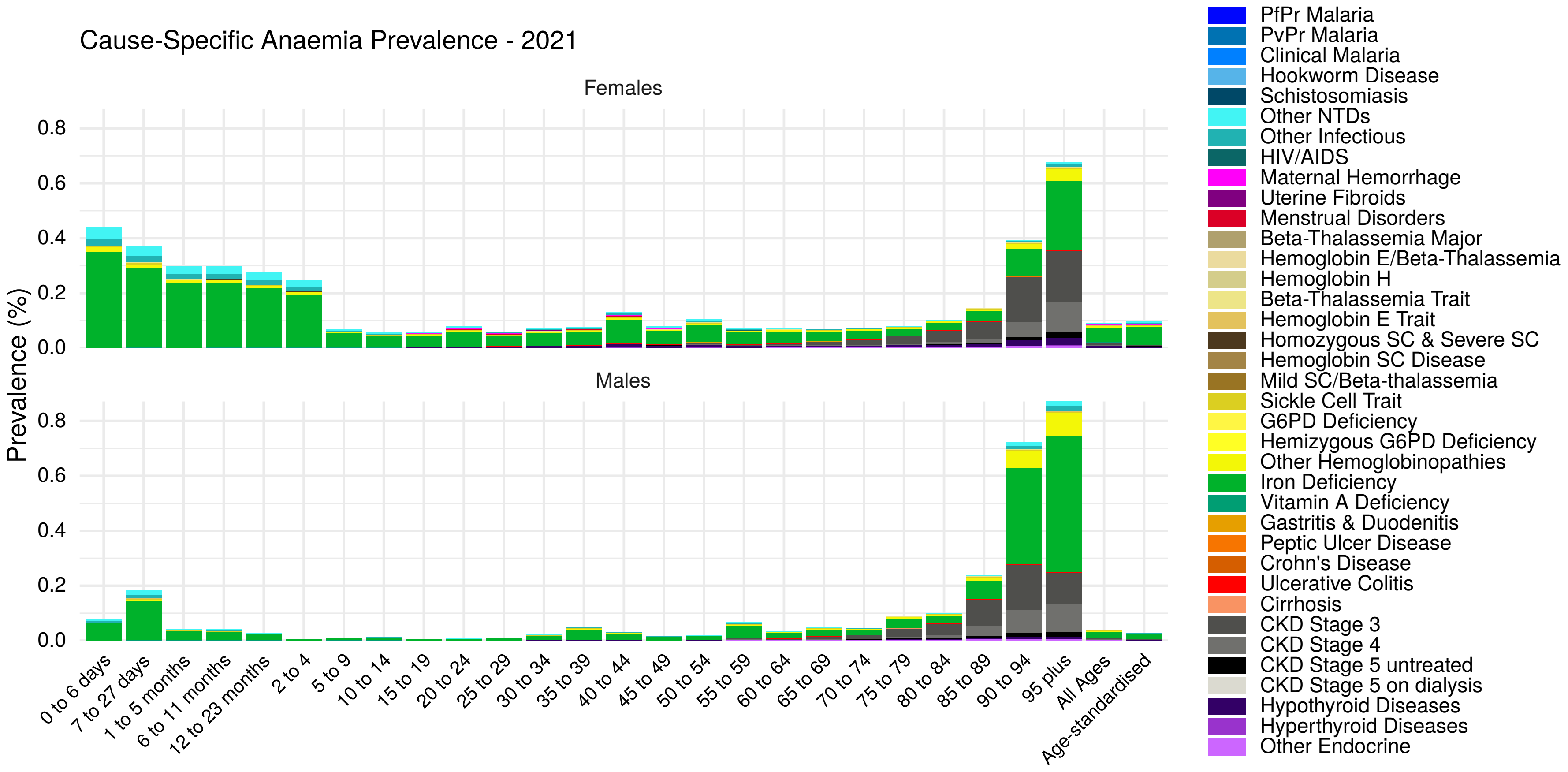

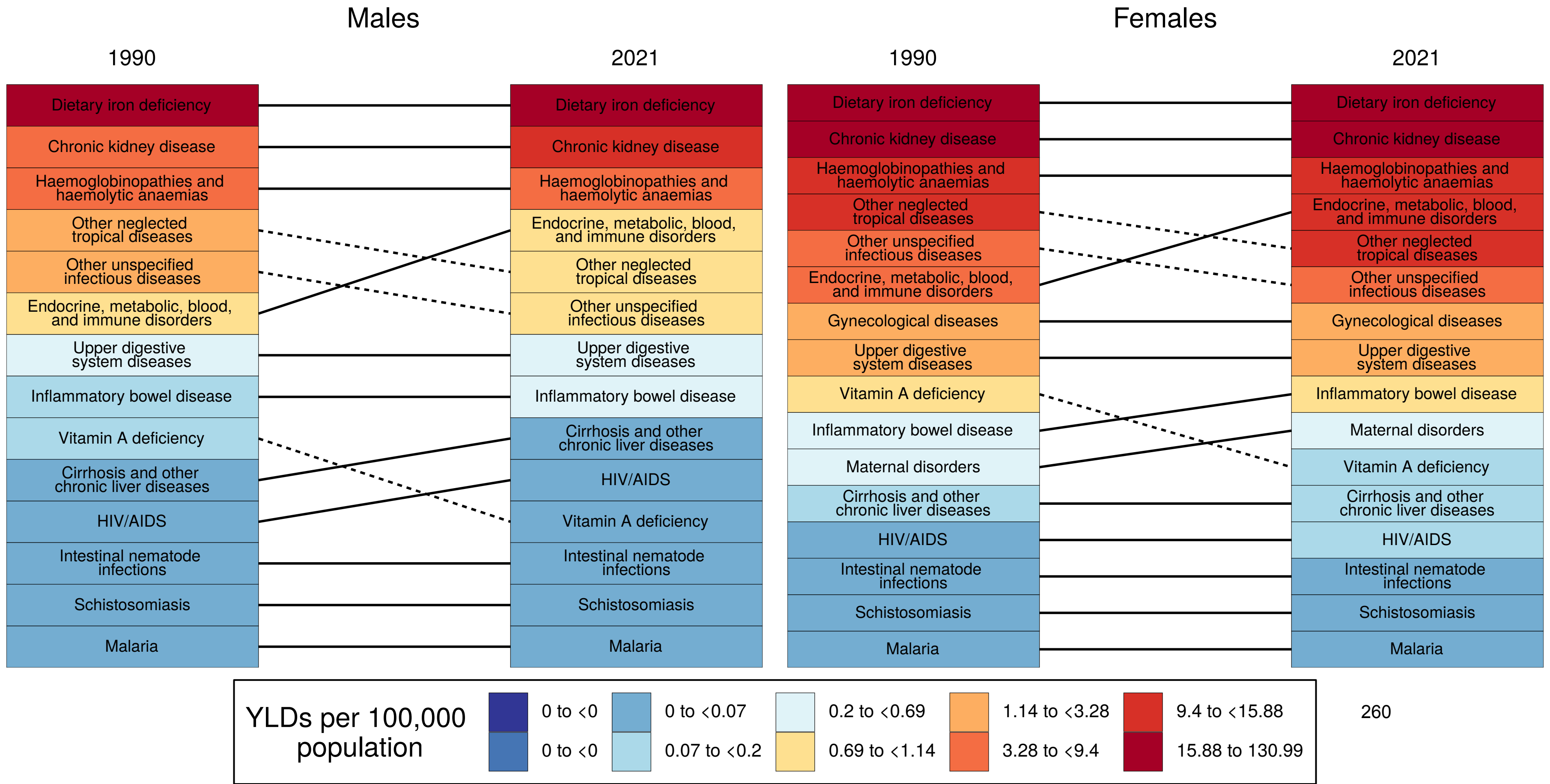

Leicester

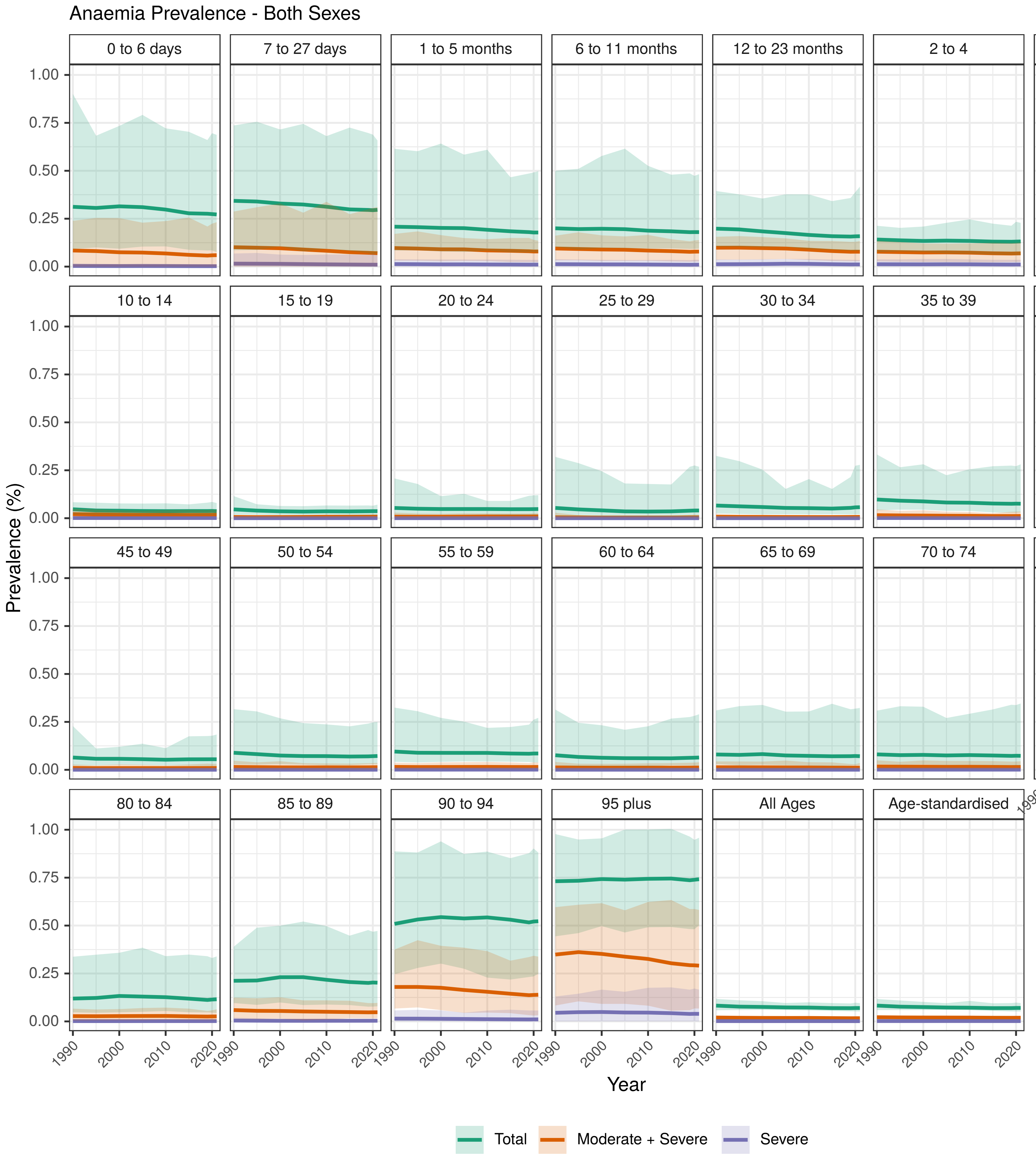

Anaemia Prevalence - Both Sexes
0 to 6 days
7 to 27 days
1 to 5 months
6 to 11 months
12 to 23 months
2 to 4
5 to 9
10 to 14
15 to 19
20 to 24
25 to 29
30 to 34
35 to 39
40 to 44
45 to 49
50 to 54
55 to 59
60 to 64
65 to 69
70 to 74
75 to 79
80 to 84
85 to 89
90 to 94
95 plus
All Ages
Age-standardised
Prevalence (%)
Year
Total
Moderate + Severe
Severe

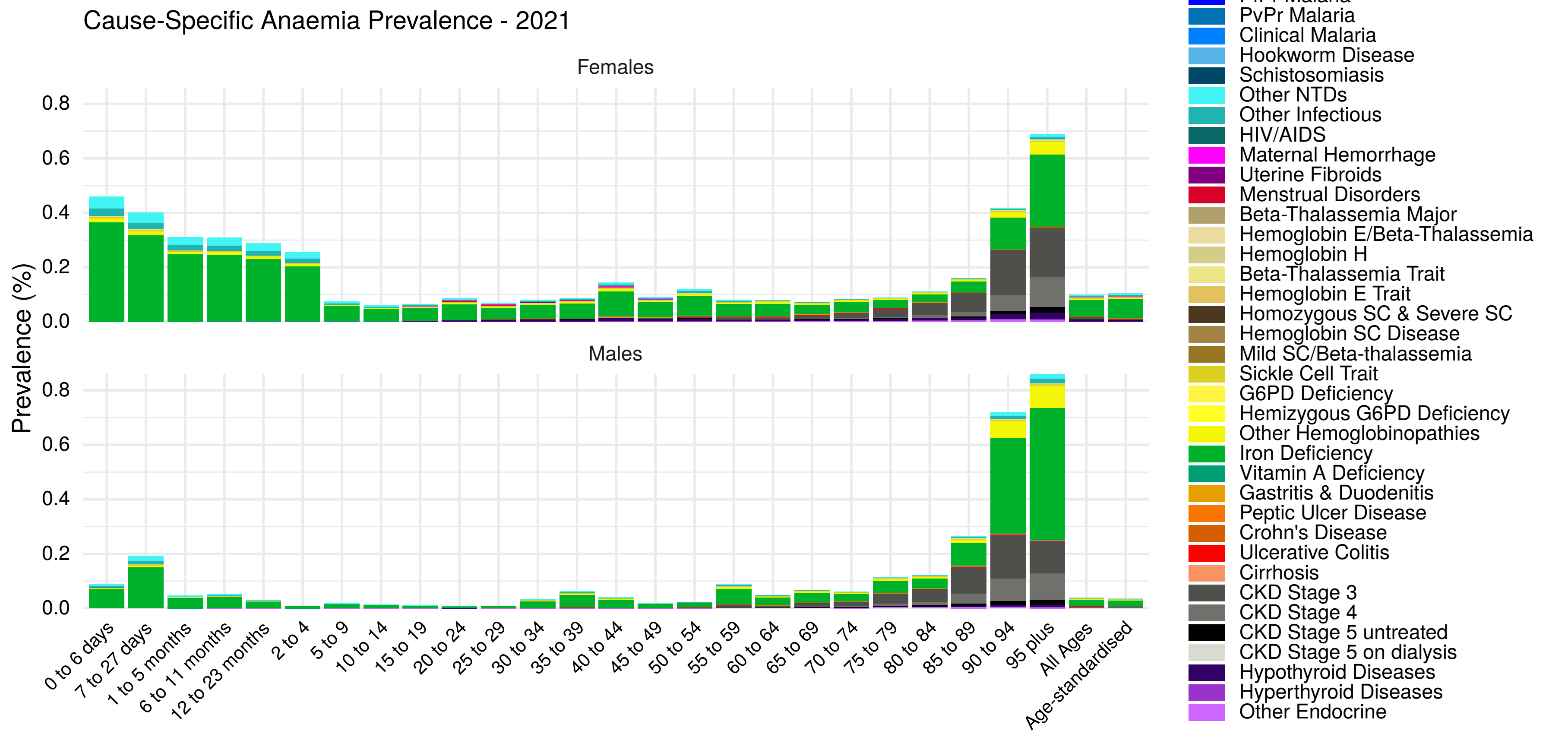

Cause-Specific Anaemia Prevalence - 2021
Females
Males
Prevalence (%)
PfPr Malaria
PvPr Malaria
Clinical Malaria
Hookworm Disease
Schistosomiasis
Other NTDs
Other Infectious
HIV/AIDS
Maternal Hemorrhage
Uterine Fibroids
Menstrual Disorders
Beta-Thalassemia Major
Hemoglobin E/Beta-Thalassemia
Hemoglobin H
Beta-Thalassemia Trait
Hemoglobin E Trait
Homozygous SC & Severe SC
Hemoglobin SC Disease
Mild SC/Beta-thalassemia
Sickle Cell Trait
G6PD Deficiency
Hemizygous G6PD Deficiency
Other Hemoglobinopathies
Iron Deficiency
Vitamin A Deficiency
Gastritis & Duodenitis
Peptic Ulcer Disease
Crohn's Disease
Ulcerative Colitis
Cirrhosis
CKD Stage 3
CKD Stage 4
CKD Stage 5 untreated
CKD Stage 5 on dialysis
Hypothyroid Diseases
Hyperthyroid Diseases
Other Endocrine

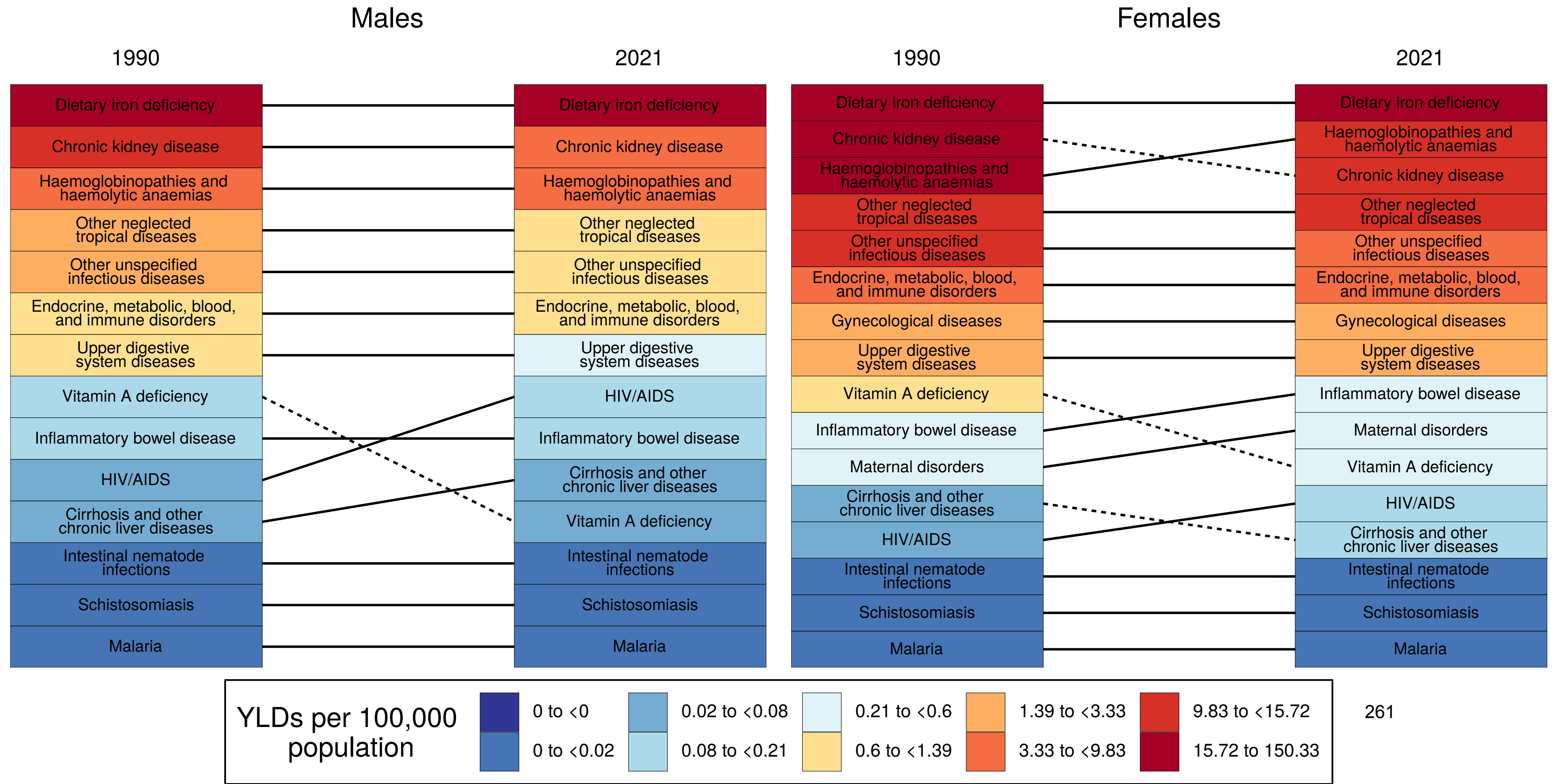

Males
Females
1990
2021
Dietary iron deficiency
Chronic kidney disease
Haemoglobinopathies and haemolytic anaemias
Other neglected tropical diseases
Other unspecified infectious diseases
Endocrine, metabolic, blood, and immune disorders
Upper digestive system diseases
Vitamin A deficiency
Inflammatory bowel disease
HIV/AIDS
Cirrhosis and other chronic liver diseases
Intestinal nematode infections
Schistosomiasis
Malaria
Gynecological diseases
Maternal disorders
YLDs per 100,000 population
0 to <0
0 to <0.02
0.02 to <0.08
0.08 to <0.21
0.21 to <0.6
0.6 to <1.39
1.39 to <3.33
3.33 to <9.83
9.83 to <15.72
15.72 to 150.33

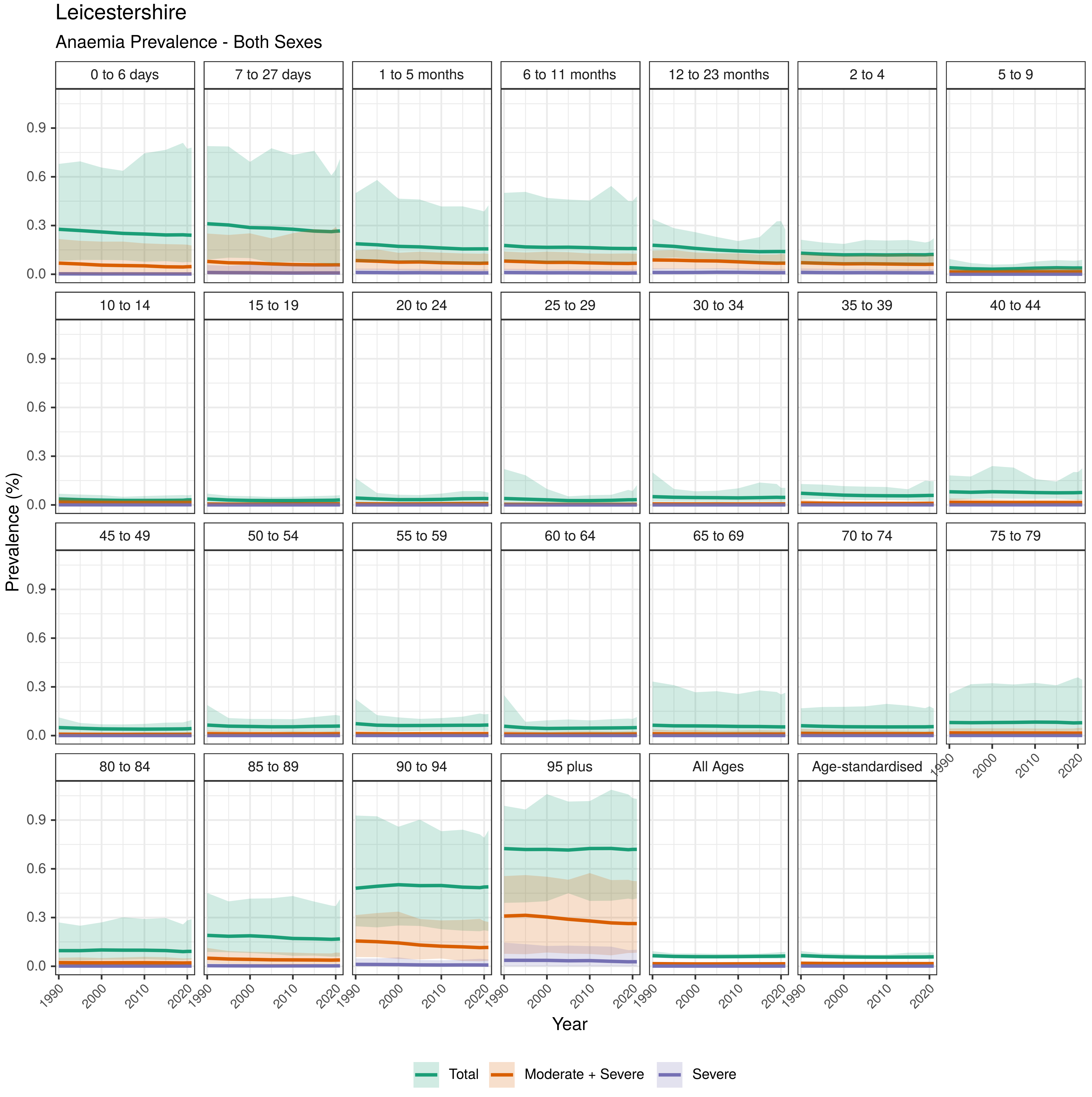
Leicestershire
Anaemia Prevalence - Both Sexes
0 to 6 days
7 to 27 days
1 to 5 months
6 to 11 months
12 to 23 months
2 to 4
5 to 9
10 to 14
15 to 19
20 to 24
25 to 29
30 to 34
35 to 39
40 to 44
45 to 49
50 to 54
55 to 59
60 to 64
65 to 69
70 to 74
75 to 79
80 to 84
85 to 89
90 to 94
95 plus
All Ages
Age-standardised
Prevalence (%)
Year
Total
Moderate + Severe
Severe

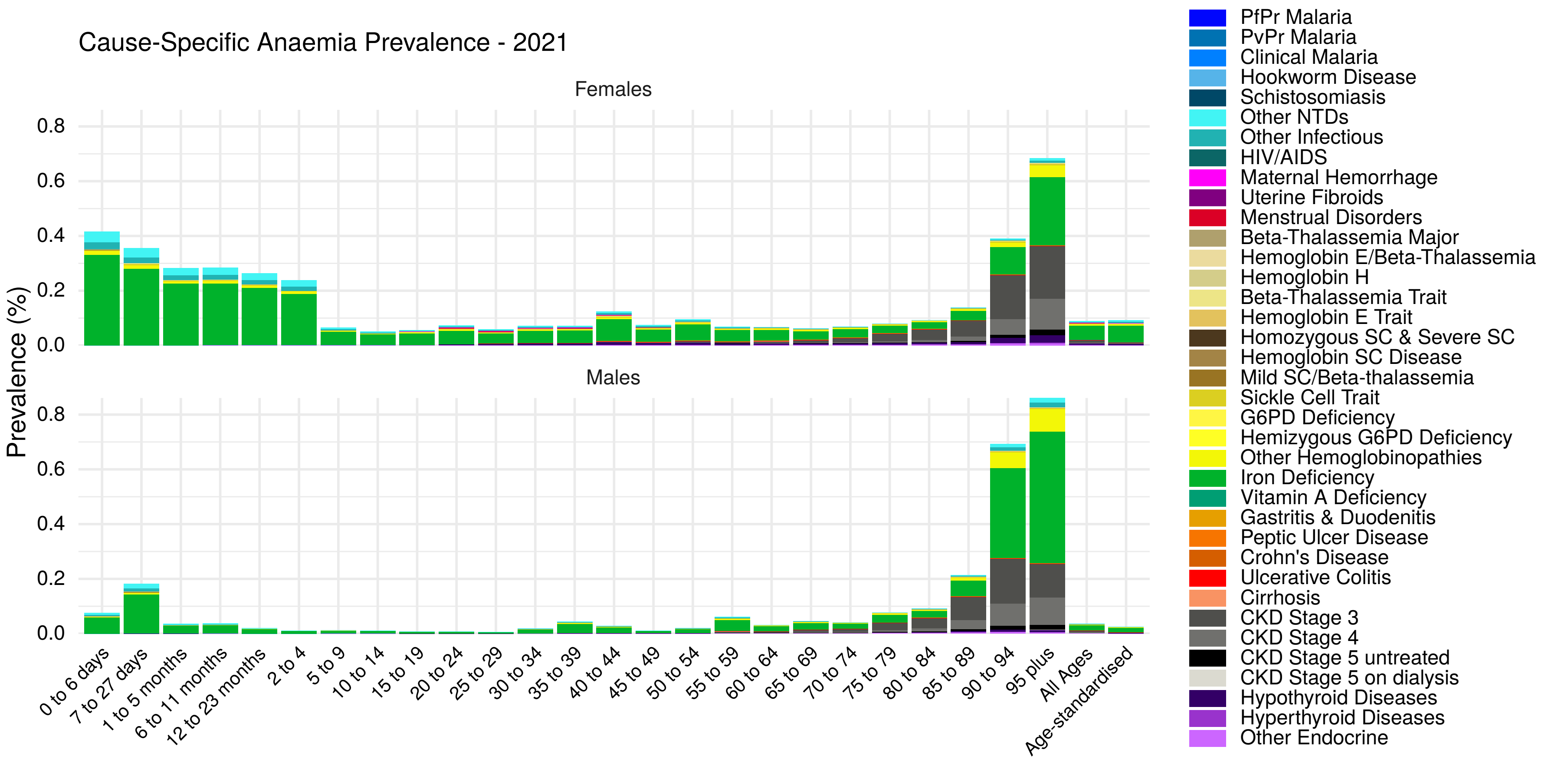
Cause-Specific Anaemia Prevalence - 2021
Females
Males
Prevalence (%)
PfPr Malaria
PvPr Malaria
Clinical Malaria
Hookworm Disease
Schistosomiasis
Other NTDs
Other Infectious
HIV/AIDS
Maternal Hemorrhage
Uterine Fibroids
Menstrual Disorders
Beta-Thalassemia Major
Hemoglobin E/Beta-Thalassemia
Hemoglobin H
Beta-Thalassemia Trait
Hemoglobin E Trait
Homozygous SC & Severe SC
Hemoglobin SC Disease
Mild SC/Beta-thalassemia
Sickle Cell Trait
G6PD Deficiency
Hemizygous G6PD Deficiency
Other Hemoglobinopathies
Iron Deficiency
Vitamin A Deficiency
Gastritis & Duodenitis
Peptic Ulcer Disease
Crohn's Disease
Ulcerative Colitis
Cirrhosis
CKD Stage 3
CKD Stage 4
CKD Stage 5 untreated
CKD Stage 5 on dialysis
Hypothyroid Diseases
Hyperthyroid Diseases
Other Endocrine

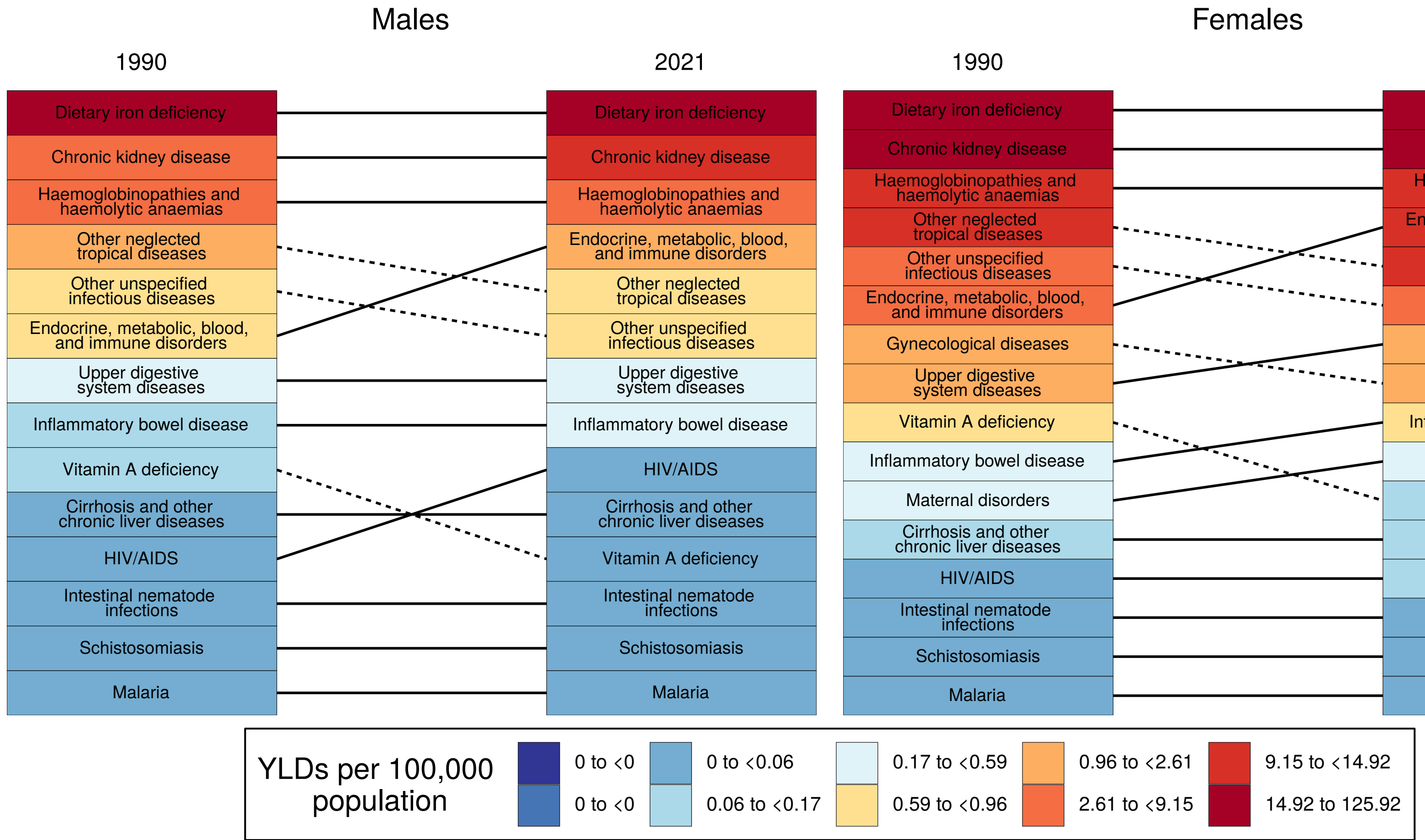
Males
Females
1990
2021
Dietary iron deficiency
Chronic kidney disease
Haemoglobinopathies and haemolytic anaemias
Other neglected tropical diseases
Other unspecified infectious diseases
Endocrine, metabolic, blood, and immune disorders
Upper digestive system diseases
Inflammatory bowel disease
Vitamin A deficiency
Cirrhosis and other chronic liver diseases
HIV/AIDS
Intestinal nematode infections
Schistosomiasis
Malaria
Gynecological diseases
Maternal disorders
YLDs per 100,000 population
0 to <0
0 to <0.06
0.06 to <0.17
0.17 to <0.59
0.59 to <0.96
0.96 to <2.61
2.61 to <9.15
9.15 to <14.92
14.92 to 125.92

Lincolnshire

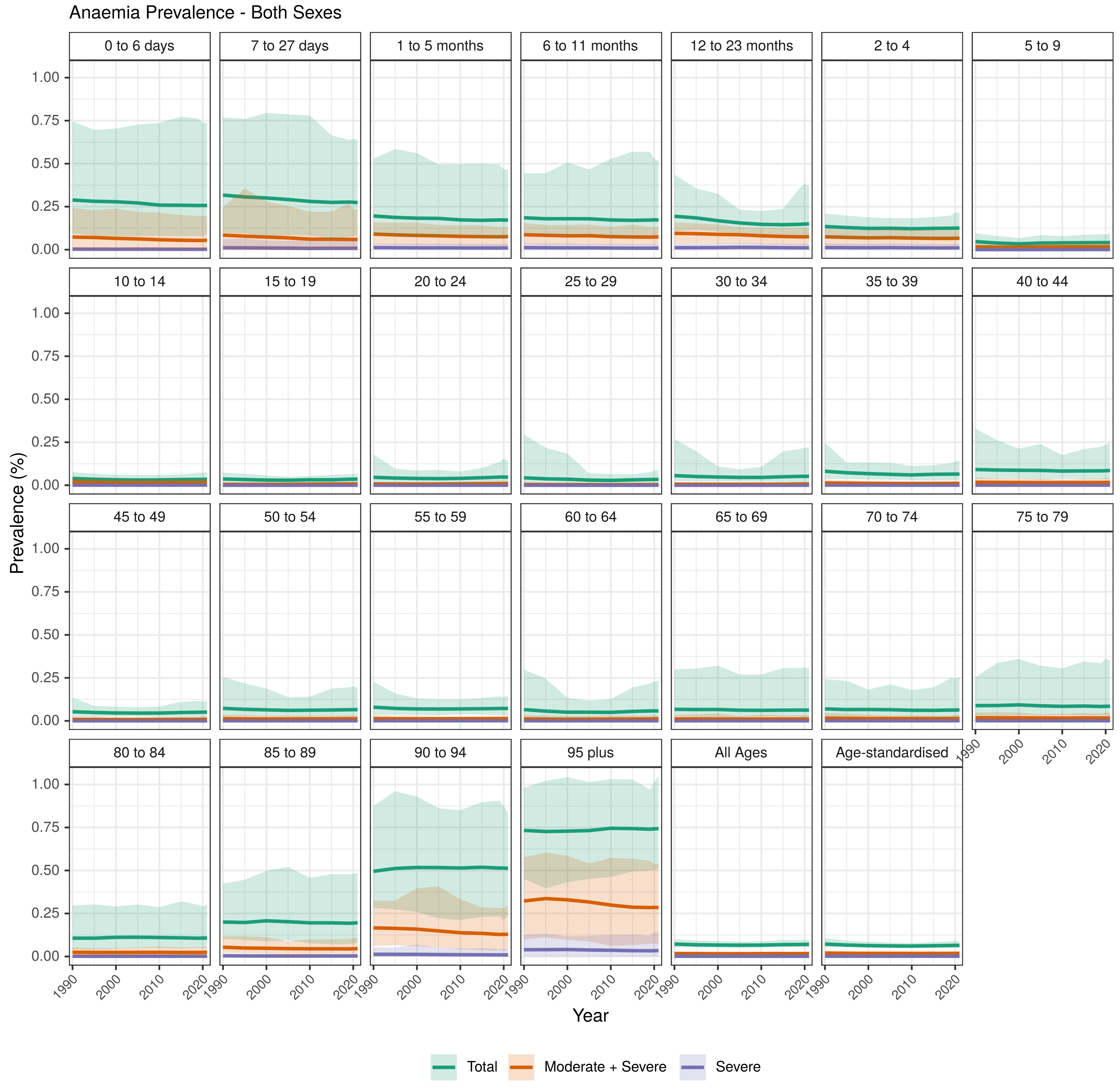

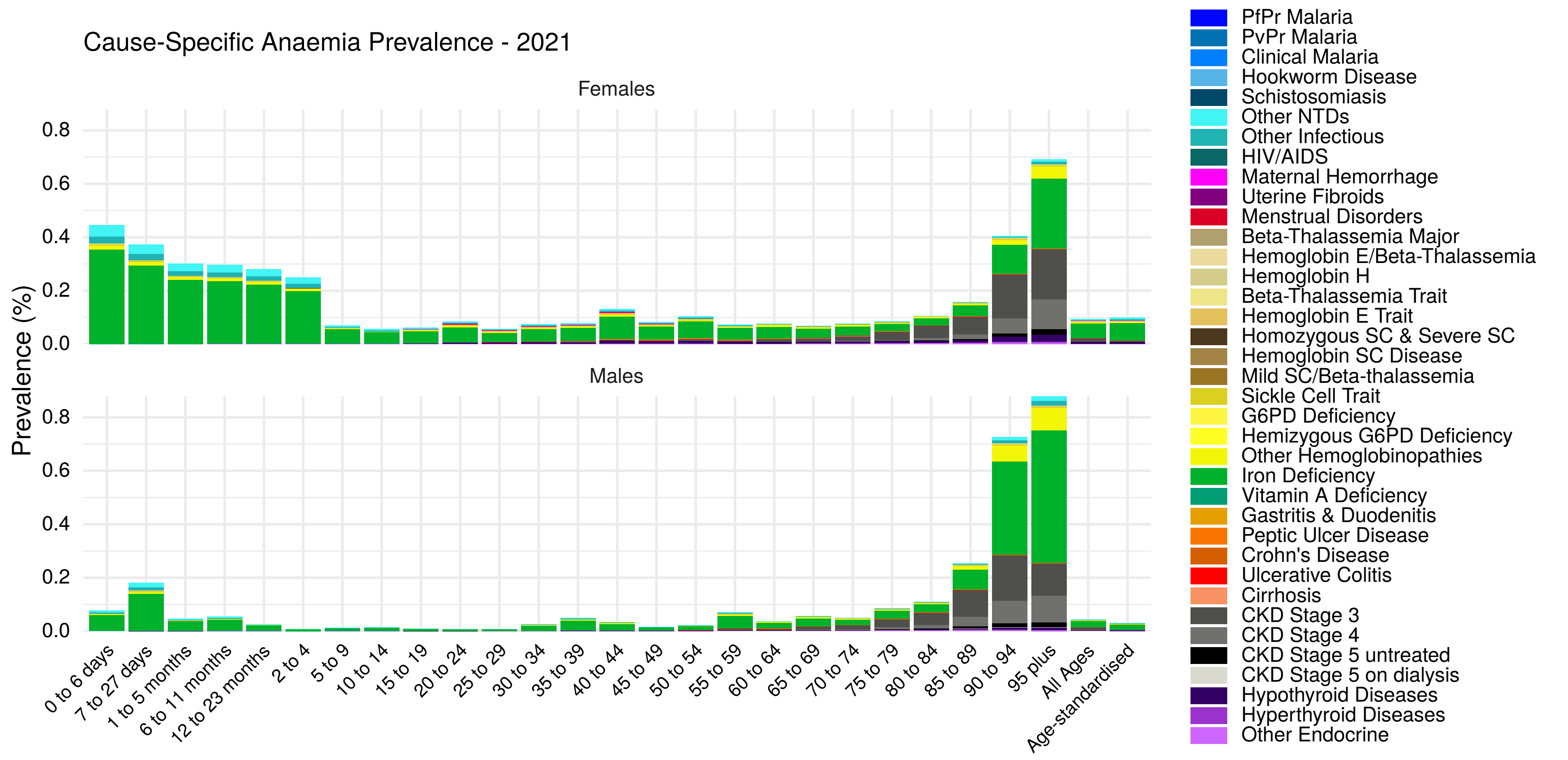

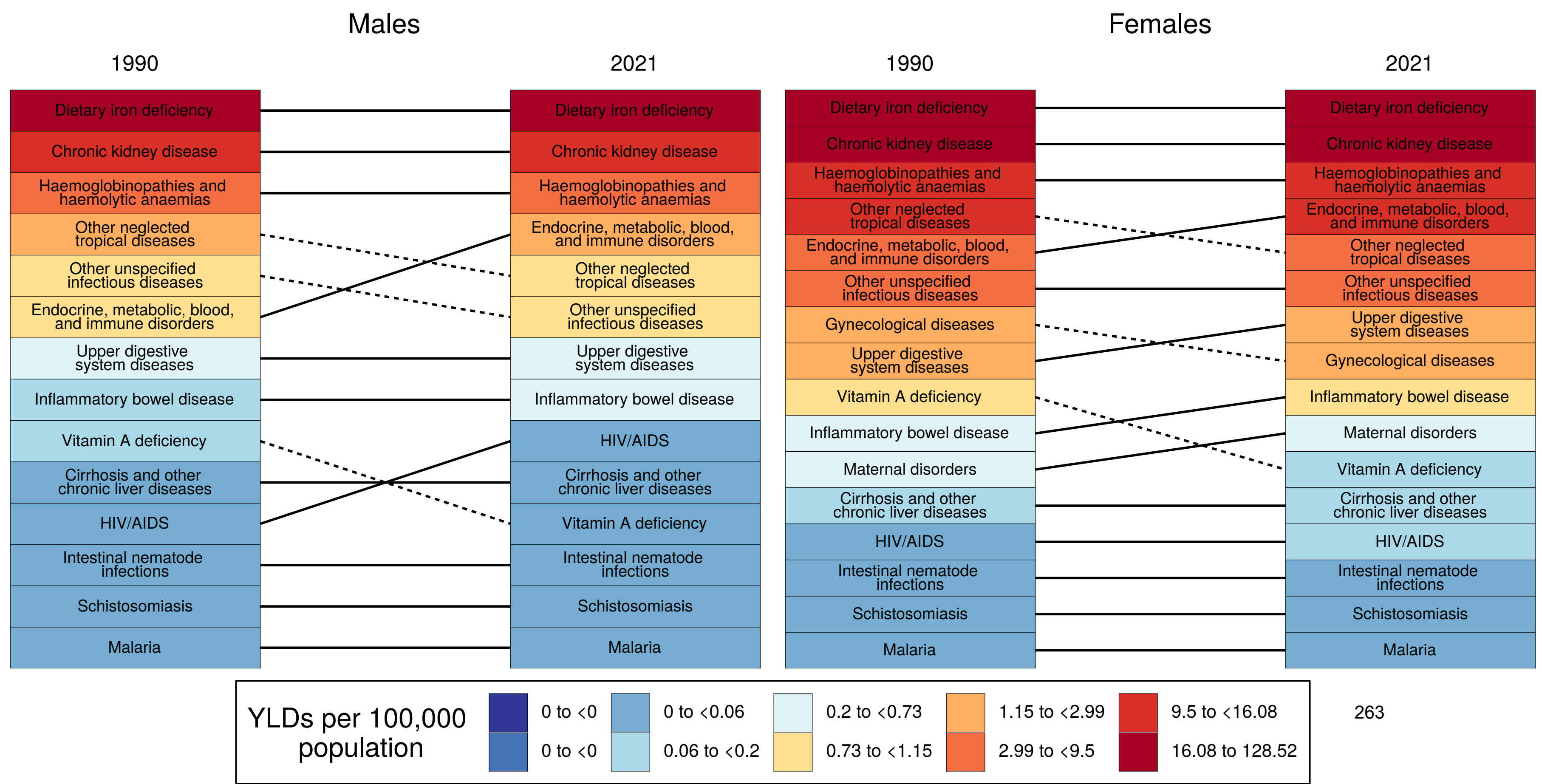

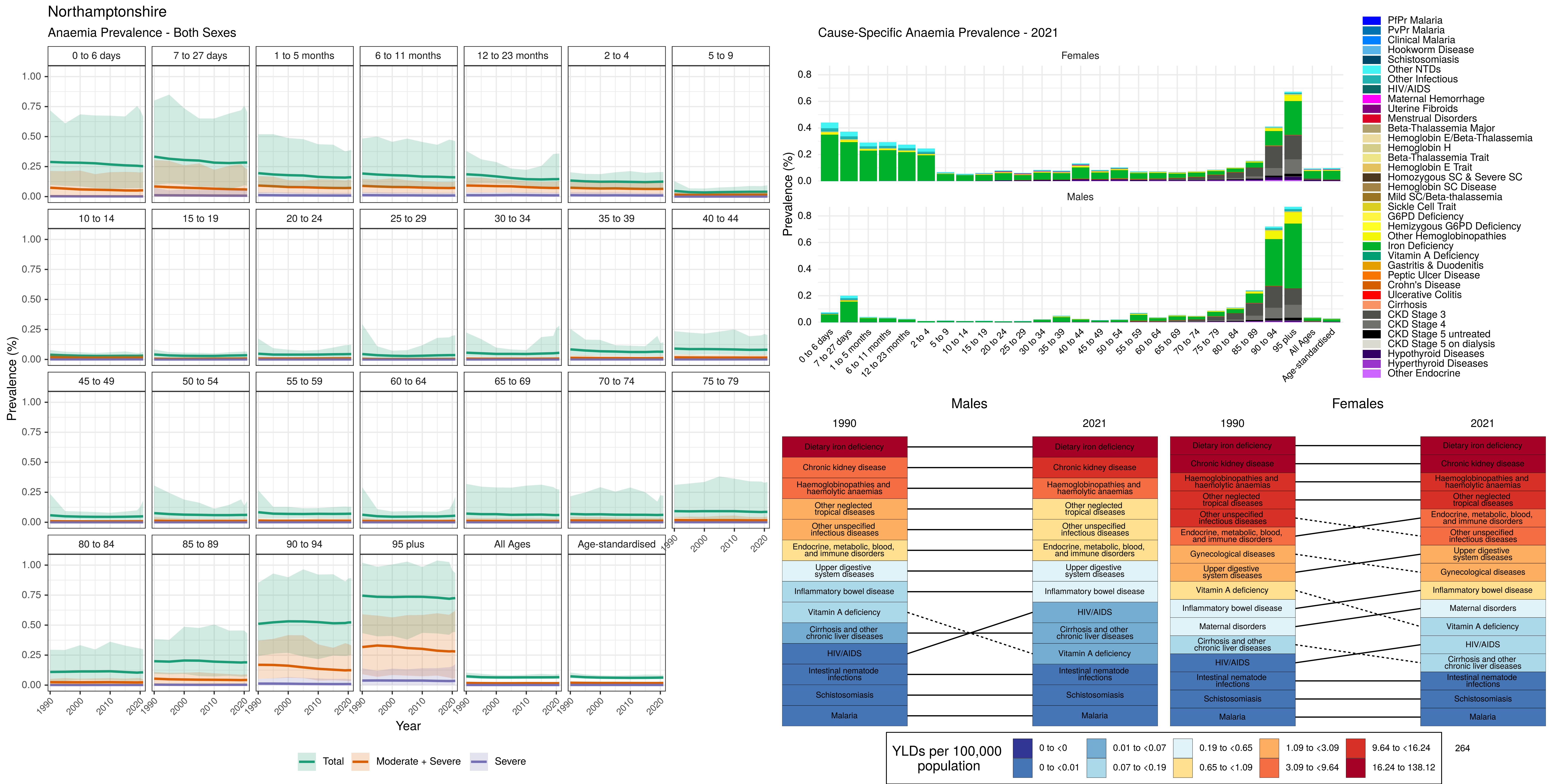
Northamptonshire
Anaemia Prevalence - Both Sexes
0 to 6 days
7 to 27 days
1 to 5 months
6 to 11 months
12 to 23 months
2 to 4
5 to 9
10 to 14
15 to 19
20 to 24
25 to 29
30 to 34
35 to 39
40 to 44
45 to 49
50 to 54
55 to 59
60 to 64
65 to 69
70 to 74
75 to 79
80 to 84
85 to 89
90 to 94
95 plus
All Ages
Age-standardised
Prevalence (%)
Year
Total
Moderate + Severe
Severe
Cause-Specific Anaemia Prevalence - 2021
Females
Males
PfPr Malaria
PvPr Malaria
Clinical Malaria
Hookworm Disease
Schistosomiasis
Other NTDs
Other Infectious
HIV/AIDS
Maternal Hemorrhage
Uterine Fibroids
Menstrual Disorders
Beta-Thalassemia Major
Hemoglobin E/Beta-Thalassemia
Hemoglobin H
Beta-Thalassemia Trait
Hemoglobin E Trait
Homozygous SC & Severe SC
Hemoglobin SC Disease
Mild SC/Beta-thalassemia
Sickle Cell Trait
G6PD Deficiency
Hemizygous G6PD Deficiency
Other Hemoglobinopathies
Iron Deficiency
Vitamin A Deficiency
Gastritis & Duodenitis
Peptic Ulcer Disease
Crohn's Disease
Ulcerative Colitis
Cirrhosis
CKD Stage 3
CKD Stage 4
CKD Stage 5 untreated
CKD Stage 5 on dialysis
Hypothyroid Diseases
Hyperthyroid Diseases
Other Endocrine
Males
Females
1990
2021
Dietary iron deficiency
Chronic kidney disease
Haemoglobinopathies and haemolytic anaemias
Other neglected tropical diseases
Other unspecified infectious diseases
Endocrine, metabolic, blood, and immune disorders
Upper digestive system diseases
Inflammatory bowel disease
Vitamin A deficiency
Cirrhosis and other chronic liver diseases
HIV/AIDS
Intestinal nematode infections
Schistosomiasis
Malaria
Gynecological diseases
Maternal disorders
YLDs per 100,000 population
0 to <0
0 to <0.01
0.01 to <0.07
0.07 to <0.19
0.19 to <0.65
0.65 to <1.09
1.09 to <3.09
3.09 to <9.64
9.64 to <16.24
16.24 to 138.12

Nottingham

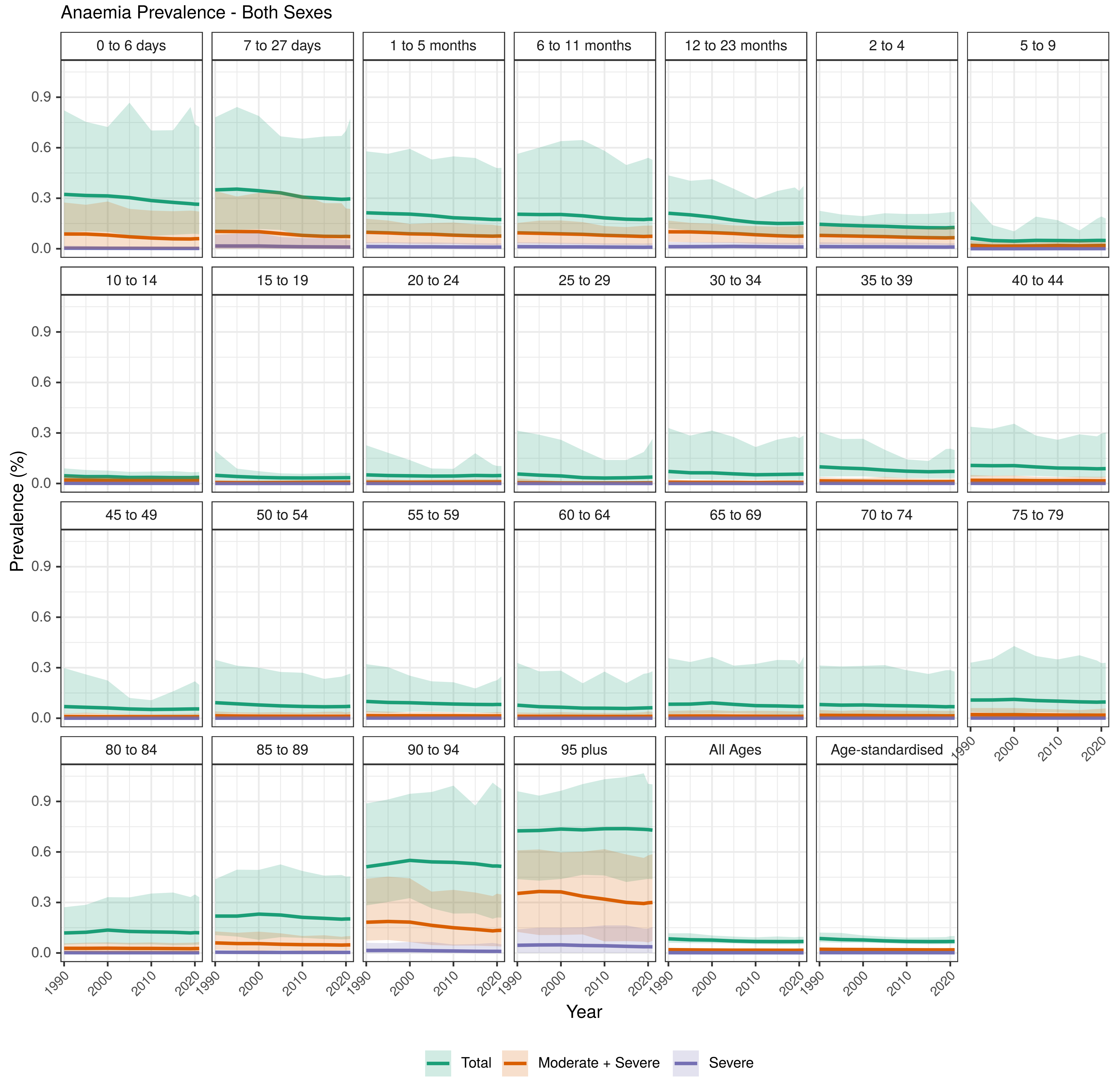
Anaemia Prevalence - Both Sexes
0 to 6 days
7 to 27 days
1 to 5 months
6 to 11 months
12 to 23 months
2 to 4
5 to 9
10 to 14
15 to 19
20 to 24
25 to 29
30 to 34
35 to 39
40 to 44
45 to 49
50 to 54
55 to 59
60 to 64
65 to 69
70 to 74
75 to 79
80 to 84
85 to 89
90 to 94
95 plus
All Ages
Age-standardised
Prevalence (%)
Year
Total
Moderate + Severe
Severe

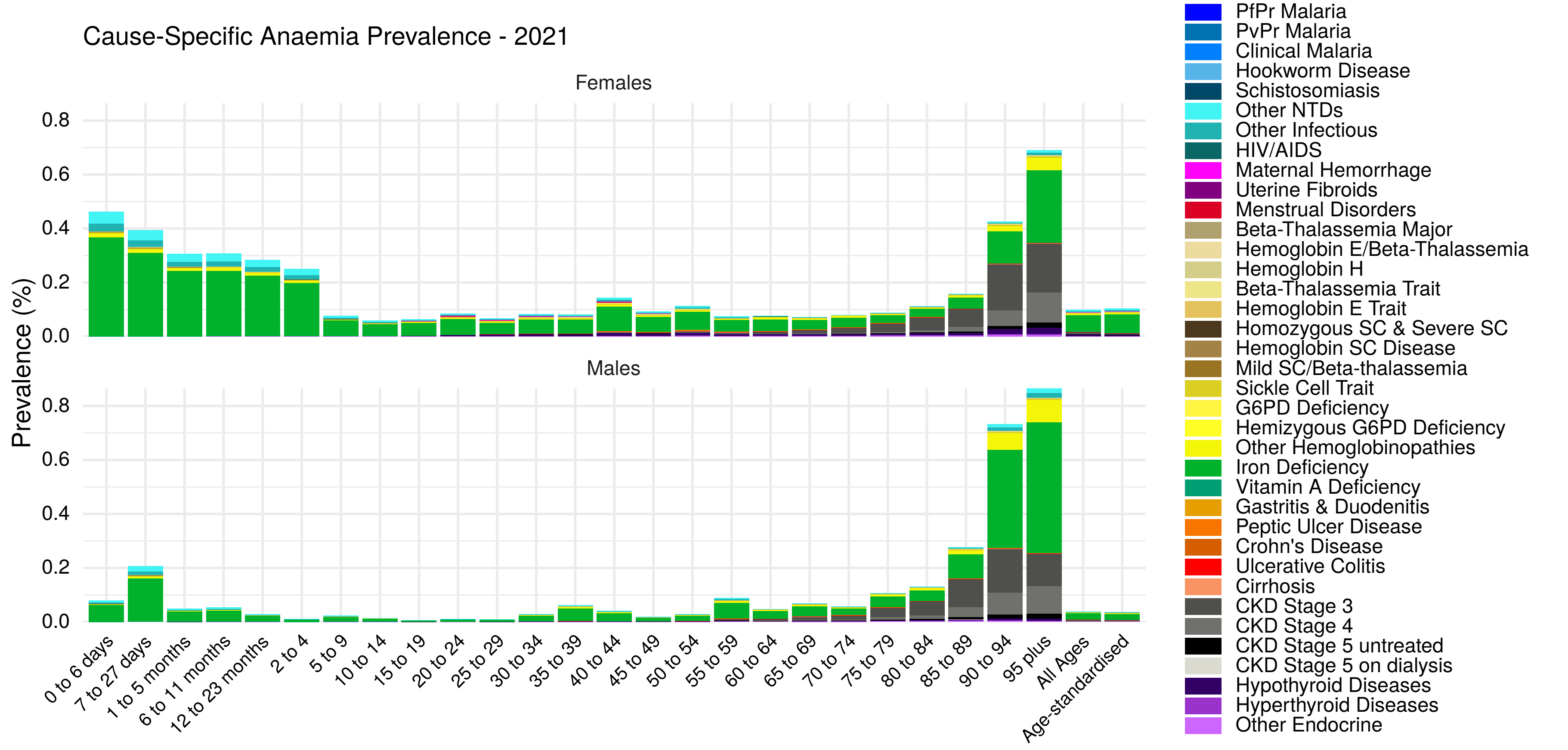
Cause-Specific Anaemia Prevalence - 2021
Females
Males
Prevalence (%)
PfPr Malaria
PvPr Malaria
Clinical Malaria
Hookworm Disease
Schistosomiasis
Other NTDs
Other Infectious
HIV/AIDS
Maternal Hemorrhage
Uterine Fibroids
Menstrual Disorders
Beta-Thalassemia Major
Hemoglobin E/Beta-Thalassemia
Hemoglobin H
Beta-Thalassemia Trait
Hemoglobin E Trait
Homozygous SC & Severe SC
Hemoglobin SC Disease
Mild SC/Beta-thalassemia
Sickle Cell Trait
G6PD Deficiency
Hemizygous G6PD Deficiency
Other Hemoglobinopathies
Iron Deficiency
Vitamin A Deficiency
Gastritis & Duodenitis
Peptic Ulcer Disease
Crohn's Disease
Ulcerative Colitis
Cirrhosis
CKD Stage 3
CKD Stage 4
CKD Stage 5 untreated
CKD Stage 5 on dialysis
Hypothyroid Diseases
Hyperthyroid Diseases
Other Endocrine

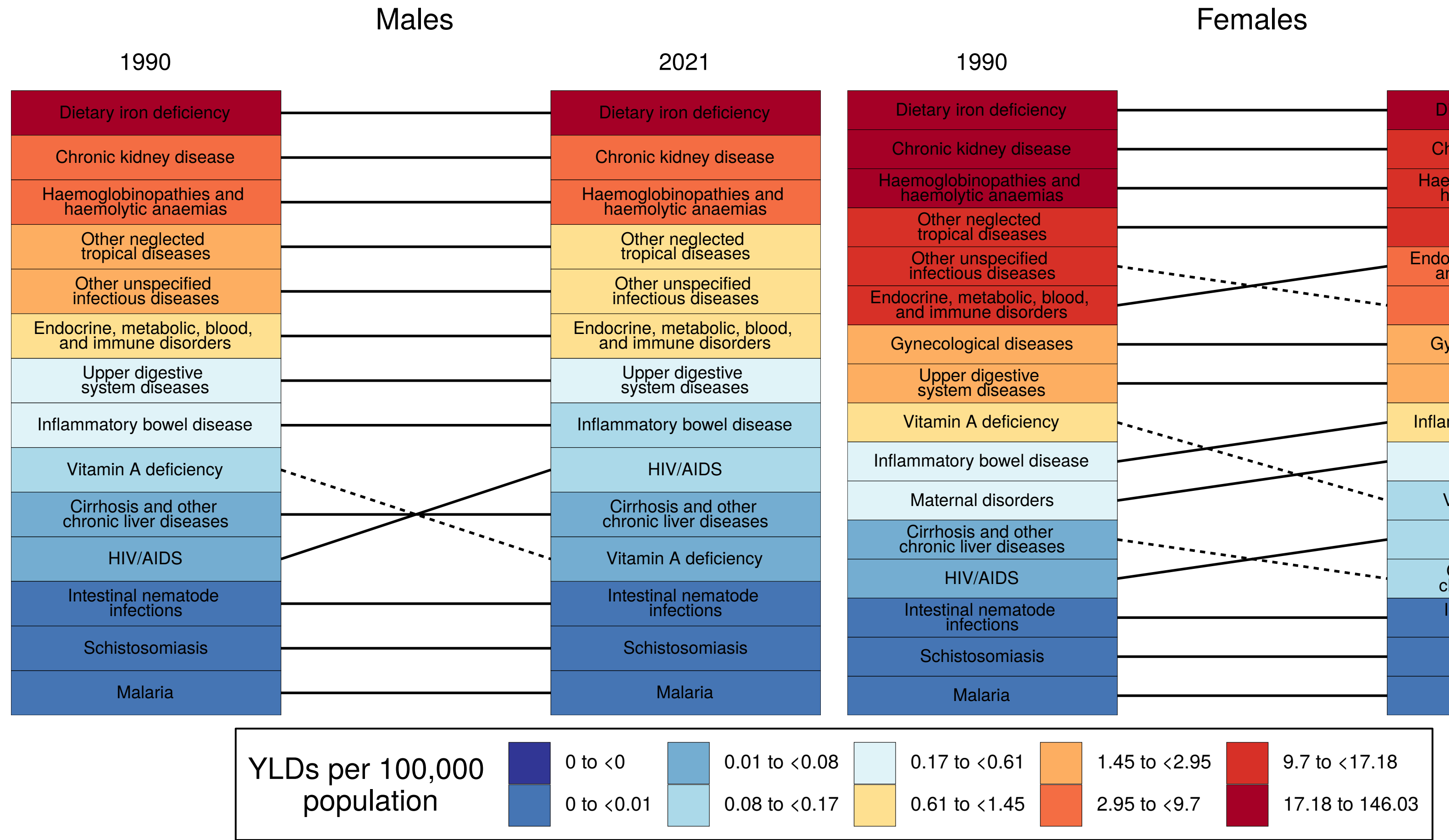
Males
Females
1990
2021
Dietary iron deficiency
Chronic kidney disease
Haemoglobinopathies and haemolytic anaemias
Other neglected tropical diseases
Other unspecified infectious diseases
Endocrine, metabolic, blood, and immune disorders
Gynecological diseases
Upper digestive system diseases
Vitamin A deficiency
Inflammatory bowel disease
Maternal disorders
HIV/AIDS
Cirrhosis and other chronic liver diseases
Intestinal nematode infections
Schistosomiasis
Malaria
YLDs per 100,000 population
0 to <0
0 to <0.01
0.01 to <0.08
0.08 to <0.17
0.17 to <0.61
0.61 to <1.45
1.45 to <2.95
2.95 to <9.7
9.7 to <17.18
17.18 to 146.03

Nottinghamshire

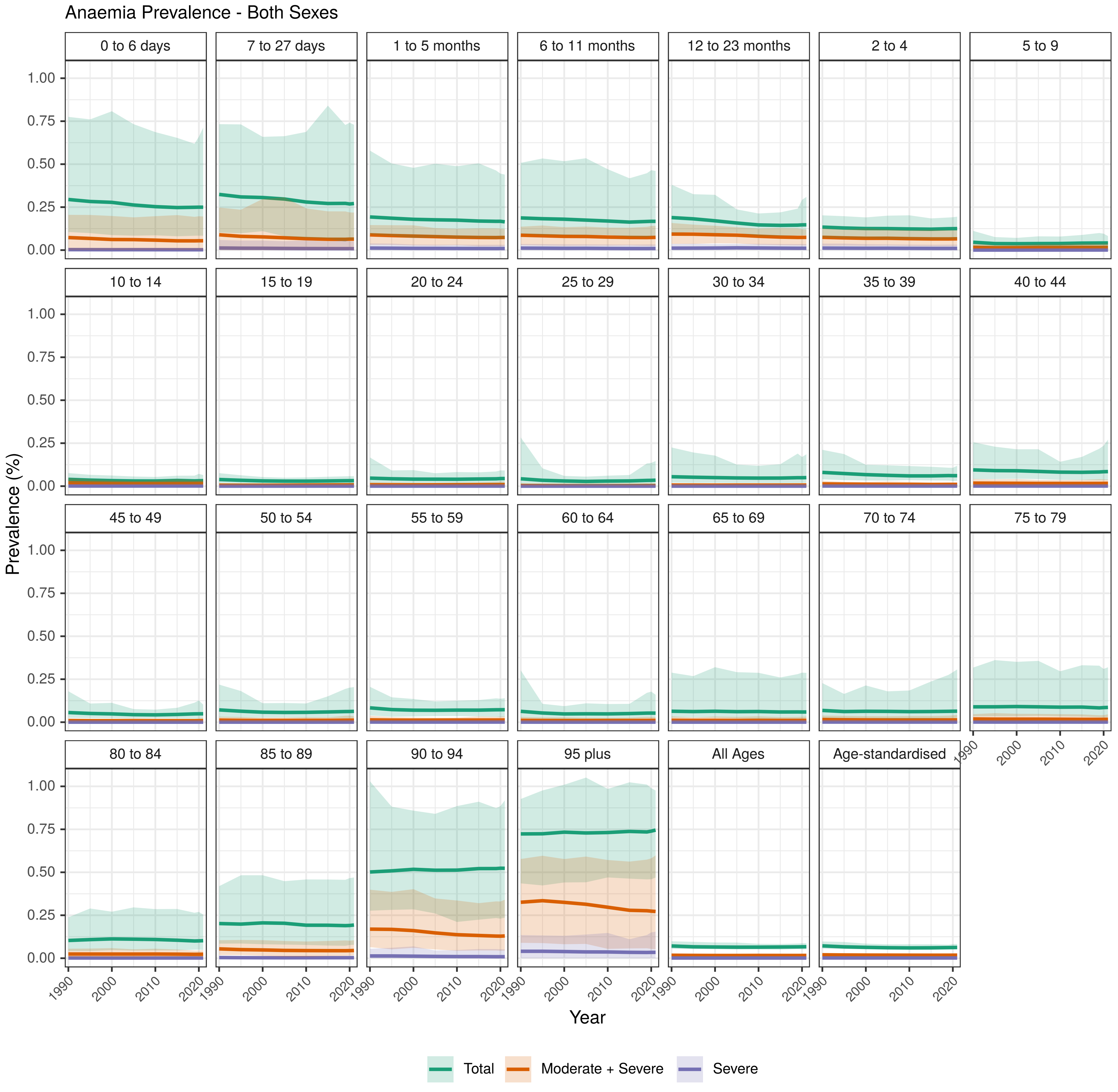

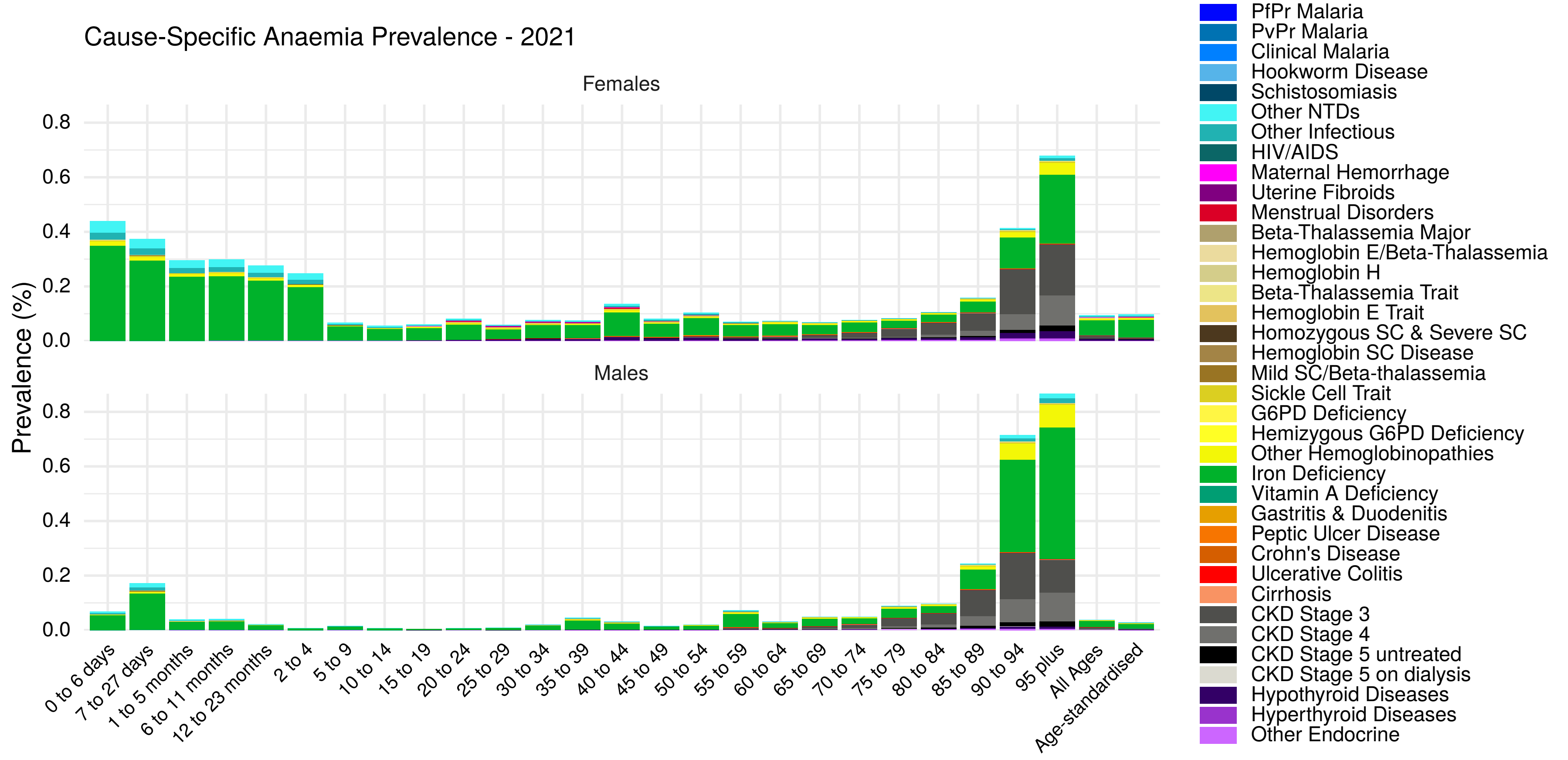

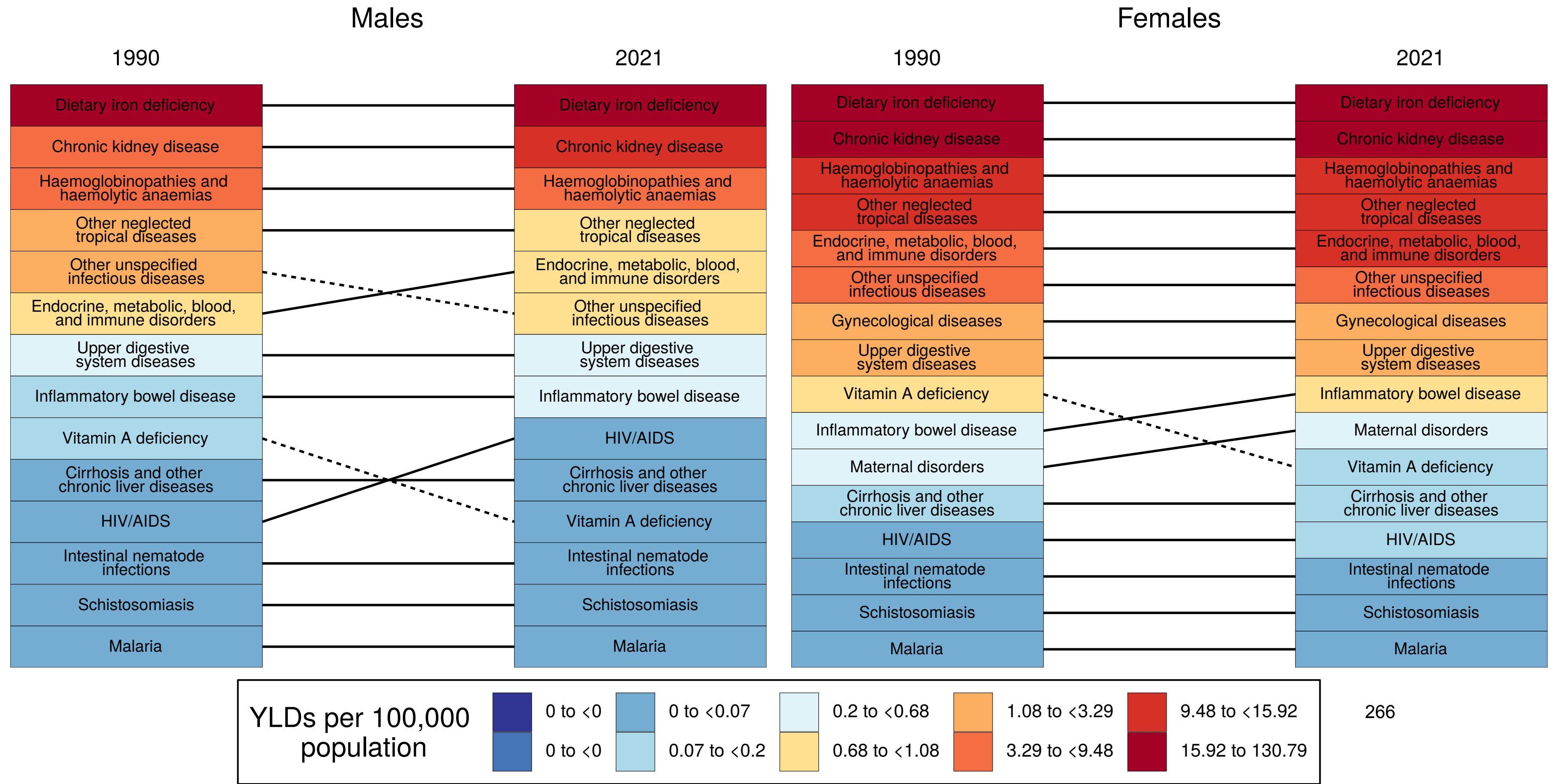

Rutland

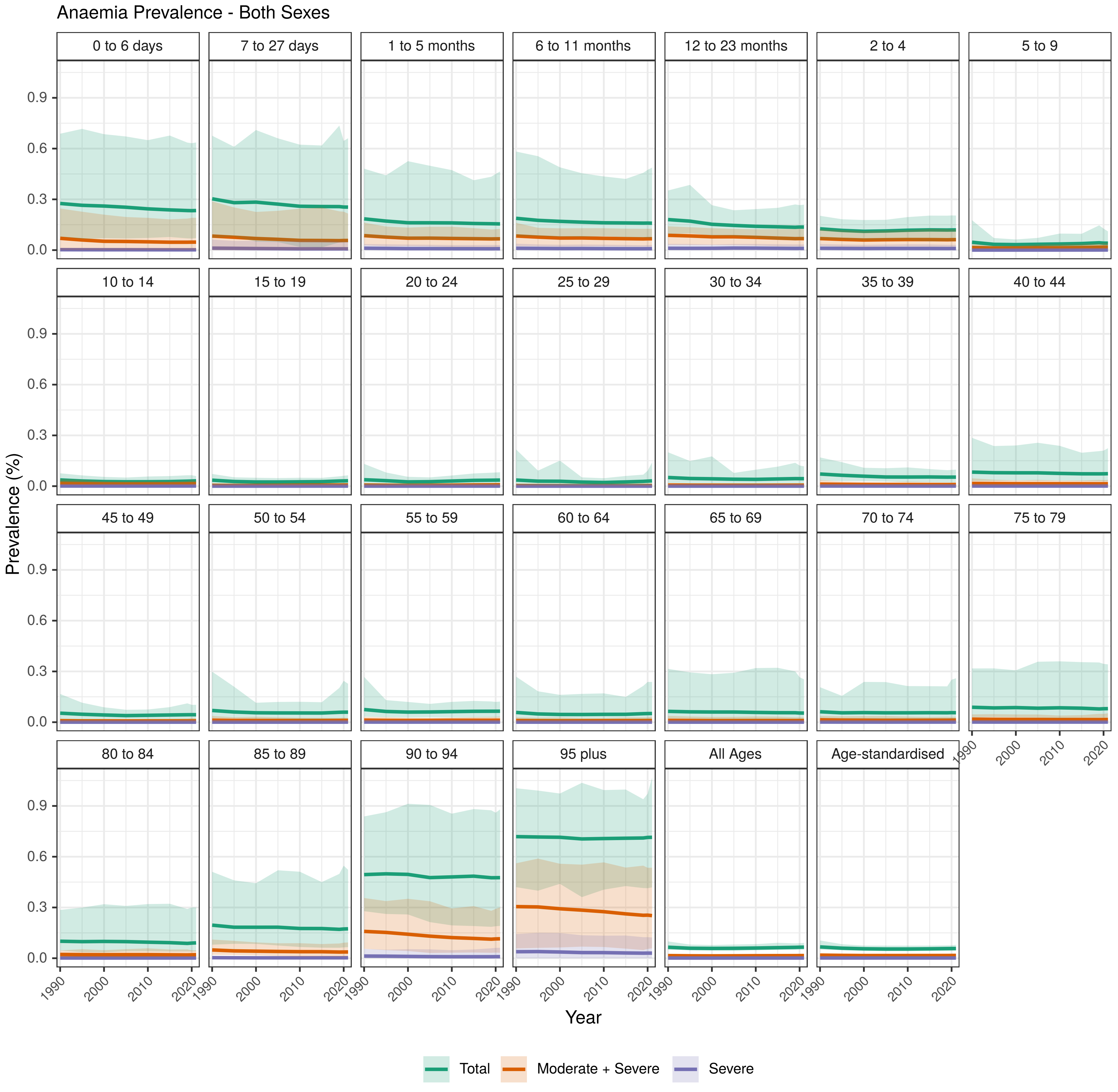
Anaemia Prevalence - Both Sexes
0 to 6 days
7 to 27 days
1 to 5 months
6 to 11 months
12 to 23 months
2 to 4
5 to 9
10 to 14
15 to 19
20 to 24
25 to 29
30 to 34
35 to 39
40 to 44
45 to 49
50 to 54
55 to 59
60 to 64
65 to 69
70 to 74
75 to 79
80 to 84
85 to 89
90 to 94
95 plus
All Ages
Age-standardised
Prevalence (%)
Year
Total
Moderate + Severe
Severe

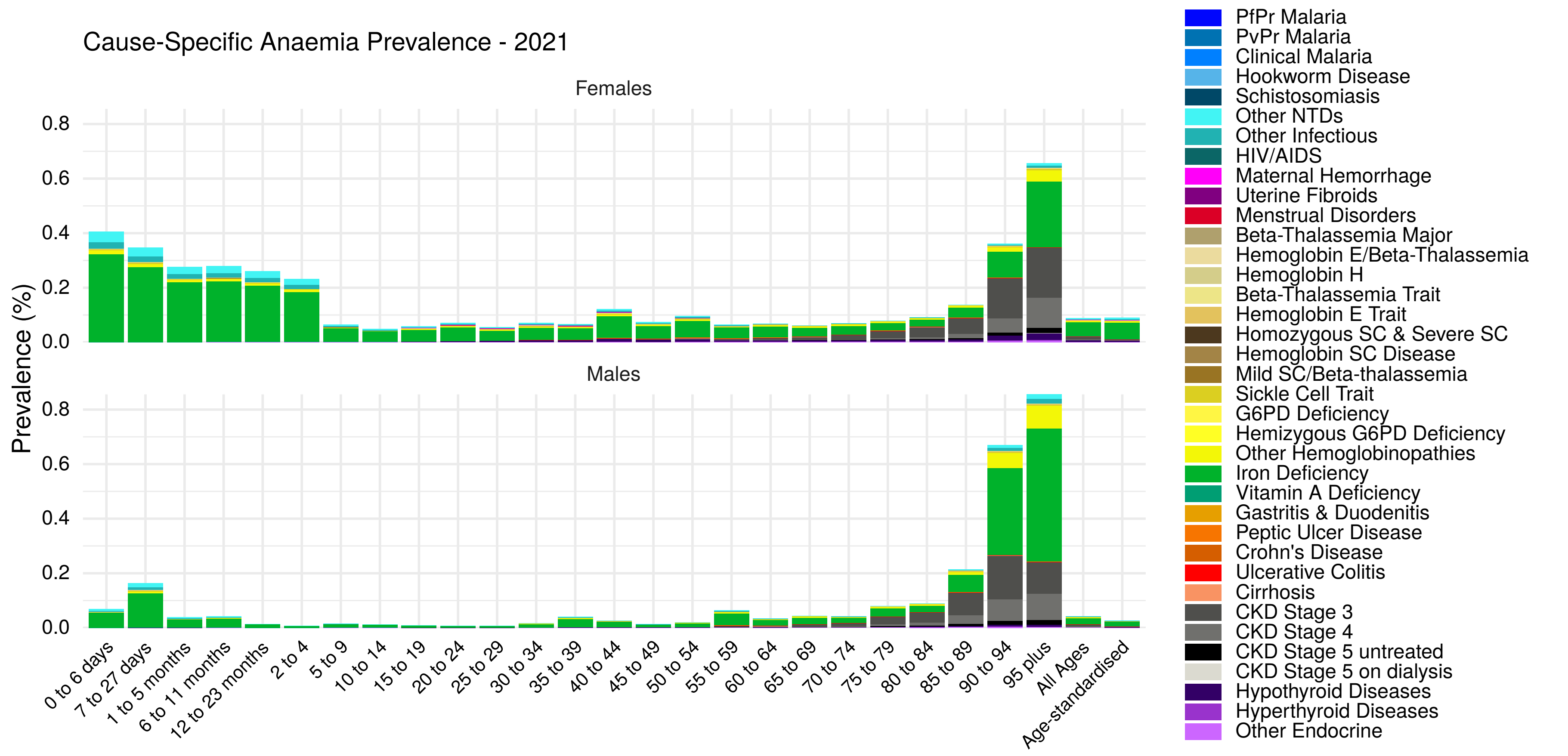
Cause-Specific Anaemia Prevalence - 2021
Females
Males
Prevalence (%)
PfPr Malaria
PvPr Malaria
Clinical Malaria
Hookworm Disease
Schistosomiasis
Other NTDs
Other Infectious
HIV/AIDS
Maternal Hemorrhage
Uterine Fibroids
Menstrual Disorders
Beta-Thalassemia Major
Hemoglobin E/Beta-Thalassemia
Hemoglobin H
Beta-Thalassemia Trait
Hemoglobin E Trait
Homozygous SC & Severe SC
Hemoglobin SC Disease
Mild SC/Beta-thalassemia
Sickle Cell Trait
G6PD Deficiency
Hemizygous G6PD Deficiency
Other Hemoglobinopathies
Iron Deficiency
Vitamin A Deficiency
Gastritis & Duodenitis
Peptic Ulcer Disease
Crohn's Disease
Ulcerative Colitis
Cirrhosis
CKD Stage 3
CKD Stage 4
CKD Stage 5 untreated
CKD Stage 5 on dialysis
Hypothyroid Diseases
Hyperthyroid Diseases
Other Endocrine

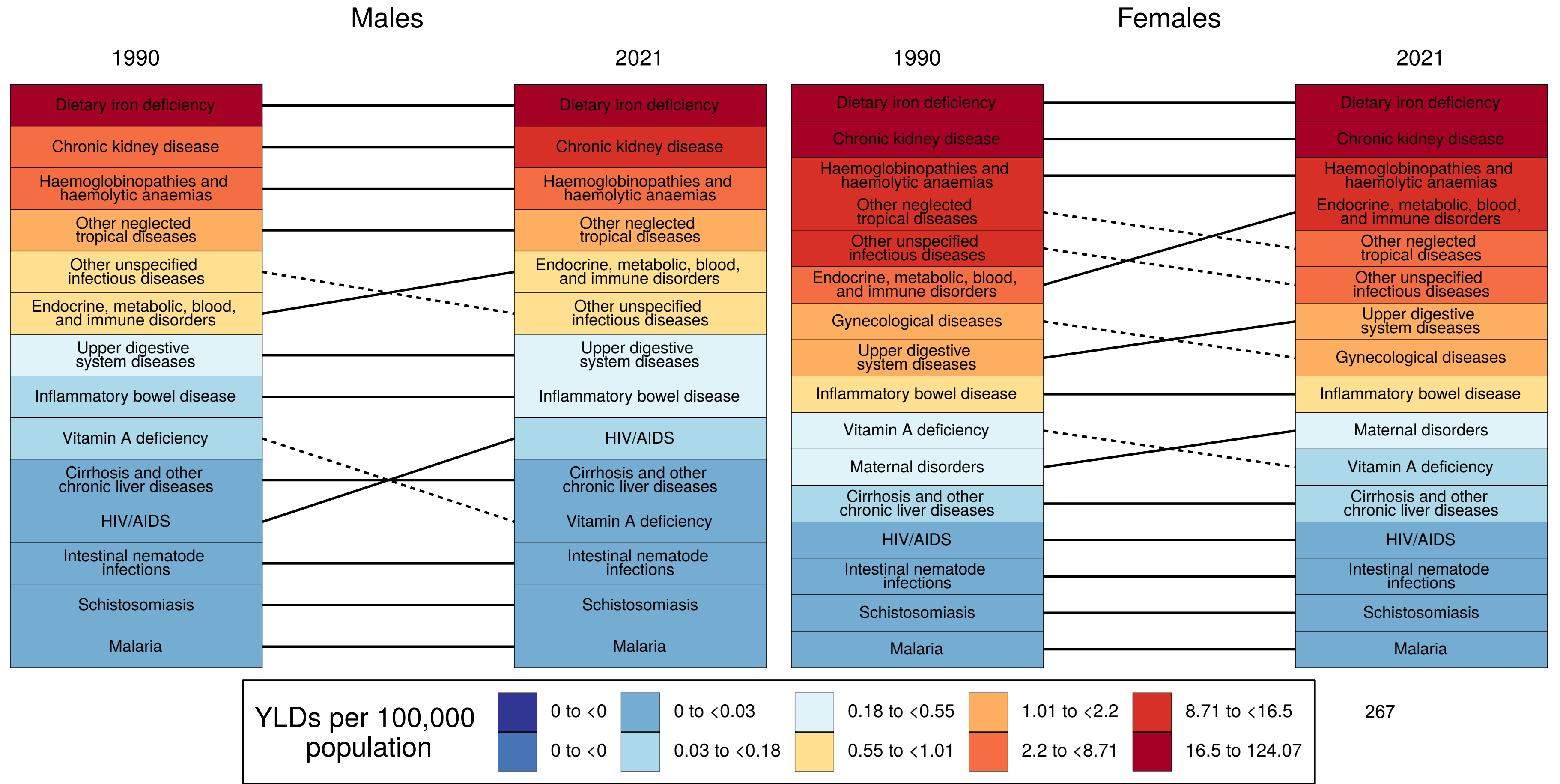
Males
Females
1990
2021
Dietary iron deficiency
Chronic kidney disease
Haemoglobinopathies and haemolytic anaemias
Other neglected tropical diseases
Other unspecified infectious diseases
Endocrine, metabolic, blood, and immune disorders
Upper digestive system diseases
Inflammatory bowel disease
Vitamin A deficiency
Cirrhosis and other chronic liver diseases
HIV/AIDS
Intestinal nematode infections
Schistosomiasis
Malaria
Gynecological diseases
Maternal disorders
YLDs per 100,000 population
0 to <0
0 to <0.03
0.03 to <0.18
0.18 to <0.55
0.55 to <1.01
1.01 to <2.2
2.2 to <8.71
8.71 to <16.5
16.5 to 124.07

East of England

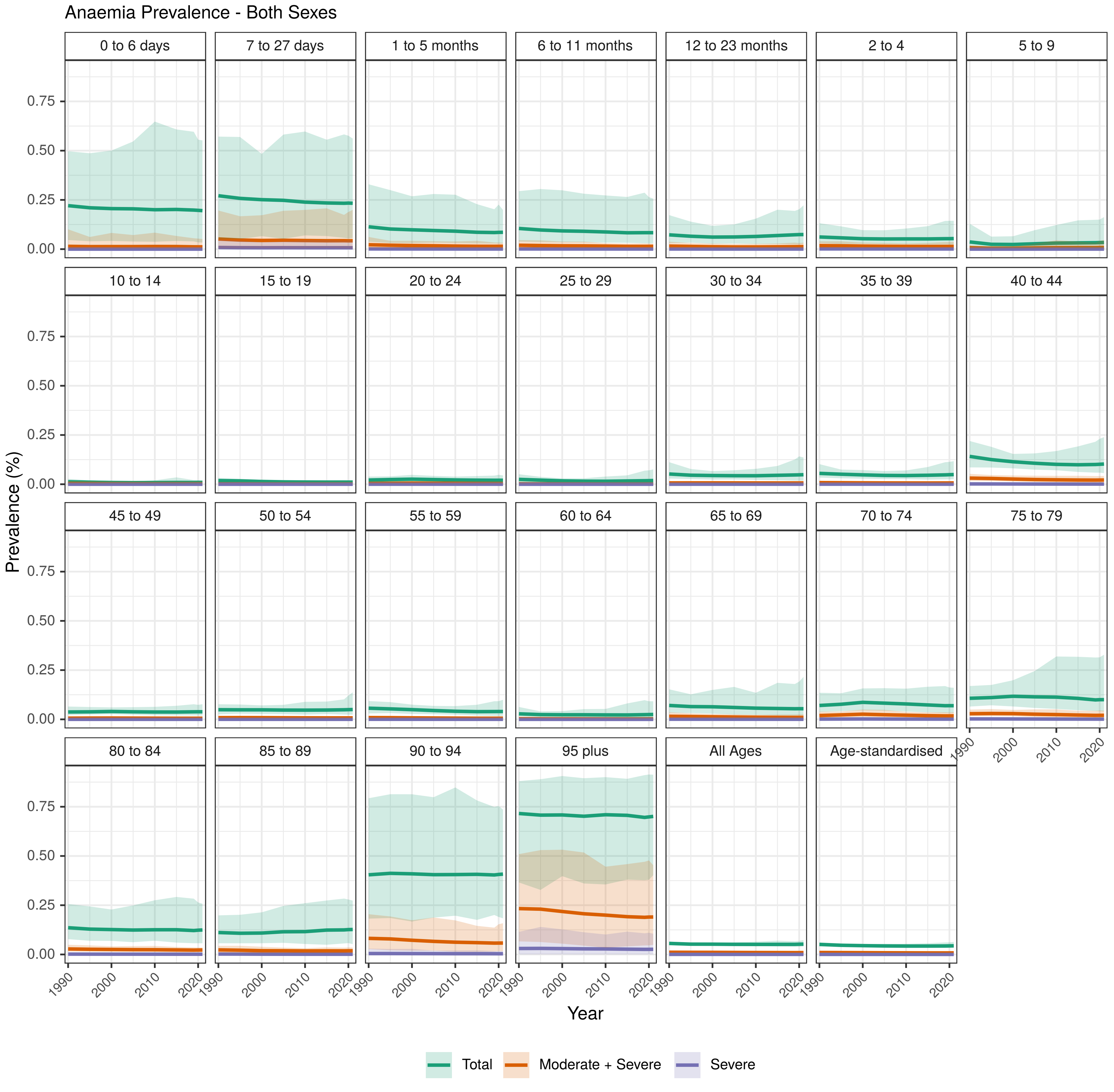

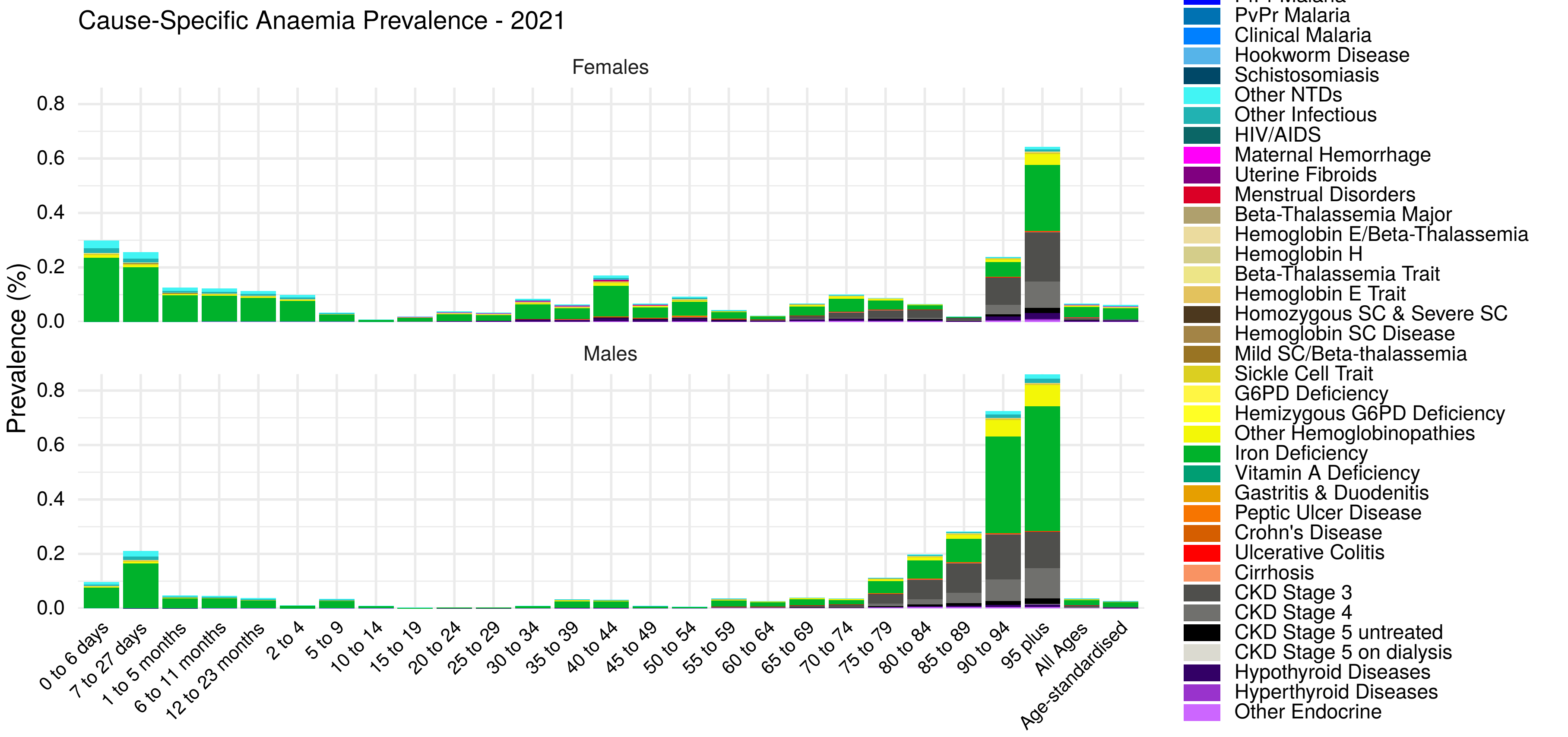

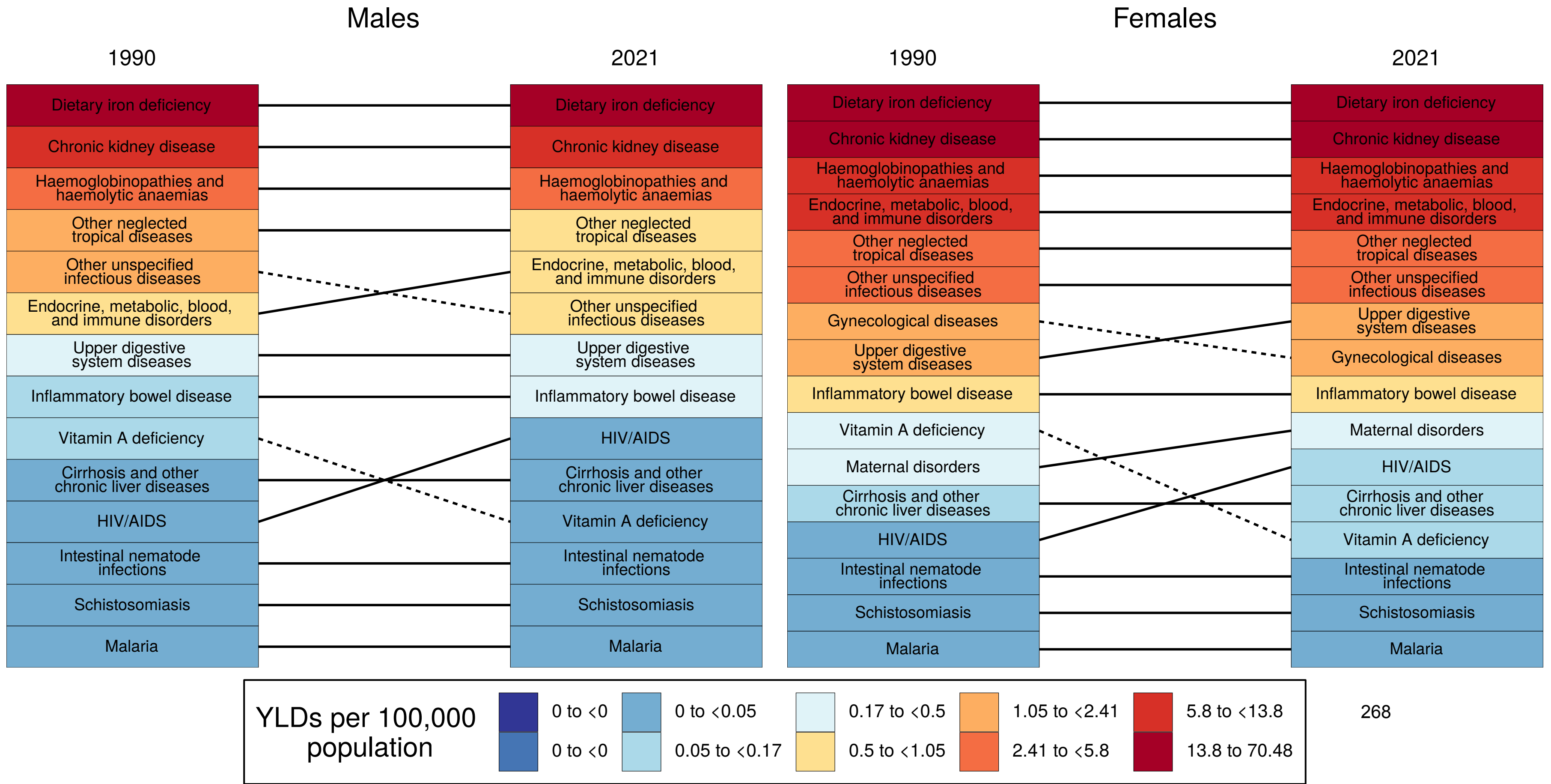

Bedford

Anaemia Prevalence - Both Sexes

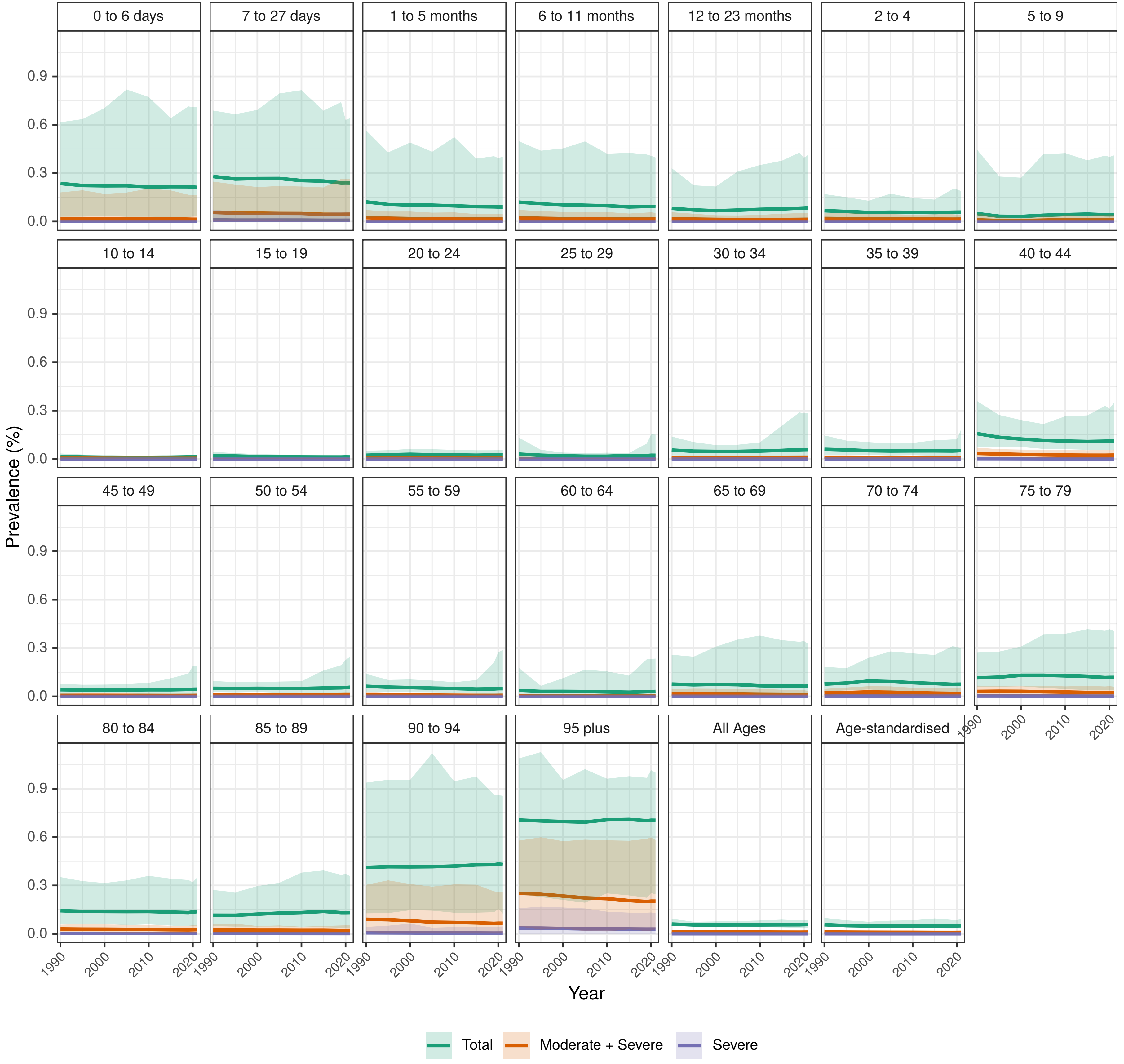

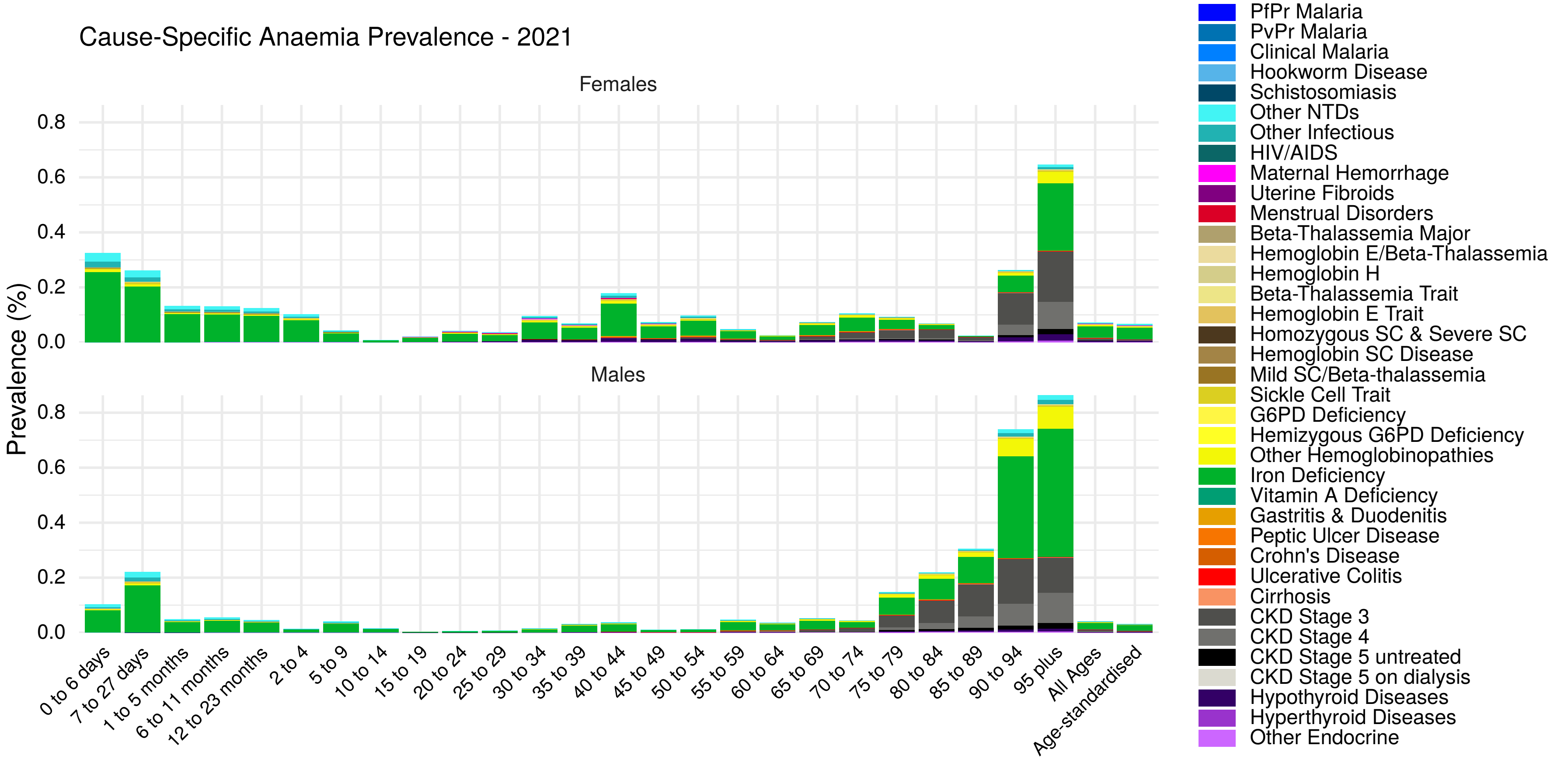

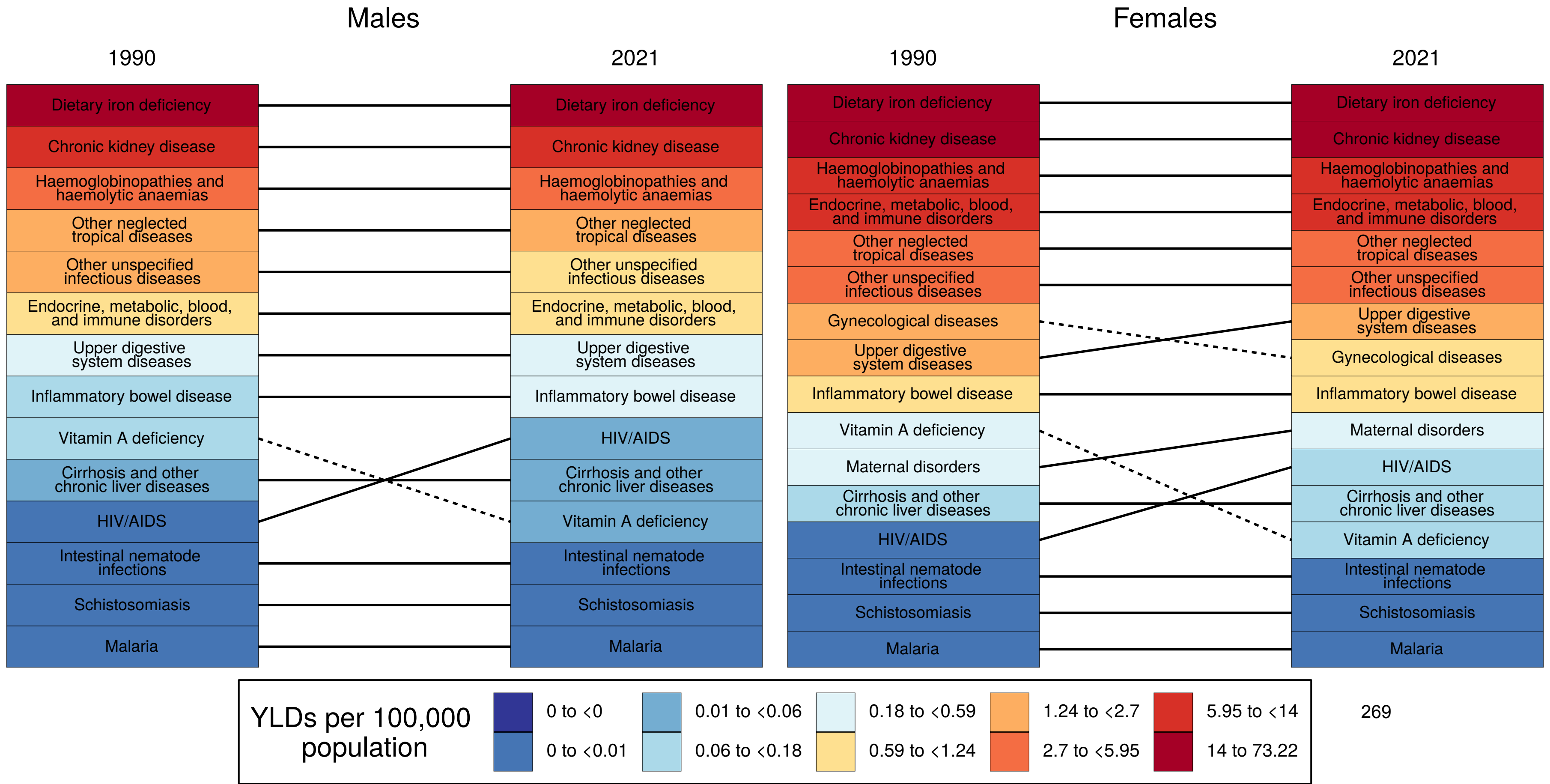

Cambridgeshire

Anaemia Prevalence - Both Sexes

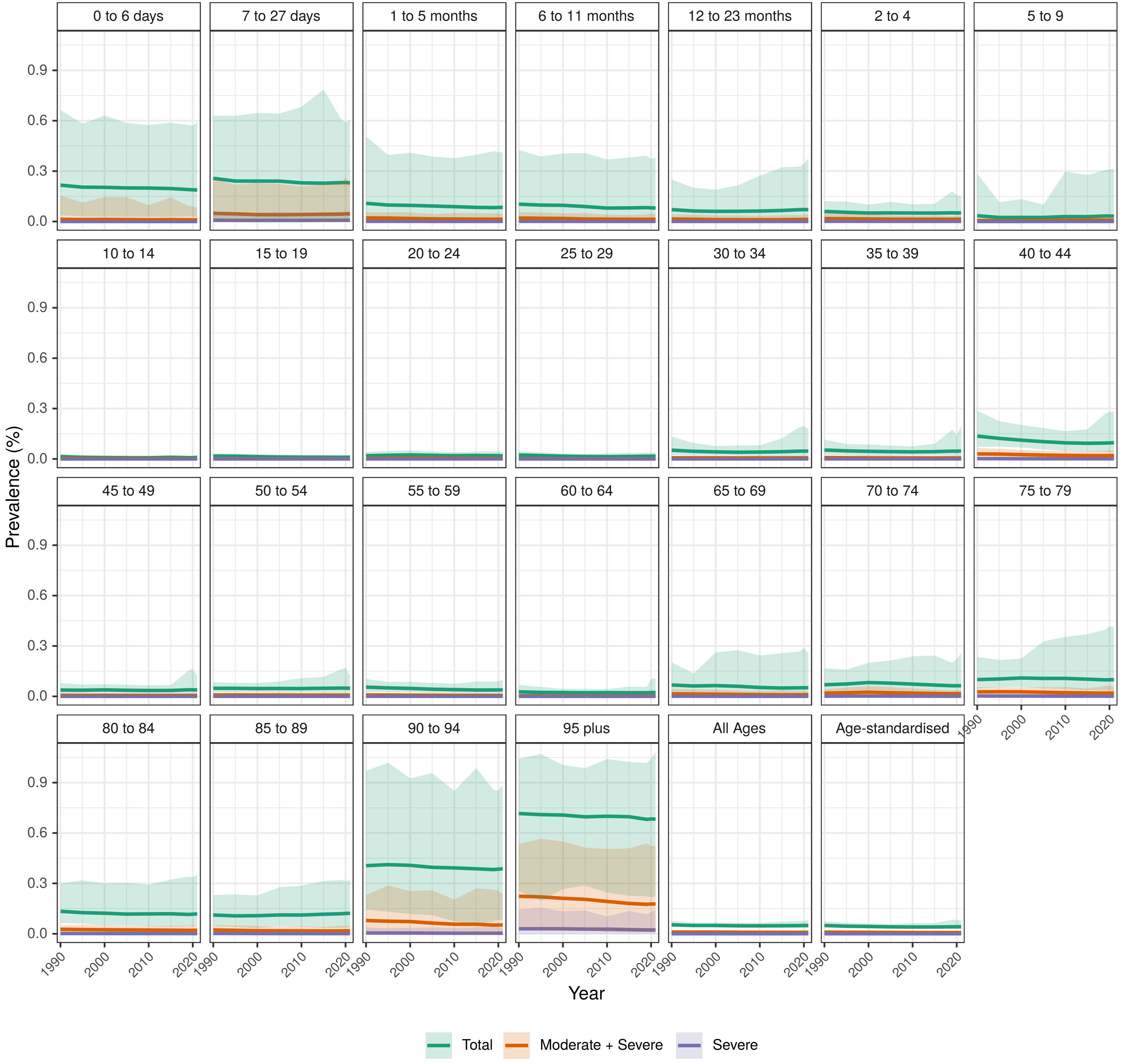

Cause-Specific Anaemia Prevalence - 2021

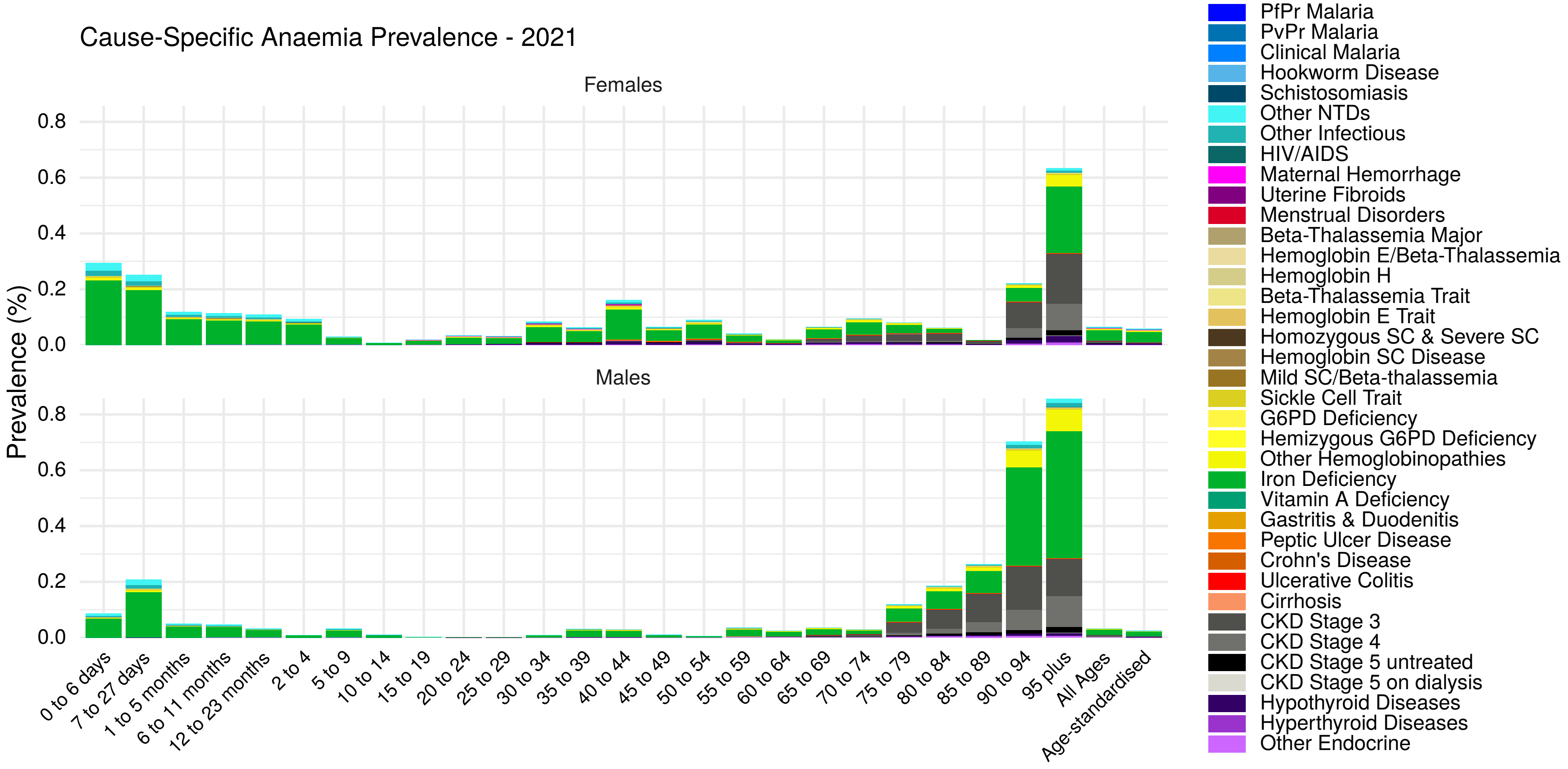

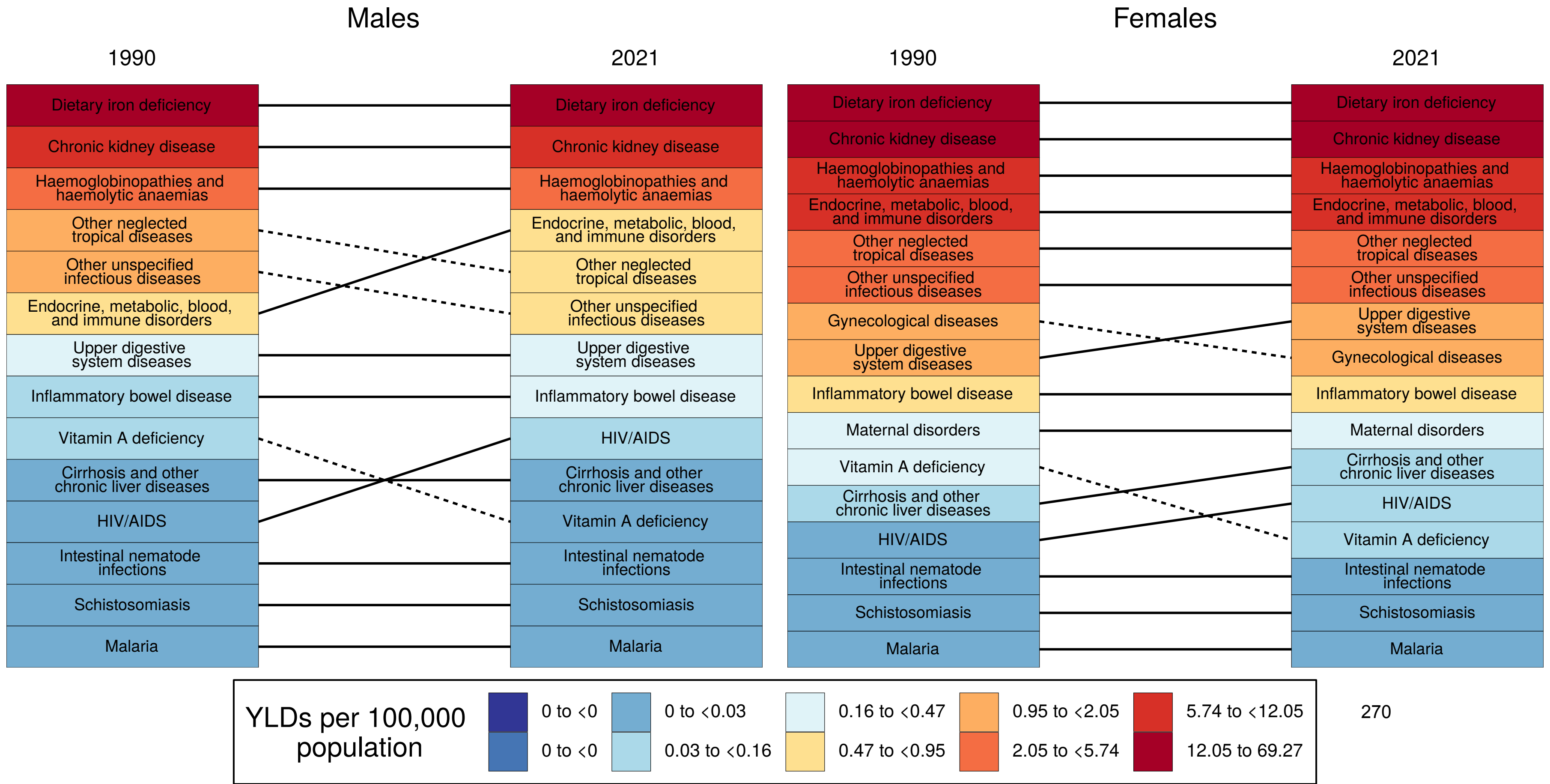

Central Bedfordshire

Anaemia Prevalence - Both Sexes

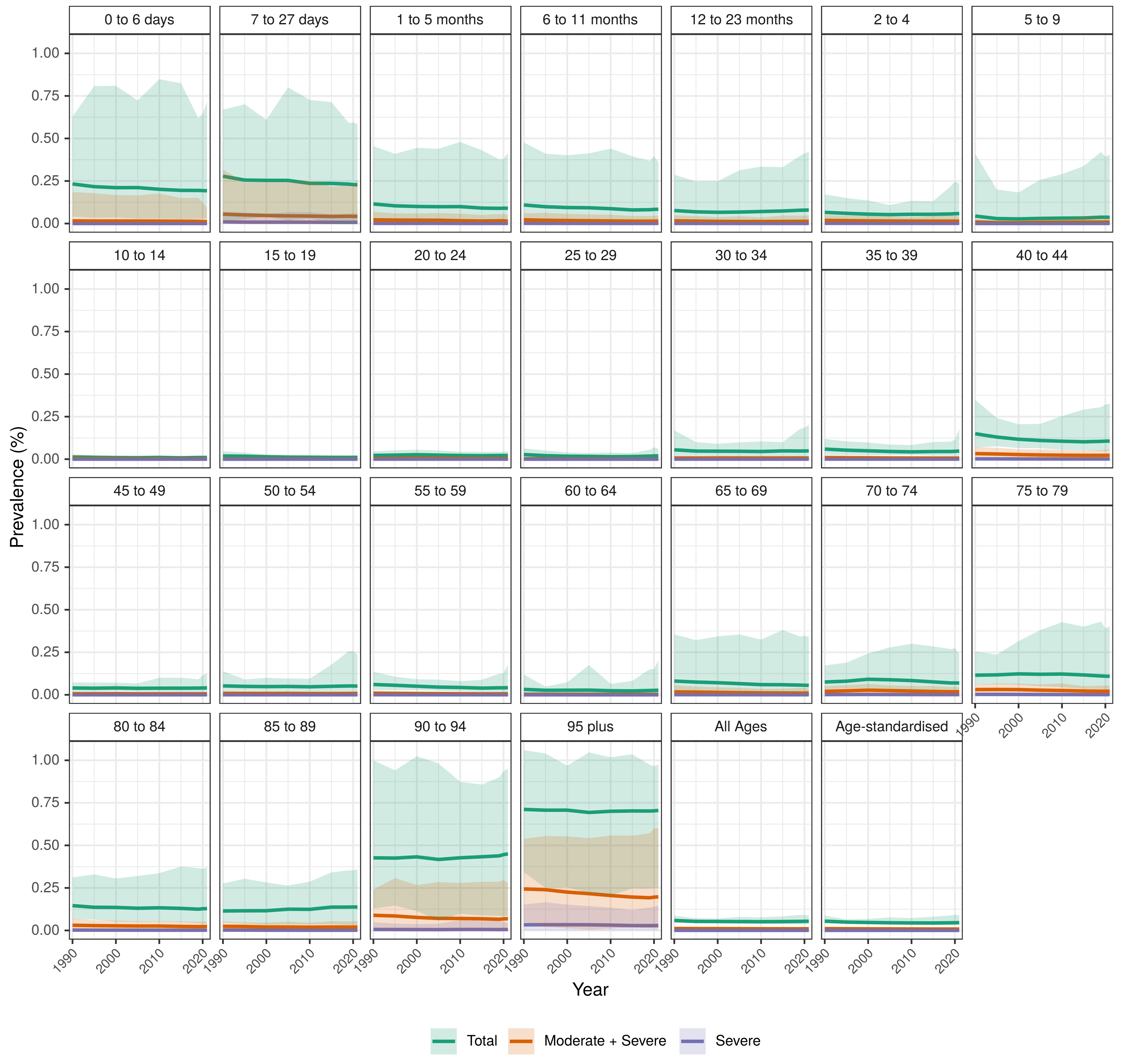

Cause-Specific Anaemia Prevalence - 2021

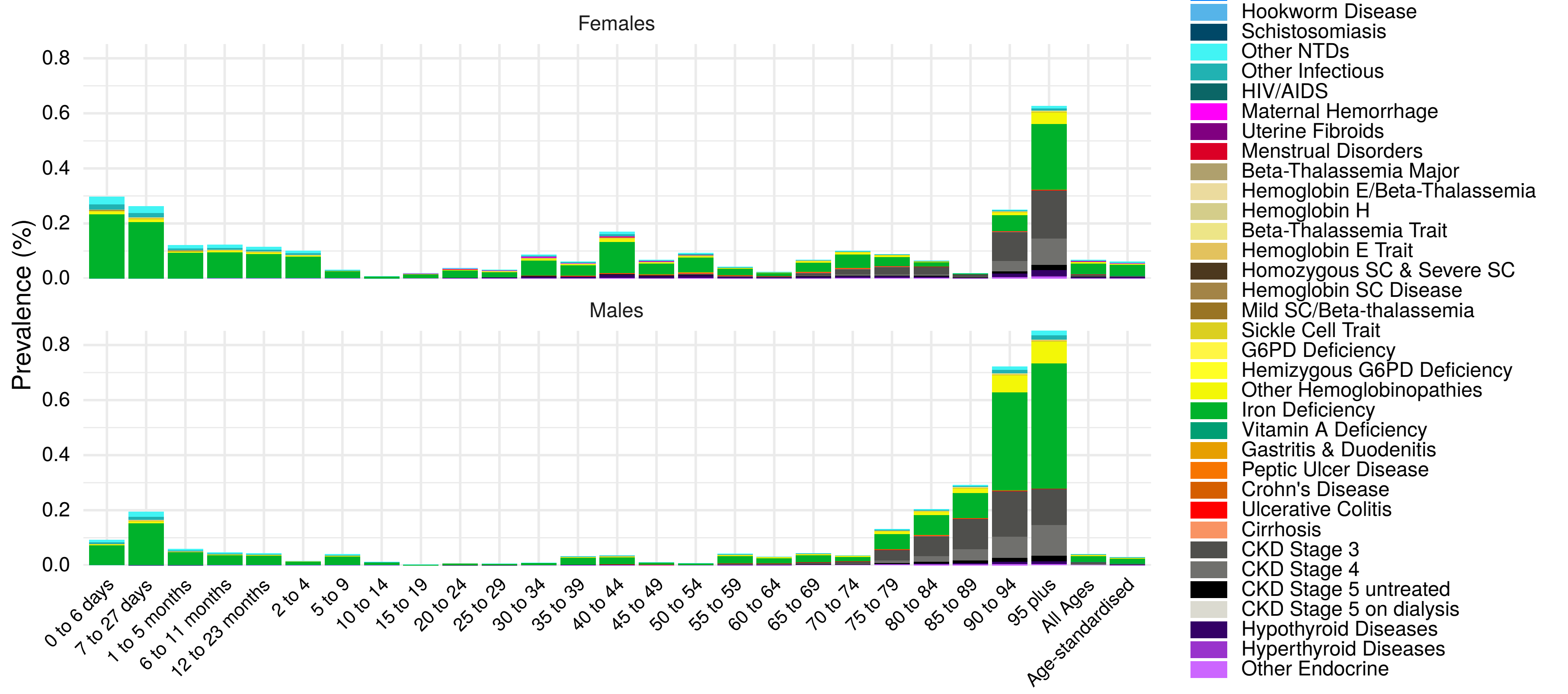

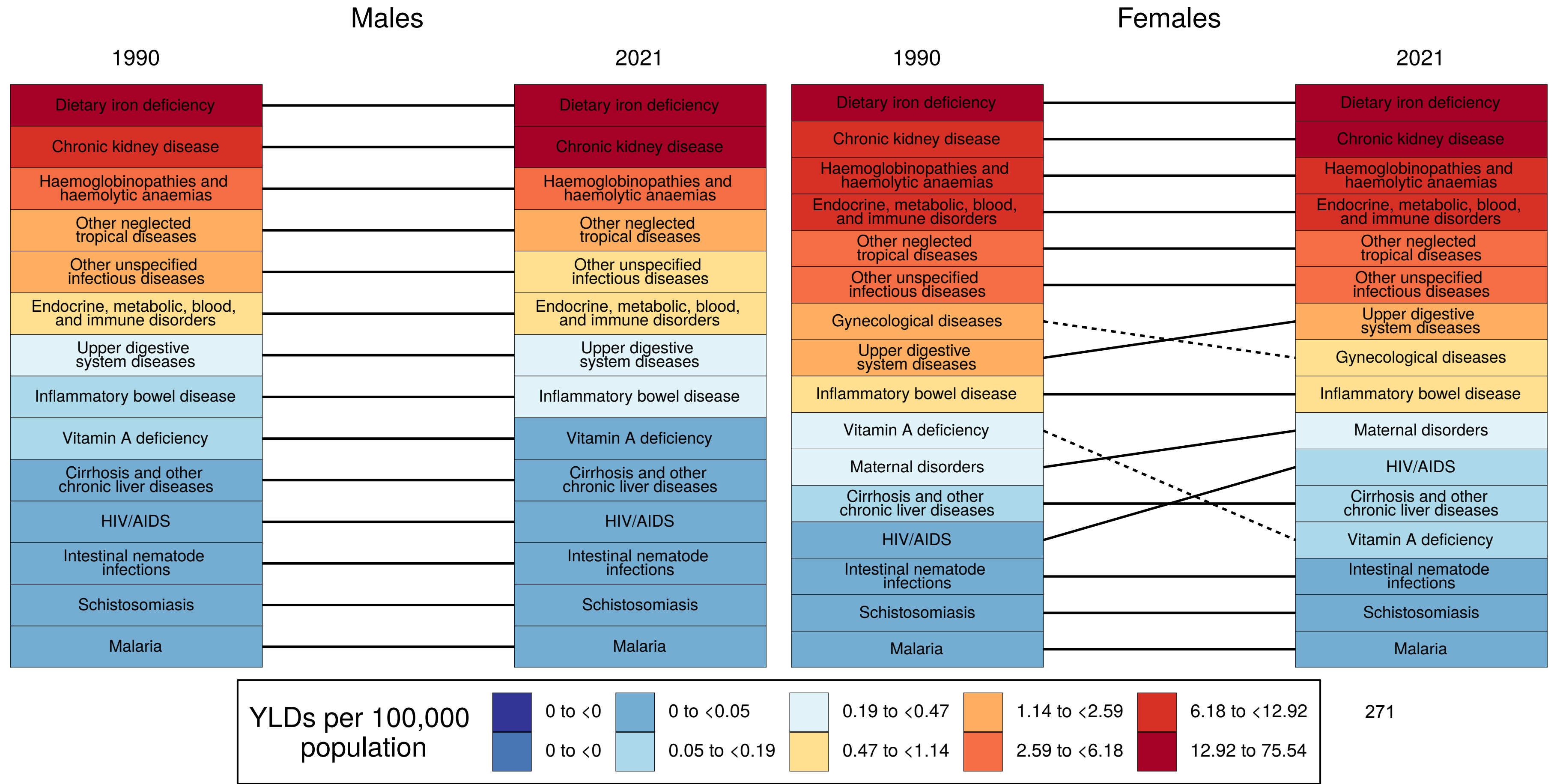

Essex

Anaemia Prevalence - Both Sexes

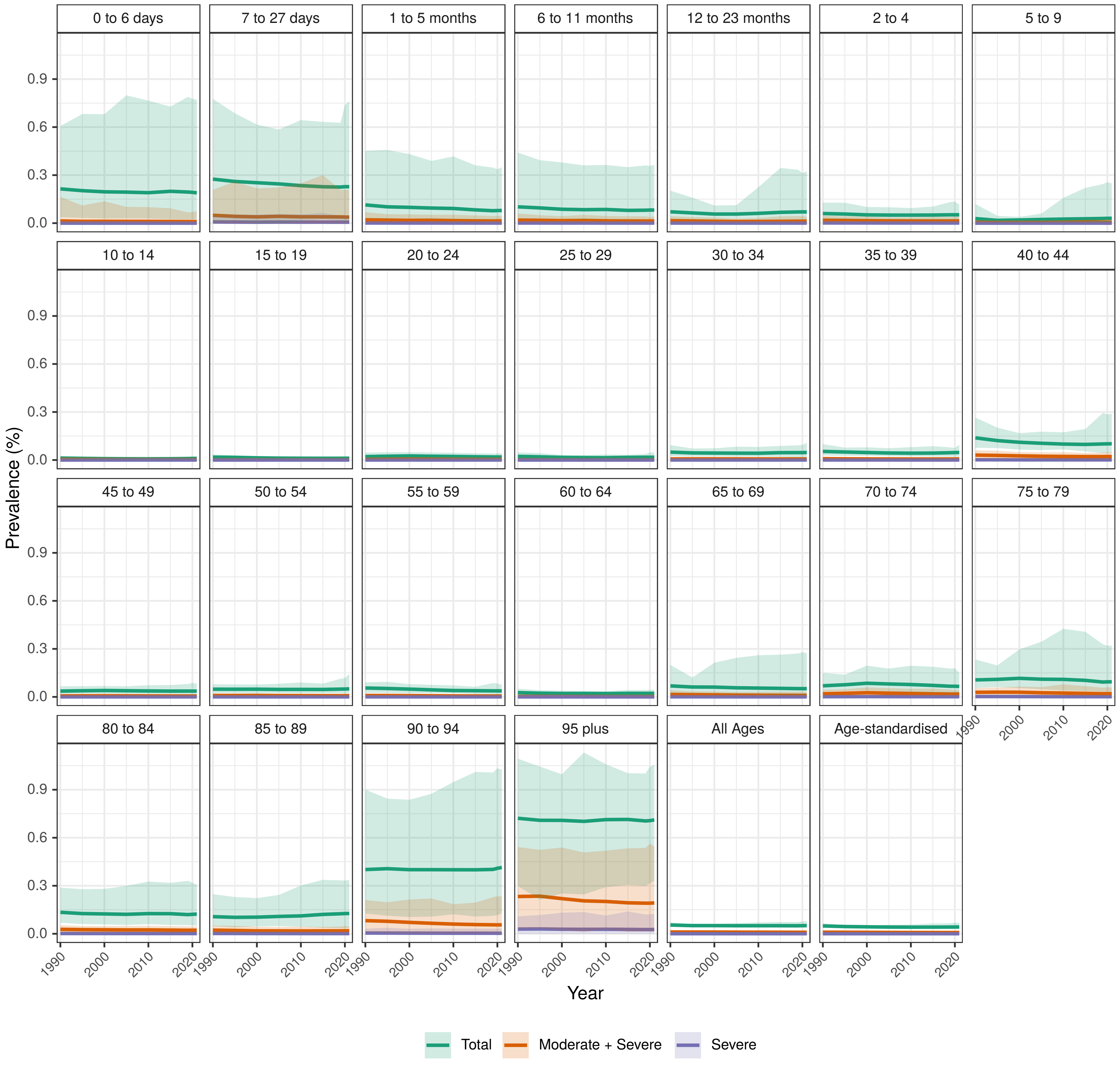

Cause-Specific Anaemia Prevalence - 2021

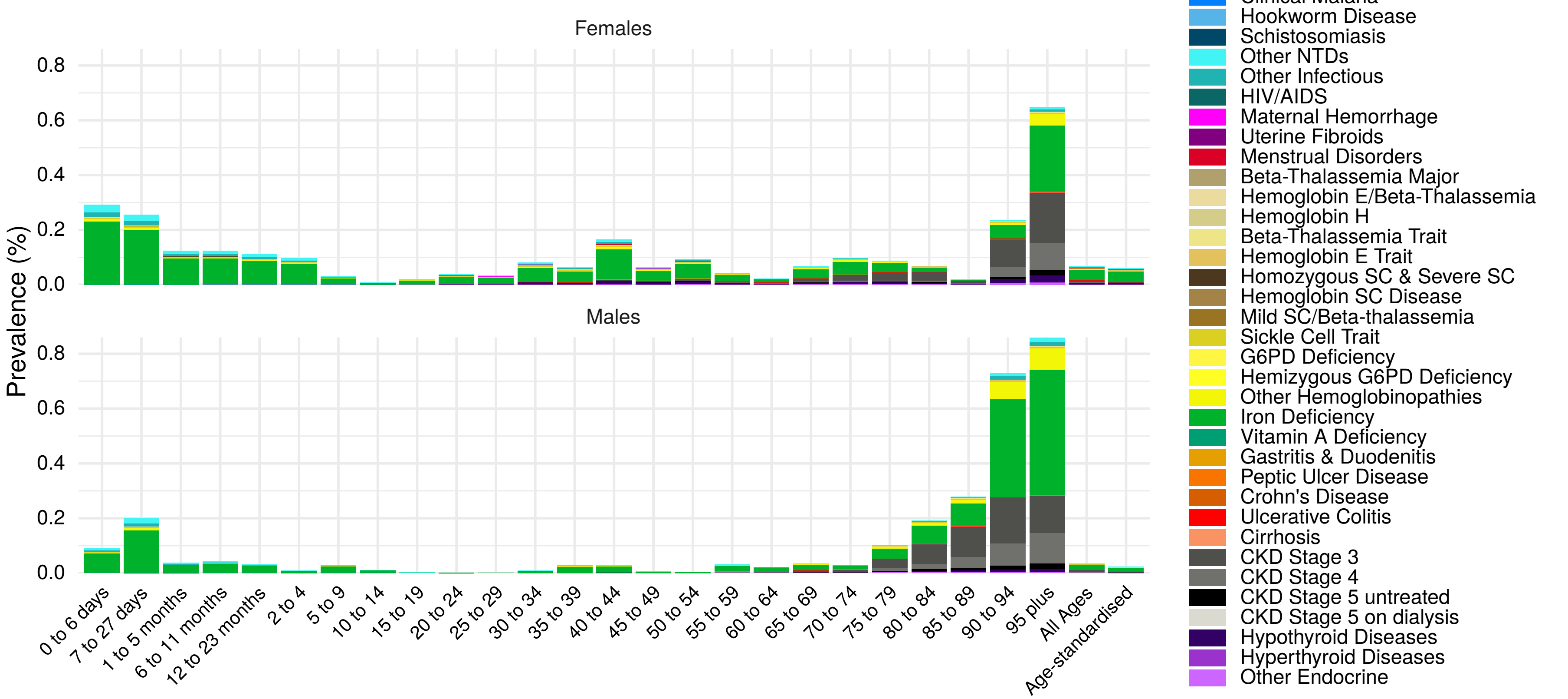

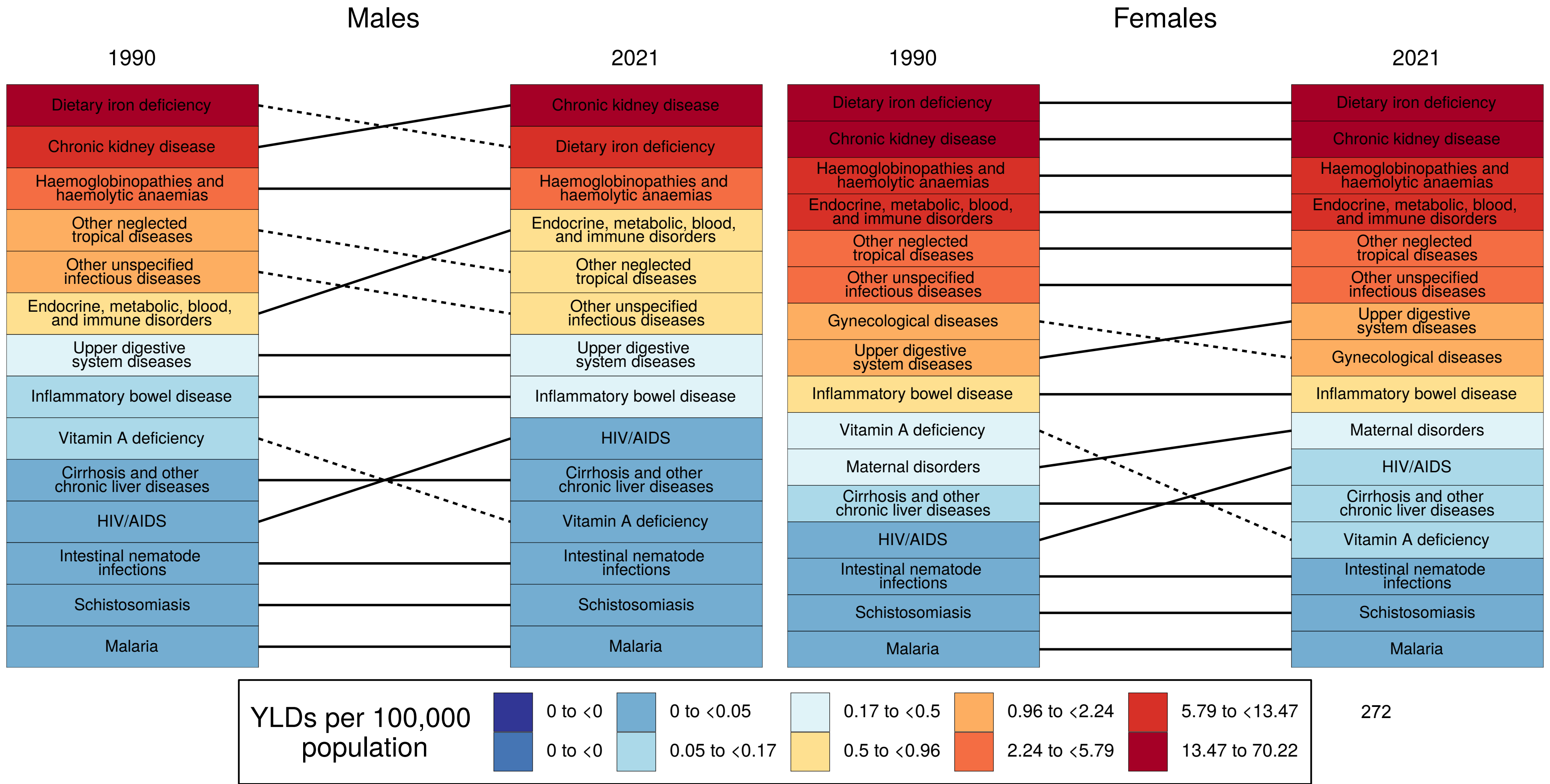

Hertfordshire

Anaemia Prevalence - Both Sexes

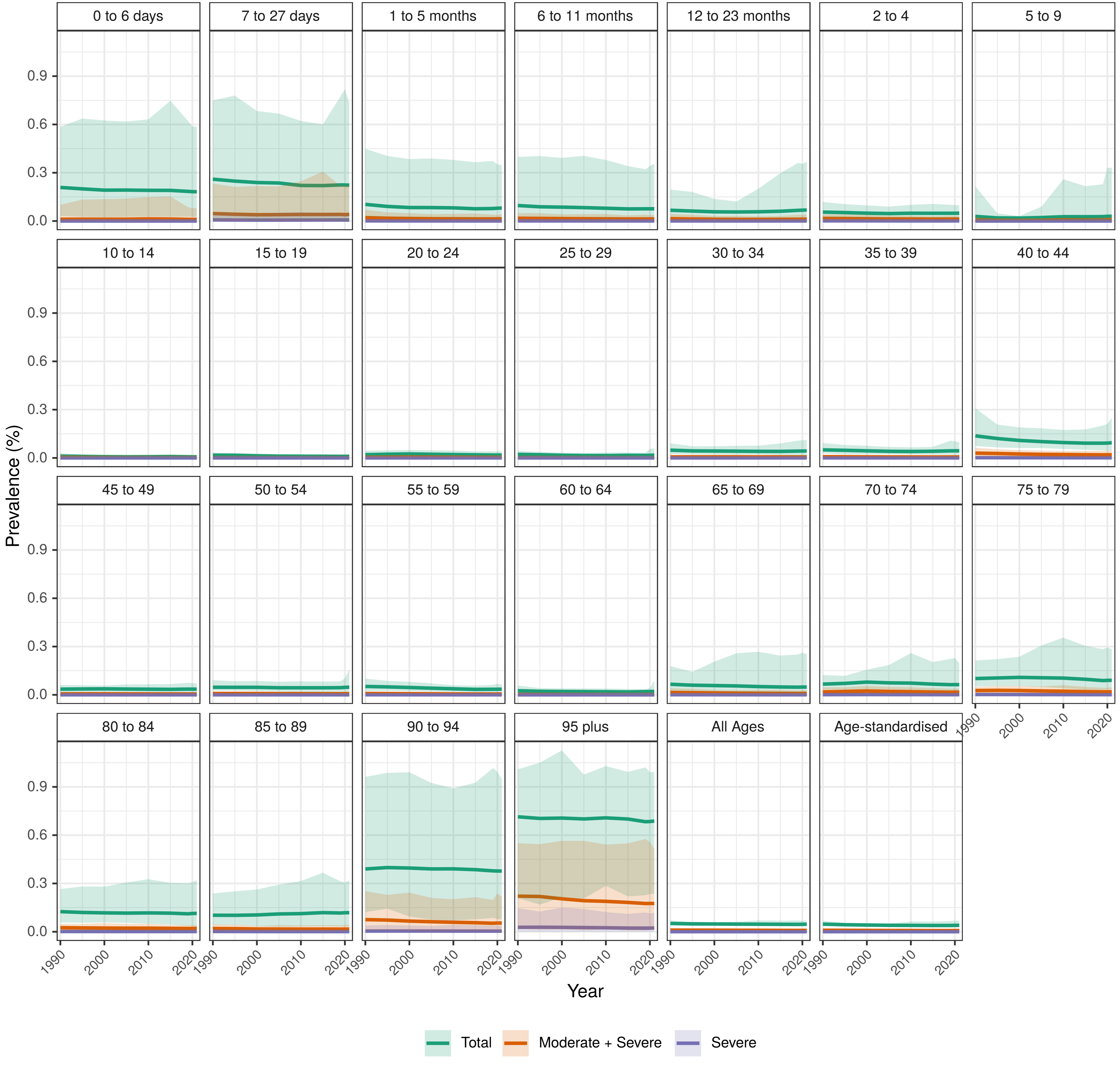

Cause-Specific Anaemia Prevalence - 2021

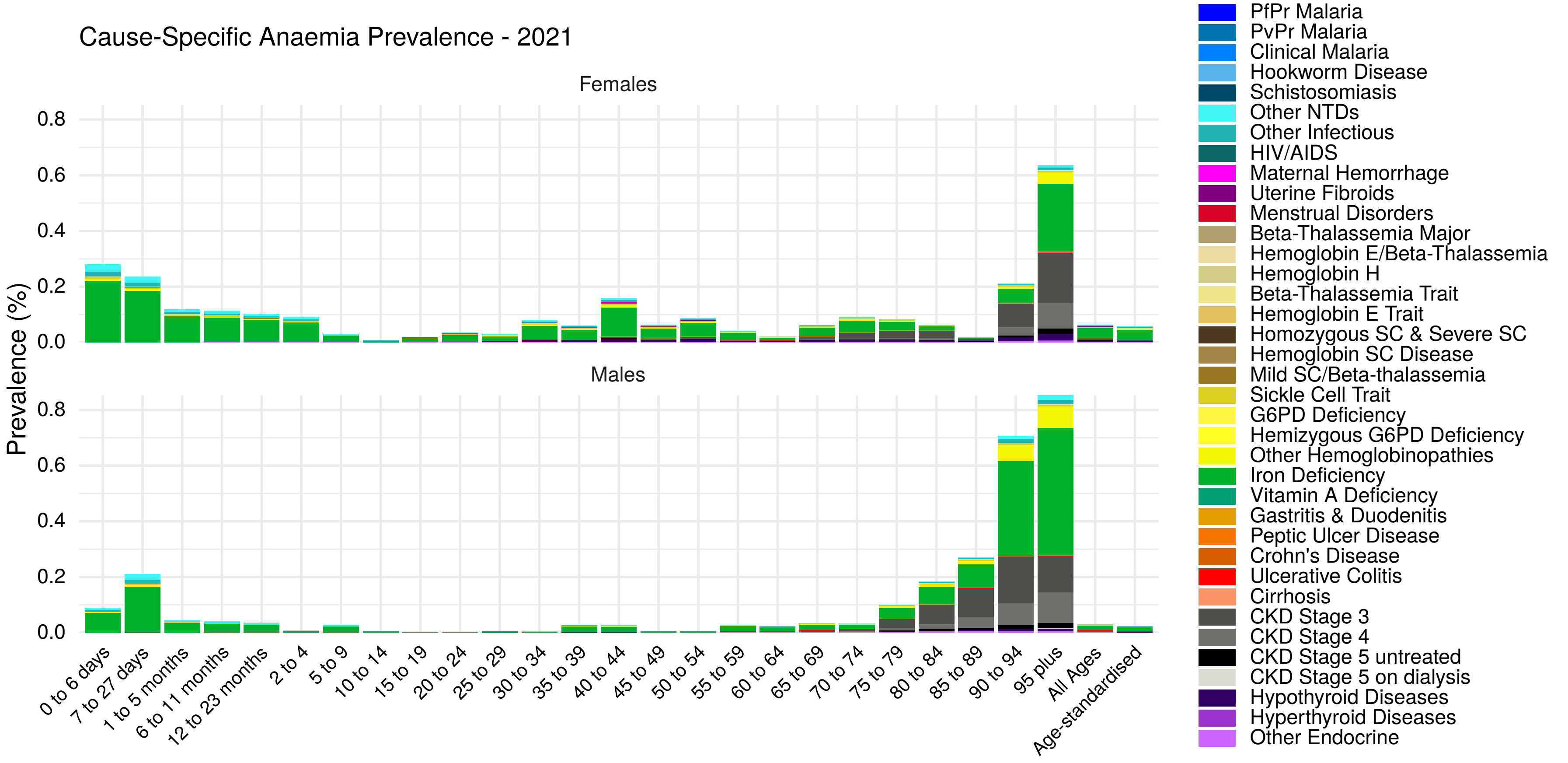

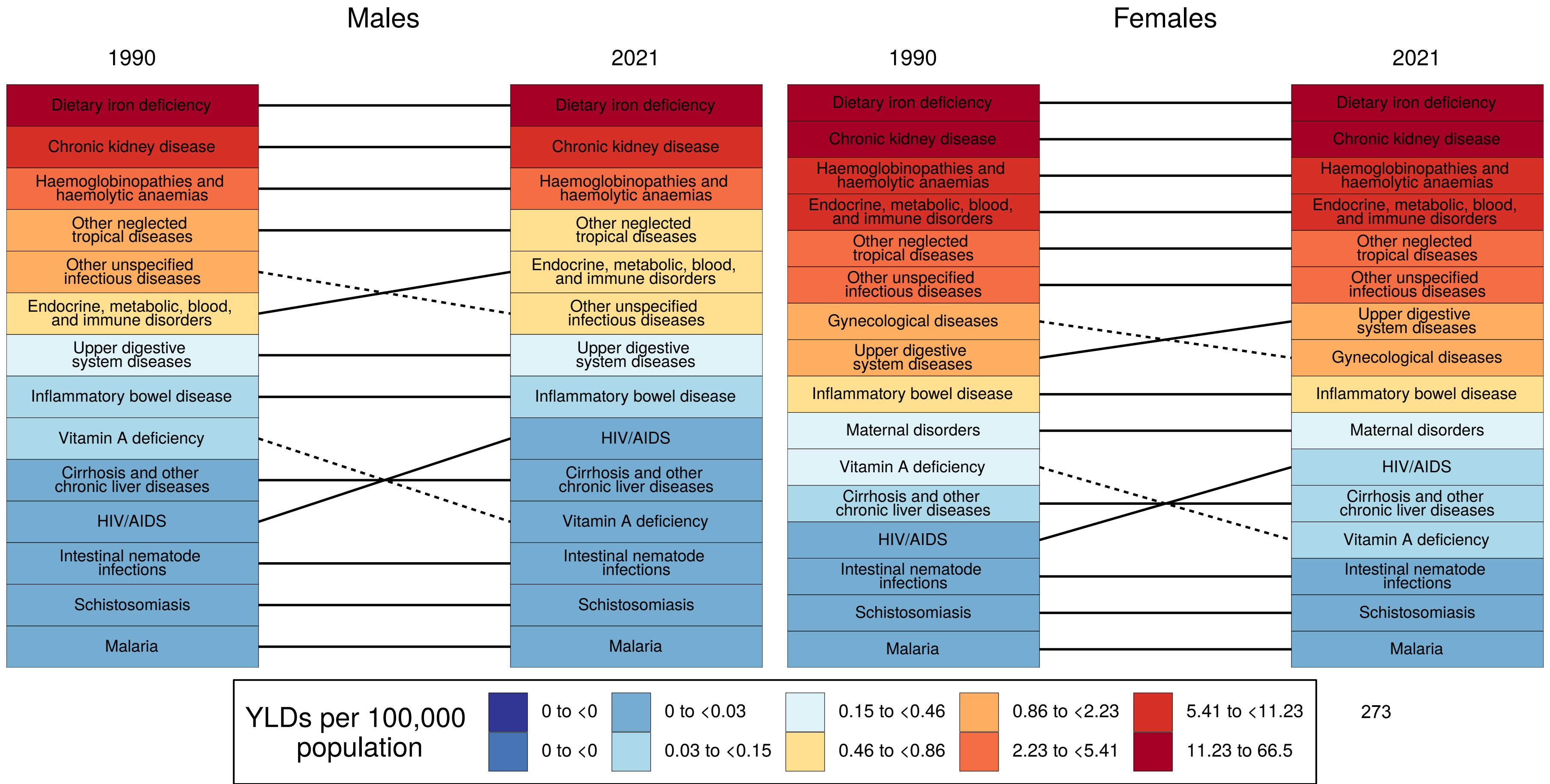

Luton

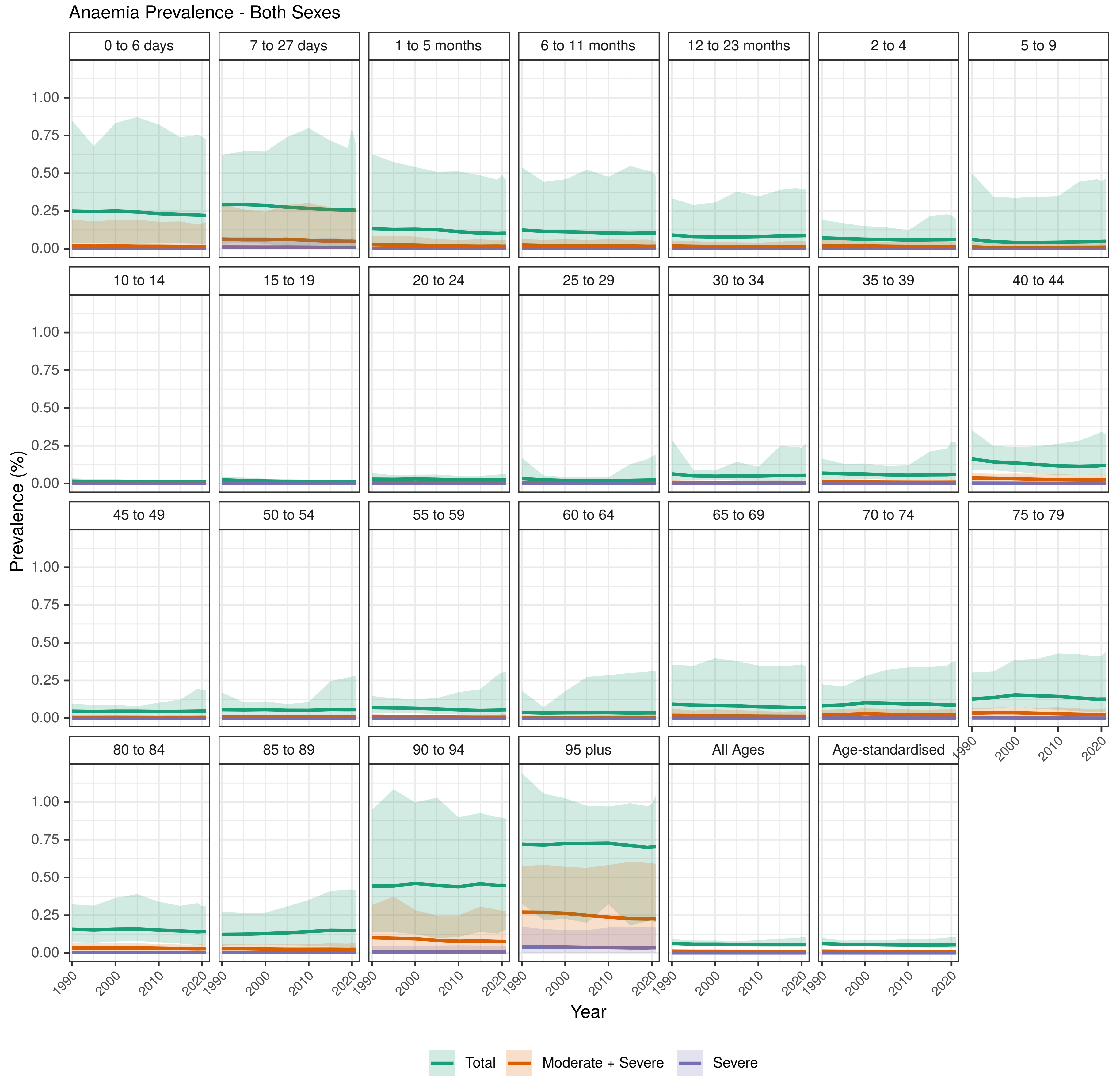

Anaemia Prevalence - Both Sexes
0 to 6 days
7 to 27 days
1 to 5 months
6 to 11 months
12 to 23 months
2 to 4
5 to 9
10 to 14
15 to 19
20 to 24
25 to 29
30 to 34
35 to 39
40 to 44
45 to 49
50 to 54
55 to 59
60 to 64
65 to 69
70 to 74
75 to 79
80 to 84
85 to 89
90 to 94
95 plus
All Ages
Age-standardised
Prevalence (%)
Year
Total
Moderate + Severe
Severe

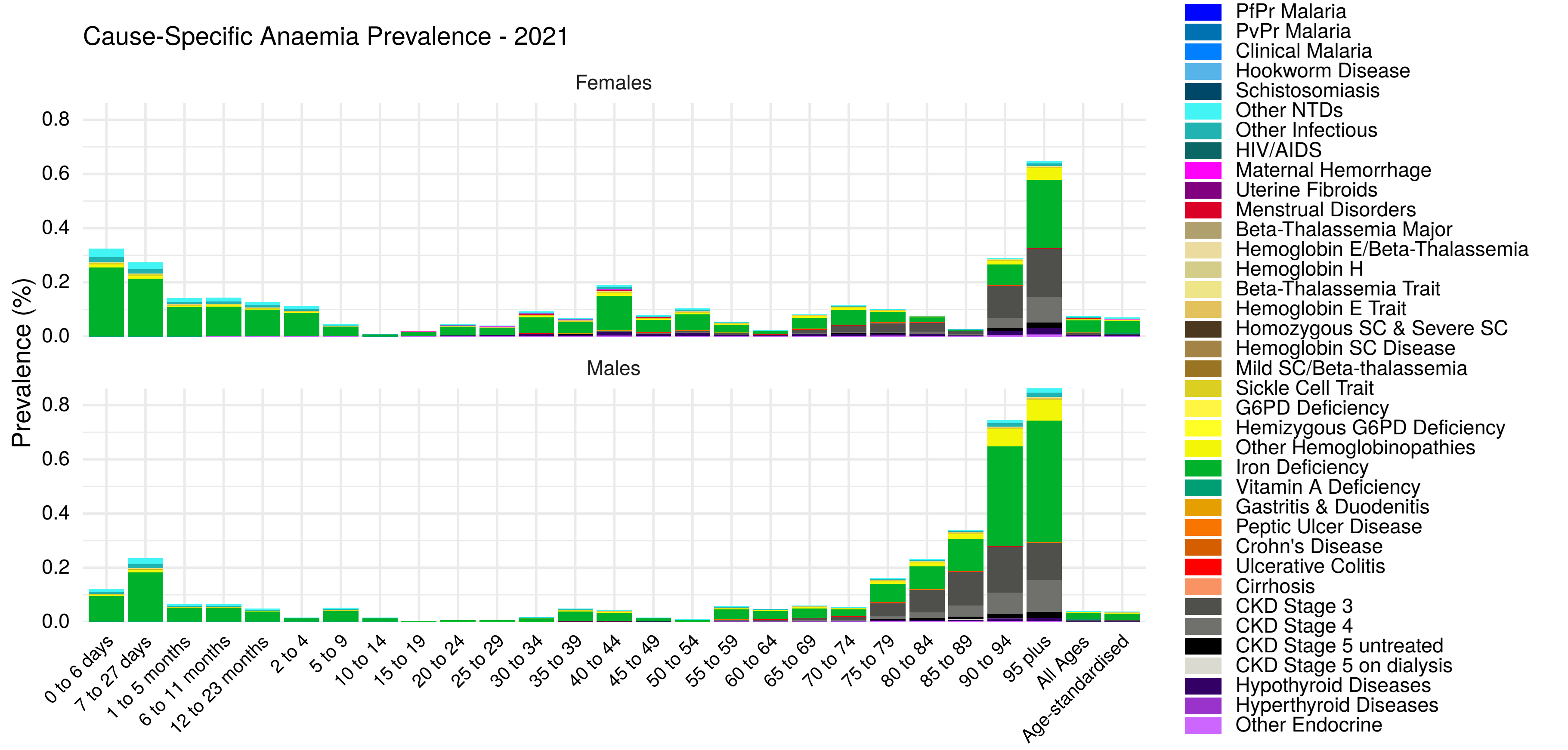

Cause-Specific Anaemia Prevalence - 2021
Females
Males
Prevalence (%)
PfPr Malaria
PvPr Malaria
Clinical Malaria
Hookworm Disease
Schistosomiasis
Other NTDs
Other Infectious
HIV/AIDS
Maternal Hemorrhage
Uterine Fibroids
Menstrual Disorders
Beta-Thalassemia Major
Hemoglobin E/Beta-Thalassemia
Hemoglobin H
Beta-Thalassemia Trait
Hemoglobin E Trait
Homozygous SC & Severe SC
Hemoglobin SC Disease
Mild SC/Beta-thalassemia
Sickle Cell Trait
G6PD Deficiency
Hemizygous G6PD Deficiency
Other Hemoglobinopathies
Iron Deficiency
Vitamin A Deficiency
Gastritis & Duodenitis
Peptic Ulcer Disease
Crohn's Disease
Ulcerative Colitis
Cirrhosis
CKD Stage 3
CKD Stage 4
CKD Stage 5 untreated
CKD Stage 5 on dialysis
Hypothyroid Diseases
Hyperthyroid Diseases
Other Endocrine

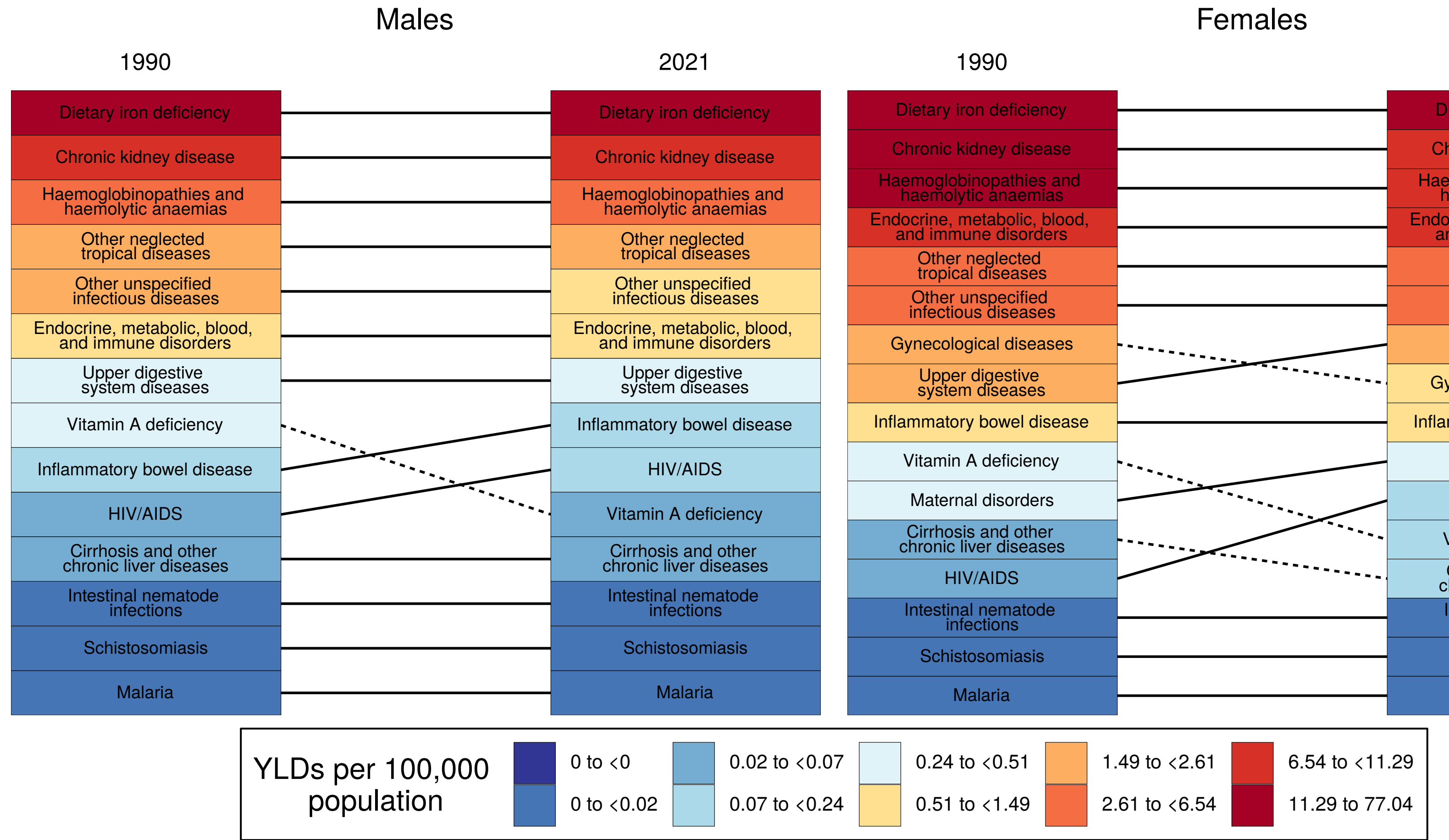

Males
Females
1990
2021
Dietary iron deficiency
Chronic kidney disease
Haemoglobinopathies and haemolytic anaemias
Other neglected tropical diseases
Other unspecified infectious diseases
Endocrine, metabolic, blood, and immune disorders
Upper digestive system diseases
Vitamin A deficiency
Inflammatory bowel disease
HIV/AIDS
Cirrhosis and other chronic liver diseases
Intestinal nematode infections
Schistosomiasis
Malaria
Gynecological diseases
Maternal disorders
YLDs per 100,000 population
0 to <0
0 to <0.02
0.02 to <0.07
0.07 to <0.24
0.24 to <0.51
0.51 to <1.49
1.49 to <2.61
2.61 to <6.54
6.54 to <11.29
11.29 to 77.04

Norfolk

Anaemia Prevalence - Both Sexes

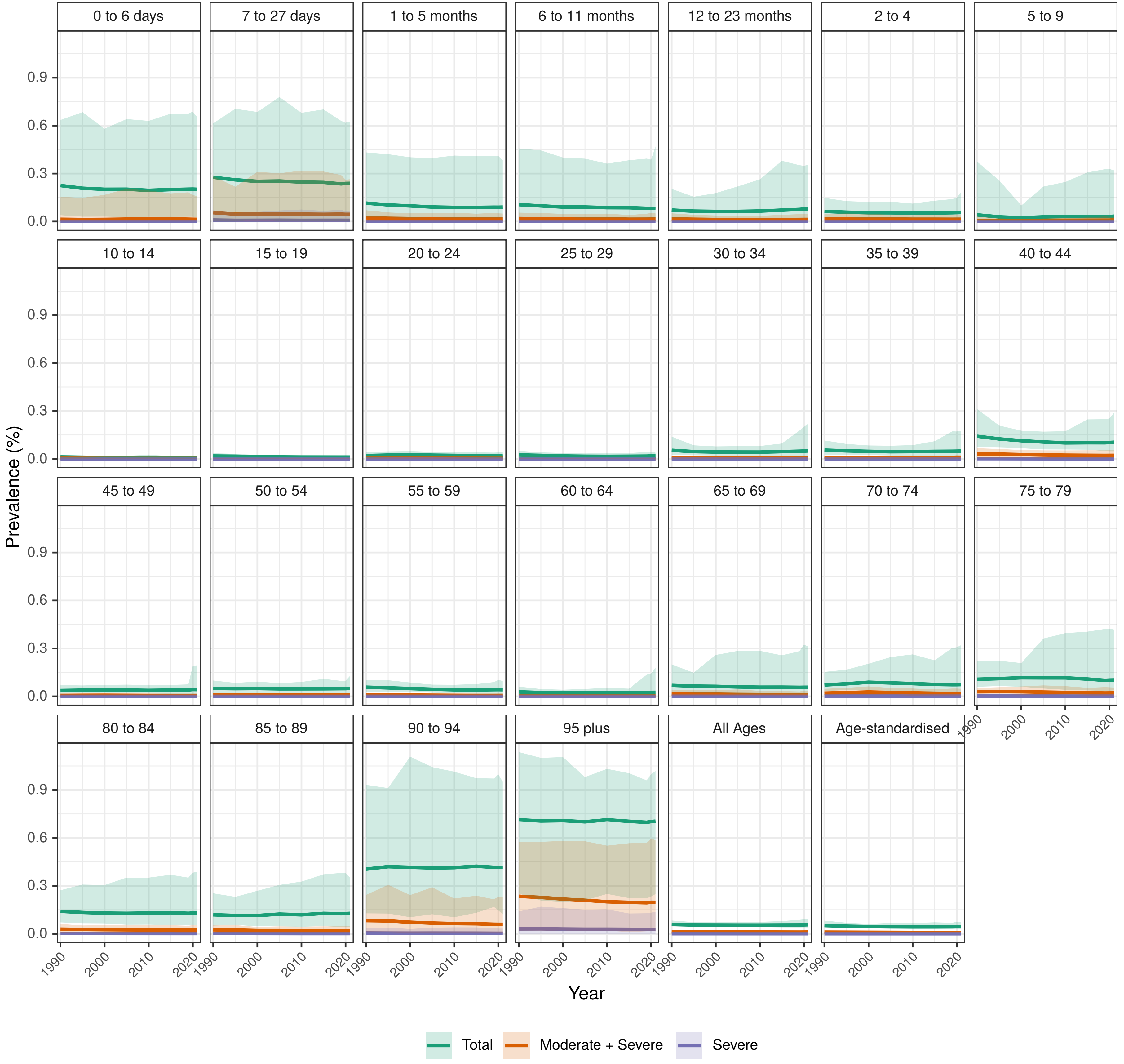

Cause-Specific Anaemia Prevalence - 2021

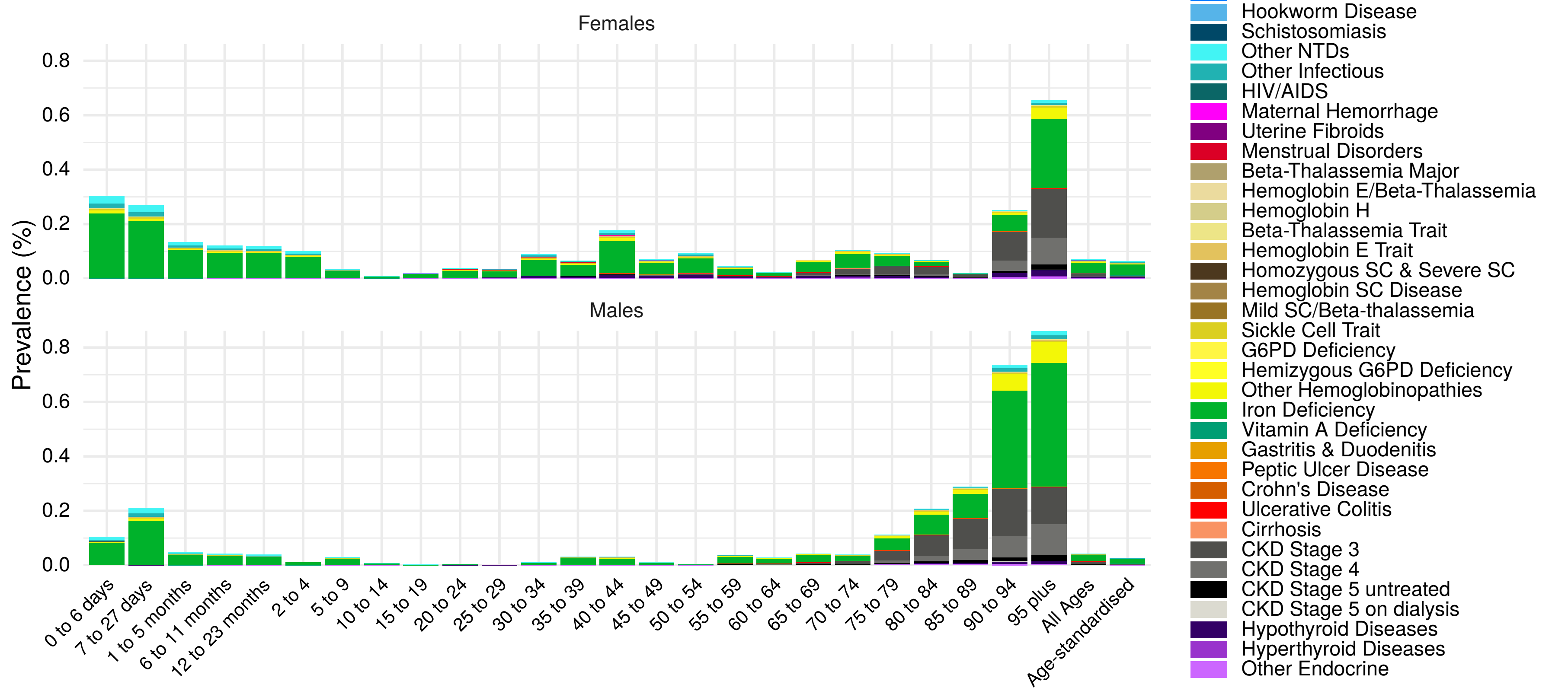

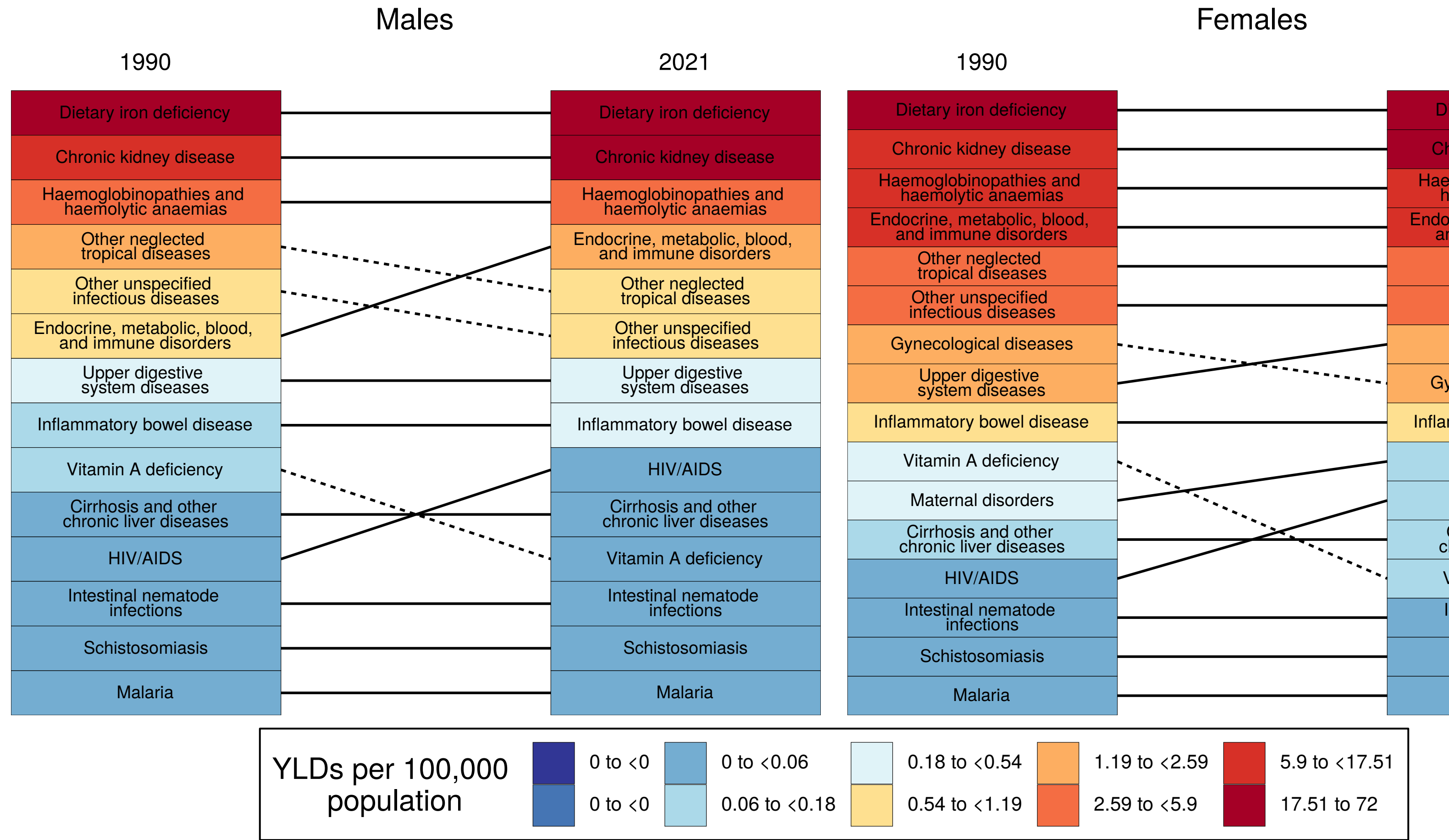

Peterborough

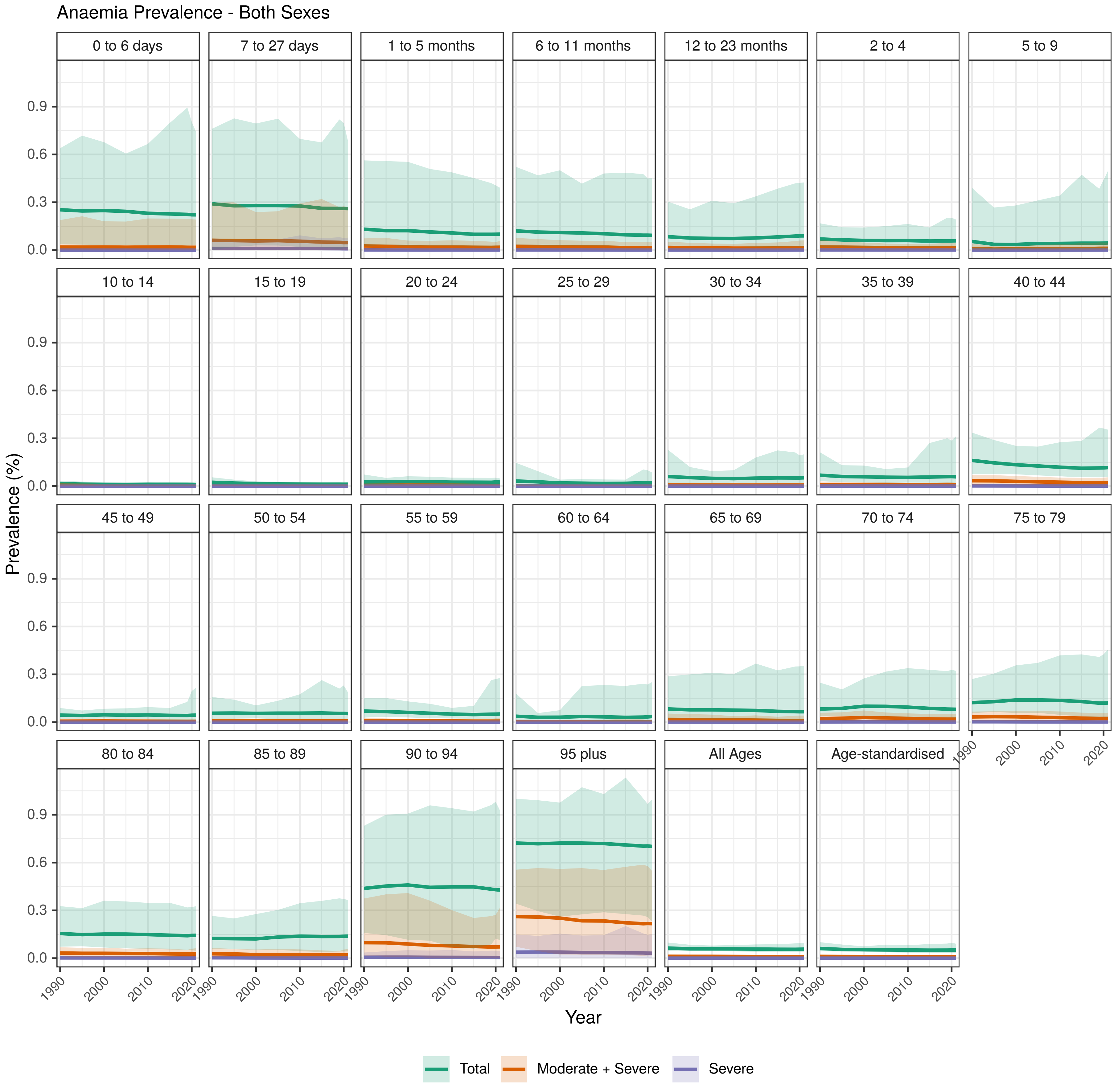

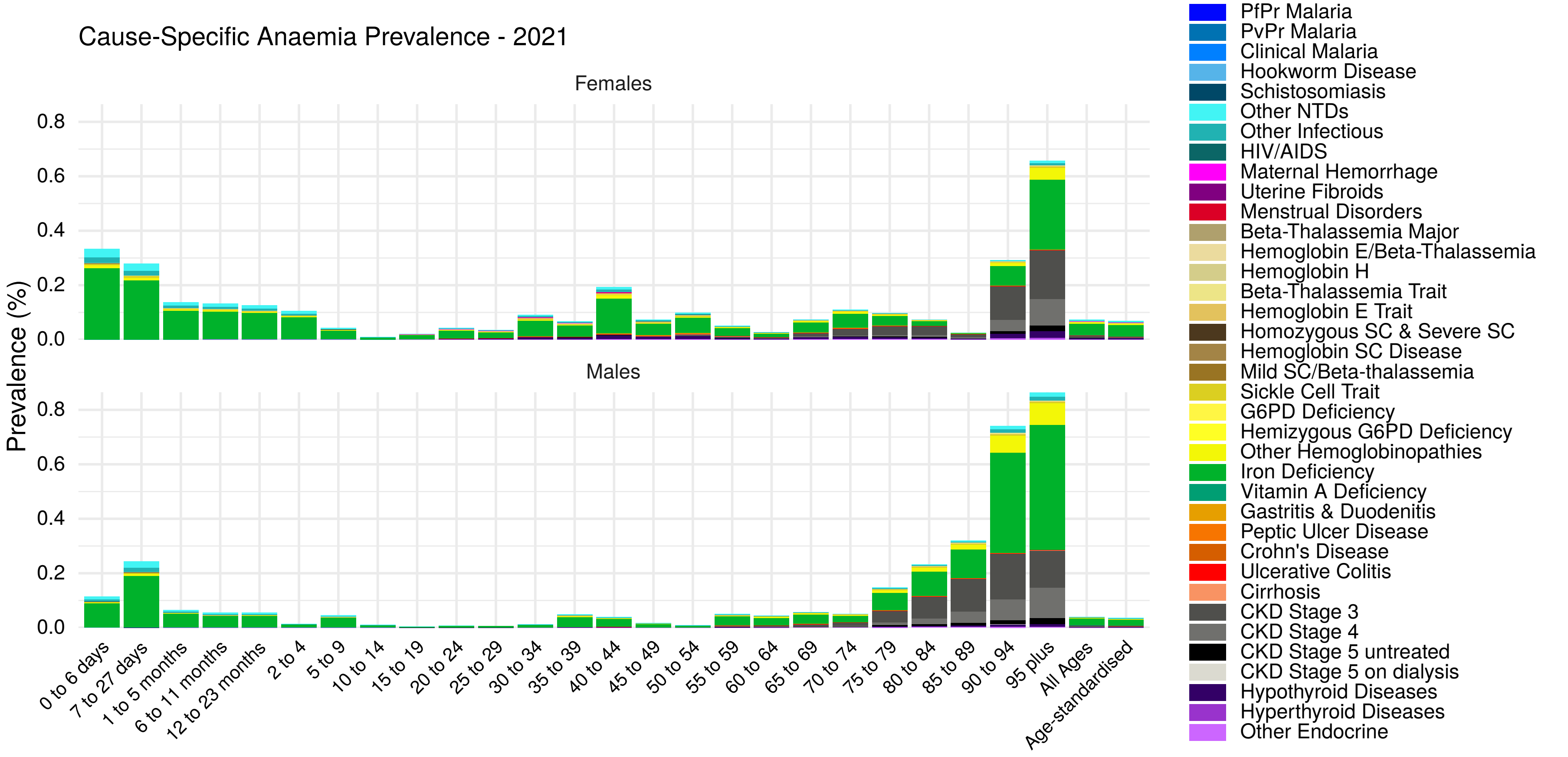

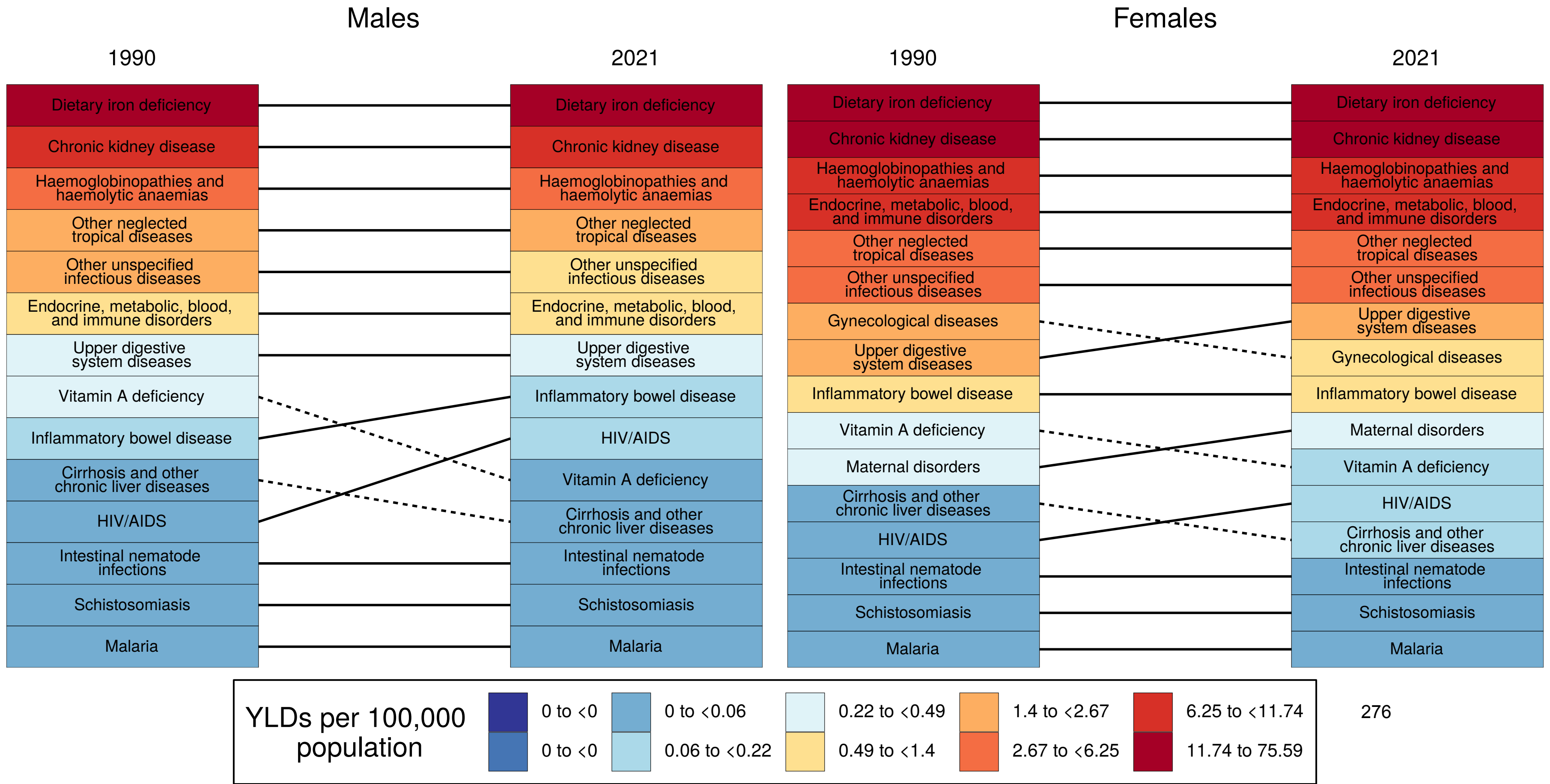

Southend-on-Sea

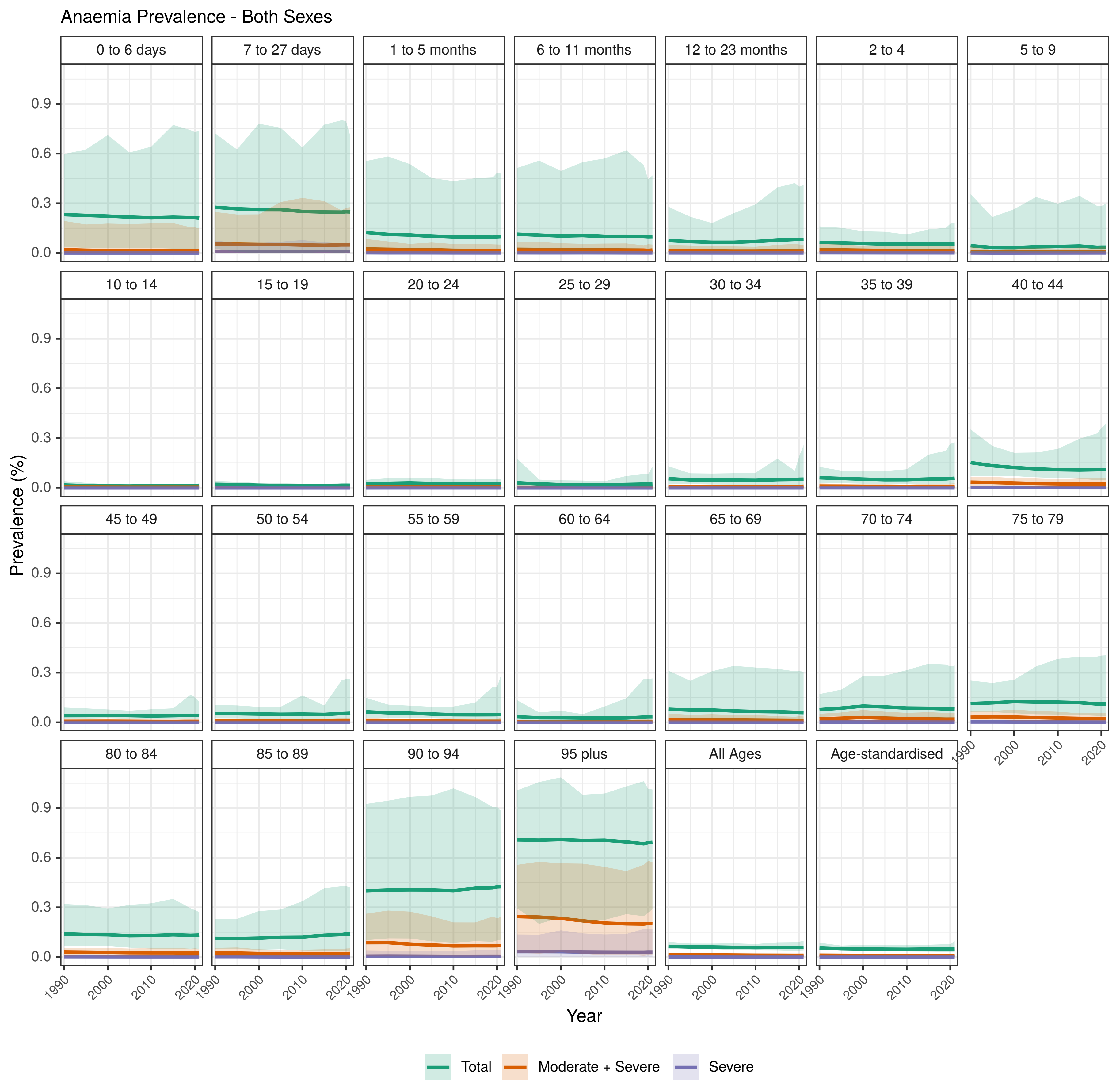

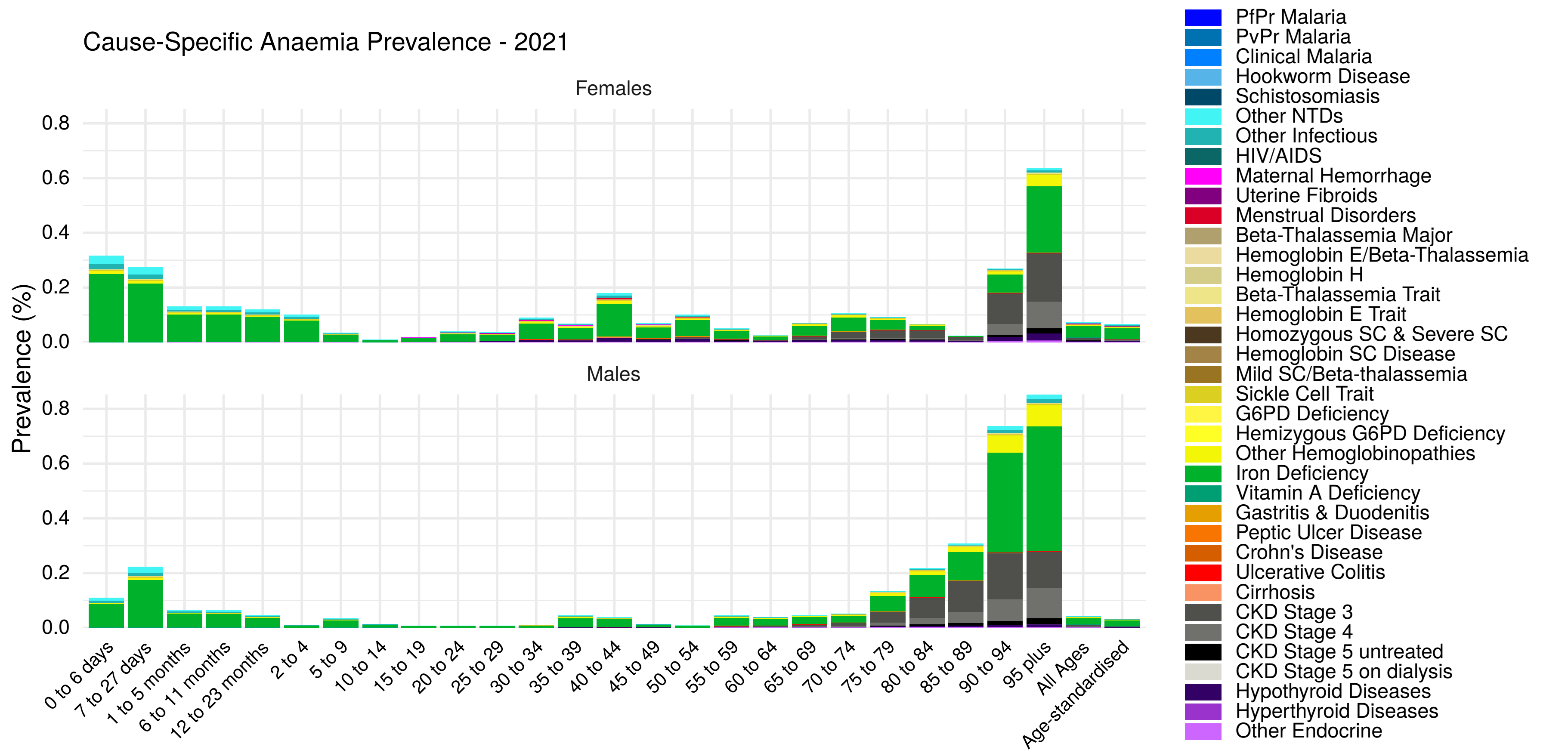

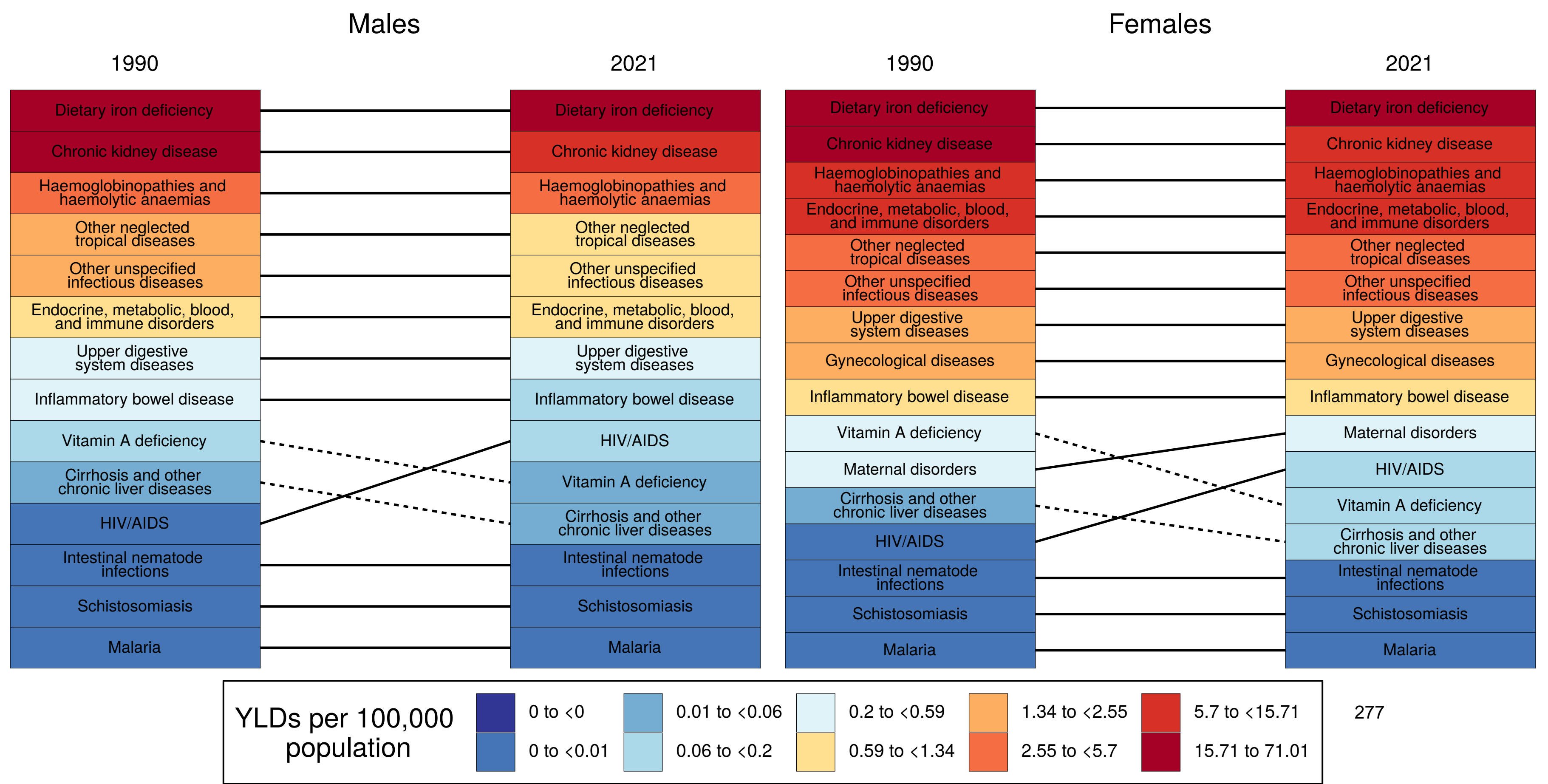

Suffolk

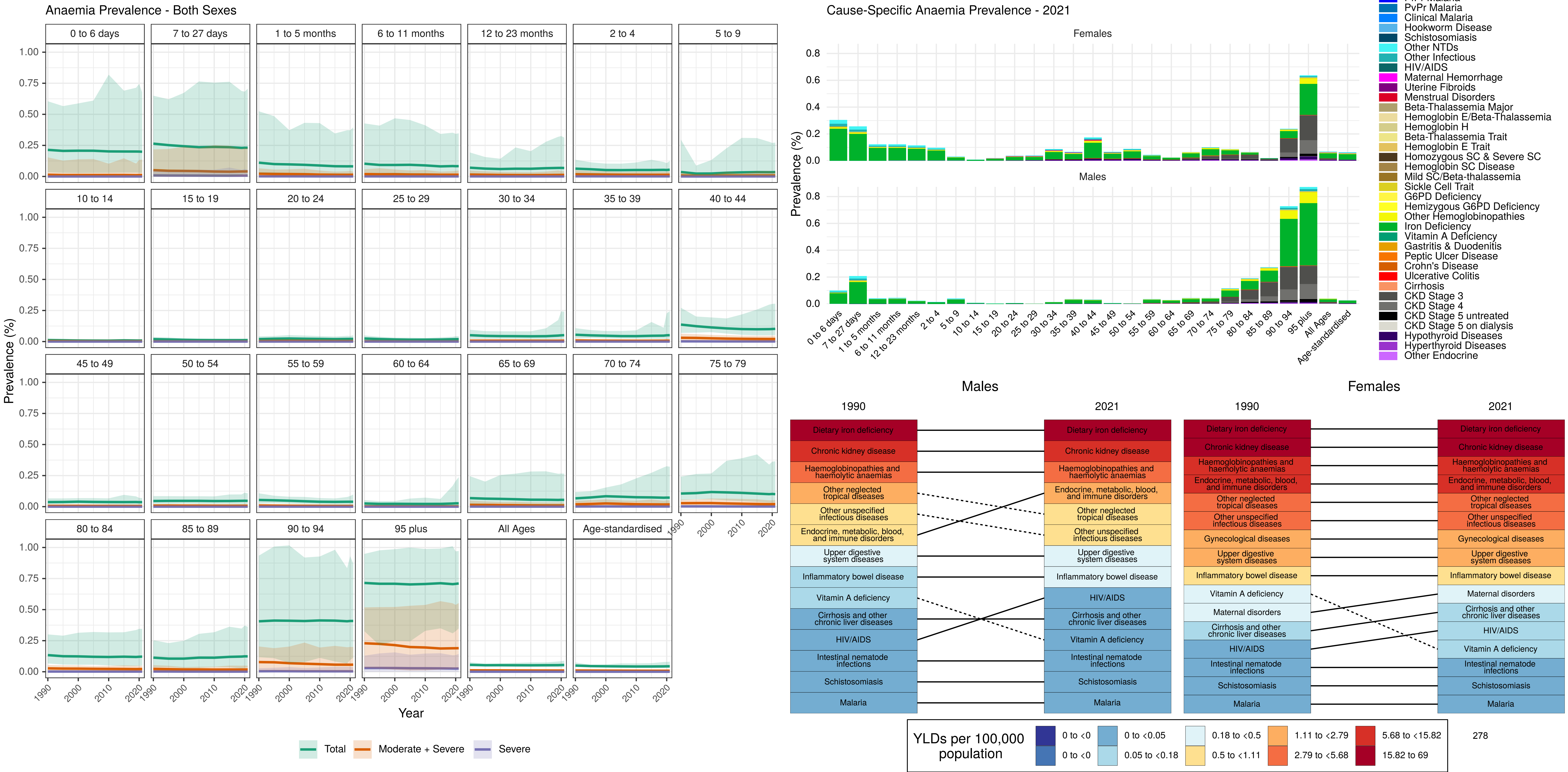

Anaemia Prevalence - Both Sexes
0 to 6 days
7 to 27 days
1 to 5 months
6 to 11 months
12 to 23 months
2 to 4
5 to 9
10 to 14
15 to 19
20 to 24
25 to 29
30 to 34
35 to 39
40 to 44
45 to 49
50 to 54
55 to 59
60 to 64
65 to 69
70 to 74
75 to 79
80 to 84
85 to 89
90 to 94
95 plus
All Ages
Age-standardised
Prevalence (%)
Year
Total
Moderate + Severe
Severe
Cause-Specific Anaemia Prevalence - 2021
Females
Males
PfPr Malaria
PvPr Malaria
Clinical Malaria
Hookworm Disease
Schistosomiasis
Other NTDs
Other Infectious
HIV/AIDS
Maternal Hemorrhage
Uterine Fibroids
Menstrual Disorders
Beta-Thalassemia Major
Hemoglobin E/Beta-Thalassemia
Hemoglobin H
Beta-Thalassemia Trait
Hemoglobin E Trait
Homozygous SC & Severe SC
Hemoglobin SC Disease
Mild SC/Beta-thalassemia
Sickle Cell Trait
G6PD Deficiency
Hemizygous G6PD Deficiency
Other Hemoglobinopathies
Iron Deficiency
Vitamin A Deficiency
Gastritis & Duodenitis
Peptic Ulcer Disease
Crohn's Disease
Ulcerative Colitis
Cirrhosis
CKD Stage 3
CKD Stage 4
CKD Stage 5 untreated
CKD Stage 5 on dialysis
Hypothyroid Diseases
Hyperthyroid Diseases
Other Endocrine
Males
Females
1990
2021
Dietary iron deficiency
Chronic kidney disease
Haemoglobinopathies and haemolytic anaemias
Other neglected tropical diseases
Other unspecified infectious diseases
Endocrine, metabolic, blood, and immune disorders
Upper digestive system diseases
Inflammatory bowel disease
Vitamin A deficiency
Cirrhosis and other chronic liver diseases
HIV/AIDS
Intestinal nematode infections
Schistosomiasis
Malaria
Gynecological diseases
Maternal disorders
YLDs per 100,000 population
0 to <0
0 to <0
0 to <0.05
0.05 to <0.18
0.18 to <0.5
0.5 to <1.11
1.11 to <2.79
2.79 to <5.68
5.68 to <15.82
15.82 to 69

Thurrock

Anaemia Prevalence - Both Sexes

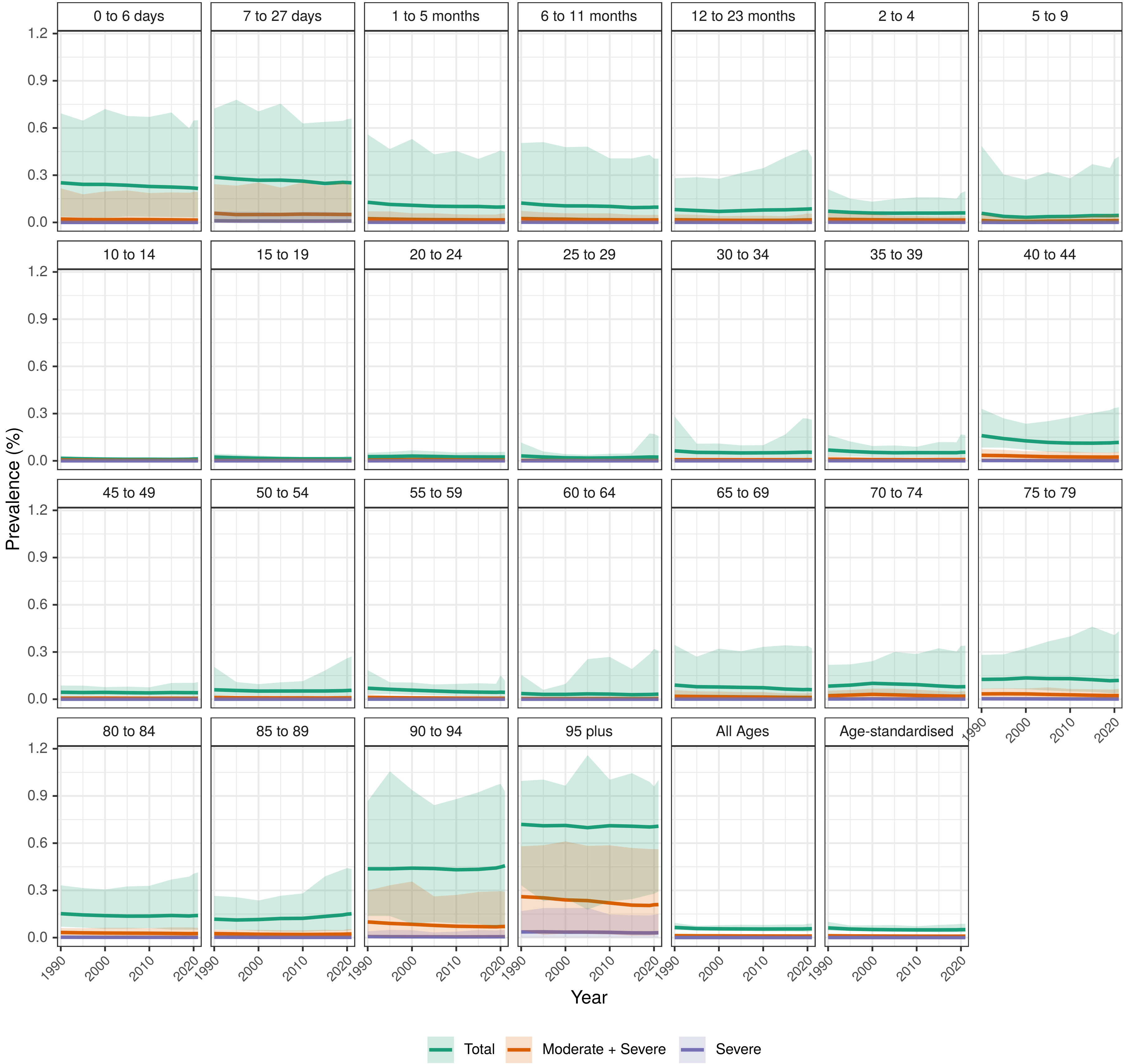

Cause-Specific Anaemia Prevalence - 2021

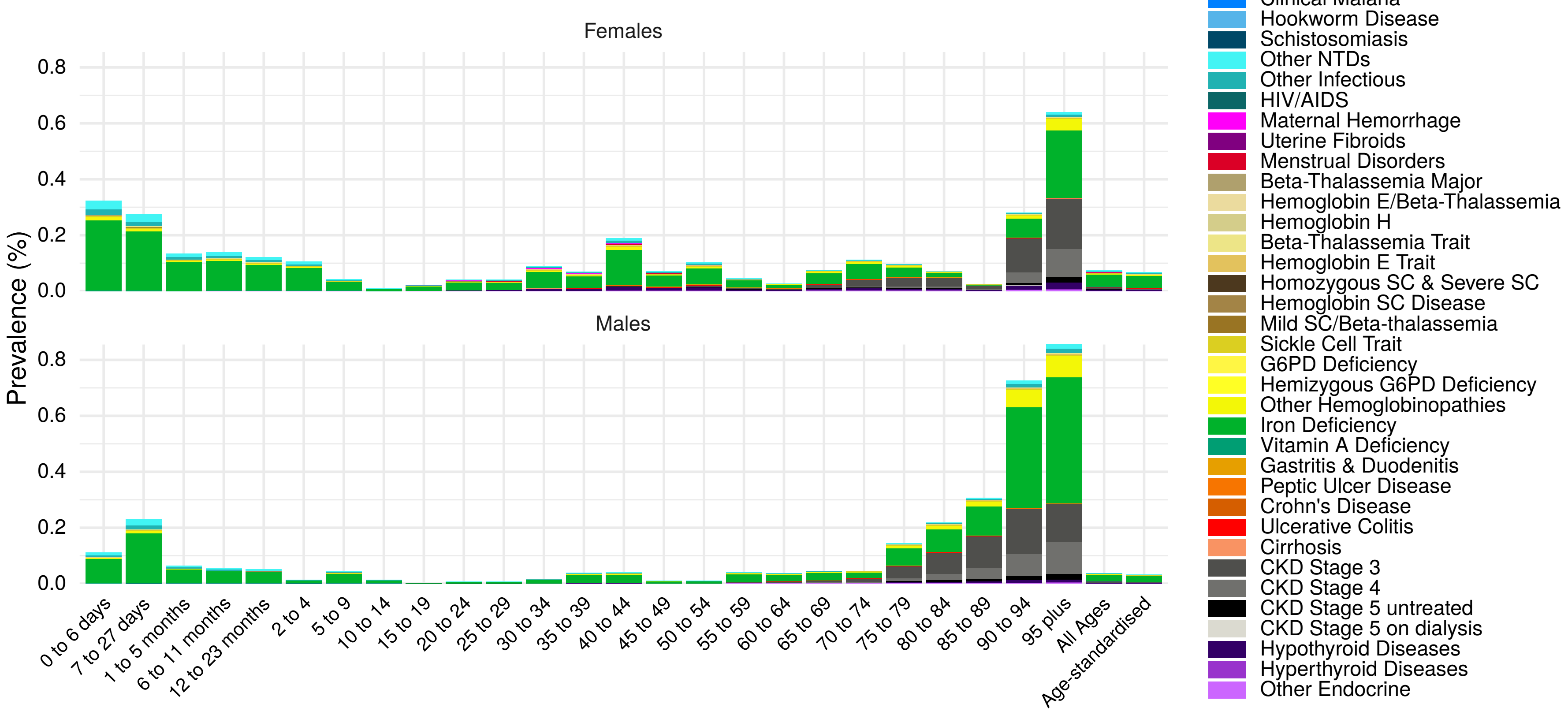

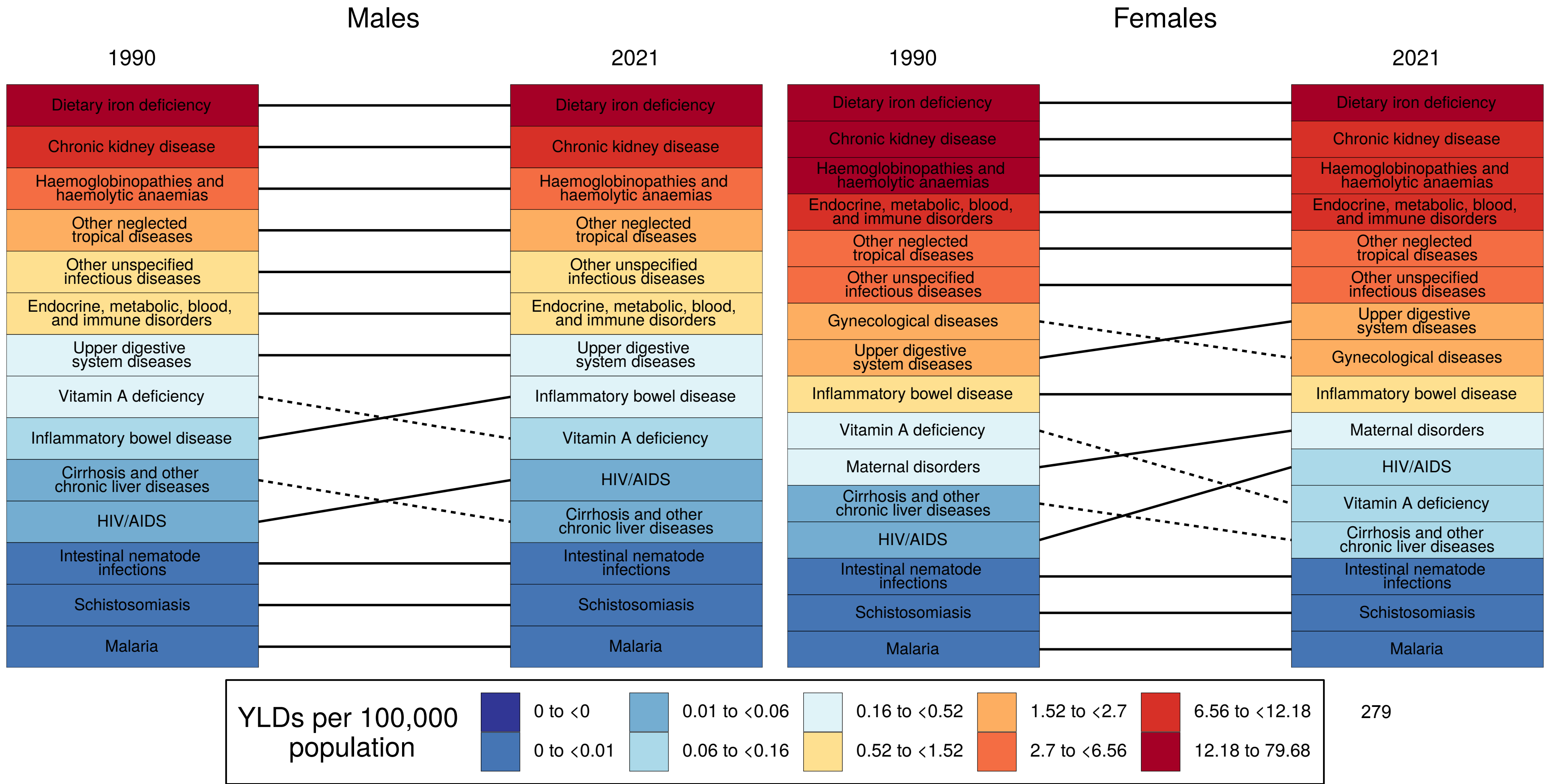

Greater London

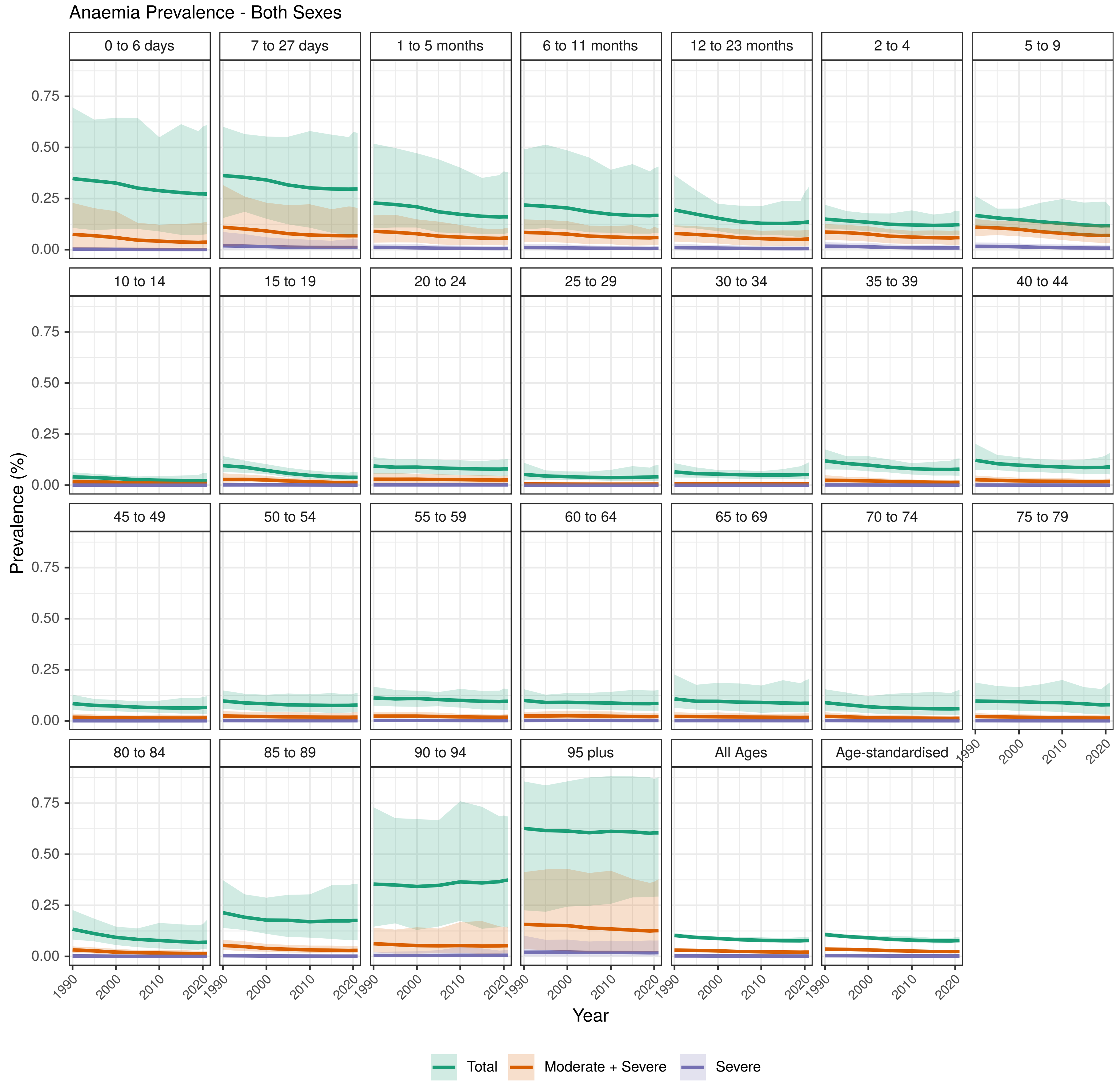

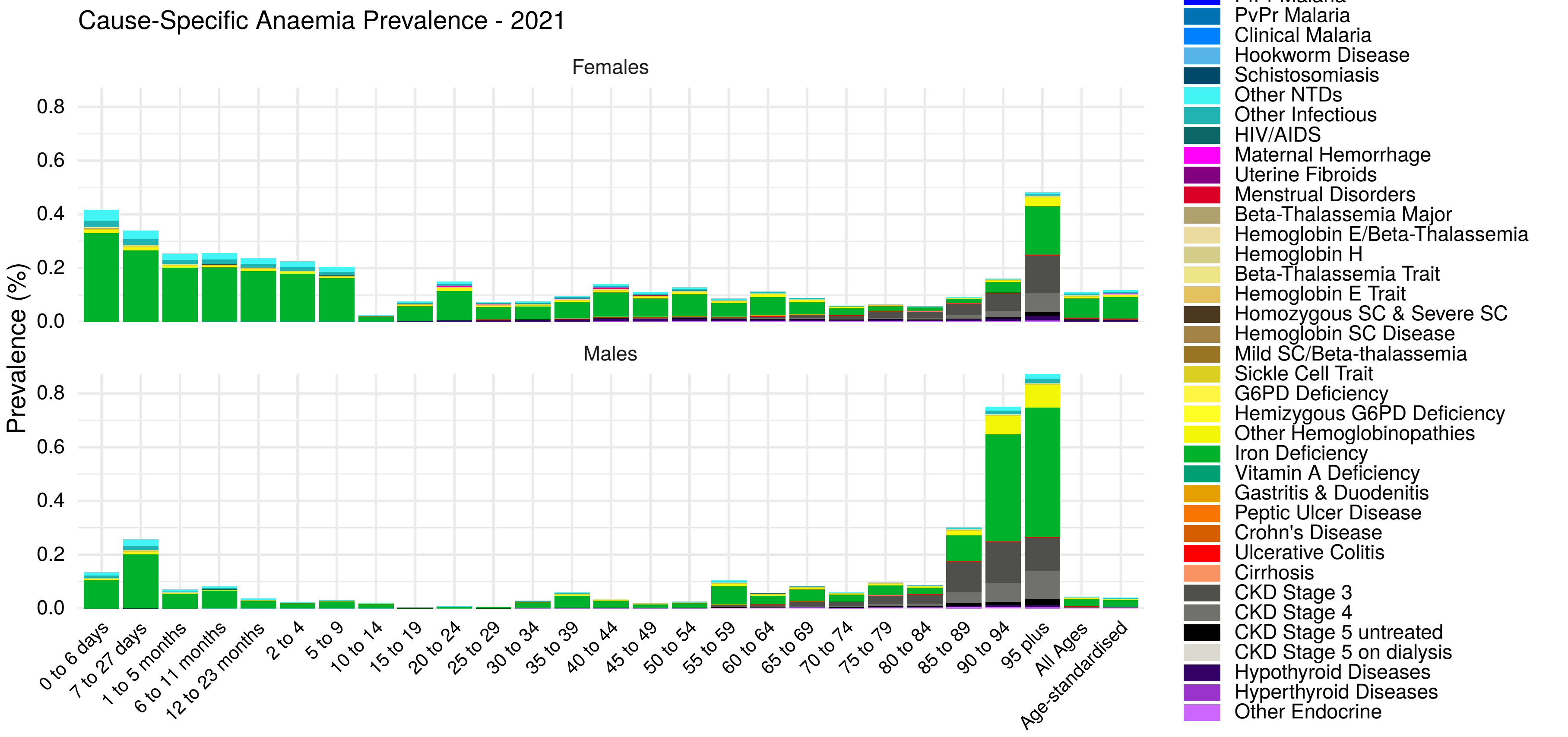

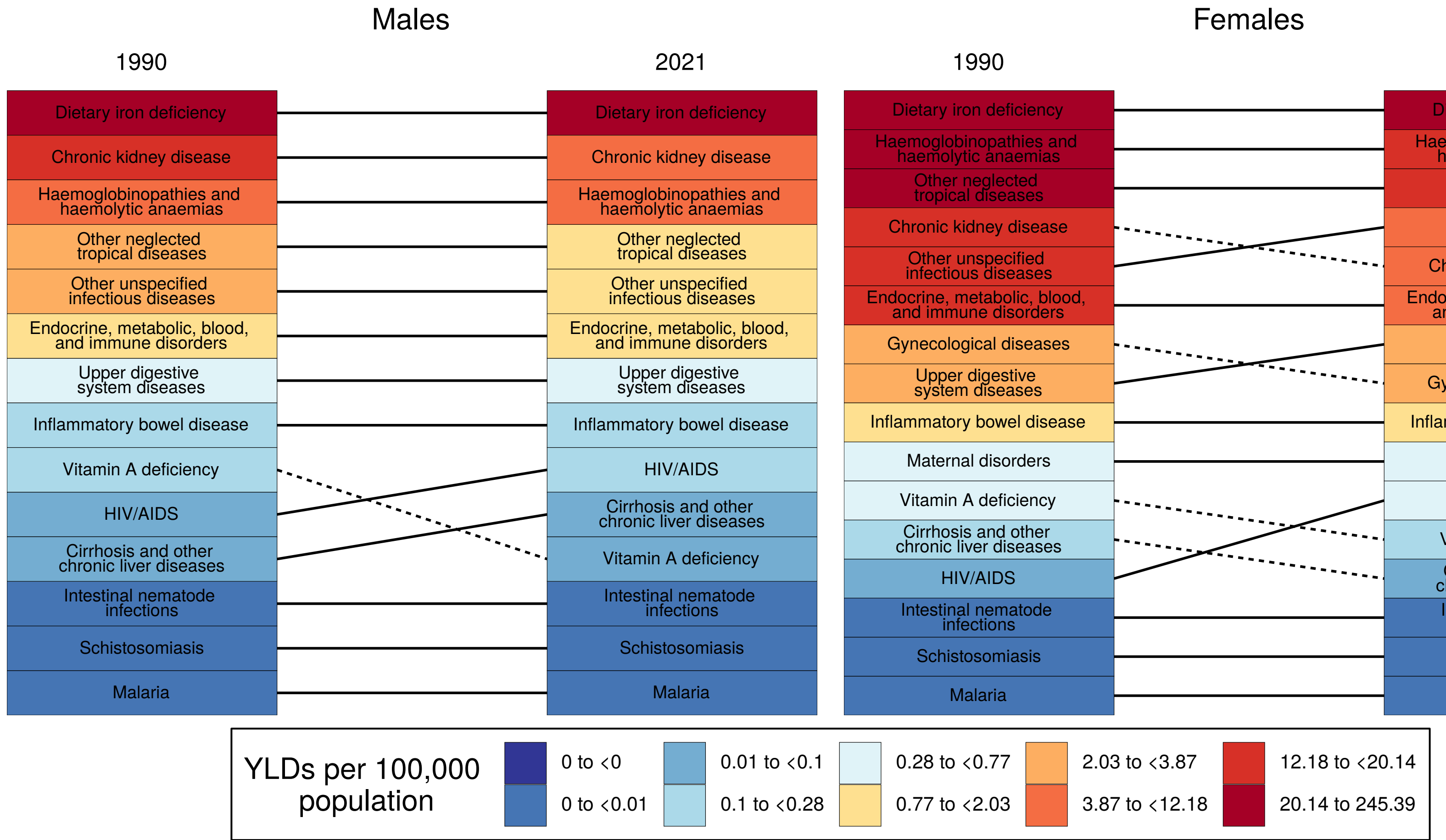

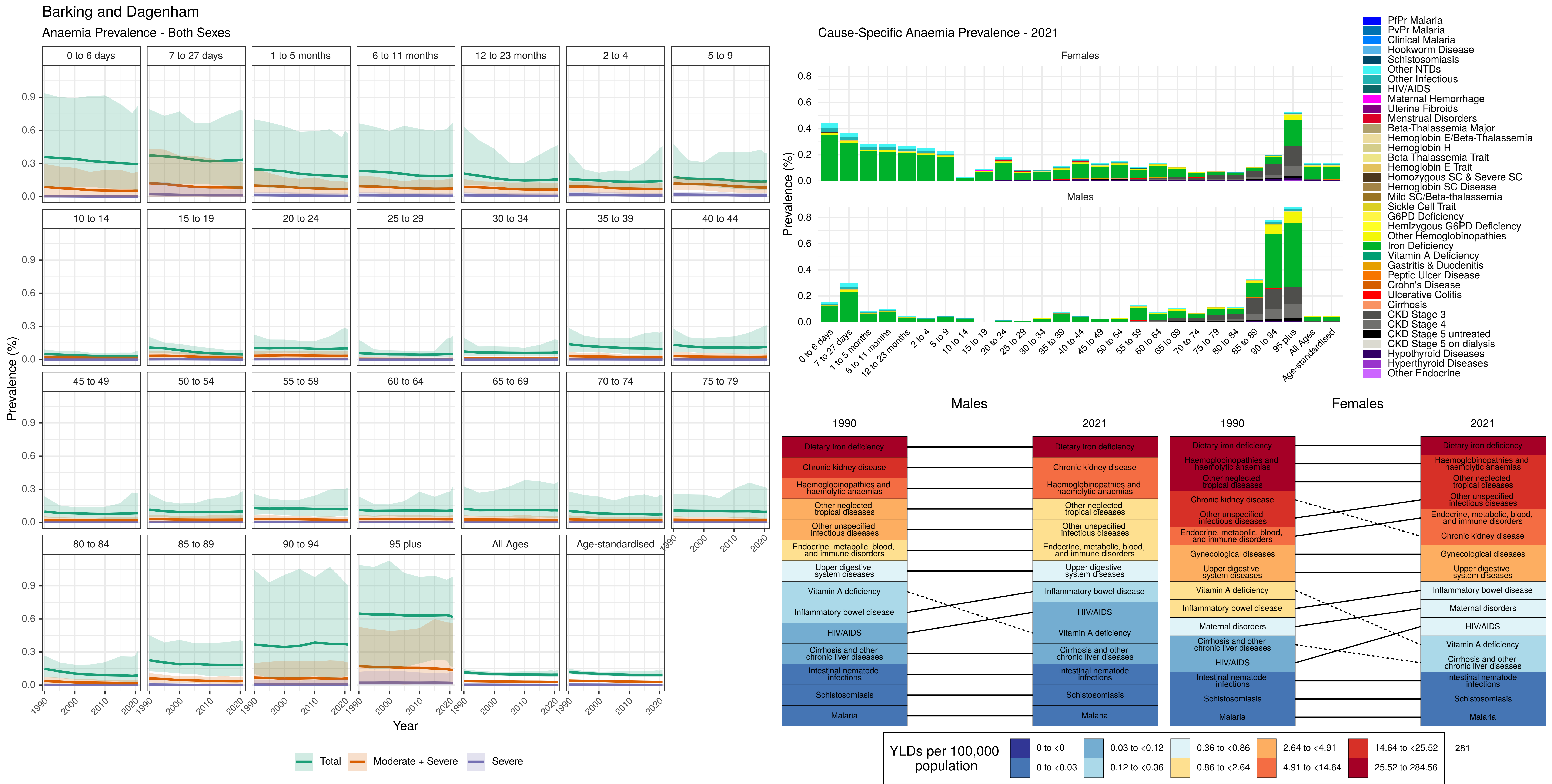
Barking and Dagenham
Anaemia Prevalence - Both Sexes
0 to 6 days
7 to 27 days
1 to 5 months
6 to 11 months
12 to 23 months
2 to 4
5 to 9
10 to 14
15 to 19
20 to 24
25 to 29
30 to 34
35 to 39
40 to 44
45 to 49
50 to 54
55 to 59
60 to 64
65 to 69
70 to 74
75 to 79
80 to 84
85 to 89
90 to 94
95 plus
All Ages
Age-standardised
Prevalence (%)
Year
Total
Moderate + Severe
Severe
Cause-Specific Anaemia Prevalence - 2021
Females
Males
Prevalence (%)
PfPr Malaria
PvPr Malaria
Clinical Malaria
Hookworm Disease
Schistosomiasis
Other NTDs
Other Infectious
HIV/AIDS
Maternal Hemorrhage
Uterine Fibroids
Menstrual Disorders
Beta-Thalassemia Major
Hemoglobin E/Beta-Thalassemia
Hemoglobin H
Beta-Thalassemia Trait
Hemoglobin E Trait
Homozygous SC & Severe SC
Hemoglobin SC Disease
Mild SC/Beta-thalassemia
Sickle Cell Trait
G6PD Deficiency
Hemizygous G6PD Deficiency
Other Hemoglobinopathies
Iron Deficiency
Vitamin A Deficiency
Gastritis & Duodenitis
Peptic Ulcer Disease
Crohn's Disease
Ulcerative Colitis
Cirrhosis
CKD Stage 3
CKD Stage 4
CKD Stage 5 untreated
CKD Stage 5 on dialysis
Hypothyroid Diseases
Hyperthyroid Diseases
Other Endocrine
Males
Females
1990
2021
Dietary iron deficiency
Chronic kidney disease
Haemoglobinopathies and haemolytic anaemias
Other neglected tropical diseases
Other unspecified infectious diseases
Endocrine, metabolic, blood, and immune disorders
Upper digestive system diseases
Vitamin A deficiency
Inflammatory bowel disease
HIV/AIDS
Cirrhosis and other chronic liver diseases
Intestinal nematode infections
Schistosomiasis
Malaria
Gynecological diseases
Maternal disorders
YLDs per 100,000 population
0 to <0
0 to <0.03
0.03 to <0.12
0.12 to <0.36
0.36 to <0.86
0.86 to <2.64
2.64 to <4.91
4.91 to <14.64
14.64 to <25.52
25.52 to 284.56

Barnet

Anaemia Prevalence - Both Sexes

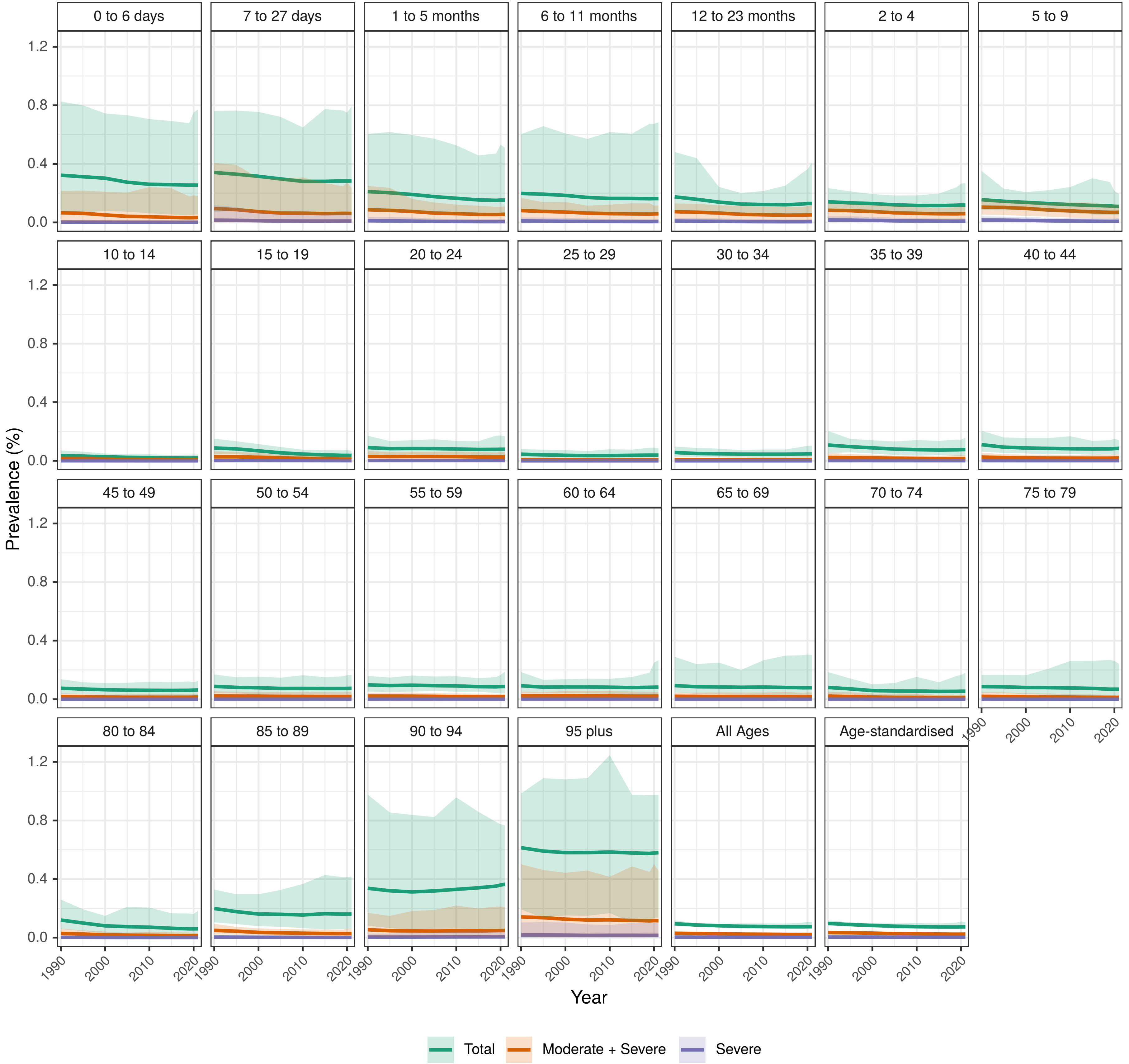

Cause-Specific Anaemia Prevalence - 2021

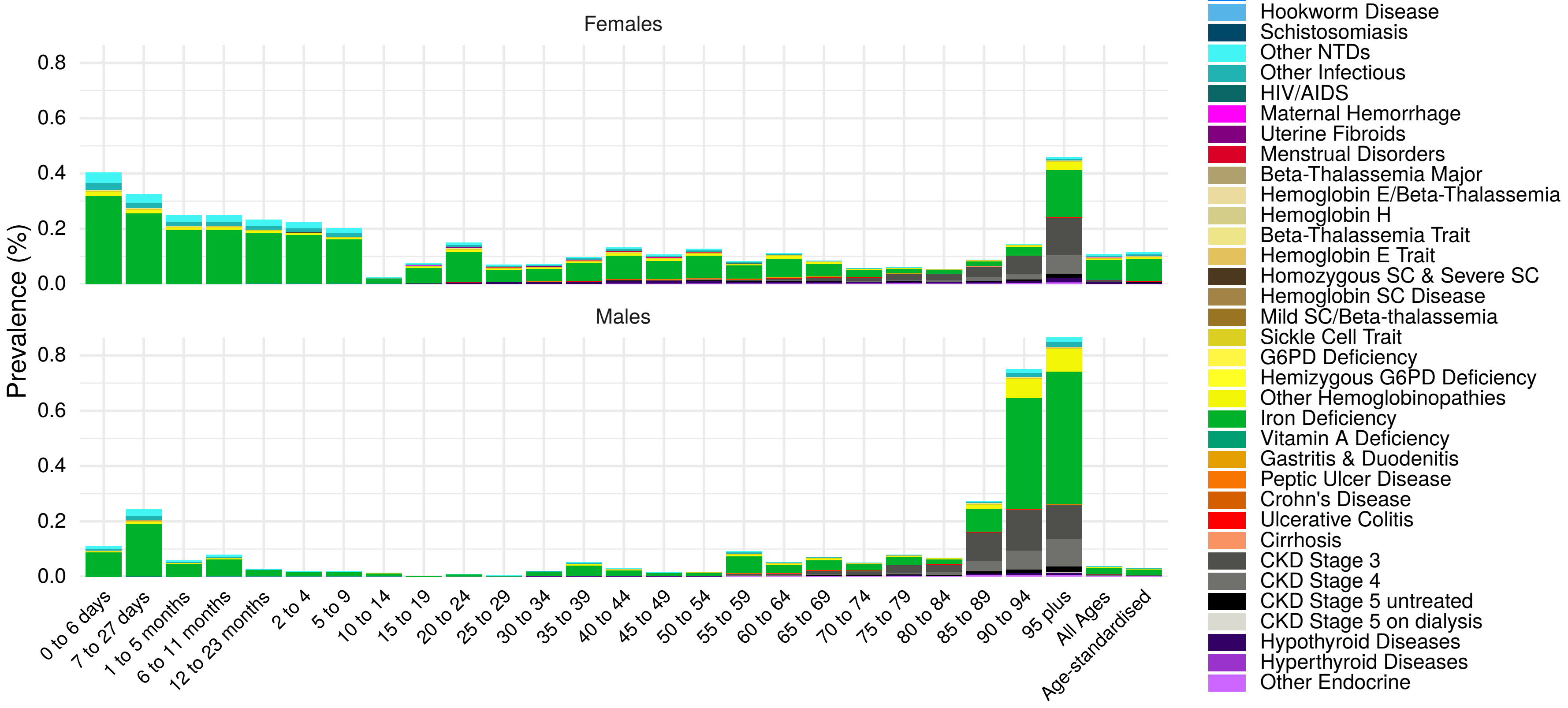

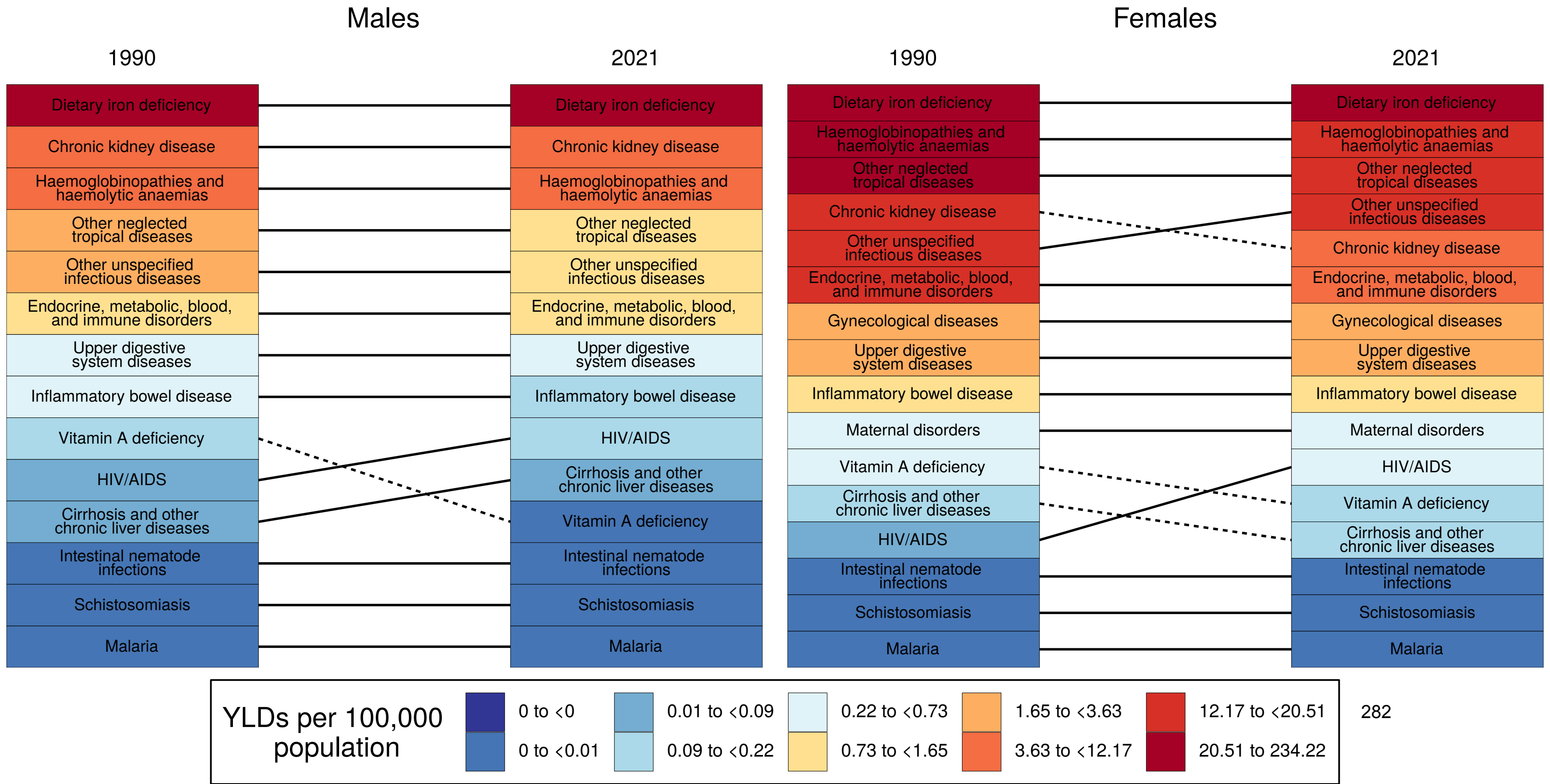

Bexley

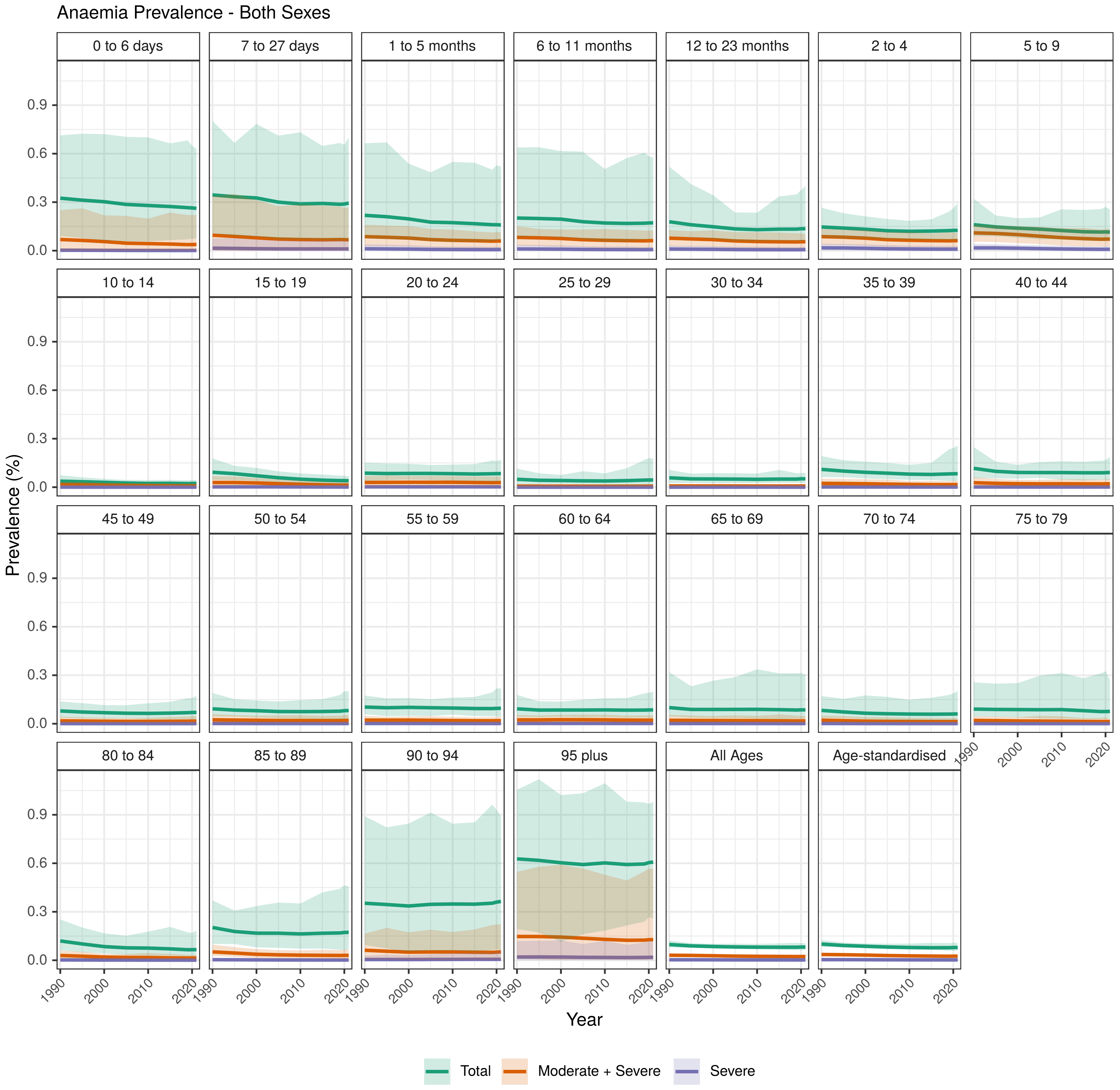

Anaemia Prevalence - Both Sexes
0 to 6 days
7 to 27 days
1 to 5 months
6 to 11 months
12 to 23 months
2 to 4
5 to 9
10 to 14
15 to 19
20 to 24
25 to 29
30 to 34
35 to 39
40 to 44
45 to 49
50 to 54
55 to 59
60 to 64
65 to 69
70 to 74
75 to 79
80 to 84
85 to 89
90 to 94
95 plus
All Ages
Age-standardised
Prevalence (%)
0.0
0.3
0.6
0.9
1990
2000
2010
2020
Year
Total
Moderate + Severe
Severe

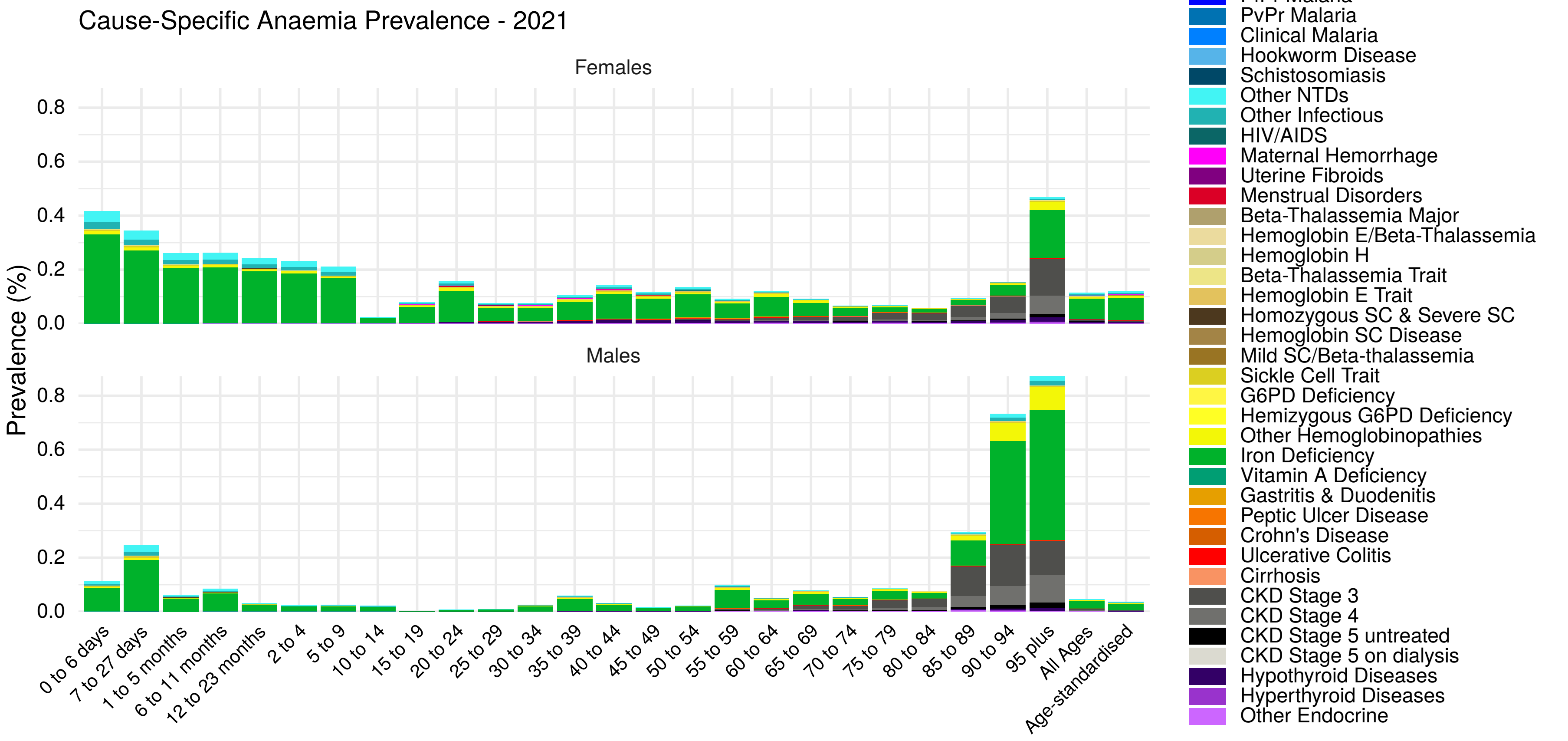

Cause-Specific Anaemia Prevalence - 2021
Females
Males
Prevalence (%)
0.0
0.2
0.4
0.6
0.8
0 to 6 days
7 to 27 days
1 to 5 months
6 to 11 months
12 to 23 months
2 to 4
5 to 9
10 to 14
15 to 19
20 to 24
25 to 29
30 to 34
35 to 39
40 to 44
45 to 49
50 to 54
55 to 59
60 to 64
65 to 69
70 to 74
75 to 79
80 to 84
85 to 89
90 to 94
95 plus
All Ages
Age-standardised
PfPr Malaria
PvPr Malaria
Clinical Malaria
Hookworm Disease
Schistosomiasis
Other NTDs
Other Infectious
HIV/AIDS
Maternal Hemorrhage
Uterine Fibroids
Menstrual Disorders
Beta-Thalassemia Major
Hemoglobin E/Beta-Thalassemia
Hemoglobin H
Beta-Thalassemia Trait
Hemoglobin E Trait
Homozygous SC & Severe SC
Hemoglobin SC Disease
Mild SC/Beta-thalassemia
Sickle Cell Trait
G6PD Deficiency
Hemizygous G6PD Deficiency
Other Hemoglobinopathies
Iron Deficiency
Vitamin A Deficiency
Gastritis & Duodenitis
Peptic Ulcer Disease
Crohn's Disease
Ulcerative Colitis
Cirrhosis
CKD Stage 3
CKD Stage 4
CKD Stage 5 untreated
CKD Stage 5 on dialysis
Hypothyroid Diseases
Hyperthyroid Diseases
Other Endocrine

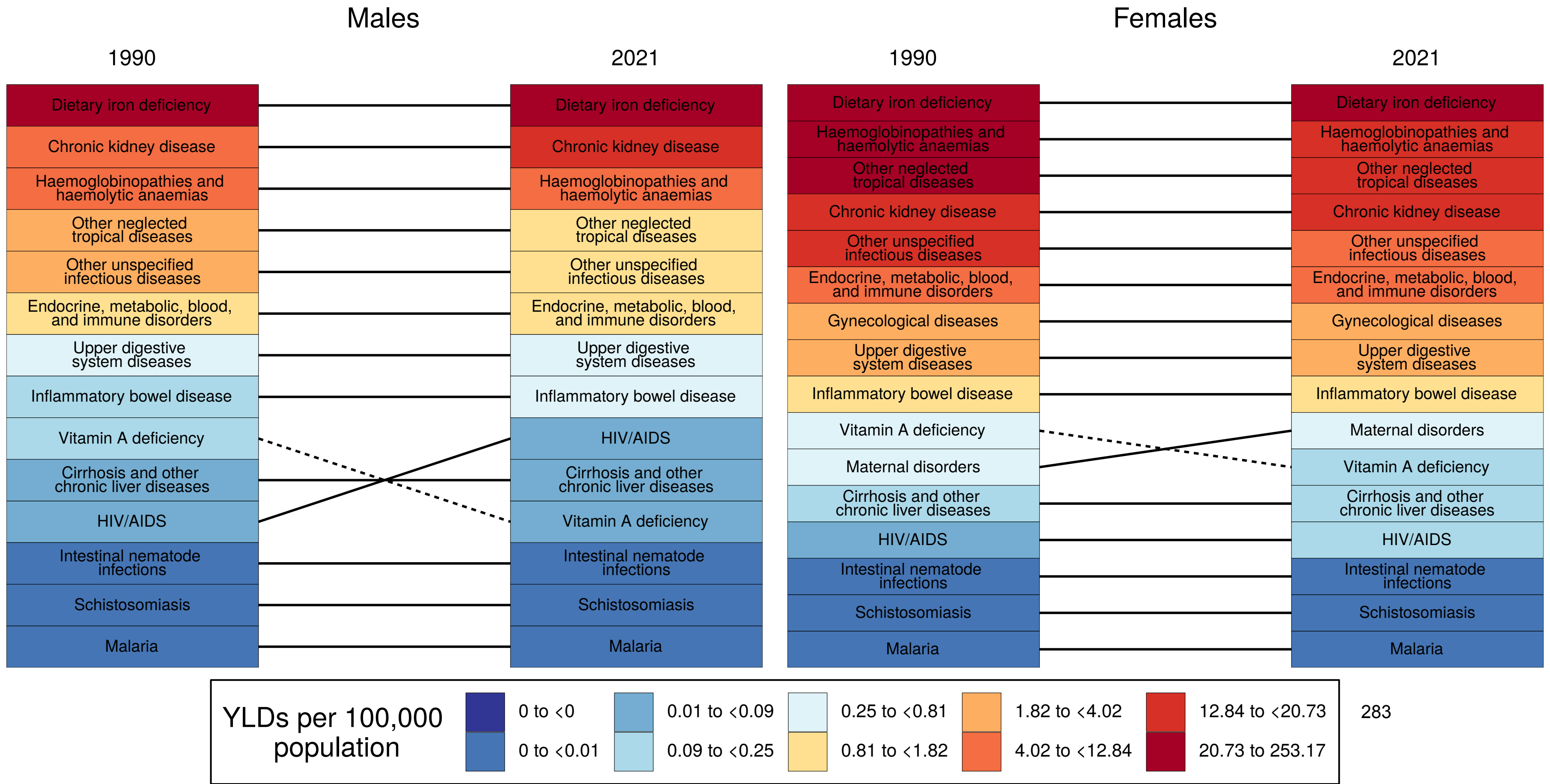

Males
Females
1990
2021
Dietary iron deficiency
Chronic kidney disease
Haemoglobinopathies and haemolytic anaemias
Other neglected tropical diseases
Other unspecified infectious diseases
Endocrine, metabolic, blood, and immune disorders
Upper digestive system diseases
Inflammatory bowel disease
Vitamin A deficiency
Cirrhosis and other chronic liver diseases
HIV/AIDS
Intestinal nematode infections
Schistosomiasis
Malaria
Gynecological diseases
Maternal disorders
YLDs per 100,000 population
0 to <0
0 to <0.01
0.01 to <0.09
0.09 to <0.25
0.25 to <0.81
0.81 to <1.82
1.82 to <4.02
4.02 to <12.84
12.84 to <20.73
20.73 to 253.17
283

Brent

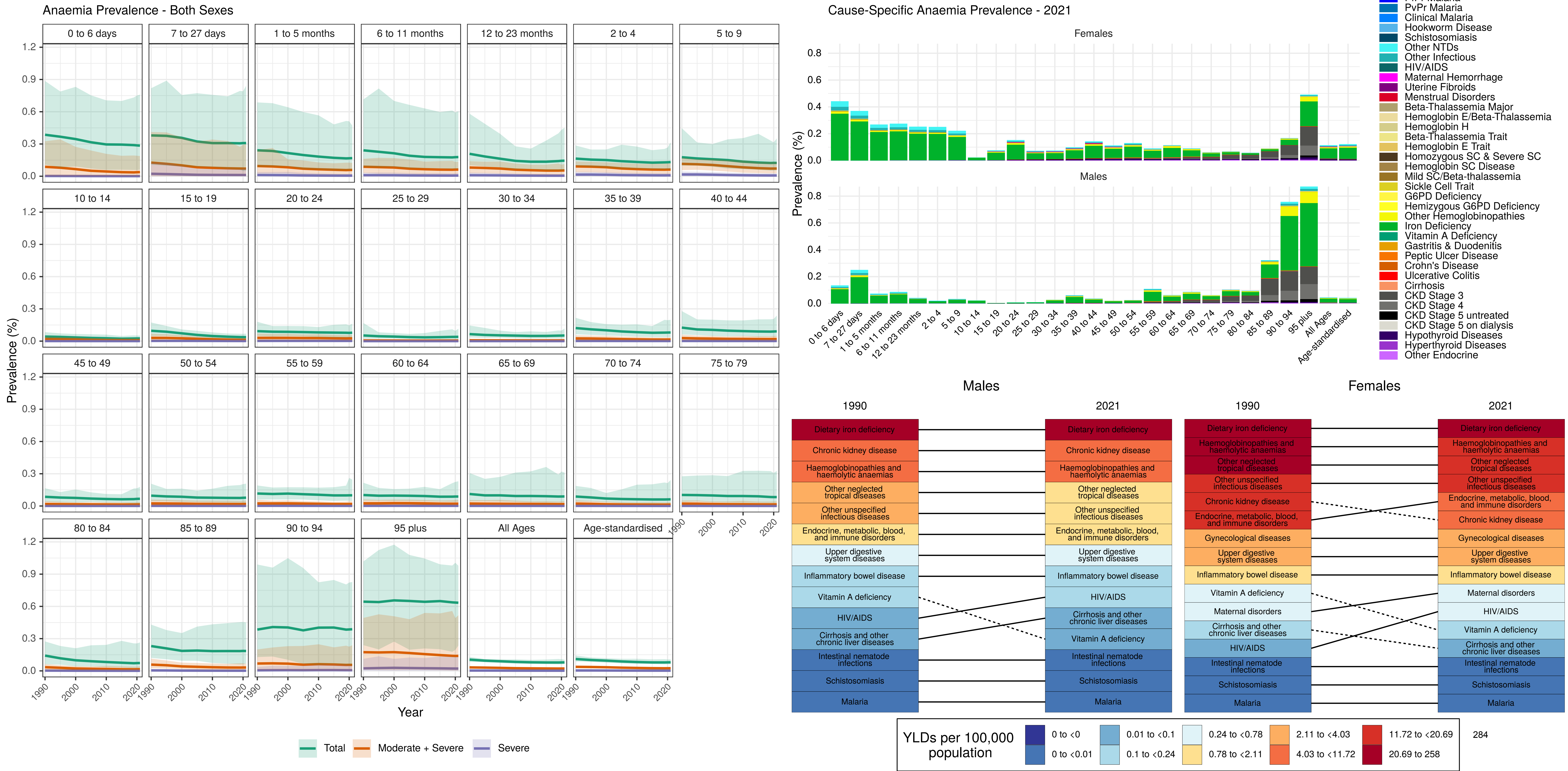

Anaemia Prevalence - Both Sexes
0 to 6 days
7 to 27 days
1 to 5 months
6 to 11 months
12 to 23 months
2 to 4
5 to 9
10 to 14
15 to 19
20 to 24
25 to 29
30 to 34
35 to 39
40 to 44
45 to 49
50 to 54
55 to 59
60 to 64
65 to 69
70 to 74
75 to 79
80 to 84
85 to 89
90 to 94
95 plus
All Ages
Age-standardised
Prevalence (%)
Year
Total
Moderate + Severe
Severe
Cause-Specific Anaemia Prevalence - 2021
Females
Males
PfPr Malaria
PvPr Malaria
Clinical Malaria
Hookworm Disease
Schistosomiasis
Other NTDs
Other Infectious
HIV/AIDS
Maternal Hemorrhage
Uterine Fibroids
Menstrual Disorders
Beta-Thalassemia Major
Hemoglobin E/Beta-Thalassemia
Hemoglobin H
Beta-Thalassemia Trait
Hemoglobin E Trait
Homozygous SC & Severe SC
Hemoglobin SC Disease
Mild SC/Beta-thalassemia
Sickle Cell Trait
G6PD Deficiency
Hemizygous G6PD Deficiency
Other Hemoglobinopathies
Iron Deficiency
Vitamin A Deficiency
Gastritis & Duodenitis
Peptic Ulcer Disease
Crohn's Disease
Ulcerative Colitis
Cirrhosis
CKD Stage 3
CKD Stage 4
CKD Stage 5 untreated
CKD Stage 5 on dialysis
Hypothyroid Diseases
Hyperthyroid Diseases
Other Endocrine
Males
Females
1990
2021
Dietary iron deficiency
Chronic kidney disease
Haemoglobinopathies and haemolytic anaemias
Other neglected tropical diseases
Other unspecified infectious diseases
Endocrine, metabolic, blood, and immune disorders
Upper digestive system diseases
Inflammatory bowel disease
Vitamin A deficiency
HIV/AIDS
Cirrhosis and other chronic liver diseases
Intestinal nematode infections
Schistosomiasis
Malaria
Gynecological diseases
Maternal disorders
YLDs per 100,000 population
0 to <0
0 to <0.01
0.01 to <0.1
0.1 to <0.24
0.24 to <0.78
0.78 to <2.11
2.11 to <4.03
4.03 to <11.72
11.72 to <20.69
20.69 to 258

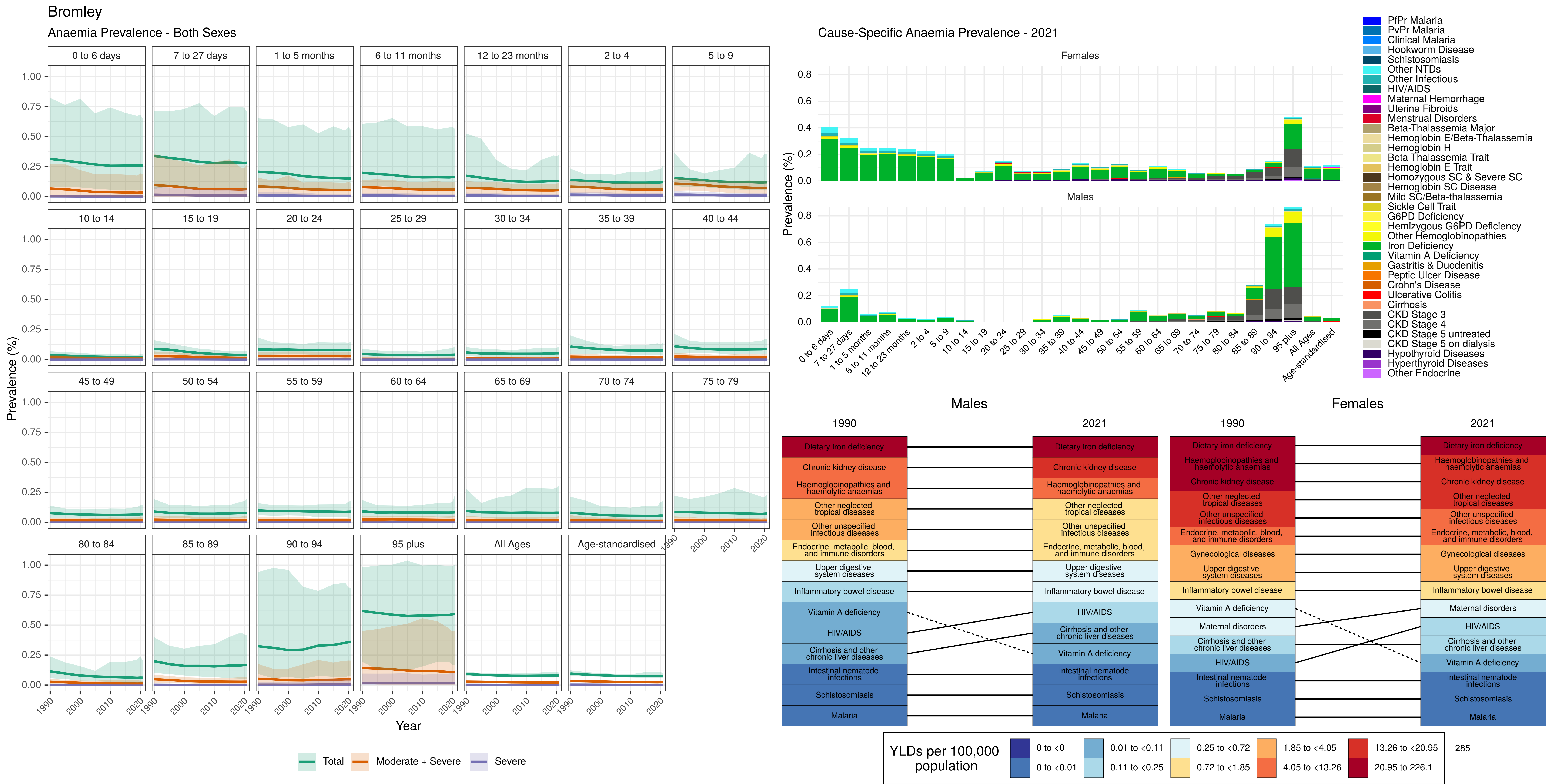
Bromley
Anaemia Prevalence - Both Sexes
0 to 6 days
7 to 27 days
1 to 5 months
6 to 11 months
12 to 23 months
2 to 4
5 to 9
10 to 14
15 to 19
20 to 24
25 to 29
30 to 34
35 to 39
40 to 44
45 to 49
50 to 54
55 to 59
60 to 64
65 to 69
70 to 74
75 to 79
80 to 84
85 to 89
90 to 94
95 plus
All Ages
Age-standardised
Prevalence (%)
Year
Total
Moderate + Severe
Severe
Cause-Specific Anaemia Prevalence - 2021
Females
Males
PfPr Malaria
PvPr Malaria
Clinical Malaria
Hookworm Disease
Schistosomiasis
Other NTDs
Other Infectious
HIV/AIDS
Maternal Hemorrhage
Uterine Fibroids
Menstrual Disorders
Beta-Thalassemia Major
Hemoglobin E/Beta-Thalassemia
Hemoglobin H
Beta-Thalassemia Trait
Hemoglobin E Trait
Homozygous SC & Severe SC
Hemoglobin SC Disease
Mild SC/Beta-thalassemia
Sickle Cell Trait
G6PD Deficiency
Hemizygous G6PD Deficiency
Other Hemoglobinopathies
Iron Deficiency
Vitamin A Deficiency
Gastritis & Duodenitis
Peptic Ulcer Disease
Crohn's Disease
Ulcerative Colitis
Cirrhosis
CKD Stage 3
CKD Stage 4
CKD Stage 5 untreated
CKD Stage 5 on dialysis
Hypothyroid Diseases
Hyperthyroid Diseases
Other Endocrine
Males
Females
1990
2021
Dietary iron deficiency
Chronic kidney disease
Haemoglobinopathies and haemolytic anaemias
Other neglected tropical diseases
Other unspecified infectious diseases
Endocrine, metabolic, blood, and immune disorders
Upper digestive system diseases
Inflammatory bowel disease
Vitamin A deficiency
HIV/AIDS
Cirrhosis and other chronic liver diseases
Intestinal nematode infections
Schistosomiasis
Malaria
Gynecological diseases
Maternal disorders
YLDs per 100,000 population
0 to <0
0 to <0.01
0.01 to <0.11
0.11 to <0.25
0.25 to <0.72
0.72 to <1.85
1.85 to <4.05
4.05 to <13.26
13.26 to <20.95
20.95 to 226.1

Camden

Anaemia Prevalence - Both Sexes

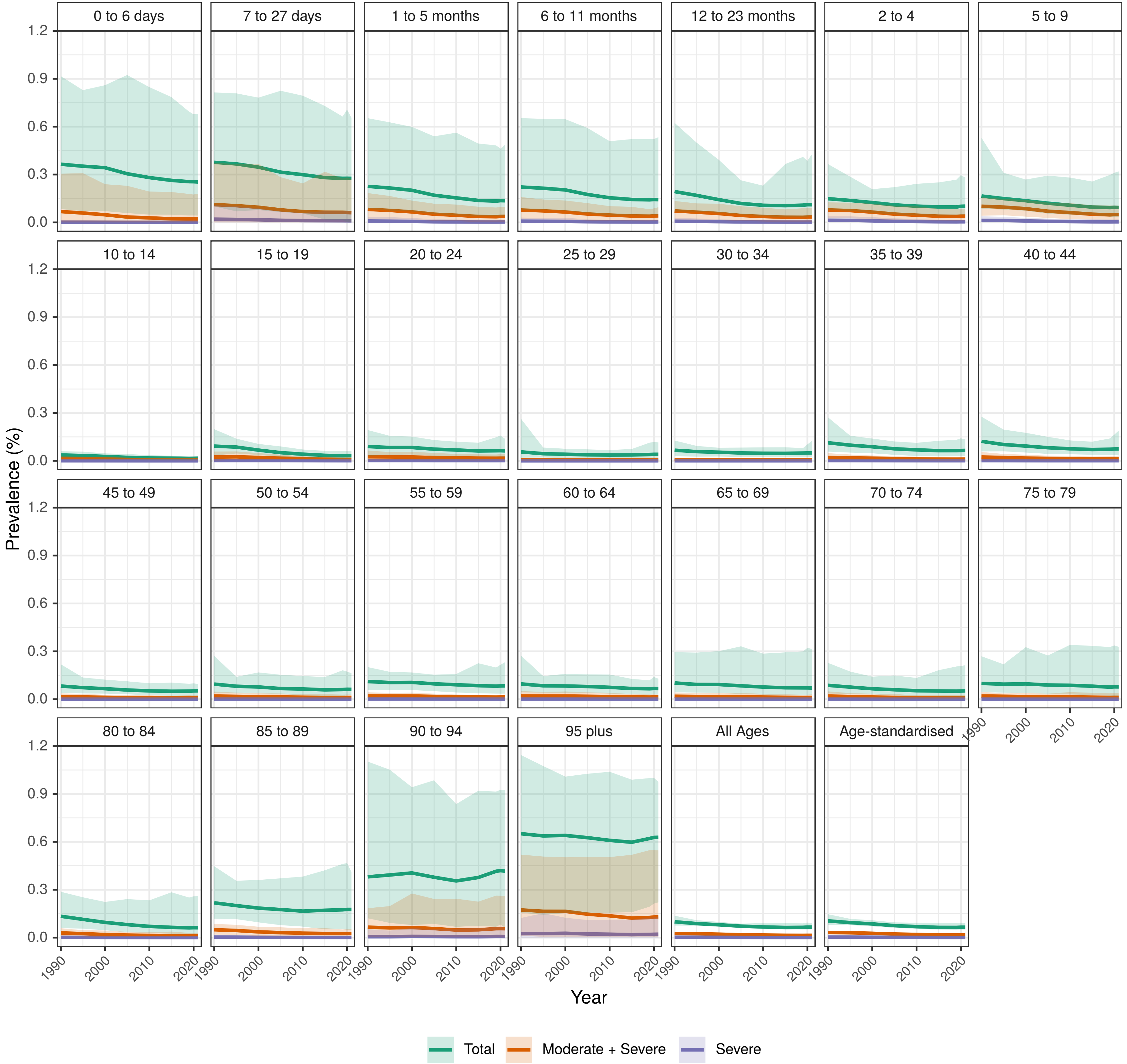

Cause-Specific Anaemia Prevalence - 2021

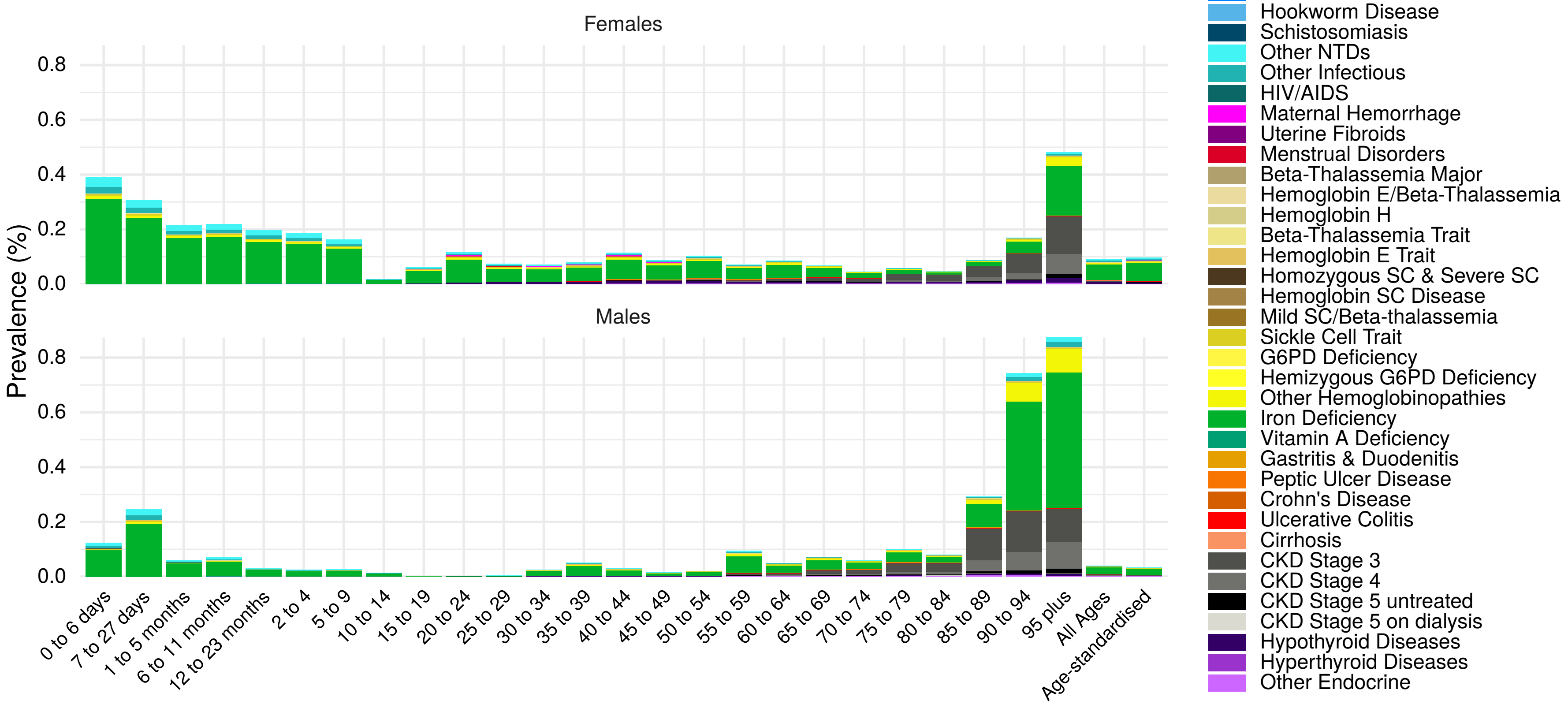

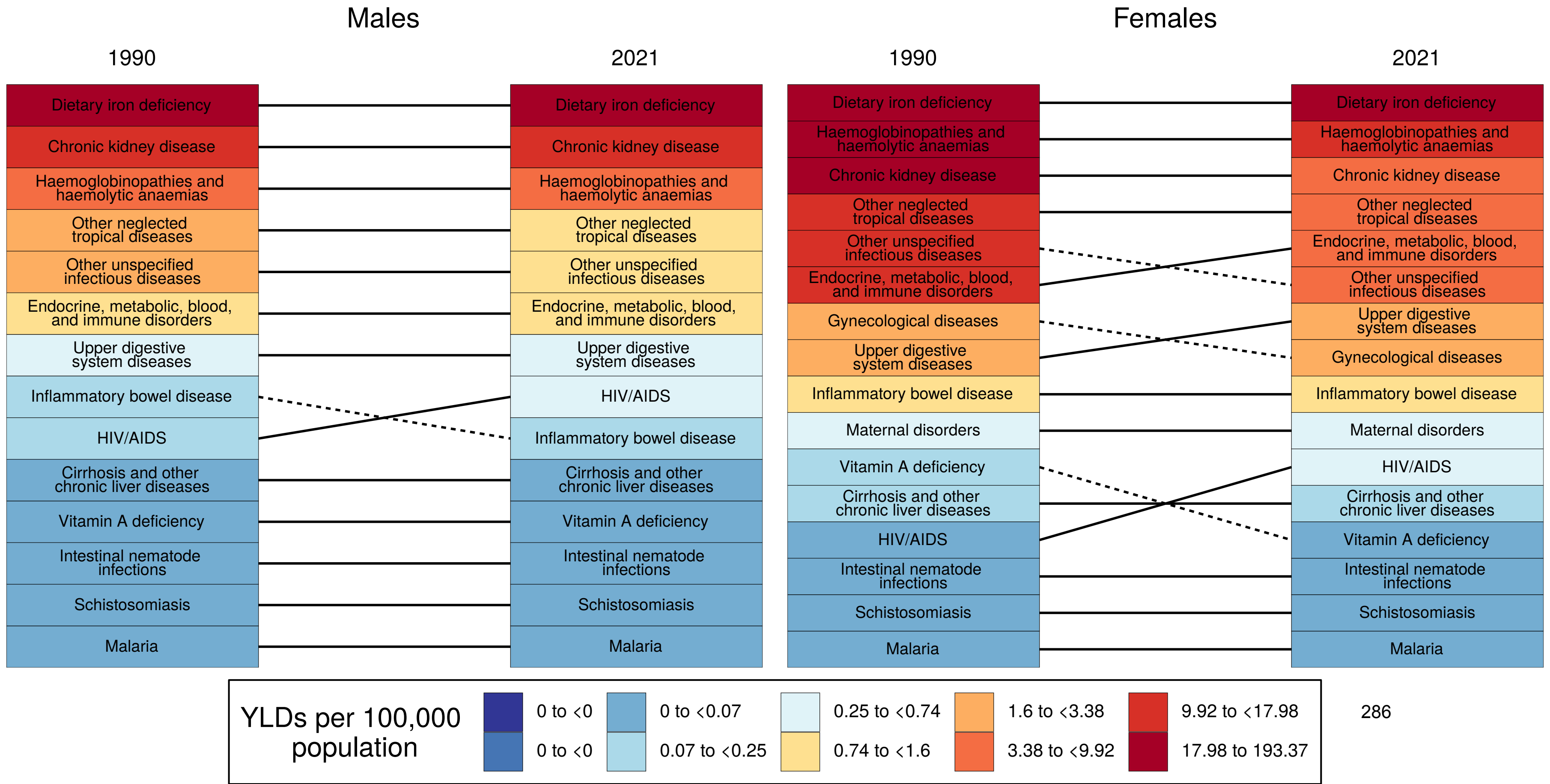

Croydon

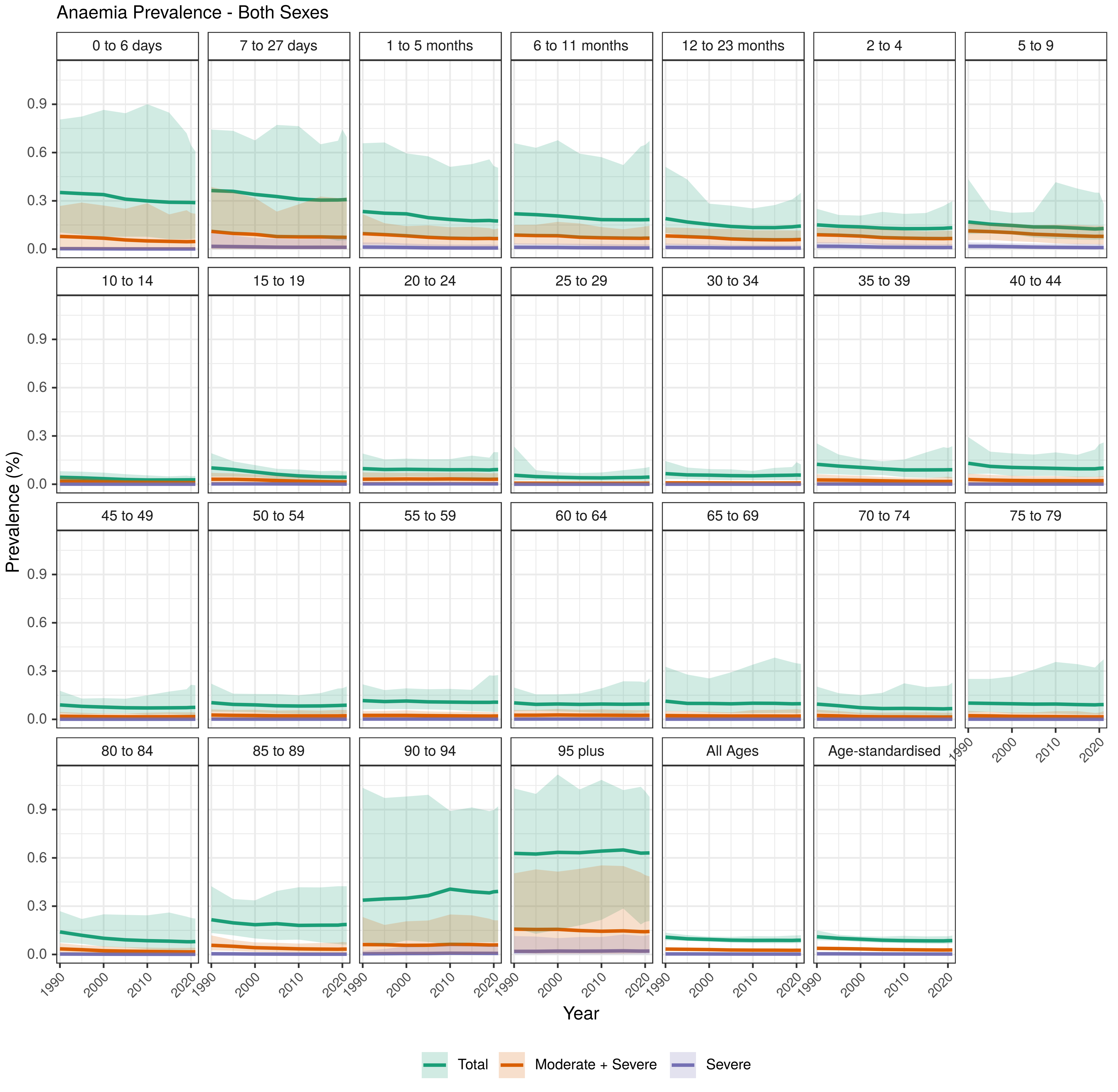
Anaemia Prevalence - Both Sexes
0 to 6 days
7 to 27 days
1 to 5 months
6 to 11 months
12 to 23 months
2 to 4
5 to 9
10 to 14
15 to 19
20 to 24
25 to 29
30 to 34
35 to 39
40 to 44
45 to 49
50 to 54
55 to 59
60 to 64
65 to 69
70 to 74
75 to 79
80 to 84
85 to 89
90 to 94
95 plus
All Ages
Age-standardised
Prevalence (%)
0.0
0.3
0.6
0.9
1990
2000
2010
2020
Year
Total
Moderate + Severe
Severe

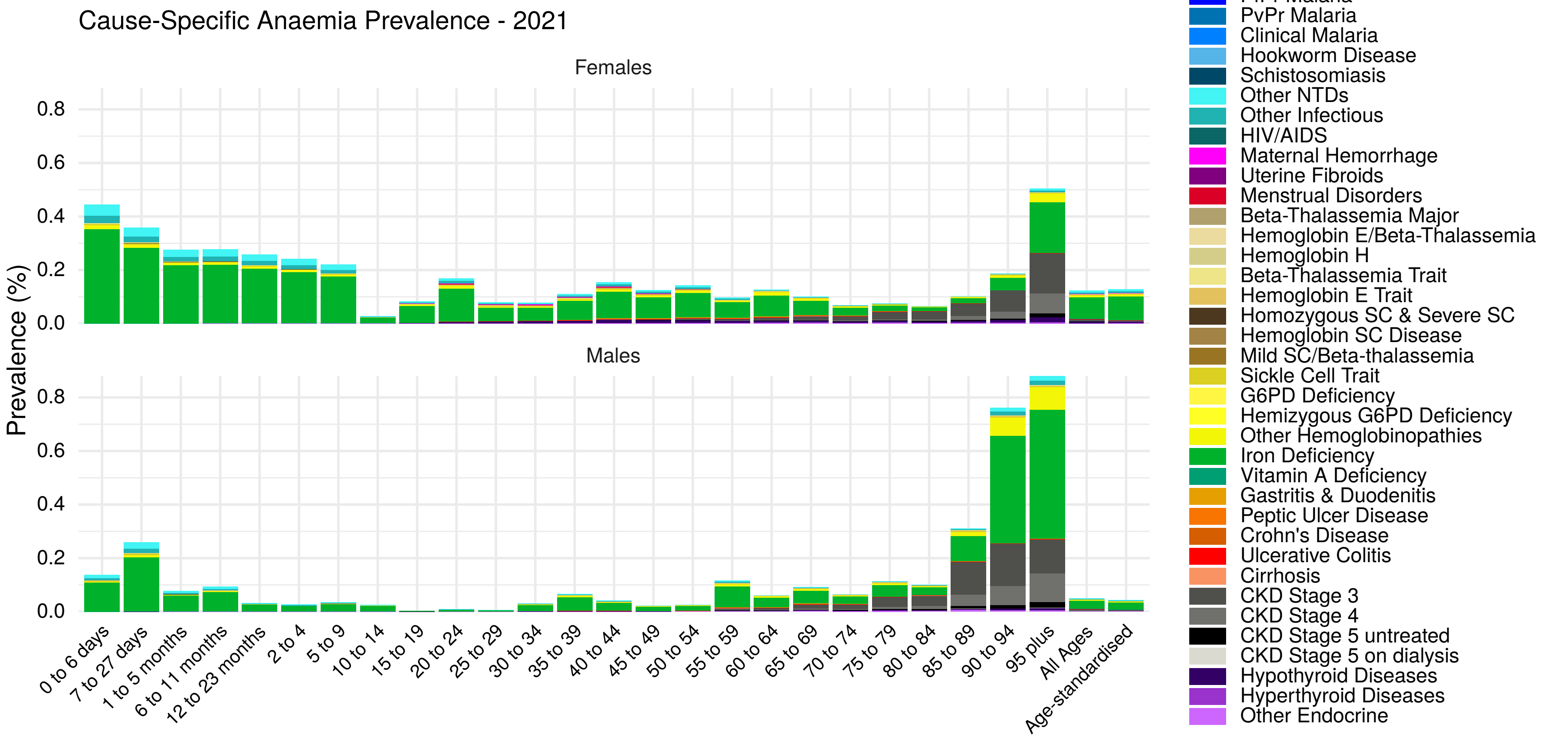
Cause-Specific Anaemia Prevalence - 2021
Females
Males
Prevalence (%)
0.0
0.2
0.4
0.6
0.8
0 to 6 days
7 to 27 days
1 to 5 months
6 to 11 months
12 to 23 months
2 to 4
5 to 9
10 to 14
15 to 19
20 to 24
25 to 29
30 to 34
35 to 39
40 to 44
45 to 49
50 to 54
55 to 59
60 to 64
65 to 69
70 to 74
75 to 79
80 to 84
85 to 89
90 to 94
95 plus
All Ages
Age-standardised
PfPr Malaria
PvPr Malaria
Clinical Malaria
Hookworm Disease
Schistosomiasis
Other NTDs
Other Infectious
HIV/AIDS
Maternal Hemorrhage
Uterine Fibroids
Menstrual Disorders
Beta-Thalassemia Major
Hemoglobin E/Beta-Thalassemia
Hemoglobin H
Beta-Thalassemia Trait
Hemoglobin E Trait
Homozygous SC & Severe SC
Hemoglobin SC Disease
Mild SC/Beta-thalassemia
Sickle Cell Trait
G6PD Deficiency
Hemizygous G6PD Deficiency
Other Hemoglobinopathies
Iron Deficiency
Vitamin A Deficiency
Gastritis & Duodenitis
Peptic Ulcer Disease
Crohn's Disease
Ulcerative Colitis
Cirrhosis
CKD Stage 3
CKD Stage 4
CKD Stage 5 untreated
CKD Stage 5 on dialysis
Hypothyroid Diseases
Hyperthyroid Diseases
Other Endocrine

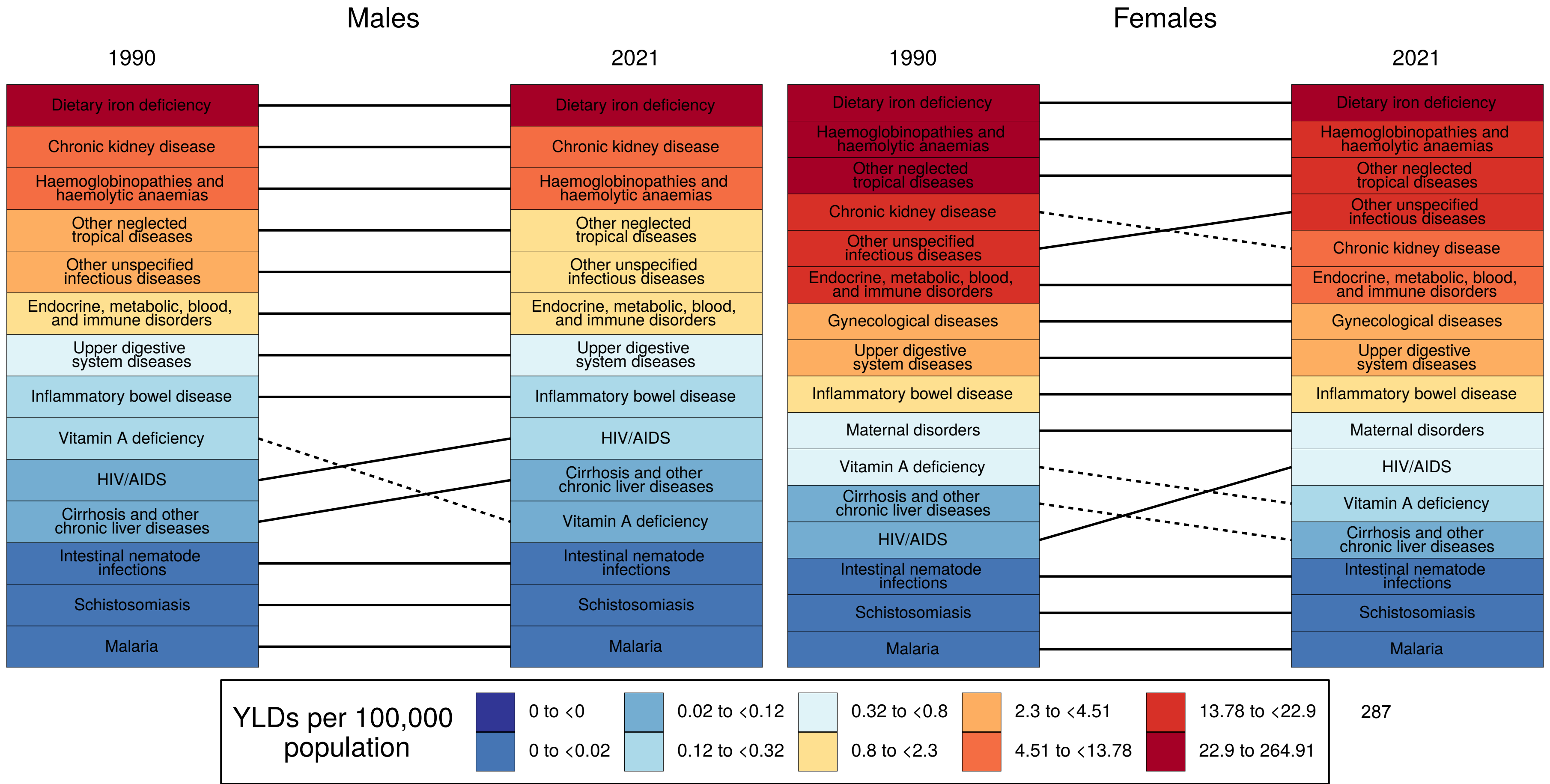
Males
Females
1990
2021
Dietary iron deficiency
Chronic kidney disease
Haemoglobinopathies and haemolytic anaemias
Other neglected tropical diseases
Other unspecified infectious diseases
Endocrine, metabolic, blood, and immune disorders
Upper digestive system diseases
Inflammatory bowel disease
Vitamin A deficiency
HIV/AIDS
Cirrhosis and other chronic liver diseases
Intestinal nematode infections
Schistosomiasis
Malaria
Gynecological diseases
Maternal disorders
YLDs per 100,000 population
0 to <0
0 to <0.02
0.02 to <0.12
0.12 to <0.32
0.32 to <0.8
0.8 to <2.3
2.3 to <4.51
4.51 to <13.78
13.78 to <22.9
22.9 to 264.91

Ealing

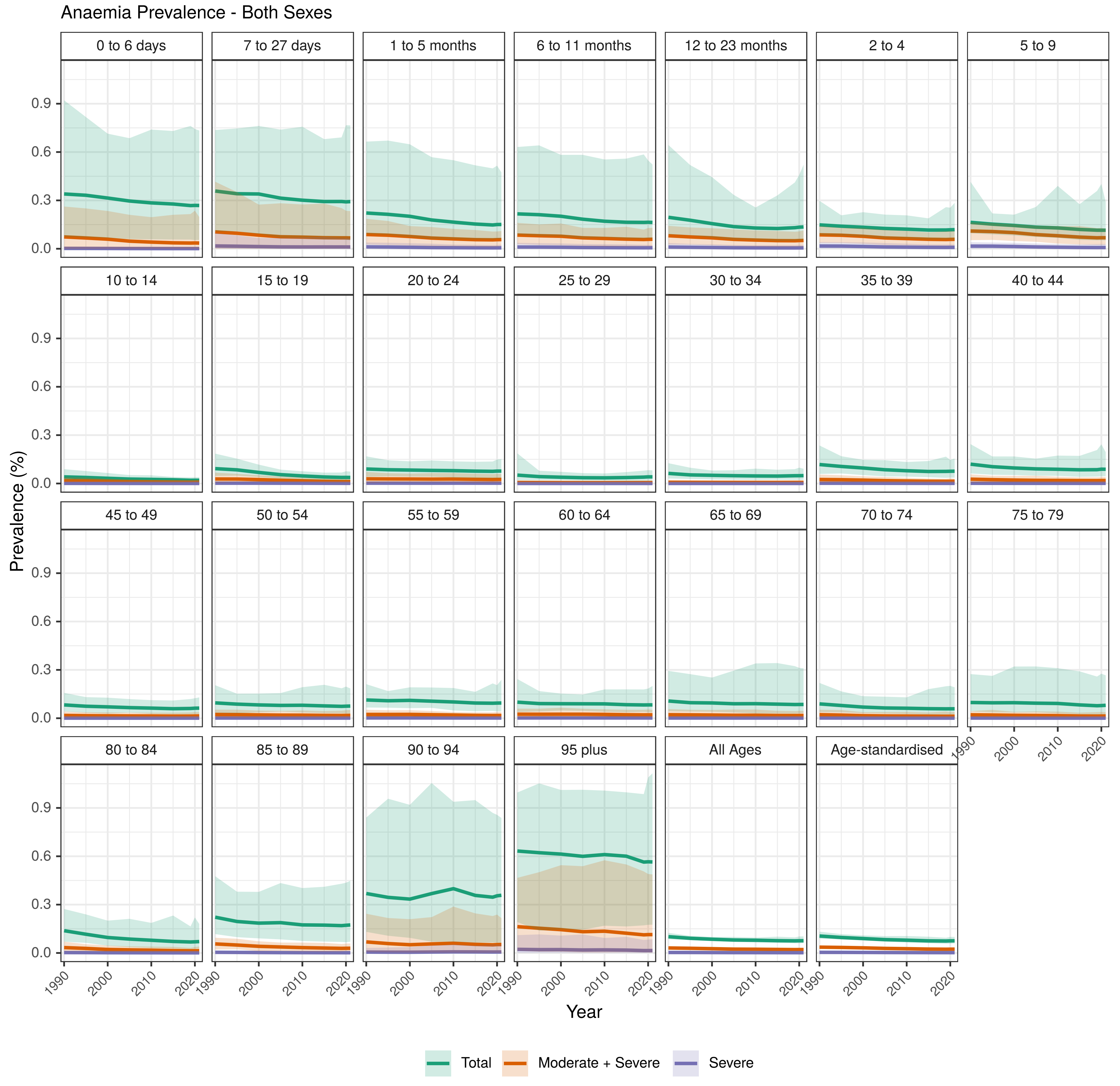

Anaemia Prevalence - Both Sexes
0 to 6 days
7 to 27 days
1 to 5 months
6 to 11 months
12 to 23 months
2 to 4
5 to 9
10 to 14
15 to 19
20 to 24
25 to 29
30 to 34
35 to 39
40 to 44
45 to 49
50 to 54
55 to 59
60 to 64
65 to 69
70 to 74
75 to 79
80 to 84
85 to 89
90 to 94
95 plus
All Ages
Age-standardised
0.0
0.3
0.6
0.9
Prevalence (%)
1990
2000
2010
2020
Year
Total
Moderate + Severe
Severe

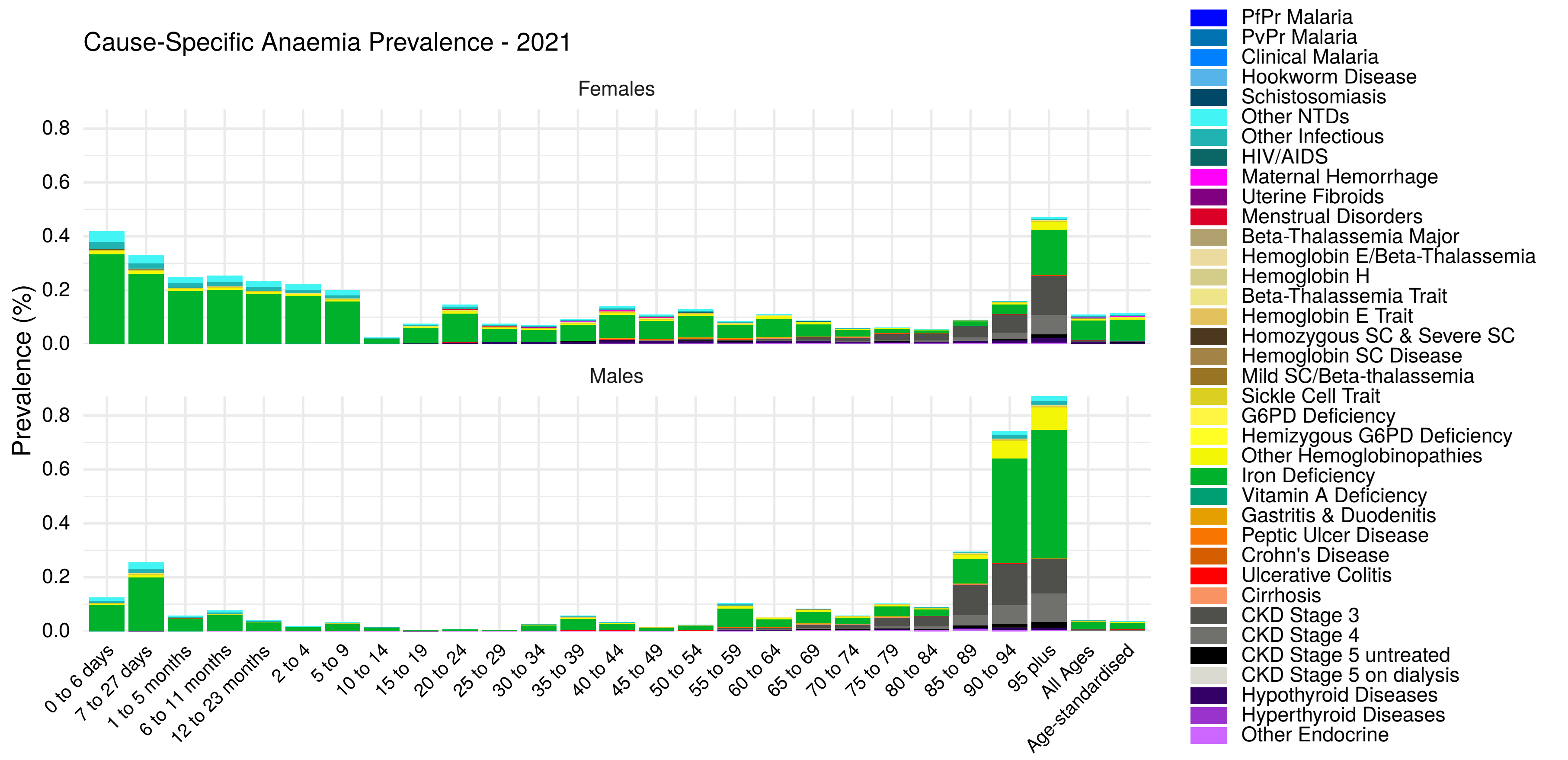

Cause-Specific Anaemia Prevalence - 2021
Females
Males
Prevalence (%)
0.0
0.2
0.4
0.6
0.8
0 to 6 days
7 to 27 days
1 to 5 months
6 to 11 months
12 to 23 months
2 to 4
5 to 9
10 to 14
15 to 19
20 to 24
25 to 29
30 to 34
35 to 39
40 to 44
45 to 49
50 to 54
55 to 59
60 to 64
65 to 69
70 to 74
75 to 79
80 to 84
85 to 89
90 to 94
95 plus
All Ages
Age-standardised
PfPr Malaria
PvPr Malaria
Clinical Malaria
Hookworm Disease
Schistosomiasis
Other NTDs
Other Infectious
HIV/AIDS
Maternal Hemorrhage
Uterine Fibroids
Menstrual Disorders
Beta-Thalassemia Major
Hemoglobin E/Beta-Thalassemia
Hemoglobin H
Beta-Thalassemia Trait
Hemoglobin E Trait
Homozygous SC & Severe SC
Hemoglobin SC Disease
Mild SC/Beta-thalassemia
Sickle Cell Trait
G6PD Deficiency
Hemizygous G6PD Deficiency
Other Hemoglobinopathies
Iron Deficiency
Vitamin A Deficiency
Gastritis & Duodenitis
Peptic Ulcer Disease
Crohn's Disease
Ulcerative Colitis
Cirrhosis
CKD Stage 3
CKD Stage 4
CKD Stage 5 untreated
CKD Stage 5 on dialysis
Hypothyroid Diseases
Hyperthyroid Diseases
Other Endocrine

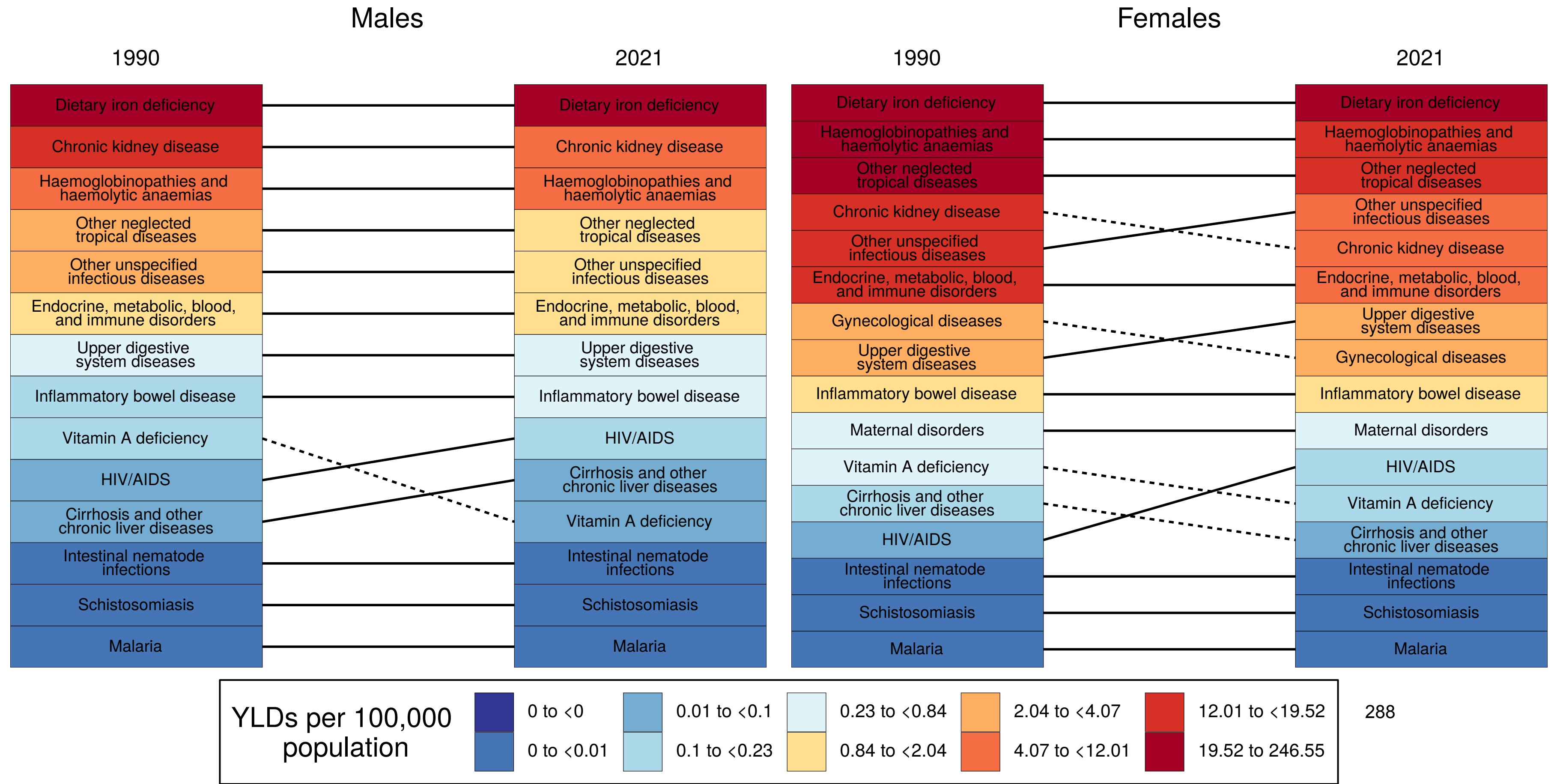

Males
Females
1990
2021
Dietary iron deficiency
Chronic kidney disease
Haemoglobinopathies and haemolytic anaemias
Other neglected tropical diseases
Other unspecified infectious diseases
Endocrine, metabolic, blood, and immune disorders
Upper digestive system diseases
Inflammatory bowel disease
Vitamin A deficiency
HIV/AIDS
Cirrhosis and other chronic liver diseases
Intestinal nematode infections
Schistosomiasis
Malaria
Gynecological diseases
Maternal disorders
YLDs per 100,000 population
0 to <0
0 to <0.01
0.01 to <0.1
0.1 to <0.23
0.23 to <0.84
0.84 to <2.04
2.04 to <4.07
4.07 to <12.01
12.01 to <19.52
19.52 to 246.55

Enfield

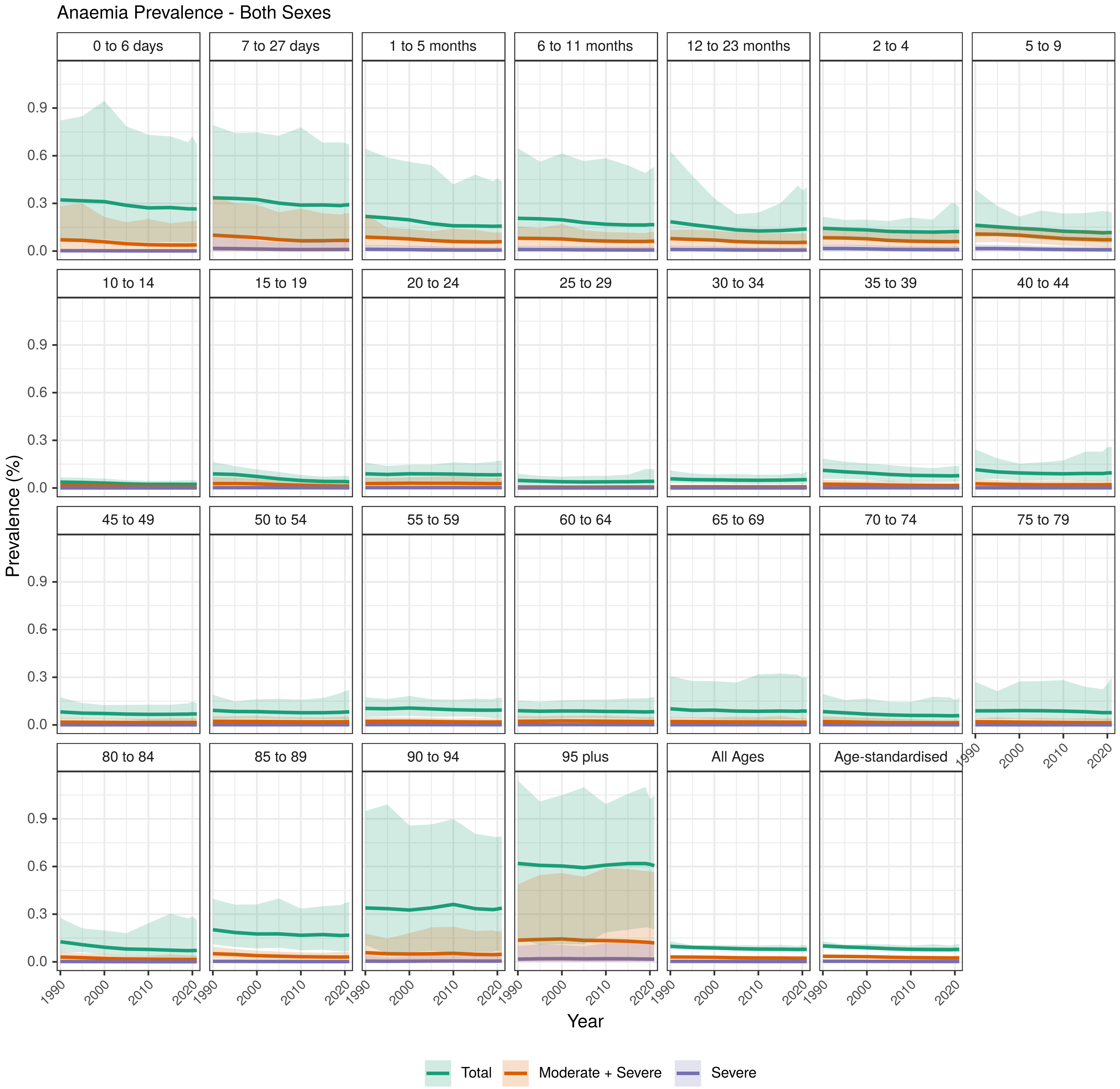

Anaemia Prevalence - Both Sexes
0 to 6 days
7 to 27 days
1 to 5 months
6 to 11 months
12 to 23 months
2 to 4
5 to 9
10 to 14
15 to 19
20 to 24
25 to 29
30 to 34
35 to 39
40 to 44
45 to 49
50 to 54
55 to 59
60 to 64
65 to 69
70 to 74
75 to 79
80 to 84
85 to 89
90 to 94
95 plus
All Ages
Age-standardised
Prevalence (%)
Year
Total
Moderate + Severe
Severe

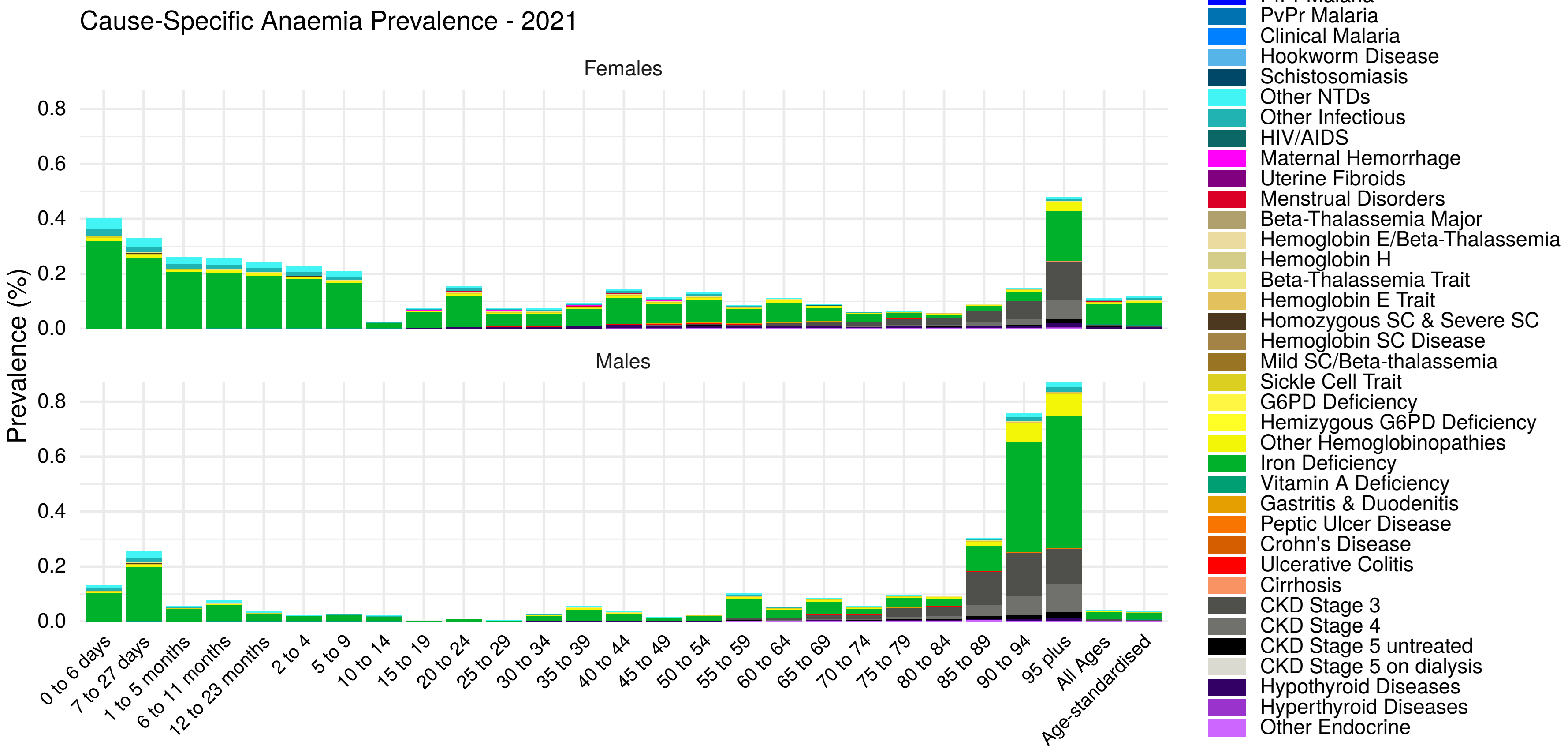

Cause-Specific Anaemia Prevalence - 2021
Females
Males
Prevalence (%)
PfPr Malaria
PvPr Malaria
Clinical Malaria
Hookworm Disease
Schistosomiasis
Other NTDs
Other Infectious
HIV/AIDS
Maternal Hemorrhage
Uterine Fibroids
Menstrual Disorders
Beta-Thalassemia Major
Hemoglobin E/Beta-Thalassemia
Hemoglobin H
Beta-Thalassemia Trait
Hemoglobin E Trait
Homozygous SC & Severe SC
Hemoglobin SC Disease
Mild SC/Beta-thalassemia
Sickle Cell Trait
G6PD Deficiency
Hemizygous G6PD Deficiency
Other Hemoglobinopathies
Iron Deficiency
Vitamin A Deficiency
Gastritis & Duodenitis
Peptic Ulcer Disease
Crohn's Disease
Ulcerative Colitis
Cirrhosis
CKD Stage 3
CKD Stage 4
CKD Stage 5 untreated
CKD Stage 5 on dialysis
Hypothyroid Diseases
Hyperthyroid Diseases
Other Endocrine

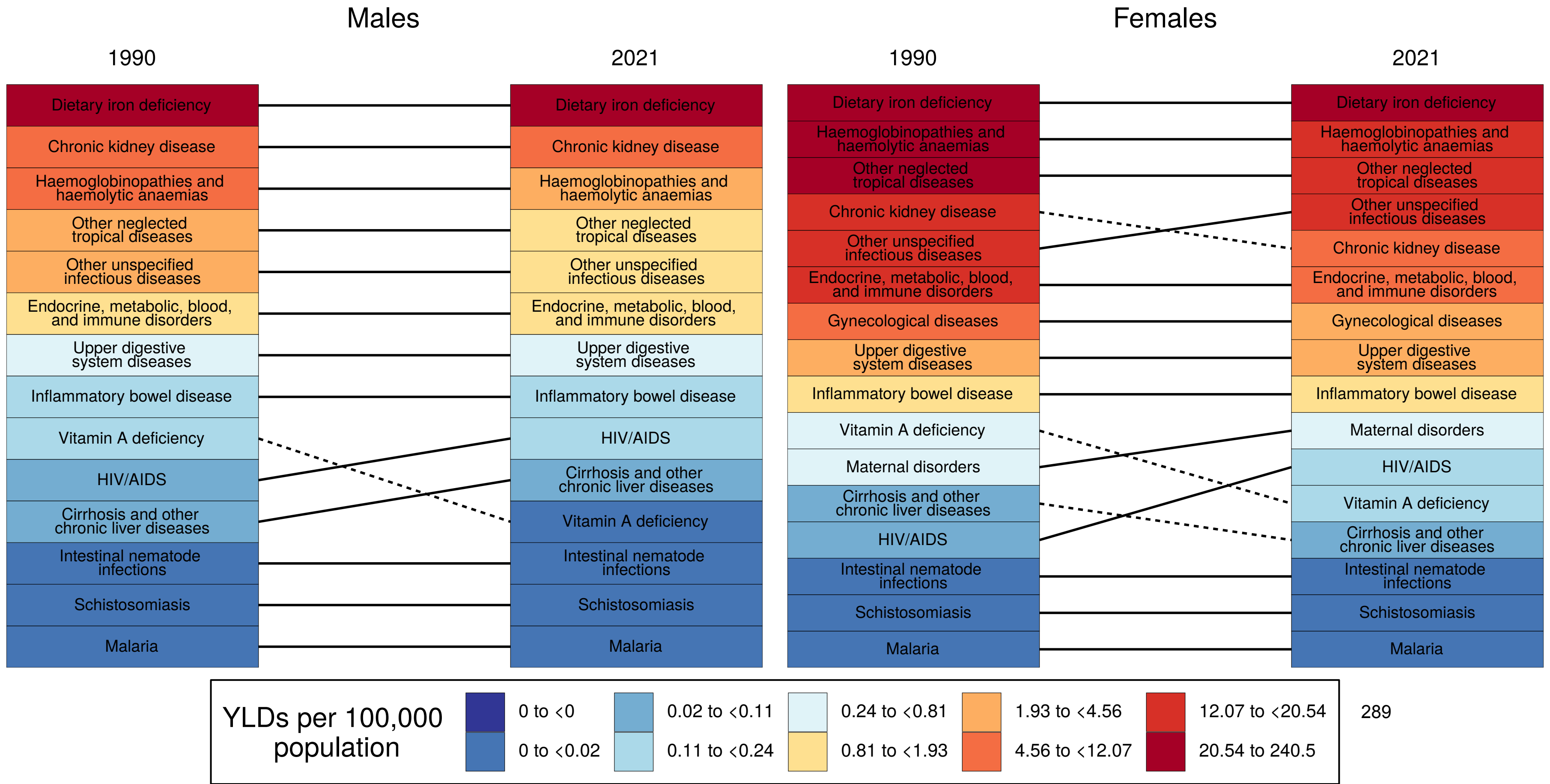

Males
Females
1990
2021
Dietary iron deficiency
Chronic kidney disease
Haemoglobinopathies and haemolytic anaemias
Other neglected tropical diseases
Other unspecified infectious diseases
Endocrine, metabolic, blood, and immune disorders
Upper digestive system diseases
Inflammatory bowel disease
Vitamin A deficiency
HIV/AIDS
Cirrhosis and other chronic liver diseases
Intestinal nematode infections
Schistosomiasis
Malaria
Gynecological diseases
Maternal disorders
YLDs per 100,000 population
0 to <0
0 to <0.02
0.02 to <0.11
0.11 to <0.24
0.24 to <0.81
0.81 to <1.93
1.93 to <4.56
4.56 to <12.07
12.07 to <20.54
20.54 to 240.5

Greenwich

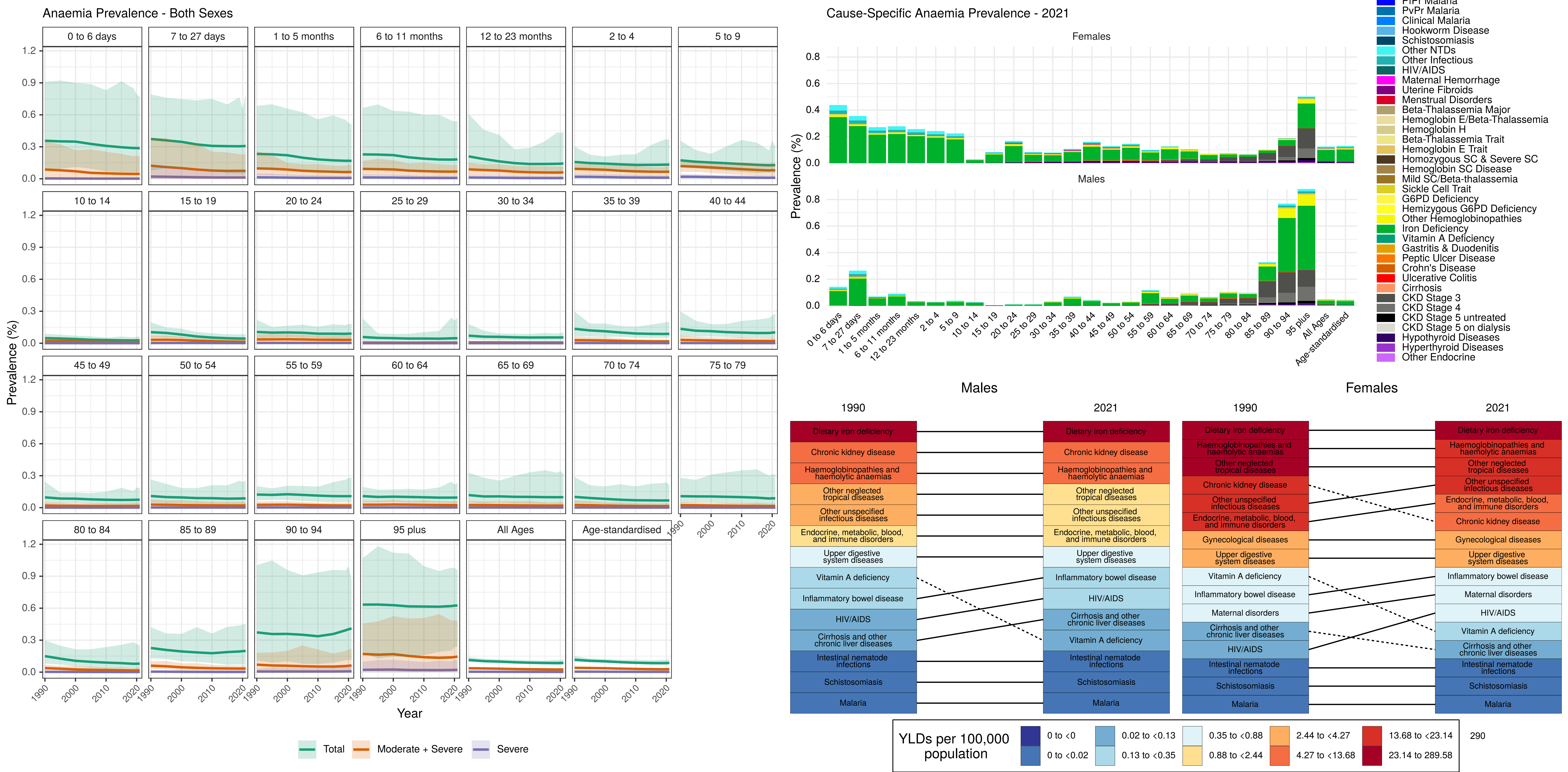

Anaemia Prevalence - Both Sexes
0 to 6 days
7 to 27 days
1 to 5 months
6 to 11 months
12 to 23 months
2 to 4
5 to 9
10 to 14
15 to 19
20 to 24
25 to 29
30 to 34
35 to 39
40 to 44
45 to 49
50 to 54
55 to 59
60 to 64
65 to 69
70 to 74
75 to 79
80 to 84
85 to 89
90 to 94
95 plus
All Ages
Age-standardised
Prevalence (%)
Year
Total
Moderate + Severe
Severe
Cause-Specific Anaemia Prevalence - 2021
Females
Males
PfPr Malaria
PvPr Malaria
Clinical Malaria
Hookworm Disease
Schistosomiasis
Other NTDs
Other Infectious
HIV/AIDS
Maternal Hemorrhage
Uterine Fibroids
Menstrual Disorders
Beta-Thalassemia Major
Hemoglobin E/Beta-Thalassemia
Hemoglobin H
Beta-Thalassemia Trait
Hemoglobin E Trait
Homozygous SC & Severe SC
Hemoglobin SC Disease
Mild SC/Beta-thalassemia
Sickle Cell Trait
G6PD Deficiency
Hemizygous G6PD Deficiency
Other Hemoglobinopathies
Iron Deficiency
Vitamin A Deficiency
Gastritis & Duodenitis
Peptic Ulcer Disease
Crohn's Disease
Ulcerative Colitis
Cirrhosis
CKD Stage 3
CKD Stage 4
CKD Stage 5 untreated
CKD Stage 5 on dialysis
Hypothyroid Diseases
Hyperthyroid Diseases
Other Endocrine
Males
Females
1990
2021
Dietary iron deficiency
Chronic kidney disease
Haemoglobinopathies and haemolytic anaemias
Other neglected tropical diseases
Other unspecified infectious diseases
Endocrine, metabolic, blood, and immune disorders
Upper digestive system diseases
Vitamin A deficiency
Inflammatory bowel disease
HIV/AIDS
Cirrhosis and other chronic liver diseases
Intestinal nematode infections
Schistosomiasis
Malaria
Gynecological diseases
Maternal disorders
YLDs per 100,000 population
0 to <0
0 to <0.02
0.02 to <0.13
0.13 to <0.35
0.35 to <0.88
0.88 to <2.44
2.44 to <4.27
4.27 to <13.68
13.68 to <23.14
23.14 to 289.58

Hackney

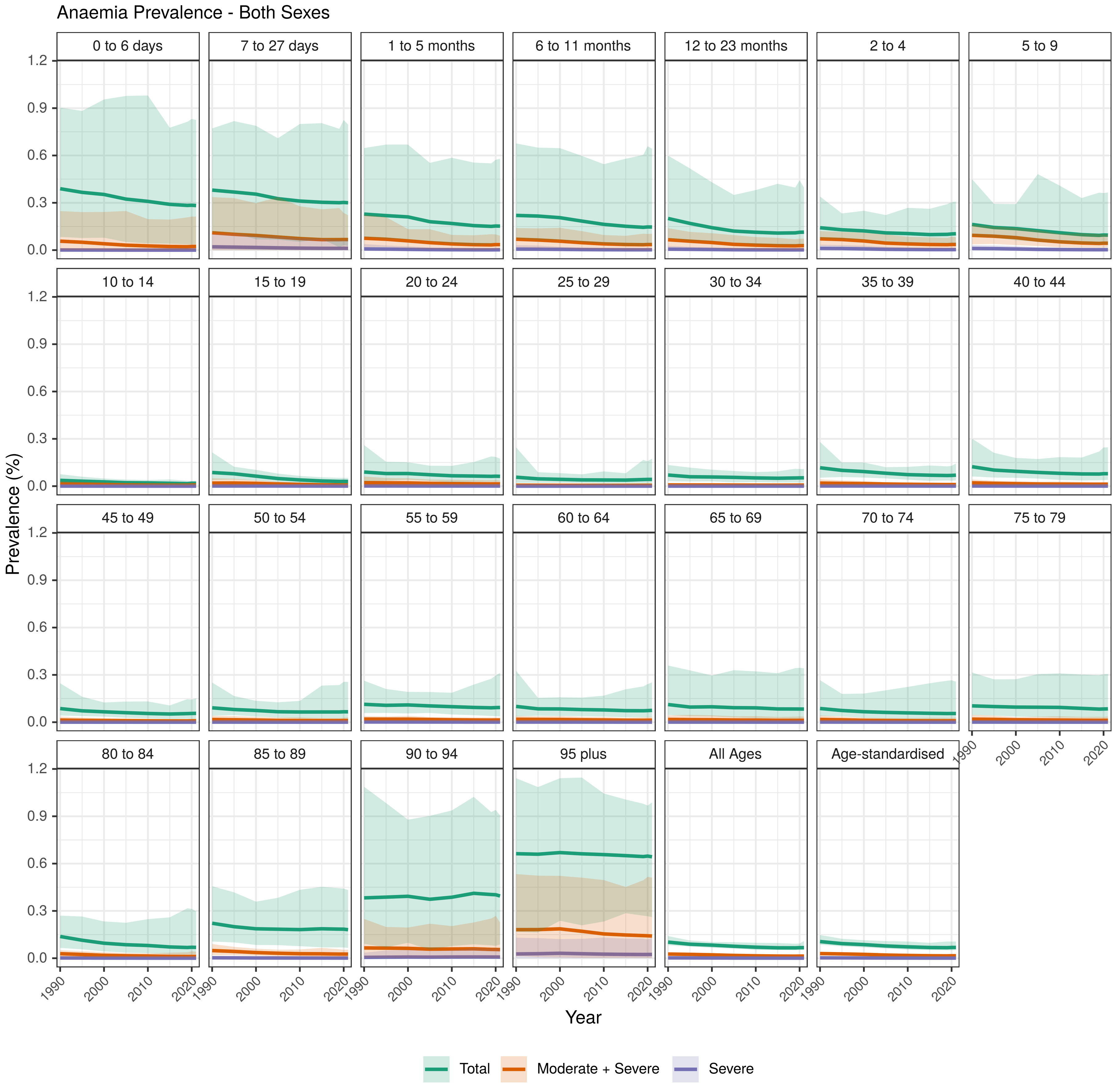

Anaemia Prevalence - Both Sexes
0 to 6 days
7 to 27 days
1 to 5 months
6 to 11 months
12 to 23 months
2 to 4
5 to 9
10 to 14
15 to 19
20 to 24
25 to 29
30 to 34
35 to 39
40 to 44
45 to 49
50 to 54
55 to 59
60 to 64
65 to 69
70 to 74
75 to 79
80 to 84
85 to 89
90 to 94
95 plus
All Ages
Age-standardised
Prevalence (%)
Year
Total
Moderate + Severe
Severe

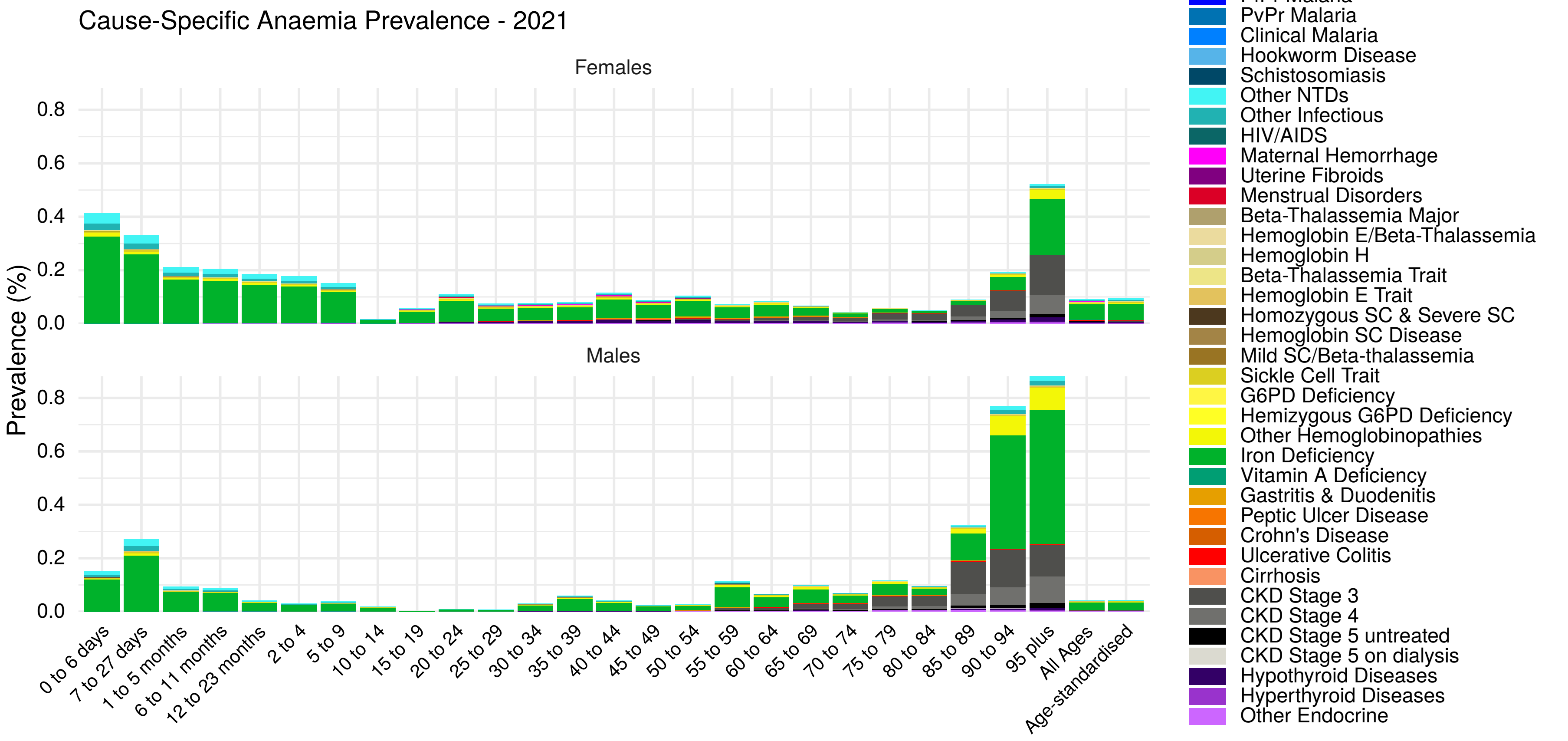

Cause-Specific Anaemia Prevalence - 2021
Females
Males
Prevalence (%)
PfPr Malaria
PvPr Malaria
Clinical Malaria
Hookworm Disease
Schistosomiasis
Other NTDs
Other Infectious
HIV/AIDS
Maternal Hemorrhage
Uterine Fibroids
Menstrual Disorders
Beta-Thalassemia Major
Hemoglobin E/Beta-Thalassemia
Hemoglobin H
Beta-Thalassemia Trait
Hemoglobin E Trait
Homozygous SC & Severe SC
Hemoglobin SC Disease
Mild SC/Beta-thalassemia
Sickle Cell Trait
G6PD Deficiency
Hemizygous G6PD Deficiency
Other Hemoglobinopathies
Iron Deficiency
Vitamin A Deficiency
Gastritis & Duodenitis
Peptic Ulcer Disease
Crohn's Disease
Ulcerative Colitis
Cirrhosis
CKD Stage 3
CKD Stage 4
CKD Stage 5 untreated
CKD Stage 5 on dialysis
Hypothyroid Diseases
Hyperthyroid Diseases
Other Endocrine

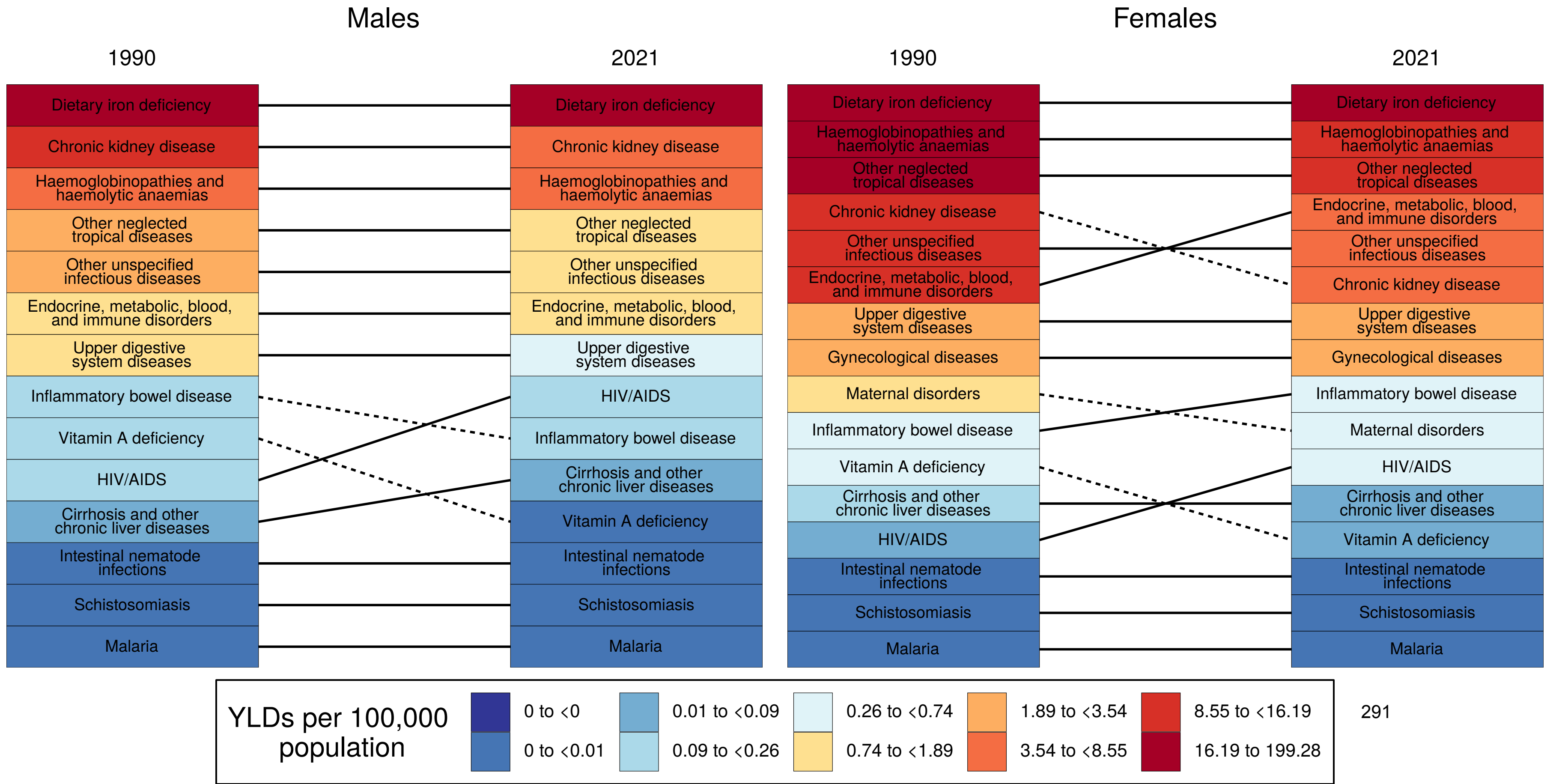

Males
Females
1990
2021
Dietary iron deficiency
Chronic kidney disease
Haemoglobinopathies and haemolytic anaemias
Other neglected tropical diseases
Other unspecified infectious diseases
Endocrine, metabolic, blood, and immune disorders
Upper digestive system diseases
Inflammatory bowel disease
Vitamin A deficiency
HIV/AIDS
Cirrhosis and other chronic liver diseases
Intestinal nematode infections
Schistosomiasis
Malaria
Gynecological diseases
Maternal disorders
YLDs per 100,000 population
0 to <0
0 to <0.01
0.01 to <0.09
0.09 to <0.26
0.26 to <0.74
0.74 to <1.89
1.89 to <3.54
3.54 to <8.55
8.55 to <16.19
16.19 to 199.28

Hammersmith and Fulham

Anaemia Prevalence - Both Sexes

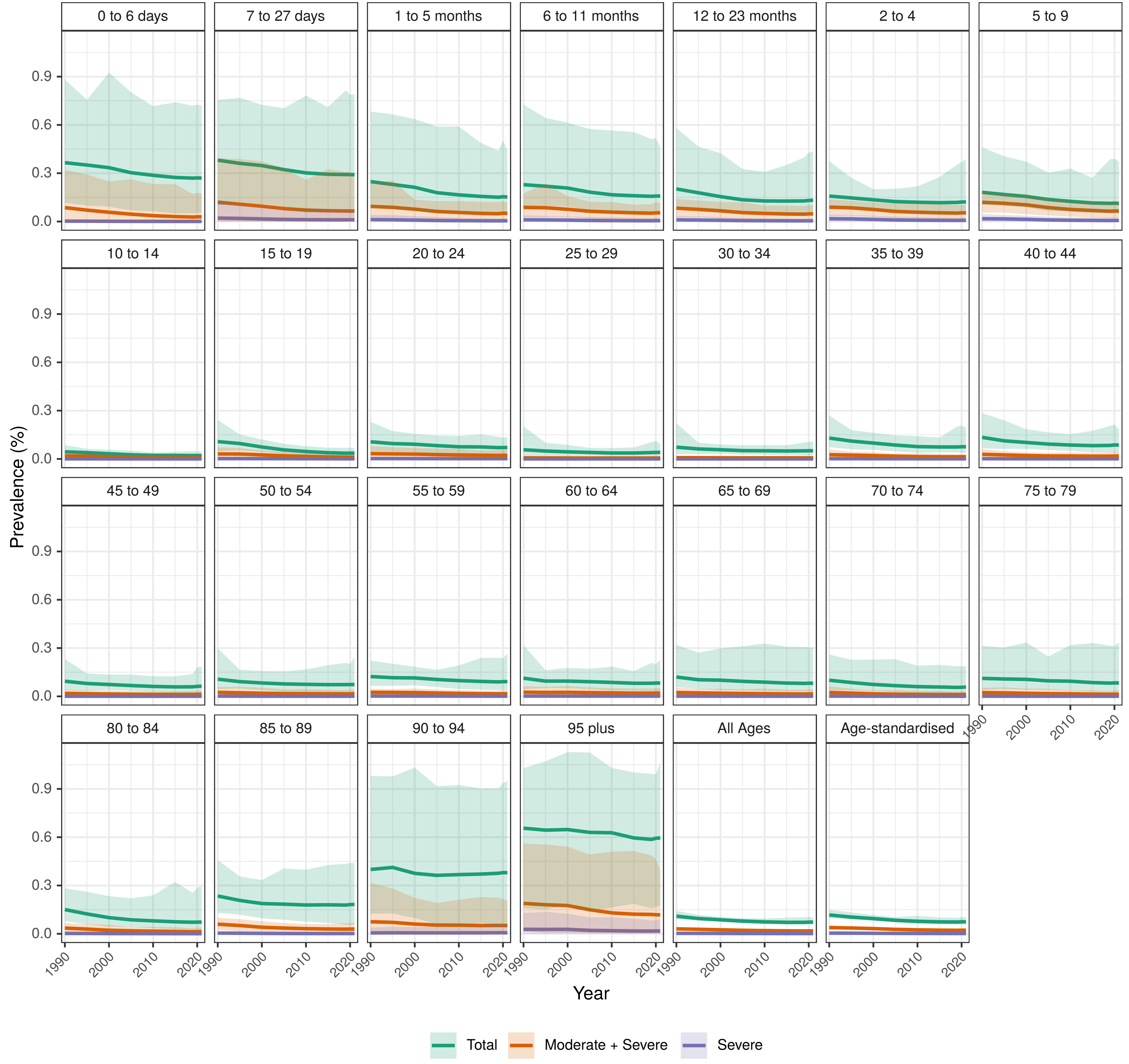

Cause-Specific Anaemia Prevalence - 2021

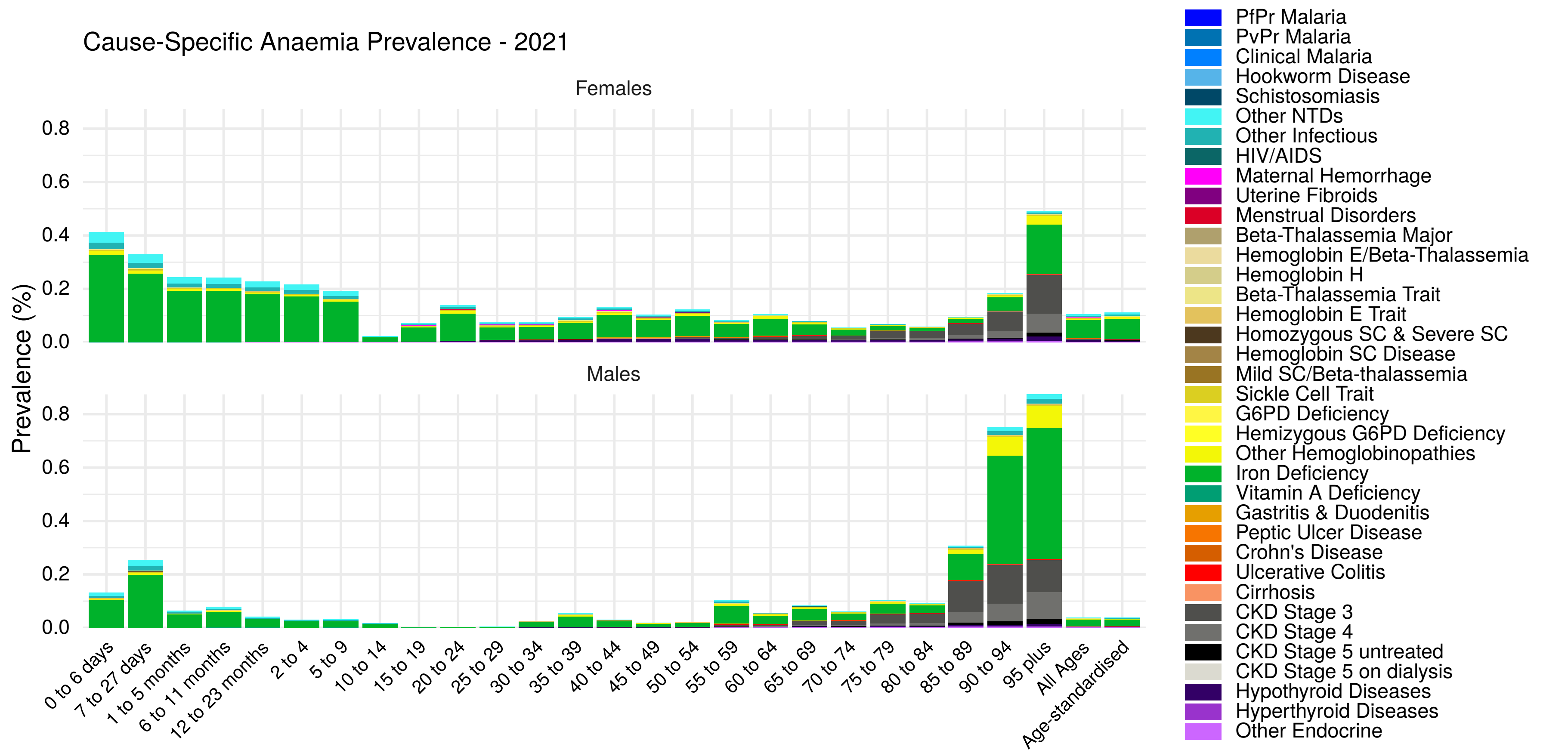

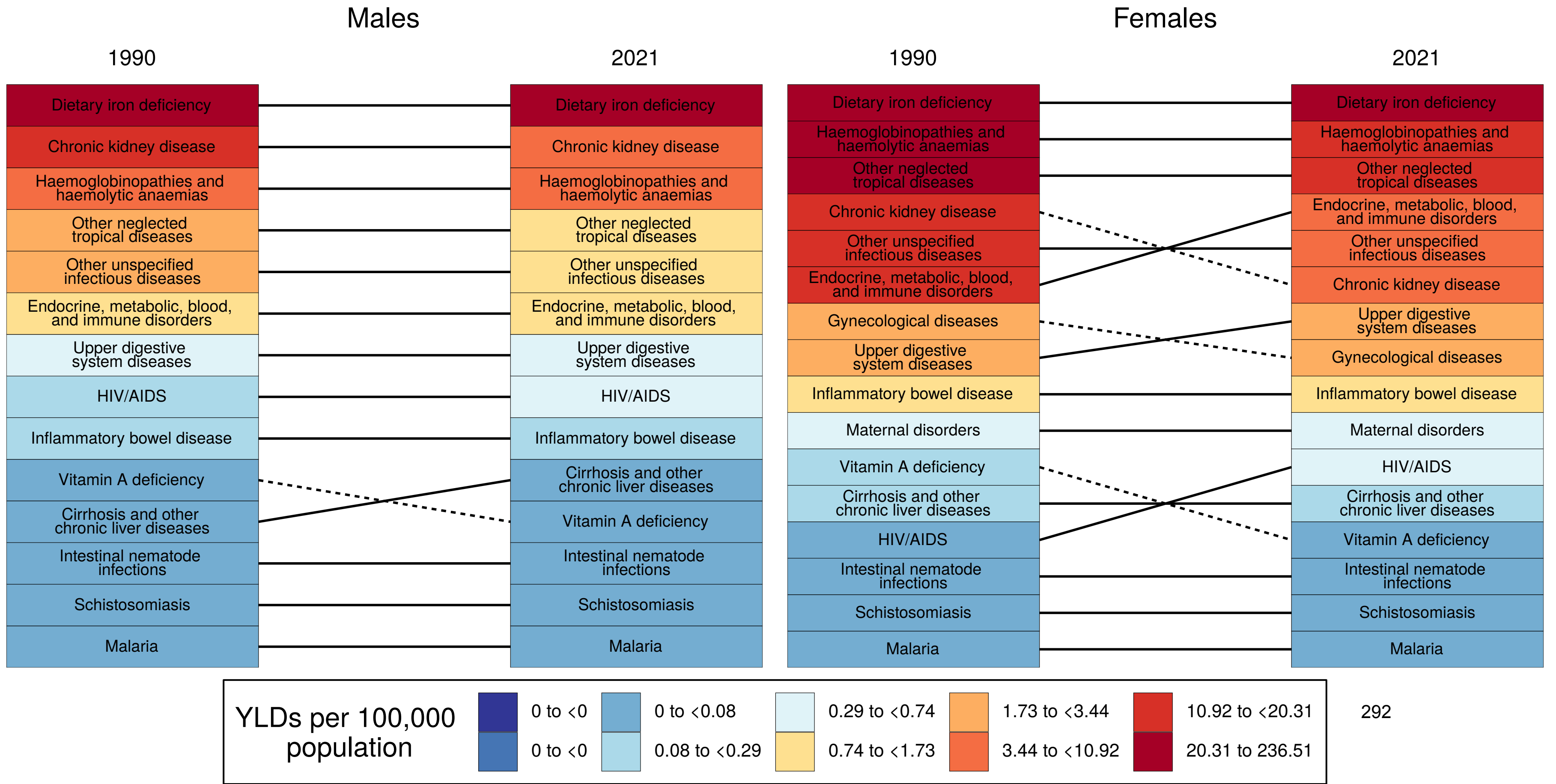

Haringey

Anaemia Prevalence - Both Sexes

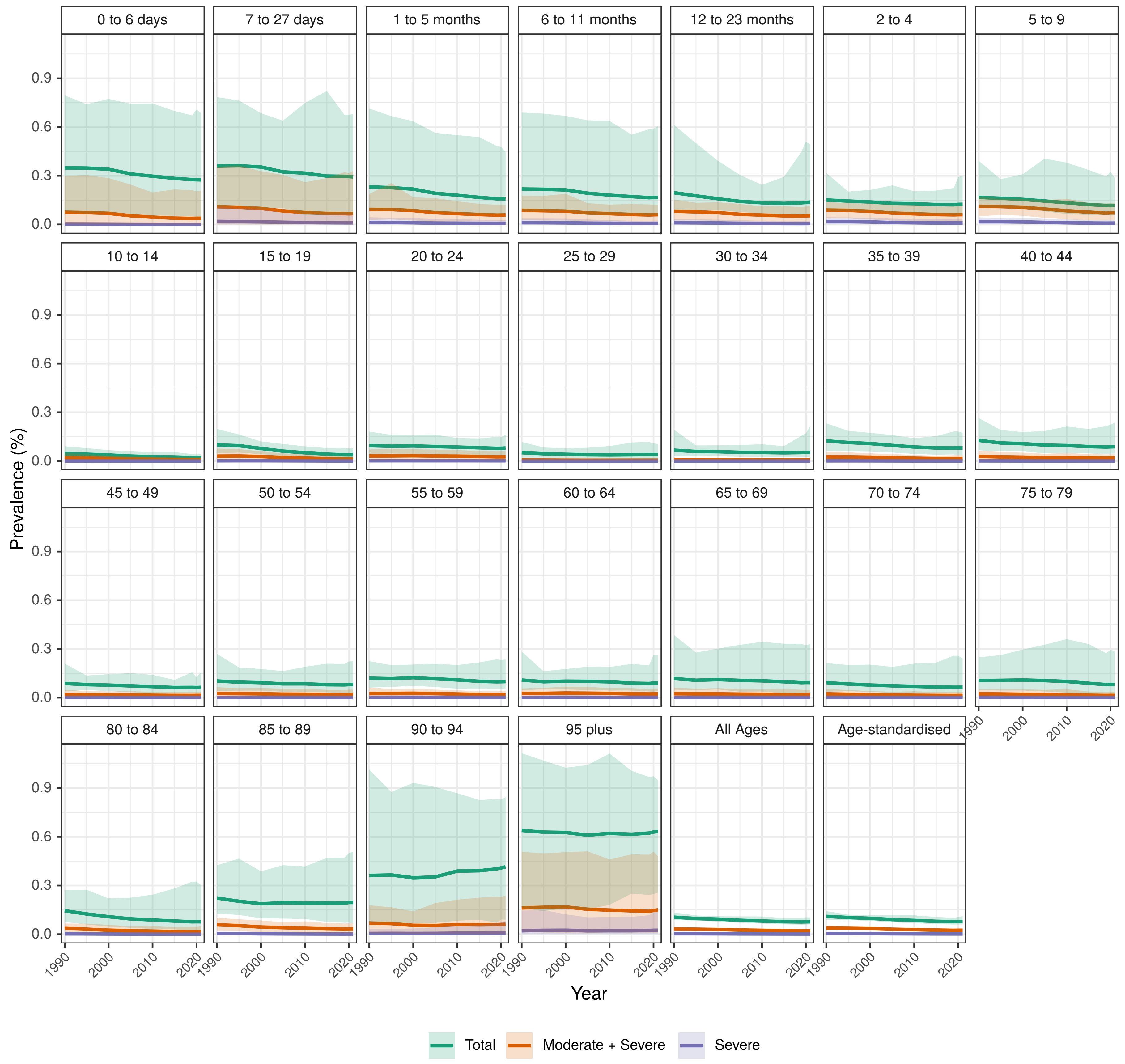

Cause-Specific Anaemia Prevalence - 2021

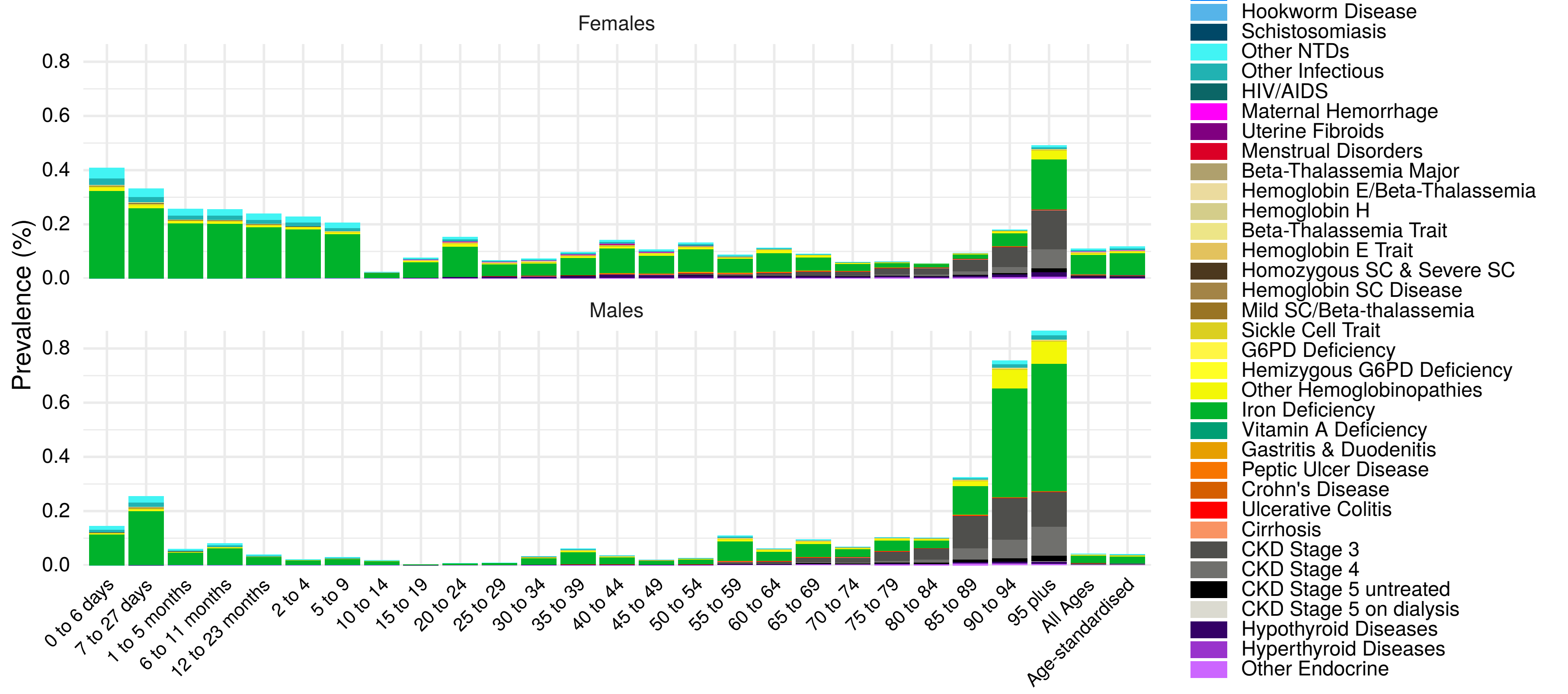

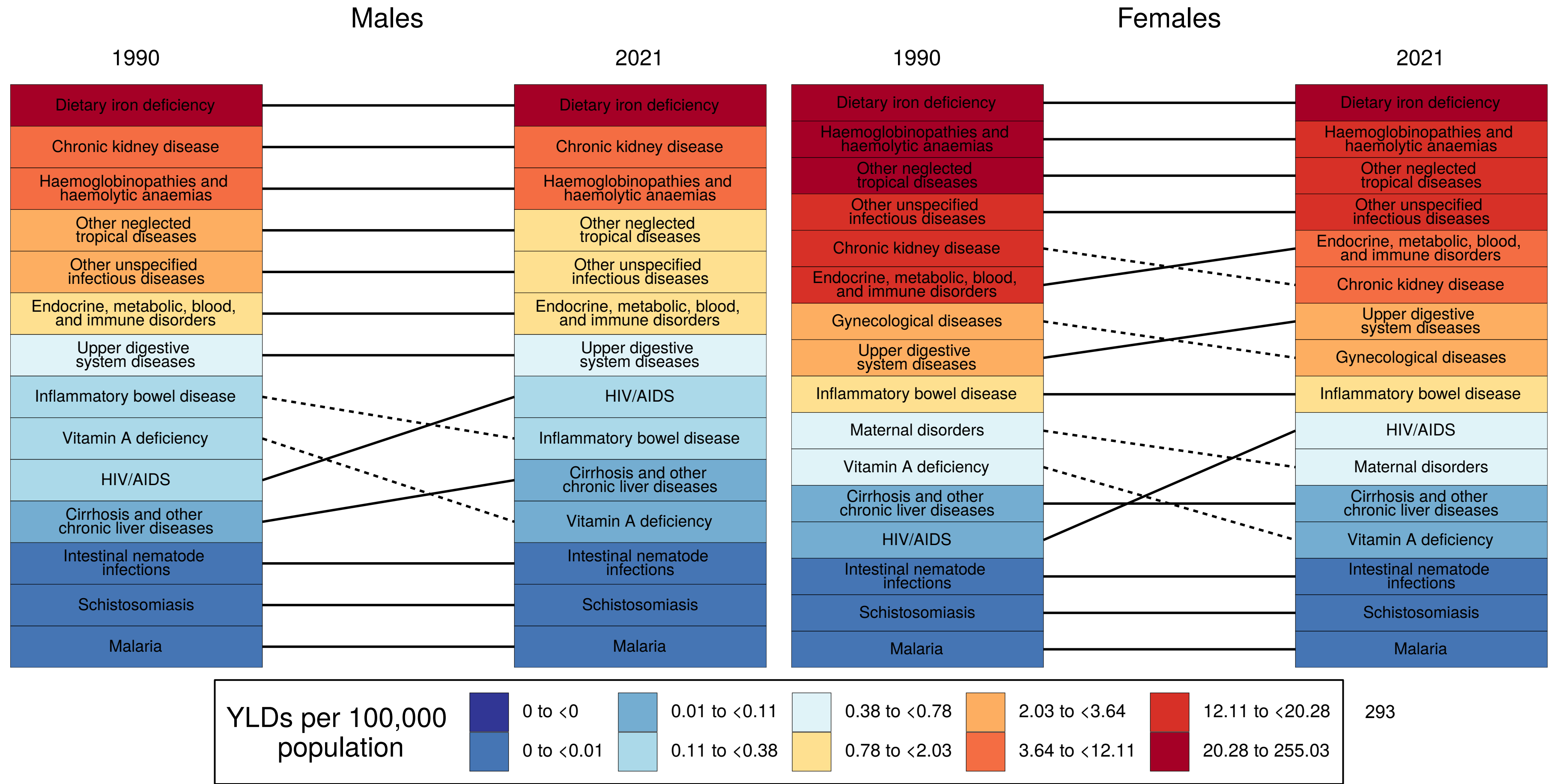

Harrow

Anaemia Prevalence - Both Sexes

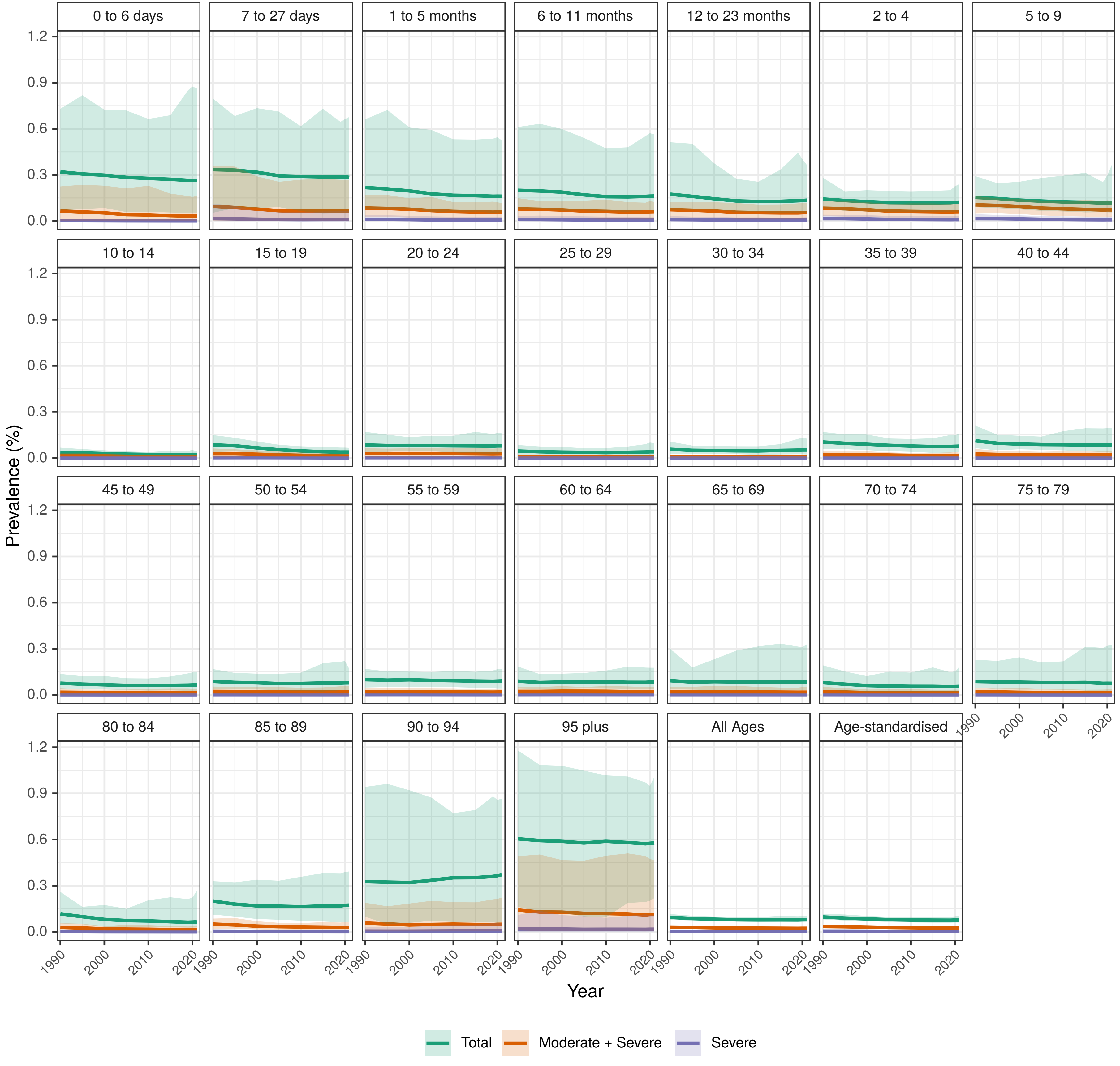

Cause-Specific Anaemia Prevalence - 2021

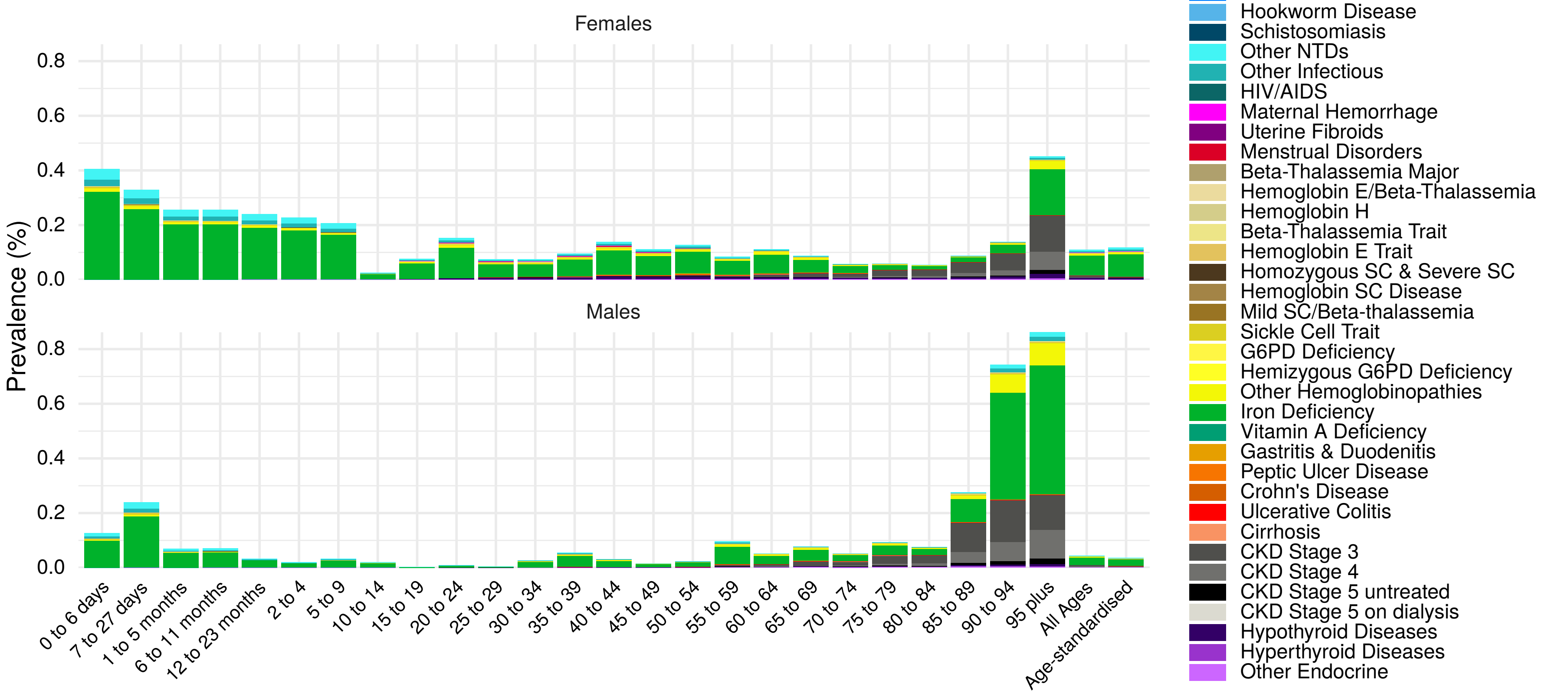

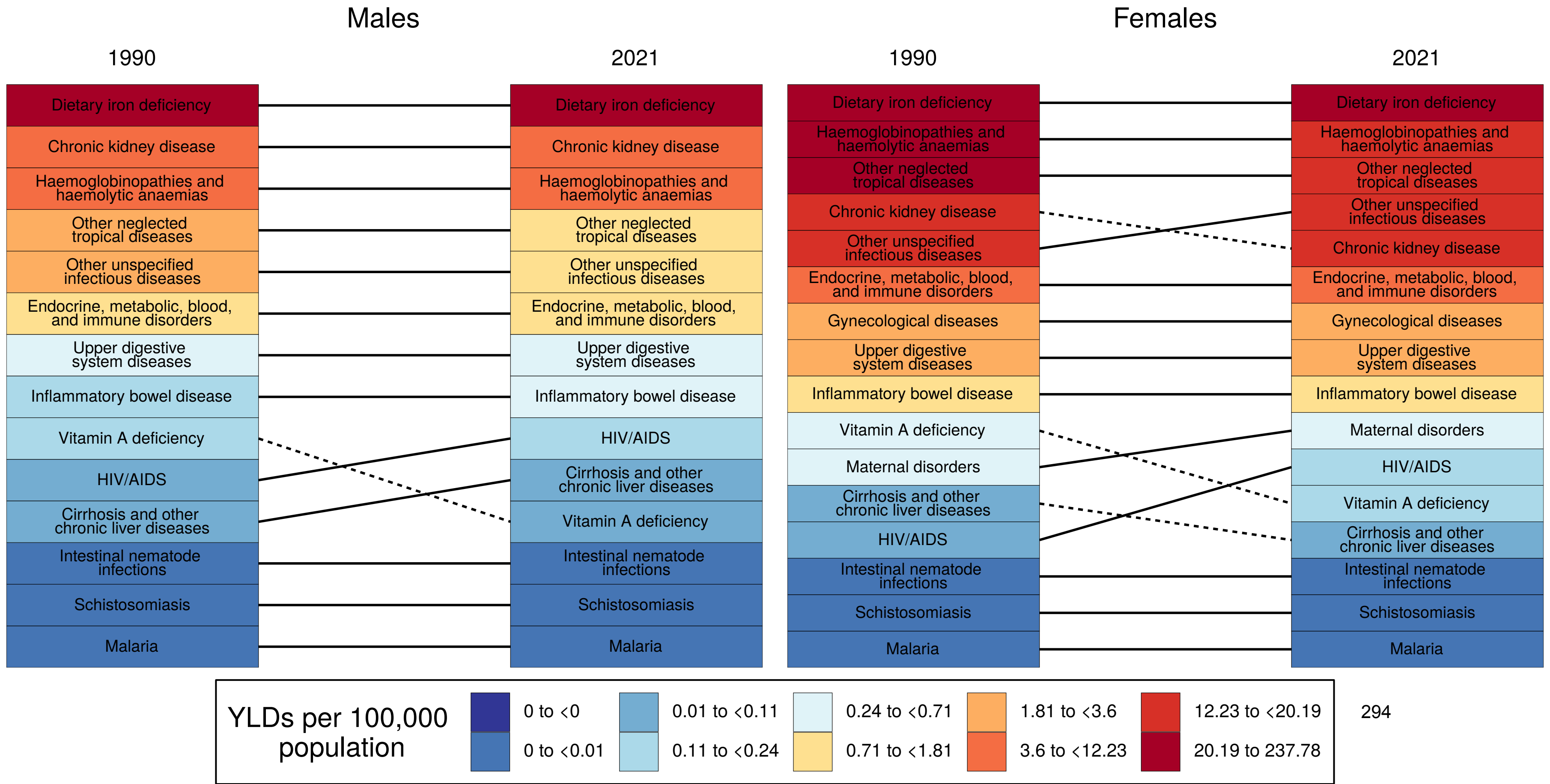

Havering

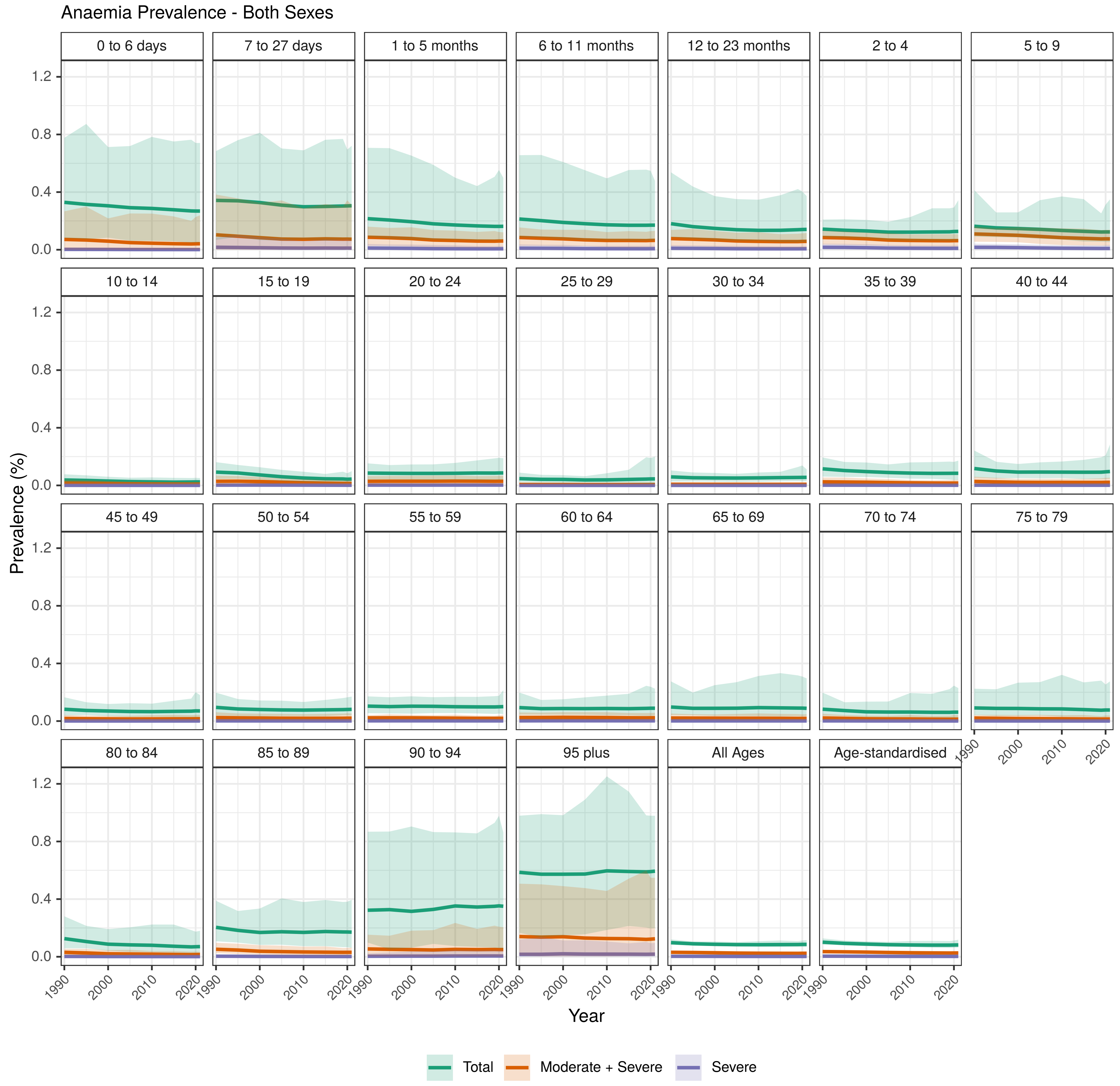

Anaemia Prevalence - Both Sexes
0 to 6 days
7 to 27 days
1 to 5 months
6 to 11 months
12 to 23 months
2 to 4
5 to 9
10 to 14
15 to 19
20 to 24
25 to 29
30 to 34
35 to 39
40 to 44
45 to 49
50 to 54
55 to 59
60 to 64
65 to 69
70 to 74
75 to 79
80 to 84
85 to 89
90 to 94
95 plus
All Ages
Age-standardised
Prevalence (%)
0.0
0.4
0.8
1.2
1990
2000
2010
2020
Year
Total
Moderate + Severe
Severe

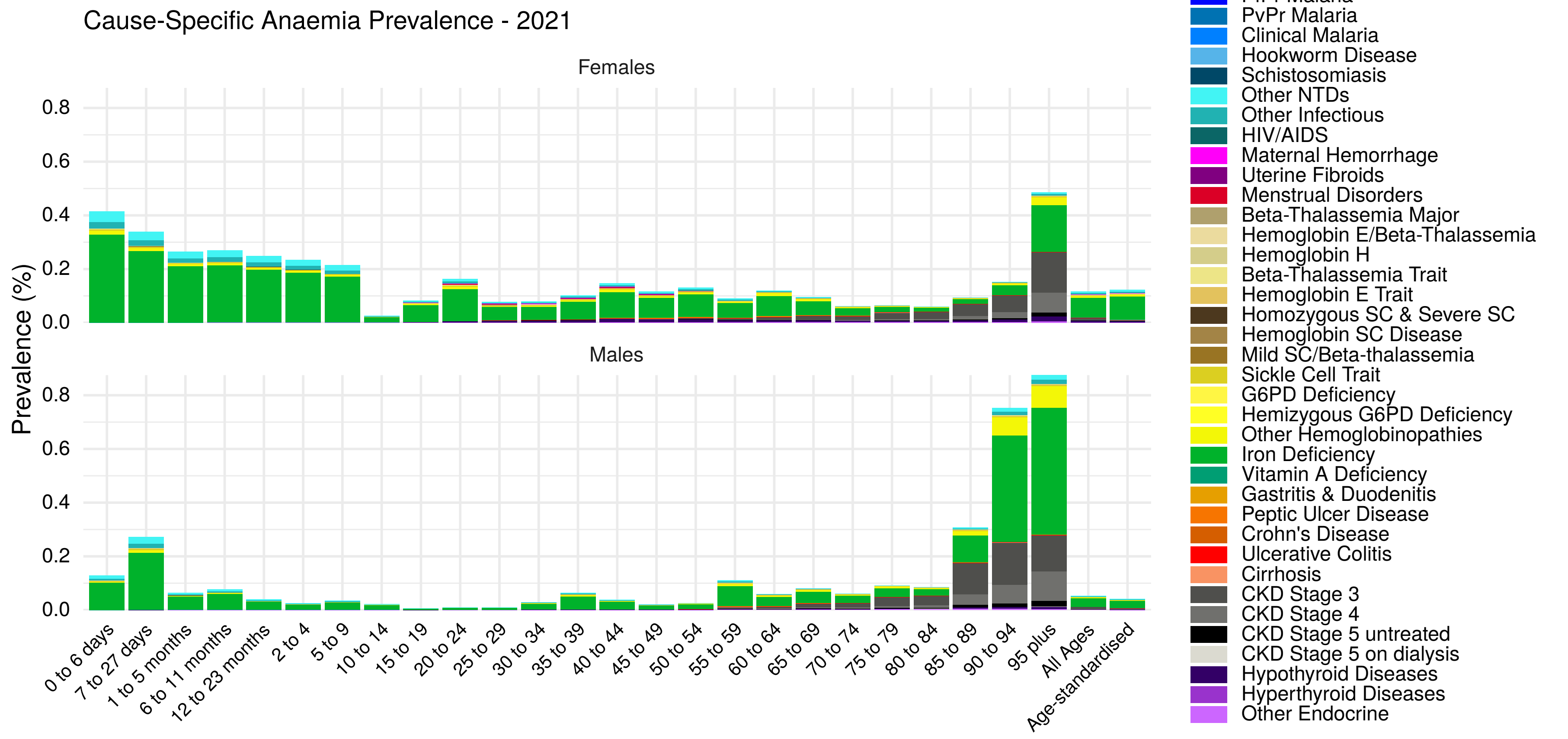

Cause-Specific Anaemia Prevalence - 2021
Females
Males
Prevalence (%)
0.0
0.2
0.4
0.6
0.8
0 to 6 days
7 to 27 days
1 to 5 months
6 to 11 months
12 to 23 months
2 to 4
5 to 9
10 to 14
15 to 19
20 to 24
25 to 29
30 to 34
35 to 39
40 to 44
45 to 49
50 to 54
55 to 59
60 to 64
65 to 69
70 to 74
75 to 79
80 to 84
85 to 89
90 to 94
95 plus
All Ages
Age-standardised
PfPr Malaria
PvPr Malaria
Clinical Malaria
Hookworm Disease
Schistosomiasis
Other NTDs
Other Infectious
HIV/AIDS
Maternal Hemorrhage
Uterine Fibroids
Menstrual Disorders
Beta-Thalassemia Major
Hemoglobin E/Beta-Thalassemia
Hemoglobin H
Beta-Thalassemia Trait
Hemoglobin E Trait
Homozygous SC & Severe SC
Hemoglobin SC Disease
Mild SC/Beta-thalassemia
Sickle Cell Trait
G6PD Deficiency
Hemizygous G6PD Deficiency
Other Hemoglobinopathies
Iron Deficiency
Vitamin A Deficiency
Gastritis & Duodenitis
Peptic Ulcer Disease
Crohn's Disease
Ulcerative Colitis
Cirrhosis
CKD Stage 3
CKD Stage 4
CKD Stage 5 untreated
CKD Stage 5 on dialysis
Hypothyroid Diseases
Hyperthyroid Diseases
Other Endocrine

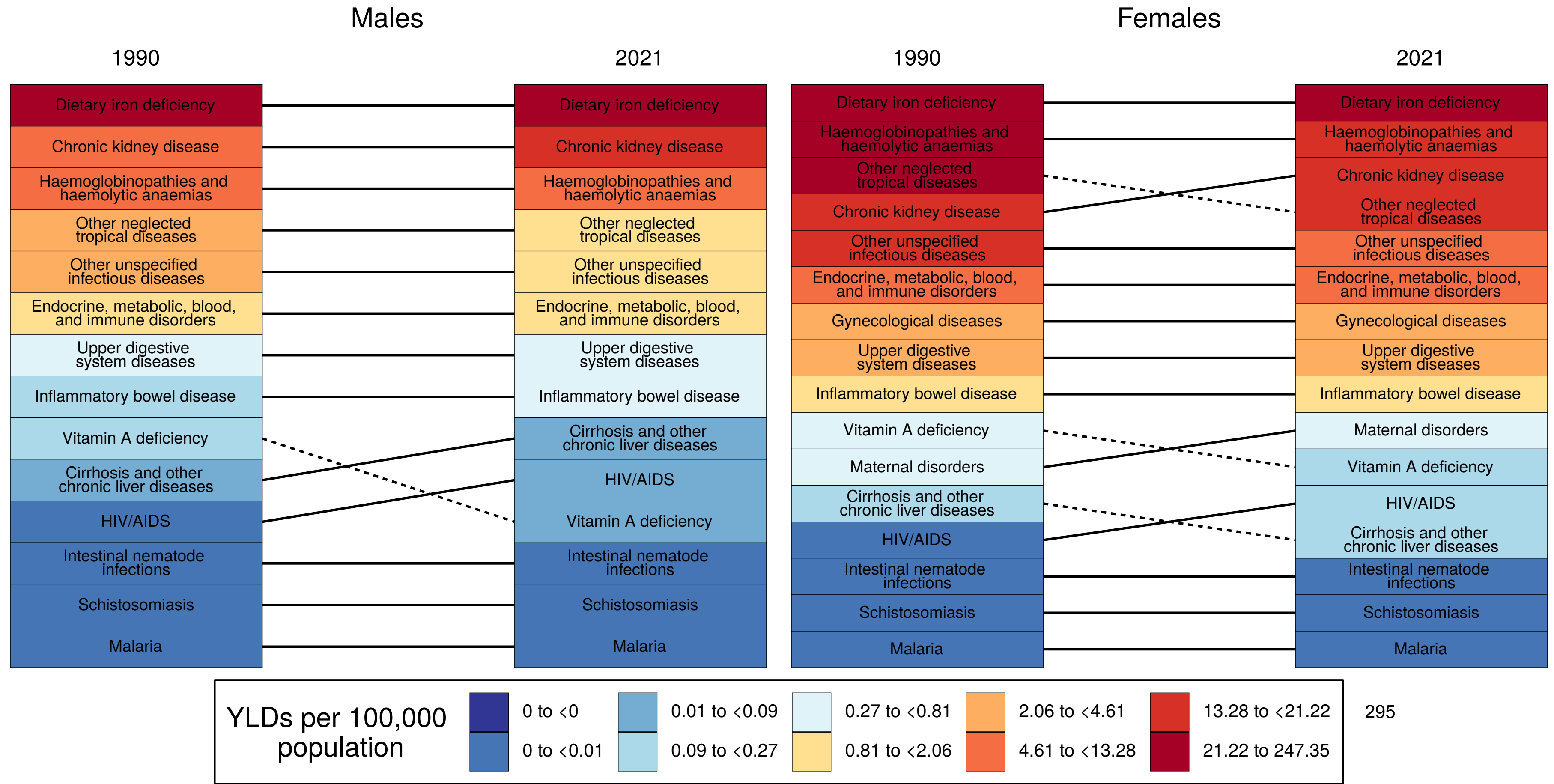

Males
Females
1990
2021
Dietary iron deficiency
Chronic kidney disease
Haemoglobinopathies and haemolytic anaemias
Other neglected tropical diseases
Other unspecified infectious diseases
Endocrine, metabolic, blood, and immune disorders
Upper digestive system diseases
Inflammatory bowel disease
Vitamin A deficiency
Cirrhosis and other chronic liver diseases
HIV/AIDS
Intestinal nematode infections
Schistosomiasis
Malaria
Gynecological diseases
Maternal disorders
YLDs per 100,000 population
0 to <0
0 to <0.01
0.01 to <0.09
0.09 to <0.27
0.27 to <0.81
0.81 to <2.06
2.06 to <4.61
4.61 to <13.28
13.28 to <21.22
21.22 to 247.35

Hillingdon

Anaemia Prevalence - Both Sexes

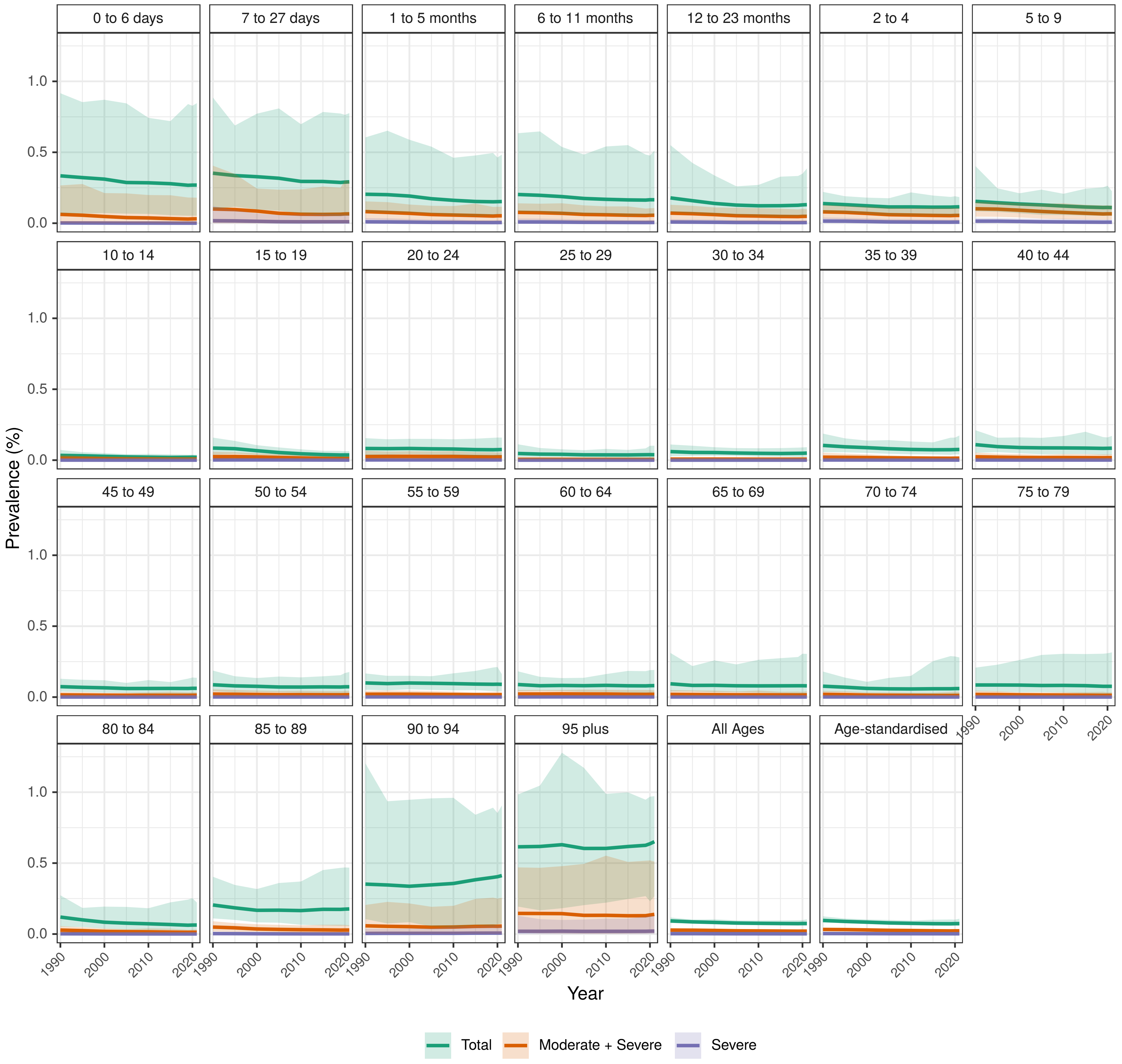

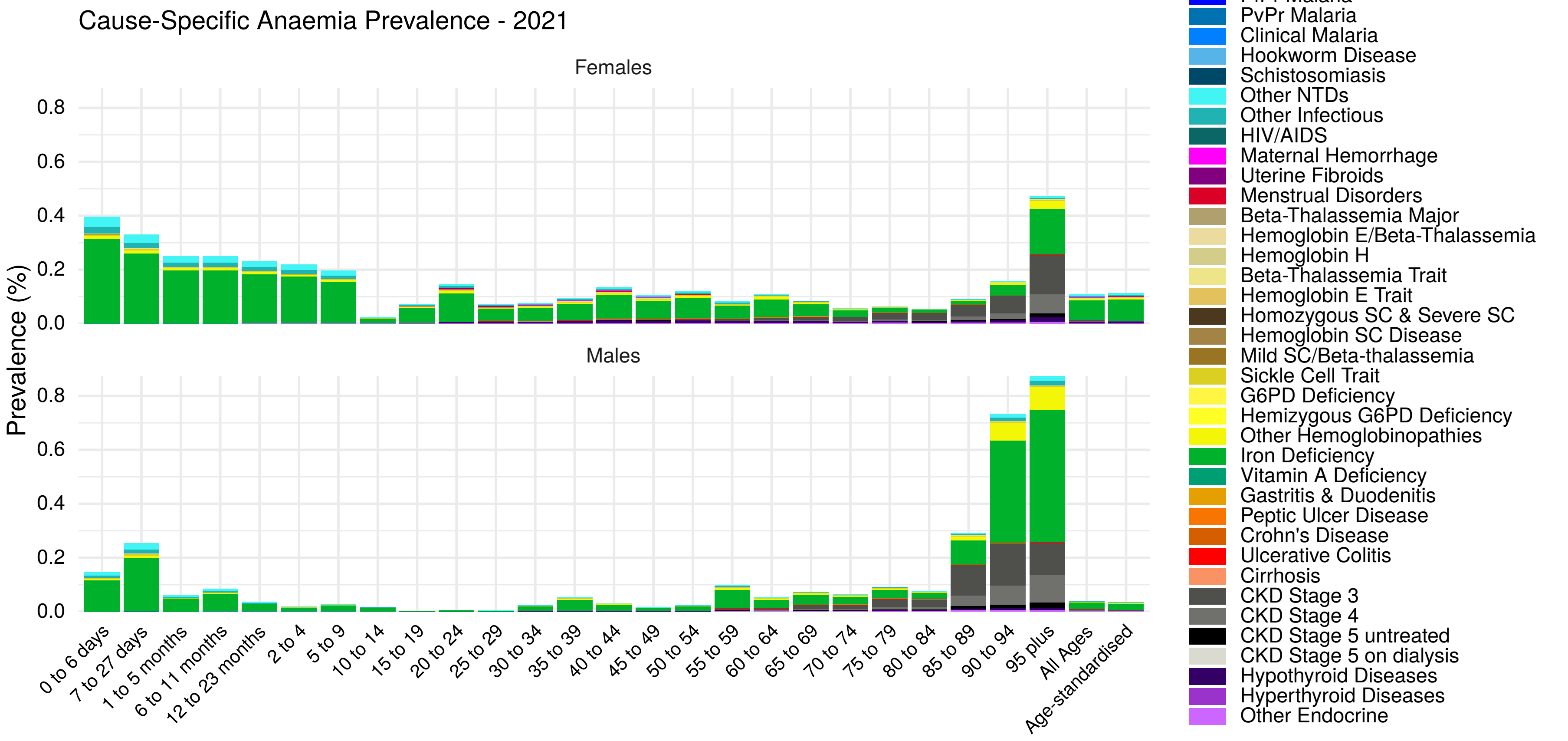

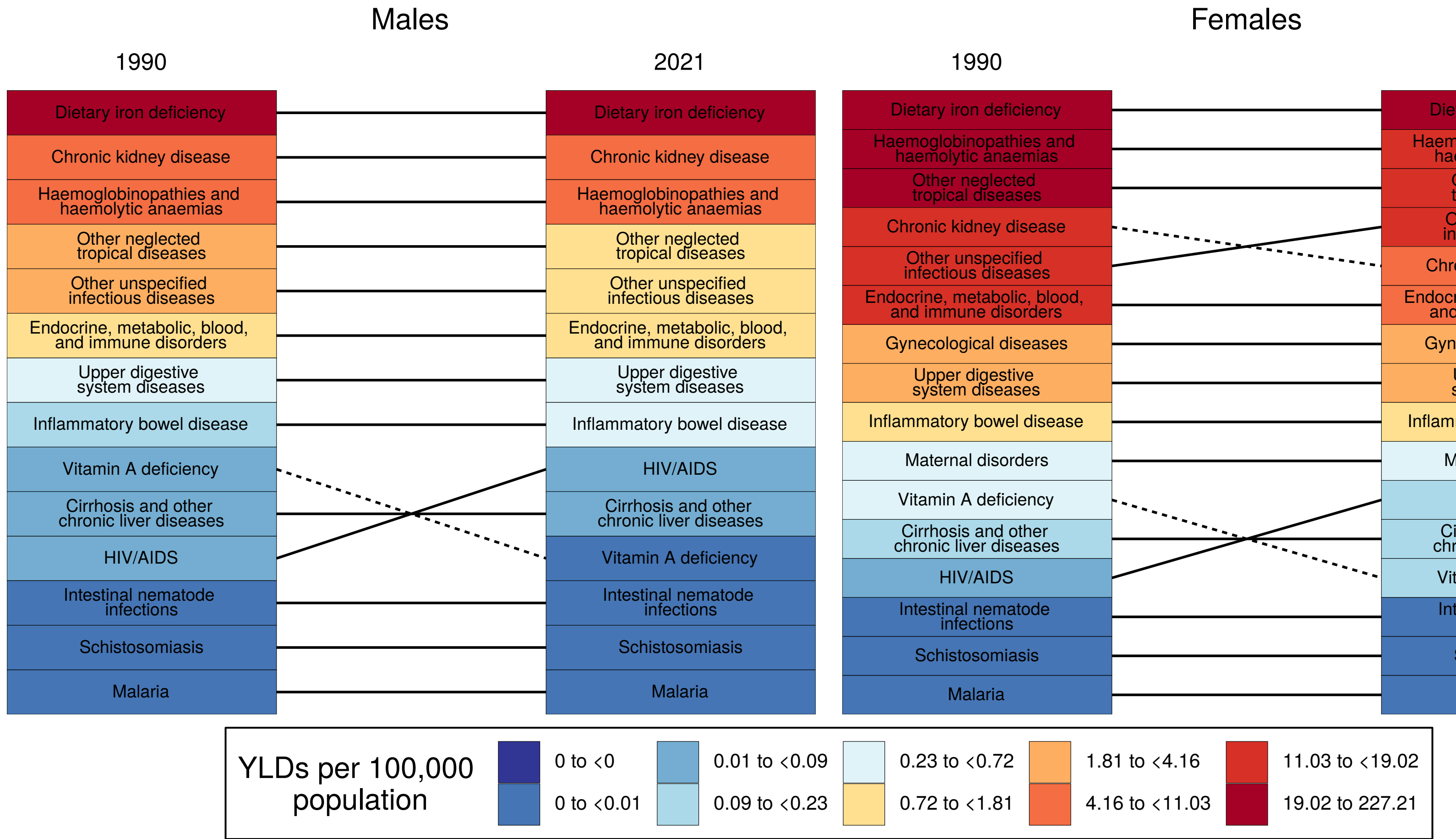

Hounslow

Anaemia Prevalence - Both Sexes

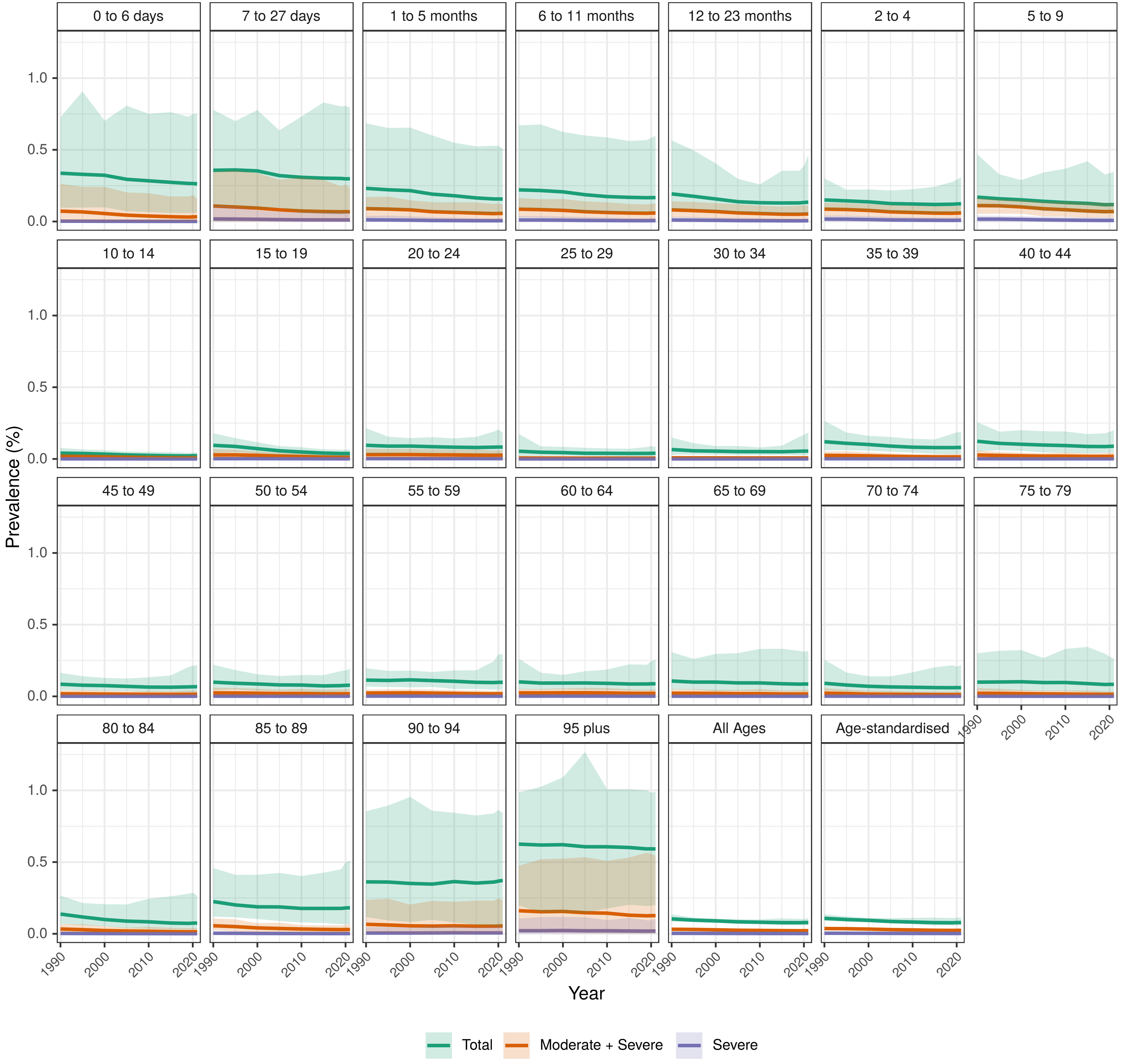

Cause-Specific Anaemia Prevalence - 2021

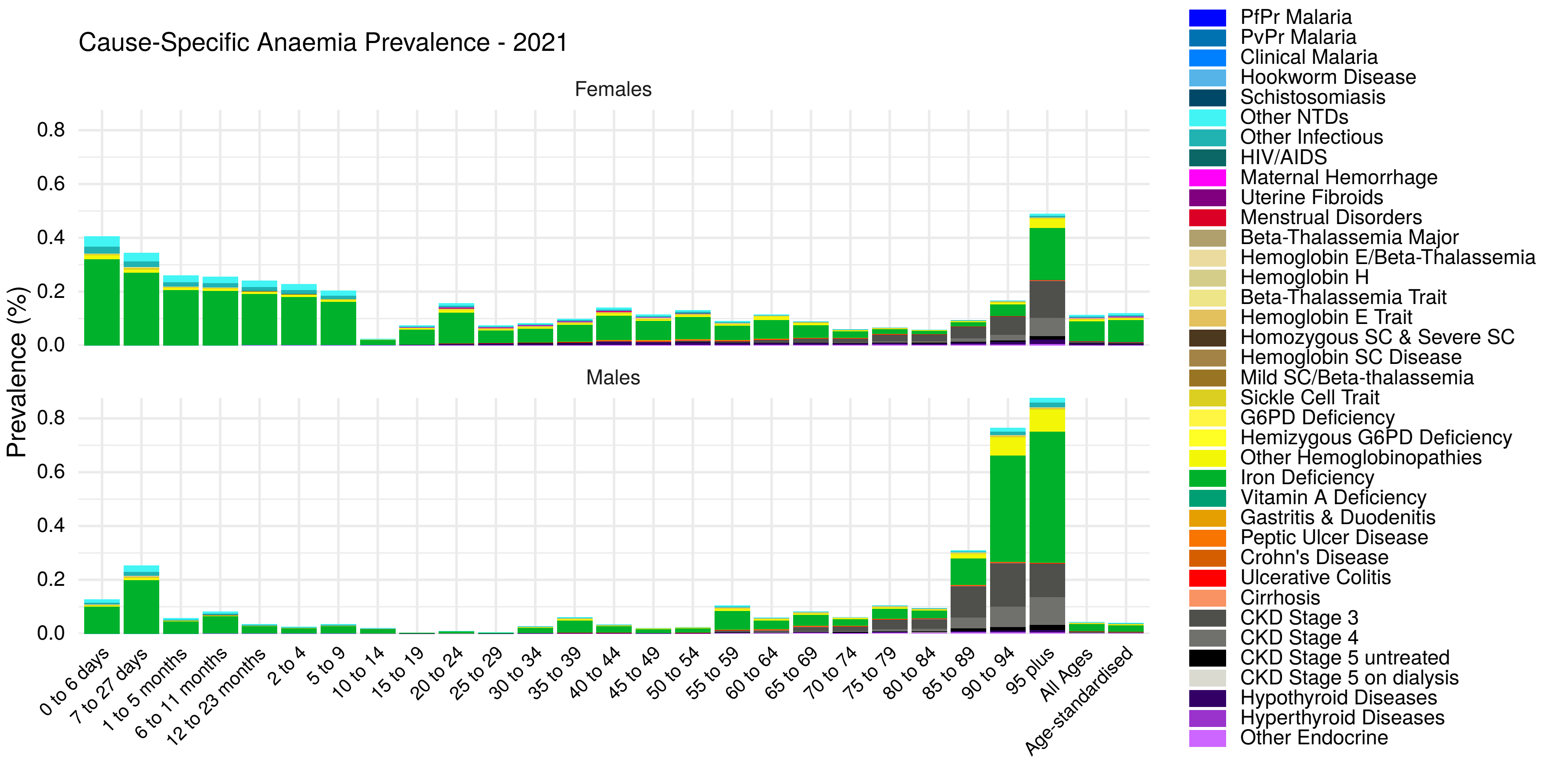

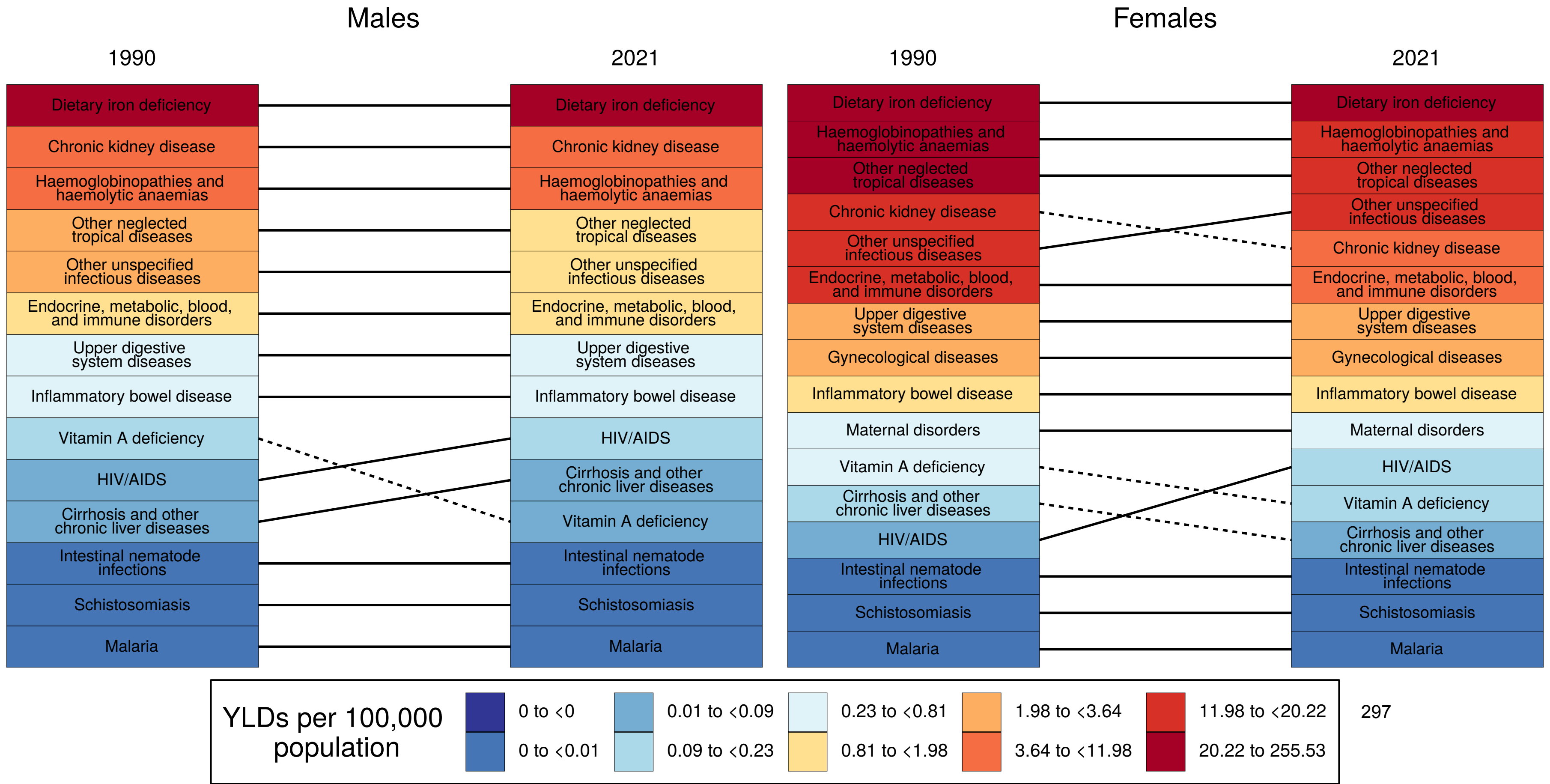

Islington

Anaemia Prevalence - Both Sexes

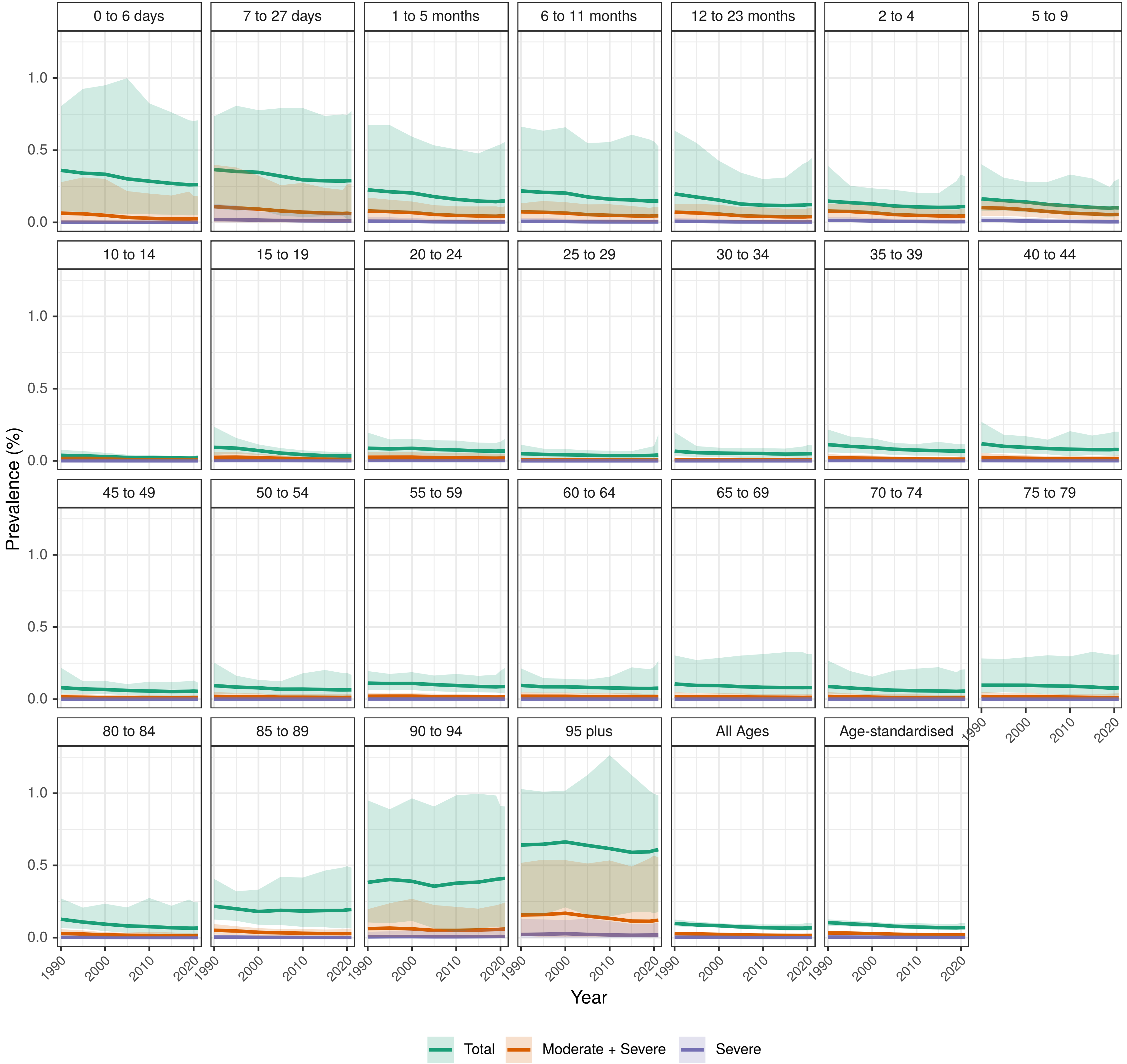

Cause-Specific Anaemia Prevalence - 2021

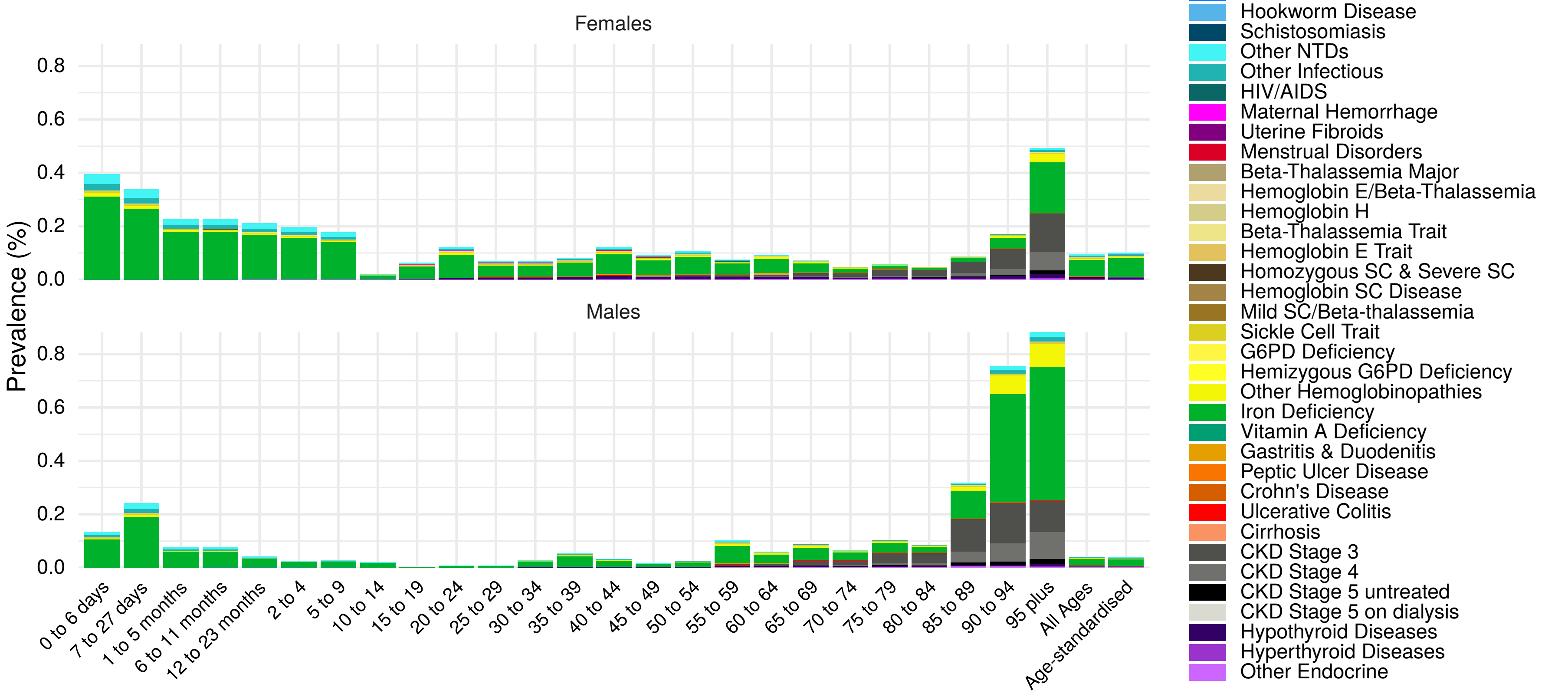

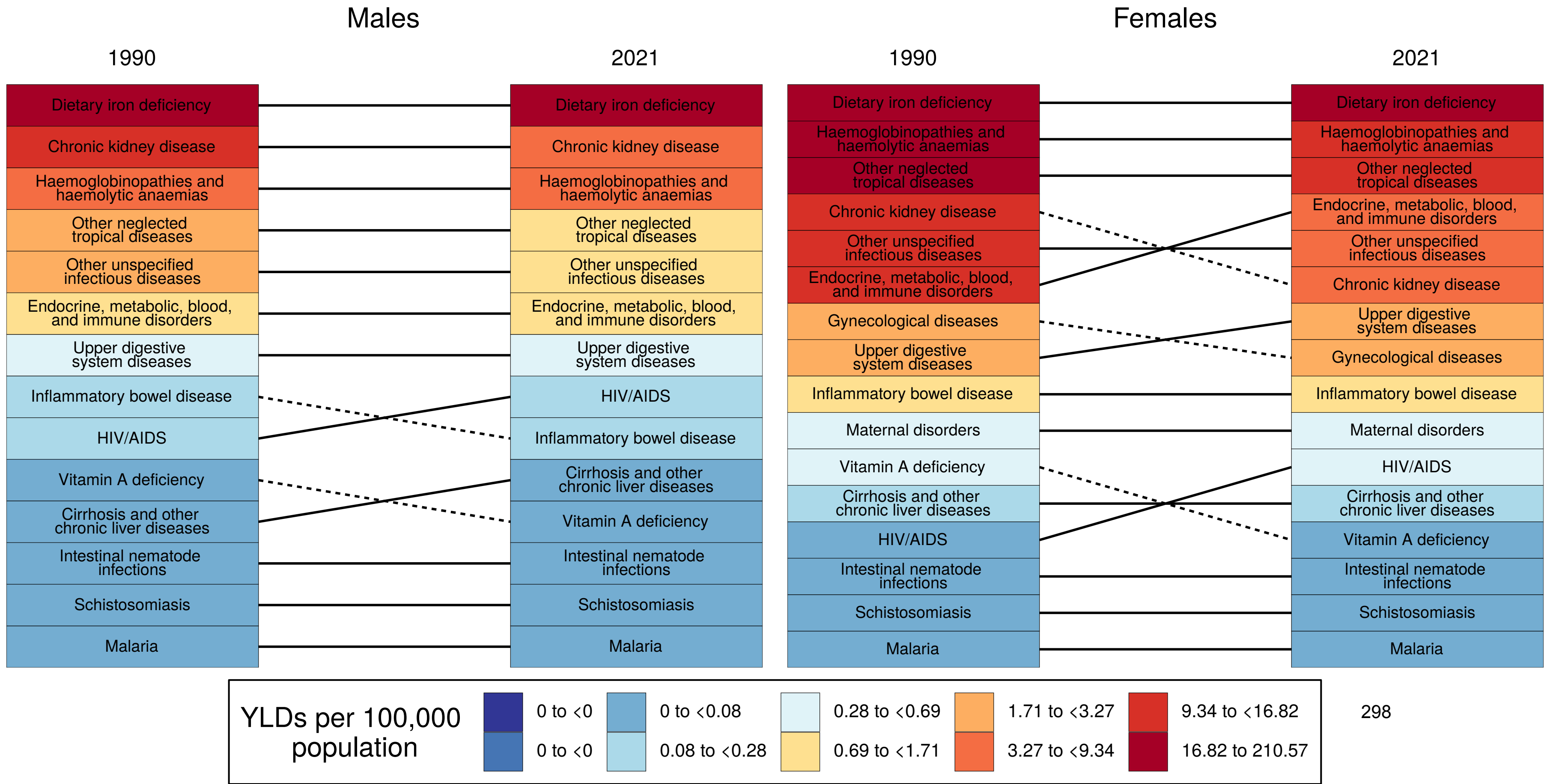

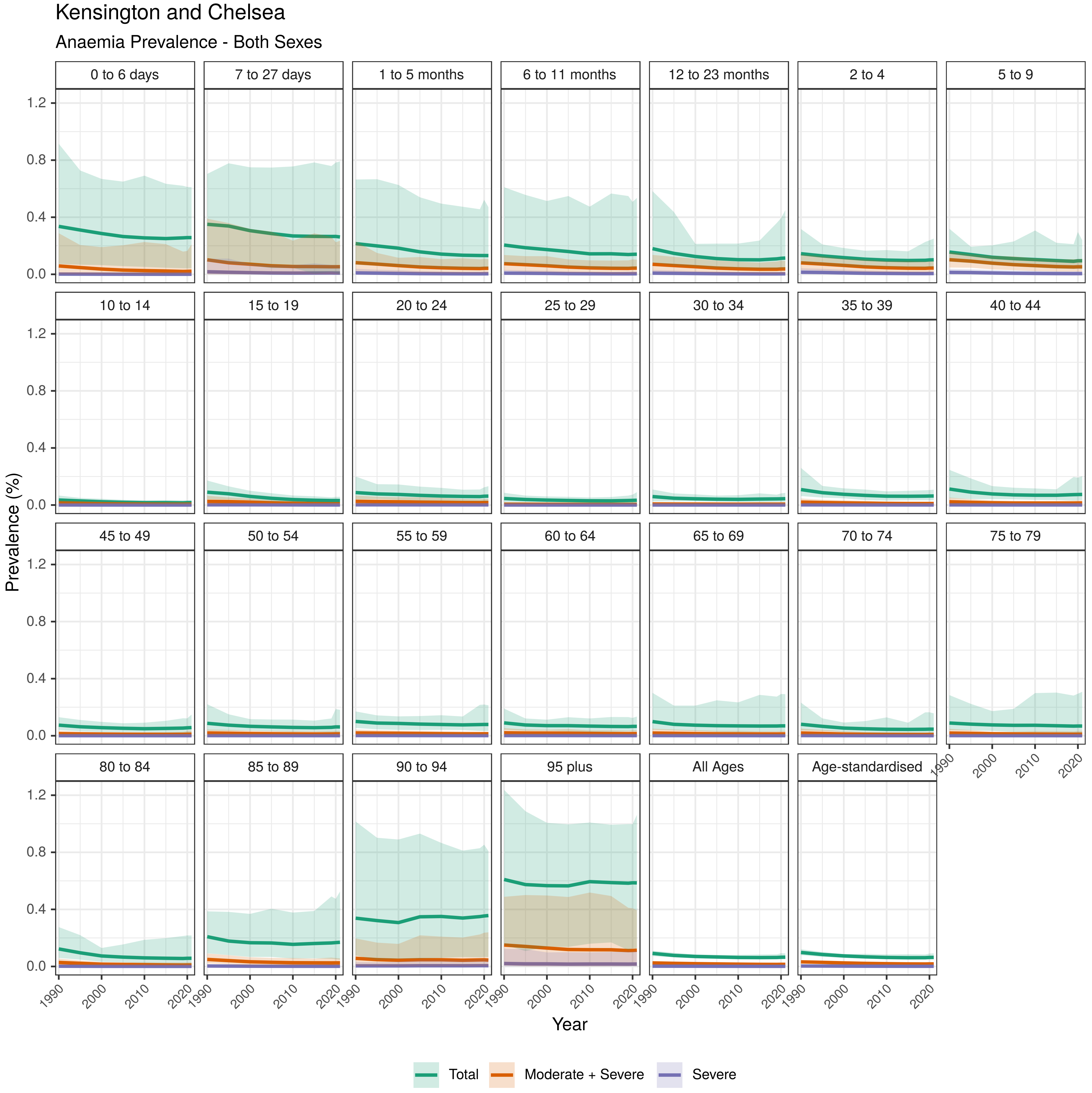

Kensington and Chelsea
Anaemia Prevalence - Both Sexes
0 to 6 days
7 to 27 days
1 to 5 months
6 to 11 months
12 to 23 months
2 to 4
5 to 9
10 to 14
15 to 19
20 to 24
25 to 29
30 to 34
35 to 39
40 to 44
45 to 49
50 to 54
55 to 59
60 to 64
65 to 69
70 to 74
75 to 79
80 to 84
85 to 89
90 to 94
95 plus
All Ages
Age-standardised
Prevalence (%)
Year
Total
Moderate + Severe
Severe

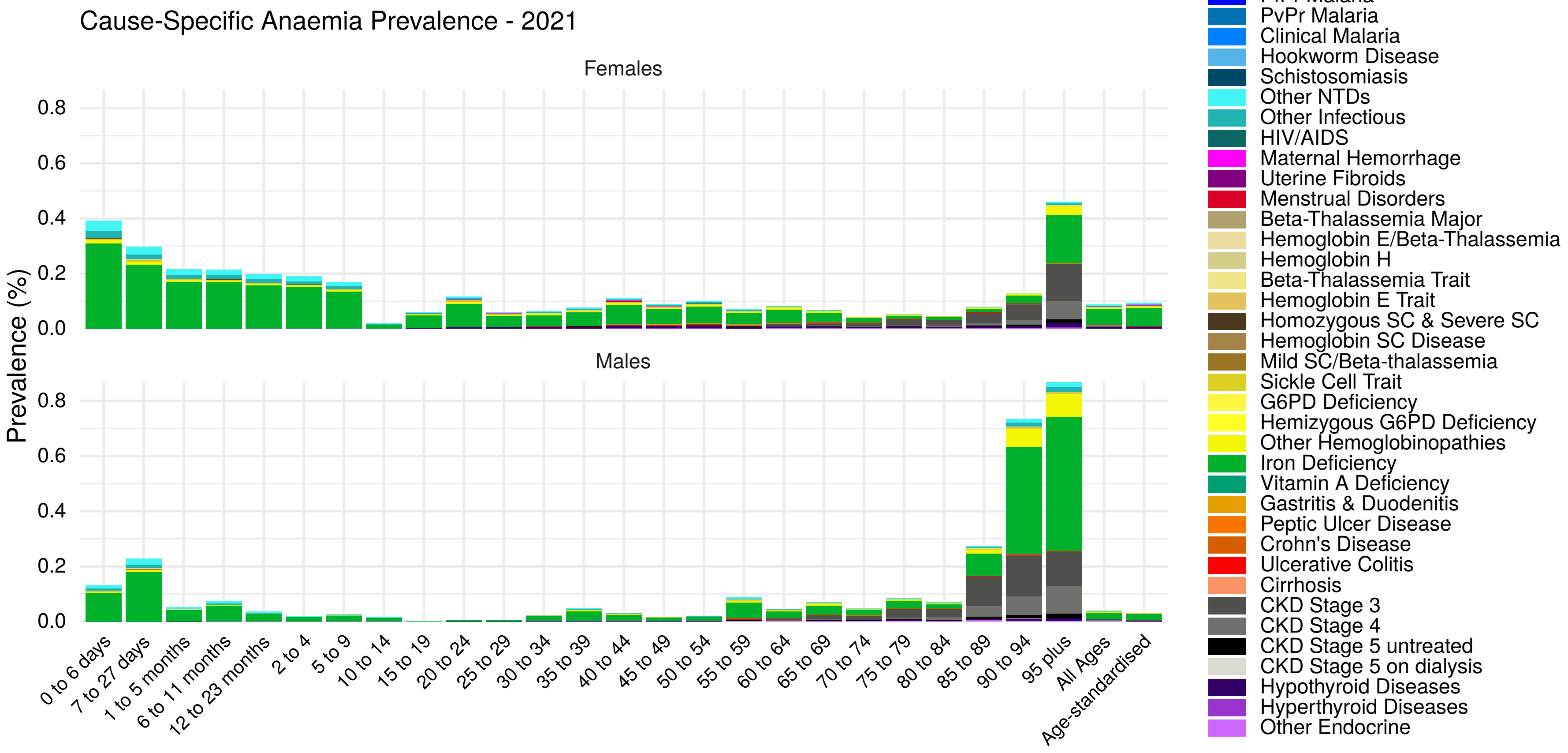

Cause-Specific Anaemia Prevalence - 2021
Females
Males
Prevalence (%)
PfPr Malaria
PvPr Malaria
Clinical Malaria
Hookworm Disease
Schistosomiasis
Other NTDs
Other Infectious
HIV/AIDS
Maternal Hemorrhage
Uterine Fibroids
Menstrual Disorders
Beta-Thalassemia Major
Hemoglobin E/Beta-Thalassemia
Hemoglobin H
Beta-Thalassemia Trait
Hemoglobin E Trait
Homozygous SC & Severe SC
Hemoglobin SC Disease
Mild SC/Beta-thalassemia
Sickle Cell Trait
G6PD Deficiency
Hemizygous G6PD Deficiency
Other Hemoglobinopathies
Iron Deficiency
Vitamin A Deficiency
Gastritis & Duodenitis
Peptic Ulcer Disease
Crohn's Disease
Ulcerative Colitis
Cirrhosis
CKD Stage 3
CKD Stage 4
CKD Stage 5 untreated
CKD Stage 5 on dialysis
Hypothyroid Diseases
Hyperthyroid Diseases
Other Endocrine

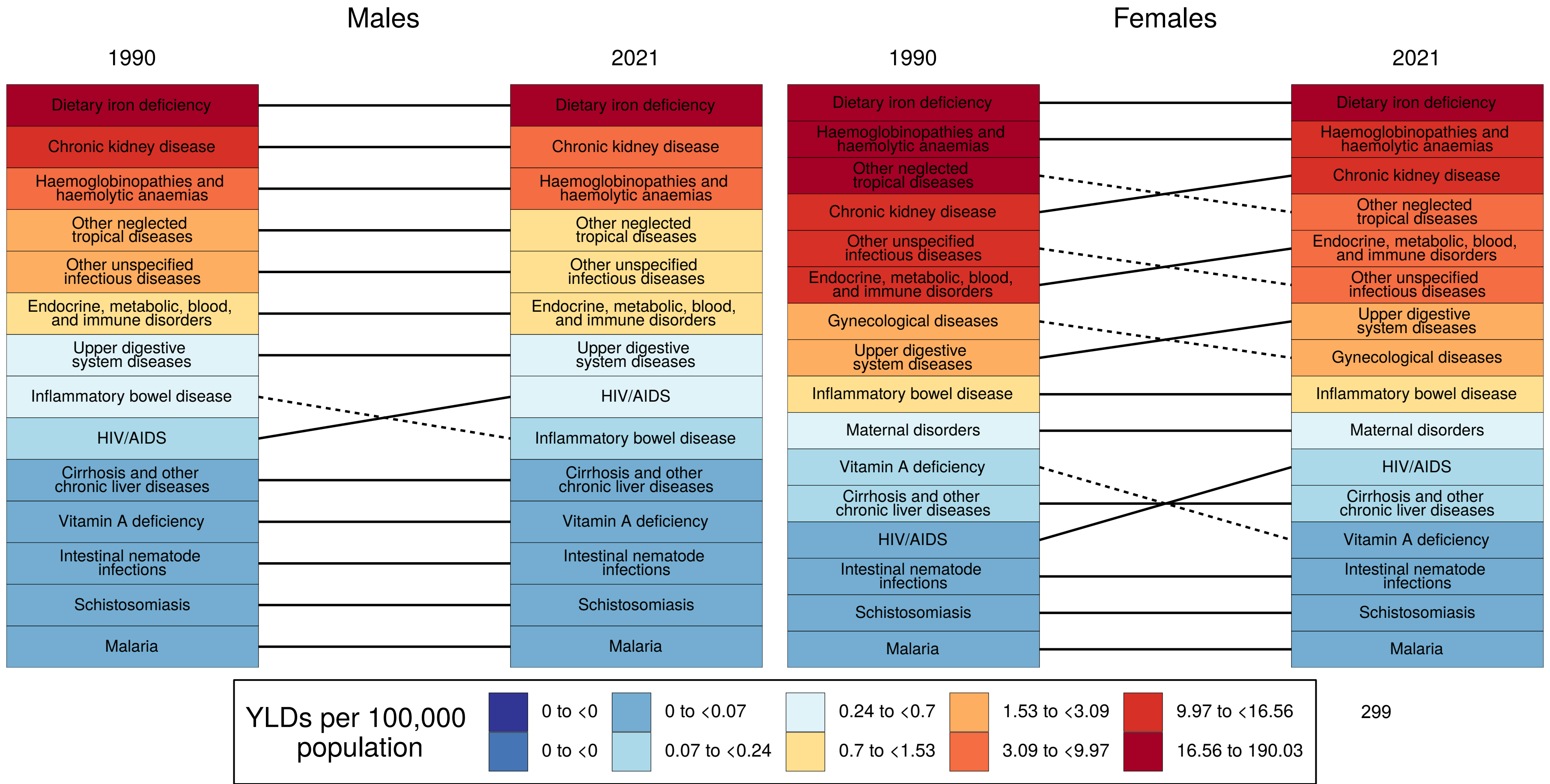

Males
Females
1990
2021
Dietary iron deficiency
Chronic kidney disease
Haemoglobinopathies and haemolytic anaemias
Other neglected tropical diseases
Other unspecified infectious diseases
Endocrine, metabolic, blood, and immune disorders
Upper digestive system diseases
Inflammatory bowel disease
HIV/AIDS
Cirrhosis and other chronic liver diseases
Vitamin A deficiency
Intestinal nematode infections
Schistosomiasis
Malaria
Gynecological diseases
Maternal disorders
YLDs per 100,000 population
0 to <0
0 to <0.07
0.07 to <0.24
0.24 to <0.7
0.7 to <1.53
1.53 to <3.09
3.09 to <9.97
9.97 to <16.56
16.56 to 190.03

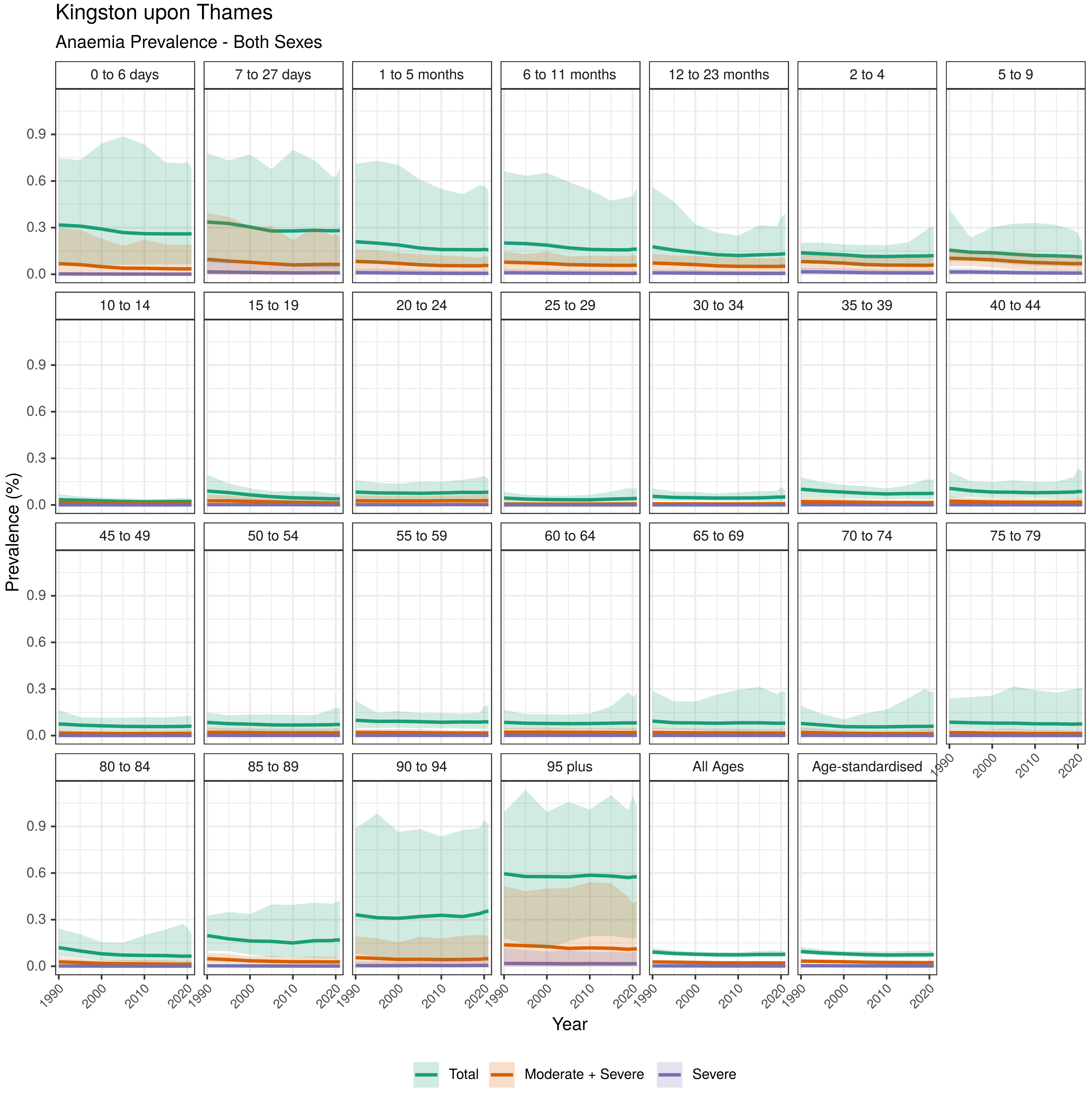
Kingston upon Thames
Anaemia Prevalence - Both Sexes
0 to 6 days
7 to 27 days
1 to 5 months
6 to 11 months
12 to 23 months
2 to 4
5 to 9
10 to 14
15 to 19
20 to 24
25 to 29
30 to 34
35 to 39
40 to 44
45 to 49
50 to 54
55 to 59
60 to 64
65 to 69
70 to 74
75 to 79
80 to 84
85 to 89
90 to 94
95 plus
All Ages
Age-standardised
Prevalence (%)
0.0
0.3
0.6
0.9
1990
2000
2010
2020
Year
Total
Moderate + Severe
Severe

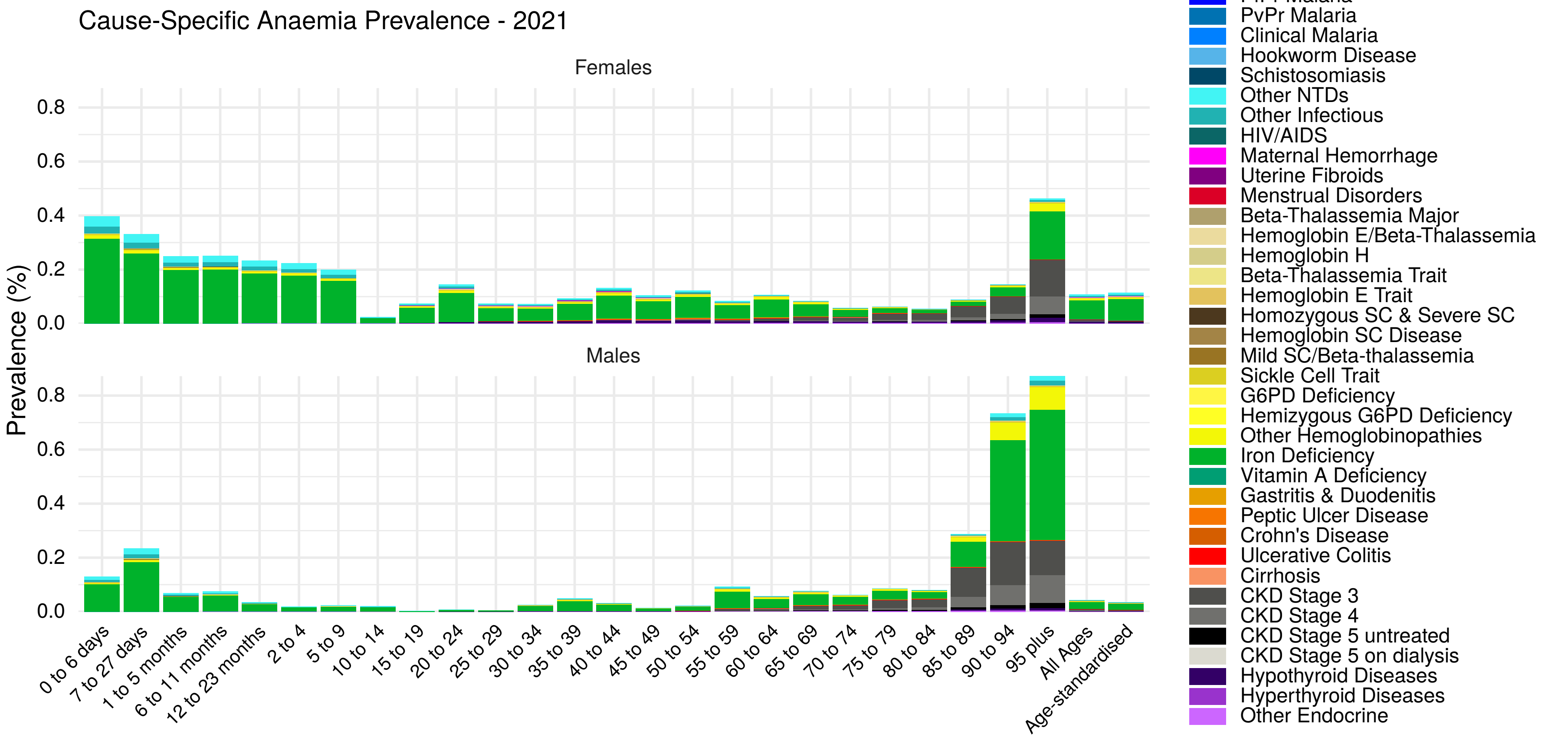
Cause-Specific Anaemia Prevalence - 2021
Females
Males
Prevalence (%)
0.0
0.2
0.4
0.6
0.8
0 to 6 days
7 to 27 days
1 to 5 months
6 to 11 months
12 to 23 months
2 to 4
5 to 9
10 to 14
15 to 19
20 to 24
25 to 29
30 to 34
35 to 39
40 to 44
45 to 49
50 to 54
55 to 59
60 to 64
65 to 69
70 to 74
75 to 79
80 to 84
85 to 89
90 to 94
95 plus
All Ages
Age-standardised
PfPr Malaria
PvPr Malaria
Clinical Malaria
Hookworm Disease
Schistosomiasis
Other NTDs
Other Infectious
HIV/AIDS
Maternal Hemorrhage
Uterine Fibroids
Menstrual Disorders
Beta-Thalassemia Major
Hemoglobin E/Beta-Thalassemia
Hemoglobin H
Beta-Thalassemia Trait
Hemoglobin E Trait
Homozygous SC & Severe SC
Hemoglobin SC Disease
Mild SC/Beta-thalassemia
Sickle Cell Trait
G6PD Deficiency
Hemizygous G6PD Deficiency
Other Hemoglobinopathies
Iron Deficiency
Vitamin A Deficiency
Gastritis & Duodenitis
Peptic Ulcer Disease
Crohn's Disease
Ulcerative Colitis
Cirrhosis
CKD Stage 3
CKD Stage 4
CKD Stage 5 untreated
CKD Stage 5 on dialysis
Hypothyroid Diseases
Hyperthyroid Diseases
Other Endocrine

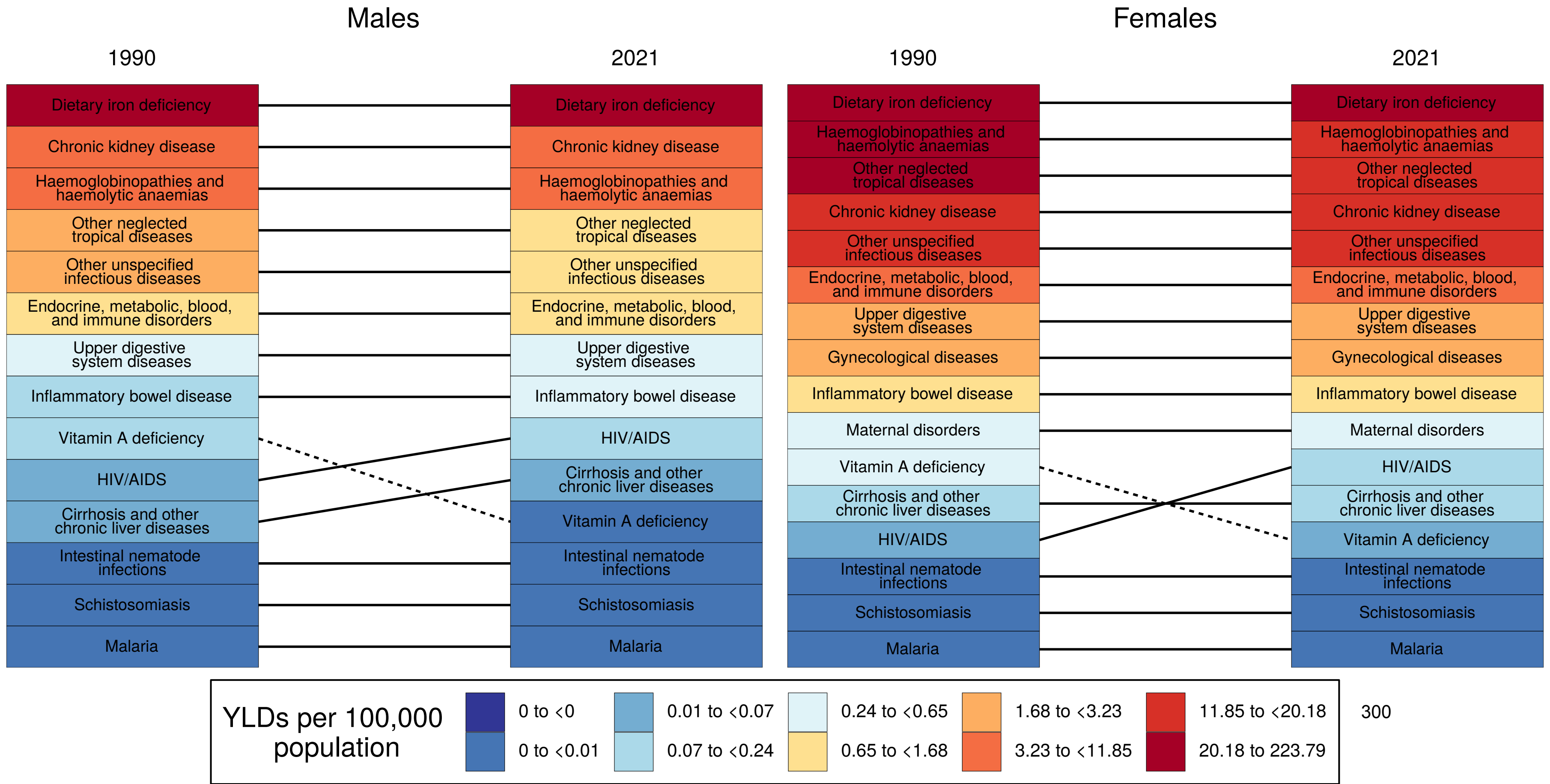
Males
Females
1990
2021
Dietary iron deficiency
Chronic kidney disease
Haemoglobinopathies and haemolytic anaemias
Other neglected tropical diseases
Other unspecified infectious diseases
Endocrine, metabolic, blood, and immune disorders
Upper digestive system diseases
Inflammatory bowel disease
Vitamin A deficiency
HIV/AIDS
Cirrhosis and other chronic liver diseases
Intestinal nematode infections
Schistosomiasis
Malaria
Gynecological diseases
Maternal disorders
YLDs per 100,000 population
0 to <0
0 to <0.01
0.01 to <0.07
0.07 to <0.24
0.24 to <0.65
0.65 to <1.68
1.68 to <3.23
3.23 to <11.85
11.85 to <20.18
20.18 to 223.79
300

Lambeth

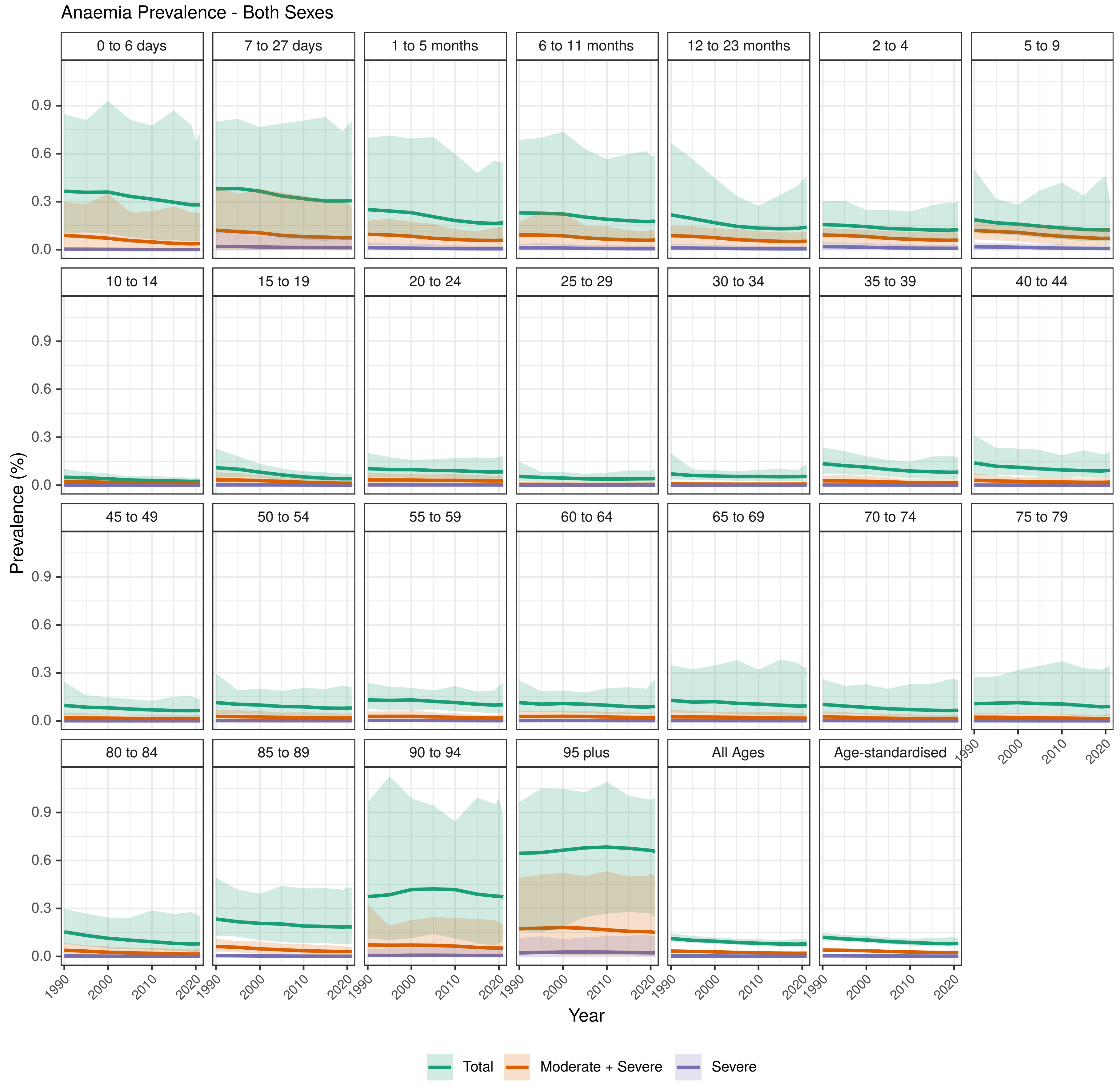

Anaemia Prevalence - Both Sexes
0 to 6 days
7 to 27 days
1 to 5 months
6 to 11 months
12 to 23 months
2 to 4
5 to 9
10 to 14
15 to 19
20 to 24
25 to 29
30 to 34
35 to 39
40 to 44
45 to 49
50 to 54
55 to 59
60 to 64
65 to 69
70 to 74
75 to 79
80 to 84
85 to 89
90 to 94
95 plus
All Ages
Age-standardised
Prevalence (%)
Year
Total
Moderate + Severe
Severe

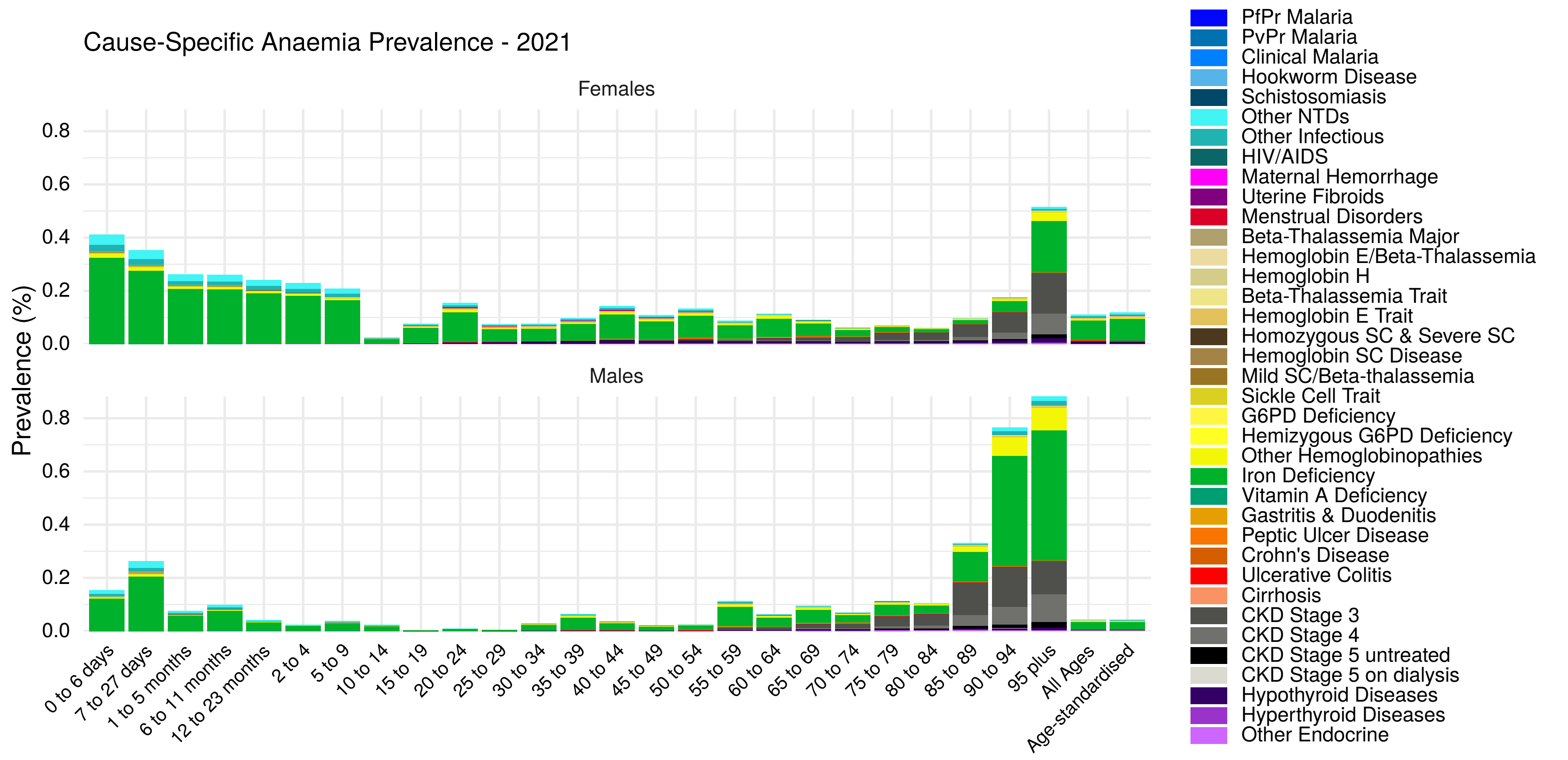

Cause-Specific Anaemia Prevalence - 2021
Females
Males
Prevalence (%)
PfPr Malaria
PvPr Malaria
Clinical Malaria
Hookworm Disease
Schistosomiasis
Other NTDs
Other Infectious
HIV/AIDS
Maternal Hemorrhage
Uterine Fibroids
Menstrual Disorders
Beta-Thalassemia Major
Hemoglobin E/Beta-Thalassemia
Hemoglobin H
Beta-Thalassemia Trait
Hemoglobin E Trait
Homozygous SC & Severe SC
Hemoglobin SC Disease
Mild SC/Beta-thalassemia
Sickle Cell Trait
G6PD Deficiency
Hemizygous G6PD Deficiency
Other Hemoglobinopathies
Iron Deficiency
Vitamin A Deficiency
Gastritis & Duodenitis
Peptic Ulcer Disease
Crohn's Disease
Ulcerative Colitis
Cirrhosis
CKD Stage 3
CKD Stage 4
CKD Stage 5 untreated
CKD Stage 5 on dialysis
Hypothyroid Diseases
Hyperthyroid Diseases
Other Endocrine

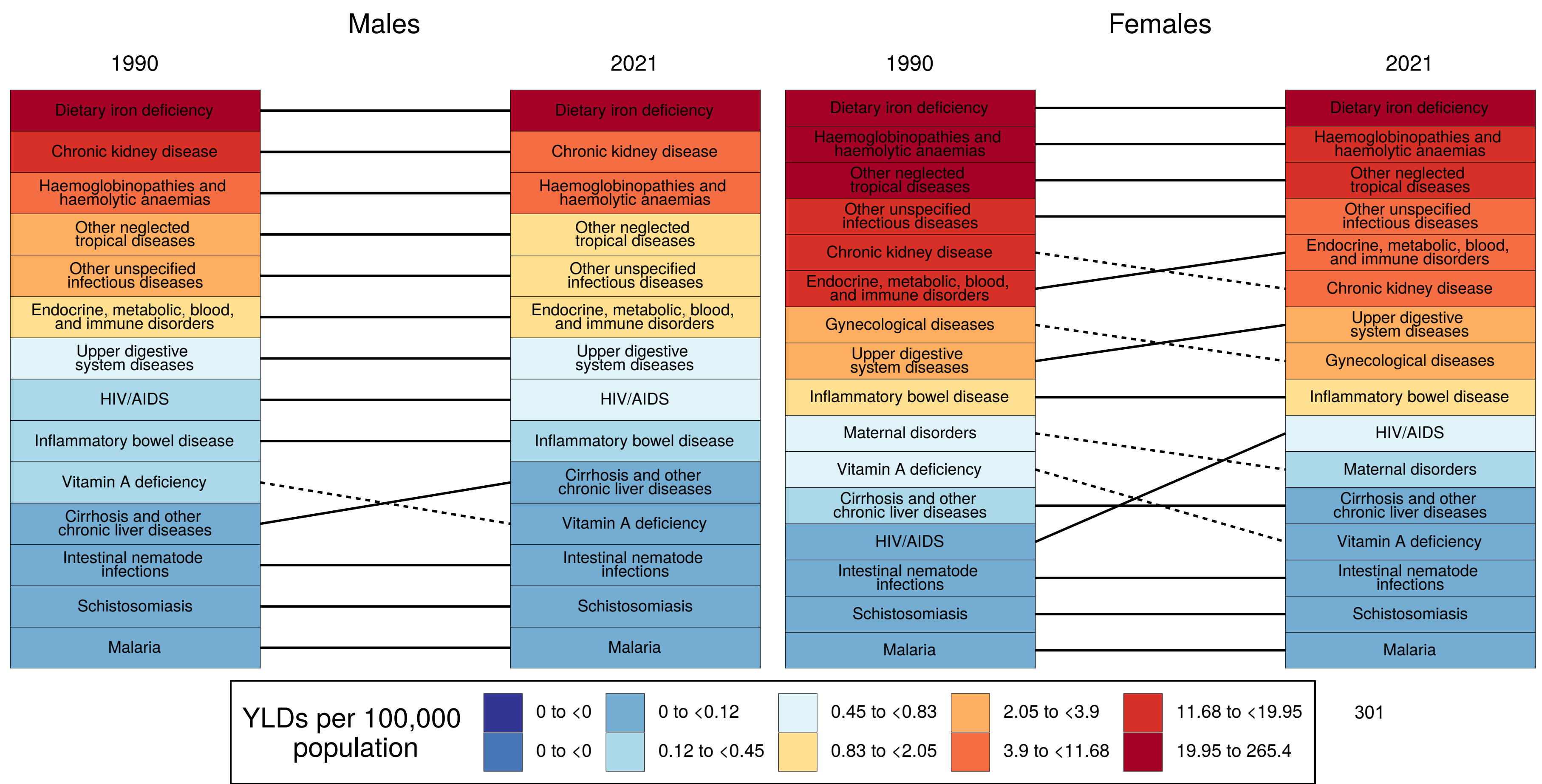

Males
Females
1990
2021
Dietary iron deficiency
Chronic kidney disease
Haemoglobinopathies and haemolytic anaemias
Other neglected tropical diseases
Other unspecified infectious diseases
Endocrine, metabolic, blood, and immune disorders
Upper digestive system diseases
HIV/AIDS
Inflammatory bowel disease
Vitamin A deficiency
Cirrhosis and other chronic liver diseases
Intestinal nematode infections
Schistosomiasis
Malaria
Gynecological diseases
Maternal disorders
YLDs per 100,000 population
0 to <0
0 to <0.12
0.12 to <0.45
0.45 to <0.83
0.83 to <2.05
2.05 to <3.9
3.9 to <11.68
11.68 to <19.95
19.95 to 265.4

Lewisham

Anaemia Prevalence - Both Sexes

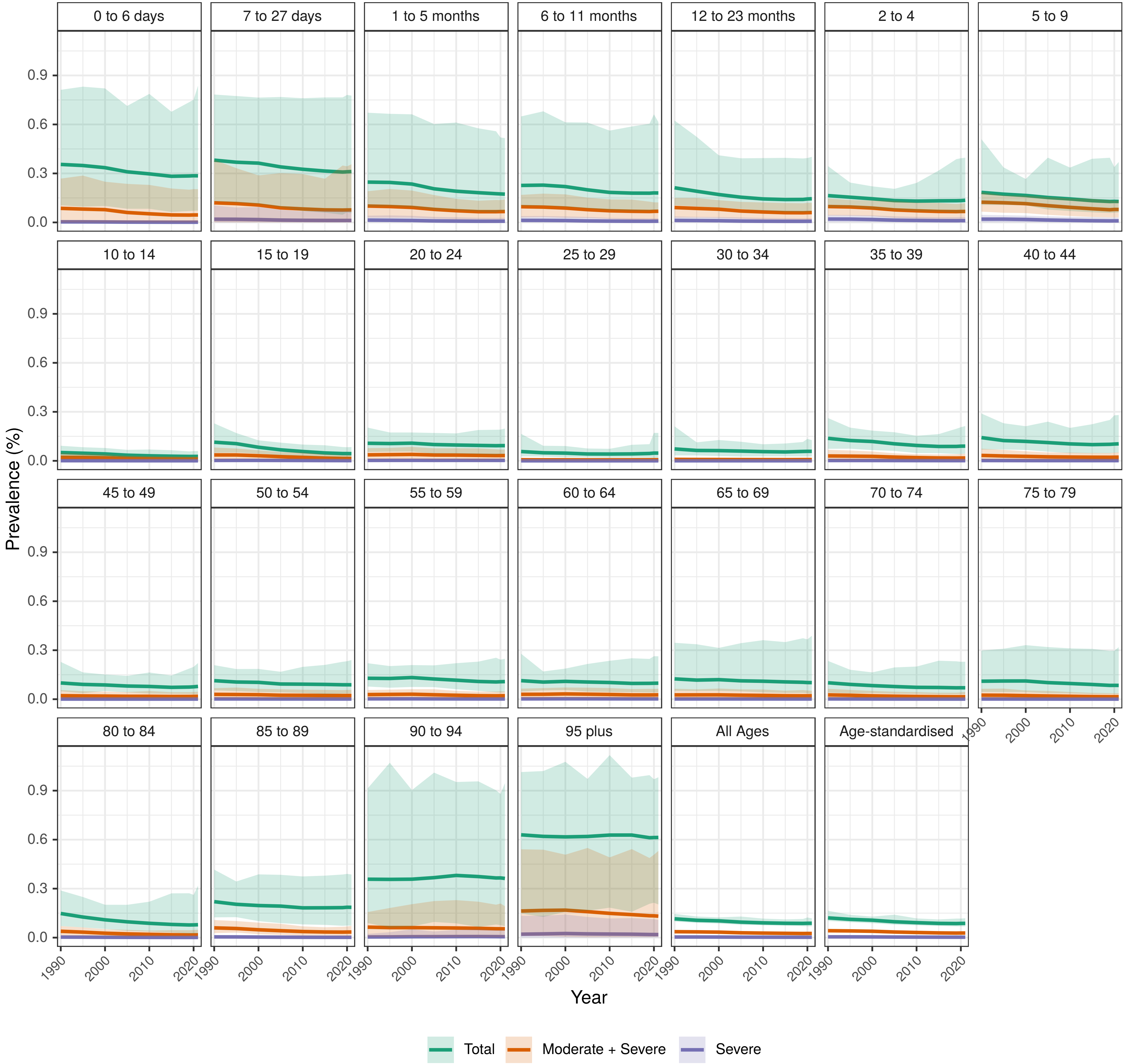

Cause-Specific Anaemia Prevalence - 2021

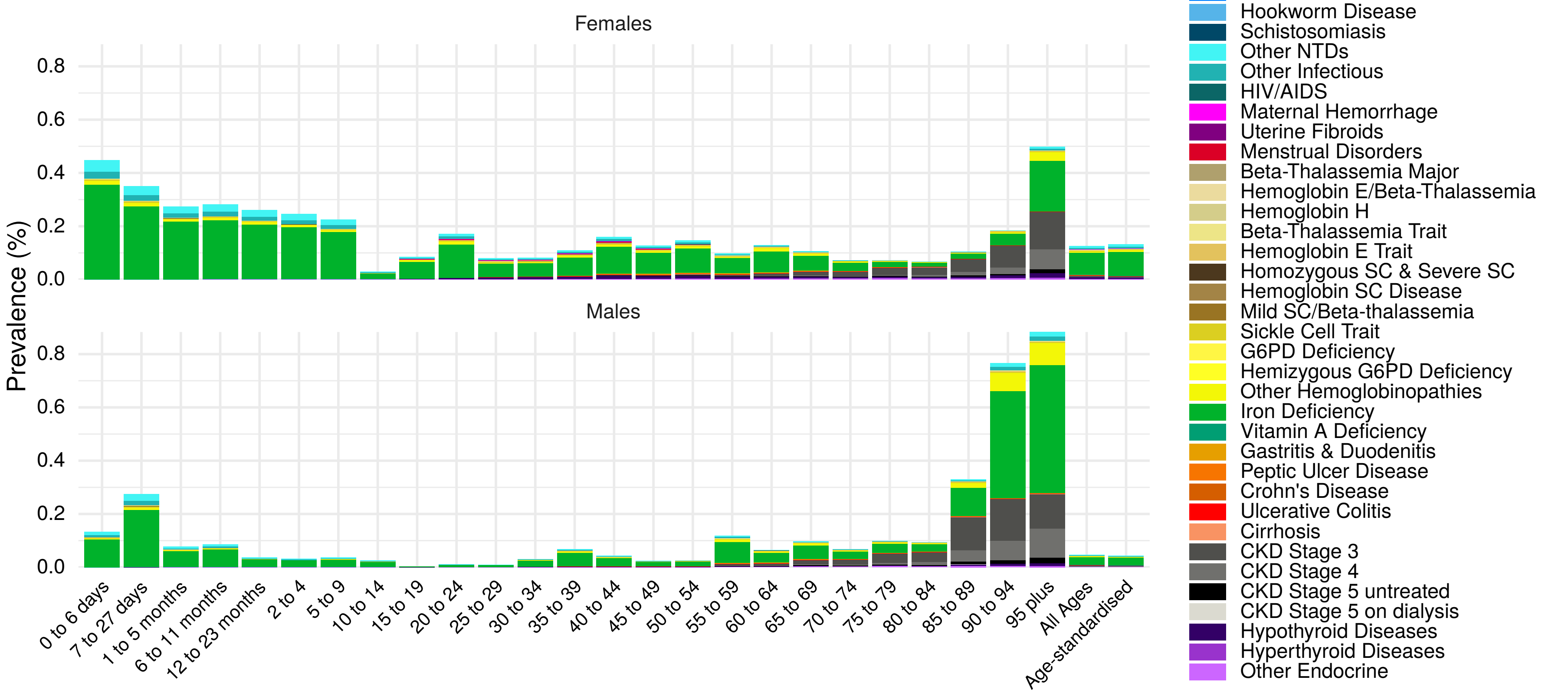

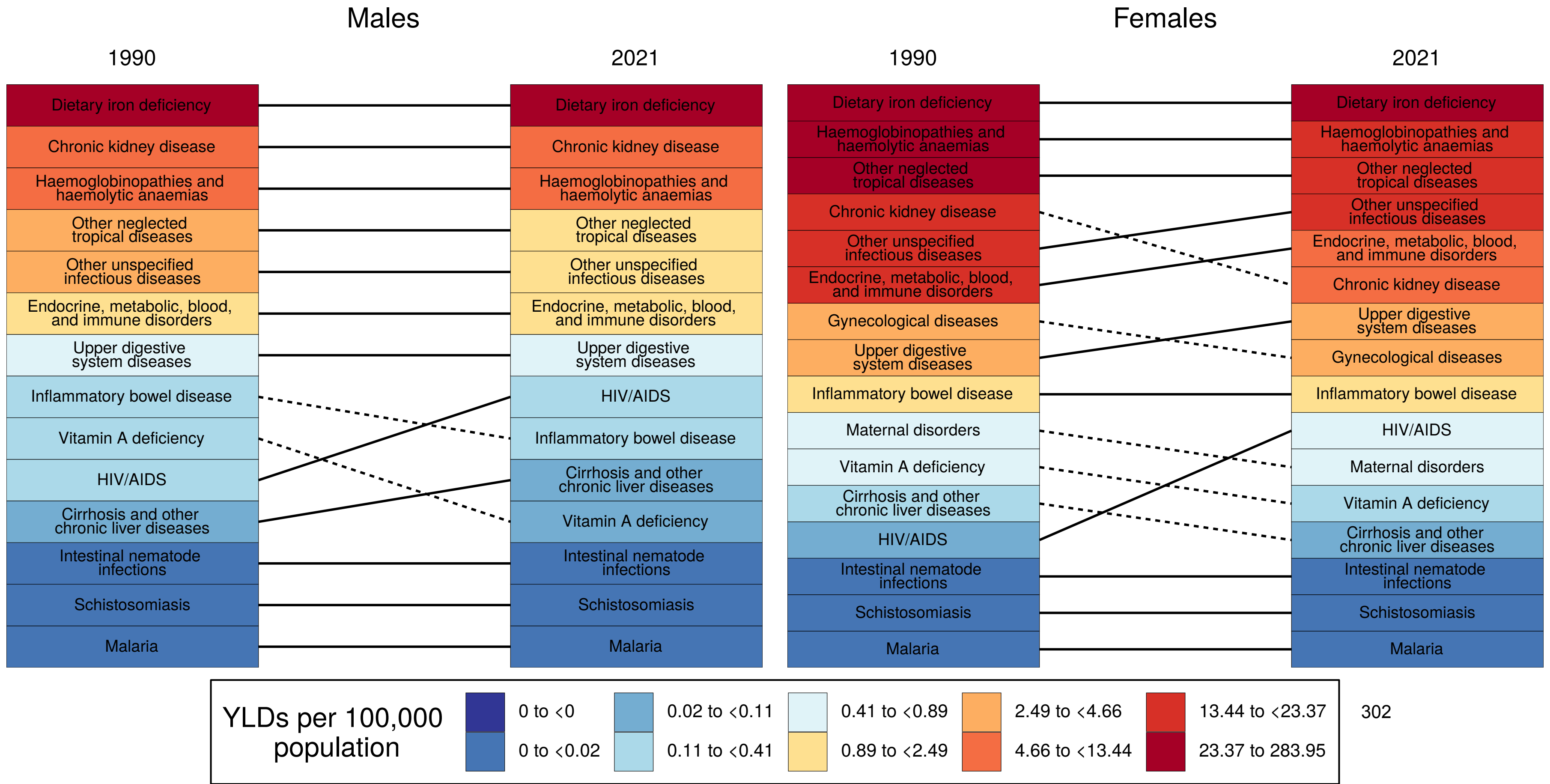

Merton

Anaemia Prevalence - Both Sexes

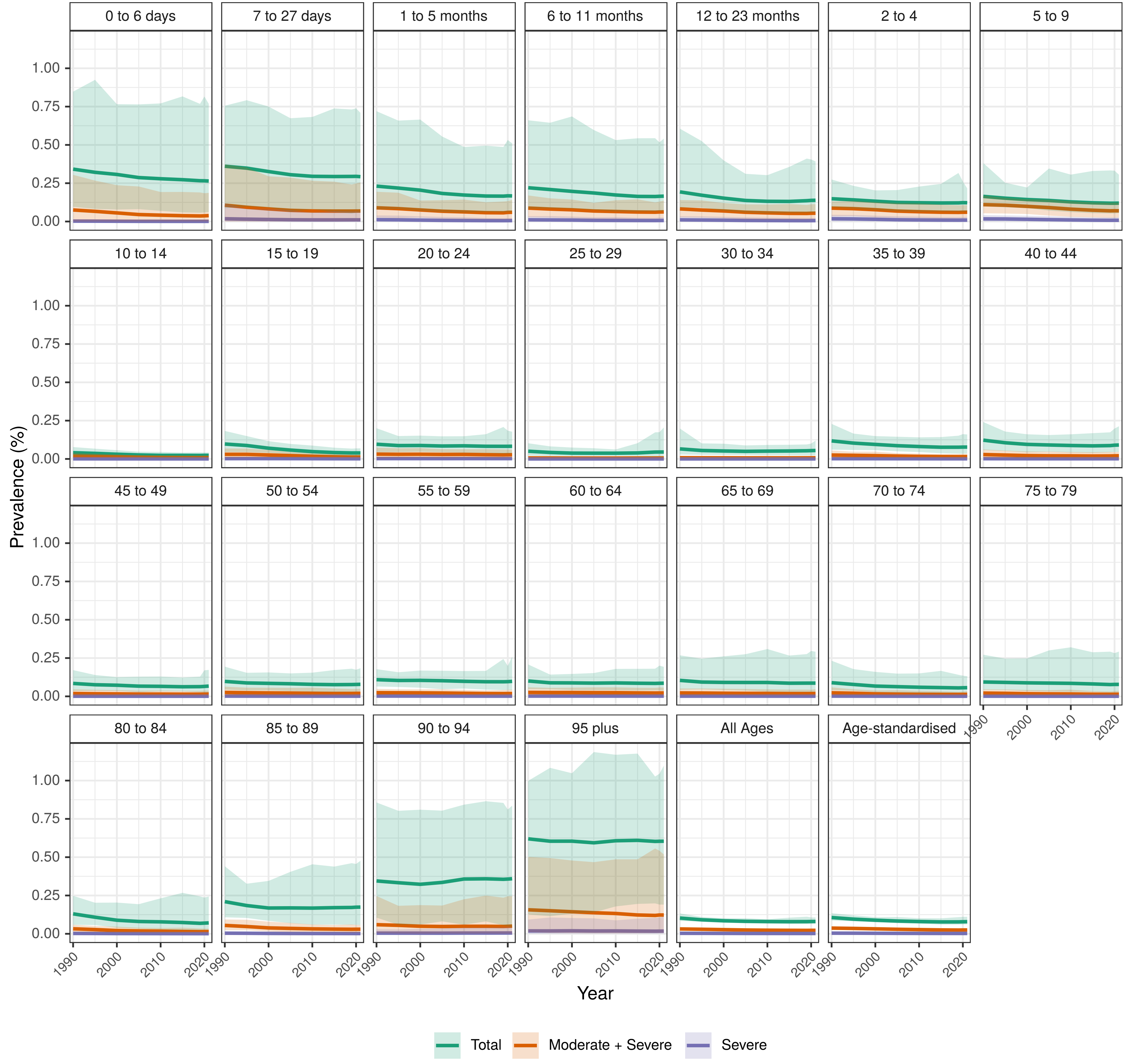

Cause-Specific Anaemia Prevalence - 2021

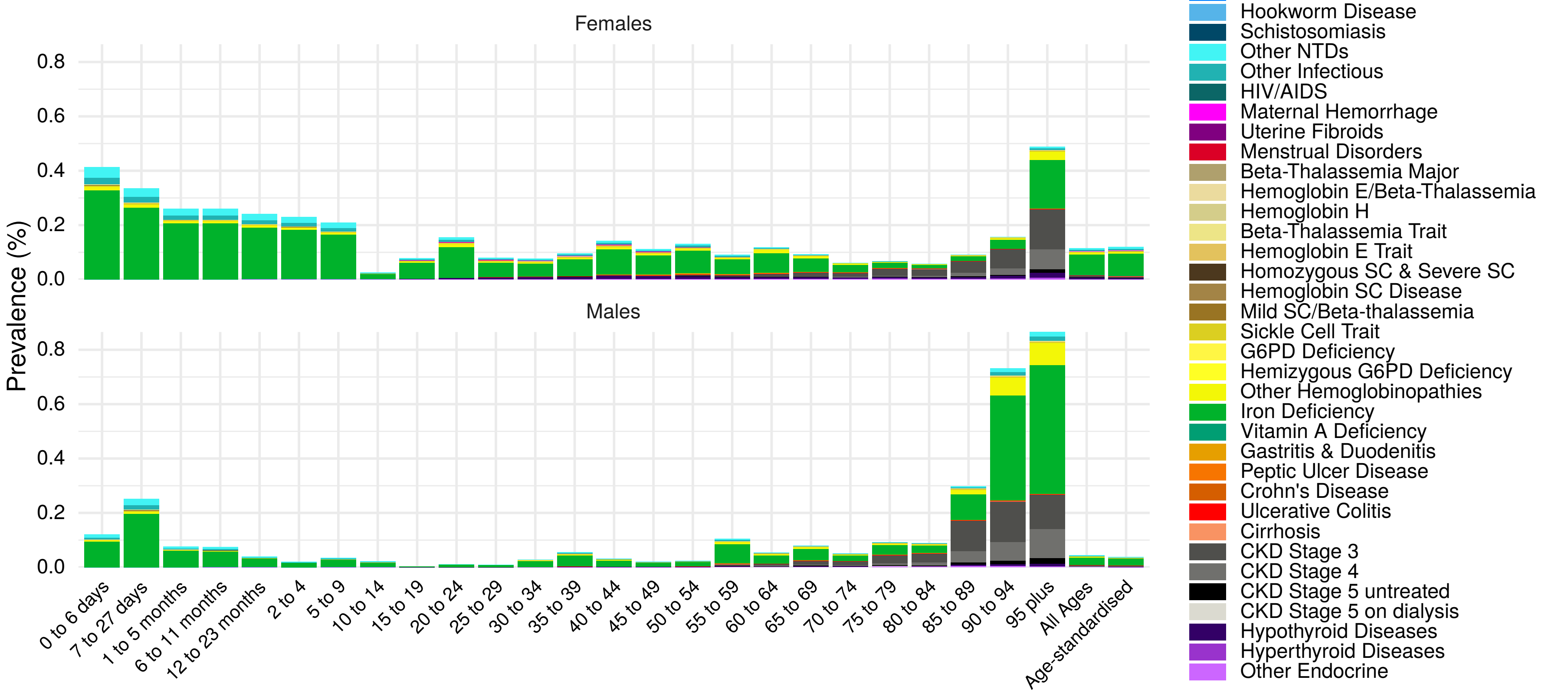

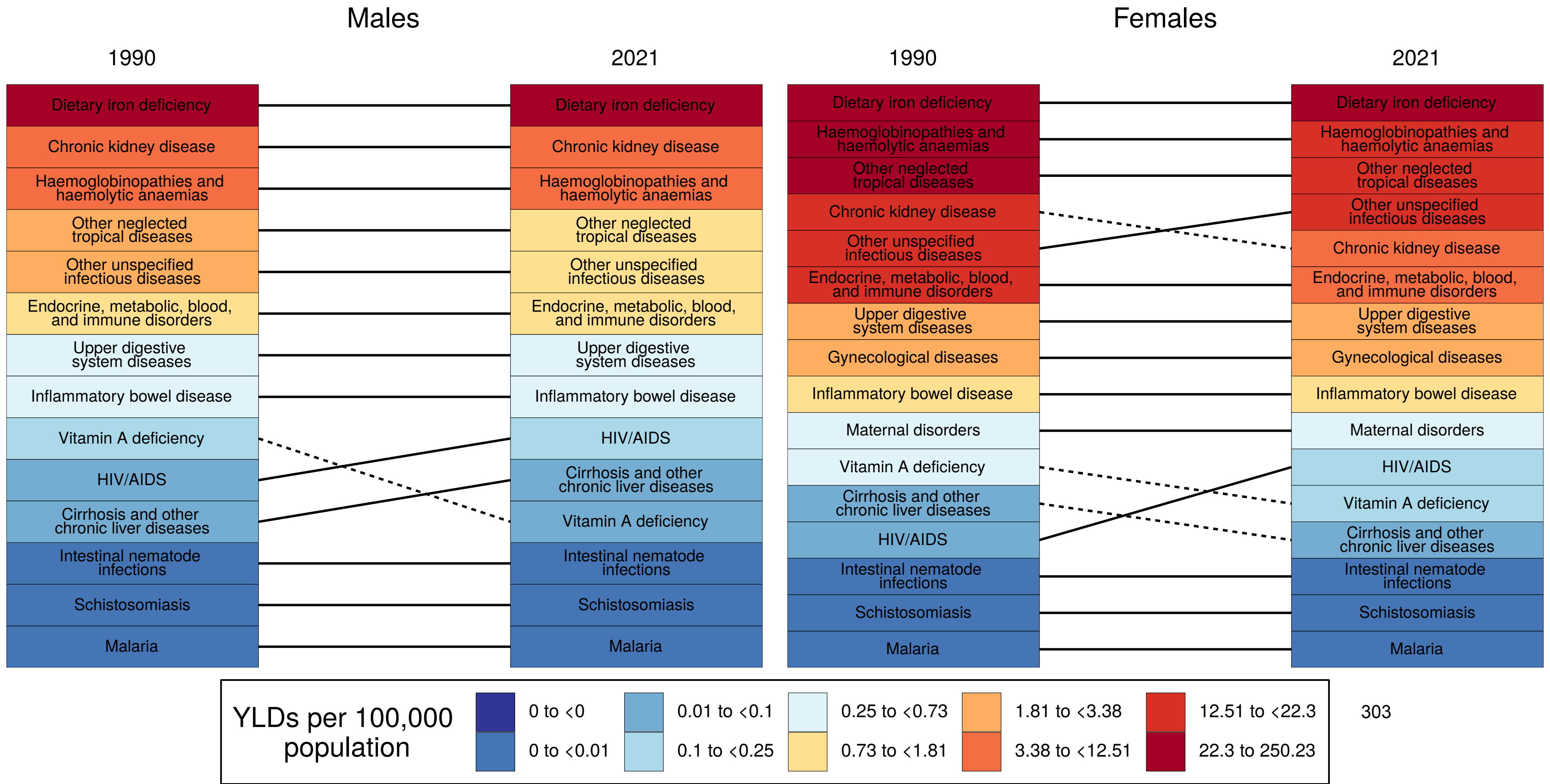

Newham

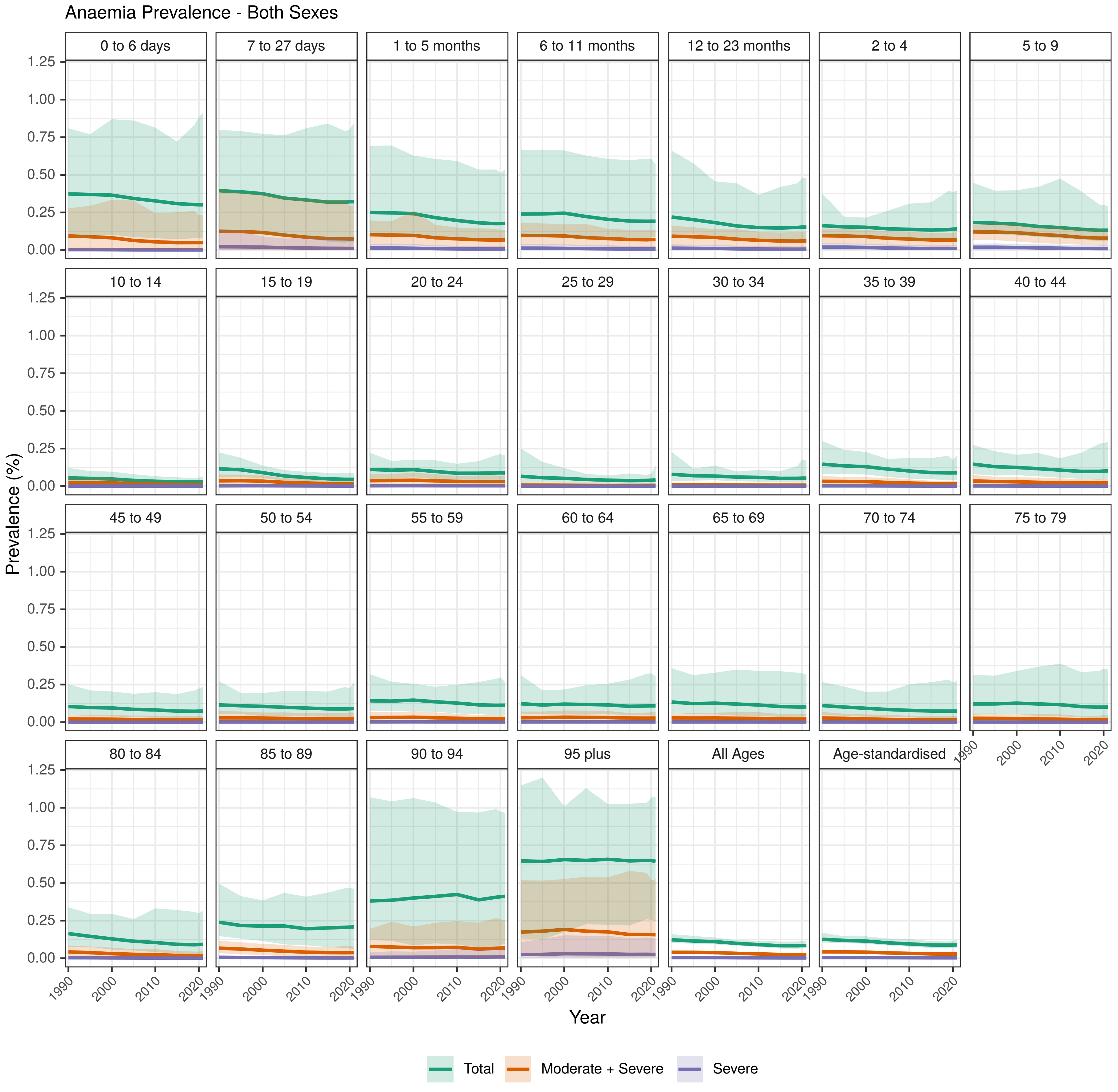

Anaemia Prevalence - Both Sexes
0 to 6 days
7 to 27 days
1 to 5 months
6 to 11 months
12 to 23 months
2 to 4
5 to 9
10 to 14
15 to 19
20 to 24
25 to 29
30 to 34
35 to 39
40 to 44
45 to 49
50 to 54
55 to 59
60 to 64
65 to 69
70 to 74
75 to 79
80 to 84
85 to 89
90 to 94
95 plus
All Ages
Age-standardised
Prevalence (%)
Year
Total
Moderate + Severe
Severe

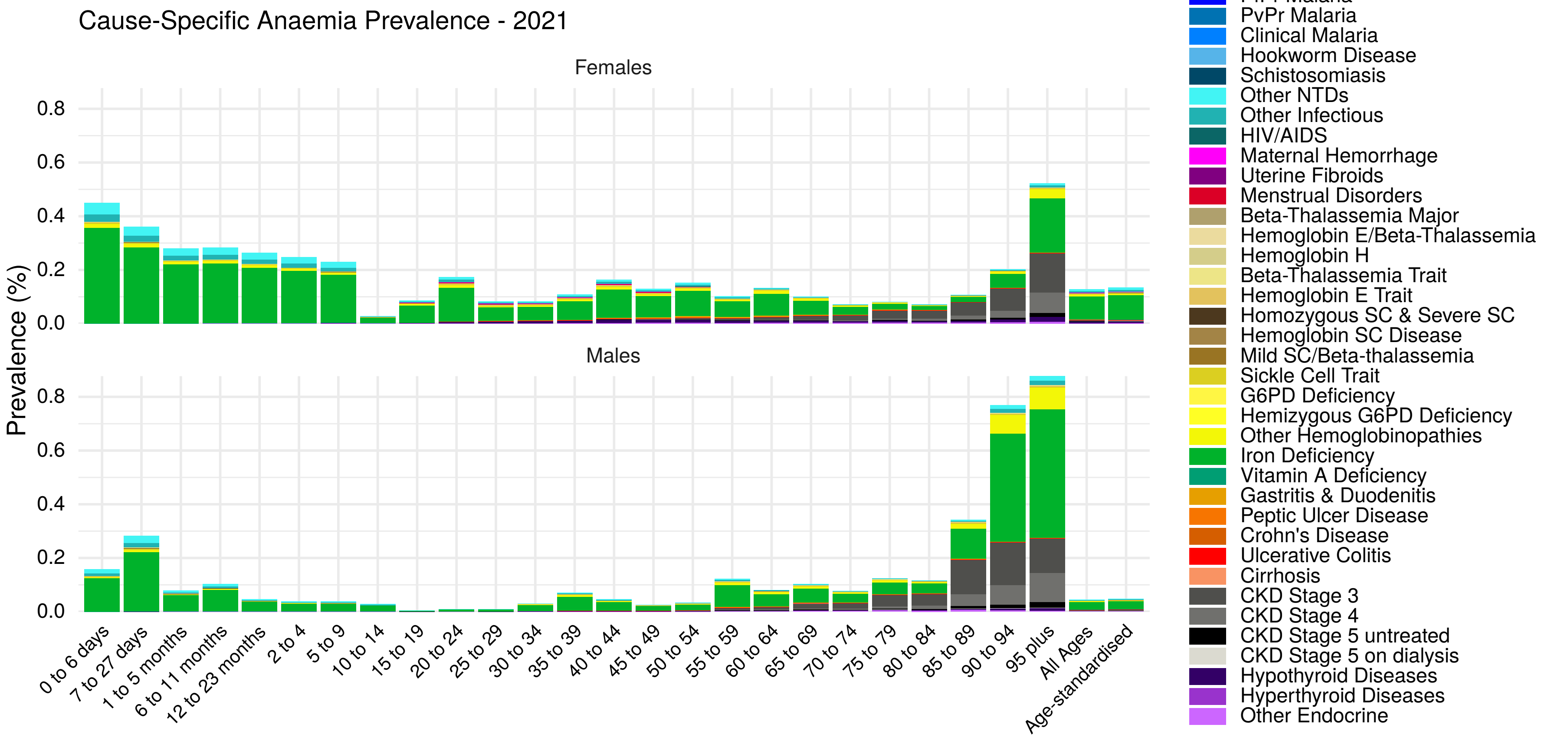

Cause-Specific Anaemia Prevalence - 2021
Females
Males
Prevalence (%)
PfPr Malaria
PvPr Malaria
Clinical Malaria
Hookworm Disease
Schistosomiasis
Other NTDs
Other Infectious
HIV/AIDS
Maternal Hemorrhage
Uterine Fibroids
Menstrual Disorders
Beta-Thalassemia Major
Hemoglobin E/Beta-Thalassemia
Hemoglobin H
Beta-Thalassemia Trait
Hemoglobin E Trait
Homozygous SC & Severe SC
Hemoglobin SC Disease
Mild SC/Beta-thalassemia
Sickle Cell Trait
G6PD Deficiency
Hemizygous G6PD Deficiency
Other Hemoglobinopathies
Iron Deficiency
Vitamin A Deficiency
Gastritis & Duodenitis
Peptic Ulcer Disease
Crohn's Disease
Ulcerative Colitis
Cirrhosis
CKD Stage 3
CKD Stage 4
CKD Stage 5 untreated
CKD Stage 5 on dialysis
Hypothyroid Diseases
Hyperthyroid Diseases
Other Endocrine

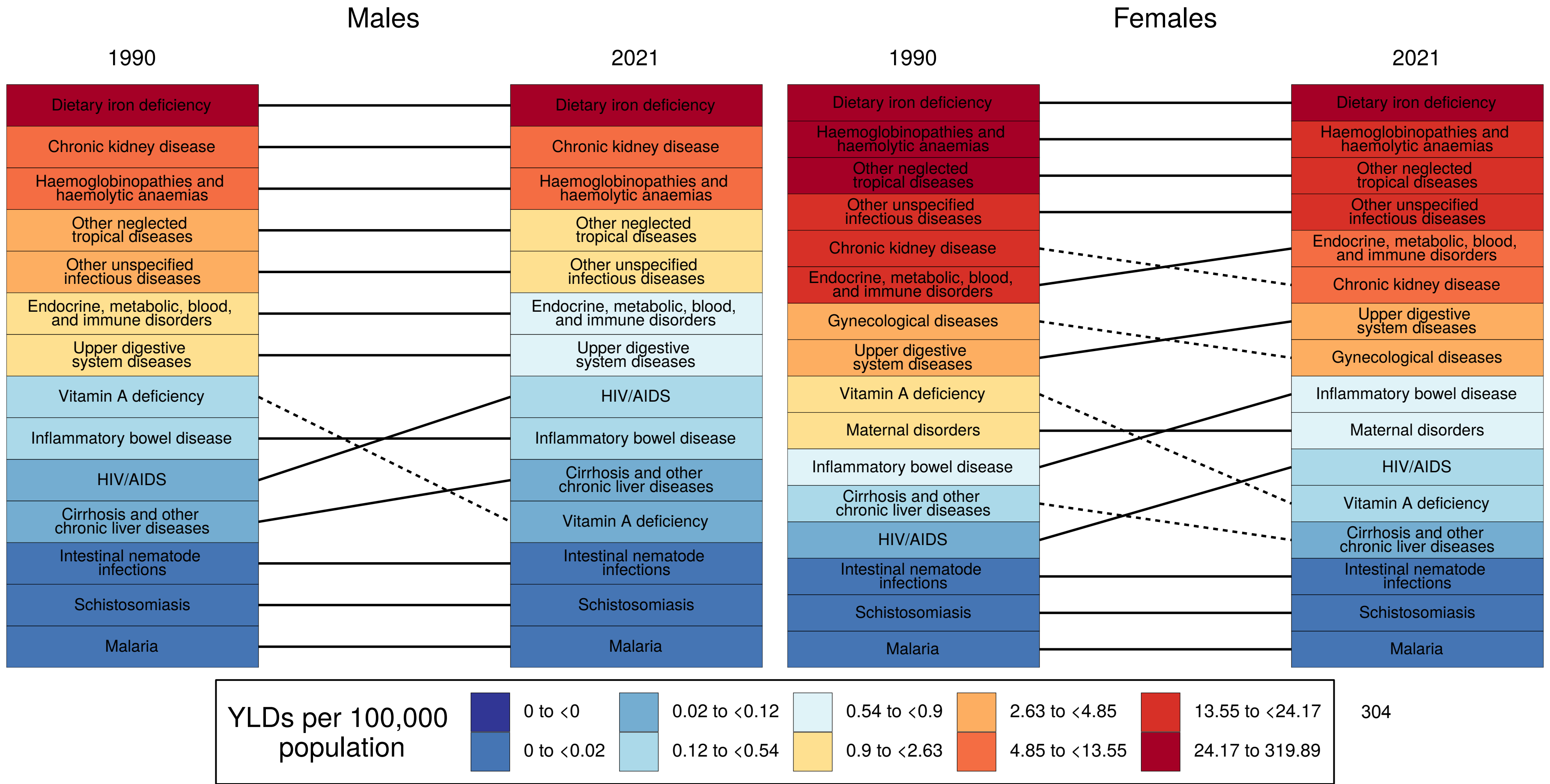

Males
Females
1990
2021
Dietary iron deficiency
Chronic kidney disease
Haemoglobinopathies and haemolytic anaemias
Other neglected tropical diseases
Other unspecified infectious diseases
Endocrine, metabolic, blood, and immune disorders
Upper digestive system diseases
Vitamin A deficiency
Inflammatory bowel disease
HIV/AIDS
Cirrhosis and other chronic liver diseases
Intestinal nematode infections
Schistosomiasis
Malaria
Gynecological diseases
Maternal disorders
YLDs per 100,000 population
0 to <0
0 to <0.02
0.02 to <0.12
0.12 to <0.54
0.54 to <0.9
0.9 to <2.63
2.63 to <4.85
4.85 to <13.55
13.55 to <24.17
24.17 to 319.89

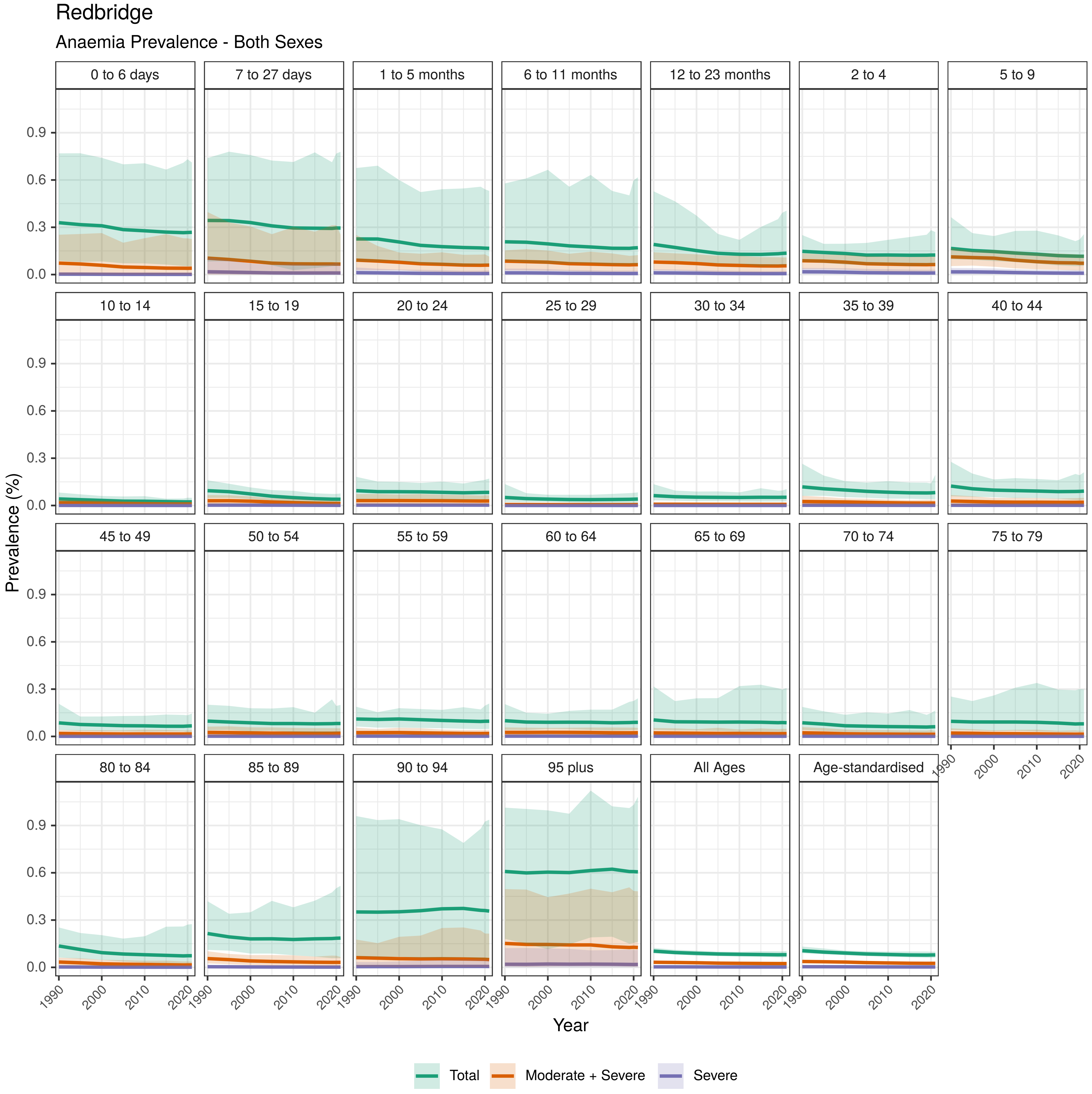
Redbridge
Anaemia Prevalence - Both Sexes
0 to 6 days
7 to 27 days
1 to 5 months
6 to 11 months
12 to 23 months
2 to 4
5 to 9
10 to 14
15 to 19
20 to 24
25 to 29
30 to 34
35 to 39
40 to 44
45 to 49
50 to 54
55 to 59
60 to 64
65 to 69
70 to 74
75 to 79
80 to 84
85 to 89
90 to 94
95 plus
All Ages
Age-standardised
Prevalence (%)
Year
Total
Moderate + Severe
Severe

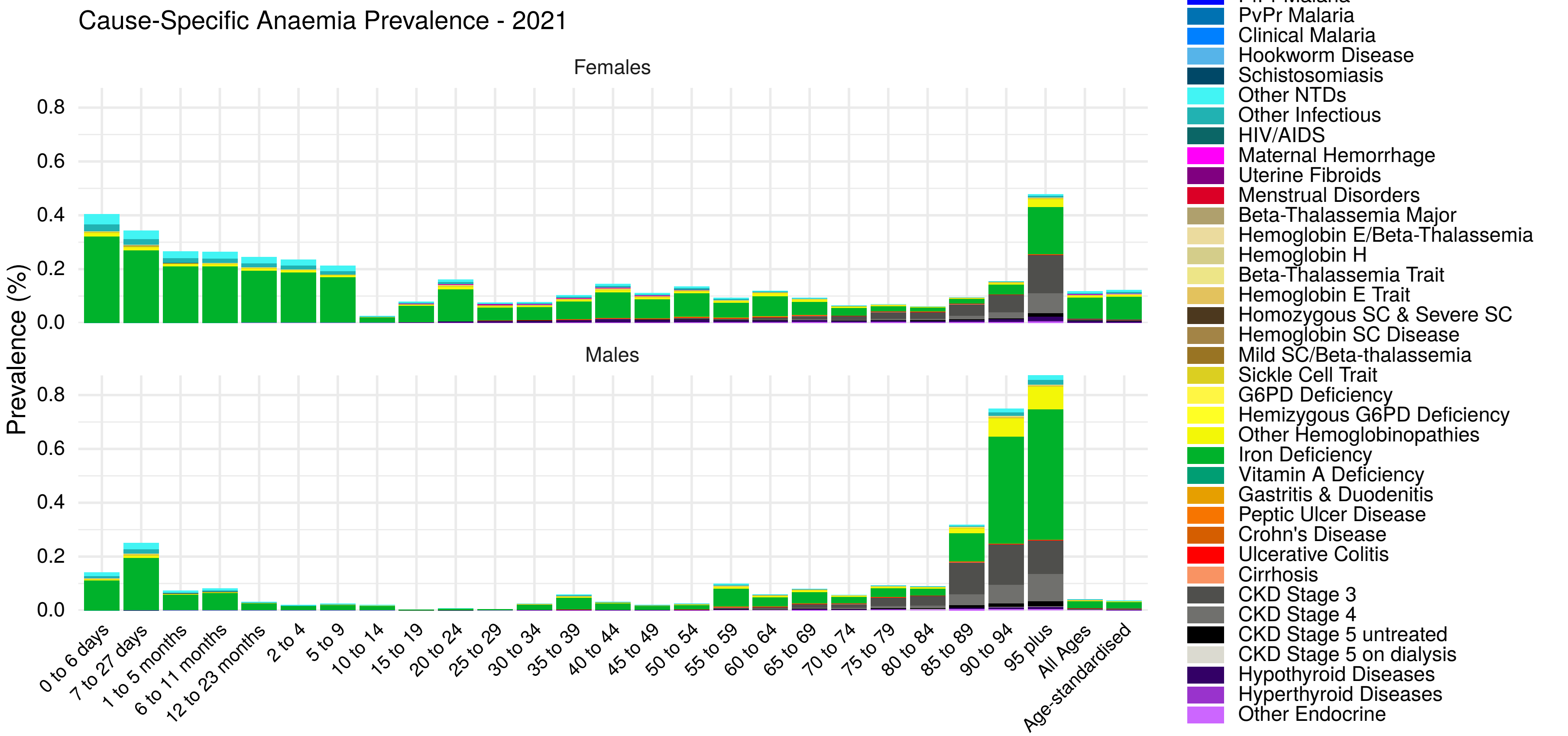
Cause-Specific Anaemia Prevalence - 2021
Females
Males
Prevalence (%)
PfPr Malaria
PvPr Malaria
Clinical Malaria
Hookworm Disease
Schistosomiasis
Other NTDs
Other Infectious
HIV/AIDS
Maternal Hemorrhage
Uterine Fibroids
Menstrual Disorders
Beta-Thalassemia Major
Hemoglobin E/Beta-Thalassemia
Hemoglobin H
Beta-Thalassemia Trait
Hemoglobin E Trait
Homozygous SC & Severe SC
Hemoglobin SC Disease
Mild SC/Beta-thalassemia
Sickle Cell Trait
G6PD Deficiency
Hemizygous G6PD Deficiency
Other Hemoglobinopathies
Iron Deficiency
Vitamin A Deficiency
Gastritis & Duodenitis
Peptic Ulcer Disease
Crohn's Disease
Ulcerative Colitis
Cirrhosis
CKD Stage 3
CKD Stage 4
CKD Stage 5 untreated
CKD Stage 5 on dialysis
Hypothyroid Diseases
Hyperthyroid Diseases
Other Endocrine

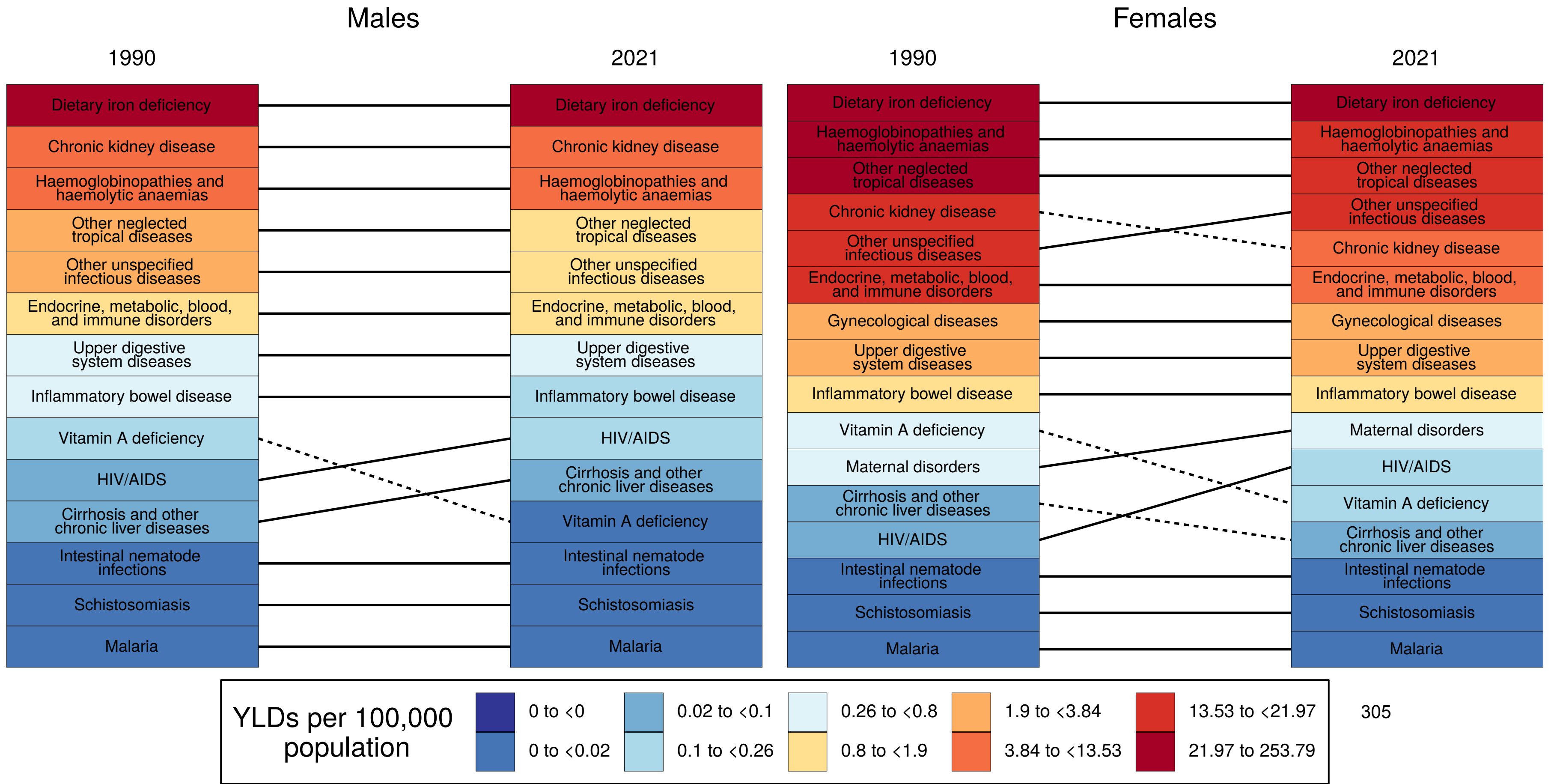
Males
Females
1990
2021
Dietary iron deficiency
Chronic kidney disease
Haemoglobinopathies and haemolytic anaemias
Other neglected tropical diseases
Other unspecified infectious diseases
Endocrine, metabolic, blood, and immune disorders
Upper digestive system diseases
Inflammatory bowel disease
Vitamin A deficiency
HIV/AIDS
Cirrhosis and other chronic liver diseases
Intestinal nematode infections
Schistosomiasis
Malaria
Gynecological diseases
Maternal disorders
YLDs per 100,000 population
0 to <0
0 to <0.02
0.02 to <0.1
0.1 to <0.26
0.26 to <0.8
0.8 to <1.9
1.9 to <3.84
3.84 to <13.53
13.53 to <21.97
21.97 to 253.79

Richmond upon Thames

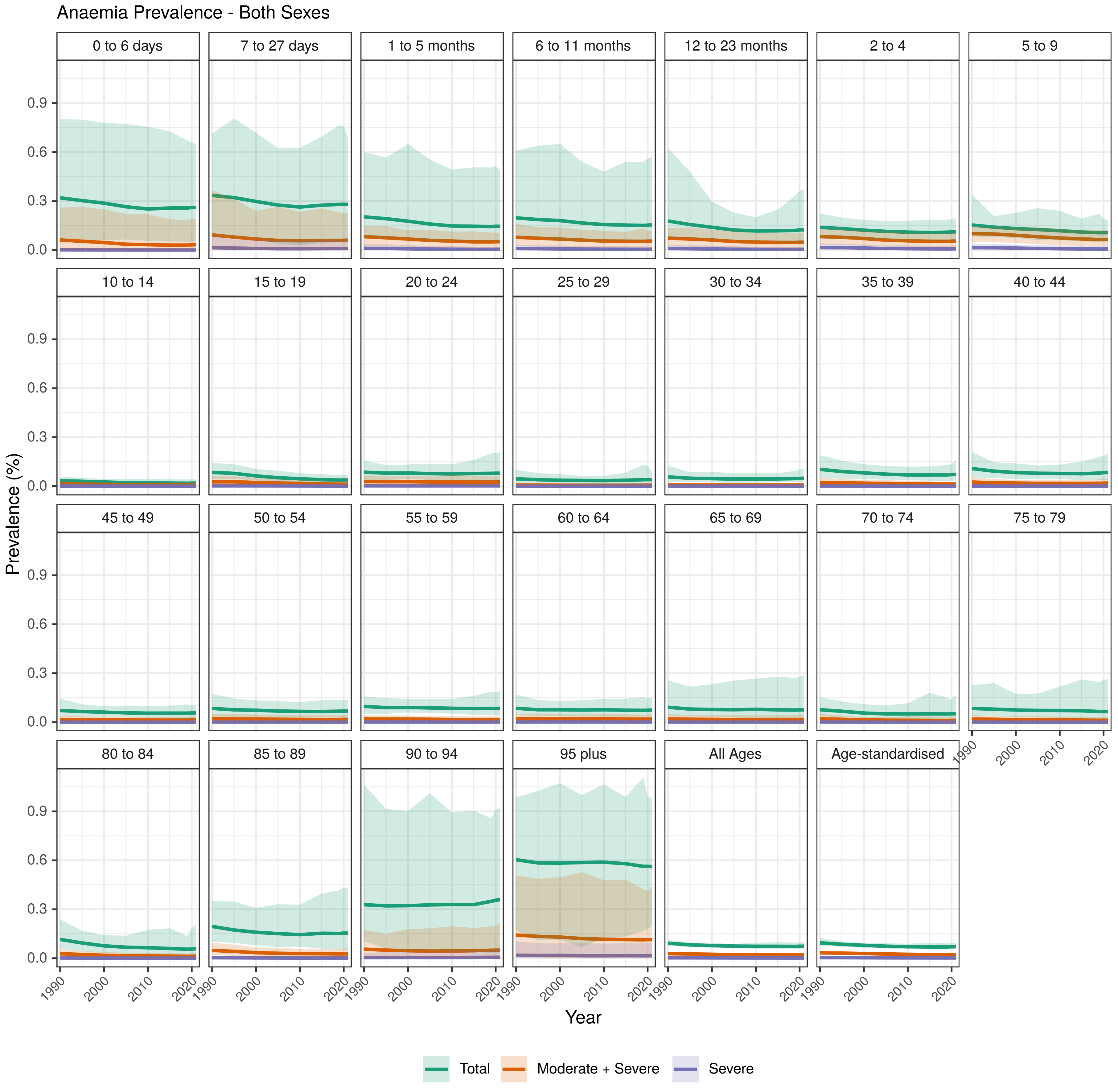

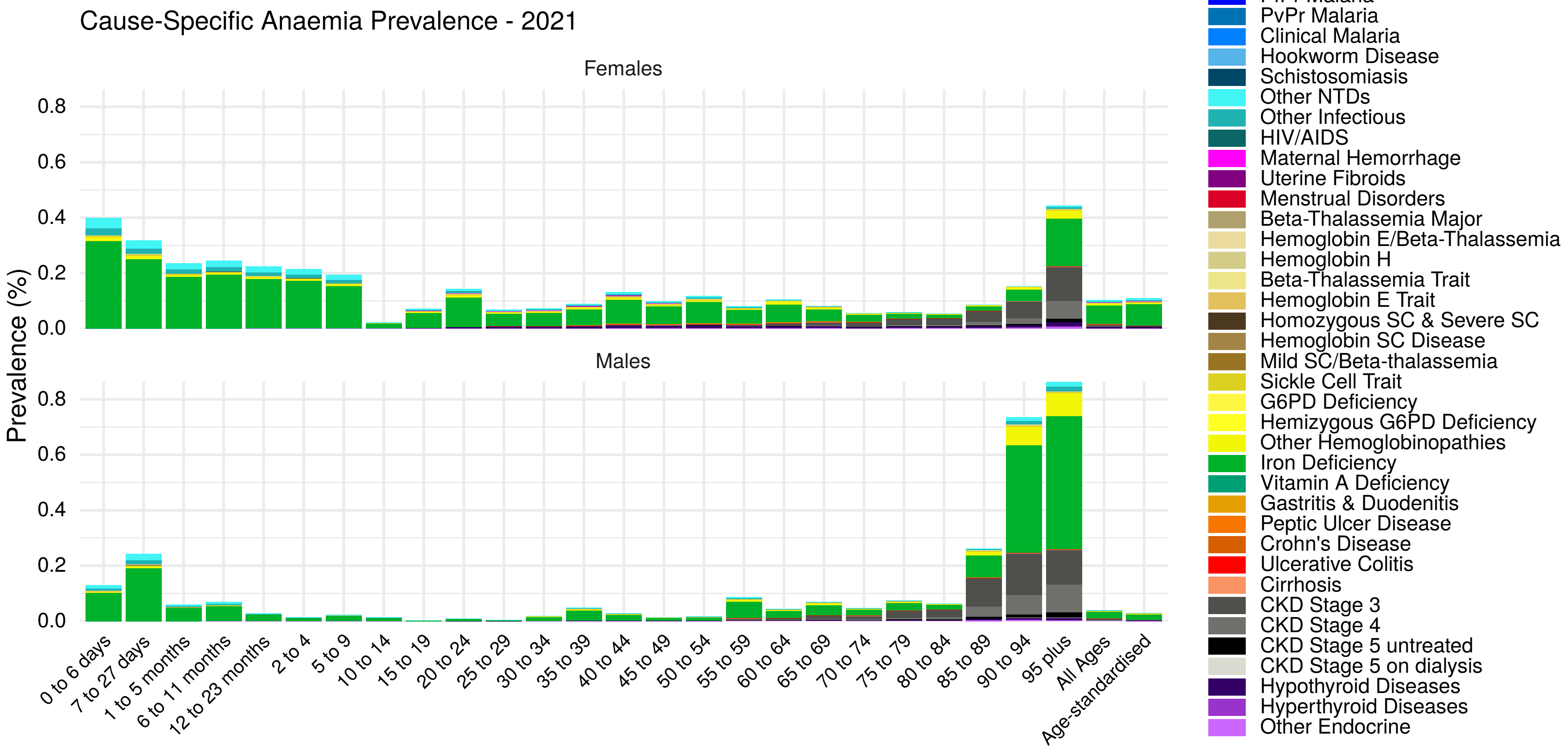

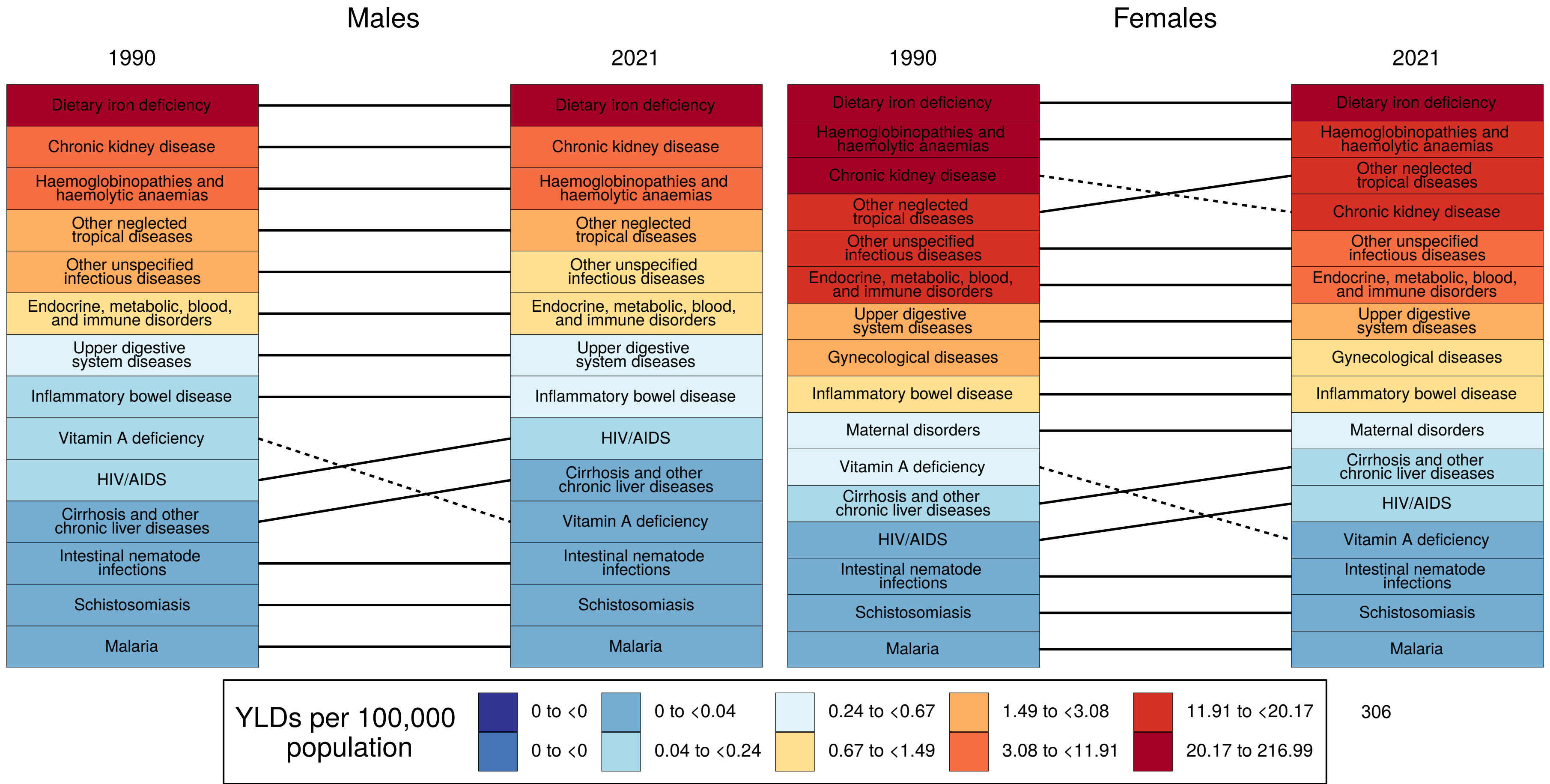

Southwark

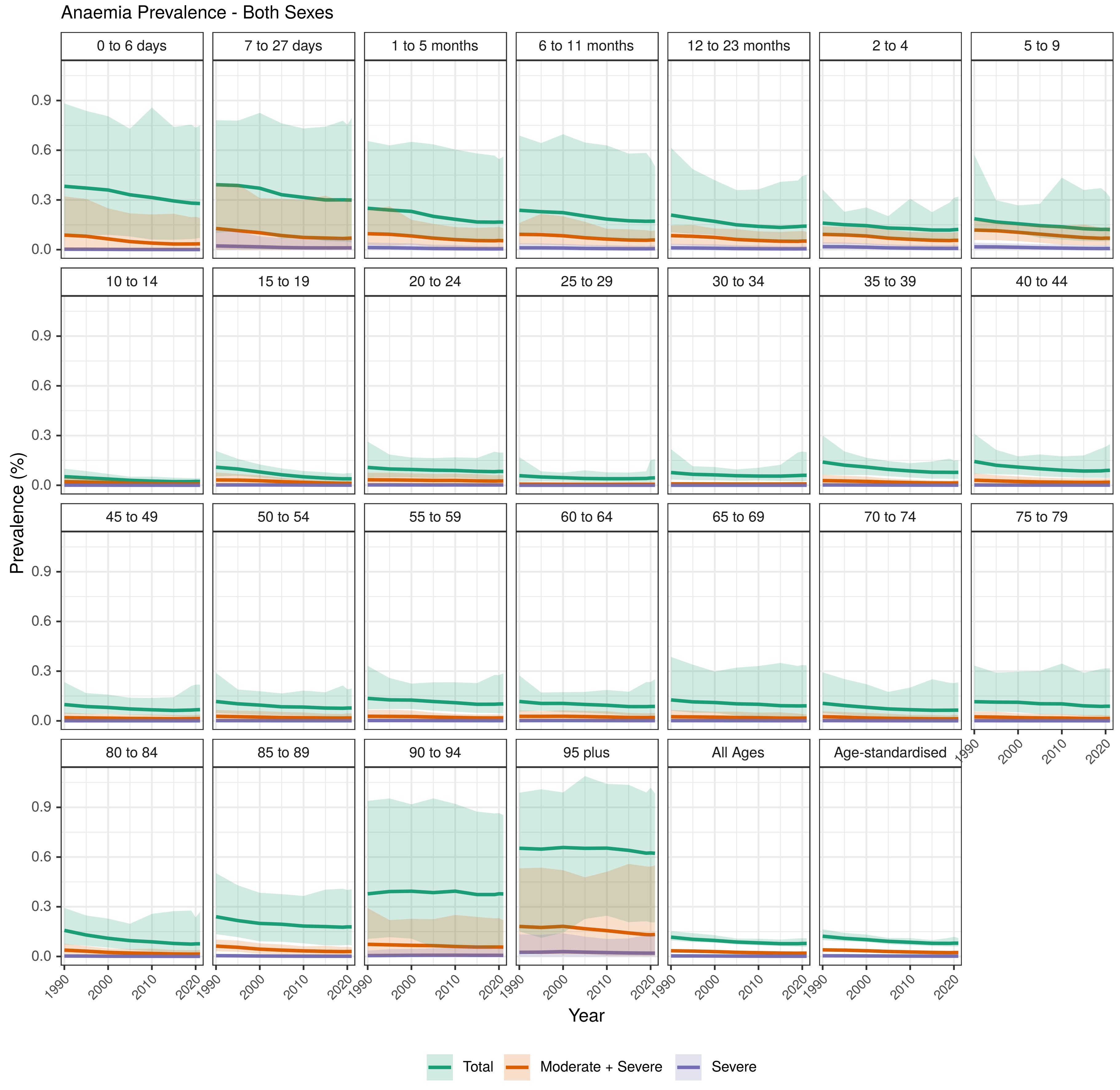
Anaemia Prevalence - Both Sexes
0 to 6 days
7 to 27 days
1 to 5 months
6 to 11 months
12 to 23 months
2 to 4
5 to 9
10 to 14
15 to 19
20 to 24
25 to 29
30 to 34
35 to 39
40 to 44
45 to 49
50 to 54
55 to 59
60 to 64
65 to 69
70 to 74
75 to 79
80 to 84
85 to 89
90 to 94
95 plus
All Ages
Age-standardised
Prevalence (%)
Year
Total
Moderate + Severe
Severe

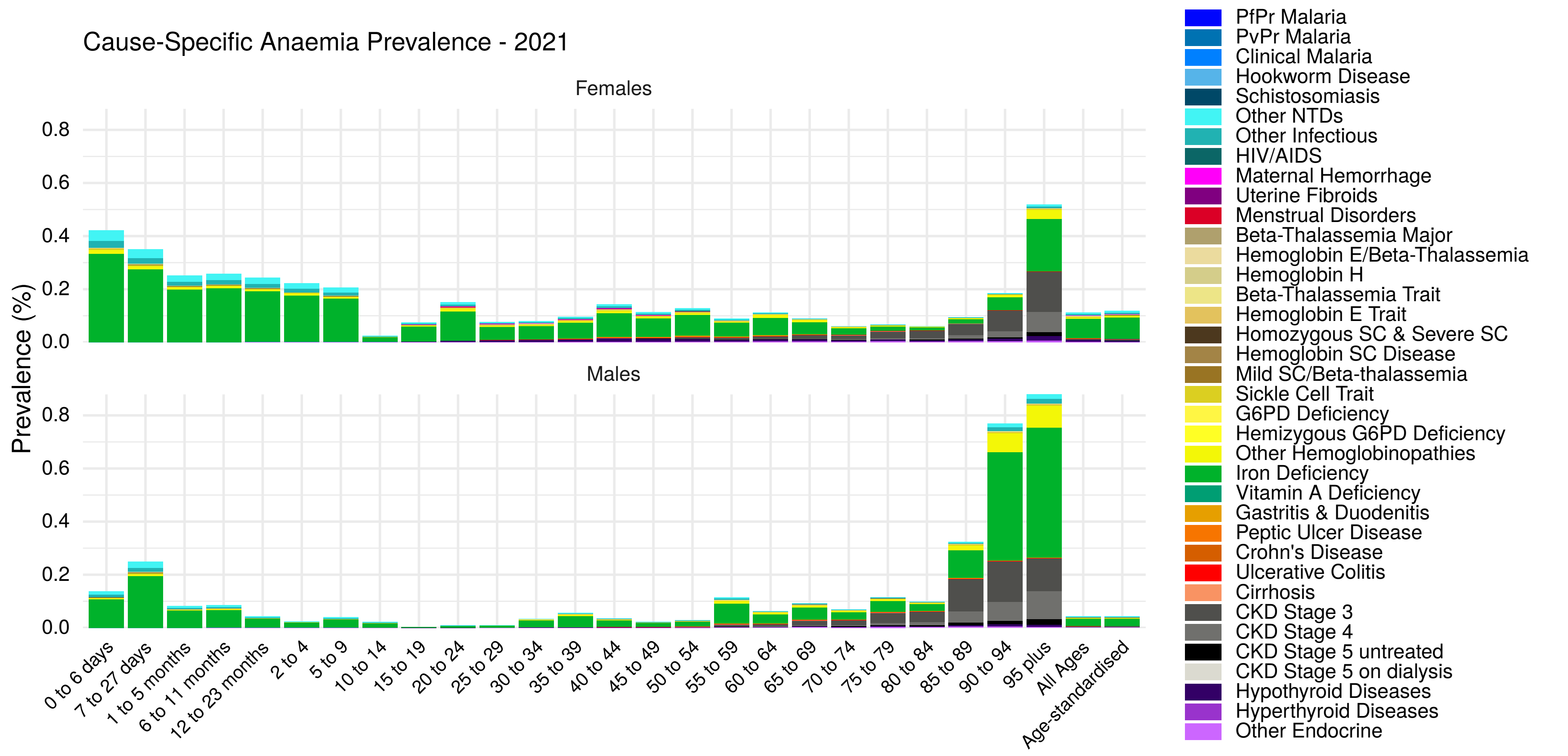
Cause-Specific Anaemia Prevalence - 2021
Females
Males
Prevalence (%)
PfPr Malaria
PvPr Malaria
Clinical Malaria
Hookworm Disease
Schistosomiasis
Other NTDs
Other Infectious
HIV/AIDS
Maternal Hemorrhage
Uterine Fibroids
Menstrual Disorders
Beta-Thalassemia Major
Hemoglobin E/Beta-Thalassemia
Hemoglobin H
Beta-Thalassemia Trait
Hemoglobin E Trait
Homozygous SC & Severe SC
Hemoglobin SC Disease
Mild SC/Beta-thalassemia
Sickle Cell Trait
G6PD Deficiency
Hemizygous G6PD Deficiency
Other Hemoglobinopathies
Iron Deficiency
Vitamin A Deficiency
Gastritis & Duodenitis
Peptic Ulcer Disease
Crohn's Disease
Ulcerative Colitis
Cirrhosis
CKD Stage 3
CKD Stage 4
CKD Stage 5 untreated
CKD Stage 5 on dialysis
Hypothyroid Diseases
Hyperthyroid Diseases
Other Endocrine

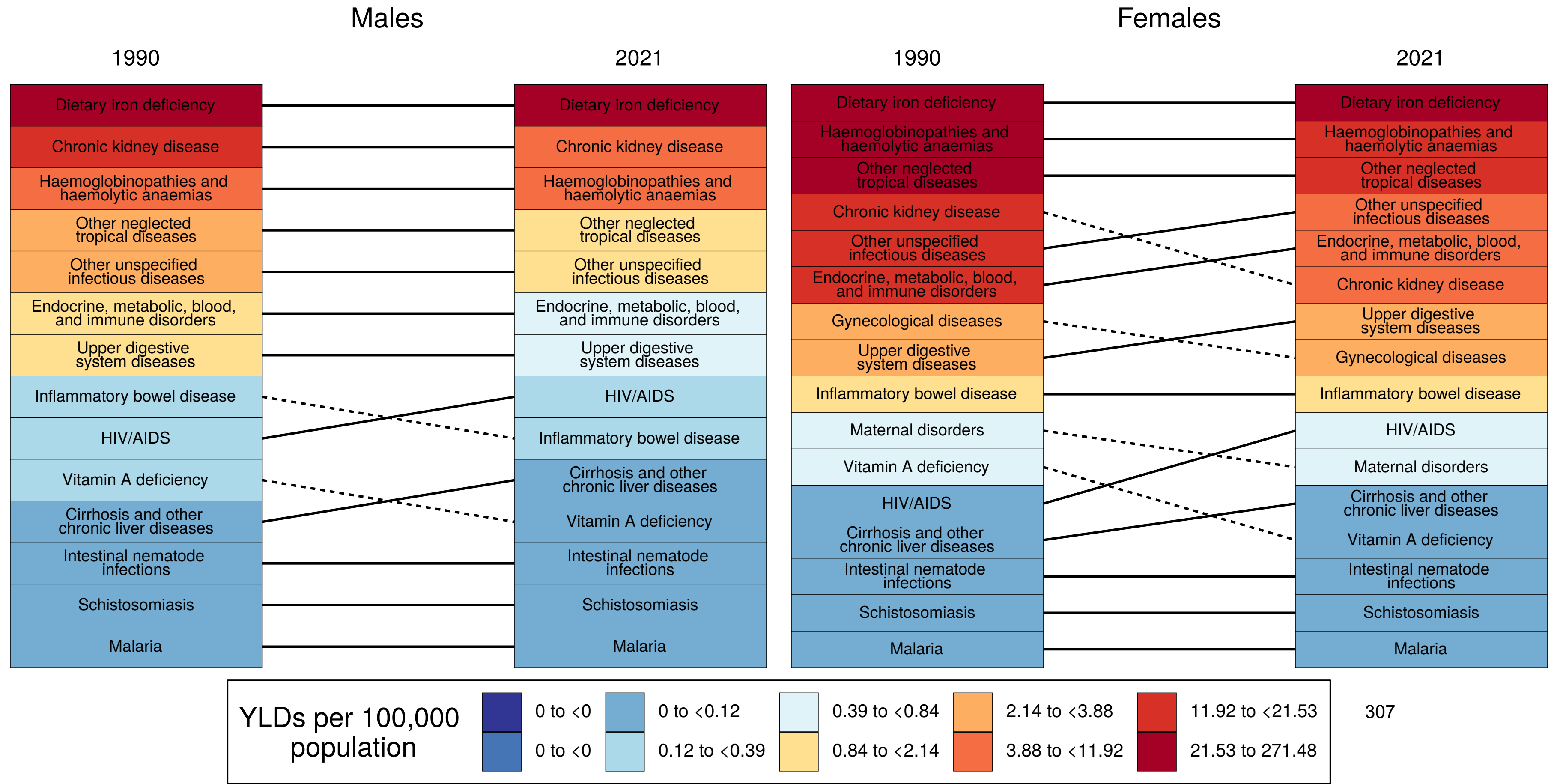
Males
Females
1990
2021
Dietary iron deficiency
Chronic kidney disease
Haemoglobinopathies and haemolytic anaemias
Other neglected tropical diseases
Other unspecified infectious diseases
Endocrine, metabolic, blood, and immune disorders
Upper digestive system diseases
Inflammatory bowel disease
HIV/AIDS
Vitamin A deficiency
Cirrhosis and other chronic liver diseases
Intestinal nematode infections
Schistosomiasis
Malaria
Gynecological diseases
Maternal disorders
YLDs per 100,000 population
0 to <0
0 to <0
0 to <0.12
0.12 to <0.39
0.39 to <0.84
0.84 to <2.14
2.14 to <3.88
3.88 to <11.92
11.92 to <21.53
21.53 to 271.48

Sutton

Anaemia Prevalence - Both Sexes

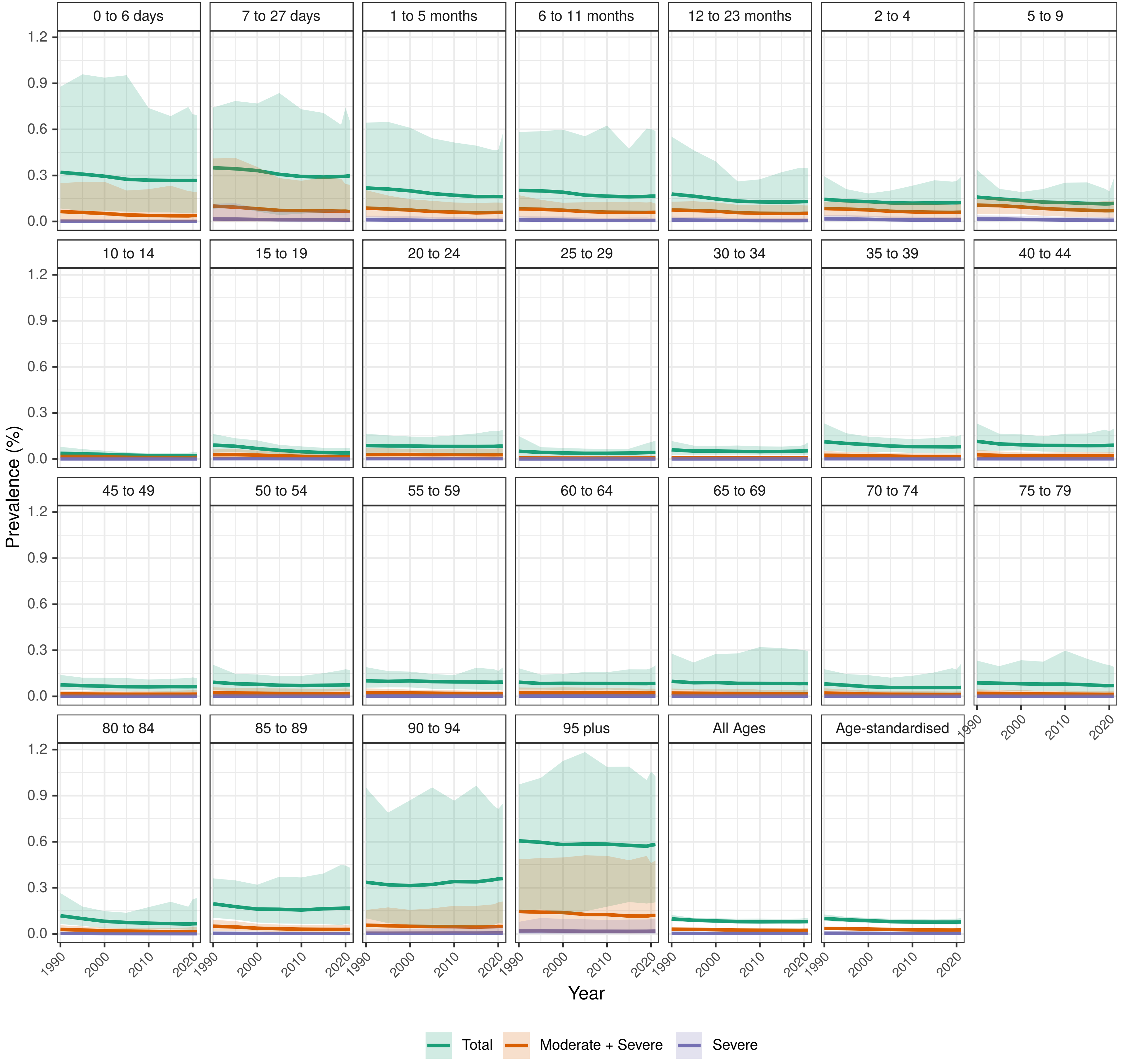

Cause-Specific Anaemia Prevalence - 2021

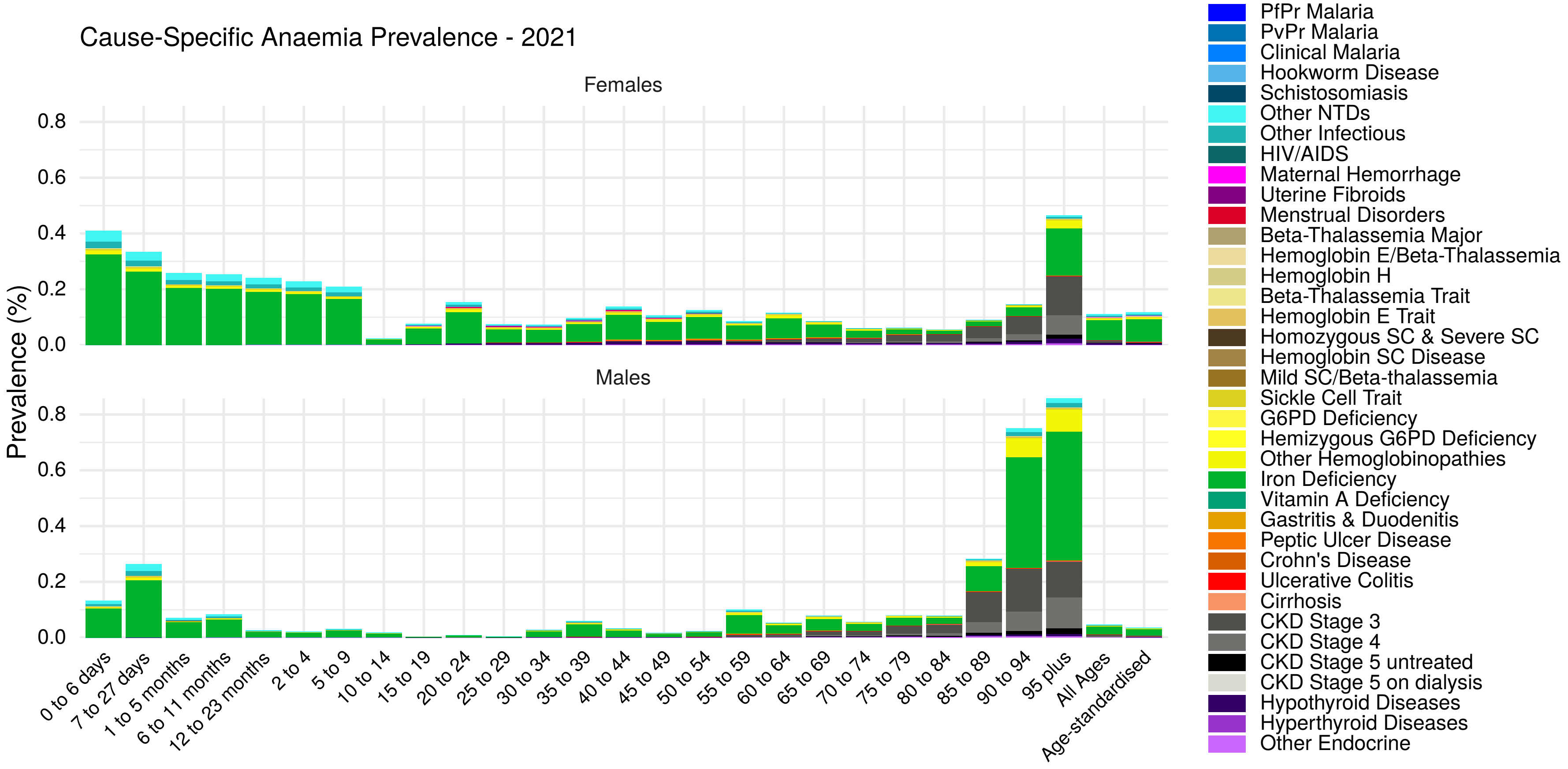

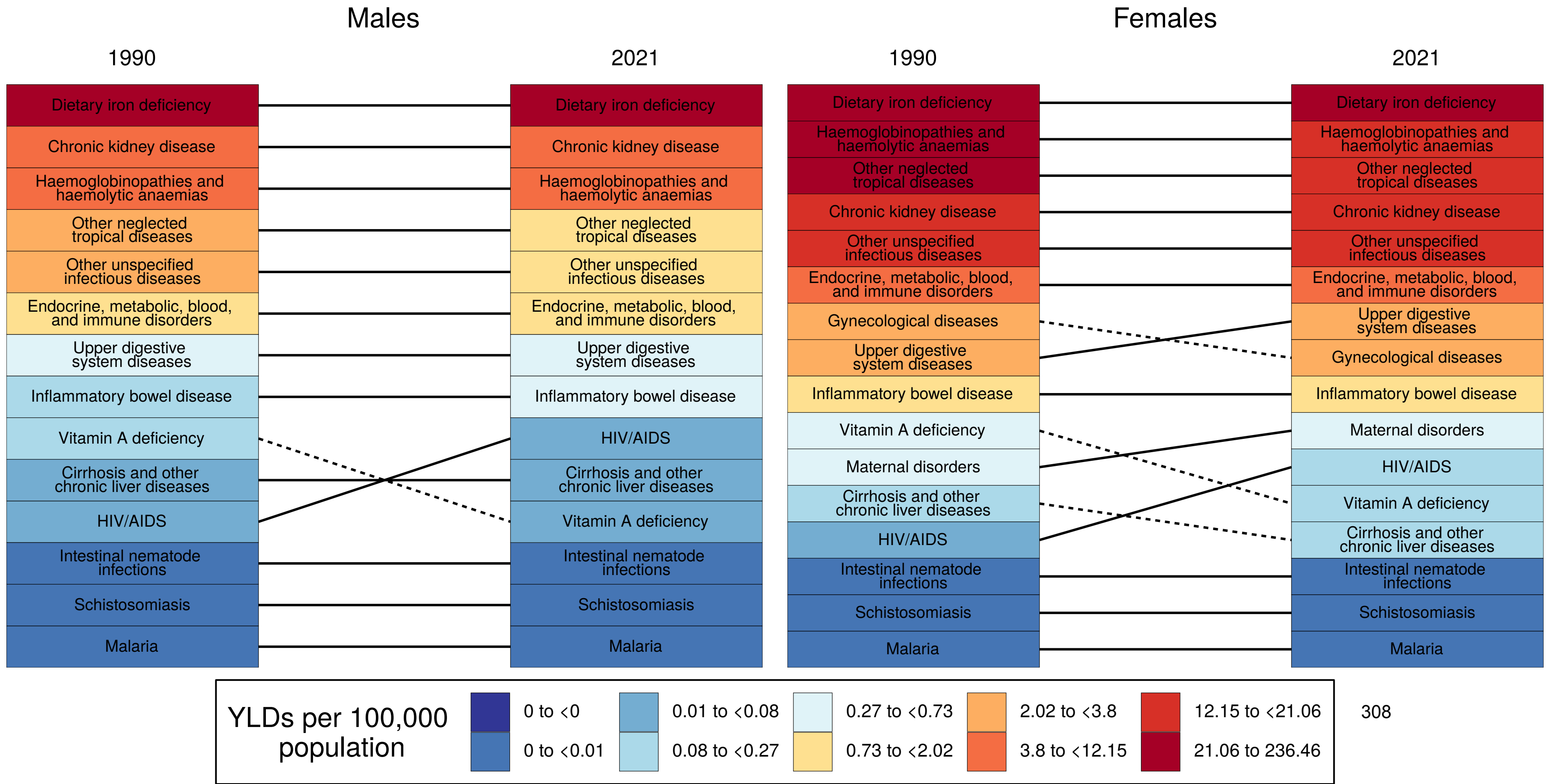

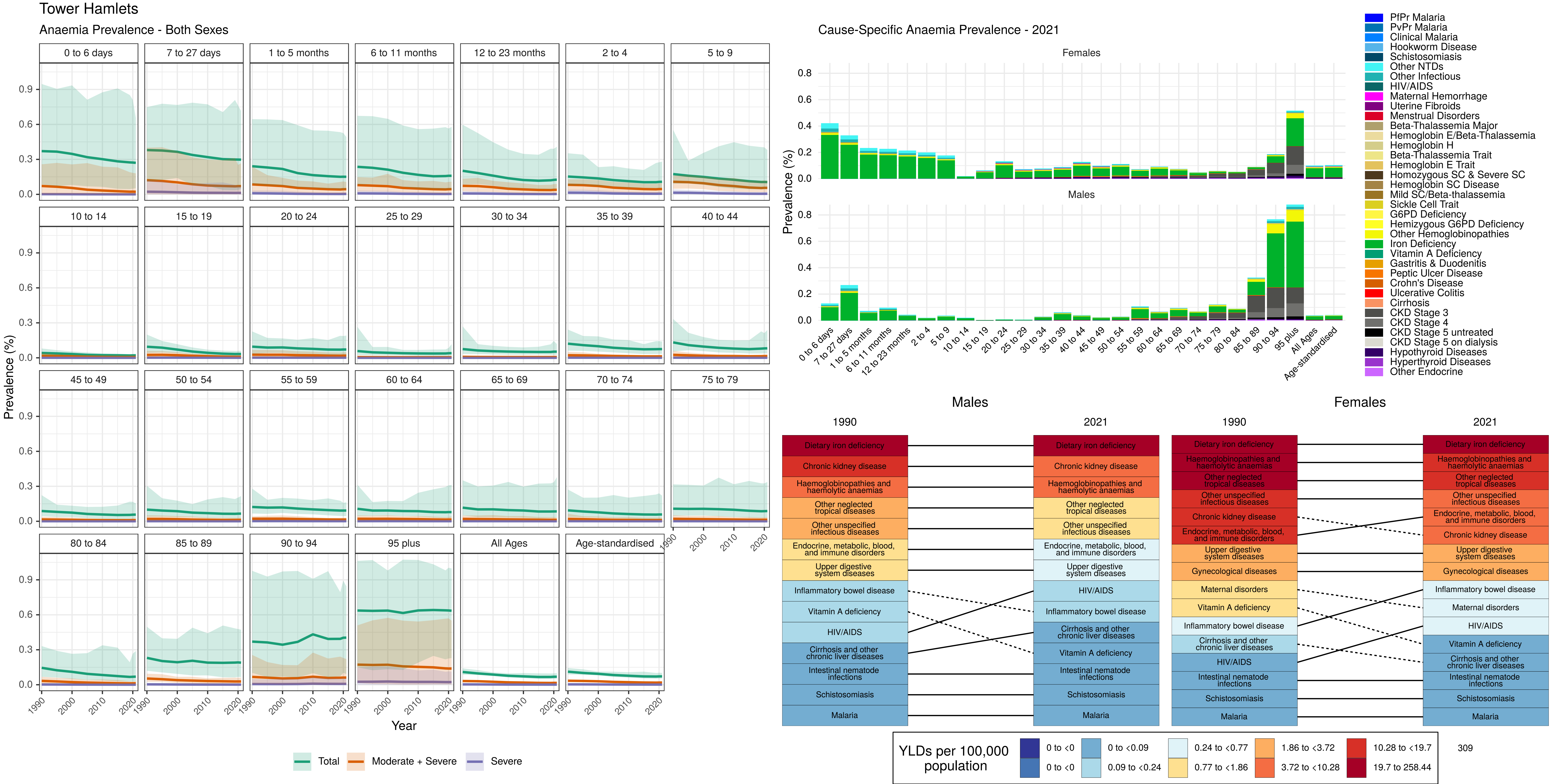
Tower Hamlets
Anaemia Prevalence - Both Sexes
0 to 6 days
7 to 27 days
1 to 5 months
6 to 11 months
12 to 23 months
2 to 4
5 to 9
10 to 14
15 to 19
20 to 24
25 to 29
30 to 34
35 to 39
40 to 44
45 to 49
50 to 54
55 to 59
60 to 64
65 to 69
70 to 74
75 to 79
80 to 84
85 to 89
90 to 94
95 plus
All Ages
Age-standardised
Prevalence (%)
Year
Total
Moderate + Severe
Severe
Cause-Specific Anaemia Prevalence - 2021
Females
Males
PfPr Malaria
PvPr Malaria
Clinical Malaria
Hookworm Disease
Schistosomiasis
Other NTDs
Other Infectious
HIV/AIDS
Maternal Hemorrhage
Uterine Fibroids
Menstrual Disorders
Beta-Thalassemia Major
Hemoglobin E/Beta-Thalassemia
Hemoglobin H
Beta-Thalassemia Trait
Hemoglobin E Trait
Homozygous SC & Severe SC
Hemoglobin SC Disease
Mild SC/Beta-thalassemia
Sickle Cell Trait
G6PD Deficiency
Hemizygous G6PD Deficiency
Other Hemoglobinopathies
Iron Deficiency
Vitamin A Deficiency
Gastritis & Duodenitis
Peptic Ulcer Disease
Crohn's Disease
Ulcerative Colitis
Cirrhosis
CKD Stage 3
CKD Stage 4
CKD Stage 5 untreated
CKD Stage 5 on dialysis
Hypothyroid Diseases
Hyperthyroid Diseases
Other Endocrine
Males
Females
1990
2021
Dietary iron deficiency
Chronic kidney disease
Haemoglobinopathies and haemolytic anaemias
Other neglected tropical diseases
Other unspecified infectious diseases
Endocrine, metabolic, blood, and immune disorders
Upper digestive system diseases
Inflammatory bowel disease
Vitamin A deficiency
HIV/AIDS
Cirrhosis and other chronic liver diseases
Intestinal nematode infections
Schistosomiasis
Malaria
Gynecological diseases
Maternal disorders
YLDs per 100,000 population
0 to <0
0 to <0.09
0.09 to <0.24
0.24 to <0.77
0.77 to <1.86
1.86 to <3.72
3.72 to <10.28
10.28 to <19.7
19.7 to 258.44

Waltham Forest

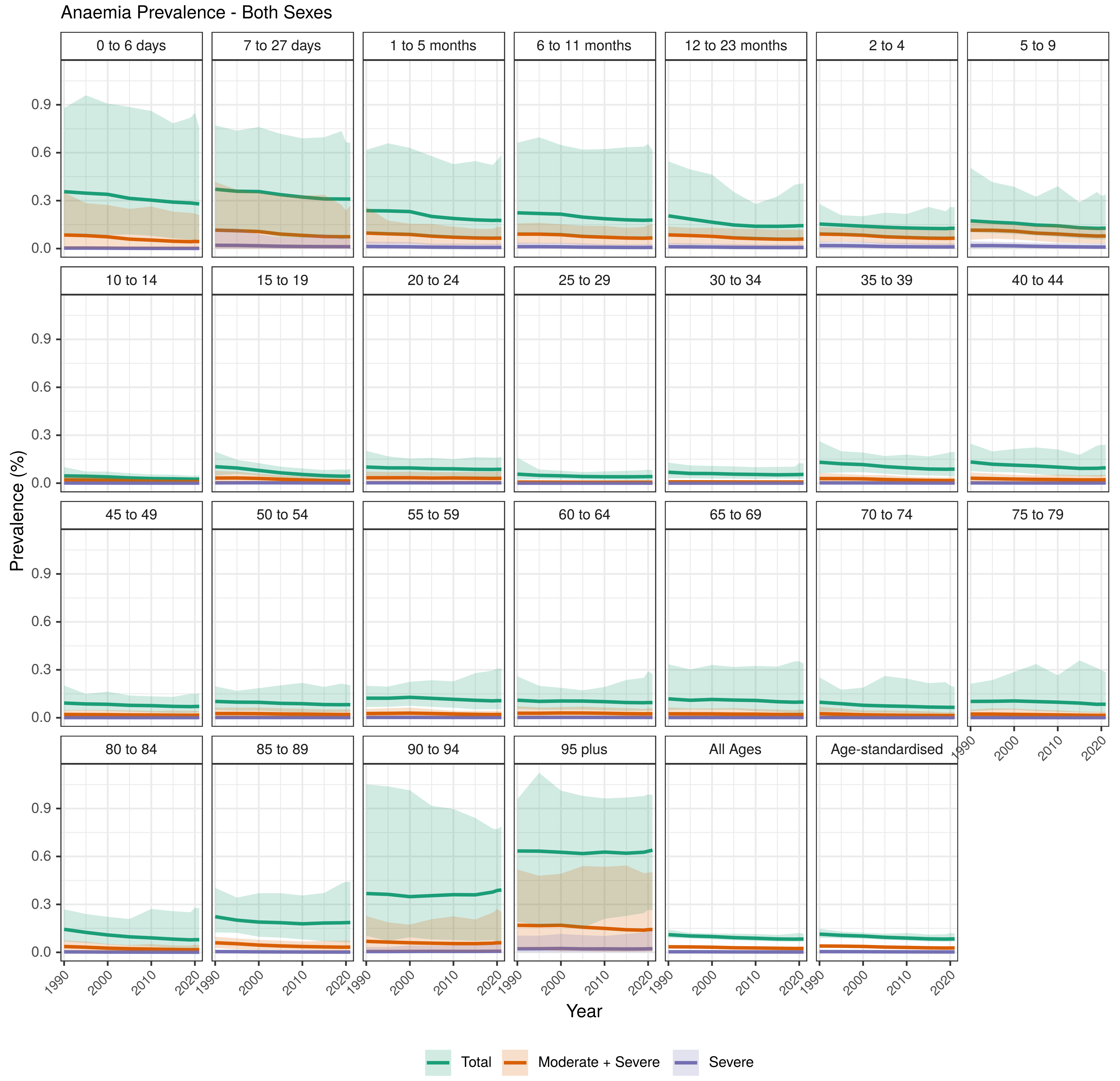

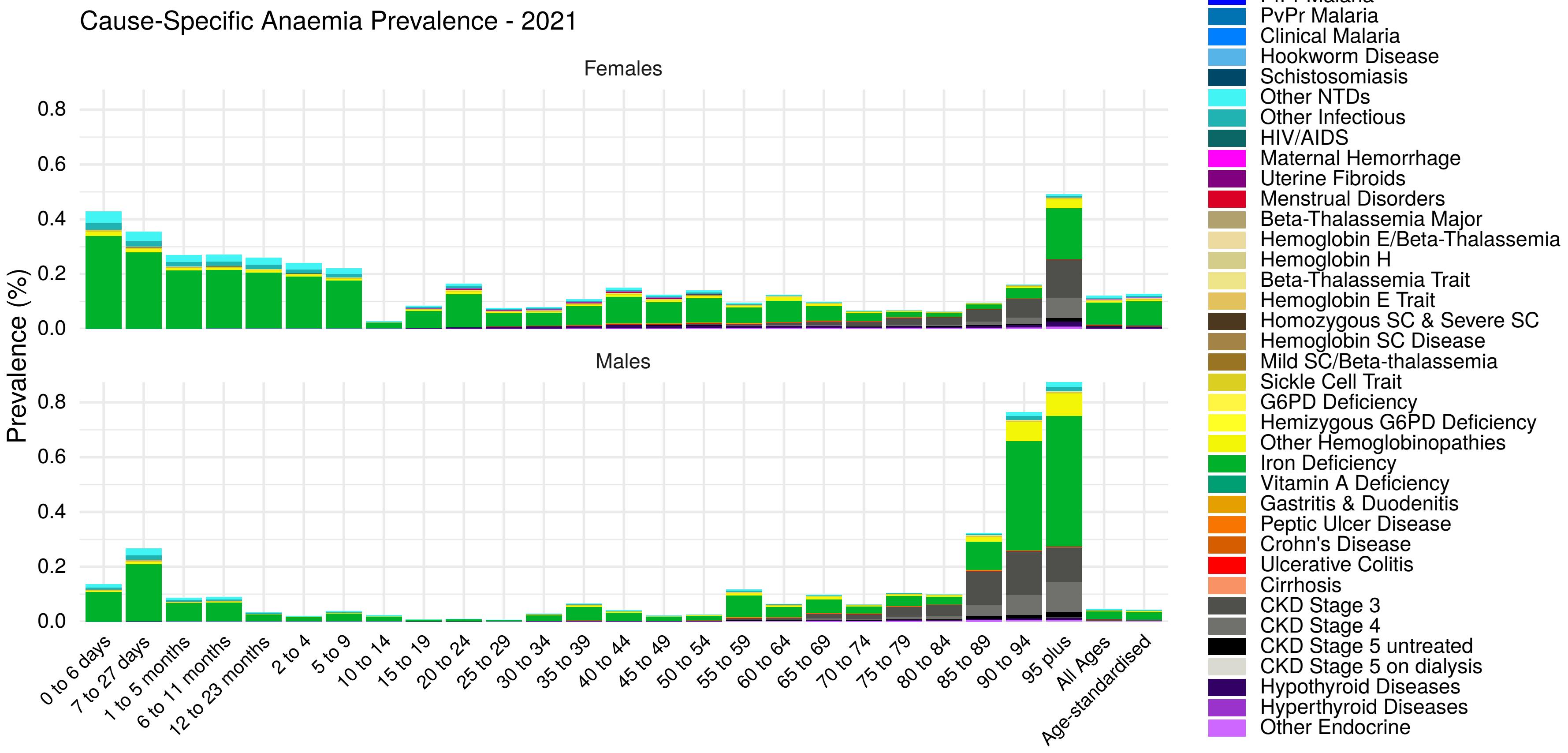

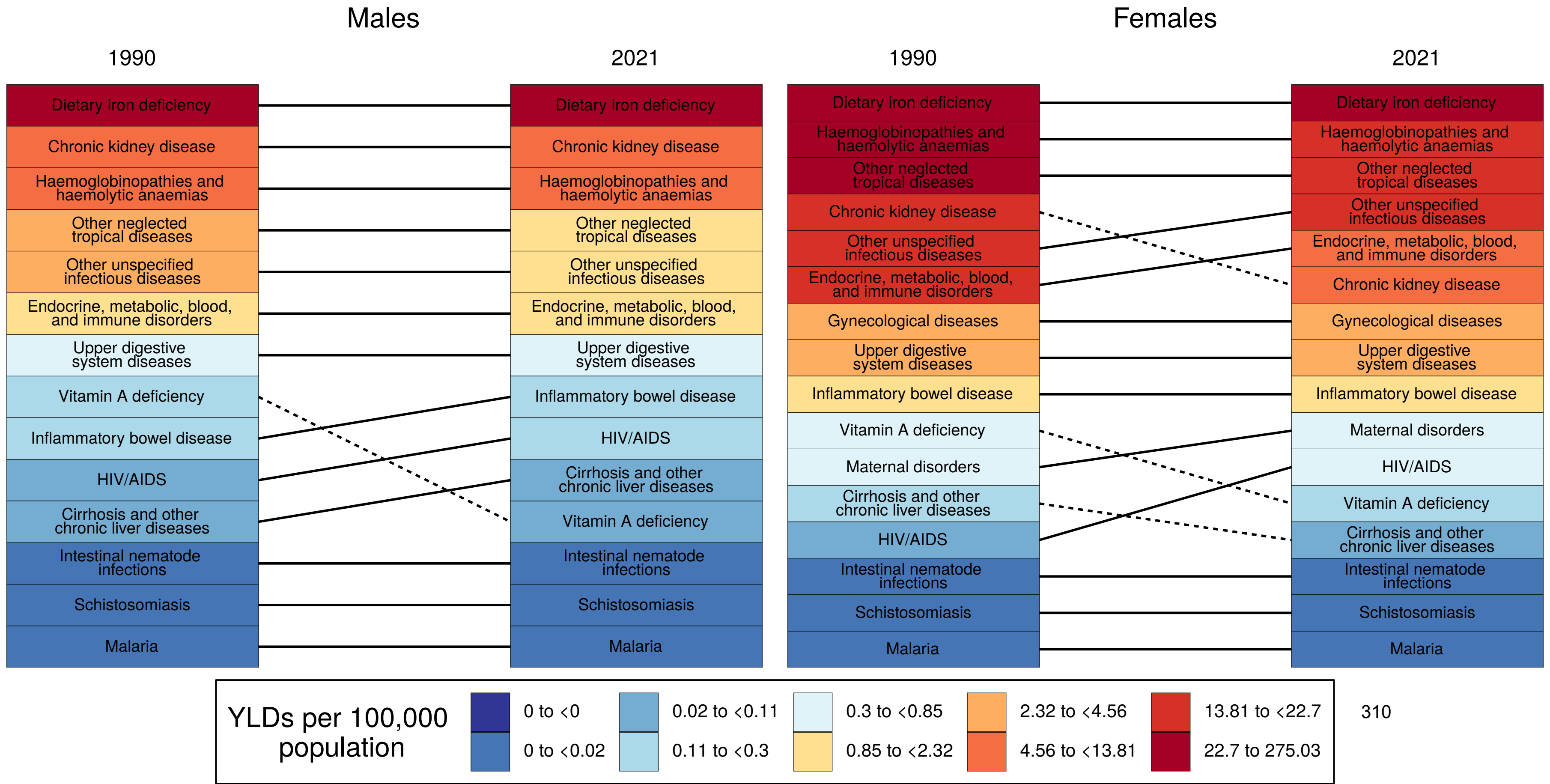

Wandsworth

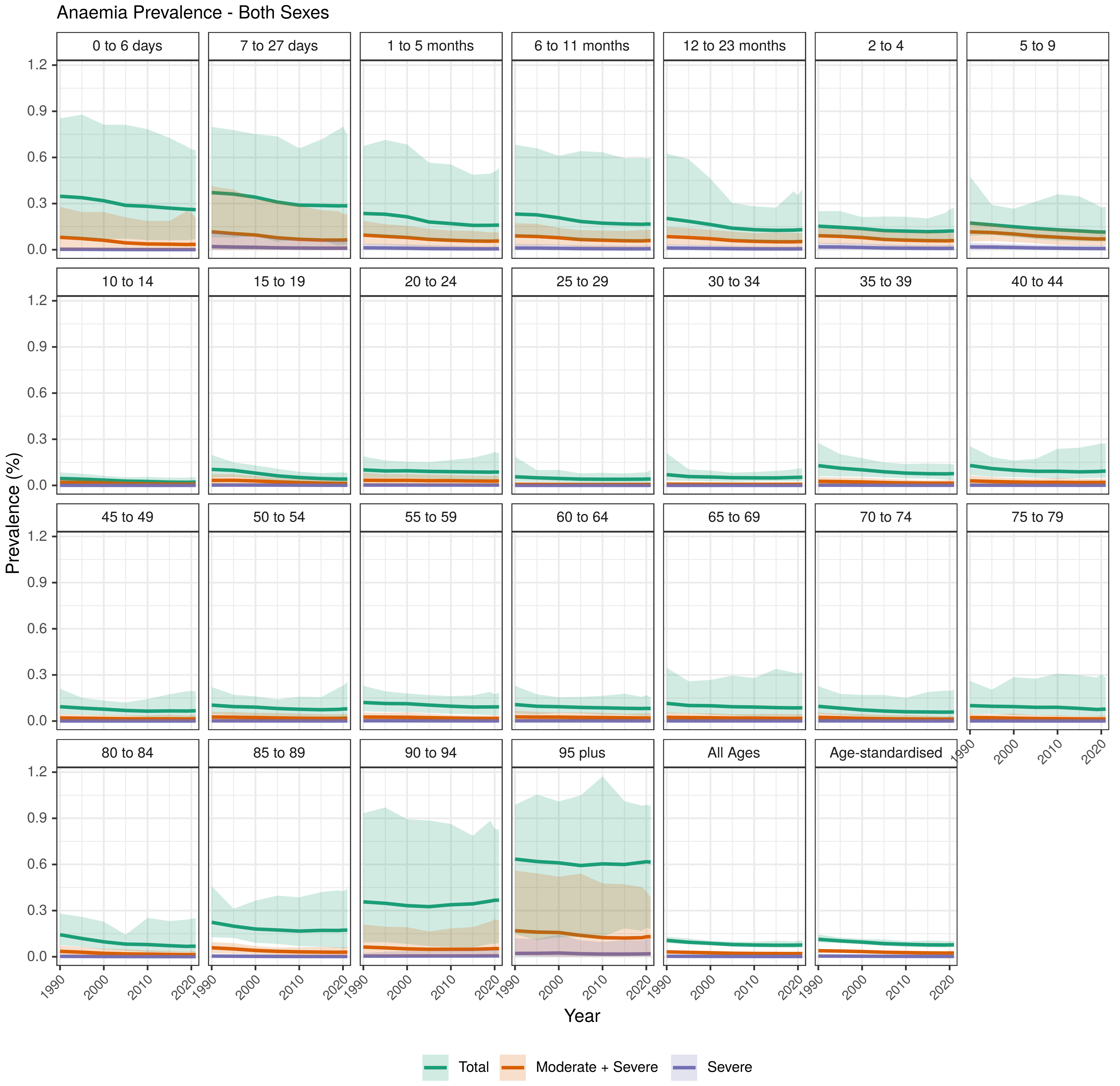

Anaemia Prevalence - Both Sexes
0 to 6 days
7 to 27 days
1 to 5 months
6 to 11 months
12 to 23 months
2 to 4
5 to 9
10 to 14
15 to 19
20 to 24
25 to 29
30 to 34
35 to 39
40 to 44
45 to 49
50 to 54
55 to 59
60 to 64
65 to 69
70 to 74
75 to 79
80 to 84
85 to 89
90 to 94
95 plus
All Ages
Age-standardised
Prevalence (%)
Year
Total
Moderate + Severe
Severe

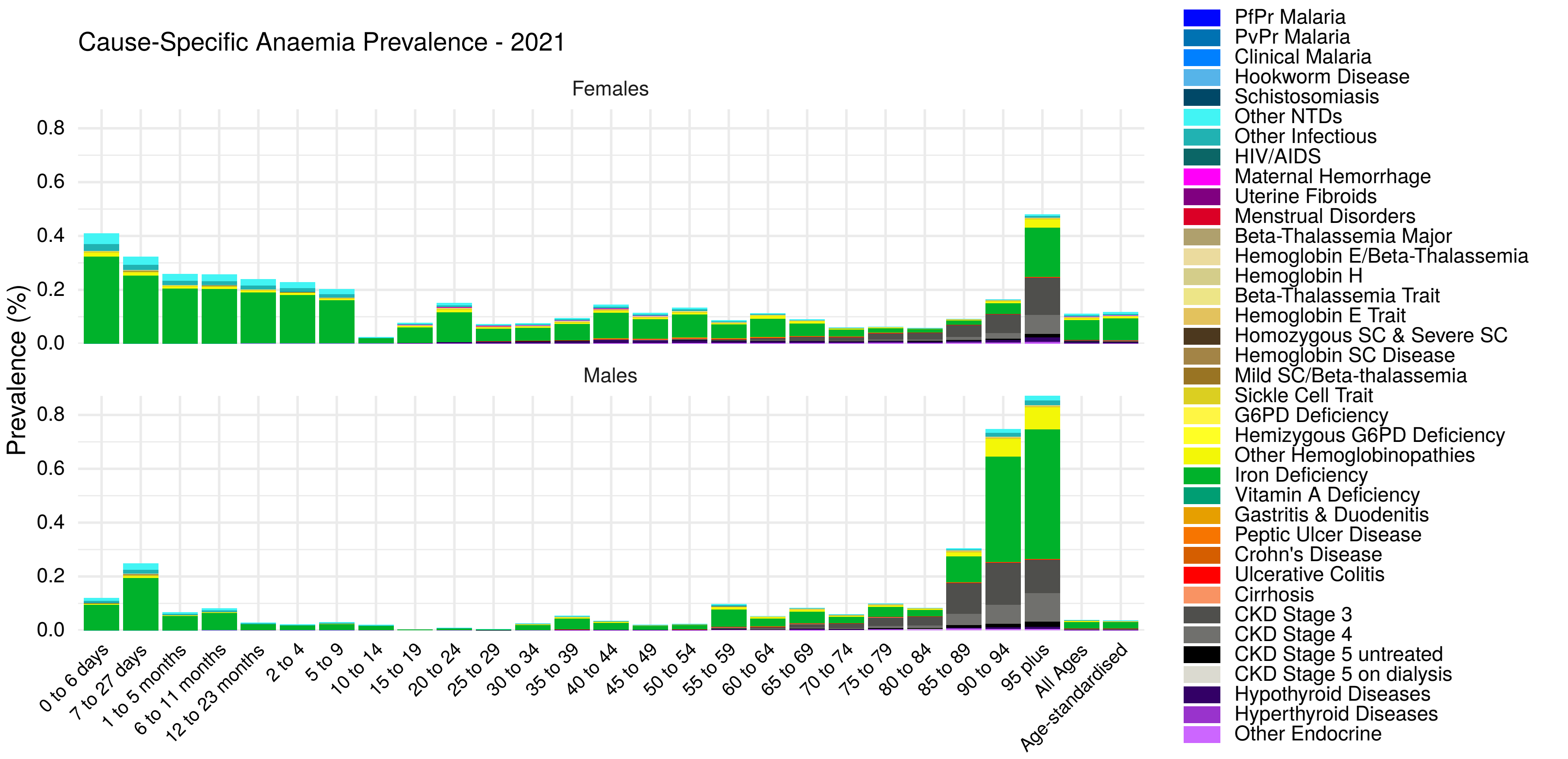

Cause-Specific Anaemia Prevalence - 2021
Females
Males
Prevalence (%)
PfPr Malaria
PvPr Malaria
Clinical Malaria
Hookworm Disease
Schistosomiasis
Other NTDs
Other Infectious
HIV/AIDS
Maternal Hemorrhage
Uterine Fibroids
Menstrual Disorders
Beta-Thalassemia Major
Hemoglobin E/Beta-Thalassemia
Hemoglobin H
Beta-Thalassemia Trait
Hemoglobin E Trait
Homozygous SC & Severe SC
Hemoglobin SC Disease
Mild SC/Beta-thalassemia
Sickle Cell Trait
G6PD Deficiency
Hemizygous G6PD Deficiency
Other Hemoglobinopathies
Iron Deficiency
Vitamin A Deficiency
Gastritis & Duodenitis
Peptic Ulcer Disease
Crohn's Disease
Ulcerative Colitis
Cirrhosis
CKD Stage 3
CKD Stage 4
CKD Stage 5 untreated
CKD Stage 5 on dialysis
Hypothyroid Diseases
Hyperthyroid Diseases
Other Endocrine

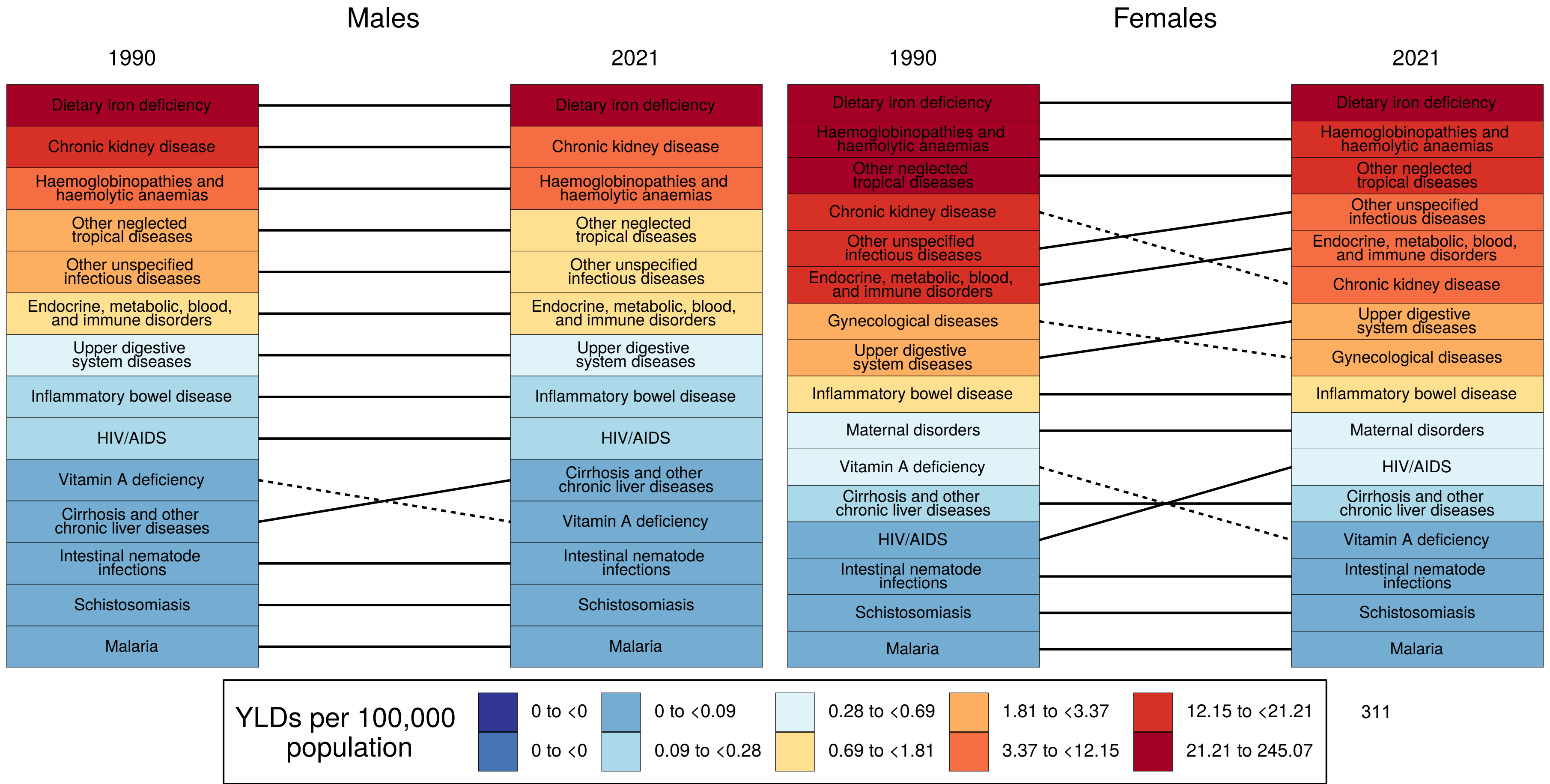

Males
Females
1990
2021
Dietary iron deficiency
Chronic kidney disease
Haemoglobinopathies and haemolytic anaemias
Other neglected tropical diseases
Other unspecified infectious diseases
Endocrine, metabolic, blood, and immune disorders
Upper digestive system diseases
Inflammatory bowel disease
HIV/AIDS
Vitamin A deficiency
Cirrhosis and other chronic liver diseases
Intestinal nematode infections
Schistosomiasis
Malaria
Gynecological diseases
Maternal disorders
YLDs per 100,000 population
0 to <0
0 to <0.09
0.09 to <0.28
0.28 to <0.69
0.69 to <1.81
1.81 to <3.37
3.37 to <12.15
12.15 to <21.21
21.21 to 245.07

Westminster

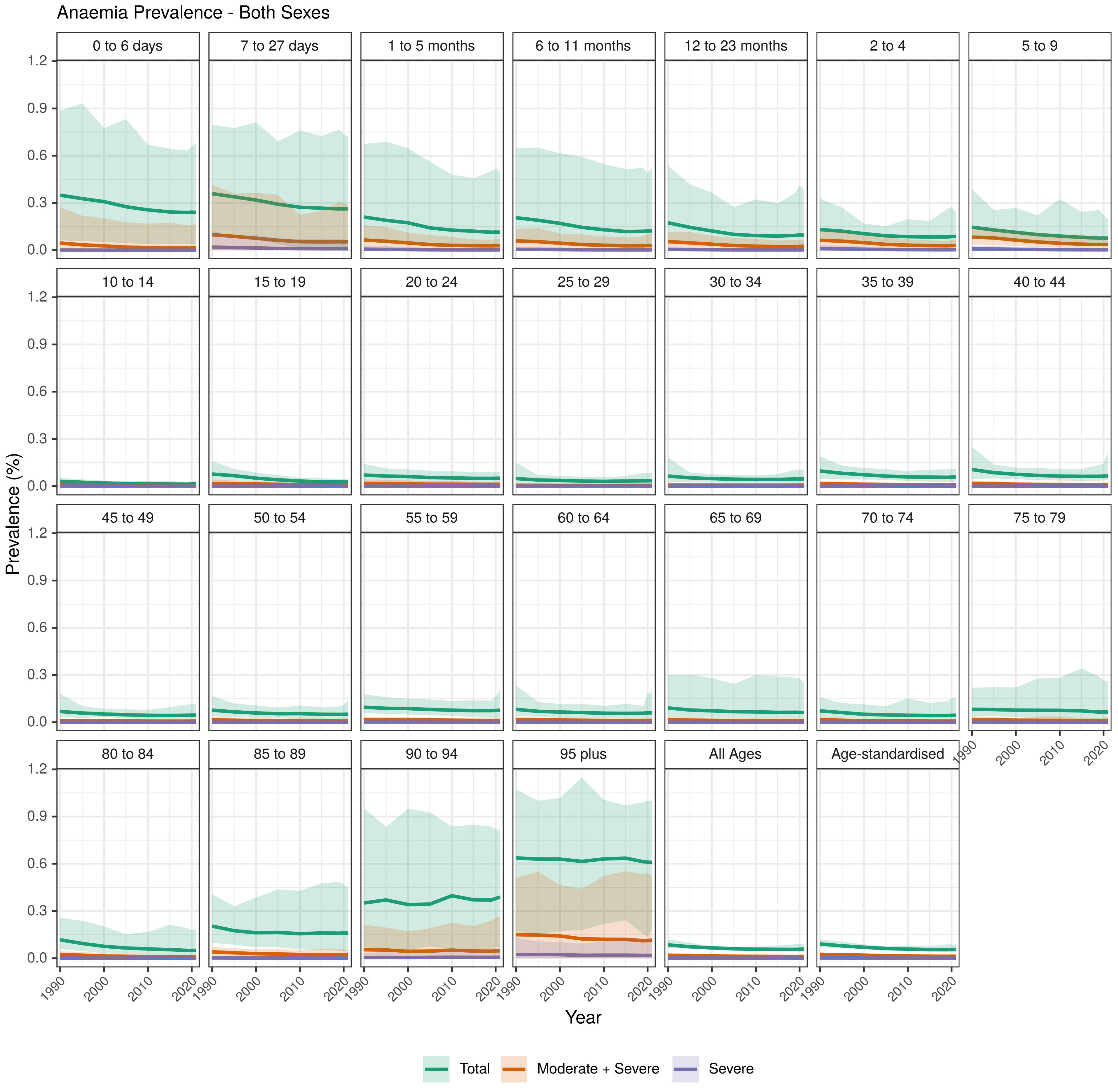

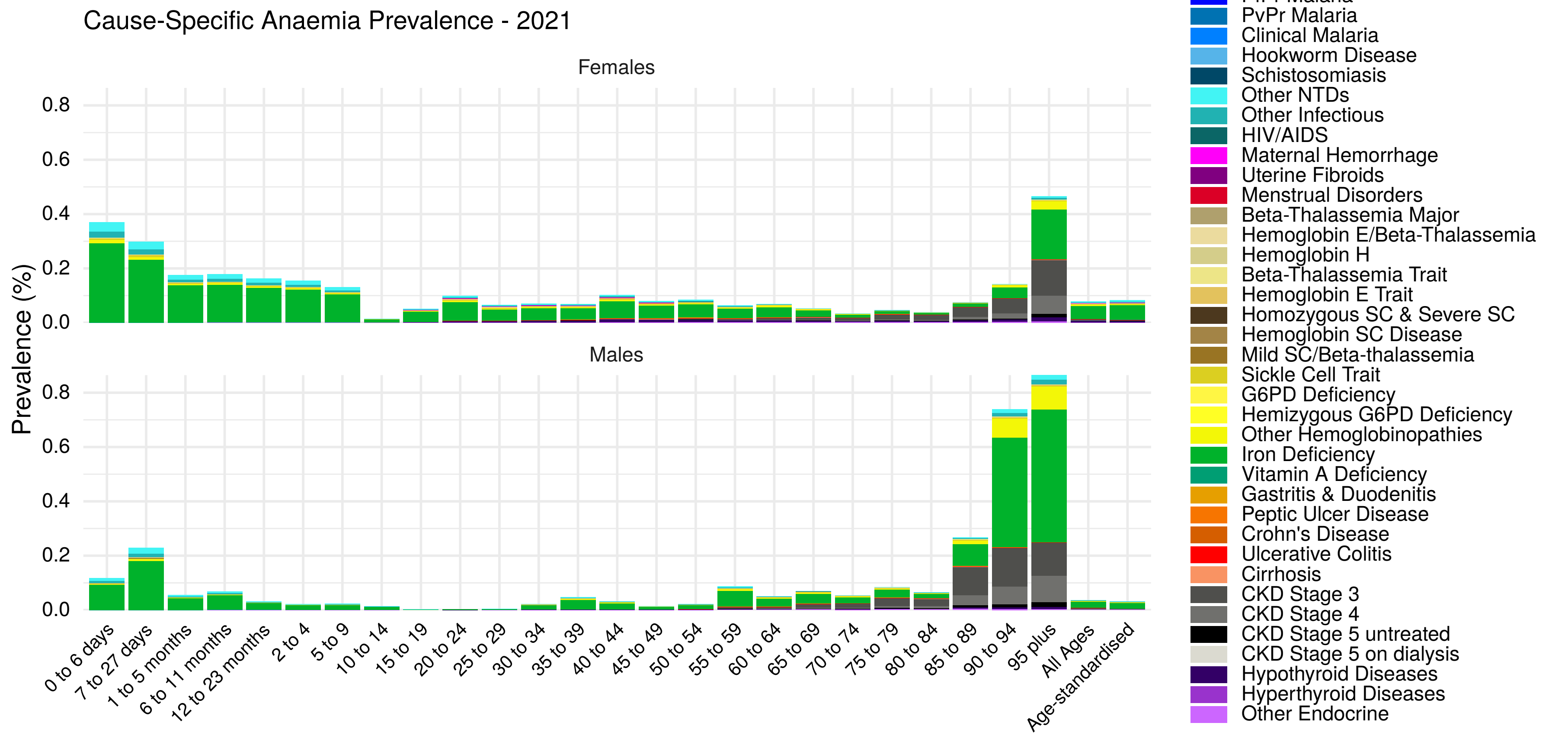

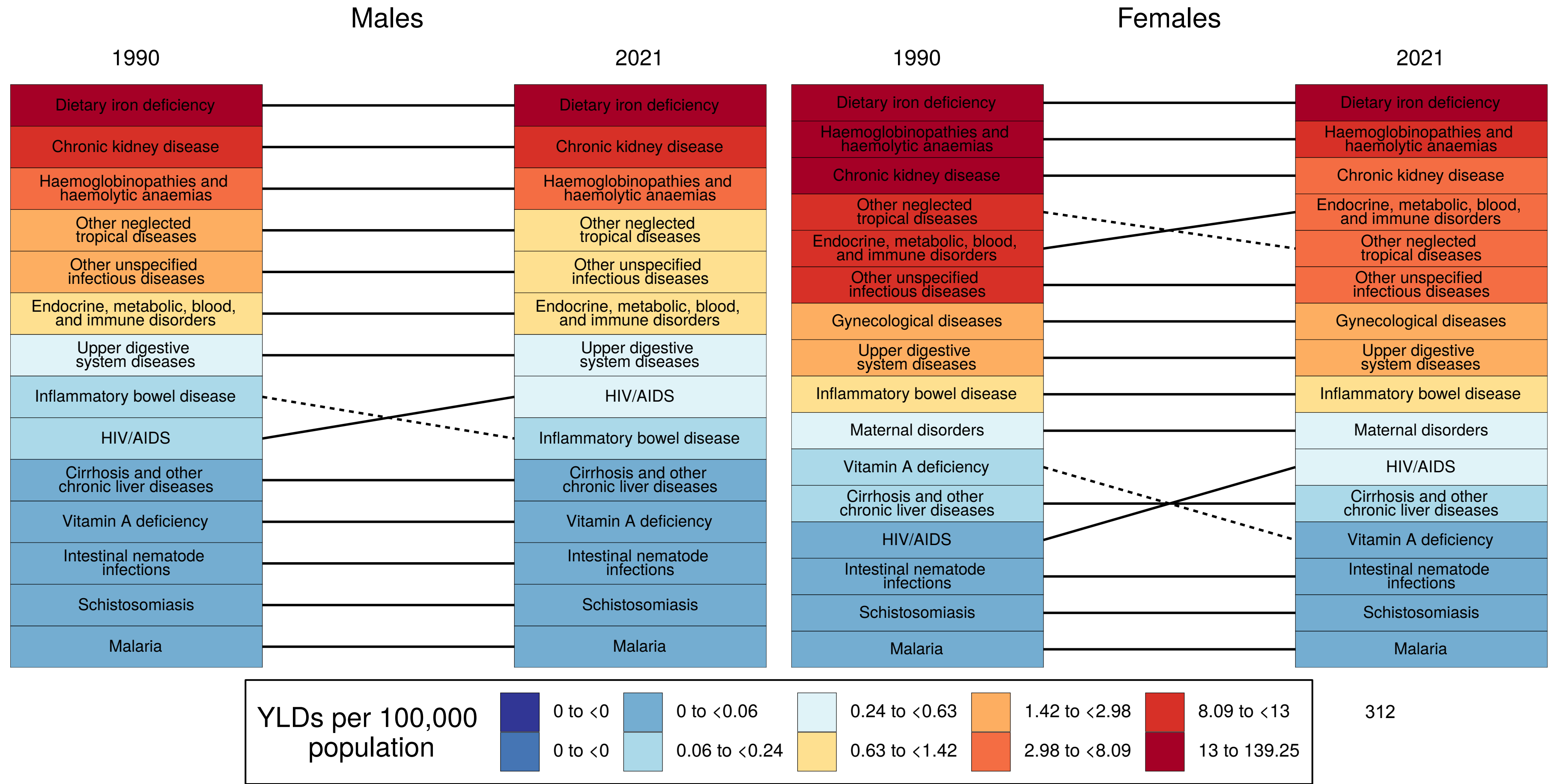

North East England

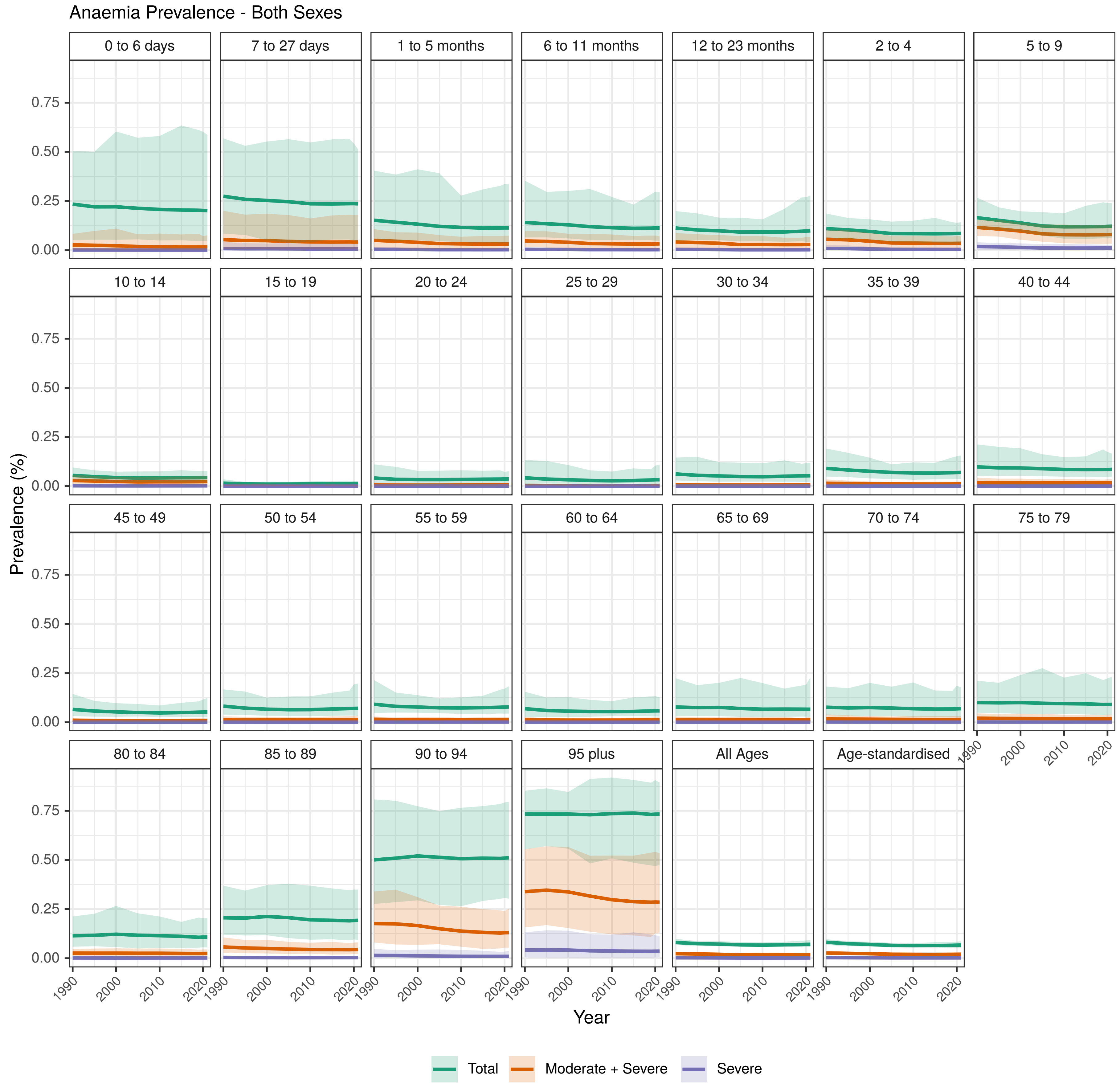

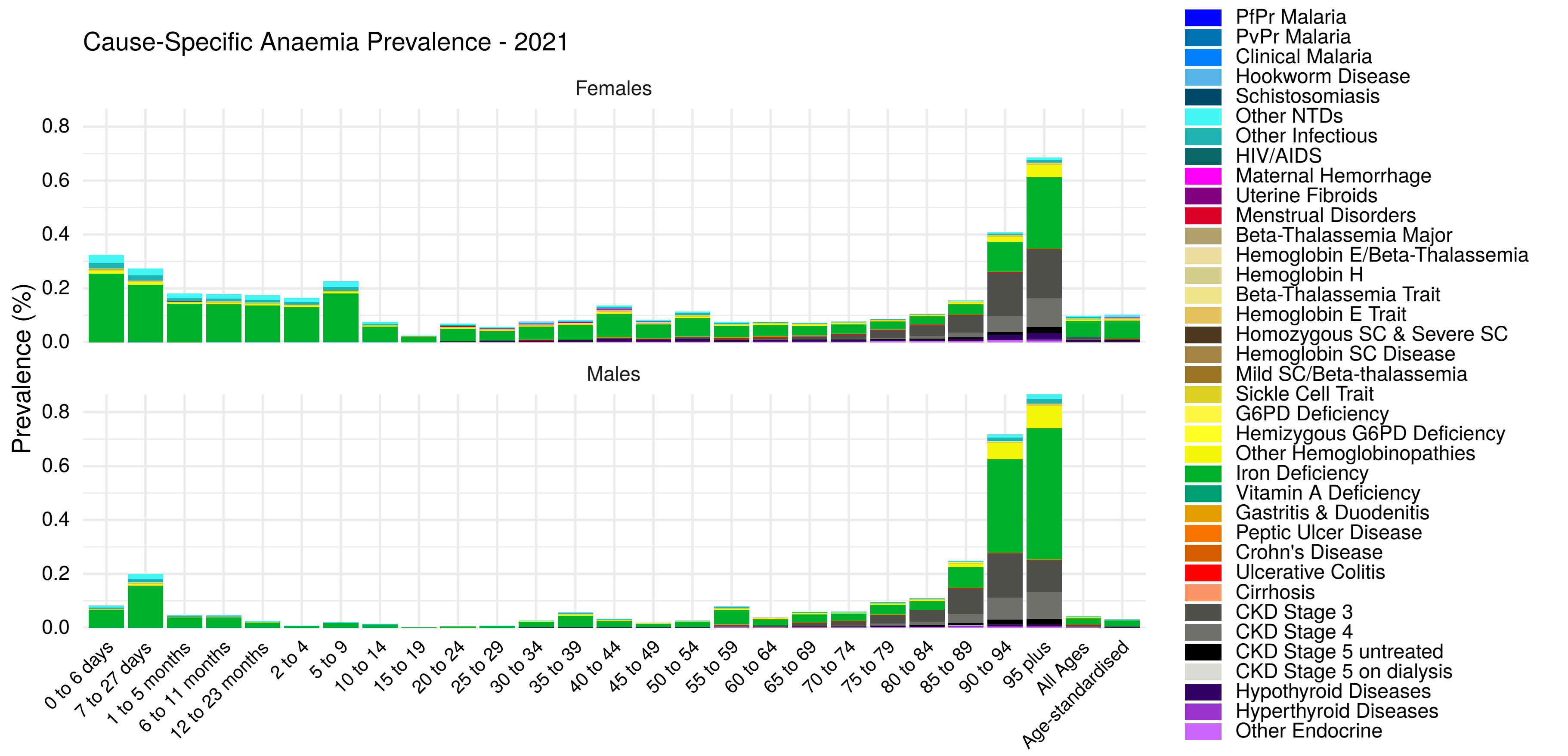

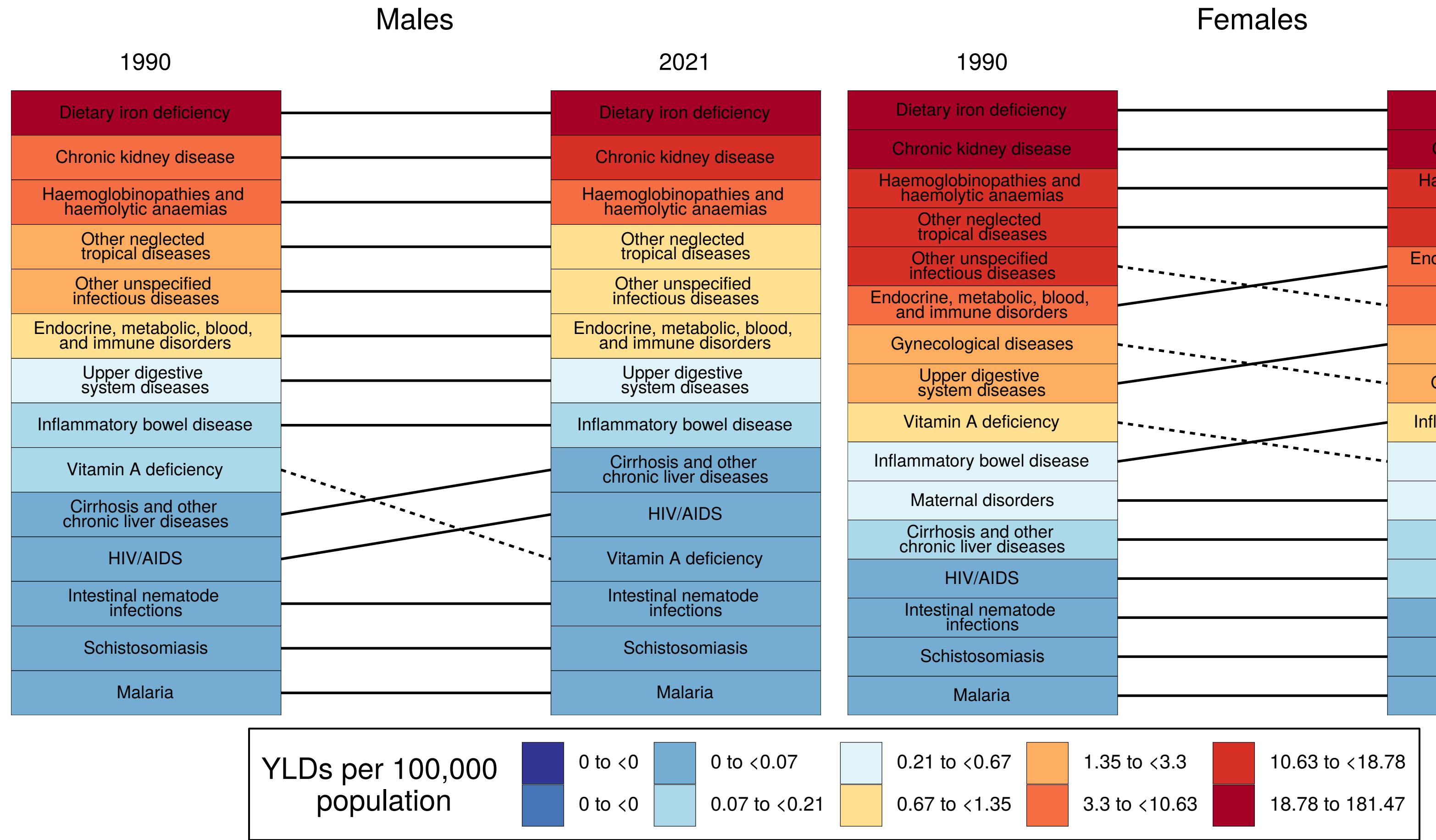

County Durham

Anaemia Prevalence - Both Sexes

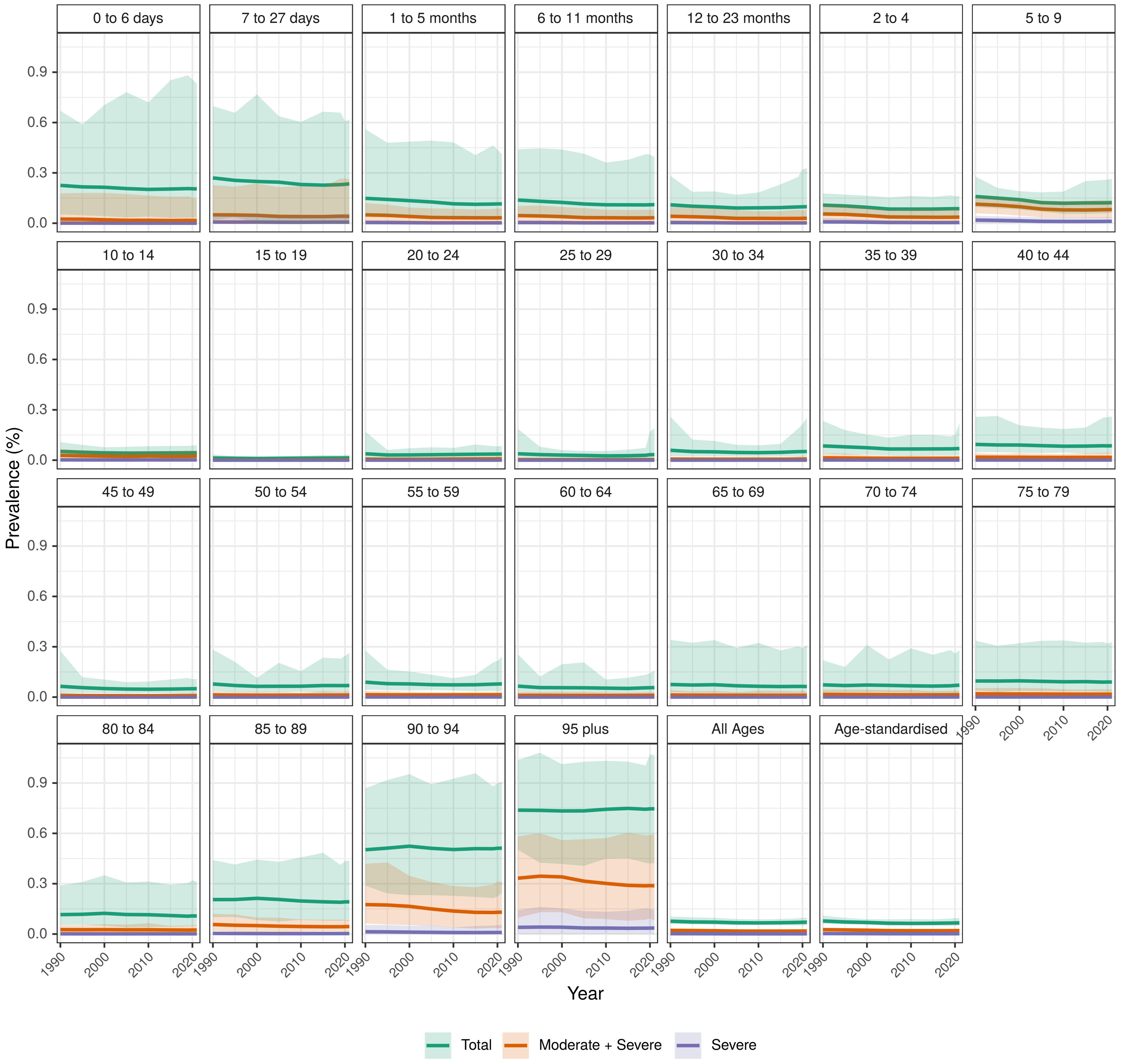

Cause-Specific Anaemia Prevalence - 2021

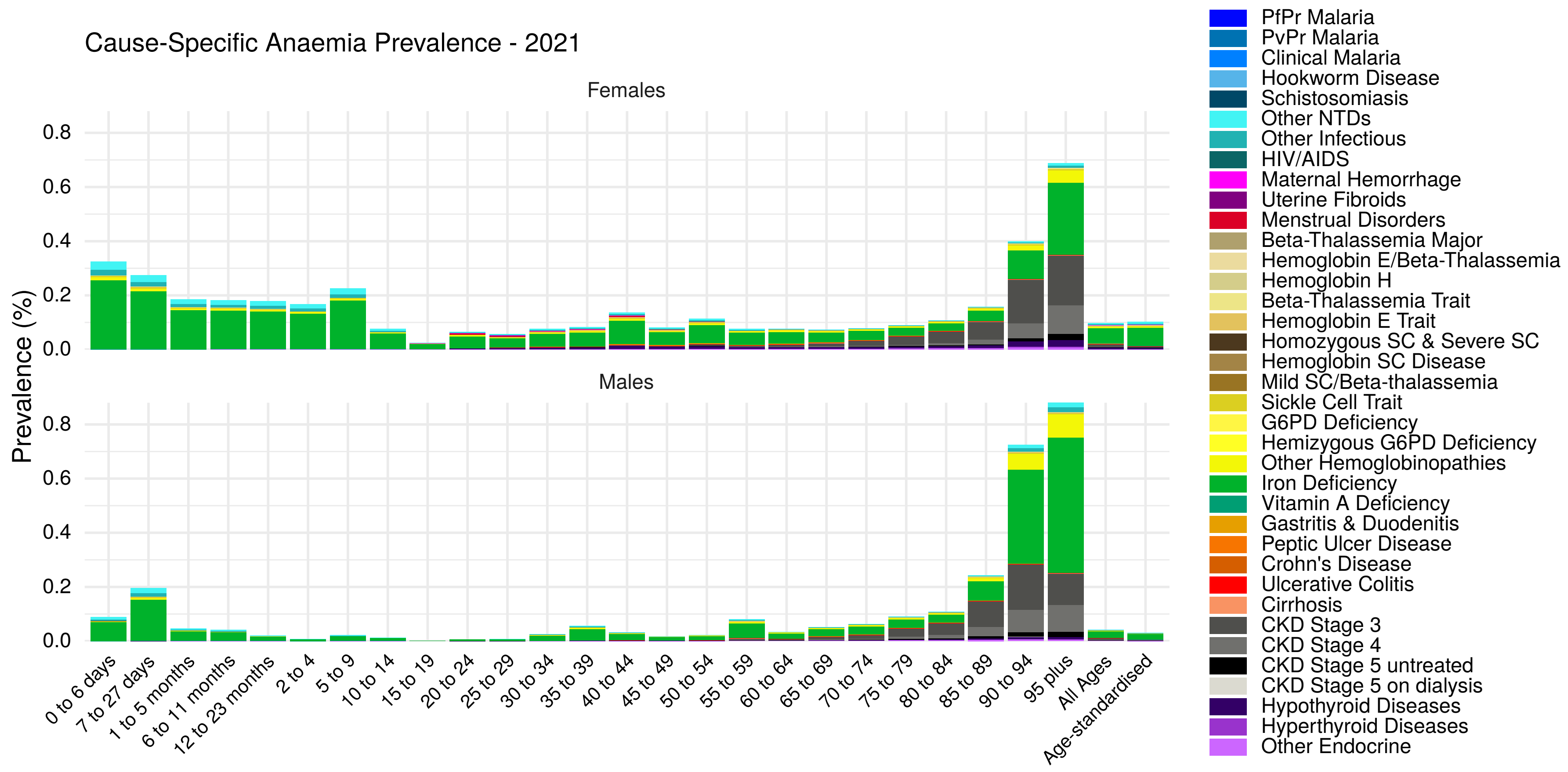

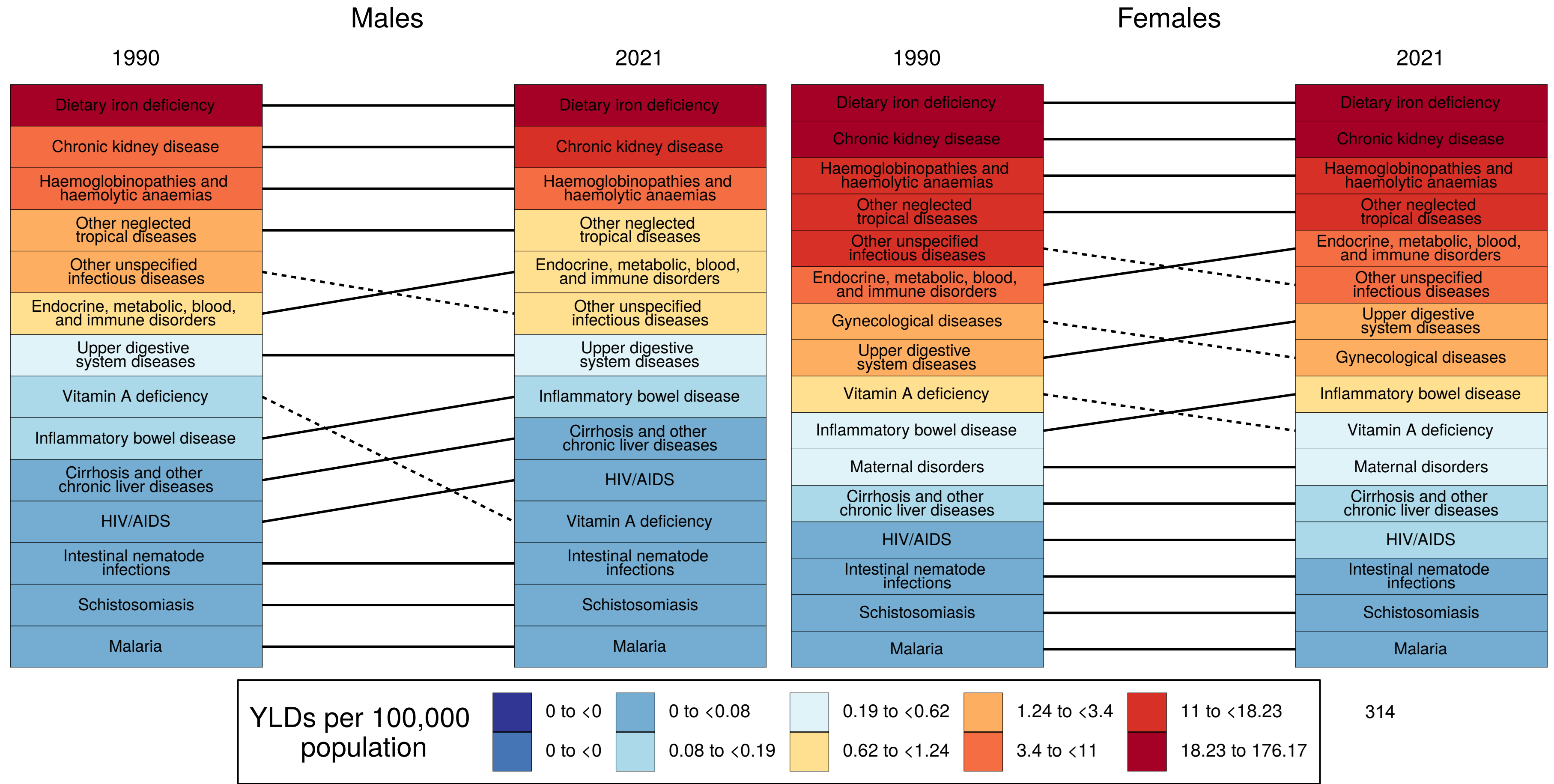

Darlington

Anaemia Prevalence - Both Sexes

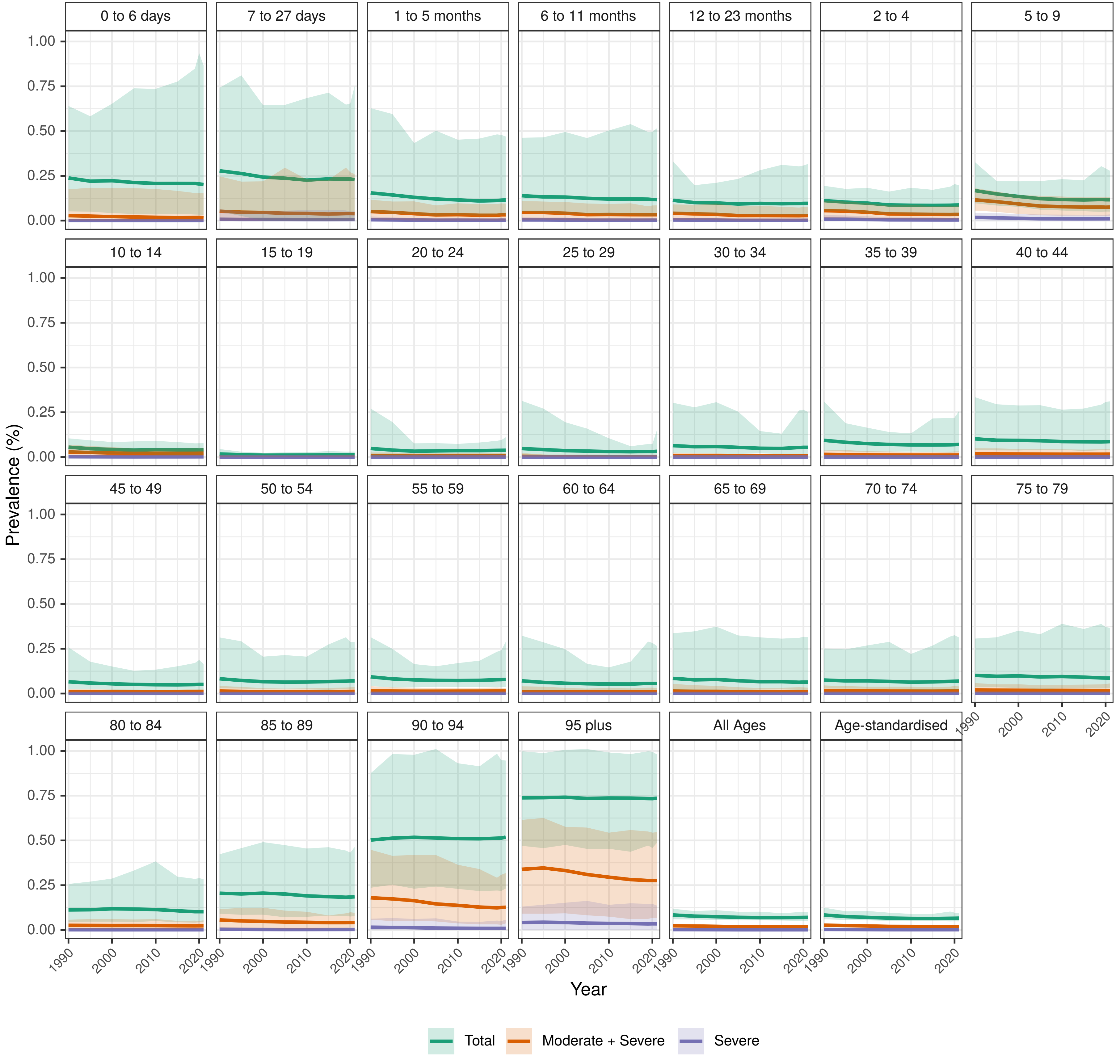

Cause-Specific Anaemia Prevalence - 2021

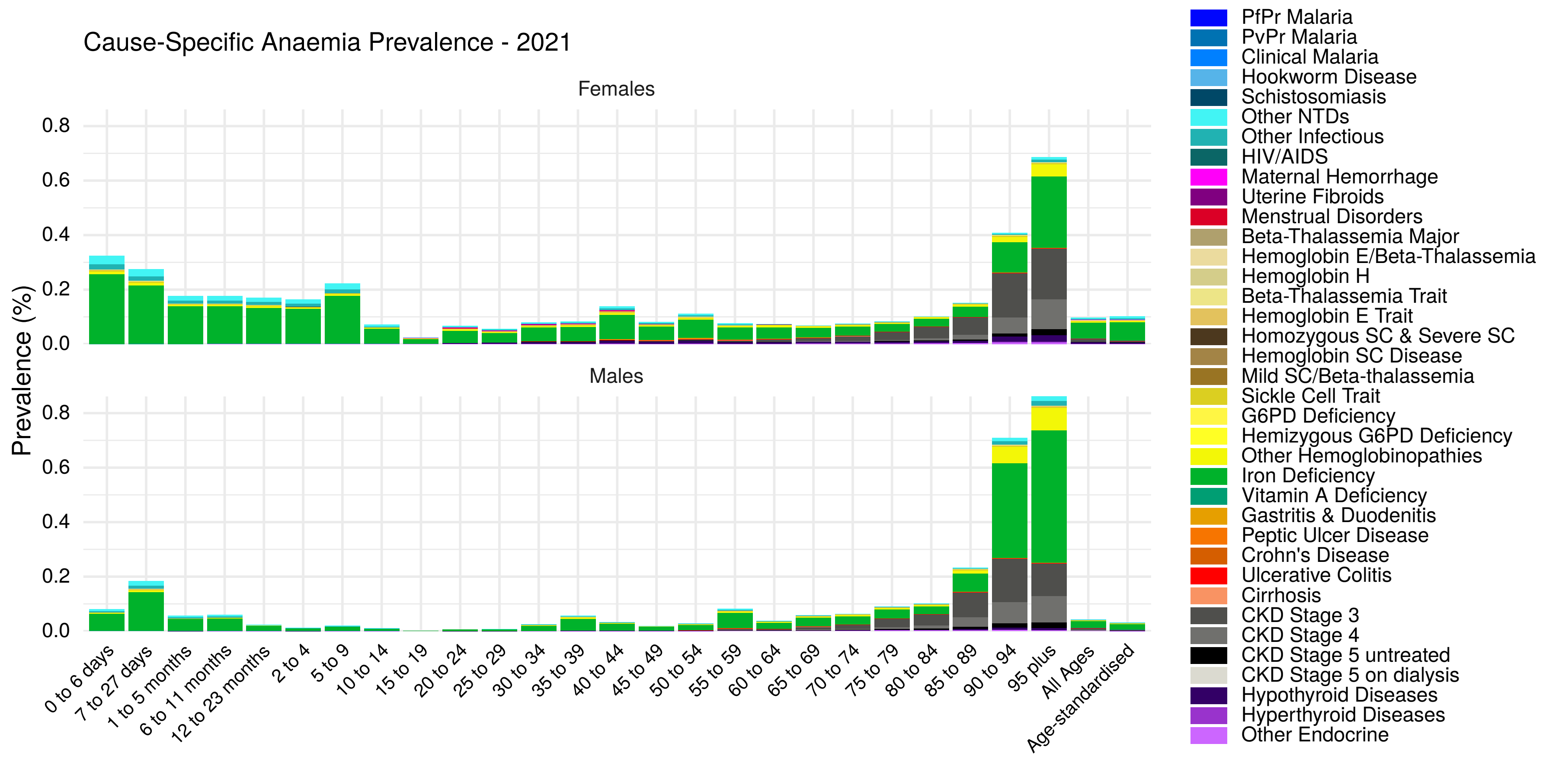

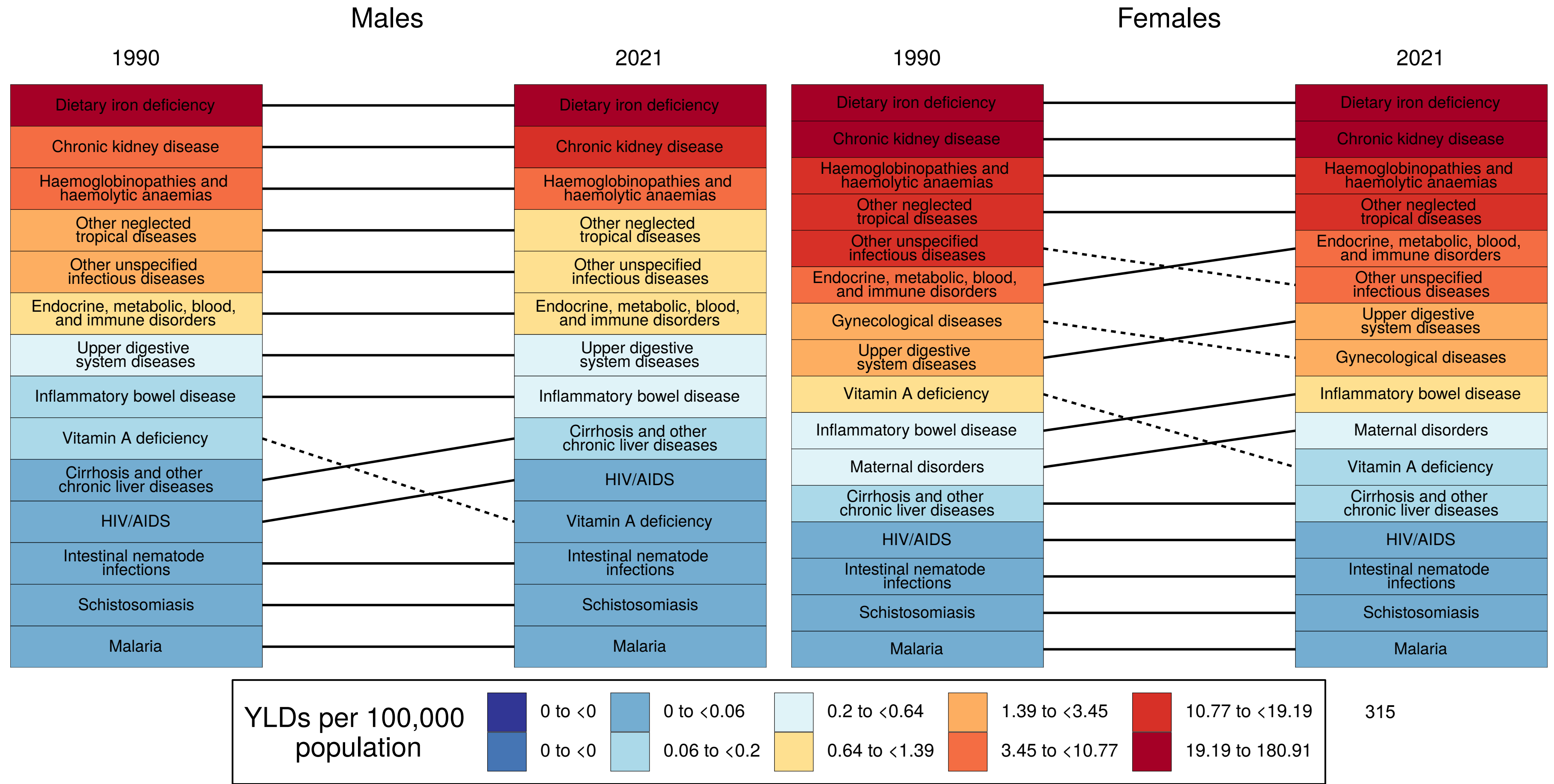

Gateshead

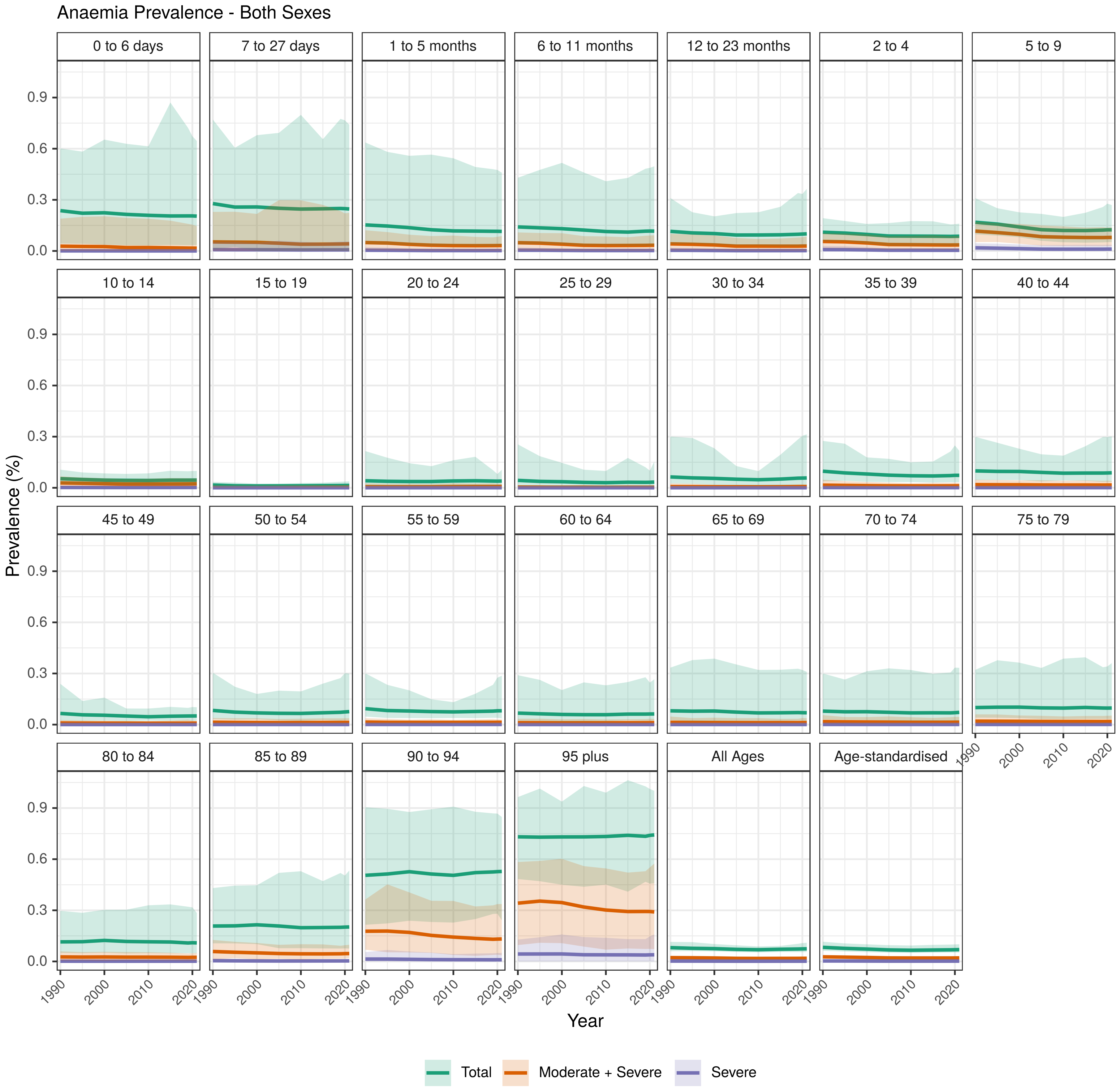

Anaemia Prevalence - Both Sexes
0 to 6 days
7 to 27 days
1 to 5 months
6 to 11 months
12 to 23 months
2 to 4
5 to 9
10 to 14
15 to 19
20 to 24
25 to 29
30 to 34
35 to 39
40 to 44
45 to 49
50 to 54
55 to 59
60 to 64
65 to 69
70 to 74
75 to 79
80 to 84
85 to 89
90 to 94
95 plus
All Ages
Age-standardised
Prevalence (%)
0.0
0.3
0.6
0.9
1990
2000
2010
2020
Year
Total
Moderate + Severe
Severe

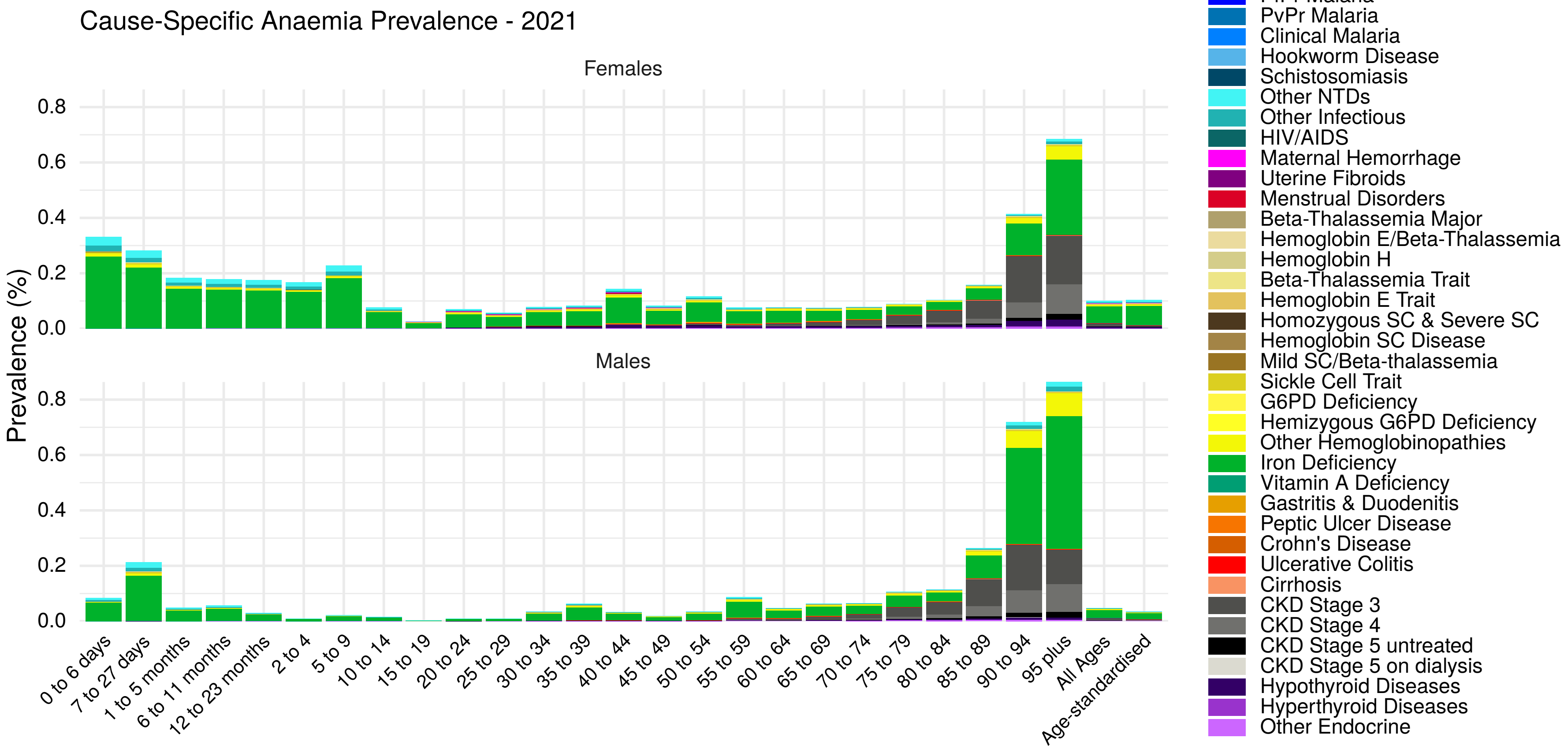

Cause-Specific Anaemia Prevalence - 2021
Females
Males
Prevalence (%)
0.0
0.2
0.4
0.6
0.8
0 to 6 days
7 to 27 days
1 to 5 months
6 to 11 months
12 to 23 months
2 to 4
5 to 9
10 to 14
15 to 19
20 to 24
25 to 29
30 to 34
35 to 39
40 to 44
45 to 49
50 to 54
55 to 59
60 to 64
65 to 69
70 to 74
75 to 79
80 to 84
85 to 89
90 to 94
95 plus
All Ages
Age-standardised
PfPr Malaria
PvPr Malaria
Clinical Malaria
Hookworm Disease
Schistosomiasis
Other NTDs
Other Infectious
HIV/AIDS
Maternal Hemorrhage
Uterine Fibroids
Menstrual Disorders
Beta-Thalassemia Major
Hemoglobin E/Beta-Thalassemia
Hemoglobin H
Beta-Thalassemia Trait
Hemoglobin E Trait
Homozygous SC & Severe SC
Hemoglobin SC Disease
Mild SC/Beta-thalassemia
Sickle Cell Trait
G6PD Deficiency
Hemizygous G6PD Deficiency
Other Hemoglobinopathies
Iron Deficiency
Vitamin A Deficiency
Gastritis & Duodenitis
Peptic Ulcer Disease
Crohn's Disease
Ulcerative Colitis
Cirrhosis
CKD Stage 3
CKD Stage 4
CKD Stage 5 untreated
CKD Stage 5 on dialysis
Hypothyroid Diseases
Hyperthyroid Diseases
Other Endocrine

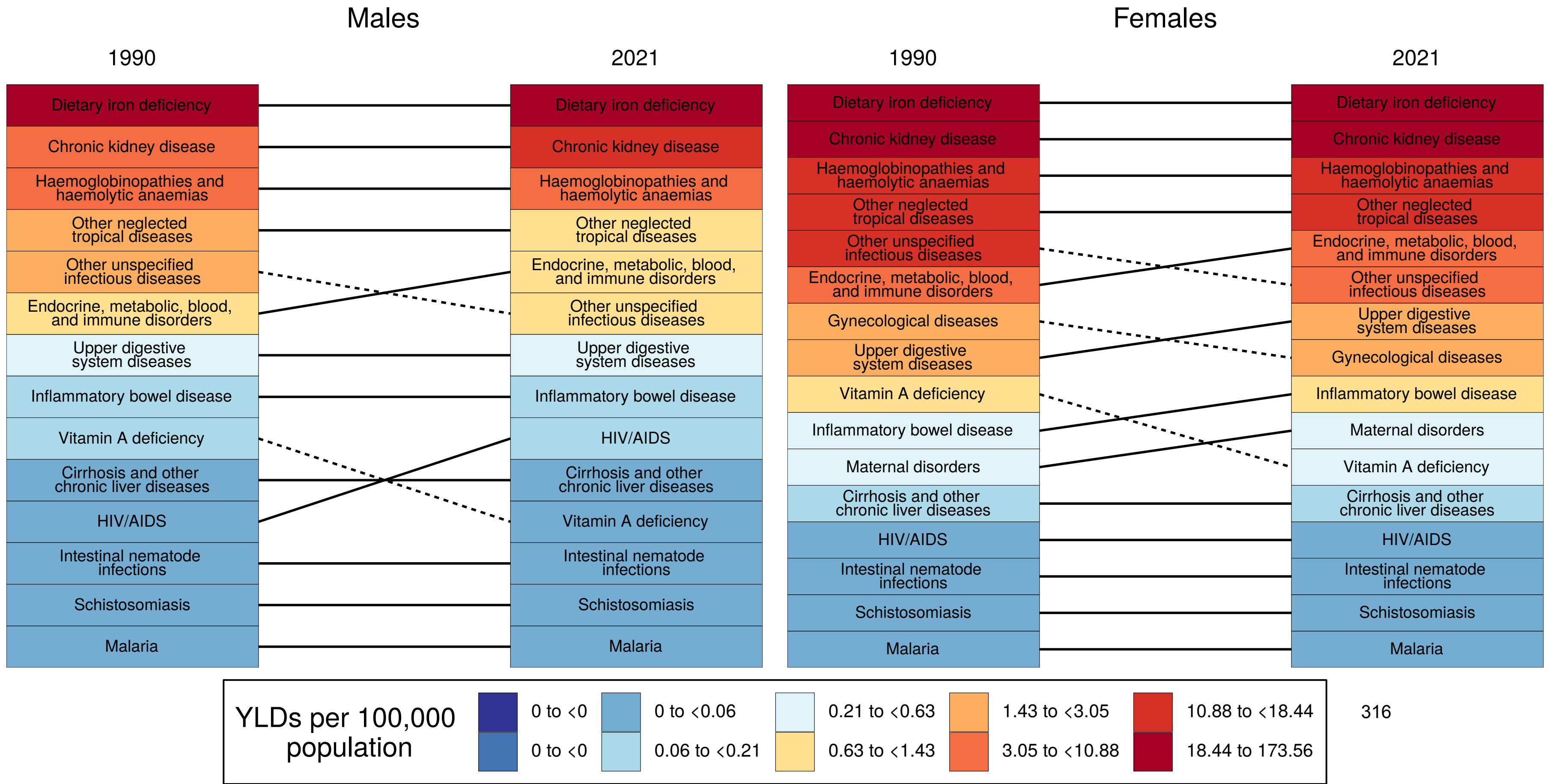

Males
1990
Dietary iron deficiency
Chronic kidney disease
Haemoglobinopathies and haemolytic anaemias
Other neglected tropical diseases
Other unspecified infectious diseases
Endocrine, metabolic, blood, and immune disorders
Upper digestive system diseases
Inflammatory bowel disease
Vitamin A deficiency
Cirrhosis and other chronic liver diseases
HIV/AIDS
Intestinal nematode infections
Schistosomiasis
Malaria
2021
Dietary iron deficiency
Chronic kidney disease
Haemoglobinopathies and haemolytic anaemias
Other neglected tropical diseases
Endocrine, metabolic, blood, and immune disorders
Other unspecified infectious diseases
Upper digestive system diseases
Inflammatory bowel disease
HIV/AIDS
Cirrhosis and other chronic liver diseases
Vitamin A deficiency
Intestinal nematode infections
Schistosomiasis
Malaria
Females
1990
Dietary iron deficiency
Chronic kidney disease
Haemoglobinopathies and haemolytic anaemias
Other neglected tropical diseases
Other unspecified infectious diseases
Endocrine, metabolic, blood, and immune disorders
Gynecological diseases
Upper digestive system diseases
Vitamin A deficiency
Inflammatory bowel disease
Maternal disorders
Cirrhosis and other chronic liver diseases
HIV/AIDS
Intestinal nematode infections
Schistosomiasis
Malaria
2021
Dietary iron deficiency
Chronic kidney disease
Haemoglobinopathies and haemolytic anaemias
Other neglected tropical diseases
Endocrine, metabolic, blood, and immune disorders
Other unspecified infectious diseases
Upper digestive system diseases
Gynecological diseases
Inflammatory bowel disease
Maternal disorders
Vitamin A deficiency
Cirrhosis and other chronic liver diseases
HIV/AIDS
Intestinal nematode infections
Schistosomiasis
Malaria
YLDs per 100,000 population
0 to <0
0 to <0
0 to <0.06
0.06 to <0.21
0.21 to <0.63
0.63 to <1.43
1.43 to <3.05
3.05 to <10.88
10.88 to <18.44
18.44 to 173.56

Hartlepool

Anaemia Prevalence - Both Sexes

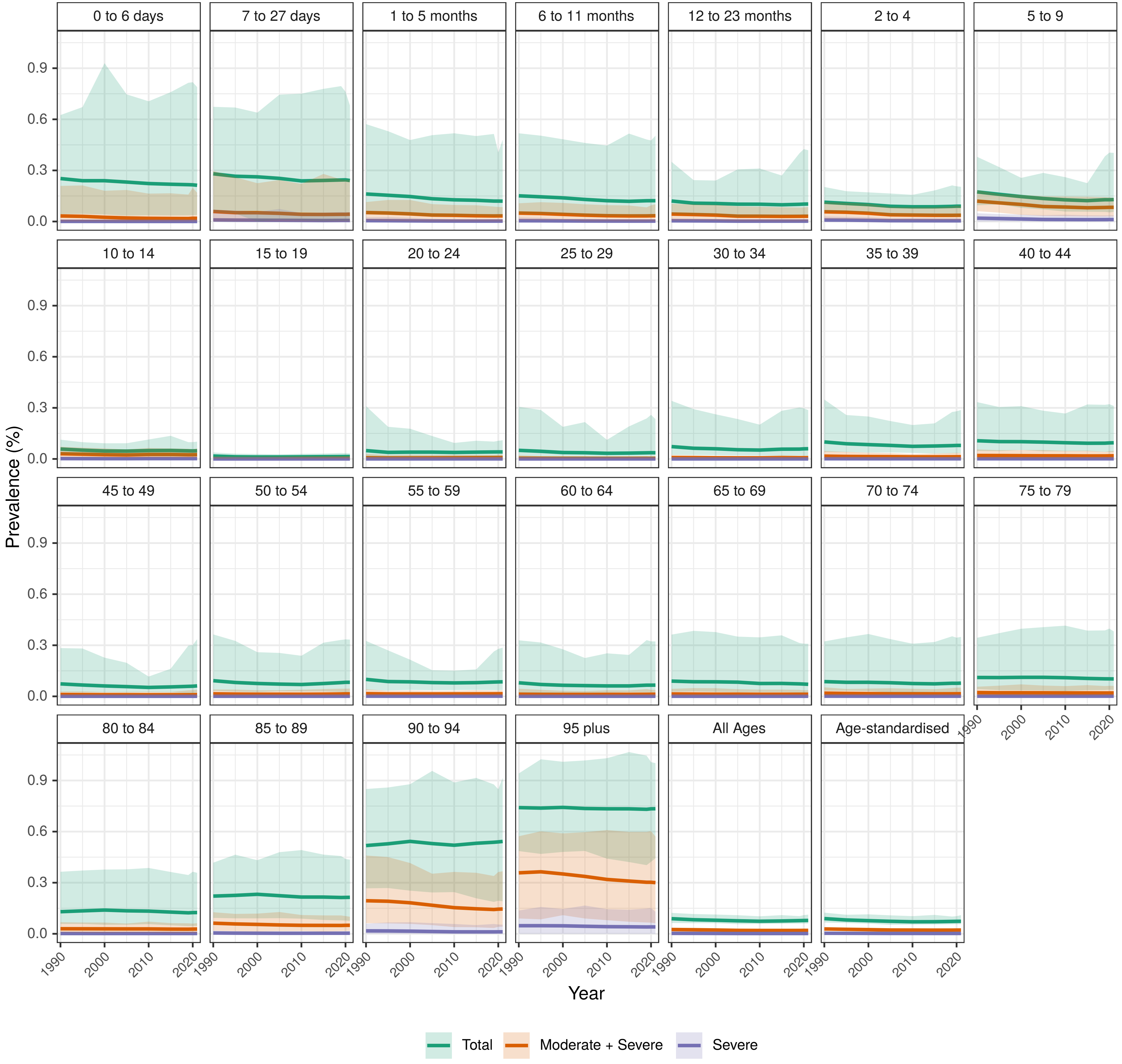

Cause-Specific Anaemia Prevalence - 2021

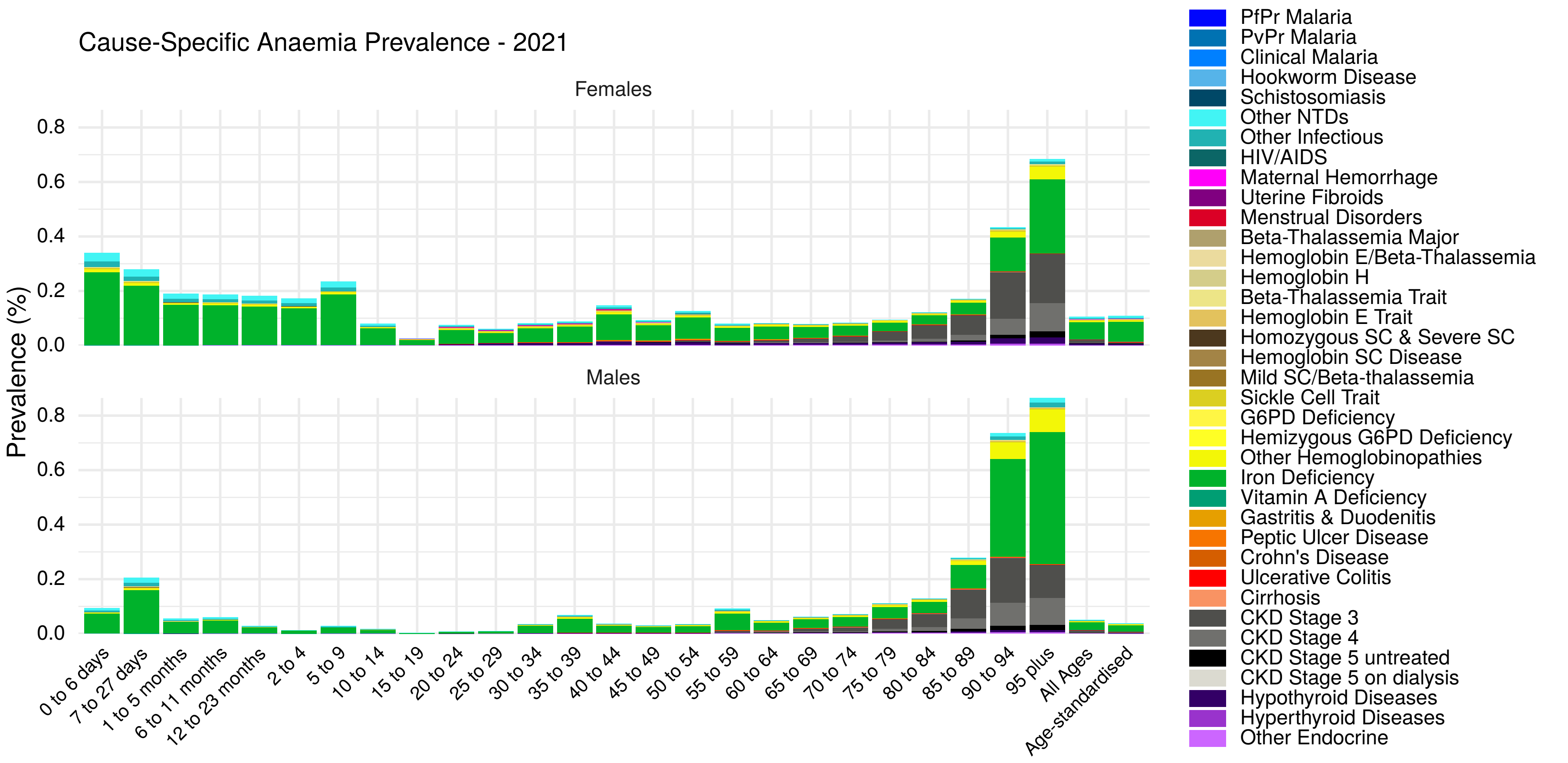

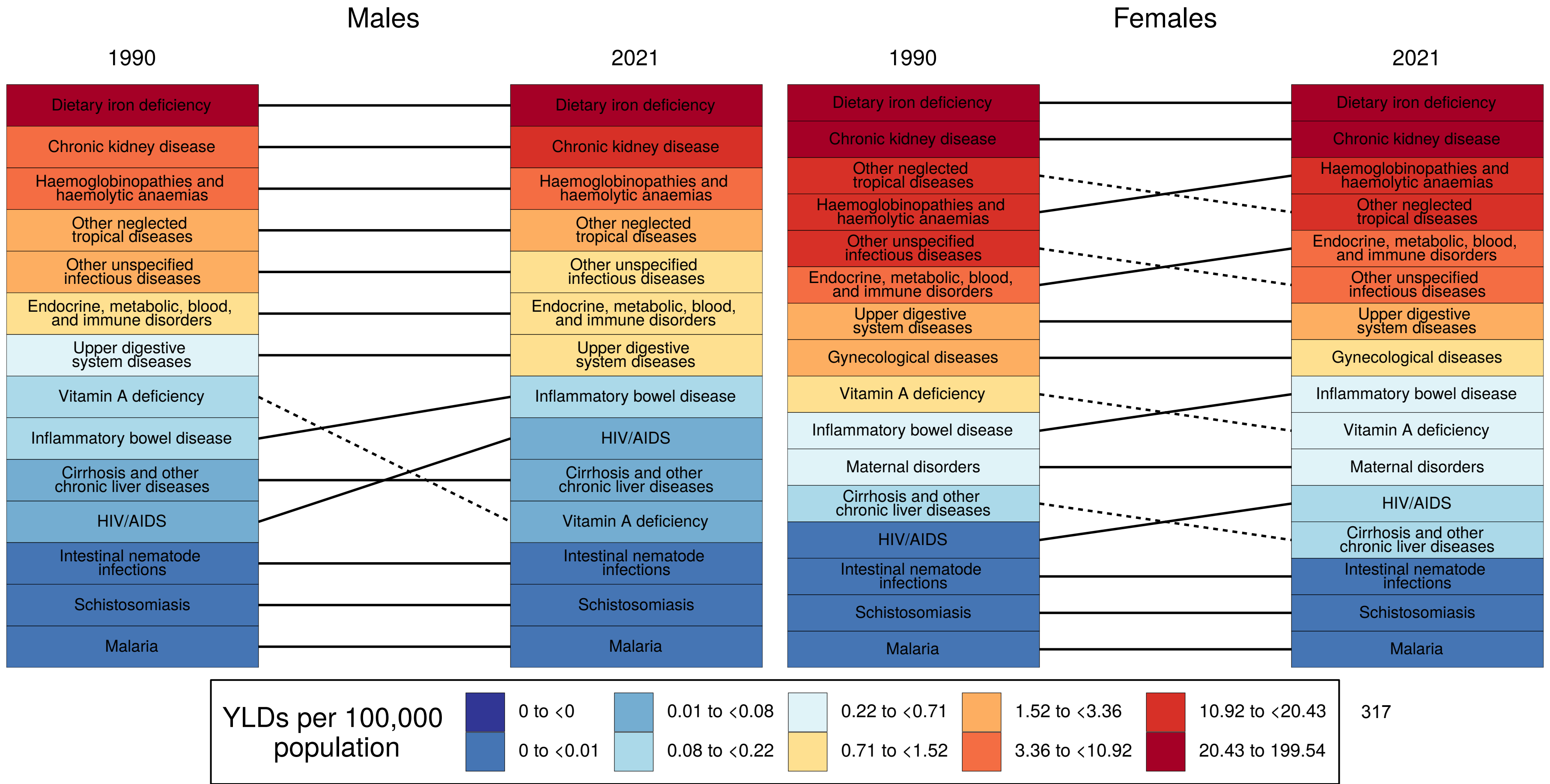

Middlesbrough

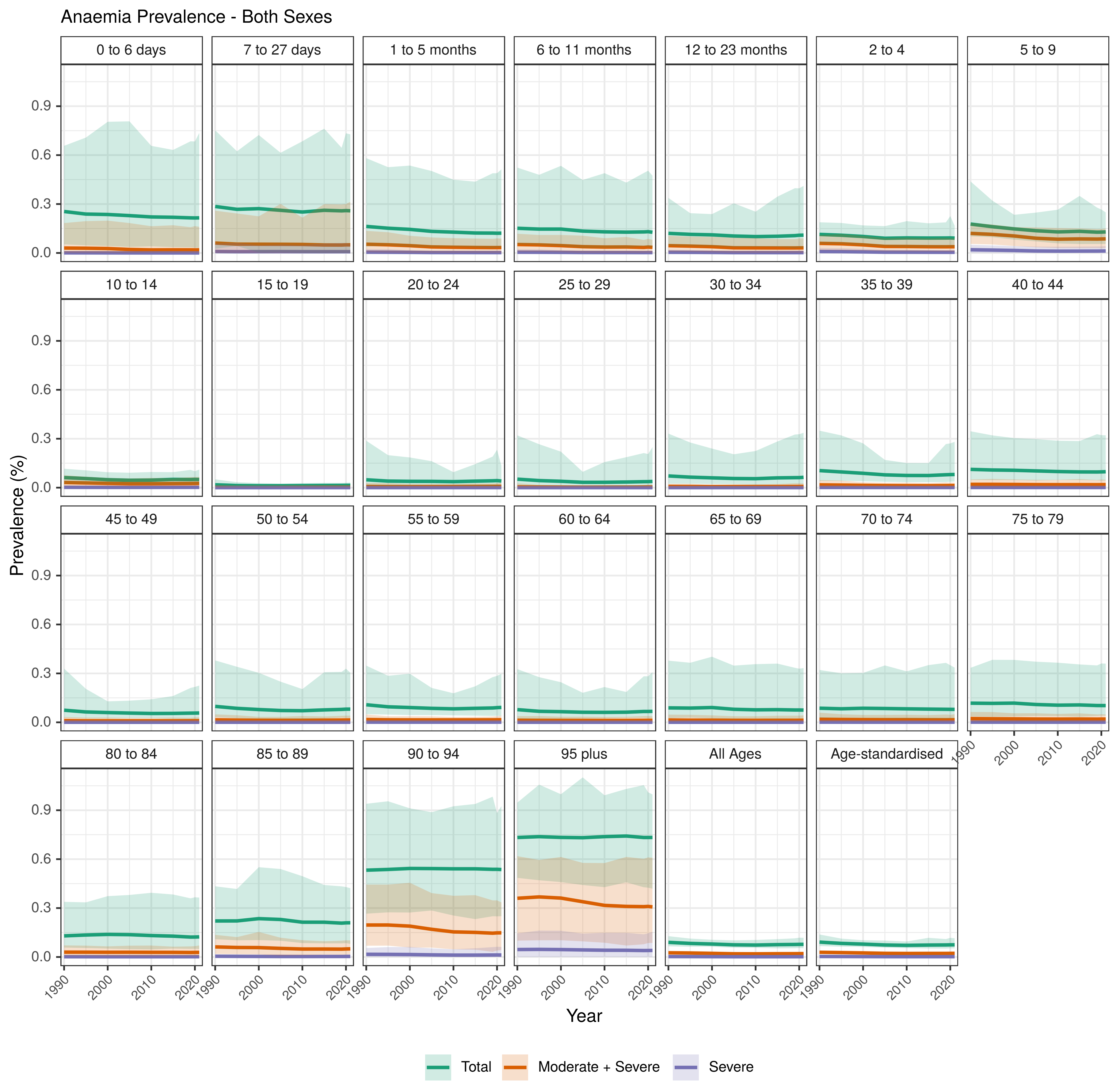

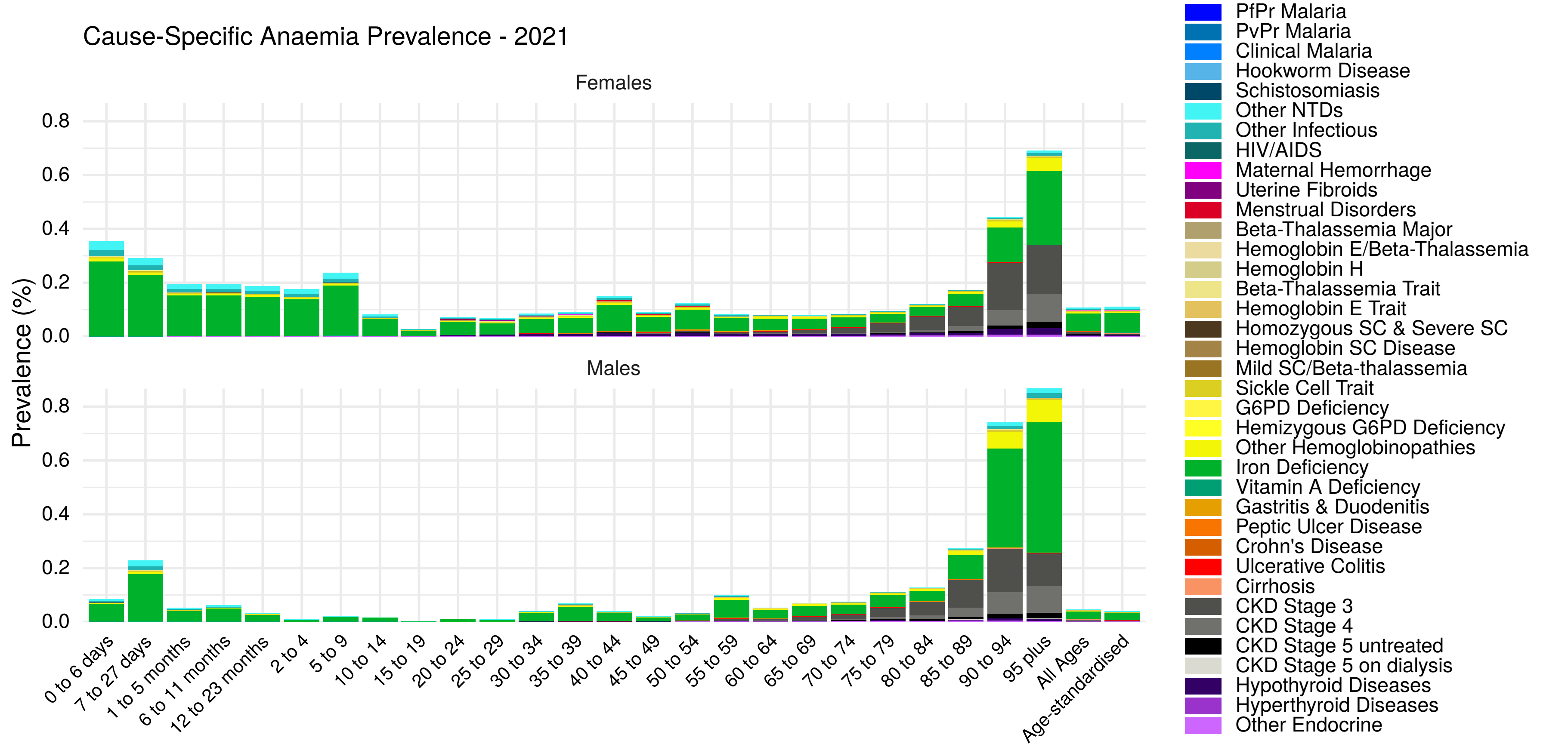

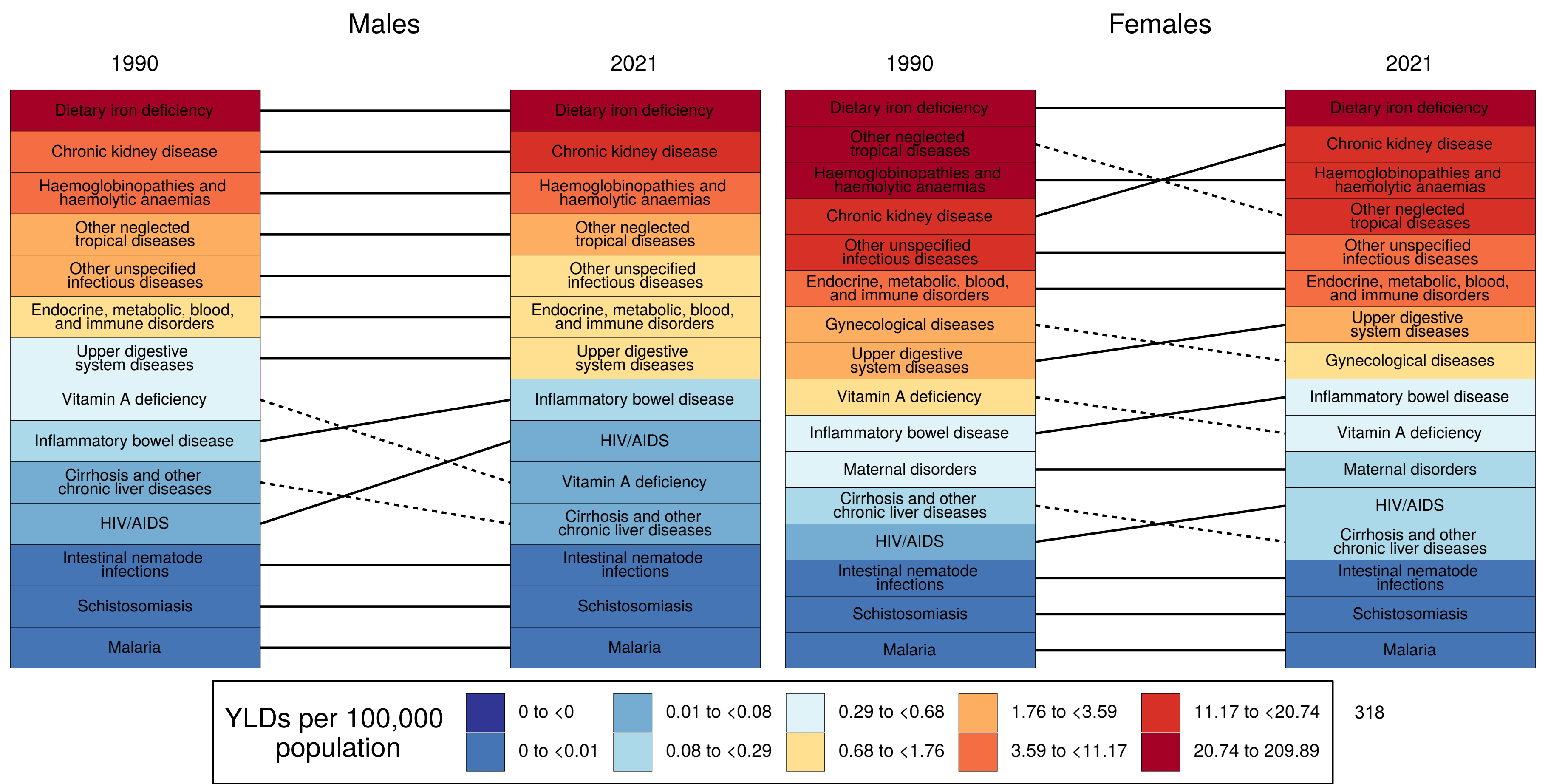

Newcastle upon Tyne

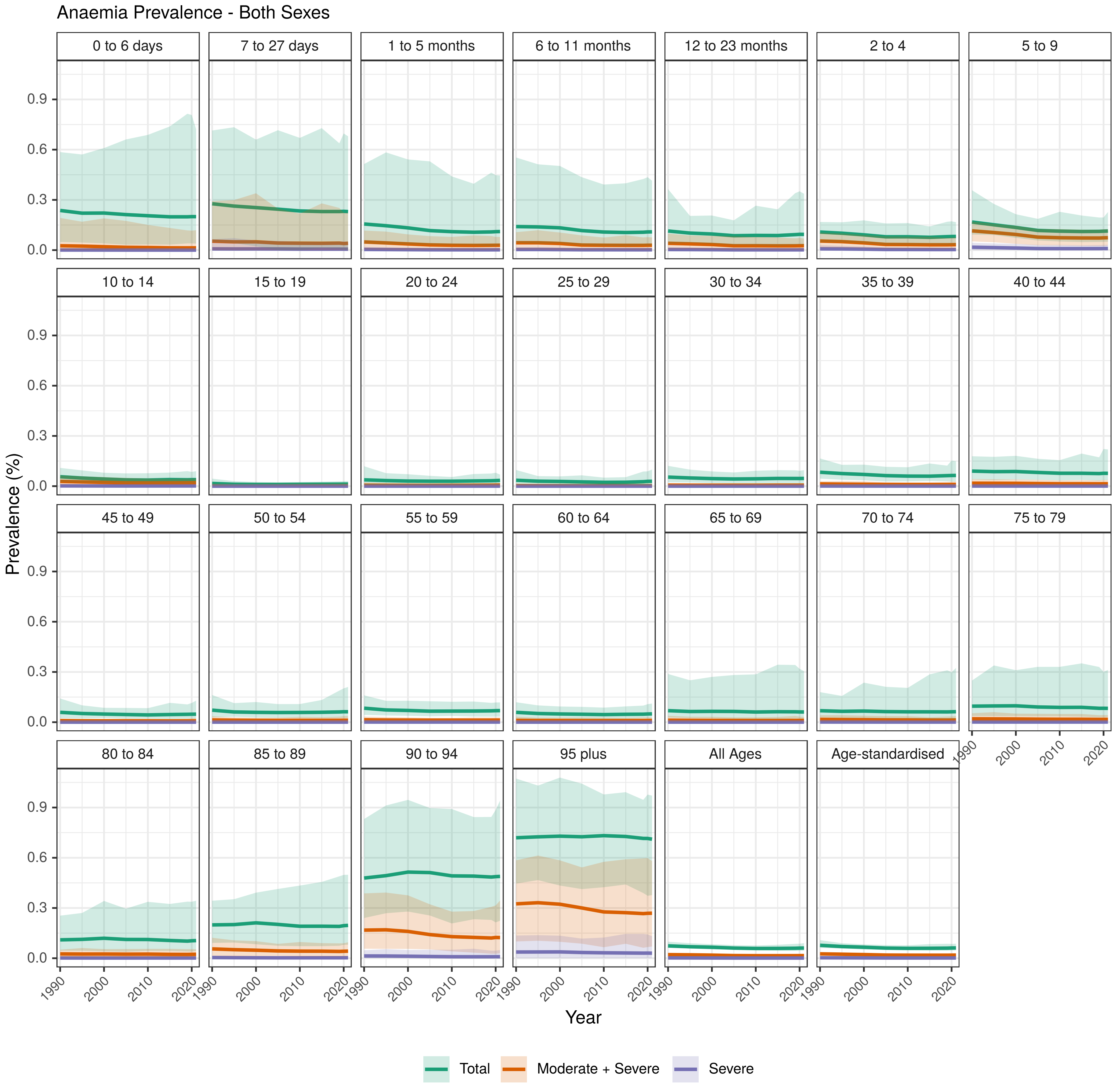

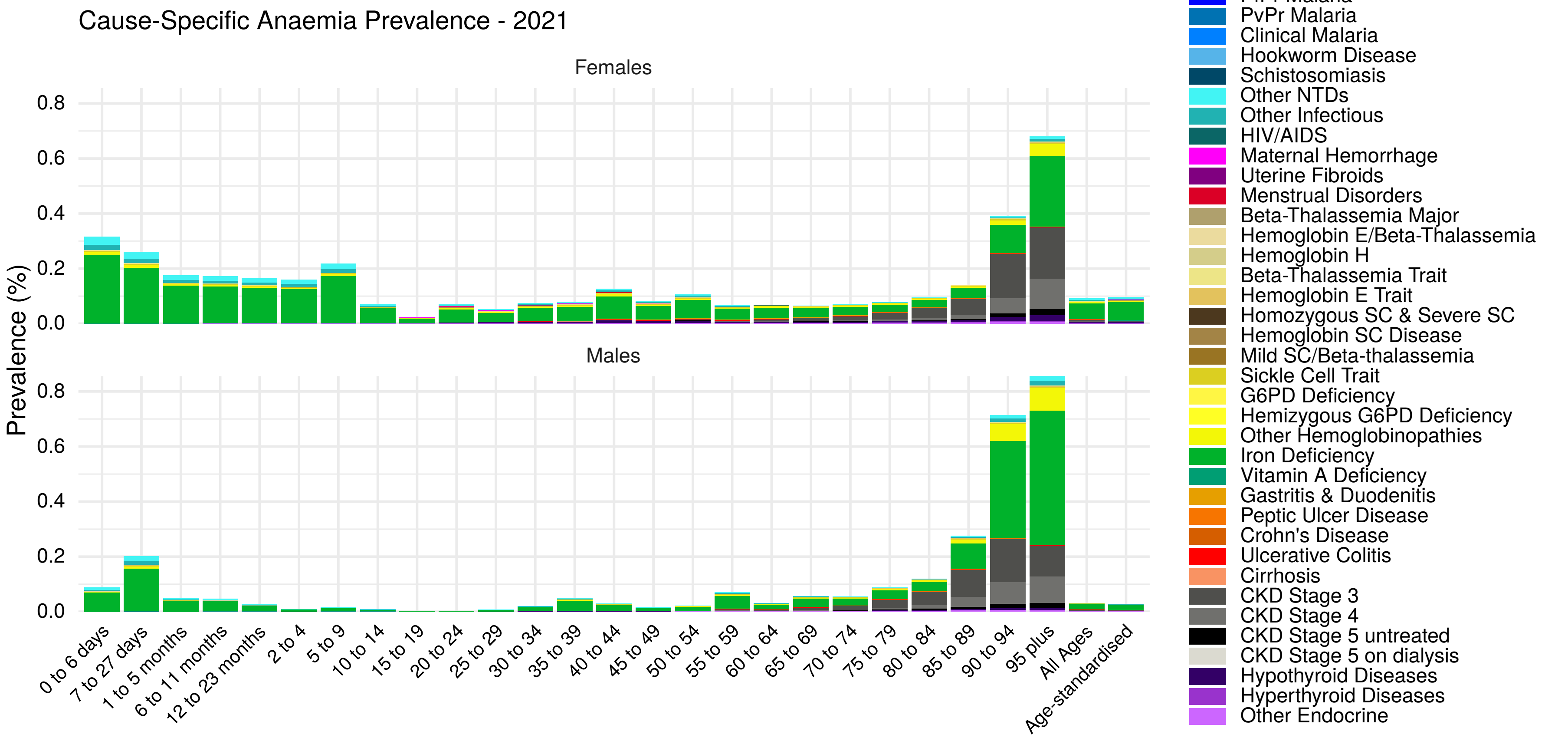

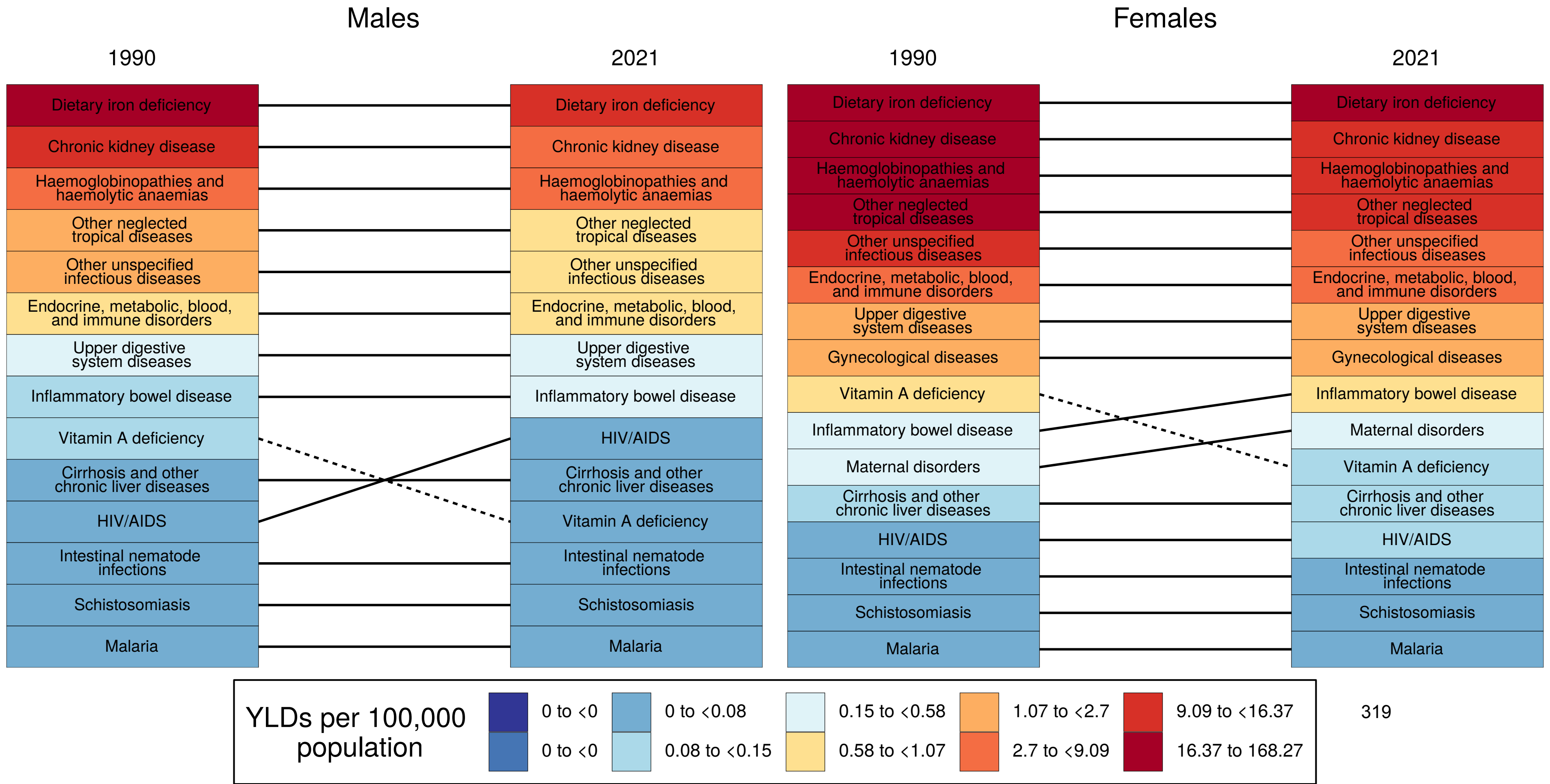

North Tyneside

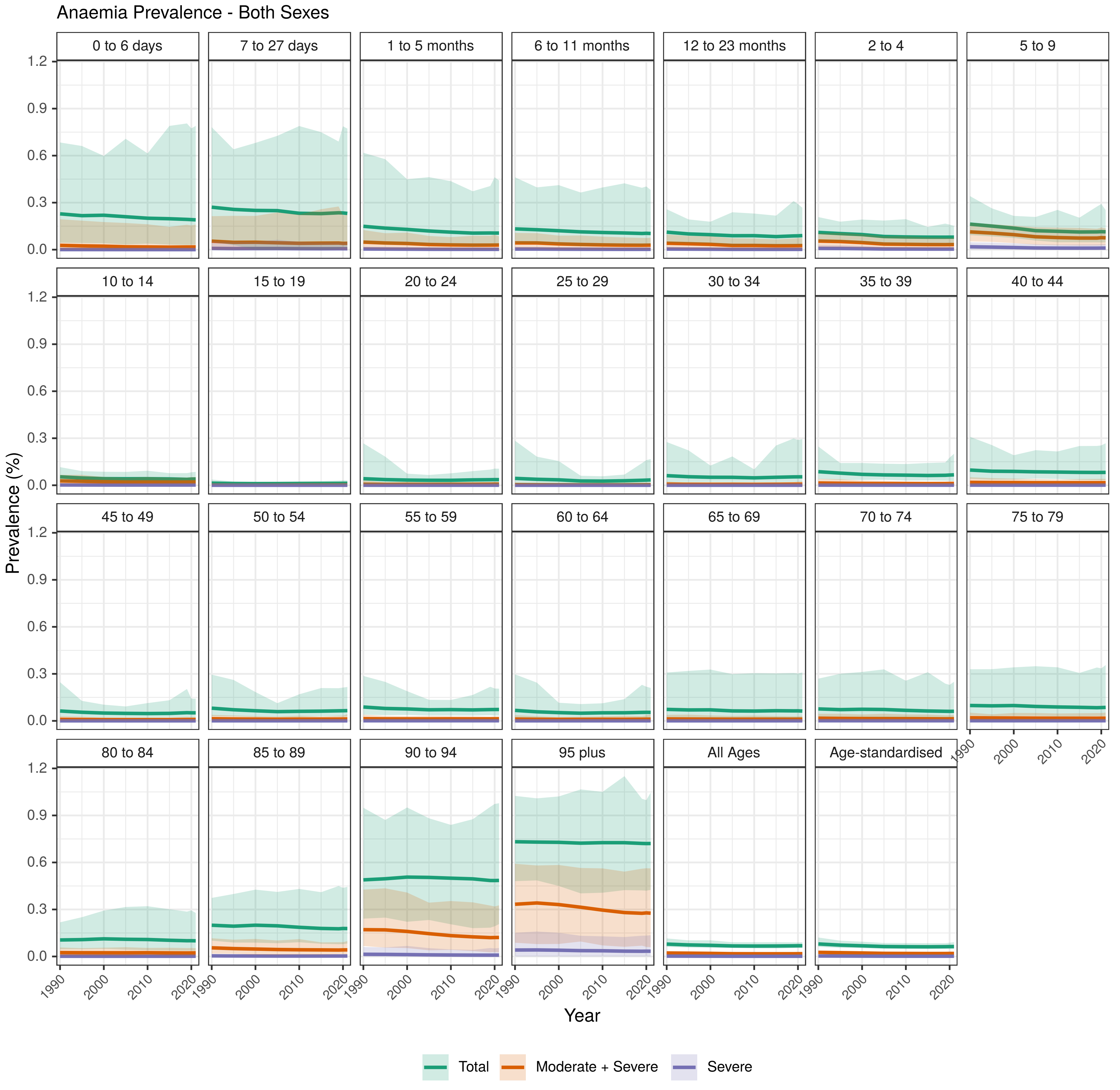

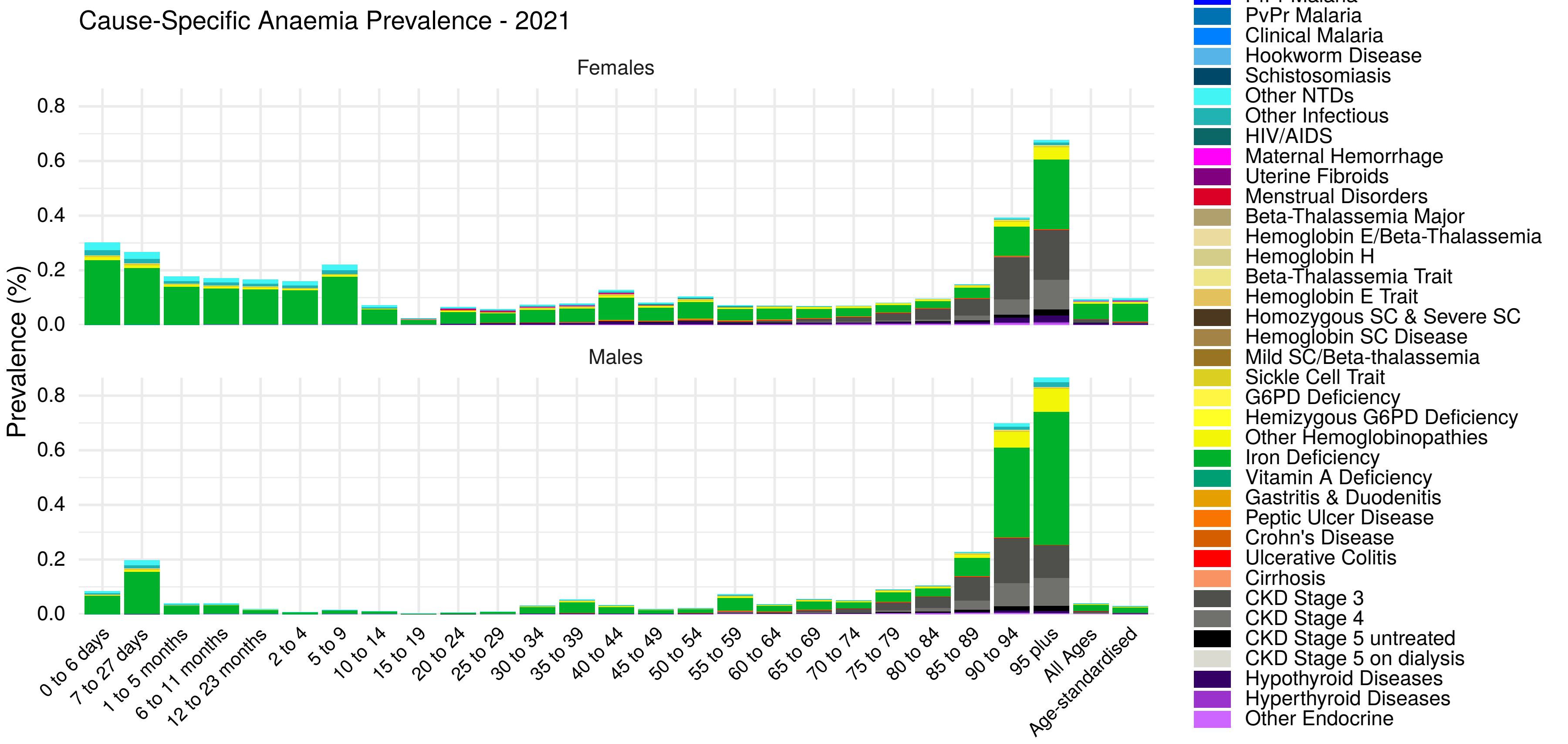

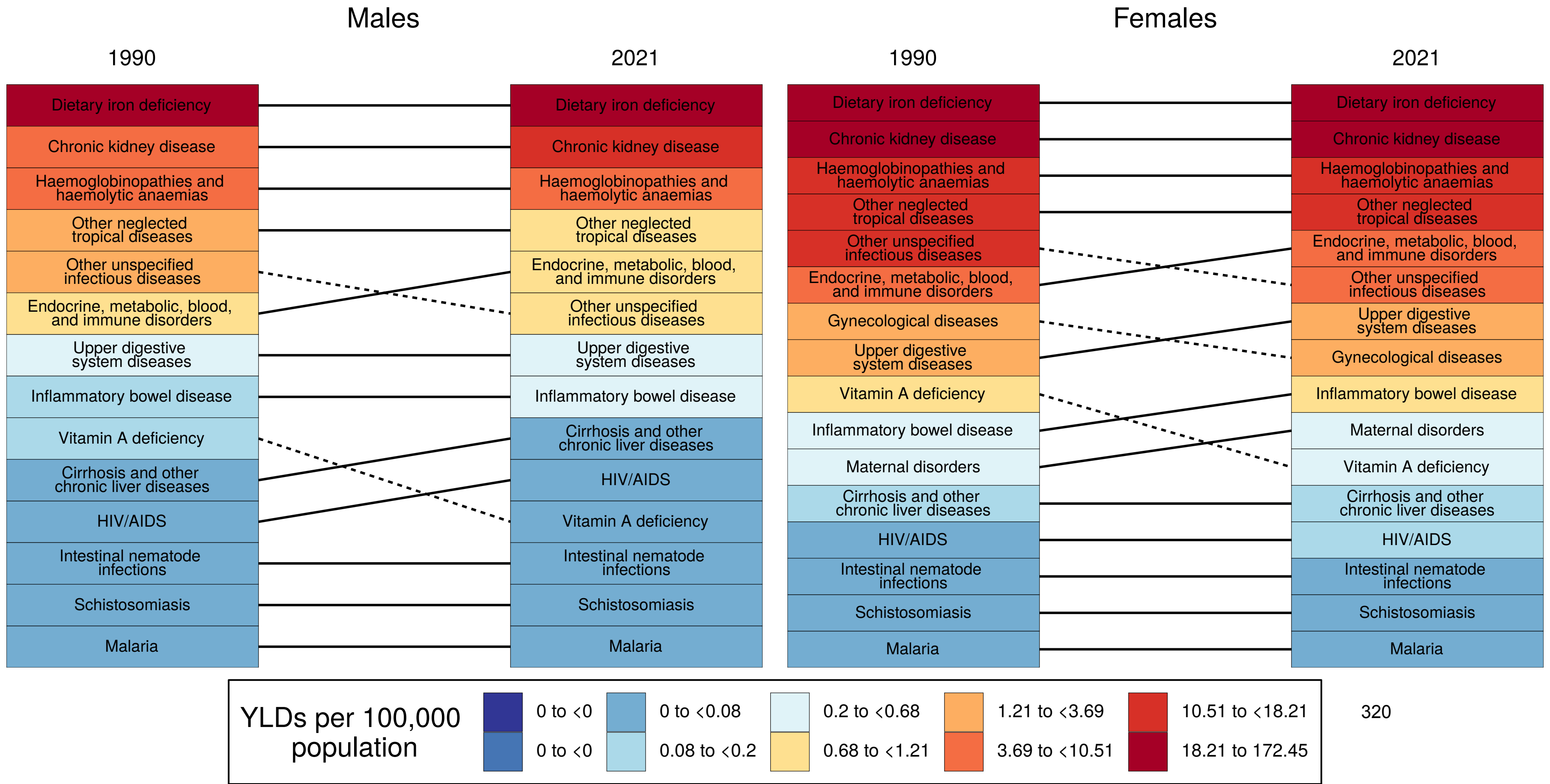

Northumberland

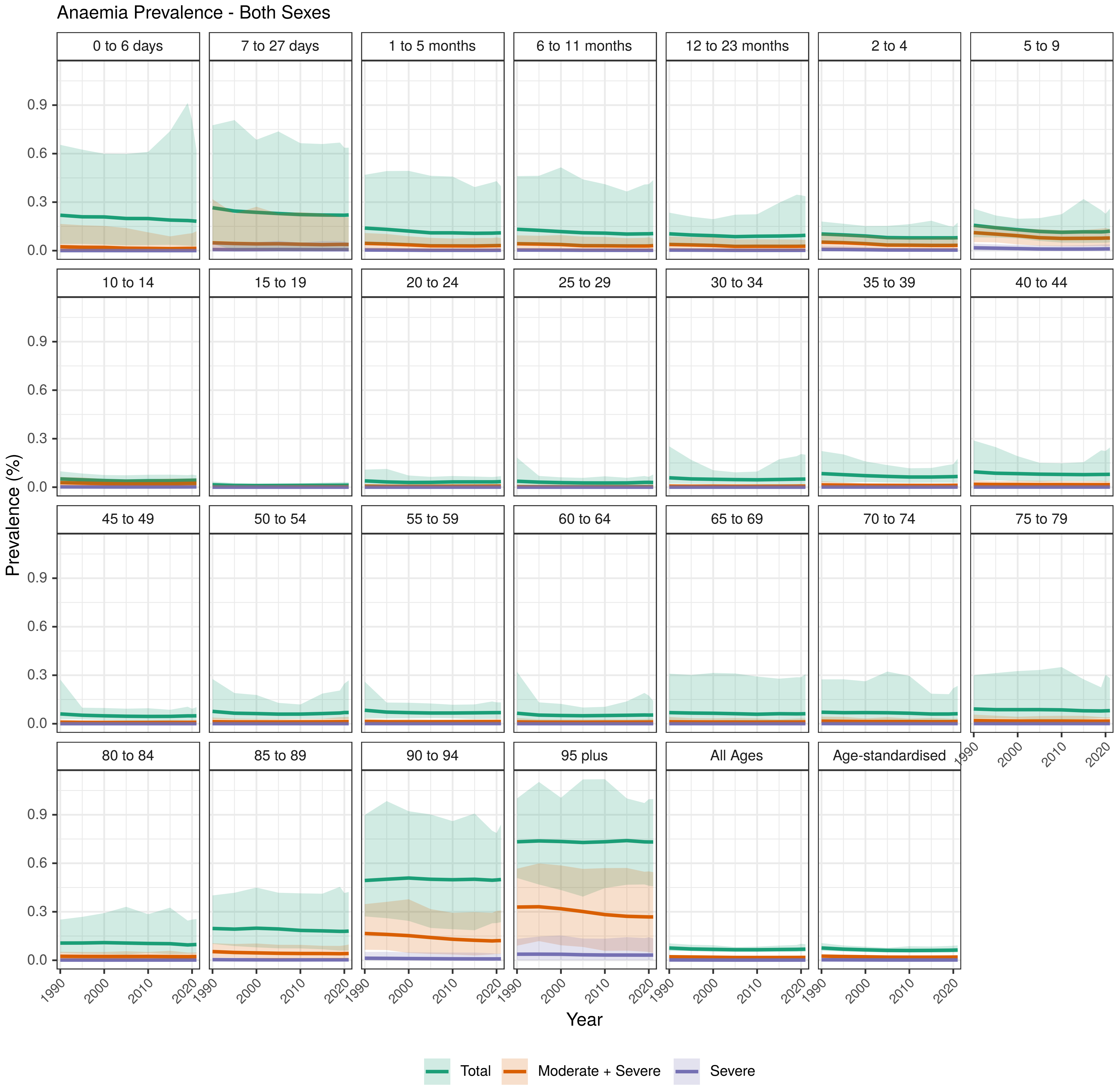

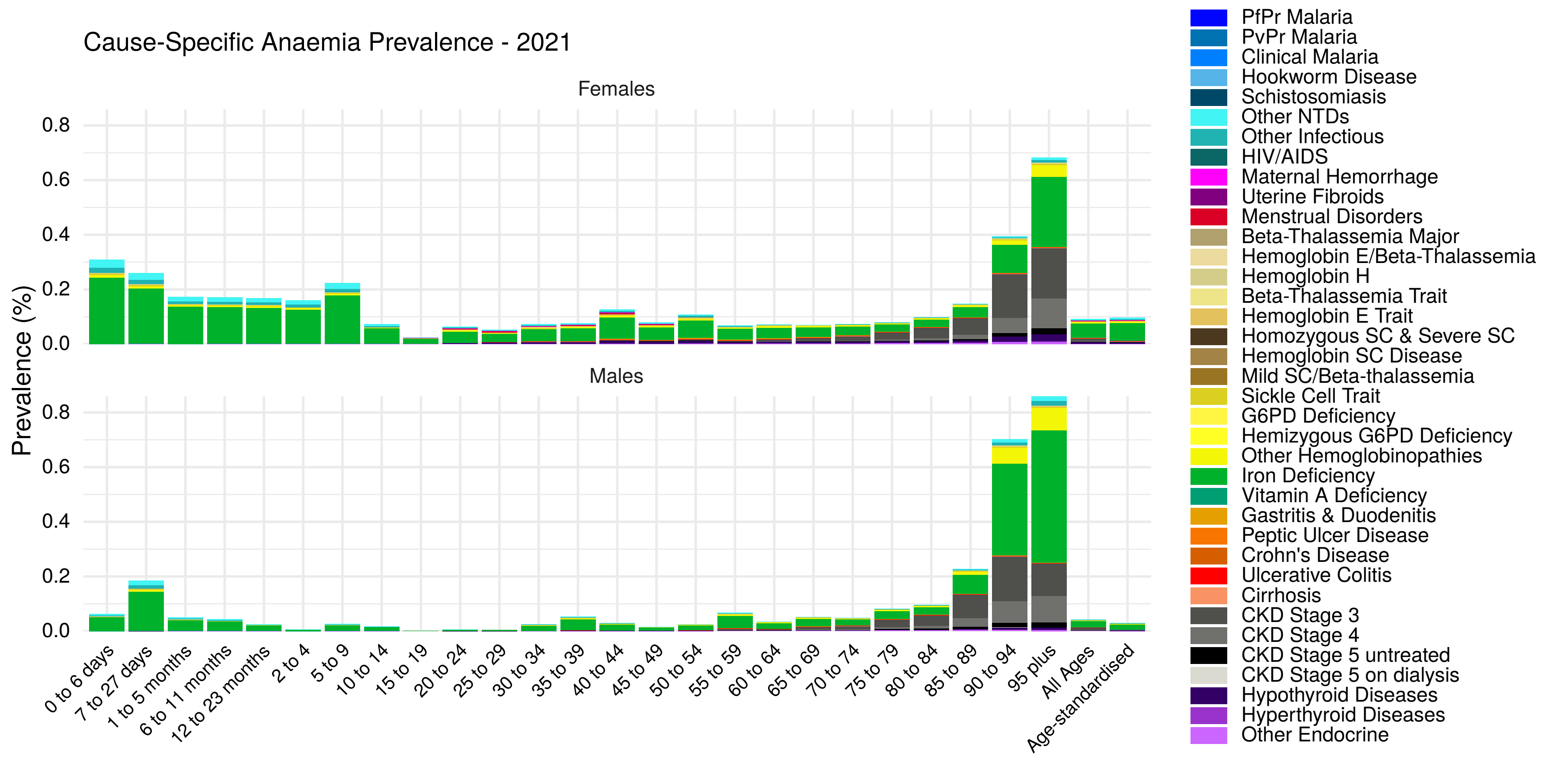

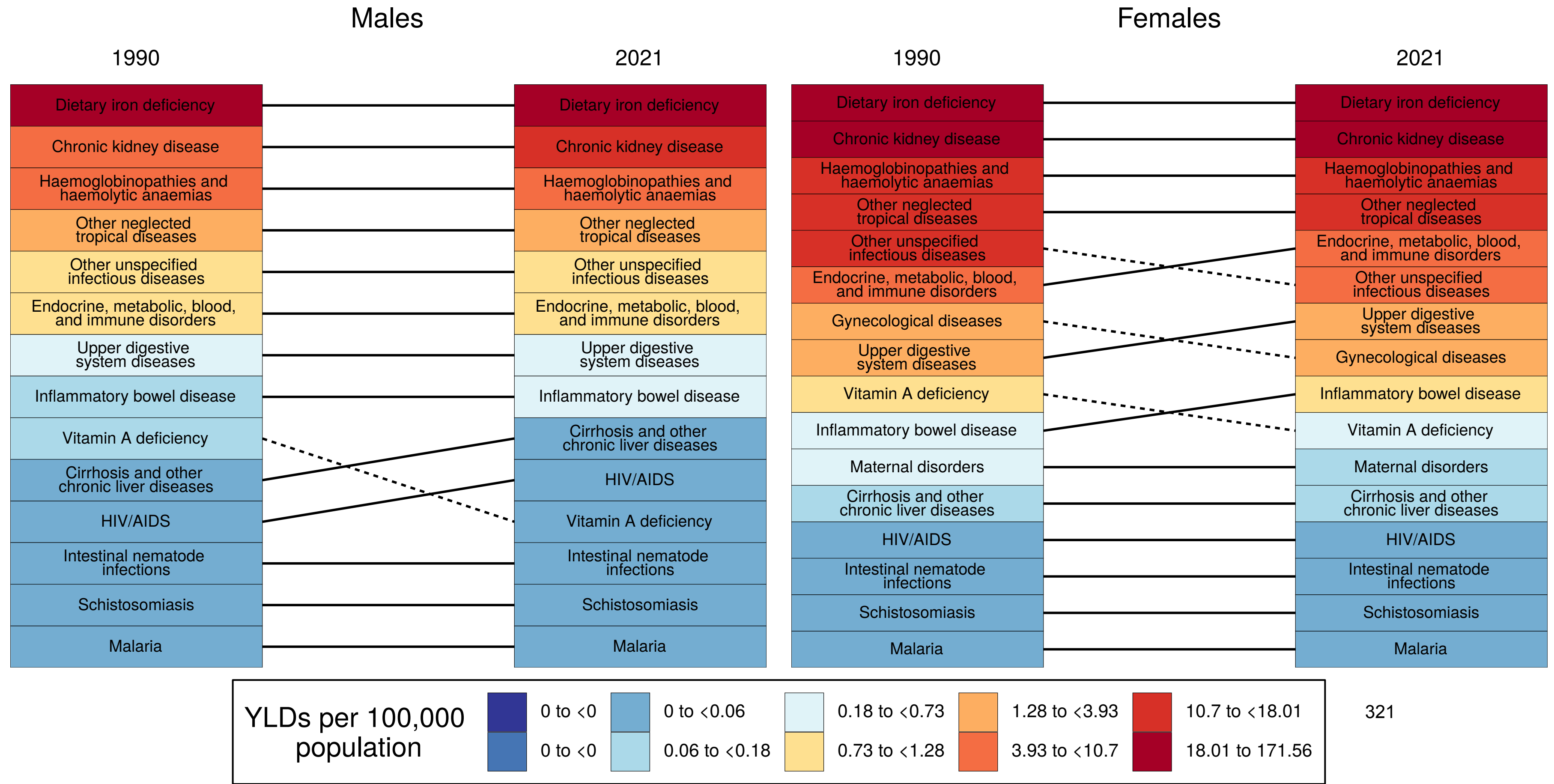

Redcar and Cleveland

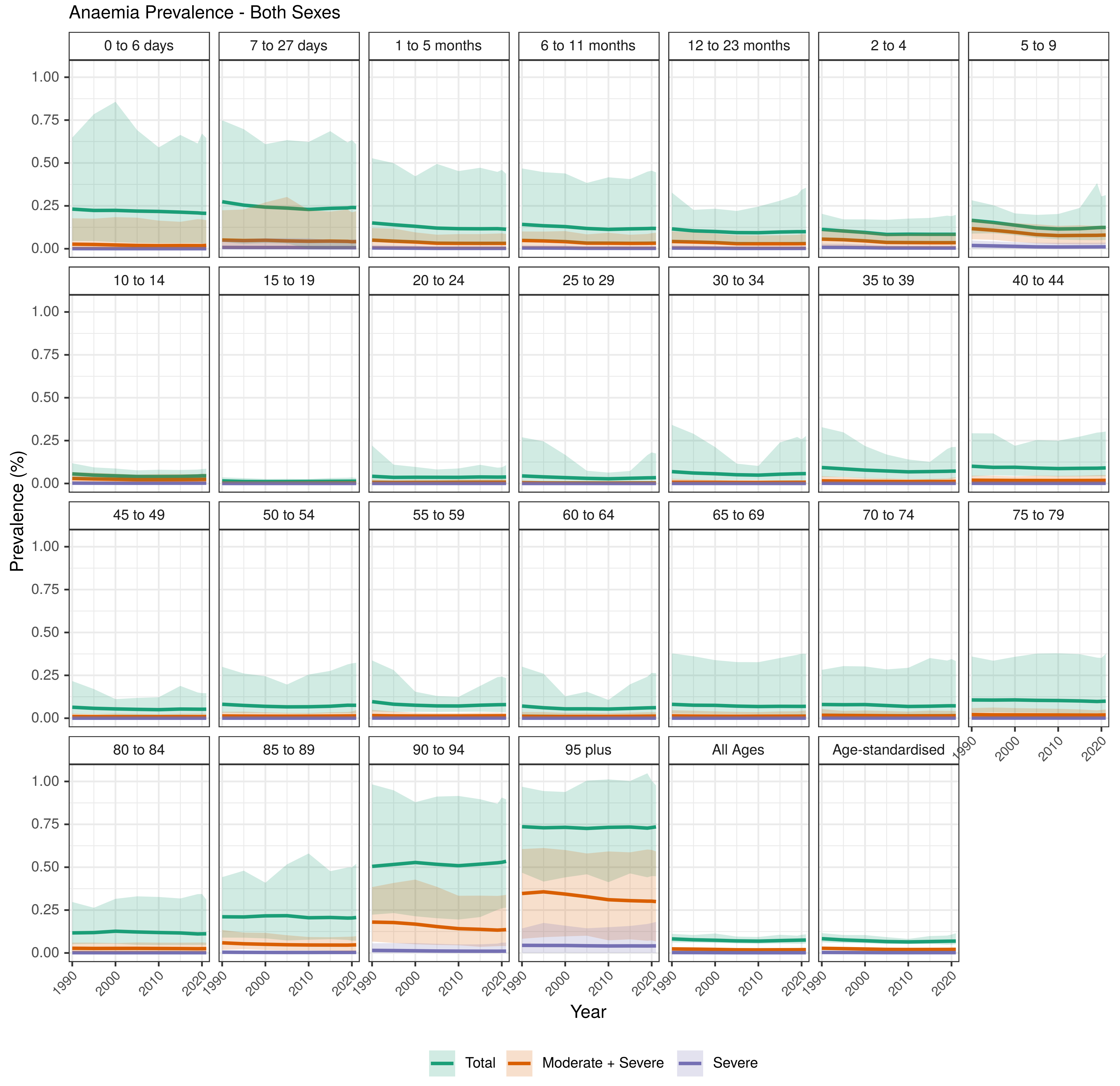

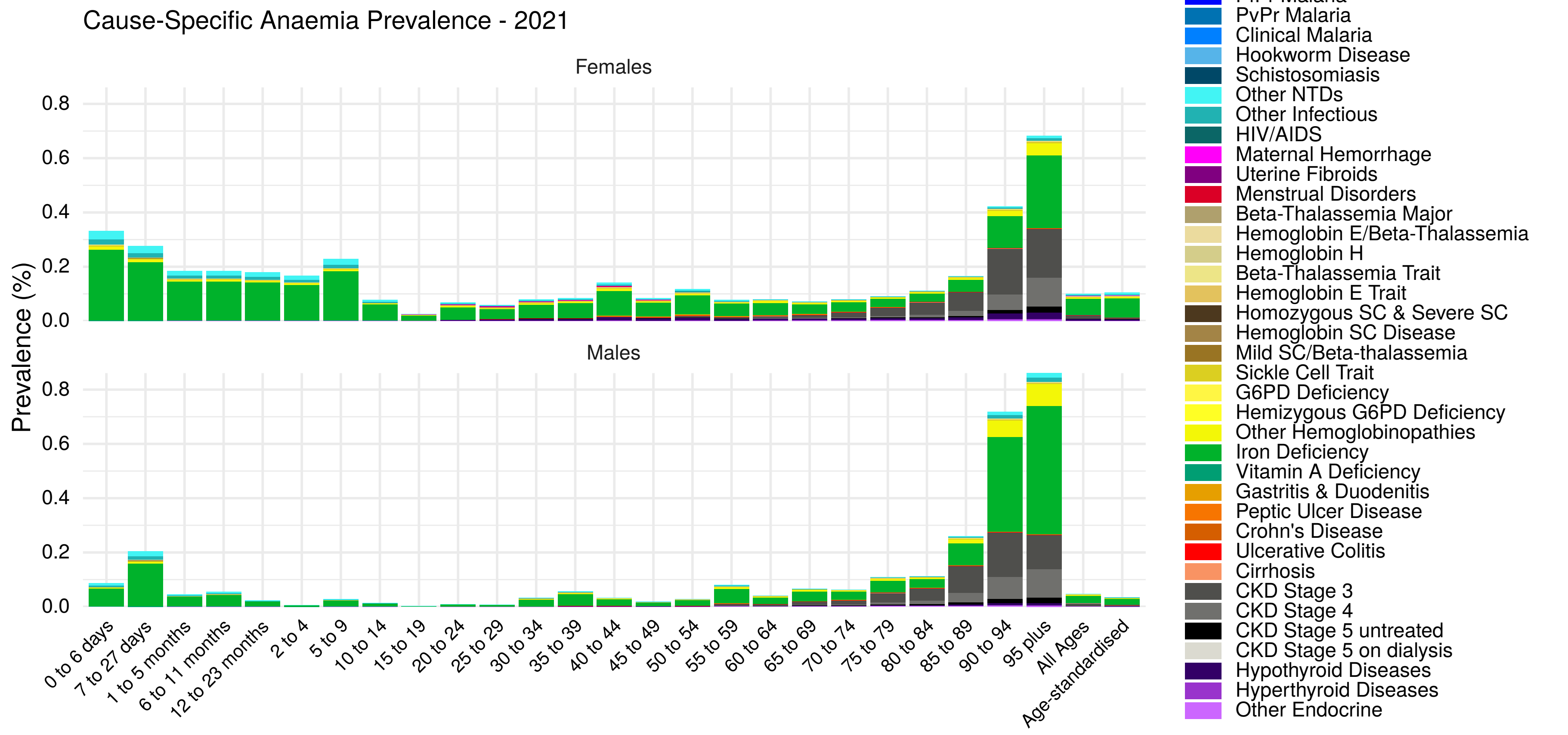

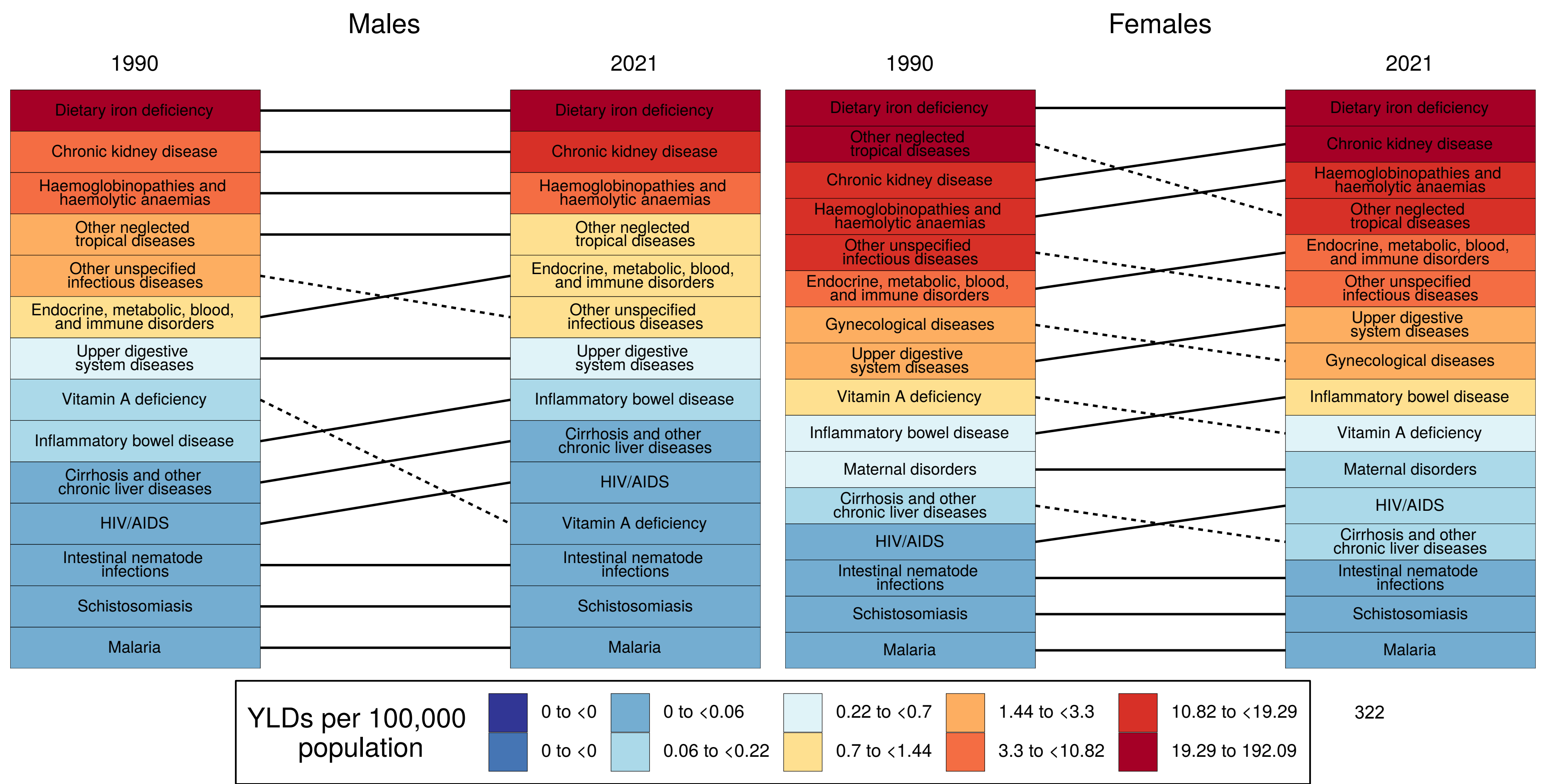

South Tyneside

Anaemia Prevalence - Both Sexes

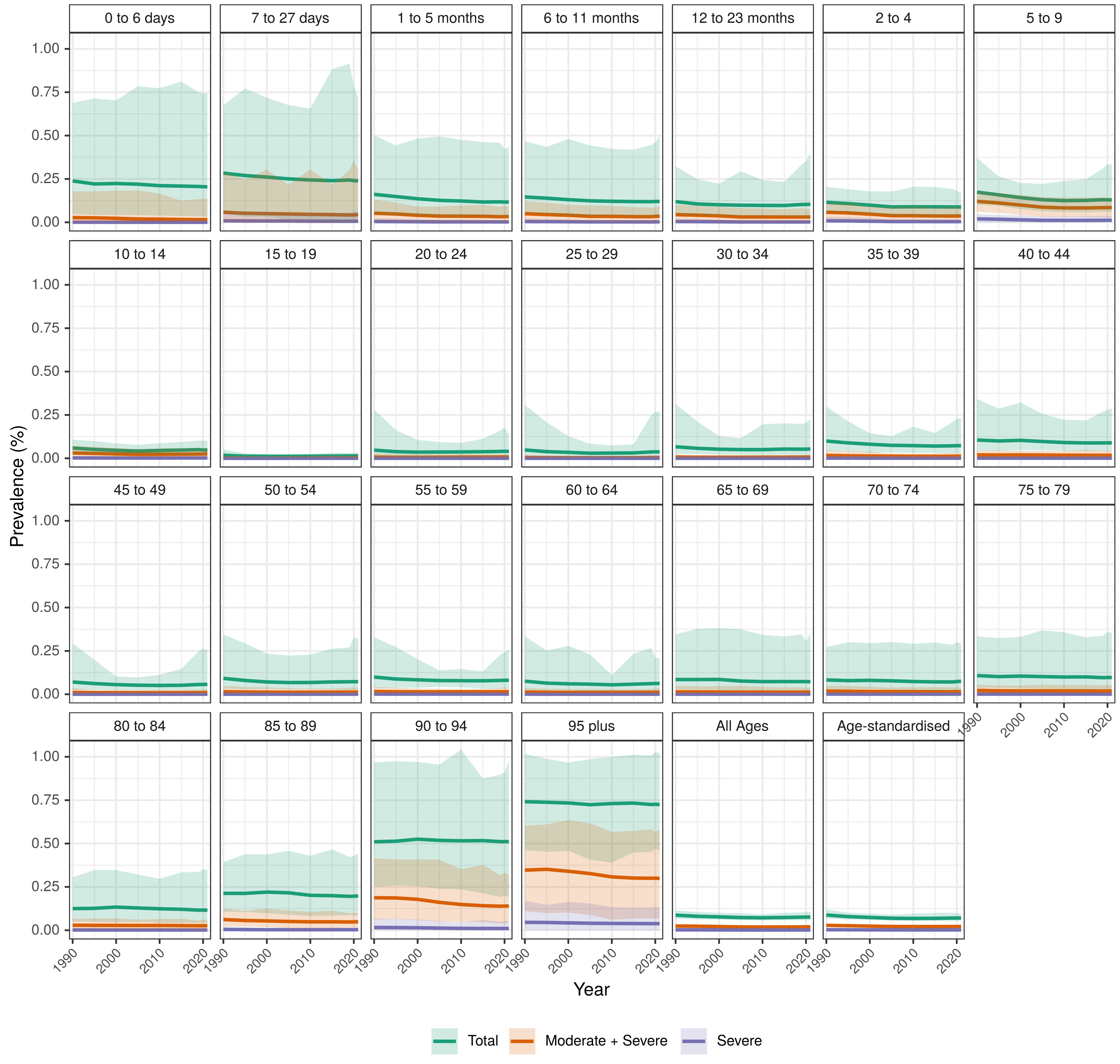

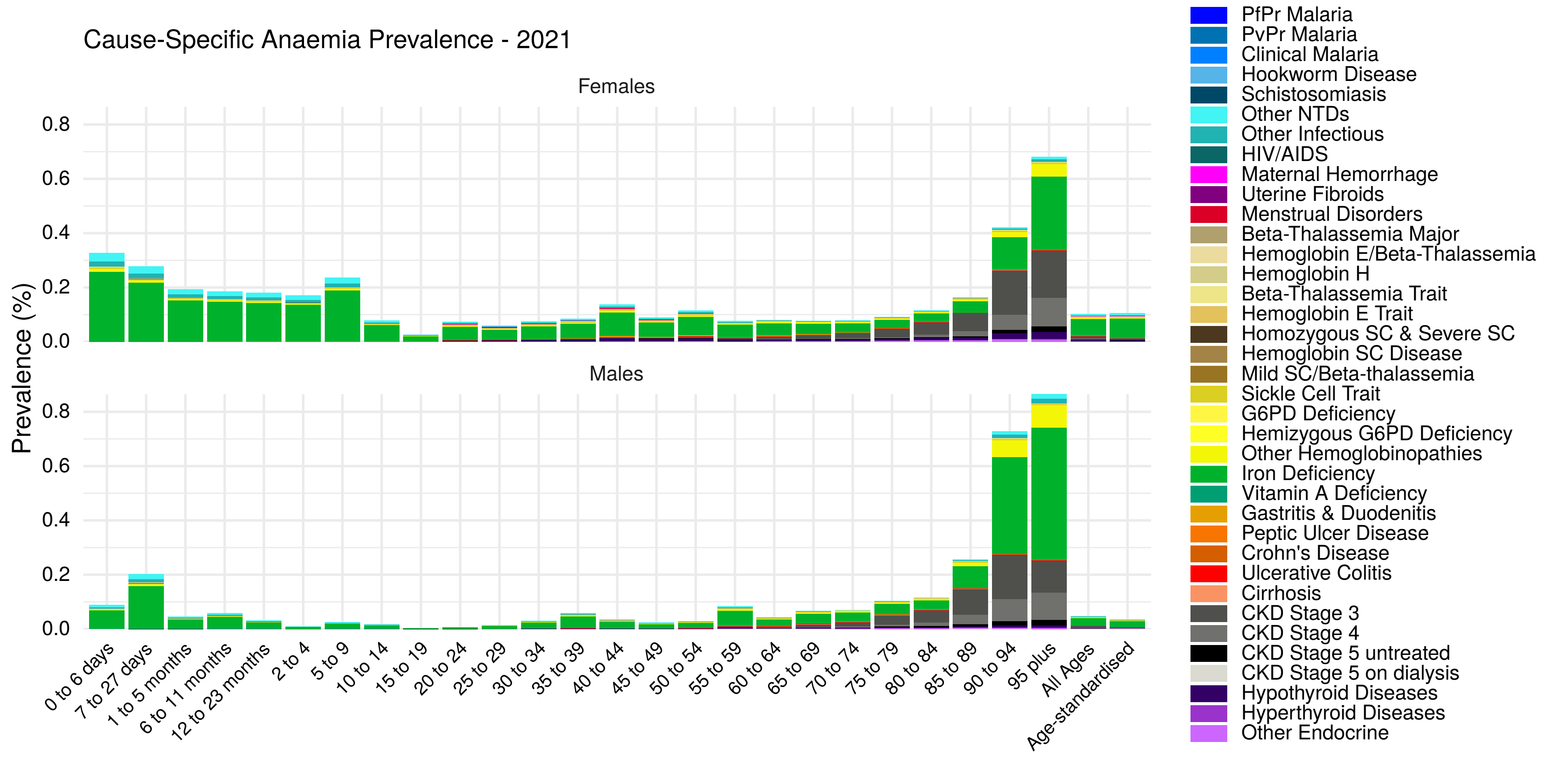

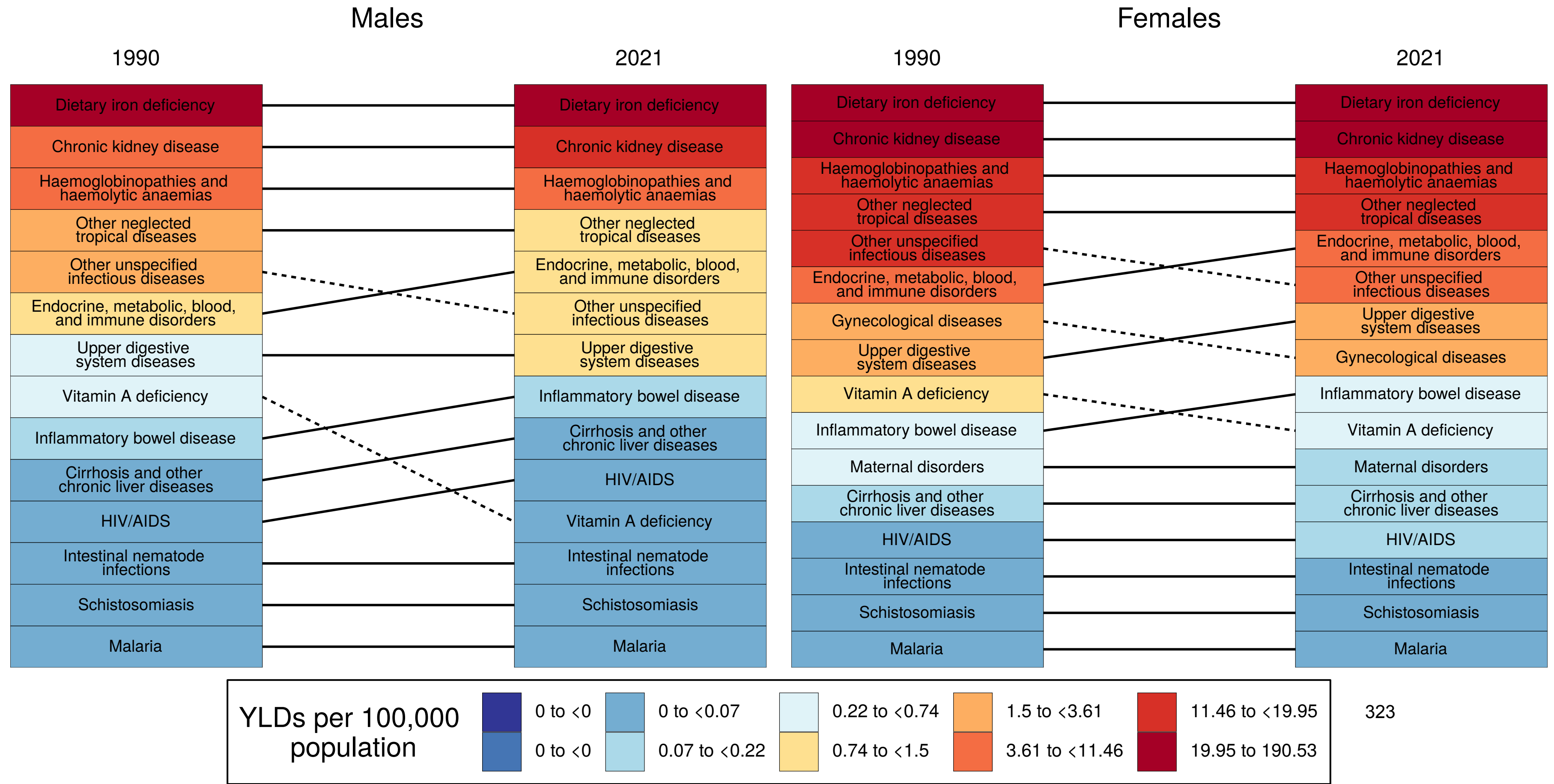

Stockton-on-Tees

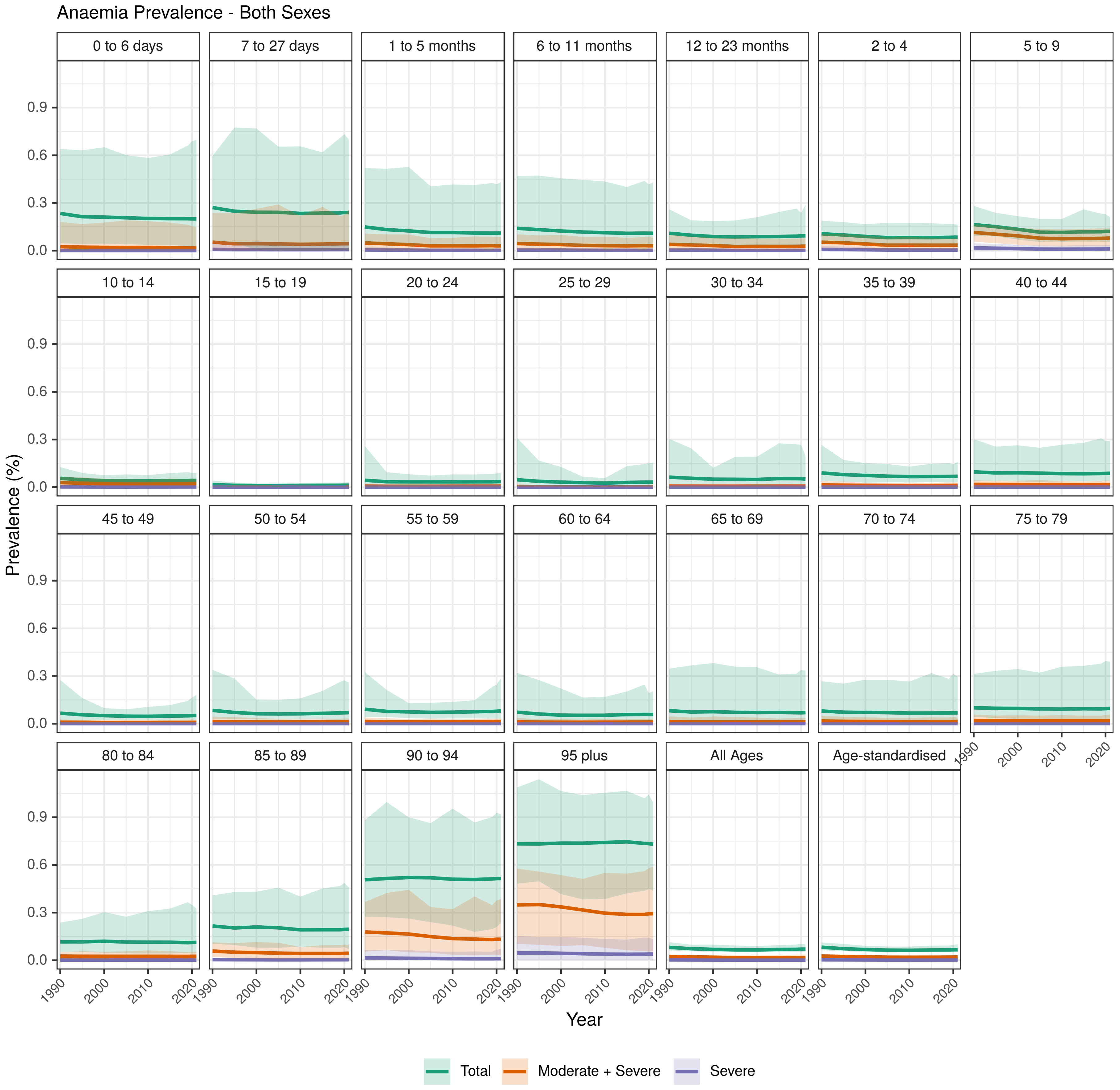

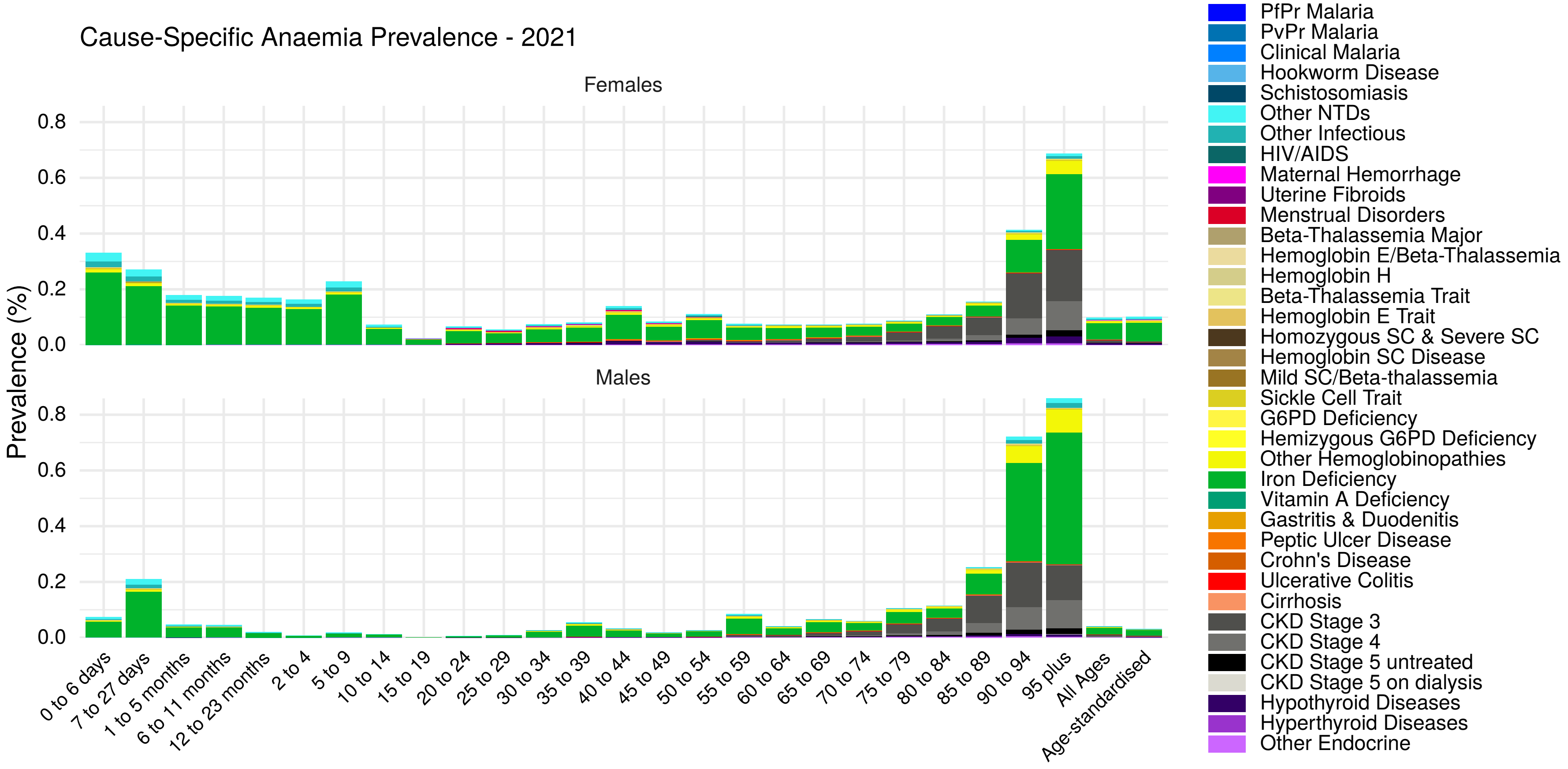

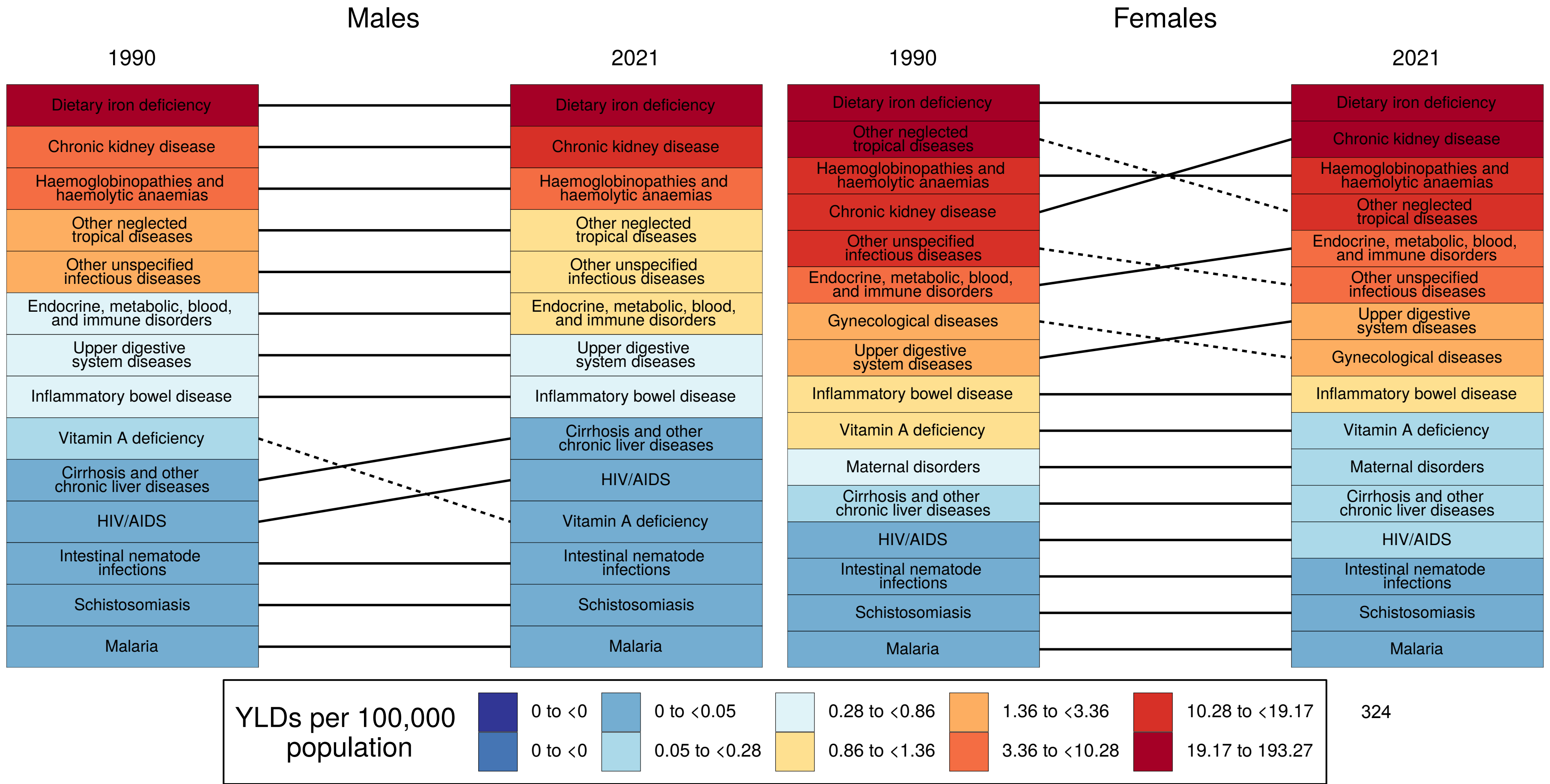

Sunderland

Anaemia Prevalence - Both Sexes

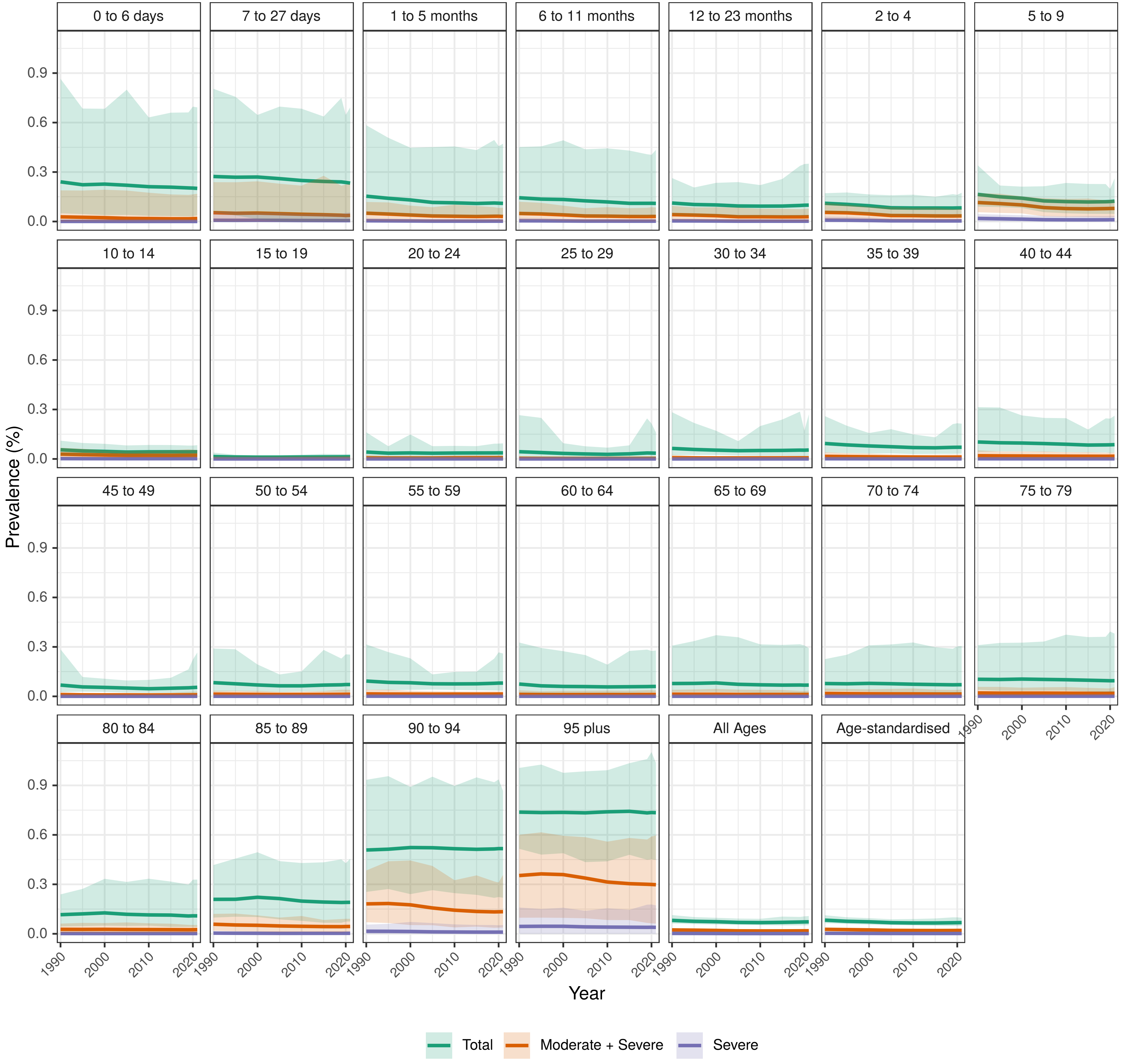

Cause-Specific Anaemia Prevalence - 2021

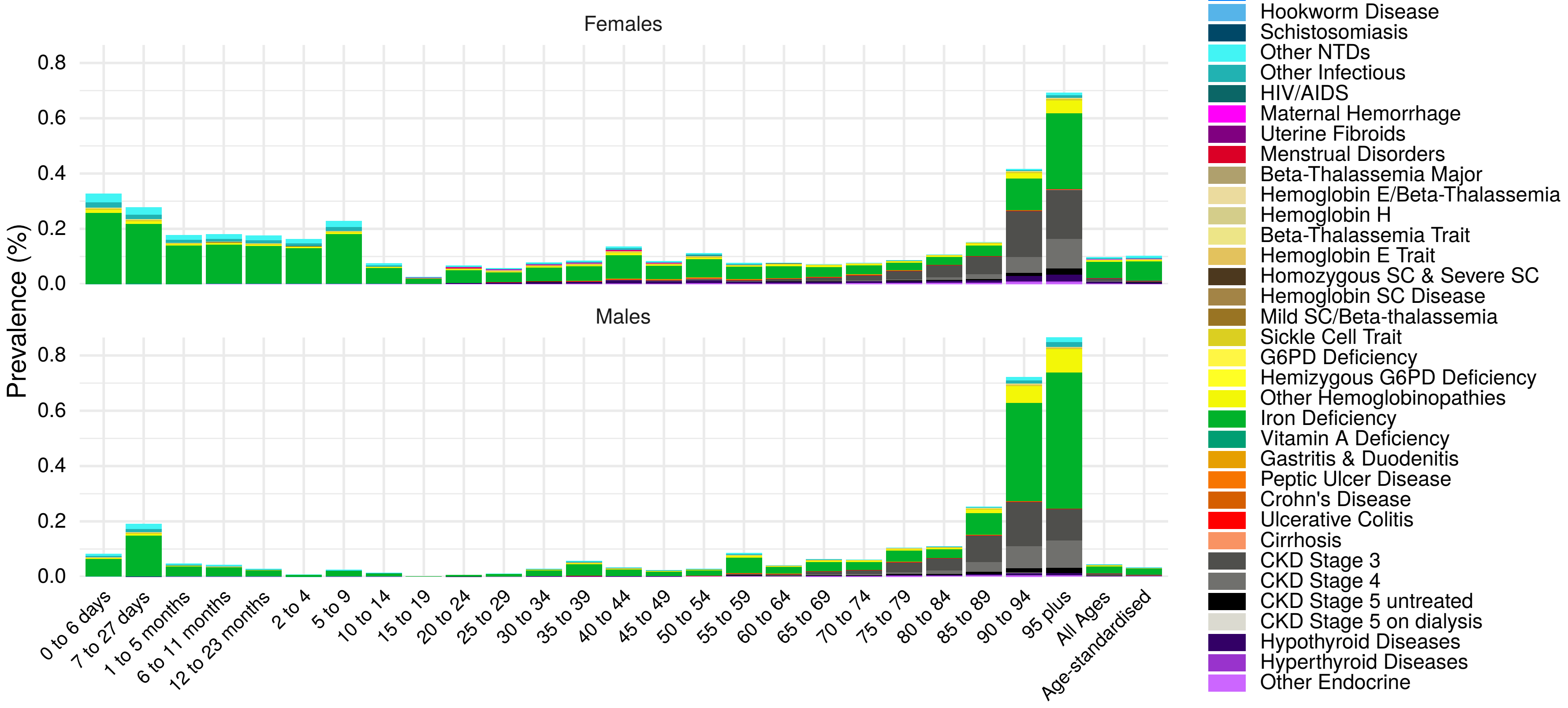

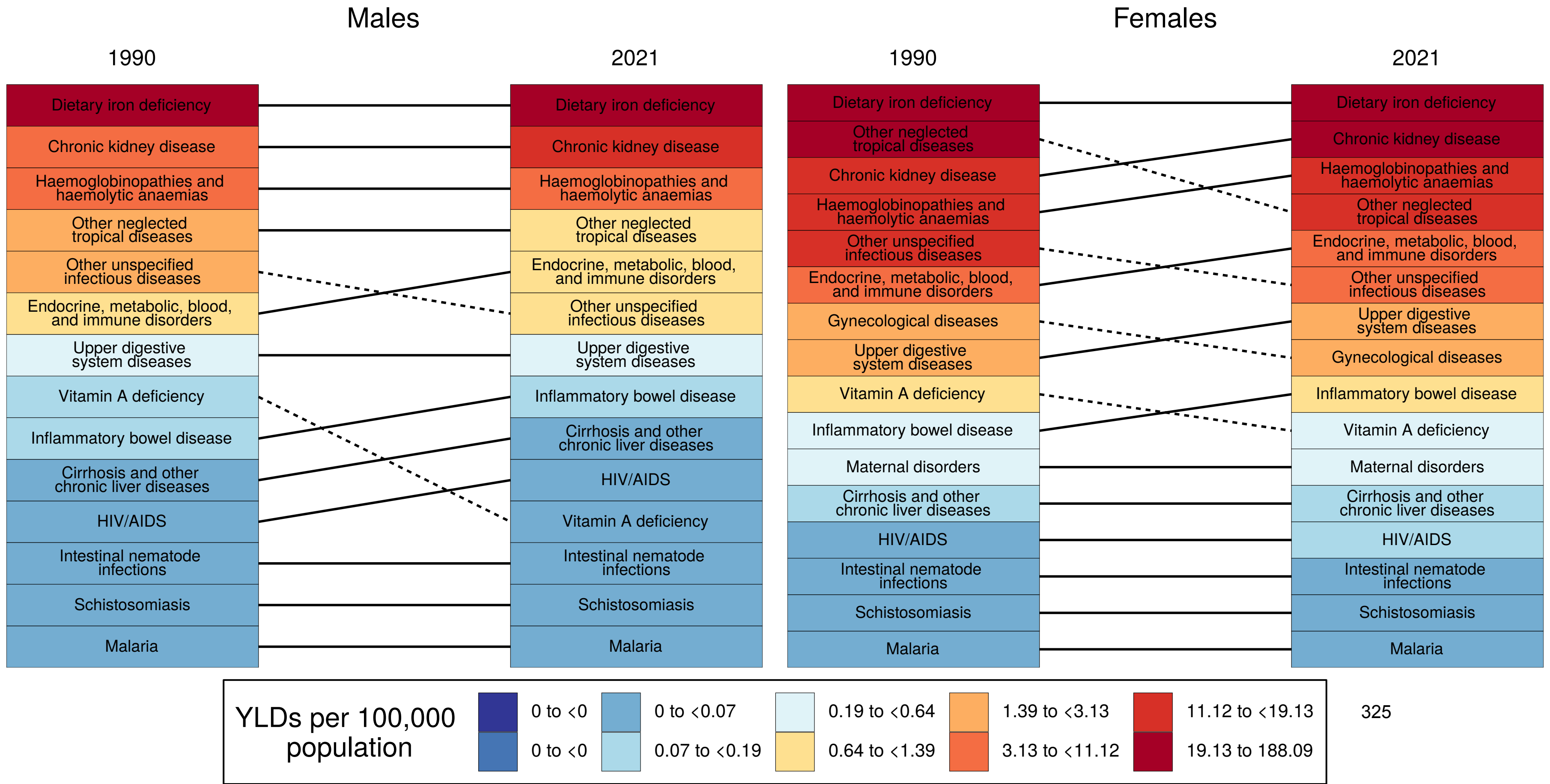

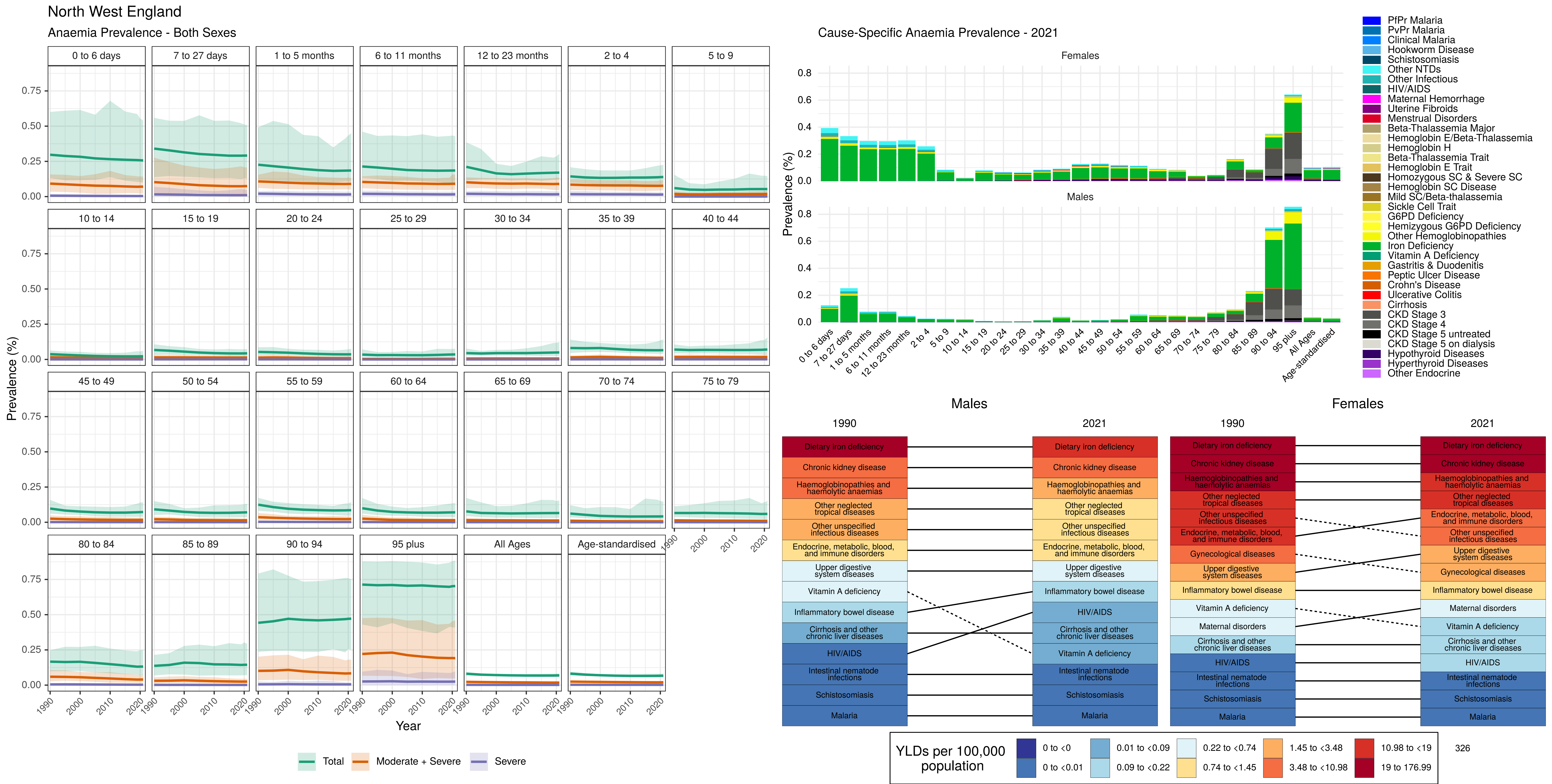

North West England
Anaemia Prevalence - Both Sexes
0 to 6 days
7 to 27 days
1 to 5 months
6 to 11 months
12 to 23 months
2 to 4
5 to 9
10 to 14
15 to 19
20 to 24
25 to 29
30 to 34
35 to 39
40 to 44
45 to 49
50 to 54
55 to 59
60 to 64
65 to 69
70 to 74
75 to 79
80 to 84
85 to 89
90 to 94
95 plus
All Ages
Age-standardised
Prevalence (%)
Year
Total
Moderate + Severe
Severe
Cause-Specific Anaemia Prevalence - 2021
Females
Males
PfPr Malaria
PvPr Malaria
Clinical Malaria
Hookworm Disease
Schistosomiasis
Other NTDs
Other Infectious
HIV/AIDS
Maternal Hemorrhage
Uterine Fibroids
Menstrual Disorders
Beta-Thalassemia Major
Hemoglobin E/Beta-Thalassemia
Hemoglobin H
Beta-Thalassemia Trait
Hemoglobin E Trait
Homozygous SC & Severe SC
Hemoglobin SC Disease
Mild SC/Beta-thalassemia
Sickle Cell Trait
G6PD Deficiency
Hemizygous G6PD Deficiency
Other Hemoglobinopathies
Iron Deficiency
Vitamin A Deficiency
Gastritis & Duodenitis
Peptic Ulcer Disease
Crohn's Disease
Ulcerative Colitis
Cirrhosis
CKD Stage 3
CKD Stage 4
CKD Stage 5 untreated
CKD Stage 5 on dialysis
Hypothyroid Diseases
Hyperthyroid Diseases
Other Endocrine
Males
Females
1990
2021
Dietary iron deficiency
Chronic kidney disease
Haemoglobinopathies and haemolytic anaemias
Other neglected tropical diseases
Other unspecified infectious diseases
Endocrine, metabolic, blood, and immune disorders
Upper digestive system diseases
Vitamin A deficiency
Inflammatory bowel disease
Cirrhosis and other chronic liver diseases
HIV/AIDS
Intestinal nematode infections
Schistosomiasis
Malaria
Gynecological diseases
Maternal disorders
YLDs per 100,000 population
0 to <0
0 to <0.01
0.01 to <0.09
0.09 to <0.22
0.22 to <0.74
0.74 to <1.45
1.45 to <3.48
3.48 to <10.98
10.98 to <19
19 to 176.99

Blackburn with Darwen

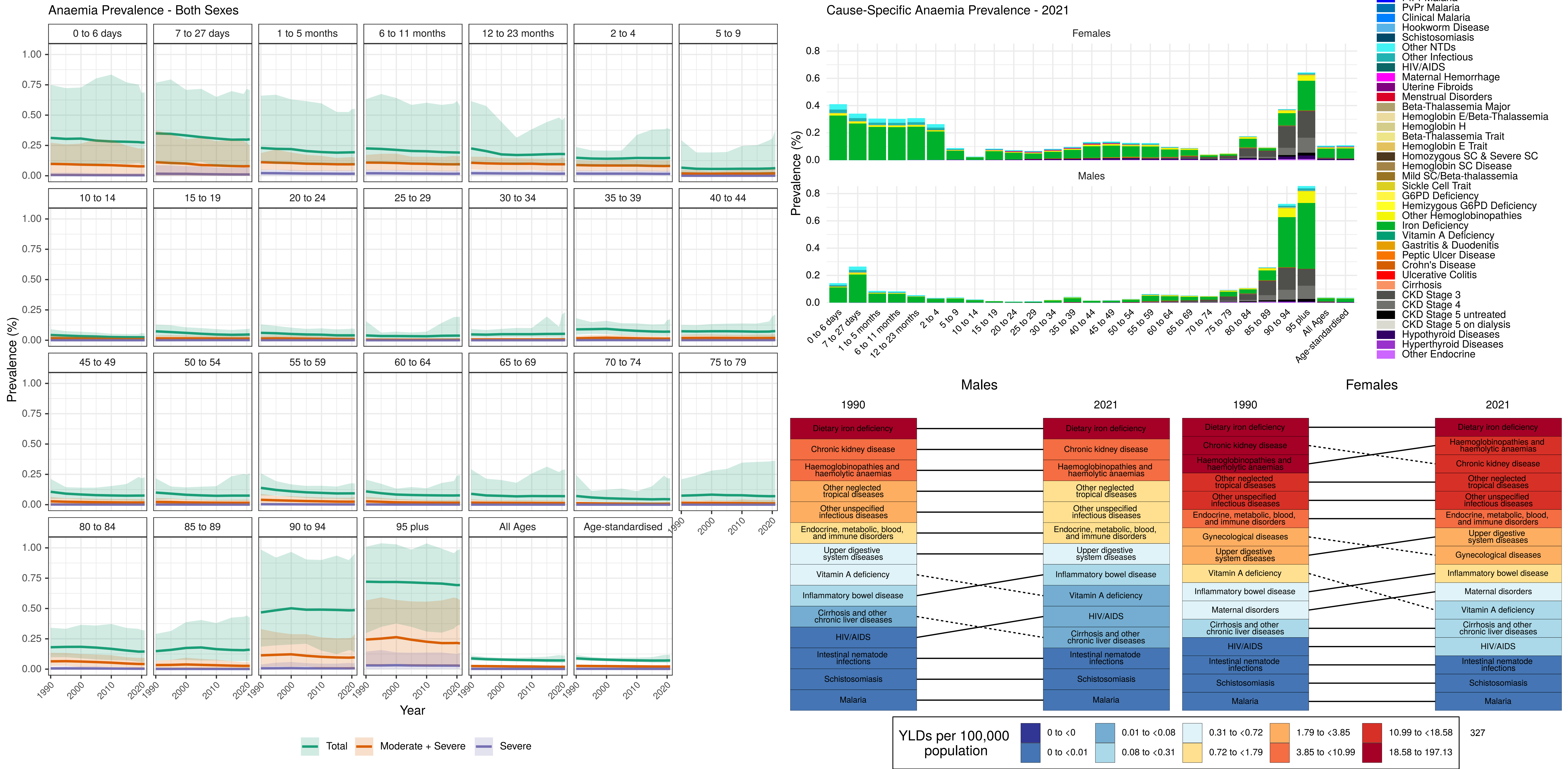

Blackpool

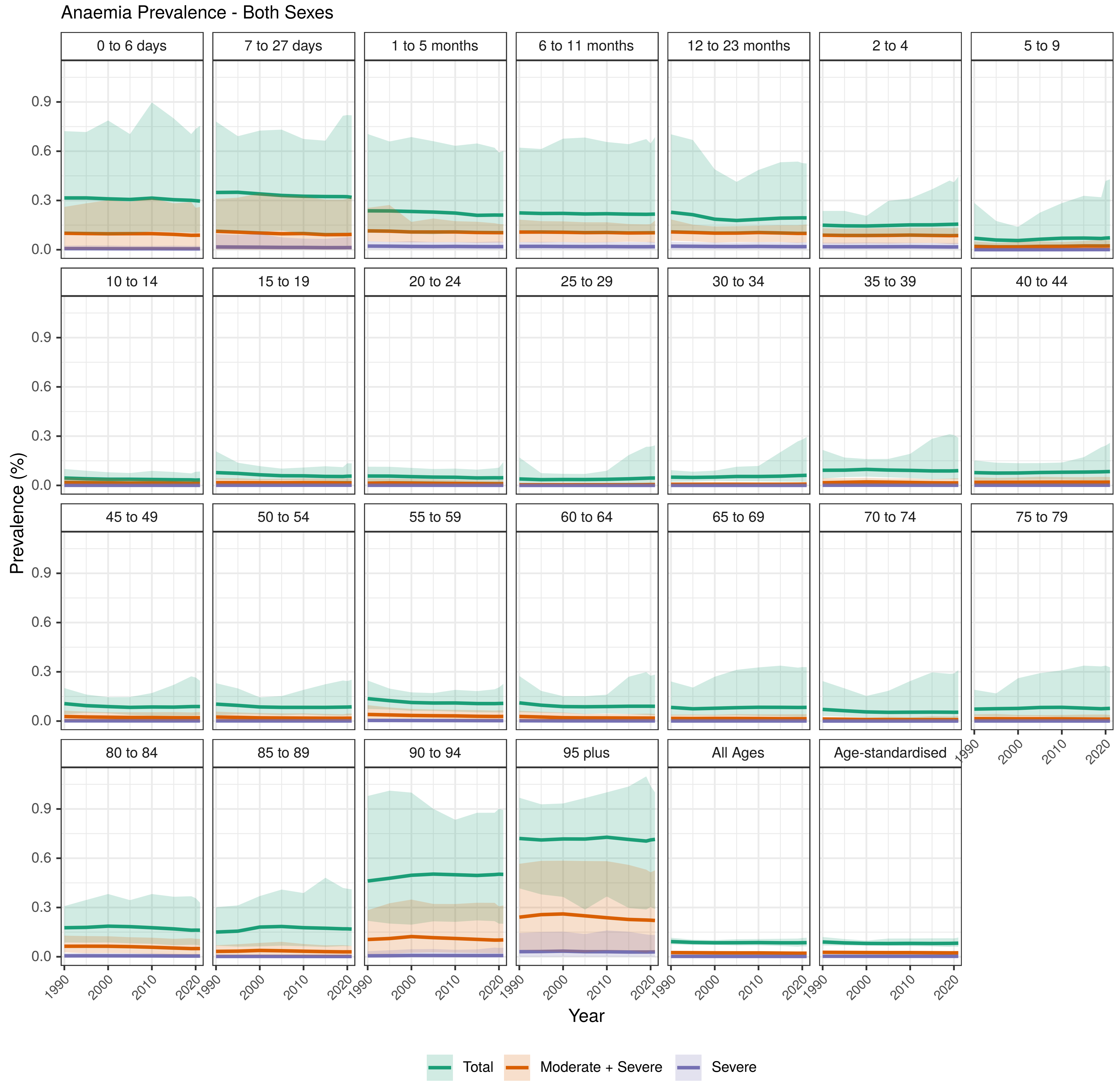

Anaemia Prevalence - Both Sexes
0 to 6 days
7 to 27 days
1 to 5 months
6 to 11 months
12 to 23 months
2 to 4
5 to 9
10 to 14
15 to 19
20 to 24
25 to 29
30 to 34
35 to 39
40 to 44
45 to 49
50 to 54
55 to 59
60 to 64
65 to 69
70 to 74
75 to 79
80 to 84
85 to 89
90 to 94
95 plus
All Ages
Age-standardised
Prevalence (%)
Year
Total
Moderate + Severe
Severe

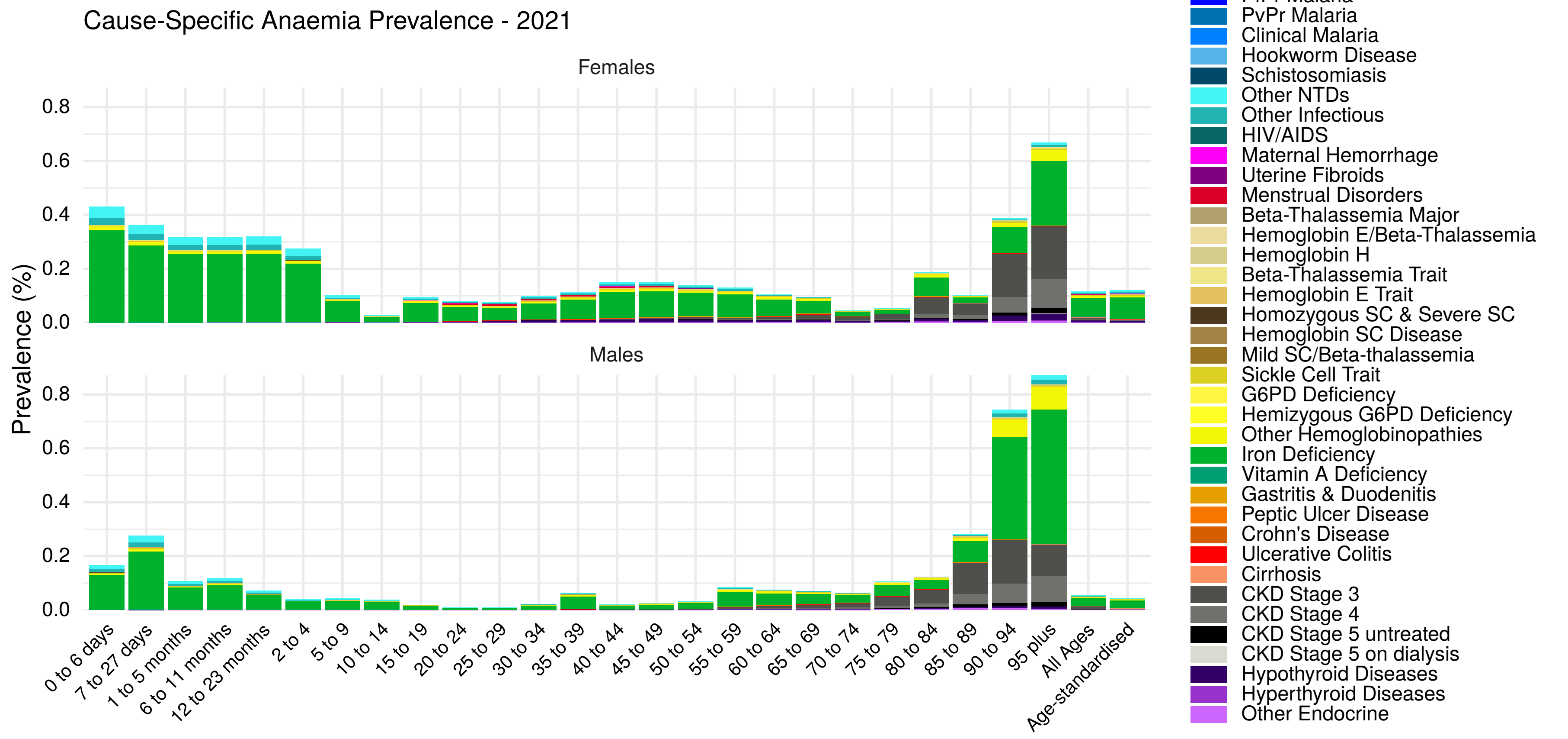

Cause-Specific Anaemia Prevalence - 2021
Females
Males
Prevalence (%)
PfPr Malaria
PvPr Malaria
Clinical Malaria
Hookworm Disease
Schistosomiasis
Other NTDs
Other Infectious
HIV/AIDS
Maternal Hemorrhage
Uterine Fibroids
Menstrual Disorders
Beta-Thalassemia Major
Hemoglobin E/Beta-Thalassemia
Hemoglobin H
Beta-Thalassemia Trait
Hemoglobin E Trait
Homozygous SC & Severe SC
Hemoglobin SC Disease
Mild SC/Beta-thalassemia
Sickle Cell Trait
G6PD Deficiency
Hemizygous G6PD Deficiency
Other Hemoglobinopathies
Iron Deficiency
Vitamin A Deficiency
Gastritis & Duodenitis
Peptic Ulcer Disease
Crohn's Disease
Ulcerative Colitis
Cirrhosis
CKD Stage 3
CKD Stage 4
CKD Stage 5 untreated
CKD Stage 5 on dialysis
Hypothyroid Diseases
Hyperthyroid Diseases
Other Endocrine

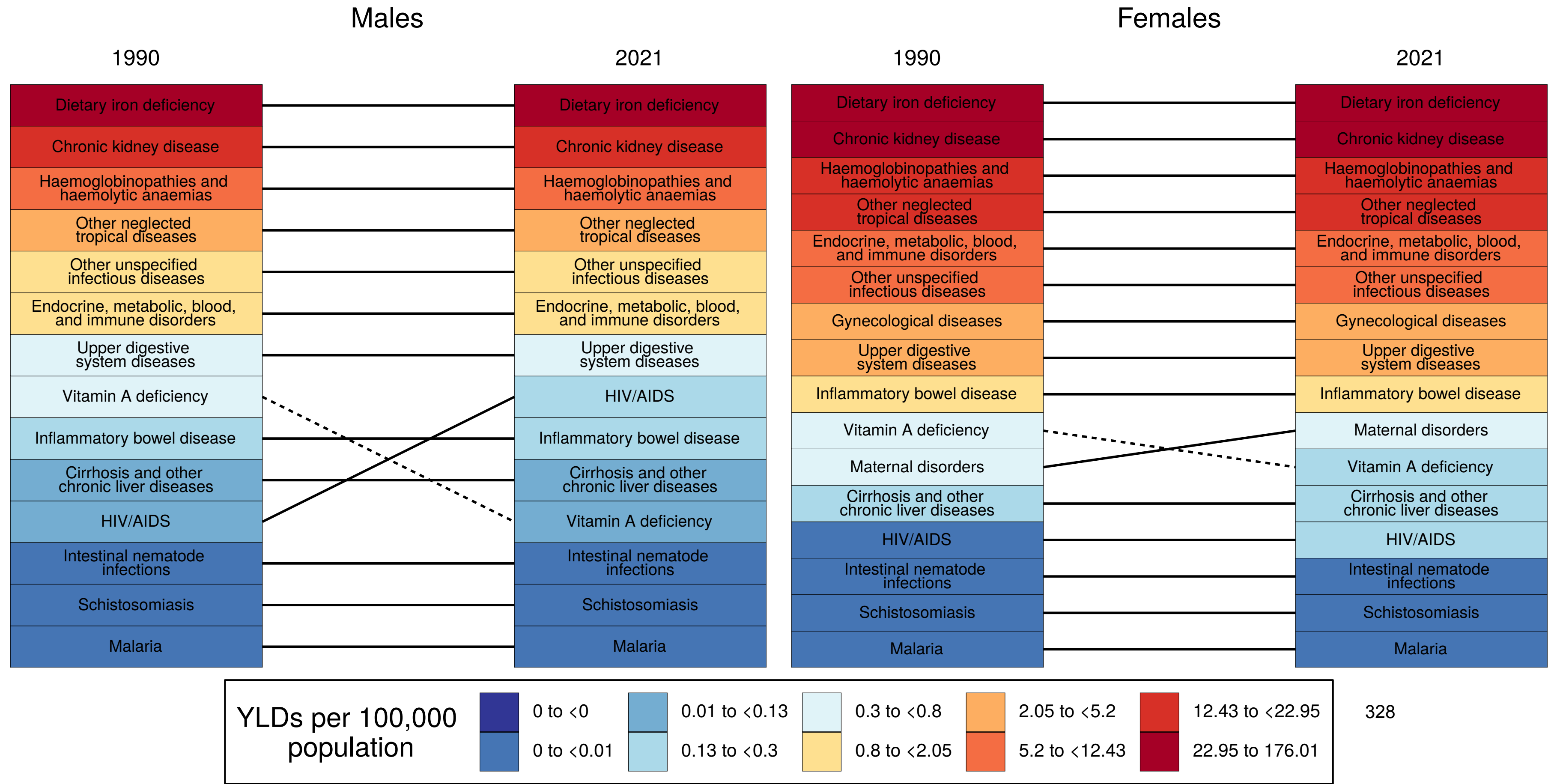

Males
Females
1990
2021
Dietary iron deficiency
Chronic kidney disease
Haemoglobinopathies and haemolytic anaemias
Other neglected tropical diseases
Other unspecified infectious diseases
Endocrine, metabolic, blood, and immune disorders
Upper digestive system diseases
Vitamin A deficiency
Inflammatory bowel disease
Cirrhosis and other chronic liver diseases
HIV/AIDS
Intestinal nematode infections
Schistosomiasis
Malaria
Gynecological diseases
Maternal disorders
YLDs per 100,000 population
0 to <0
0 to <0.01
0.01 to <0.13
0.13 to <0.3
0.3 to <0.8
0.8 to <2.05
2.05 to <5.2
5.2 to <12.43
12.43 to <22.95
22.95 to 176.01

Bolton

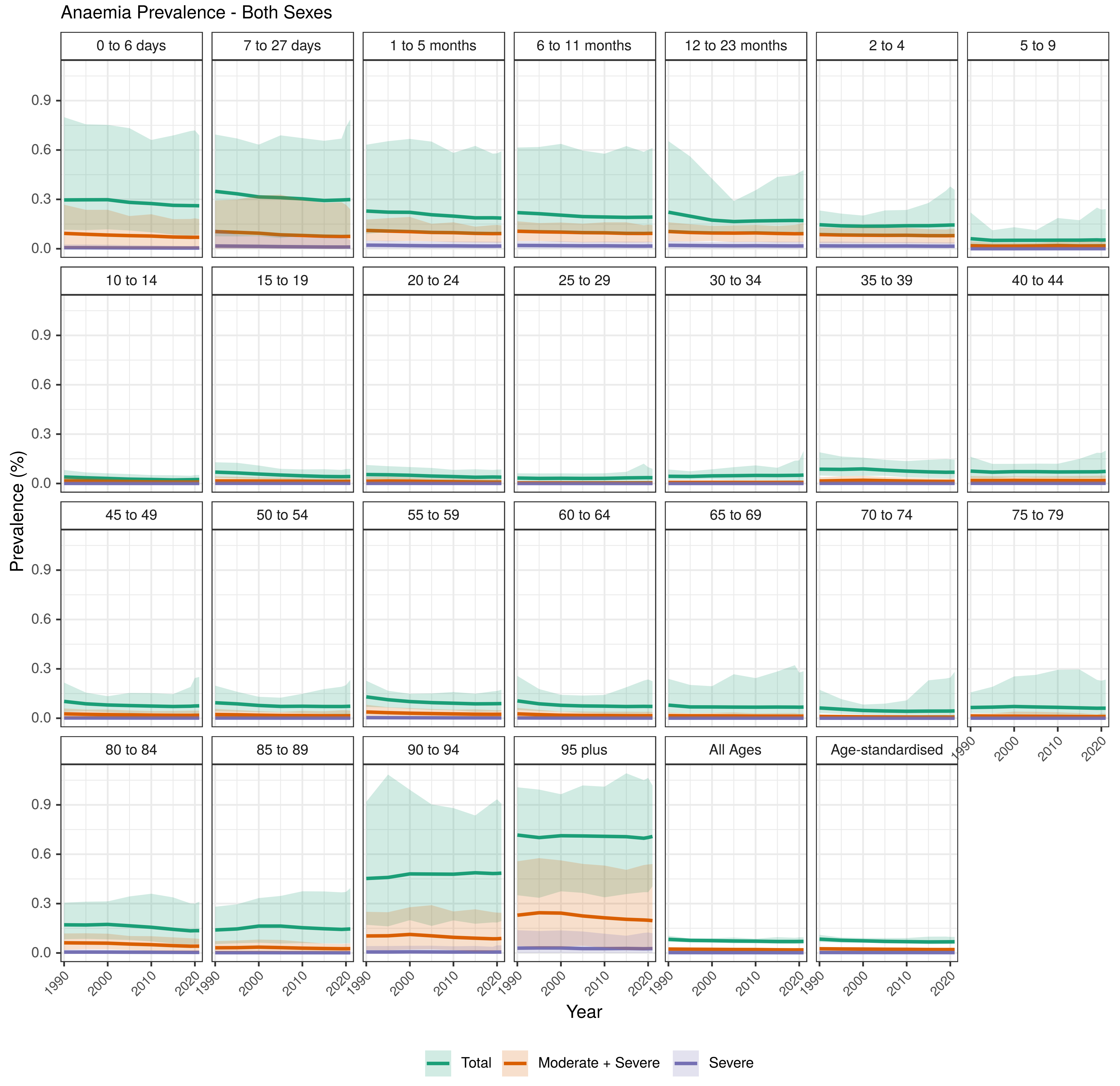

Anaemia Prevalence - Both Sexes
0 to 6 days
7 to 27 days
1 to 5 months
6 to 11 months
12 to 23 months
2 to 4
5 to 9
10 to 14
15 to 19
20 to 24
25 to 29
30 to 34
35 to 39
40 to 44
45 to 49
50 to 54
55 to 59
60 to 64
65 to 69
70 to 74
75 to 79
80 to 84
85 to 89
90 to 94
95 plus
All Ages
Age-standardised
Prevalence (%)
Year
Total
Moderate + Severe
Severe

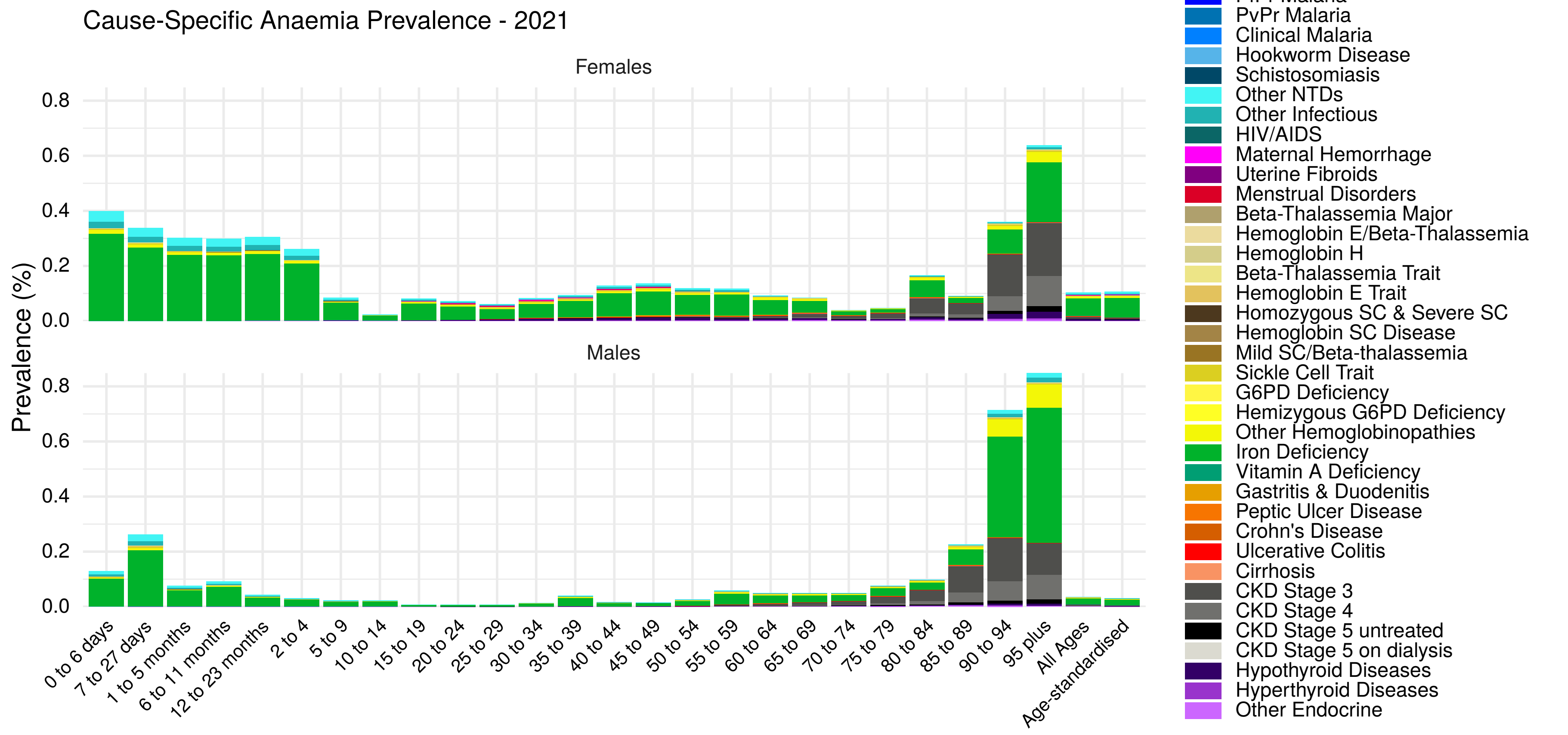

Cause-Specific Anaemia Prevalence - 2021
Females
Males
Prevalence (%)
PfPr Malaria
PvPr Malaria
Clinical Malaria
Hookworm Disease
Schistosomiasis
Other NTDs
Other Infectious
HIV/AIDS
Maternal Hemorrhage
Uterine Fibroids
Menstrual Disorders
Beta-Thalassemia Major
Hemoglobin E/Beta-Thalassemia
Hemoglobin H
Beta-Thalassemia Trait
Hemoglobin E Trait
Homozygous SC & Severe SC
Hemoglobin SC Disease
Mild SC/Beta-thalassemia
Sickle Cell Trait
G6PD Deficiency
Hemizygous G6PD Deficiency
Other Hemoglobinopathies
Iron Deficiency
Vitamin A Deficiency
Gastritis & Duodenitis
Peptic Ulcer Disease
Crohn's Disease
Ulcerative Colitis
Cirrhosis
CKD Stage 3
CKD Stage 4
CKD Stage 5 untreated
CKD Stage 5 on dialysis
Hypothyroid Diseases
Hyperthyroid Diseases
Other Endocrine

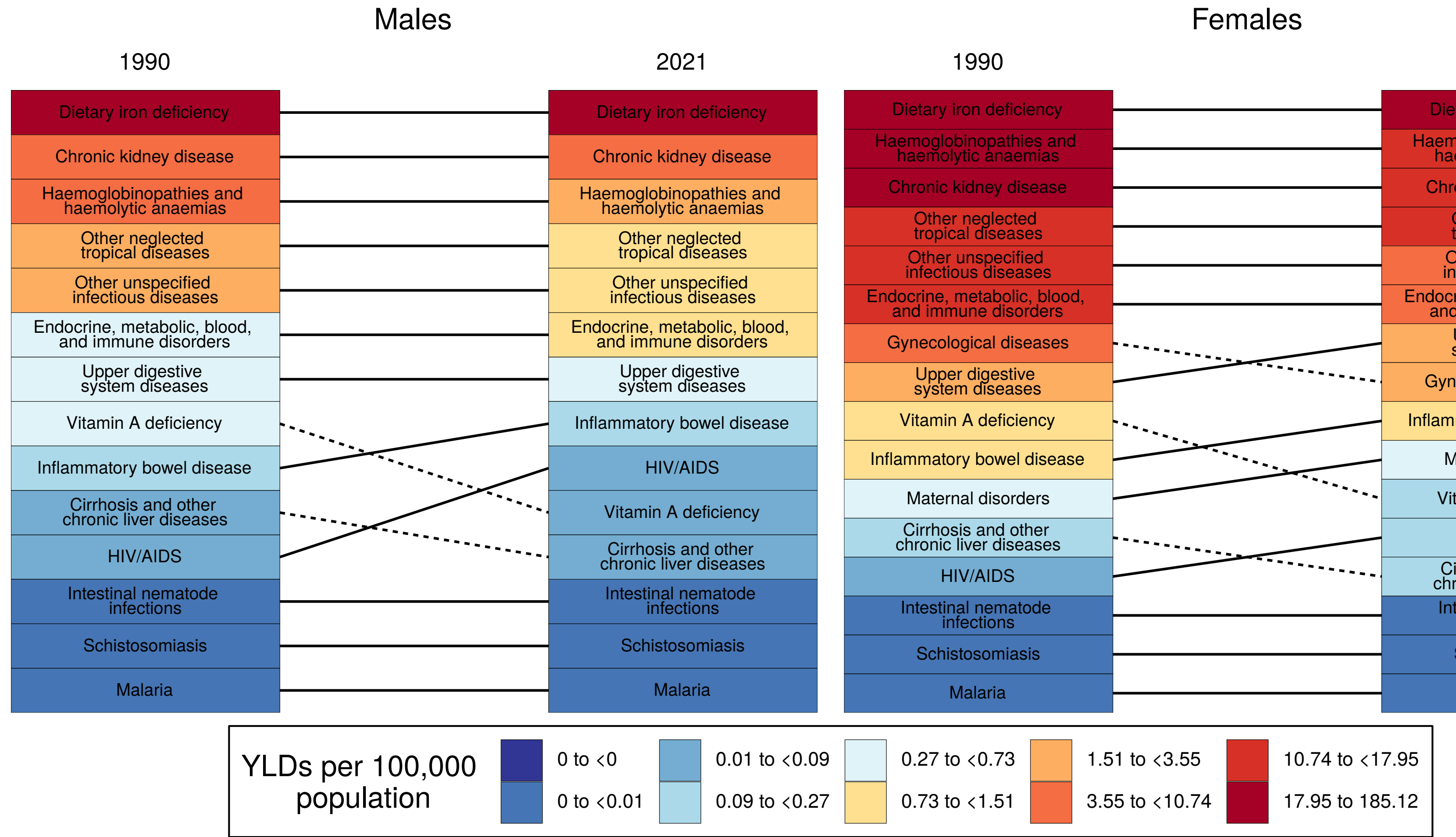

Males
Females
1990
2021
Dietary iron deficiency
Chronic kidney disease
Haemoglobinopathies and haemolytic anaemias
Other neglected tropical diseases
Other unspecified infectious diseases
Endocrine, metabolic, blood, and immune disorders
Upper digestive system diseases
Vitamin A deficiency
Inflammatory bowel disease
Cirrhosis and other chronic liver diseases
HIV/AIDS
Intestinal nematode infections
Schistosomiasis
Malaria
Gynecological diseases
Maternal disorders
YLDs per 100,000 population
0 to <0
0 to <0.01
0.01 to <0.09
0.09 to <0.27
0.27 to <0.73
0.73 to <1.51
1.51 to <3.55
3.55 to <10.74
10.74 to <17.95
17.95 to 185.12

Bury

Anaemia Prevalence - Both Sexes

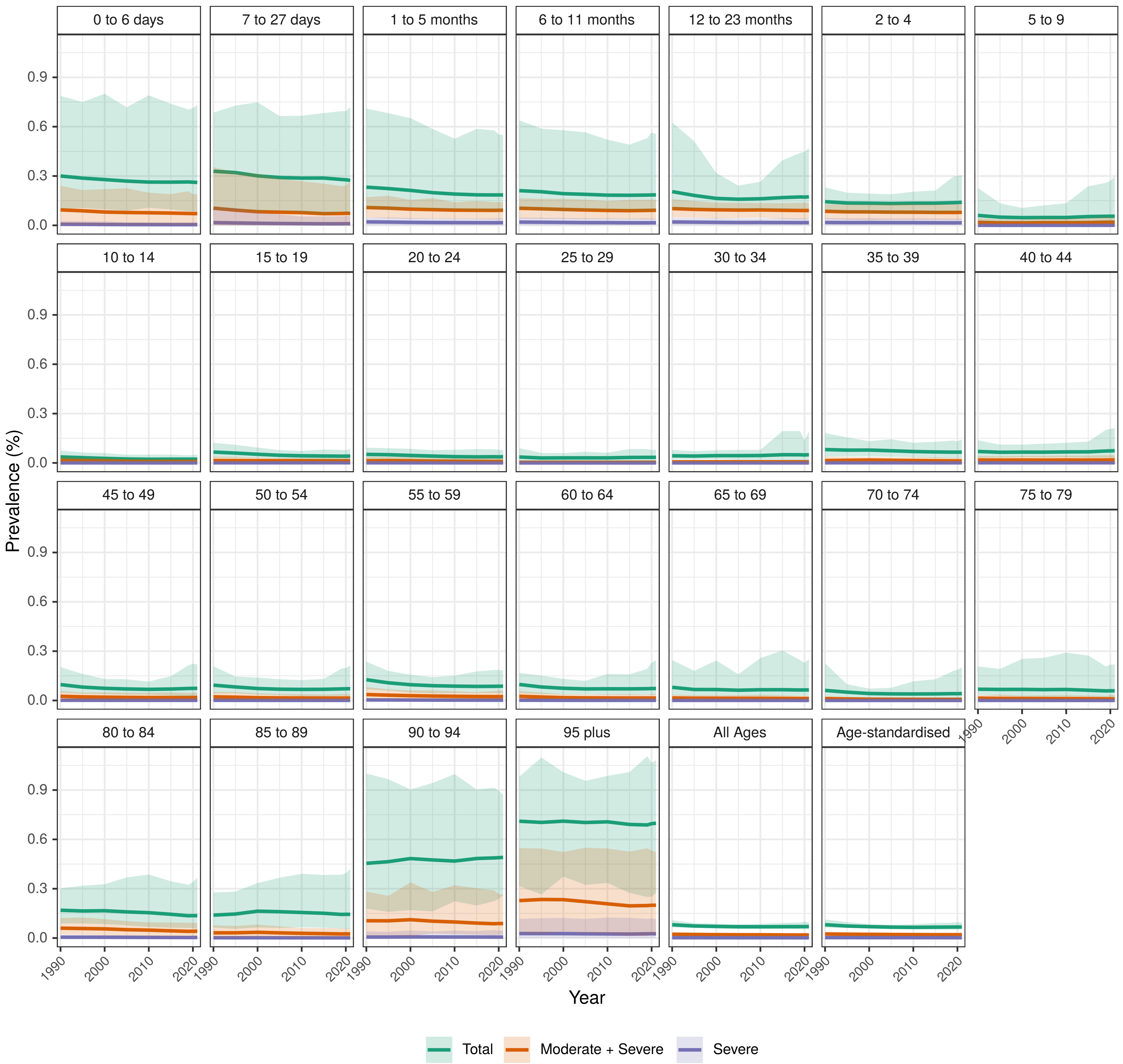

Cause-Specific Anaemia Prevalence - 2021

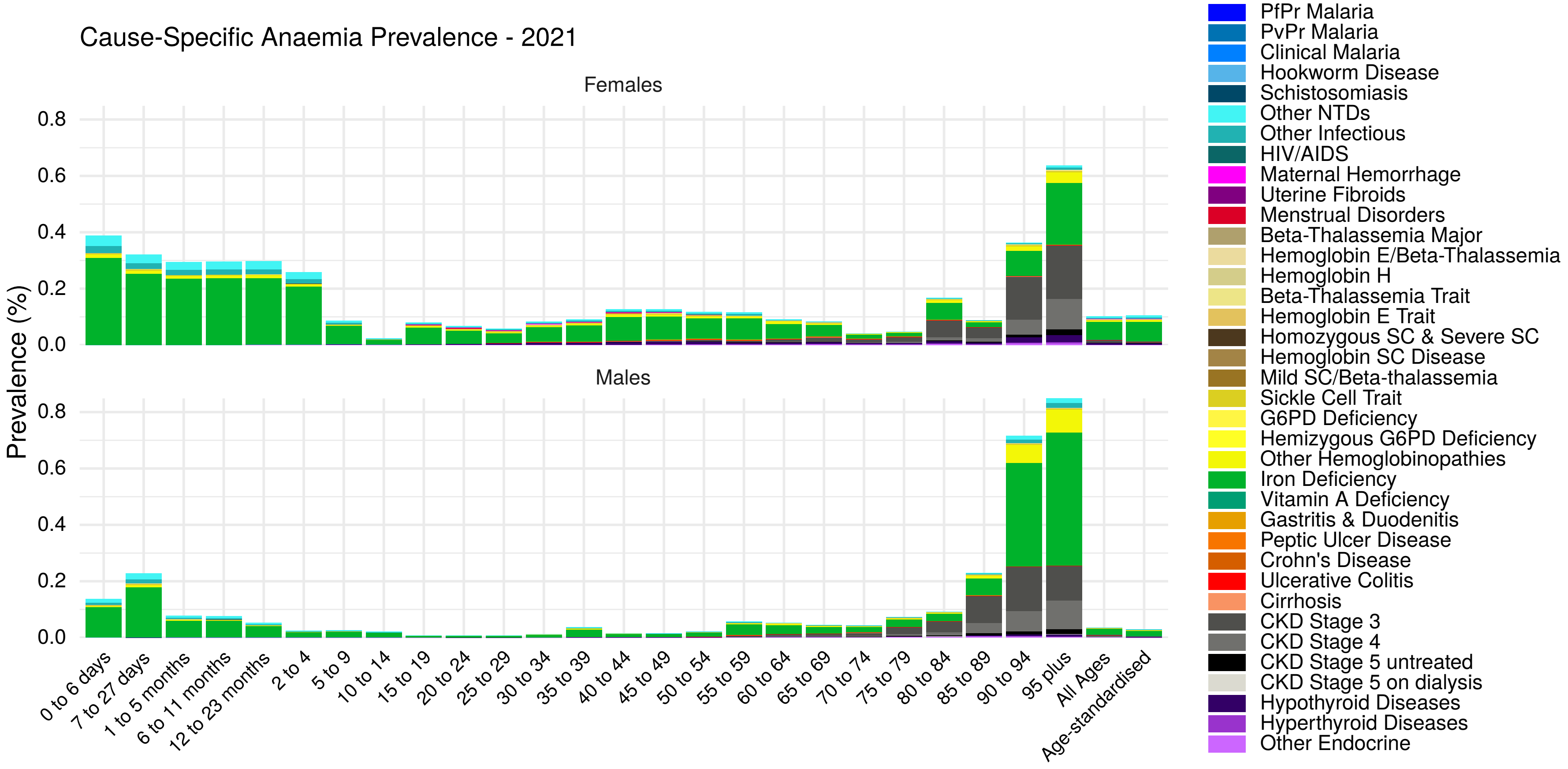

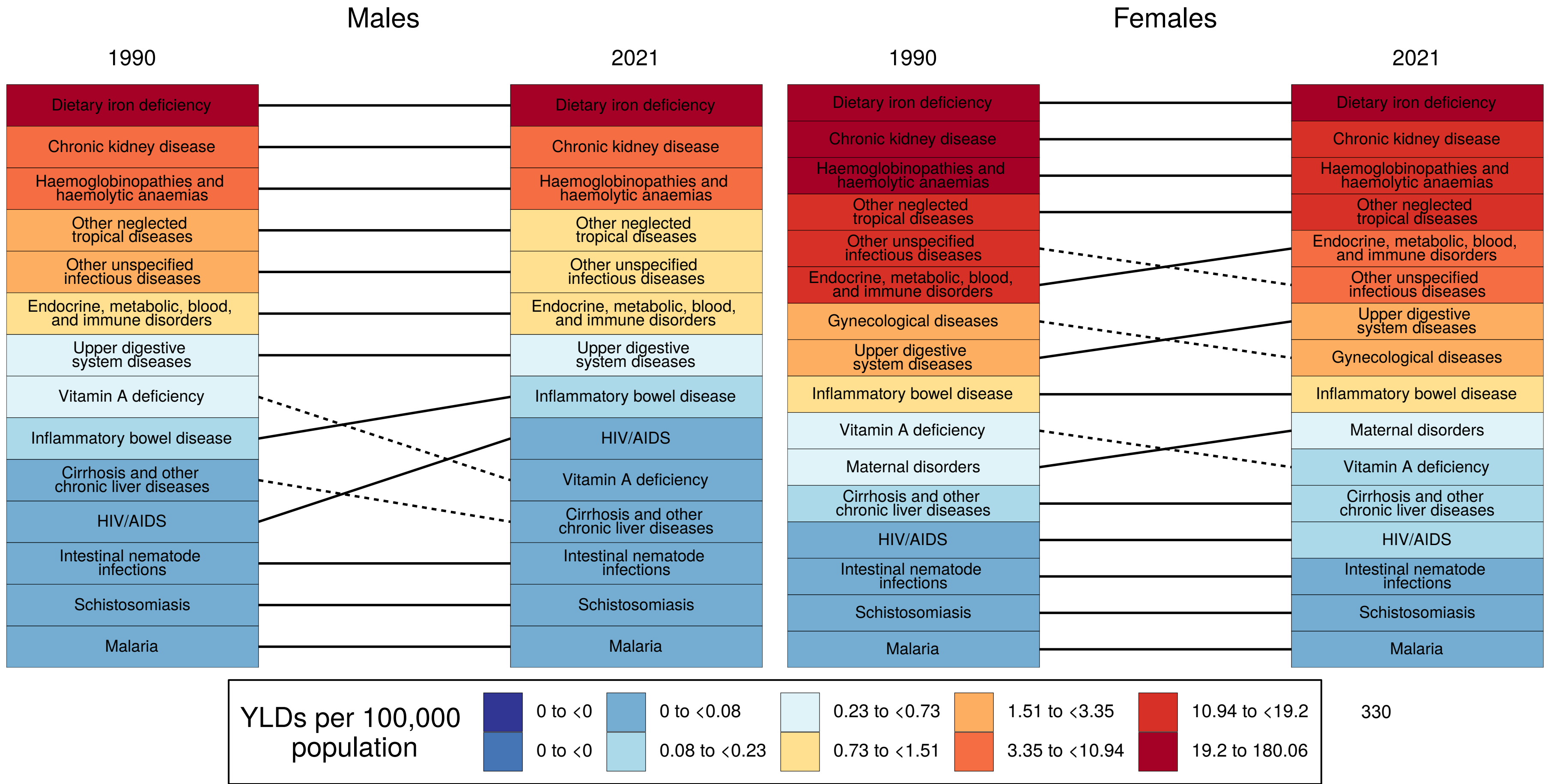

Cheshire East

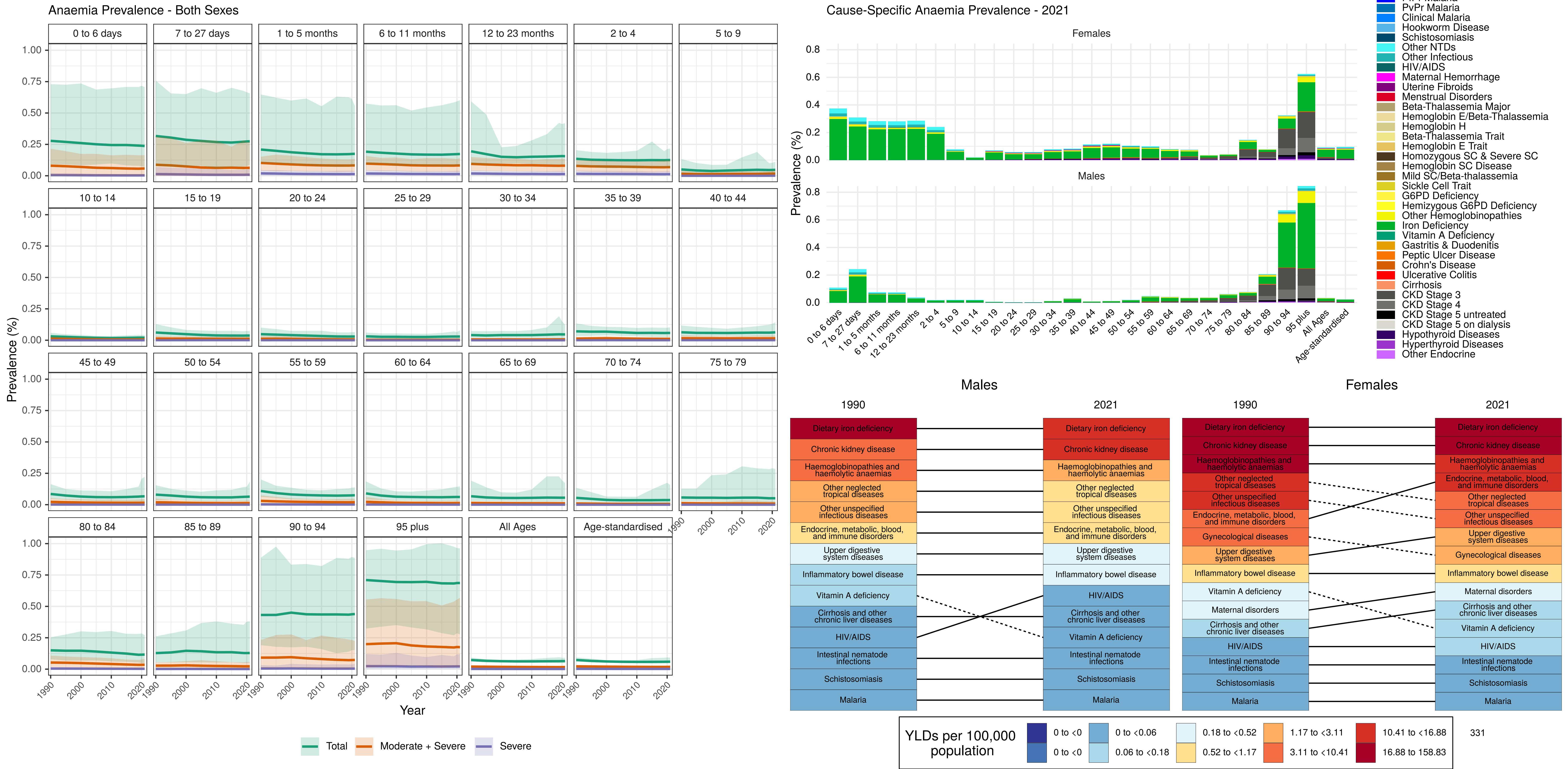

Cheshire West and Chester

Anaemia Prevalence - Both Sexes

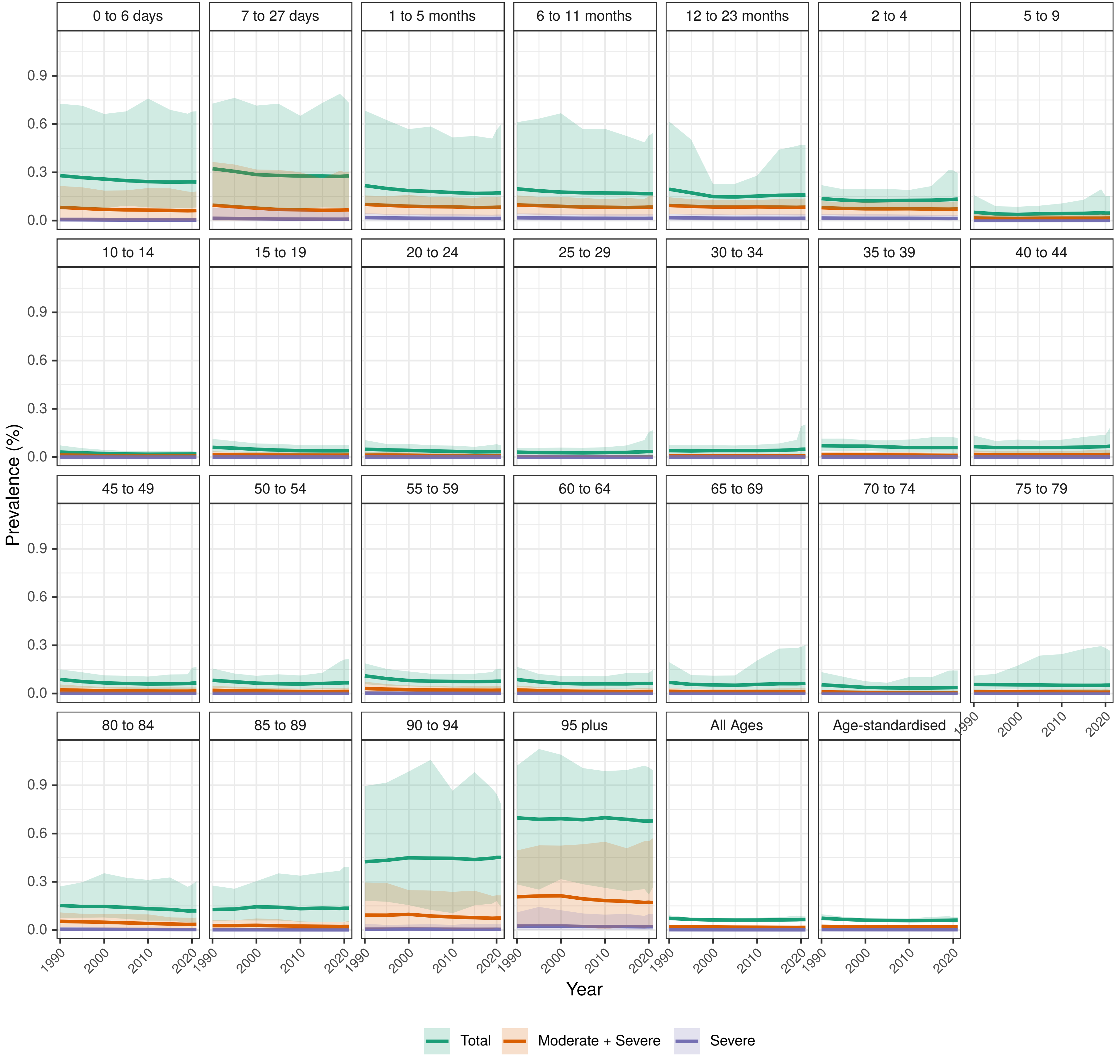

Cause-Specific Anaemia Prevalence - 2021

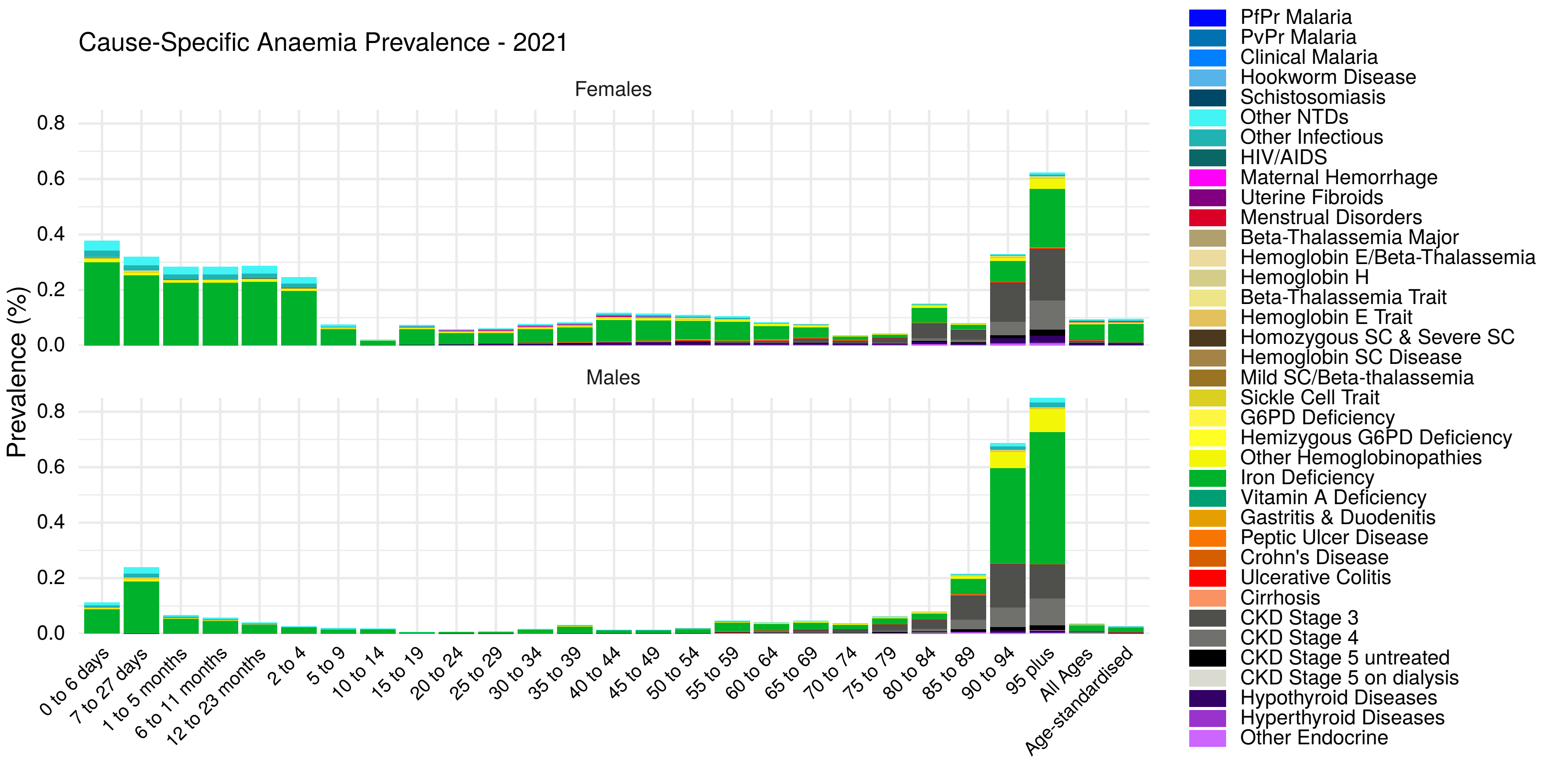

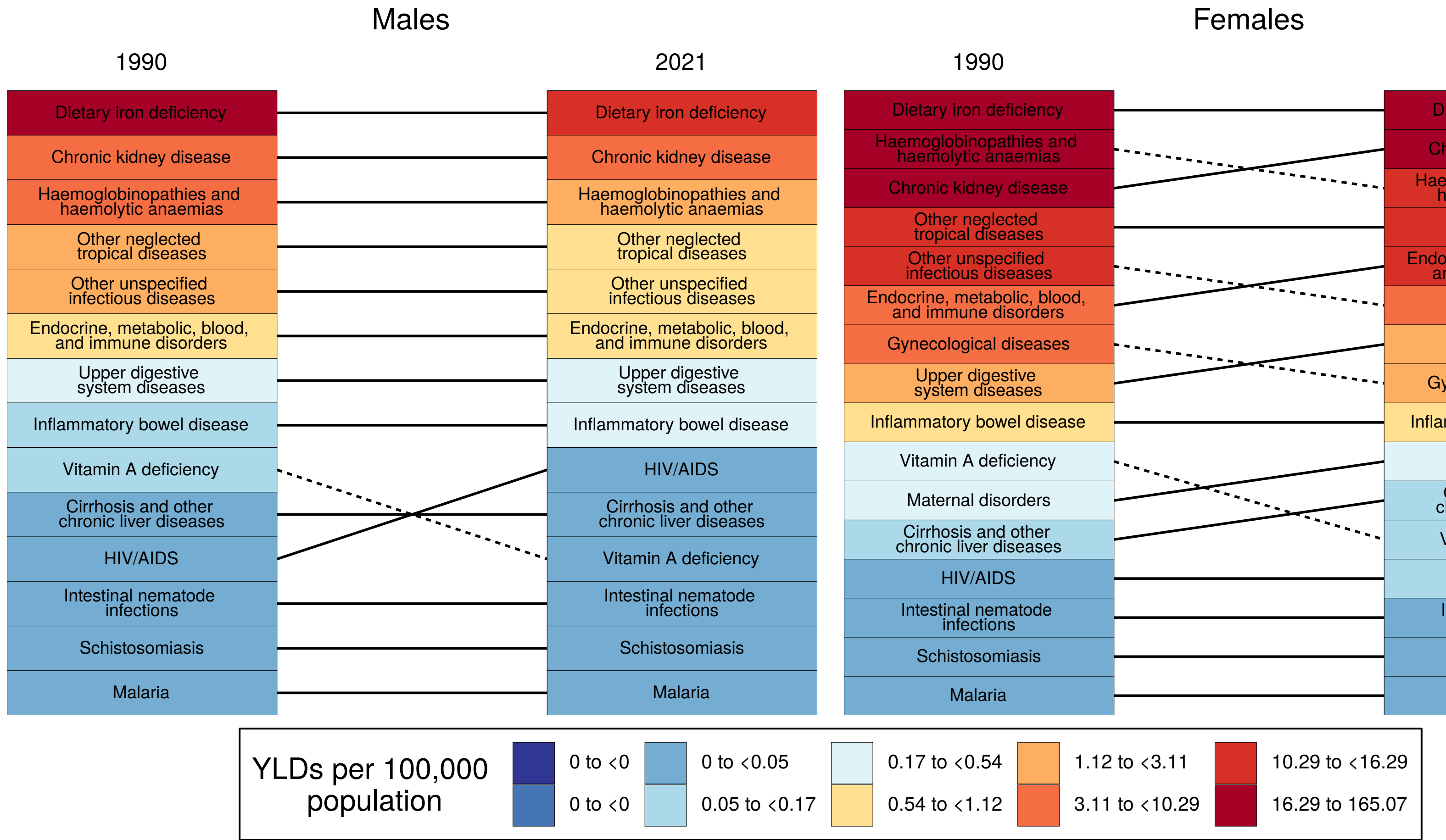

Cumbria

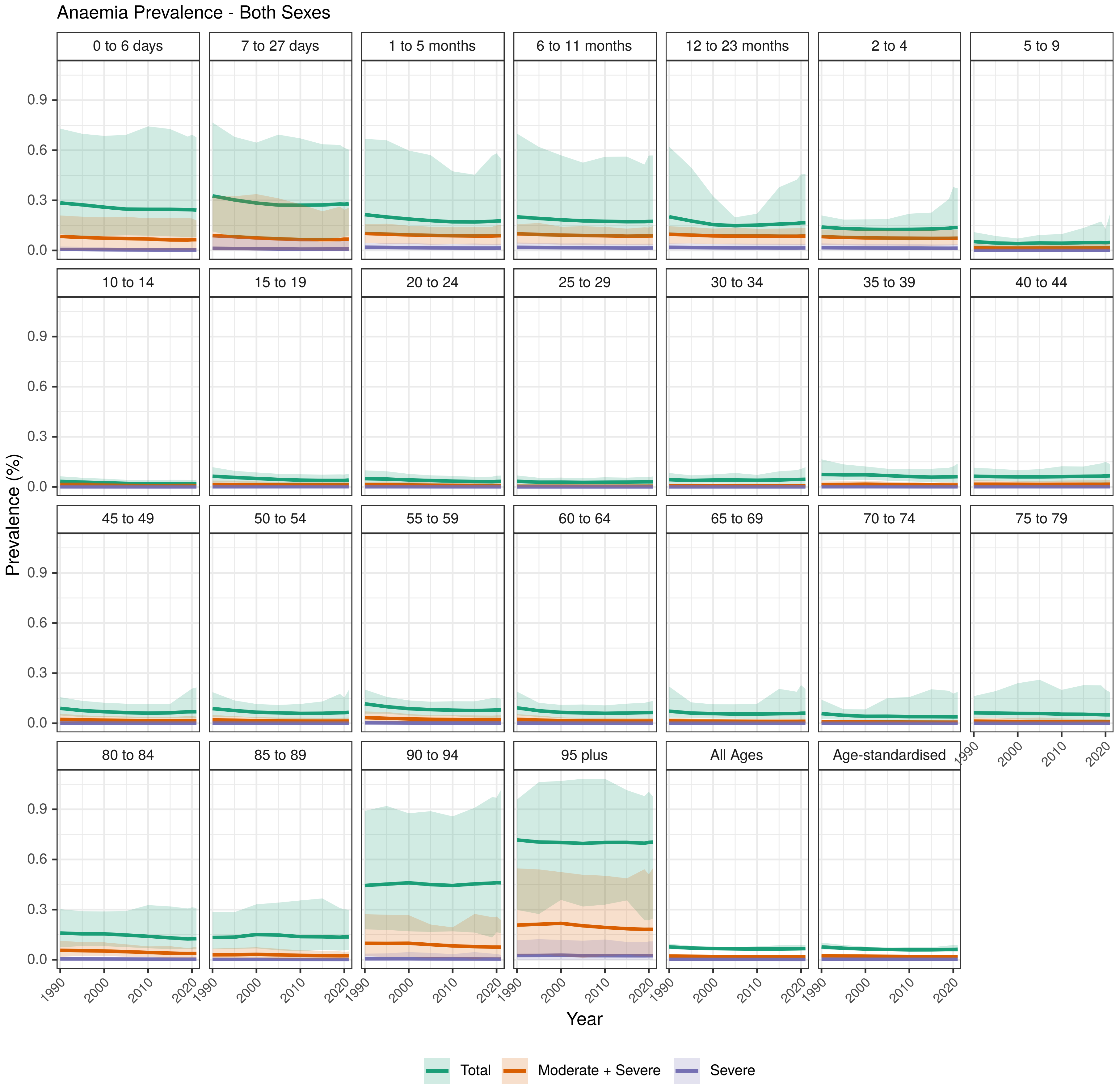

Anaemia Prevalence - Both Sexes
0 to 6 days
7 to 27 days
1 to 5 months
6 to 11 months
12 to 23 months
2 to 4
5 to 9
10 to 14
15 to 19
20 to 24
25 to 29
30 to 34
35 to 39
40 to 44
45 to 49
50 to 54
55 to 59
60 to 64
65 to 69
70 to 74
75 to 79
80 to 84
85 to 89
90 to 94
95 plus
All Ages
Age-standardised
Prevalence (%)
Year
Total
Moderate + Severe
Severe

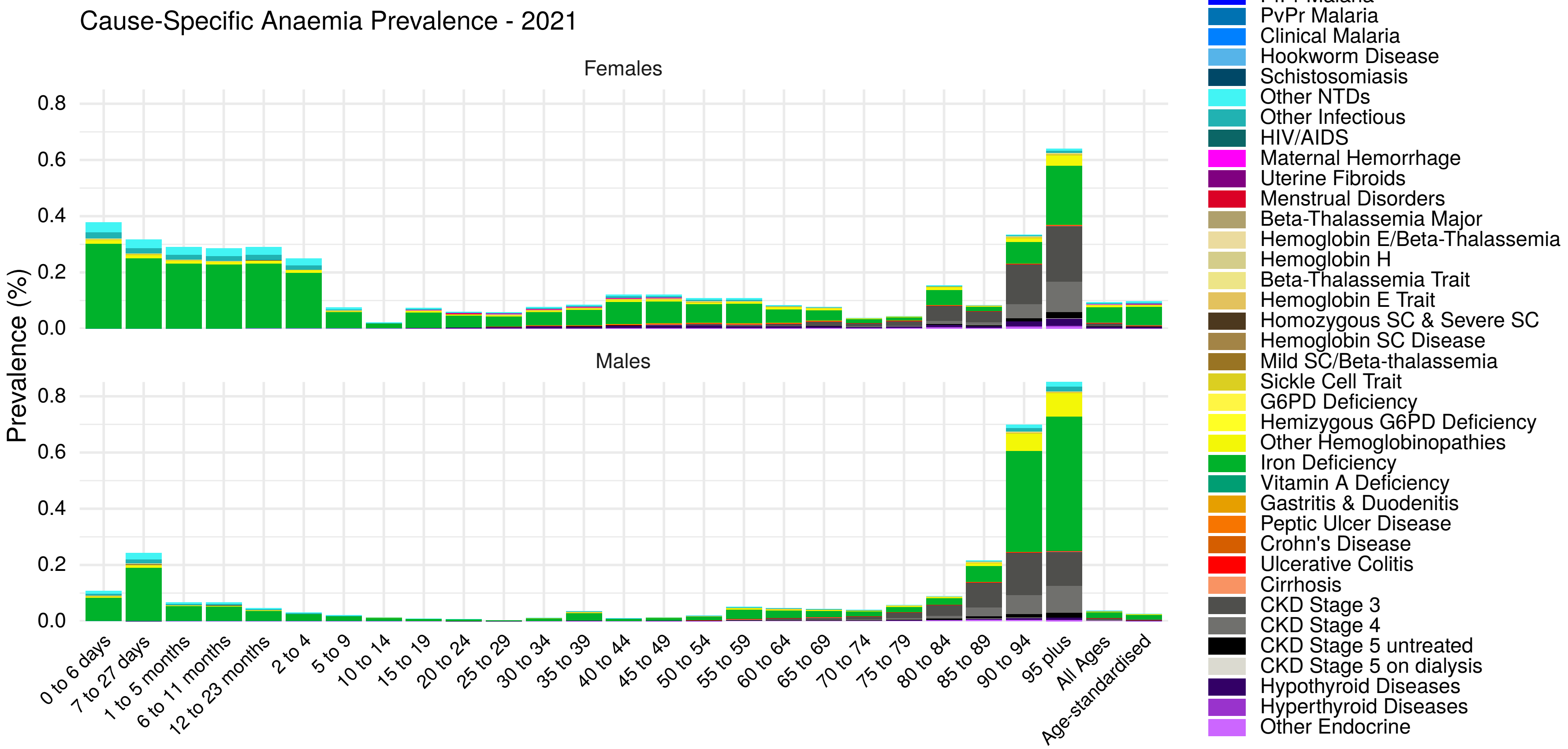

Cause-Specific Anaemia Prevalence - 2021
Females
Males
Prevalence (%)
PfPr Malaria
PvPr Malaria
Clinical Malaria
Hookworm Disease
Schistosomiasis
Other NTDs
Other Infectious
HIV/AIDS
Maternal Hemorrhage
Uterine Fibroids
Menstrual Disorders
Beta-Thalassemia Major
Hemoglobin E/Beta-Thalassemia
Hemoglobin H
Beta-Thalassemia Trait
Hemoglobin E Trait
Homozygous SC & Severe SC
Hemoglobin SC Disease
Mild SC/Beta-thalassemia
Sickle Cell Trait
G6PD Deficiency
Hemizygous G6PD Deficiency
Other Hemoglobinopathies
Iron Deficiency
Vitamin A Deficiency
Gastritis & Duodenitis
Peptic Ulcer Disease
Crohn's Disease
Ulcerative Colitis
Cirrhosis
CKD Stage 3
CKD Stage 4
CKD Stage 5 untreated
CKD Stage 5 on dialysis
Hypothyroid Diseases
Hyperthyroid Diseases
Other Endocrine

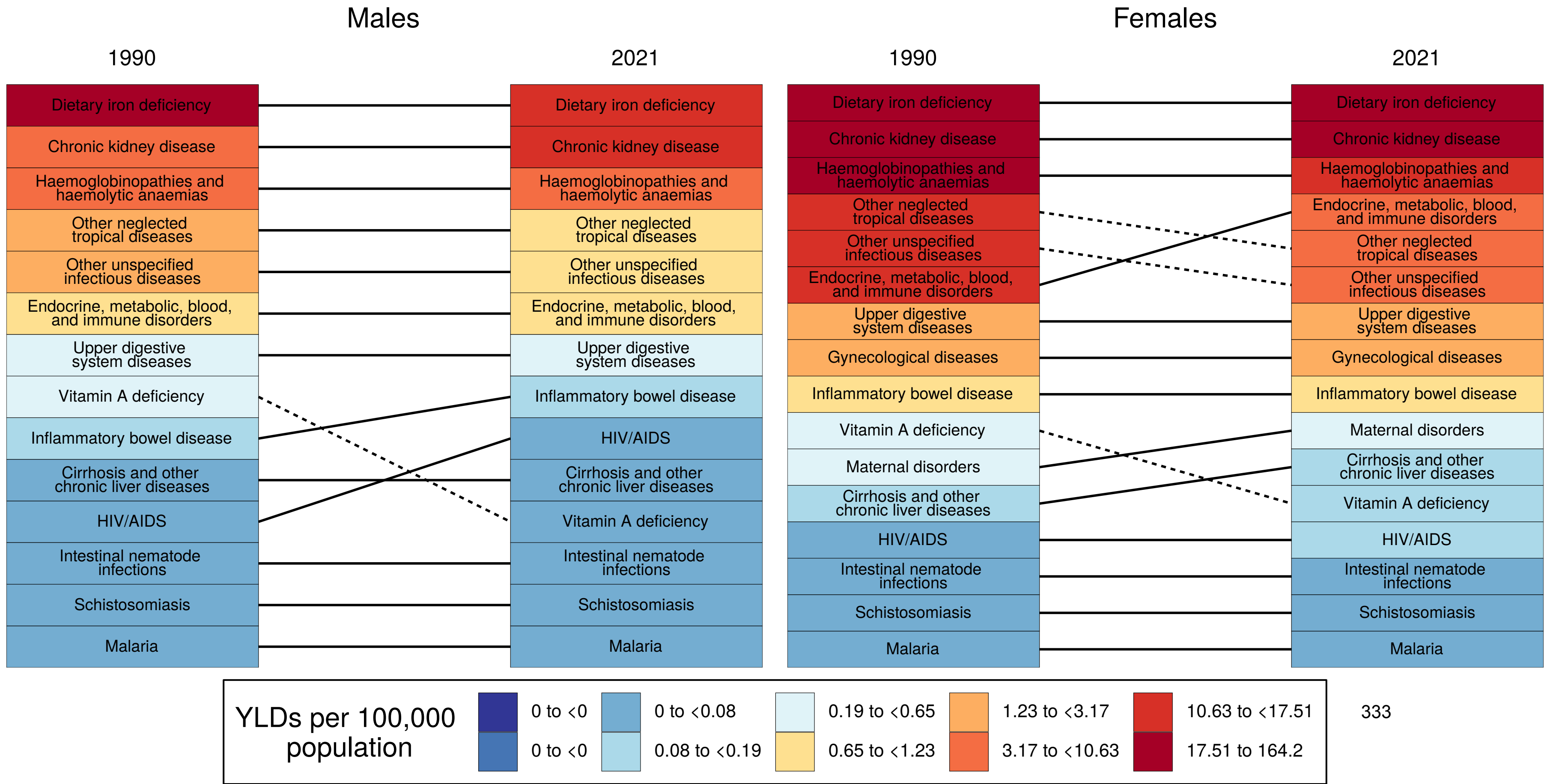

Males
Females
1990
2021
Dietary iron deficiency
Chronic kidney disease
Haemoglobinopathies and haemolytic anaemias
Other neglected tropical diseases
Other unspecified infectious diseases
Endocrine, metabolic, blood, and immune disorders
Upper digestive system diseases
Vitamin A deficiency
Inflammatory bowel disease
Cirrhosis and other chronic liver diseases
HIV/AIDS
Intestinal nematode infections
Schistosomiasis
Malaria
Gynecological diseases
Maternal disorders
YLDs per 100,000 population
0 to <0
0 to <0.08
0.08 to <0.19
0.19 to <0.65
0.65 to <1.23
1.23 to <3.17
3.17 to <10.63
10.63 to <17.51
17.51 to 164.2

Halton

Anaemia Prevalence - Both Sexes

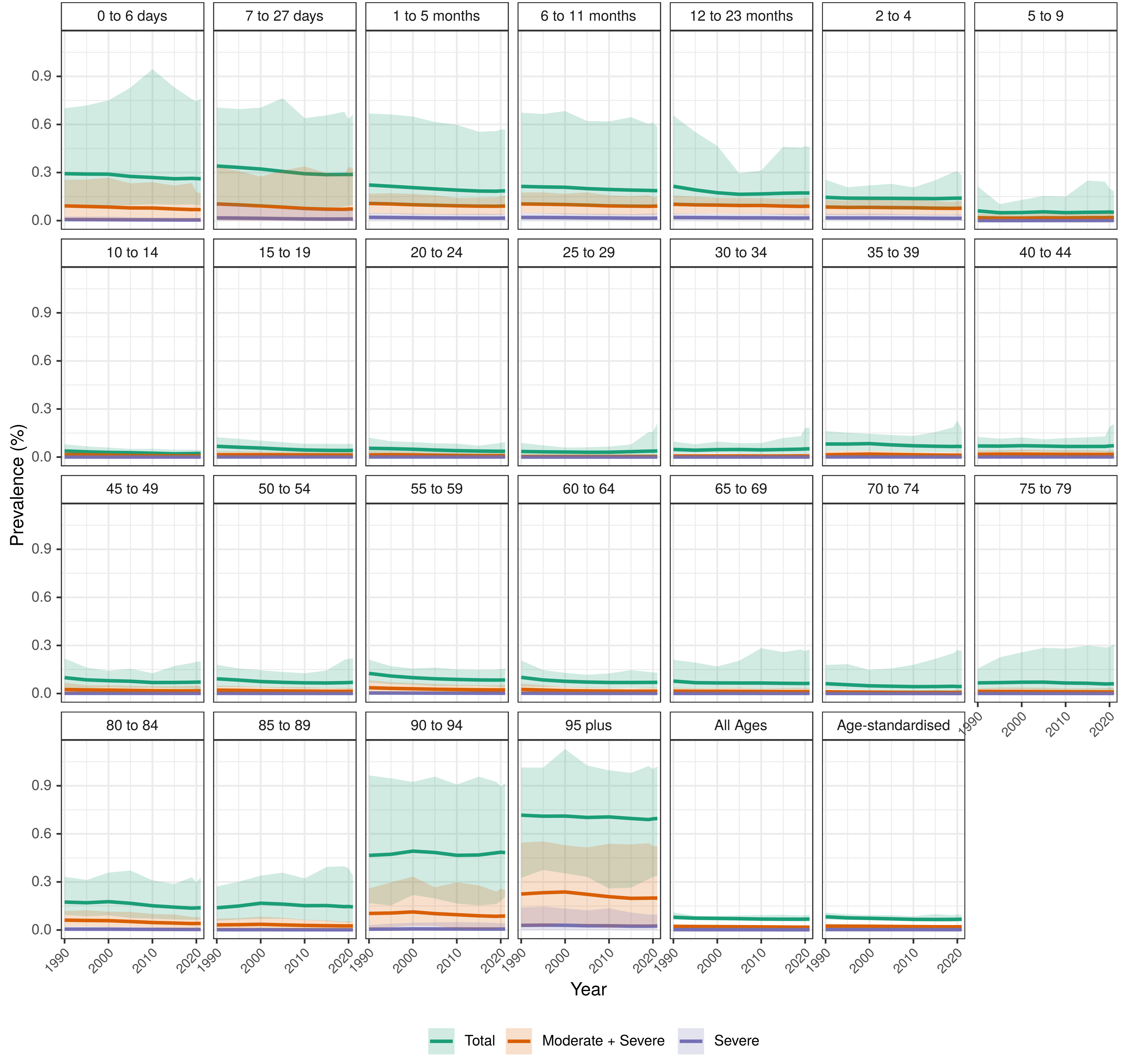

Cause-Specific Anaemia Prevalence - 2021

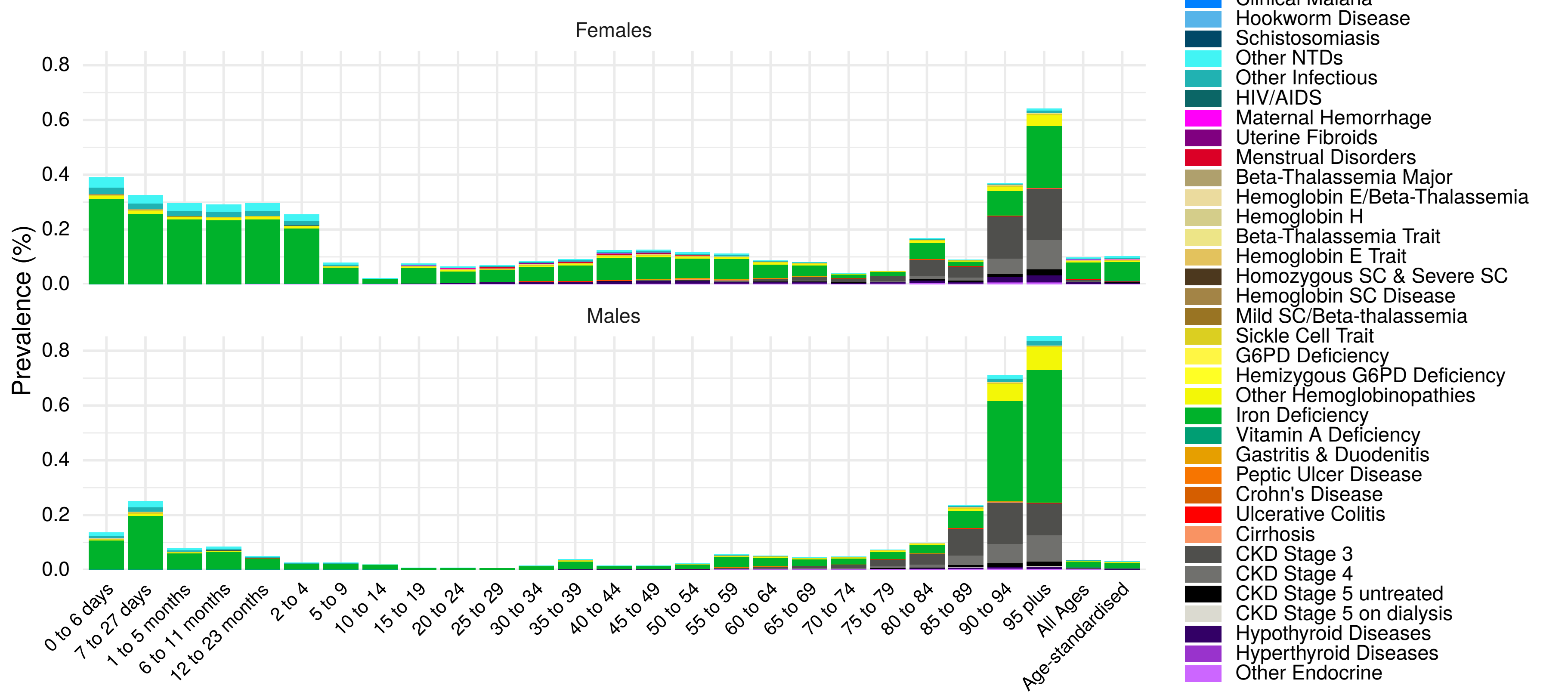

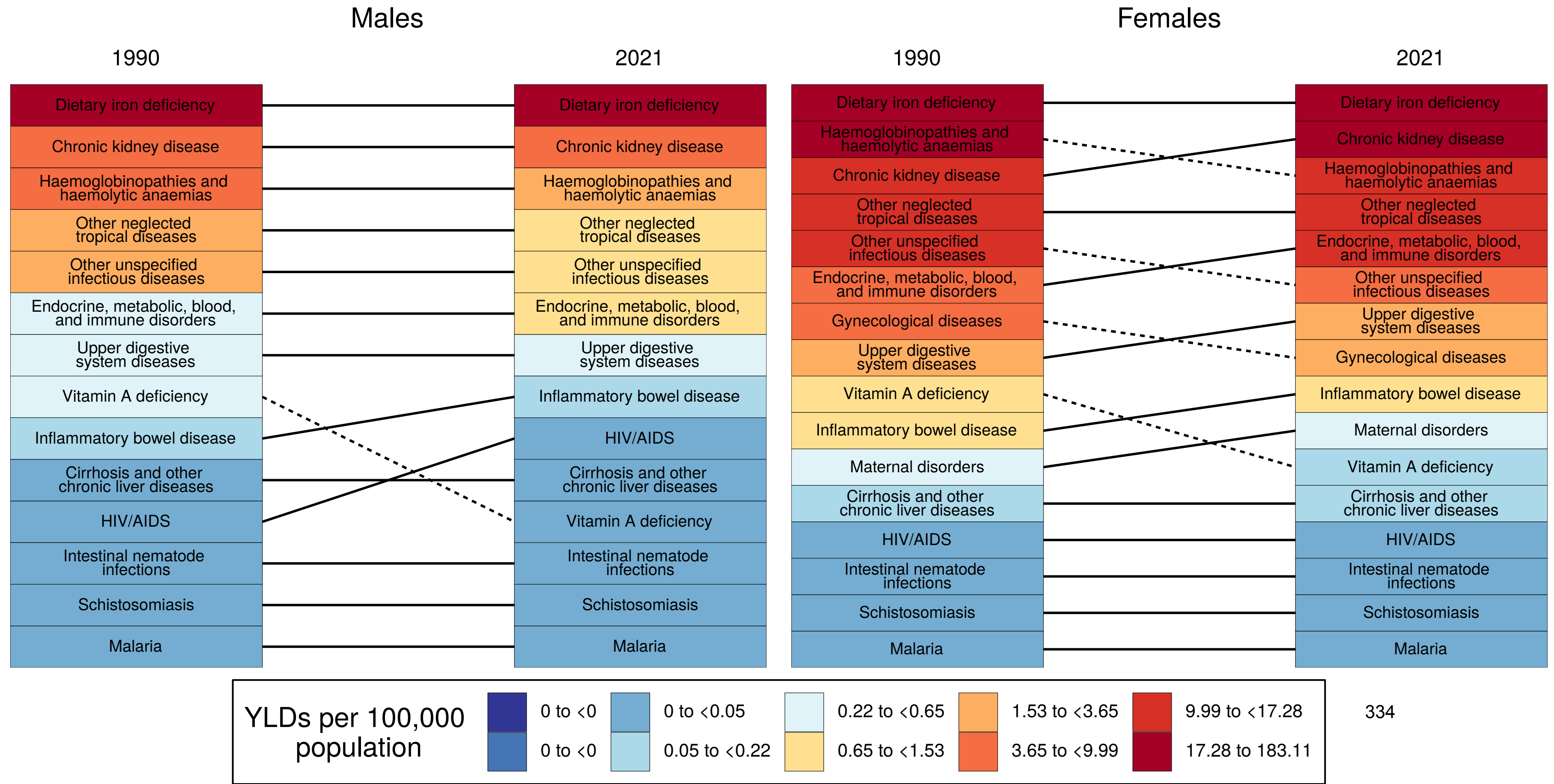

Knowsley

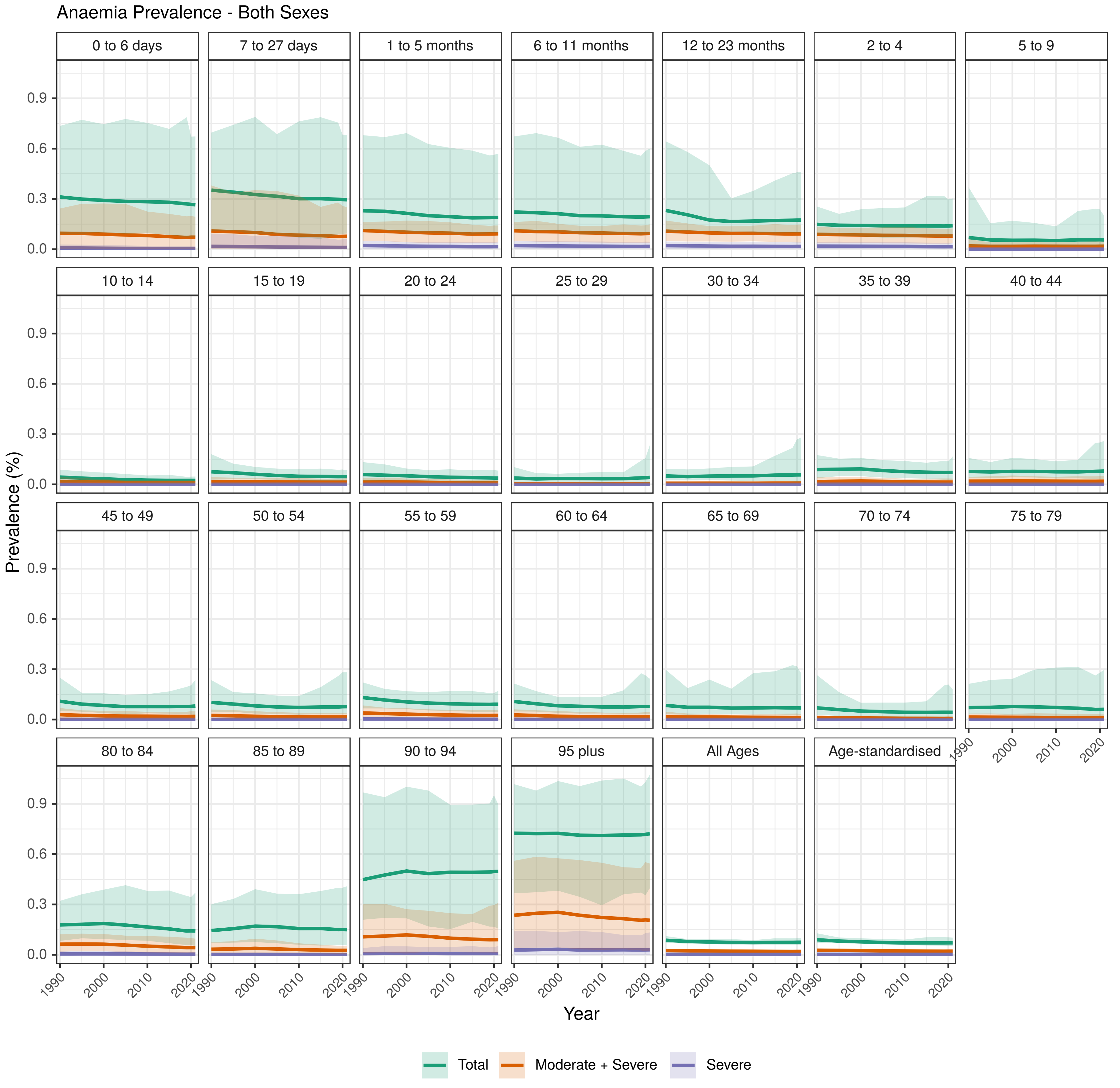
Anaemia Prevalence - Both Sexes
0 to 6 days
7 to 27 days
1 to 5 months
6 to 11 months
12 to 23 months
2 to 4
5 to 9
10 to 14
15 to 19
20 to 24
25 to 29
30 to 34
35 to 39
40 to 44
45 to 49
50 to 54
55 to 59
60 to 64
65 to 69
70 to 74
75 to 79
80 to 84
85 to 89
90 to 94
95 plus
All Ages
Age-standardised
Prevalence (%)
0.0
0.3
0.6
0.9
1990
2000
2010
2020
Year
Total
Moderate + Severe
Severe

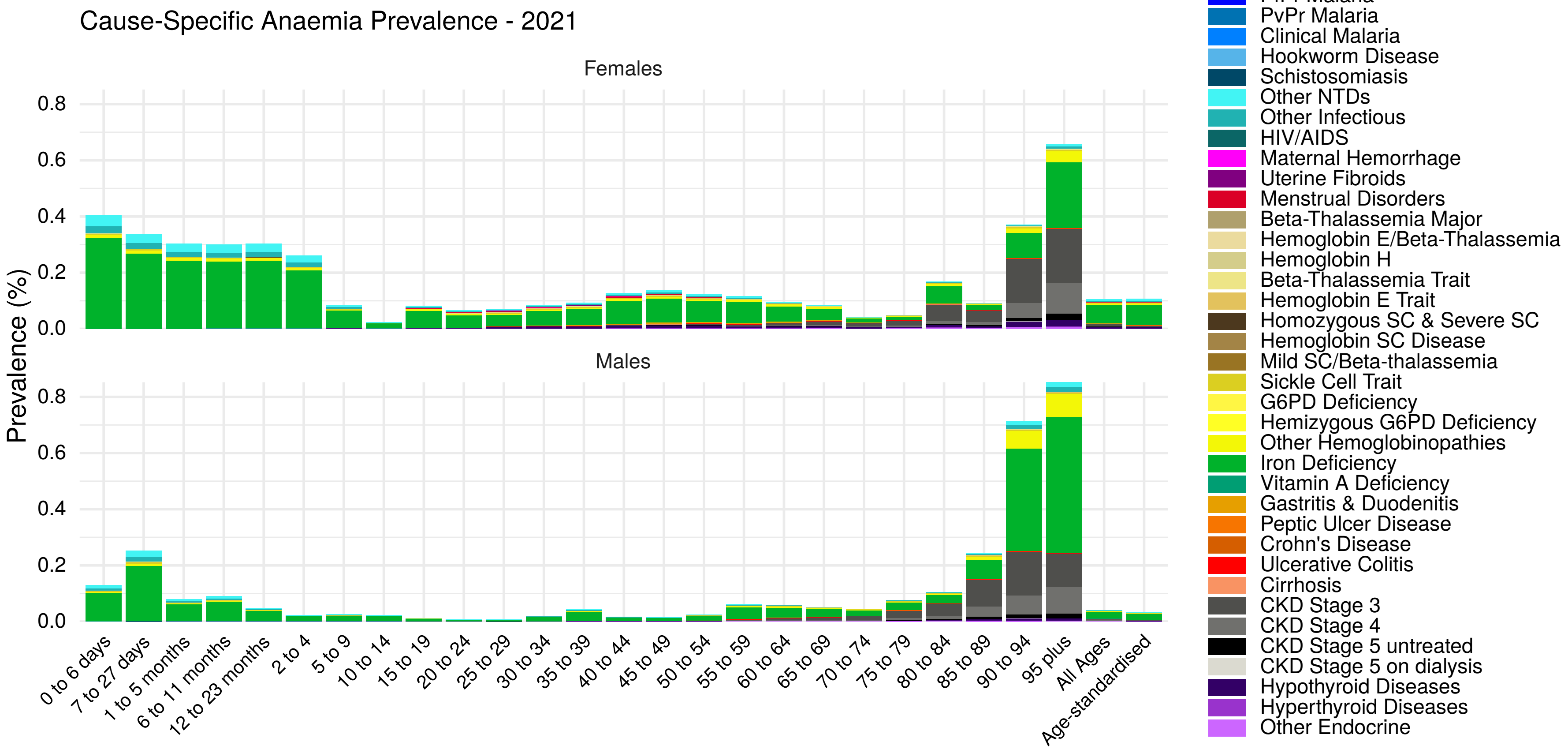
Cause-Specific Anaemia Prevalence - 2021
Females
Males
Prevalence (%)
0.0
0.2
0.4
0.6
0.8
0 to 6 days
7 to 27 days
1 to 5 months
6 to 11 months
12 to 23 months
2 to 4
5 to 9
10 to 14
15 to 19
20 to 24
25 to 29
30 to 34
35 to 39
40 to 44
45 to 49
50 to 54
55 to 59
60 to 64
65 to 69
70 to 74
75 to 79
80 to 84
85 to 89
90 to 94
95 plus
All Ages
Age-standardised
PfPr Malaria
PvPr Malaria
Clinical Malaria
Hookworm Disease
Schistosomiasis
Other NTDs
Other Infectious
HIV/AIDS
Maternal Hemorrhage
Uterine Fibroids
Menstrual Disorders
Beta-Thalassemia Major
Hemoglobin E/Beta-Thalassemia
Hemoglobin H
Beta-Thalassemia Trait
Hemoglobin E Trait
Homozygous SC & Severe SC
Hemoglobin SC Disease
Mild SC/Beta-thalassemia
Sickle Cell Trait
G6PD Deficiency
Hemizygous G6PD Deficiency
Other Hemoglobinopathies
Iron Deficiency
Vitamin A Deficiency
Gastritis & Duodenitis
Peptic Ulcer Disease
Crohn's Disease
Ulcerative Colitis
Cirrhosis
CKD Stage 3
CKD Stage 4
CKD Stage 5 untreated
CKD Stage 5 on dialysis
Hypothyroid Diseases
Hyperthyroid Diseases
Other Endocrine

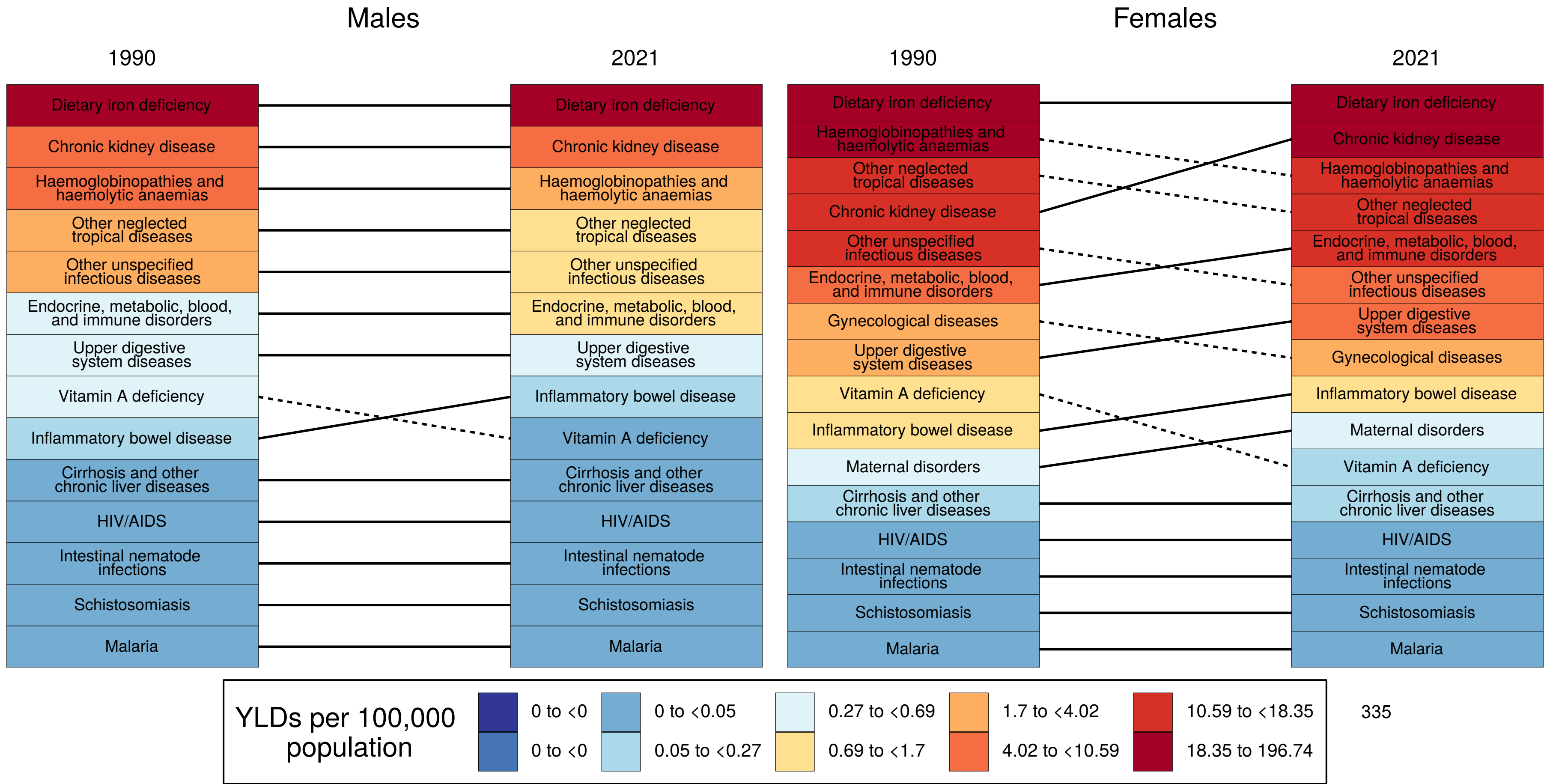
Males
Females
1990
2021
Dietary iron deficiency
Chronic kidney disease
Haemoglobinopathies and haemolytic anaemias
Other neglected tropical diseases
Other unspecified infectious diseases
Endocrine, metabolic, blood, and immune disorders
Upper digestive system diseases
Vitamin A deficiency
Inflammatory bowel disease
Cirrhosis and other chronic liver diseases
HIV/AIDS
Intestinal nematode infections
Schistosomiasis
Malaria
Gynecological diseases
Maternal disorders
YLDs per 100,000 population
0 to <0
0 to <0
0 to <0.05
0.05 to <0.27
0.27 to <0.69
0.69 to <1.7
1.7 to <4.02
4.02 to <10.59
10.59 to <18.35
18.35 to 196.74

Lancashire

Anaemia Prevalence - Both Sexes

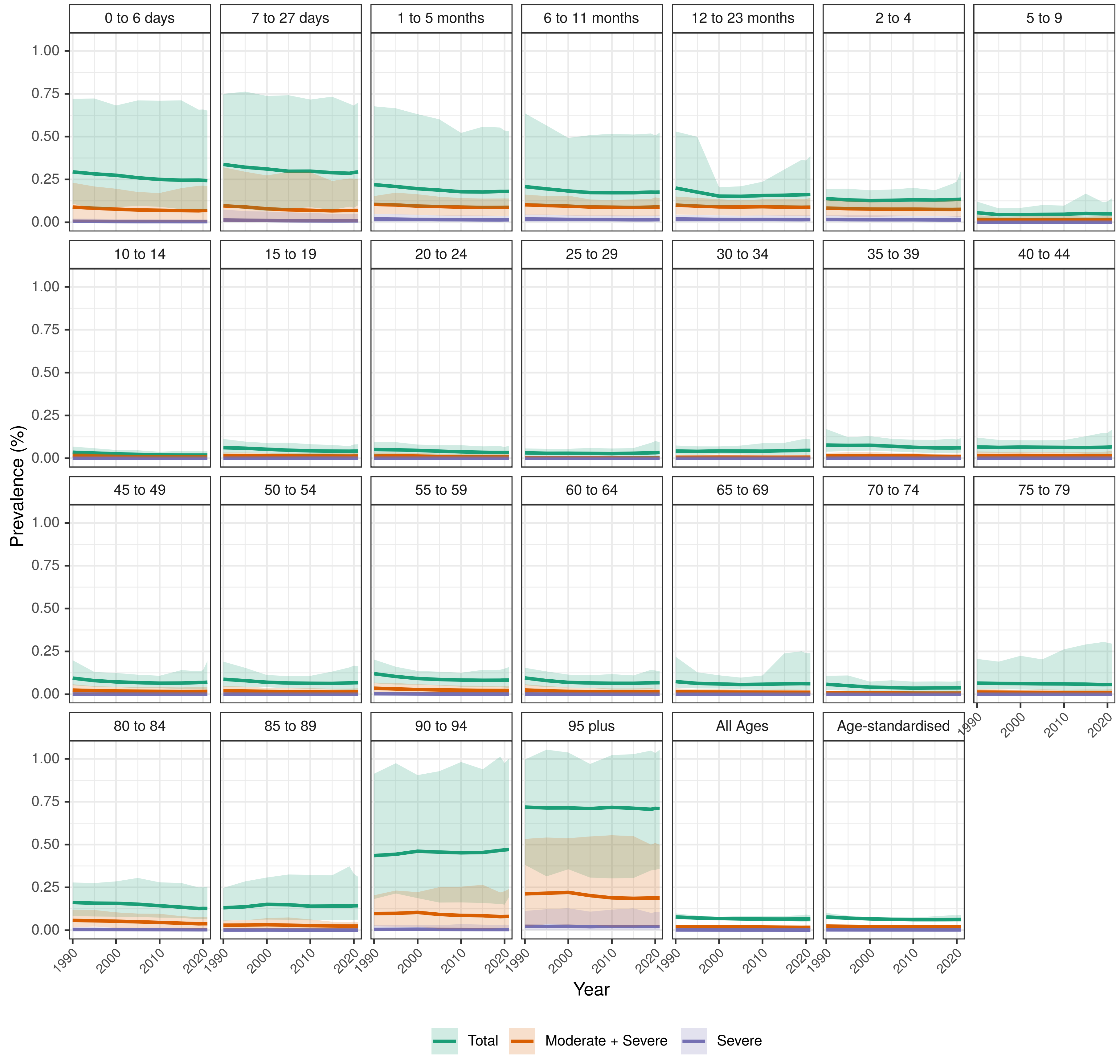

Cause-Specific Anaemia Prevalence - 2021

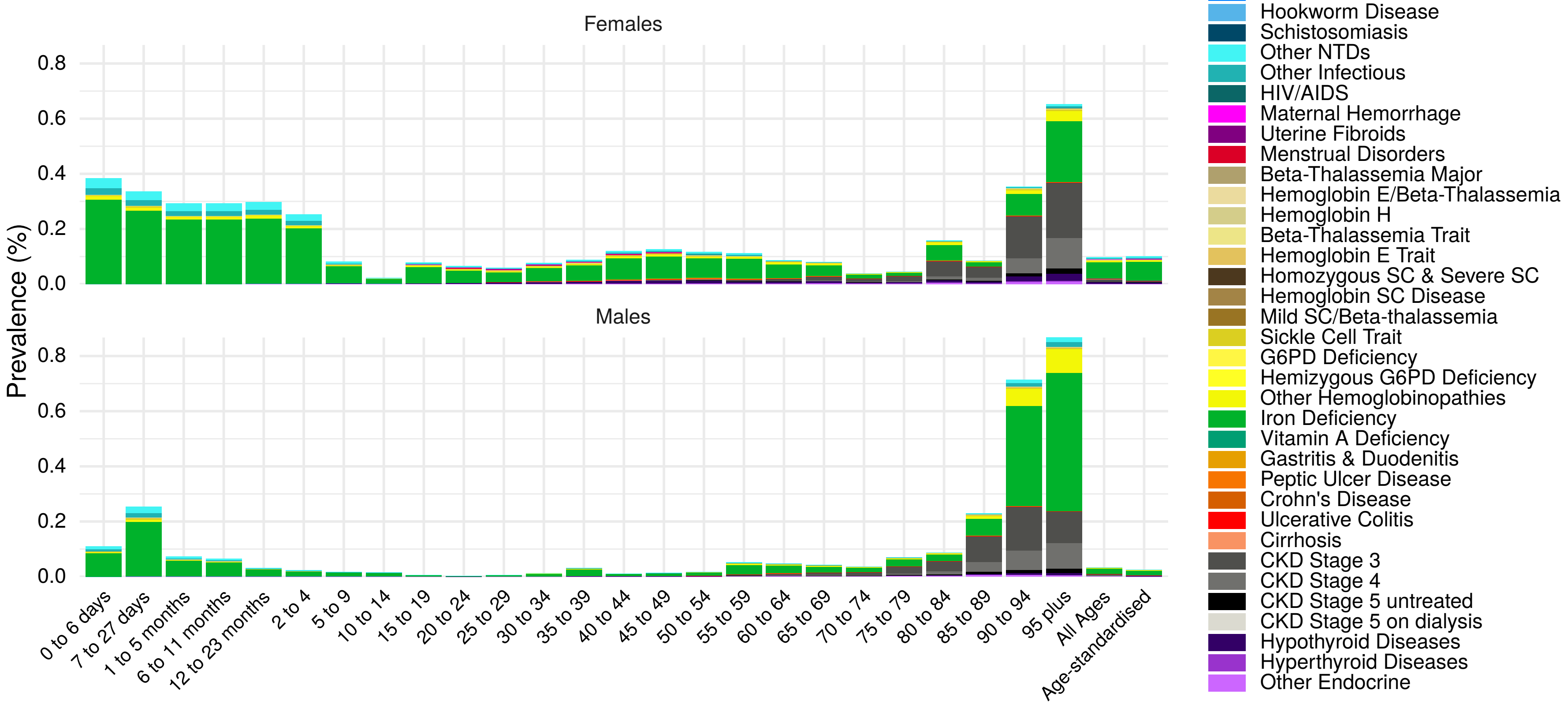

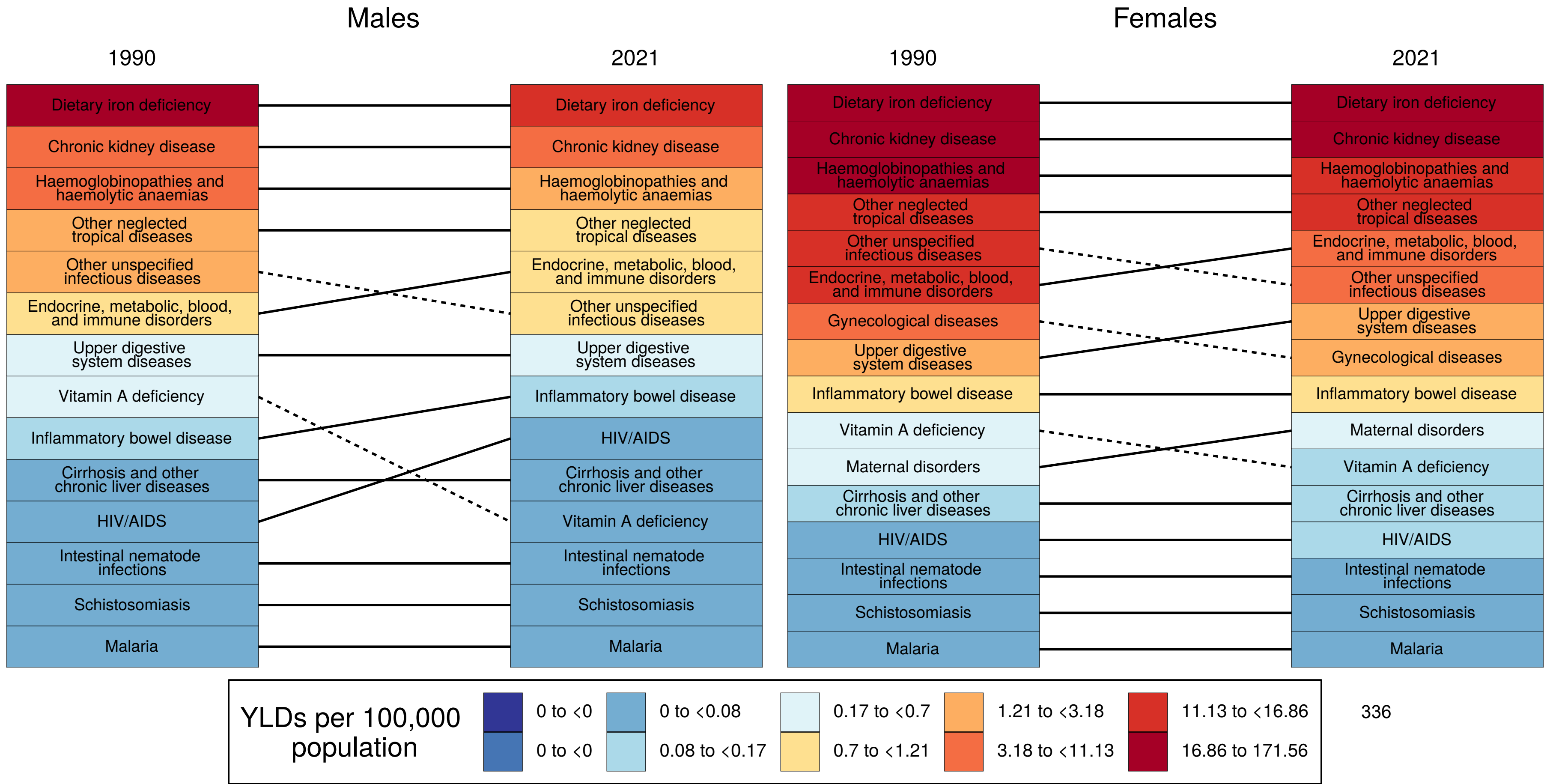

Liverpool

Anaemia Prevalence - Both Sexes

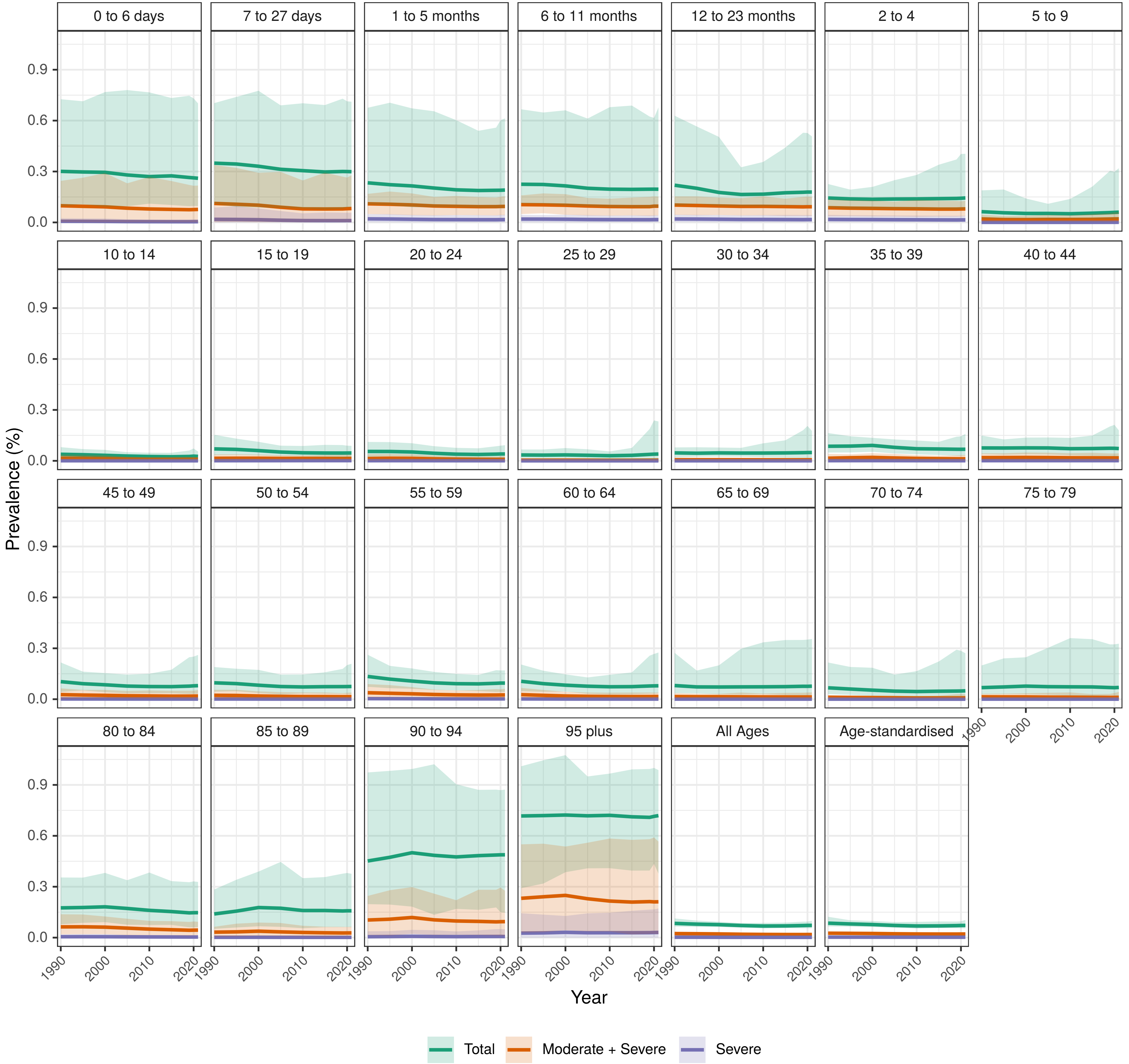

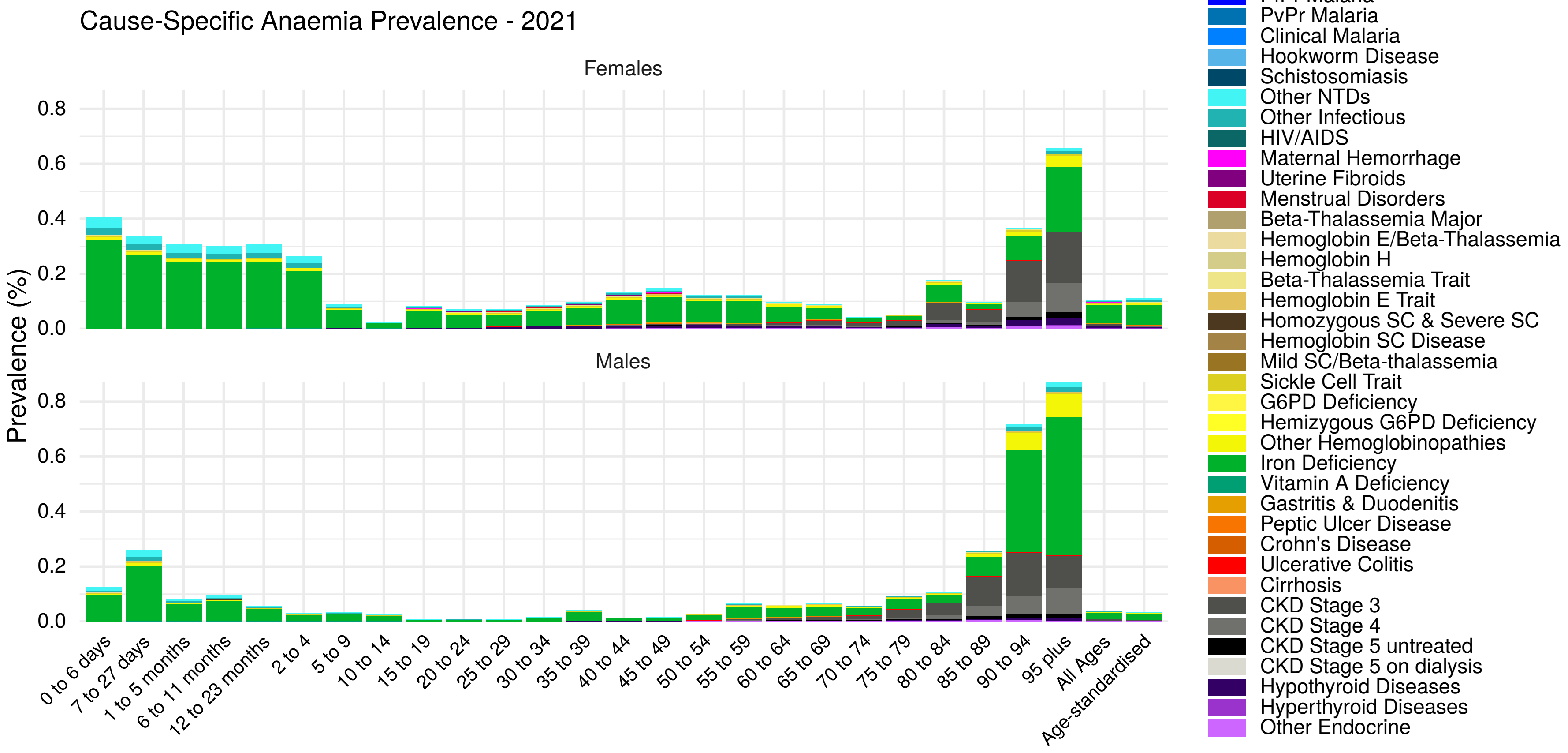

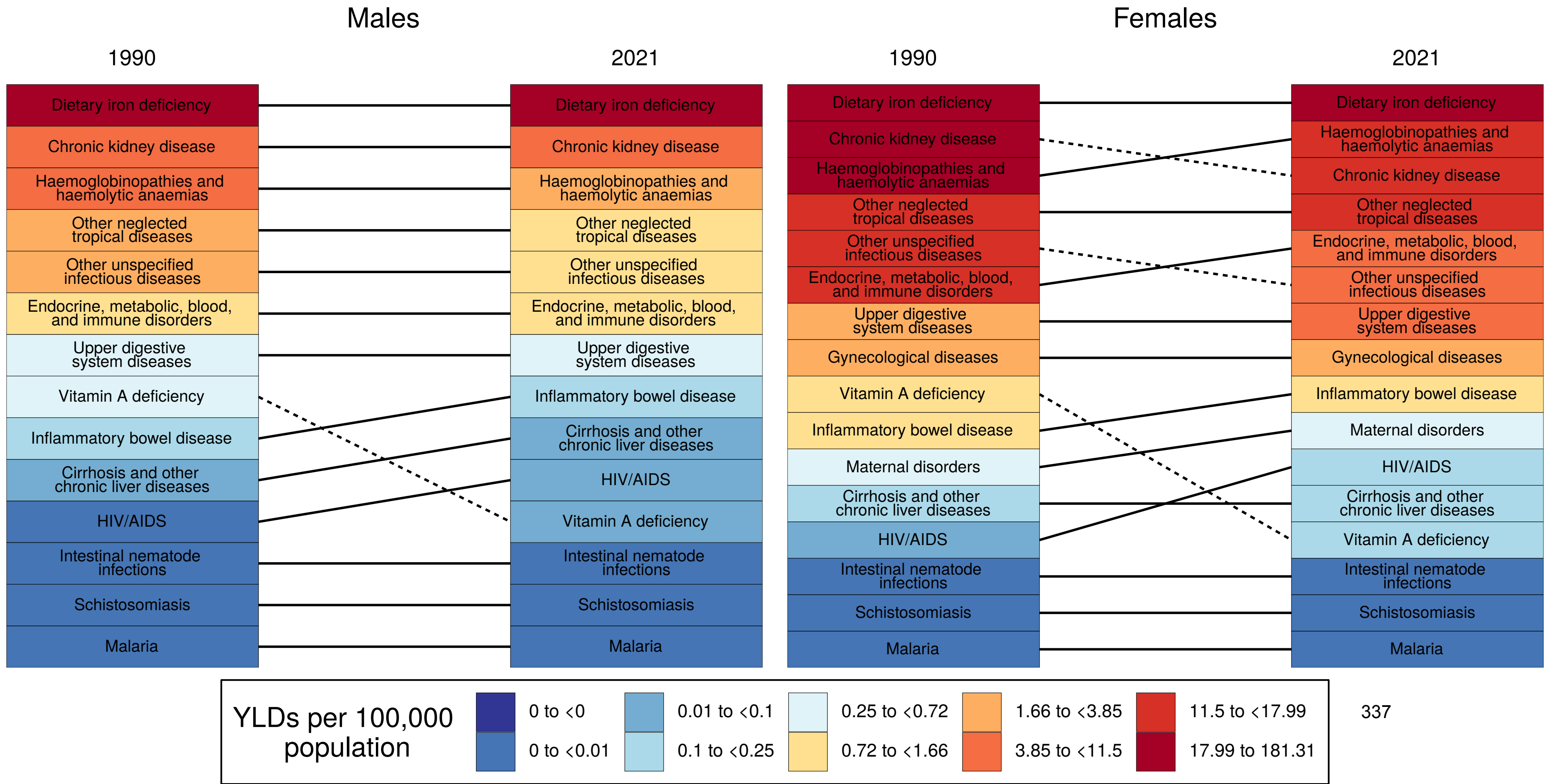

Manchester

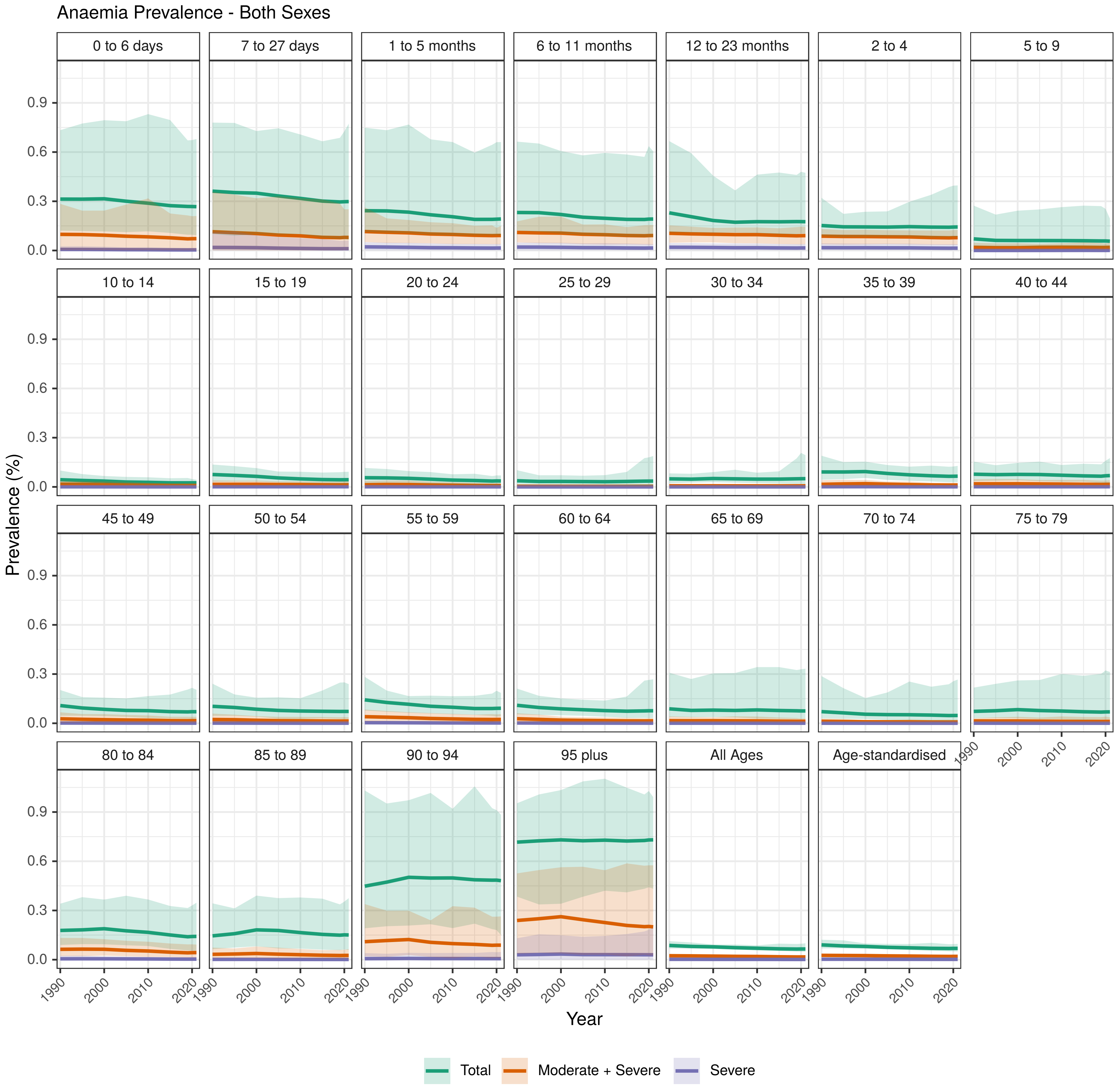
Anaemia Prevalence - Both Sexes
0 to 6 days
7 to 27 days
1 to 5 months
6 to 11 months
12 to 23 months
2 to 4
5 to 9
10 to 14
15 to 19
20 to 24
25 to 29
30 to 34
35 to 39
40 to 44
45 to 49
50 to 54
55 to 59
60 to 64
65 to 69
70 to 74
75 to 79
80 to 84
85 to 89
90 to 94
95 plus
All Ages
Age-standardised
Prevalence (%)
0.0
0.3
0.6
0.9
1990
2000
2010
2020
Year
Total
Moderate + Severe
Severe

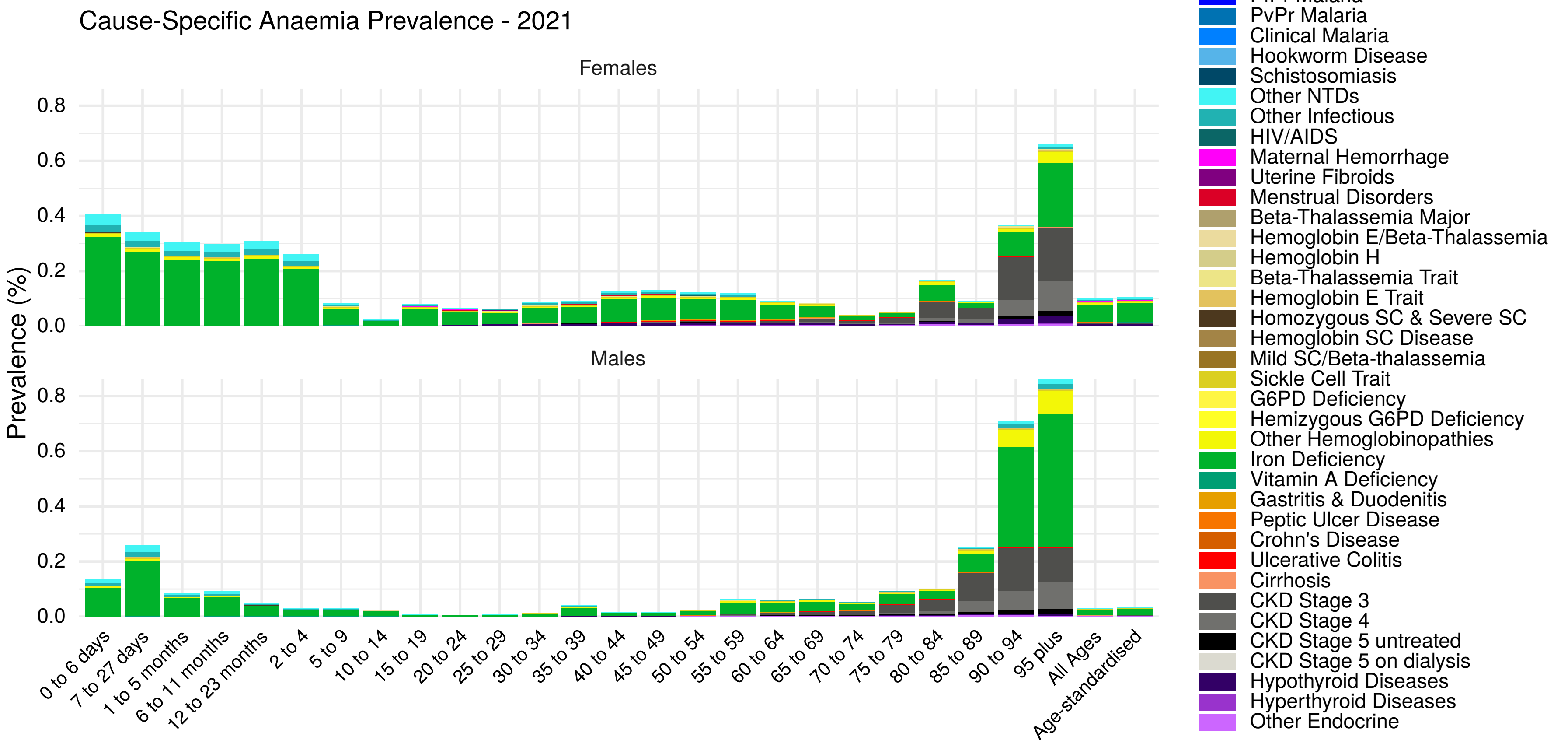
Cause-Specific Anaemia Prevalence - 2021
Females
Males
Prevalence (%)
0.0
0.2
0.4
0.6
0.8
0 to 6 days
7 to 27 days
1 to 5 months
6 to 11 months
12 to 23 months
2 to 4
5 to 9
10 to 14
15 to 19
20 to 24
25 to 29
30 to 34
35 to 39
40 to 44
45 to 49
50 to 54
55 to 59
60 to 64
65 to 69
70 to 74
75 to 79
80 to 84
85 to 89
90 to 94
95 plus
All Ages
Age-standardised
PfPr Malaria
PvPr Malaria
Clinical Malaria
Hookworm Disease
Schistosomiasis
Other NTDs
Other Infectious
HIV/AIDS
Maternal Hemorrhage
Uterine Fibroids
Menstrual Disorders
Beta-Thalassemia Major
Hemoglobin E/Beta-Thalassemia
Hemoglobin H
Beta-Thalassemia Trait
Hemoglobin E Trait
Homozygous SC & Severe SC
Hemoglobin SC Disease
Mild SC/Beta-thalassemia
Sickle Cell Trait
G6PD Deficiency
Hemizygous G6PD Deficiency
Other Hemoglobinopathies
Iron Deficiency
Vitamin A Deficiency
Gastritis & Duodenitis
Peptic Ulcer Disease
Crohn's Disease
Ulcerative Colitis
Cirrhosis
CKD Stage 3
CKD Stage 4
CKD Stage 5 untreated
CKD Stage 5 on dialysis
Hypothyroid Diseases
Hyperthyroid Diseases
Other Endocrine

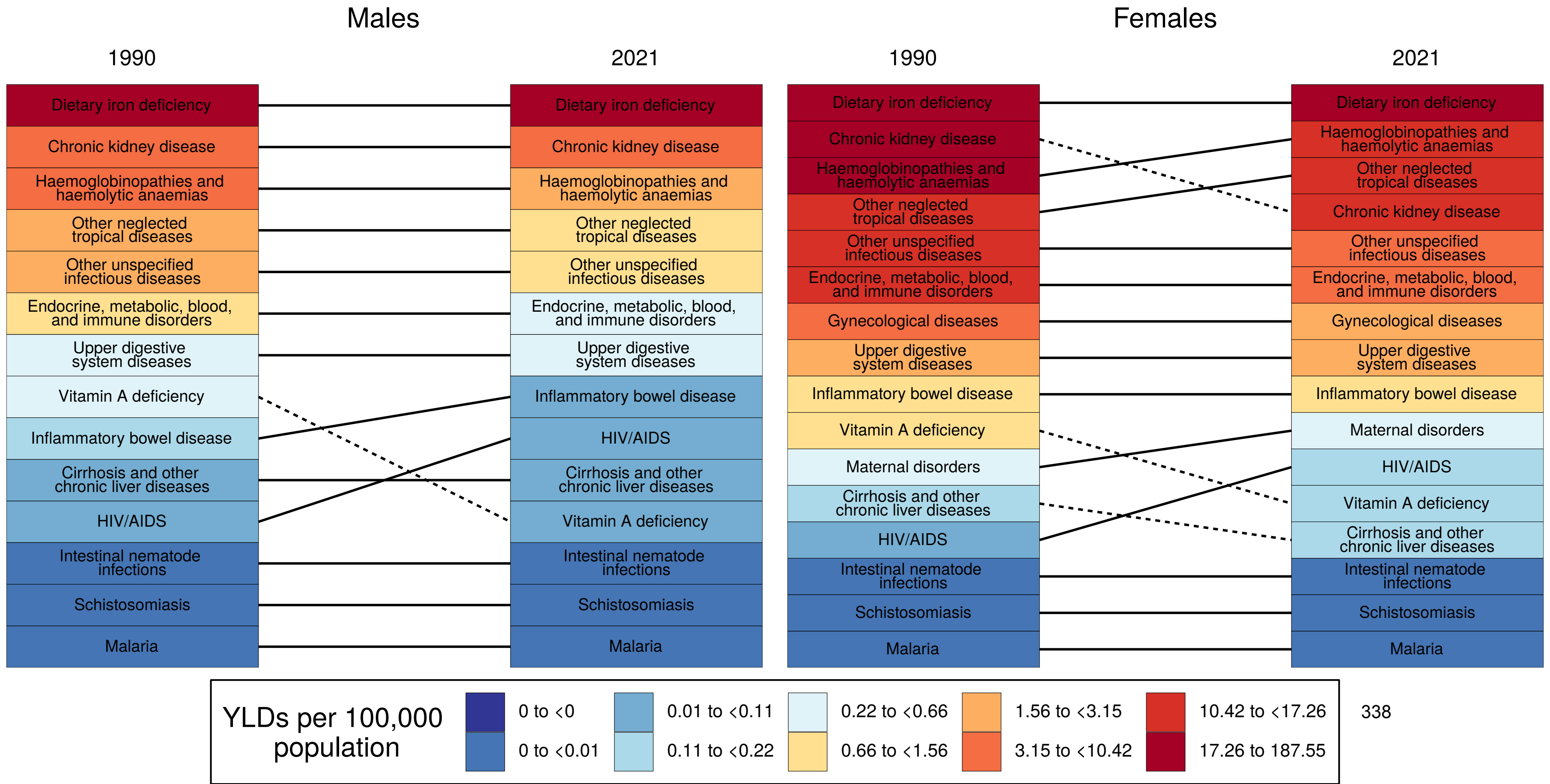
Males
Females
1990
2021
Dietary iron deficiency
Chronic kidney disease
Haemoglobinopathies and haemolytic anaemias
Other neglected tropical diseases
Other unspecified infectious diseases
Endocrine, metabolic, blood, and immune disorders
Upper digestive system diseases
Vitamin A deficiency
Inflammatory bowel disease
Cirrhosis and other chronic liver diseases
HIV/AIDS
Intestinal nematode infections
Schistosomiasis
Malaria
Gynecological diseases
Maternal disorders
YLDs per 100,000 population
0 to <0
0 to <0.01
0.01 to <0.11
0.11 to <0.22
0.22 to <0.66
0.66 to <1.56
1.56 to <3.15
3.15 to <10.42
10.42 to <17.26
17.26 to 187.55

Oldham

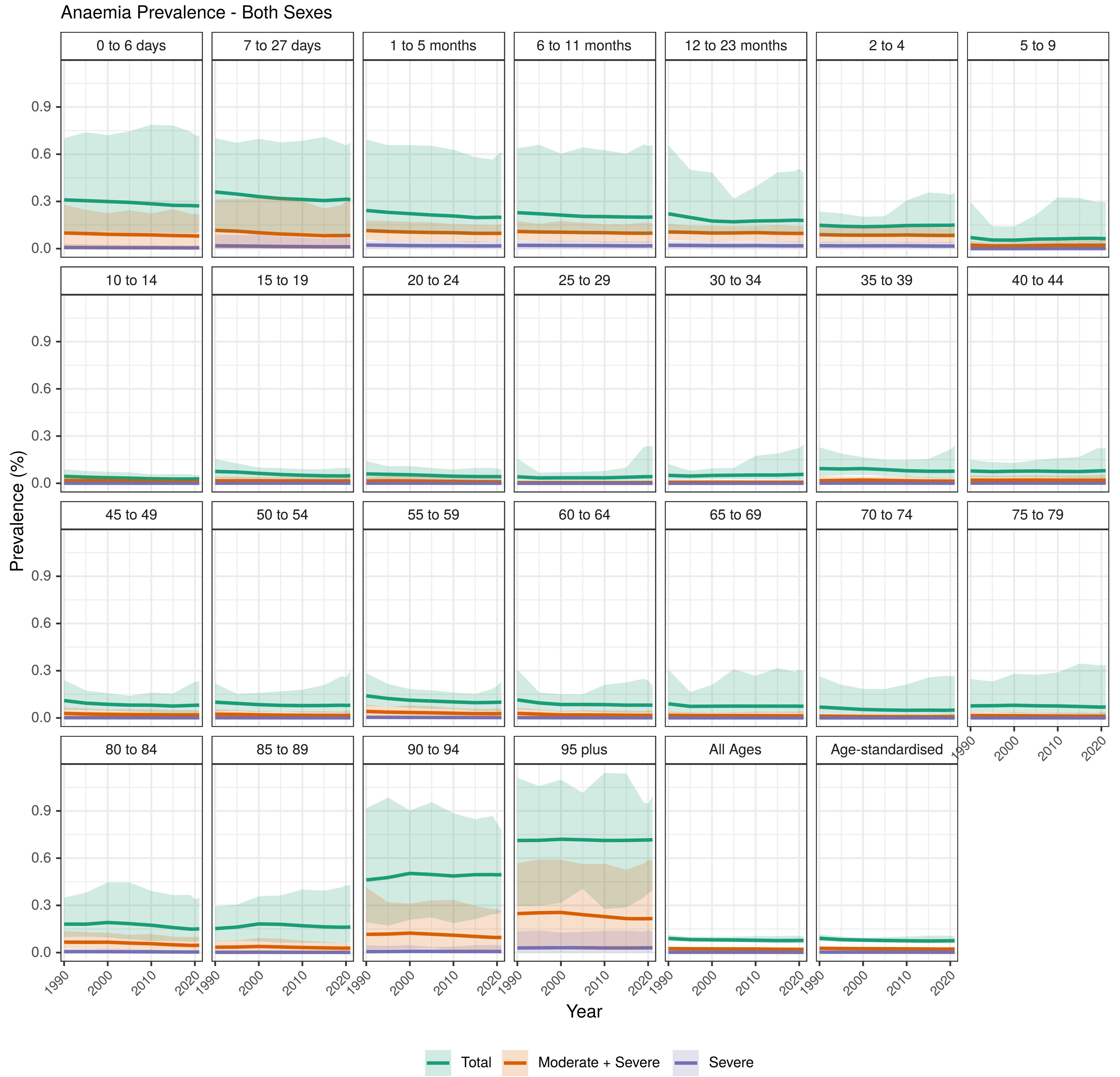

Anaemia Prevalence - Both Sexes
0 to 6 days
7 to 27 days
1 to 5 months
6 to 11 months
12 to 23 months
2 to 4
5 to 9
10 to 14
15 to 19
20 to 24
25 to 29
30 to 34
35 to 39
40 to 44
45 to 49
50 to 54
55 to 59
60 to 64
65 to 69
70 to 74
75 to 79
80 to 84
85 to 89
90 to 94
95 plus
All Ages
Age-standardised
Prevalence (%)
Year
Total
Moderate + Severe
Severe

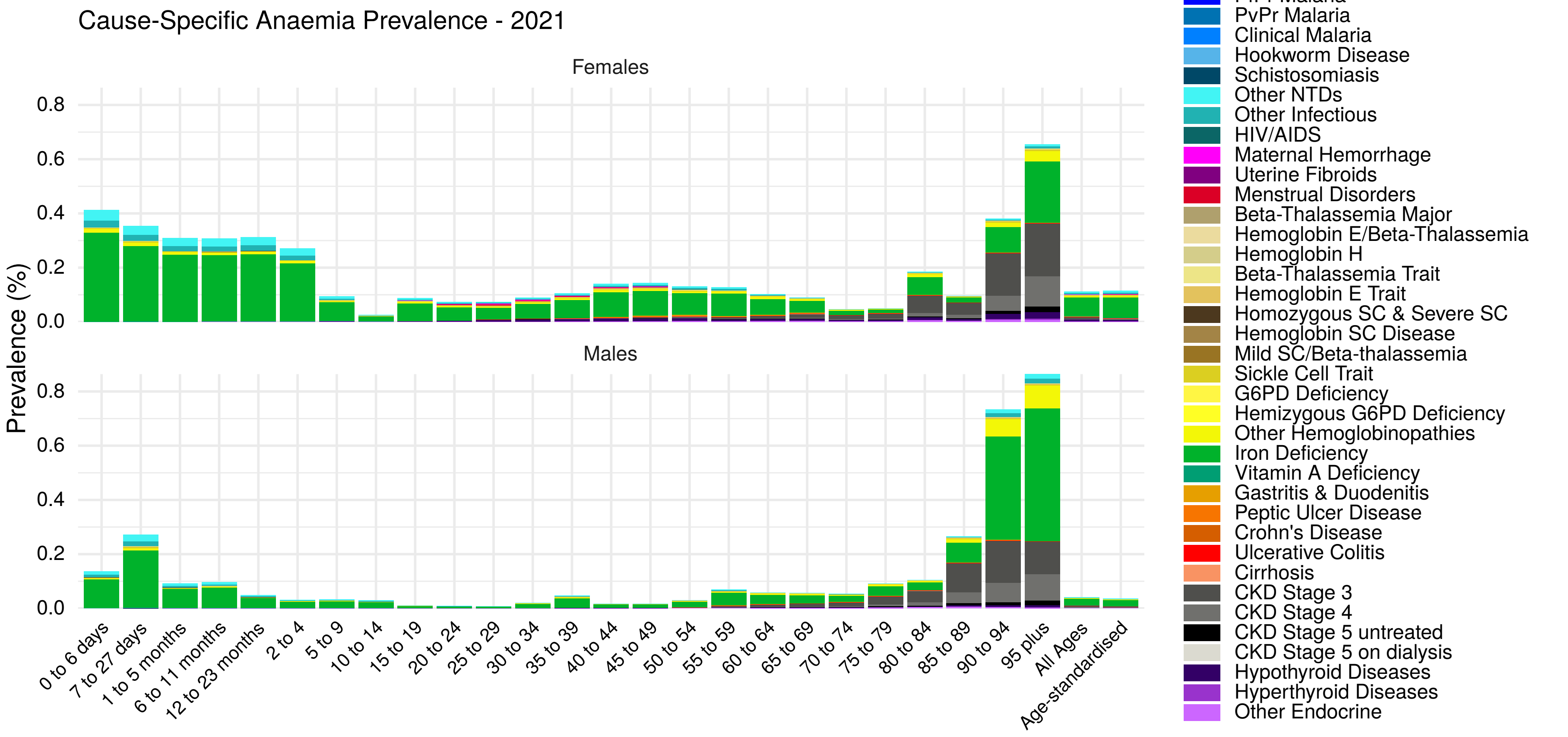

Cause-Specific Anaemia Prevalence - 2021
Females
Males
Prevalence (%)
PfPr Malaria
PvPr Malaria
Clinical Malaria
Hookworm Disease
Schistosomiasis
Other NTDs
Other Infectious
HIV/AIDS
Maternal Hemorrhage
Uterine Fibroids
Menstrual Disorders
Beta-Thalassemia Major
Hemoglobin E/Beta-Thalassemia
Hemoglobin H
Beta-Thalassemia Trait
Hemoglobin E Trait
Homozygous SC & Severe SC
Hemoglobin SC Disease
Mild SC/Beta-thalassemia
Sickle Cell Trait
G6PD Deficiency
Hemizygous G6PD Deficiency
Other Hemoglobinopathies
Iron Deficiency
Vitamin A Deficiency
Gastritis & Duodenitis
Peptic Ulcer Disease
Crohn's Disease
Ulcerative Colitis
Cirrhosis
CKD Stage 3
CKD Stage 4
CKD Stage 5 untreated
CKD Stage 5 on dialysis
Hypothyroid Diseases
Hyperthyroid Diseases
Other Endocrine

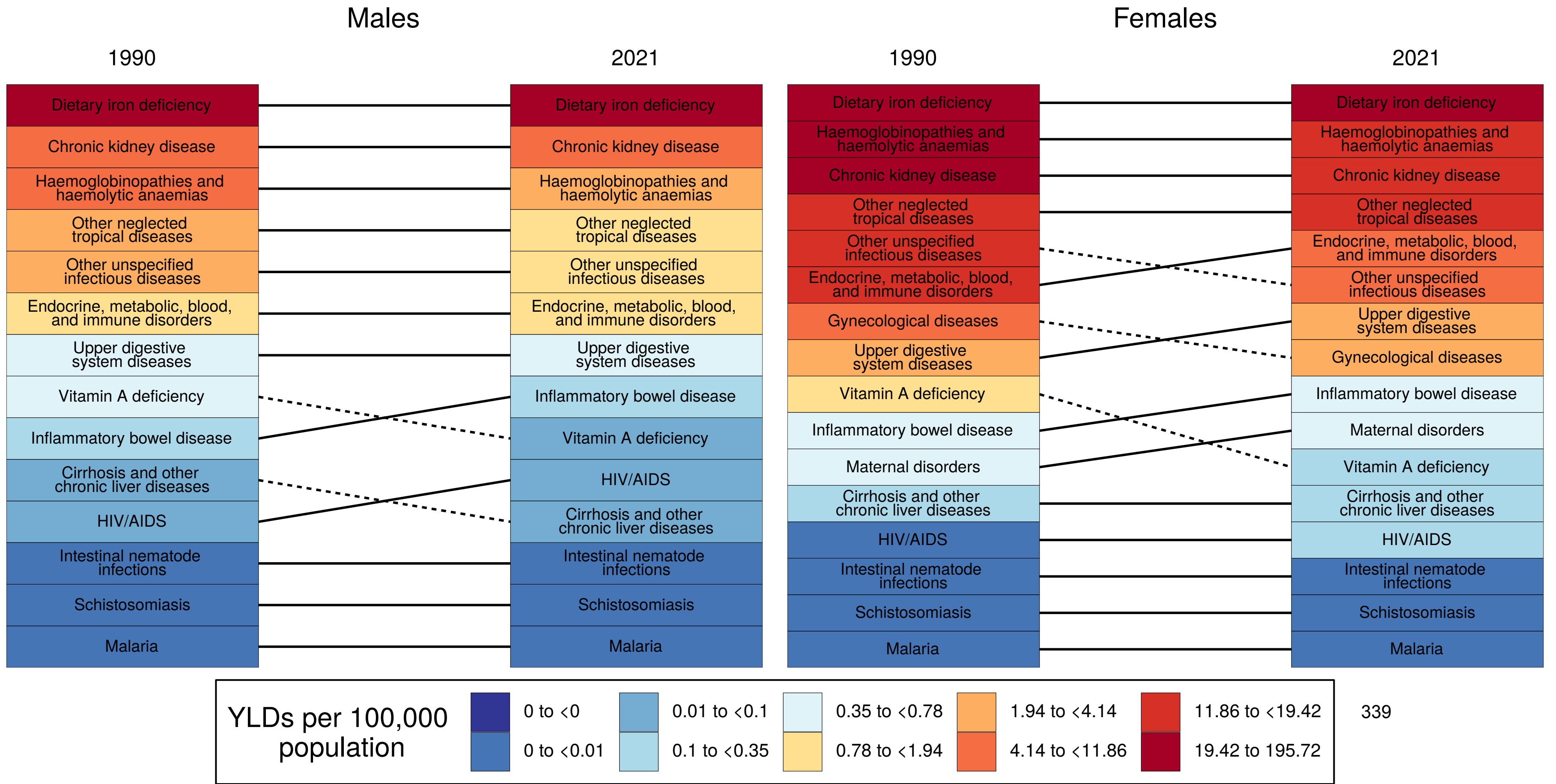

Males
Females
1990
2021
Dietary iron deficiency
Chronic kidney disease
Haemoglobinopathies and haemolytic anaemias
Other neglected tropical diseases
Other unspecified infectious diseases
Endocrine, metabolic, blood, and immune disorders
Upper digestive system diseases
Vitamin A deficiency
Inflammatory bowel disease
Cirrhosis and other chronic liver diseases
HIV/AIDS
Intestinal nematode infections
Schistosomiasis
Malaria
Gynecological diseases
Maternal disorders
YLDs per 100,000 population
0 to <0
0 to <0.01
0.01 to <0.1
0.1 to <0.35
0.35 to <0.78
0.78 to <1.94
1.94 to <4.14
4.14 to <11.86
11.86 to <19.42
19.42 to 195.72

Rochdale

Anaemia Prevalence - Both Sexes

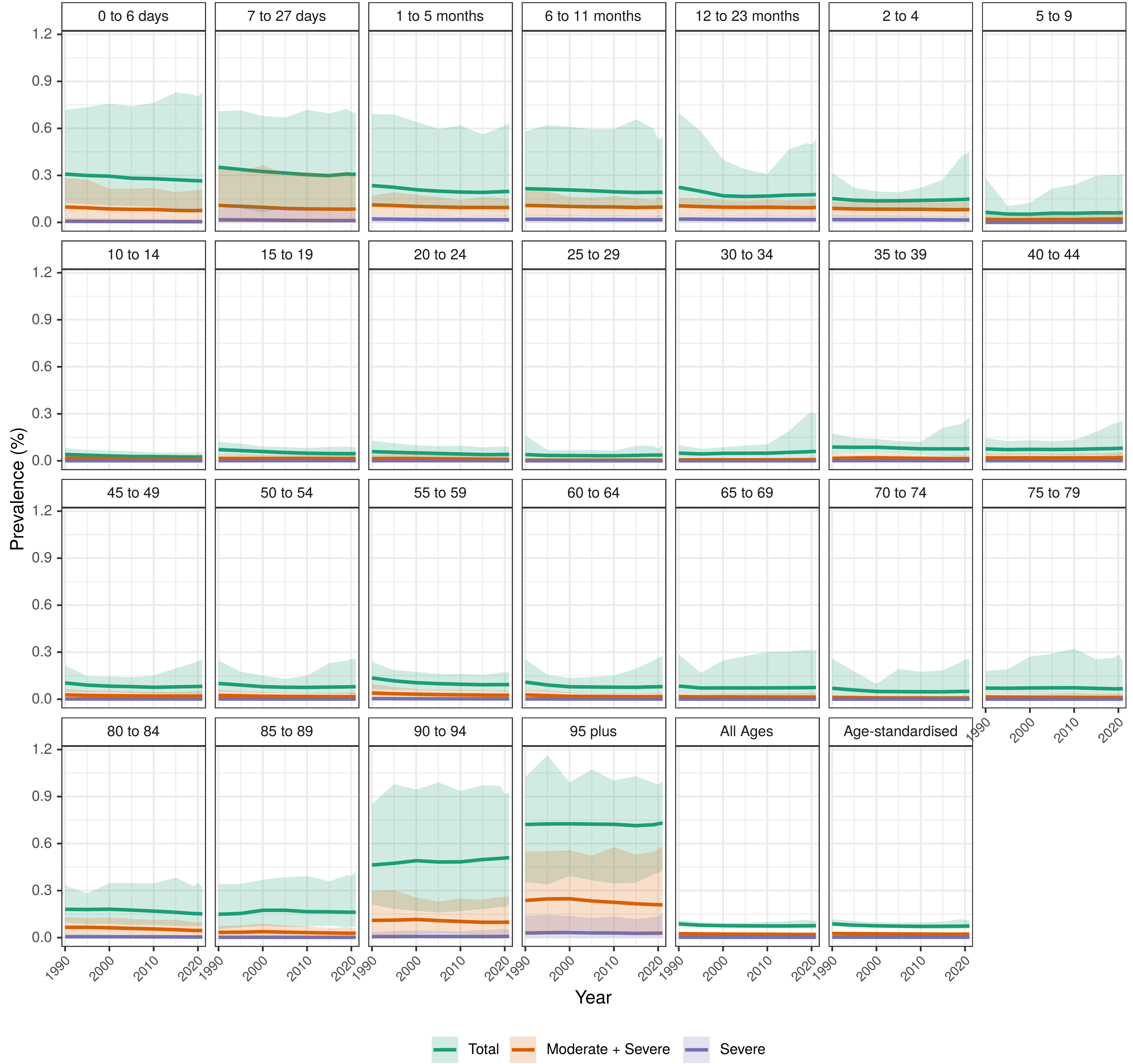

Cause-Specific Anaemia Prevalence - 2021

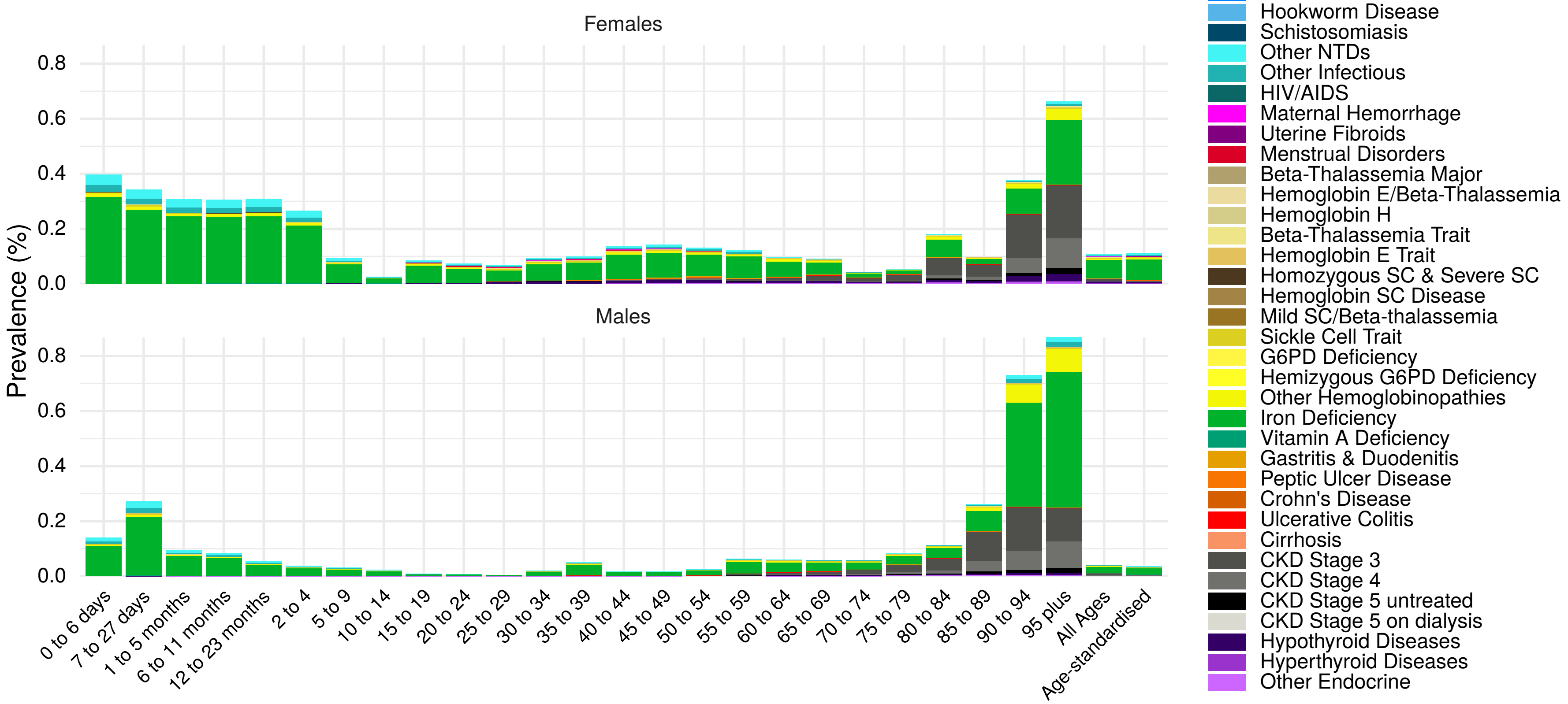

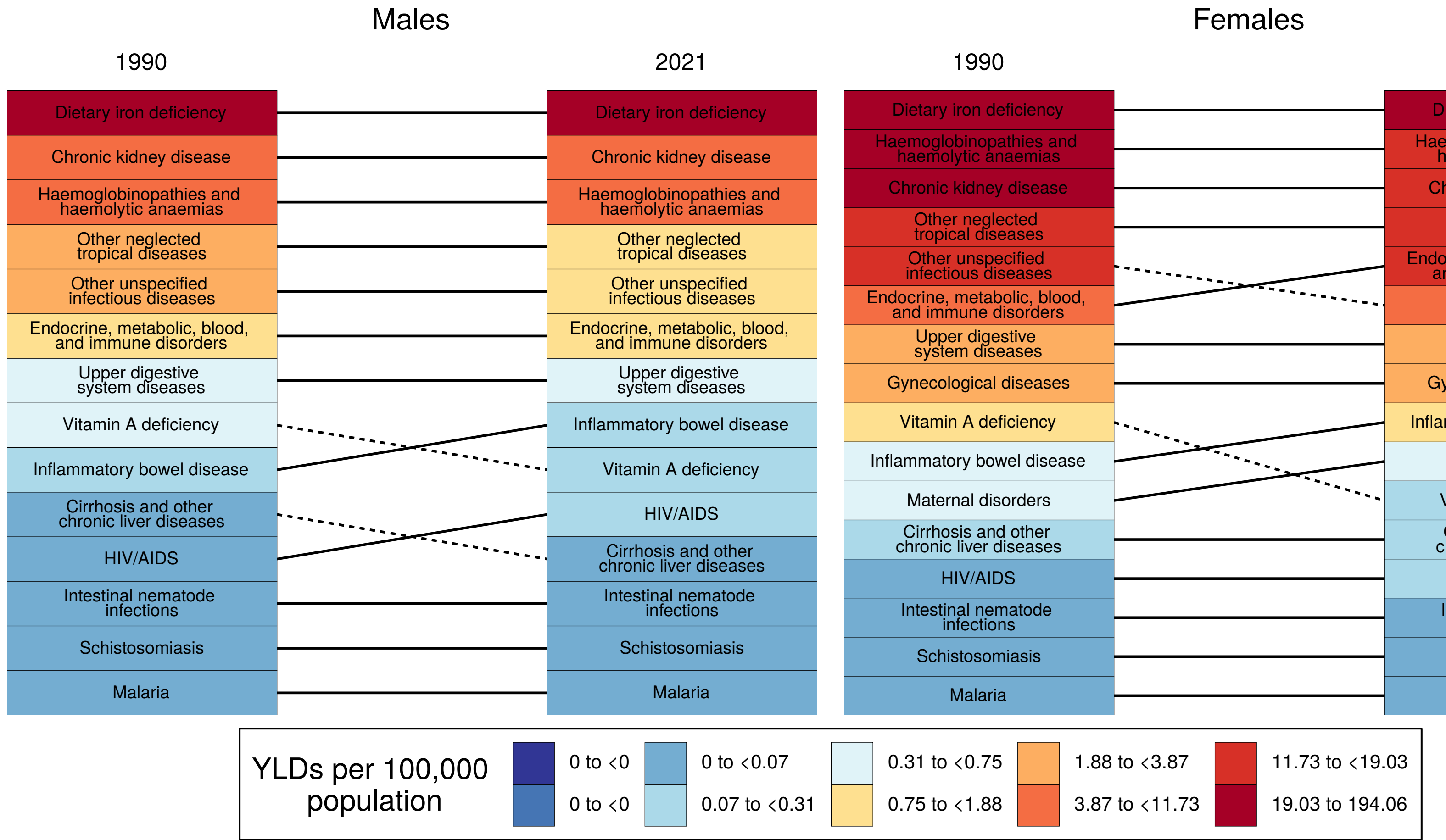

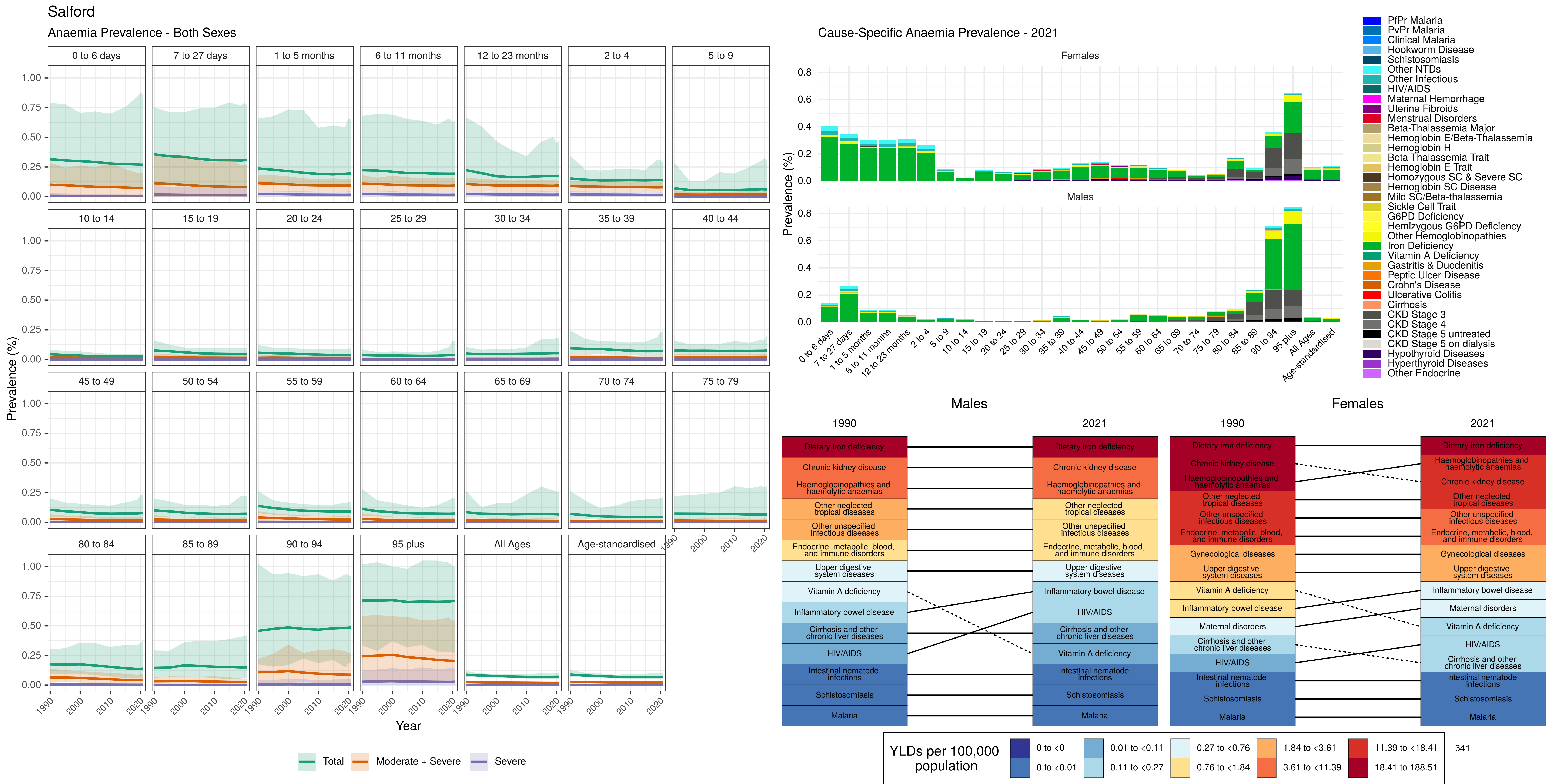

Salford
Anaemia Prevalence - Both Sexes
0 to 6 days
7 to 27 days
1 to 5 months
6 to 11 months
12 to 23 months
2 to 4
5 to 9
10 to 14
15 to 19
20 to 24
25 to 29
30 to 34
35 to 39
40 to 44
45 to 49
50 to 54
55 to 59
60 to 64
65 to 69
70 to 74
75 to 79
80 to 84
85 to 89
90 to 94
95 plus
All Ages
Age-standardised
Prevalence (%)
Year
Total
Moderate + Severe
Severe
Cause-Specific Anaemia Prevalence - 2021
Females
Males
Prevalence (%)
PfPr Malaria
PvPr Malaria
Clinical Malaria
Hookworm Disease
Schistosomiasis
Other NTDs
Other Infectious
HIV/AIDS
Maternal Hemorrhage
Uterine Fibroids
Menstrual Disorders
Beta-Thalassemia Major
Hemoglobin E/Beta-Thalassemia
Hemoglobin H
Beta-Thalassemia Trait
Hemoglobin E Trait
Homozygous SC & Severe SC
Hemoglobin SC Disease
Mild SC/Beta-thalassemia
Sickle Cell Trait
G6PD Deficiency
Hemizygous G6PD Deficiency
Other Hemoglobinopathies
Iron Deficiency
Vitamin A Deficiency
Gastritis & Duodenitis
Peptic Ulcer Disease
Crohn's Disease
Ulcerative Colitis
Cirrhosis
CKD Stage 3
CKD Stage 4
CKD Stage 5 untreated
CKD Stage 5 on dialysis
Hypothyroid Diseases
Hyperthyroid Diseases
Other Endocrine
Males
Females
1990
2021
Dietary iron deficiency
Chronic kidney disease
Haemoglobinopathies and haemolytic anaemias
Other neglected tropical diseases
Other unspecified infectious diseases
Endocrine, metabolic, blood, and immune disorders
Upper digestive system diseases
Vitamin A deficiency
Inflammatory bowel disease
Cirrhosis and other chronic liver diseases
HIV/AIDS
Intestinal nematode infections
Schistosomiasis
Malaria
Gynecological diseases
Maternal disorders
YLDs per 100,000 population
0 to <0
0 to <0.01
0.01 to <0.11
0.11 to <0.27
0.27 to <0.76
0.76 to <1.84
1.84 to <3.61
3.61 to <11.39
11.39 to <18.41
18.41 to 188.51

Sefton

Anaemia Prevalence - Both Sexes

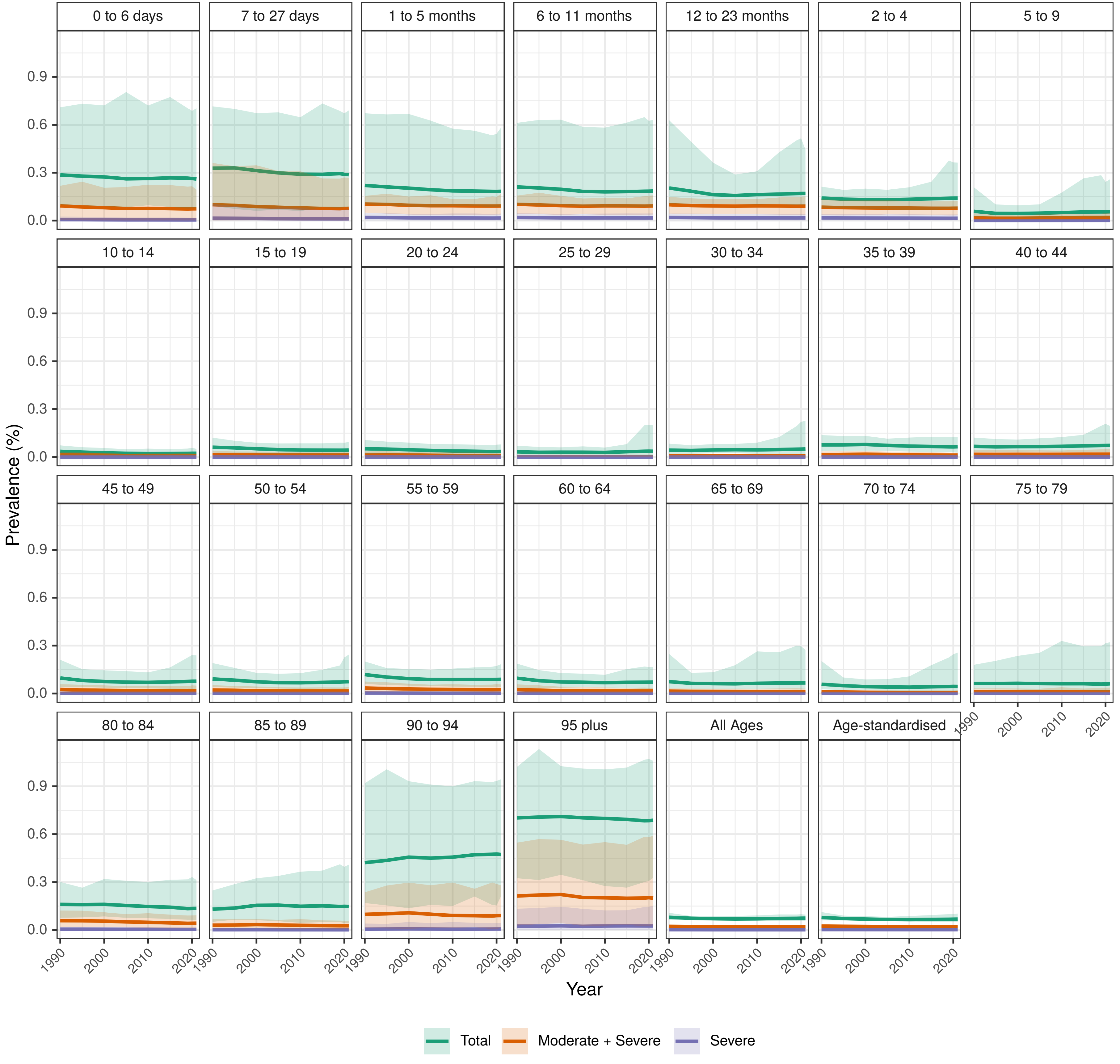

Cause-Specific Anaemia Prevalence - 2021

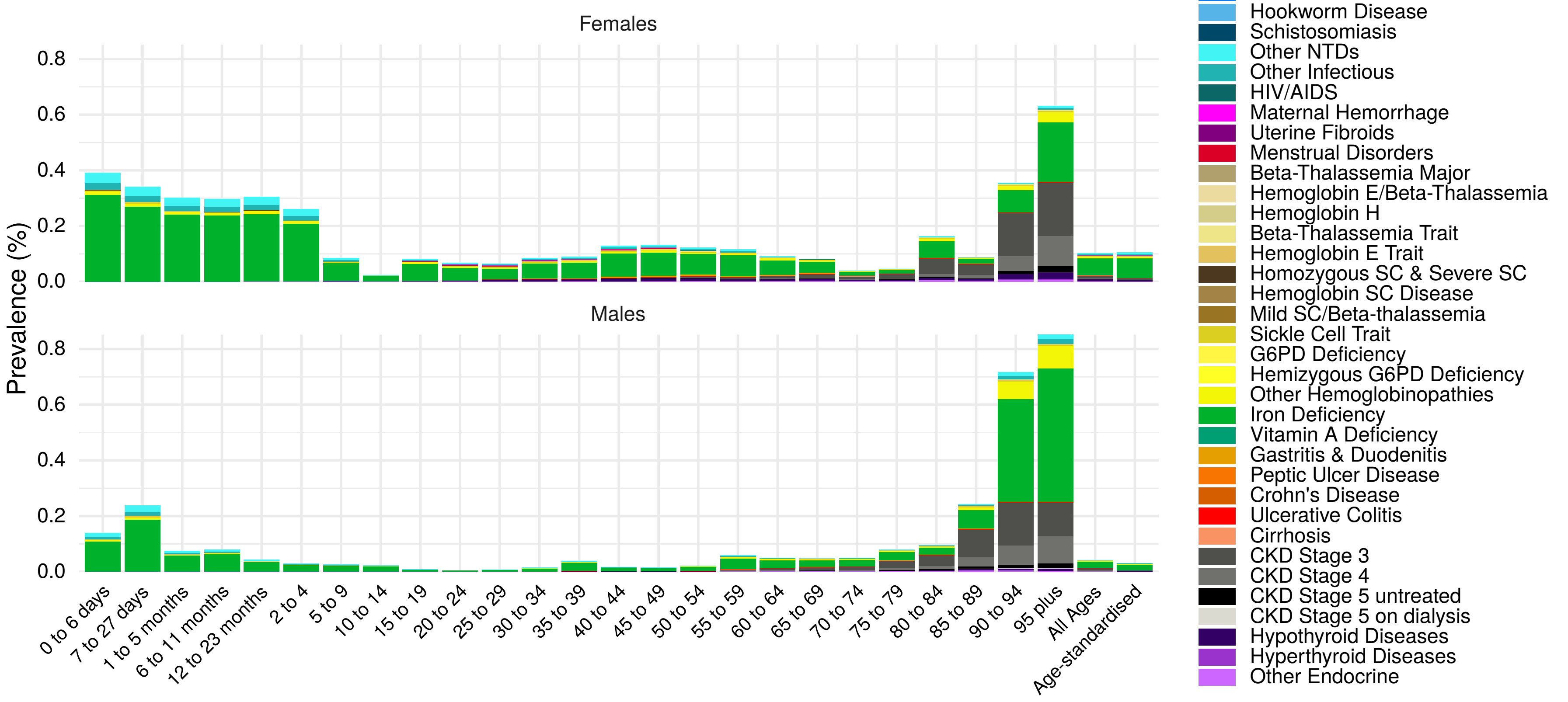

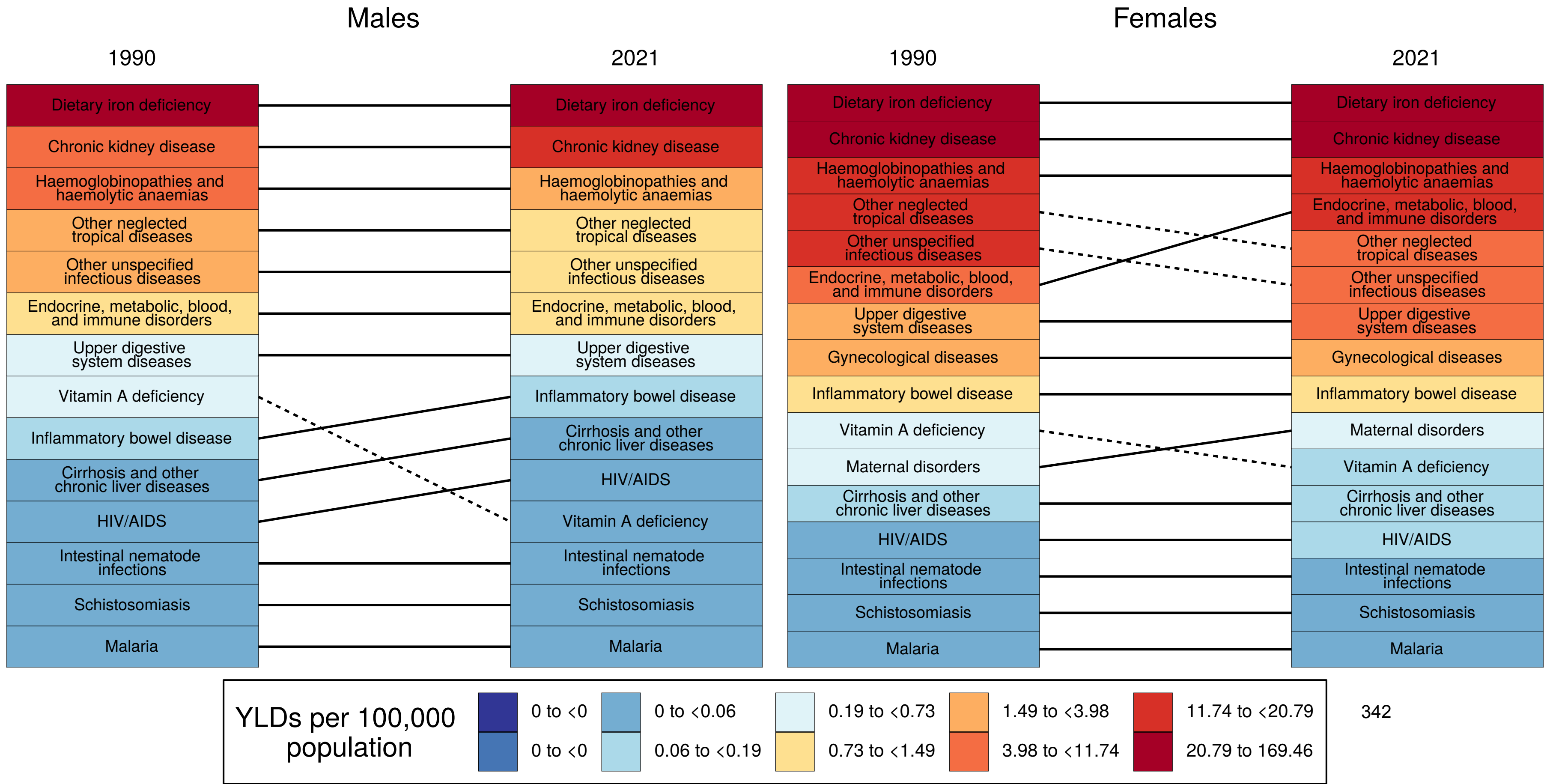

342

St Helens

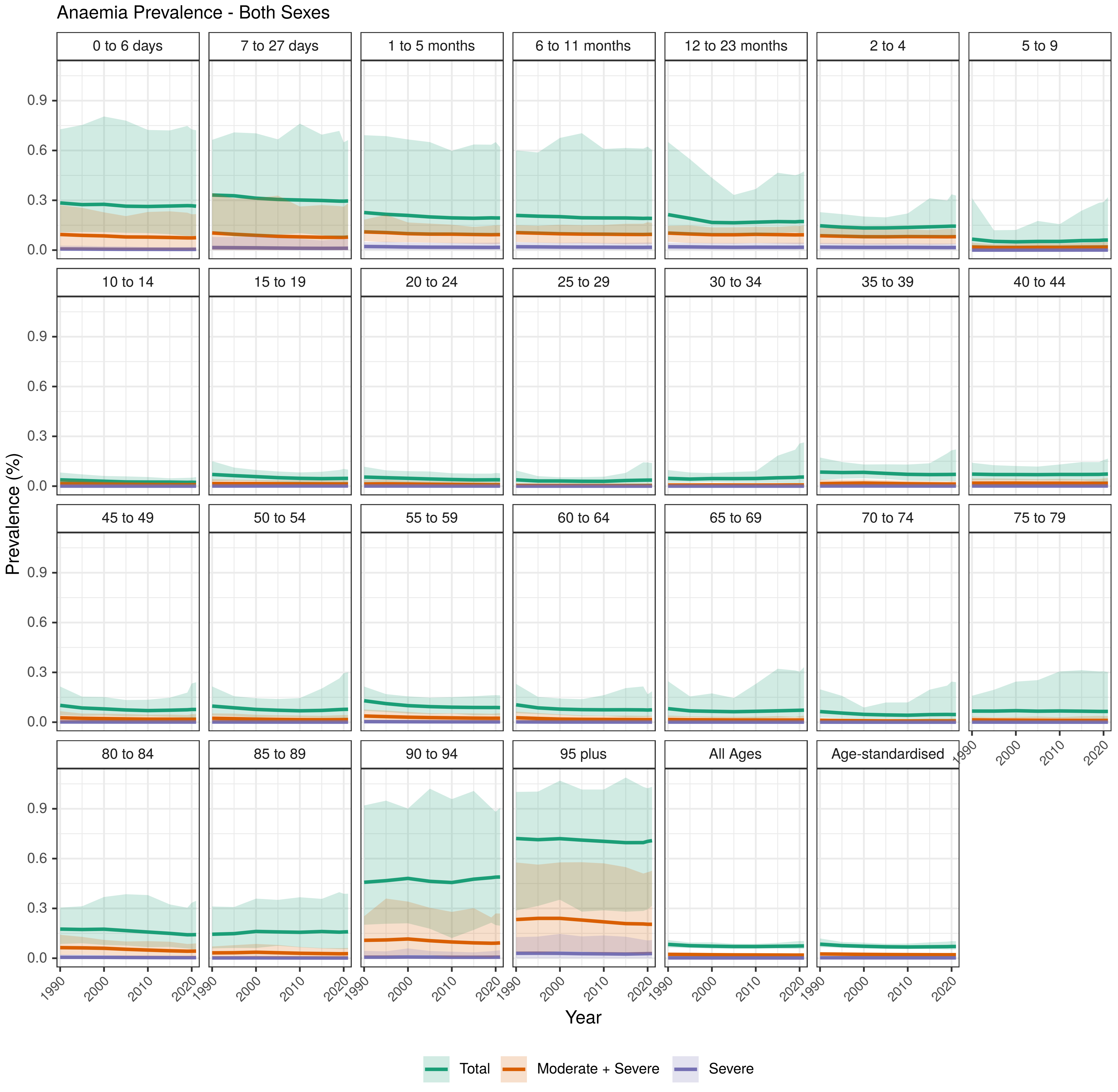

Anaemia Prevalence - Both Sexes
0 to 6 days
7 to 27 days
1 to 5 months
6 to 11 months
12 to 23 months
2 to 4
5 to 9
10 to 14
15 to 19
20 to 24
25 to 29
30 to 34
35 to 39
40 to 44
45 to 49
50 to 54
55 to 59
60 to 64
65 to 69
70 to 74
75 to 79
80 to 84
85 to 89
90 to 94
95 plus
All Ages
Age-standardised
Prevalence (%)
Year
Total
Moderate + Severe
Severe

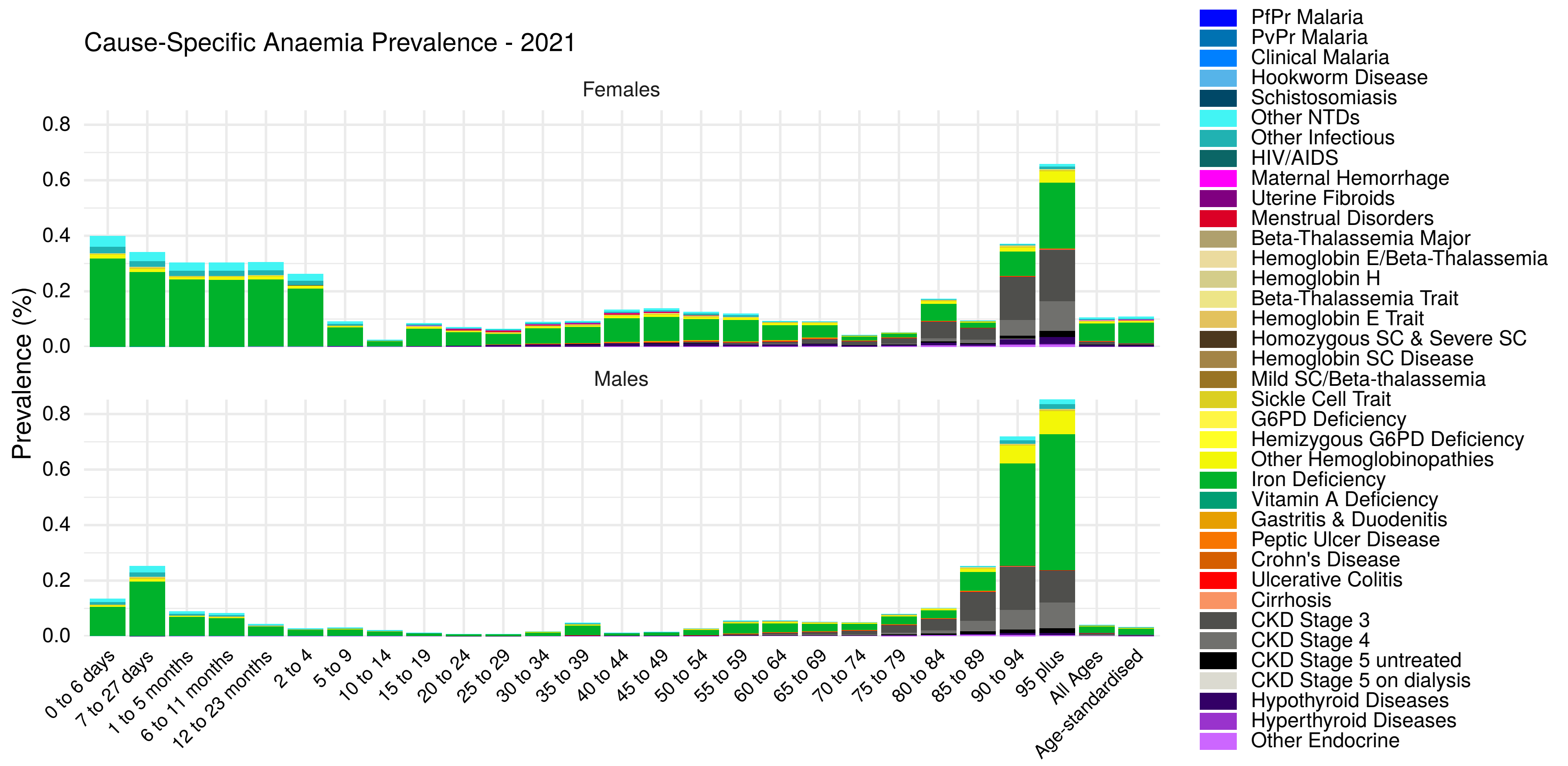

Cause-Specific Anaemia Prevalence - 2021
Females
Males
Prevalence (%)
PfPr Malaria
PvPr Malaria
Clinical Malaria
Hookworm Disease
Schistosomiasis
Other NTDs
Other Infectious
HIV/AIDS
Maternal Hemorrhage
Uterine Fibroids
Menstrual Disorders
Beta-Thalassemia Major
Hemoglobin E/Beta-Thalassemia
Hemoglobin H
Beta-Thalassemia Trait
Hemoglobin E Trait
Homozygous SC & Severe SC
Hemoglobin SC Disease
Mild SC/Beta-thalassemia
Sickle Cell Trait
G6PD Deficiency
Hemizygous G6PD Deficiency
Other Hemoglobinopathies
Iron Deficiency
Vitamin A Deficiency
Gastritis & Duodenitis
Peptic Ulcer Disease
Crohn's Disease
Ulcerative Colitis
Cirrhosis
CKD Stage 3
CKD Stage 4
CKD Stage 5 untreated
CKD Stage 5 on dialysis
Hypothyroid Diseases
Hyperthyroid Diseases
Other Endocrine

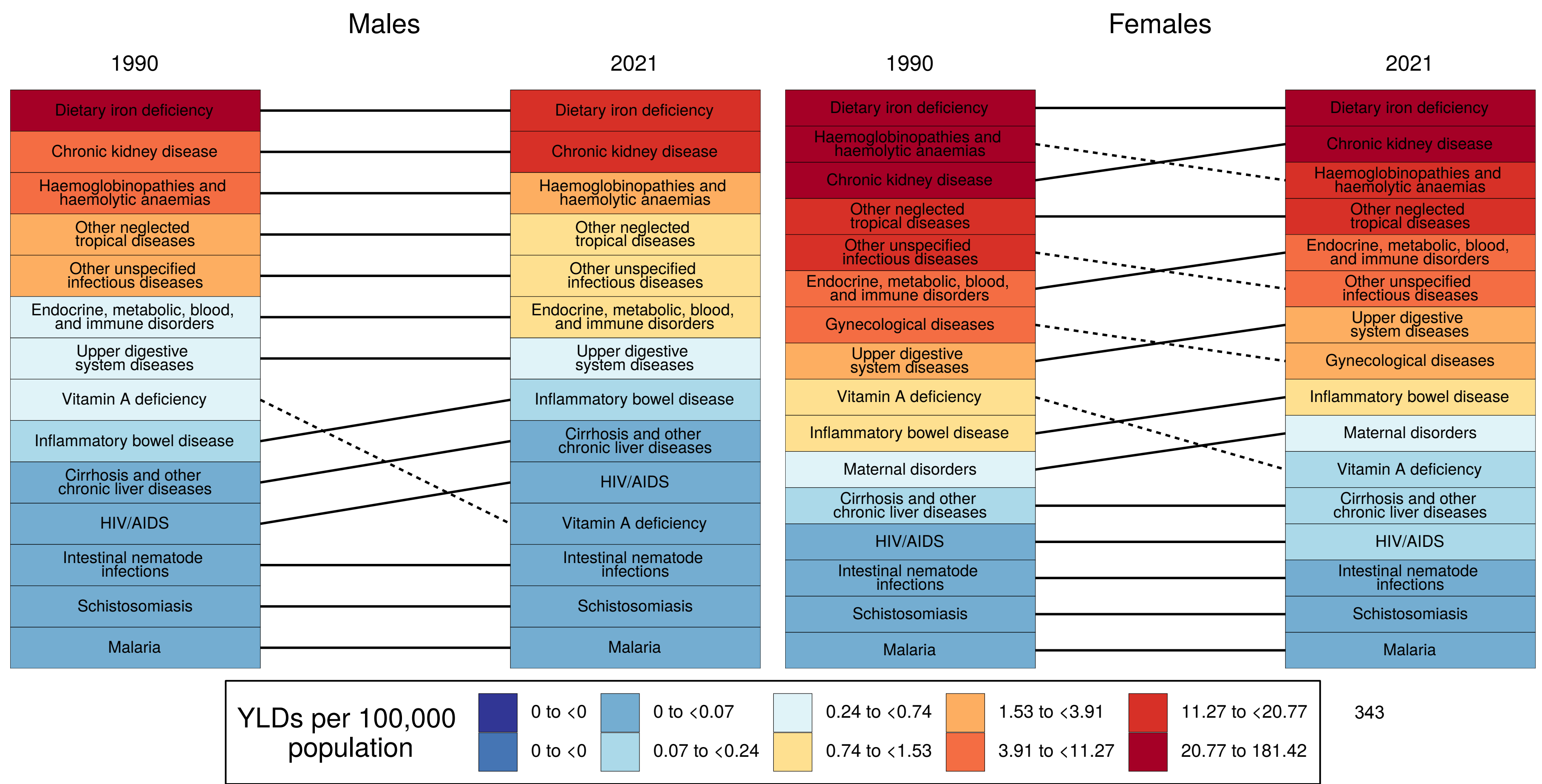

Males
Females
1990
2021
Dietary iron deficiency
Chronic kidney disease
Haemoglobinopathies and haemolytic anaemias
Other neglected tropical diseases
Other unspecified infectious diseases
Endocrine, metabolic, blood, and immune disorders
Upper digestive system diseases
Vitamin A deficiency
Inflammatory bowel disease
Cirrhosis and other chronic liver diseases
HIV/AIDS
Intestinal nematode infections
Schistosomiasis
Malaria
Gynecological diseases
Maternal disorders
YLDs per 100,000 population
0 to <0
0 to <0
0 to <0.07
0.07 to <0.24
0.24 to <0.74
0.74 to <1.53
1.53 to <3.91
3.91 to <11.27
11.27 to <20.77
20.77 to 181.42

Stockport

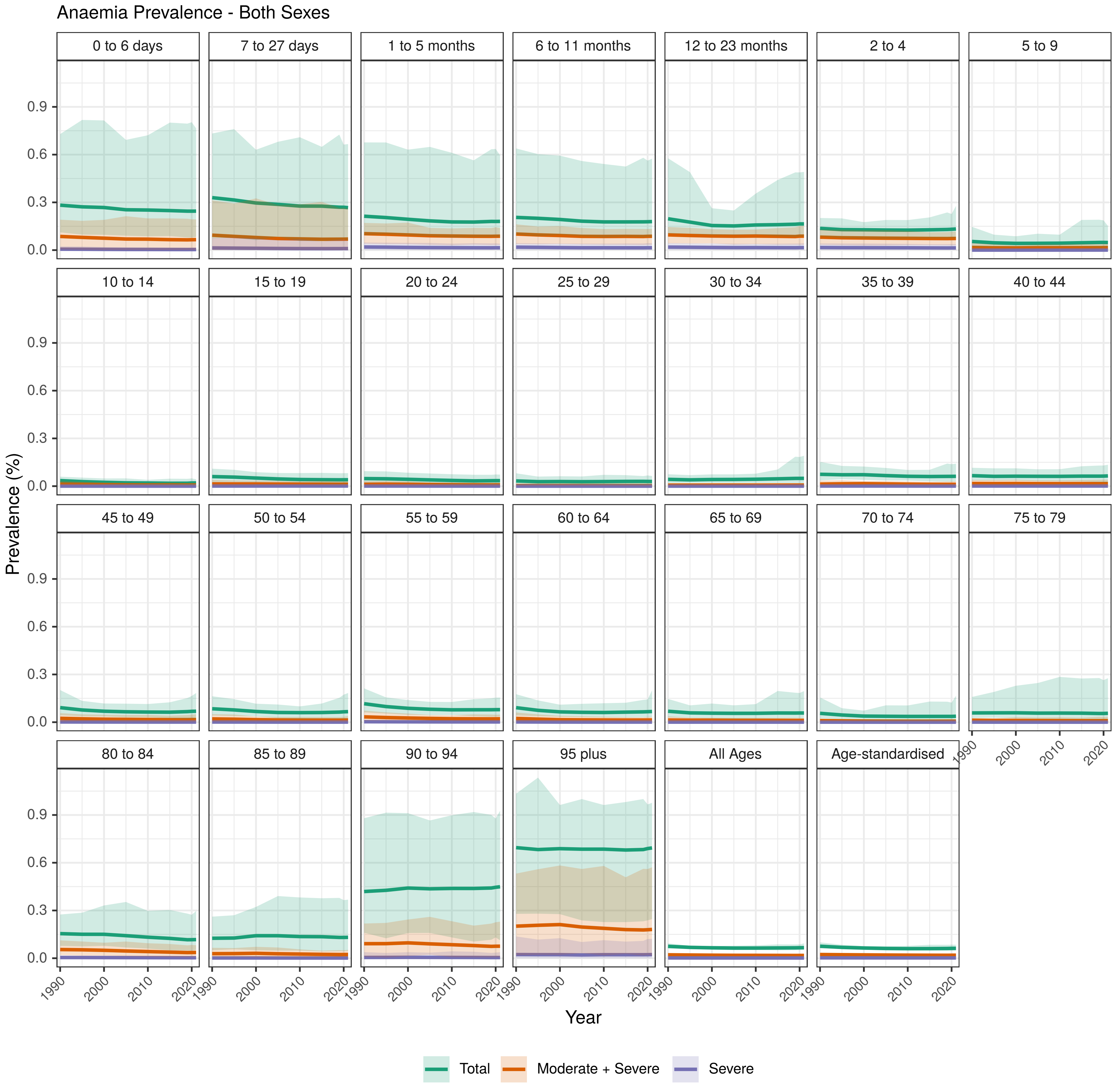

Anaemia Prevalence - Both Sexes
0 to 6 days
7 to 27 days
1 to 5 months
6 to 11 months
12 to 23 months
2 to 4
5 to 9
10 to 14
15 to 19
20 to 24
25 to 29
30 to 34
35 to 39
40 to 44
45 to 49
50 to 54
55 to 59
60 to 64
65 to 69
70 to 74
75 to 79
80 to 84
85 to 89
90 to 94
95 plus
All Ages
Age-standardised
Prevalence (%)
0.0
0.3
0.6
0.9
1990
2000
2010
2020
Year
Total
Moderate + Severe
Severe

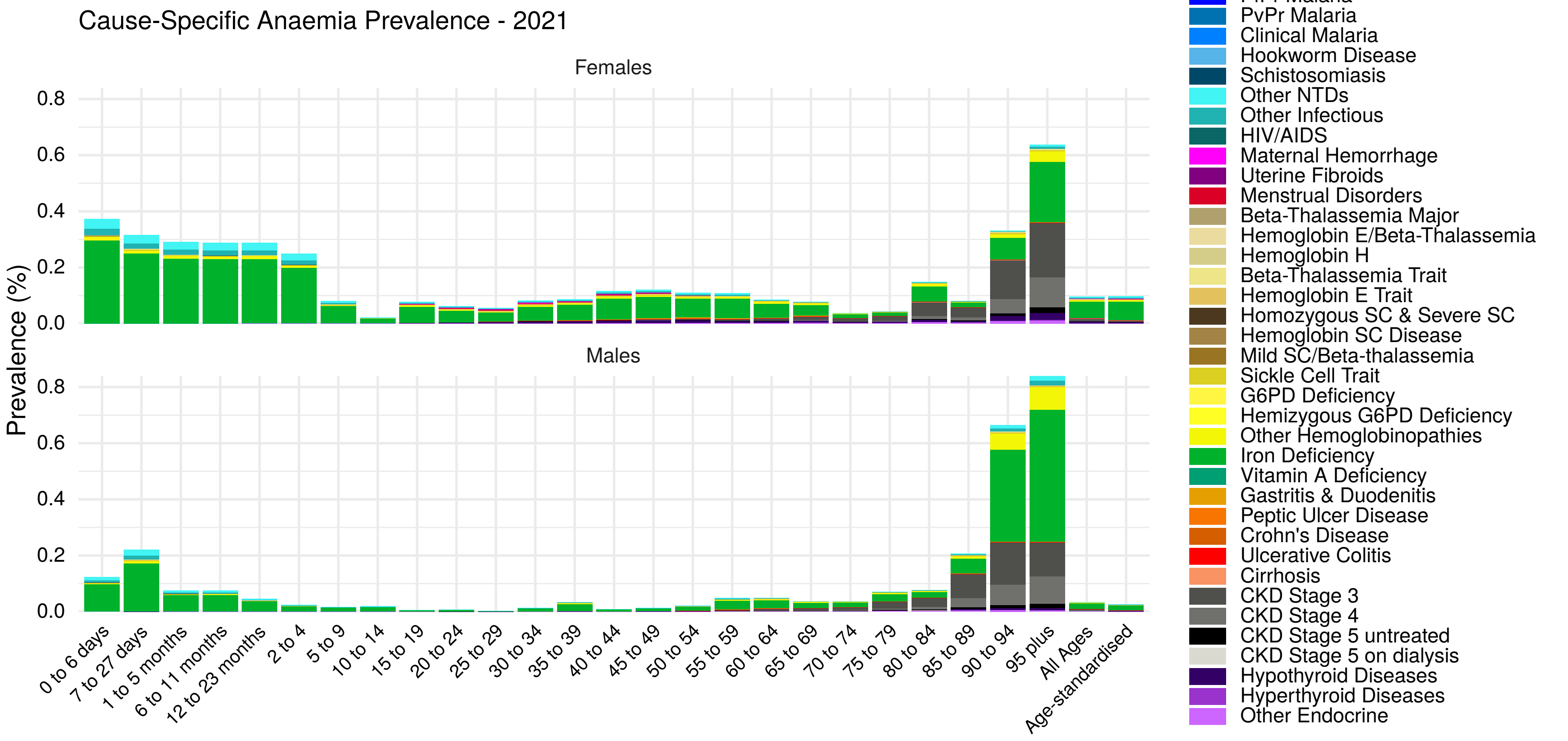

Cause-Specific Anaemia Prevalence - 2021
Females
Males
Prevalence (%)
0.0
0.2
0.4
0.6
0.8
0 to 6 days
7 to 27 days
1 to 5 months
6 to 11 months
12 to 23 months
2 to 4
5 to 9
10 to 14
15 to 19
20 to 24
25 to 29
30 to 34
35 to 39
40 to 44
45 to 49
50 to 54
55 to 59
60 to 64
65 to 69
70 to 74
75 to 79
80 to 84
85 to 89
90 to 94
95 plus
All Ages
Age-standardised
PfPr Malaria
PvPr Malaria
Clinical Malaria
Hookworm Disease
Schistosomiasis
Other NTDs
Other Infectious
HIV/AIDS
Maternal Hemorrhage
Uterine Fibroids
Menstrual Disorders
Beta-Thalassemia Major
Hemoglobin E/Beta-Thalassemia
Hemoglobin H
Beta-Thalassemia Trait
Hemoglobin E Trait
Homozygous SC & Severe SC
Hemoglobin SC Disease
Mild SC/Beta-thalassemia
Sickle Cell Trait
G6PD Deficiency
Hemizygous G6PD Deficiency
Other Hemoglobinopathies
Iron Deficiency
Vitamin A Deficiency
Gastritis & Duodenitis
Peptic Ulcer Disease
Crohn's Disease
Ulcerative Colitis
Cirrhosis
CKD Stage 3
CKD Stage 4
CKD Stage 5 untreated
CKD Stage 5 on dialysis
Hypothyroid Diseases
Hyperthyroid Diseases
Other Endocrine

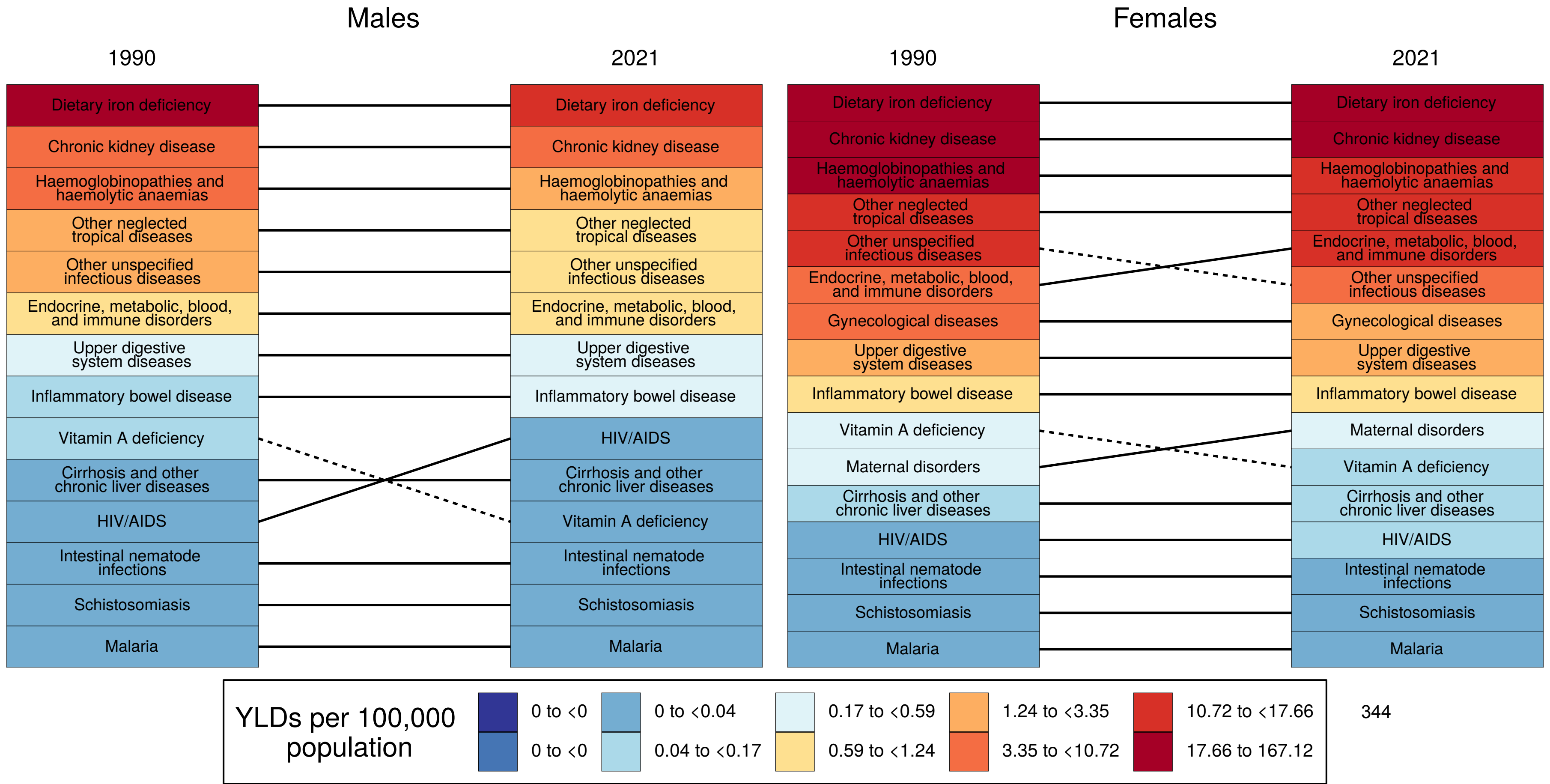

Males
Females
1990
2021
Dietary iron deficiency
Chronic kidney disease
Haemoglobinopathies and haemolytic anaemias
Other neglected tropical diseases
Other unspecified infectious diseases
Endocrine, metabolic, blood, and immune disorders
Upper digestive system diseases
Inflammatory bowel disease
Vitamin A deficiency
Cirrhosis and other chronic liver diseases
HIV/AIDS
Intestinal nematode infections
Schistosomiasis
Malaria
Gynecological diseases
Maternal disorders
YLDs per 100,000 population
0 to <0
0 to <0
0 to <0.04
0.04 to <0.17
0.17 to <0.59
0.59 to <1.24
1.24 to <3.35
3.35 to <10.72
10.72 to <17.66
17.66 to 167.12
344

Tameside

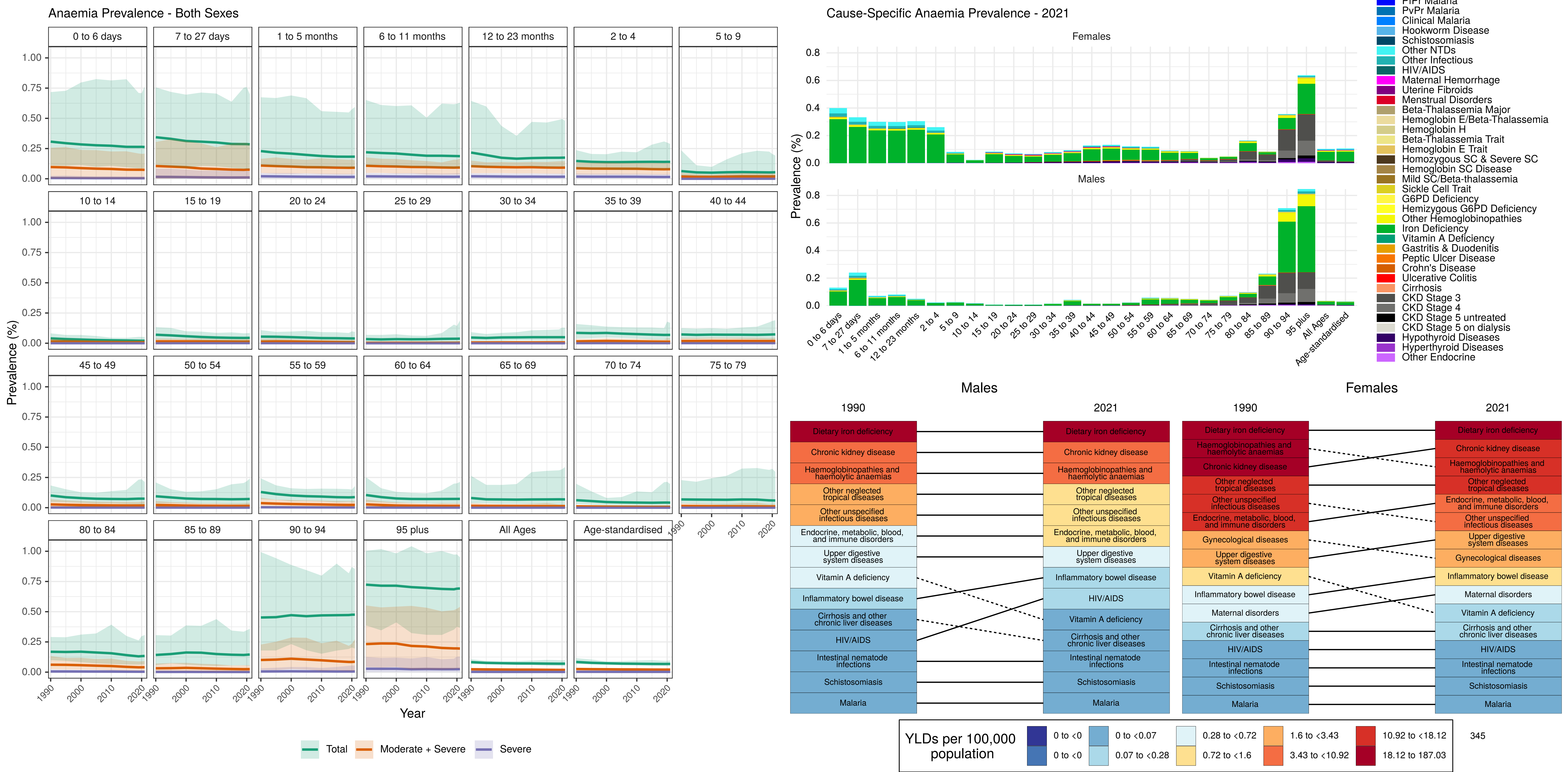

Anaemia Prevalence - Both Sexes
0 to 6 days
7 to 27 days
1 to 5 months
6 to 11 months
12 to 23 months
2 to 4
5 to 9
10 to 14
15 to 19
20 to 24
25 to 29
30 to 34
35 to 39
40 to 44
45 to 49
50 to 54
55 to 59
60 to 64
65 to 69
70 to 74
75 to 79
80 to 84
85 to 89
90 to 94
95 plus
All Ages
Age-standardised
Prevalence (%)
Year
Total
Moderate + Severe
Severe
Cause-Specific Anaemia Prevalence - 2021
Females
Males
PfPr Malaria
PvPr Malaria
Clinical Malaria
Hookworm Disease
Schistosomiasis
Other NTDs
Other Infectious
HIV/AIDS
Maternal Hemorrhage
Uterine Fibroids
Menstrual Disorders
Beta-Thalassemia Major
Hemoglobin E/Beta-Thalassemia
Hemoglobin H
Beta-Thalassemia Trait
Hemoglobin E Trait
Homozygous SC & Severe SC
Hemoglobin SC Disease
Mild SC/Beta-thalassemia
Sickle Cell Trait
G6PD Deficiency
Hemizygous G6PD Deficiency
Other Hemoglobinopathies
Iron Deficiency
Vitamin A Deficiency
Gastritis & Duodenitis
Peptic Ulcer Disease
Crohn's Disease
Ulcerative Colitis
Cirrhosis
CKD Stage 3
CKD Stage 4
CKD Stage 5 untreated
CKD Stage 5 on dialysis
Hypothyroid Diseases
Hyperthyroid Diseases
Other Endocrine
Males
Females
1990
2021
Dietary iron deficiency
Chronic kidney disease
Haemoglobinopathies and haemolytic anaemias
Other neglected tropical diseases
Other unspecified infectious diseases
Endocrine, metabolic, blood, and immune disorders
Upper digestive system diseases
Vitamin A deficiency
Inflammatory bowel disease
Cirrhosis and other chronic liver diseases
HIV/AIDS
Intestinal nematode infections
Schistosomiasis
Malaria
Gynecological diseases
Maternal disorders
YLDs per 100,000 population
0 to <0
0 to <0
0 to <0.07
0.07 to <0.28
0.28 to <0.72
0.72 to <1.6
1.6 to <3.43
3.43 to <10.92
10.92 to <18.12
18.12 to 187.03

Trafford

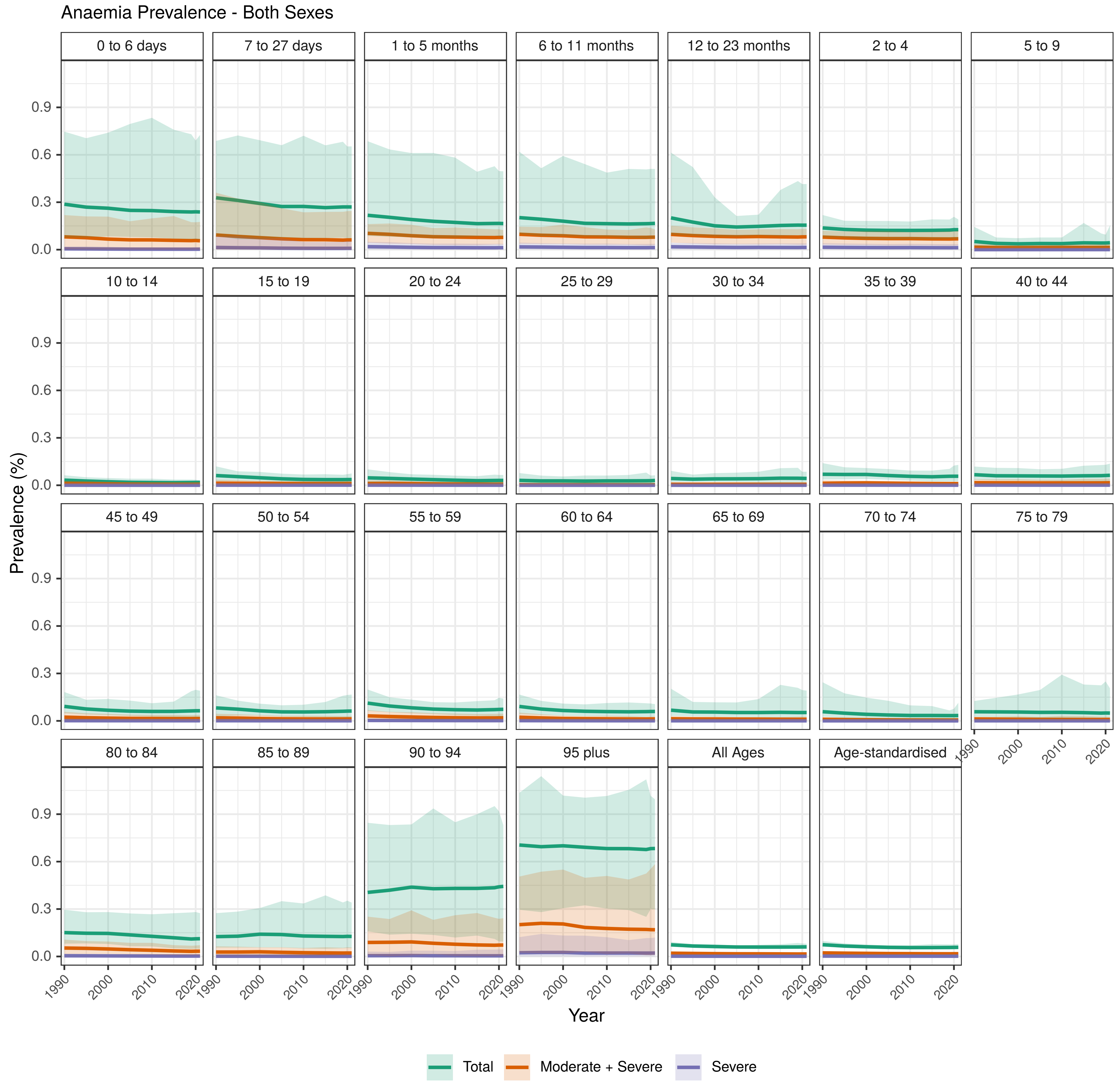

Anaemia Prevalence - Both Sexes
0 to 6 days
7 to 27 days
1 to 5 months
6 to 11 months
12 to 23 months
2 to 4
5 to 9
10 to 14
15 to 19
20 to 24
25 to 29
30 to 34
35 to 39
40 to 44
45 to 49
50 to 54
55 to 59
60 to 64
65 to 69
70 to 74
75 to 79
80 to 84
85 to 89
90 to 94
95 plus
All Ages
Age-standardised
Prevalence (%)
Year
Total
Moderate + Severe
Severe

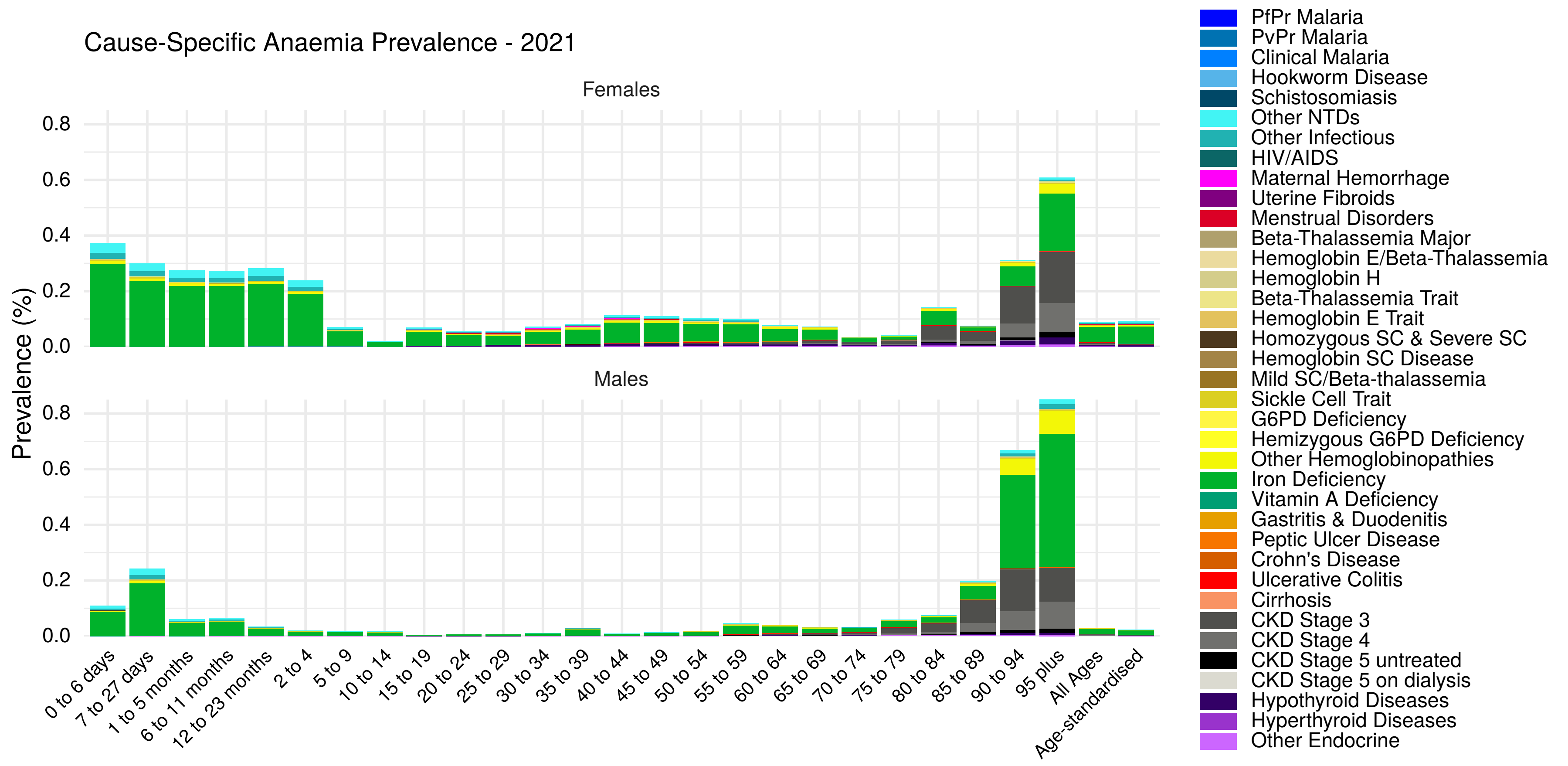

Cause-Specific Anaemia Prevalence - 2021
Females
Males
Prevalence (%)
PfPr Malaria
PvPr Malaria
Clinical Malaria
Hookworm Disease
Schistosomiasis
Other NTDs
Other Infectious
HIV/AIDS
Maternal Hemorrhage
Uterine Fibroids
Menstrual Disorders
Beta-Thalassemia Major
Hemoglobin E/Beta-Thalassemia
Hemoglobin H
Beta-Thalassemia Trait
Hemoglobin E Trait
Homozygous SC & Severe SC
Hemoglobin SC Disease
Mild SC/Beta-thalassemia
Sickle Cell Trait
G6PD Deficiency
Hemizygous G6PD Deficiency
Other Hemoglobinopathies
Iron Deficiency
Vitamin A Deficiency
Gastritis & Duodenitis
Peptic Ulcer Disease
Crohn's Disease
Ulcerative Colitis
Cirrhosis
CKD Stage 3
CKD Stage 4
CKD Stage 5 untreated
CKD Stage 5 on dialysis
Hypothyroid Diseases
Hyperthyroid Diseases
Other Endocrine

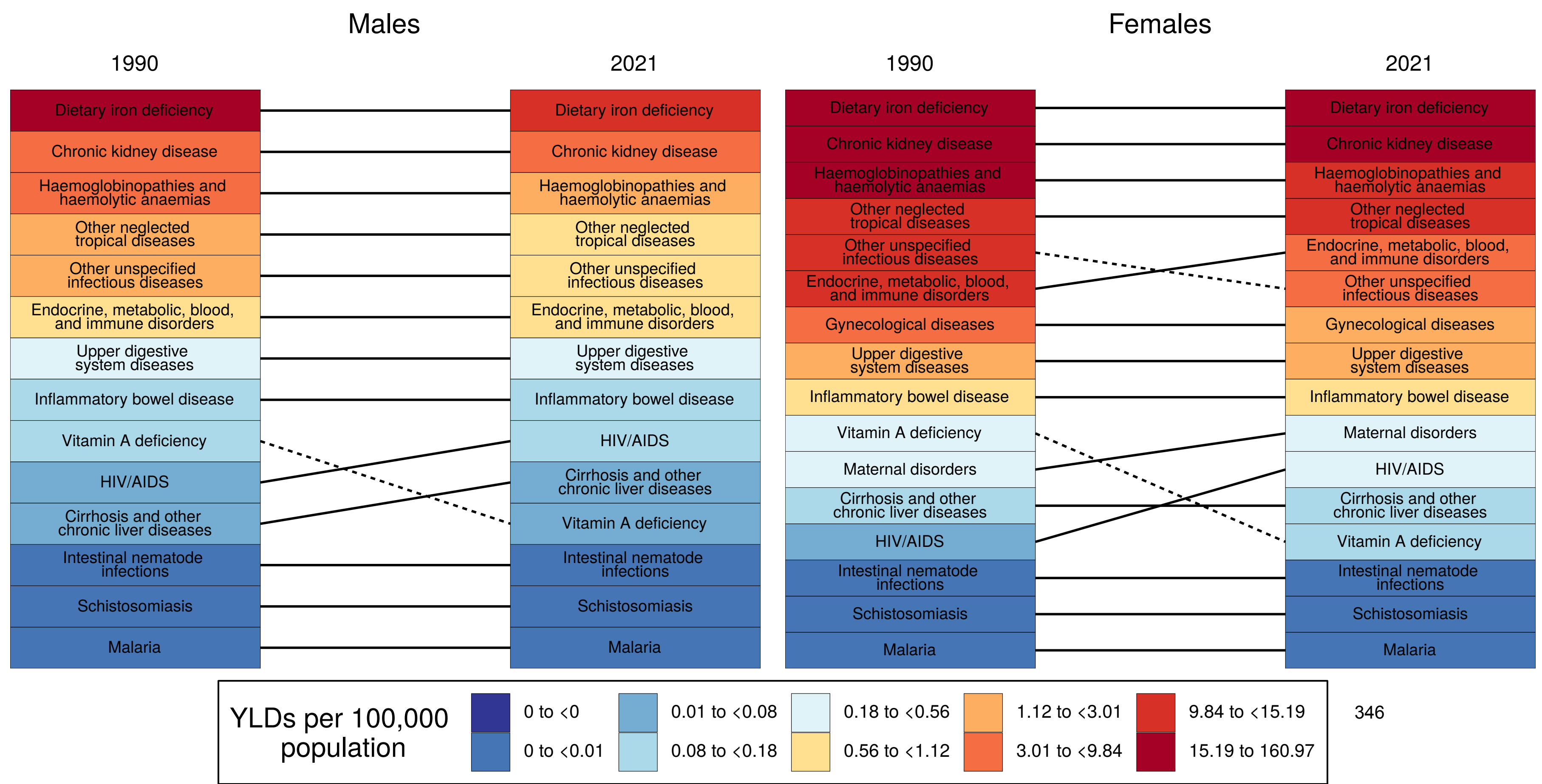

Males
Females
1990
2021
Dietary iron deficiency
Chronic kidney disease
Haemoglobinopathies and haemolytic anaemias
Other neglected tropical diseases
Other unspecified infectious diseases
Endocrine, metabolic, blood, and immune disorders
Upper digestive system diseases
Inflammatory bowel disease
Vitamin A deficiency
HIV/AIDS
Cirrhosis and other chronic liver diseases
Intestinal nematode infections
Schistosomiasis
Malaria
Gynecological diseases
Maternal disorders
YLDs per 100,000 population
0 to <0
0 to <0.01
0.01 to <0.08
0.08 to <0.18
0.18 to <0.56
0.56 to <1.12
1.12 to <3.01
3.01 to <9.84
9.84 to <15.19
15.19 to 160.97

Warrington

Anaemia Prevalence - Both Sexes

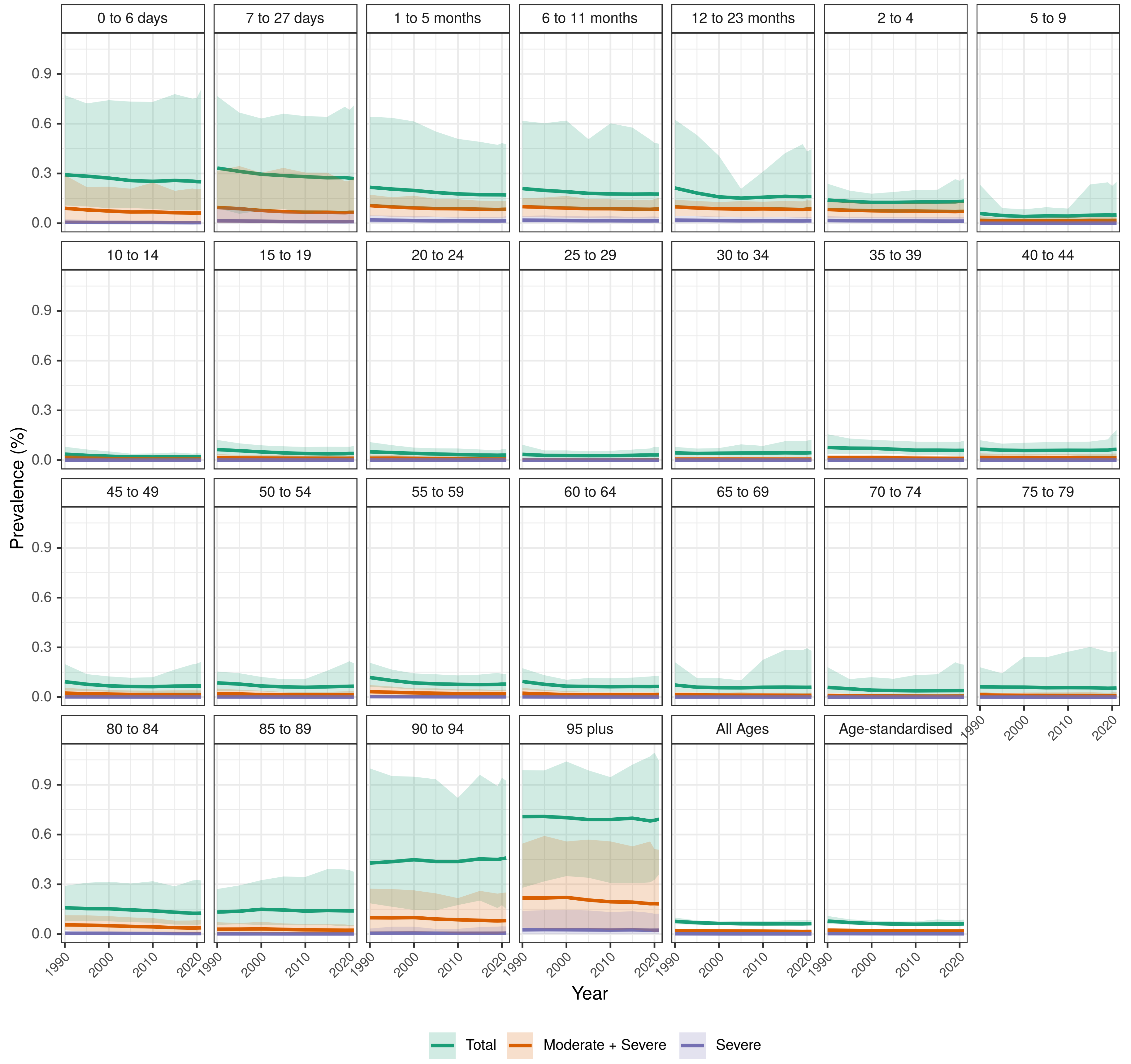

Cause-Specific Anaemia Prevalence - 2021

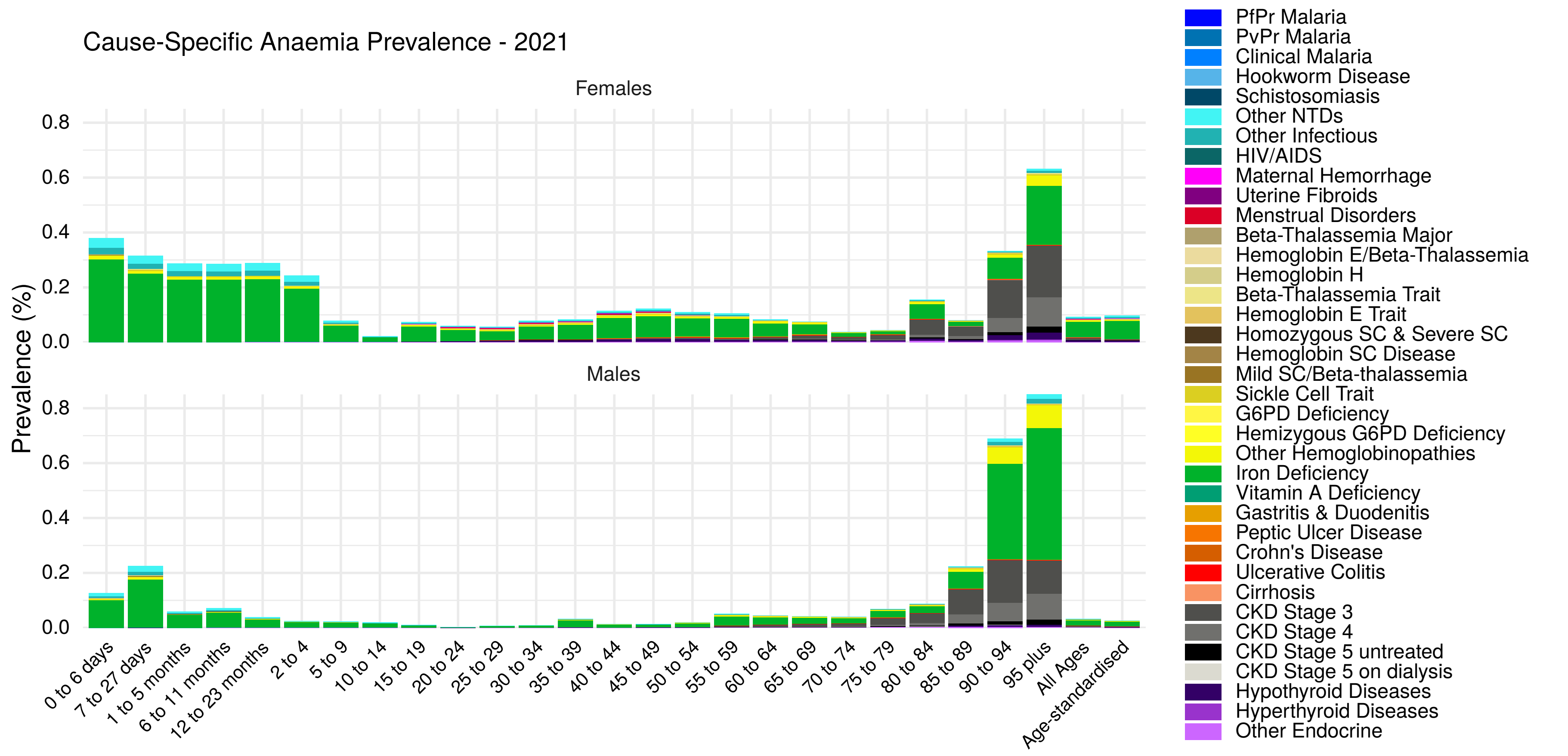

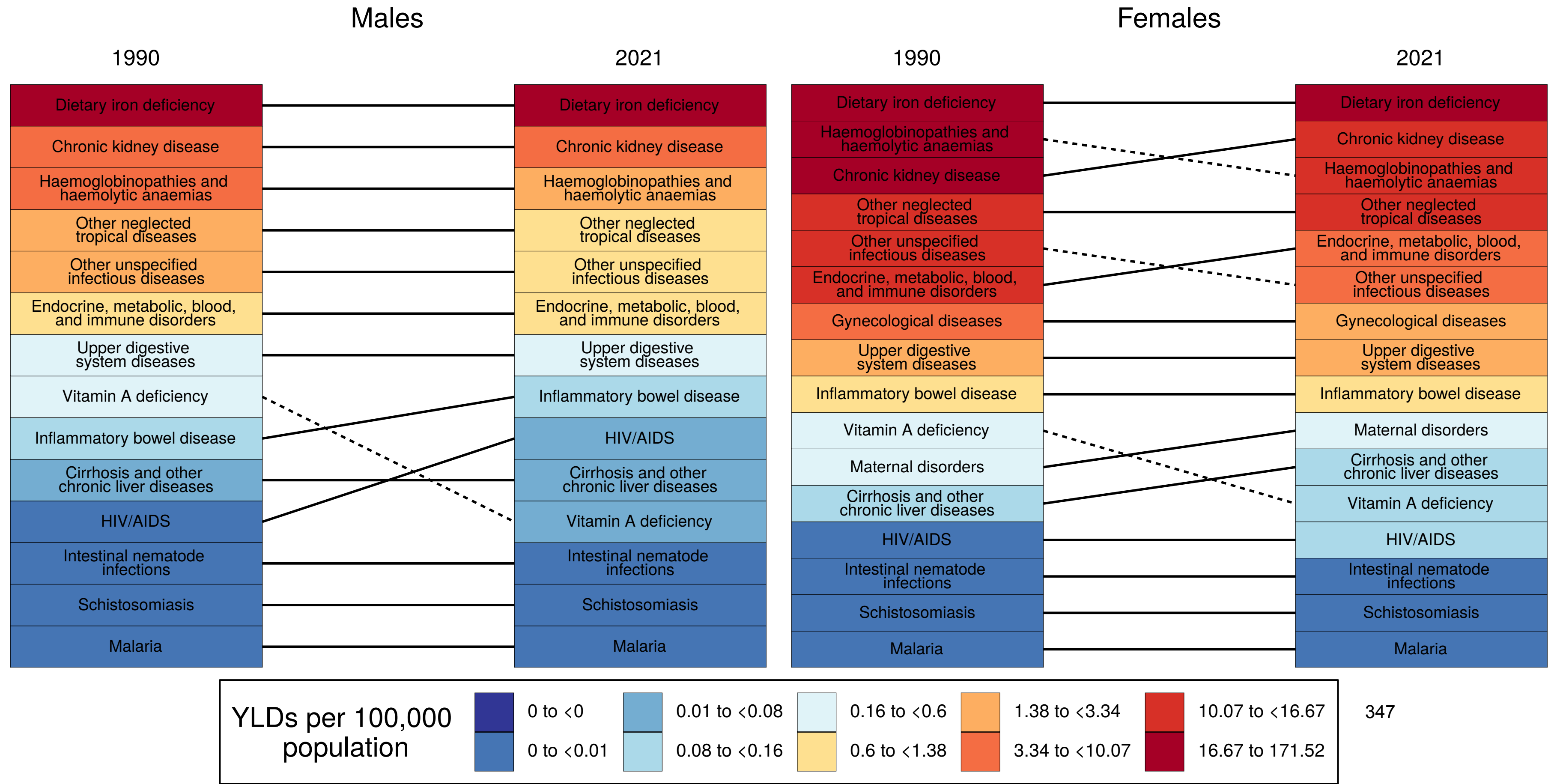

Wigan

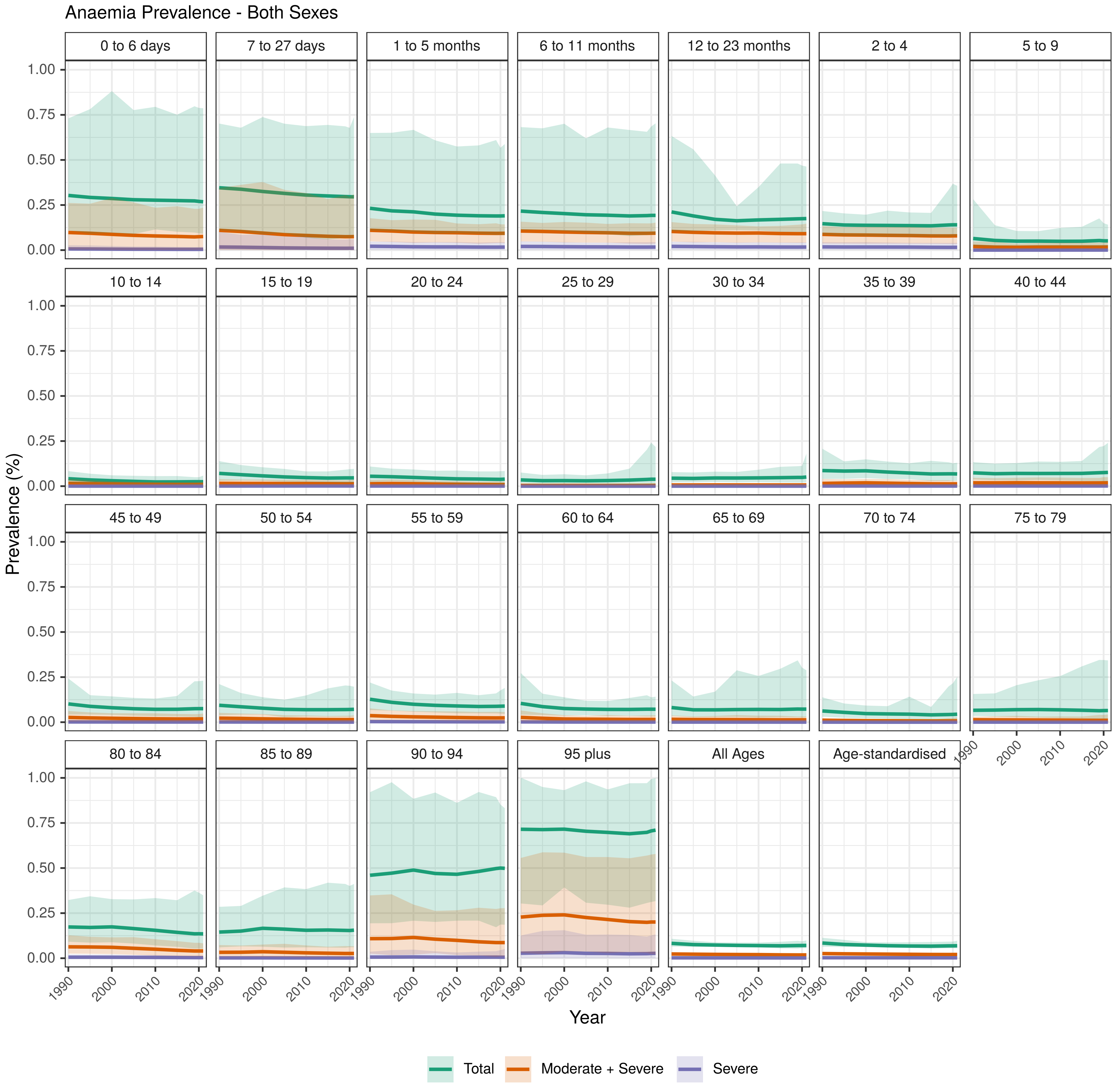

Anaemia Prevalence - Both Sexes
0 to 6 days
7 to 27 days
1 to 5 months
6 to 11 months
12 to 23 months
2 to 4
5 to 9
10 to 14
15 to 19
20 to 24
25 to 29
30 to 34
35 to 39
40 to 44
45 to 49
50 to 54
55 to 59
60 to 64
65 to 69
70 to 74
75 to 79
80 to 84
85 to 89
90 to 94
95 plus
All Ages
Age-standardised
Prevalence (%)
Year
Total
Moderate + Severe
Severe

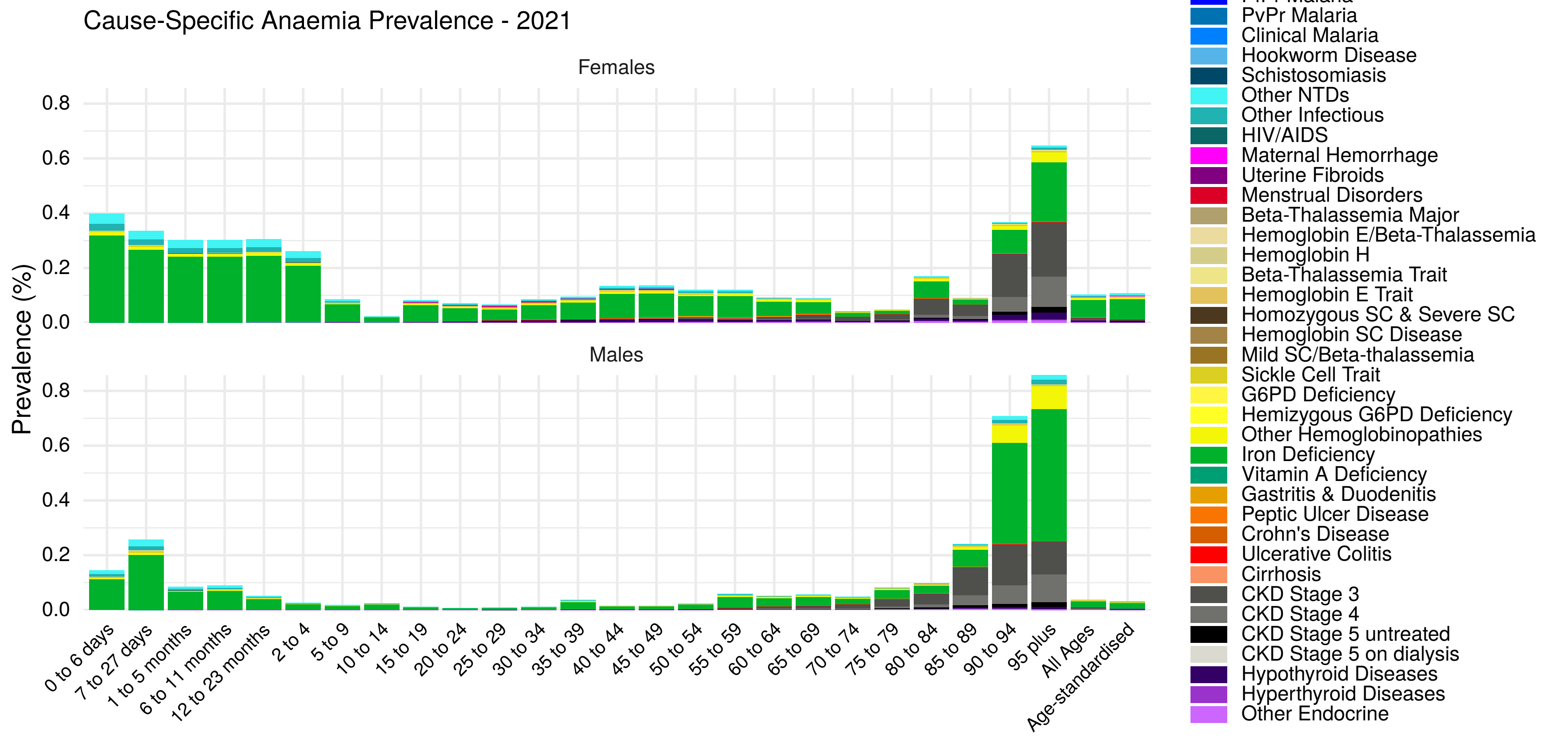

Cause-Specific Anaemia Prevalence - 2021
Females
Males
Prevalence (%)
PfPr Malaria
PvPr Malaria
Clinical Malaria
Hookworm Disease
Schistosomiasis
Other NTDs
Other Infectious
HIV/AIDS
Maternal Hemorrhage
Uterine Fibroids
Menstrual Disorders
Beta-Thalassemia Major
Hemoglobin E/Beta-Thalassemia
Hemoglobin H
Beta-Thalassemia Trait
Hemoglobin E Trait
Homozygous SC & Severe SC
Hemoglobin SC Disease
Mild SC/Beta-thalassemia
Sickle Cell Trait
G6PD Deficiency
Hemizygous G6PD Deficiency
Other Hemoglobinopathies
Iron Deficiency
Vitamin A Deficiency
Gastritis & Duodenitis
Peptic Ulcer Disease
Crohn's Disease
Ulcerative Colitis
Cirrhosis
CKD Stage 3
CKD Stage 4
CKD Stage 5 untreated
CKD Stage 5 on dialysis
Hypothyroid Diseases
Hyperthyroid Diseases
Other Endocrine

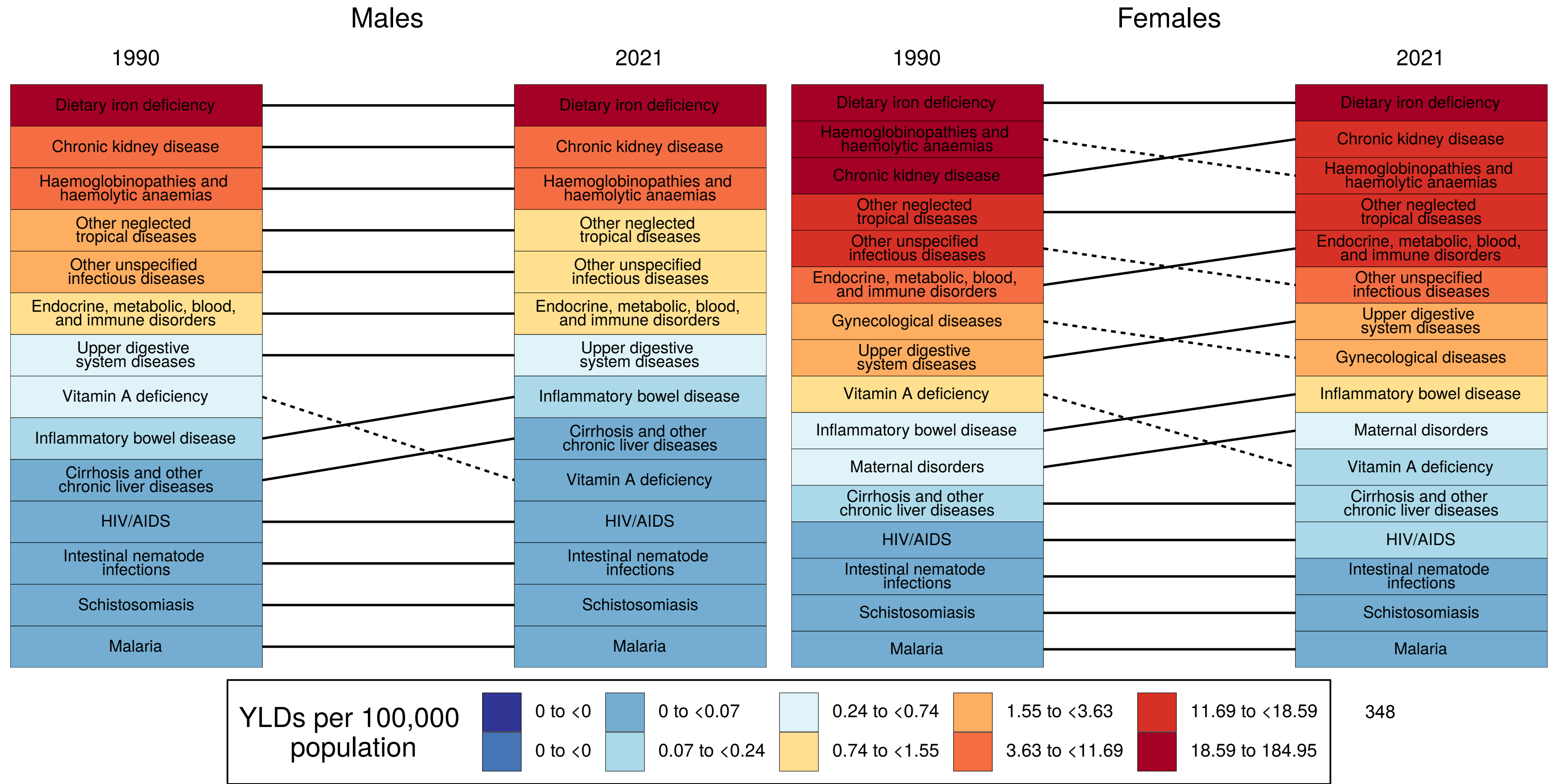

Males
Females
1990
2021
Dietary iron deficiency
Chronic kidney disease
Haemoglobinopathies and haemolytic anaemias
Other neglected tropical diseases
Other unspecified infectious diseases
Endocrine, metabolic, blood, and immune disorders
Upper digestive system diseases
Vitamin A deficiency
Inflammatory bowel disease
Cirrhosis and other chronic liver diseases
HIV/AIDS
Intestinal nematode infections
Schistosomiasis
Malaria
Gynecological diseases
Maternal disorders
YLDs per 100,000 population
0 to <0
0 to <0.07
0.07 to <0.24
0.24 to <0.74
0.74 to <1.55
1.55 to <3.63
3.63 to <11.69
11.69 to <18.59
18.59 to 184.95

Wirral

Anaemia Prevalence - Both Sexes

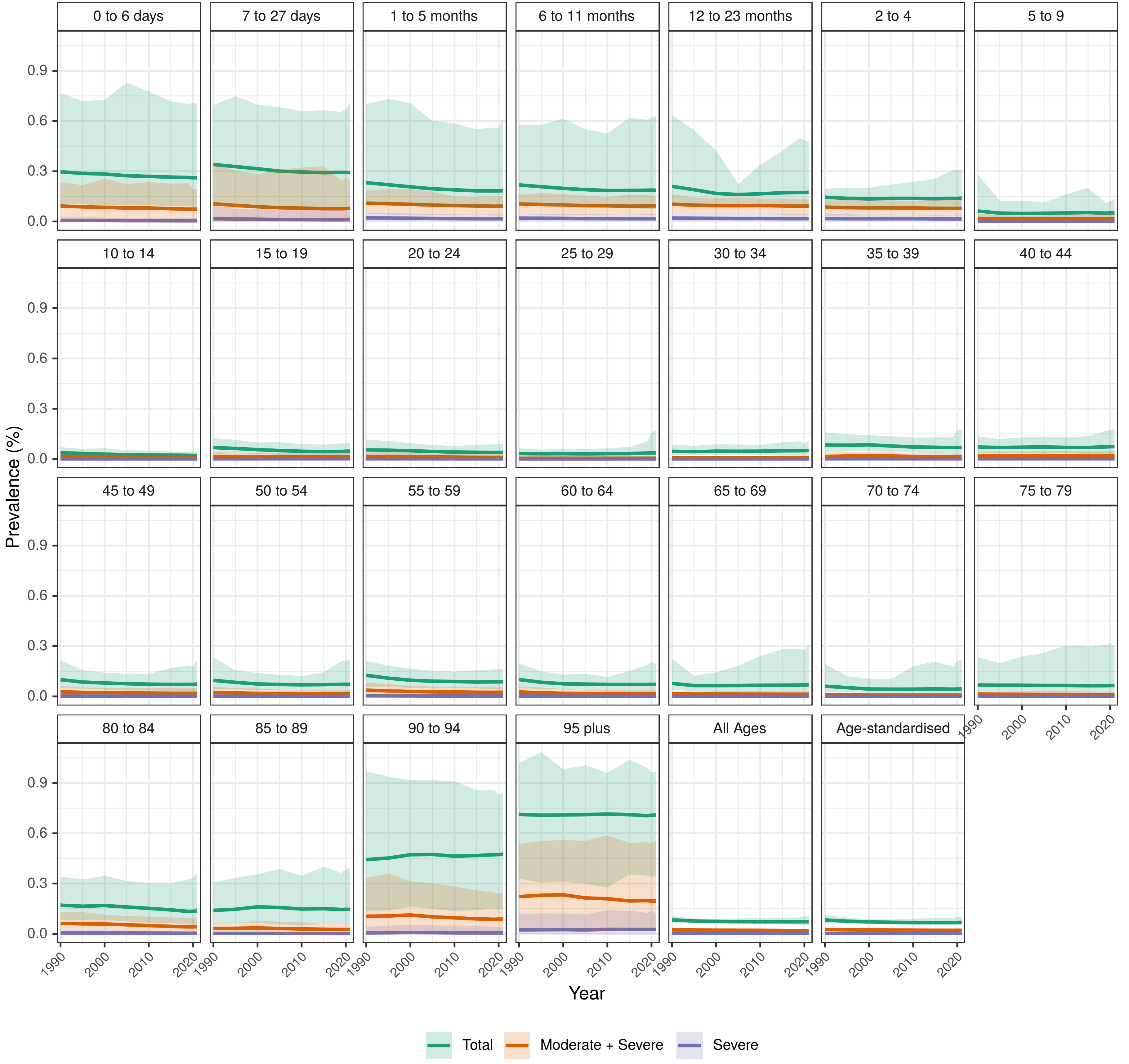

Cause-Specific Anaemia Prevalence - 2021

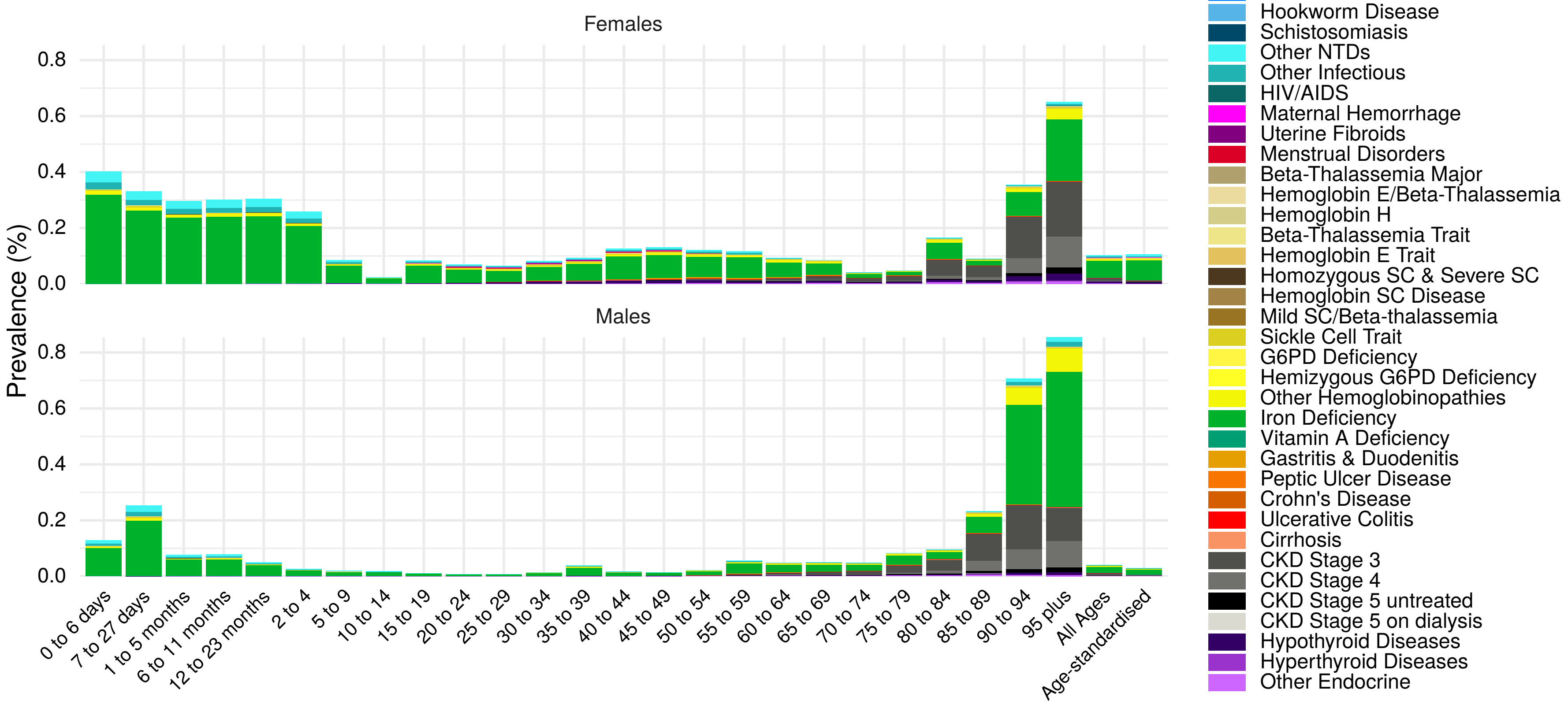

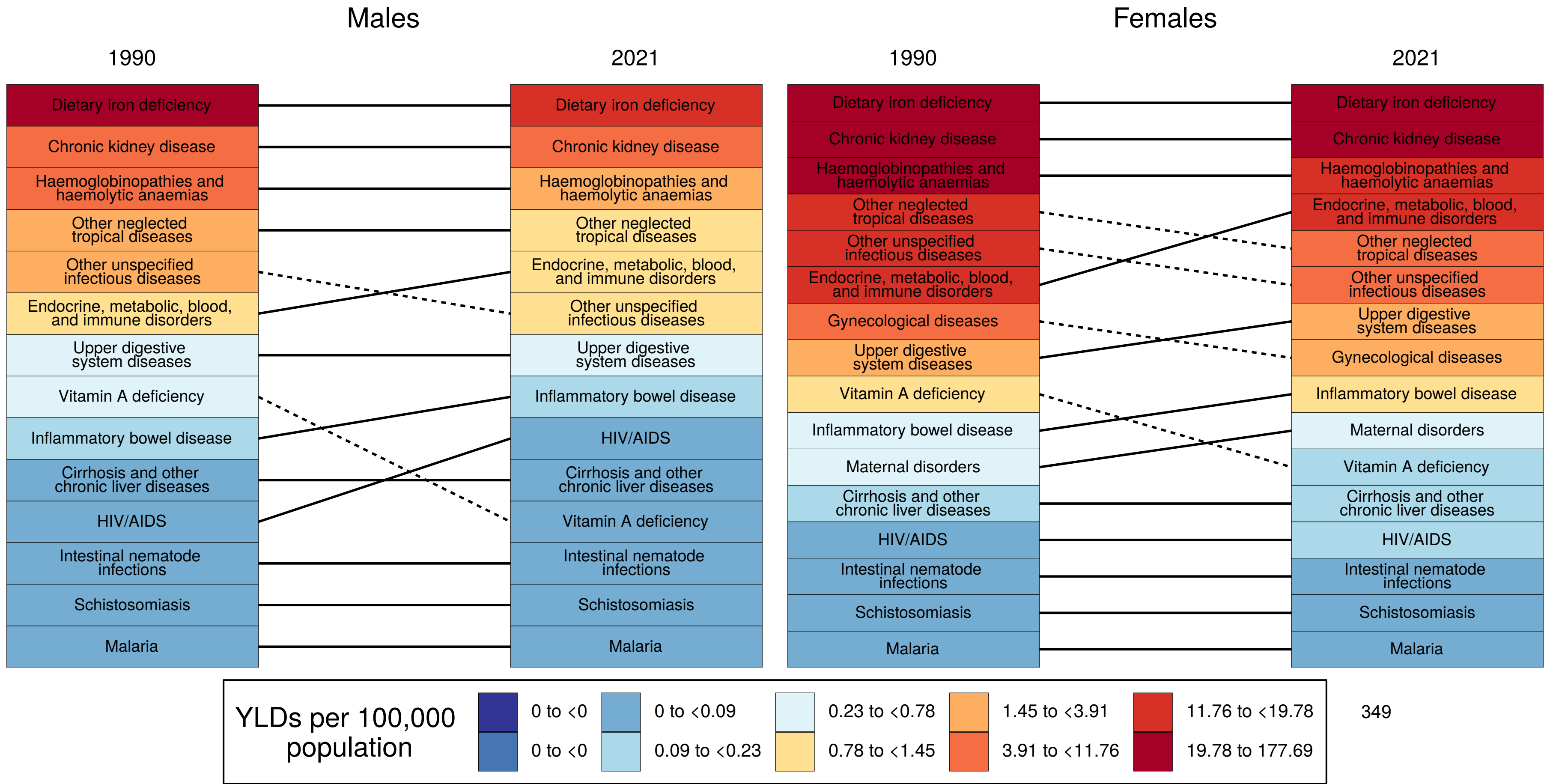

South East England

Anaemia Prevalence - Both Sexes

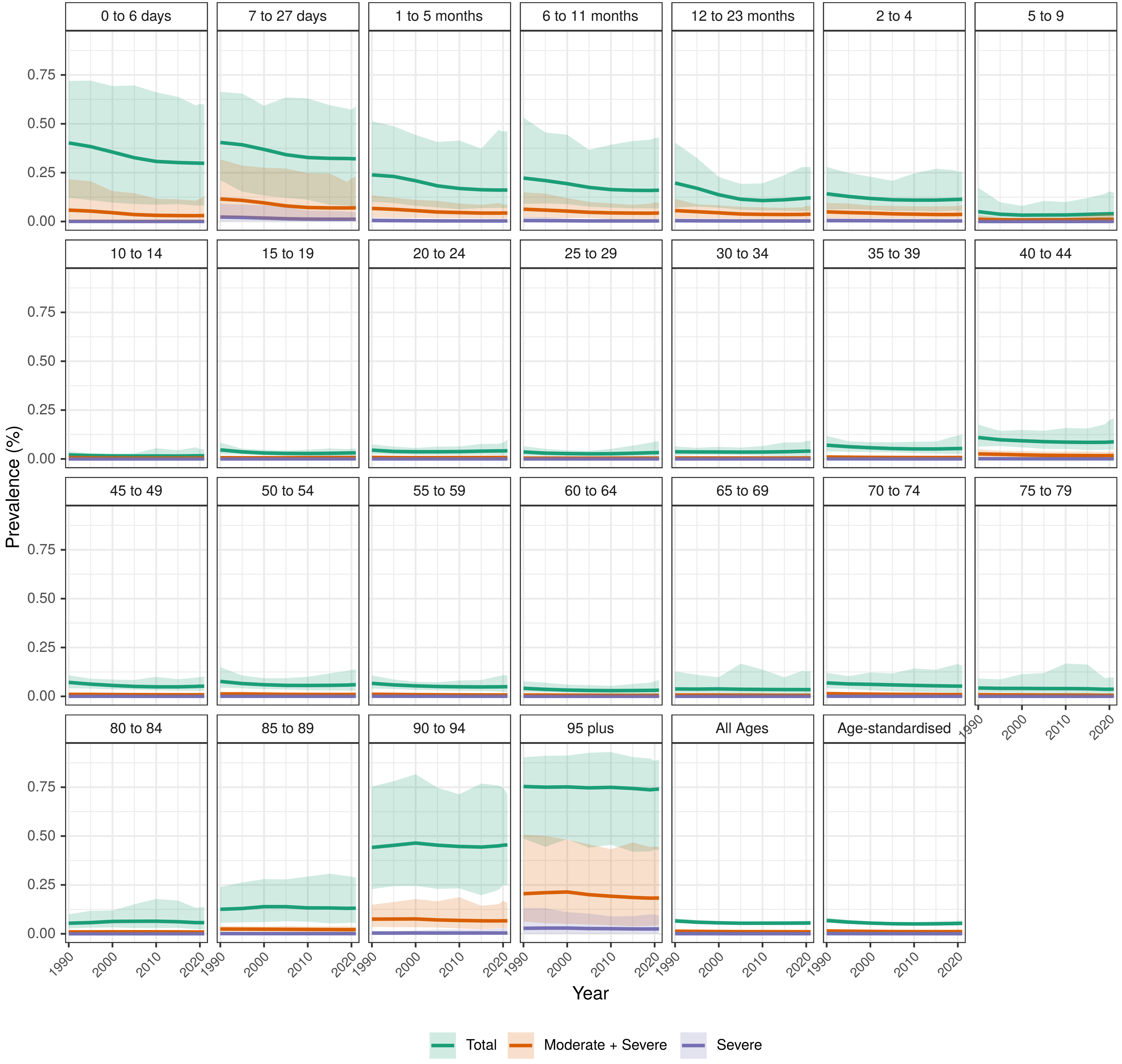

Cause-Specific Anaemia Prevalence - 2021

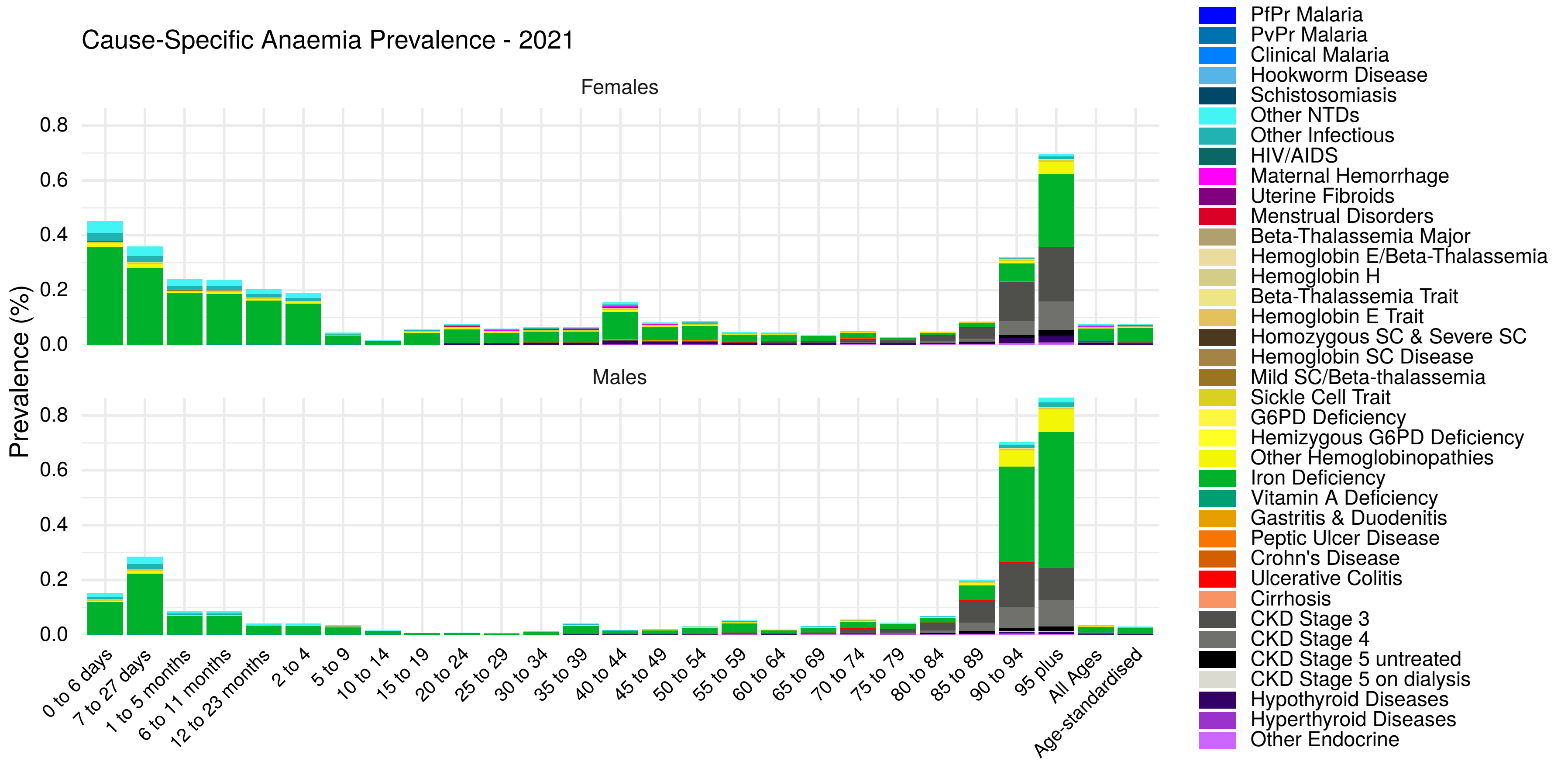

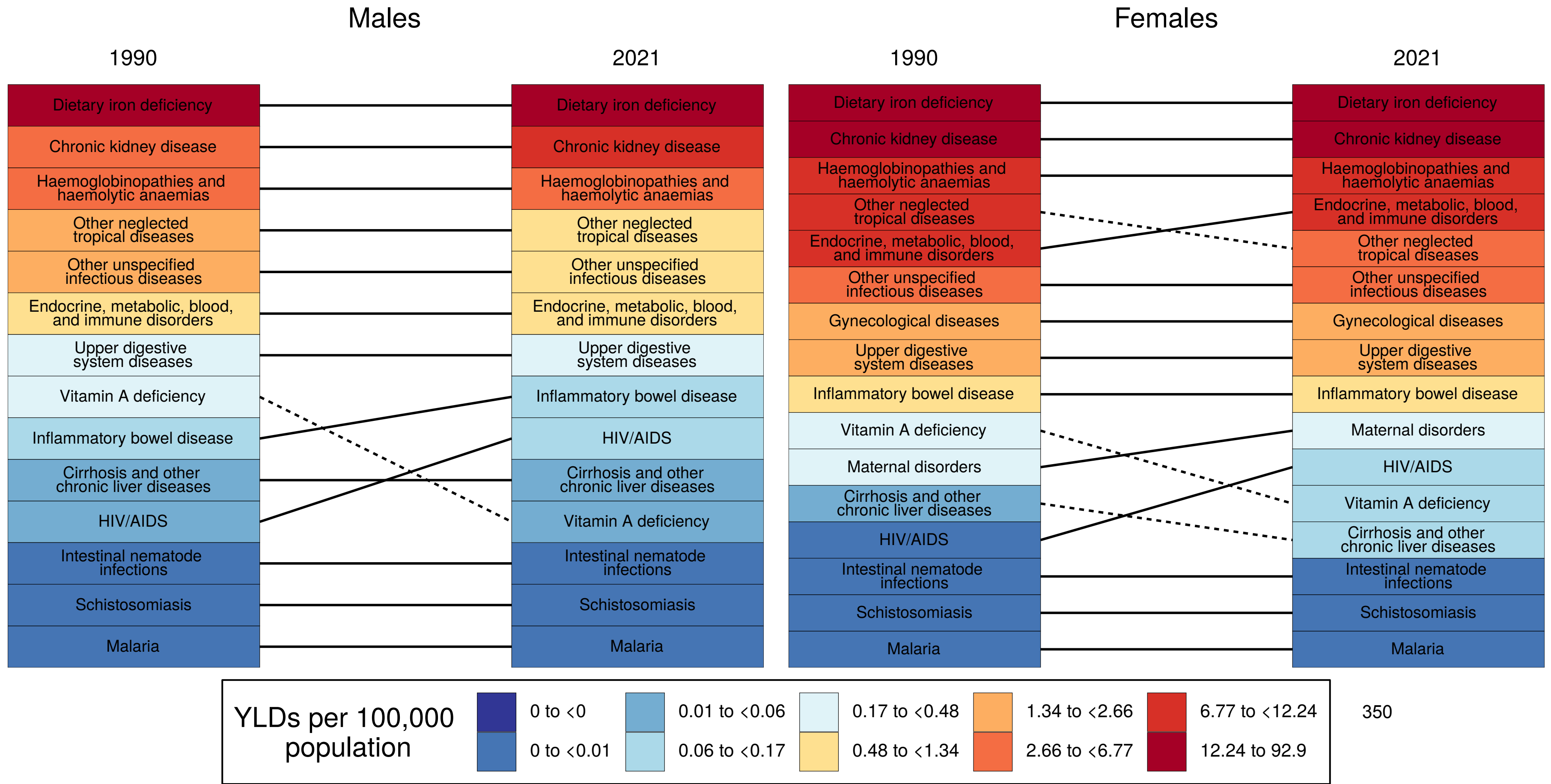

350

Bracknell Forest

Anaemia Prevalence - Both Sexes

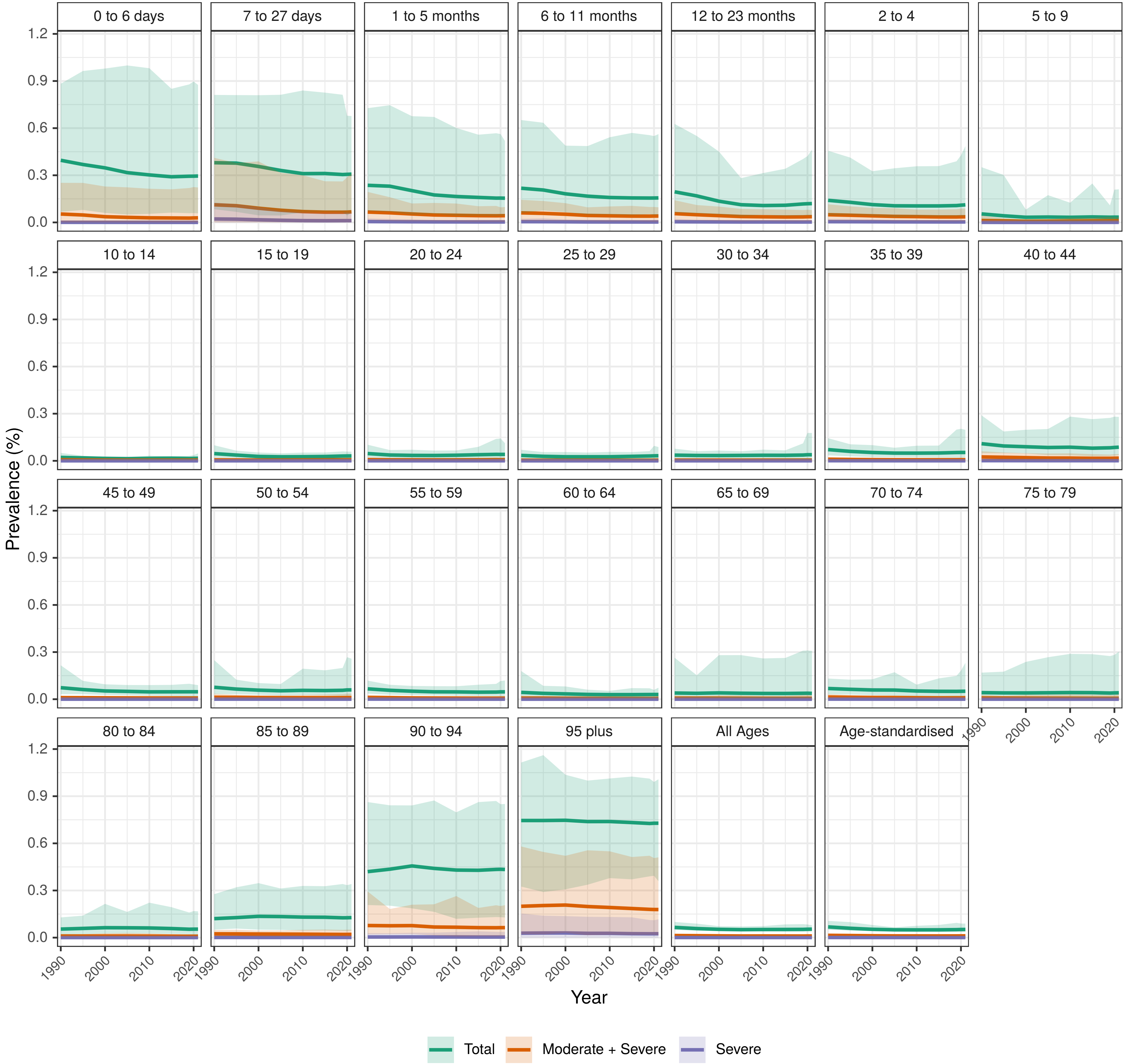

Cause-Specific Anaemia Prevalence - 2021

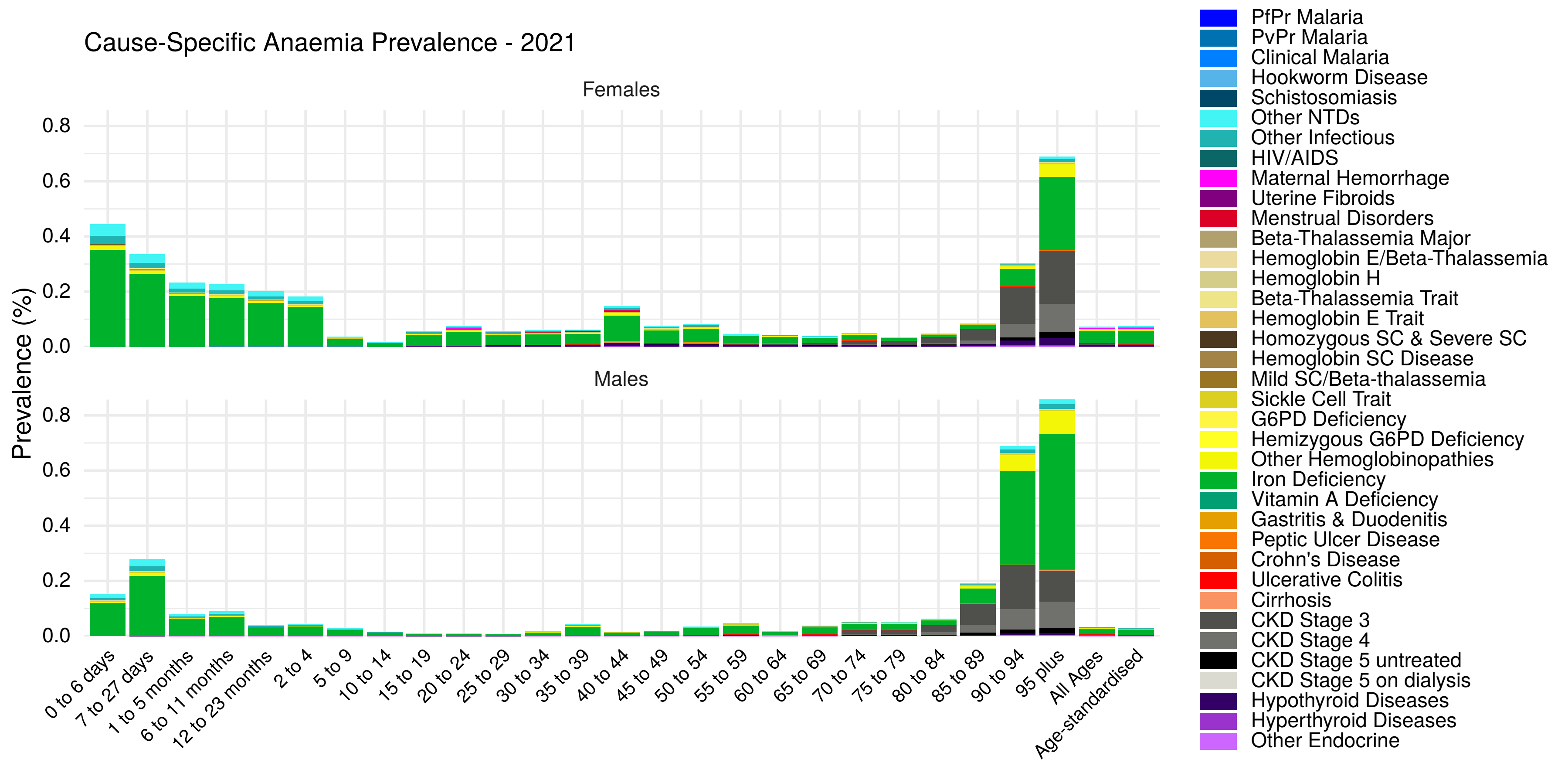

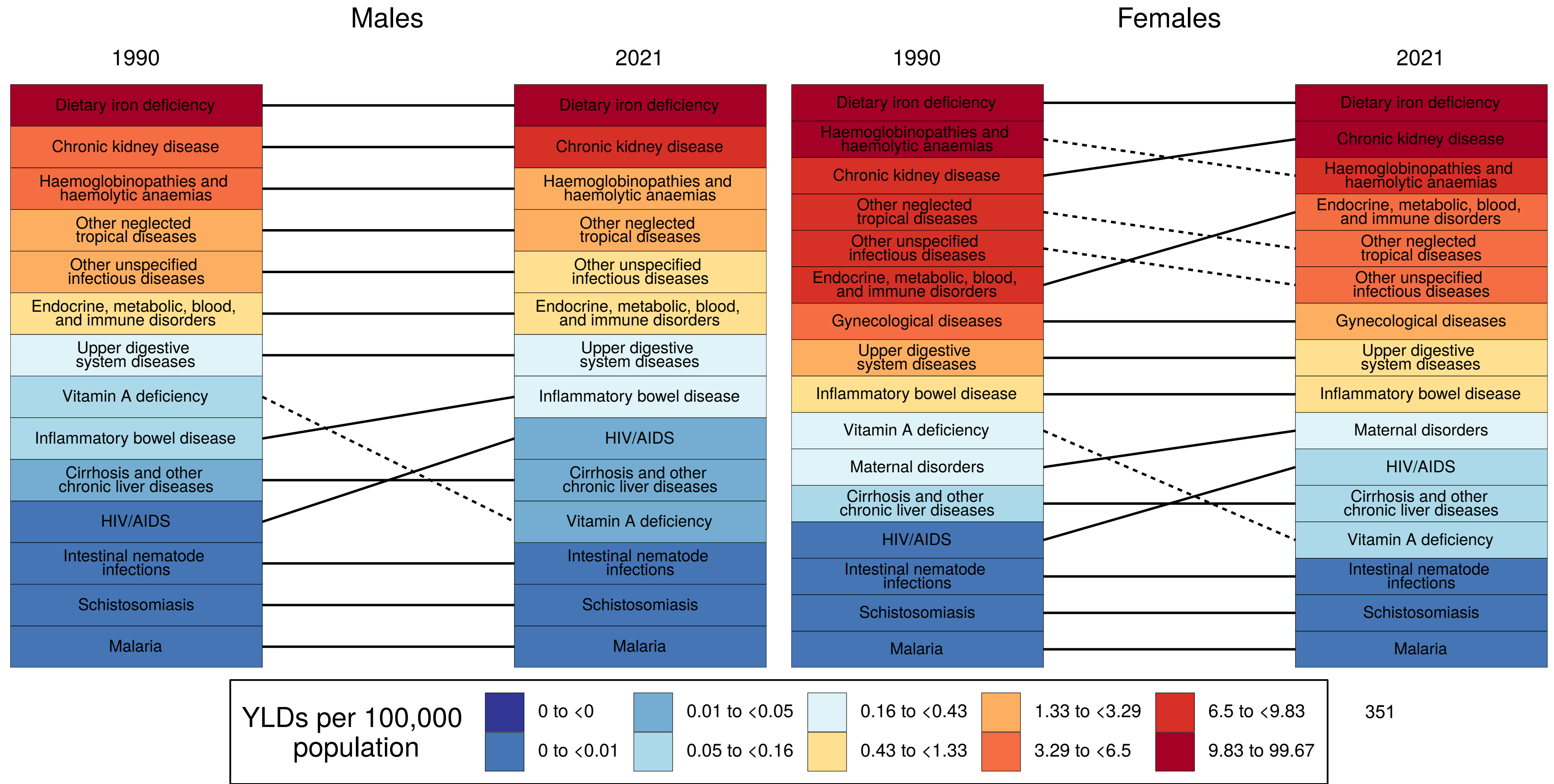

Brighton and Hove

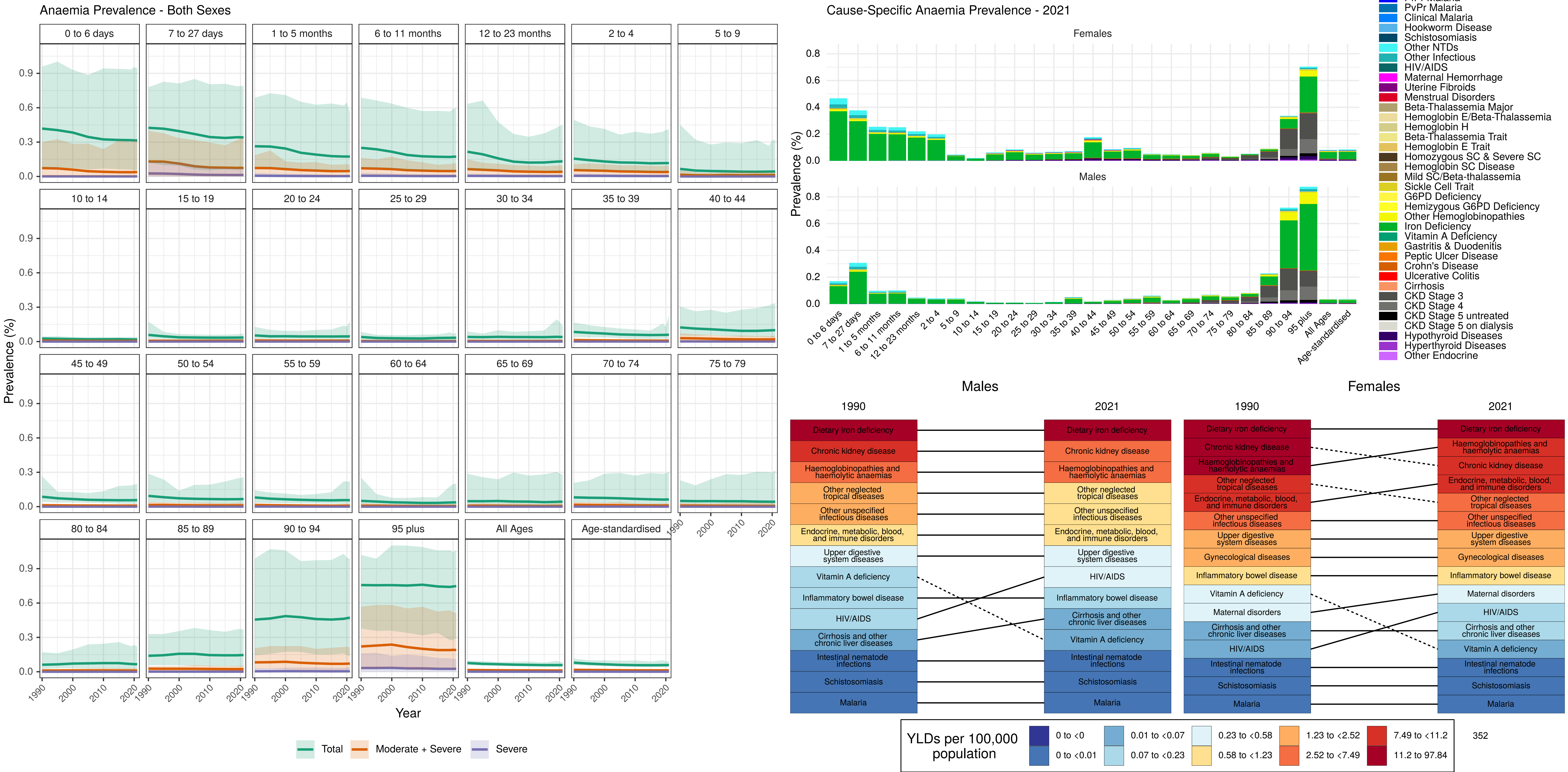

Buckinghamshire

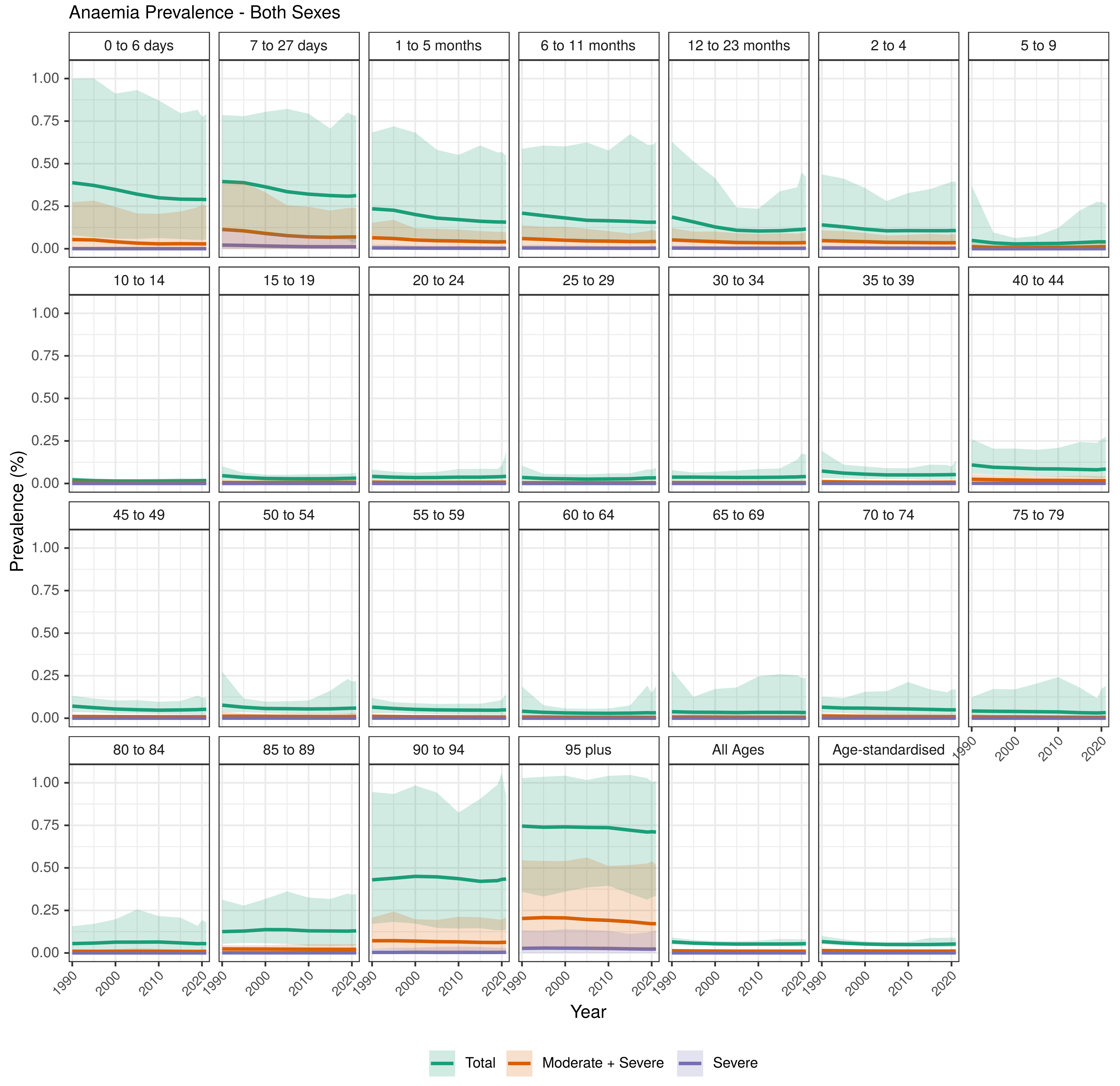

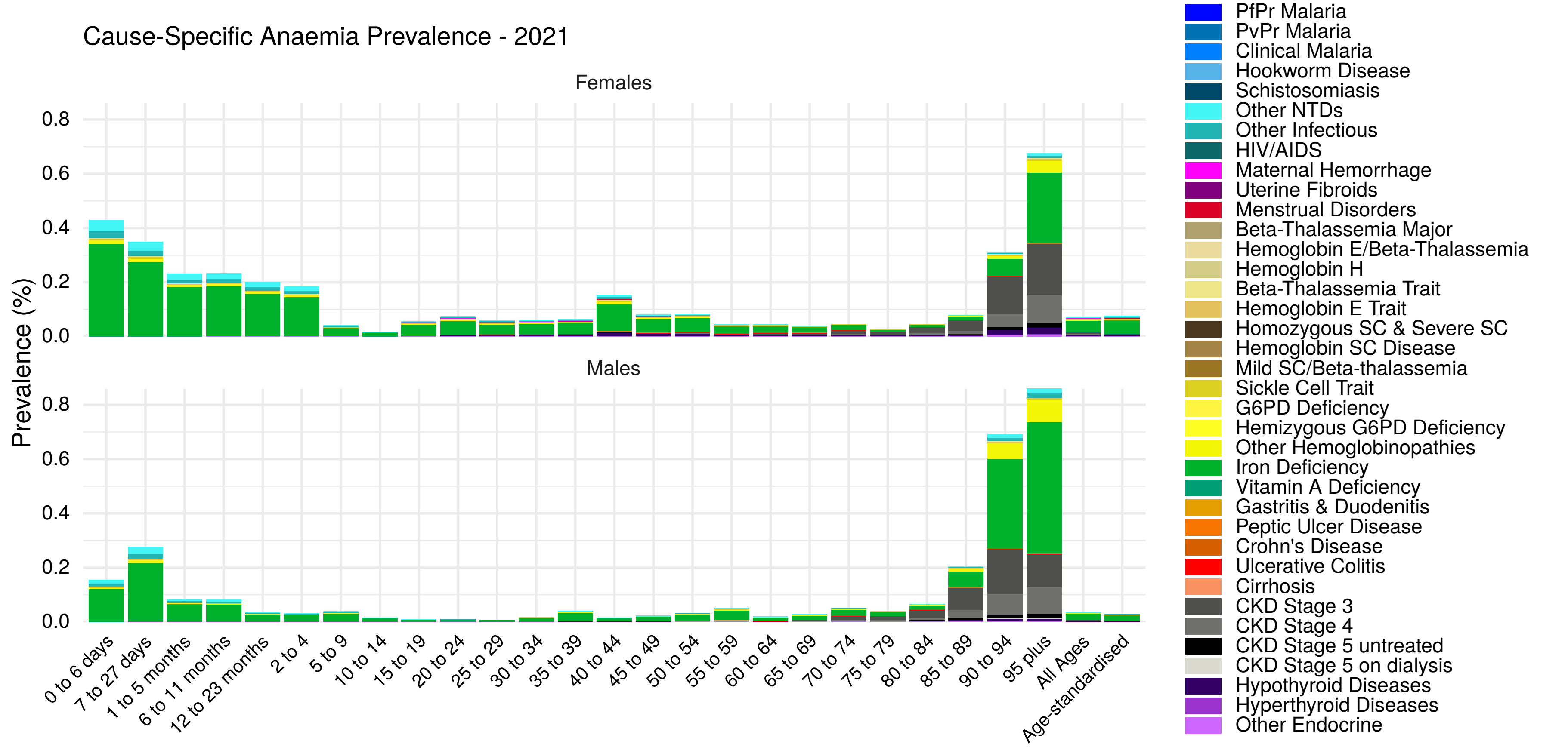

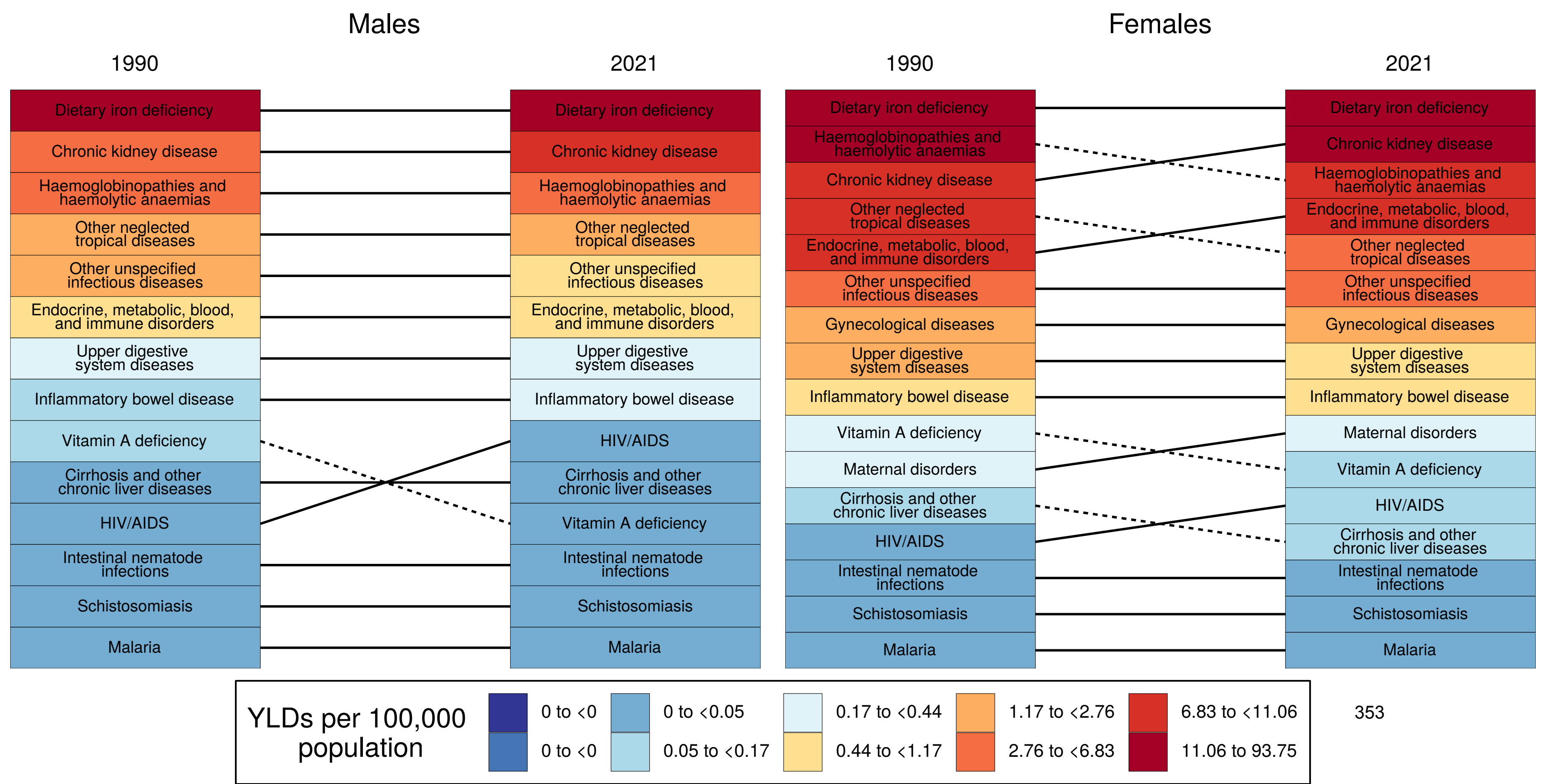

East Sussex

Anaemia Prevalence - Both Sexes

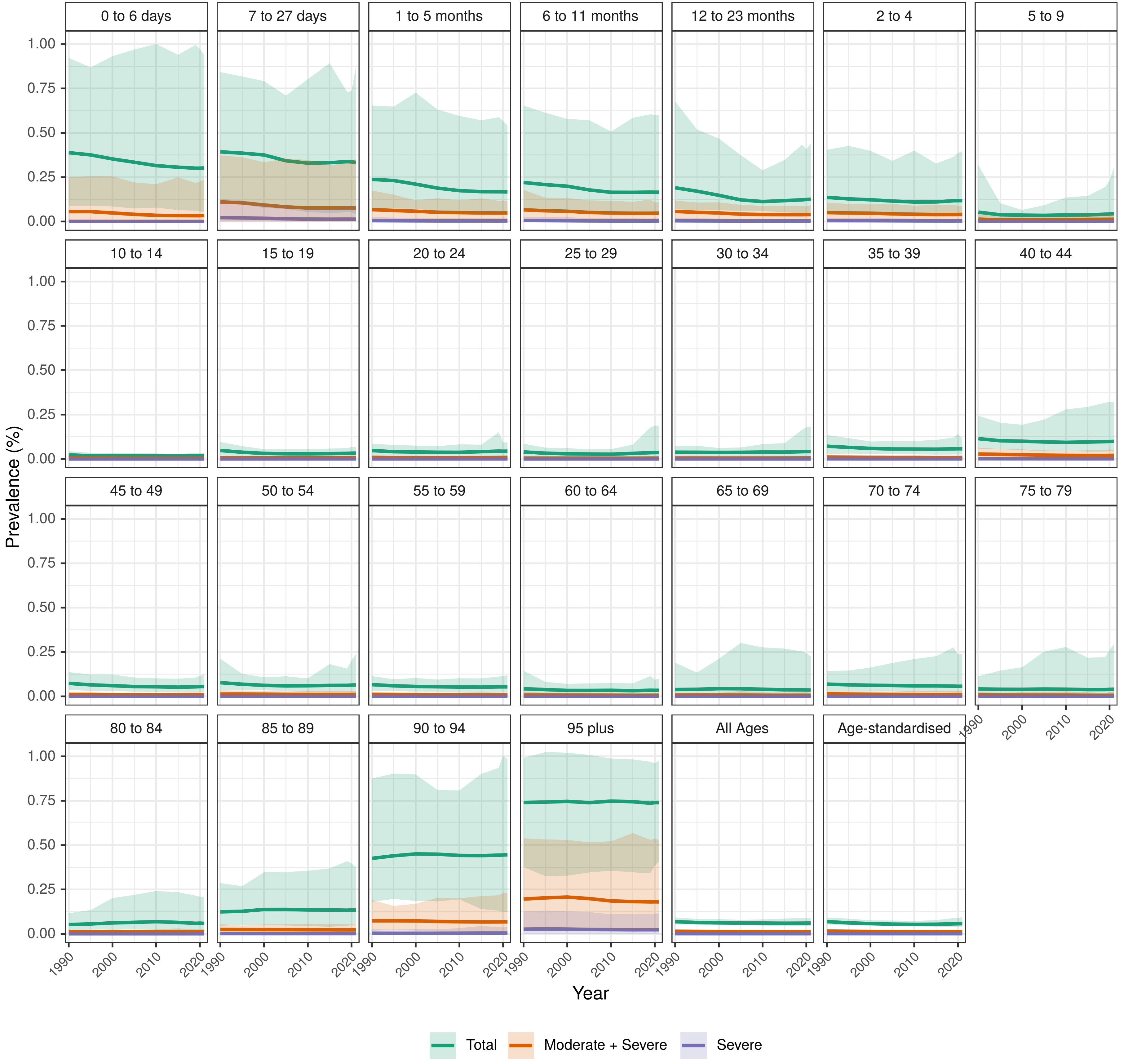

Cause-Specific Anaemia Prevalence - 2021

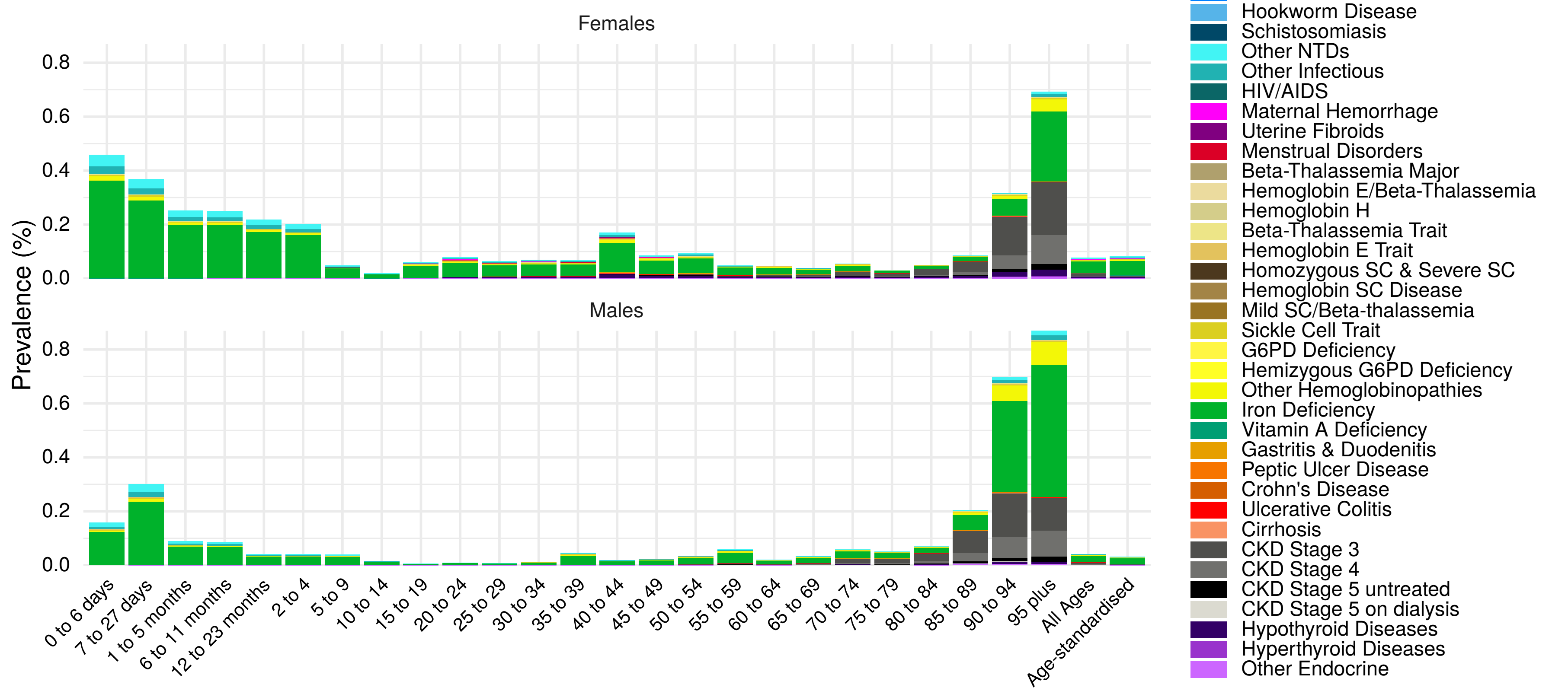

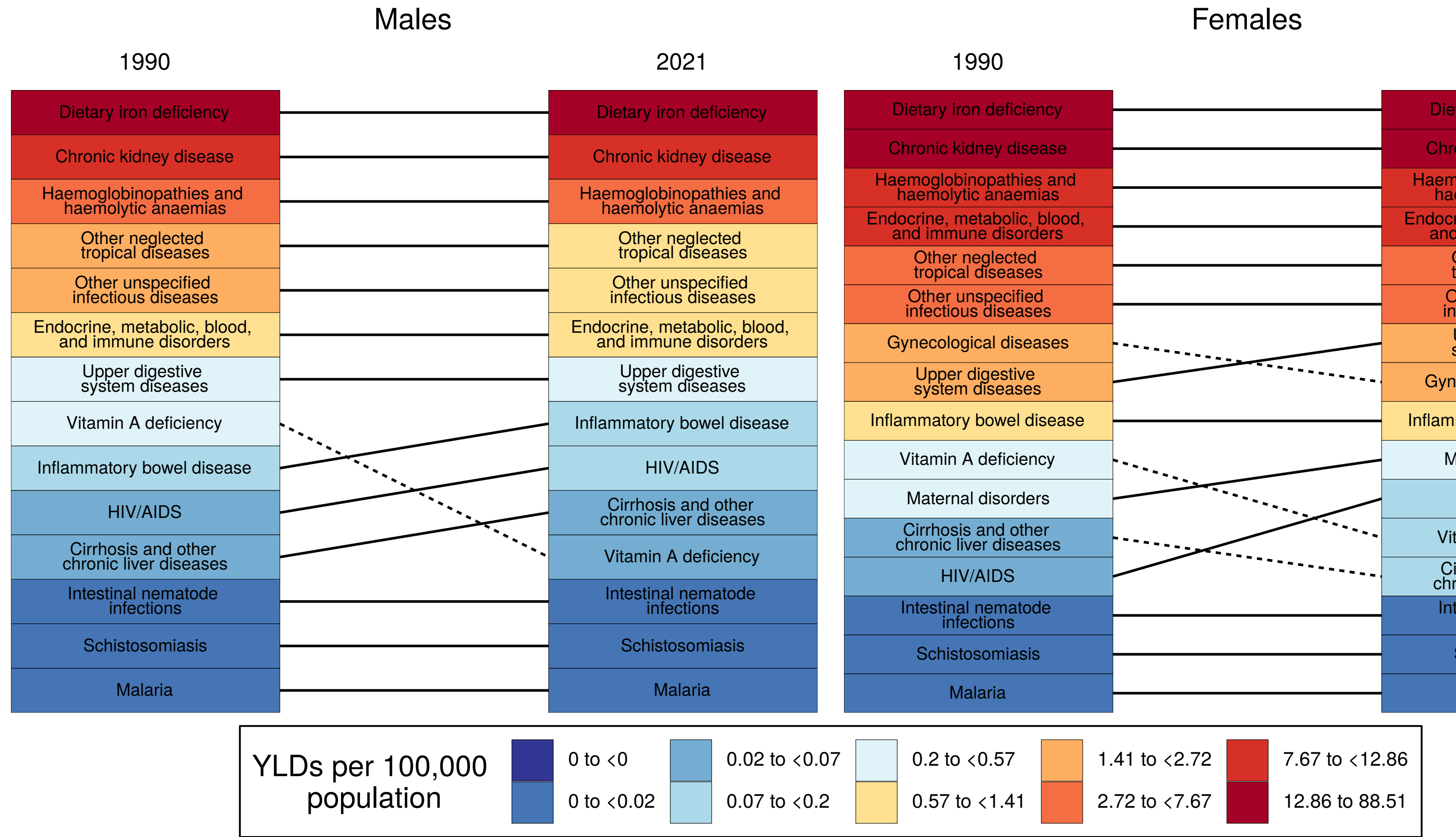

Hampshire

Anaemia Prevalence - Both Sexes

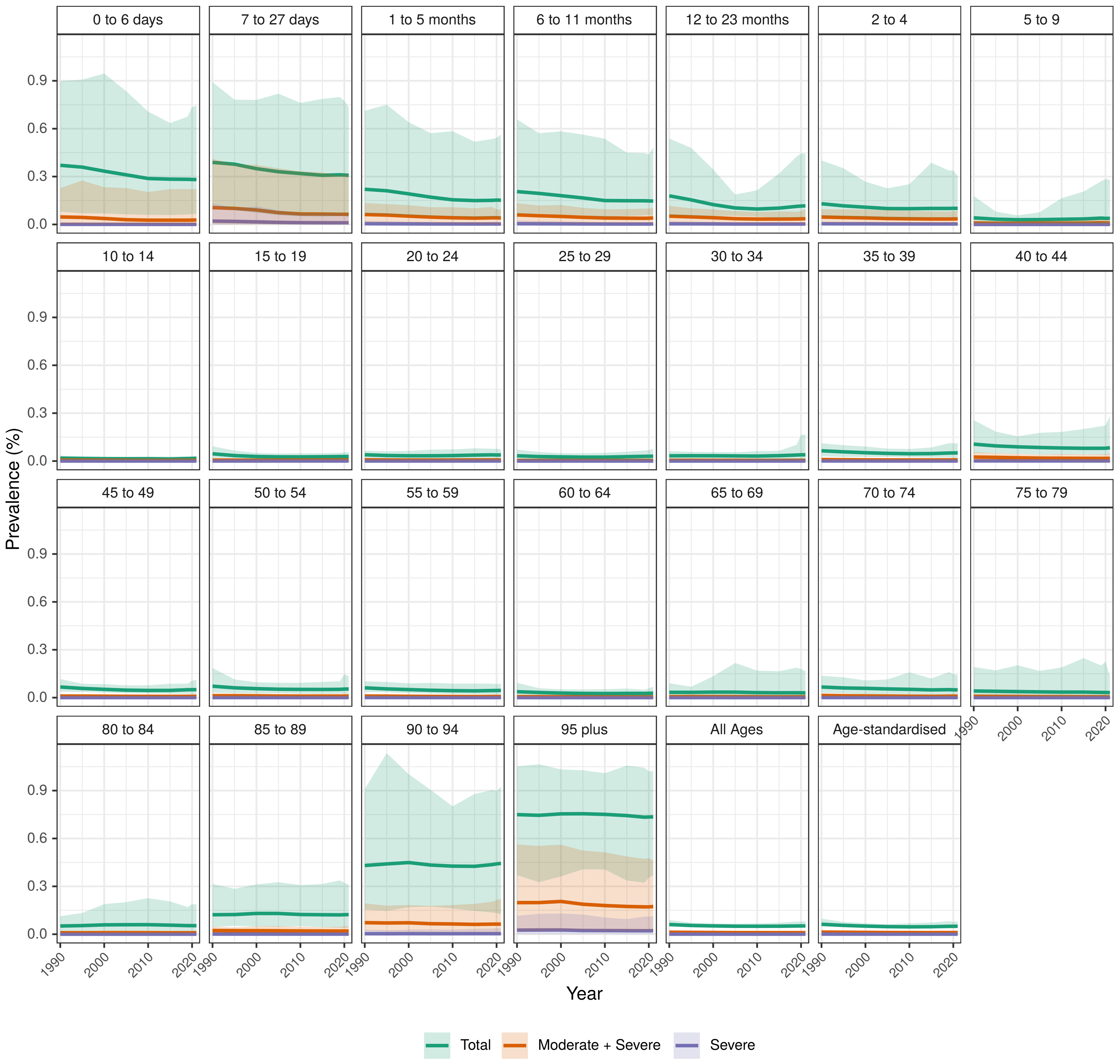

Cause-Specific Anaemia Prevalence - 2021

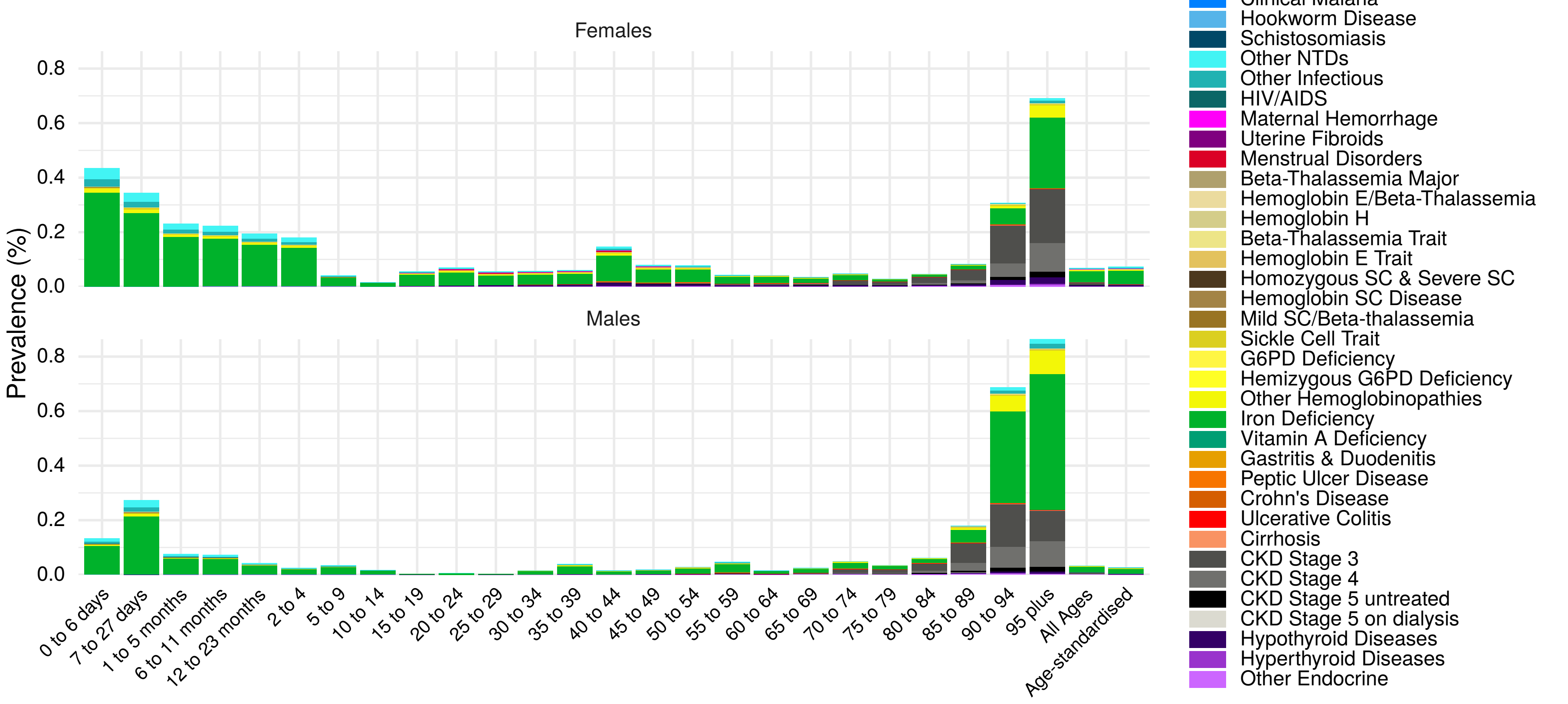

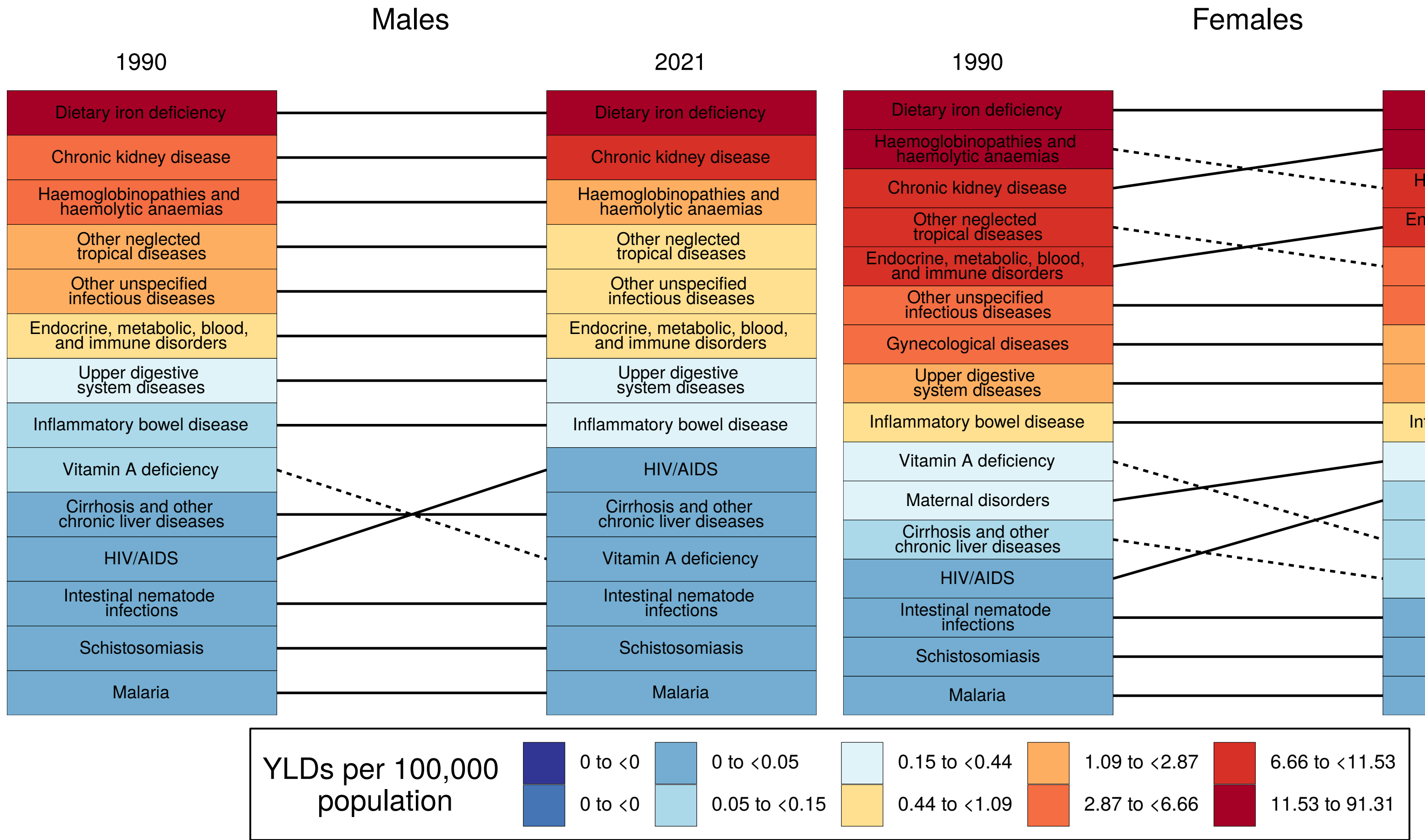

Isle of Wight

Anaemia Prevalence - Both Sexes

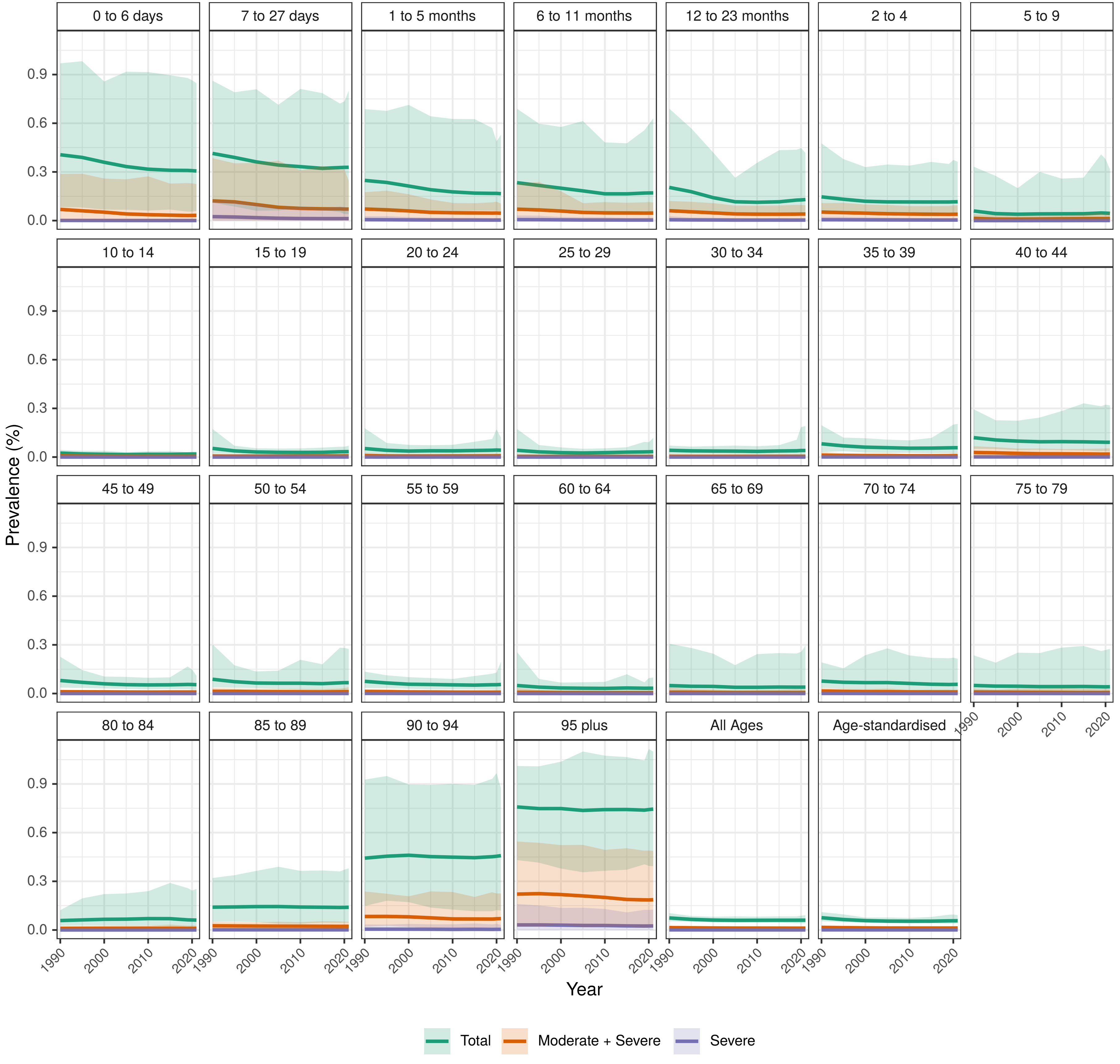

Cause-Specific Anaemia Prevalence - 2021

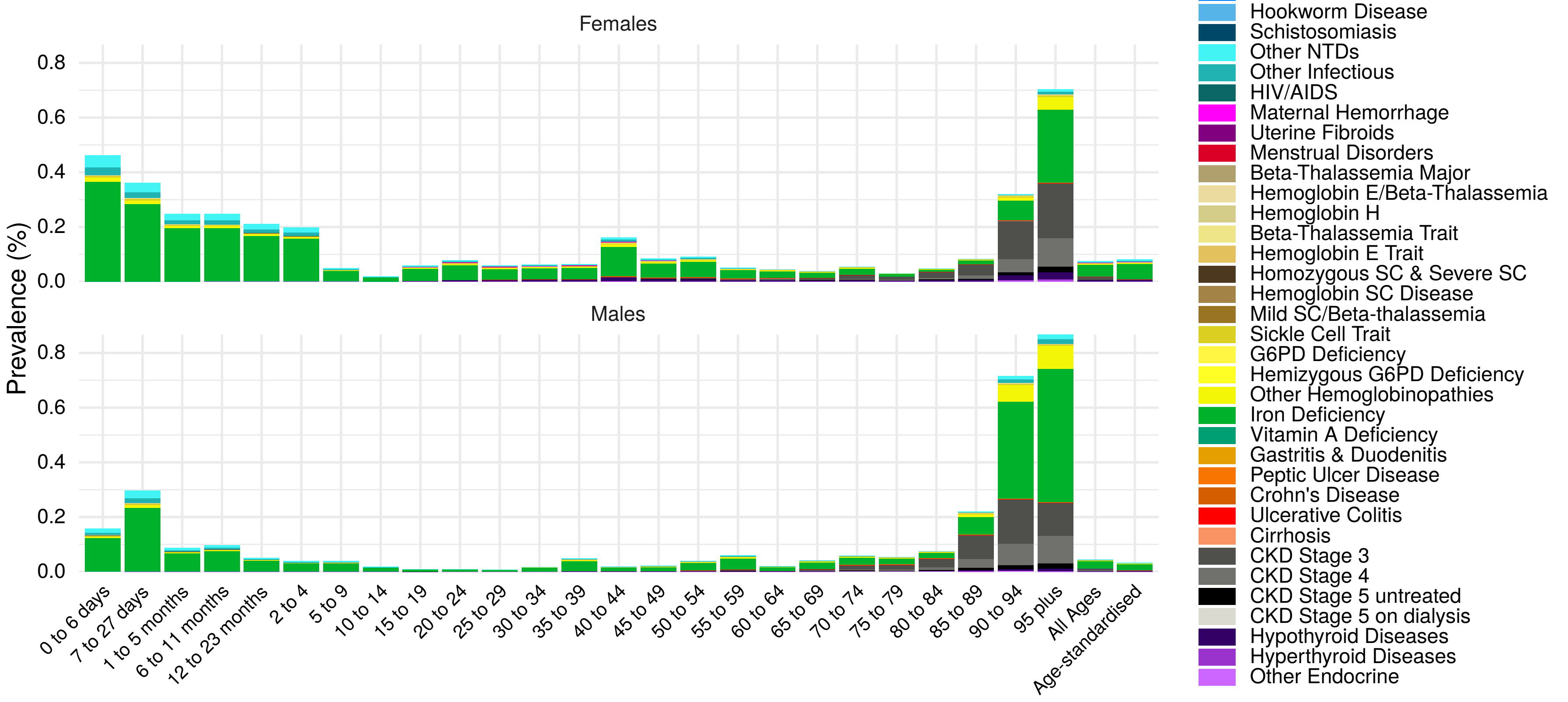

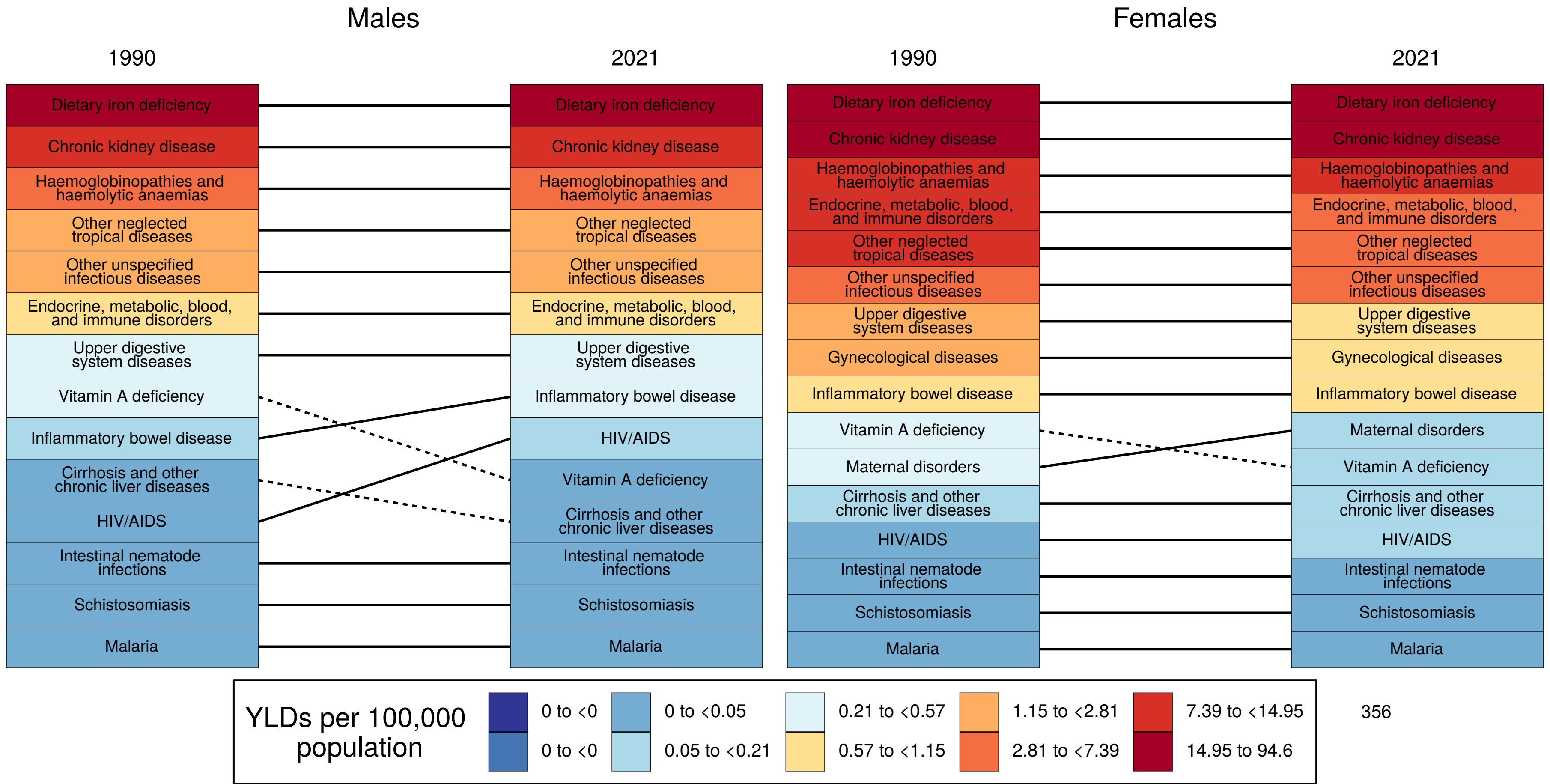

Kent

Anaemia Prevalence - Both Sexes

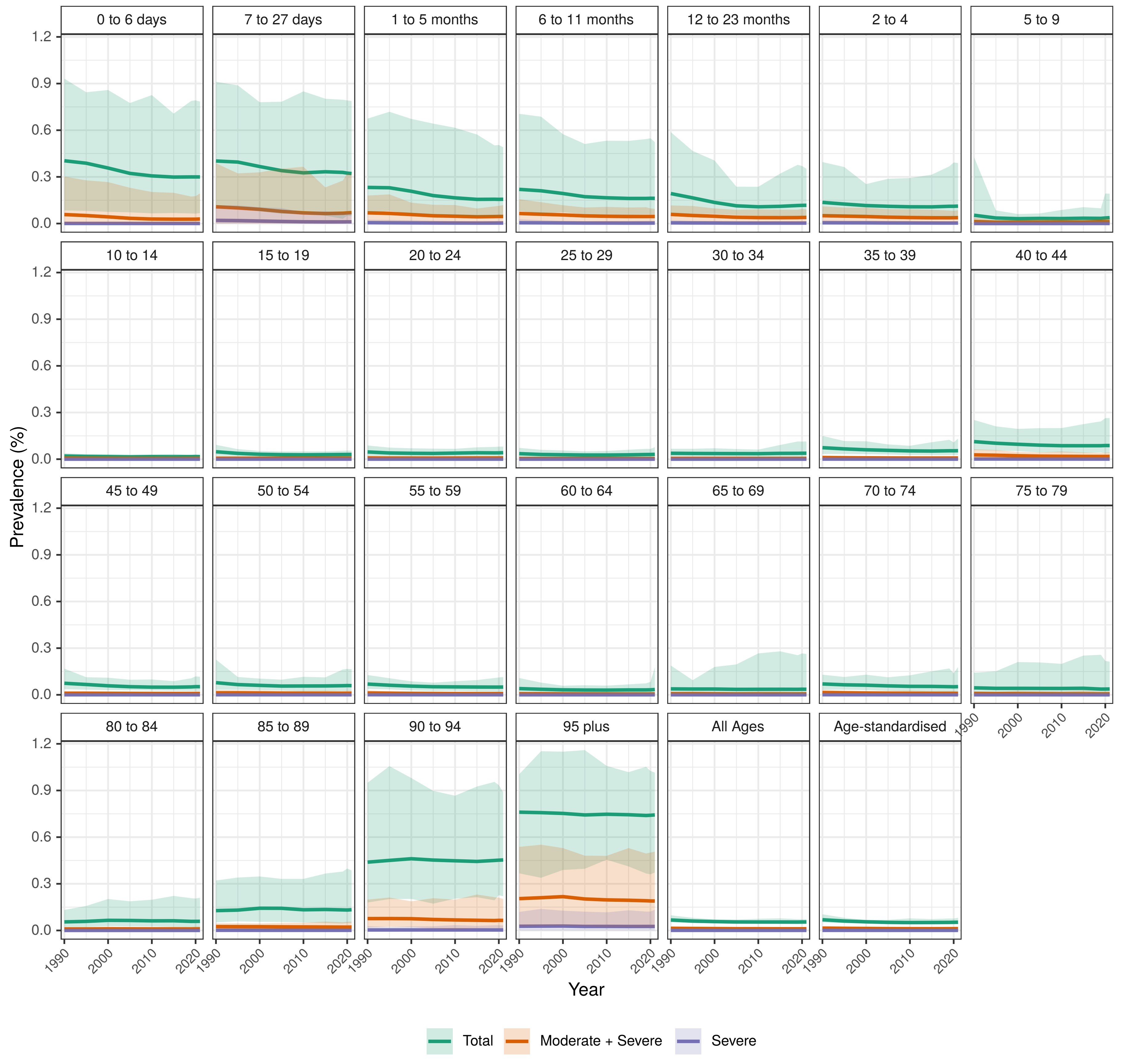

Cause-Specific Anaemia Prevalence - 2021

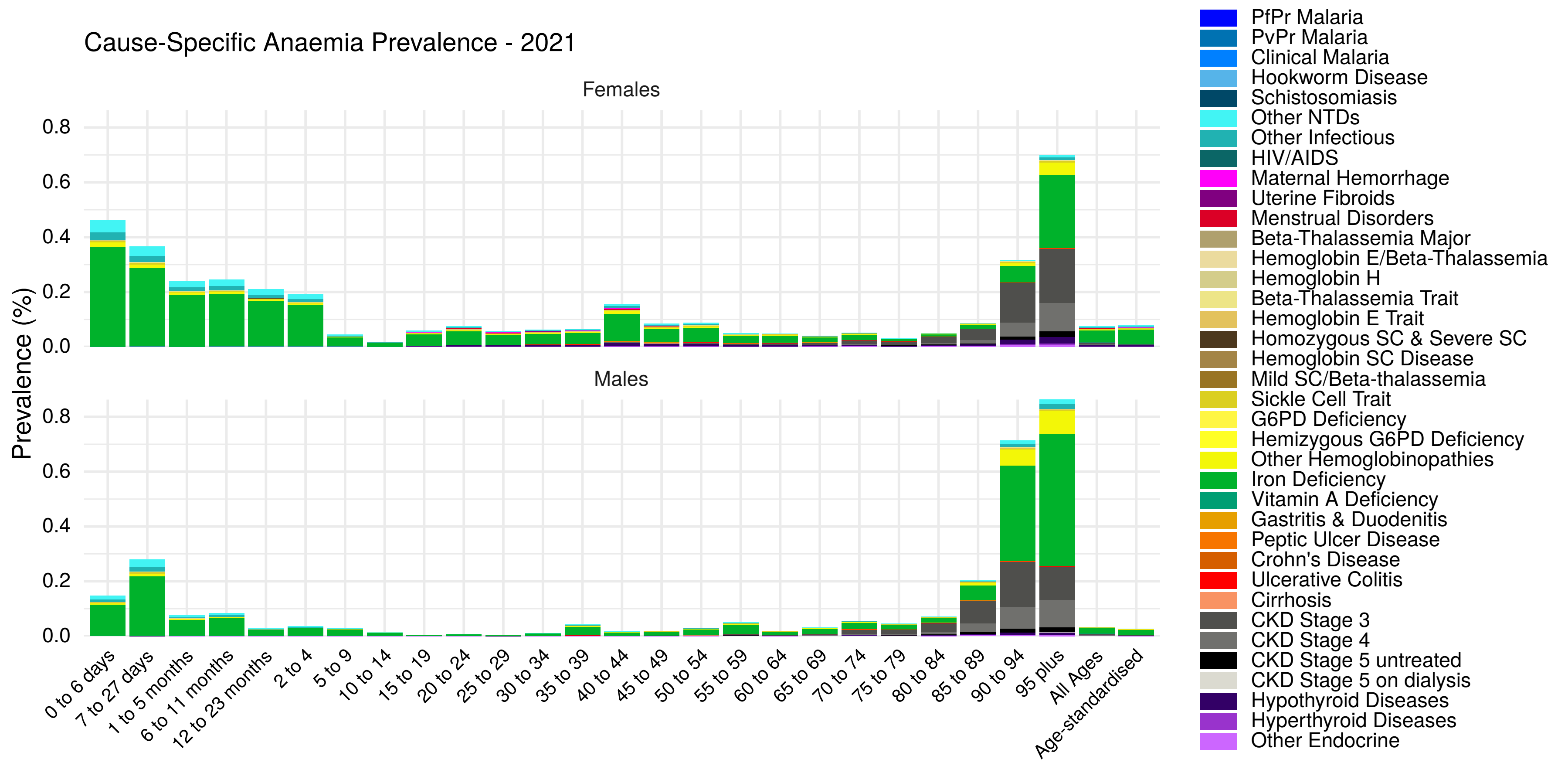

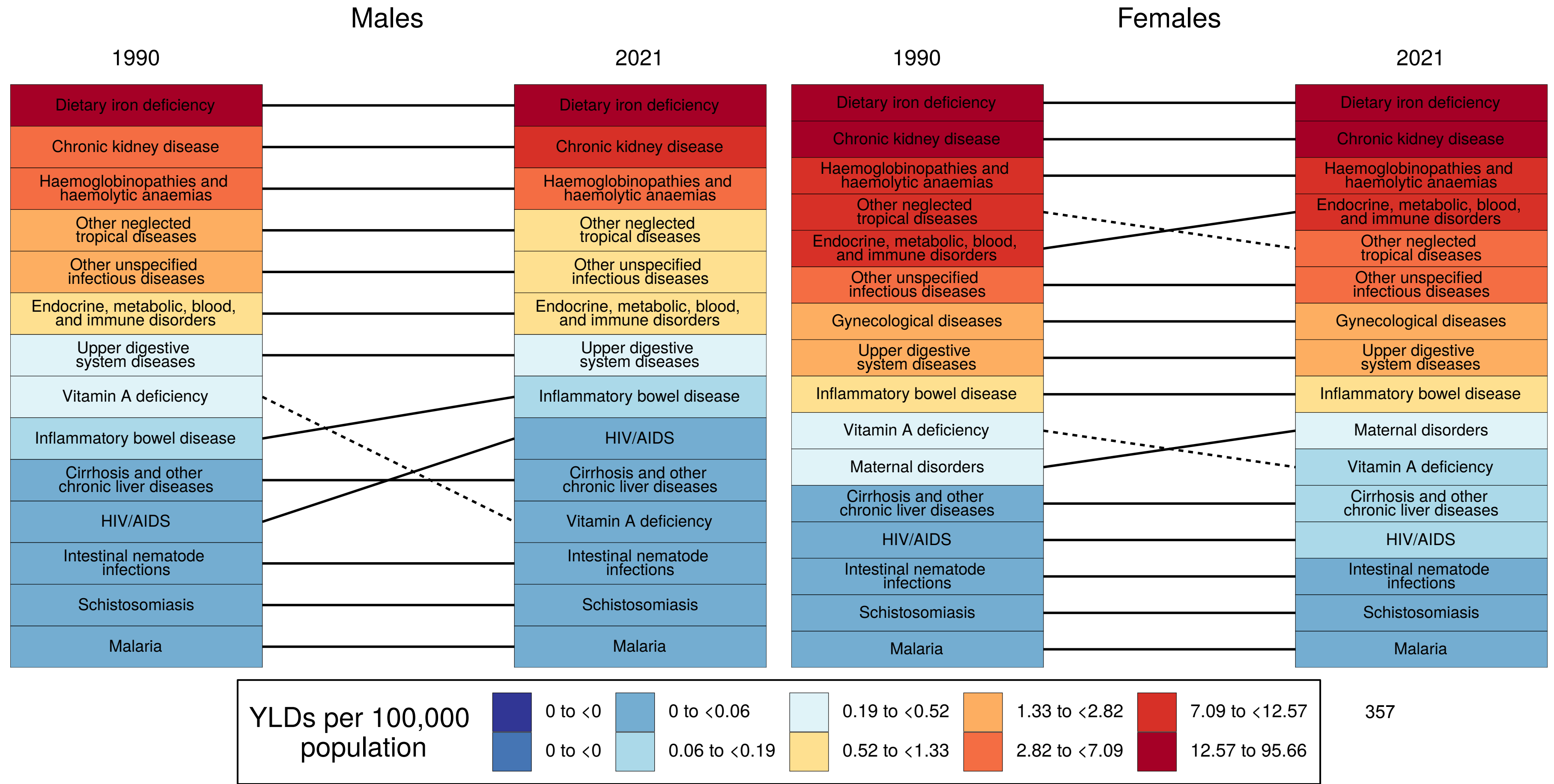

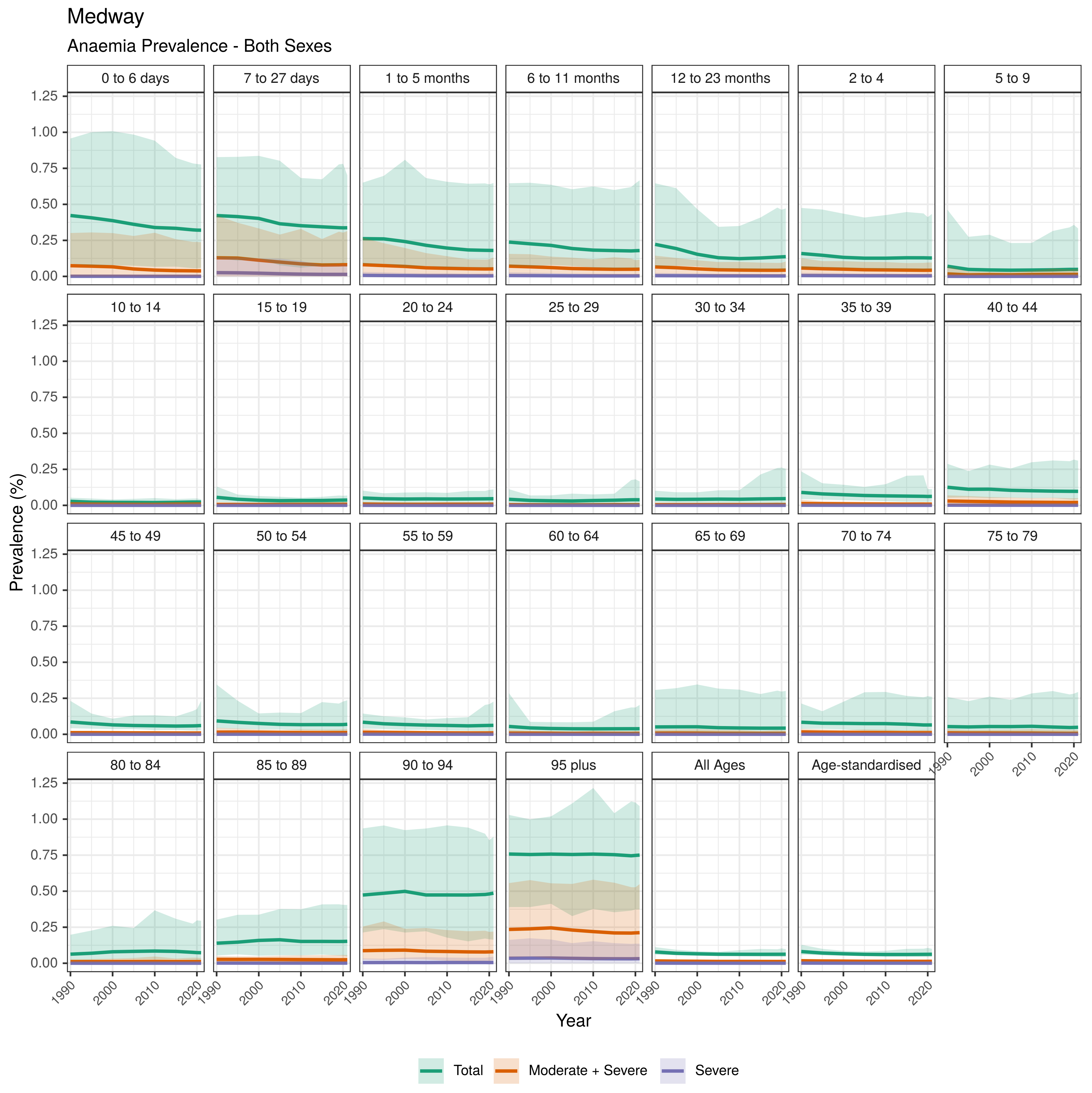
Medway
Anaemia Prevalence - Both Sexes
0 to 6 days
7 to 27 days
1 to 5 months
6 to 11 months
12 to 23 months
2 to 4
5 to 9
10 to 14
15 to 19
20 to 24
25 to 29
30 to 34
35 to 39
40 to 44
45 to 49
50 to 54
55 to 59
60 to 64
65 to 69
70 to 74
75 to 79
80 to 84
85 to 89
90 to 94
95 plus
All Ages
Age-standardised
Prevalence (%)
Year
Total
Moderate + Severe
Severe

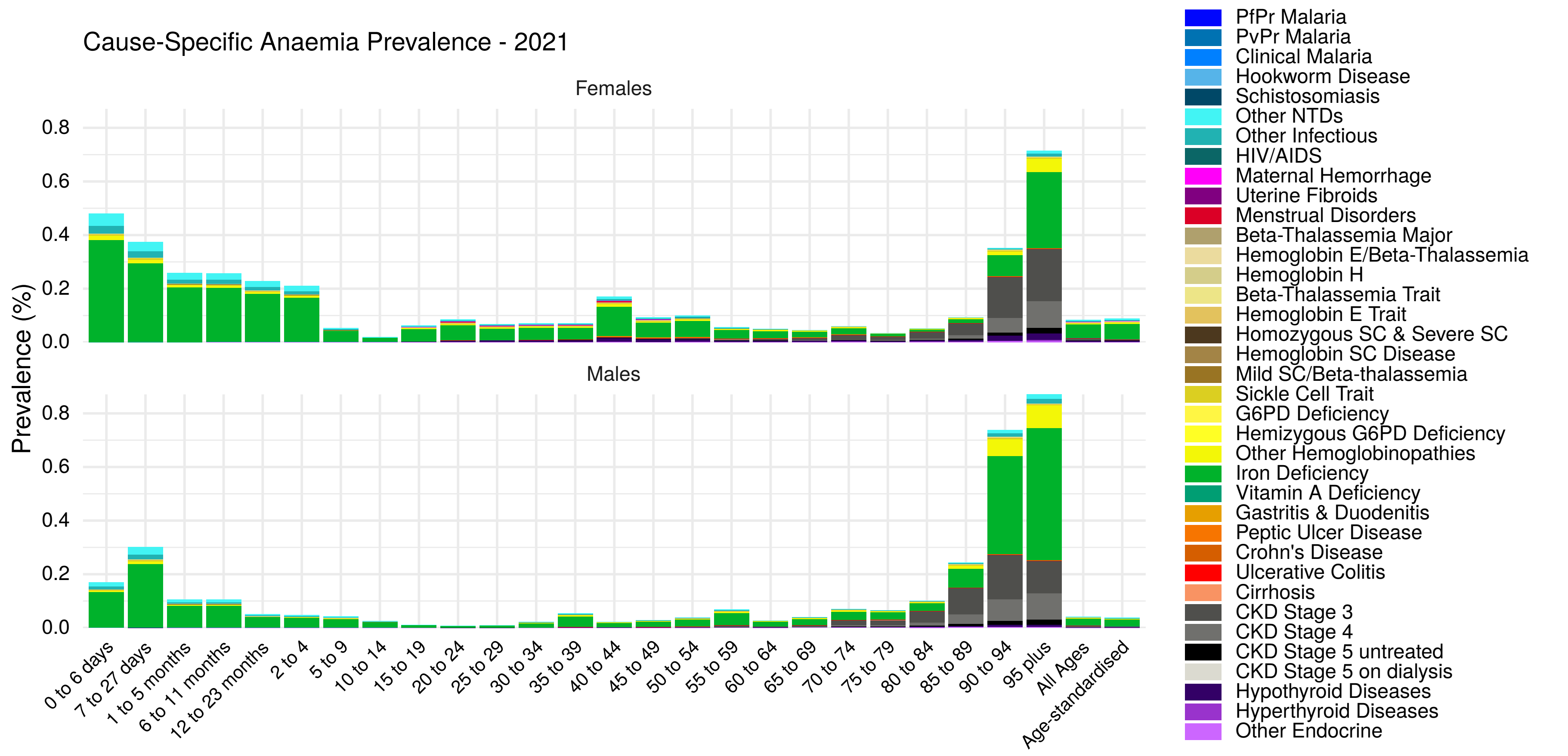
Cause-Specific Anaemia Prevalence - 2021
Females
Males
Prevalence (%)
PfPr Malaria
PvPr Malaria
Clinical Malaria
Hookworm Disease
Schistosomiasis
Other NTDs
Other Infectious
HIV/AIDS
Maternal Hemorrhage
Uterine Fibroids
Menstrual Disorders
Beta-Thalassemia Major
Hemoglobin E/Beta-Thalassemia
Hemoglobin H
Beta-Thalassemia Trait
Hemoglobin E Trait
Homozygous SC & Severe SC
Hemoglobin SC Disease
Mild SC/Beta-thalassemia
Sickle Cell Trait
G6PD Deficiency
Hemizygous G6PD Deficiency
Other Hemoglobinopathies
Iron Deficiency
Vitamin A Deficiency
Gastritis & Duodenitis
Peptic Ulcer Disease
Crohn's Disease
Ulcerative Colitis
Cirrhosis
CKD Stage 3
CKD Stage 4
CKD Stage 5 untreated
CKD Stage 5 on dialysis
Hypothyroid Diseases
Hyperthyroid Diseases
Other Endocrine

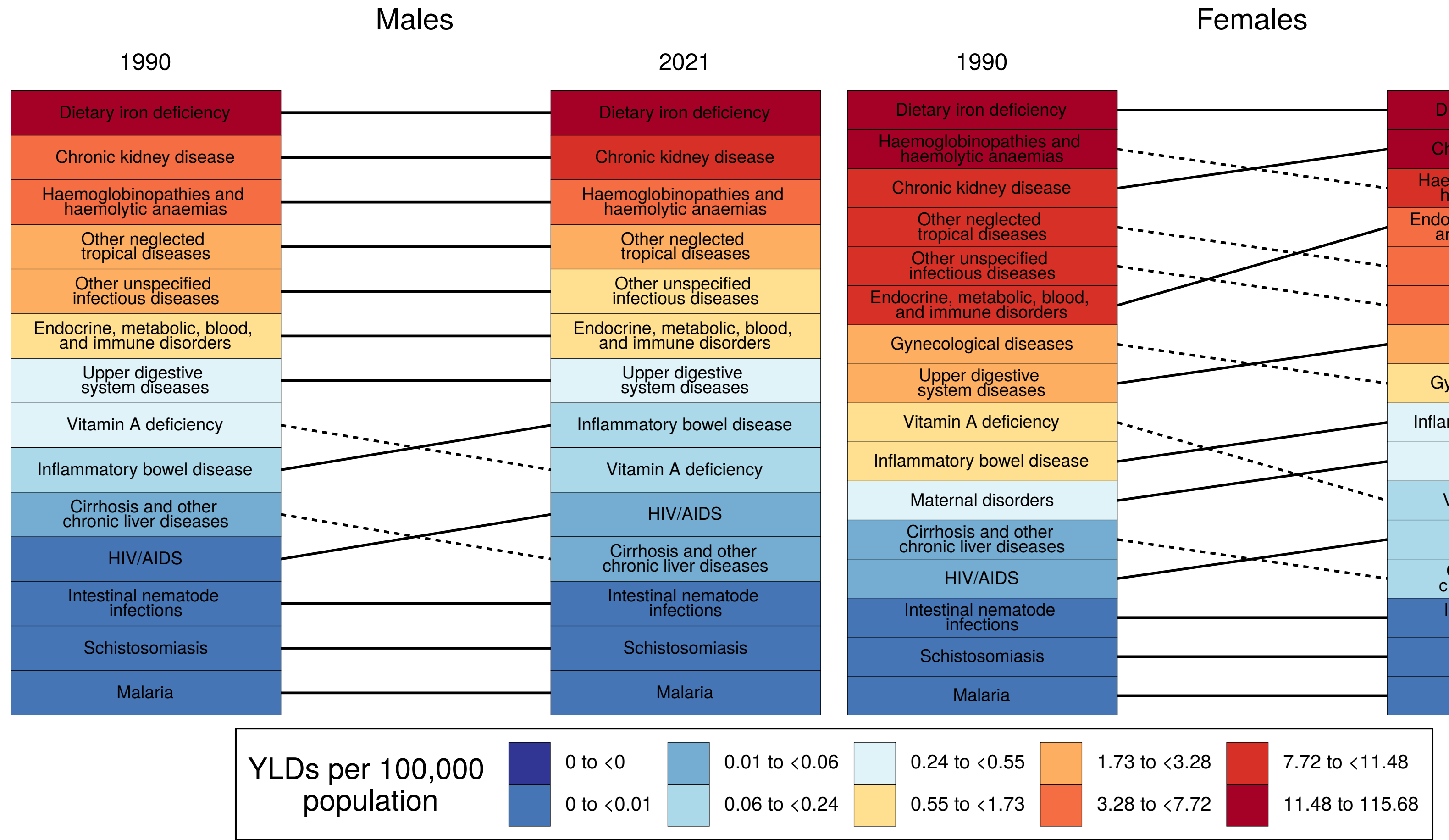
Males
Females
1990
2021
Dietary iron deficiency
Chronic kidney disease
Haemoglobinopathies and haemolytic anaemias
Other neglected tropical diseases
Other unspecified infectious diseases
Endocrine, metabolic, blood, and immune disorders
Upper digestive system diseases
Vitamin A deficiency
Inflammatory bowel disease
Cirrhosis and other chronic liver diseases
HIV/AIDS
Intestinal nematode infections
Schistosomiasis
Malaria
Gynecological diseases
Maternal disorders
YLDs per 100,000 population
0 to <0
0 to <0.01
0.01 to <0.06
0.06 to <0.24
0.24 to <0.55
0.55 to <1.73
1.73 to <3.28
3.28 to <7.72
7.72 to <11.48
11.48 to 115.68

Milton Keynes

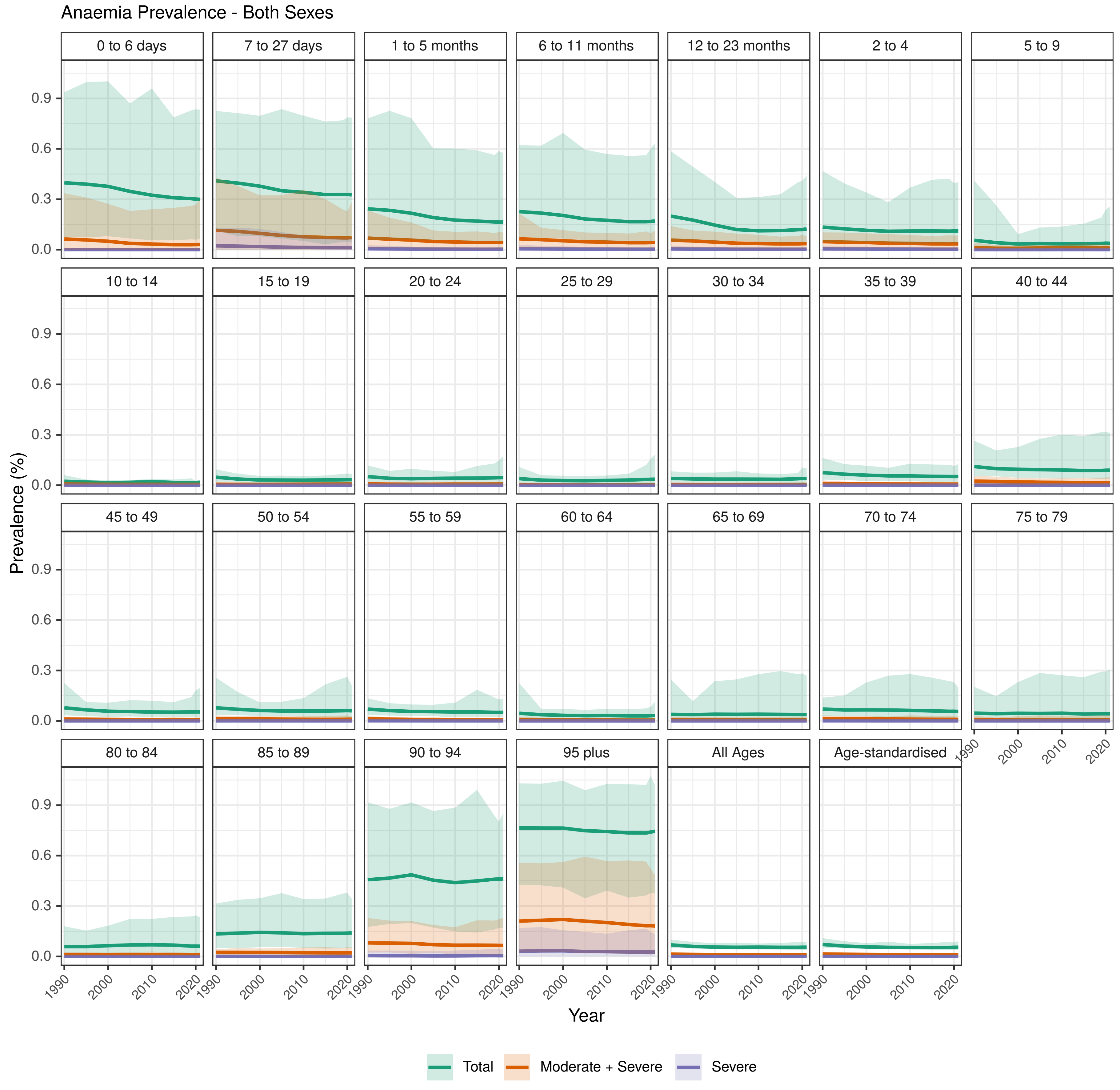

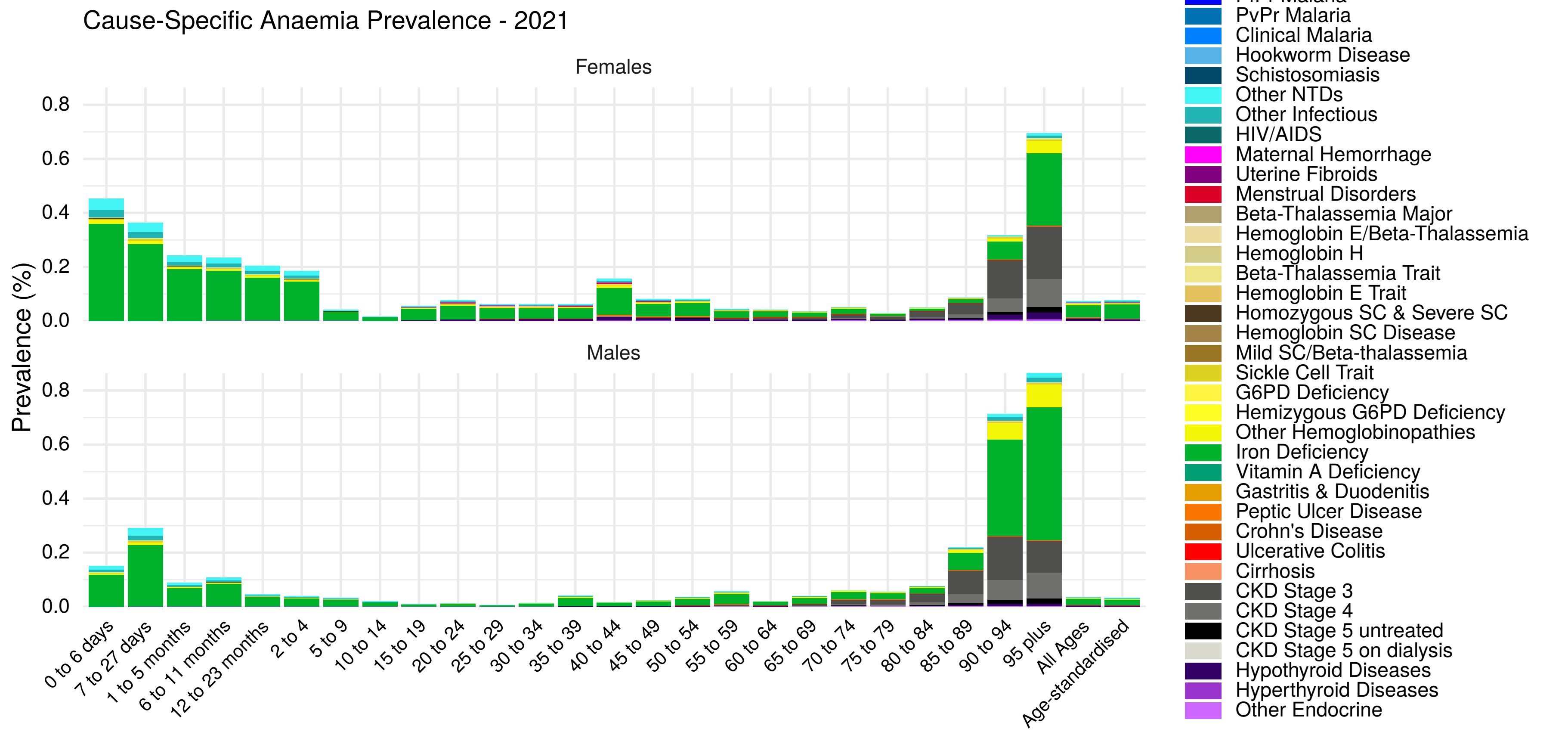

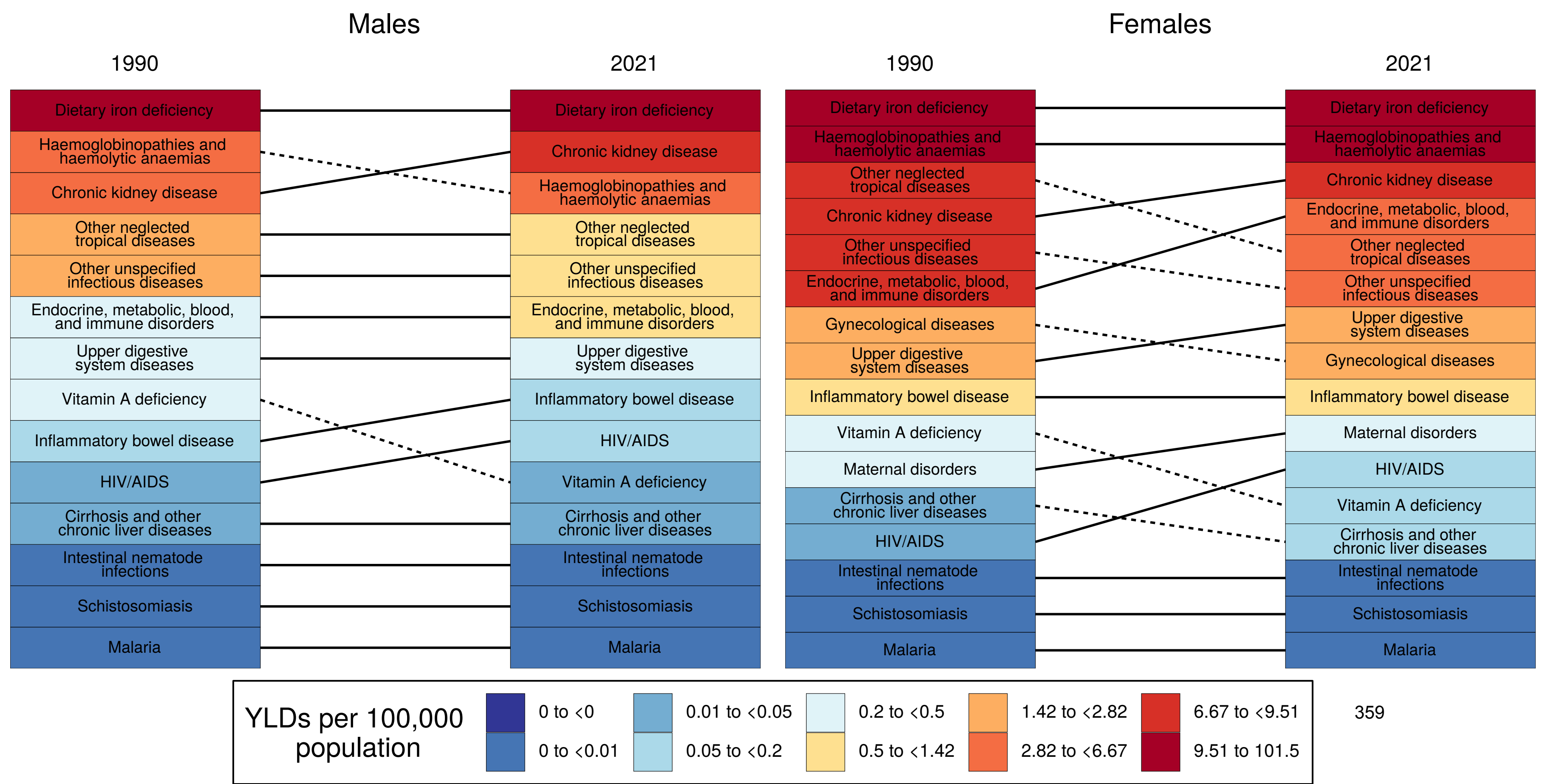

Oxfordshire

Anaemia Prevalence - Both Sexes

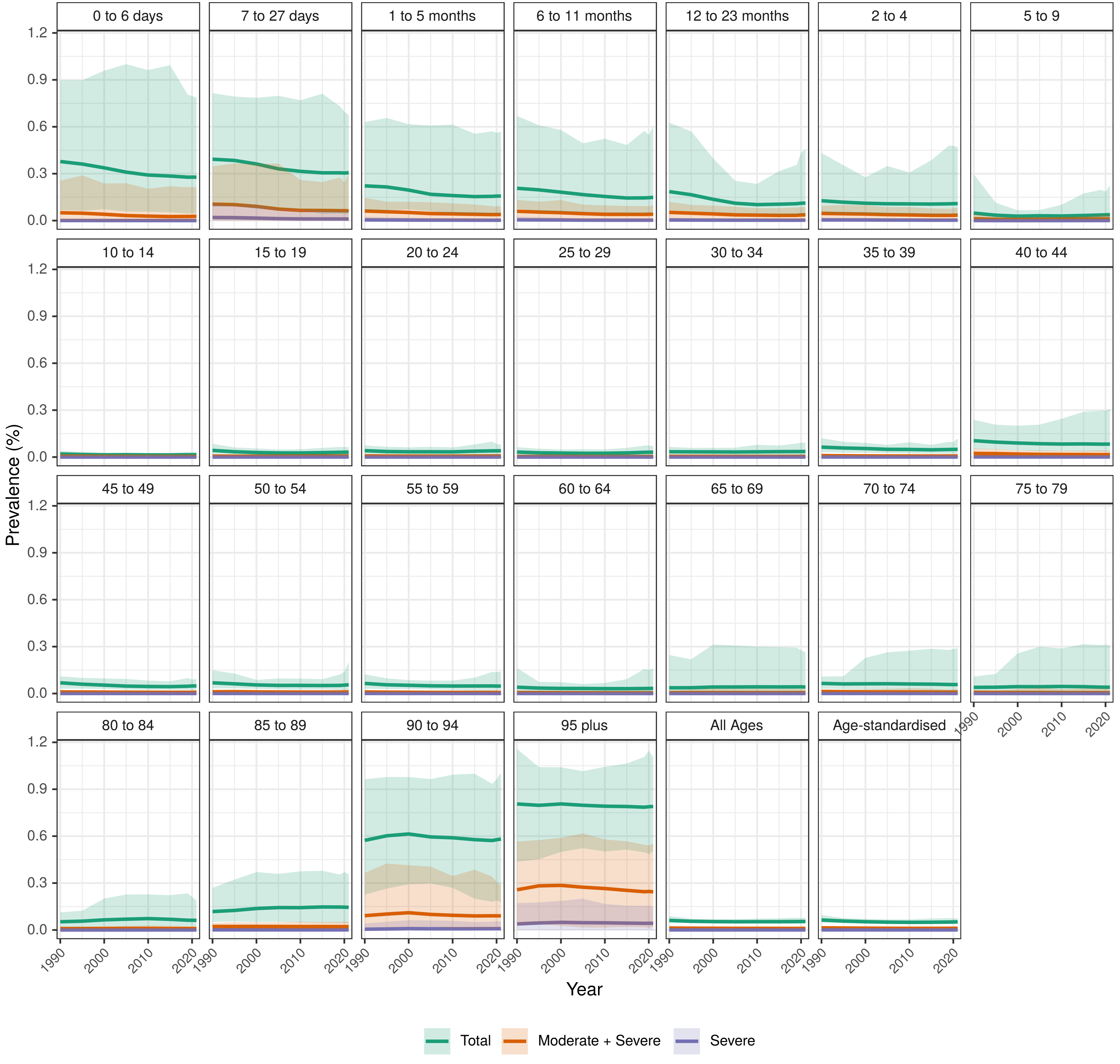

Cause-Specific Anaemia Prevalence - 2021

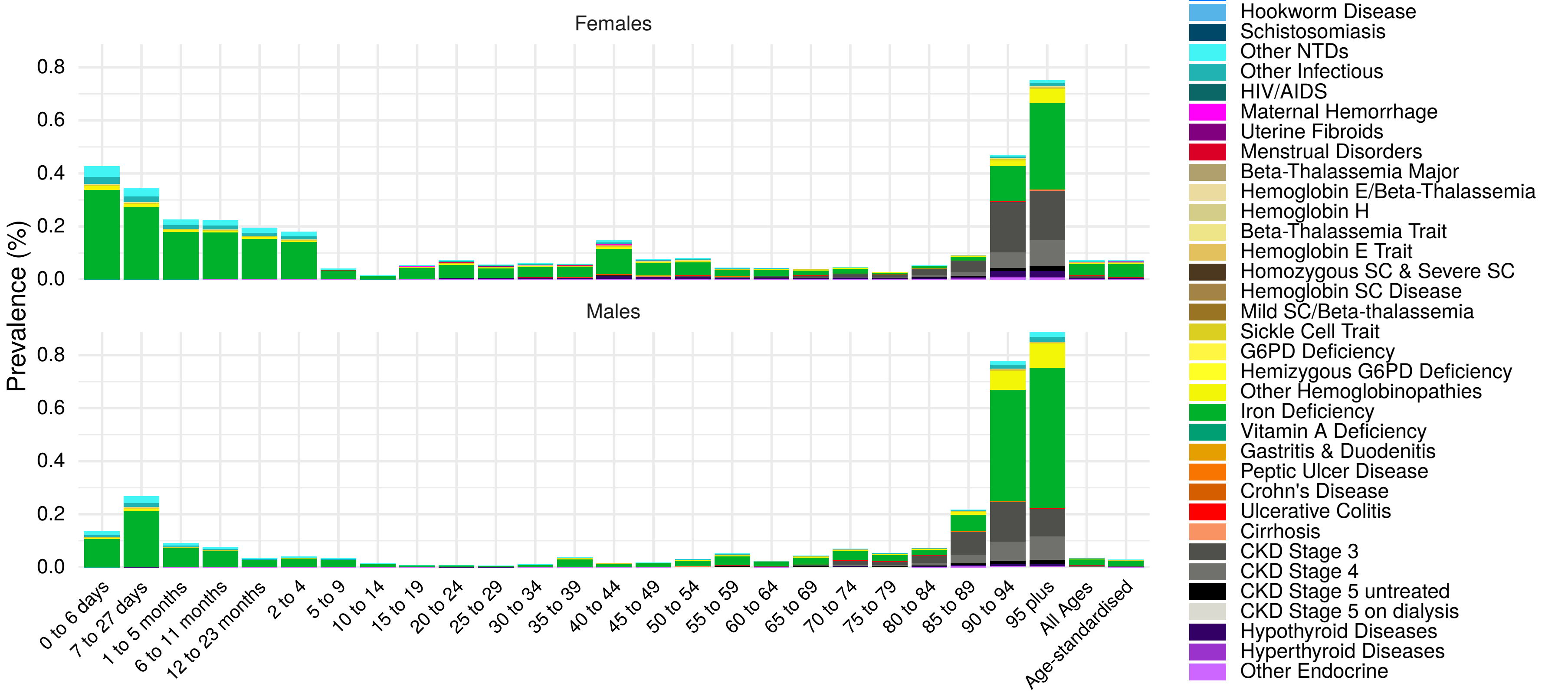

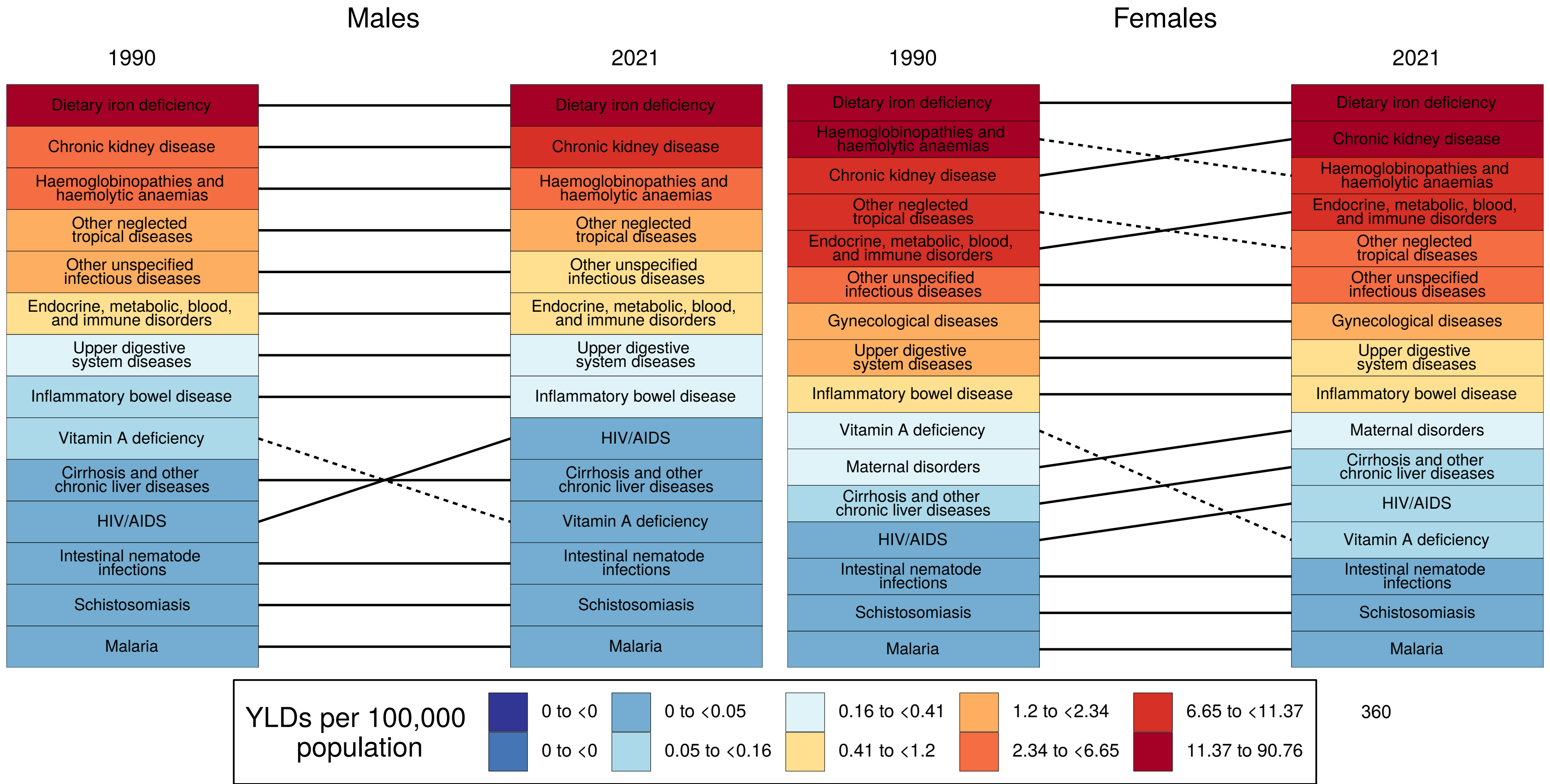

Portsmouth

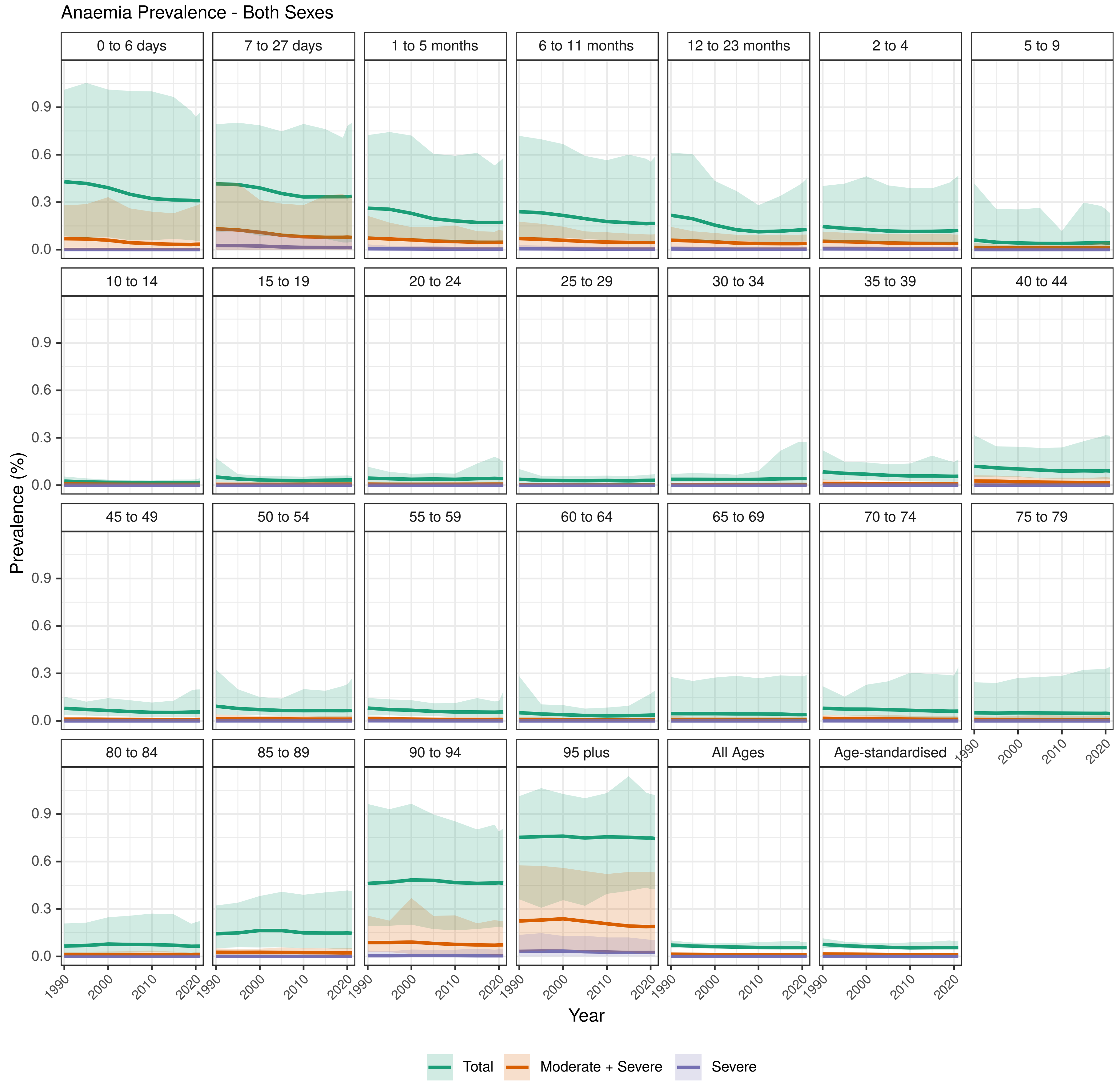

Anaemia Prevalence - Both Sexes
0 to 6 days
7 to 27 days
1 to 5 months
6 to 11 months
12 to 23 months
2 to 4
5 to 9
10 to 14
15 to 19
20 to 24
25 to 29
30 to 34
35 to 39
40 to 44
45 to 49
50 to 54
55 to 59
60 to 64
65 to 69
70 to 74
75 to 79
80 to 84
85 to 89
90 to 94
95 plus
All Ages
Age-standardised
Prevalence (%)
Year
Total
Moderate + Severe
Severe

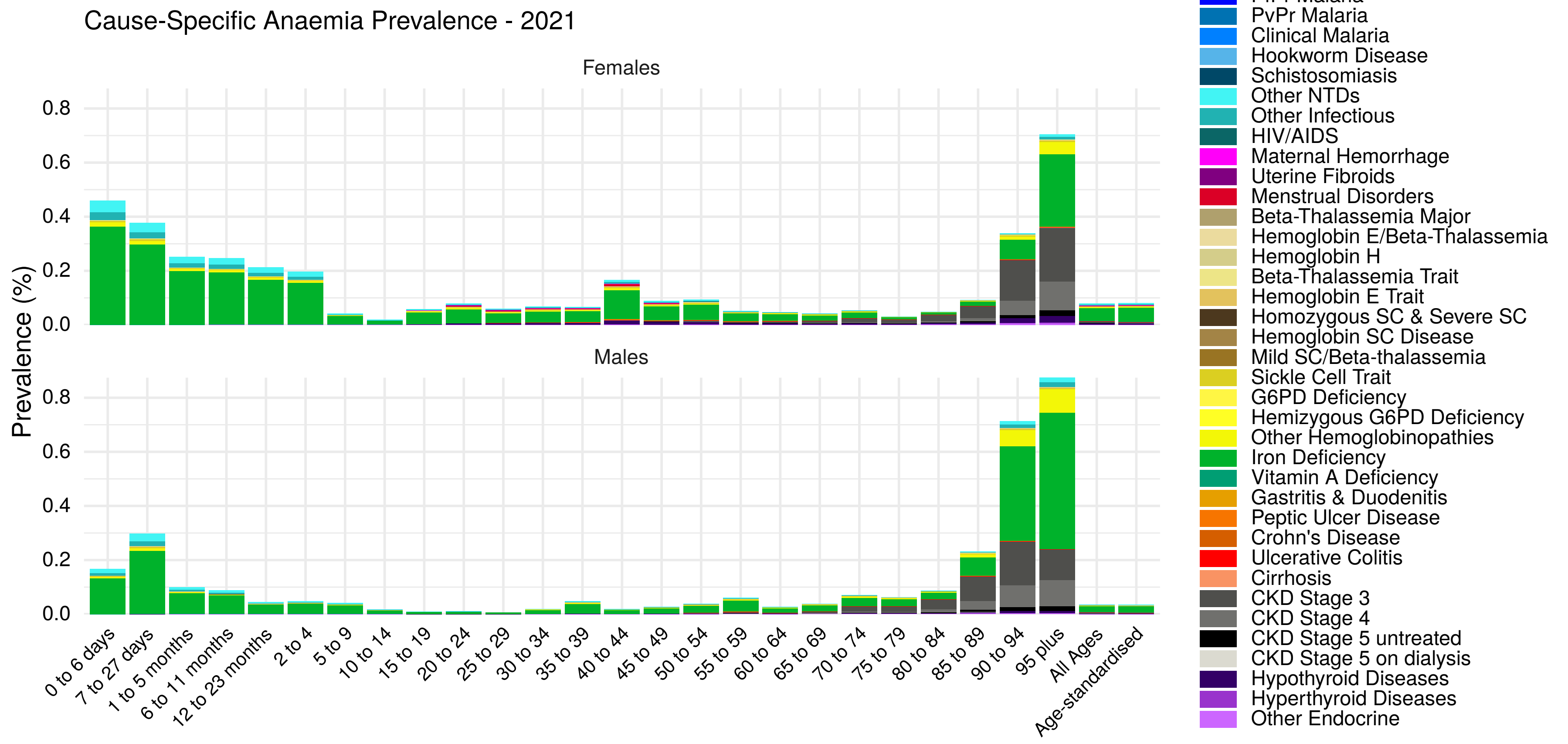

Cause-Specific Anaemia Prevalence - 2021
Females
Males
Prevalence (%)
PfPr Malaria
PvPr Malaria
Clinical Malaria
Hookworm Disease
Schistosomiasis
Other NTDs
Other Infectious
HIV/AIDS
Maternal Hemorrhage
Uterine Fibroids
Menstrual Disorders
Beta-Thalassemia Major
Hemoglobin E/Beta-Thalassemia
Hemoglobin H
Beta-Thalassemia Trait
Hemoglobin E Trait
Homozygous SC & Severe SC
Hemoglobin SC Disease
Mild SC/Beta-thalassemia
Sickle Cell Trait
G6PD Deficiency
Hemizygous G6PD Deficiency
Other Hemoglobinopathies
Iron Deficiency
Vitamin A Deficiency
Gastritis & Duodenitis
Peptic Ulcer Disease
Crohn's Disease
Ulcerative Colitis
Cirrhosis
CKD Stage 3
CKD Stage 4
CKD Stage 5 untreated
CKD Stage 5 on dialysis
Hypothyroid Diseases
Hyperthyroid Diseases
Other Endocrine

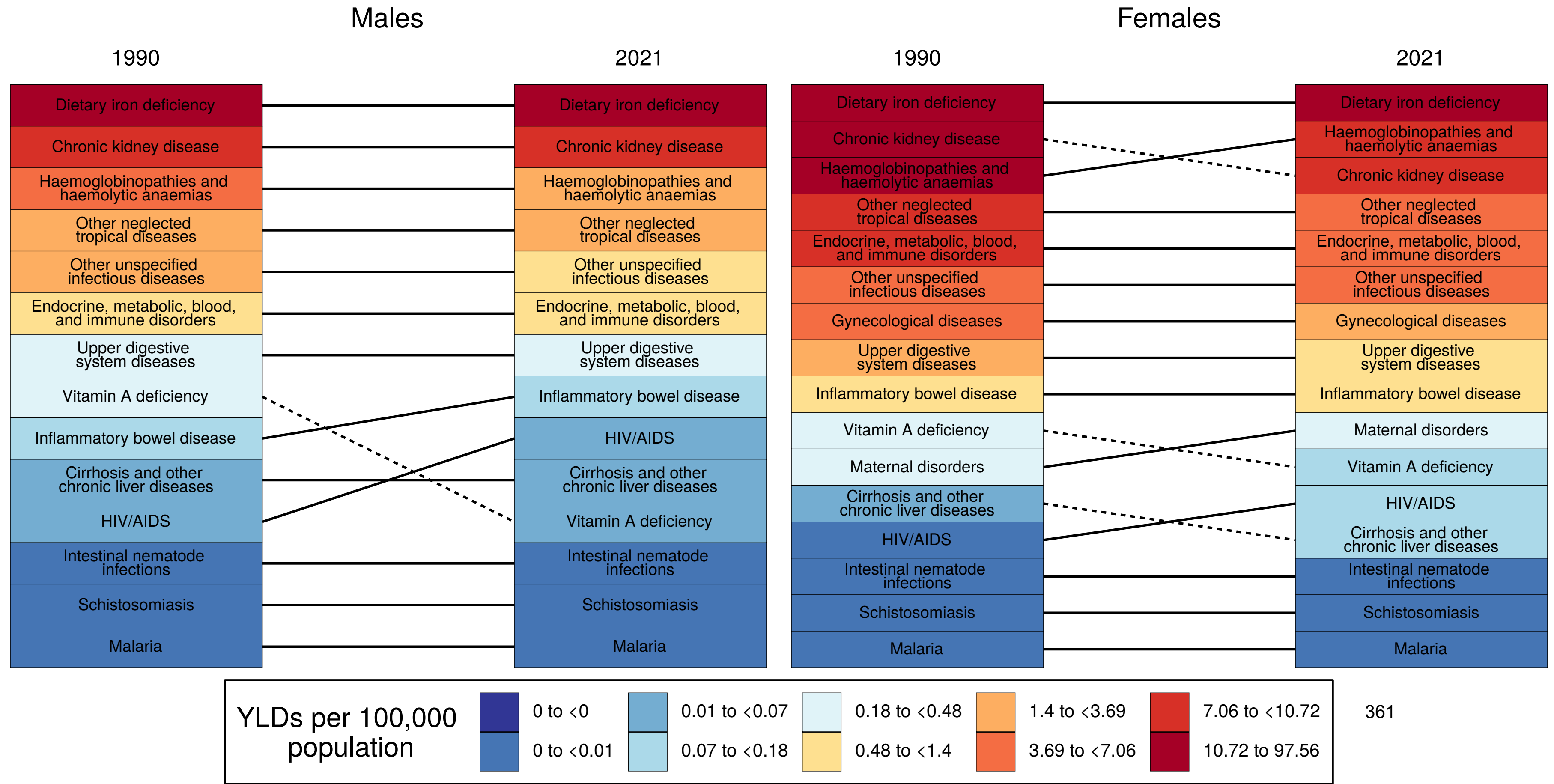

Males
Females
1990
2021
Dietary iron deficiency
Chronic kidney disease
Haemoglobinopathies and haemolytic anaemias
Other neglected tropical diseases
Other unspecified infectious diseases
Endocrine, metabolic, blood, and immune disorders
Upper digestive system diseases
Vitamin A deficiency
Inflammatory bowel disease
Cirrhosis and other chronic liver diseases
HIV/AIDS
Intestinal nematode infections
Schistosomiasis
Malaria
Gynecological diseases
Maternal disorders
YLDs per 100,000 population
0 to <0
0 to <0.01
0.01 to <0.07
0.07 to <0.18
0.18 to <0.48
0.48 to <1.4
1.4 to <3.69
3.69 to <7.06
7.06 to <10.72
10.72 to 97.56

Reading

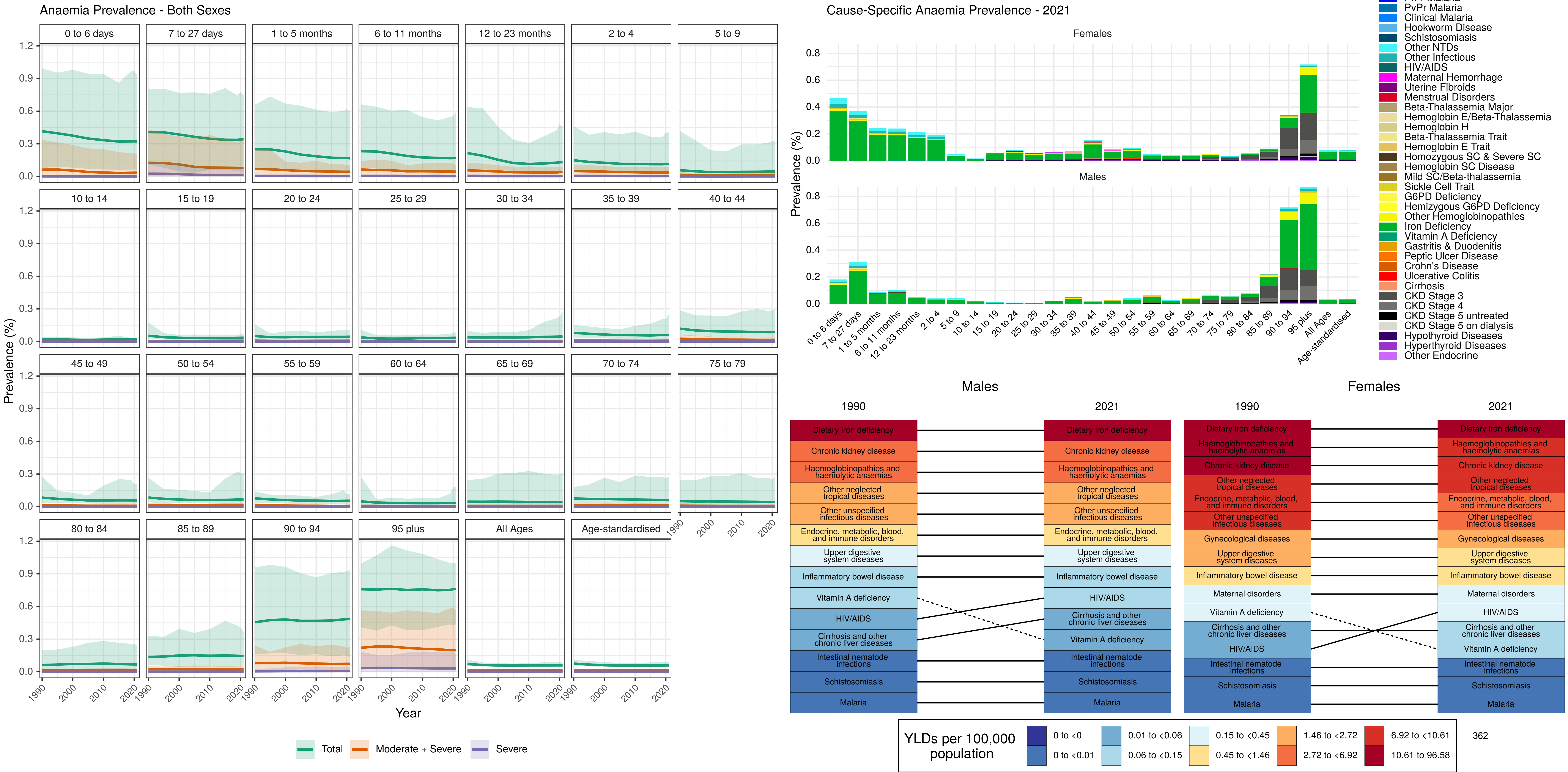

Anaemia Prevalence - Both Sexes
0 to 6 days
7 to 27 days
1 to 5 months
6 to 11 months
12 to 23 months
2 to 4
5 to 9
10 to 14
15 to 19
20 to 24
25 to 29
30 to 34
35 to 39
40 to 44
45 to 49
50 to 54
55 to 59
60 to 64
65 to 69
70 to 74
75 to 79
80 to 84
85 to 89
90 to 94
95 plus
All Ages
Age-standardised
Prevalence (%)
Year
Total
Moderate + Severe
Severe
Cause-Specific Anaemia Prevalence - 2021
Females
Males
PfPr Malaria
PvPr Malaria
Clinical Malaria
Hookworm Disease
Schistosomiasis
Other NTDs
Other Infectious
HIV/AIDS
Maternal Hemorrhage
Uterine Fibroids
Menstrual Disorders
Beta-Thalassemia Major
Hemoglobin E/Beta-Thalassemia
Hemoglobin H
Beta-Thalassemia Trait
Hemoglobin E Trait
Homozygous SC & Severe SC
Hemoglobin SC Disease
Mild SC/Beta-thalassemia
Sickle Cell Trait
G6PD Deficiency
Hemizygous G6PD Deficiency
Other Hemoglobinopathies
Iron Deficiency
Vitamin A Deficiency
Gastritis & Duodenitis
Peptic Ulcer Disease
Crohn's Disease
Ulcerative Colitis
Cirrhosis
CKD Stage 3
CKD Stage 4
CKD Stage 5 untreated
CKD Stage 5 on dialysis
Hypothyroid Diseases
Hyperthyroid Diseases
Other Endocrine
Males
Females
1990
2021
Dietary iron deficiency
Chronic kidney disease
Haemoglobinopathies and haemolytic anaemias
Other neglected tropical diseases
Other unspecified infectious diseases
Endocrine, metabolic, blood, and immune disorders
Upper digestive system diseases
Inflammatory bowel disease
Vitamin A deficiency
HIV/AIDS
Cirrhosis and other chronic liver diseases
Intestinal nematode infections
Schistosomiasis
Malaria
Gynecological diseases
Maternal disorders
YLDs per 100,000 population
0 to <0
0 to <0.01
0.01 to <0.06
0.06 to <0.15
0.15 to <0.45
0.45 to <1.46
1.46 to <2.72
2.72 to <6.92
6.92 to <10.61
10.61 to 96.58

Slough

Anaemia Prevalence - Both Sexes

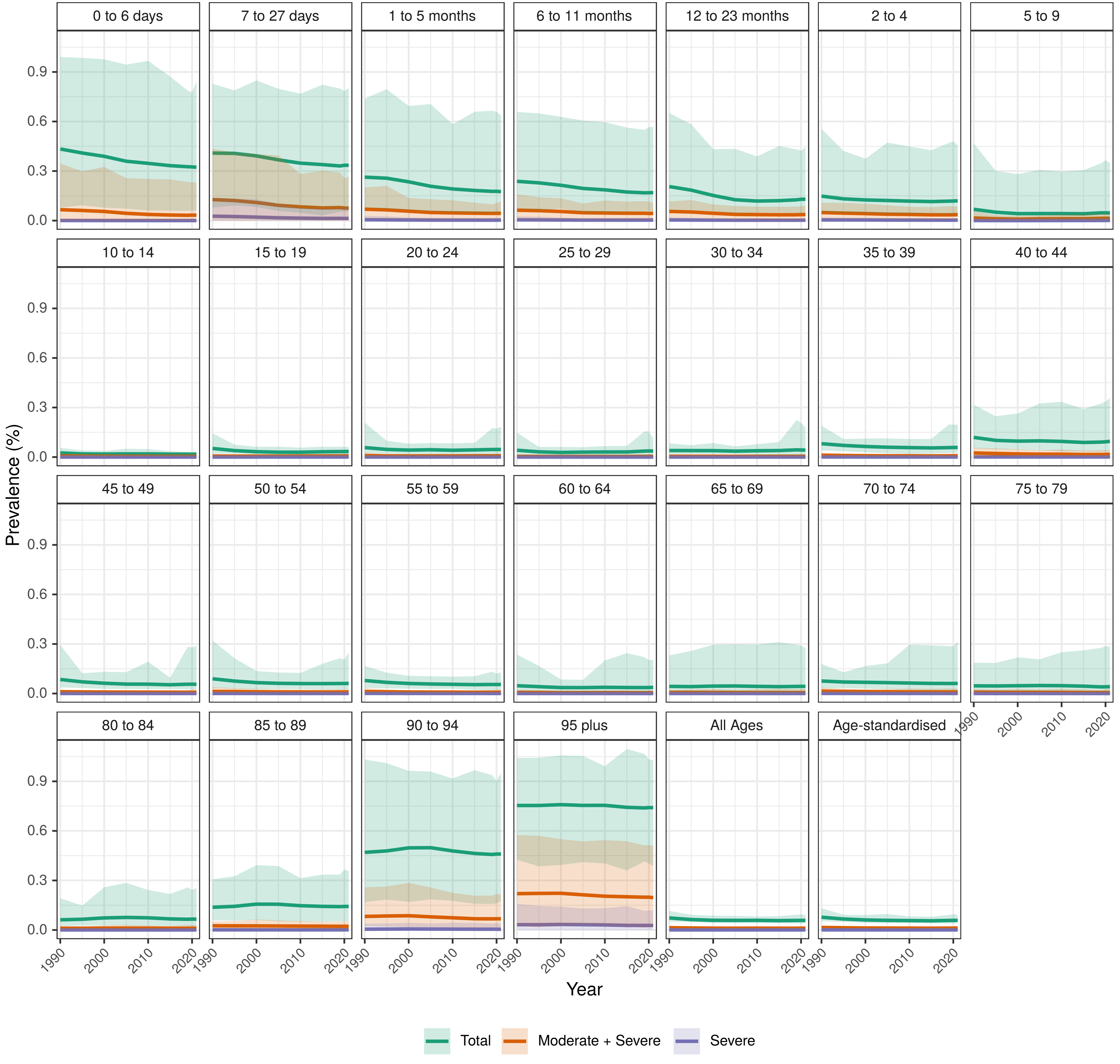

Cause-Specific Anaemia Prevalence - 2021

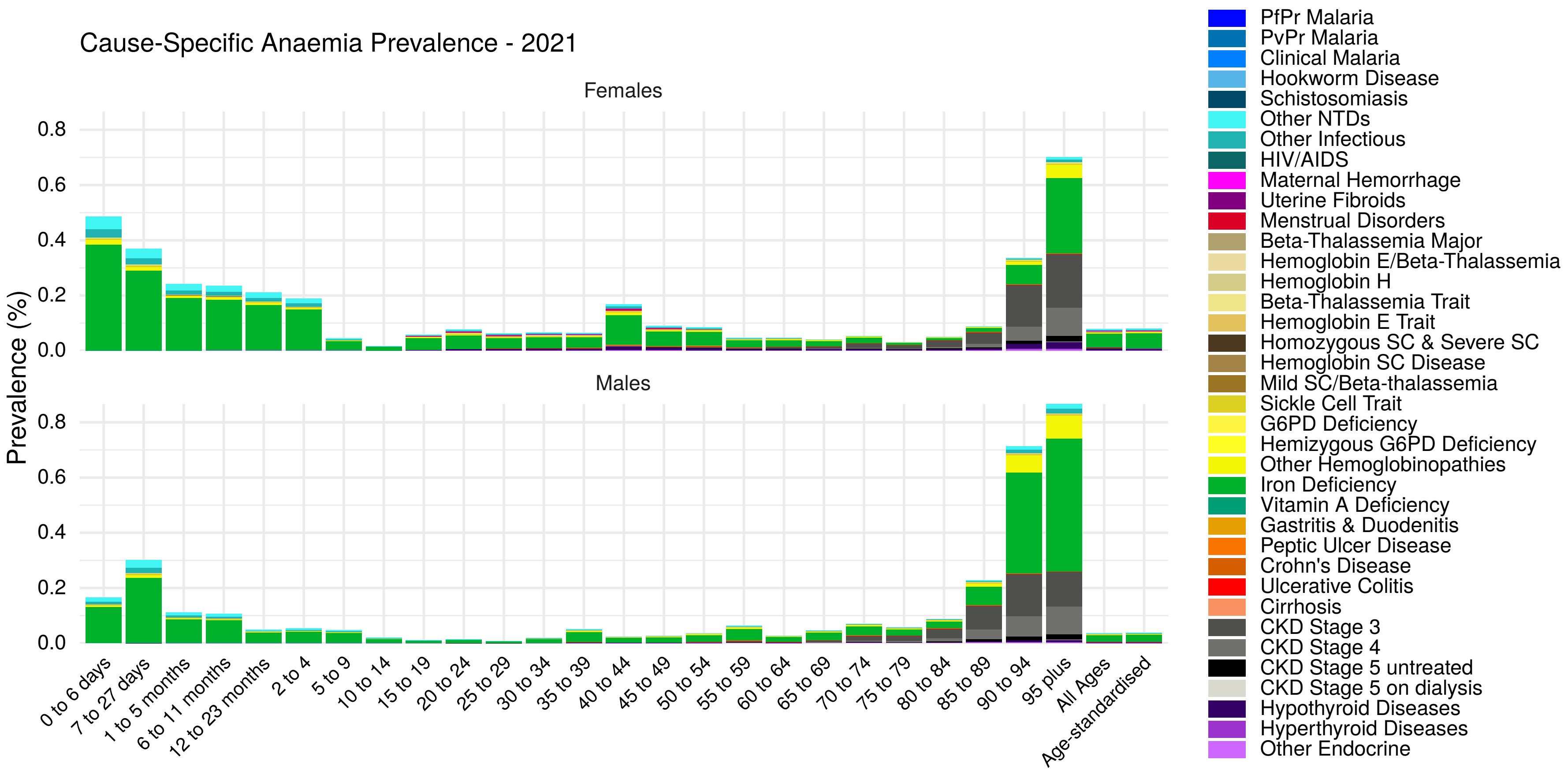

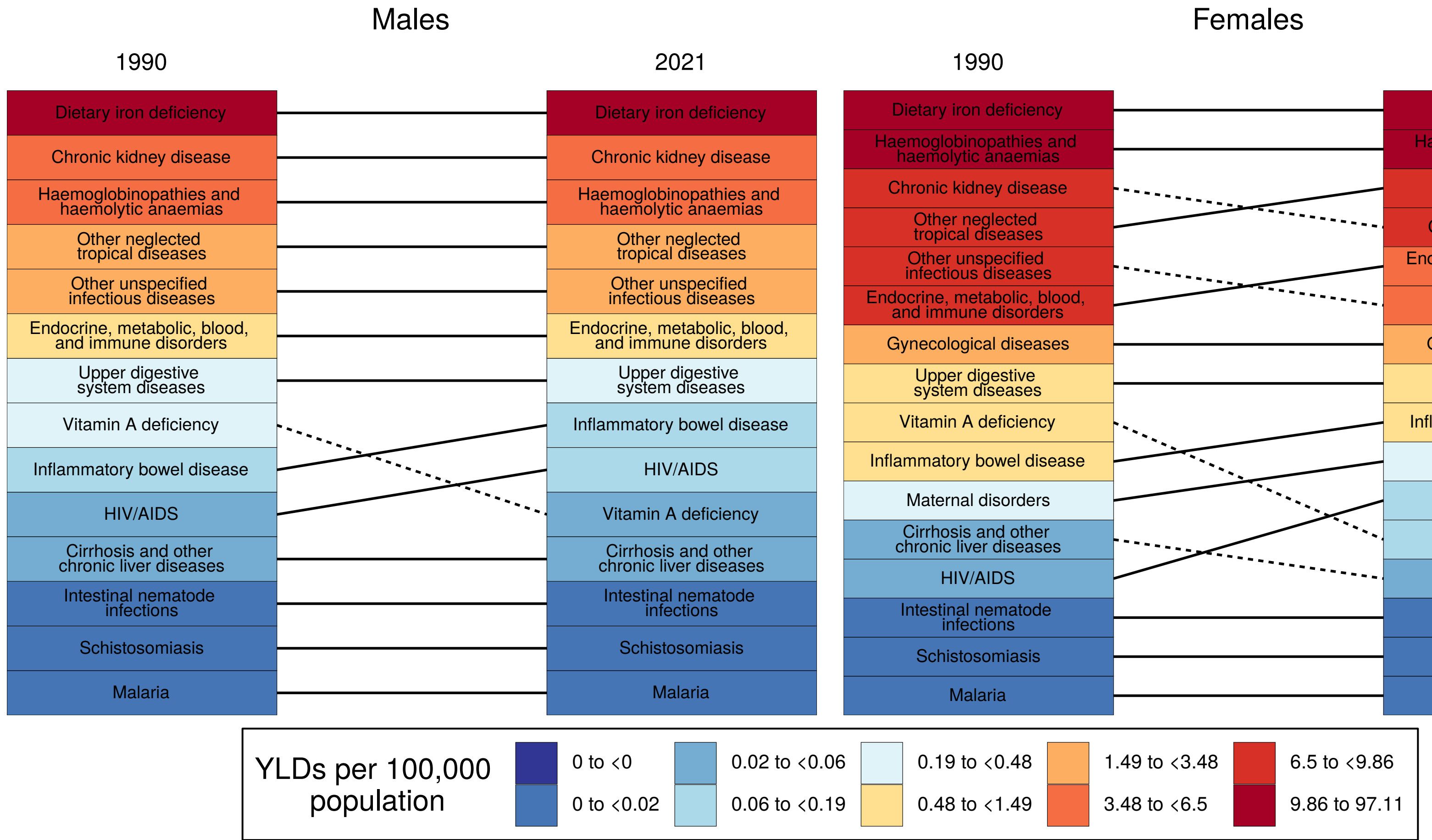

Southampton

Anaemia Prevalence - Both Sexes

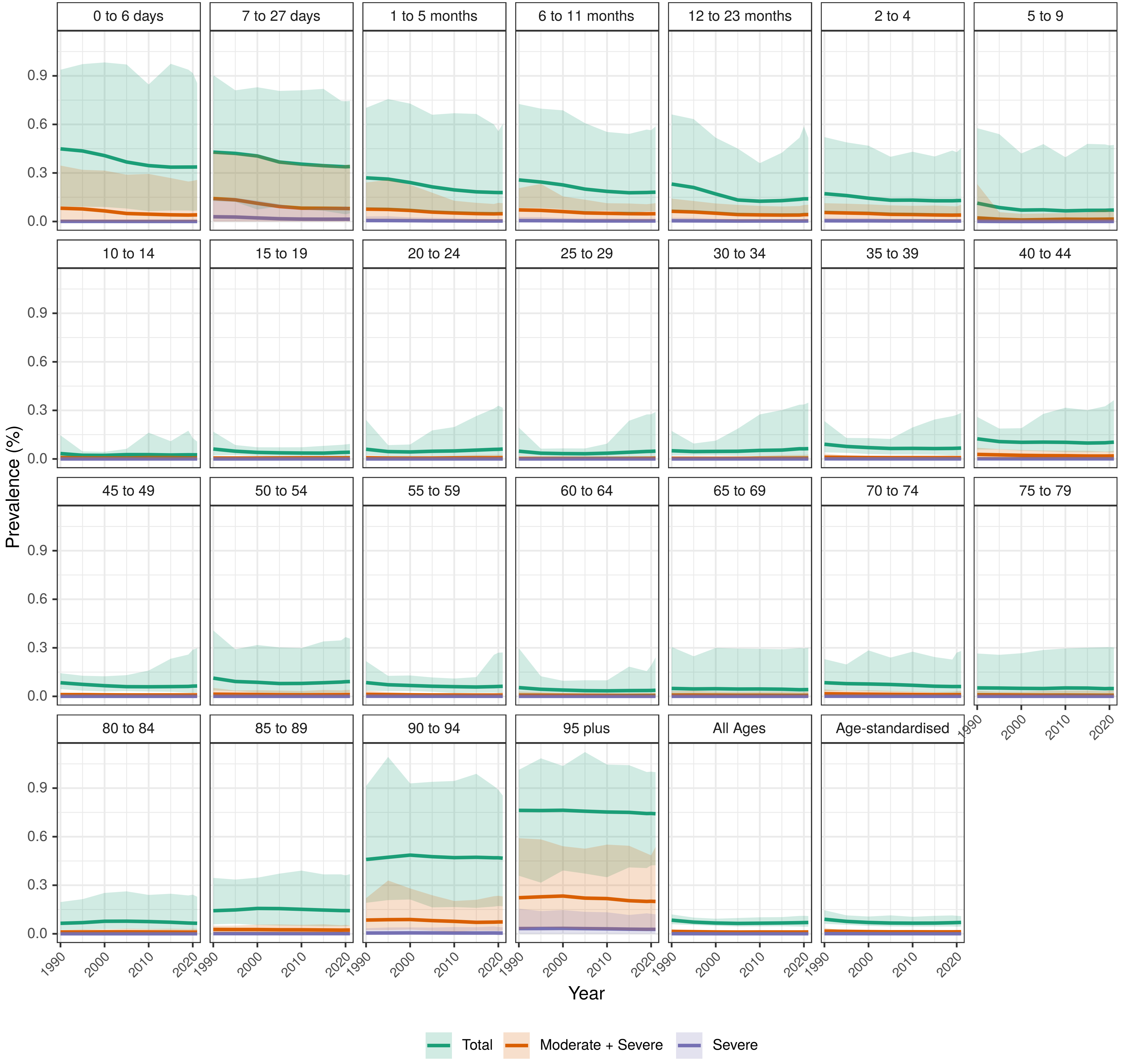

Cause-Specific Anaemia Prevalence - 2021

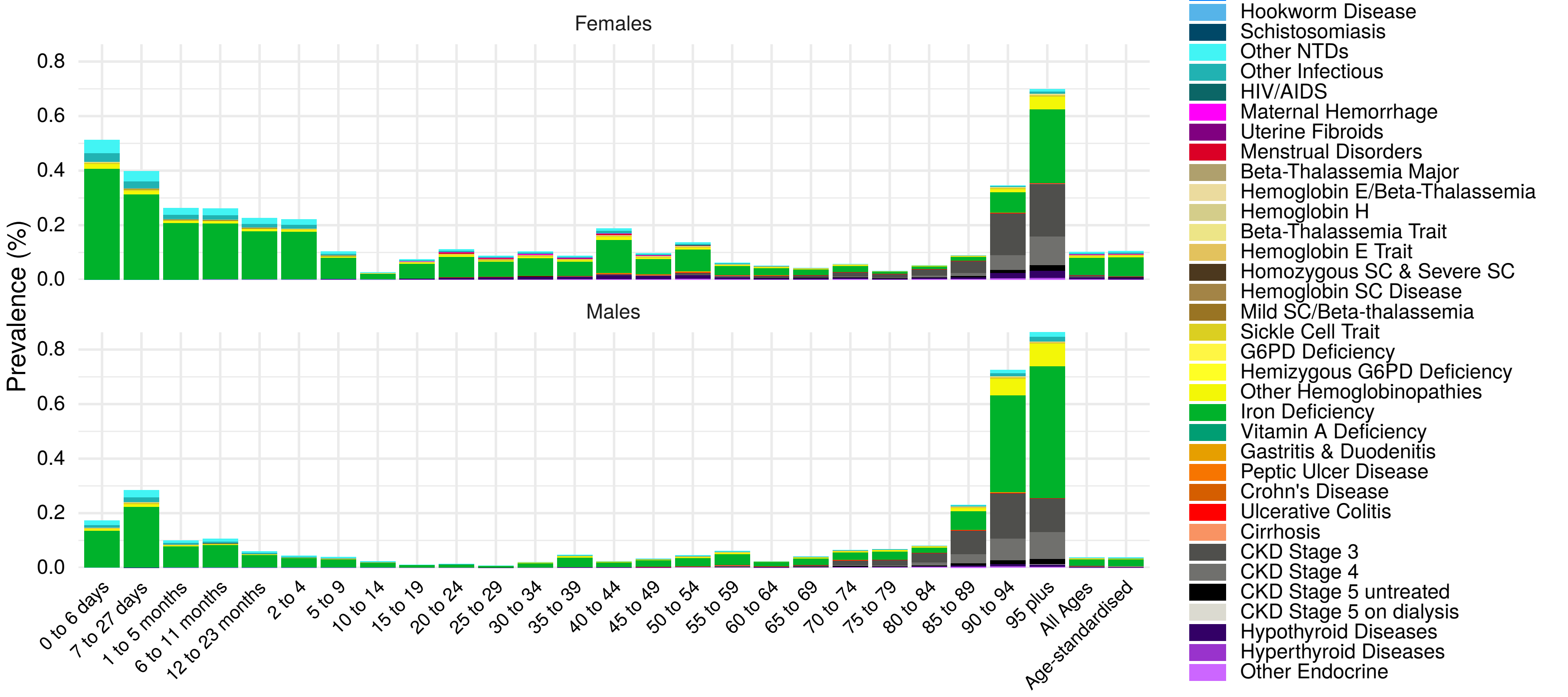

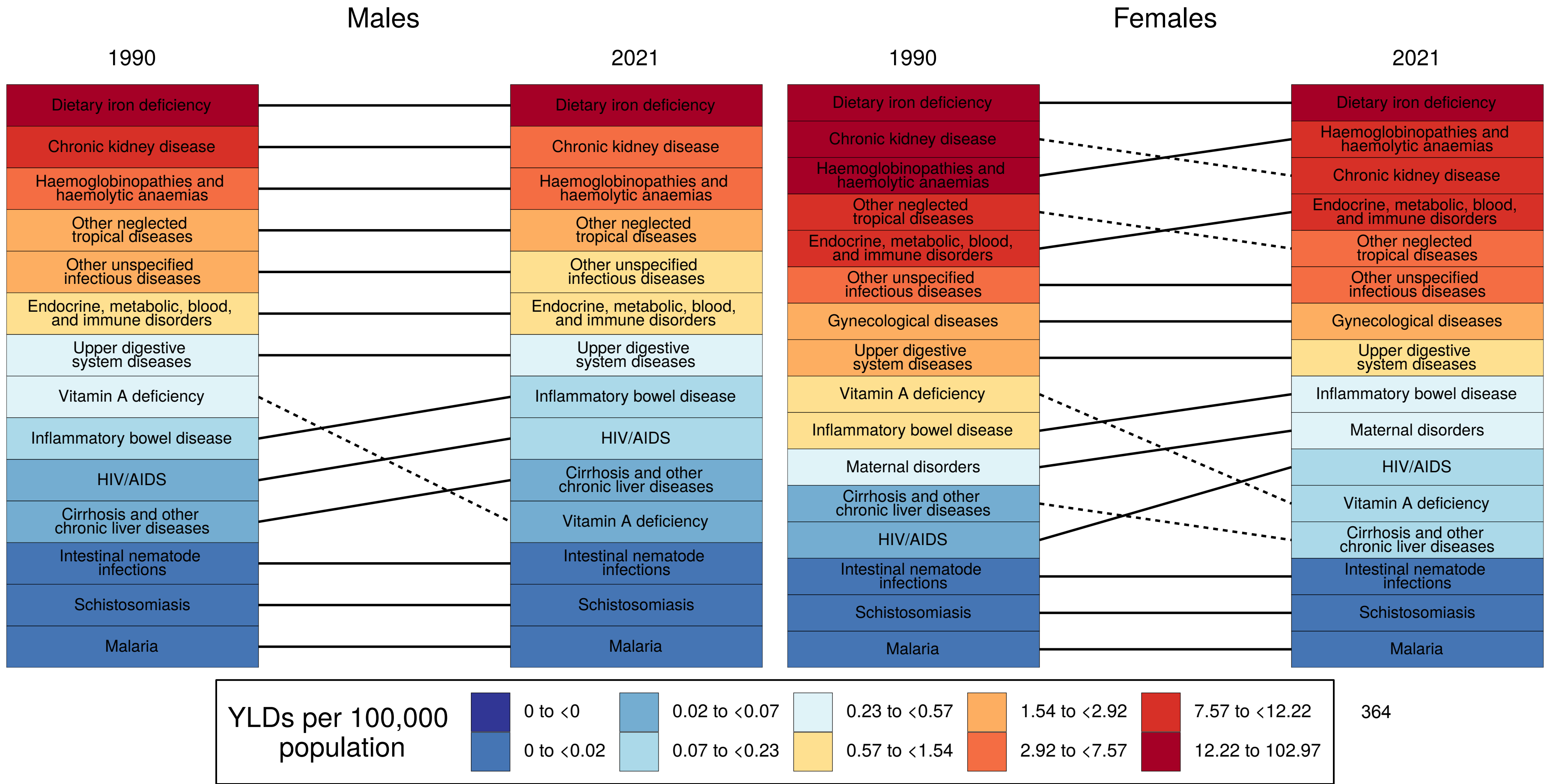

364

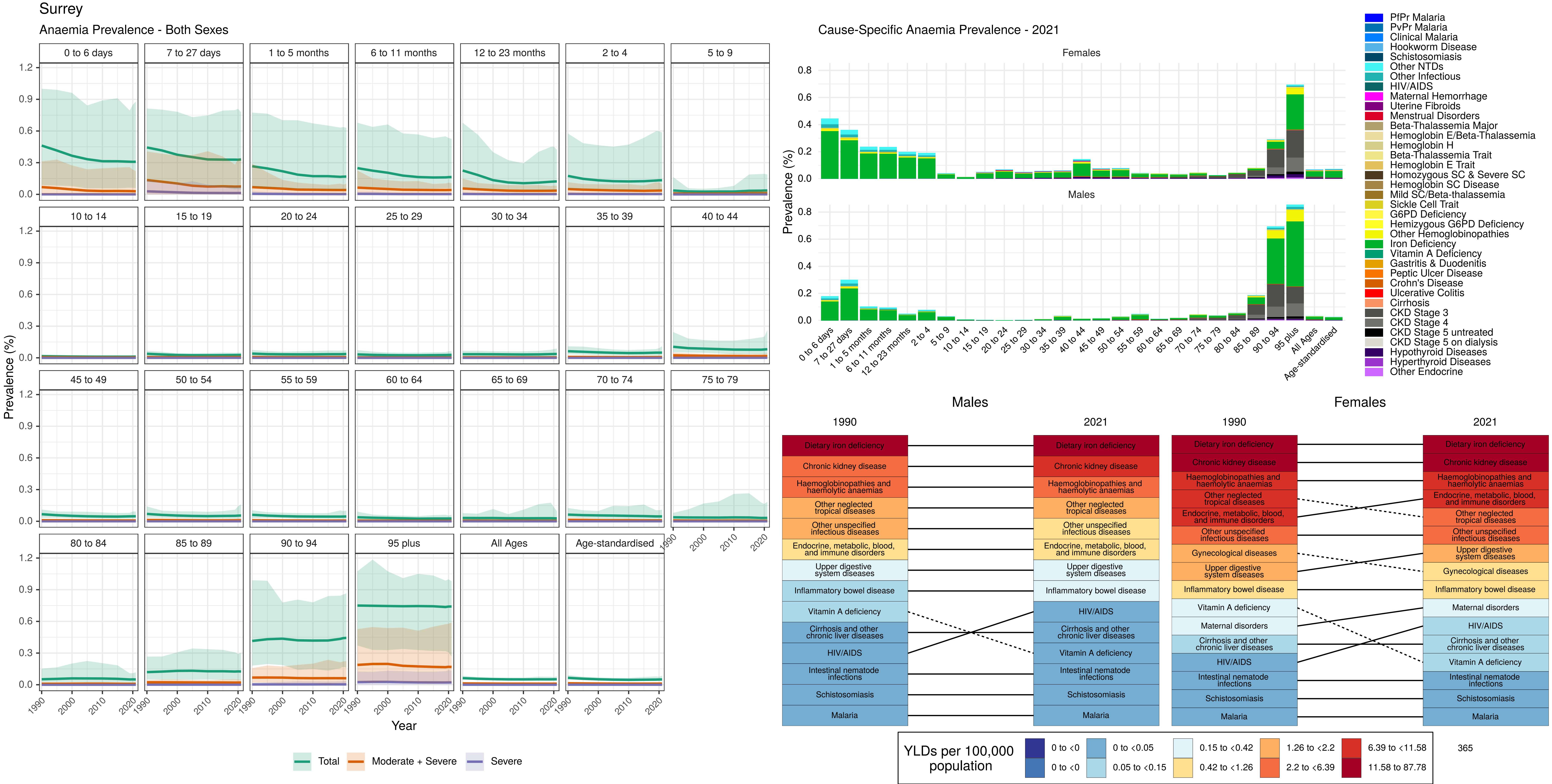
Surrey
Anaemia Prevalence - Both Sexes
0 to 6 days
7 to 27 days
1 to 5 months
6 to 11 months
12 to 23 months
2 to 4
5 to 9
10 to 14
15 to 19
20 to 24
25 to 29
30 to 34
35 to 39
40 to 44
45 to 49
50 to 54
55 to 59
60 to 64
65 to 69
70 to 74
75 to 79
80 to 84
85 to 89
90 to 94
95 plus
All Ages
Age-standardised
Prevalence (%)
Year
Total
Moderate + Severe
Severe
Cause-Specific Anaemia Prevalence - 2021
Females
Males
PfPr Malaria
PvPr Malaria
Clinical Malaria
Hookworm Disease
Schistosomiasis
Other NTDs
Other Infectious
HIV/AIDS
Maternal Hemorrhage
Uterine Fibroids
Menstrual Disorders
Beta-Thalassemia Major
Hemoglobin E/Beta-Thalassemia
Hemoglobin H
Beta-Thalassemia Trait
Hemoglobin E Trait
Homozygous SC & Severe SC
Hemoglobin SC Disease
Mild SC/Beta-thalassemia
Sickle Cell Trait
G6PD Deficiency
Hemizygous G6PD Deficiency
Other Hemoglobinopathies
Iron Deficiency
Vitamin A Deficiency
Gastritis & Duodenitis
Peptic Ulcer Disease
Crohn's Disease
Ulcerative Colitis
Cirrhosis
CKD Stage 3
CKD Stage 4
CKD Stage 5 untreated
CKD Stage 5 on dialysis
Hypothyroid Diseases
Hyperthyroid Diseases
Other Endocrine
Males
Females
1990
2021
Dietary iron deficiency
Chronic kidney disease
Haemoglobinopathies and haemolytic anaemias
Other neglected tropical diseases
Other unspecified infectious diseases
Endocrine, metabolic, blood, and immune disorders
Upper digestive system diseases
Inflammatory bowel disease
Vitamin A deficiency
Cirrhosis and other chronic liver diseases
HIV/AIDS
Intestinal nematode infections
Schistosomiasis
Malaria
Gynecological diseases
Maternal disorders
YLDs per 100,000 population
0 to <0
0 to <0.05
0.05 to <0.15
0.15 to <0.42
0.42 to <1.26
1.26 to <2.2
2.2 to <6.39
6.39 to <11.58
11.58 to 87.78

West Berkshire

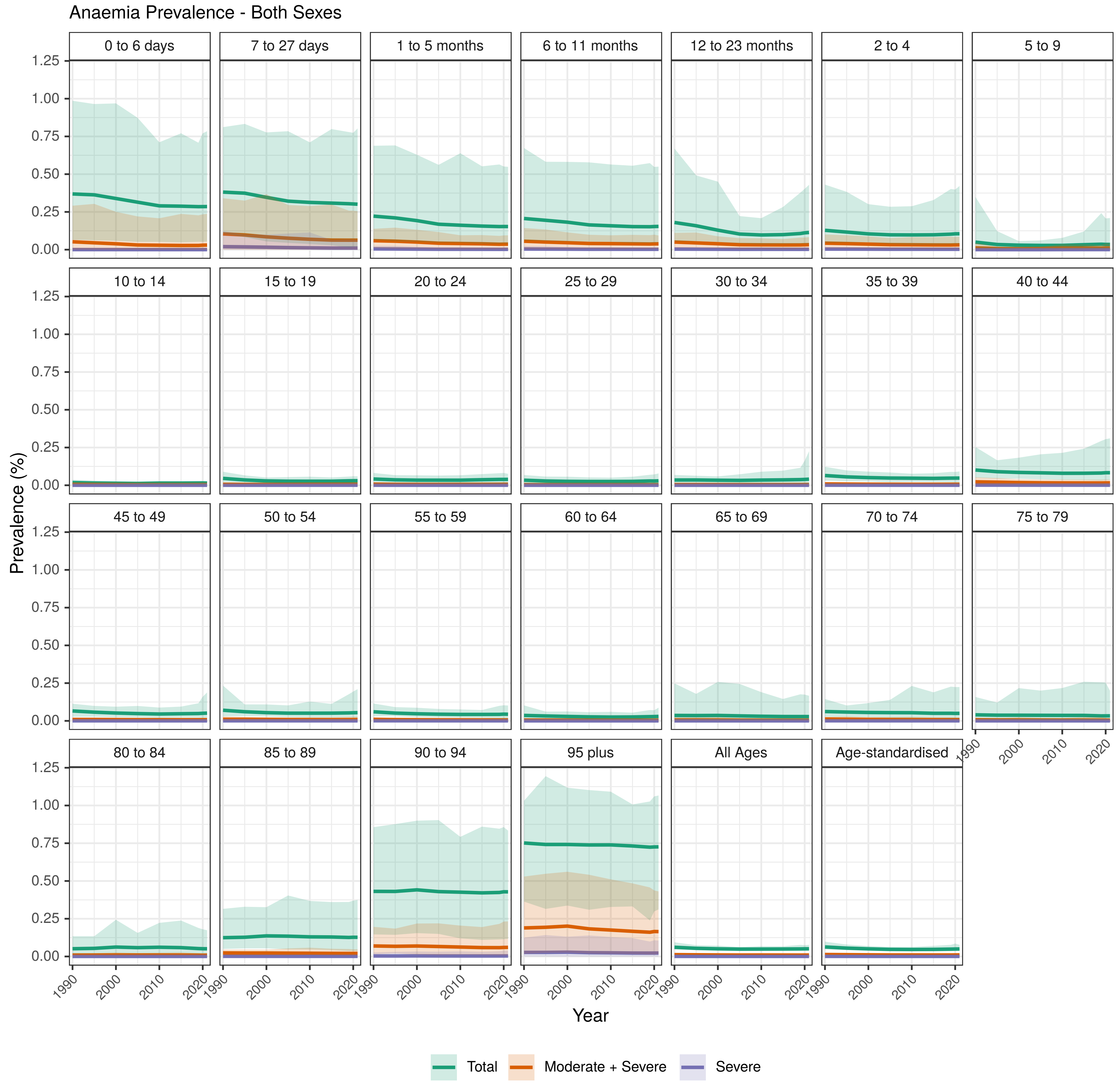

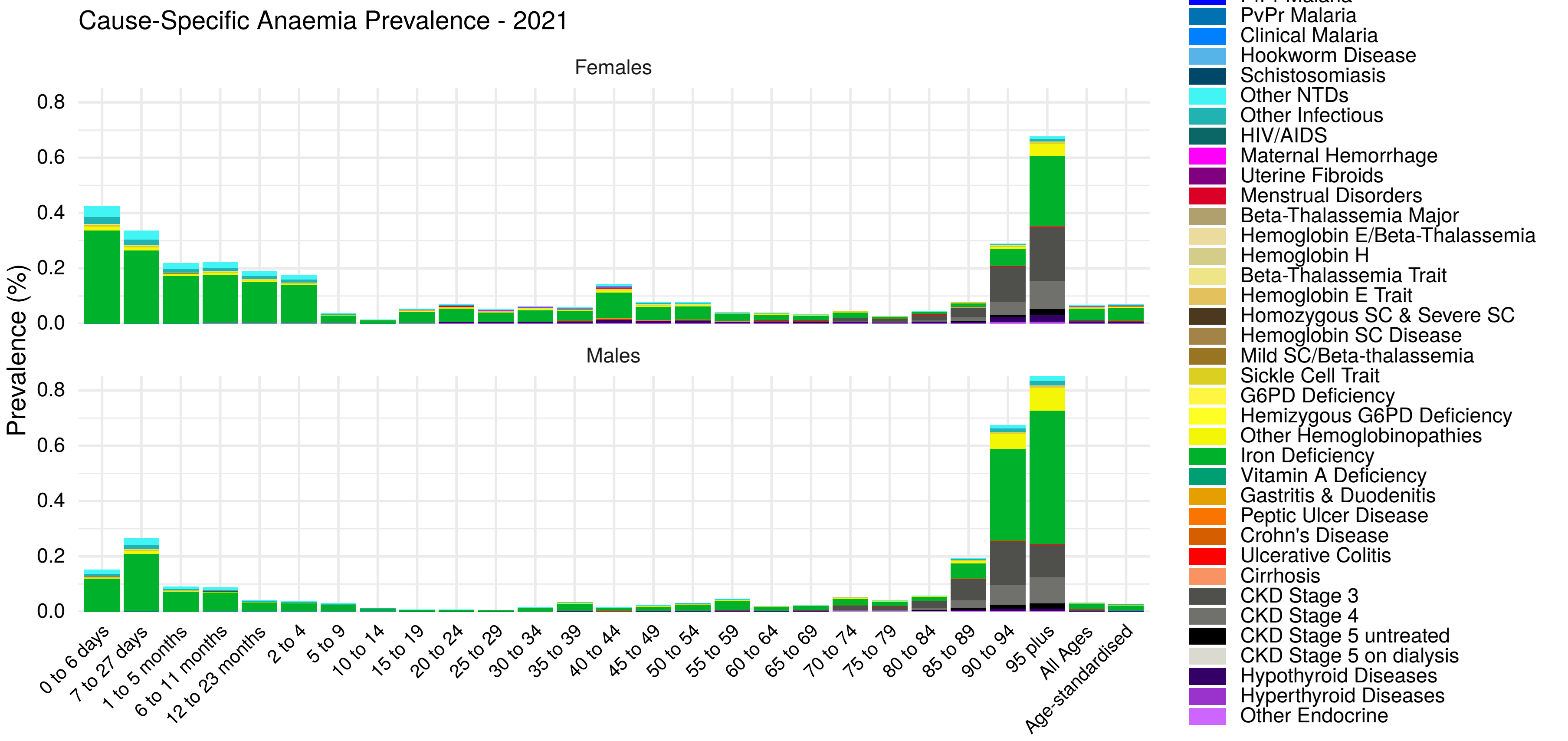

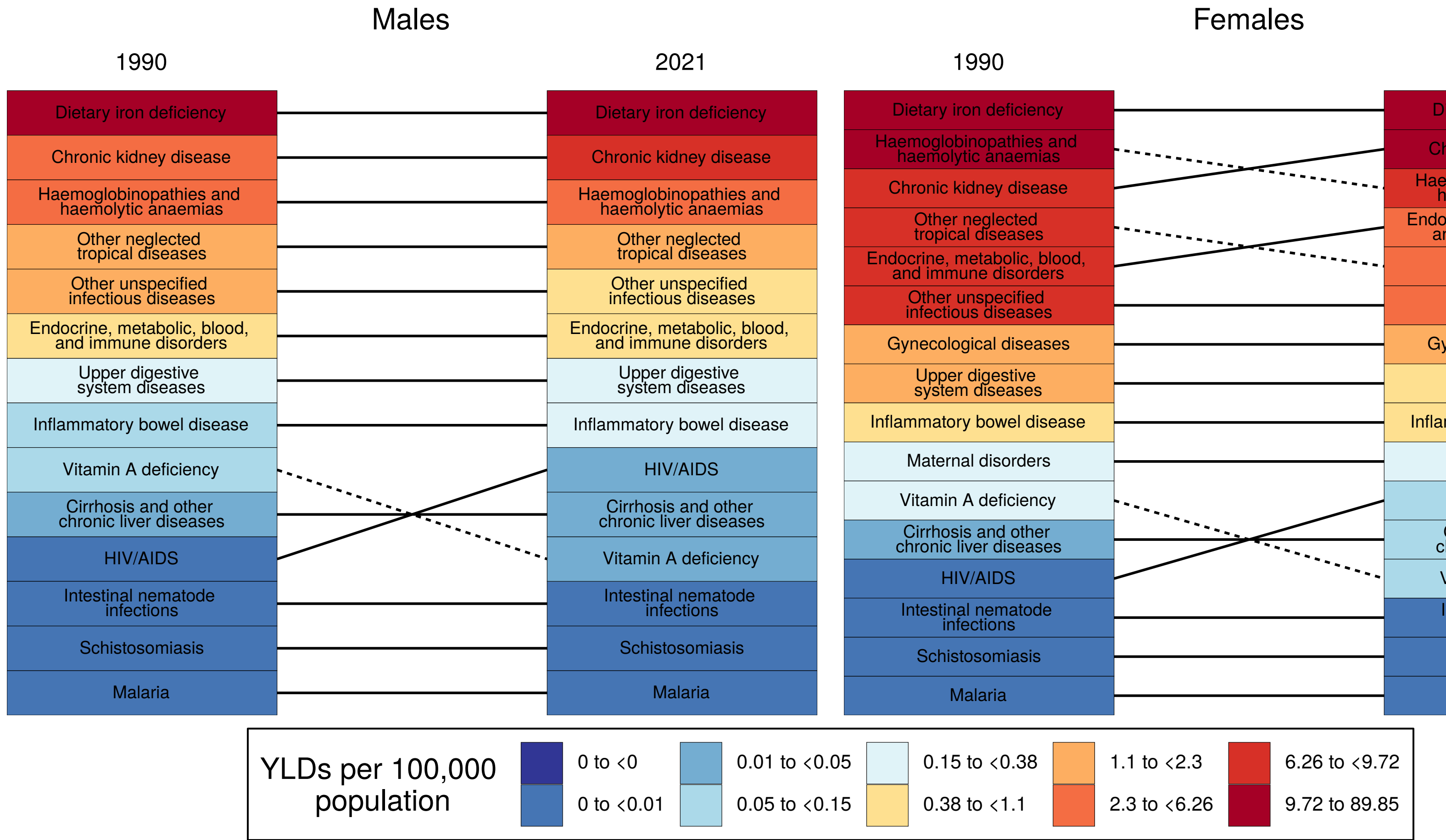

West Sussex

Anaemia Prevalence - Both Sexes

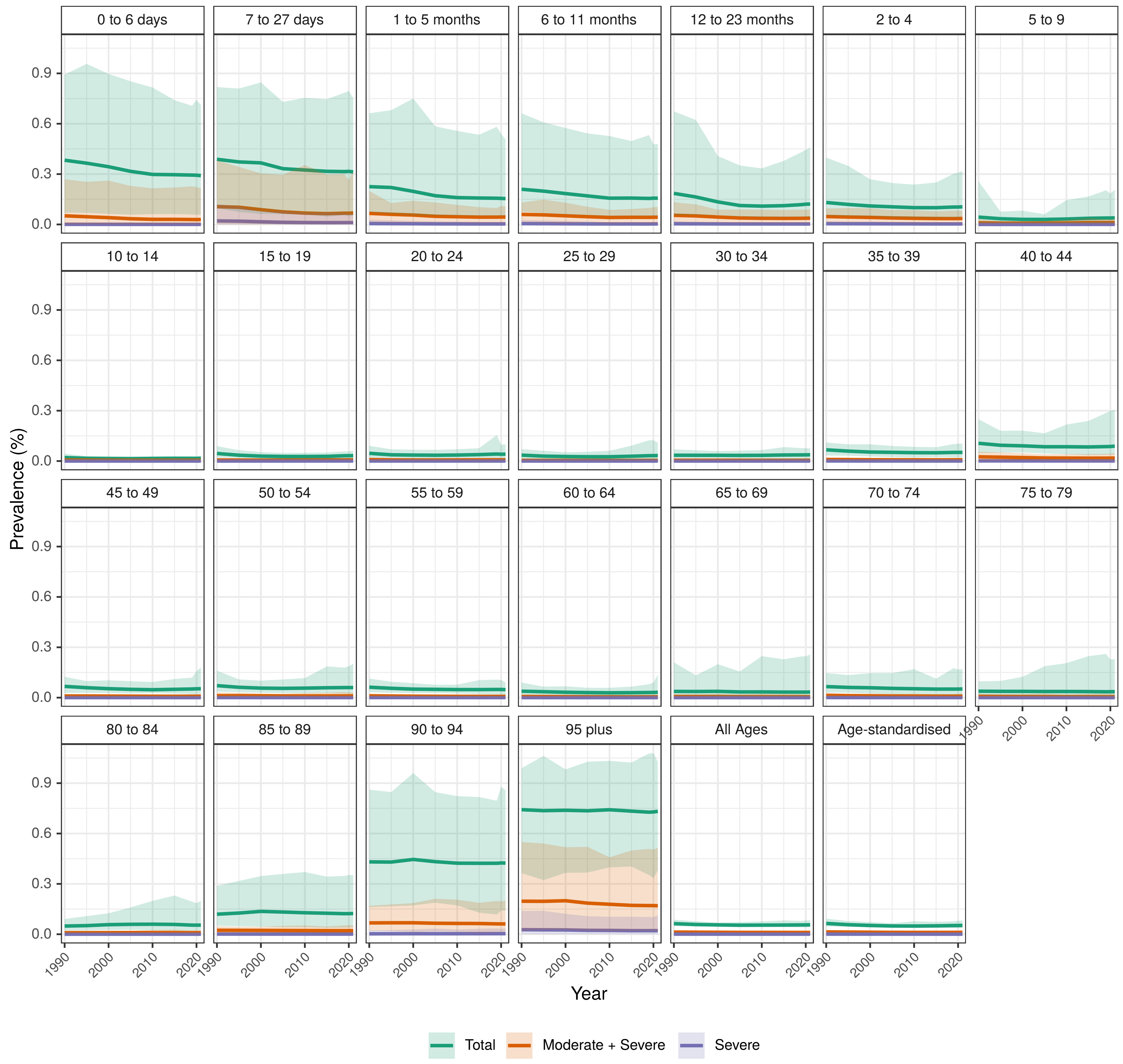

Cause-Specific Anaemia Prevalence - 2021

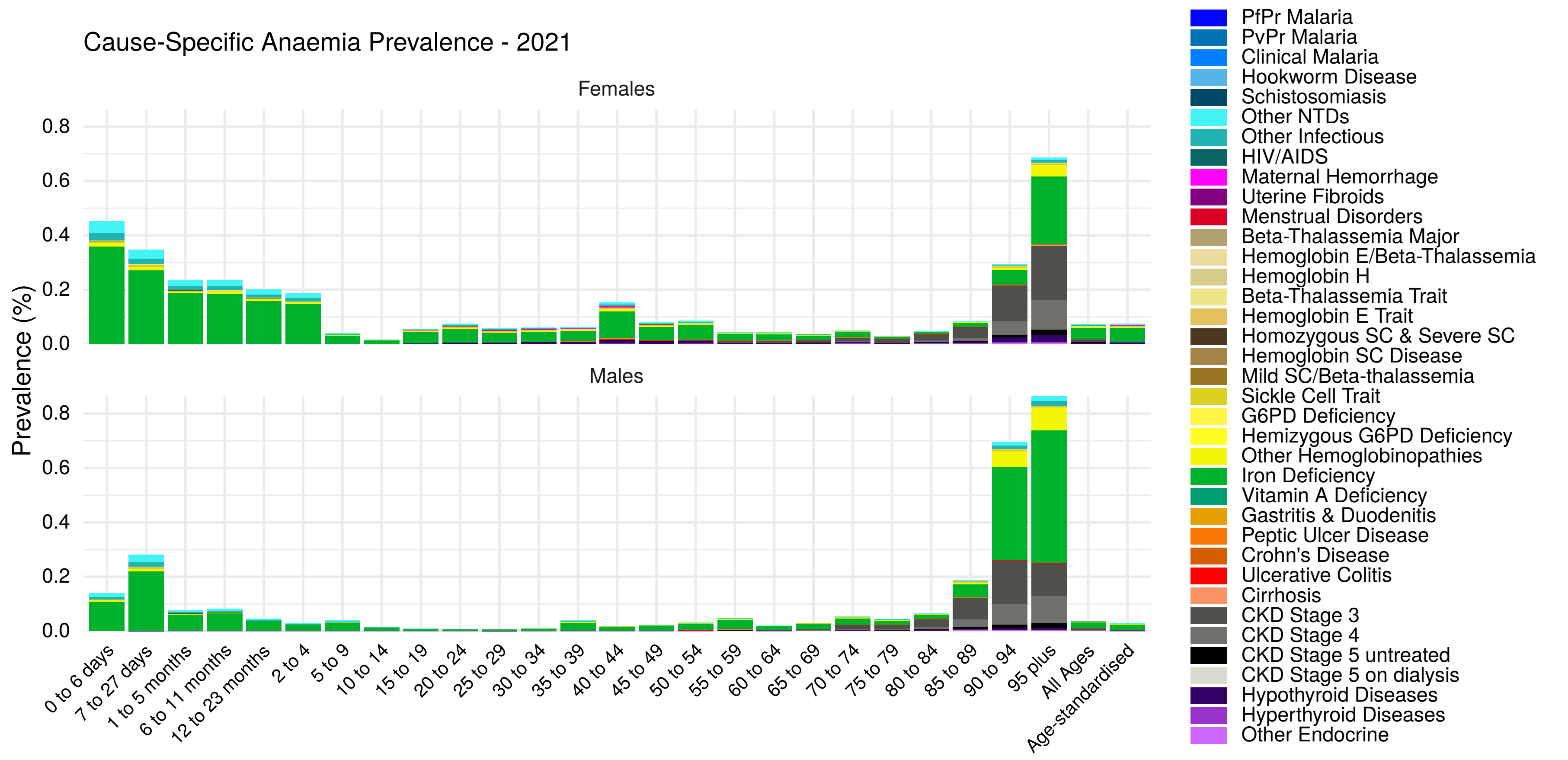

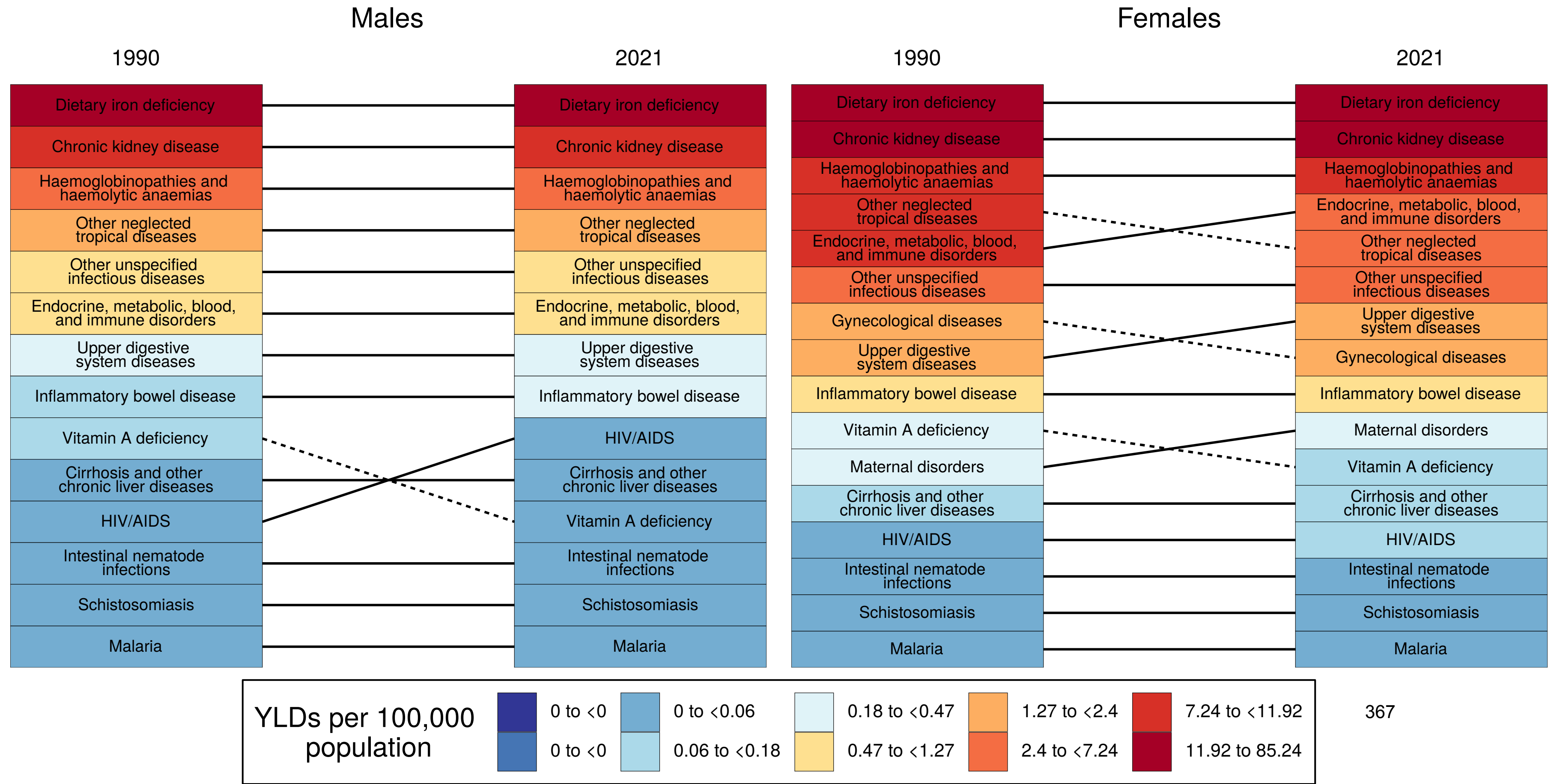

Windsor and Maidenhead

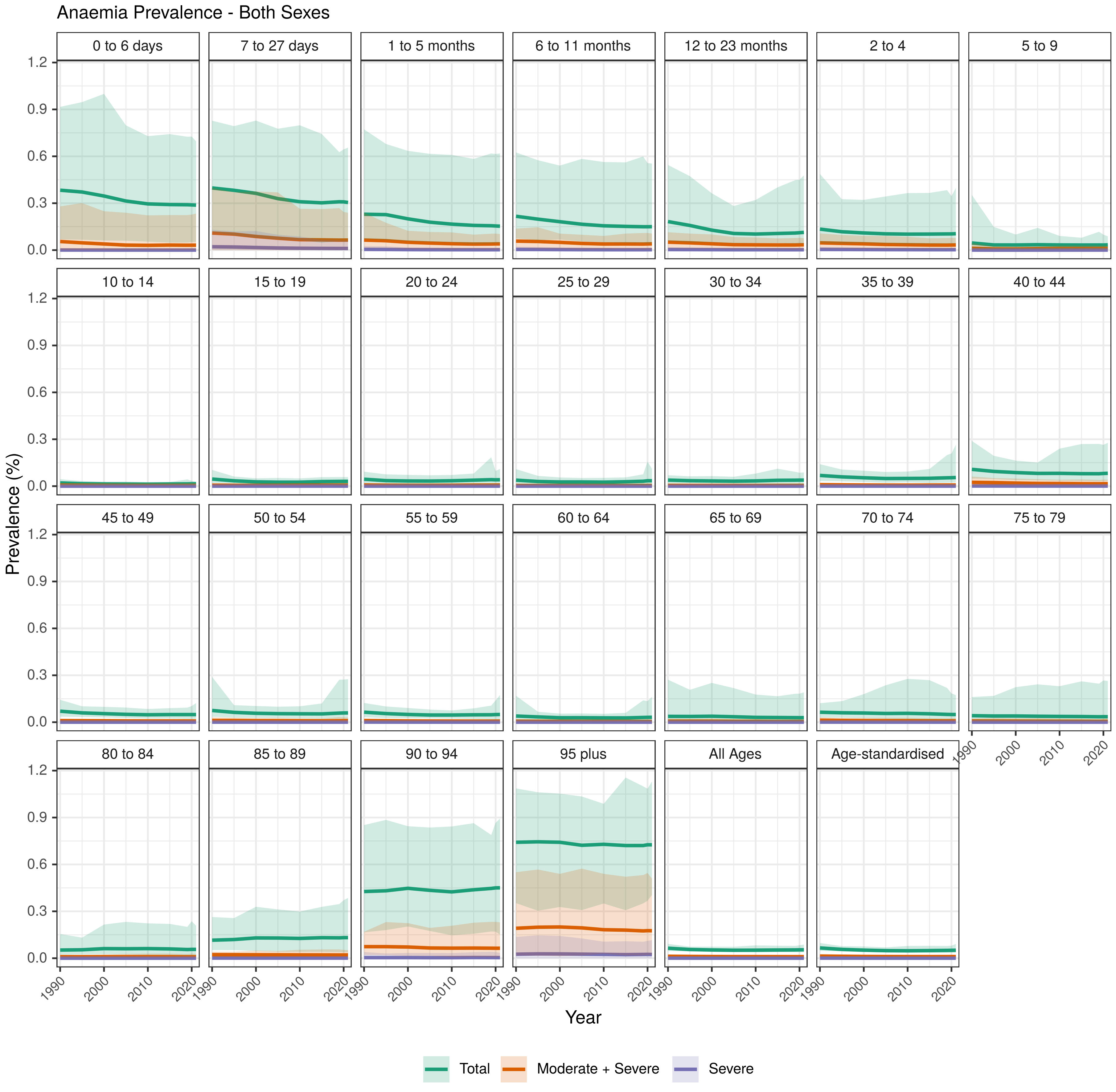

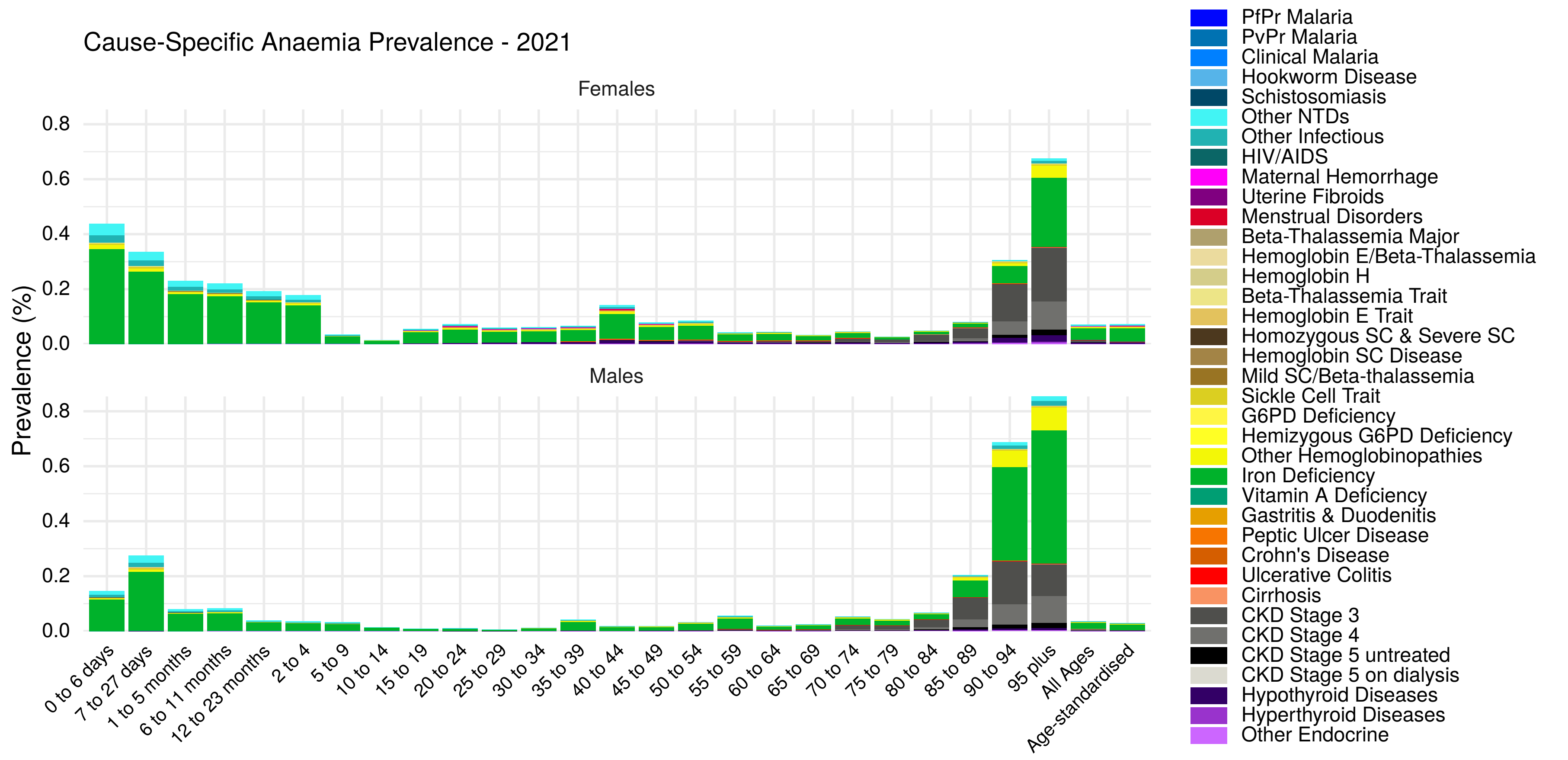

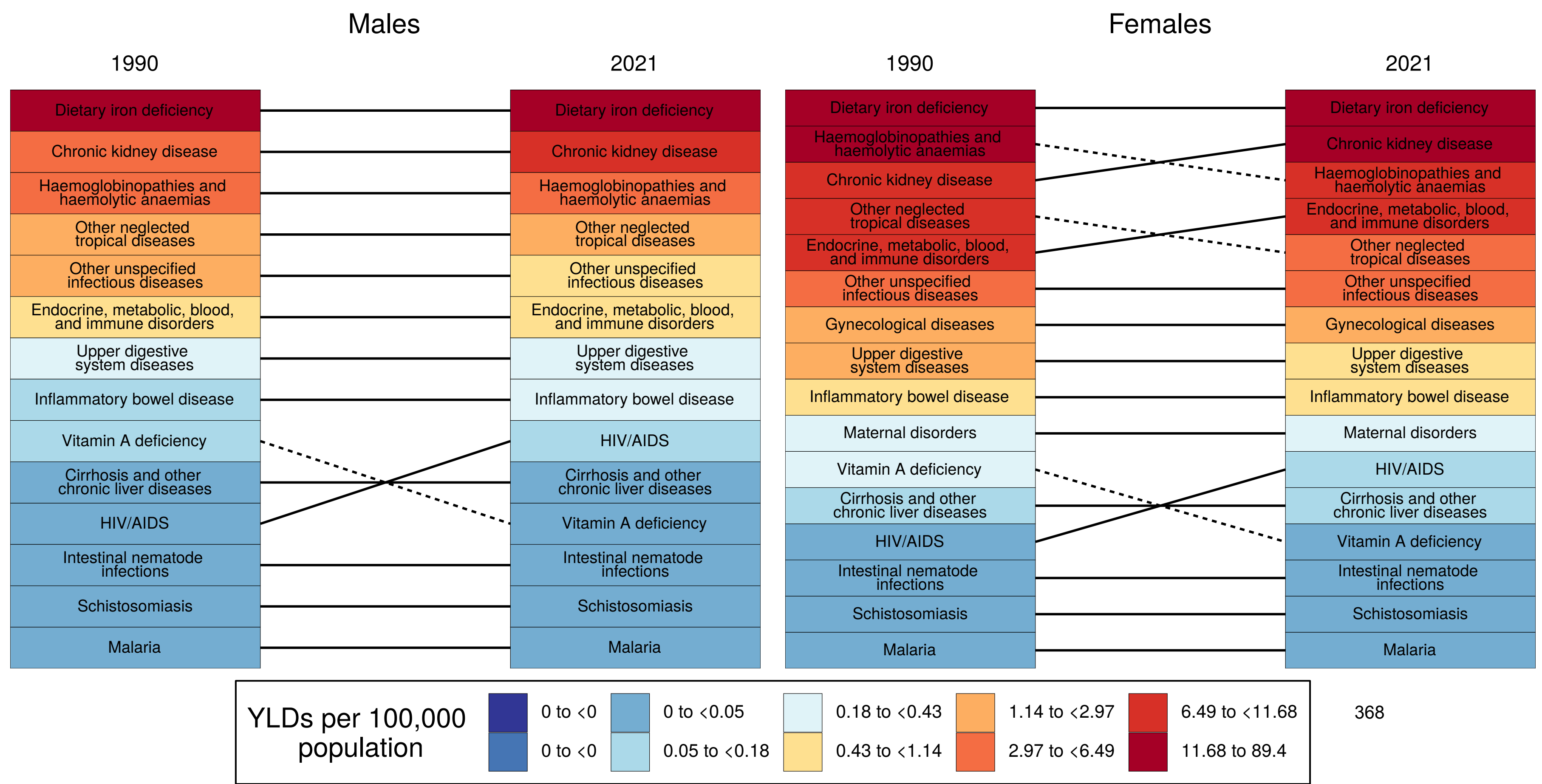

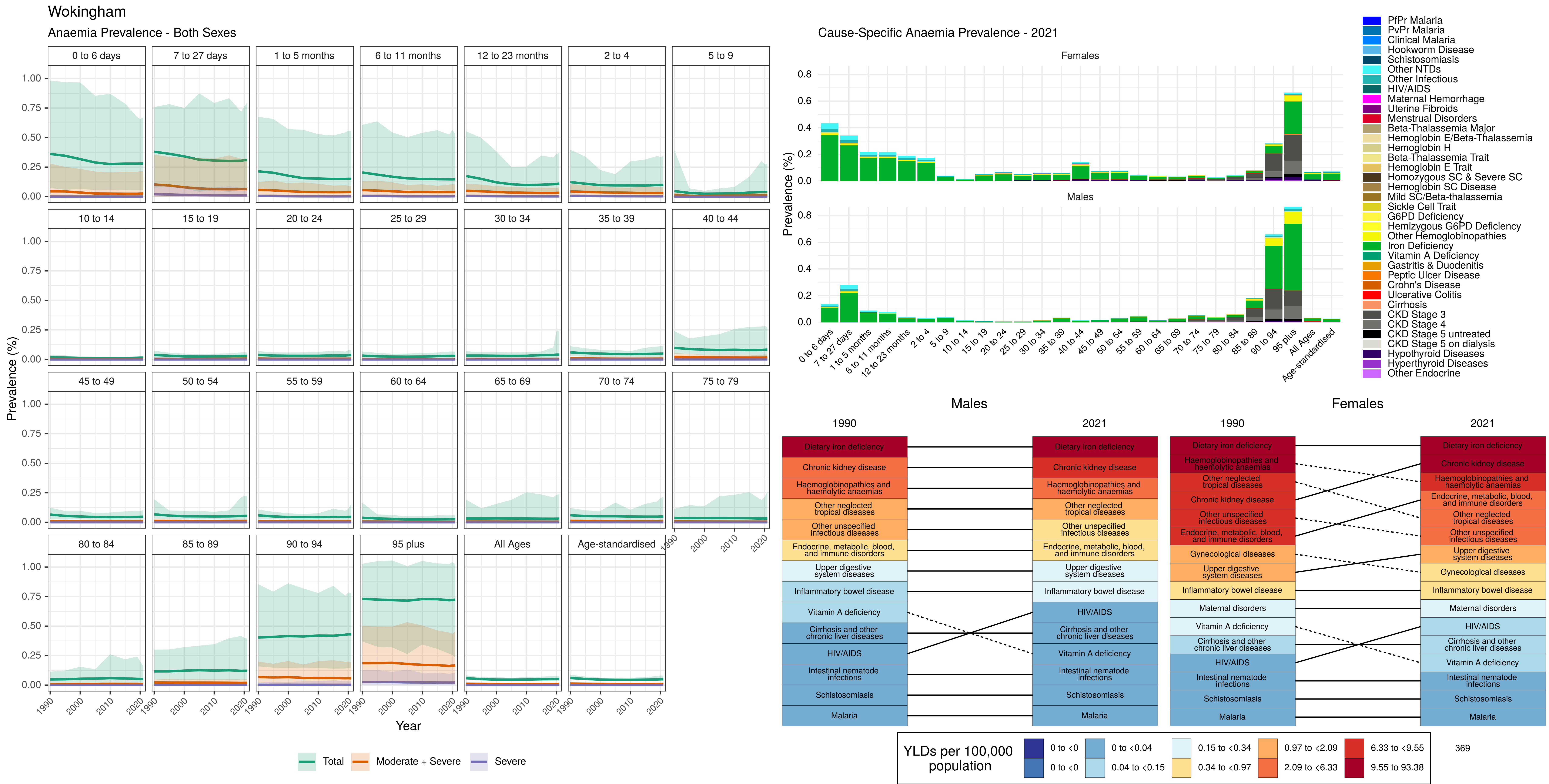

Wokingham
Anaemia Prevalence - Both Sexes
0 to 6 days
7 to 27 days
1 to 5 months
6 to 11 months
12 to 23 months
2 to 4
5 to 9
10 to 14
15 to 19
20 to 24
25 to 29
30 to 34
35 to 39
40 to 44
45 to 49
50 to 54
55 to 59
60 to 64
65 to 69
70 to 74
75 to 79
80 to 84
85 to 89
90 to 94
95 plus
All Ages
Age-standardised
Prevalence (%)
Year
Total
Moderate + Severe
Severe
Cause-Specific Anaemia Prevalence - 2021
Females
Males
Prevalence (%)
PfPr Malaria
PvPr Malaria
Clinical Malaria
Hookworm Disease
Schistosomiasis
Other NTDs
Other Infectious
HIV/AIDS
Maternal Hemorrhage
Uterine Fibroids
Menstrual Disorders
Beta-Thalassemia Major
Hemoglobin E/Beta-Thalassemia
Hemoglobin H
Beta-Thalassemia Trait
Hemoglobin E Trait
Homozygous SC & Severe SC
Hemoglobin SC Disease
Mild SC/Beta-thalassemia
Sickle Cell Trait
G6PD Deficiency
Hemizygous G6PD Deficiency
Other Hemoglobinopathies
Iron Deficiency
Vitamin A Deficiency
Gastritis & Duodenitis
Peptic Ulcer Disease
Crohn's Disease
Ulcerative Colitis
Cirrhosis
CKD Stage 3
CKD Stage 4
CKD Stage 5 untreated
CKD Stage 5 on dialysis
Hypothyroid Diseases
Hyperthyroid Diseases
Other Endocrine
Males
Females
1990
2021
Dietary iron deficiency
Chronic kidney disease
Haemoglobinopathies and haemolytic anaemias
Other neglected tropical diseases
Other unspecified infectious diseases
Endocrine, metabolic, blood, and immune disorders
Upper digestive system diseases
Inflammatory bowel disease
Vitamin A deficiency
Cirrhosis and other chronic liver diseases
HIV/AIDS
Intestinal nematode infections
Schistosomiasis
Malaria
Gynecological diseases
Maternal disorders
YLDs per 100,000 population
0 to <0
0 to <0.04
0.04 to <0.15
0.15 to <0.34
0.34 to <0.97
0.97 to <2.09
2.09 to <6.33
6.33 to <9.55
9.55 to 93.38

South West England

Anaemia Prevalence - Both Sexes

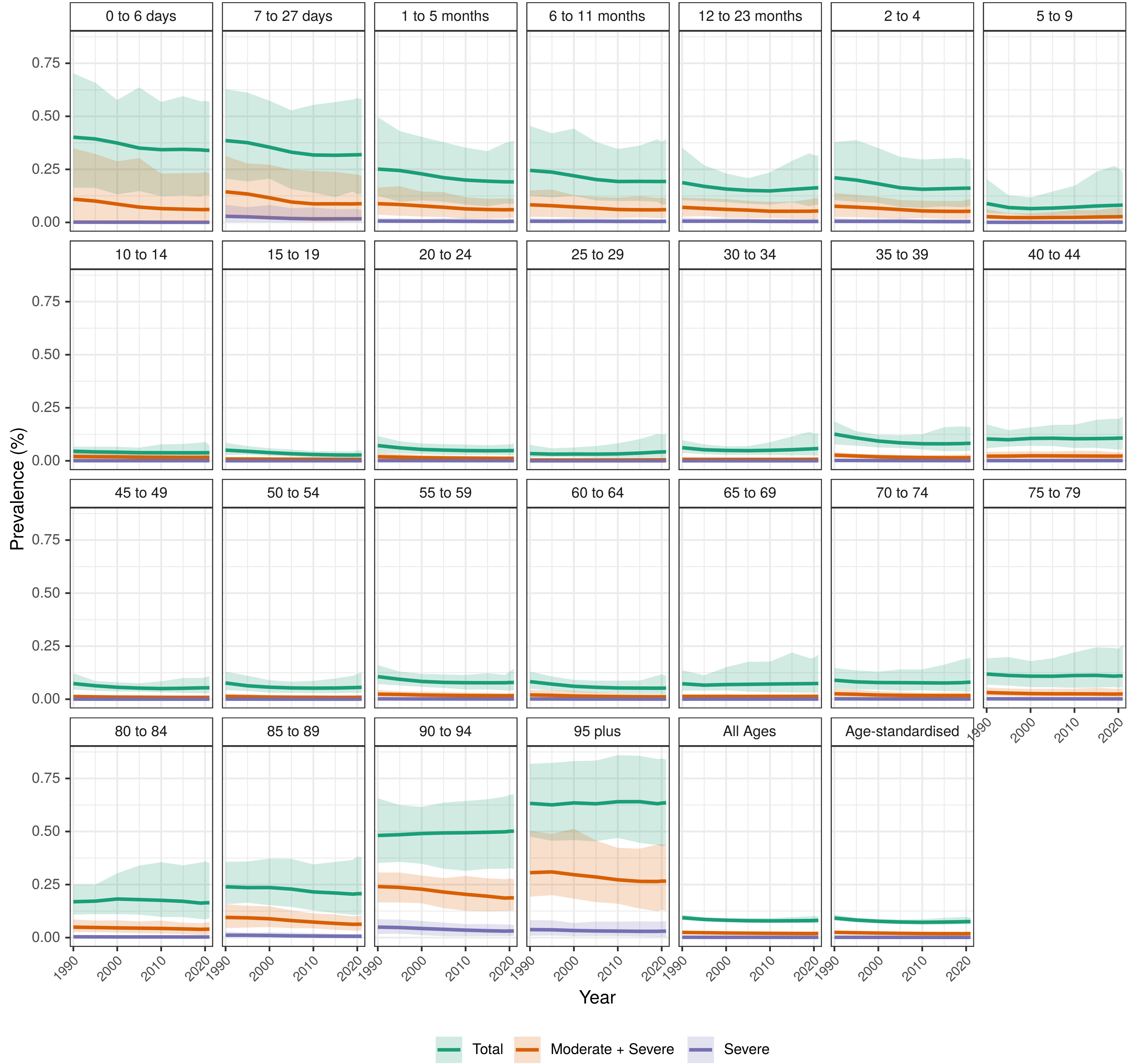

Cause-Specific Anaemia Prevalence - 2021

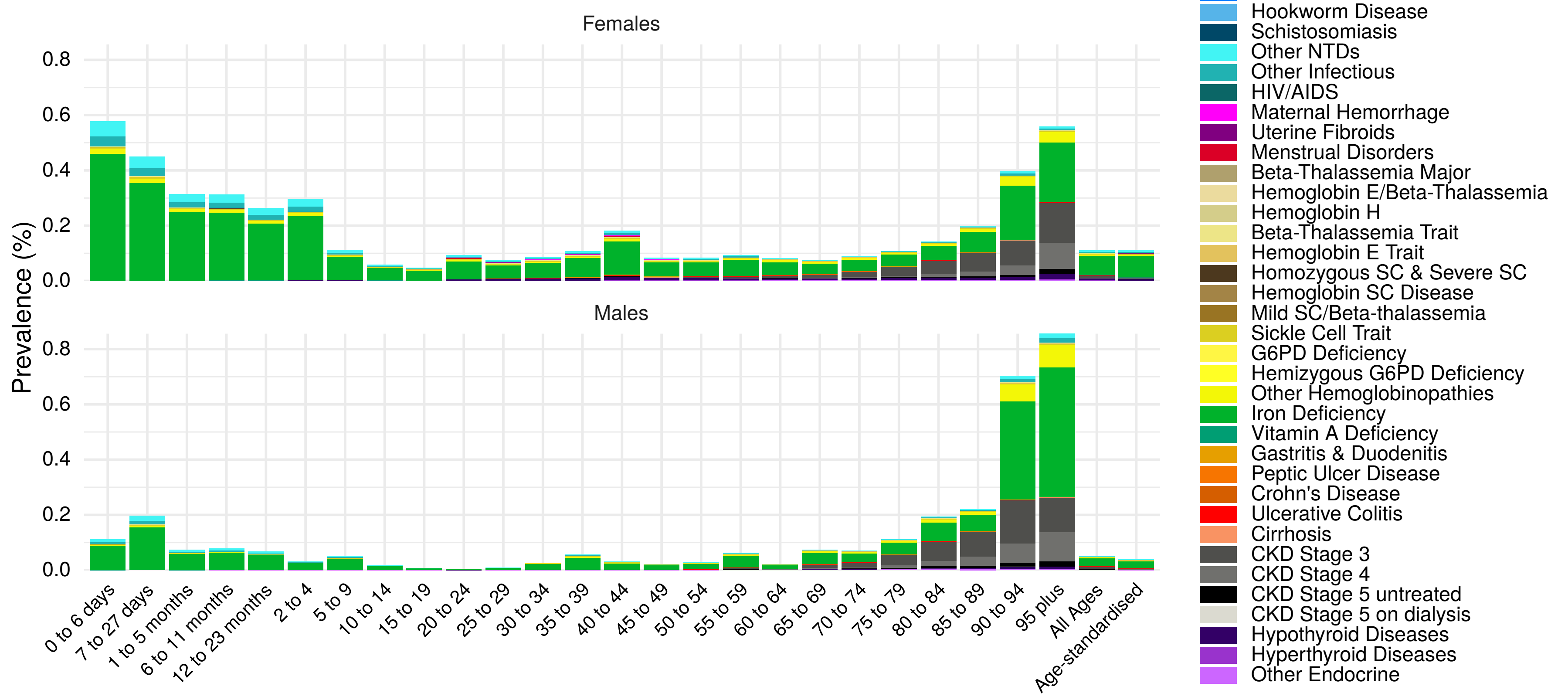

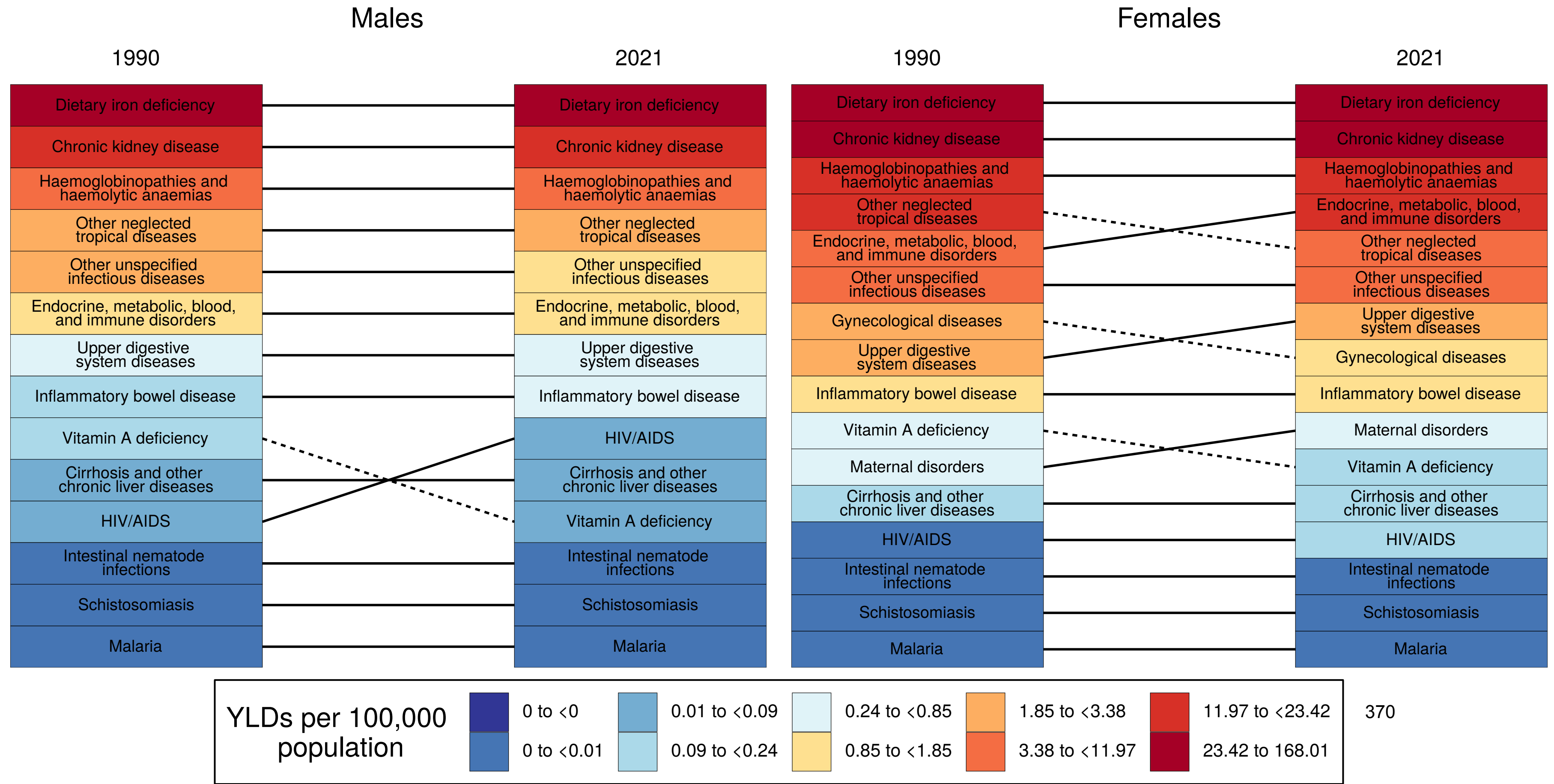

Bath and North East Somerset

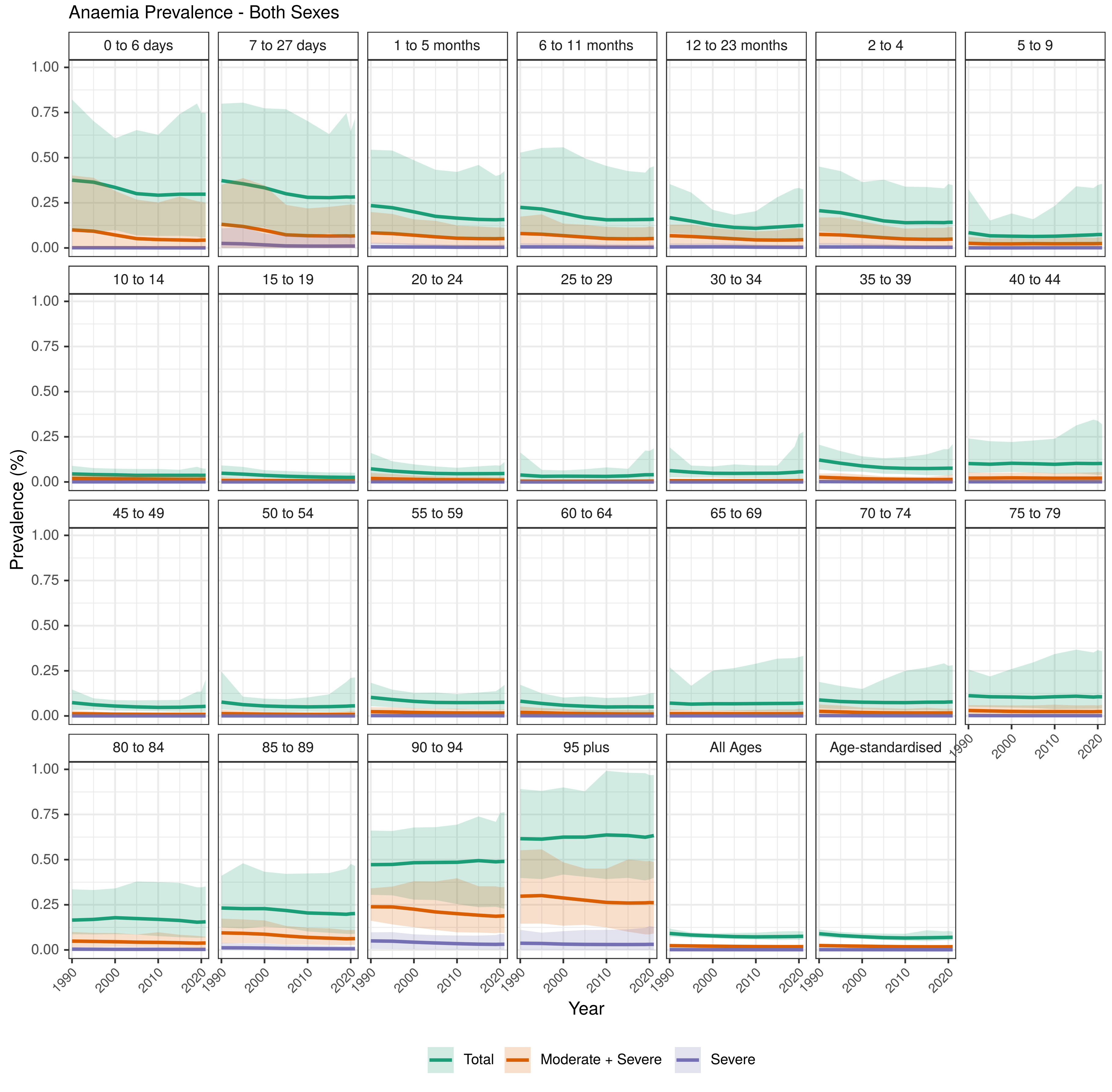

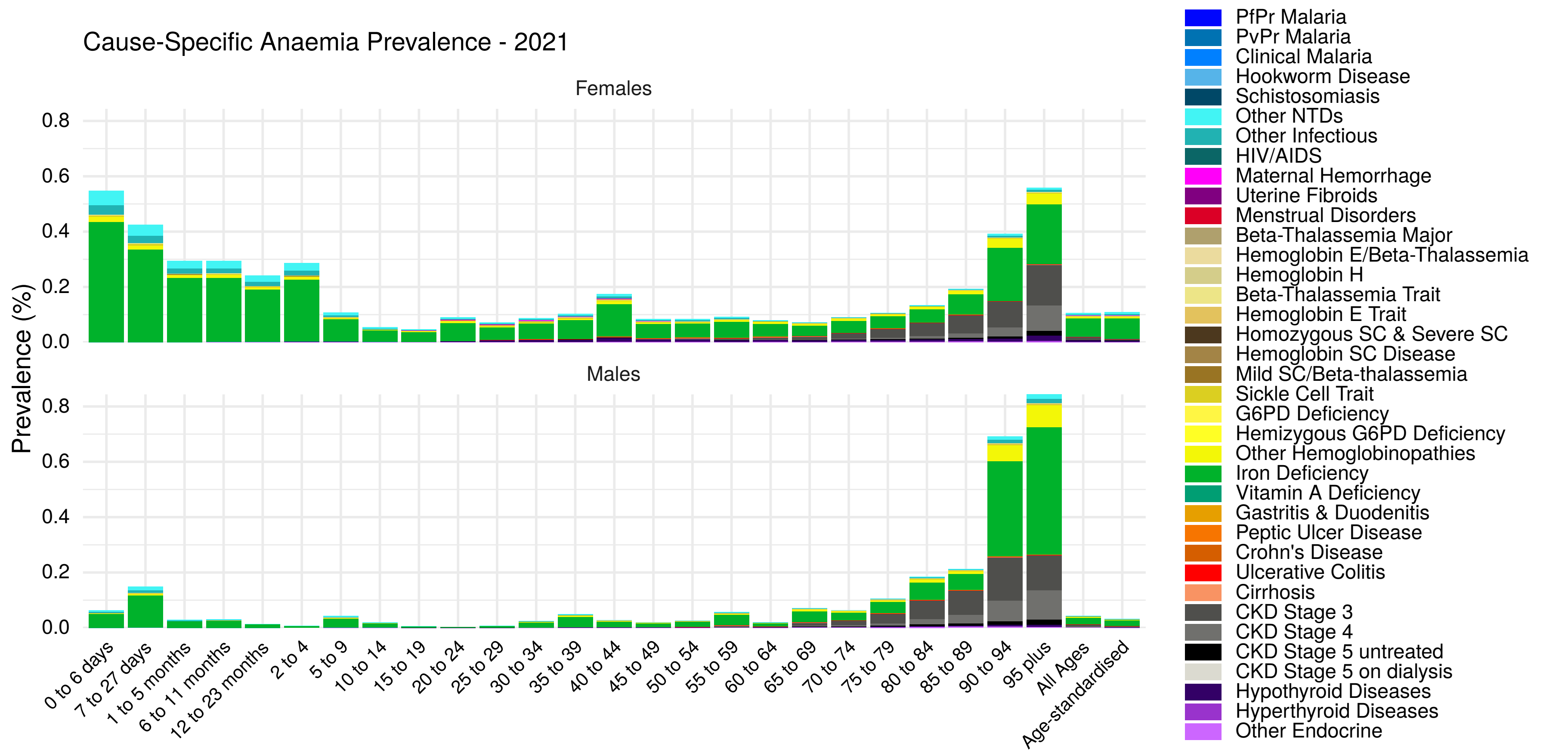

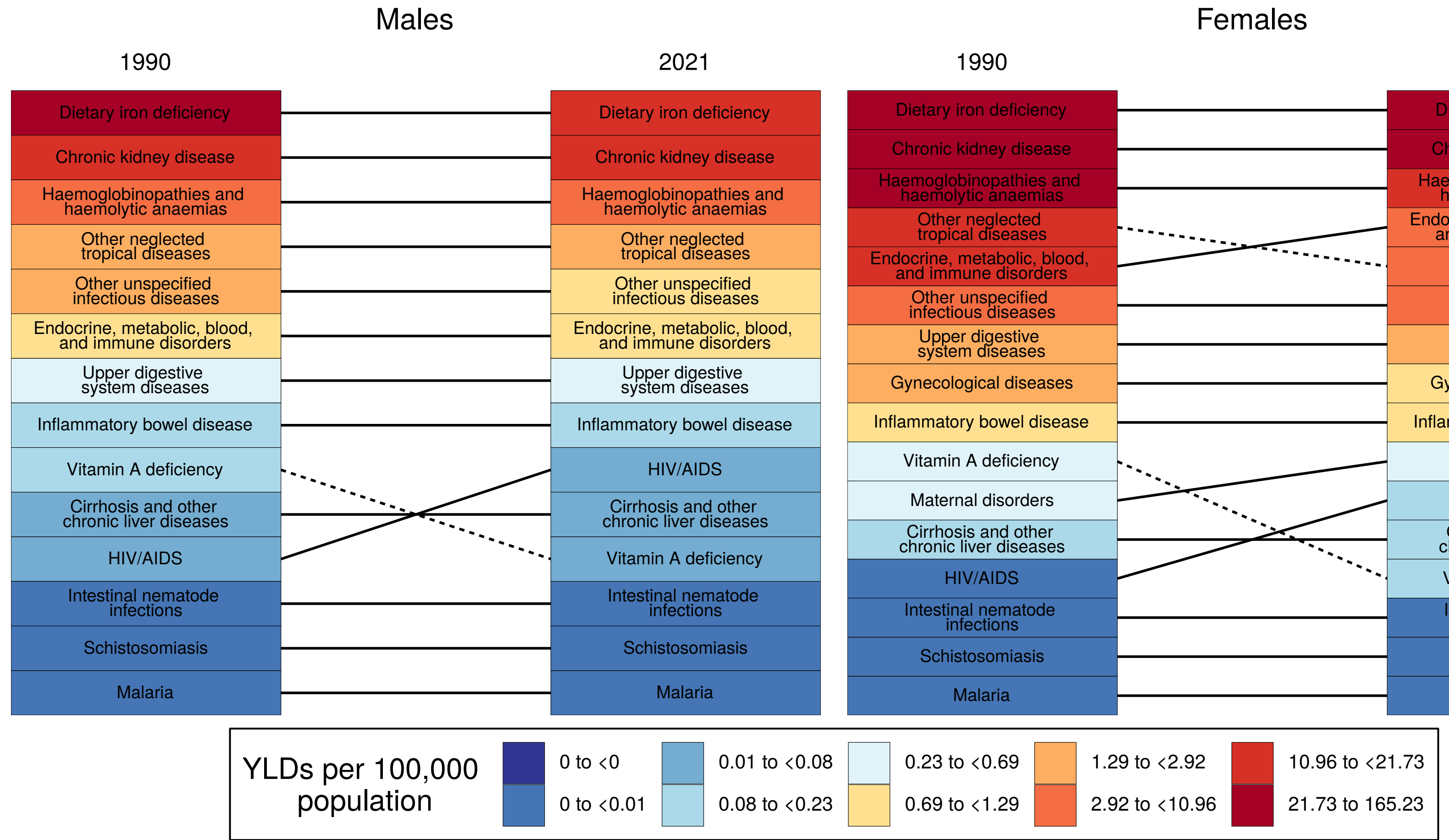

Bournemouth

Anaemia Prevalence - Both Sexes

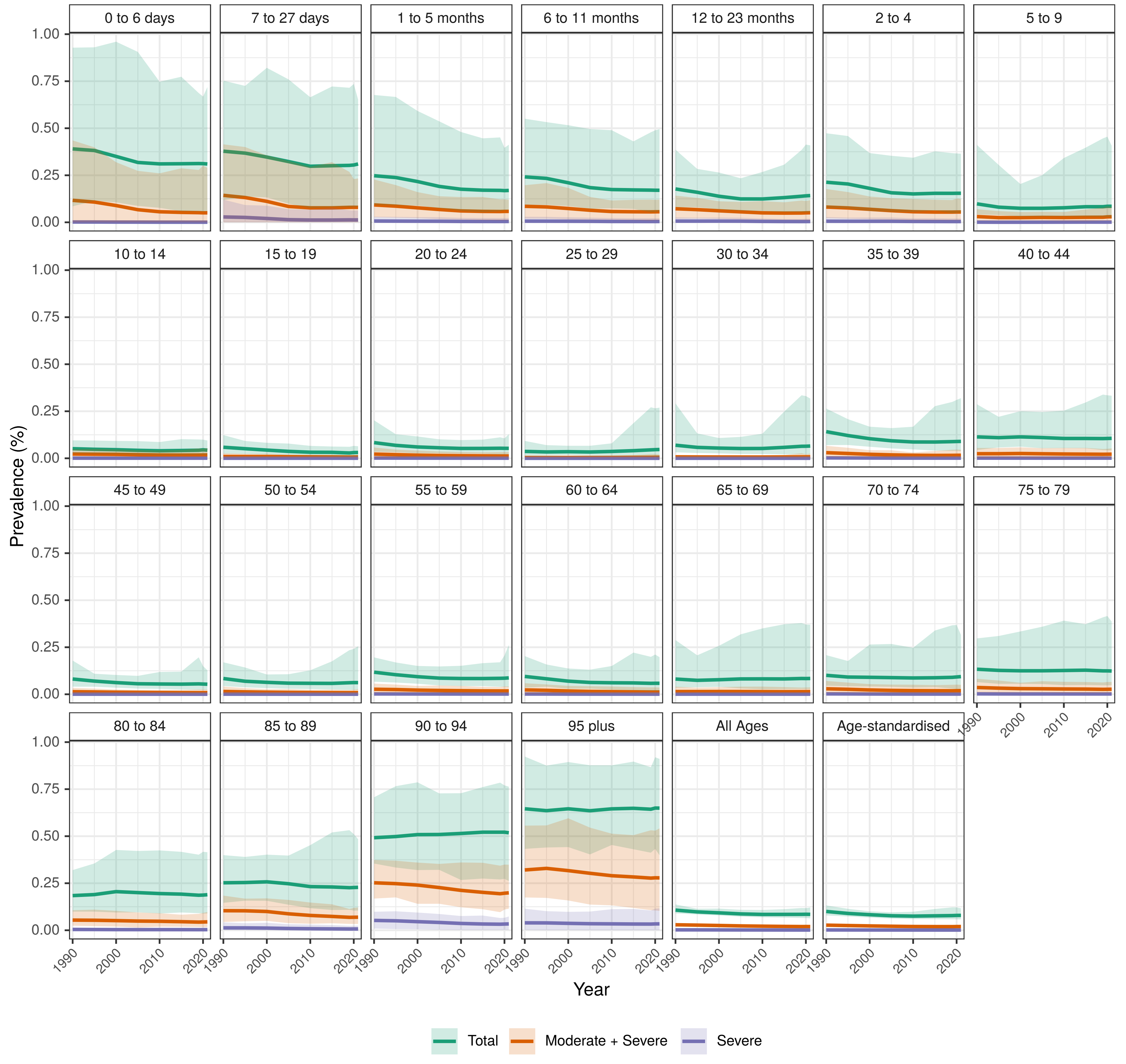

Cause-Specific Anaemia Prevalence - 2021

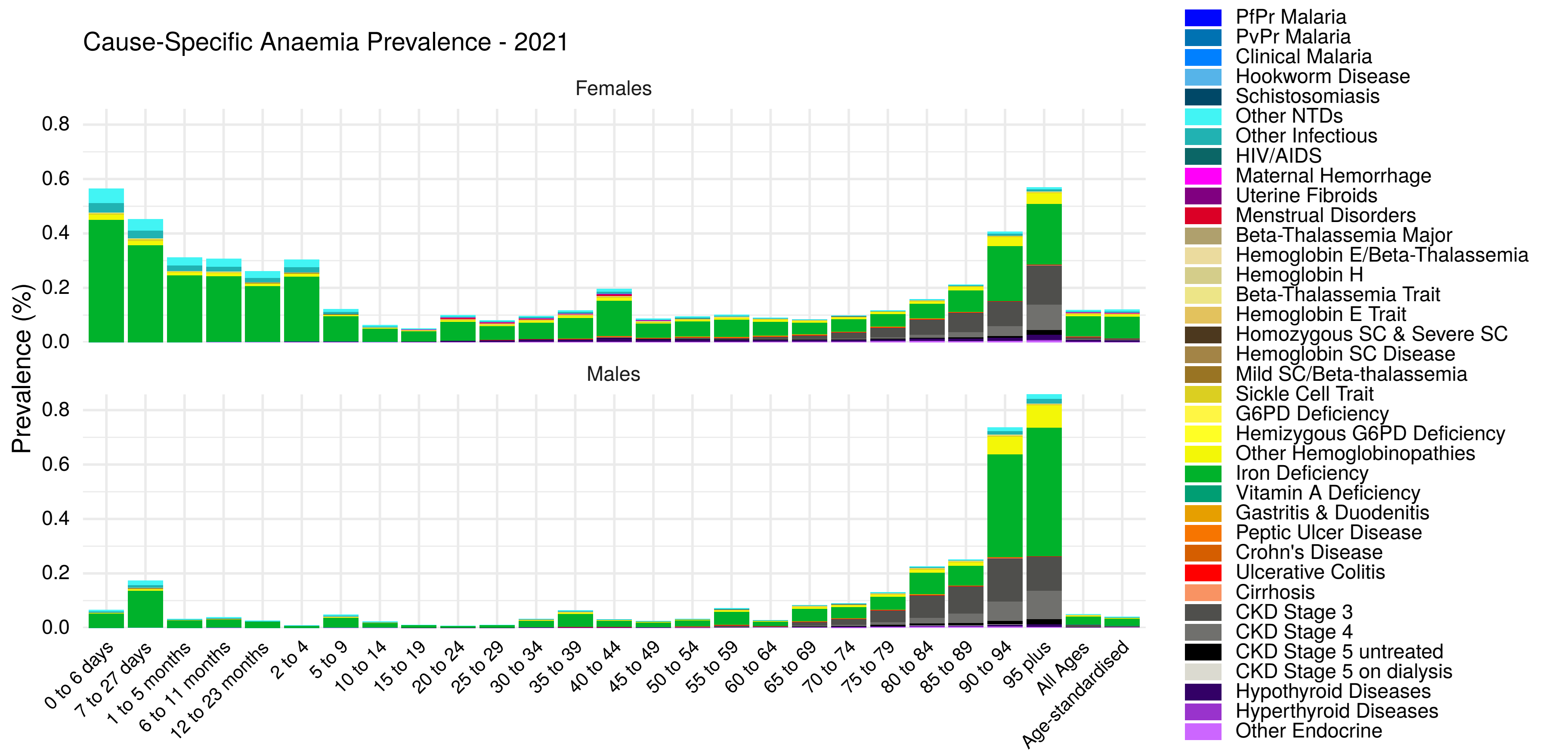

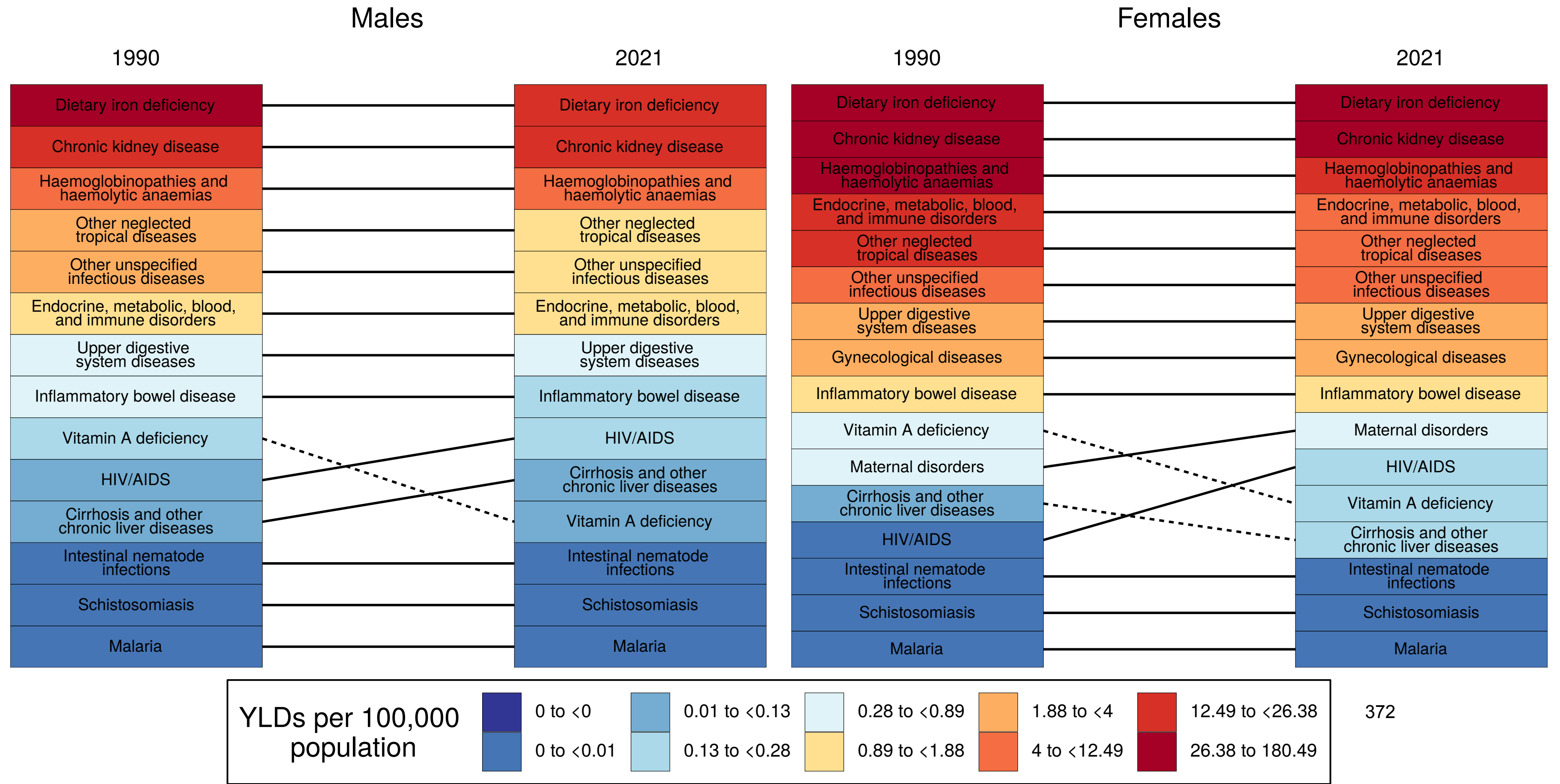

Bristol, City of

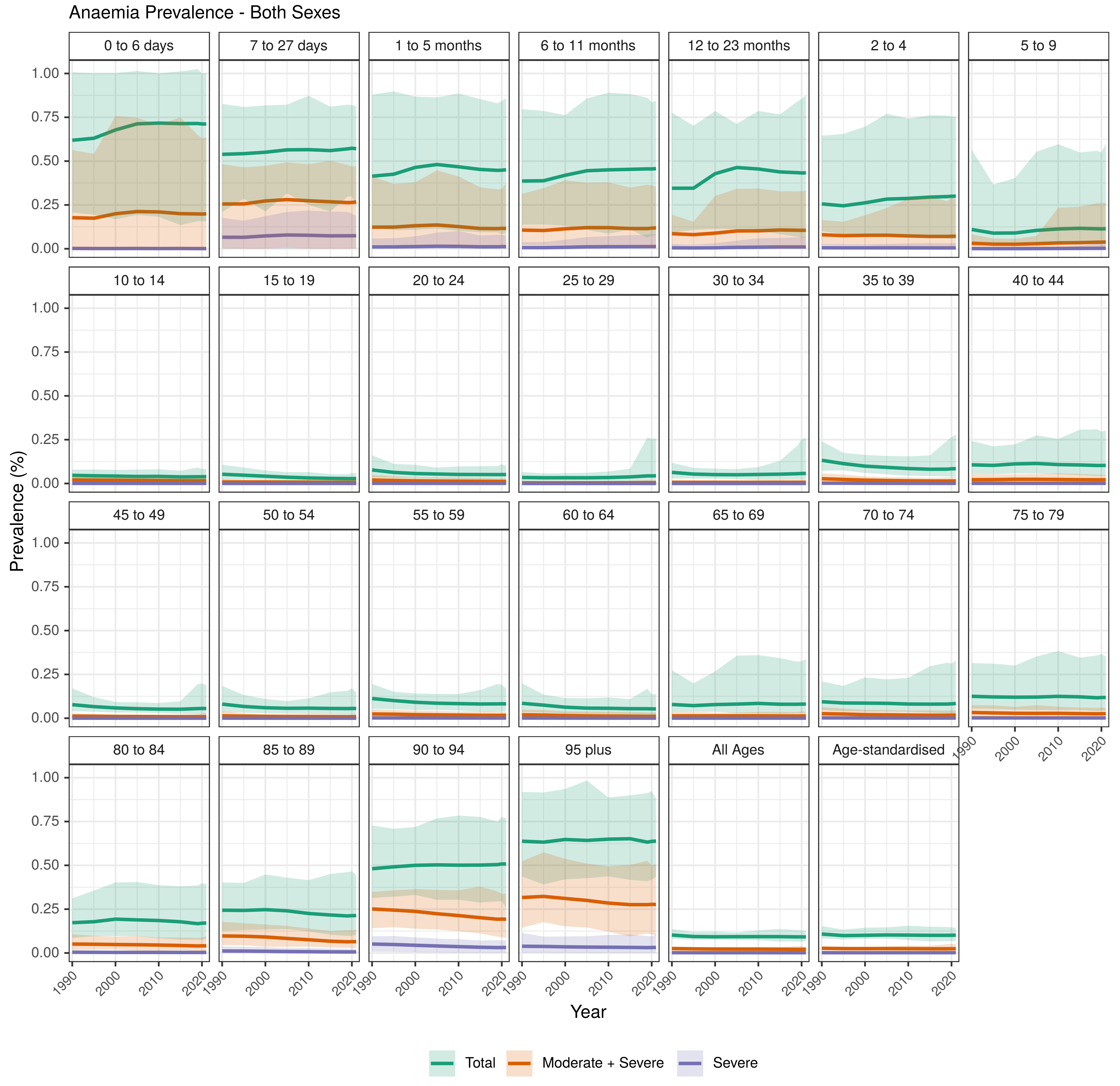

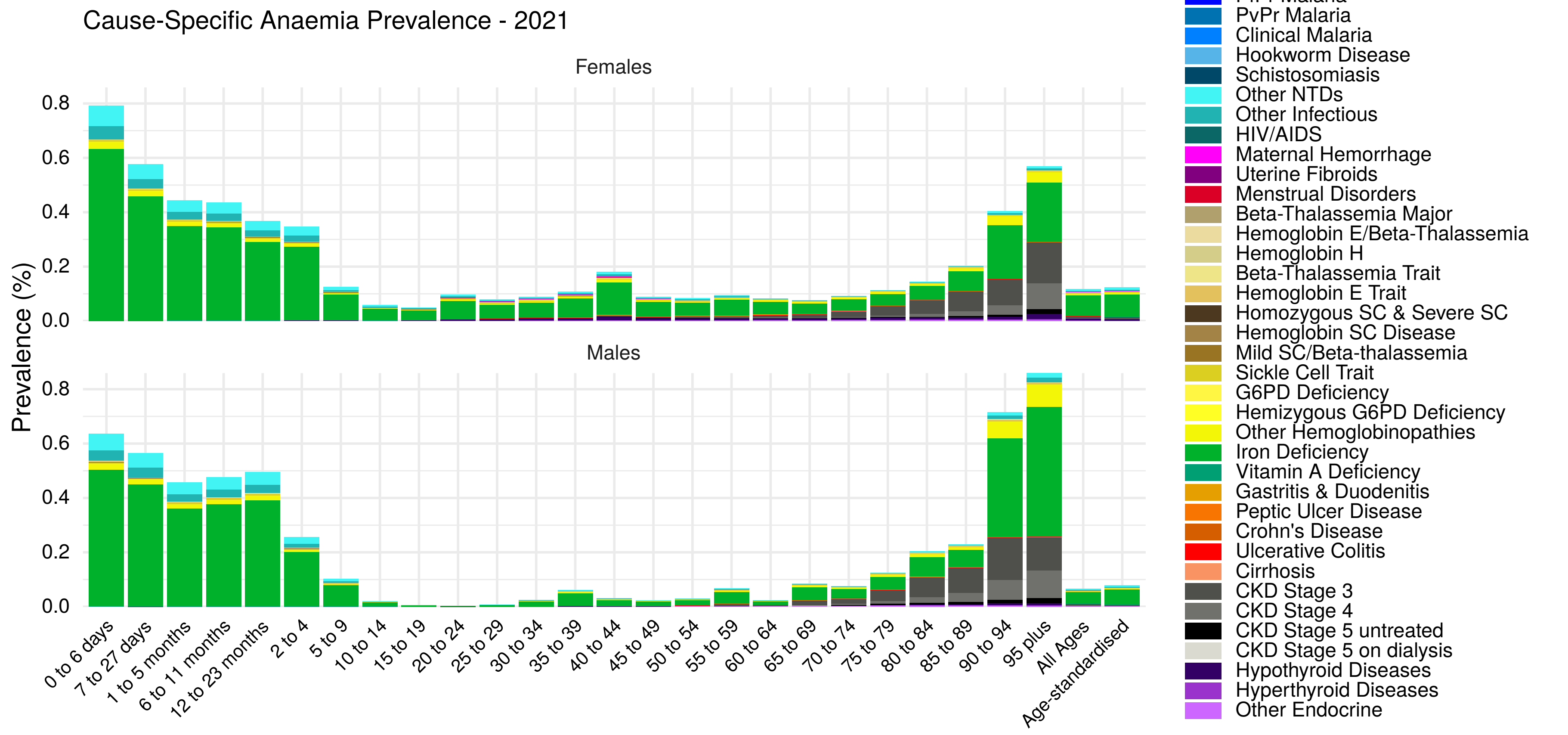

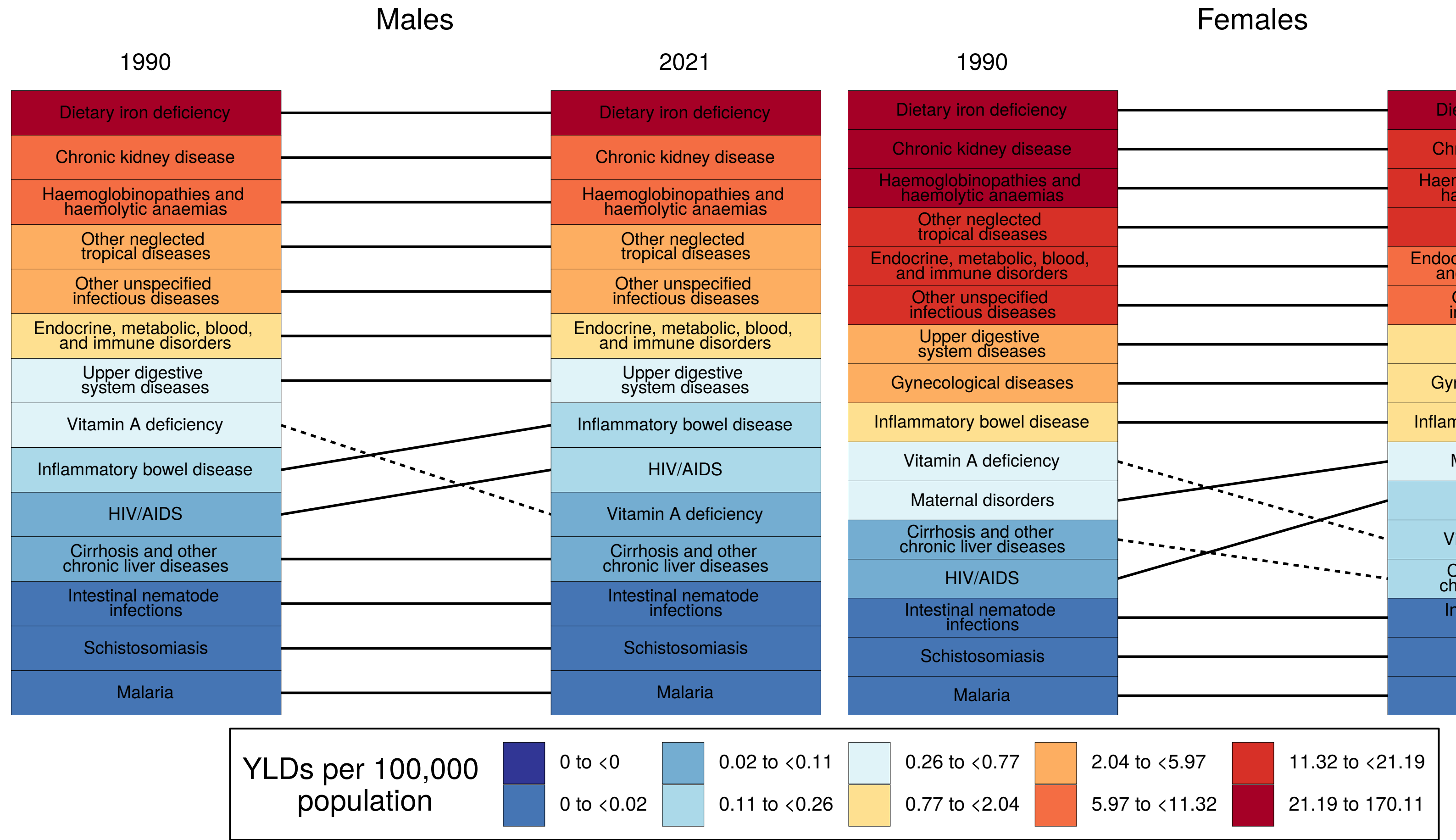

Cornwall

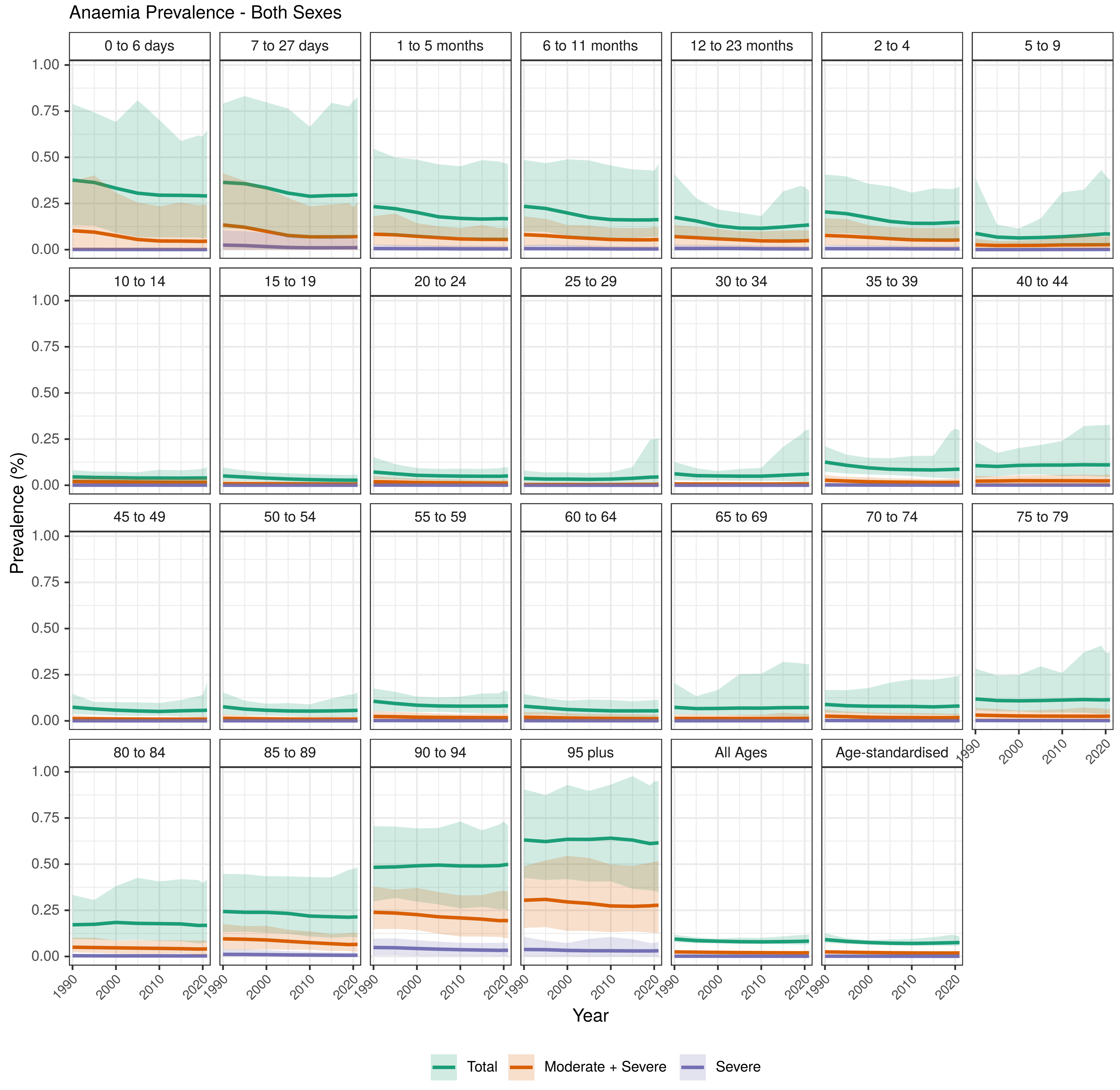

Anaemia Prevalence - Both Sexes
0 to 6 days
7 to 27 days
1 to 5 months
6 to 11 months
12 to 23 months
2 to 4
5 to 9
10 to 14
15 to 19
20 to 24
25 to 29
30 to 34
35 to 39
40 to 44
45 to 49
50 to 54
55 to 59
60 to 64
65 to 69
70 to 74
75 to 79
80 to 84
85 to 89
90 to 94
95 plus
All Ages
Age-standardised
Prevalence (%)
1.00
0.75
0.50
0.25
0.00
1990
2000
2010
2020
Year
Total
Moderate + Severe
Severe

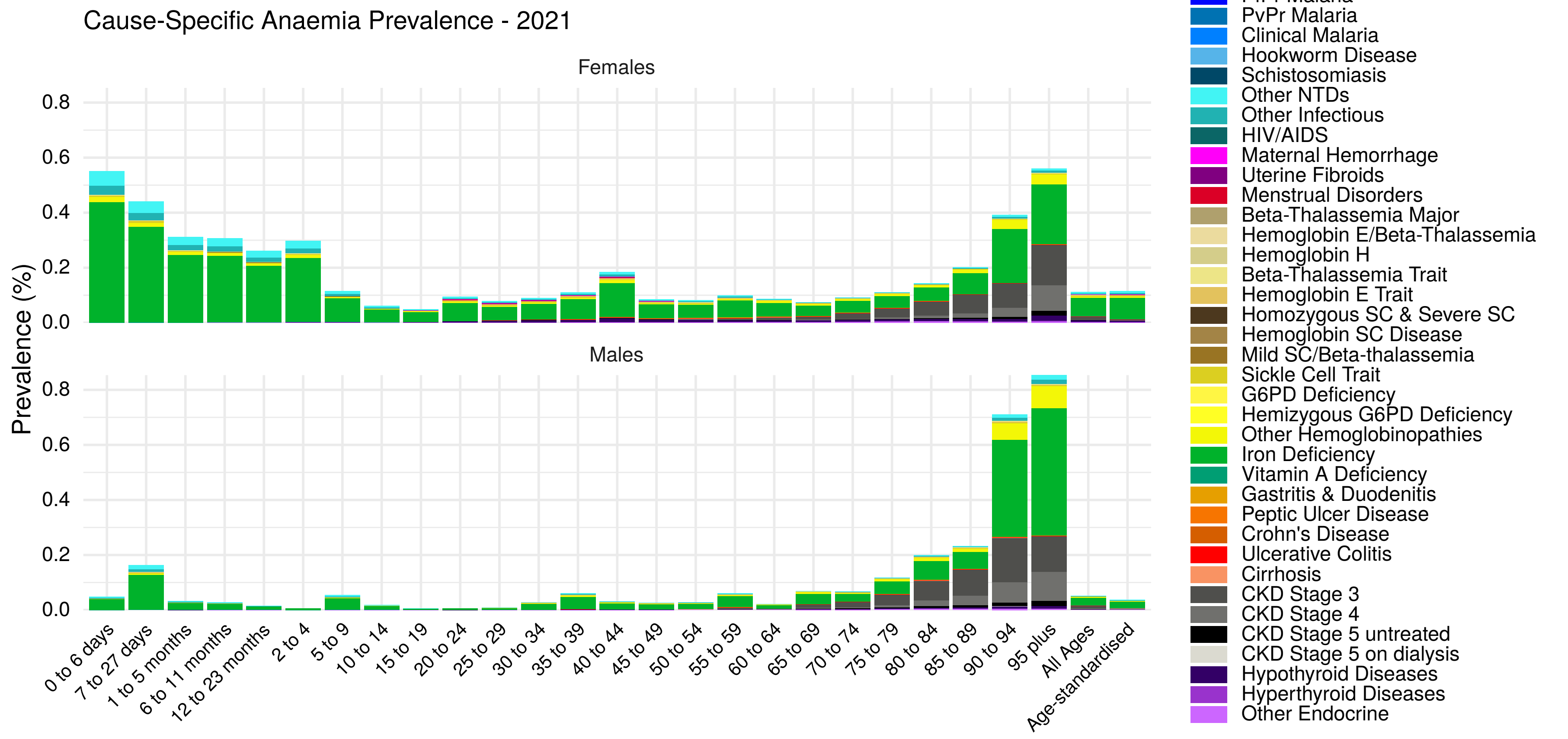

Cause-Specific Anaemia Prevalence - 2021
Females
Males
Prevalence (%)
0.8
0.6
0.4
0.2
0.0
0 to 6 days
7 to 27 days
1 to 5 months
6 to 11 months
12 to 23 months
2 to 4
5 to 9
10 to 14
15 to 19
20 to 24
25 to 29
30 to 34
35 to 39
40 to 44
45 to 49
50 to 54
55 to 59
60 to 64
65 to 69
70 to 74
75 to 79
80 to 84
85 to 89
90 to 94
95 plus
All Ages
Age-standardised
PfPr Malaria
PvPr Malaria
Clinical Malaria
Hookworm Disease
Schistosomiasis
Other NTDs
Other Infectious
HIV/AIDS
Maternal Hemorrhage
Uterine Fibroids
Menstrual Disorders
Beta-Thalassemia Major
Hemoglobin E/Beta-Thalassemia
Hemoglobin H
Beta-Thalassemia Trait
Hemoglobin E Trait
Homozygous SC & Severe SC
Hemoglobin SC Disease
Mild SC/Beta-thalassemia
Sickle Cell Trait
G6PD Deficiency
Hemizygous G6PD Deficiency
Other Hemoglobinopathies
Iron Deficiency
Vitamin A Deficiency
Gastritis & Duodenitis
Peptic Ulcer Disease
Crohn's Disease
Ulcerative Colitis
Cirrhosis
CKD Stage 3
CKD Stage 4
CKD Stage 5 untreated
CKD Stage 5 on dialysis
Hypothyroid Diseases
Hyperthyroid Diseases
Other Endocrine

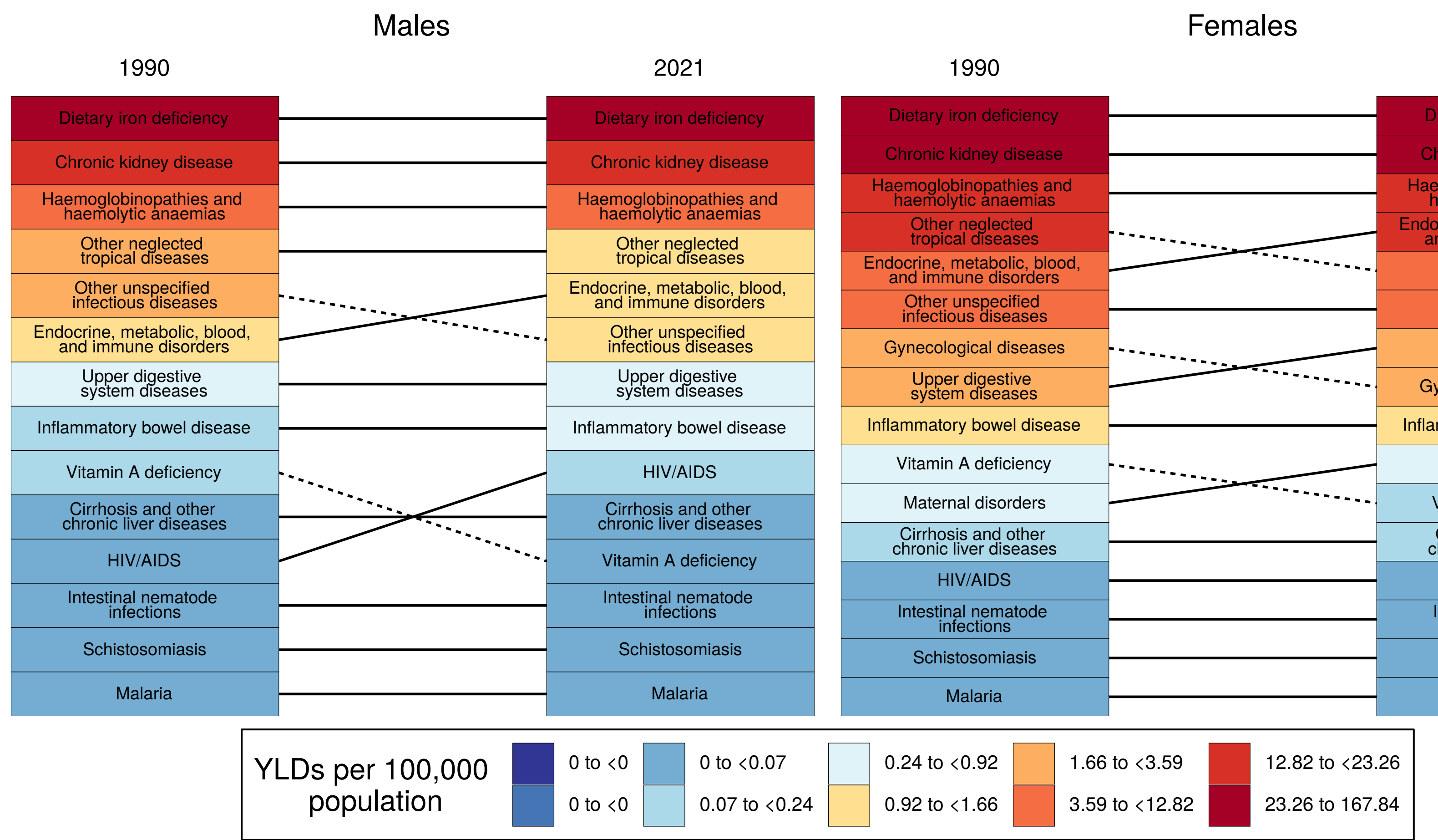

Males
1990
Dietary iron deficiency
Chronic kidney disease
Haemoglobinopathies and haemolytic anaemias
Other neglected tropical diseases
Other unspecified infectious diseases
Endocrine, metabolic, blood, and immune disorders
Upper digestive system diseases
Inflammatory bowel disease
Vitamin A deficiency
Cirrhosis and other chronic liver diseases
HIV/AIDS
Intestinal nematode infections
Schistosomiasis
Malaria
2021
Dietary iron deficiency
Chronic kidney disease
Haemoglobinopathies and haemolytic anaemias
Other neglected tropical diseases
Endocrine, metabolic, blood, and immune disorders
Other unspecified infectious diseases
Upper digestive system diseases
Inflammatory bowel disease
HIV/AIDS
Cirrhosis and other chronic liver diseases
Vitamin A deficiency
Intestinal nematode infections
Schistosomiasis
Malaria
Females
1990
Dietary iron deficiency
Chronic kidney disease
Haemoglobinopathies and haemolytic anaemias
Other neglected tropical diseases
Endocrine, metabolic, blood, and immune disorders
Other unspecified infectious diseases
Gynecological diseases
Upper digestive system diseases
Inflammatory bowel disease
Vitamin A deficiency
Maternal disorders
Cirrhosis and other chronic liver diseases
HIV/AIDS
Intestinal nematode infections
Schistosomiasis
Malaria
2021
Dietary iron deficiency
Chronic kidney disease
Haemoglobinopathies and haemolytic anaemias
Endocrine, metabolic, blood, and immune disorders
Other neglected tropical diseases
Other unspecified infectious diseases
Upper digestive system diseases
Gynecological diseases
Inflammatory bowel disease
Maternal disorders
Vitamin A deficiency
Cirrhosis and other chronic liver diseases
HIV/AIDS
Intestinal nematode infections
Schistosomiasis
Malaria
YLDs per 100,000 population
0 to <0
0 to <0
0 to <0.07
0.07 to <0.24
0.24 to <0.92
0.92 to <1.66
1.66 to <3.59
3.59 to <12.82
12.82 to <23.26
23.26 to 167.84

Devon

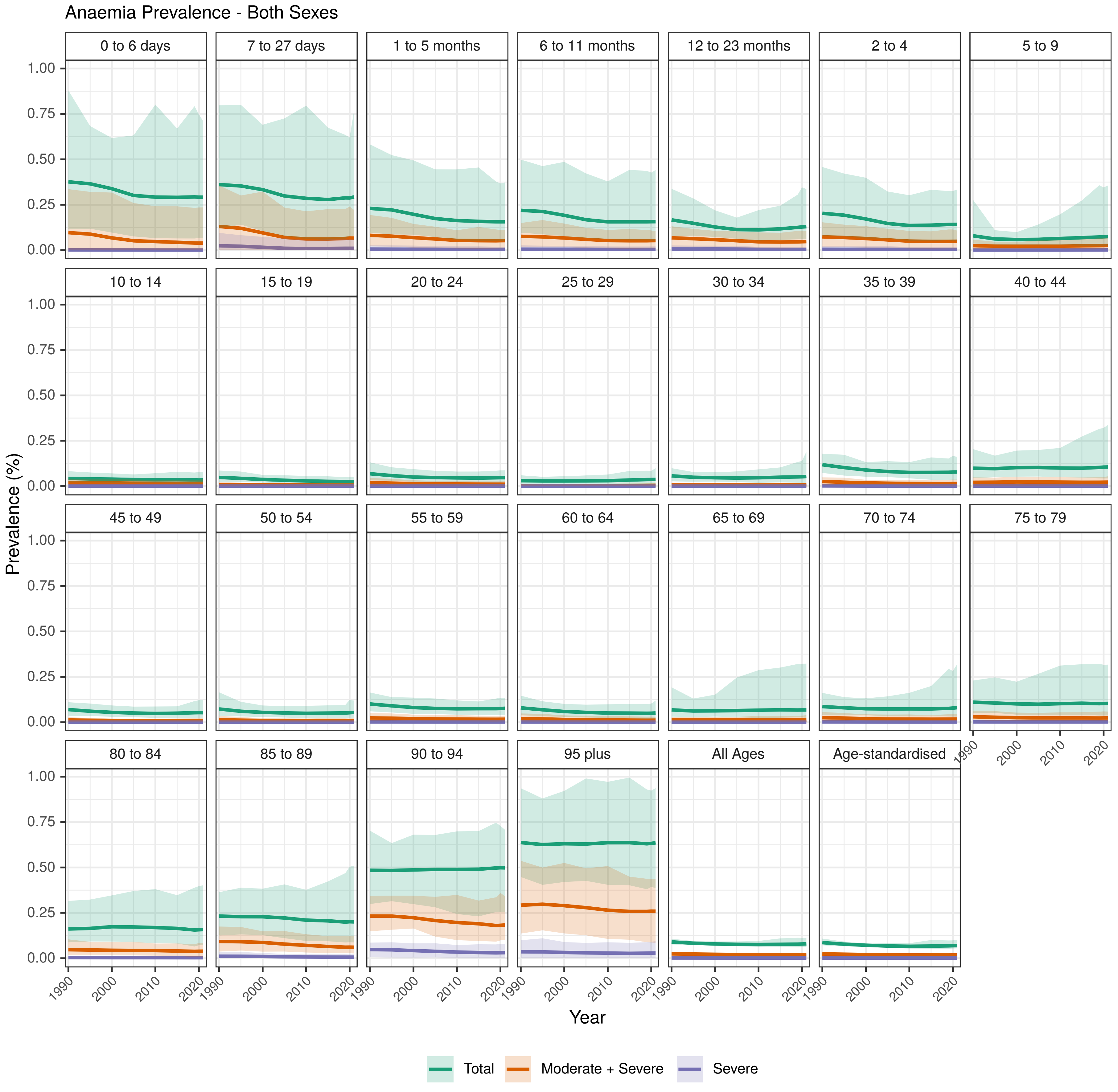

Anaemia Prevalence - Both Sexes
0 to 6 days
7 to 27 days
1 to 5 months
6 to 11 months
12 to 23 months
2 to 4
5 to 9
10 to 14
15 to 19
20 to 24
25 to 29
30 to 34
35 to 39
40 to 44
45 to 49
50 to 54
55 to 59
60 to 64
65 to 69
70 to 74
75 to 79
80 to 84
85 to 89
90 to 94
95 plus
All Ages
Age-standardised
Prevalence (%)
Year
Total
Moderate + Severe
Severe

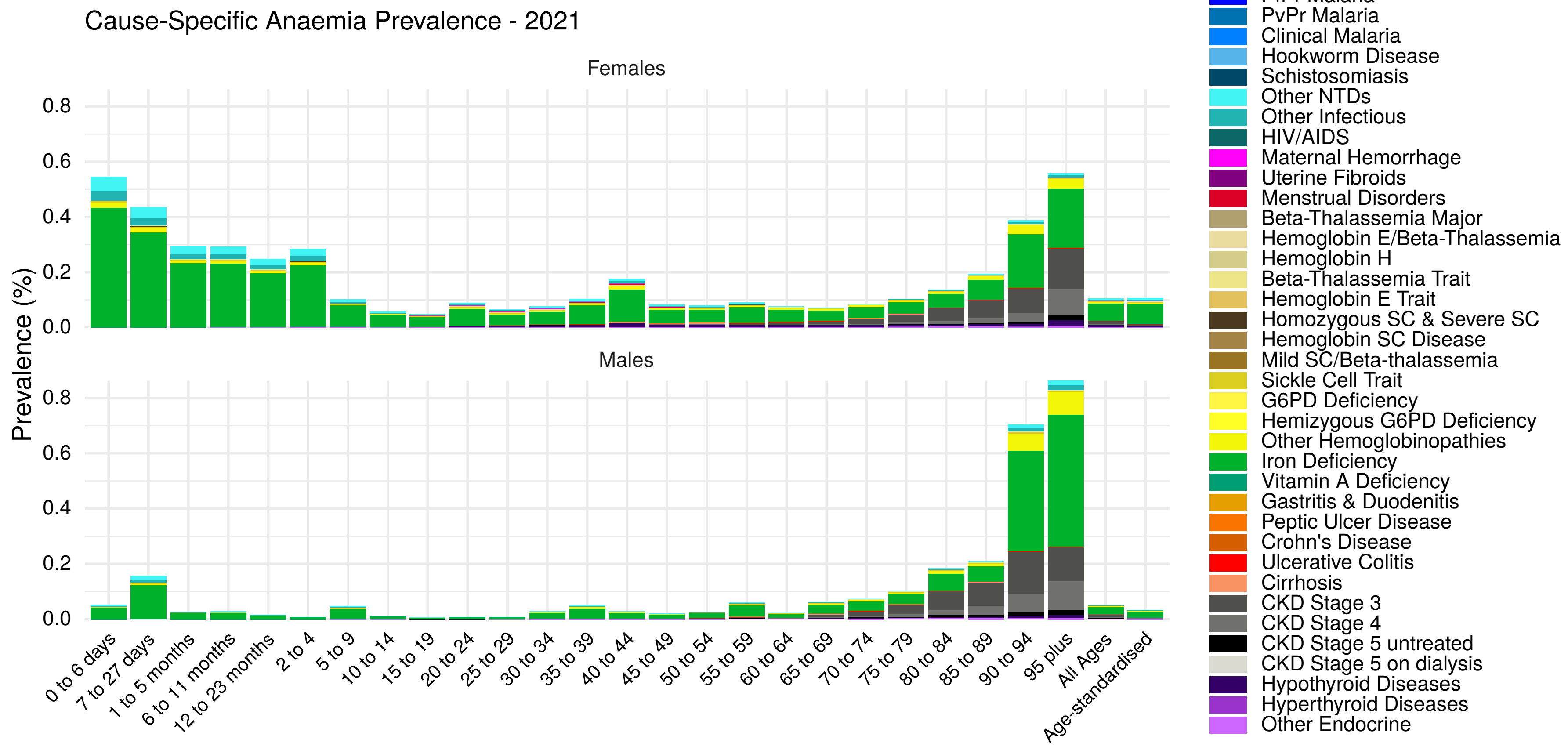

Cause-Specific Anaemia Prevalence - 2021
Females
Males
Prevalence (%)
PfPr Malaria
PvPr Malaria
Clinical Malaria
Hookworm Disease
Schistosomiasis
Other NTDs
Other Infectious
HIV/AIDS
Maternal Hemorrhage
Uterine Fibroids
Menstrual Disorders
Beta-Thalassemia Major
Hemoglobin E/Beta-Thalassemia
Hemoglobin H
Beta-Thalassemia Trait
Hemoglobin E Trait
Homozygous SC & Severe SC
Hemoglobin SC Disease
Mild SC/Beta-thalassemia
Sickle Cell Trait
G6PD Deficiency
Hemizygous G6PD Deficiency
Other Hemoglobinopathies
Iron Deficiency
Vitamin A Deficiency
Gastritis & Duodenitis
Peptic Ulcer Disease
Crohn's Disease
Ulcerative Colitis
Cirrhosis
CKD Stage 3
CKD Stage 4
CKD Stage 5 untreated
CKD Stage 5 on dialysis
Hypothyroid Diseases
Hyperthyroid Diseases
Other Endocrine

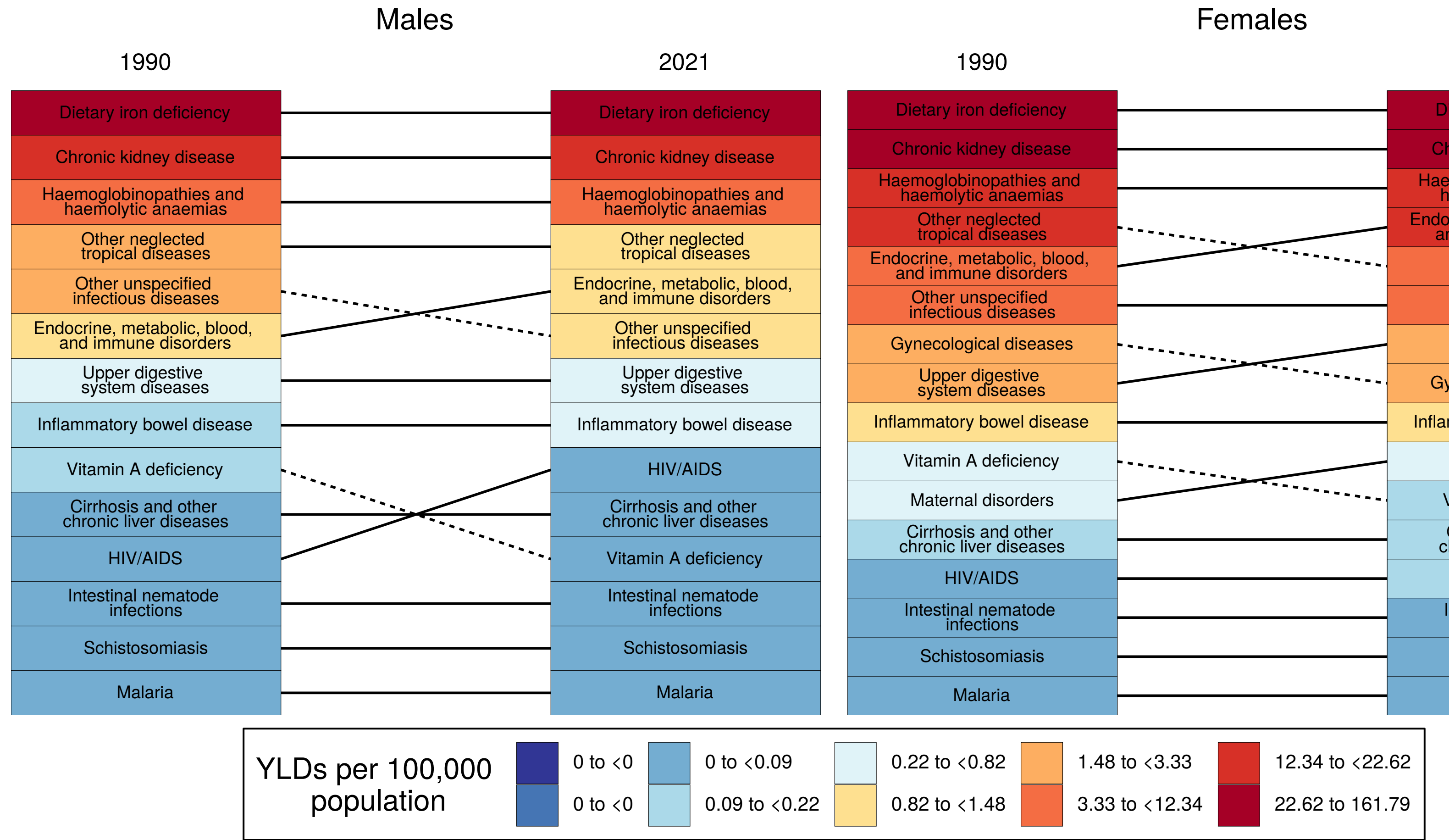

Males
Females
1990
2021
Dietary iron deficiency
Chronic kidney disease
Haemoglobinopathies and haemolytic anaemias
Other neglected tropical diseases
Other unspecified infectious diseases
Endocrine, metabolic, blood, and immune disorders
Upper digestive system diseases
Inflammatory bowel disease
Vitamin A deficiency
Cirrhosis and other chronic liver diseases
HIV/AIDS
Intestinal nematode infections
Schistosomiasis
Malaria
Gynecological diseases
Maternal disorders
YLDs per 100,000 population
0 to <0
0 to <0.09
0.09 to <0.22
0.22 to <0.82
0.82 to <1.48
1.48 to <3.33
3.33 to <12.34
12.34 to <22.62
22.62 to 161.79

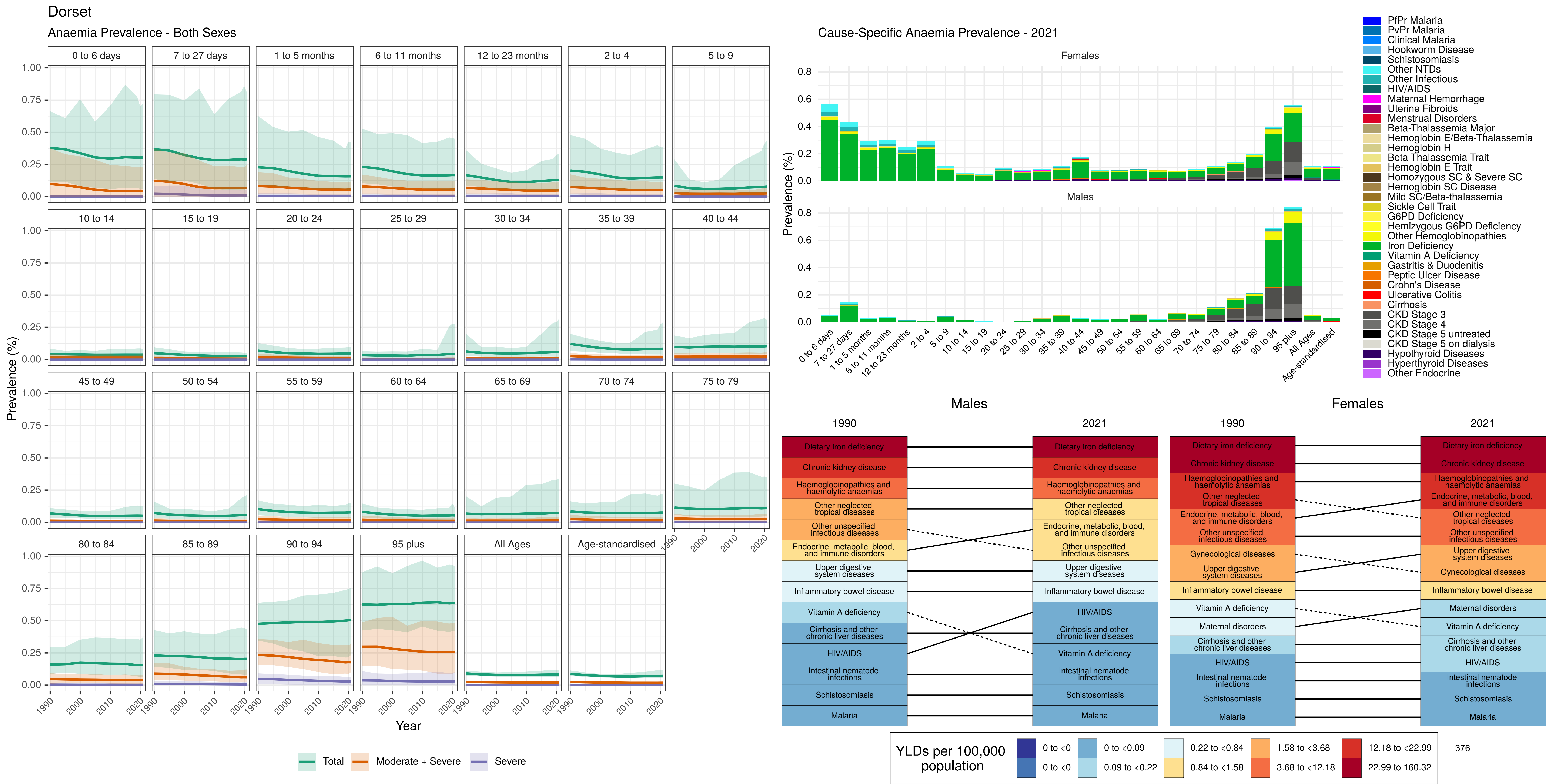
Dorset
Anaemia Prevalence - Both Sexes
0 to 6 days
7 to 27 days
1 to 5 months
6 to 11 months
12 to 23 months
2 to 4
5 to 9
10 to 14
15 to 19
20 to 24
25 to 29
30 to 34
35 to 39
40 to 44
45 to 49
50 to 54
55 to 59
60 to 64
65 to 69
70 to 74
75 to 79
80 to 84
85 to 89
90 to 94
95 plus
All Ages
Age-standardised
Prevalence (%)
Year
Total
Moderate + Severe
Severe
Cause-Specific Anaemia Prevalence - 2021
Females
Males
PfPr Malaria
PvPr Malaria
Clinical Malaria
Hookworm Disease
Schistosomiasis
Other NTDs
Other Infectious
HIV/AIDS
Maternal Hemorrhage
Uterine Fibroids
Menstrual Disorders
Beta-Thalassemia Major
Hemoglobin E/Beta-Thalassemia
Hemoglobin H
Beta-Thalassemia Trait
Hemoglobin E Trait
Homozygous SC & Severe SC
Hemoglobin SC Disease
Mild SC/Beta-thalassemia
Sickle Cell Trait
G6PD Deficiency
Hemizygous G6PD Deficiency
Other Hemoglobinopathies
Iron Deficiency
Vitamin A Deficiency
Gastritis & Duodenitis
Peptic Ulcer Disease
Crohn's Disease
Ulcerative Colitis
Cirrhosis
CKD Stage 3
CKD Stage 4
CKD Stage 5 untreated
CKD Stage 5 on dialysis
Hypothyroid Diseases
Hyperthyroid Diseases
Other Endocrine
Males
Females
1990
2021
Dietary iron deficiency
Chronic kidney disease
Haemoglobinopathies and haemolytic anaemias
Other neglected tropical diseases
Other unspecified infectious diseases
Endocrine, metabolic, blood, and immune disorders
Upper digestive system diseases
Inflammatory bowel disease
Vitamin A deficiency
Cirrhosis and other chronic liver diseases
HIV/AIDS
Intestinal nematode infections
Schistosomiasis
Malaria
Gynecological diseases
Maternal disorders
YLDs per 100,000 population
0 to <0
0 to <0
0 to <0.09
0.09 to <0.22
0.22 to <0.84
0.84 to <1.58
1.58 to <3.68
3.68 to <12.18
12.18 to <22.99
22.99 to 160.32

Gloucestershire

Anaemia Prevalence - Both Sexes

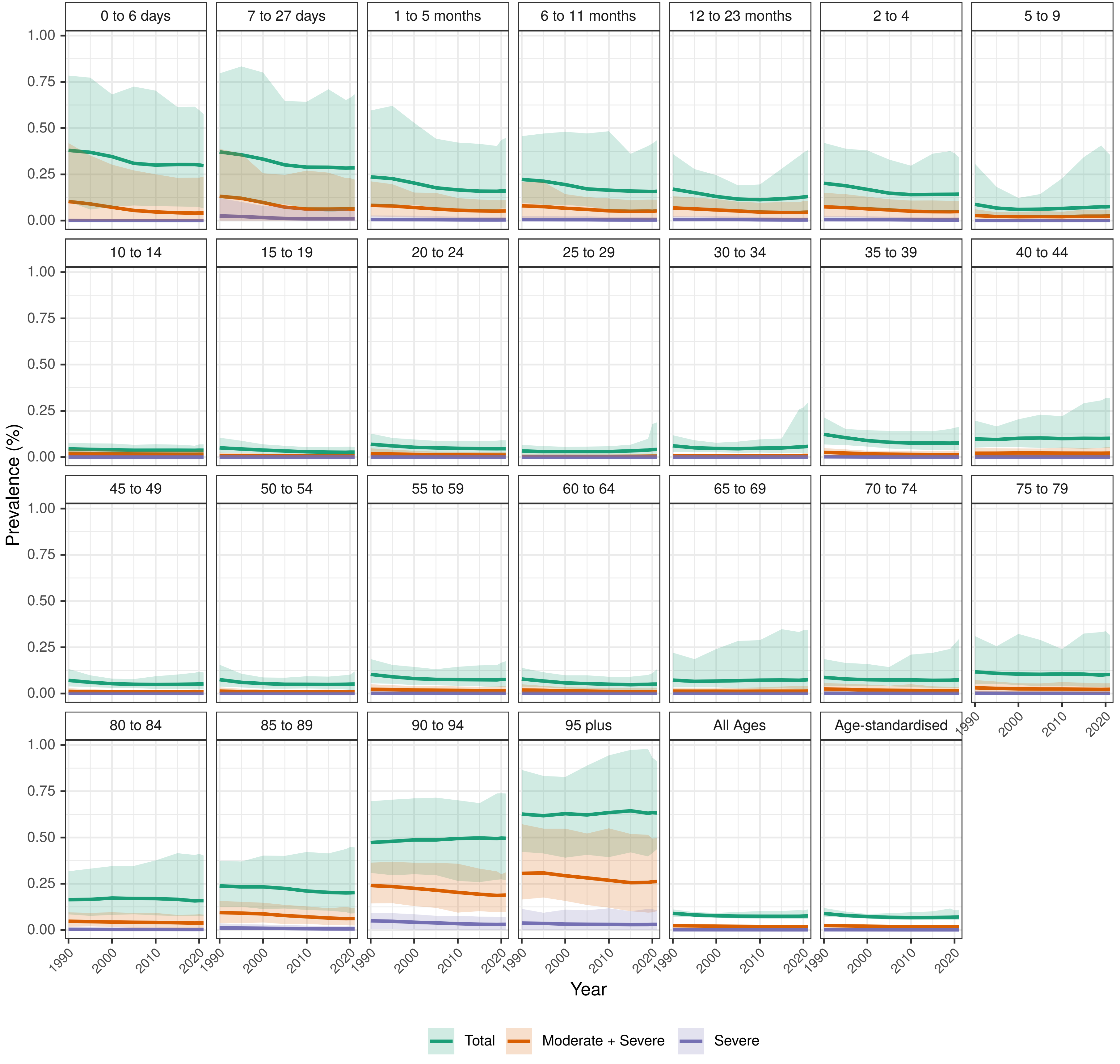

Cause-Specific Anaemia Prevalence - 2021

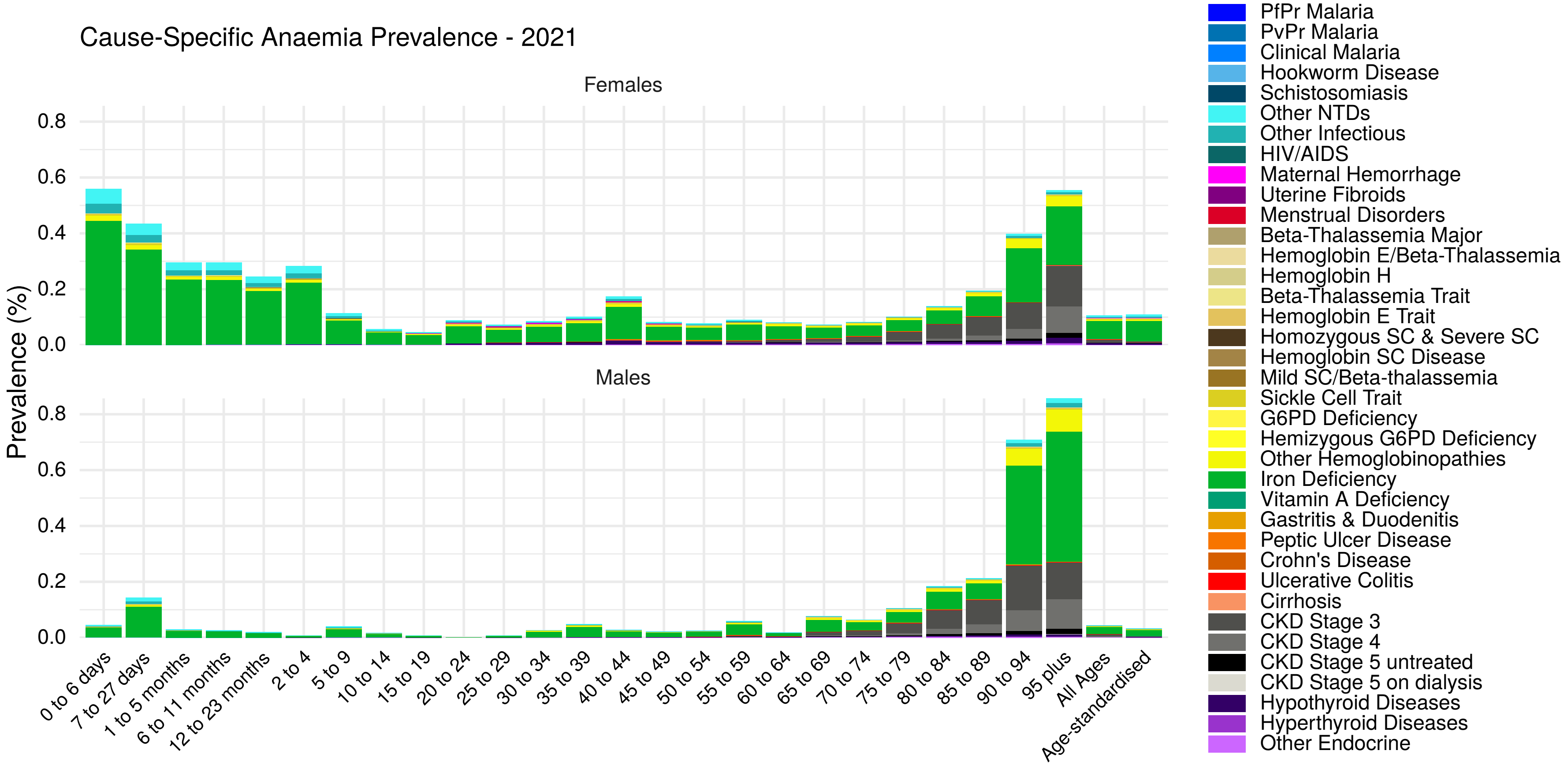

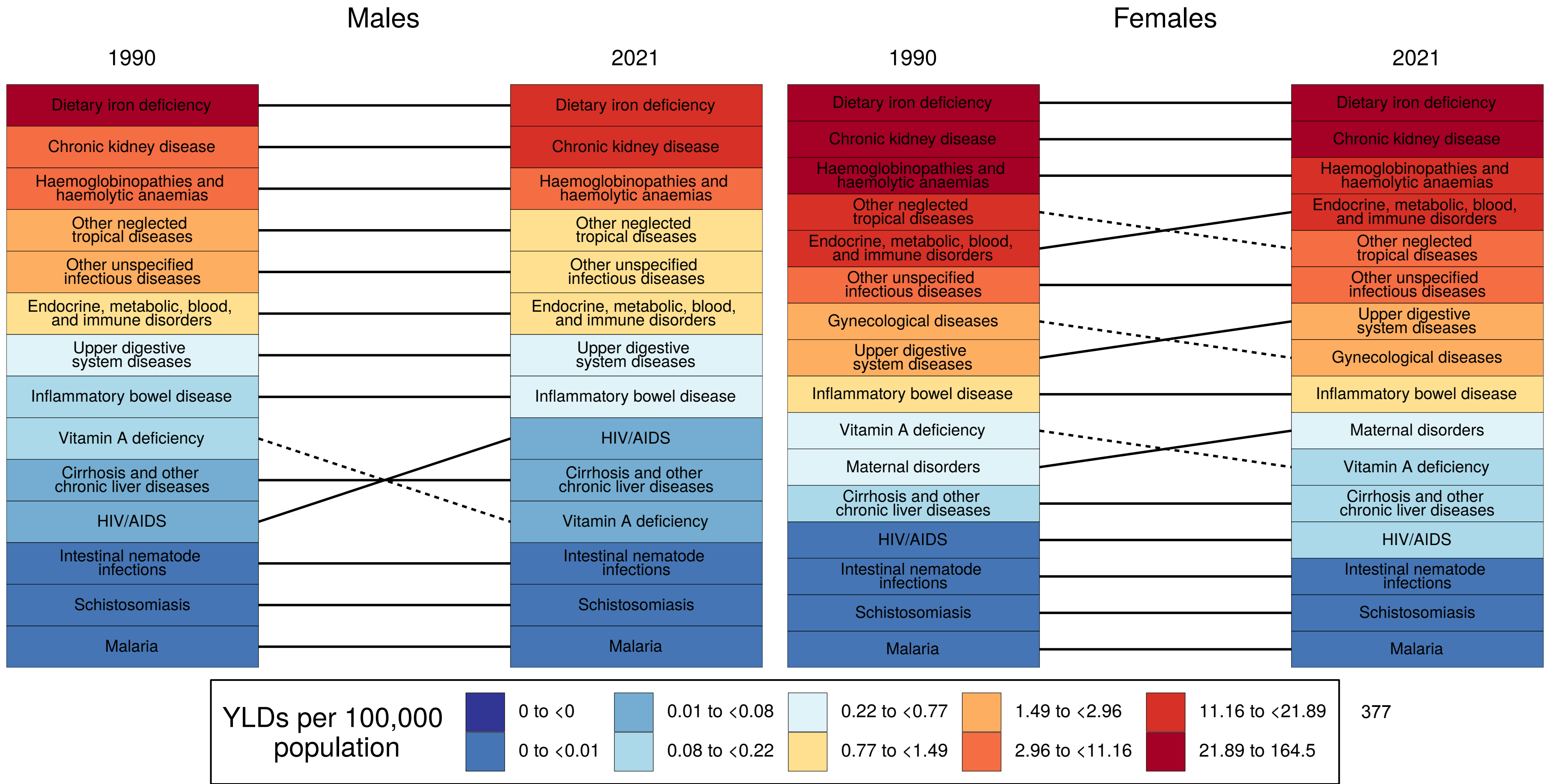

North Somerset

Anaemia Prevalence - Both Sexes

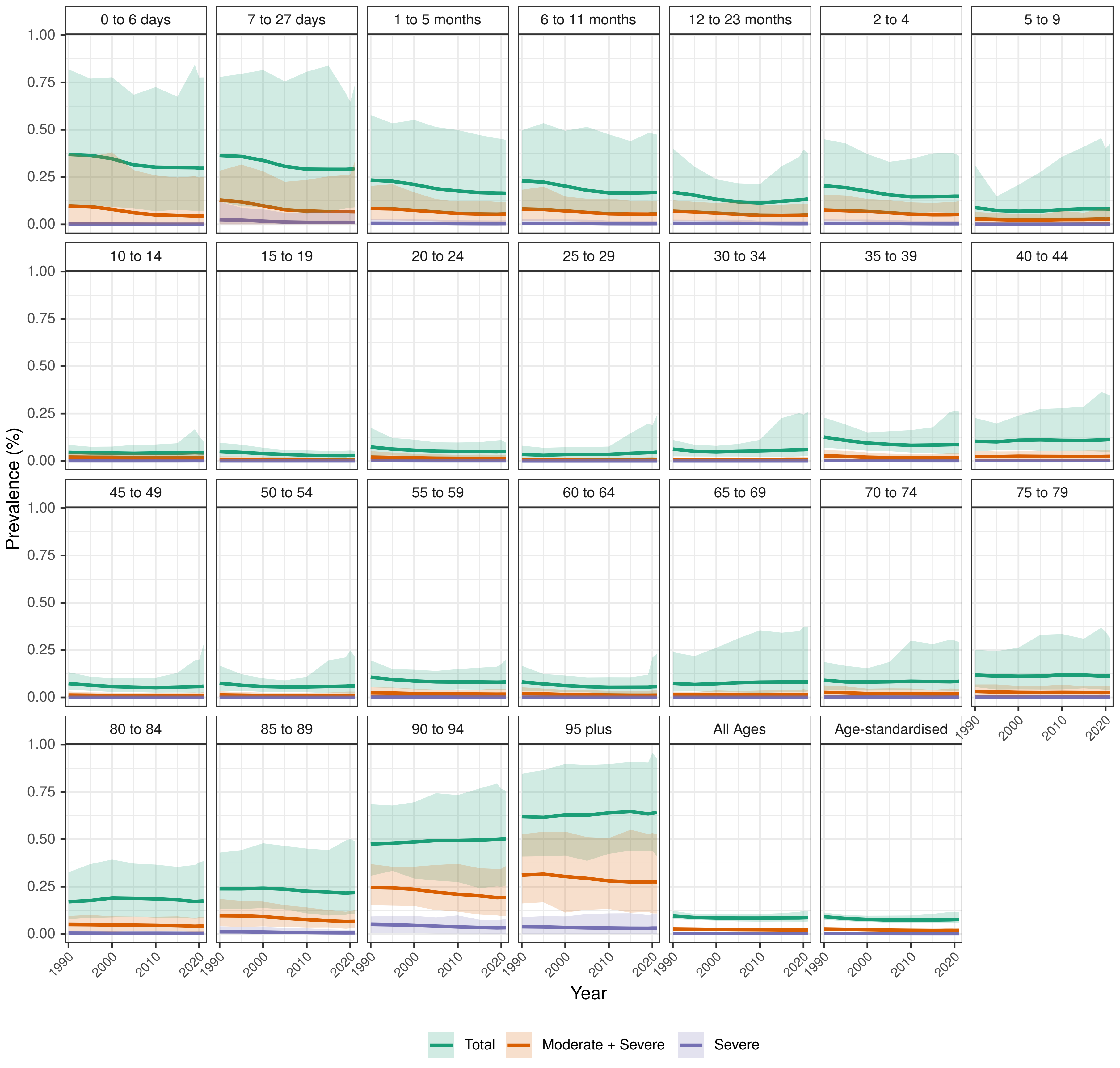

Cause-Specific Anaemia Prevalence - 2021

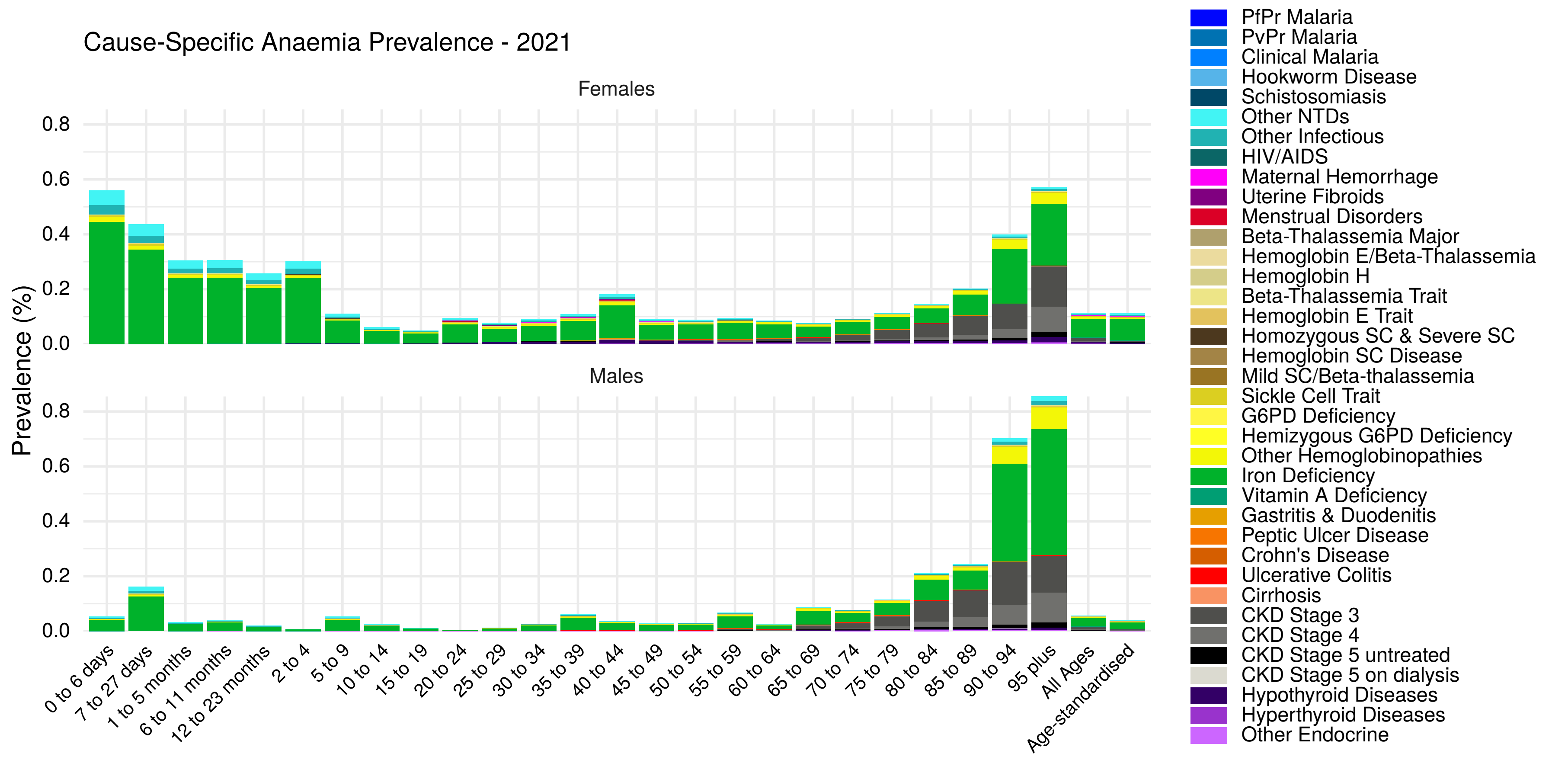

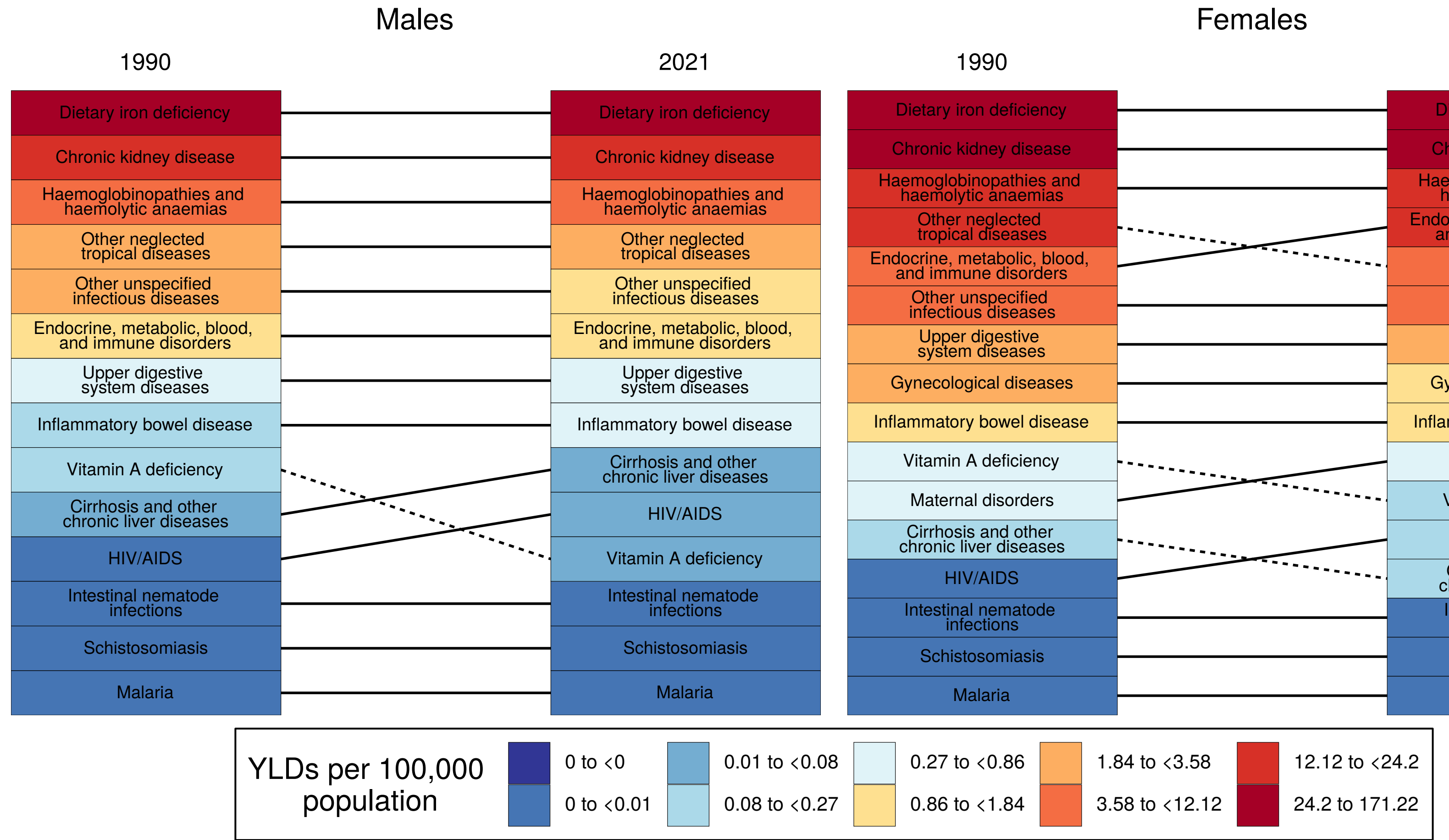

Plymouth

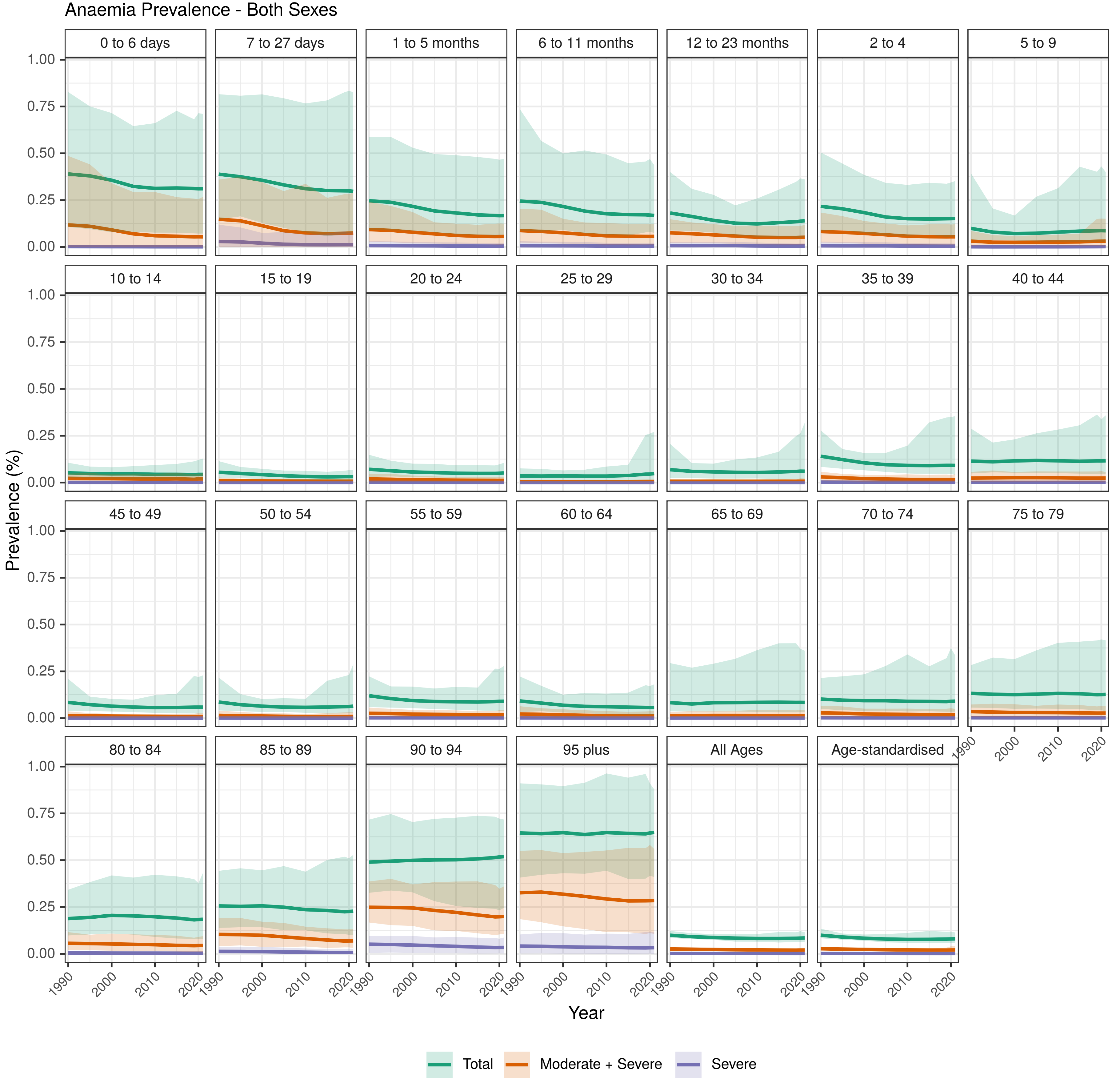
Anaemia Prevalence - Both Sexes
0 to 6 days
7 to 27 days
1 to 5 months
6 to 11 months
12 to 23 months
2 to 4
5 to 9
10 to 14
15 to 19
20 to 24
25 to 29
30 to 34
35 to 39
40 to 44
45 to 49
50 to 54
55 to 59
60 to 64
65 to 69
70 to 74
75 to 79
80 to 84
85 to 89
90 to 94
95 plus
All Ages
Age-standardised
Prevalence (%)
Year
Total
Moderate + Severe
Severe

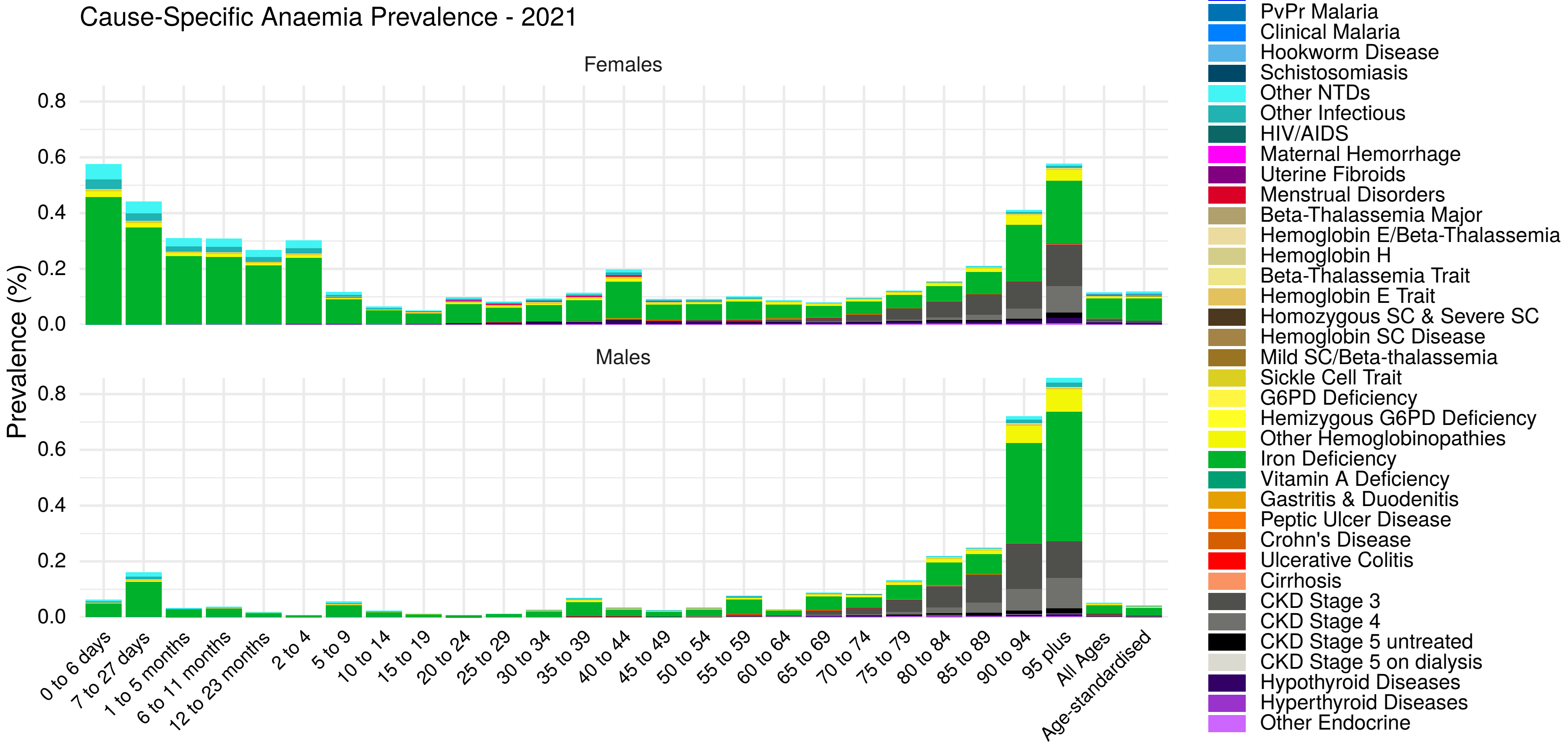
Cause-Specific Anaemia Prevalence - 2021
Females
Males
Prevalence (%)
PfPr Malaria
PvPr Malaria
Clinical Malaria
Hookworm Disease
Schistosomiasis
Other NTDs
Other Infectious
HIV/AIDS
Maternal Hemorrhage
Uterine Fibroids
Menstrual Disorders
Beta-Thalassemia Major
Hemoglobin E/Beta-Thalassemia
Hemoglobin H
Beta-Thalassemia Trait
Hemoglobin E Trait
Homozygous SC & Severe SC
Hemoglobin SC Disease
Mild SC/Beta-thalassemia
Sickle Cell Trait
G6PD Deficiency
Hemizygous G6PD Deficiency
Other Hemoglobinopathies
Iron Deficiency
Vitamin A Deficiency
Gastritis & Duodenitis
Peptic Ulcer Disease
Crohn's Disease
Ulcerative Colitis
Cirrhosis
CKD Stage 3
CKD Stage 4
CKD Stage 5 untreated
CKD Stage 5 on dialysis
Hypothyroid Diseases
Hyperthyroid Diseases
Other Endocrine

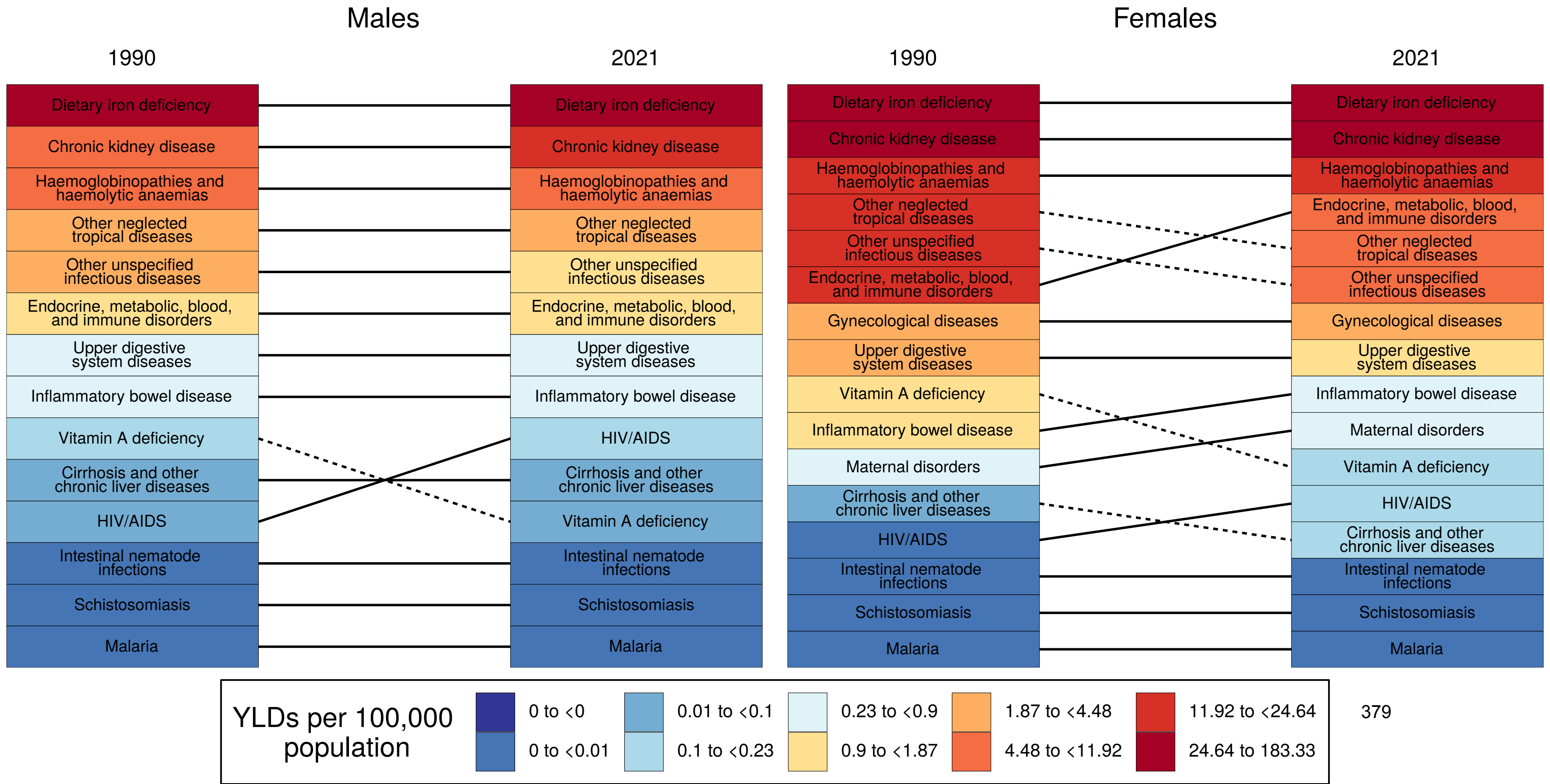
Males
Females
1990
2021
Dietary iron deficiency
Chronic kidney disease
Haemoglobinopathies and haemolytic anaemias
Other neglected tropical diseases
Other unspecified infectious diseases
Endocrine, metabolic, blood, and immune disorders
Upper digestive system diseases
Inflammatory bowel disease
Vitamin A deficiency
Cirrhosis and other chronic liver diseases
HIV/AIDS
Intestinal nematode infections
Schistosomiasis
Malaria
Gynecological diseases
Maternal disorders
YLDs per 100,000 population
0 to <0
0 to <0.01
0.01 to <0.1
0.1 to <0.23
0.23 to <0.9
0.9 to <1.87
1.87 to <4.48
4.48 to <11.92
11.92 to <24.64
24.64 to 183.33

Poole

Anaemia Prevalence - Both Sexes

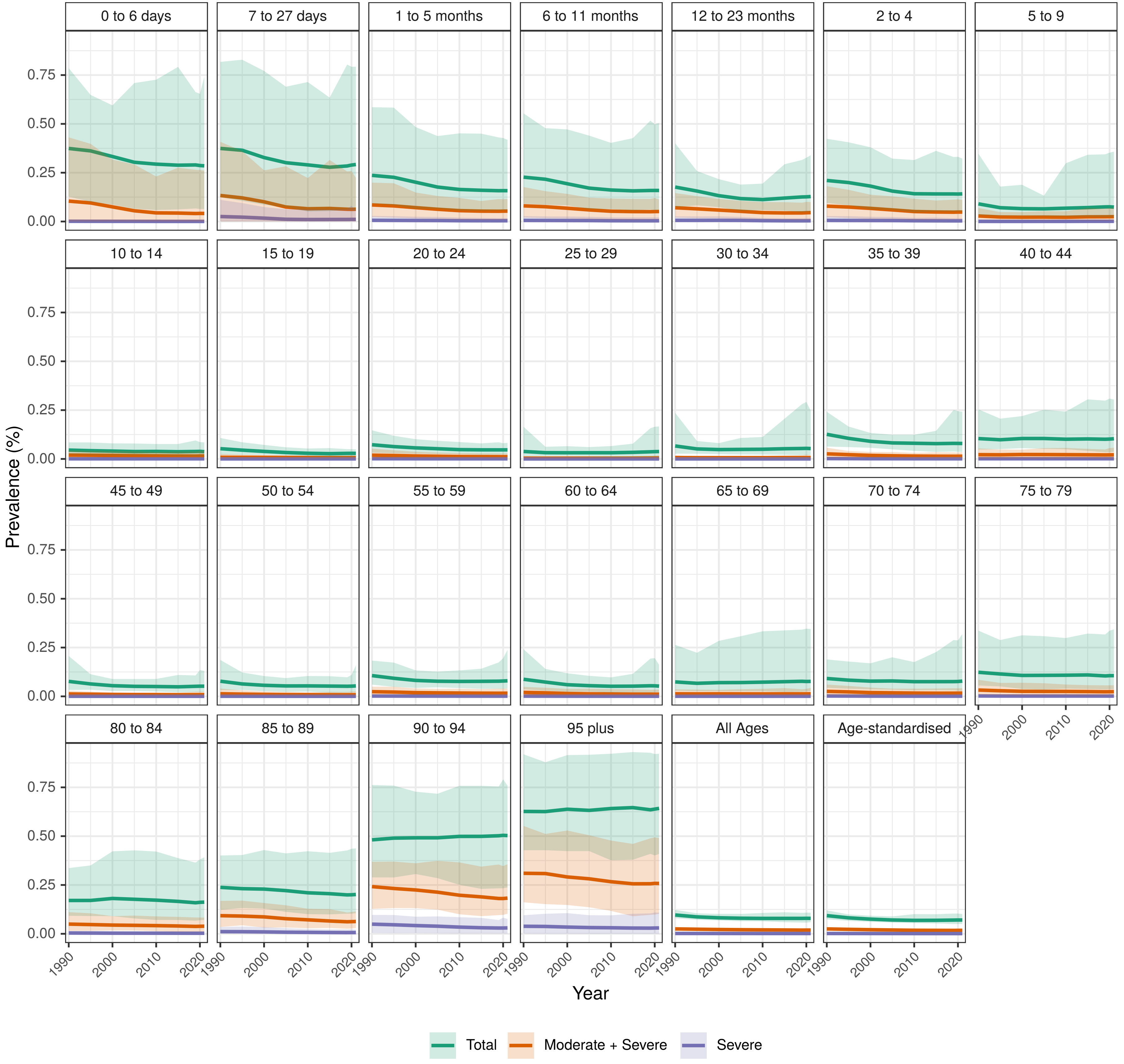

Cause-Specific Anaemia Prevalence - 2021

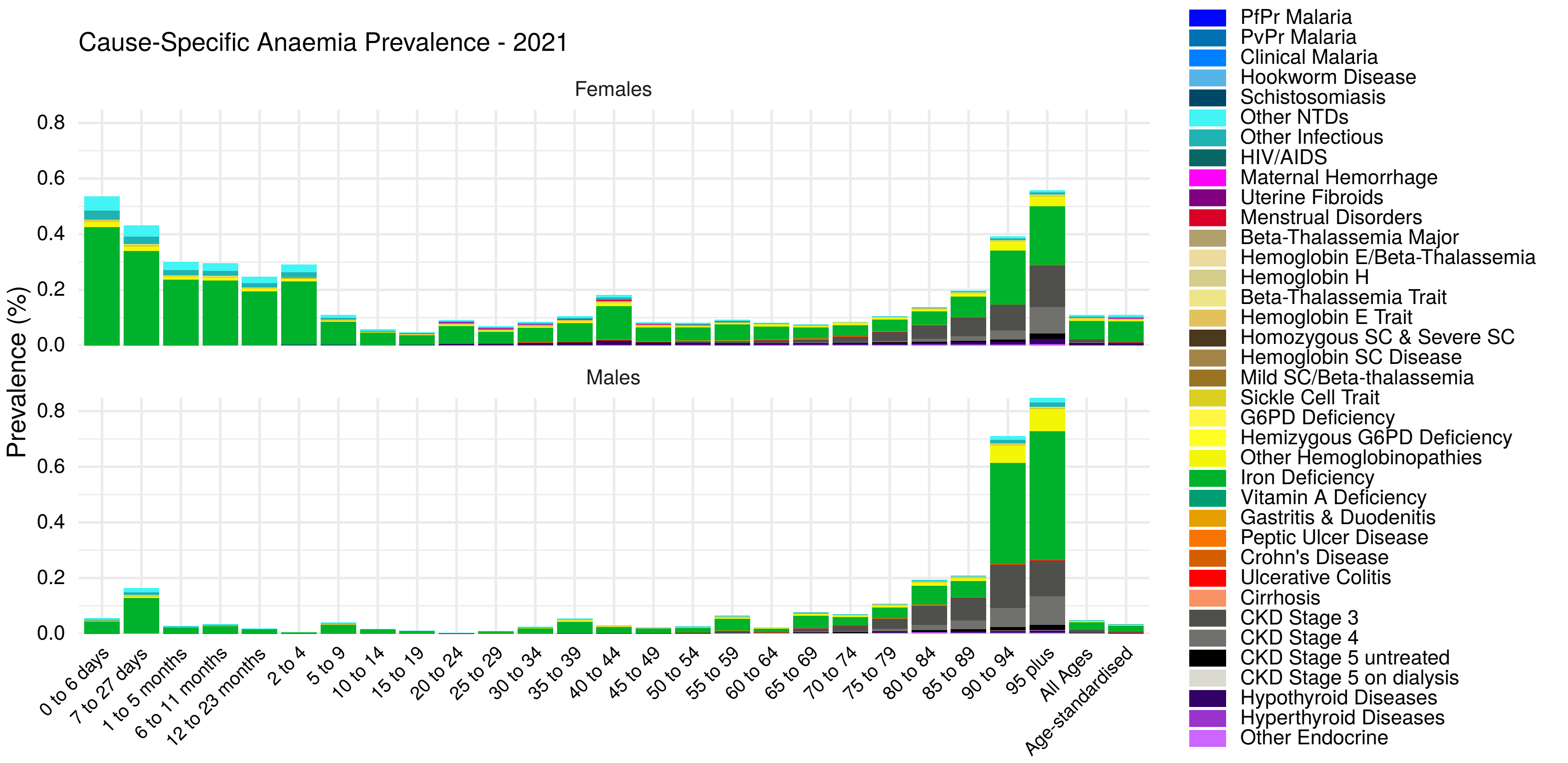

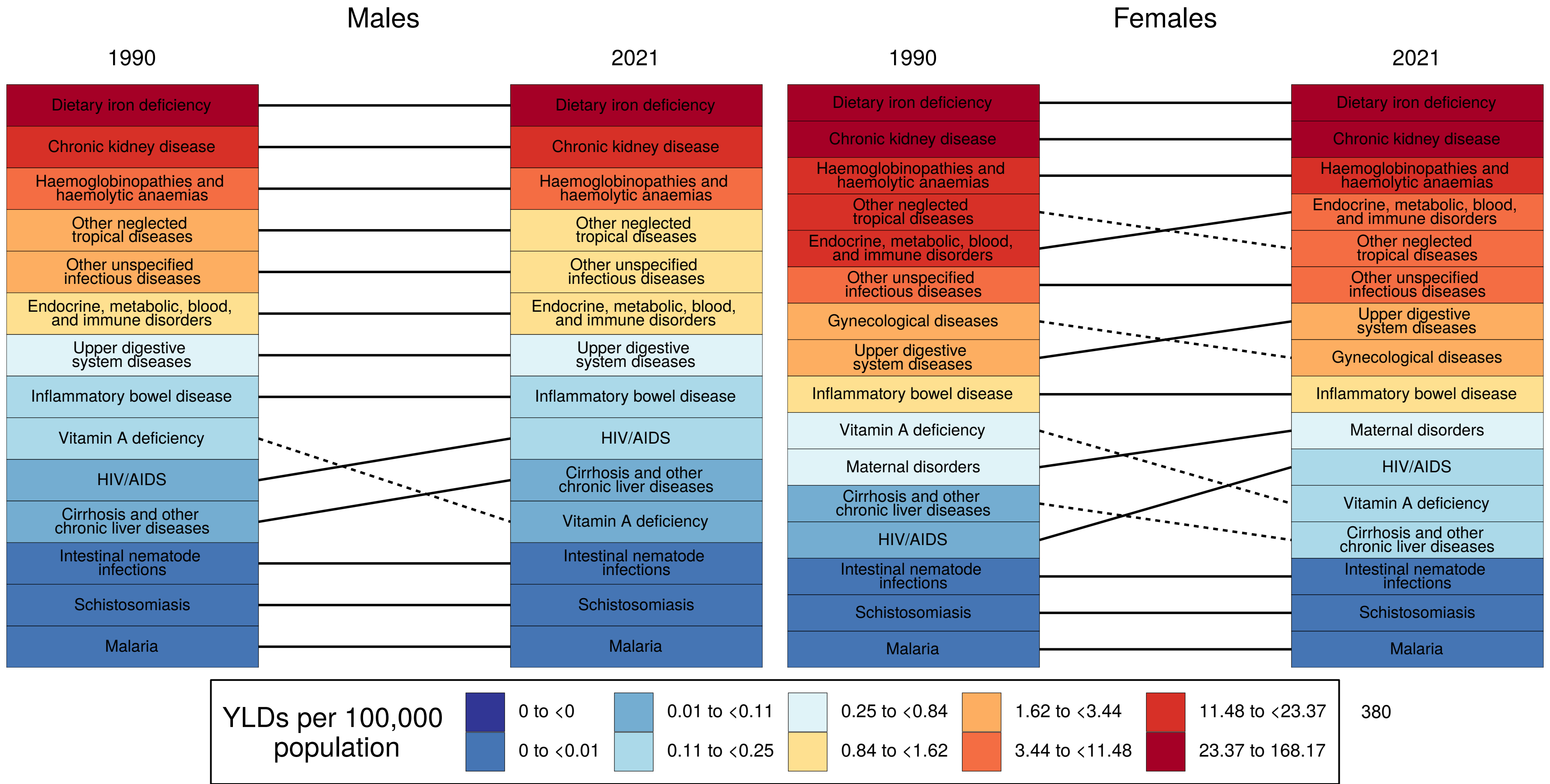

380

Somerset

Anaemia Prevalence - Both Sexes

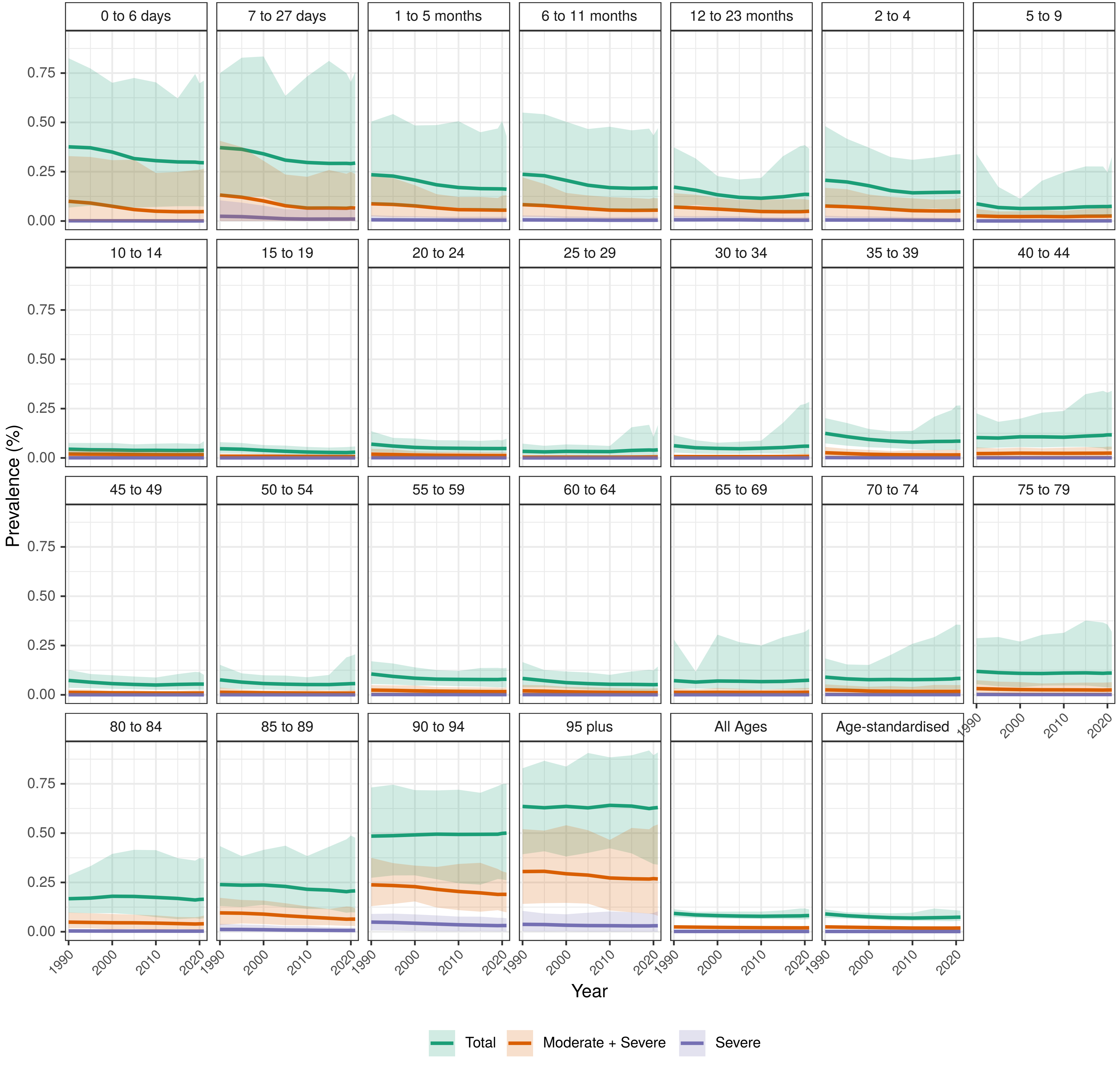

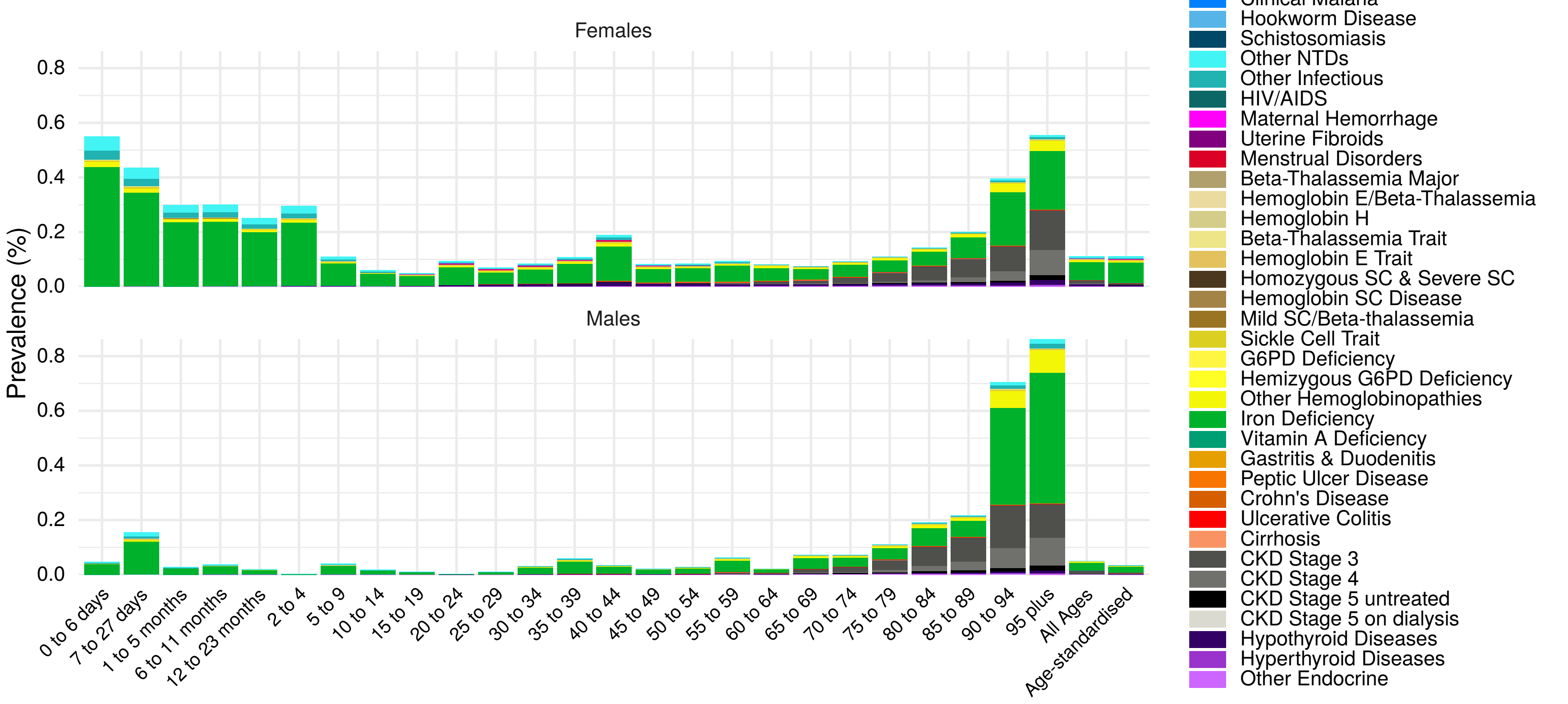

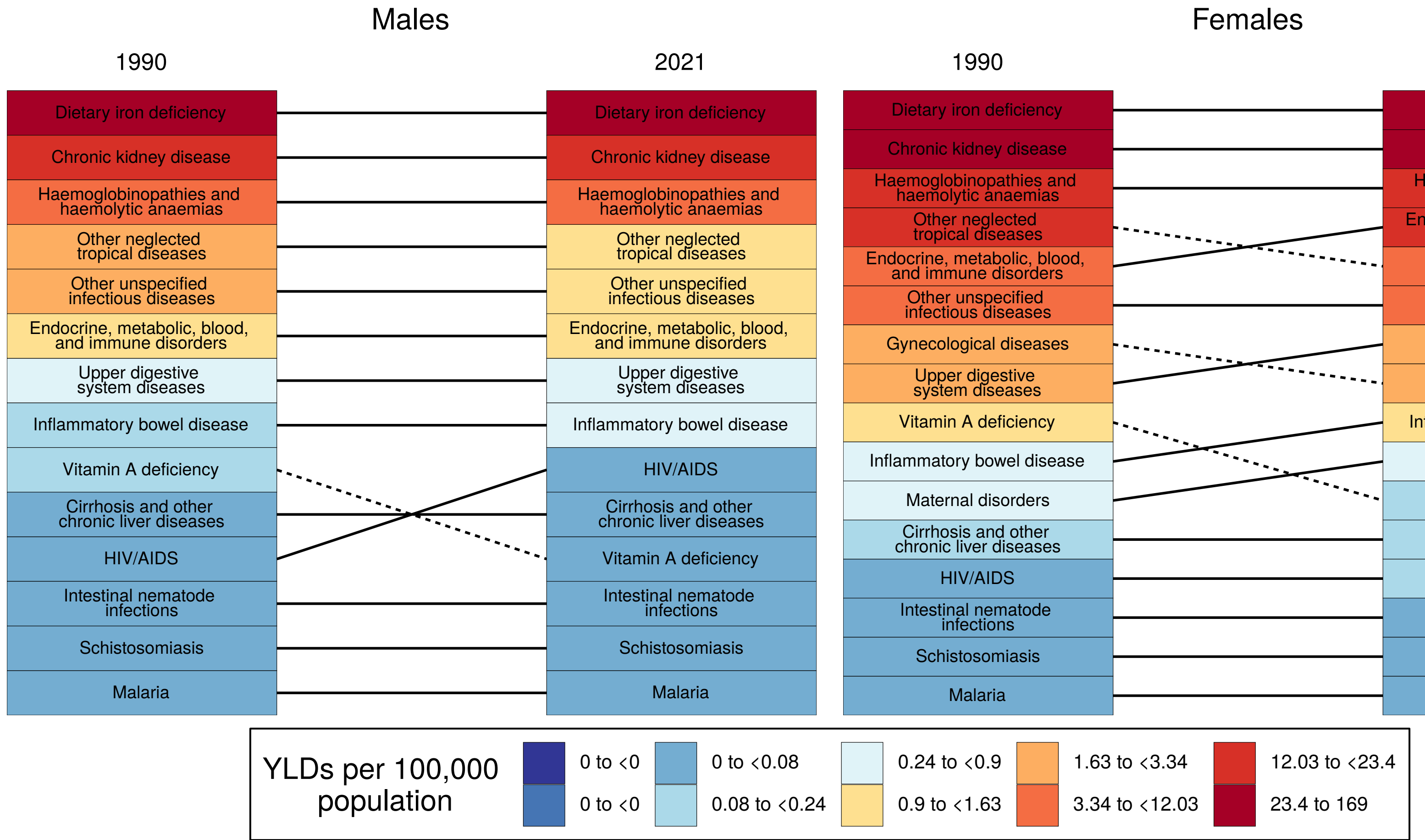

South Gloucestershire

Anaemia Prevalence - Both Sexes

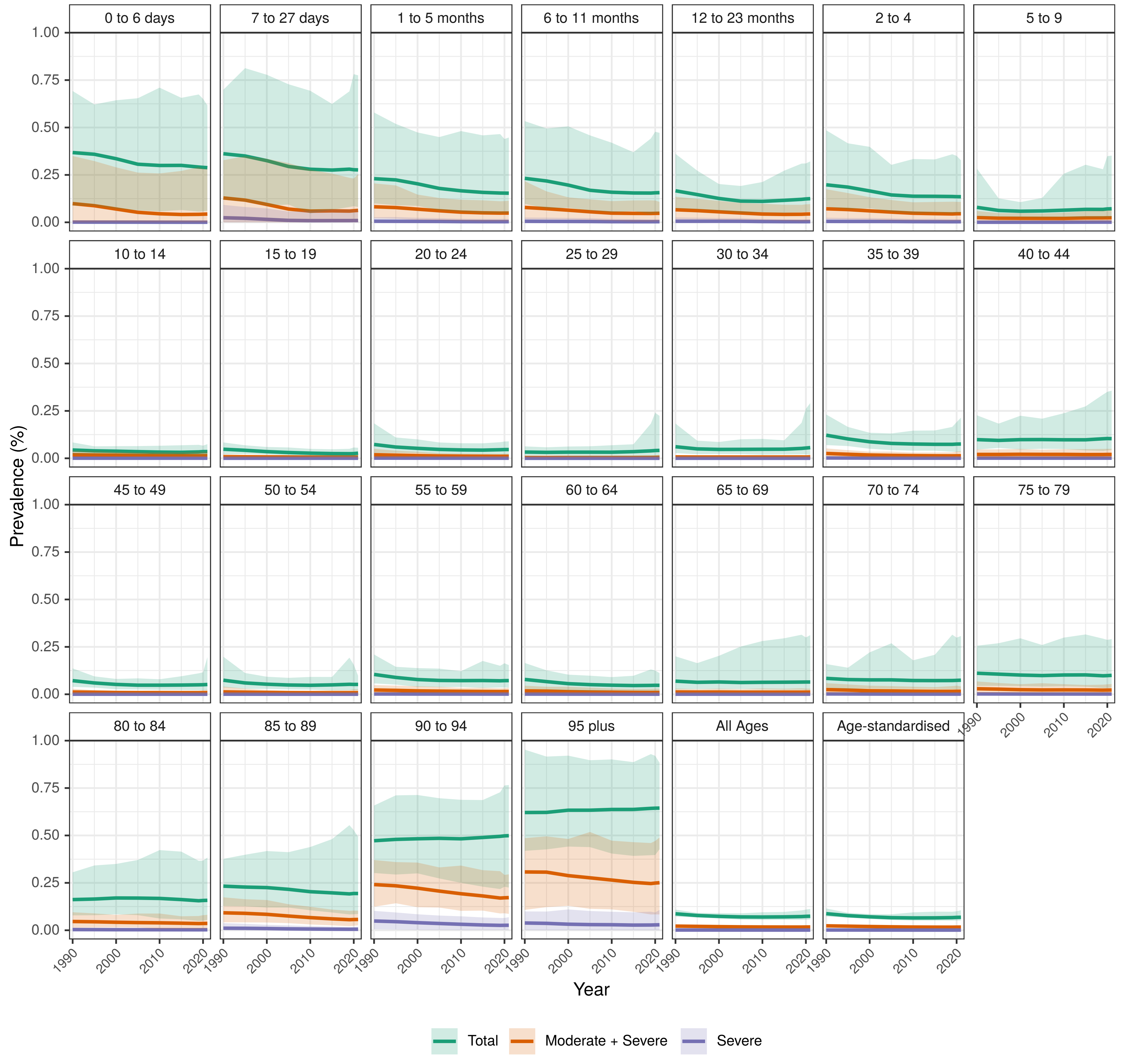

Cause-Specific Anaemia Prevalence - 2021

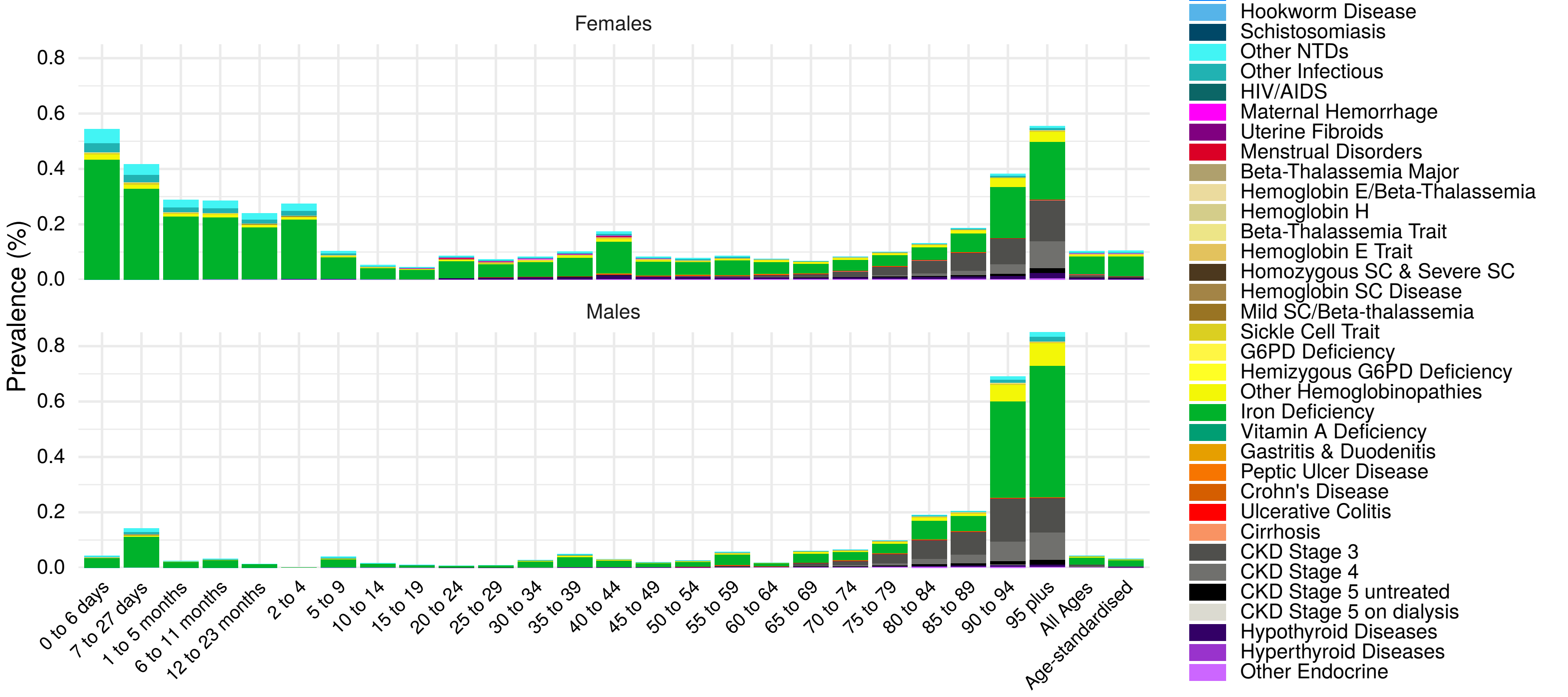

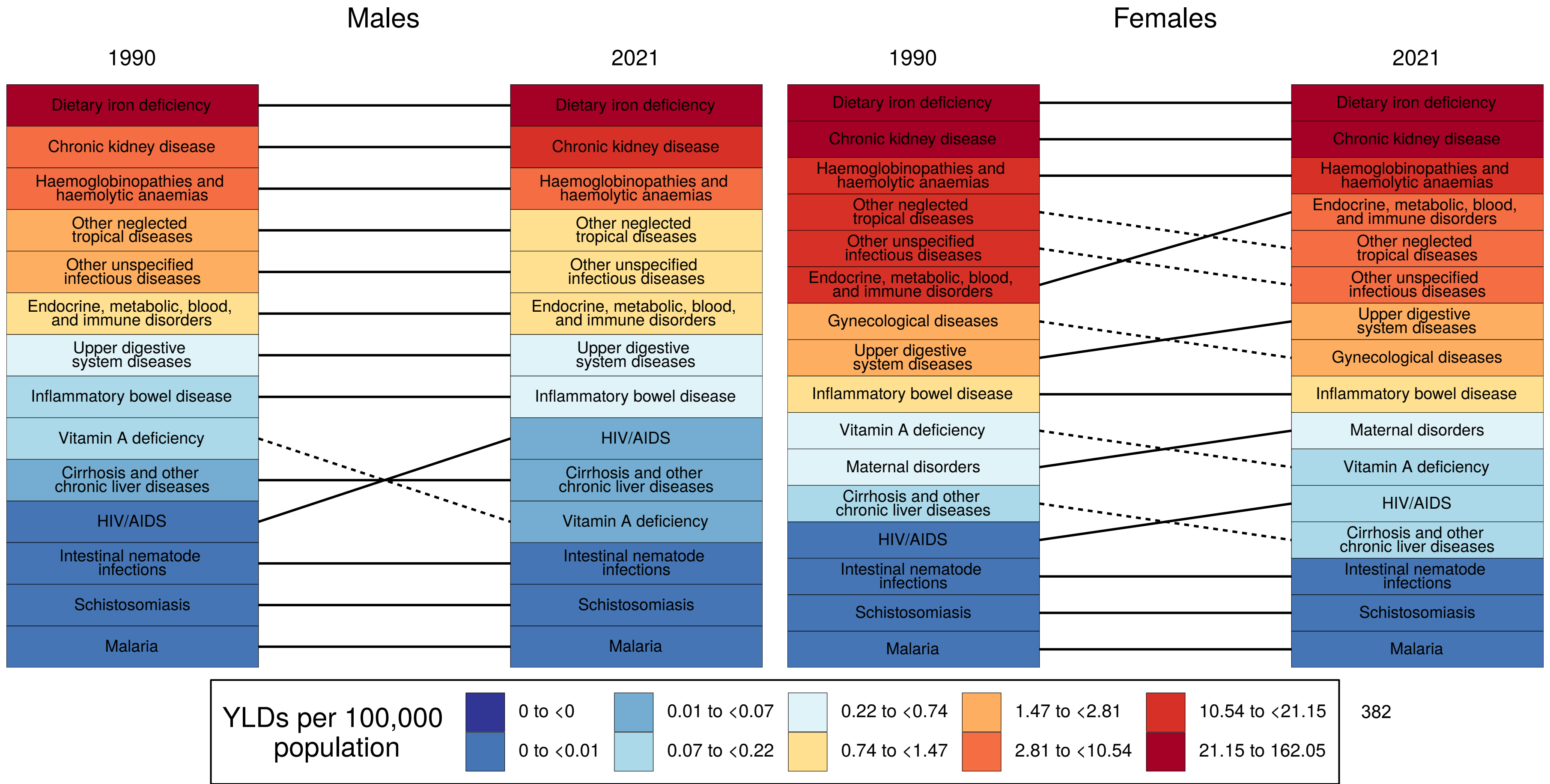

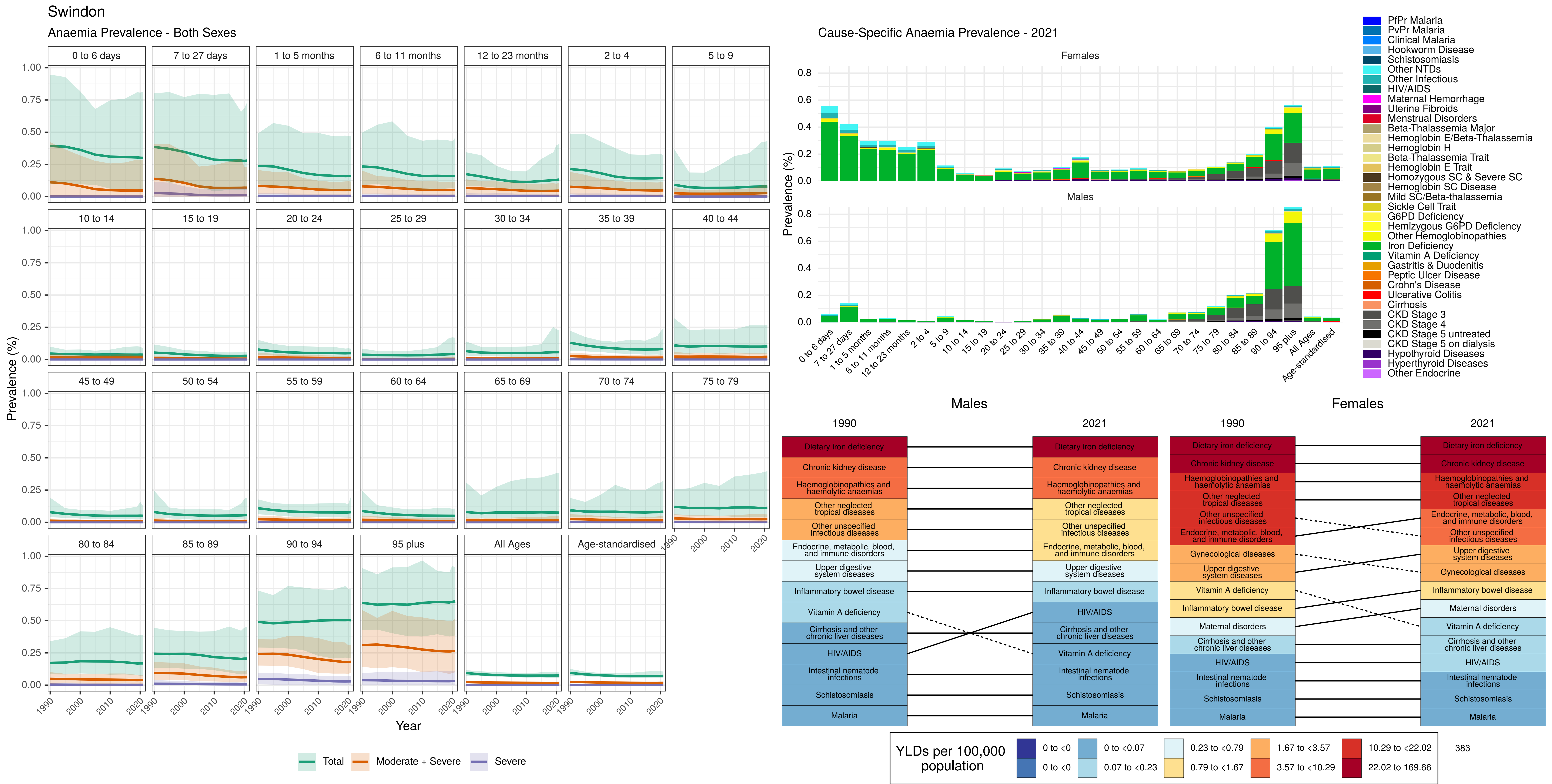
Swindon
Anaemia Prevalence - Both Sexes
0 to 6 days
7 to 27 days
1 to 5 months
6 to 11 months
12 to 23 months
2 to 4
5 to 9
10 to 14
15 to 19
20 to 24
25 to 29
30 to 34
35 to 39
40 to 44
45 to 49
50 to 54
55 to 59
60 to 64
65 to 69
70 to 74
75 to 79
80 to 84
85 to 89
90 to 94
95 plus
All Ages
Age-standardised
Prevalence (%)
Year
Total
Moderate + Severe
Severe
Cause-Specific Anaemia Prevalence - 2021
Females
Males
Prevalence (%)
PfPr Malaria
PvPr Malaria
Clinical Malaria
Hookworm Disease
Schistosomiasis
Other NTDs
Other Infectious
HIV/AIDS
Maternal Hemorrhage
Uterine Fibroids
Menstrual Disorders
Beta-Thalassemia Major
Hemoglobin E/Beta-Thalassemia
Hemoglobin H
Beta-Thalassemia Trait
Hemoglobin E Trait
Homozygous SC & Severe SC
Hemoglobin SC Disease
Mild SC/Beta-thalassemia
Sickle Cell Trait
G6PD Deficiency
Hemizygous G6PD Deficiency
Other Hemoglobinopathies
Iron Deficiency
Vitamin A Deficiency
Gastritis & Duodenitis
Peptic Ulcer Disease
Crohn's Disease
Ulcerative Colitis
Cirrhosis
CKD Stage 3
CKD Stage 4
CKD Stage 5 untreated
CKD Stage 5 on dialysis
Hypothyroid Diseases
Hyperthyroid Diseases
Other Endocrine
Males
Females
1990
2021
Dietary iron deficiency
Chronic kidney disease
Haemoglobinopathies and haemolytic anaemias
Other neglected tropical diseases
Other unspecified infectious diseases
Endocrine, metabolic, blood, and immune disorders
Upper digestive system diseases
Inflammatory bowel disease
Vitamin A deficiency
Cirrhosis and other chronic liver diseases
HIV/AIDS
Intestinal nematode infections
Schistosomiasis
Malaria
Gynecological diseases
Maternal disorders
YLDs per 100,000 population
0 to <0
0 to <0.07
0.07 to <0.23
0.23 to <0.79
0.79 to <1.67
1.67 to <3.57
3.57 to <10.29
10.29 to <22.02
22.02 to 169.66

Torbay

Anaemia Prevalence - Both Sexes

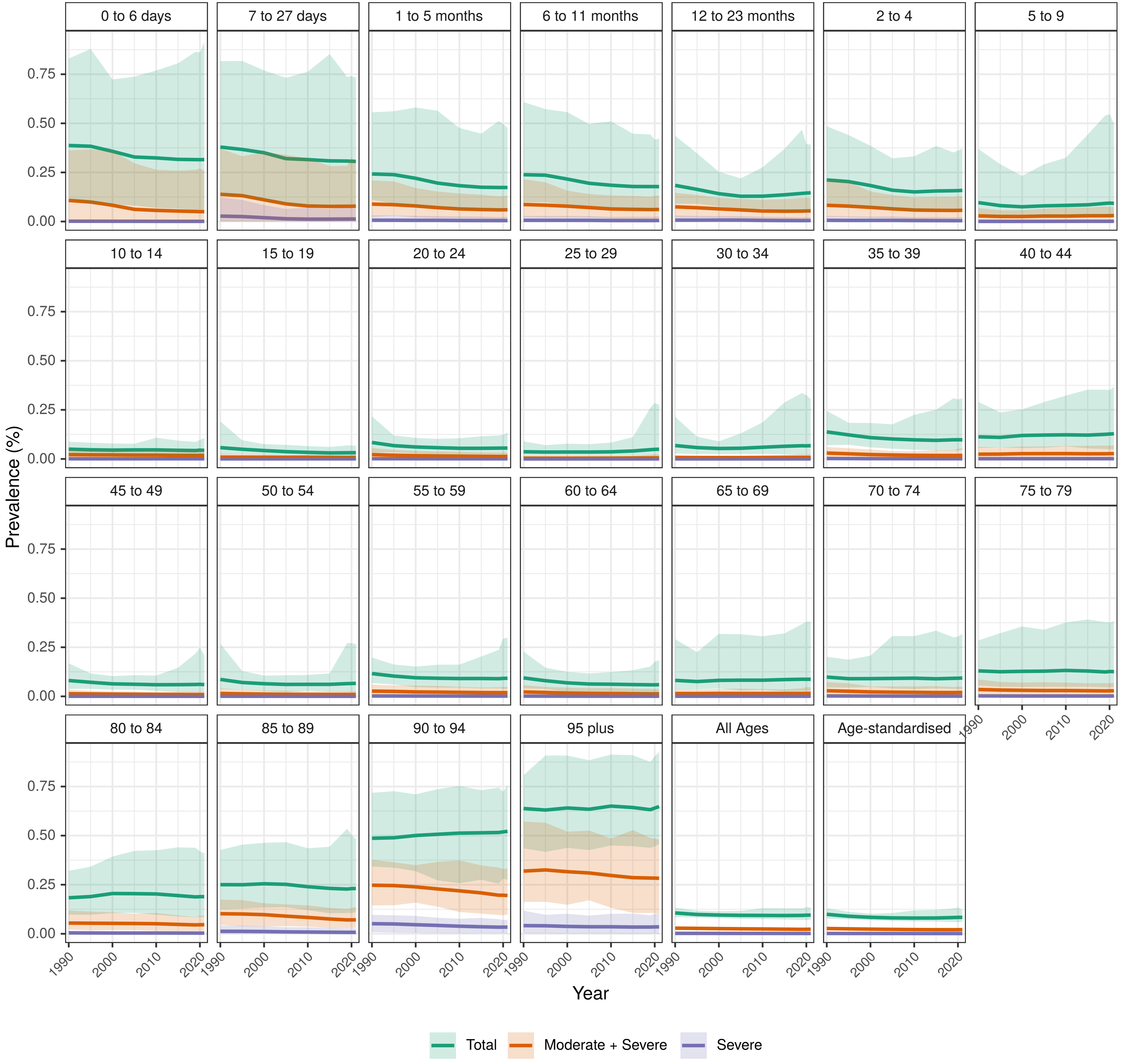

Cause-Specific Anaemia Prevalence - 2021

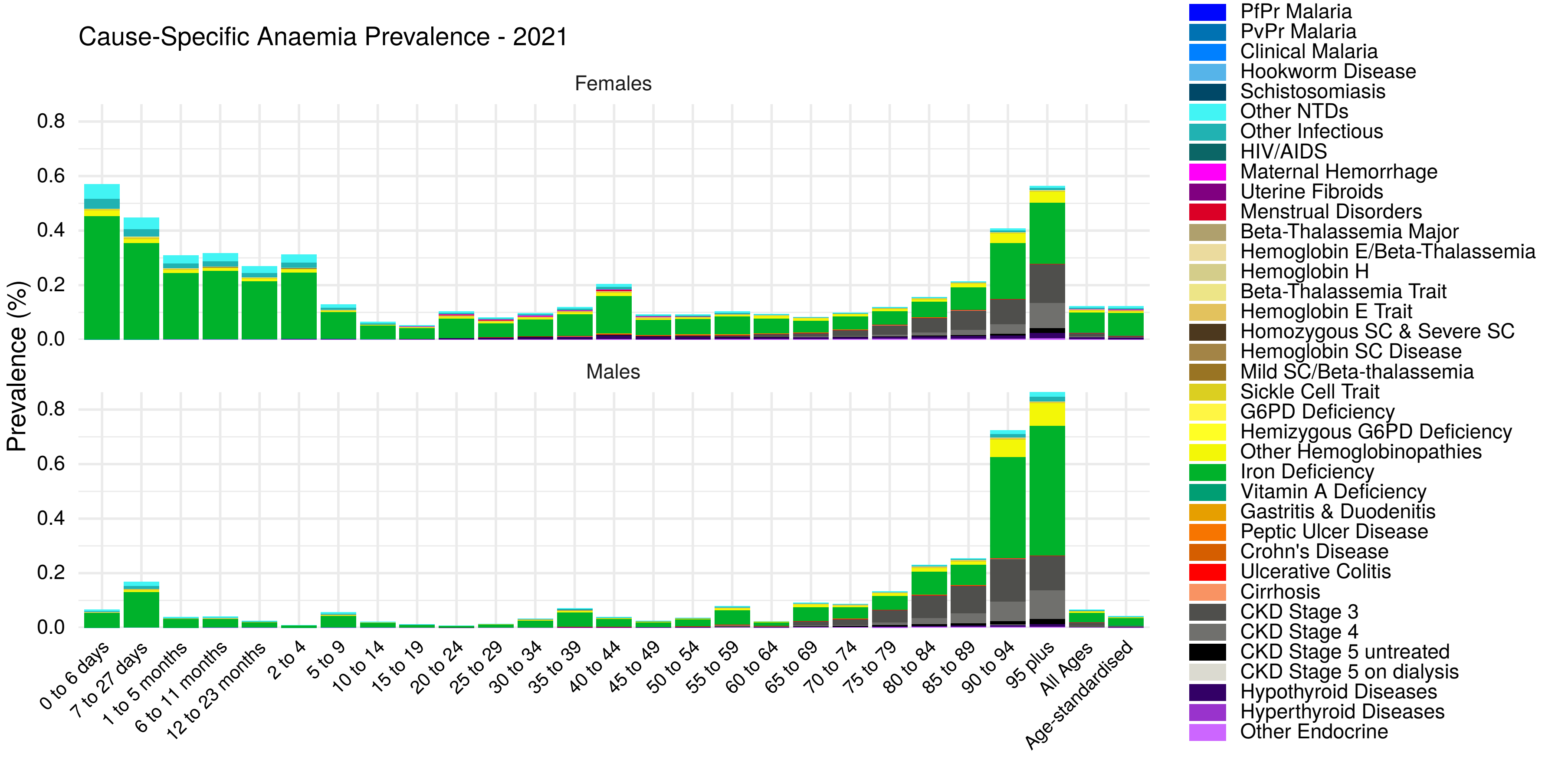

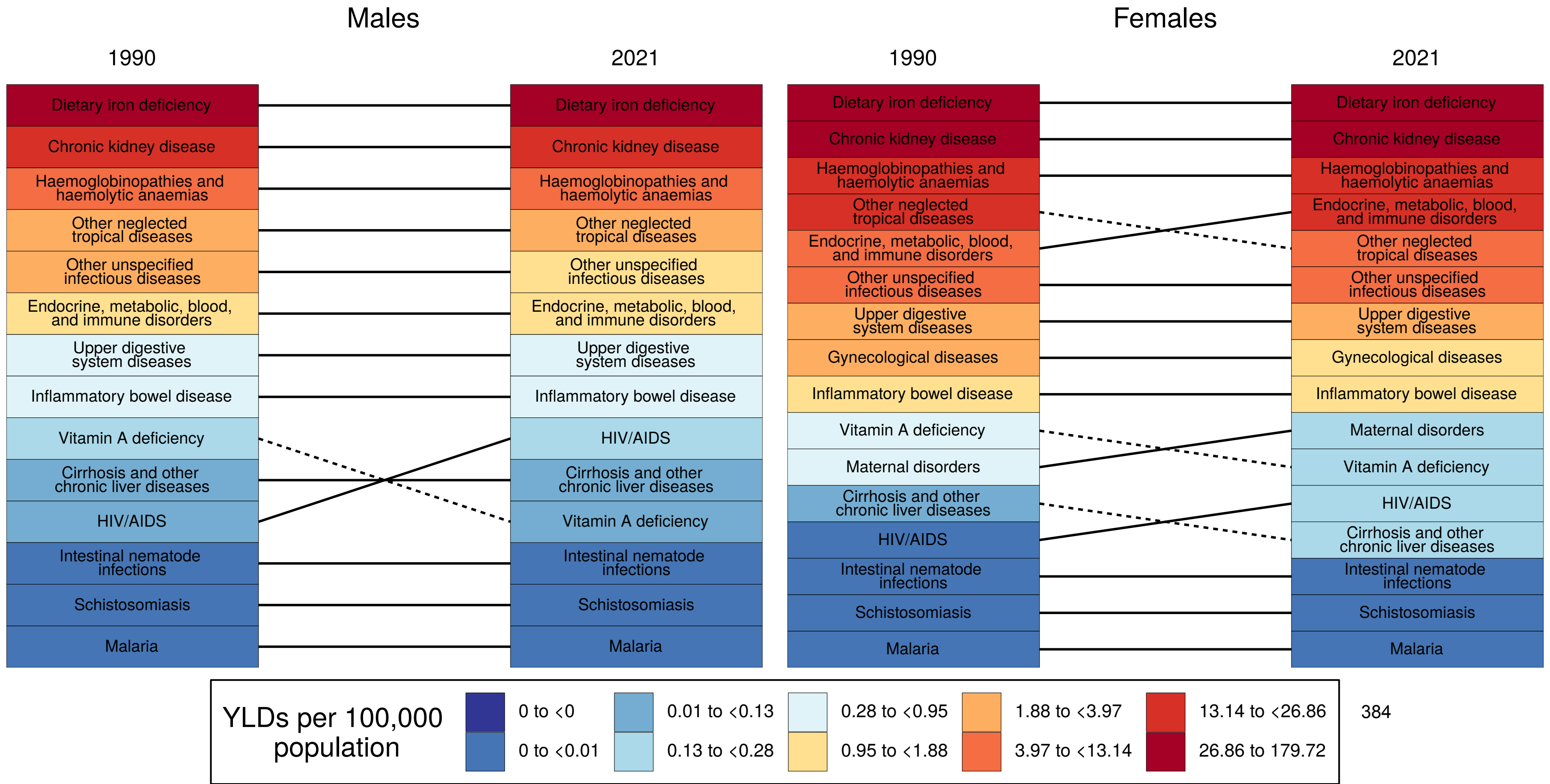

Wiltshire

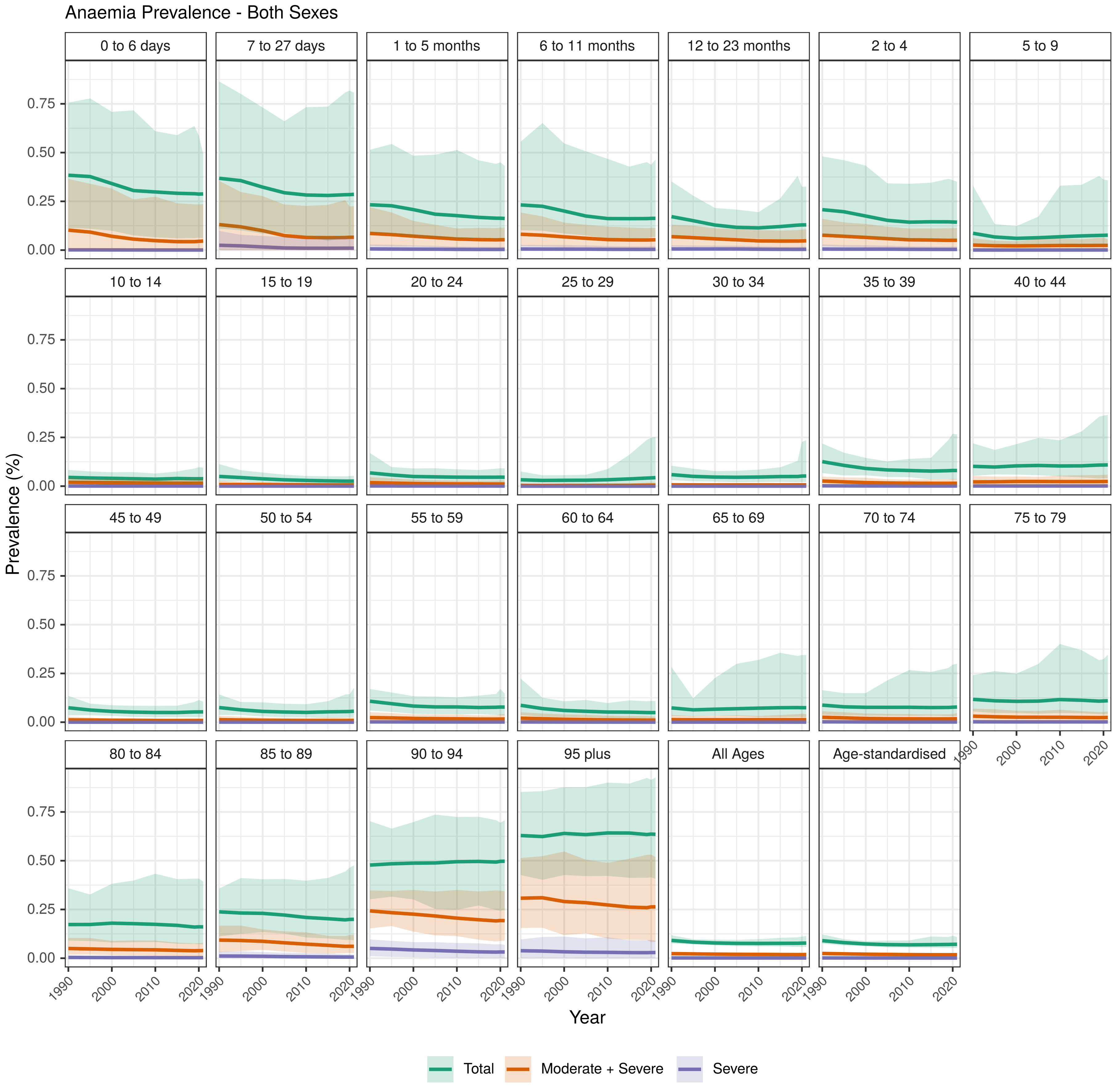

Anaemia Prevalence - Both Sexes
0 to 6 days
7 to 27 days
1 to 5 months
6 to 11 months
12 to 23 months
2 to 4
5 to 9
10 to 14
15 to 19
20 to 24
25 to 29
30 to 34
35 to 39
40 to 44
45 to 49
50 to 54
55 to 59
60 to 64
65 to 69
70 to 74
75 to 79
80 to 84
85 to 89
90 to 94
95 plus
All Ages
Age-standardised
Prevalence (%)
Year
Total
Moderate + Severe
Severe

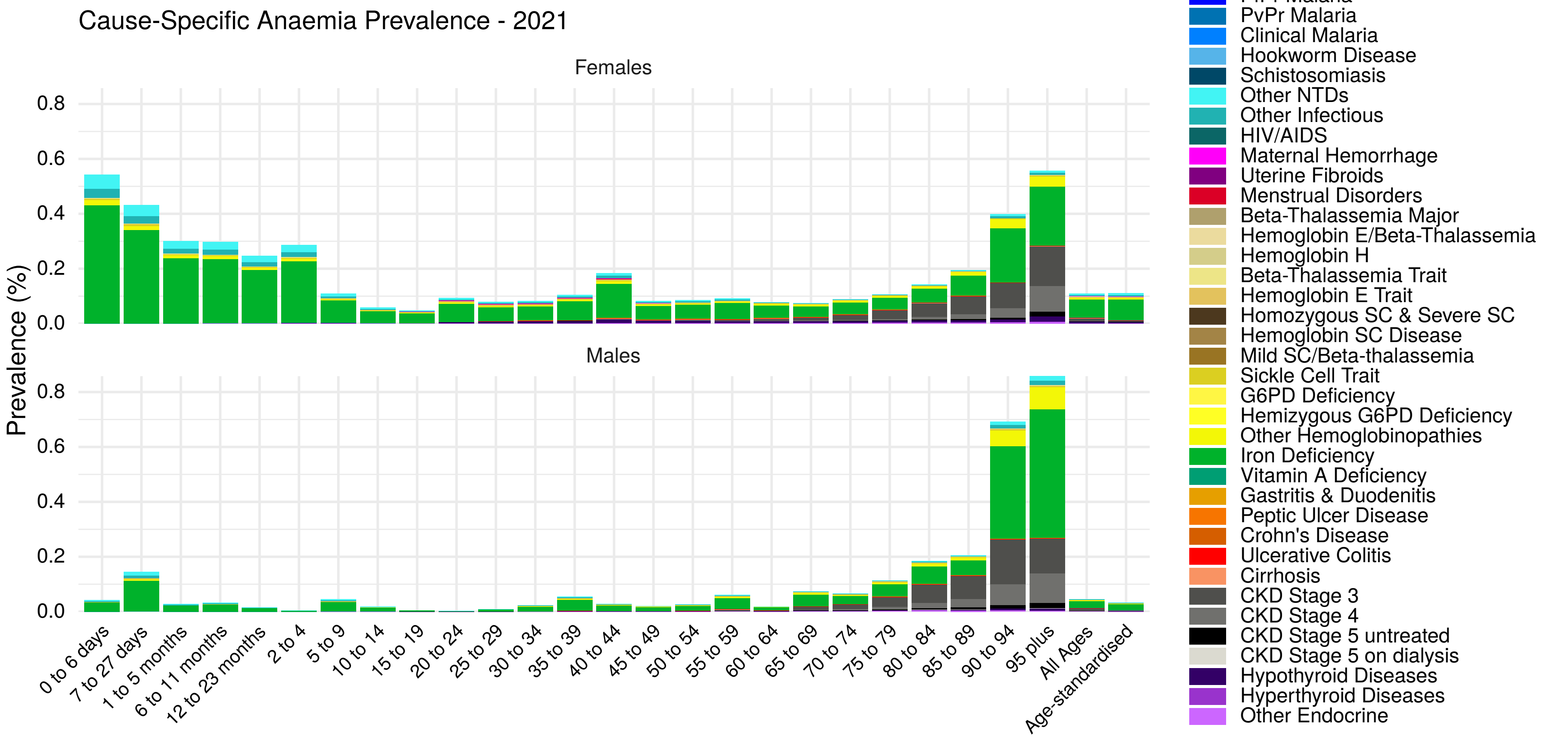

Cause-Specific Anaemia Prevalence - 2021
Females
Males
Prevalence (%)
PfPr Malaria
PvPr Malaria
Clinical Malaria
Hookworm Disease
Schistosomiasis
Other NTDs
Other Infectious
HIV/AIDS
Maternal Hemorrhage
Uterine Fibroids
Menstrual Disorders
Beta-Thalassemia Major
Hemoglobin E/Beta-Thalassemia
Hemoglobin H
Beta-Thalassemia Trait
Hemoglobin E Trait
Homozygous SC & Severe SC
Hemoglobin SC Disease
Mild SC/Beta-thalassemia
Sickle Cell Trait
G6PD Deficiency
Hemizygous G6PD Deficiency
Other Hemoglobinopathies
Iron Deficiency
Vitamin A Deficiency
Gastritis & Duodenitis
Peptic Ulcer Disease
Crohn's Disease
Ulcerative Colitis
Cirrhosis
CKD Stage 3
CKD Stage 4
CKD Stage 5 untreated
CKD Stage 5 on dialysis
Hypothyroid Diseases
Hyperthyroid Diseases
Other Endocrine

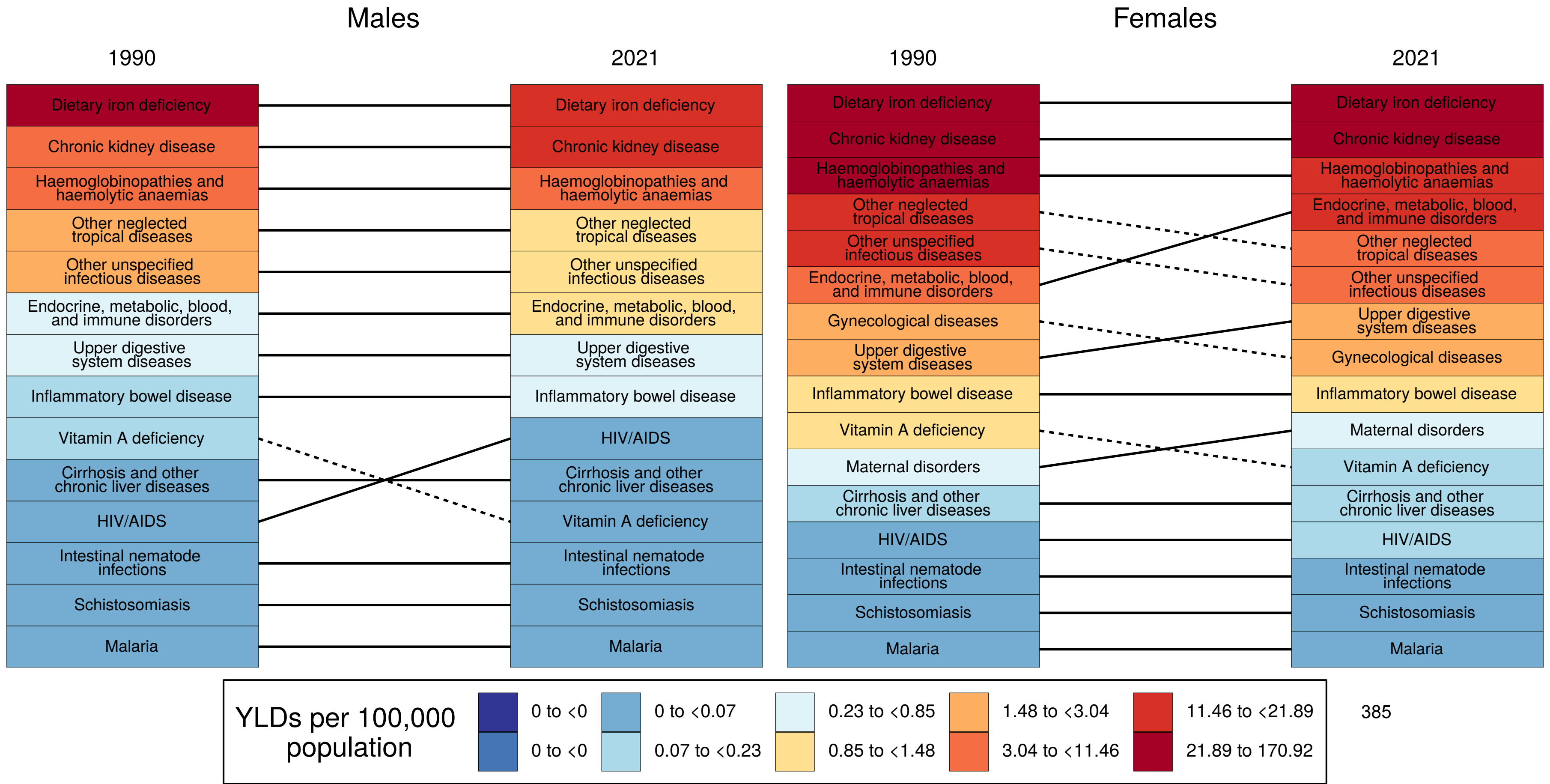

Males
Females
1990
2021
Dietary iron deficiency
Chronic kidney disease
Haemoglobinopathies and haemolytic anaemias
Other neglected tropical diseases
Other unspecified infectious diseases
Endocrine, metabolic, blood, and immune disorders
Upper digestive system diseases
Inflammatory bowel disease
Vitamin A deficiency
Cirrhosis and other chronic liver diseases
HIV/AIDS
Intestinal nematode infections
Schistosomiasis
Malaria
Gynecological diseases
Maternal disorders
YLDs per 100,000 population
0 to <0
0 to <0.07
0.07 to <0.23
0.23 to <0.85
0.85 to <1.48
1.48 to <3.04
3.04 to <11.46
11.46 to <21.89
21.89 to 170.92

West Midlands

Anaemia Prevalence - Both Sexes

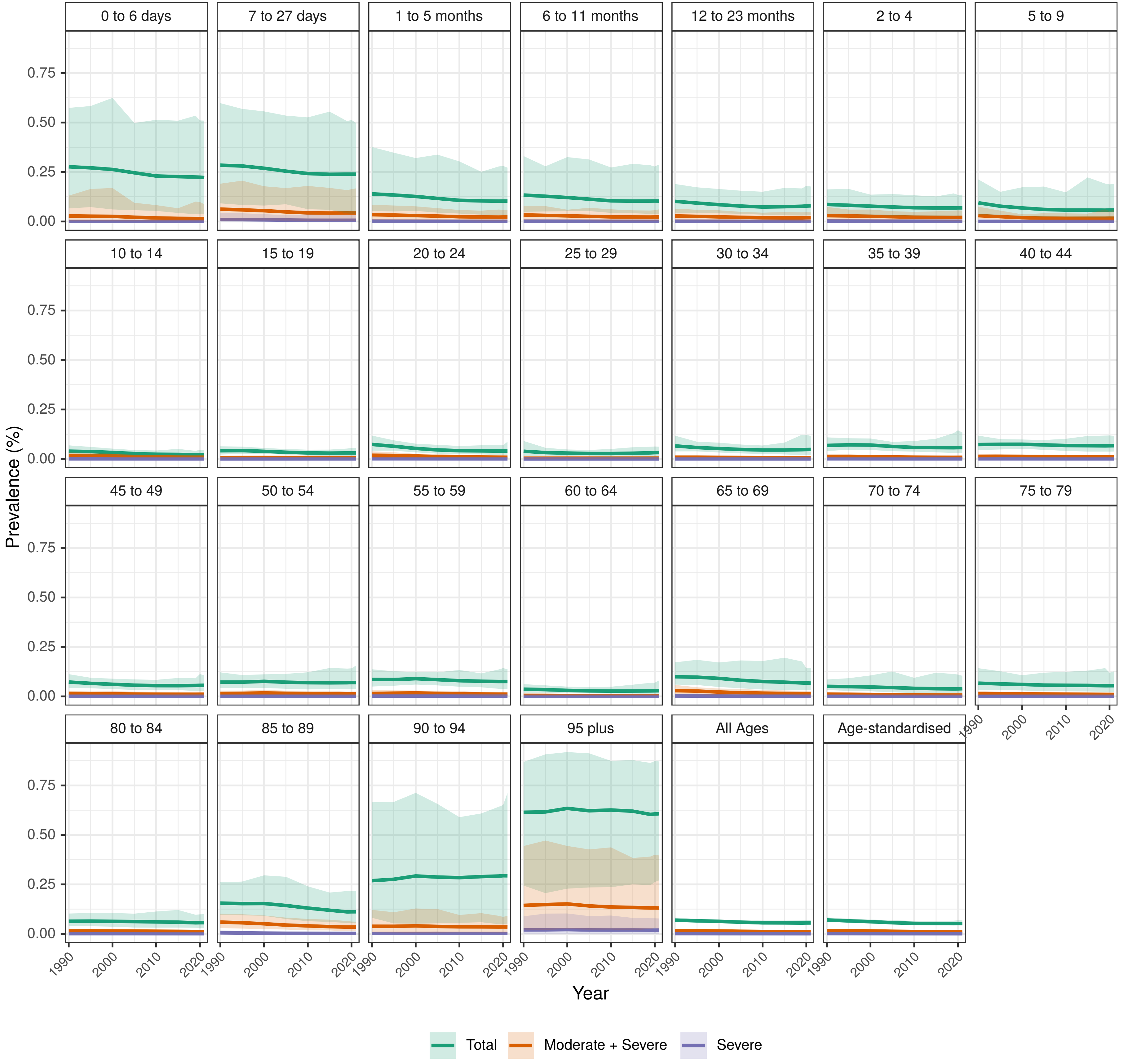

Cause-Specific Anaemia Prevalence - 2021

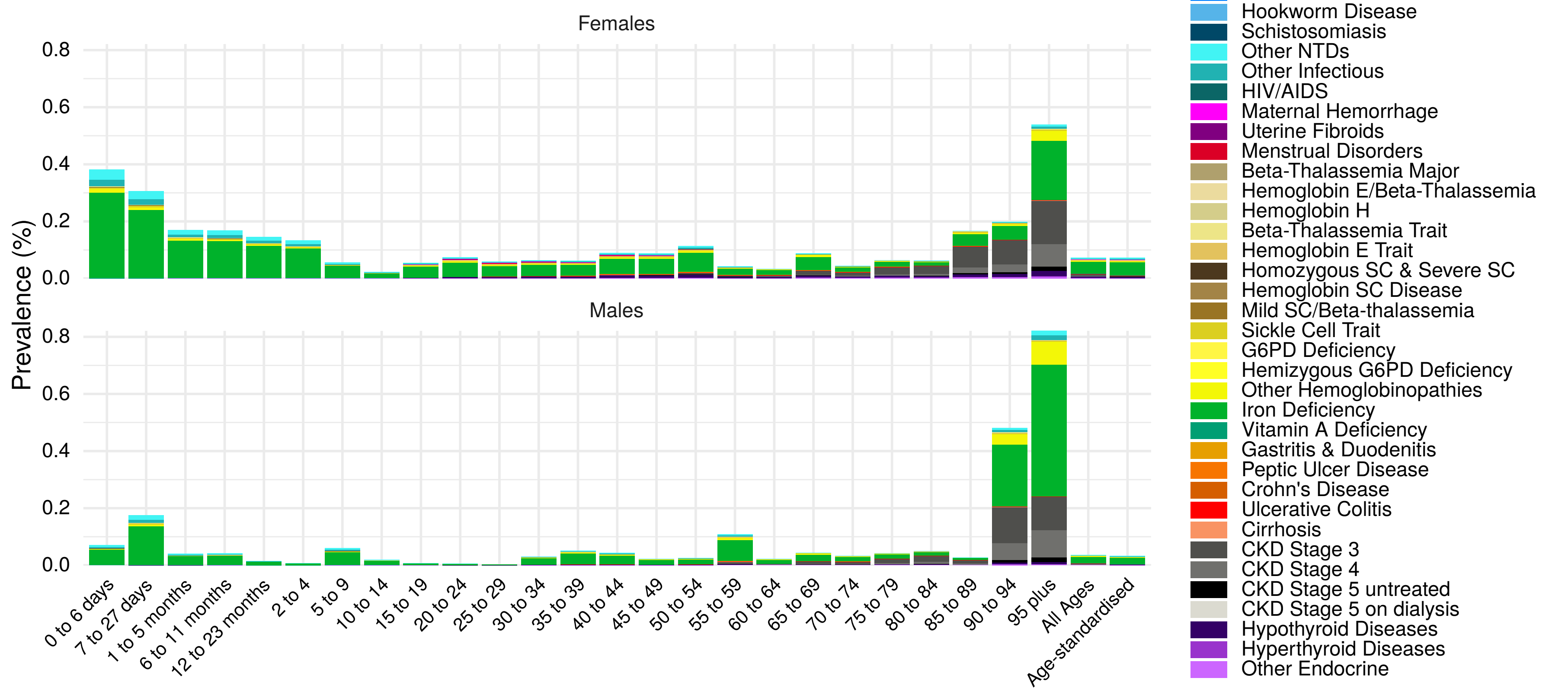

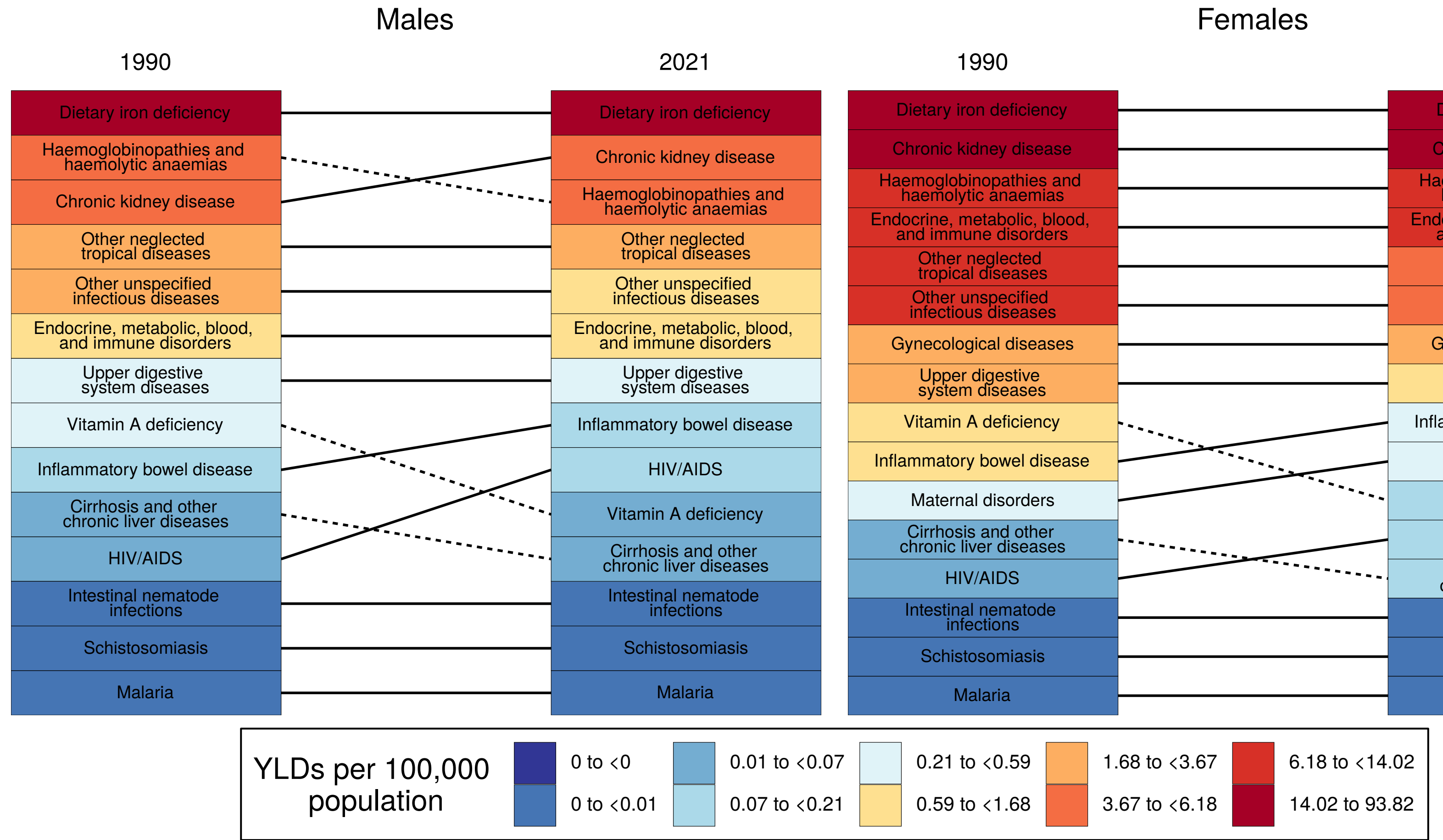

Birmingham

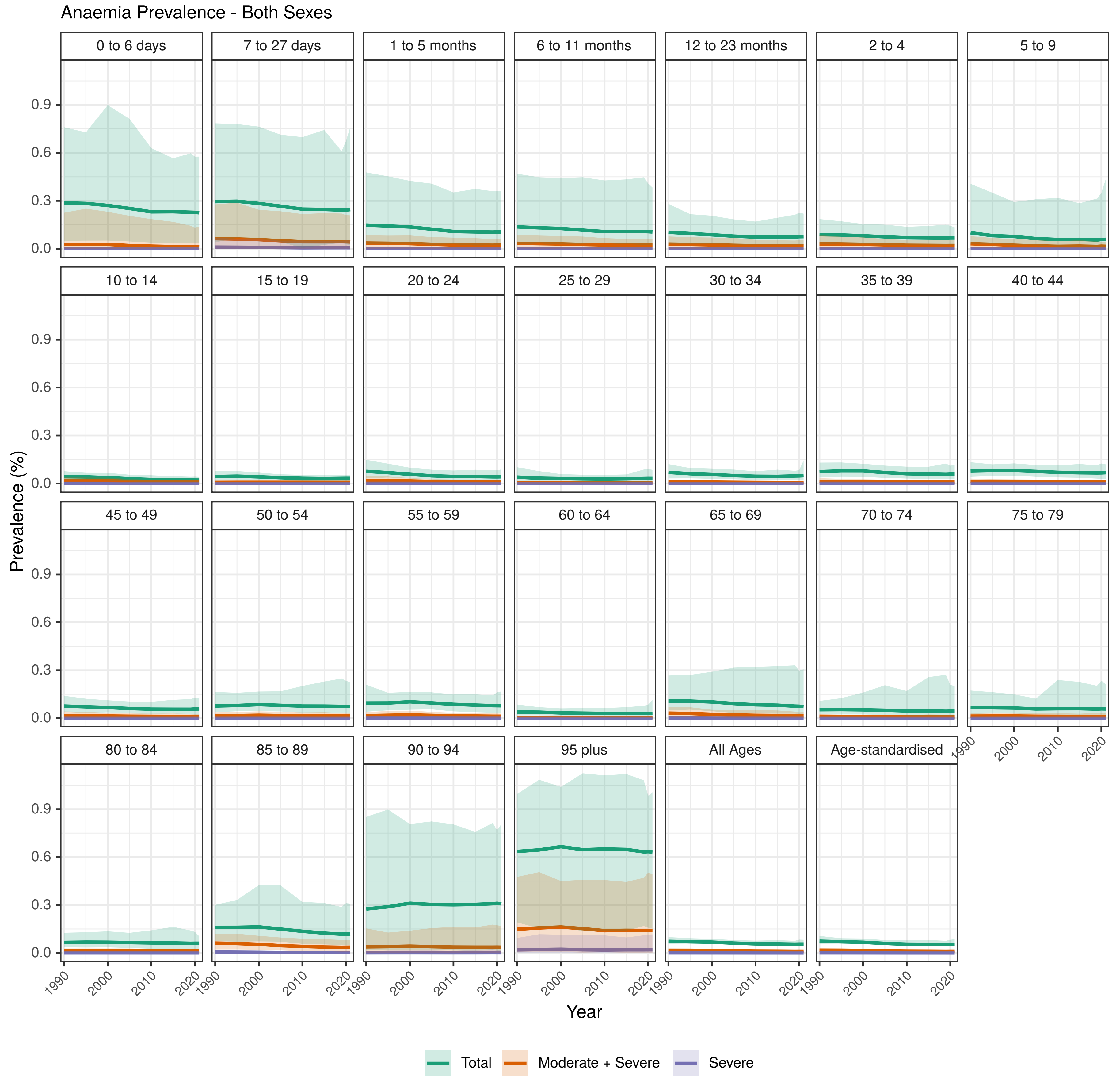
Anaemia Prevalence - Both Sexes
0 to 6 days
7 to 27 days
1 to 5 months
6 to 11 months
12 to 23 months
2 to 4
5 to 9
10 to 14
15 to 19
20 to 24
25 to 29
30 to 34
35 to 39
40 to 44
45 to 49
50 to 54
55 to 59
60 to 64
65 to 69
70 to 74
75 to 79
80 to 84
85 to 89
90 to 94
95 plus
All Ages
Age-standardised
Prevalence (%)
Year
Total
Moderate + Severe
Severe

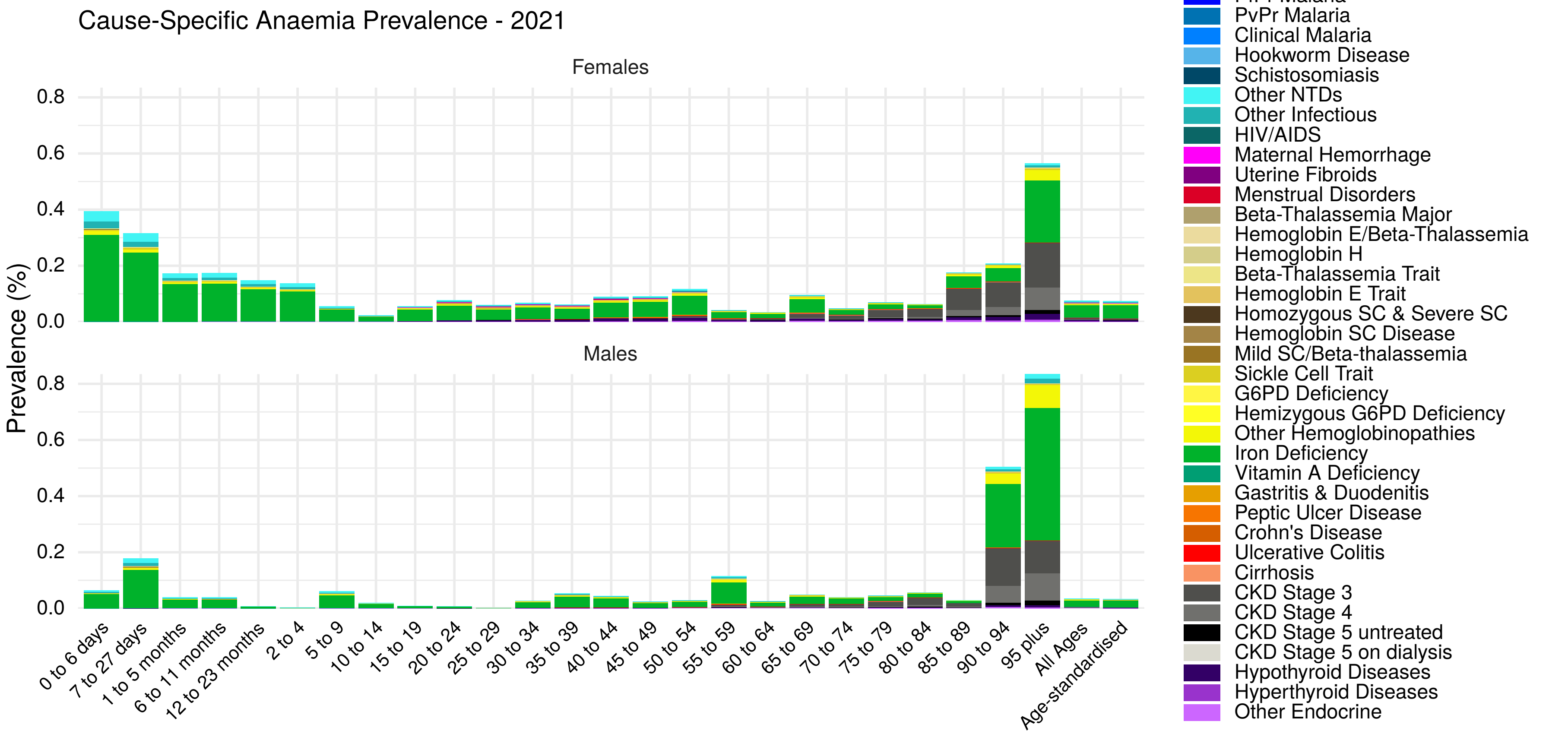
Cause-Specific Anaemia Prevalence - 2021
Females
Males
Prevalence (%)
0 to 6 days
7 to 27 days
1 to 5 months
6 to 11 months
12 to 23 months
2 to 4
5 to 9
10 to 14
15 to 19
20 to 24
25 to 29
30 to 34
35 to 39
40 to 44
45 to 49
50 to 54
55 to 59
60 to 64
65 to 69
70 to 74
75 to 79
80 to 84
85 to 89
90 to 94
95 plus
All Ages
Age-standardised
PfPr Malaria
PvPr Malaria
Clinical Malaria
Hookworm Disease
Schistosomiasis
Other NTDs
Other Infectious
HIV/AIDS
Maternal Hemorrhage
Uterine Fibroids
Menstrual Disorders
Beta-Thalassemia Major
Hemoglobin E/Beta-Thalassemia
Hemoglobin H
Beta-Thalassemia Trait
Hemoglobin E Trait
Homozygous SC & Severe SC
Hemoglobin SC Disease
Mild SC/Beta-thalassemia
Sickle Cell Trait
G6PD Deficiency
Hemizygous G6PD Deficiency
Other Hemoglobinopathies
Iron Deficiency
Vitamin A Deficiency
Gastritis & Duodenitis
Peptic Ulcer Disease
Crohn's Disease
Ulcerative Colitis
Cirrhosis
CKD Stage 3
CKD Stage 4
CKD Stage 5 untreated
CKD Stage 5 on dialysis
Hypothyroid Diseases
Hyperthyroid Diseases
Other Endocrine

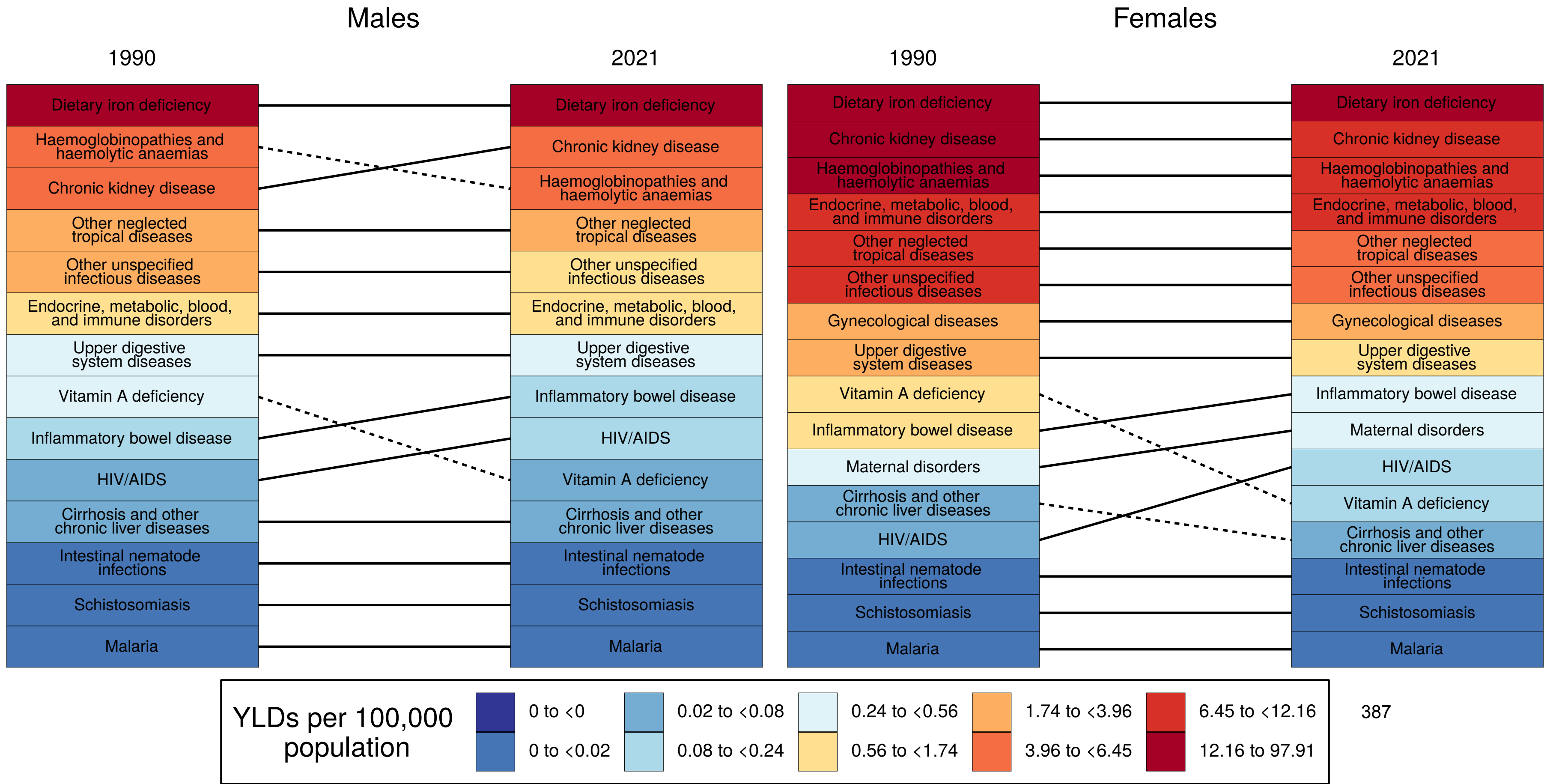
Males
Females
1990
2021
Dietary iron deficiency
Haemoglobinopathies and haemolytic anaemias
Chronic kidney disease
Other neglected tropical diseases
Other unspecified infectious diseases
Endocrine, metabolic, blood, and immune disorders
Upper digestive system diseases
Vitamin A deficiency
Inflammatory bowel disease
HIV/AIDS
Cirrhosis and other chronic liver diseases
Intestinal nematode infections
Schistosomiasis
Malaria
Gynecological diseases
Maternal disorders
YLDs per 100,000 population
0 to <0
0 to <0.02
0.02 to <0.08
0.08 to <0.24
0.24 to <0.56
0.56 to <1.74
1.74 to <3.96
3.96 to <6.45
6.45 to <12.16
12.16 to 97.91

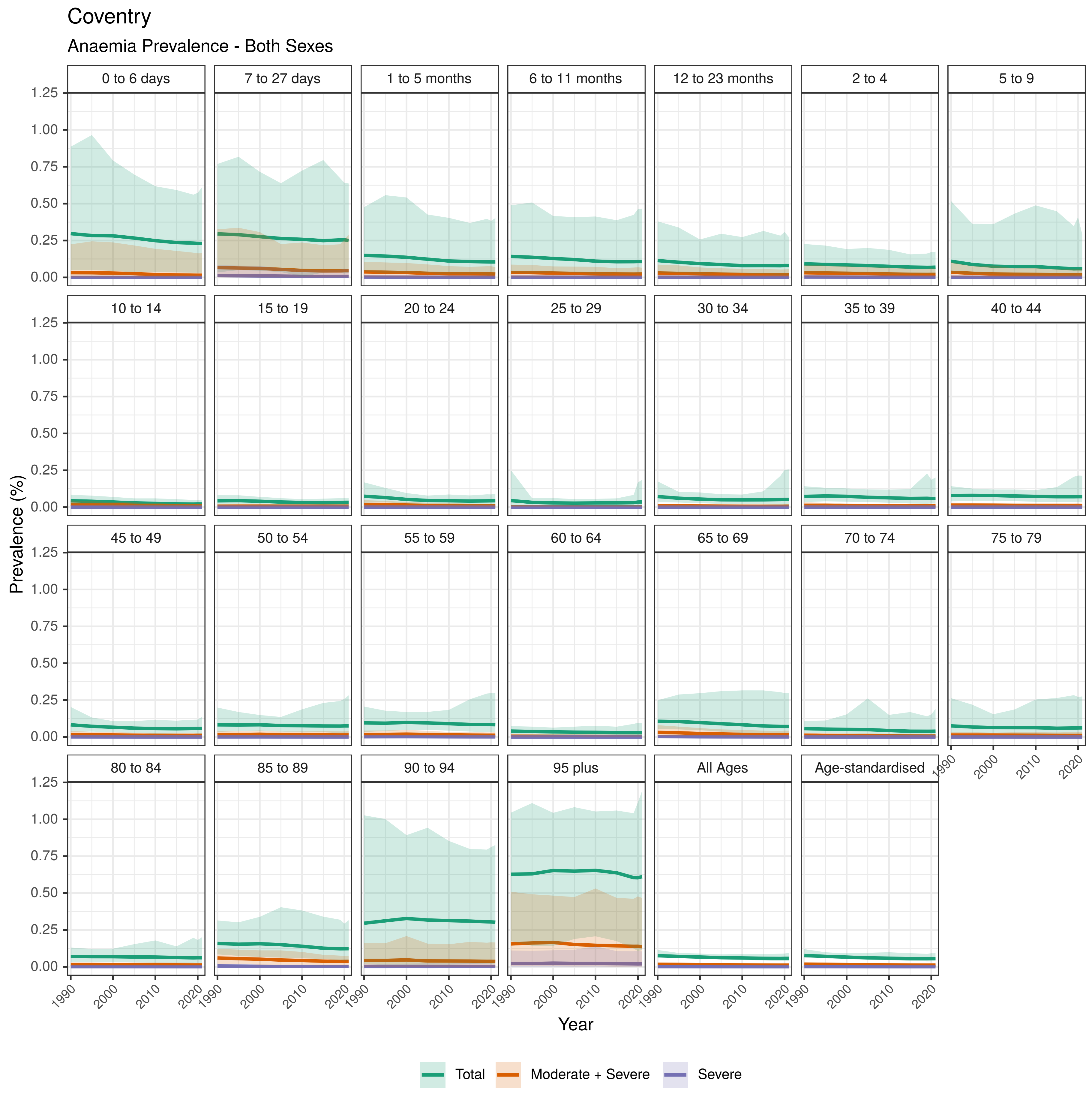

Coventry
Anaemia Prevalence - Both Sexes
0 to 6 days
7 to 27 days
1 to 5 months
6 to 11 months
12 to 23 months
2 to 4
5 to 9
10 to 14
15 to 19
20 to 24
25 to 29
30 to 34
35 to 39
40 to 44
45 to 49
50 to 54
55 to 59
60 to 64
65 to 69
70 to 74
75 to 79
80 to 84
85 to 89
90 to 94
95 plus
All Ages
Age-standardised
Prevalence (%)
Year
Total
Moderate + Severe
Severe

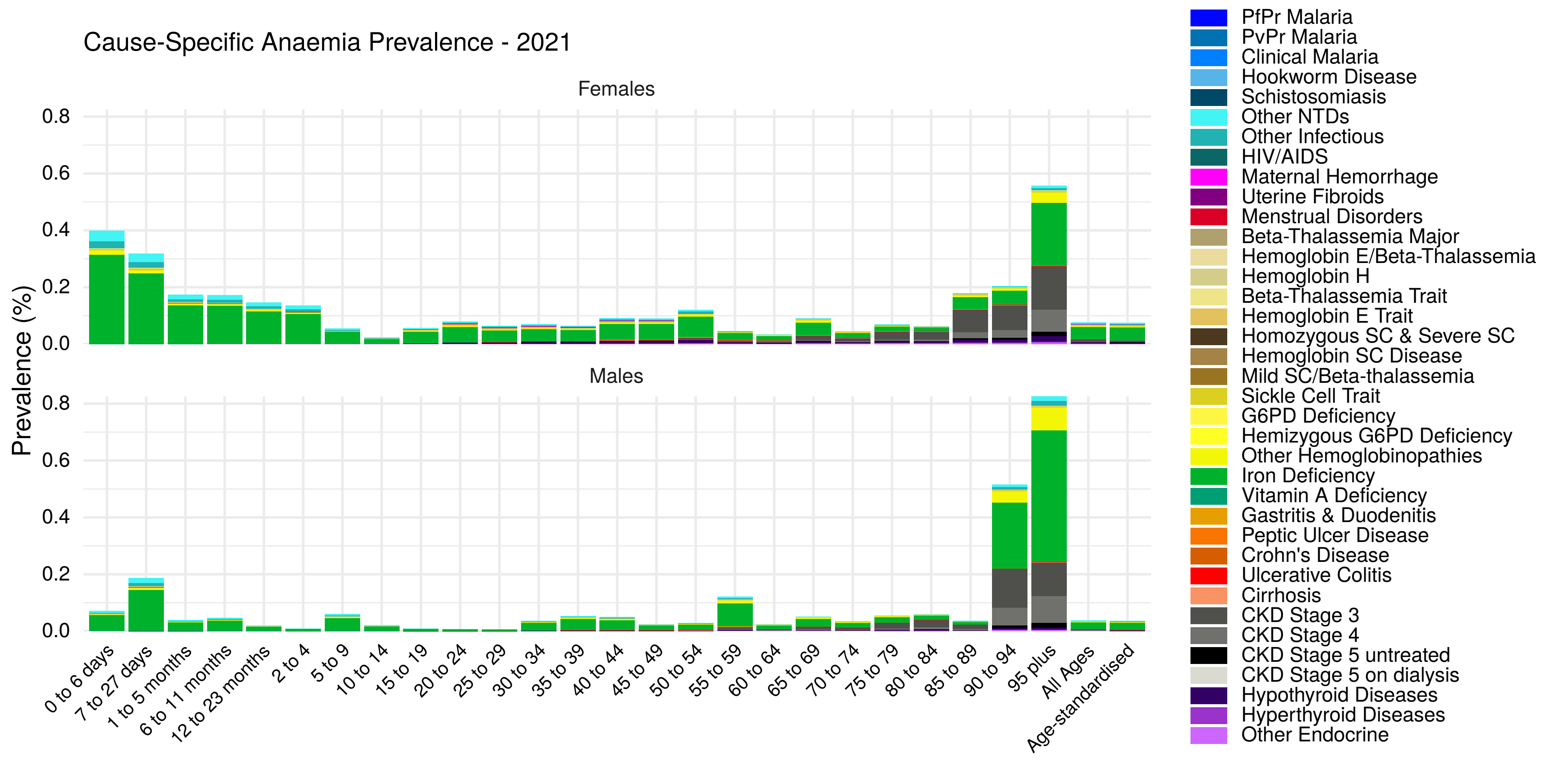

Cause-Specific Anaemia Prevalence - 2021
Females
Males
Prevalence (%)
PfPr Malaria
PvPr Malaria
Clinical Malaria
Hookworm Disease
Schistosomiasis
Other NTDs
Other Infectious
HIV/AIDS
Maternal Hemorrhage
Uterine Fibroids
Menstrual Disorders
Beta-Thalassemia Major
Hemoglobin E/Beta-Thalassemia
Hemoglobin H
Beta-Thalassemia Trait
Hemoglobin E Trait
Homozygous SC & Severe SC
Hemoglobin SC Disease
Mild SC/Beta-thalassemia
Sickle Cell Trait
G6PD Deficiency
Hemizygous G6PD Deficiency
Other Hemoglobinopathies
Iron Deficiency
Vitamin A Deficiency
Gastritis & Duodenitis
Peptic Ulcer Disease
Crohn's Disease
Ulcerative Colitis
Cirrhosis
CKD Stage 3
CKD Stage 4
CKD Stage 5 untreated
CKD Stage 5 on dialysis
Hypothyroid Diseases
Hyperthyroid Diseases
Other Endocrine

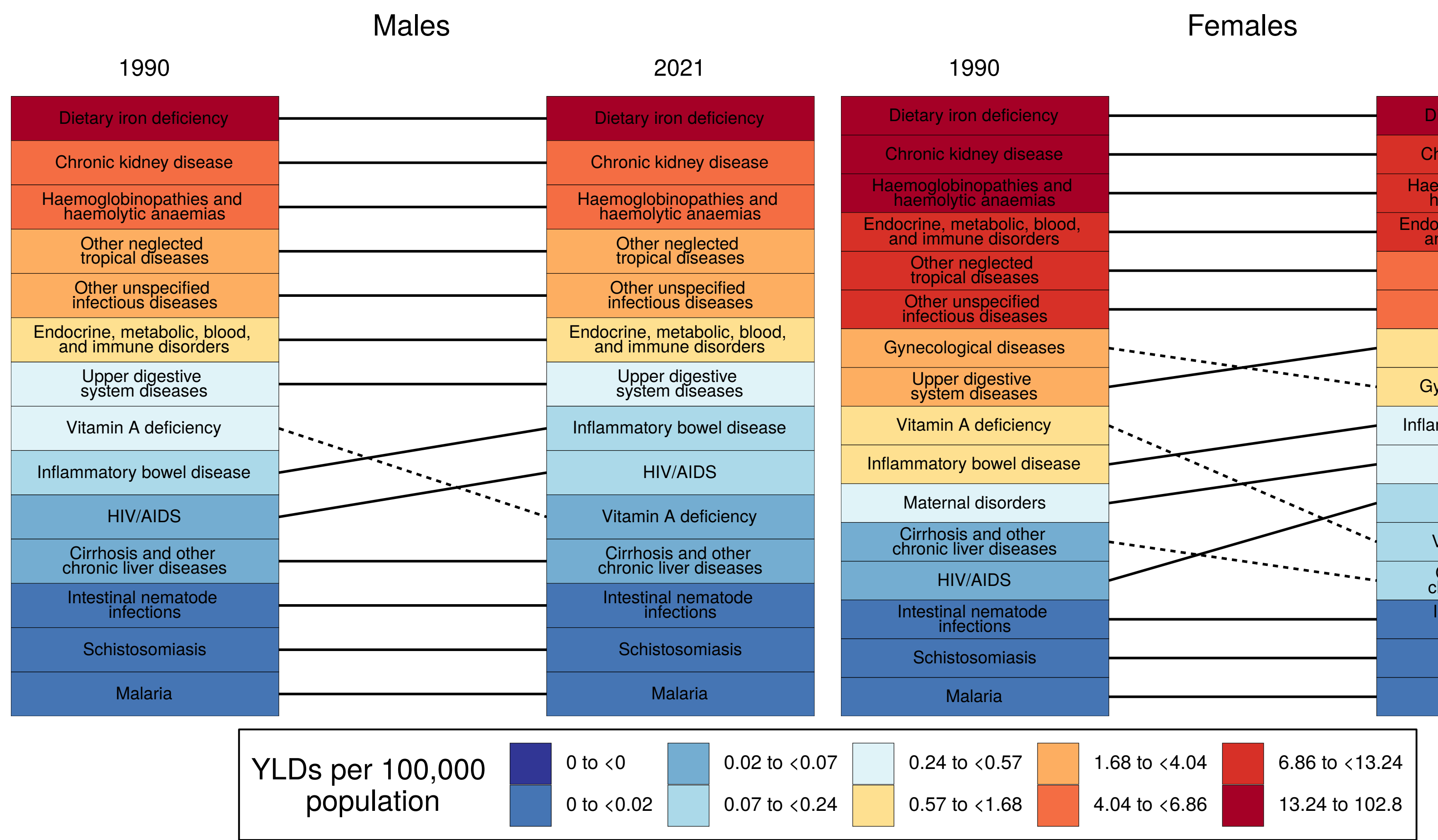

Males
Females
1990
2021
Dietary iron deficiency
Chronic kidney disease
Haemoglobinopathies and haemolytic anaemias
Other neglected tropical diseases
Other unspecified infectious diseases
Endocrine, metabolic, blood, and immune disorders
Upper digestive system diseases
Vitamin A deficiency
Inflammatory bowel disease
HIV/AIDS
Cirrhosis and other chronic liver diseases
Intestinal nematode infections
Schistosomiasis
Malaria
Gynecological diseases
Maternal disorders
YLDs per 100,000 population
0 to <0
0 to <0.02
0.02 to <0.07
0.07 to <0.24
0.24 to <0.57
0.57 to <1.68
1.68 to <4.04
4.04 to <6.86
6.86 to <13.24
13.24 to 102.8

Dudley

Anaemia Prevalence - Both Sexes

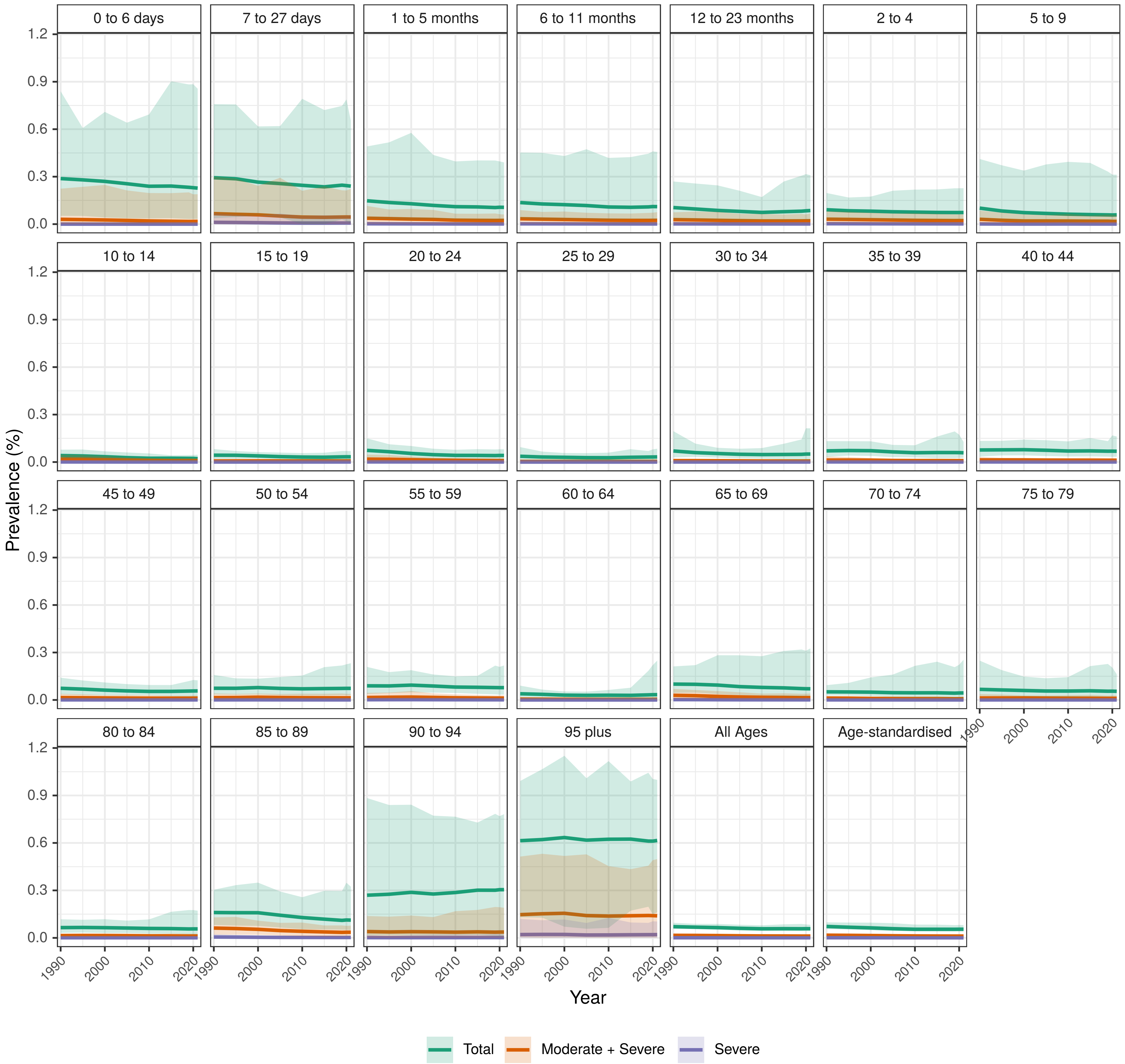

Cause-Specific Anaemia Prevalence - 2021

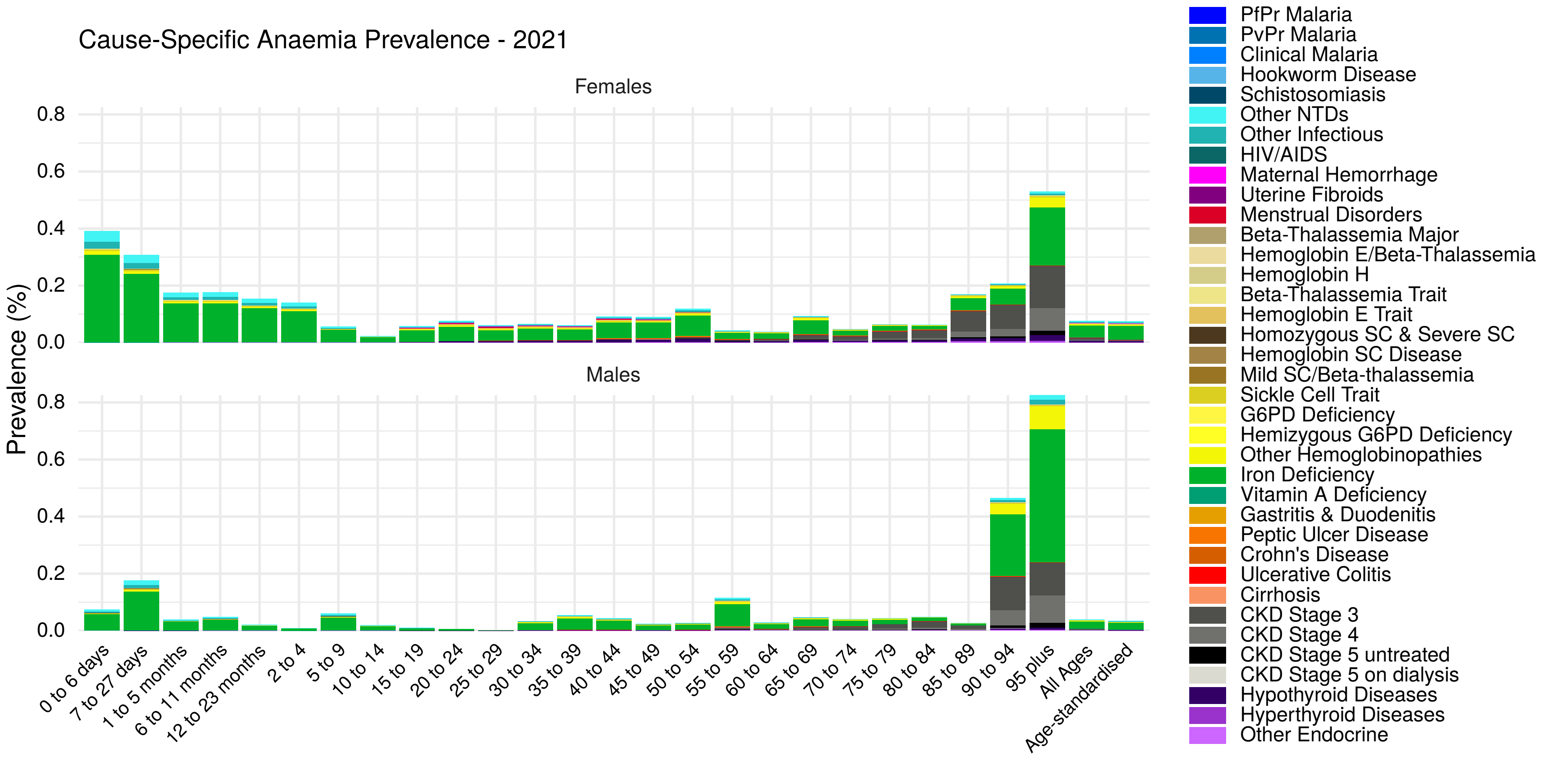

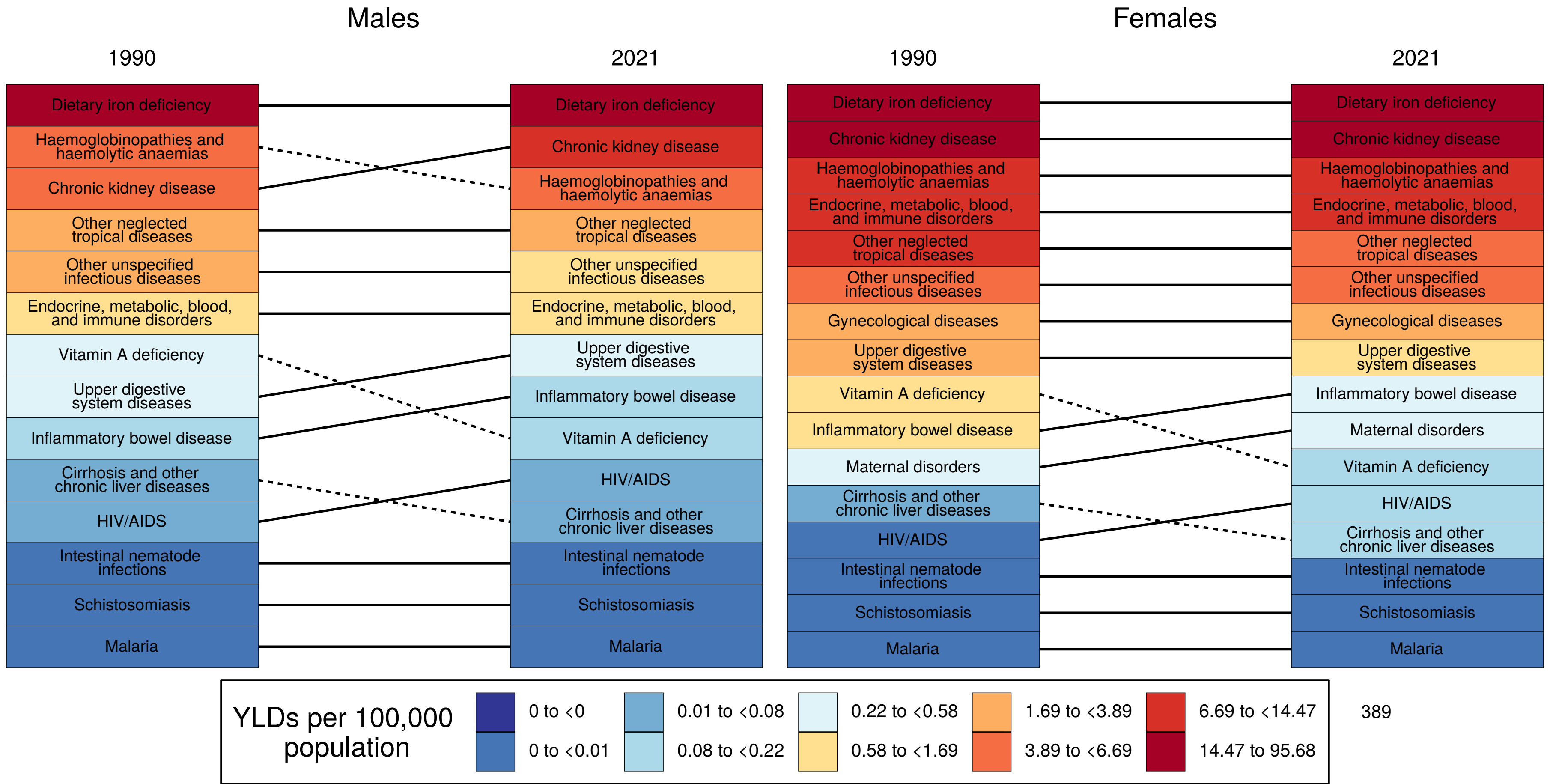

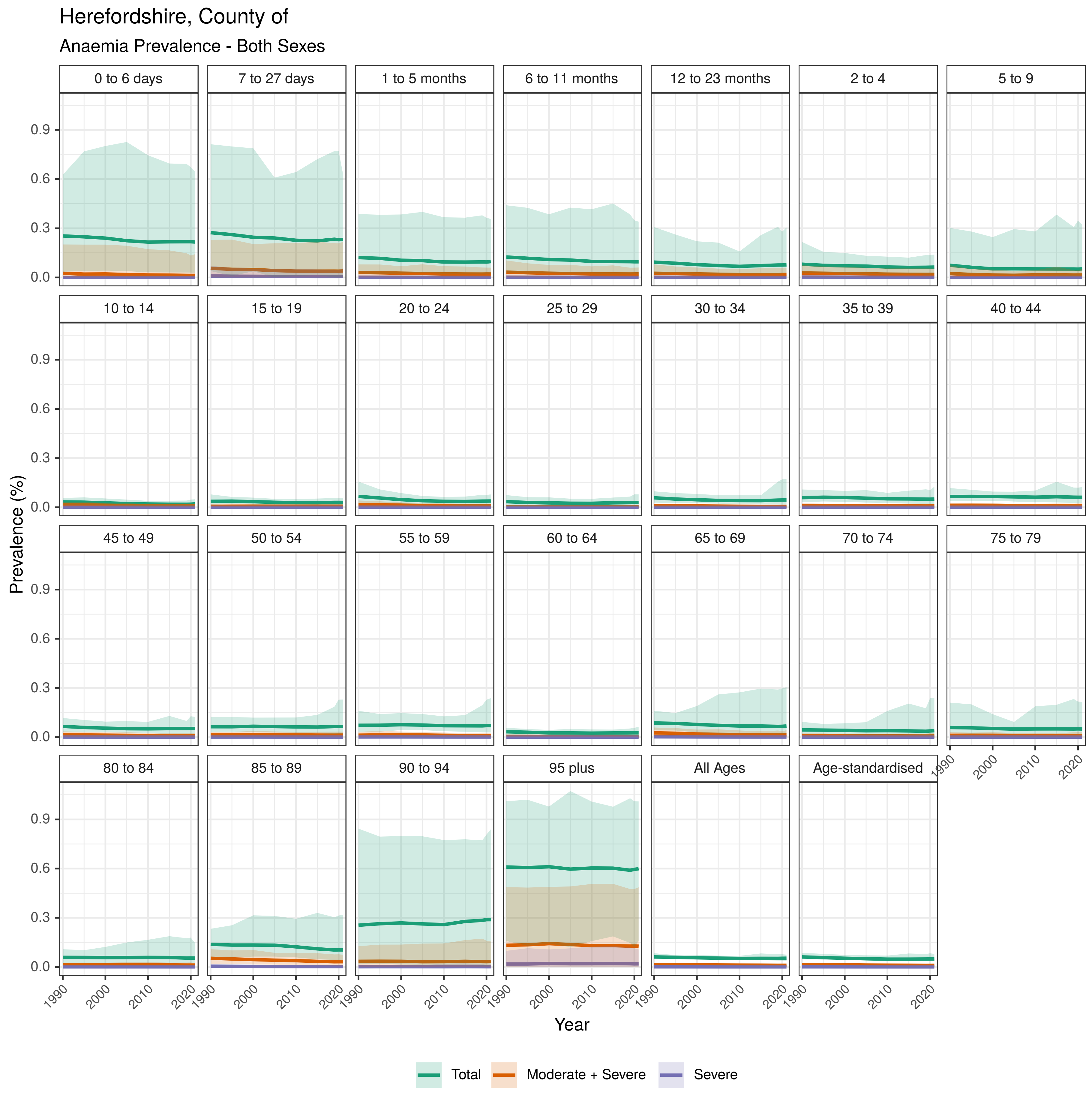
Herefordshire, County of
Anaemia Prevalence - Both Sexes
0 to 6 days
7 to 27 days
1 to 5 months
6 to 11 months
12 to 23 months
2 to 4
5 to 9
10 to 14
15 to 19
20 to 24
25 to 29
30 to 34
35 to 39
40 to 44
45 to 49
50 to 54
55 to 59
60 to 64
65 to 69
70 to 74
75 to 79
80 to 84
85 to 89
90 to 94
95 plus
All Ages
Age-standardised
Prevalence (%)
Year
Total
Moderate + Severe
Severe

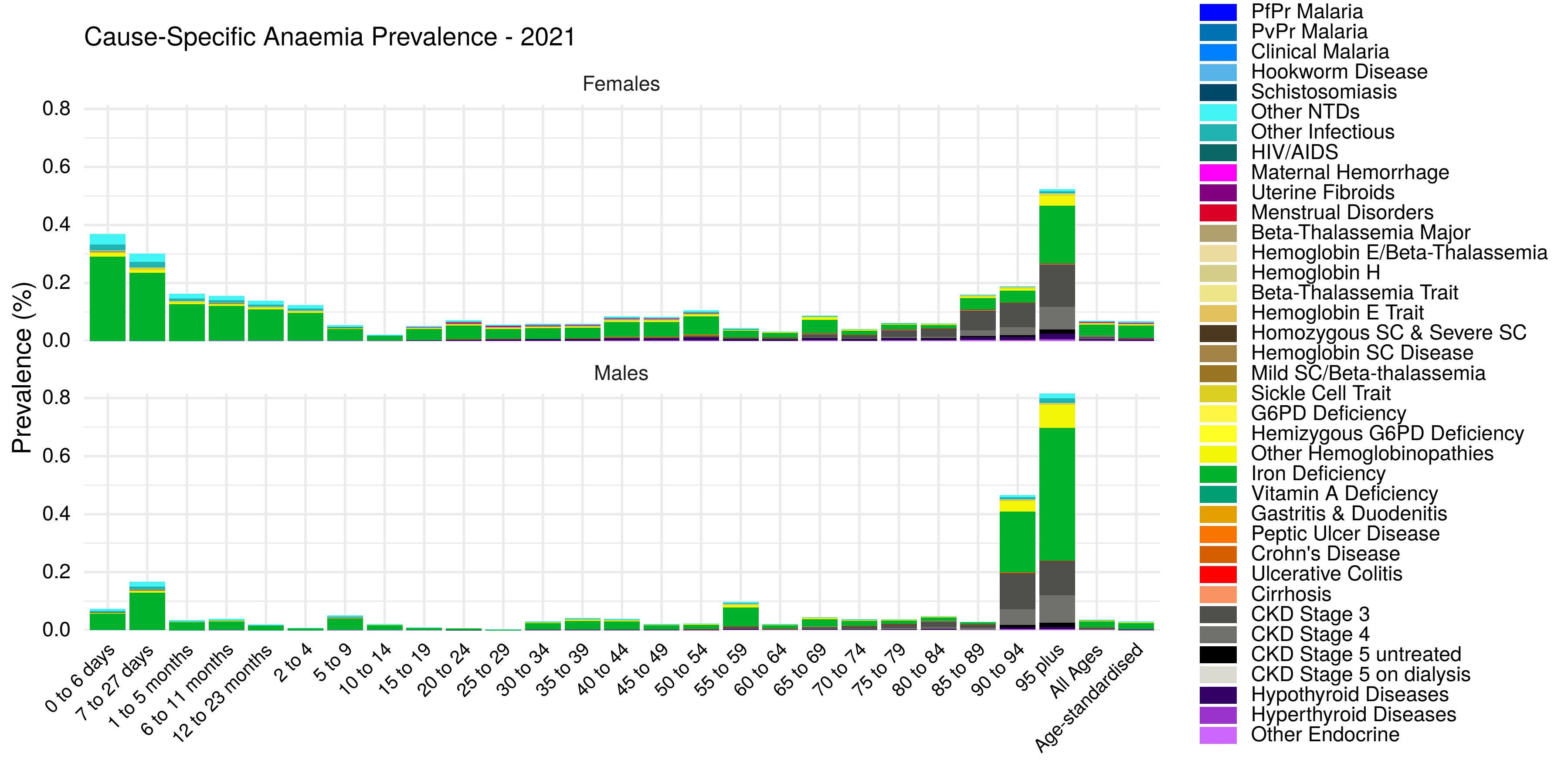
Cause-Specific Anaemia Prevalence - 2021
Females
Males
Prevalence (%)
PfPr Malaria
PvPr Malaria
Clinical Malaria
Hookworm Disease
Schistosomiasis
Other NTDs
Other Infectious
HIV/AIDS
Maternal Hemorrhage
Uterine Fibroids
Menstrual Disorders
Beta-Thalassemia Major
Hemoglobin E/Beta-Thalassemia
Hemoglobin H
Beta-Thalassemia Trait
Hemoglobin E Trait
Homozygous SC & Severe SC
Hemoglobin SC Disease
Mild SC/Beta-thalassemia
Sickle Cell Trait
G6PD Deficiency
Hemizygous G6PD Deficiency
Other Hemoglobinopathies
Iron Deficiency
Vitamin A Deficiency
Gastritis & Duodenitis
Peptic Ulcer Disease
Crohn's Disease
Ulcerative Colitis
Cirrhosis
CKD Stage 3
CKD Stage 4
CKD Stage 5 untreated
CKD Stage 5 on dialysis
Hypothyroid Diseases
Hyperthyroid Diseases
Other Endocrine

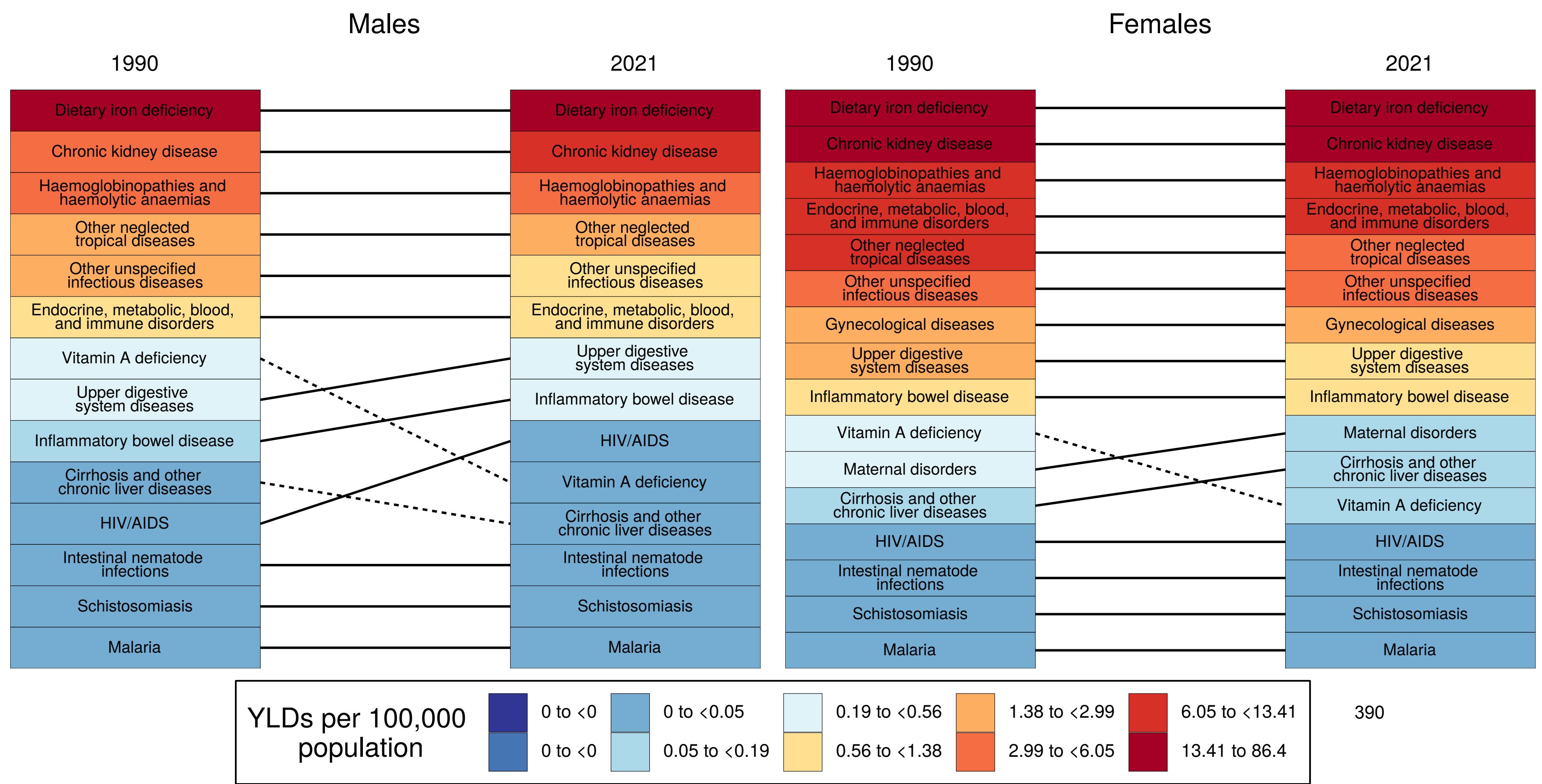
Males
Females
1990
2021
Dietary iron deficiency
Chronic kidney disease
Haemoglobinopathies and haemolytic anaemias
Other neglected tropical diseases
Other unspecified infectious diseases
Endocrine, metabolic, blood, and immune disorders
Vitamin A deficiency
Upper digestive system diseases
Inflammatory bowel disease
Cirrhosis and other chronic liver diseases
HIV/AIDS
Intestinal nematode infections
Schistosomiasis
Malaria
Gynecological diseases
Maternal disorders
YLDs per 100,000 population
0 to <0
0 to <0.05
0.05 to <0.19
0.19 to <0.56
0.56 to <1.38
1.38 to <2.99
2.99 to <6.05
6.05 to <13.41
13.41 to 86.4

Sandwell

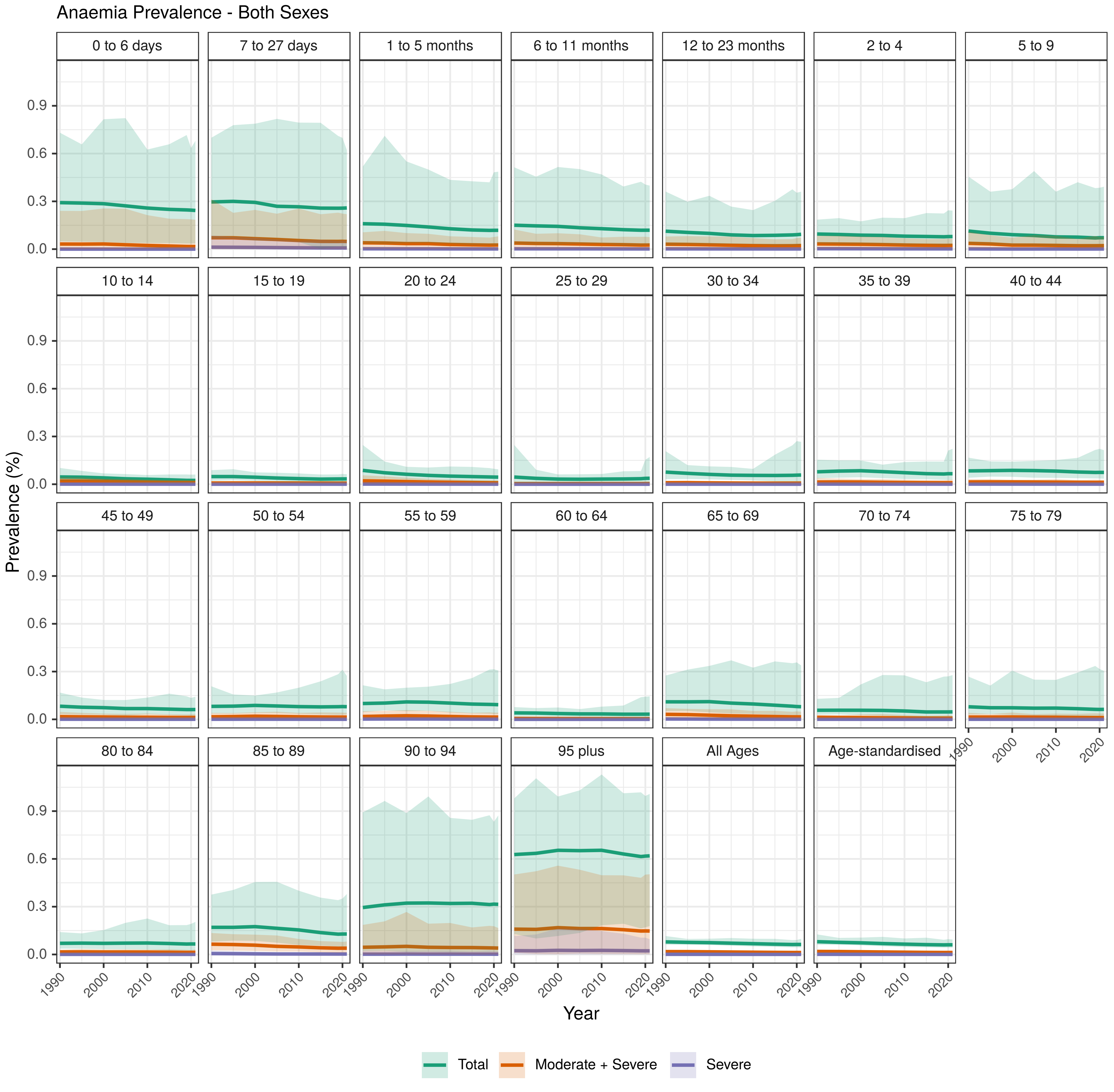

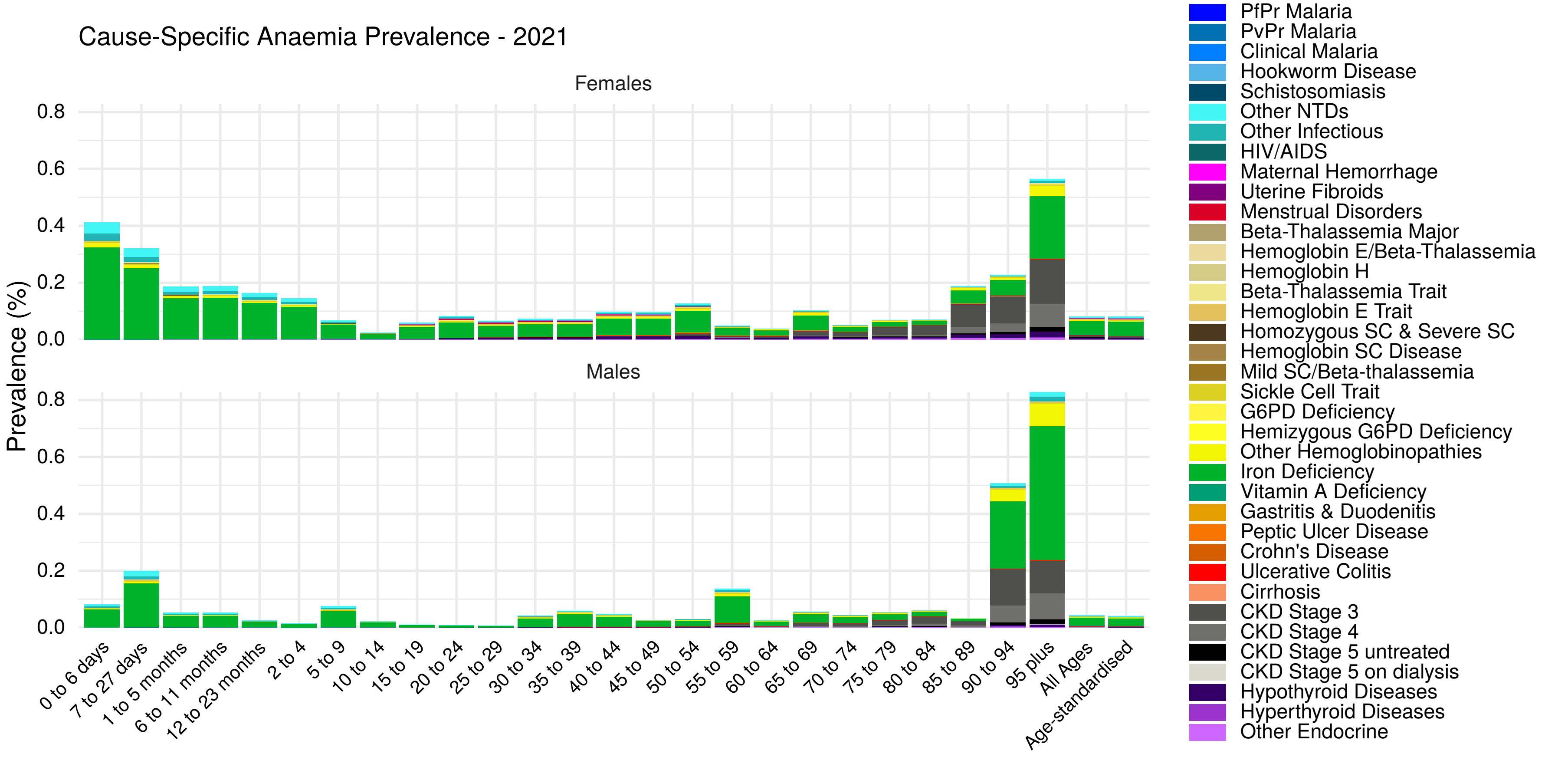

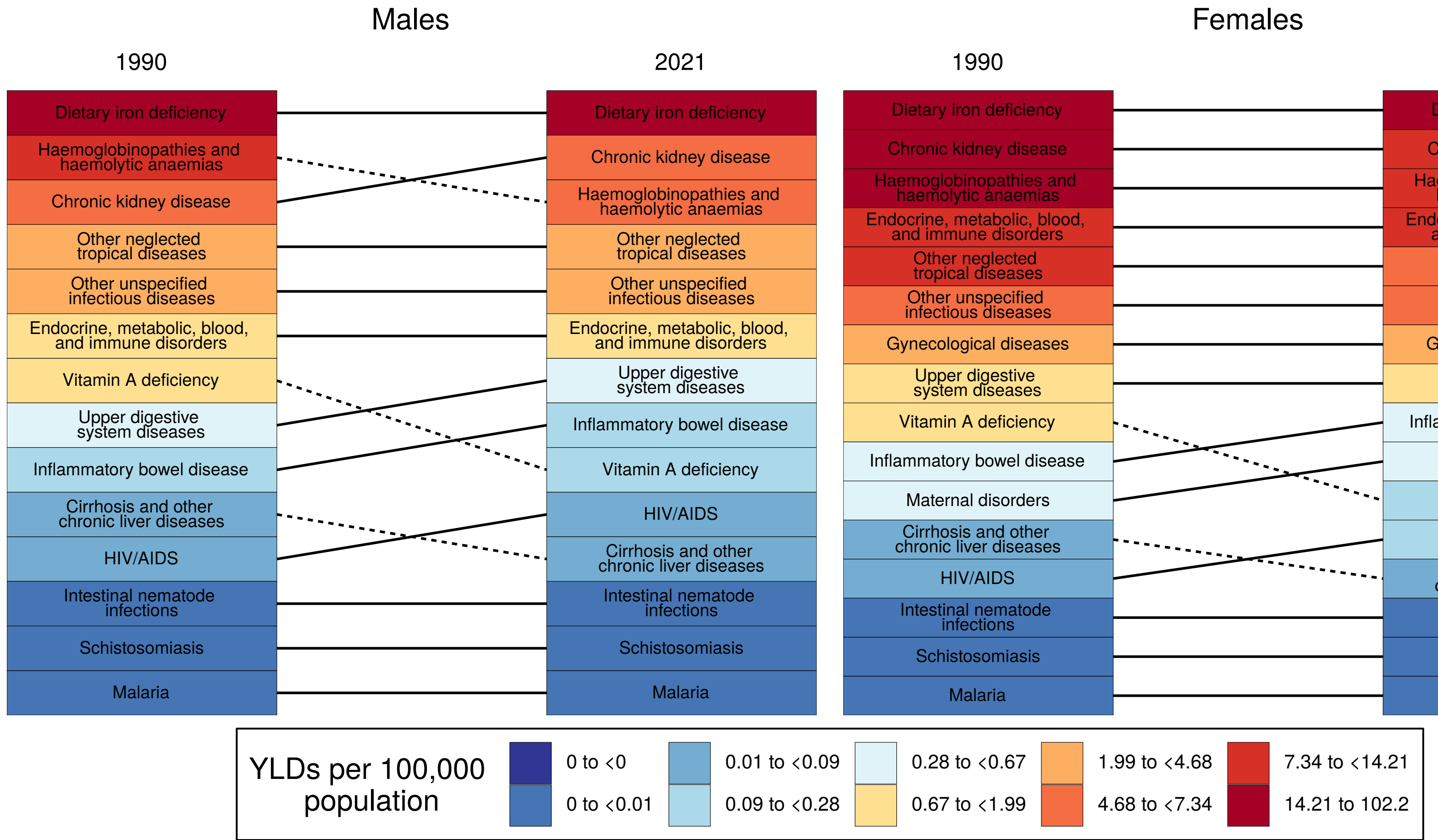

391

Shropshire

Anaemia Prevalence - Both Sexes

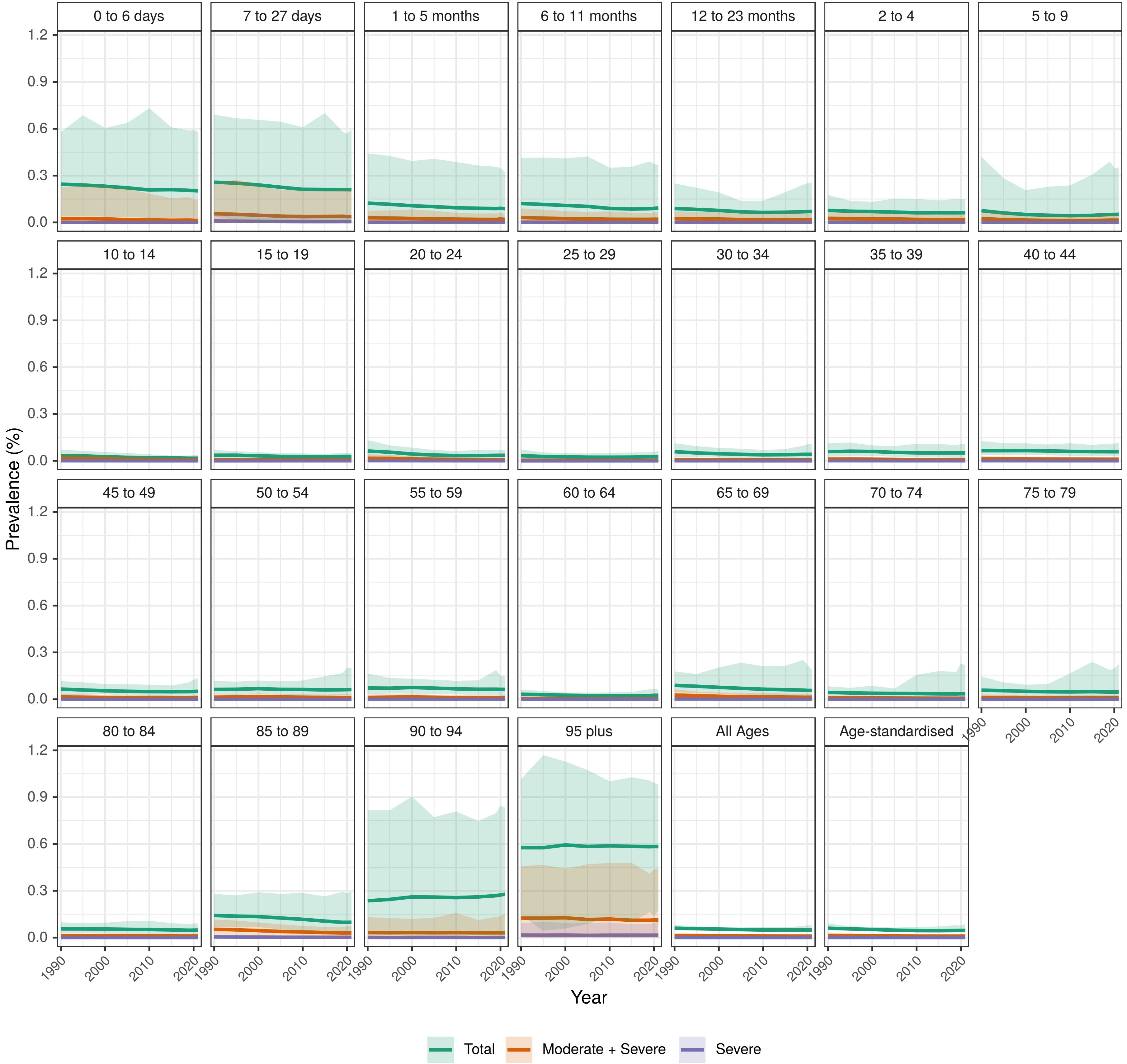

Cause-Specific Anaemia Prevalence - 2021

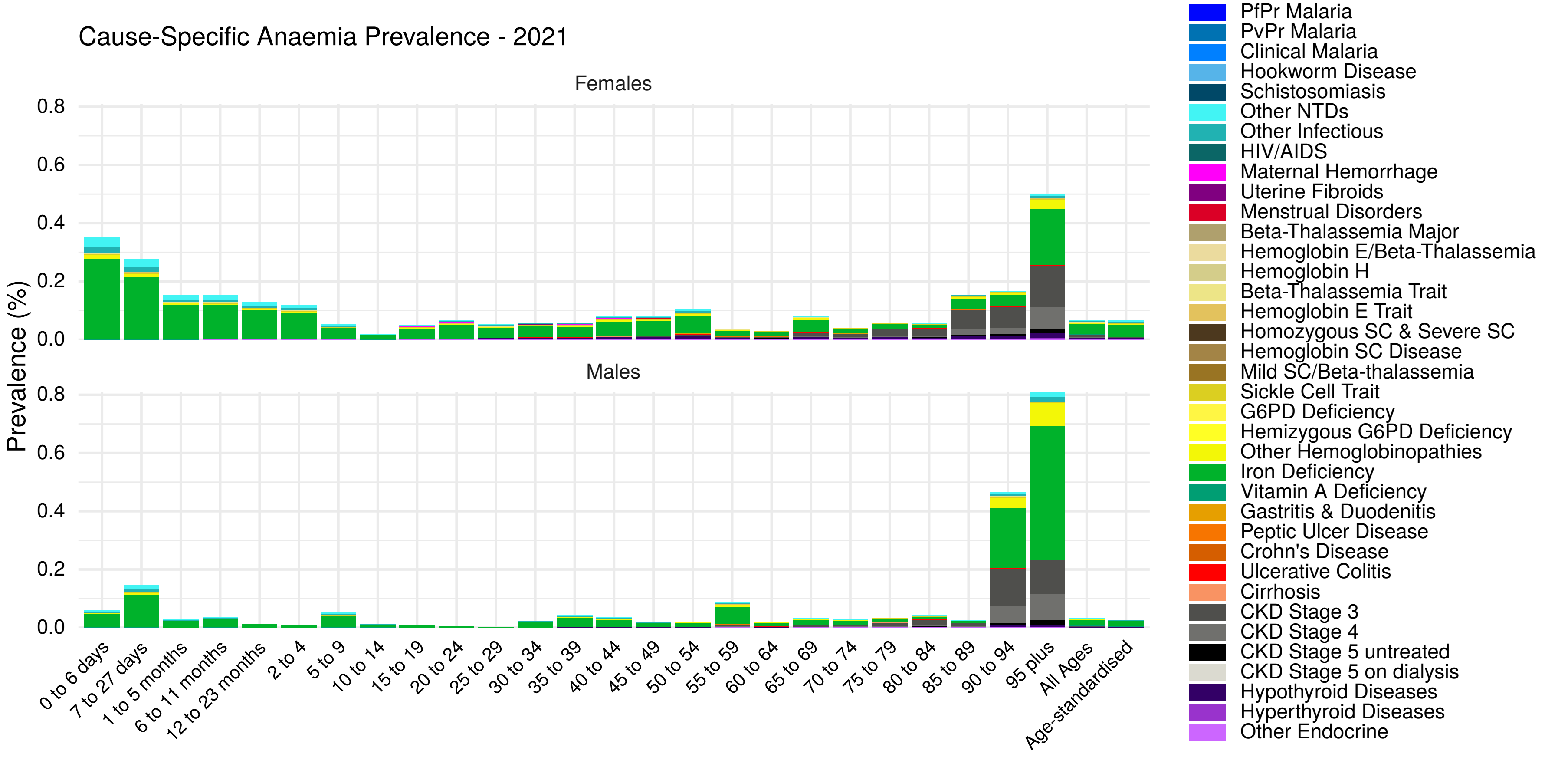

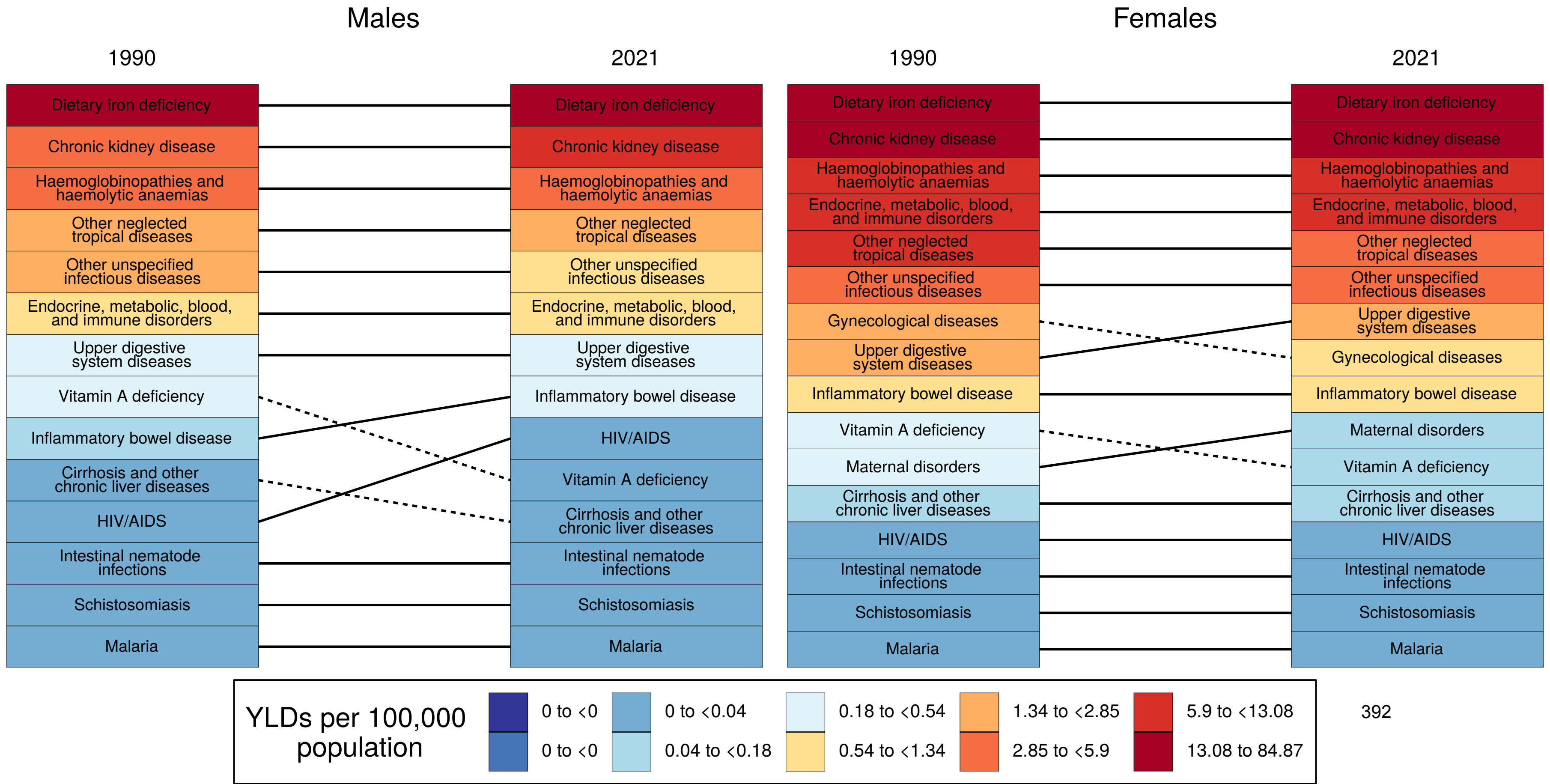

Solihull

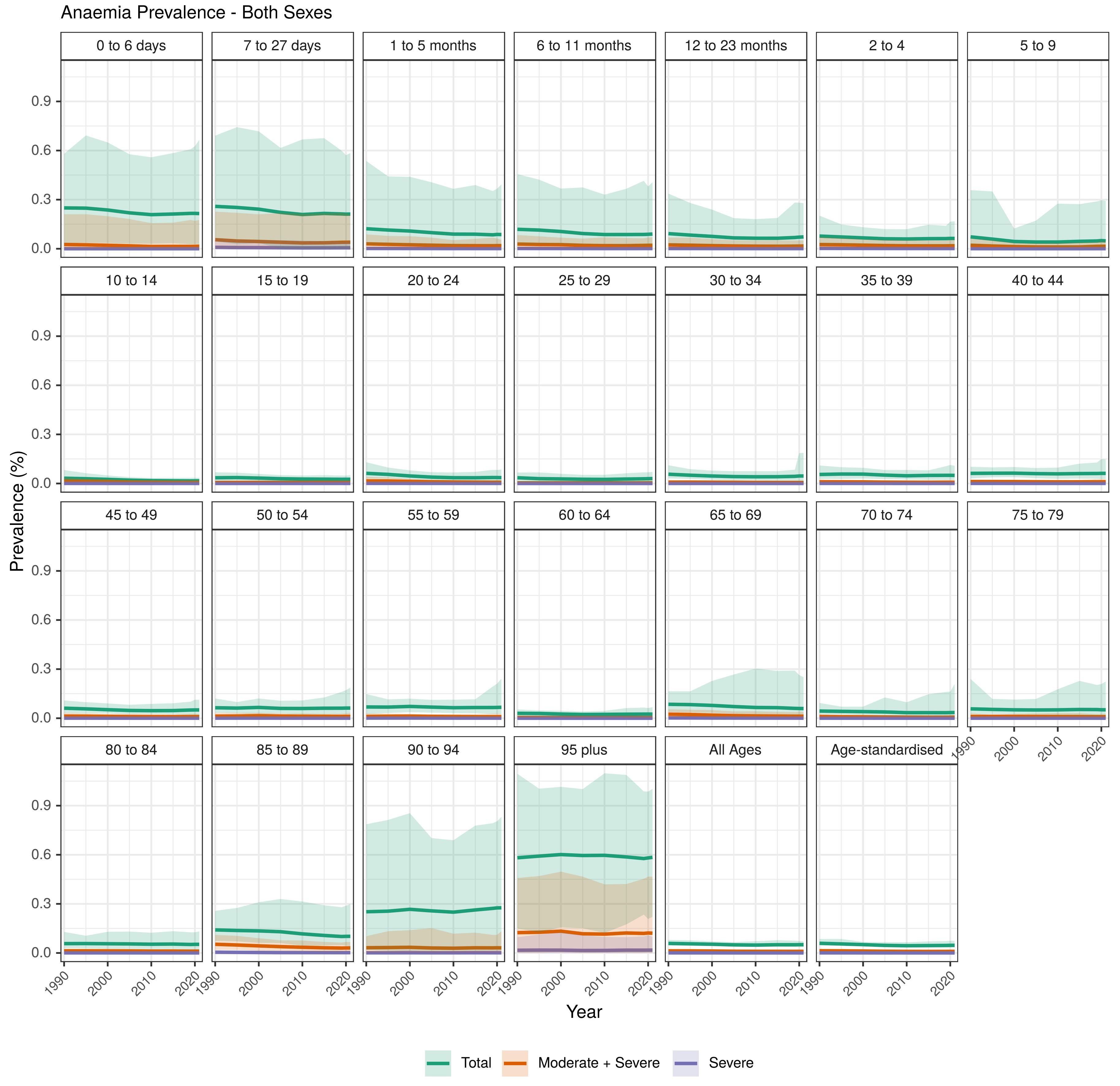

Anaemia Prevalence - Both Sexes
0 to 6 days
7 to 27 days
1 to 5 months
6 to 11 months
12 to 23 months
2 to 4
5 to 9
10 to 14
15 to 19
20 to 24
25 to 29
30 to 34
35 to 39
40 to 44
45 to 49
50 to 54
55 to 59
60 to 64
65 to 69
70 to 74
75 to 79
80 to 84
85 to 89
90 to 94
95 plus
All Ages
Age-standardised
Prevalence (%)
0.0
0.3
0.6
0.9
1990
2000
2010
2020
Year
Total
Moderate + Severe
Severe

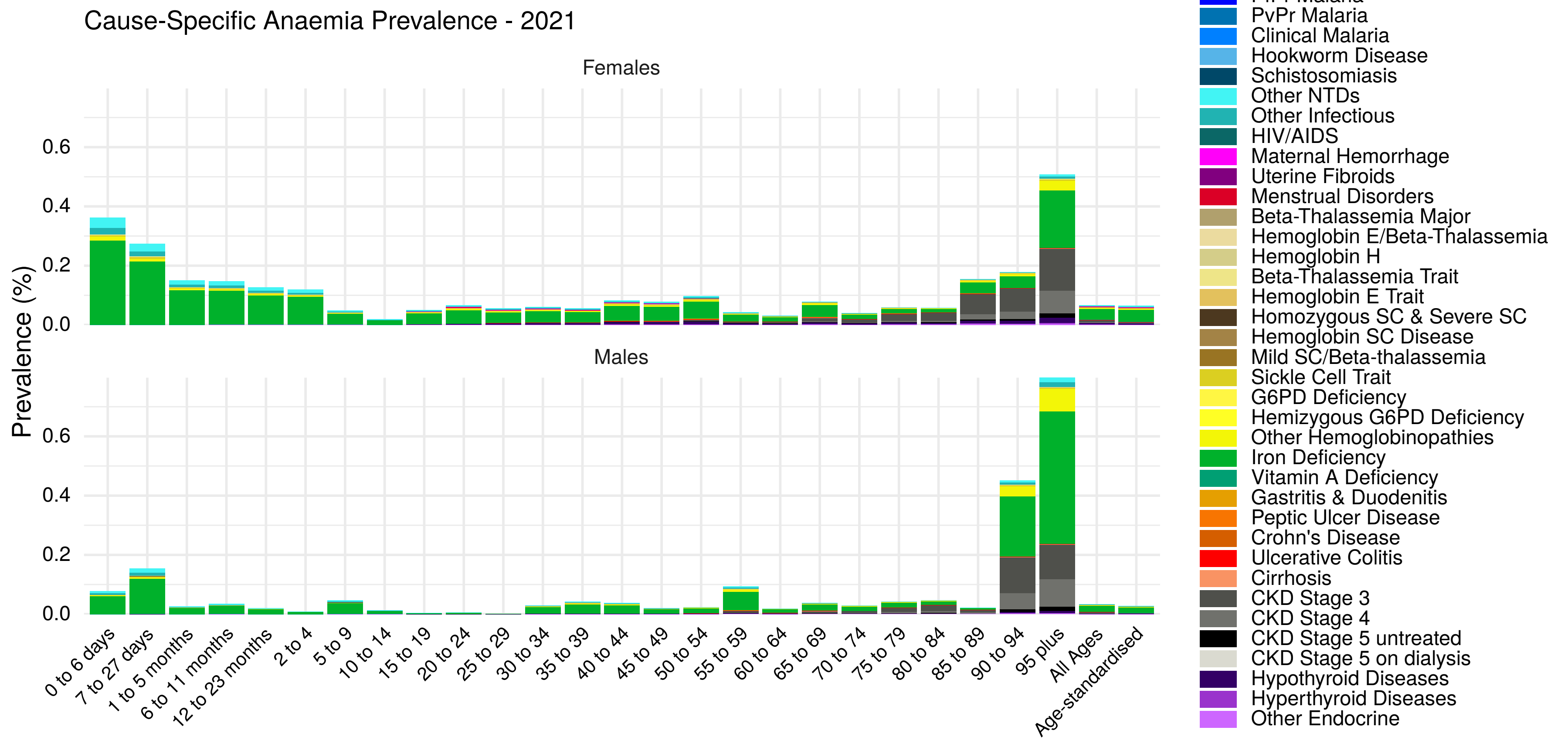

Cause-Specific Anaemia Prevalence - 2021
Females
Males
Prevalence (%)
0.0
0.2
0.4
0.6
0 to 6 days
7 to 27 days
1 to 5 months
6 to 11 months
12 to 23 months
2 to 4
5 to 9
10 to 14
15 to 19
20 to 24
25 to 29
30 to 34
35 to 39
40 to 44
45 to 49
50 to 54
55 to 59
60 to 64
65 to 69
70 to 74
75 to 79
80 to 84
85 to 89
90 to 94
95 plus
All Ages
Age-standardised
PfPr Malaria
PvPr Malaria
Clinical Malaria
Hookworm Disease
Schistosomiasis
Other NTDs
Other Infectious
HIV/AIDS
Maternal Hemorrhage
Uterine Fibroids
Menstrual Disorders
Beta-Thalassemia Major
Hemoglobin E/Beta-Thalassemia
Hemoglobin H
Beta-Thalassemia Trait
Hemoglobin E Trait
Homozygous SC & Severe SC
Hemoglobin SC Disease
Mild SC/Beta-thalassemia
Sickle Cell Trait
G6PD Deficiency
Hemizygous G6PD Deficiency
Other Hemoglobinopathies
Iron Deficiency
Vitamin A Deficiency
Gastritis & Duodenitis
Peptic Ulcer Disease
Crohn's Disease
Ulcerative Colitis
Cirrhosis
CKD Stage 3
CKD Stage 4
CKD Stage 5 untreated
CKD Stage 5 on dialysis
Hypothyroid Diseases
Hyperthyroid Diseases
Other Endocrine

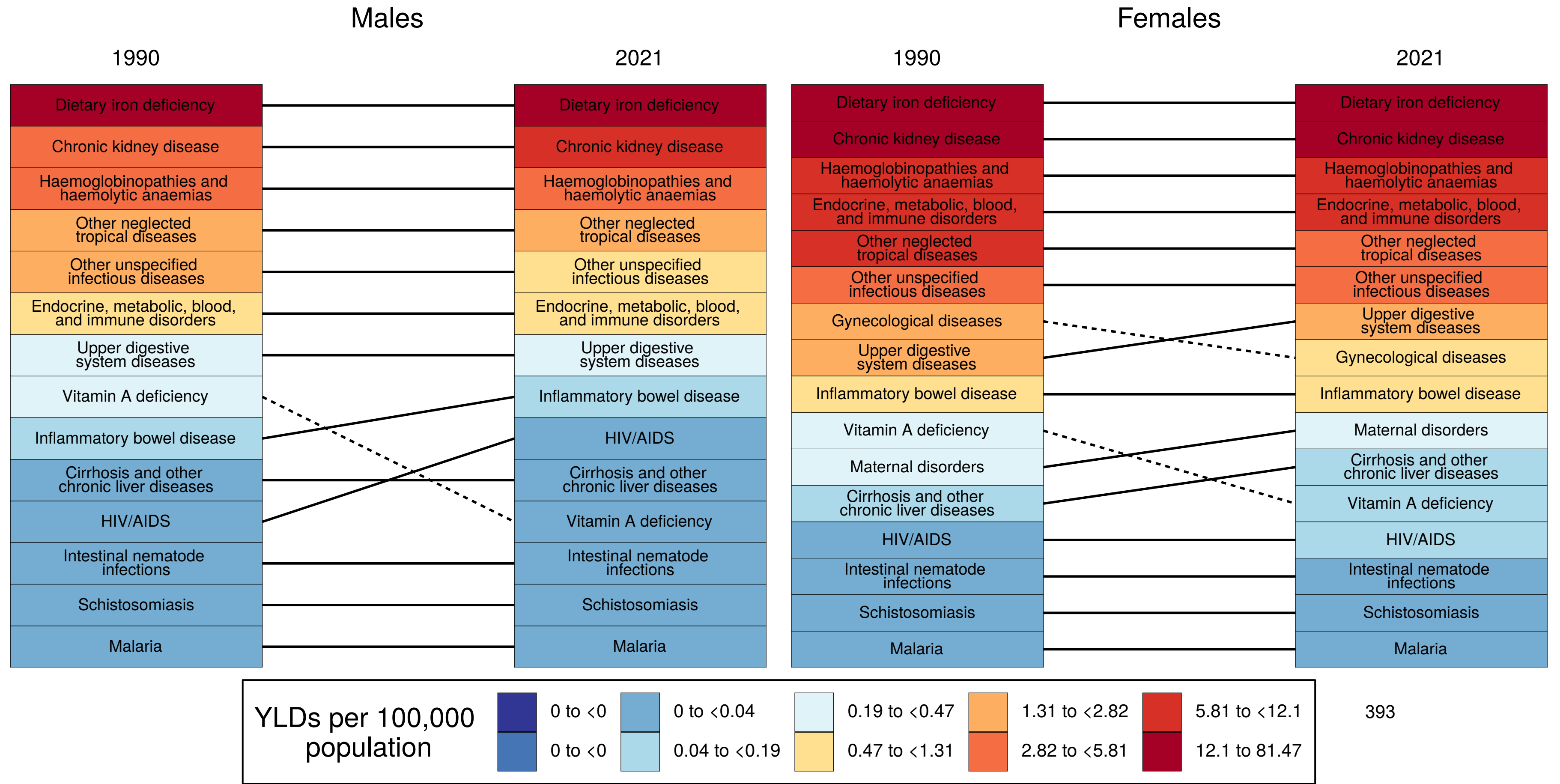

Males
Females
1990
2021
Dietary iron deficiency
Chronic kidney disease
Haemoglobinopathies and haemolytic anaemias
Other neglected tropical diseases
Other unspecified infectious diseases
Endocrine, metabolic, blood, and immune disorders
Upper digestive system diseases
Vitamin A deficiency
Inflammatory bowel disease
Cirrhosis and other chronic liver diseases
HIV/AIDS
Intestinal nematode infections
Schistosomiasis
Malaria
Gynecological diseases
Maternal disorders
YLDs per 100,000 population
0 to <0
0 to <0
0 to <0.04
0.04 to <0.19
0.19 to <0.47
0.47 to <1.31
1.31 to <2.82
2.82 to <5.81
5.81 to <12.1
12.1 to 81.47

Staffordshire

Anaemia Prevalence - Both Sexes

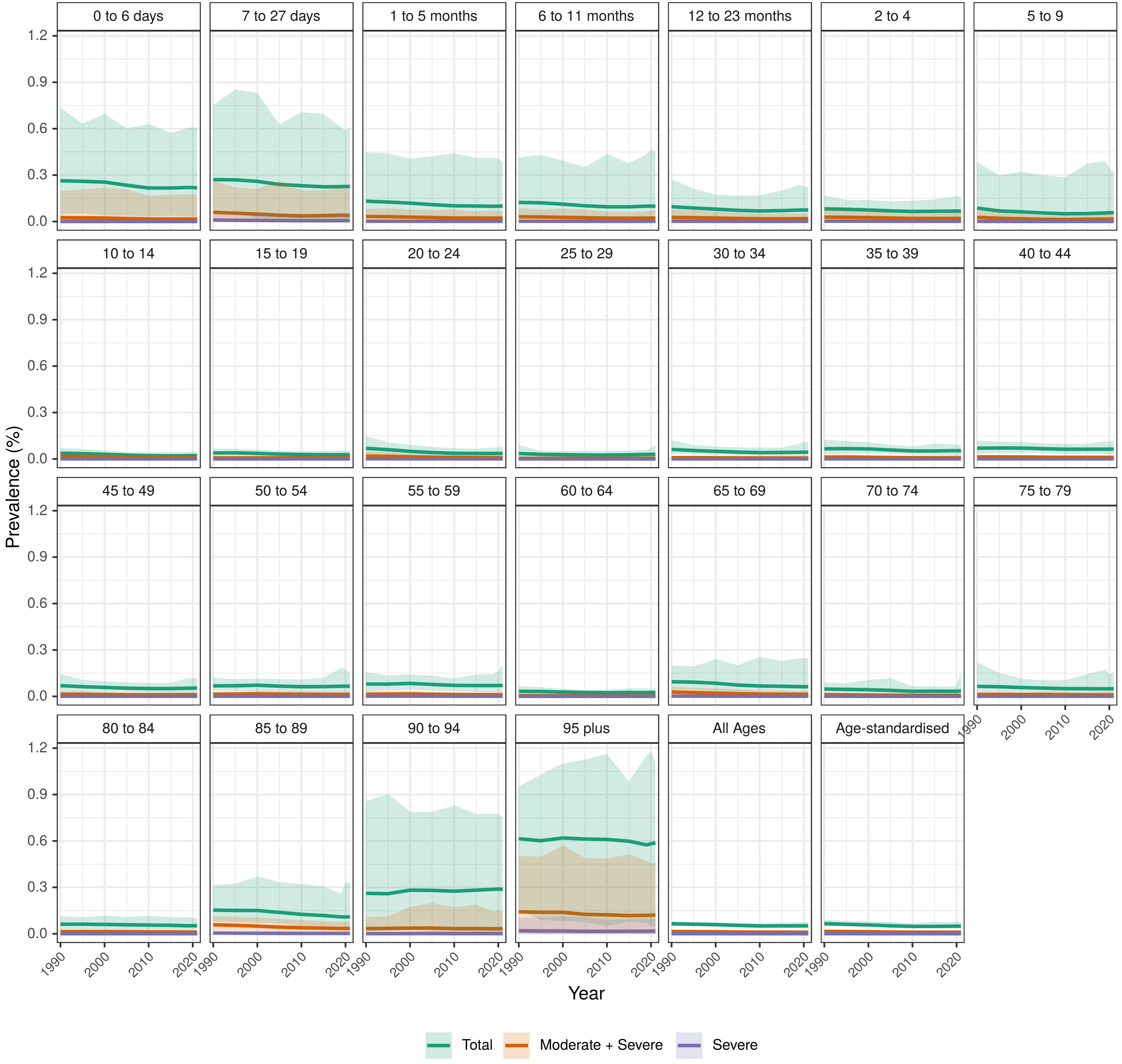

Cause-Specific Anaemia Prevalence - 2021

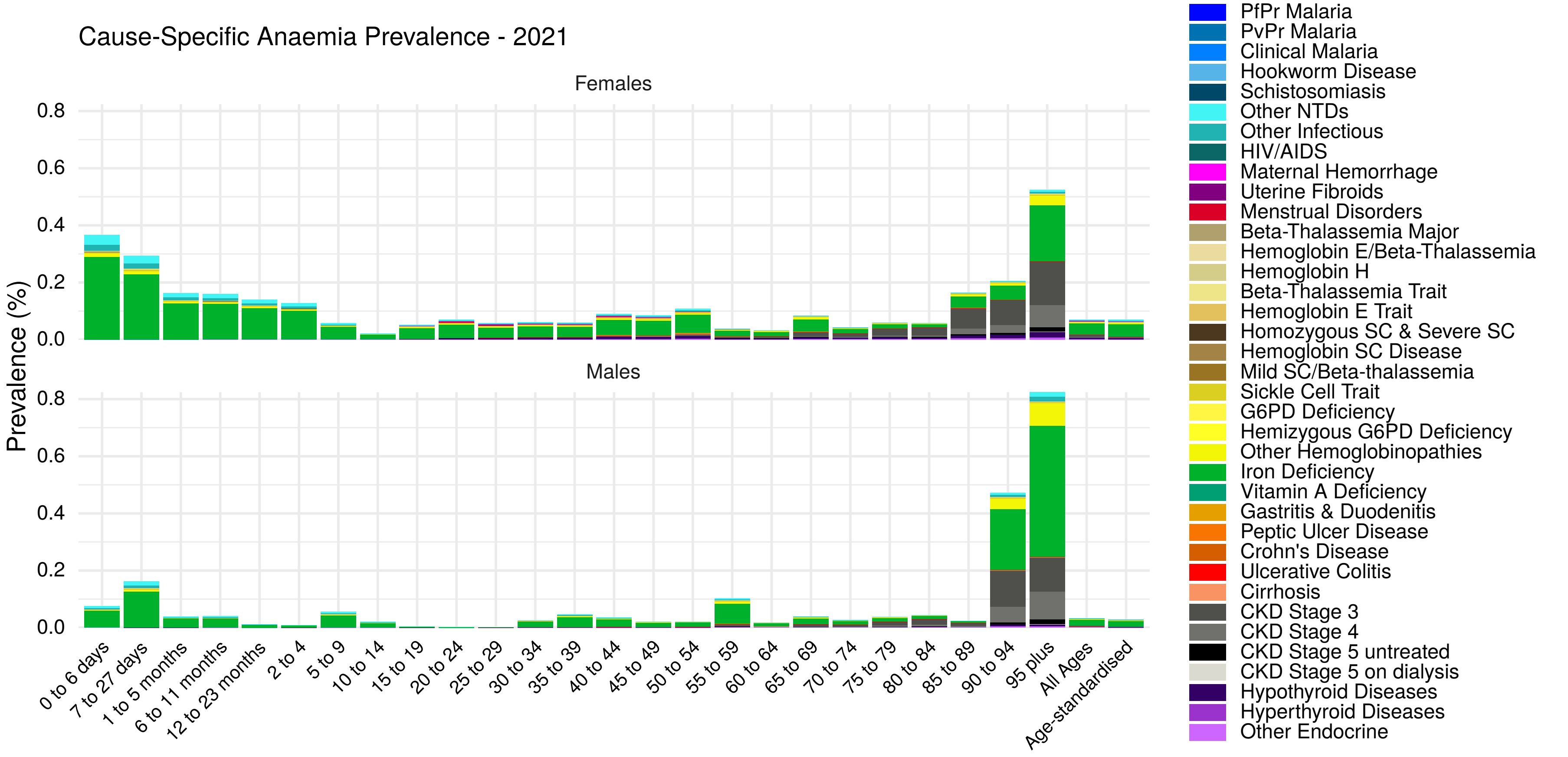

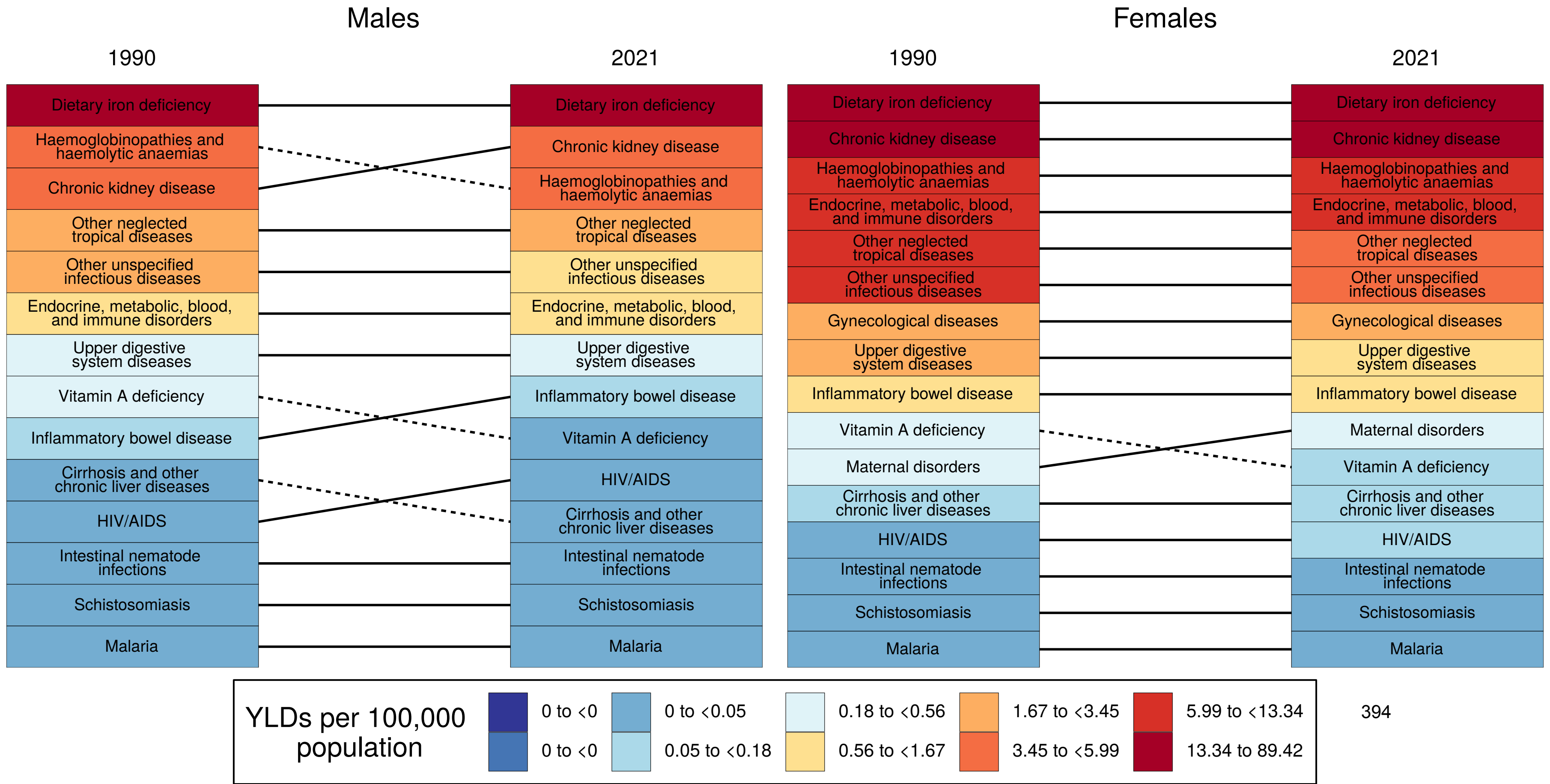

Stoke-on-Trent

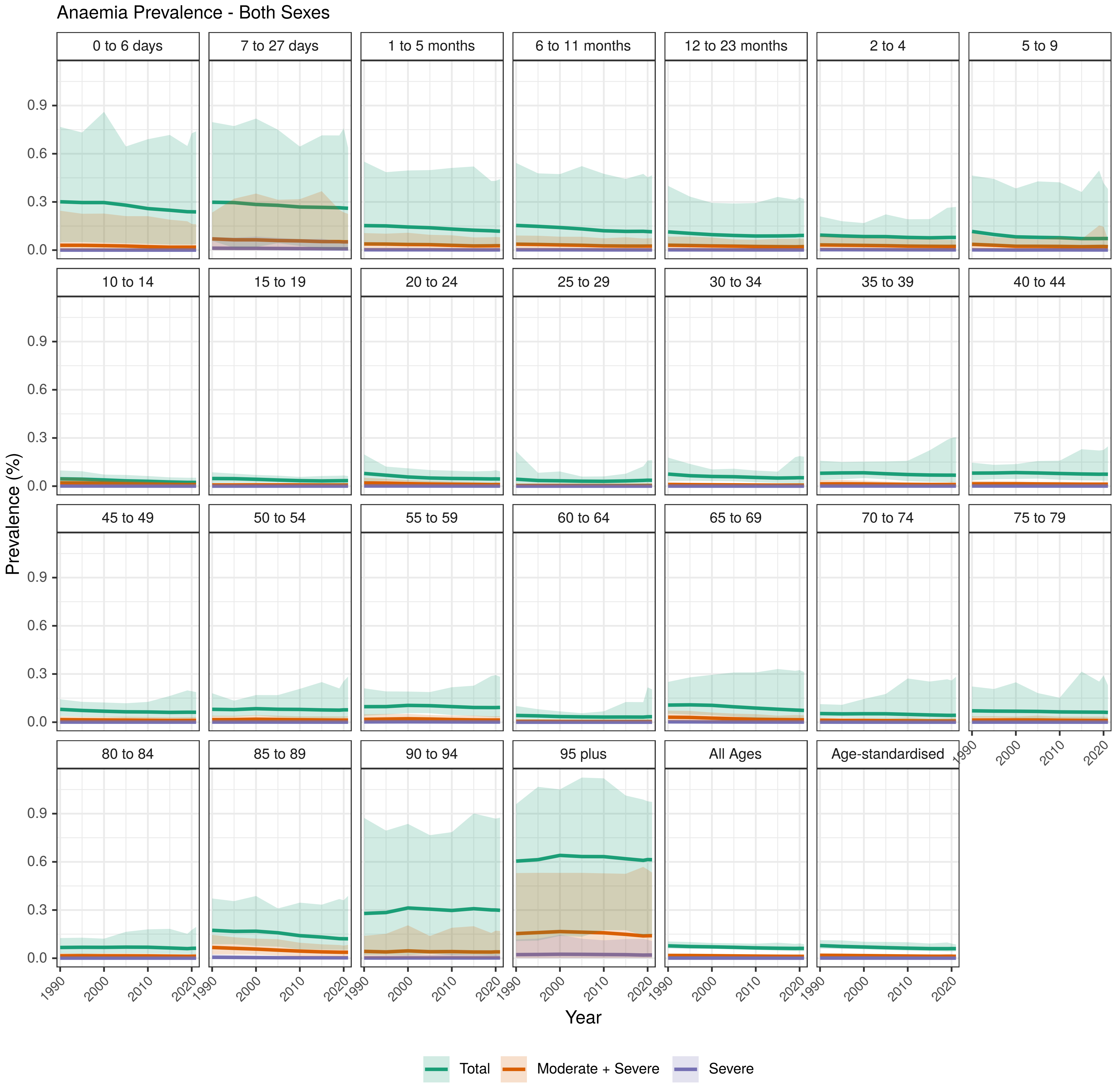

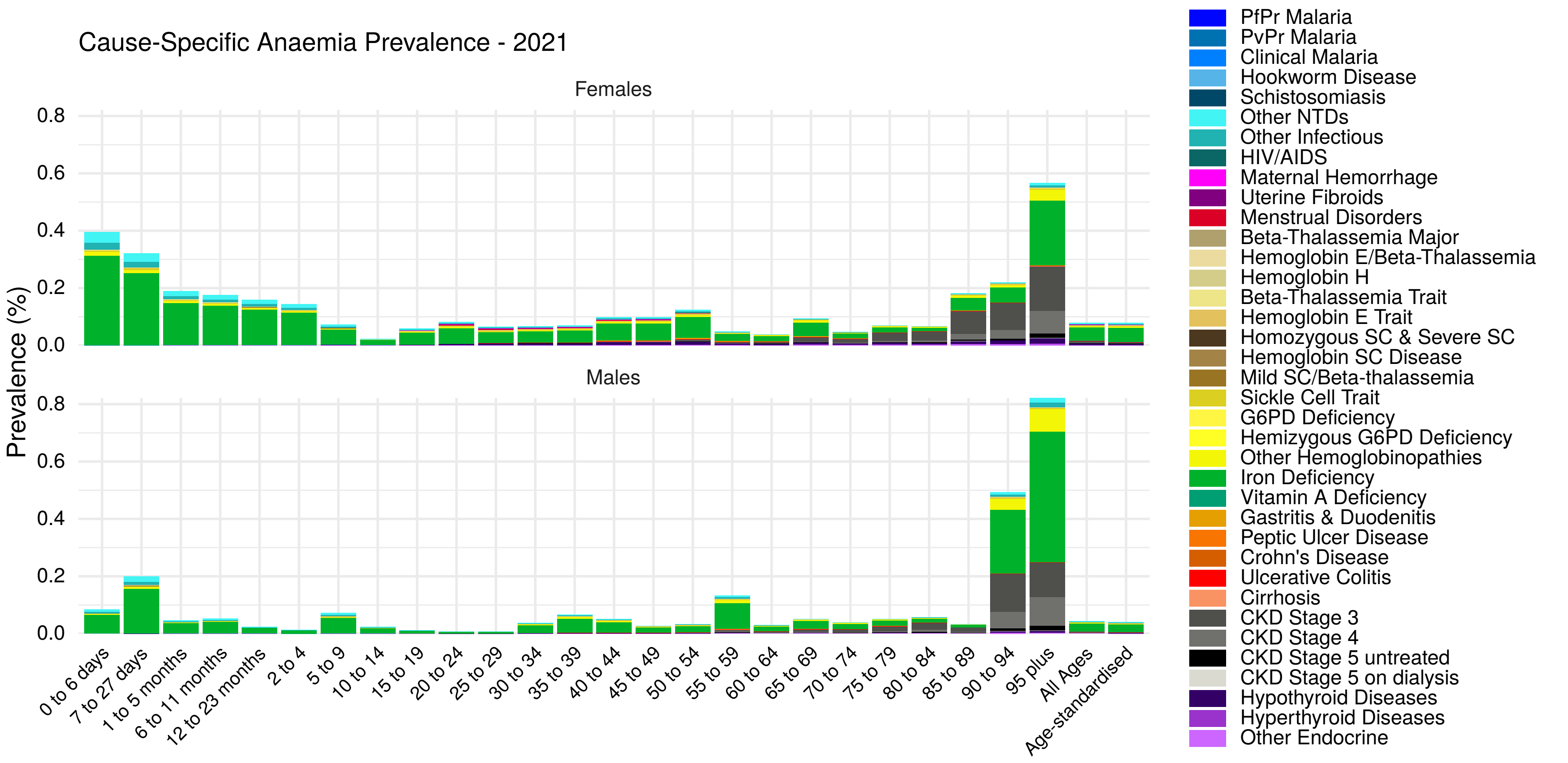

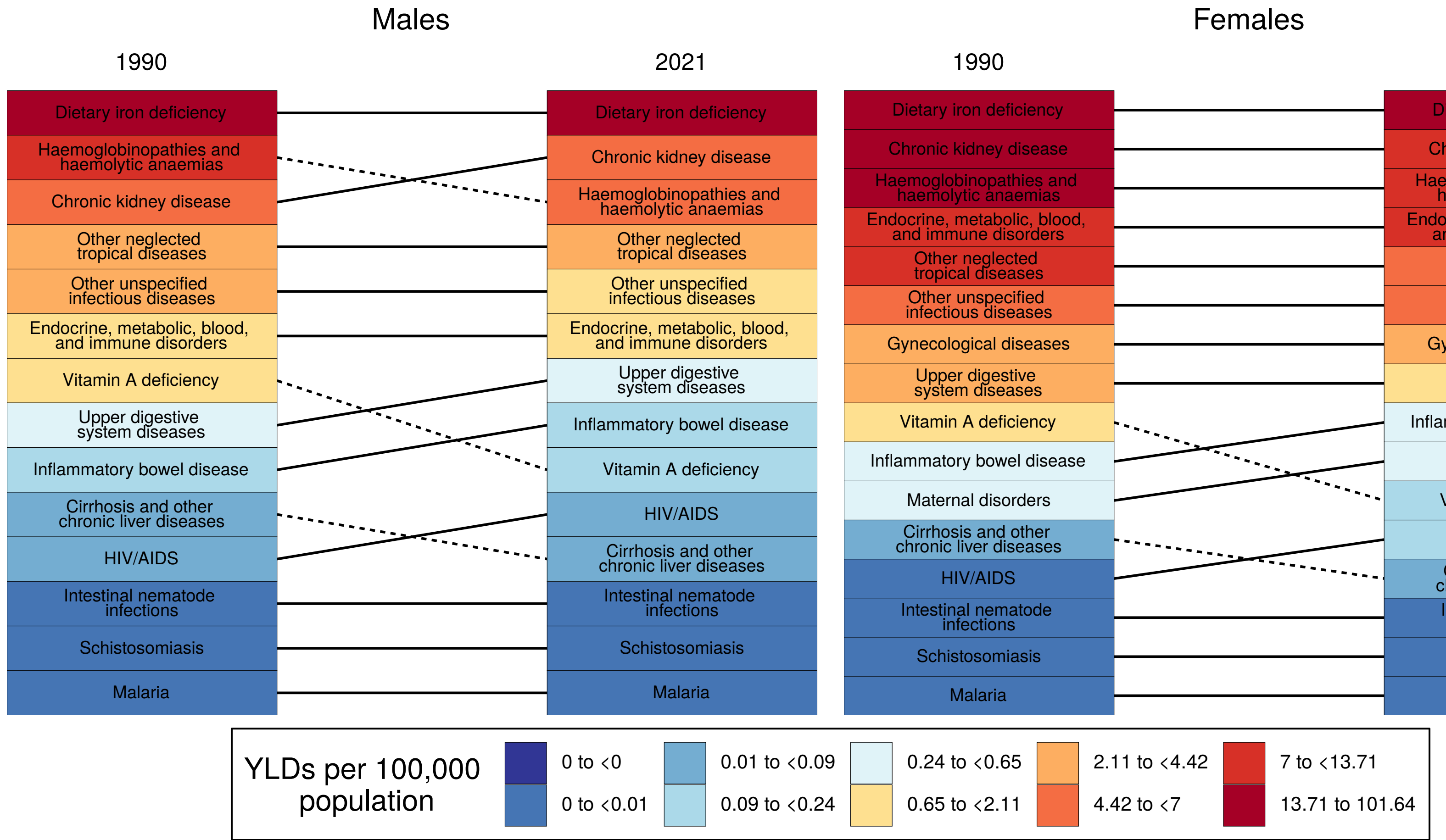

Telford and Wrekin

Anaemia Prevalence - Both Sexes

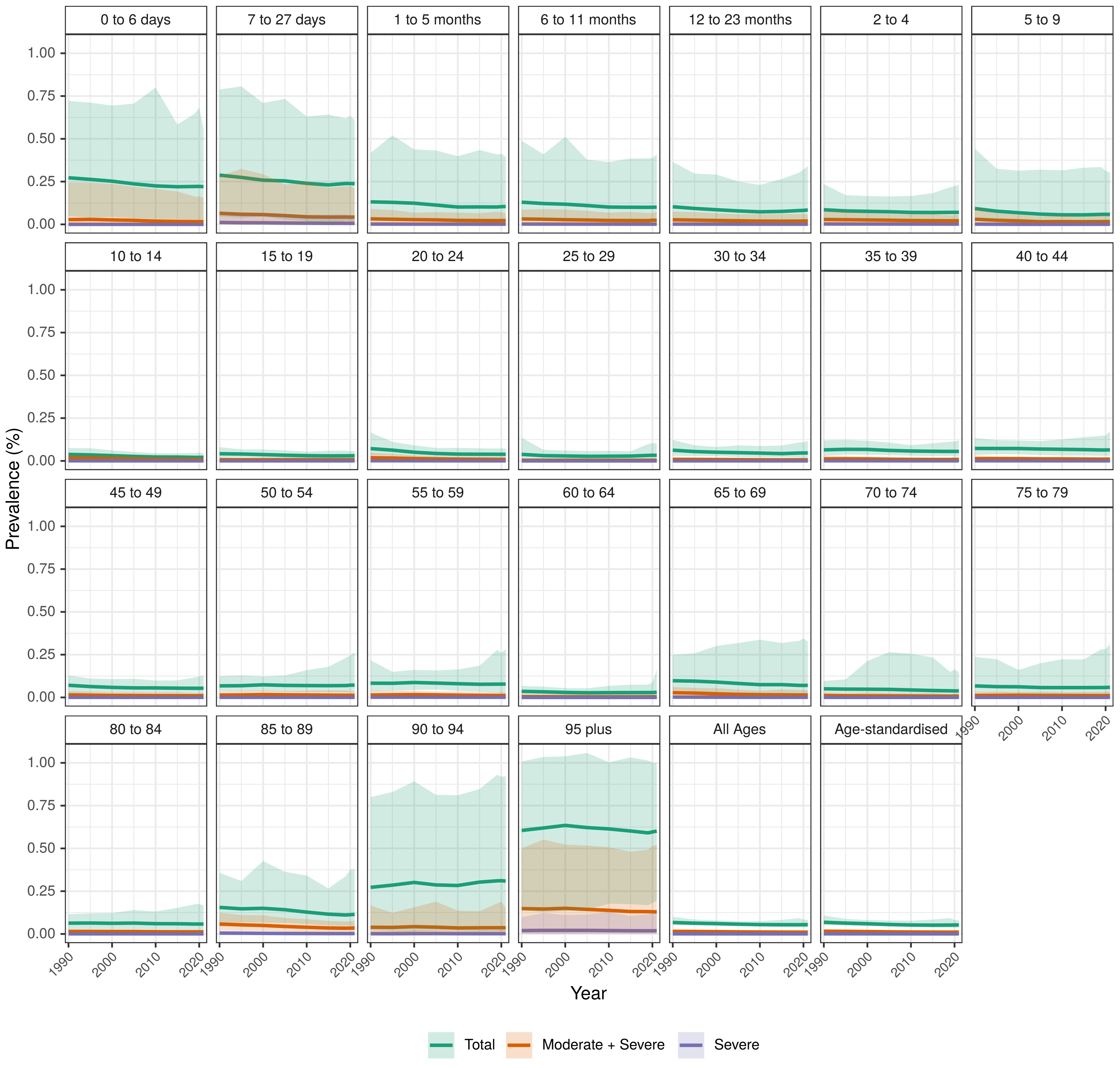

Cause-Specific Anaemia Prevalence - 2021

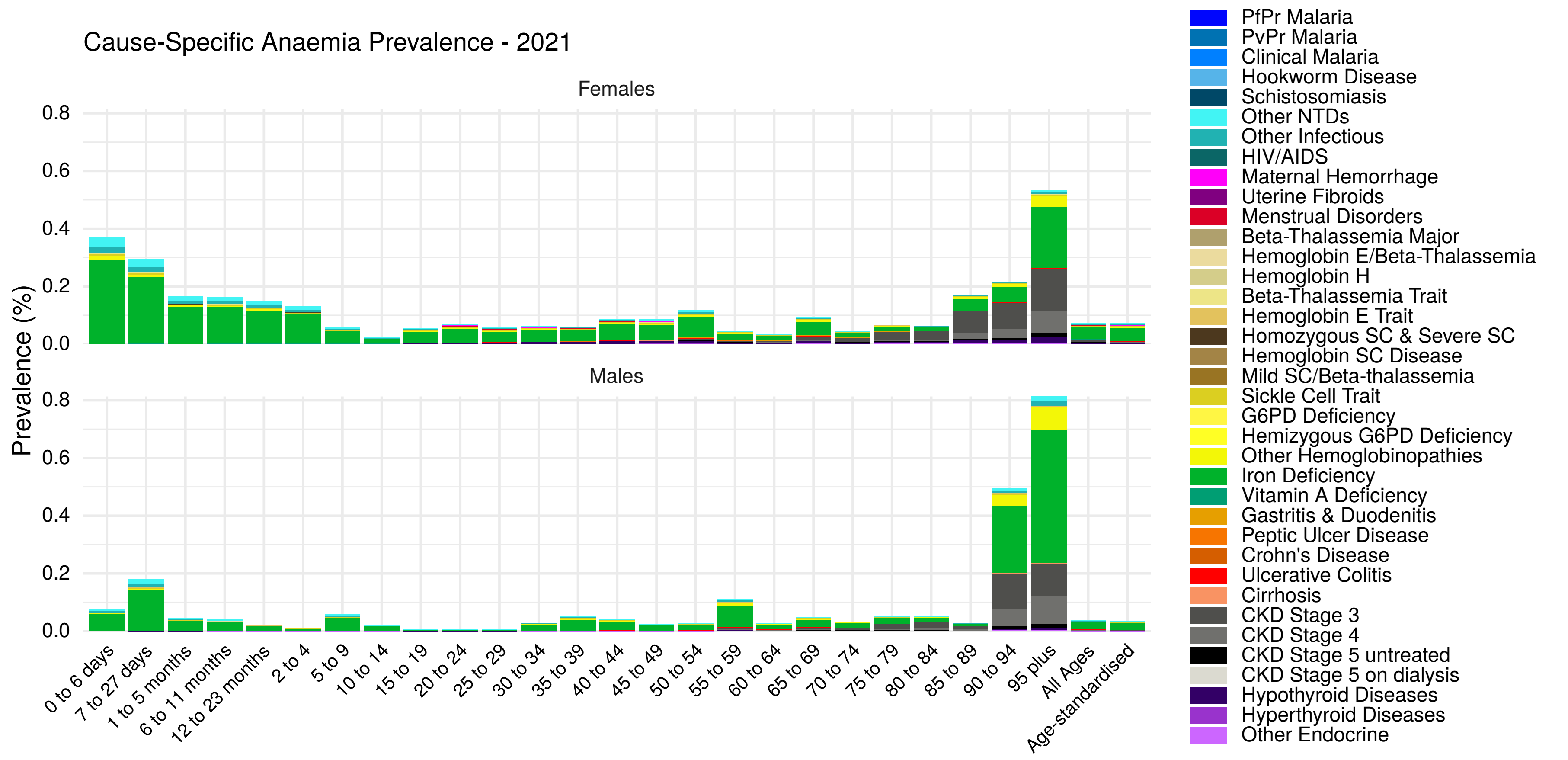

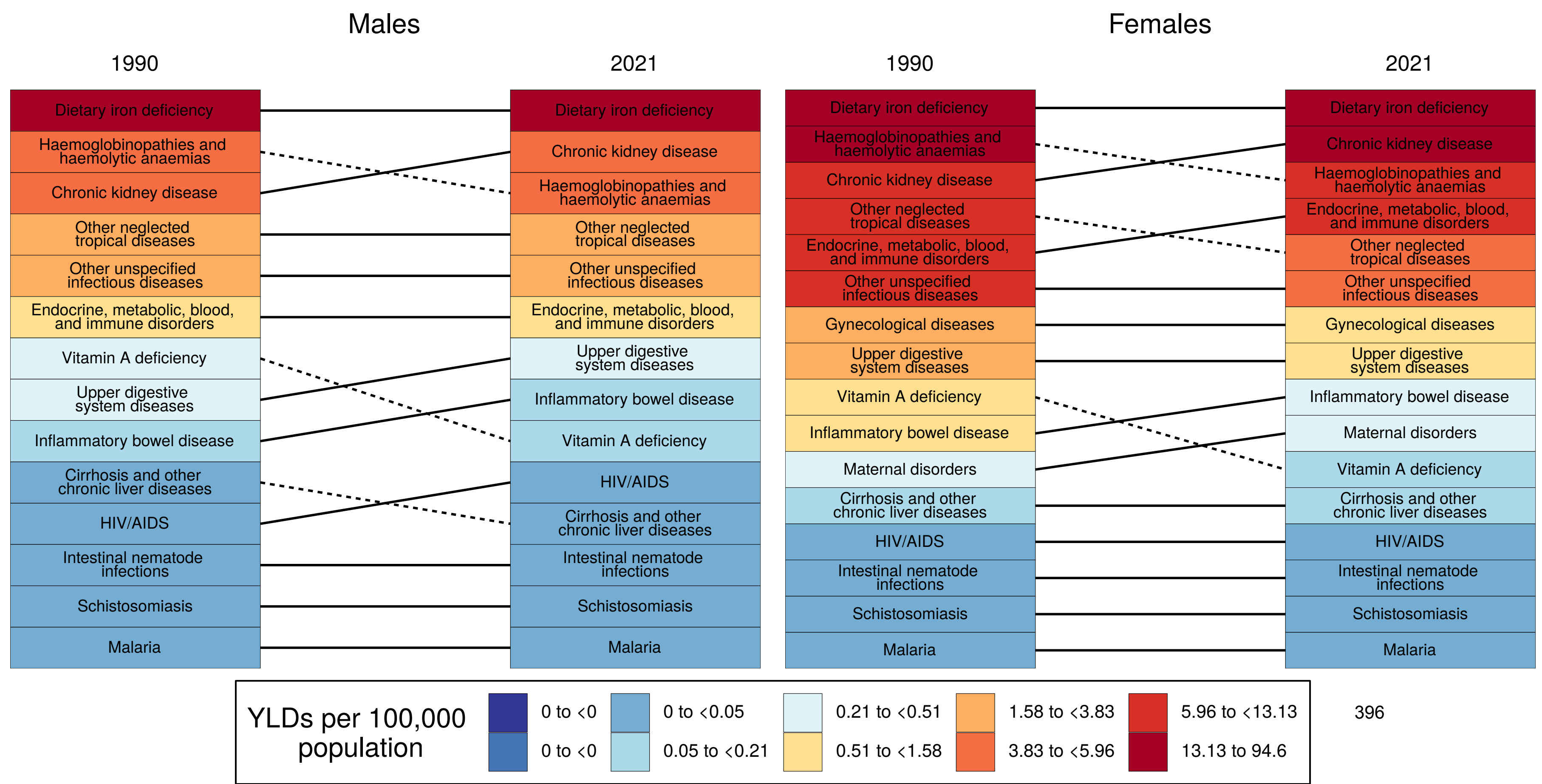

Walsall

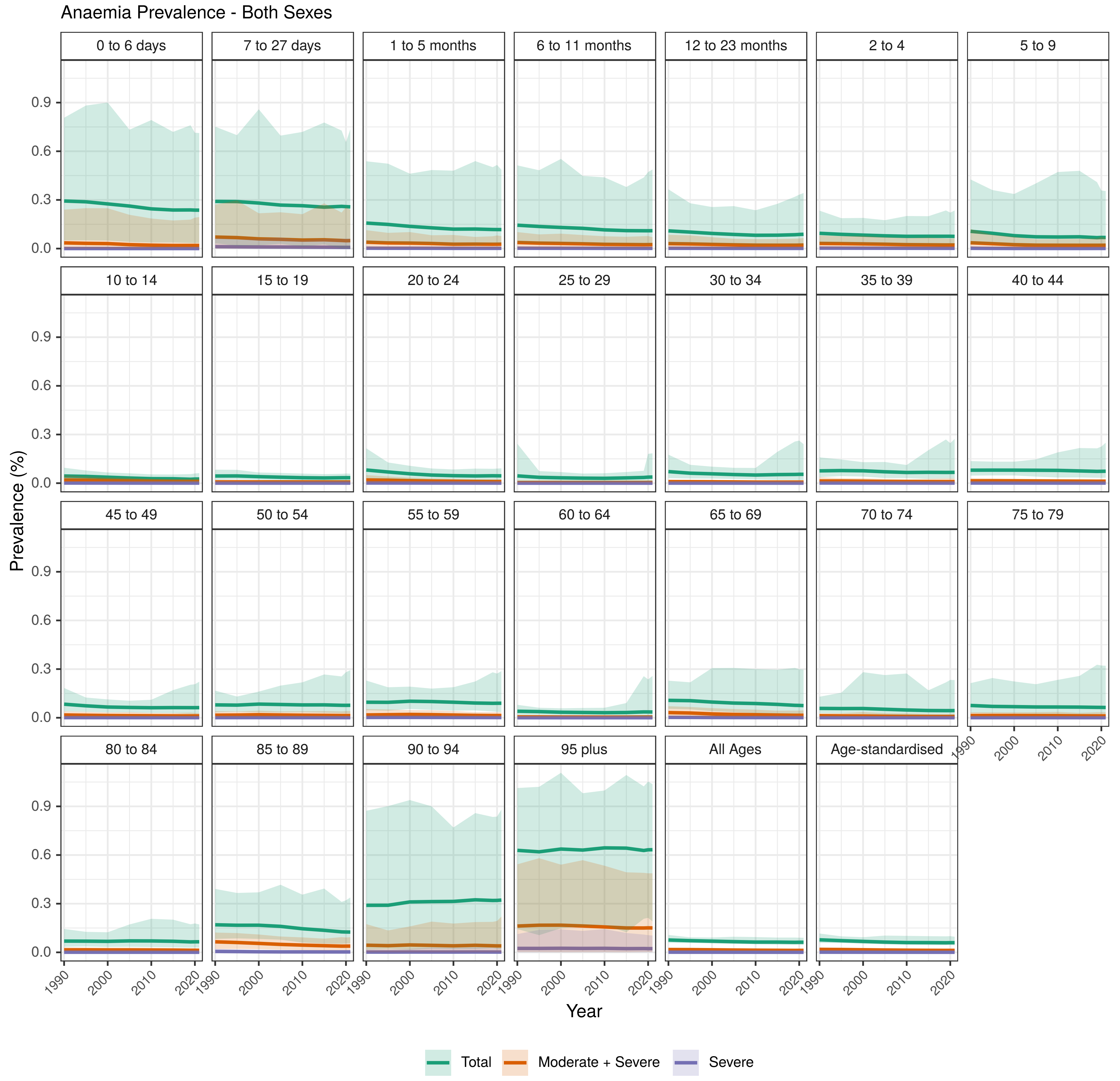

Anaemia Prevalence - Both Sexes
0 to 6 days
7 to 27 days
1 to 5 months
6 to 11 months
12 to 23 months
2 to 4
5 to 9
10 to 14
15 to 19
20 to 24
25 to 29
30 to 34
35 to 39
40 to 44
45 to 49
50 to 54
55 to 59
60 to 64
65 to 69
70 to 74
75 to 79
80 to 84
85 to 89
90 to 94
95 plus
All Ages
Age-standardised
Prevalence (%)
0.0
0.3
0.6
0.9
1990
2000
2010
2020
Year
Total
Moderate + Severe
Severe

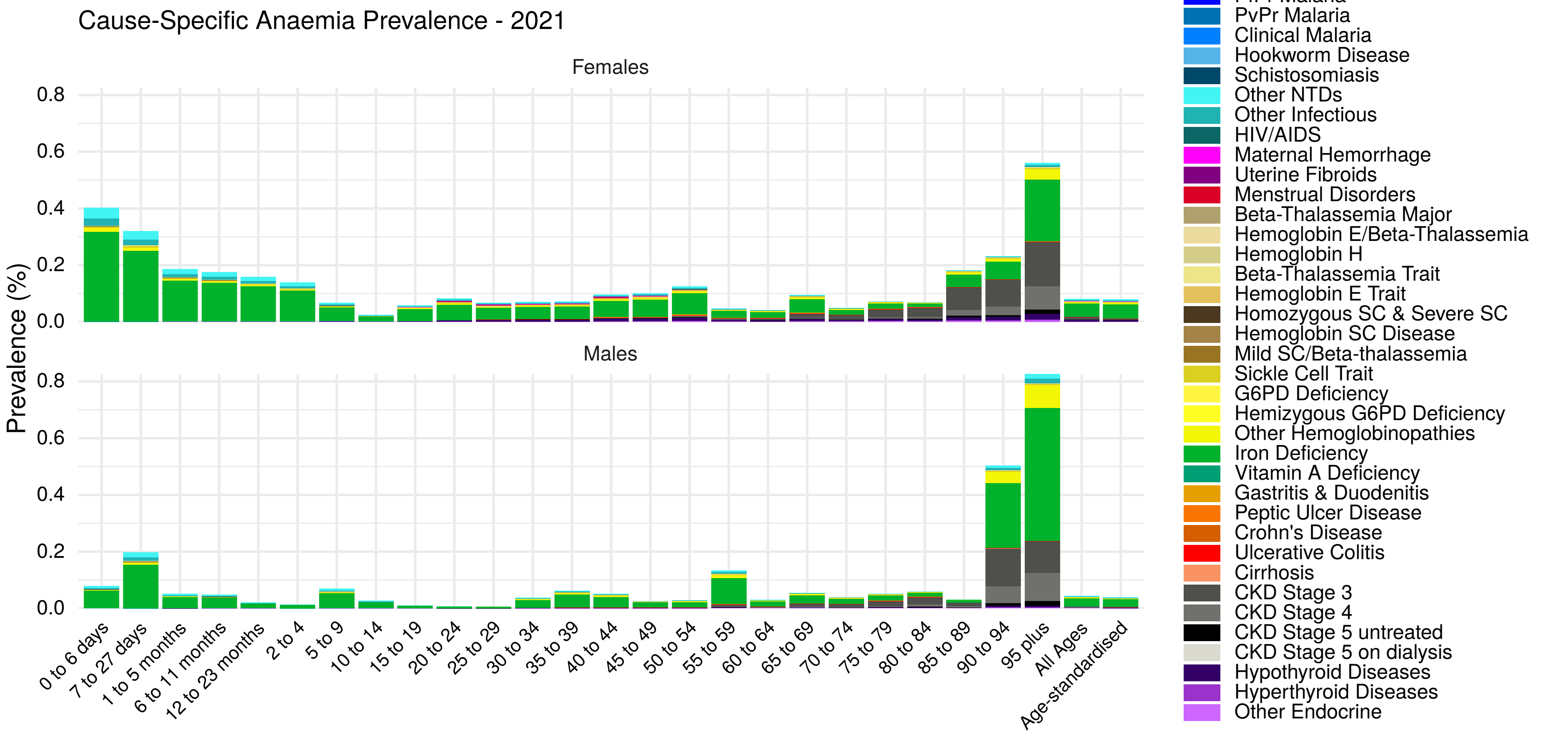

Cause-Specific Anaemia Prevalence - 2021
Females
Males
Prevalence (%)
0.0
0.2
0.4
0.6
0.8
0 to 6 days
7 to 27 days
1 to 5 months
6 to 11 months
12 to 23 months
2 to 4
5 to 9
10 to 14
15 to 19
20 to 24
25 to 29
30 to 34
35 to 39
40 to 44
45 to 49
50 to 54
55 to 59
60 to 64
65 to 69
70 to 74
75 to 79
80 to 84
85 to 89
90 to 94
95 plus
All Ages
Age-standardised
PfPr Malaria
PvPr Malaria
Clinical Malaria
Hookworm Disease
Schistosomiasis
Other NTDs
Other Infectious
HIV/AIDS
Maternal Hemorrhage
Uterine Fibroids
Menstrual Disorders
Beta-Thalassemia Major
Hemoglobin E/Beta-Thalassemia
Hemoglobin H
Beta-Thalassemia Trait
Hemoglobin E Trait
Homozygous SC & Severe SC
Hemoglobin SC Disease
Mild SC/Beta-thalassemia
Sickle Cell Trait
G6PD Deficiency
Hemizygous G6PD Deficiency
Other Hemoglobinopathies
Iron Deficiency
Vitamin A Deficiency
Gastritis & Duodenitis
Peptic Ulcer Disease
Crohn's Disease
Ulcerative Colitis
Cirrhosis
CKD Stage 3
CKD Stage 4
CKD Stage 5 untreated
CKD Stage 5 on dialysis
Hypothyroid Diseases
Hyperthyroid Diseases
Other Endocrine

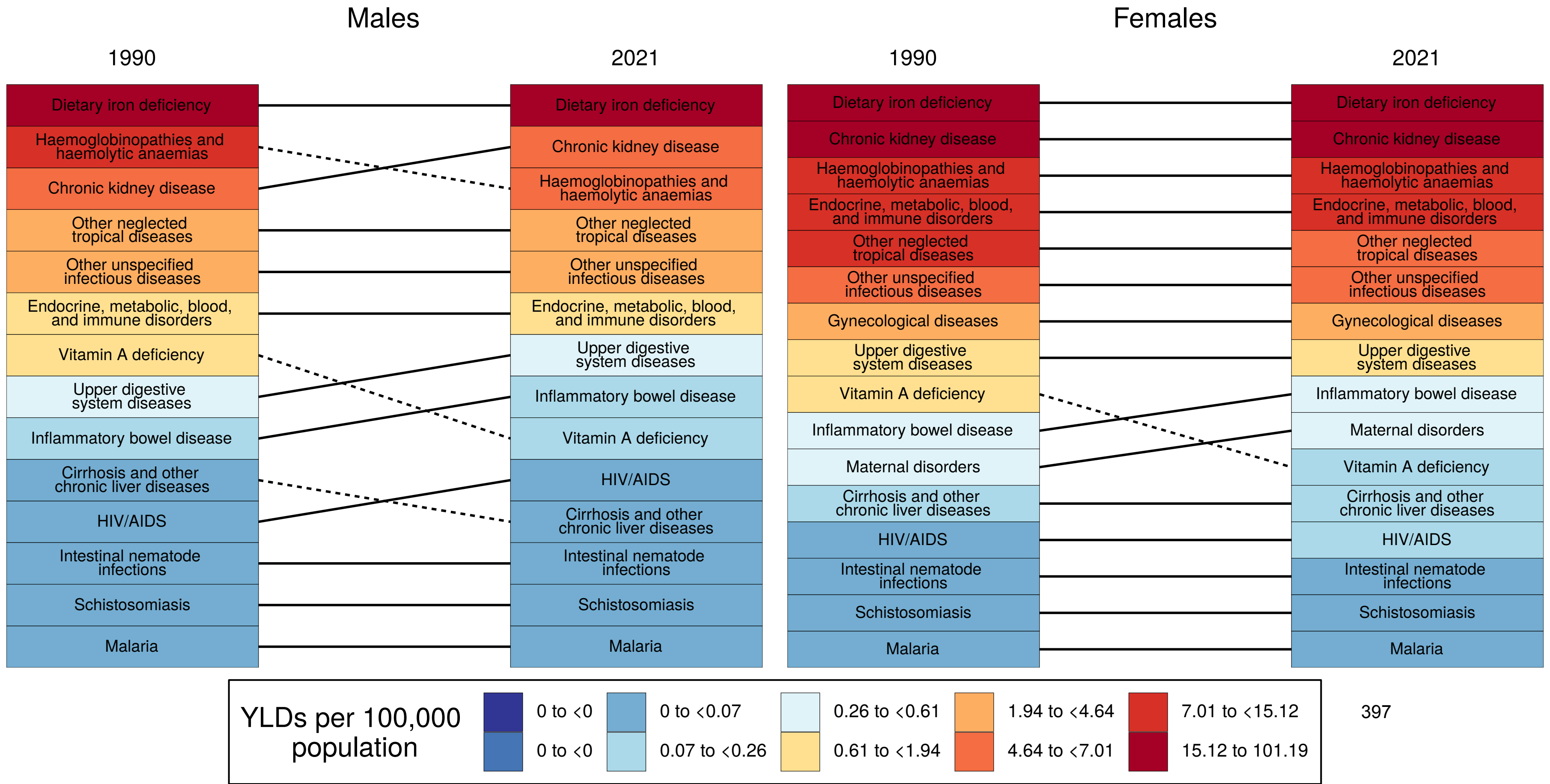

Males
Females
1990
2021
Dietary iron deficiency
Haemoglobinopathies and haemolytic anaemias
Chronic kidney disease
Other neglected tropical diseases
Other unspecified infectious diseases
Endocrine, metabolic, blood, and immune disorders
Vitamin A deficiency
Upper digestive system diseases
Inflammatory bowel disease
Cirrhosis and other chronic liver diseases
HIV/AIDS
Intestinal nematode infections
Schistosomiasis
Malaria
Gynecological diseases
Maternal disorders
YLDs per 100,000 population
0 to <0
0 to <0.07
0.07 to <0.26
0.26 to <0.61
0.61 to <1.94
1.94 to <4.64
4.64 to <7.01
7.01 to <15.12
15.12 to 101.19

Warwickshire

Anaemia Prevalence - Both Sexes

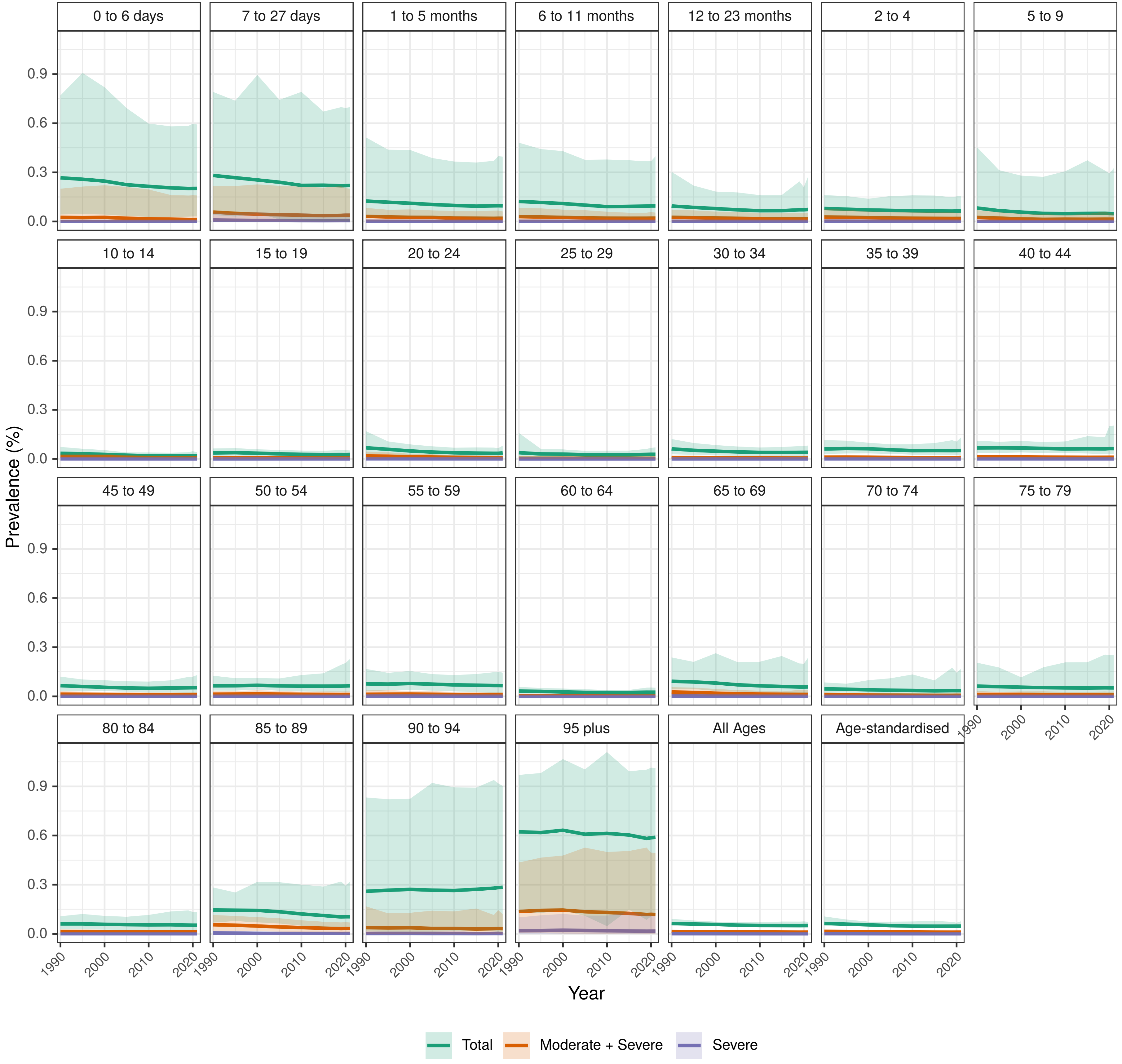

Cause-Specific Anaemia Prevalence - 2021

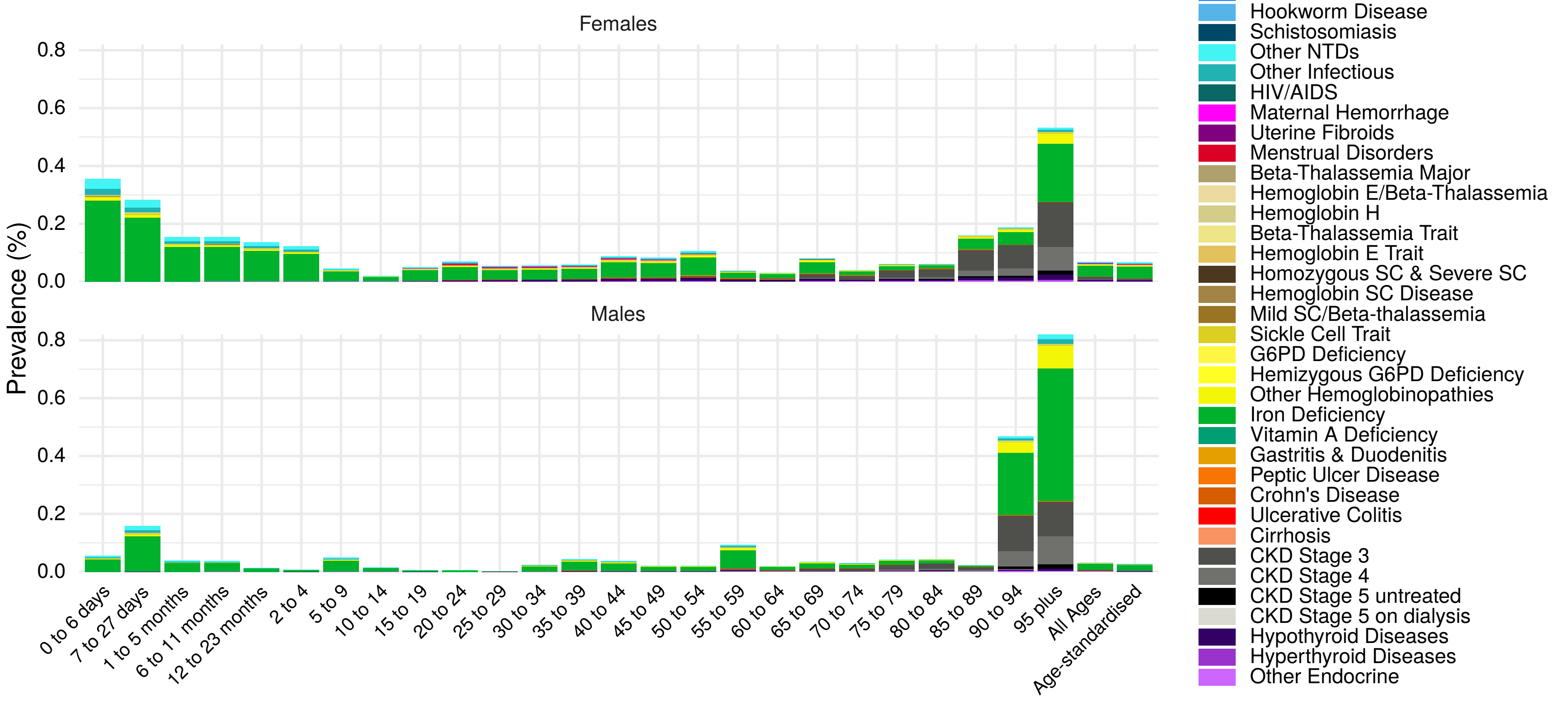

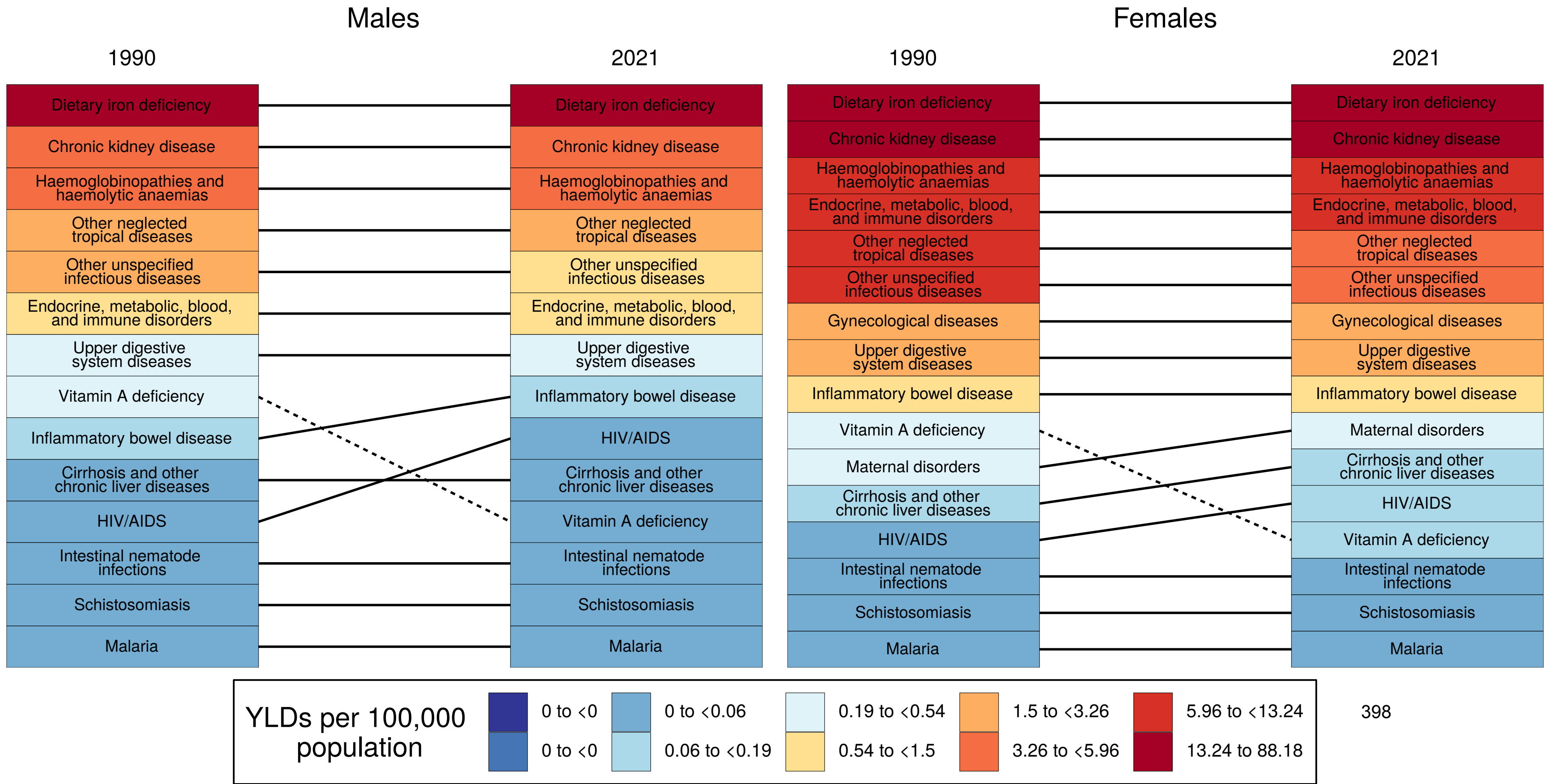

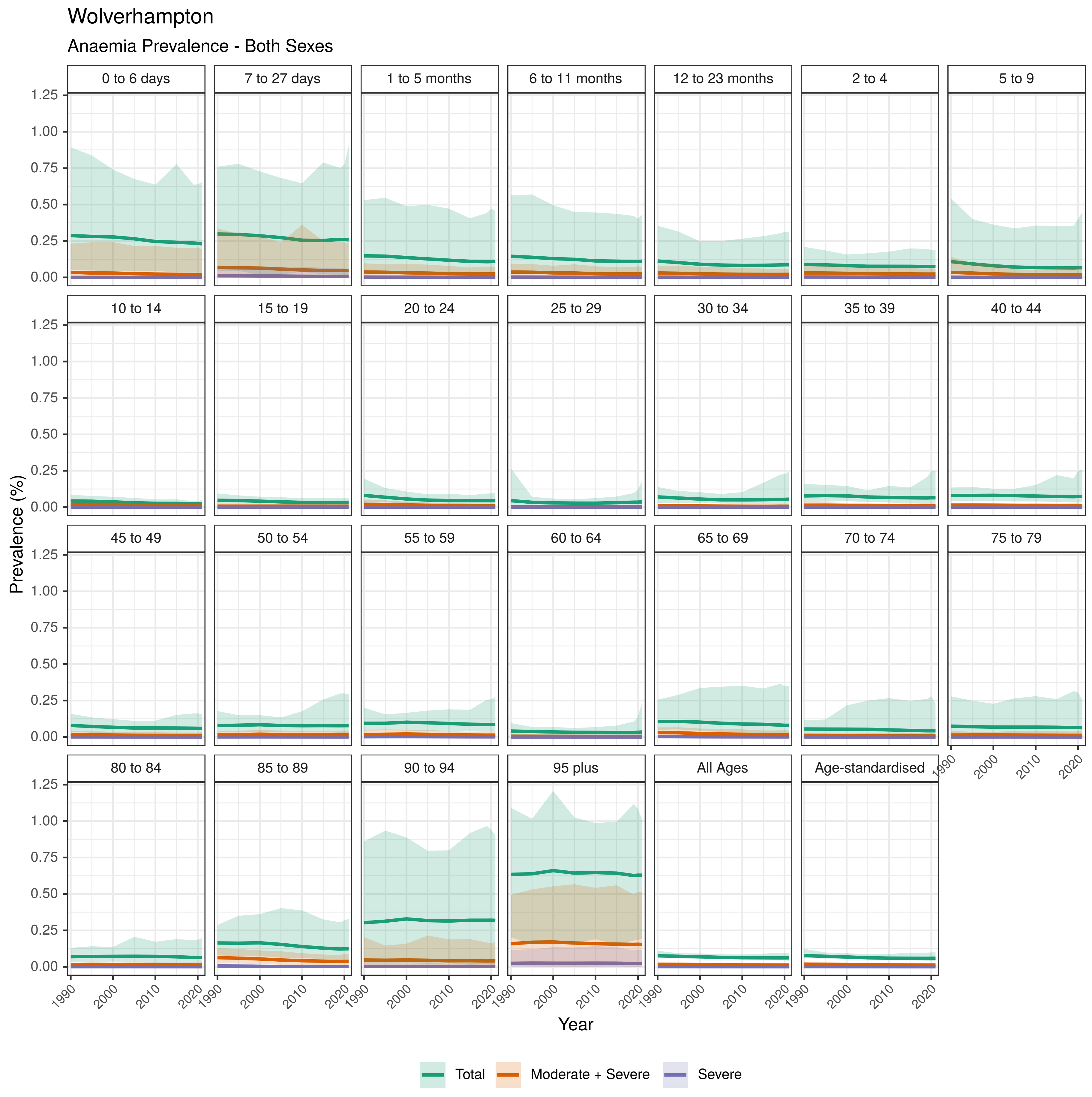
Wolverhampton
Anaemia Prevalence - Both Sexes
0 to 6 days
7 to 27 days
1 to 5 months
6 to 11 months
12 to 23 months
2 to 4
5 to 9
10 to 14
15 to 19
20 to 24
25 to 29
30 to 34
35 to 39
40 to 44
45 to 49
50 to 54
55 to 59
60 to 64
65 to 69
70 to 74
75 to 79
80 to 84
85 to 89
90 to 94
95 plus
All Ages
Age-standardised
Prevalence (%)
0.00
0.25
0.50
0.75
1.00
1.25
1990
2000
2010
2020
Year
Total
Moderate + Severe
Severe

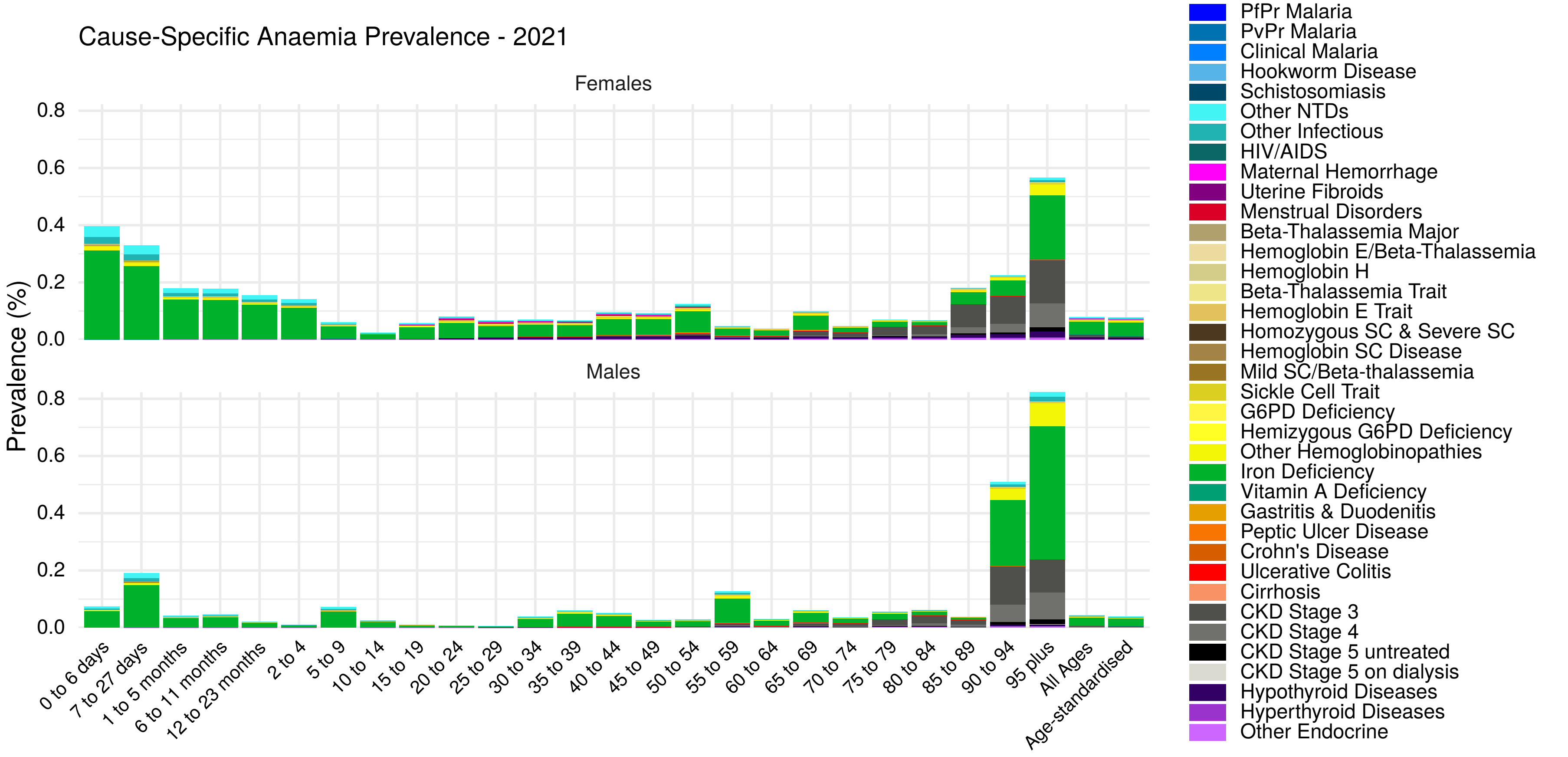
Cause-Specific Anaemia Prevalence - 2021
Females
Males
Prevalence (%)
0.0
0.2
0.4
0.6
0.8
0 to 6 days
7 to 27 days
1 to 5 months
6 to 11 months
12 to 23 months
2 to 4
5 to 9
10 to 14
15 to 19
20 to 24
25 to 29
30 to 34
35 to 39
40 to 44
45 to 49
50 to 54
55 to 59
60 to 64
65 to 69
70 to 74
75 to 79
80 to 84
85 to 89
90 to 94
95 plus
All Ages
Age-standardised
PfPr Malaria
PvPr Malaria
Clinical Malaria
Hookworm Disease
Schistosomiasis
Other NTDs
Other Infectious
HIV/AIDS
Maternal Hemorrhage
Uterine Fibroids
Menstrual Disorders
Beta-Thalassemia Major
Hemoglobin E/Beta-Thalassemia
Hemoglobin H
Beta-Thalassemia Trait
Hemoglobin E Trait
Homozygous SC & Severe SC
Hemoglobin SC Disease
Mild SC/Beta-thalassemia
Sickle Cell Trait
G6PD Deficiency
Hemizygous G6PD Deficiency
Other Hemoglobinopathies
Iron Deficiency
Vitamin A Deficiency
Gastritis & Duodenitis
Peptic Ulcer Disease
Crohn's Disease
Ulcerative Colitis
Cirrhosis
CKD Stage 3
CKD Stage 4
CKD Stage 5 untreated
CKD Stage 5 on dialysis
Hypothyroid Diseases
Hyperthyroid Diseases
Other Endocrine

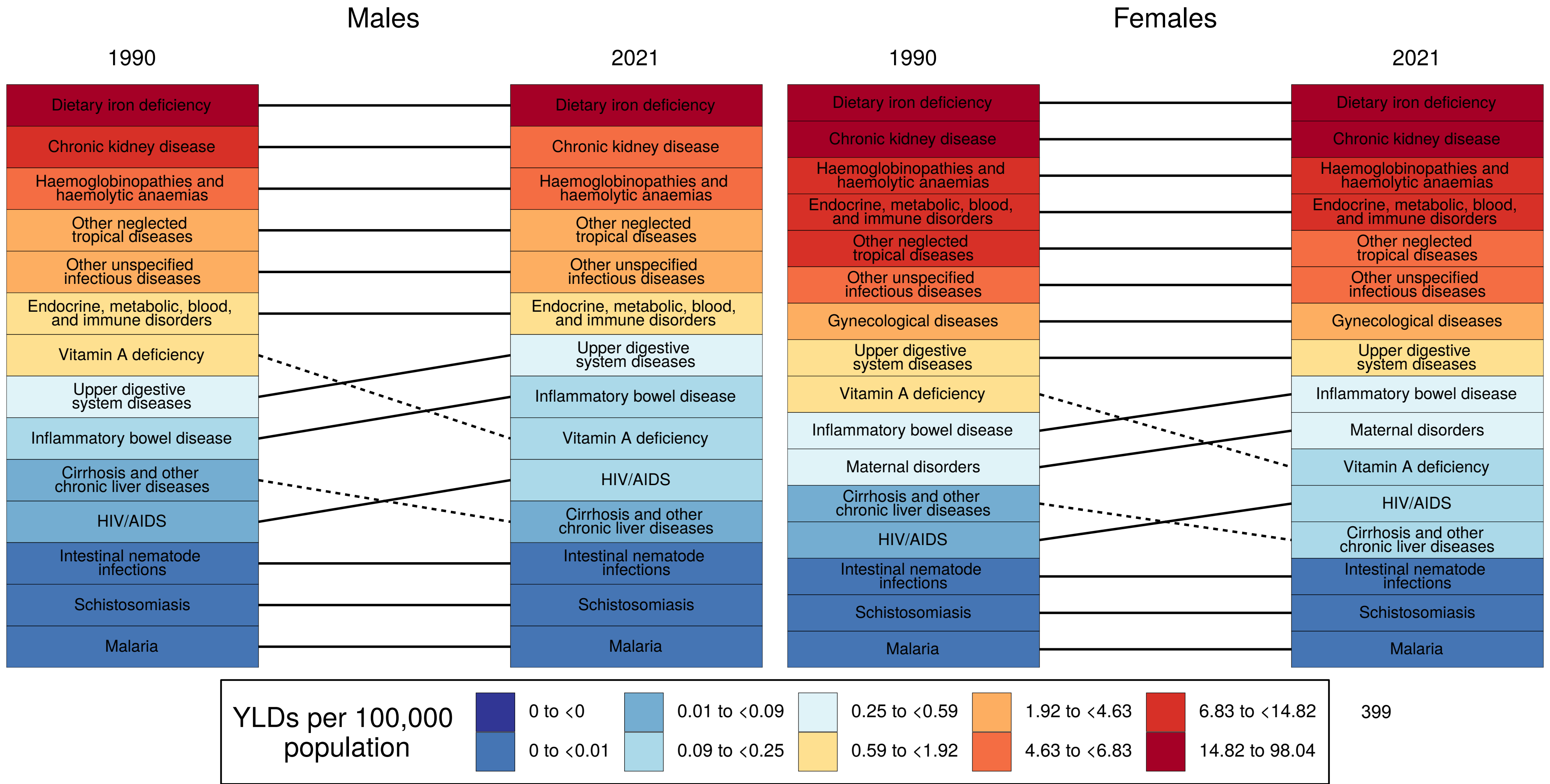
Males
Females
1990
2021
Dietary iron deficiency
Chronic kidney disease
Haemoglobinopathies and haemolytic anaemias
Other neglected tropical diseases
Other unspecified infectious diseases
Endocrine, metabolic, blood, and immune disorders
Vitamin A deficiency
Upper digestive system diseases
Inflammatory bowel disease
Cirrhosis and other chronic liver diseases
HIV/AIDS
Intestinal nematode infections
Schistosomiasis
Malaria
Gynecological diseases
Maternal disorders
YLDs per 100,000 population
0 to <0
0 to <0.01
0.01 to <0.09
0.09 to <0.25
0.25 to <0.59
0.59 to <1.92
1.92 to <4.63
4.63 to <6.83
6.83 to <14.82
14.82 to 98.04

Worcestershire

Anaemia Prevalence - Both Sexes

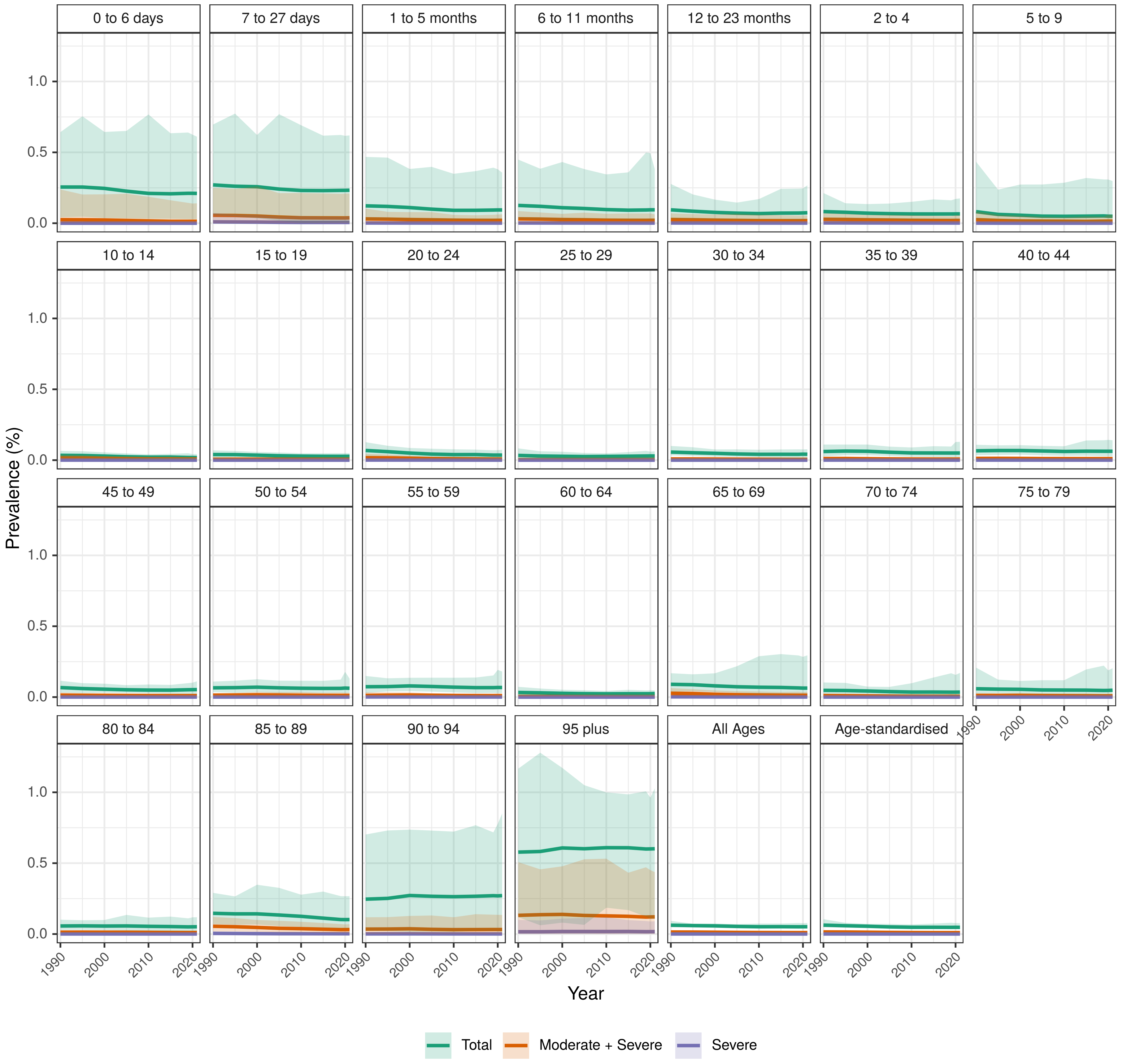

Cause-Specific Anaemia Prevalence - 2021

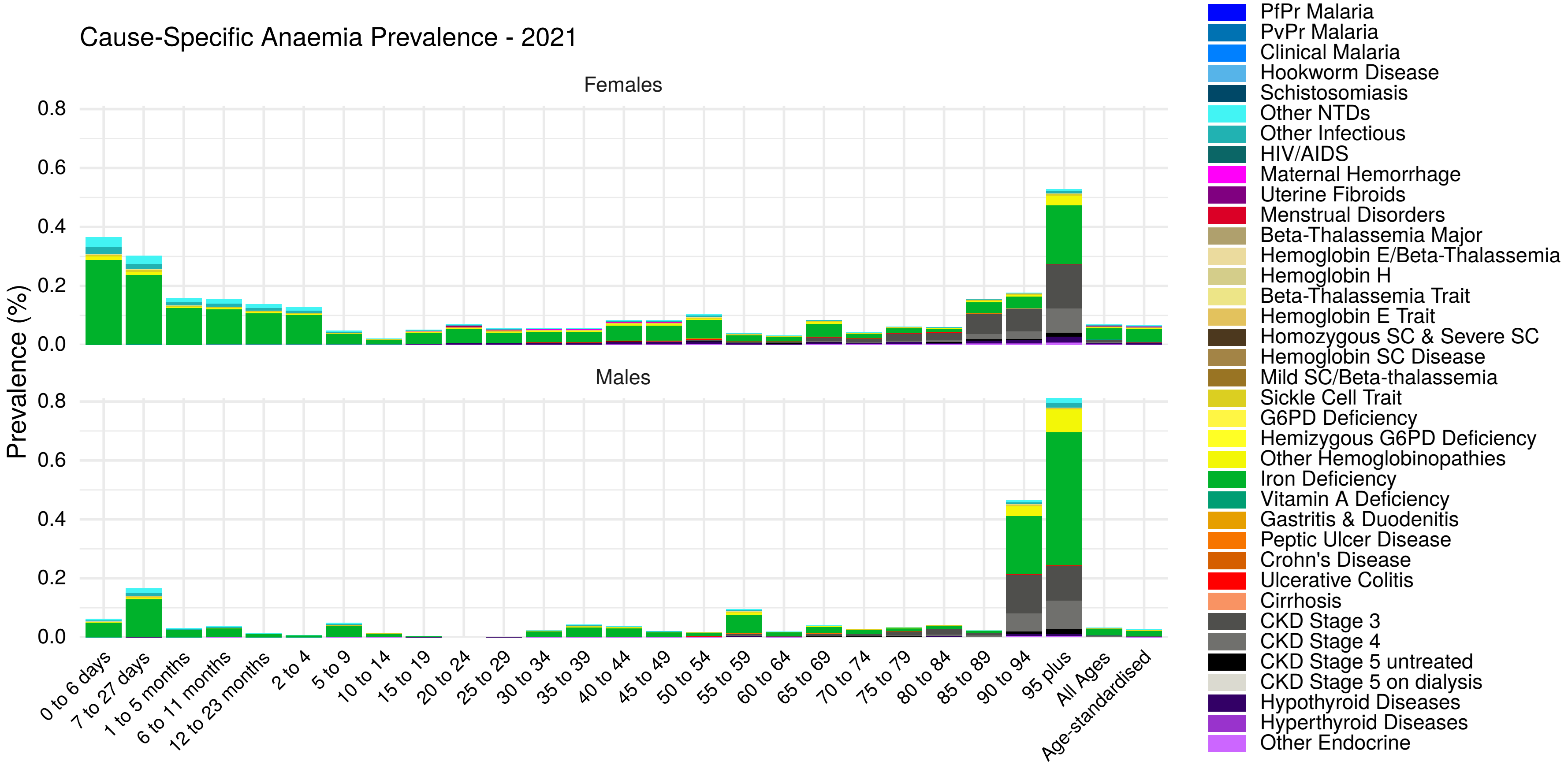

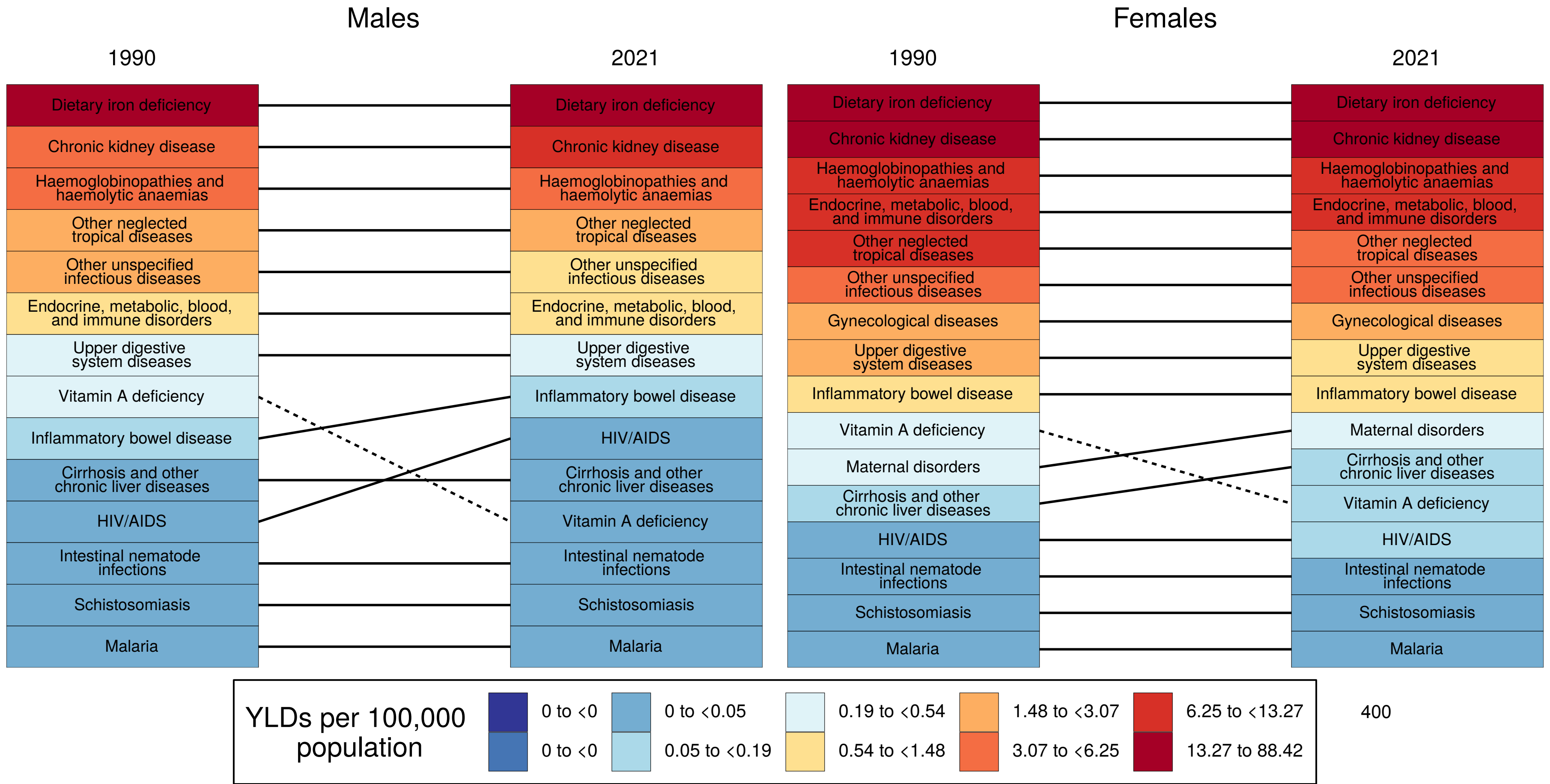

Yorkshire and the Humber

Anaemia Prevalence - Both Sexes

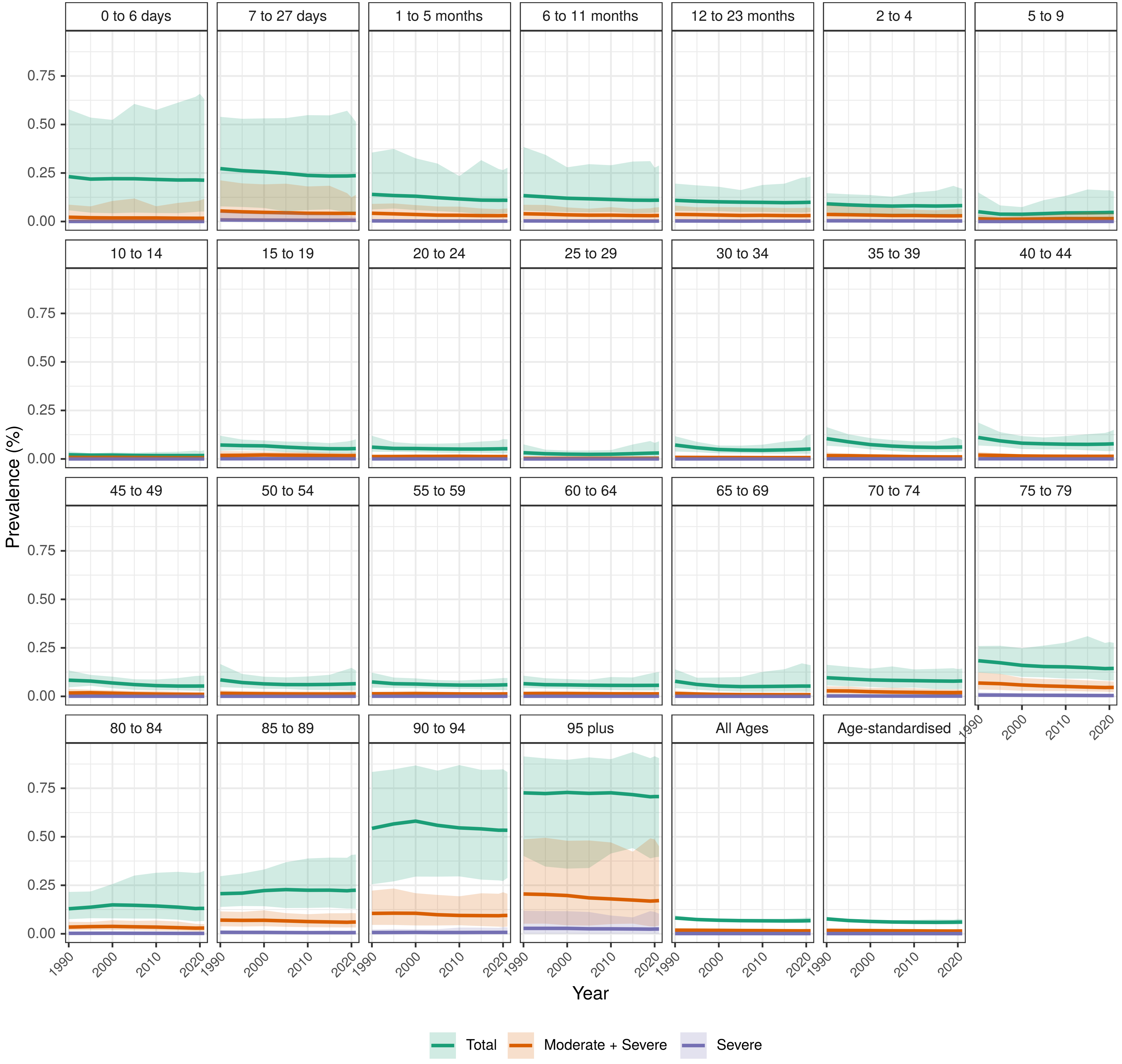

Cause-Specific Anaemia Prevalence - 2021

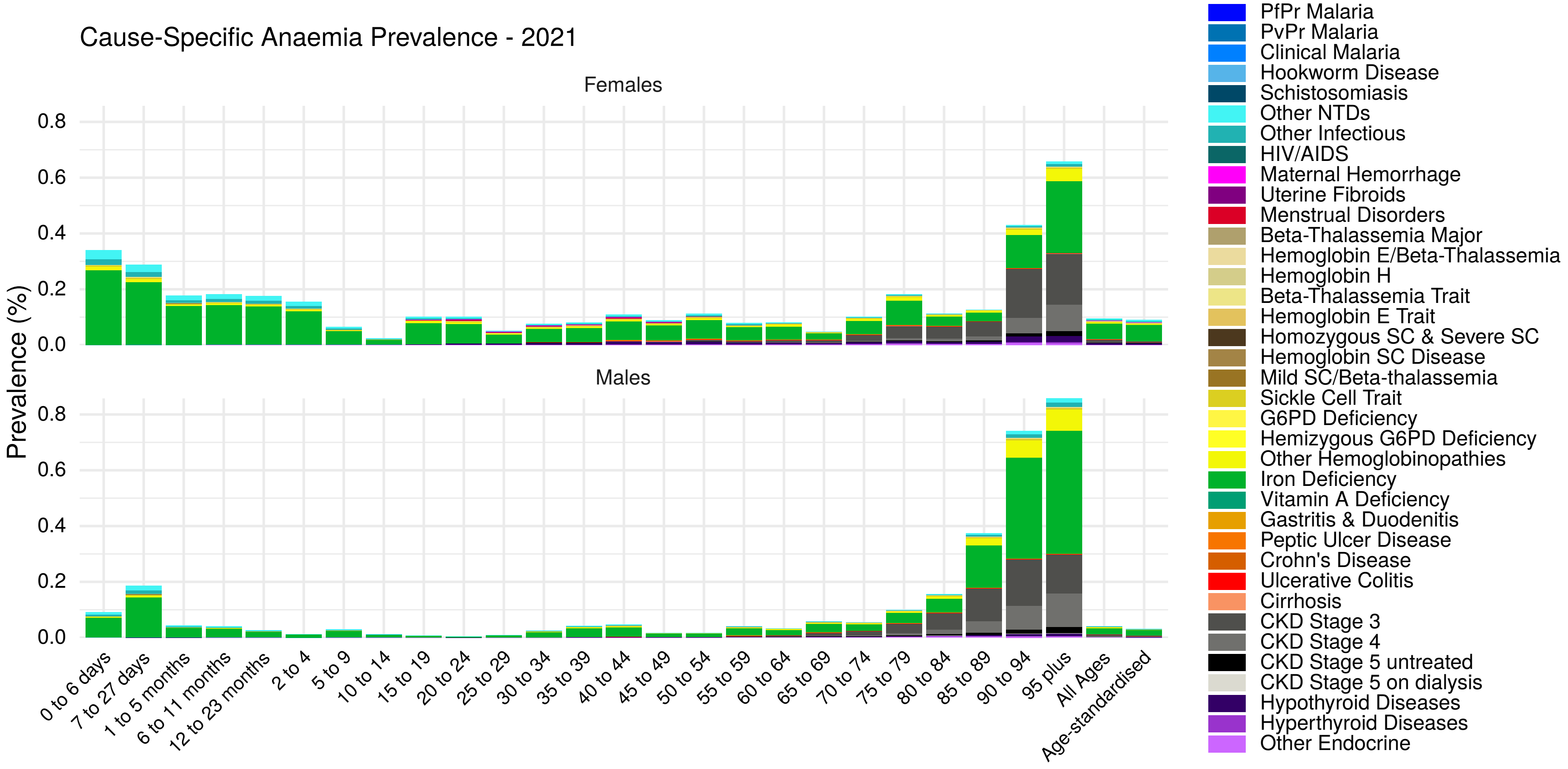

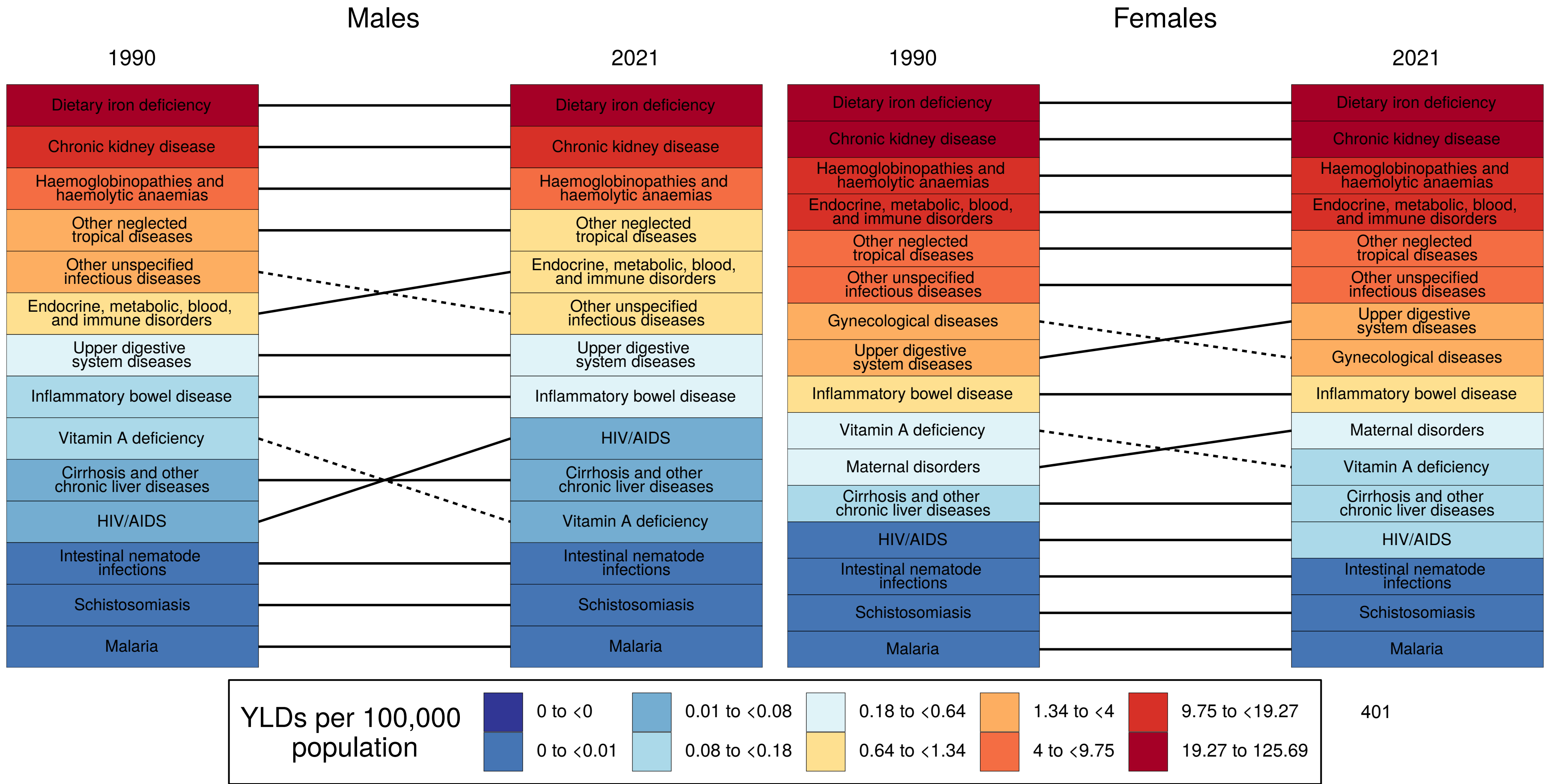

Barnsley

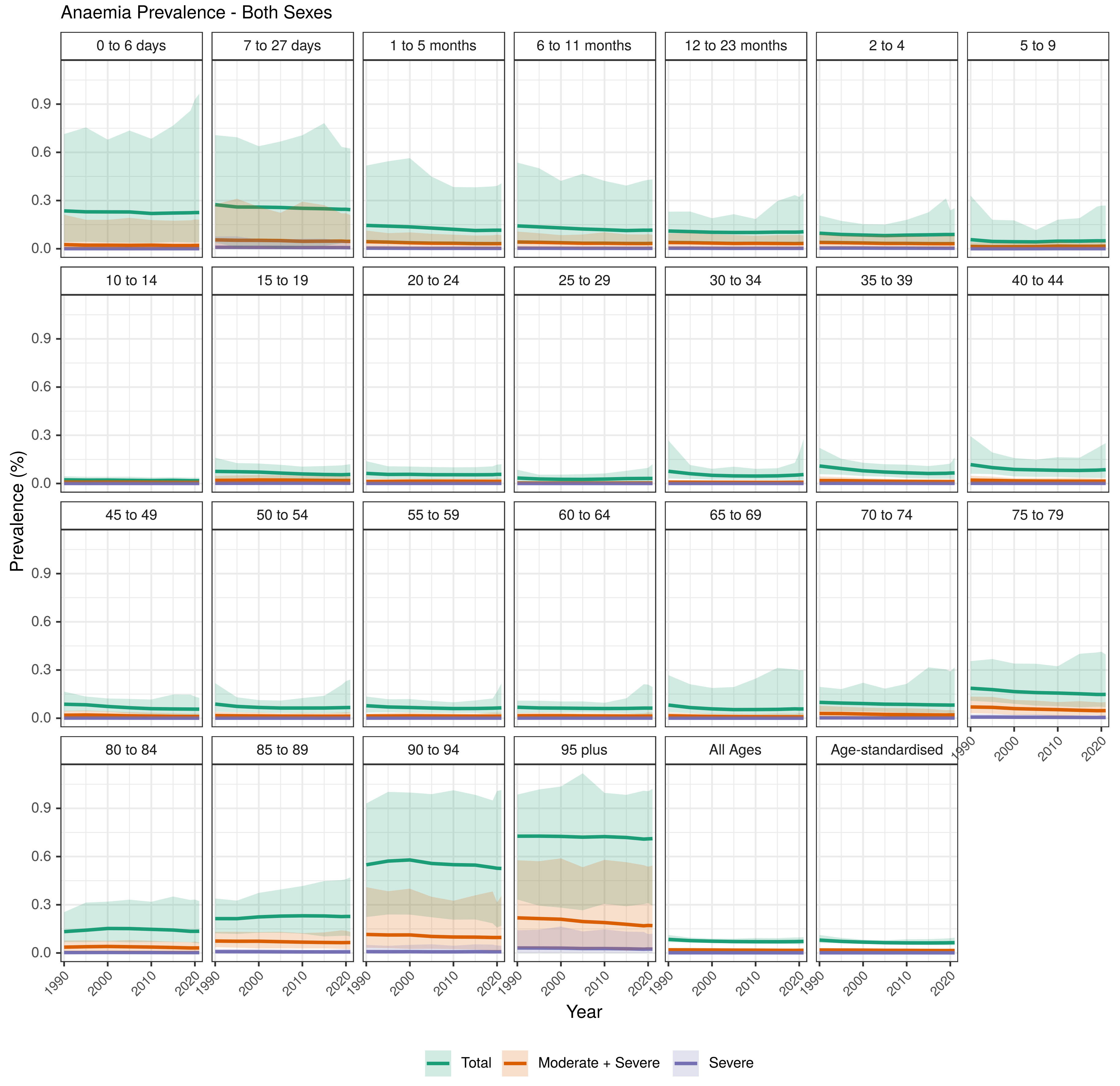

Anaemia Prevalence - Both Sexes
0 to 6 days
7 to 27 days
1 to 5 months
6 to 11 months
12 to 23 months
2 to 4
5 to 9
10 to 14
15 to 19
20 to 24
25 to 29
30 to 34
35 to 39
40 to 44
45 to 49
50 to 54
55 to 59
60 to 64
65 to 69
70 to 74
75 to 79
80 to 84
85 to 89
90 to 94
95 plus
All Ages
Age-standardised
0.0
0.3
0.6
0.9
1990
2000
2010
2020
Prevalence (%)
Year
Total
Moderate + Severe
Severe

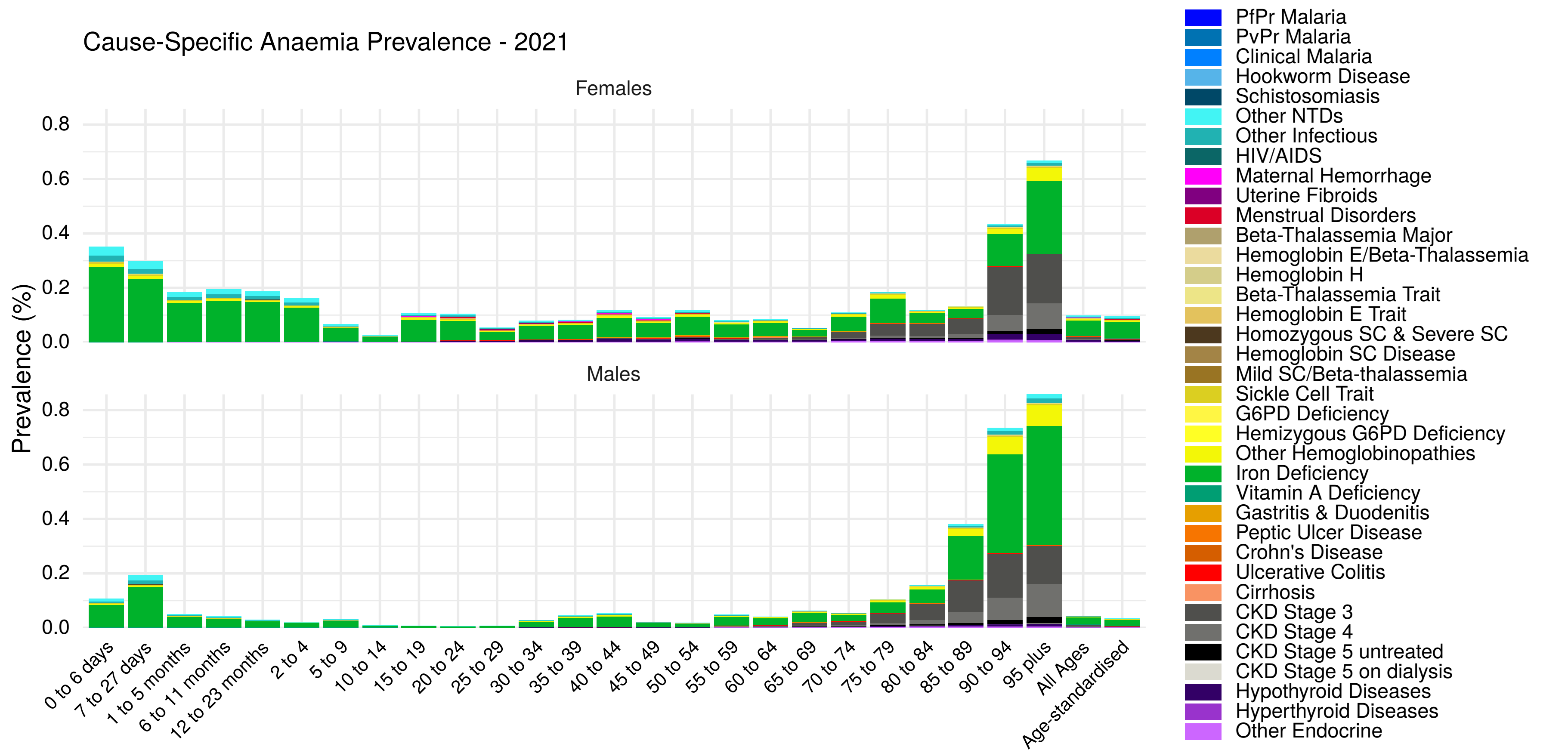

Cause-Specific Anaemia Prevalence - 2021
Females
Males
Prevalence (%)
0.0
0.2
0.4
0.6
0.8
0 to 6 days
7 to 27 days
1 to 5 months
6 to 11 months
12 to 23 months
2 to 4
5 to 9
10 to 14
15 to 19
20 to 24
25 to 29
30 to 34
35 to 39
40 to 44
45 to 49
50 to 54
55 to 59
60 to 64
65 to 69
70 to 74
75 to 79
80 to 84
85 to 89
90 to 94
95 plus
All Ages
Age-standardised
PfPr Malaria
PvPr Malaria
Clinical Malaria
Hookworm Disease
Schistosomiasis
Other NTDs
Other Infectious
HIV/AIDS
Maternal Hemorrhage
Uterine Fibroids
Menstrual Disorders
Beta-Thalassemia Major
Hemoglobin E/Beta-Thalassemia
Hemoglobin H
Beta-Thalassemia Trait
Hemoglobin E Trait
Homozygous SC & Severe SC
Hemoglobin SC Disease
Mild SC/Beta-thalassemia
Sickle Cell Trait
G6PD Deficiency
Hemizygous G6PD Deficiency
Other Hemoglobinopathies
Iron Deficiency
Vitamin A Deficiency
Gastritis & Duodenitis
Peptic Ulcer Disease
Crohn's Disease
Ulcerative Colitis
Cirrhosis
CKD Stage 3
CKD Stage 4
CKD Stage 5 untreated
CKD Stage 5 on dialysis
Hypothyroid Diseases
Hyperthyroid Diseases
Other Endocrine

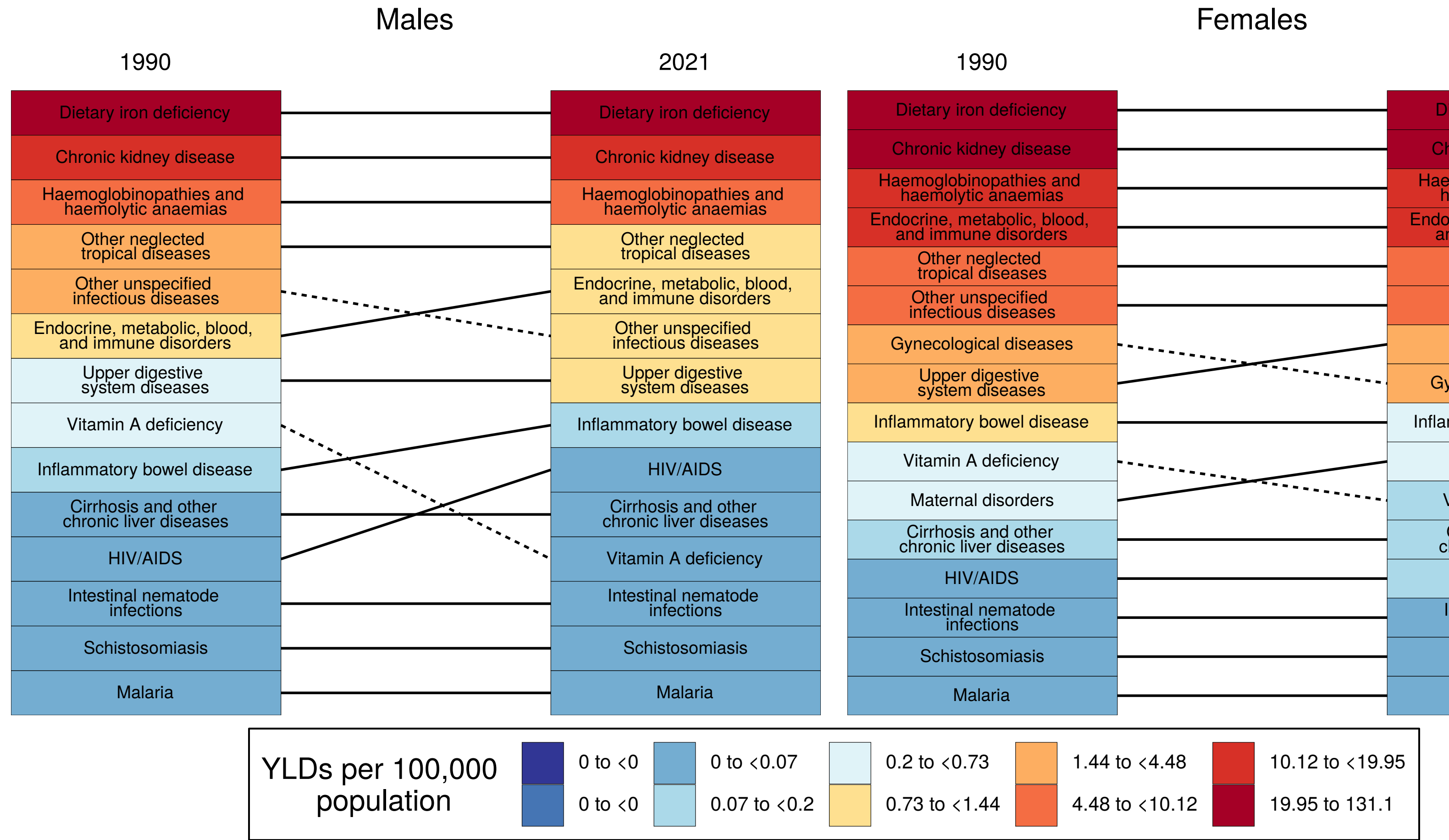

Males
Females
1990
2021
1990
2021
Dietary iron deficiency
Chronic kidney disease
Haemoglobinopathies and haemolytic anaemias
Other neglected tropical diseases
Other unspecified infectious diseases
Endocrine, metabolic, blood, and immune disorders
Upper digestive system diseases
Vitamin A deficiency
Inflammatory bowel disease
Cirrhosis and other chronic liver diseases
HIV/AIDS
Intestinal nematode infections
Schistosomiasis
Malaria
Dietary iron deficiency
Chronic kidney disease
Haemoglobinopathies and haemolytic anaemias
Other neglected tropical diseases
Endocrine, metabolic, blood, and immune disorders
Other unspecified infectious diseases
Upper digestive system diseases
Inflammatory bowel disease
HIV/AIDS
Cirrhosis and other chronic liver diseases
Vitamin A deficiency
Intestinal nematode infections
Schistosomiasis
Malaria
Dietary iron deficiency
Chronic kidney disease
Haemoglobinopathies and haemolytic anaemias
Endocrine, metabolic, blood, and immune disorders
Other neglected tropical diseases
Other unspecified infectious diseases
Gynecological diseases
Upper digestive system diseases
Inflammatory bowel disease
Vitamin A deficiency
Maternal disorders
Cirrhosis and other chronic liver diseases
HIV/AIDS
Intestinal nematode infections
Schistosomiasis
Malaria
Dietary iron deficiency
Chronic kidney disease
Haemoglobinopathies and haemolytic anaemias
Endocrine, metabolic, blood, and immune disorders
Other neglected tropical diseases
Other unspecified infectious diseases
Upper digestive system diseases
Gynecological diseases
Inflammatory bowel disease
Maternal disorders
Vitamin A deficiency
Cirrhosis and other chronic liver diseases
HIV/AIDS
Intestinal nematode infections
Schistosomiasis
Malaria
YLDs per 100,000 population
0 to <0
0 to <0
0 to <0.07
0.07 to <0.2
0.2 to <0.73
0.73 to <1.44
1.44 to <4.48
4.48 to <10.12
10.12 to <19.95
19.95 to 131.1

Bradford

Anaemia Prevalence - Both Sexes

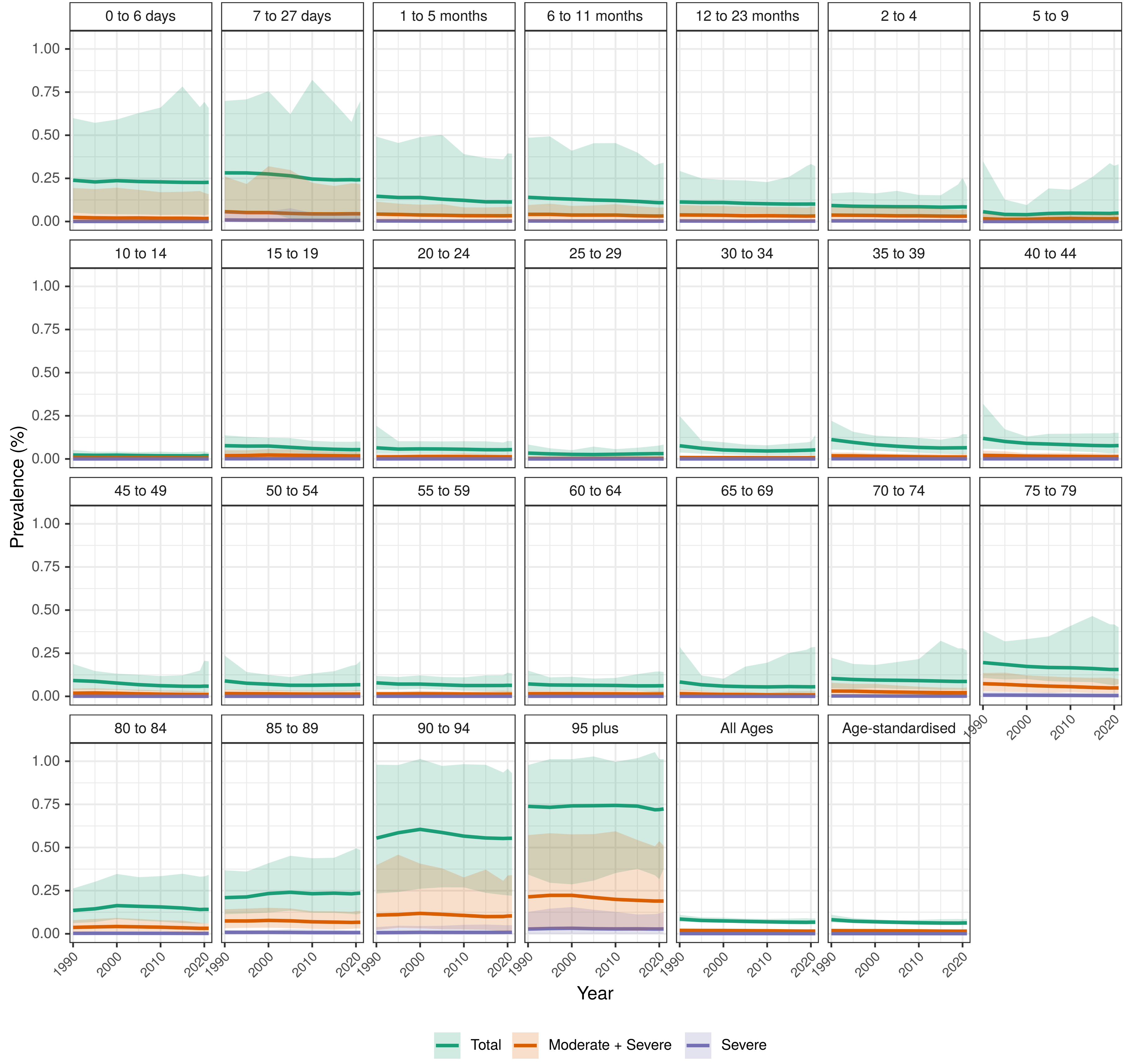

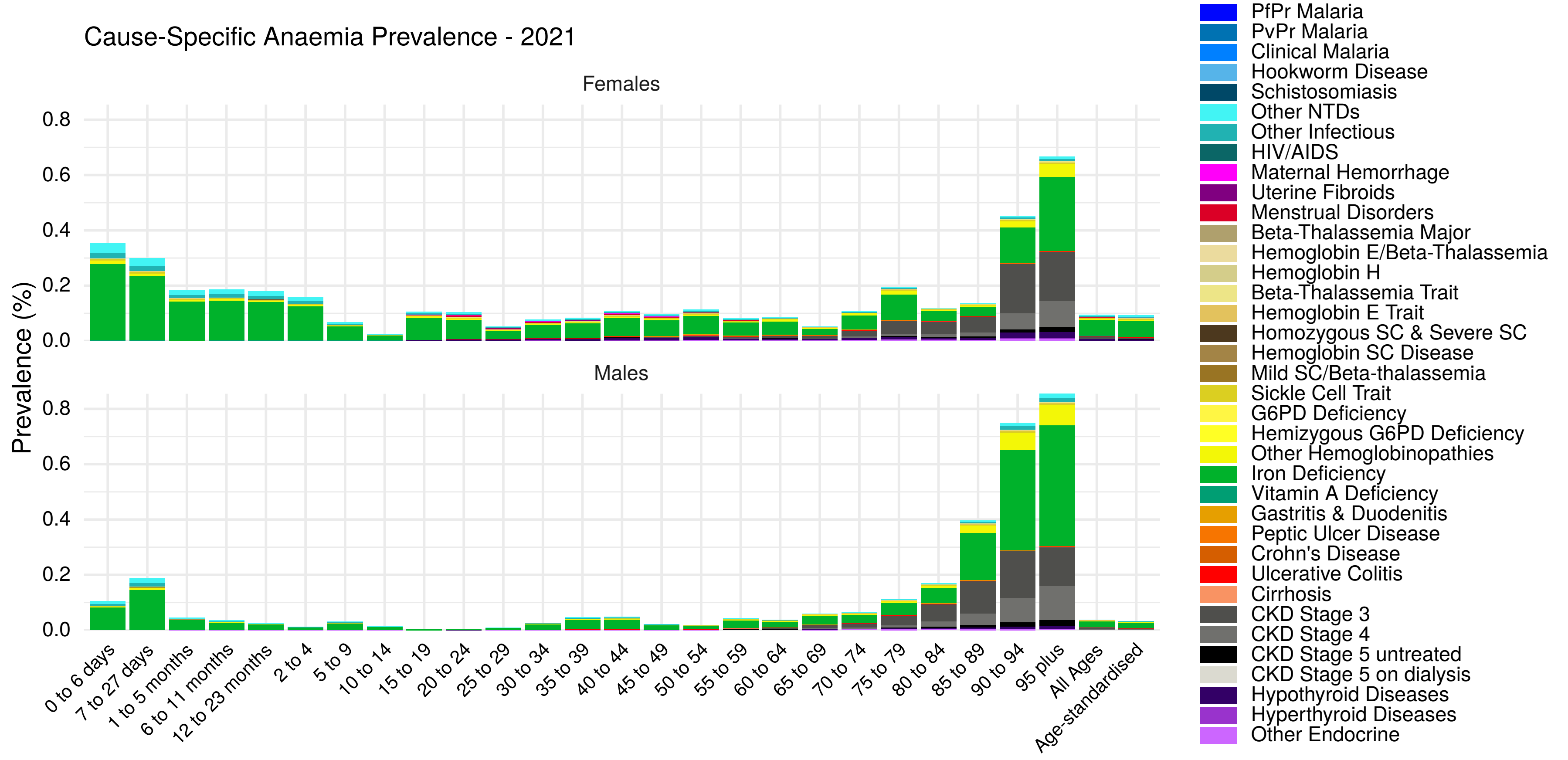

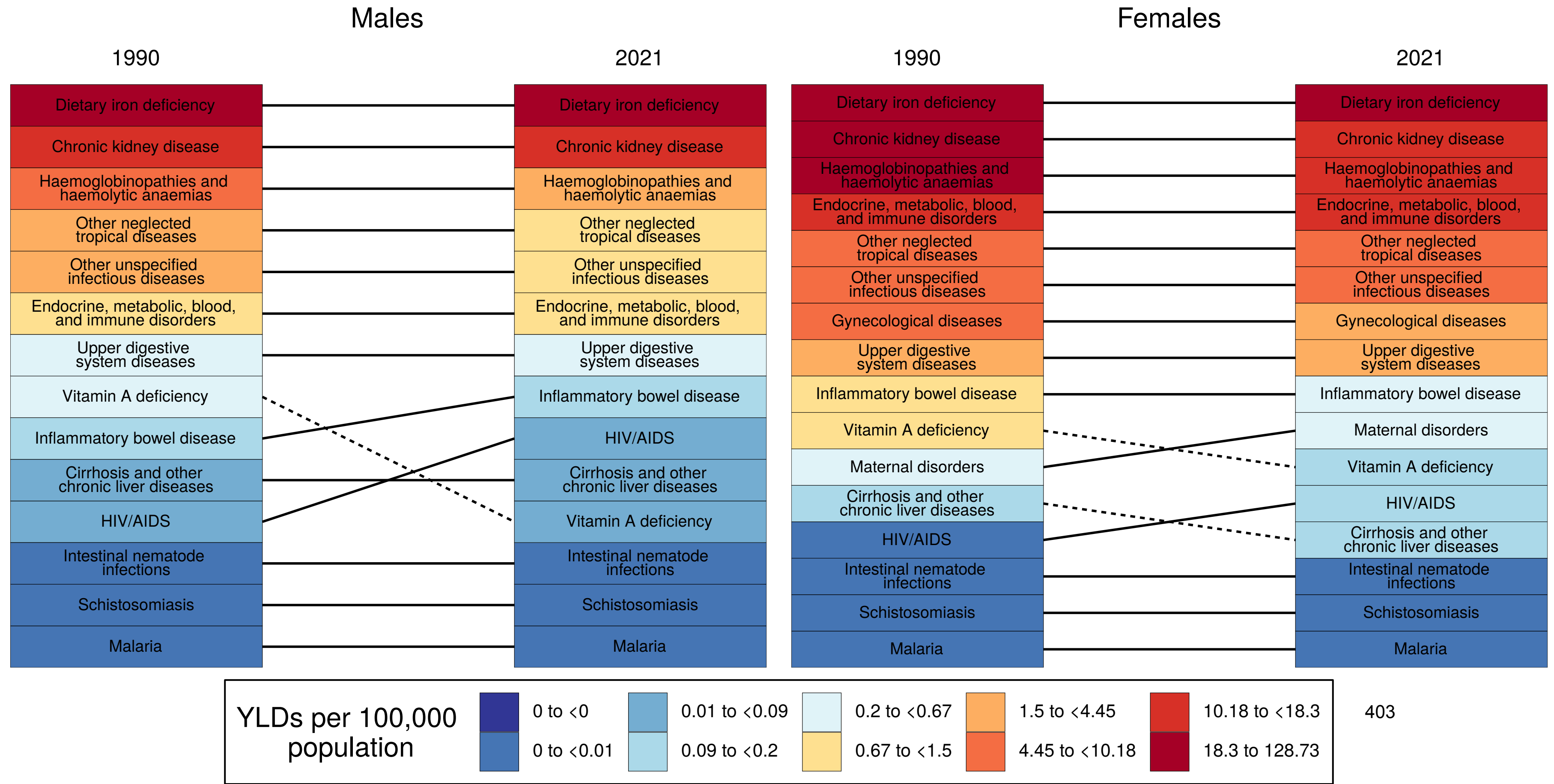

Calderdale

Anaemia Prevalence - Both Sexes

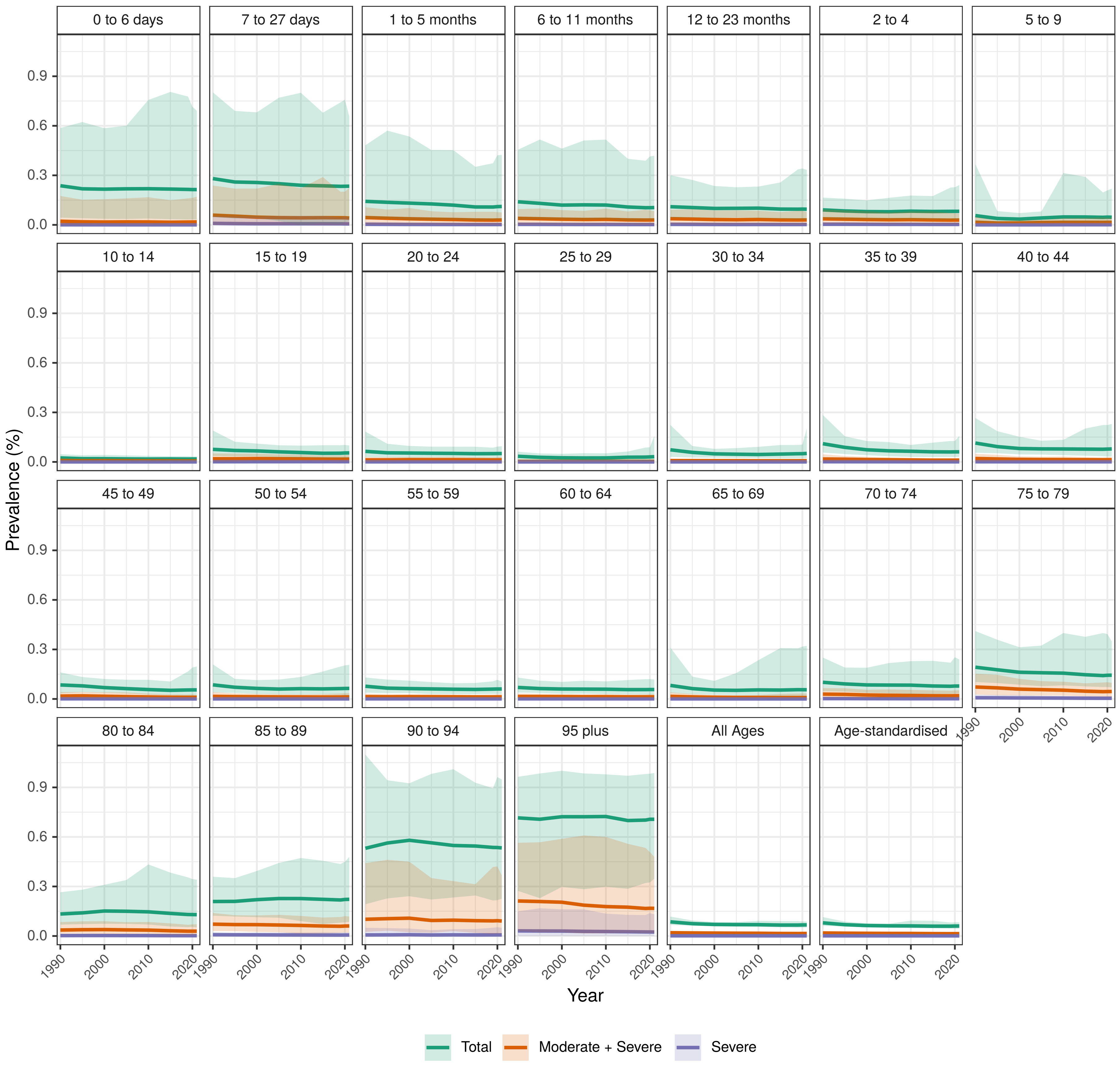

Cause-Specific Anaemia Prevalence - 2021

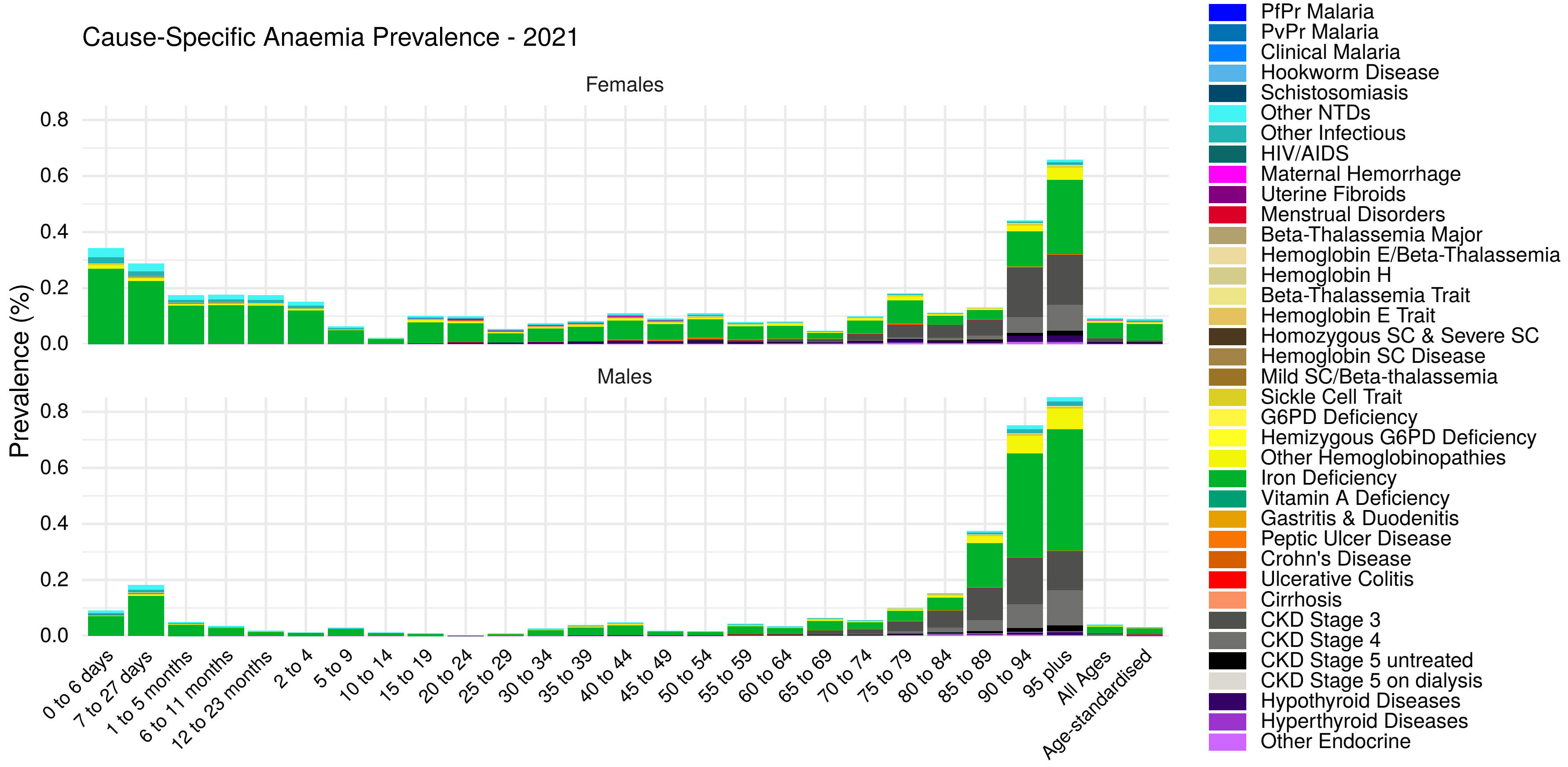

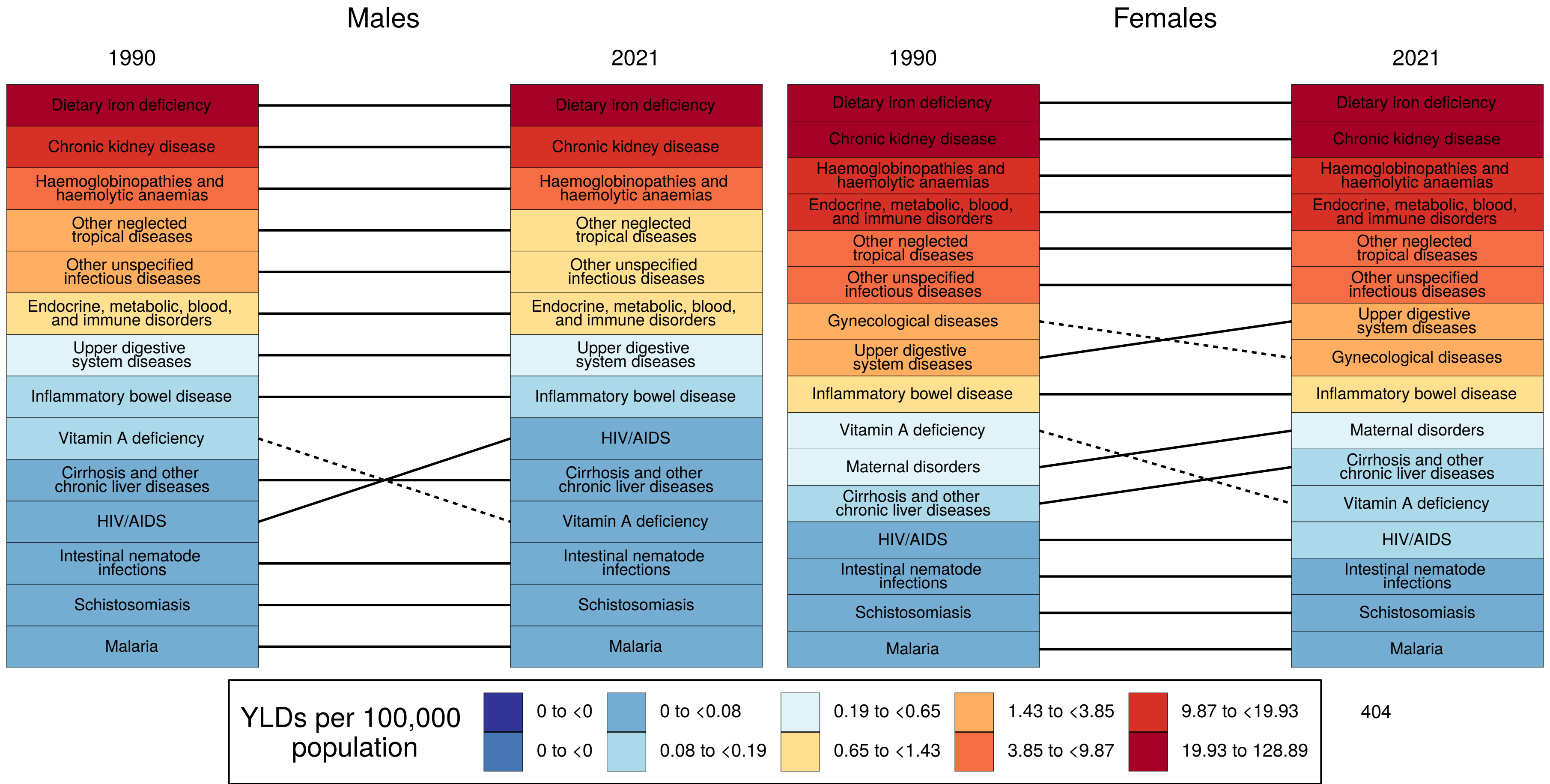

Doncaster

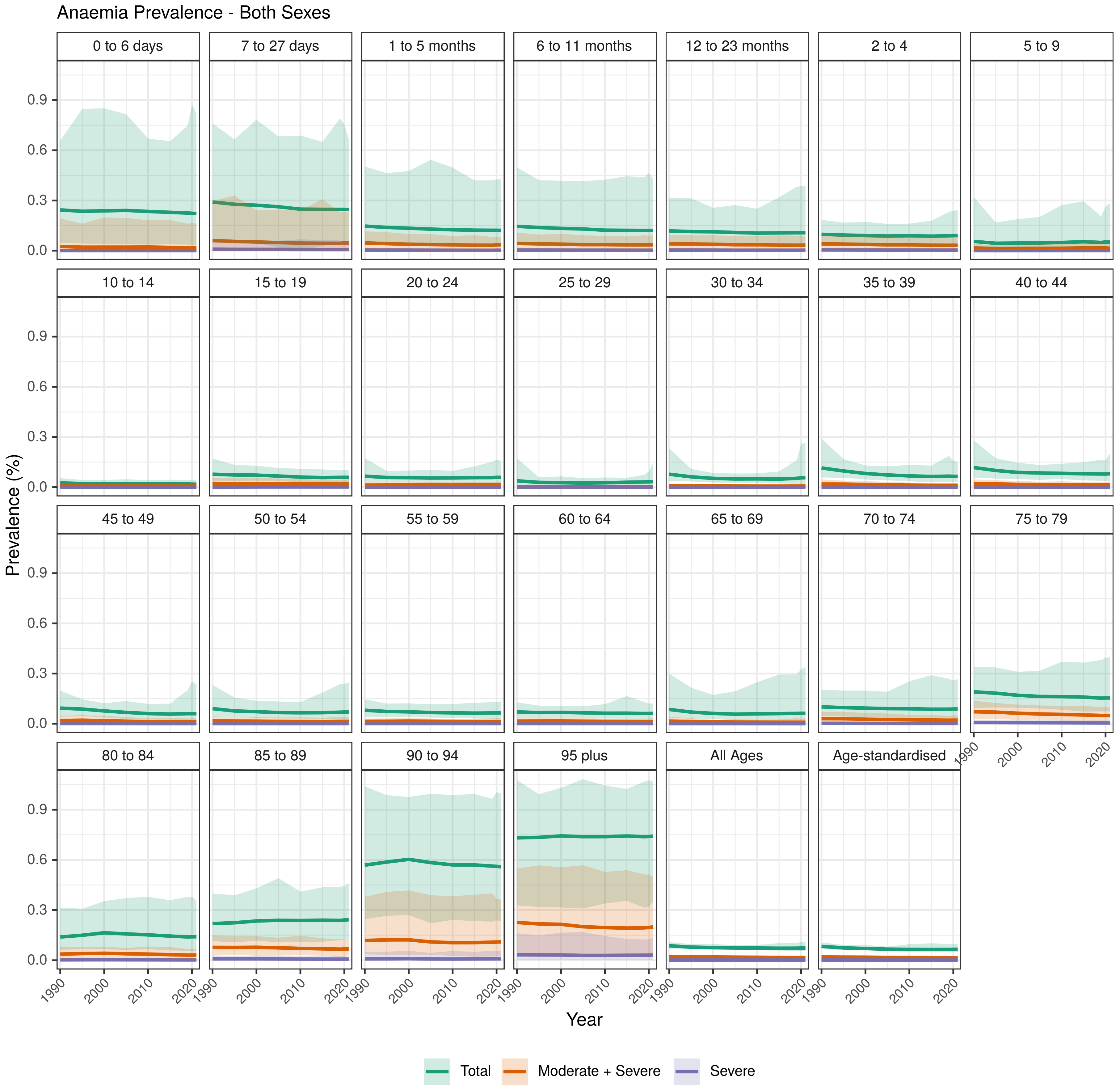
Anaemia Prevalence - Both Sexes
0 to 6 days
7 to 27 days
1 to 5 months
6 to 11 months
12 to 23 months
2 to 4
5 to 9
10 to 14
15 to 19
20 to 24
25 to 29
30 to 34
35 to 39
40 to 44
45 to 49
50 to 54
55 to 59
60 to 64
65 to 69
70 to 74
75 to 79
80 to 84
85 to 89
90 to 94
95 plus
All Ages
Age-standardised
Prevalence (%)
Year
Total
Moderate + Severe
Severe

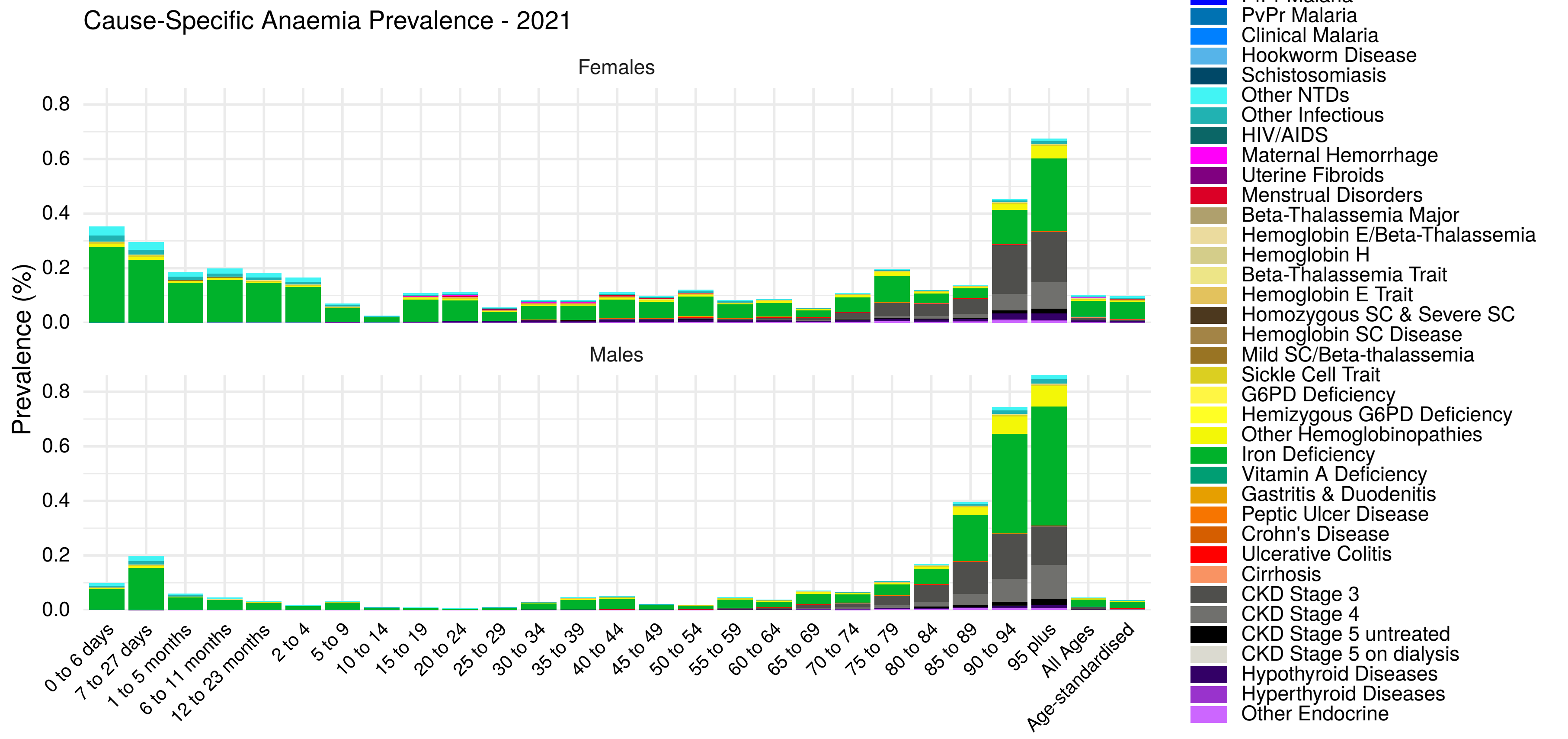
Cause-Specific Anaemia Prevalence - 2021
Females
Males
Prevalence (%)
PfPr Malaria
PvPr Malaria
Clinical Malaria
Hookworm Disease
Schistosomiasis
Other NTDs
Other Infectious
HIV/AIDS
Maternal Hemorrhage
Uterine Fibroids
Menstrual Disorders
Beta-Thalassemia Major
Hemoglobin E/Beta-Thalassemia
Hemoglobin H
Beta-Thalassemia Trait
Hemoglobin E Trait
Homozygous SC & Severe SC
Hemoglobin SC Disease
Mild SC/Beta-thalassemia
Sickle Cell Trait
G6PD Deficiency
Hemizygous G6PD Deficiency
Other Hemoglobinopathies
Iron Deficiency
Vitamin A Deficiency
Gastritis & Duodenitis
Peptic Ulcer Disease
Crohn's Disease
Ulcerative Colitis
Cirrhosis
CKD Stage 3
CKD Stage 4
CKD Stage 5 untreated
CKD Stage 5 on dialysis
Hypothyroid Diseases
Hyperthyroid Diseases
Other Endocrine

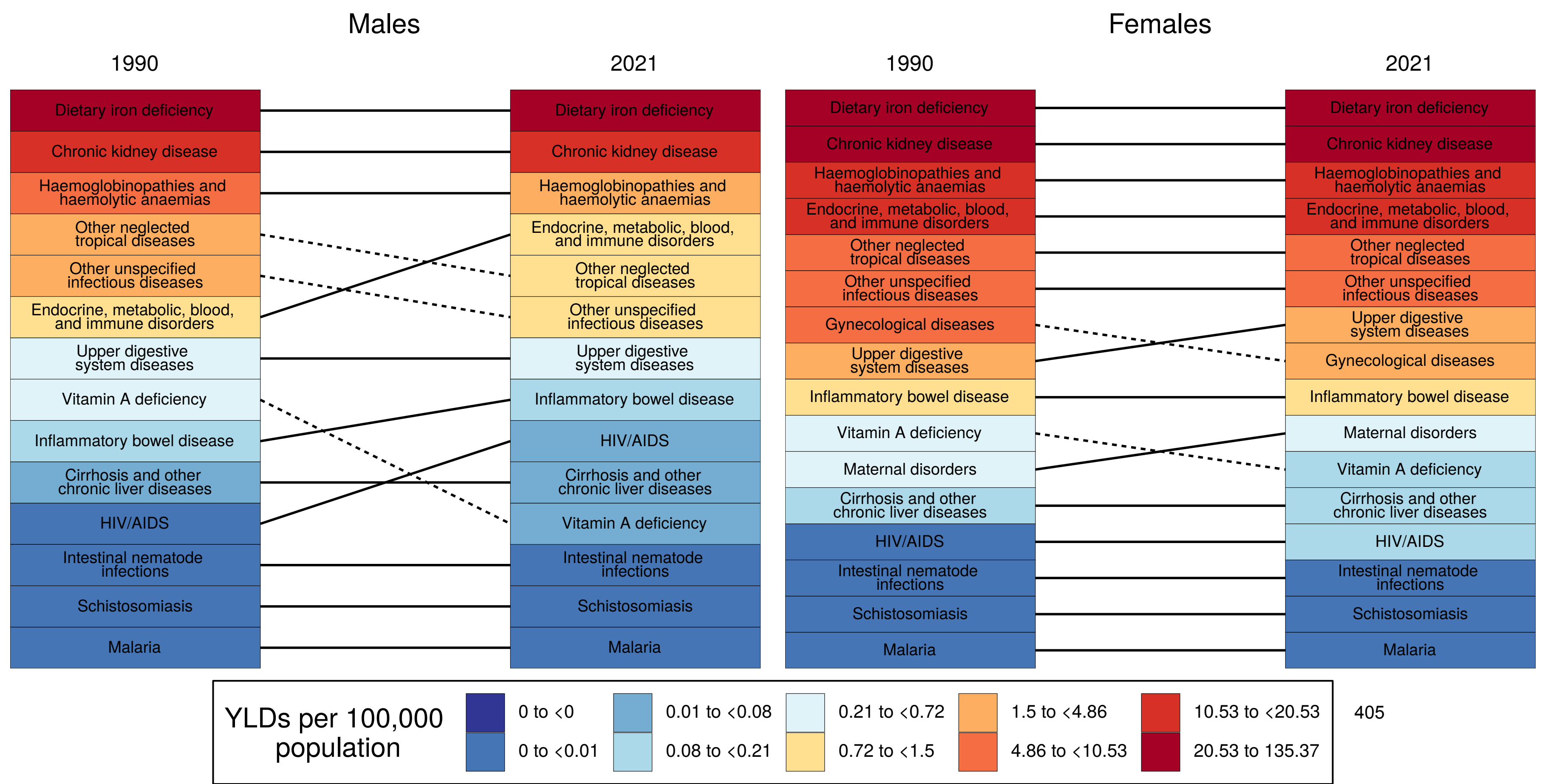
Males
Females
1990
2021
Dietary iron deficiency
Chronic kidney disease
Haemoglobinopathies and haemolytic anaemias
Other neglected tropical diseases
Other unspecified infectious diseases
Endocrine, metabolic, blood, and immune disorders
Upper digestive system diseases
Vitamin A deficiency
Inflammatory bowel disease
Cirrhosis and other chronic liver diseases
HIV/AIDS
Intestinal nematode infections
Schistosomiasis
Malaria
Gynecological diseases
Maternal disorders
YLDs per 100,000 population
0 to <0
0 to <0.01
0.01 to <0.08
0.08 to <0.21
0.21 to <0.72
0.72 to <1.5
1.5 to <4.86
4.86 to <10.53
10.53 to <20.53
20.53 to 135.37

East Riding of Yorkshire

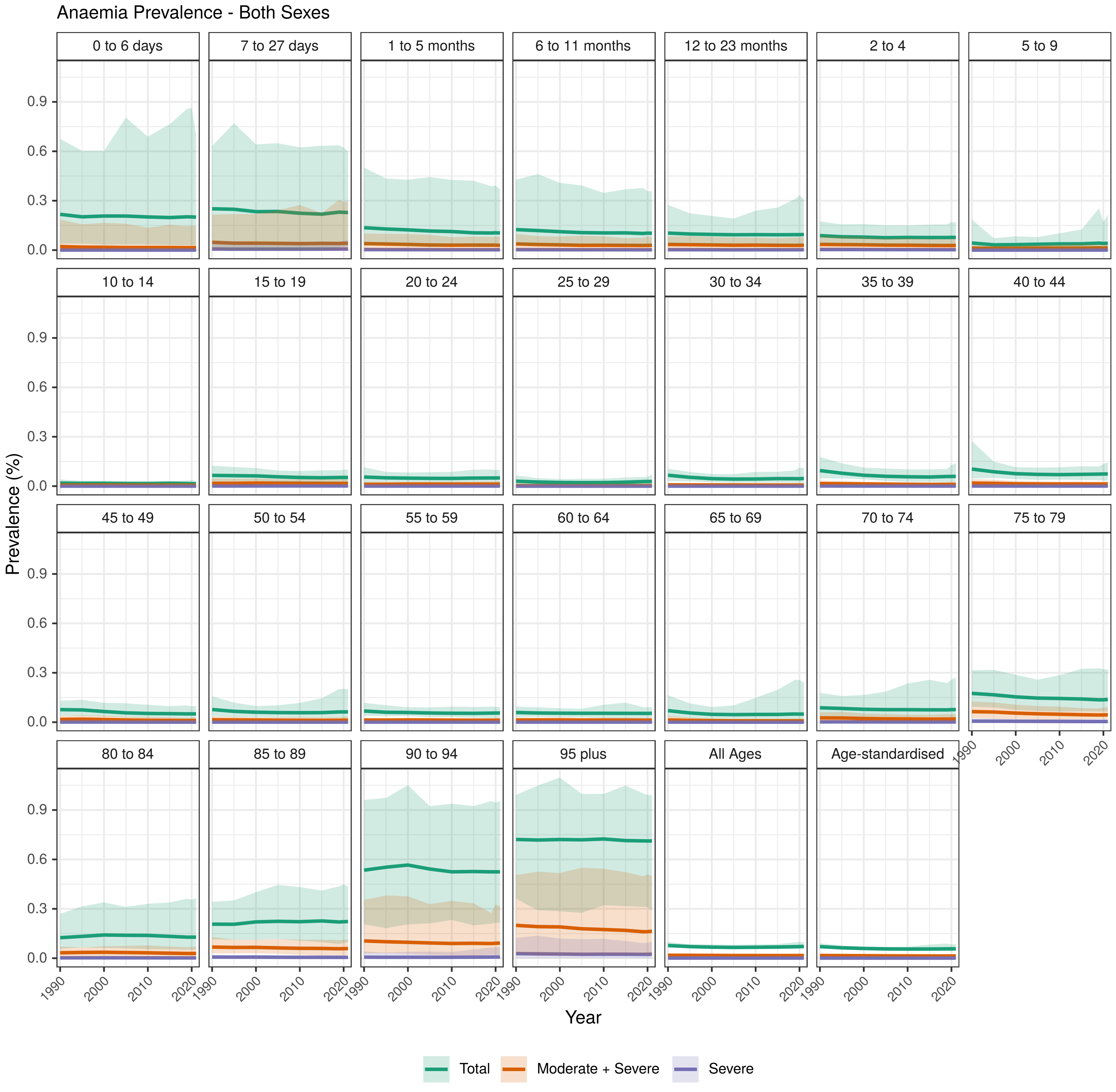

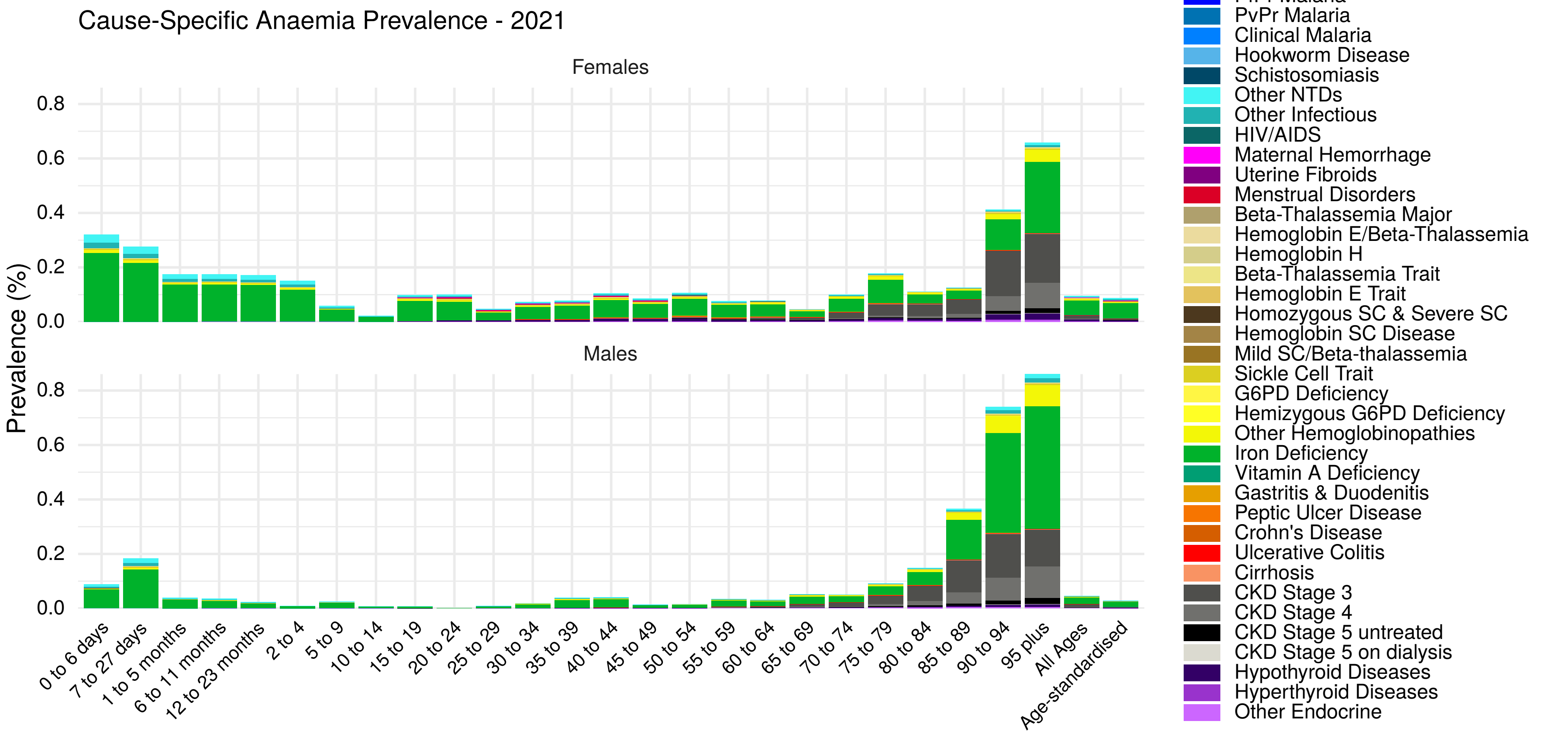

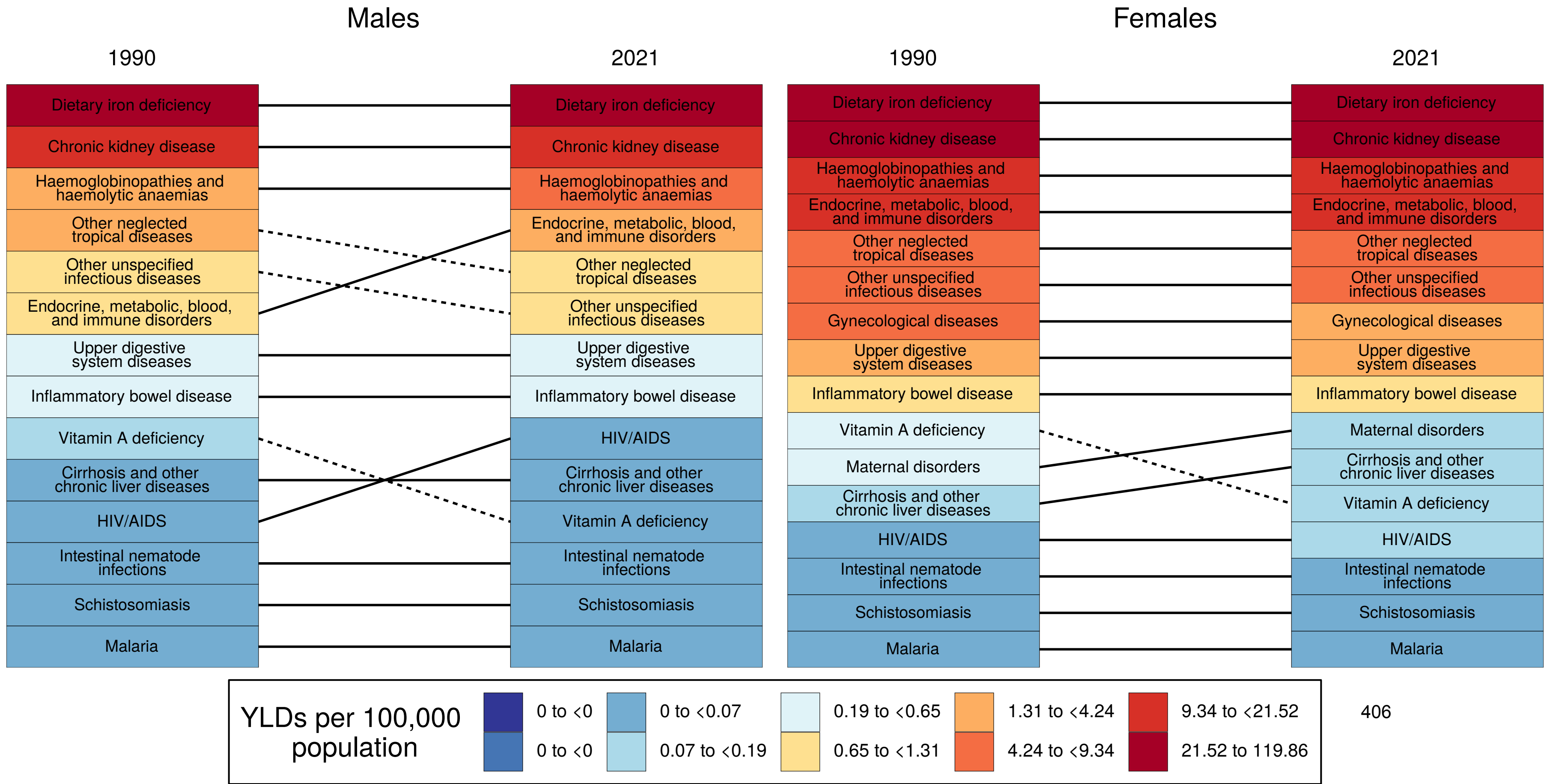

Kingston upon Hull, City of

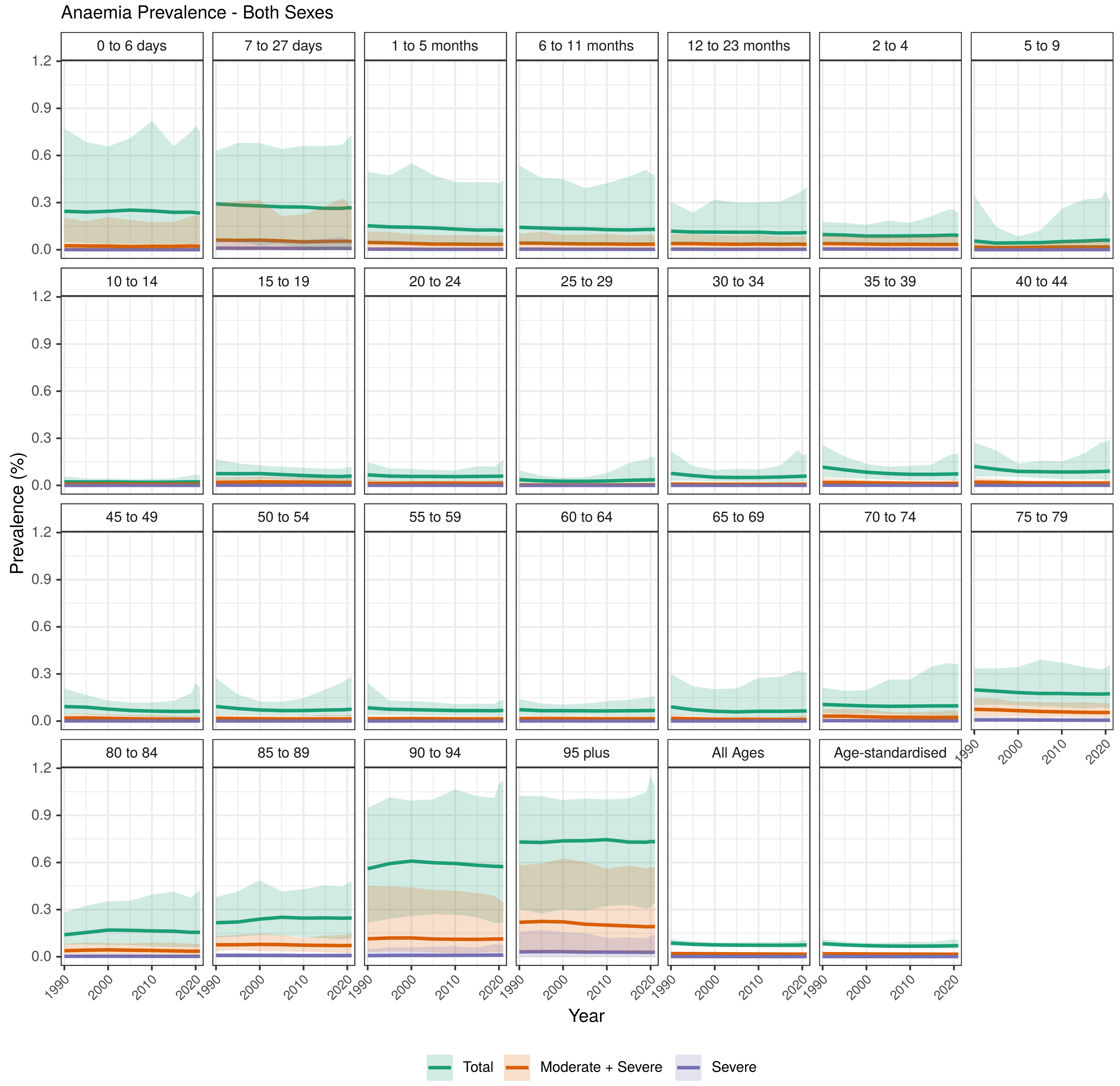

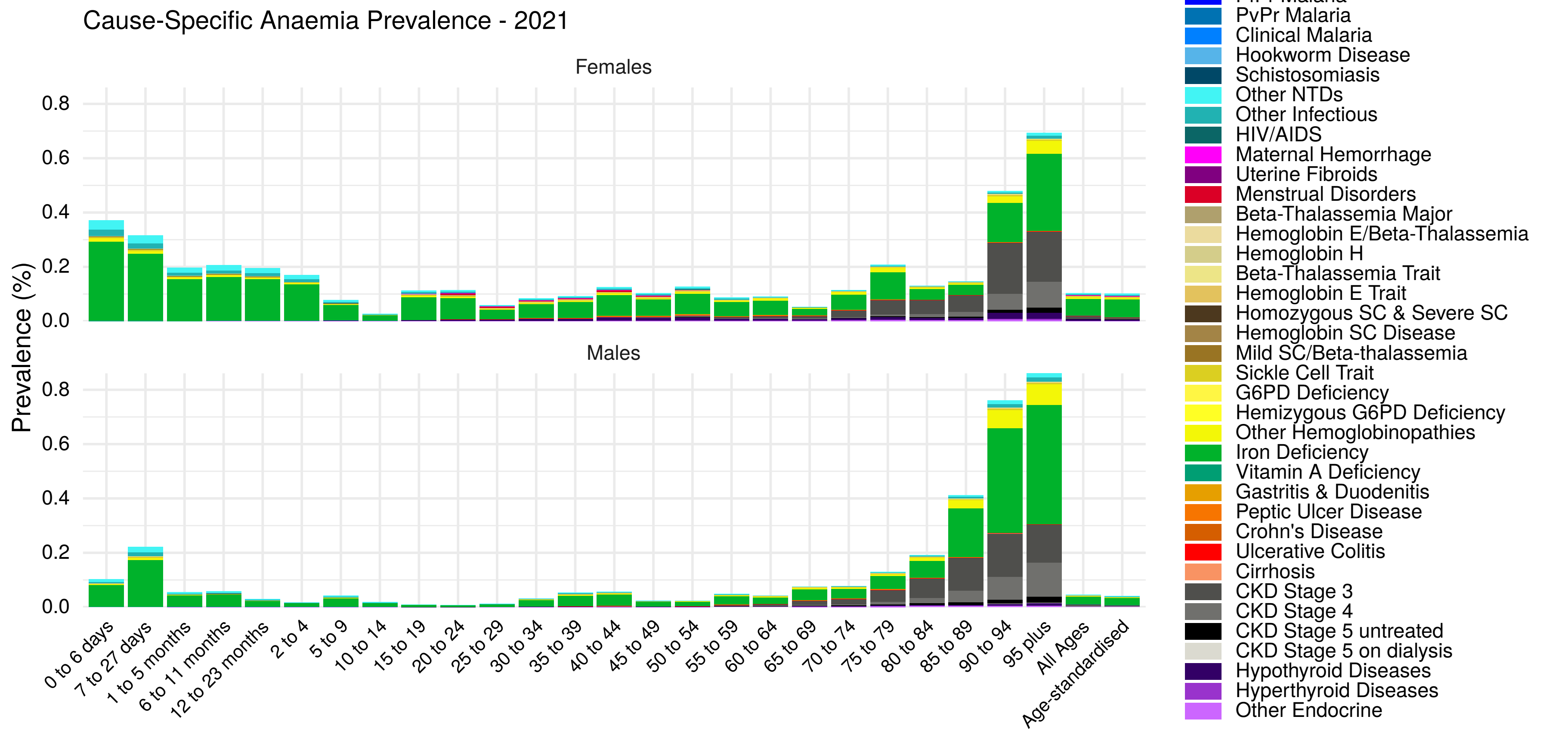

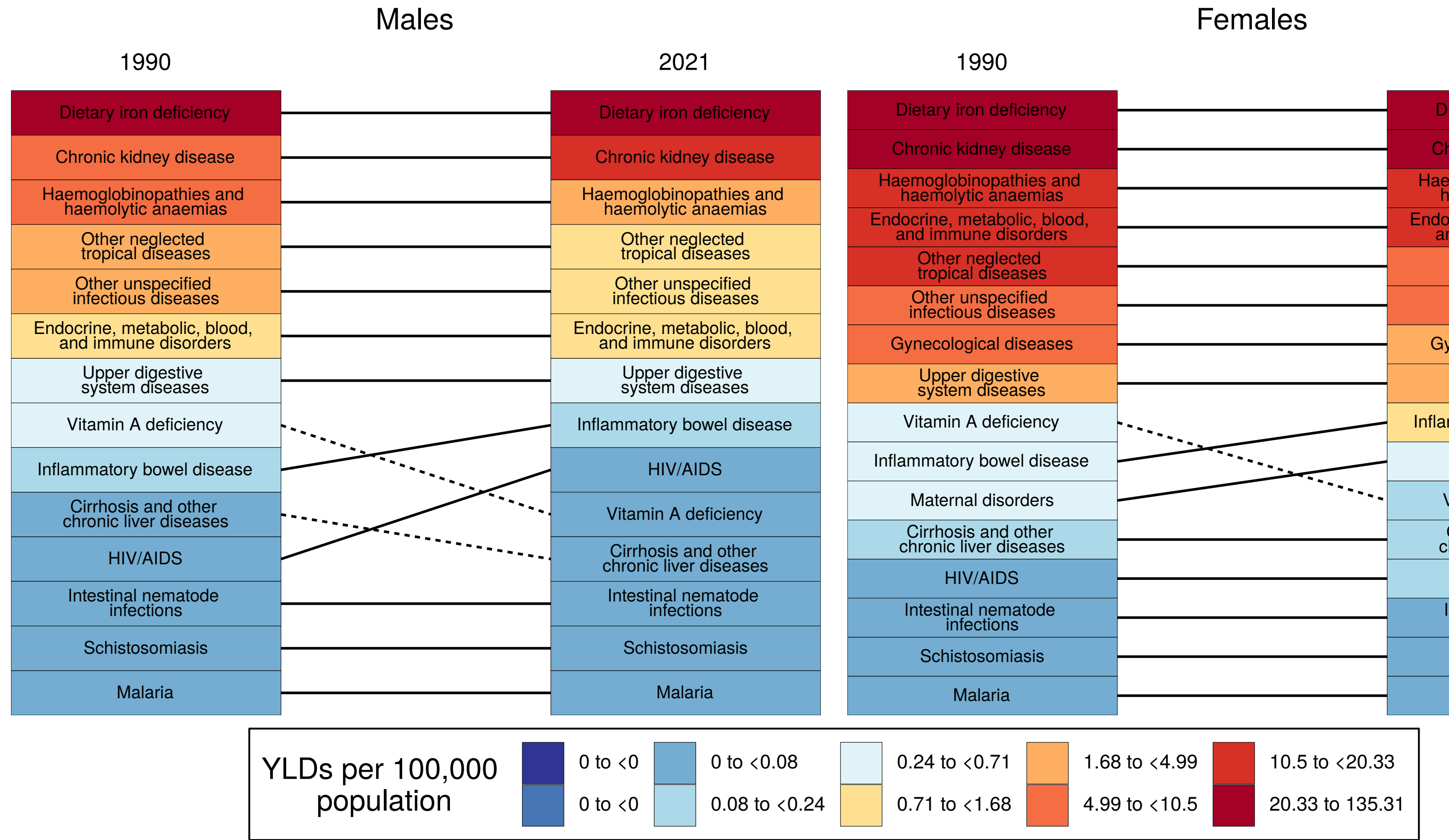

Kirklees

Anaemia Prevalence - Both Sexes

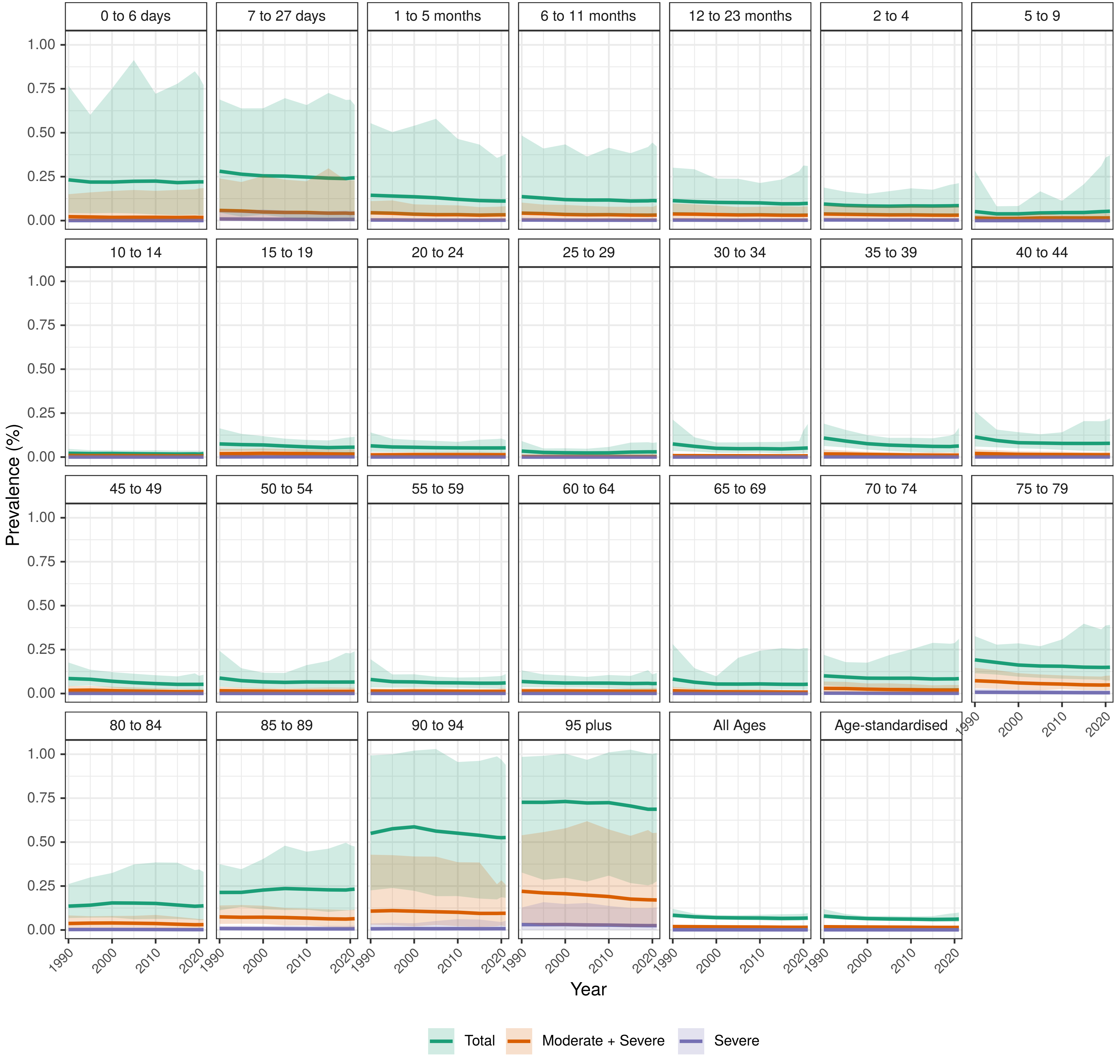

Cause-Specific Anaemia Prevalence - 2021

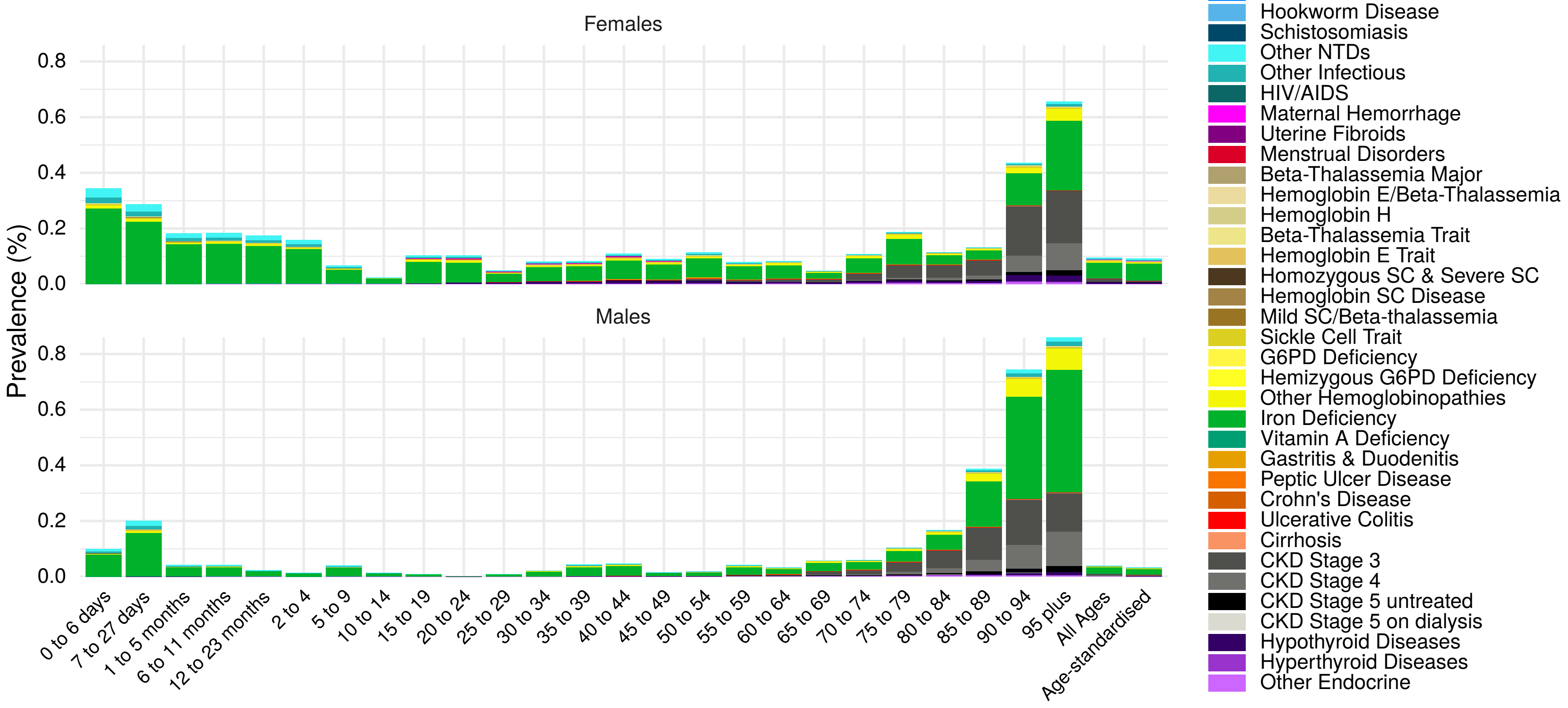

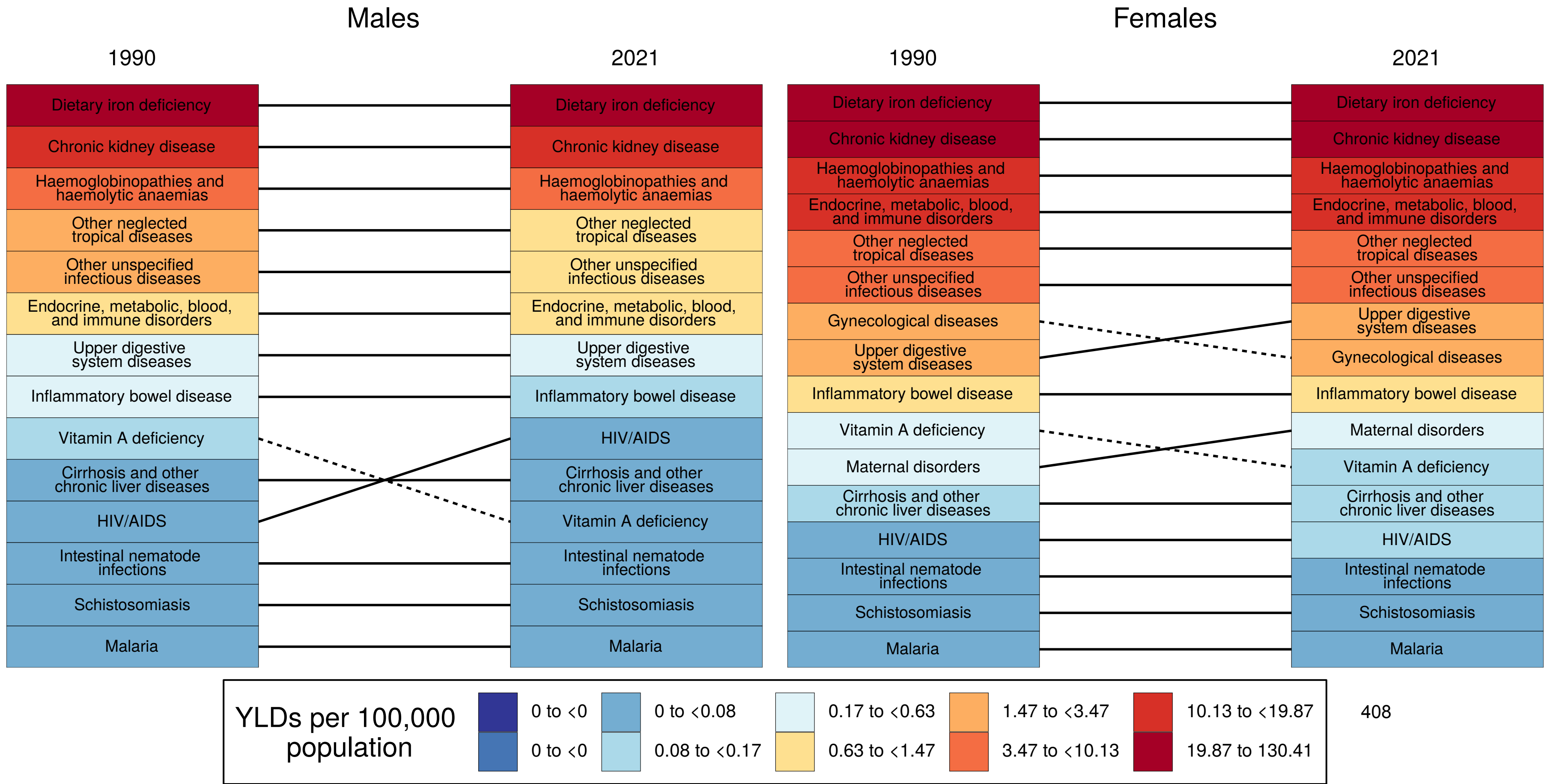

Leeds

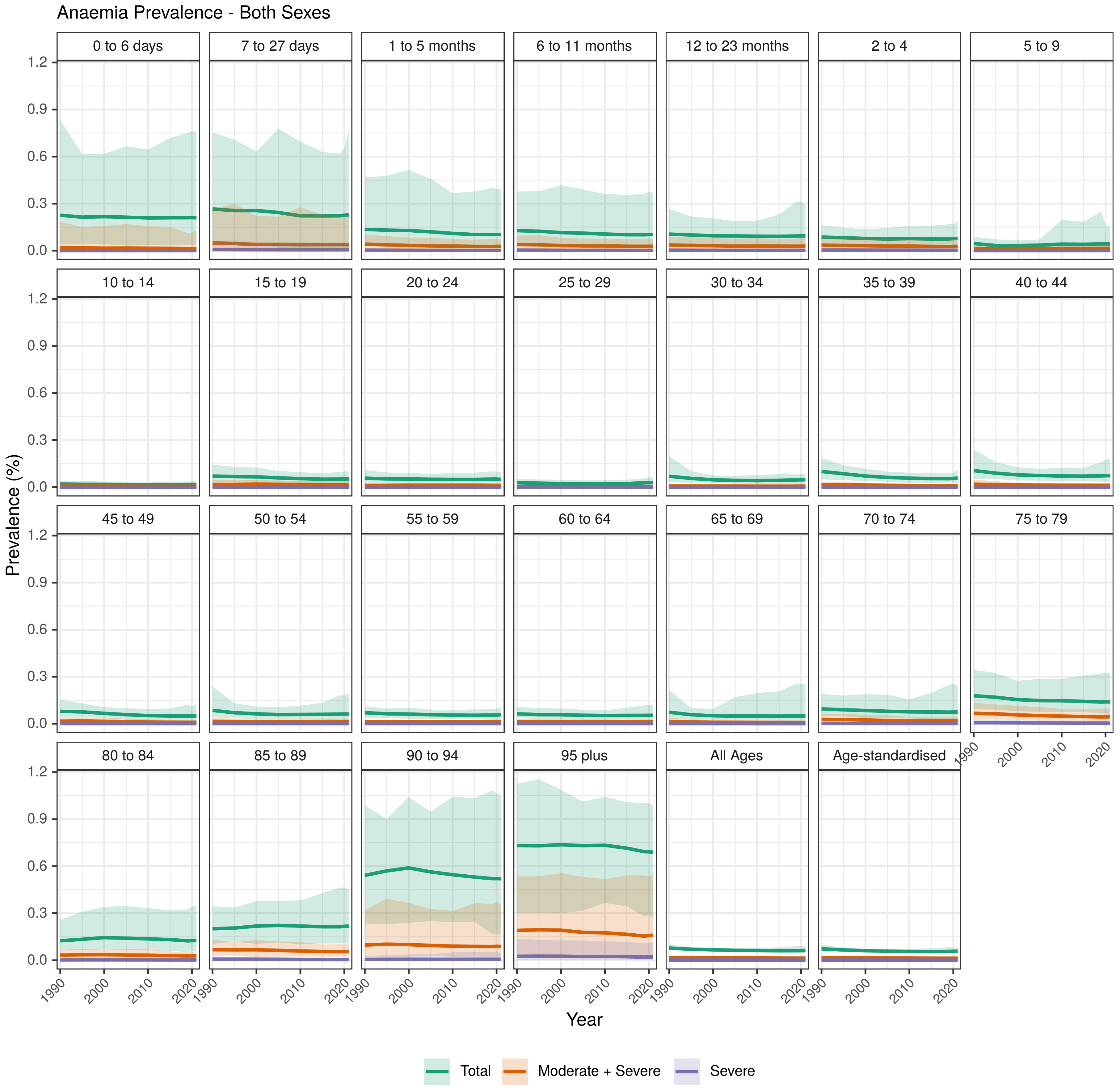
Anaemia Prevalence - Both Sexes
0 to 6 days
7 to 27 days
1 to 5 months
6 to 11 months
12 to 23 months
2 to 4
5 to 9
10 to 14
15 to 19
20 to 24
25 to 29
30 to 34
35 to 39
40 to 44
45 to 49
50 to 54
55 to 59
60 to 64
65 to 69
70 to 74
75 to 79
80 to 84
85 to 89
90 to 94
95 plus
All Ages
Age-standardised
Prevalence (%)
Year
Total
Moderate + Severe
Severe

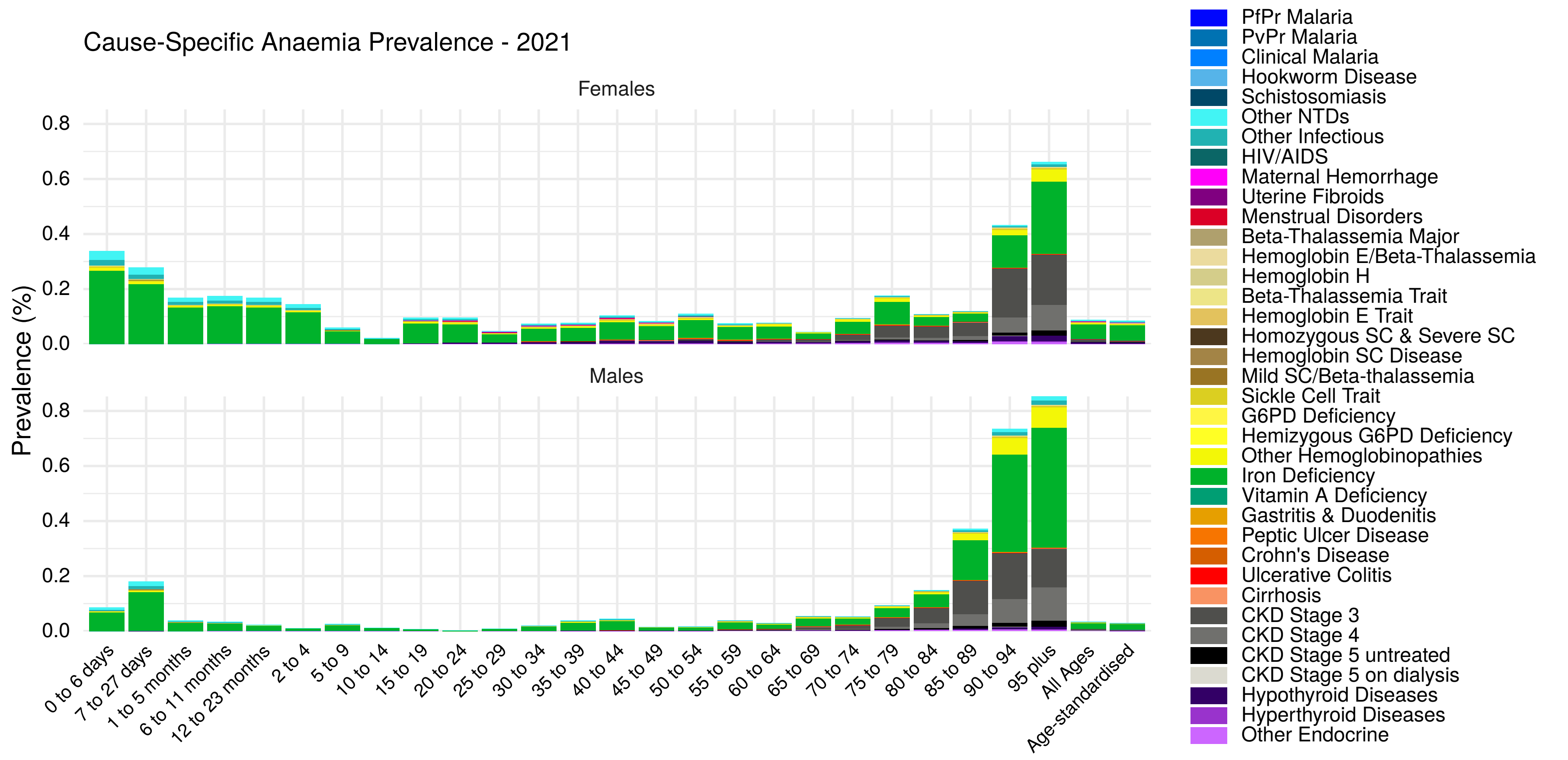
Cause-Specific Anaemia Prevalence - 2021
Females
Males
Prevalence (%)
PfPr Malaria
PvPr Malaria
Clinical Malaria
Hookworm Disease
Schistosomiasis
Other NTDs
Other Infectious
HIV/AIDS
Maternal Hemorrhage
Uterine Fibroids
Menstrual Disorders
Beta-Thalassemia Major
Hemoglobin E/Beta-Thalassemia
Hemoglobin H
Beta-Thalassemia Trait
Hemoglobin E Trait
Homozygous SC & Severe SC
Hemoglobin SC Disease
Mild SC/Beta-thalassemia
Sickle Cell Trait
G6PD Deficiency
Hemizygous G6PD Deficiency
Other Hemoglobinopathies
Iron Deficiency
Vitamin A Deficiency
Gastritis & Duodenitis
Peptic Ulcer Disease
Crohn's Disease
Ulcerative Colitis
Cirrhosis
CKD Stage 3
CKD Stage 4
CKD Stage 5 untreated
CKD Stage 5 on dialysis
Hypothyroid Diseases
Hyperthyroid Diseases
Other Endocrine

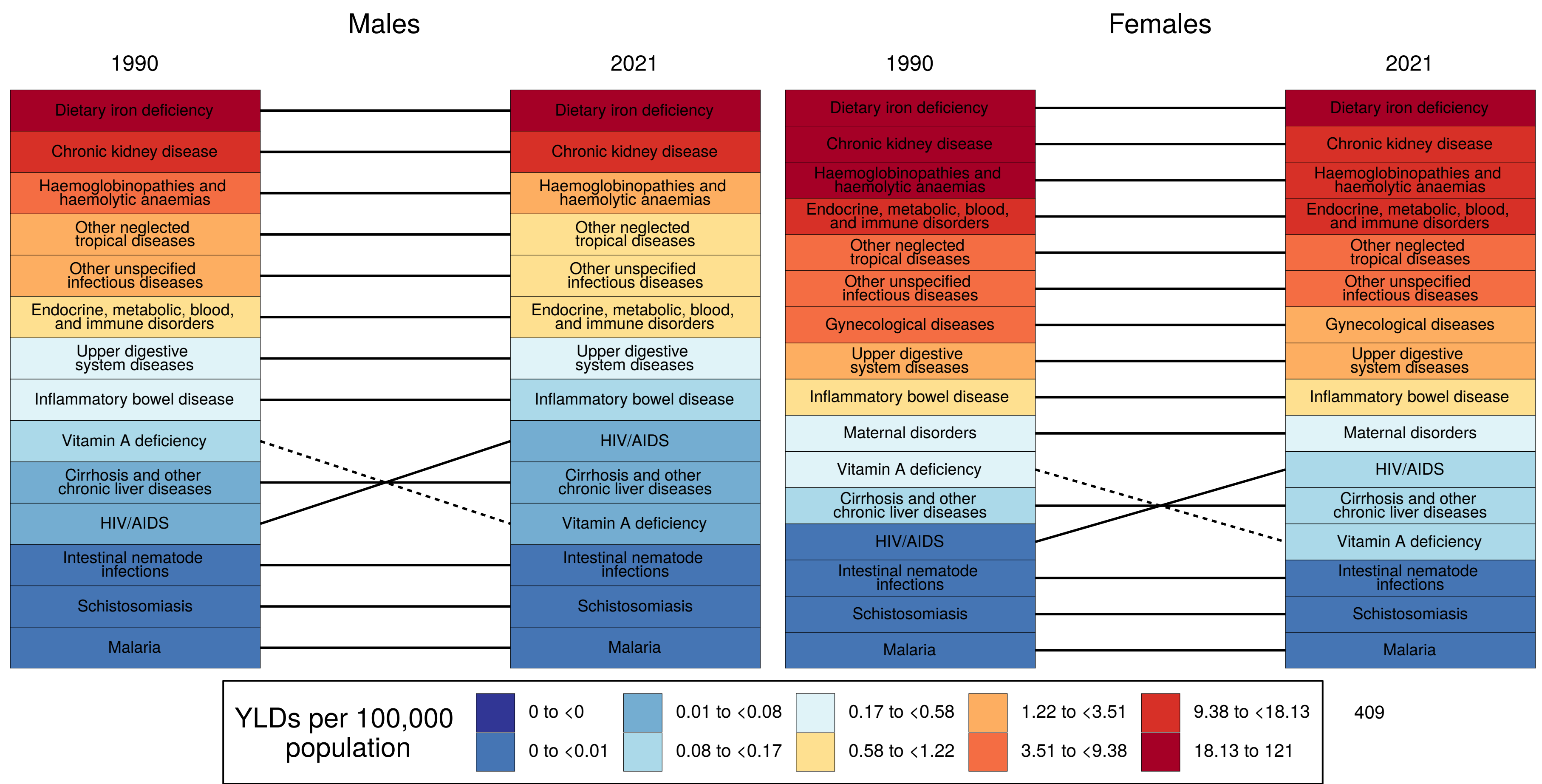
Males
Females
1990
2021
Dietary iron deficiency
Chronic kidney disease
Haemoglobinopathies and haemolytic anaemias
Other neglected tropical diseases
Other unspecified infectious diseases
Endocrine, metabolic, blood, and immune disorders
Upper digestive system diseases
Inflammatory bowel disease
Vitamin A deficiency
Cirrhosis and other chronic liver diseases
HIV/AIDS
Intestinal nematode infections
Schistosomiasis
Malaria
Gynecological diseases
Maternal disorders
YLDs per 100,000 population
0 to <0
0 to <0.01
0.01 to <0.08
0.08 to <0.17
0.17 to <0.58
0.58 to <1.22
1.22 to <3.51
3.51 to <9.38
9.38 to <18.13
18.13 to 121

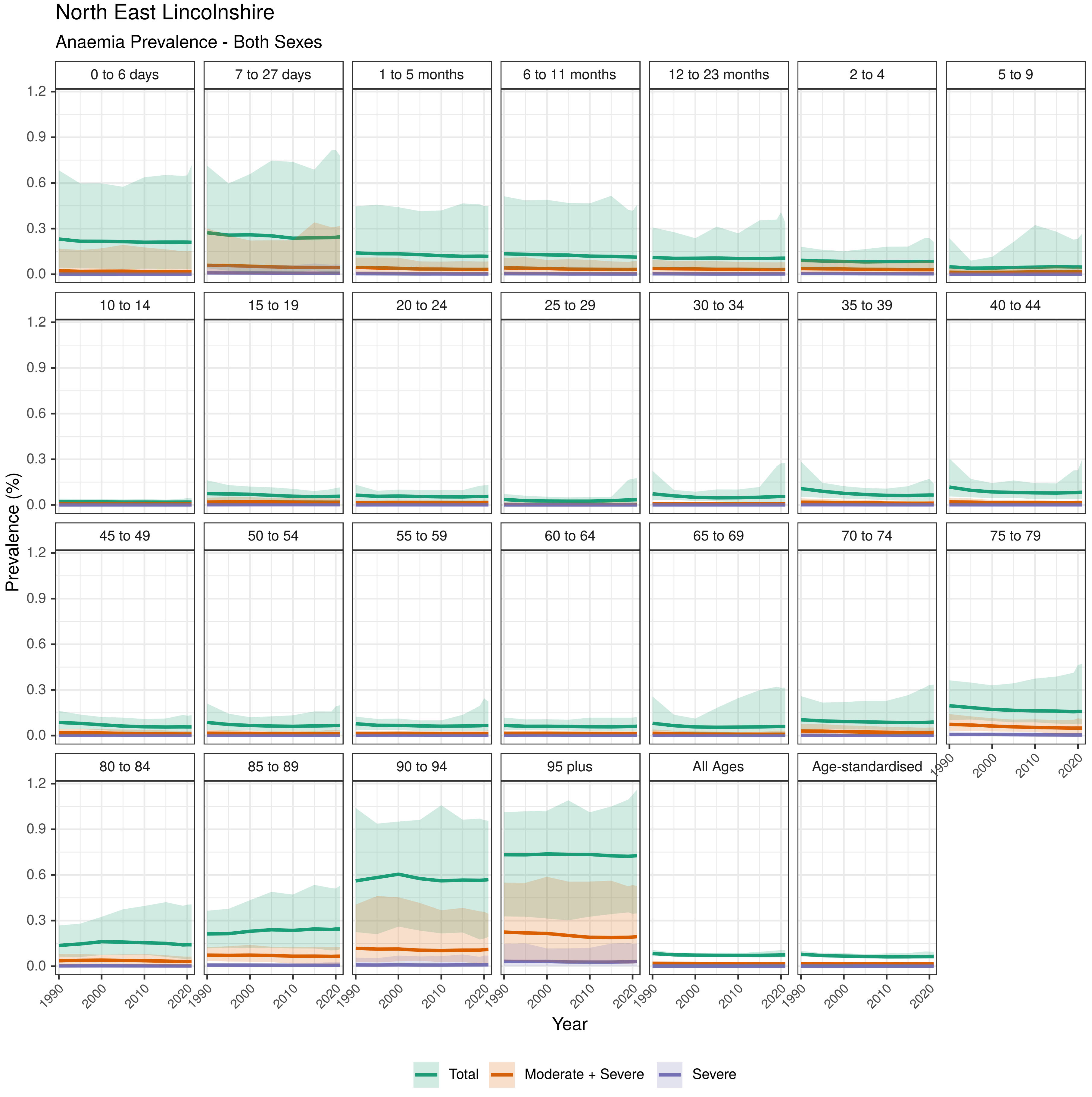
North East Lincolnshire
Anaemia Prevalence - Both Sexes
0 to 6 days
7 to 27 days
1 to 5 months
6 to 11 months
12 to 23 months
2 to 4
5 to 9
10 to 14
15 to 19
20 to 24
25 to 29
30 to 34
35 to 39
40 to 44
45 to 49
50 to 54
55 to 59
60 to 64
65 to 69
70 to 74
75 to 79
80 to 84
85 to 89
90 to 94
95 plus
All Ages
Age-standardised
Prevalence (%)
Year
Total
Moderate + Severe
Severe

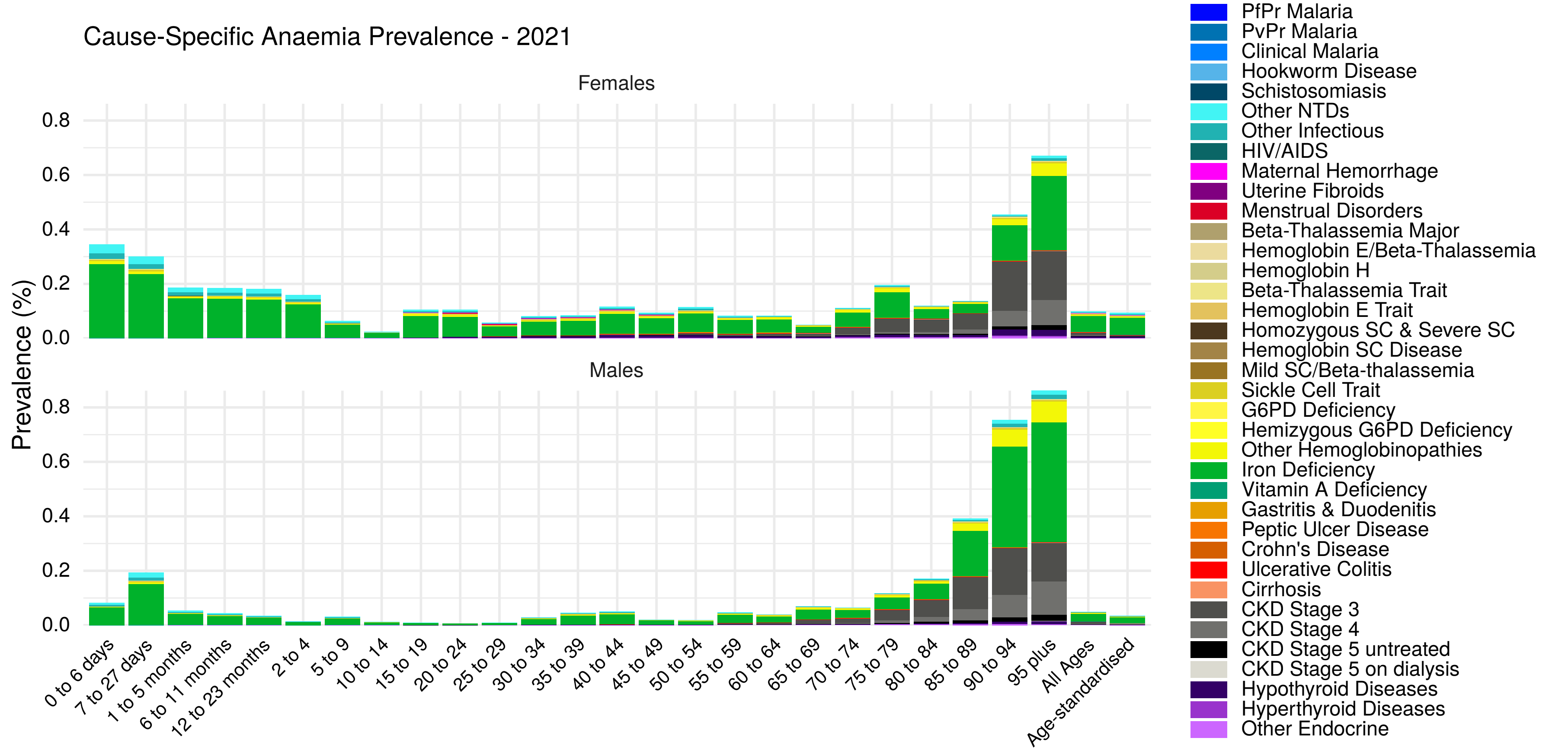
Cause-Specific Anaemia Prevalence - 2021
Females
Males
Prevalence (%)
PfPr Malaria
PvPr Malaria
Clinical Malaria
Hookworm Disease
Schistosomiasis
Other NTDs
Other Infectious
HIV/AIDS
Maternal Hemorrhage
Uterine Fibroids
Menstrual Disorders
Beta-Thalassemia Major
Hemoglobin E/Beta-Thalassemia
Hemoglobin H
Beta-Thalassemia Trait
Hemoglobin E Trait
Homozygous SC & Severe SC
Hemoglobin SC Disease
Mild SC/Beta-thalassemia
Sickle Cell Trait
G6PD Deficiency
Hemizygous G6PD Deficiency
Other Hemoglobinopathies
Iron Deficiency
Vitamin A Deficiency
Gastritis & Duodenitis
Peptic Ulcer Disease
Crohn's Disease
Ulcerative Colitis
Cirrhosis
CKD Stage 3
CKD Stage 4
CKD Stage 5 untreated
CKD Stage 5 on dialysis
Hypothyroid Diseases
Hyperthyroid Diseases
Other Endocrine

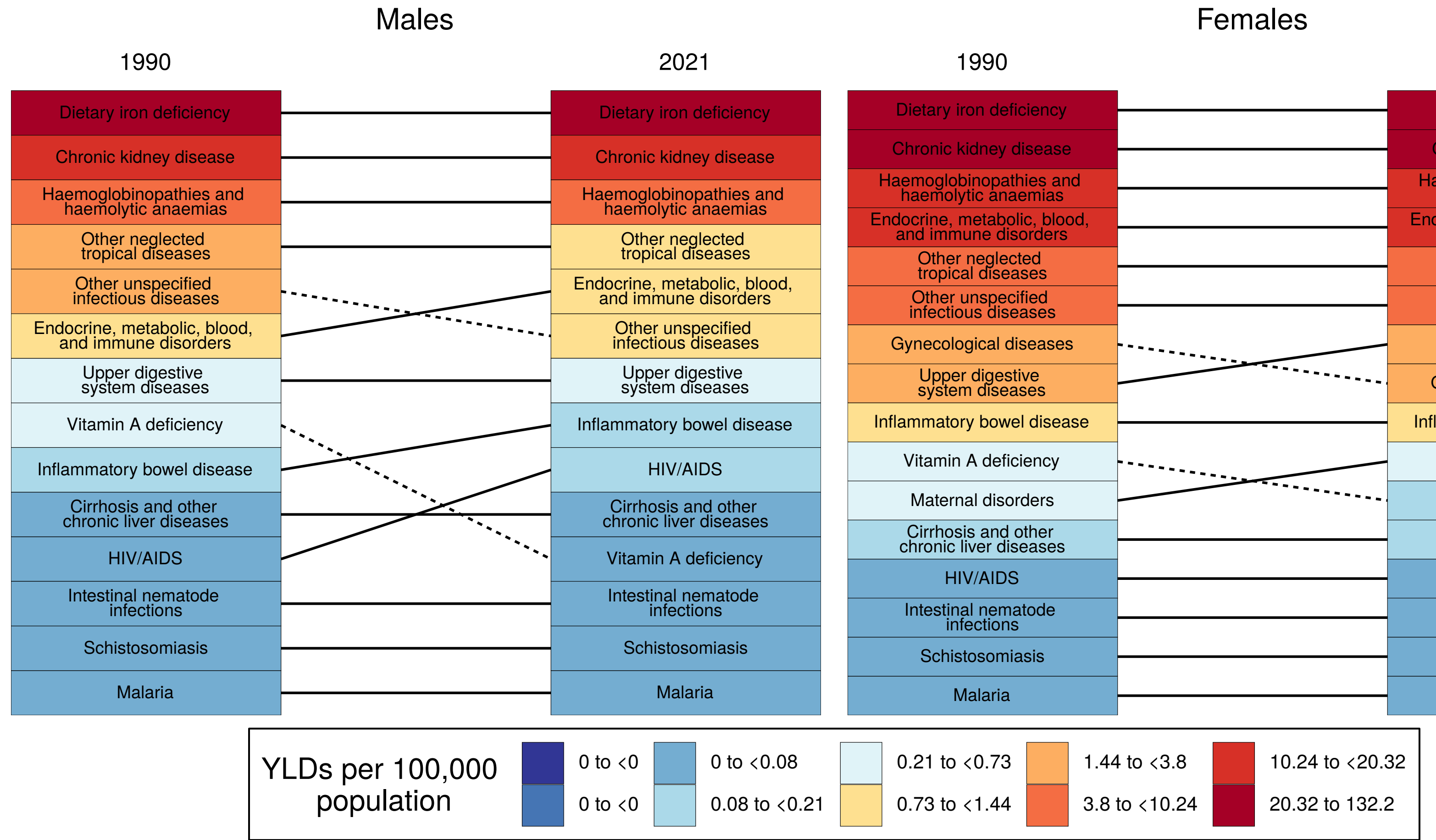
Males
Females
1990
2021
Dietary iron deficiency
Chronic kidney disease
Haemoglobinopathies and haemolytic anaemias
Other neglected tropical diseases
Other unspecified infectious diseases
Endocrine, metabolic, blood, and immune disorders
Upper digestive system diseases
Vitamin A deficiency
Inflammatory bowel disease
Cirrhosis and other chronic liver diseases
HIV/AIDS
Intestinal nematode infections
Schistosomiasis
Malaria
Gynecological diseases
Maternal disorders
YLDs per 100,000 population
0 to <0
0 to <0.08
0.08 to <0.21
0.21 to <0.73
0.73 to <1.44
1.44 to <3.8
3.8 to <10.24
10.24 to <20.32
20.32 to 132.2

North Lincolnshire

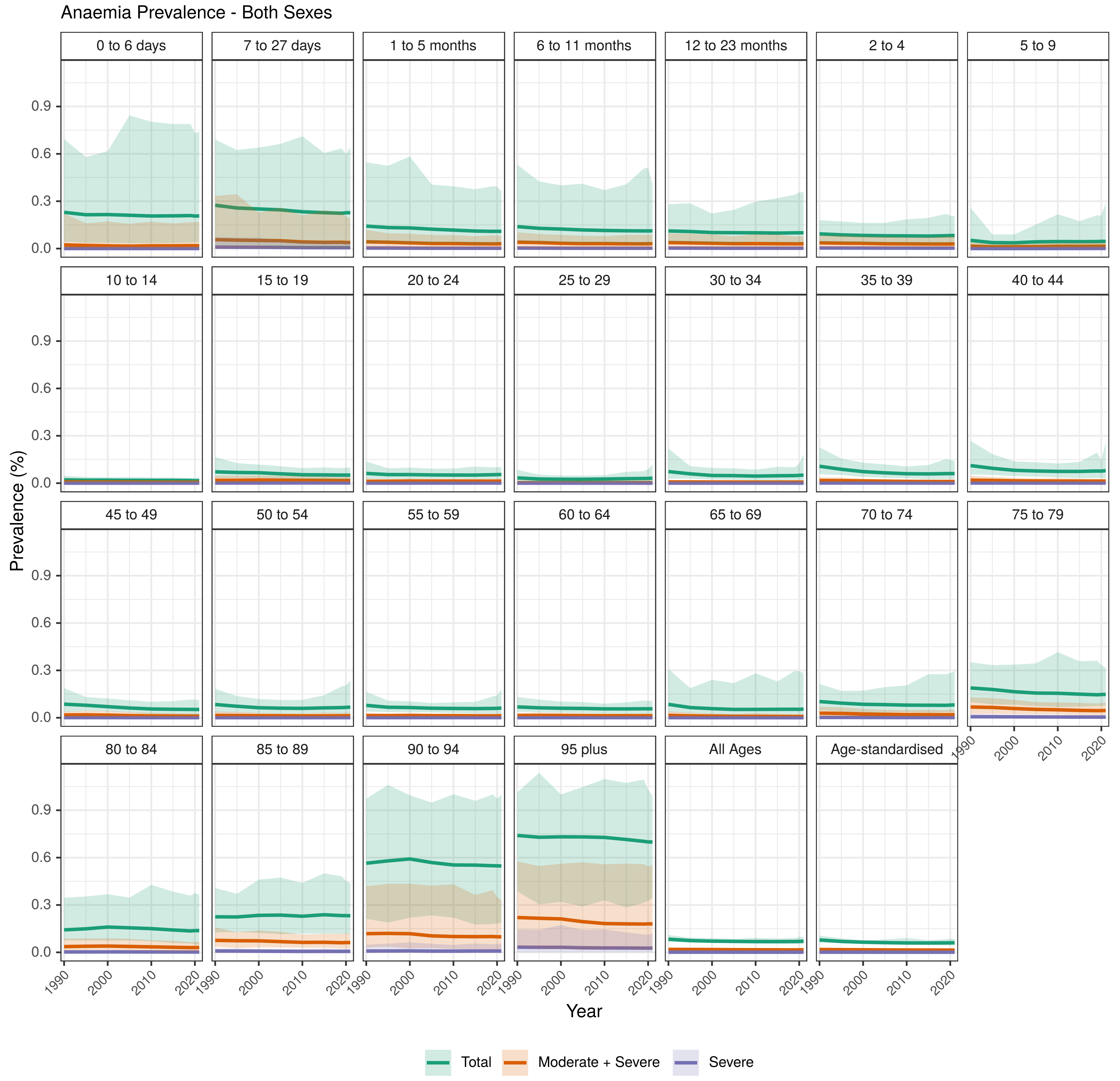

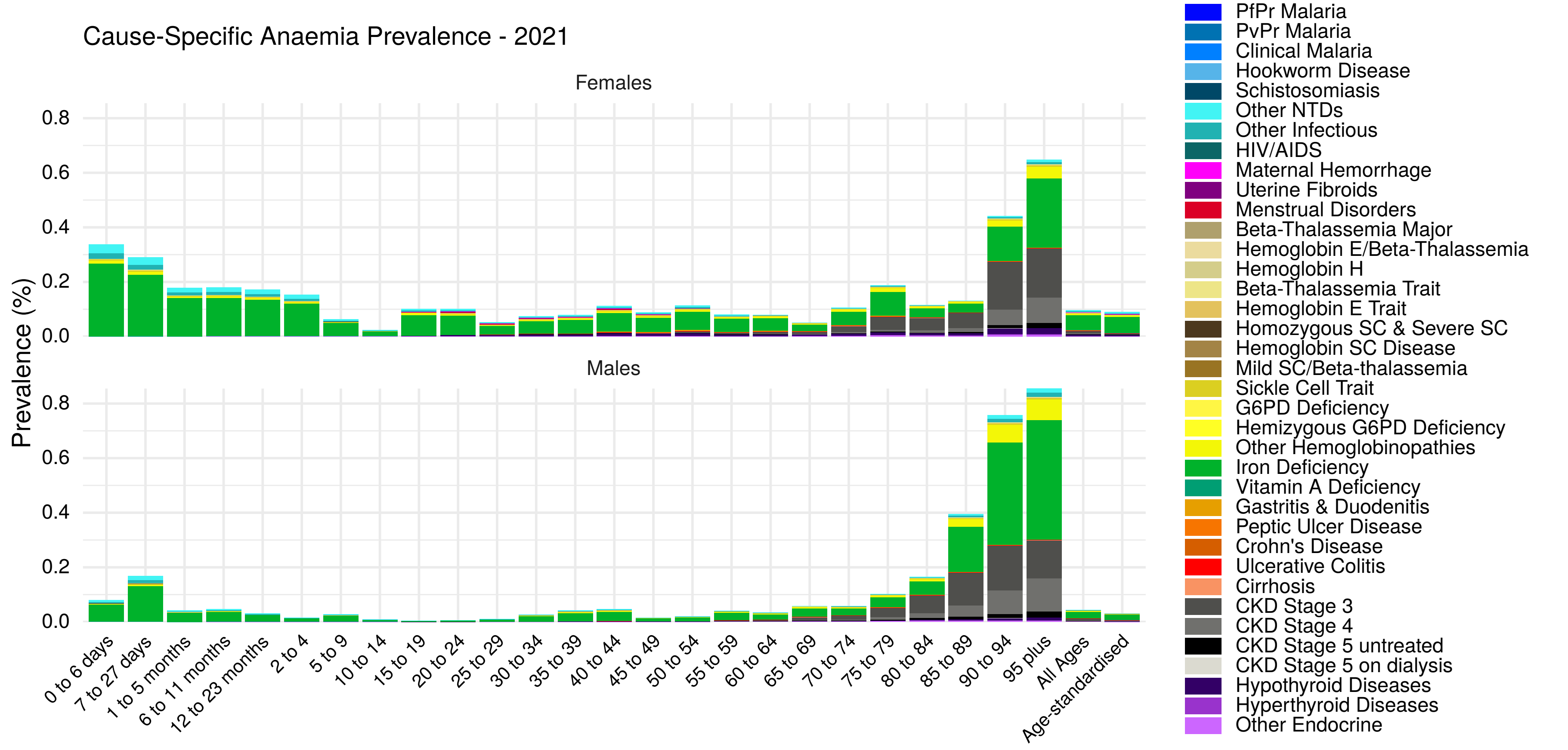

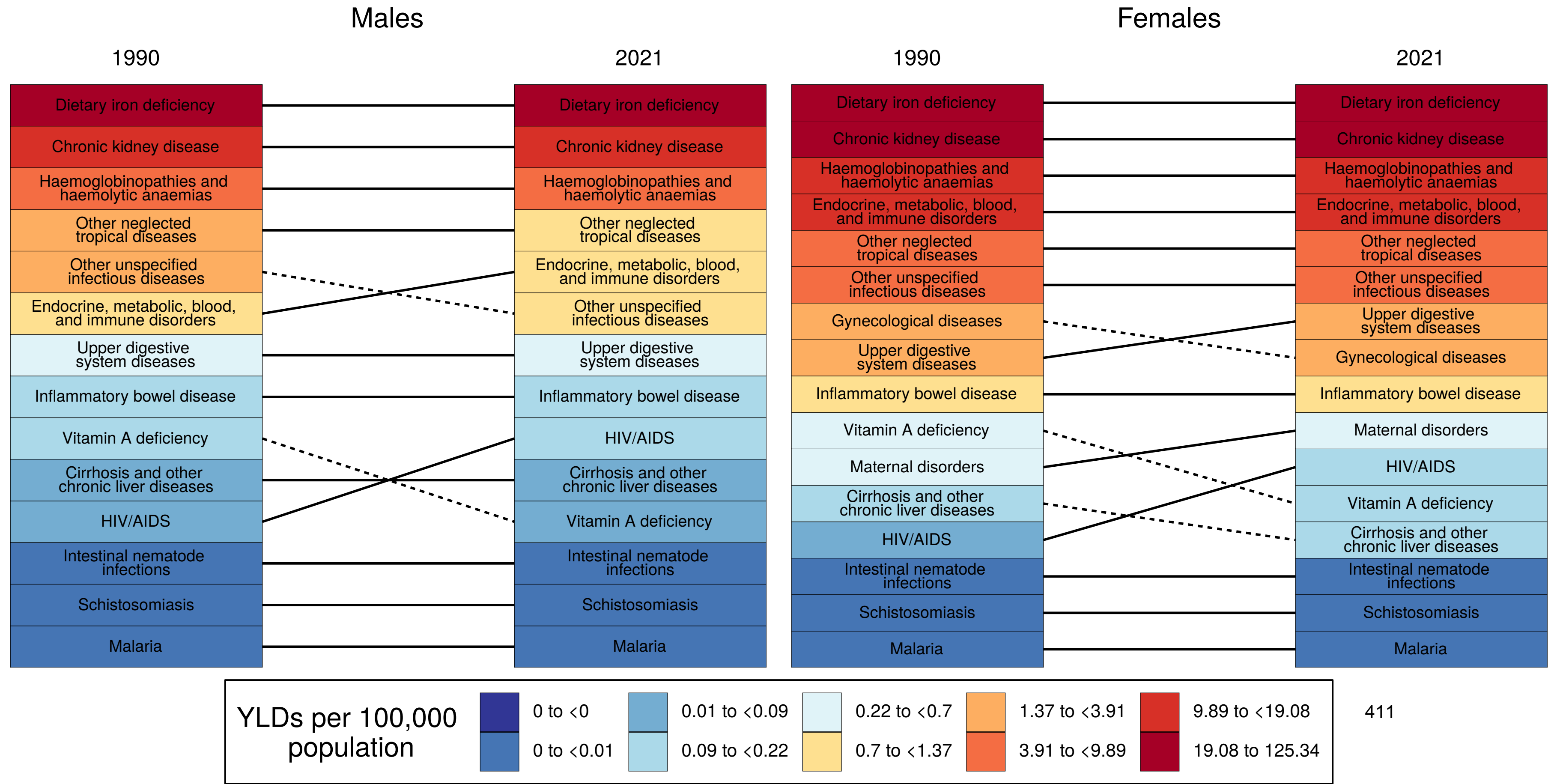

North Yorkshire

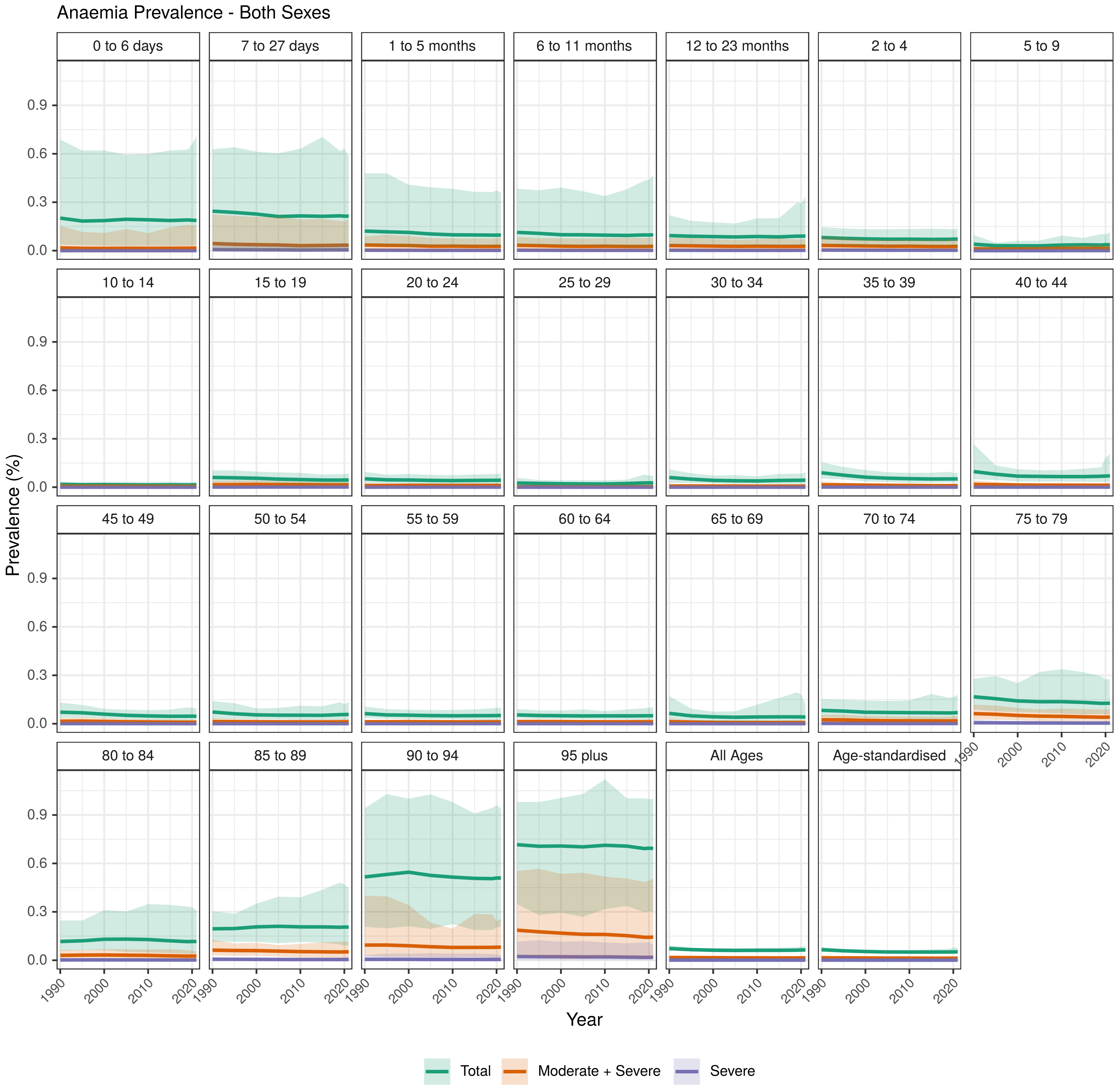

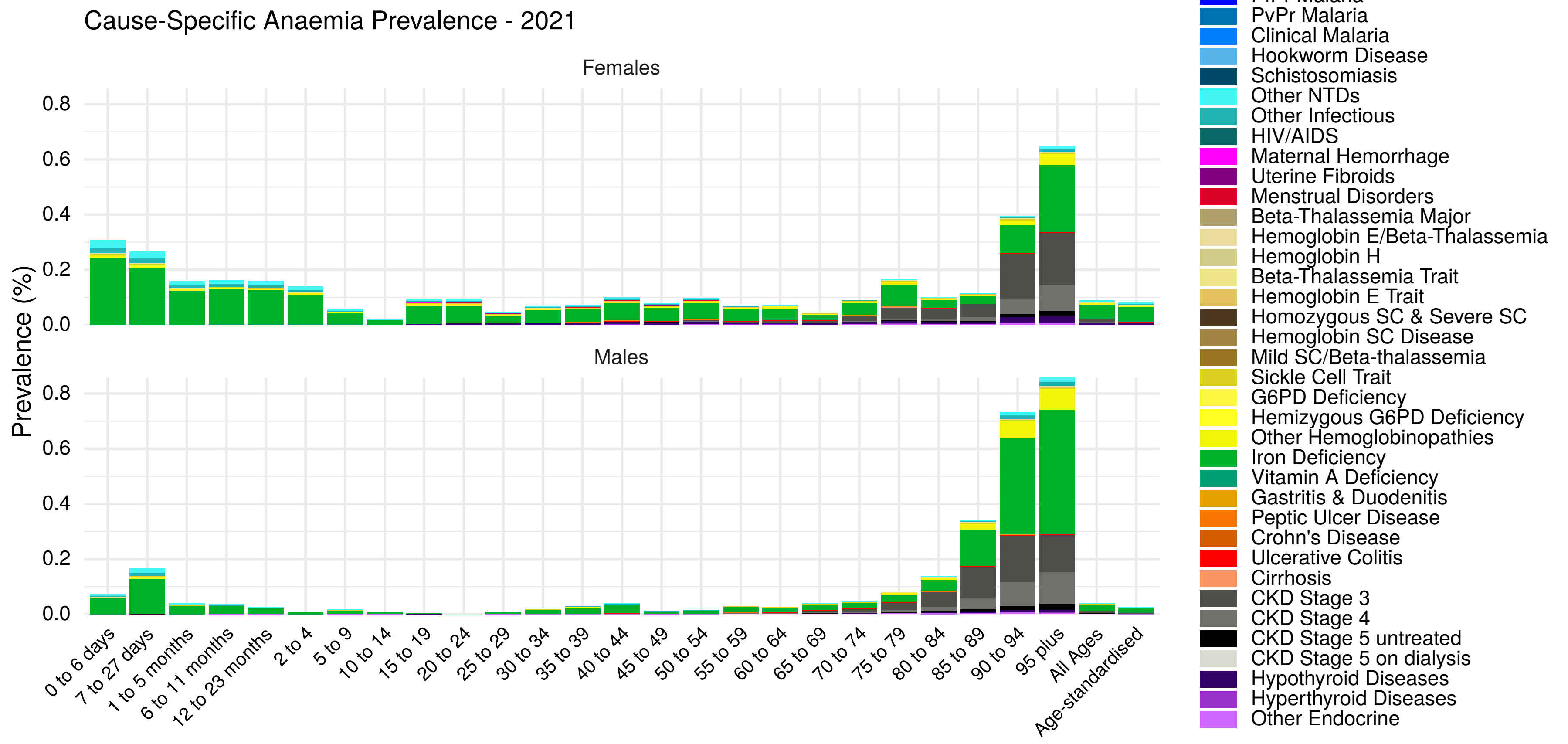

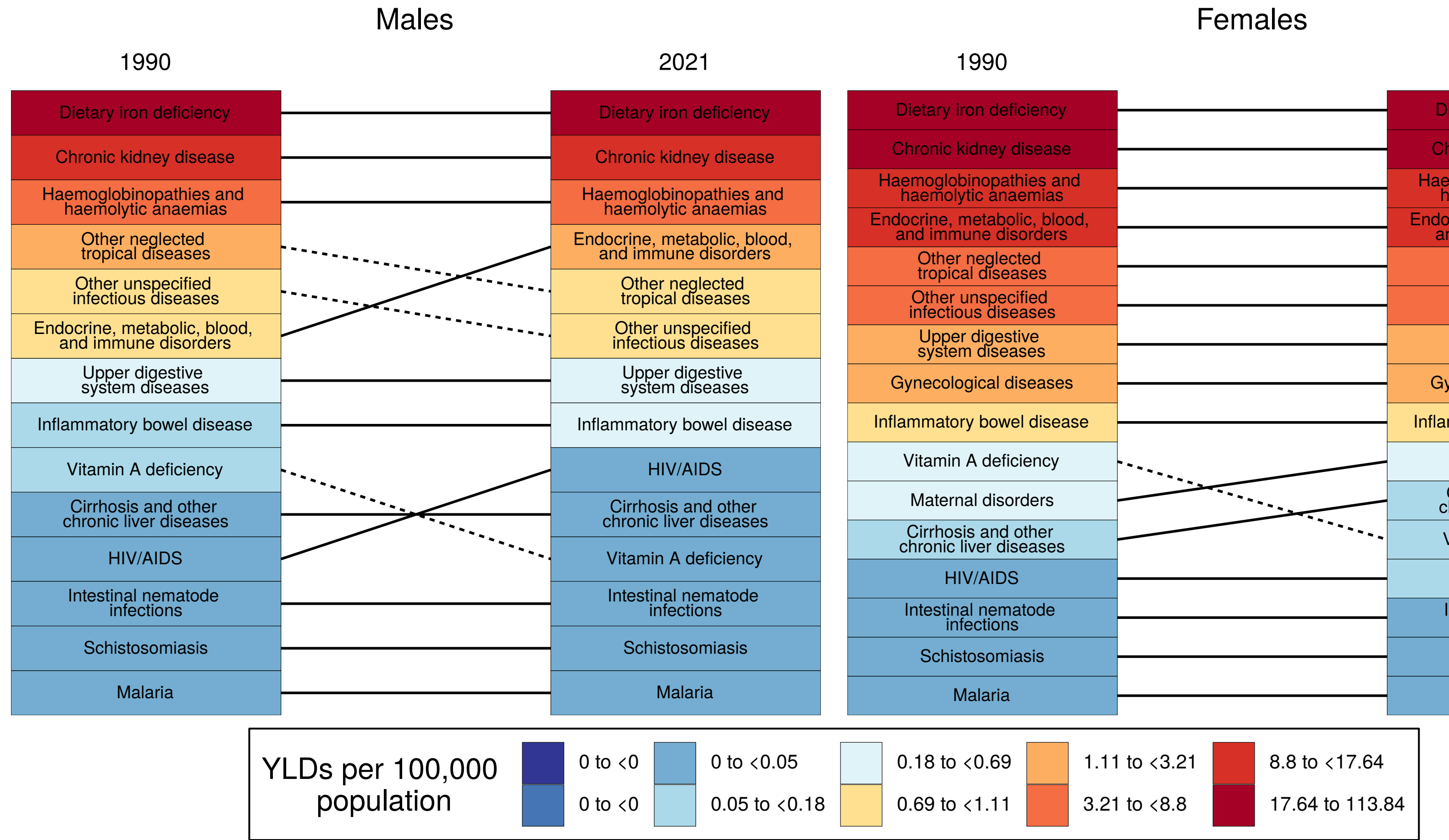

Rotherham

Anaemia Prevalence - Both Sexes

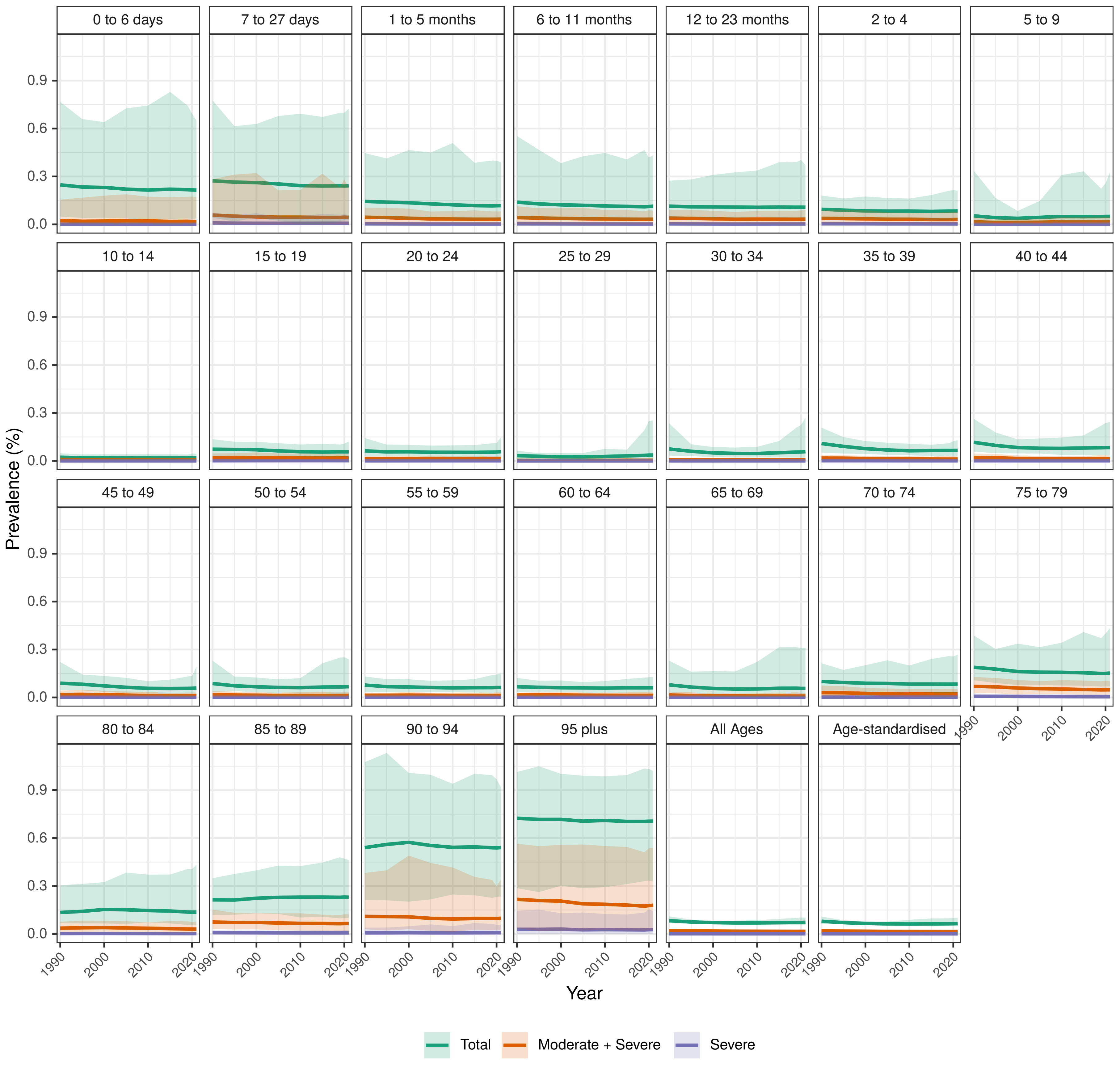

Cause-Specific Anaemia Prevalence - 2021

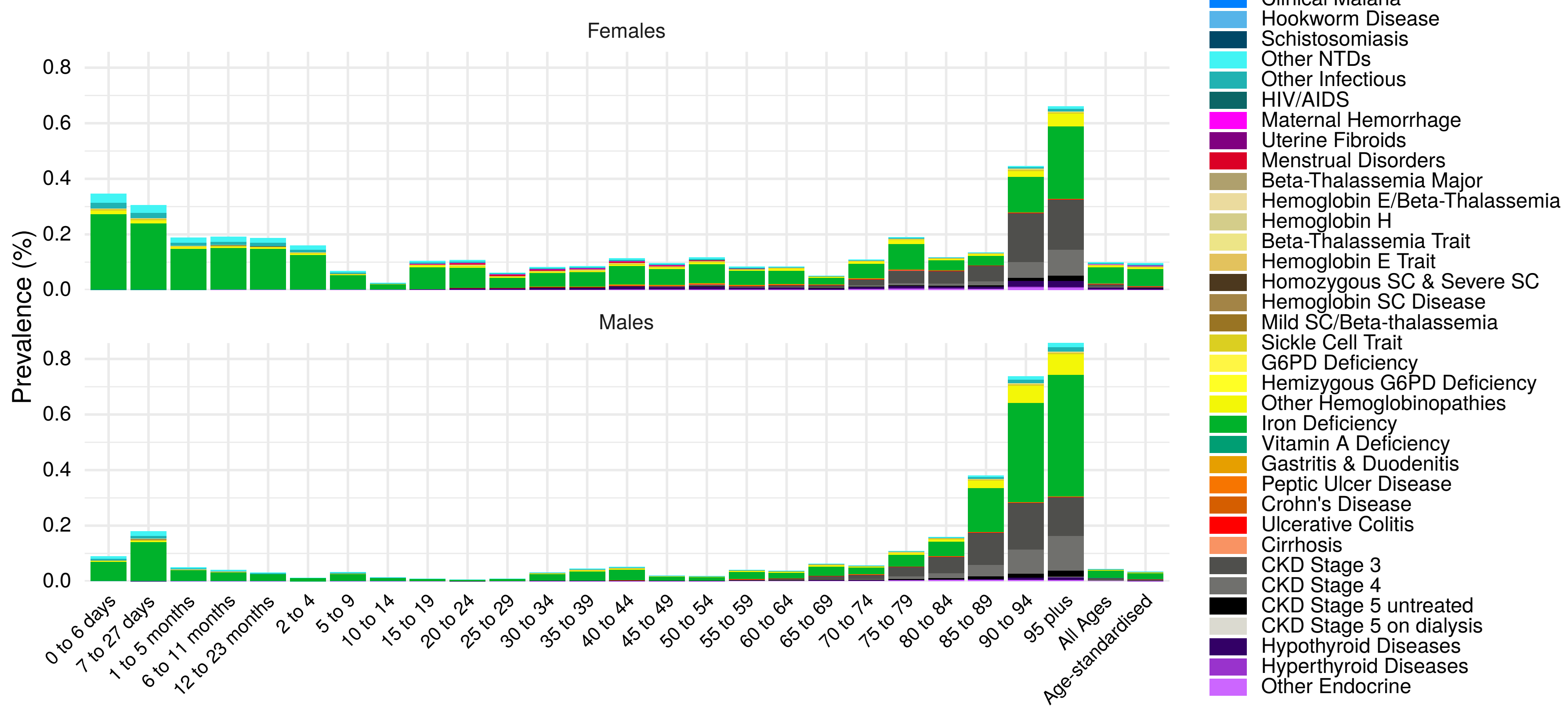

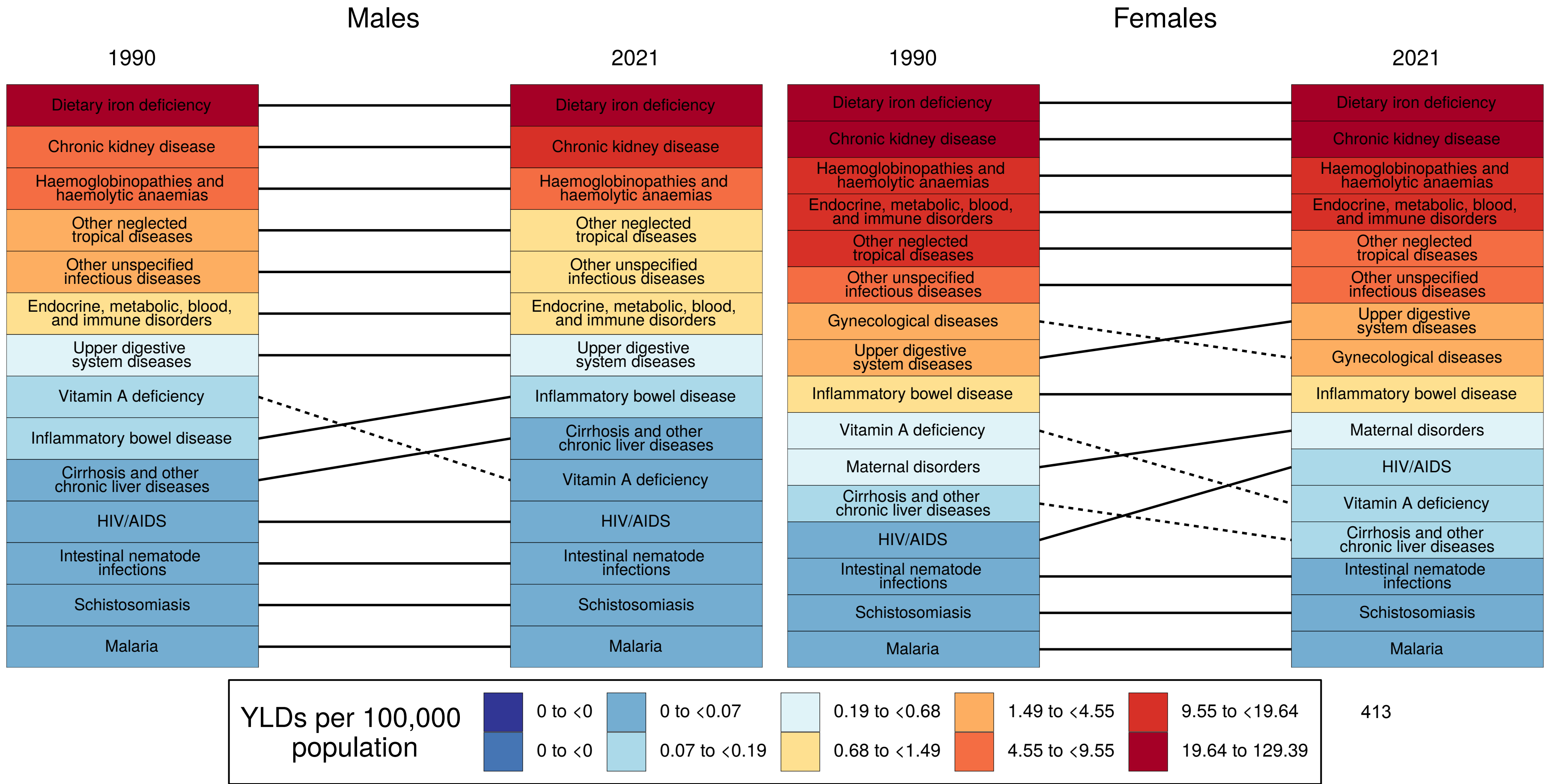

Sheffield

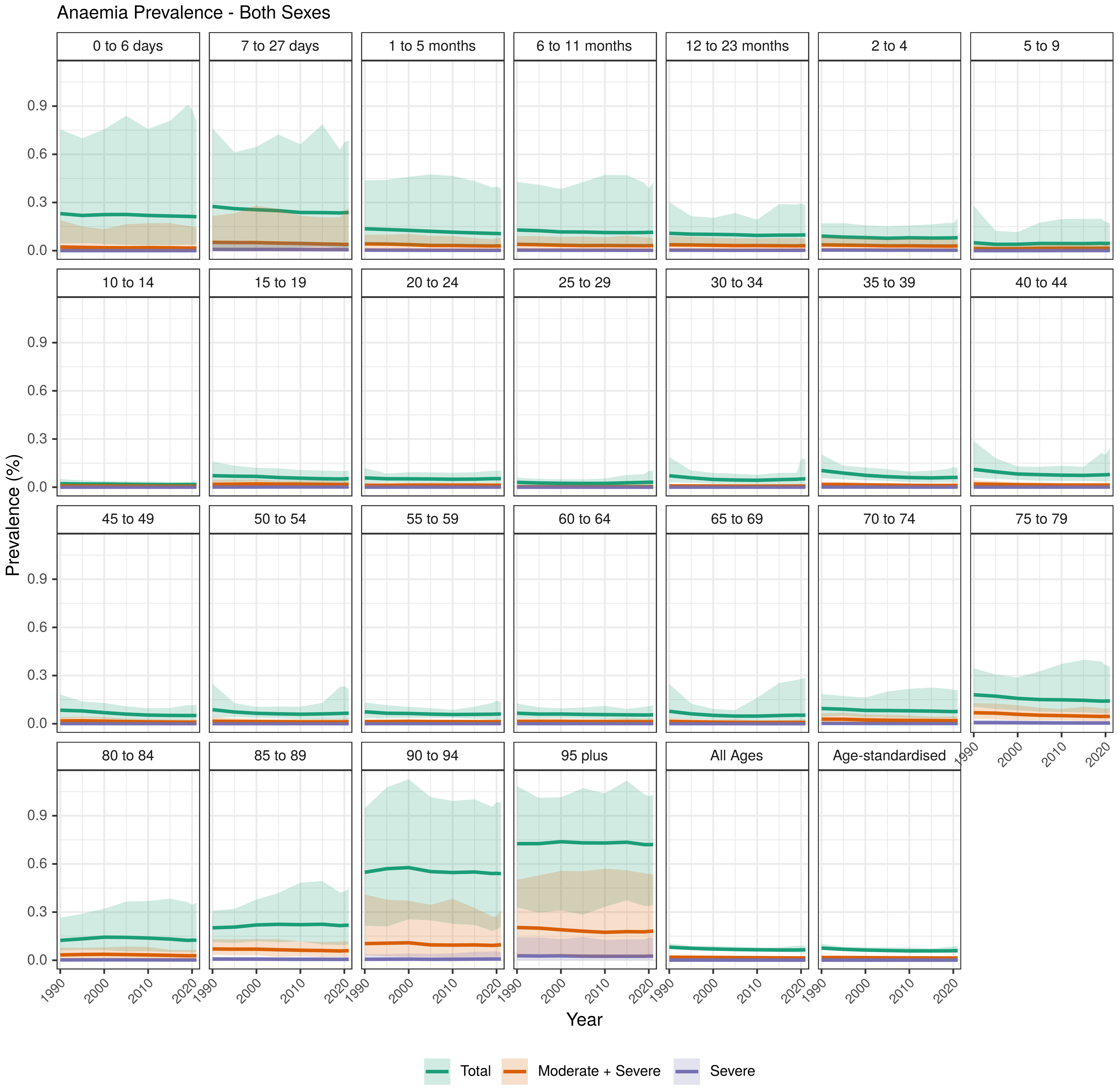
Anaemia Prevalence - Both Sexes
0 to 6 days
7 to 27 days
1 to 5 months
6 to 11 months
12 to 23 months
2 to 4
5 to 9
10 to 14
15 to 19
20 to 24
25 to 29
30 to 34
35 to 39
40 to 44
45 to 49
50 to 54
55 to 59
60 to 64
65 to 69
70 to 74
75 to 79
80 to 84
85 to 89
90 to 94
95 plus
All Ages
Age-standardised
0.0
0.3
0.6
0.9
Prevalence (%)
1990
2000
2010
2020
Year
Total
Moderate + Severe
Severe

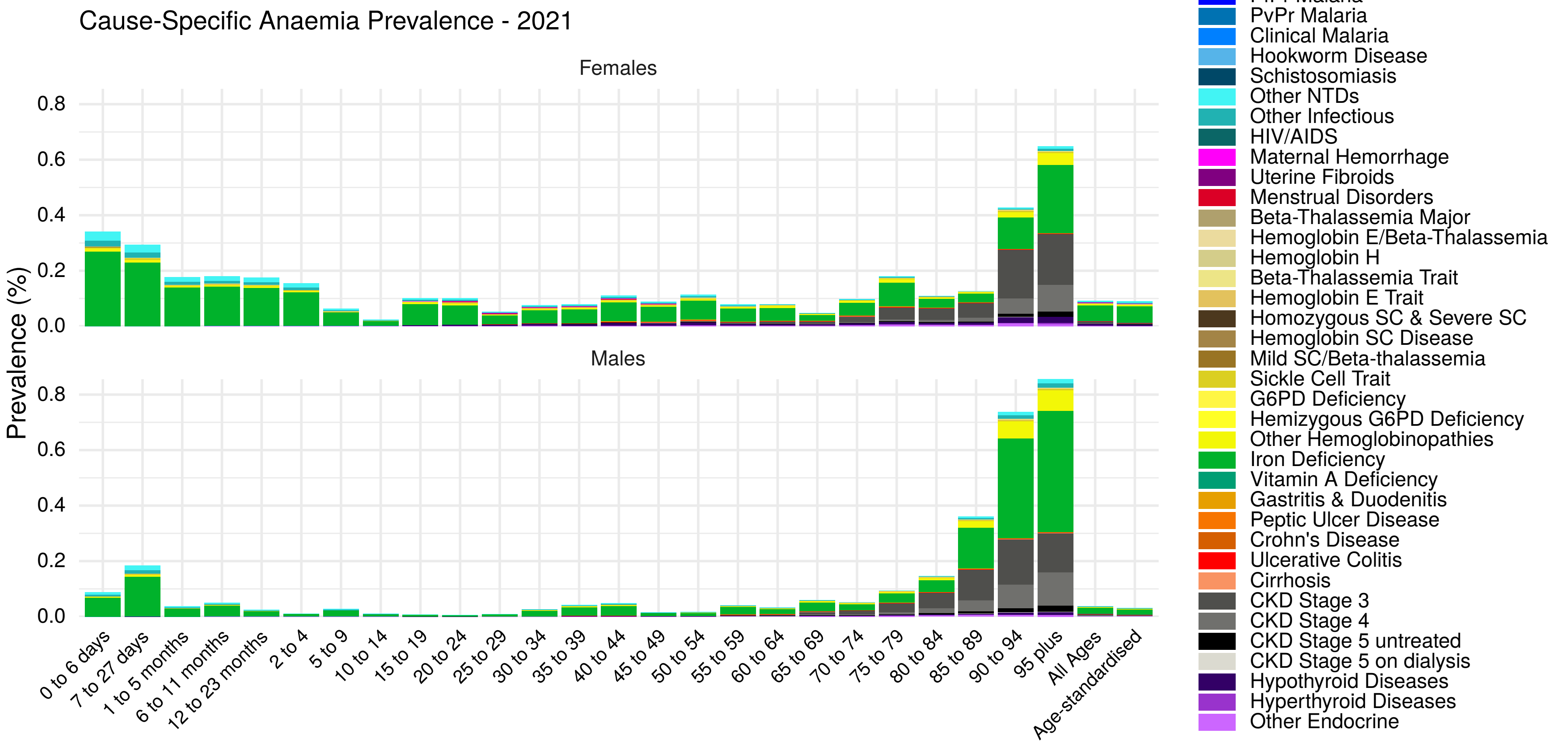
Cause-Specific Anaemia Prevalence - 2021
Females
Males
0.0
0.2
0.4
0.6
0.8
Prevalence (%)
0 to 6 days
7 to 27 days
1 to 5 months
6 to 11 months
12 to 23 months
2 to 4
5 to 9
10 to 14
15 to 19
20 to 24
25 to 29
30 to 34
35 to 39
40 to 44
45 to 49
50 to 54
55 to 59
60 to 64
65 to 69
70 to 74
75 to 79
80 to 84
85 to 89
90 to 94
95 plus
All Ages
Age-standardised
PfPr Malaria
PvPr Malaria
Clinical Malaria
Hookworm Disease
Schistosomiasis
Other NTDs
Other Infectious
HIV/AIDS
Maternal Hemorrhage
Uterine Fibroids
Menstrual Disorders
Beta-Thalassemia Major
Hemoglobin E/Beta-Thalassemia
Hemoglobin H
Beta-Thalassemia Trait
Hemoglobin E Trait
Homozygous SC & Severe SC
Hemoglobin SC Disease
Mild SC/Beta-thalassemia
Sickle Cell Trait
G6PD Deficiency
Hemizygous G6PD Deficiency
Other Hemoglobinopathies
Iron Deficiency
Vitamin A Deficiency
Gastritis & Duodenitis
Peptic Ulcer Disease
Crohn's Disease
Ulcerative Colitis
Cirrhosis
CKD Stage 3
CKD Stage 4
CKD Stage 5 untreated
CKD Stage 5 on dialysis
Hypothyroid Diseases
Hyperthyroid Diseases
Other Endocrine

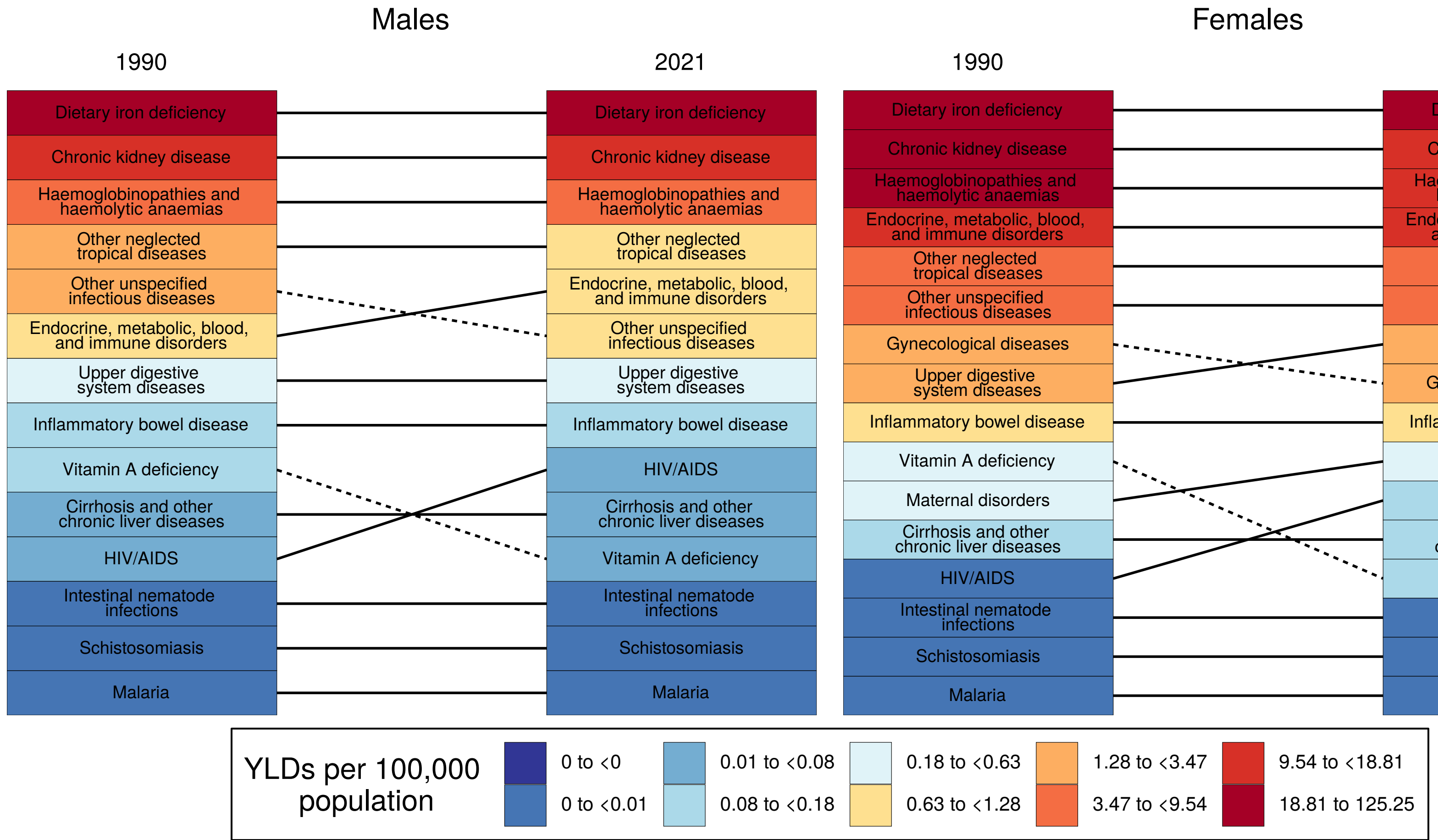
Males
Females
1990
2021
Dietary iron deficiency
Chronic kidney disease
Haemoglobinopathies and haemolytic anaemias
Other neglected tropical diseases
Other unspecified infectious diseases
Endocrine, metabolic, blood, and immune disorders
Upper digestive system diseases
Inflammatory bowel disease
Vitamin A deficiency
Cirrhosis and other chronic liver diseases
HIV/AIDS
Intestinal nematode infections
Schistosomiasis
Malaria
Gynecological diseases
Maternal disorders
YLDs per 100,000 population
0 to <0
0 to <0.01
0.01 to <0.08
0.08 to <0.18
0.18 to <0.63
0.63 to <1.28
1.28 to <3.47
3.47 to <9.54
9.54 to <18.81
18.81 to 125.25

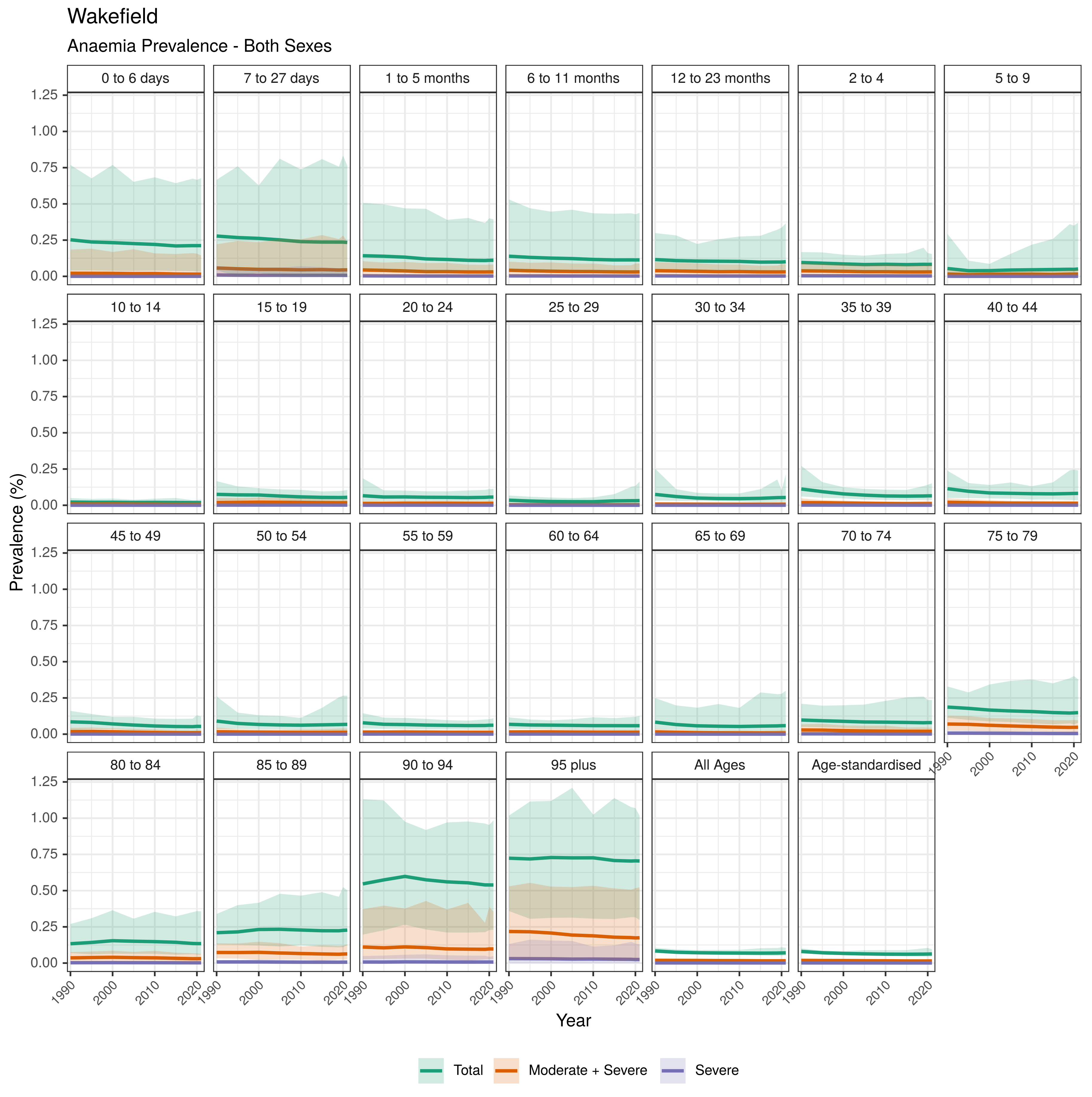
Wakefield
Anaemia Prevalence - Both Sexes
0 to 6 days
7 to 27 days
1 to 5 months
6 to 11 months
12 to 23 months
2 to 4
5 to 9
10 to 14
15 to 19
20 to 24
25 to 29
30 to 34
35 to 39
40 to 44
45 to 49
50 to 54
55 to 59
60 to 64
65 to 69
70 to 74
75 to 79
80 to 84
85 to 89
90 to 94
95 plus
All Ages
Age-standardised
Prevalence (%)
Year
Total
Moderate + Severe
Severe

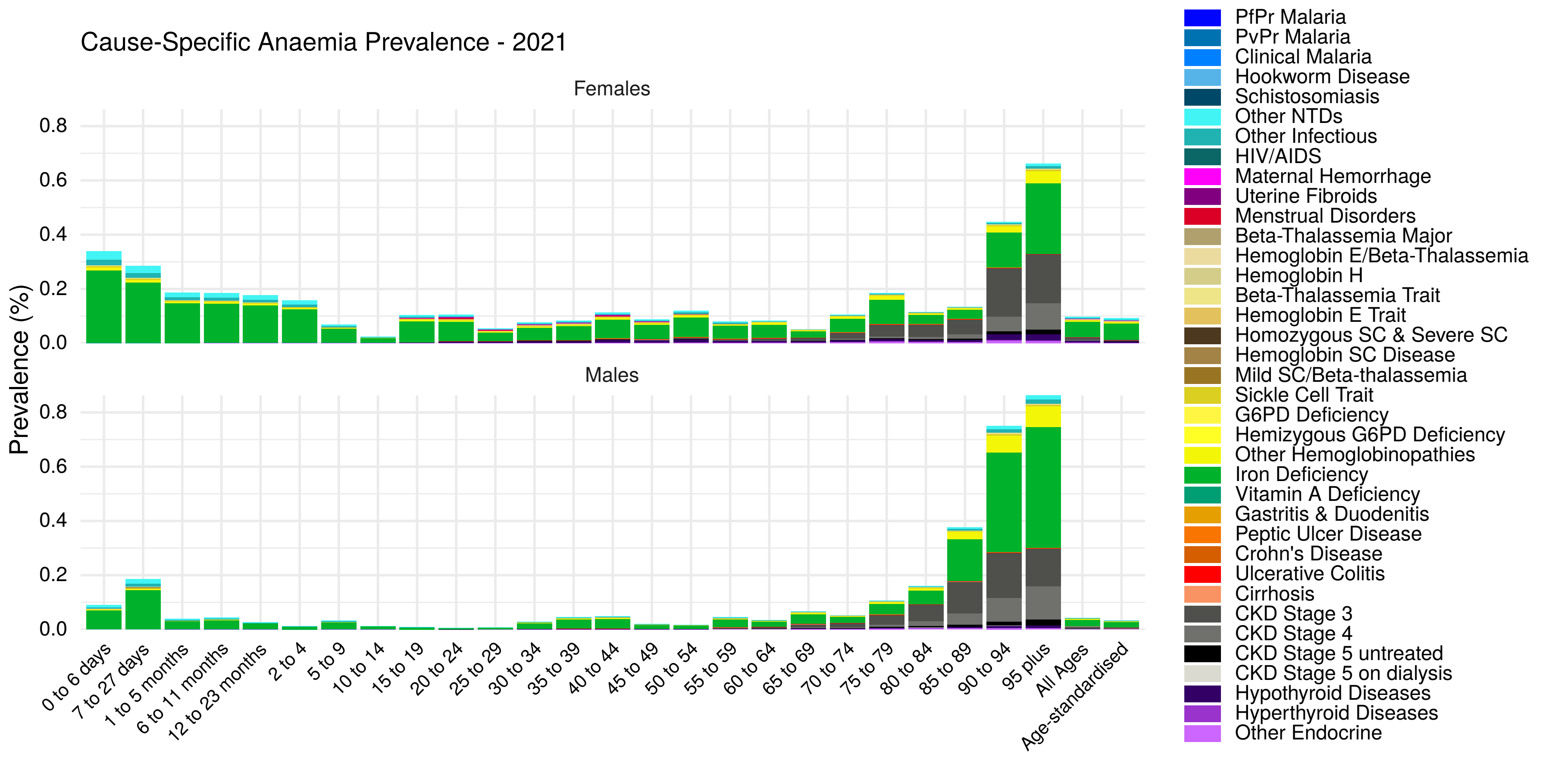
Cause-Specific Anaemia Prevalence - 2021
Females
Males
Prevalence (%)
PfPr Malaria
PvPr Malaria
Clinical Malaria
Hookworm Disease
Schistosomiasis
Other NTDs
Other Infectious
HIV/AIDS
Maternal Hemorrhage
Uterine Fibroids
Menstrual Disorders
Beta-Thalassemia Major
Hemoglobin E/Beta-Thalassemia
Hemoglobin H
Beta-Thalassemia Trait
Hemoglobin E Trait
Homozygous SC & Severe SC
Hemoglobin SC Disease
Mild SC/Beta-thalassemia
Sickle Cell Trait
G6PD Deficiency
Hemizygous G6PD Deficiency
Other Hemoglobinopathies
Iron Deficiency
Vitamin A Deficiency
Gastritis & Duodenitis
Peptic Ulcer Disease
Crohn's Disease
Ulcerative Colitis
Cirrhosis
CKD Stage 3
CKD Stage 4
CKD Stage 5 untreated
CKD Stage 5 on dialysis
Hypothyroid Diseases
Hyperthyroid Diseases
Other Endocrine

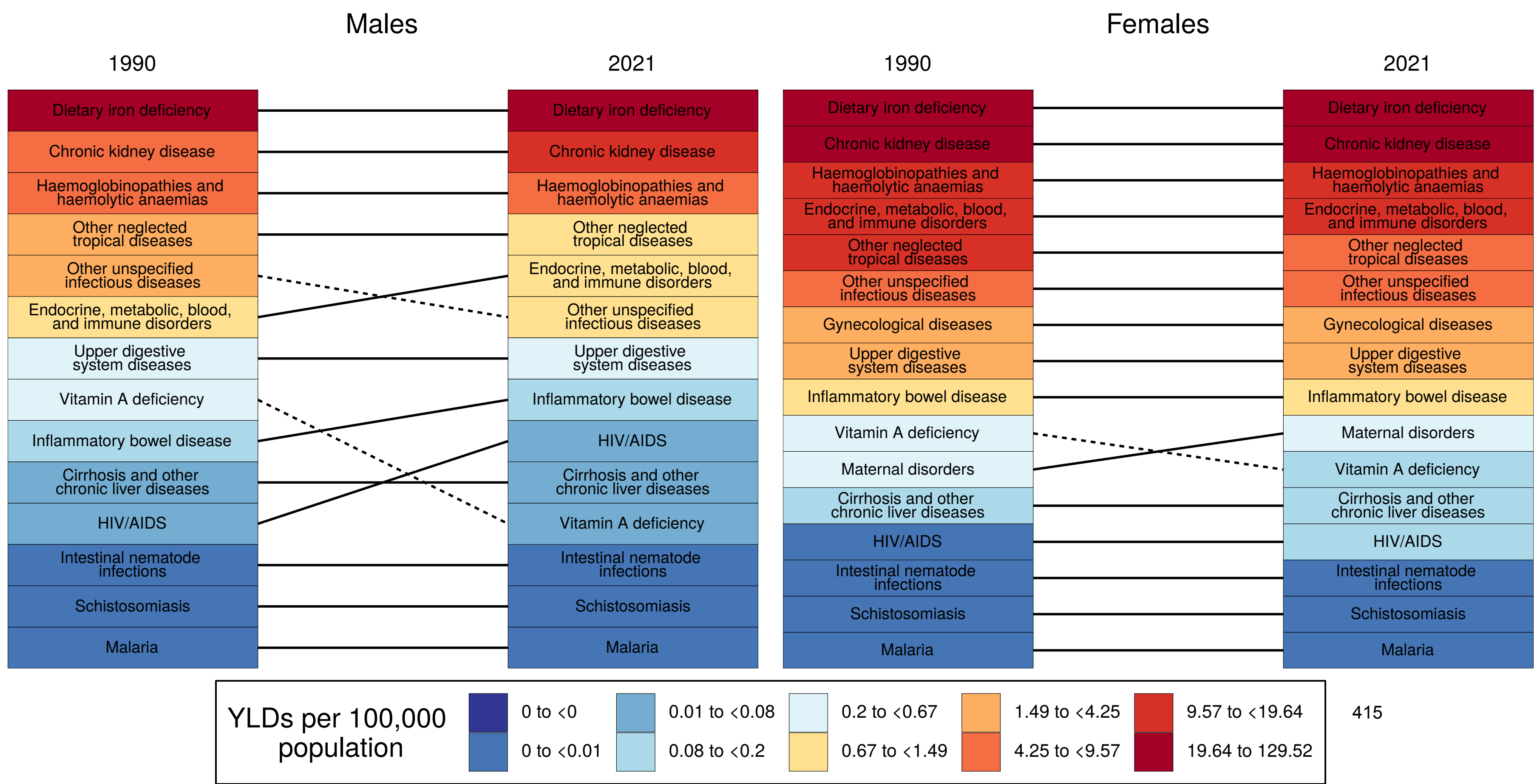
Males
Females
1990
2021
Dietary iron deficiency
Chronic kidney disease
Haemoglobinopathies and haemolytic anaemias
Other neglected tropical diseases
Other unspecified infectious diseases
Endocrine, metabolic, blood, and immune disorders
Upper digestive system diseases
Vitamin A deficiency
Inflammatory bowel disease
Cirrhosis and other chronic liver diseases
HIV/AIDS
Intestinal nematode infections
Schistosomiasis
Malaria
Gynecological diseases
Maternal disorders
YLDs per 100,000 population
0 to <0
0 to <0.01
0.01 to <0.08
0.08 to <0.2
0.2 to <0.67
0.67 to <1.49
1.49 to <4.25
4.25 to <9.57
9.57 to <19.64
19.64 to 129.52

York

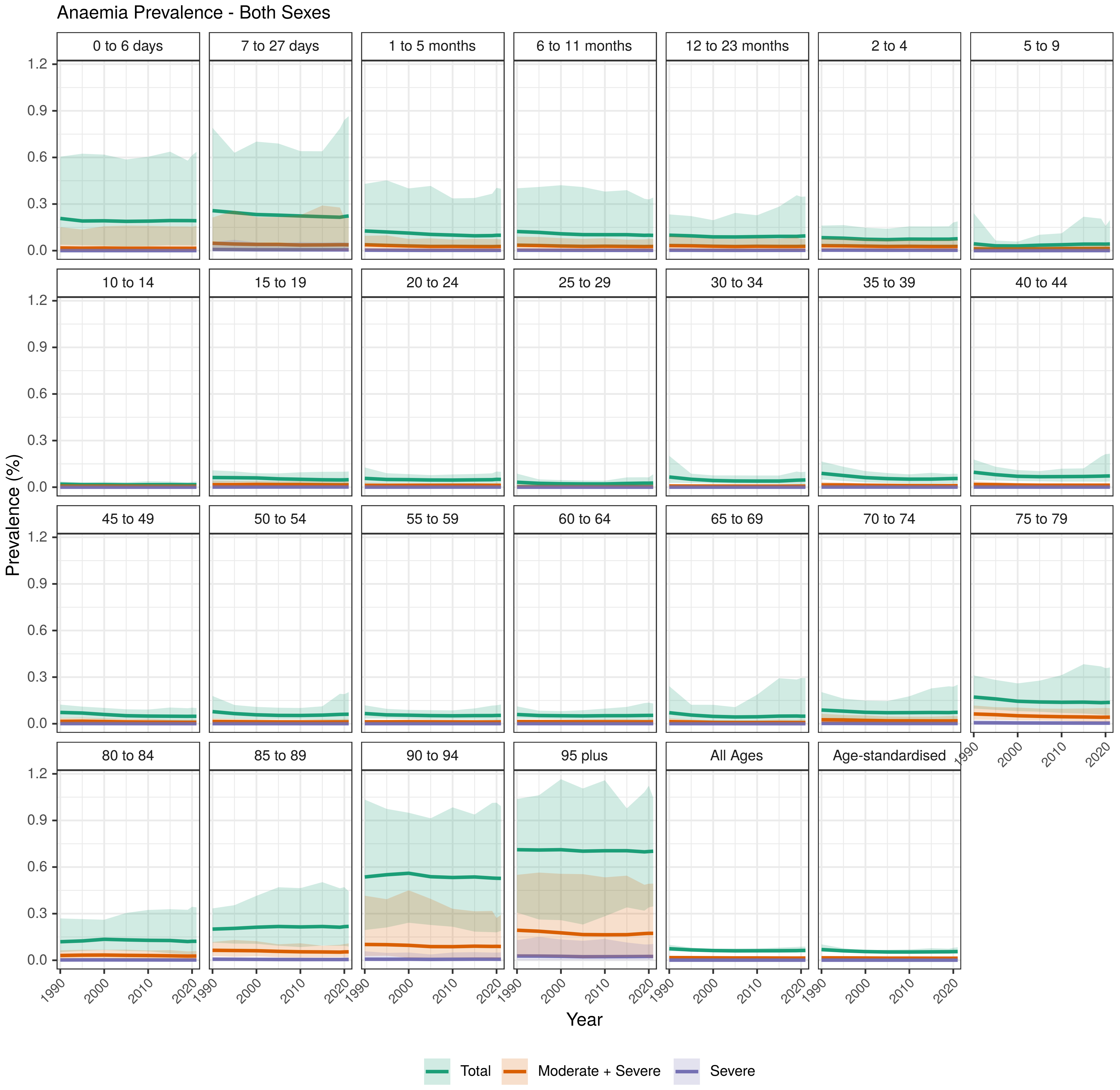
Anaemia Prevalence - Both Sexes
0 to 6 days
7 to 27 days
1 to 5 months
6 to 11 months
12 to 23 months
2 to 4
5 to 9
10 to 14
15 to 19
20 to 24
25 to 29
30 to 34
35 to 39
40 to 44
45 to 49
50 to 54
55 to 59
60 to 64
65 to 69
70 to 74
75 to 79
80 to 84
85 to 89
90 to 94
95 plus
All Ages
Age-standardised
Prevalence (%)
Year
Total
Moderate + Severe
Severe

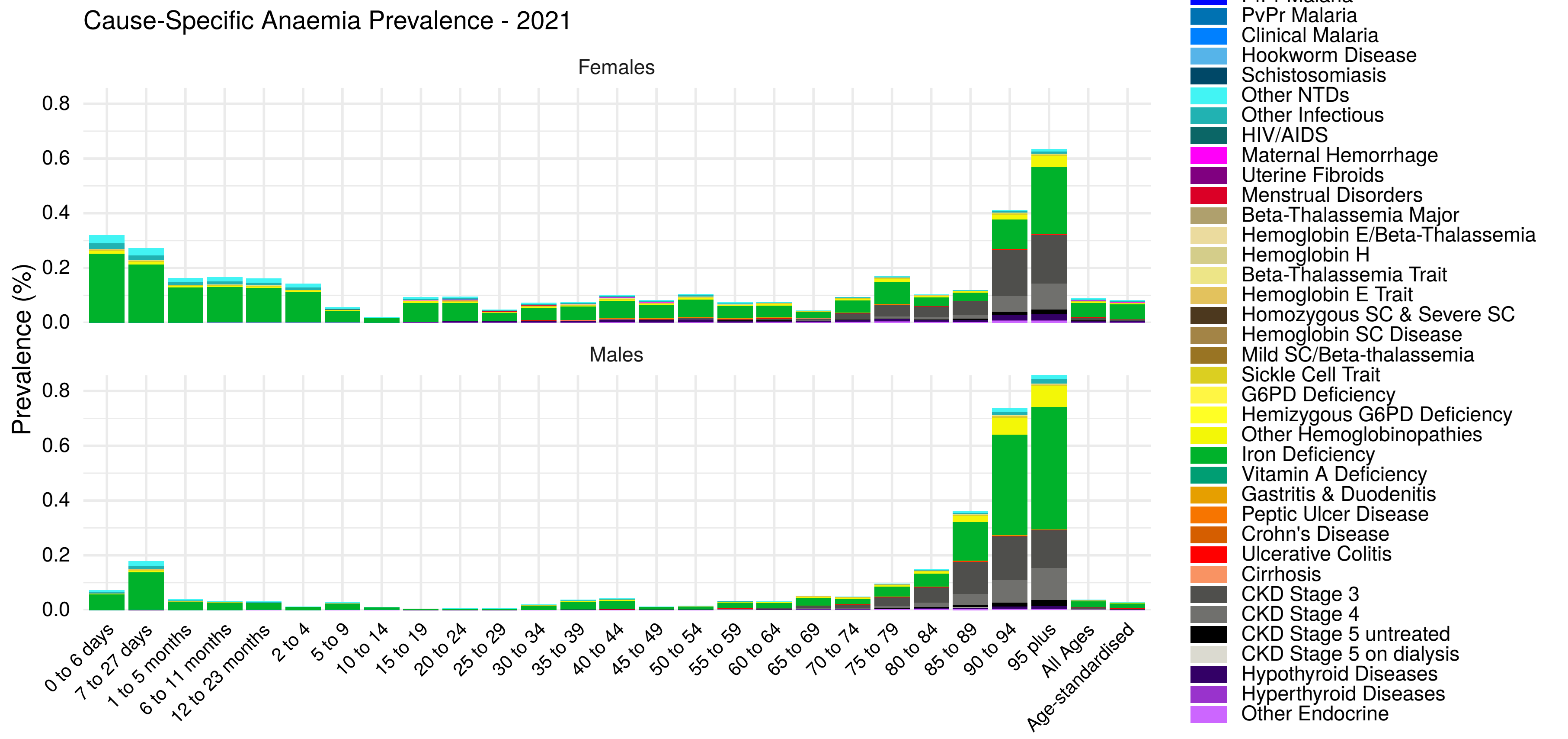
Cause-Specific Anaemia Prevalence - 2021
Females
Males
Prevalence (%)
PfPr Malaria
PvPr Malaria
Clinical Malaria
Hookworm Disease
Schistosomiasis
Other NTDs
Other Infectious
HIV/AIDS
Maternal Hemorrhage
Uterine Fibroids
Menstrual Disorders
Beta-Thalassemia Major
Hemoglobin E/Beta-Thalassemia
Hemoglobin H
Beta-Thalassemia Trait
Hemoglobin E Trait
Homozygous SC & Severe SC
Hemoglobin SC Disease
Mild SC/Beta-thalassemia
Sickle Cell Trait
G6PD Deficiency
Hemizygous G6PD Deficiency
Other Hemoglobinopathies
Iron Deficiency
Vitamin A Deficiency
Gastritis & Duodenitis
Peptic Ulcer Disease
Crohn's Disease
Ulcerative Colitis
Cirrhosis
CKD Stage 3
CKD Stage 4
CKD Stage 5 untreated
CKD Stage 5 on dialysis
Hypothyroid Diseases
Hyperthyroid Diseases
Other Endocrine

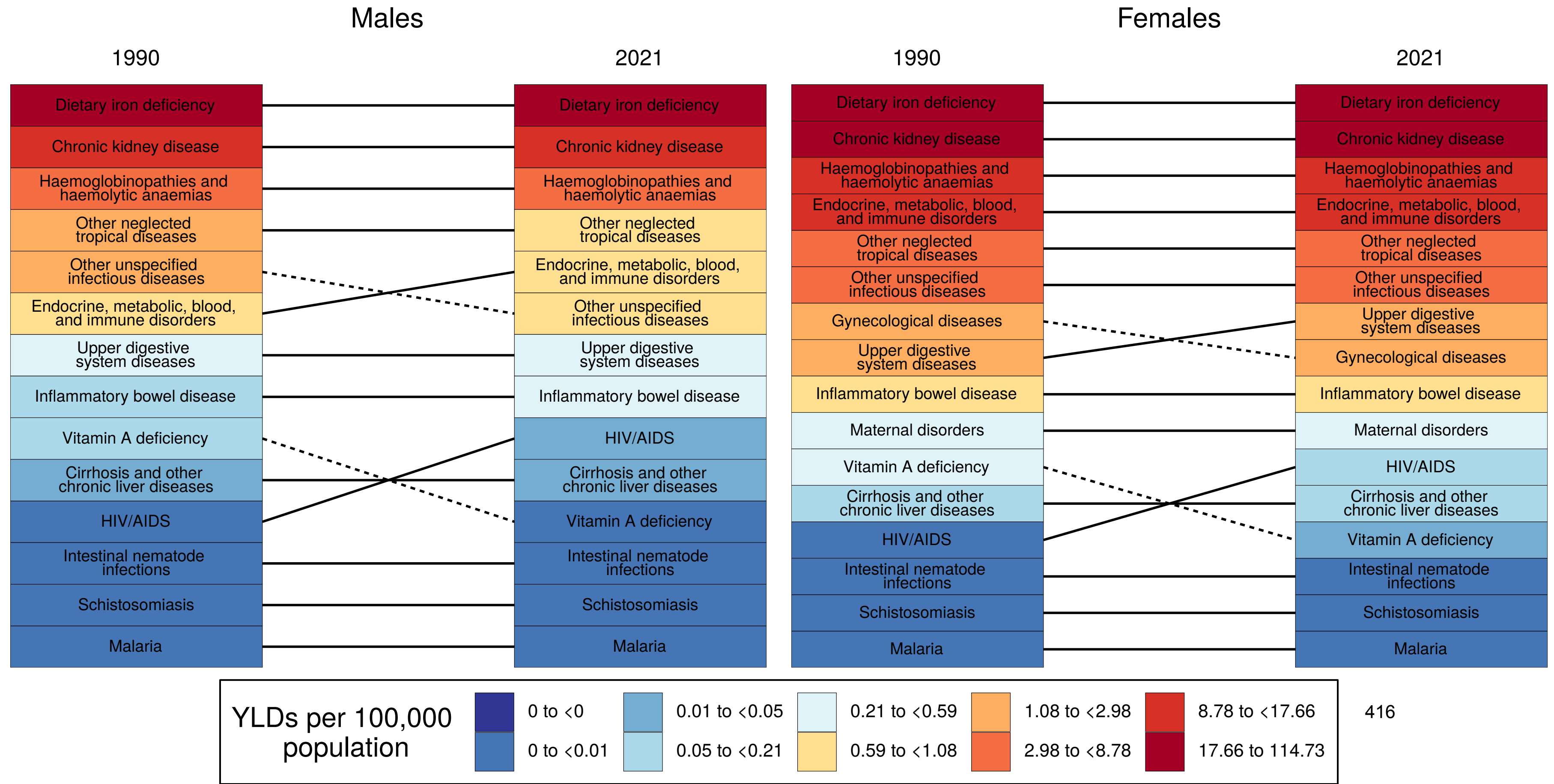
Males
Females
1990
2021
Dietary iron deficiency
Chronic kidney disease
Haemoglobinopathies and haemolytic anaemias
Other neglected tropical diseases
Other unspecified infectious diseases
Endocrine, metabolic, blood, and immune disorders
Upper digestive system diseases
Inflammatory bowel disease
Vitamin A deficiency
Cirrhosis and other chronic liver diseases
HIV/AIDS
Intestinal nematode infections
Schistosomiasis
Malaria
Gynecological diseases
Maternal disorders
YLDs per 100,000 population
0 to <0
0 to <0.01
0.01 to <0.05
0.05 to <0.21
0.21 to <0.59
0.59 to <1.08
1.08 to <2.98
2.98 to <8.78
8.78 to <17.66
17.66 to 114.73

Northern Ireland

Anaemia Prevalence - Both Sexes

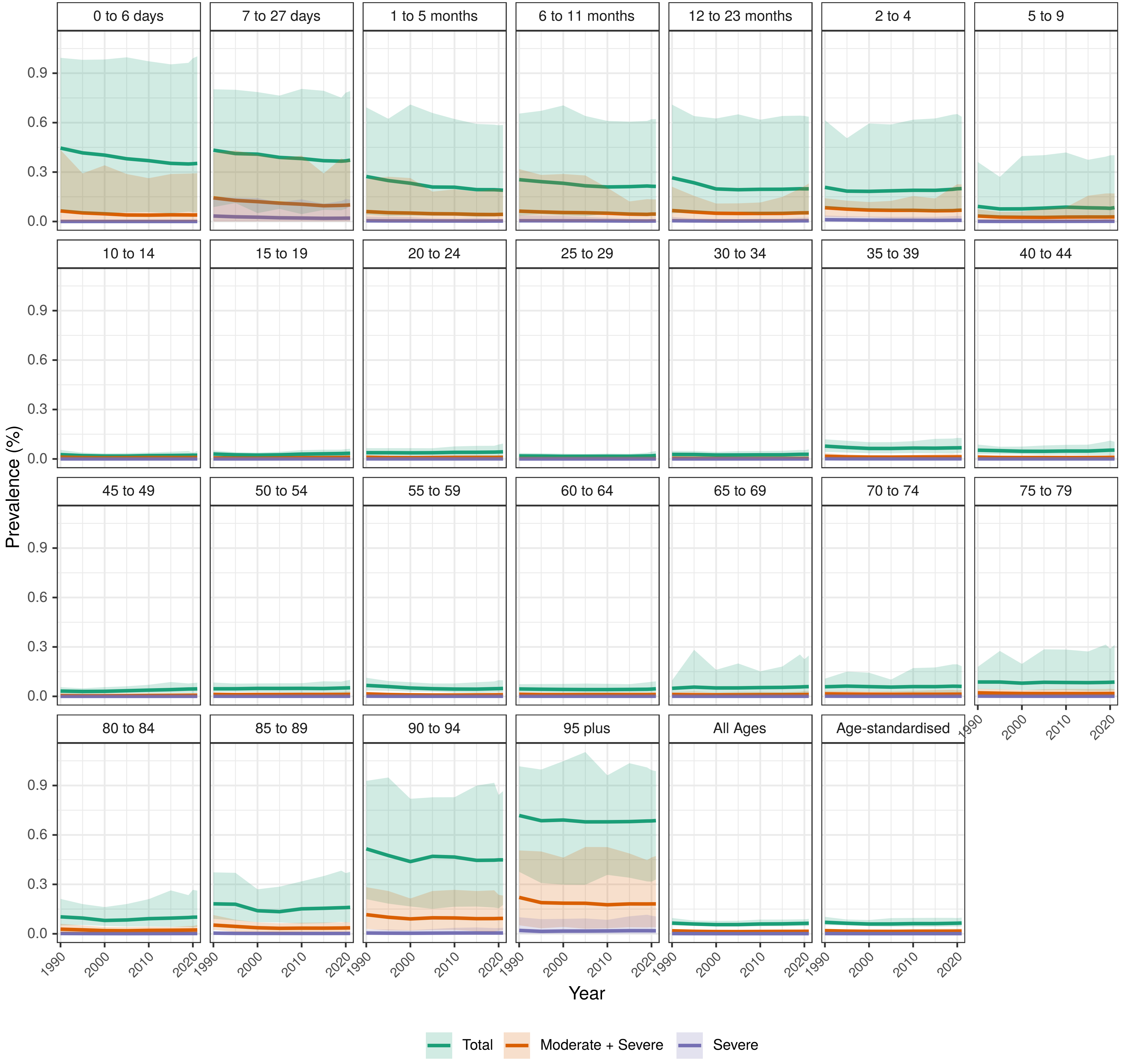

Cause-Specific Anaemia Prevalence - 2021

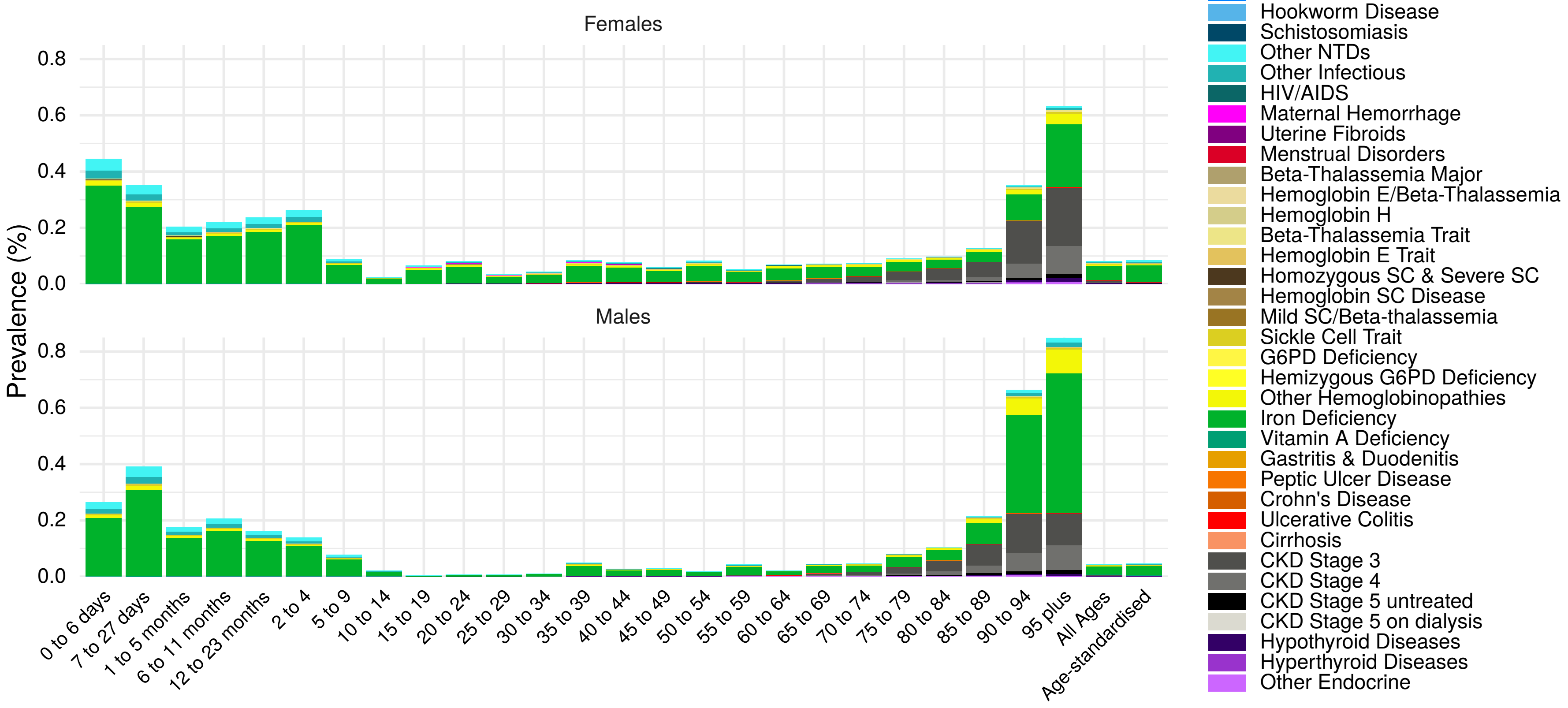

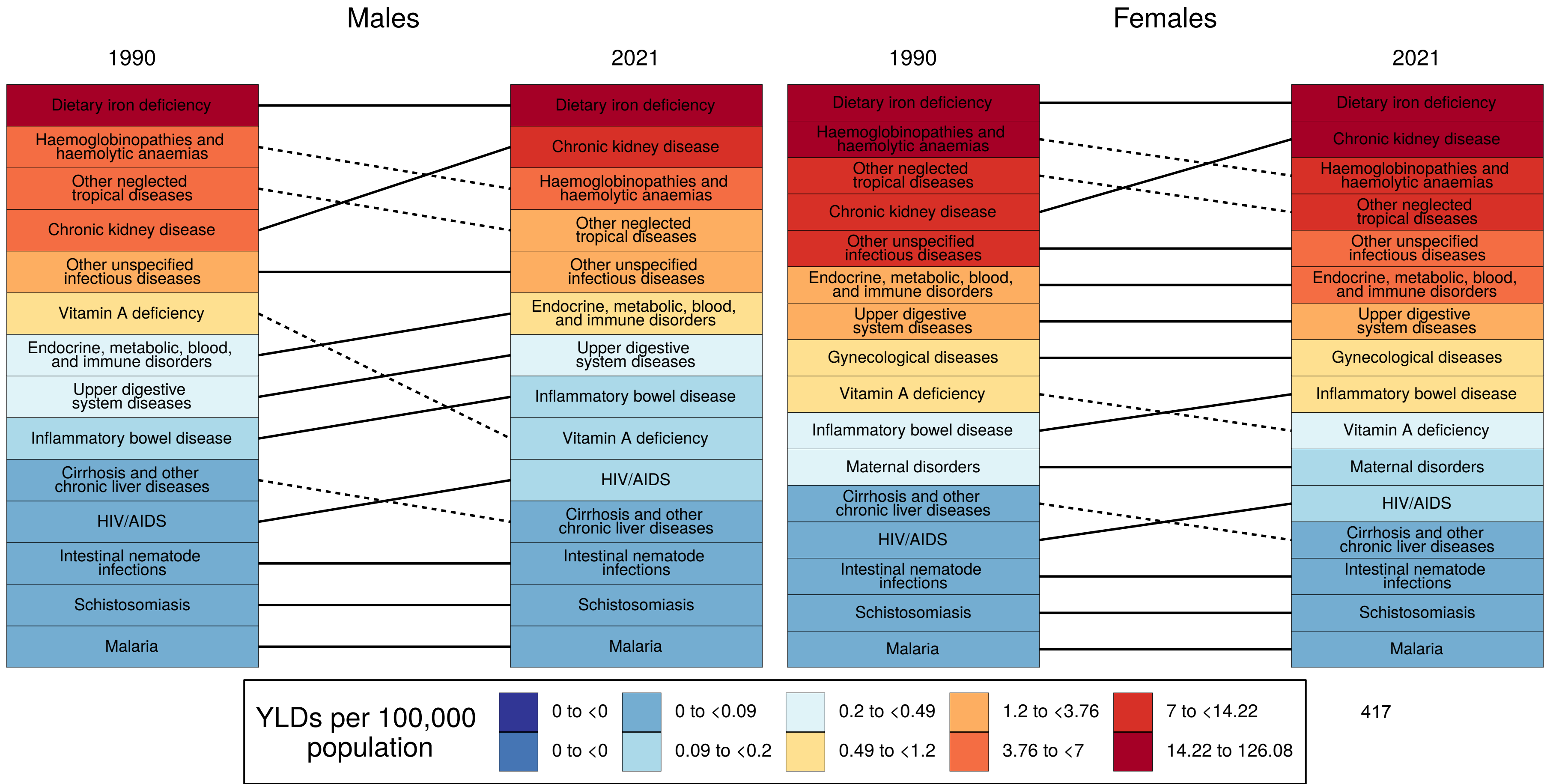

Scotland

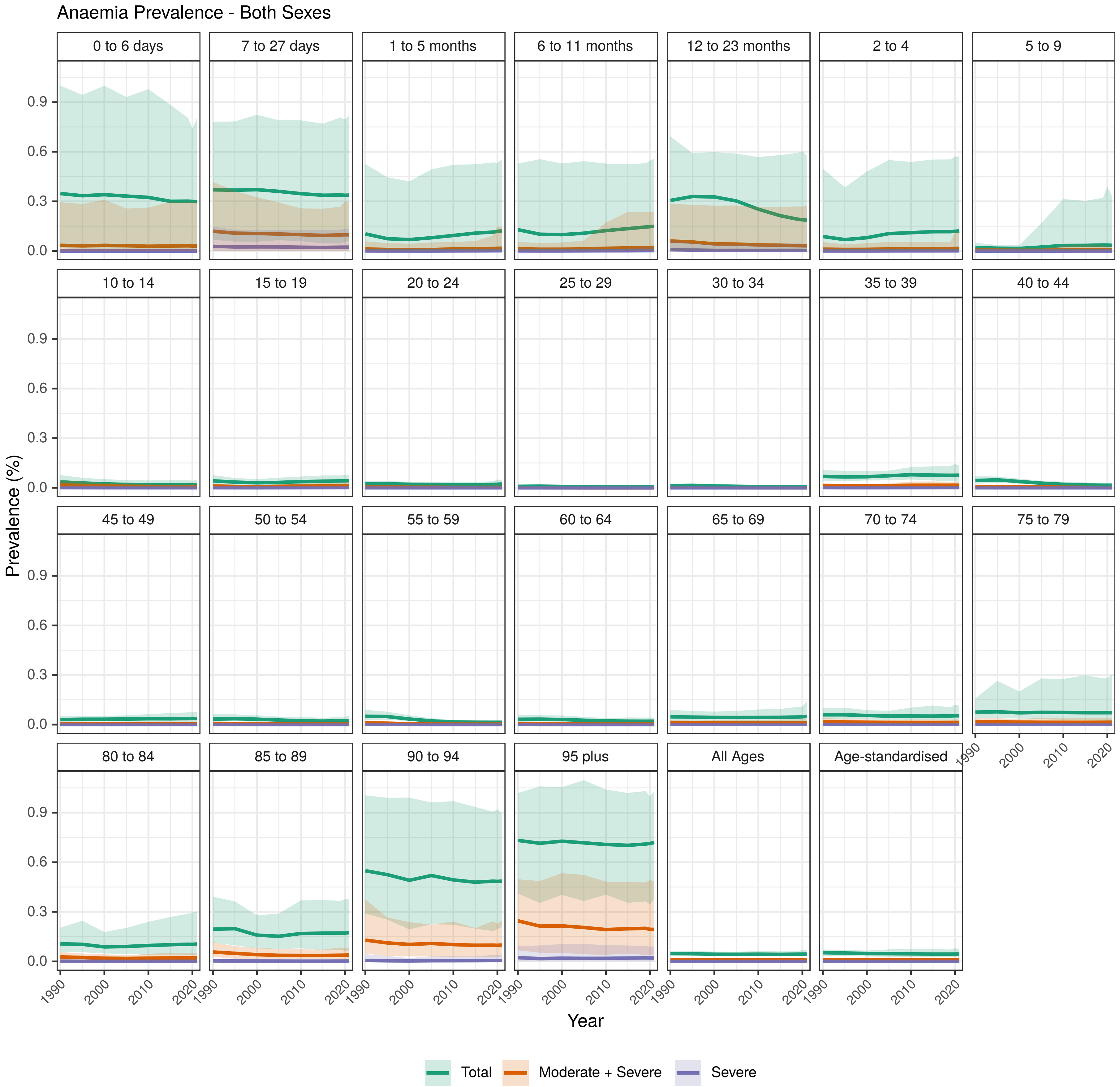
Anaemia Prevalence - Both Sexes
0 to 6 days
7 to 27 days
1 to 5 months
6 to 11 months
12 to 23 months
2 to 4
5 to 9
10 to 14
15 to 19
20 to 24
25 to 29
30 to 34
35 to 39
40 to 44
45 to 49
50 to 54
55 to 59
60 to 64
65 to 69
70 to 74
75 to 79
80 to 84
85 to 89
90 to 94
95 plus
All Ages
Age-standardised
Prevalence (%)
Year
Total
Moderate + Severe
Severe

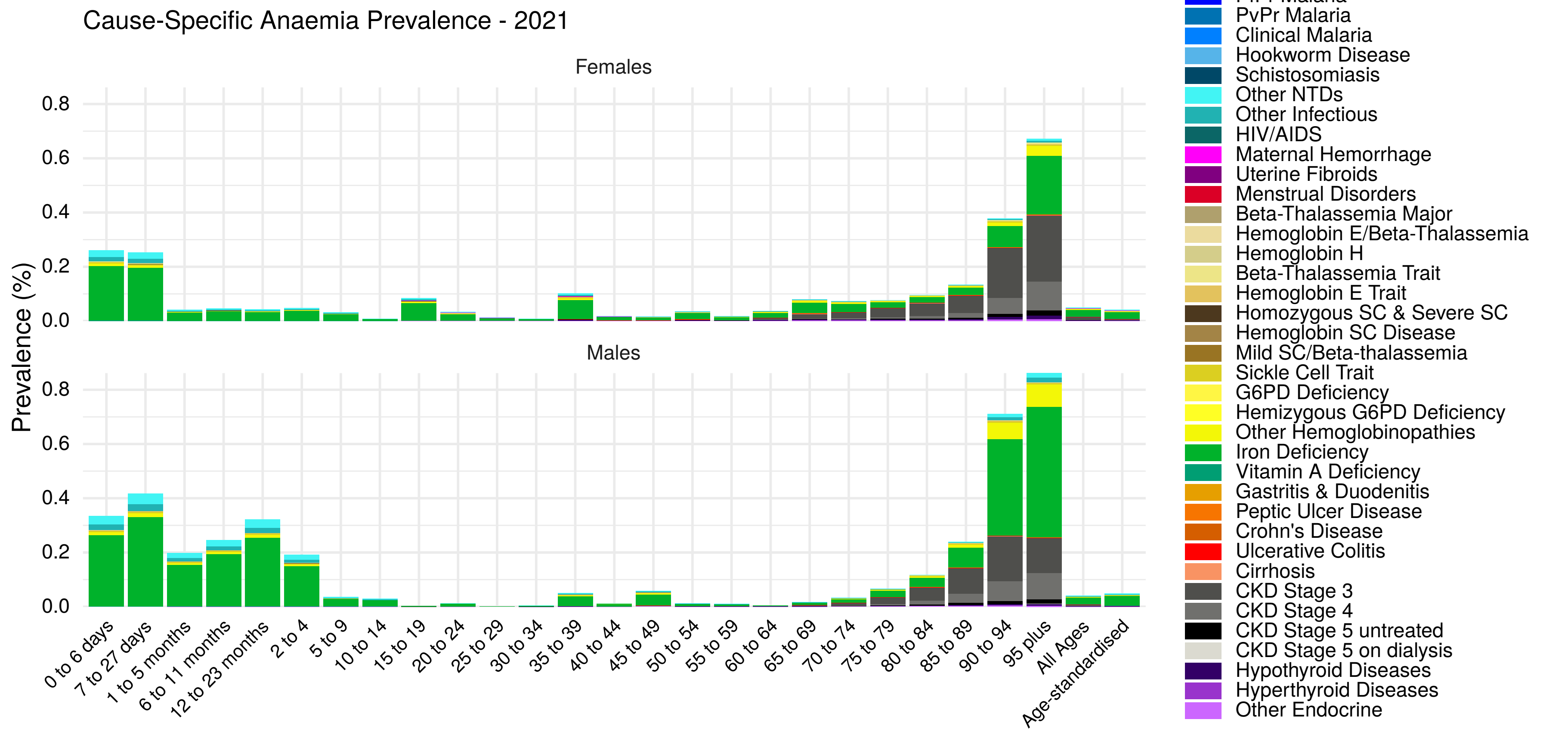
Cause-Specific Anaemia Prevalence - 2021
Females
Males
Prevalence (%)
PfPr Malaria
PvPr Malaria
Clinical Malaria
Hookworm Disease
Schistosomiasis
Other NTDs
Other Infectious
HIV/AIDS
Maternal Hemorrhage
Uterine Fibroids
Menstrual Disorders
Beta-Thalassemia Major
Hemoglobin E/Beta-Thalassemia
Hemoglobin H
Beta-Thalassemia Trait
Hemoglobin E Trait
Homozygous SC & Severe SC
Hemoglobin SC Disease
Mild SC/Beta-thalassemia
Sickle Cell Trait
G6PD Deficiency
Hemizygous G6PD Deficiency
Other Hemoglobinopathies
Iron Deficiency
Vitamin A Deficiency
Gastritis & Duodenitis
Peptic Ulcer Disease
Crohn's Disease
Ulcerative Colitis
Cirrhosis
CKD Stage 3
CKD Stage 4
CKD Stage 5 untreated
CKD Stage 5 on dialysis
Hypothyroid Diseases
Hyperthyroid Diseases
Other Endocrine

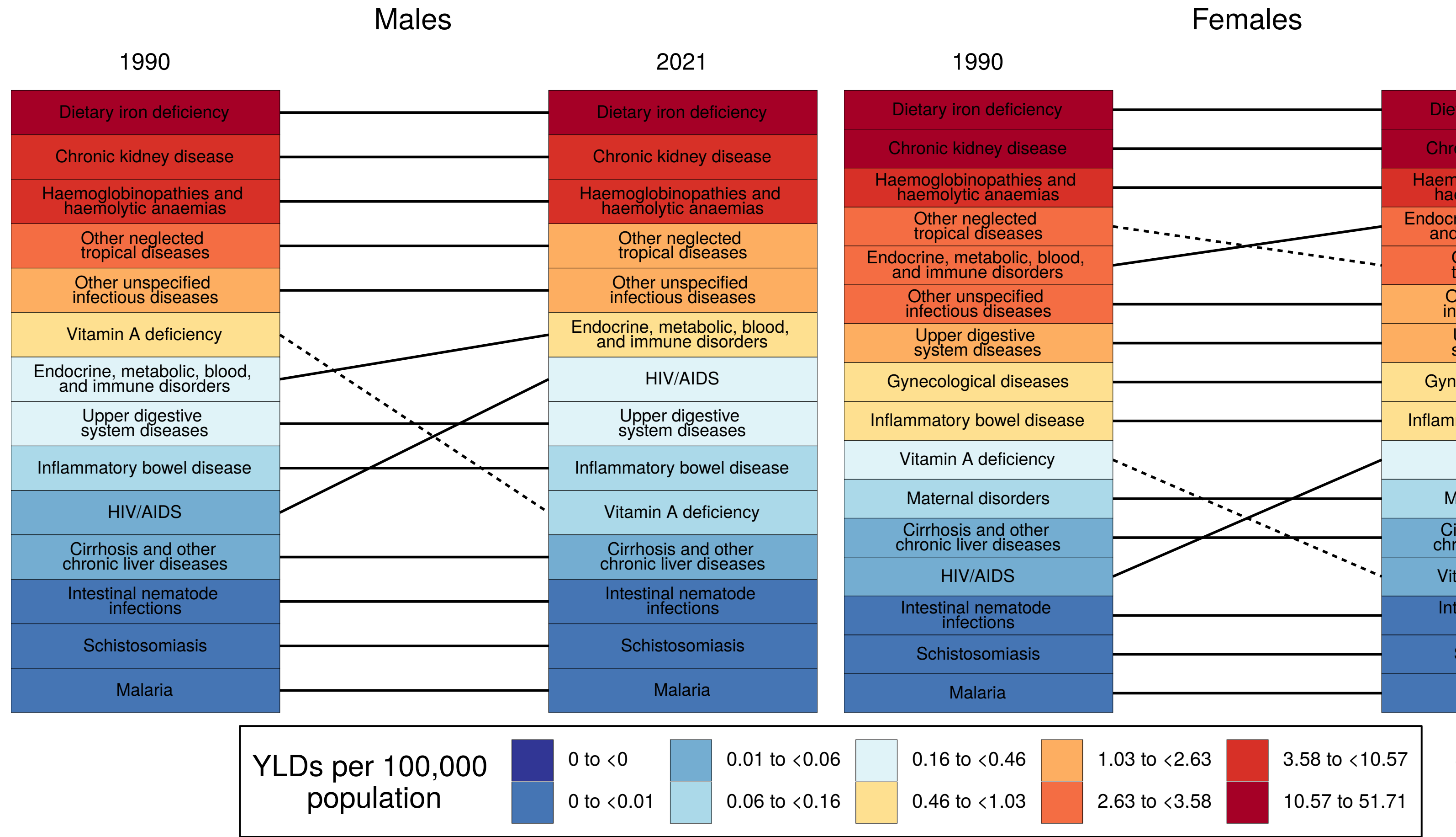
Males
Females
1990
2021
Dietary iron deficiency
Chronic kidney disease
Haemoglobinopathies and haemolytic anaemias
Other neglected tropical diseases
Other unspecified infectious diseases
Vitamin A deficiency
Endocrine, metabolic, blood, and immune disorders
Upper digestive system diseases
Inflammatory bowel disease
HIV/AIDS
Cirrhosis and other chronic liver diseases
Intestinal nematode infections
Schistosomiasis
Malaria
Gynecological diseases
Maternal disorders
YLDs per 100,000 population
0 to <0
0 to <0.01
0.01 to <0.06
0.06 to <0.16
0.16 to <0.46
0.46 to <1.03
1.03 to <2.63
2.63 to <3.58
3.58 to <10.57
10.57 to 51.71

Wales

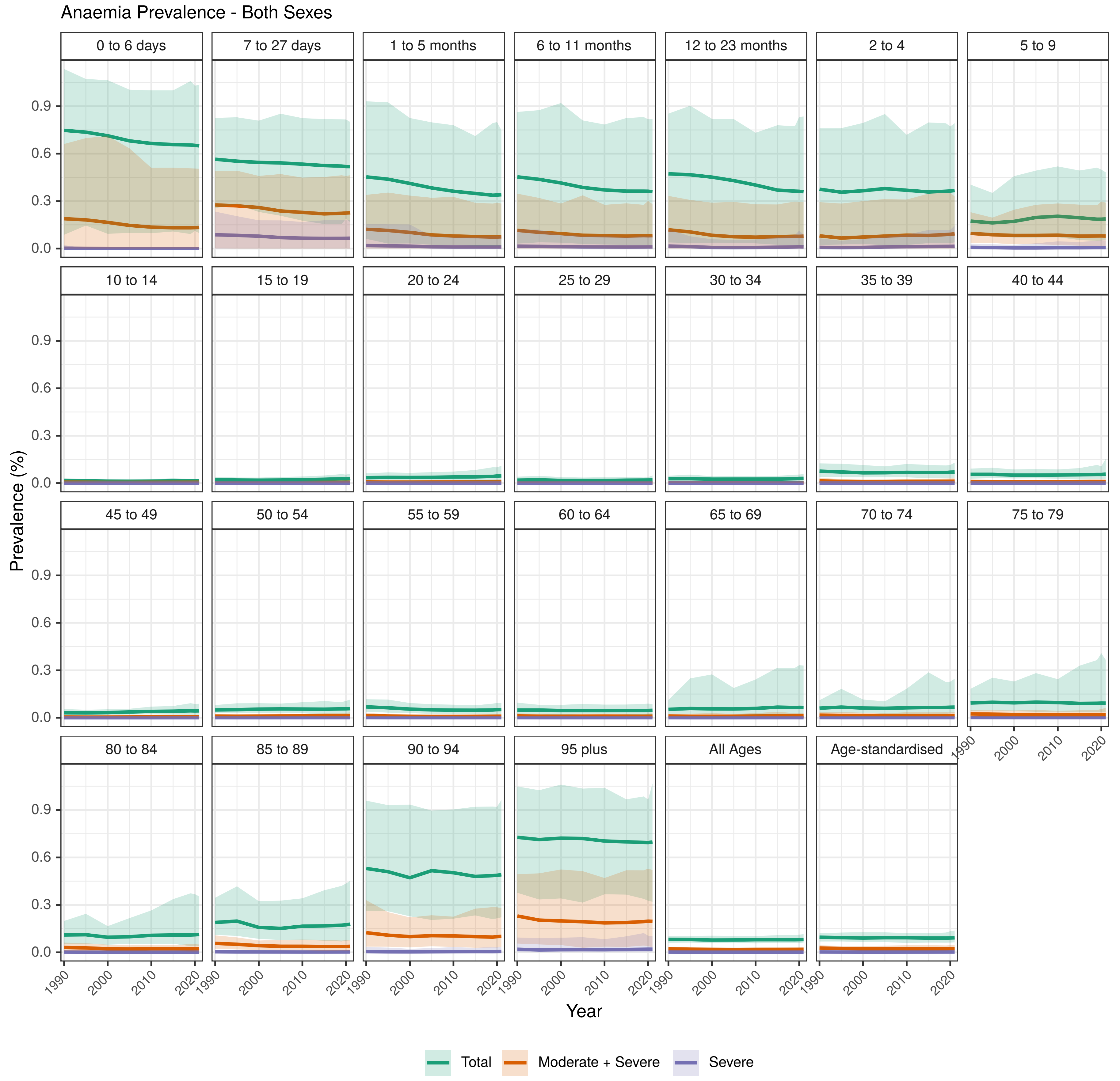
Anaemia Prevalence - Both Sexes
0 to 6 days
7 to 27 days
1 to 5 months
6 to 11 months
12 to 23 months
2 to 4
5 to 9
10 to 14
15 to 19
20 to 24
25 to 29
30 to 34
35 to 39
40 to 44
45 to 49
50 to 54
55 to 59
60 to 64
65 to 69
70 to 74
75 to 79
80 to 84
85 to 89
90 to 94
95 plus
All Ages
Age-standardised
Prevalence (%)
0.0
0.3
0.6
0.9
1990
2000
2010
2020
Year
Total
Moderate + Severe
Severe

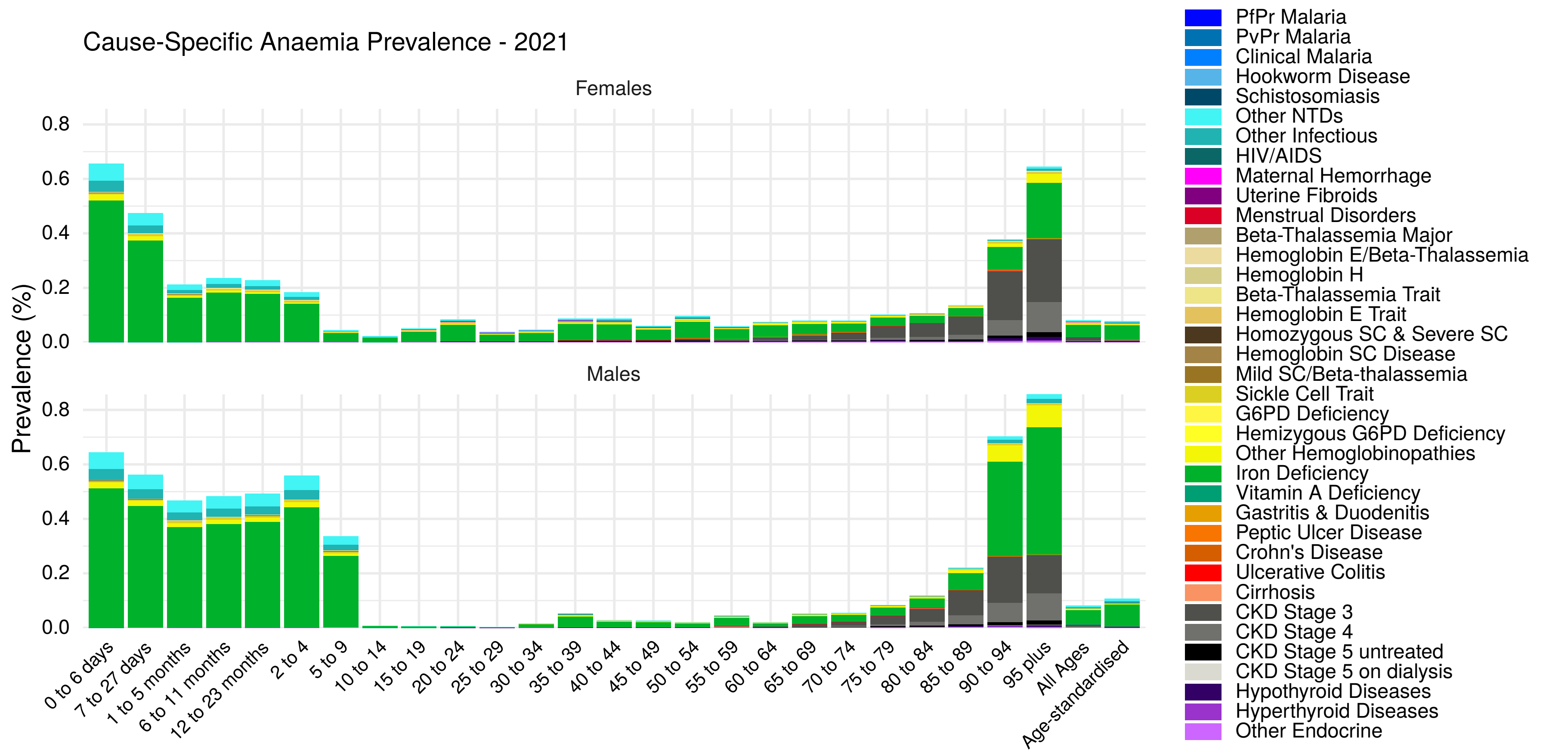
Cause-Specific Anaemia Prevalence - 2021
Females
Males
Prevalence (%)
0.0
0.2
0.4
0.6
0.8
0 to 6 days
7 to 27 days
1 to 5 months
6 to 11 months
12 to 23 months
2 to 4
5 to 9
10 to 14
15 to 19
20 to 24
25 to 29
30 to 34
35 to 39
40 to 44
45 to 49
50 to 54
55 to 59
60 to 64
65 to 69
70 to 74
75 to 79
80 to 84
85 to 89
90 to 94
95 plus
All Ages
Age-standardised
PfPr Malaria
PvPr Malaria
Clinical Malaria
Hookworm Disease
Schistosomiasis
Other NTDs
Other Infectious
HIV/AIDS
Maternal Hemorrhage
Uterine Fibroids
Menstrual Disorders
Beta-Thalassemia Major
Hemoglobin E/Beta-Thalassemia
Hemoglobin H
Beta-Thalassemia Trait
Hemoglobin E Trait
Homozygous SC & Severe SC
Hemoglobin SC Disease
Mild SC/Beta-thalassemia
Sickle Cell Trait
G6PD Deficiency
Hemizygous G6PD Deficiency
Other Hemoglobinopathies
Iron Deficiency
Vitamin A Deficiency
Gastritis & Duodenitis
Peptic Ulcer Disease
Crohn's Disease
Ulcerative Colitis
Cirrhosis
CKD Stage 3
CKD Stage 4
CKD Stage 5 untreated
CKD Stage 5 on dialysis
Hypothyroid Diseases
Hyperthyroid Diseases
Other Endocrine

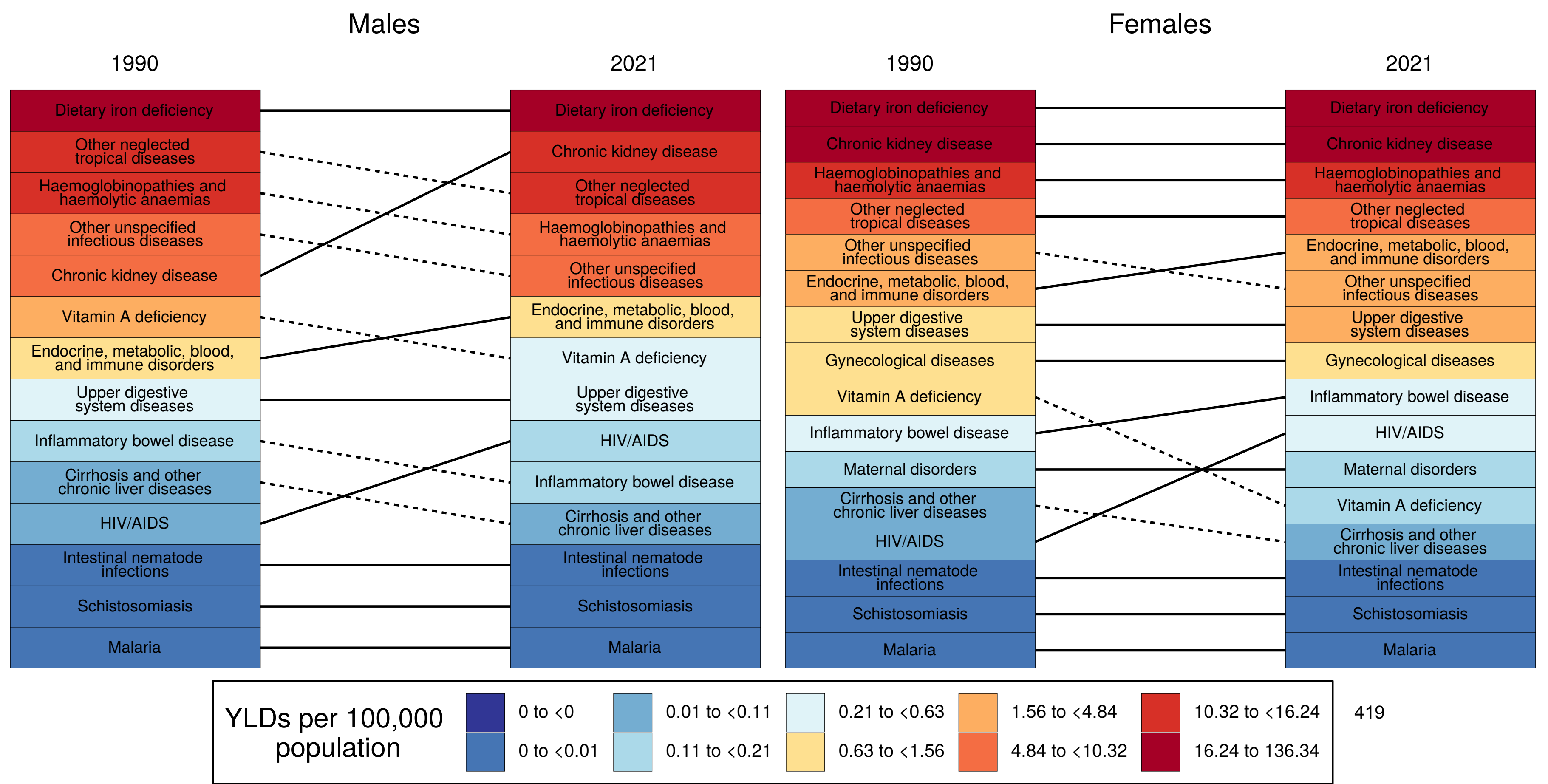
Males
Females
1990
2021
Dietary iron deficiency
Other neglected tropical diseases
Haemoglobinopathies and haemolytic anaemias
Other unspecified infectious diseases
Chronic kidney disease
Vitamin A deficiency
Endocrine, metabolic, blood, and immune disorders
Upper digestive system diseases
Inflammatory bowel disease
Cirrhosis and other chronic liver diseases
HIV/AIDS
Intestinal nematode infections
Schistosomiasis
Malaria
Gynecological diseases
Maternal disorders
YLDs per 100,000 population
0 to <0
0 to <0.01
0.01 to <0.11
0.11 to <0.21
0.21 to <0.63
0.63 to <1.56
1.56 to <4.84
4.84 to <10.32
10.32 to <16.24
16.24 to 136.34

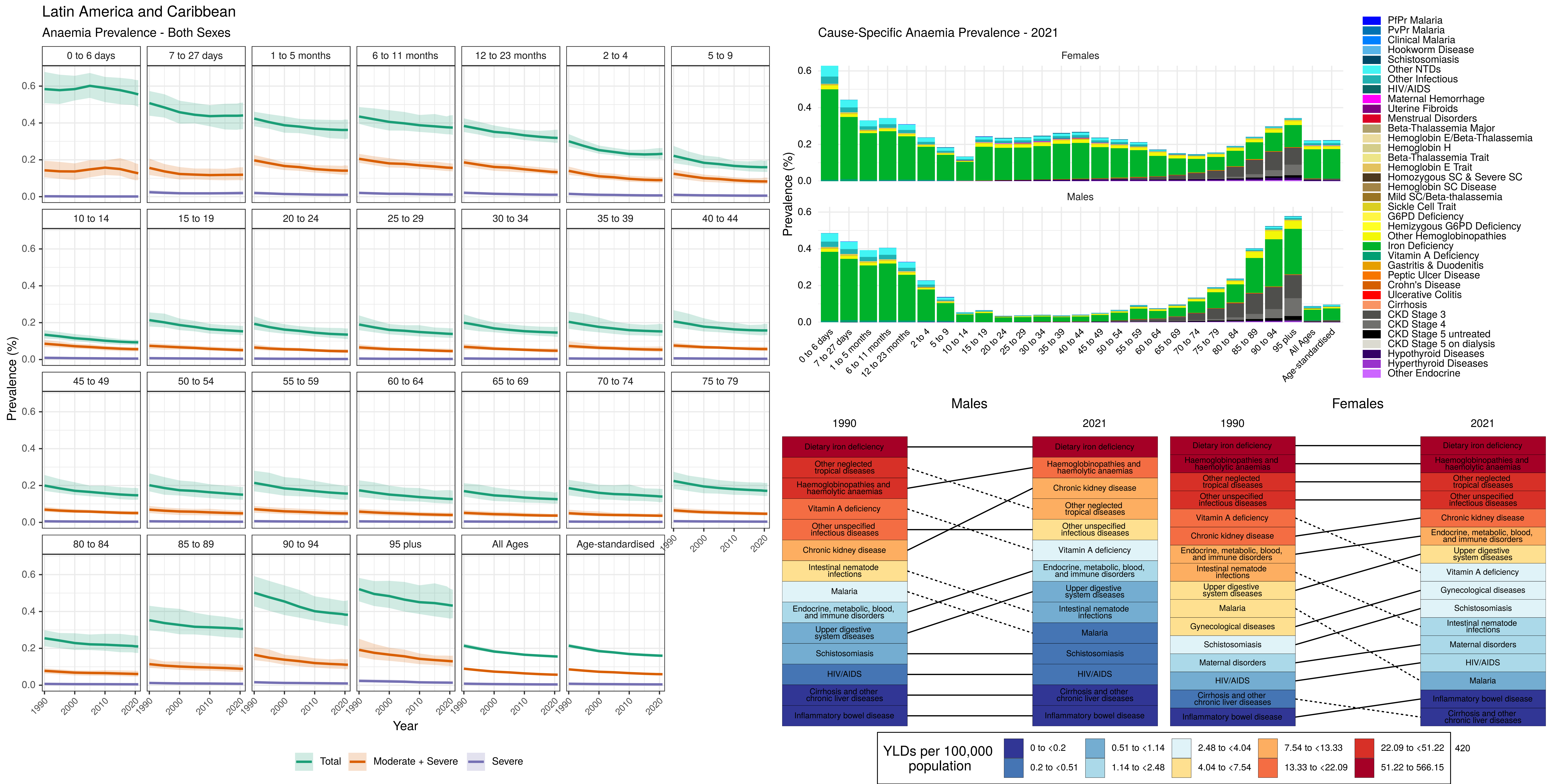

Latin America and Caribbean
Anaemia Prevalence - Both Sexes
0 to 6 days
7 to 27 days
1 to 5 months
6 to 11 months
12 to 23 months
2 to 4
5 to 9
10 to 14
15 to 19
20 to 24
25 to 29
30 to 34
35 to 39
40 to 44
45 to 49
50 to 54
55 to 59
60 to 64
65 to 69
70 to 74
75 to 79
80 to 84
85 to 89
90 to 94
95 plus
All Ages
Age-standardised
Prevalence (%)
Year
Total
Moderate + Severe
Severe
Cause-Specific Anaemia Prevalence - 2021
Females
Males
PfPr Malaria
PvPr Malaria
Clinical Malaria
Hookworm Disease
Schistosomiasis
Other NTDs
Other Infectious
HIV/AIDS
Maternal Hemorrhage
Uterine Fibroids
Menstrual Disorders
Beta-Thalassemia Major
Hemoglobin E/Beta-Thalassemia
Hemoglobin H
Beta-Thalassemia Trait
Hemoglobin E Trait
Homozygous SC & Severe SC
Hemoglobin SC Disease
Mild SC/Beta-thalassemia
Sickle Cell Trait
G6PD Deficiency
Hemizygous G6PD Deficiency
Other Hemoglobinopathies
Iron Deficiency
Vitamin A Deficiency
Gastritis & Duodenitis
Peptic Ulcer Disease
Crohn's Disease
Ulcerative Colitis
Cirrhosis
CKD Stage 3
CKD Stage 4
CKD Stage 5 untreated
CKD Stage 5 on dialysis
Hypothyroid Diseases
Hyperthyroid Diseases
Other Endocrine
Males
Females
1990
2021
Dietary iron deficiency
Other neglected tropical diseases
Haemoglobinopathies and haemolytic anaemias
Vitamin A deficiency
Other unspecified infectious diseases
Chronic kidney disease
Intestinal nematode infections
Malaria
Endocrine, metabolic, blood, and immune disorders
Upper digestive system diseases
Schistosomiasis
HIV/AIDS
Cirrhosis and other chronic liver diseases
Inflammatory bowel disease
Gynecological diseases
Maternal disorders
YLDs per 100,000 population
0 to <0.2
0.2 to <0.51
0.51 to <1.14
1.14 to <2.48
2.48 to <4.04
4.04 to <7.54
7.54 to <13.33
13.33 to <22.09
22.09 to <51.22
51.22 to 566.15

420

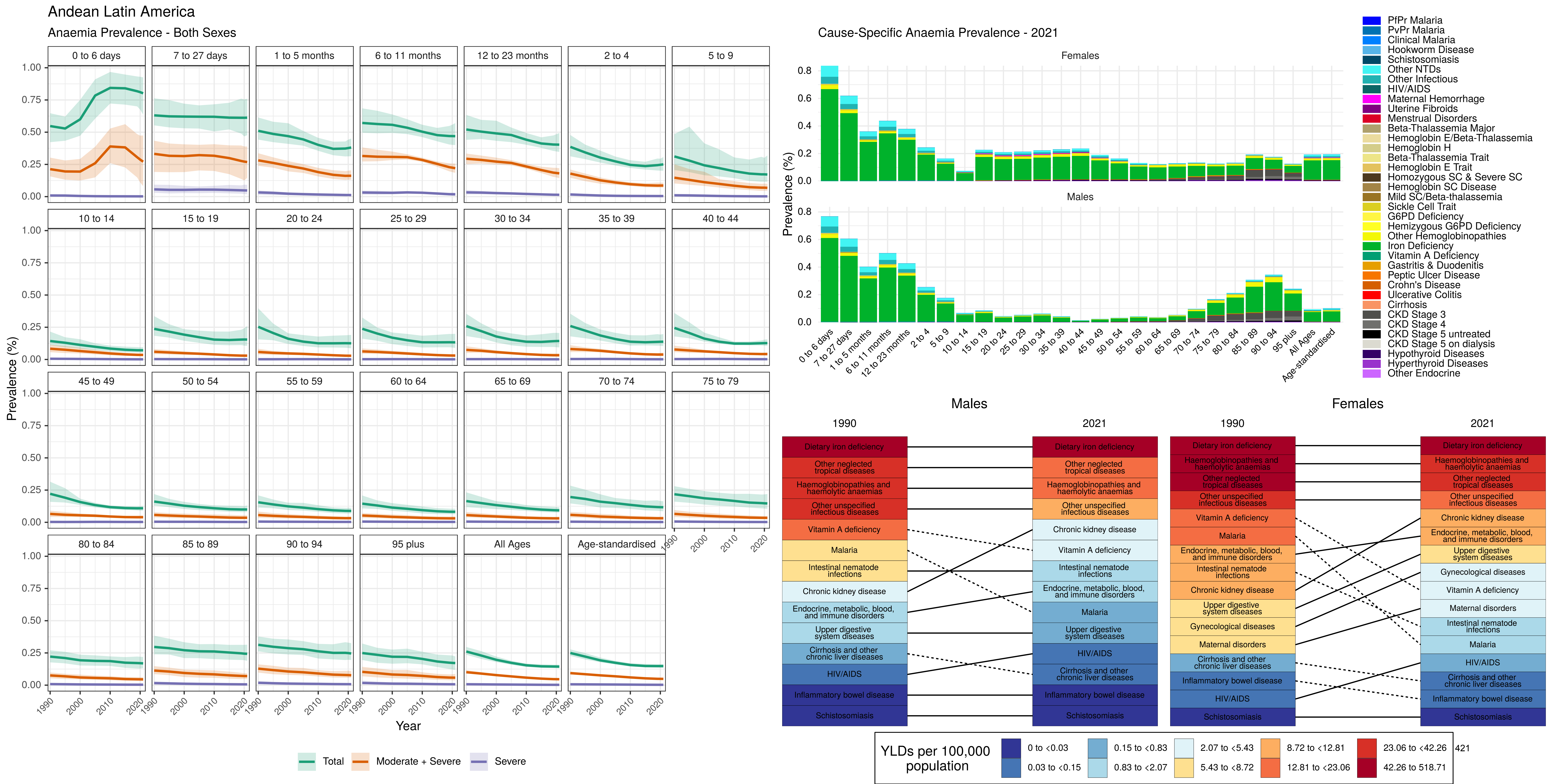

Andean Latin America
Anaemia Prevalence - Both Sexes
0 to 6 days
7 to 27 days
1 to 5 months
6 to 11 months
12 to 23 months
2 to 4
5 to 9
10 to 14
15 to 19
20 to 24
25 to 29
30 to 34
35 to 39
40 to 44
45 to 49
50 to 54
55 to 59
60 to 64
65 to 69
70 to 74
75 to 79
80 to 84
85 to 89
90 to 94
95 plus
All Ages
Age-standardised
Prevalence (%)
Year
Total
Moderate + Severe
Severe
Cause-Specific Anaemia Prevalence - 2021
Females
Males
Prevalence (%)
PfPr Malaria
PvPr Malaria
Clinical Malaria
Hookworm Disease
Schistosomiasis
Other NTDs
Other Infectious
HIV/AIDS
Maternal Hemorrhage
Uterine Fibroids
Menstrual Disorders
Beta-Thalassemia Major
Hemoglobin E/Beta-Thalassemia
Hemoglobin H
Beta-Thalassemia Trait
Hemoglobin E Trait
Homozygous SC & Severe SC
Hemoglobin SC Disease
Mild SC/Beta-thalassemia
Sickle Cell Trait
G6PD Deficiency
Hemizygous G6PD Deficiency
Other Hemoglobinopathies
Iron Deficiency
Vitamin A Deficiency
Gastritis & Duodenitis
Peptic Ulcer Disease
Crohn's Disease
Ulcerative Colitis
Cirrhosis
CKD Stage 3
CKD Stage 4
CKD Stage 5 untreated
CKD Stage 5 on dialysis
Hypothyroid Diseases
Hyperthyroid Diseases
Other Endocrine
Males
Females
1990
2021
Dietary iron deficiency
Other neglected tropical diseases
Haemoglobinopathies and haemolytic anaemias
Other unspecified infectious diseases
Vitamin A deficiency
Malaria
Intestinal nematode infections
Chronic kidney disease
Endocrine, metabolic, blood, and immune disorders
Upper digestive system diseases
Cirrhosis and other chronic liver diseases
HIV/AIDS
Inflammatory bowel disease
Schistosomiasis
Gynecological diseases
Maternal disorders
YLDs per 100,000 population
0 to <0.03
0.03 to <0.15
0.15 to <0.83
0.83 to <2.07
2.07 to <5.43
5.43 to <8.72
8.72 to <12.81
12.81 to <23.06
23.06 to <42.26
42.26 to 518.71

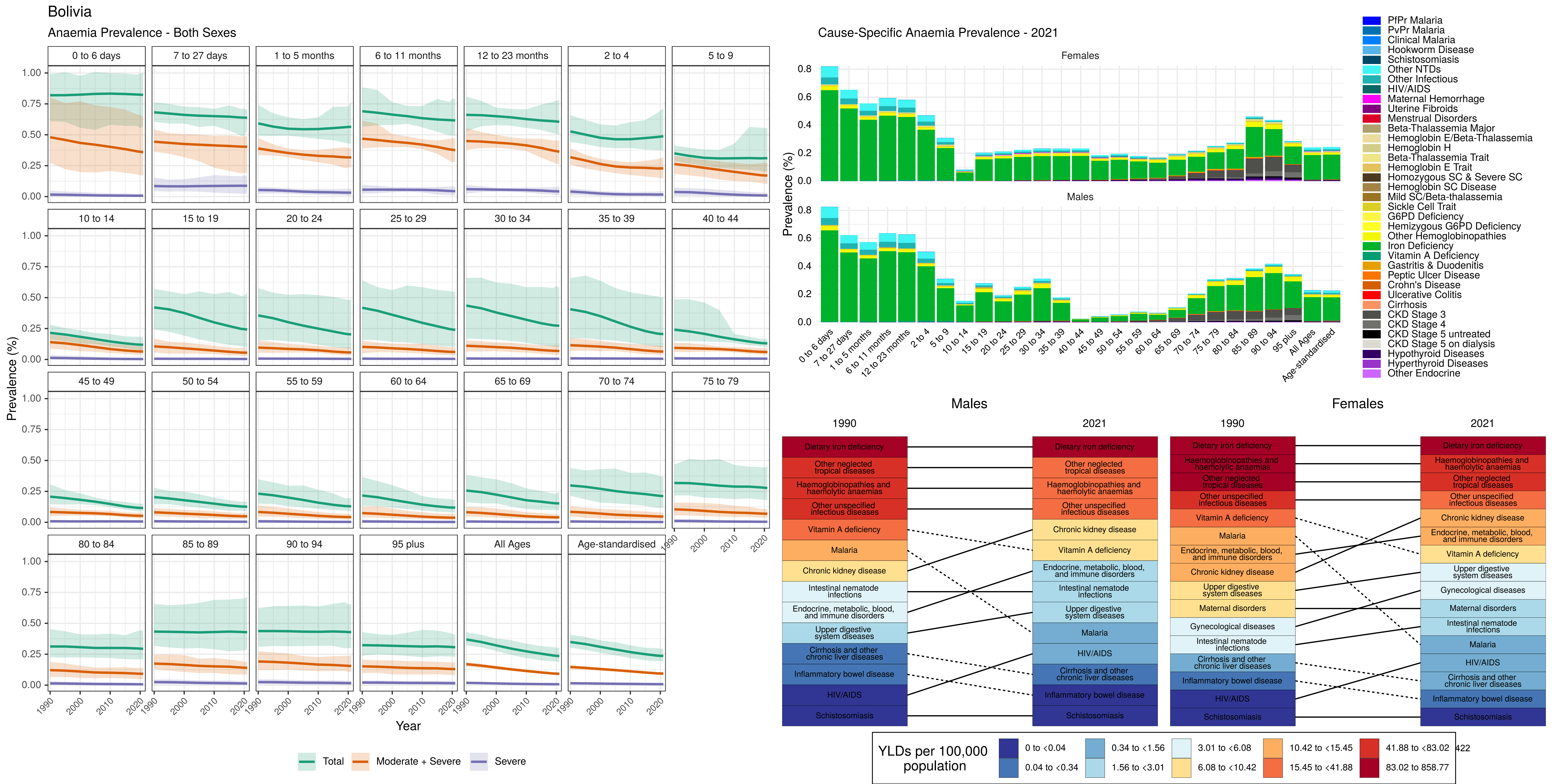

Bolivia
Anaemia Prevalence - Both Sexes
0 to 6 days
7 to 27 days
1 to 5 months
6 to 11 months
12 to 23 months
2 to 4
5 to 9
10 to 14
15 to 19
20 to 24
25 to 29
30 to 34
35 to 39
40 to 44
45 to 49
50 to 54
55 to 59
60 to 64
65 to 69
70 to 74
75 to 79
80 to 84
85 to 89
90 to 94
95 plus
All Ages
Age-standardised
Prevalence (%)
Year
Total
Moderate + Severe
Severe
Cause-Specific Anaemia Prevalence - 2021
Females
Males
Prevalence (%)
PfPr Malaria
PvPr Malaria
Clinical Malaria
Hookworm Disease
Schistosomiasis
Other NTDs
Other Infectious
HIV/AIDS
Maternal Hemorrhage
Uterine Fibroids
Menstrual Disorders
Beta-Thalassemia Major
Hemoglobin E/Beta-Thalassemia
Hemoglobin H
Beta-Thalassemia Trait
Hemoglobin E Trait
Homozygous SC & Severe SC
Hemoglobin SC Disease
Mild SC/Beta-thalassemia
Sickle Cell Trait
G6PD Deficiency
Hemizygous G6PD Deficiency
Other Hemoglobinopathies
Iron Deficiency
Vitamin A Deficiency
Gastritis & Duodenitis
Peptic Ulcer Disease
Crohn's Disease
Ulcerative Colitis
Cirrhosis
CKD Stage 3
CKD Stage 4
CKD Stage 5 untreated
CKD Stage 5 on dialysis
Hypothyroid Diseases
Hyperthyroid Diseases
Other Endocrine
Males
Females
1990
2021
Dietary iron deficiency
Other neglected tropical diseases
Haemoglobinopathies and haemolytic anaemias
Other unspecified infectious diseases
Vitamin A deficiency
Malaria
Chronic kidney disease
Intestinal nematode infections
Endocrine, metabolic, blood, and immune disorders
Upper digestive system diseases
Cirrhosis and other chronic liver diseases
Inflammatory bowel disease
HIV/AIDS
Schistosomiasis
Maternal disorders
Gynecological diseases
YLDs per 100,000 population
0 to <0.04
0.04 to <0.34
0.34 to <1.56
1.56 to <3.01
3.01 to <6.08
6.08 to <10.42
10.42 to <15.45
15.45 to <41.88
41.88 to <83.02
83.02 to 858.77

Ecuador

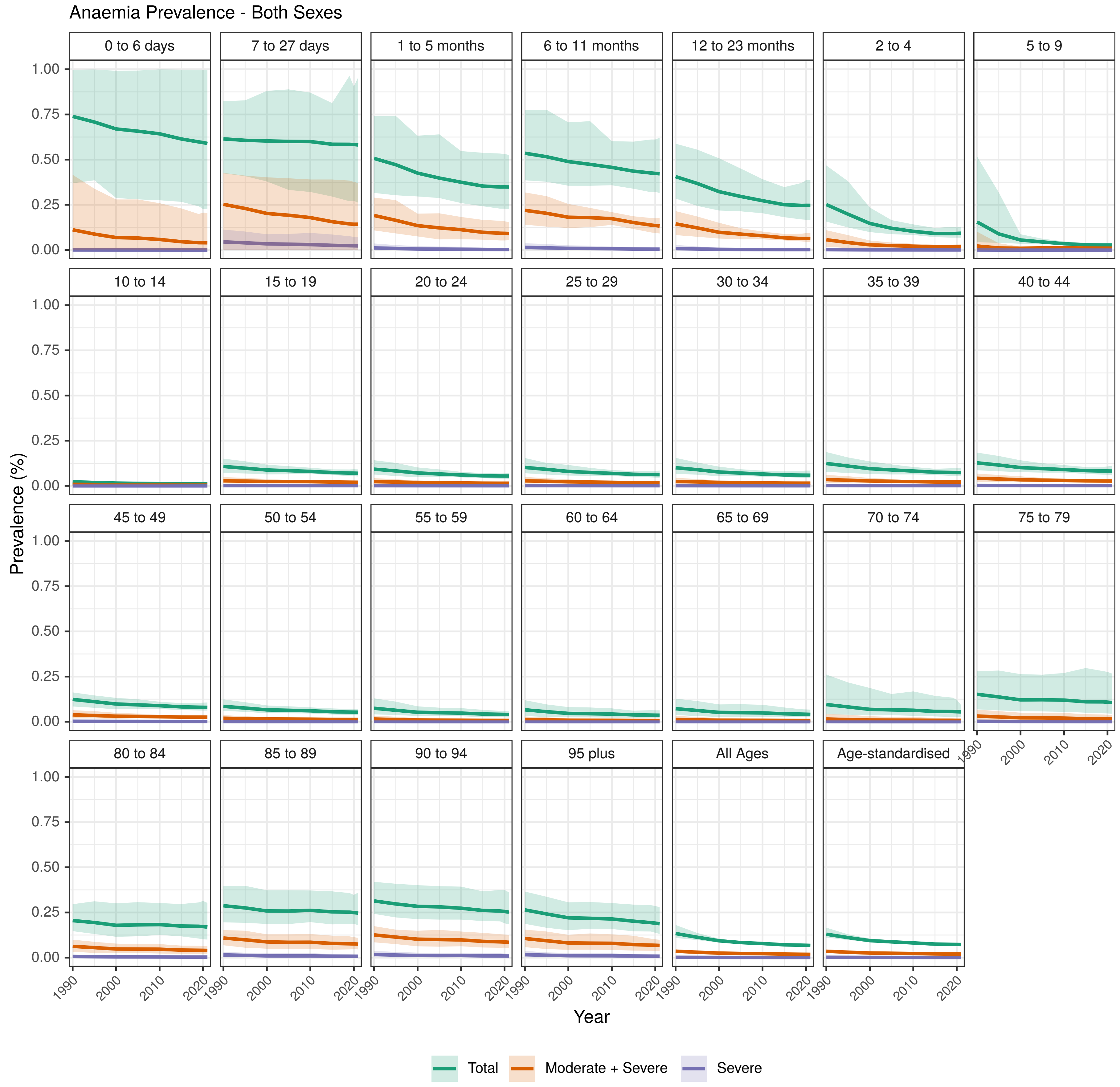
Anaemia Prevalence - Both Sexes
0 to 6 days
7 to 27 days
1 to 5 months
6 to 11 months
12 to 23 months
2 to 4
5 to 9
10 to 14
15 to 19
20 to 24
25 to 29
30 to 34
35 to 39
40 to 44
45 to 49
50 to 54
55 to 59
60 to 64
65 to 69
70 to 74
75 to 79
80 to 84
85 to 89
90 to 94
95 plus
All Ages
Age-standardised
Prevalence (%)
0.00
0.25
0.50
0.75
1.00
1990
2000
2010
2020
Year
Total
Moderate + Severe
Severe

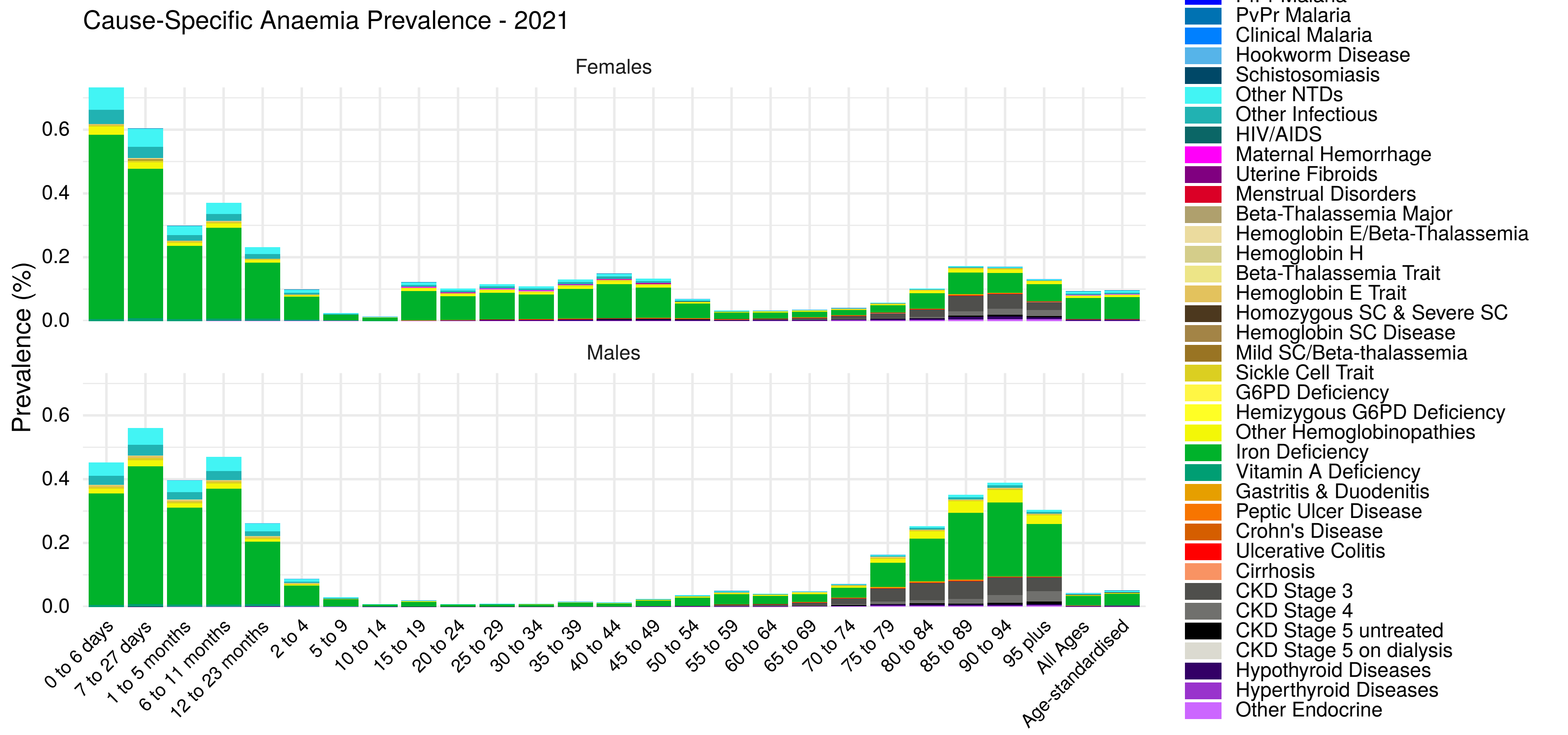
Cause-Specific Anaemia Prevalence - 2021
Females
Males
Prevalence (%)
0.0
0.2
0.4
0.6
0 to 6 days
7 to 27 days
1 to 5 months
6 to 11 months
12 to 23 months
2 to 4
5 to 9
10 to 14
15 to 19
20 to 24
25 to 29
30 to 34
35 to 39
40 to 44
45 to 49
50 to 54
55 to 59
60 to 64
65 to 69
70 to 74
75 to 79
80 to 84
85 to 89
90 to 94
95 plus
All Ages
Age-standardised
PfPr Malaria
PvPr Malaria
Clinical Malaria
Hookworm Disease
Schistosomiasis
Other NTDs
Other Infectious
HIV/AIDS
Maternal Hemorrhage
Uterine Fibroids
Menstrual Disorders
Beta-Thalassemia Major
Hemoglobin E/Beta-Thalassemia
Hemoglobin H
Beta-Thalassemia Trait
Hemoglobin E Trait
Homozygous SC & Severe SC
Hemoglobin SC Disease
Mild SC/Beta-thalassemia
Sickle Cell Trait
G6PD Deficiency
Hemizygous G6PD Deficiency
Other Hemoglobinopathies
Iron Deficiency
Vitamin A Deficiency
Gastritis & Duodenitis
Peptic Ulcer Disease
Crohn's Disease
Ulcerative Colitis
Cirrhosis
CKD Stage 3
CKD Stage 4
CKD Stage 5 untreated
CKD Stage 5 on dialysis
Hypothyroid Diseases
Hyperthyroid Diseases
Other Endocrine

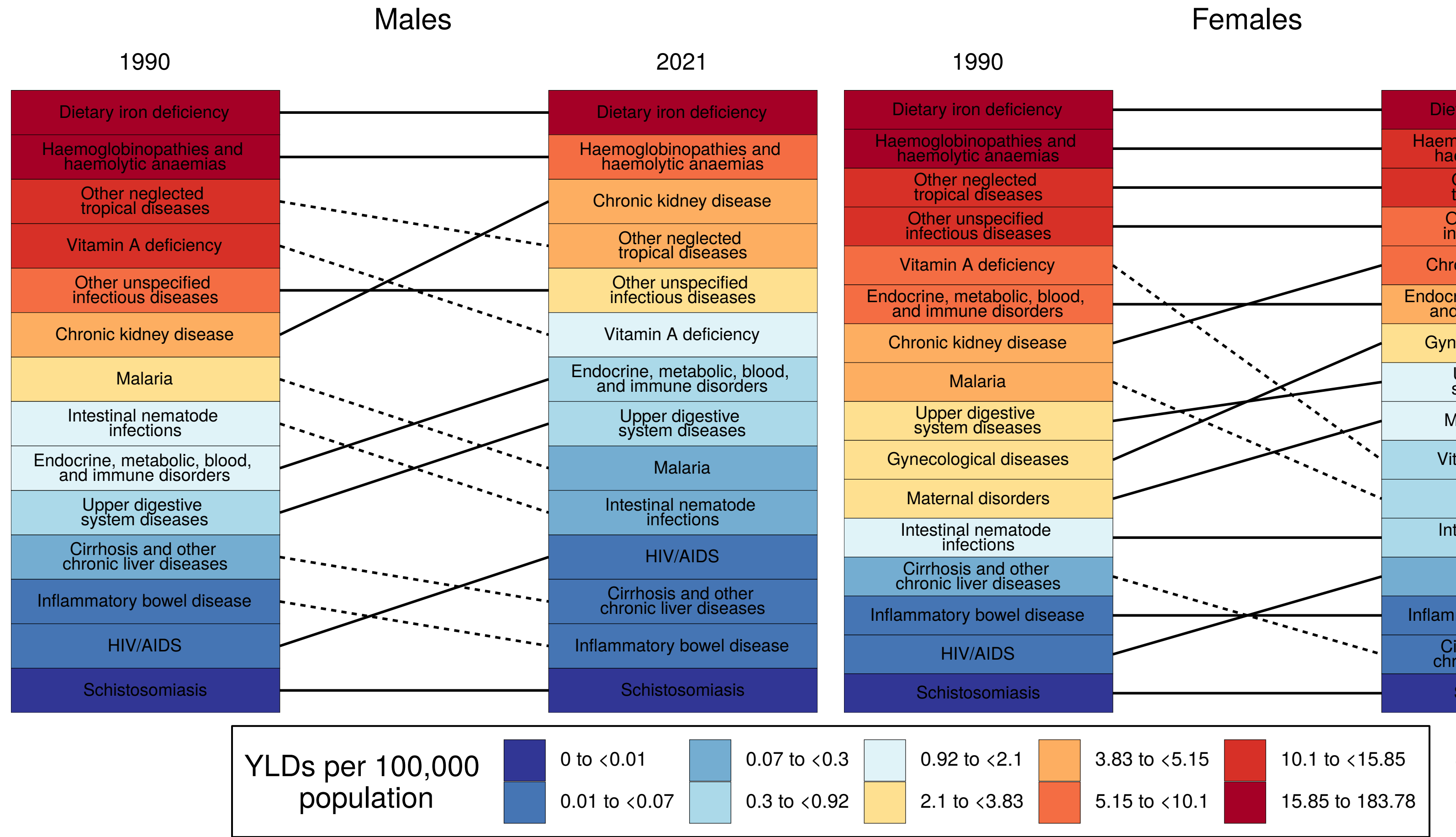
Males
Females
1990
2021
Dietary iron deficiency
Haemoglobinopathies and haemolytic anaemias
Other neglected tropical diseases
Vitamin A deficiency
Other unspecified infectious diseases
Chronic kidney disease
Malaria
Intestinal nematode infections
Endocrine, metabolic, blood, and immune disorders
Upper digestive system diseases
Cirrhosis and other chronic liver diseases
Inflammatory bowel disease
HIV/AIDS
Schistosomiasis
Gynecological diseases
Maternal disorders
YLDs per 100,000 population
0 to <0.01
0.01 to <0.07
0.07 to <0.3
0.3 to <0.92
0.92 to <2.1
2.1 to <3.83
3.83 to <5.15
5.15 to <10.1
10.1 to <15.85
15.85 to 183.78

Peru

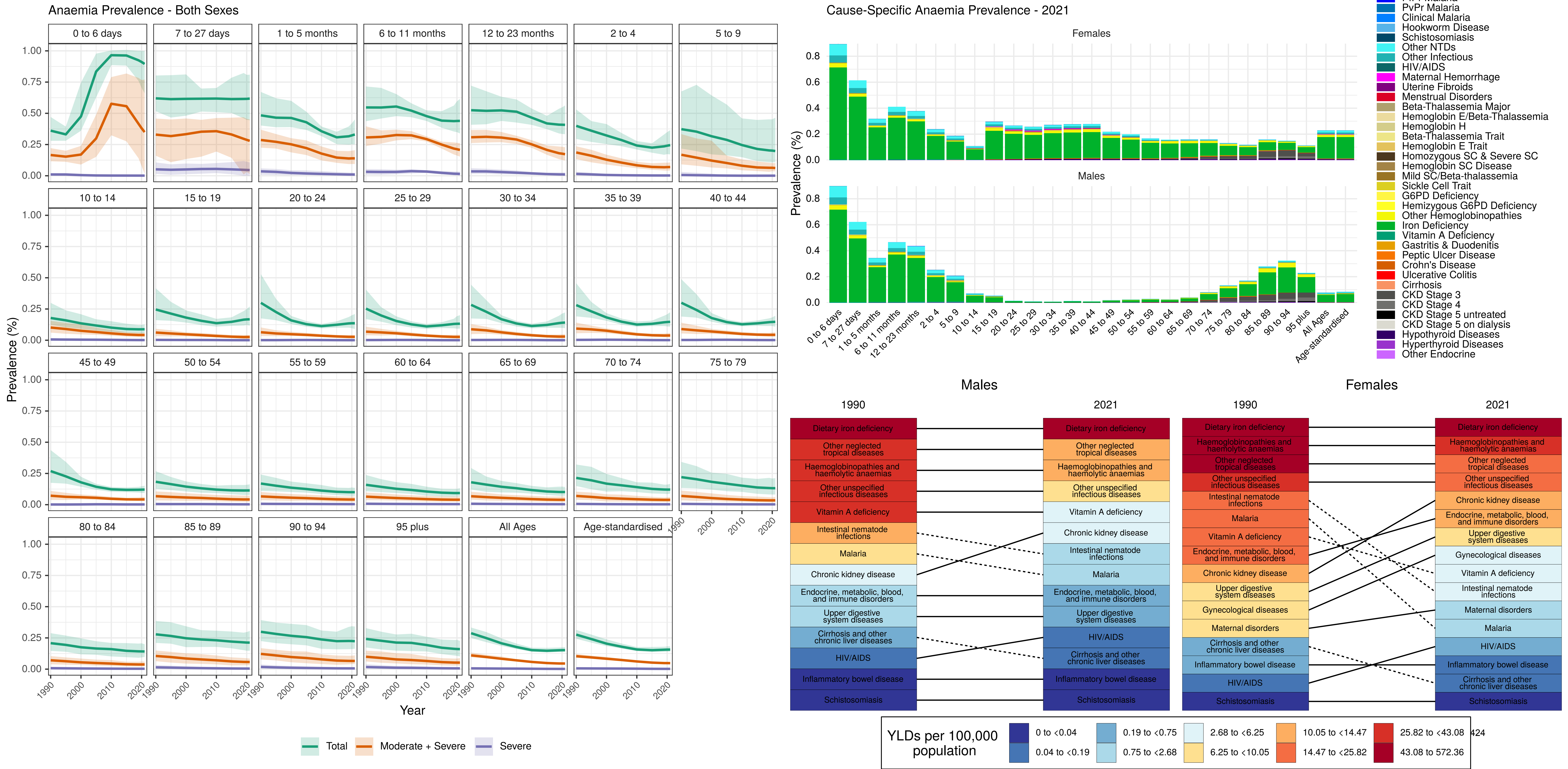

Anaemia Prevalence - Both Sexes
0 to 6 days
7 to 27 days
1 to 5 months
6 to 11 months
12 to 23 months
2 to 4
5 to 9
10 to 14
15 to 19
20 to 24
25 to 29
30 to 34
35 to 39
40 to 44
45 to 49
50 to 54
55 to 59
60 to 64
65 to 69
70 to 74
75 to 79
80 to 84
85 to 89
90 to 94
95 plus
All Ages
Age-standardised
Prevalence (%)
Year
Total
Moderate + Severe
Severe
Cause-Specific Anaemia Prevalence - 2021
Females
Males
Prevalence (%)
PfPr Malaria
PvPr Malaria
Clinical Malaria
Hookworm Disease
Schistosomiasis
Other NTDs
Other Infectious
HIV/AIDS
Maternal Hemorrhage
Uterine Fibroids
Menstrual Disorders
Beta-Thalassemia Major
Hemoglobin E/Beta-Thalassemia
Hemoglobin H
Beta-Thalassemia Trait
Hemoglobin E Trait
Homozygous SC & Severe SC
Hemoglobin SC Disease
Mild SC/Beta-thalassemia
Sickle Cell Trait
G6PD Deficiency
Hemizygous G6PD Deficiency
Other Hemoglobinopathies
Iron Deficiency
Vitamin A Deficiency
Gastritis & Duodenitis
Peptic Ulcer Disease
Crohn's Disease
Ulcerative Colitis
Cirrhosis
CKD Stage 3
CKD Stage 4
CKD Stage 5 untreated
CKD Stage 5 on dialysis
Hypothyroid Diseases
Hyperthyroid Diseases
Other Endocrine
Males
1990
2021
Dietary iron deficiency
Other neglected tropical diseases
Haemoglobinopathies and haemolytic anaemias
Other unspecified infectious diseases
Vitamin A deficiency
Intestinal nematode infections
Malaria
Chronic kidney disease
Endocrine, metabolic, blood, and immune disorders
Upper digestive system diseases
Cirrhosis and other chronic liver diseases
HIV/AIDS
Inflammatory bowel disease
Schistosomiasis
Females
1990
2021
Dietary iron deficiency
Haemoglobinopathies and haemolytic anaemias
Other neglected tropical diseases
Other unspecified infectious diseases
Intestinal nematode infections
Malaria
Vitamin A deficiency
Endocrine, metabolic, blood, and immune disorders
Chronic kidney disease
Upper digestive system diseases
Gynecological diseases
Maternal disorders
Cirrhosis and other chronic liver diseases
Inflammatory bowel disease
HIV/AIDS
Schistosomiasis
YLDs per 100,000 population
0 to <0.04
0.04 to <0.19
0.19 to <0.75
0.75 to <2.68
2.68 to <6.25
6.25 to <10.05
10.05 to <14.47
14.47 to <25.82
25.82 to <43.08
43.08 to 572.36

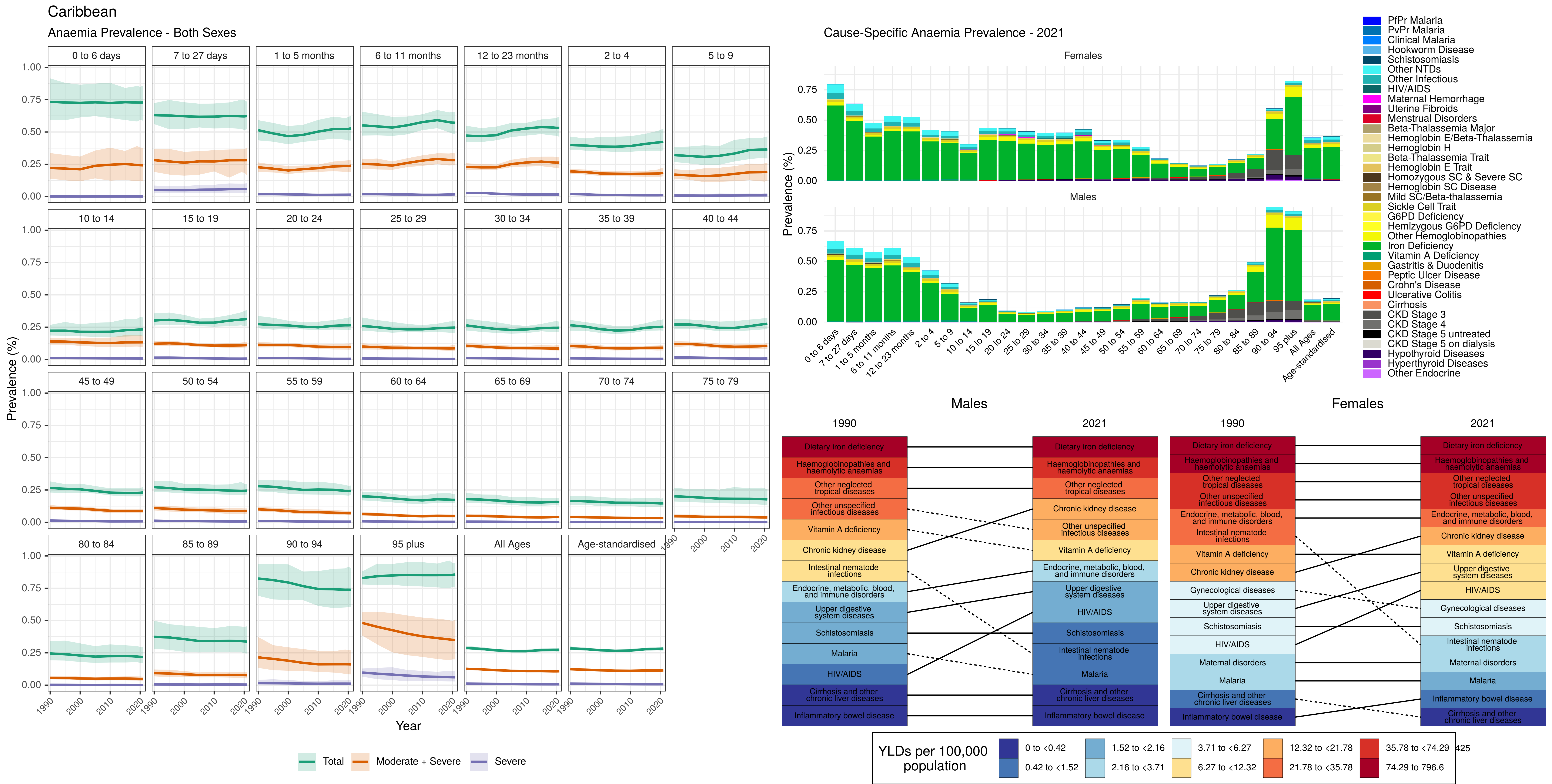
Caribbean
Anaemia Prevalence - Both Sexes
0 to 6 days
7 to 27 days
1 to 5 months
6 to 11 months
12 to 23 months
2 to 4
5 to 9
10 to 14
15 to 19
20 to 24
25 to 29
30 to 34
35 to 39
40 to 44
45 to 49
50 to 54
55 to 59
60 to 64
65 to 69
70 to 74
75 to 79
80 to 84
85 to 89
90 to 94
95 plus
All Ages
Age-standardised
Prevalence (%)
Year
Total
Moderate + Severe
Severe
Cause-Specific Anaemia Prevalence - 2021
Females
Males
PfPr Malaria
PvPr Malaria
Clinical Malaria
Hookworm Disease
Schistosomiasis
Other NTDs
Other Infectious
HIV/AIDS
Maternal Hemorrhage
Uterine Fibroids
Menstrual Disorders
Beta-Thalassemia Major
Hemoglobin E/Beta-Thalassemia
Hemoglobin H
Beta-Thalassemia Trait
Hemoglobin E Trait
Homozygous SC & Severe SC
Hemoglobin SC Disease
Mild SC/Beta-thalassemia
Sickle Cell Trait
G6PD Deficiency
Hemizygous G6PD Deficiency
Other Hemoglobinopathies
Iron Deficiency
Vitamin A Deficiency
Gastritis & Duodenitis
Peptic Ulcer Disease
Crohn's Disease
Ulcerative Colitis
Cirrhosis
CKD Stage 3
CKD Stage 4
CKD Stage 5 untreated
CKD Stage 5 on dialysis
Hypothyroid Diseases
Hyperthyroid Diseases
Other Endocrine
Males
1990
Dietary iron deficiency
Haemoglobinopathies and haemolytic anaemias
Other neglected tropical diseases
Other unspecified infectious diseases
Vitamin A deficiency
Chronic kidney disease
Intestinal nematode infections
Endocrine, metabolic, blood, and immune disorders
Upper digestive system diseases
Schistosomiasis
Malaria
HIV/AIDS
Cirrhosis and other chronic liver diseases
Inflammatory bowel disease
2021
Dietary iron deficiency
Haemoglobinopathies and haemolytic anaemias
Other neglected tropical diseases
Chronic kidney disease
Other unspecified infectious diseases
Vitamin A deficiency
Endocrine, metabolic, blood, and immune disorders
Upper digestive system diseases
HIV/AIDS
Schistosomiasis
Intestinal nematode infections
Malaria
Cirrhosis and other chronic liver diseases
Inflammatory bowel disease
Females
1990
Dietary iron deficiency
Haemoglobinopathies and haemolytic anaemias
Other neglected tropical diseases
Other unspecified infectious diseases
Endocrine, metabolic, blood, and immune disorders
Intestinal nematode infections
Vitamin A deficiency
Chronic kidney disease
Gynecological diseases
Upper digestive system diseases
Schistosomiasis
HIV/AIDS
Maternal disorders
Malaria
Cirrhosis and other chronic liver diseases
Inflammatory bowel disease
2021
Dietary iron deficiency
Haemoglobinopathies and haemolytic anaemias
Other neglected tropical diseases
Other unspecified infectious diseases
Endocrine, metabolic, blood, and immune disorders
Chronic kidney disease
Vitamin A deficiency
Upper digestive system diseases
HIV/AIDS
Gynecological diseases
Schistosomiasis
Intestinal nematode infections
Maternal disorders
Malaria
Inflammatory bowel disease
Cirrhosis and other chronic liver diseases
YLDs per 100,000 population
0 to <0.42
0.42 to <1.52
1.52 to <2.16
2.16 to <3.71
3.71 to <6.27
6.27 to <12.32
12.32 to <21.78
21.78 to <35.78
35.78 to <74.29
74.29 to 796.6

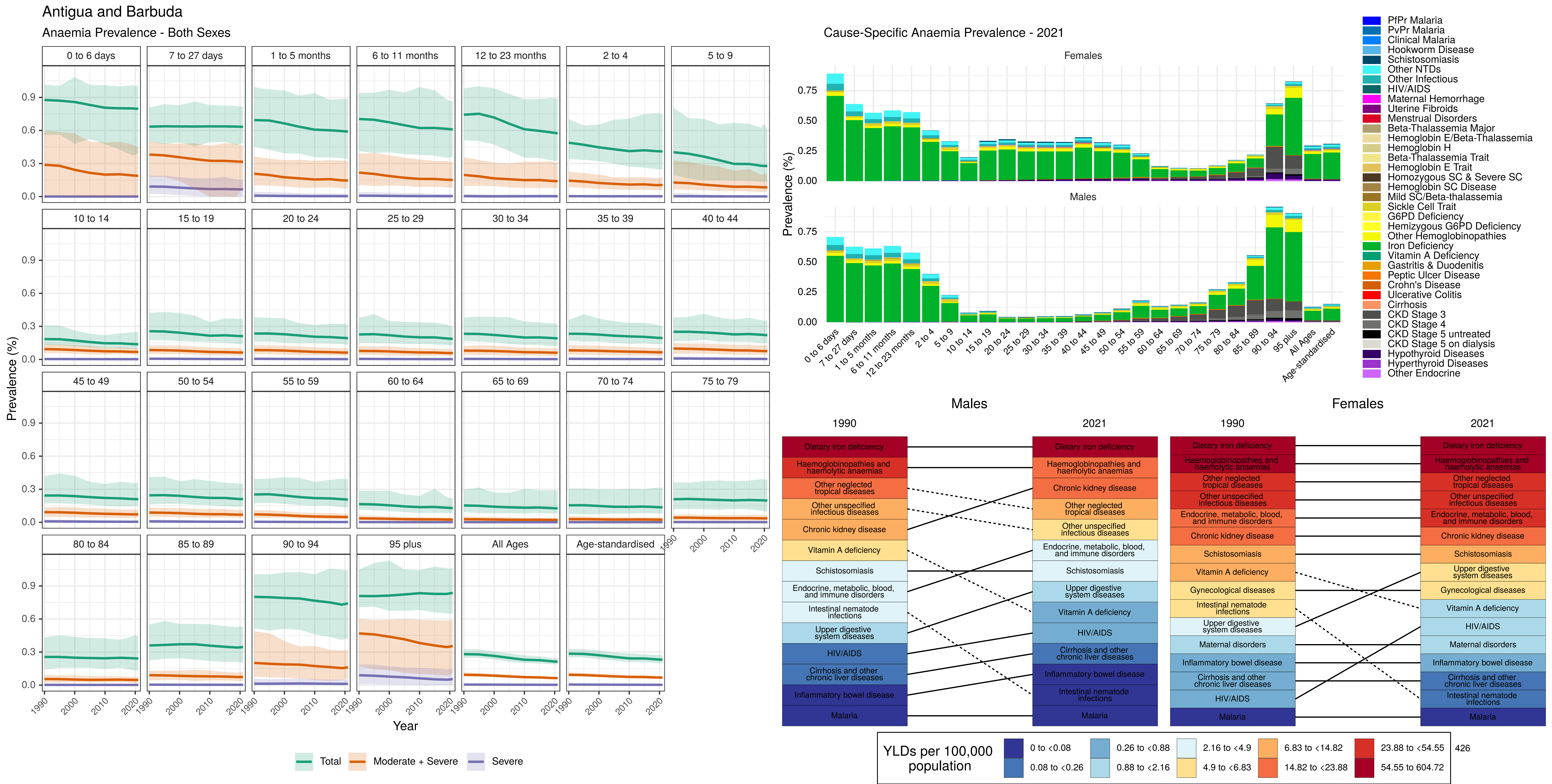
Antigua and Barbuda
Anaemia Prevalence - Both Sexes
0 to 6 days
7 to 27 days
1 to 5 months
6 to 11 months
12 to 23 months
2 to 4
5 to 9
10 to 14
15 to 19
20 to 24
25 to 29
30 to 34
35 to 39
40 to 44
45 to 49
50 to 54
55 to 59
60 to 64
65 to 69
70 to 74
75 to 79
80 to 84
85 to 89
90 to 94
95 plus
All Ages
Age-standardised
Prevalence (%)
Year
Total
Moderate + Severe
Severe
Cause-Specific Anaemia Prevalence - 2021
Females
Males
Prevalence (%)
PfPr Malaria
PvPr Malaria
Clinical Malaria
Hookworm Disease
Schistosomiasis
Other NTDs
Other Infectious
HIV/AIDS
Maternal Hemorrhage
Uterine Fibroids
Menstrual Disorders
Beta-Thalassemia Major
Hemoglobin E/Beta-Thalassemia
Hemoglobin H
Beta-Thalassemia Trait
Hemoglobin E Trait
Homozygous SC & Severe SC
Hemoglobin SC Disease
Mild SC/Beta-thalassemia
Sickle Cell Trait
G6PD Deficiency
Hemizygous G6PD Deficiency
Other Hemoglobinopathies
Iron Deficiency
Vitamin A Deficiency
Gastritis & Duodenitis
Peptic Ulcer Disease
Crohn's Disease
Ulcerative Colitis
Cirrhosis
CKD Stage 3
CKD Stage 4
CKD Stage 5 untreated
CKD Stage 5 on dialysis
Hypothyroid Diseases
Hyperthyroid Diseases
Other Endocrine
Males
Females
1990
2021
Dietary iron deficiency
Haemoglobinopathies and haemolytic anaemias
Other neglected tropical diseases
Other unspecified infectious diseases
Chronic kidney disease
Vitamin A deficiency
Schistosomiasis
Endocrine, metabolic, blood, and immune disorders
Intestinal nematode infections
Upper digestive system diseases
HIV/AIDS
Cirrhosis and other chronic liver diseases
Inflammatory bowel disease
Malaria
Gynecological diseases
Maternal disorders
YLDs per 100,000 population
0 to <0.08
0.08 to <0.26
0.26 to <0.88
0.88 to <2.16
2.16 to <4.9
4.9 to <6.83
6.83 to <14.82
14.82 to <23.88
23.88 to <54.55
54.55 to 604.72

The Bahamas

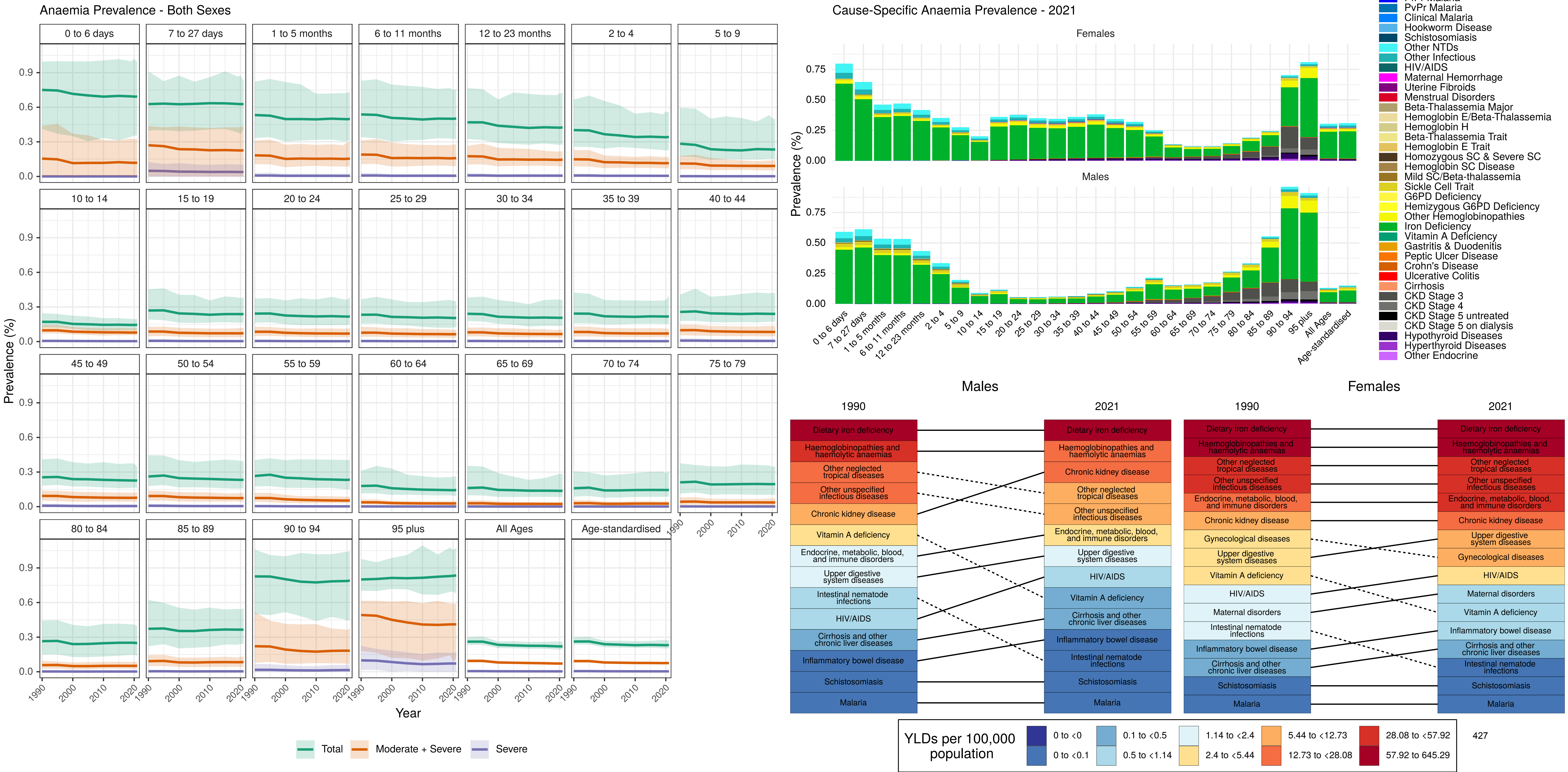

427

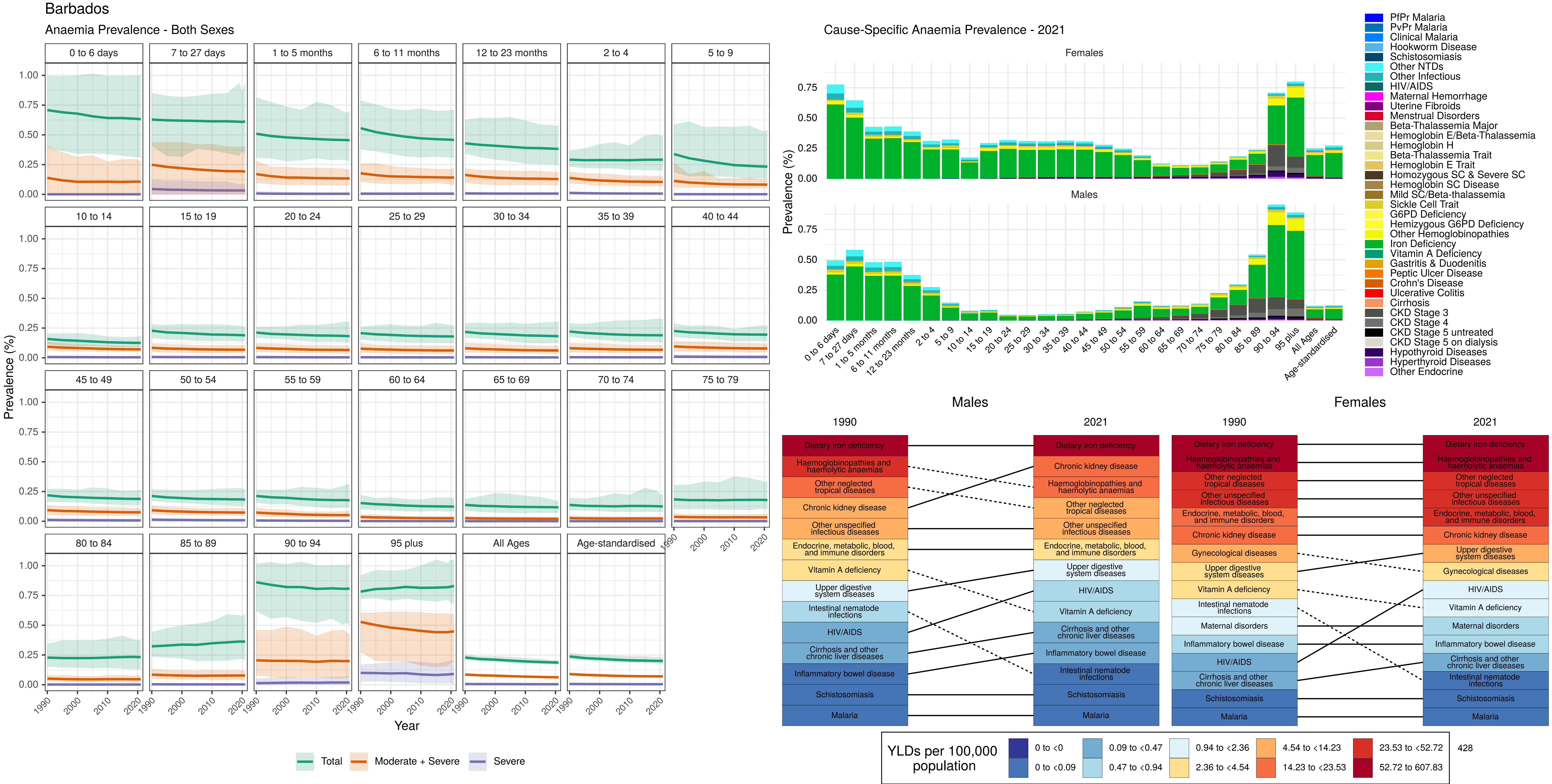

Barbados
Anaemia Prevalence - Both Sexes
0 to 6 days
7 to 27 days
1 to 5 months
6 to 11 months
12 to 23 months
2 to 4
5 to 9
10 to 14
15 to 19
20 to 24
25 to 29
30 to 34
35 to 39
40 to 44
45 to 49
50 to 54
55 to 59
60 to 64
65 to 69
70 to 74
75 to 79
80 to 84
85 to 89
90 to 94
95 plus
All Ages
Age-standardised
Prevalence (%)
Year
Total
Moderate + Severe
Severe
Cause-Specific Anaemia Prevalence - 2021
Females
Males
PfPr Malaria
PvPr Malaria
Clinical Malaria
Hookworm Disease
Schistosomiasis
Other NTDs
Other Infectious
HIV/AIDS
Maternal Hemorrhage
Uterine Fibroids
Menstrual Disorders
Beta-Thalassemia Major
Hemoglobin E/Beta-Thalassemia
Hemoglobin H
Beta-Thalassemia Trait
Hemoglobin E Trait
Homozygous SC & Severe SC
Hemoglobin SC Disease
Mild SC/Beta-thalassemia
Sickle Cell Trait
G6PD Deficiency
Hemizygous G6PD Deficiency
Other Hemoglobinopathies
Iron Deficiency
Vitamin A Deficiency
Gastritis & Duodenitis
Peptic Ulcer Disease
Crohn's Disease
Ulcerative Colitis
Cirrhosis
CKD Stage 3
CKD Stage 4
CKD Stage 5 untreated
CKD Stage 5 on dialysis
Hypothyroid Diseases
Hyperthyroid Diseases
Other Endocrine
Males
Females
1990
2021
Males 1990: Dietary iron deficiency; Haemoglobinopathies and haemolytic anaemias; Other neglected tropical diseases; Chronic kidney disease; Other unspecified infectious diseases; Endocrine, metabolic, blood, and immune disorders; Vitamin A deficiency; Upper digestive system diseases; Intestinal nematode infections; HIV/AIDS; Cirrhosis and other chronic liver diseases; Inflammatory bowel disease; Schistosomiasis; Malaria
Males 2021: Dietary iron deficiency; Chronic kidney disease; Haemoglobinopathies and haemolytic anaemias; Other neglected tropical diseases; Other unspecified infectious diseases; Endocrine, metabolic, blood, and immune disorders; Upper digestive system diseases; HIV/AIDS; Vitamin A deficiency; Cirrhosis and other chronic liver diseases; Inflammatory bowel disease; Intestinal nematode infections; Schistosomiasis; Malaria
Females 1990: Dietary iron deficiency; Haemoglobinopathies and haemolytic anaemias; Other neglected tropical diseases; Other unspecified infectious diseases; Endocrine, metabolic, blood, and immune disorders; Chronic kidney disease; Gynecological diseases; Upper digestive system diseases; Vitamin A deficiency; Intestinal nematode infections; Maternal disorders; Inflammatory bowel disease; HIV/AIDS; Cirrhosis and other chronic liver diseases; Schistosomiasis; Malaria
Females 2021: Dietary iron deficiency; Haemoglobinopathies and haemolytic anaemias; Other neglected tropical diseases; Other unspecified infectious diseases; Endocrine, metabolic, blood, and immune disorders; Chronic kidney disease; Upper digestive system diseases; Gynecological diseases; HIV/AIDS; Vitamin A deficiency; Maternal disorders; Inflammatory bowel disease; Cirrhosis and other chronic liver diseases; Intestinal nematode infections; Schistosomiasis; Malaria
YLDs per 100,000 population
0 to <0
0 to <0.09
0.09 to <0.47
0.47 to <0.94
0.94 to <2.36
2.36 to <4.54
4.54 to <14.23
14.23 to <23.53
23.53 to <52.72
52.72 to 607.83

Belize

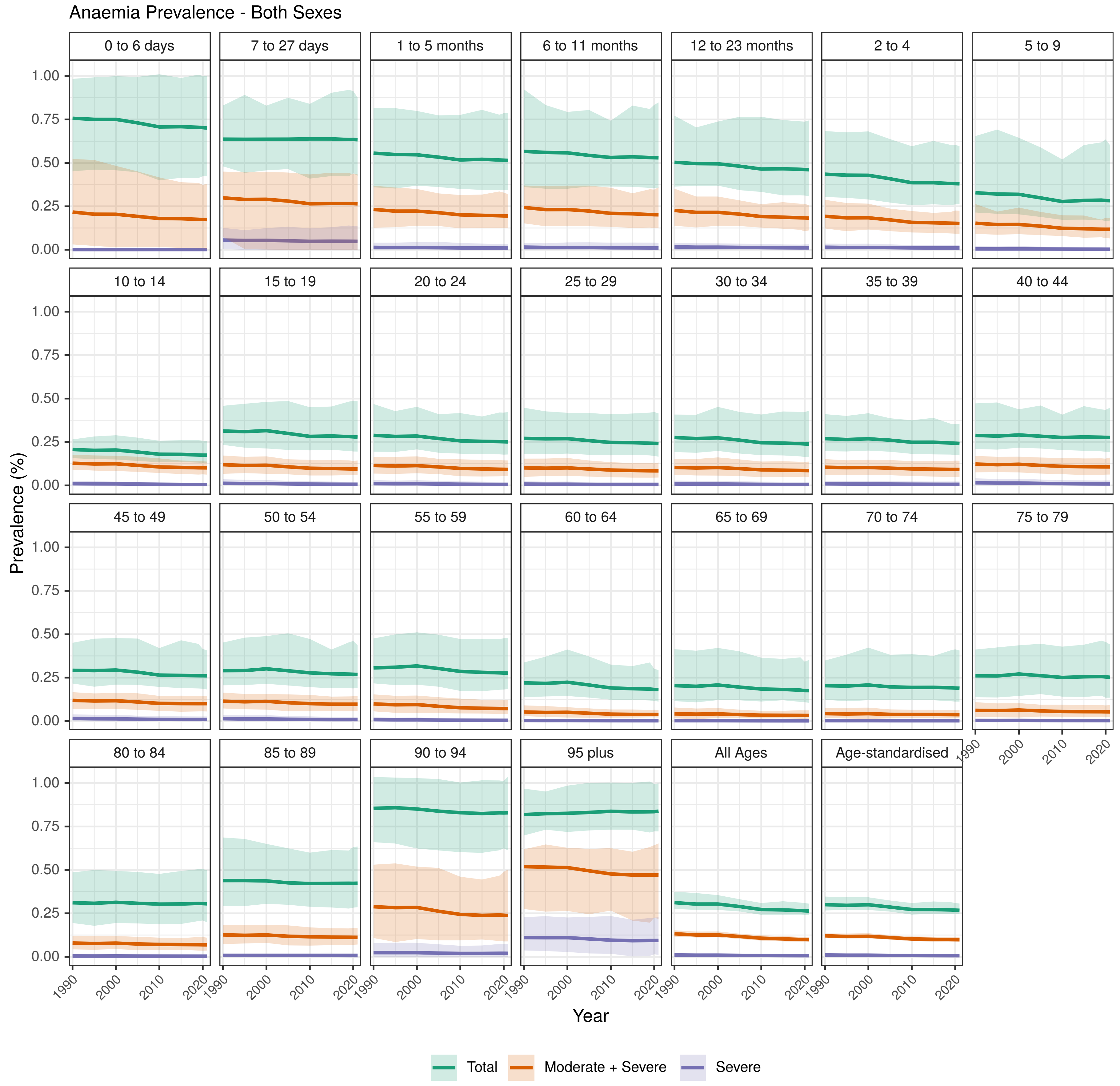

Anaemia Prevalence - Both Sexes
0 to 6 days
7 to 27 days
1 to 5 months
6 to 11 months
12 to 23 months
2 to 4
5 to 9
10 to 14
15 to 19
20 to 24
25 to 29
30 to 34
35 to 39
40 to 44
45 to 49
50 to 54
55 to 59
60 to 64
65 to 69
70 to 74
75 to 79
80 to 84
85 to 89
90 to 94
95 plus
All Ages
Age-standardised
Prevalence (%)
Year
Total
Moderate + Severe
Severe

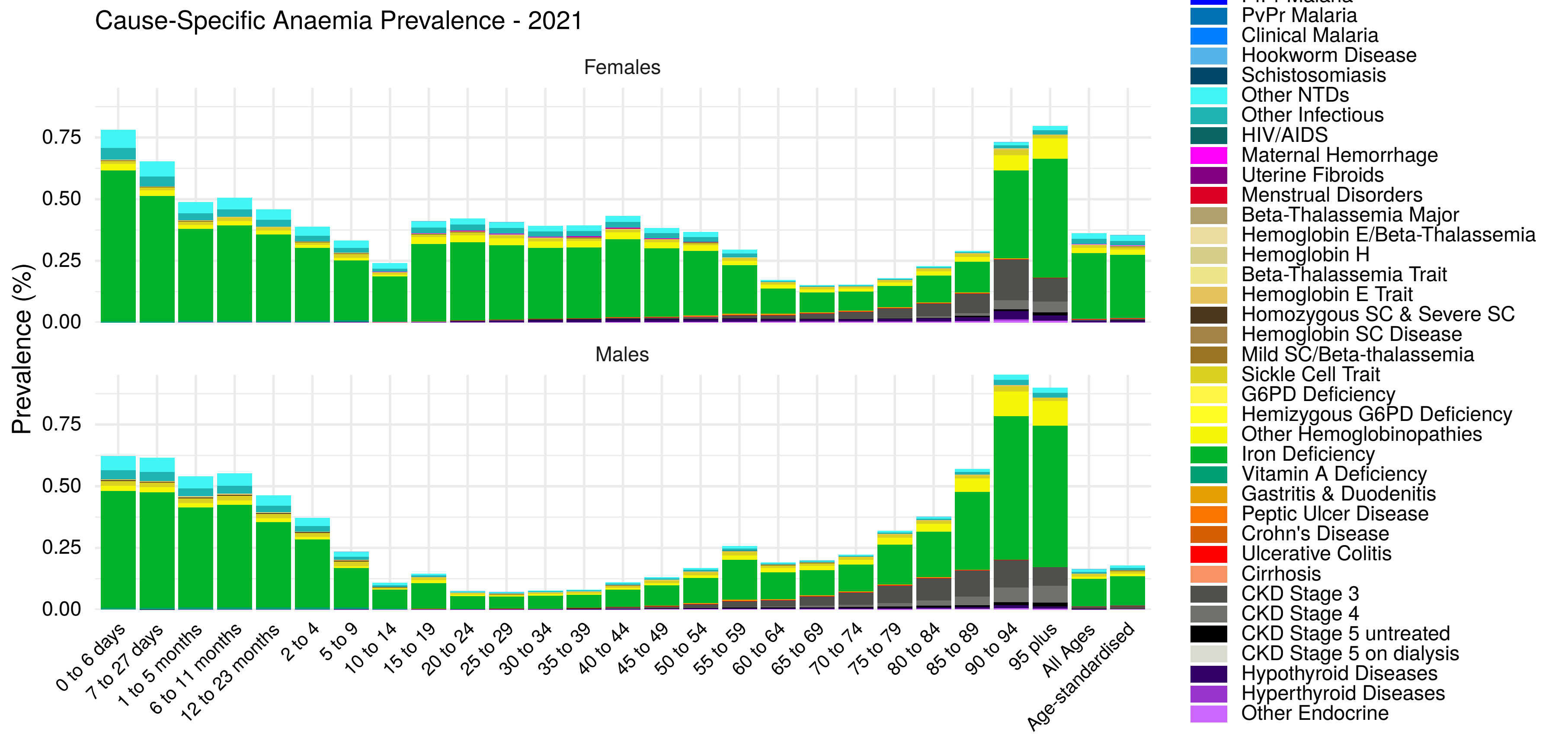

Cause-Specific Anaemia Prevalence - 2021
Females
Males
Prevalence (%)
PfPr Malaria
PvPr Malaria
Clinical Malaria
Hookworm Disease
Schistosomiasis
Other NTDs
Other Infectious
HIV/AIDS
Maternal Hemorrhage
Uterine Fibroids
Menstrual Disorders
Beta-Thalassemia Major
Hemoglobin E/Beta-Thalassemia
Hemoglobin H
Beta-Thalassemia Trait
Hemoglobin E Trait
Homozygous SC & Severe SC
Hemoglobin SC Disease
Mild SC/Beta-thalassemia
Sickle Cell Trait
G6PD Deficiency
Hemizygous G6PD Deficiency
Other Hemoglobinopathies
Iron Deficiency
Vitamin A Deficiency
Gastritis & Duodenitis
Peptic Ulcer Disease
Crohn's Disease
Ulcerative Colitis
Cirrhosis
CKD Stage 3
CKD Stage 4
CKD Stage 5 untreated
CKD Stage 5 on dialysis
Hypothyroid Diseases
Hyperthyroid Diseases
Other Endocrine

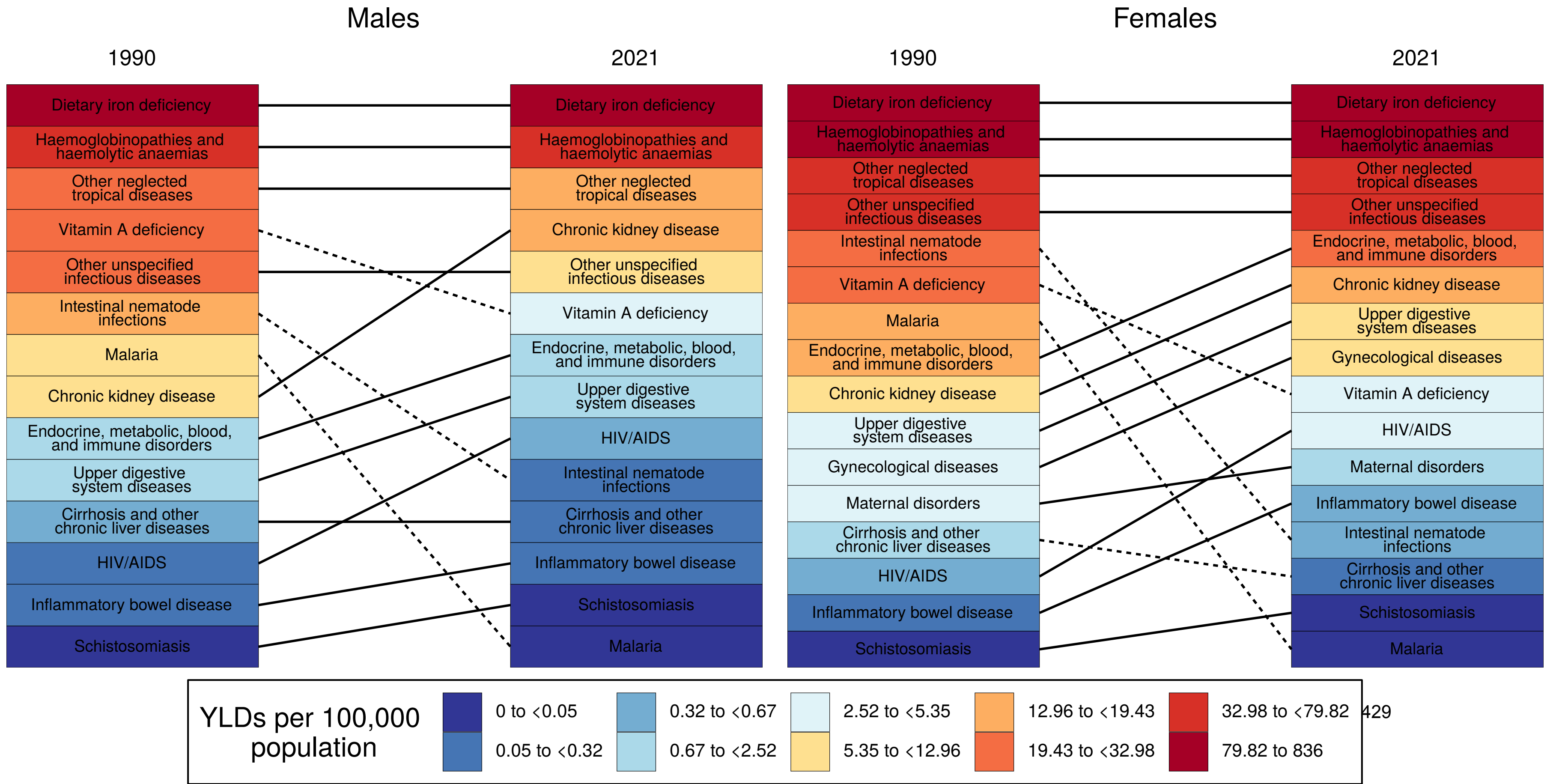

Males
Females
1990
2021
Dietary iron deficiency
Haemoglobinopathies and haemolytic anaemias
Other neglected tropical diseases
Vitamin A deficiency
Other unspecified infectious diseases
Intestinal nematode infections
Malaria
Chronic kidney disease
Endocrine, metabolic, blood, and immune disorders
Upper digestive system diseases
Cirrhosis and other chronic liver diseases
HIV/AIDS
Inflammatory bowel disease
Schistosomiasis
Gynecological diseases
Maternal disorders
YLDs per 100,000 population
0 to <0.05
0.05 to <0.32
0.32 to <0.67
0.67 to <2.52
2.52 to <5.35
5.35 to <12.96
12.96 to <19.43
19.43 to <32.98
32.98 to <79.82
79.82 to 836

Bermuda

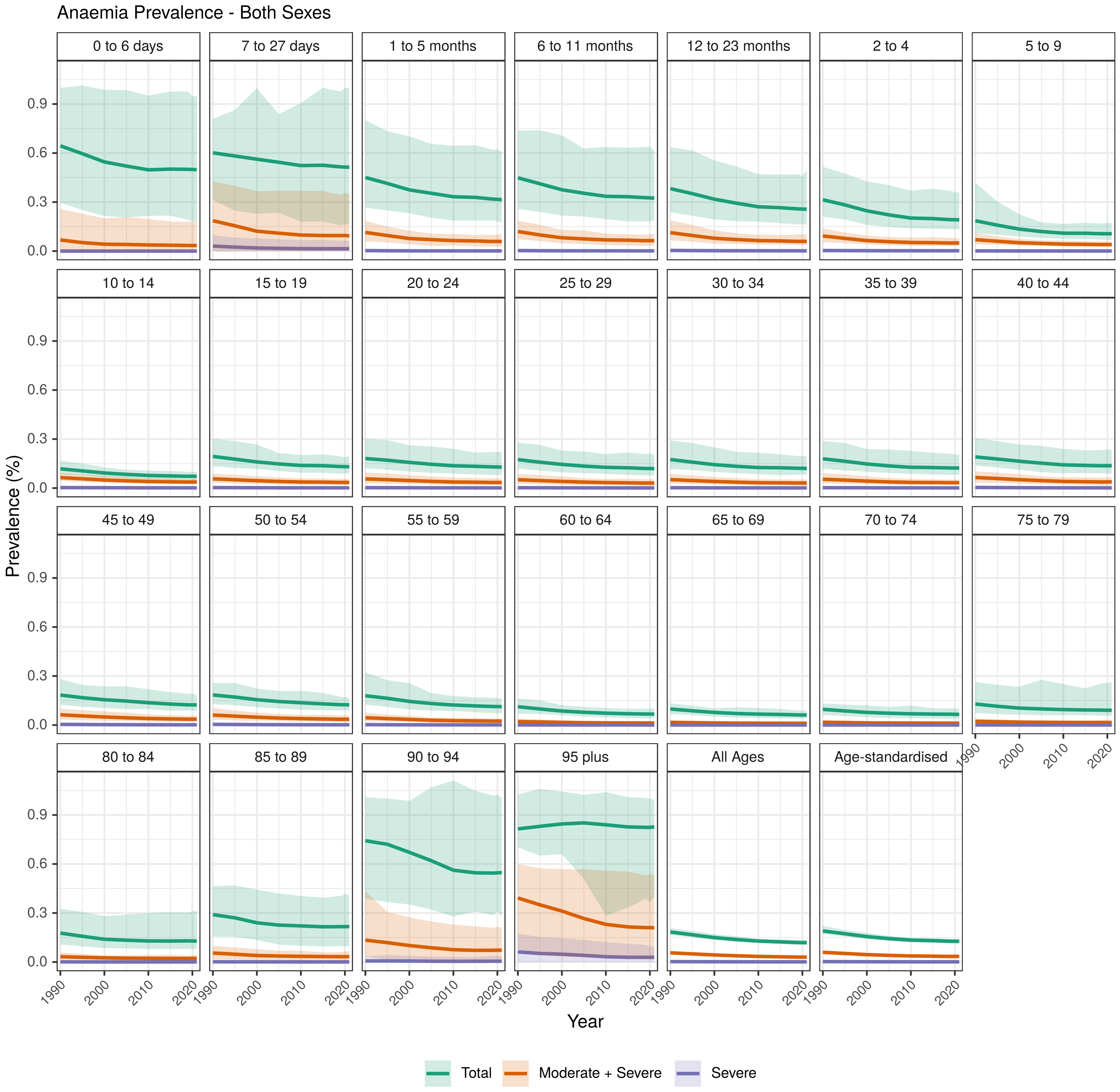

Anaemia Prevalence - Both Sexes
0 to 6 days
7 to 27 days
1 to 5 months
6 to 11 months
12 to 23 months
2 to 4
5 to 9
10 to 14
15 to 19
20 to 24
25 to 29
30 to 34
35 to 39
40 to 44
45 to 49
50 to 54
55 to 59
60 to 64
65 to 69
70 to 74
75 to 79
80 to 84
85 to 89
90 to 94
95 plus
All Ages
Age-standardised
Prevalence (%)
Year
Total
Moderate + Severe
Severe

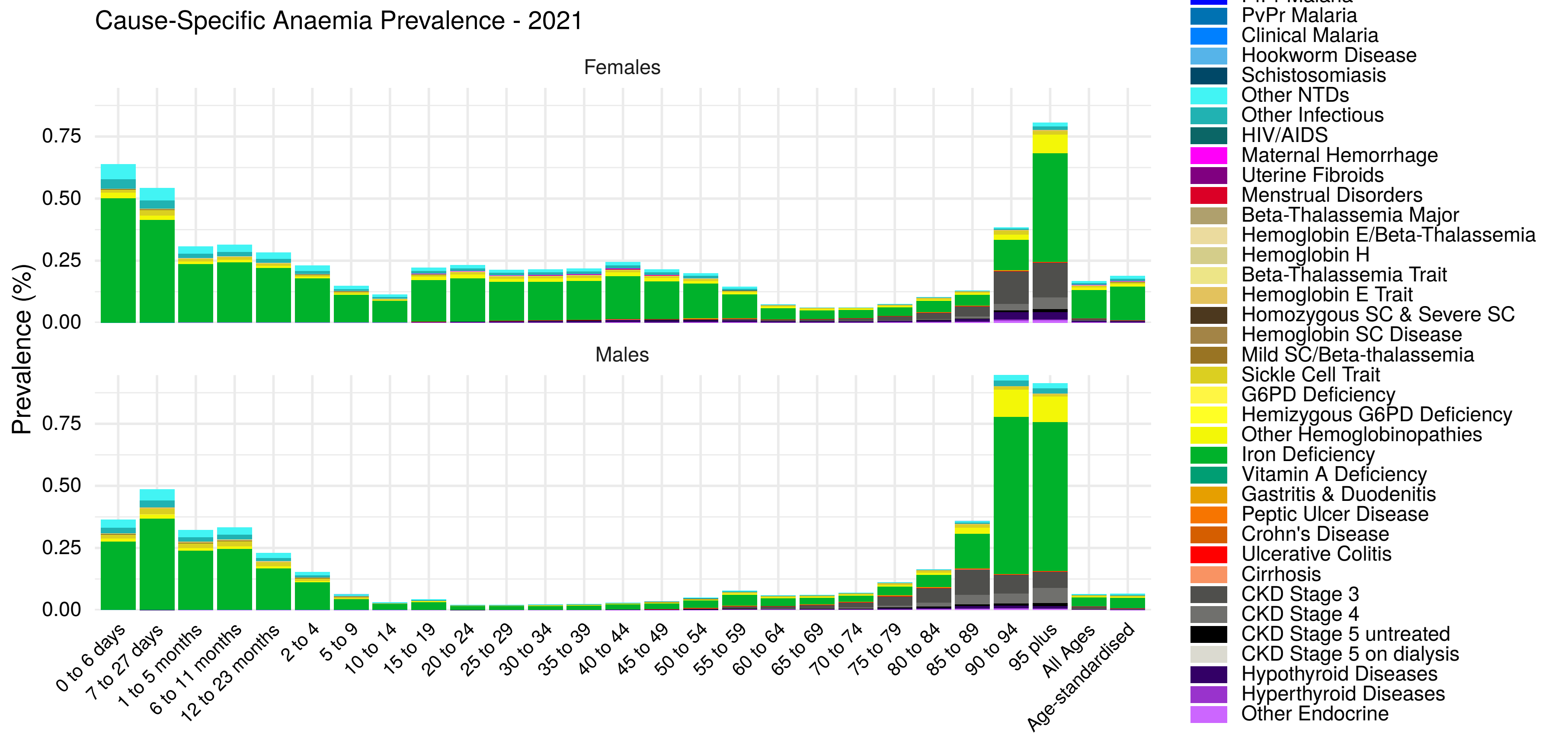

Cause-Specific Anaemia Prevalence - 2021
Females
Males
Prevalence (%)
PfPr Malaria
PvPr Malaria
Clinical Malaria
Hookworm Disease
Schistosomiasis
Other NTDs
Other Infectious
HIV/AIDS
Maternal Hemorrhage
Uterine Fibroids
Menstrual Disorders
Beta-Thalassemia Major
Hemoglobin E/Beta-Thalassemia
Hemoglobin H
Beta-Thalassemia Trait
Hemoglobin E Trait
Homozygous SC & Severe SC
Hemoglobin SC Disease
Mild SC/Beta-thalassemia
Sickle Cell Trait
G6PD Deficiency
Hemizygous G6PD Deficiency
Other Hemoglobinopathies
Iron Deficiency
Vitamin A Deficiency
Gastritis & Duodenitis
Peptic Ulcer Disease
Crohn's Disease
Ulcerative Colitis
Cirrhosis
CKD Stage 3
CKD Stage 4
CKD Stage 5 untreated
CKD Stage 5 on dialysis
Hypothyroid Diseases
Hyperthyroid Diseases
Other Endocrine

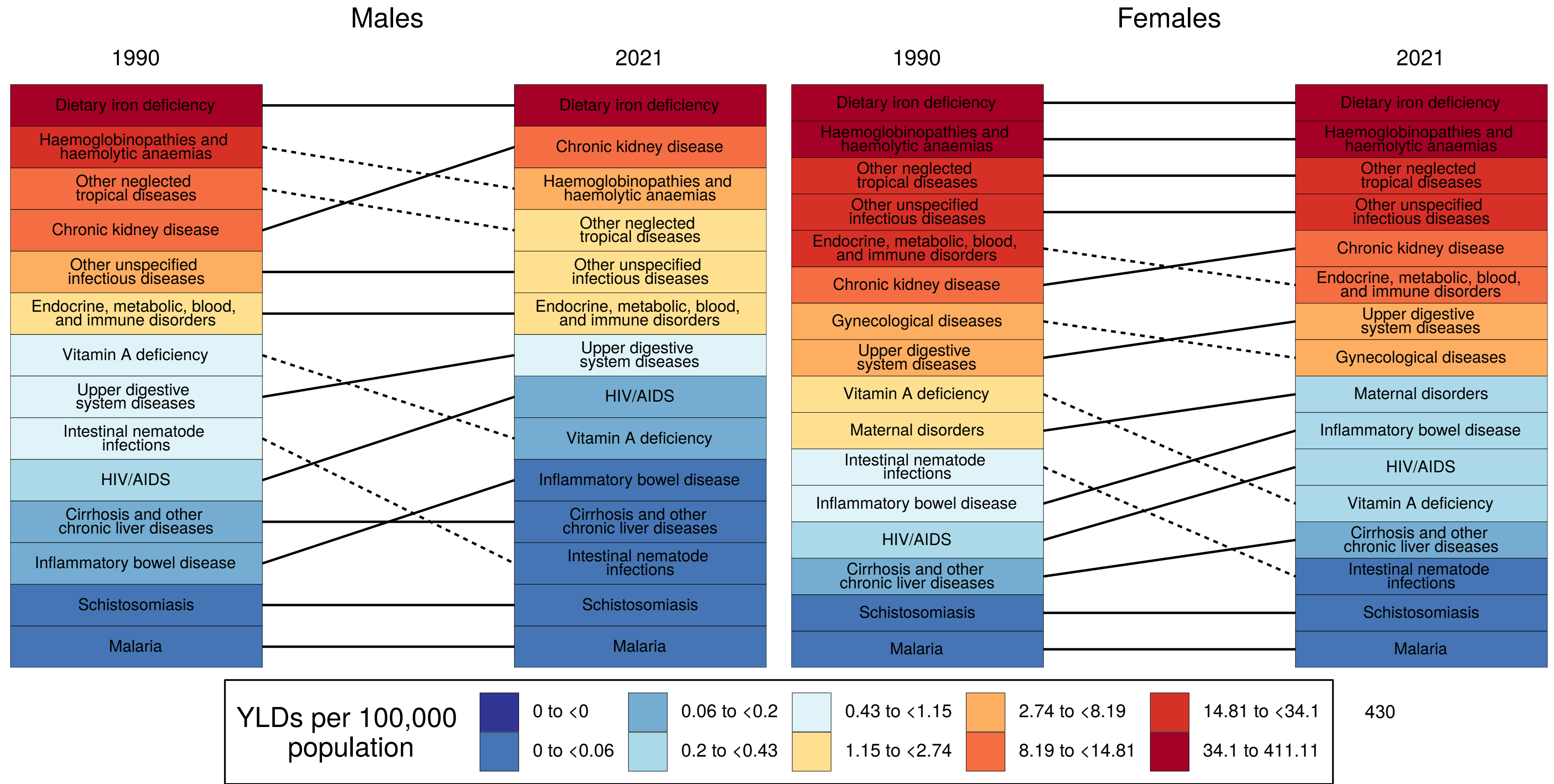

Males
Females
1990
2021
Dietary iron deficiency
Haemoglobinopathies and haemolytic anaemias
Other neglected tropical diseases
Chronic kidney disease
Other unspecified infectious diseases
Endocrine, metabolic, blood, and immune disorders
Vitamin A deficiency
Upper digestive system diseases
Intestinal nematode infections
HIV/AIDS
Cirrhosis and other chronic liver diseases
Inflammatory bowel disease
Schistosomiasis
Malaria
Gynecological diseases
Maternal disorders
YLDs per 100,000 population
0 to <0
0 to <0.06
0.06 to <0.2
0.2 to <0.43
0.43 to <1.15
1.15 to <2.74
2.74 to <8.19
8.19 to <14.81
14.81 to <34.1
34.1 to 411.11
430

Cuba

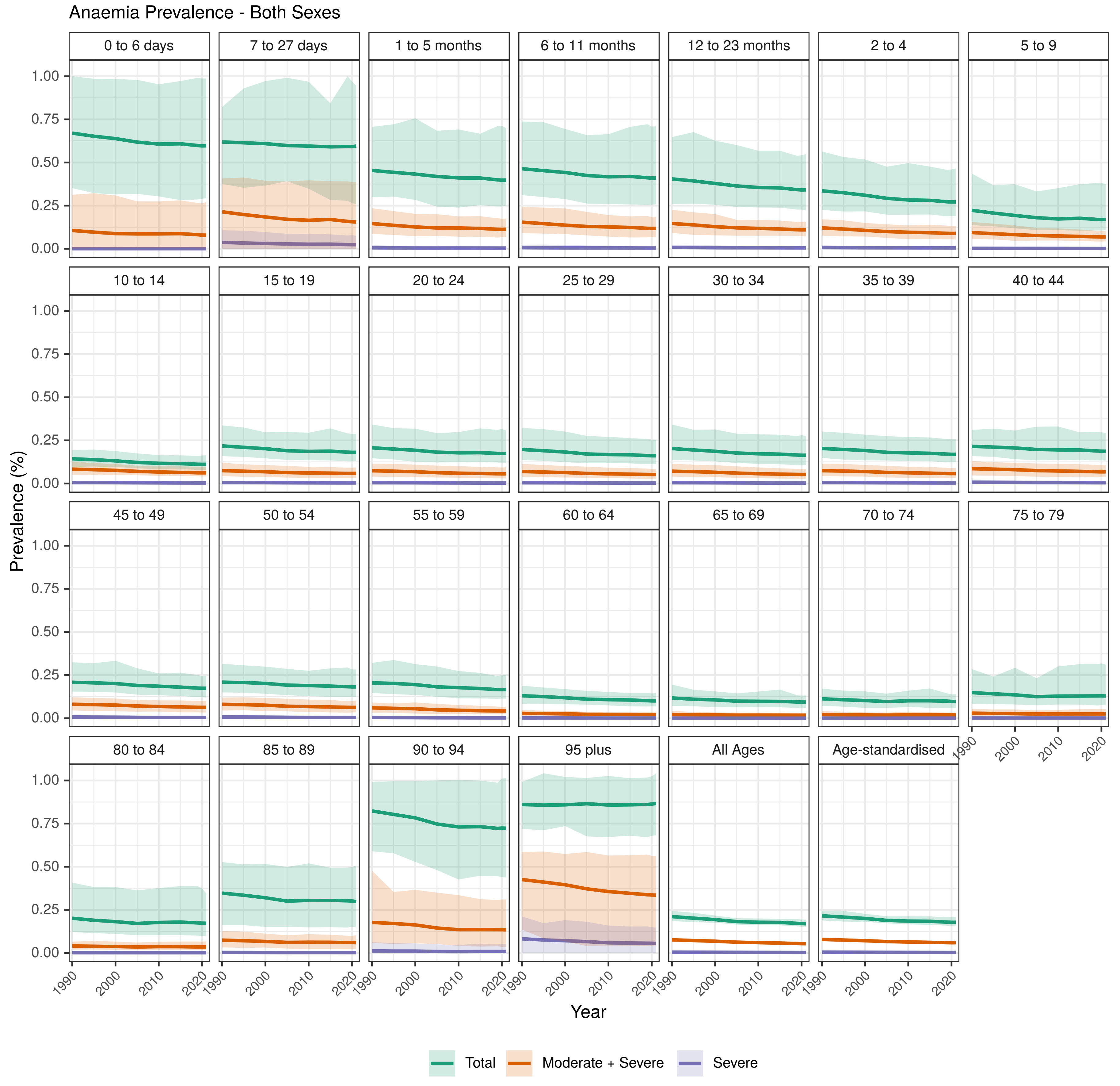

Anaemia Prevalence - Both Sexes
0 to 6 days
7 to 27 days
1 to 5 months
6 to 11 months
12 to 23 months
2 to 4
5 to 9
10 to 14
15 to 19
20 to 24
25 to 29
30 to 34
35 to 39
40 to 44
45 to 49
50 to 54
55 to 59
60 to 64
65 to 69
70 to 74
75 to 79
80 to 84
85 to 89
90 to 94
95 plus
All Ages
Age-standardised
Prevalence (%)
Year
Total
Moderate + Severe
Severe

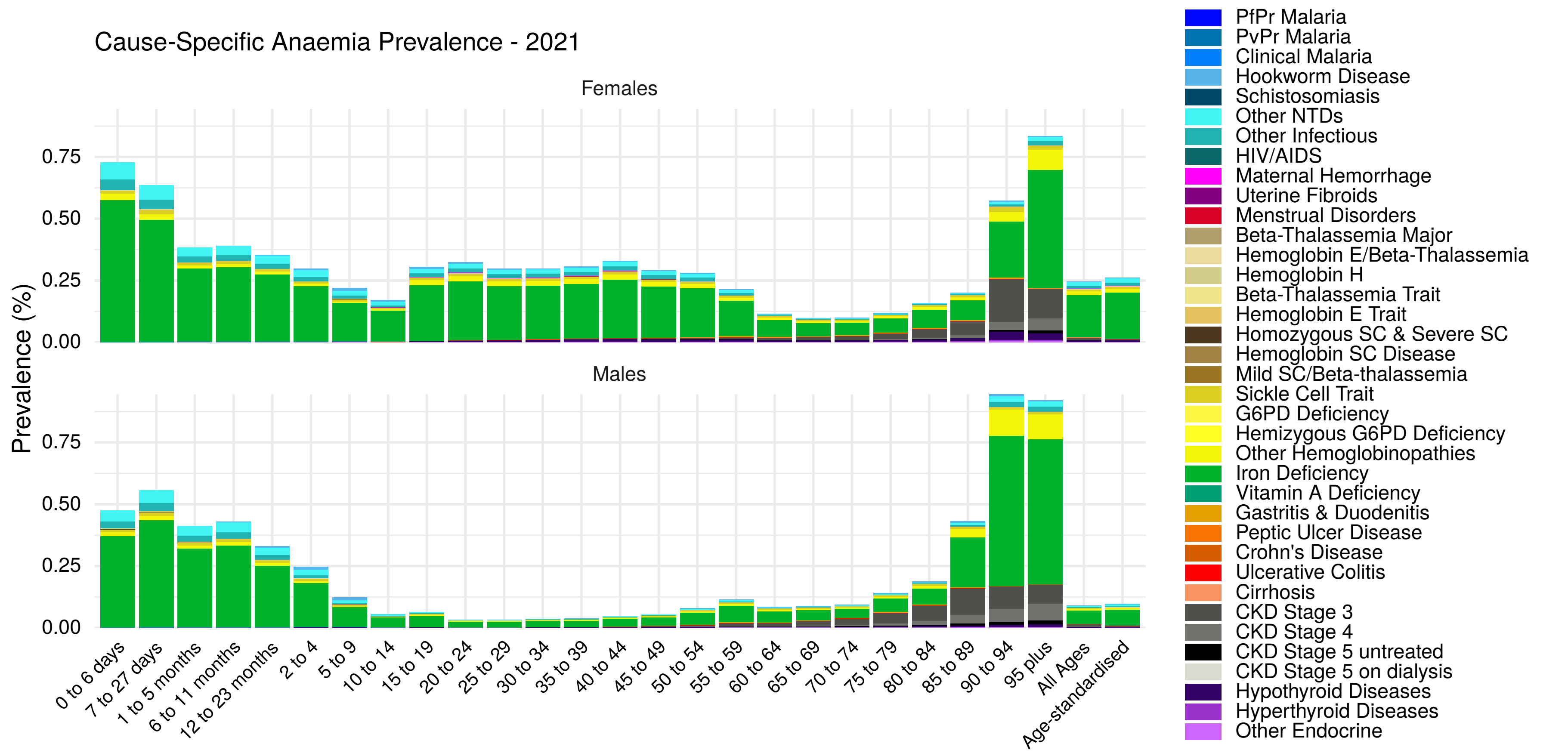

Cause-Specific Anaemia Prevalence - 2021
Females
Males
Prevalence (%)
PfPr Malaria
PvPr Malaria
Clinical Malaria
Hookworm Disease
Schistosomiasis
Other NTDs
Other Infectious
HIV/AIDS
Maternal Hemorrhage
Uterine Fibroids
Menstrual Disorders
Beta-Thalassemia Major
Hemoglobin E/Beta-Thalassemia
Hemoglobin H
Beta-Thalassemia Trait
Hemoglobin E Trait
Homozygous SC & Severe SC
Hemoglobin SC Disease
Mild SC/Beta-thalassemia
Sickle Cell Trait
G6PD Deficiency
Hemizygous G6PD Deficiency
Other Hemoglobinopathies
Iron Deficiency
Vitamin A Deficiency
Gastritis & Duodenitis
Peptic Ulcer Disease
Crohn's Disease
Cirrhosis
Ulcerative Colitis
CKD Stage 3
CKD Stage 4
CKD Stage 5 untreated
CKD Stage 5 on dialysis
Hypothyroid Diseases
Hyperthyroid Diseases
Other Endocrine

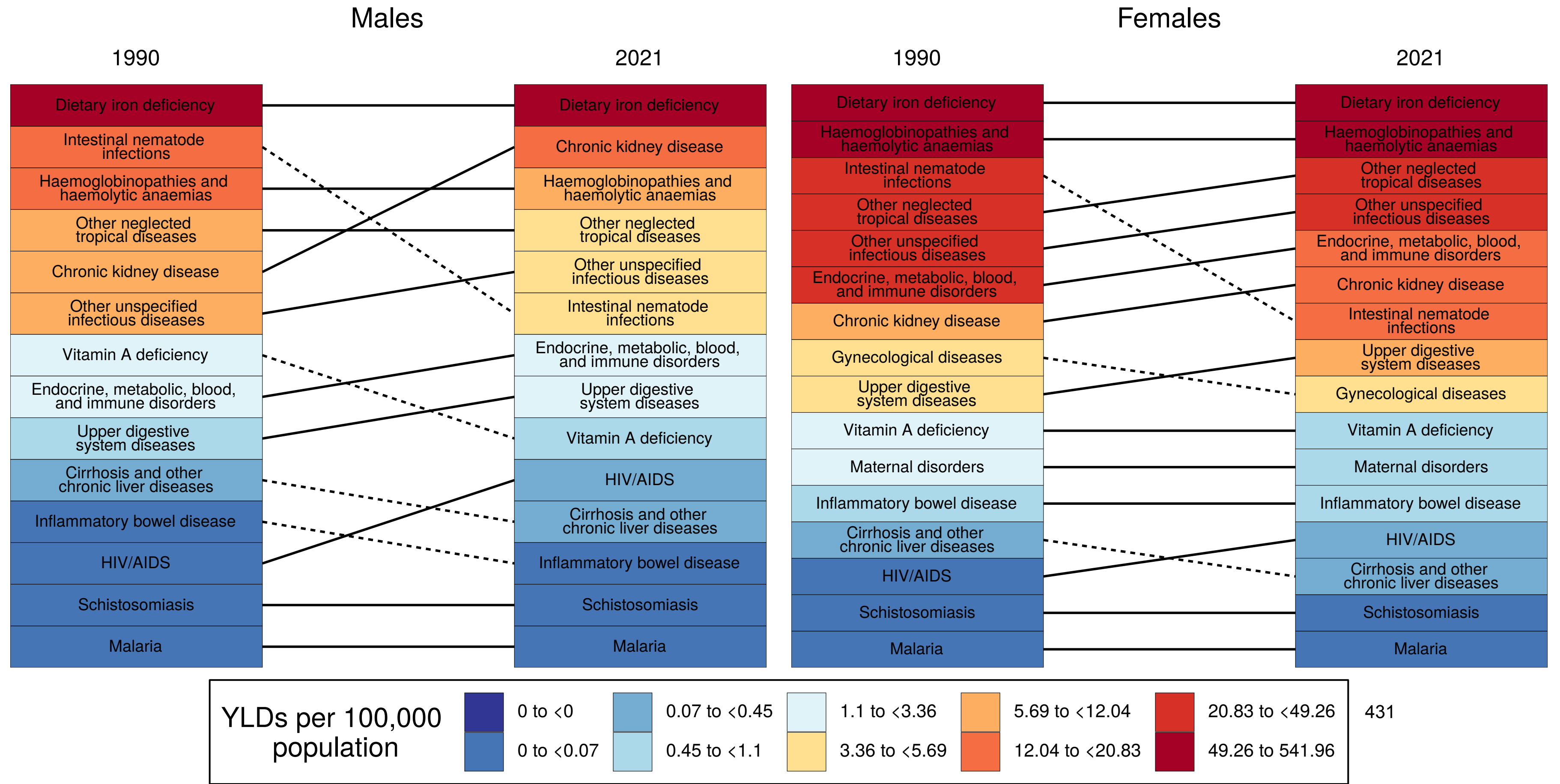

Males
Females
1990
2021
Dietary iron deficiency
Intestinal nematode infections
Haemoglobinopathies and haemolytic anaemias
Other neglected tropical diseases
Chronic kidney disease
Other unspecified infectious diseases
Vitamin A deficiency
Endocrine, metabolic, blood, and immune disorders
Upper digestive system diseases
Cirrhosis and other chronic liver diseases
Inflammatory bowel disease
HIV/AIDS
Schistosomiasis
Malaria
Gynecological diseases
Maternal disorders
YLDs per 100,000 population
0 to <0
0 to <0.07
0.07 to <0.45
0.45 to <1.1
1.1 to <3.36
3.36 to <5.69
5.69 to <12.04
12.04 to <20.83
20.83 to <49.26
49.26 to 541.96
431

Dominica

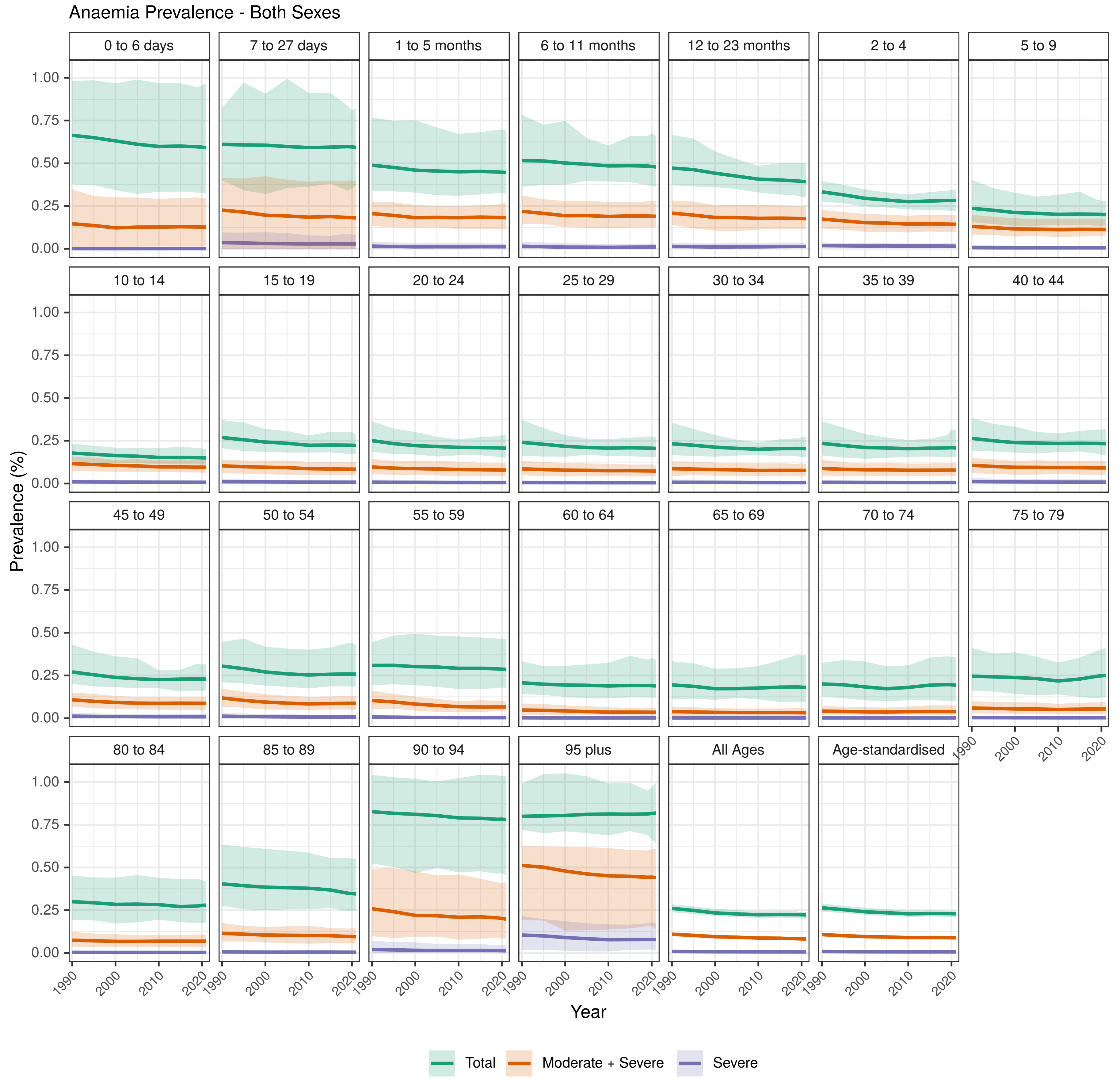
Anaemia Prevalence - Both Sexes
0 to 6 days
7 to 27 days
1 to 5 months
6 to 11 months
12 to 23 months
2 to 4
5 to 9
10 to 14
15 to 19
20 to 24
25 to 29
30 to 34
35 to 39
40 to 44
45 to 49
50 to 54
55 to 59
60 to 64
65 to 69
70 to 74
75 to 79
80 to 84
85 to 89
90 to 94
95 plus
All Ages
Age-standardised
Prevalence (%)
0.00
0.25
0.50
0.75
1.00
1990
2000
2010
2020
Year
Total
Moderate + Severe
Severe

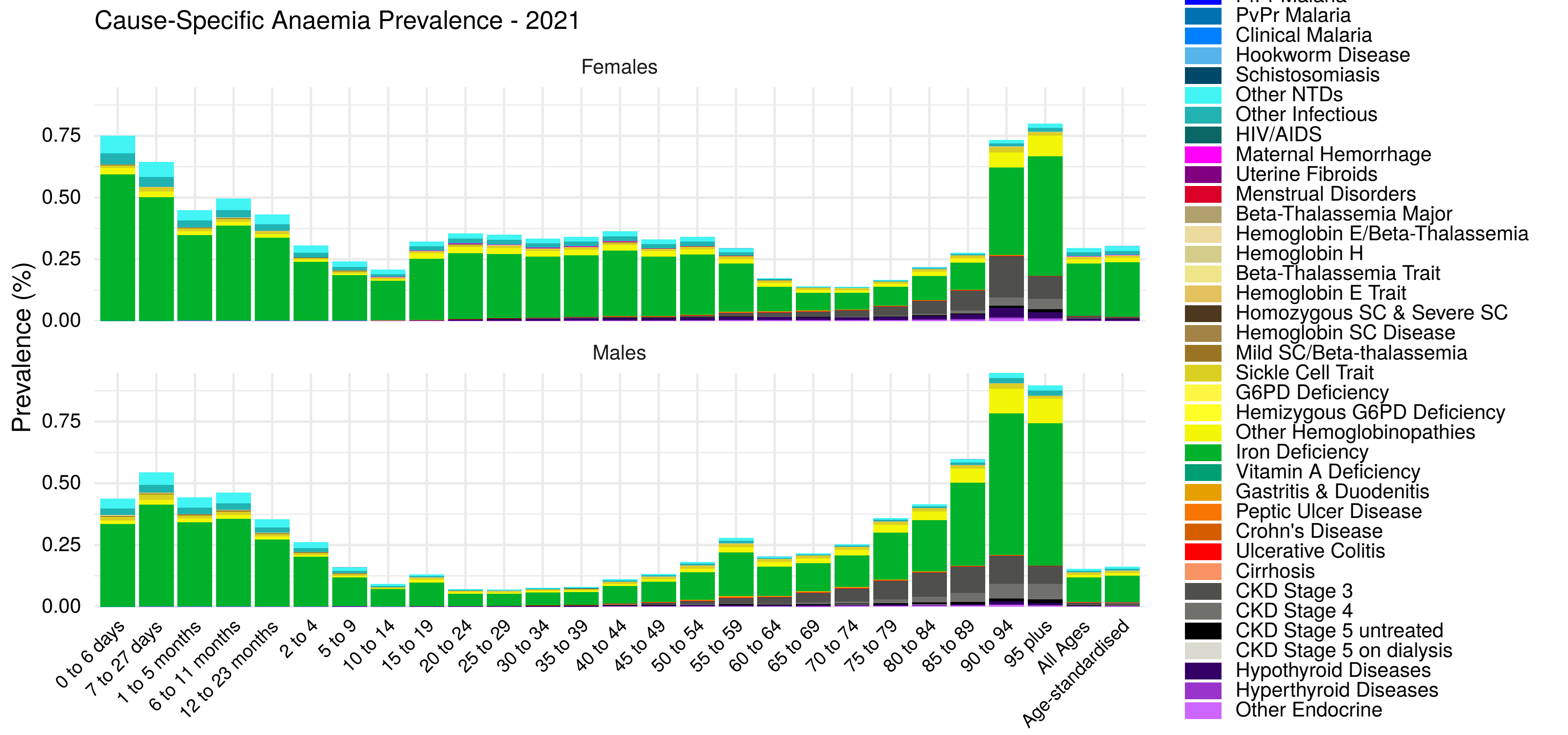
Cause-Specific Anaemia Prevalence - 2021
Females
Males
Prevalence (%)
0.00
0.25
0.50
0.75
0 to 6 days
7 to 27 days
1 to 5 months
6 to 11 months
12 to 23 months
2 to 4
5 to 9
10 to 14
15 to 19
20 to 24
25 to 29
30 to 34
35 to 39
40 to 44
45 to 49
50 to 54
55 to 59
60 to 64
65 to 69
70 to 74
75 to 79
80 to 84
85 to 89
90 to 94
95 plus
All Ages
Age-standardised
PfPr Malaria
PvPr Malaria
Clinical Malaria
Hookworm Disease
Schistosomiasis
Other NTDs
Other Infectious
HIV/AIDS
Maternal Hemorrhage
Uterine Fibroids
Menstrual Disorders
Beta-Thalassemia Major
Hemoglobin E/Beta-Thalassemia
Hemoglobin H
Beta-Thalassemia Trait
Hemoglobin E Trait
Homozygous SC & Severe SC
Hemoglobin SC Disease
Mild SC/Beta-thalassemia
Sickle Cell Trait
G6PD Deficiency
Hemizygous G6PD Deficiency
Other Hemoglobinopathies
Iron Deficiency
Vitamin A Deficiency
Gastritis & Duodenitis
Peptic Ulcer Disease
Crohn's Disease
Ulcerative Colitis
Cirrhosis
CKD Stage 3
CKD Stage 4
CKD Stage 5 untreated
CKD Stage 5 on dialysis
Hypothyroid Diseases
Hyperthyroid Diseases
Other Endocrine

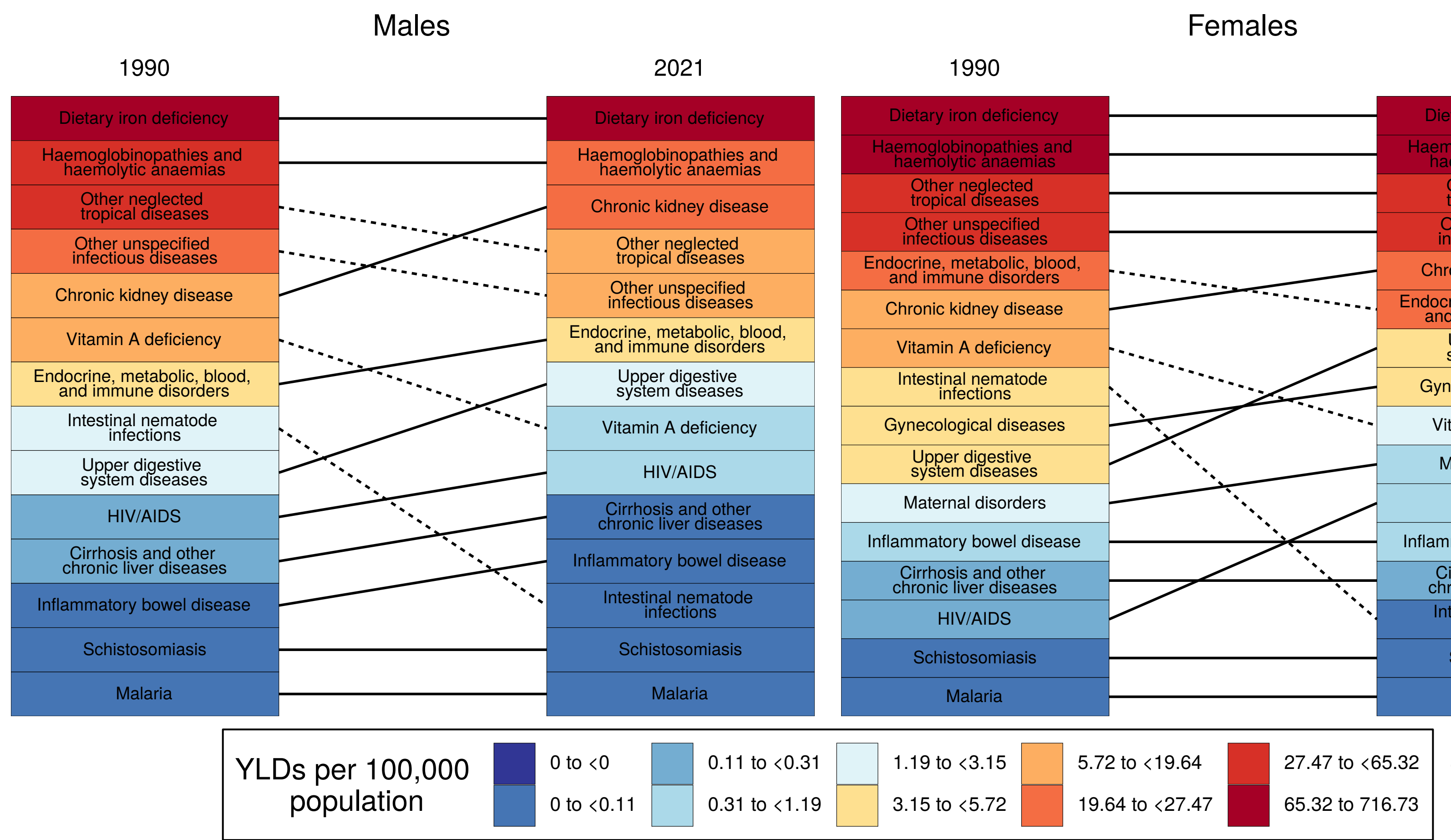
Males
1990
Dietary iron deficiency
Haemoglobinopathies and haemolytic anaemias
Other neglected tropical diseases
Other unspecified infectious diseases
Chronic kidney disease
Vitamin A deficiency
Endocrine, metabolic, blood, and immune disorders
Intestinal nematode infections
Upper digestive system diseases
HIV/AIDS
Cirrhosis and other chronic liver diseases
Inflammatory bowel disease
Schistosomiasis
Malaria
2021
Dietary iron deficiency
Haemoglobinopathies and haemolytic anaemias
Chronic kidney disease
Other neglected tropical diseases
Other unspecified infectious diseases
Endocrine, metabolic, blood, and immune disorders
Upper digestive system diseases
Vitamin A deficiency
HIV/AIDS
Cirrhosis and other chronic liver diseases
Inflammatory bowel disease
Intestinal nematode infections
Schistosomiasis
Malaria
Females
1990
Dietary iron deficiency
Haemoglobinopathies and haemolytic anaemias
Other neglected tropical diseases
Other unspecified infectious diseases
Endocrine, metabolic, blood, and immune disorders
Chronic kidney disease
Vitamin A deficiency
Intestinal nematode infections
Gynecological diseases
Upper digestive system diseases
Maternal disorders
Inflammatory bowel disease
Cirrhosis and other chronic liver diseases
HIV/AIDS
Schistosomiasis
Malaria
2021
Dietary iron deficiency
Haemoglobinopathies and haemolytic anaemias
Other neglected tropical diseases
Other unspecified infectious diseases
Chronic kidney disease
Endocrine, metabolic, blood, and immune disorders
Upper digestive system diseases
Gynecological diseases
Vitamin A deficiency
Maternal disorders
HIV/AIDS
Inflammatory bowel disease
Cirrhosis and other chronic liver diseases
Intestinal nematode infections
Schistosomiasis
Malaria
YLDs per 100,000 population
0 to <0
0 to <0.11
0.11 to <0.31
0.31 to <1.19
1.19 to <3.15
3.15 to <5.72
5.72 to <19.64
19.64 to <27.47
27.47 to <65.32
65.32 to 716.73

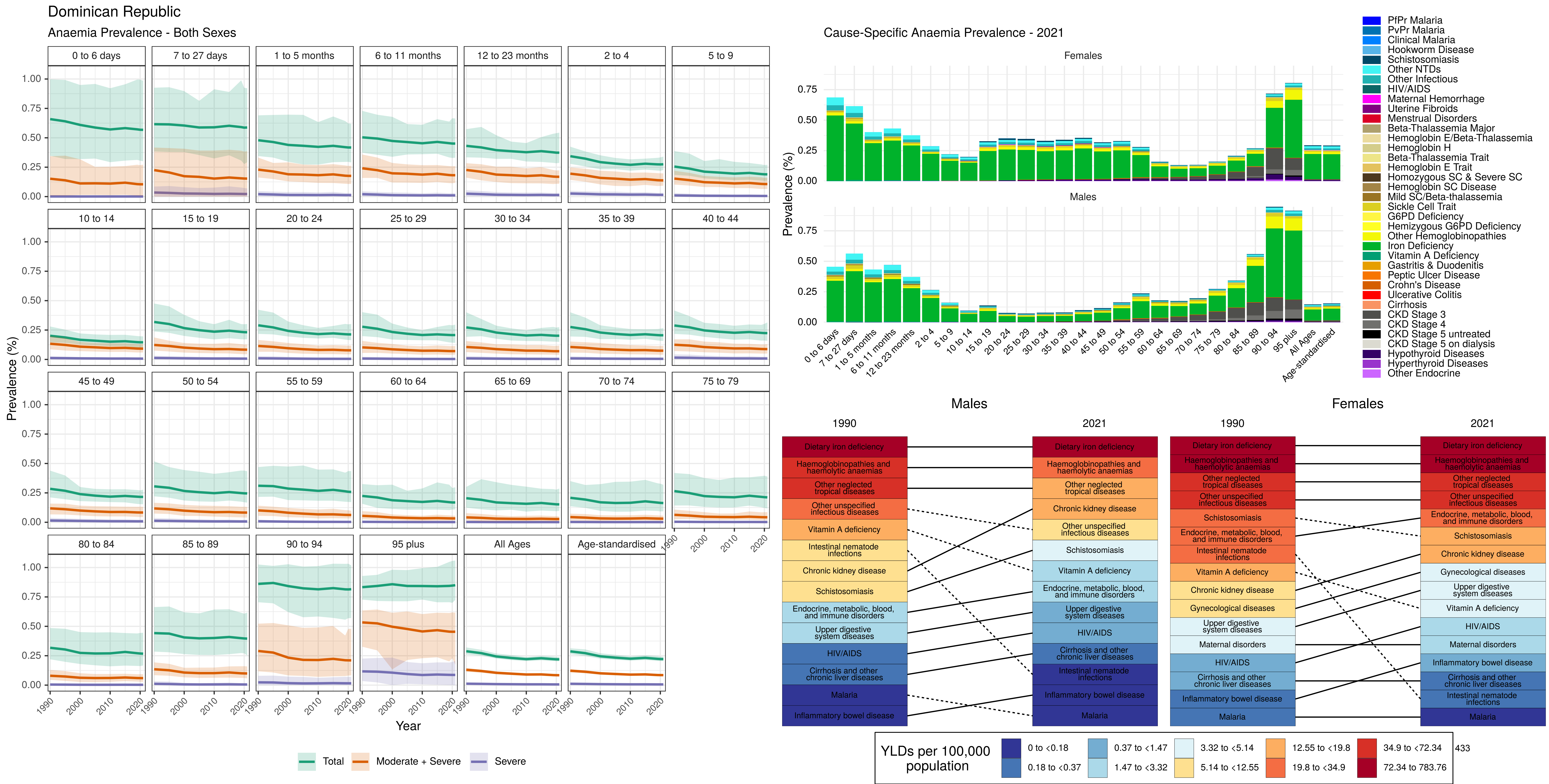

Dominican Republic
Anaemia Prevalence - Both Sexes
0 to 6 days
7 to 27 days
1 to 5 months
6 to 11 months
12 to 23 months
2 to 4
5 to 9
10 to 14
15 to 19
20 to 24
25 to 29
30 to 34
35 to 39
40 to 44
45 to 49
50 to 54
55 to 59
60 to 64
65 to 69
70 to 74
75 to 79
80 to 84
85 to 89
90 to 94
95 plus
All Ages
Age-standardised
Prevalence (%)
Year
Total
Moderate + Severe
Severe
Cause-Specific Anaemia Prevalence - 2021
Females
Males
PfPr Malaria
PvPr Malaria
Clinical Malaria
Hookworm Disease
Schistosomiasis
Other NTDs
Other Infectious
HIV/AIDS
Maternal Hemorrhage
Uterine Fibroids
Menstrual Disorders
Beta-Thalassemia Major
Hemoglobin E/Beta-Thalassemia
Hemoglobin H
Beta-Thalassemia Trait
Hemoglobin E Trait
Homozygous SC & Severe SC
Hemoglobin SC Disease
Mild SC/Beta-thalassemia
Sickle Cell Trait
G6PD Deficiency
Hemizygous G6PD Deficiency
Other Hemoglobinopathies
Iron Deficiency
Vitamin A Deficiency
Gastritis & Duodenitis
Peptic Ulcer Disease
Crohn's Disease
Ulcerative Colitis
Cirrhosis
CKD Stage 3
CKD Stage 4
CKD Stage 5 untreated
CKD Stage 5 on dialysis
Hypothyroid Diseases
Hyperthyroid Diseases
Other Endocrine
Males
1990
2021
Dietary iron deficiency
Haemoglobinopathies and haemolytic anaemias
Other neglected tropical diseases
Other unspecified infectious diseases
Vitamin A deficiency
Intestinal nematode infections
Chronic kidney disease
Schistosomiasis
Endocrine, metabolic, blood, and immune disorders
Upper digestive system diseases
HIV/AIDS
Cirrhosis and other chronic liver diseases
Malaria
Inflammatory bowel disease
Females
1990
2021
Dietary iron deficiency
Haemoglobinopathies and haemolytic anaemias
Other neglected tropical diseases
Other unspecified infectious diseases
Schistosomiasis
Endocrine, metabolic, blood, and immune disorders
Intestinal nematode infections
Vitamin A deficiency
Chronic kidney disease
Gynecological diseases
Upper digestive system diseases
Maternal disorders
HIV/AIDS
Cirrhosis and other chronic liver diseases
Inflammatory bowel disease
Malaria
YLDs per 100,000 population
0 to <0.18
0.18 to <0.37
0.37 to <1.47
1.47 to <3.32
3.32 to <5.14
5.14 to <12.55
12.55 to <19.8
19.8 to <34.9
34.9 to <72.34
72.34 to 783.76

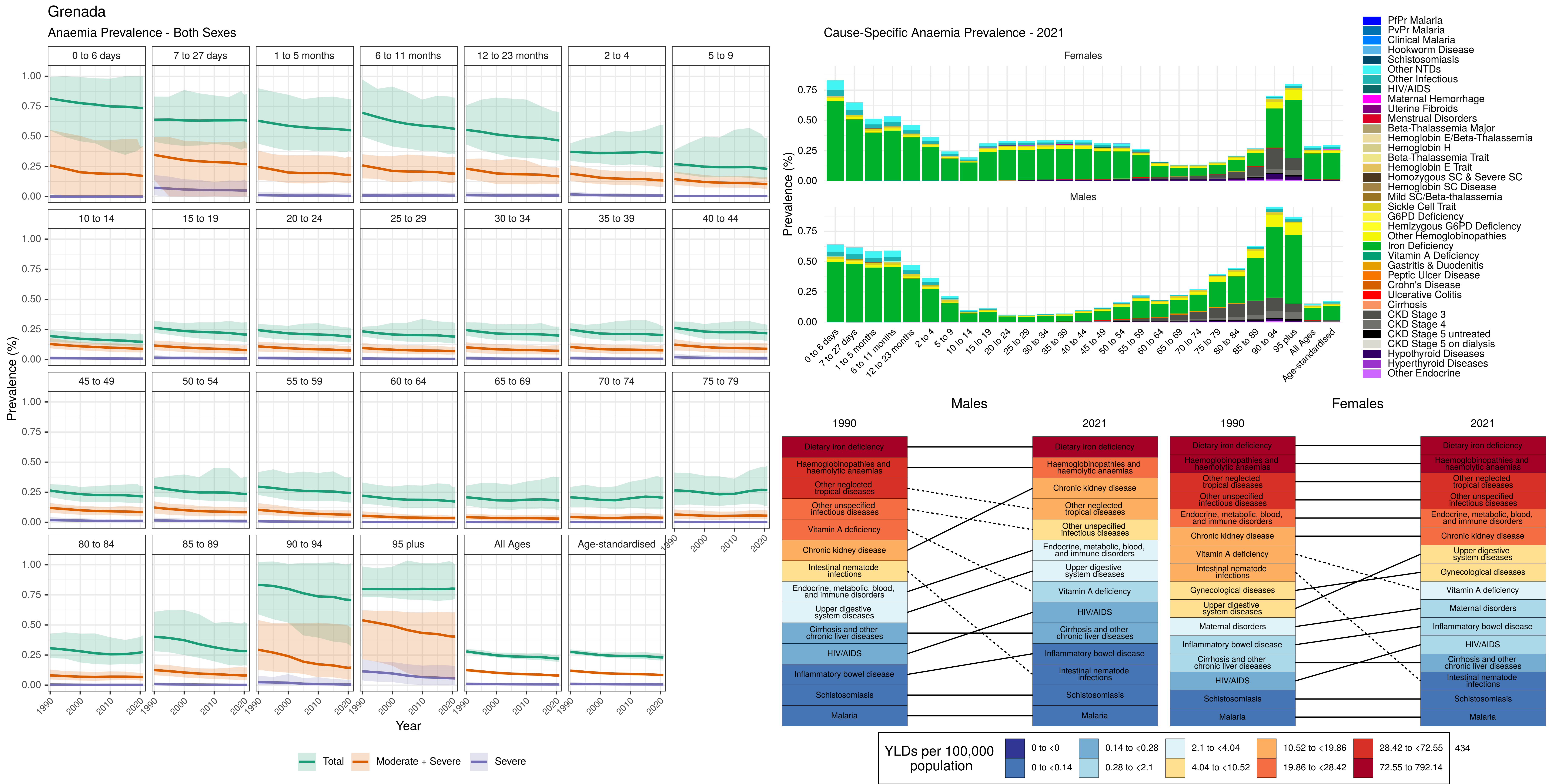
Grenada
Anaemia Prevalence - Both Sexes
0 to 6 days
7 to 27 days
1 to 5 months
6 to 11 months
12 to 23 months
2 to 4
5 to 9
10 to 14
15 to 19
20 to 24
25 to 29
30 to 34
35 to 39
40 to 44
45 to 49
50 to 54
55 to 59
60 to 64
65 to 69
70 to 74
75 to 79
80 to 84
85 to 89
90 to 94
95 plus
All Ages
Age-standardised
Prevalence (%)
1.00
0.75
0.50
0.25
0.00
1990
2000
2010
2020
Year
Total
Moderate + Severe
Severe
Cause-Specific Anaemia Prevalence - 2021
Females
Males
Prevalence (%)
PfPr Malaria
PvPr Malaria
Clinical Malaria
Hookworm Disease
Schistosomiasis
Other NTDs
Other Infectious
HIV/AIDS
Maternal Hemorrhage
Uterine Fibroids
Menstrual Disorders
Beta-Thalassemia Major
Hemoglobin E/Beta-Thalassemia
Hemoglobin H
Beta-Thalassemia Trait
Hemoglobin E Trait
Homozygous SC & Severe SC
Hemoglobin SC Disease
Mild SC/Beta-thalassemia
Sickle Cell Trait
G6PD Deficiency
Hemizygous G6PD Deficiency
Other Hemoglobinopathies
Iron Deficiency
Vitamin A Deficiency
Gastritis & Duodenitis
Peptic Ulcer Disease
Crohn's Disease
Ulcerative Colitis
Cirrhosis
CKD Stage 3
CKD Stage 4
CKD Stage 5 untreated
CKD Stage 5 on dialysis
Hypothyroid Diseases
Hyperthyroid Diseases
Other Endocrine
Males
Females
1990
2021
Dietary iron deficiency
Haemoglobinopathies and haemolytic anaemias
Other neglected tropical diseases
Other unspecified infectious diseases
Vitamin A deficiency
Chronic kidney disease
Intestinal nematode infections
Endocrine, metabolic, blood, and immune disorders
Upper digestive system diseases
Cirrhosis and other chronic liver diseases
HIV/AIDS
Inflammatory bowel disease
Schistosomiasis
Malaria
Gynecological diseases
Maternal disorders
YLDs per 100,000 population
0 to <0
0 to <0.14
0.14 to <0.28
0.28 to <2.1
2.1 to <4.04
4.04 to <10.52
10.52 to <19.86
19.86 to <28.42
28.42 to <72.55
72.55 to 792.14
434

Guyana

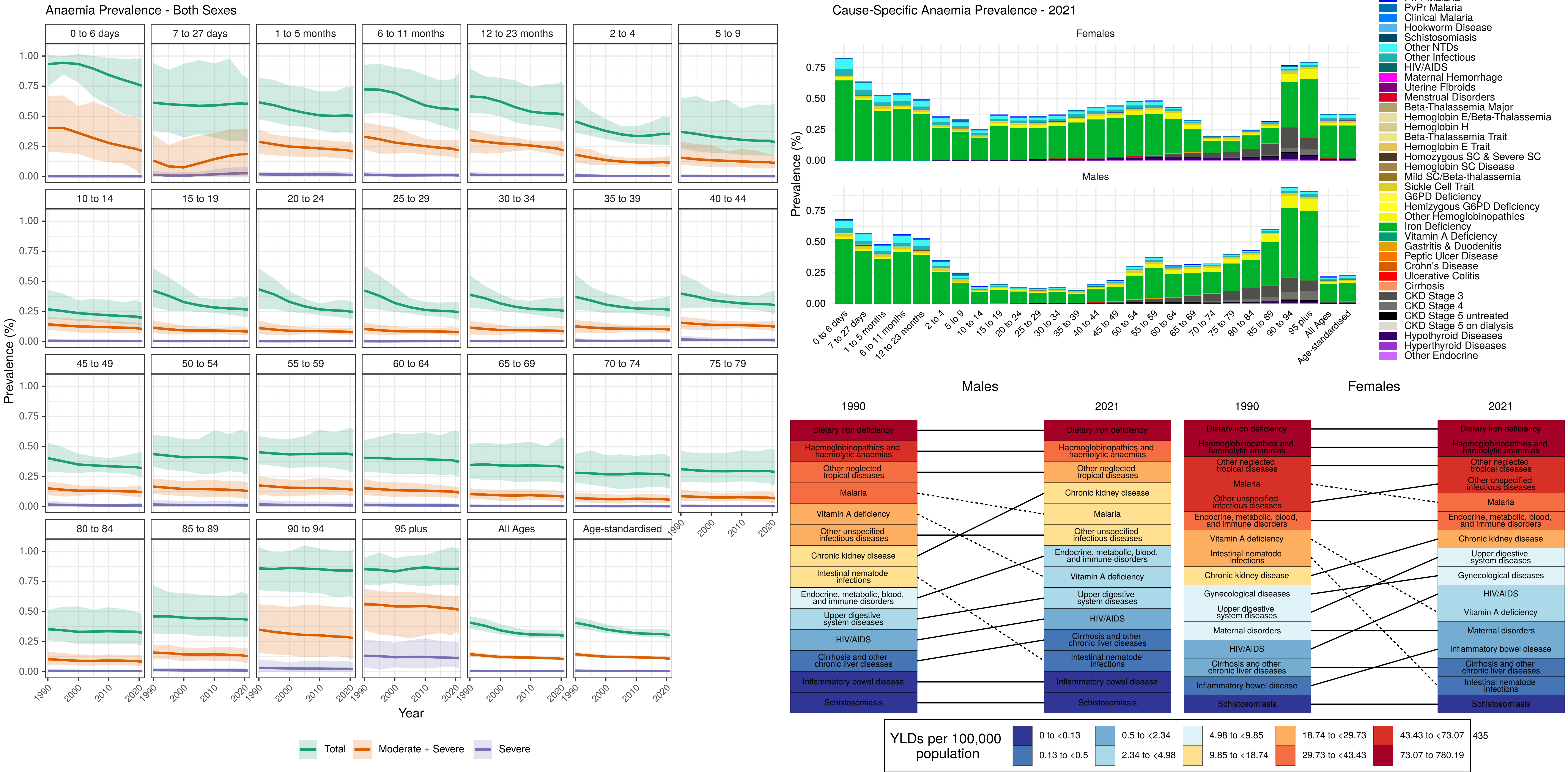

Anaemia Prevalence - Both Sexes
0 to 6 days
7 to 27 days
1 to 5 months
6 to 11 months
12 to 23 months
2 to 4
5 to 9
10 to 14
15 to 19
20 to 24
25 to 29
30 to 34
35 to 39
40 to 44
45 to 49
50 to 54
55 to 59
60 to 64
65 to 69
70 to 74
75 to 79
80 to 84
85 to 89
90 to 94
95 plus
All Ages
Age-standardised
Prevalence (%)
Year
Total
Moderate + Severe
Severe
Cause-Specific Anaemia Prevalence - 2021
Females
Males
Prevalence (%)
PfPr Malaria
PvPr Malaria
Clinical Malaria
Hookworm Disease
Schistosomiasis
Other NTDs
Other Infectious
HIV/AIDS
Maternal Hemorrhage
Uterine Fibroids
Menstrual Disorders
Beta-Thalassemia Major
Hemoglobin E/Beta-Thalassemia
Hemoglobin H
Beta-Thalassemia Trait
Hemoglobin E Trait
Homozygous SC & Severe SC
Hemoglobin SC Disease
Mild SC/Beta-thalassemia
Sickle Cell Trait
G6PD Deficiency
Hemizygous G6PD Deficiency
Other Hemoglobinopathies
Iron Deficiency
Vitamin A Deficiency
Gastritis & Duodenitis
Peptic Ulcer Disease
Crohn's Disease
Ulcerative Colitis
Cirrhosis
CKD Stage 3
CKD Stage 4
CKD Stage 5 untreated
CKD Stage 5 on dialysis
Hypothyroid Diseases
Hyperthyroid Diseases
Other Endocrine
Males
Females
1990
2021
Dietary iron deficiency
Haemoglobinopathies and haemolytic anaemias
Other neglected tropical diseases
Malaria
Vitamin A deficiency
Other unspecified infectious diseases
Chronic kidney disease
Intestinal nematode infections
Endocrine, metabolic, blood, and immune disorders
Upper digestive system diseases
HIV/AIDS
Cirrhosis and other chronic liver diseases
Inflammatory bowel disease
Schistosomiasis
Gynecological diseases
Maternal disorders
YLDs per 100,000 population
0 to <0.13
0.13 to <0.5
0.5 to <2.34
2.34 to <4.98
4.98 to <9.85
9.85 to <18.74
18.74 to <29.73
29.73 to <43.43
43.43 to <73.07
73.07 to 780.19

Haiti

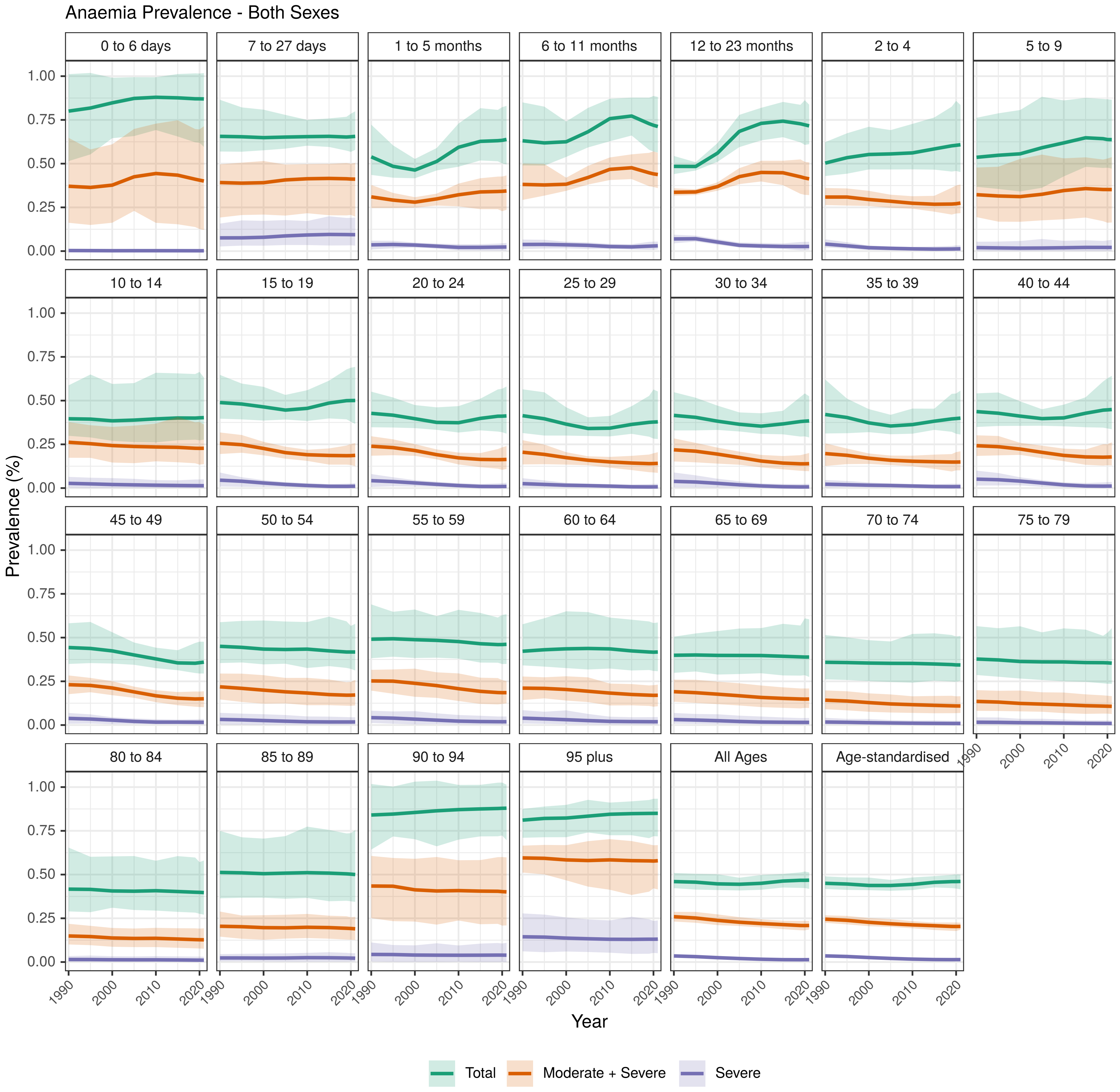

Anaemia Prevalence - Both Sexes
0 to 6 days
7 to 27 days
1 to 5 months
6 to 11 months
12 to 23 months
2 to 4
5 to 9
10 to 14
15 to 19
20 to 24
25 to 29
30 to 34
35 to 39
40 to 44
45 to 49
50 to 54
55 to 59
60 to 64
65 to 69
70 to 74
75 to 79
80 to 84
85 to 89
90 to 94
95 plus
All Ages
Age-standardised
Prevalence (%)
Year
Total
Moderate + Severe
Severe

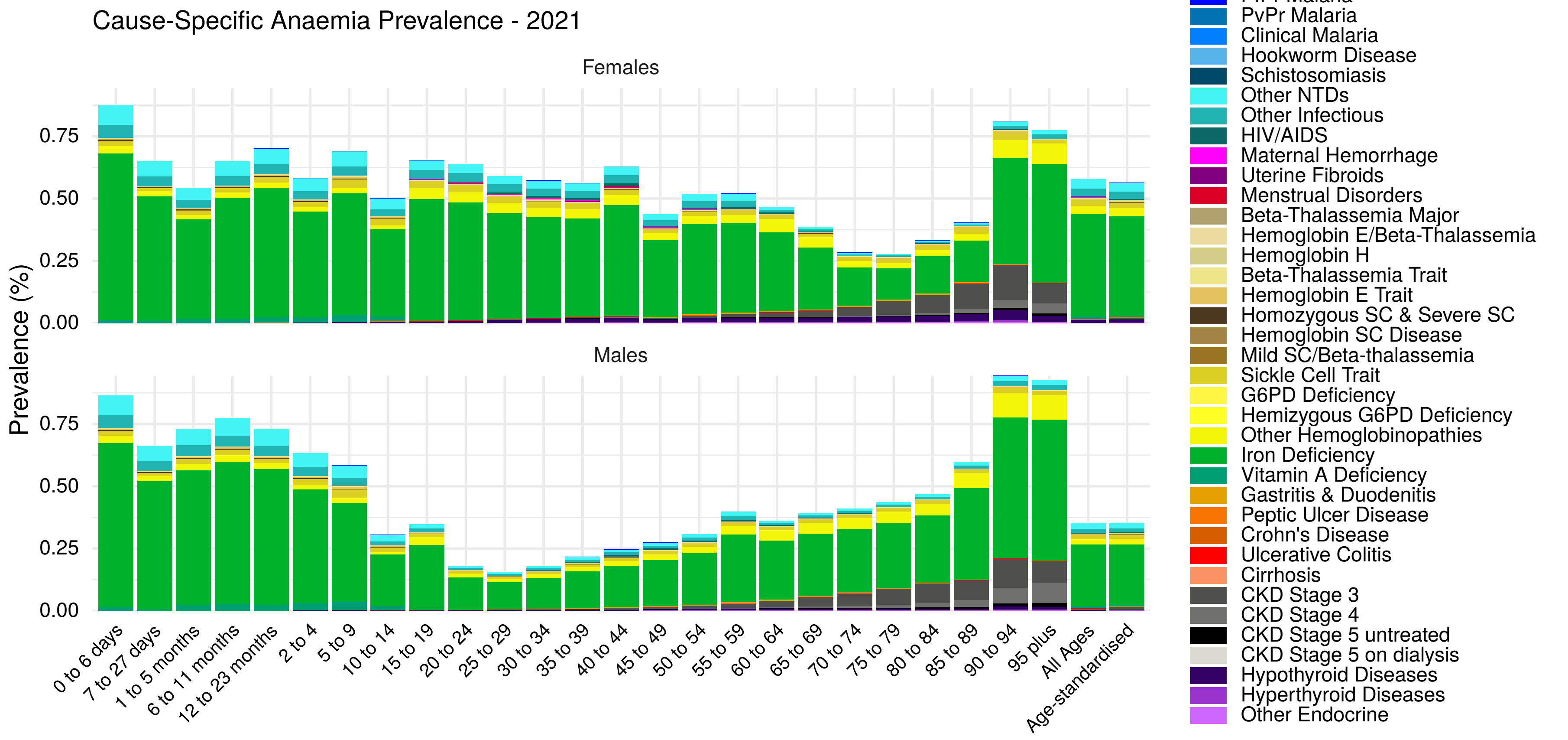

Cause-Specific Anaemia Prevalence - 2021
Females
Males
Prevalence (%)
PfPr Malaria
PvPr Malaria
Clinical Malaria
Hookworm Disease
Schistosomiasis
Other NTDs
Other Infectious
HIV/AIDS
Maternal Hemorrhage
Uterine Fibroids
Menstrual Disorders
Beta-Thalassemia Major
Hemoglobin E/Beta-Thalassemia
Hemoglobin H
Beta-Thalassemia Trait
Hemoglobin E Trait
Homozygous SC & Severe SC
Hemoglobin SC Disease
Mild SC/Beta-thalassemia
Sickle Cell Trait
G6PD Deficiency
Hemizygous G6PD Deficiency
Other Hemoglobinopathies
Iron Deficiency
Vitamin A Deficiency
Gastritis & Duodenitis
Peptic Ulcer Disease
Crohn's Disease
Ulcerative Colitis
Cirrhosis
CKD Stage 3
CKD Stage 4
CKD Stage 5 untreated
CKD Stage 5 on dialysis
Hypothyroid Diseases
Hyperthyroid Diseases
Other Endocrine

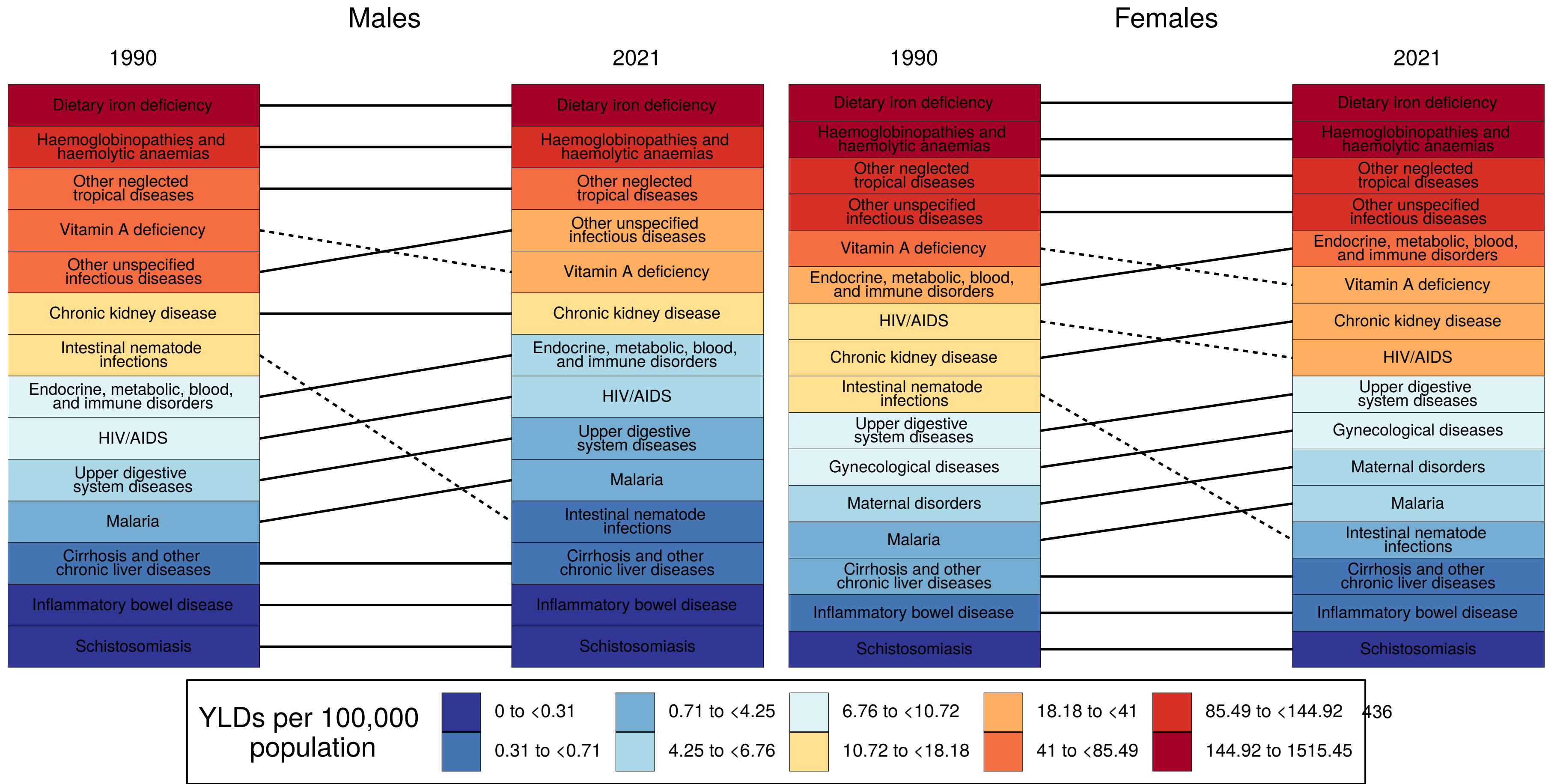

Males
Females
1990
2021
Dietary iron deficiency
Haemoglobinopathies and haemolytic anaemias
Other neglected tropical diseases
Vitamin A deficiency
Other unspecified infectious diseases
Chronic kidney disease
Intestinal nematode infections
Endocrine, metabolic, blood, and immune disorders
HIV/AIDS
Upper digestive system diseases
Malaria
Cirrhosis and other chronic liver diseases
Inflammatory bowel disease
Schistosomiasis
Gynecological diseases
Maternal disorders
YLDs per 100,000 population
0 to <0.31
0.31 to <0.71
0.71 to <4.25
4.25 to <6.76
6.76 to <10.72
10.72 to <18.18
18.18 to <41
41 to <85.49
85.49 to <144.92
144.92 to 1515.45

Jamaica

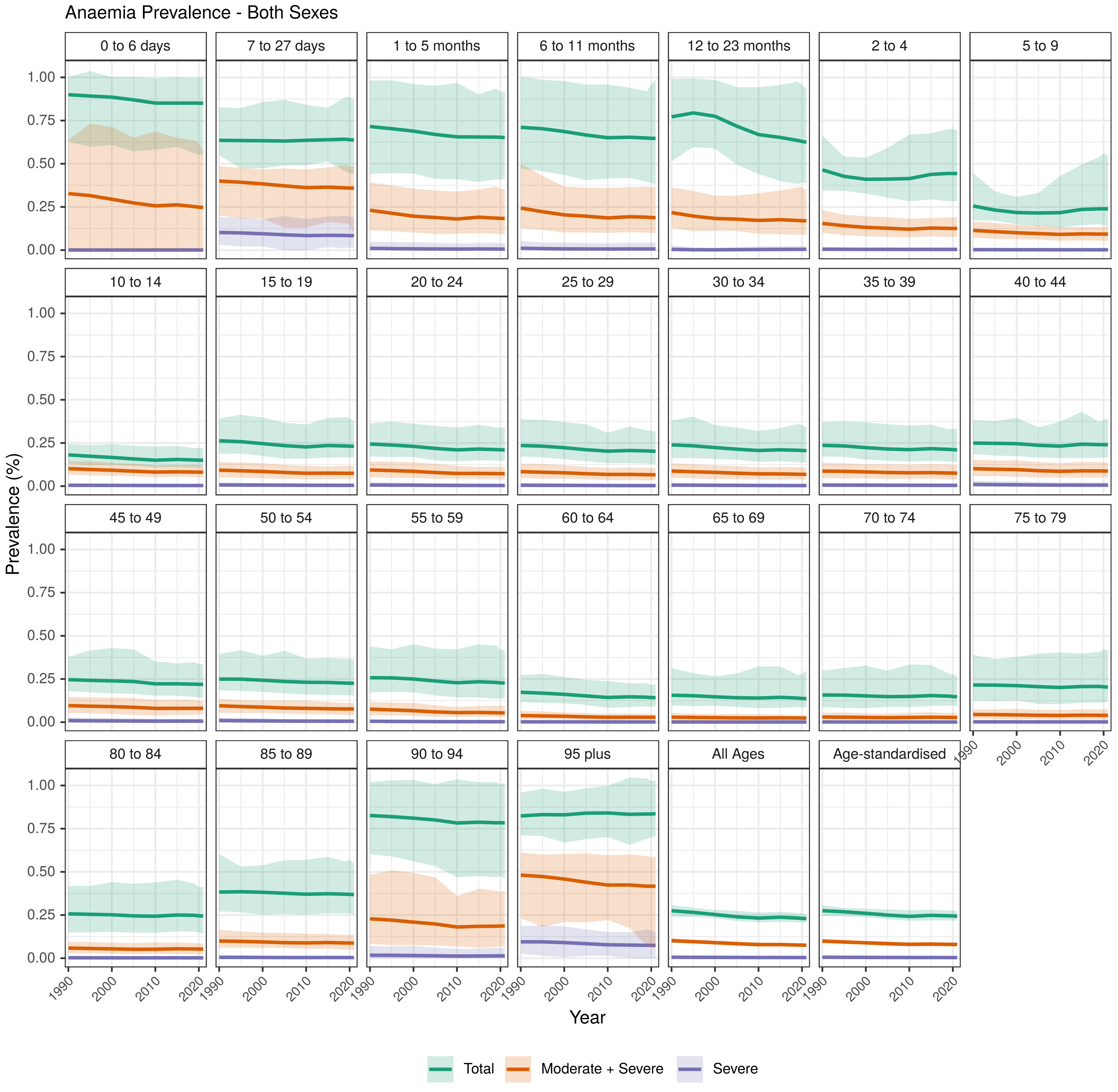

Anaemia Prevalence - Both Sexes
0 to 6 days
7 to 27 days
1 to 5 months
6 to 11 months
12 to 23 months
2 to 4
5 to 9
10 to 14
15 to 19
20 to 24
25 to 29
30 to 34
35 to 39
40 to 44
45 to 49
50 to 54
55 to 59
60 to 64
65 to 69
70 to 74
75 to 79
80 to 84
85 to 89
90 to 94
95 plus
All Ages
Age-standardised
Prevalence (%)
1.00
0.75
0.50
0.25
0.00
1990
2000
2010
2020
Year
Total
Moderate + Severe
Severe

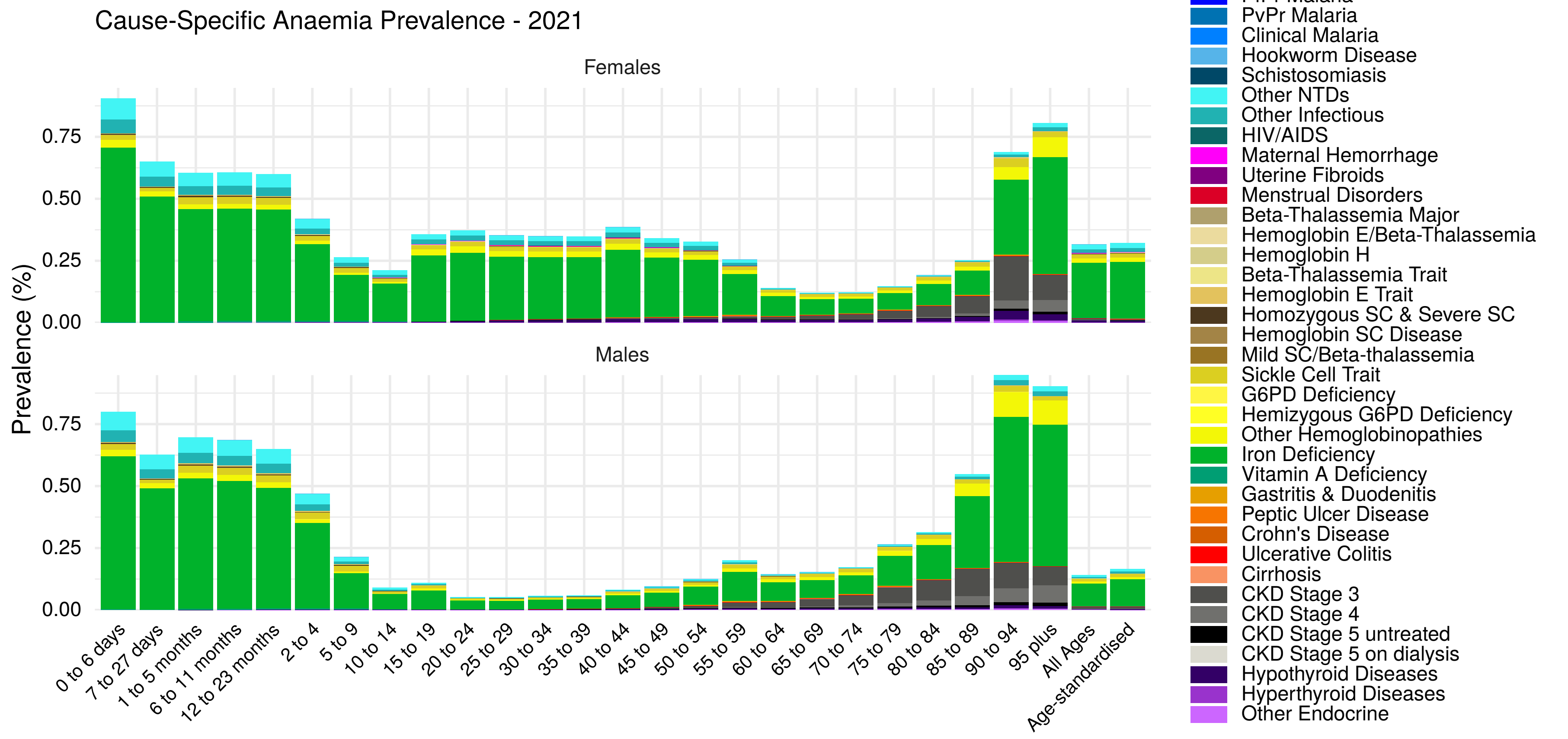

Cause-Specific Anaemia Prevalence - 2021
Females
Males
Prevalence (%)
0.75
0.50
0.25
0.00
0 to 6 days
7 to 27 days
1 to 5 months
6 to 11 months
12 to 23 months
2 to 4
5 to 9
10 to 14
15 to 19
20 to 24
25 to 29
30 to 34
35 to 39
40 to 44
45 to 49
50 to 54
55 to 59
60 to 64
65 to 69
70 to 74
75 to 79
80 to 84
85 to 89
90 to 94
95 plus
All Ages
Age-standardised
PfPr Malaria
PvPr Malaria
Clinical Malaria
Hookworm Disease
Schistosomiasis
Other NTDs
Other Infectious
HIV/AIDS
Maternal Hemorrhage
Uterine Fibroids
Menstrual Disorders
Beta-Thalassemia Major
Hemoglobin E/Beta-Thalassemia
Hemoglobin H
Beta-Thalassemia Trait
Hemoglobin E Trait
Homozygous SC & Severe SC
Hemoglobin SC Disease
Mild SC/Beta-thalassemia
Sickle Cell Trait
G6PD Deficiency
Hemizygous G6PD Deficiency
Other Hemoglobinopathies
Iron Deficiency
Vitamin A Deficiency
Gastritis & Duodenitis
Peptic Ulcer Disease
Crohn's Disease
Ulcerative Colitis
Cirrhosis
CKD Stage 3
CKD Stage 4
CKD Stage 5 untreated
CKD Stage 5 on dialysis
Hypothyroid Diseases
Hyperthyroid Diseases
Other Endocrine

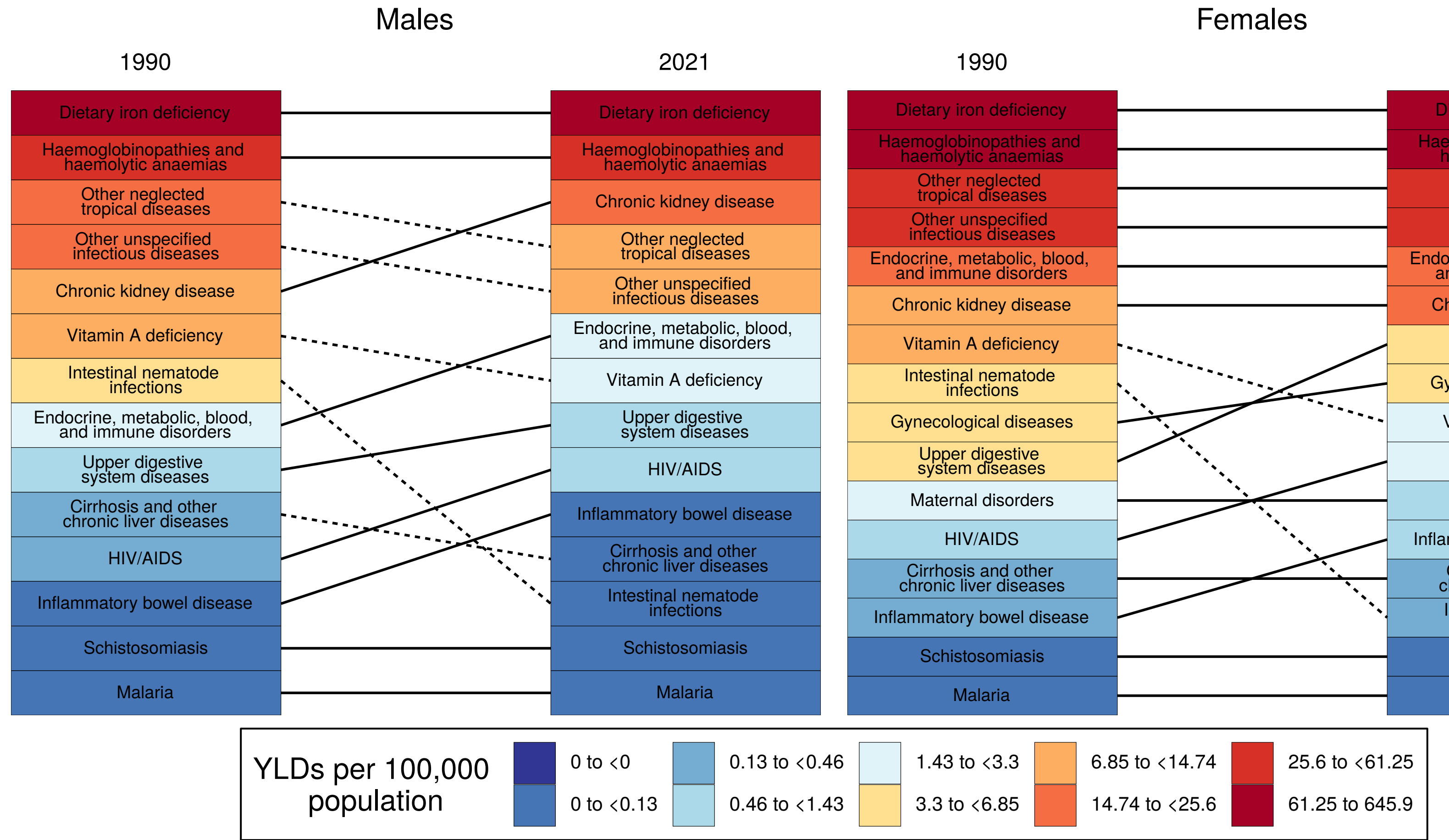

Males
1990
Dietary iron deficiency
Haemoglobinopathies and haemolytic anaemias
Other neglected tropical diseases
Other unspecified infectious diseases
Chronic kidney disease
Vitamin A deficiency
Intestinal nematode infections
Endocrine, metabolic, blood, and immune disorders
Upper digestive system diseases
Cirrhosis and other chronic liver diseases
HIV/AIDS
Inflammatory bowel disease
Schistosomiasis
Malaria
2021
Dietary iron deficiency
Haemoglobinopathies and haemolytic anaemias
Chronic kidney disease
Other neglected tropical diseases
Other unspecified infectious diseases
Endocrine, metabolic, blood, and immune disorders
Vitamin A deficiency
Upper digestive system diseases
HIV/AIDS
Inflammatory bowel disease
Cirrhosis and other chronic liver diseases
Intestinal nematode infections
Schistosomiasis
Malaria
Females
1990
Dietary iron deficiency
Haemoglobinopathies and haemolytic anaemias
Other neglected tropical diseases
Other unspecified infectious diseases
Endocrine, metabolic, blood, and immune disorders
Chronic kidney disease
Vitamin A deficiency
Intestinal nematode infections
Gynecological diseases
Upper digestive system diseases
Maternal disorders
HIV/AIDS
Cirrhosis and other chronic liver diseases
Inflammatory bowel disease
Schistosomiasis
Malaria
2021
Dietary iron deficiency
Haemoglobinopathies and haemolytic anaemias
Other neglected tropical diseases
Other unspecified infectious diseases
Endocrine, metabolic, blood, and immune disorders
Chronic kidney disease
Upper digestive system diseases
Gynecological diseases
Vitamin A deficiency
HIV/AIDS
Maternal disorders
Inflammatory bowel disease
Cirrhosis and other chronic liver diseases
Intestinal nematode infections
Schistosomiasis
Malaria
YLDs per 100,000 population
0 to <0
0 to <0.13
0.13 to <0.46
0.46 to <1.43
1.43 to <3.3
3.3 to <6.85
6.85 to <14.74
14.74 to <25.6
25.6 to <61.25
61.25 to 645.9

Puerto Rico

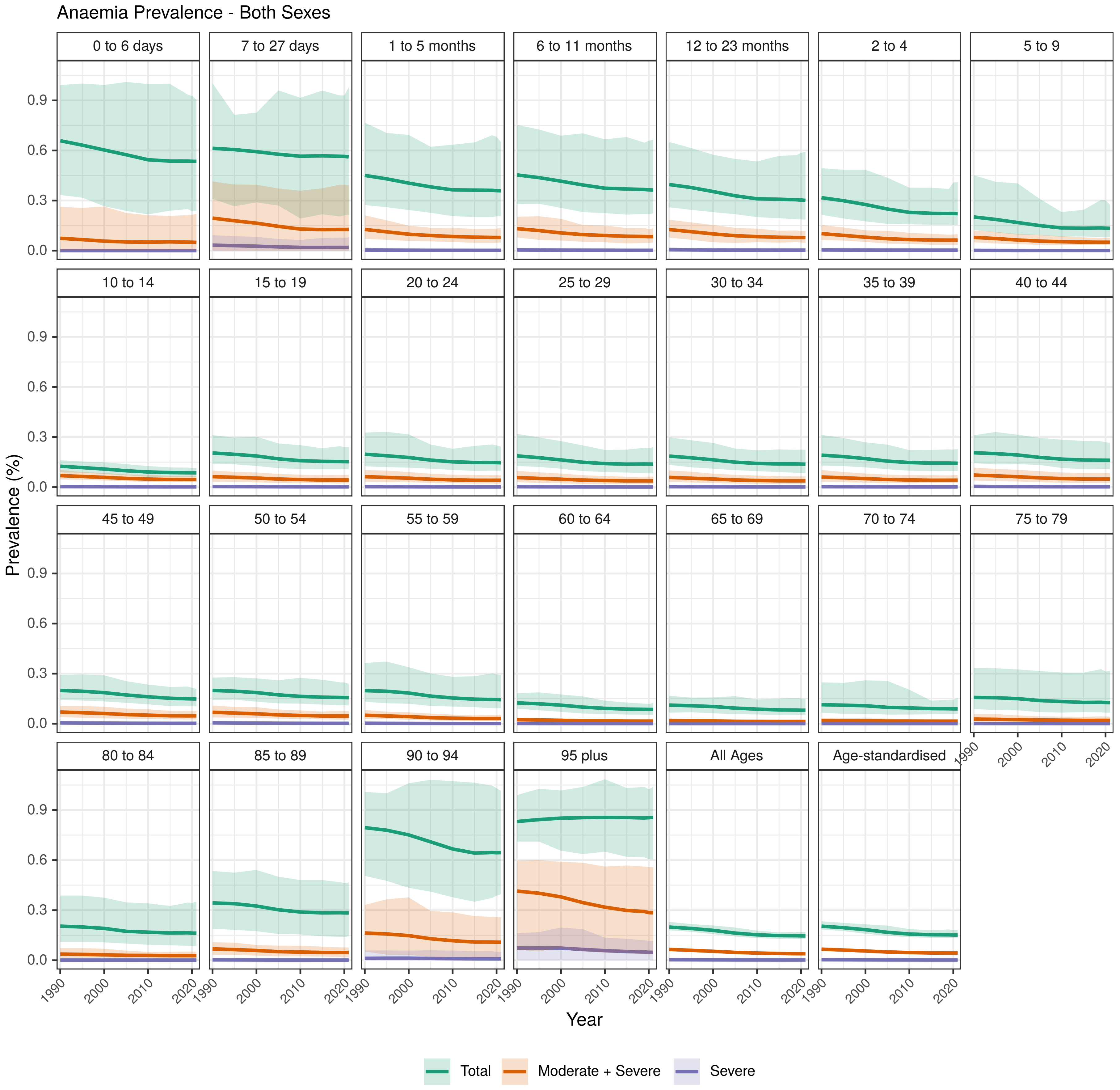

Anaemia Prevalence - Both Sexes
0 to 6 days
7 to 27 days
1 to 5 months
6 to 11 months
12 to 23 months
2 to 4
5 to 9
10 to 14
15 to 19
20 to 24
25 to 29
30 to 34
35 to 39
40 to 44
45 to 49
50 to 54
55 to 59
60 to 64
65 to 69
70 to 74
75 to 79
80 to 84
85 to 89
90 to 94
95 plus
All Ages
Age-standardised
0.0
0.3
0.6
0.9
Prevalence (%)
1990
2000
2010
2020
Year
Total
Moderate + Severe
Severe

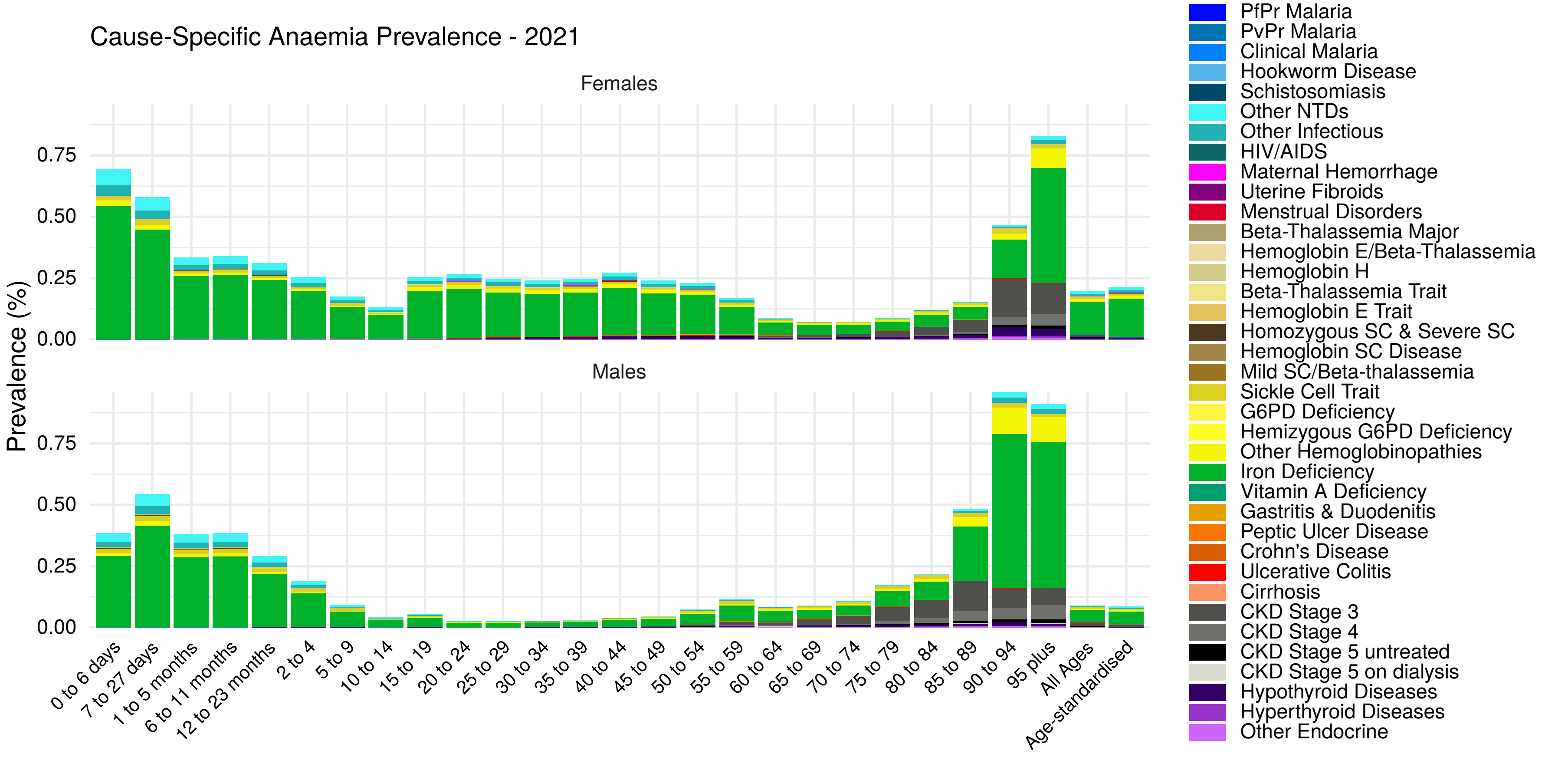

Cause-Specific Anaemia Prevalence - 2021
Females
Males
Prevalence (%)
0.00
0.25
0.50
0.75
0 to 6 days
7 to 27 days
1 to 5 months
6 to 11 months
12 to 23 months
2 to 4
5 to 9
10 to 14
15 to 19
20 to 24
25 to 29
30 to 34
35 to 39
40 to 44
45 to 49
50 to 54
55 to 59
60 to 64
65 to 69
70 to 74
75 to 79
80 to 84
85 to 89
90 to 94
95 plus
All Ages
Age-standardised
PfPr Malaria
PvPr Malaria
Clinical Malaria
Hookworm Disease
Schistosomiasis
Other NTDs
Other Infectious
HIV/AIDS
Maternal Hemorrhage
Uterine Fibroids
Menstrual Disorders
Beta-Thalassemia Major
Hemoglobin E/Beta-Thalassemia
Hemoglobin H
Beta-Thalassemia Trait
Hemoglobin E Trait
Homozygous SC & Severe SC
Hemoglobin SC Disease
Mild SC/Beta-thalassemia
Sickle Cell Trait
G6PD Deficiency
Hemizygous G6PD Deficiency
Other Hemoglobinopathies
Iron Deficiency
Vitamin A Deficiency
Gastritis & Duodenitis
Peptic Ulcer Disease
Crohn's Disease
Ulcerative Colitis
Cirrhosis
CKD Stage 3
CKD Stage 4
CKD Stage 5 untreated
CKD Stage 5 on dialysis
Hypothyroid Diseases
Hyperthyroid Diseases
Other Endocrine

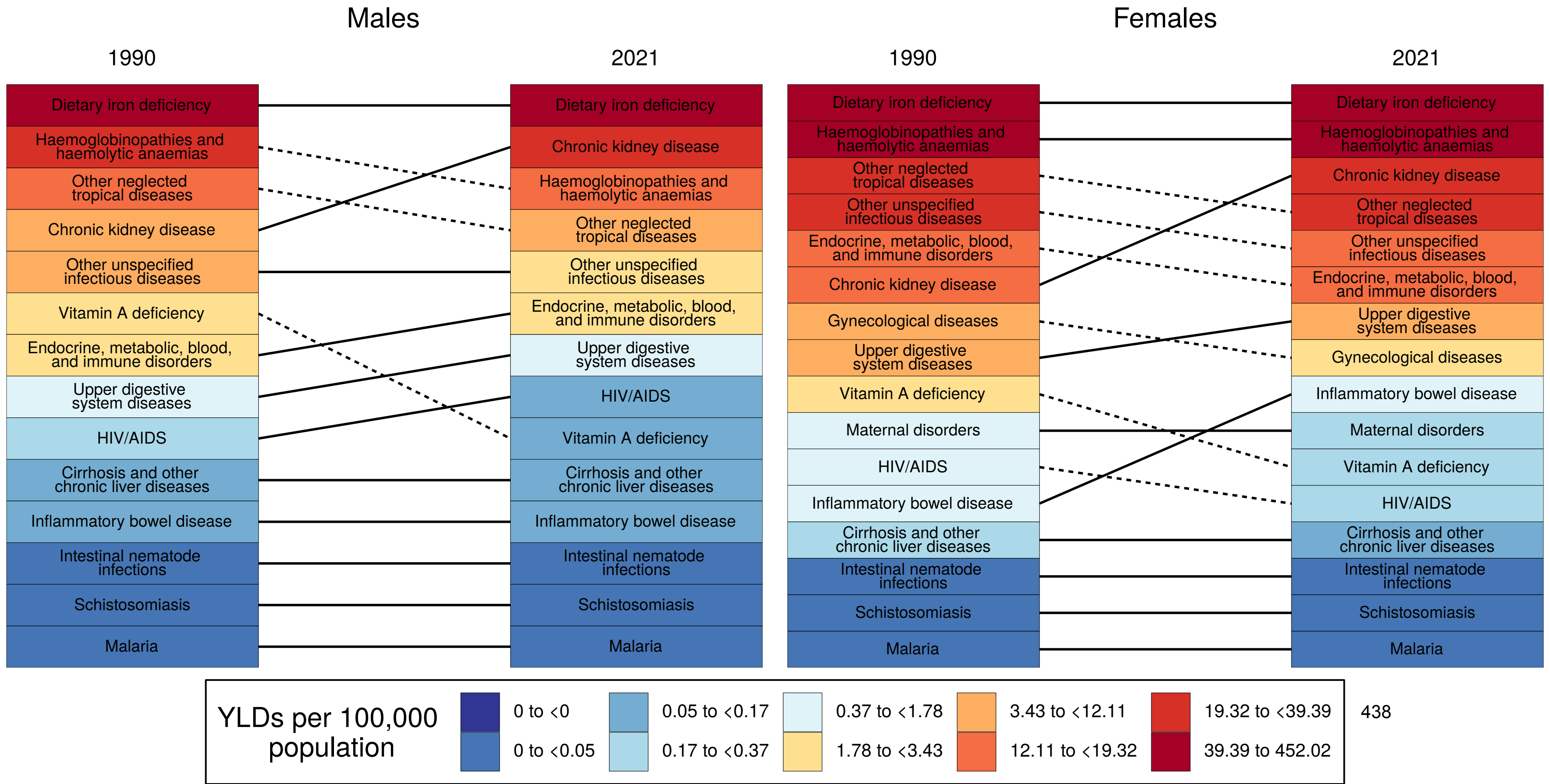

Males
Females
1990
2021
Dietary iron deficiency
Haemoglobinopathies and haemolytic anaemias
Other neglected tropical diseases
Chronic kidney disease
Other unspecified infectious diseases
Vitamin A deficiency
Endocrine, metabolic, blood, and immune disorders
Upper digestive system diseases
HIV/AIDS
Cirrhosis and other chronic liver diseases
Inflammatory bowel disease
Intestinal nematode infections
Schistosomiasis
Malaria
Gynecological diseases
Maternal disorders
YLDs per 100,000 population
0 to <0
0 to <0.05
0.05 to <0.17
0.17 to <0.37
0.37 to <1.78
1.78 to <3.43
3.43 to <12.11
12.11 to <19.32
19.32 to <39.39
39.39 to 452.02

Saint Kitts and Nevis

Anaemia Prevalence - Both Sexes

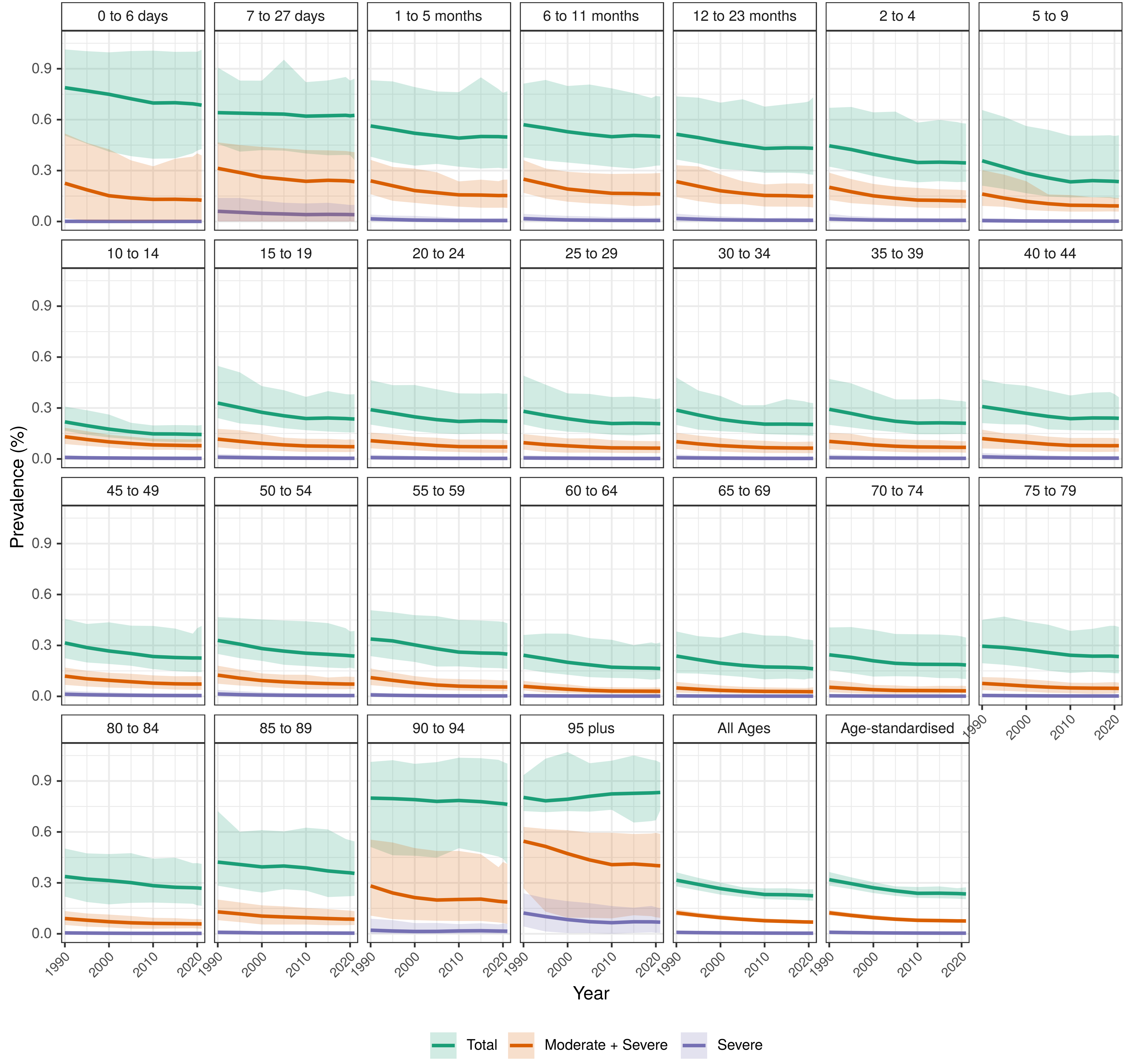

Cause-Specific Anaemia Prevalence - 2021

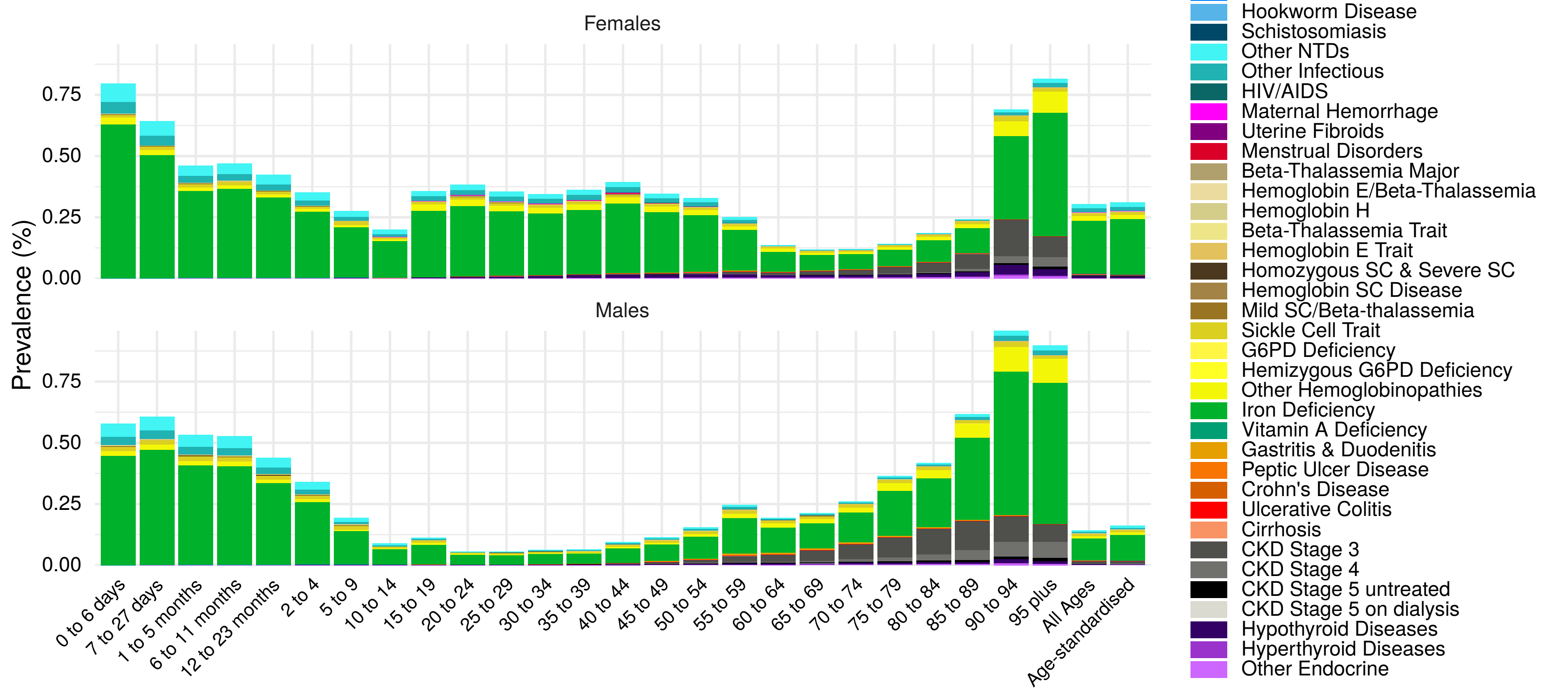

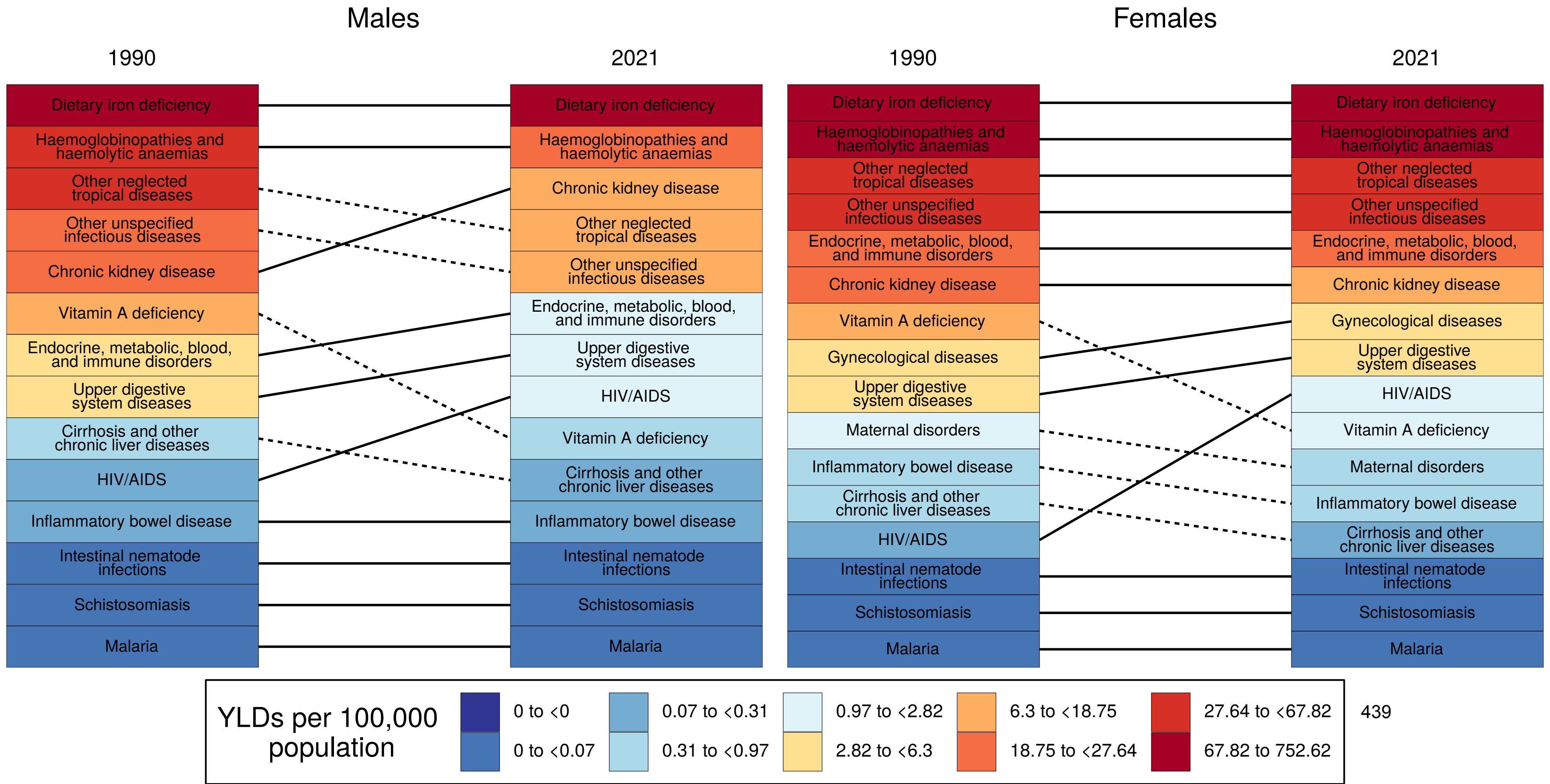

439

Saint Lucia

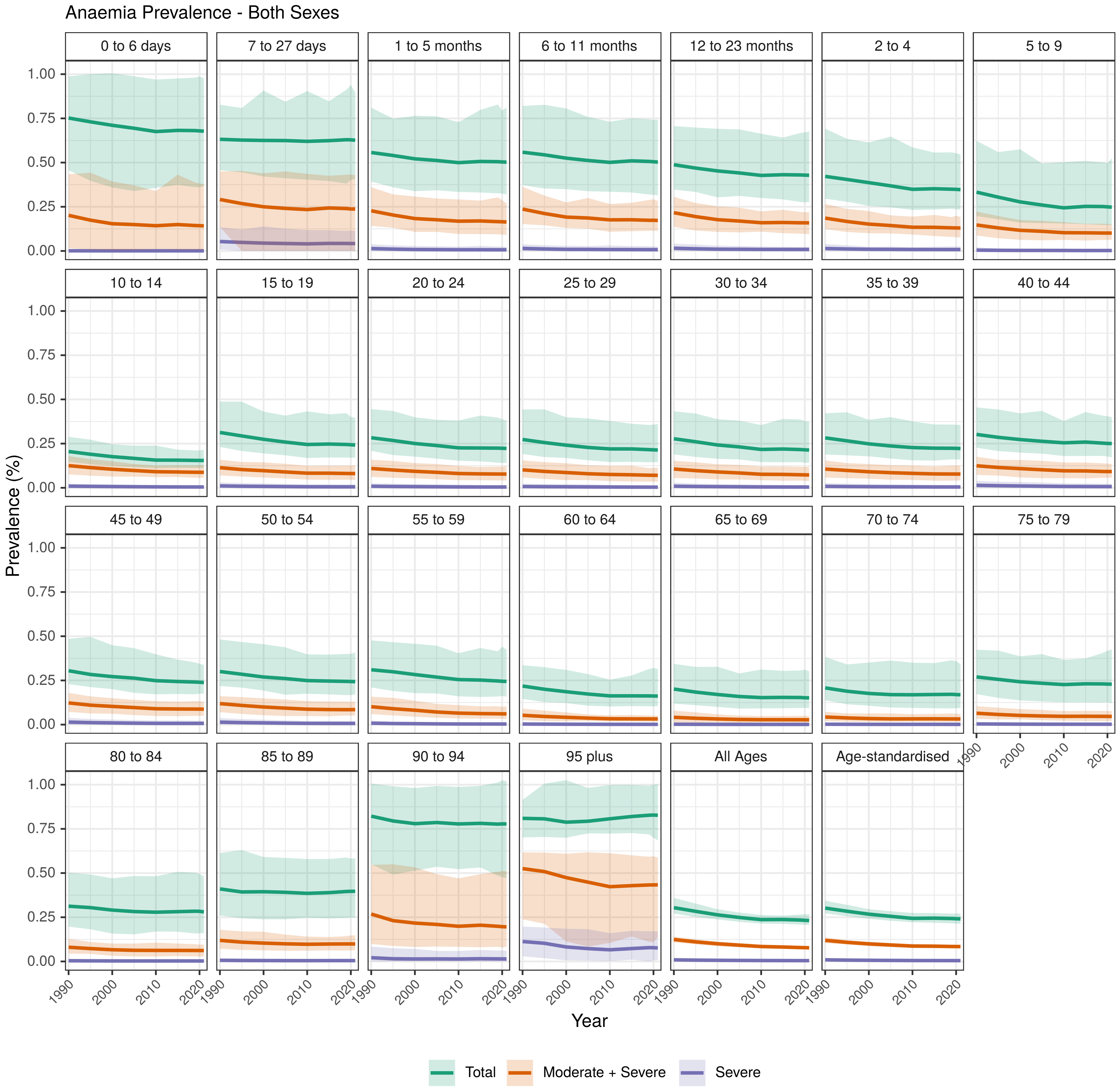
Anaemia Prevalence - Both Sexes
0 to 6 days
7 to 27 days
1 to 5 months
6 to 11 months
12 to 23 months
2 to 4
5 to 9
10 to 14
15 to 19
20 to 24
25 to 29
30 to 34
35 to 39
40 to 44
45 to 49
50 to 54
55 to 59
60 to 64
65 to 69
70 to 74
75 to 79
80 to 84
85 to 89
90 to 94
95 plus
All Ages
Age-standardised
Prevalence (%)
Year
Total
Moderate + Severe
Severe

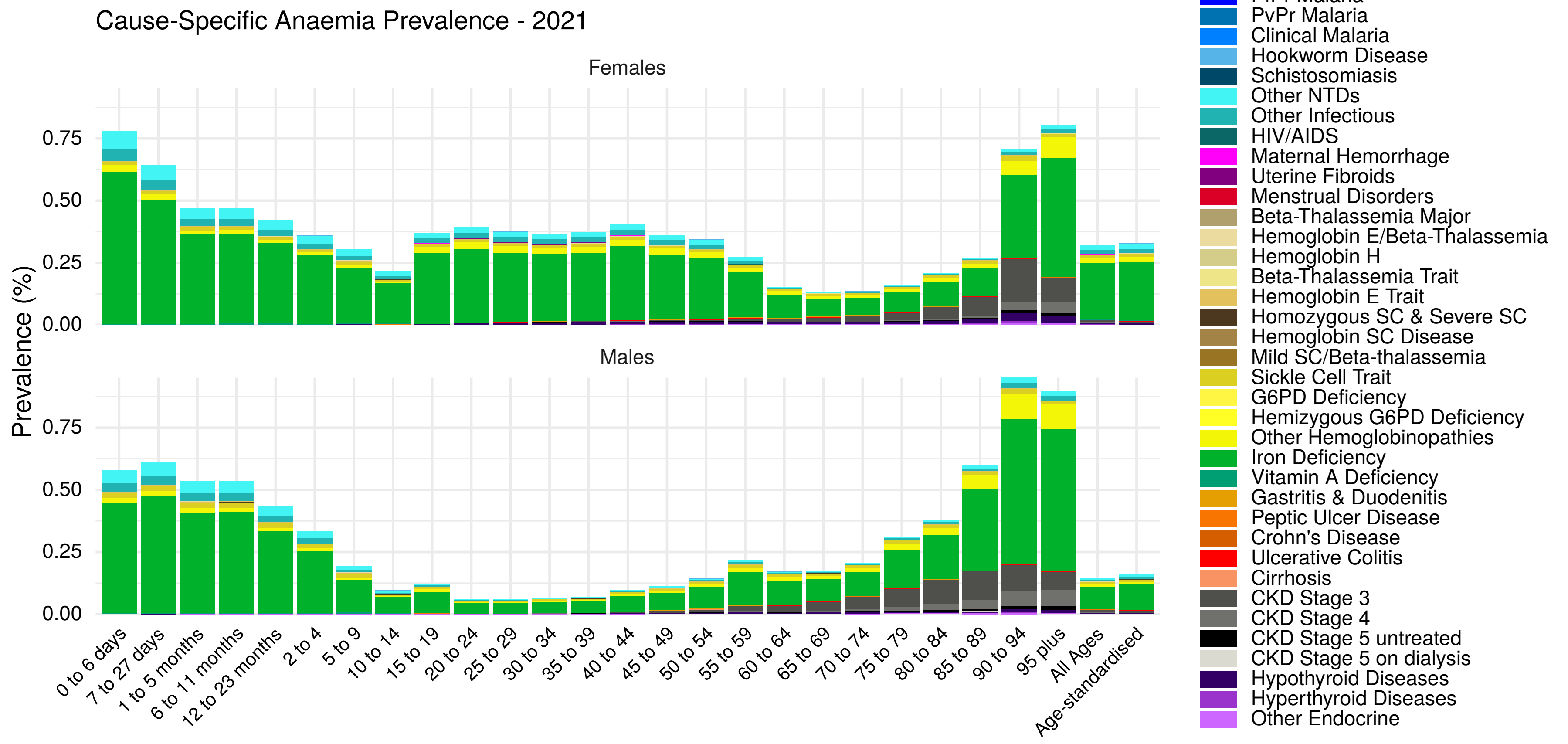
Cause-Specific Anaemia Prevalence - 2021
Females
Males
Prevalence (%)
PfPr Malaria
PvPr Malaria
Clinical Malaria
Hookworm Disease
Schistosomiasis
Other NTDs
Other Infectious
HIV/AIDS
Maternal Hemorrhage
Uterine Fibroids
Menstrual Disorders
Beta-Thalassemia Major
Hemoglobin E/Beta-Thalassemia
Hemoglobin H
Beta-Thalassemia Trait
Hemoglobin E Trait
Homozygous SC & Severe SC
Hemoglobin SC Disease
Mild SC/Beta-thalassemia
Sickle Cell Trait
G6PD Deficiency
Hemizygous G6PD Deficiency
Other Hemoglobinopathies
Iron Deficiency
Vitamin A Deficiency
Gastritis & Duodenitis
Peptic Ulcer Disease
Crohn's Disease
Ulcerative Colitis
Cirrhosis
CKD Stage 3
CKD Stage 4
CKD Stage 5 untreated
CKD Stage 5 on dialysis
Hypothyroid Diseases
Hyperthyroid Diseases
Other Endocrine

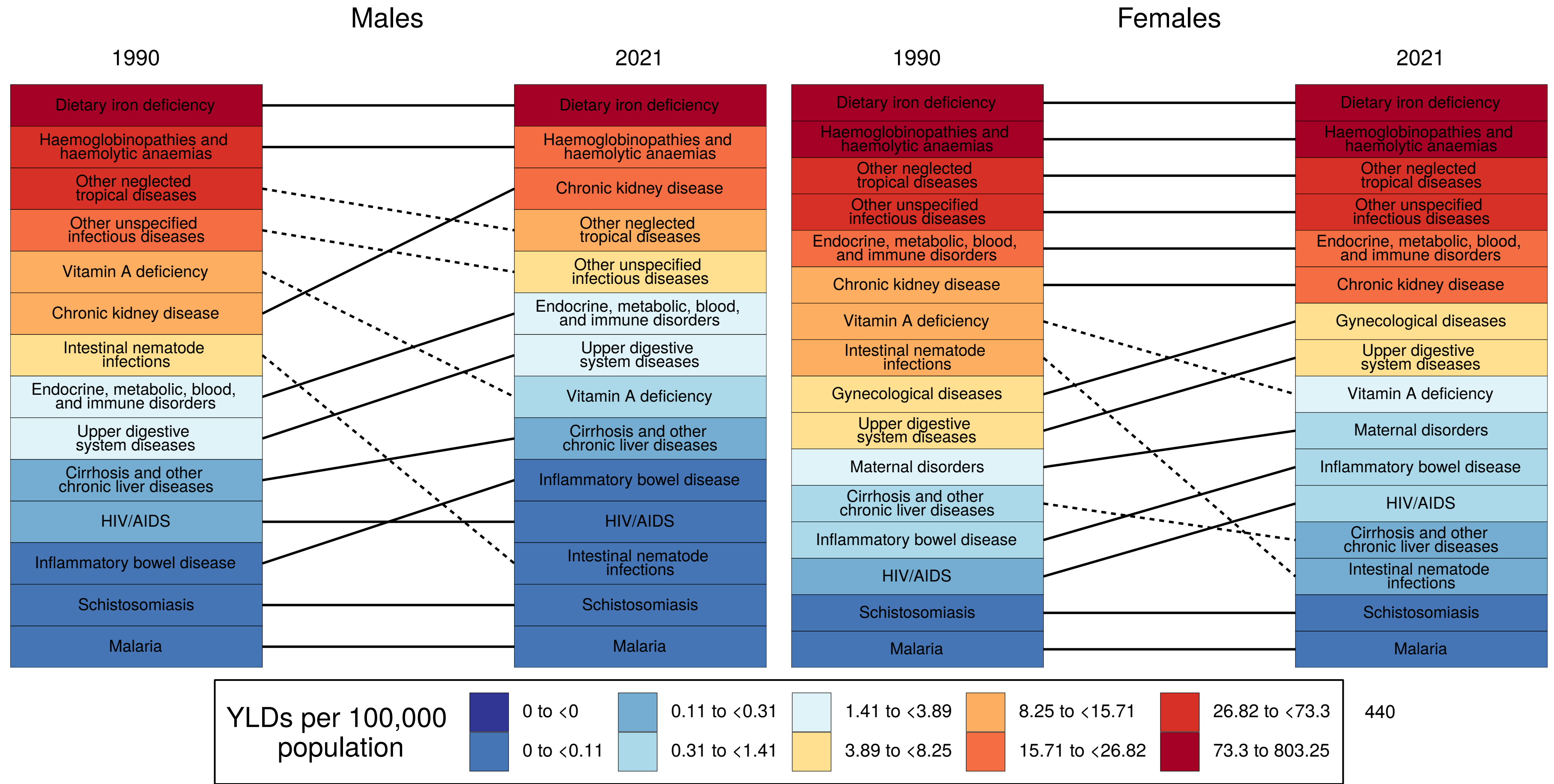
Males
Females
1990
2021
Dietary iron deficiency
Haemoglobinopathies and haemolytic anaemias
Other neglected tropical diseases
Other unspecified infectious diseases
Vitamin A deficiency
Chronic kidney disease
Intestinal nematode infections
Endocrine, metabolic, blood, and immune disorders
Upper digestive system diseases
Cirrhosis and other chronic liver diseases
HIV/AIDS
Inflammatory bowel disease
Schistosomiasis
Malaria
Gynecological diseases
Maternal disorders
YLDs per 100,000 population
0 to <0
0 to <0.11
0.11 to <0.31
0.31 to <1.41
1.41 to <3.89
3.89 to <8.25
8.25 to <15.71
15.71 to <26.82
26.82 to <73.3
73.3 to 803.25
440

Saint Vincent and the Grenadines

Anaemia Prevalence - Both Sexes

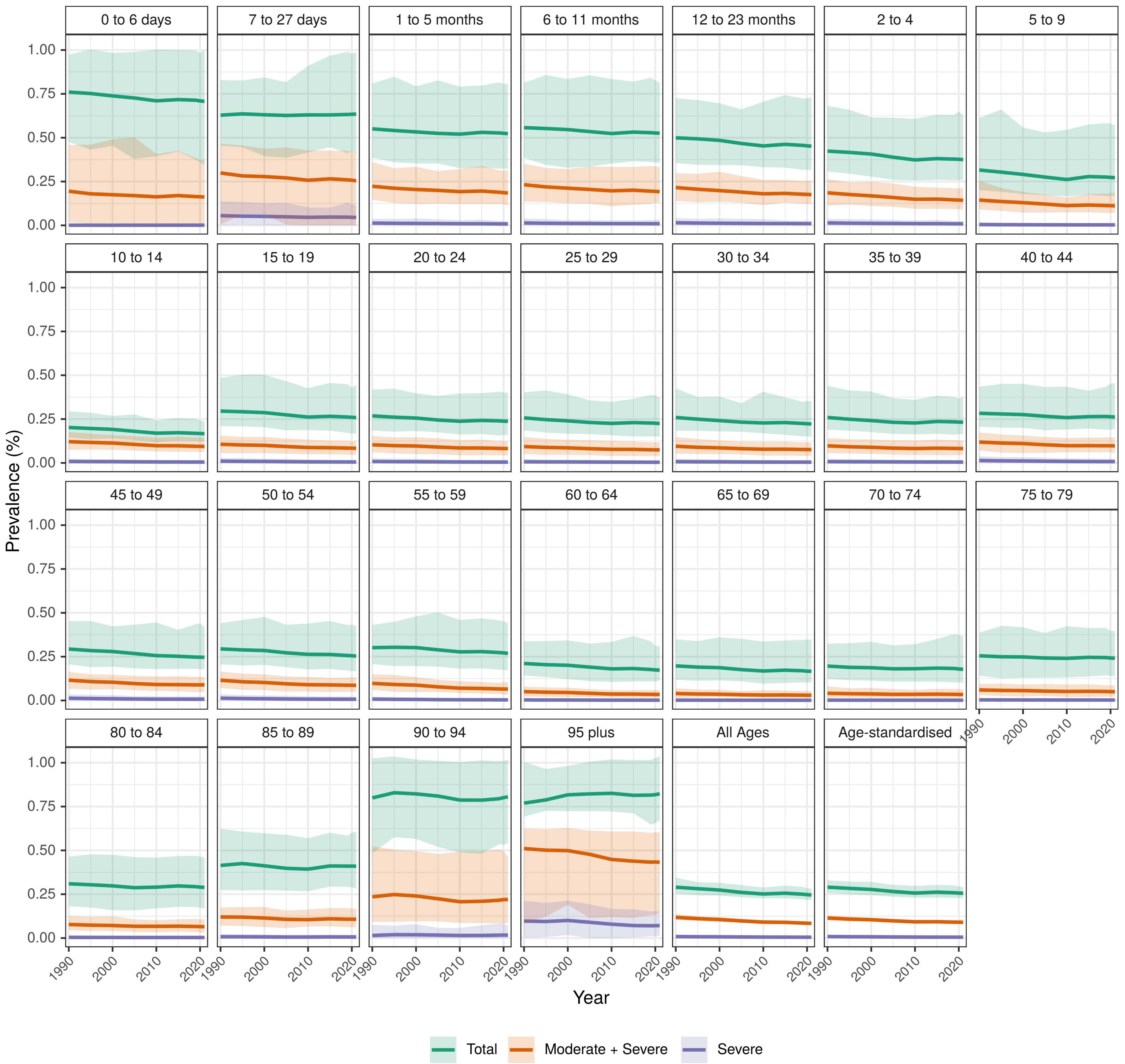

Cause-Specific Anaemia Prevalence - 2021

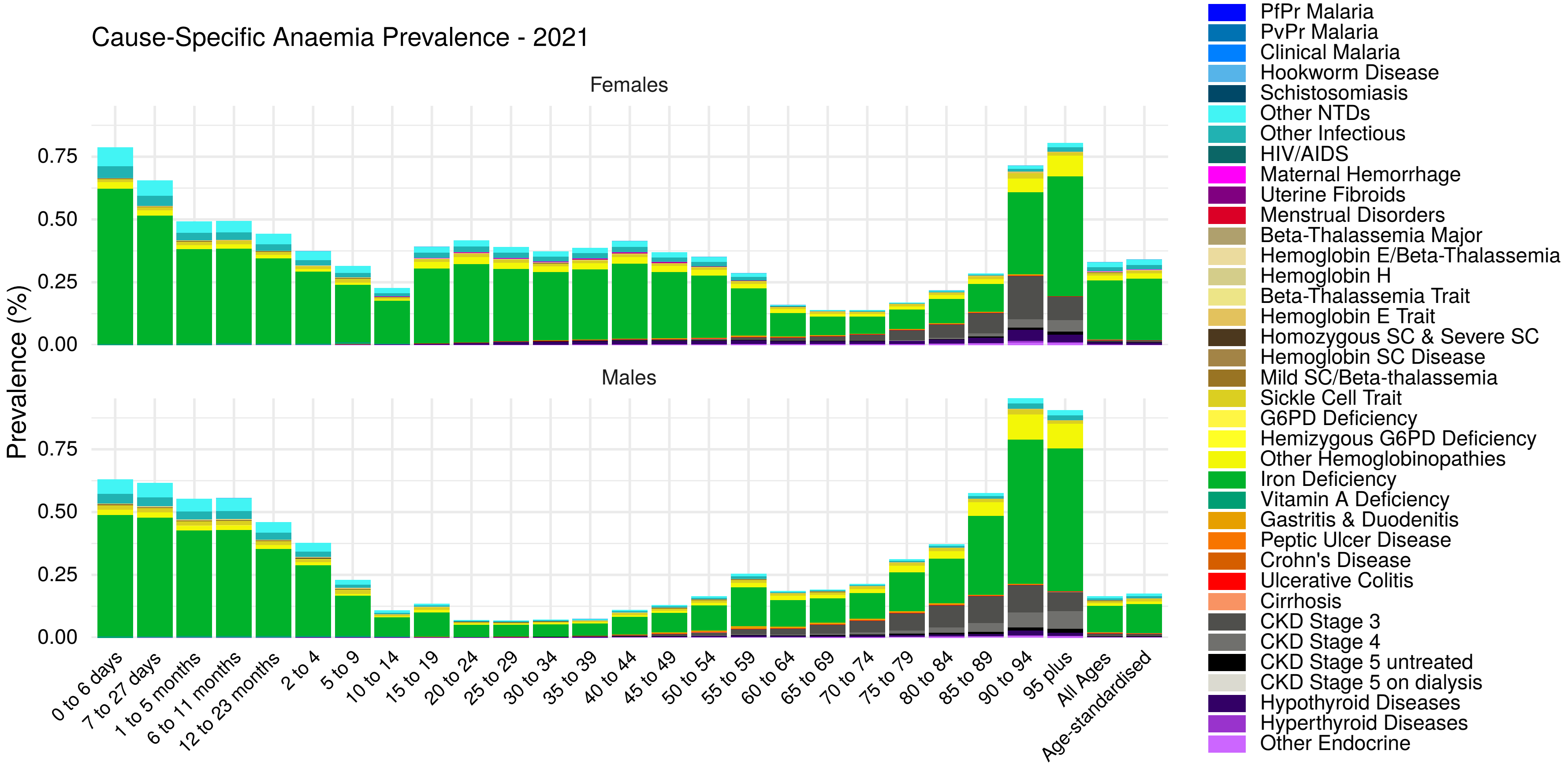

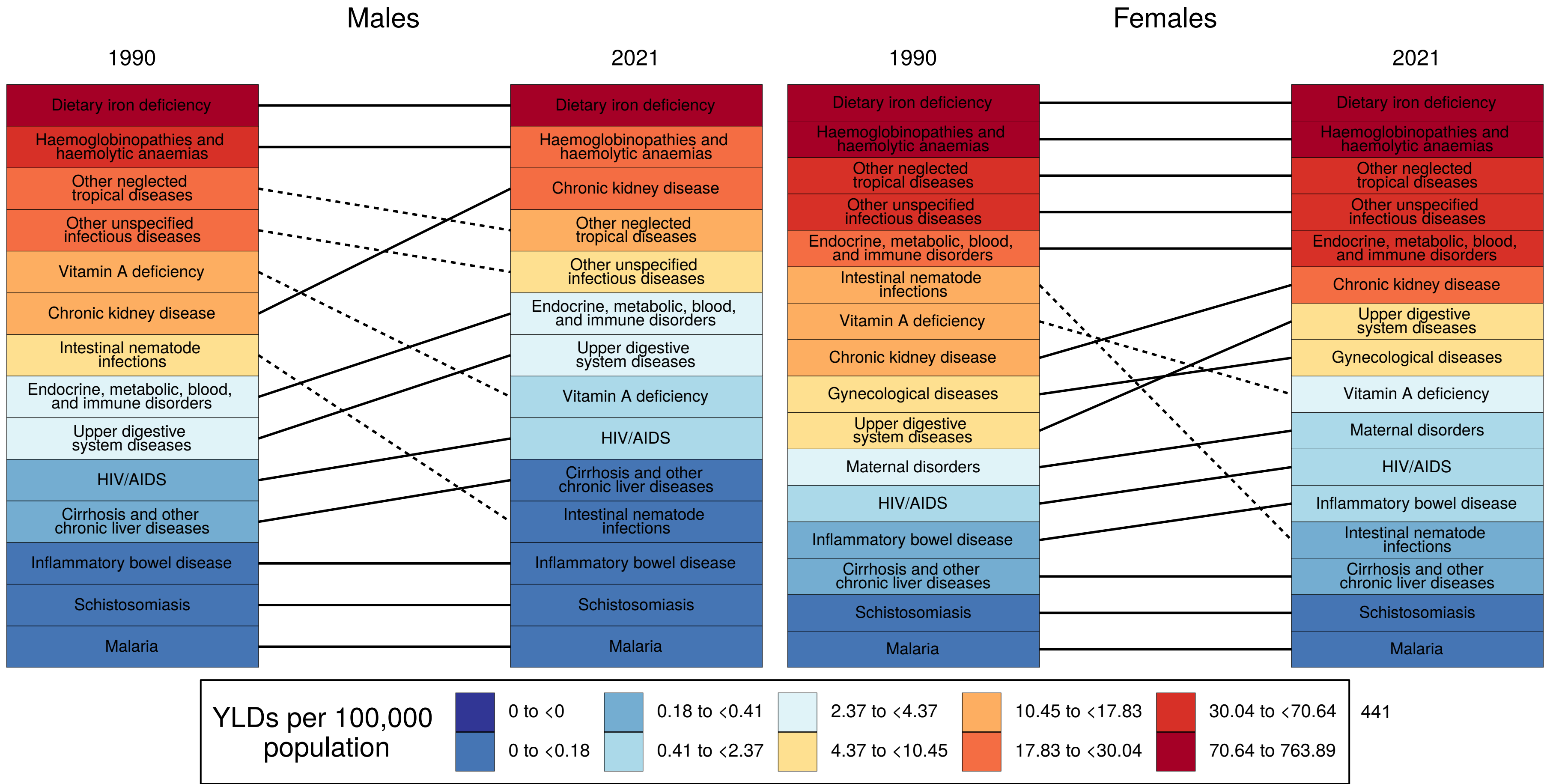

Suriname

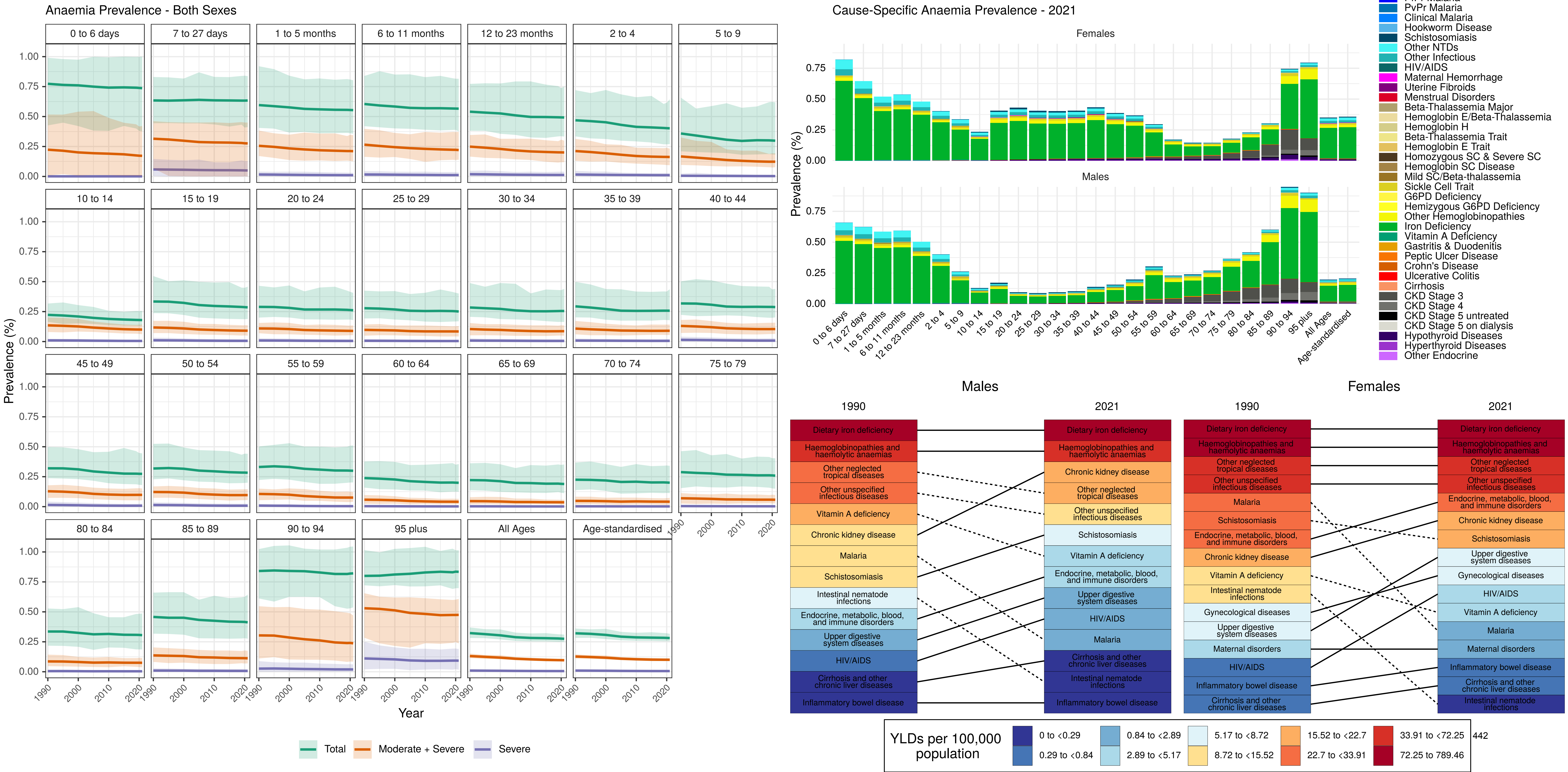
Anaemia Prevalence - Both Sexes
0 to 6 days
7 to 27 days
1 to 5 months
6 to 11 months
12 to 23 months
2 to 4
5 to 9
10 to 14
15 to 19
20 to 24
25 to 29
30 to 34
35 to 39
40 to 44
45 to 49
50 to 54
55 to 59
60 to 64
65 to 69
70 to 74
75 to 79
80 to 84
85 to 89
90 to 94
95 plus
All Ages
Age-standardised
Prevalence (%)
Year
Total
Moderate + Severe
Severe
Cause-Specific Anaemia Prevalence - 2021
Females
Males
PfPr Malaria
PvPr Malaria
Clinical Malaria
Hookworm Disease
Schistosomiasis
Other NTDs
Other Infectious
HIV/AIDS
Maternal Hemorrhage
Uterine Fibroids
Menstrual Disorders
Beta-Thalassemia Major
Hemoglobin E/Beta-Thalassemia
Hemoglobin H
Beta-Thalassemia Trait
Hemoglobin E Trait
Homozygous SC & Severe SC
Hemoglobin SC Disease
Mild SC/Beta-thalassemia
Sickle Cell Trait
G6PD Deficiency
Hemizygous G6PD Deficiency
Other Hemoglobinopathies
Iron Deficiency
Vitamin A Deficiency
Gastritis & Duodenitis
Peptic Ulcer Disease
Crohn's Disease
Ulcerative Colitis
Cirrhosis
CKD Stage 3
CKD Stage 4
CKD Stage 5 untreated
CKD Stage 5 on dialysis
Hypothyroid Diseases
Hyperthyroid Diseases
Other Endocrine
Males
Females
1990
2021
Dietary iron deficiency
Haemoglobinopathies and haemolytic anaemias
Other neglected tropical diseases
Other unspecified infectious diseases
Vitamin A deficiency
Chronic kidney disease
Malaria
Schistosomiasis
Intestinal nematode infections
Endocrine, metabolic, blood, and immune disorders
Upper digestive system diseases
HIV/AIDS
Cirrhosis and other chronic liver diseases
Inflammatory bowel disease
Gynecological diseases
Maternal disorders
YLDs per 100,000 population
0 to <0.29
0.29 to <0.84
0.84 to <2.89
2.89 to <5.17
5.17 to <8.72
8.72 to <15.52
15.52 to <22.7
22.7 to <33.91
33.91 to <72.25
72.25 to 789.46

Trinidad and Tobago

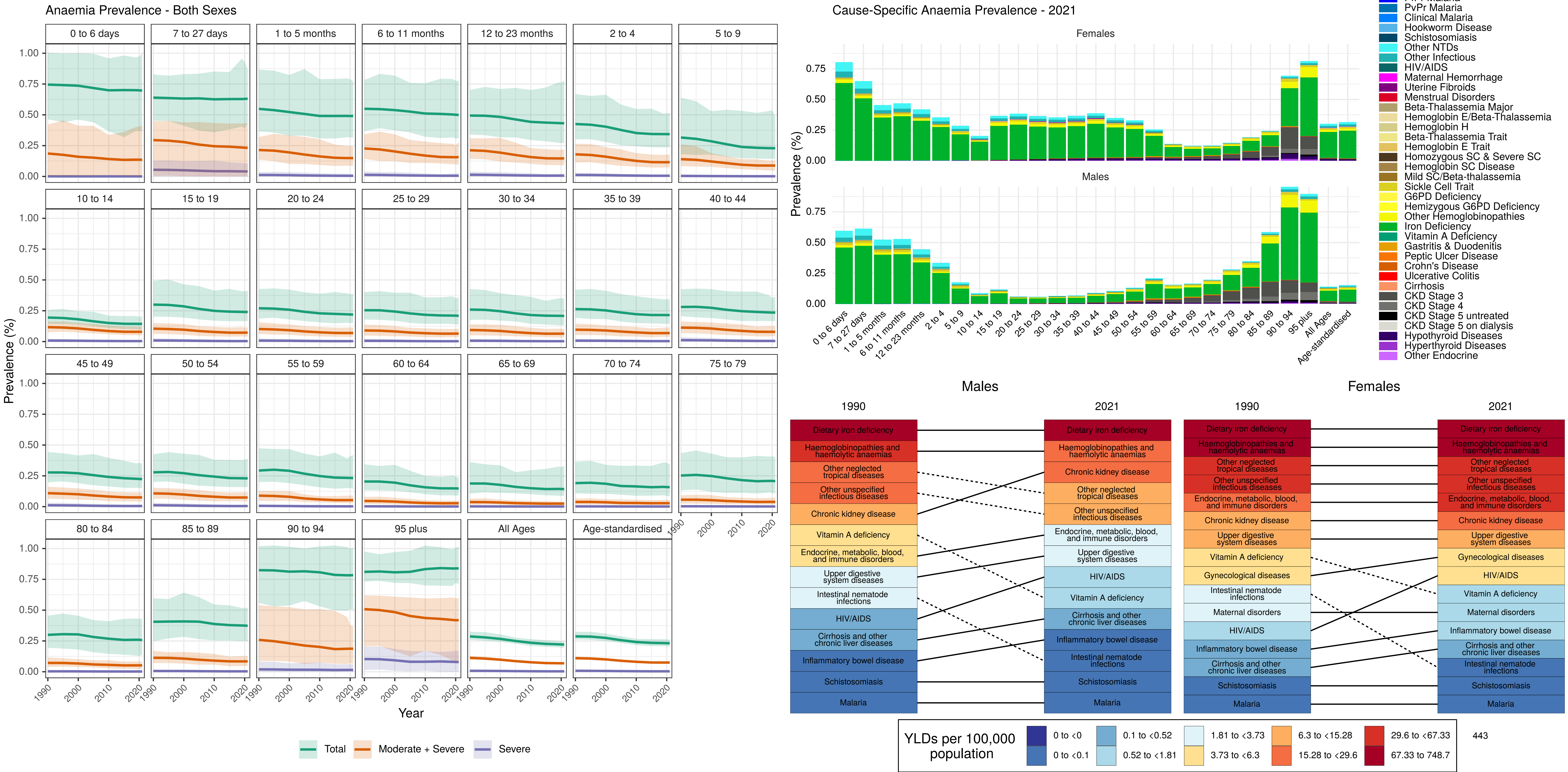

Virgin Islands

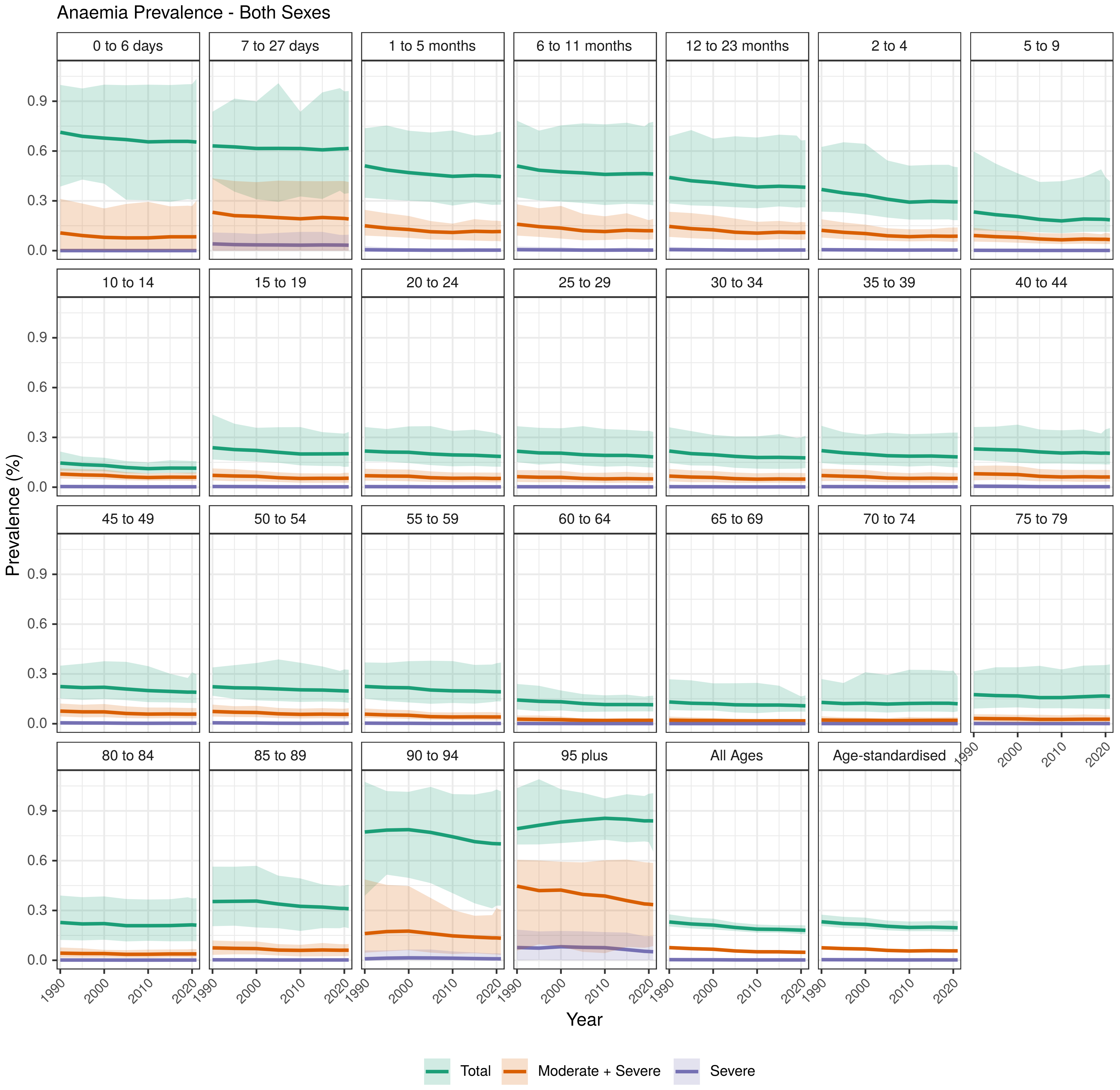

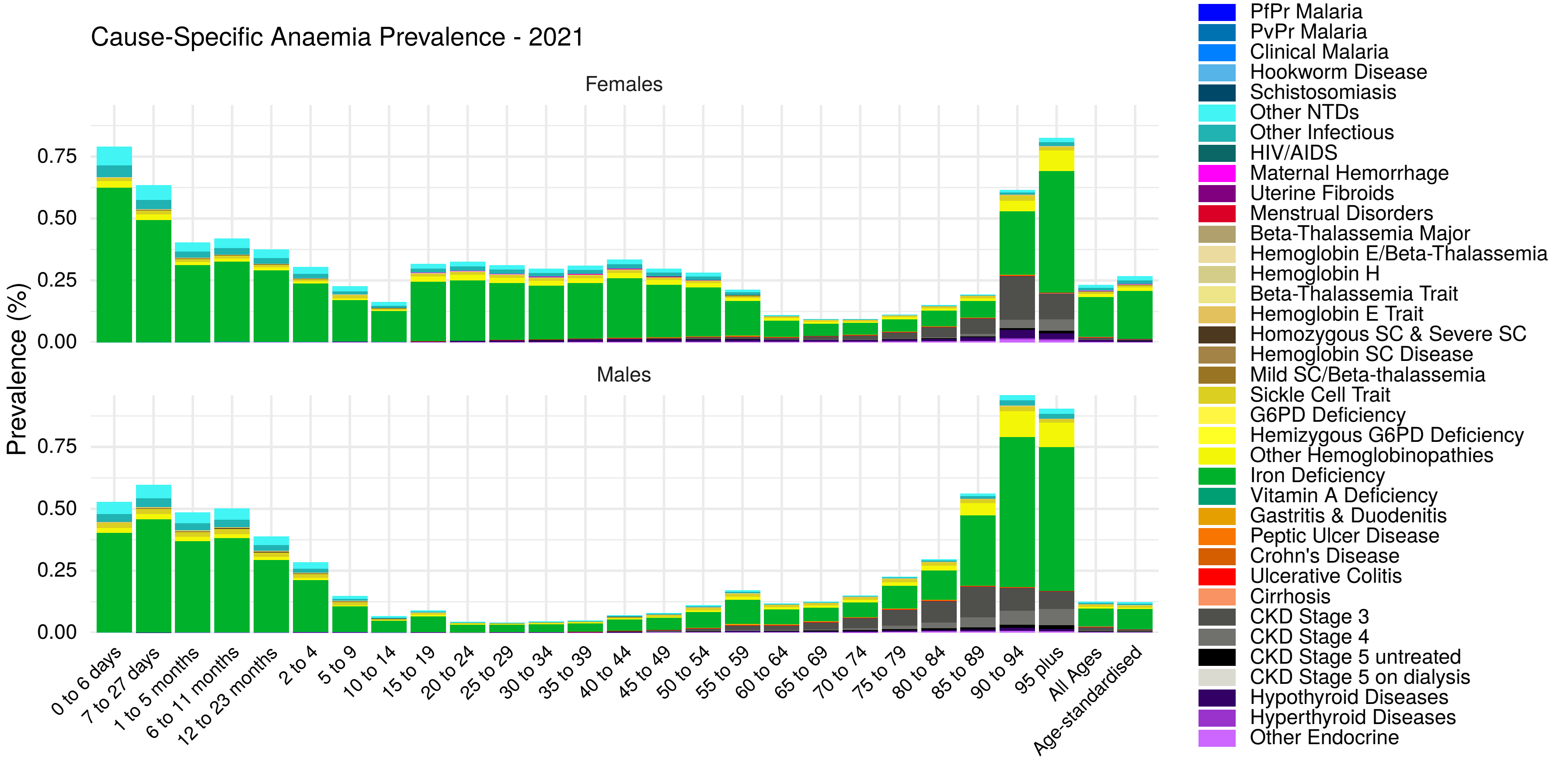

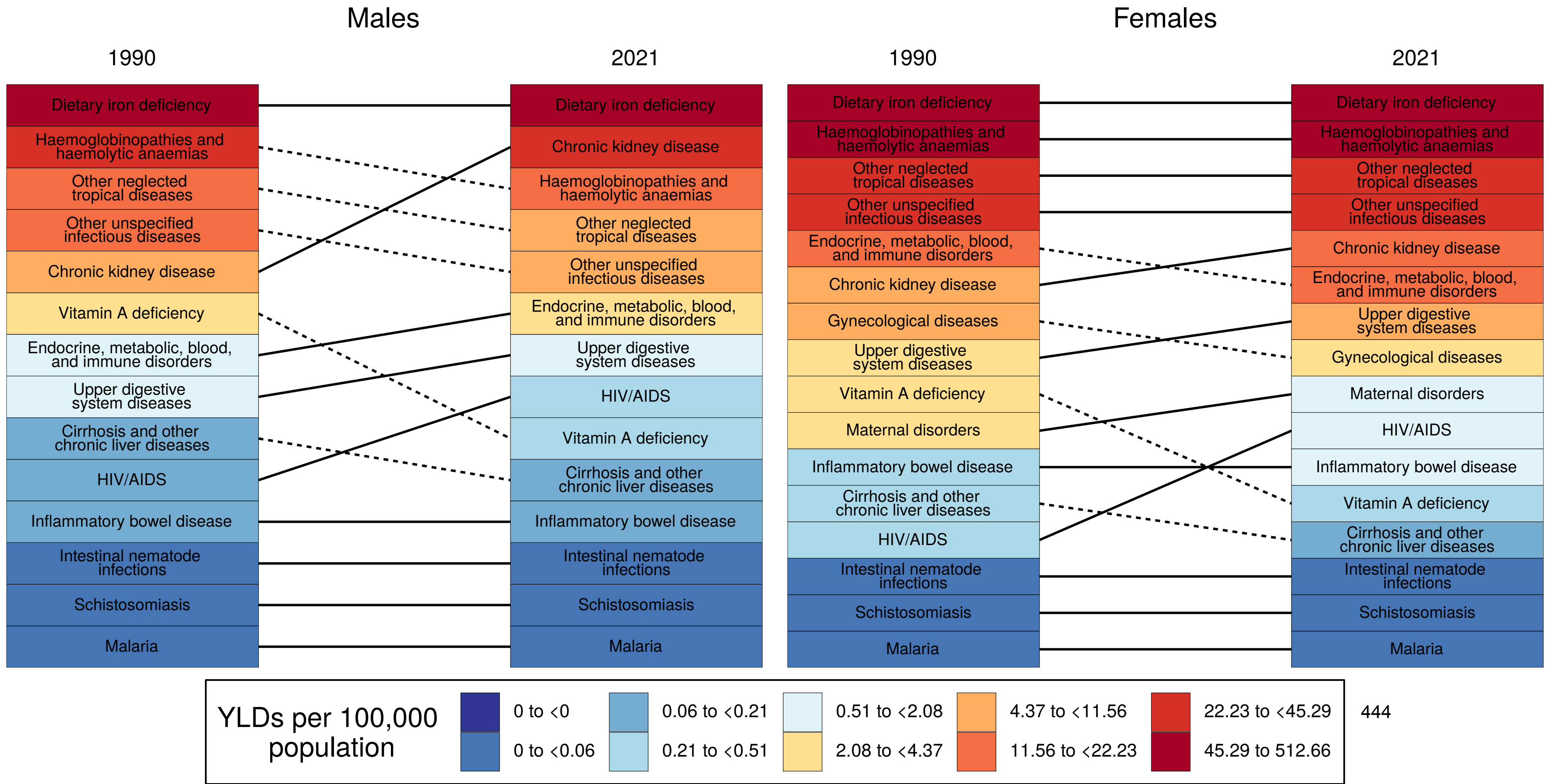

444

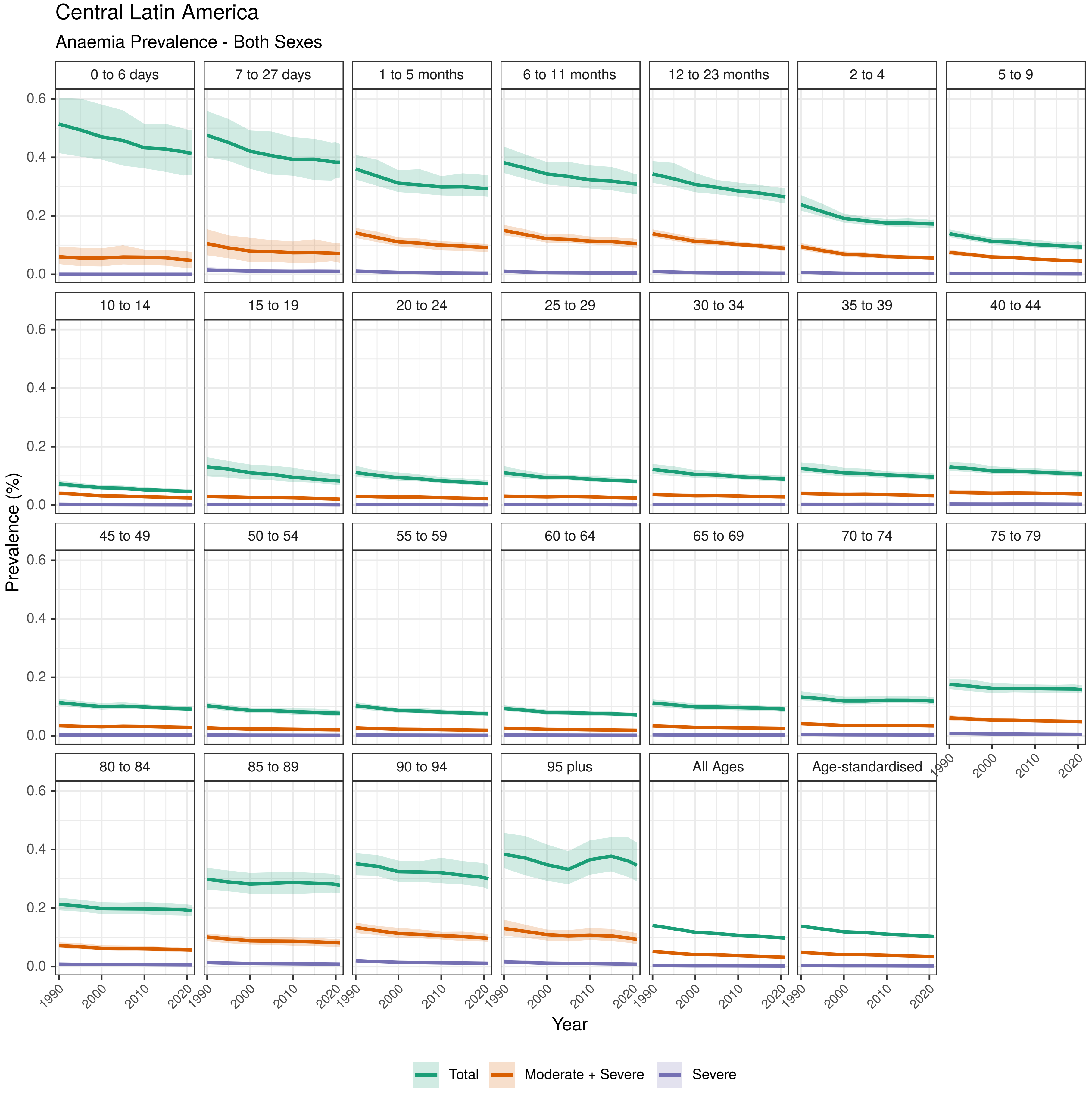

Central Latin America
Anaemia Prevalence - Both Sexes
0 to 6 days
7 to 27 days
1 to 5 months
6 to 11 months
12 to 23 months
2 to 4
5 to 9
10 to 14
15 to 19
20 to 24
25 to 29
30 to 34
35 to 39
40 to 44
45 to 49
50 to 54
55 to 59
60 to 64
65 to 69
70 to 74
75 to 79
80 to 84
85 to 89
90 to 94
95 plus
All Ages
Age-standardised
Prevalence (%)
Year
Total
Moderate + Severe
Severe

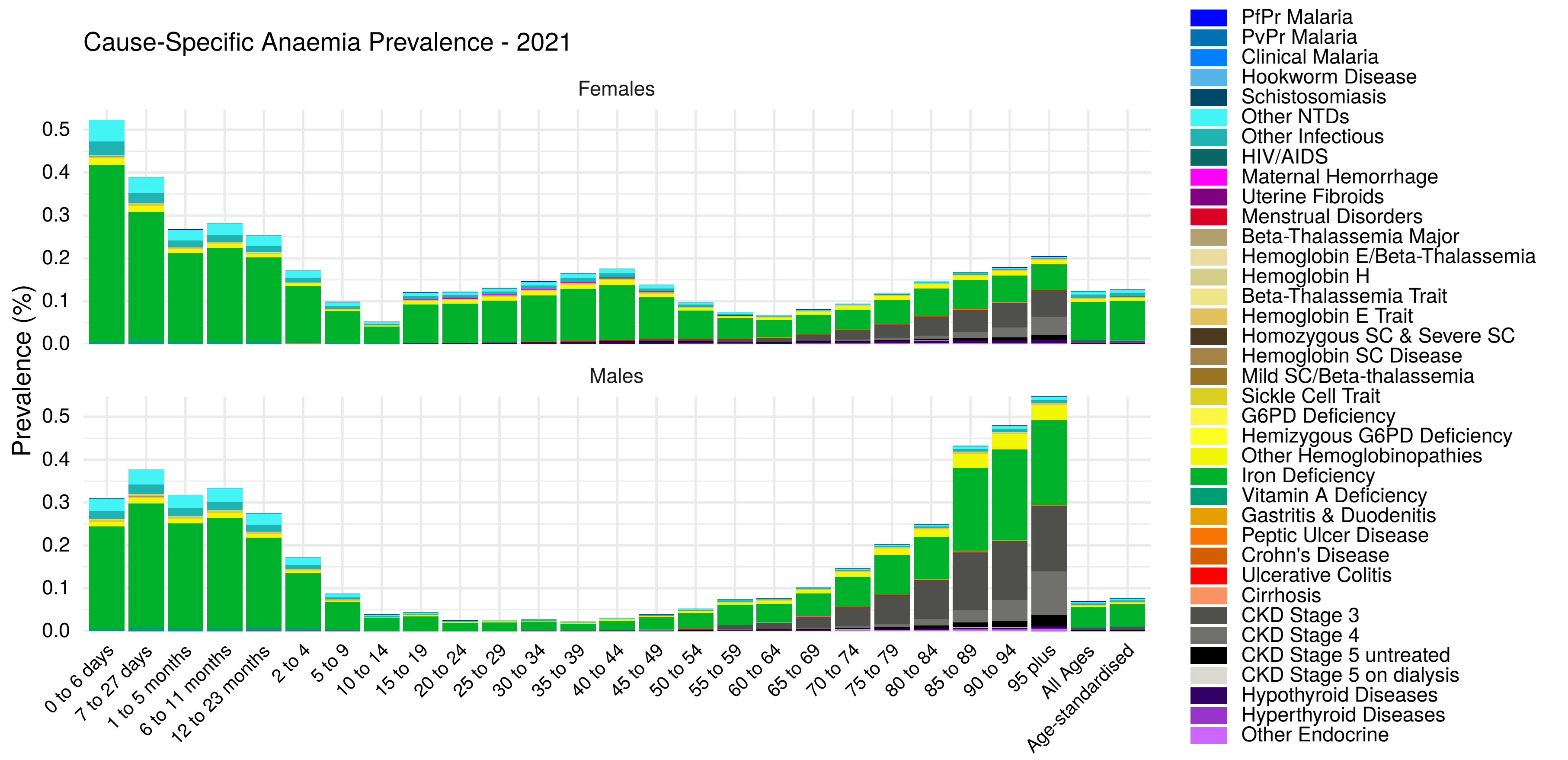

Cause-Specific Anaemia Prevalence - 2021
Females
Males
Prevalence (%)
PfPr Malaria
PvPr Malaria
Clinical Malaria
Hookworm Disease
Schistosomiasis
Other NTDs
Other Infectious
HIV/AIDS
Maternal Hemorrhage
Uterine Fibroids
Menstrual Disorders
Beta-Thalassemia Major
Hemoglobin E/Beta-Thalassemia
Hemoglobin H
Beta-Thalassemia Trait
Hemoglobin E Trait
Homozygous SC & Severe SC
Hemoglobin SC Disease
Mild SC/Beta-thalassemia
Sickle Cell Trait
G6PD Deficiency
Hemizygous G6PD Deficiency
Other Hemoglobinopathies
Iron Deficiency
Vitamin A Deficiency
Gastritis & Duodenitis
Peptic Ulcer Disease
Crohn's Disease
Ulcerative Colitis
Cirrhosis
CKD Stage 3
CKD Stage 4
CKD Stage 5 untreated
CKD Stage 5 on dialysis
Hypothyroid Diseases
Hyperthyroid Diseases
Other Endocrine

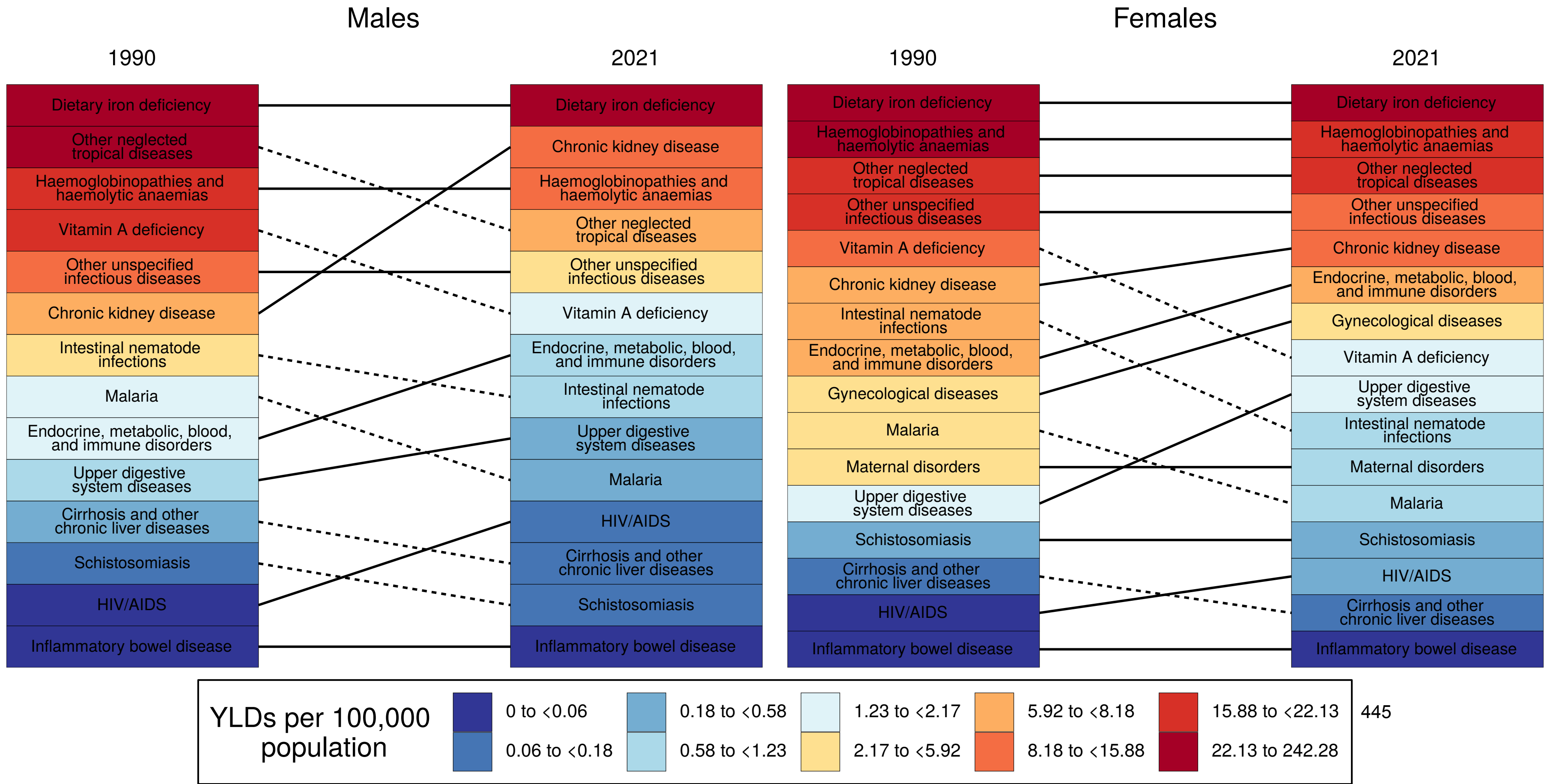

Males
Females
1990
2021
Dietary iron deficiency
Other neglected tropical diseases
Haemoglobinopathies and haemolytic anaemias
Vitamin A deficiency
Other unspecified infectious diseases
Chronic kidney disease
Intestinal nematode infections
Malaria
Endocrine, metabolic, blood, and immune disorders
Upper digestive system diseases
Cirrhosis and other chronic liver diseases
Schistosomiasis
HIV/AIDS
Inflammatory bowel disease
Gynecological diseases
Maternal disorders
YLDs per 100,000 population
0 to <0.06
0.06 to <0.18
0.18 to <0.58
0.58 to <1.23
1.23 to <2.17
2.17 to <5.92
5.92 to <8.18
8.18 to <15.88
15.88 to <22.13
22.13 to 242.28

Colombia

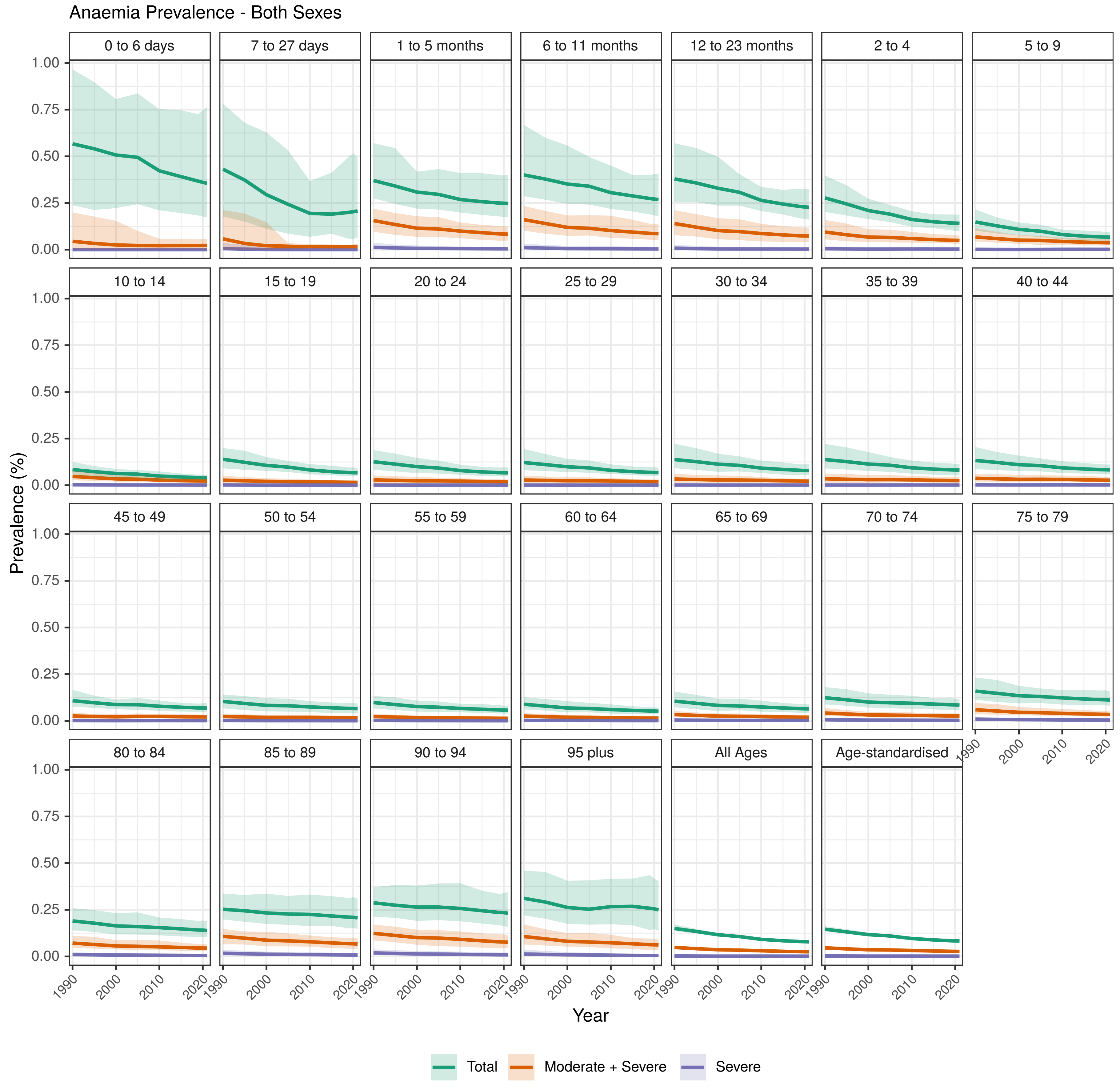

Anaemia Prevalence - Both Sexes
0 to 6 days
7 to 27 days
1 to 5 months
6 to 11 months
12 to 23 months
2 to 4
5 to 9
10 to 14
15 to 19
20 to 24
25 to 29
30 to 34
35 to 39
40 to 44
45 to 49
50 to 54
55 to 59
60 to 64
65 to 69
70 to 74
75 to 79
80 to 84
85 to 89
90 to 94
95 plus
All Ages
Age-standardised
Prevalence (%)
Year
Total
Moderate + Severe
Severe

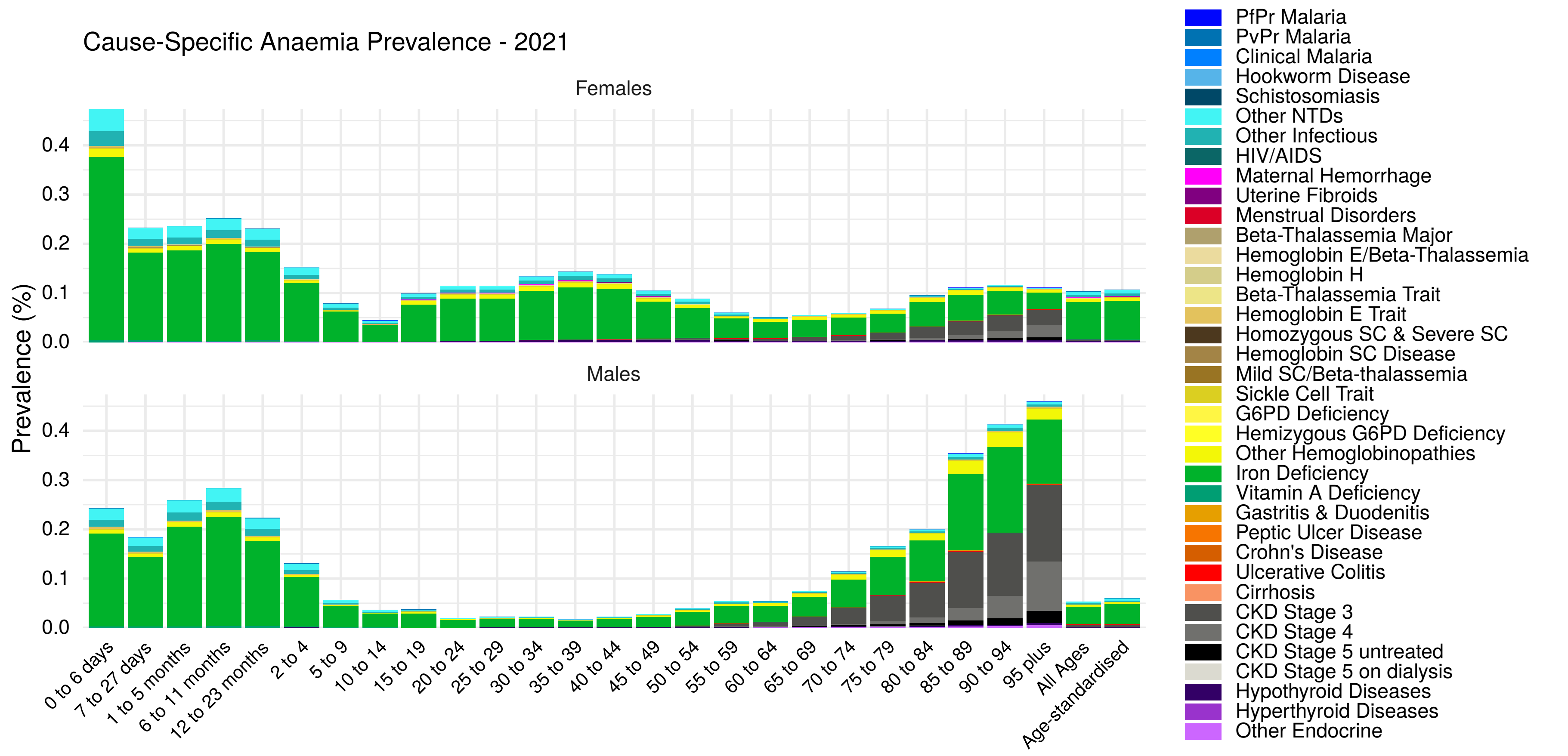

Cause-Specific Anaemia Prevalence - 2021
Females
Males
Prevalence (%)
PfPr Malaria
PvPr Malaria
Clinical Malaria
Hookworm Disease
Schistosomiasis
Other NTDs
Other Infectious
HIV/AIDS
Maternal Hemorrhage
Uterine Fibroids
Menstrual Disorders
Beta-Thalassemia Major
Hemoglobin E/Beta-Thalassemia
Hemoglobin H
Beta-Thalassemia Trait
Hemoglobin E Trait
Homozygous SC & Severe SC
Hemoglobin SC Disease
Mild SC/Beta-thalassemia
Sickle Cell Trait
G6PD Deficiency
Hemizygous G6PD Deficiency
Other Hemoglobinopathies
Iron Deficiency
Vitamin A Deficiency
Gastritis & Duodenitis
Peptic Ulcer Disease
Crohn's Disease
Ulcerative Colitis
Cirrhosis
CKD Stage 3
CKD Stage 4
CKD Stage 5 untreated
CKD Stage 5 on dialysis
Hypothyroid Diseases
Hyperthyroid Diseases
Other Endocrine

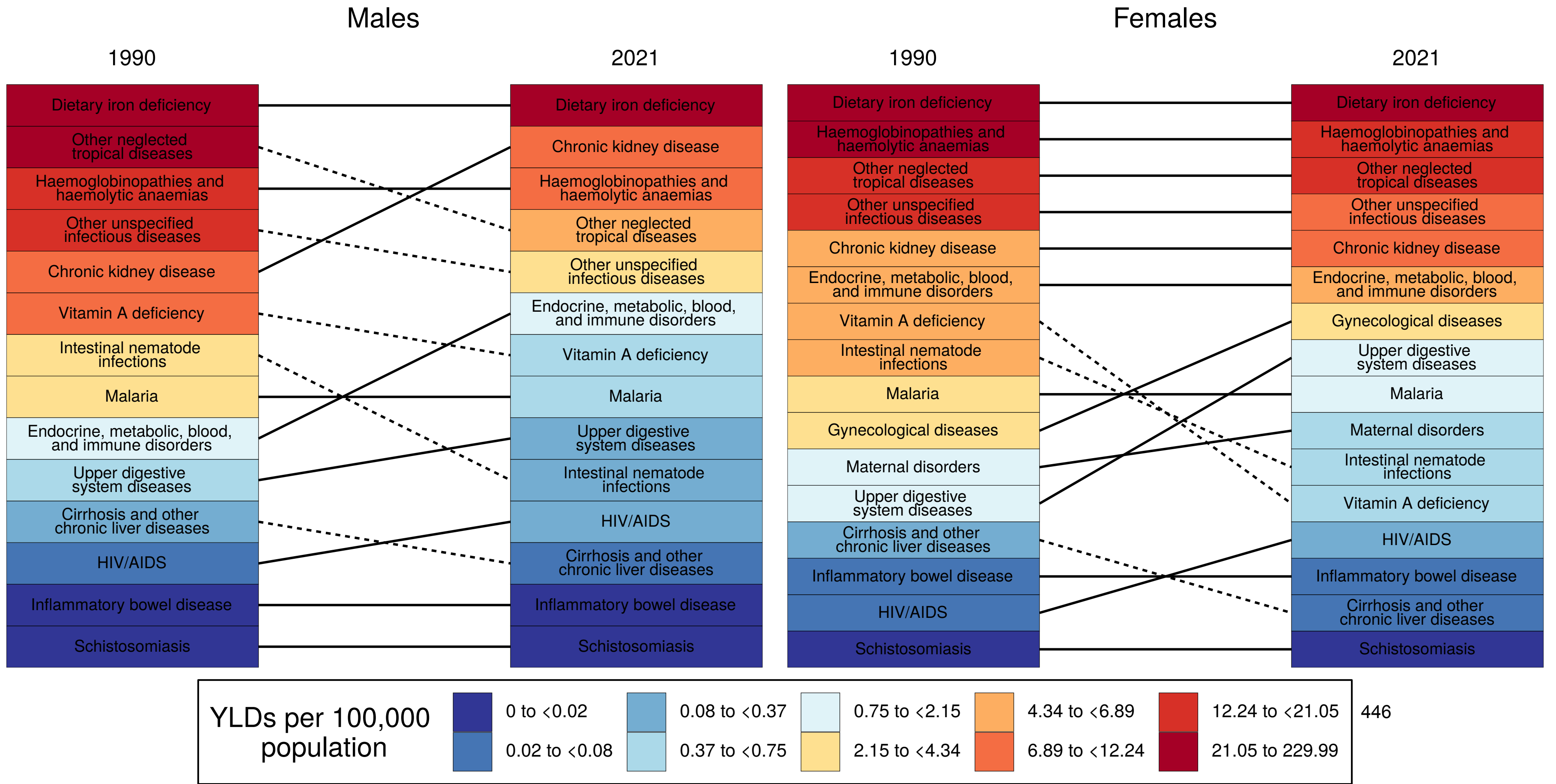

Males
Females
1990
2021
Dietary iron deficiency
Other neglected tropical diseases
Haemoglobinopathies and haemolytic anaemias
Other unspecified infectious diseases
Chronic kidney disease
Vitamin A deficiency
Intestinal nematode infections
Malaria
Endocrine, metabolic, blood, and immune disorders
Upper digestive system diseases
Cirrhosis and other chronic liver diseases
HIV/AIDS
Inflammatory bowel disease
Schistosomiasis
Gynecological diseases
Maternal disorders
YLDs per 100,000 population
0 to <0.02
0.02 to <0.08
0.08 to <0.37
0.37 to <0.75
0.75 to <2.15
2.15 to <4.34
4.34 to <6.89
6.89 to <12.24
12.24 to <21.05
21.05 to 229.99

Costa Rica

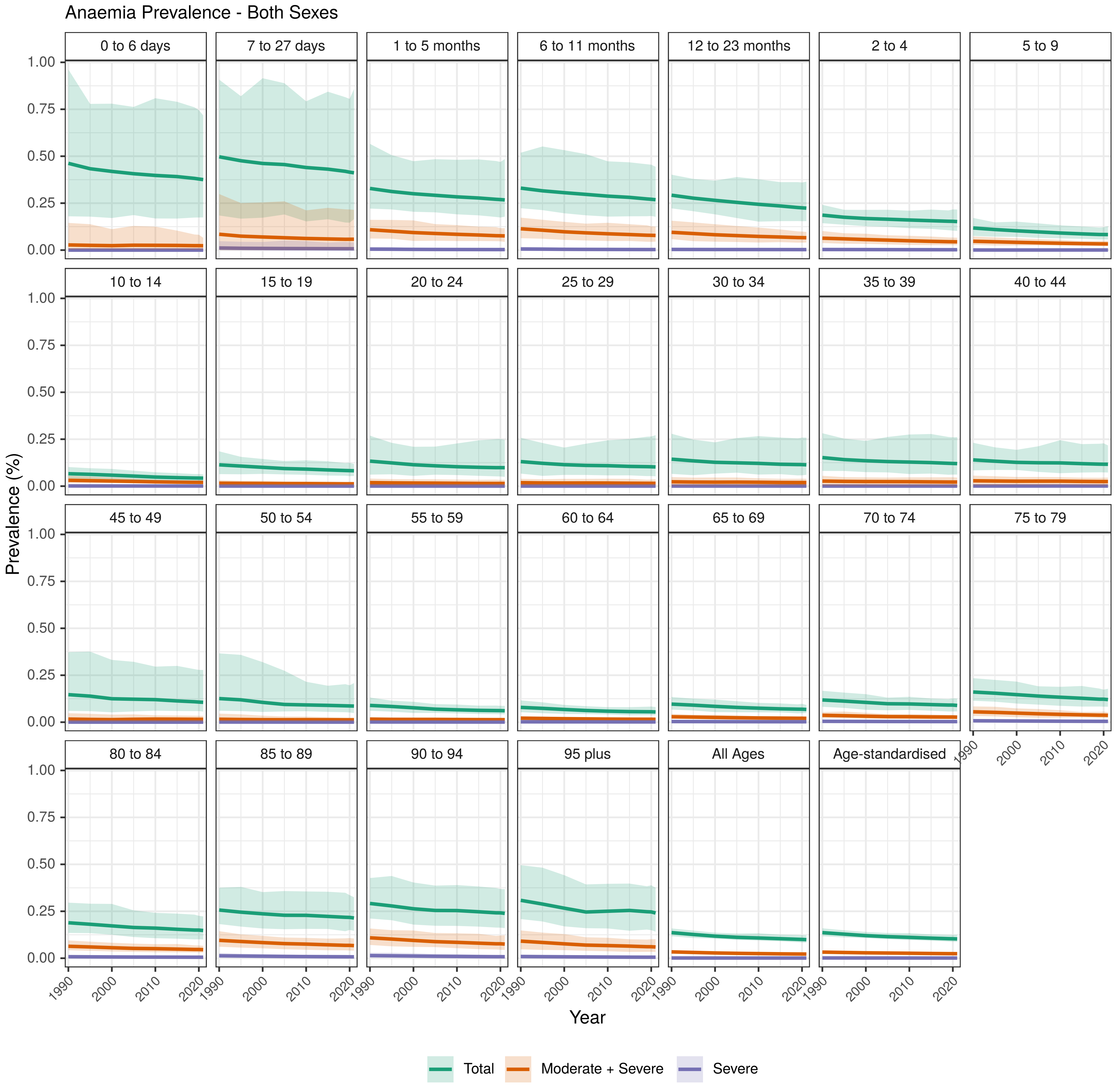

Anaemia Prevalence - Both Sexes
0 to 6 days
7 to 27 days
1 to 5 months
6 to 11 months
12 to 23 months
2 to 4
5 to 9
10 to 14
15 to 19
20 to 24
25 to 29
30 to 34
35 to 39
40 to 44
45 to 49
50 to 54
55 to 59
60 to 64
65 to 69
70 to 74
75 to 79
80 to 84
85 to 89
90 to 94
95 plus
All Ages
Age-standardised
Prevalence (%)
Year
Total
Moderate + Severe
Severe

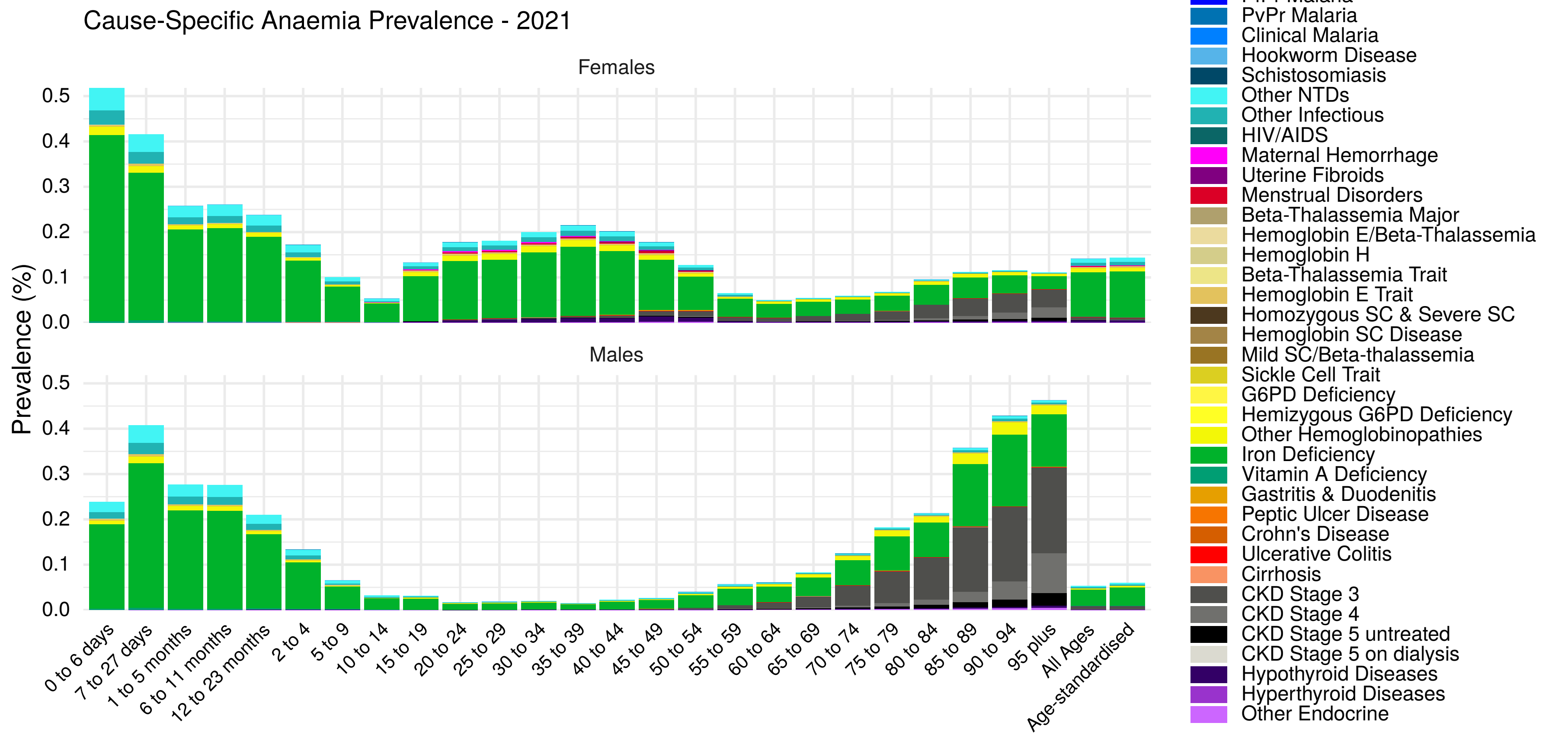

Cause-Specific Anaemia Prevalence - 2021
Females
Males
Prevalence (%)
PfPr Malaria
PvPr Malaria
Clinical Malaria
Hookworm Disease
Schistosomiasis
Other NTDs
Other Infectious
HIV/AIDS
Maternal Hemorrhage
Uterine Fibroids
Menstrual Disorders
Beta-Thalassemia Major
Hemoglobin E/Beta-Thalassemia
Hemoglobin H
Beta-Thalassemia Trait
Hemoglobin E Trait
Homozygous SC & Severe SC
Hemoglobin SC Disease
Mild SC/Beta-thalassemia
Sickle Cell Trait
G6PD Deficiency
Hemizygous G6PD Deficiency
Other Hemoglobinopathies
Iron Deficiency
Vitamin A Deficiency
Gastritis & Duodenitis
Peptic Ulcer Disease
Crohn's Disease
Ulcerative Colitis
Cirrhosis
CKD Stage 3
CKD Stage 4
CKD Stage 5 untreated
CKD Stage 5 on dialysis
Hypothyroid Diseases
Hyperthyroid Diseases
Other Endocrine

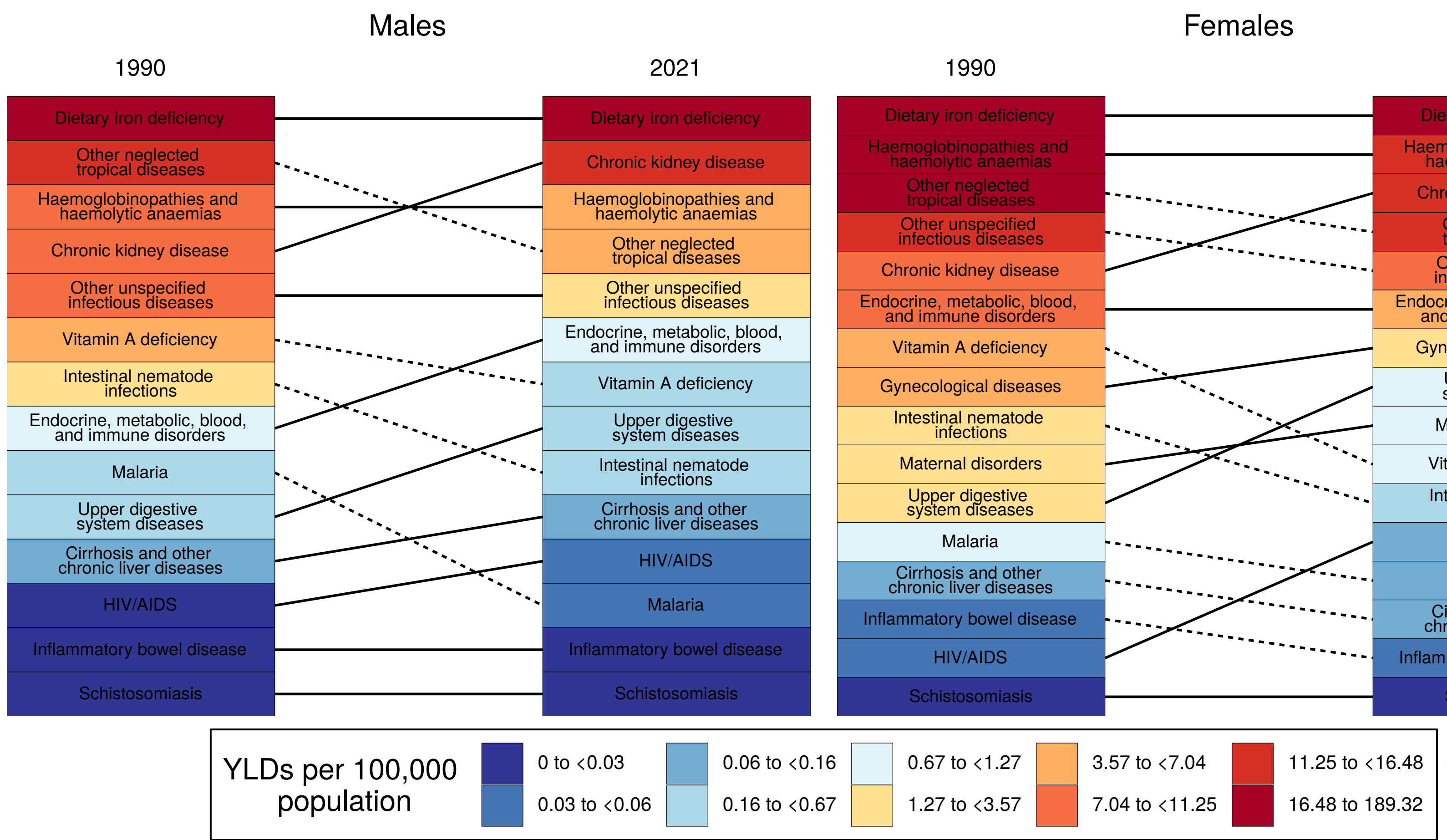

Males
Females
1990
2021
Dietary iron deficiency
Other neglected tropical diseases
Haemoglobinopathies and haemolytic anaemias
Chronic kidney disease
Other unspecified infectious diseases
Vitamin A deficiency
Intestinal nematode infections
Endocrine, metabolic, blood, and immune disorders
Malaria
Upper digestive system diseases
Cirrhosis and other chronic liver diseases
HIV/AIDS
Inflammatory bowel disease
Schistosomiasis
Gynecological diseases
Maternal disorders
YLDs per 100,000 population
0 to <0.03
0.03 to <0.06
0.06 to <0.16
0.16 to <0.67
0.67 to <1.27
1.27 to <3.57
3.57 to <7.04
7.04 to <11.25
11.25 to <16.48
16.48 to 189.32

El Salvador

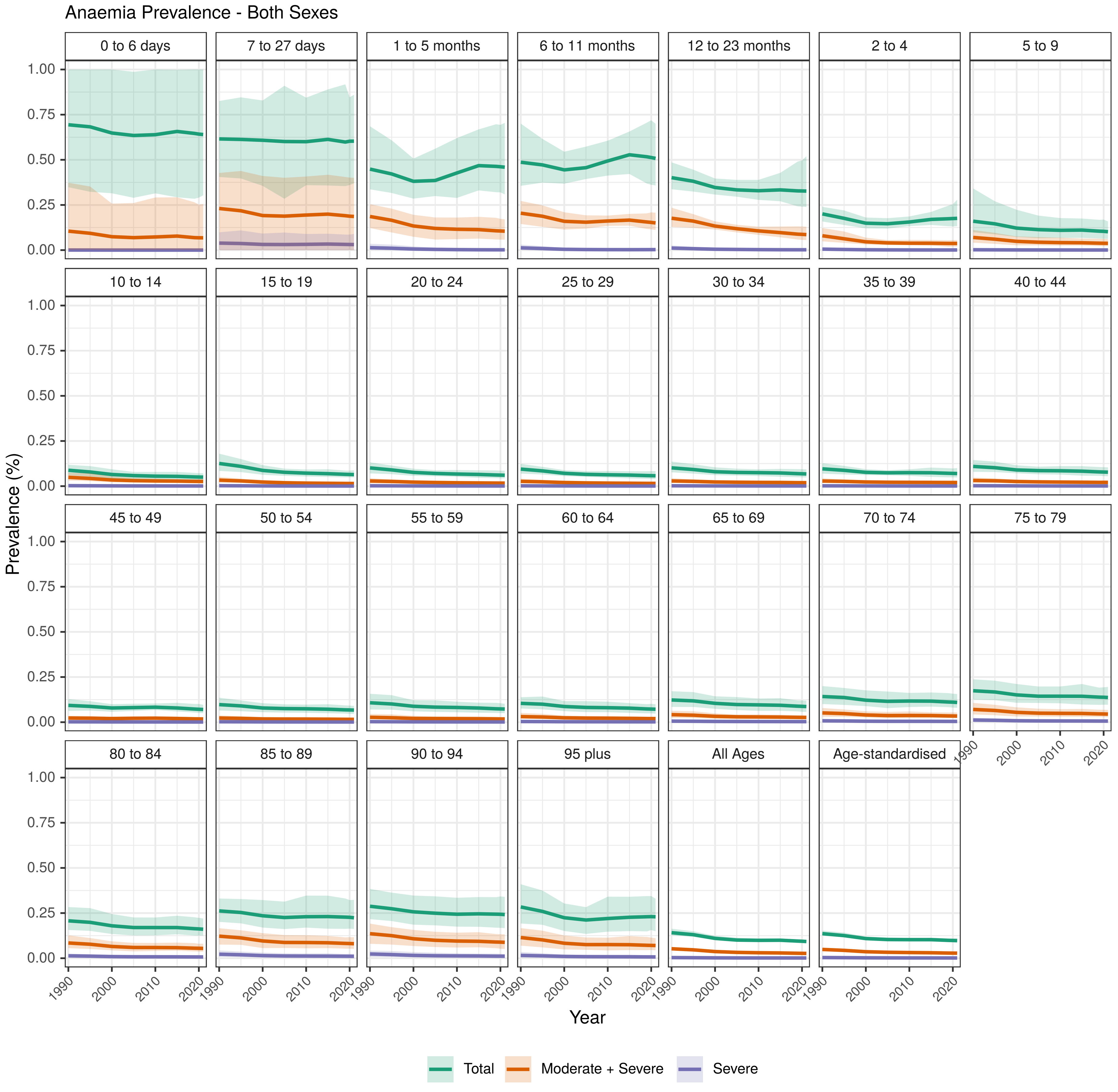

Anaemia Prevalence - Both Sexes
0 to 6 days
7 to 27 days
1 to 5 months
6 to 11 months
12 to 23 months
2 to 4
5 to 9
10 to 14
15 to 19
20 to 24
25 to 29
30 to 34
35 to 39
40 to 44
45 to 49
50 to 54
55 to 59
60 to 64
65 to 69
70 to 74
75 to 79
80 to 84
85 to 89
90 to 94
95 plus
All Ages
Age-standardised
Prevalence (%)
Year
Total
Moderate + Severe
Severe

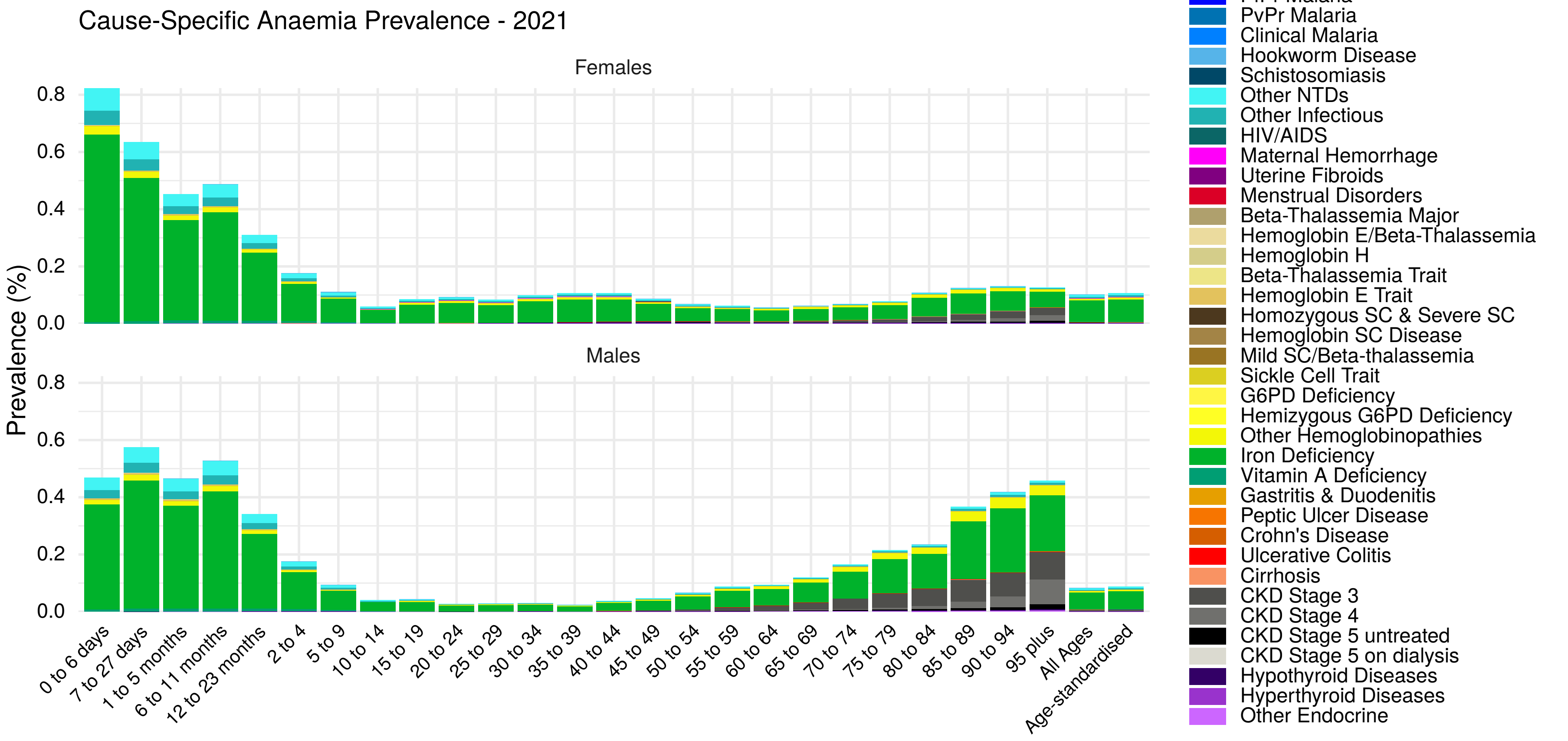

Cause-Specific Anaemia Prevalence - 2021
Females
Males
Prevalence (%)
PfPr Malaria
PvPr Malaria
Clinical Malaria
Hookworm Disease
Schistosomiasis
Other NTDs
Other Infectious
HIV/AIDS
Maternal Hemorrhage
Uterine Fibroids
Menstrual Disorders
Beta-Thalassemia Major
Hemoglobin E/Beta-Thalassemia
Hemoglobin H
Beta-Thalassemia Trait
Hemoglobin E Trait
Homozygous SC & Severe SC
Hemoglobin SC Disease
Mild SC/Beta-thalassemia
Sickle Cell Trait
G6PD Deficiency
Hemizygous G6PD Deficiency
Other Hemoglobinopathies
Iron Deficiency
Vitamin A Deficiency
Gastritis & Duodenitis
Peptic Ulcer Disease
Crohn's Disease
Ulcerative Colitis
Cirrhosis
CKD Stage 3
CKD Stage 4
CKD Stage 5 untreated
CKD Stage 5 on dialysis
Hypothyroid Diseases
Hyperthyroid Diseases
Other Endocrine

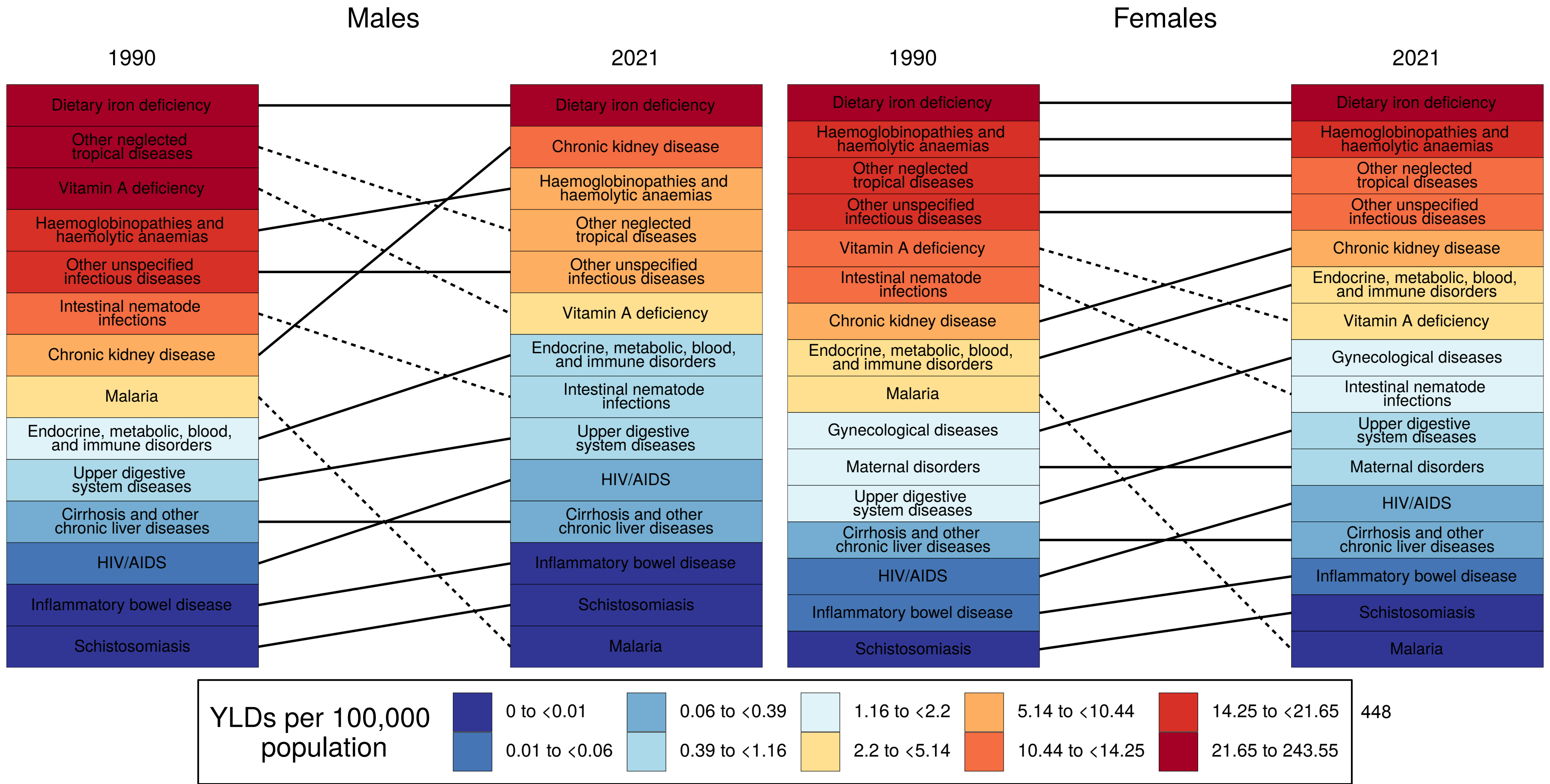

Males
Females
1990
2021
Dietary iron deficiency
Other neglected tropical diseases
Vitamin A deficiency
Haemoglobinopathies and haemolytic anaemias
Other unspecified infectious diseases
Intestinal nematode infections
Chronic kidney disease
Malaria
Endocrine, metabolic, blood, and immune disorders
Upper digestive system diseases
Cirrhosis and other chronic liver diseases
HIV/AIDS
Inflammatory bowel disease
Schistosomiasis
Gynecological diseases
Maternal disorders
YLDs per 100,000 population
0 to <0.01
0.01 to <0.06
0.06 to <0.39
0.39 to <1.16
1.16 to <2.2
2.2 to <5.14
5.14 to <10.44
10.44 to <14.25
14.25 to <21.65
21.65 to 243.55

Guatemala

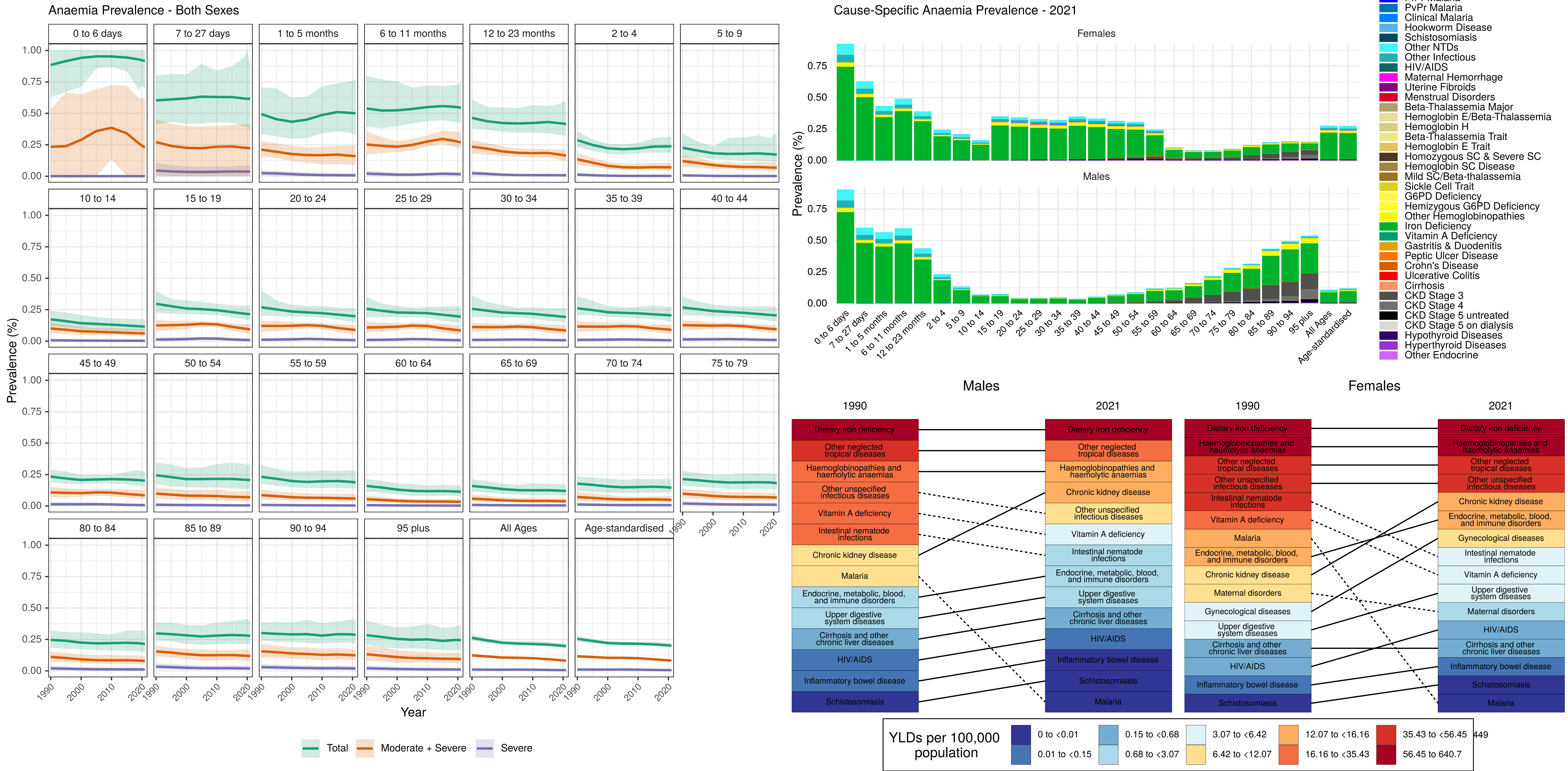

Anaemia Prevalence - Both Sexes
0 to 6 days
7 to 27 days
1 to 5 months
6 to 11 months
12 to 23 months
2 to 4
5 to 9
10 to 14
15 to 19
20 to 24
25 to 29
30 to 34
35 to 39
40 to 44
45 to 49
50 to 54
55 to 59
60 to 64
65 to 69
70 to 74
75 to 79
80 to 84
85 to 89
90 to 94
95 plus
All Ages
Age-standardised
Prevalence (%)
Year
Total
Moderate + Severe
Severe
Cause-Specific Anaemia Prevalence - 2021
Females
Males
Prevalence (%)
PfPr Malaria
PvPr Malaria
Clinical Malaria
Hookworm Disease
Schistosomiasis
Other NTDs
Other Infectious
HIV/AIDS
Maternal Hemorrhage
Uterine Fibroids
Menstrual Disorders
Beta-Thalassemia Major
Hemoglobin E/Beta-Thalassemia
Hemoglobin H
Beta-Thalassemia Trait
Hemoglobin E Trait
Homozygous SC & Severe SC
Hemoglobin SC Disease
Mild SC/Beta-thalassemia
Sickle Cell Trait
G6PD Deficiency
Hemizygous G6PD Deficiency
Other Hemoglobinopathies
Iron Deficiency
Vitamin A Deficiency
Gastritis & Duodenitis
Peptic Ulcer Disease
Crohn's Disease
Ulcerative Colitis
Cirrhosis
CKD Stage 3
CKD Stage 4
CKD Stage 5 untreated
CKD Stage 5 on dialysis
Hypothyroid Diseases
Hyperthyroid Diseases
Other Endocrine
Males
Females
1990
2021
Dietary iron deficiency
Other neglected tropical diseases
Haemoglobinopathies and haemolytic anaemias
Other unspecified infectious diseases
Vitamin A deficiency
Intestinal nematode infections
Chronic kidney disease
Malaria
Endocrine, metabolic, blood, and immune disorders
Upper digestive system diseases
Cirrhosis and other chronic liver diseases
HIV/AIDS
Inflammatory bowel disease
Schistosomiasis
Maternal disorders
Gynecological diseases
YLDs per 100,000 population
0 to <0.01
0.01 to <0.15
0.15 to <0.68
0.68 to <3.07
3.07 to <6.42
6.42 to <12.07
12.07 to <16.16
16.16 to <35.43
35.43 to <56.45
56.45 to 640.7

Honduras

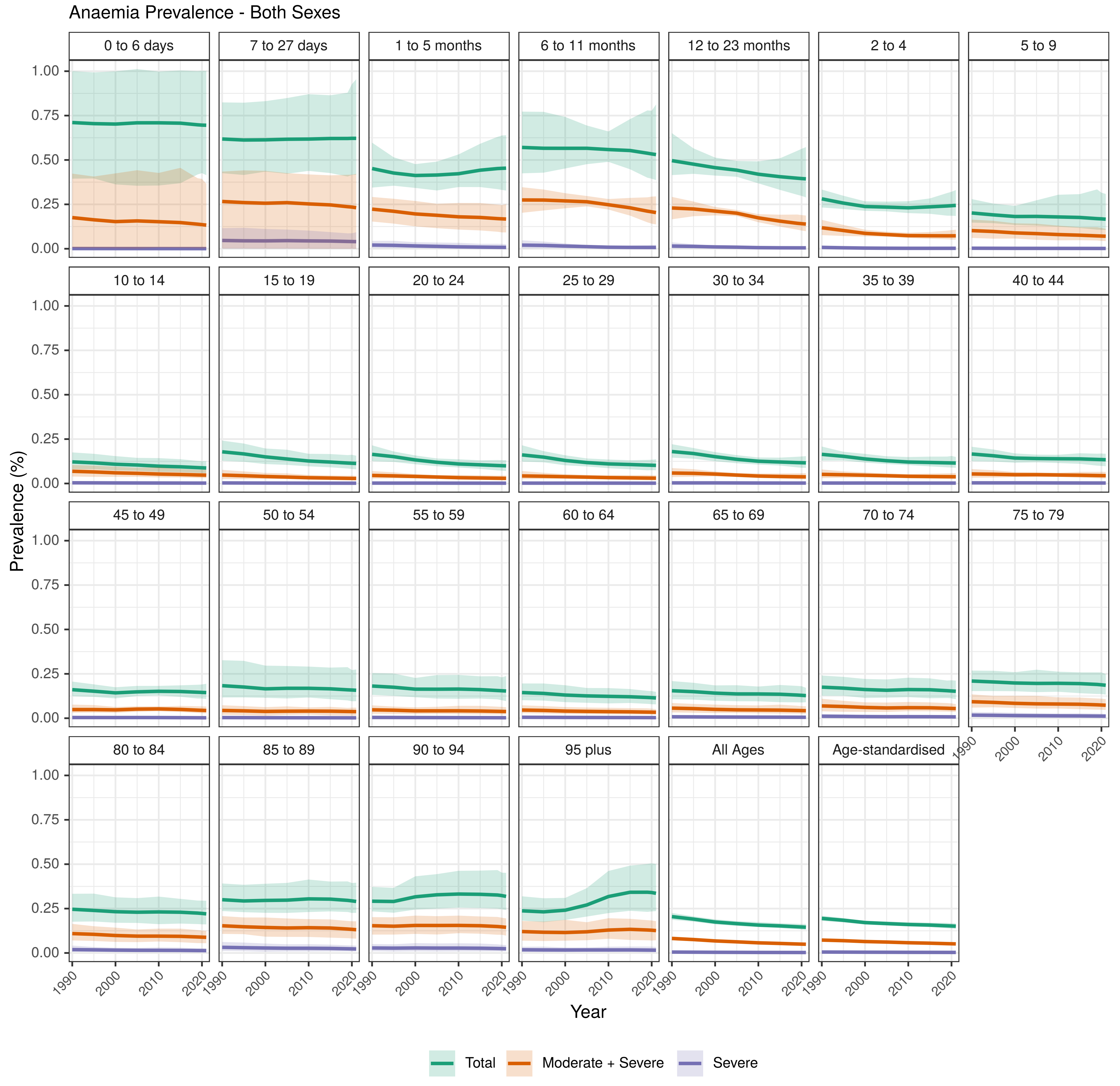

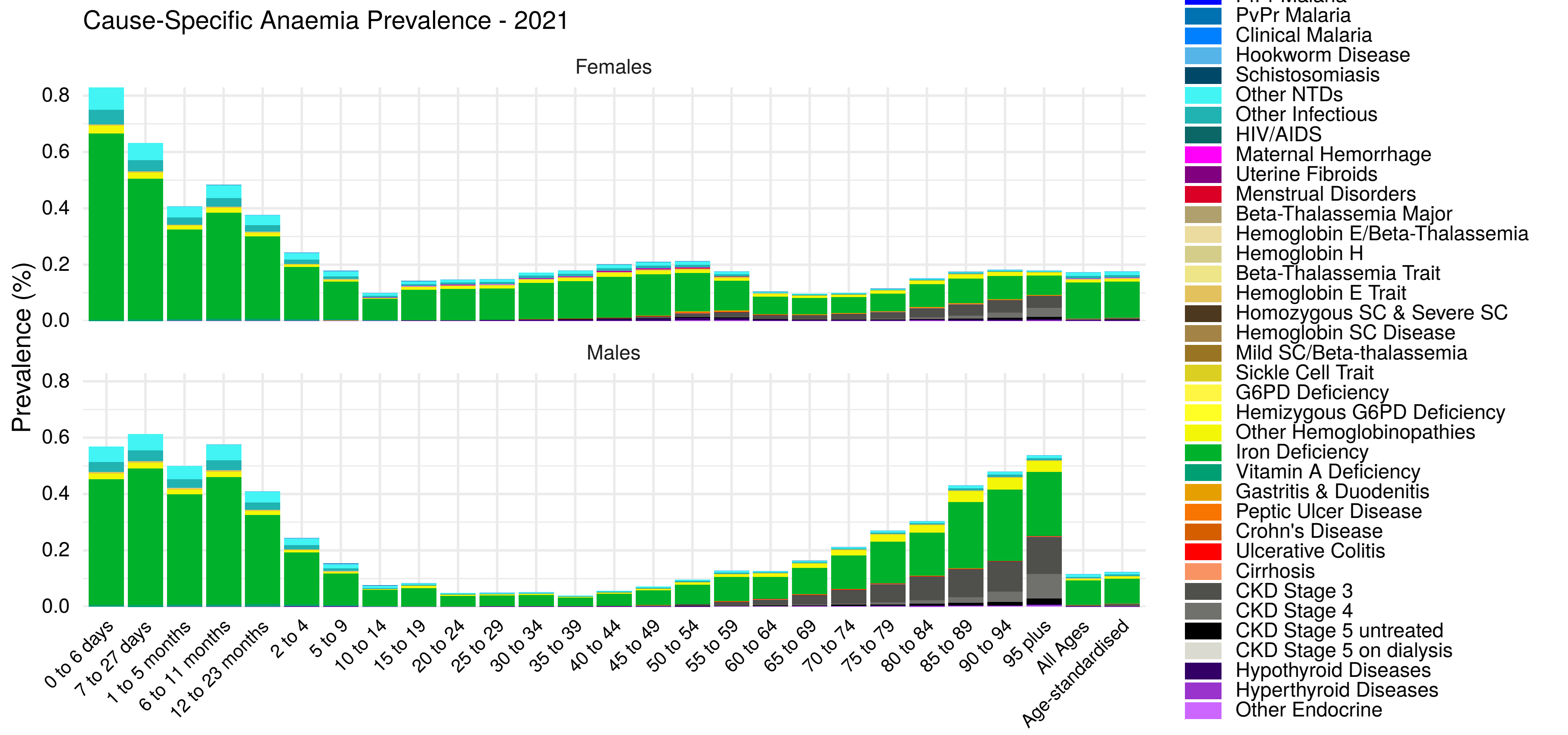

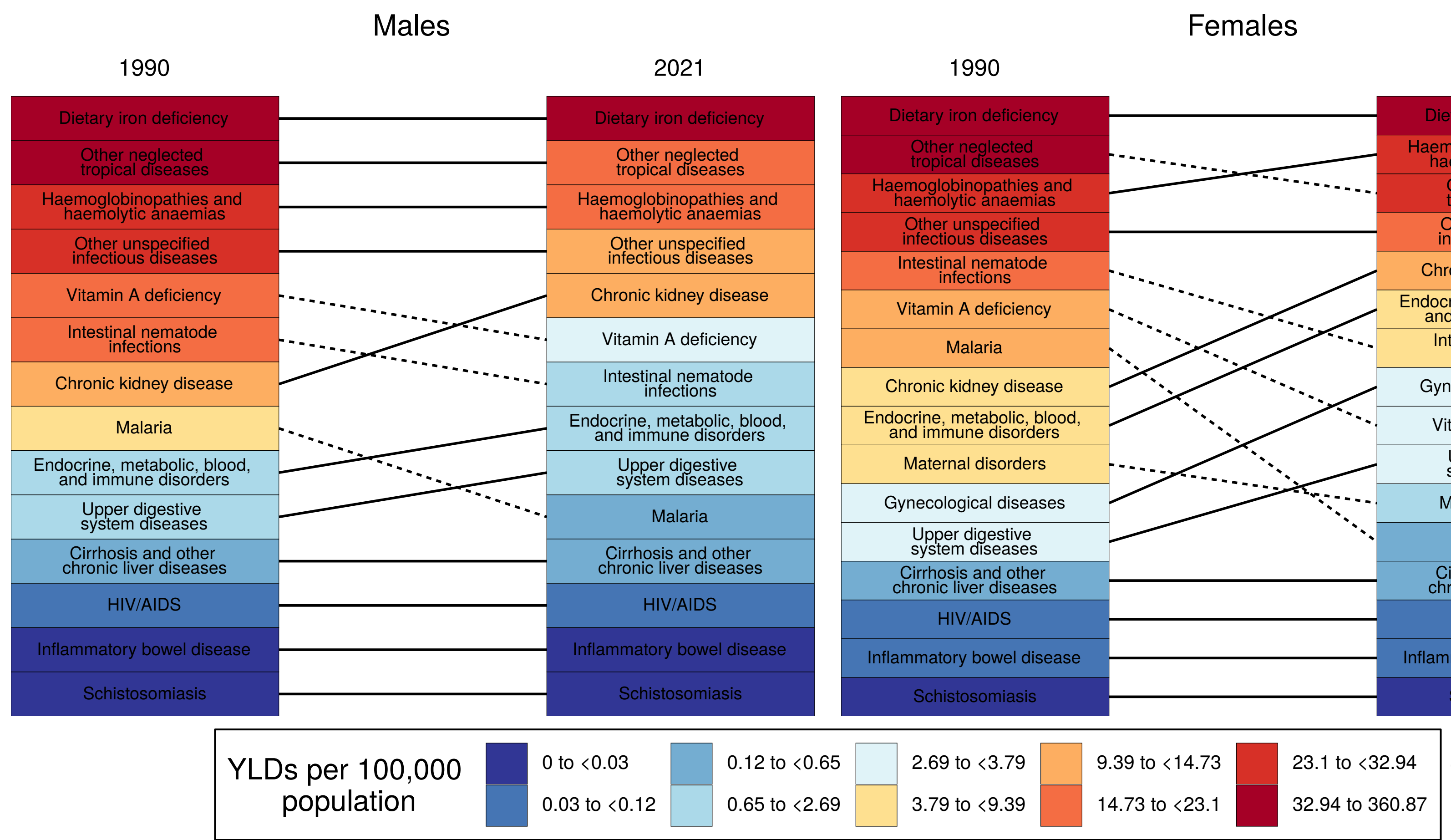

450

Mexico

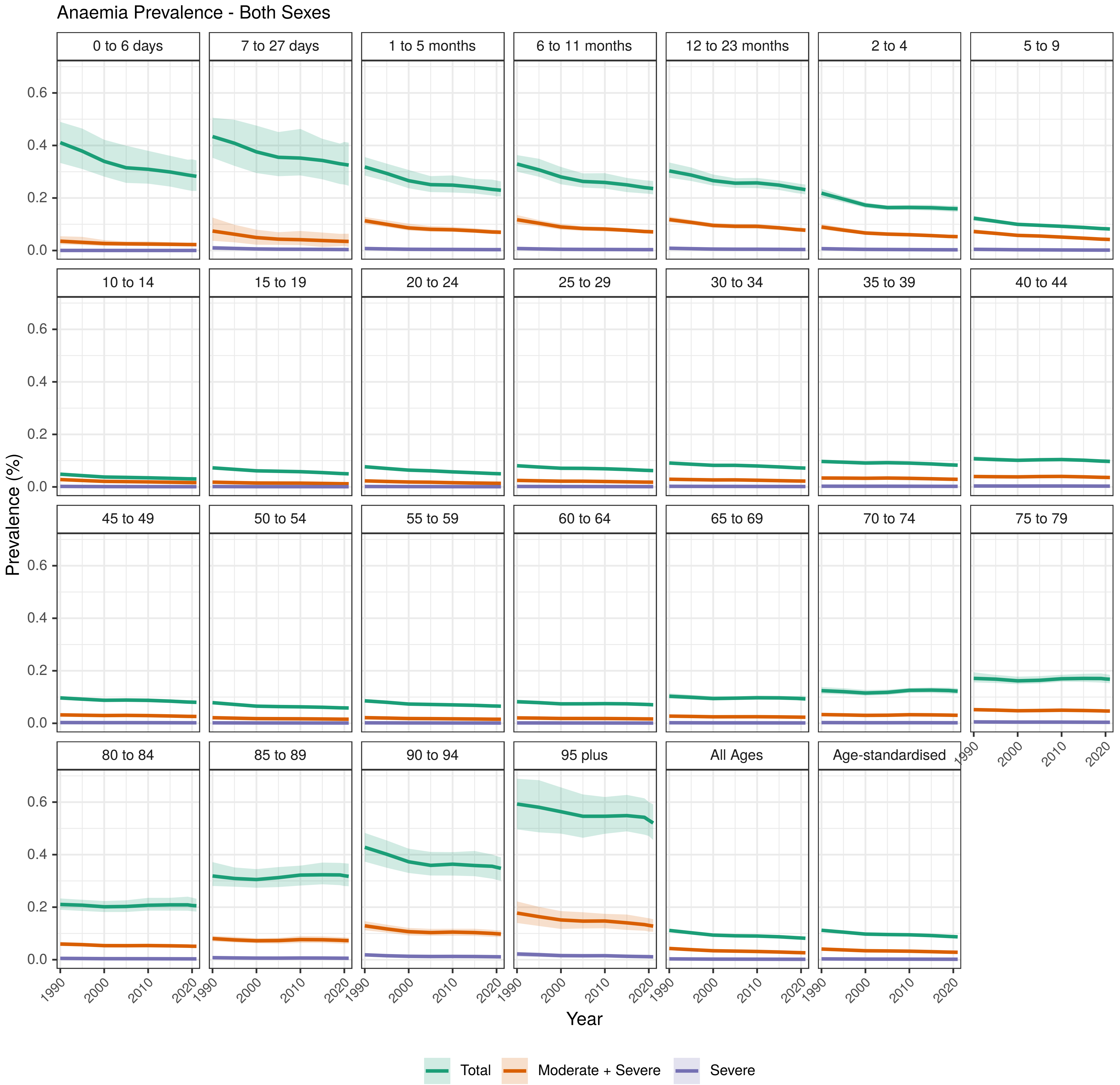

Anaemia Prevalence - Both Sexes
0 to 6 days
7 to 27 days
1 to 5 months
6 to 11 months
12 to 23 months
2 to 4
5 to 9
10 to 14
15 to 19
20 to 24
25 to 29
30 to 34
35 to 39
40 to 44
45 to 49
50 to 54
55 to 59
60 to 64
65 to 69
70 to 74
75 to 79
80 to 84
85 to 89
90 to 94
95 plus
All Ages
Age-standardised
Prevalence (%)
Year
Total
Moderate + Severe
Severe

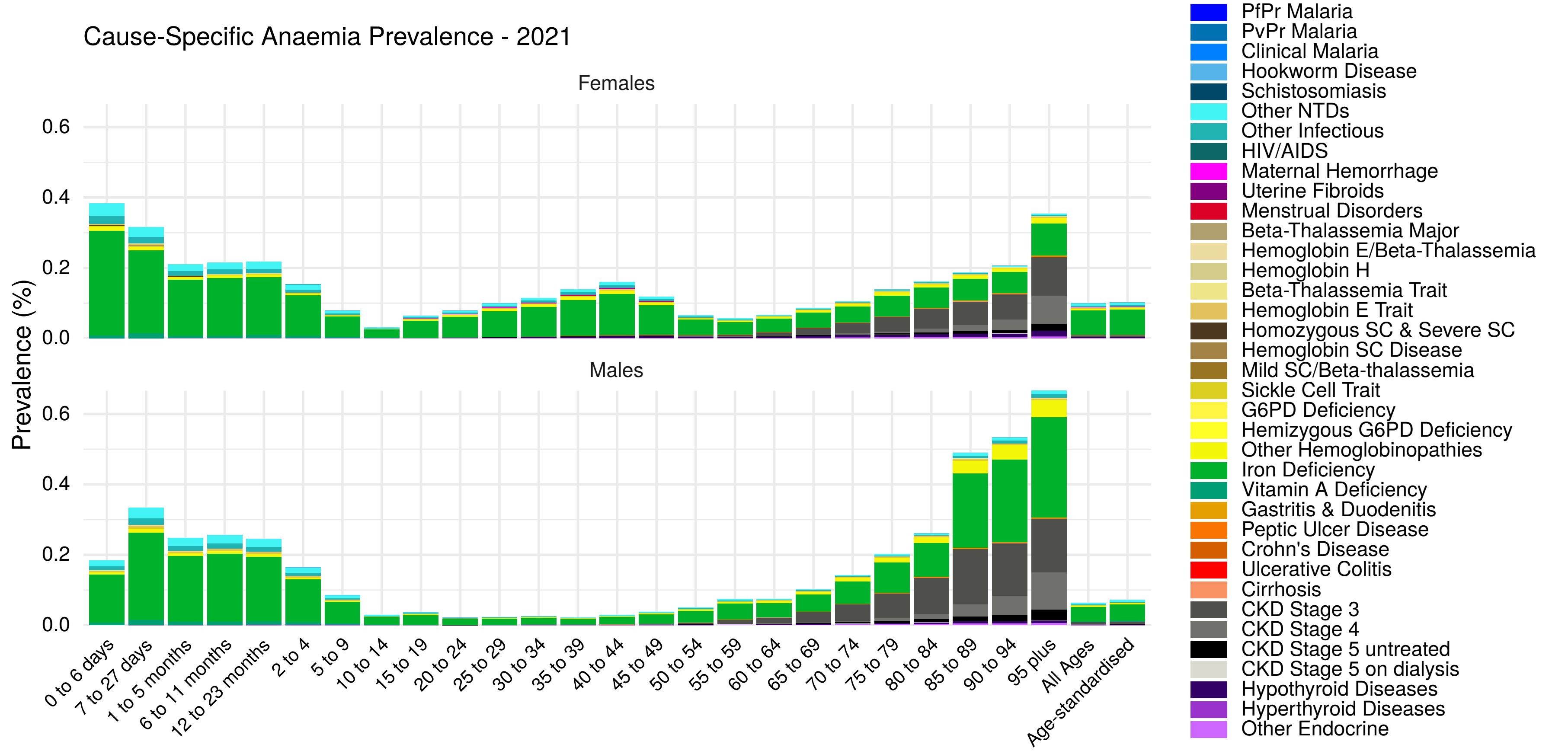

Cause-Specific Anaemia Prevalence - 2021
Females
Males
Prevalence (%)
PfPr Malaria
PvPr Malaria
Clinical Malaria
Hookworm Disease
Schistosomiasis
Other NTDs
Other Infectious
HIV/AIDS
Maternal Hemorrhage
Uterine Fibroids
Menstrual Disorders
Beta-Thalassemia Major
Hemoglobin E/Beta-Thalassemia
Hemoglobin H
Beta-Thalassemia Trait
Hemoglobin E Trait
Homozygous SC & Severe SC
Hemoglobin SC Disease
Mild SC/Beta-thalassemia
Sickle Cell Trait
G6PD Deficiency
Hemizygous G6PD Deficiency
Other Hemoglobinopathies
Iron Deficiency
Vitamin A Deficiency
Gastritis & Duodenitis
Peptic Ulcer Disease
Crohn's Disease
Ulcerative Colitis
Cirrhosis
CKD Stage 3
CKD Stage 4
CKD Stage 5 untreated
CKD Stage 5 on dialysis
Hypothyroid Diseases
Hyperthyroid Diseases
Other Endocrine

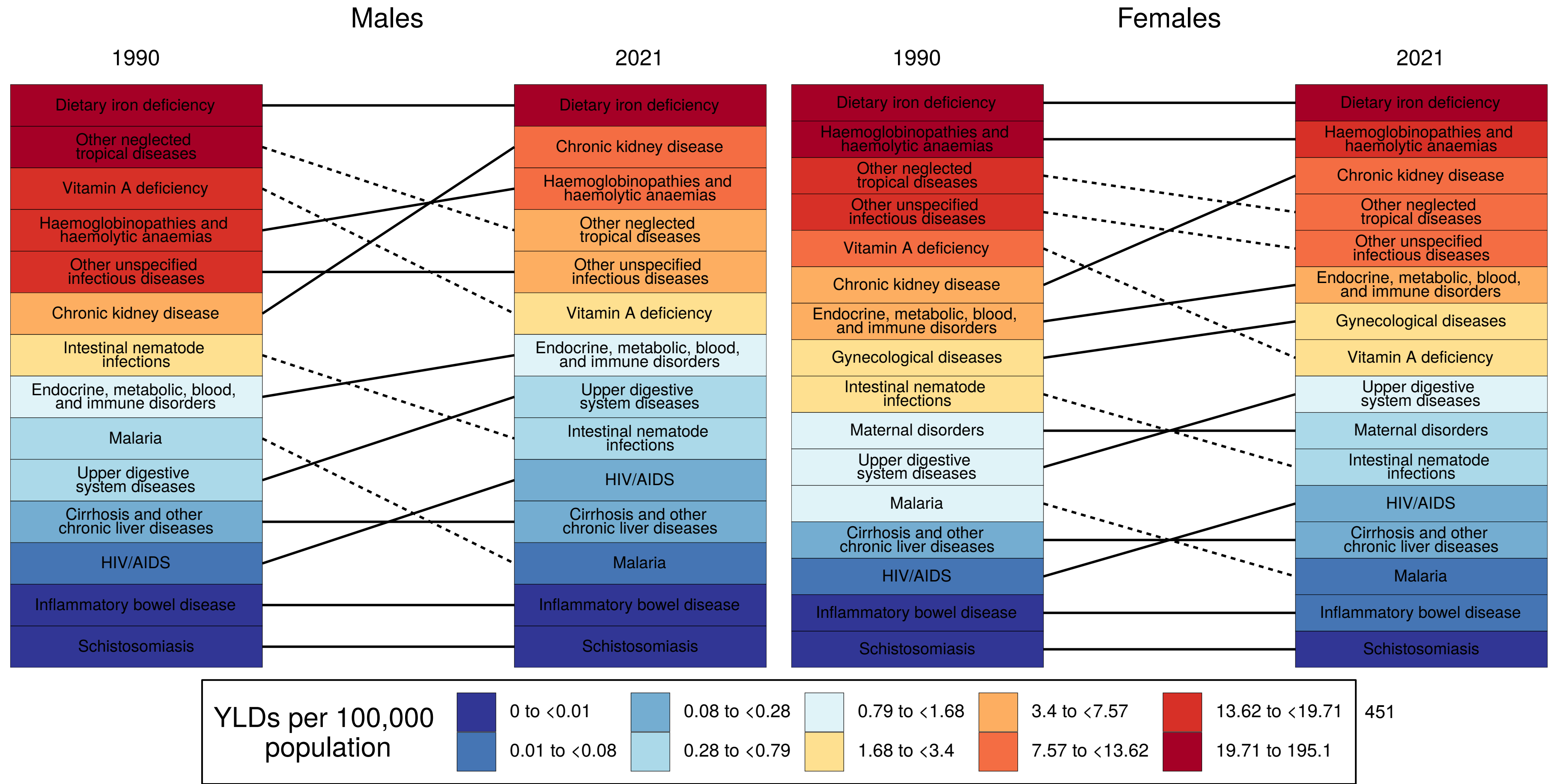

Males
Females
1990
2021
Dietary iron deficiency
Other neglected tropical diseases
Vitamin A deficiency
Haemoglobinopathies and haemolytic anaemias
Other unspecified infectious diseases
Chronic kidney disease
Intestinal nematode infections
Endocrine, metabolic, blood, and immune disorders
Malaria
Upper digestive system diseases
Cirrhosis and other chronic liver diseases
HIV/AIDS
Inflammatory bowel disease
Schistosomiasis
Gynecological diseases
Maternal disorders
YLDs per 100,000 population
0 to <0.01
0.01 to <0.08
0.08 to <0.28
0.28 to <0.79
0.79 to <1.68
1.68 to <3.4
3.4 to <7.57
7.57 to <13.62
13.62 to <19.71
19.71 to 195.1

Aguascalientes

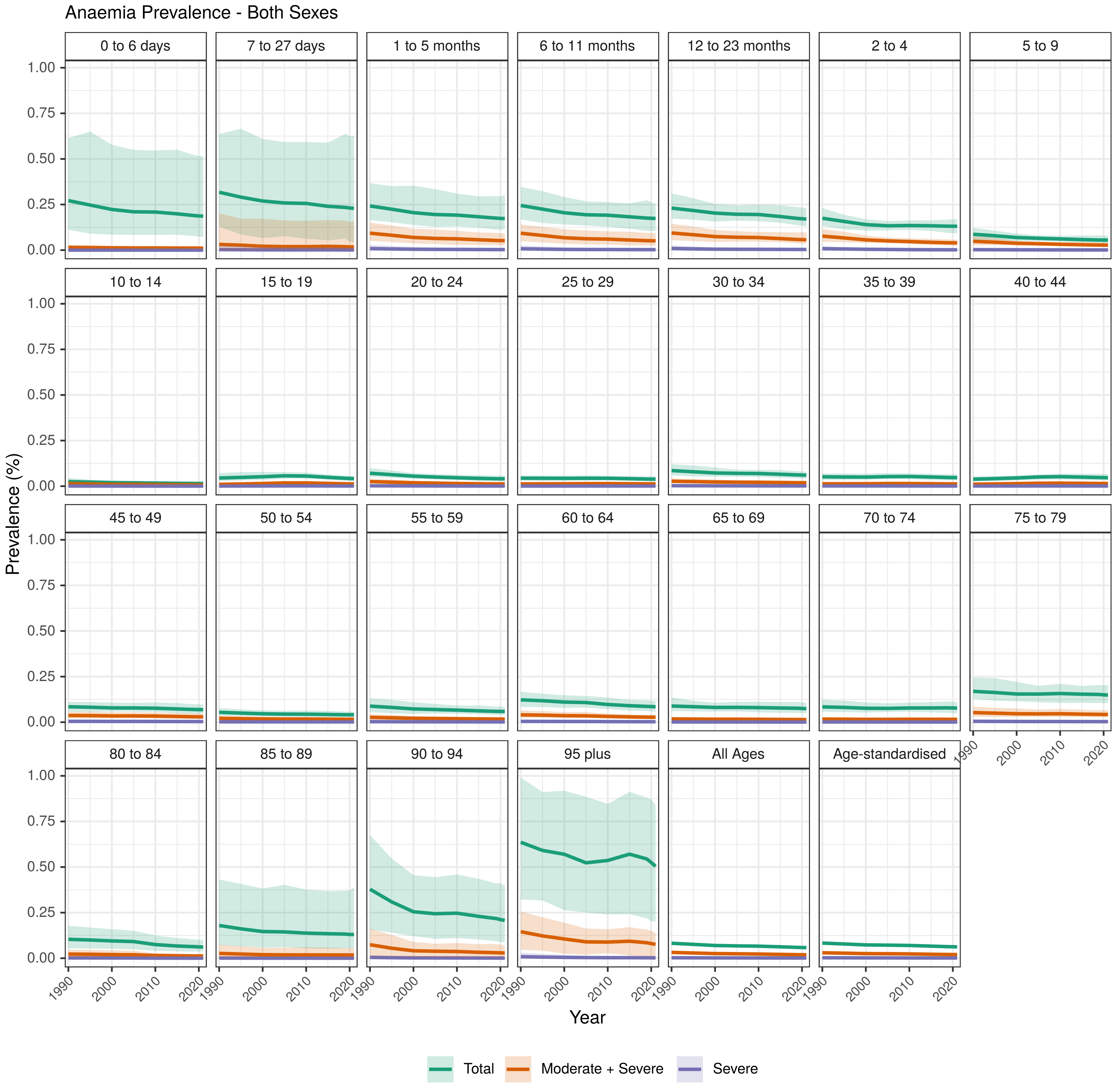

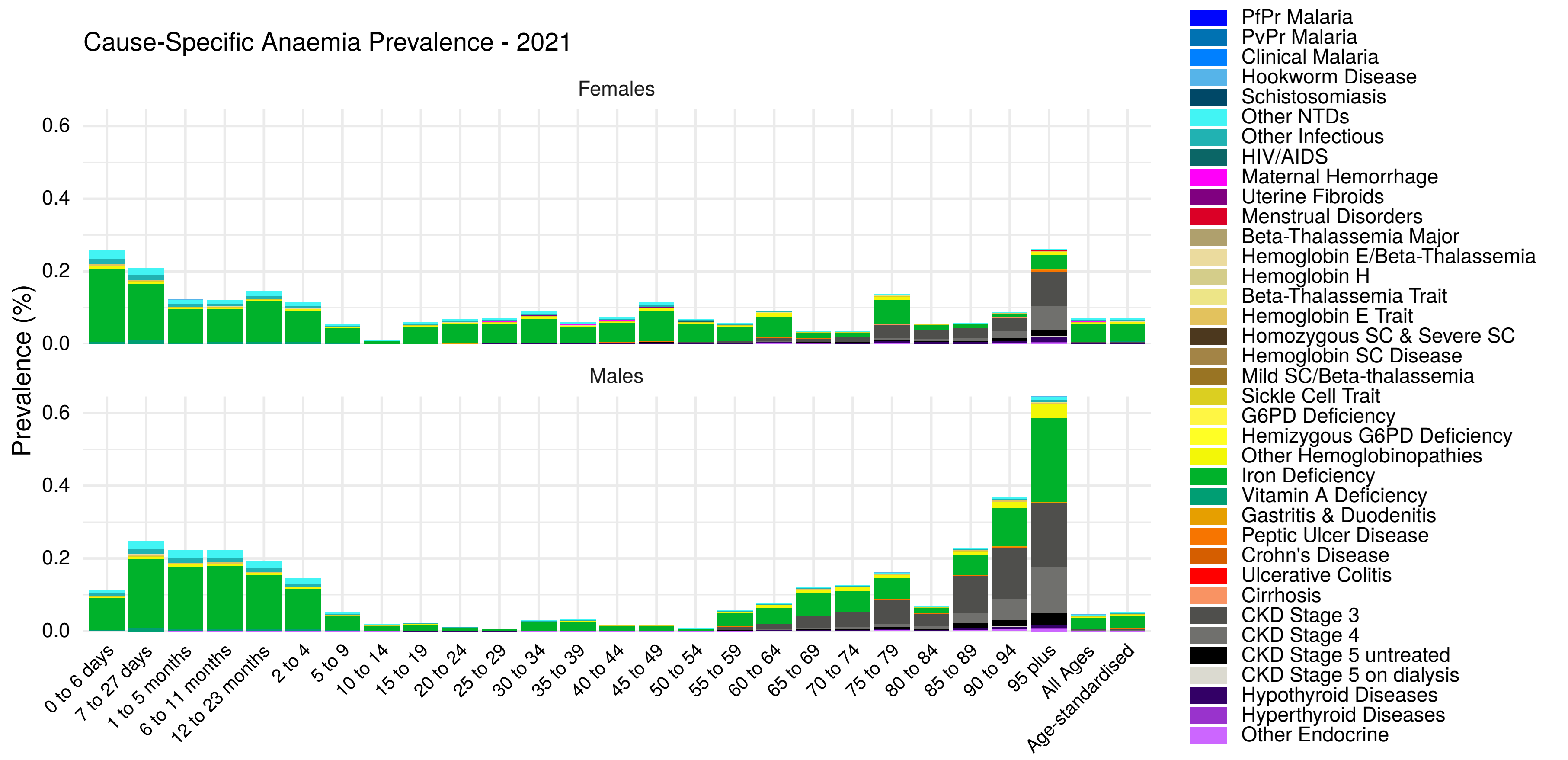

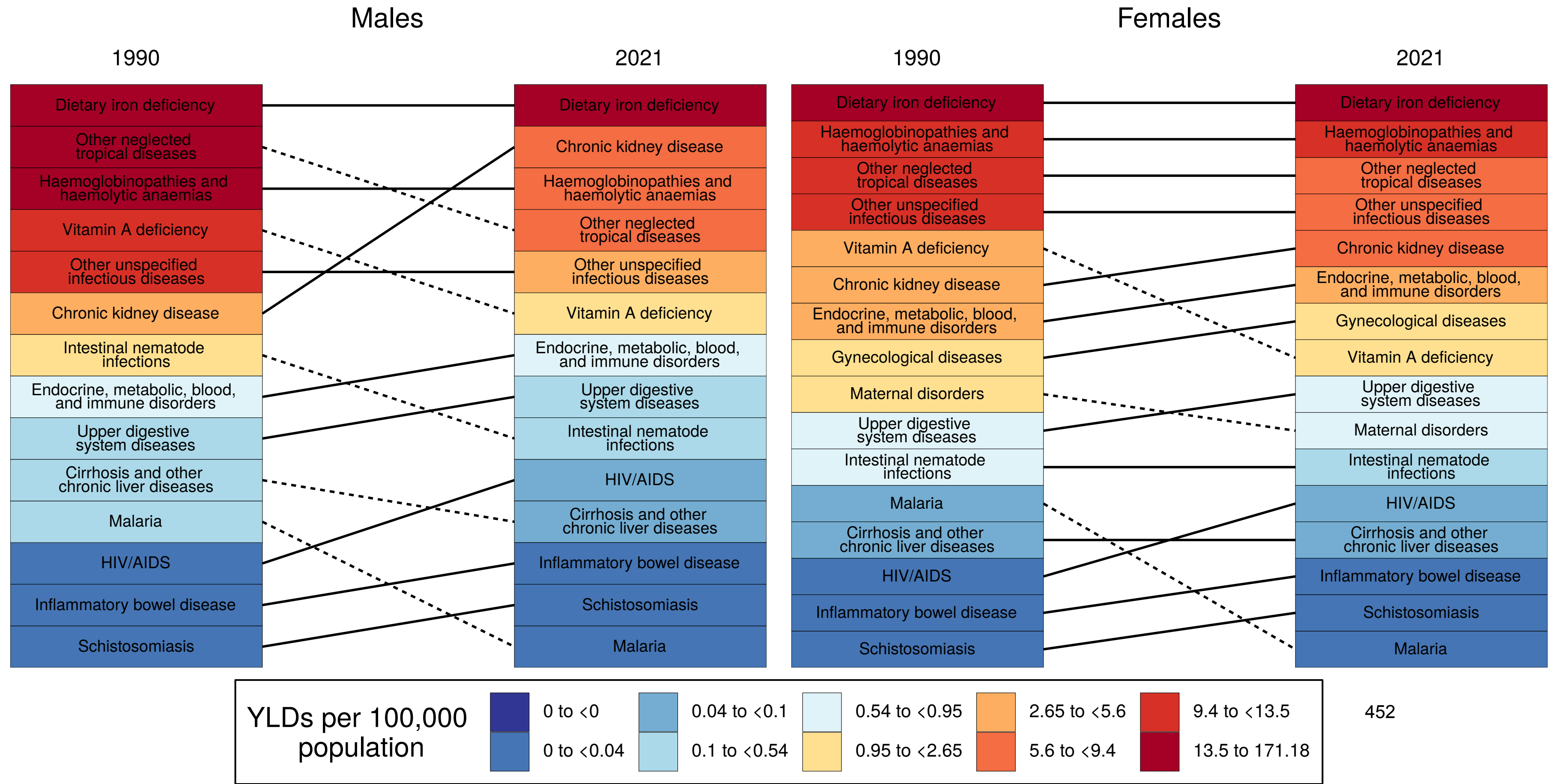

Baja California

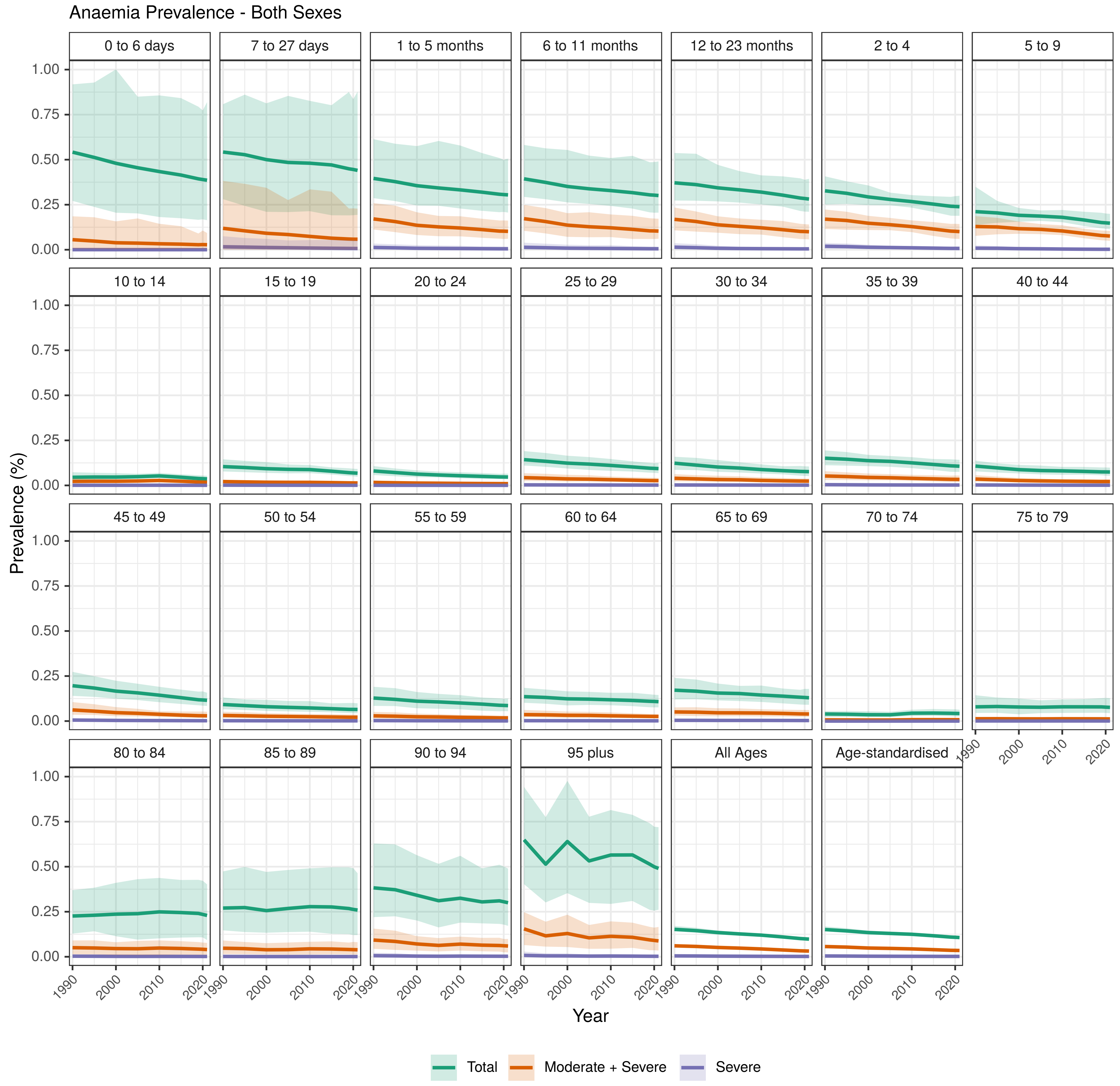

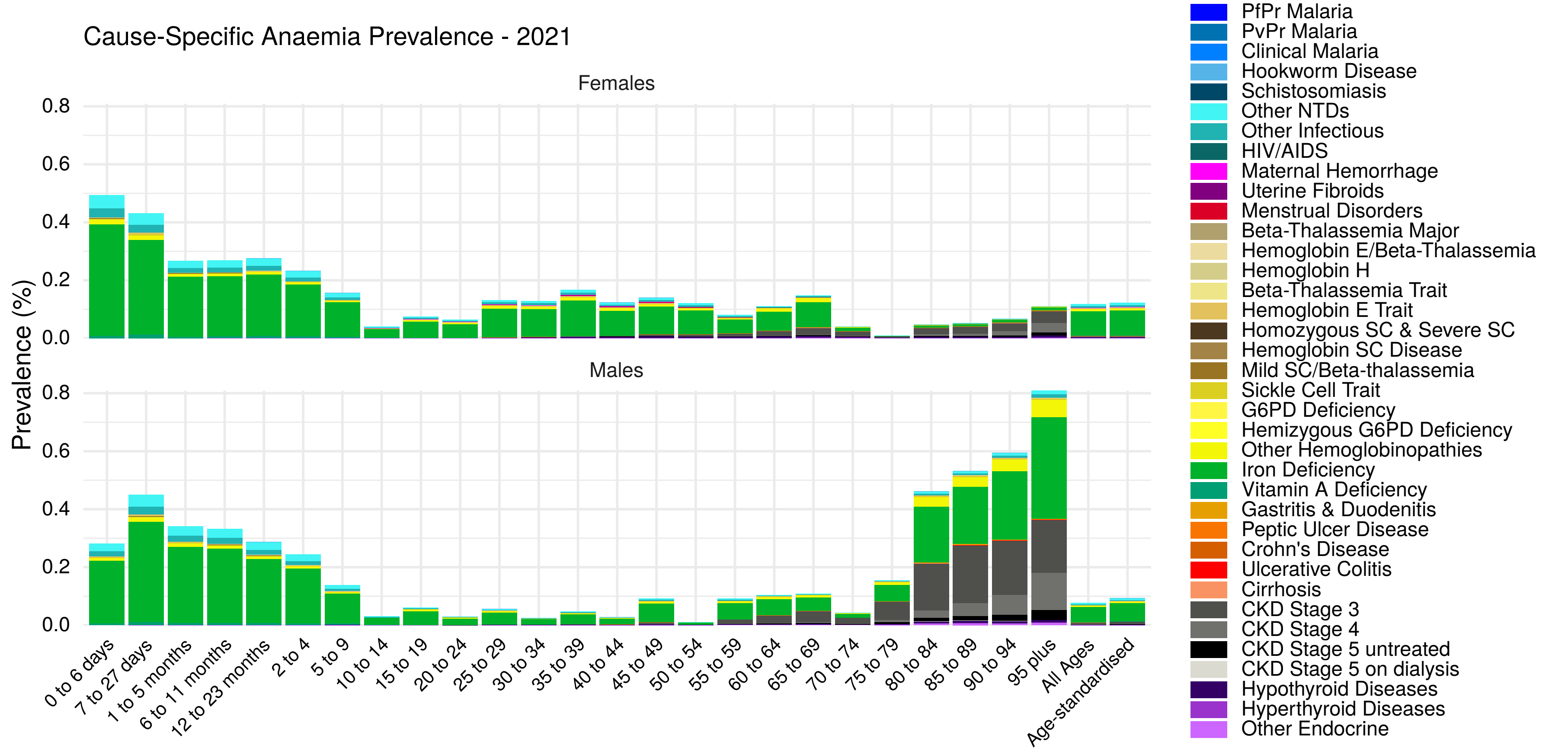

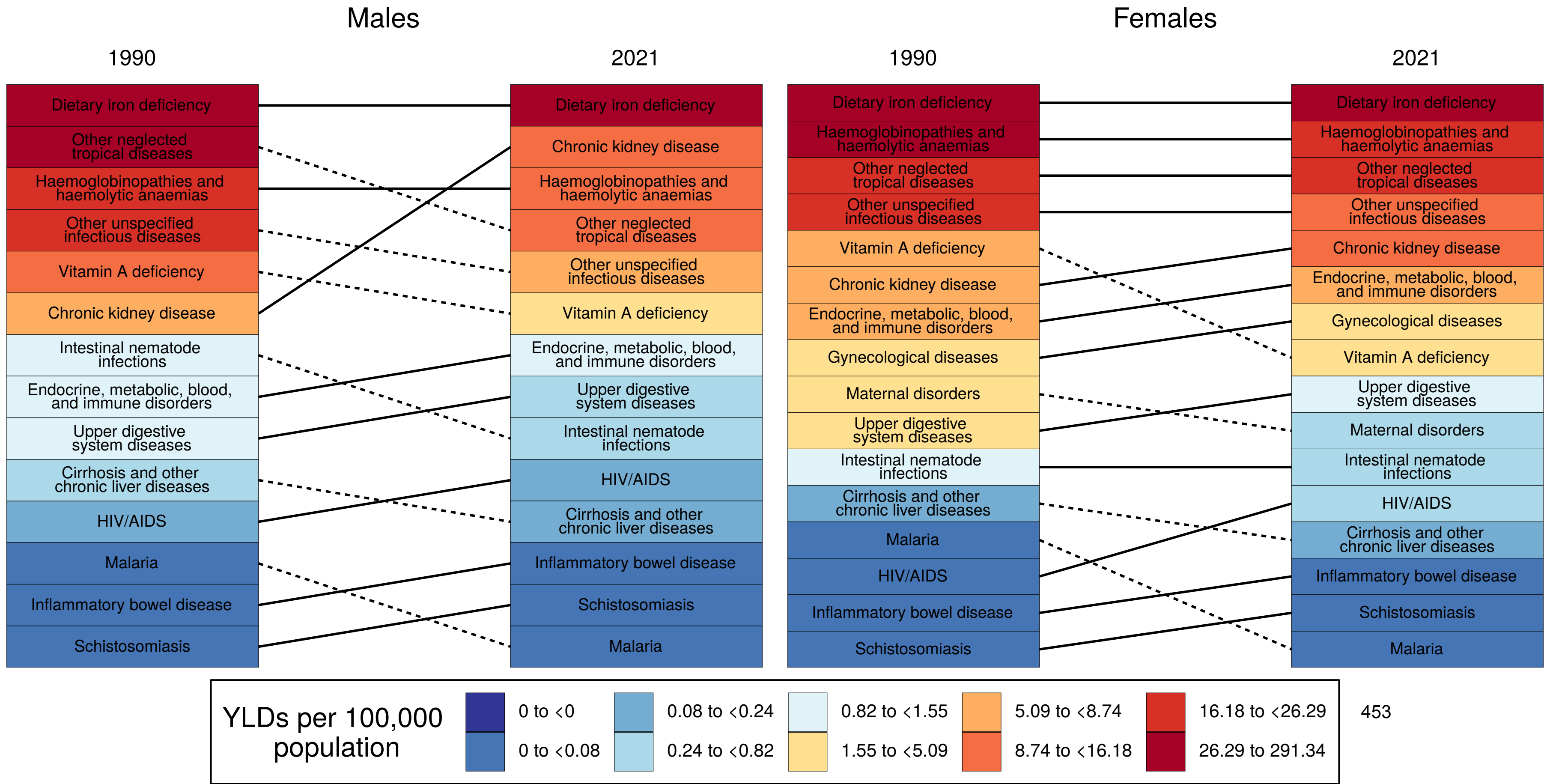

Baja California Sur

Anaemia Prevalence - Both Sexes

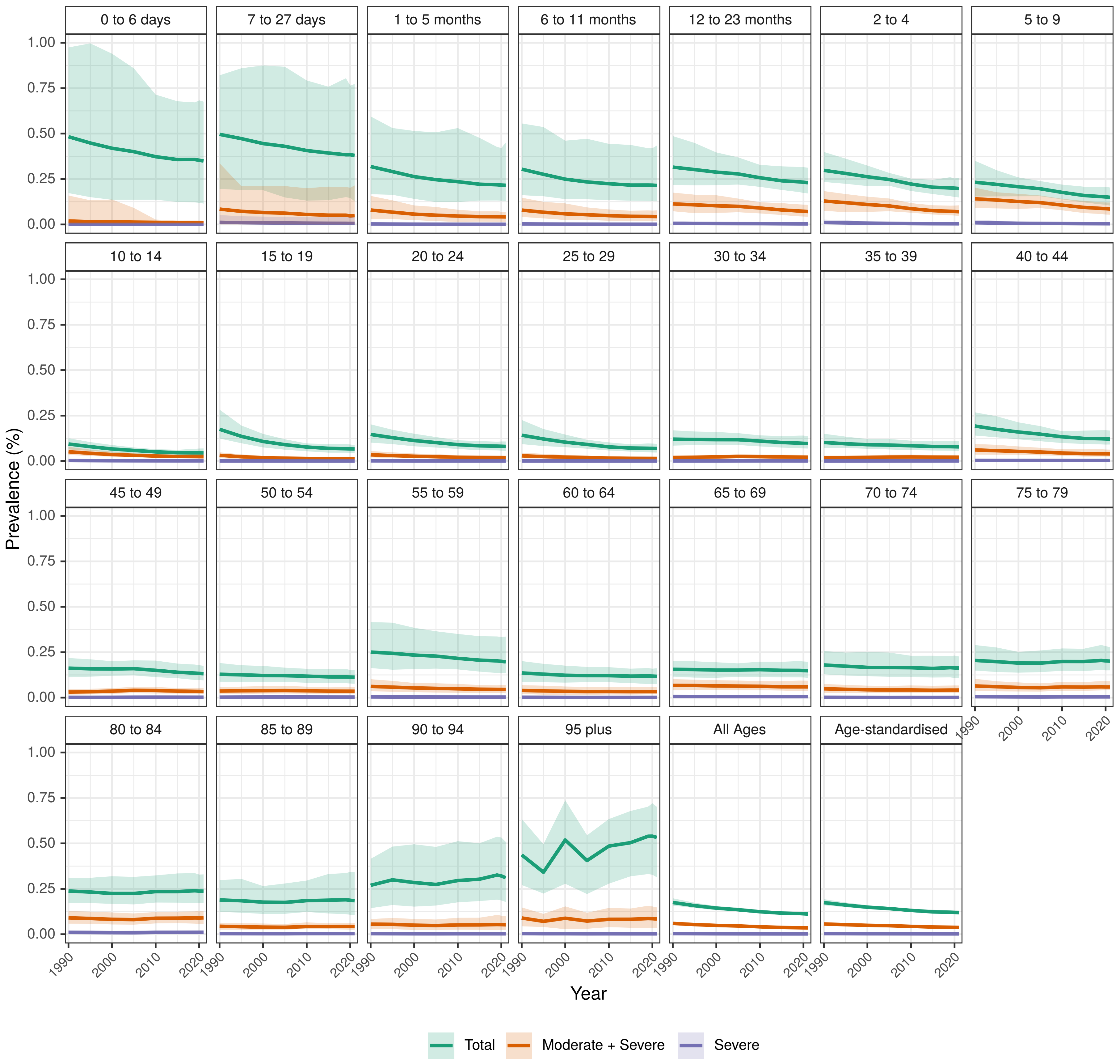

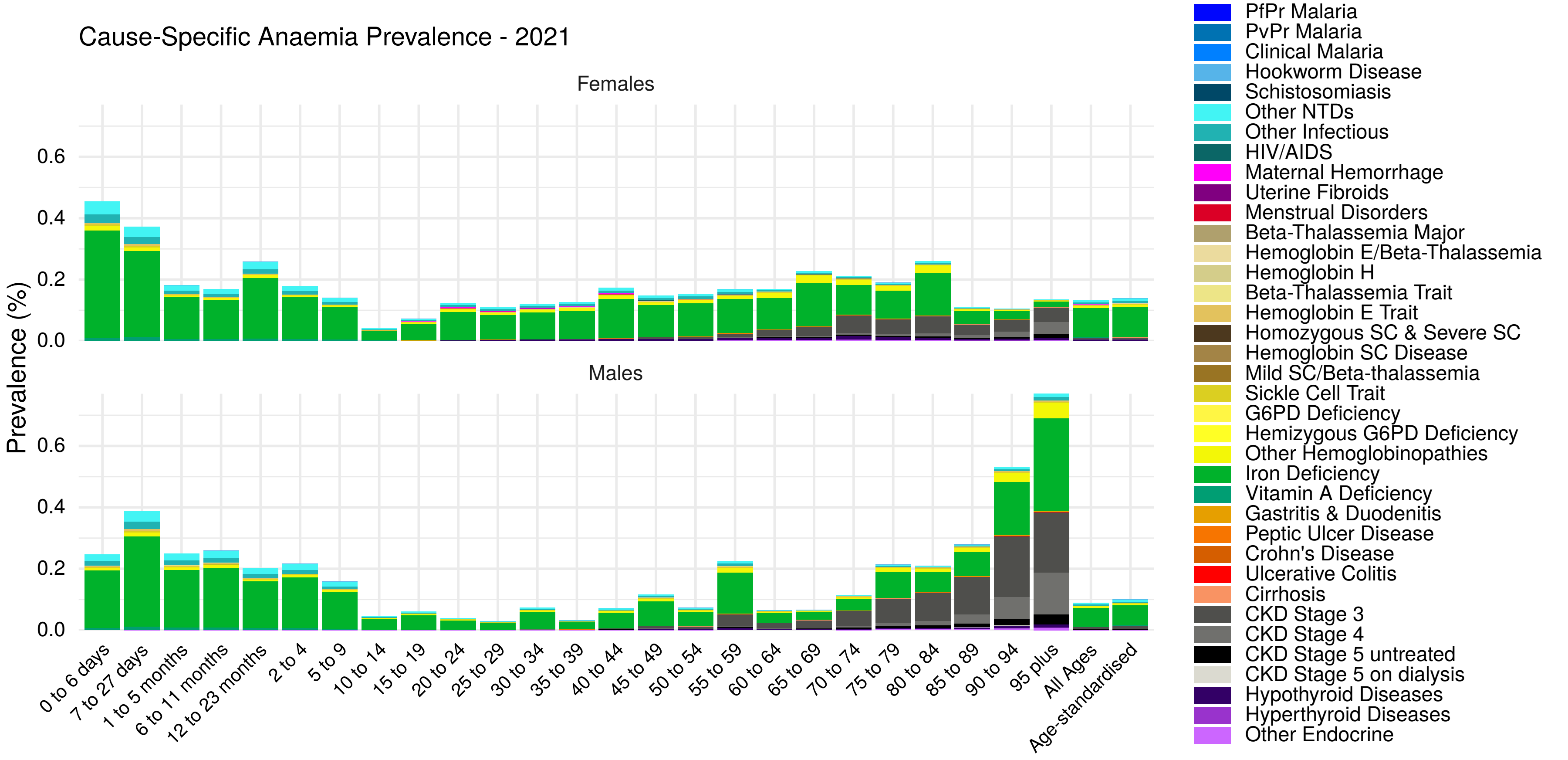

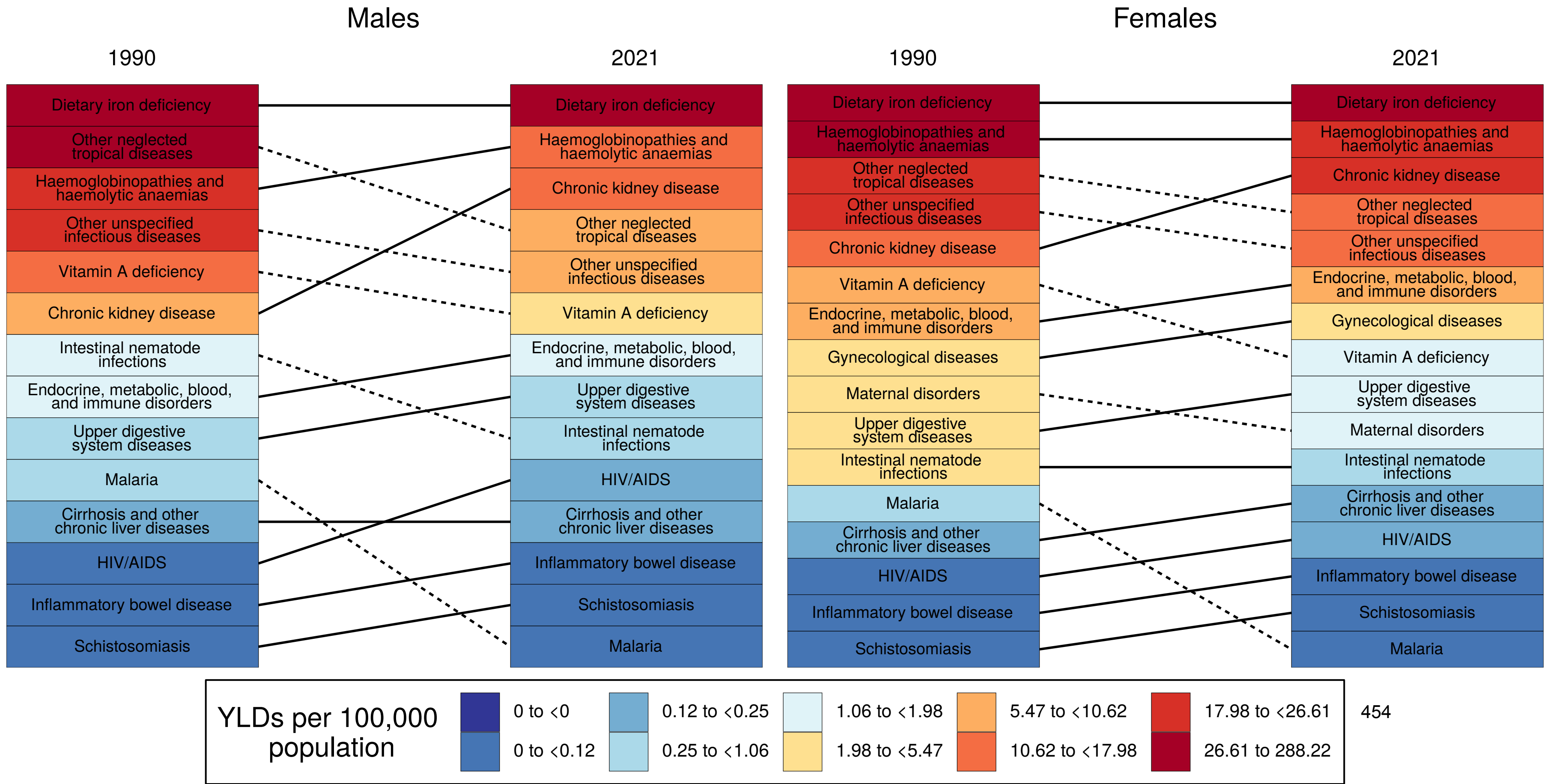

Campeche

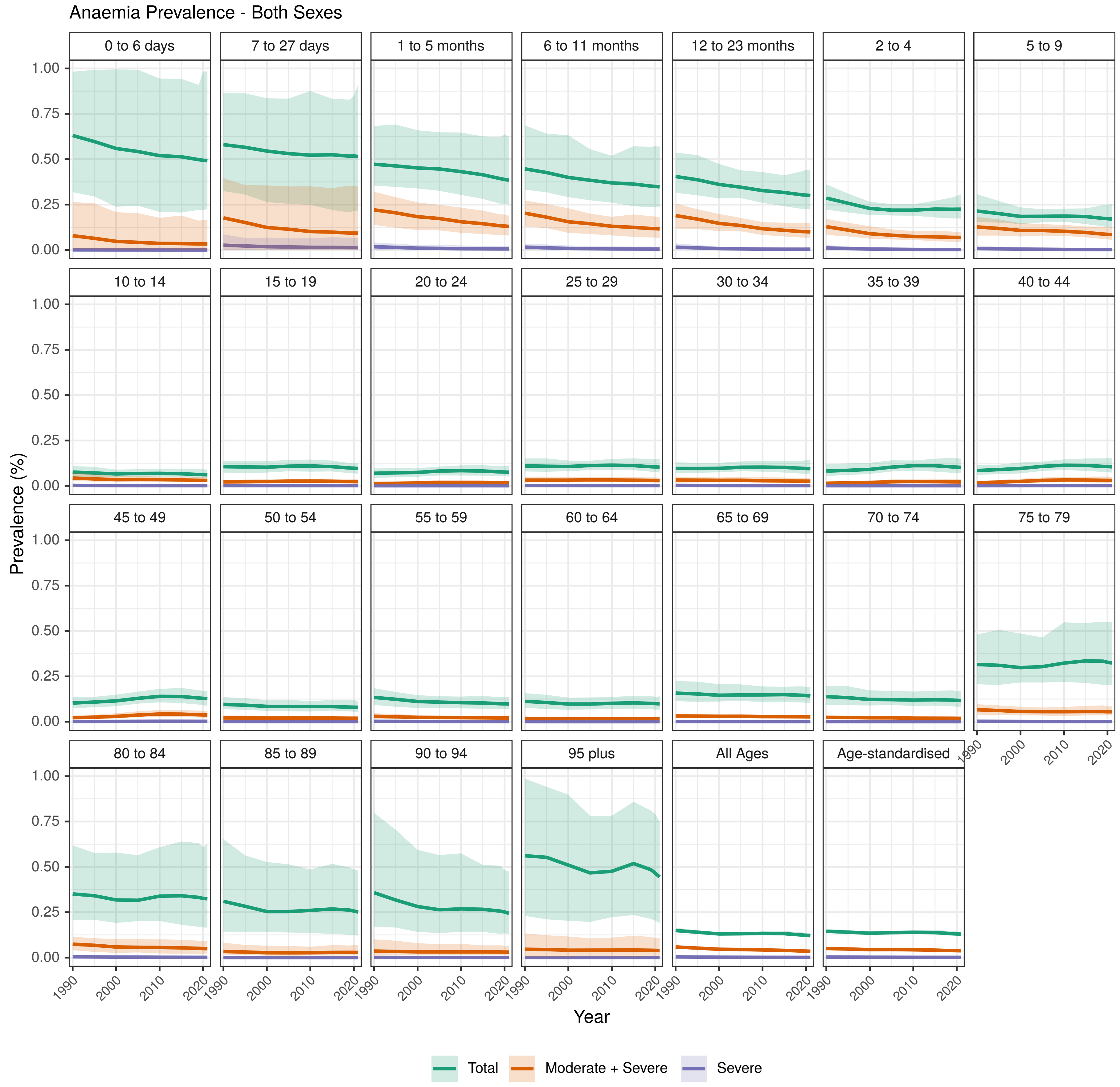
Anaemia Prevalence - Both Sexes
0 to 6 days
7 to 27 days
1 to 5 months
6 to 11 months
12 to 23 months
2 to 4
5 to 9
10 to 14
15 to 19
20 to 24
25 to 29
30 to 34
35 to 39
40 to 44
45 to 49
50 to 54
55 to 59
60 to 64
65 to 69
70 to 74
75 to 79
80 to 84
85 to 89
90 to 94
95 plus
All Ages
Age-standardised
Prevalence (%)
0.00
0.25
0.50
0.75
1.00
1990
2000
2010
2020
Year
Total
Moderate + Severe
Severe

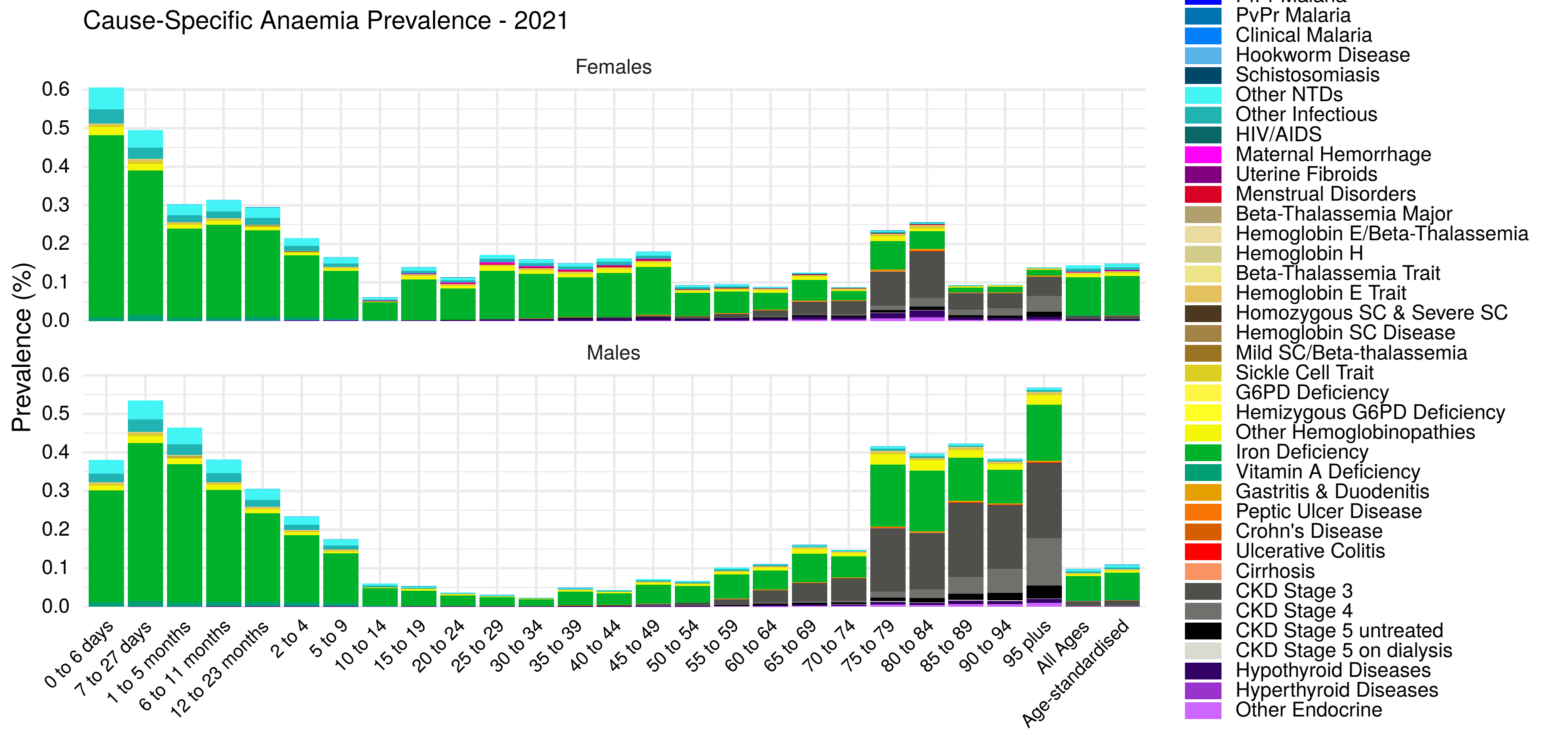
Cause-Specific Anaemia Prevalence - 2021
Females
Males
Prevalence (%)
0.0
0.1
0.2
0.3
0.4
0.5
0.6
0 to 6 days
7 to 27 days
1 to 5 months
6 to 11 months
12 to 23 months
2 to 4
5 to 9
10 to 14
15 to 19
20 to 24
25 to 29
30 to 34
35 to 39
40 to 44
45 to 49
50 to 54
55 to 59
60 to 64
65 to 69
70 to 74
75 to 79
80 to 84
85 to 89
90 to 94
95 plus
All Ages
Age-standardised
PfPr Malaria
PvPr Malaria
Clinical Malaria
Hookworm Disease
Schistosomiasis
Other NTDs
Other Infectious
HIV/AIDS
Maternal Hemorrhage
Uterine Fibroids
Menstrual Disorders
Beta-Thalassemia Major
Hemoglobin E/Beta-Thalassemia
Hemoglobin H
Beta-Thalassemia Trait
Hemoglobin E Trait
Homozygous SC & Severe SC
Hemoglobin SC Disease
Mild SC/Beta-thalassemia
Sickle Cell Trait
G6PD Deficiency
Hemizygous G6PD Deficiency
Other Hemoglobinopathies
Iron Deficiency
Vitamin A Deficiency
Gastritis & Duodenitis
Peptic Ulcer Disease
Crohn's Disease
Ulcerative Colitis
Cirrhosis
CKD Stage 3
CKD Stage 4
CKD Stage 5 untreated
CKD Stage 5 on dialysis
Hypothyroid Diseases
Hyperthyroid Diseases
Other Endocrine

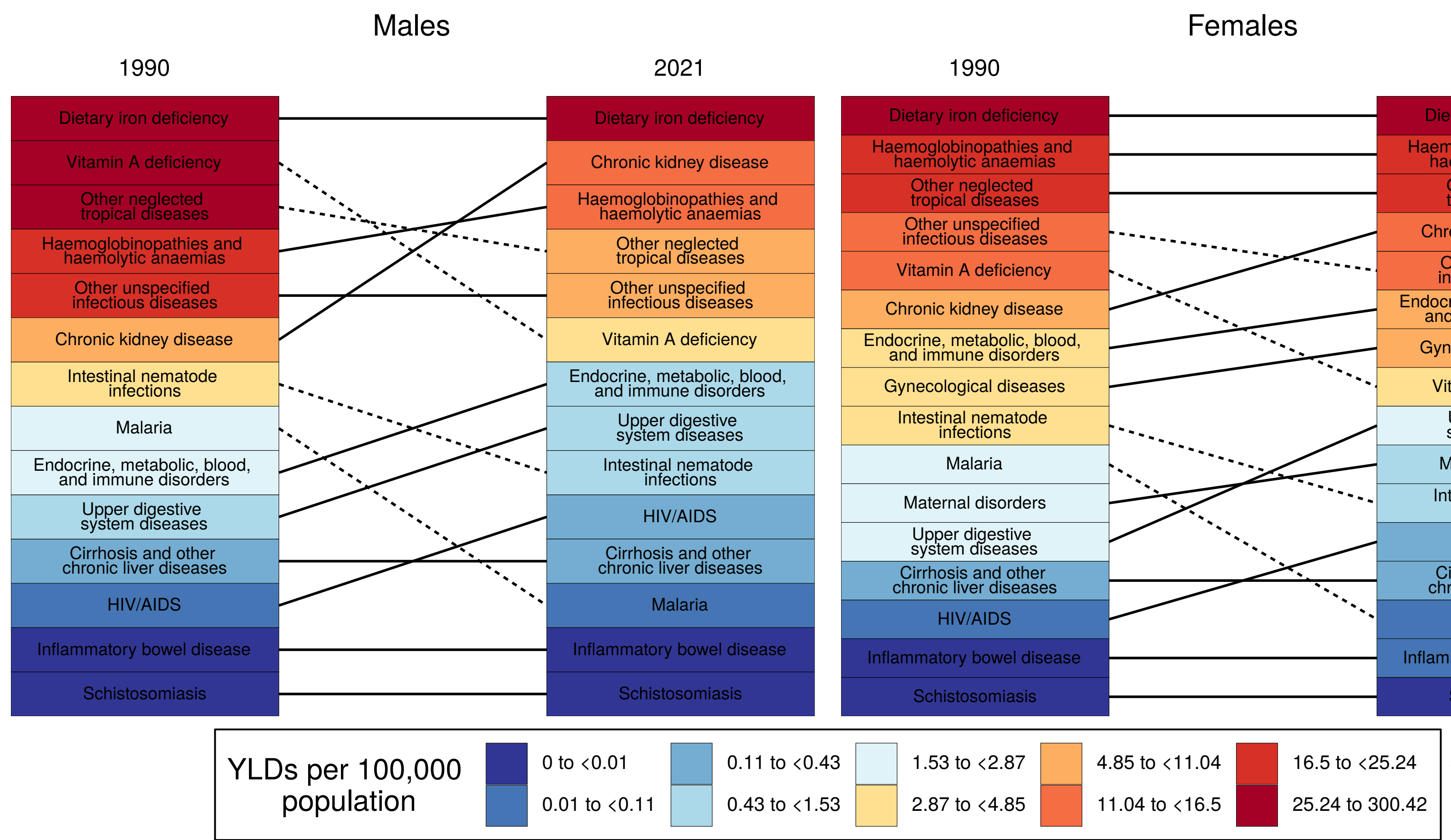
Males
Females
1990
2021
Dietary iron deficiency
Vitamin A deficiency
Other neglected tropical diseases
Haemoglobinopathies and haemolytic anaemias
Other unspecified infectious diseases
Chronic kidney disease
Intestinal nematode infections
Malaria
Endocrine, metabolic, blood, and immune disorders
Upper digestive system diseases
Cirrhosis and other chronic liver diseases
HIV/AIDS
Inflammatory bowel disease
Schistosomiasis
Gynecological diseases
Maternal disorders
YLDs per 100,000 population
0 to <0.01
0.01 to <0.11
0.11 to <0.43
0.43 to <1.53
1.53 to <2.87
2.87 to <4.85
4.85 to <11.04
11.04 to <16.5
16.5 to <25.24
25.24 to 300.42

Chiapas

Anaemia Prevalence - Both Sexes

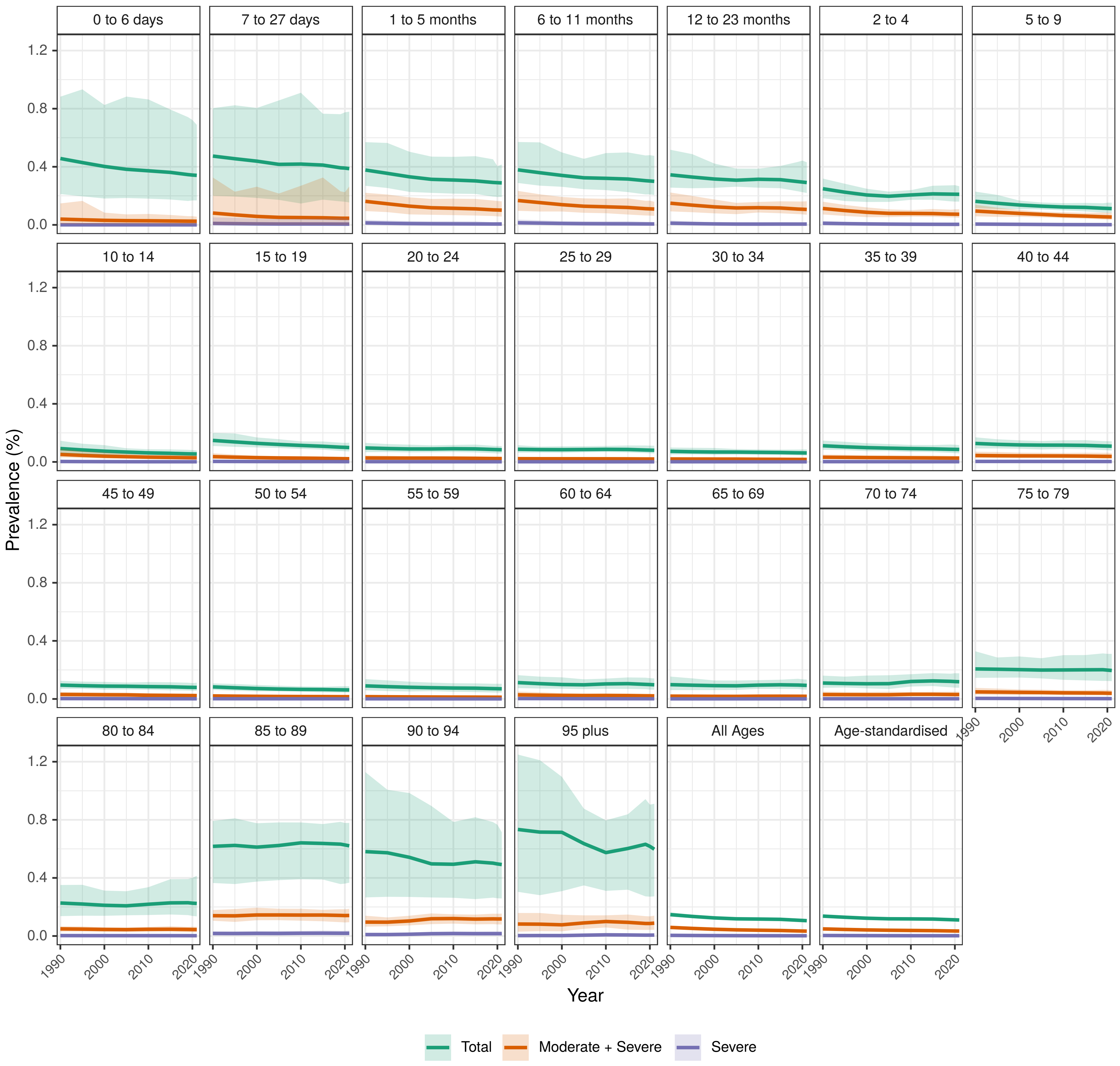

Cause-Specific Anaemia Prevalence - 2021

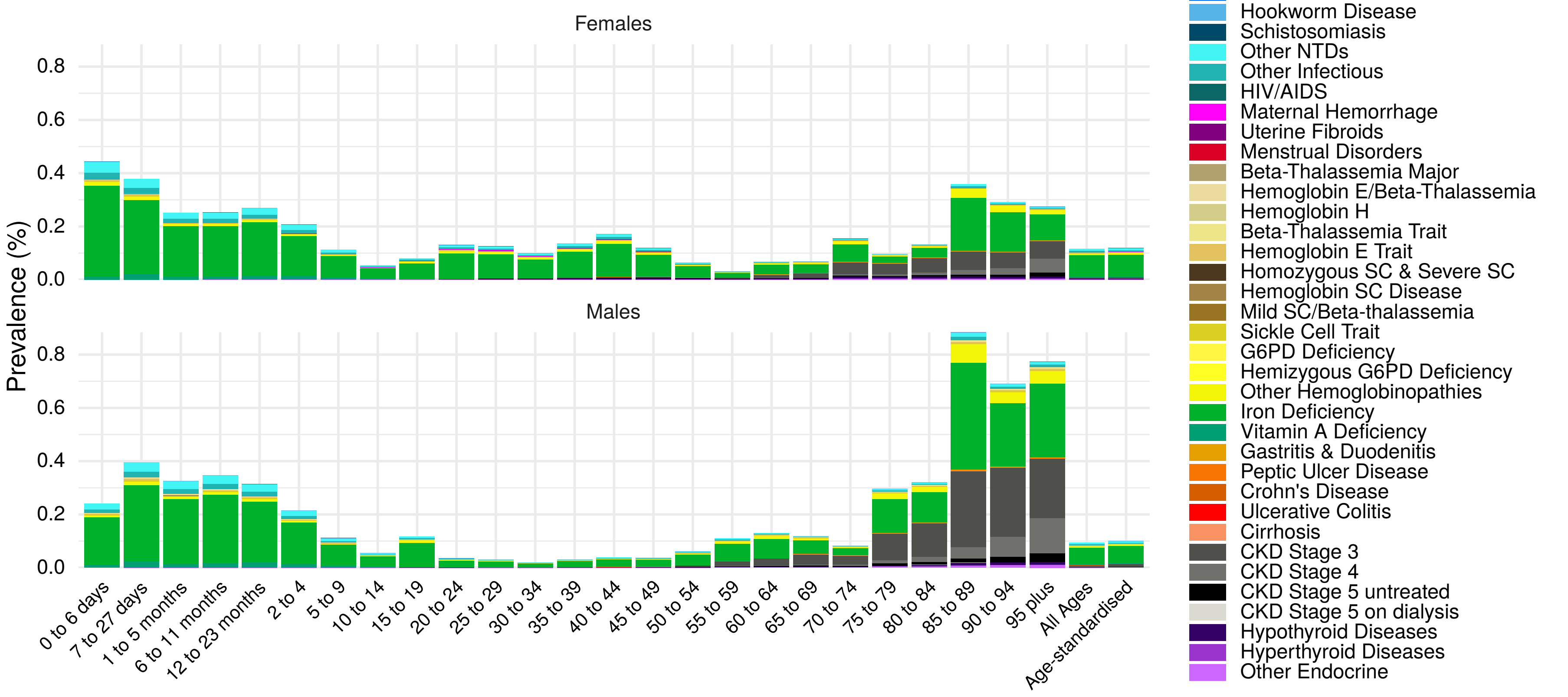

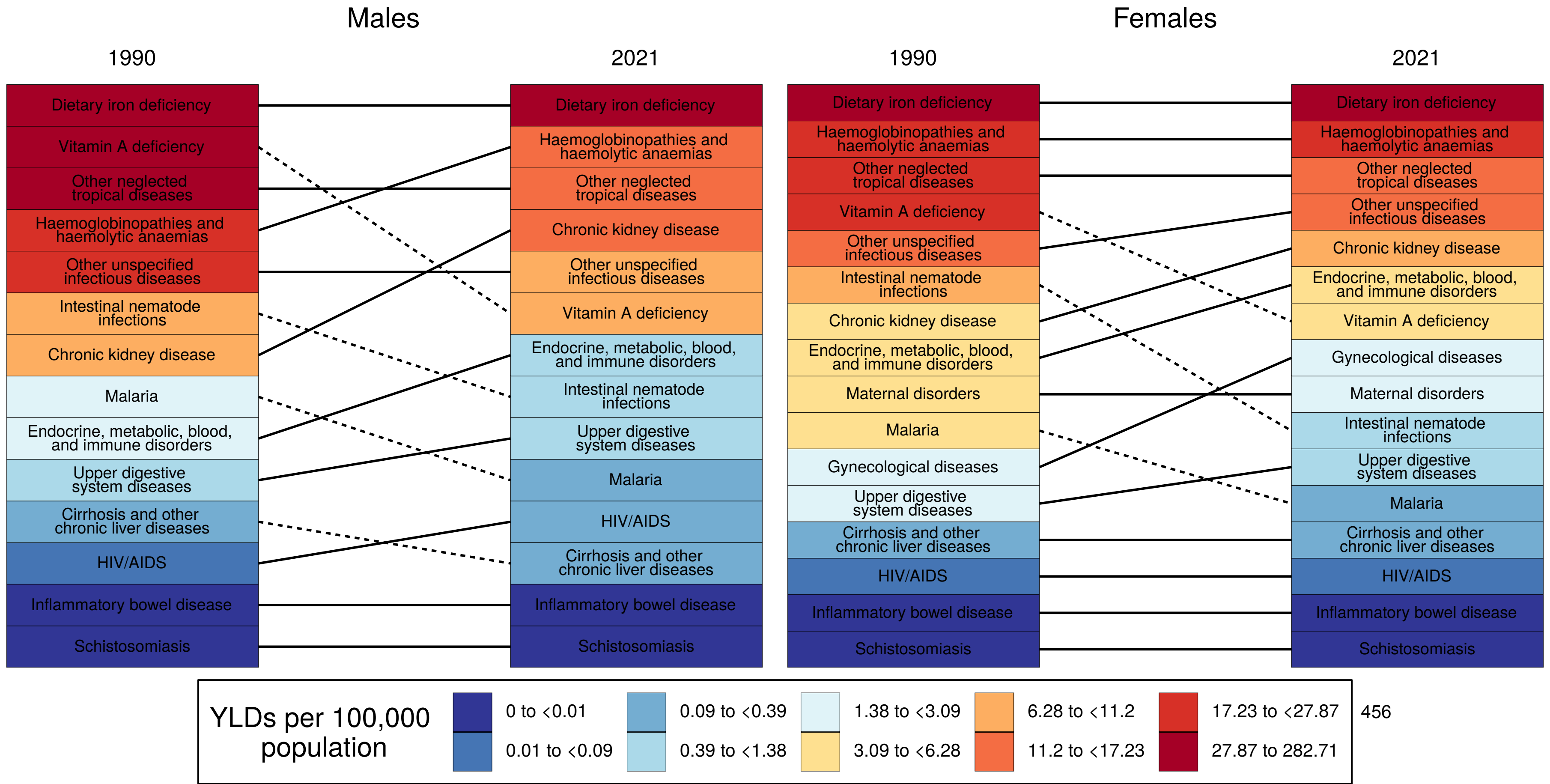

Chihuahua

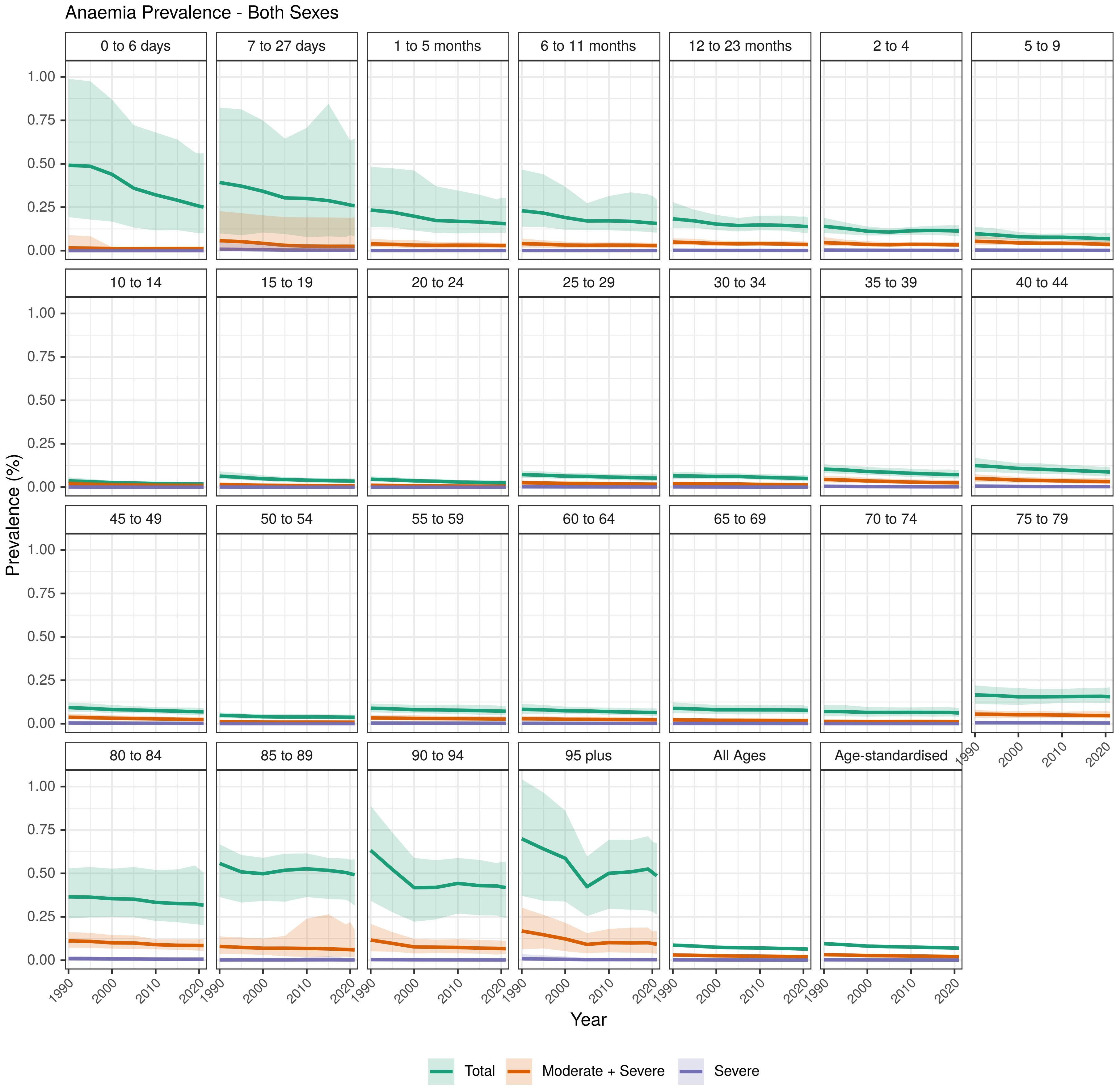
Anaemia Prevalence - Both Sexes
0 to 6 days
7 to 27 days
1 to 5 months
6 to 11 months
12 to 23 months
2 to 4
5 to 9
10 to 14
15 to 19
20 to 24
25 to 29
30 to 34
35 to 39
40 to 44
45 to 49
50 to 54
55 to 59
60 to 64
65 to 69
70 to 74
75 to 79
80 to 84
85 to 89
90 to 94
95 plus
All Ages
Age-standardised
Prevalence (%)
0.00
0.25
0.50
0.75
1.00
1990
2000
2010
2020
Year
Total
Moderate + Severe
Severe

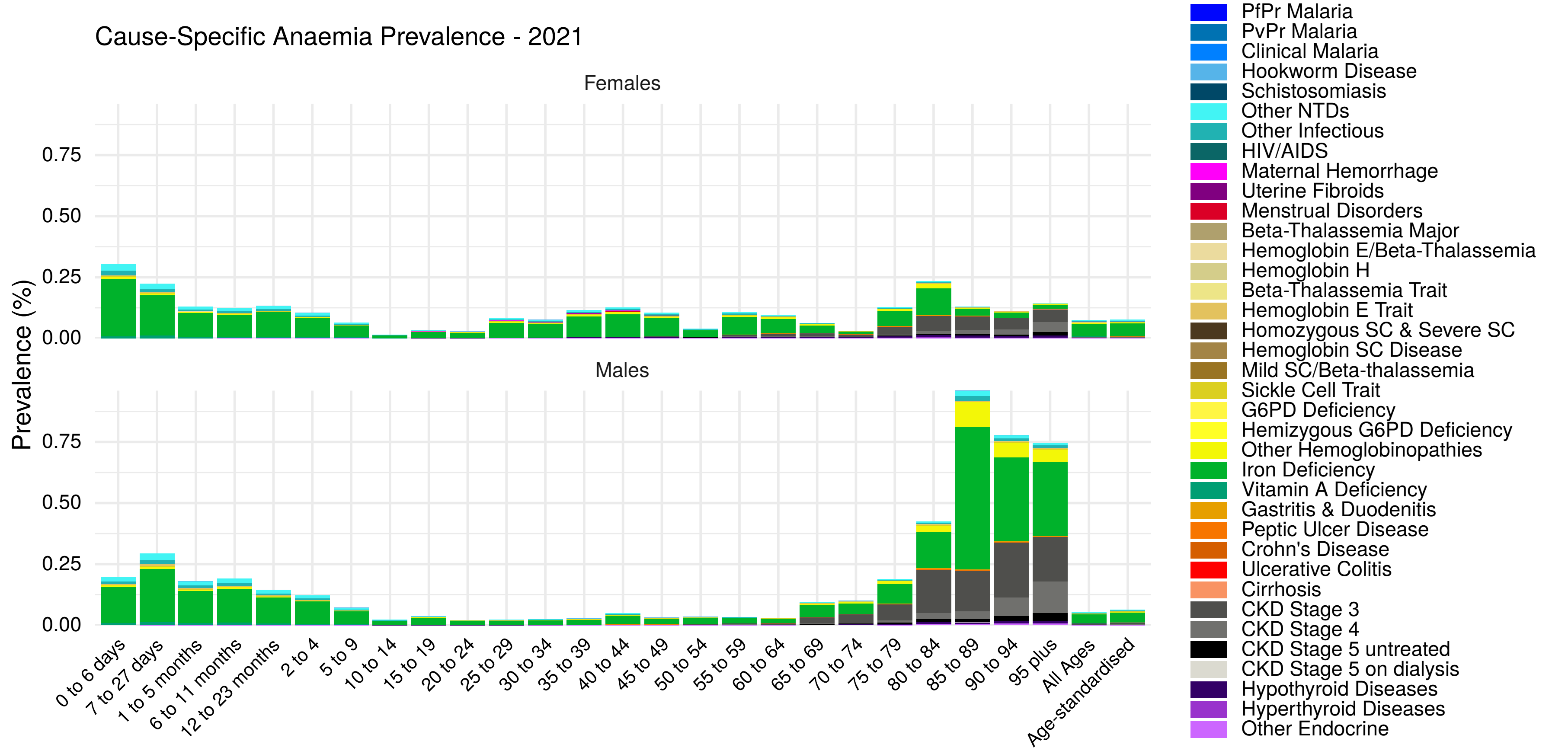
Cause-Specific Anaemia Prevalence - 2021
Females
Males
Prevalence (%)
0.00
0.25
0.50
0.75
0 to 6 days
7 to 27 days
1 to 5 months
6 to 11 months
12 to 23 months
2 to 4
5 to 9
10 to 14
15 to 19
20 to 24
25 to 29
30 to 34
35 to 39
40 to 44
45 to 49
50 to 54
55 to 59
60 to 64
65 to 69
70 to 74
75 to 79
80 to 84
85 to 89
90 to 94
95 plus
All Ages
Age-standardised
PfPr Malaria
PvPr Malaria
Clinical Malaria
Hookworm Disease
Schistosomiasis
Other NTDs
Other Infectious
HIV/AIDS
Maternal Hemorrhage
Uterine Fibroids
Menstrual Disorders
Beta-Thalassemia Major
Hemoglobin E/Beta-Thalassemia
Hemoglobin H
Beta-Thalassemia Trait
Hemoglobin E Trait
Homozygous SC & Severe SC
Hemoglobin SC Disease
Mild SC/Beta-thalassemia
Sickle Cell Trait
G6PD Deficiency
Hemizygous G6PD Deficiency
Other Hemoglobinopathies
Iron Deficiency
Vitamin A Deficiency
Gastritis & Duodenitis
Peptic Ulcer Disease
Crohn's Disease
Ulcerative Colitis
Cirrhosis
CKD Stage 3
CKD Stage 4
CKD Stage 5 untreated
CKD Stage 5 on dialysis
Hypothyroid Diseases
Hyperthyroid Diseases
Other Endocrine

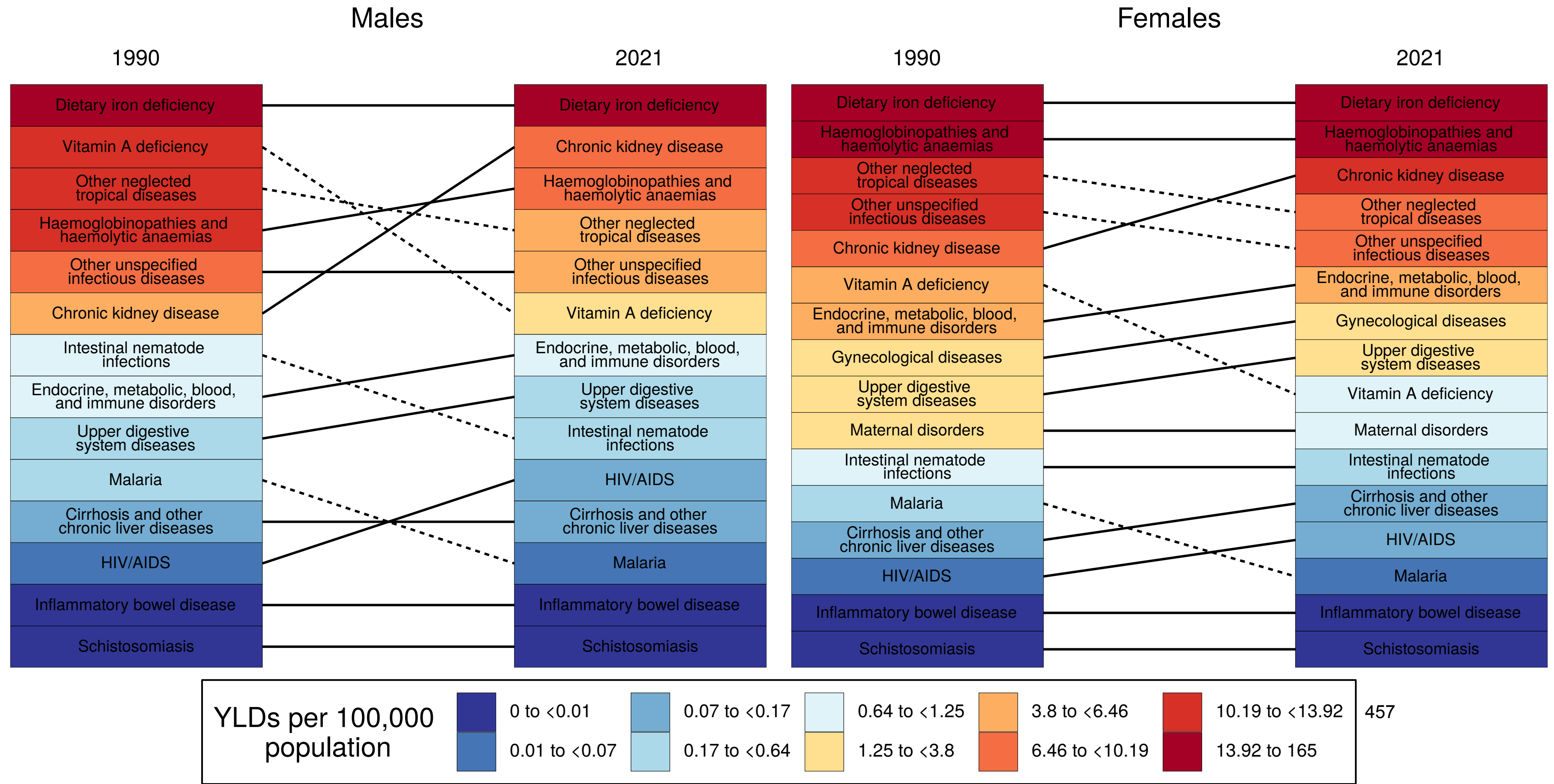
Males
1990
Dietary iron deficiency
Vitamin A deficiency
Other neglected tropical diseases
Haemoglobinopathies and haemolytic anaemias
Other unspecified infectious diseases
Chronic kidney disease
Intestinal nematode infections
Endocrine, metabolic, blood, and immune disorders
Upper digestive system diseases
Malaria
Cirrhosis and other chronic liver diseases
HIV/AIDS
Inflammatory bowel disease
Schistosomiasis
2021
Dietary iron deficiency
Chronic kidney disease
Haemoglobinopathies and haemolytic anaemias
Other neglected tropical diseases
Other unspecified infectious diseases
Vitamin A deficiency
Endocrine, metabolic, blood, and immune disorders
Upper digestive system diseases
Intestinal nematode infections
HIV/AIDS
Cirrhosis and other chronic liver diseases
Malaria
Inflammatory bowel disease
Schistosomiasis
Females
1990
Dietary iron deficiency
Haemoglobinopathies and haemolytic anaemias
Other neglected tropical diseases
Other unspecified infectious diseases
Chronic kidney disease
Vitamin A deficiency
Endocrine, metabolic, blood, and immune disorders
Gynecological diseases
Upper digestive system diseases
Maternal disorders
Intestinal nematode infections
Malaria
Cirrhosis and other chronic liver diseases
HIV/AIDS
Inflammatory bowel disease
Schistosomiasis
2021
Dietary iron deficiency
Haemoglobinopathies and haemolytic anaemias
Chronic kidney disease
Other neglected tropical diseases
Other unspecified infectious diseases
Endocrine, metabolic, blood, and immune disorders
Gynecological diseases
Upper digestive system diseases
Vitamin A deficiency
Maternal disorders
Intestinal nematode infections
Cirrhosis and other chronic liver diseases
HIV/AIDS
Malaria
Inflammatory bowel disease
Schistosomiasis
YLDs per 100,000 population
0 to <0.01
0.01 to <0.07
0.07 to <0.17
0.17 to <0.64
0.64 to <1.25
1.25 to <3.8
3.8 to <6.46
6.46 to <10.19
10.19 to <13.92
13.92 to 165

Coahuila

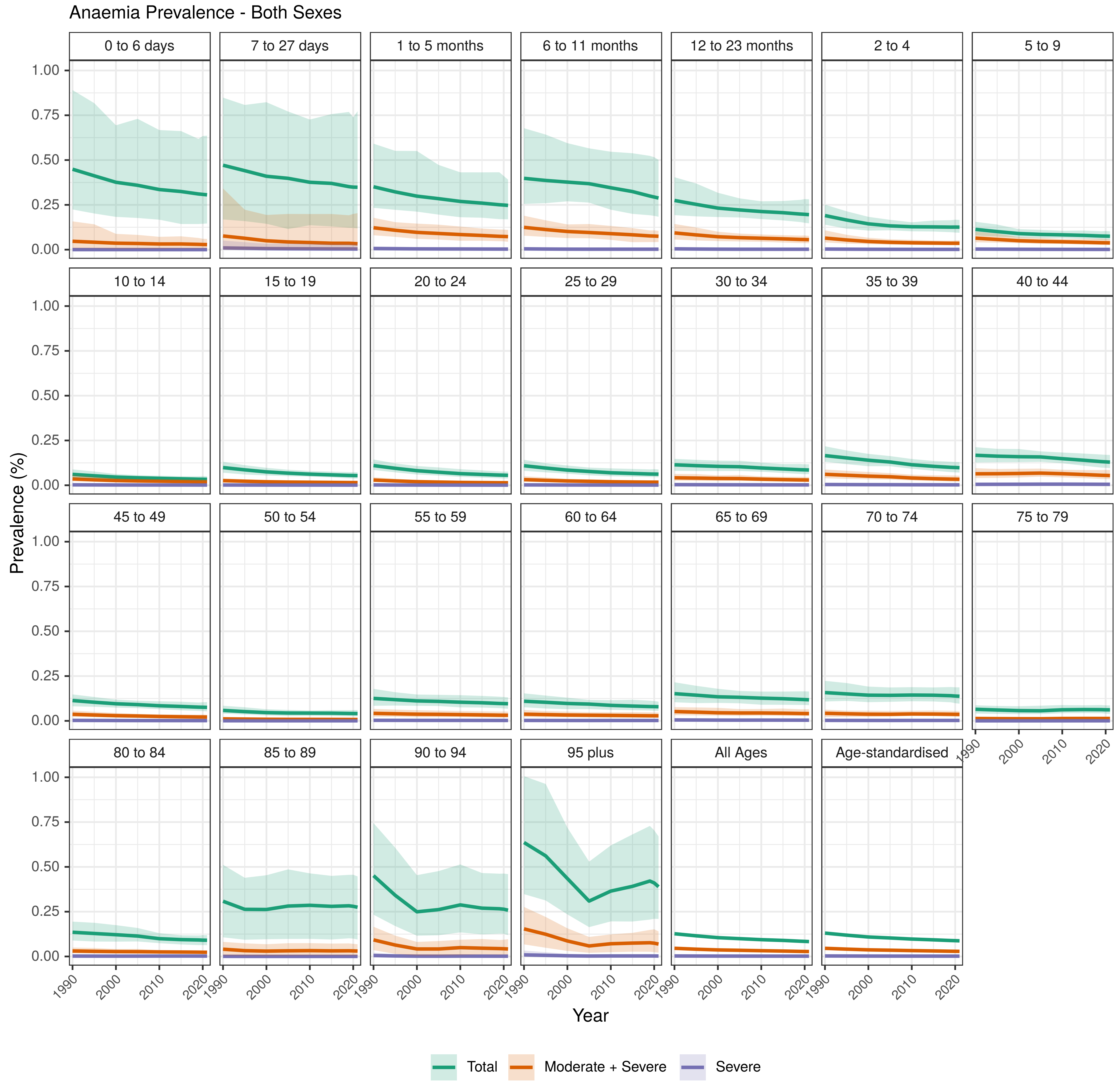
Anaemia Prevalence - Both Sexes
0 to 6 days
7 to 27 days
1 to 5 months
6 to 11 months
12 to 23 months
2 to 4
5 to 9
10 to 14
15 to 19
20 to 24
25 to 29
30 to 34
35 to 39
40 to 44
45 to 49
50 to 54
55 to 59
60 to 64
65 to 69
70 to 74
75 to 79
80 to 84
85 to 89
90 to 94
95 plus
All Ages
Age-standardised
Prevalence (%)
0.00
0.25
0.50
0.75
1.00
1990
2000
2010
2020
Year
Total
Moderate + Severe
Severe

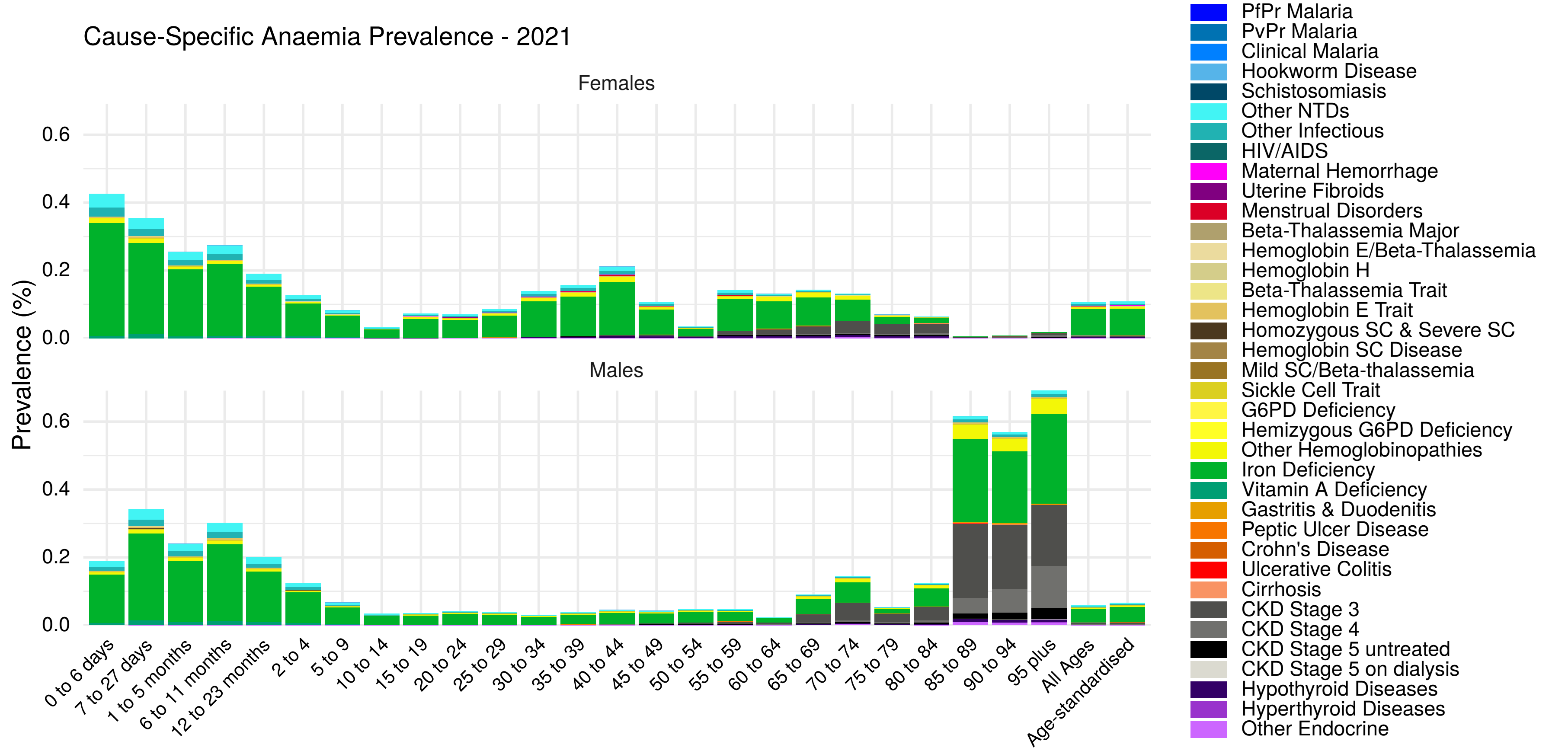
Cause-Specific Anaemia Prevalence - 2021
Females
Males
Prevalence (%)
0.0
0.2
0.4
0.6
0 to 6 days
7 to 27 days
1 to 5 months
6 to 11 months
12 to 23 months
2 to 4
5 to 9
10 to 14
15 to 19
20 to 24
25 to 29
30 to 34
35 to 39
40 to 44
45 to 49
50 to 54
55 to 59
60 to 64
65 to 69
70 to 74
75 to 79
80 to 84
85 to 89
90 to 94
95 plus
All Ages
Age-standardised
PfPr Malaria
PvPr Malaria
Clinical Malaria
Hookworm Disease
Schistosomiasis
Other NTDs
Other Infectious
HIV/AIDS
Maternal Hemorrhage
Uterine Fibroids
Menstrual Disorders
Beta-Thalassemia Major
Hemoglobin E/Beta-Thalassemia
Hemoglobin H
Beta-Thalassemia Trait
Hemoglobin E Trait
Homozygous SC & Severe SC
Hemoglobin SC Disease
Mild SC/Beta-thalassemia
Sickle Cell Trait
G6PD Deficiency
Hemizygous G6PD Deficiency
Other Hemoglobinopathies
Iron Deficiency
Vitamin A Deficiency
Gastritis & Duodenitis
Peptic Ulcer Disease
Crohn's Disease
Ulcerative Colitis
Cirrhosis
CKD Stage 3
CKD Stage 4
CKD Stage 5 untreated
CKD Stage 5 on dialysis
Hypothyroid Diseases
Hyperthyroid Diseases
Other Endocrine

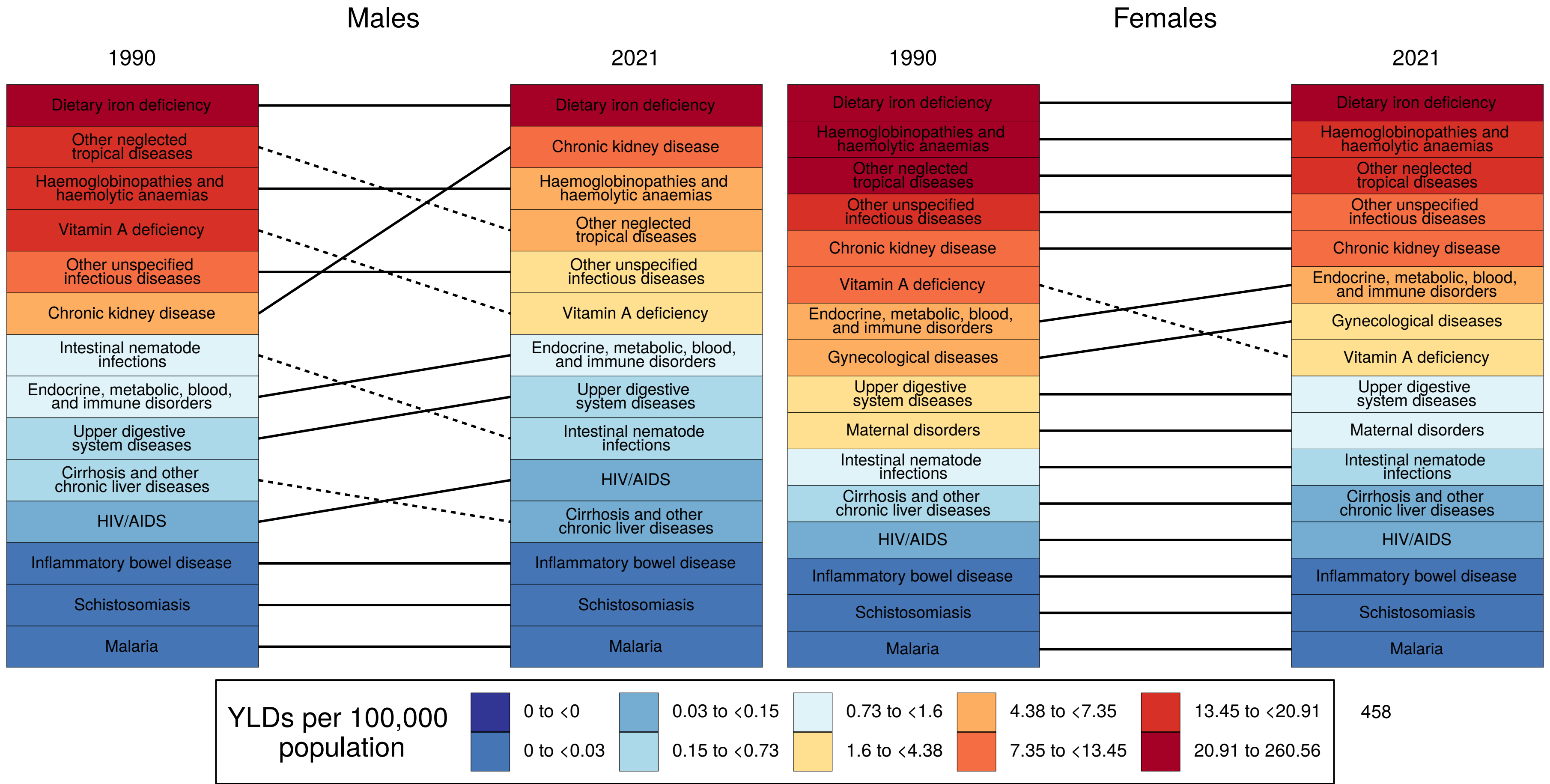
Males
1990
Dietary iron deficiency
Other neglected tropical diseases
Haemoglobinopathies and haemolytic anaemias
Vitamin A deficiency
Other unspecified infectious diseases
Chronic kidney disease
Intestinal nematode infections
Endocrine, metabolic, blood, and immune disorders
Upper digestive system diseases
Cirrhosis and other chronic liver diseases
HIV/AIDS
Inflammatory bowel disease
Schistosomiasis
Malaria
2021
Dietary iron deficiency
Chronic kidney disease
Haemoglobinopathies and haemolytic anaemias
Other neglected tropical diseases
Other unspecified infectious diseases
Vitamin A deficiency
Endocrine, metabolic, blood, and immune disorders
Upper digestive system diseases
Intestinal nematode infections
HIV/AIDS
Cirrhosis and other chronic liver diseases
Inflammatory bowel disease
Schistosomiasis
Malaria
Females
1990
Dietary iron deficiency
Haemoglobinopathies and haemolytic anaemias
Other neglected tropical diseases
Other unspecified infectious diseases
Chronic kidney disease
Vitamin A deficiency
Endocrine, metabolic, blood, and immune disorders
Gynecological diseases
Upper digestive system diseases
Maternal disorders
Intestinal nematode infections
Cirrhosis and other chronic liver diseases
HIV/AIDS
Inflammatory bowel disease
Schistosomiasis
Malaria
2021
Dietary iron deficiency
Haemoglobinopathies and haemolytic anaemias
Other neglected tropical diseases
Other unspecified infectious diseases
Chronic kidney disease
Endocrine, metabolic, blood, and immune disorders
Gynecological diseases
Vitamin A deficiency
Upper digestive system diseases
Maternal disorders
Intestinal nematode infections
Cirrhosis and other chronic liver diseases
HIV/AIDS
Inflammatory bowel disease
Schistosomiasis
Malaria
YLDs per 100,000 population
0 to <0
0 to <0.03
0.03 to <0.15
0.15 to <0.73
0.73 to <1.6
1.6 to <4.38
4.38 to <7.35
7.35 to <13.45
13.45 to <20.91
20.91 to 260.56

Colima

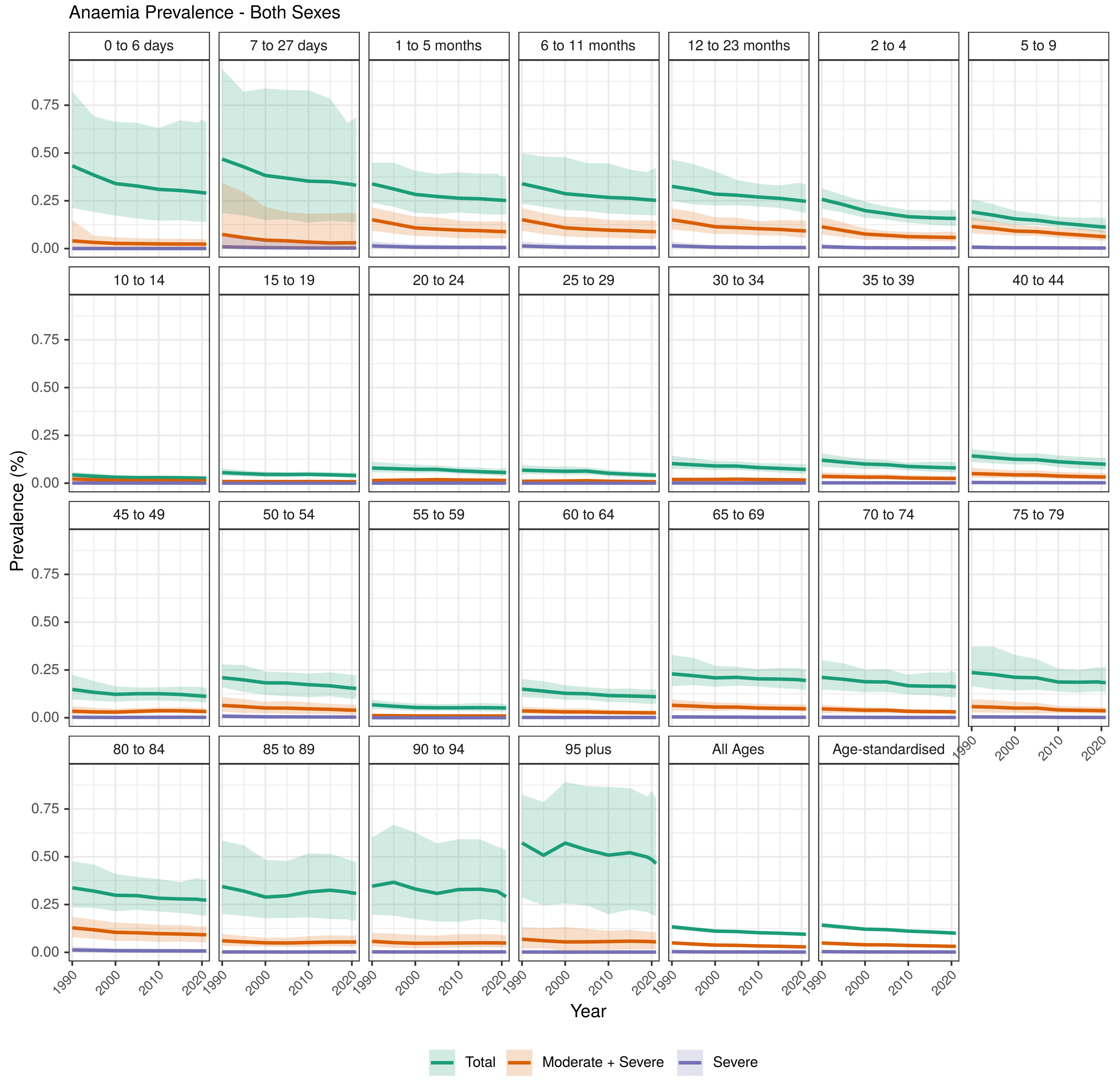

Anaemia Prevalence - Both Sexes
0 to 6 days
7 to 27 days
1 to 5 months
6 to 11 months
12 to 23 months
2 to 4
5 to 9
10 to 14
15 to 19
20 to 24
25 to 29
30 to 34
35 to 39
40 to 44
45 to 49
50 to 54
55 to 59
60 to 64
65 to 69
70 to 74
75 to 79
80 to 84
85 to 89
90 to 94
95 plus
All Ages
Age-standardised
Prevalence (%)
0.00
0.25
0.50
0.75
1990
2000
2010
2020
Year
Total
Moderate + Severe
Severe

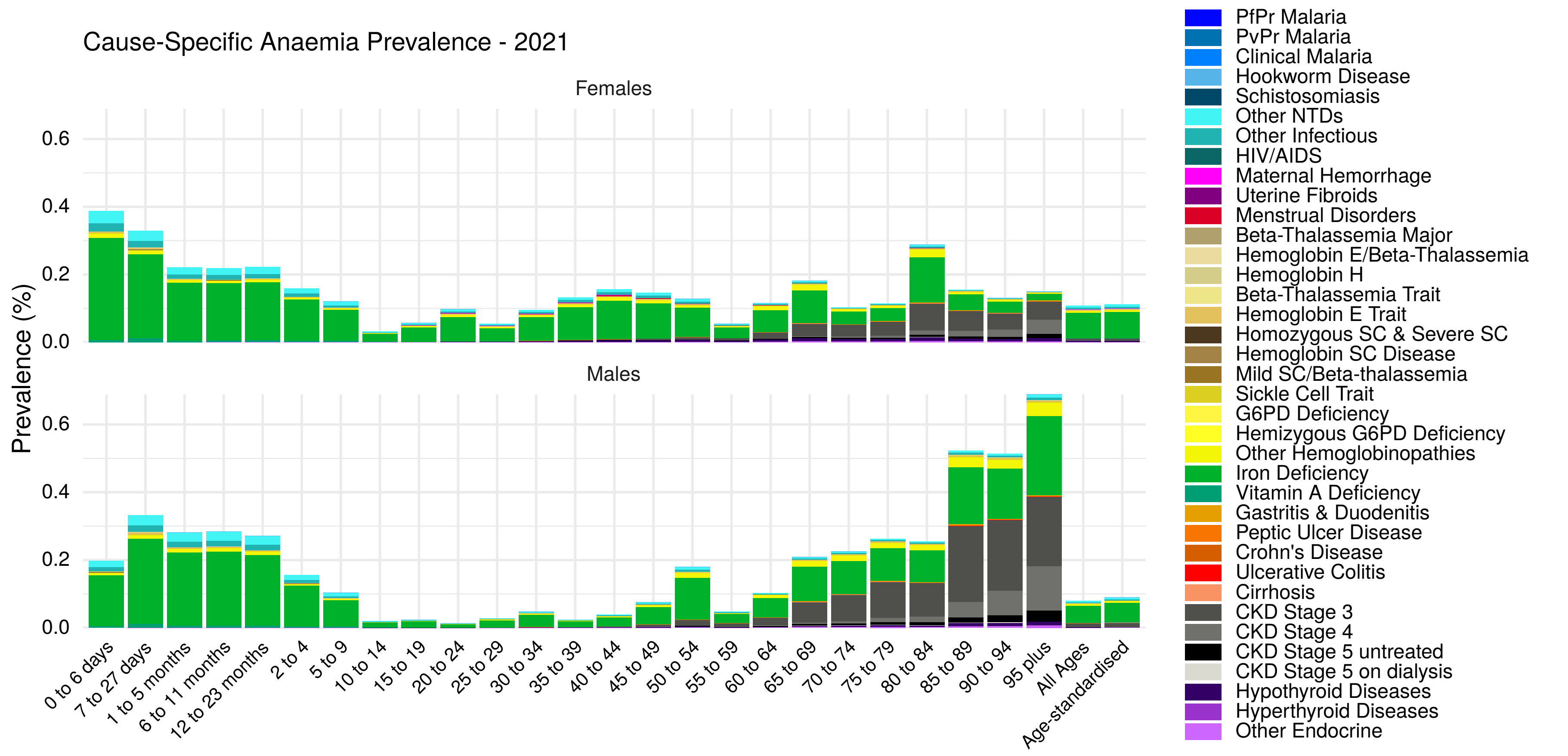

Cause-Specific Anaemia Prevalence - 2021
Females
Males
Prevalence (%)
0.0
0.2
0.4
0.6
0 to 6 days
7 to 27 days
1 to 5 months
6 to 11 months
12 to 23 months
2 to 4
5 to 9
10 to 14
15 to 19
20 to 24
25 to 29
30 to 34
35 to 39
40 to 44
45 to 49
50 to 54
55 to 59
60 to 64
65 to 69
70 to 74
75 to 79
80 to 84
85 to 89
90 to 94
95 plus
All Ages
Age-standardised
PfPr Malaria
PvPr Malaria
Clinical Malaria
Hookworm Disease
Schistosomiasis
Other NTDs
Other Infectious
HIV/AIDS
Maternal Hemorrhage
Uterine Fibroids
Menstrual Disorders
Beta-Thalassemia Major
Hemoglobin E/Beta-Thalassemia
Hemoglobin H
Beta-Thalassemia Trait
Hemoglobin E Trait
Homozygous SC & Severe SC
Hemoglobin SC Disease
Mild SC/Beta-thalassemia
Sickle Cell Trait
G6PD Deficiency
Hemizygous G6PD Deficiency
Other Hemoglobinopathies
Iron Deficiency
Vitamin A Deficiency
Gastritis & Duodenitis
Peptic Ulcer Disease
Crohn's Disease
Ulcerative Colitis
Cirrhosis
CKD Stage 3
CKD Stage 4
CKD Stage 5 untreated
CKD Stage 5 on dialysis
Hypothyroid Diseases
Hyperthyroid Diseases
Other Endocrine

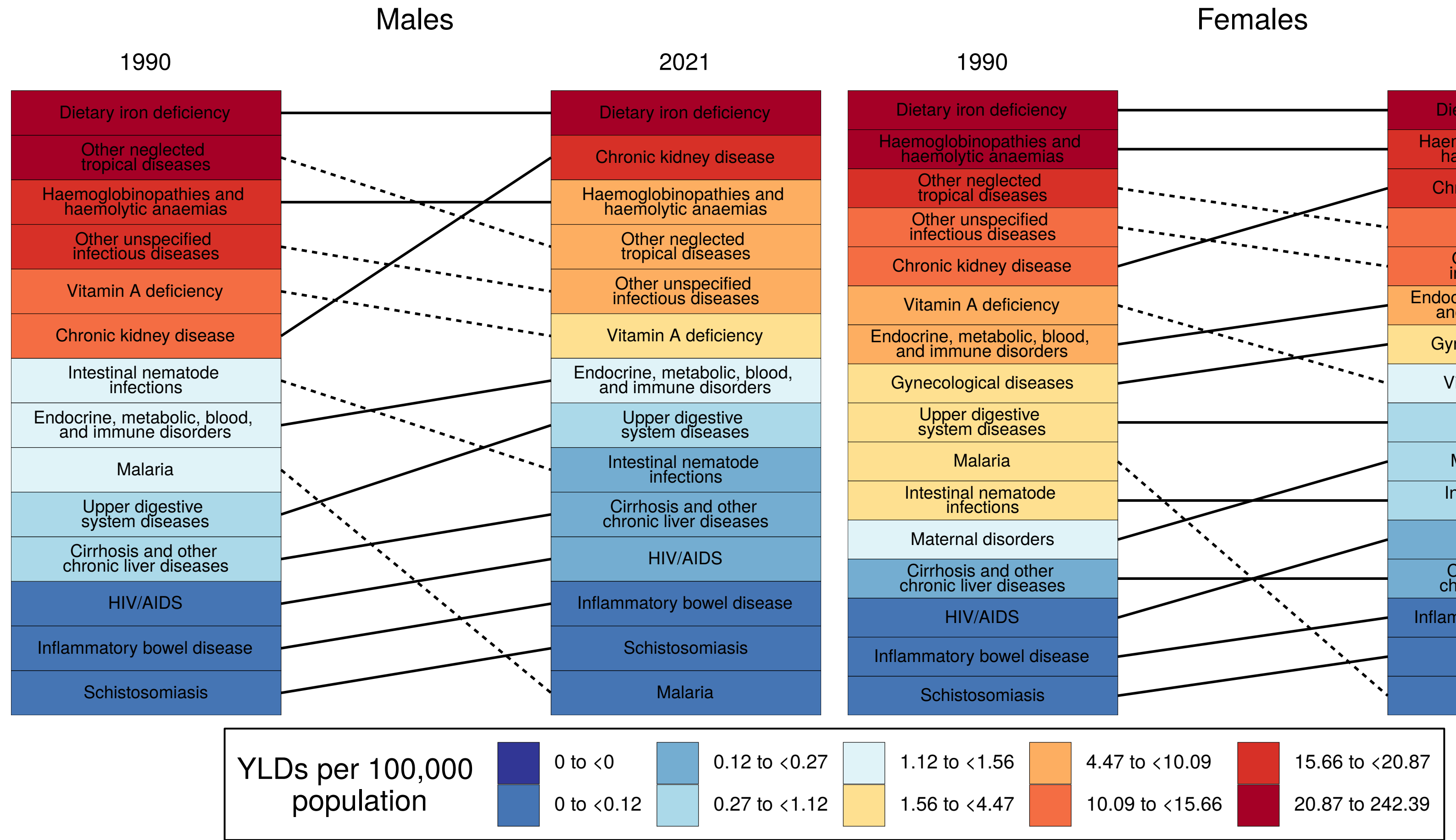

Males
Females
1990
2021
Dietary iron deficiency
Other neglected tropical diseases
Haemoglobinopathies and haemolytic anaemias
Other unspecified infectious diseases
Vitamin A deficiency
Chronic kidney disease
Intestinal nematode infections
Endocrine, metabolic, blood, and immune disorders
Malaria
Upper digestive system diseases
Cirrhosis and other chronic liver diseases
HIV/AIDS
Inflammatory bowel disease
Schistosomiasis
Gynecological diseases
Maternal disorders
YLDs per 100,000 population
0 to <0
0 to <0.12
0.12 to <0.27
0.27 to <1.12
1.12 to <1.56
1.56 to <4.47
4.47 to <10.09
10.09 to <15.66
15.66 to <20.87
20.87 to 242.39

Durango

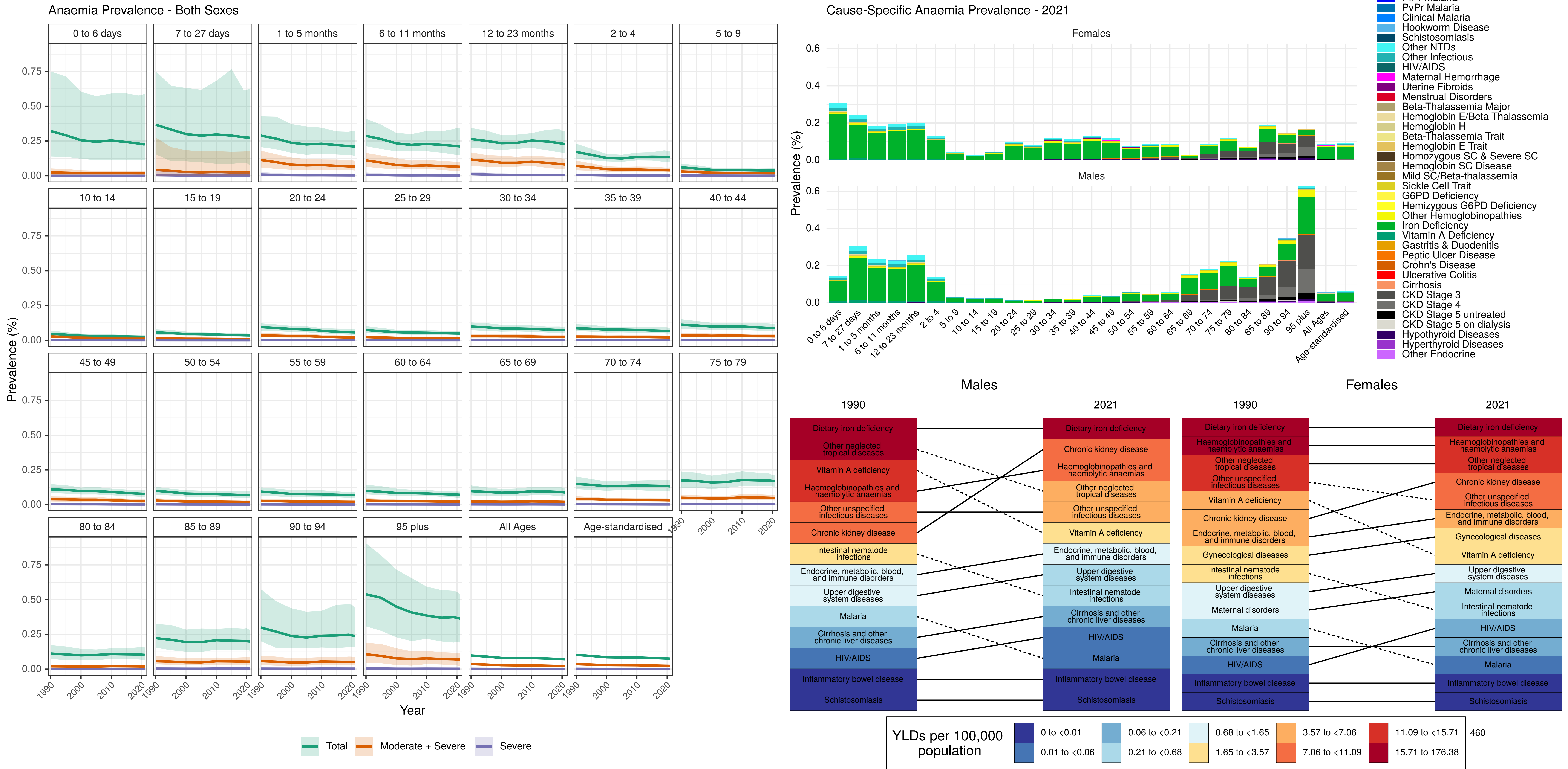

Anaemia Prevalence - Both Sexes
0 to 6 days
7 to 27 days
1 to 5 months
6 to 11 months
12 to 23 months
2 to 4
5 to 9
10 to 14
15 to 19
20 to 24
25 to 29
30 to 34
35 to 39
40 to 44
45 to 49
50 to 54
55 to 59
60 to 64
65 to 69
70 to 74
75 to 79
80 to 84
85 to 89
90 to 94
95 plus
All Ages
Age-standardised
Prevalence (%)
Year
Total
Moderate + Severe
Severe
Cause-Specific Anaemia Prevalence - 2021
Females
Males
PfPr Malaria
PvPr Malaria
Clinical Malaria
Hookworm Disease
Schistosomiasis
Other NTDs
Other Infectious
HIV/AIDS
Maternal Hemorrhage
Uterine Fibroids
Menstrual Disorders
Beta-Thalassemia Major
Hemoglobin E/Beta-Thalassemia
Hemoglobin H
Beta-Thalassemia Trait
Hemoglobin E Trait
Homozygous SC & Severe SC
Hemoglobin SC Disease
Mild SC/Beta-thalassemia
Sickle Cell Trait
G6PD Deficiency
Hemizygous G6PD Deficiency
Other Hemoglobinopathies
Iron Deficiency
Vitamin A Deficiency
Gastritis & Duodenitis
Peptic Ulcer Disease
Crohn's Disease
Ulcerative Colitis
Cirrhosis
CKD Stage 3
CKD Stage 4
CKD Stage 5 untreated
CKD Stage 5 on dialysis
Hypothyroid Diseases
Hyperthyroid Diseases
Other Endocrine
Males
Females
1990
2021
Dietary iron deficiency
Other neglected tropical diseases
Vitamin A deficiency
Haemoglobinopathies and haemolytic anaemias
Other unspecified infectious diseases
Chronic kidney disease
Intestinal nematode infections
Endocrine, metabolic, blood, and immune disorders
Upper digestive system diseases
Malaria
Cirrhosis and other chronic liver diseases
HIV/AIDS
Inflammatory bowel disease
Schistosomiasis
Gynecological diseases
Maternal disorders
YLDs per 100,000 population
0 to <0.01
0.01 to <0.06
0.06 to <0.21
0.21 to <0.68
0.68 to <1.65
1.65 to <3.57
3.57 to <7.06
7.06 to <11.09
11.09 to <15.71
15.71 to 176.38

460

Guanajuato

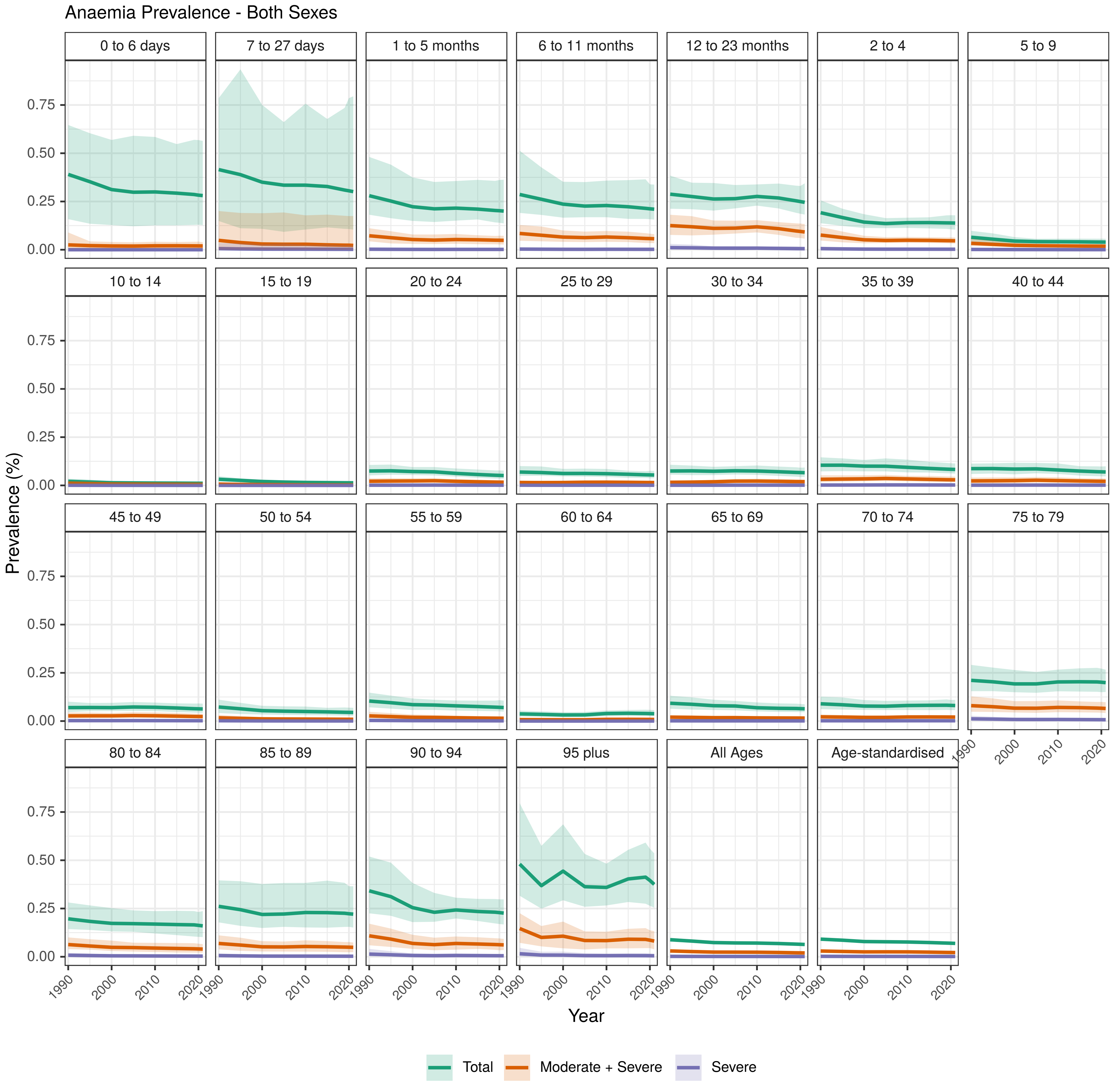
Anaemia Prevalence - Both Sexes
0 to 6 days
7 to 27 days
1 to 5 months
6 to 11 months
12 to 23 months
2 to 4
5 to 9
10 to 14
15 to 19
20 to 24
25 to 29
30 to 34
35 to 39
40 to 44
45 to 49
50 to 54
55 to 59
60 to 64
65 to 69
70 to 74
75 to 79
80 to 84
85 to 89
90 to 94
95 plus
All Ages
Age-standardised
Prevalence (%)
Year
Total
Moderate + Severe
Severe

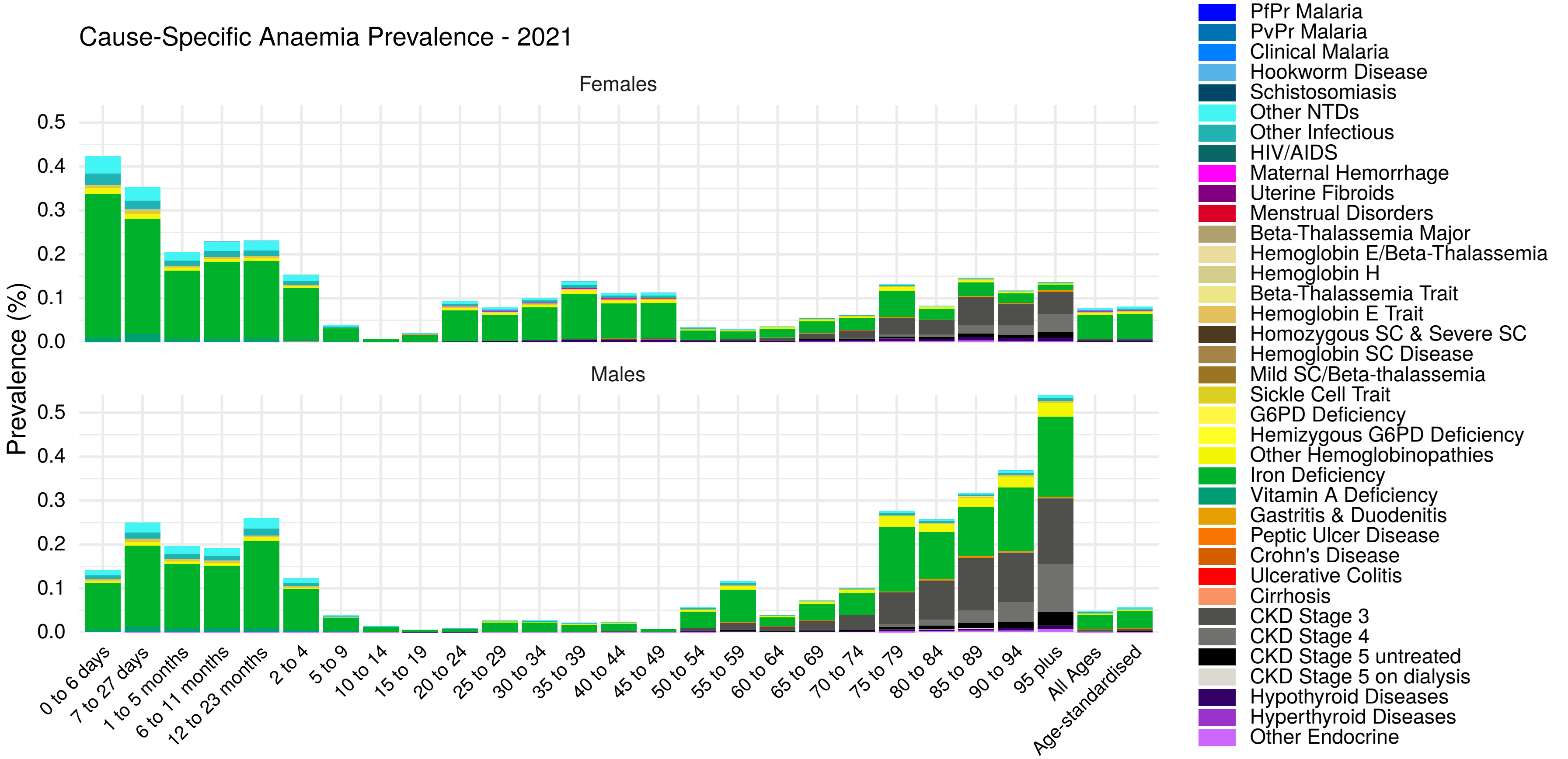
Cause-Specific Anaemia Prevalence - 2021
Females
Males
Prevalence (%)
PfPr Malaria
PvPr Malaria
Clinical Malaria
Hookworm Disease
Schistosomiasis
Other NTDs
Other Infectious
HIV/AIDS
Maternal Hemorrhage
Uterine Fibroids
Menstrual Disorders
Beta-Thalassemia Major
Hemoglobin E/Beta-Thalassemia
Hemoglobin H
Beta-Thalassemia Trait
Hemoglobin E Trait
Homozygous SC & Severe SC
Hemoglobin SC Disease
Mild SC/Beta-thalassemia
Sickle Cell Trait
G6PD Deficiency
Hemizygous G6PD Deficiency
Other Hemoglobinopathies
Iron Deficiency
Vitamin A Deficiency
Gastritis & Duodenitis
Peptic Ulcer Disease
Crohn's Disease
Ulcerative Colitis
Cirrhosis
CKD Stage 3
CKD Stage 4
CKD Stage 5 untreated
CKD Stage 5 on dialysis
Hypothyroid Diseases
Hyperthyroid Diseases
Other Endocrine

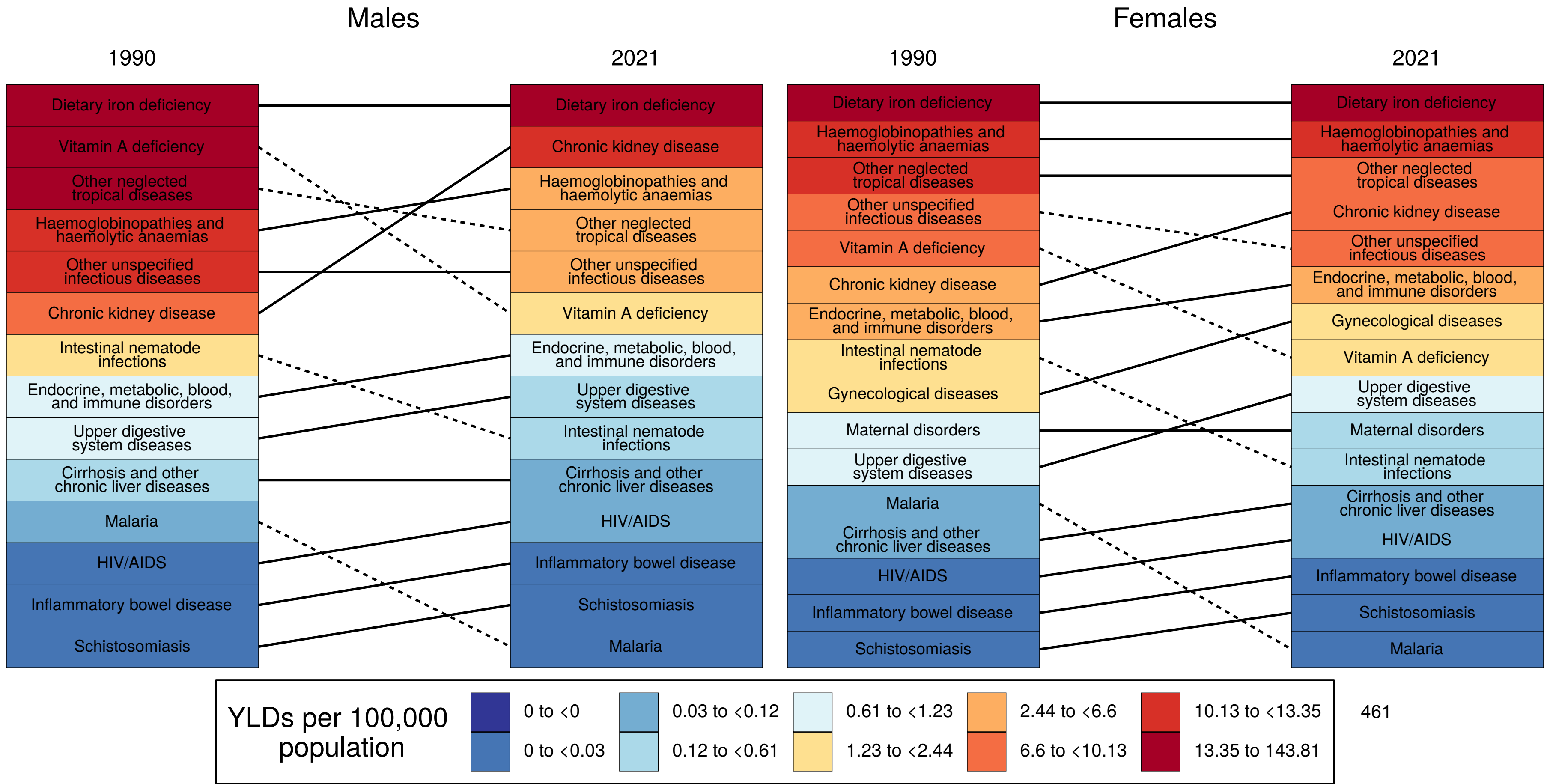
Males
Females
1990
2021
Dietary iron deficiency
Vitamin A deficiency
Other neglected tropical diseases
Haemoglobinopathies and haemolytic anaemias
Other unspecified infectious diseases
Chronic kidney disease
Intestinal nematode infections
Endocrine, metabolic, blood, and immune disorders
Upper digestive system diseases
Cirrhosis and other chronic liver diseases
Malaria
HIV/AIDS
Inflammatory bowel disease
Schistosomiasis
Gynecological diseases
Maternal disorders
YLDs per 100,000 population
0 to <0
0 to <0.03
0.03 to <0.12
0.12 to <0.61
0.61 to <1.23
1.23 to <2.44
2.44 to <6.6
6.6 to <10.13
10.13 to <13.35
13.35 to 143.81

Guerrero

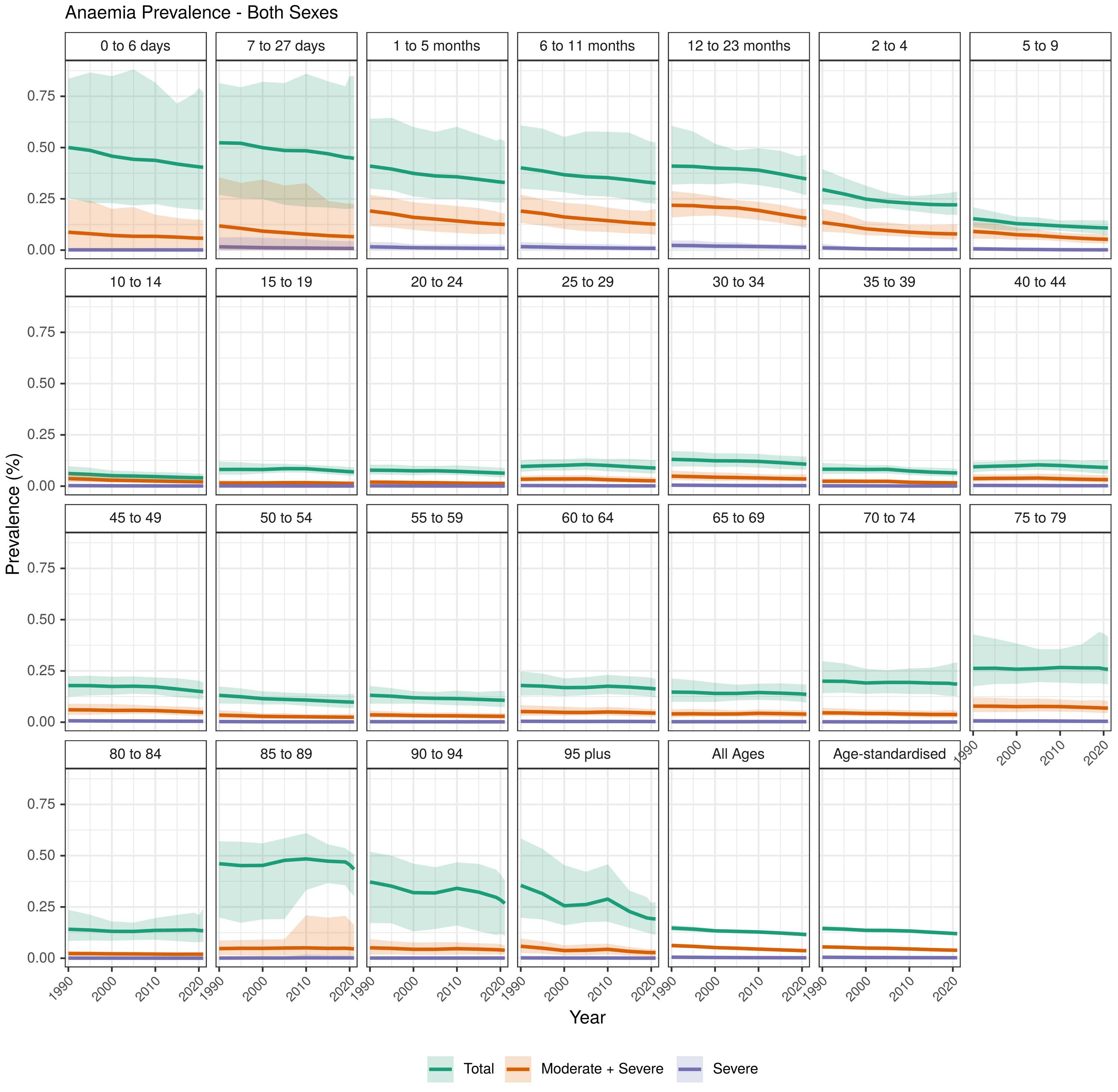

Anaemia Prevalence - Both Sexes
0 to 6 days
7 to 27 days
1 to 5 months
6 to 11 months
12 to 23 months
2 to 4
5 to 9
10 to 14
15 to 19
20 to 24
25 to 29
30 to 34
35 to 39
40 to 44
45 to 49
50 to 54
55 to 59
60 to 64
65 to 69
70 to 74
75 to 79
80 to 84
85 to 89
90 to 94
95 plus
All Ages
Age-standardised
Prevalence (%)
Year
Total
Moderate + Severe
Severe

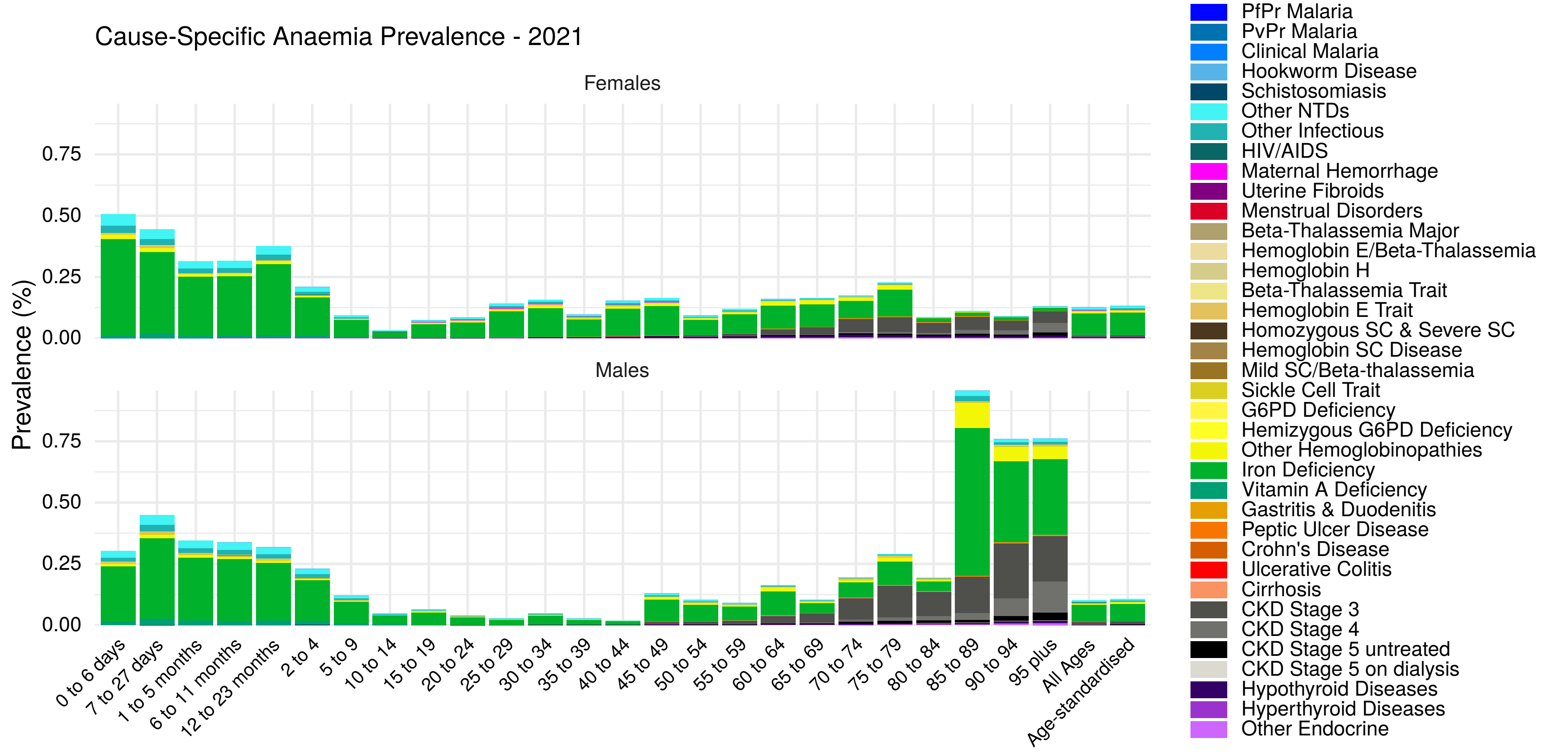

Cause-Specific Anaemia Prevalence - 2021
Females
Males
Prevalence (%)
PfPr Malaria
PvPr Malaria
Clinical Malaria
Hookworm Disease
Schistosomiasis
Other NTDs
Other Infectious
HIV/AIDS
Maternal Hemorrhage
Uterine Fibroids
Menstrual Disorders
Beta-Thalassemia Major
Hemoglobin E/Beta-Thalassemia
Hemoglobin H
Beta-Thalassemia Trait
Hemoglobin E Trait
Homozygous SC & Severe SC
Hemoglobin SC Disease
Mild SC/Beta-thalassemia
Sickle Cell Trait
G6PD Deficiency
Hemizygous G6PD Deficiency
Other Hemoglobinopathies
Iron Deficiency
Vitamin A Deficiency
Gastritis & Duodenitis
Peptic Ulcer Disease
Crohn's Disease
Ulcerative Colitis
Cirrhosis
CKD Stage 3
CKD Stage 4
CKD Stage 5 untreated
CKD Stage 5 on dialysis
Hypothyroid Diseases
Hyperthyroid Diseases
Other Endocrine

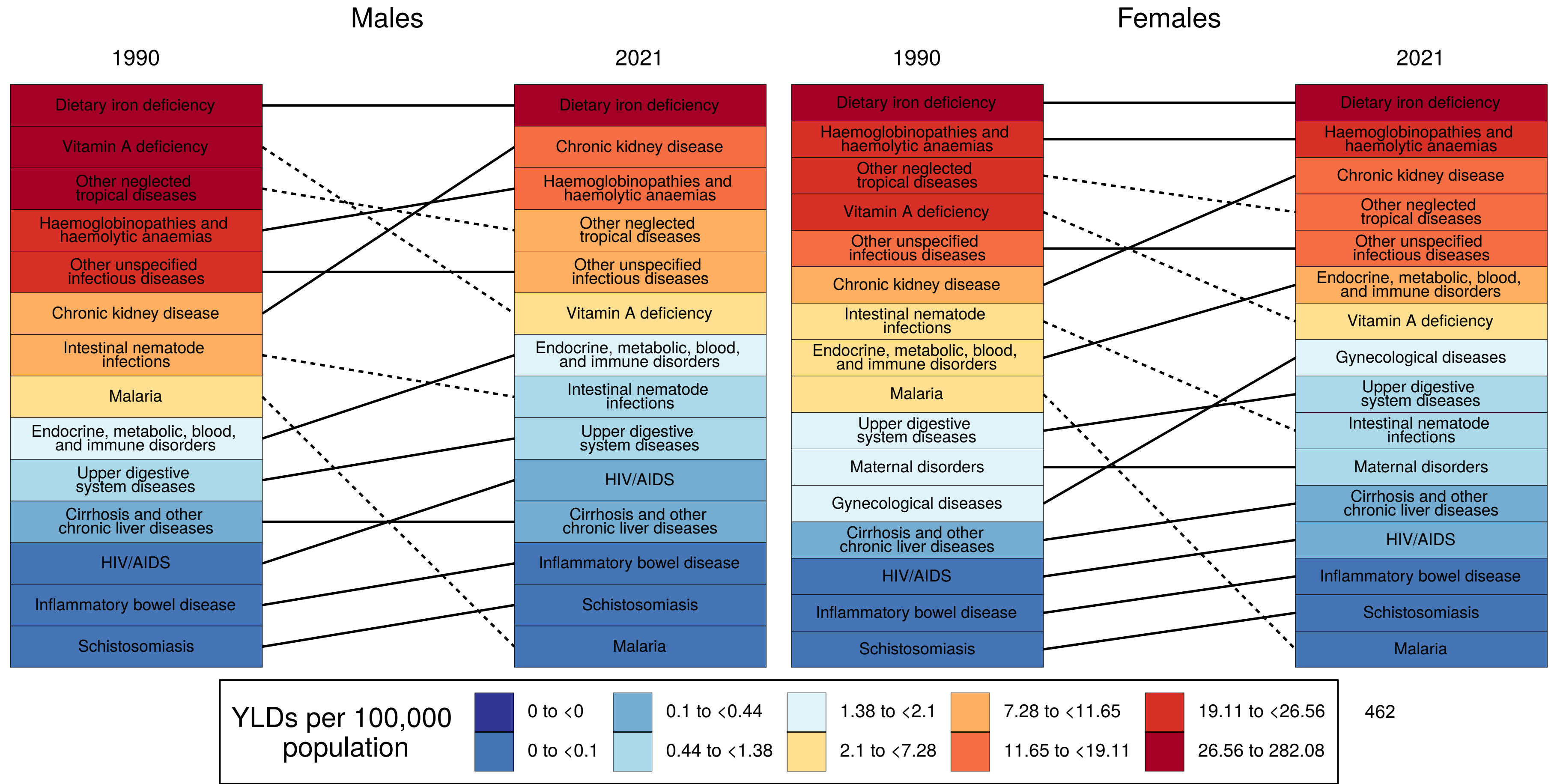

Males
Females
1990
2021
Dietary iron deficiency
Vitamin A deficiency
Other neglected tropical diseases
Haemoglobinopathies and haemolytic anaemias
Other unspecified infectious diseases
Chronic kidney disease
Intestinal nematode infections
Malaria
Endocrine, metabolic, blood, and immune disorders
Upper digestive system diseases
Cirrhosis and other chronic liver diseases
HIV/AIDS
Inflammatory bowel disease
Schistosomiasis
Maternal disorders
Gynecological diseases
YLDs per 100,000 population
0 to <0
0 to <0.1
0.1 to <0.44
0.44 to <1.38
1.38 to <2.1
2.1 to <7.28
7.28 to <11.65
11.65 to <19.11
19.11 to <26.56
26.56 to 282.08

Hidalgo

Anaemia Prevalence - Both Sexes

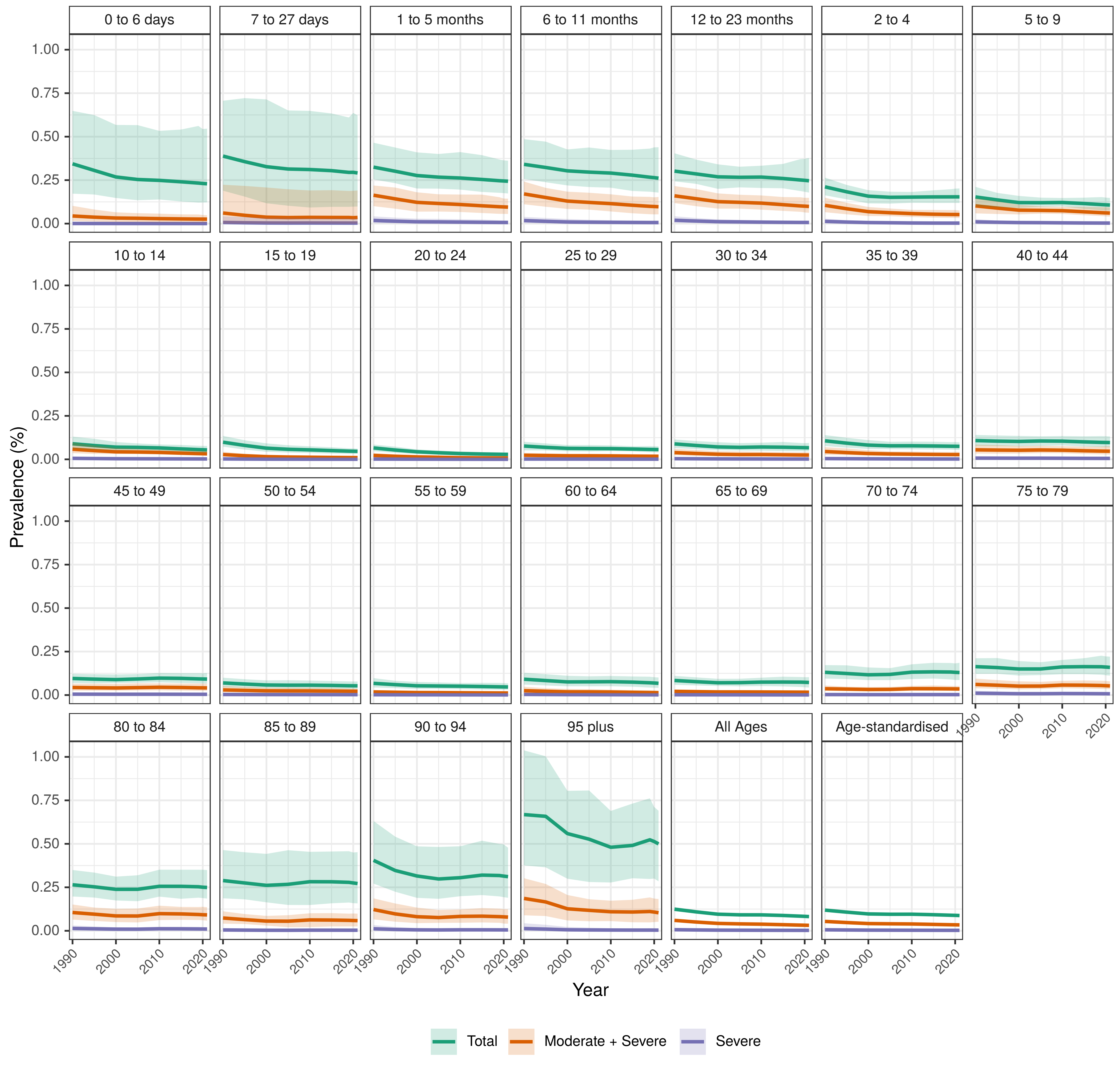

Cause-Specific Anaemia Prevalence - 2021

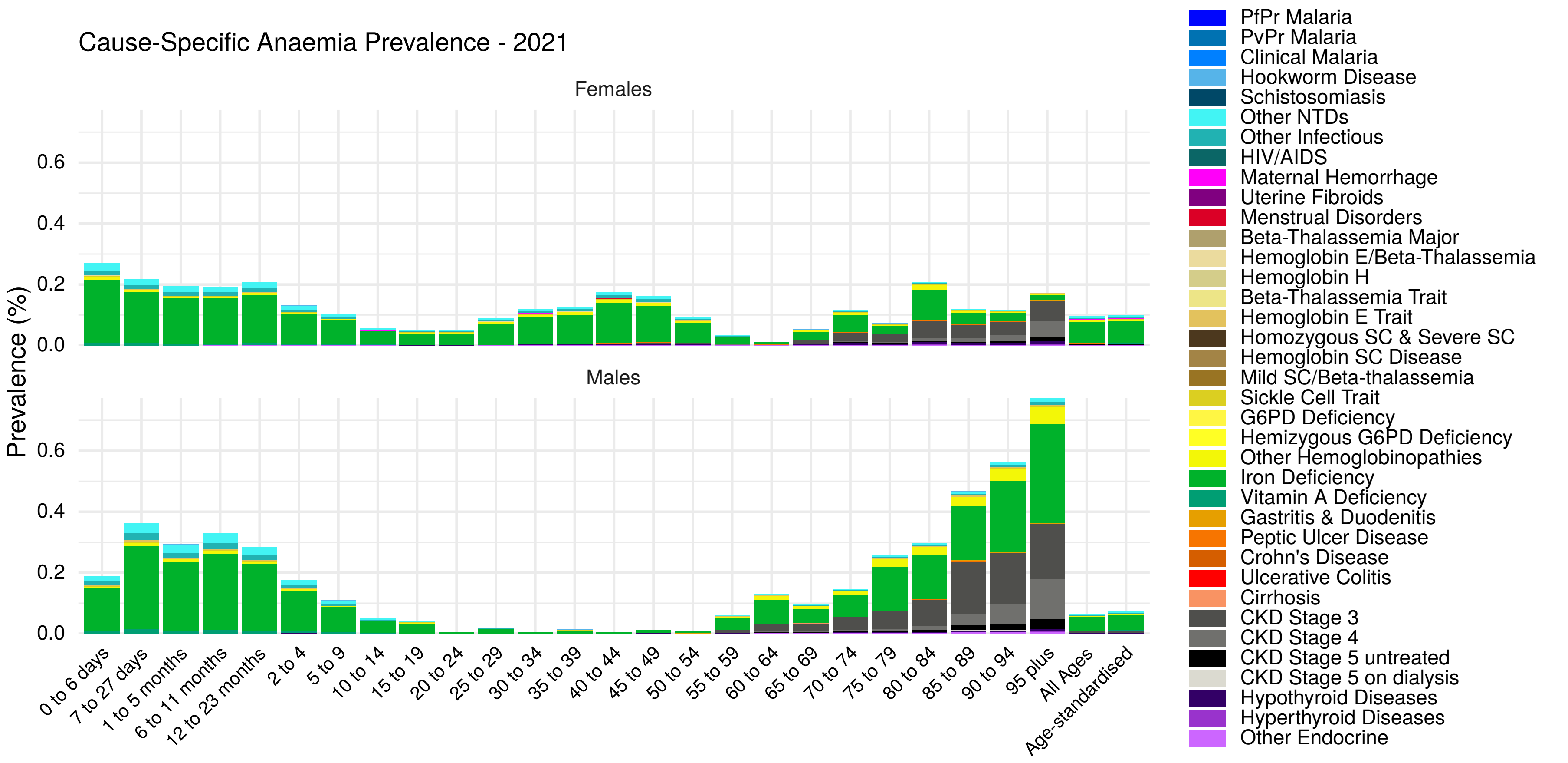

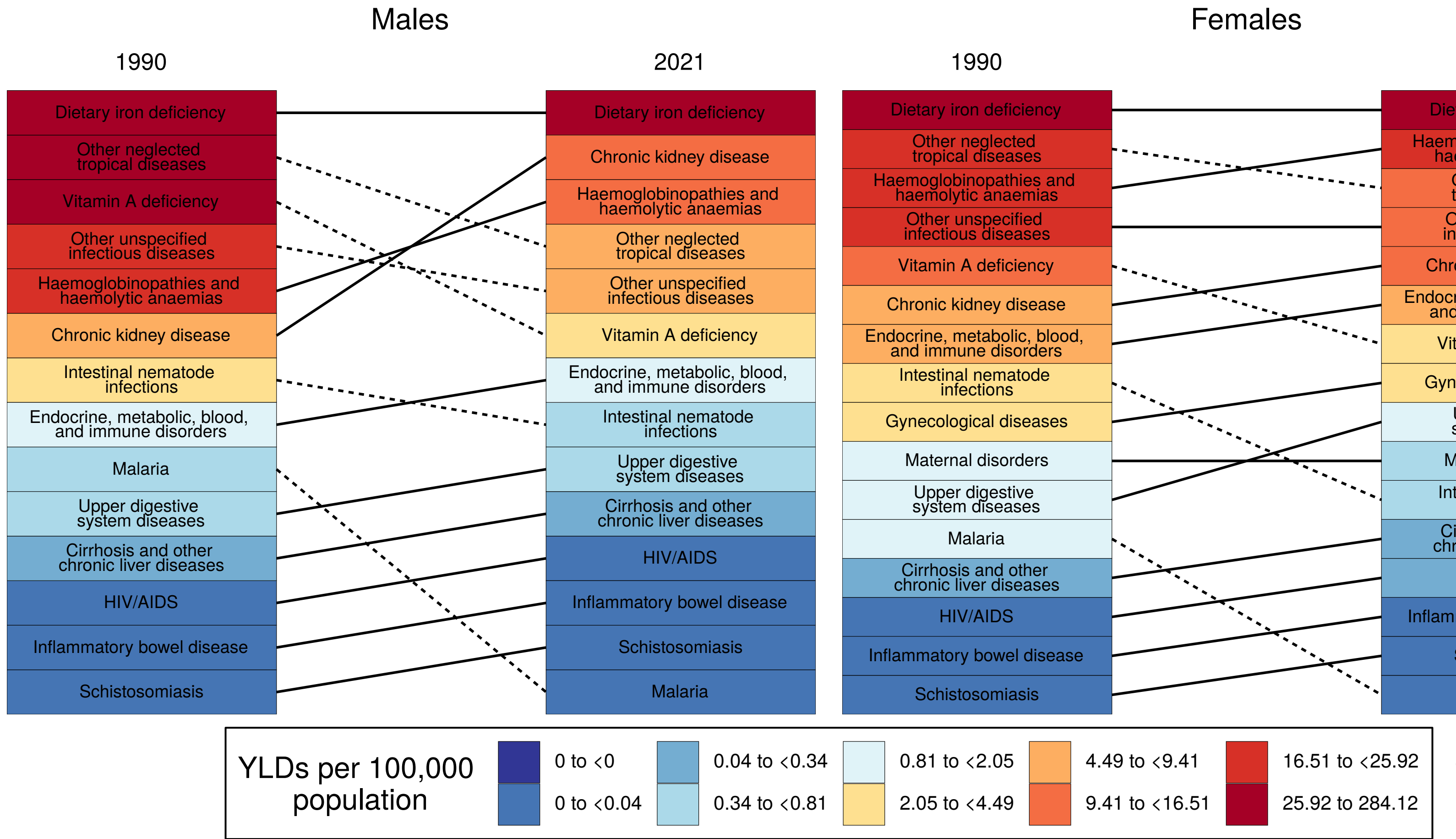

463

Jalisco

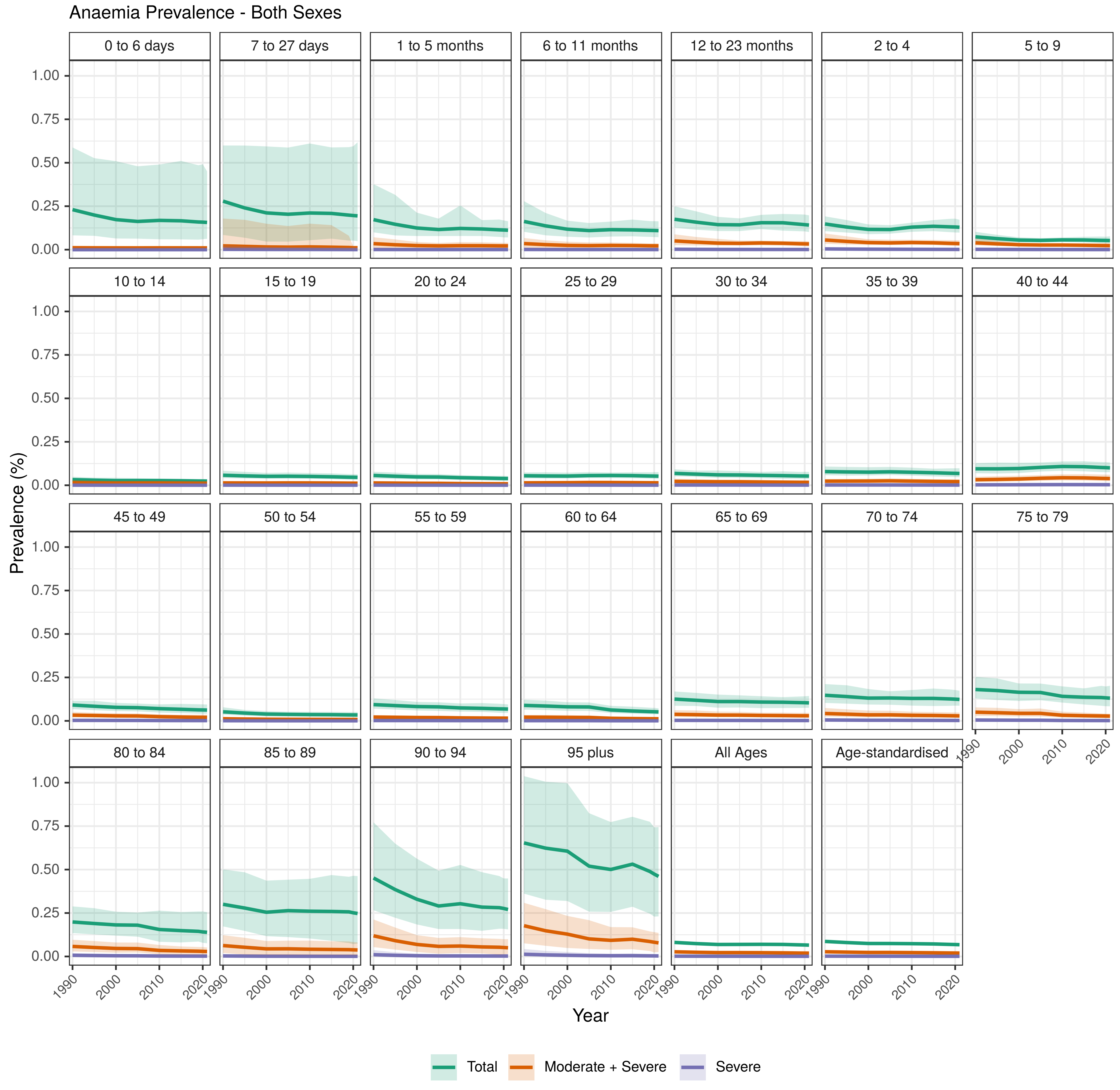
Anaemia Prevalence - Both Sexes
0 to 6 days
7 to 27 days
1 to 5 months
6 to 11 months
12 to 23 months
2 to 4
5 to 9
10 to 14
15 to 19
20 to 24
25 to 29
30 to 34
35 to 39
40 to 44
45 to 49
50 to 54
55 to 59
60 to 64
65 to 69
70 to 74
75 to 79
80 to 84
85 to 89
90 to 94
95 plus
All Ages
Age-standardised
Prevalence (%)
Year
Total
Moderate + Severe
Severe

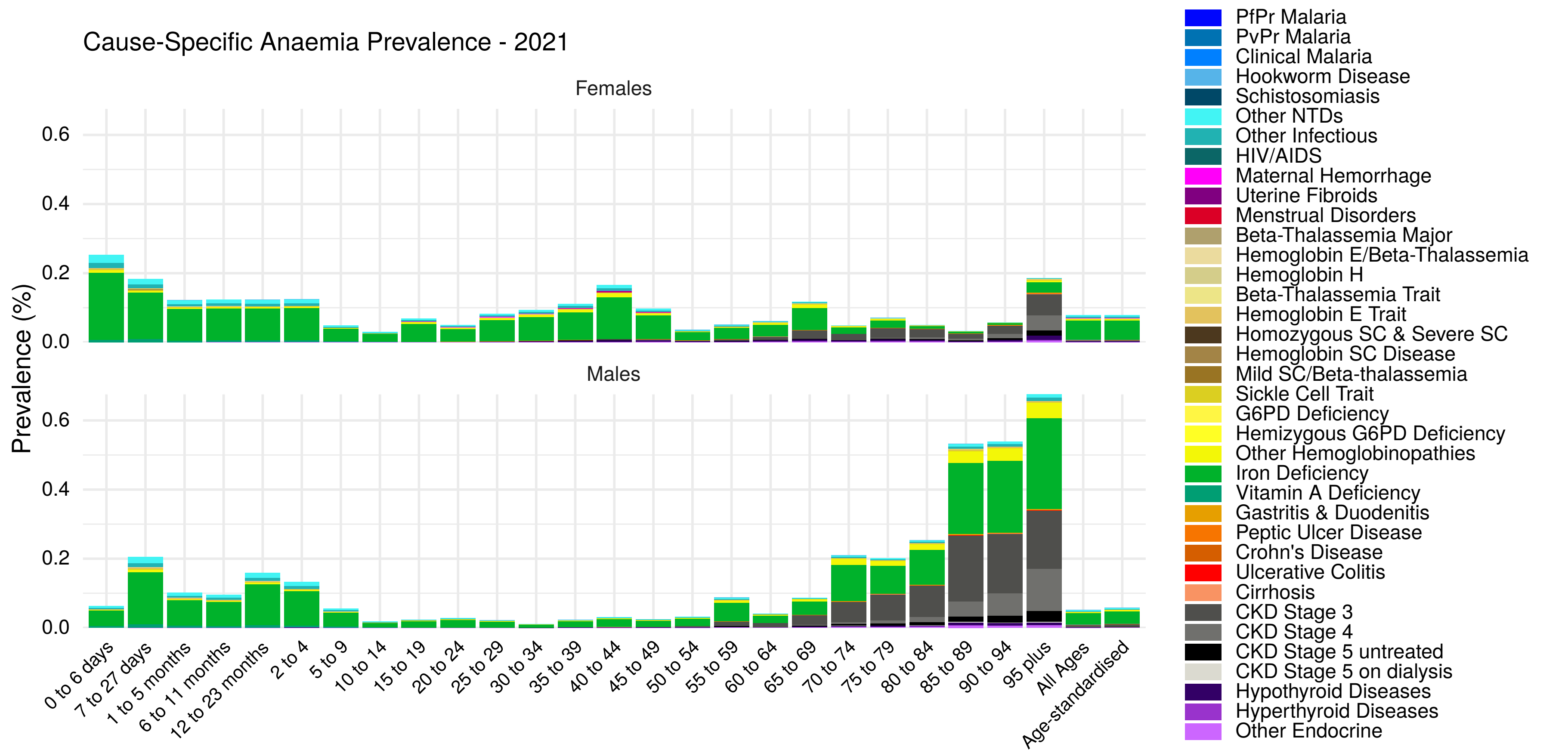
Cause-Specific Anaemia Prevalence - 2021
Females
Males
Prevalence (%)
PfPr Malaria
PvPr Malaria
Clinical Malaria
Hookworm Disease
Schistosomiasis
Other NTDs
Other Infectious
HIV/AIDS
Maternal Hemorrhage
Uterine Fibroids
Menstrual Disorders
Beta-Thalassemia Major
Hemoglobin E/Beta-Thalassemia
Hemoglobin H
Beta-Thalassemia Trait
Hemoglobin E Trait
Homozygous SC & Severe SC
Hemoglobin SC Disease
Mild SC/Beta-thalassemia
Sickle Cell Trait
G6PD Deficiency
Hemizygous G6PD Deficiency
Other Hemoglobinopathies
Iron Deficiency
Vitamin A Deficiency
Gastritis & Duodenitis
Peptic Ulcer Disease
Crohn's Disease
Ulcerative Colitis
Cirrhosis
CKD Stage 3
CKD Stage 4
CKD Stage 5 untreated
CKD Stage 5 on dialysis
Hypothyroid Diseases
Hyperthyroid Diseases
Other Endocrine

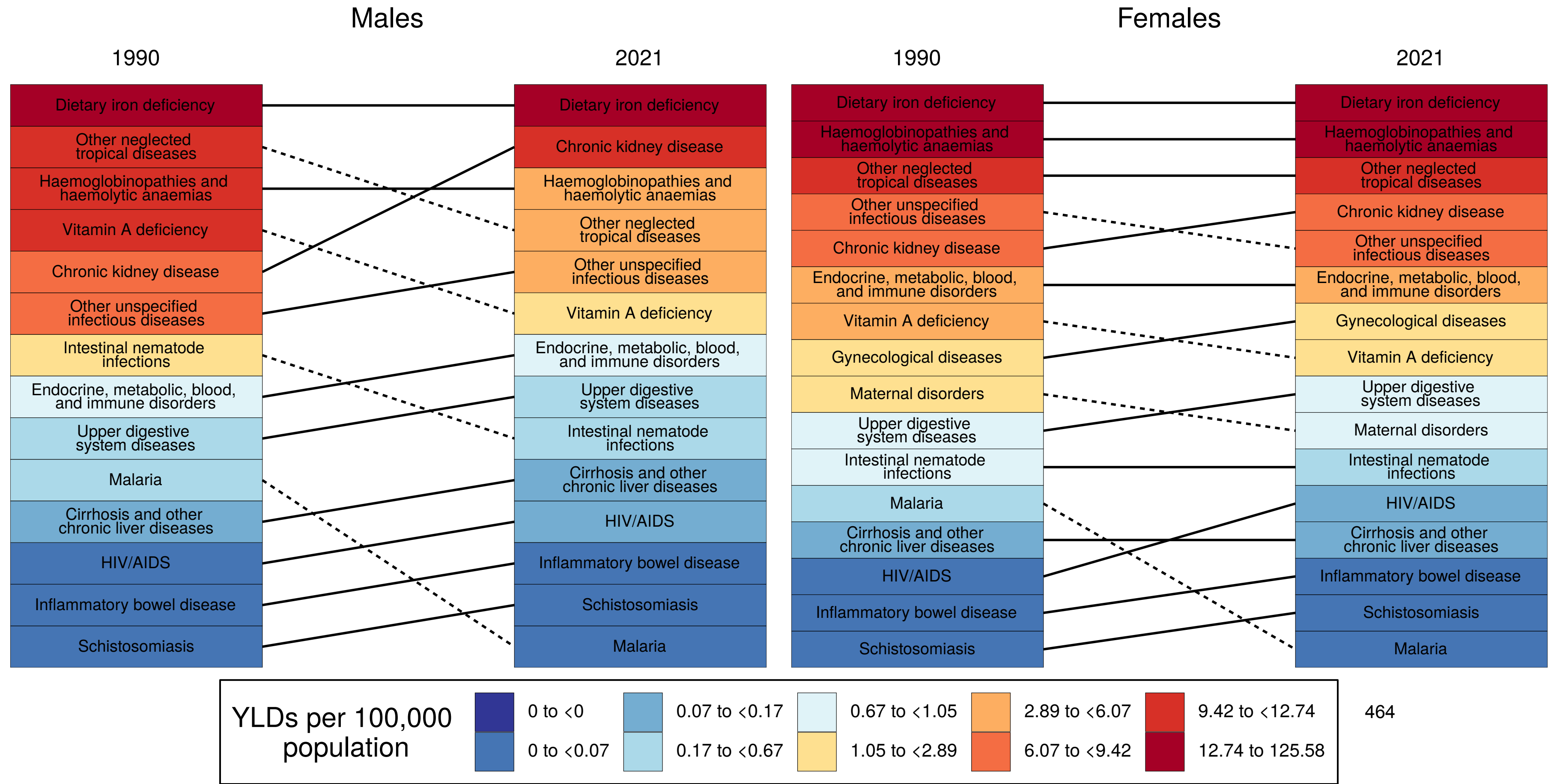
Males
Females
1990
2021
Dietary iron deficiency
Other neglected tropical diseases
Haemoglobinopathies and haemolytic anaemias
Vitamin A deficiency
Chronic kidney disease
Other unspecified infectious diseases
Intestinal nematode infections
Endocrine, metabolic, blood, and immune disorders
Upper digestive system diseases
Malaria
Cirrhosis and other chronic liver diseases
HIV/AIDS
Inflammatory bowel disease
Schistosomiasis
Gynecological diseases
Maternal disorders
YLDs per 100,000 population
0 to <0
0 to <0.07
0.07 to <0.17
0.17 to <0.67
0.67 to <1.05
1.05 to <2.89
2.89 to <6.07
6.07 to <9.42
9.42 to <12.74
12.74 to 125.58

México

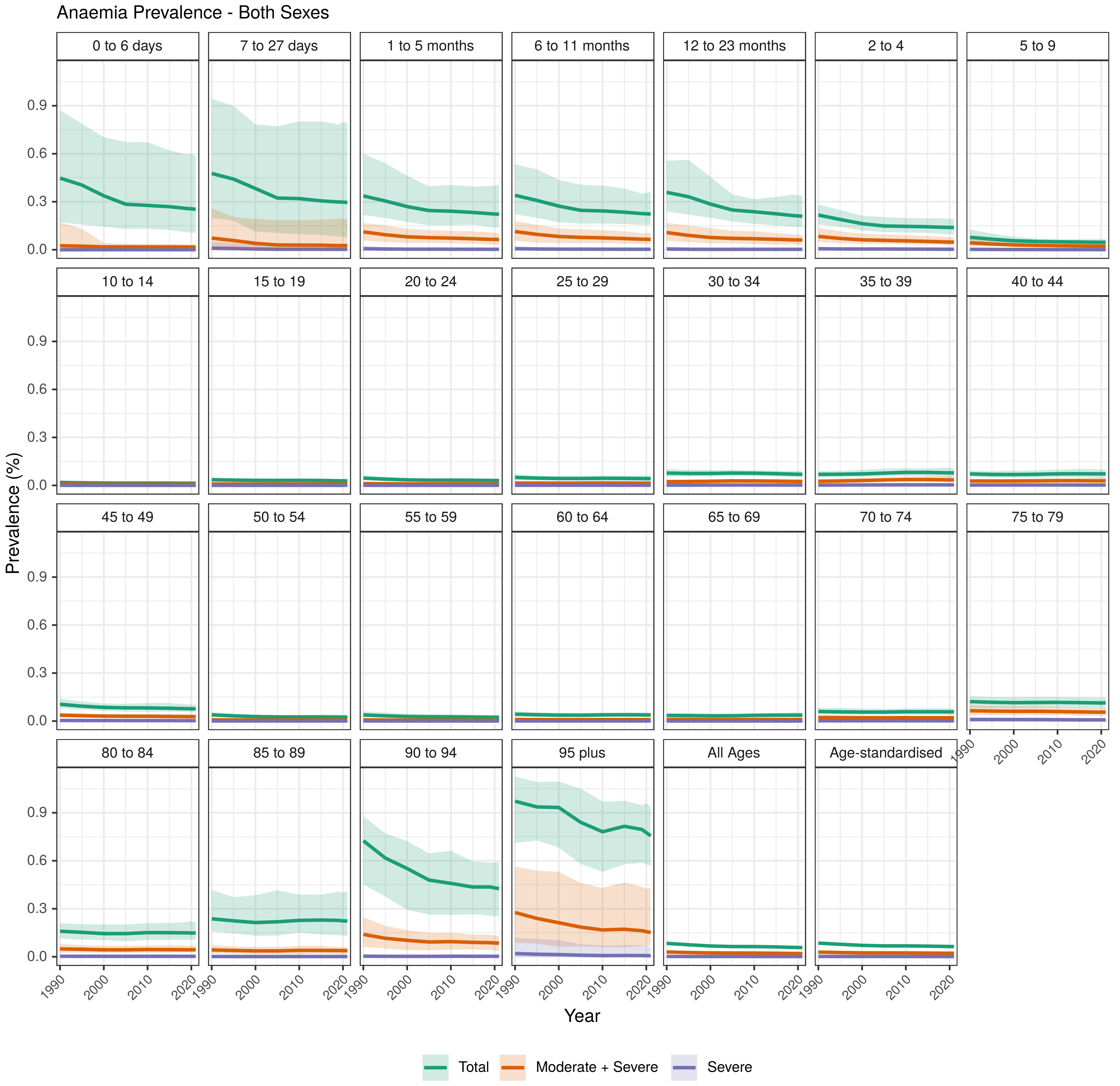
Anaemia Prevalence - Both Sexes
0 to 6 days
7 to 27 days
1 to 5 months
6 to 11 months
12 to 23 months
2 to 4
5 to 9
10 to 14
15 to 19
20 to 24
25 to 29
30 to 34
35 to 39
40 to 44
45 to 49
50 to 54
55 to 59
60 to 64
65 to 69
70 to 74
75 to 79
80 to 84
85 to 89
90 to 94
95 plus
All Ages
Age-standardised
Prevalence (%)
Year
Total
Moderate + Severe
Severe

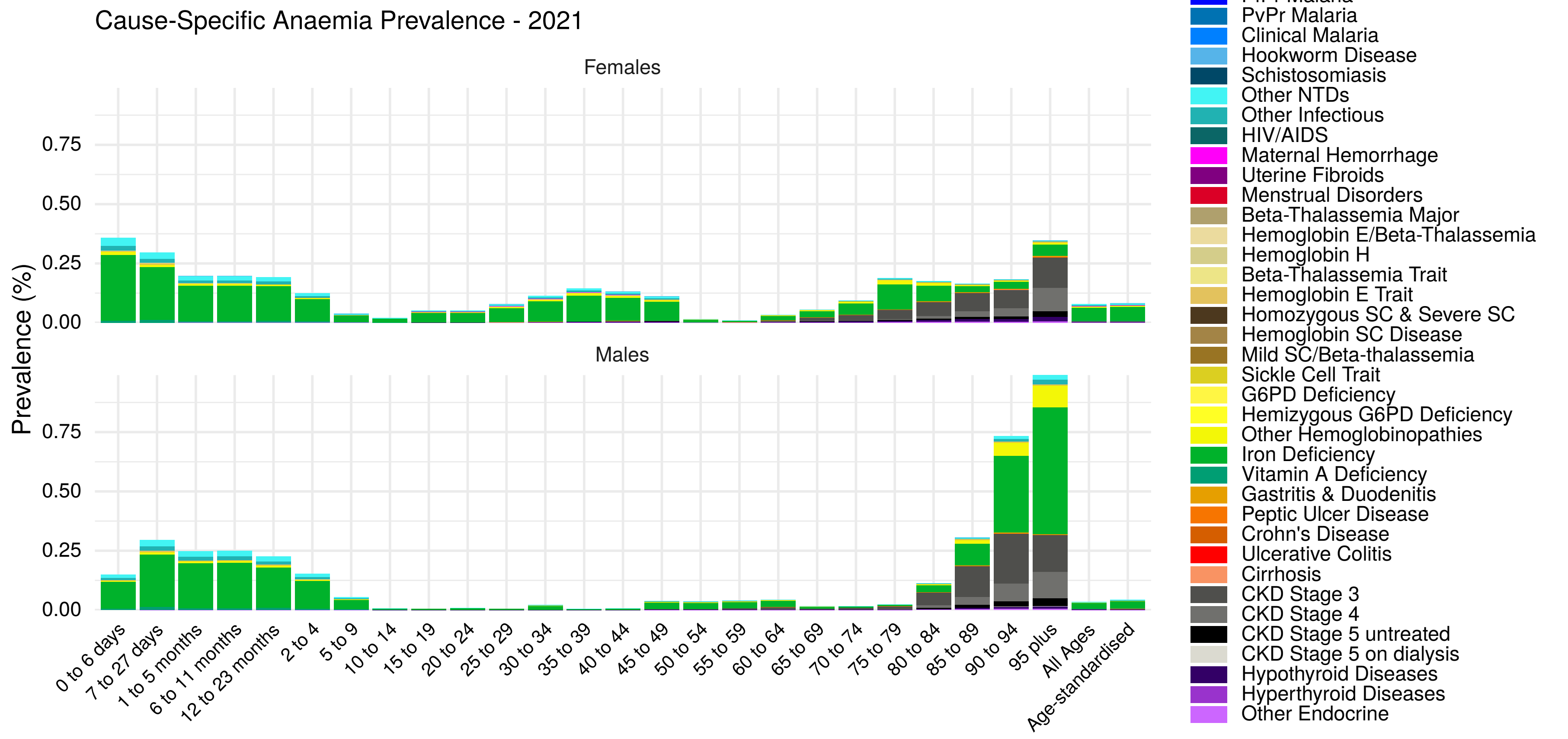
Cause-Specific Anaemia Prevalence - 2021
Females
Males
Prevalence (%)
PfPr Malaria
PvPr Malaria
Clinical Malaria
Hookworm Disease
Schistosomiasis
Other NTDs
Other Infectious
HIV/AIDS
Maternal Hemorrhage
Uterine Fibroids
Menstrual Disorders
Beta-Thalassemia Major
Hemoglobin E/Beta-Thalassemia
Hemoglobin H
Beta-Thalassemia Trait
Hemoglobin E Trait
Homozygous SC & Severe SC
Hemoglobin SC Disease
Mild SC/Beta-thalassemia
Sickle Cell Trait
G6PD Deficiency
Hemizygous G6PD Deficiency
Other Hemoglobinopathies
Iron Deficiency
Vitamin A Deficiency
Gastritis & Duodenitis
Peptic Ulcer Disease
Crohn's Disease
Ulcerative Colitis
Cirrhosis
CKD Stage 3
CKD Stage 4
CKD Stage 5 untreated
CKD Stage 5 on dialysis
Hypothyroid Diseases
Hyperthyroid Diseases
Other Endocrine

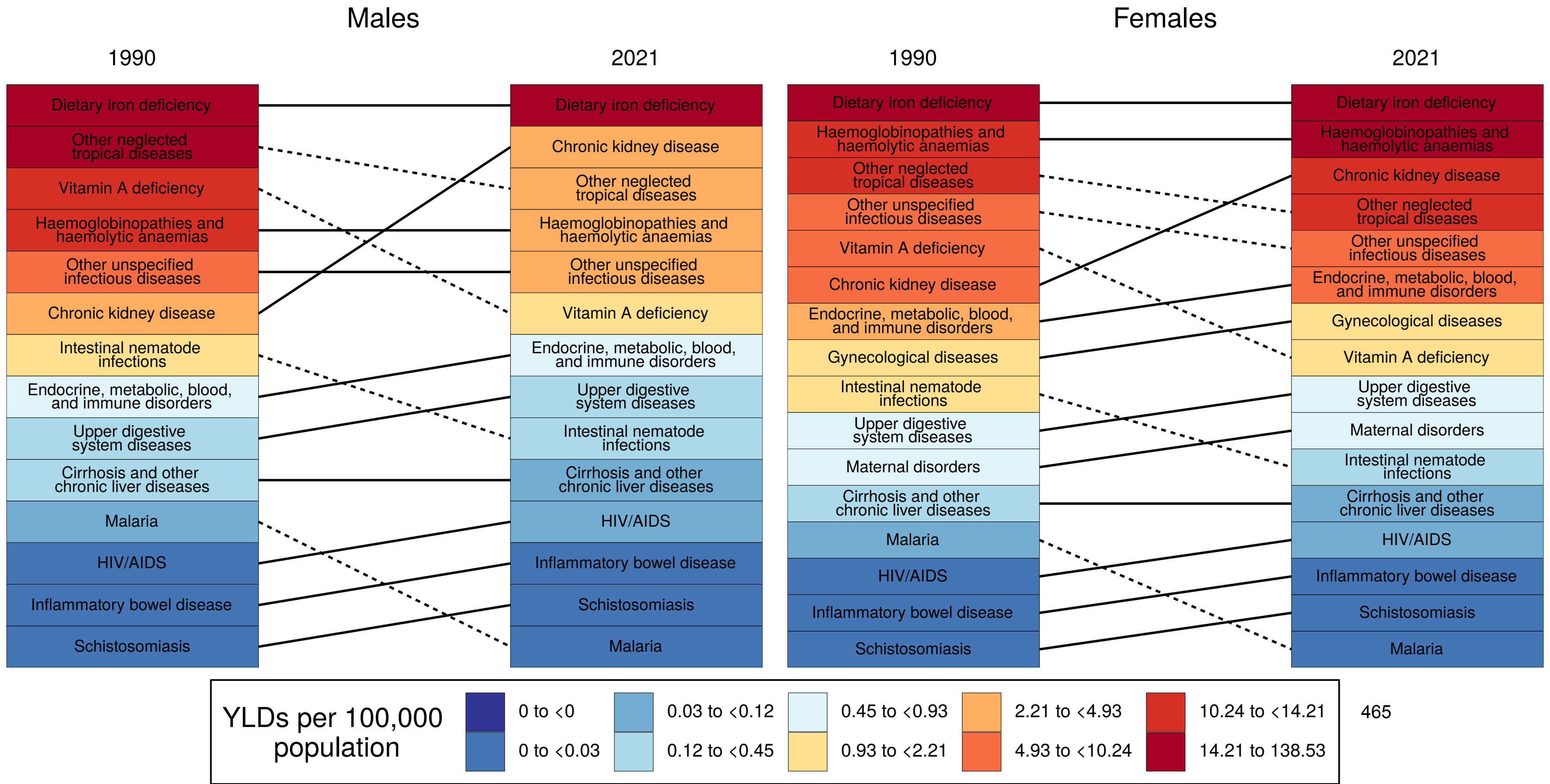
Males
Females
1990
2021
Dietary iron deficiency
Other neglected tropical diseases
Vitamin A deficiency
Haemoglobinopathies and haemolytic anaemias
Other unspecified infectious diseases
Chronic kidney disease
Intestinal nematode infections
Endocrine, metabolic, blood, and immune disorders
Upper digestive system diseases
Cirrhosis and other chronic liver diseases
Malaria
HIV/AIDS
Inflammatory bowel disease
Schistosomiasis
Gynecological diseases
Maternal disorders
YLDs per 100,000 population
0 to <0
0 to <0.03
0.03 to <0.12
0.12 to <0.45
0.45 to <0.93
0.93 to <2.21
2.21 to <4.93
4.93 to <10.24
10.24 to <14.21
14.21 to 138.53
465

Mexico City

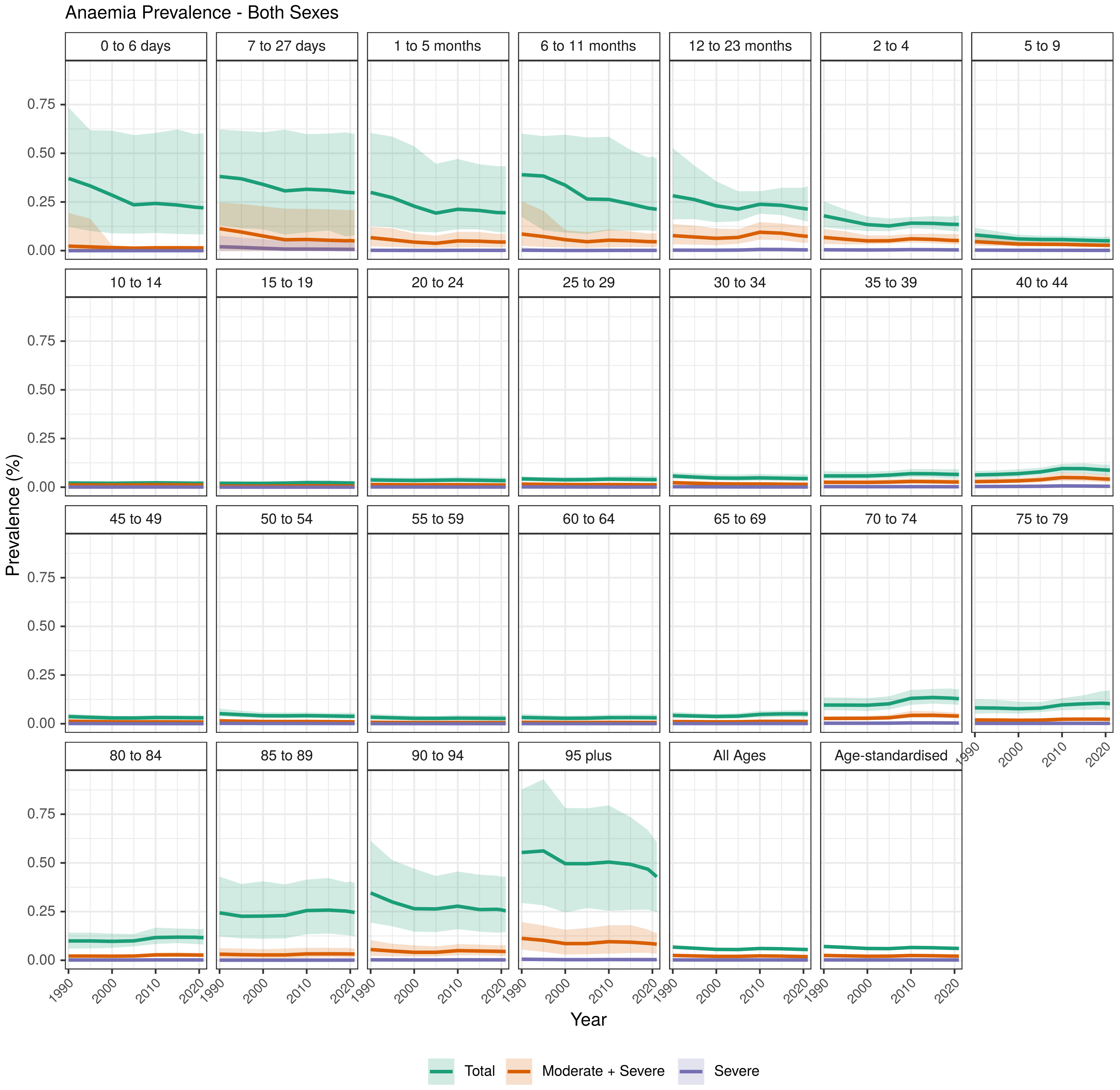

Anaemia Prevalence - Both Sexes
0 to 6 days
7 to 27 days
1 to 5 months
6 to 11 months
12 to 23 months
2 to 4
5 to 9
10 to 14
15 to 19
20 to 24
25 to 29
30 to 34
35 to 39
40 to 44
45 to 49
50 to 54
55 to 59
60 to 64
65 to 69
70 to 74
75 to 79
80 to 84
85 to 89
90 to 94
95 plus
All Ages
Age-standardised
Prevalence (%)
Year
Total
Moderate + Severe
Severe

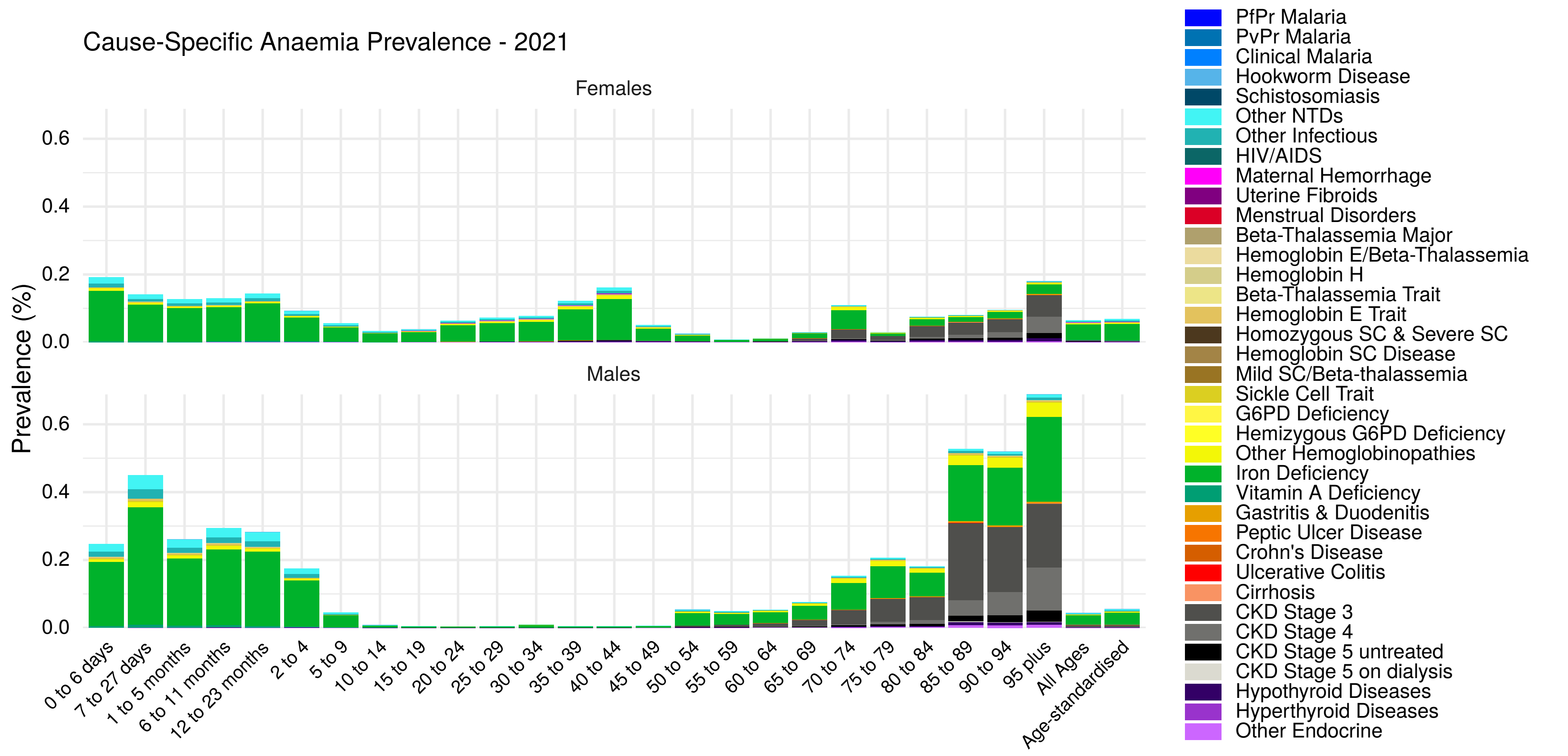

Cause-Specific Anaemia Prevalence - 2021
Females
Males
Prevalence (%)
PfPr Malaria
PvPr Malaria
Clinical Malaria
Hookworm Disease
Schistosomiasis
Other NTDs
Other Infectious
HIV/AIDS
Maternal Hemorrhage
Uterine Fibroids
Menstrual Disorders
Beta-Thalassemia Major
Hemoglobin E/Beta-Thalassemia
Hemoglobin H
Beta-Thalassemia Trait
Hemoglobin E Trait
Homozygous SC & Severe SC
Hemoglobin SC Disease
Mild SC/Beta-thalassemia
Sickle Cell Trait
G6PD Deficiency
Hemizygous G6PD Deficiency
Other Hemoglobinopathies
Iron Deficiency
Vitamin A Deficiency
Gastritis & Duodenitis
Peptic Ulcer Disease
Crohn's Disease
Ulcerative Colitis
Cirrhosis
CKD Stage 3
CKD Stage 4
CKD Stage 5 untreated
CKD Stage 5 on dialysis
Hypothyroid Diseases
Hyperthyroid Diseases
Other Endocrine

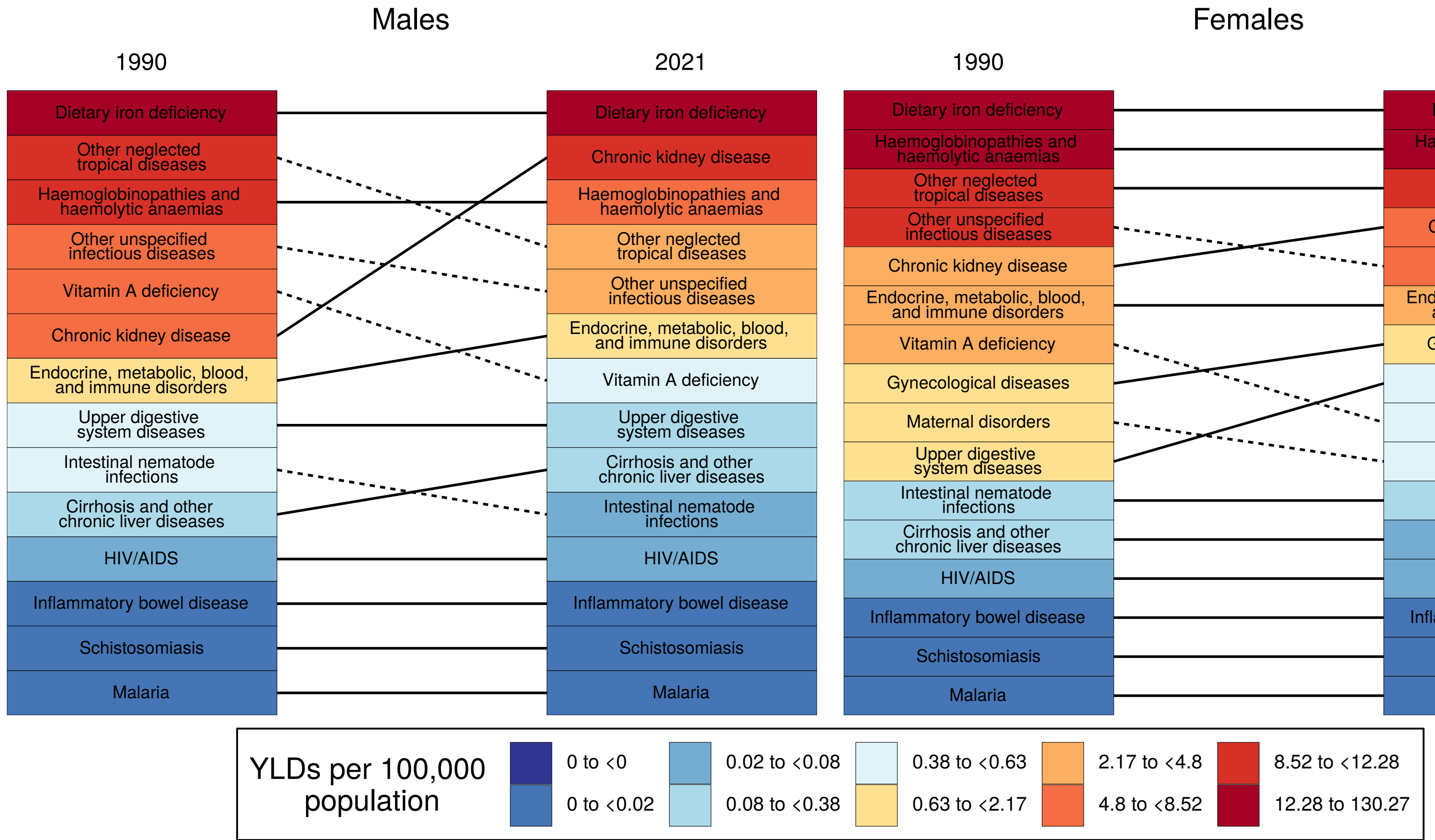

Males
Females
1990
2021
Dietary iron deficiency
Other neglected tropical diseases
Haemoglobinopathies and haemolytic anaemias
Other unspecified infectious diseases
Vitamin A deficiency
Chronic kidney disease
Endocrine, metabolic, blood, and immune disorders
Upper digestive system diseases
Intestinal nematode infections
Cirrhosis and other chronic liver diseases
HIV/AIDS
Inflammatory bowel disease
Schistosomiasis
Malaria
Gynecological diseases
Maternal disorders
YLDs per 100,000 population
0 to <0
0 to <0.02
0.02 to <0.08
0.08 to <0.38
0.38 to <0.63
0.63 to <2.17
2.17 to <4.8
4.8 to <8.52
8.52 to <12.28
12.28 to 130.27

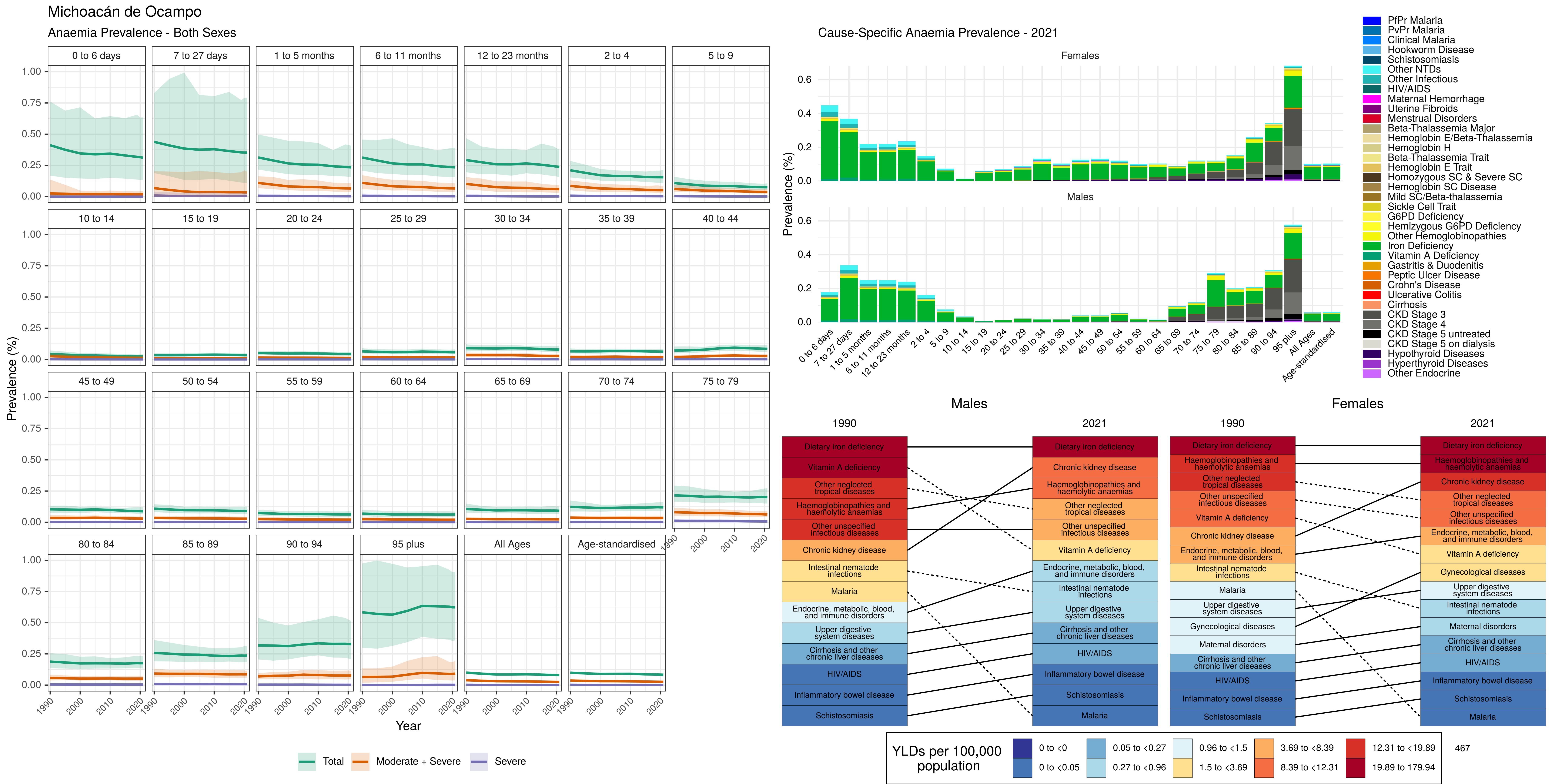
Michoacán de Ocampo
Anaemia Prevalence - Both Sexes
0 to 6 days
7 to 27 days
1 to 5 months
6 to 11 months
12 to 23 months
2 to 4
5 to 9
10 to 14
15 to 19
20 to 24
25 to 29
30 to 34
35 to 39
40 to 44
45 to 49
50 to 54
55 to 59
60 to 64
65 to 69
70 to 74
75 to 79
80 to 84
85 to 89
90 to 94
95 plus
All Ages
Age-standardised
Prevalence (%)
Year
Total
Moderate + Severe
Severe
Cause-Specific Anaemia Prevalence - 2021
Females
Males
Prevalence (%)
PfPr Malaria
PvPr Malaria
Clinical Malaria
Hookworm Disease
Schistosomiasis
Other NTDs
Other Infectious
HIV/AIDS
Maternal Hemorrhage
Uterine Fibroids
Menstrual Disorders
Beta-Thalassemia Major
Hemoglobin E/Beta-Thalassemia
Hemoglobin H
Beta-Thalassemia Trait
Hemoglobin E Trait
Homozygous SC & Severe SC
Hemoglobin SC Disease
Mild SC/Beta-thalassemia
Sickle Cell Trait
G6PD Deficiency
Hemizygous G6PD Deficiency
Other Hemoglobinopathies
Iron Deficiency
Vitamin A Deficiency
Gastritis & Duodenitis
Peptic Ulcer Disease
Crohn's Disease
Ulcerative Colitis
Cirrhosis
CKD Stage 3
CKD Stage 4
CKD Stage 5 untreated
CKD Stage 5 on dialysis
Hypothyroid Diseases
Hyperthyroid Diseases
Other Endocrine
Males
Females
1990
2021
Males 1990: Dietary iron deficiency; Vitamin A deficiency; Other neglected tropical diseases; Haemoglobinopathies and haemolytic anaemias; Other unspecified infectious diseases; Chronic kidney disease; Intestinal nematode infections; Malaria; Endocrine, metabolic, blood, and immune disorders; Upper digestive system diseases; Cirrhosis and other chronic liver diseases; HIV/AIDS; Inflammatory bowel disease; Schistosomiasis
Males 2021: Dietary iron deficiency; Chronic kidney disease; Haemoglobinopathies and haemolytic anaemias; Other neglected tropical diseases; Other unspecified infectious diseases; Vitamin A deficiency; Endocrine, metabolic, blood, and immune disorders; Intestinal nematode infections; Upper digestive system diseases; Cirrhosis and other chronic liver diseases; HIV/AIDS; Inflammatory bowel disease; Schistosomiasis; Malaria
Females 1990: Dietary iron deficiency; Haemoglobinopathies and haemolytic anaemias; Other neglected tropical diseases; Other unspecified infectious diseases; Vitamin A deficiency; Chronic kidney disease; Endocrine, metabolic, blood, and immune disorders; Intestinal nematode infections; Malaria; Upper digestive system diseases; Gynecological diseases; Maternal disorders; Cirrhosis and other chronic liver diseases; HIV/AIDS; Inflammatory bowel disease; Schistosomiasis
Females 2021: Dietary iron deficiency; Haemoglobinopathies and haemolytic anaemias; Chronic kidney disease; Other neglected tropical diseases; Other unspecified infectious diseases; Endocrine, metabolic, blood, and immune disorders; Vitamin A deficiency; Gynecological diseases; Upper digestive system diseases; Intestinal nematode infections; Maternal disorders; Cirrhosis and other chronic liver diseases; HIV/AIDS; Inflammatory bowel disease; Schistosomiasis; Malaria
YLDs per 100,000 population
0 to <0
0 to <0.05
0.05 to <0.27
0.27 to <0.96
0.96 to <1.5
1.5 to <3.69
3.69 to <8.39
8.39 to <12.31
12.31 to <19.89
19.89 to 179.94

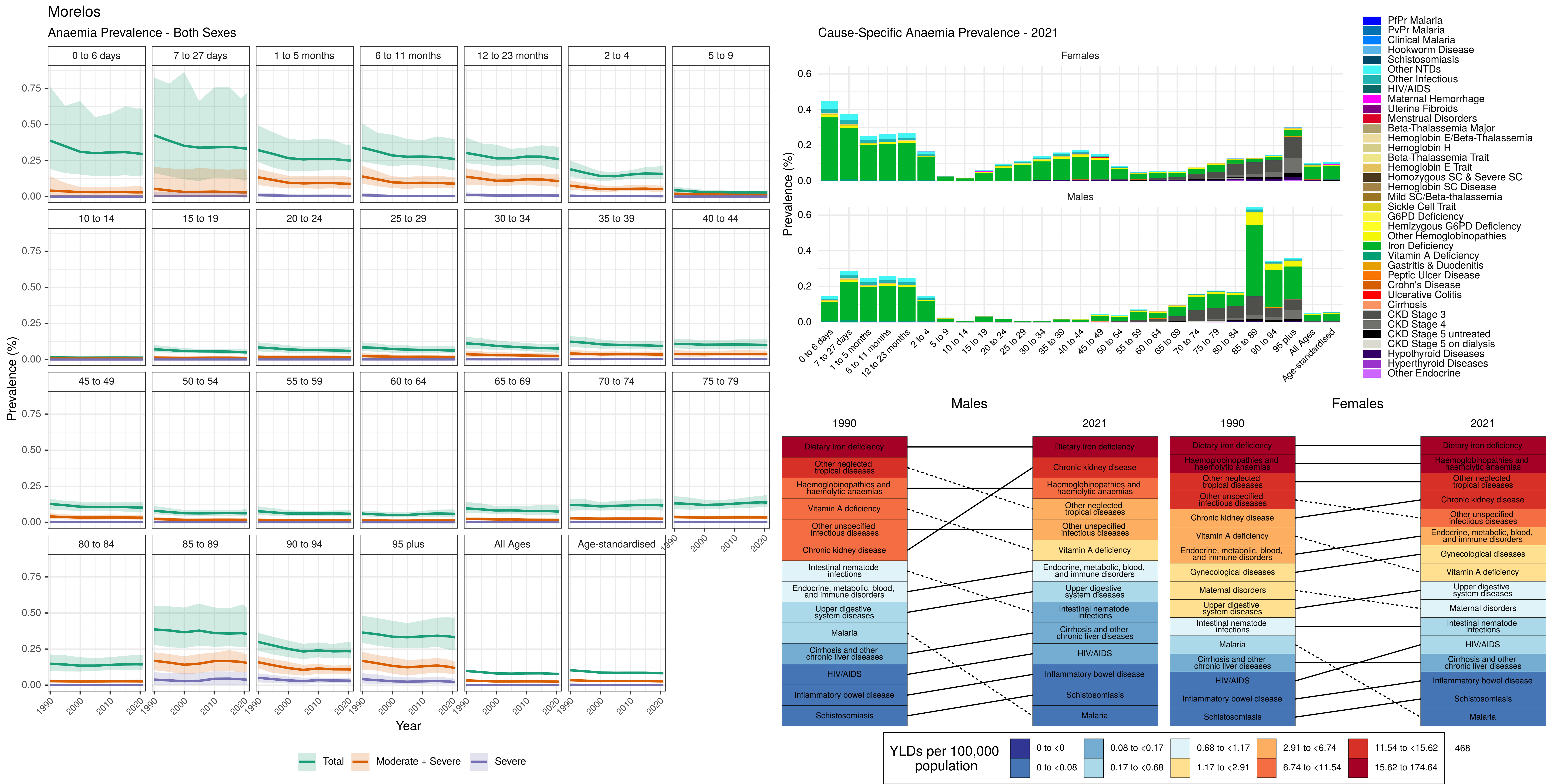
Morelos
Anaemia Prevalence - Both Sexes
0 to 6 days
7 to 27 days
1 to 5 months
6 to 11 months
12 to 23 months
2 to 4
5 to 9
10 to 14
15 to 19
20 to 24
25 to 29
30 to 34
35 to 39
40 to 44
45 to 49
50 to 54
55 to 59
60 to 64
65 to 69
70 to 74
75 to 79
80 to 84
85 to 89
90 to 94
95 plus
All Ages
Age-standardised
0.00
0.25
0.50
0.75
Prevalence (%)
1990
2000
2010
2020
Year
Total
Moderate + Severe
Severe
Cause-Specific Anaemia Prevalence - 2021
Females
Males
Prevalence (%)
0.0
0.2
0.4
0.6
PfPr Malaria
PvPr Malaria
Clinical Malaria
Hookworm Disease
Schistosomiasis
Other NTDs
Other Infectious
HIV/AIDS
Maternal Hemorrhage
Uterine Fibroids
Menstrual Disorders
Beta-Thalassemia Major
Hemoglobin E/Beta-Thalassemia
Hemoglobin H
Beta-Thalassemia Trait
Hemoglobin E Trait
Homozygous SC & Severe SC
Hemoglobin SC Disease
Mild SC/Beta-thalassemia
Sickle Cell Trait
G6PD Deficiency
Hemizygous G6PD Deficiency
Other Hemoglobinopathies
Iron Deficiency
Vitamin A Deficiency
Gastritis & Duodenitis
Peptic Ulcer Disease
Crohn's Disease
Ulcerative Colitis
Cirrhosis
CKD Stage 3
CKD Stage 4
CKD Stage 5 untreated
CKD Stage 5 on dialysis
Hypothyroid Diseases
Hyperthyroid Diseases
Other Endocrine
Males
Females
1990
2021
Males 1990: Dietary iron deficiency; Other neglected tropical diseases; Haemoglobinopathies and haemolytic anaemias; Vitamin A deficiency; Other unspecified infectious diseases; Chronic kidney disease; Intestinal nematode infections; Endocrine, metabolic, blood, and immune disorders; Upper digestive system diseases; Malaria; Cirrhosis and other chronic liver diseases; HIV/AIDS; Inflammatory bowel disease; Schistosomiasis
Males 2021: Dietary iron deficiency; Chronic kidney disease; Haemoglobinopathies and haemolytic anaemias; Other neglected tropical diseases; Other unspecified infectious diseases; Vitamin A deficiency; Endocrine, metabolic, blood, and immune disorders; Upper digestive system diseases; Intestinal nematode infections; Cirrhosis and other chronic liver diseases; HIV/AIDS; Inflammatory bowel disease; Schistosomiasis; Malaria
Females 1990: Dietary iron deficiency; Haemoglobinopathies and haemolytic anaemias; Other neglected tropical diseases; Other unspecified infectious diseases; Chronic kidney disease; Vitamin A deficiency; Endocrine, metabolic, blood, and immune disorders; Gynecological diseases; Maternal disorders; Upper digestive system diseases; Intestinal nematode infections; Malaria; Cirrhosis and other chronic liver diseases; HIV/AIDS; Inflammatory bowel disease; Schistosomiasis
Females 2021: Dietary iron deficiency; Haemoglobinopathies and haemolytic anaemias; Other neglected tropical diseases; Chronic kidney disease; Other unspecified infectious diseases; Endocrine, metabolic, blood, and immune disorders; Gynecological diseases; Vitamin A deficiency; Upper digestive system diseases; Maternal disorders; Intestinal nematode infections; HIV/AIDS; Cirrhosis and other chronic liver diseases; Inflammatory bowel disease; Schistosomiasis; Malaria
YLDs per 100,000 population
0 to <0
0 to <0.08
0.08 to <0.17
0.17 to <0.68
0.68 to <1.17
1.17 to <2.91
2.91 to <6.74
6.74 to <11.54
11.54 to <15.62
15.62 to 174.64

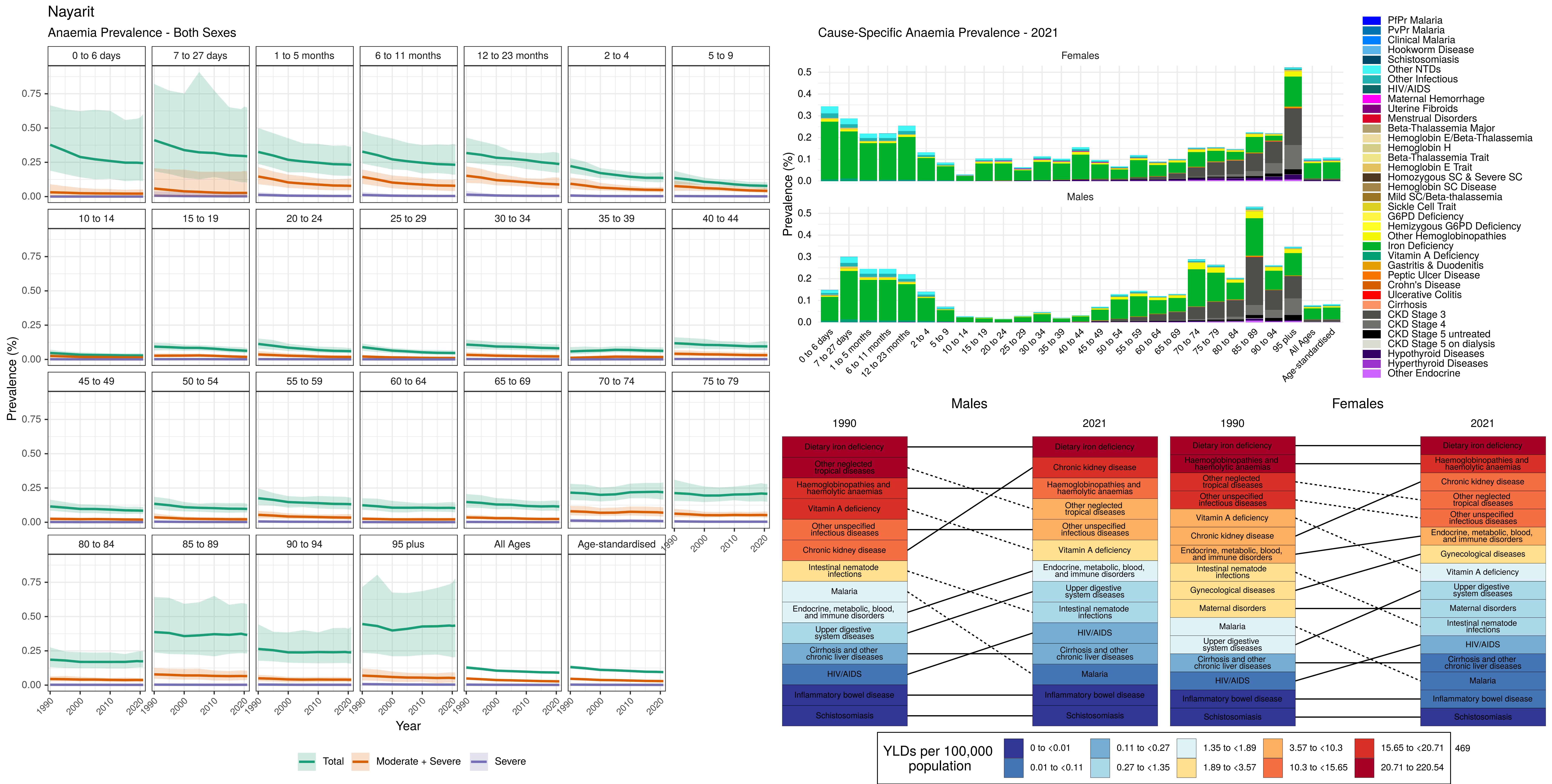
Nayarit
Anaemia Prevalence - Both Sexes
0 to 6 days
7 to 27 days
1 to 5 months
6 to 11 months
12 to 23 months
2 to 4
5 to 9
10 to 14
15 to 19
20 to 24
25 to 29
30 to 34
35 to 39
40 to 44
45 to 49
50 to 54
55 to 59
60 to 64
65 to 69
70 to 74
75 to 79
80 to 84
85 to 89
90 to 94
95 plus
All Ages
Age-standardised
Prevalence (%)
Year
Total
Moderate + Severe
Severe
Cause-Specific Anaemia Prevalence - 2021
Females
Males
Prevalence (%)
PfPr Malaria
PvPr Malaria
Clinical Malaria
Hookworm Disease
Schistosomiasis
Other NTDs
Other Infectious
HIV/AIDS
Maternal Hemorrhage
Uterine Fibroids
Menstrual Disorders
Beta-Thalassemia Major
Hemoglobin E/Beta-Thalassemia
Hemoglobin H
Beta-Thalassemia Trait
Hemoglobin E Trait
Homozygous SC & Severe SC
Hemoglobin SC Disease
Mild SC/Beta-thalassemia
Sickle Cell Trait
G6PD Deficiency
Hemizygous G6PD Deficiency
Other Hemoglobinopathies
Iron Deficiency
Vitamin A Deficiency
Gastritis & Duodenitis
Peptic Ulcer Disease
Crohn's Disease
Ulcerative Colitis
Cirrhosis
CKD Stage 3
CKD Stage 4
CKD Stage 5 untreated
CKD Stage 5 on dialysis
Hypothyroid Diseases
Hyperthyroid Diseases
Other Endocrine
Males
Females
1990
2021
Dietary iron deficiency
Other neglected tropical diseases
Haemoglobinopathies and haemolytic anaemias
Vitamin A deficiency
Other unspecified infectious diseases
Chronic kidney disease
Intestinal nematode infections
Malaria
Endocrine, metabolic, blood, and immune disorders
Upper digestive system diseases
Cirrhosis and other chronic liver diseases
HIV/AIDS
Inflammatory bowel disease
Schistosomiasis
Gynecological diseases
Maternal disorders
YLDs per 100,000 population
0 to <0.01
0.01 to <0.11
0.11 to <0.27
0.27 to <1.35
1.35 to <1.89
1.89 to <3.57
3.57 to <10.3
10.3 to <15.65
15.65 to <20.71
20.71 to 220.54
469

Nuevo León

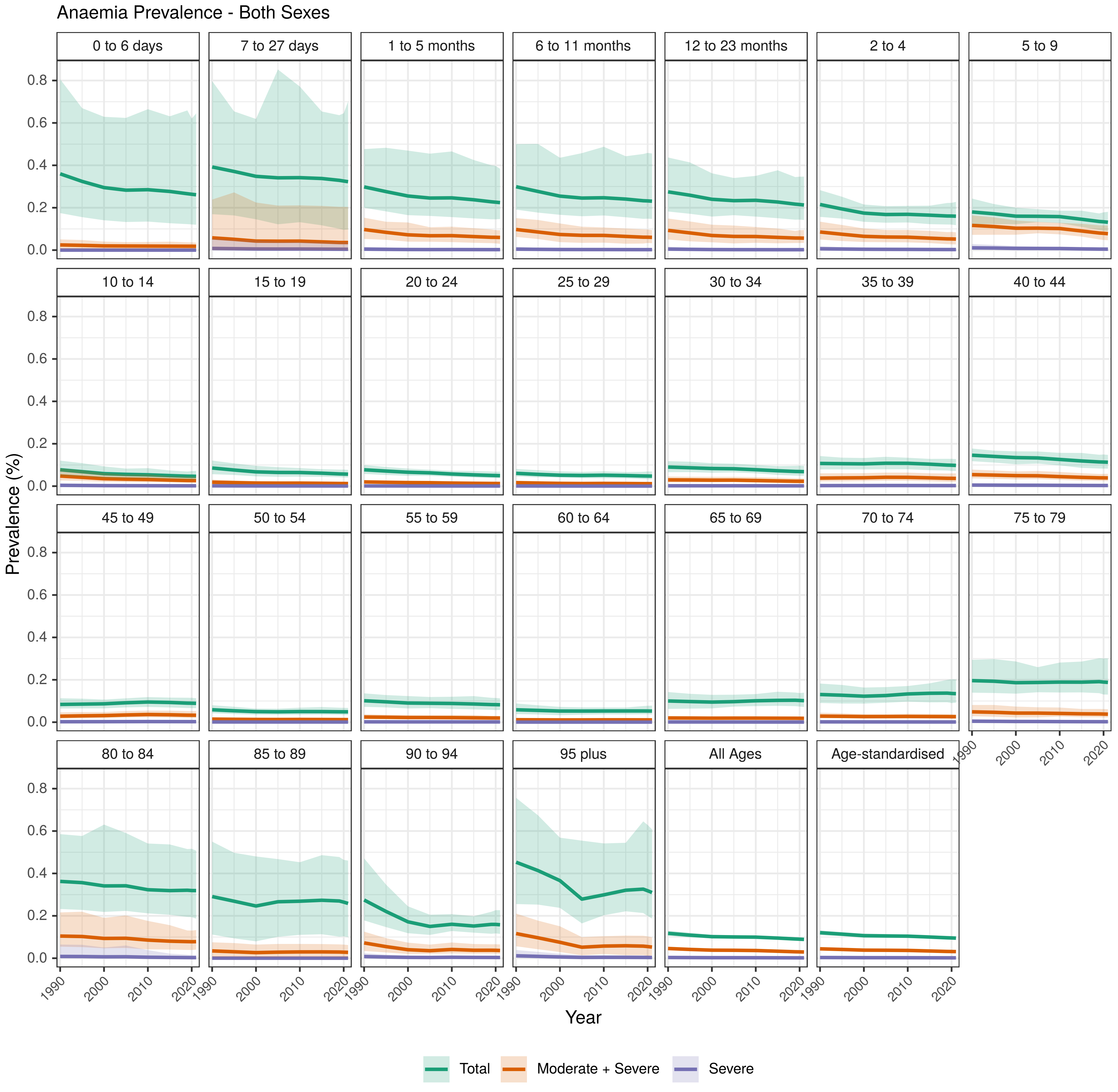
Anaemia Prevalence - Both Sexes
0 to 6 days
7 to 27 days
1 to 5 months
6 to 11 months
12 to 23 months
2 to 4
5 to 9
10 to 14
15 to 19
20 to 24
25 to 29
30 to 34
35 to 39
40 to 44
45 to 49
50 to 54
55 to 59
60 to 64
65 to 69
70 to 74
75 to 79
80 to 84
85 to 89
90 to 94
95 plus
All Ages
Age-standardised
Prevalence (%)
Year
Total
Moderate + Severe
Severe

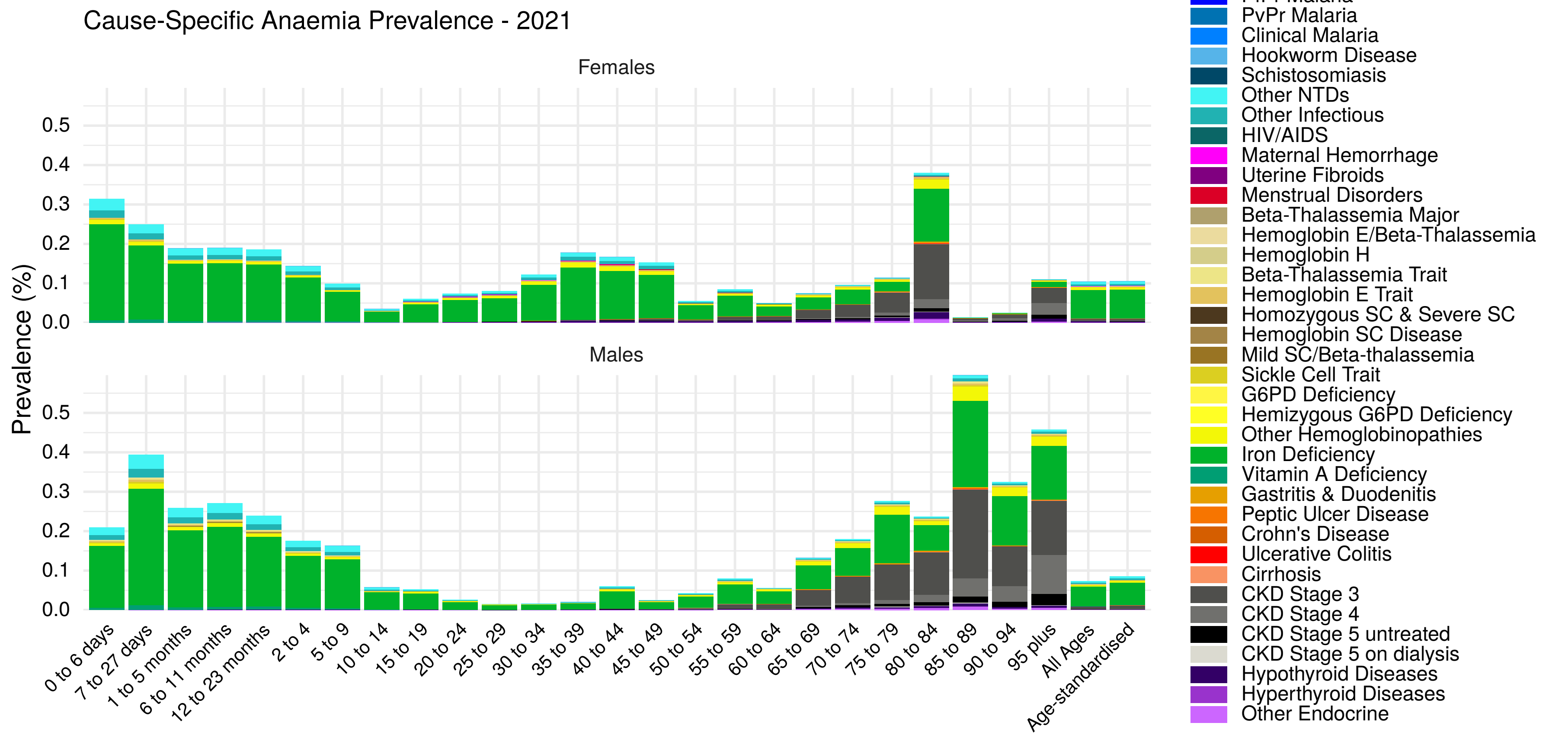
Cause-Specific Anaemia Prevalence - 2021
Females
Males
Prevalence (%)
PfPr Malaria
PvPr Malaria
Clinical Malaria
Hookworm Disease
Schistosomiasis
Other NTDs
Other Infectious
HIV/AIDS
Maternal Hemorrhage
Uterine Fibroids
Menstrual Disorders
Beta-Thalassemia Major
Hemoglobin E/Beta-Thalassemia
Hemoglobin H
Beta-Thalassemia Trait
Hemoglobin E Trait
Homozygous SC & Severe SC
Hemoglobin SC Disease
Mild SC/Beta-thalassemia
Sickle Cell Trait
G6PD Deficiency
Hemizygous G6PD Deficiency
Other Hemoglobinopathies
Iron Deficiency
Vitamin A Deficiency
Gastritis & Duodenitis
Peptic Ulcer Disease
Crohn's Disease
Ulcerative Colitis
Cirrhosis
CKD Stage 3
CKD Stage 4
CKD Stage 5 untreated
CKD Stage 5 on dialysis
Hypothyroid Diseases
Hyperthyroid Diseases
Other Endocrine

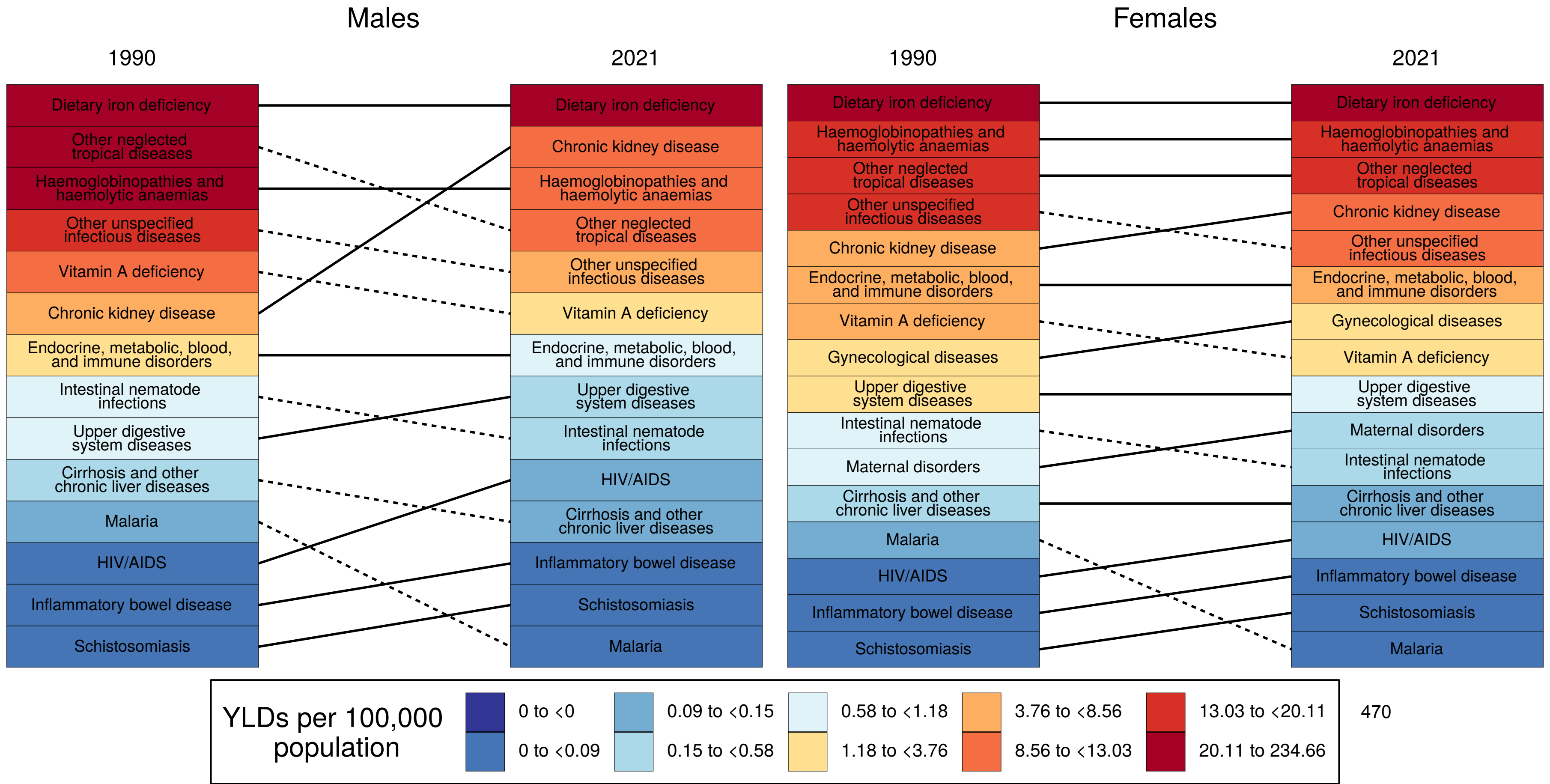
Males
Females
1990
2021
Dietary iron deficiency
Other neglected tropical diseases
Haemoglobinopathies and haemolytic anaemias
Other unspecified infectious diseases
Vitamin A deficiency
Chronic kidney disease
Endocrine, metabolic, blood, and immune disorders
Intestinal nematode infections
Upper digestive system diseases
Cirrhosis and other chronic liver diseases
Malaria
HIV/AIDS
Inflammatory bowel disease
Schistosomiasis
Gynecological diseases
Maternal disorders
YLDs per 100,000 population
0 to <0
0 to <0.09
0.09 to <0.15
0.15 to <0.58
0.58 to <1.18
1.18 to <3.76
3.76 to <8.56
8.56 to <13.03
13.03 to <20.11
20.11 to 234.66
470

Oaxaca

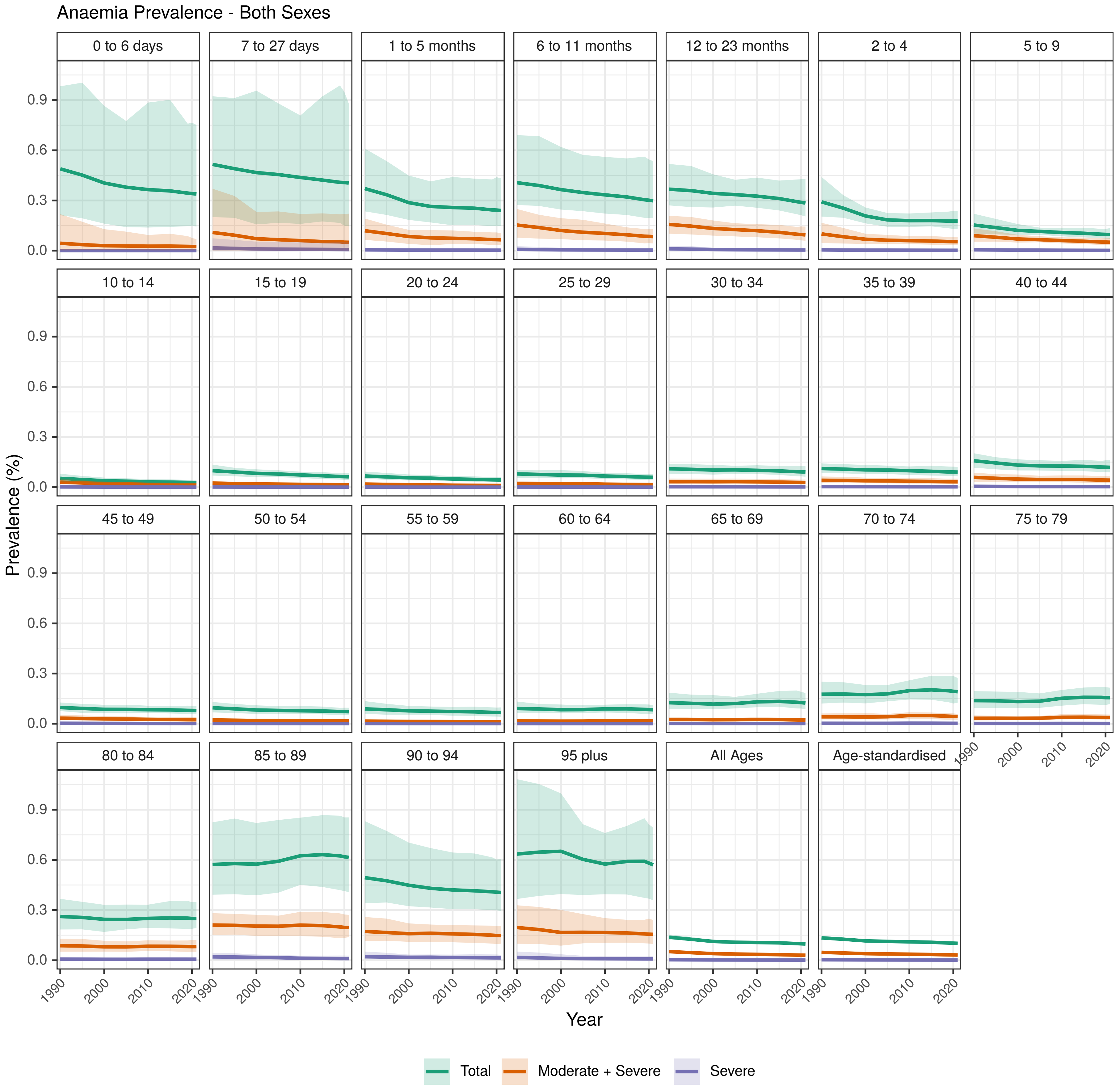

Anaemia Prevalence - Both Sexes
0 to 6 days
7 to 27 days
1 to 5 months
6 to 11 months
12 to 23 months
2 to 4
5 to 9
10 to 14
15 to 19
20 to 24
25 to 29
30 to 34
35 to 39
40 to 44
45 to 49
50 to 54
55 to 59
60 to 64
65 to 69
70 to 74
75 to 79
80 to 84
85 to 89
90 to 94
95 plus
All Ages
Age-standardised
Prevalence (%)
0.0
0.3
0.6
0.9
1990
2000
2010
2020
Year
Total
Moderate + Severe
Severe

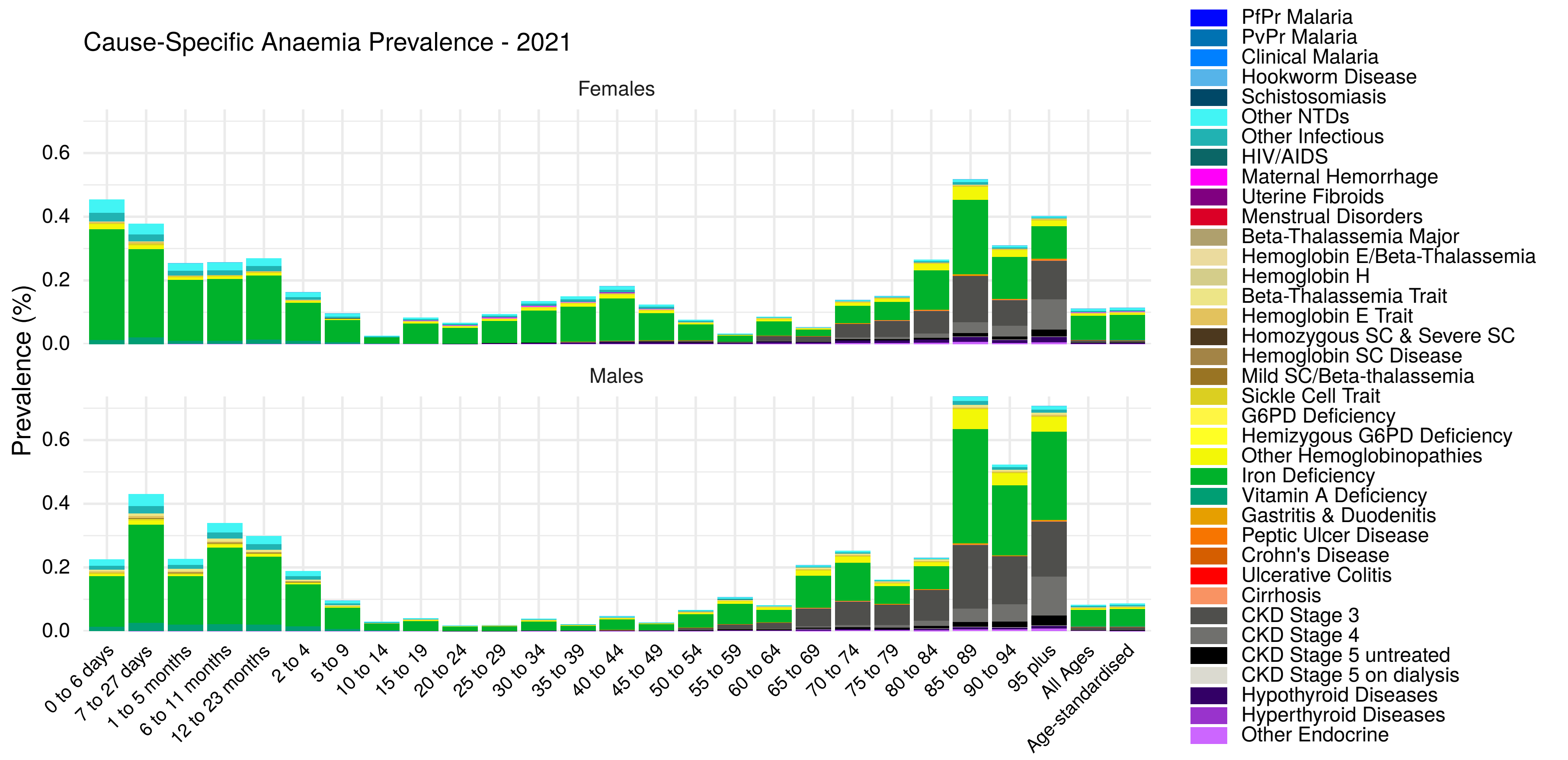

Cause-Specific Anaemia Prevalence - 2021
Females
Males
Prevalence (%)
0.0
0.2
0.4
0.6
0 to 6 days
7 to 27 days
1 to 5 months
6 to 11 months
12 to 23 months
2 to 4
5 to 9
10 to 14
15 to 19
20 to 24
25 to 29
30 to 34
35 to 39
40 to 44
45 to 49
50 to 54
55 to 59
60 to 64
65 to 69
70 to 74
75 to 79
80 to 84
85 to 89
90 to 94
95 plus
All Ages
Age-standardised
PfPr Malaria
PvPr Malaria
Clinical Malaria
Hookworm Disease
Schistosomiasis
Other NTDs
Other Infectious
HIV/AIDS
Maternal Hemorrhage
Uterine Fibroids
Menstrual Disorders
Beta-Thalassemia Major
Hemoglobin E/Beta-Thalassemia
Hemoglobin H
Beta-Thalassemia Trait
Hemoglobin E Trait
Homozygous SC & Severe SC
Hemoglobin SC Disease
Mild SC/Beta-thalassemia
Sickle Cell Trait
G6PD Deficiency
Hemizygous G6PD Deficiency
Other Hemoglobinopathies
Iron Deficiency
Vitamin A Deficiency
Gastritis & Duodenitis
Peptic Ulcer Disease
Crohn's Disease
Ulcerative Colitis
Cirrhosis
CKD Stage 3
CKD Stage 4
CKD Stage 5 untreated
CKD Stage 5 on dialysis
Hypothyroid Diseases
Hyperthyroid Diseases
Other Endocrine

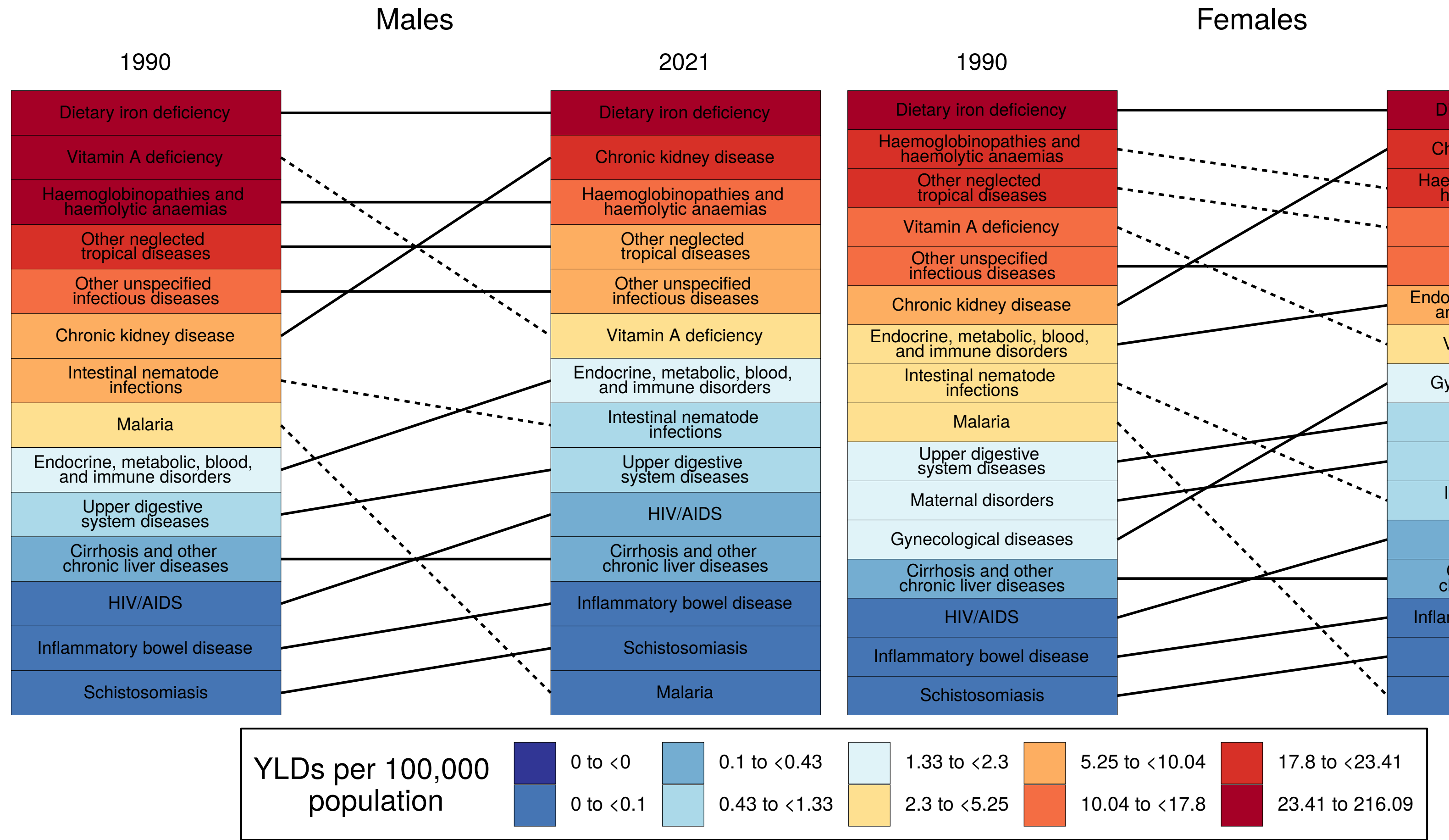

Males
Females
1990
2021
Dietary iron deficiency
Vitamin A deficiency
Haemoglobinopathies and haemolytic anaemias
Other neglected tropical diseases
Other unspecified infectious diseases
Chronic kidney disease
Intestinal nematode infections
Malaria
Endocrine, metabolic, blood, and immune disorders
Upper digestive system diseases
Cirrhosis and other chronic liver diseases
HIV/AIDS
Inflammatory bowel disease
Schistosomiasis
Maternal disorders
Gynecological diseases
YLDs per 100,000 population
0 to <0
0 to <0.1
0.1 to <0.43
0.43 to <1.33
1.33 to <2.3
2.3 to <5.25
5.25 to <10.04
10.04 to <17.8
17.8 to <23.41
23.41 to 216.09

Puebla

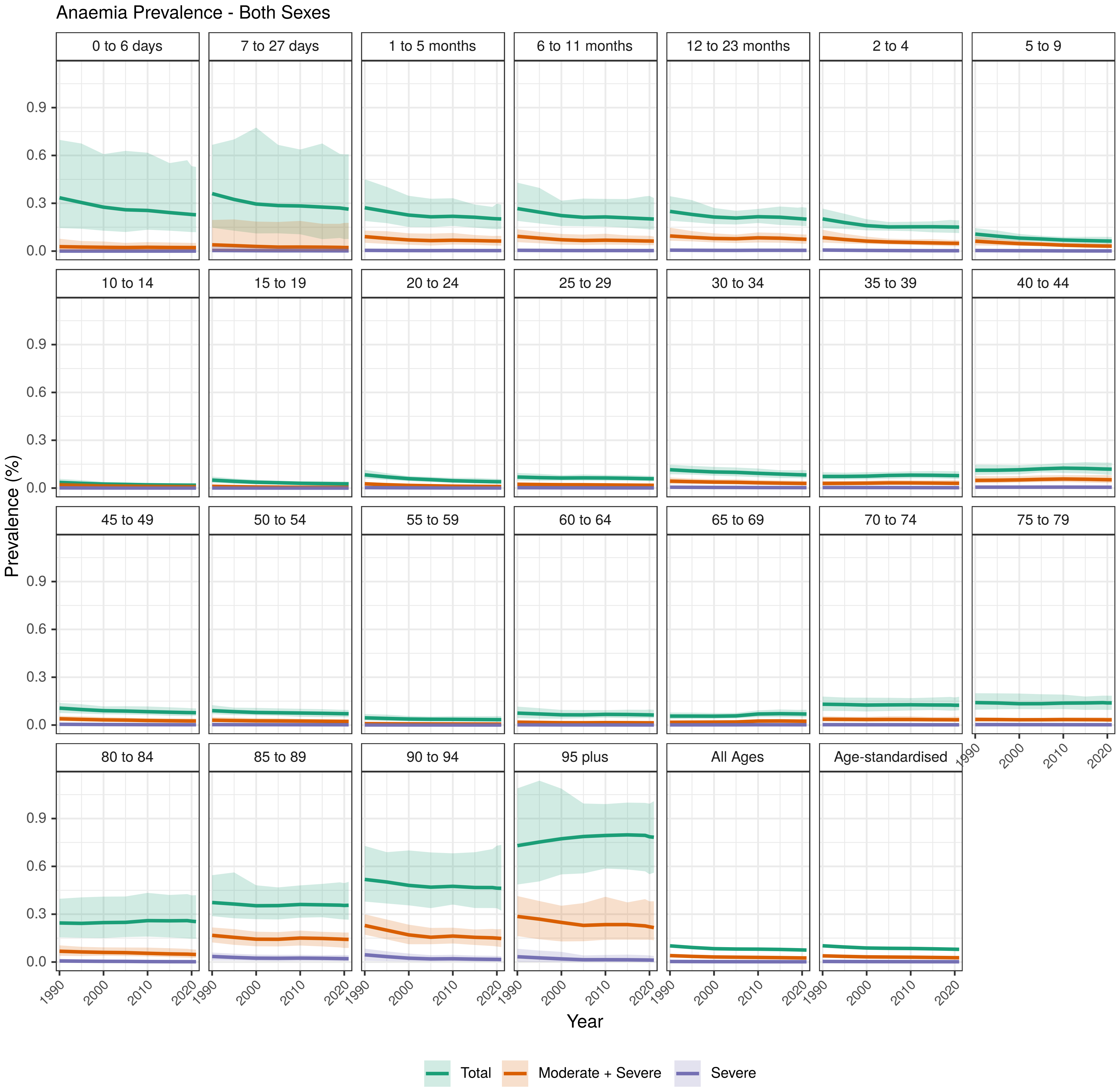
Anaemia Prevalence - Both Sexes
0 to 6 days
7 to 27 days
1 to 5 months
6 to 11 months
12 to 23 months
2 to 4
5 to 9
10 to 14
15 to 19
20 to 24
25 to 29
30 to 34
35 to 39
40 to 44
45 to 49
50 to 54
55 to 59
60 to 64
65 to 69
70 to 74
75 to 79
80 to 84
85 to 89
90 to 94
95 plus
All Ages
Age-standardised
Prevalence (%)
0.0
0.3
0.6
0.9
1990
2000
2010
2020
Year
Total
Moderate + Severe
Severe

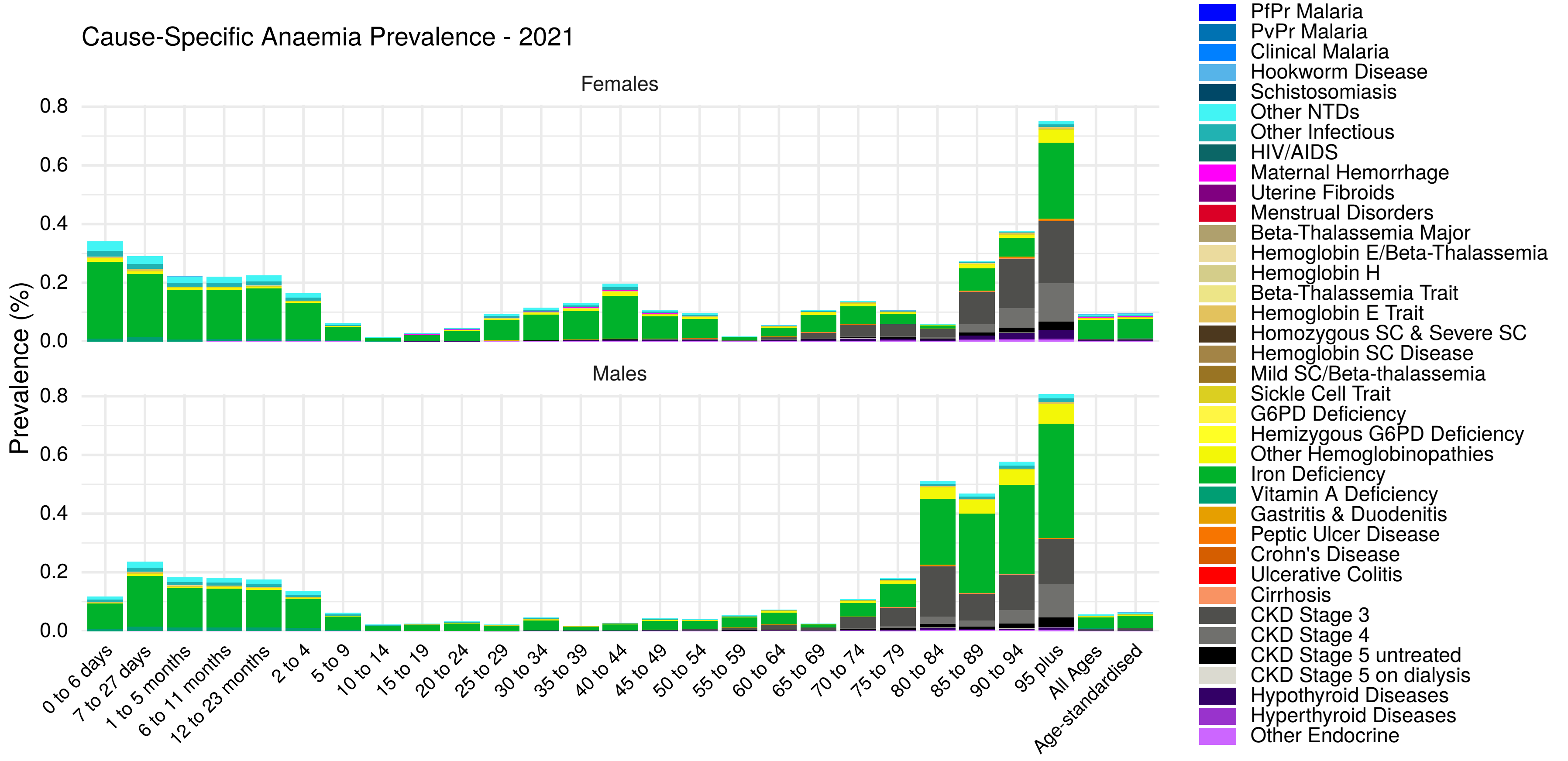
Cause-Specific Anaemia Prevalence - 2021
Females
Males
Prevalence (%)
0.0
0.2
0.4
0.6
0.8
0 to 6 days
7 to 27 days
1 to 5 months
6 to 11 months
12 to 23 months
2 to 4
5 to 9
10 to 14
15 to 19
20 to 24
25 to 29
30 to 34
35 to 39
40 to 44
45 to 49
50 to 54
55 to 59
60 to 64
65 to 69
70 to 74
75 to 79
80 to 84
85 to 89
90 to 94
95 plus
All Ages
Age-standardised
PfPr Malaria
PvPr Malaria
Clinical Malaria
Hookworm Disease
Schistosomiasis
Other NTDs
Other Infectious
HIV/AIDS
Maternal Hemorrhage
Uterine Fibroids
Menstrual Disorders
Beta-Thalassemia Major
Hemoglobin E/Beta-Thalassemia
Hemoglobin H
Beta-Thalassemia Trait
Hemoglobin E Trait
Homozygous SC & Severe SC
Hemoglobin SC Disease
Mild SC/Beta-thalassemia
Sickle Cell Trait
G6PD Deficiency
Hemizygous G6PD Deficiency
Other Hemoglobinopathies
Iron Deficiency
Vitamin A Deficiency
Gastritis & Duodenitis
Peptic Ulcer Disease
Crohn's Disease
Ulcerative Colitis
Cirrhosis
CKD Stage 3
CKD Stage 4
CKD Stage 5 untreated
CKD Stage 5 on dialysis
Hypothyroid Diseases
Hyperthyroid Diseases
Other Endocrine

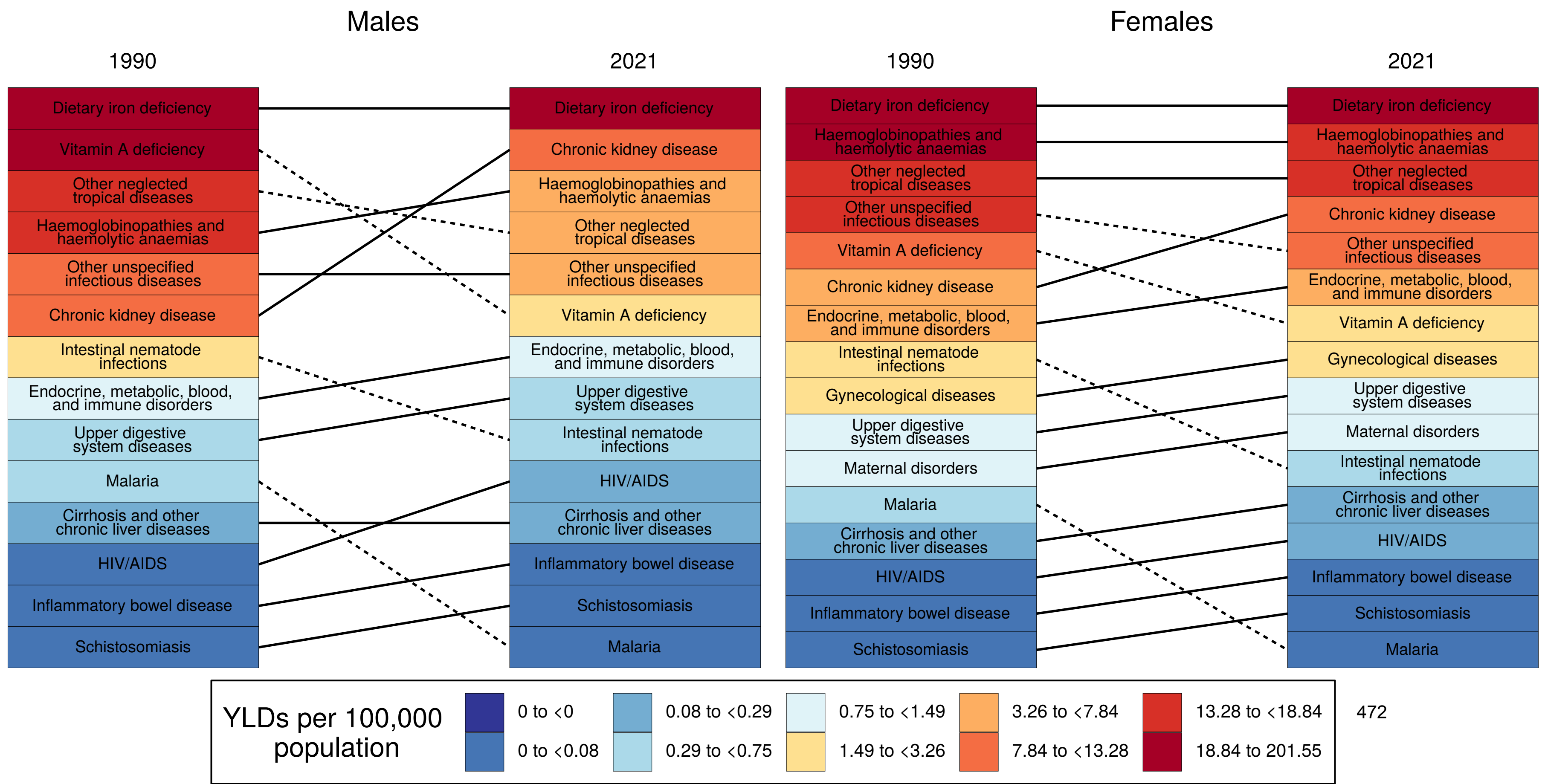
Males
Females
1990
2021
Dietary iron deficiency
Vitamin A deficiency
Other neglected tropical diseases
Haemoglobinopathies and haemolytic anaemias
Other unspecified infectious diseases
Chronic kidney disease
Intestinal nematode infections
Endocrine, metabolic, blood, and immune disorders
Upper digestive system diseases
Malaria
Cirrhosis and other chronic liver diseases
HIV/AIDS
Inflammatory bowel disease
Schistosomiasis
Gynecological diseases
Maternal disorders
YLDs per 100,000 population
0 to <0
0 to <0.08
0.08 to <0.29
0.29 to <0.75
0.75 to <1.49
1.49 to <3.26
3.26 to <7.84
7.84 to <13.28
13.28 to <18.84
18.84 to 201.55

Querétaro

Anaemia Prevalence - Both Sexes

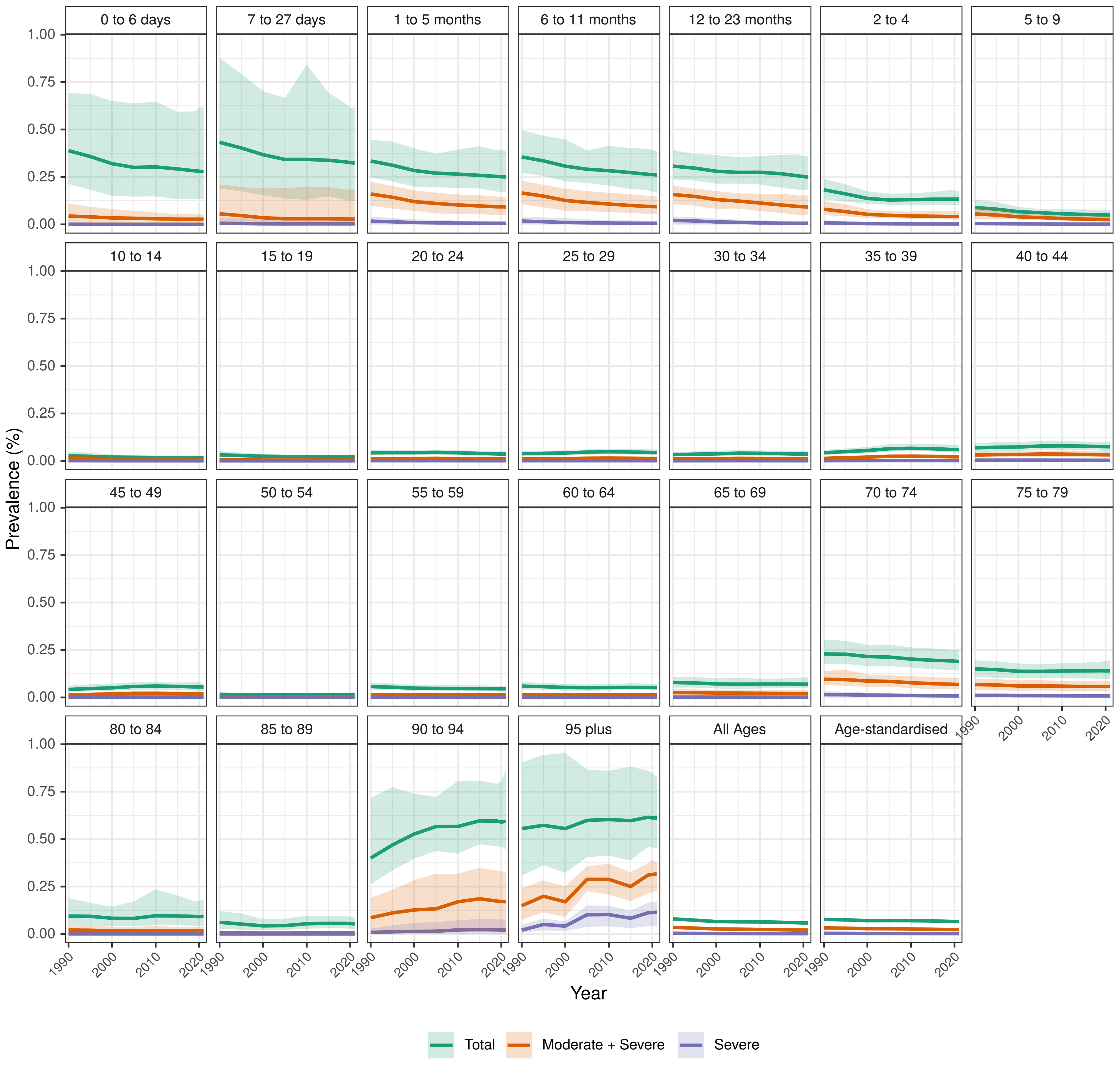

Cause-Specific Anaemia Prevalence - 2021

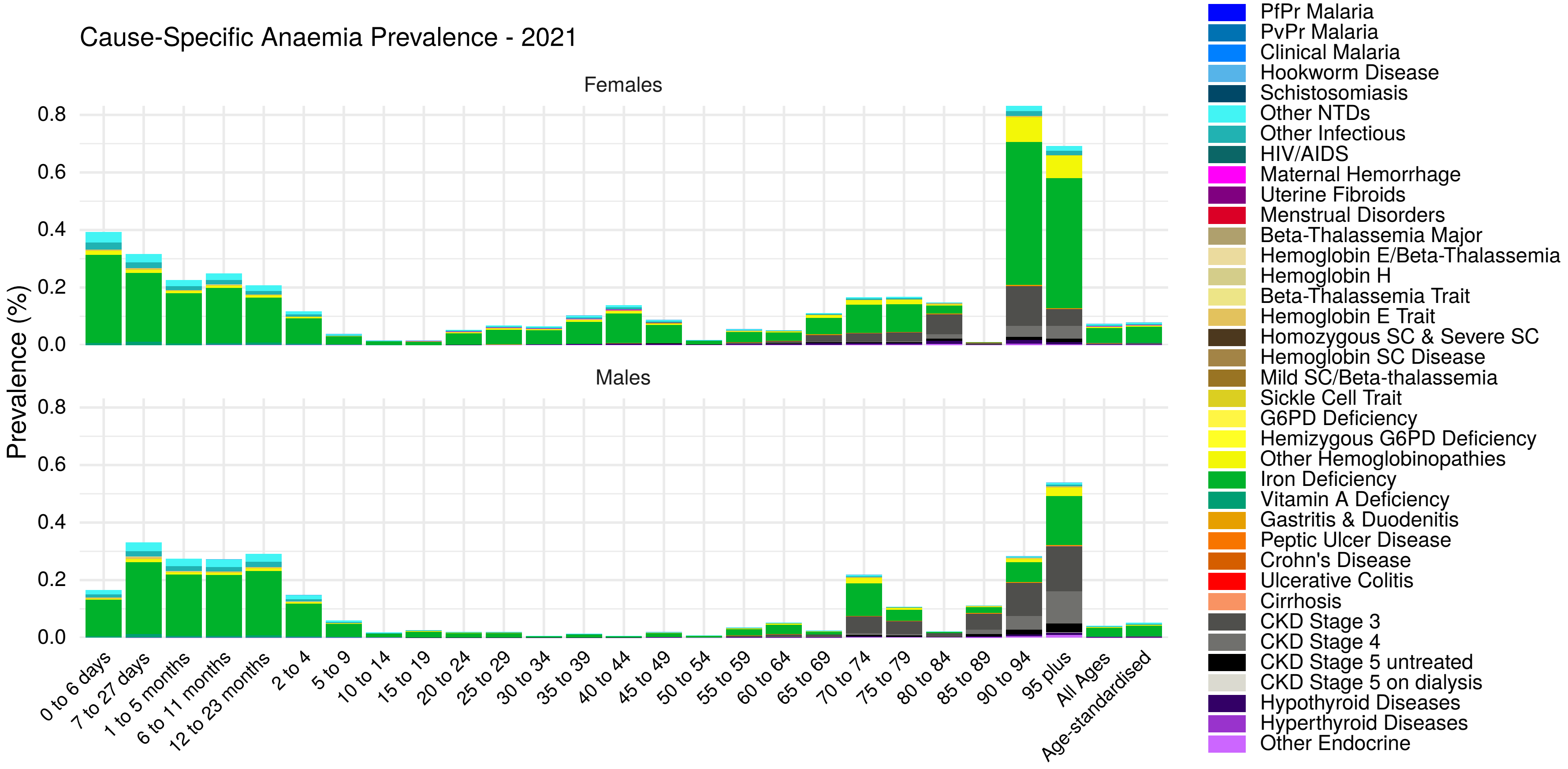

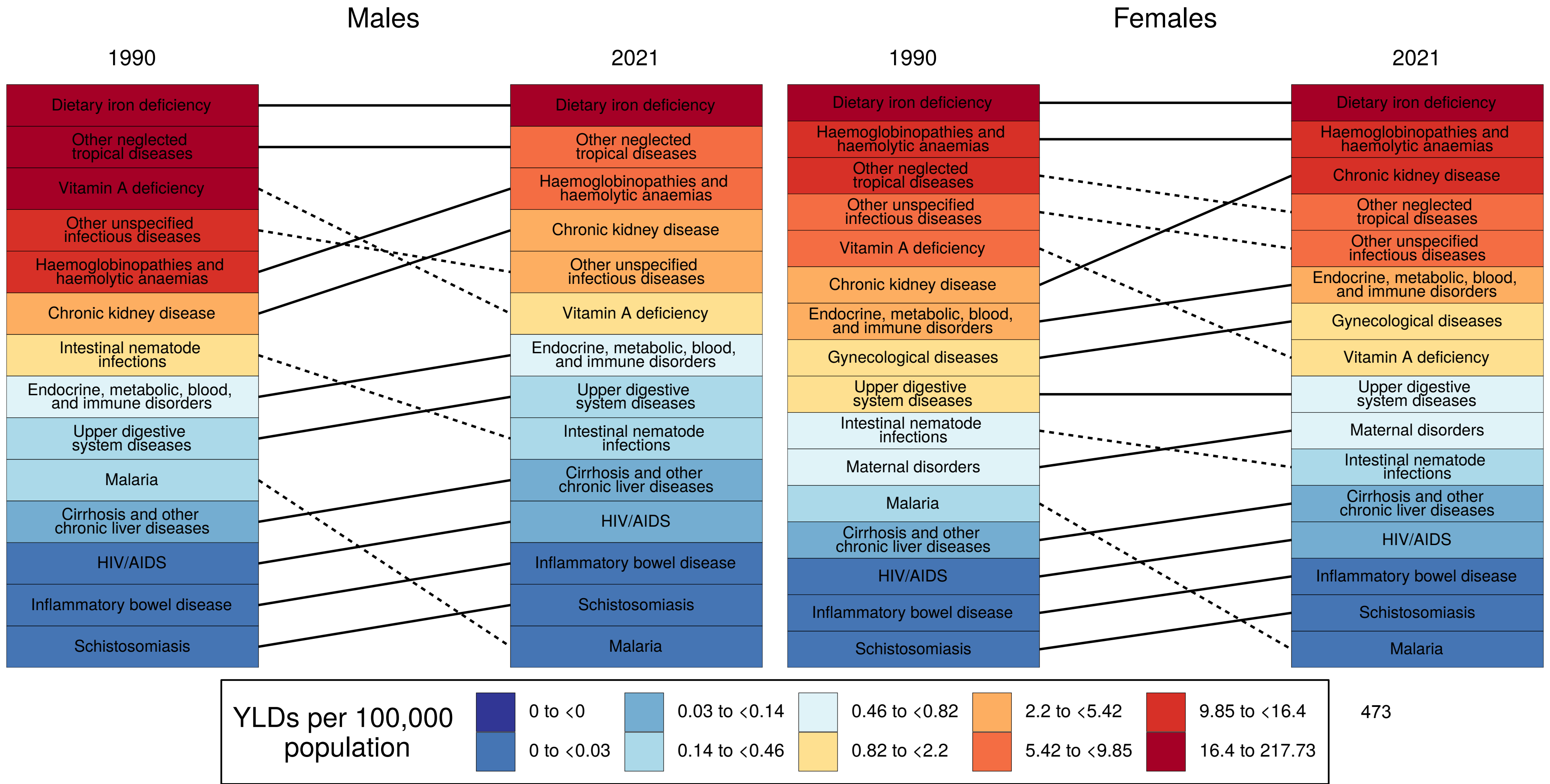

Quintana Roo

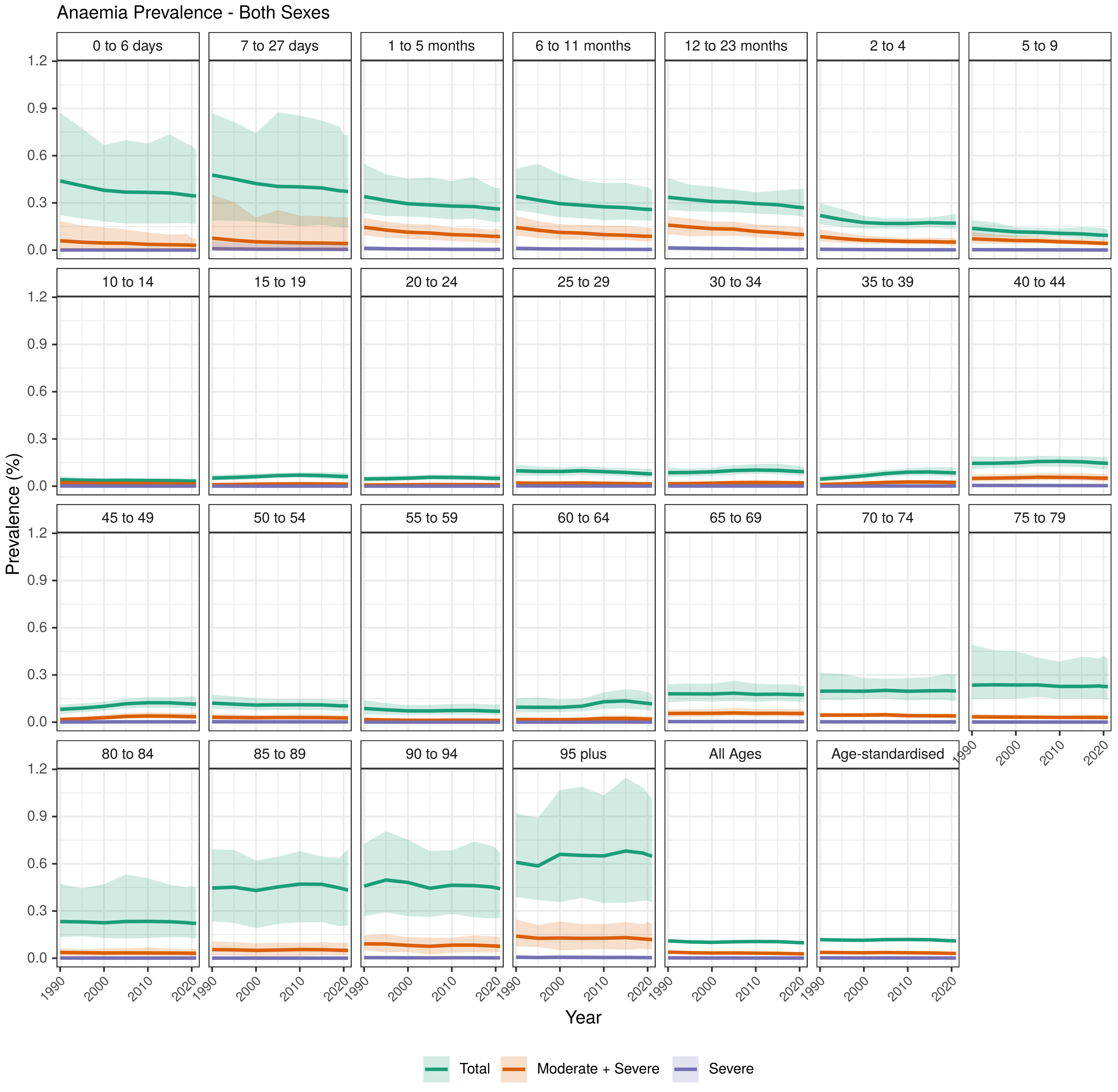

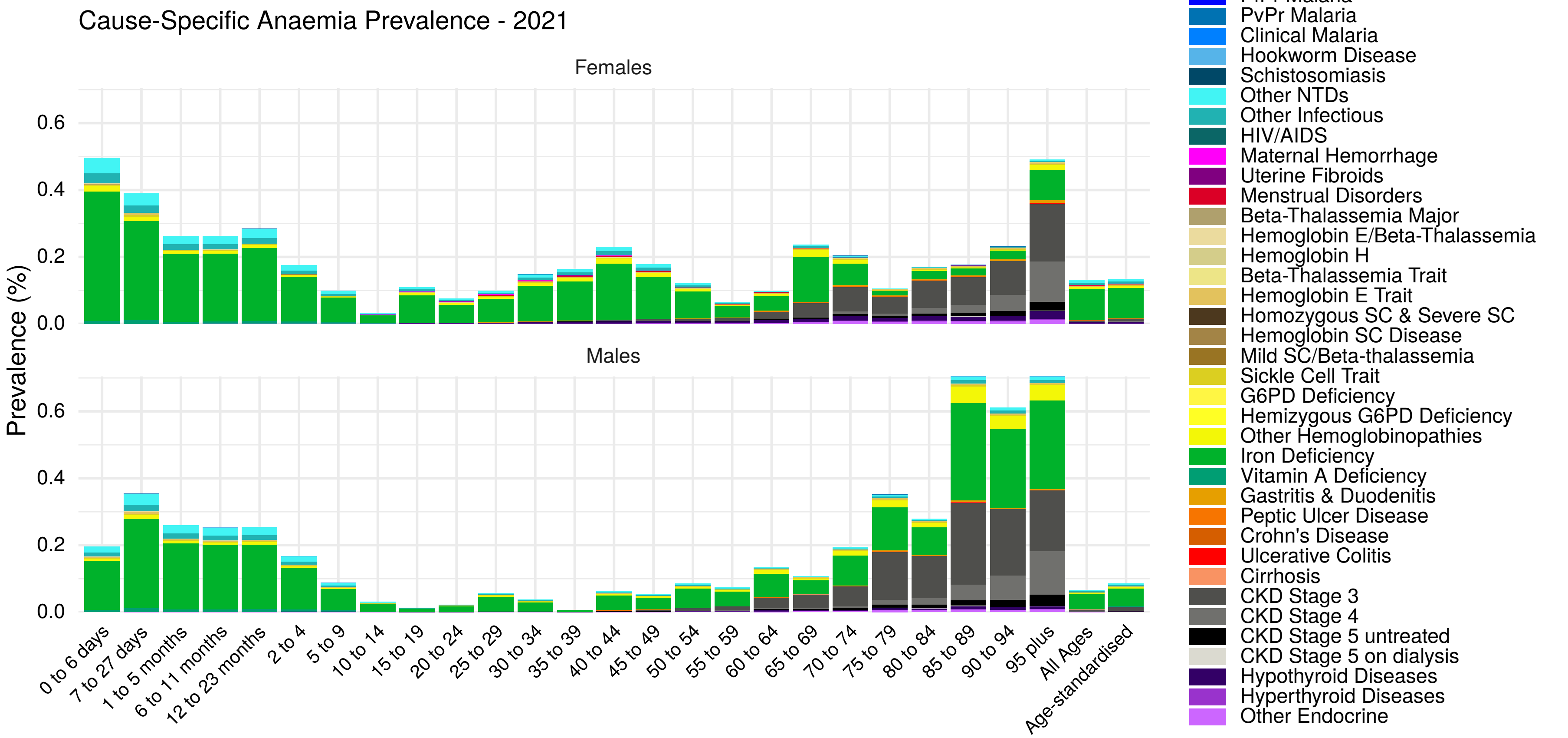

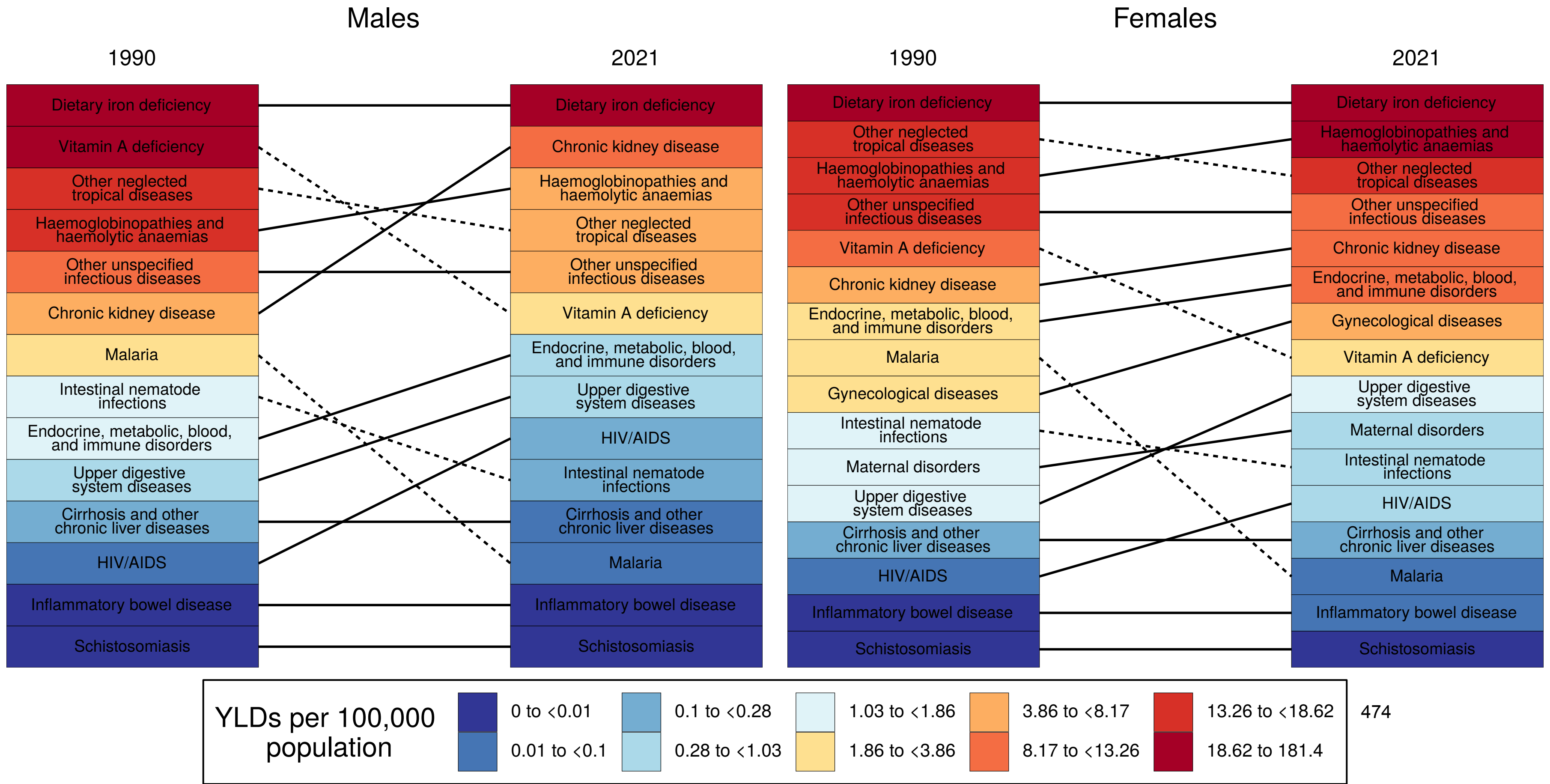

San Luis Potosí

Anaemia Prevalence - Both Sexes

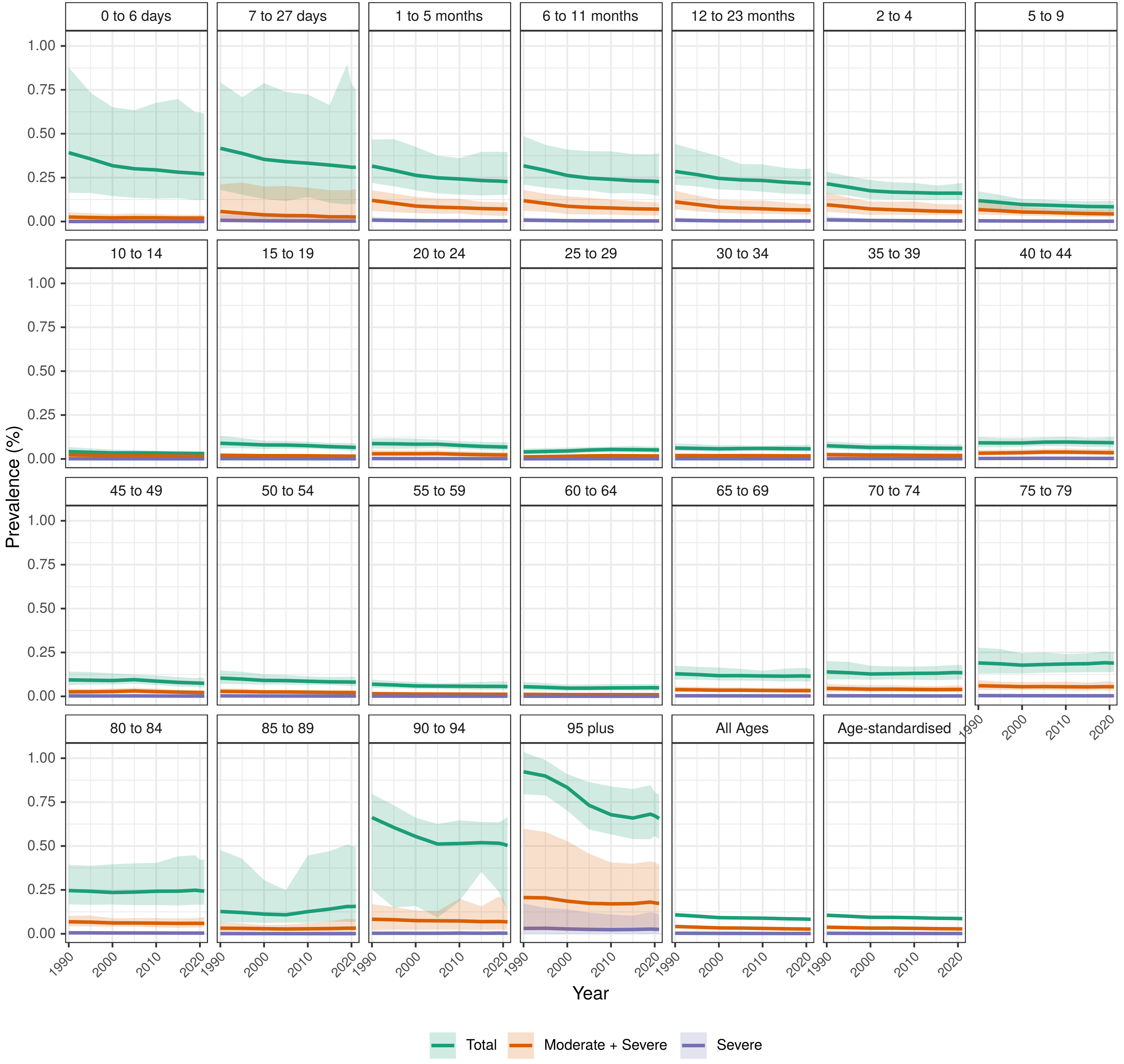

Cause-Specific Anaemia Prevalence - 2021

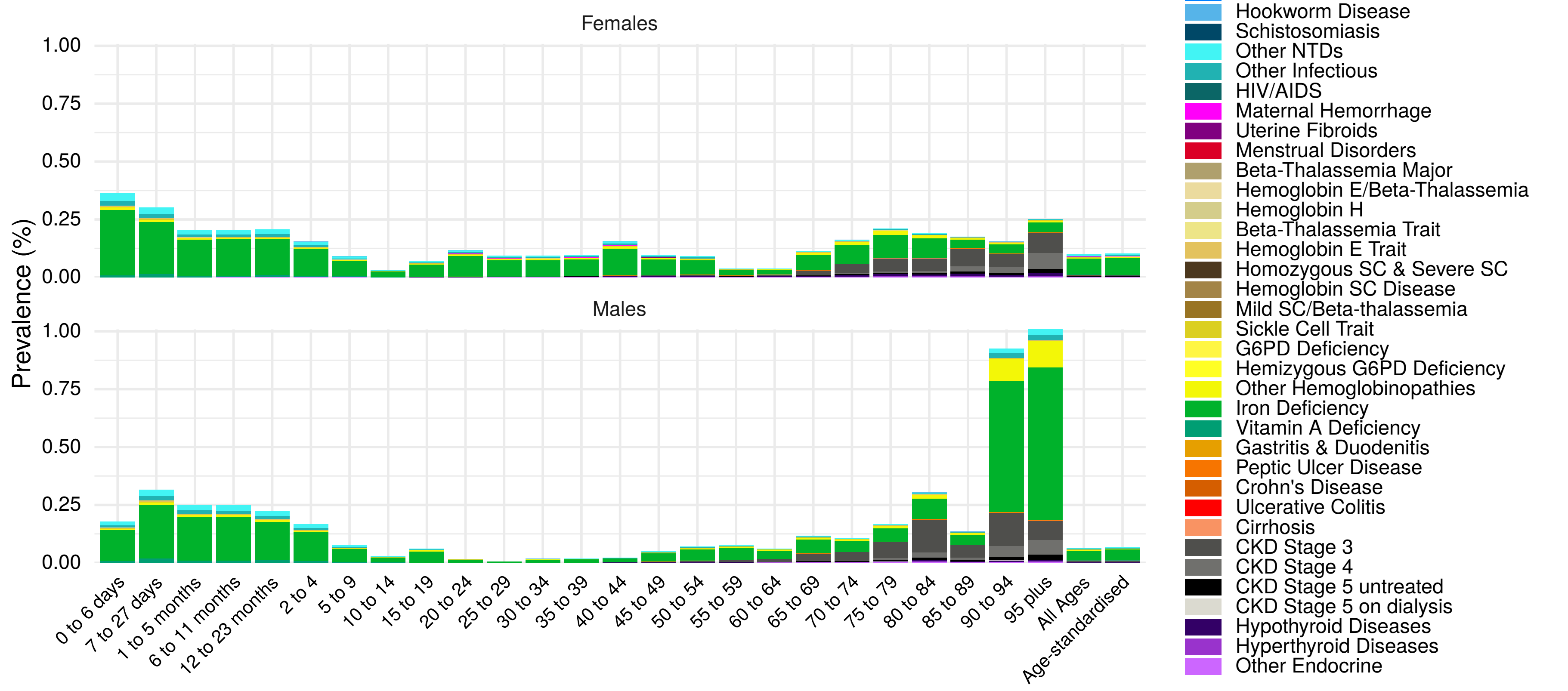

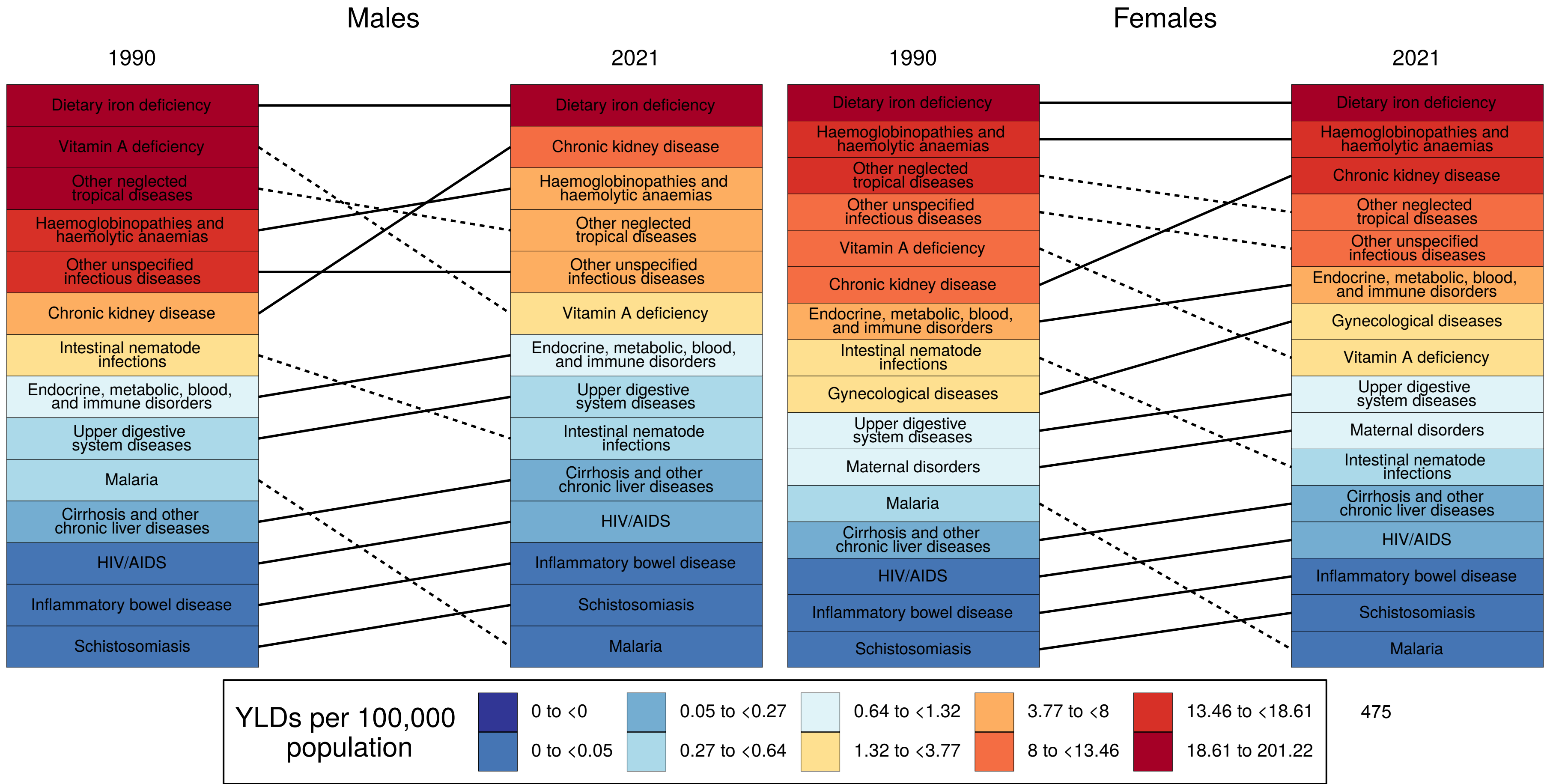

Sinaloa

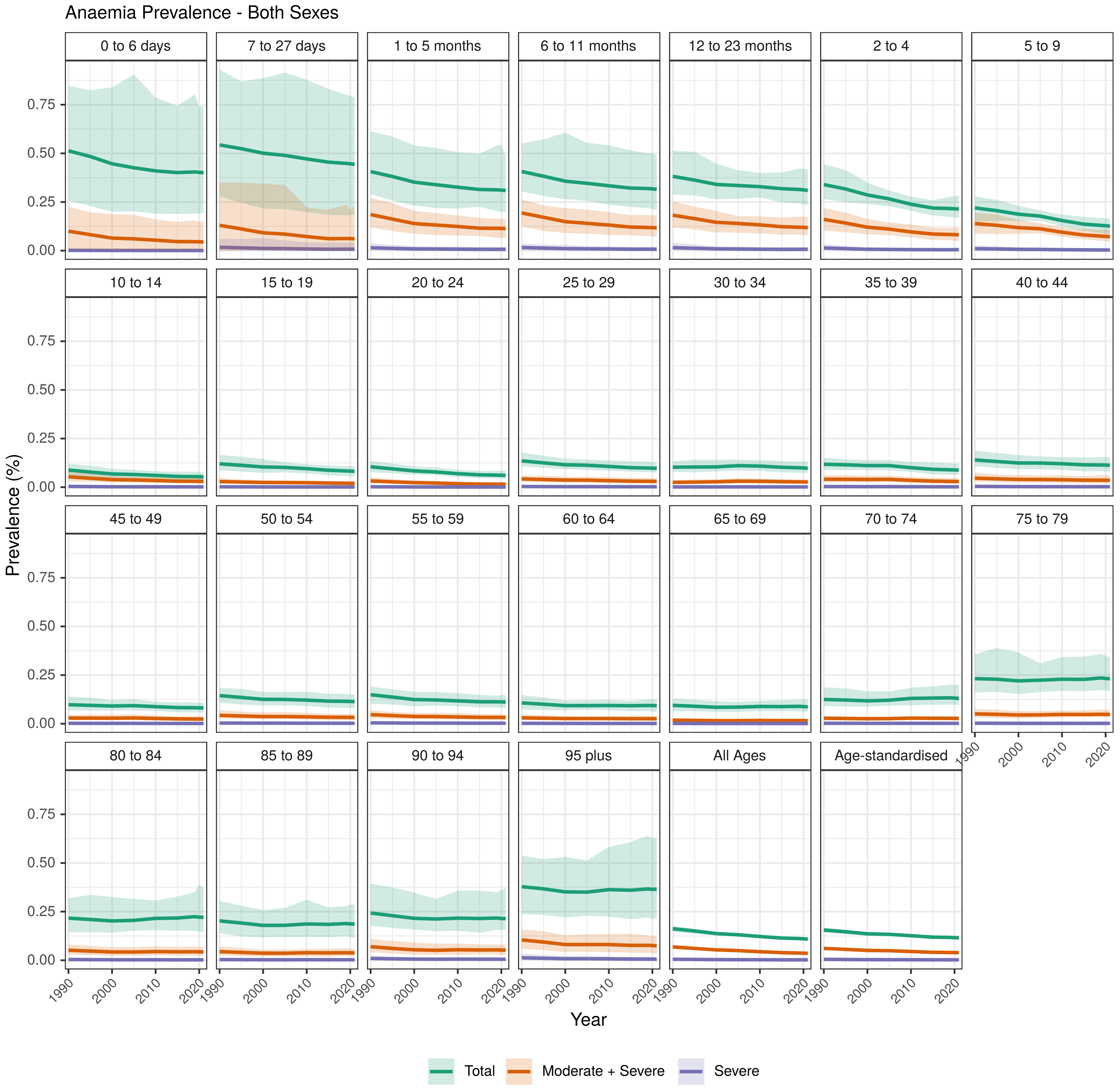
Anaemia Prevalence - Both Sexes
0 to 6 days
7 to 27 days
1 to 5 months
6 to 11 months
12 to 23 months
2 to 4
5 to 9
10 to 14
15 to 19
20 to 24
25 to 29
30 to 34
35 to 39
40 to 44
45 to 49
50 to 54
55 to 59
60 to 64
65 to 69
70 to 74
75 to 79
80 to 84
85 to 89
90 to 94
95 plus
All Ages
Age-standardised
0.00
0.25
0.50
0.75
Prevalence (%)
1990
2000
2010
2020
Year
Total
Moderate + Severe
Severe

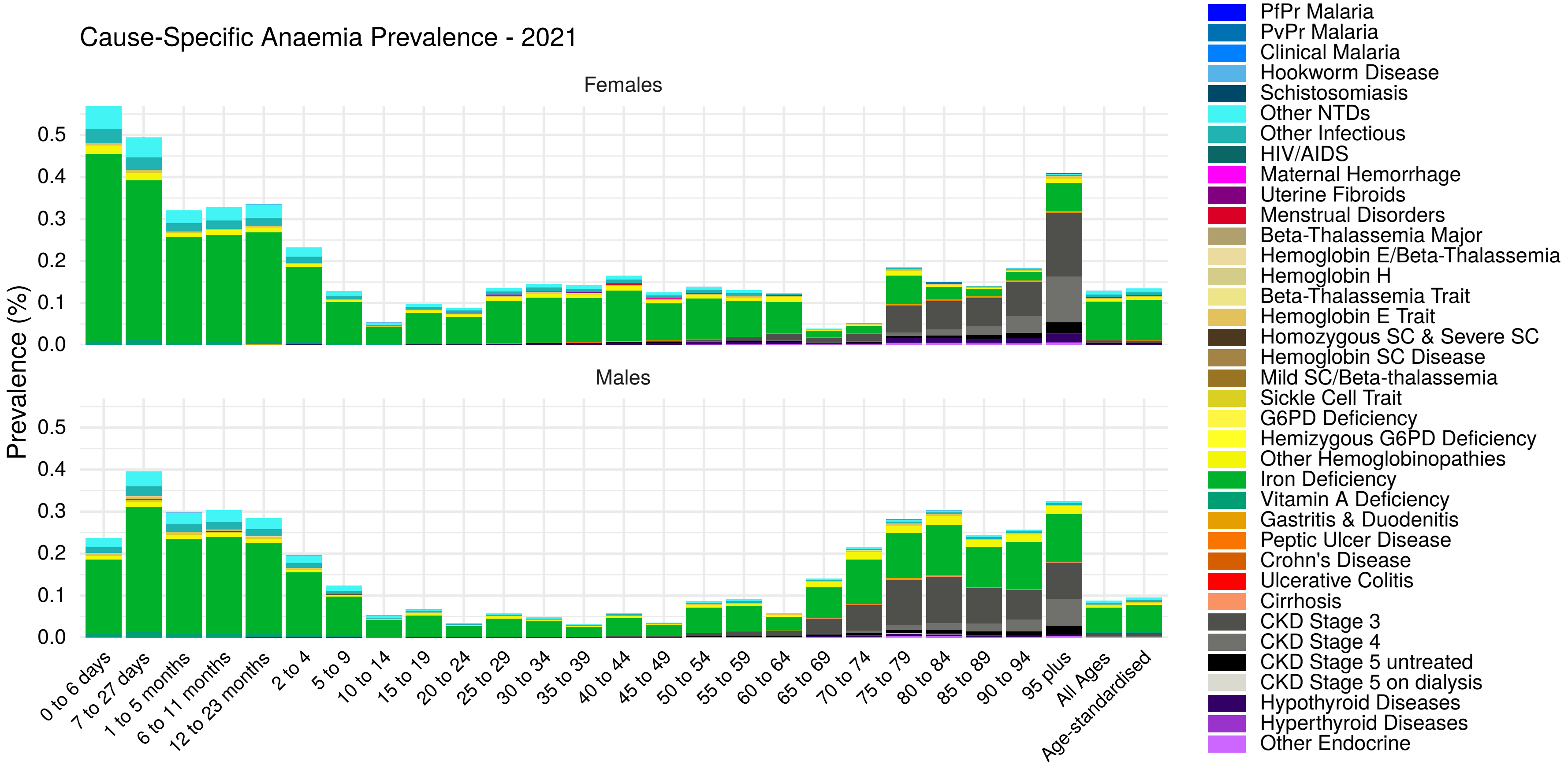
Cause-Specific Anaemia Prevalence - 2021
Females
Males
Prevalence (%)
0.0
0.1
0.2
0.3
0.4
0.5
0 to 6 days
7 to 27 days
1 to 5 months
6 to 11 months
12 to 23 months
2 to 4
5 to 9
10 to 14
15 to 19
20 to 24
25 to 29
30 to 34
35 to 39
40 to 44
45 to 49
50 to 54
55 to 59
60 to 64
65 to 69
70 to 74
75 to 79
80 to 84
85 to 89
90 to 94
95 plus
All Ages
Age-standardised
PfPr Malaria
PvPr Malaria
Clinical Malaria
Hookworm Disease
Schistosomiasis
Other NTDs
Other Infectious
HIV/AIDS
Maternal Hemorrhage
Uterine Fibroids
Menstrual Disorders
Beta-Thalassemia Major
Hemoglobin E/Beta-Thalassemia
Hemoglobin H
Beta-Thalassemia Trait
Hemoglobin E Trait
Homozygous SC & Severe SC
Hemoglobin SC Disease
Mild SC/Beta-thalassemia
Sickle Cell Trait
G6PD Deficiency
Hemizygous G6PD Deficiency
Other Hemoglobinopathies
Iron Deficiency
Vitamin A Deficiency
Gastritis & Duodenitis
Peptic Ulcer Disease
Crohn's Disease
Ulcerative Colitis
Cirrhosis
CKD Stage 3
CKD Stage 4
CKD Stage 5 untreated
CKD Stage 5 on dialysis
Hypothyroid Diseases
Hyperthyroid Diseases
Other Endocrine

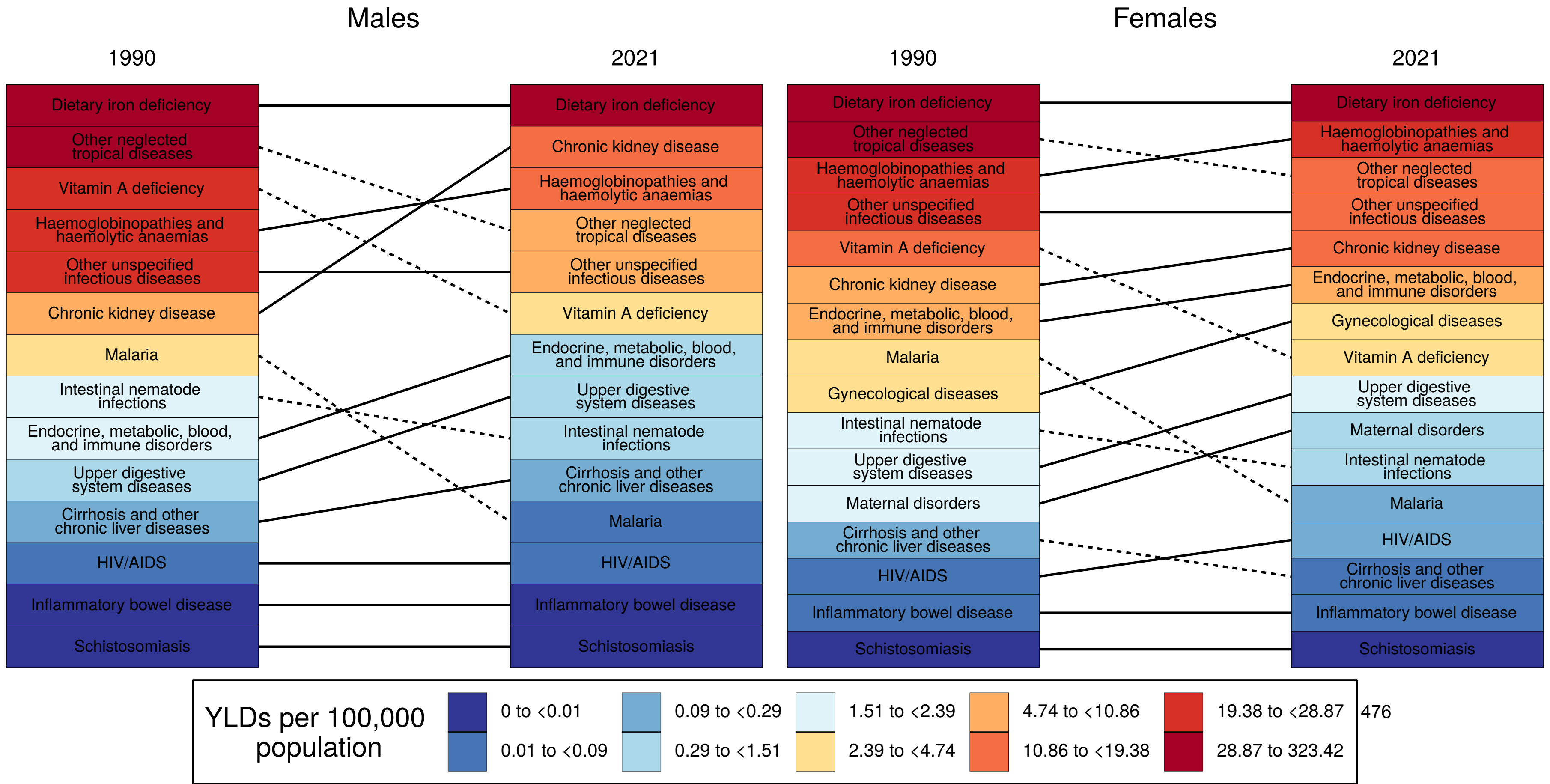
Males
Females
1990
2021
Dietary iron deficiency
Other neglected tropical diseases
Vitamin A deficiency
Haemoglobinopathies and haemolytic anaemias
Other unspecified infectious diseases
Chronic kidney disease
Malaria
Intestinal nematode infections
Endocrine, metabolic, blood, and immune disorders
Upper digestive system diseases
Cirrhosis and other chronic liver diseases
HIV/AIDS
Inflammatory bowel disease
Schistosomiasis
Gynecological diseases
Maternal disorders
YLDs per 100,000 population
0 to <0.01
0.01 to <0.09
0.09 to <0.29
0.29 to <1.51
1.51 to <2.39
2.39 to <4.74
4.74 to <10.86
10.86 to <19.38
19.38 to <28.87
28.87 to 323.42

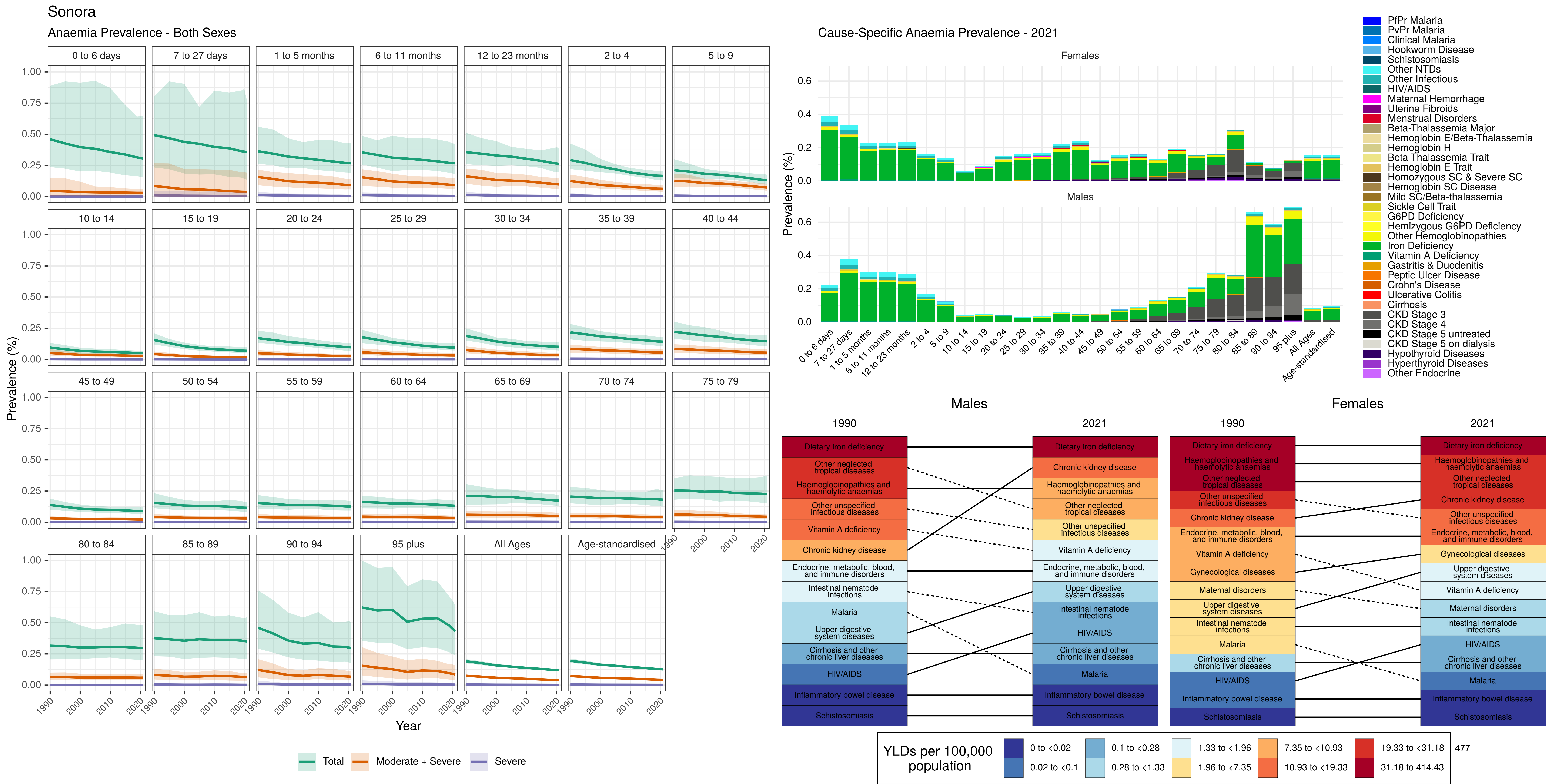
Sonora
Anaemia Prevalence - Both Sexes
0 to 6 days
7 to 27 days
1 to 5 months
6 to 11 months
12 to 23 months
2 to 4
5 to 9
10 to 14
15 to 19
20 to 24
25 to 29
30 to 34
35 to 39
40 to 44
45 to 49
50 to 54
55 to 59
60 to 64
65 to 69
70 to 74
75 to 79
80 to 84
85 to 89
90 to 94
95 plus
All Ages
Age-standardised
Prevalence (%)
Year
Total
Moderate + Severe
Severe
Cause-Specific Anaemia Prevalence - 2021
Females
Males
PfPr Malaria
PvPr Malaria
Clinical Malaria
Hookworm Disease
Schistosomiasis
Other NTDs
Other Infectious
HIV/AIDS
Maternal Hemorrhage
Uterine Fibroids
Menstrual Disorders
Beta-Thalassemia Major
Hemoglobin E/Beta-Thalassemia
Hemoglobin H
Beta-Thalassemia Trait
Hemoglobin E Trait
Homozygous SC & Severe SC
Hemoglobin SC Disease
Mild SC/Beta-thalassemia
Sickle Cell Trait
G6PD Deficiency
Hemizygous G6PD Deficiency
Other Hemoglobinopathies
Iron Deficiency
Vitamin A Deficiency
Gastritis & Duodenitis
Peptic Ulcer Disease
Crohn's Disease
Ulcerative Colitis
Cirrhosis
CKD Stage 3
CKD Stage 4
CKD Stage 5 untreated
CKD Stage 5 on dialysis
Hypothyroid Diseases
Hyperthyroid Diseases
Other Endocrine
Males
Females
1990
2021
Dietary iron deficiency
Other neglected tropical diseases
Haemoglobinopathies and haemolytic anaemias
Other unspecified infectious diseases
Vitamin A deficiency
Chronic kidney disease
Endocrine, metabolic, blood, and immune disorders
Intestinal nematode infections
Malaria
Upper digestive system diseases
Cirrhosis and other chronic liver diseases
HIV/AIDS
Inflammatory bowel disease
Schistosomiasis
Gynecological diseases
Maternal disorders
YLDs per 100,000 population
0 to <0.02
0.02 to <0.1
0.1 to <0.28
0.28 to <1.33
1.33 to <1.96
1.96 to <7.35
7.35 to <10.93
10.93 to <19.33
19.33 to <31.18
31.18 to 414.43
477

Tabasco

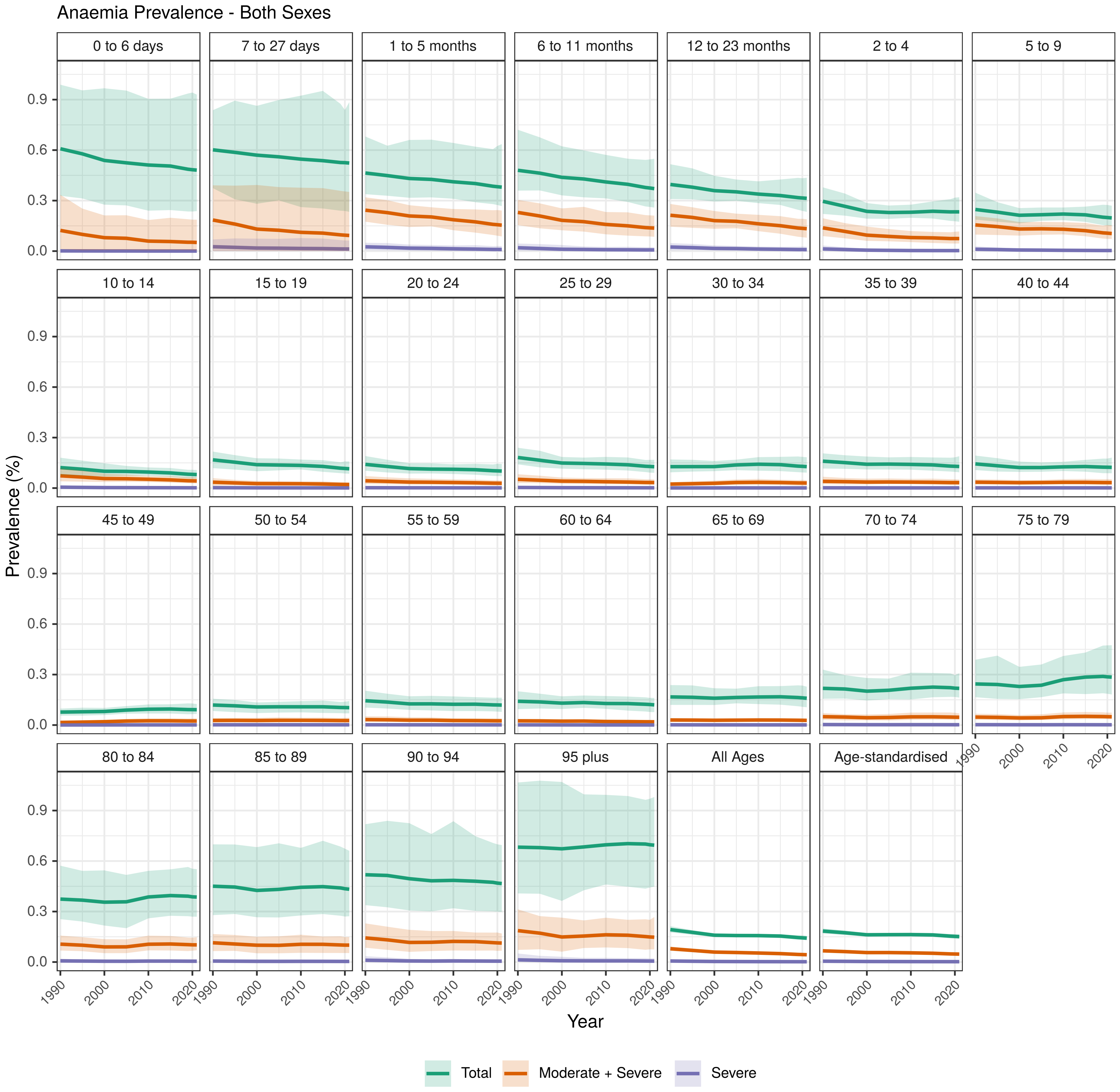

Anaemia Prevalence - Both Sexes
0 to 6 days
7 to 27 days
1 to 5 months
6 to 11 months
12 to 23 months
2 to 4
5 to 9
10 to 14
15 to 19
20 to 24
25 to 29
30 to 34
35 to 39
40 to 44
45 to 49
50 to 54
55 to 59
60 to 64
65 to 69
70 to 74
75 to 79
80 to 84
85 to 89
90 to 94
95 plus
All Ages
Age-standardised
Prevalence (%)
Year
Total
Moderate + Severe
Severe

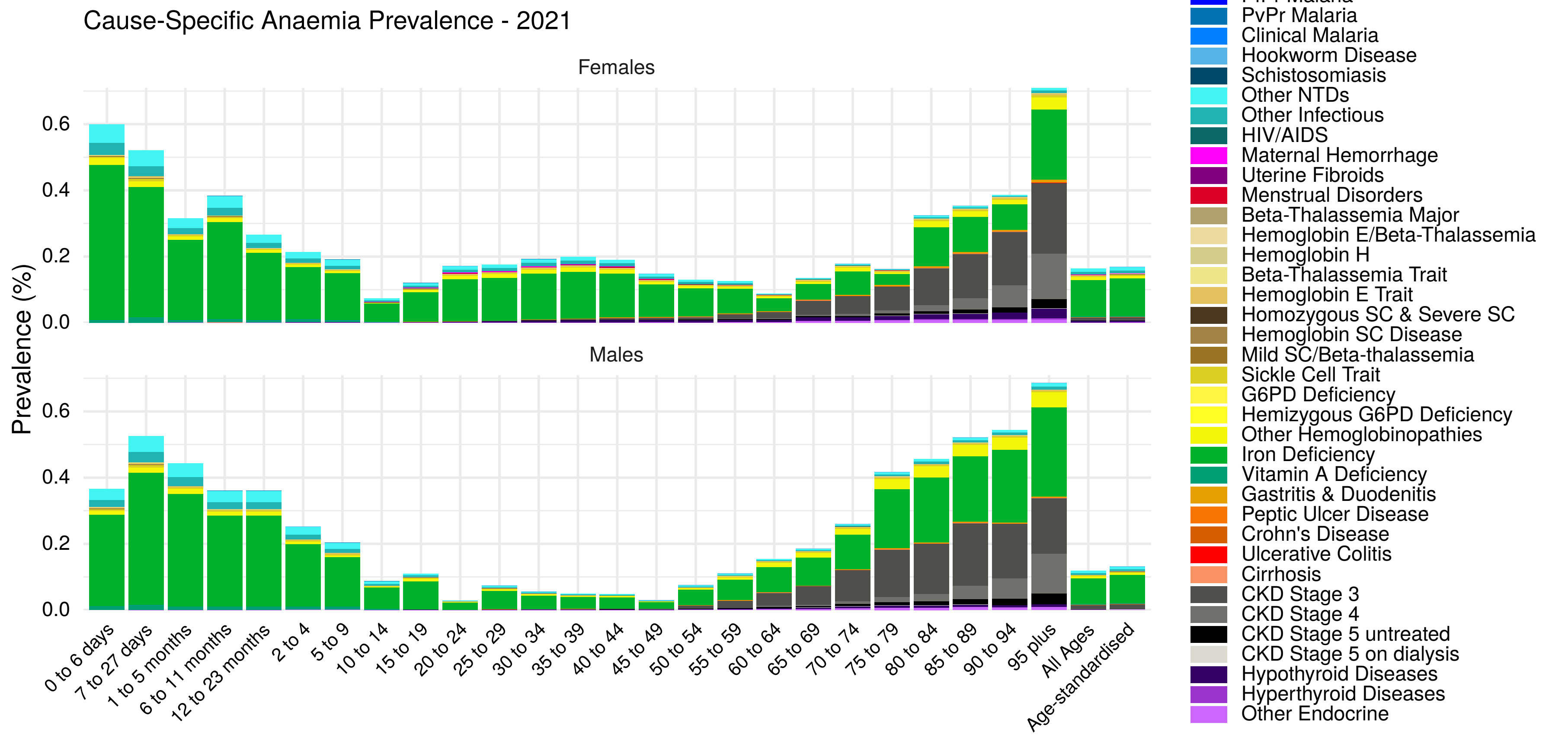

Cause-Specific Anaemia Prevalence - 2021
Females
Males
Prevalence (%)
PfPr Malaria
PvPr Malaria
Clinical Malaria
Hookworm Disease
Schistosomiasis
Other NTDs
Other Infectious
HIV/AIDS
Maternal Hemorrhage
Uterine Fibroids
Menstrual Disorders
Beta-Thalassemia Major
Hemoglobin E/Beta-Thalassemia
Hemoglobin H
Beta-Thalassemia Trait
Hemoglobin E Trait
Homozygous SC & Severe SC
Hemoglobin SC Disease
Mild SC/Beta-thalassemia
Sickle Cell Trait
G6PD Deficiency
Hemizygous G6PD Deficiency
Other Hemoglobinopathies
Iron Deficiency
Vitamin A Deficiency
Gastritis & Duodenitis
Peptic Ulcer Disease
Crohn's Disease
Ulcerative Colitis
Cirrhosis
CKD Stage 3
CKD Stage 4
CKD Stage 5 untreated
CKD Stage 5 on dialysis
Hypothyroid Diseases
Hyperthyroid Diseases
Other Endocrine

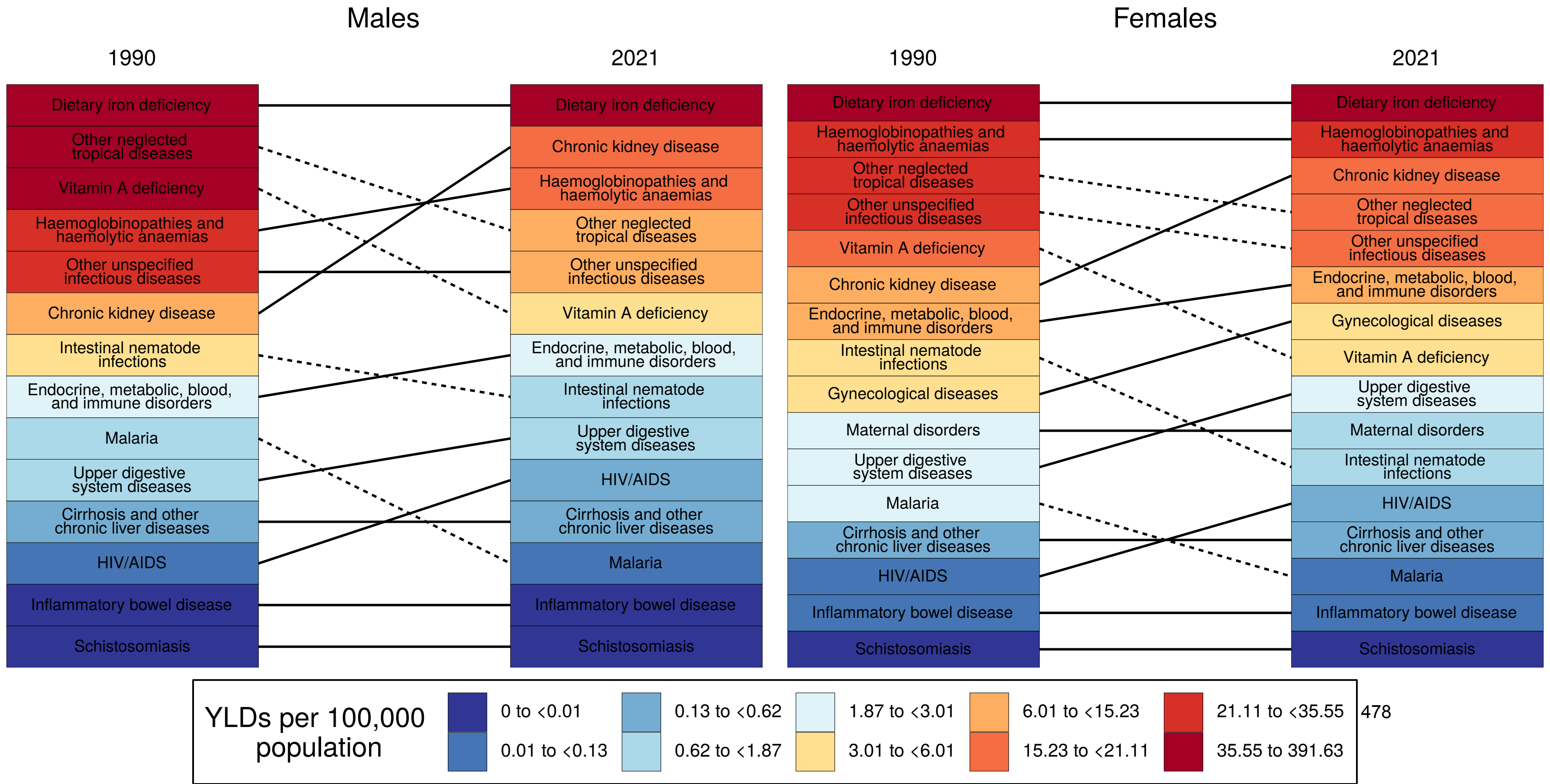

Males
Females
1990
2021
Dietary iron deficiency
Other neglected tropical diseases
Vitamin A deficiency
Haemoglobinopathies and haemolytic anaemias
Other unspecified infectious diseases
Chronic kidney disease
Intestinal nematode infections
Endocrine, metabolic, blood, and immune disorders
Malaria
Upper digestive system diseases
Cirrhosis and other chronic liver diseases
HIV/AIDS
Inflammatory bowel disease
Schistosomiasis
Maternal disorders
Gynecological diseases
YLDs per 100,000 population
0 to <0.01
0.01 to <0.13
0.13 to <0.62
0.62 to <1.87
1.87 to <3.01
3.01 to <6.01
6.01 to <15.23
15.23 to <21.11
21.11 to <35.55
35.55 to 391.63

478

Tamaulipas

Anaemia Prevalence - Both Sexes

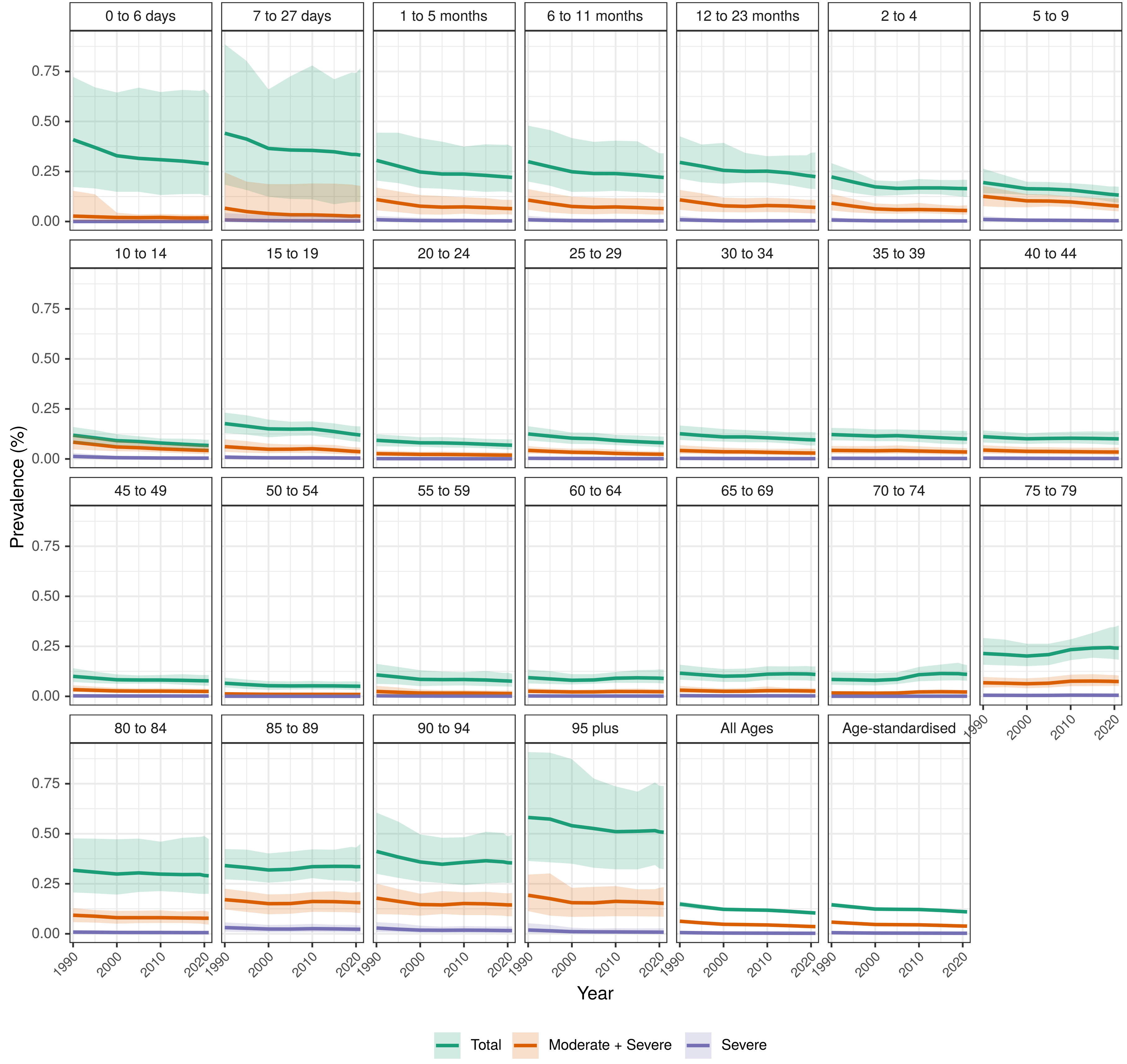

Cause-Specific Anaemia Prevalence - 2021

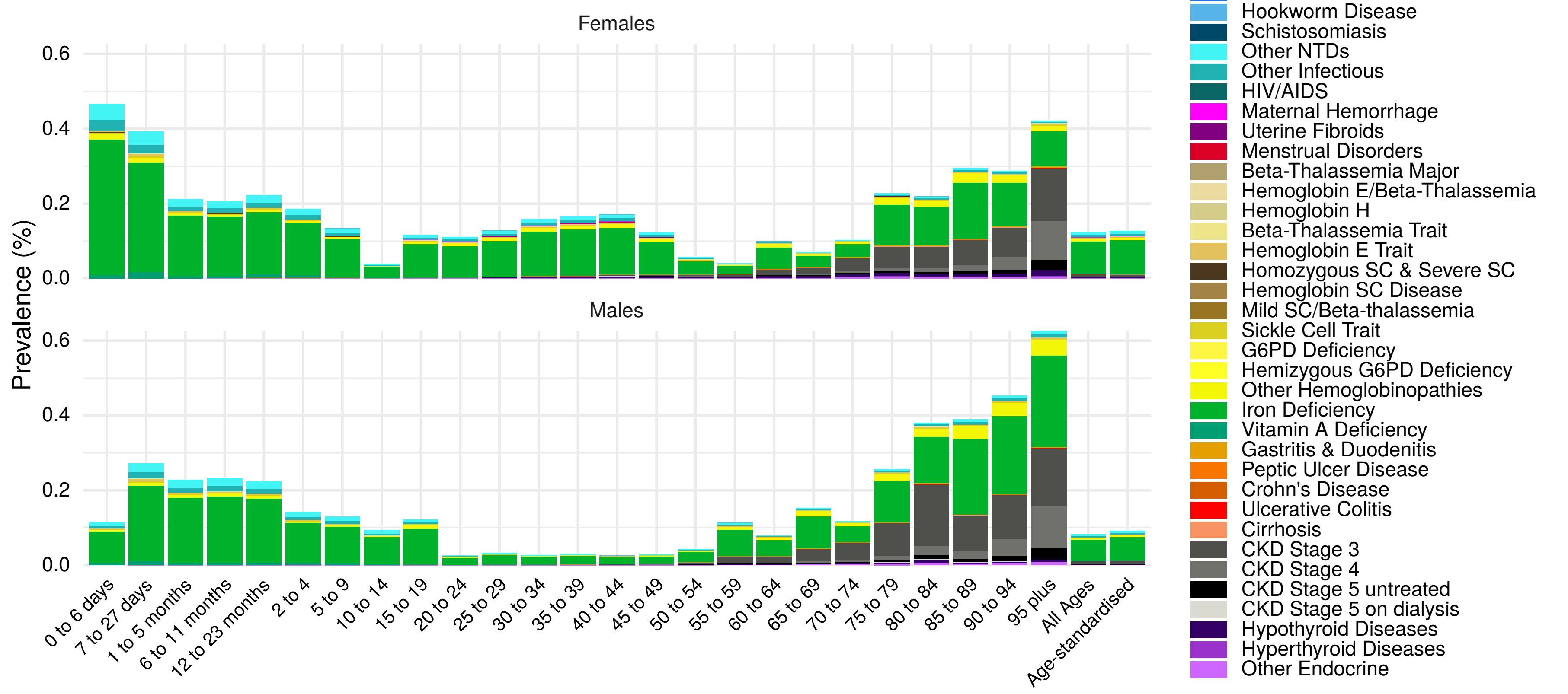

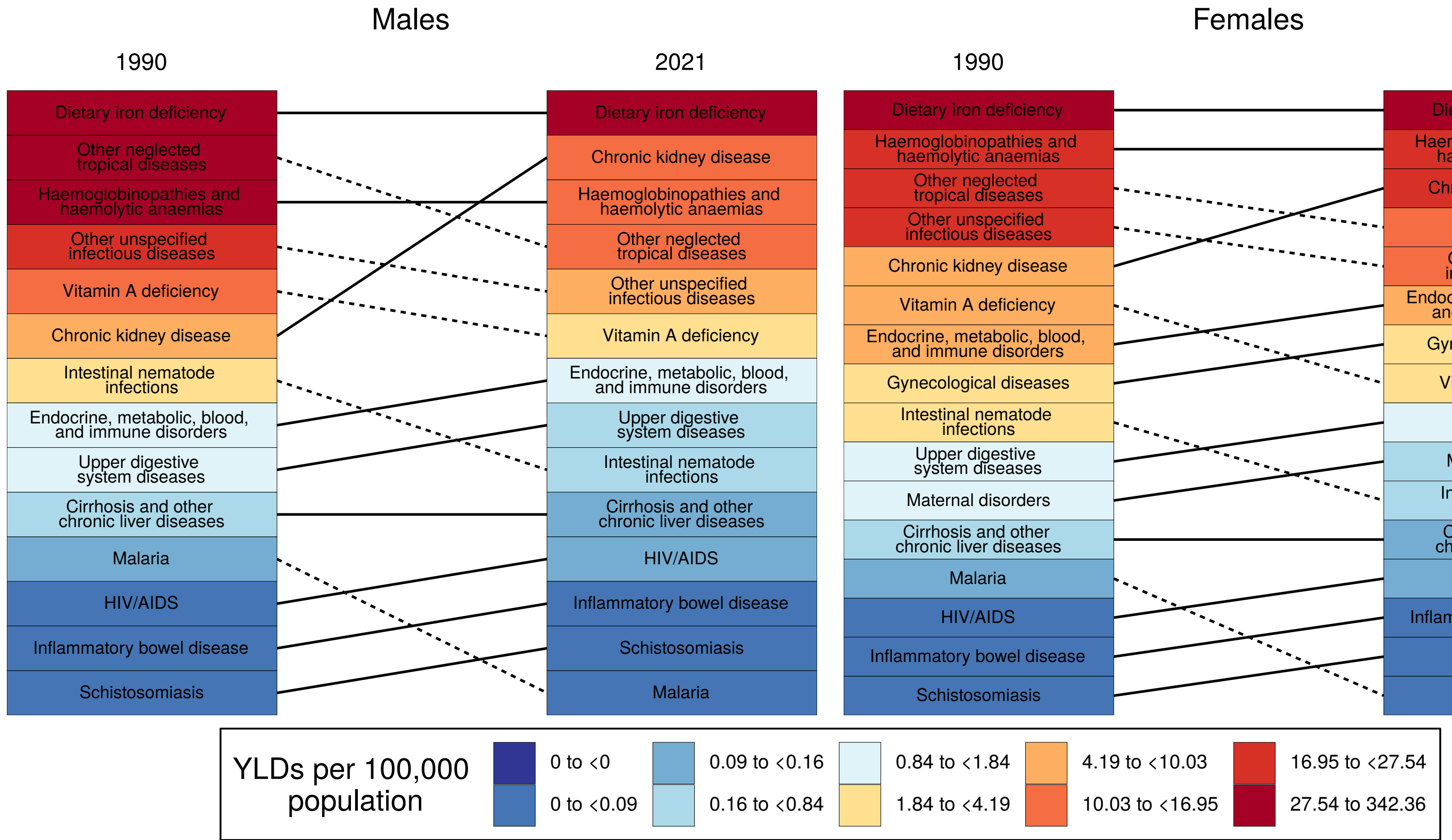

479

Tlaxcala

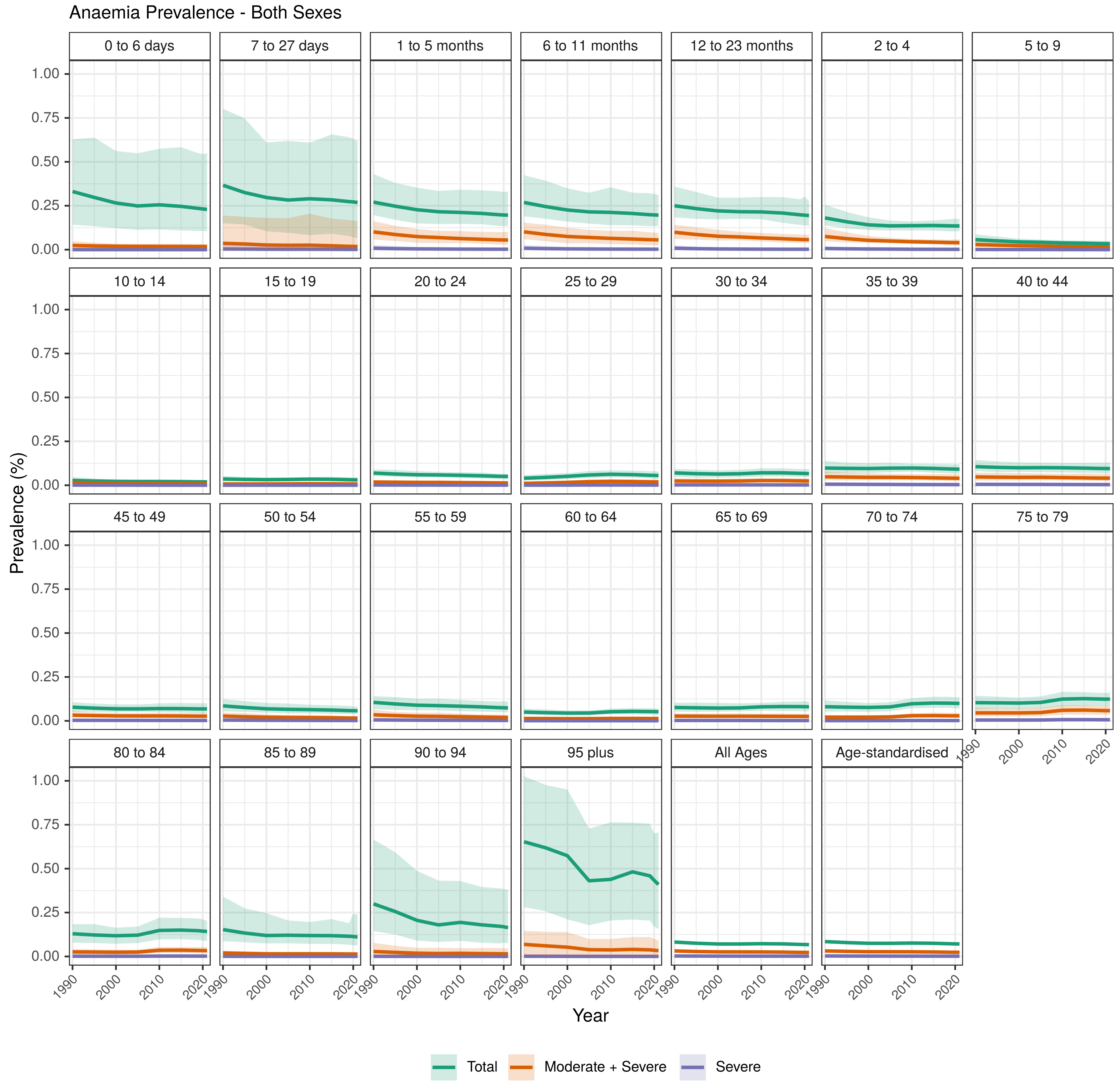

Anaemia Prevalence - Both Sexes
0 to 6 days
7 to 27 days
1 to 5 months
6 to 11 months
12 to 23 months
2 to 4
5 to 9
10 to 14
15 to 19
20 to 24
25 to 29
30 to 34
35 to 39
40 to 44
45 to 49
50 to 54
55 to 59
60 to 64
65 to 69
70 to 74
75 to 79
80 to 84
85 to 89
90 to 94
95 plus
All Ages
Age-standardised
Prevalence (%)
Year
Total
Moderate + Severe
Severe

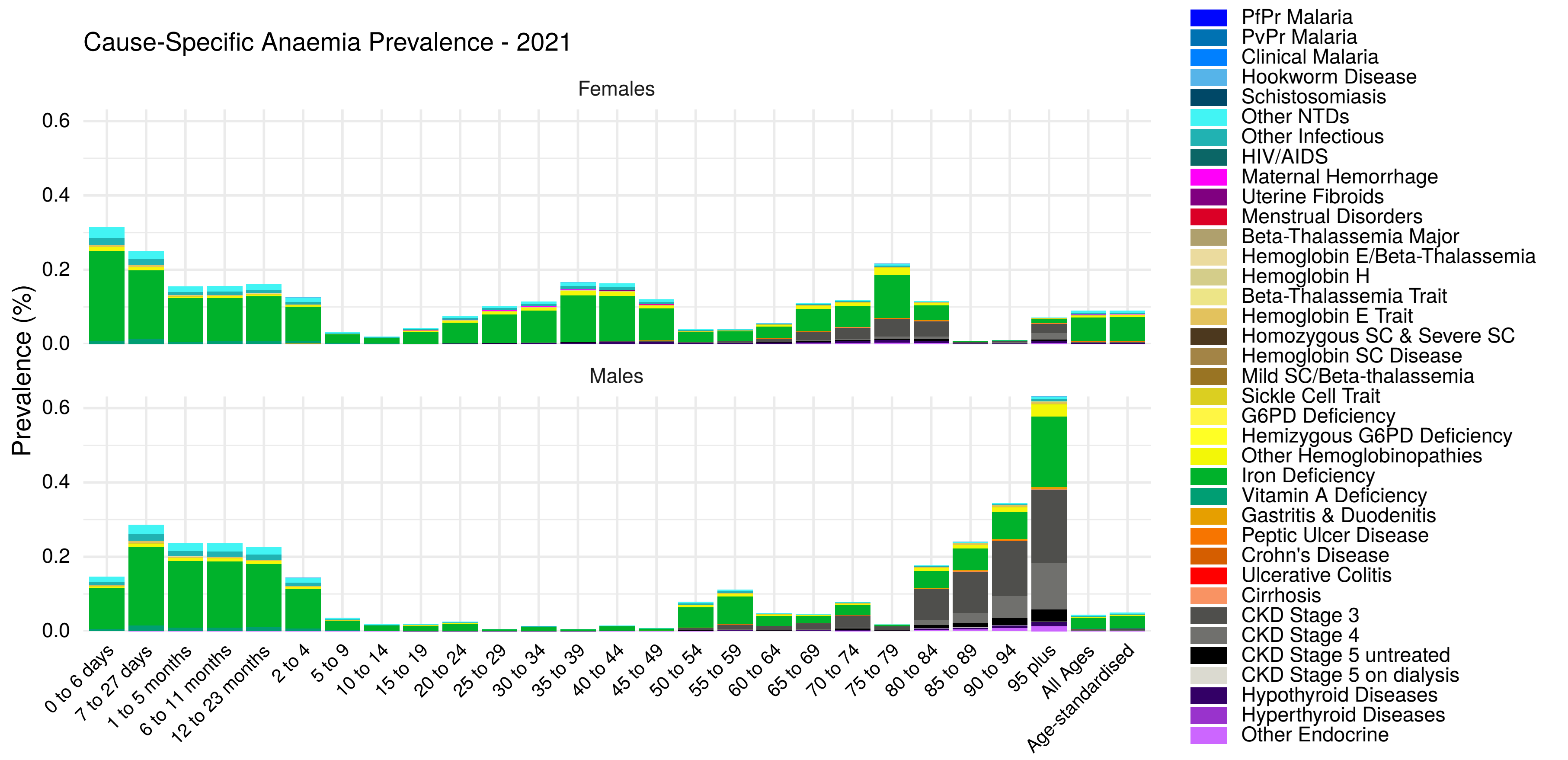

Cause-Specific Anaemia Prevalence - 2021
Females
Males
Prevalence (%)
PfPr Malaria
PvPr Malaria
Clinical Malaria
Hookworm Disease
Schistosomiasis
Other NTDs
Other Infectious
HIV/AIDS
Maternal Hemorrhage
Uterine Fibroids
Menstrual Disorders
Beta-Thalassemia Major
Hemoglobin E/Beta-Thalassemia
Hemoglobin H
Beta-Thalassemia Trait
Hemoglobin E Trait
Homozygous SC & Severe SC
Hemoglobin SC Disease
Mild SC/Beta-thalassemia
Sickle Cell Trait
G6PD Deficiency
Hemizygous G6PD Deficiency
Other Hemoglobinopathies
Iron Deficiency
Vitamin A Deficiency
Gastritis & Duodenitis
Peptic Ulcer Disease
Crohn's Disease
Ulcerative Colitis
Cirrhosis
CKD Stage 3
CKD Stage 4
CKD Stage 5 untreated
CKD Stage 5 on dialysis
Hypothyroid Diseases
Hyperthyroid Diseases
Other Endocrine

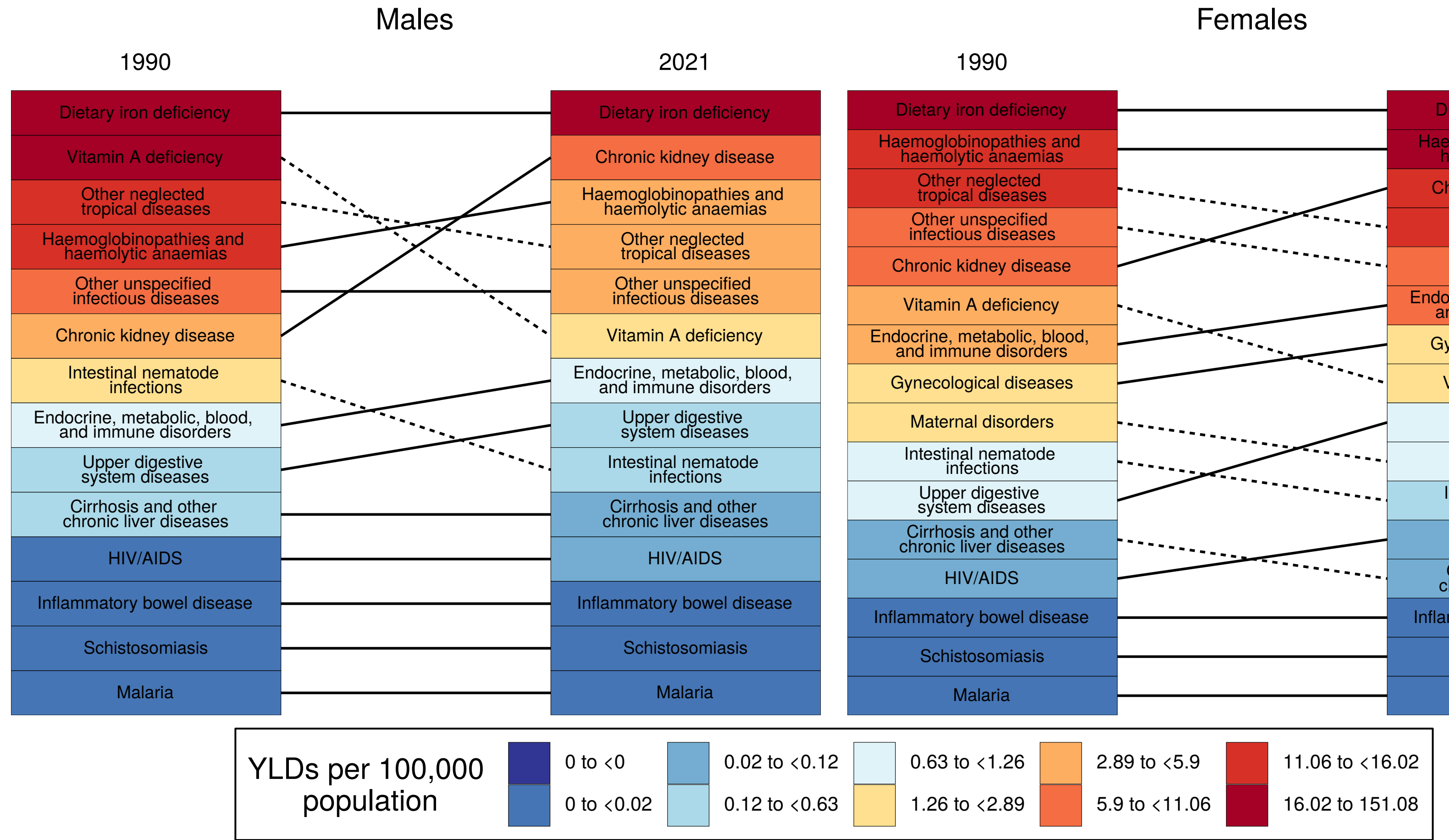

Males
Females
1990
2021
Dietary iron deficiency
Vitamin A deficiency
Other neglected tropical diseases
Haemoglobinopathies and haemolytic anaemias
Other unspecified infectious diseases
Chronic kidney disease
Intestinal nematode infections
Endocrine, metabolic, blood, and immune disorders
Upper digestive system diseases
Cirrhosis and other chronic liver diseases
HIV/AIDS
Inflammatory bowel disease
Schistosomiasis
Malaria
Gynecological diseases
Maternal disorders
YLDs per 100,000 population
0 to <0
0 to <0.02
0.02 to <0.12
0.12 to <0.63
0.63 to <1.26
1.26 to <2.89
2.89 to <5.9
5.9 to <11.06
11.06 to <16.02
16.02 to 151.08

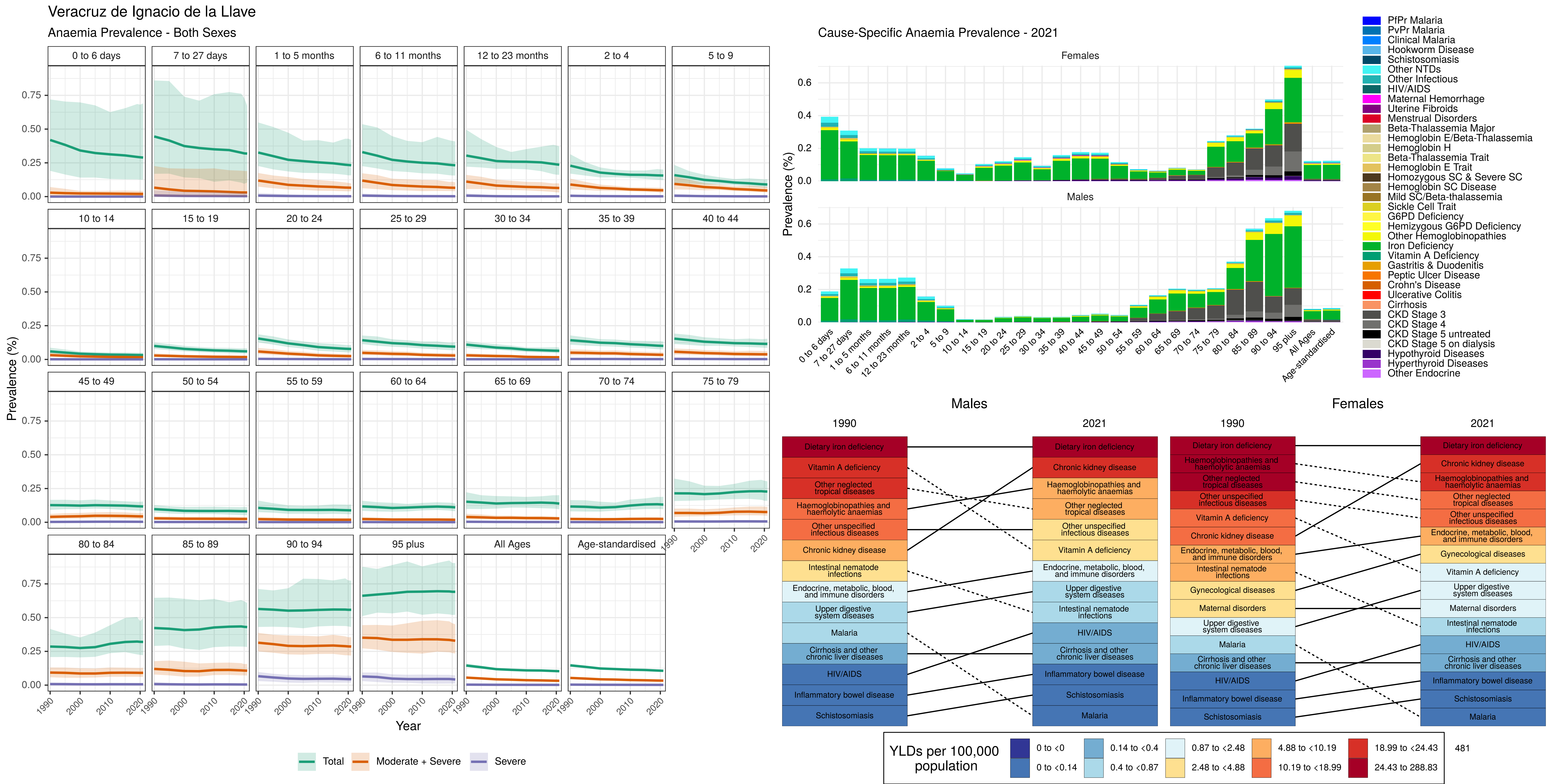

Veracruz de Ignacio de la Llave
Anaemia Prevalence - Both Sexes
0 to 6 days
7 to 27 days
1 to 5 months
6 to 11 months
12 to 23 months
2 to 4
5 to 9
10 to 14
15 to 19
20 to 24
25 to 29
30 to 34
35 to 39
40 to 44
45 to 49
50 to 54
55 to 59
60 to 64
65 to 69
70 to 74
75 to 79
80 to 84
85 to 89
90 to 94
95 plus
All Ages
Age-standardised
Prevalence (%)
Year
Total
Moderate + Severe
Severe
Cause-Specific Anaemia Prevalence - 2021
Females
Males
Prevalence (%)
PfPr Malaria
PvPr Malaria
Clinical Malaria
Hookworm Disease
Schistosomiasis
Other NTDs
Other Infectious
HIV/AIDS
Maternal Hemorrhage
Uterine Fibroids
Menstrual Disorders
Beta-Thalassemia Major
Hemoglobin E/Beta-Thalassemia
Hemoglobin H
Beta-Thalassemia Trait
Hemoglobin E Trait
Homozygous SC & Severe SC
Hemoglobin SC Disease
Mild SC/Beta-thalassemia
Sickle Cell Trait
G6PD Deficiency
Hemizygous G6PD Deficiency
Other Hemoglobinopathies
Iron Deficiency
Vitamin A Deficiency
Gastritis & Duodenitis
Peptic Ulcer Disease
Crohn's Disease
Ulcerative Colitis
Cirrhosis
CKD Stage 3
CKD Stage 4
CKD Stage 5 untreated
CKD Stage 5 on dialysis
Hypothyroid Diseases
Hyperthyroid Diseases
Other Endocrine
Males
Females
1990
2021
Dietary iron deficiency
Vitamin A deficiency
Other neglected tropical diseases
Haemoglobinopathies and haemolytic anaemias
Other unspecified infectious diseases
Chronic kidney disease
Intestinal nematode infections
Endocrine, metabolic, blood, and immune disorders
Upper digestive system diseases
Malaria
Cirrhosis and other chronic liver diseases
HIV/AIDS
Inflammatory bowel disease
Schistosomiasis
Gynecological diseases
Maternal disorders
YLDs per 100,000 population
0 to <0
0 to <0.14
0.14 to <0.4
0.4 to <0.87
0.87 to <2.48
2.48 to <4.88
4.88 to <10.19
10.19 to <18.99
18.99 to <24.43
24.43 to 288.83

Yucatán

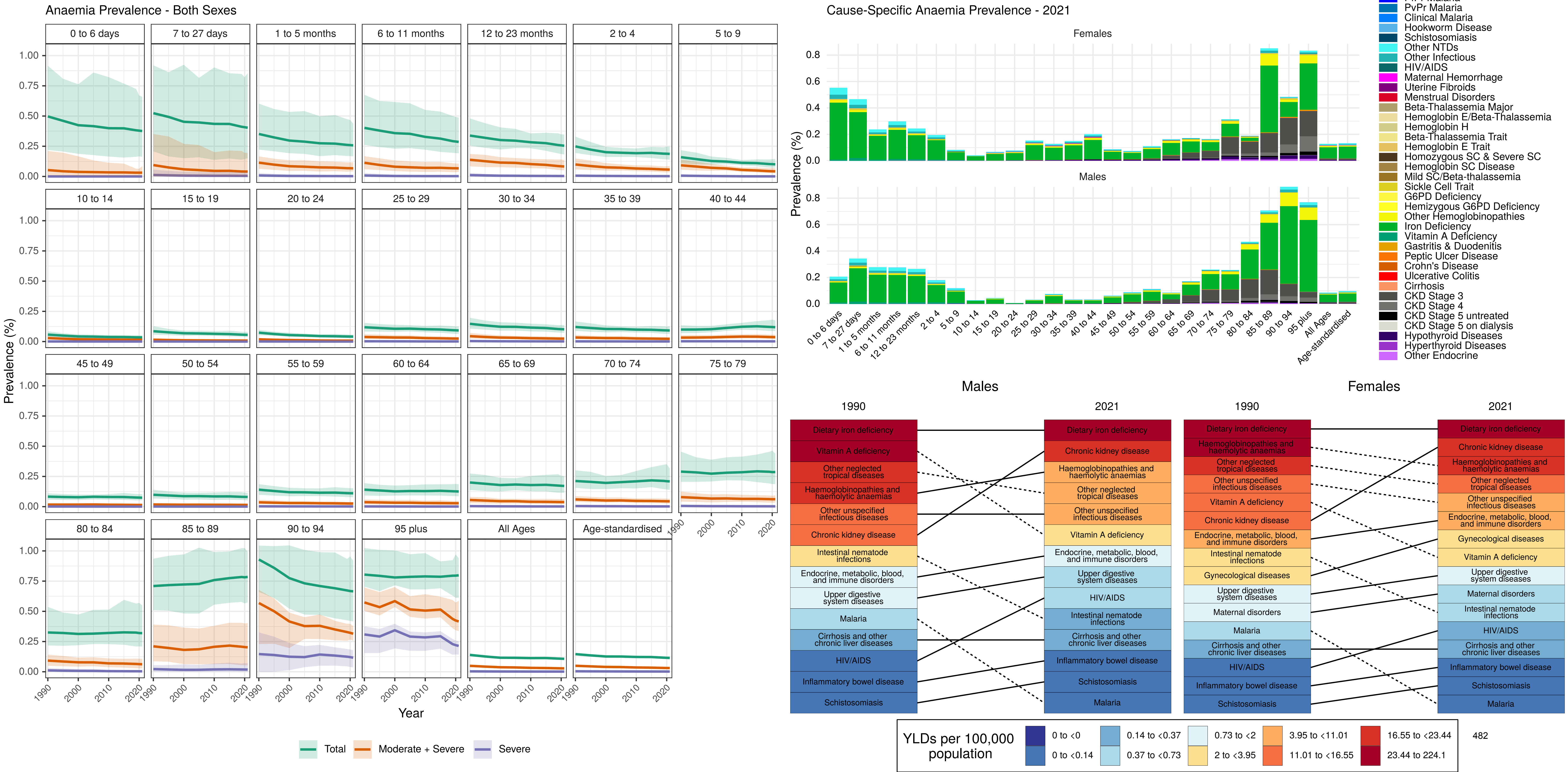

Anaemia Prevalence - Both Sexes
0 to 6 days
7 to 27 days
1 to 5 months
6 to 11 months
12 to 23 months
2 to 4
5 to 9
10 to 14
15 to 19
20 to 24
25 to 29
30 to 34
35 to 39
40 to 44
45 to 49
50 to 54
55 to 59
60 to 64
65 to 69
70 to 74
75 to 79
80 to 84
85 to 89
90 to 94
95 plus
All Ages
Age-standardised
Prevalence (%)
Year
Total
Moderate + Severe
Severe
Cause-Specific Anaemia Prevalence - 2021
Females
Males
PfPr Malaria
PvPr Malaria
Clinical Malaria
Hookworm Disease
Schistosomiasis
Other NTDs
Other Infectious
HIV/AIDS
Maternal Hemorrhage
Uterine Fibroids
Menstrual Disorders
Beta-Thalassemia Major
Hemoglobin E/Beta-Thalassemia
Hemoglobin H
Beta-Thalassemia Trait
Hemoglobin E Trait
Homozygous SC & Severe SC
Hemoglobin SC Disease
Mild SC/Beta-thalassemia
Sickle Cell Trait
G6PD Deficiency
Hemizygous G6PD Deficiency
Other Hemoglobinopathies
Iron Deficiency
Vitamin A Deficiency
Gastritis & Duodenitis
Peptic Ulcer Disease
Crohn's Disease
Ulcerative Colitis
Cirrhosis
CKD Stage 3
CKD Stage 4
CKD Stage 5 untreated
CKD Stage 5 on dialysis
Hypothyroid Diseases
Hyperthyroid Diseases
Other Endocrine
Males
Females
1990
2021
Dietary iron deficiency
Vitamin A deficiency
Other neglected tropical diseases
Haemoglobinopathies and haemolytic anaemias
Other unspecified infectious diseases
Chronic kidney disease
Intestinal nematode infections
Endocrine, metabolic, blood, and immune disorders
Upper digestive system diseases
Malaria
Cirrhosis and other chronic liver diseases
HIV/AIDS
Inflammatory bowel disease
Schistosomiasis
Gynecological diseases
Maternal disorders
YLDs per 100,000 population
0 to <0
0 to <0.14
0.14 to <0.37
0.37 to <0.73
0.73 to <2
2 to <3.95
3.95 to <11.01
11.01 to <16.55
16.55 to <23.44
23.44 to 224.1

Zacatecas

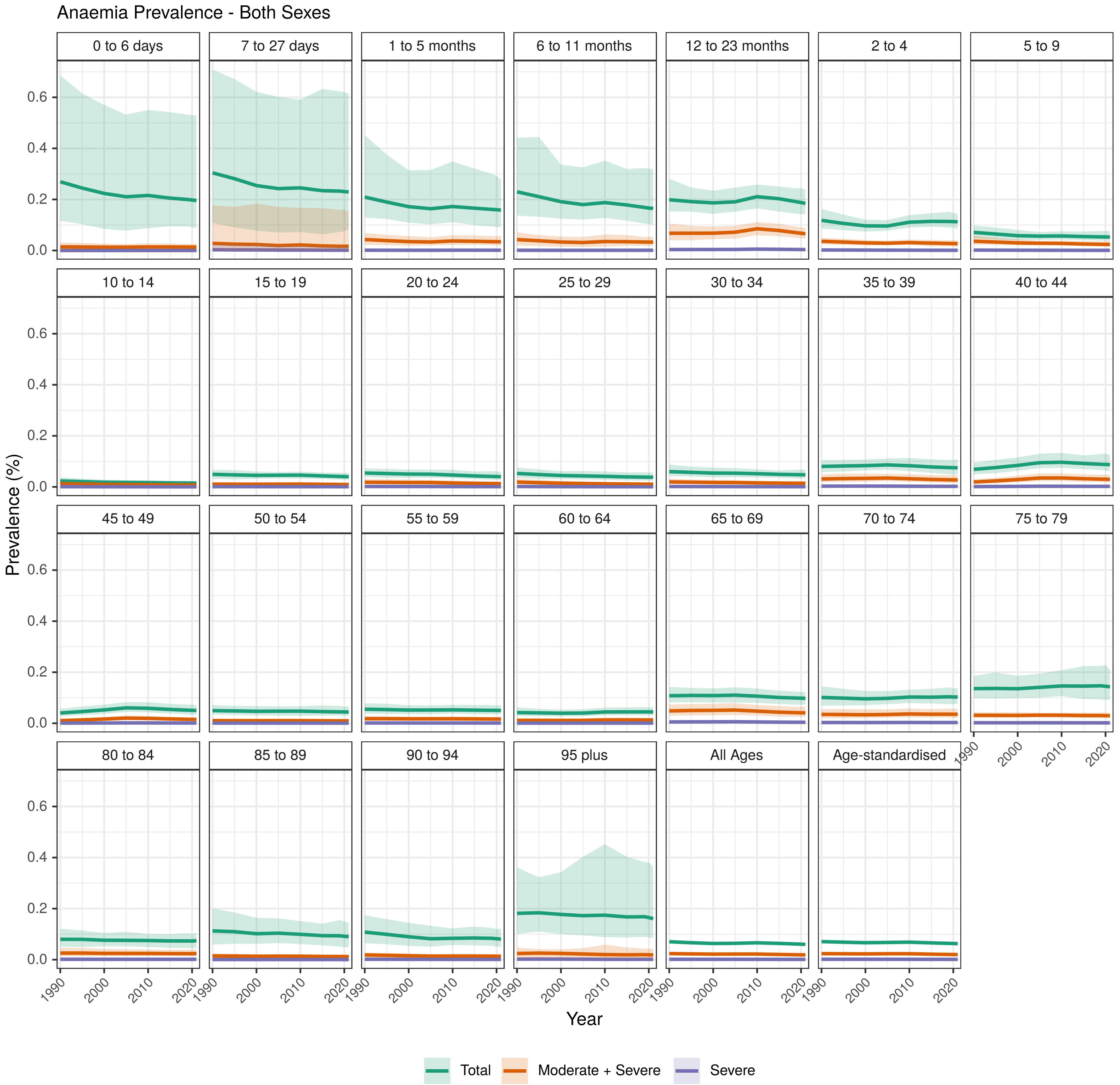
Anaemia Prevalence - Both Sexes
0 to 6 days
7 to 27 days
1 to 5 months
6 to 11 months
12 to 23 months
2 to 4
5 to 9
10 to 14
15 to 19
20 to 24
25 to 29
30 to 34
35 to 39
40 to 44
45 to 49
50 to 54
55 to 59
60 to 64
65 to 69
70 to 74
75 to 79
80 to 84
85 to 89
90 to 94
95 plus
All Ages
Age-standardised
Prevalence (%)
0.0
0.2
0.4
0.6
1990
2000
2010
2020
Year
Total
Moderate + Severe
Severe

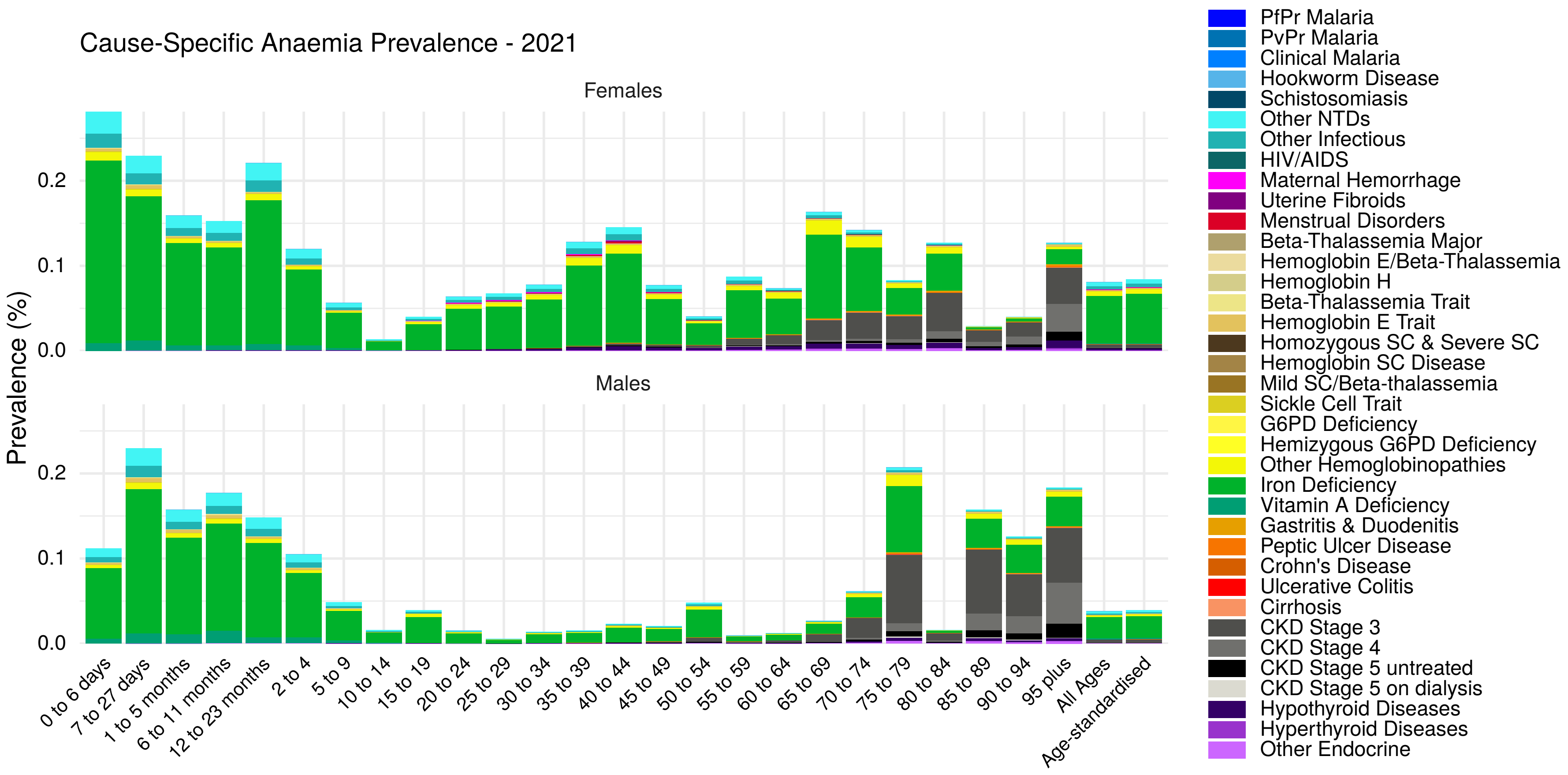
Cause-Specific Anaemia Prevalence - 2021
Females
Males
Prevalence (%)
0.0
0.1
0.2
0 to 6 days
7 to 27 days
1 to 5 months
6 to 11 months
12 to 23 months
2 to 4
5 to 9
10 to 14
15 to 19
20 to 24
25 to 29
30 to 34
35 to 39
40 to 44
45 to 49
50 to 54
55 to 59
60 to 64
65 to 69
70 to 74
75 to 79
80 to 84
85 to 89
90 to 94
95 plus
All Ages
Age-standardised
PfPr Malaria
PvPr Malaria
Clinical Malaria
Hookworm Disease
Schistosomiasis
Other NTDs
Other Infectious
HIV/AIDS
Maternal Hemorrhage
Uterine Fibroids
Menstrual Disorders
Beta-Thalassemia Major
Hemoglobin E/Beta-Thalassemia
Hemoglobin H
Beta-Thalassemia Trait
Hemoglobin E Trait
Homozygous SC & Severe SC
Hemoglobin SC Disease
Mild SC/Beta-thalassemia
Sickle Cell Trait
G6PD Deficiency
Hemizygous G6PD Deficiency
Other Hemoglobinopathies
Iron Deficiency
Vitamin A Deficiency
Gastritis & Duodenitis
Peptic Ulcer Disease
Crohn's Disease
Ulcerative Colitis
Cirrhosis
CKD Stage 3
CKD Stage 4
CKD Stage 5 untreated
CKD Stage 5 on dialysis
Hypothyroid Diseases
Hyperthyroid Diseases
Other Endocrine

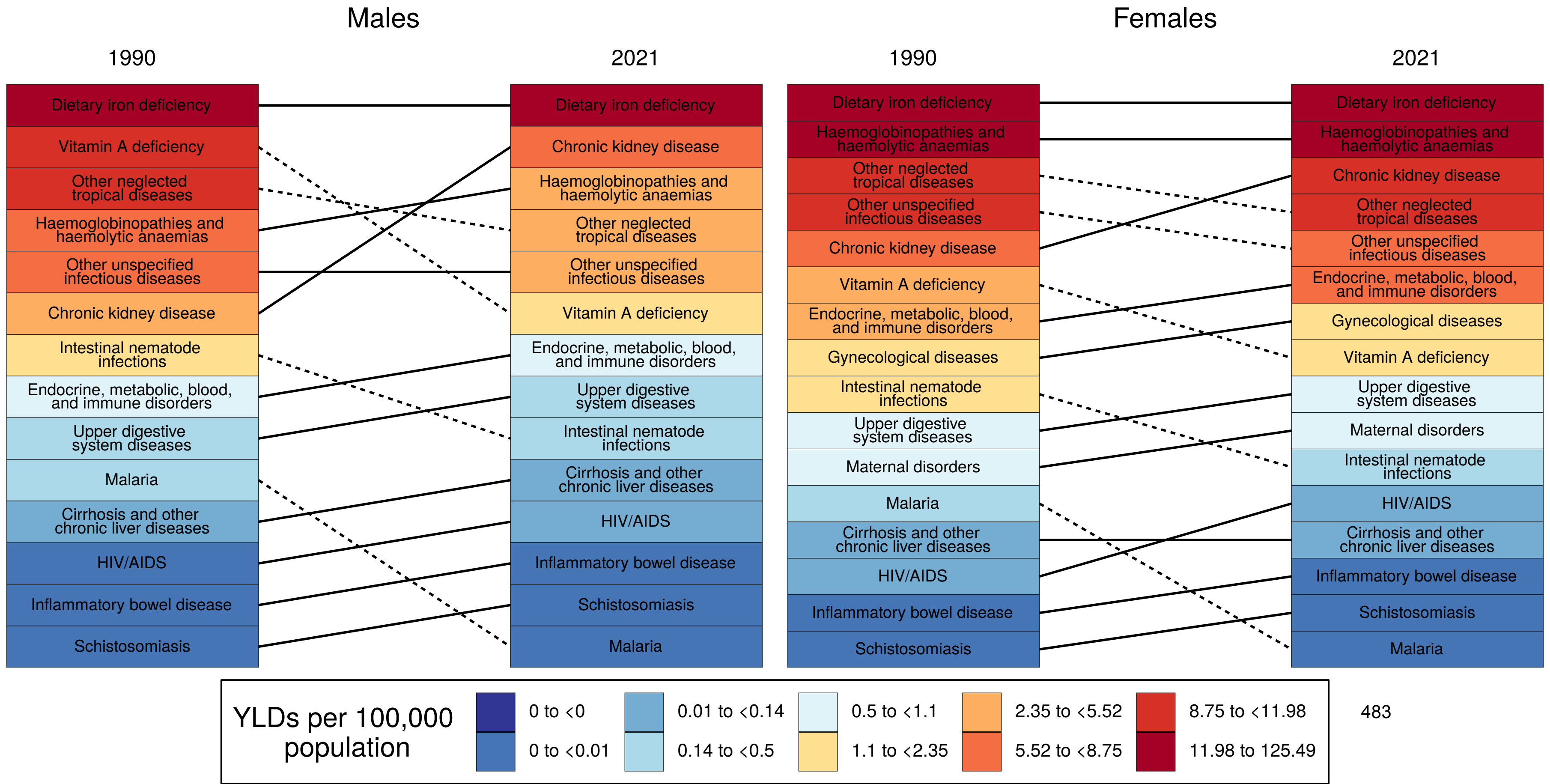
Males
Females
1990
2021
Dietary iron deficiency
Vitamin A deficiency
Other neglected tropical diseases
Haemoglobinopathies and haemolytic anaemias
Other unspecified infectious diseases
Chronic kidney disease
Intestinal nematode infections
Endocrine, metabolic, blood, and immune disorders
Upper digestive system diseases
Malaria
Cirrhosis and other chronic liver diseases
HIV/AIDS
Inflammatory bowel disease
Schistosomiasis
Gynecological diseases
Maternal disorders
YLDs per 100,000 population
0 to <0
0 to <0.01
0.01 to <0.14
0.14 to <0.5
0.5 to <1.1
1.1 to <2.35
2.35 to <5.52
5.52 to <8.75
8.75 to <11.98
11.98 to 125.49

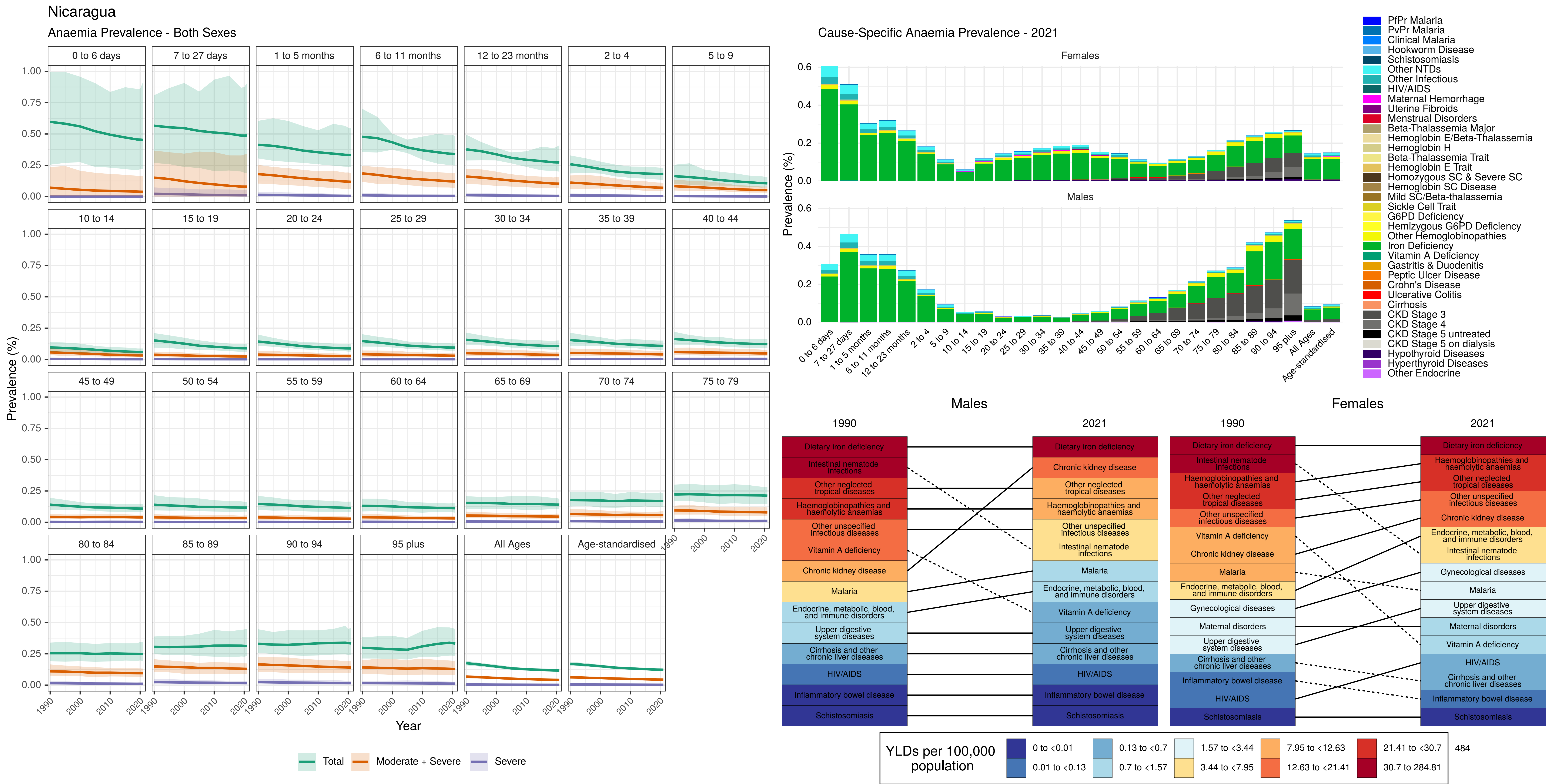
Nicaragua
Anaemia Prevalence - Both Sexes
0 to 6 days
7 to 27 days
1 to 5 months
6 to 11 months
12 to 23 months
2 to 4
5 to 9
10 to 14
15 to 19
20 to 24
25 to 29
30 to 34
35 to 39
40 to 44
45 to 49
50 to 54
55 to 59
60 to 64
65 to 69
70 to 74
75 to 79
80 to 84
85 to 89
90 to 94
95 plus
All Ages
Age-standardised
Prevalence (%)
Year
Total
Moderate + Severe
Severe
Cause-Specific Anaemia Prevalence - 2021
Females
Males
Prevalence (%)
PfPr Malaria
PvPr Malaria
Clinical Malaria
Hookworm Disease
Schistosomiasis
Other NTDs
Other Infectious
HIV/AIDS
Maternal Hemorrhage
Uterine Fibroids
Menstrual Disorders
Beta-Thalassemia Major
Hemoglobin E/Beta-Thalassemia
Hemoglobin H
Beta-Thalassemia Trait
Hemoglobin E Trait
Homozygous SC & Severe SC
Hemoglobin SC Disease
Mild SC/Beta-thalassemia
Sickle Cell Trait
G6PD Deficiency
Hemizygous G6PD Deficiency
Other Hemoglobinopathies
Iron Deficiency
Vitamin A Deficiency
Gastritis & Duodenitis
Peptic Ulcer Disease
Crohn's Disease
Ulcerative Colitis
Cirrhosis
CKD Stage 3
CKD Stage 4
CKD Stage 5 untreated
CKD Stage 5 on dialysis
Hypothyroid Diseases
Hyperthyroid Diseases
Other Endocrine
Males
Females
1990
2021
Dietary iron deficiency
Intestinal nematode infections
Other neglected tropical diseases
Haemoglobinopathies and haemolytic anaemias
Other unspecified infectious diseases
Vitamin A deficiency
Chronic kidney disease
Malaria
Endocrine, metabolic, blood, and immune disorders
Upper digestive system diseases
Cirrhosis and other chronic liver diseases
HIV/AIDS
Inflammatory bowel disease
Schistosomiasis
Gynecological diseases
Maternal disorders
YLDs per 100,000 population
0 to <0.01
0.01 to <0.13
0.13 to <0.7
0.7 to <1.57
1.57 to <3.44
3.44 to <7.95
7.95 to <12.63
12.63 to <21.41
21.41 to <30.7
30.7 to 284.81

Panama

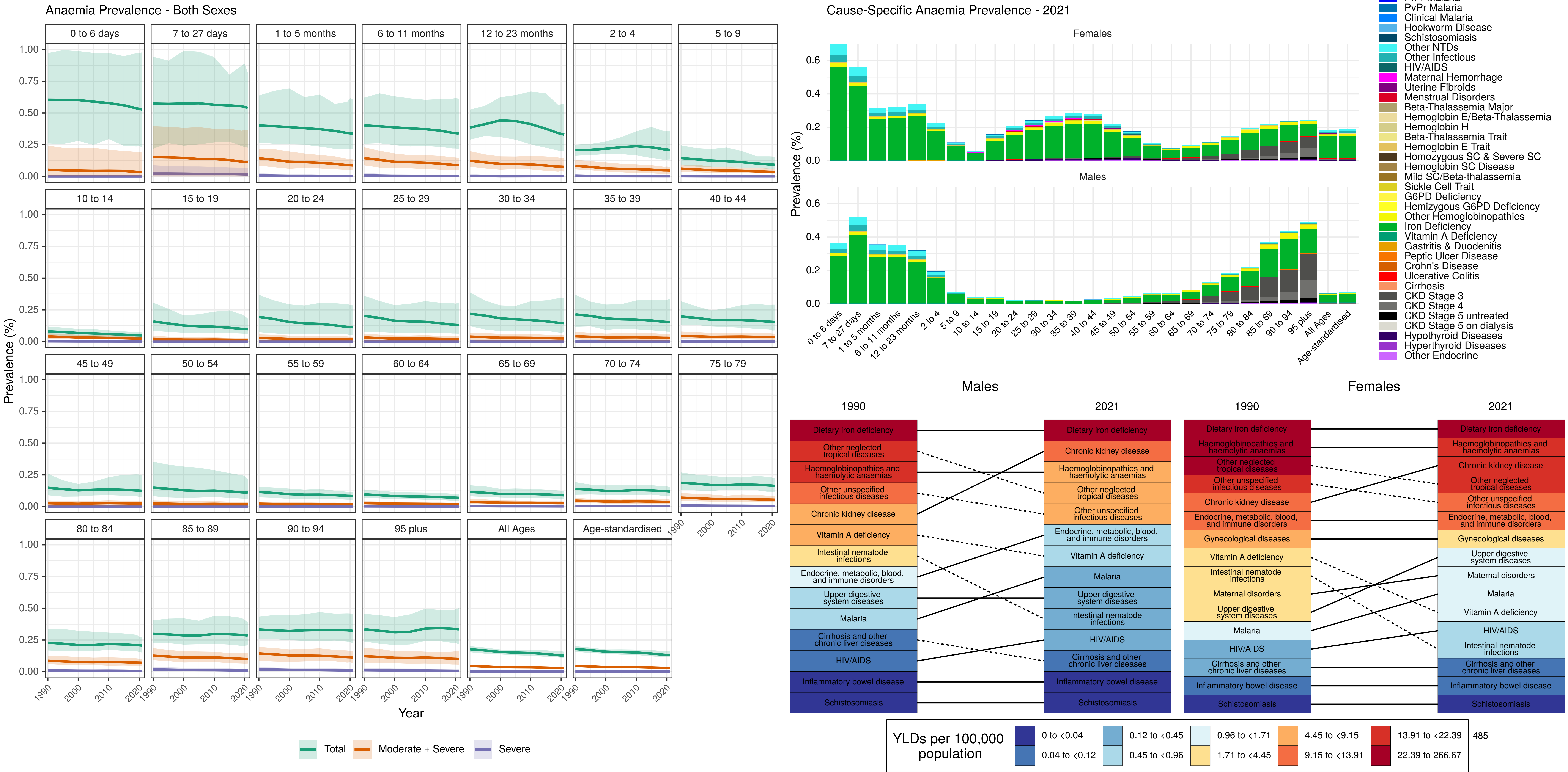

Anaemia Prevalence - Both Sexes
0 to 6 days
7 to 27 days
1 to 5 months
6 to 11 months
12 to 23 months
2 to 4
5 to 9
10 to 14
15 to 19
20 to 24
25 to 29
30 to 34
35 to 39
40 to 44
45 to 49
50 to 54
55 to 59
60 to 64
65 to 69
70 to 74
75 to 79
80 to 84
85 to 89
90 to 94
95 plus
All Ages
Age-standardised
Prevalence (%)
Year
Total
Moderate + Severe
Severe
Cause-Specific Anaemia Prevalence - 2021
Females
Males
PfPr Malaria
PvPr Malaria
Clinical Malaria
Hookworm Disease
Schistosomiasis
Other NTDs
Other Infectious
HIV/AIDS
Maternal Hemorrhage
Uterine Fibroids
Menstrual Disorders
Beta-Thalassemia Major
Hemoglobin E/Beta-Thalassemia
Hemoglobin H
Beta-Thalassemia Trait
Hemoglobin E Trait
Homozygous SC & Severe SC
Hemoglobin SC Disease
Mild SC/Beta-thalassemia
Sickle Cell Trait
G6PD Deficiency
Hemizygous G6PD Deficiency
Other Hemoglobinopathies
Iron Deficiency
Vitamin A Deficiency
Gastritis & Duodenitis
Peptic Ulcer Disease
Crohn's Disease
Ulcerative Colitis
Cirrhosis
CKD Stage 3
CKD Stage 4
CKD Stage 5 untreated
CKD Stage 5 on dialysis
Hypothyroid Diseases
Hyperthyroid Diseases
Other Endocrine
Males
Females
1990
2021
Dietary iron deficiency
Other neglected tropical diseases
Haemoglobinopathies and haemolytic anaemias
Other unspecified infectious diseases
Chronic kidney disease
Vitamin A deficiency
Intestinal nematode infections
Endocrine, metabolic, blood, and immune disorders
Upper digestive system diseases
Malaria
Cirrhosis and other chronic liver diseases
HIV/AIDS
Inflammatory bowel disease
Schistosomiasis
Gynecological diseases
Maternal disorders
YLDs per 100,000 population
0 to <0.04
0.04 to <0.12
0.12 to <0.45
0.45 to <0.96
0.96 to <1.71
1.71 to <4.45
4.45 to <9.15
9.15 to <13.91
13.91 to <22.39
22.39 to 266.67

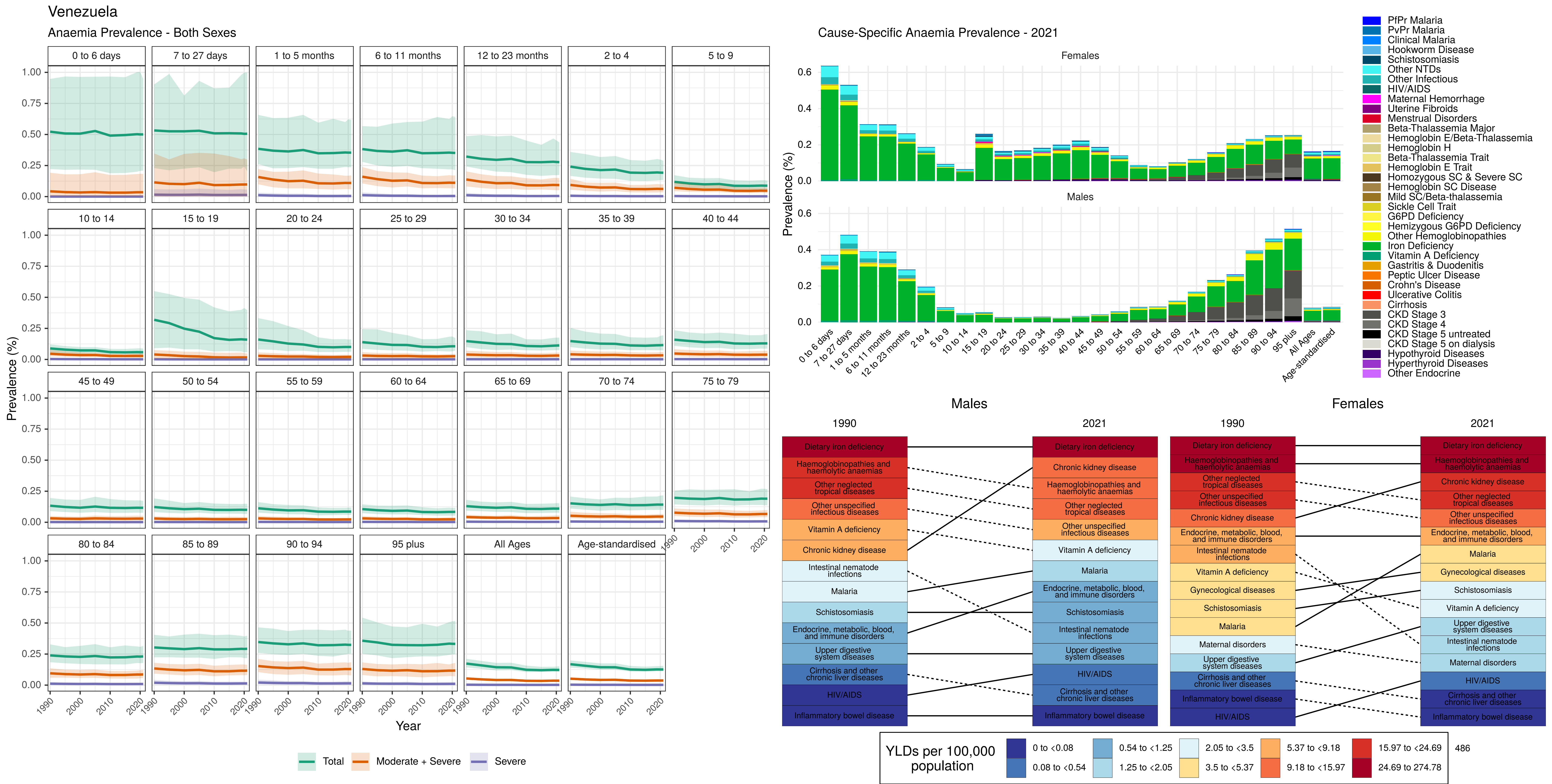

Venezuela
Anaemia Prevalence - Both Sexes
0 to 6 days
7 to 27 days
1 to 5 months
6 to 11 months
12 to 23 months
2 to 4
5 to 9
10 to 14
15 to 19
20 to 24
25 to 29
30 to 34
35 to 39
40 to 44
45 to 49
50 to 54
55 to 59
60 to 64
65 to 69
70 to 74
75 to 79
80 to 84
85 to 89
90 to 94
95 plus
All Ages
Age-standardised
Prevalence (%)
1.00
0.75
0.50
0.25
0.00
1990
2000
2010
2020
Year
Total
Moderate + Severe
Severe
Cause-Specific Anaemia Prevalence - 2021
Females
Males
Prevalence (%)
0.6
0.4
0.2
0.0
PfPr Malaria
PvPr Malaria
Clinical Malaria
Hookworm Disease
Schistosomiasis
Other NTDs
Other Infectious
HIV/AIDS
Maternal Hemorrhage
Uterine Fibroids
Menstrual Disorders
Beta-Thalassemia Major
Hemoglobin E/Beta-Thalassemia
Hemoglobin H
Beta-Thalassemia Trait
Hemoglobin E Trait
Homozygous SC & Severe SC
Hemoglobin SC Disease
Mild SC/Beta-thalassemia
Sickle Cell Trait
G6PD Deficiency
Hemizygous G6PD Deficiency
Other Hemoglobinopathies
Iron Deficiency
Vitamin A Deficiency
Gastritis & Duodenitis
Peptic Ulcer Disease
Crohn's Disease
Ulcerative Colitis
Cirrhosis
CKD Stage 3
CKD Stage 4
CKD Stage 5 untreated
CKD Stage 5 on dialysis
Hypothyroid Diseases
Hyperthyroid Diseases
Other Endocrine
Males
Females
1990
2021
Dietary iron deficiency
Haemoglobinopathies and haemolytic anaemias
Other neglected tropical diseases
Other unspecified infectious diseases
Vitamin A deficiency
Chronic kidney disease
Intestinal nematode infections
Malaria
Schistosomiasis
Endocrine, metabolic, blood, and immune disorders
Upper digestive system diseases
Cirrhosis and other chronic liver diseases
HIV/AIDS
Inflammatory bowel disease
Gynecological diseases
Maternal disorders
YLDs per 100,000 population
0 to <0.08
0.08 to <0.54
0.54 to <1.25
1.25 to <2.05
2.05 to <3.5
3.5 to <5.37
5.37 to <9.18
9.18 to <15.97
15.97 to <24.69
24.69 to 274.78
486

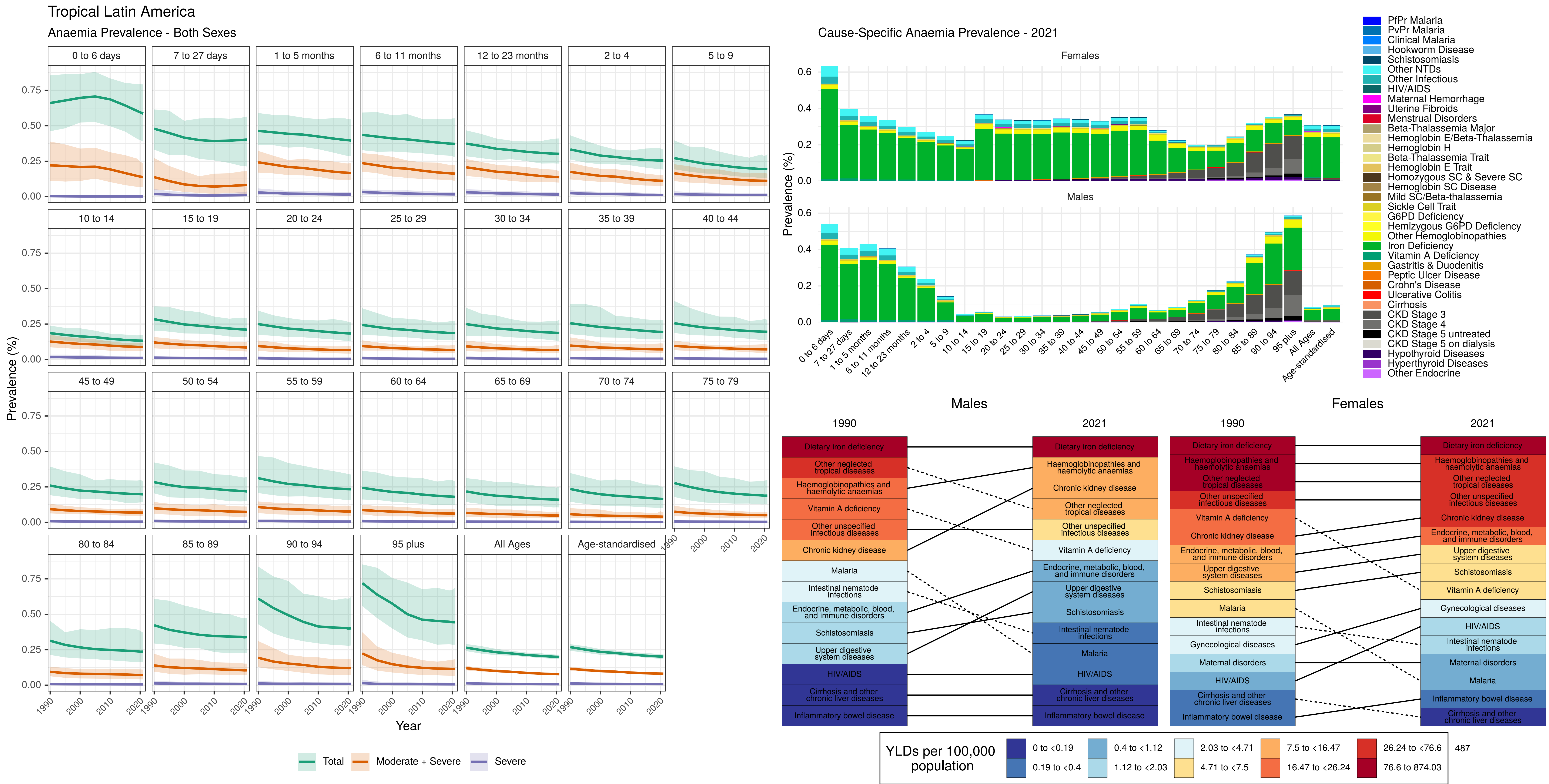
Tropical Latin America
Anaemia Prevalence - Both Sexes
0 to 6 days
7 to 27 days
1 to 5 months
6 to 11 months
12 to 23 months
2 to 4
5 to 9
10 to 14
15 to 19
20 to 24
25 to 29
30 to 34
35 to 39
40 to 44
45 to 49
50 to 54
55 to 59
60 to 64
65 to 69
70 to 74
75 to 79
80 to 84
85 to 89
90 to 94
95 plus
All Ages
Age-standardised
Prevalence (%)
Year
Total
Moderate + Severe
Severe
Cause-Specific Anaemia Prevalence - 2021
Females
Males
Prevalence (%)
PfPr Malaria
PvPr Malaria
Clinical Malaria
Hookworm Disease
Schistosomiasis
Other NTDs
Other Infectious
HIV/AIDS
Maternal Hemorrhage
Uterine Fibroids
Menstrual Disorders
Beta-Thalassemia Major
Hemoglobin E/Beta-Thalassemia
Hemoglobin H
Beta-Thalassemia Trait
Hemoglobin E Trait
Homozygous SC & Severe SC
Hemoglobin SC Disease
Mild SC/Beta-thalassemia
Sickle Cell Trait
G6PD Deficiency
Hemizygous G6PD Deficiency
Other Hemoglobinopathies
Iron Deficiency
Vitamin A Deficiency
Gastritis & Duodenitis
Peptic Ulcer Disease
Crohn's Disease
Ulcerative Colitis
Cirrhosis
CKD Stage 3
CKD Stage 4
CKD Stage 5 untreated
CKD Stage 5 on dialysis
Hypothyroid Diseases
Hyperthyroid Diseases
Other Endocrine
Males
Females
1990
2021
Dietary iron deficiency
Other neglected tropical diseases
Haemoglobinopathies and haemolytic anaemias
Vitamin A deficiency
Other unspecified infectious diseases
Chronic kidney disease
Malaria
Intestinal nematode infections
Endocrine, metabolic, blood, and immune disorders
Schistosomiasis
Upper digestive system diseases
HIV/AIDS
Cirrhosis and other chronic liver diseases
Inflammatory bowel disease
Gynecological diseases
Maternal disorders
YLDs per 100,000 population
0 to <0.19
0.19 to <0.4
0.4 to <1.12
1.12 to <2.03
2.03 to <4.71
4.71 to <7.5
7.5 to <16.47
16.47 to <26.24
26.24 to <76.6
76.6 to 874.03

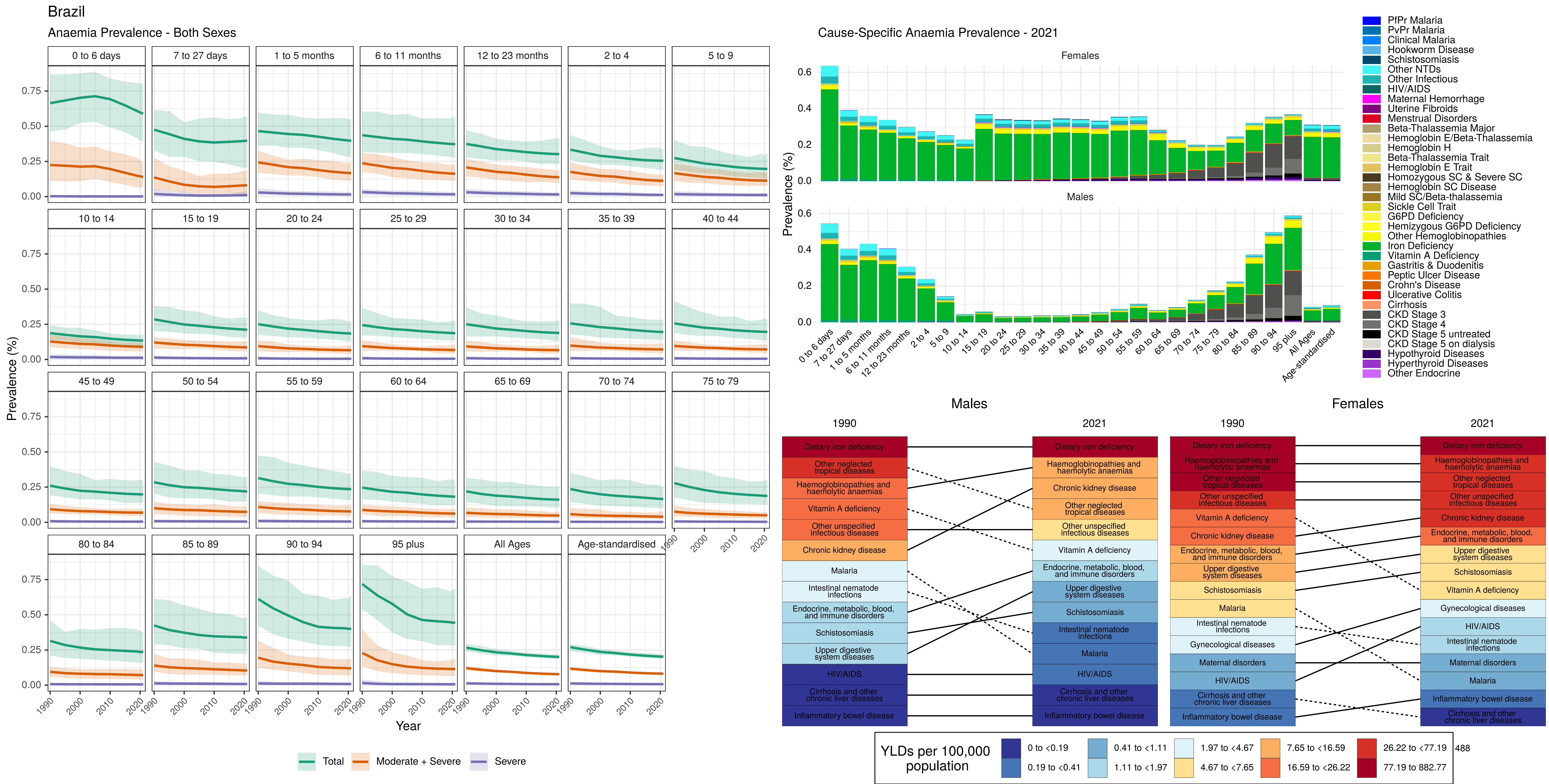
Brazil
Anaemia Prevalence - Both Sexes
0 to 6 days
7 to 27 days
1 to 5 months
6 to 11 months
12 to 23 months
2 to 4
5 to 9
10 to 14
15 to 19
20 to 24
25 to 29
30 to 34
35 to 39
40 to 44
45 to 49
50 to 54
55 to 59
60 to 64
65 to 69
70 to 74
75 to 79
80 to 84
85 to 89
90 to 94
95 plus
All Ages
Age-standardised
Prevalence (%)
Year
Total
Moderate + Severe
Severe
Cause-Specific Anaemia Prevalence - 2021
Females
Males
PfPr Malaria
PvPr Malaria
Clinical Malaria
Hookworm Disease
Schistosomiasis
Other NTDs
Other Infectious
HIV/AIDS
Maternal Hemorrhage
Uterine Fibroids
Menstrual Disorders
Beta-Thalassemia Major
Hemoglobin E/Beta-Thalassemia
Hemoglobin H
Beta-Thalassemia Trait
Hemoglobin E Trait
Homozygous SC & Severe SC
Hemoglobin SC Disease
Mild SC/Beta-thalassemia
Sickle Cell Trait
G6PD Deficiency
Hemizygous G6PD Deficiency
Other Hemoglobinopathies
Iron Deficiency
Vitamin A Deficiency
Gastritis & Duodenitis
Peptic Ulcer Disease
Crohn's Disease
Ulcerative Colitis
Cirrhosis
CKD Stage 3
CKD Stage 4
CKD Stage 5 untreated
CKD Stage 5 on dialysis
Hypothyroid Diseases
Hyperthyroid Diseases
Other Endocrine
Males
Females
1990
2021
Dietary iron deficiency
Other neglected tropical diseases
Haemoglobinopathies and haemolytic anaemias
Vitamin A deficiency
Other unspecified infectious diseases
Chronic kidney disease
Malaria
Intestinal nematode infections
Endocrine, metabolic, blood, and immune disorders
Schistosomiasis
Upper digestive system diseases
HIV/AIDS
Cirrhosis and other chronic liver diseases
Inflammatory bowel disease
Gynecological diseases
Maternal disorders
YLDs per 100,000 population
0 to <0.19
0.19 to <0.41
0.41 to <1.11
1.11 to <1.97
1.97 to <4.67
4.67 to <7.65
7.65 to <16.59
16.59 to <26.22
26.22 to <77.19
77.19 to 882.77

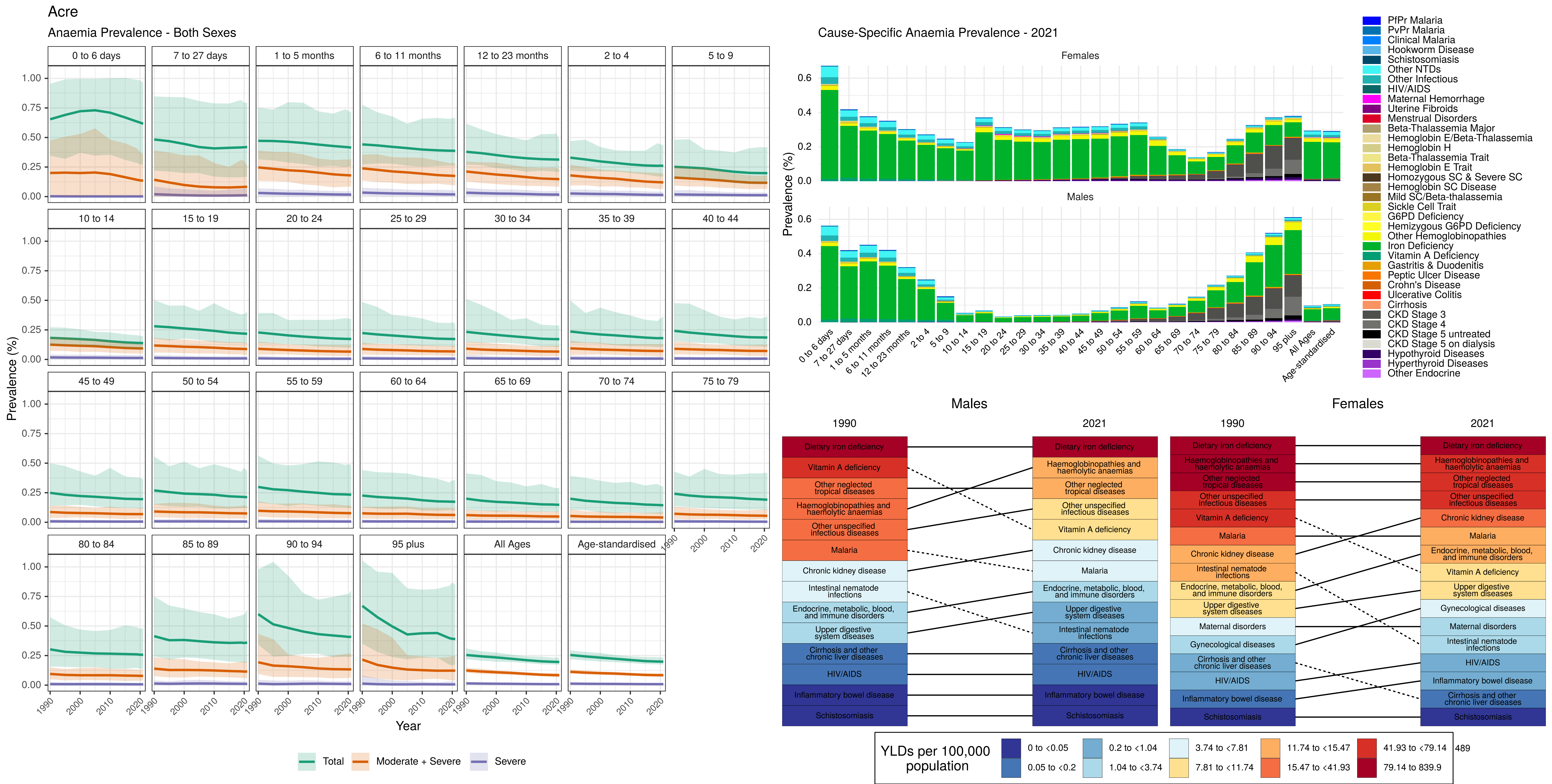

Acre
Anaemia Prevalence - Both Sexes
0 to 6 days
7 to 27 days
1 to 5 months
6 to 11 months
12 to 23 months
2 to 4
5 to 9
10 to 14
15 to 19
20 to 24
25 to 29
30 to 34
35 to 39
40 to 44
45 to 49
50 to 54
55 to 59
60 to 64
65 to 69
70 to 74
75 to 79
80 to 84
85 to 89
90 to 94
95 plus
All Ages
Age-standardised
Prevalence (%)
Year
Total
Moderate + Severe
Severe
Cause-Specific Anaemia Prevalence - 2021
Females
Males
PfPr Malaria
PvPr Malaria
Clinical Malaria
Hookworm Disease
Schistosomiasis
Other NTDs
Other Infectious
HIV/AIDS
Maternal Hemorrhage
Uterine Fibroids
Menstrual Disorders
Beta-Thalassemia Major
Hemoglobin E/Beta-Thalassemia
Hemoglobin H
Beta-Thalassemia Trait
Hemoglobin E Trait
Homozygous SC & Severe SC
Hemoglobin SC Disease
Mild SC/Beta-thalassemia
Sickle Cell Trait
G6PD Deficiency
Hemizygous G6PD Deficiency
Other Hemoglobinopathies
Iron Deficiency
Vitamin A Deficiency
Gastritis & Duodenitis
Peptic Ulcer Disease
Crohn's Disease
Ulcerative Colitis
Cirrhosis
CKD Stage 3
CKD Stage 4
CKD Stage 5 untreated
CKD Stage 5 on dialysis
Hypothyroid Diseases
Hyperthyroid Diseases
Other Endocrine
Males
1990
2021
Dietary iron deficiency
Vitamin A deficiency
Other neglected tropical diseases
Haemoglobinopathies and haemolytic anaemias
Other unspecified infectious diseases
Malaria
Chronic kidney disease
Intestinal nematode infections
Endocrine, metabolic, blood, and immune disorders
Upper digestive system diseases
Cirrhosis and other chronic liver diseases
HIV/AIDS
Inflammatory bowel disease
Schistosomiasis
Females
1990
2021
Maternal disorders
Gynecological diseases
YLDs per 100,000 population
0 to <0.05
0.05 to <0.2
0.2 to <1.04
1.04 to <3.74
3.74 to <7.81
7.81 to <11.74
11.74 to <15.47
15.47 to <41.93
41.93 to <79.14
79.14 to 839.9

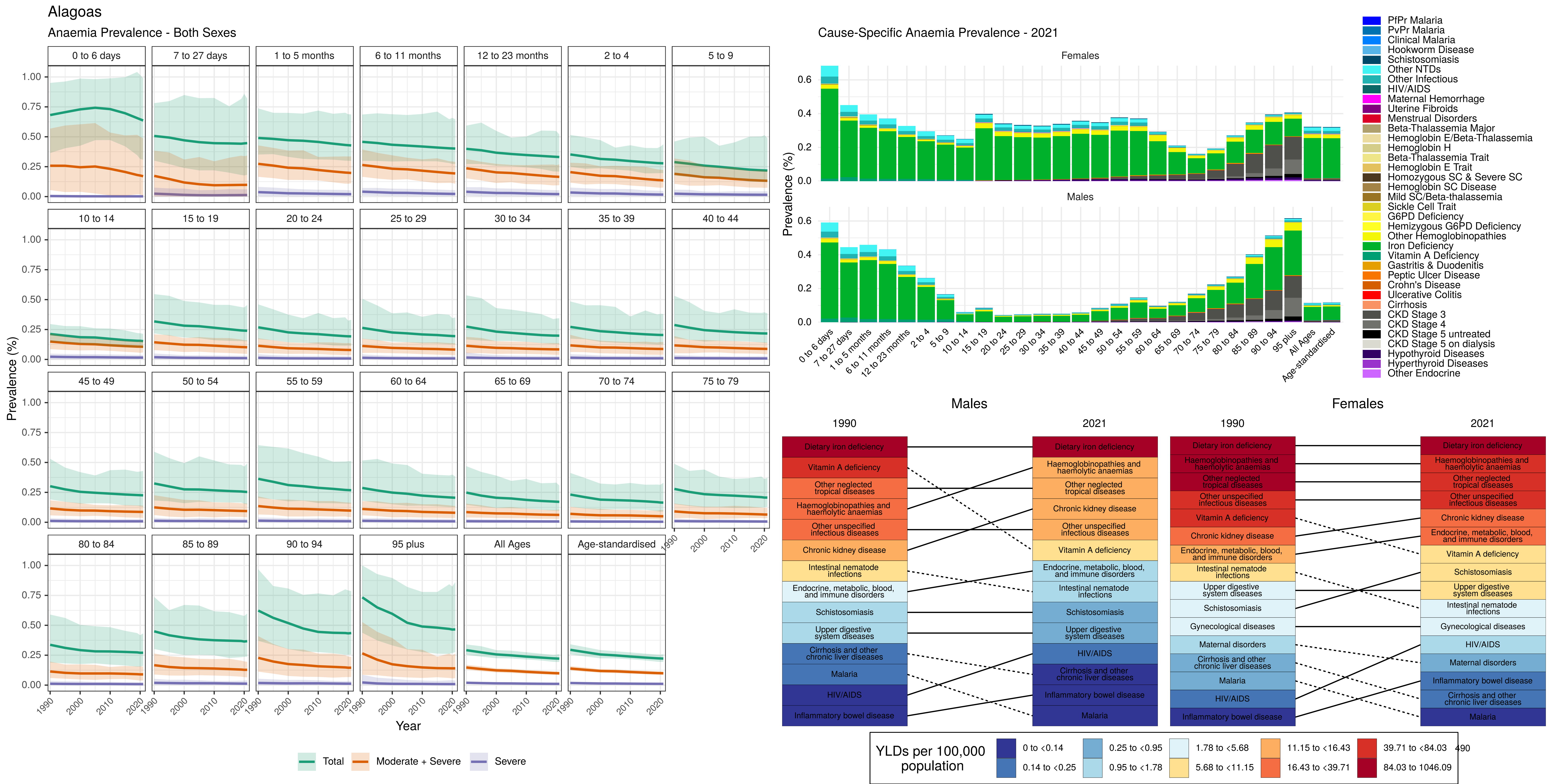
Alagoas
Anaemia Prevalence - Both Sexes
0 to 6 days
7 to 27 days
1 to 5 months
6 to 11 months
12 to 23 months
2 to 4
5 to 9
10 to 14
15 to 19
20 to 24
25 to 29
30 to 34
35 to 39
40 to 44
45 to 49
50 to 54
55 to 59
60 to 64
65 to 69
70 to 74
75 to 79
80 to 84
85 to 89
90 to 94
95 plus
All Ages
Age-standardised
Prevalence (%)
Year
Total
Moderate + Severe
Severe
Cause-Specific Anaemia Prevalence - 2021
Females
Males
Prevalence (%)
PfPr Malaria
PvPr Malaria
Clinical Malaria
Hookworm Disease
Schistosomiasis
Other NTDs
Other Infectious
HIV/AIDS
Maternal Hemorrhage
Uterine Fibroids
Menstrual Disorders
Beta-Thalassemia Major
Hemoglobin E/Beta-Thalassemia
Hemoglobin H
Beta-Thalassemia Trait
Hemoglobin E Trait
Homozygous SC & Severe SC
Hemoglobin SC Disease
Mild SC/Beta-thalassemia
Sickle Cell Trait
G6PD Deficiency
Hemizygous G6PD Deficiency
Other Hemoglobinopathies
Iron Deficiency
Vitamin A Deficiency
Gastritis & Duodenitis
Peptic Ulcer Disease
Crohn's Disease
Ulcerative Colitis
Cirrhosis
CKD Stage 3
CKD Stage 4
CKD Stage 5 untreated
CKD Stage 5 on dialysis
Hypothyroid Diseases
Hyperthyroid Diseases
Other Endocrine
Males
Females
1990
2021
Males 1990: Dietary iron deficiency; Vitamin A deficiency; Other neglected tropical diseases; Haemoglobinopathies and haemolytic anaemias; Other unspecified infectious diseases; Chronic kidney disease; Intestinal nematode infections; Endocrine, metabolic, blood, and immune disorders; Schistosomiasis; Upper digestive system diseases; Cirrhosis and other chronic liver diseases; Malaria; HIV/AIDS; Inflammatory bowel disease
Males 2021: Dietary iron deficiency; Haemoglobinopathies and haemolytic anaemias; Other neglected tropical diseases; Chronic kidney disease; Other unspecified infectious diseases; Vitamin A deficiency; Endocrine, metabolic, blood, and immune disorders; Intestinal nematode infections; Schistosomiasis; Upper digestive system diseases; HIV/AIDS; Cirrhosis and other chronic liver diseases; Inflammatory bowel disease; Malaria
Females 1990: Dietary iron deficiency; Haemoglobinopathies and haemolytic anaemias; Other neglected tropical diseases; Other unspecified infectious diseases; Vitamin A deficiency; Chronic kidney disease; Endocrine, metabolic, blood, and immune disorders; Intestinal nematode infections; Upper digestive system diseases; Schistosomiasis; Gynecological diseases; Maternal disorders; Cirrhosis and other chronic liver diseases; Malaria; HIV/AIDS; Inflammatory bowel disease
Females 2021: Dietary iron deficiency; Haemoglobinopathies and haemolytic anaemias; Other neglected tropical diseases; Other unspecified infectious diseases; Chronic kidney disease; Endocrine, metabolic, blood, and immune disorders; Vitamin A deficiency; Schistosomiasis; Upper digestive system diseases; Intestinal nematode infections; Gynecological diseases; HIV/AIDS; Maternal disorders; Inflammatory bowel disease; Cirrhosis and other chronic liver diseases; Malaria
YLDs per 100,000 population
0 to <0.14
0.14 to <0.25
0.25 to <0.95
0.95 to <1.78
1.78 to <5.68
5.68 to <11.15
11.15 to <16.43
16.43 to <39.71
39.71 to <84.03
84.03 to 1046.09

Amapá

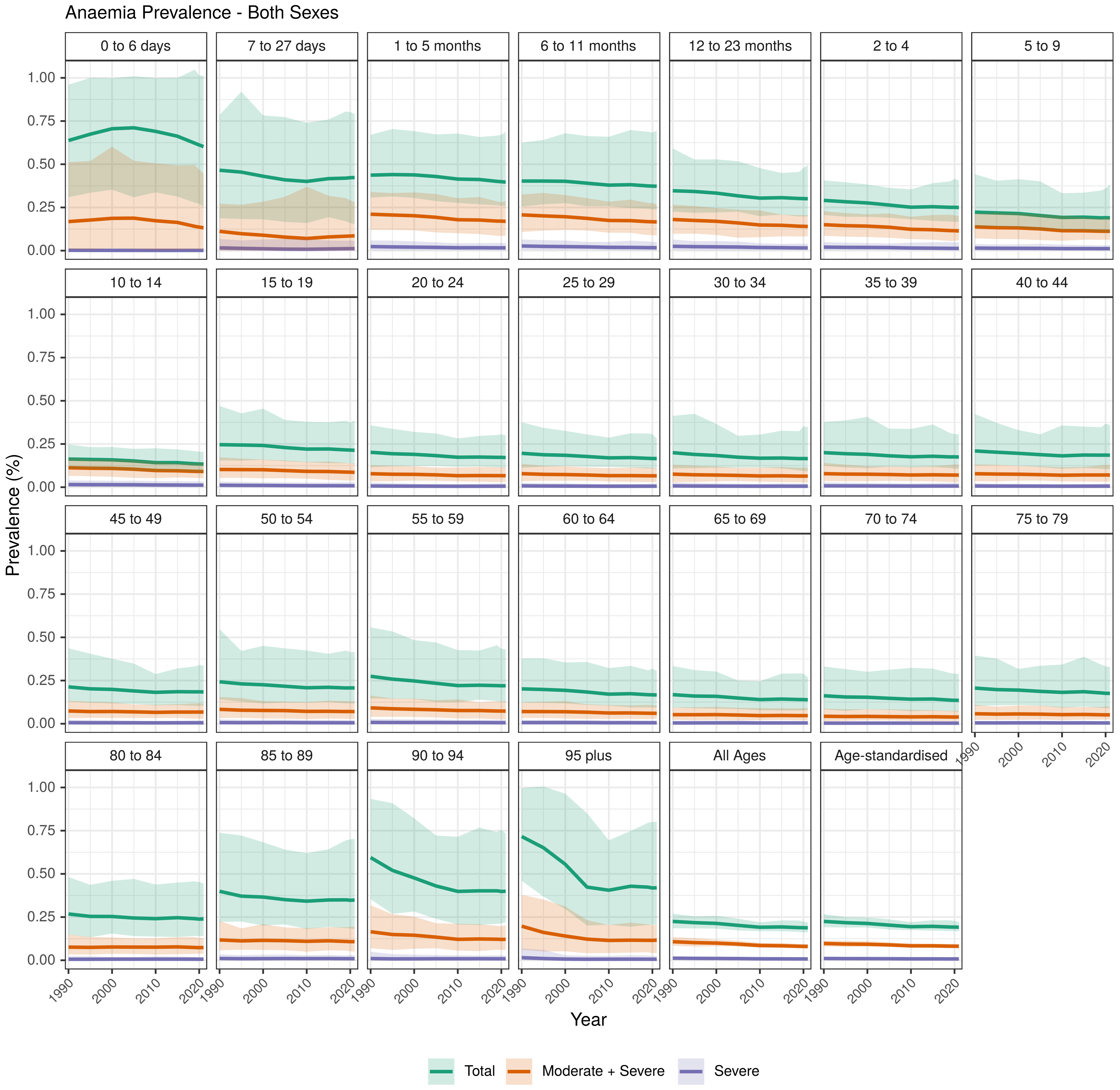
Anaemia Prevalence - Both Sexes
0 to 6 days
7 to 27 days
1 to 5 months
6 to 11 months
12 to 23 months
2 to 4
5 to 9
10 to 14
15 to 19
20 to 24
25 to 29
30 to 34
35 to 39
40 to 44
45 to 49
50 to 54
55 to 59
60 to 64
65 to 69
70 to 74
75 to 79
80 to 84
85 to 89
90 to 94
95 plus
All Ages
Age-standardised
Prevalence (%)
Year
Total
Moderate + Severe
Severe

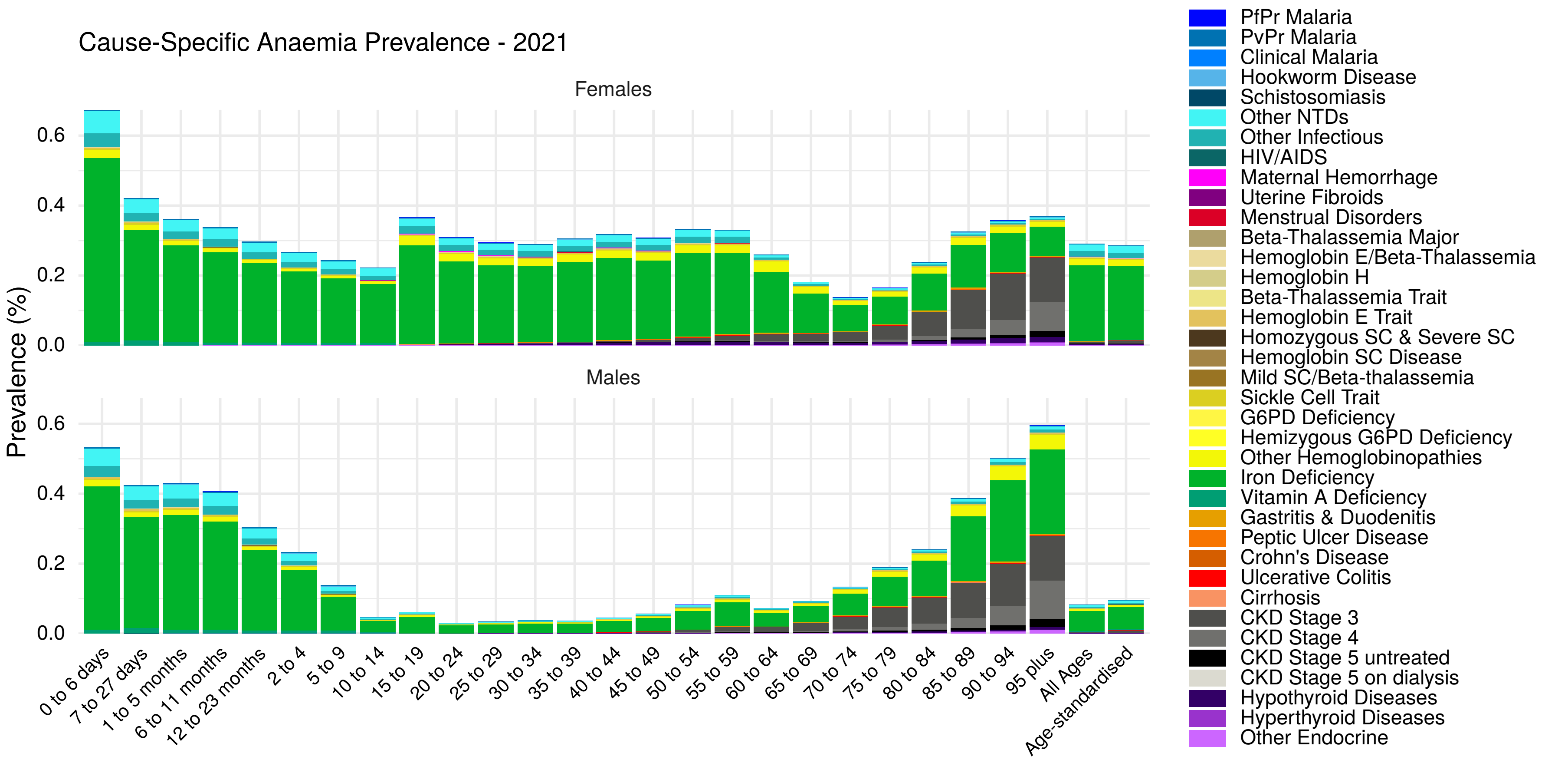
Cause-Specific Anaemia Prevalence - 2021
Females
Males
Prevalence (%)
PfPr Malaria
PvPr Malaria
Clinical Malaria
Hookworm Disease
Schistosomiasis
Other NTDs
Other Infectious
HIV/AIDS
Maternal Hemorrhage
Uterine Fibroids
Menstrual Disorders
Beta-Thalassemia Major
Hemoglobin E/Beta-Thalassemia
Hemoglobin H
Beta-Thalassemia Trait
Hemoglobin E Trait
Homozygous SC & Severe SC
Hemoglobin SC Disease
Mild SC/Beta-thalassemia
Sickle Cell Trait
G6PD Deficiency
Hemizygous G6PD Deficiency
Other Hemoglobinopathies
Iron Deficiency
Vitamin A Deficiency
Gastritis & Duodenitis
Peptic Ulcer Disease
Crohn's Disease
Ulcerative Colitis
Cirrhosis
CKD Stage 3
CKD Stage 4
CKD Stage 5 untreated
CKD Stage 5 on dialysis
Hypothyroid Diseases
Hyperthyroid Diseases
Other Endocrine

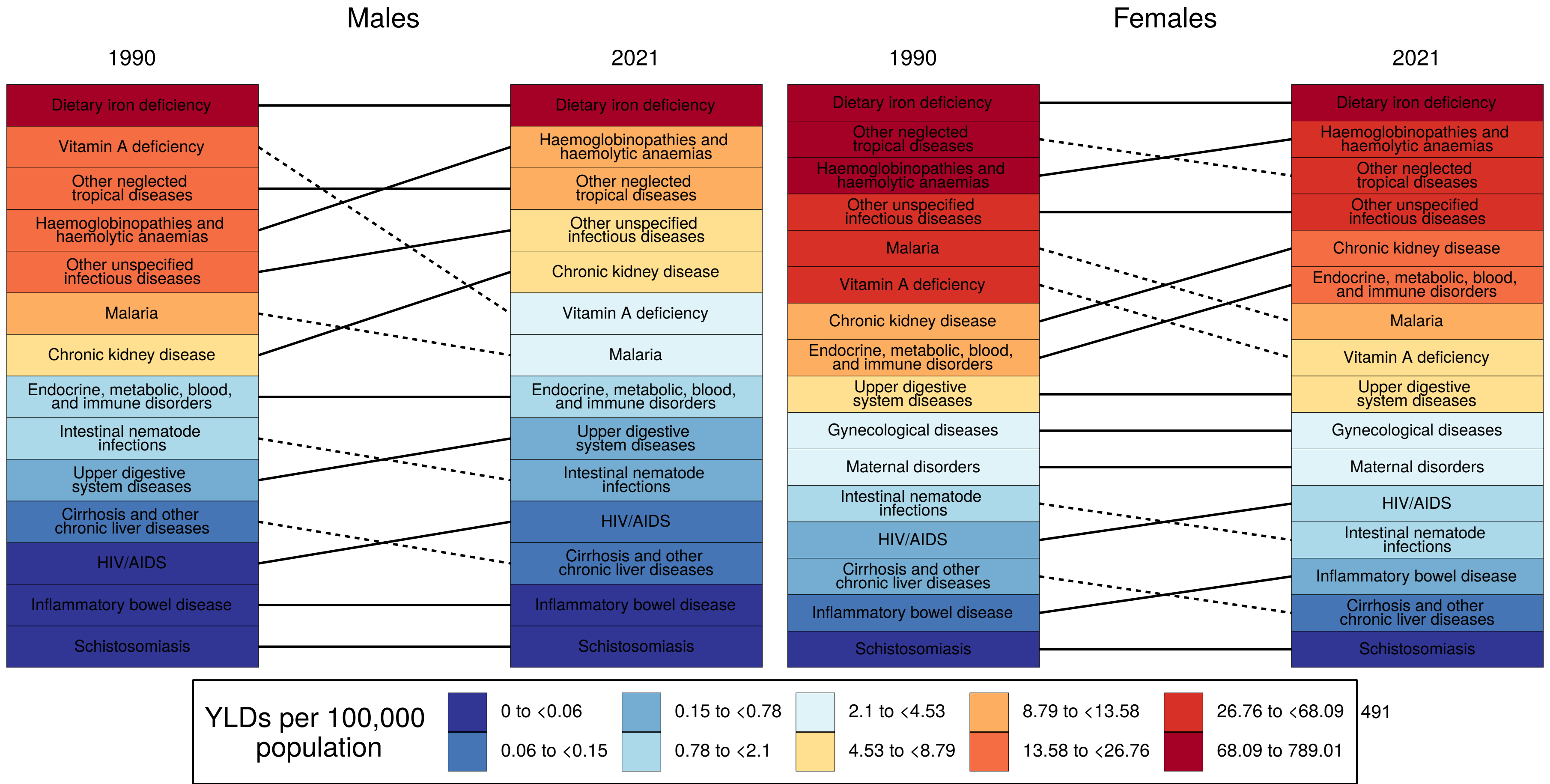
Males
Females
1990
2021
Dietary iron deficiency
Vitamin A deficiency
Other neglected tropical diseases
Haemoglobinopathies and haemolytic anaemias
Other unspecified infectious diseases
Malaria
Chronic kidney disease
Endocrine, metabolic, blood, and immune disorders
Intestinal nematode infections
Upper digestive system diseases
Cirrhosis and other chronic liver diseases
HIV/AIDS
Inflammatory bowel disease
Schistosomiasis
Gynecological diseases
Maternal disorders
YLDs per 100,000 population
0 to <0.06
0.06 to <0.15
0.15 to <0.78
0.78 to <2.1
2.1 to <4.53
4.53 to <8.79
8.79 to <13.58
13.58 to <26.76
26.76 to <68.09
68.09 to 789.01

Amazonas

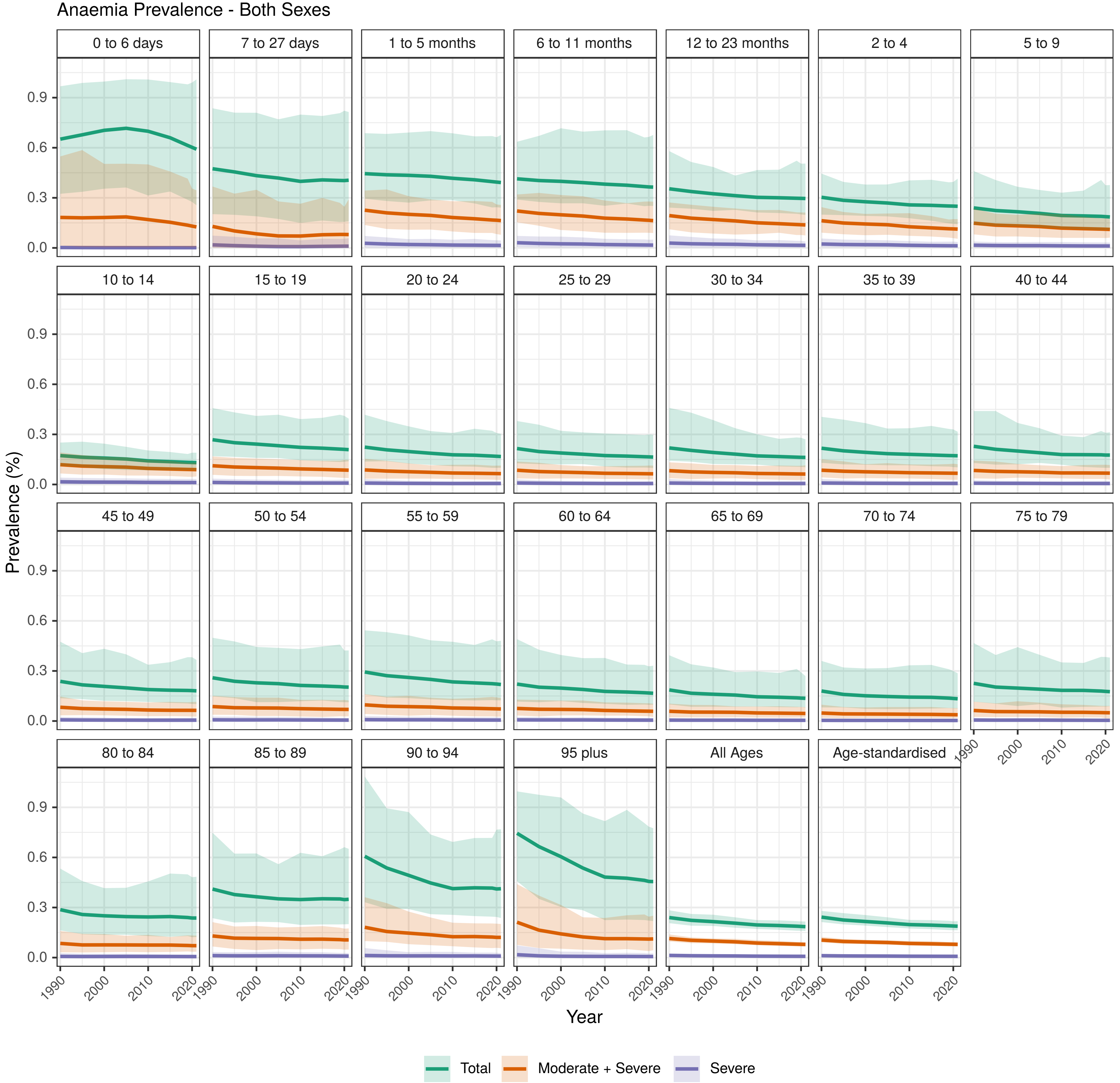

Anaemia Prevalence - Both Sexes
0 to 6 days
7 to 27 days
1 to 5 months
6 to 11 months
12 to 23 months
2 to 4
5 to 9
10 to 14
15 to 19
20 to 24
25 to 29
30 to 34
35 to 39
40 to 44
45 to 49
50 to 54
55 to 59
60 to 64
65 to 69
70 to 74
75 to 79
80 to 84
85 to 89
90 to 94
95 plus
All Ages
Age-standardised
Prevalence (%)
0.0
0.3
0.6
0.9
1990
2000
2010
2020
Year
Total
Moderate + Severe
Severe

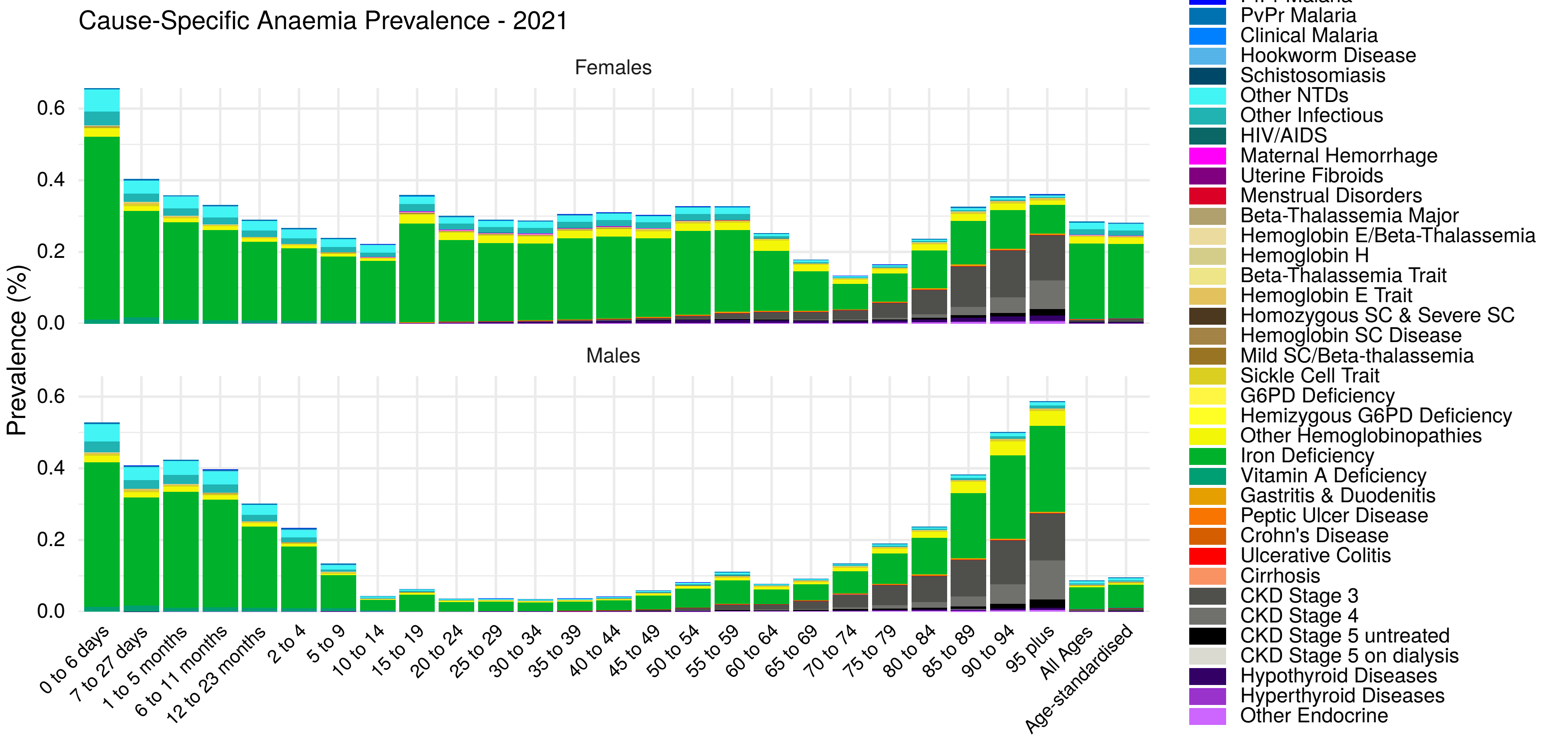

Cause-Specific Anaemia Prevalence - 2021
Females
Males
Prevalence (%)
0.0
0.2
0.4
0.6
0 to 6 days
7 to 27 days
1 to 5 months
6 to 11 months
12 to 23 months
2 to 4
5 to 9
10 to 14
15 to 19
20 to 24
25 to 29
30 to 34
35 to 39
40 to 44
45 to 49
50 to 54
55 to 59
60 to 64
65 to 69
70 to 74
75 to 79
80 to 84
85 to 89
90 to 94
95 plus
All Ages
Age-standardised
PfPr Malaria
PvPr Malaria
Clinical Malaria
Hookworm Disease
Schistosomiasis
Other NTDs
Other Infectious
HIV/AIDS
Maternal Hemorrhage
Uterine Fibroids
Menstrual Disorders
Beta-Thalassemia Major
Hemoglobin E/Beta-Thalassemia
Hemoglobin H
Beta-Thalassemia Trait
Hemoglobin E Trait
Homozygous SC & Severe SC
Hemoglobin SC Disease
Mild SC/Beta-thalassemia
Sickle Cell Trait
G6PD Deficiency
Hemizygous G6PD Deficiency
Other Hemoglobinopathies
Iron Deficiency
Vitamin A Deficiency
Gastritis & Duodenitis
Peptic Ulcer Disease
Crohn's Disease
Ulcerative Colitis
Cirrhosis
CKD Stage 3
CKD Stage 4
CKD Stage 5 untreated
CKD Stage 5 on dialysis
Hypothyroid Diseases
Hyperthyroid Diseases
Other Endocrine

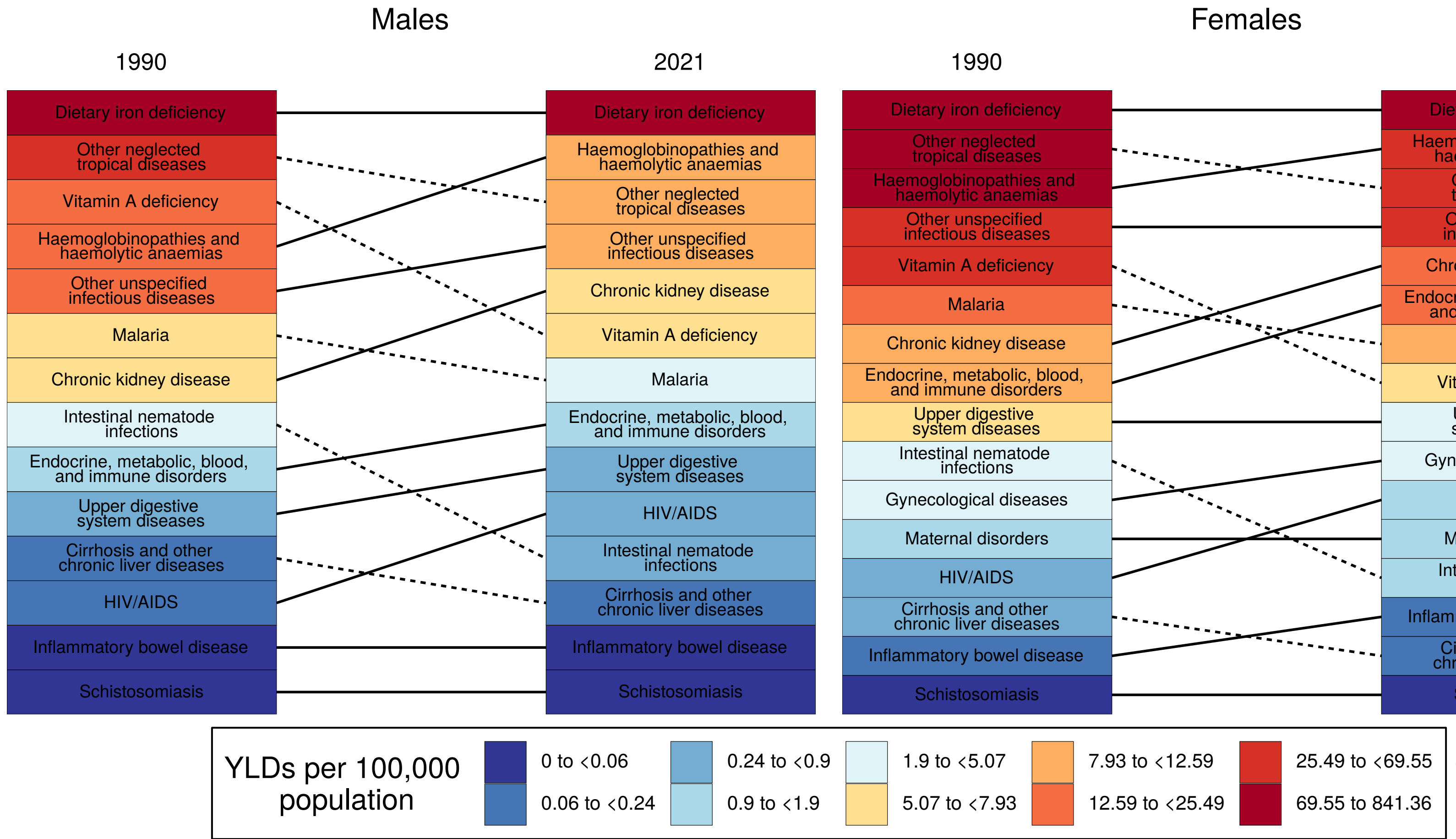

Males
Females
1990
2021
Dietary iron deficiency
Other neglected tropical diseases
Vitamin A deficiency
Haemoglobinopathies and haemolytic anaemias
Other unspecified infectious diseases
Malaria
Chronic kidney disease
Intestinal nematode infections
Endocrine, metabolic, blood, and immune disorders
Upper digestive system diseases
Cirrhosis and other chronic liver diseases
HIV/AIDS
Inflammatory bowel disease
Schistosomiasis
Gynecological diseases
Maternal disorders
YLDs per 100,000 population
0 to <0.06
0.06 to <0.24
0.24 to <0.9
0.9 to <1.9
1.9 to <5.07
5.07 to <7.93
7.93 to <12.59
12.59 to <25.49
25.49 to <69.55
69.55 to 841.36

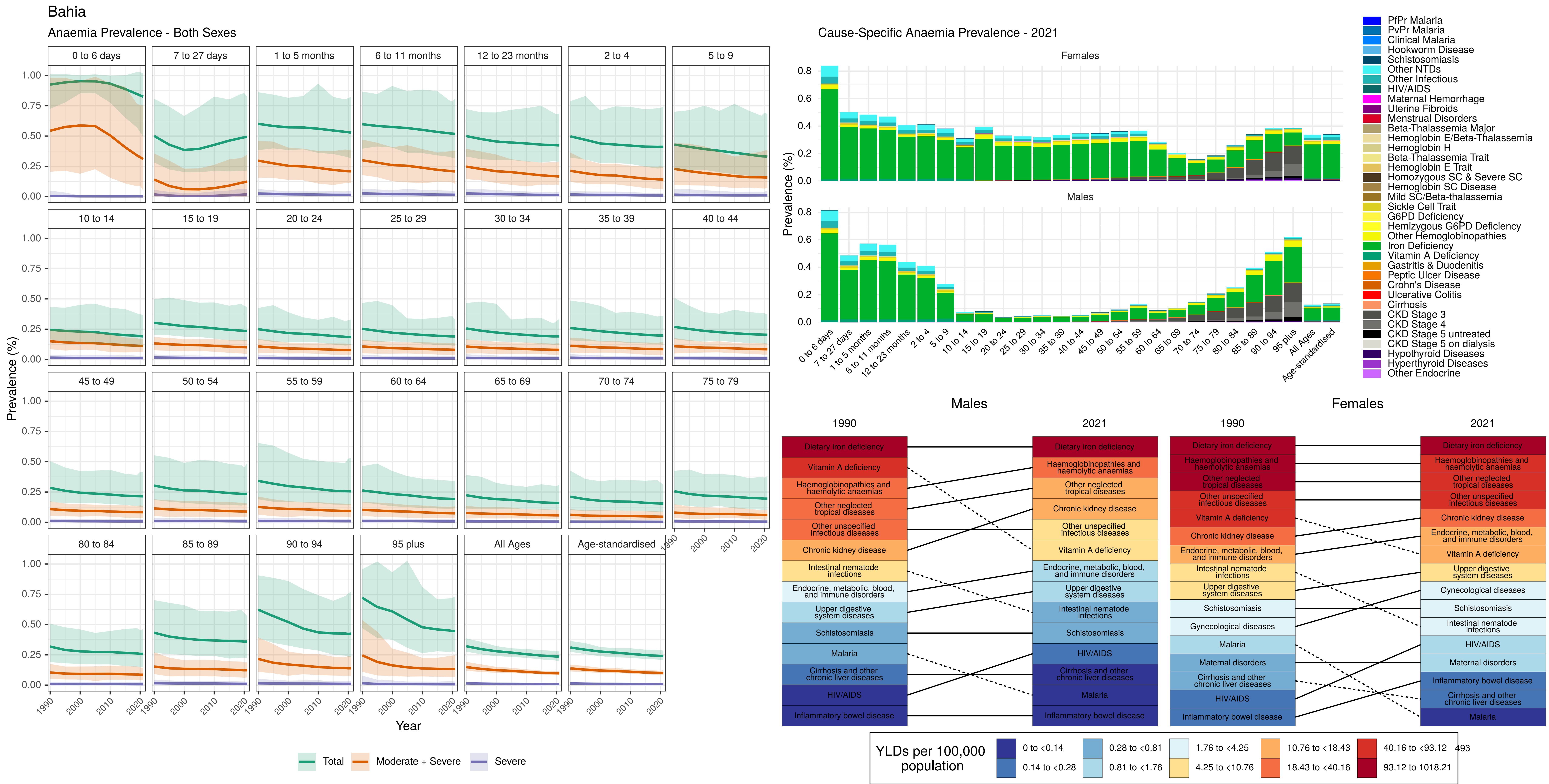

Bahia
Anaemia Prevalence - Both Sexes
0 to 6 days
7 to 27 days
1 to 5 months
6 to 11 months
12 to 23 months
2 to 4
5 to 9
10 to 14
15 to 19
20 to 24
25 to 29
30 to 34
35 to 39
40 to 44
45 to 49
50 to 54
55 to 59
60 to 64
65 to 69
70 to 74
75 to 79
80 to 84
85 to 89
90 to 94
95 plus
All Ages
Age-standardised
Prevalence (%)
Year
Total
Moderate + Severe
Severe
Cause-Specific Anaemia Prevalence - 2021
Females
Males
Prevalence (%)
PfPr Malaria
PvPr Malaria
Clinical Malaria
Hookworm Disease
Schistosomiasis
Other NTDs
Other Infectious
HIV/AIDS
Maternal Hemorrhage
Uterine Fibroids
Menstrual Disorders
Beta-Thalassemia Major
Hemoglobin E/Beta-Thalassemia
Hemoglobin H
Beta-Thalassemia Trait
Hemoglobin E Trait
Homozygous SC & Severe SC
Hemoglobin SC Disease
Mild SC/Beta-thalassemia
Sickle Cell Trait
G6PD Deficiency
Hemizygous G6PD Deficiency
Other Hemoglobinopathies
Iron Deficiency
Vitamin A Deficiency
Gastritis & Duodenitis
Peptic Ulcer Disease
Crohn's Disease
Ulcerative Colitis
Cirrhosis
CKD Stage 3
CKD Stage 4
CKD Stage 5 untreated
CKD Stage 5 on dialysis
Hypothyroid Diseases
Hyperthyroid Diseases
Other Endocrine
Males
Females
1990
2021
Dietary iron deficiency
Vitamin A deficiency
Haemoglobinopathies and haemolytic anaemias
Other neglected tropical diseases
Other unspecified infectious diseases
Chronic kidney disease
Intestinal nematode infections
Endocrine, metabolic, blood, and immune disorders
Upper digestive system diseases
Schistosomiasis
Malaria
Cirrhosis and other chronic liver diseases
HIV/AIDS
Inflammatory bowel disease
Gynecological diseases
Maternal disorders
YLDs per 100,000 population
0 to <0.14
0.14 to <0.28
0.28 to <0.81
0.81 to <1.76
1.76 to <4.25
4.25 to <10.76
10.76 to <18.43
18.43 to <40.16
40.16 to <93.12
93.12 to 1018.21

Ceará

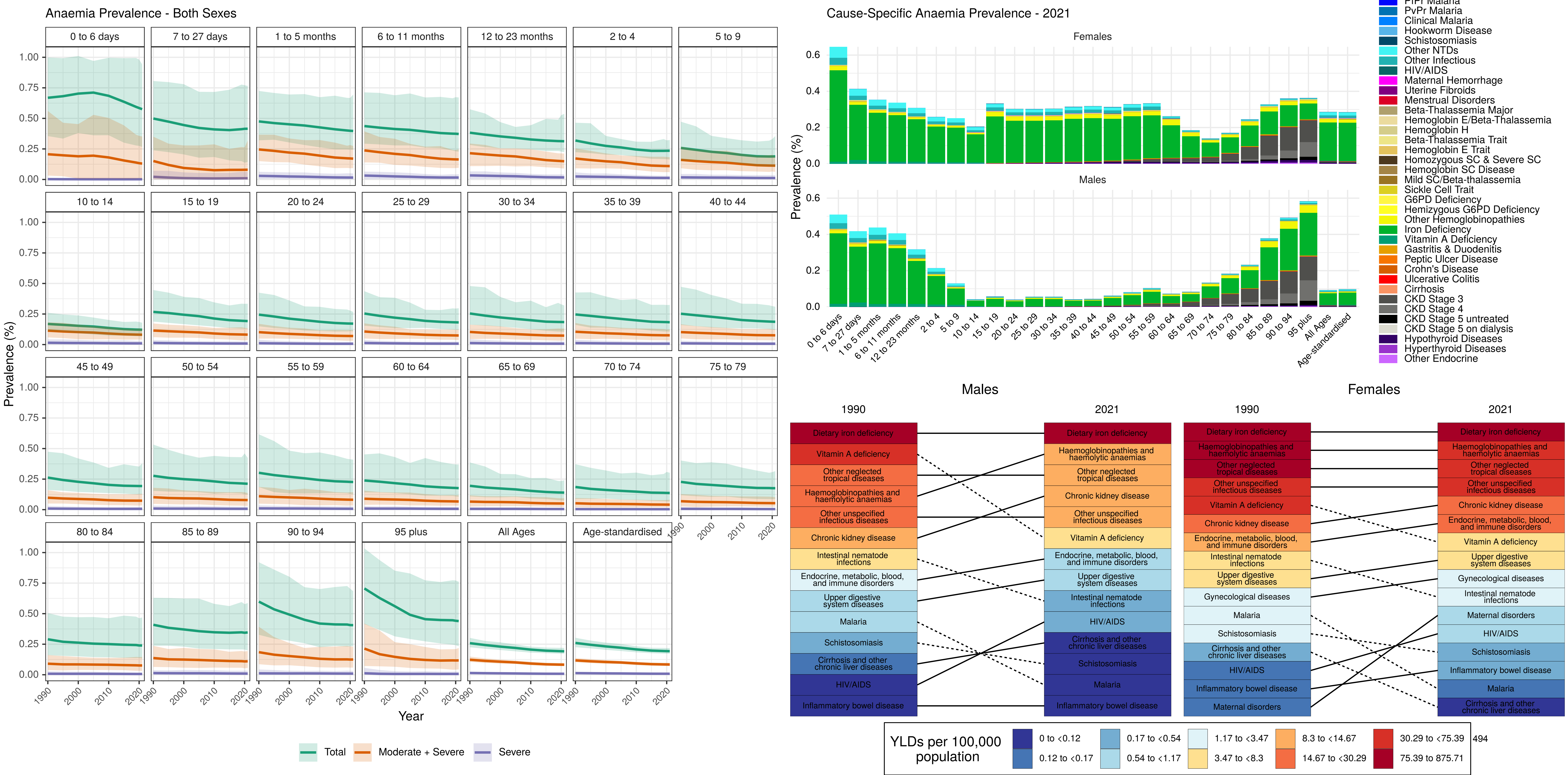

Anaemia Prevalence - Both Sexes
0 to 6 days
7 to 27 days
1 to 5 months
6 to 11 months
12 to 23 months
2 to 4
5 to 9
10 to 14
15 to 19
20 to 24
25 to 29
30 to 34
35 to 39
40 to 44
45 to 49
50 to 54
55 to 59
60 to 64
65 to 69
70 to 74
75 to 79
80 to 84
85 to 89
90 to 94
95 plus
All Ages
Age-standardised
Prevalence (%)
Year
Total
Moderate + Severe
Severe
Cause-Specific Anaemia Prevalence - 2021
Females
Males
PfPr Malaria
PvPr Malaria
Clinical Malaria
Hookworm Disease
Schistosomiasis
Other NTDs
Other Infectious
HIV/AIDS
Maternal Hemorrhage
Uterine Fibroids
Menstrual Disorders
Beta-Thalassemia Major
Hemoglobin E/Beta-Thalassemia
Hemoglobin H
Beta-Thalassemia Trait
Hemoglobin E Trait
Homozygous SC & Severe SC
Hemoglobin SC Disease
Mild SC/Beta-thalassemia
Sickle Cell Trait
G6PD Deficiency
Hemizygous G6PD Deficiency
Other Hemoglobinopathies
Iron Deficiency
Vitamin A Deficiency
Gastritis & Duodenitis
Peptic Ulcer Disease
Crohn's Disease
Ulcerative Colitis
Cirrhosis
CKD Stage 3
CKD Stage 4
CKD Stage 5 untreated
CKD Stage 5 on dialysis
Hypothyroid Diseases
Hyperthyroid Diseases
Other Endocrine
Males
Females
1990
2021
Dietary iron deficiency
Vitamin A deficiency
Other neglected tropical diseases
Haemoglobinopathies and haemolytic anaemias
Other unspecified infectious diseases
Chronic kidney disease
Intestinal nematode infections
Endocrine, metabolic, blood, and immune disorders
Upper digestive system diseases
Malaria
Schistosomiasis
Cirrhosis and other chronic liver diseases
HIV/AIDS
Inflammatory bowel disease
Gynecological diseases
Maternal disorders
YLDs per 100,000 population
0 to <0.12
0.12 to <0.17
0.17 to <0.54
0.54 to <1.17
1.17 to <3.47
3.47 to <8.3
8.3 to <14.67
14.67 to <30.29
30.29 to <75.39
75.39 to 875.71

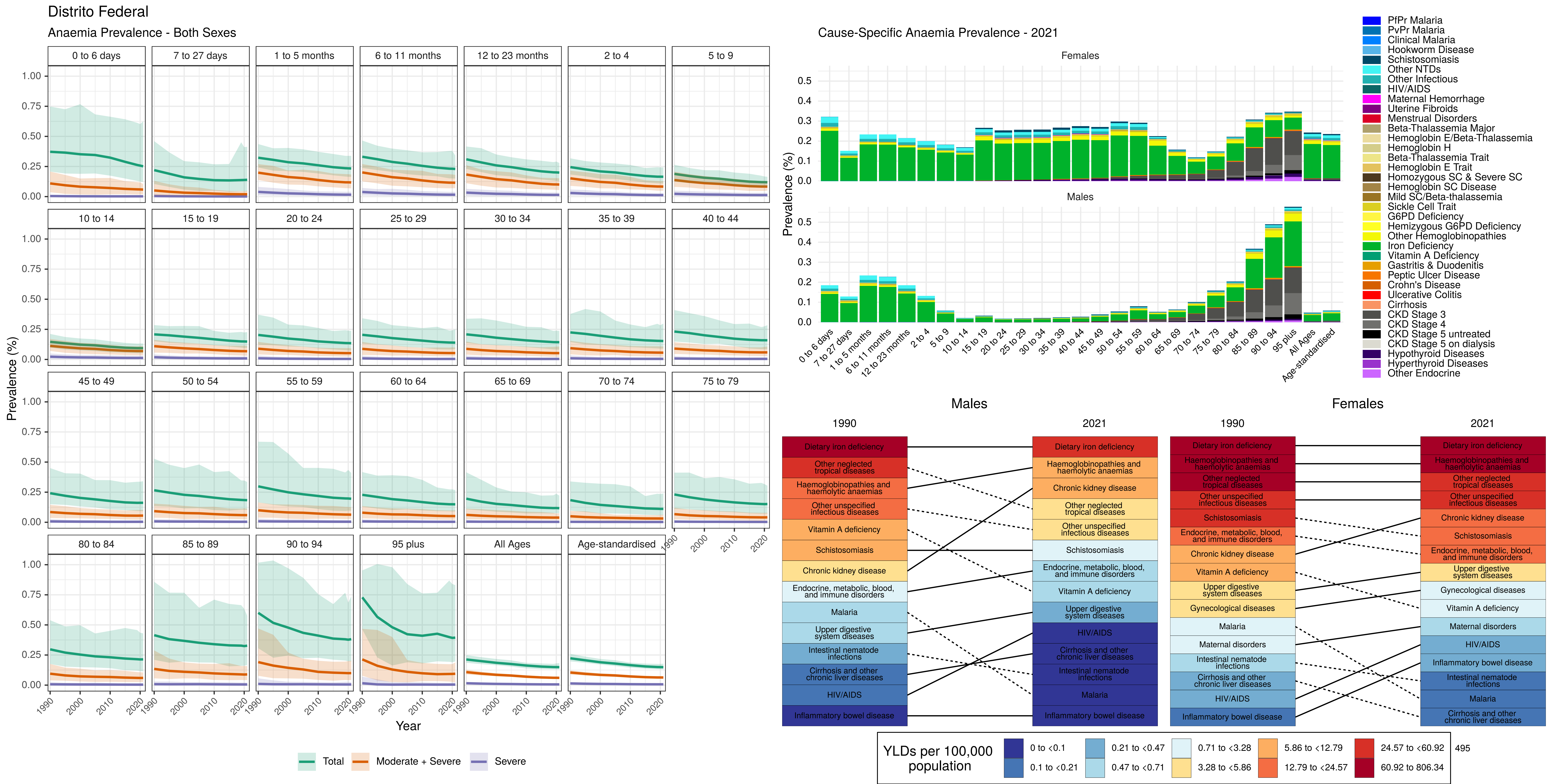

Distrito Federal
Anaemia Prevalence - Both Sexes
0 to 6 days
7 to 27 days
1 to 5 months
6 to 11 months
12 to 23 months
2 to 4
5 to 9
10 to 14
15 to 19
20 to 24
25 to 29
30 to 34
35 to 39
40 to 44
45 to 49
50 to 54
55 to 59
60 to 64
65 to 69
70 to 74
75 to 79
80 to 84
85 to 89
90 to 94
95 plus
All Ages
Age-standardised
Prevalence (%)
Year
Total
Moderate + Severe
Severe
Cause-Specific Anaemia Prevalence - 2021
Females
Males
PfPr Malaria
PvPr Malaria
Clinical Malaria
Hookworm Disease
Schistosomiasis
Other NTDs
Other Infectious
HIV/AIDS
Maternal Hemorrhage
Uterine Fibroids
Menstrual Disorders
Beta-Thalassemia Major
Hemoglobin E/Beta-Thalassemia
Hemoglobin H
Beta-Thalassemia Trait
Hemoglobin E Trait
Homozygous SC & Severe SC
Hemoglobin SC Disease
Mild SC/Beta-thalassemia
Sickle Cell Trait
G6PD Deficiency
Hemizygous G6PD Deficiency
Other Hemoglobinopathies
Iron Deficiency
Vitamin A Deficiency
Gastritis & Duodenitis
Peptic Ulcer Disease
Crohn's Disease
Ulcerative Colitis
Cirrhosis
CKD Stage 3
CKD Stage 4
CKD Stage 5 untreated
CKD Stage 5 on dialysis
Hypothyroid Diseases
Hyperthyroid Diseases
Other Endocrine
Males
Females
1990
2021
Dietary iron deficiency
Other neglected tropical diseases
Haemoglobinopathies and haemolytic anaemias
Other unspecified infectious diseases
Vitamin A deficiency
Schistosomiasis
Chronic kidney disease
Endocrine, metabolic, blood, and immune disorders
Malaria
Upper digestive system diseases
Intestinal nematode infections
Cirrhosis and other chronic liver diseases
HIV/AIDS
Inflammatory bowel disease
Gynecological diseases
Maternal disorders
YLDs per 100,000 population
0 to <0.1
0.1 to <0.21
0.21 to <0.47
0.47 to <0.71
0.71 to <3.28
3.28 to <5.86
5.86 to <12.79
12.79 to <24.57
24.57 to <60.92
60.92 to 806.34

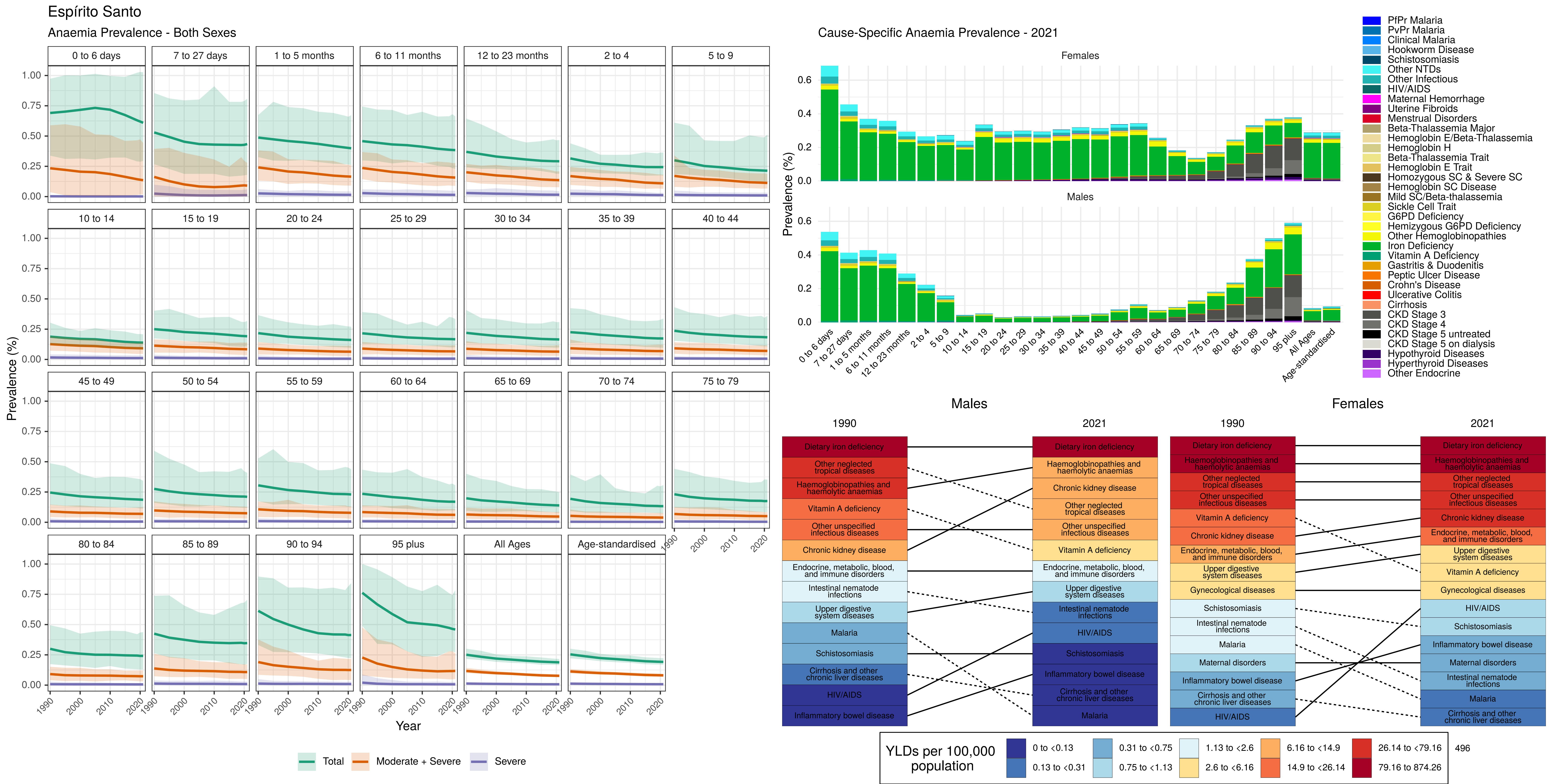

Espírito Santo
Anaemia Prevalence - Both Sexes
0 to 6 days
7 to 27 days
1 to 5 months
6 to 11 months
12 to 23 months
2 to 4
5 to 9
10 to 14
15 to 19
20 to 24
25 to 29
30 to 34
35 to 39
40 to 44
45 to 49
50 to 54
55 to 59
60 to 64
65 to 69
70 to 74
75 to 79
80 to 84
85 to 89
90 to 94
95 plus
All Ages
Age-standardised
Prevalence (%)
Year
Total
Moderate + Severe
Severe
Cause-Specific Anaemia Prevalence - 2021
Females
Males
Prevalence (%)
PfPr Malaria
PvPr Malaria
Clinical Malaria
Hookworm Disease
Schistosomiasis
Other NTDs
Other Infectious
HIV/AIDS
Maternal Hemorrhage
Uterine Fibroids
Menstrual Disorders
Beta-Thalassemia Major
Hemoglobin E/Beta-Thalassemia
Hemoglobin H
Beta-Thalassemia Trait
Hemoglobin E Trait
Homozygous SC & Severe SC
Hemoglobin SC Disease
Mild SC/Beta-thalassemia
Sickle Cell Trait
G6PD Deficiency
Hemizygous G6PD Deficiency
Other Hemoglobinopathies
Iron Deficiency
Vitamin A Deficiency
Gastritis & Duodenitis
Peptic Ulcer Disease
Crohn's Disease
Ulcerative Colitis
Cirrhosis
CKD Stage 3
CKD Stage 4
CKD Stage 5 untreated
CKD Stage 5 on dialysis
Hypothyroid Diseases
Hyperthyroid Diseases
Other Endocrine
Males
Females
1990
2021
Dietary iron deficiency
Other neglected tropical diseases
Haemoglobinopathies and haemolytic anaemias
Vitamin A deficiency
Other unspecified infectious diseases
Chronic kidney disease
Endocrine, metabolic, blood, and immune disorders
Intestinal nematode infections
Upper digestive system diseases
Malaria
Schistosomiasis
Cirrhosis and other chronic liver diseases
HIV/AIDS
Inflammatory bowel disease
Gynecological diseases
Maternal disorders
YLDs per 100,000 population
0 to <0.13
0.13 to <0.31
0.31 to <0.75
0.75 to <1.13
1.13 to <2.6
2.6 to <6.16
6.16 to <14.9
14.9 to <26.14
26.14 to <79.16
79.16 to 874.26
496

Goiás

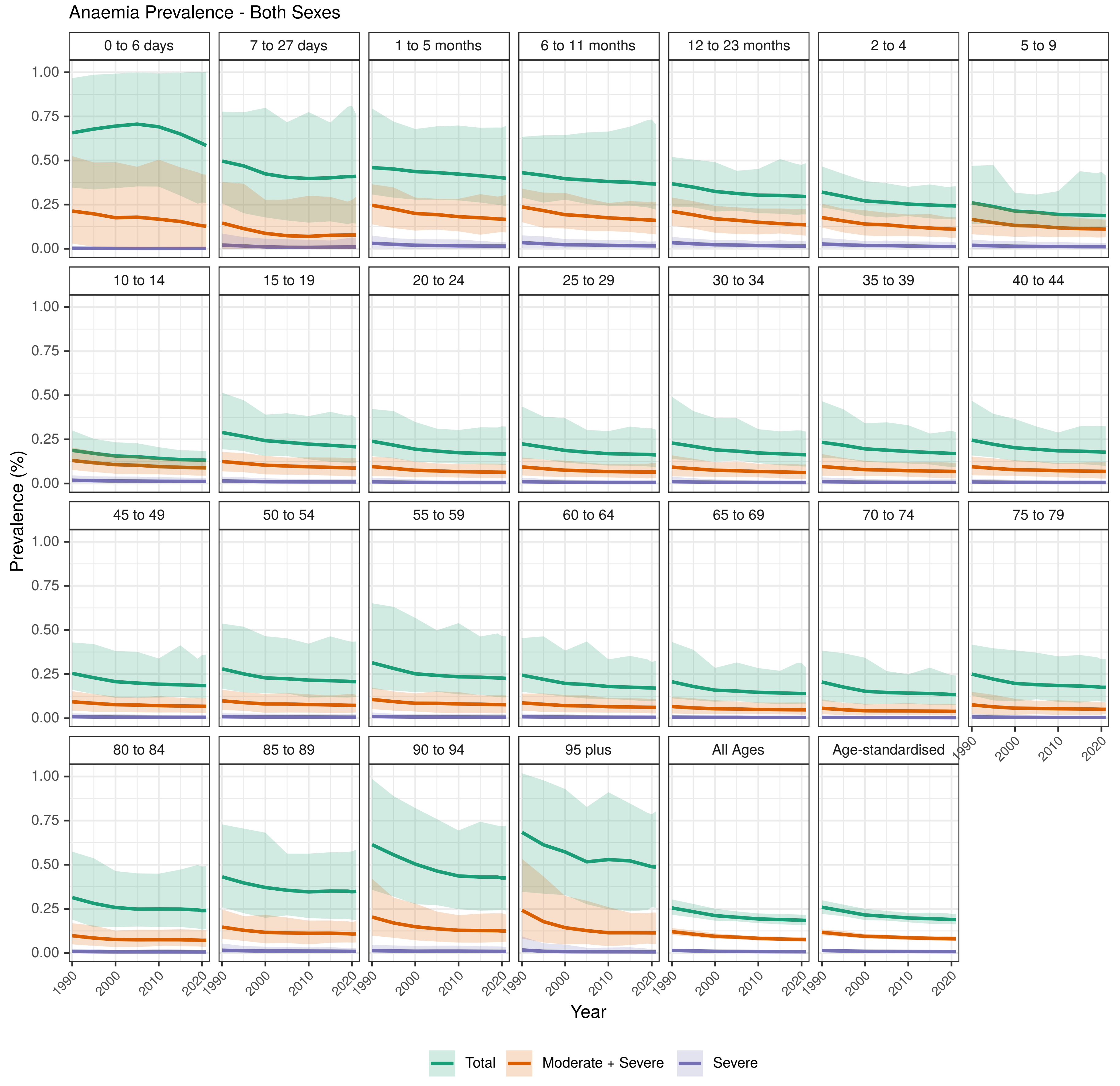
Anaemia Prevalence - Both Sexes
0 to 6 days
7 to 27 days
1 to 5 months
6 to 11 months
12 to 23 months
2 to 4
5 to 9
10 to 14
15 to 19
20 to 24
25 to 29
30 to 34
35 to 39
40 to 44
45 to 49
50 to 54
55 to 59
60 to 64
65 to 69
70 to 74
75 to 79
80 to 84
85 to 89
90 to 94
95 plus
All Ages
Age-standardised
Prevalence (%)
1.00
0.75
0.50
0.25
0.00
1990
2000
2010
2020
Year
Total
Moderate + Severe
Severe

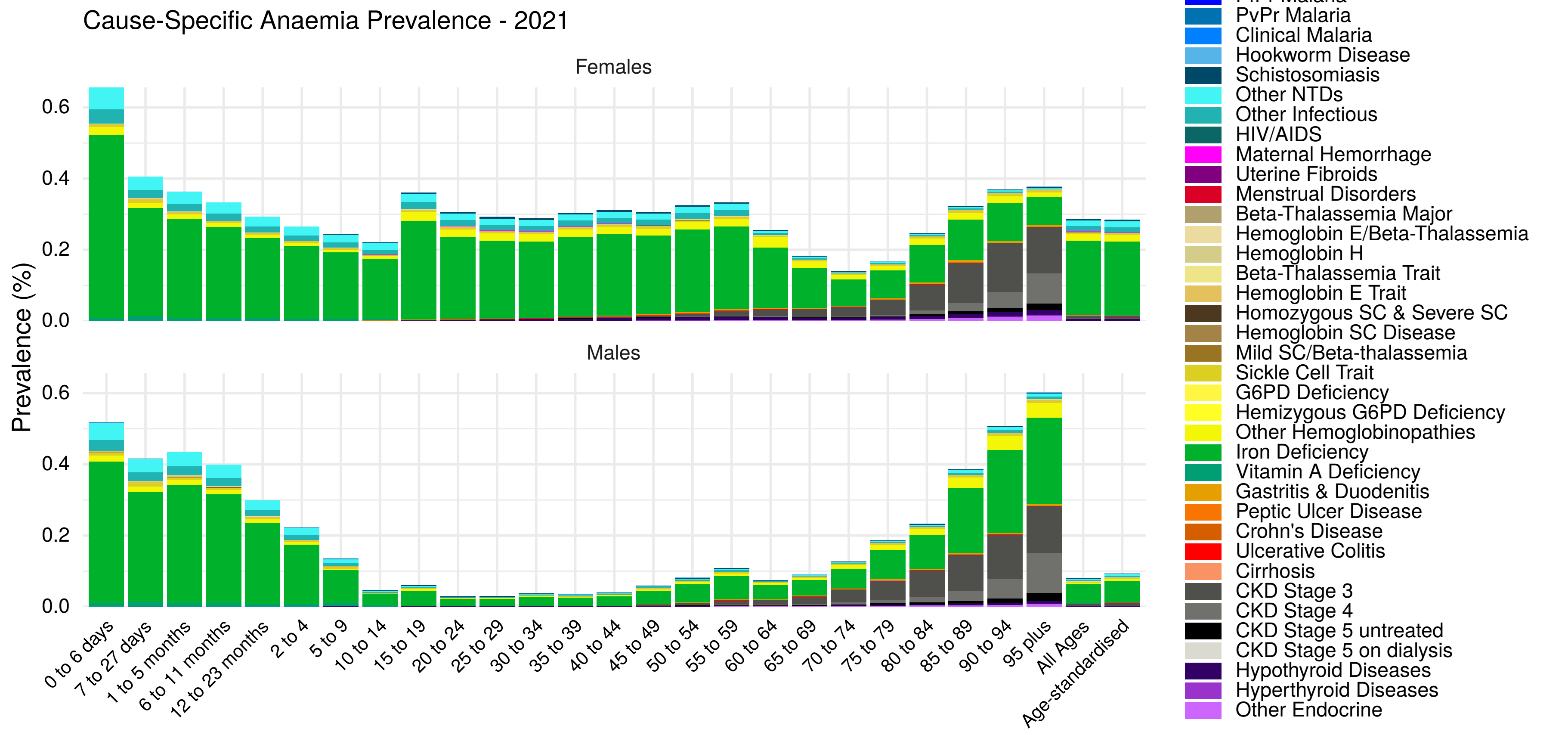
Cause-Specific Anaemia Prevalence - 2021
Females
Males
Prevalence (%)
0.6
0.4
0.2
0.0
0 to 6 days
7 to 27 days
1 to 5 months
6 to 11 months
12 to 23 months
2 to 4
5 to 9
10 to 14
15 to 19
20 to 24
25 to 29
30 to 34
35 to 39
40 to 44
45 to 49
50 to 54
55 to 59
60 to 64
65 to 69
70 to 74
75 to 79
80 to 84
85 to 89
90 to 94
95 plus
All Ages
Age-standardised
PfPr Malaria
PvPr Malaria
Clinical Malaria
Hookworm Disease
Schistosomiasis
Other NTDs
Other Infectious
HIV/AIDS
Maternal Hemorrhage
Uterine Fibroids
Menstrual Disorders
Beta-Thalassemia Major
Hemoglobin E/Beta-Thalassemia
Hemoglobin H
Beta-Thalassemia Trait
Hemoglobin E Trait
Homozygous SC & Severe SC
Hemoglobin SC Disease
Mild SC/Beta-thalassemia
Sickle Cell Trait
G6PD Deficiency
Hemizygous G6PD Deficiency
Other Hemoglobinopathies
Iron Deficiency
Vitamin A Deficiency
Gastritis & Duodenitis
Peptic Ulcer Disease
Crohn's Disease
Ulcerative Colitis
Cirrhosis
CKD Stage 3
CKD Stage 4
CKD Stage 5 untreated
CKD Stage 5 on dialysis
Hypothyroid Diseases
Hyperthyroid Diseases
Other Endocrine

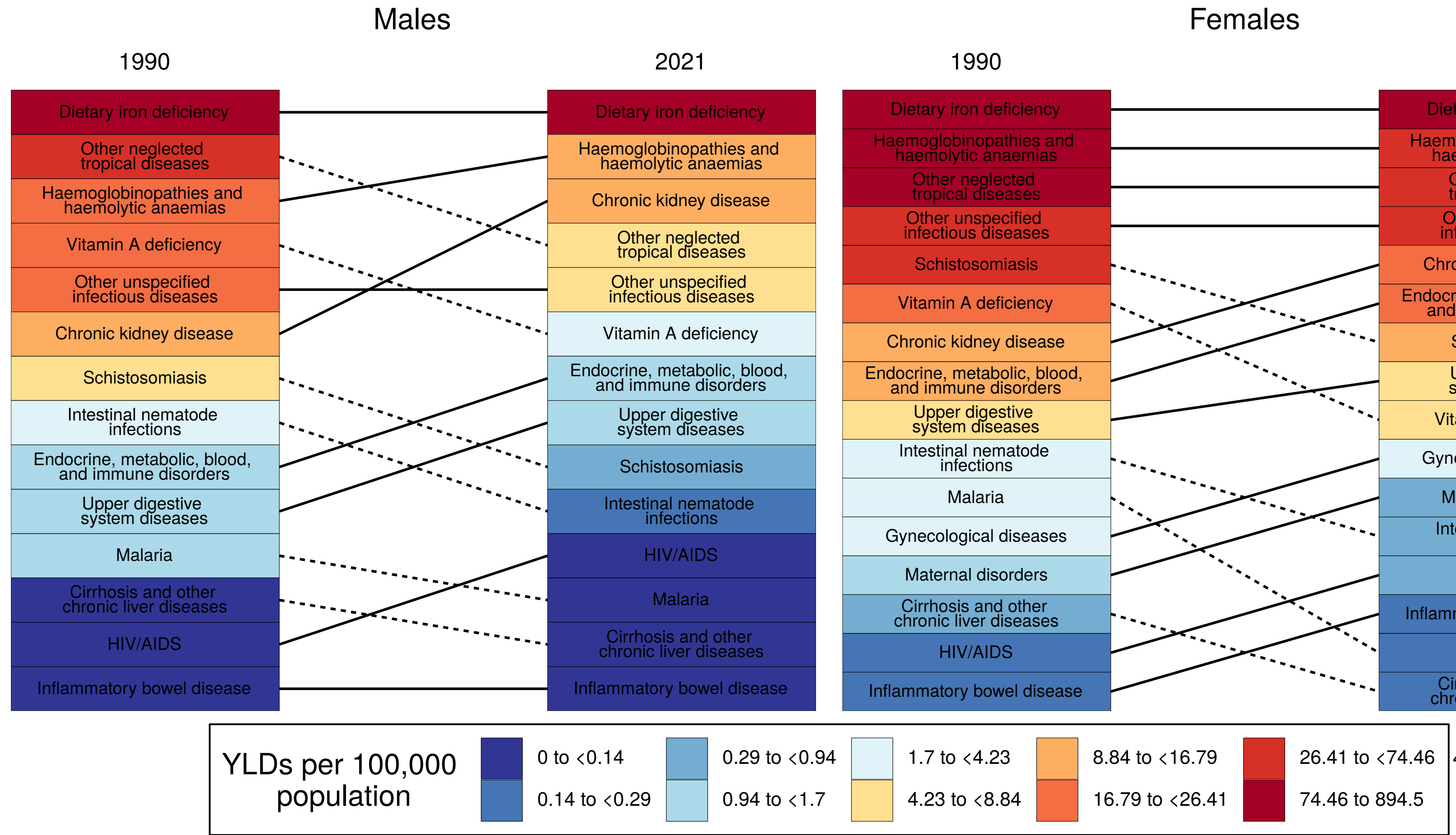
Males
Females
1990
2021
Dietary iron deficiency
Other neglected tropical diseases
Haemoglobinopathies and haemolytic anaemias
Vitamin A deficiency
Other unspecified infectious diseases
Chronic kidney disease
Schistosomiasis
Intestinal nematode infections
Endocrine, metabolic, blood, and immune disorders
Upper digestive system diseases
Malaria
Cirrhosis and other chronic liver diseases
HIV/AIDS
Inflammatory bowel disease
Gynecological diseases
Maternal disorders
YLDs per 100,000 population
0 to <0.14
0.14 to <0.29
0.29 to <0.94
0.94 to <1.7
1.7 to <4.23
4.23 to <8.84
8.84 to <16.79
16.79 to <26.41
26.41 to <74.46
74.46 to 894.5

Maranhão

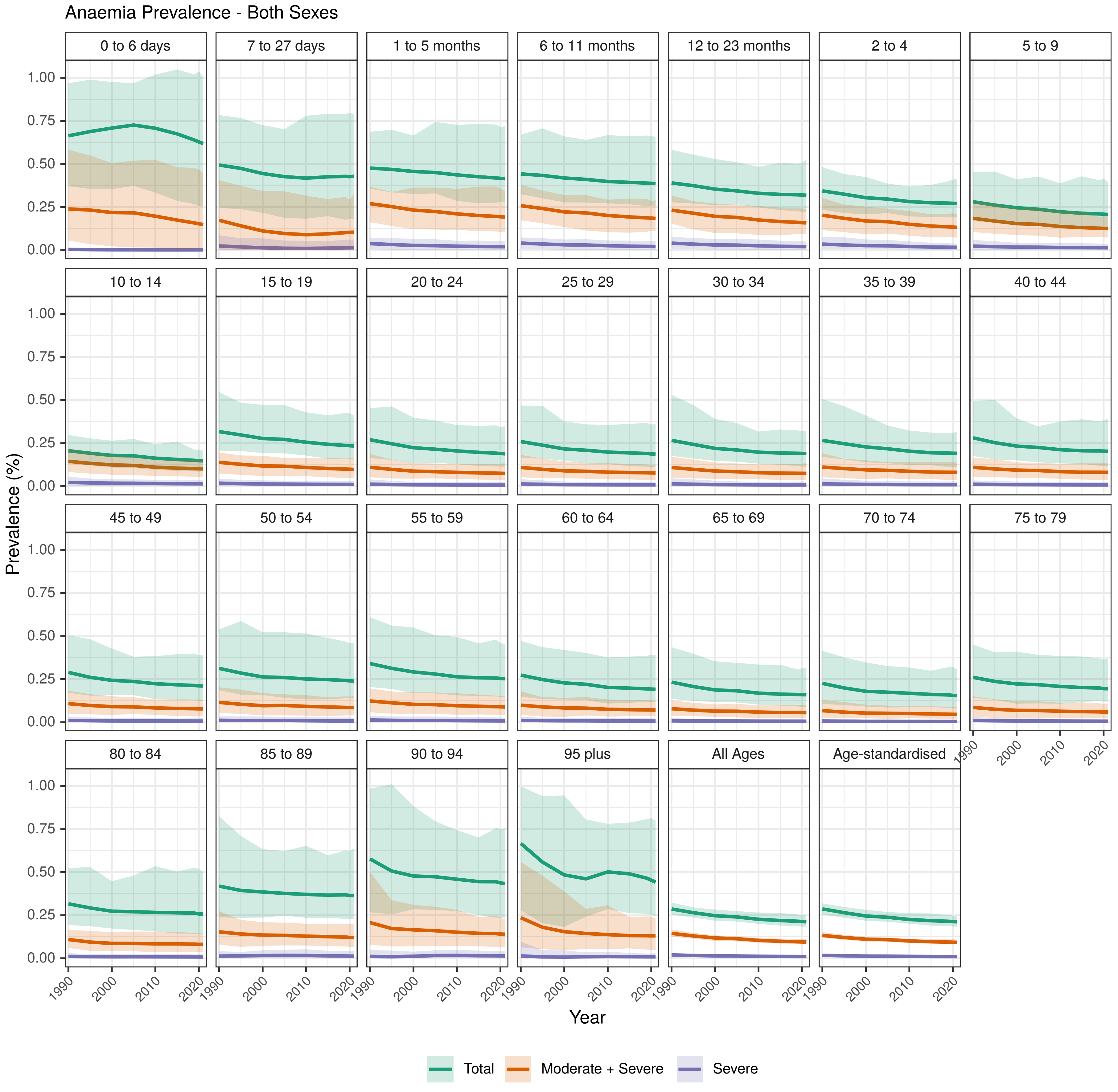
Anaemia Prevalence - Both Sexes
0 to 6 days
7 to 27 days
1 to 5 months
6 to 11 months
12 to 23 months
2 to 4
5 to 9
10 to 14
15 to 19
20 to 24
25 to 29
30 to 34
35 to 39
40 to 44
45 to 49
50 to 54
55 to 59
60 to 64
65 to 69
70 to 74
75 to 79
80 to 84
85 to 89
90 to 94
95 plus
All Ages
Age-standardised
Prevalence (%)
0.00
0.25
0.50
0.75
1.00
1990
2000
2010
2020
Year
Total
Moderate + Severe
Severe

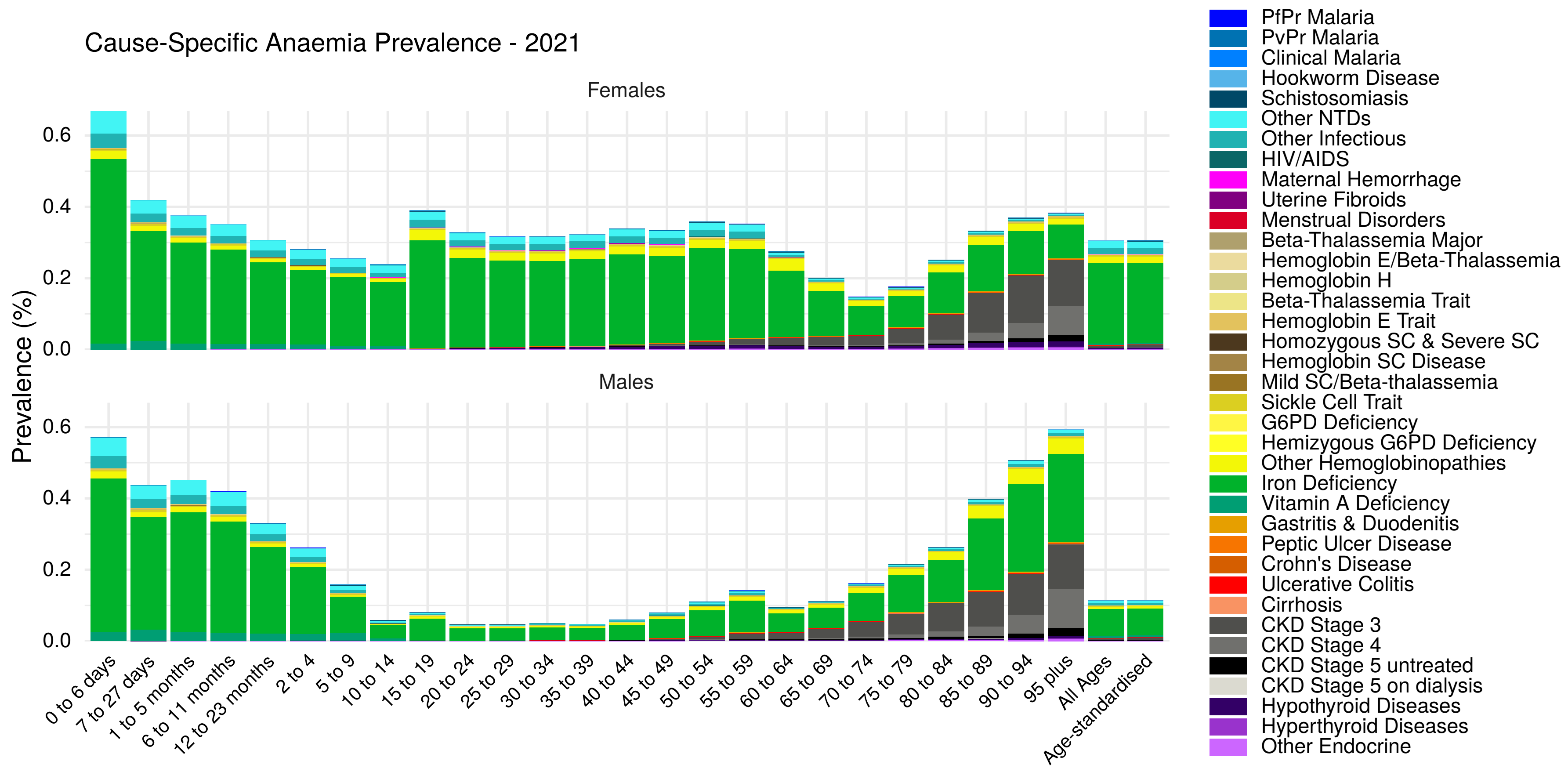
Cause-Specific Anaemia Prevalence - 2021
Females
Males
Prevalence (%)
0.0
0.2
0.4
0.6
0 to 6 days
7 to 27 days
1 to 5 months
6 to 11 months
12 to 23 months
2 to 4
5 to 9
10 to 14
15 to 19
20 to 24
25 to 29
30 to 34
35 to 39
40 to 44
45 to 49
50 to 54
55 to 59
60 to 64
65 to 69
70 to 74
75 to 79
80 to 84
85 to 89
90 to 94
95 plus
All Ages
Age-standardised
PfPr Malaria
PvPr Malaria
Clinical Malaria
Hookworm Disease
Schistosomiasis
Other NTDs
Other Infectious
HIV/AIDS
Maternal Hemorrhage
Uterine Fibroids
Menstrual Disorders
Beta-Thalassemia Major
Hemoglobin E/Beta-Thalassemia
Hemoglobin H
Beta-Thalassemia Trait
Hemoglobin E Trait
Homozygous SC & Severe SC
Hemoglobin SC Disease
Mild SC/Beta-thalassemia
Sickle Cell Trait
G6PD Deficiency
Hemizygous G6PD Deficiency
Other Hemoglobinopathies
Iron Deficiency
Vitamin A Deficiency
Gastritis & Duodenitis
Peptic Ulcer Disease
Crohn's Disease
Ulcerative Colitis
Cirrhosis
CKD Stage 3
CKD Stage 4
CKD Stage 5 untreated
CKD Stage 5 on dialysis
Hypothyroid Diseases
Hyperthyroid Diseases
Other Endocrine

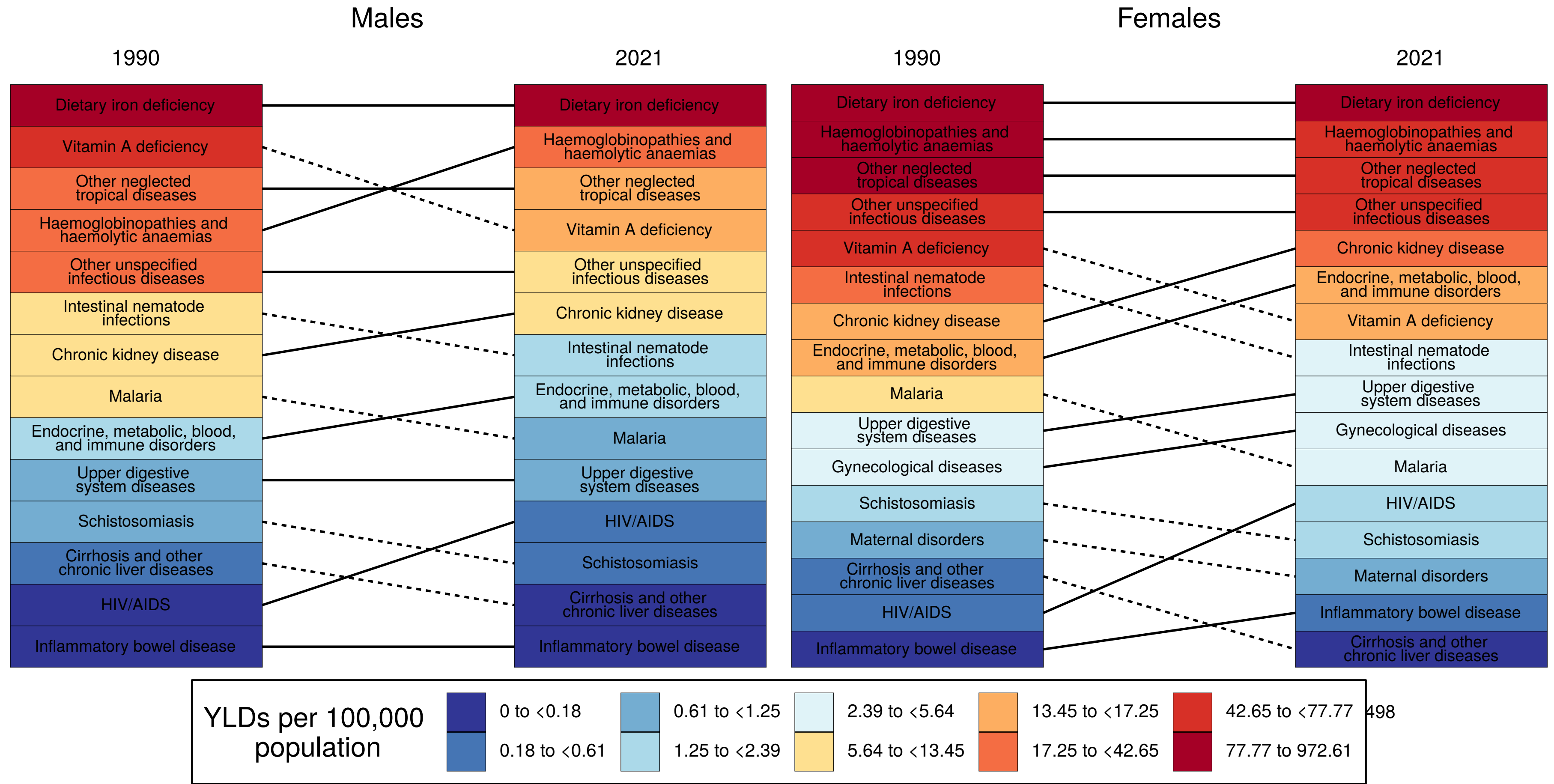
Males
Females
1990
2021
Dietary iron deficiency
Vitamin A deficiency
Other neglected tropical diseases
Haemoglobinopathies and haemolytic anaemias
Other unspecified infectious diseases
Intestinal nematode infections
Chronic kidney disease
Malaria
Endocrine, metabolic, blood, and immune disorders
Upper digestive system diseases
Schistosomiasis
Cirrhosis and other chronic liver diseases
HIV/AIDS
Inflammatory bowel disease
Gynecological diseases
Maternal disorders
YLDs per 100,000 population
0 to <0.18
0.18 to <0.61
0.61 to <1.25
1.25 to <2.39
2.39 to <5.64
5.64 to <13.45
13.45 to <17.25
17.25 to <42.65
42.65 to <77.77
77.77 to 972.61

Mato Grosso

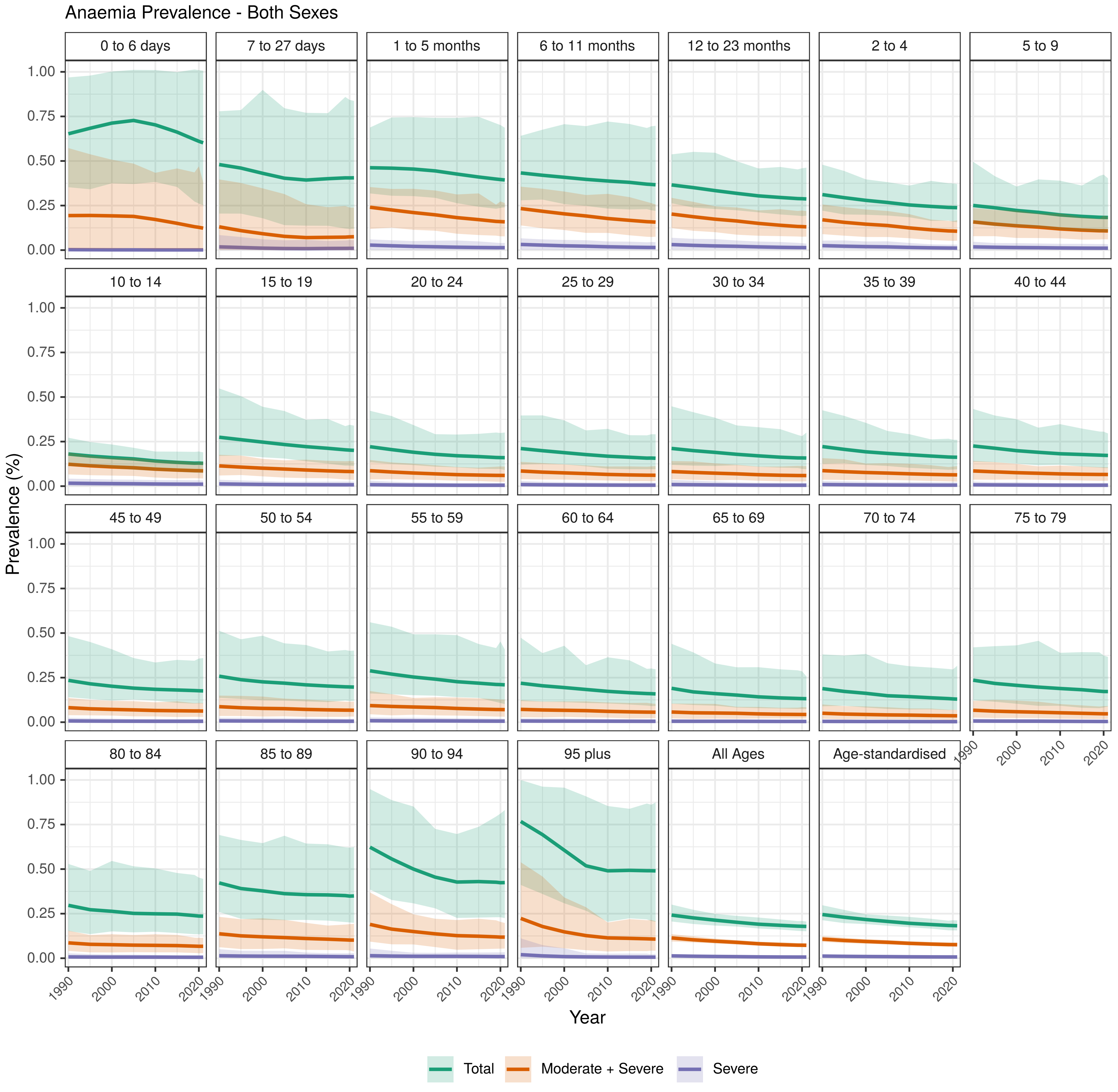

Anaemia Prevalence - Both Sexes
0 to 6 days
7 to 27 days
1 to 5 months
6 to 11 months
12 to 23 months
2 to 4
5 to 9
10 to 14
15 to 19
20 to 24
25 to 29
30 to 34
35 to 39
40 to 44
45 to 49
50 to 54
55 to 59
60 to 64
65 to 69
70 to 74
75 to 79
80 to 84
85 to 89
90 to 94
95 plus
All Ages
Age-standardised
Prevalence (%)
0.00
0.25
0.50
0.75
1.00
1990
2000
2010
2020
Year
Total
Moderate + Severe
Severe

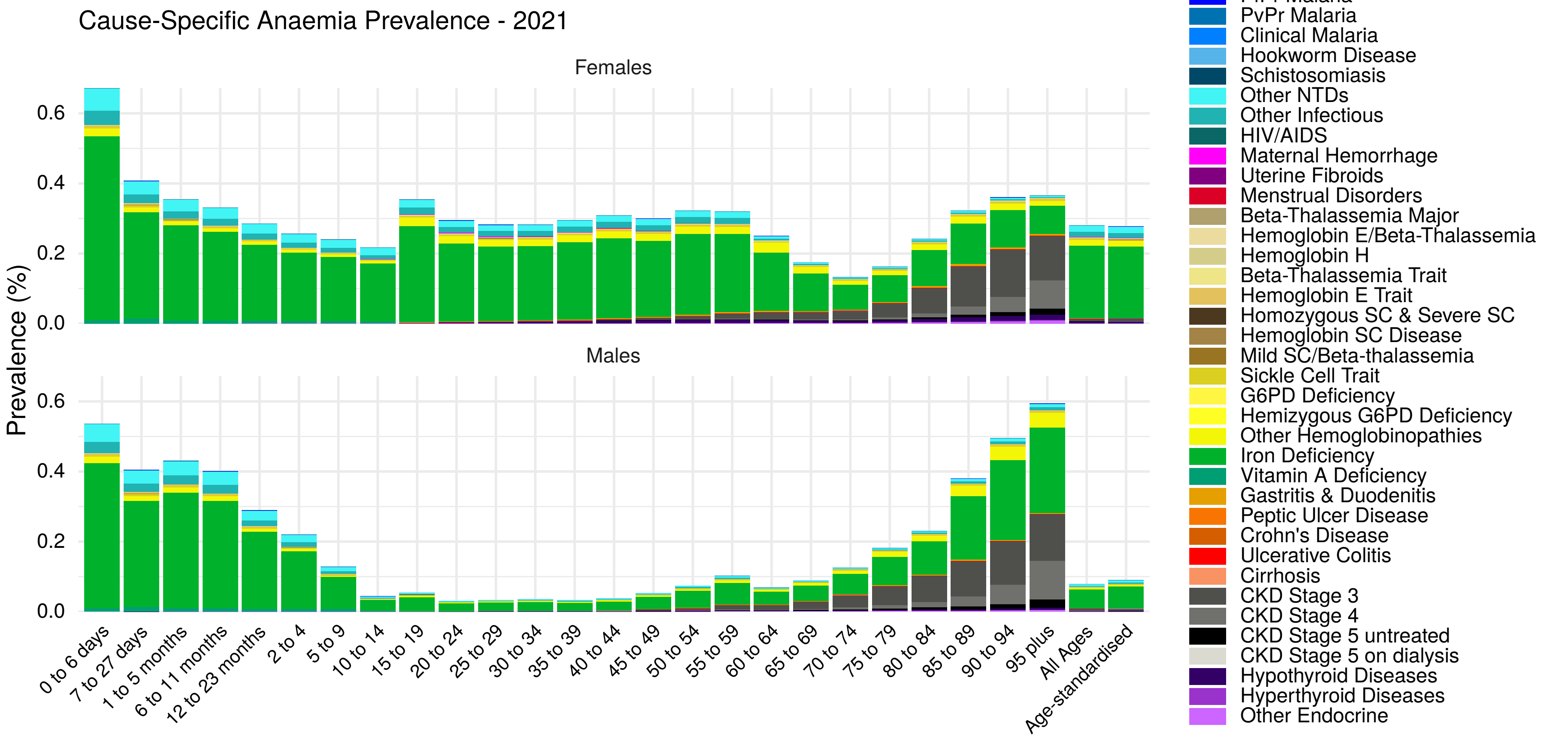

Cause-Specific Anaemia Prevalence - 2021
Females
Males
Prevalence (%)
0.0
0.2
0.4
0.6
0 to 6 days
7 to 27 days
1 to 5 months
6 to 11 months
12 to 23 months
2 to 4
5 to 9
10 to 14
15 to 19
20 to 24
25 to 29
30 to 34
35 to 39
40 to 44
45 to 49
50 to 54
55 to 59
60 to 64
65 to 69
70 to 74
75 to 79
80 to 84
85 to 89
90 to 94
95 plus
All Ages
Age-standardised
PfPr Malaria
PvPr Malaria
Clinical Malaria
Hookworm Disease
Schistosomiasis
Other NTDs
Other Infectious
HIV/AIDS
Maternal Hemorrhage
Uterine Fibroids
Menstrual Disorders
Beta-Thalassemia Major
Hemoglobin E/Beta-Thalassemia
Hemoglobin H
Beta-Thalassemia Trait
Hemoglobin E Trait
Homozygous SC & Severe SC
Hemoglobin SC Disease
Mild SC/Beta-thalassemia
Sickle Cell Trait
G6PD Deficiency
Hemizygous G6PD Deficiency
Other Hemoglobinopathies
Iron Deficiency
Vitamin A Deficiency
Gastritis & Duodenitis
Peptic Ulcer Disease
Crohn's Disease
Ulcerative Colitis
Cirrhosis
CKD Stage 3
CKD Stage 4
CKD Stage 5 untreated
CKD Stage 5 on dialysis
Hypothyroid Diseases
Hyperthyroid Diseases
Other Endocrine

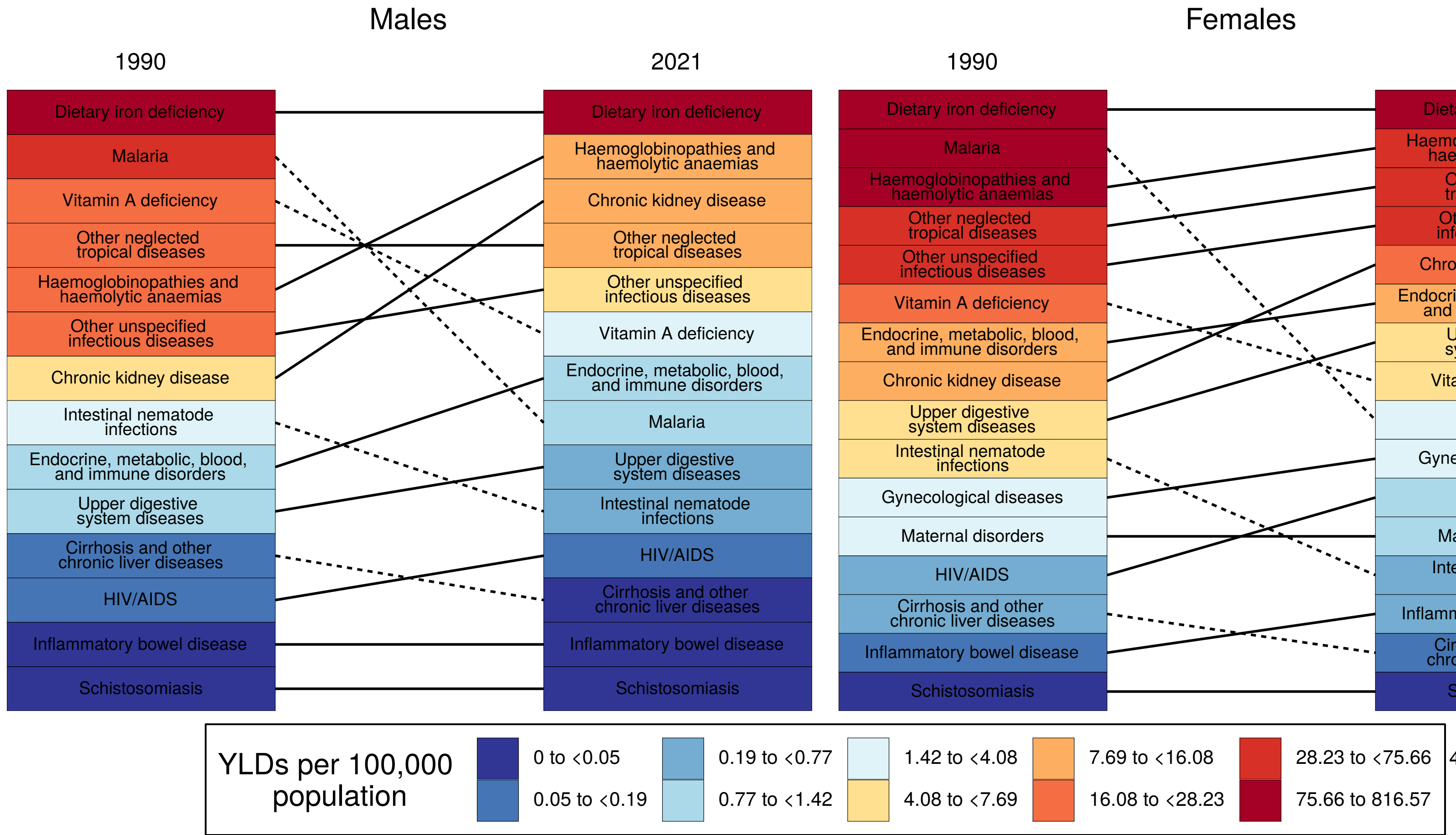

Males
Females
1990
2021
Dietary iron deficiency
Malaria
Vitamin A deficiency
Other neglected tropical diseases
Haemoglobinopathies and haemolytic anaemias
Other unspecified infectious diseases
Chronic kidney disease
Intestinal nematode infections
Endocrine, metabolic, blood, and immune disorders
Upper digestive system diseases
Cirrhosis and other chronic liver diseases
HIV/AIDS
Inflammatory bowel disease
Schistosomiasis
Gynecological diseases
Maternal disorders
YLDs per 100,000 population
0 to <0.05
0.05 to <0.19
0.19 to <0.77
0.77 to <1.42
1.42 to <4.08
4.08 to <7.69
7.69 to <16.08
16.08 to <28.23
28.23 to <75.66
75.66 to 816.57

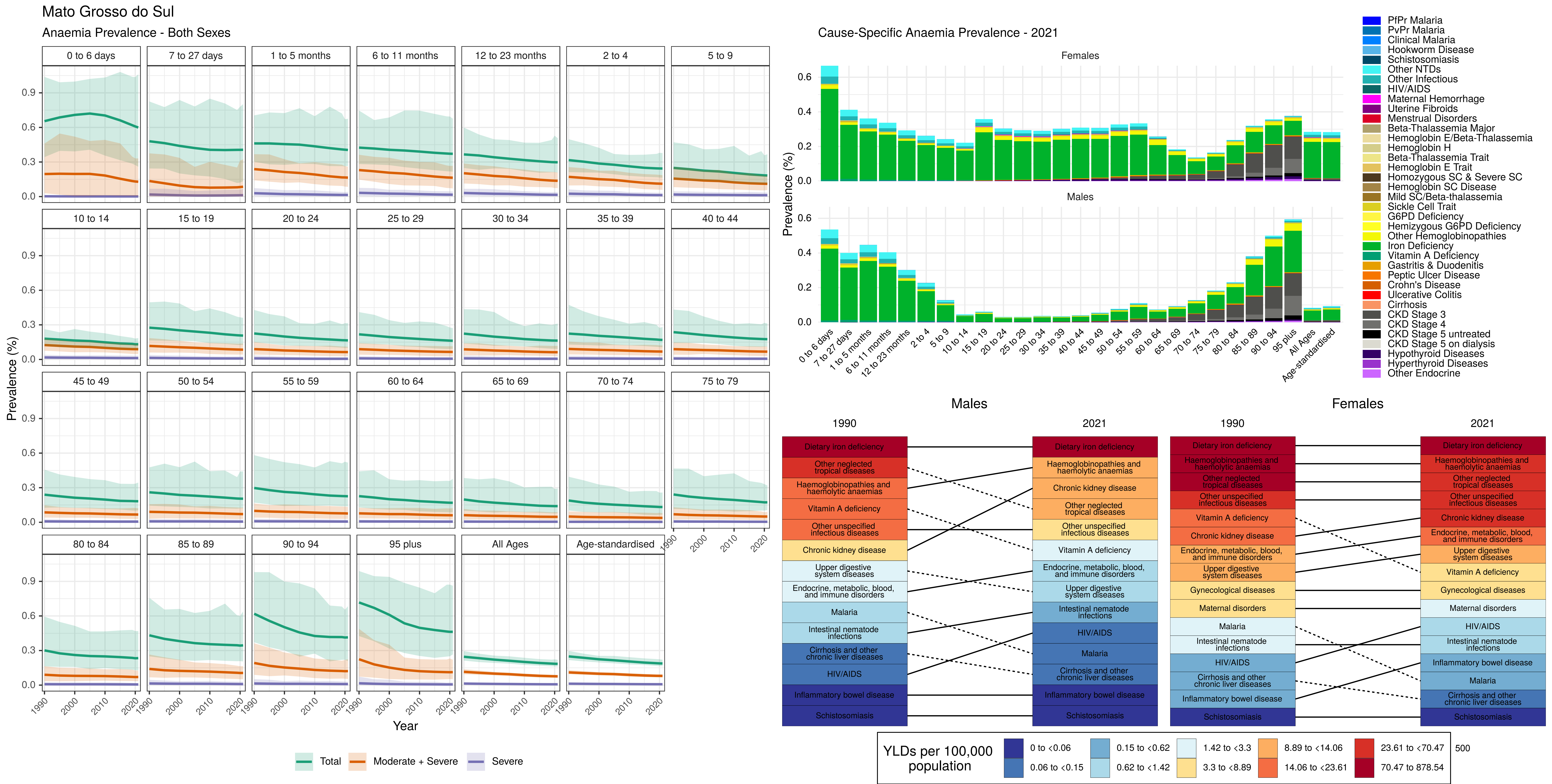

Mato Grosso do Sul
Anaemia Prevalence - Both Sexes
0 to 6 days
7 to 27 days
1 to 5 months
6 to 11 months
12 to 23 months
2 to 4
5 to 9
10 to 14
15 to 19
20 to 24
25 to 29
30 to 34
35 to 39
40 to 44
45 to 49
50 to 54
55 to 59
60 to 64
65 to 69
70 to 74
75 to 79
80 to 84
85 to 89
90 to 94
95 plus
All Ages
Age-standardised
Prevalence (%)
Year
Total
Moderate + Severe
Severe
Cause-Specific Anaemia Prevalence - 2021
Females
Males
PfPr Malaria
PvPr Malaria
Clinical Malaria
Hookworm Disease
Schistosomiasis
Other NTDs
Other Infectious
HIV/AIDS
Maternal Hemorrhage
Uterine Fibroids
Menstrual Disorders
Beta-Thalassemia Major
Hemoglobin E/Beta-Thalassemia
Hemoglobin H
Beta-Thalassemia Trait
Hemoglobin E Trait
Homozygous SC & Severe SC
Hemoglobin SC Disease
Mild SC/Beta-thalassemia
Sickle Cell Trait
G6PD Deficiency
Hemizygous G6PD Deficiency
Other Hemoglobinopathies
Iron Deficiency
Vitamin A Deficiency
Gastritis & Duodenitis
Peptic Ulcer Disease
Crohn's Disease
Ulcerative Colitis
Cirrhosis
CKD Stage 3
CKD Stage 4
CKD Stage 5 untreated
CKD Stage 5 on dialysis
Hypothyroid Diseases
Hyperthyroid Diseases
Other Endocrine
Males
Females
1990
2021
Dietary iron deficiency
Other neglected tropical diseases
Haemoglobinopathies and haemolytic anaemias
Vitamin A deficiency
Other unspecified infectious diseases
Chronic kidney disease
Upper digestive system diseases
Endocrine, metabolic, blood, and immune disorders
Malaria
Intestinal nematode infections
Cirrhosis and other chronic liver diseases
HIV/AIDS
Inflammatory bowel disease
Schistosomiasis
Gynecological diseases
Maternal disorders
YLDs per 100,000 population
0 to <0.06
0.06 to <0.15
0.15 to <0.62
0.62 to <1.42
1.42 to <3.3
3.3 to <8.89
8.89 to <14.06
14.06 to <23.61
23.61 to <70.47
70.47 to 878.54
500

Minas Gerais

Anaemia Prevalence - Both Sexes

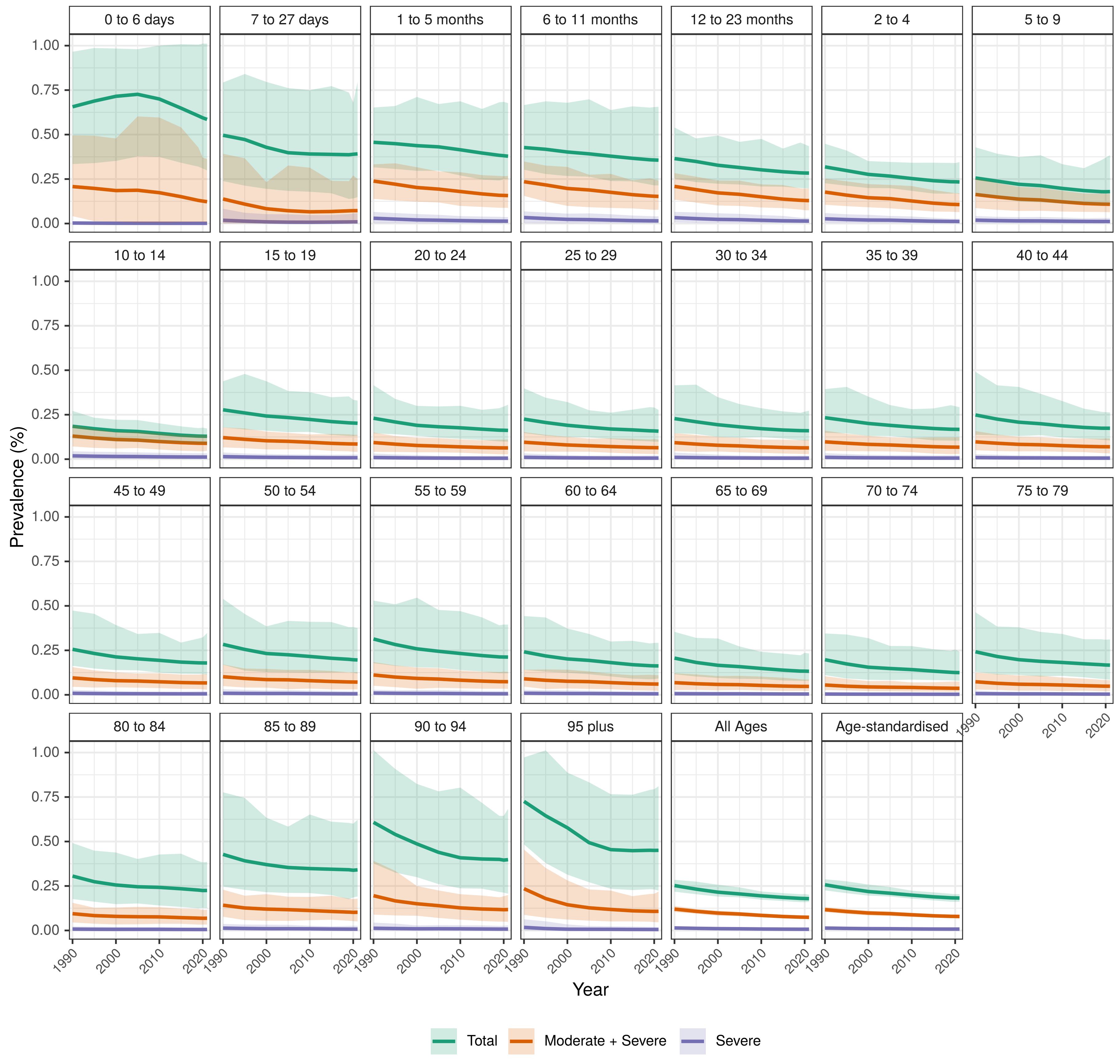

Cause-Specific Anaemia Prevalence - 2021

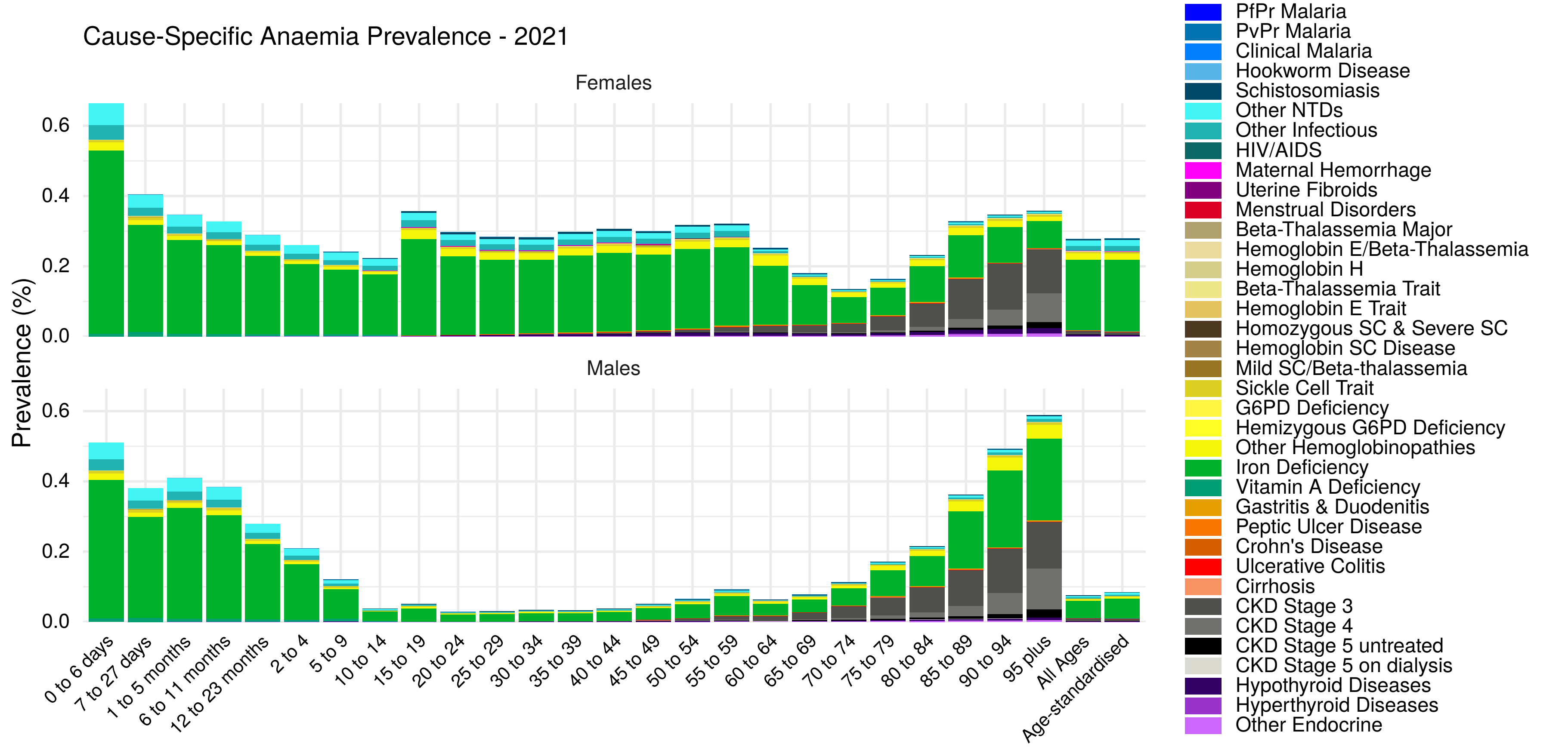

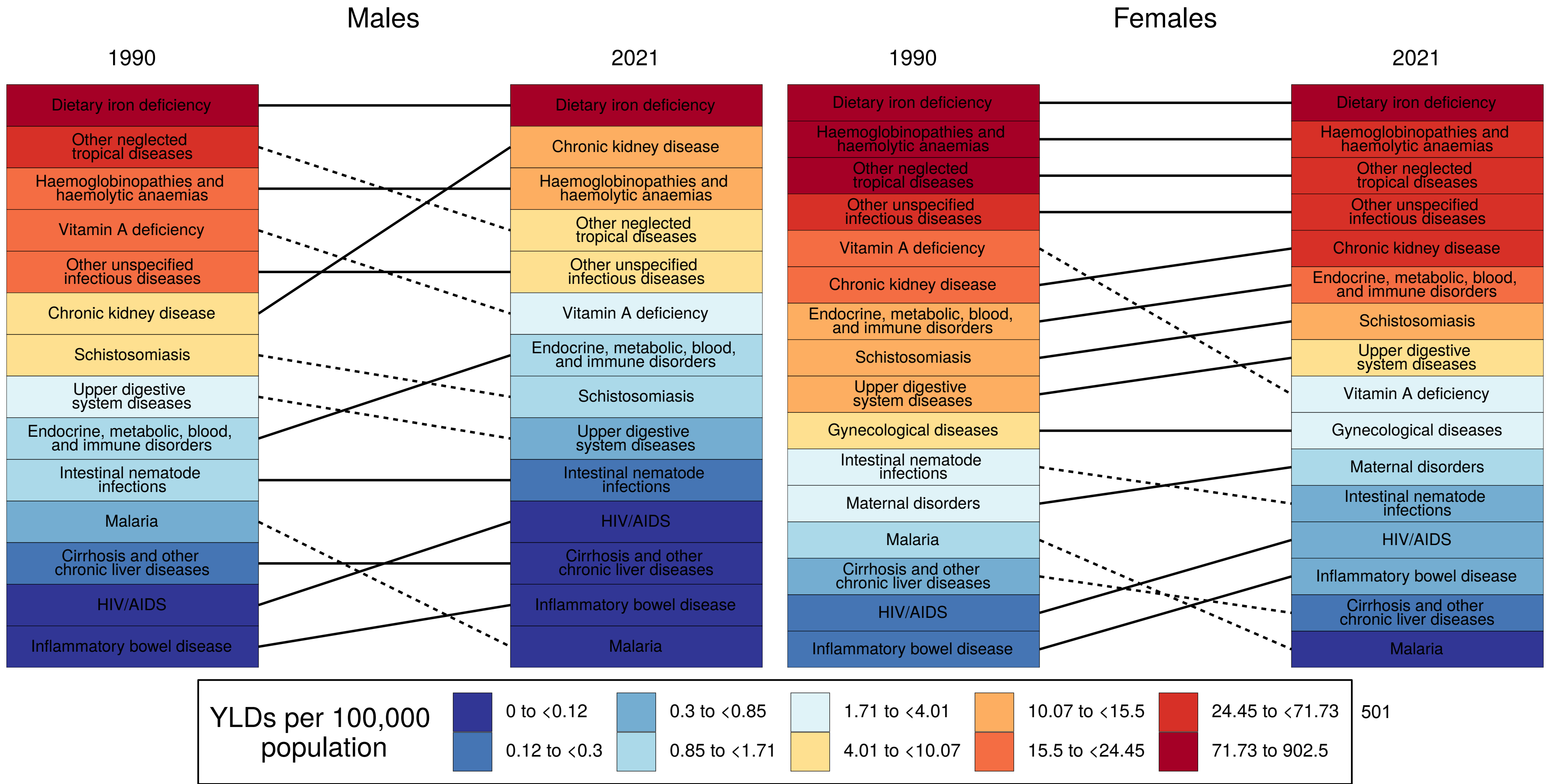

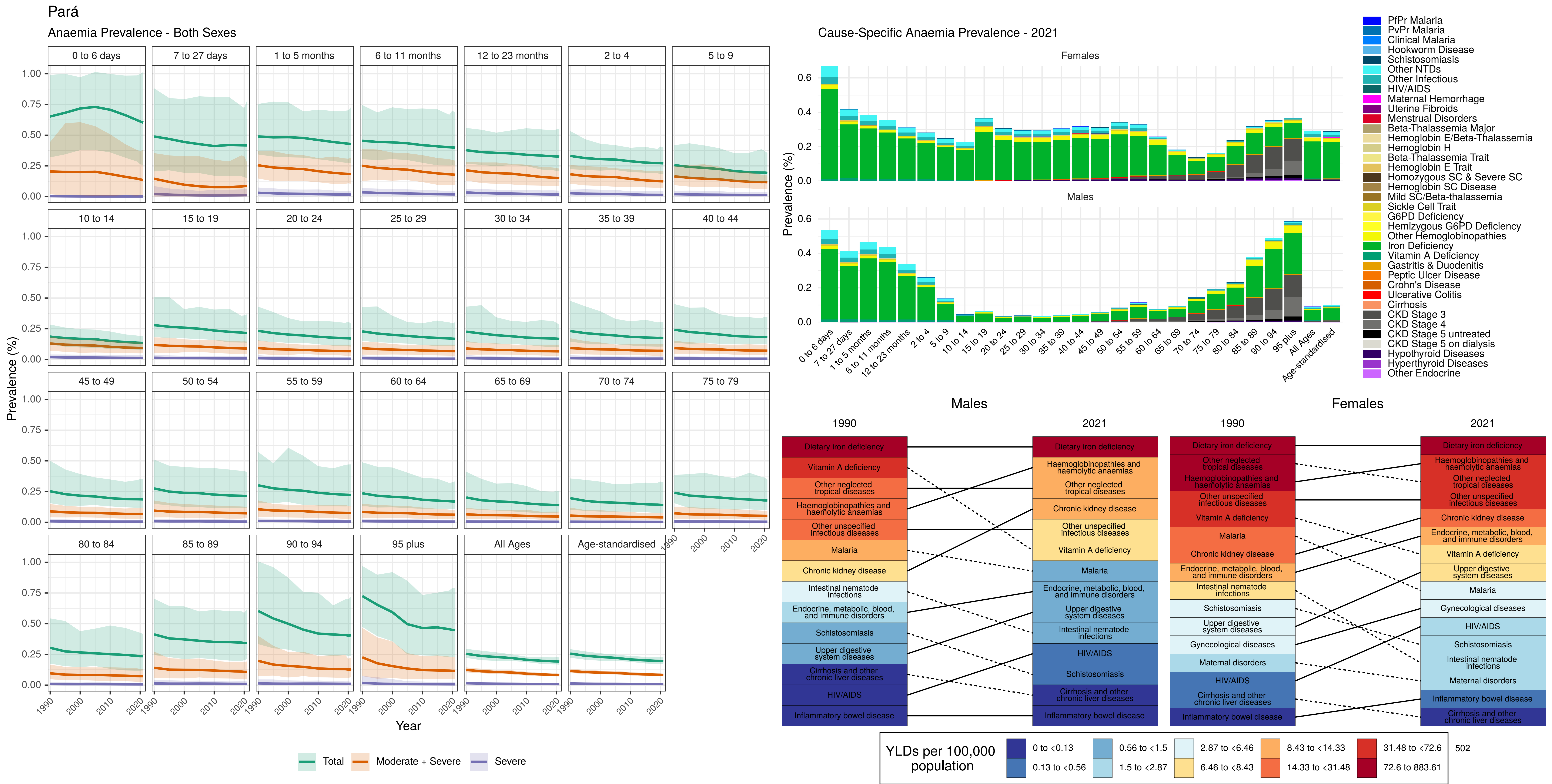

Pará
Anaemia Prevalence - Both Sexes
0 to 6 days
7 to 27 days
1 to 5 months
6 to 11 months
12 to 23 months
2 to 4
5 to 9
10 to 14
15 to 19
20 to 24
25 to 29
30 to 34
35 to 39
40 to 44
45 to 49
50 to 54
55 to 59
60 to 64
65 to 69
70 to 74
75 to 79
80 to 84
85 to 89
90 to 94
95 plus
All Ages
Age-standardised
Prevalence (%)
Year
Total
Moderate + Severe
Severe
Cause-Specific Anaemia Prevalence - 2021
Females
Males
PfPr Malaria
PvPr Malaria
Clinical Malaria
Hookworm Disease
Schistosomiasis
Other NTDs
Other Infectious
HIV/AIDS
Maternal Hemorrhage
Uterine Fibroids
Menstrual Disorders
Beta-Thalassemia Major
Hemoglobin E/Beta-Thalassemia
Hemoglobin H
Beta-Thalassemia Trait
Hemoglobin E Trait
Homozygous SC & Severe SC
Hemoglobin SC Disease
Mild SC/Beta-thalassemia
Sickle Cell Trait
G6PD Deficiency
Hemizygous G6PD Deficiency
Other Hemoglobinopathies
Iron Deficiency
Vitamin A Deficiency
Gastritis & Duodenitis
Peptic Ulcer Disease
Crohn's Disease
Ulcerative Colitis
Cirrhosis
CKD Stage 3
CKD Stage 4
CKD Stage 5 untreated
CKD Stage 5 on dialysis
Hypothyroid Diseases
Hyperthyroid Diseases
Other Endocrine
Males
Females
1990
2021
Dietary iron deficiency
Vitamin A deficiency
Other neglected tropical diseases
Haemoglobinopathies and haemolytic anaemias
Other unspecified infectious diseases
Malaria
Chronic kidney disease
Intestinal nematode infections
Endocrine, metabolic, blood, and immune disorders
Schistosomiasis
Upper digestive system diseases
Cirrhosis and other chronic liver diseases
HIV/AIDS
Inflammatory bowel disease
Gynecological diseases
Maternal disorders
YLDs per 100,000 population
0 to <0.13
0.13 to <0.56
0.56 to <1.5
1.5 to <2.87
2.87 to <6.46
6.46 to <8.43
8.43 to <14.33
14.33 to <31.48
31.48 to <72.6
72.6 to 883.61

Paraíba

Anaemia Prevalence - Both Sexes

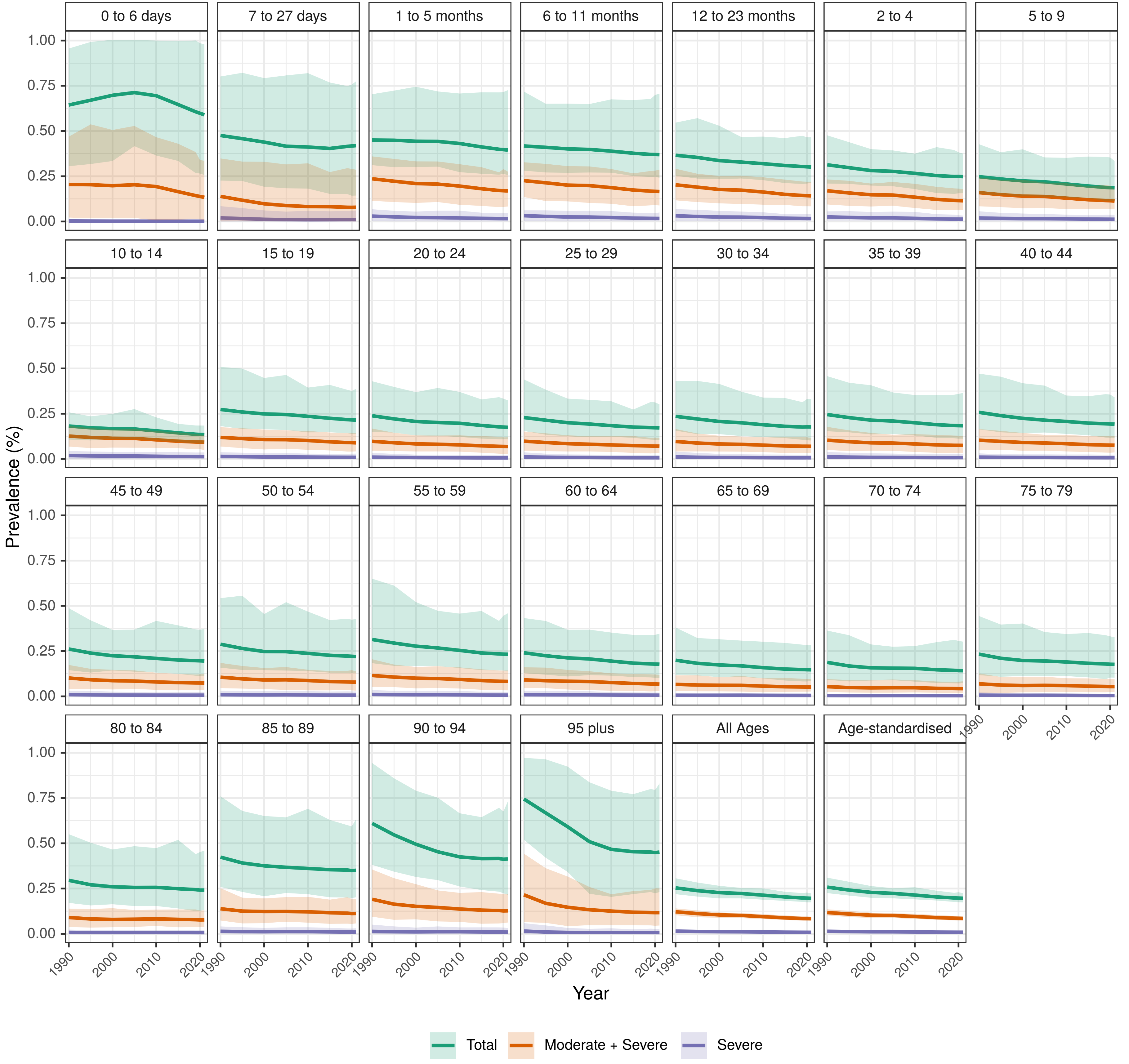

Cause-Specific Anaemia Prevalence - 2021

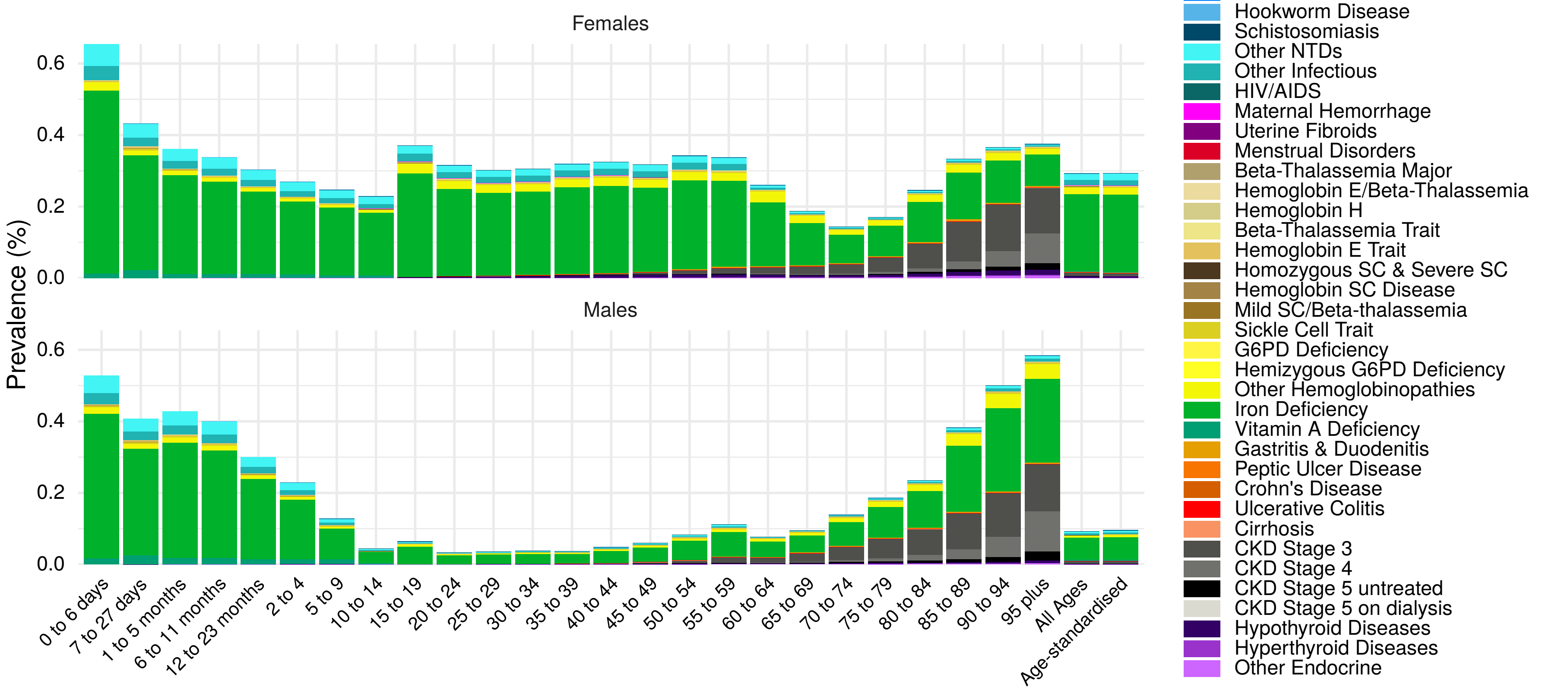

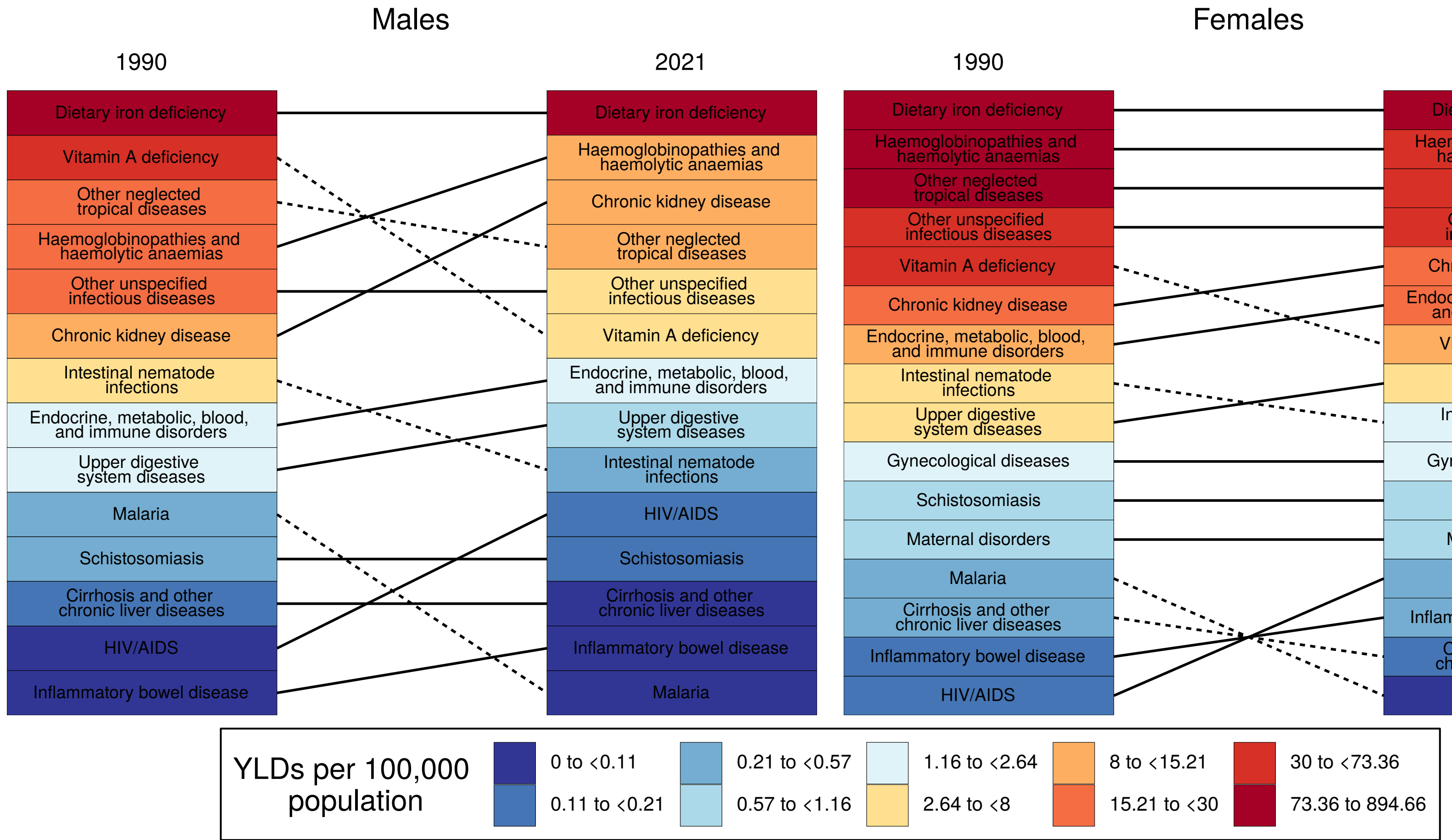

Paraná

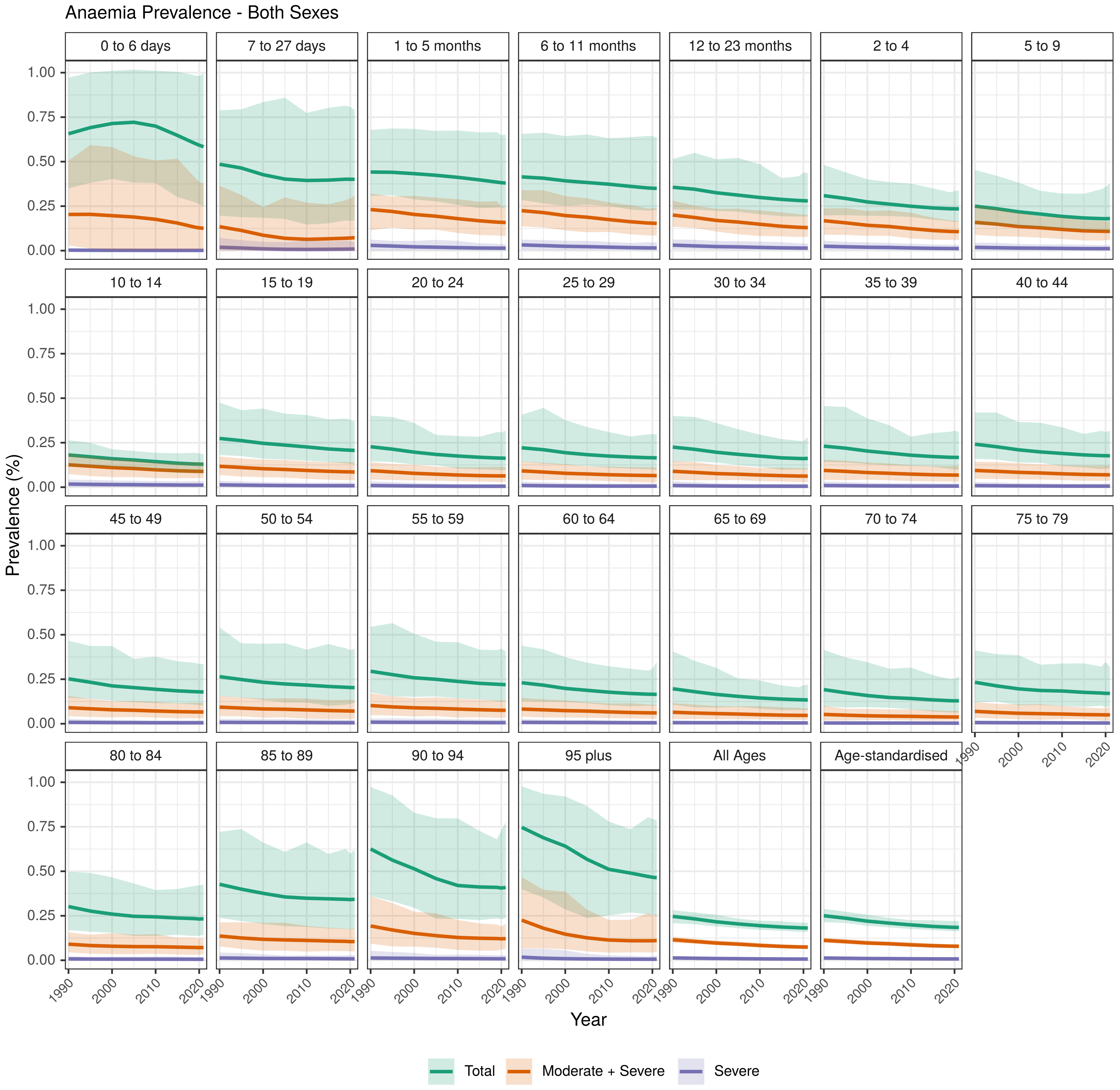
Anaemia Prevalence - Both Sexes
0 to 6 days
7 to 27 days
1 to 5 months
6 to 11 months
12 to 23 months
2 to 4
5 to 9
10 to 14
15 to 19
20 to 24
25 to 29
30 to 34
35 to 39
40 to 44
45 to 49
50 to 54
55 to 59
60 to 64
65 to 69
70 to 74
75 to 79
80 to 84
85 to 89
90 to 94
95 plus
All Ages
Age-standardised
Prevalence (%)
1.00
0.75
0.50
0.25
0.00
1990
2000
2010
2020
Year
Total
Moderate + Severe
Severe

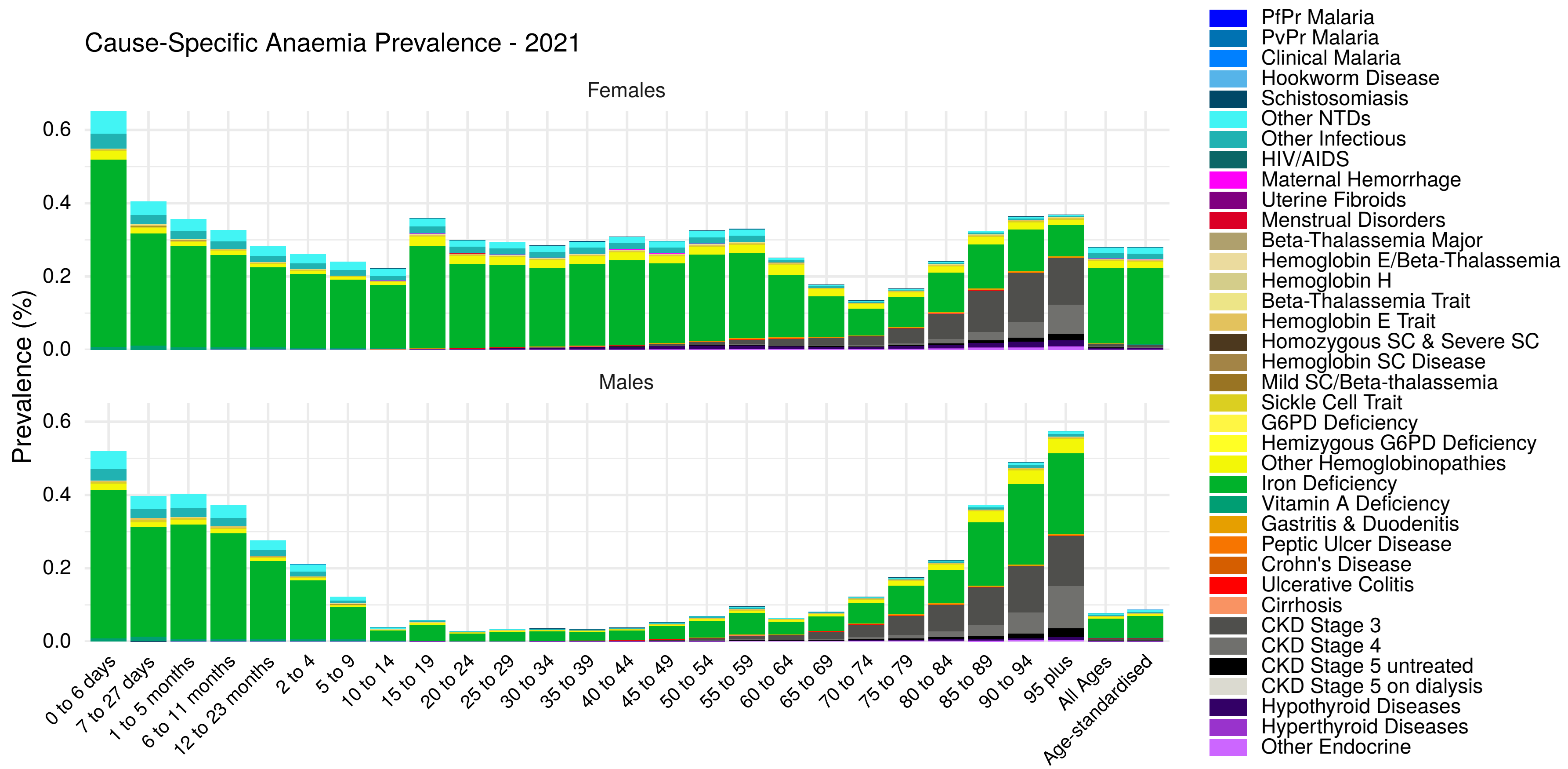
Cause-Specific Anaemia Prevalence - 2021
Females
Males
Prevalence (%)
0.6
0.4
0.2
0.0
0 to 6 days
7 to 27 days
1 to 5 months
6 to 11 months
12 to 23 months
2 to 4
5 to 9
10 to 14
15 to 19
20 to 24
25 to 29
30 to 34
35 to 39
40 to 44
45 to 49
50 to 54
55 to 59
60 to 64
65 to 69
70 to 74
75 to 79
80 to 84
85 to 89
90 to 94
95 plus
All Ages
Age-standardised
PfPr Malaria
PvPr Malaria
Clinical Malaria
Hookworm Disease
Schistosomiasis
Other NTDs
Other Infectious
HIV/AIDS
Maternal Hemorrhage
Uterine Fibroids
Menstrual Disorders
Beta-Thalassemia Major
Hemoglobin E/Beta-Thalassemia
Hemoglobin H
Beta-Thalassemia Trait
Hemoglobin E Trait
Homozygous SC & Severe SC
Hemoglobin SC Disease
Mild SC/Beta-thalassemia
Sickle Cell Trait
G6PD Deficiency
Hemizygous G6PD Deficiency
Other Hemoglobinopathies
Iron Deficiency
Vitamin A Deficiency
Gastritis & Duodenitis
Peptic Ulcer Disease
Crohn's Disease
Ulcerative Colitis
Cirrhosis
CKD Stage 3
CKD Stage 4
CKD Stage 5 untreated
CKD Stage 5 on dialysis
Hypothyroid Diseases
Hyperthyroid Diseases
Other Endocrine

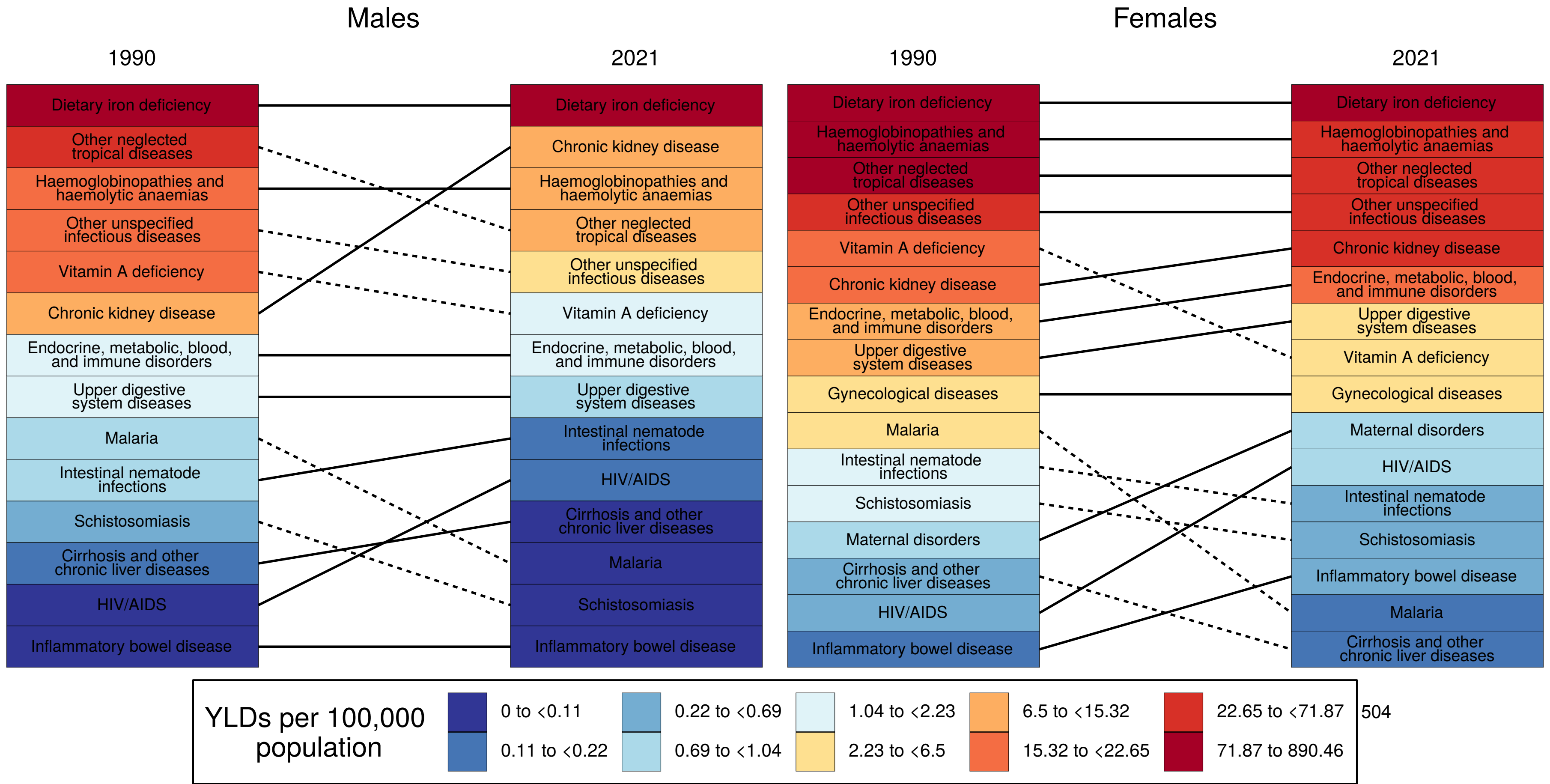
Males
Females
1990
2021
Dietary iron deficiency
Other neglected tropical diseases
Haemoglobinopathies and haemolytic anaemias
Other unspecified infectious diseases
Vitamin A deficiency
Chronic kidney disease
Endocrine, metabolic, blood, and immune disorders
Upper digestive system diseases
Malaria
Intestinal nematode infections
Schistosomiasis
Cirrhosis and other chronic liver diseases
HIV/AIDS
Inflammatory bowel disease
Gynecological diseases
Maternal disorders
YLDs per 100,000 population
0 to <0.11
0.11 to <0.22
0.22 to <0.69
0.69 to <1.04
1.04 to <2.23
2.23 to <6.5
6.5 to <15.32
15.32 to <22.65
22.65 to <71.87
71.87 to 890.46

Pernambuco

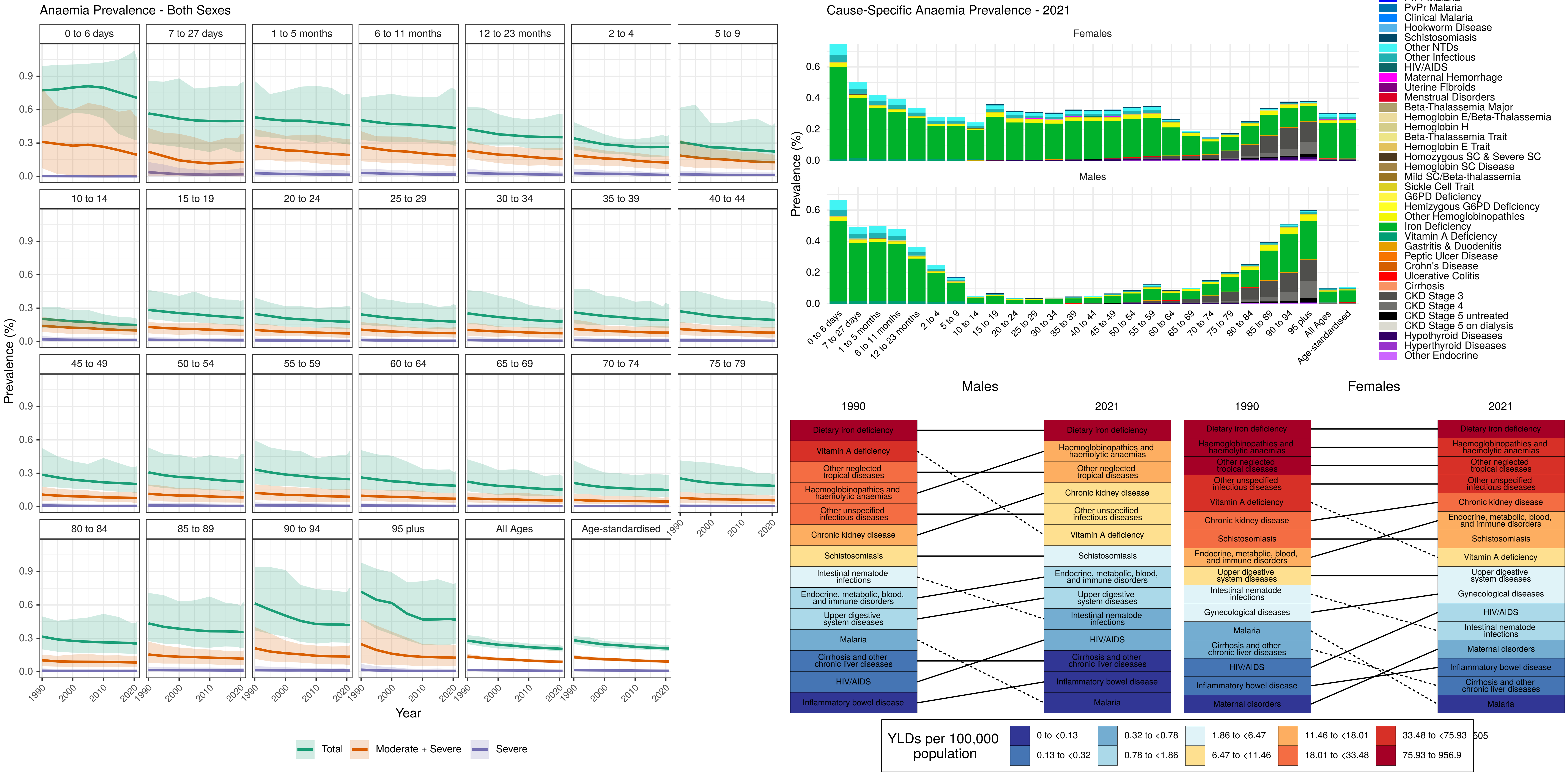

Anaemia Prevalence - Both Sexes
0 to 6 days
7 to 27 days
1 to 5 months
6 to 11 months
12 to 23 months
2 to 4
5 to 9
10 to 14
15 to 19
20 to 24
25 to 29
30 to 34
35 to 39
40 to 44
45 to 49
50 to 54
55 to 59
60 to 64
65 to 69
70 to 74
75 to 79
80 to 84
85 to 89
90 to 94
95 plus
All Ages
Age-standardised
Prevalence (%)
Year
Total
Moderate + Severe
Severe
Cause-Specific Anaemia Prevalence - 2021
Females
Males
Prevalence (%)
PfPr Malaria
PvPr Malaria
Clinical Malaria
Hookworm Disease
Schistosomiasis
Other NTDs
Other Infectious
HIV/AIDS
Maternal Hemorrhage
Uterine Fibroids
Menstrual Disorders
Beta-Thalassemia Major
Hemoglobin E/Beta-Thalassemia
Hemoglobin H
Beta-Thalassemia Trait
Hemoglobin E Trait
Homozygous SC & Severe SC
Hemoglobin SC Disease
Mild SC/Beta-thalassemia
Sickle Cell Trait
G6PD Deficiency
Hemizygous G6PD Deficiency
Other Hemoglobinopathies
Iron Deficiency
Vitamin A Deficiency
Gastritis & Duodenitis
Peptic Ulcer Disease
Crohn's Disease
Ulcerative Colitis
Cirrhosis
CKD Stage 3
CKD Stage 4
CKD Stage 5 untreated
CKD Stage 5 on dialysis
Hypothyroid Diseases
Hyperthyroid Diseases
Other Endocrine
Males
Females
1990
2021
Dietary iron deficiency
Vitamin A deficiency
Other neglected tropical diseases
Haemoglobinopathies and haemolytic anaemias
Other unspecified infectious diseases
Chronic kidney disease
Schistosomiasis
Intestinal nematode infections
Endocrine, metabolic, blood, and immune disorders
Upper digestive system diseases
Malaria
Cirrhosis and other chronic liver diseases
HIV/AIDS
Inflammatory bowel disease
Gynecological diseases
Maternal disorders
YLDs per 100,000 population
0 to <0.13
0.13 to <0.32
0.32 to <0.78
0.78 to <1.86
1.86 to <6.47
6.47 to <11.46
11.46 to <18.01
18.01 to <33.48
33.48 to <75.93
75.93 to 956.9

Piauí

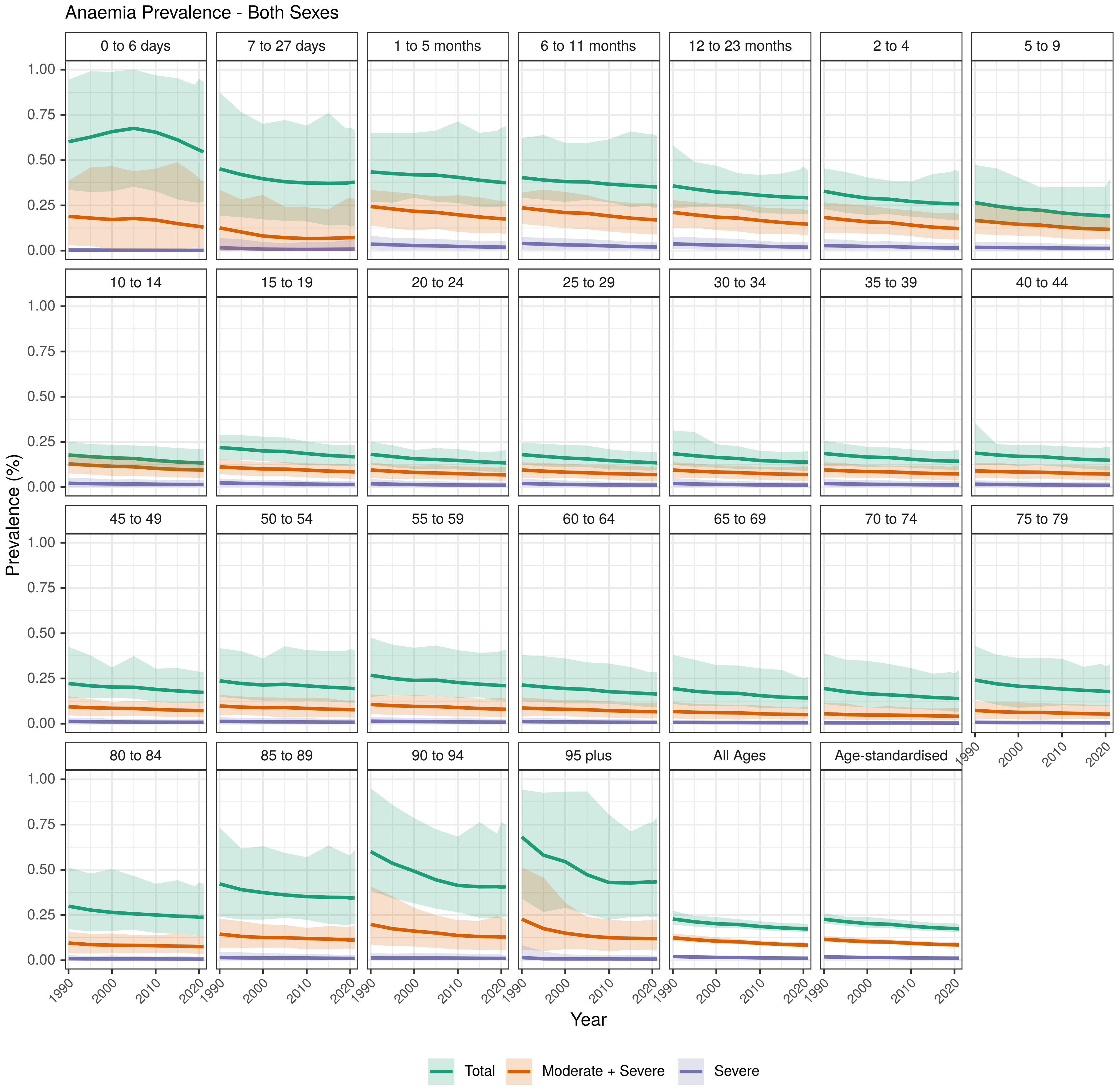

Anaemia Prevalence - Both Sexes
0 to 6 days
7 to 27 days
1 to 5 months
6 to 11 months
12 to 23 months
2 to 4
5 to 9
10 to 14
15 to 19
20 to 24
25 to 29
30 to 34
35 to 39
40 to 44
45 to 49
50 to 54
55 to 59
60 to 64
65 to 69
70 to 74
75 to 79
80 to 84
85 to 89
90 to 94
95 plus
All Ages
Age-standardised
Prevalence (%)
Year
Total
Moderate + Severe
Severe

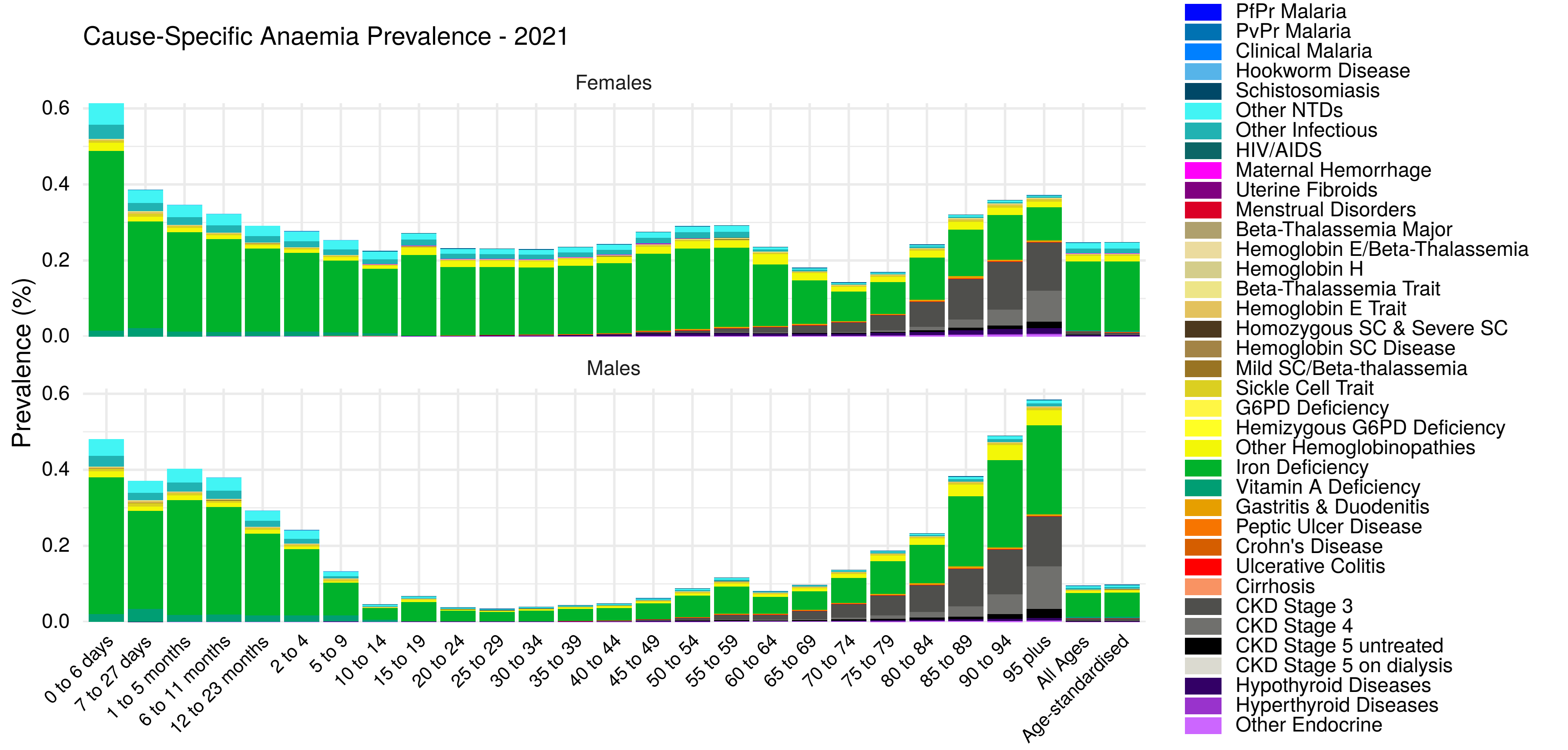

Cause-Specific Anaemia Prevalence - 2021
Females
Males
Prevalence (%)
PfPr Malaria
PvPr Malaria
Clinical Malaria
Hookworm Disease
Schistosomiasis
Other NTDs
Other Infectious
HIV/AIDS
Maternal Hemorrhage
Uterine Fibroids
Menstrual Disorders
Beta-Thalassemia Major
Hemoglobin E/Beta-Thalassemia
Hemoglobin H
Beta-Thalassemia Trait
Hemoglobin E Trait
Homozygous SC & Severe SC
Hemoglobin SC Disease
Mild SC/Beta-thalassemia
Sickle Cell Trait
G6PD Deficiency
Hemizygous G6PD Deficiency
Other Hemoglobinopathies
Iron Deficiency
Vitamin A Deficiency
Gastritis & Duodenitis
Peptic Ulcer Disease
Crohn's Disease
Ulcerative Colitis
Cirrhosis
CKD Stage 3
CKD Stage 4
CKD Stage 5 untreated
CKD Stage 5 on dialysis
Hypothyroid Diseases
Hyperthyroid Diseases
Other Endocrine

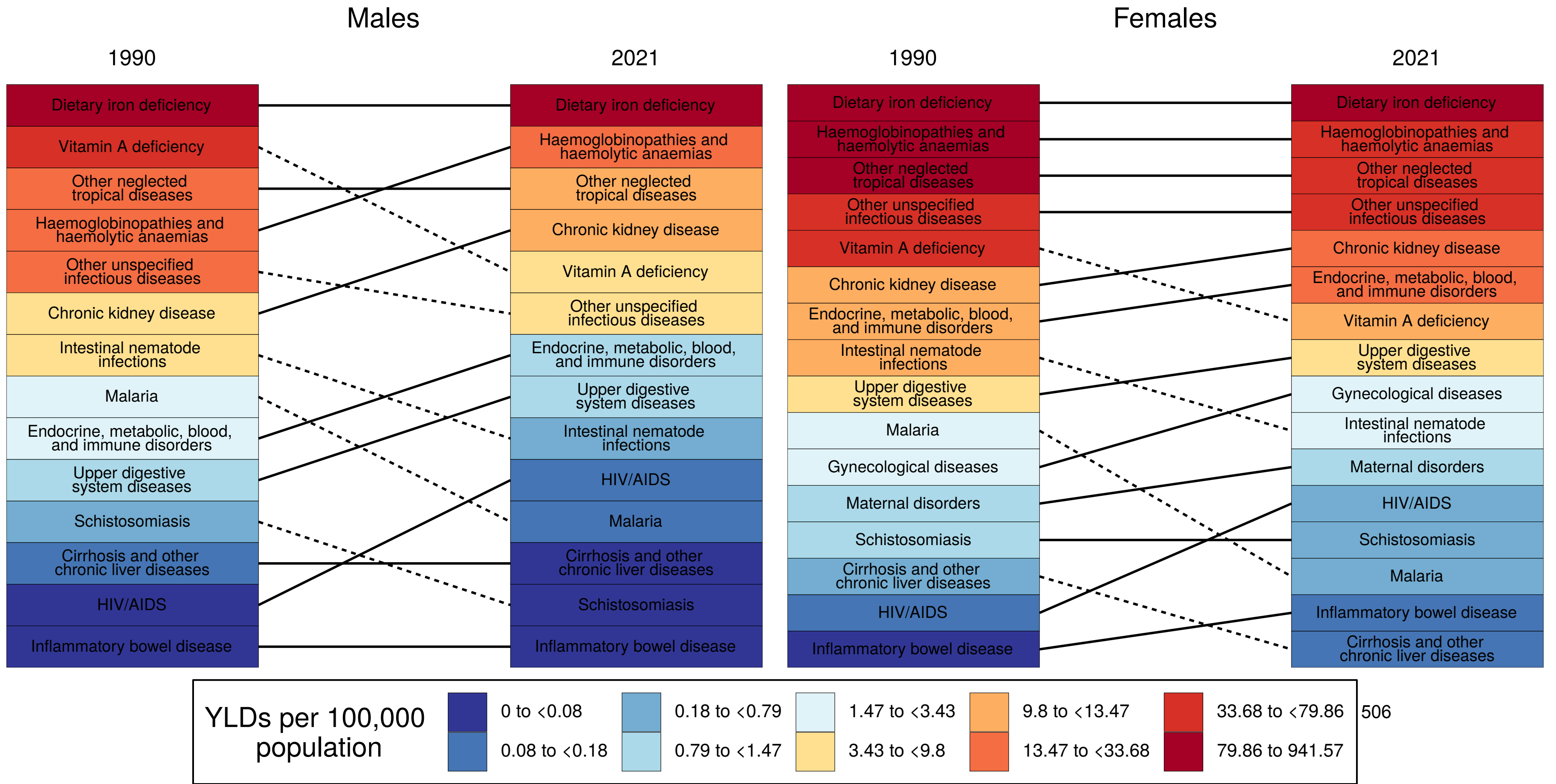

Males
Females
1990
2021
Dietary iron deficiency
Vitamin A deficiency
Other neglected tropical diseases
Haemoglobinopathies and haemolytic anaemias
Other unspecified infectious diseases
Chronic kidney disease
Intestinal nematode infections
Malaria
Endocrine, metabolic, blood, and immune disorders
Upper digestive system diseases
Schistosomiasis
Cirrhosis and other chronic liver diseases
HIV/AIDS
Inflammatory bowel disease
Gynecological diseases
Maternal disorders
YLDs per 100,000 population
0 to <0.08
0.08 to <0.18
0.18 to <0.79
0.79 to <1.47
1.47 to <3.43
3.43 to <9.8
9.8 to <13.47
13.47 to <33.68
33.68 to <79.86
79.86 to 941.57

Rio de Janeiro

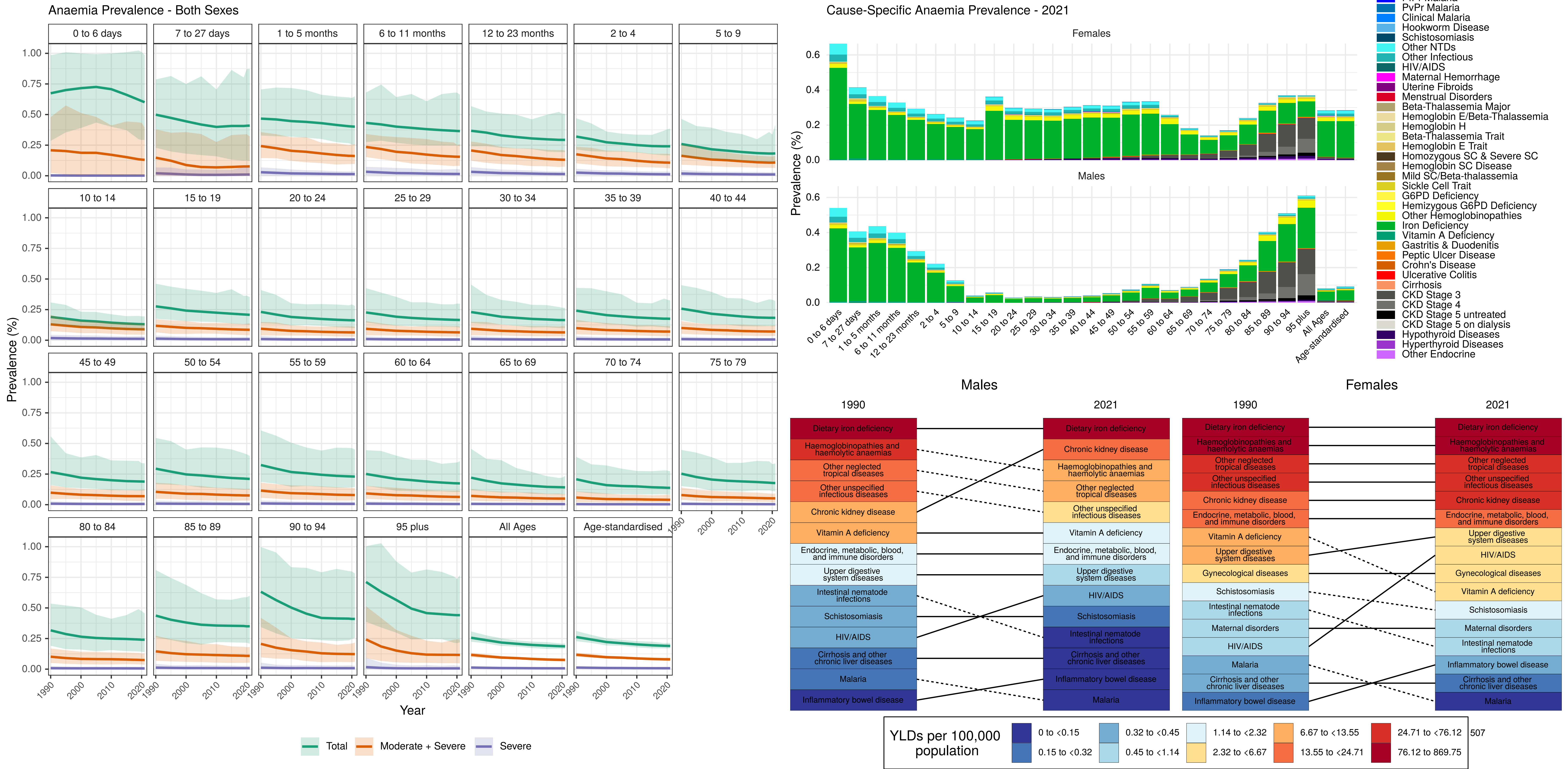

Rio Grande do Norte

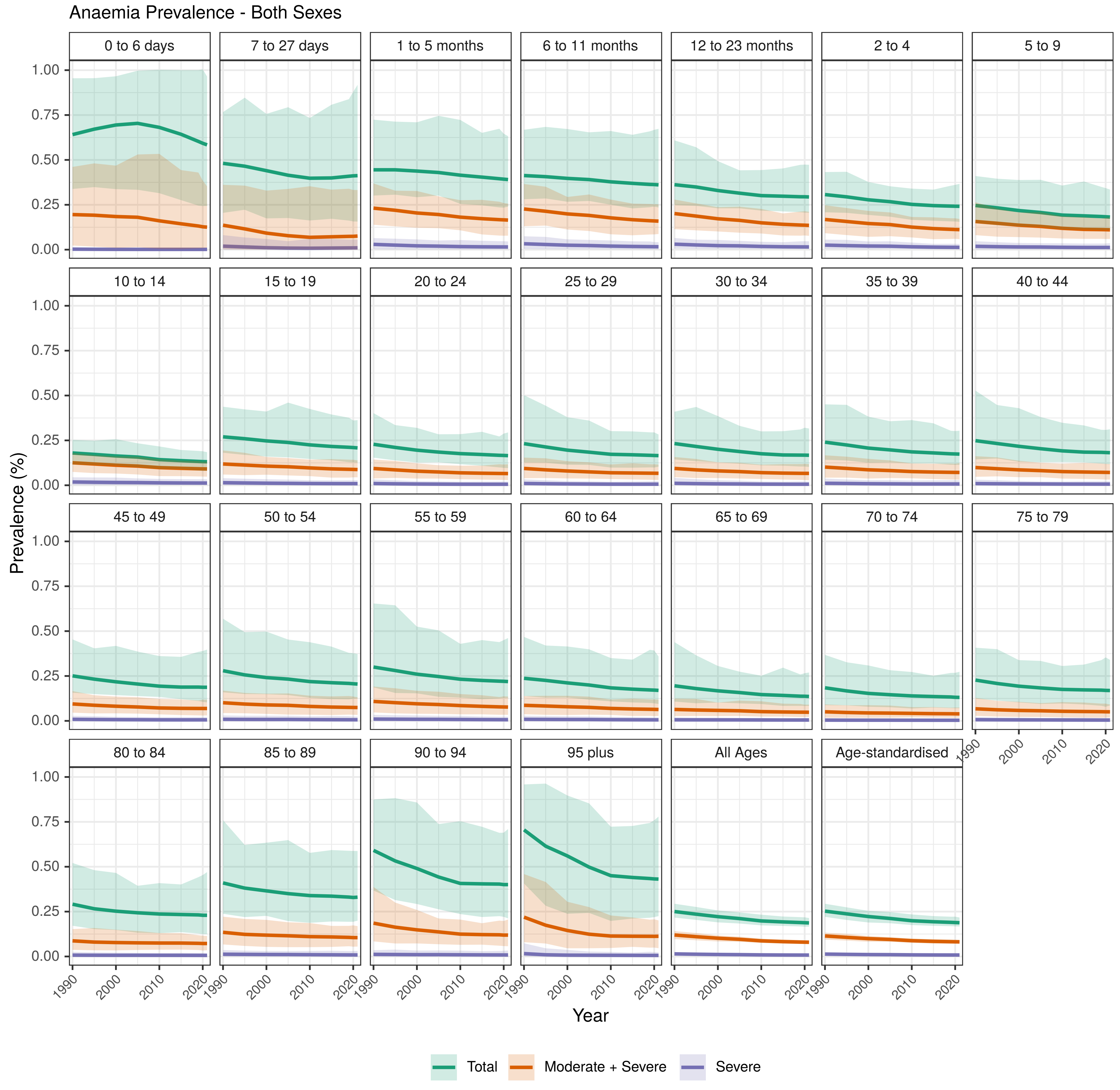

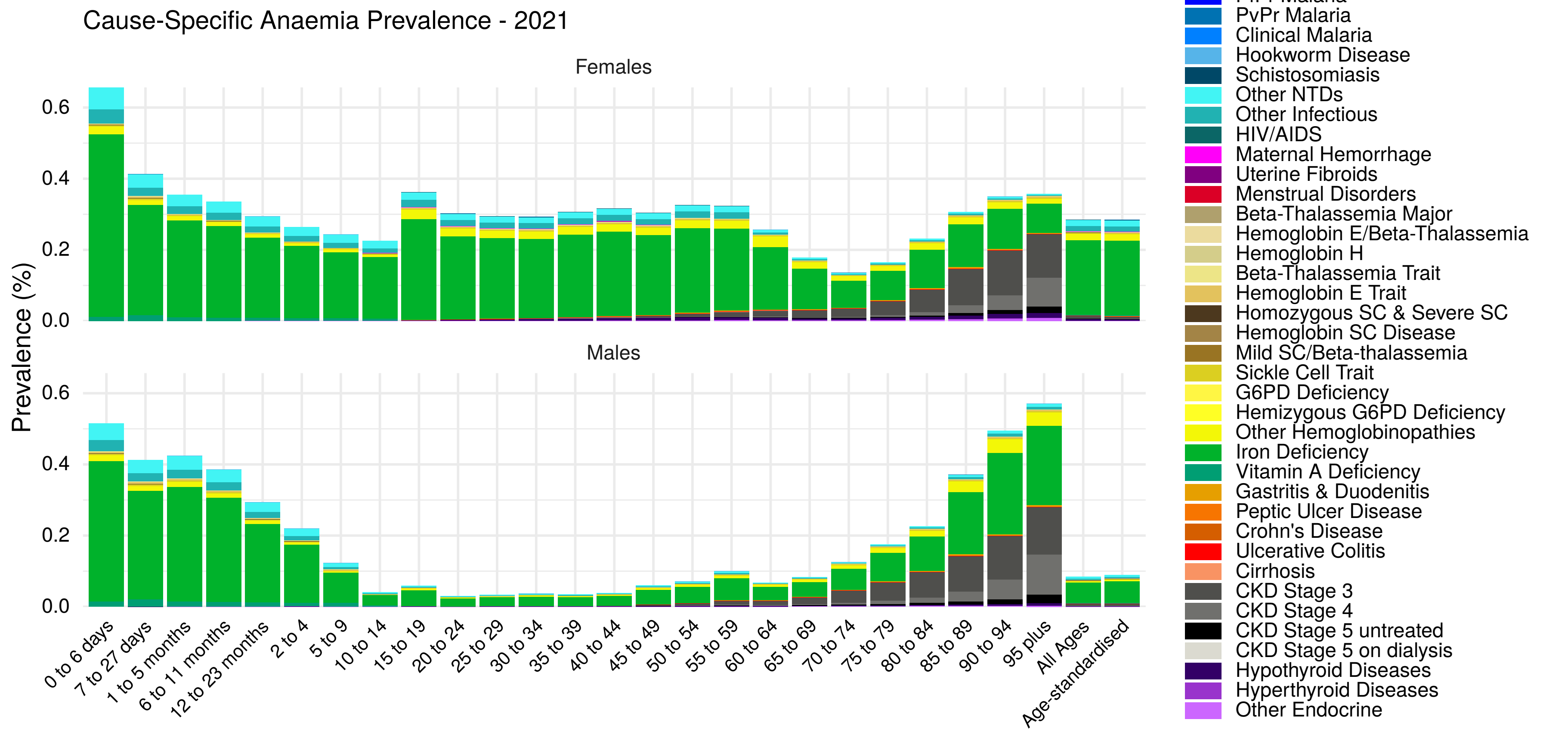

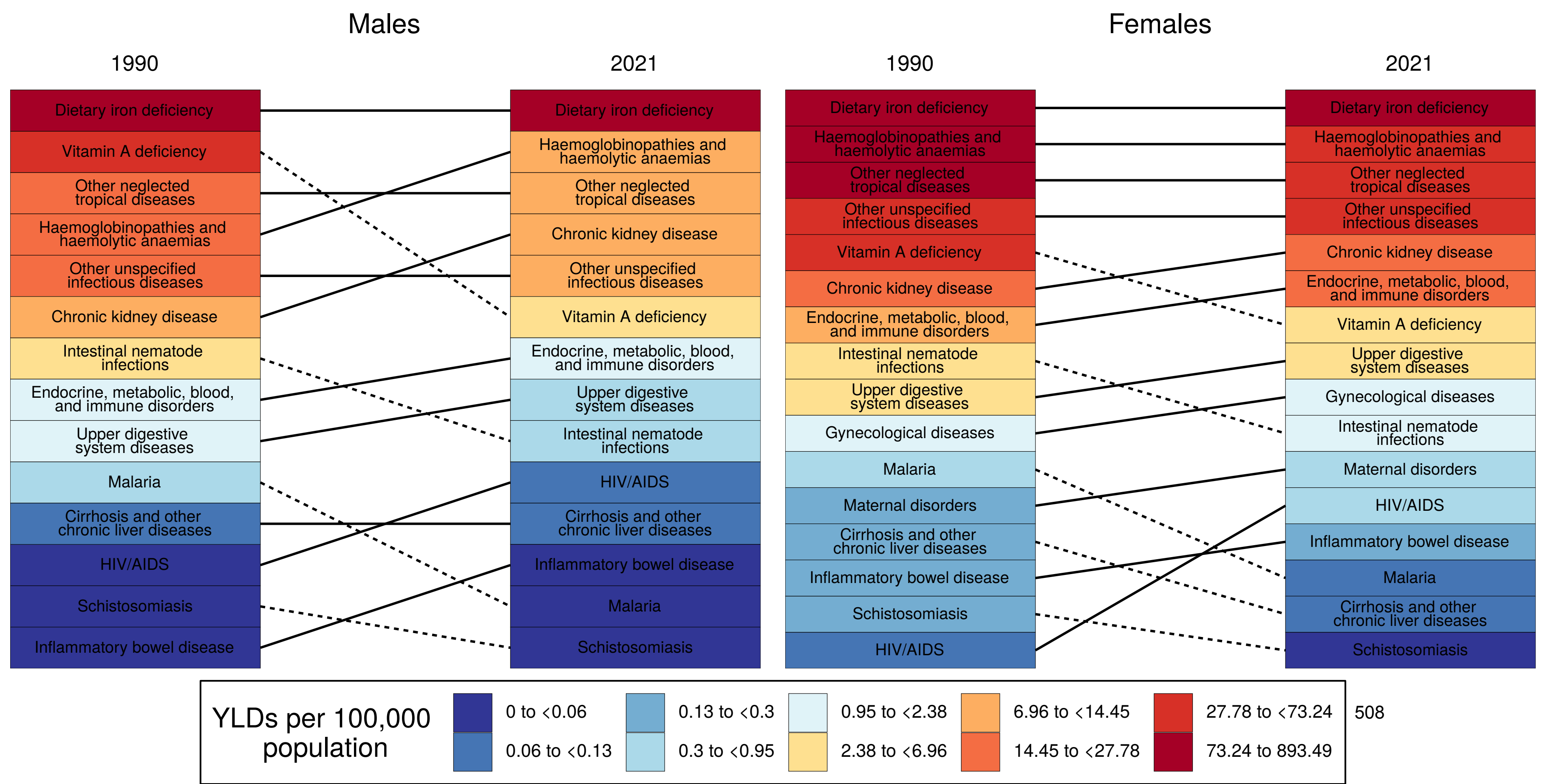

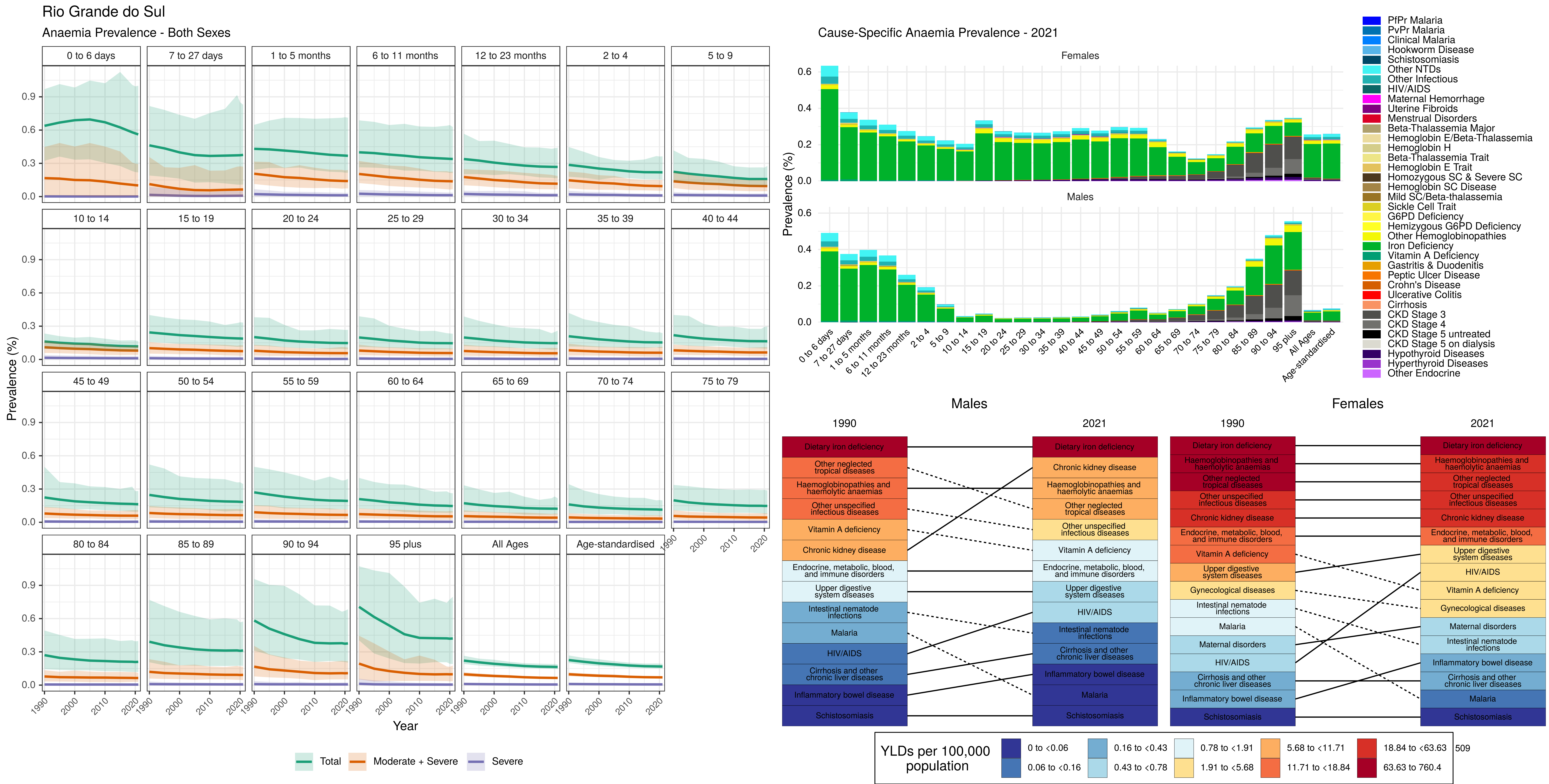
Rio Grande do Sul
Anaemia Prevalence - Both Sexes
0 to 6 days
7 to 27 days
1 to 5 months
6 to 11 months
12 to 23 months
2 to 4
5 to 9
10 to 14
15 to 19
20 to 24
25 to 29
30 to 34
35 to 39
40 to 44
45 to 49
50 to 54
55 to 59
60 to 64
65 to 69
70 to 74
75 to 79
80 to 84
85 to 89
90 to 94
95 plus
All Ages
Age-standardised
Prevalence (%)
Year
Total
Moderate + Severe
Severe
Cause-Specific Anaemia Prevalence - 2021
Females
Males
PfPr Malaria
PvPr Malaria
Clinical Malaria
Hookworm Disease
Schistosomiasis
Other NTDs
Other Infectious
HIV/AIDS
Maternal Hemorrhage
Uterine Fibroids
Menstrual Disorders
Beta-Thalassemia Major
Hemoglobin E/Beta-Thalassemia
Hemoglobin H
Beta-Thalassemia Trait
Hemoglobin E Trait
Homozygous SC & Severe SC
Hemoglobin SC Disease
Mild SC/Beta-thalassemia
Sickle Cell Trait
G6PD Deficiency
Hemizygous G6PD Deficiency
Other Hemoglobinopathies
Iron Deficiency
Vitamin A Deficiency
Gastritis & Duodenitis
Peptic Ulcer Disease
Crohn's Disease
Ulcerative Colitis
Cirrhosis
CKD Stage 3
CKD Stage 4
CKD Stage 5 untreated
CKD Stage 5 on dialysis
Hypothyroid Diseases
Hyperthyroid Diseases
Other Endocrine
Males
Females
1990
2021
Dietary iron deficiency
Other neglected tropical diseases
Haemoglobinopathies and haemolytic anaemias
Other unspecified infectious diseases
Vitamin A deficiency
Chronic kidney disease
Endocrine, metabolic, blood, and immune disorders
Upper digestive system diseases
Intestinal nematode infections
Malaria
HIV/AIDS
Cirrhosis and other chronic liver diseases
Inflammatory bowel disease
Schistosomiasis
Gynecological diseases
Maternal disorders
YLDs per 100,000 population
0 to <0.06
0.06 to <0.16
0.16 to <0.43
0.43 to <0.78
0.78 to <1.91
1.91 to <5.68
5.68 to <11.71
11.71 to <18.84
18.84 to <63.63
63.63 to 760.4

Rondônia

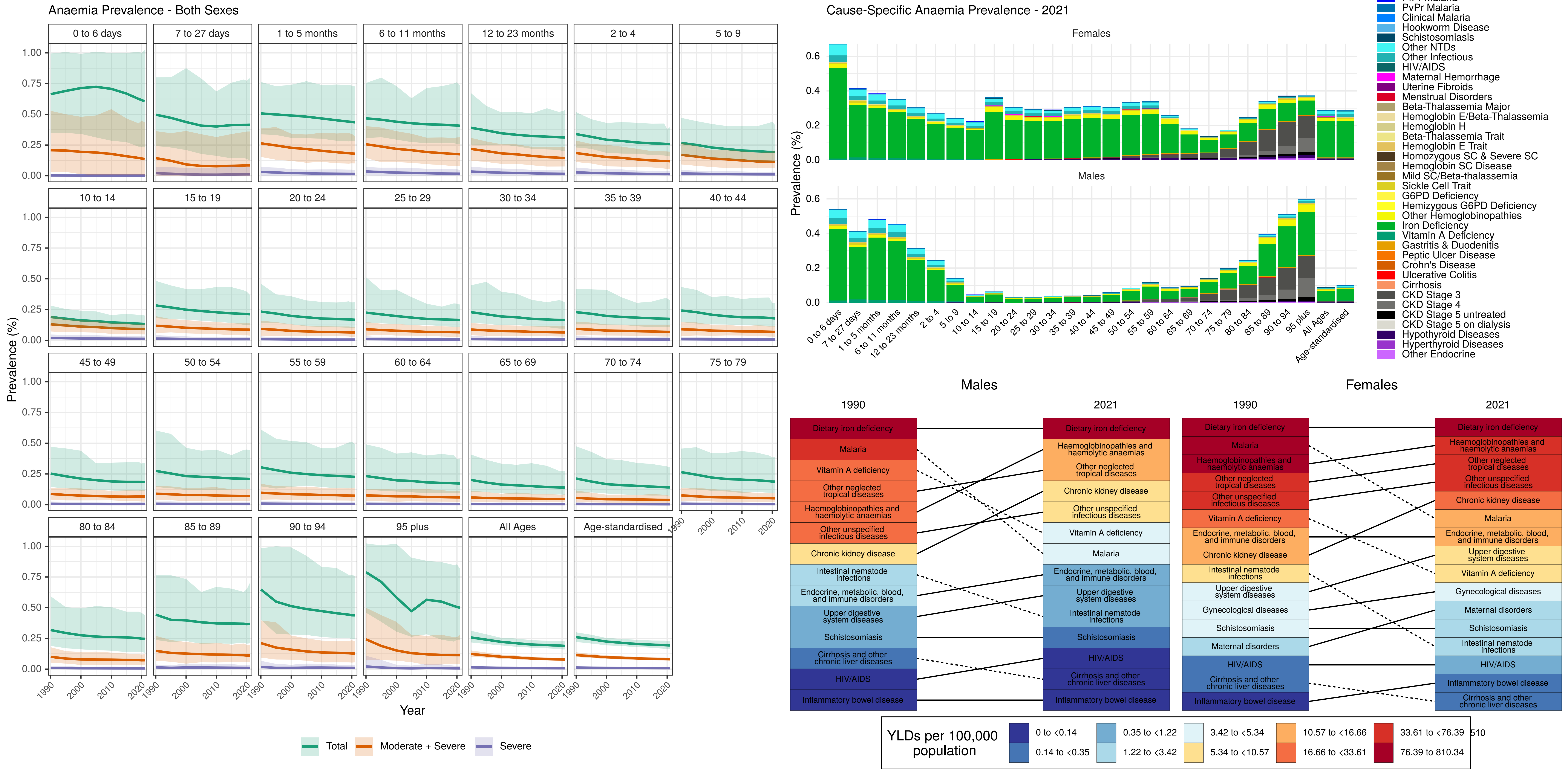

Anaemia Prevalence - Both Sexes
0 to 6 days
7 to 27 days
1 to 5 months
6 to 11 months
12 to 23 months
2 to 4
5 to 9
10 to 14
15 to 19
20 to 24
25 to 29
30 to 34
35 to 39
40 to 44
45 to 49
50 to 54
55 to 59
60 to 64
65 to 69
70 to 74
75 to 79
80 to 84
85 to 89
90 to 94
95 plus
All Ages
Age-standardised
Prevalence (%)
Year
Total
Moderate + Severe
Severe
Cause-Specific Anaemia Prevalence - 2021
Females
Males
Prevalence (%)
PfPr Malaria
PvPr Malaria
Clinical Malaria
Hookworm Disease
Schistosomiasis
Other NTDs
Other Infectious
HIV/AIDS
Maternal Hemorrhage
Uterine Fibroids
Menstrual Disorders
Beta-Thalassemia Major
Hemoglobin E/Beta-Thalassemia
Hemoglobin H
Beta-Thalassemia Trait
Hemoglobin E Trait
Homozygous SC & Severe SC
Hemoglobin SC Disease
Mild SC/Beta-thalassemia
Sickle Cell Trait
G6PD Deficiency
Hemizygous G6PD Deficiency
Other Hemoglobinopathies
Iron Deficiency
Vitamin A Deficiency
Gastritis & Duodenitis
Peptic Ulcer Disease
Crohn's Disease
Ulcerative Colitis
Cirrhosis
CKD Stage 3
CKD Stage 4
CKD Stage 5 untreated
CKD Stage 5 on dialysis
Hypothyroid Diseases
Hyperthyroid Diseases
Other Endocrine
Males
Females
1990
2021
Dietary iron deficiency
Malaria
Vitamin A deficiency
Other neglected tropical diseases
Haemoglobinopathies and haemolytic anaemias
Other unspecified infectious diseases
Chronic kidney disease
Intestinal nematode infections
Endocrine, metabolic, blood, and immune disorders
Upper digestive system diseases
Schistosomiasis
Cirrhosis and other chronic liver diseases
HIV/AIDS
Inflammatory bowel disease
Gynecological diseases
Maternal disorders
YLDs per 100,000 population
0 to <0.14
0.14 to <0.35
0.35 to <1.22
1.22 to <3.42
3.42 to <5.34
5.34 to <10.57
10.57 to <16.66
16.66 to <33.61
33.61 to <76.39
76.39 to 810.34

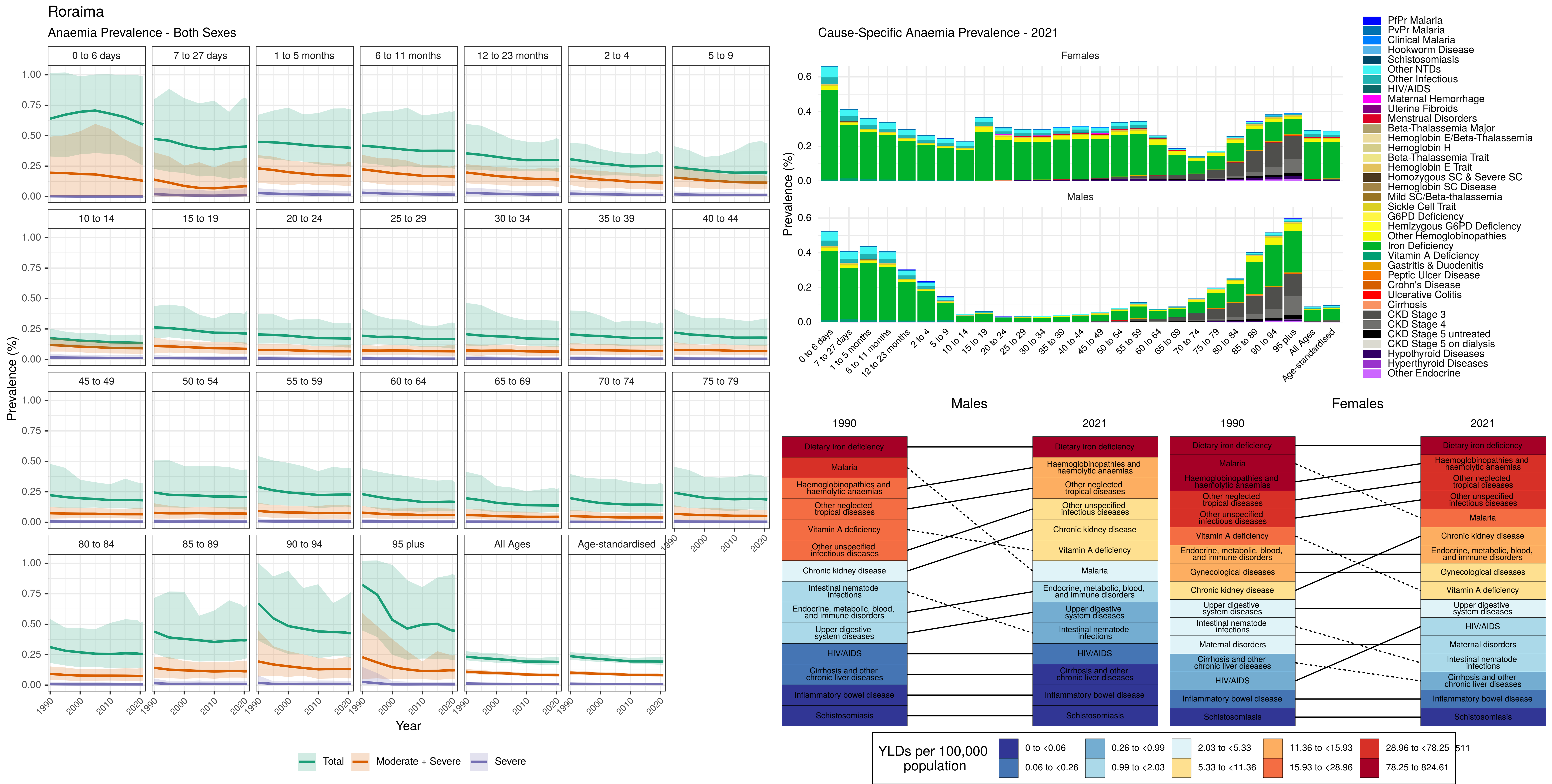
Roraima
Anaemia Prevalence - Both Sexes
0 to 6 days
7 to 27 days
1 to 5 months
6 to 11 months
12 to 23 months
2 to 4
5 to 9
10 to 14
15 to 19
20 to 24
25 to 29
30 to 34
35 to 39
40 to 44
45 to 49
50 to 54
55 to 59
60 to 64
65 to 69
70 to 74
75 to 79
80 to 84
85 to 89
90 to 94
95 plus
All Ages
Age-standardised
Prevalence (%)
Year
Total
Moderate + Severe
Severe
Cause-Specific Anaemia Prevalence - 2021
Females
Males
PfPr Malaria
PvPr Malaria
Clinical Malaria
Hookworm Disease
Schistosomiasis
Other NTDs
Other Infectious
HIV/AIDS
Maternal Hemorrhage
Uterine Fibroids
Menstrual Disorders
Beta-Thalassemia Major
Hemoglobin E/Beta-Thalassemia
Hemoglobin H
Beta-Thalassemia Trait
Hemoglobin E Trait
Homozygous SC & Severe SC
Hemoglobin SC Disease
Mild SC/Beta-thalassemia
Sickle Cell Trait
G6PD Deficiency
Hemizygous G6PD Deficiency
Other Hemoglobinopathies
Iron Deficiency
Vitamin A Deficiency
Gastritis & Duodenitis
Peptic Ulcer Disease
Crohn's Disease
Ulcerative Colitis
Cirrhosis
CKD Stage 3
CKD Stage 4
CKD Stage 5 untreated
CKD Stage 5 on dialysis
Hypothyroid Diseases
Hyperthyroid Diseases
Other Endocrine
Males
Females
1990
2021
Dietary iron deficiency
Malaria
Haemoglobinopathies and haemolytic anaemias
Other neglected tropical diseases
Vitamin A deficiency
Other unspecified infectious diseases
Chronic kidney disease
Intestinal nematode infections
Endocrine, metabolic, blood, and immune disorders
Upper digestive system diseases
HIV/AIDS
Cirrhosis and other chronic liver diseases
Inflammatory bowel disease
Schistosomiasis
Gynecological diseases
Maternal disorders
YLDs per 100,000 population
0 to <0.06
0.06 to <0.26
0.26 to <0.99
0.99 to <2.03
2.03 to <5.33
5.33 to <11.36
11.36 to <15.93
15.93 to <28.96
28.96 to <78.25
78.25 to 824.61

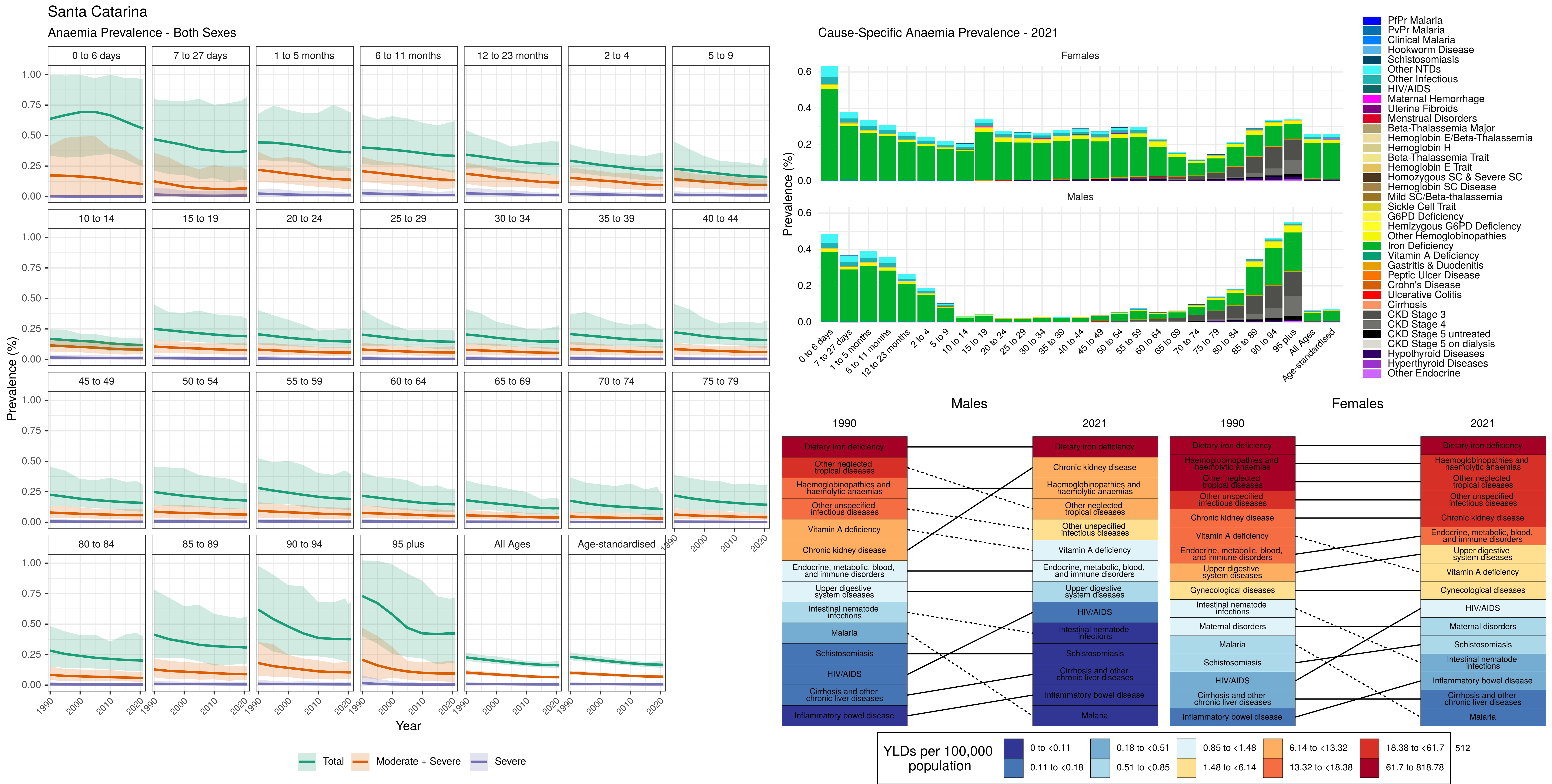

Santa Catarina
Anaemia Prevalence - Both Sexes
0 to 6 days
7 to 27 days
1 to 5 months
6 to 11 months
12 to 23 months
2 to 4
5 to 9
10 to 14
15 to 19
20 to 24
25 to 29
30 to 34
35 to 39
40 to 44
45 to 49
50 to 54
55 to 59
60 to 64
65 to 69
70 to 74
75 to 79
80 to 84
85 to 89
90 to 94
95 plus
All Ages
Age-standardised
Prevalence (%)
Year
Total
Moderate + Severe
Severe
Cause-Specific Anaemia Prevalence - 2021
Females
Males
PfPr Malaria
PvPr Malaria
Clinical Malaria
Hookworm Disease
Schistosomiasis
Other NTDs
Other Infectious
HIV/AIDS
Maternal Hemorrhage
Uterine Fibroids
Menstrual Disorders
Beta-Thalassemia Major
Hemoglobin E/Beta-Thalassemia
Hemoglobin H
Beta-Thalassemia Trait
Hemoglobin E Trait
Homozygous SC & Severe SC
Hemoglobin SC Disease
Mild SC/Beta-thalassemia
Sickle Cell Trait
G6PD Deficiency
Hemizygous G6PD Deficiency
Other Hemoglobinopathies
Iron Deficiency
Vitamin A Deficiency
Gastritis & Duodenitis
Peptic Ulcer Disease
Crohn's Disease
Ulcerative Colitis
Cirrhosis
CKD Stage 3
CKD Stage 4
CKD Stage 5 untreated
CKD Stage 5 on dialysis
Hypothyroid Diseases
Hyperthyroid Diseases
Other Endocrine
Males
Females
1990
2021
Dietary iron deficiency
Other neglected tropical diseases
Haemoglobinopathies and haemolytic anaemias
Other unspecified infectious diseases
Vitamin A deficiency
Chronic kidney disease
Endocrine, metabolic, blood, and immune disorders
Upper digestive system diseases
Intestinal nematode infections
Malaria
Schistosomiasis
HIV/AIDS
Cirrhosis and other chronic liver diseases
Inflammatory bowel disease
Gynecological diseases
Maternal disorders
YLDs per 100,000 population
0 to <0.11
0.11 to <0.18
0.18 to <0.51
0.51 to <0.85
0.85 to <1.48
1.48 to <6.14
6.14 to <13.32
13.32 to <18.38
18.38 to <61.7
61.7 to 818.78

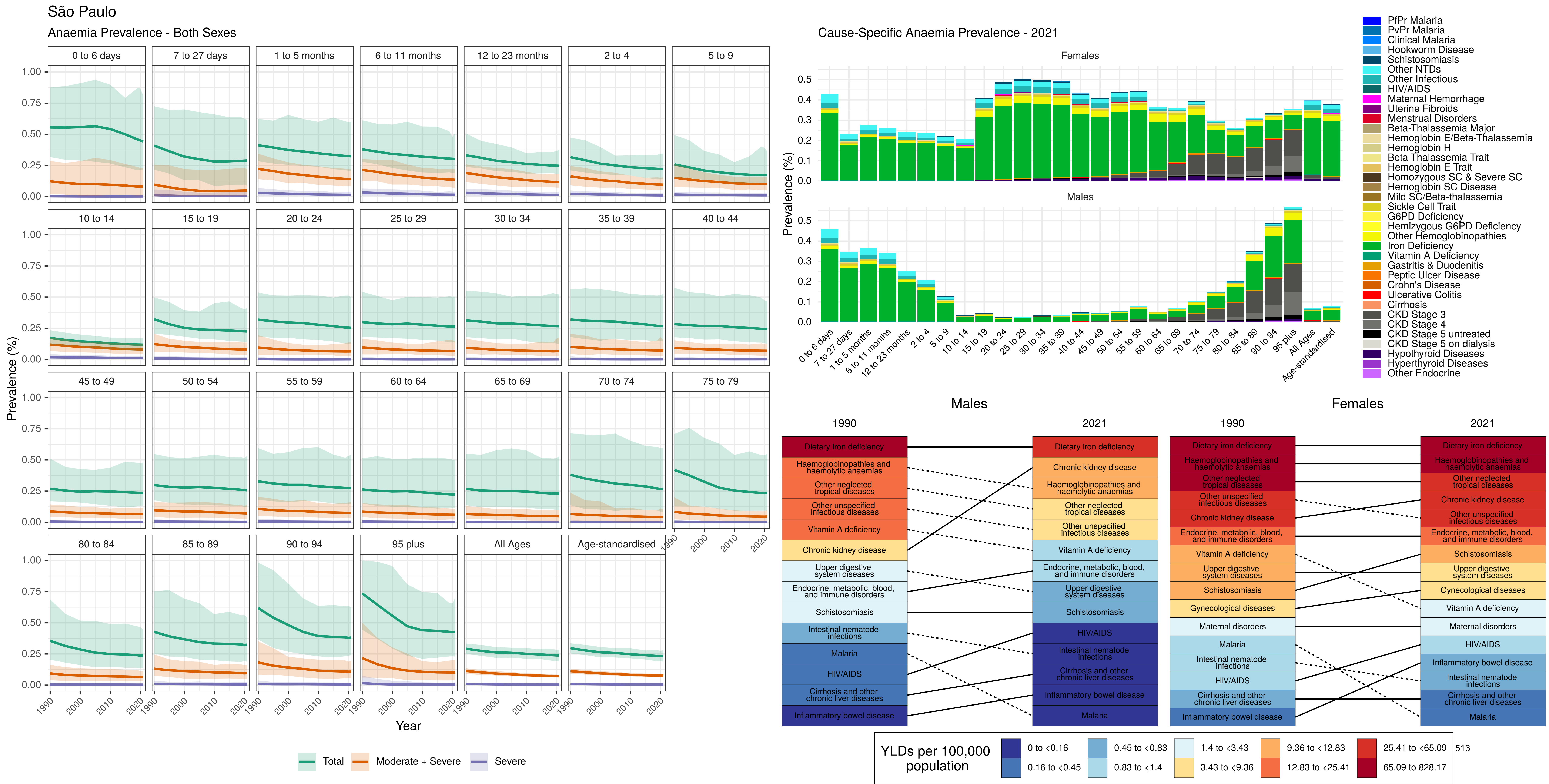

São Paulo
Anaemia Prevalence - Both Sexes
0 to 6 days
7 to 27 days
1 to 5 months
6 to 11 months
12 to 23 months
2 to 4
5 to 9
10 to 14
15 to 19
20 to 24
25 to 29
30 to 34
35 to 39
40 to 44
45 to 49
50 to 54
55 to 59
60 to 64
65 to 69
70 to 74
75 to 79
80 to 84
85 to 89
90 to 94
95 plus
All Ages
Age-standardised
Prevalence (%)
Year
Total
Moderate + Severe
Severe
Cause-Specific Anaemia Prevalence - 2021
Females
Males
Prevalence (%)
PfPr Malaria
PvPr Malaria
Clinical Malaria
Hookworm Disease
Schistosomiasis
Other NTDs
Other Infectious
HIV/AIDS
Maternal Hemorrhage
Uterine Fibroids
Menstrual Disorders
Beta-Thalassemia Major
Hemoglobin E/Beta-Thalassemia
Hemoglobin H
Beta-Thalassemia Trait
Hemoglobin E Trait
Homozygous SC & Severe SC
Hemoglobin SC Disease
Mild SC/Beta-thalassemia
Sickle Cell Trait
G6PD Deficiency
Hemizygous G6PD Deficiency
Other Hemoglobinopathies
Iron Deficiency
Vitamin A Deficiency
Gastritis & Duodenitis
Peptic Ulcer Disease
Crohn's Disease
Ulcerative Colitis
Cirrhosis
CKD Stage 3
CKD Stage 4
CKD Stage 5 untreated
CKD Stage 5 on dialysis
Hypothyroid Diseases
Hyperthyroid Diseases
Other Endocrine
Males
1990
Dietary iron deficiency
Haemoglobinopathies and haemolytic anaemias
Other neglected tropical diseases
Other unspecified infectious diseases
Vitamin A deficiency
Chronic kidney disease
Upper digestive system diseases
Endocrine, metabolic, blood, and immune disorders
Schistosomiasis
Intestinal nematode infections
Malaria
HIV/AIDS
Cirrhosis and other chronic liver diseases
Inflammatory bowel disease
2021
Dietary iron deficiency
Chronic kidney disease
Haemoglobinopathies and haemolytic anaemias
Other neglected tropical diseases
Other unspecified infectious diseases
Vitamin A deficiency
Endocrine, metabolic, blood, and immune disorders
Upper digestive system diseases
Schistosomiasis
HIV/AIDS
Intestinal nematode infections
Cirrhosis and other chronic liver diseases
Inflammatory bowel disease
Malaria
Females
1990
Dietary iron deficiency
Haemoglobinopathies and haemolytic anaemias
Other neglected tropical diseases
Other unspecified infectious diseases
Chronic kidney disease
Endocrine, metabolic, blood, and immune disorders
Vitamin A deficiency
Upper digestive system diseases
Schistosomiasis
Gynecological diseases
Maternal disorders
Malaria
Intestinal nematode infections
HIV/AIDS
Cirrhosis and other chronic liver diseases
Inflammatory bowel disease
2021
Dietary iron deficiency
Haemoglobinopathies and haemolytic anaemias
Other neglected tropical diseases
Chronic kidney disease
Other unspecified infectious diseases
Endocrine, metabolic, blood, and immune disorders
Schistosomiasis
Upper digestive system diseases
Gynecological diseases
Vitamin A deficiency
Maternal disorders
HIV/AIDS
Inflammatory bowel disease
Intestinal nematode infections
Cirrhosis and other chronic liver diseases
Malaria
YLDs per 100,000 population
0 to <0.16
0.16 to <0.45
0.45 to <0.83
0.83 to <1.4
1.4 to <3.43
3.43 to <9.36
9.36 to <12.83
12.83 to <25.41
25.41 to <65.09
65.09 to 828.17

Sergipe

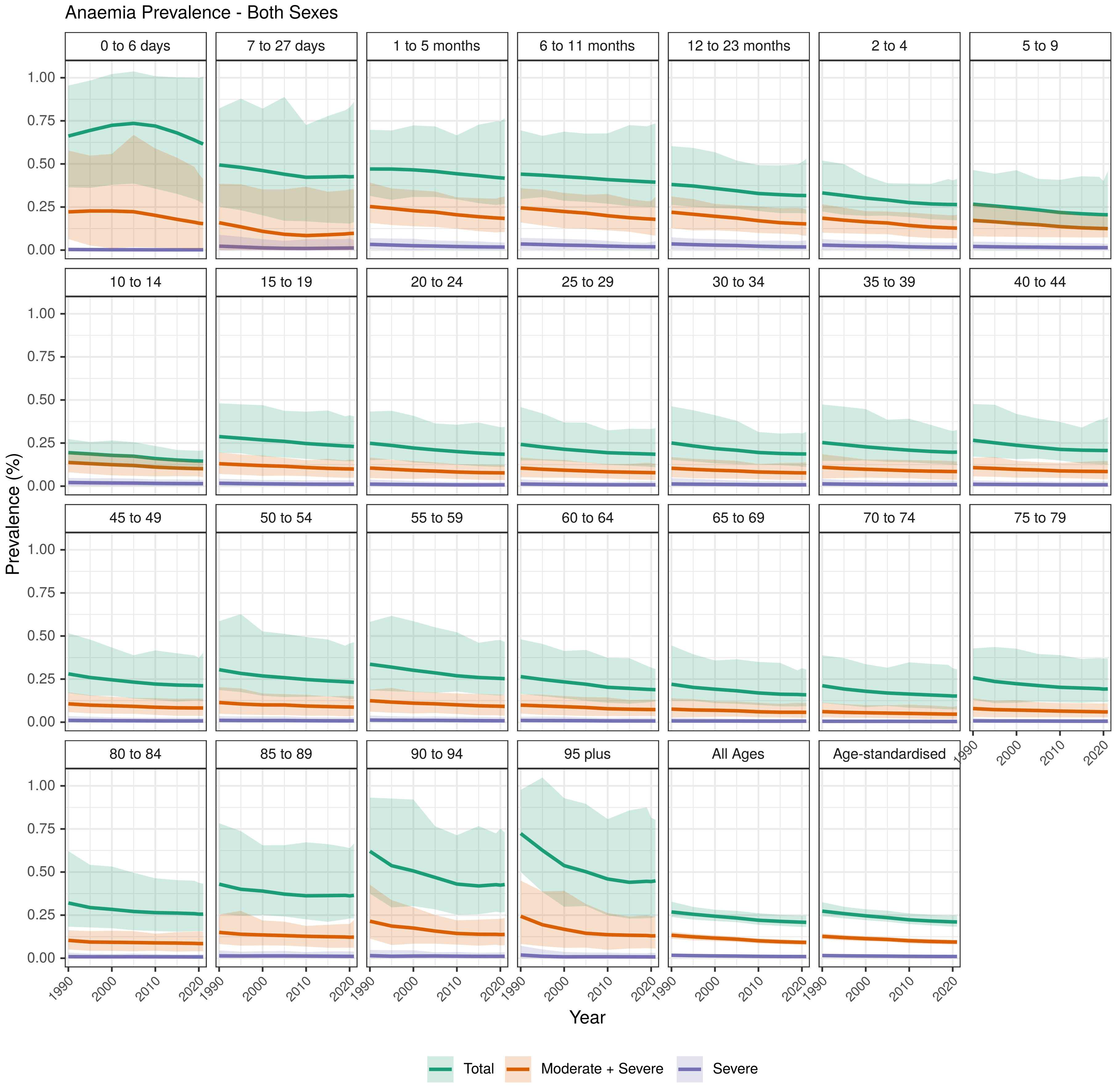

Anaemia Prevalence - Both Sexes
0 to 6 days
7 to 27 days
1 to 5 months
6 to 11 months
12 to 23 months
2 to 4
5 to 9
10 to 14
15 to 19
20 to 24
25 to 29
30 to 34
35 to 39
40 to 44
45 to 49
50 to 54
55 to 59
60 to 64
65 to 69
70 to 74
75 to 79
80 to 84
85 to 89
90 to 94
95 plus
All Ages
Age-standardised
Prevalence (%)
Year
Total
Moderate + Severe
Severe

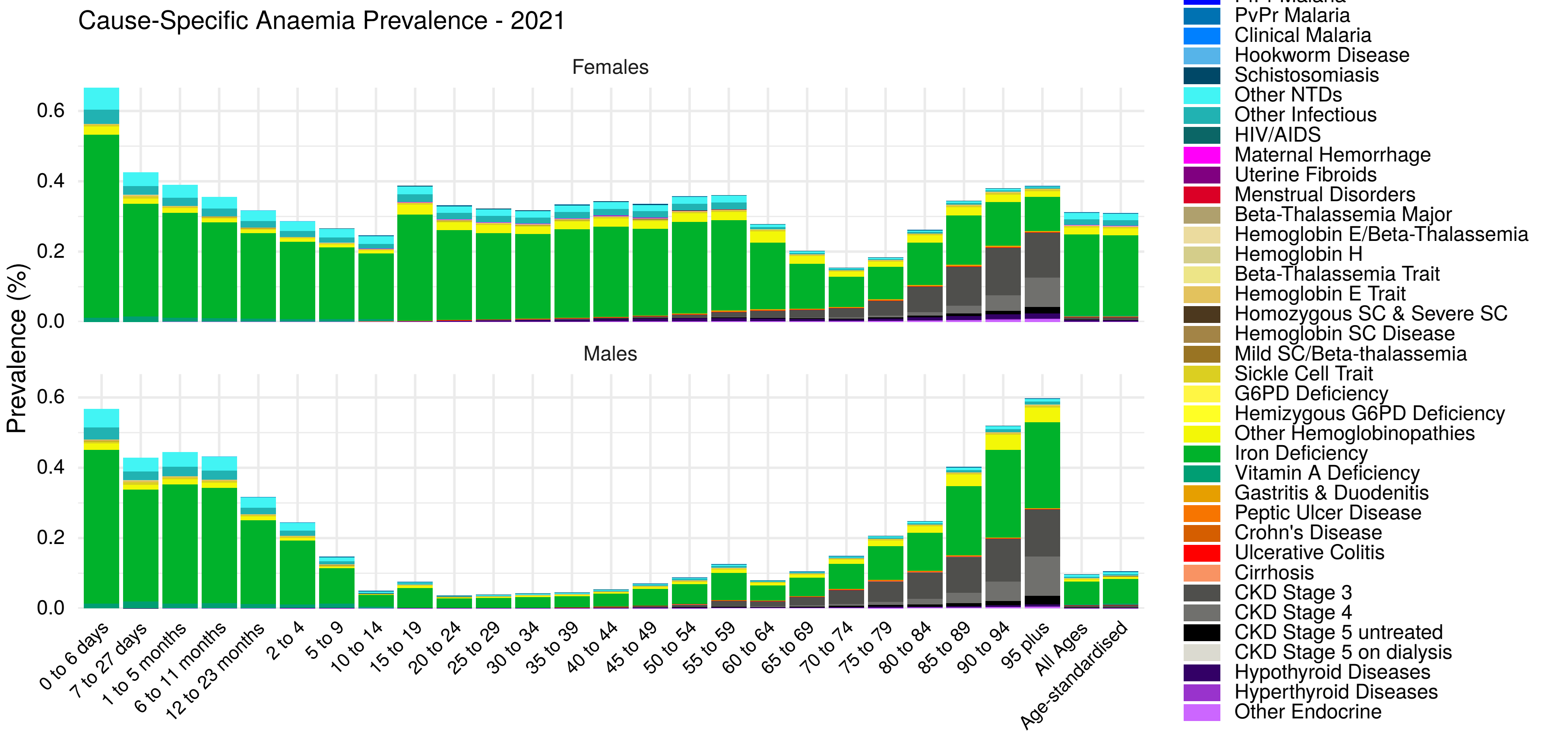

Cause-Specific Anaemia Prevalence - 2021
Females
Males
Prevalence (%)
PfPr Malaria
PvPr Malaria
Clinical Malaria
Hookworm Disease
Schistosomiasis
Other NTDs
Other Infectious
HIV/AIDS
Maternal Hemorrhage
Uterine Fibroids
Menstrual Disorders
Beta-Thalassemia Major
Hemoglobin E/Beta-Thalassemia
Hemoglobin H
Beta-Thalassemia Trait
Hemoglobin E Trait
Homozygous SC & Severe SC
Hemoglobin SC Disease
Mild SC/Beta-thalassemia
Sickle Cell Trait
G6PD Deficiency
Hemizygous G6PD Deficiency
Other Hemoglobinopathies
Iron Deficiency
Vitamin A Deficiency
Gastritis & Duodenitis
Peptic Ulcer Disease
Crohn's Disease
Ulcerative Colitis
Cirrhosis
CKD Stage 3
CKD Stage 4
CKD Stage 5 untreated
CKD Stage 5 on dialysis
Hypothyroid Diseases
Hyperthyroid Diseases
Other Endocrine

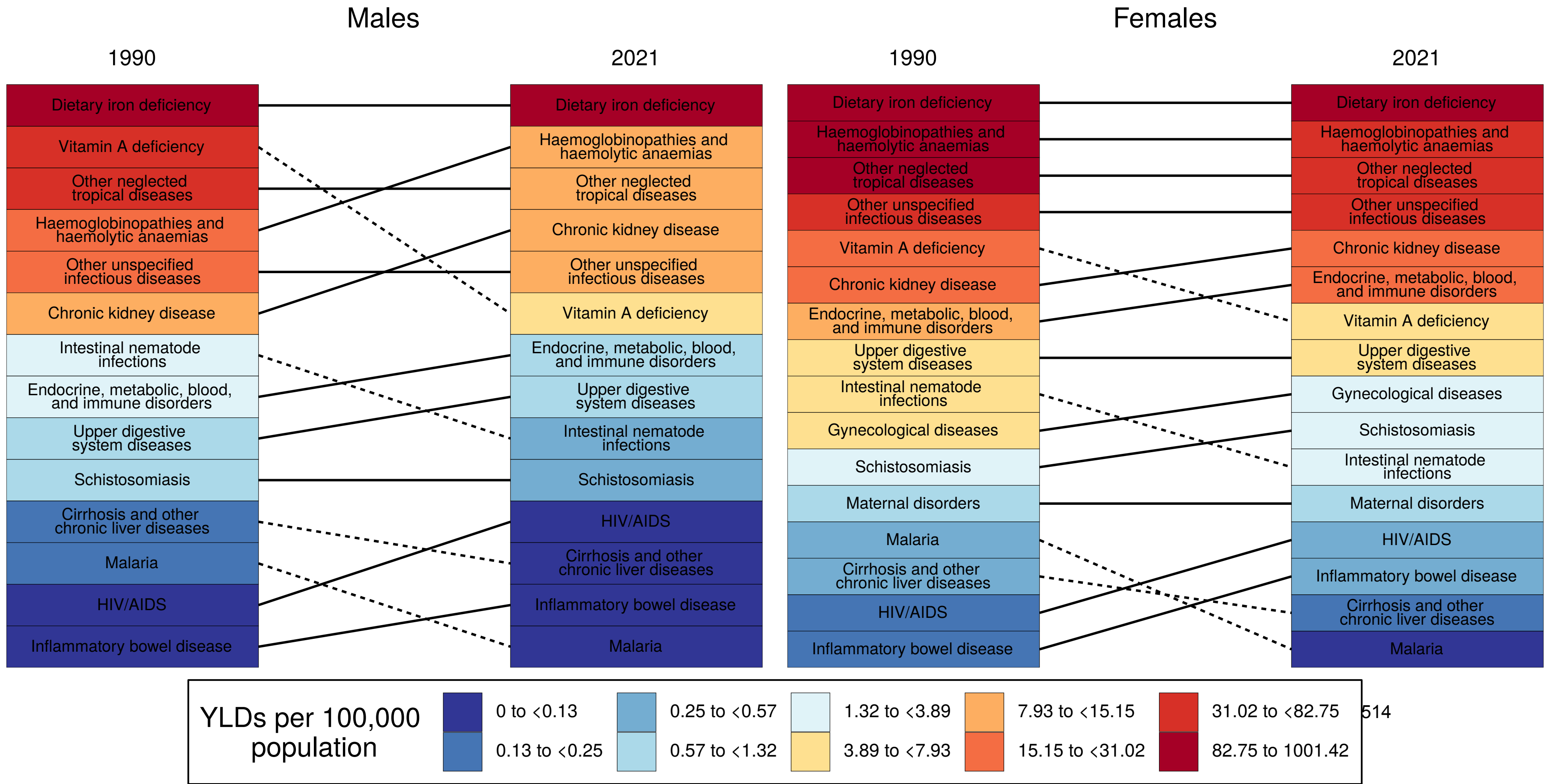

Males
Females
1990
2021
Dietary iron deficiency
Vitamin A deficiency
Other neglected tropical diseases
Haemoglobinopathies and haemolytic anaemias
Other unspecified infectious diseases
Chronic kidney disease
Intestinal nematode infections
Endocrine, metabolic, blood, and immune disorders
Upper digestive system diseases
Schistosomiasis
Cirrhosis and other chronic liver diseases
Malaria
HIV/AIDS
Inflammatory bowel disease
Gynecological diseases
Maternal disorders
YLDs per 100,000 population
0 to <0.13
0.13 to <0.25
0.25 to <0.57
0.57 to <1.32
1.32 to <3.89
3.89 to <7.93
7.93 to <15.15
15.15 to <31.02
31.02 to <82.75
82.75 to 1001.42

Tocantins

Anaemia Prevalence - Both Sexes

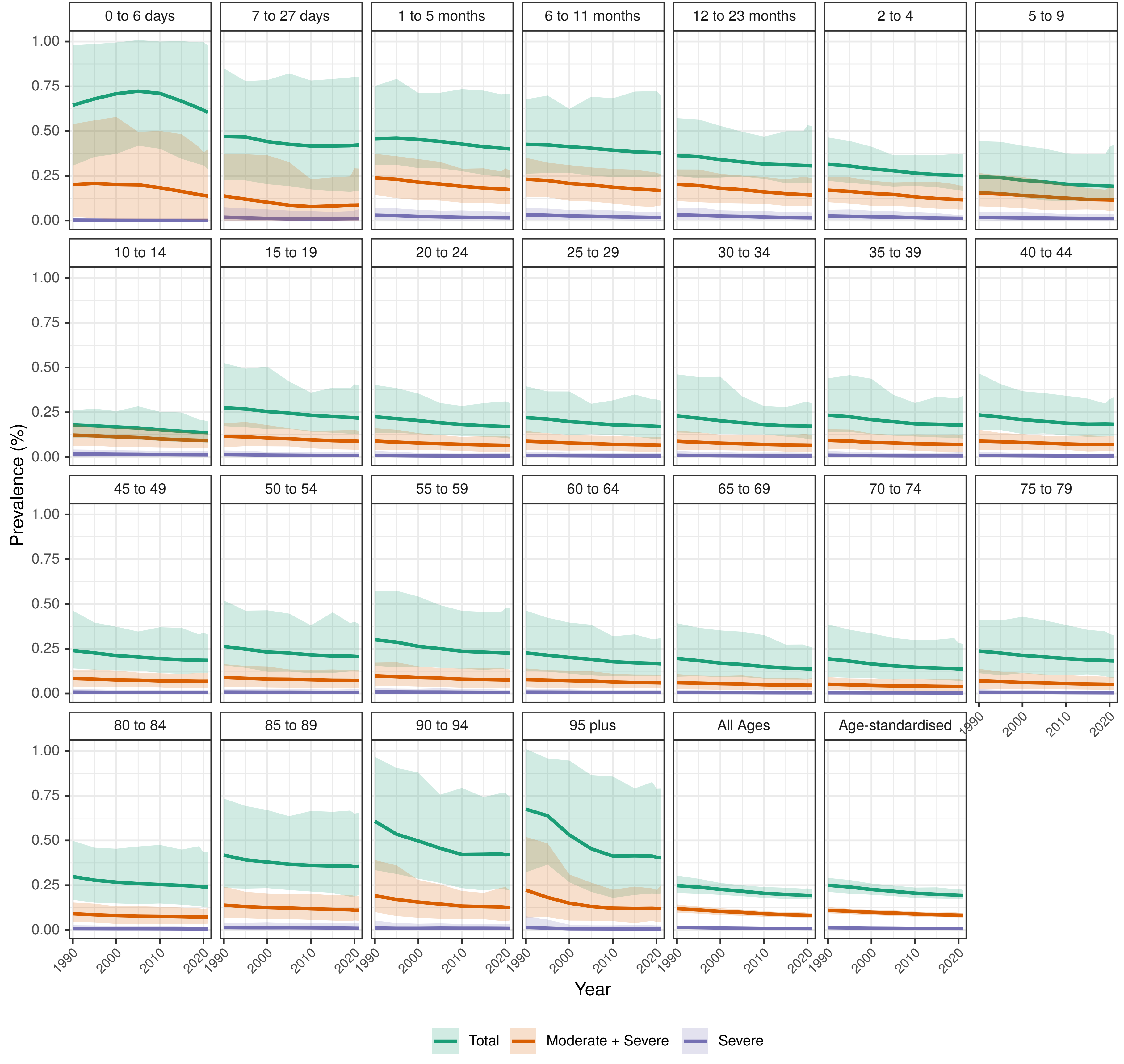

Cause-Specific Anaemia Prevalence - 2021

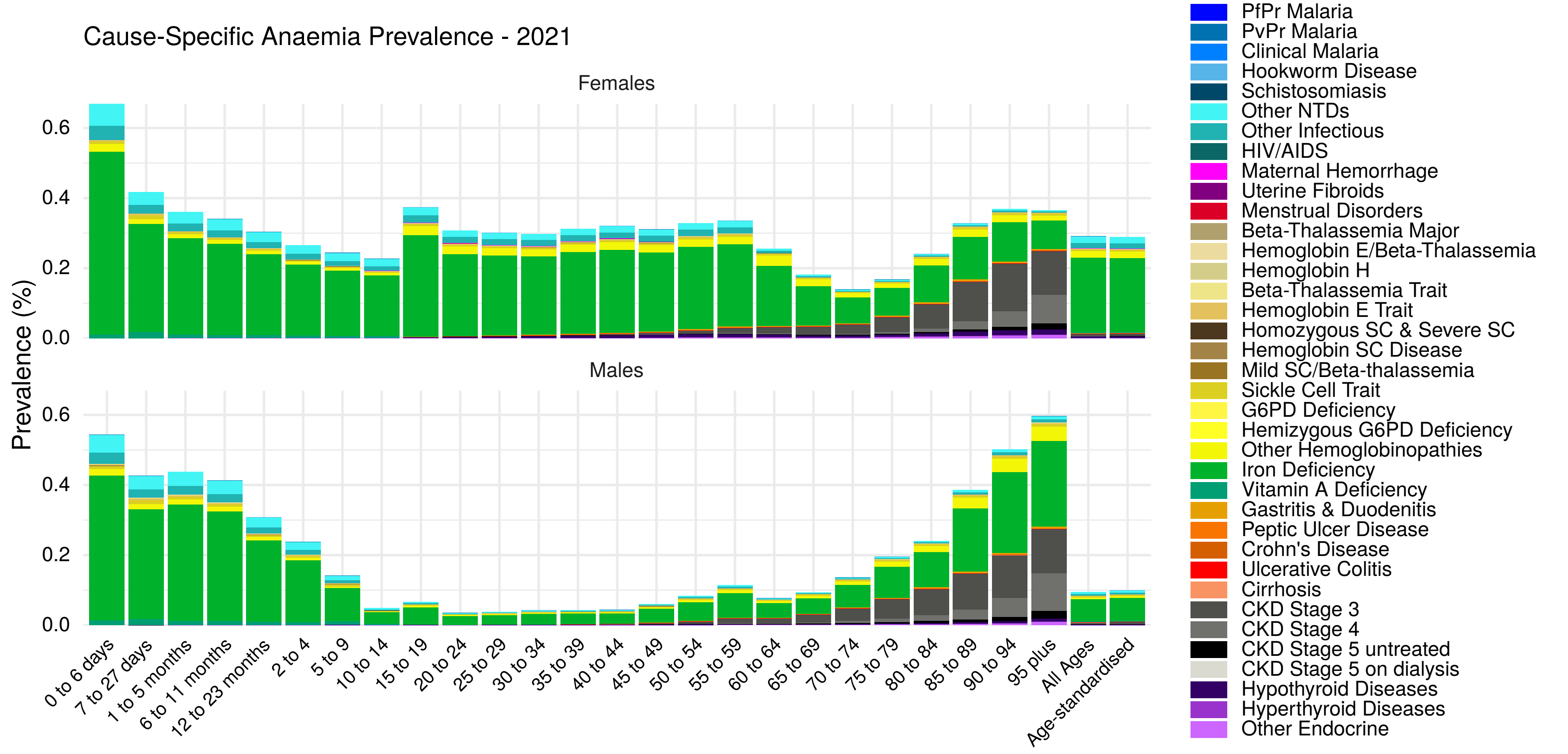

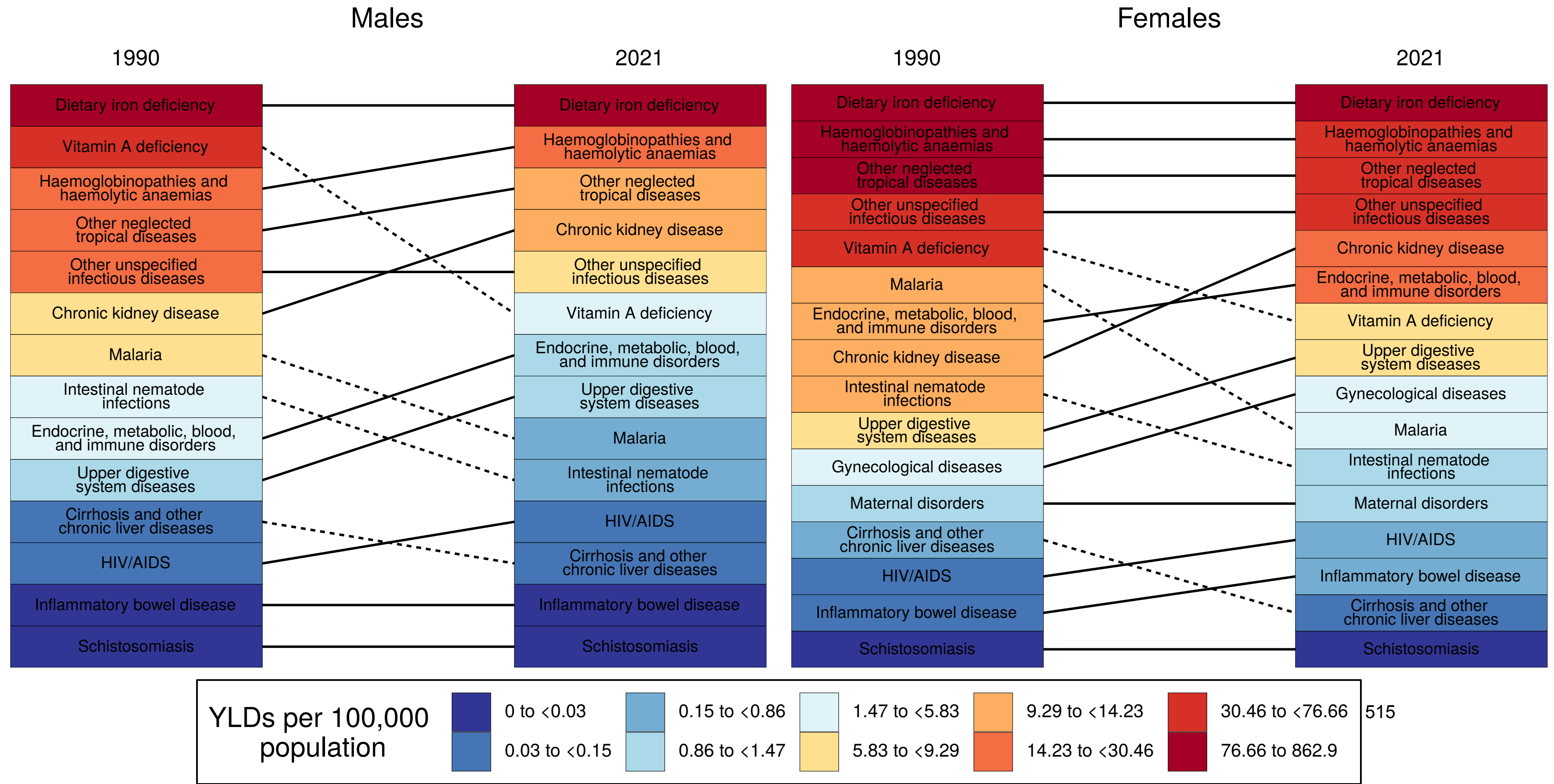

Paraguay

Anaemia Prevalence - Both Sexes

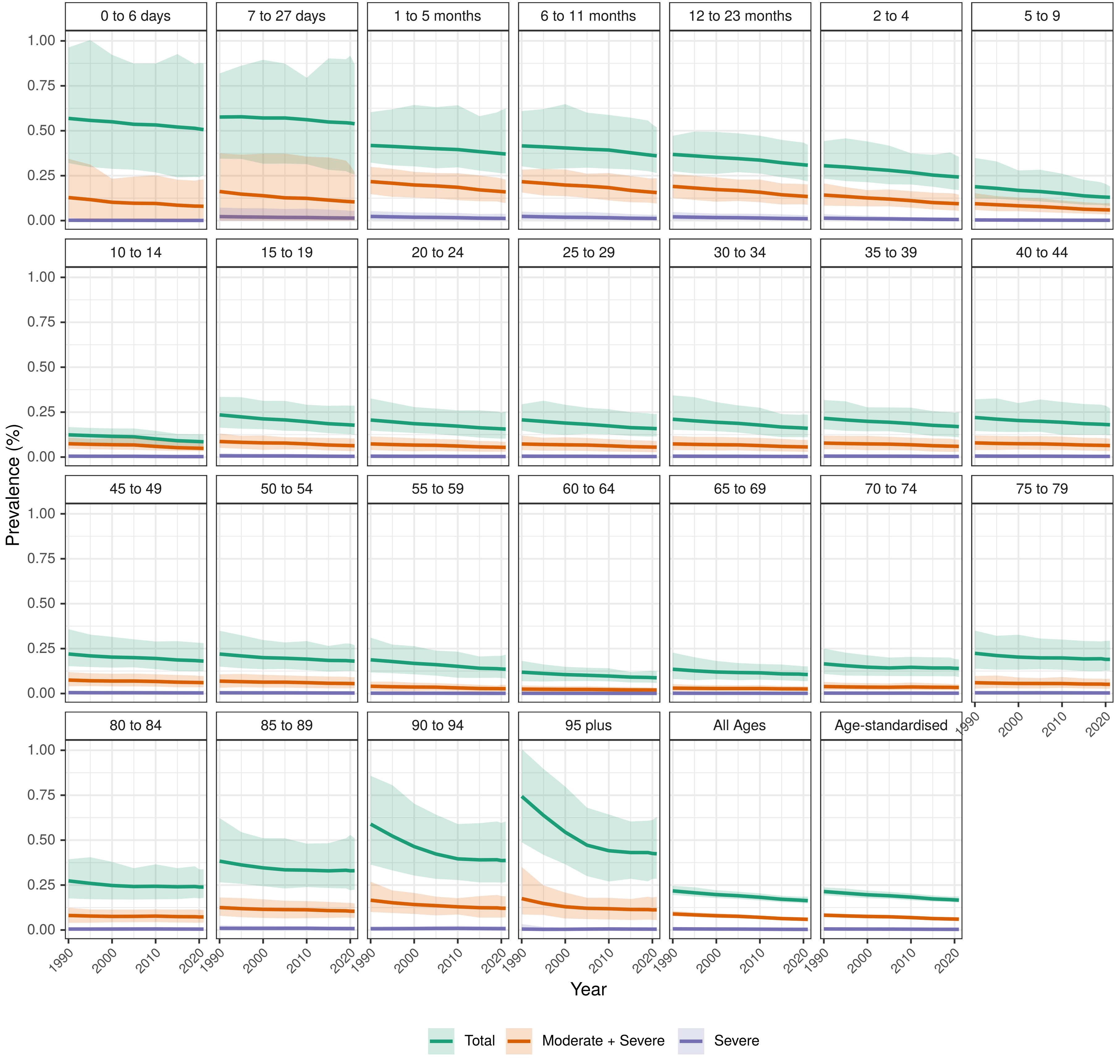

Cause-Specific Anaemia Prevalence - 2021

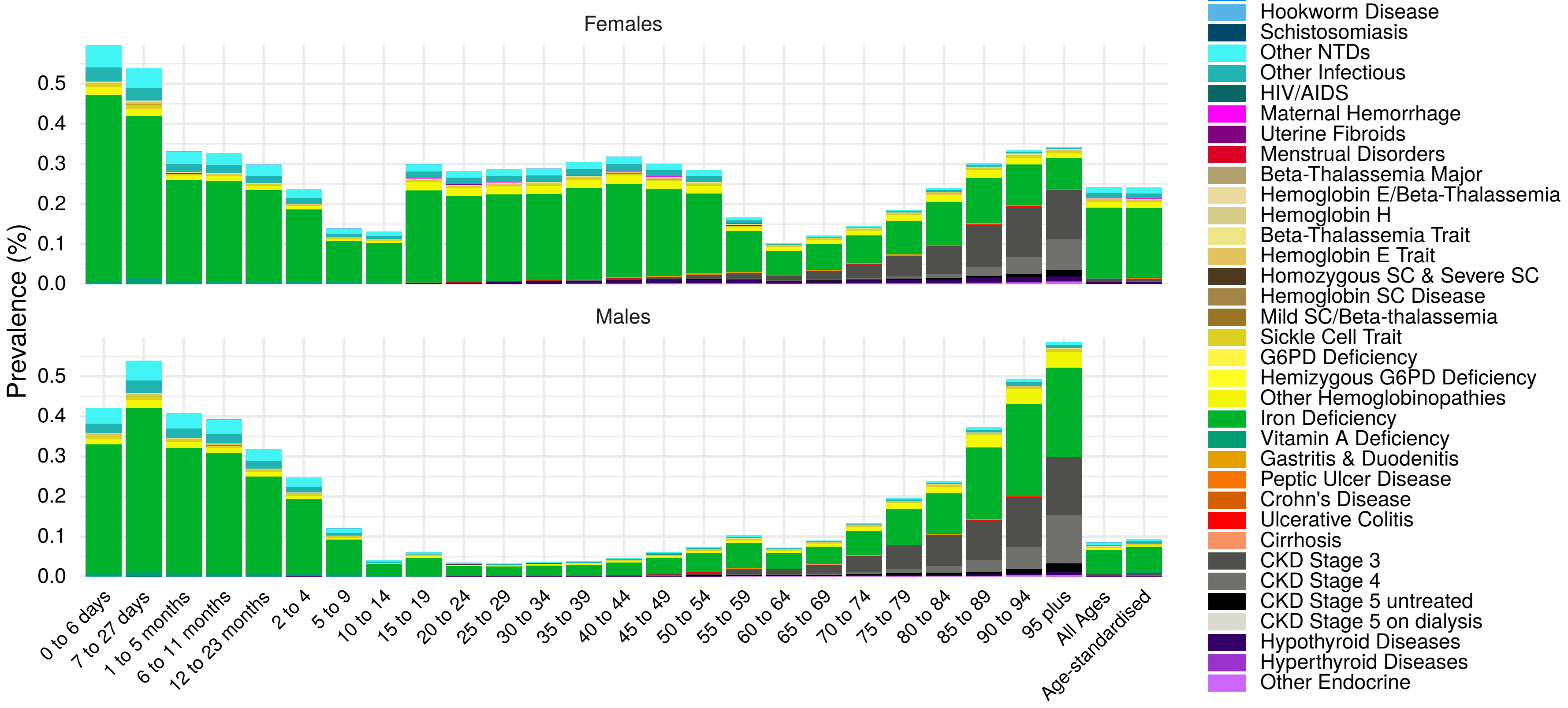

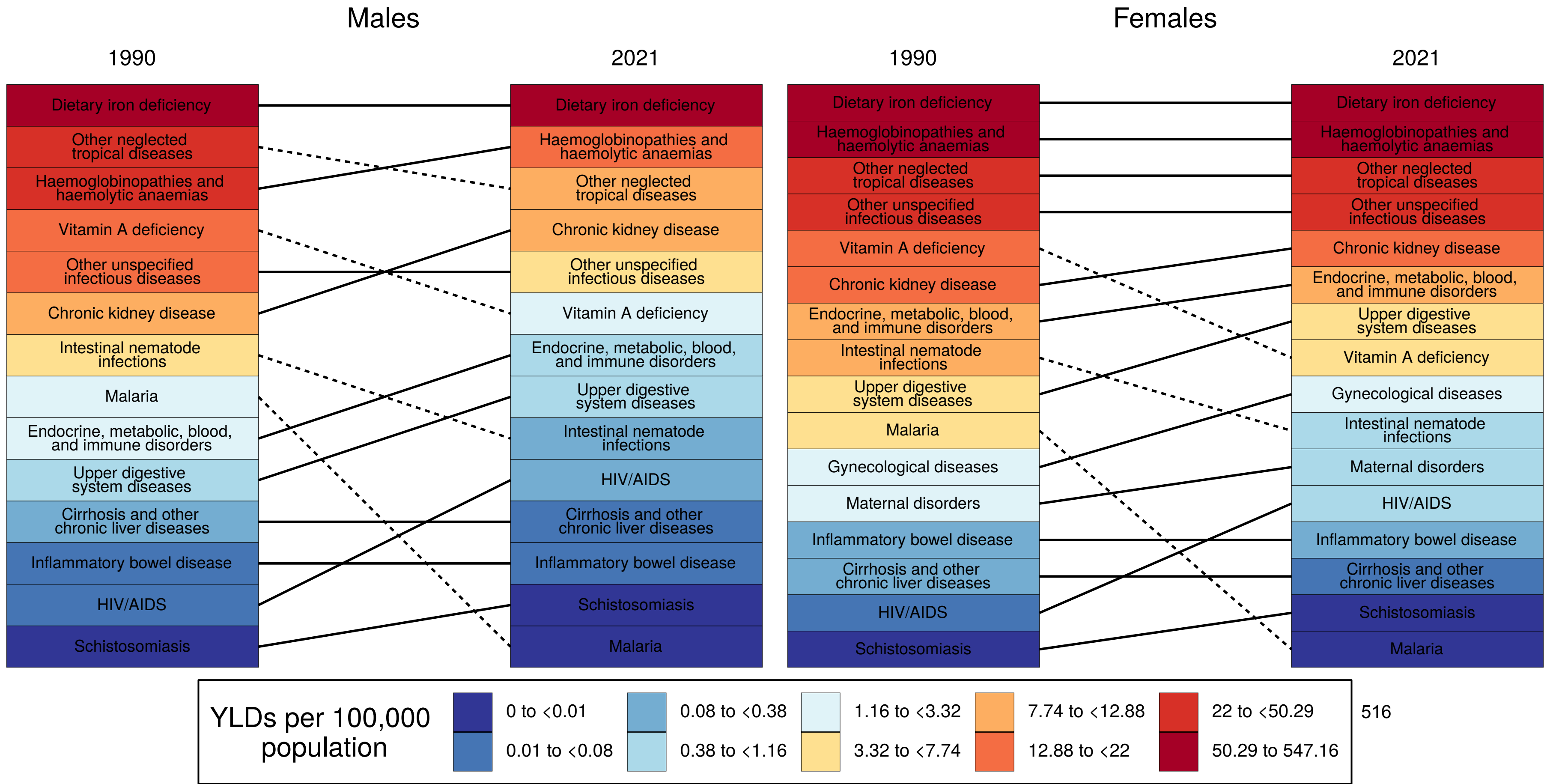

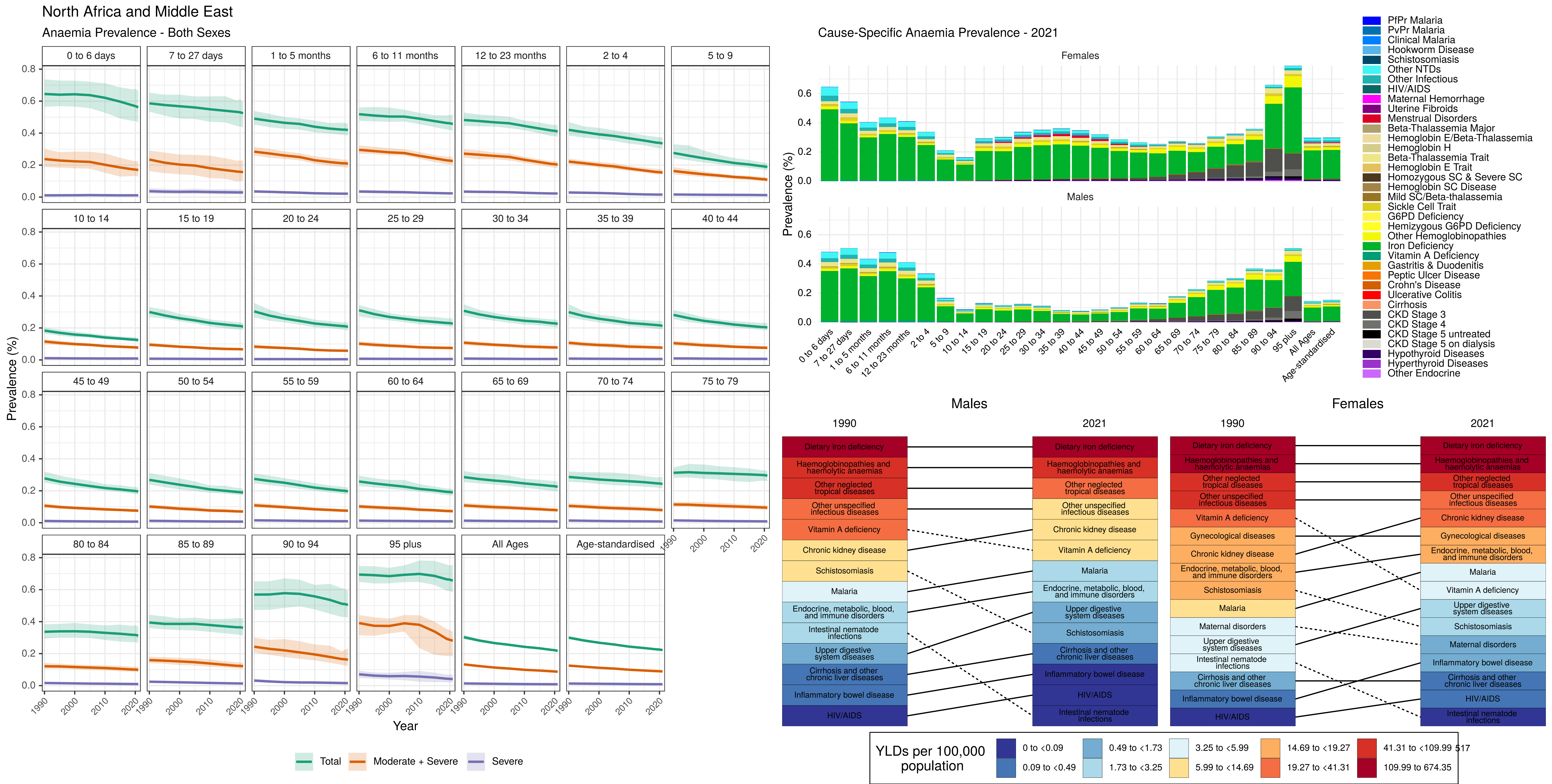

North Africa and Middle East
Anaemia Prevalence - Both Sexes
0 to 6 days
7 to 27 days
1 to 5 months
6 to 11 months
12 to 23 months
2 to 4
5 to 9
10 to 14
15 to 19
20 to 24
25 to 29
30 to 34
35 to 39
40 to 44
45 to 49
50 to 54
55 to 59
60 to 64
65 to 69
70 to 74
75 to 79
80 to 84
85 to 89
90 to 94
95 plus
All Ages
Age-standardised
Prevalence (%)
Year
Total
Moderate + Severe
Severe
Cause-Specific Anaemia Prevalence - 2021
Females
Males
PfPr Malaria
PvPr Malaria
Clinical Malaria
Hookworm Disease
Schistosomiasis
Other NTDs
Other Infectious
HIV/AIDS
Maternal Hemorrhage
Uterine Fibroids
Menstrual Disorders
Beta-Thalassemia Major
Hemoglobin E/Beta-Thalassemia
Hemoglobin H
Beta-Thalassemia Trait
Hemoglobin E Trait
Homozygous SC & Severe SC
Hemoglobin SC Disease
Mild SC/Beta-thalassemia
Sickle Cell Trait
G6PD Deficiency
Hemizygous G6PD Deficiency
Other Hemoglobinopathies
Iron Deficiency
Vitamin A Deficiency
Gastritis & Duodenitis
Peptic Ulcer Disease
Crohn's Disease
Ulcerative Colitis
Cirrhosis
CKD Stage 3
CKD Stage 4
CKD Stage 5 untreated
CKD Stage 5 on dialysis
Hypothyroid Diseases
Hyperthyroid Diseases
Other Endocrine
Males
Females
1990
2021
Dietary iron deficiency
Haemoglobinopathies and haemolytic anaemias
Other neglected tropical diseases
Other unspecified infectious diseases
Vitamin A deficiency
Chronic kidney disease
Schistosomiasis
Malaria
Endocrine, metabolic, blood, and immune disorders
Intestinal nematode infections
Upper digestive system diseases
Cirrhosis and other chronic liver diseases
Inflammatory bowel disease
HIV/AIDS
Gynecological diseases
Maternal disorders
YLDs per 100,000 population
0 to <0.09
0.09 to <0.49
0.49 to <1.73
1.73 to <3.25
3.25 to <5.99
5.99 to <14.69
14.69 to <19.27
19.27 to <41.31
41.31 to <109.99
109.99 to 674.35
517

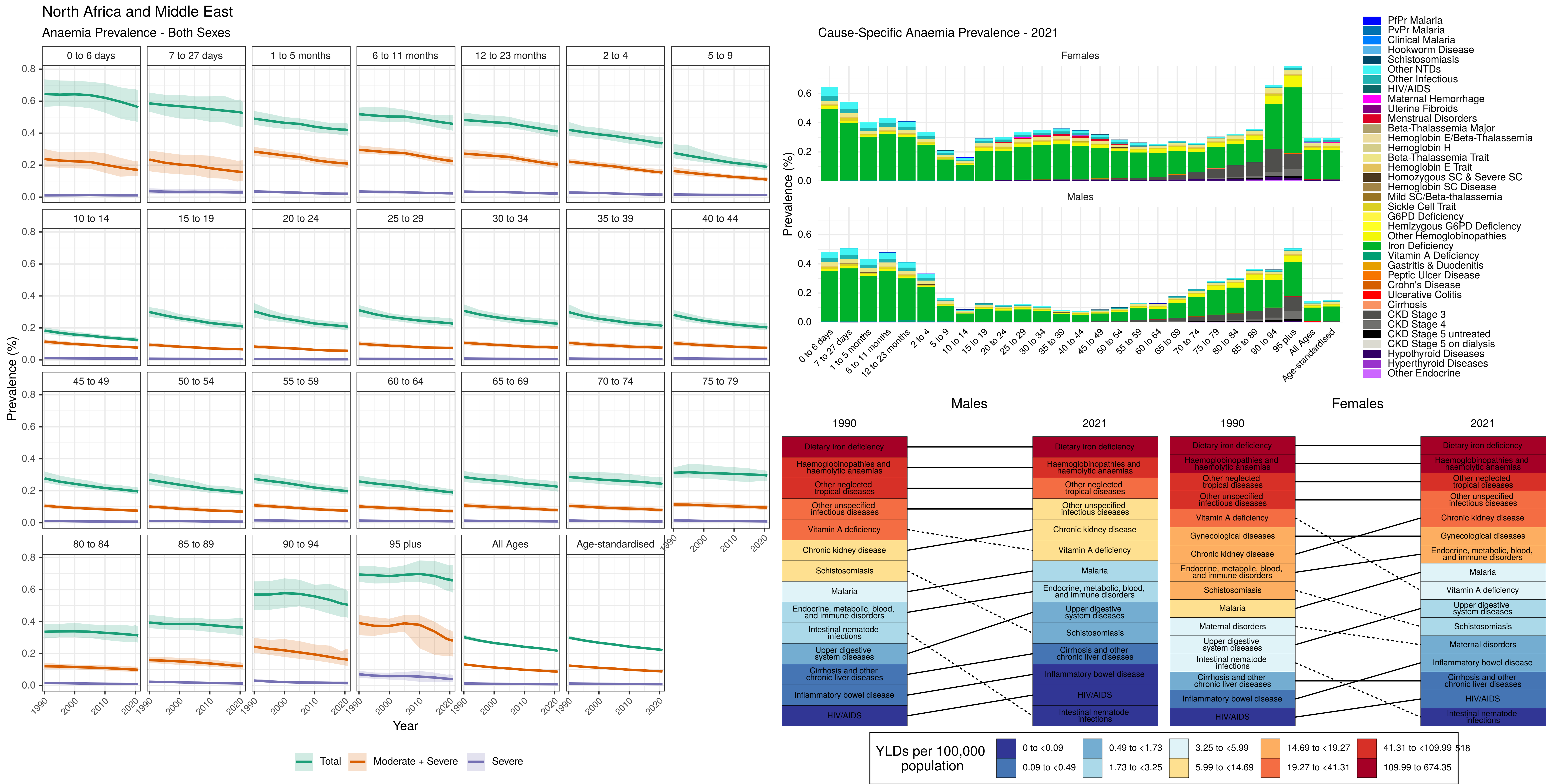
North Africa and Middle East
Anaemia Prevalence - Both Sexes
0 to 6 days
7 to 27 days
1 to 5 months
6 to 11 months
12 to 23 months
2 to 4
5 to 9
10 to 14
15 to 19
20 to 24
25 to 29
30 to 34
35 to 39
40 to 44
45 to 49
50 to 54
55 to 59
60 to 64
65 to 69
70 to 74
75 to 79
80 to 84
85 to 89
90 to 94
95 plus
All Ages
Age-standardised
Prevalence (%)
Year
0.0
0.2
0.4
0.6
0.8
1990
2000
2010
2020
Total
Moderate + Severe
Severe
Cause-Specific Anaemia Prevalence - 2021
Females
Males
Prevalence (%)
PfPr Malaria
PvPr Malaria
Clinical Malaria
Hookworm Disease
Schistosomiasis
Other NTDs
Other Infectious
HIV/AIDS
Maternal Hemorrhage
Uterine Fibroids
Menstrual Disorders
Beta-Thalassemia Major
Hemoglobin E/Beta-Thalassemia
Hemoglobin H
Beta-Thalassemia Trait
Hemoglobin E Trait
Homozygous SC & Severe SC
Hemoglobin SC Disease
Mild SC/Beta-thalassemia
Sickle Cell Trait
G6PD Deficiency
Hemizygous G6PD Deficiency
Other Hemoglobinopathies
Iron Deficiency
Vitamin A Deficiency
Gastritis & Duodenitis
Peptic Ulcer Disease
Crohn's Disease
Ulcerative Colitis
Cirrhosis
CKD Stage 3
CKD Stage 4
CKD Stage 5 untreated
CKD Stage 5 on dialysis
Hypothyroid Diseases
Hyperthyroid Diseases
Other Endocrine
Males
Females
1990
2021
Dietary iron deficiency
Haemoglobinopathies and haemolytic anaemias
Other neglected tropical diseases
Other unspecified infectious diseases
Vitamin A deficiency
Chronic kidney disease
Schistosomiasis
Malaria
Endocrine, metabolic, blood, and immune disorders
Intestinal nematode infections
Upper digestive system diseases
Cirrhosis and other chronic liver diseases
Inflammatory bowel disease
HIV/AIDS
Gynecological diseases
Maternal disorders
YLDs per 100,000 population
0 to <0.09
0.09 to <0.49
0.49 to <1.73
1.73 to <3.25
3.25 to <5.99
5.99 to <14.69
14.69 to <19.27
19.27 to <41.31
41.31 to <109.99
109.99 to 674.35

Afghanistan

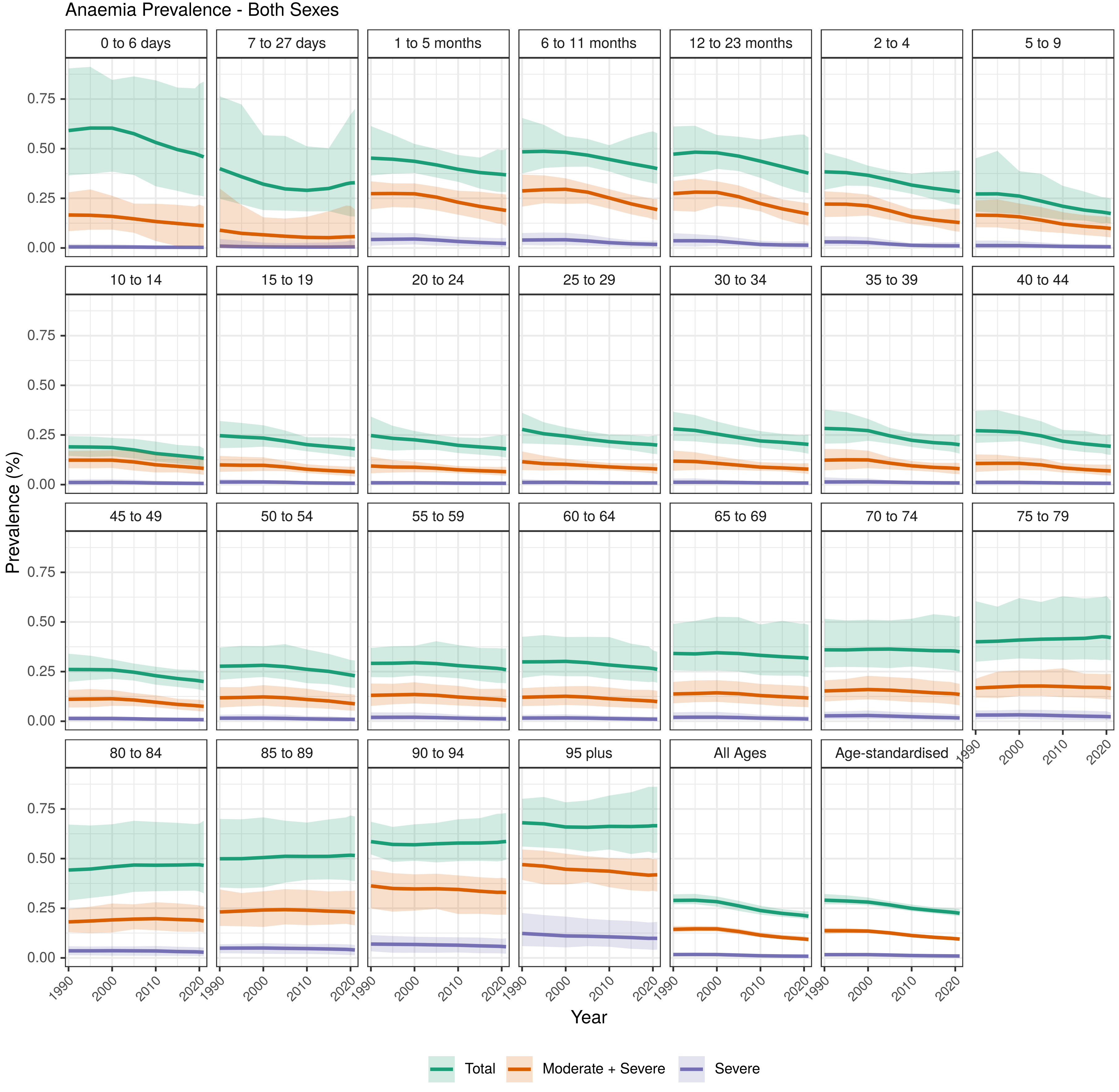

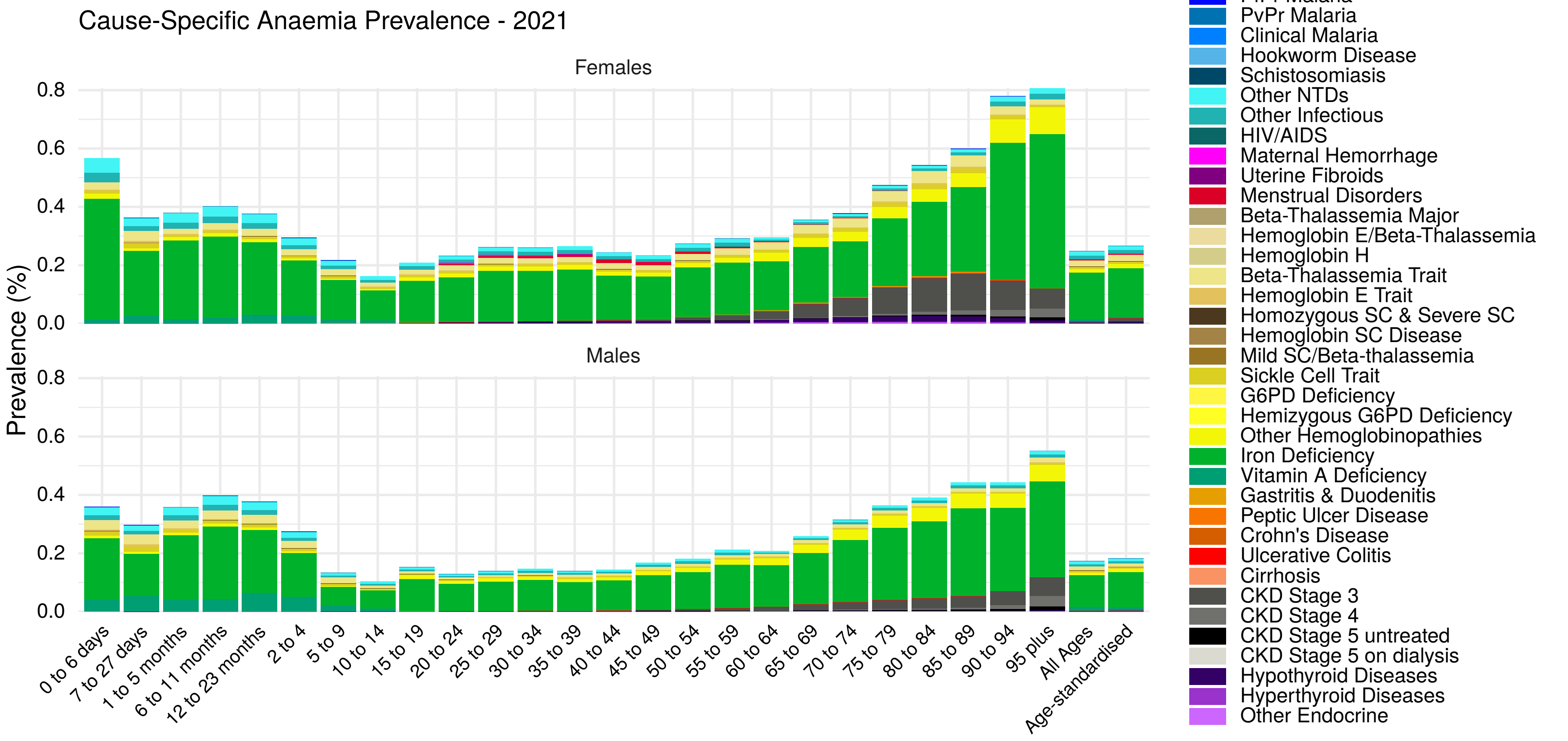

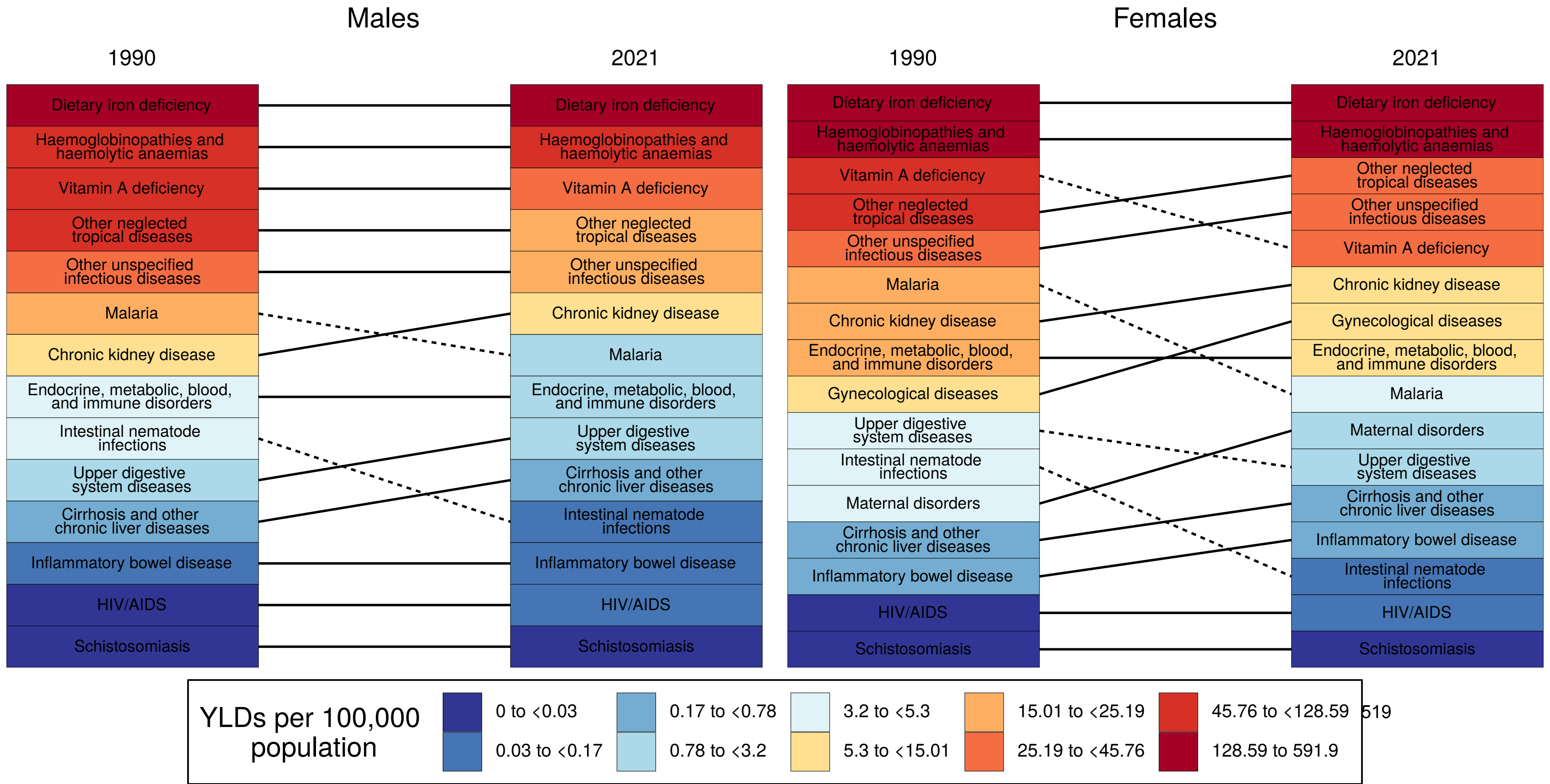

Algeria

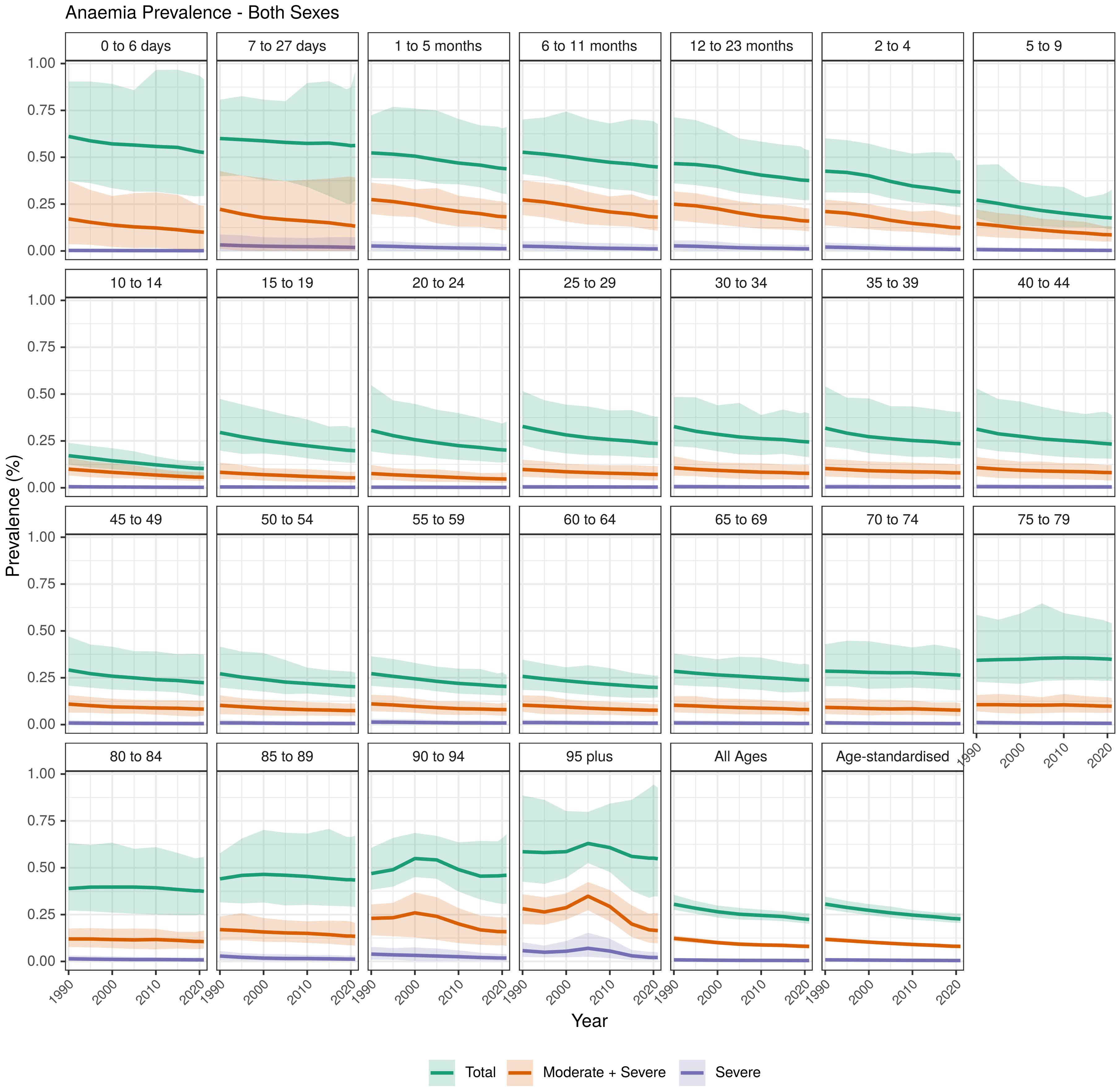
Anaemia Prevalence - Both Sexes
0 to 6 days
7 to 27 days
1 to 5 months
6 to 11 months
12 to 23 months
2 to 4
5 to 9
10 to 14
15 to 19
20 to 24
25 to 29
30 to 34
35 to 39
40 to 44
45 to 49
50 to 54
55 to 59
60 to 64
65 to 69
70 to 74
75 to 79
80 to 84
85 to 89
90 to 94
95 plus
All Ages
Age-standardised
Prevalence (%)
Year
Total
Moderate + Severe
Severe

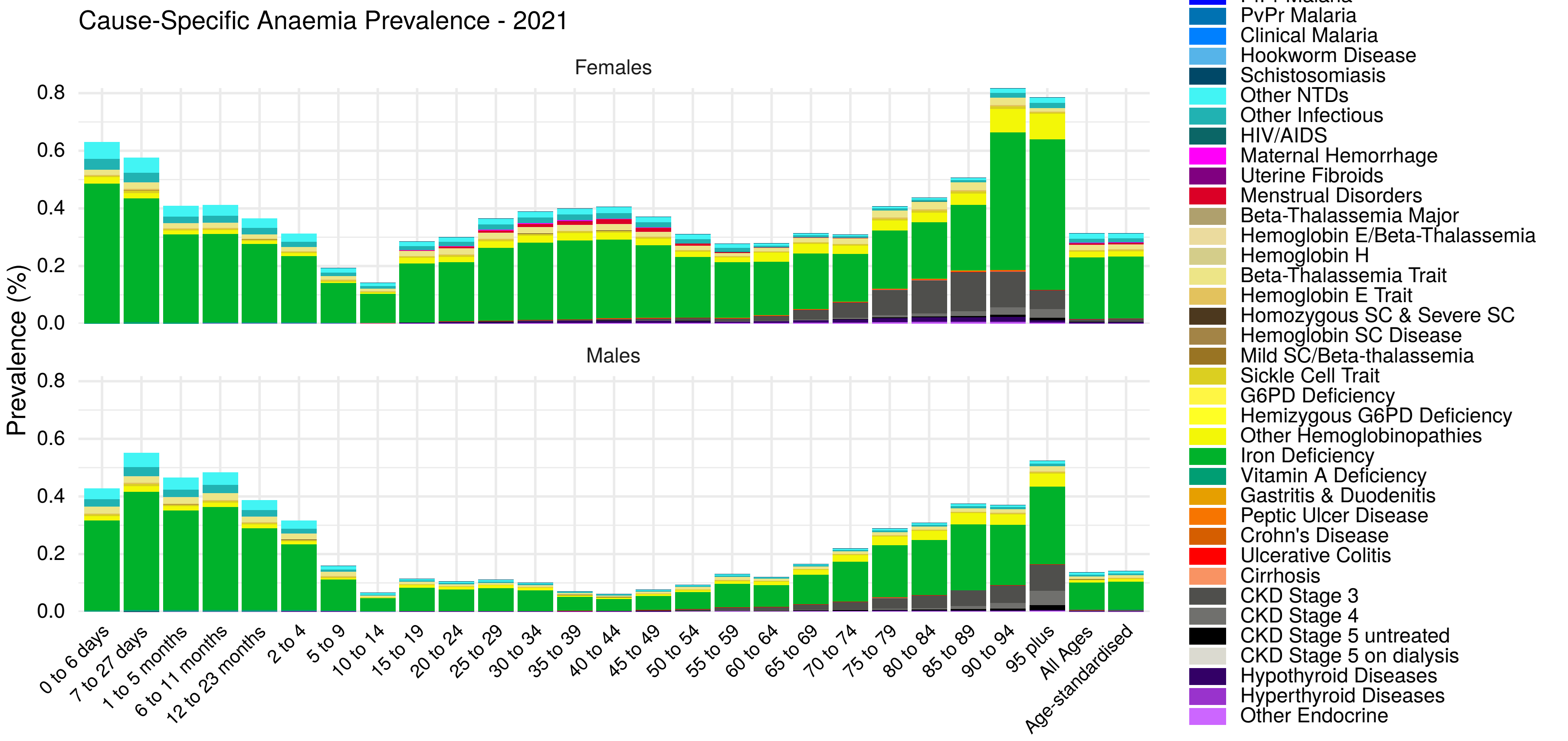
Cause-Specific Anaemia Prevalence - 2021
Females
Males
Prevalence (%)
PfPr Malaria
PvPr Malaria
Clinical Malaria
Hookworm Disease
Schistosomiasis
Other NTDs
Other Infectious
HIV/AIDS
Maternal Hemorrhage
Uterine Fibroids
Menstrual Disorders
Beta-Thalassemia Major
Hemoglobin E/Beta-Thalassemia
Hemoglobin H
Beta-Thalassemia Trait
Hemoglobin E Trait
Homozygous SC & Severe SC
Hemoglobin SC Disease
Mild SC/Beta-thalassemia
Sickle Cell Trait
G6PD Deficiency
Hemizygous G6PD Deficiency
Other Hemoglobinopathies
Iron Deficiency
Vitamin A Deficiency
Gastritis & Duodenitis
Peptic Ulcer Disease
Crohn's Disease
Ulcerative Colitis
Cirrhosis
CKD Stage 3
CKD Stage 4
CKD Stage 5 untreated
CKD Stage 5 on dialysis
Hypothyroid Diseases
Hyperthyroid Diseases
Other Endocrine

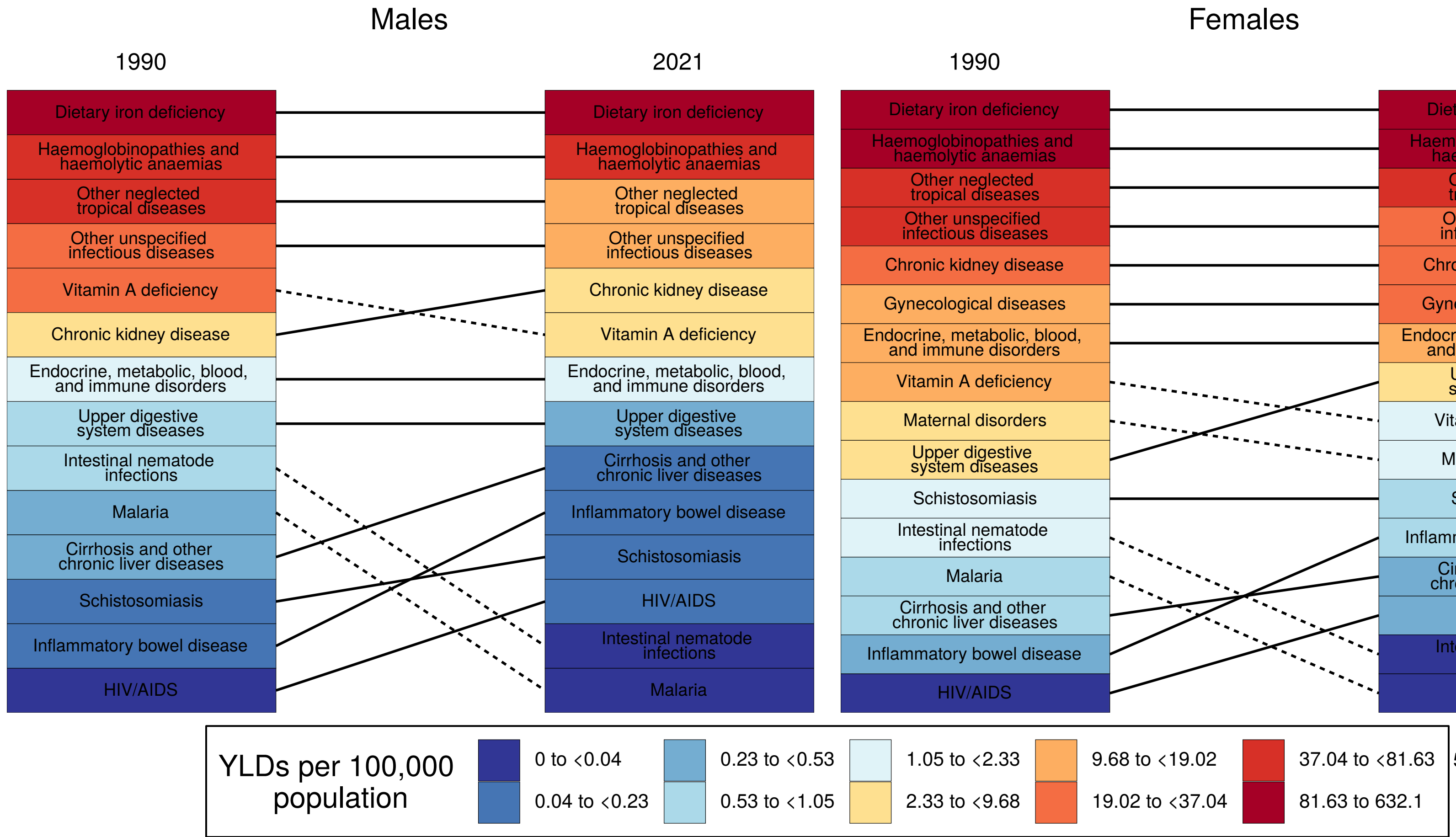
Males
Females
1990
2021
Dietary iron deficiency
Haemoglobinopathies and haemolytic anaemias
Other neglected tropical diseases
Other unspecified infectious diseases
Vitamin A deficiency
Chronic kidney disease
Endocrine, metabolic, blood, and immune disorders
Upper digestive system diseases
Intestinal nematode infections
Malaria
Cirrhosis and other chronic liver diseases
Schistosomiasis
Inflammatory bowel disease
HIV/AIDS
Gynecological diseases
Maternal disorders
YLDs per 100,000 population
0 to <0.04
0.04 to <0.23
0.23 to <0.53
0.53 to <1.05
1.05 to <2.33
2.33 to <9.68
9.68 to <19.02
19.02 to <37.04
37.04 to <81.63
81.63 to 632.1

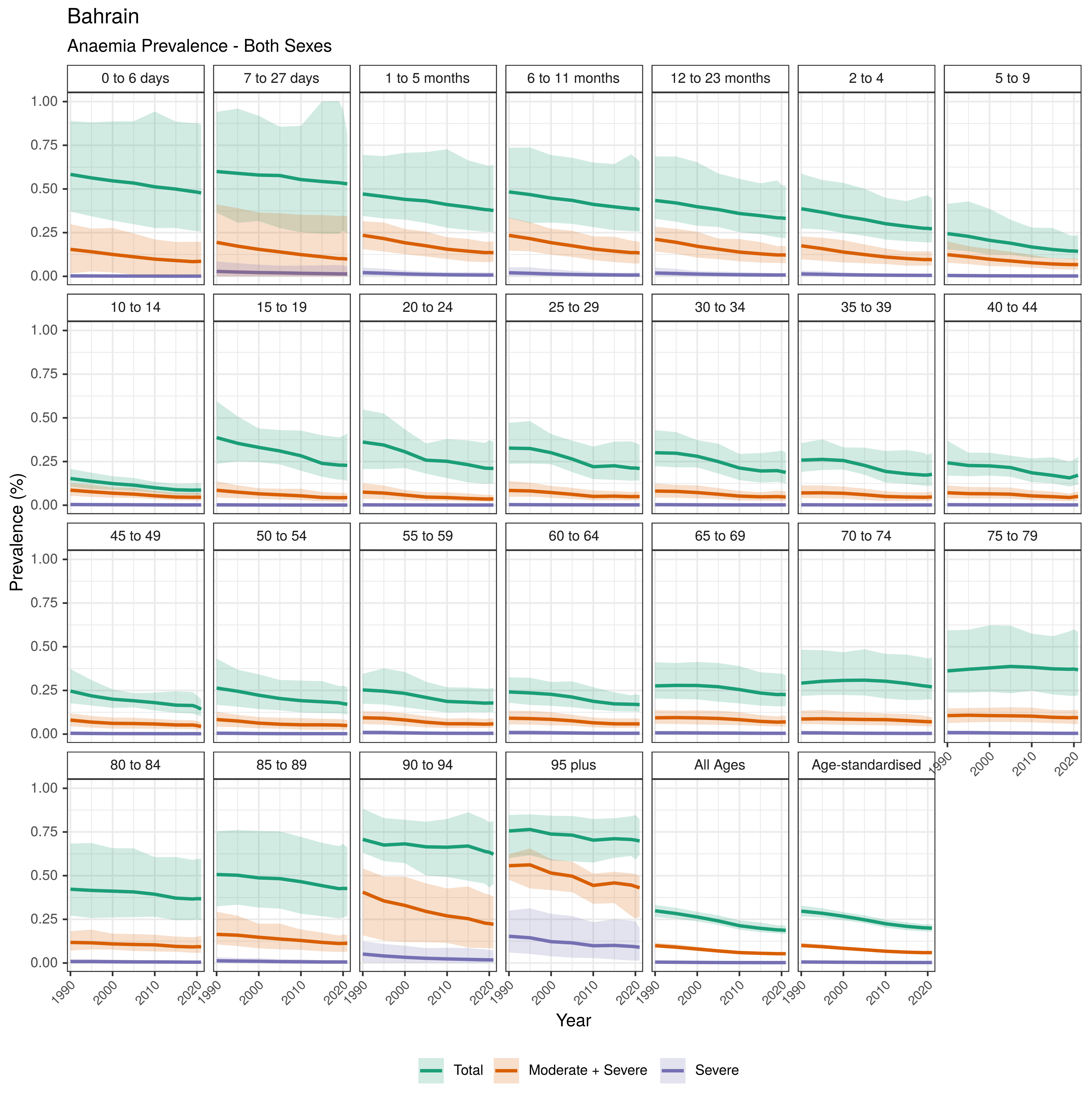
Bahrain
Anaemia Prevalence - Both Sexes
0 to 6 days
7 to 27 days
1 to 5 months
6 to 11 months
12 to 23 months
2 to 4
5 to 9
10 to 14
15 to 19
20 to 24
25 to 29
30 to 34
35 to 39
40 to 44
45 to 49
50 to 54
55 to 59
60 to 64
65 to 69
70 to 74
75 to 79
80 to 84
85 to 89
90 to 94
95 plus
All Ages
Age-standardised
0.00
0.25
0.50
0.75
1.00
1990
2000
2010
2020
Prevalence (%)
Year
Total
Moderate + Severe
Severe

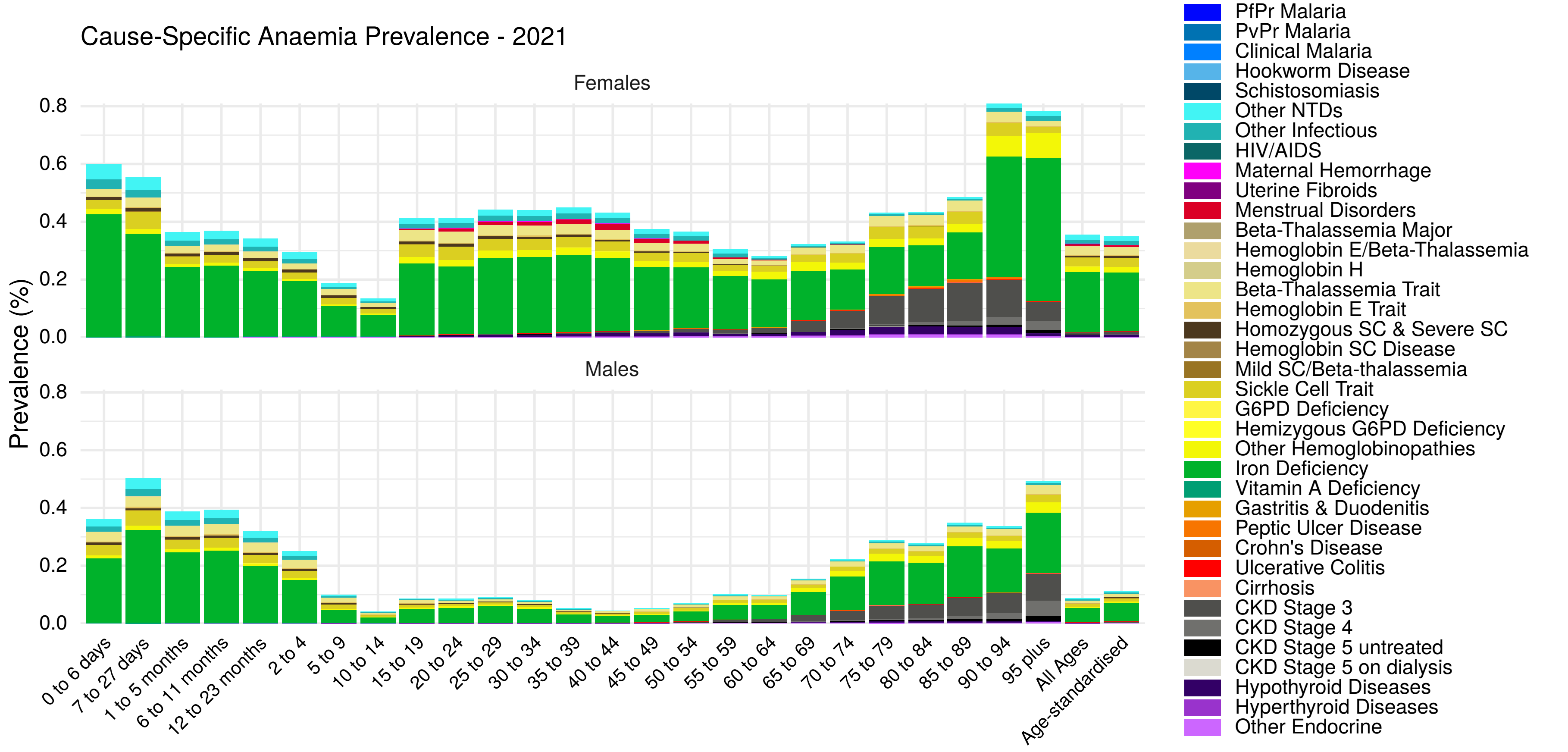
Cause-Specific Anaemia Prevalence - 2021
Females
Males
Prevalence (%)
0.0
0.2
0.4
0.6
0.8
0 to 6 days
7 to 27 days
1 to 5 months
6 to 11 months
12 to 23 months
2 to 4
5 to 9
10 to 14
15 to 19
20 to 24
25 to 29
30 to 34
35 to 39
40 to 44
45 to 49
50 to 54
55 to 59
60 to 64
65 to 69
70 to 74
75 to 79
80 to 84
85 to 89
90 to 94
95 plus
All Ages
Age-standardised
PfPr Malaria
PvPr Malaria
Clinical Malaria
Hookworm Disease
Schistosomiasis
Other NTDs
Other Infectious
HIV/AIDS
Maternal Hemorrhage
Uterine Fibroids
Menstrual Disorders
Beta-Thalassemia Major
Hemoglobin E/Beta-Thalassemia
Hemoglobin H
Beta-Thalassemia Trait
Hemoglobin E Trait
Homozygous SC & Severe SC
Hemoglobin SC Disease
Mild SC/Beta-thalassemia
Sickle Cell Trait
G6PD Deficiency
Hemizygous G6PD Deficiency
Other Hemoglobinopathies
Iron Deficiency
Vitamin A Deficiency
Gastritis & Duodenitis
Peptic Ulcer Disease
Crohn's Disease
Ulcerative Colitis
Cirrhosis
CKD Stage 3
CKD Stage 4
CKD Stage 5 untreated
CKD Stage 5 on dialysis
Hypothyroid Diseases
Hyperthyroid Diseases
Other Endocrine

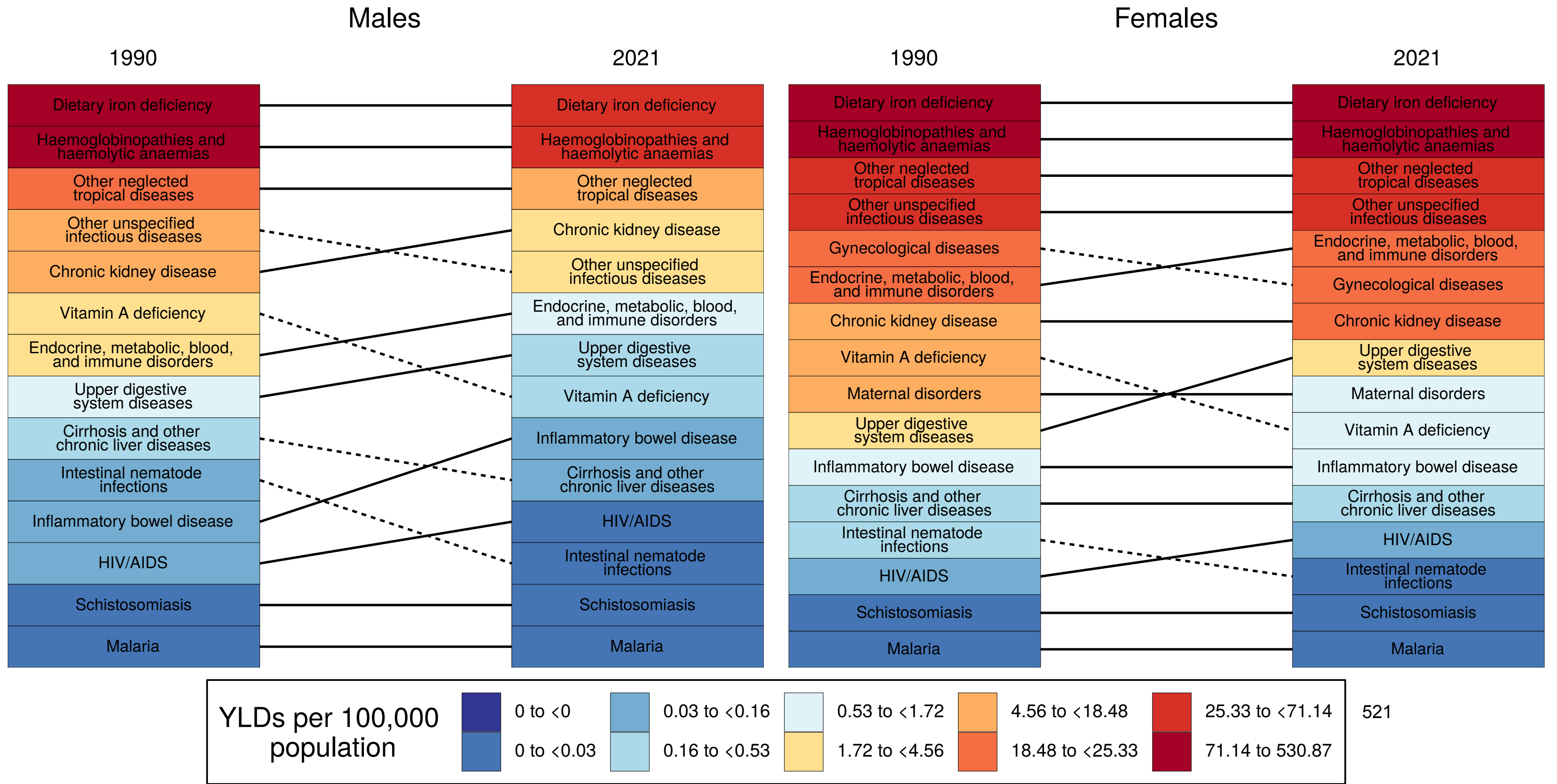
Males
Females
1990
2021
Dietary iron deficiency
Haemoglobinopathies and haemolytic anaemias
Other neglected tropical diseases
Other unspecified infectious diseases
Chronic kidney disease
Vitamin A deficiency
Endocrine, metabolic, blood, and immune disorders
Upper digestive system diseases
Cirrhosis and other chronic liver diseases
Intestinal nematode infections
Inflammatory bowel disease
HIV/AIDS
Schistosomiasis
Malaria
Gynecological diseases
Maternal disorders
YLDs per 100,000 population
0 to <0
0 to <0.03
0.03 to <0.16
0.16 to <0.53
0.53 to <1.72
1.72 to <4.56
4.56 to <18.48
18.48 to <25.33
25.33 to <71.14
71.14 to 530.87
521

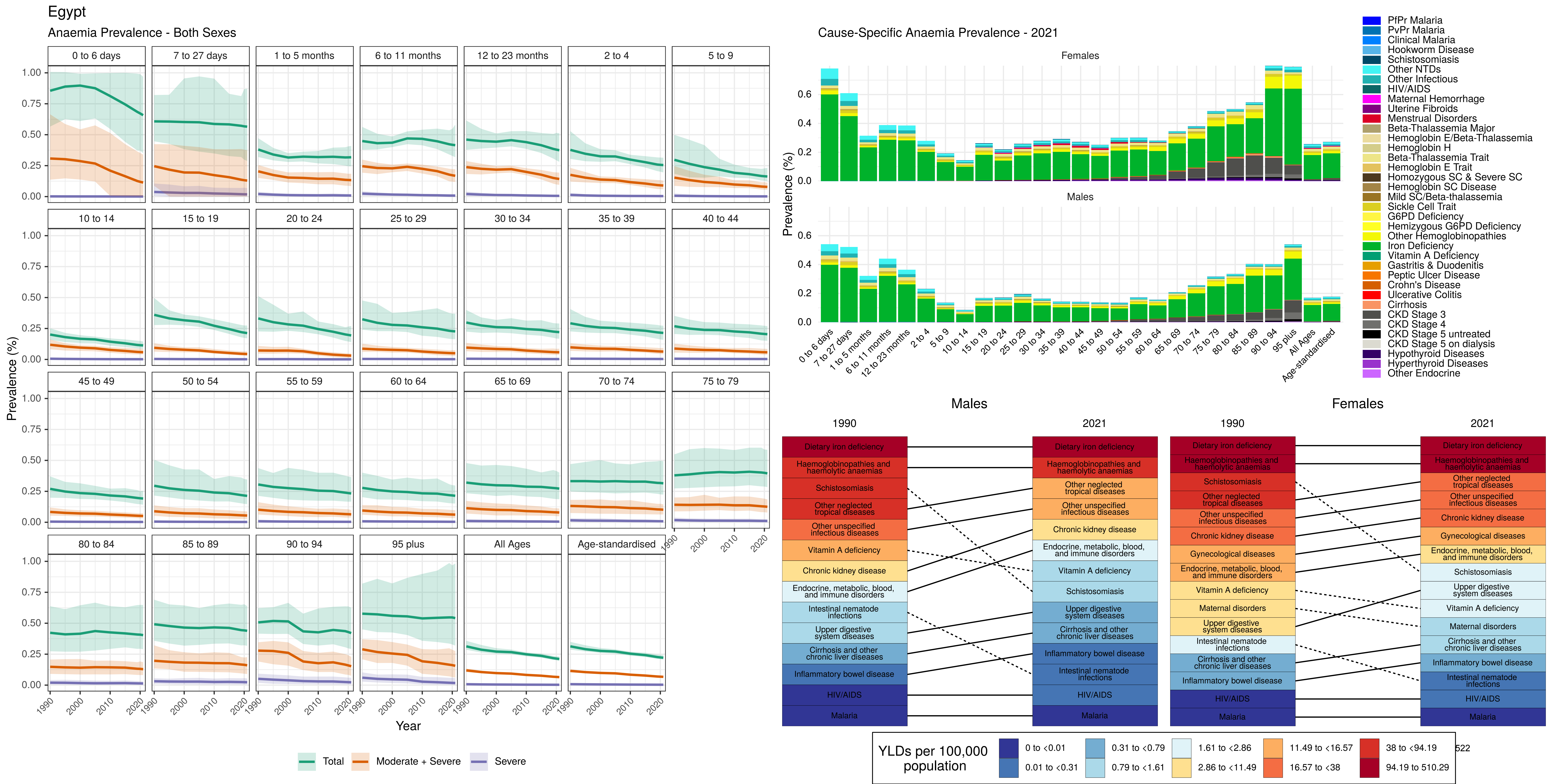

Egypt
Anaemia Prevalence - Both Sexes
0 to 6 days
7 to 27 days
1 to 5 months
6 to 11 months
12 to 23 months
2 to 4
5 to 9
10 to 14
15 to 19
20 to 24
25 to 29
30 to 34
35 to 39
40 to 44
45 to 49
50 to 54
55 to 59
60 to 64
65 to 69
70 to 74
75 to 79
80 to 84
85 to 89
90 to 94
95 plus
All Ages
Age-standardised
Prevalence (%)
Year
Total
Moderate + Severe
Severe
Cause-Specific Anaemia Prevalence - 2021
Females
Males
Prevalence (%)
PfPr Malaria
PvPr Malaria
Clinical Malaria
Hookworm Disease
Schistosomiasis
Other NTDs
Other Infectious
HIV/AIDS
Maternal Hemorrhage
Uterine Fibroids
Menstrual Disorders
Beta-Thalassemia Major
Hemoglobin E/Beta-Thalassemia
Hemoglobin H
Beta-Thalassemia Trait
Hemoglobin E Trait
Homozygous SC & Severe SC
Hemoglobin SC Disease
Mild SC/Beta-thalassemia
Sickle Cell Trait
G6PD Deficiency
Hemizygous G6PD Deficiency
Other Hemoglobinopathies
Iron Deficiency
Vitamin A Deficiency
Gastritis & Duodenitis
Peptic Ulcer Disease
Crohn's Disease
Ulcerative Colitis
Cirrhosis
CKD Stage 3
CKD Stage 4
CKD Stage 5 untreated
CKD Stage 5 on dialysis
Hypothyroid Diseases
Hyperthyroid Diseases
Other Endocrine
Males
Females
1990
2021
Dietary iron deficiency
Haemoglobinopathies and haemolytic anaemias
Schistosomiasis
Other neglected tropical diseases
Other unspecified infectious diseases
Vitamin A deficiency
Chronic kidney disease
Endocrine, metabolic, blood, and immune disorders
Intestinal nematode infections
Upper digestive system diseases
Cirrhosis and other chronic liver diseases
Inflammatory bowel disease
HIV/AIDS
Malaria
Gynecological diseases
Maternal disorders
YLDs per 100,000 population
0 to <0.01
0.01 to <0.31
0.31 to <0.79
0.79 to <1.61
1.61 to <2.86
2.86 to <11.49
11.49 to <16.57
16.57 to <38
38 to <94.19
94.19 to 510.29
522

Iran

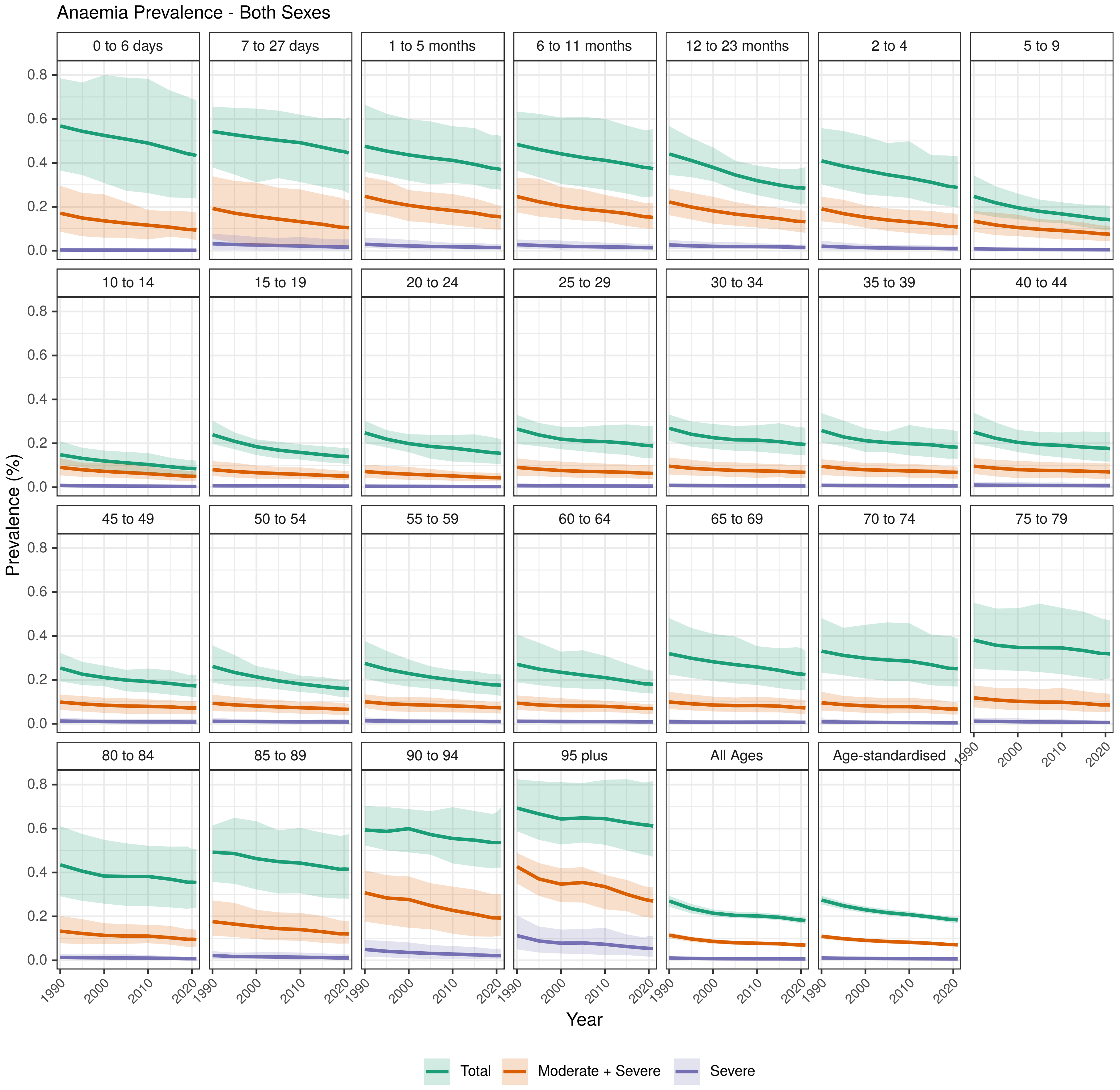
Anaemia Prevalence - Both Sexes
0 to 6 days
7 to 27 days
1 to 5 months
6 to 11 months
12 to 23 months
2 to 4
5 to 9
10 to 14
15 to 19
20 to 24
25 to 29
30 to 34
35 to 39
40 to 44
45 to 49
50 to 54
55 to 59
60 to 64
65 to 69
70 to 74
75 to 79
80 to 84
85 to 89
90 to 94
95 plus
All Ages
Age-standardised
Prevalence (%)
0.0
0.2
0.4
0.6
0.8
1990
2000
2010
2020
Year
Total
Moderate + Severe
Severe

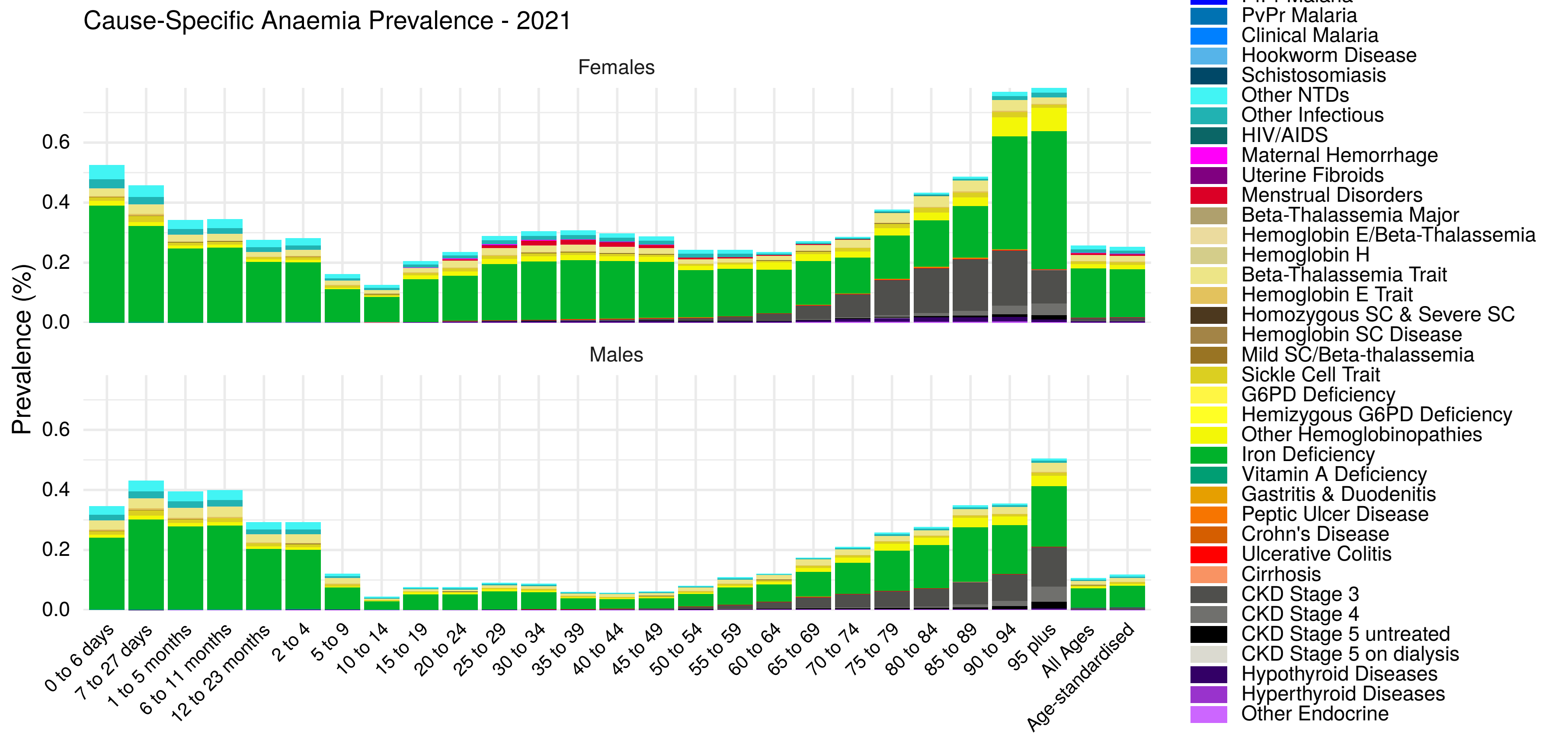
Cause-Specific Anaemia Prevalence - 2021
Females
Males
Prevalence (%)
0.0
0.2
0.4
0.6
0 to 6 days
7 to 27 days
1 to 5 months
6 to 11 months
12 to 23 months
2 to 4
5 to 9
10 to 14
15 to 19
20 to 24
25 to 29
30 to 34
35 to 39
40 to 44
45 to 49
50 to 54
55 to 59
60 to 64
65 to 69
70 to 74
75 to 79
80 to 84
85 to 89
90 to 94
95 plus
All Ages
Age-standardised
PfPr Malaria
PvPr Malaria
Clinical Malaria
Hookworm Disease
Schistosomiasis
Other NTDs
Other Infectious
HIV/AIDS
Maternal Hemorrhage
Uterine Fibroids
Menstrual Disorders
Beta-Thalassemia Major
Hemoglobin E/Beta-Thalassemia
Hemoglobin H
Beta-Thalassemia Trait
Hemoglobin E Trait
Homozygous SC & Severe SC
Hemoglobin SC Disease
Mild SC/Beta-thalassemia
Sickle Cell Trait
G6PD Deficiency
Hemizygous G6PD Deficiency
Other Hemoglobinopathies
Iron Deficiency
Vitamin A Deficiency
Gastritis & Duodenitis
Peptic Ulcer Disease
Crohn's Disease
Ulcerative Colitis
Cirrhosis
CKD Stage 3
CKD Stage 4
CKD Stage 5 untreated
CKD Stage 5 on dialysis
Hypothyroid Diseases
Hyperthyroid Diseases
Other Endocrine

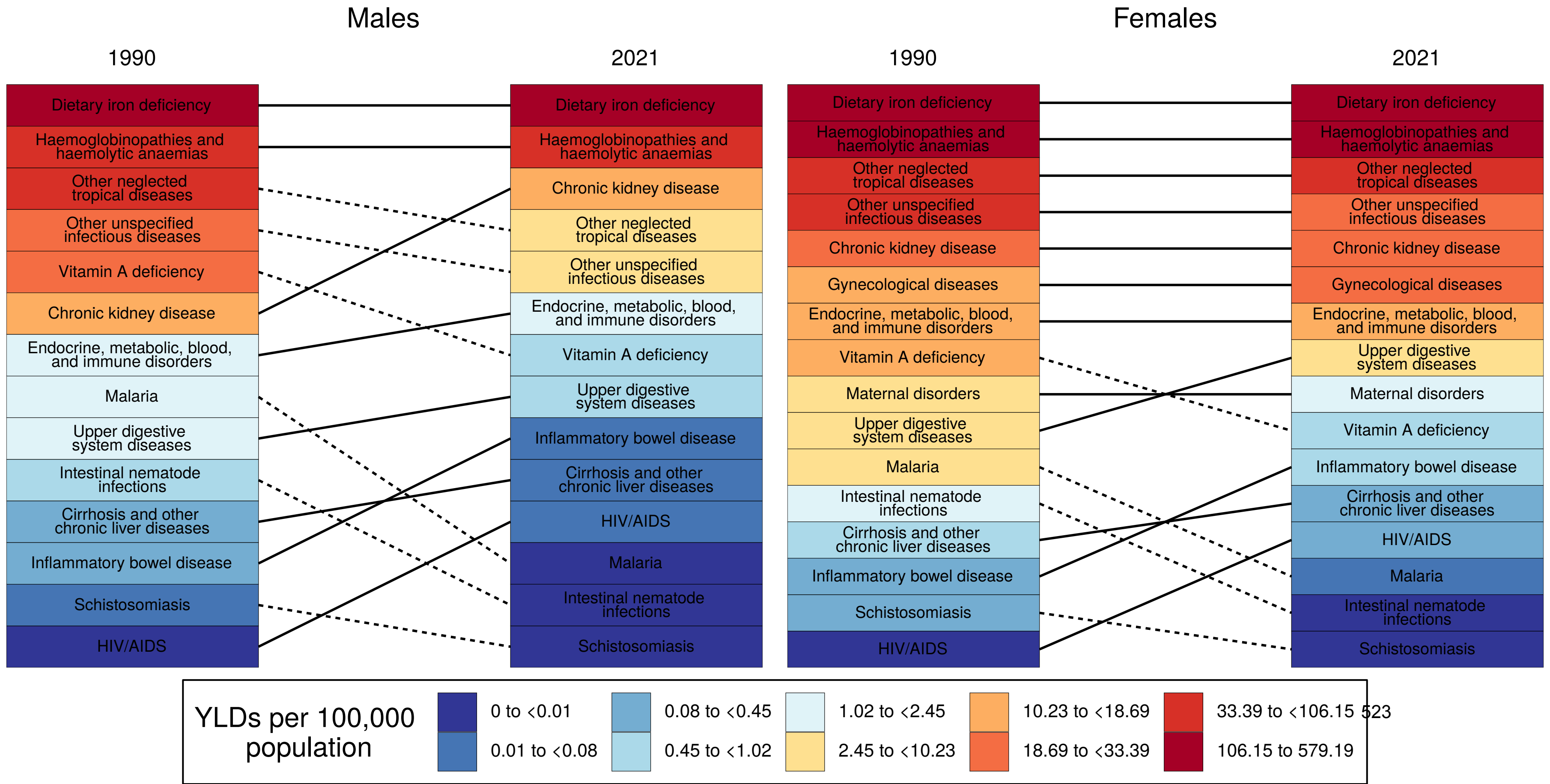
Males
1990
2021
Dietary iron deficiency
Haemoglobinopathies and haemolytic anaemias
Other neglected tropical diseases
Other unspecified infectious diseases
Vitamin A deficiency
Chronic kidney disease
Endocrine, metabolic, blood, and immune disorders
Malaria
Upper digestive system diseases
Intestinal nematode infections
Cirrhosis and other chronic liver diseases
Inflammatory bowel disease
Schistosomiasis
HIV/AIDS
Dietary iron deficiency
Haemoglobinopathies and haemolytic anaemias
Chronic kidney disease
Other neglected tropical diseases
Other unspecified infectious diseases
Endocrine, metabolic, blood, and immune disorders
Vitamin A deficiency
Upper digestive system diseases
Inflammatory bowel disease
Cirrhosis and other chronic liver diseases
HIV/AIDS
Malaria
Intestinal nematode infections
Schistosomiasis
Females
1990
2021
Dietary iron deficiency
Haemoglobinopathies and haemolytic anaemias
Other neglected tropical diseases
Other unspecified infectious diseases
Chronic kidney disease
Gynecological diseases
Endocrine, metabolic, blood, and immune disorders
Vitamin A deficiency
Maternal disorders
Upper digestive system diseases
Malaria
Intestinal nematode infections
Cirrhosis and other chronic liver diseases
Inflammatory bowel disease
Schistosomiasis
HIV/AIDS
Dietary iron deficiency
Haemoglobinopathies and haemolytic anaemias
Other neglected tropical diseases
Other unspecified infectious diseases
Chronic kidney disease
Gynecological diseases
Endocrine, metabolic, blood, and immune disorders
Upper digestive system diseases
Maternal disorders
Vitamin A deficiency
Inflammatory bowel disease
Cirrhosis and other chronic liver diseases
HIV/AIDS
Malaria
Intestinal nematode infections
Schistosomiasis
YLDs per 100,000 population
0 to <0.01
0.01 to <0.08
0.08 to <0.45
0.45 to <1.02
1.02 to <2.45
2.45 to <10.23
10.23 to <18.69
18.69 to <33.39
33.39 to <106.15
106.15 to 579.19
523

Alborz

Anaemia Prevalence - Both Sexes

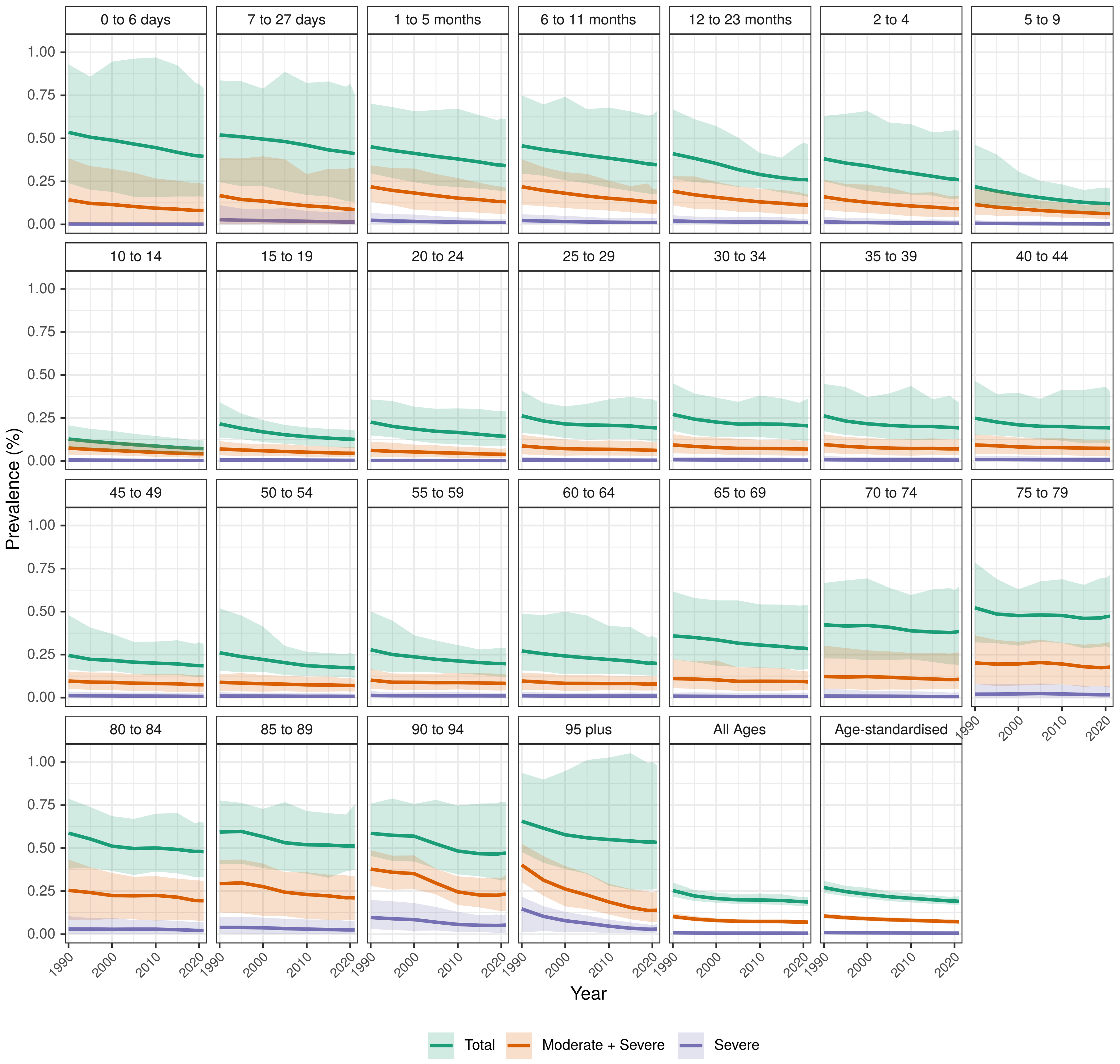

Cause-Specific Anaemia Prevalence - 2021

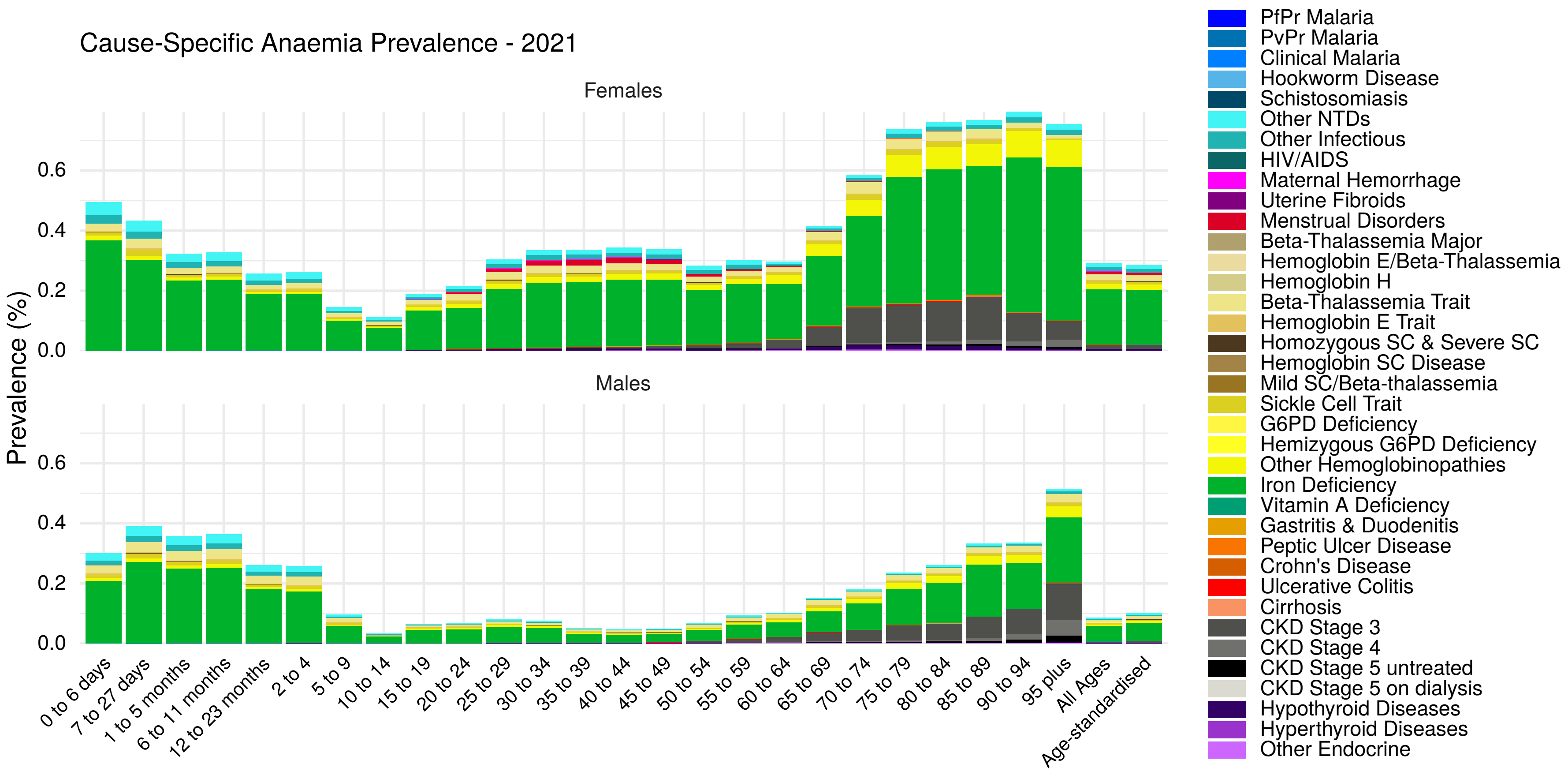

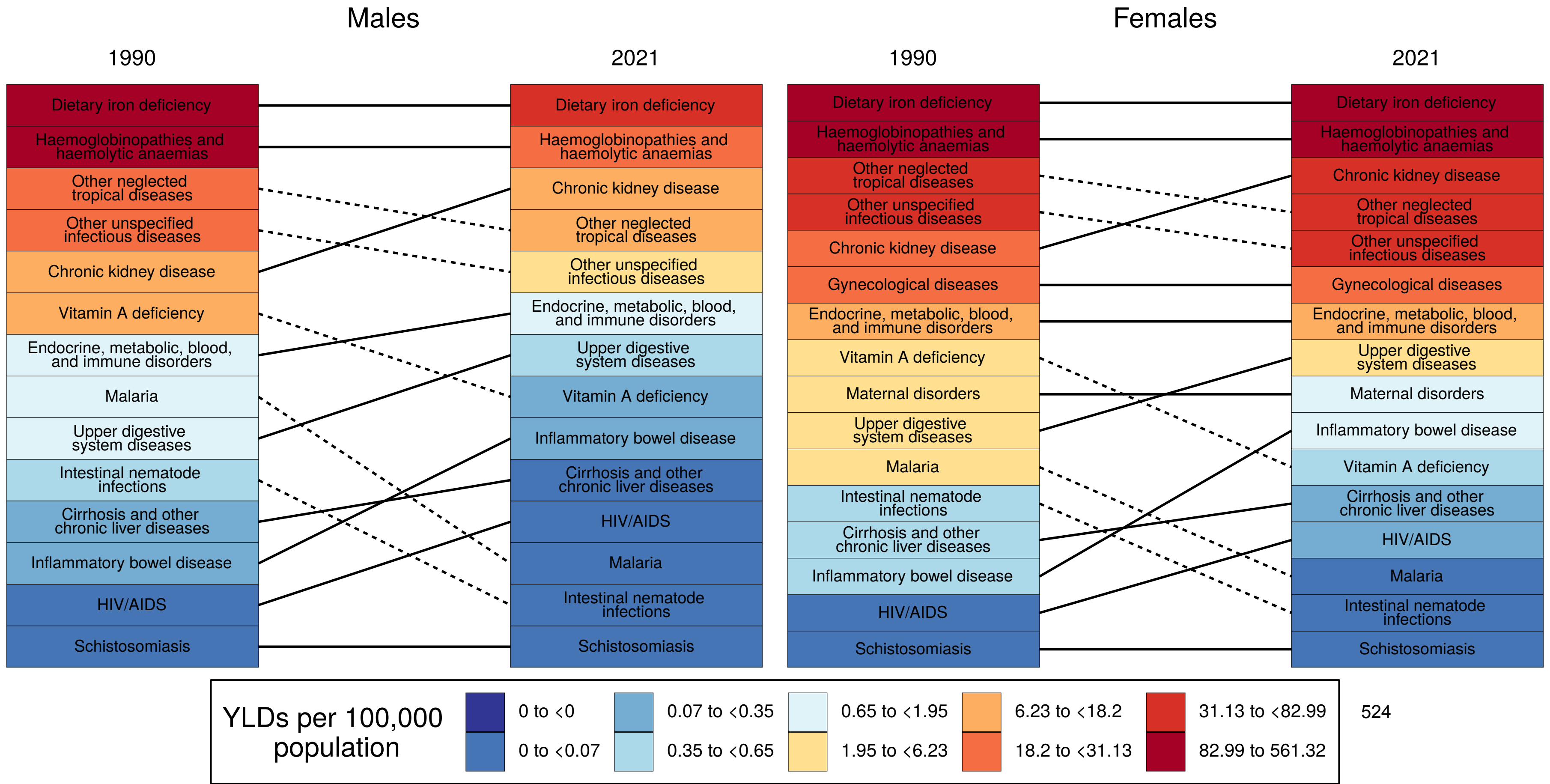

Ardebil

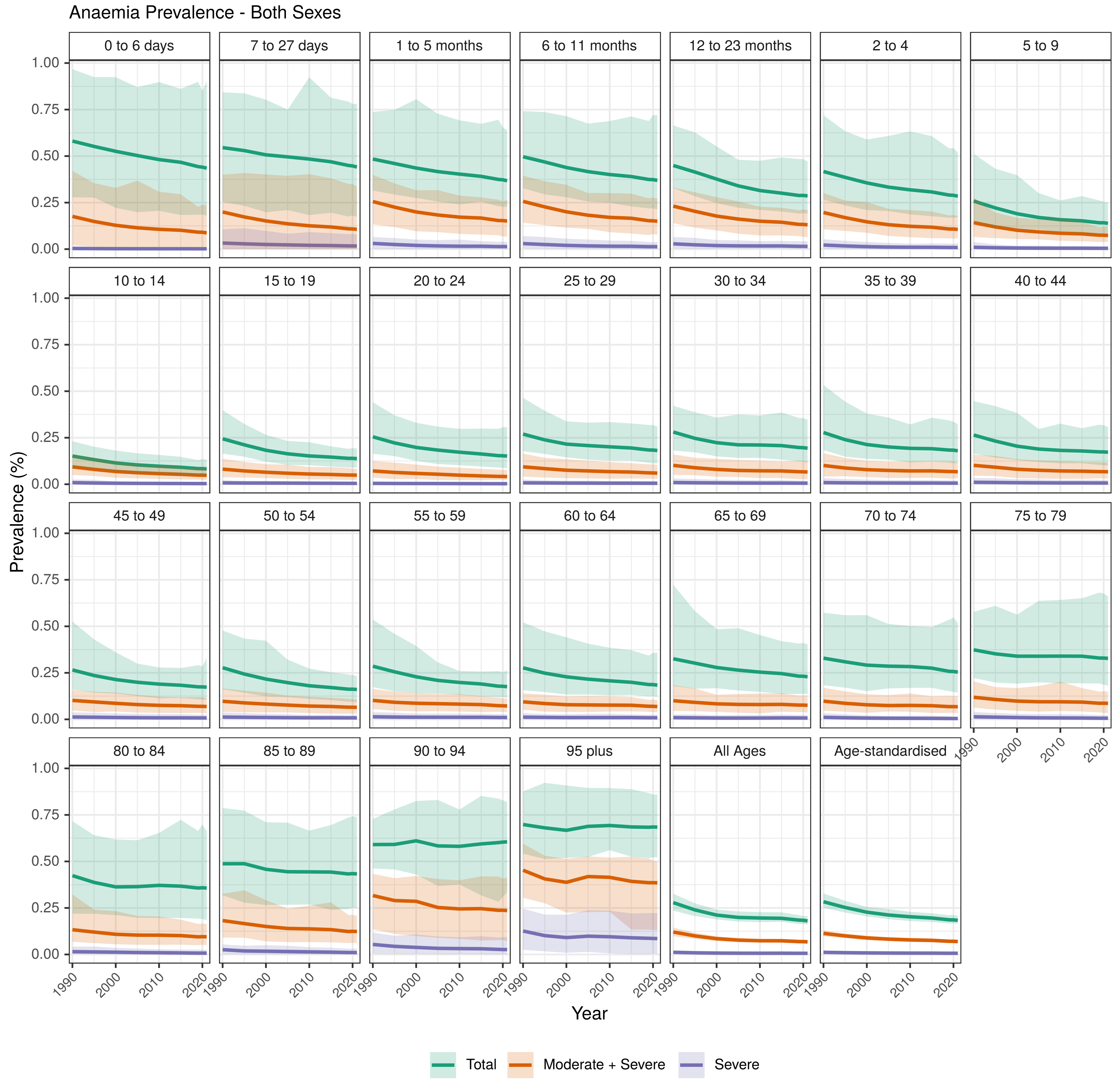

Anaemia Prevalence - Both Sexes
0 to 6 days
7 to 27 days
1 to 5 months
6 to 11 months
12 to 23 months
2 to 4
5 to 9
10 to 14
15 to 19
20 to 24
25 to 29
30 to 34
35 to 39
40 to 44
45 to 49
50 to 54
55 to 59
60 to 64
65 to 69
70 to 74
75 to 79
80 to 84
85 to 89
90 to 94
95 plus
All Ages
Age-standardised
Prevalence (%)
0.00
0.25
0.50
0.75
1.00
1990
2000
2010
2020
Year
Total
Moderate + Severe
Severe

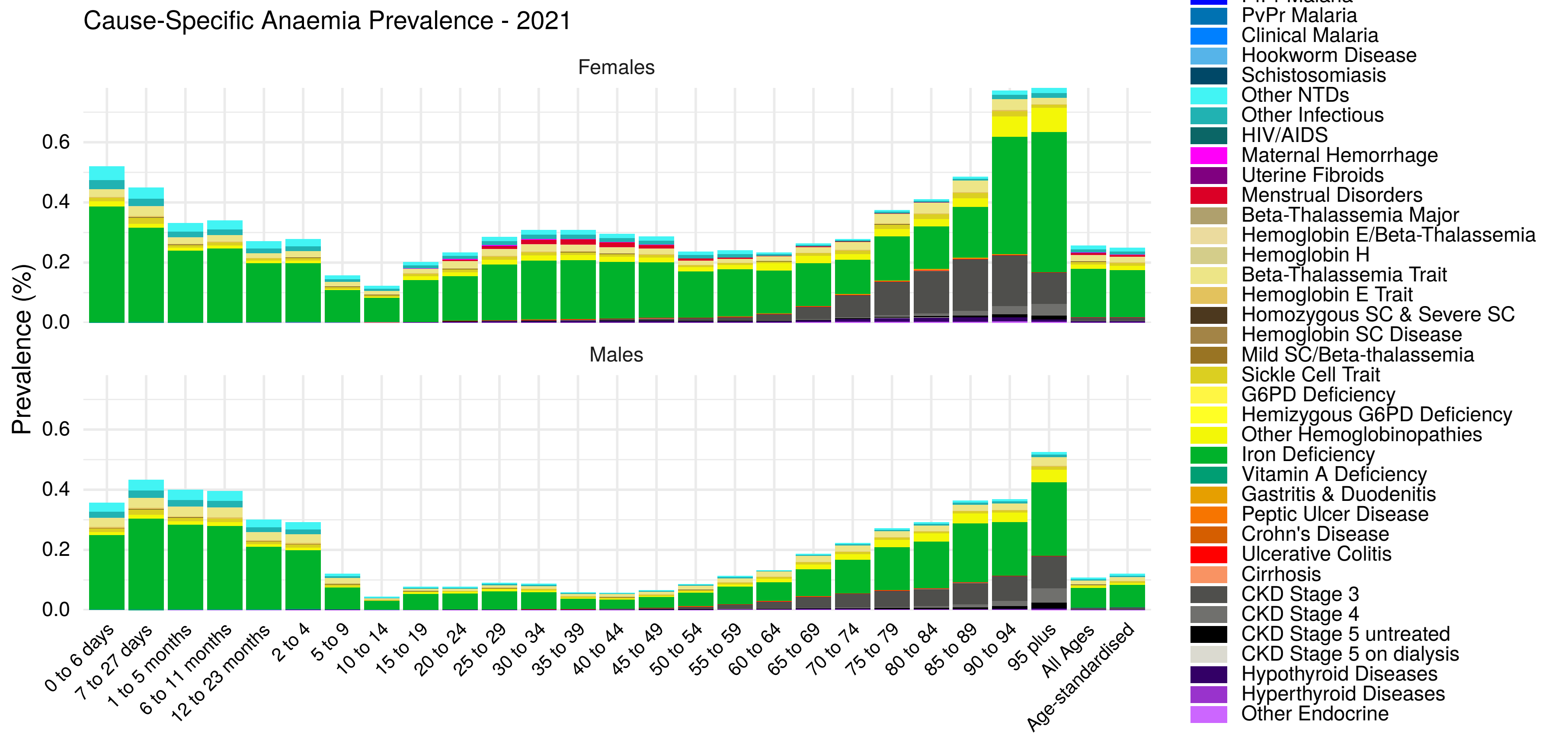

Cause-Specific Anaemia Prevalence - 2021
Females
Males
Prevalence (%)
0.0
0.2
0.4
0.6
0 to 6 days
7 to 27 days
1 to 5 months
6 to 11 months
12 to 23 months
2 to 4
5 to 9
10 to 14
15 to 19
20 to 24
25 to 29
30 to 34
35 to 39
40 to 44
45 to 49
50 to 54
55 to 59
60 to 64
65 to 69
70 to 74
75 to 79
80 to 84
85 to 89
90 to 94
95 plus
All Ages
Age-standardised
PfPr Malaria
PvPr Malaria
Clinical Malaria
Hookworm Disease
Schistosomiasis
Other NTDs
Other Infectious
HIV/AIDS
Maternal Hemorrhage
Uterine Fibroids
Menstrual Disorders
Beta-Thalassemia Major
Hemoglobin E/Beta-Thalassemia
Hemoglobin H
Beta-Thalassemia Trait
Hemoglobin E Trait
Homozygous SC & Severe SC
Hemoglobin SC Disease
Mild SC/Beta-thalassemia
Sickle Cell Trait
G6PD Deficiency
Hemizygous G6PD Deficiency
Other Hemoglobinopathies
Iron Deficiency
Vitamin A Deficiency
Gastritis & Duodenitis
Peptic Ulcer Disease
Crohn's Disease
Ulcerative Colitis
Cirrhosis
CKD Stage 3
CKD Stage 4
CKD Stage 5 untreated
CKD Stage 5 on dialysis
Hypothyroid Diseases
Hyperthyroid Diseases
Other Endocrine

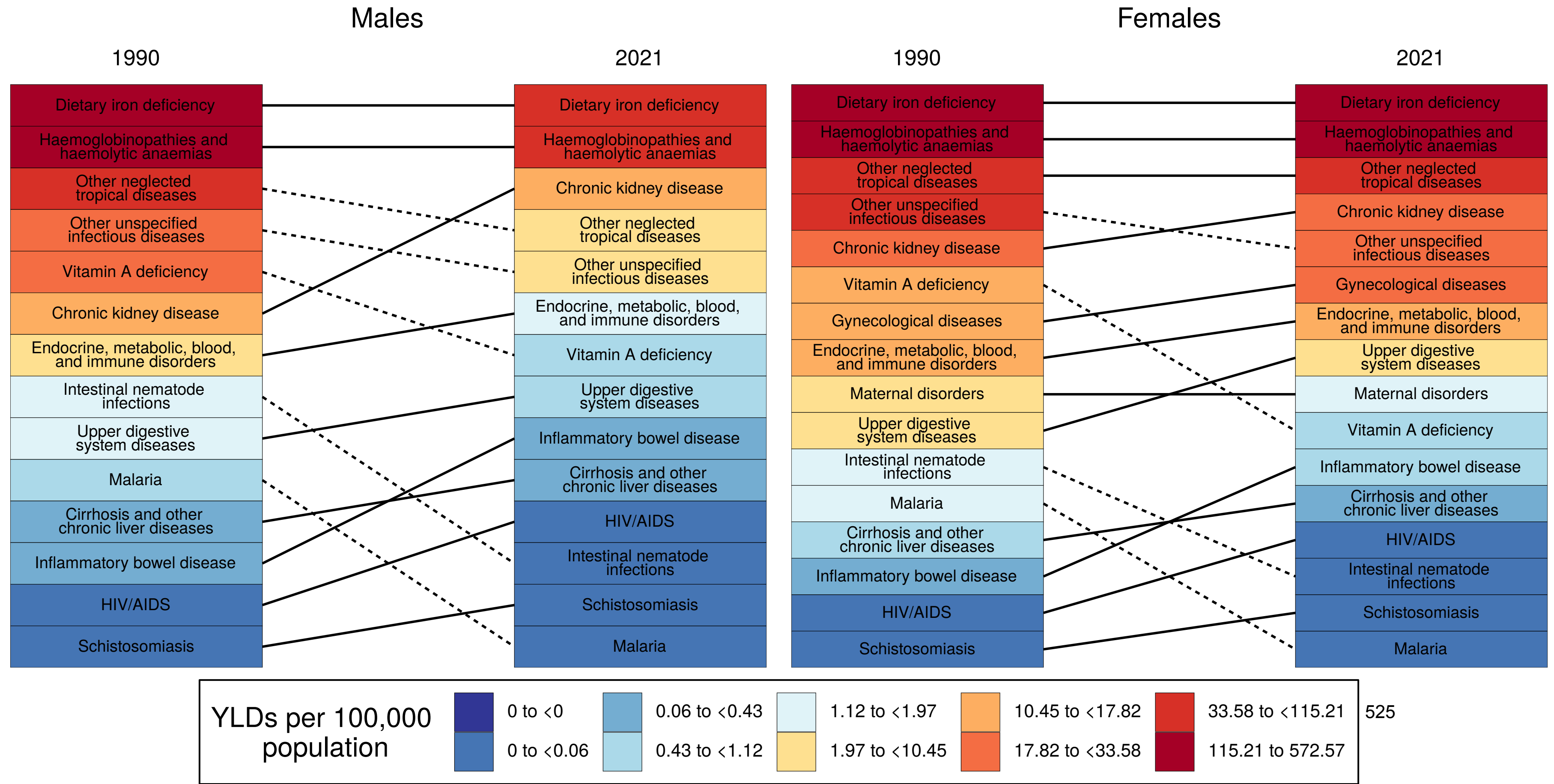

Males
Females
1990
2021
Dietary iron deficiency
Haemoglobinopathies and haemolytic anaemias
Other neglected tropical diseases
Other unspecified infectious diseases
Vitamin A deficiency
Chronic kidney disease
Endocrine, metabolic, blood, and immune disorders
Intestinal nematode infections
Upper digestive system diseases
Malaria
Cirrhosis and other chronic liver diseases
Inflammatory bowel disease
HIV/AIDS
Schistosomiasis
Gynecological diseases
Maternal disorders
YLDs per 100,000 population
0 to <0
0 to <0.06
0.06 to <0.43
0.43 to <1.12
1.12 to <1.97
1.97 to <10.45
10.45 to <17.82
17.82 to <33.58
33.58 to <115.21
115.21 to 572.57

525

Bushehr

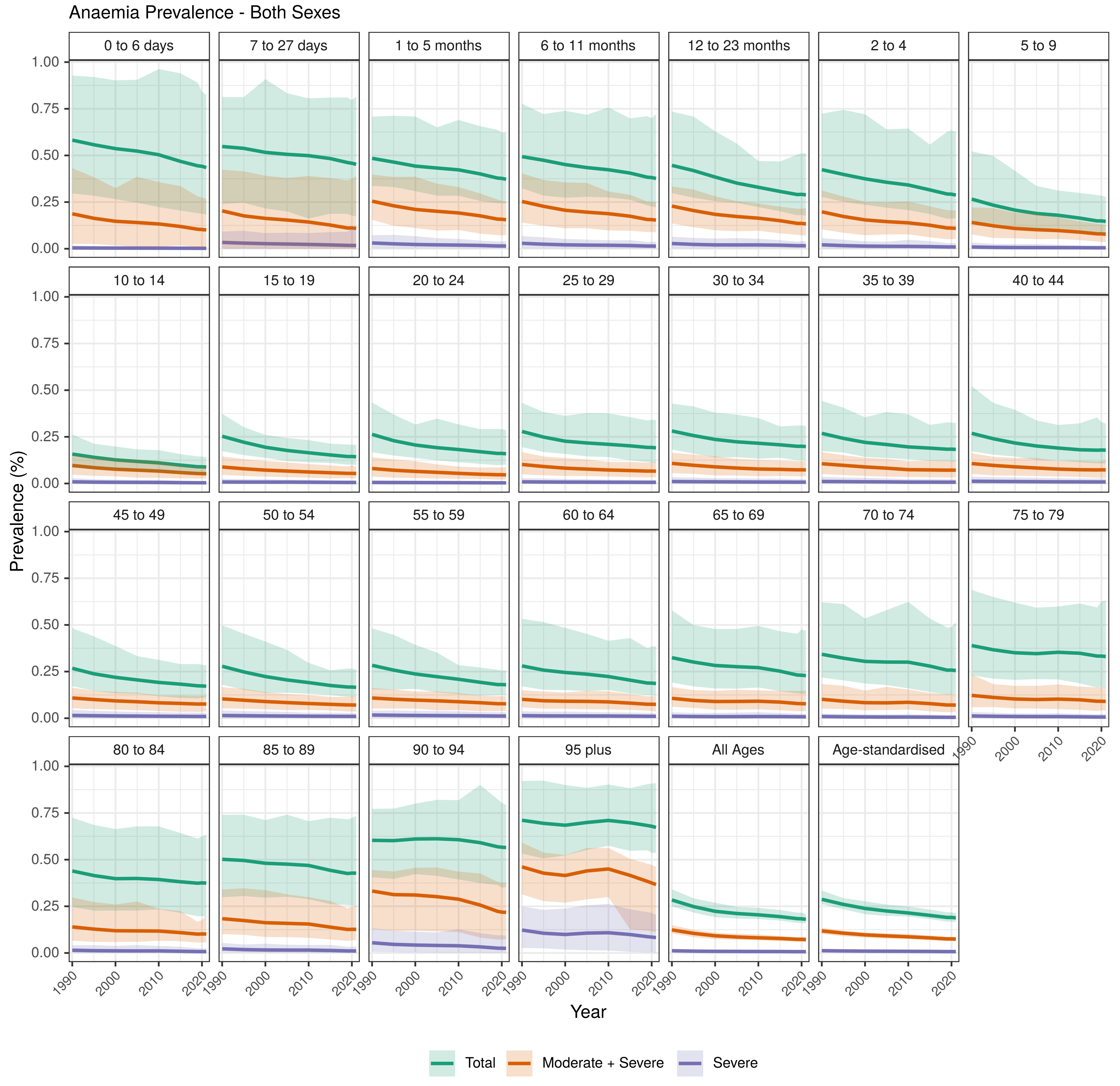

Anaemia Prevalence - Both Sexes
0 to 6 days
7 to 27 days
1 to 5 months
6 to 11 months
12 to 23 months
2 to 4
5 to 9
10 to 14
15 to 19
20 to 24
25 to 29
30 to 34
35 to 39
40 to 44
45 to 49
50 to 54
55 to 59
60 to 64
65 to 69
70 to 74
75 to 79
80 to 84
85 to 89
90 to 94
95 plus
All Ages
Age-standardised
Prevalence (%)
1.00
0.75
0.50
0.25
0.00
1990
2000
2010
2020
Year
Total
Moderate + Severe
Severe

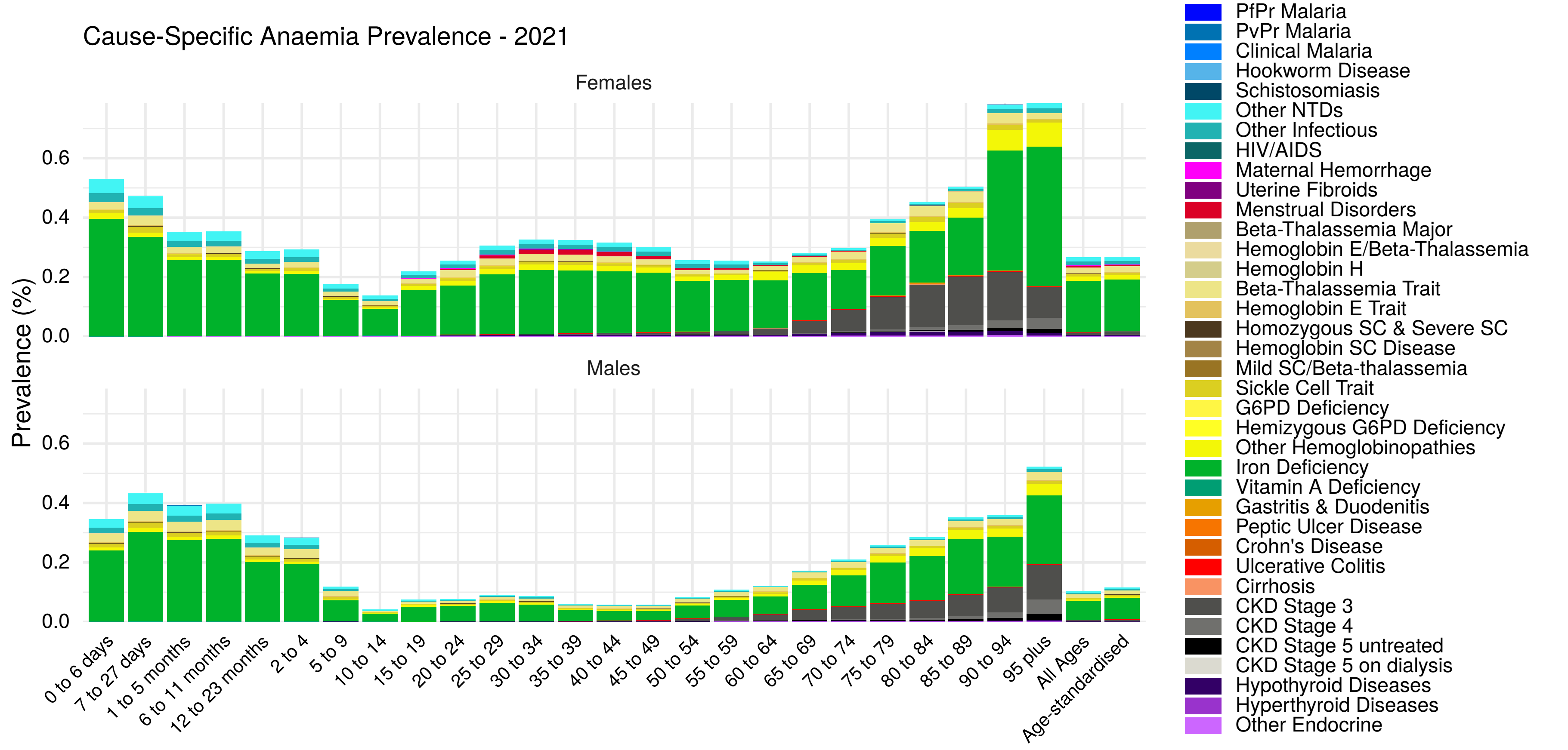

Cause-Specific Anaemia Prevalence - 2021
Females
Males
Prevalence (%)
0.6
0.4
0.2
0.0
0 to 6 days
7 to 27 days
1 to 5 months
6 to 11 months
12 to 23 months
2 to 4
5 to 9
10 to 14
15 to 19
20 to 24
25 to 29
30 to 34
35 to 39
40 to 44
45 to 49
50 to 54
55 to 59
60 to 64
65 to 69
70 to 74
75 to 79
80 to 84
85 to 89
90 to 94
95 plus
All Ages
Age-standardised
PfPr Malaria
PvPr Malaria
Clinical Malaria
Hookworm Disease
Schistosomiasis
Other NTDs
Other Infectious
HIV/AIDS
Maternal Hemorrhage
Uterine Fibroids
Menstrual Disorders
Beta-Thalassemia Major
Hemoglobin E/Beta-Thalassemia
Hemoglobin H
Beta-Thalassemia Trait
Hemoglobin E Trait
Homozygous SC & Severe SC
Hemoglobin SC Disease
Mild SC/Beta-thalassemia
Sickle Cell Trait
G6PD Deficiency
Hemizygous G6PD Deficiency
Other Hemoglobinopathies
Iron Deficiency
Vitamin A Deficiency
Gastritis & Duodenitis
Peptic Ulcer Disease
Crohn's Disease
Ulcerative Colitis
Cirrhosis
CKD Stage 3
CKD Stage 4
CKD Stage 5 untreated
CKD Stage 5 on dialysis
Hypothyroid Diseases
Hyperthyroid Diseases
Other Endocrine

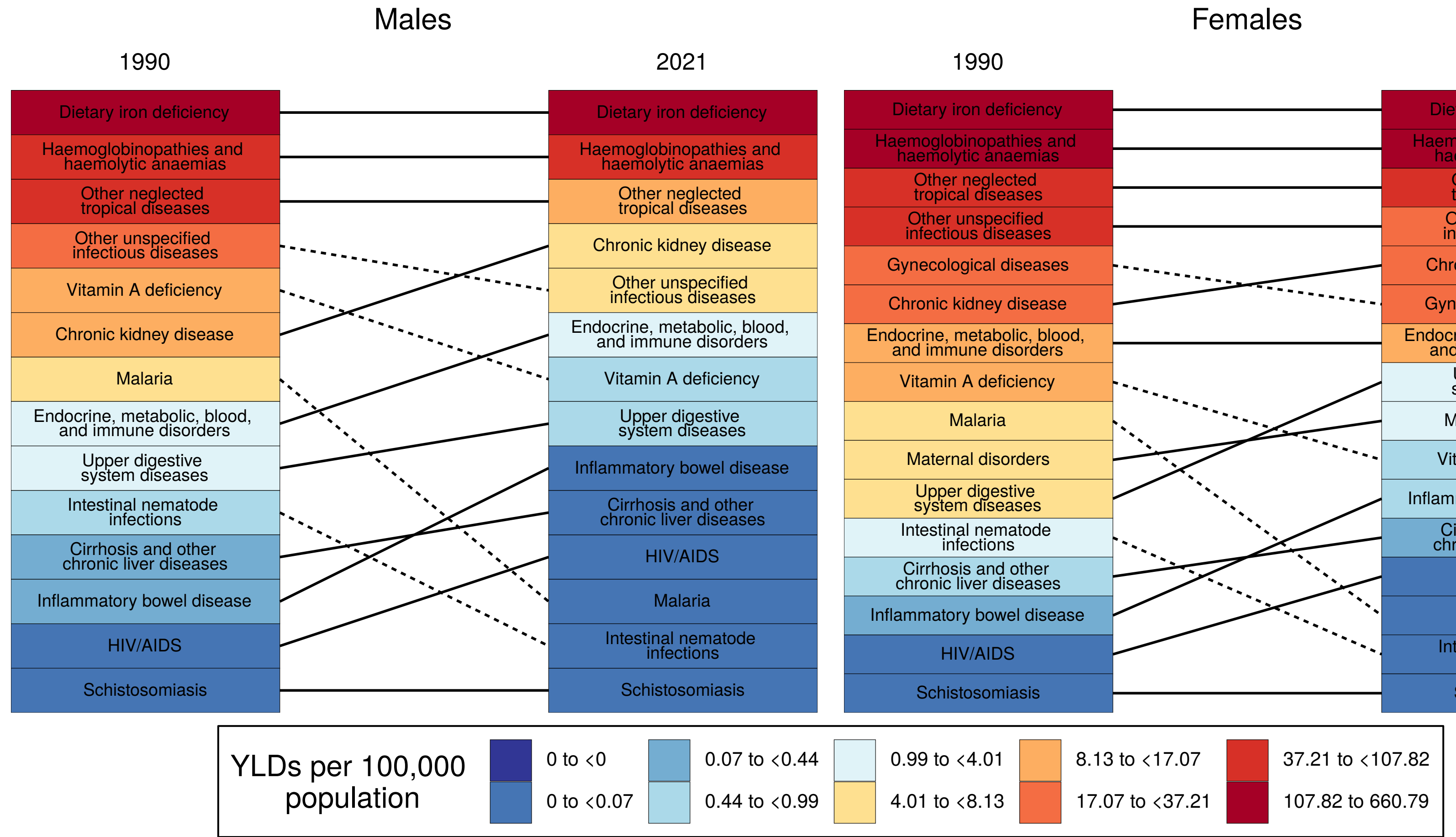

Males
1990
Dietary iron deficiency
Haemoglobinopathies and haemolytic anaemias
Other neglected tropical diseases
Other unspecified infectious diseases
Vitamin A deficiency
Chronic kidney disease
Malaria
Endocrine, metabolic, blood, and immune disorders
Upper digestive system diseases
Intestinal nematode infections
Cirrhosis and other chronic liver diseases
Inflammatory bowel disease
HIV/AIDS
Schistosomiasis
2021
Dietary iron deficiency
Haemoglobinopathies and haemolytic anaemias
Other neglected tropical diseases
Chronic kidney disease
Other unspecified infectious diseases
Endocrine, metabolic, blood, and immune disorders
Vitamin A deficiency
Upper digestive system diseases
Inflammatory bowel disease
Cirrhosis and other chronic liver diseases
HIV/AIDS
Malaria
Intestinal nematode infections
Schistosomiasis
Females
1990
Dietary iron deficiency
Haemoglobinopathies and haemolytic anaemias
Other neglected tropical diseases
Other unspecified infectious diseases
Gynecological diseases
Chronic kidney disease
Endocrine, metabolic, blood, and immune disorders
Vitamin A deficiency
Malaria
Maternal disorders
Upper digestive system diseases
Intestinal nematode infections
Cirrhosis and other chronic liver diseases
Inflammatory bowel disease
HIV/AIDS
Schistosomiasis
2021
Dietary iron deficiency
Haemoglobinopathies and haemolytic anaemias
Other neglected tropical diseases
Other unspecified infectious diseases
Chronic kidney disease
Gynecological diseases
Endocrine, metabolic, blood, and immune disorders
Upper digestive system diseases
Maternal disorders
Vitamin A deficiency
Inflammatory bowel disease
Cirrhosis and other chronic liver diseases
HIV/AIDS
Malaria
Intestinal nematode infections
Schistosomiasis
YLDs per 100,000 population
0 to <0
0 to <0.07
0.07 to <0.44
0.44 to <0.99
0.99 to <4.01
4.01 to <8.13
8.13 to <17.07
17.07 to <37.21
37.21 to <107.82
107.82 to 660.79

Chahar Mahaal and Bakhtiari

Anaemia Prevalence - Both Sexes

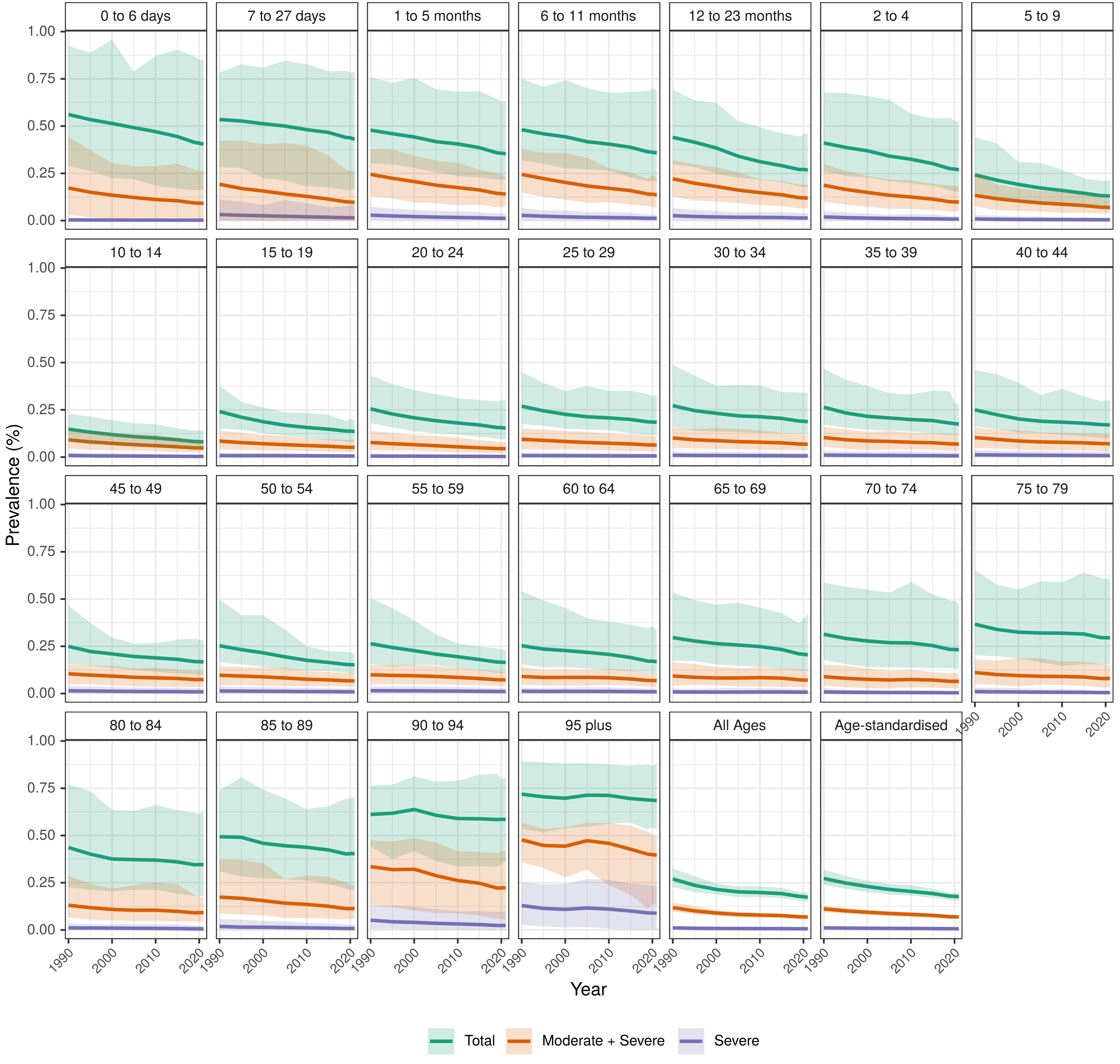

Cause-Specific Anaemia Prevalence - 2021

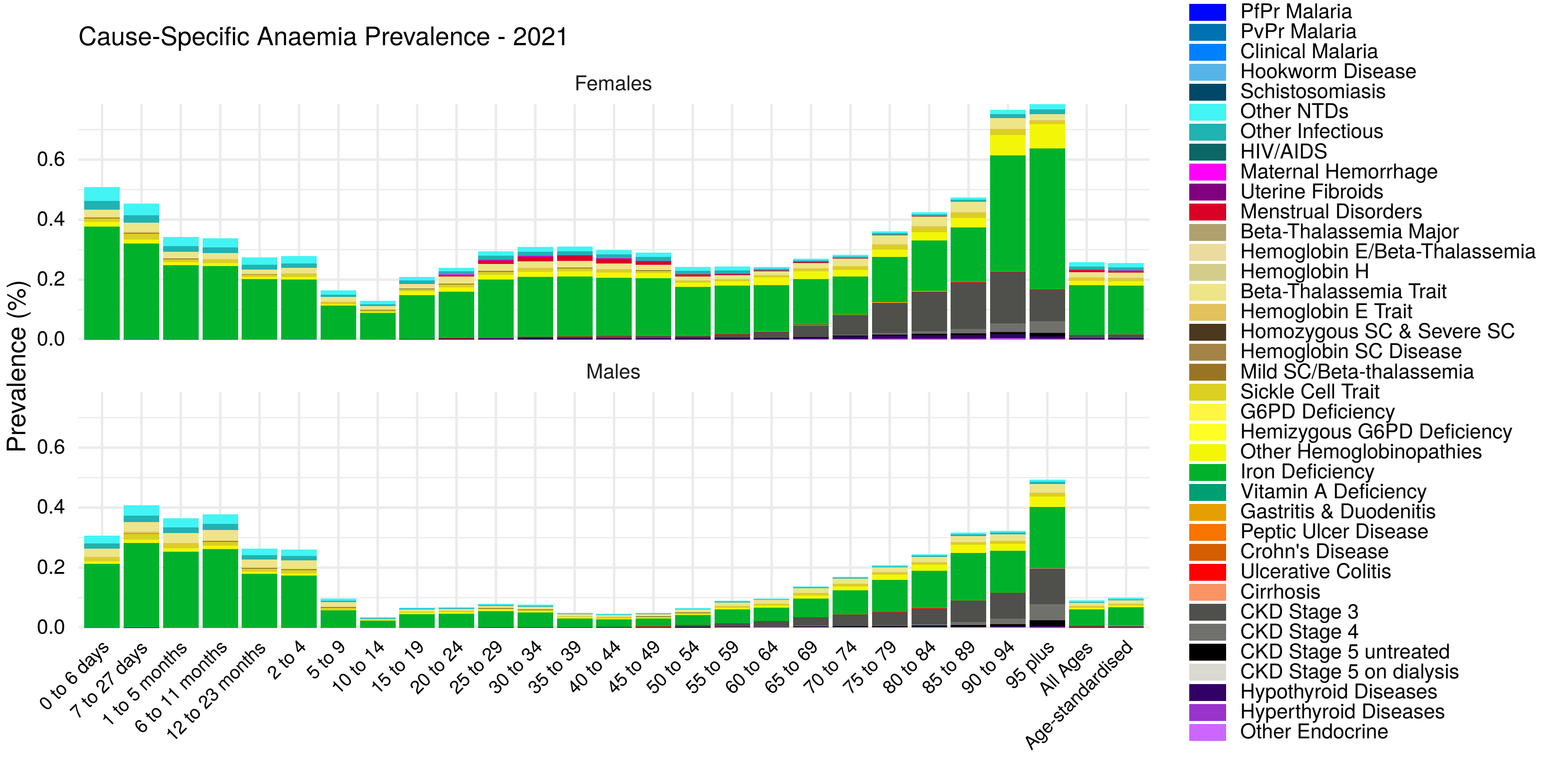

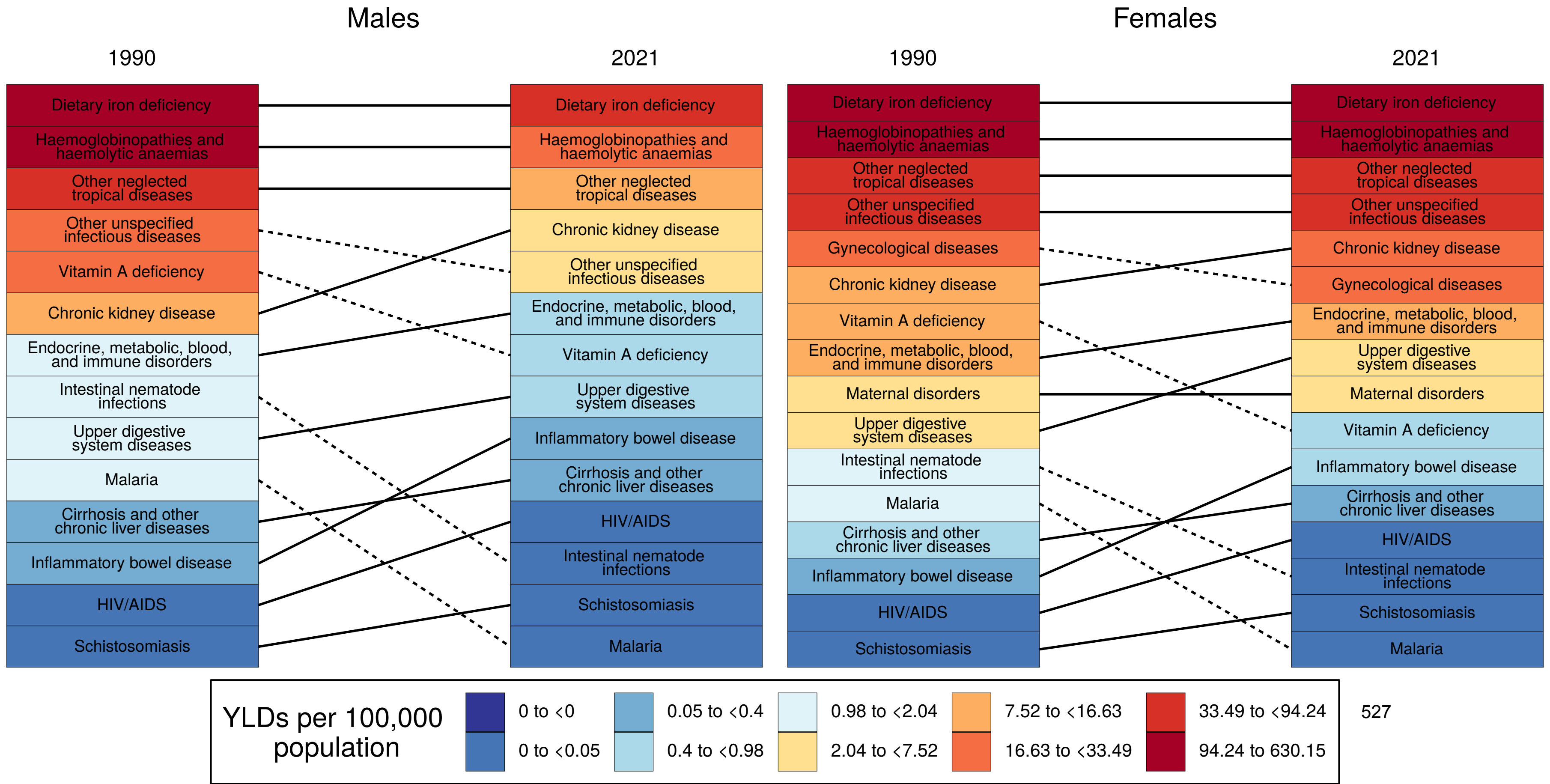

East Azarbayejan

Anaemia Prevalence - Both Sexes

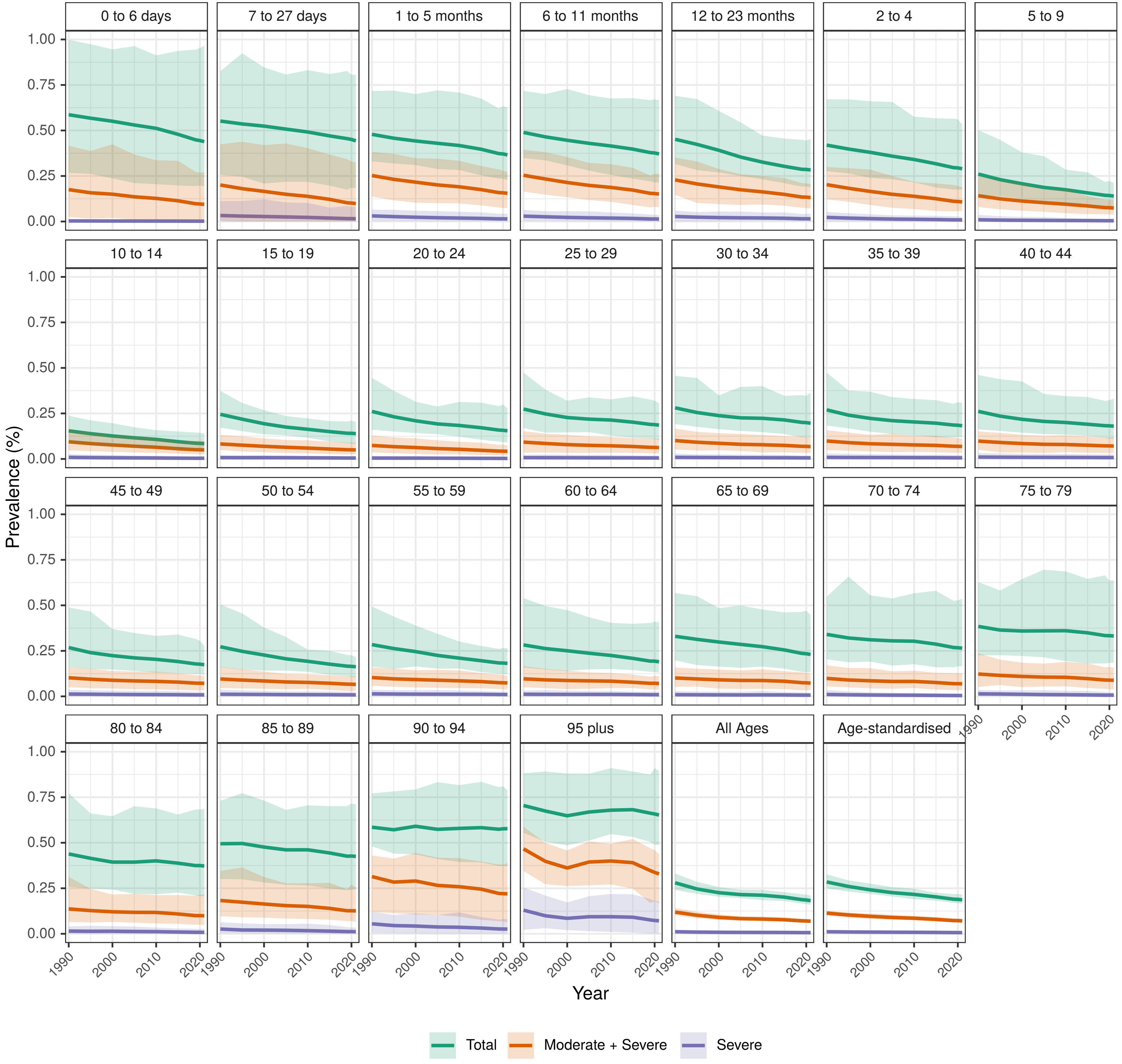

Cause-Specific Anaemia Prevalence - 2021

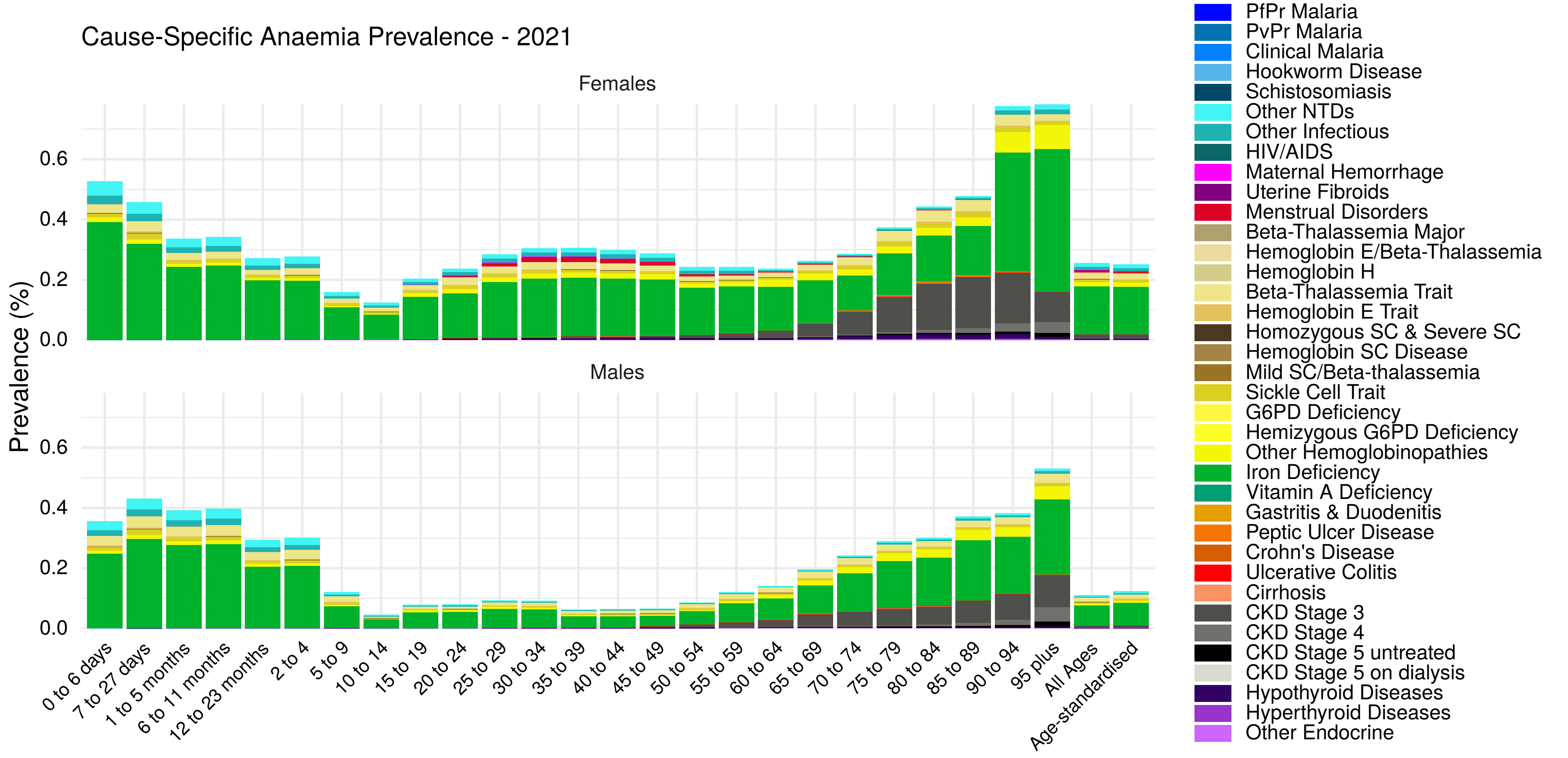

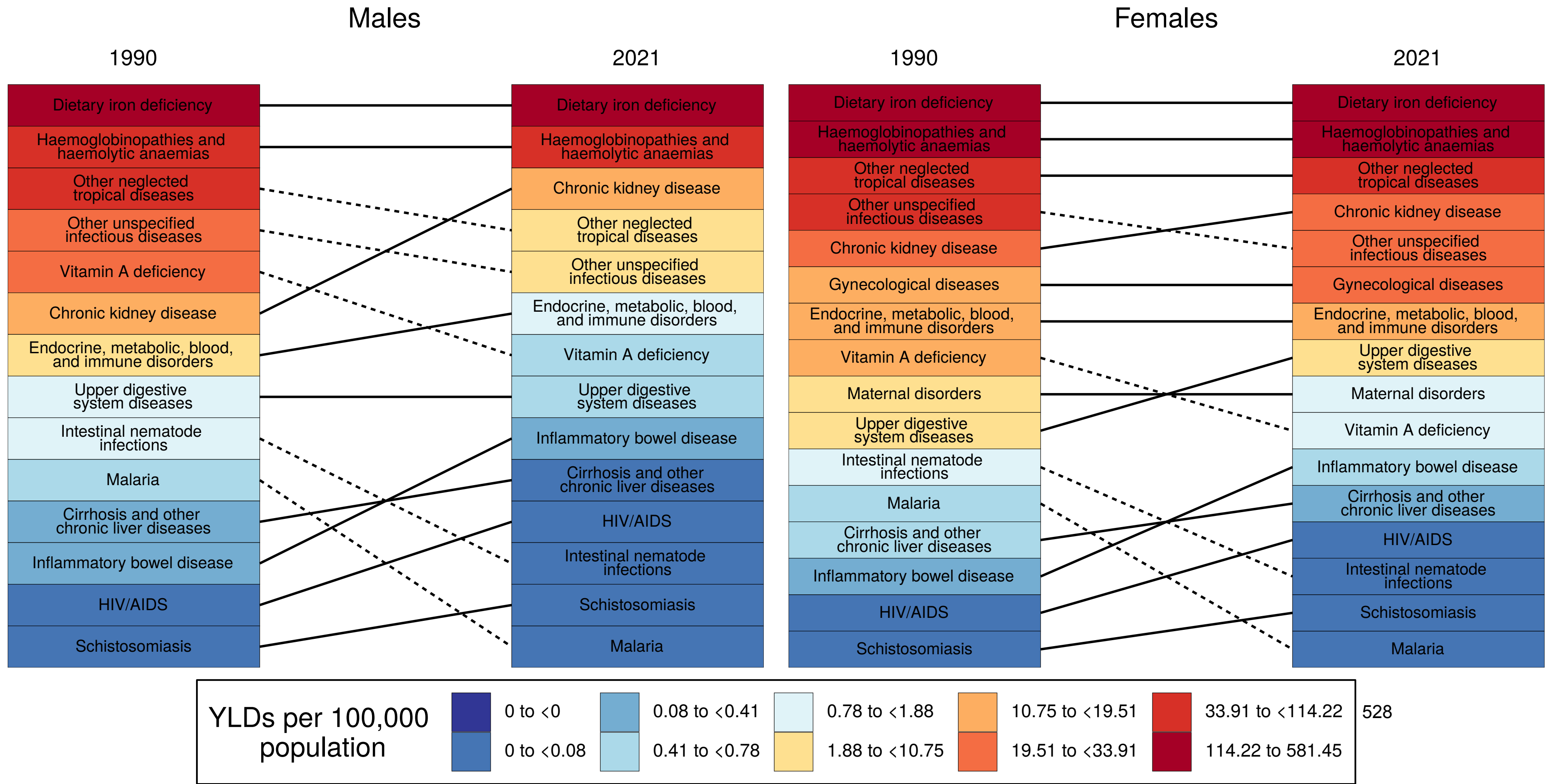

Fars

Anaemia Prevalence - Both Sexes

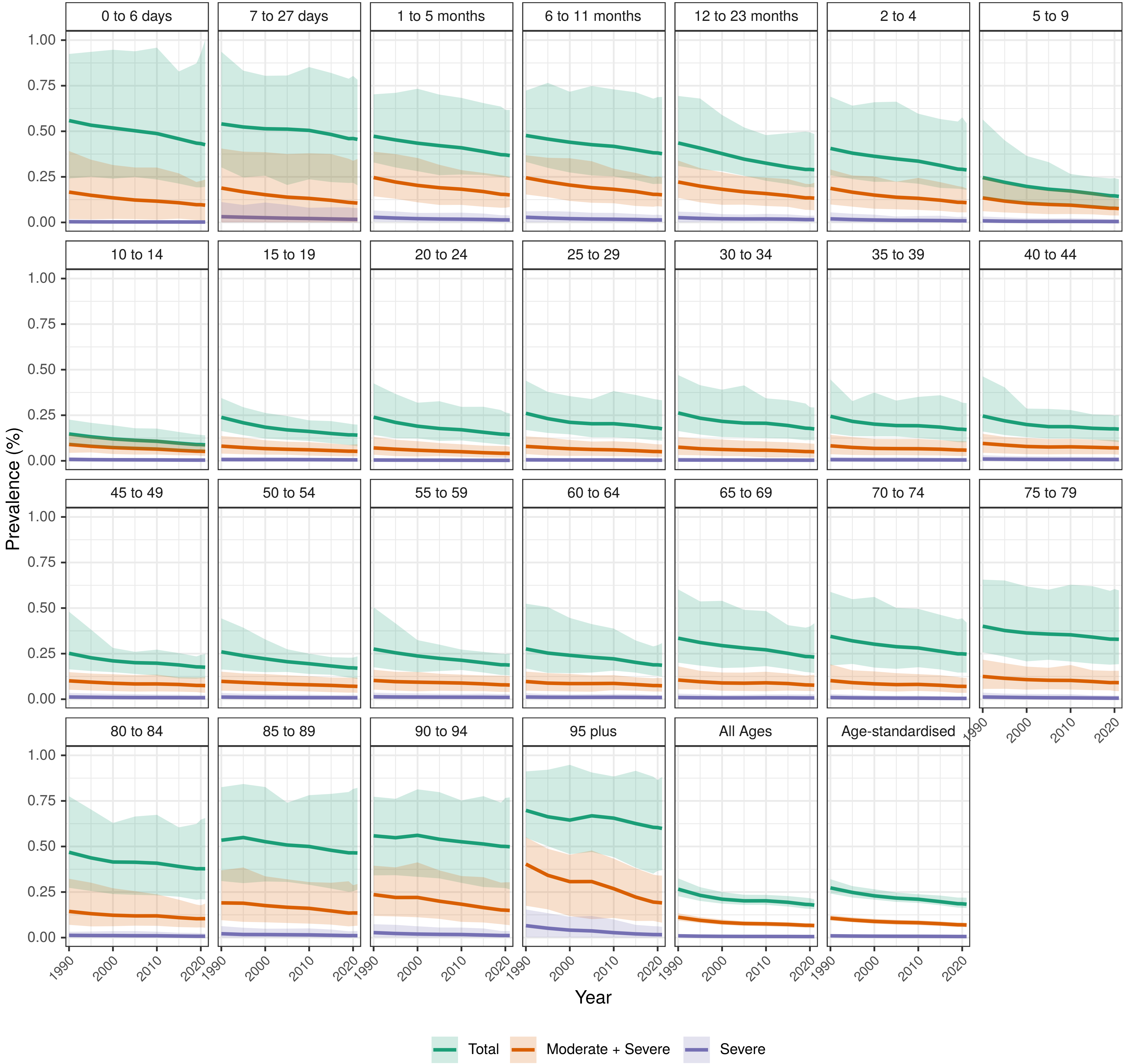

Cause-Specific Anaemia Prevalence - 2021

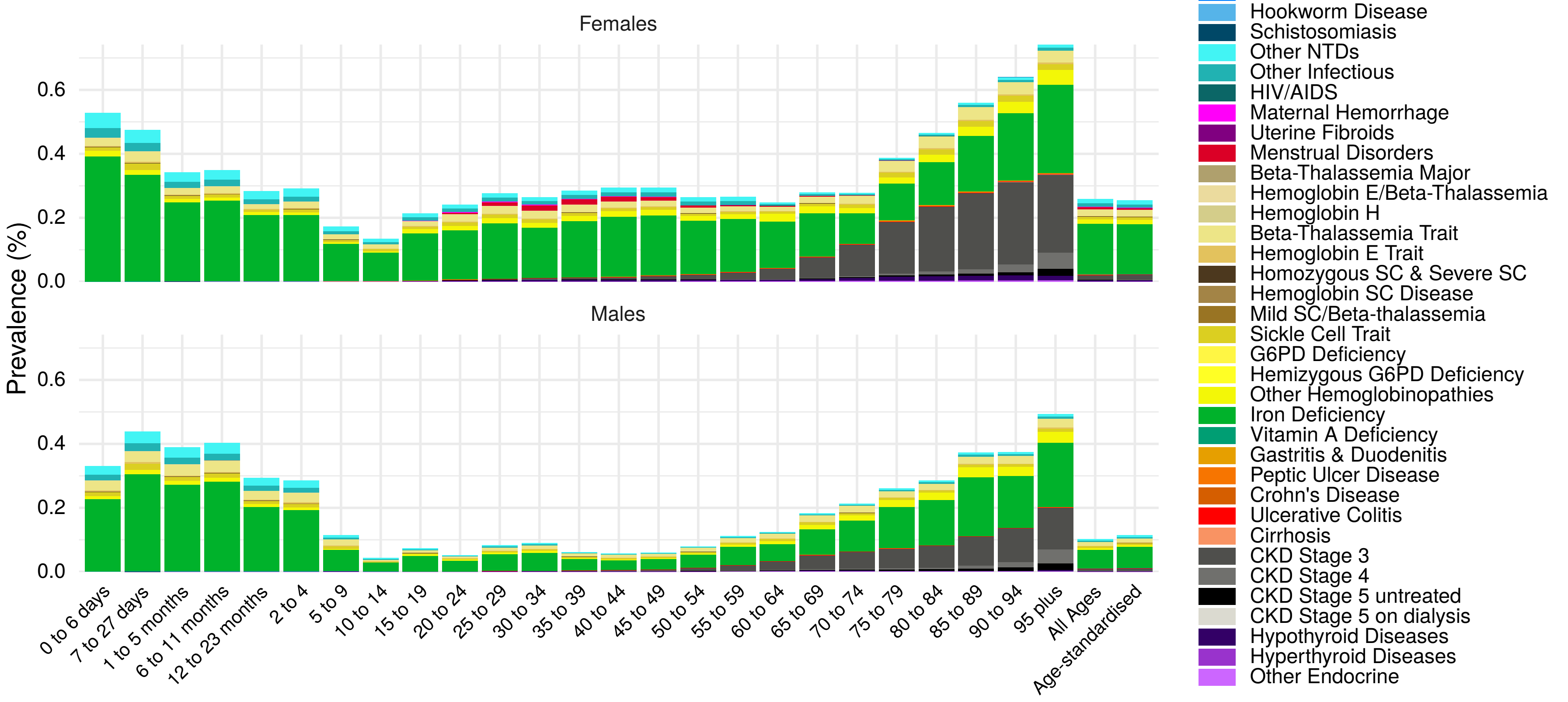

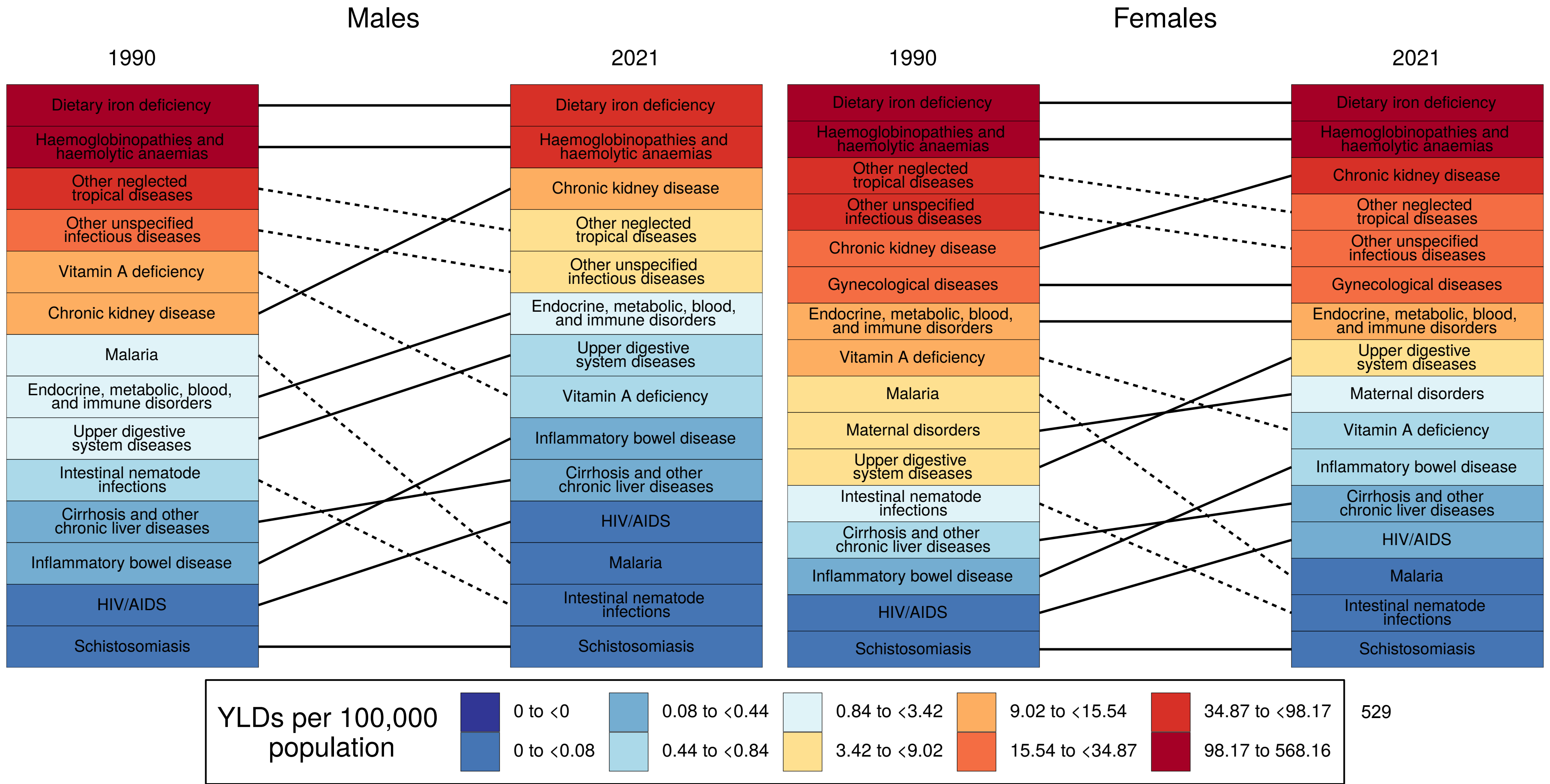

Gilan

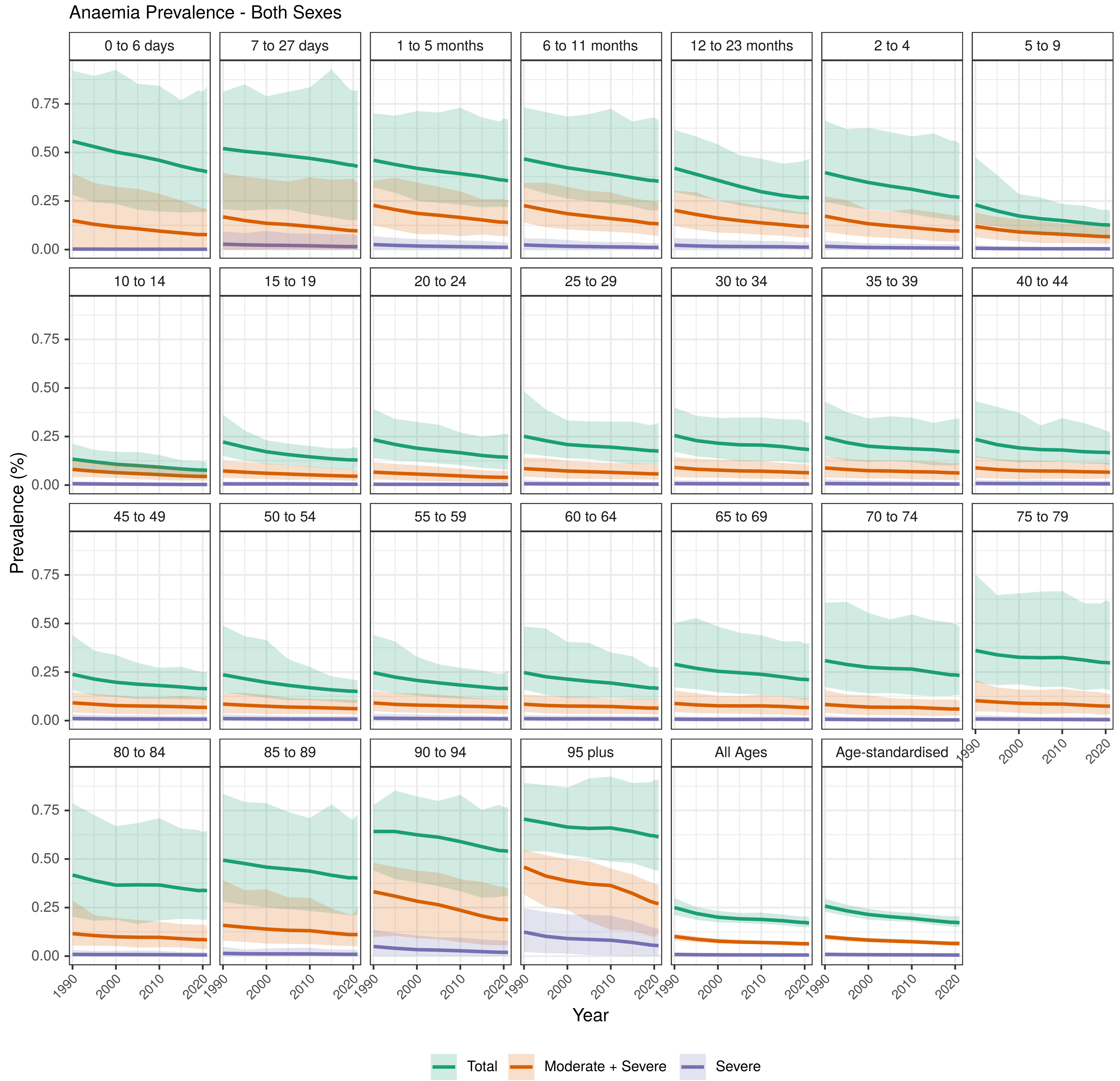
Anaemia Prevalence - Both Sexes
0 to 6 days
7 to 27 days
1 to 5 months
6 to 11 months
12 to 23 months
2 to 4
5 to 9
10 to 14
15 to 19
20 to 24
25 to 29
30 to 34
35 to 39
40 to 44
45 to 49
50 to 54
55 to 59
60 to 64
65 to 69
70 to 74
75 to 79
80 to 84
85 to 89
90 to 94
95 plus
All Ages
Age-standardised
Prevalence (%)
Year
Total
Moderate + Severe
Severe

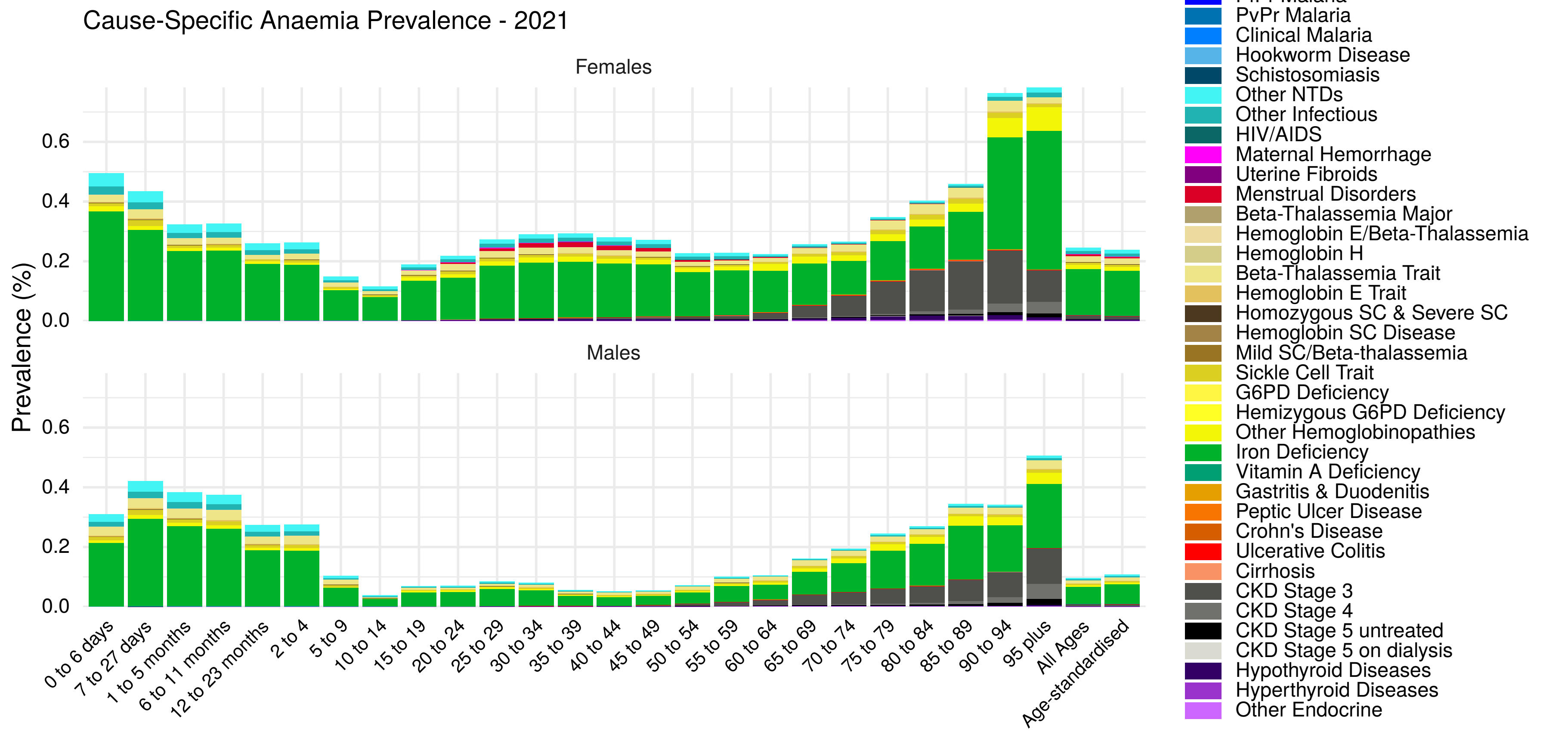
Cause-Specific Anaemia Prevalence - 2021
Females
Males
Prevalence (%)
PfPr Malaria
PvPr Malaria
Clinical Malaria
Hookworm Disease
Schistosomiasis
Other NTDs
Other Infectious
HIV/AIDS
Maternal Hemorrhage
Uterine Fibroids
Menstrual Disorders
Beta-Thalassemia Major
Hemoglobin E/Beta-Thalassemia
Hemoglobin H
Beta-Thalassemia Trait
Hemoglobin E Trait
Homozygous SC & Severe SC
Hemoglobin SC Disease
Mild SC/Beta-thalassemia
Sickle Cell Trait
G6PD Deficiency
Hemizygous G6PD Deficiency
Other Hemoglobinopathies
Iron Deficiency
Vitamin A Deficiency
Gastritis & Duodenitis
Peptic Ulcer Disease
Crohn's Disease
Ulcerative Colitis
Cirrhosis
CKD Stage 3
CKD Stage 4
CKD Stage 5 untreated
CKD Stage 5 on dialysis
Hypothyroid Diseases
Hyperthyroid Diseases
Other Endocrine

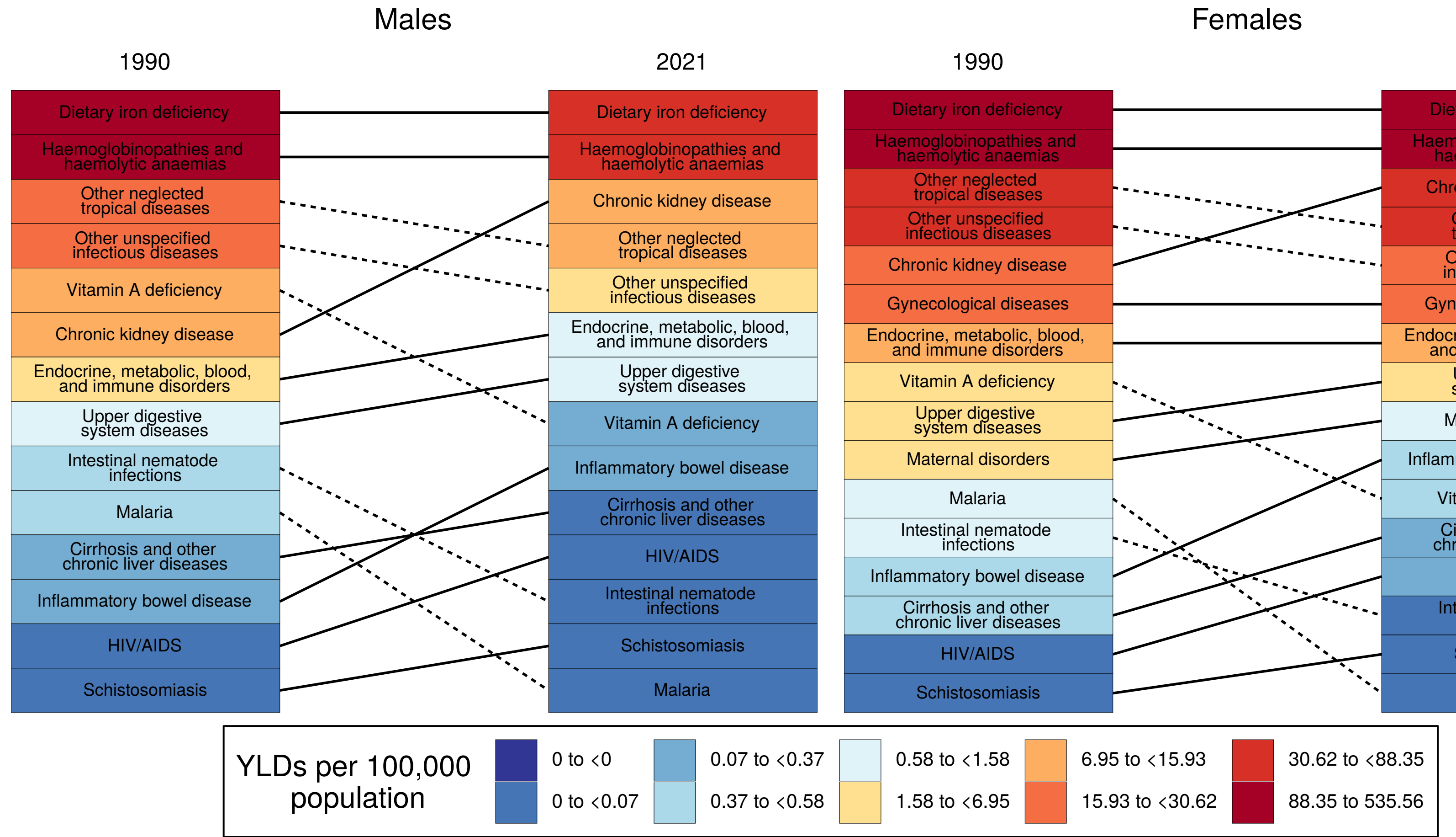
Males
Females
1990
2021
Males 1990: Dietary iron deficiency; Haemoglobinopathies and haemolytic anaemias; Other neglected tropical diseases; Other unspecified infectious diseases; Vitamin A deficiency; Chronic kidney disease; Endocrine, metabolic, blood, and immune disorders; Upper digestive system diseases; Intestinal nematode infections; Malaria; Cirrhosis and other chronic liver diseases; Inflammatory bowel disease; HIV/AIDS; Schistosomiasis
Males 2021: Dietary iron deficiency; Haemoglobinopathies and haemolytic anaemias; Chronic kidney disease; Other neglected tropical diseases; Other unspecified infectious diseases; Endocrine, metabolic, blood, and immune disorders; Upper digestive system diseases; Vitamin A deficiency; Inflammatory bowel disease; Cirrhosis and other chronic liver diseases; HIV/AIDS; Intestinal nematode infections; Schistosomiasis; Malaria
Females 1990: Dietary iron deficiency; Haemoglobinopathies and haemolytic anaemias; Other neglected tropical diseases; Other unspecified infectious diseases; Chronic kidney disease; Gynecological diseases; Endocrine, metabolic, blood, and immune disorders; Vitamin A deficiency; Upper digestive system diseases; Maternal disorders; Malaria; Intestinal nematode infections; Inflammatory bowel disease; Cirrhosis and other chronic liver diseases; HIV/AIDS; Schistosomiasis
Females 2021: Dietary iron deficiency; Haemoglobinopathies and haemolytic anaemias; Chronic kidney disease; Other neglected tropical diseases; Other unspecified infectious diseases; Gynecological diseases; Endocrine, metabolic, blood, and immune disorders; Upper digestive system diseases; Maternal disorders; Inflammatory bowel disease; Vitamin A deficiency; Cirrhosis and other chronic liver diseases; HIV/AIDS; Intestinal nematode infections; Schistosomiasis; Malaria
YLDs per 100,000 population
0 to <0
0 to <0.07
0.07 to <0.37
0.37 to <0.58
0.58 to <1.58
1.58 to <6.95
6.95 to <15.93
15.93 to <30.62
30.62 to <88.35
88.35 to 535.56

530

Golestan

Anaemia Prevalence - Both Sexes

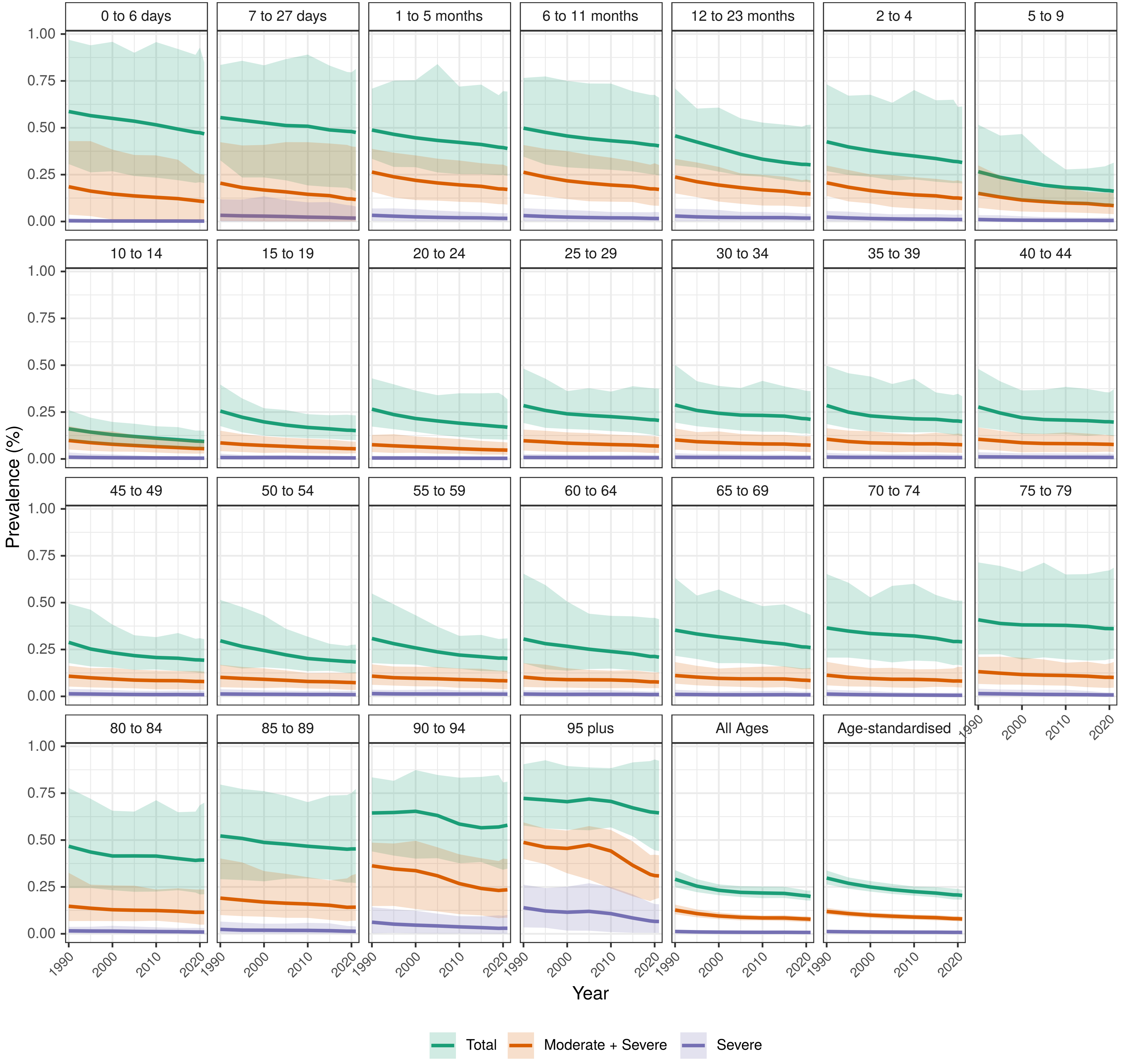

Cause-Specific Anaemia Prevalence - 2021

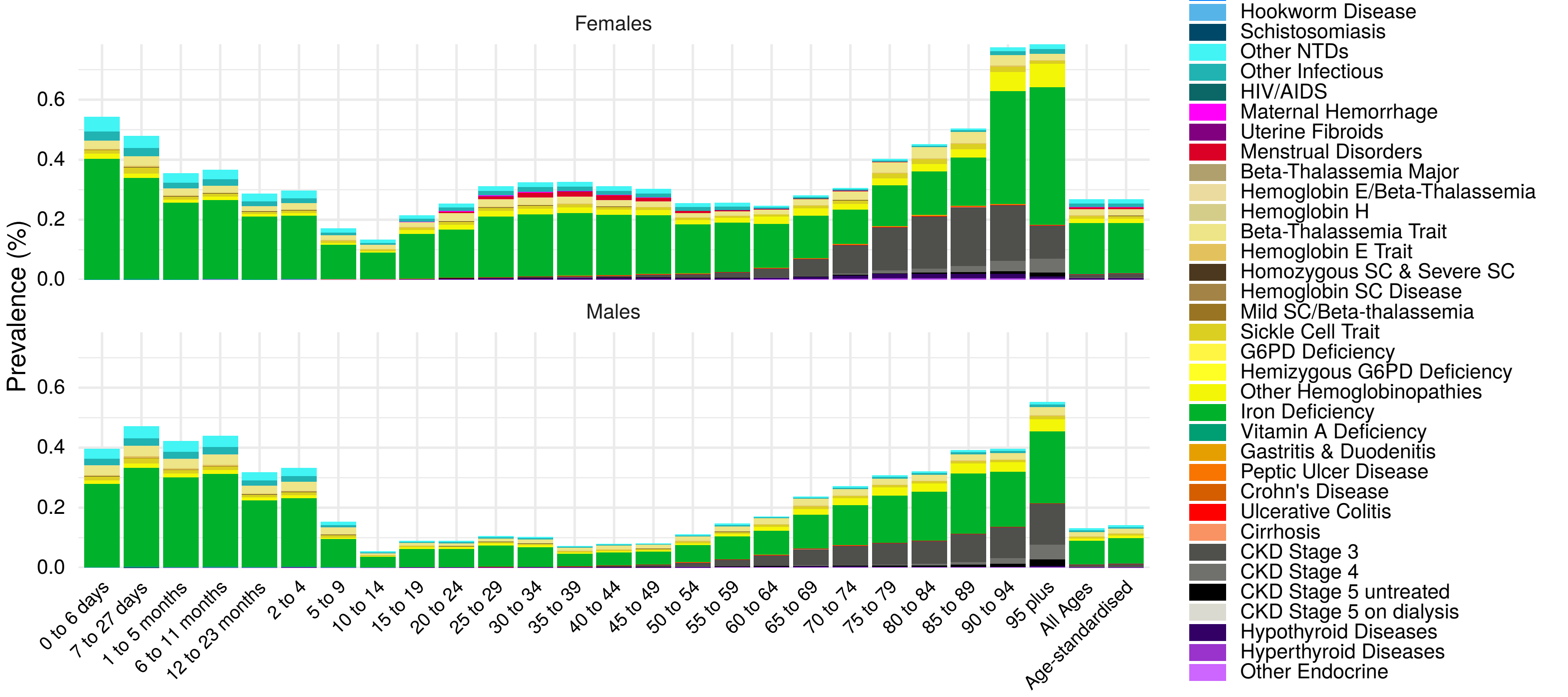

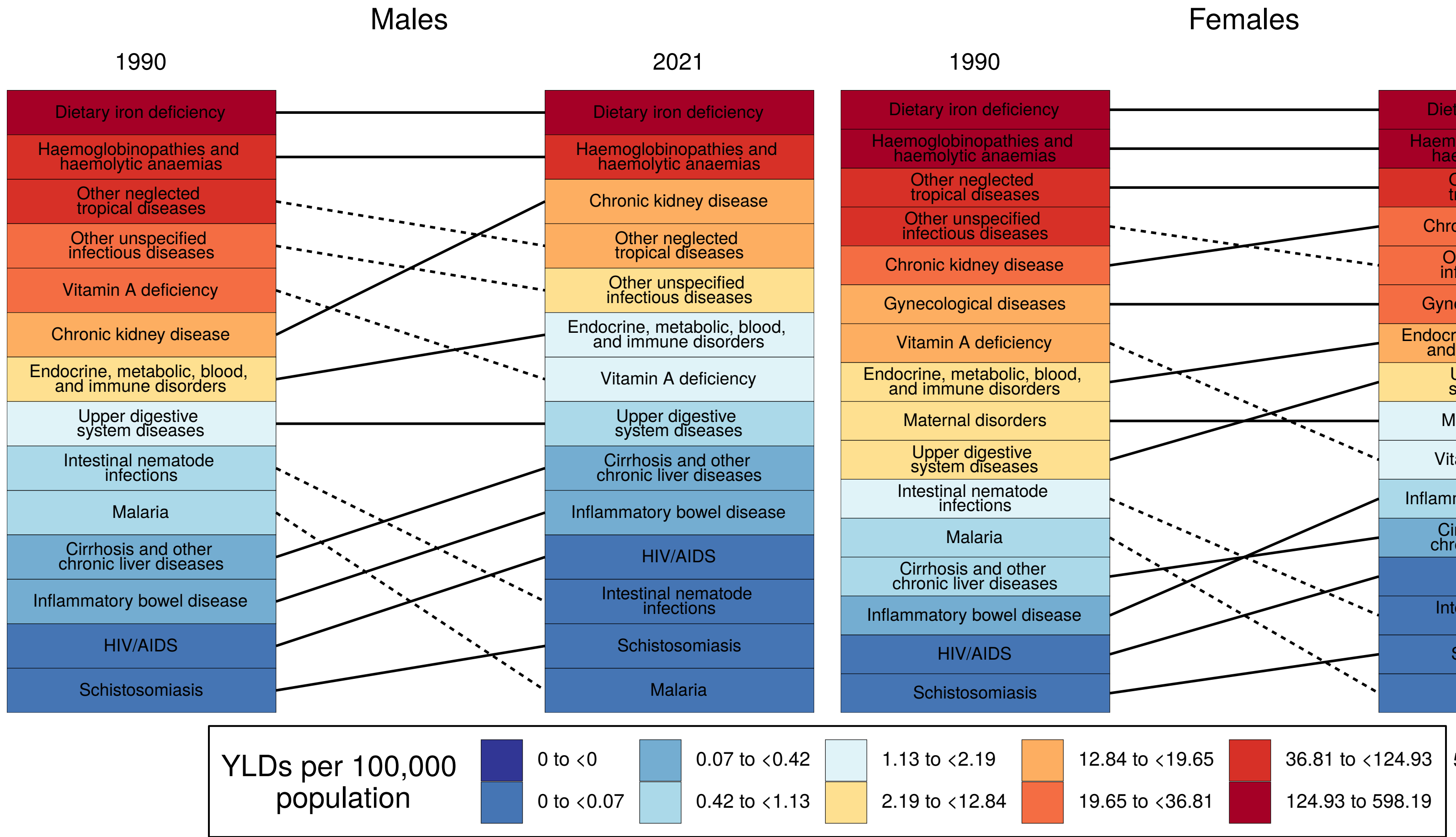

Hamadan

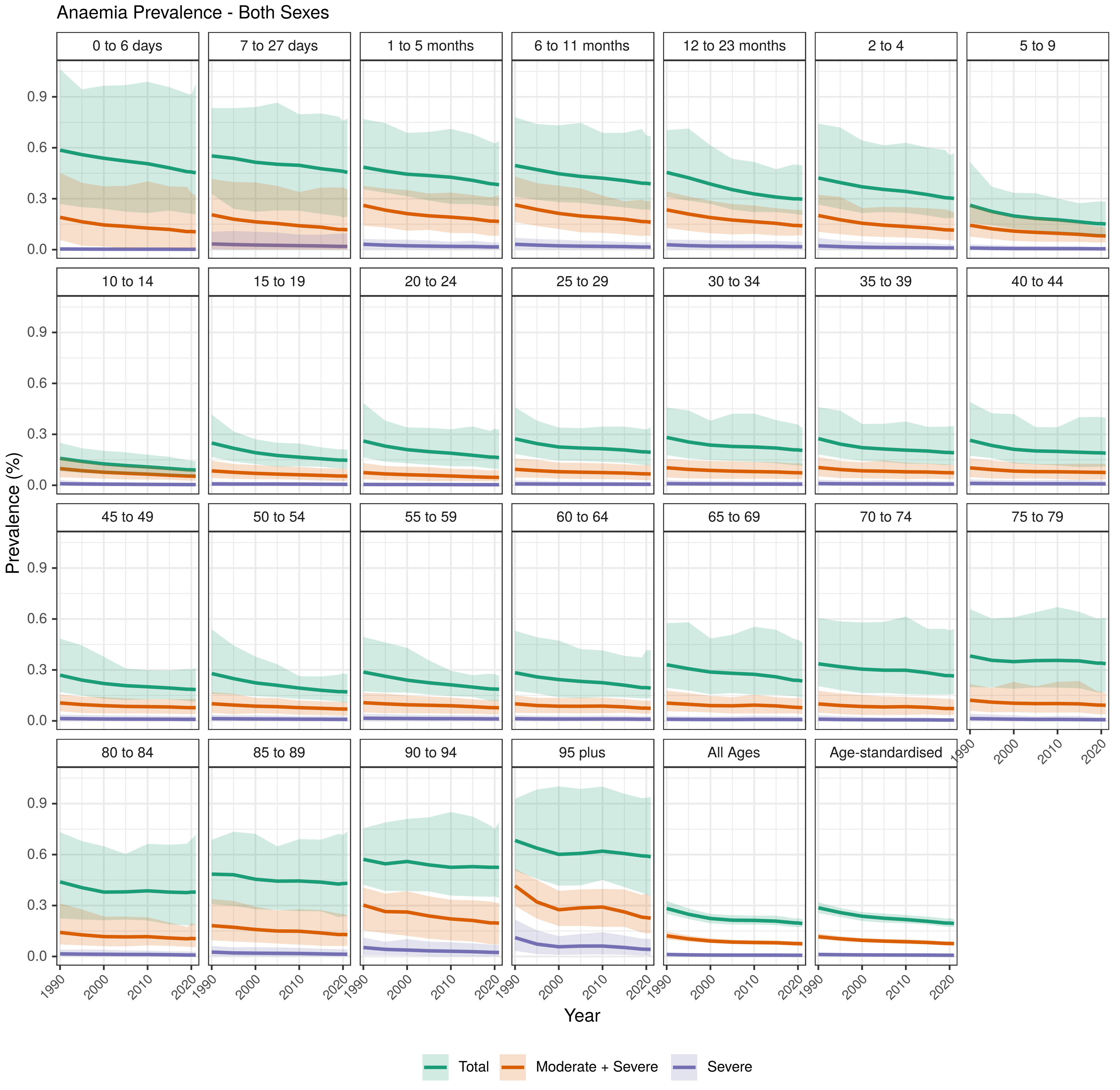

Anaemia Prevalence - Both Sexes
0 to 6 days
7 to 27 days
1 to 5 months
6 to 11 months
12 to 23 months
2 to 4
5 to 9
10 to 14
15 to 19
20 to 24
25 to 29
30 to 34
35 to 39
40 to 44
45 to 49
50 to 54
55 to 59
60 to 64
65 to 69
70 to 74
75 to 79
80 to 84
85 to 89
90 to 94
95 plus
All Ages
Age-standardised
Prevalence (%)
0.0
0.3
0.6
0.9
1990
2000
2010
2020
Year
Total
Moderate + Severe
Severe

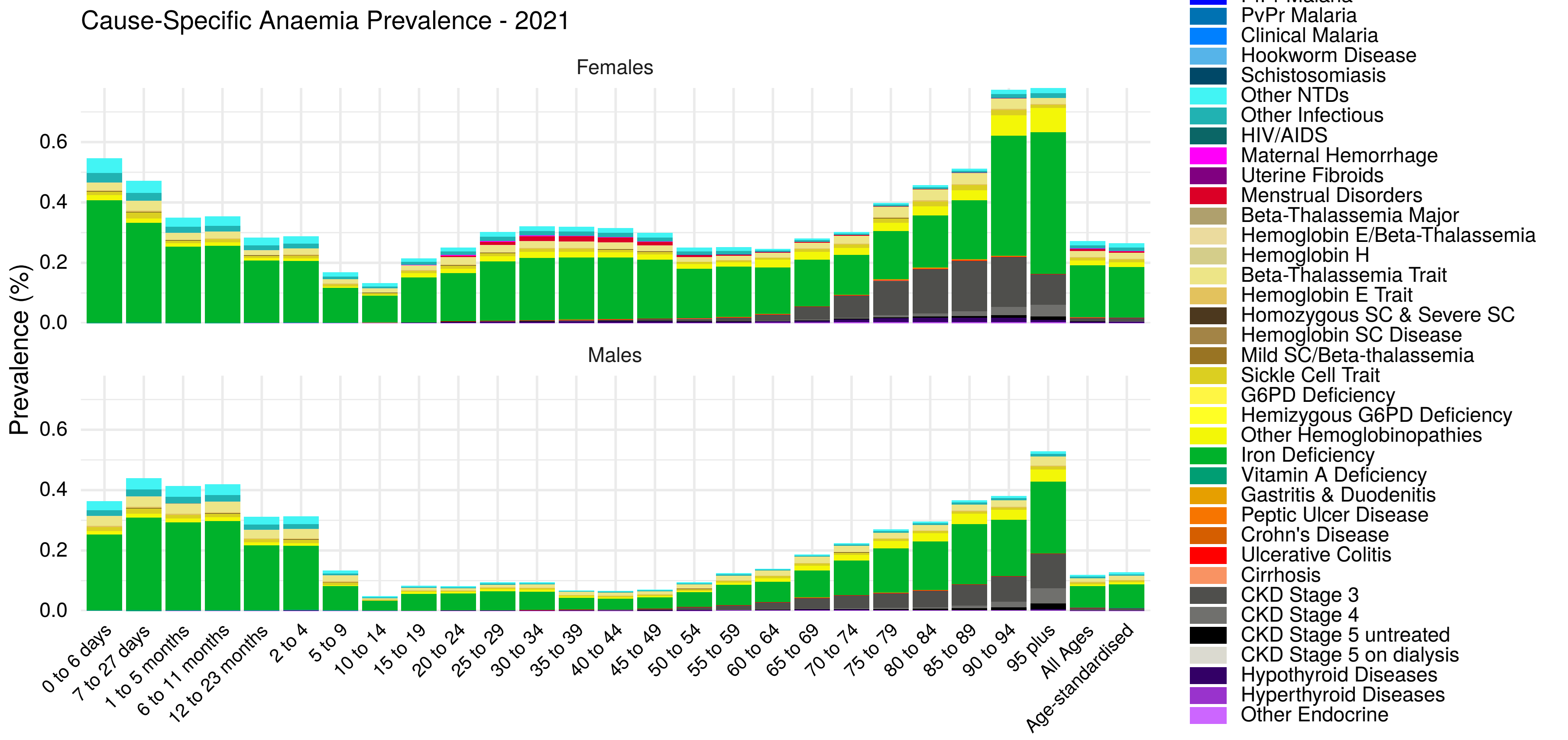

Cause-Specific Anaemia Prevalence - 2021
Females
Males
Prevalence (%)
0.0
0.2
0.4
0.6
0 to 6 days
7 to 27 days
1 to 5 months
6 to 11 months
12 to 23 months
2 to 4
5 to 9
10 to 14
15 to 19
20 to 24
25 to 29
30 to 34
35 to 39
40 to 44
45 to 49
50 to 54
55 to 59
60 to 64
65 to 69
70 to 74
75 to 79
80 to 84
85 to 89
90 to 94
95 plus
All Ages
Age-standardised
PfPr Malaria
PvPr Malaria
Clinical Malaria
Hookworm Disease
Schistosomiasis
Other NTDs
Other Infectious
HIV/AIDS
Maternal Hemorrhage
Uterine Fibroids
Menstrual Disorders
Beta-Thalassemia Major
Hemoglobin E/Beta-Thalassemia
Hemoglobin H
Beta-Thalassemia Trait
Hemoglobin E Trait
Homozygous SC & Severe SC
Hemoglobin SC Disease
Mild SC/Beta-thalassemia
Sickle Cell Trait
G6PD Deficiency
Hemizygous G6PD Deficiency
Other Hemoglobinopathies
Iron Deficiency
Vitamin A Deficiency
Gastritis & Duodenitis
Peptic Ulcer Disease
Crohn's Disease
Ulcerative Colitis
Cirrhosis
CKD Stage 3
CKD Stage 4
CKD Stage 5 untreated
CKD Stage 5 on dialysis
Hypothyroid Diseases
Hyperthyroid Diseases
Other Endocrine

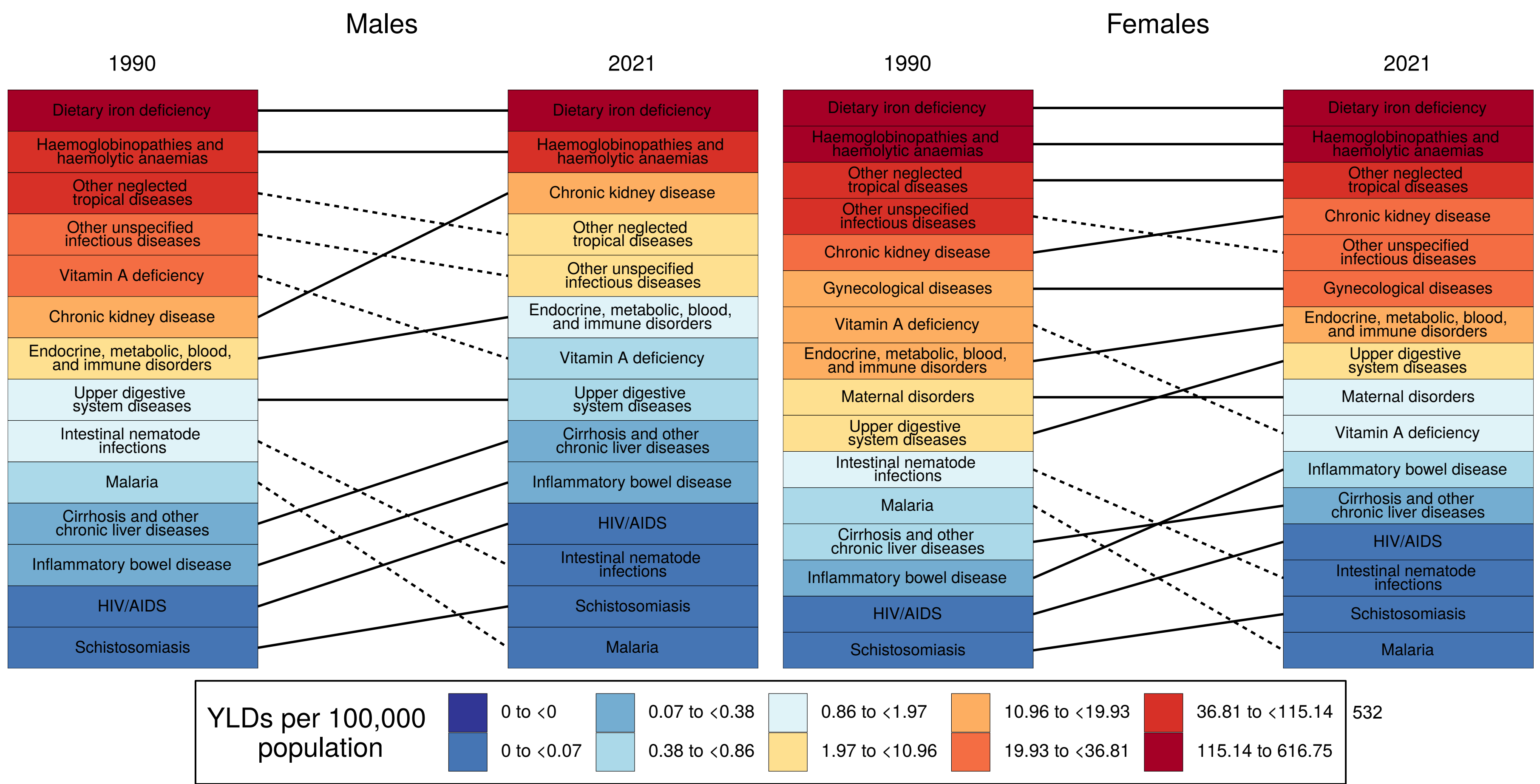

Males
Females
1990
2021
Dietary iron deficiency
Haemoglobinopathies and haemolytic anaemias
Other neglected tropical diseases
Other unspecified infectious diseases
Vitamin A deficiency
Chronic kidney disease
Endocrine, metabolic, blood, and immune disorders
Upper digestive system diseases
Intestinal nematode infections
Malaria
Cirrhosis and other chronic liver diseases
Inflammatory bowel disease
HIV/AIDS
Schistosomiasis
Gynecological diseases
Maternal disorders
YLDs per 100,000 population
0 to <0
0 to <0.07
0.07 to <0.38
0.38 to <0.86
0.86 to <1.97
1.97 to <10.96
10.96 to <19.93
19.93 to <36.81
36.81 to <115.14
115.14 to 616.75
532

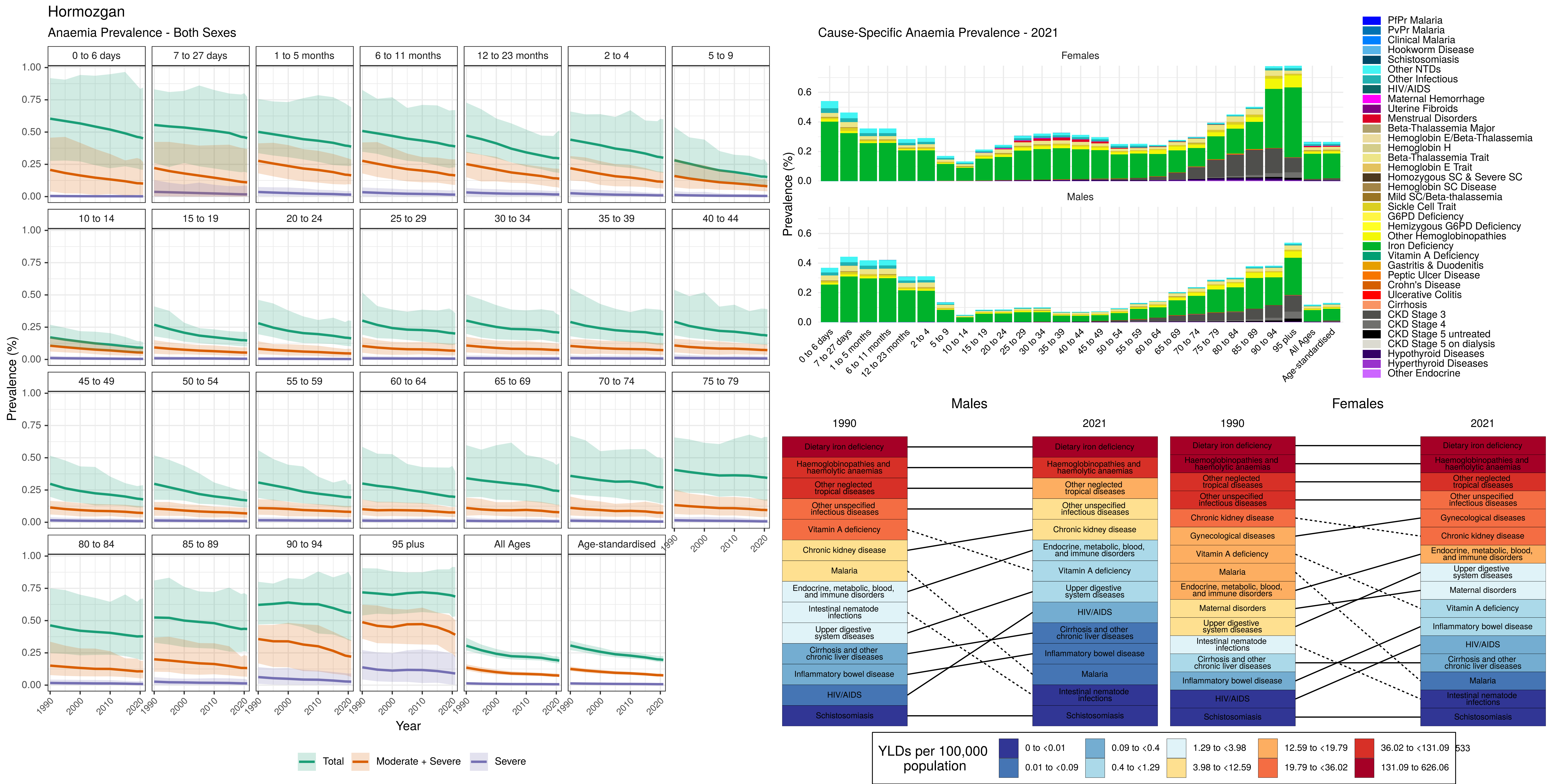

Hormozgan
Anaemia Prevalence - Both Sexes
0 to 6 days
7 to 27 days
1 to 5 months
6 to 11 months
12 to 23 months
2 to 4
5 to 9
10 to 14
15 to 19
20 to 24
25 to 29
30 to 34
35 to 39
40 to 44
45 to 49
50 to 54
55 to 59
60 to 64
65 to 69
70 to 74
75 to 79
80 to 84
85 to 89
90 to 94
95 plus
All Ages
Age-standardised
Prevalence (%)
Year
Total
Moderate + Severe
Severe
Cause-Specific Anaemia Prevalence - 2021
Females
Males
Prevalence (%)
PfPr Malaria
PvPr Malaria
Clinical Malaria
Hookworm Disease
Schistosomiasis
Other NTDs
Other Infectious
HIV/AIDS
Maternal Hemorrhage
Uterine Fibroids
Menstrual Disorders
Beta-Thalassemia Major
Hemoglobin E/Beta-Thalassemia
Hemoglobin H
Beta-Thalassemia Trait
Hemoglobin E Trait
Homozygous SC & Severe SC
Hemoglobin SC Disease
Mild SC/Beta-thalassemia
Sickle Cell Trait
G6PD Deficiency
Hemizygous G6PD Deficiency
Other Hemoglobinopathies
Iron Deficiency
Vitamin A Deficiency
Gastritis & Duodenitis
Peptic Ulcer Disease
Crohn's Disease
Ulcerative Colitis
Cirrhosis
CKD Stage 3
CKD Stage 4
CKD Stage 5 untreated
CKD Stage 5 on dialysis
Hypothyroid Diseases
Hyperthyroid Diseases
Other Endocrine
Males
Females
1990
2021
Dietary iron deficiency
Haemoglobinopathies and haemolytic anaemias
Other neglected tropical diseases
Other unspecified infectious diseases
Vitamin A deficiency
Chronic kidney disease
Malaria
Endocrine, metabolic, blood, and immune disorders
Intestinal nematode infections
Upper digestive system diseases
Cirrhosis and other chronic liver diseases
Inflammatory bowel disease
HIV/AIDS
Schistosomiasis
Gynecological diseases
Maternal disorders
YLDs per 100,000 population
0 to <0.01
0.01 to <0.09
0.09 to <0.4
0.4 to <1.29
1.29 to <3.98
3.98 to <12.59
12.59 to <19.79
19.79 to <36.02
36.02 to <131.09
131.09 to 626.06

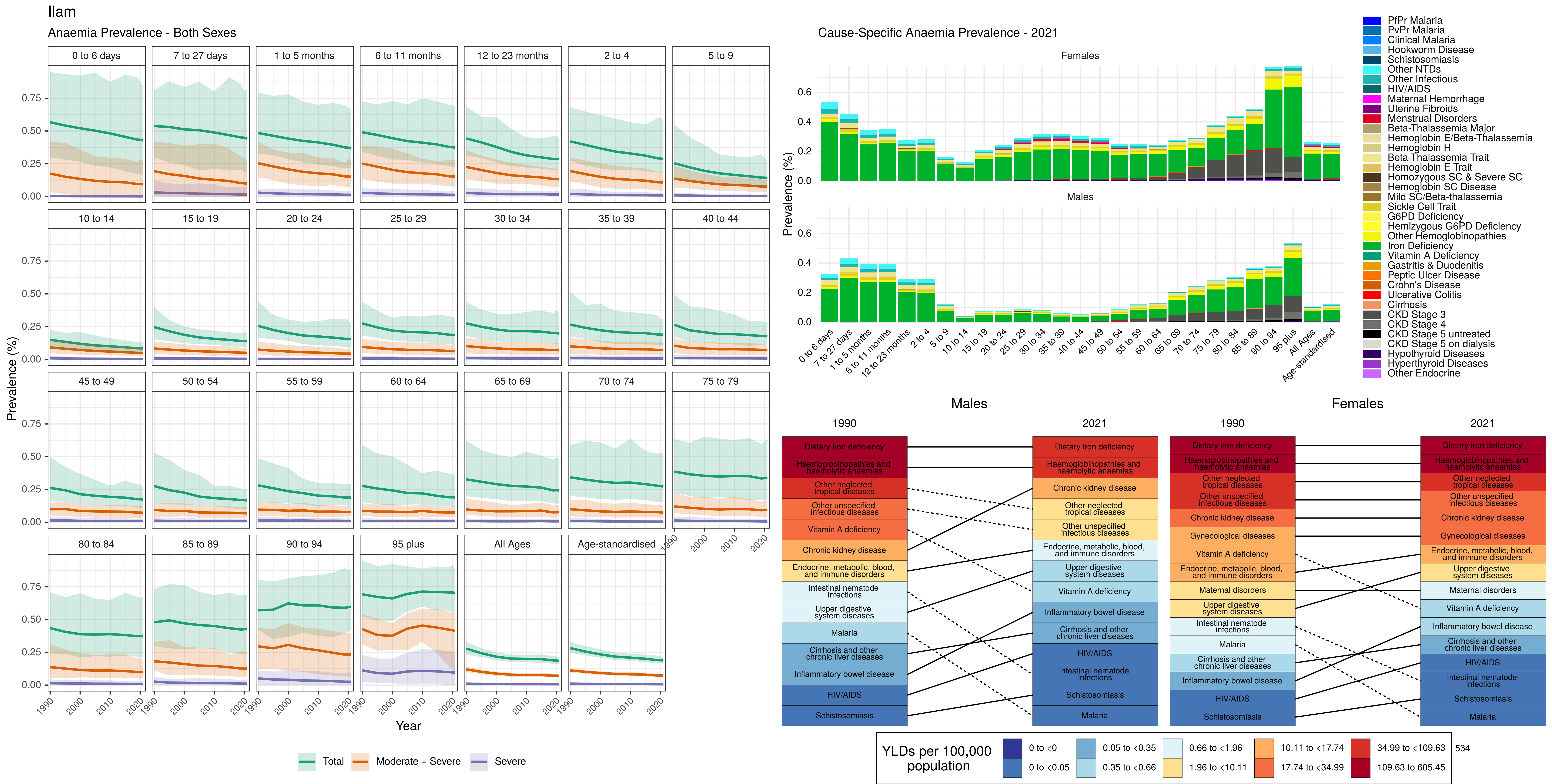

Ilam
Anaemia Prevalence - Both Sexes
0 to 6 days
7 to 27 days
1 to 5 months
6 to 11 months
12 to 23 months
2 to 4
5 to 9
10 to 14
15 to 19
20 to 24
25 to 29
30 to 34
35 to 39
40 to 44
45 to 49
50 to 54
55 to 59
60 to 64
65 to 69
70 to 74
75 to 79
80 to 84
85 to 89
90 to 94
95 plus
All Ages
Age-standardised
Prevalence (%)
Year
Total
Moderate + Severe
Severe
Cause-Specific Anaemia Prevalence - 2021
Females
Males
Prevalence (%)
PfPr Malaria
PvPr Malaria
Clinical Malaria
Hookworm Disease
Schistosomiasis
Other NTDs
Other Infectious
HIV/AIDS
Maternal Hemorrhage
Uterine Fibroids
Menstrual Disorders
Beta-Thalassemia Major
Hemoglobin E/Beta-Thalassemia
Hemoglobin H
Beta-Thalassemia Trait
Hemoglobin E Trait
Homozygous SC & Severe SC
Hemoglobin SC Disease
Mild SC/Beta-thalassemia
Sickle Cell Trait
G6PD Deficiency
Hemizygous G6PD Deficiency
Other Hemoglobinopathies
Iron Deficiency
Vitamin A Deficiency
Gastritis & Duodenitis
Peptic Ulcer Disease
Crohn's Disease
Ulcerative Colitis
Cirrhosis
CKD Stage 3
CKD Stage 4
CKD Stage 5 untreated
CKD Stage 5 on dialysis
Hypothyroid Diseases
Hyperthyroid Diseases
Other Endocrine
Males
Females
1990
2021
Dietary iron deficiency
Haemoglobinopathies and haemolytic anaemias
Other neglected tropical diseases
Other unspecified infectious diseases
Vitamin A deficiency
Chronic kidney disease
Endocrine, metabolic, blood, and immune disorders
Intestinal nematode infections
Upper digestive system diseases
Malaria
Cirrhosis and other chronic liver diseases
Inflammatory bowel disease
HIV/AIDS
Schistosomiasis
Gynecological diseases
Maternal disorders
YLDs per 100,000 population
0 to <0
0 to <0.05
0.05 to <0.35
0.35 to <0.66
0.66 to <1.96
1.96 to <10.11
10.11 to <17.74
17.74 to <34.99
34.99 to <109.63
109.63 to 605.45

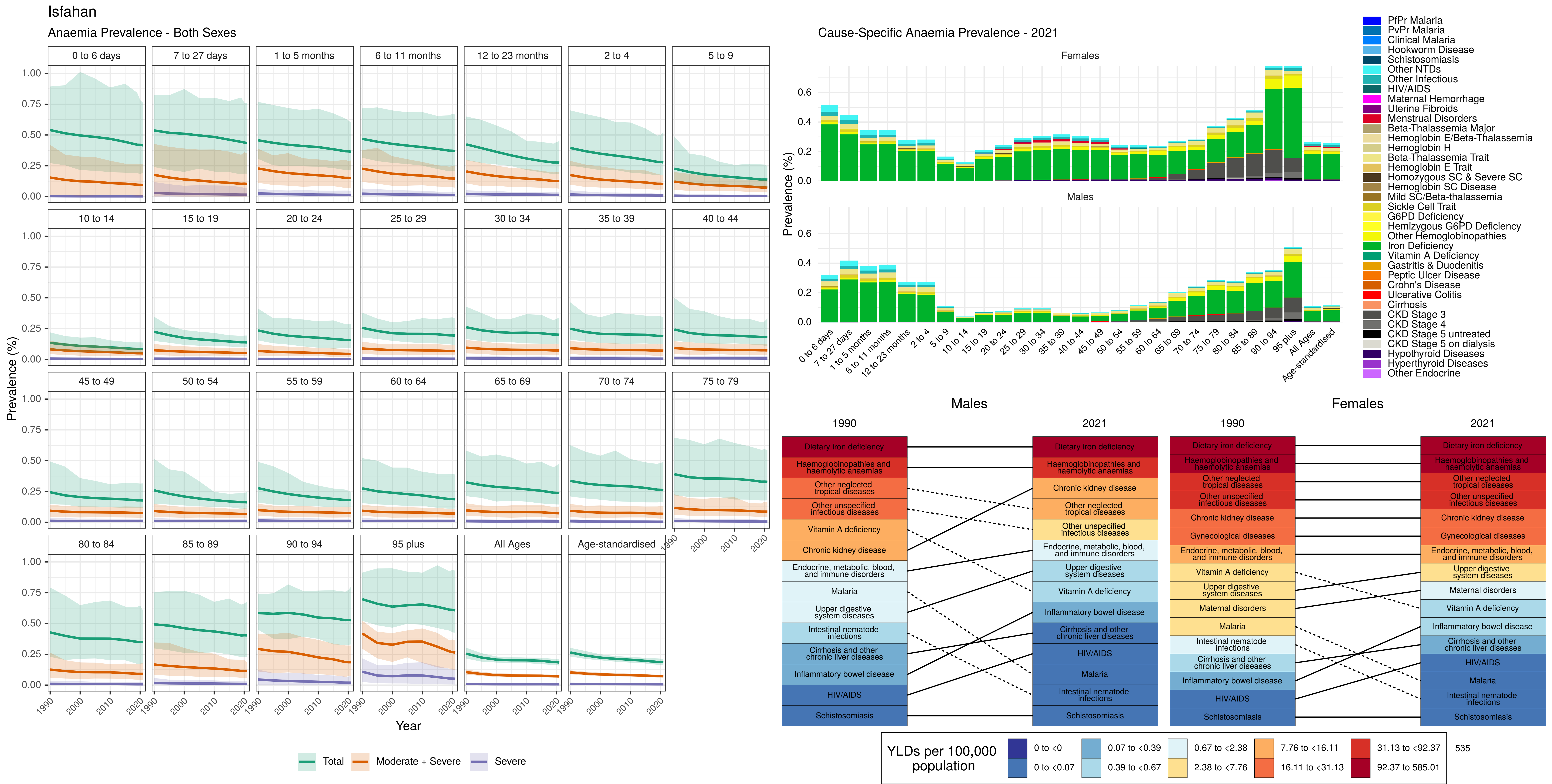

Isfahan
Anaemia Prevalence - Both Sexes
0 to 6 days
7 to 27 days
1 to 5 months
6 to 11 months
12 to 23 months
2 to 4
5 to 9
10 to 14
15 to 19
20 to 24
25 to 29
30 to 34
35 to 39
40 to 44
45 to 49
50 to 54
55 to 59
60 to 64
65 to 69
70 to 74
75 to 79
80 to 84
85 to 89
90 to 94
95 plus
All Ages
Age-standardised
Prevalence (%)
Year
Total
Moderate + Severe
Severe
Cause-Specific Anaemia Prevalence - 2021
Females
Males
PfPr Malaria
PvPr Malaria
Clinical Malaria
Hookworm Disease
Schistosomiasis
Other NTDs
Other Infectious
HIV/AIDS
Maternal Hemorrhage
Uterine Fibroids
Menstrual Disorders
Beta-Thalassemia Major
Hemoglobin E/Beta-Thalassemia
Hemoglobin H
Beta-Thalassemia Trait
Hemoglobin E Trait
Homozygous SC & Severe SC
Hemoglobin SC Disease
Mild SC/Beta-thalassemia
Sickle Cell Trait
G6PD Deficiency
Hemizygous G6PD Deficiency
Other Hemoglobinopathies
Iron Deficiency
Vitamin A Deficiency
Gastritis & Duodenitis
Peptic Ulcer Disease
Crohn's Disease
Ulcerative Colitis
Cirrhosis
CKD Stage 3
CKD Stage 4
CKD Stage 5 untreated
CKD Stage 5 on dialysis
Hypothyroid Diseases
Hyperthyroid Diseases
Other Endocrine
Males
1990
2021
Dietary iron deficiency
Haemoglobinopathies and haemolytic anaemias
Other neglected tropical diseases
Other unspecified infectious diseases
Vitamin A deficiency
Chronic kidney disease
Endocrine, metabolic, blood, and immune disorders
Malaria
Upper digestive system diseases
Intestinal nematode infections
Cirrhosis and other chronic liver diseases
Inflammatory bowel disease
HIV/AIDS
Schistosomiasis
Females
Gynecological diseases
Maternal disorders
YLDs per 100,000 population
0 to <0
0 to <0.07
0.07 to <0.39
0.39 to <0.67
0.67 to <2.38
2.38 to <7.76
7.76 to <16.11
16.11 to <31.13
31.13 to <92.37
92.37 to 585.01

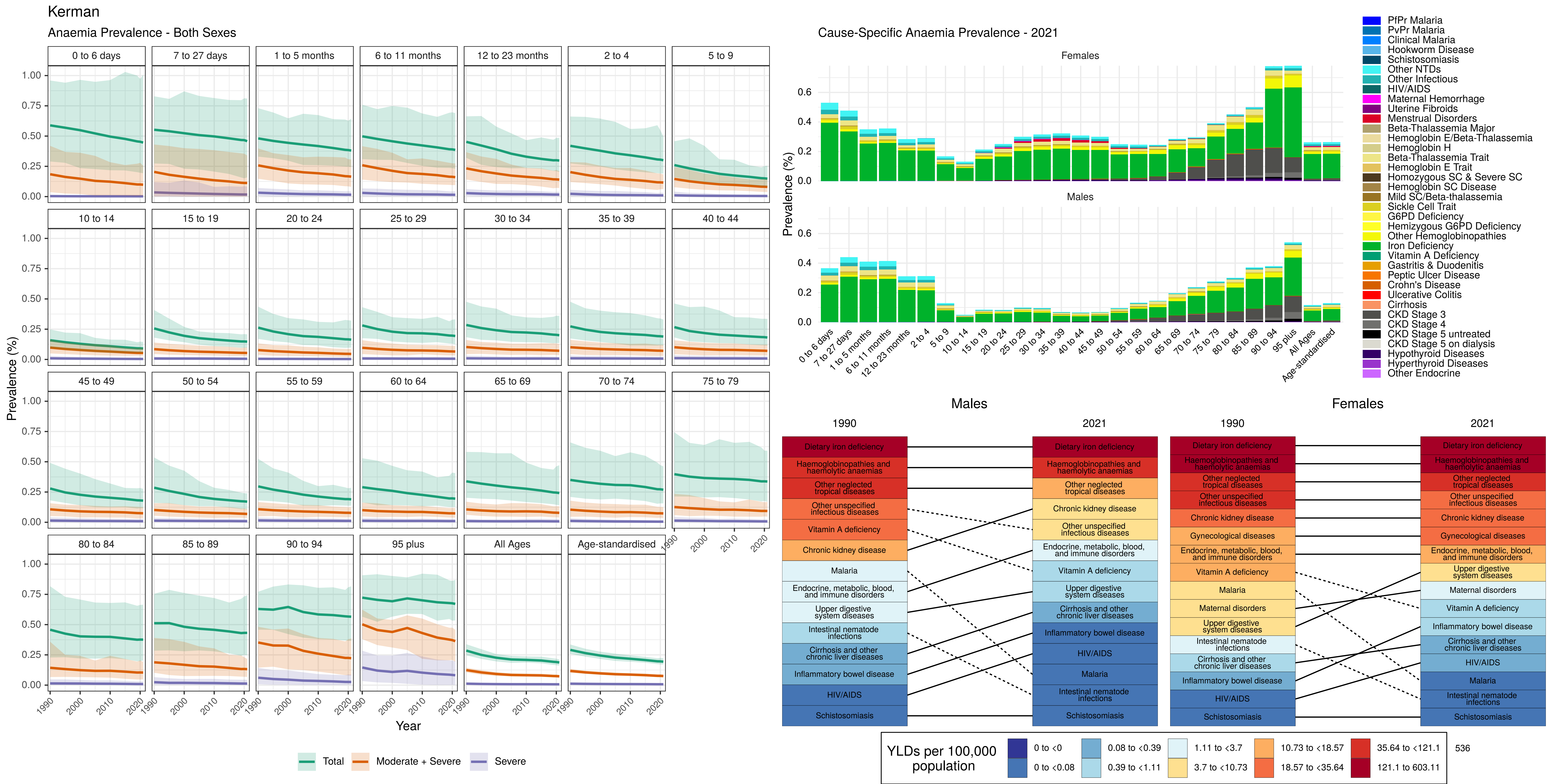
Kerman
Anaemia Prevalence - Both Sexes
0 to 6 days
7 to 27 days
1 to 5 months
6 to 11 months
12 to 23 months
2 to 4
5 to 9
10 to 14
15 to 19
20 to 24
25 to 29
30 to 34
35 to 39
40 to 44
45 to 49
50 to 54
55 to 59
60 to 64
65 to 69
70 to 74
75 to 79
80 to 84
85 to 89
90 to 94
95 plus
All Ages
Age-standardised
Prevalence (%)
Year
Total
Moderate + Severe
Severe
Cause-Specific Anaemia Prevalence - 2021
Females
Males
PfPr Malaria
PvPr Malaria
Clinical Malaria
Hookworm Disease
Schistosomiasis
Other NTDs
Other Infectious
HIV/AIDS
Maternal Hemorrhage
Uterine Fibroids
Menstrual Disorders
Beta-Thalassemia Major
Hemoglobin E/Beta-Thalassemia
Hemoglobin H
Beta-Thalassemia Trait
Hemoglobin E Trait
Homozygous SC & Severe SC
Hemoglobin SC Disease
Mild SC/Beta-thalassemia
Sickle Cell Trait
G6PD Deficiency
Hemizygous G6PD Deficiency
Other Hemoglobinopathies
Iron Deficiency
Vitamin A Deficiency
Gastritis & Duodenitis
Peptic Ulcer Disease
Crohn's Disease
Ulcerative Colitis
Cirrhosis
CKD Stage 3
CKD Stage 4
CKD Stage 5 untreated
CKD Stage 5 on dialysis
Hypothyroid Diseases
Hyperthyroid Diseases
Other Endocrine
Males
Females
1990
2021
Dietary iron deficiency
Haemoglobinopathies and haemolytic anaemias
Other neglected tropical diseases
Other unspecified infectious diseases
Vitamin A deficiency
Chronic kidney disease
Malaria
Endocrine, metabolic, blood, and immune disorders
Upper digestive system diseases
Intestinal nematode infections
Cirrhosis and other chronic liver diseases
Inflammatory bowel disease
HIV/AIDS
Schistosomiasis
Gynecological diseases
Maternal disorders
YLDs per 100,000 population
0 to <0
0 to <0.08
0.08 to <0.39
0.39 to <1.11
1.11 to <3.7
3.7 to <10.73
10.73 to <18.57
18.57 to <35.64
35.64 to <121.1
121.1 to 603.11

Kermanshah

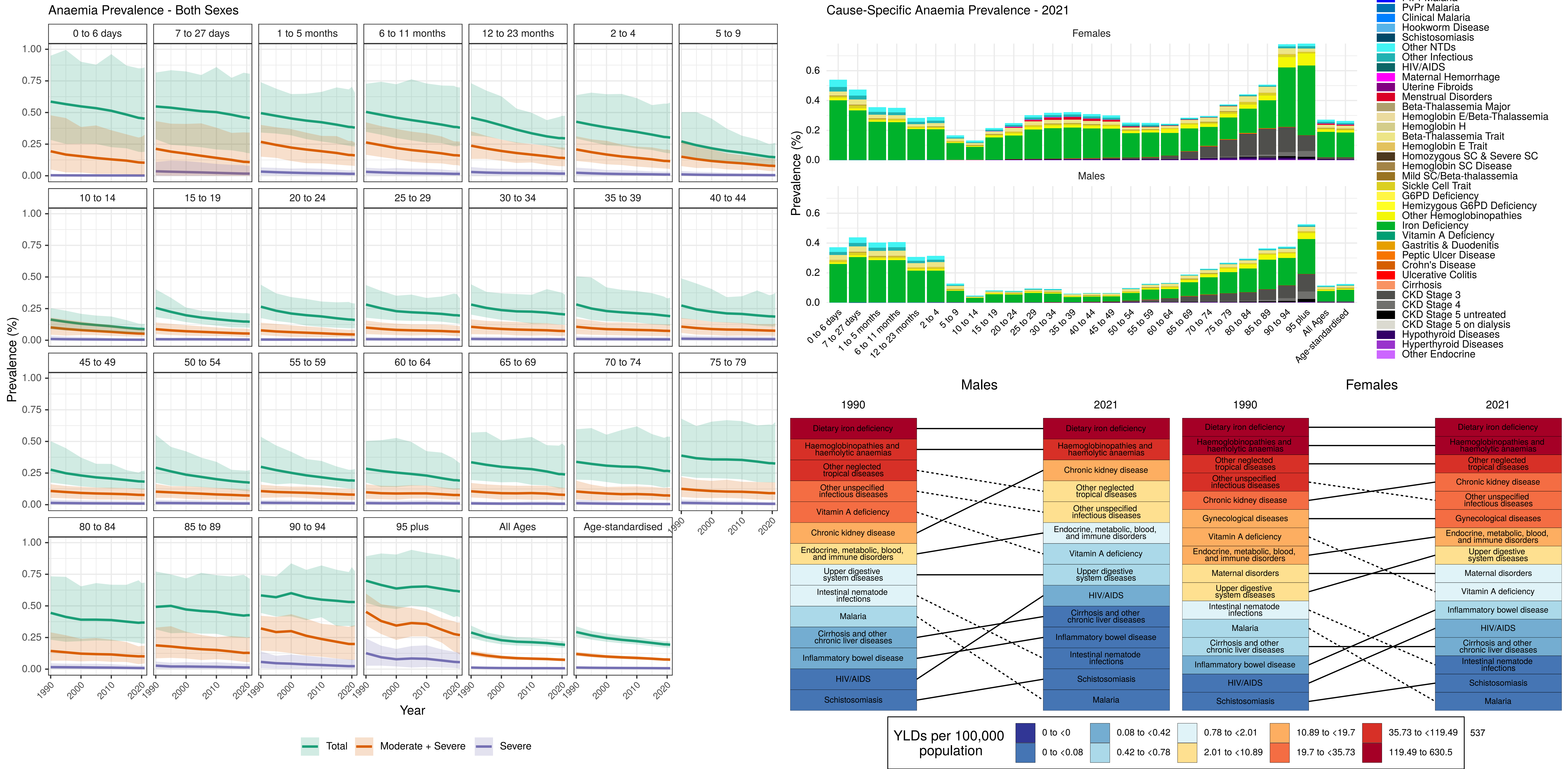

Anaemia Prevalence - Both Sexes
0 to 6 days
7 to 27 days
1 to 5 months
6 to 11 months
12 to 23 months
2 to 4
5 to 9
10 to 14
15 to 19
20 to 24
25 to 29
30 to 34
35 to 39
40 to 44
45 to 49
50 to 54
55 to 59
60 to 64
65 to 69
70 to 74
75 to 79
80 to 84
85 to 89
90 to 94
95 plus
All Ages
Age-standardised
Prevalence (%)
Year
Total
Moderate + Severe
Severe
Cause-Specific Anaemia Prevalence - 2021
Females
Males
PfPr Malaria
PvPr Malaria
Clinical Malaria
Hookworm Disease
Schistosomiasis
Other NTDs
Other Infectious
HIV/AIDS
Maternal Hemorrhage
Uterine Fibroids
Menstrual Disorders
Beta-Thalassemia Major
Hemoglobin E/Beta-Thalassemia
Hemoglobin H
Beta-Thalassemia Trait
Hemoglobin E Trait
Homozygous SC & Severe SC
Hemoglobin SC Disease
Mild SC/Beta-thalassemia
Sickle Cell Trait
G6PD Deficiency
Hemizygous G6PD Deficiency
Other Hemoglobinopathies
Iron Deficiency
Vitamin A Deficiency
Gastritis & Duodenitis
Peptic Ulcer Disease
Crohn's Disease
Ulcerative Colitis
Cirrhosis
CKD Stage 3
CKD Stage 4
CKD Stage 5 untreated
CKD Stage 5 on dialysis
Hypothyroid Diseases
Hyperthyroid Diseases
Other Endocrine
Males
Females
1990
2021
Males 1990: Dietary iron deficiency; Haemoglobinopathies and haemolytic anaemias; Other neglected tropical diseases; Other unspecified infectious diseases; Vitamin A deficiency; Chronic kidney disease; Endocrine, metabolic, blood, and immune disorders; Upper digestive system diseases; Intestinal nematode infections; Malaria; Cirrhosis and other chronic liver diseases; Inflammatory bowel disease; HIV/AIDS; Schistosomiasis
Males 2021: Dietary iron deficiency; Haemoglobinopathies and haemolytic anaemias; Chronic kidney disease; Other neglected tropical diseases; Other unspecified infectious diseases; Endocrine, metabolic, blood, and immune disorders; Vitamin A deficiency; Upper digestive system diseases; HIV/AIDS; Cirrhosis and other chronic liver diseases; Inflammatory bowel disease; Intestinal nematode infections; Schistosomiasis; Malaria
Females 1990: Dietary iron deficiency; Haemoglobinopathies and haemolytic anaemias; Other neglected tropical diseases; Other unspecified infectious diseases; Chronic kidney disease; Gynecological diseases; Vitamin A deficiency; Endocrine, metabolic, blood, and immune disorders; Maternal disorders; Upper digestive system diseases; Intestinal nematode infections; Malaria; Cirrhosis and other chronic liver diseases; Inflammatory bowel disease; HIV/AIDS; Schistosomiasis
Females 2021: Dietary iron deficiency; Haemoglobinopathies and haemolytic anaemias; Other neglected tropical diseases; Chronic kidney disease; Other unspecified infectious diseases; Gynecological diseases; Endocrine, metabolic, blood, and immune disorders; Upper digestive system diseases; Maternal disorders; Vitamin A deficiency; Inflammatory bowel disease; HIV/AIDS; Cirrhosis and other chronic liver diseases; Intestinal nematode infections; Schistosomiasis; Malaria
YLDs per 100,000 population
0 to <0
0 to <0.08
0.08 to <0.42
0.42 to <0.78
0.78 to <2.01
2.01 to <10.89
10.89 to <19.7
19.7 to <35.73
35.73 to <119.49
119.49 to 630.5

Khorasan-e-Razavi

Anaemia Prevalence - Both Sexes

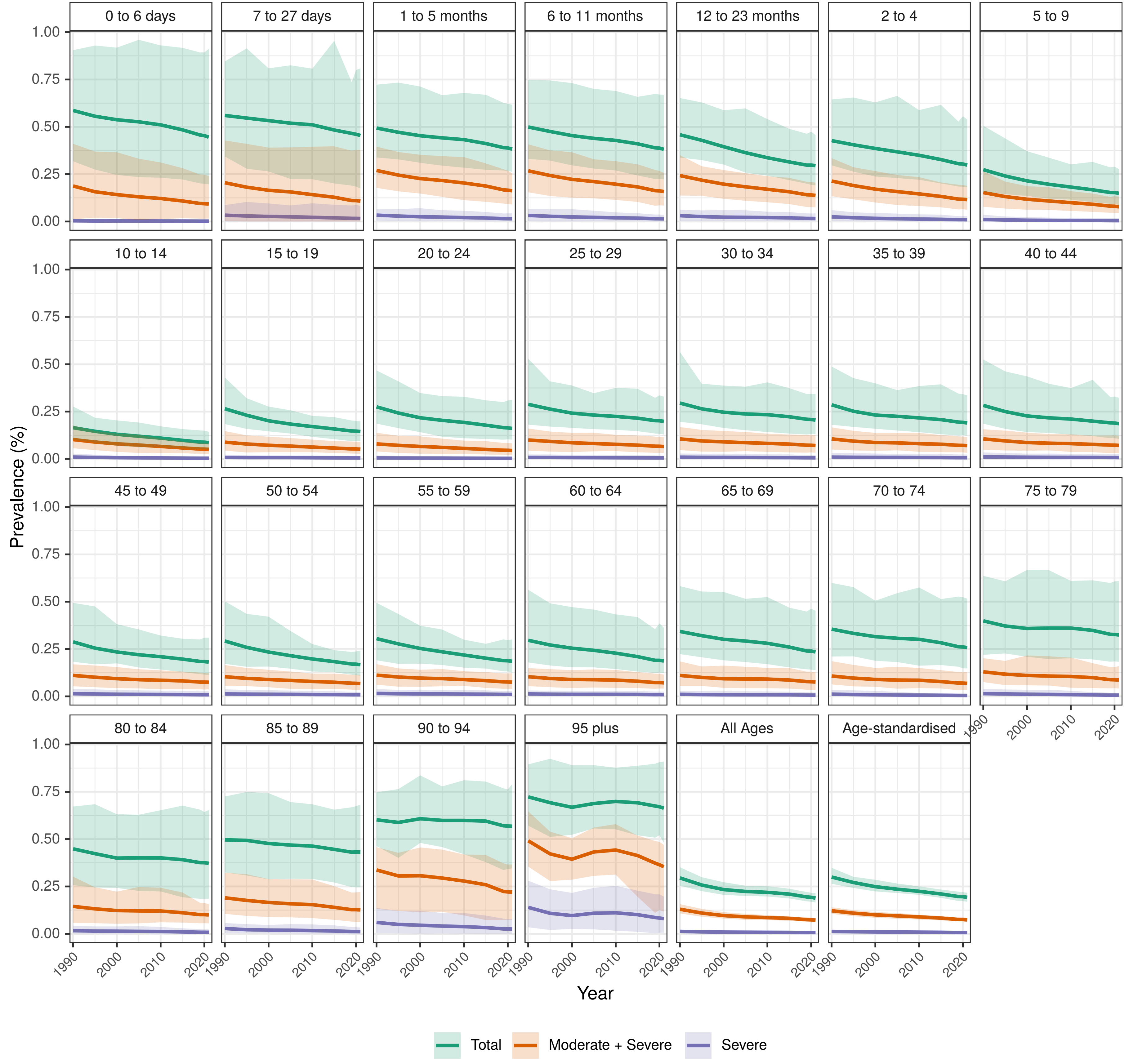

Cause-Specific Anaemia Prevalence - 2021

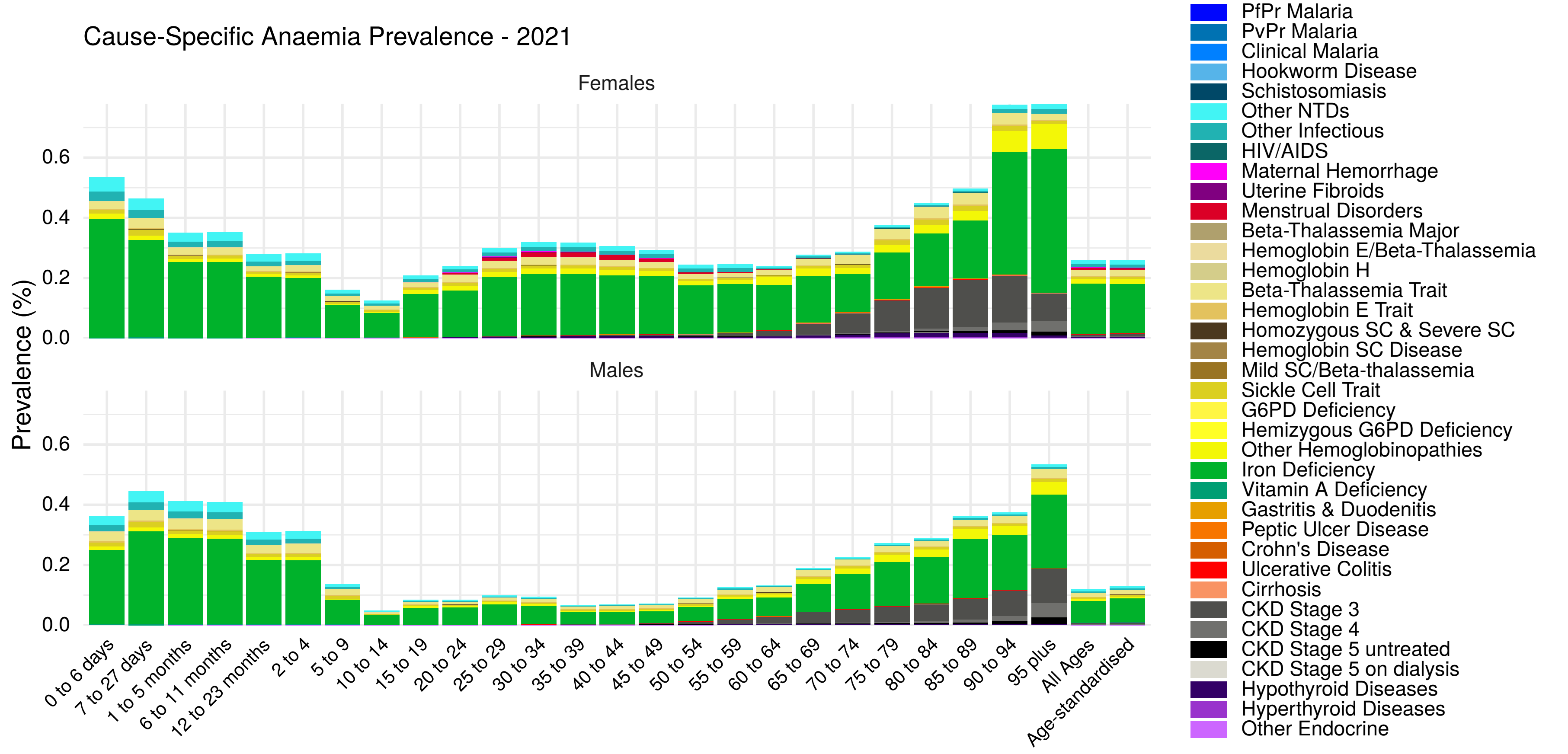

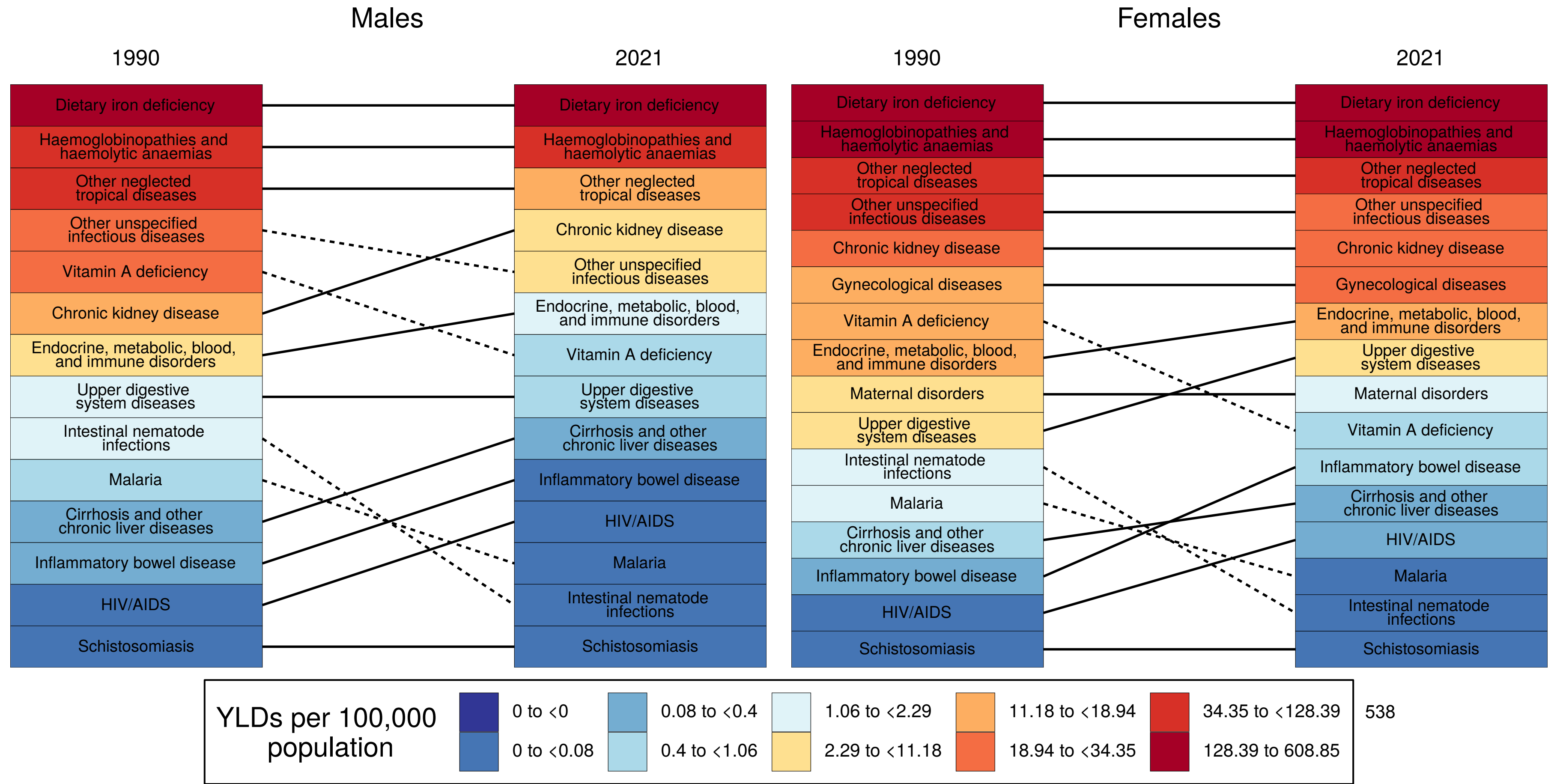

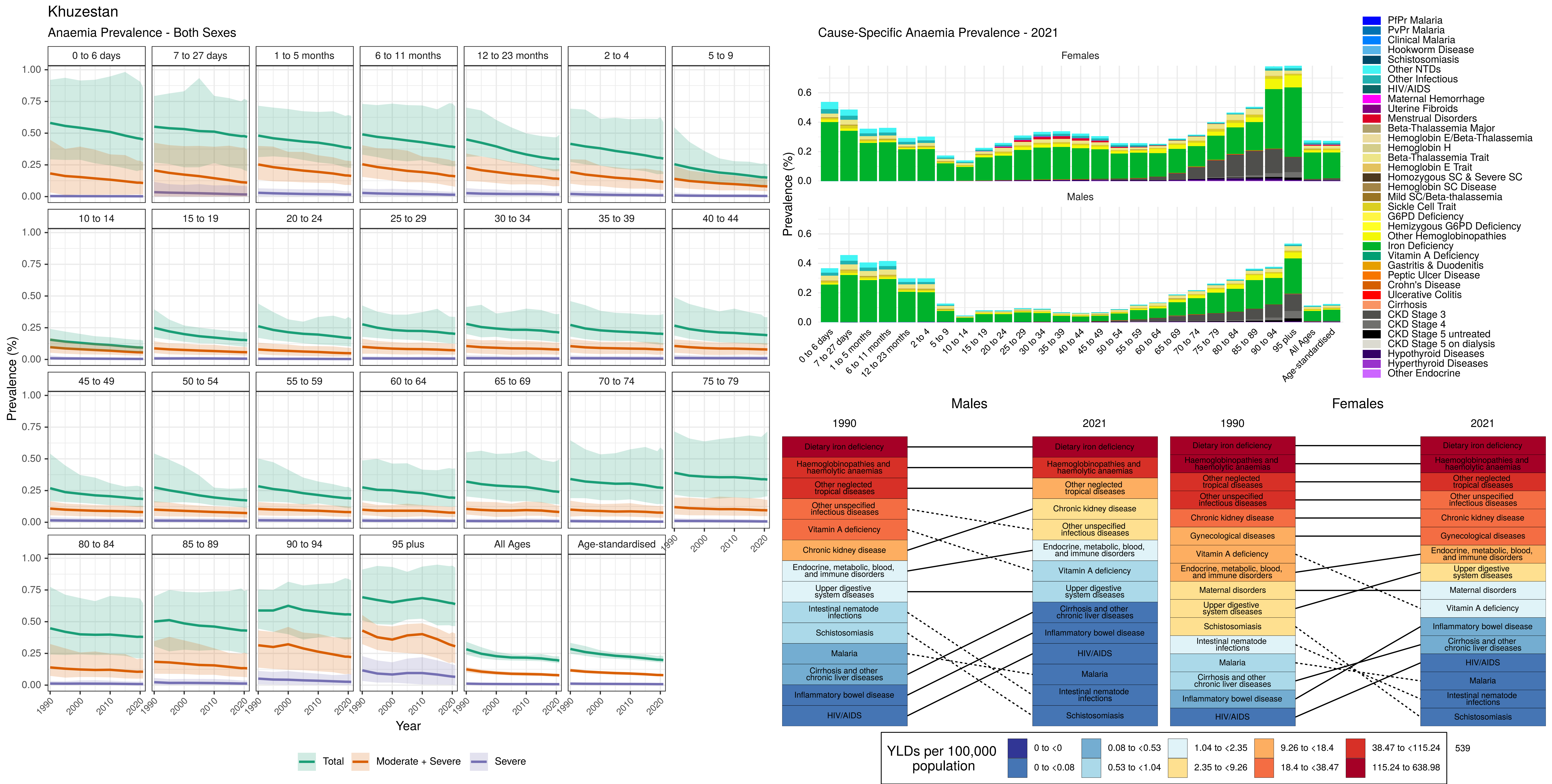
Khuzestan
Anaemia Prevalence - Both Sexes
0 to 6 days
7 to 27 days
1 to 5 months
6 to 11 months
12 to 23 months
2 to 4
5 to 9
10 to 14
15 to 19
20 to 24
25 to 29
30 to 34
35 to 39
40 to 44
45 to 49
50 to 54
55 to 59
60 to 64
65 to 69
70 to 74
75 to 79
80 to 84
85 to 89
90 to 94
95 plus
All Ages
Age-standardised
Prevalence (%)
Year
Total
Moderate + Severe
Severe
Cause-Specific Anaemia Prevalence - 2021
Females
Males
Prevalence (%)
PfPr Malaria
PvPr Malaria
Clinical Malaria
Hookworm Disease
Schistosomiasis
Other NTDs
Other Infectious
HIV/AIDS
Maternal Hemorrhage
Uterine Fibroids
Menstrual Disorders
Beta-Thalassemia Major
Hemoglobin E/Beta-Thalassemia
Hemoglobin H
Beta-Thalassemia Trait
Hemoglobin E Trait
Homozygous SC & Severe SC
Hemoglobin SC Disease
Mild SC/Beta-thalassemia
Sickle Cell Trait
G6PD Deficiency
Hemizygous G6PD Deficiency
Other Hemoglobinopathies
Iron Deficiency
Vitamin A Deficiency
Gastritis & Duodenitis
Peptic Ulcer Disease
Crohn's Disease
Ulcerative Colitis
Cirrhosis
CKD Stage 3
CKD Stage 4
CKD Stage 5 untreated
CKD Stage 5 on dialysis
Hypothyroid Diseases
Hyperthyroid Diseases
Other Endocrine
Males
1990
2021
Dietary iron deficiency
Haemoglobinopathies and haemolytic anaemias
Other neglected tropical diseases
Other unspecified infectious diseases
Vitamin A deficiency
Chronic kidney disease
Endocrine, metabolic, blood, and immune disorders
Upper digestive system diseases
Intestinal nematode infections
Schistosomiasis
Malaria
Cirrhosis and other chronic liver diseases
Inflammatory bowel disease
HIV/AIDS
Females
1990
2021
Dietary iron deficiency
Haemoglobinopathies and haemolytic anaemias
Other neglected tropical diseases
Other unspecified infectious diseases
Chronic kidney disease
Gynecological diseases
Vitamin A deficiency
Endocrine, metabolic, blood, and immune disorders
Maternal disorders
Upper digestive system diseases
Schistosomiasis
Intestinal nematode infections
Malaria
Cirrhosis and other chronic liver diseases
Inflammatory bowel disease
HIV/AIDS
YLDs per 100,000 population
0 to <0
0 to <0.08
0.08 to <0.53
0.53 to <1.04
1.04 to <2.35
2.35 to <9.26
9.26 to <18.4
18.4 to <38.47
38.47 to <115.24
115.24 to 638.98
539

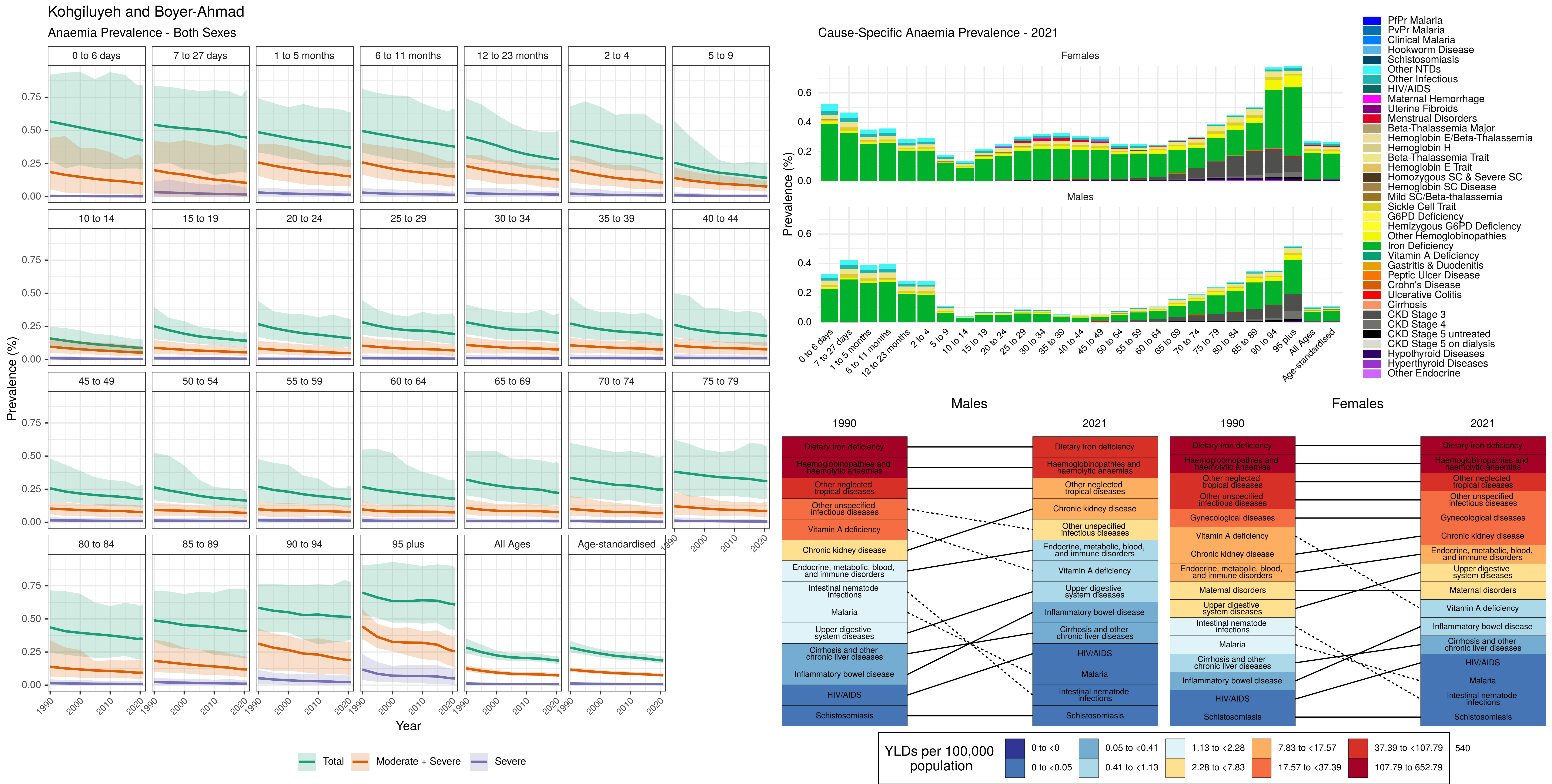

Kohgiluyeh and Boyer-Ahmad
Anaemia Prevalence - Both Sexes
0 to 6 days
7 to 27 days
1 to 5 months
6 to 11 months
12 to 23 months
2 to 4
5 to 9
10 to 14
15 to 19
20 to 24
25 to 29
30 to 34
35 to 39
40 to 44
45 to 49
50 to 54
55 to 59
60 to 64
65 to 69
70 to 74
75 to 79
80 to 84
85 to 89
90 to 94
95 plus
All Ages
Age-standardised
Prevalence (%)
Year
Total
Moderate + Severe
Severe
Cause-Specific Anaemia Prevalence - 2021
Females
Males
PfPr Malaria
PvPr Malaria
Clinical Malaria
Hookworm Disease
Schistosomiasis
Other NTDs
Other Infectious
HIV/AIDS
Maternal Hemorrhage
Uterine Fibroids
Menstrual Disorders
Beta-Thalassemia Major
Hemoglobin E/Beta-Thalassemia
Hemoglobin H
Beta-Thalassemia Trait
Hemoglobin E Trait
Homozygous SC & Severe SC
Hemoglobin SC Disease
Mild SC/Beta-thalassemia
Sickle Cell Trait
G6PD Deficiency
Hemizygous G6PD Deficiency
Other Hemoglobinopathies
Iron Deficiency
Vitamin A Deficiency
Gastritis & Duodenitis
Peptic Ulcer Disease
Crohn's Disease
Ulcerative Colitis
Cirrhosis
CKD Stage 3
CKD Stage 4
CKD Stage 5 untreated
CKD Stage 5 on dialysis
Hypothyroid Diseases
Hyperthyroid Diseases
Other Endocrine
Males
1990
2021
Dietary iron deficiency
Haemoglobinopathies and haemolytic anaemias
Other neglected tropical diseases
Other unspecified infectious diseases
Vitamin A deficiency
Chronic kidney disease
Endocrine, metabolic, blood, and immune disorders
Intestinal nematode infections
Malaria
Upper digestive system diseases
Cirrhosis and other chronic liver diseases
Inflammatory bowel disease
HIV/AIDS
Schistosomiasis
Females
1990
2021
Dietary iron deficiency
Haemoglobinopathies and haemolytic anaemias
Other neglected tropical diseases
Other unspecified infectious diseases
Gynecological diseases
Vitamin A deficiency
Chronic kidney disease
Endocrine, metabolic, blood, and immune disorders
Maternal disorders
Upper digestive system diseases
Intestinal nematode infections
Malaria
Cirrhosis and other chronic liver diseases
Inflammatory bowel disease
HIV/AIDS
Schistosomiasis
YLDs per 100,000 population
0 to <0
0 to <0.05
0.05 to <0.41
0.41 to <1.13
1.13 to <2.28
2.28 to <7.83
7.83 to <17.57
17.57 to <37.39
37.39 to <107.79
107.79 to 652.79
540

Kurdistan

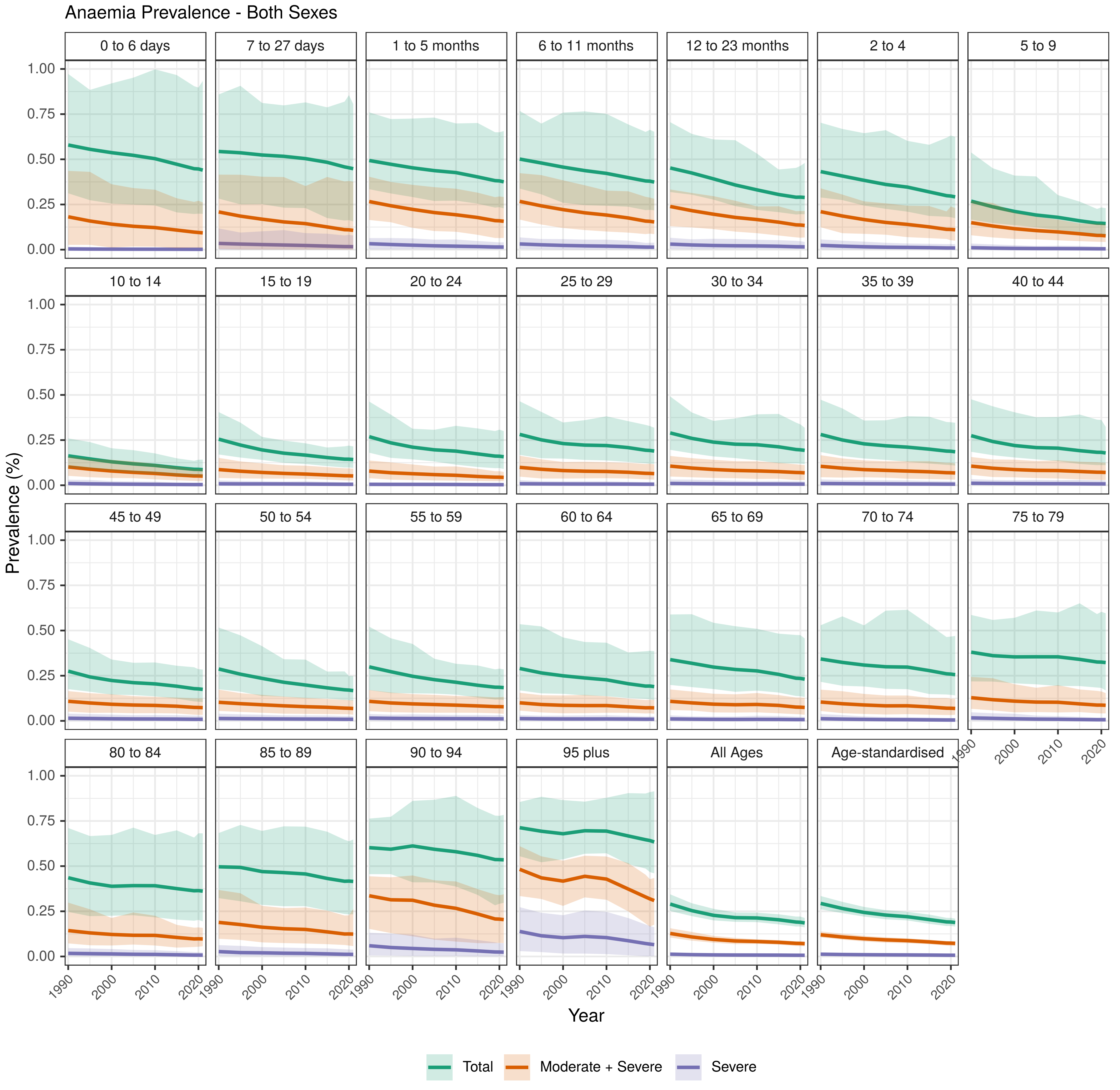

Anaemia Prevalence - Both Sexes
0 to 6 days
7 to 27 days
1 to 5 months
6 to 11 months
12 to 23 months
2 to 4
5 to 9
10 to 14
15 to 19
20 to 24
25 to 29
30 to 34
35 to 39
40 to 44
45 to 49
50 to 54
55 to 59
60 to 64
65 to 69
70 to 74
75 to 79
80 to 84
85 to 89
90 to 94
95 plus
All Ages
Age-standardised
1.00
0.75
0.50
0.25
0.00
Prevalence (%)
1990
2000
2010
2020
Year
Total
Moderate + Severe
Severe

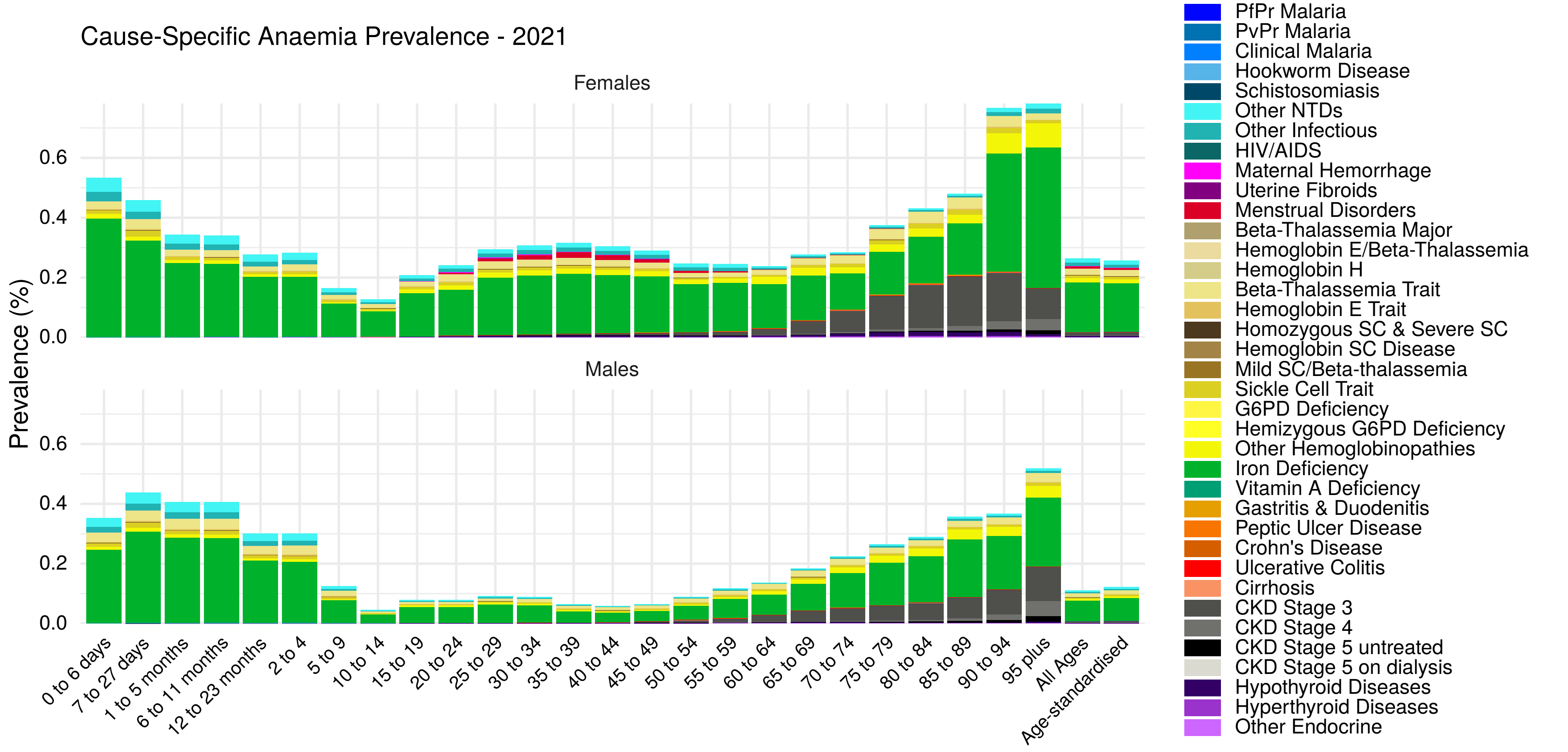

Cause-Specific Anaemia Prevalence - 2021
Females
Males
Prevalence (%)
0.6
0.4
0.2
0.0
0 to 6 days
7 to 27 days
1 to 5 months
6 to 11 months
12 to 23 months
2 to 4
5 to 9
10 to 14
15 to 19
20 to 24
25 to 29
30 to 34
35 to 39
40 to 44
45 to 49
50 to 54
55 to 59
60 to 64
65 to 69
70 to 74
75 to 79
80 to 84
85 to 89
90 to 94
95 plus
All Ages
Age-standardised
PfPr Malaria
PvPr Malaria
Clinical Malaria
Hookworm Disease
Schistosomiasis
Other NTDs
Other Infectious
HIV/AIDS
Maternal Hemorrhage
Uterine Fibroids
Menstrual Disorders
Beta-Thalassemia Major
Hemoglobin E/Beta-Thalassemia
Hemoglobin H
Beta-Thalassemia Trait
Hemoglobin E Trait
Homozygous SC & Severe SC
Hemoglobin SC Disease
Mild SC/Beta-thalassemia
Sickle Cell Trait
G6PD Deficiency
Hemizygous G6PD Deficiency
Other Hemoglobinopathies
Iron Deficiency
Vitamin A Deficiency
Gastritis & Duodenitis
Peptic Ulcer Disease
Crohn's Disease
Ulcerative Colitis
Cirrhosis
CKD Stage 3
CKD Stage 4
CKD Stage 5 untreated
CKD Stage 5 on dialysis
Hypothyroid Diseases
Hyperthyroid Diseases
Other Endocrine

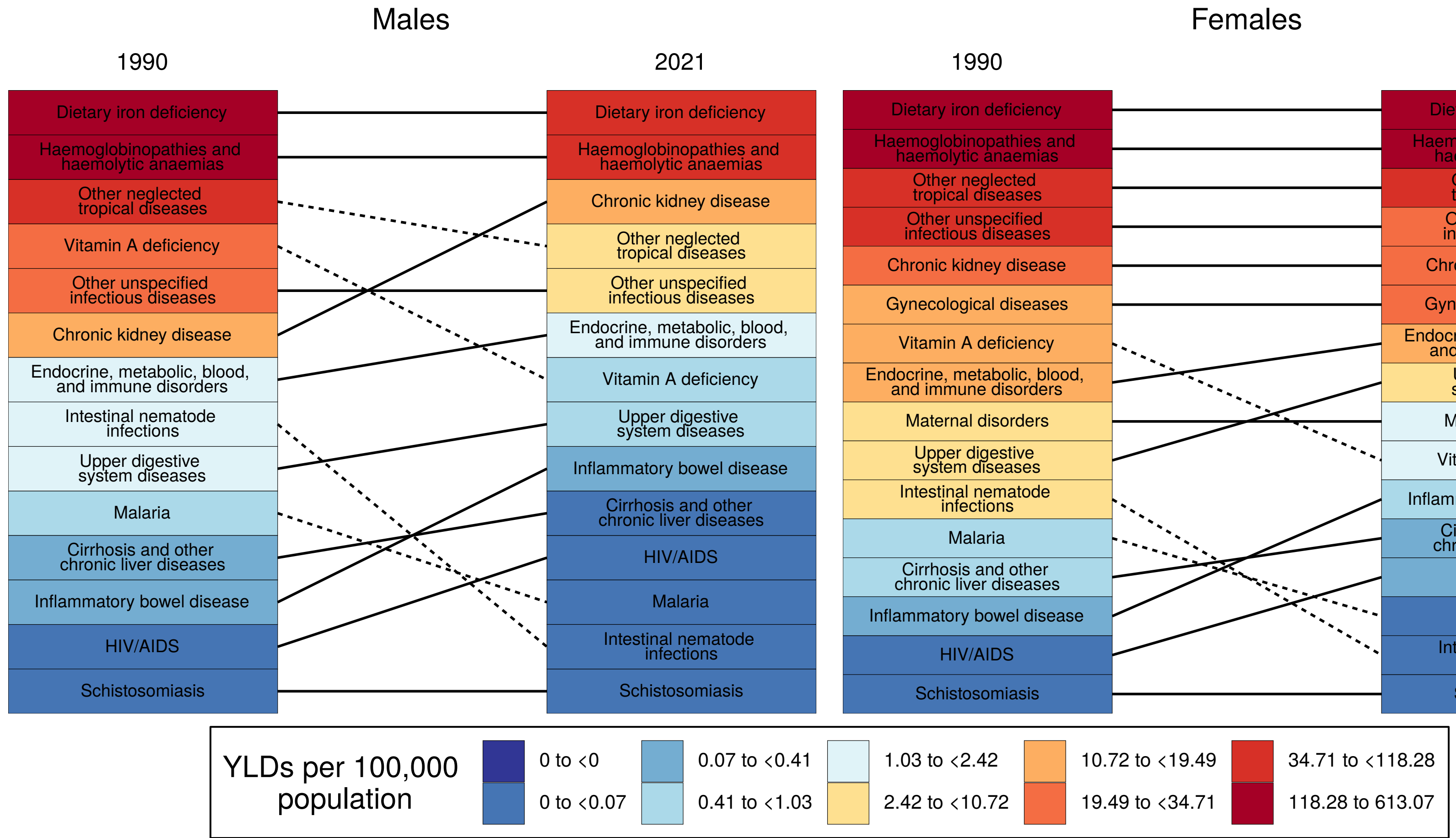

Males
Females
1990
2021
Dietary iron deficiency
Haemoglobinopathies and haemolytic anaemias
Other neglected tropical diseases
Vitamin A deficiency
Other unspecified infectious diseases
Chronic kidney disease
Endocrine, metabolic, blood, and immune disorders
Intestinal nematode infections
Upper digestive system diseases
Malaria
Cirrhosis and other chronic liver diseases
Inflammatory bowel disease
HIV/AIDS
Schistosomiasis
Gynecological diseases
Maternal disorders
YLDs per 100,000 population
0 to <0
0 to <0.07
0.07 to <0.41
0.41 to <1.03
1.03 to <2.42
2.42 to <10.72
10.72 to <19.49
19.49 to <34.71
34.71 to <118.28
118.28 to 613.07

Lorestan

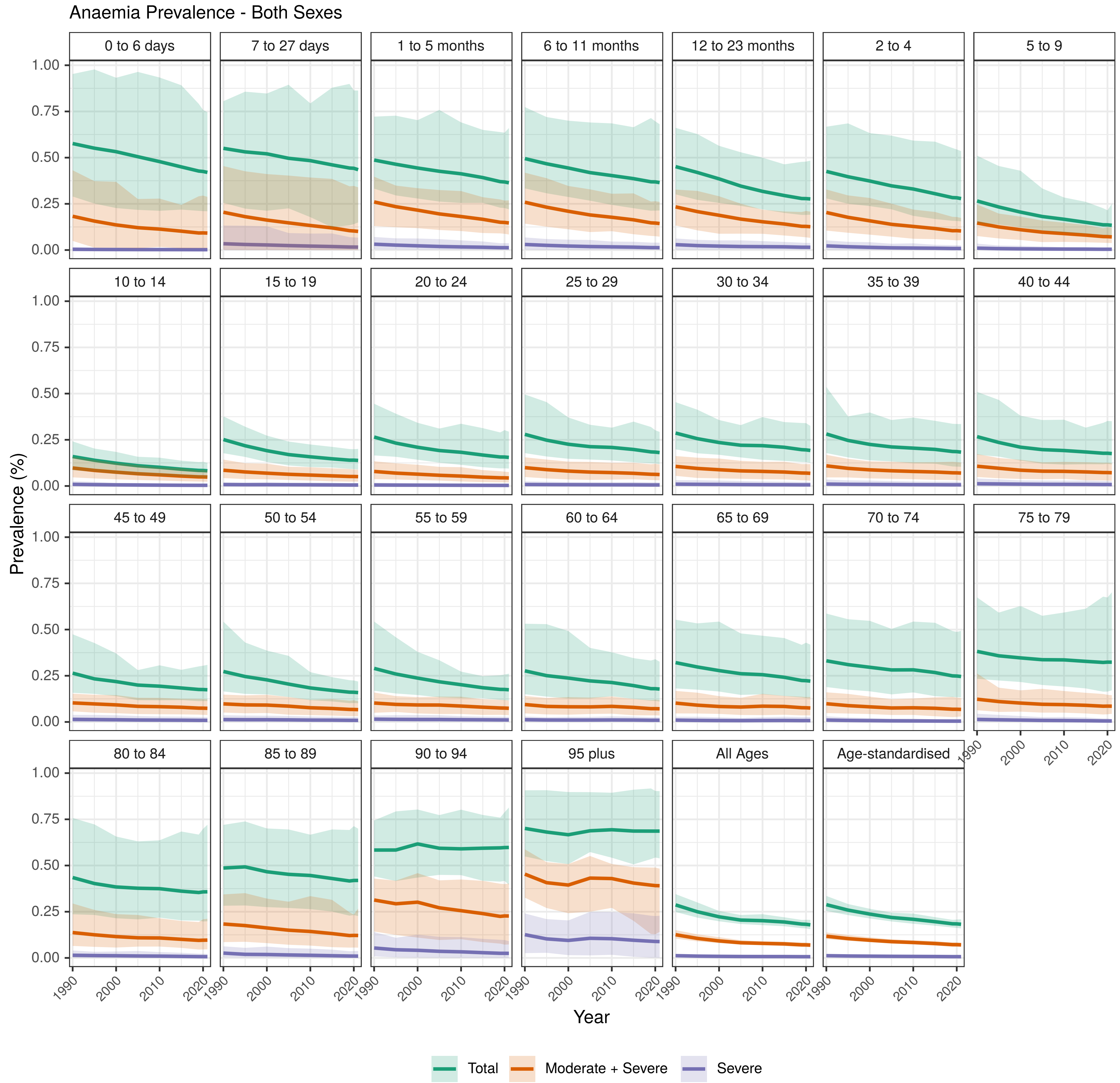

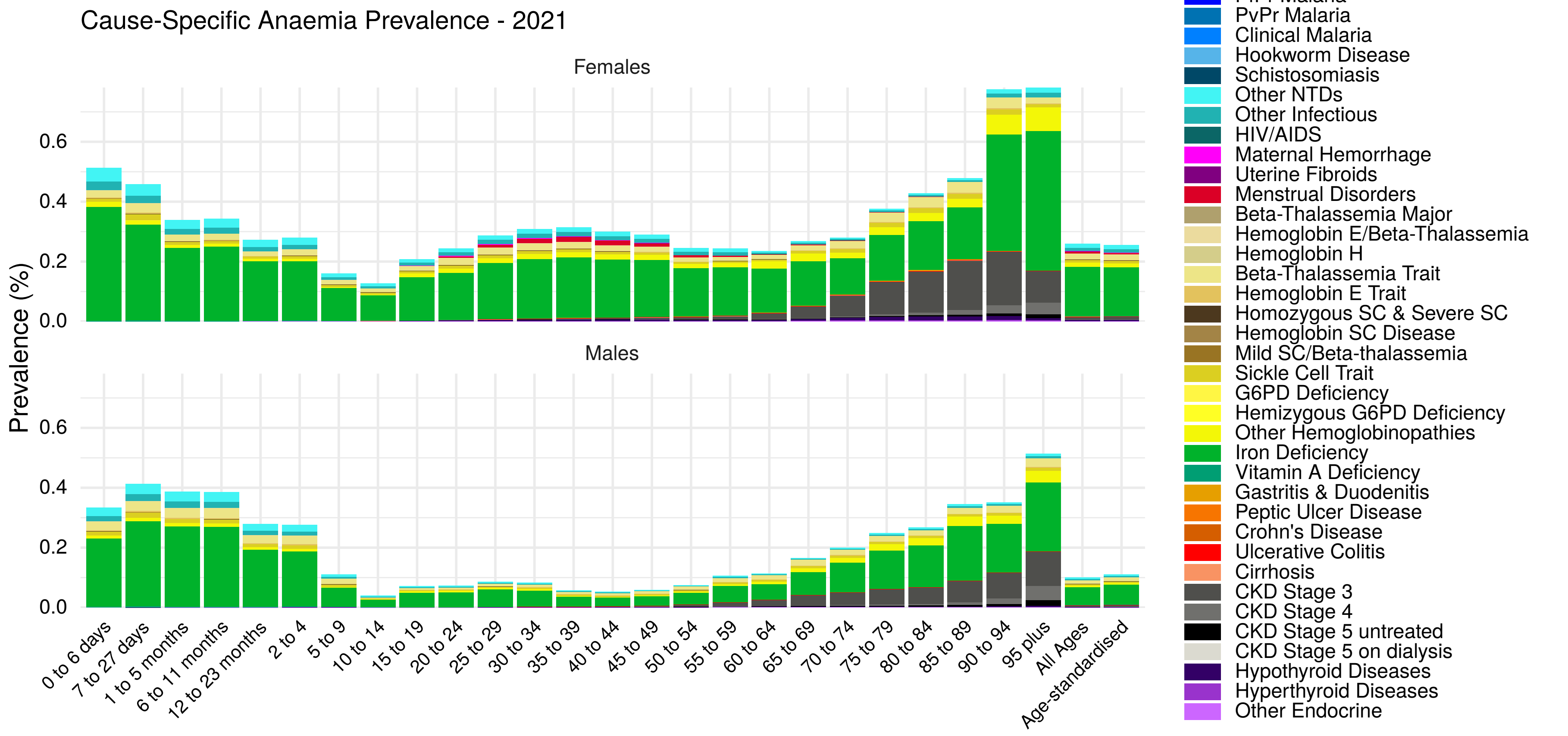

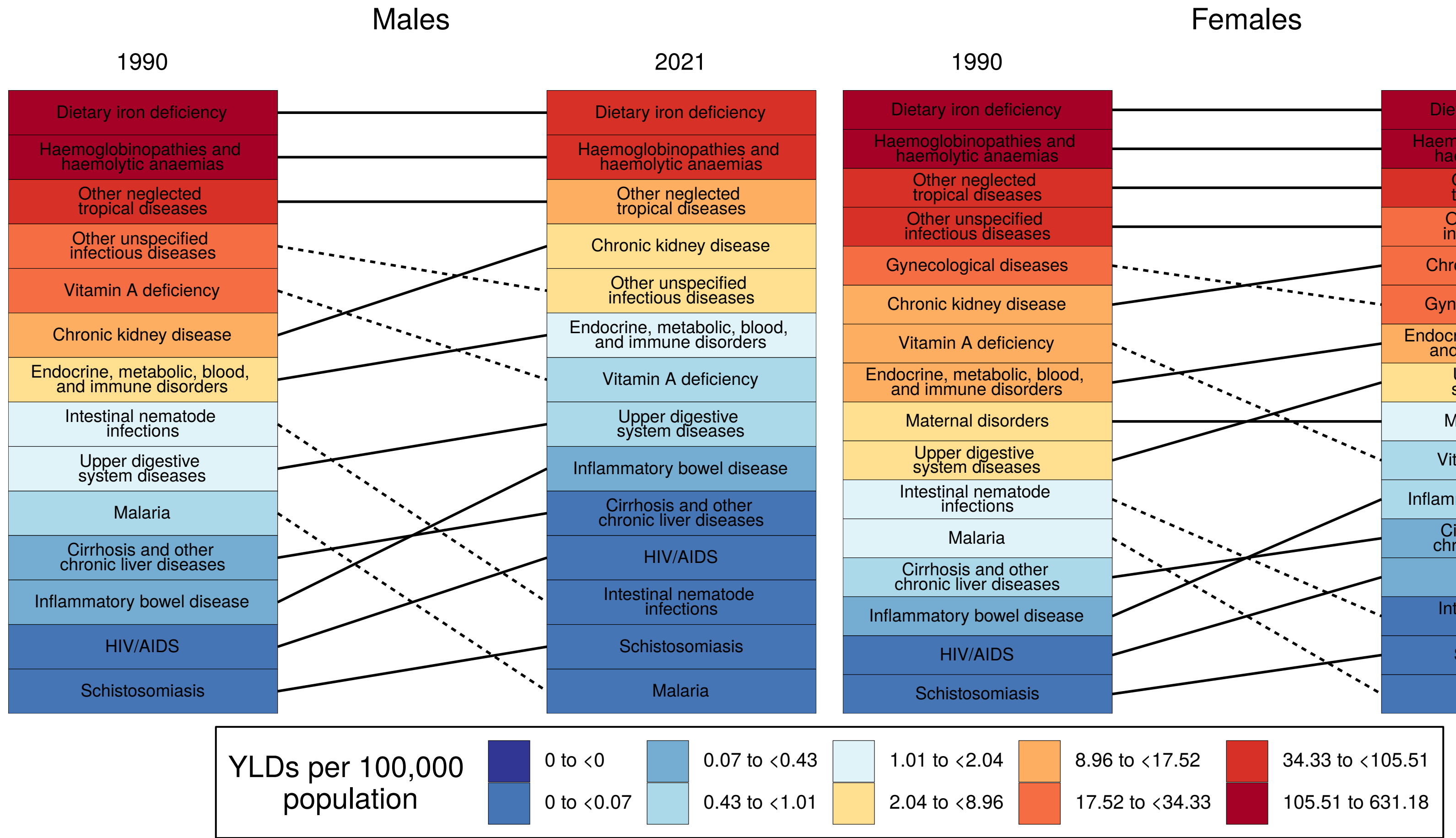

542

Markazi

Anaemia Prevalence - Both Sexes

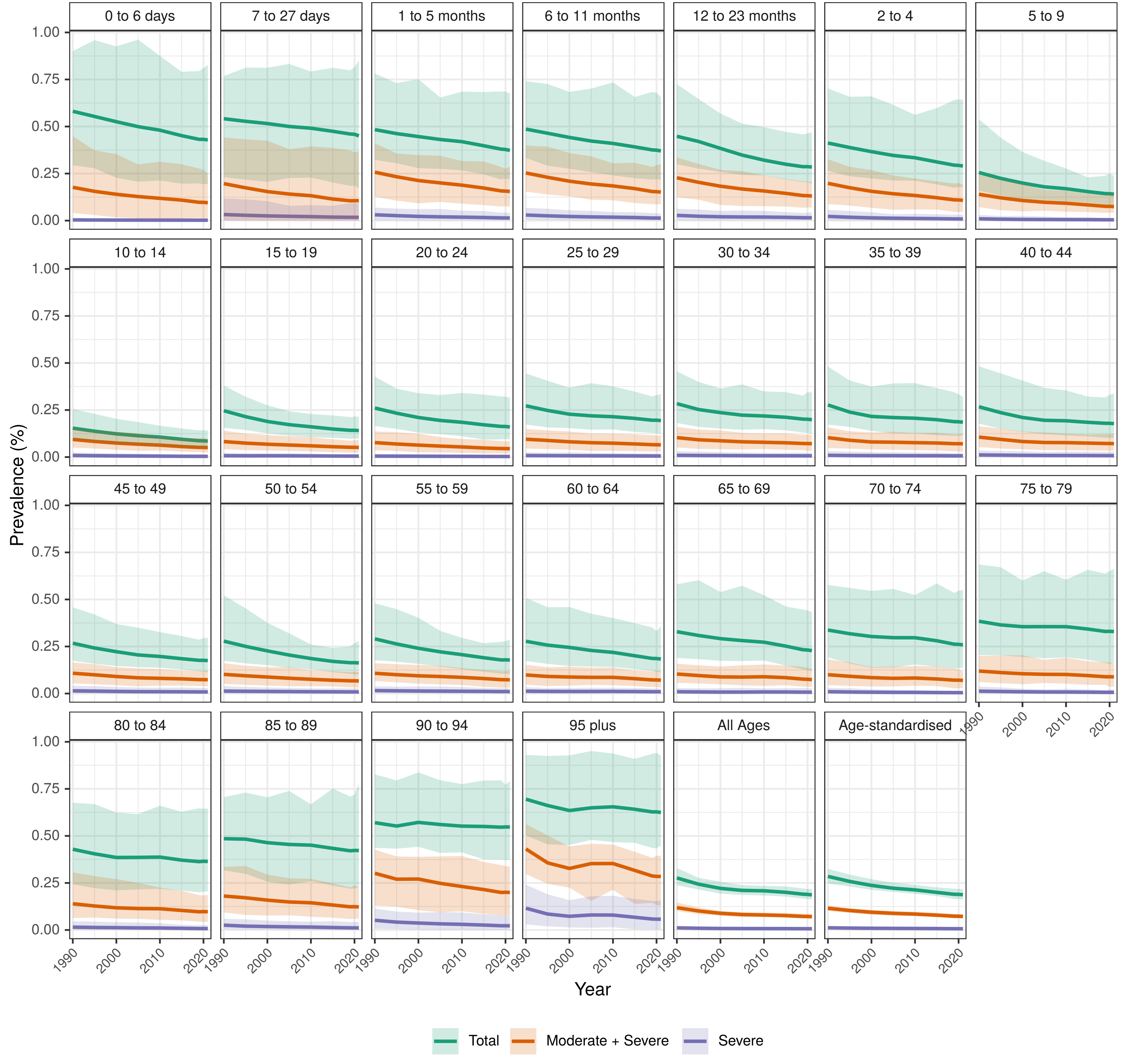

Cause-Specific Anaemia Prevalence - 2021

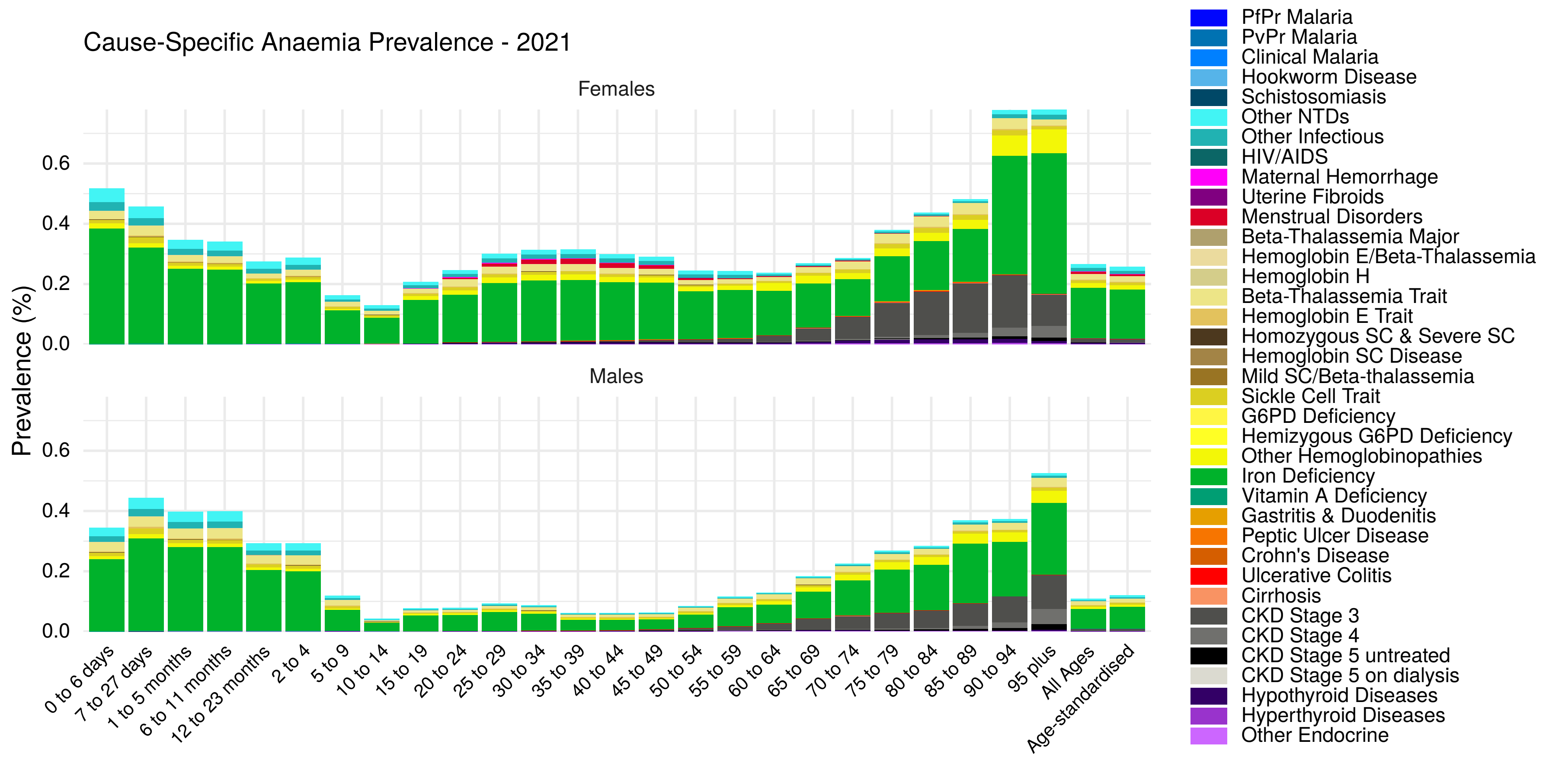

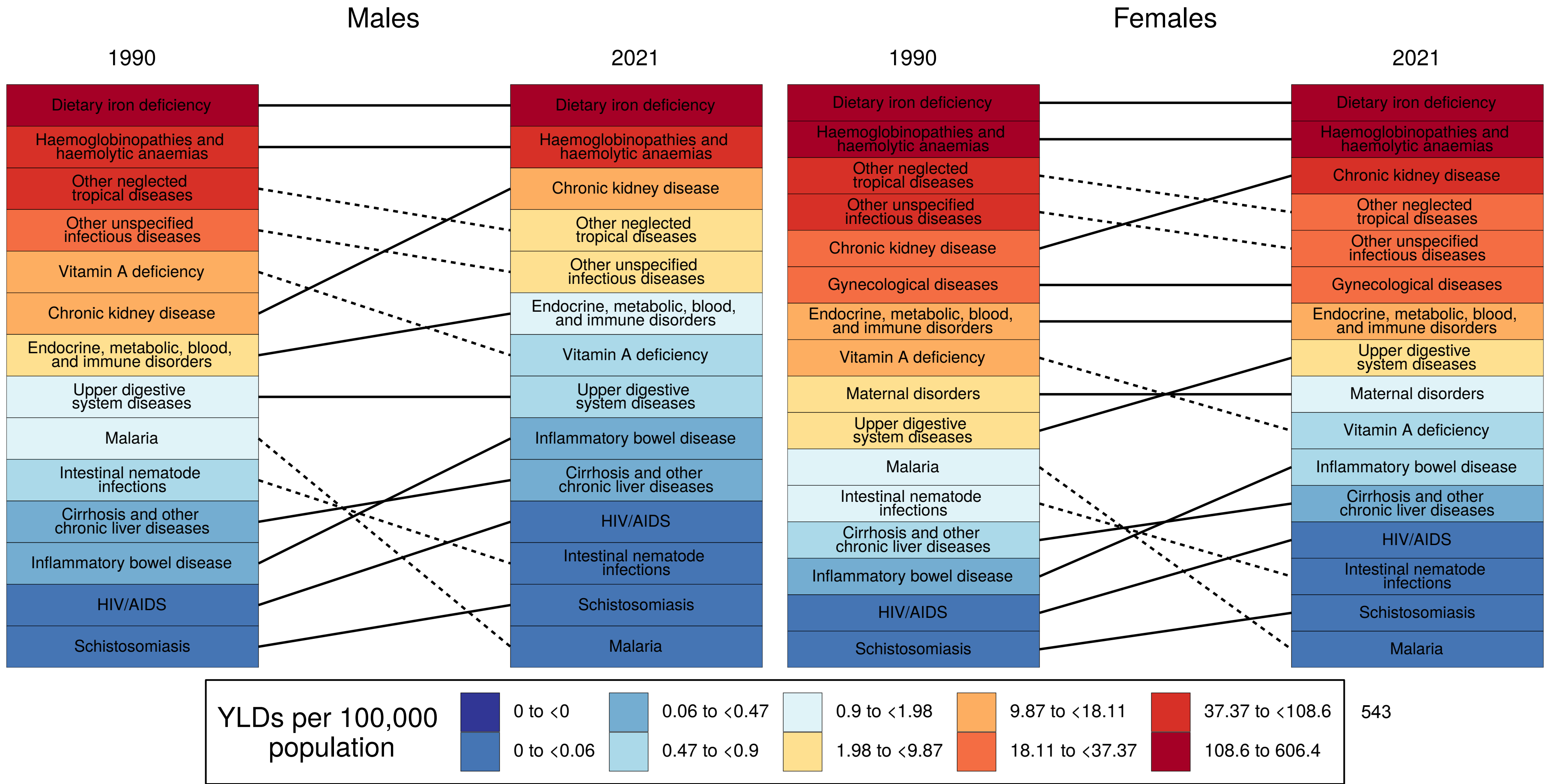

543

Mazandaran

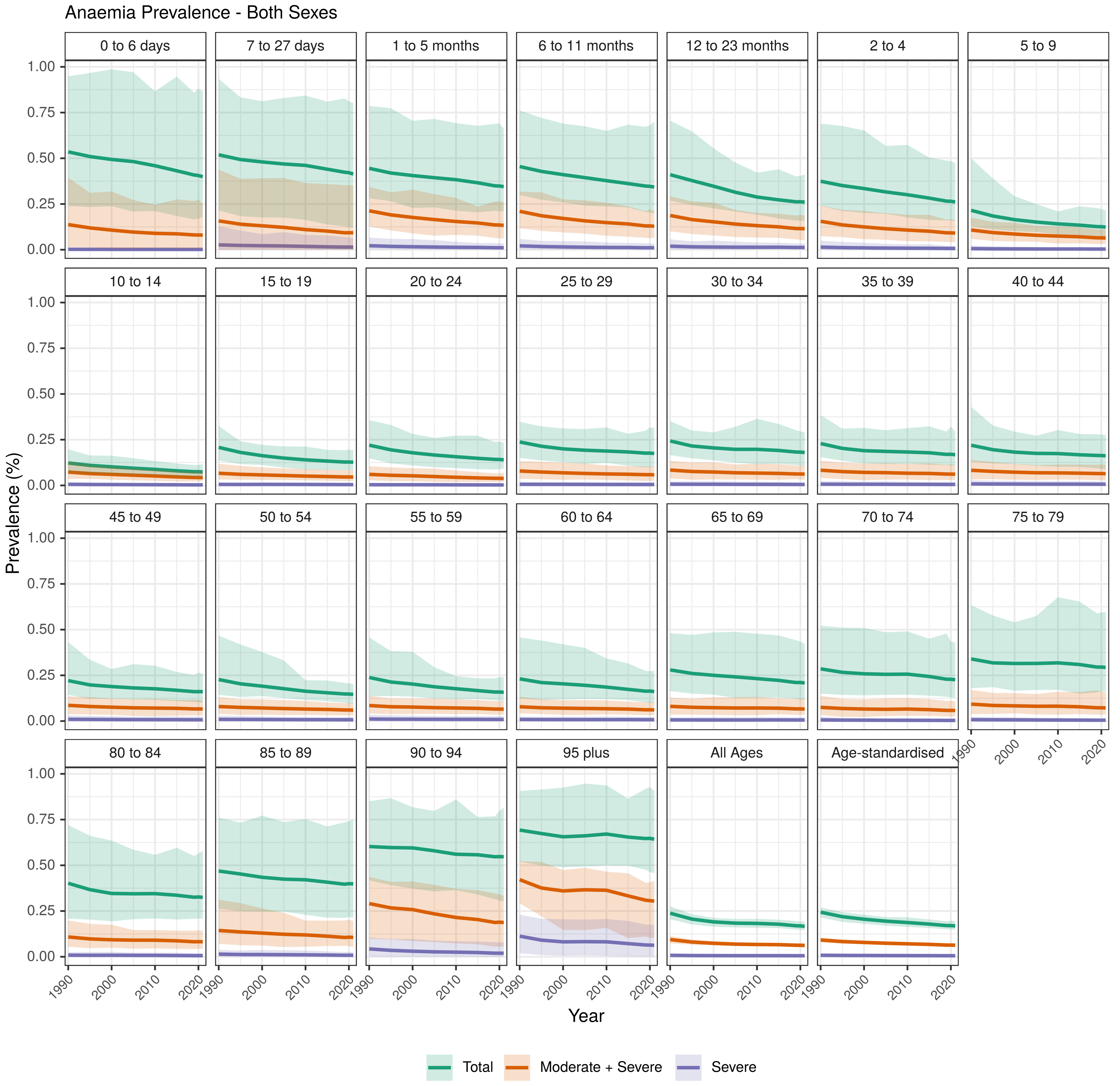

Anaemia Prevalence - Both Sexes
0 to 6 days
7 to 27 days
1 to 5 months
6 to 11 months
12 to 23 months
2 to 4
5 to 9
10 to 14
15 to 19
20 to 24
25 to 29
30 to 34
35 to 39
40 to 44
45 to 49
50 to 54
55 to 59
60 to 64
65 to 69
70 to 74
75 to 79
80 to 84
85 to 89
90 to 94
95 plus
All Ages
Age-standardised
Prevalence (%)
Year
1.00
0.75
0.50
0.25
0.00
1990
2000
2010
2020
Total
Moderate + Severe
Severe

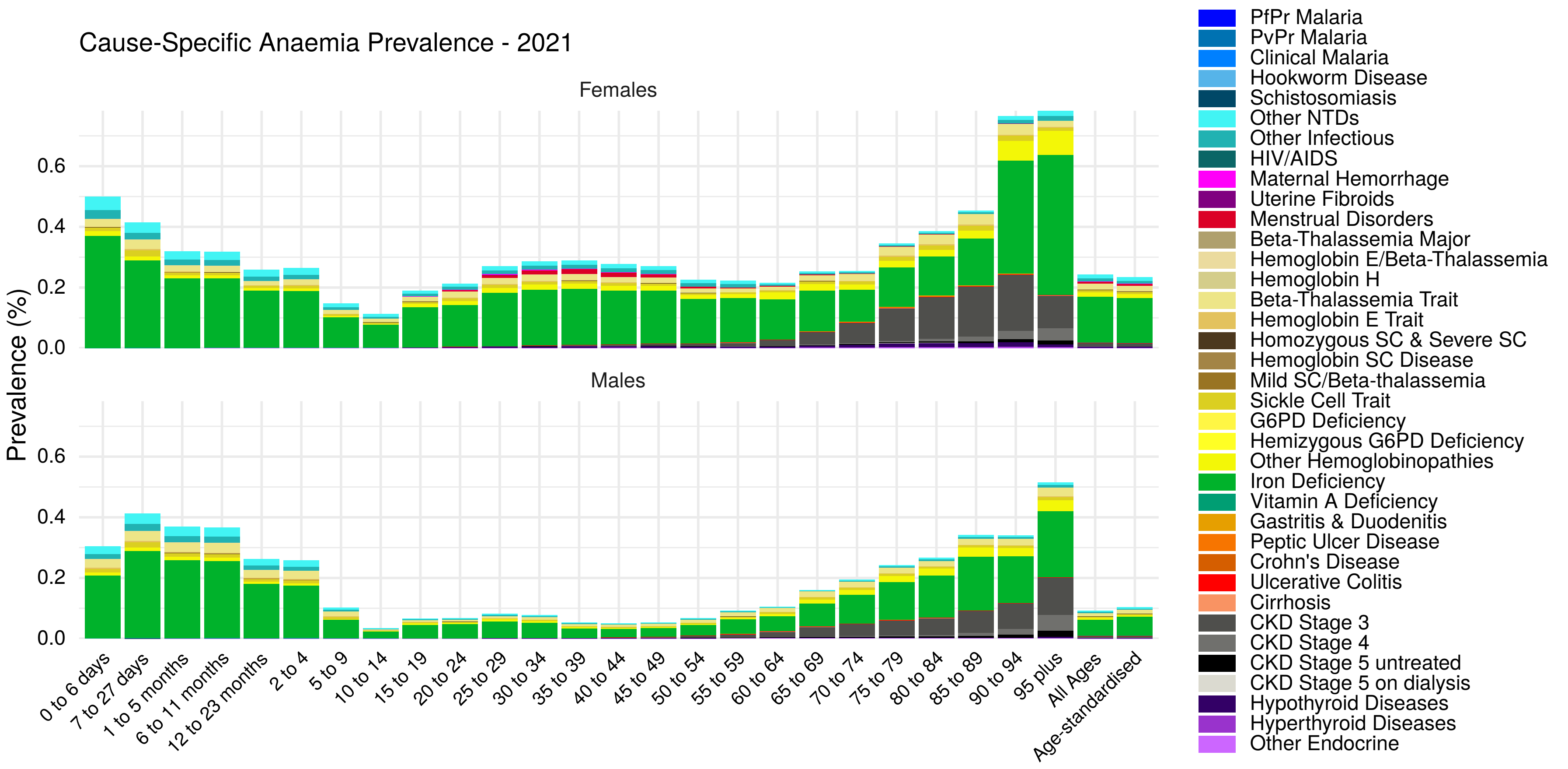

Cause-Specific Anaemia Prevalence - 2021
Females
Males
Prevalence (%)
0.6
0.4
0.2
0.0
0 to 6 days
7 to 27 days
1 to 5 months
6 to 11 months
12 to 23 months
2 to 4
5 to 9
10 to 14
15 to 19
20 to 24
25 to 29
30 to 34
35 to 39
40 to 44
45 to 49
50 to 54
55 to 59
60 to 64
65 to 69
70 to 74
75 to 79
80 to 84
85 to 89
90 to 94
95 plus
All Ages
Age-standardised
PfPr Malaria
PvPr Malaria
Clinical Malaria
Hookworm Disease
Schistosomiasis
Other NTDs
Other Infectious
HIV/AIDS
Maternal Hemorrhage
Uterine Fibroids
Menstrual Disorders
Beta-Thalassemia Major
Hemoglobin E/Beta-Thalassemia
Hemoglobin H
Beta-Thalassemia Trait
Hemoglobin E Trait
Homozygous SC & Severe SC
Hemoglobin SC Disease
Mild SC/Beta-thalassemia
Sickle Cell Trait
G6PD Deficiency
Hemizygous G6PD Deficiency
Other Hemoglobinopathies
Iron Deficiency
Vitamin A Deficiency
Gastritis & Duodenitis
Peptic Ulcer Disease
Crohn's Disease
Ulcerative Colitis
Cirrhosis
CKD Stage 3
CKD Stage 4
CKD Stage 5 untreated
CKD Stage 5 on dialysis
Hypothyroid Diseases
Hyperthyroid Diseases
Other Endocrine

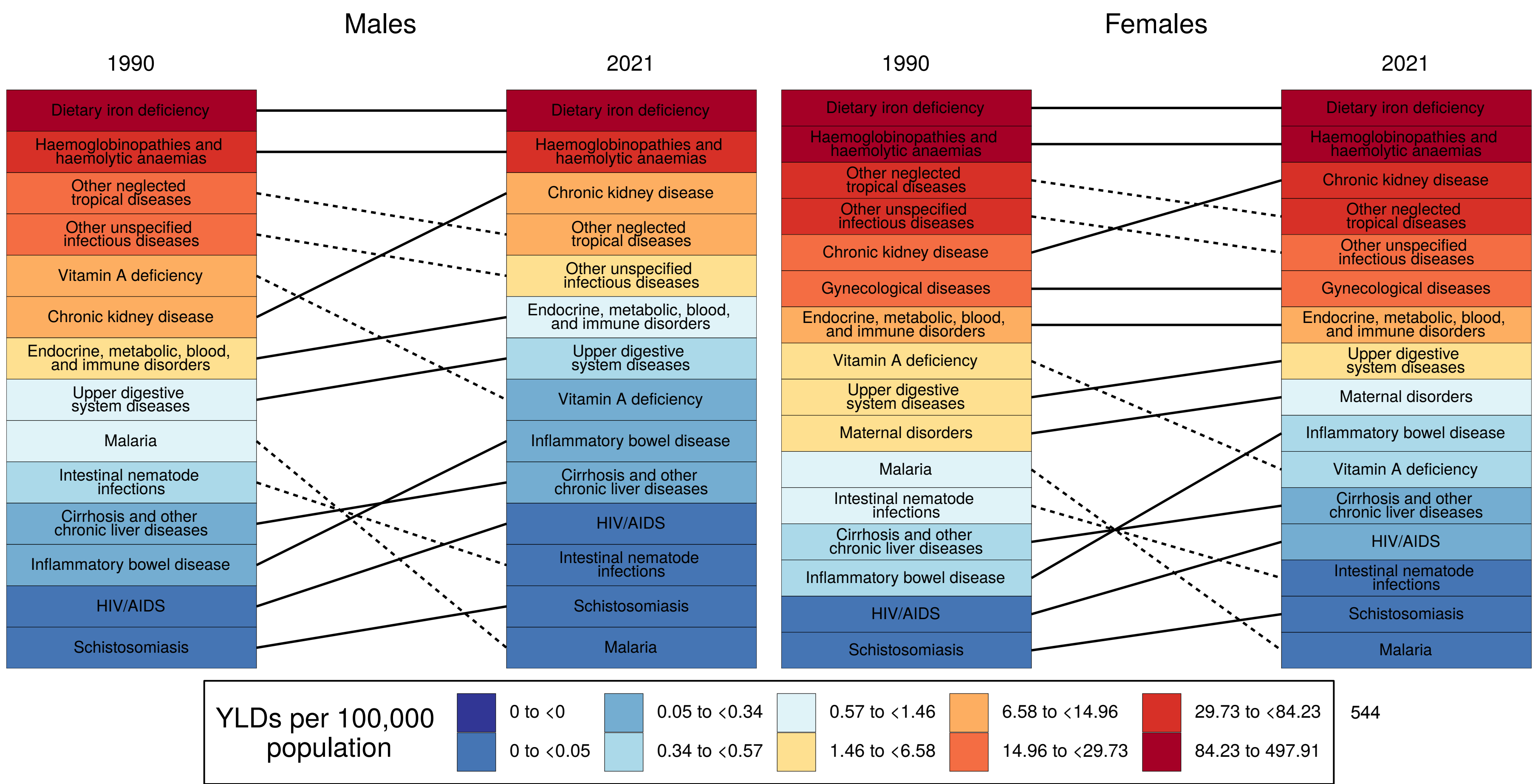

Males
Females
1990
2021
Dietary iron deficiency
Haemoglobinopathies and haemolytic anaemias
Other neglected tropical diseases
Other unspecified infectious diseases
Vitamin A deficiency
Chronic kidney disease
Endocrine, metabolic, blood, and immune disorders
Upper digestive system diseases
Malaria
Intestinal nematode infections
Cirrhosis and other chronic liver diseases
Inflammatory bowel disease
HIV/AIDS
Schistosomiasis
Gynecological diseases
Maternal disorders
YLDs per 100,000 population
0 to <0
0 to <0.05
0.05 to <0.34
0.34 to <0.57
0.57 to <1.46
1.46 to <6.58
6.58 to <14.96
14.96 to <29.73
29.73 to <84.23
84.23 to 497.91

North Khorasan

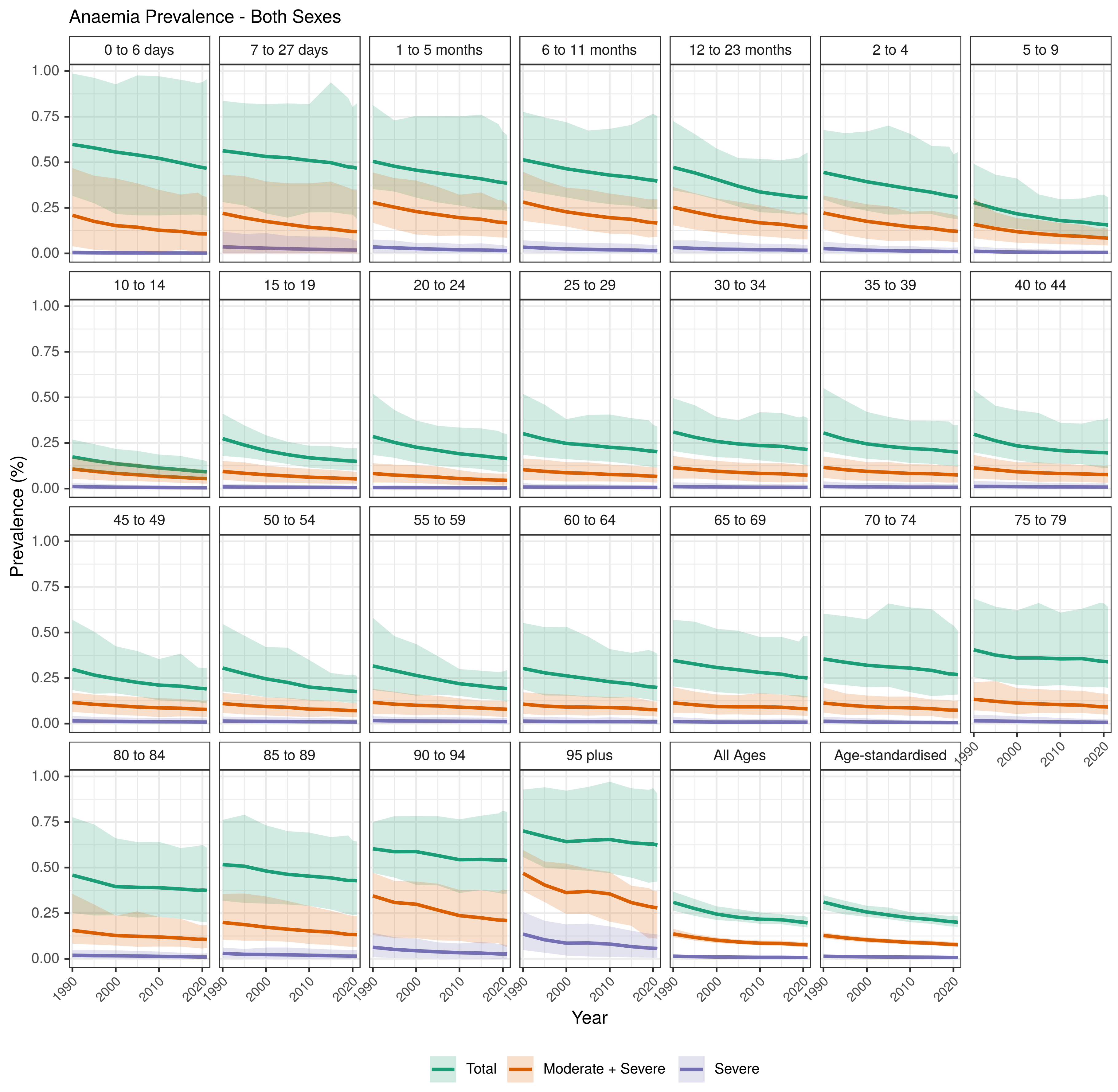

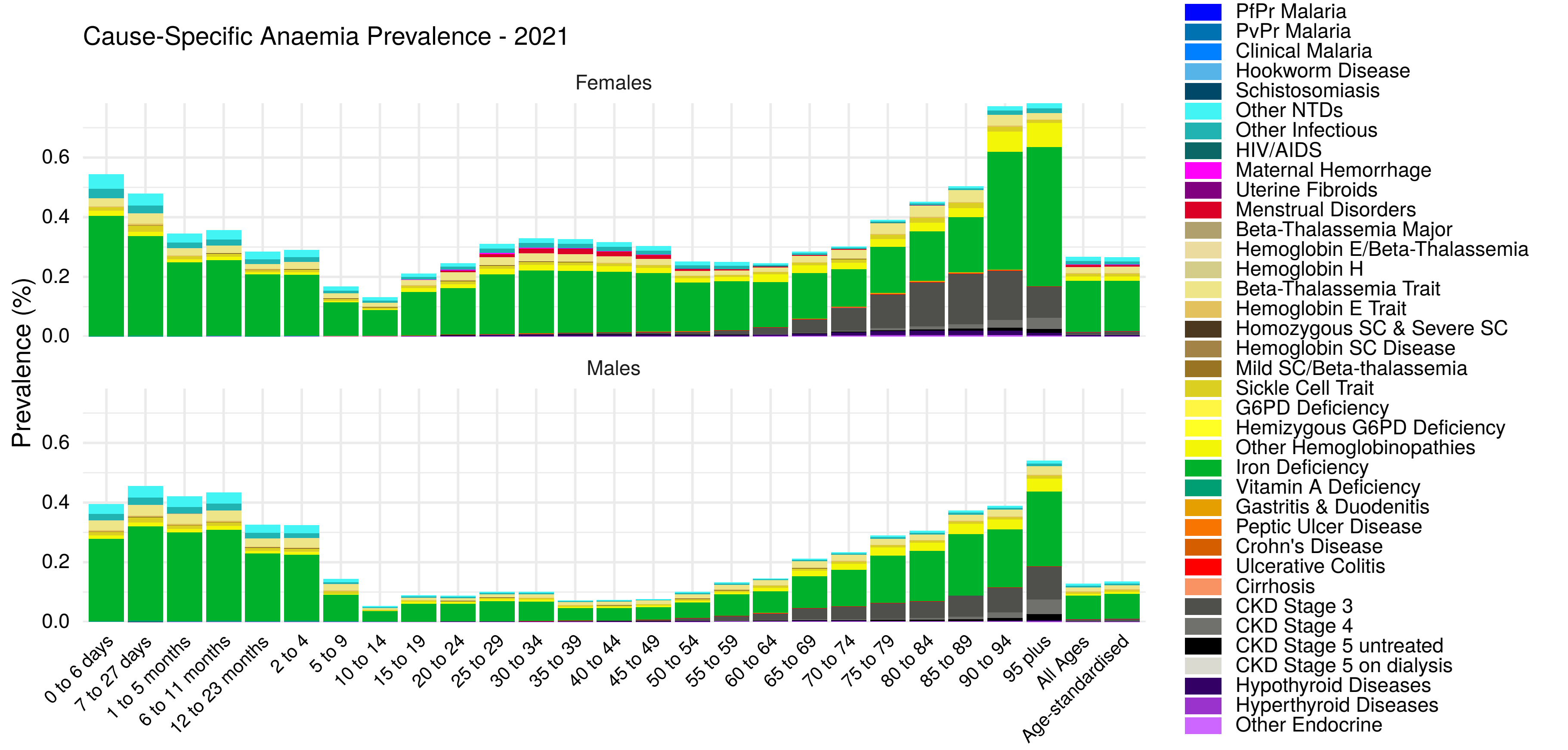

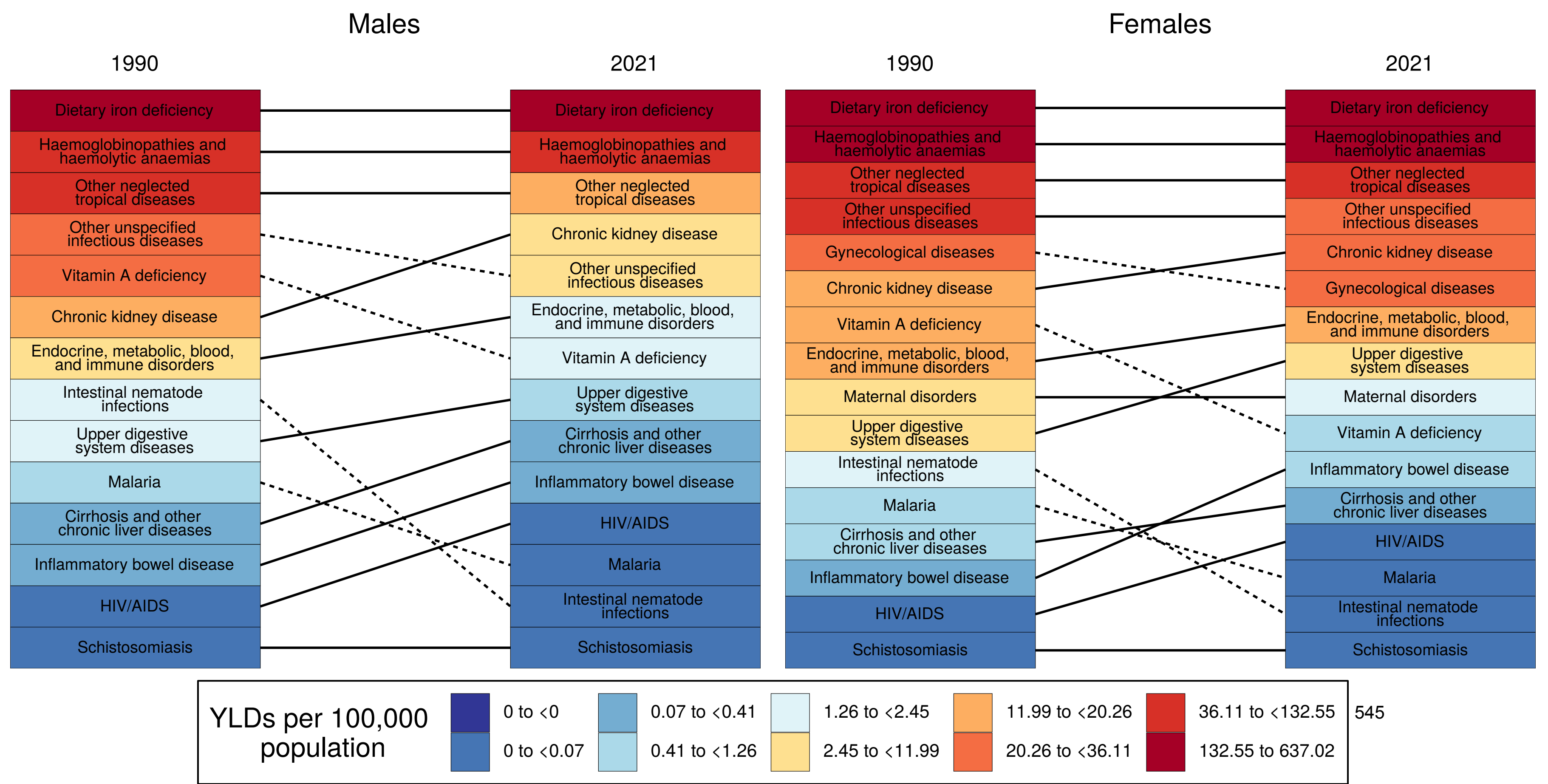

Qazvin

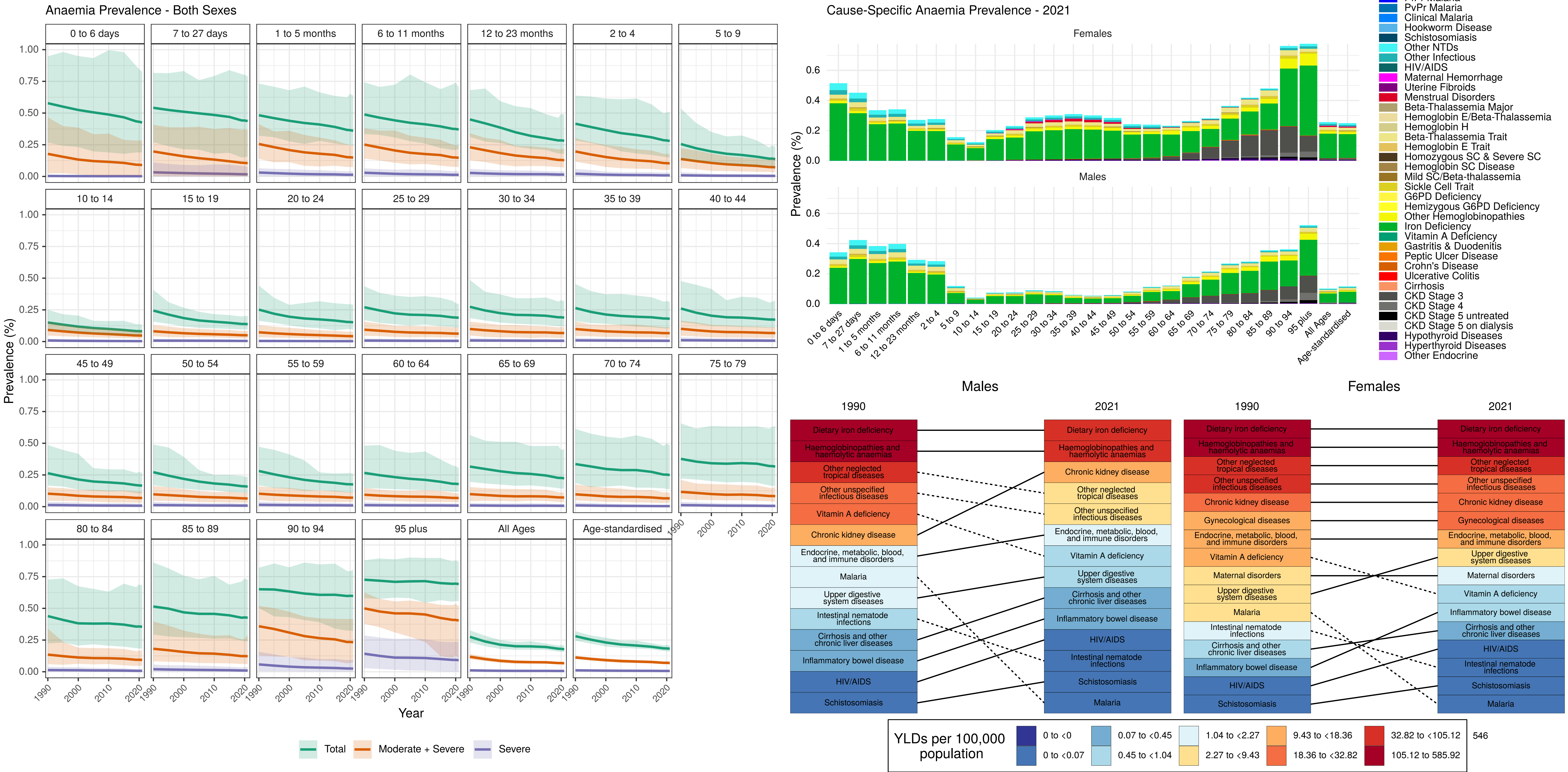
Anaemia Prevalence - Both Sexes
0 to 6 days
7 to 27 days
1 to 5 months
6 to 11 months
12 to 23 months
2 to 4
5 to 9
10 to 14
15 to 19
20 to 24
25 to 29
30 to 34
35 to 39
40 to 44
45 to 49
50 to 54
55 to 59
60 to 64
65 to 69
70 to 74
75 to 79
80 to 84
85 to 89
90 to 94
95 plus
All Ages
Age-standardised
Prevalence (%)
Year
Total
Moderate + Severe
Severe
Cause-Specific Anaemia Prevalence - 2021
Females
Males
Prevalence (%)
PfPr Malaria
PvPr Malaria
Clinical Malaria
Hookworm Disease
Schistosomiasis
Other NTDs
Other Infectious
HIV/AIDS
Maternal Hemorrhage
Uterine Fibroids
Menstrual Disorders
Beta-Thalassemia Major
Hemoglobin E/Beta-Thalassemia
Hemoglobin H
Beta-Thalassemia Trait
Hemoglobin E Trait
Homozygous SC & Severe SC
Hemoglobin SC Disease
Mild SC/Beta-thalassemia
Sickle Cell Trait
G6PD Deficiency
Hemizygous G6PD Deficiency
Other Hemoglobinopathies
Iron Deficiency
Vitamin A Deficiency
Gastritis & Duodenitis
Peptic Ulcer Disease
Crohn's Disease
Ulcerative Colitis
Cirrhosis
CKD Stage 3
CKD Stage 4
CKD Stage 5 untreated
CKD Stage 5 on dialysis
Hypothyroid Diseases
Hyperthyroid Diseases
Other Endocrine
Males
1990
Dietary iron deficiency
Haemoglobinopathies and haemolytic anaemias
Other neglected tropical diseases
Other unspecified infectious diseases
Vitamin A deficiency
Chronic kidney disease
Endocrine, metabolic, blood, and immune disorders
Malaria
Upper digestive system diseases
Intestinal nematode infections
Cirrhosis and other chronic liver diseases
Inflammatory bowel disease
HIV/AIDS
Schistosomiasis
2021
Dietary iron deficiency
Haemoglobinopathies and haemolytic anaemias
Chronic kidney disease
Other neglected tropical diseases
Other unspecified infectious diseases
Endocrine, metabolic, blood, and immune disorders
Vitamin A deficiency
Upper digestive system diseases
Cirrhosis and other chronic liver diseases
Inflammatory bowel disease
HIV/AIDS
Intestinal nematode infections
Schistosomiasis
Malaria
Females
1990
Dietary iron deficiency
Haemoglobinopathies and haemolytic anaemias
Other neglected tropical diseases
Other unspecified infectious diseases
Chronic kidney disease
Gynecological diseases
Endocrine, metabolic, blood, and immune disorders
Vitamin A deficiency
Maternal disorders
Upper digestive system diseases
Malaria
Intestinal nematode infections
Cirrhosis and other chronic liver diseases
Inflammatory bowel disease
HIV/AIDS
Schistosomiasis
2021
Dietary iron deficiency
Haemoglobinopathies and haemolytic anaemias
Other neglected tropical diseases
Other unspecified infectious diseases
Chronic kidney disease
Gynecological diseases
Endocrine, metabolic, blood, and immune disorders
Upper digestive system diseases
Maternal disorders
Vitamin A deficiency
Inflammatory bowel disease
Cirrhosis and other chronic liver diseases
HIV/AIDS
Intestinal nematode infections
Schistosomiasis
Malaria
YLDs per 100,000 population
0 to <0
0 to <0.07
0.07 to <0.45
0.45 to <1.04
1.04 to <2.27
2.27 to <9.43
9.43 to <18.36
18.36 to <32.82
32.82 to <105.12
105.12 to 585.92

Qom

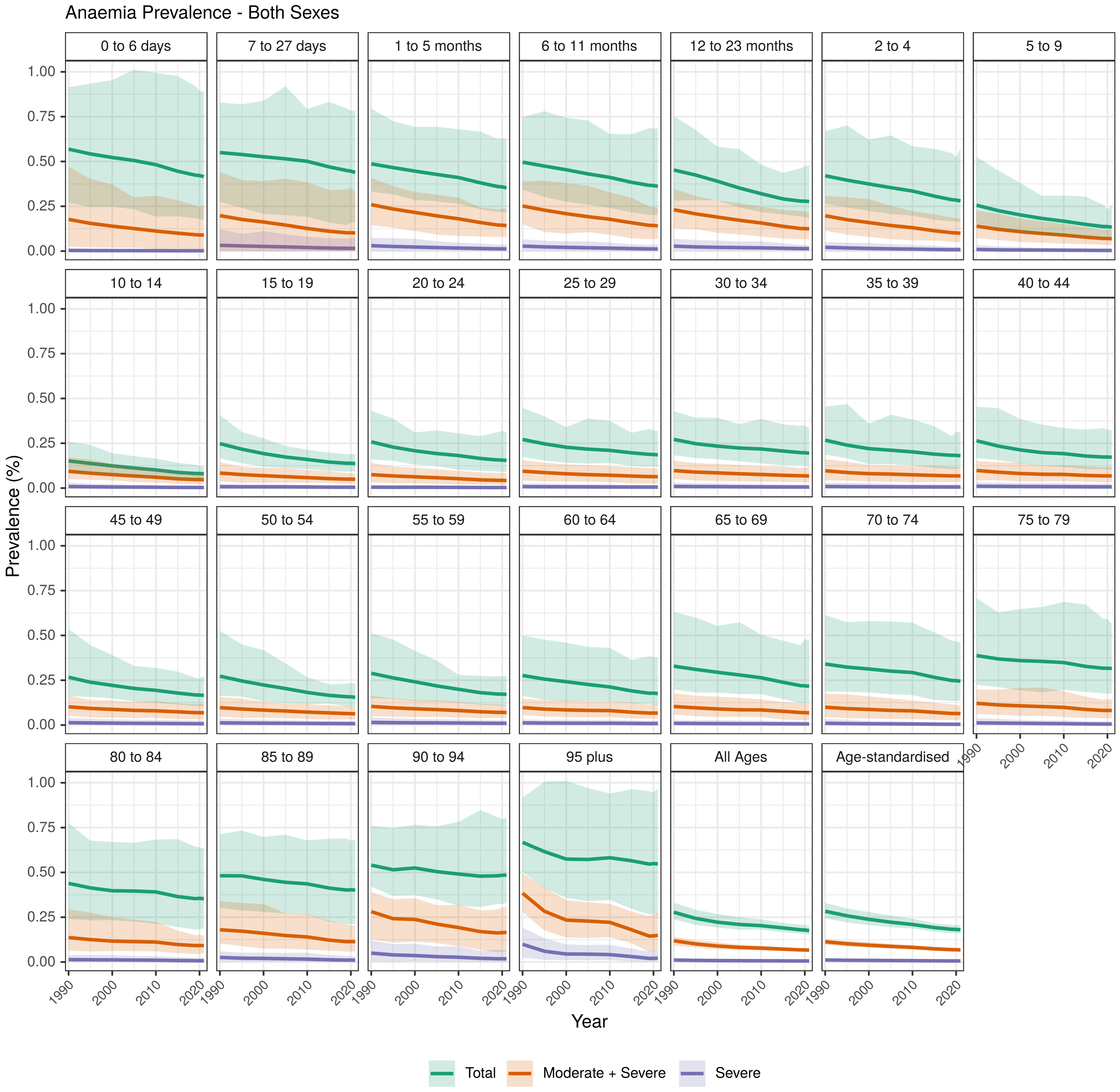
Anaemia Prevalence - Both Sexes
0 to 6 days
7 to 27 days
1 to 5 months
6 to 11 months
12 to 23 months
2 to 4
5 to 9
10 to 14
15 to 19
20 to 24
25 to 29
30 to 34
35 to 39
40 to 44
45 to 49
50 to 54
55 to 59
60 to 64
65 to 69
70 to 74
75 to 79
80 to 84
85 to 89
90 to 94
95 plus
All Ages
Age-standardised
Prevalence (%)
Year
Total
Moderate + Severe
Severe

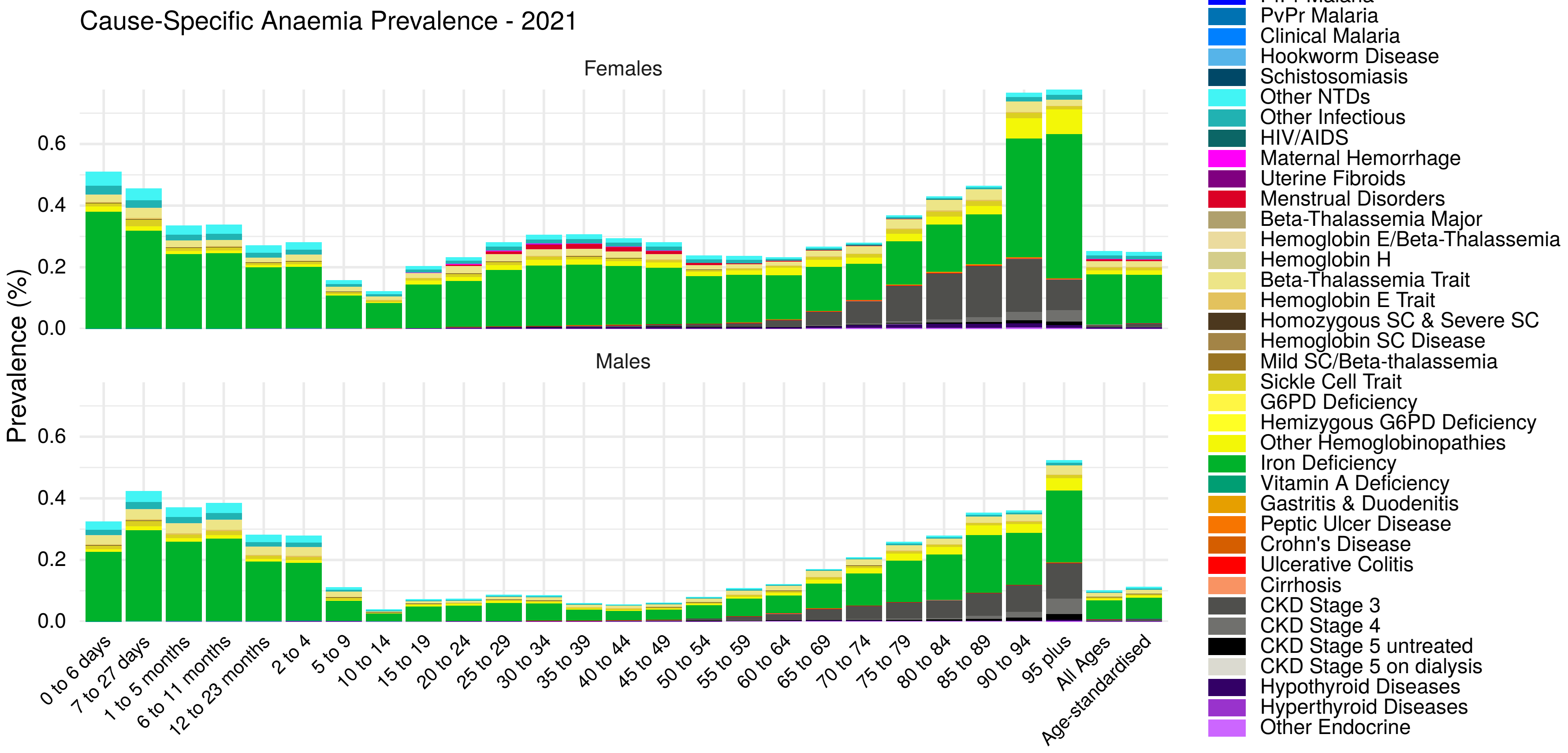
Cause-Specific Anaemia Prevalence - 2021
Females
Males
Prevalence (%)
PfPr Malaria
PvPr Malaria
Clinical Malaria
Hookworm Disease
Schistosomiasis
Other NTDs
Other Infectious
HIV/AIDS
Maternal Hemorrhage
Uterine Fibroids
Menstrual Disorders
Beta-Thalassemia Major
Hemoglobin E/Beta-Thalassemia
Hemoglobin H
Beta-Thalassemia Trait
Hemoglobin E Trait
Homozygous SC & Severe SC
Hemoglobin SC Disease
Mild SC/Beta-thalassemia
Sickle Cell Trait
G6PD Deficiency
Hemizygous G6PD Deficiency
Other Hemoglobinopathies
Iron Deficiency
Vitamin A Deficiency
Gastritis & Duodenitis
Peptic Ulcer Disease
Crohn's Disease
Ulcerative Colitis
Cirrhosis
CKD Stage 3
CKD Stage 4
CKD Stage 5 untreated
CKD Stage 5 on dialysis
Hypothyroid Diseases
Hyperthyroid Diseases
Other Endocrine

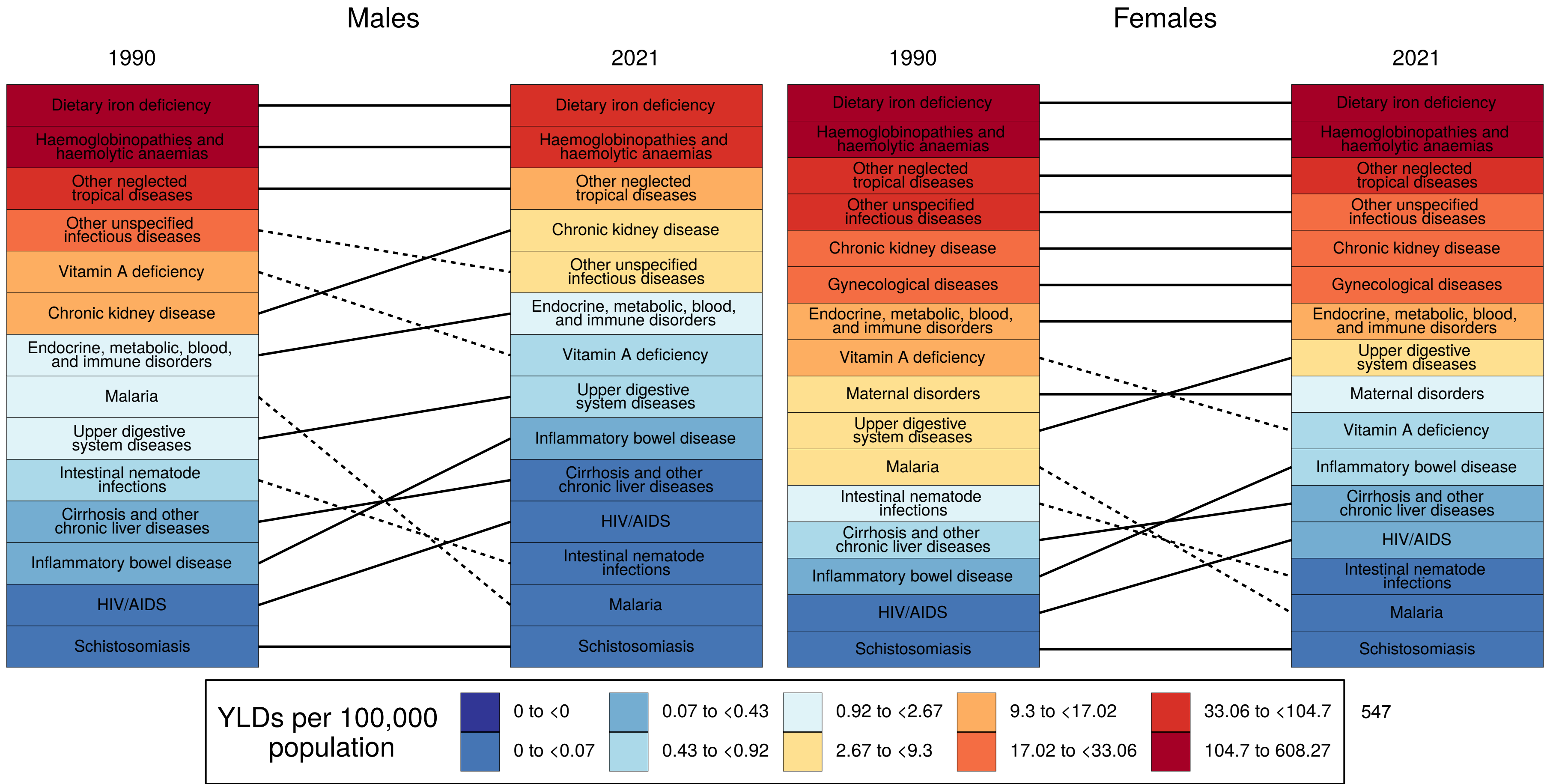
Males
Females
1990
2021
Dietary iron deficiency
Haemoglobinopathies and haemolytic anaemias
Other neglected tropical diseases
Other unspecified infectious diseases
Vitamin A deficiency
Chronic kidney disease
Endocrine, metabolic, blood, and immune disorders
Malaria
Upper digestive system diseases
Intestinal nematode infections
Cirrhosis and other chronic liver diseases
Inflammatory bowel disease
HIV/AIDS
Schistosomiasis
Gynecological diseases
Maternal disorders
YLDs per 100,000 population
0 to <0
0 to <0.07
0.07 to <0.43
0.43 to <0.92
0.92 to <2.67
2.67 to <9.3
9.3 to <17.02
17.02 to <33.06
33.06 to <104.7
104.7 to 608.27

547

Semnan

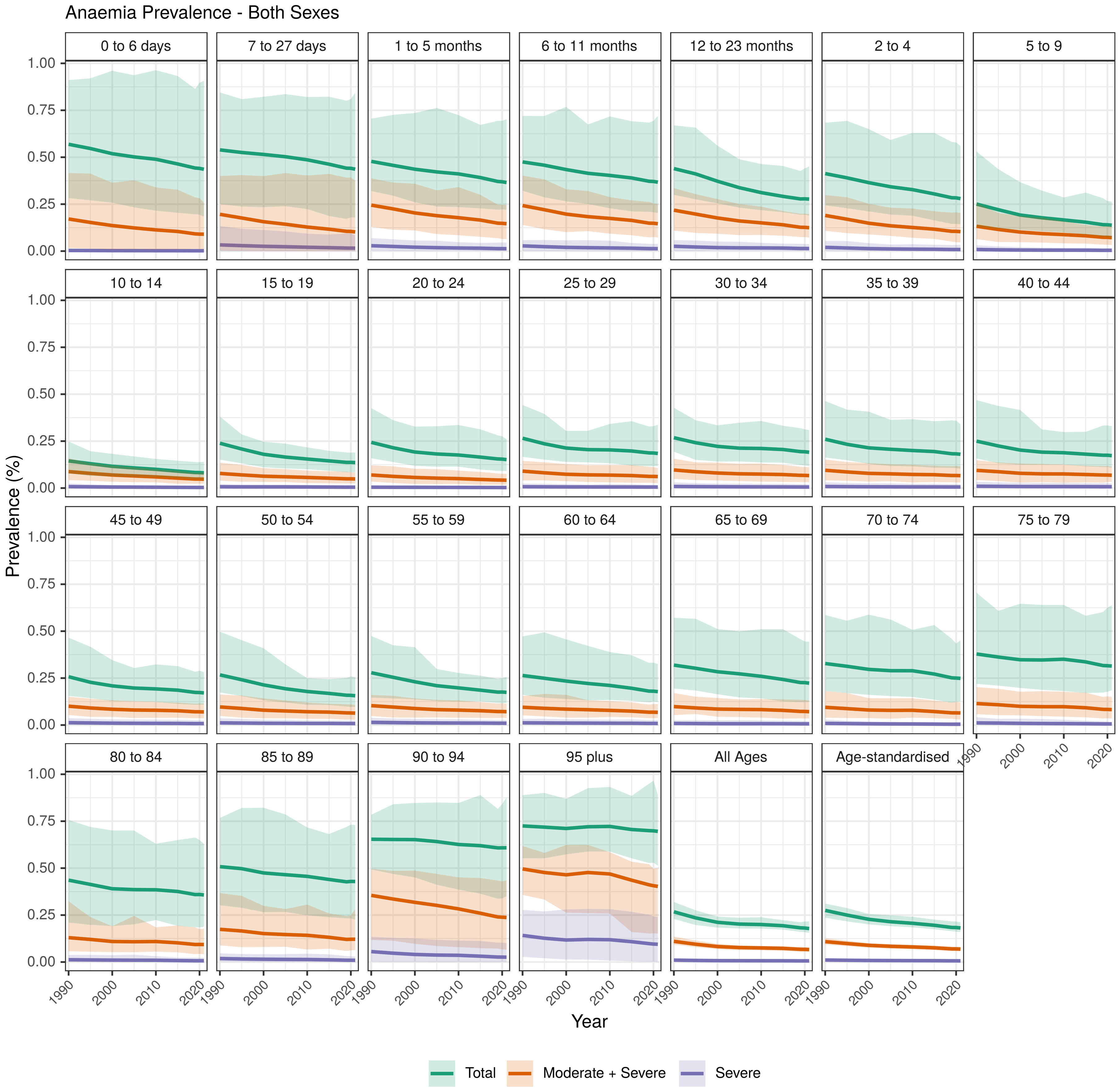

Anaemia Prevalence - Both Sexes
0 to 6 days
7 to 27 days
1 to 5 months
6 to 11 months
12 to 23 months
2 to 4
5 to 9
10 to 14
15 to 19
20 to 24
25 to 29
30 to 34
35 to 39
40 to 44
45 to 49
50 to 54
55 to 59
60 to 64
65 to 69
70 to 74
75 to 79
80 to 84
85 to 89
90 to 94
95 plus
All Ages
Age-standardised
Prevalence (%)
Year
Total
Moderate + Severe
Severe

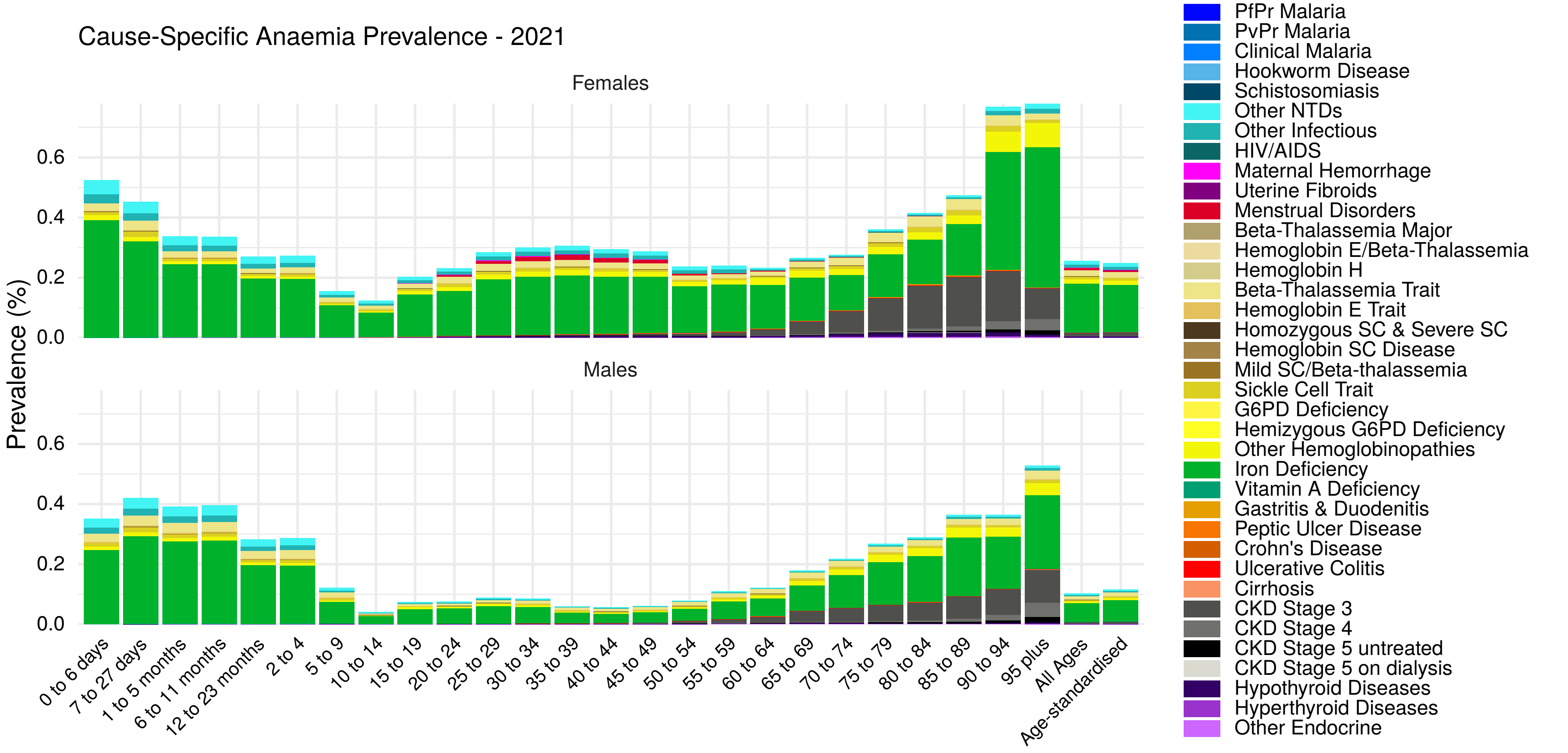

Cause-Specific Anaemia Prevalence - 2021
Females
Males
Prevalence (%)
PfPr Malaria
PvPr Malaria
Clinical Malaria
Hookworm Disease
Schistosomiasis
Other NTDs
Other Infectious
HIV/AIDS
Maternal Hemorrhage
Uterine Fibroids
Menstrual Disorders
Beta-Thalassemia Major
Hemoglobin E/Beta-Thalassemia
Hemoglobin H
Beta-Thalassemia Trait
Hemoglobin E Trait
Homozygous SC & Severe SC
Hemoglobin SC Disease
Mild SC/Beta-thalassemia
Sickle Cell Trait
G6PD Deficiency
Hemizygous G6PD Deficiency
Other Hemoglobinopathies
Iron Deficiency
Vitamin A Deficiency
Gastritis & Duodenitis
Peptic Ulcer Disease
Crohn's Disease
Ulcerative Colitis
Cirrhosis
CKD Stage 3
CKD Stage 4
CKD Stage 5 untreated
CKD Stage 5 on dialysis
Hypothyroid Diseases
Hyperthyroid Diseases
Other Endocrine

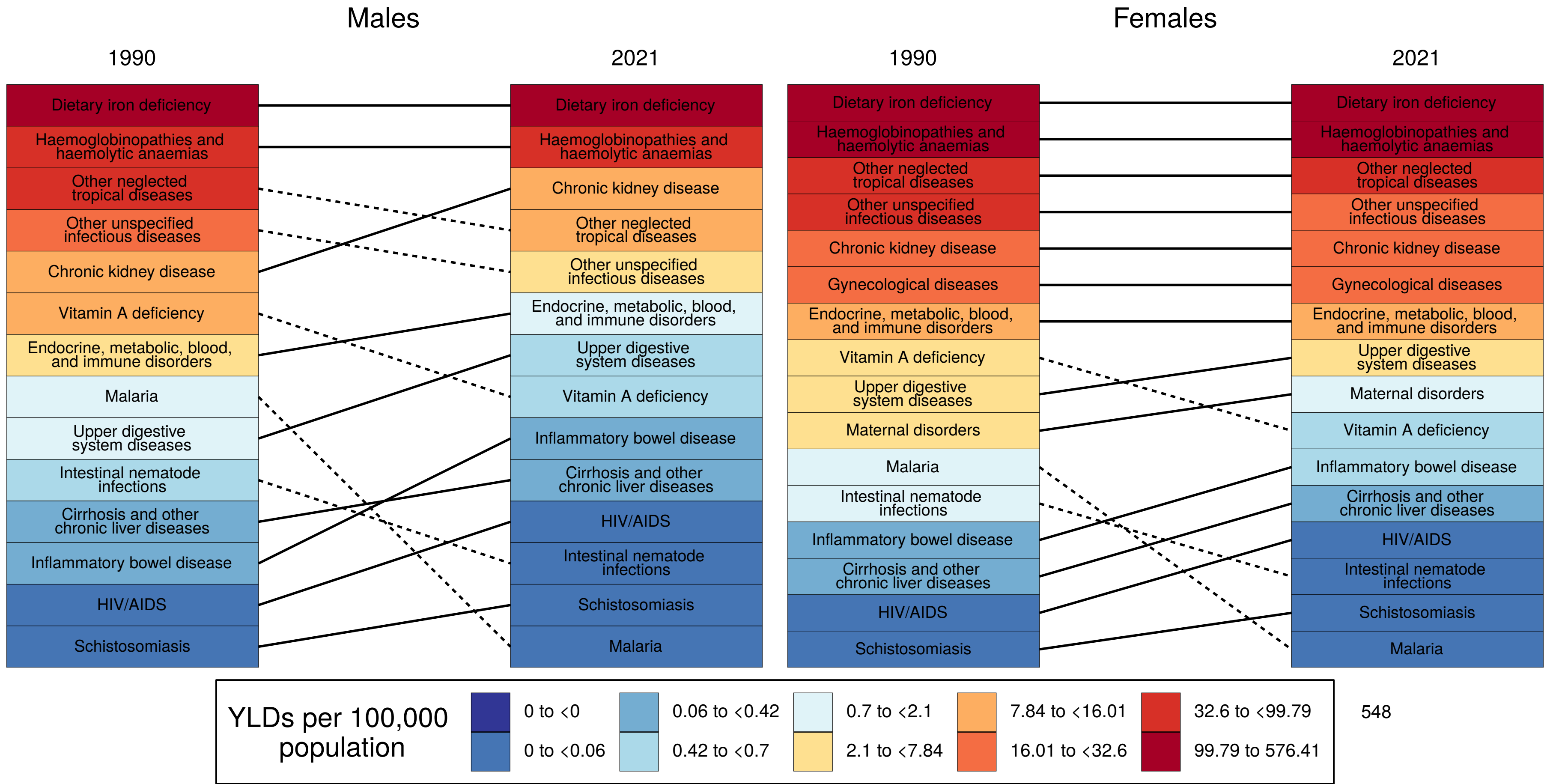

Males
Females
1990
2021
Dietary iron deficiency
Haemoglobinopathies and haemolytic anaemias
Other neglected tropical diseases
Other unspecified infectious diseases
Chronic kidney disease
Vitamin A deficiency
Endocrine, metabolic, blood, and immune disorders
Malaria
Upper digestive system diseases
Intestinal nematode infections
Cirrhosis and other chronic liver diseases
Inflammatory bowel disease
HIV/AIDS
Schistosomiasis
Gynecological diseases
Maternal disorders
YLDs per 100,000 population
0 to <0
0 to <0.06
0.06 to <0.42
0.42 to <0.7
0.7 to <2.1
2.1 to <7.84
7.84 to <16.01
16.01 to <32.6
32.6 to <99.79
99.79 to 576.41

Sistan and Baluchistan

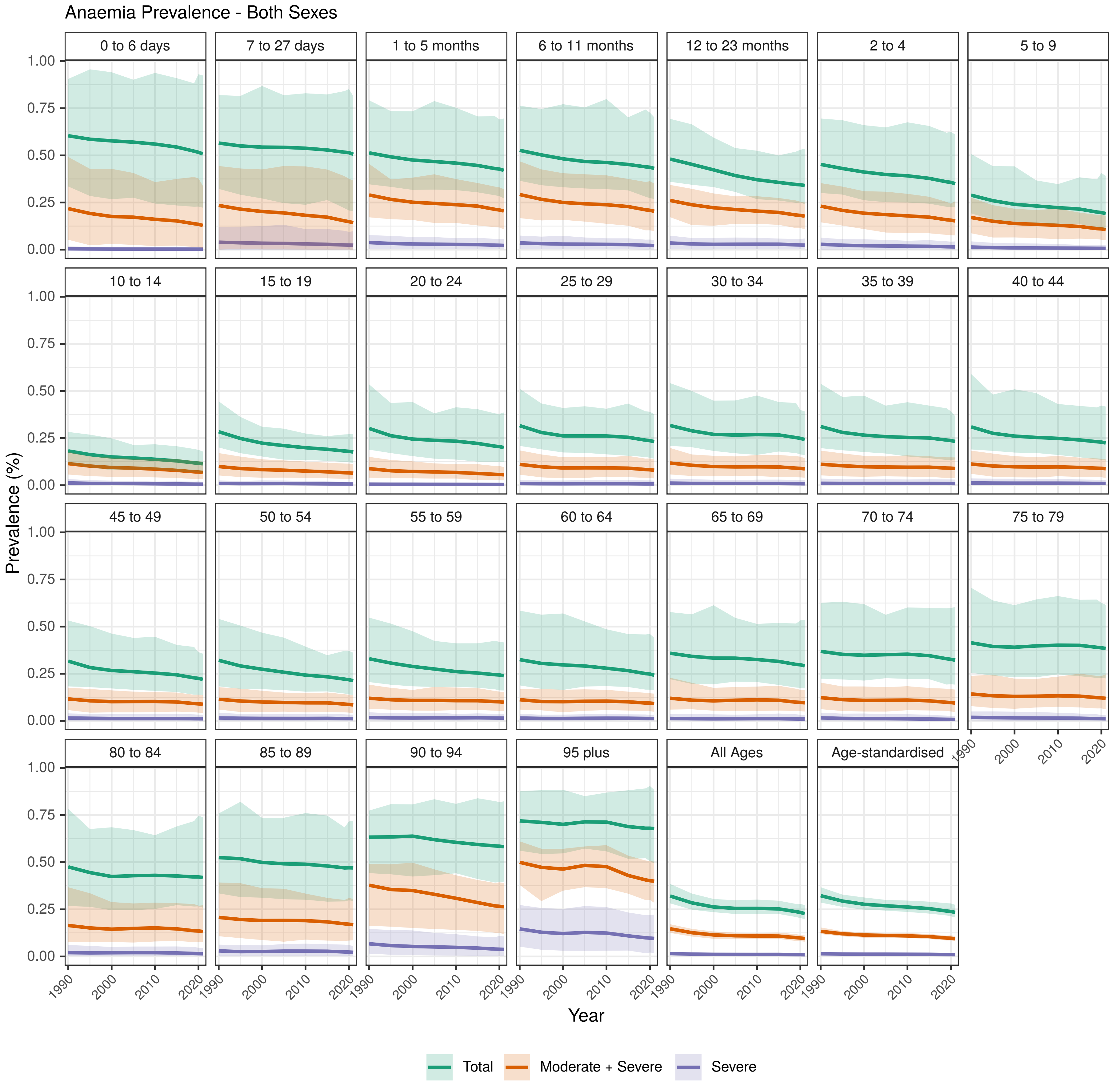

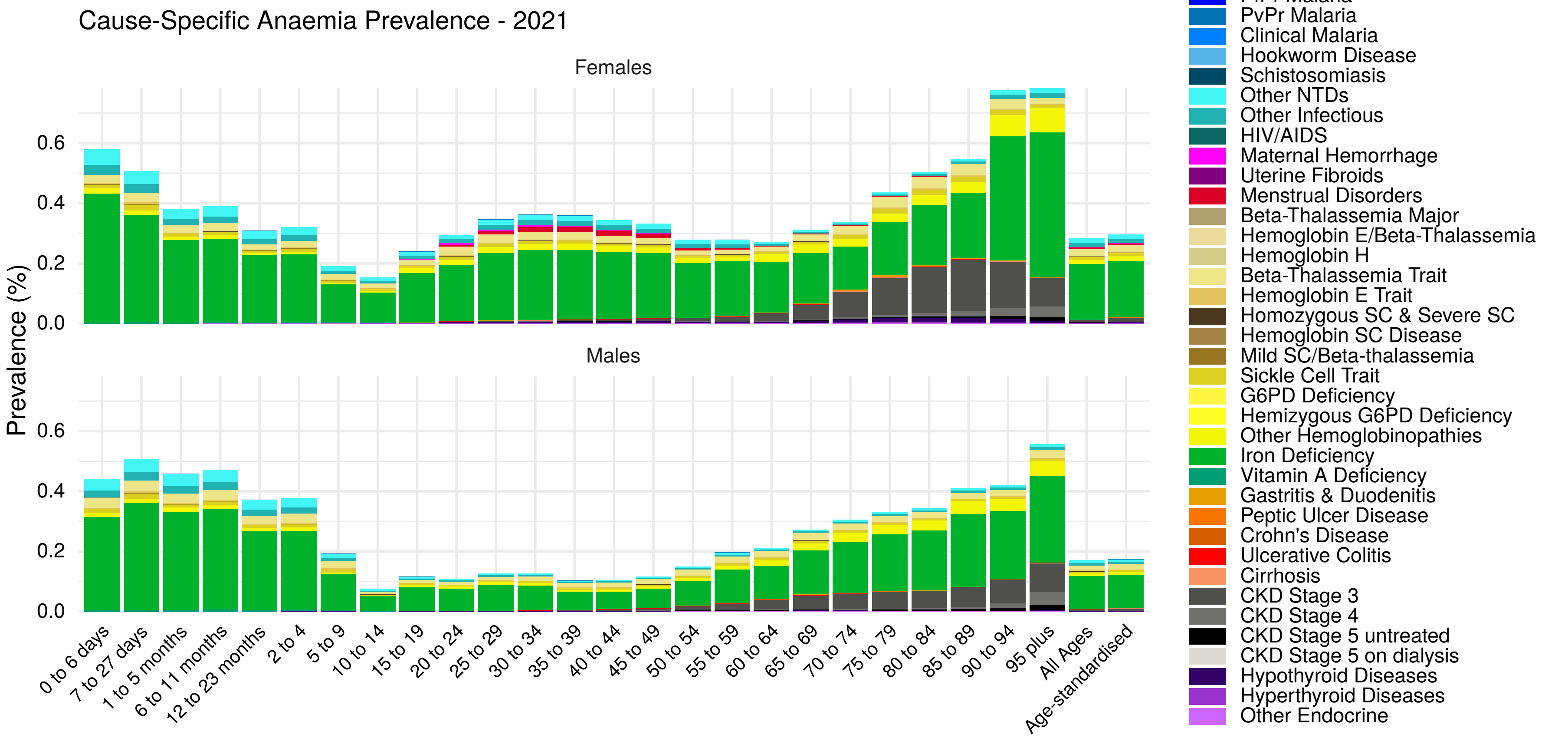

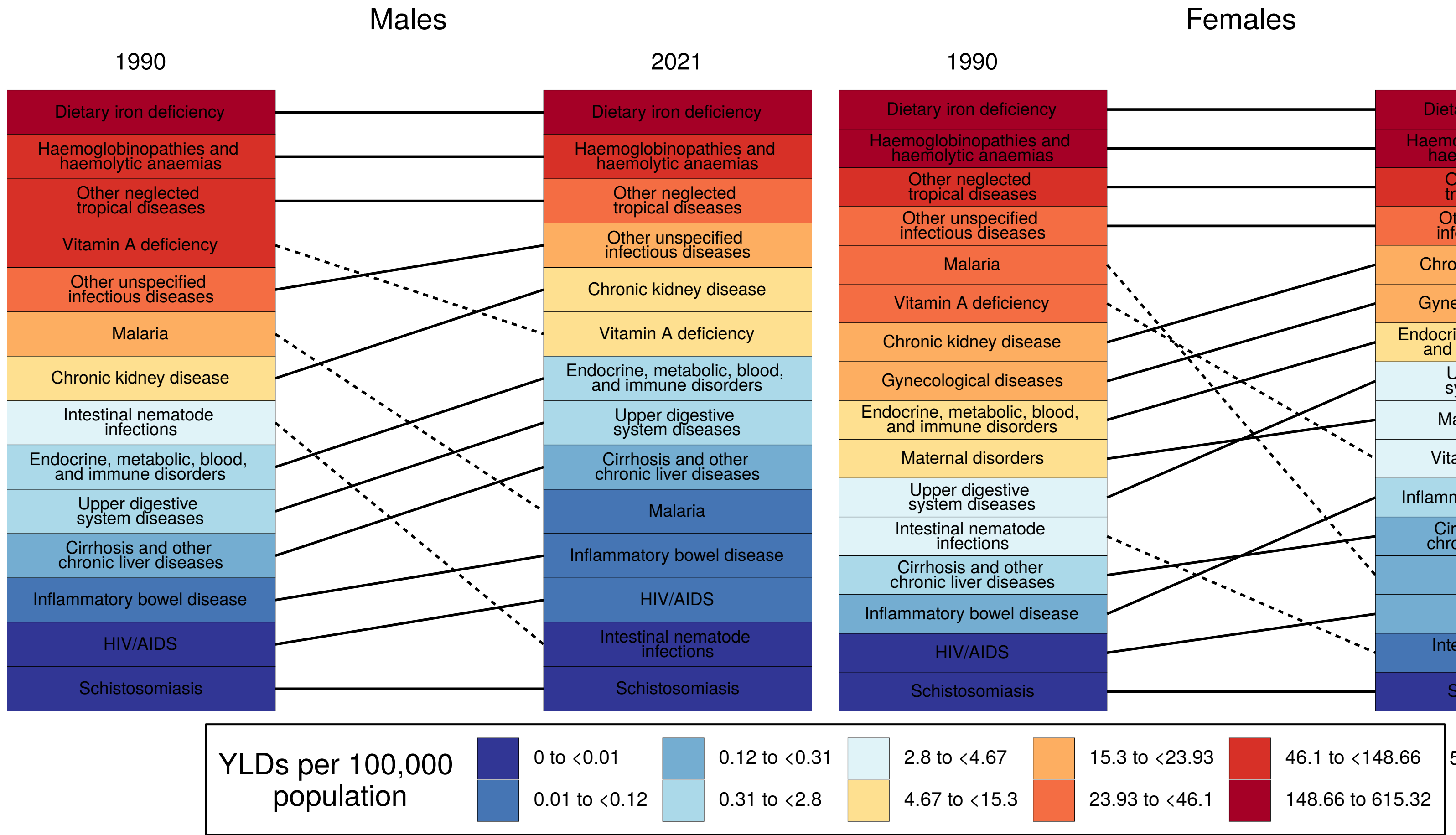

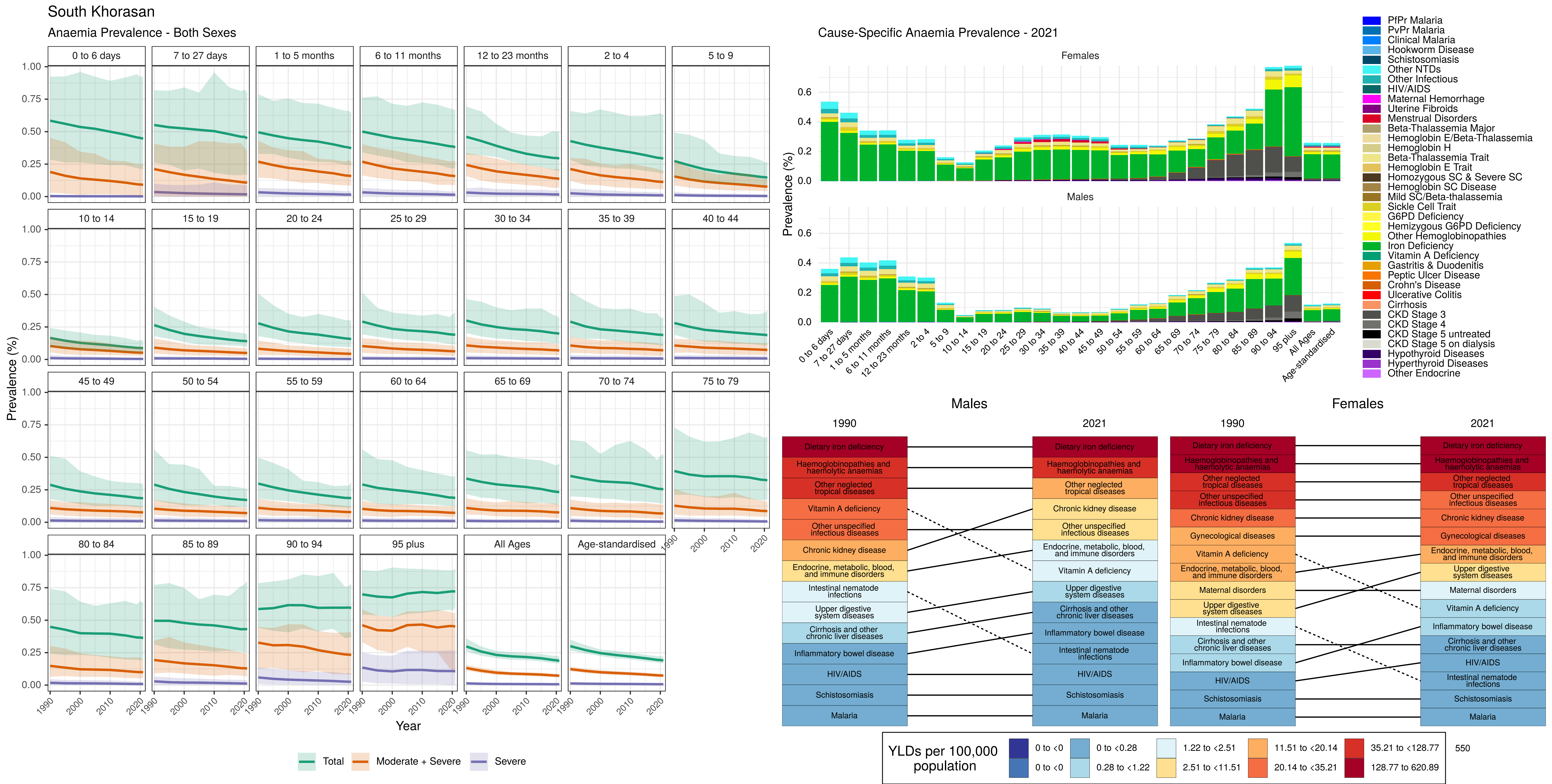

South Khorasan
Anaemia Prevalence - Both Sexes
0 to 6 days
7 to 27 days
1 to 5 months
6 to 11 months
12 to 23 months
2 to 4
5 to 9
10 to 14
15 to 19
20 to 24
25 to 29
30 to 34
35 to 39
40 to 44
45 to 49
50 to 54
55 to 59
60 to 64
65 to 69
70 to 74
75 to 79
80 to 84
85 to 89
90 to 94
95 plus
All Ages
Age-standardised
Prevalence (%)
Year
Total
Moderate + Severe
Severe
Cause-Specific Anaemia Prevalence - 2021
Females
Males
PfPr Malaria
PvPr Malaria
Clinical Malaria
Hookworm Disease
Schistosomiasis
Other NTDs
Other Infectious
HIV/AIDS
Maternal Hemorrhage
Uterine Fibroids
Menstrual Disorders
Beta-Thalassemia Major
Hemoglobin E/Beta-Thalassemia
Hemoglobin H
Beta-Thalassemia Trait
Hemoglobin E Trait
Homozygous SC & Severe SC
Hemoglobin SC Disease
Mild SC/Beta-thalassemia
Sickle Cell Trait
G6PD Deficiency
Hemizygous G6PD Deficiency
Other Hemoglobinopathies
Iron Deficiency
Vitamin A Deficiency
Gastritis & Duodenitis
Peptic Ulcer Disease
Crohn's Disease
Ulcerative Colitis
Cirrhosis
CKD Stage 3
CKD Stage 4
CKD Stage 5 untreated
CKD Stage 5 on dialysis
Hypothyroid Diseases
Hyperthyroid Diseases
Other Endocrine
Males
Females
1990
2021
Dietary iron deficiency
Haemoglobinopathies and haemolytic anaemias
Other neglected tropical diseases
Vitamin A deficiency
Other unspecified infectious diseases
Chronic kidney disease
Endocrine, metabolic, blood, and immune disorders
Intestinal nematode infections
Upper digestive system diseases
Cirrhosis and other chronic liver diseases
Inflammatory bowel disease
HIV/AIDS
Schistosomiasis
Malaria
Gynecological diseases
Maternal disorders
YLDs per 100,000 population
0 to <0
0 to <0
0 to <0.28
0.28 to <1.22
1.22 to <2.51
2.51 to <11.51
11.51 to <20.14
20.14 to <35.21
35.21 to <128.77
128.77 to 620.89

Tehran

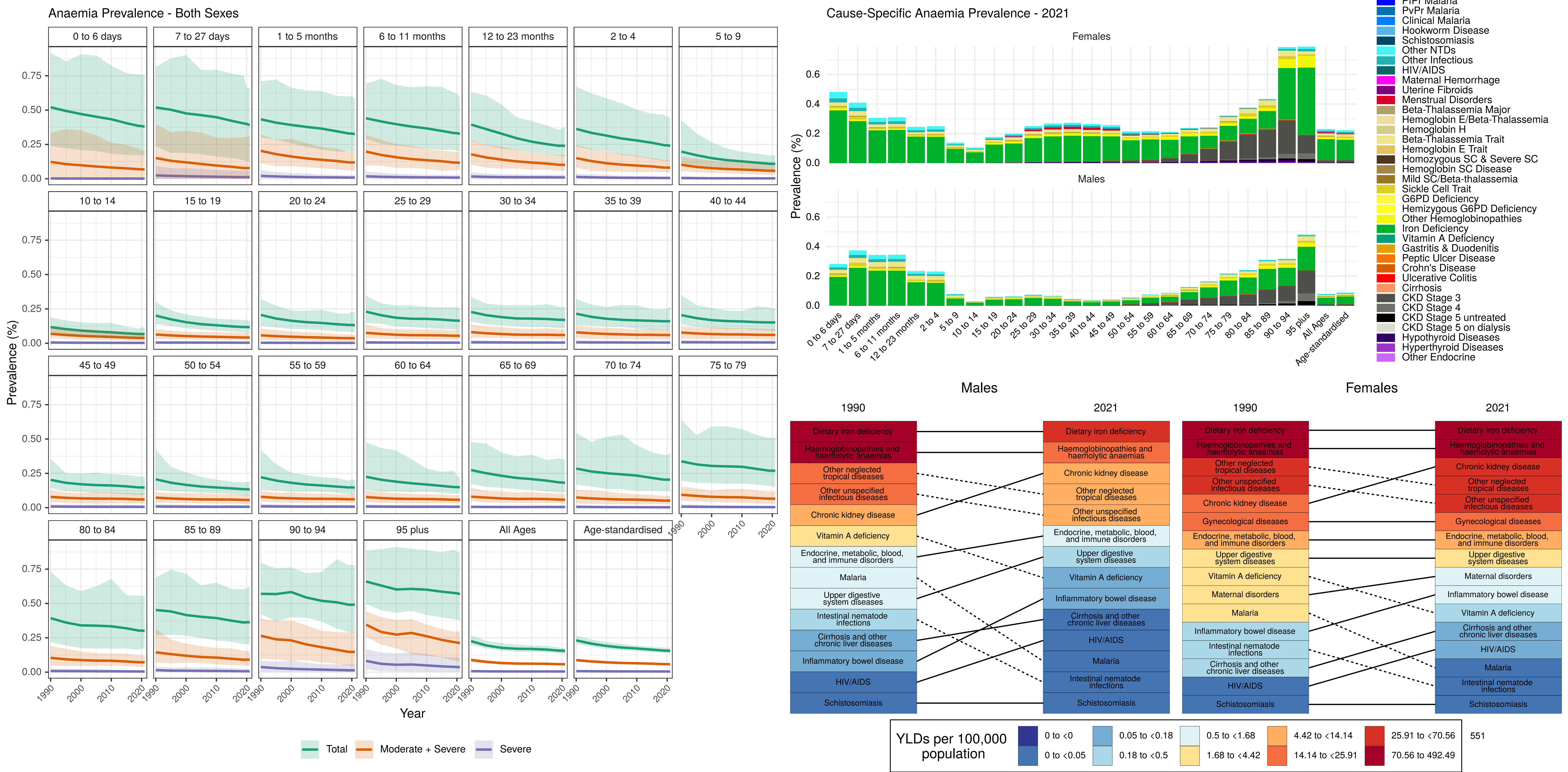

Anaemia Prevalence - Both Sexes
0 to 6 days
7 to 27 days
1 to 5 months
6 to 11 months
12 to 23 months
2 to 4
5 to 9
10 to 14
15 to 19
20 to 24
25 to 29
30 to 34
35 to 39
40 to 44
45 to 49
50 to 54
55 to 59
60 to 64
65 to 69
70 to 74
75 to 79
80 to 84
85 to 89
90 to 94
95 plus
All Ages
Age-standardised
Prevalence (%)
Year
Total
Moderate + Severe
Severe
Cause-Specific Anaemia Prevalence - 2021
Females
Males
PfPr Malaria
PvPr Malaria
Clinical Malaria
Hookworm Disease
Schistosomiasis
Other NTDs
Other Infectious
HIV/AIDS
Maternal Hemorrhage
Uterine Fibroids
Menstrual Disorders
Beta-Thalassemia Major
Hemoglobin E/Beta-Thalassemia
Hemoglobin H
Beta-Thalassemia Trait
Hemoglobin E Trait
Homozygous SC & Severe SC
Hemoglobin SC Disease
Mild SC/Beta-thalassemia
Sickle Cell Trait
G6PD Deficiency
Hemizygous G6PD Deficiency
Other Hemoglobinopathies
Iron Deficiency
Vitamin A Deficiency
Gastritis & Duodenitis
Peptic Ulcer Disease
Crohn's Disease
Ulcerative Colitis
Cirrhosis
CKD Stage 3
CKD Stage 4
CKD Stage 5 untreated
CKD Stage 5 on dialysis
Hypothyroid Diseases
Hyperthyroid Diseases
Other Endocrine
Males
Females
1990
2021
Dietary iron deficiency
Haemoglobinopathies and haemolytic anaemias
Other neglected tropical diseases
Other unspecified infectious diseases
Chronic kidney disease
Vitamin A deficiency
Endocrine, metabolic, blood, and immune disorders
Malaria
Upper digestive system diseases
Intestinal nematode infections
Cirrhosis and other chronic liver diseases
Inflammatory bowel disease
HIV/AIDS
Schistosomiasis
Gynecological diseases
Maternal disorders
YLDs per 100,000 population
0 to <0
0 to <0.05
0.05 to <0.18
0.18 to <0.5
0.5 to <1.68
1.68 to <4.42
4.42 to <14.14
14.14 to <25.91
25.91 to <70.56
70.56 to 492.49

West Azarbayejan

Anaemia Prevalence - Both Sexes

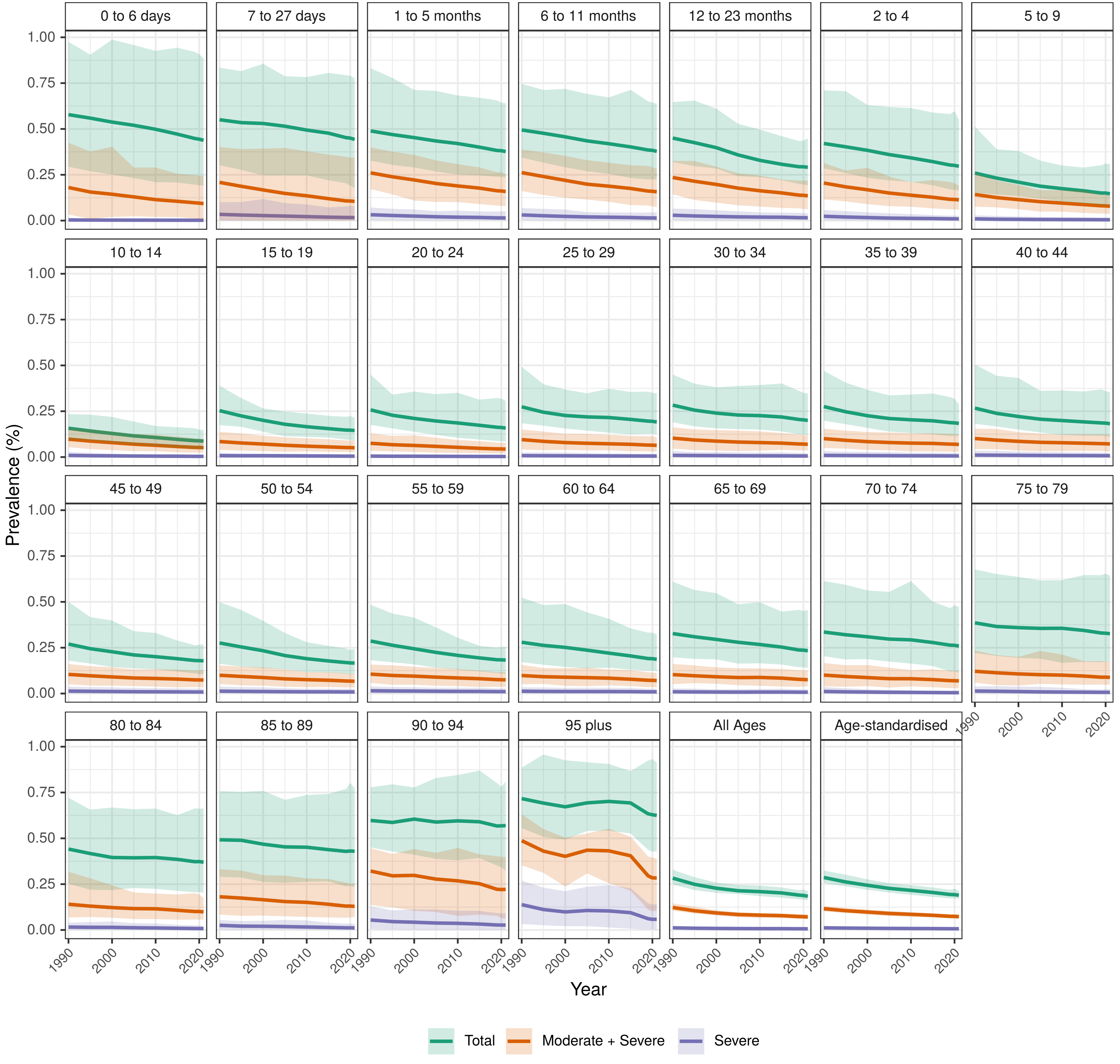

Cause-Specific Anaemia Prevalence - 2021

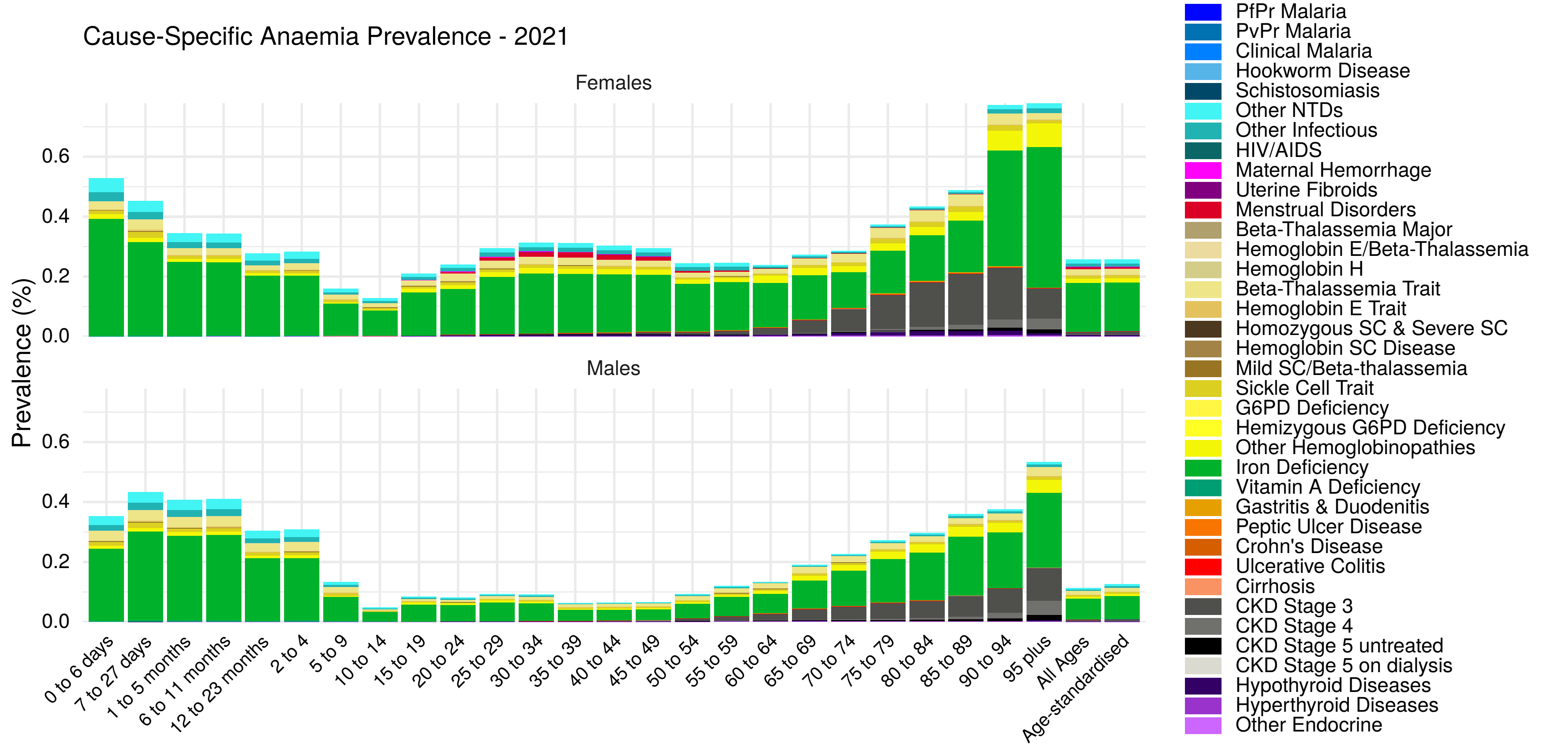

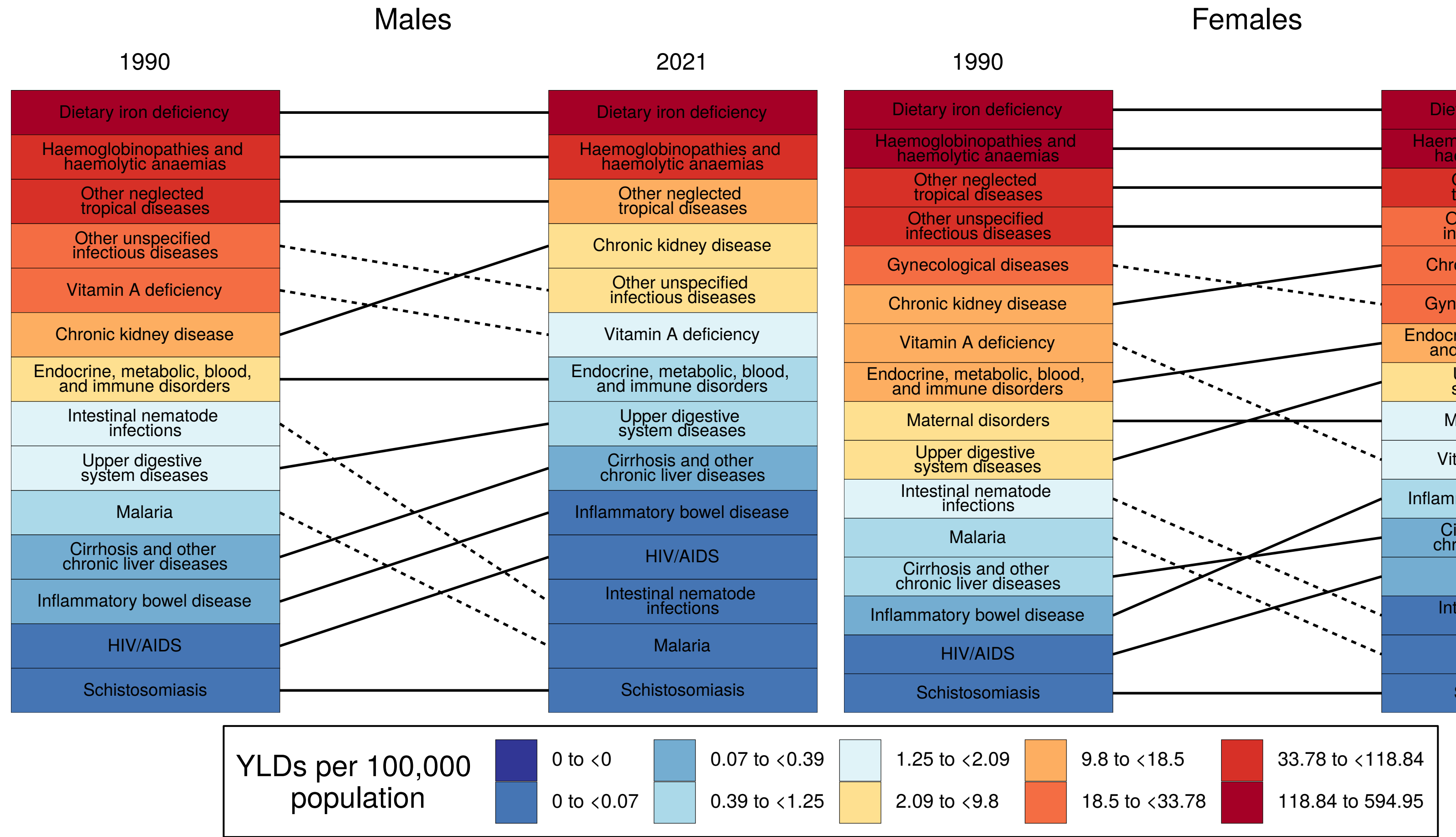

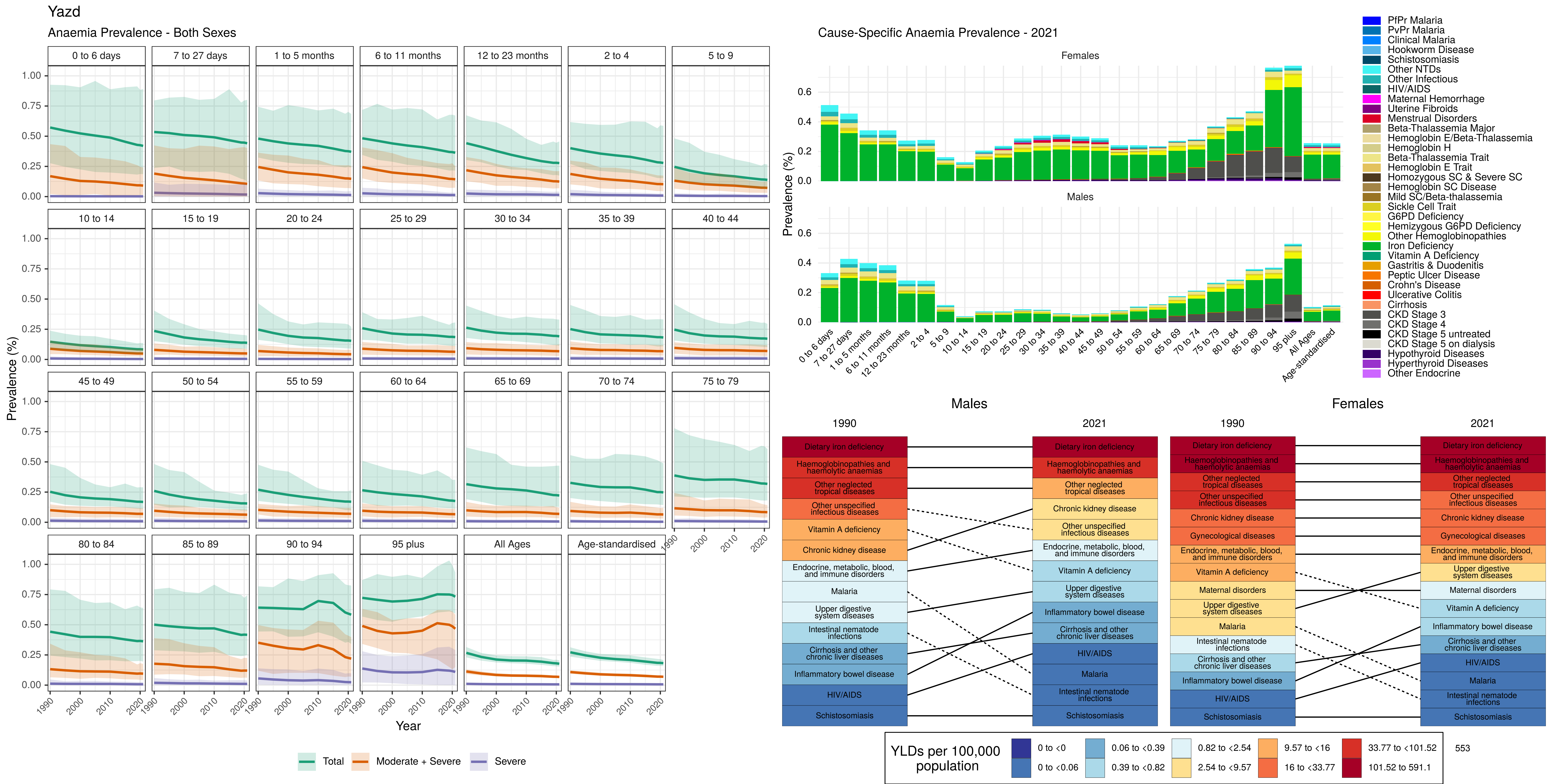

Yazd
Anaemia Prevalence - Both Sexes
0 to 6 days
7 to 27 days
1 to 5 months
6 to 11 months
12 to 23 months
2 to 4
5 to 9
10 to 14
15 to 19
20 to 24
25 to 29
30 to 34
35 to 39
40 to 44
45 to 49
50 to 54
55 to 59
60 to 64
65 to 69
70 to 74
75 to 79
80 to 84
85 to 89
90 to 94
95 plus
All Ages
Age-standardised
Prevalence (%)
Year
Total
Moderate + Severe
Severe
Cause-Specific Anaemia Prevalence - 2021
Females
Males
PfPr Malaria
PvPr Malaria
Clinical Malaria
Hookworm Disease
Schistosomiasis
Other NTDs
Other Infectious
HIV/AIDS
Maternal Hemorrhage
Uterine Fibroids
Menstrual Disorders
Beta-Thalassemia Major
Hemoglobin E/Beta-Thalassemia
Hemoglobin H
Beta-Thalassemia Trait
Hemoglobin E Trait
Homozygous SC & Severe SC
Hemoglobin SC Disease
Mild SC/Beta-thalassemia
Sickle Cell Trait
G6PD Deficiency
Hemizygous G6PD Deficiency
Other Hemoglobinopathies
Iron Deficiency
Vitamin A Deficiency
Gastritis & Duodenitis
Peptic Ulcer Disease
Crohn's Disease
Ulcerative Colitis
Cirrhosis
CKD Stage 3
CKD Stage 4
CKD Stage 5 untreated
CKD Stage 5 on dialysis
Hypothyroid Diseases
Hyperthyroid Diseases
Other Endocrine
Males
Females
1990
2021
Dietary iron deficiency
Haemoglobinopathies and haemolytic anaemias
Other neglected tropical diseases
Other unspecified infectious diseases
Vitamin A deficiency
Chronic kidney disease
Endocrine, metabolic, blood, and immune disorders
Malaria
Upper digestive system diseases
Intestinal nematode infections
Cirrhosis and other chronic liver diseases
Inflammatory bowel disease
HIV/AIDS
Schistosomiasis
Gynecological diseases
Maternal disorders
YLDs per 100,000 population
0 to <0
0 to <0.06
0.06 to <0.39
0.39 to <0.82
0.82 to <2.54
2.54 to <9.57
9.57 to <16
16 to <33.77
33.77 to <101.52
101.52 to 591.1

Zanjan

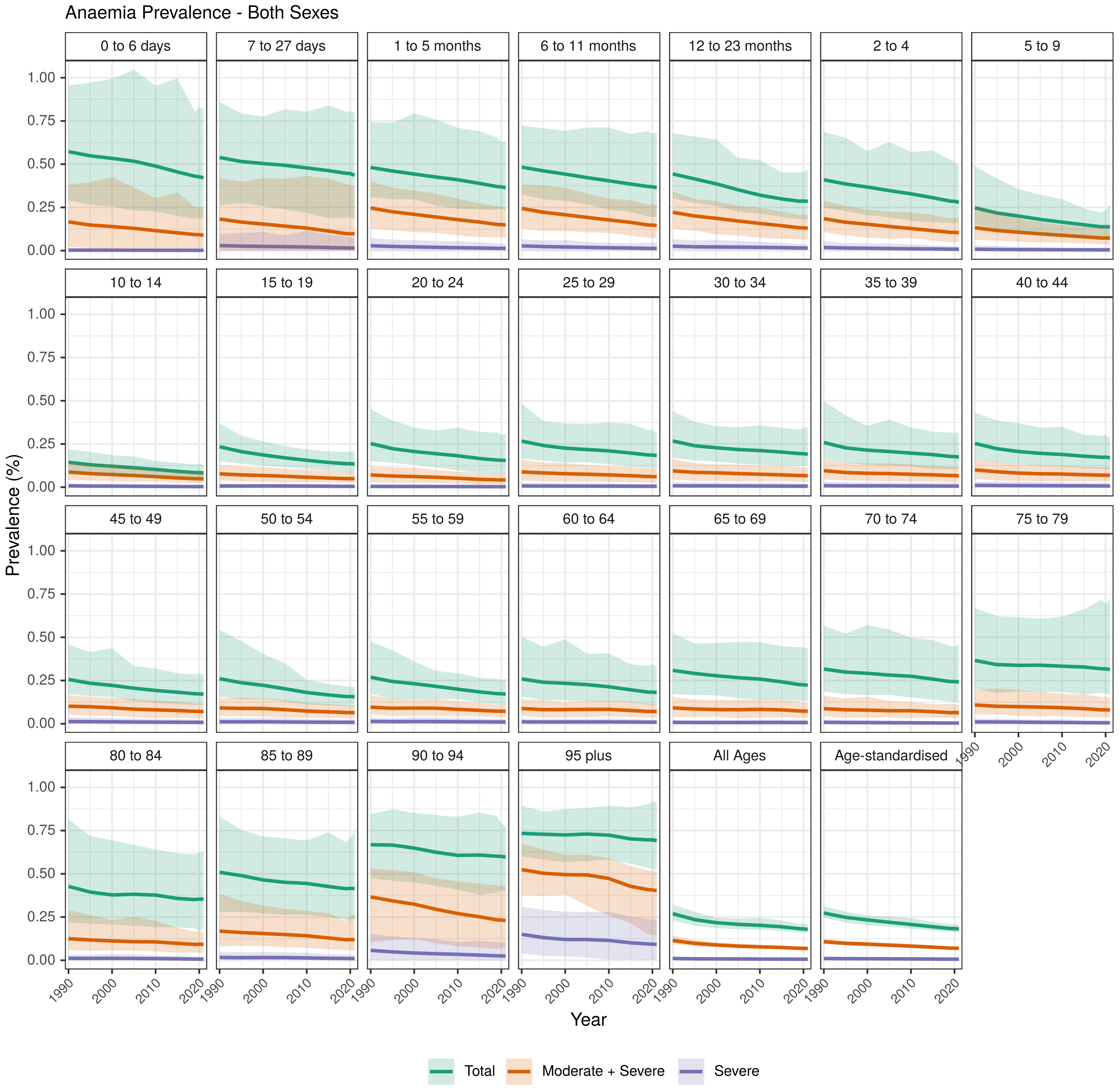

Anaemia Prevalence - Both Sexes
0 to 6 days
7 to 27 days
1 to 5 months
6 to 11 months
12 to 23 months
2 to 4
5 to 9
10 to 14
15 to 19
20 to 24
25 to 29
30 to 34
35 to 39
40 to 44
45 to 49
50 to 54
55 to 59
60 to 64
65 to 69
70 to 74
75 to 79
80 to 84
85 to 89
90 to 94
95 plus
All Ages
Age-standardised
Prevalence (%)
Year
Total
Moderate + Severe
Severe

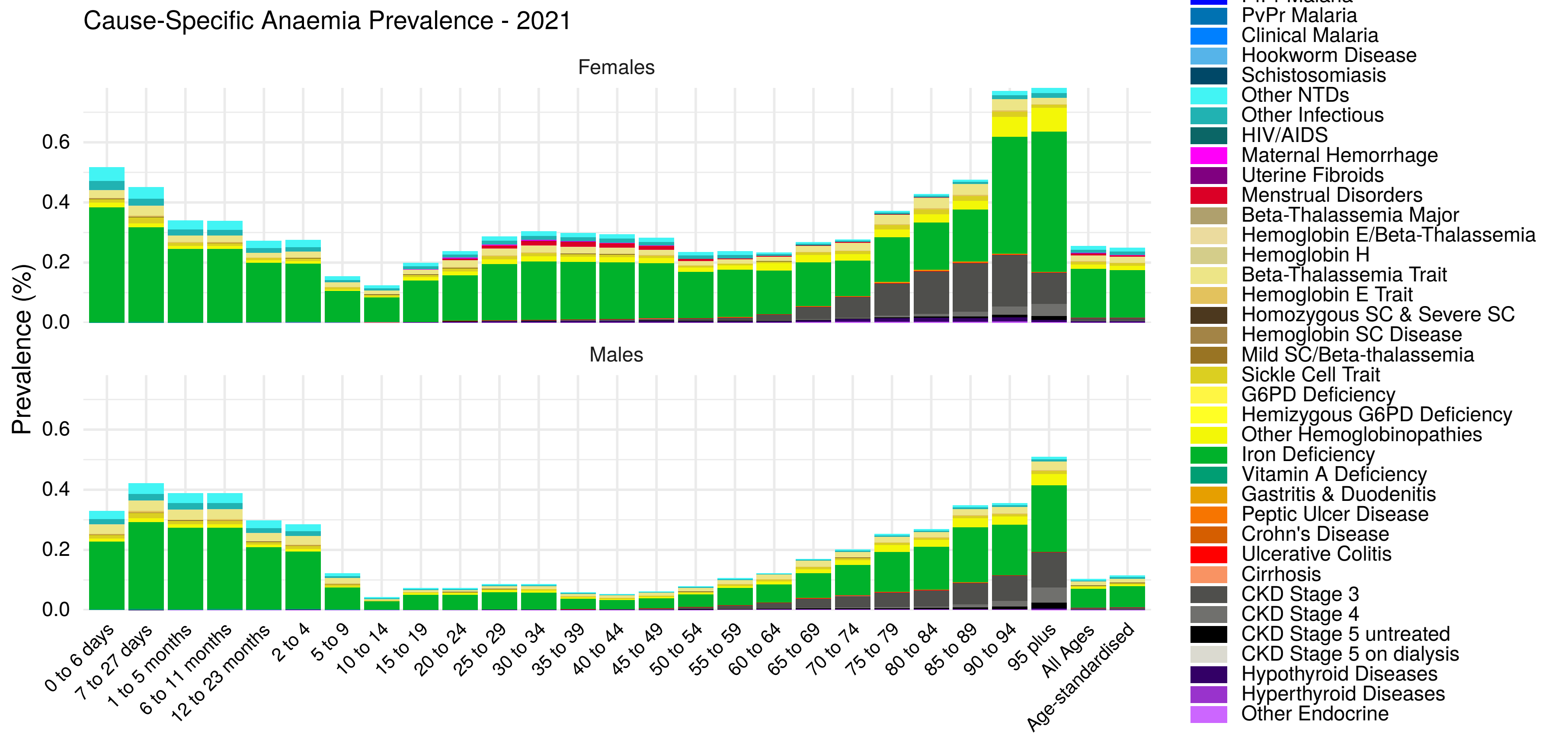

Cause-Specific Anaemia Prevalence - 2021
Females
Males
Prevalence (%)
PfPr Malaria
PvPr Malaria
Clinical Malaria
Hookworm Disease
Schistosomiasis
Other NTDs
Other Infectious
HIV/AIDS
Maternal Hemorrhage
Uterine Fibroids
Menstrual Disorders
Beta-Thalassemia Major
Hemoglobin E/Beta-Thalassemia
Hemoglobin H
Beta-Thalassemia Trait
Hemoglobin E Trait
Homozygous SC & Severe SC
Hemoglobin SC Disease
Mild SC/Beta-thalassemia
Sickle Cell Trait
G6PD Deficiency
Hemizygous G6PD Deficiency
Other Hemoglobinopathies
Iron Deficiency
Vitamin A Deficiency
Gastritis & Duodenitis
Peptic Ulcer Disease
Crohn's Disease
Ulcerative Colitis
Cirrhosis
CKD Stage 3
CKD Stage 4
CKD Stage 5 untreated
CKD Stage 5 on dialysis
Hypothyroid Diseases
Hyperthyroid Diseases
Other Endocrine

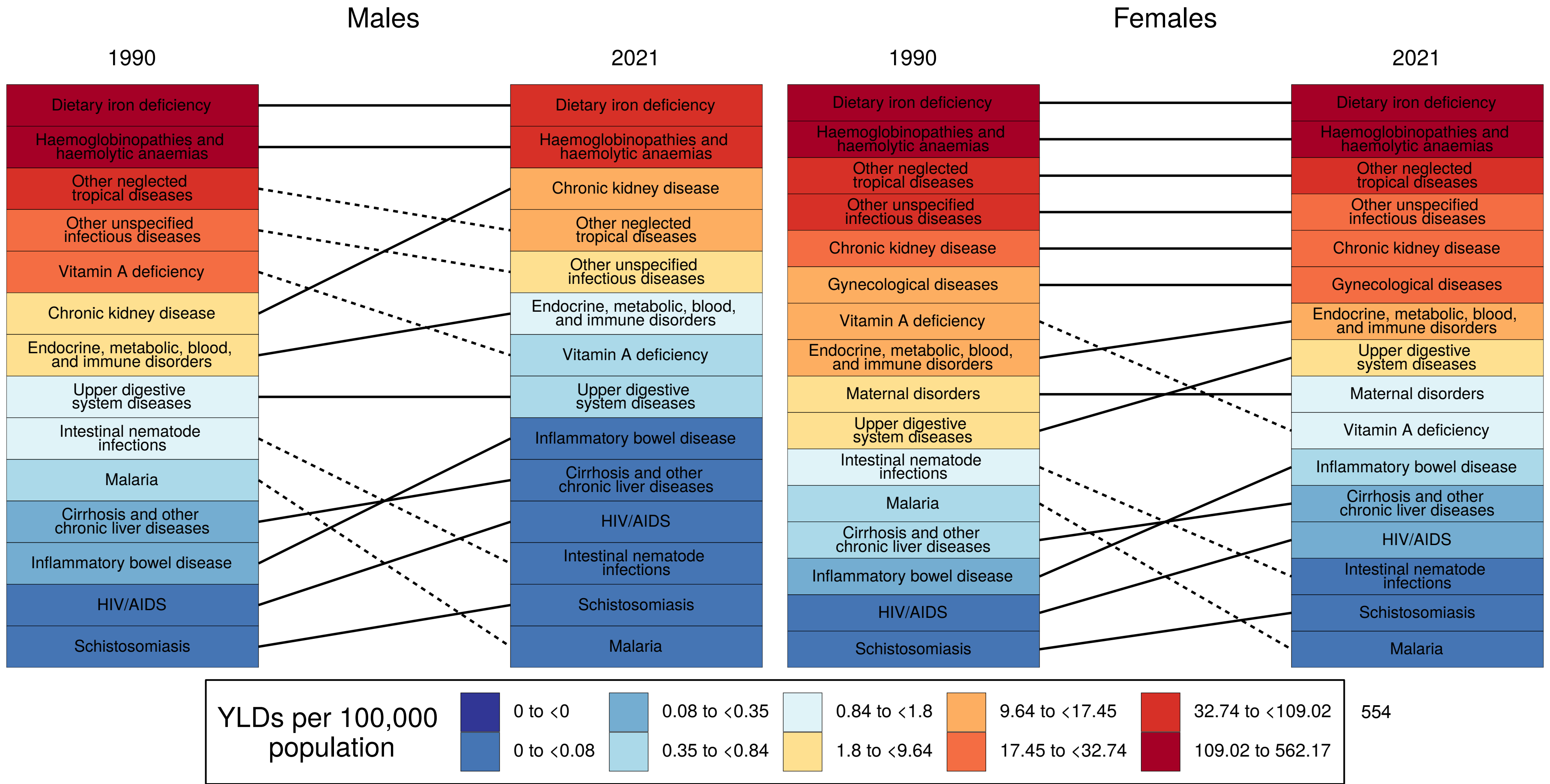

Males
Females
1990
2021
Dietary iron deficiency
Haemoglobinopathies and haemolytic anaemias
Other neglected tropical diseases
Other unspecified infectious diseases
Vitamin A deficiency
Chronic kidney disease
Endocrine, metabolic, blood, and immune disorders
Upper digestive system diseases
Intestinal nematode infections
Malaria
Cirrhosis and other chronic liver diseases
Inflammatory bowel disease
HIV/AIDS
Schistosomiasis
Gynecological diseases
Maternal disorders
YLDs per 100,000 population
0 to <0
0 to <0.08
0.08 to <0.35
0.35 to <0.84
0.84 to <1.8
1.8 to <9.64
9.64 to <17.45
17.45 to <32.74
32.74 to <109.02
109.02 to 562.17

554

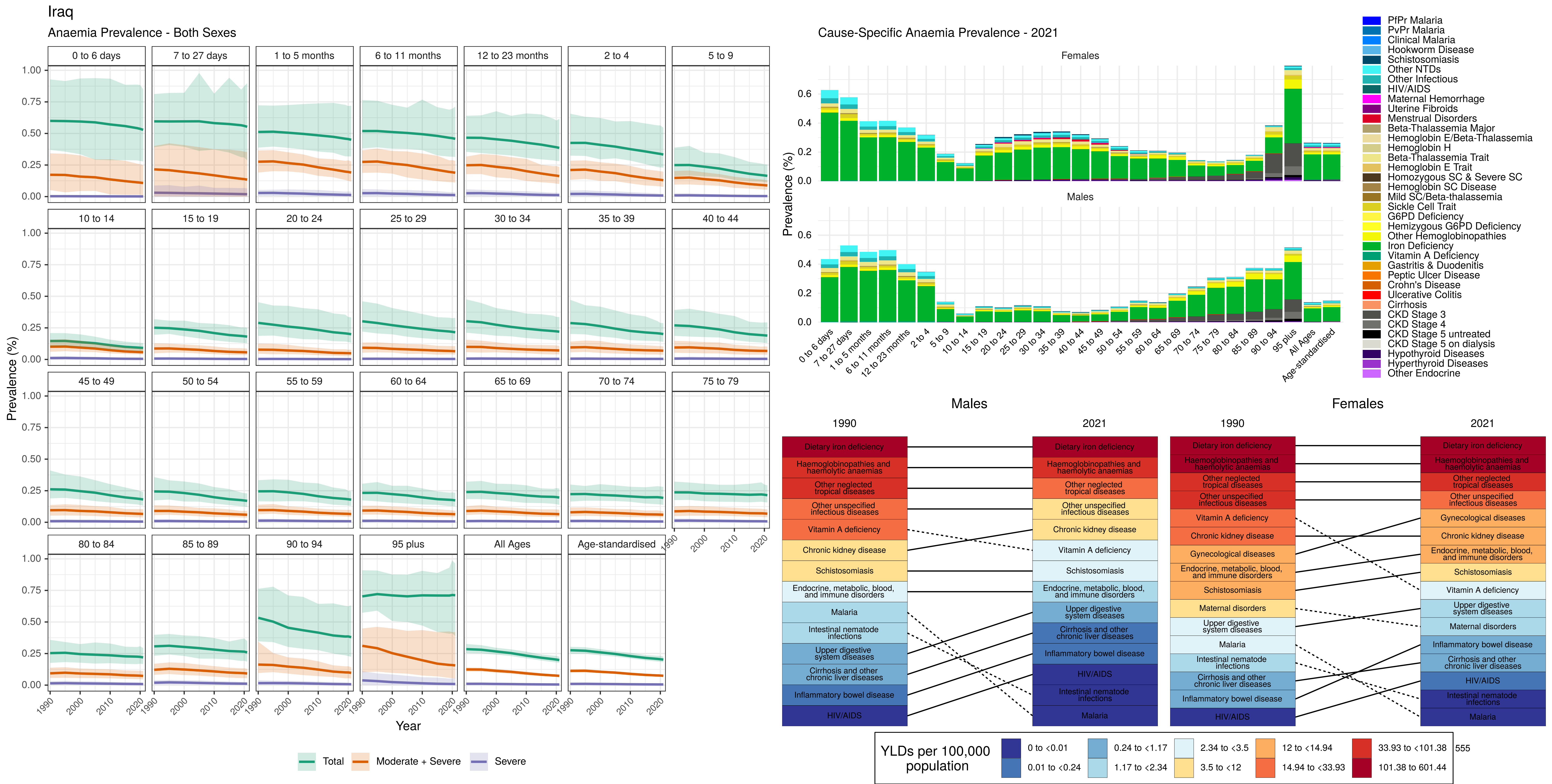
Iraq
Anaemia Prevalence - Both Sexes
0 to 6 days
7 to 27 days
1 to 5 months
6 to 11 months
12 to 23 months
2 to 4
5 to 9
10 to 14
15 to 19
20 to 24
25 to 29
30 to 34
35 to 39
40 to 44
45 to 49
50 to 54
55 to 59
60 to 64
65 to 69
70 to 74
75 to 79
80 to 84
85 to 89
90 to 94
95 plus
All Ages
Age-standardised
Prevalence (%)
Year
Total
Moderate + Severe
Severe
Cause-Specific Anaemia Prevalence - 2021
Females
Males
PfPr Malaria
PvPr Malaria
Clinical Malaria
Hookworm Disease
Schistosomiasis
Other NTDs
Other Infectious
HIV/AIDS
Maternal Hemorrhage
Uterine Fibroids
Menstrual Disorders
Beta-Thalassemia Major
Hemoglobin E/Beta-Thalassemia
Hemoglobin H
Beta-Thalassemia Trait
Hemoglobin E Trait
Homozygous SC & Severe SC
Hemoglobin SC Disease
Mild SC/Beta-thalassemia
Sickle Cell Trait
G6PD Deficiency
Hemizygous G6PD Deficiency
Other Hemoglobinopathies
Iron Deficiency
Vitamin A Deficiency
Gastritis & Duodenitis
Peptic Ulcer Disease
Crohn's Disease
Ulcerative Colitis
Cirrhosis
CKD Stage 3
CKD Stage 4
CKD Stage 5 untreated
CKD Stage 5 on dialysis
Hypothyroid Diseases
Hyperthyroid Diseases
Other Endocrine
Males
Females
1990
2021
Dietary iron deficiency
Haemoglobinopathies and haemolytic anaemias
Other neglected tropical diseases
Other unspecified infectious diseases
Vitamin A deficiency
Chronic kidney disease
Schistosomiasis
Endocrine, metabolic, blood, and immune disorders
Malaria
Intestinal nematode infections
Upper digestive system diseases
Cirrhosis and other chronic liver diseases
Inflammatory bowel disease
HIV/AIDS
Gynecological diseases
Maternal disorders
YLDs per 100,000 population
0 to <0.01
0.01 to <0.24
0.24 to <1.17
1.17 to <2.34
2.34 to <3.5
3.5 to <12
12 to <14.94
14.94 to <33.93
33.93 to <101.38
101.38 to 601.44

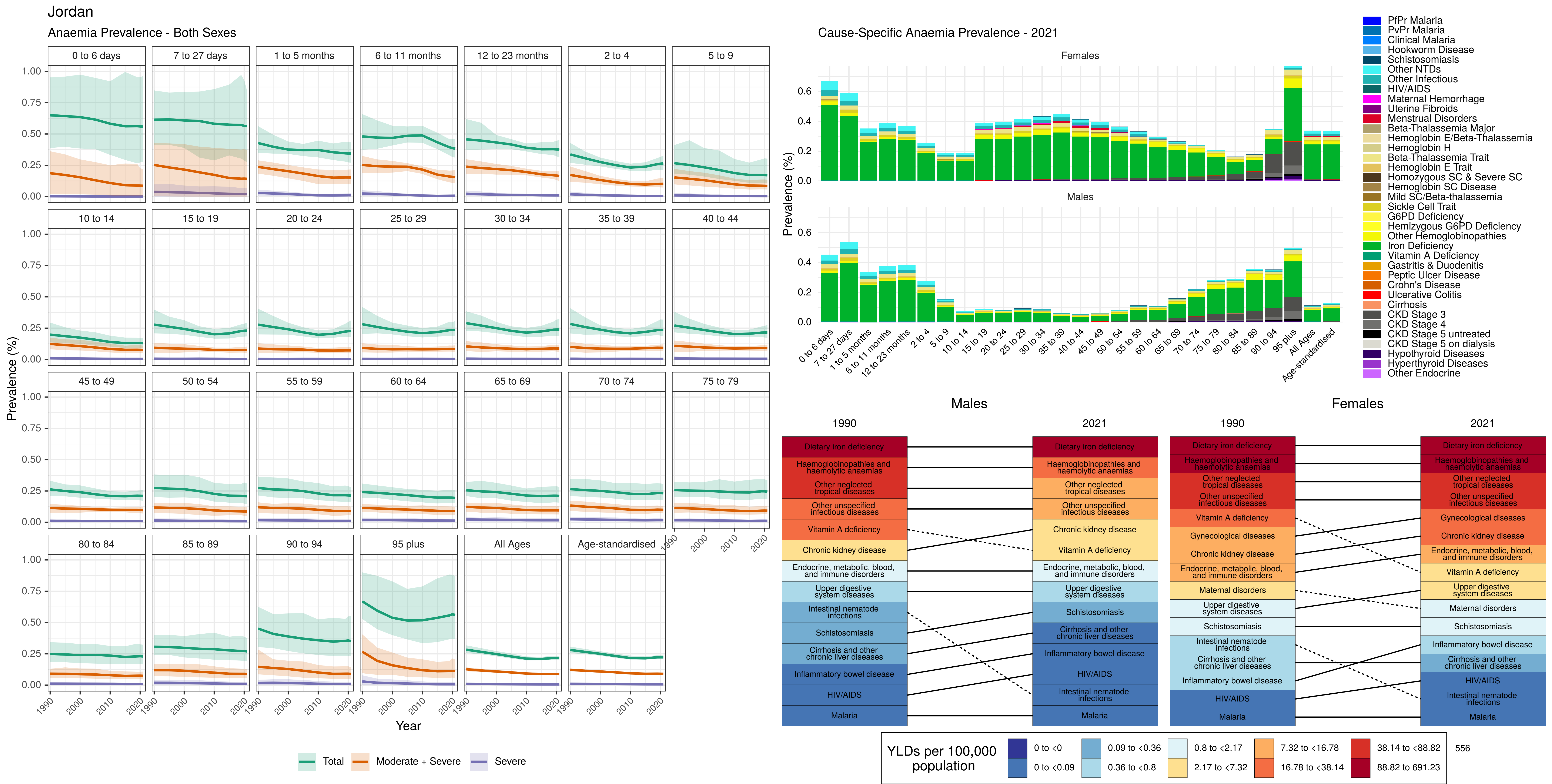

Jordan
Anaemia Prevalence - Both Sexes
0 to 6 days
7 to 27 days
1 to 5 months
6 to 11 months
12 to 23 months
2 to 4
5 to 9
10 to 14
15 to 19
20 to 24
25 to 29
30 to 34
35 to 39
40 to 44
45 to 49
50 to 54
55 to 59
60 to 64
65 to 69
70 to 74
75 to 79
80 to 84
85 to 89
90 to 94
95 plus
All Ages
Age-standardised
Prevalence (%)
Year
Total
Moderate + Severe
Severe
Cause-Specific Anaemia Prevalence - 2021
Females
Males
PfPr Malaria
PvPr Malaria
Clinical Malaria
Hookworm Disease
Schistosomiasis
Other NTDs
Other Infectious
HIV/AIDS
Maternal Hemorrhage
Uterine Fibroids
Menstrual Disorders
Beta-Thalassemia Major
Hemoglobin E/Beta-Thalassemia
Hemoglobin H
Beta-Thalassemia Trait
Hemoglobin E Trait
Homozygous SC & Severe SC
Hemoglobin SC Disease
Mild SC/Beta-thalassemia
Sickle Cell Trait
G6PD Deficiency
Hemizygous G6PD Deficiency
Other Hemoglobinopathies
Iron Deficiency
Vitamin A Deficiency
Gastritis & Duodenitis
Peptic Ulcer Disease
Crohn's Disease
Ulcerative Colitis
Cirrhosis
CKD Stage 3
CKD Stage 4
CKD Stage 5 untreated
CKD Stage 5 on dialysis
Hypothyroid Diseases
Hyperthyroid Diseases
Other Endocrine
Males
Females
1990
2021
Dietary iron deficiency
Haemoglobinopathies and haemolytic anaemias
Other neglected tropical diseases
Other unspecified infectious diseases
Vitamin A deficiency
Chronic kidney disease
Endocrine, metabolic, blood, and immune disorders
Upper digestive system diseases
Intestinal nematode infections
Schistosomiasis
Cirrhosis and other chronic liver diseases
Inflammatory bowel disease
HIV/AIDS
Malaria
Gynecological diseases
Maternal disorders
YLDs per 100,000 population
0 to <0
0 to <0.09
0.09 to <0.36
0.36 to <0.8
0.8 to <2.17
2.17 to <7.32
7.32 to <16.78
16.78 to <38.14
38.14 to <88.82
88.82 to 691.23

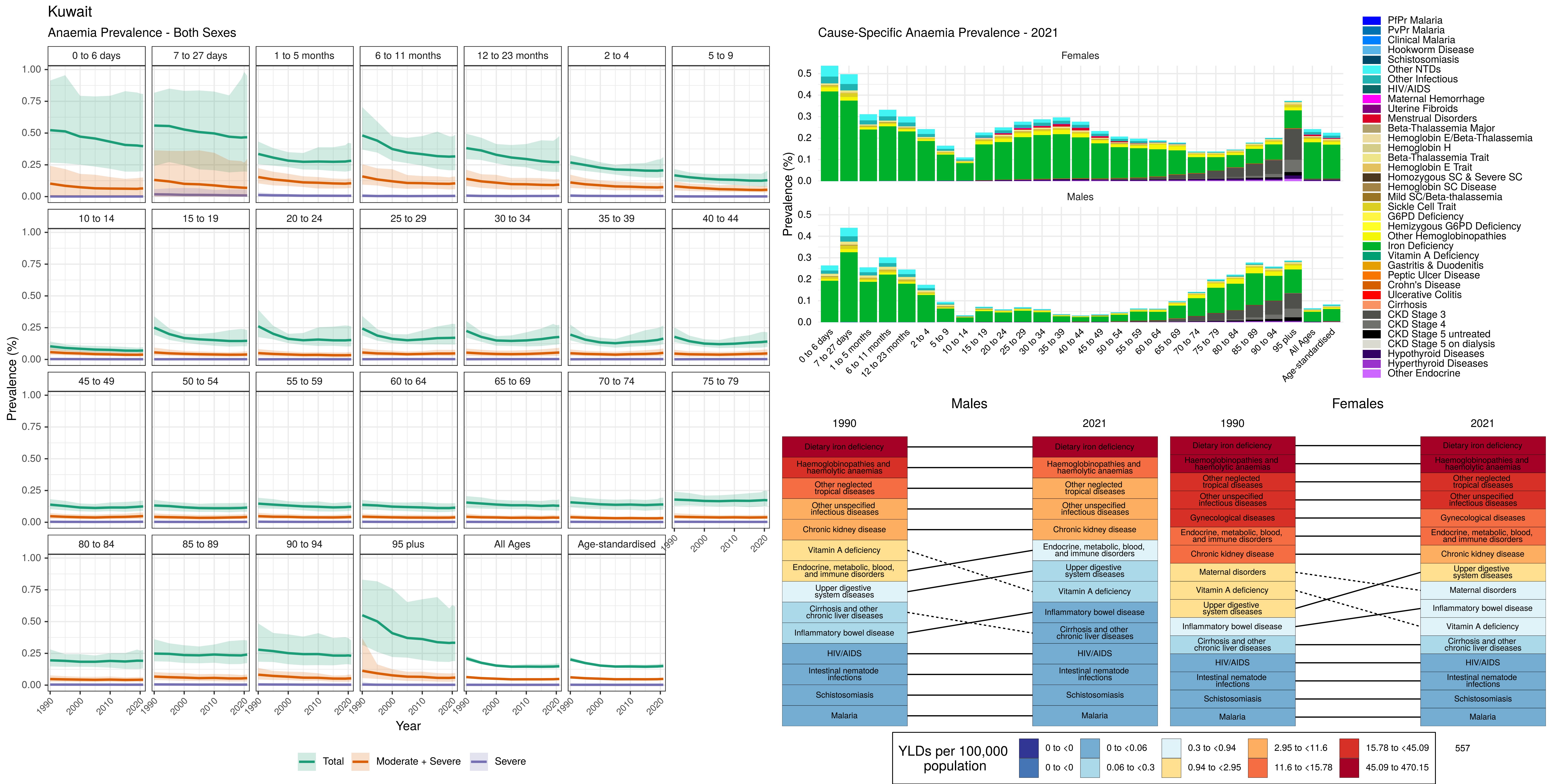
Kuwait
Anaemia Prevalence - Both Sexes
0 to 6 days
7 to 27 days
1 to 5 months
6 to 11 months
12 to 23 months
2 to 4
5 to 9
10 to 14
15 to 19
20 to 24
25 to 29
30 to 34
35 to 39
40 to 44
45 to 49
50 to 54
55 to 59
60 to 64
65 to 69
70 to 74
75 to 79
80 to 84
85 to 89
90 to 94
95 plus
All Ages
Age-standardised
Prevalence (%)
Year
Total
Moderate + Severe
Severe
Cause-Specific Anaemia Prevalence - 2021
Females
Males
PfPr Malaria
PvPr Malaria
Clinical Malaria
Hookworm Disease
Schistosomiasis
Other NTDs
Other Infectious
HIV/AIDS
Maternal Hemorrhage
Uterine Fibroids
Menstrual Disorders
Beta-Thalassemia Major
Hemoglobin E/Beta-Thalassemia
Hemoglobin H
Beta-Thalassemia Trait
Hemoglobin E Trait
Homozygous SC & Severe SC
Hemoglobin SC Disease
Mild SC/Beta-thalassemia
Sickle Cell Trait
G6PD Deficiency
Hemizygous G6PD Deficiency
Other Hemoglobinopathies
Iron Deficiency
Vitamin A Deficiency
Gastritis & Duodenitis
Peptic Ulcer Disease
Crohn's Disease
Ulcerative Colitis
Cirrhosis
CKD Stage 3
CKD Stage 4
CKD Stage 5 untreated
CKD Stage 5 on dialysis
Hypothyroid Diseases
Hyperthyroid Diseases
Other Endocrine
Males
Females
1990
2021
Dietary iron deficiency
Haemoglobinopathies and haemolytic anaemias
Other neglected tropical diseases
Other unspecified infectious diseases
Chronic kidney disease
Vitamin A deficiency
Endocrine, metabolic, blood, and immune disorders
Upper digestive system diseases
Cirrhosis and other chronic liver diseases
Inflammatory bowel disease
HIV/AIDS
Intestinal nematode infections
Schistosomiasis
Malaria
Gynecological diseases
Maternal disorders
YLDs per 100,000 population
0 to <0
0 to <0
0 to <0.06
0.06 to <0.3
0.3 to <0.94
0.94 to <2.95
2.95 to <11.6
11.6 to <15.78
15.78 to <45.09
45.09 to 470.15

Lebanon

Anaemia Prevalence - Both Sexes

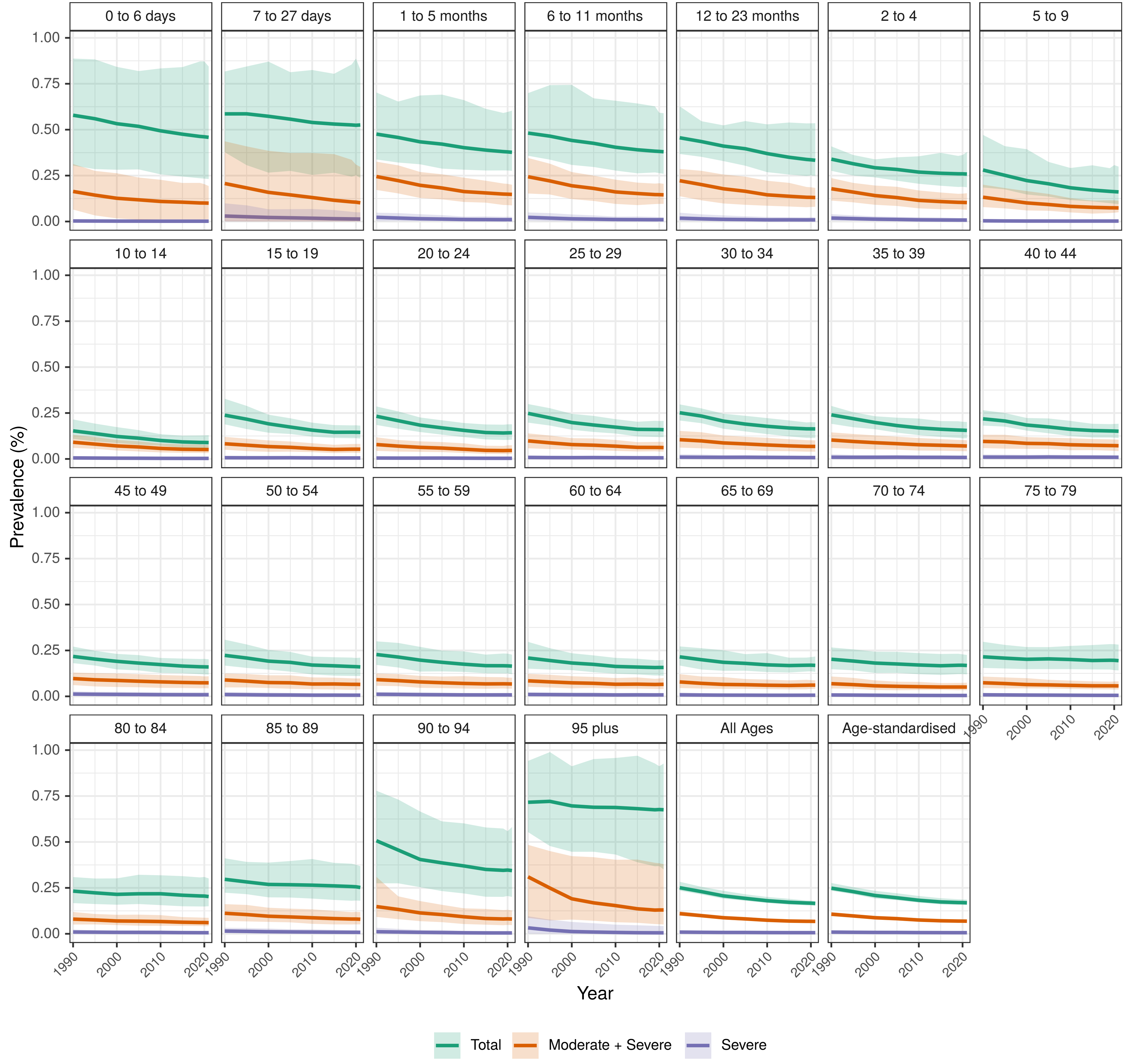

Cause-Specific Anaemia Prevalence - 2021

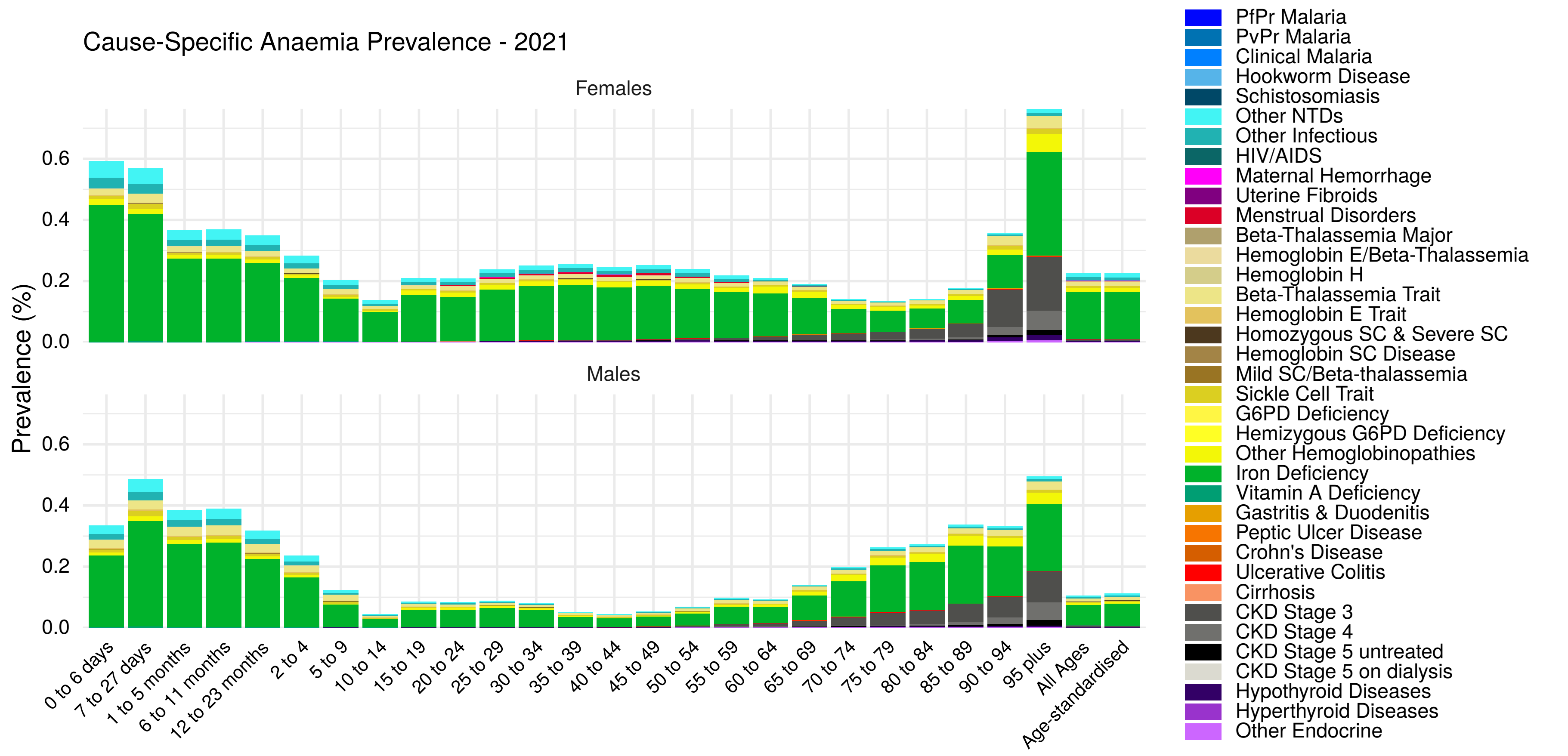

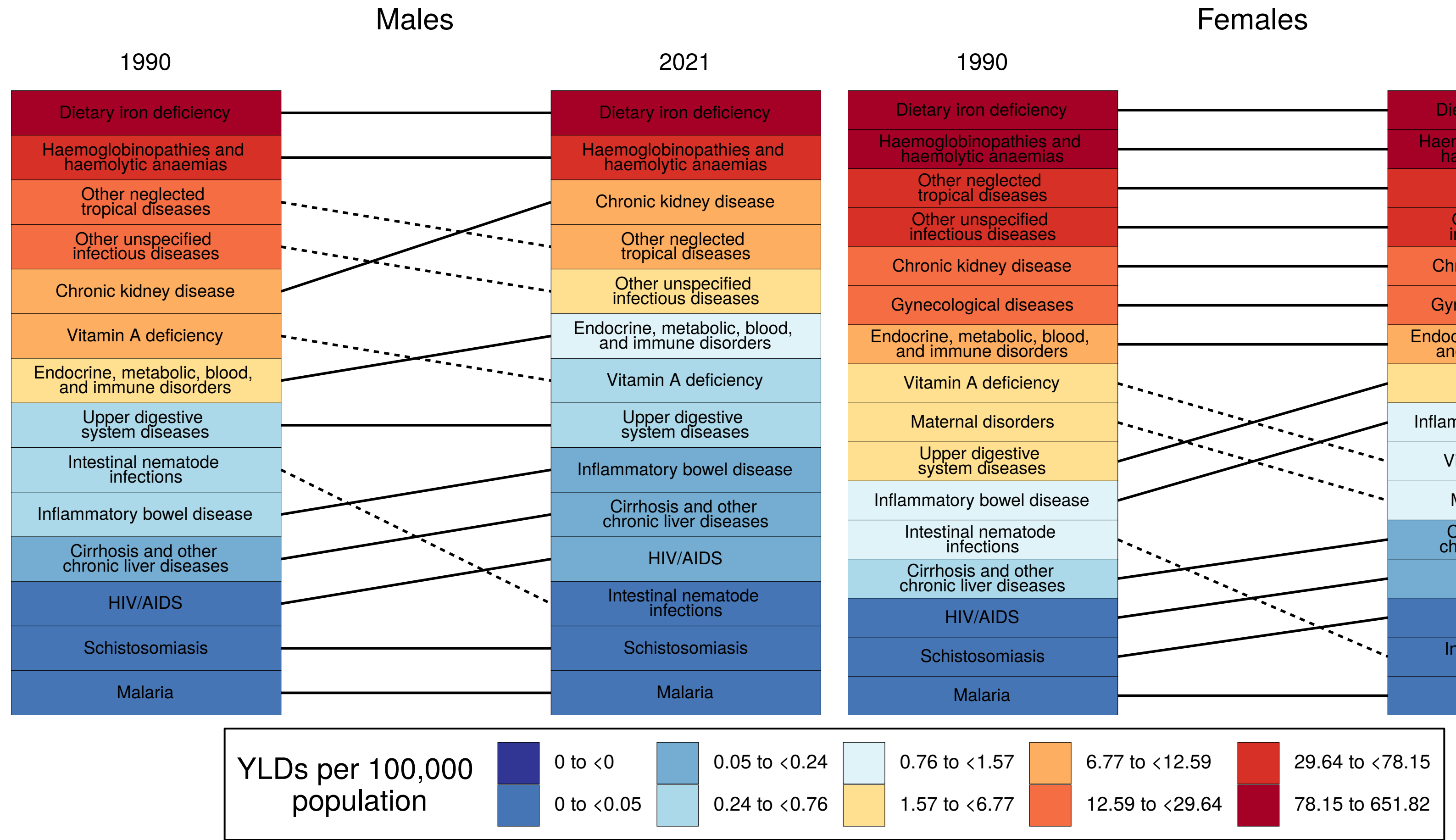

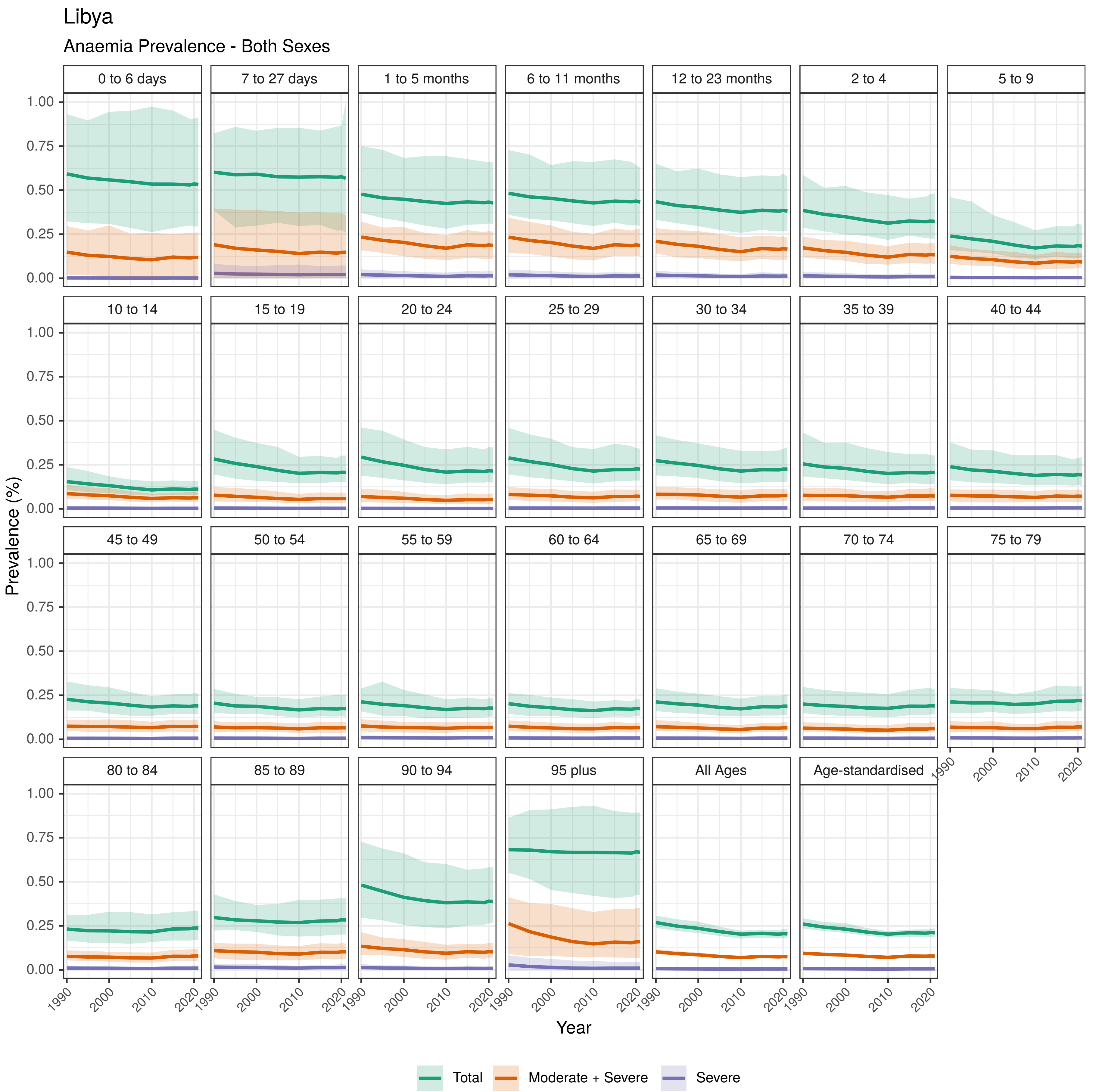
Libya
Anaemia Prevalence - Both Sexes
0 to 6 days
7 to 27 days
1 to 5 months
6 to 11 months
12 to 23 months
2 to 4
5 to 9
10 to 14
15 to 19
20 to 24
25 to 29
30 to 34
35 to 39
40 to 44
45 to 49
50 to 54
55 to 59
60 to 64
65 to 69
70 to 74
75 to 79
80 to 84
85 to 89
90 to 94
95 plus
All Ages
Age-standardised
Prevalence (%)
Year
Total
Moderate + Severe
Severe

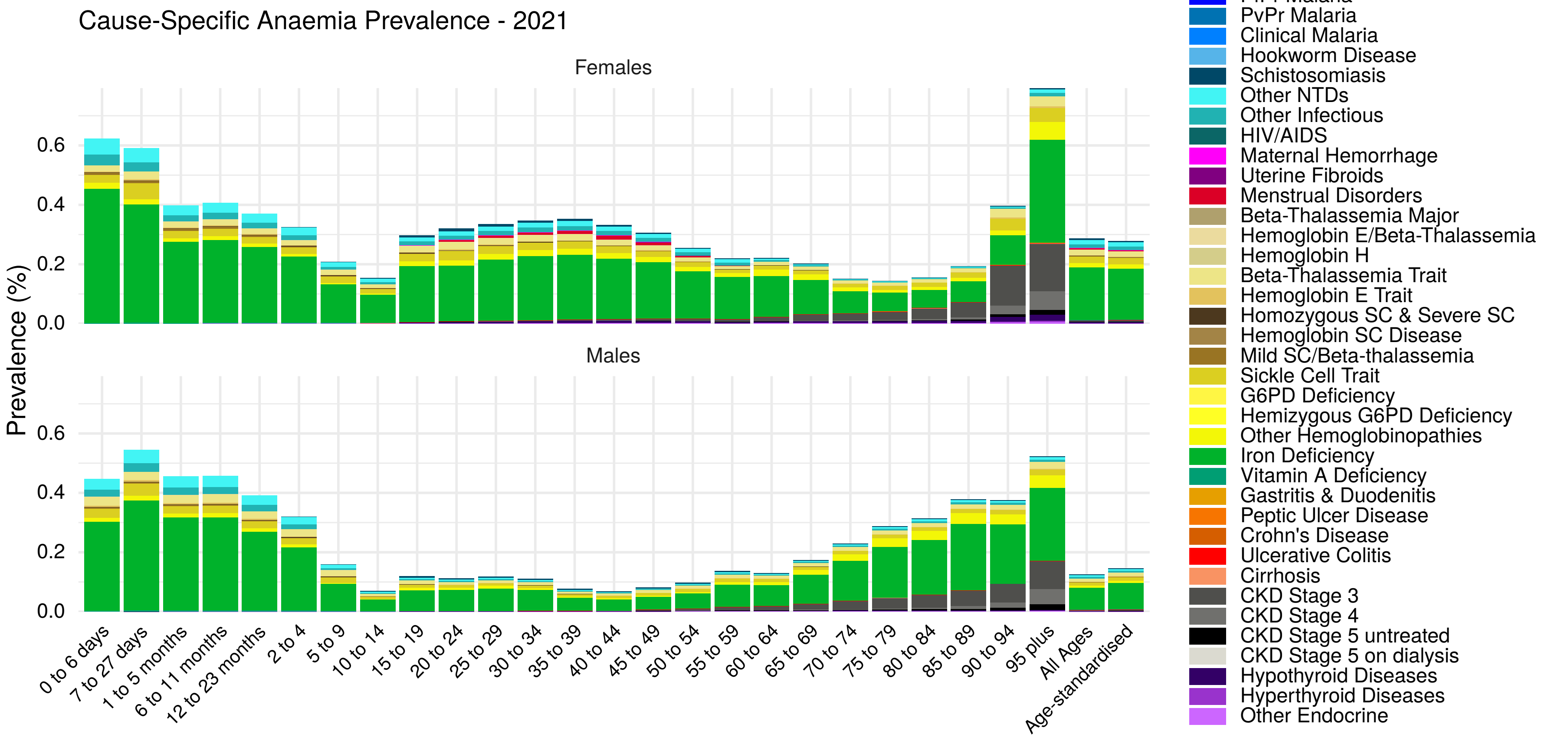
Cause-Specific Anaemia Prevalence - 2021
Females
Males
Prevalence (%)
PfPr Malaria
PvPr Malaria
Clinical Malaria
Hookworm Disease
Schistosomiasis
Other NTDs
Other Infectious
HIV/AIDS
Maternal Hemorrhage
Uterine Fibroids
Menstrual Disorders
Beta-Thalassemia Major
Hemoglobin E/Beta-Thalassemia
Hemoglobin H
Beta-Thalassemia Trait
Hemoglobin E Trait
Homozygous SC & Severe SC
Hemoglobin SC Disease
Mild SC/Beta-thalassemia
Sickle Cell Trait
G6PD Deficiency
Hemizygous G6PD Deficiency
Other Hemoglobinopathies
Iron Deficiency
Vitamin A Deficiency
Gastritis & Duodenitis
Peptic Ulcer Disease
Crohn's Disease
Ulcerative Colitis
Cirrhosis
CKD Stage 3
CKD Stage 4
CKD Stage 5 untreated
CKD Stage 5 on dialysis
Hypothyroid Diseases
Hyperthyroid Diseases
Other Endocrine

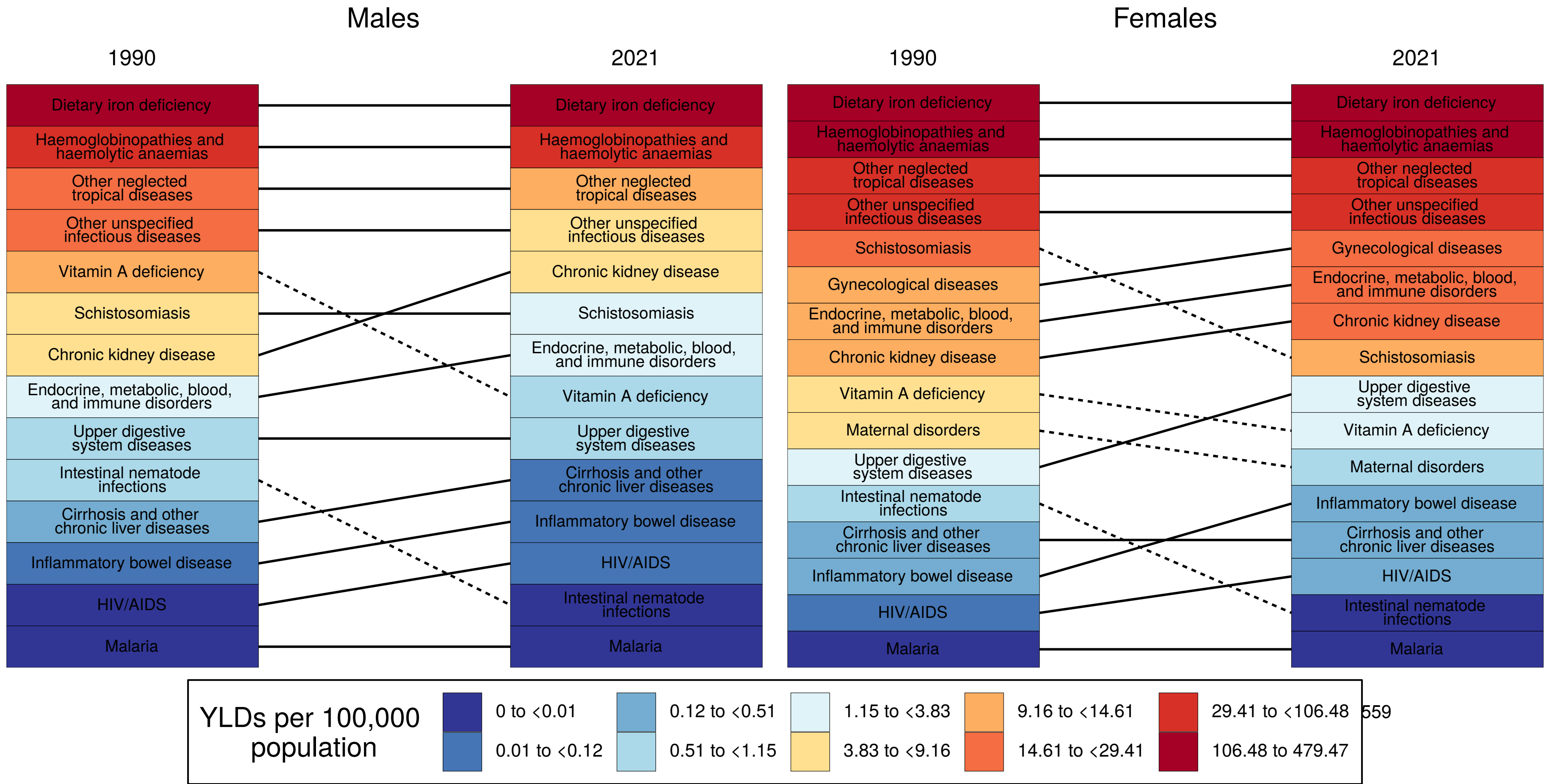
Males
Females
1990
2021
Dietary iron deficiency
Haemoglobinopathies and haemolytic anaemias
Other neglected tropical diseases
Other unspecified infectious diseases
Vitamin A deficiency
Schistosomiasis
Chronic kidney disease
Endocrine, metabolic, blood, and immune disorders
Upper digestive system diseases
Intestinal nematode infections
Cirrhosis and other chronic liver diseases
Inflammatory bowel disease
HIV/AIDS
Malaria
Gynecological diseases
Maternal disorders
YLDs per 100,000 population
0 to <0.01
0.01 to <0.12
0.12 to <0.51
0.51 to <1.15
1.15 to <3.83
3.83 to <9.16
9.16 to <14.61
14.61 to <29.41
29.41 to <106.48
106.48 to 479.47

Morocco

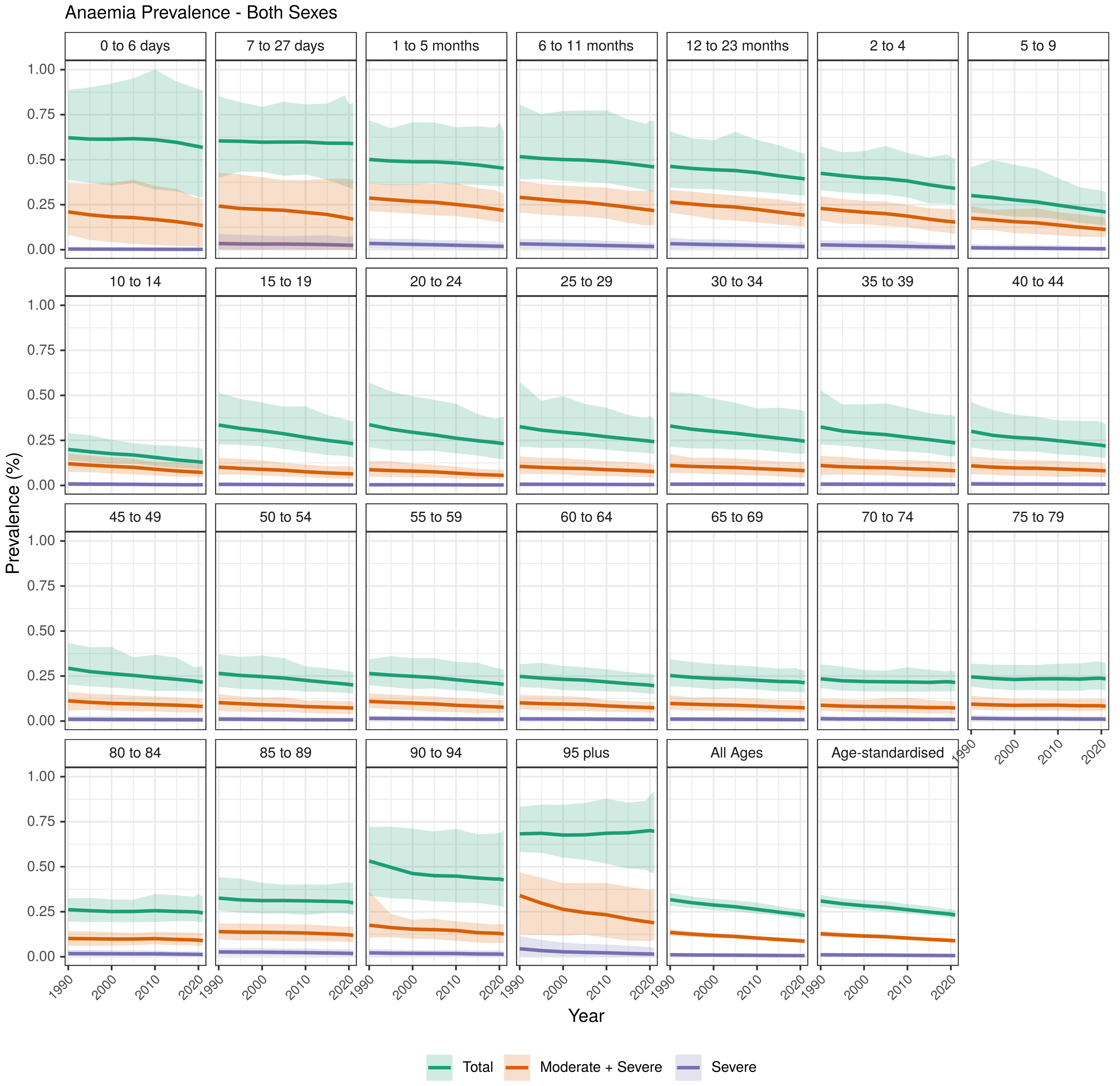
Anaemia Prevalence - Both Sexes
0 to 6 days
7 to 27 days
1 to 5 months
6 to 11 months
12 to 23 months
2 to 4
5 to 9
10 to 14
15 to 19
20 to 24
25 to 29
30 to 34
35 to 39
40 to 44
45 to 49
50 to 54
55 to 59
60 to 64
65 to 69
70 to 74
75 to 79
80 to 84
85 to 89
90 to 94
95 plus
All Ages
Age-standardised
Prevalence (%)
0.00
0.25
0.50
0.75
1.00
1990
2000
2010
2020
Year
Total
Moderate + Severe
Severe

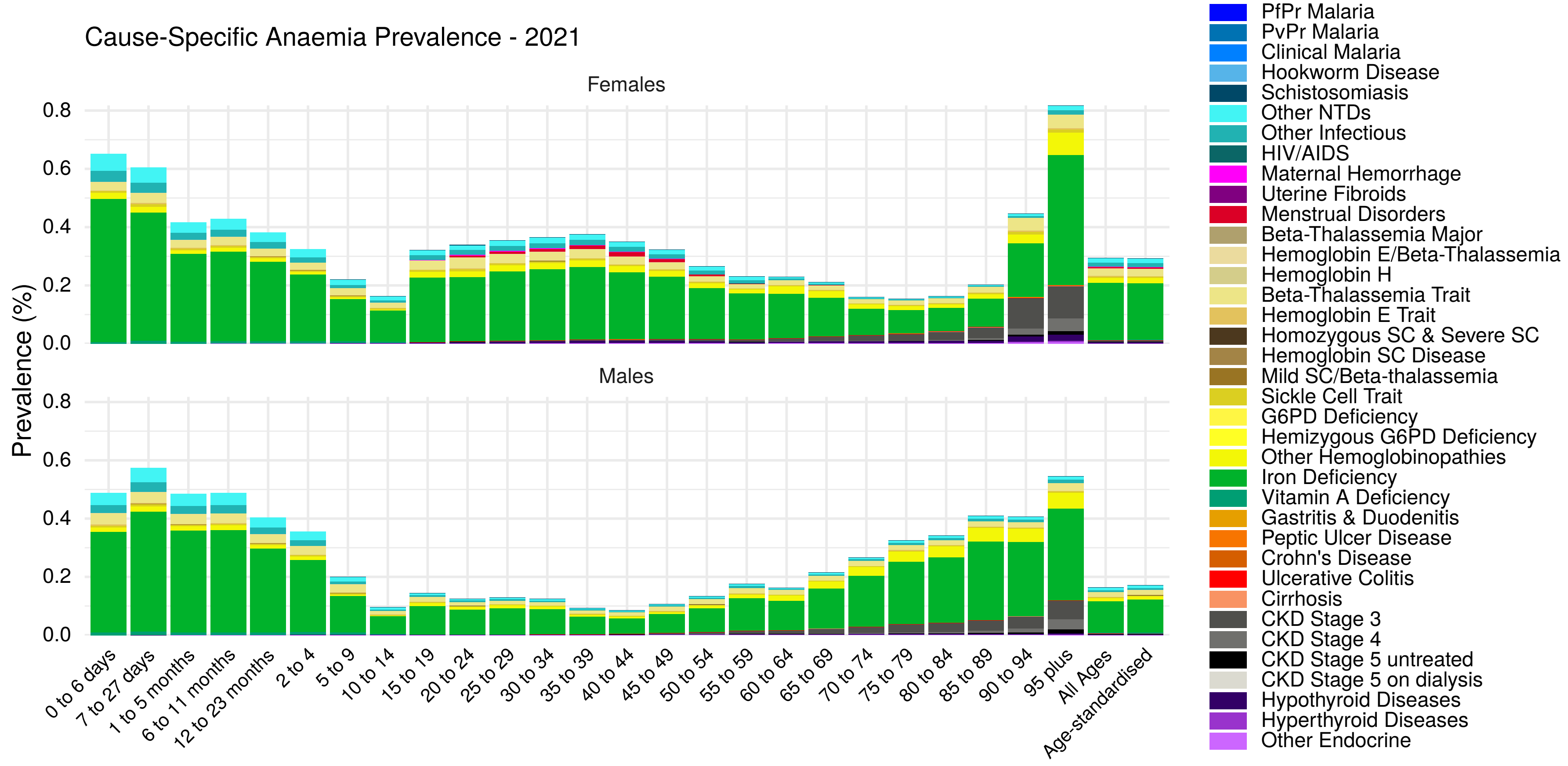
Cause-Specific Anaemia Prevalence - 2021
Females
Males
Prevalence (%)
0.0
0.2
0.4
0.6
0.8
0 to 6 days
7 to 27 days
1 to 5 months
6 to 11 months
12 to 23 months
2 to 4
5 to 9
10 to 14
15 to 19
20 to 24
25 to 29
30 to 34
35 to 39
40 to 44
45 to 49
50 to 54
55 to 59
60 to 64
65 to 69
70 to 74
75 to 79
80 to 84
85 to 89
90 to 94
95 plus
All Ages
Age-standardised
PfPr Malaria
PvPr Malaria
Clinical Malaria
Hookworm Disease
Schistosomiasis
Other NTDs
Other Infectious
HIV/AIDS
Maternal Hemorrhage
Uterine Fibroids
Menstrual Disorders
Beta-Thalassemia Major
Hemoglobin E/Beta-Thalassemia
Hemoglobin H
Beta-Thalassemia Trait
Hemoglobin E Trait
Homozygous SC & Severe SC
Hemoglobin SC Disease
Mild SC/Beta-thalassemia
Sickle Cell Trait
G6PD Deficiency
Hemizygous G6PD Deficiency
Other Hemoglobinopathies
Iron Deficiency
Vitamin A Deficiency
Gastritis & Duodenitis
Peptic Ulcer Disease
Crohn's Disease
Ulcerative Colitis
Cirrhosis
CKD Stage 3
CKD Stage 4
CKD Stage 5 untreated
CKD Stage 5 on dialysis
Hypothyroid Diseases
Hyperthyroid Diseases
Other Endocrine

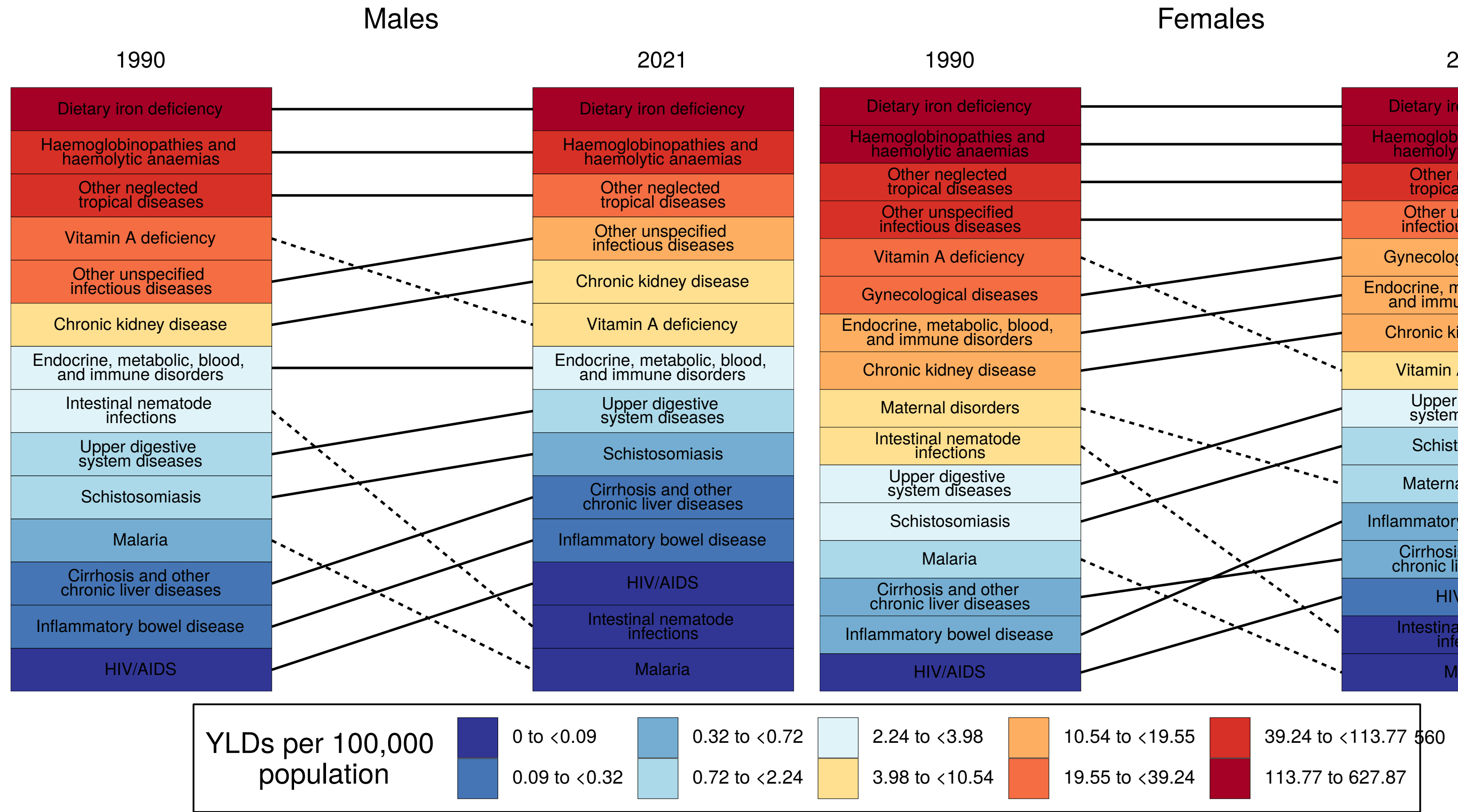
Males
Females
1990
2021
Dietary iron deficiency
Haemoglobinopathies and haemolytic anaemias
Other neglected tropical diseases
Vitamin A deficiency
Other unspecified infectious diseases
Chronic kidney disease
Endocrine, metabolic, blood, and immune disorders
Intestinal nematode infections
Upper digestive system diseases
Schistosomiasis
Malaria
Cirrhosis and other chronic liver diseases
Inflammatory bowel disease
HIV/AIDS
Gynecological diseases
Maternal disorders
YLDs per 100,000 population
0 to <0.09
0.09 to <0.32
0.32 to <0.72
0.72 to <2.24
2.24 to <3.98
3.98 to <10.54
10.54 to <19.55
19.55 to <39.24
39.24 to <113.77
113.77 to 627.87
560

Oman

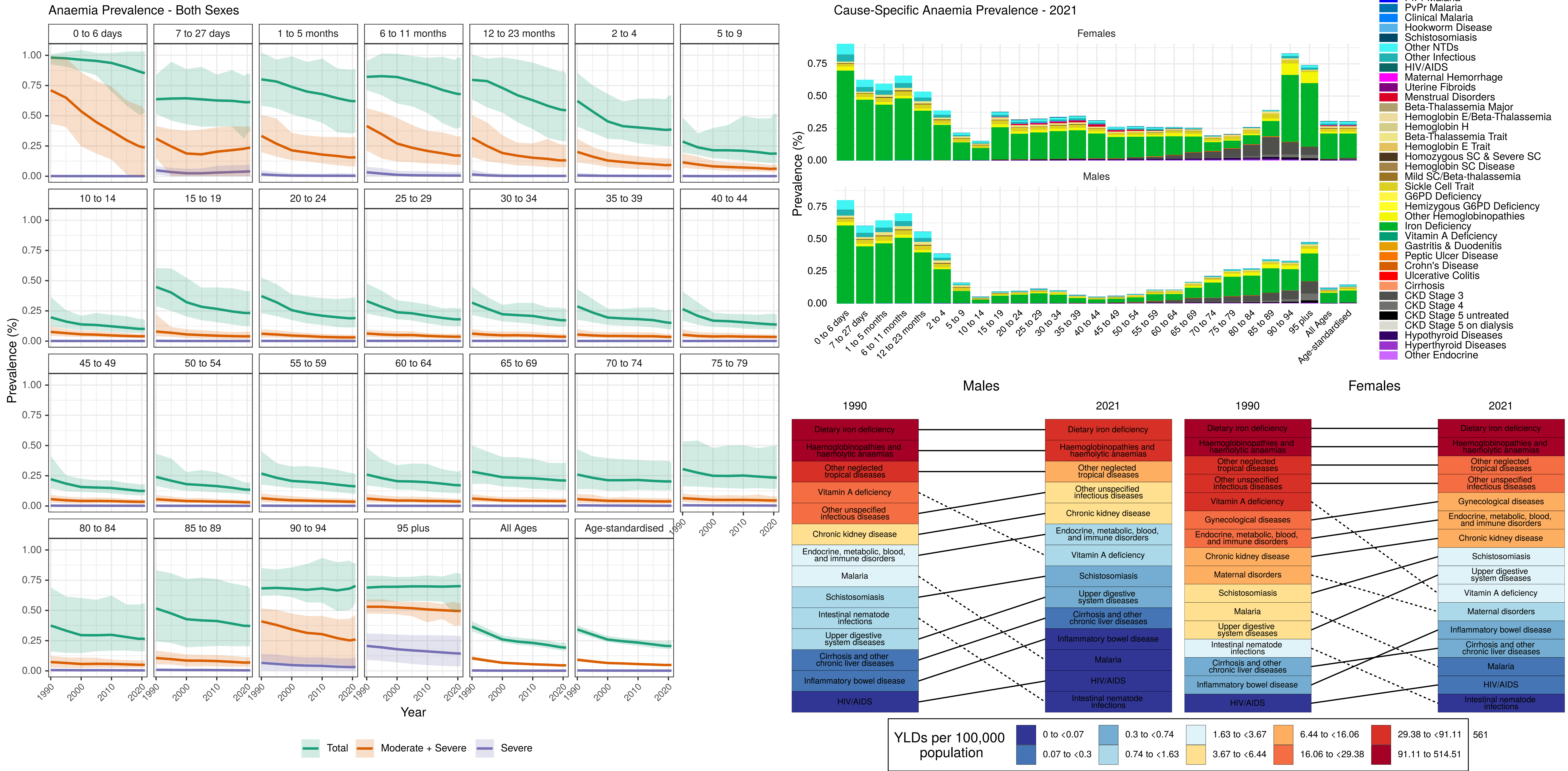

Anaemia Prevalence - Both Sexes
0 to 6 days
7 to 27 days
1 to 5 months
6 to 11 months
12 to 23 months
2 to 4
5 to 9
10 to 14
15 to 19
20 to 24
25 to 29
30 to 34
35 to 39
40 to 44
45 to 49
50 to 54
55 to 59
60 to 64
65 to 69
70 to 74
75 to 79
80 to 84
85 to 89
90 to 94
95 plus
All Ages
Age-standardised
Prevalence (%)
Year
Total
Moderate + Severe
Severe
Cause-Specific Anaemia Prevalence - 2021
Females
Males
Prevalence (%)
PfPr Malaria
PvPr Malaria
Clinical Malaria
Hookworm Disease
Schistosomiasis
Other NTDs
Other Infectious
HIV/AIDS
Maternal Hemorrhage
Uterine Fibroids
Menstrual Disorders
Beta-Thalassemia Major
Hemoglobin E/Beta-Thalassemia
Hemoglobin H
Beta-Thalassemia Trait
Hemoglobin E Trait
Homozygous SC & Severe SC
Hemoglobin SC Disease
Mild SC/Beta-thalassemia
Sickle Cell Trait
G6PD Deficiency
Hemizygous G6PD Deficiency
Other Hemoglobinopathies
Iron Deficiency
Vitamin A Deficiency
Gastritis & Duodenitis
Peptic Ulcer Disease
Crohn's Disease
Ulcerative Colitis
Cirrhosis
CKD Stage 3
CKD Stage 4
CKD Stage 5 untreated
CKD Stage 5 on dialysis
Hypothyroid Diseases
Hyperthyroid Diseases
Other Endocrine
Males
1990
Dietary iron deficiency
Haemoglobinopathies and haemolytic anaemias
Other neglected tropical diseases
Vitamin A deficiency
Other unspecified infectious diseases
Chronic kidney disease
Endocrine, metabolic, blood, and immune disorders
Malaria
Schistosomiasis
Intestinal nematode infections
Upper digestive system diseases
Cirrhosis and other chronic liver diseases
Inflammatory bowel disease
HIV/AIDS
2021
Dietary iron deficiency
Haemoglobinopathies and haemolytic anaemias
Other neglected tropical diseases
Other unspecified infectious diseases
Chronic kidney disease
Endocrine, metabolic, blood, and immune disorders
Vitamin A deficiency
Schistosomiasis
Upper digestive system diseases
Cirrhosis and other chronic liver diseases
Inflammatory bowel disease
Malaria
HIV/AIDS
Intestinal nematode infections
Females
1990
Dietary iron deficiency
Haemoglobinopathies and haemolytic anaemias
Other neglected tropical diseases
Other unspecified infectious diseases
Vitamin A deficiency
Gynecological diseases
Endocrine, metabolic, blood, and immune disorders
Chronic kidney disease
Maternal disorders
Schistosomiasis
Malaria
Upper digestive system diseases
Intestinal nematode infections
Cirrhosis and other chronic liver diseases
Inflammatory bowel disease
HIV/AIDS
2021
Dietary iron deficiency
Haemoglobinopathies and haemolytic anaemias
Other neglected tropical diseases
Other unspecified infectious diseases
Gynecological diseases
Endocrine, metabolic, blood, and immune disorders
Chronic kidney disease
Schistosomiasis
Upper digestive system diseases
Vitamin A deficiency
Maternal disorders
Inflammatory bowel disease
Cirrhosis and other chronic liver diseases
Malaria
HIV/AIDS
Intestinal nematode infections
YLDs per 100,000 population
0 to <0.07
0.07 to <0.3
0.3 to <0.74
0.74 to <1.63
1.63 to <3.67
3.67 to <6.44
6.44 to <16.06
16.06 to <29.38
29.38 to <91.11
91.11 to 514.51

Palestine

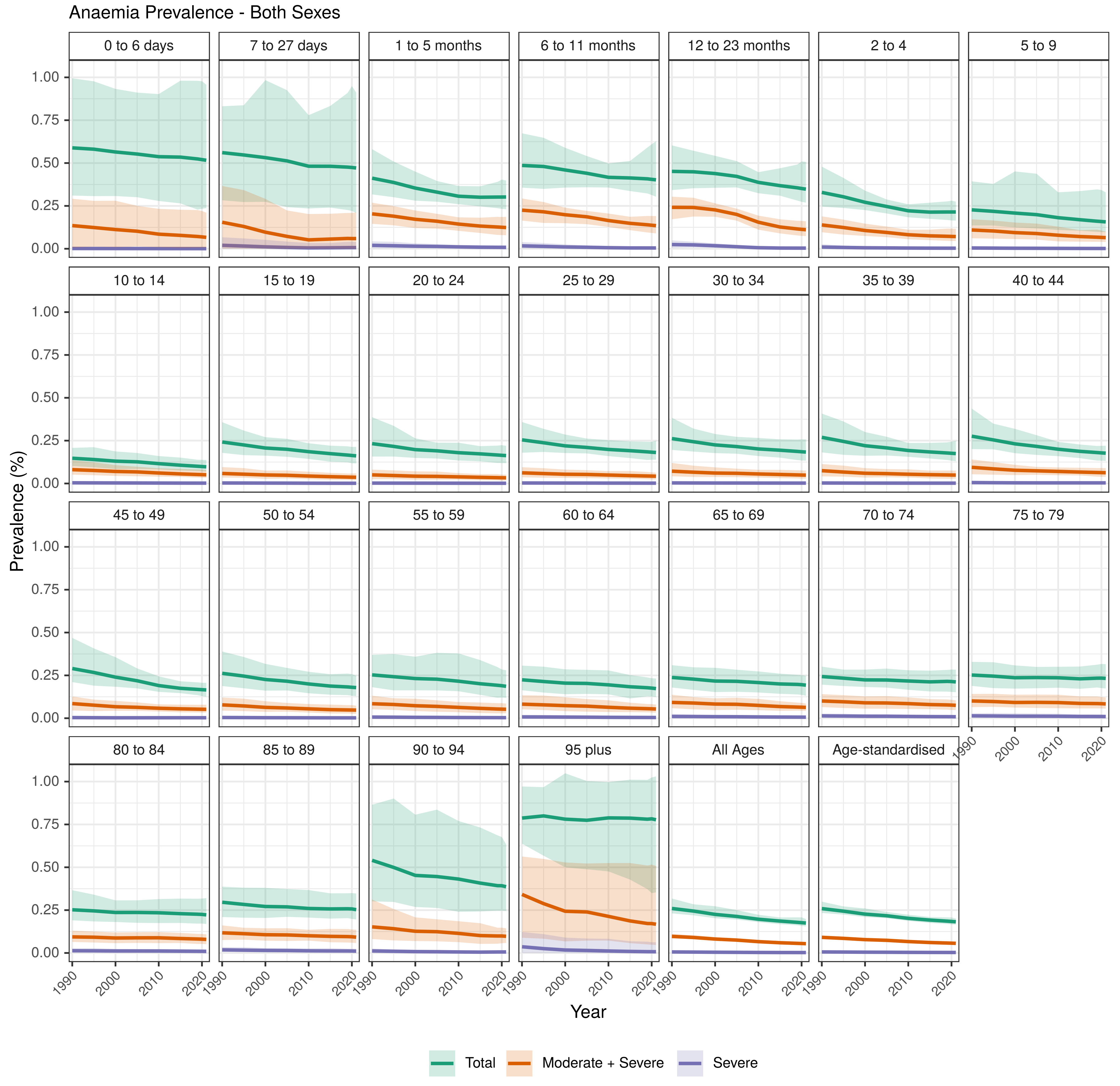

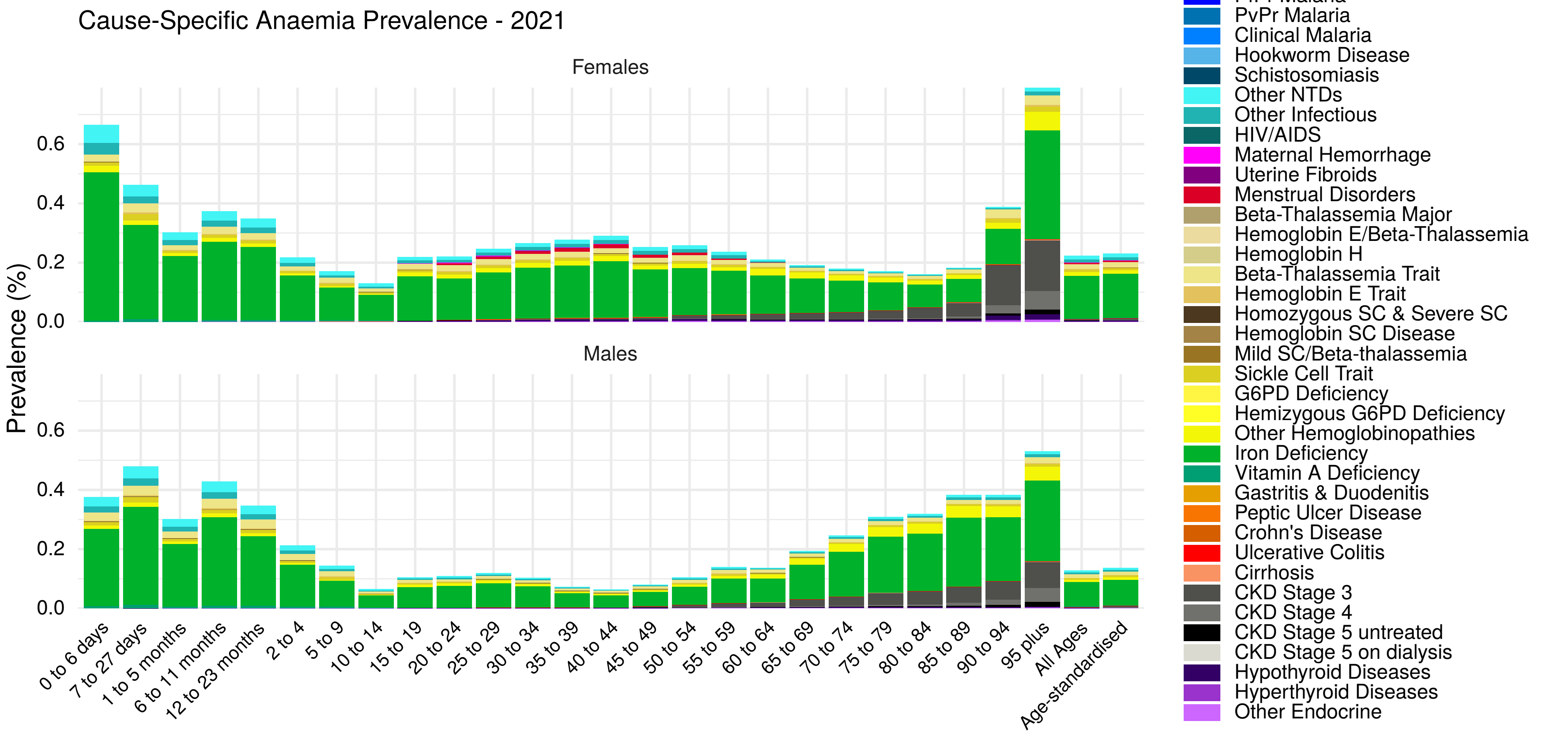

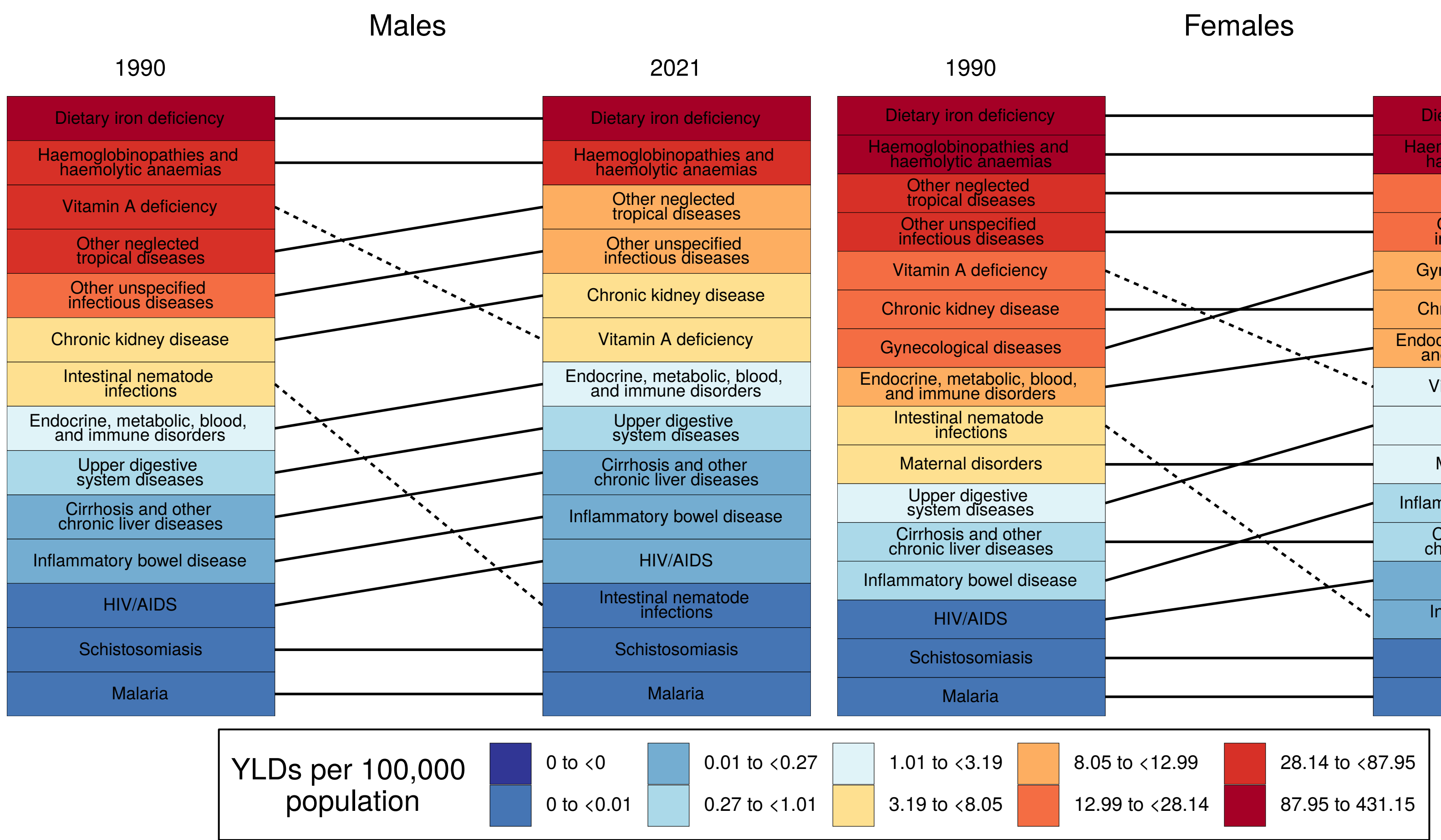

562

Qatar

Anaemia Prevalence - Both Sexes

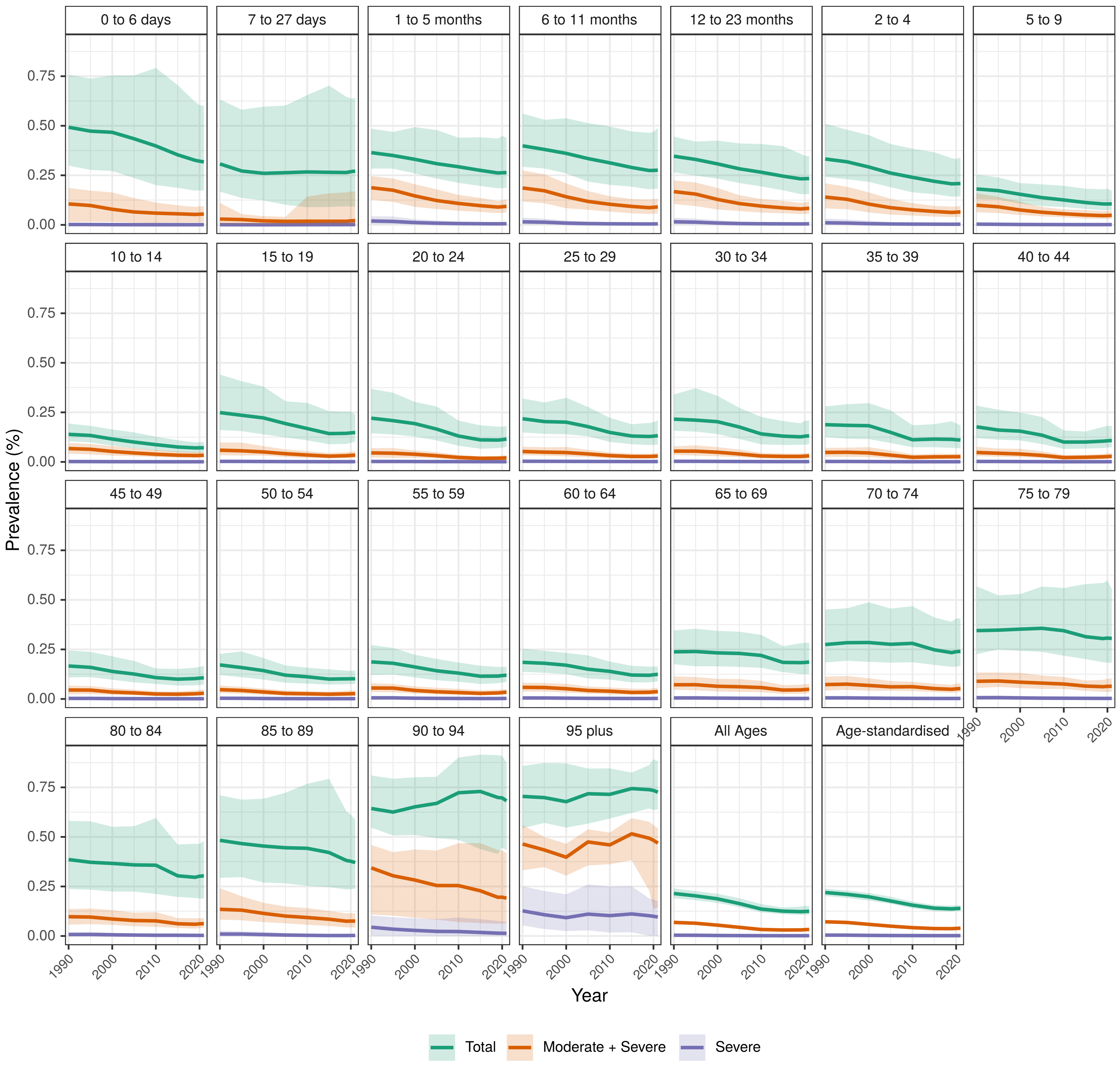

Cause-Specific Anaemia Prevalence - 2021

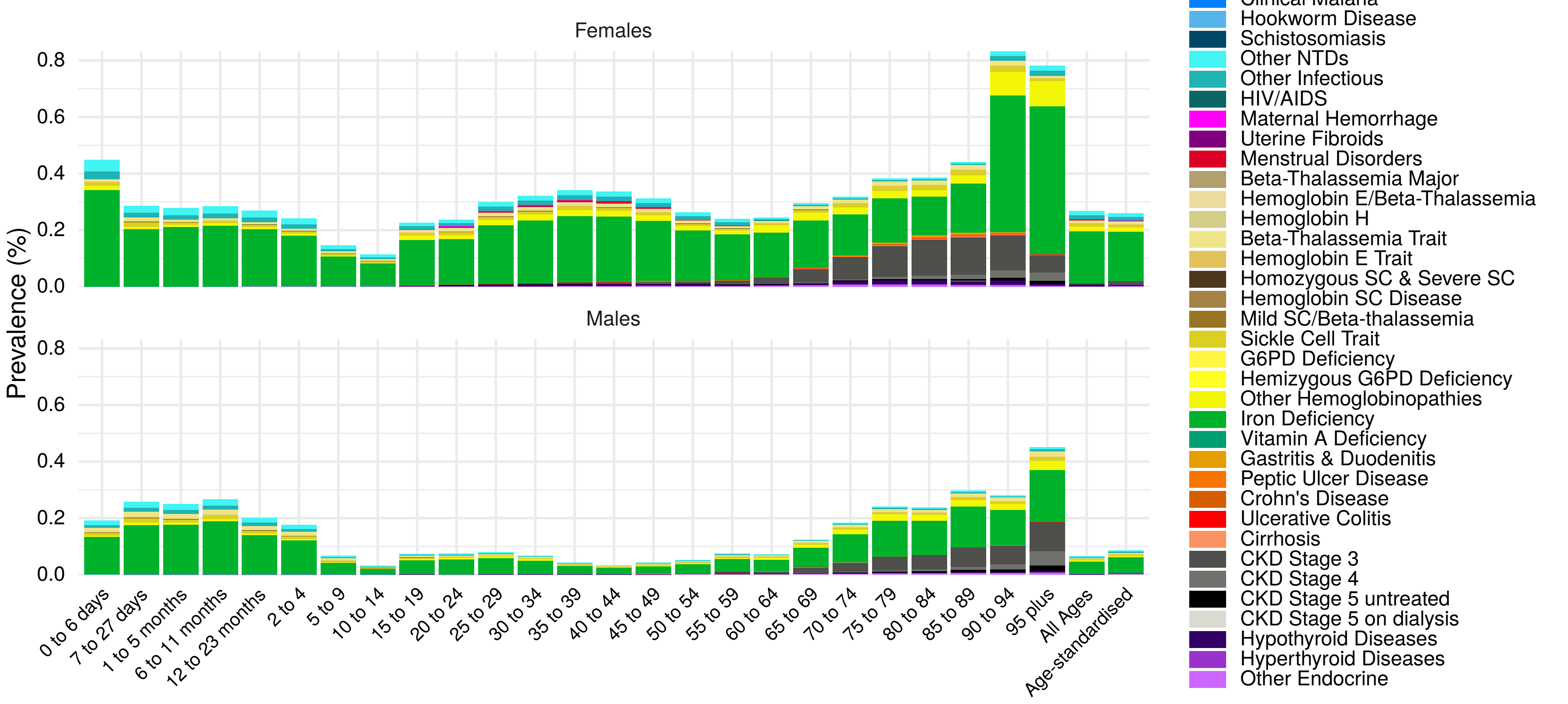

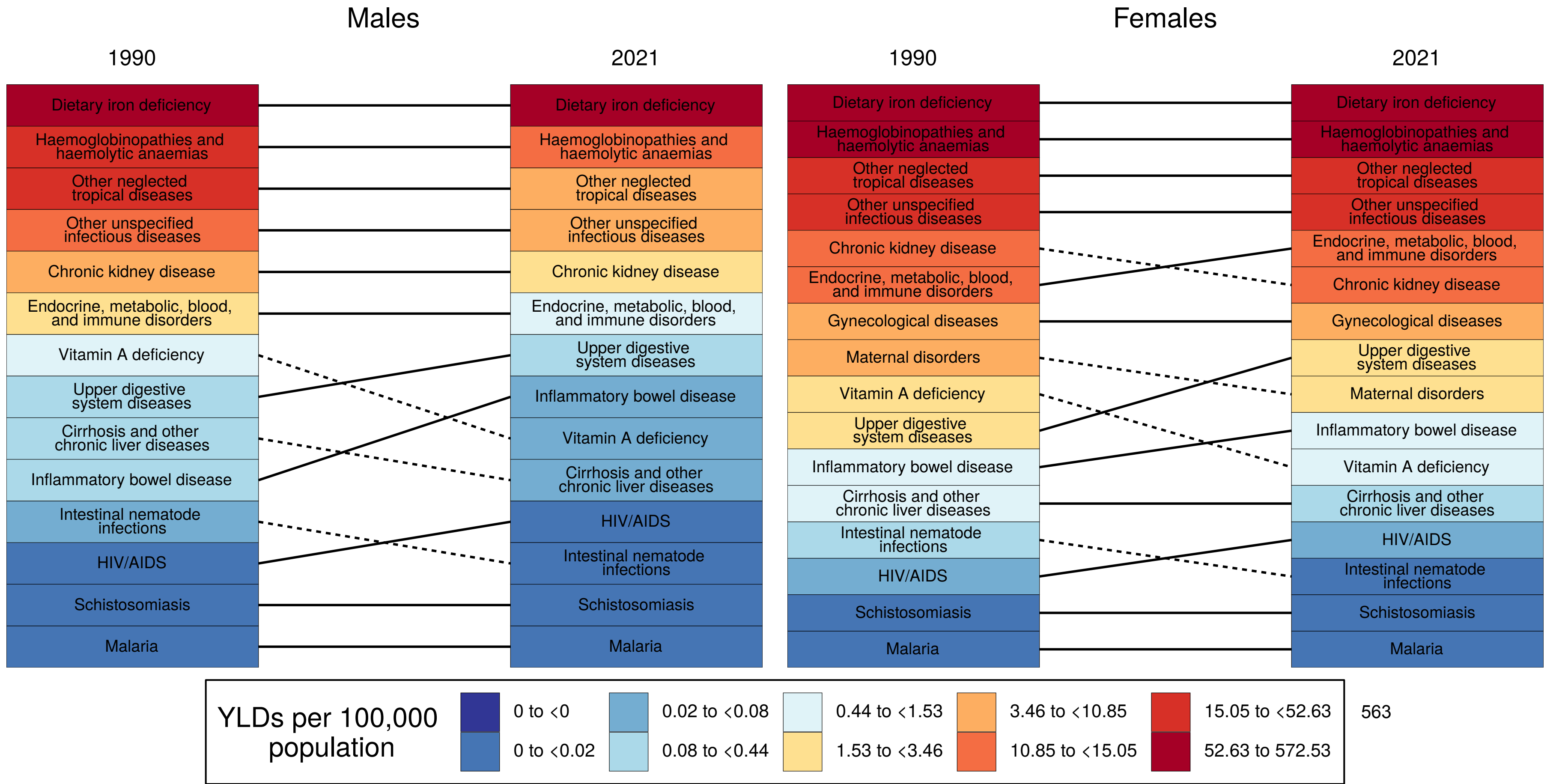

Saudi Arabia

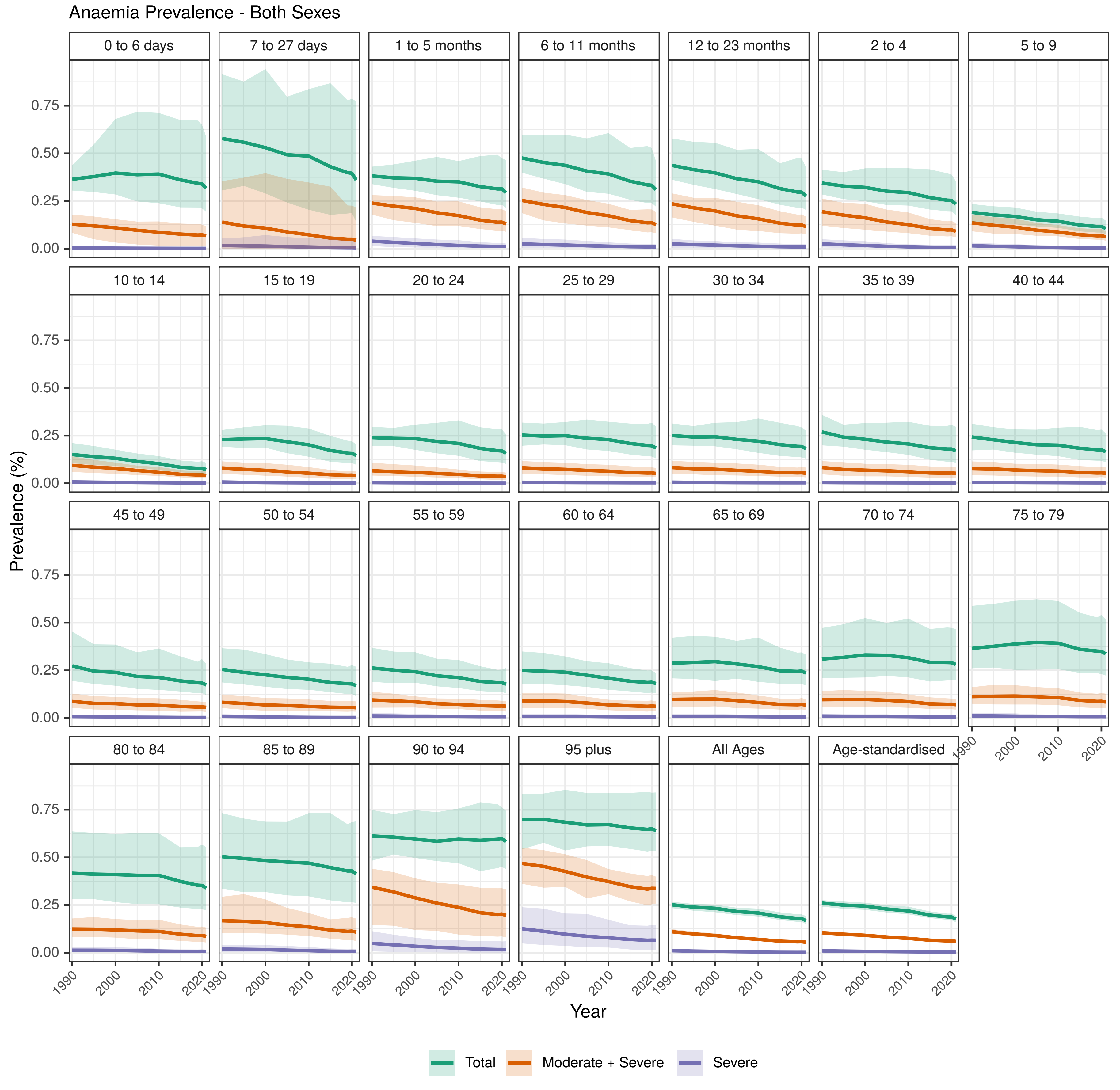

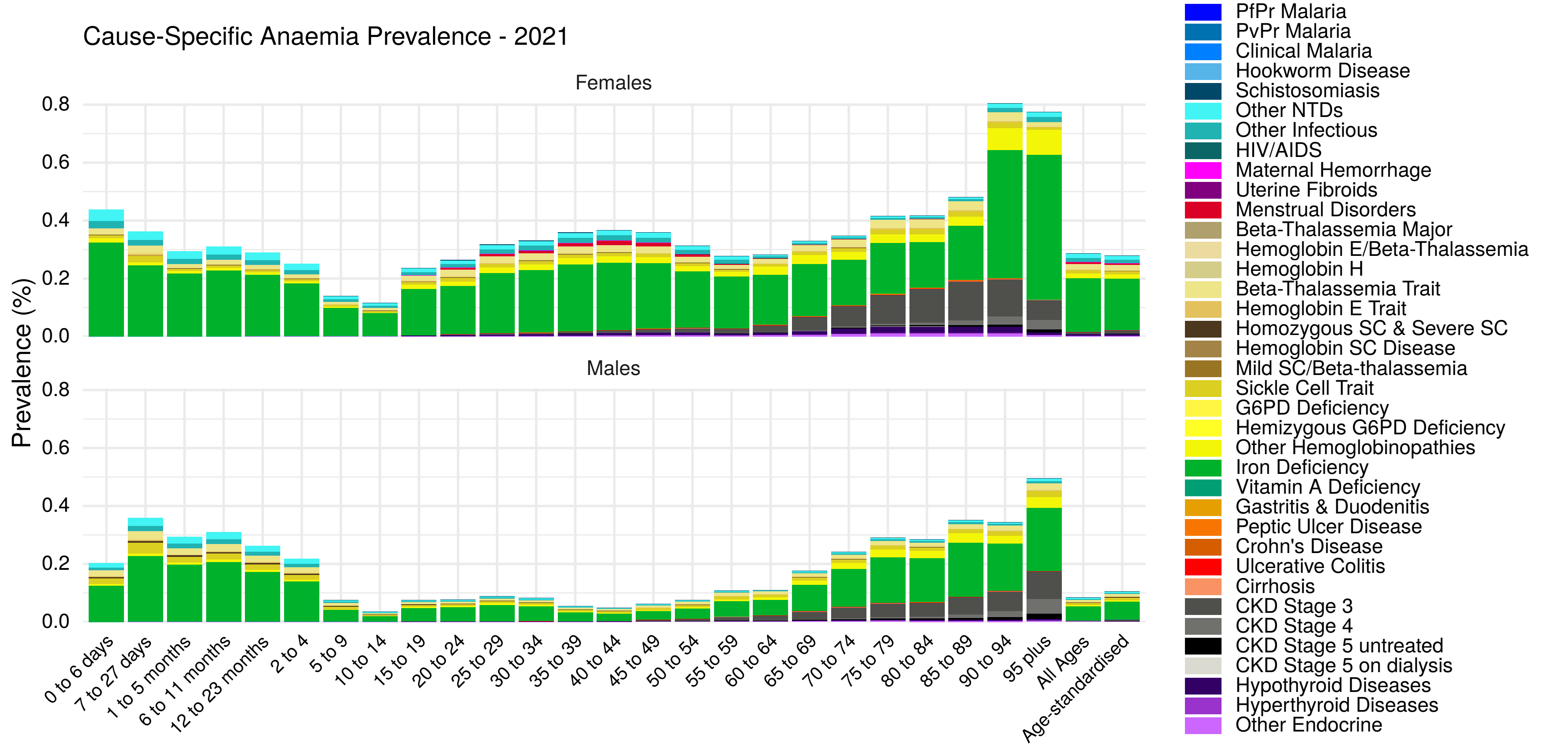

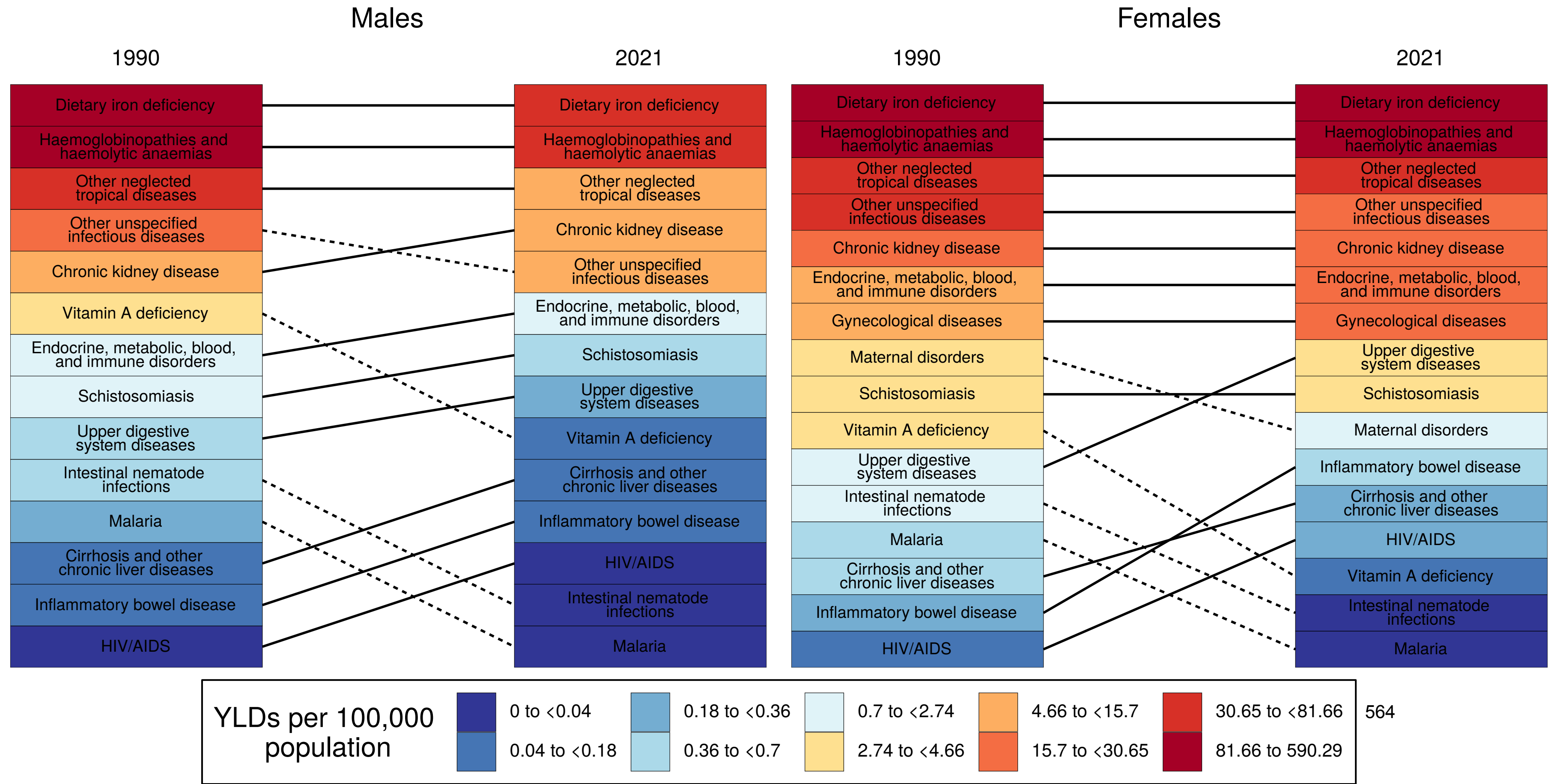

Sudan

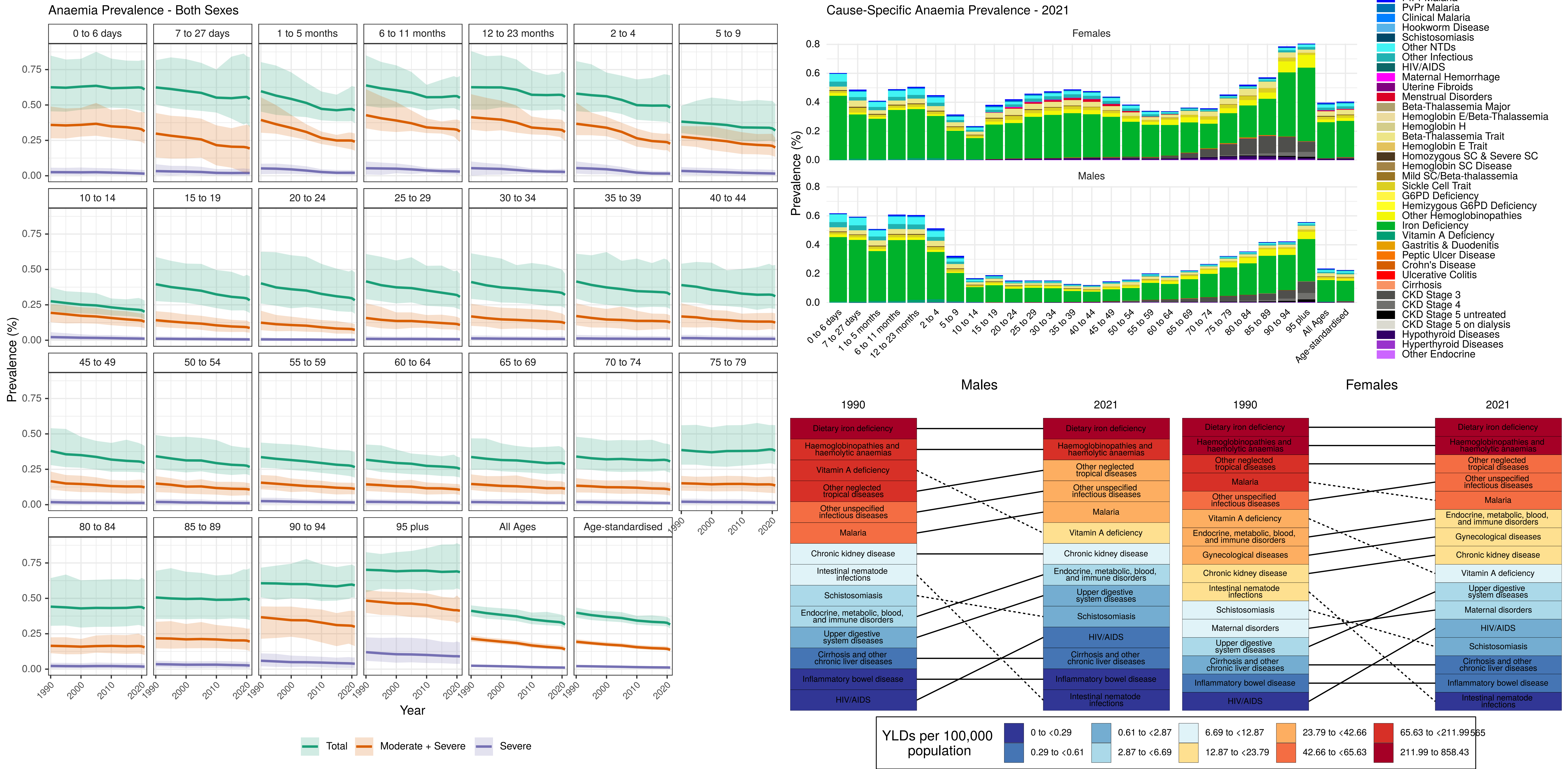

Anaemia Prevalence - Both Sexes
0 to 6 days
7 to 27 days
1 to 5 months
6 to 11 months
12 to 23 months
2 to 4
5 to 9
10 to 14
15 to 19
20 to 24
25 to 29
30 to 34
35 to 39
40 to 44
45 to 49
50 to 54
55 to 59
60 to 64
65 to 69
70 to 74
75 to 79
80 to 84
85 to 89
90 to 94
95 plus
All Ages
Age-standardised
Prevalence (%)
0.00
0.25
0.50
0.75
1990
2000
2010
2020
Year
Total
Moderate + Severe
Severe
Cause-Specific Anaemia Prevalence - 2021
Females
Males
Prevalence (%)
0.0
0.2
0.4
0.6
0.8
PfPr Malaria
PvPr Malaria
Clinical Malaria
Hookworm Disease
Schistosomiasis
Other NTDs
Other Infectious
HIV/AIDS
Maternal Hemorrhage
Uterine Fibroids
Menstrual Disorders
Beta-Thalassemia Major
Hemoglobin E/Beta-Thalassemia
Hemoglobin H
Beta-Thalassemia Trait
Hemoglobin E Trait
Homozygous SC & Severe SC
Hemoglobin SC Disease
Mild SC/Beta-thalassemia
Sickle Cell Trait
G6PD Deficiency
Hemizygous G6PD Deficiency
Other Hemoglobinopathies
Iron Deficiency
Vitamin A Deficiency
Gastritis & Duodenitis
Peptic Ulcer Disease
Crohn's Disease
Ulcerative Colitis
Cirrhosis
CKD Stage 3
CKD Stage 4
CKD Stage 5 untreated
CKD Stage 5 on dialysis
Hypothyroid Diseases
Hyperthyroid Diseases
Other Endocrine
Males
Females
1990
2021
Dietary iron deficiency
Haemoglobinopathies and haemolytic anaemias
Vitamin A deficiency
Other neglected tropical diseases
Other unspecified infectious diseases
Malaria
Chronic kidney disease
Intestinal nematode infections
Schistosomiasis
Endocrine, metabolic, blood, and immune disorders
Upper digestive system diseases
Cirrhosis and other chronic liver diseases
Inflammatory bowel disease
HIV/AIDS
Gynecological diseases
Maternal disorders
YLDs per 100,000 population
0 to <0.29
0.29 to <0.61
0.61 to <2.87
2.87 to <6.69
6.69 to <12.87
12.87 to <23.79
23.79 to <42.66
42.66 to <65.63
65.63 to <211.99
211.99 to 858.43

Syria

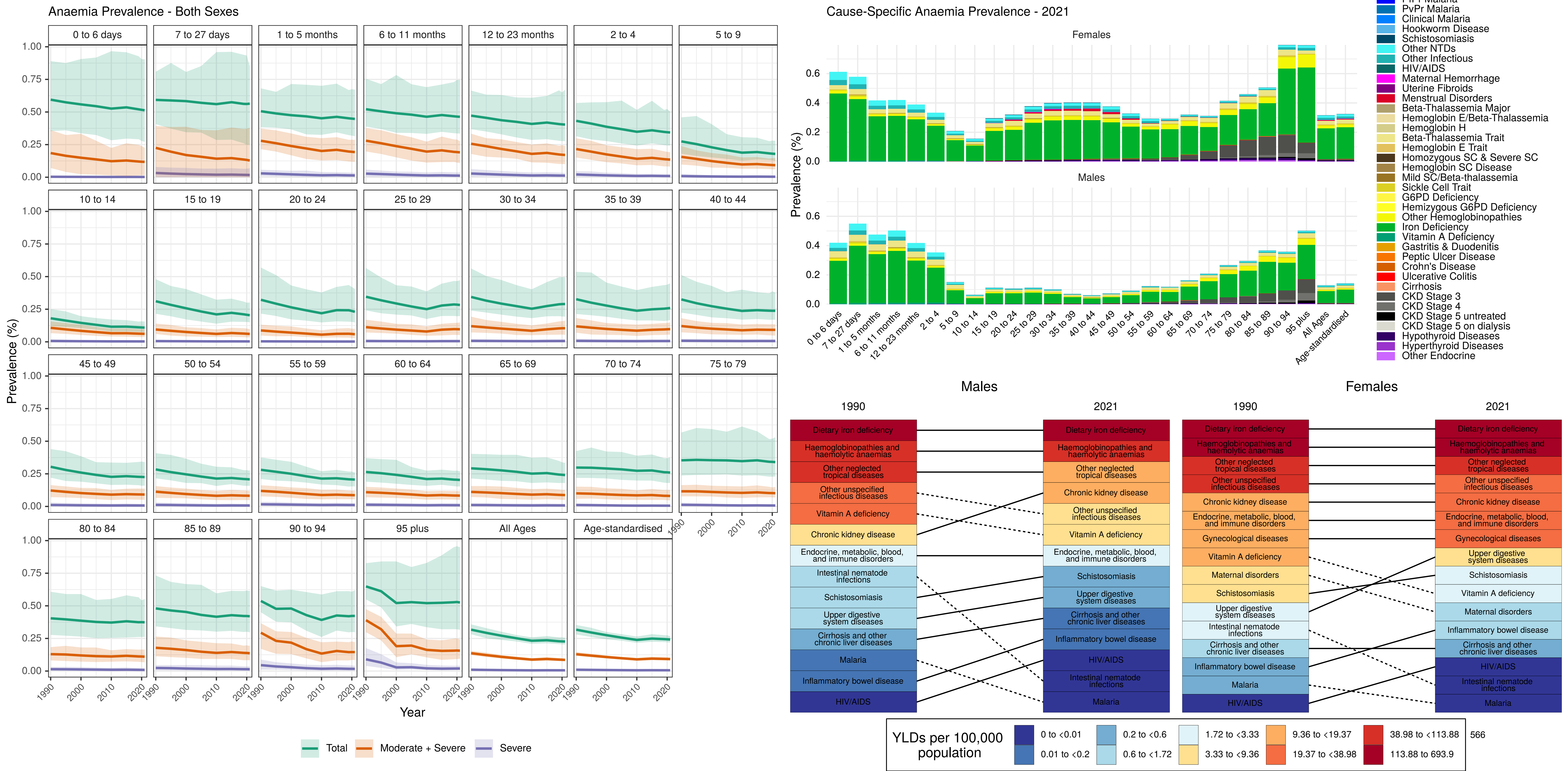

Anaemia Prevalence - Both Sexes
0 to 6 days
7 to 27 days
1 to 5 months
6 to 11 months
12 to 23 months
2 to 4
5 to 9
10 to 14
15 to 19
20 to 24
25 to 29
30 to 34
35 to 39
40 to 44
45 to 49
50 to 54
55 to 59
60 to 64
65 to 69
70 to 74
75 to 79
80 to 84
85 to 89
90 to 94
95 plus
All Ages
Age-standardised
Prevalence (%)
Year
Total
Moderate + Severe
Severe
Cause-Specific Anaemia Prevalence - 2021
Females
Males
PfPr Malaria
PvPr Malaria
Clinical Malaria
Hookworm Disease
Schistosomiasis
Other NTDs
Other Infectious
HIV/AIDS
Maternal Hemorrhage
Uterine Fibroids
Menstrual Disorders
Beta-Thalassemia Major
Hemoglobin E/Beta-Thalassemia
Hemoglobin H
Beta-Thalassemia Trait
Hemoglobin E Trait
Homozygous SC & Severe SC
Hemoglobin SC Disease
Mild SC/Beta-thalassemia
Sickle Cell Trait
G6PD Deficiency
Hemizygous G6PD Deficiency
Other Hemoglobinopathies
Iron Deficiency
Vitamin A Deficiency
Gastritis & Duodenitis
Peptic Ulcer Disease
Crohn's Disease
Ulcerative Colitis
Cirrhosis
CKD Stage 3
CKD Stage 4
CKD Stage 5 untreated
CKD Stage 5 on dialysis
Hypothyroid Diseases
Hyperthyroid Diseases
Other Endocrine
Males
1990
Dietary iron deficiency
Haemoglobinopathies and haemolytic anaemias
Other neglected tropical diseases
Other unspecified infectious diseases
Vitamin A deficiency
Chronic kidney disease
Endocrine, metabolic, blood, and immune disorders
Intestinal nematode infections
Schistosomiasis
Upper digestive system diseases
Cirrhosis and other chronic liver diseases
Malaria
Inflammatory bowel disease
HIV/AIDS
2021
Dietary iron deficiency
Haemoglobinopathies and haemolytic anaemias
Other neglected tropical diseases
Chronic kidney disease
Other unspecified infectious diseases
Vitamin A deficiency
Endocrine, metabolic, blood, and immune disorders
Schistosomiasis
Upper digestive system diseases
Cirrhosis and other chronic liver diseases
Inflammatory bowel disease
HIV/AIDS
Intestinal nematode infections
Malaria
Females
1990
Dietary iron deficiency
Haemoglobinopathies and haemolytic anaemias
Other neglected tropical diseases
Other unspecified infectious diseases
Chronic kidney disease
Endocrine, metabolic, blood, and immune disorders
Gynecological diseases
Vitamin A deficiency
Maternal disorders
Schistosomiasis
Upper digestive system diseases
Intestinal nematode infections
Cirrhosis and other chronic liver diseases
Inflammatory bowel disease
Malaria
HIV/AIDS
2021
Dietary iron deficiency
Haemoglobinopathies and haemolytic anaemias
Other neglected tropical diseases
Other unspecified infectious diseases
Chronic kidney disease
Endocrine, metabolic, blood, and immune disorders
Gynecological diseases
Upper digestive system diseases
Schistosomiasis
Vitamin A deficiency
Maternal disorders
Inflammatory bowel disease
Cirrhosis and other chronic liver diseases
HIV/AIDS
Intestinal nematode infections
Malaria
YLDs per 100,000 population
0 to <0.01
0.01 to <0.2
0.2 to <0.6
0.6 to <1.72
1.72 to <3.33
3.33 to <9.36
9.36 to <19.37
19.37 to <38.98
38.98 to <113.88
113.88 to 693.9

Tunisia

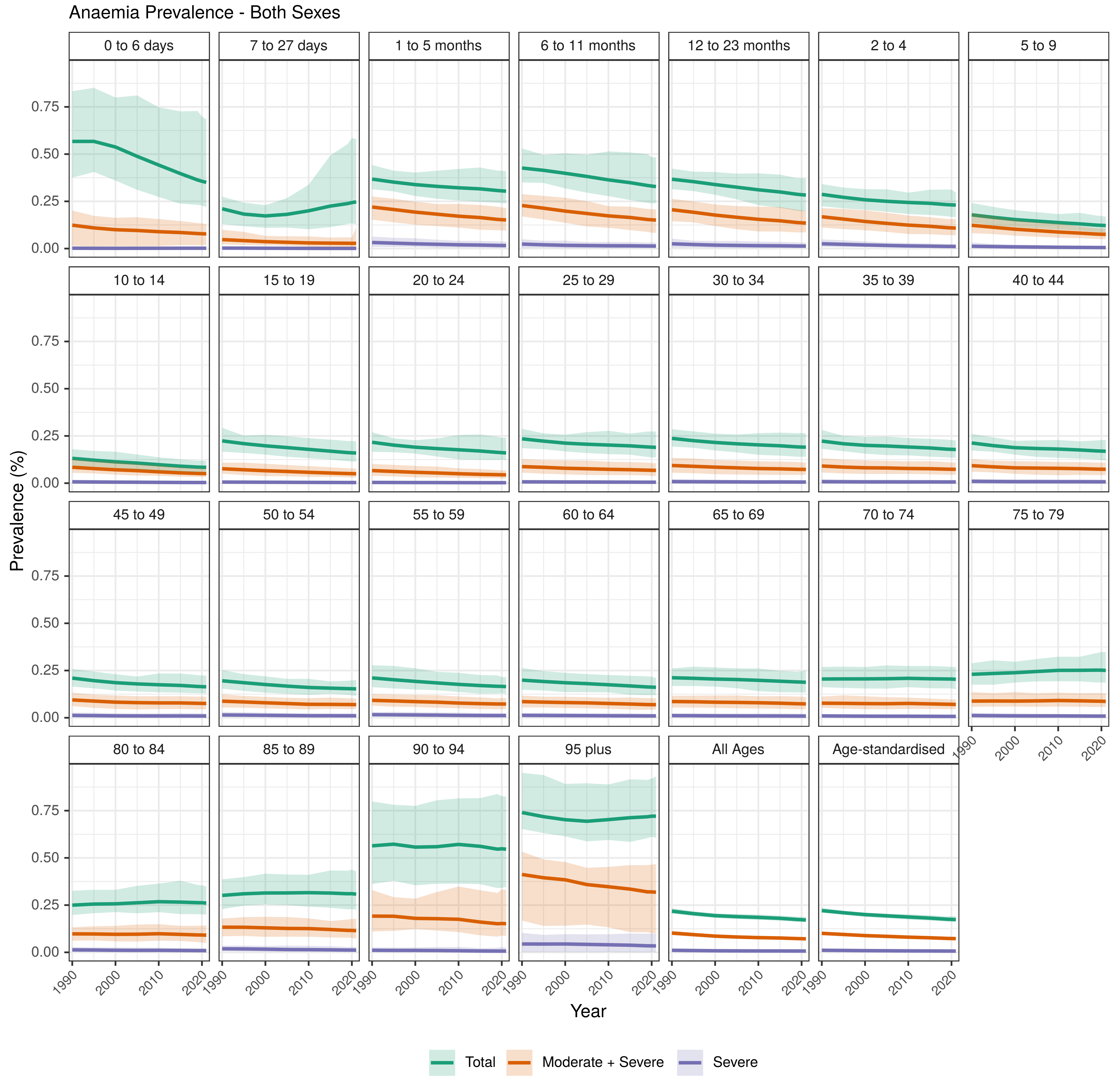

Anaemia Prevalence - Both Sexes
0 to 6 days
7 to 27 days
1 to 5 months
6 to 11 months
12 to 23 months
2 to 4
5 to 9
10 to 14
15 to 19
20 to 24
25 to 29
30 to 34
35 to 39
40 to 44
45 to 49
50 to 54
55 to 59
60 to 64
65 to 69
70 to 74
75 to 79
80 to 84
85 to 89
90 to 94
95 plus
All Ages
Age-standardised
0.00
0.25
0.50
0.75
1990
2000
2010
2020
Prevalence (%)
Year
Total
Moderate + Severe
Severe

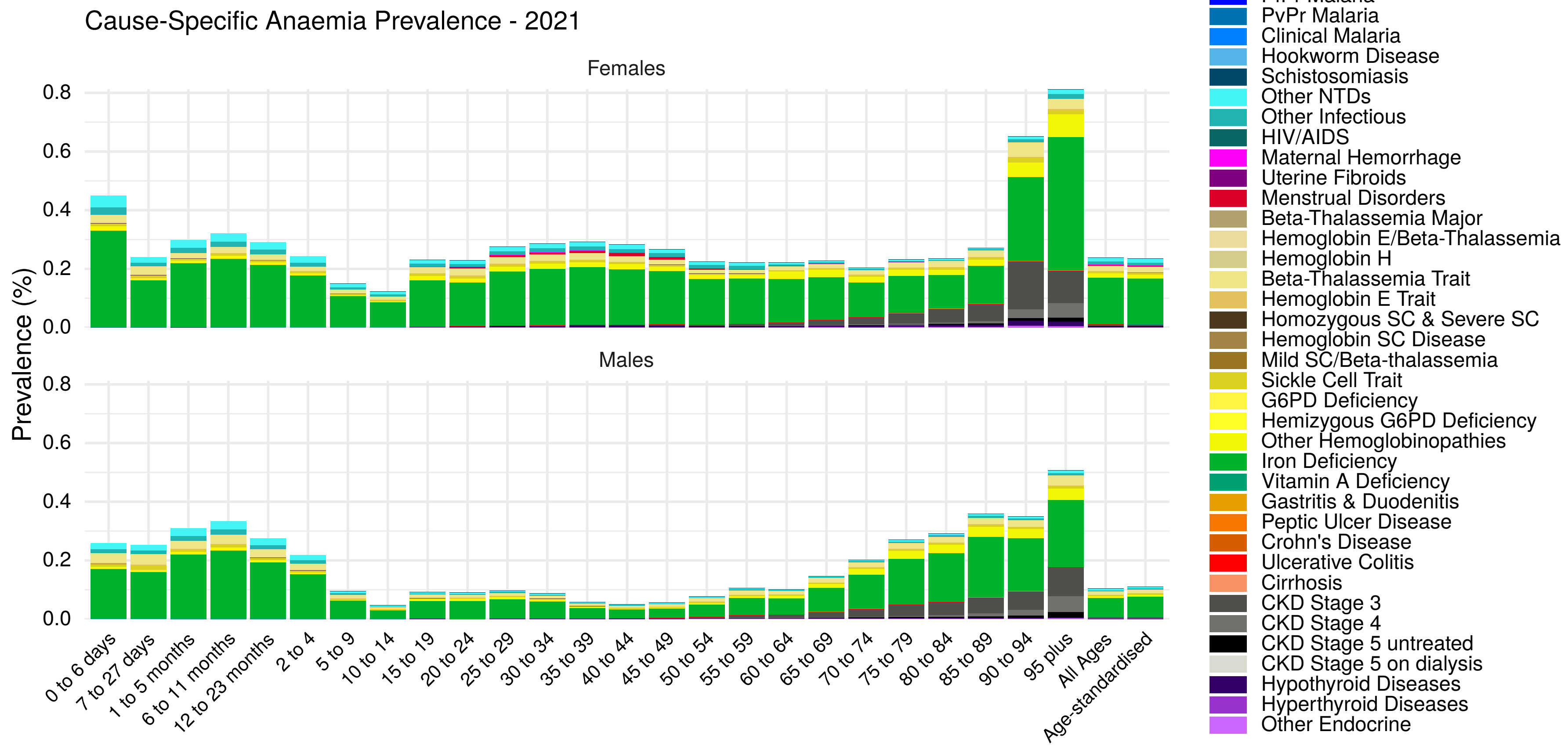

Cause-Specific Anaemia Prevalence - 2021
Females
Males
Prevalence (%)
0.0
0.2
0.4
0.6
0.8
0 to 6 days
7 to 27 days
1 to 5 months
6 to 11 months
12 to 23 months
2 to 4
5 to 9
10 to 14
15 to 19
20 to 24
25 to 29
30 to 34
35 to 39
40 to 44
45 to 49
50 to 54
55 to 59
60 to 64
65 to 69
70 to 74
75 to 79
80 to 84
85 to 89
90 to 94
95 plus
All Ages
Age-standardised
PfPr Malaria
PvPr Malaria
Clinical Malaria
Hookworm Disease
Schistosomiasis
Other NTDs
Other Infectious
HIV/AIDS
Maternal Hemorrhage
Uterine Fibroids
Menstrual Disorders
Beta-Thalassemia Major
Hemoglobin E/Beta-Thalassemia
Hemoglobin H
Beta-Thalassemia Trait
Hemoglobin E Trait
Homozygous SC & Severe SC
Hemoglobin SC Disease
Mild SC/Beta-thalassemia
Sickle Cell Trait
G6PD Deficiency
Hemizygous G6PD Deficiency
Other Hemoglobinopathies
Iron Deficiency
Vitamin A Deficiency
Gastritis & Duodenitis
Peptic Ulcer Disease
Crohn's Disease
Ulcerative Colitis
Cirrhosis
CKD Stage 3
CKD Stage 4
CKD Stage 5 untreated
CKD Stage 5 on dialysis
Hypothyroid Diseases
Hyperthyroid Diseases
Other Endocrine

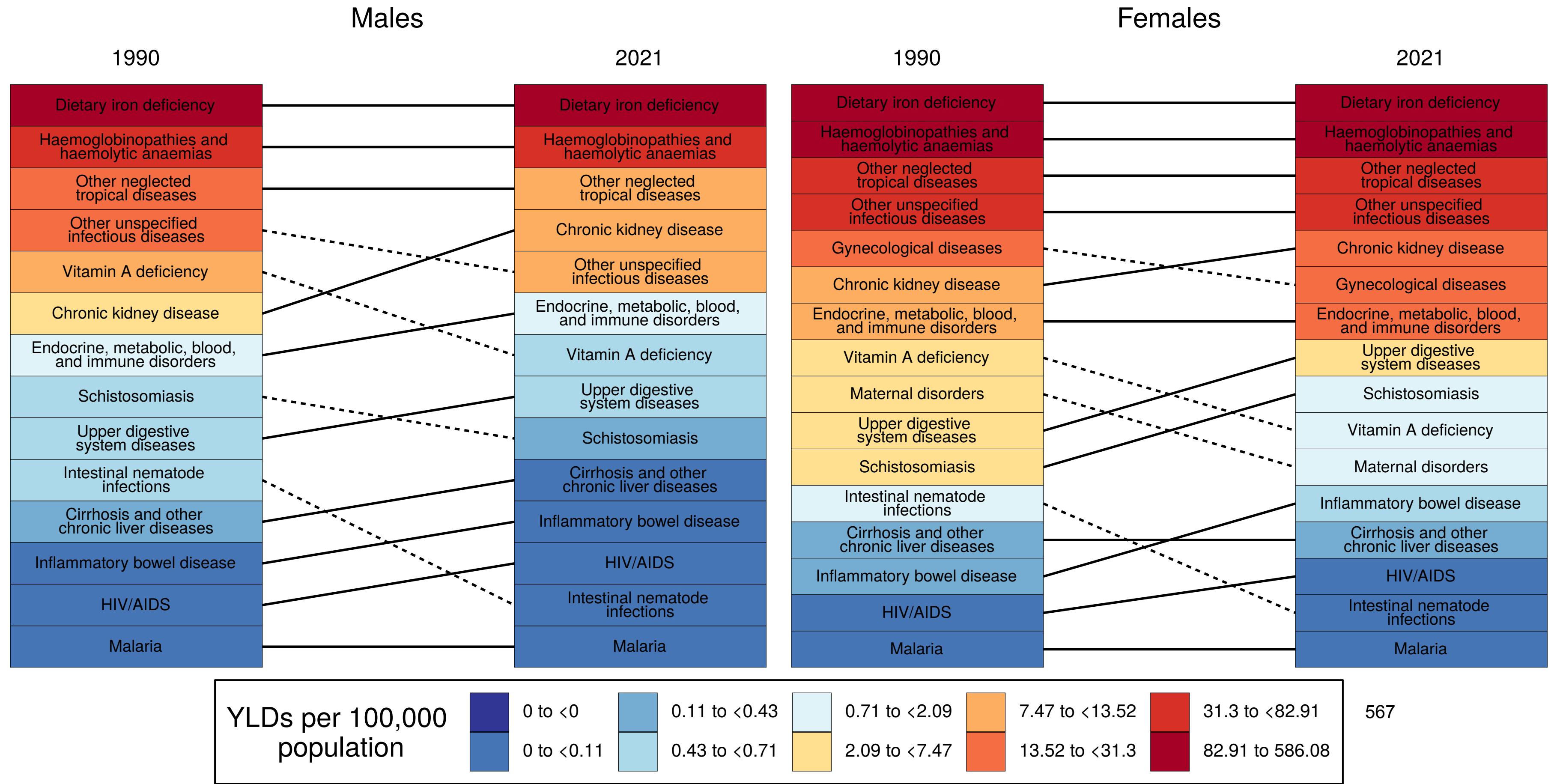

Males
Females
1990
2021
Dietary iron deficiency
Haemoglobinopathies and haemolytic anaemias
Other neglected tropical diseases
Other unspecified infectious diseases
Vitamin A deficiency
Chronic kidney disease
Endocrine, metabolic, blood, and immune disorders
Schistosomiasis
Upper digestive system diseases
Intestinal nematode infections
Cirrhosis and other chronic liver diseases
Inflammatory bowel disease
HIV/AIDS
Malaria
Gynecological diseases
Maternal disorders
YLDs per 100,000 population
0 to <0
0 to <0.11
0.11 to <0.43
0.43 to <0.71
0.71 to <2.09
2.09 to <7.47
7.47 to <13.52
13.52 to <31.3
31.3 to <82.91
82.91 to 586.08

Türkiye

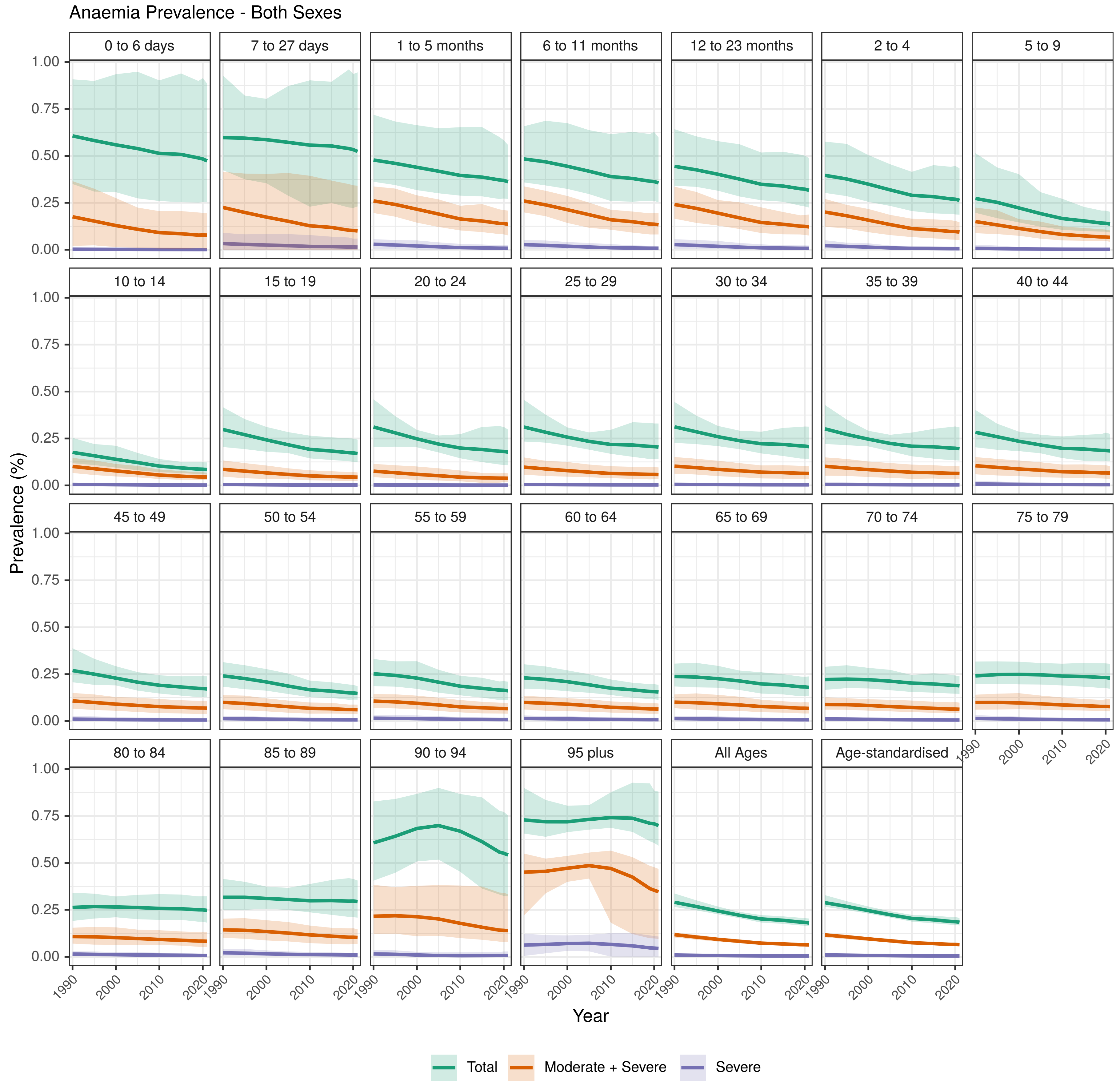

Anaemia Prevalence - Both Sexes
0 to 6 days
7 to 27 days
1 to 5 months
6 to 11 months
12 to 23 months
2 to 4
5 to 9
10 to 14
15 to 19
20 to 24
25 to 29
30 to 34
35 to 39
40 to 44
45 to 49
50 to 54
55 to 59
60 to 64
65 to 69
70 to 74
75 to 79
80 to 84
85 to 89
90 to 94
95 plus
All Ages
Age-standardised
Prevalence (%)
1.00
0.75
0.50
0.25
0.00
1990
2000
2010
2020
Year
Total
Moderate + Severe
Severe

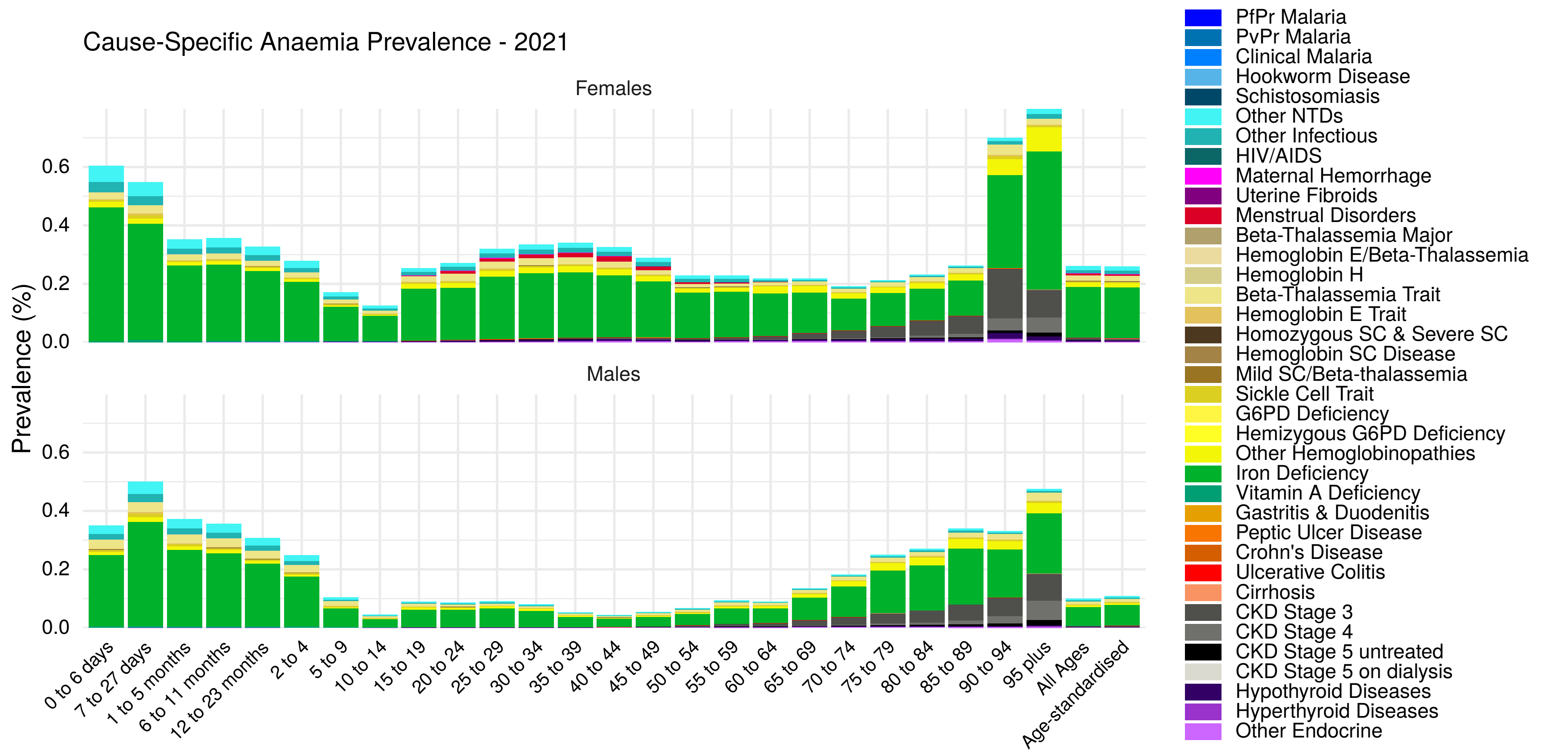

Cause-Specific Anaemia Prevalence - 2021
Females
Males
Prevalence (%)
0.6
0.4
0.2
0.0
0 to 6 days
7 to 27 days
1 to 5 months
6 to 11 months
12 to 23 months
2 to 4
5 to 9
10 to 14
15 to 19
20 to 24
25 to 29
30 to 34
35 to 39
40 to 44
45 to 49
50 to 54
55 to 59
60 to 64
65 to 69
70 to 74
75 to 79
80 to 84
85 to 89
90 to 94
95 plus
All Ages
Age-standardised
PfPr Malaria
PvPr Malaria
Clinical Malaria
Hookworm Disease
Schistosomiasis
Other NTDs
Other Infectious
HIV/AIDS
Maternal Hemorrhage
Uterine Fibroids
Menstrual Disorders
Beta-Thalassemia Major
Hemoglobin E/Beta-Thalassemia
Hemoglobin H
Beta-Thalassemia Trait
Hemoglobin E Trait
Homozygous SC & Severe SC
Hemoglobin SC Disease
Mild SC/Beta-thalassemia
Sickle Cell Trait
G6PD Deficiency
Hemizygous G6PD Deficiency
Other Hemoglobinopathies
Iron Deficiency
Vitamin A Deficiency
Gastritis & Duodenitis
Peptic Ulcer Disease
Crohn's Disease
Ulcerative Colitis
Cirrhosis
CKD Stage 3
CKD Stage 4
CKD Stage 5 untreated
CKD Stage 5 on dialysis
Hypothyroid Diseases
Hyperthyroid Diseases
Other Endocrine

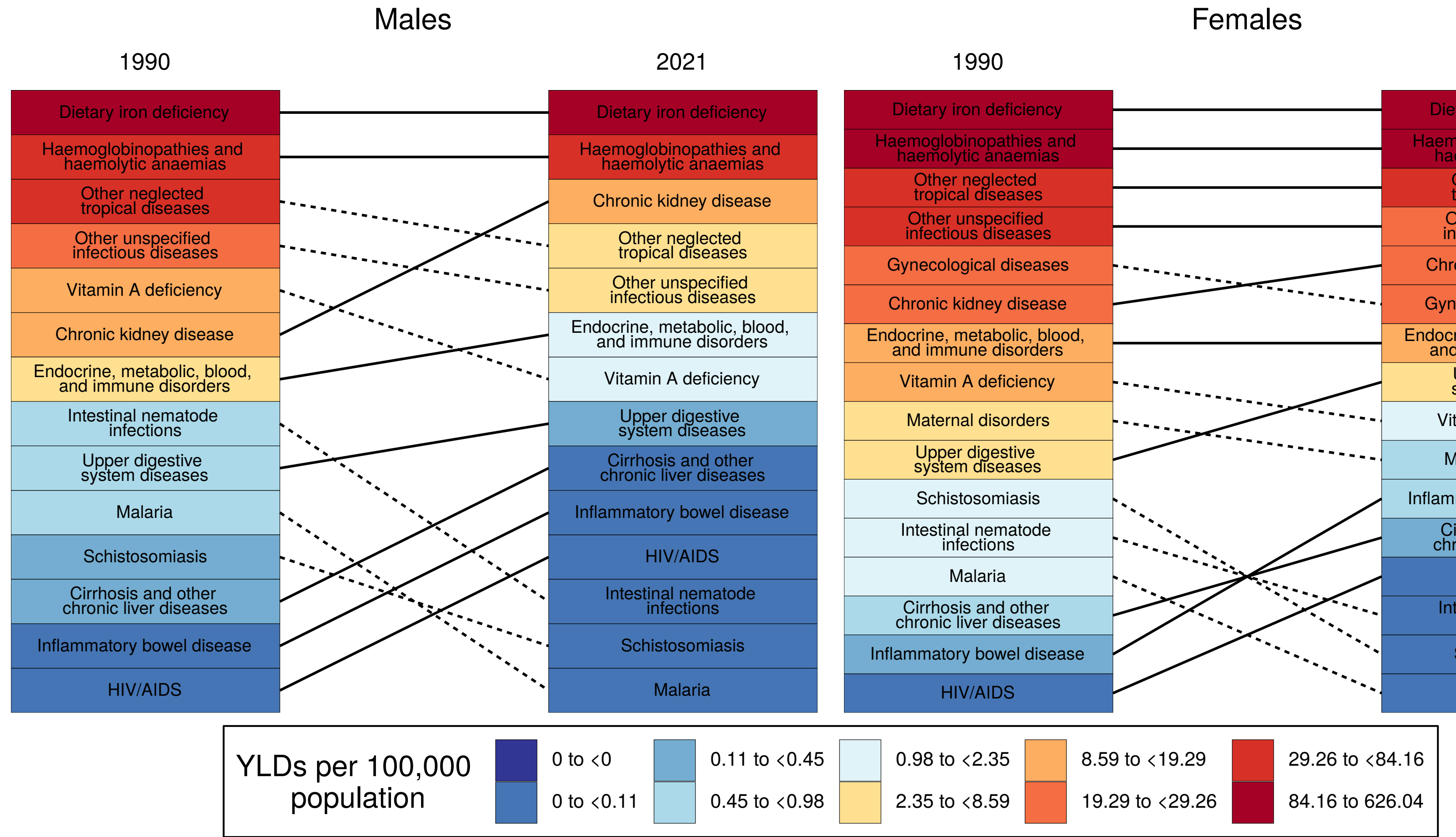

Males
Females
1990
2021
Dietary iron deficiency
Haemoglobinopathies and haemolytic anaemias
Other neglected tropical diseases
Other unspecified infectious diseases
Vitamin A deficiency
Chronic kidney disease
Endocrine, metabolic, blood, and immune disorders
Intestinal nematode infections
Upper digestive system diseases
Malaria
Schistosomiasis
Cirrhosis and other chronic liver diseases
Inflammatory bowel disease
HIV/AIDS
Gynecological diseases
Maternal disorders
YLDs per 100,000 population
0 to <0
0 to <0.11
0.11 to <0.45
0.45 to <0.98
0.98 to <2.35
2.35 to <8.59
8.59 to <19.29
19.29 to <29.26
29.26 to <84.16
84.16 to 626.04

United Arab Emirates

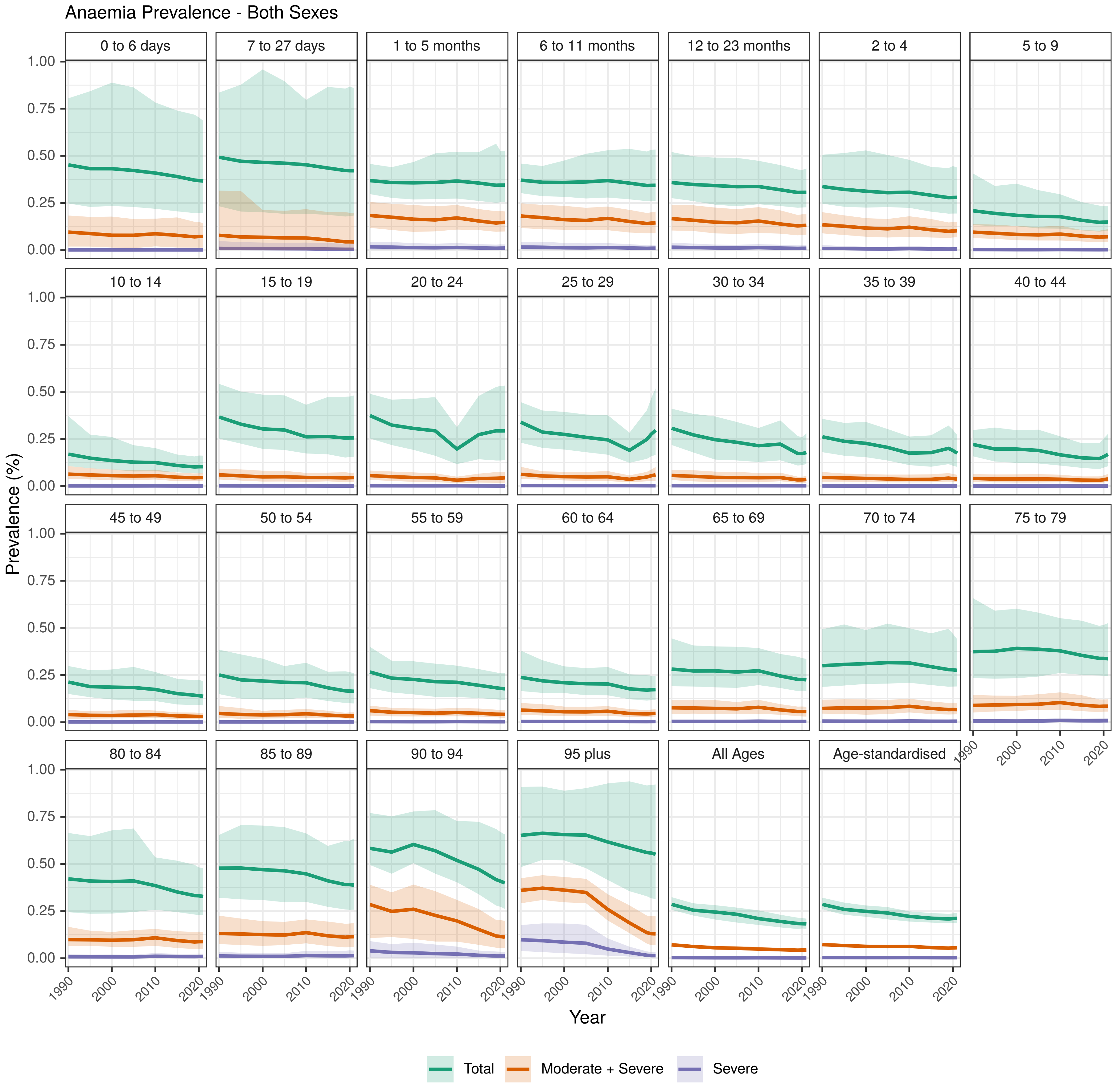

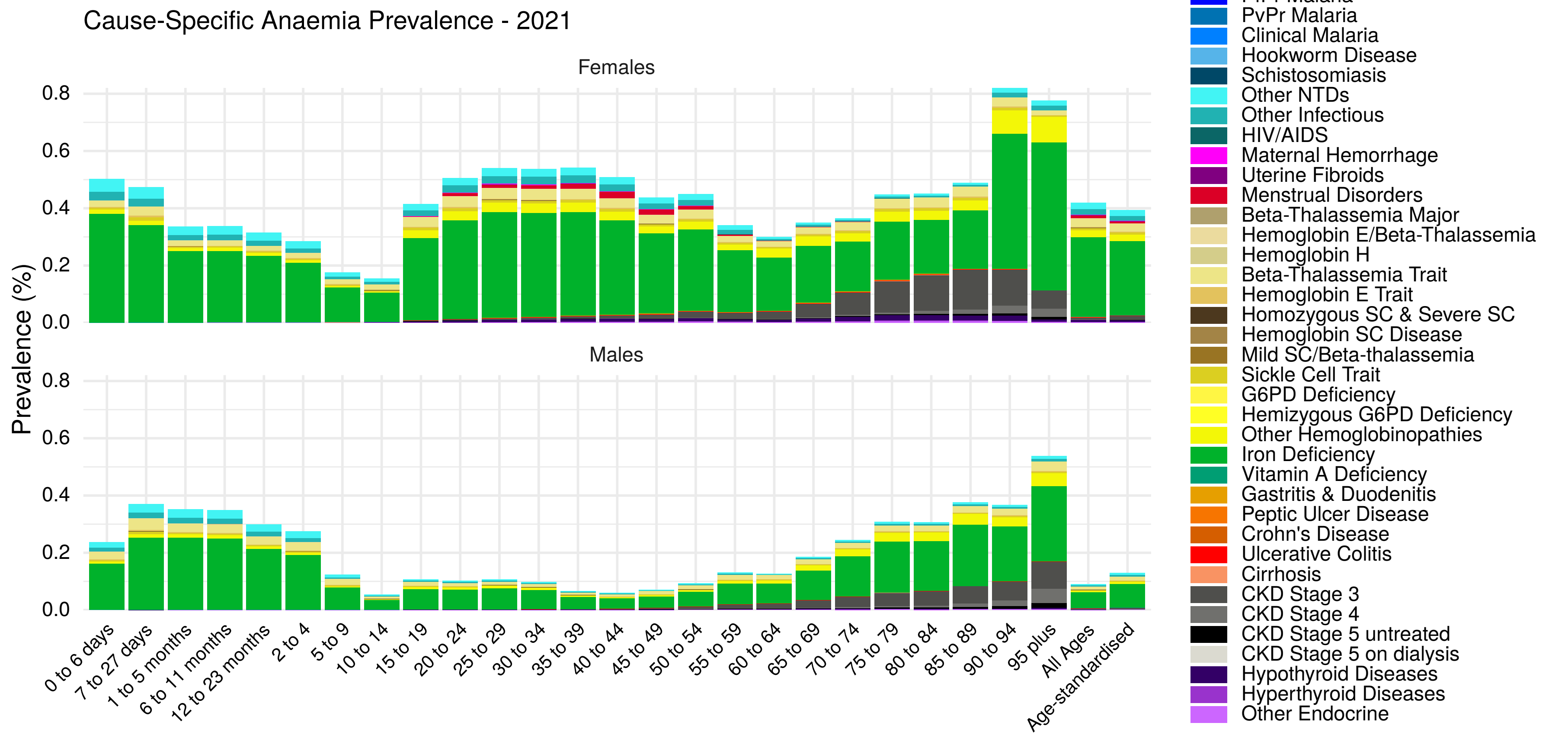

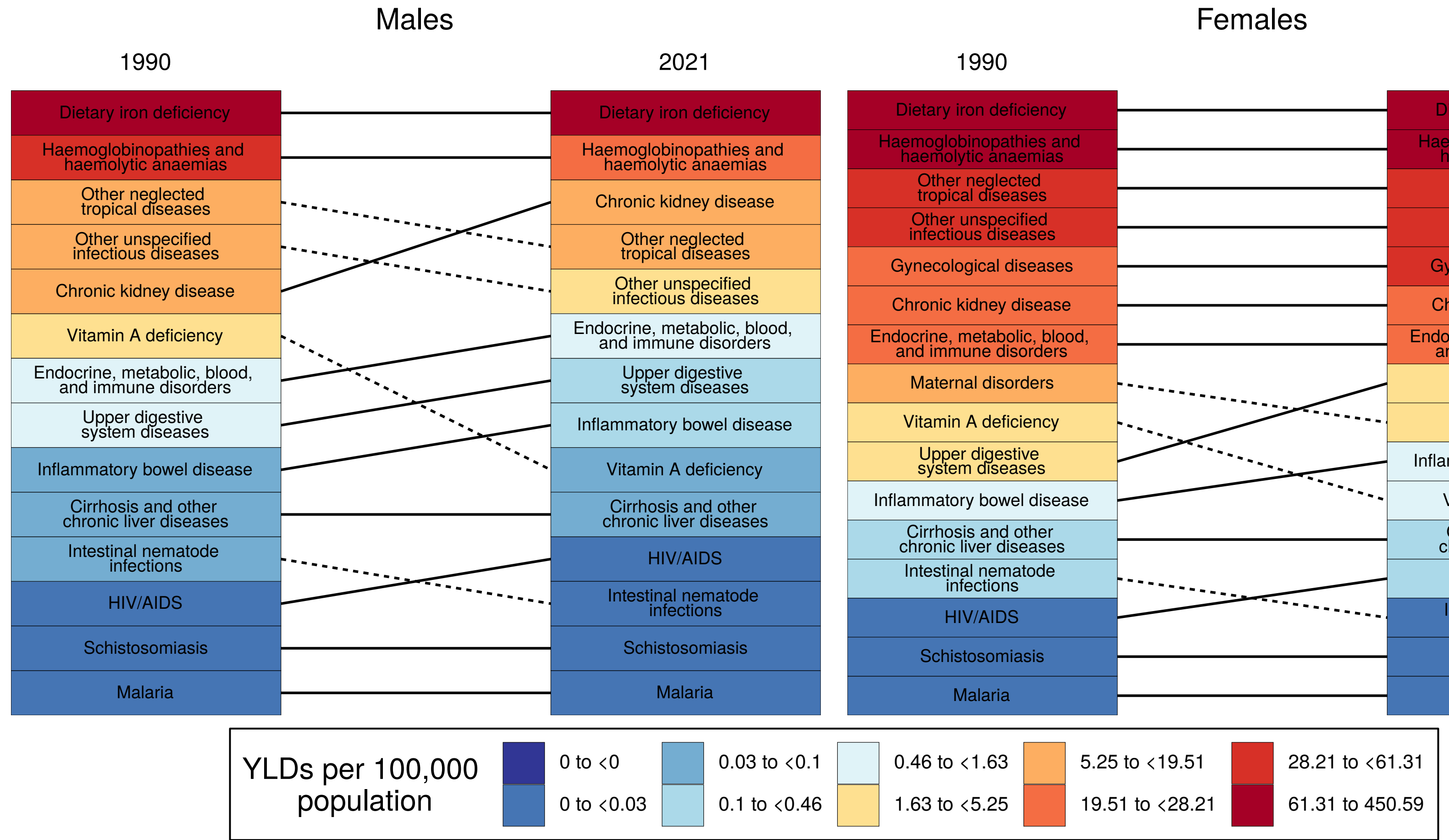

569

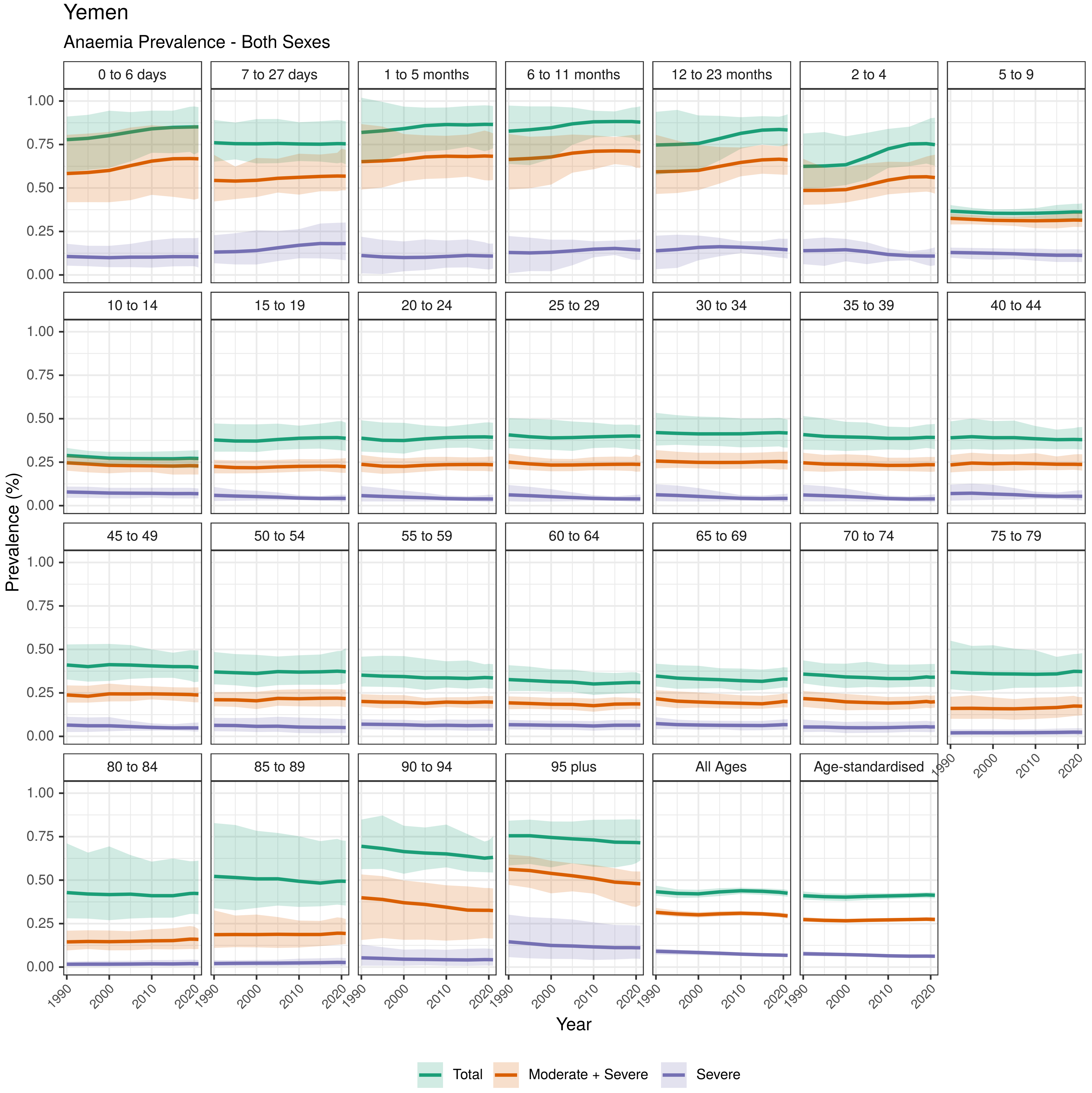
Yemen
Anaemia Prevalence - Both Sexes
0 to 6 days
7 to 27 days
1 to 5 months
6 to 11 months
12 to 23 months
2 to 4
5 to 9
10 to 14
15 to 19
20 to 24
25 to 29
30 to 34
35 to 39
40 to 44
45 to 49
50 to 54
55 to 59
60 to 64
65 to 69
70 to 74
75 to 79
80 to 84
85 to 89
90 to 94
95 plus
All Ages
Age-standardised
0.00
0.25
0.50
0.75
1.00
1990
2000
2010
2020
Prevalence (%)
Year
Total
Moderate + Severe
Severe

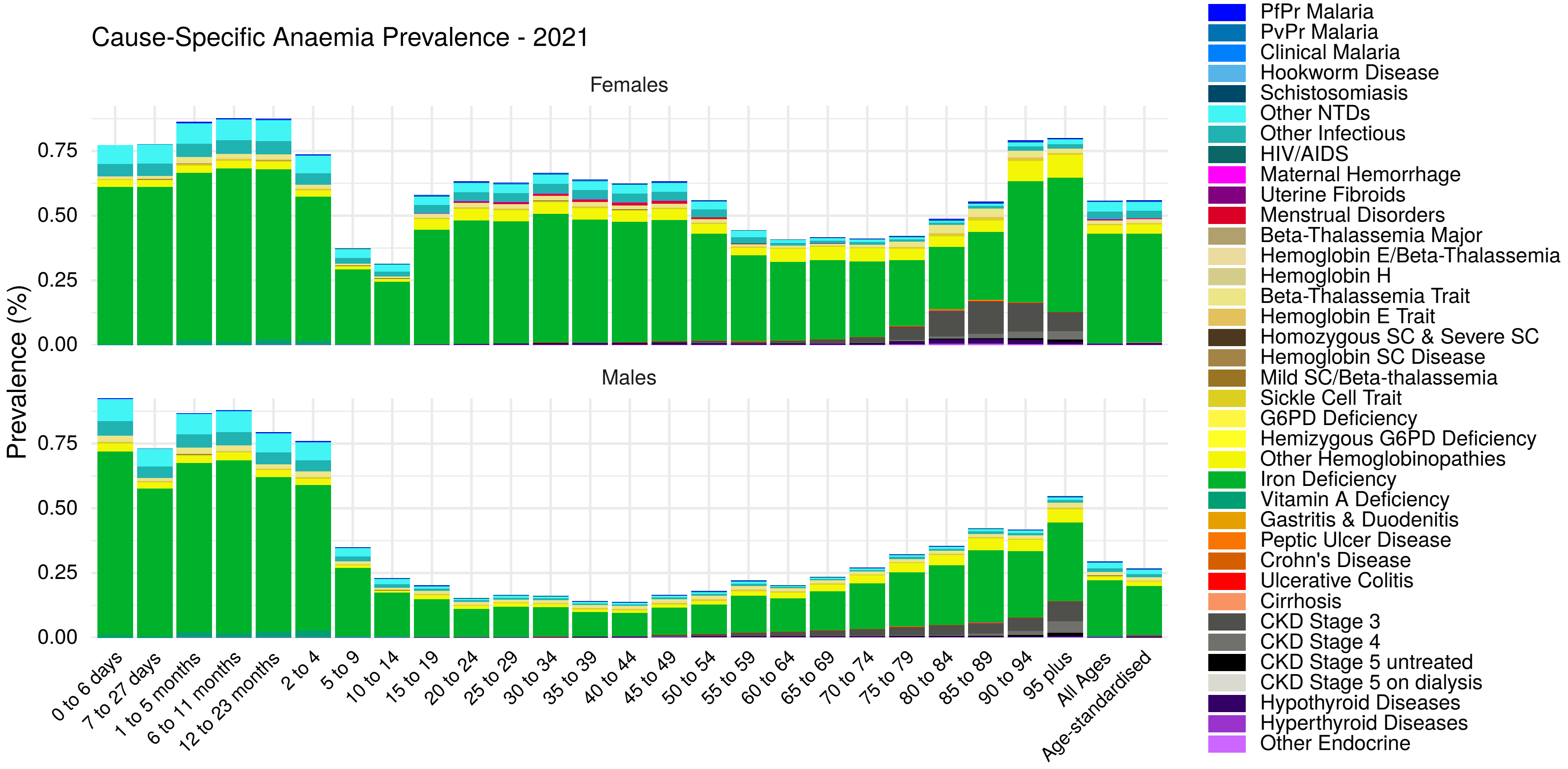
Cause-Specific Anaemia Prevalence - 2021
Females
Males
Prevalence (%)
0.00
0.25
0.50
0.75
0 to 6 days
7 to 27 days
1 to 5 months
6 to 11 months
12 to 23 months
2 to 4
5 to 9
10 to 14
15 to 19
20 to 24
25 to 29
30 to 34
35 to 39
40 to 44
45 to 49
50 to 54
55 to 59
60 to 64
65 to 69
70 to 74
75 to 79
80 to 84
85 to 89
90 to 94
95 plus
All Ages
Age-standardised
PfPr Malaria
PvPr Malaria
Clinical Malaria
Hookworm Disease
Schistosomiasis
Other NTDs
Other Infectious
HIV/AIDS
Maternal Hemorrhage
Uterine Fibroids
Menstrual Disorders
Beta-Thalassemia Major
Hemoglobin E/Beta-Thalassemia
Hemoglobin H
Beta-Thalassemia Trait
Hemoglobin E Trait
Homozygous SC & Severe SC
Hemoglobin SC Disease
Mild SC/Beta-thalassemia
Sickle Cell Trait
G6PD Deficiency
Hemizygous G6PD Deficiency
Other Hemoglobinopathies
Iron Deficiency
Vitamin A Deficiency
Gastritis & Duodenitis
Peptic Ulcer Disease
Crohn's Disease
Ulcerative Colitis
Cirrhosis
CKD Stage 3
CKD Stage 4
CKD Stage 5 untreated
CKD Stage 5 on dialysis
Hypothyroid Diseases
Hyperthyroid Diseases
Other Endocrine

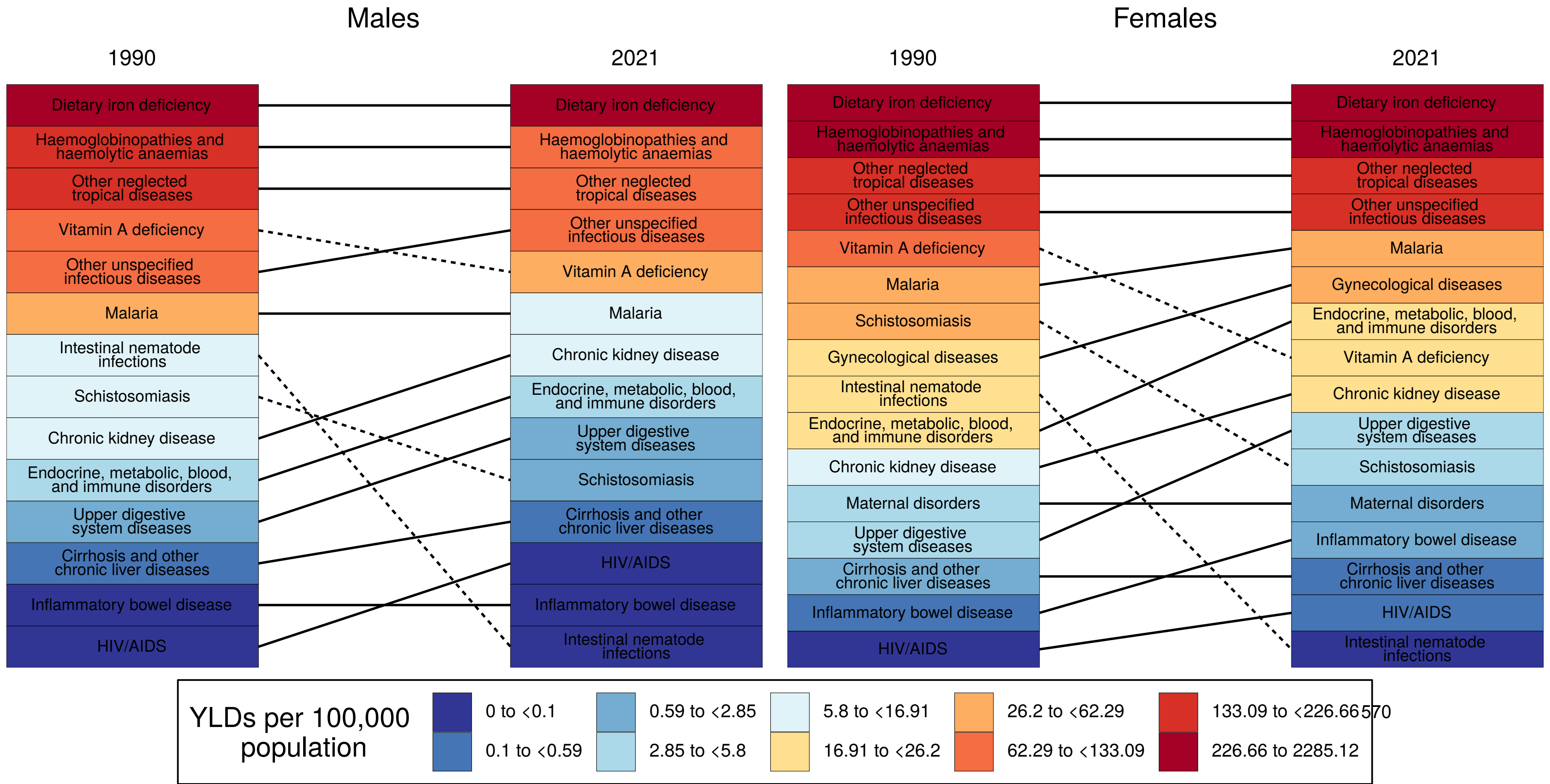
Males
Females
1990
2021
Dietary iron deficiency
Haemoglobinopathies and haemolytic anaemias
Other neglected tropical diseases
Vitamin A deficiency
Other unspecified infectious diseases
Malaria
Intestinal nematode infections
Schistosomiasis
Chronic kidney disease
Endocrine, metabolic, blood, and immune disorders
Upper digestive system diseases
Cirrhosis and other chronic liver diseases
Inflammatory bowel disease
HIV/AIDS
Gynecological diseases
Maternal disorders
YLDs per 100,000 population
0 to <0.1
0.1 to <0.59
0.59 to <2.85
2.85 to <5.8
5.8 to <16.91
16.91 to <26.2
26.2 to <62.29
62.29 to <133.09
133.09 to <226.66
226.66 to 2285.12
571

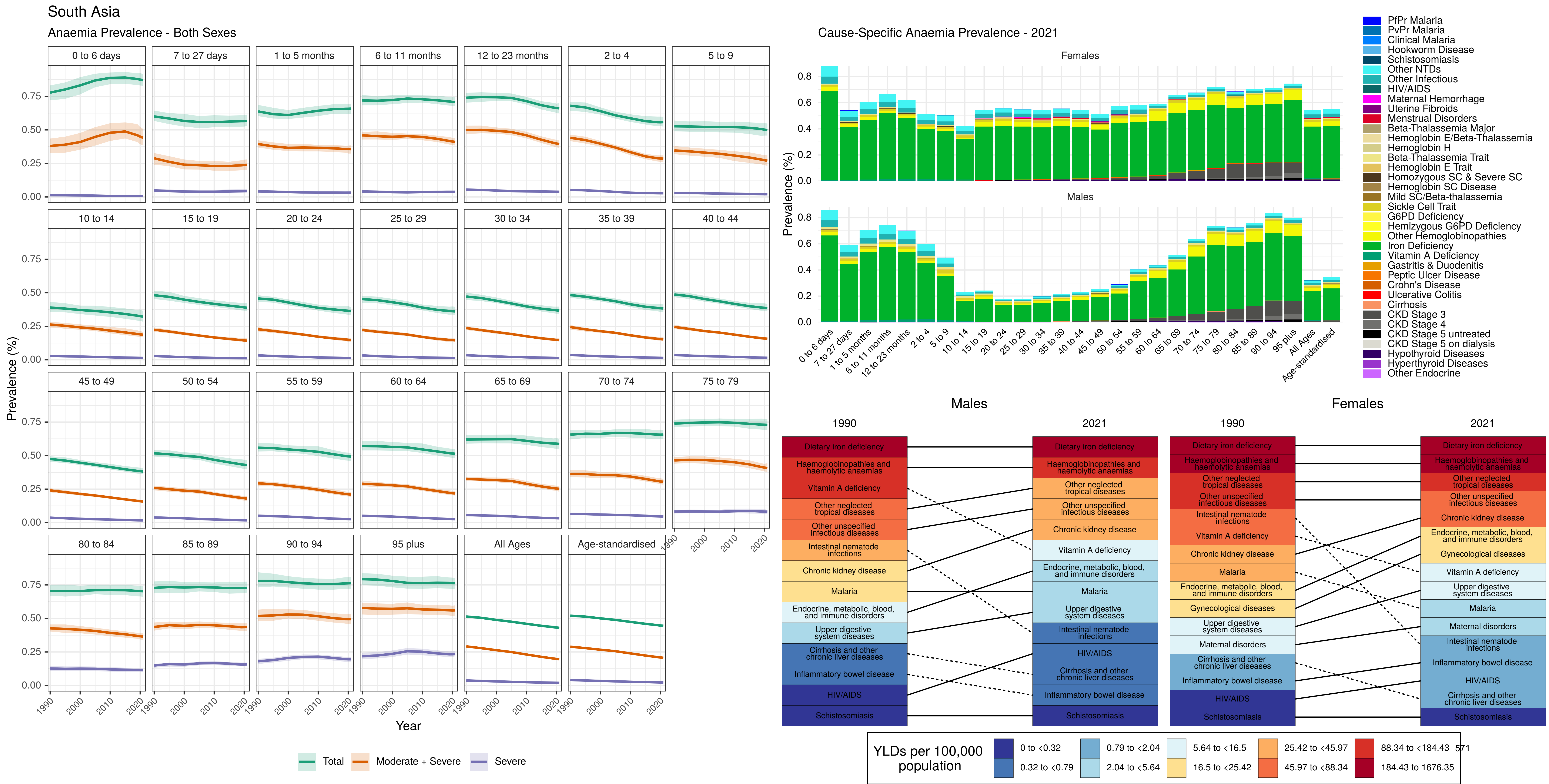
South Asia
Anaemia Prevalence - Both Sexes
0 to 6 days
7 to 27 days
1 to 5 months
6 to 11 months
12 to 23 months
2 to 4
5 to 9
10 to 14
15 to 19
20 to 24
25 to 29
30 to 34
35 to 39
40 to 44
45 to 49
50 to 54
55 to 59
60 to 64
65 to 69
70 to 74
75 to 79
80 to 84
85 to 89
90 to 94
95 plus
All Ages
Age-standardised
Prevalence (%)
Year
Total
Moderate + Severe
Severe
Cause-Specific Anaemia Prevalence - 2021
Females
Males
Prevalence (%)
PfPr Malaria
PvPr Malaria
Clinical Malaria
Hookworm Disease
Schistosomiasis
Other NTDs
Other Infectious
HIV/AIDS
Maternal Hemorrhage
Uterine Fibroids
Menstrual Disorders
Beta-Thalassemia Major
Hemoglobin E/Beta-Thalassemia
Hemoglobin H
Beta-Thalassemia Trait
Hemoglobin E Trait
Homozygous SC & Severe SC
Hemoglobin SC Disease
Mild SC/Beta-thalassemia
Sickle Cell Trait
G6PD Deficiency
Hemizygous G6PD Deficiency
Other Hemoglobinopathies
Iron Deficiency
Vitamin A Deficiency
Gastritis & Duodenitis
Peptic Ulcer Disease
Crohn's Disease
Ulcerative Colitis
Cirrhosis
CKD Stage 3
CKD Stage 4
CKD Stage 5 untreated
CKD Stage 5 on dialysis
Hypothyroid Diseases
Hyperthyroid Diseases
Other Endocrine
Males
Females
1990
2021
Dietary iron deficiency
Haemoglobinopathies and haemolytic anaemias
Vitamin A deficiency
Other neglected tropical diseases
Other unspecified infectious diseases
Intestinal nematode infections
Chronic kidney disease
Malaria
Endocrine, metabolic, blood, and immune disorders
Upper digestive system diseases
Cirrhosis and other chronic liver diseases
Inflammatory bowel disease
HIV/AIDS
Schistosomiasis
Gynecological diseases
Maternal disorders
YLDs per 100,000 population
0 to <0.32
0.32 to <0.79
0.79 to <2.04
2.04 to <5.64
5.64 to <16.5
16.5 to <25.42
25.42 to <45.97
45.97 to <88.34
88.34 to <184.43
184.43 to 1676.35

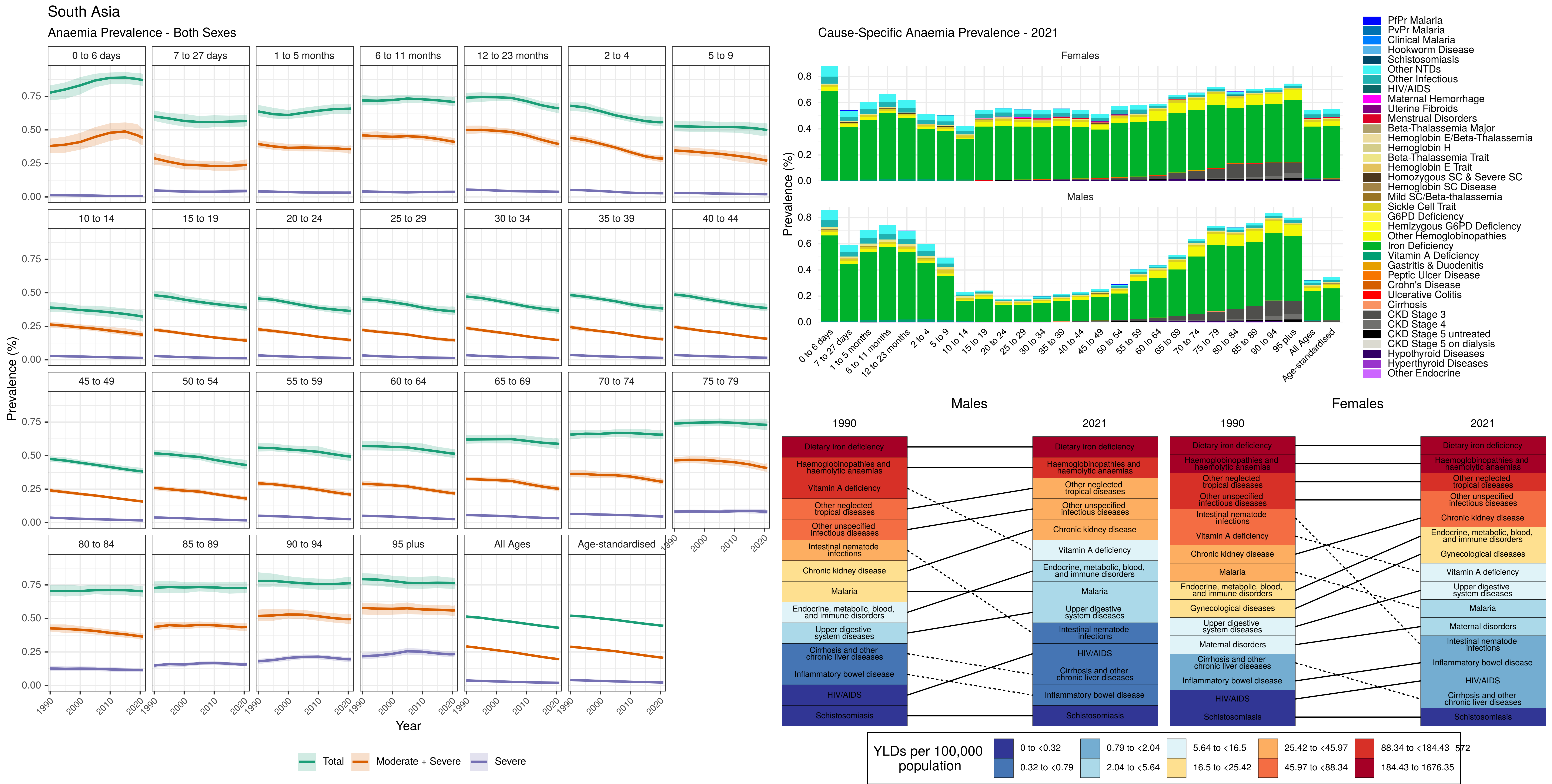
South Asia
Anaemia Prevalence - Both Sexes
0 to 6 days
7 to 27 days
1 to 5 months
6 to 11 months
12 to 23 months
2 to 4
5 to 9
10 to 14
15 to 19
20 to 24
25 to 29
30 to 34
35 to 39
40 to 44
45 to 49
50 to 54
55 to 59
60 to 64
65 to 69
70 to 74
75 to 79
80 to 84
85 to 89
90 to 94
95 plus
All Ages
Age-standardised
Prevalence (%)
Year
Total
Moderate + Severe
Severe
Cause-Specific Anaemia Prevalence - 2021
Females
Males
PfPr Malaria
PvPr Malaria
Clinical Malaria
Hookworm Disease
Schistosomiasis
Other NTDs
Other Infectious
HIV/AIDS
Maternal Hemorrhage
Uterine Fibroids
Menstrual Disorders
Beta-Thalassemia Major
Hemoglobin E/Beta-Thalassemia
Hemoglobin H
Beta-Thalassemia Trait
Hemoglobin E Trait
Homozygous SC & Severe SC
Hemoglobin SC Disease
Mild SC/Beta-thalassemia
Sickle Cell Trait
G6PD Deficiency
Hemizygous G6PD Deficiency
Other Hemoglobinopathies
Iron Deficiency
Vitamin A Deficiency
Gastritis & Duodenitis
Peptic Ulcer Disease
Crohn's Disease
Ulcerative Colitis
Cirrhosis
CKD Stage 3
CKD Stage 4
CKD Stage 5 untreated
CKD Stage 5 on dialysis
Hypothyroid Diseases
Hyperthyroid Diseases
Other Endocrine
Males
Females
1990
2021
Dietary iron deficiency
Haemoglobinopathies and haemolytic anaemias
Vitamin A deficiency
Other neglected tropical diseases
Other unspecified infectious diseases
Intestinal nematode infections
Chronic kidney disease
Malaria
Endocrine, metabolic, blood, and immune disorders
Upper digestive system diseases
Cirrhosis and other chronic liver diseases
Inflammatory bowel disease
HIV/AIDS
Schistosomiasis
Gynecological diseases
Maternal disorders
YLDs per 100,000 population
0 to <0.32
0.32 to <0.79
0.79 to <2.04
2.04 to <5.64
5.64 to <16.5
16.5 to <25.42
25.42 to <45.97
45.97 to <88.34
88.34 to <184.43
184.43 to 1676.35

Bangladesh

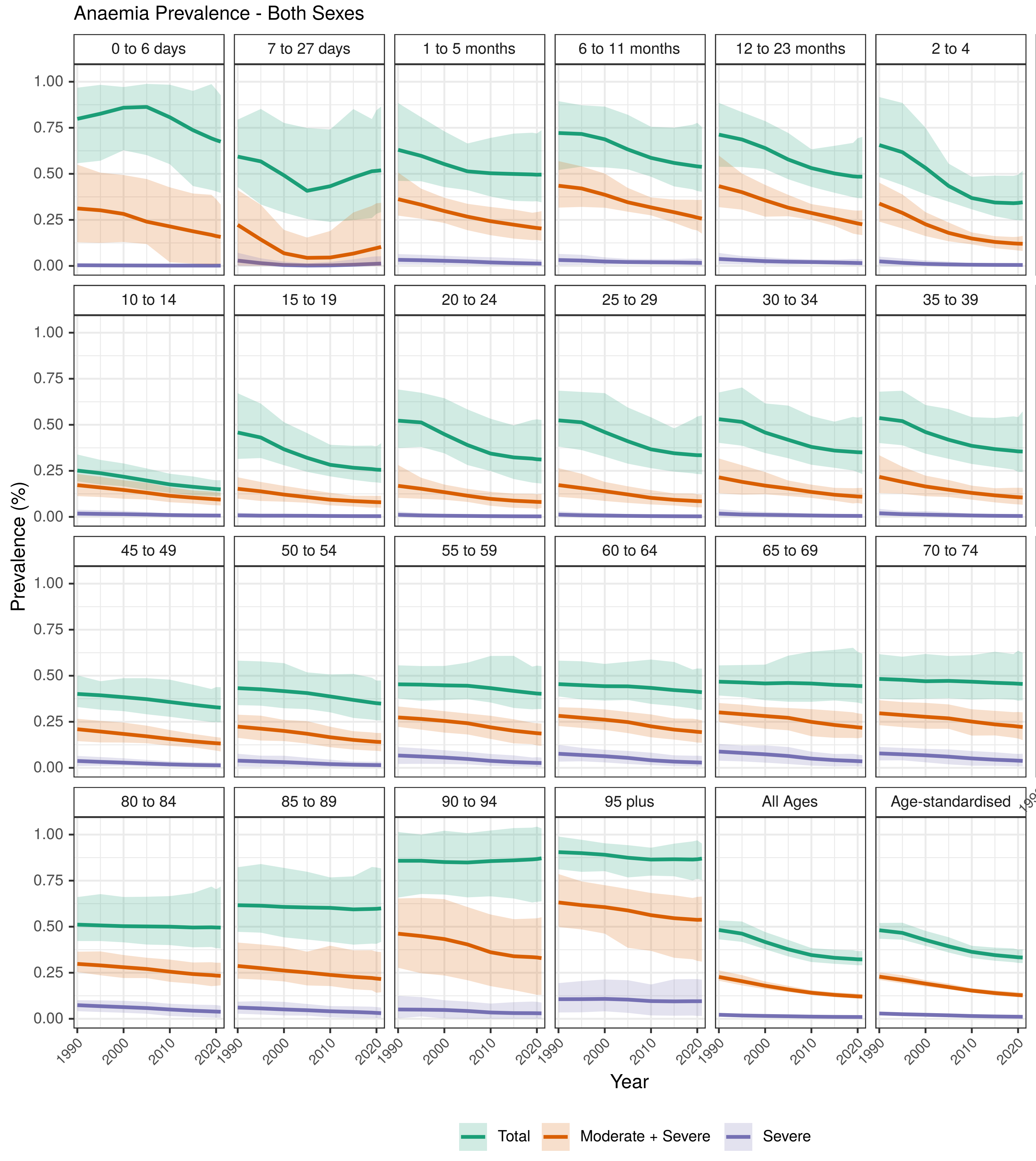
Anaemia Prevalence - Both Sexes
0 to 6 days
7 to 27 days
1 to 5 months
6 to 11 months
12 to 23 months
2 to 4
5 to 9
10 to 14
15 to 19
20 to 24
25 to 29
30 to 34
35 to 39
40 to 44
45 to 49
50 to 54
55 to 59
60 to 64
65 to 69
70 to 74
75 to 79
80 to 84
85 to 89
90 to 94
95 plus
All Ages
Age-standardised
1.00
0.75
0.50
0.25
0.00
1990
2000
2010
2020
Prevalence (%)
Year
Total
Moderate + Severe
Severe

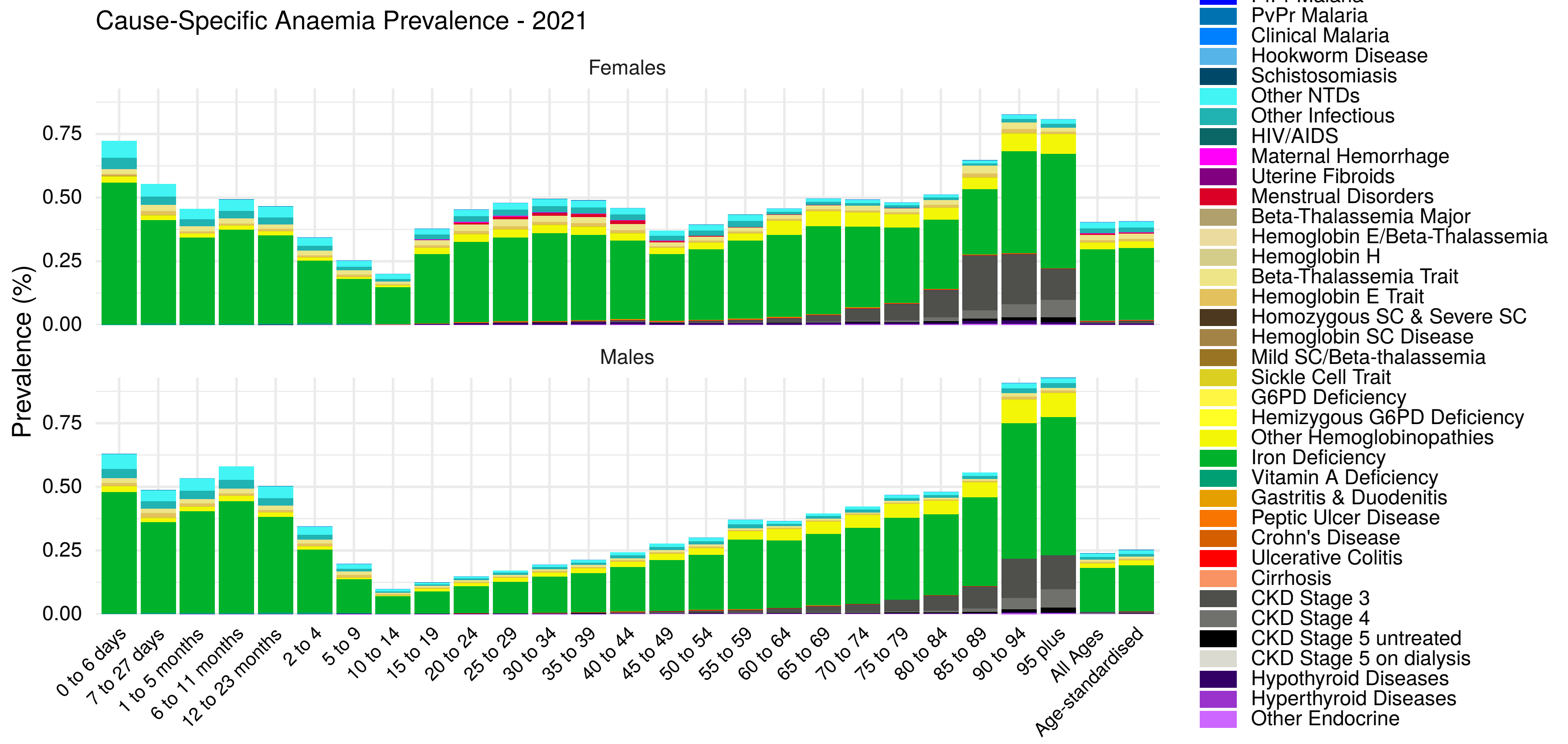
Cause-Specific Anaemia Prevalence - 2021
Females
Males
Prevalence (%)
0.75
0.50
0.25
0.00
0 to 6 days
7 to 27 days
1 to 5 months
6 to 11 months
12 to 23 months
2 to 4
5 to 9
10 to 14
15 to 19
20 to 24
25 to 29
30 to 34
35 to 39
40 to 44
45 to 49
50 to 54
55 to 59
60 to 64
65 to 69
70 to 74
75 to 79
80 to 84
85 to 89
90 to 94
95 plus
All Ages
Age-standardised
PfPr Malaria
PvPr Malaria
Clinical Malaria
Hookworm Disease
Schistosomiasis
Other NTDs
Other Infectious
HIV/AIDS
Maternal Hemorrhage
Uterine Fibroids
Menstrual Disorders
Beta-Thalassemia Major
Hemoglobin E/Beta-Thalassemia
Hemoglobin H
Beta-Thalassemia Trait
Hemoglobin E Trait
Homozygous SC & Severe SC
Hemoglobin SC Disease
Mild SC/Beta-thalassemia
Sickle Cell Trait
G6PD Deficiency
Hemizygous G6PD Deficiency
Other Hemoglobinopathies
Iron Deficiency
Vitamin A Deficiency
Gastritis & Duodenitis
Peptic Ulcer Disease
Crohn's Disease
Ulcerative Colitis
Cirrhosis
CKD Stage 3
CKD Stage 4
CKD Stage 5 untreated
CKD Stage 5 on dialysis
Hypothyroid Diseases
Hyperthyroid Diseases
Other Endocrine

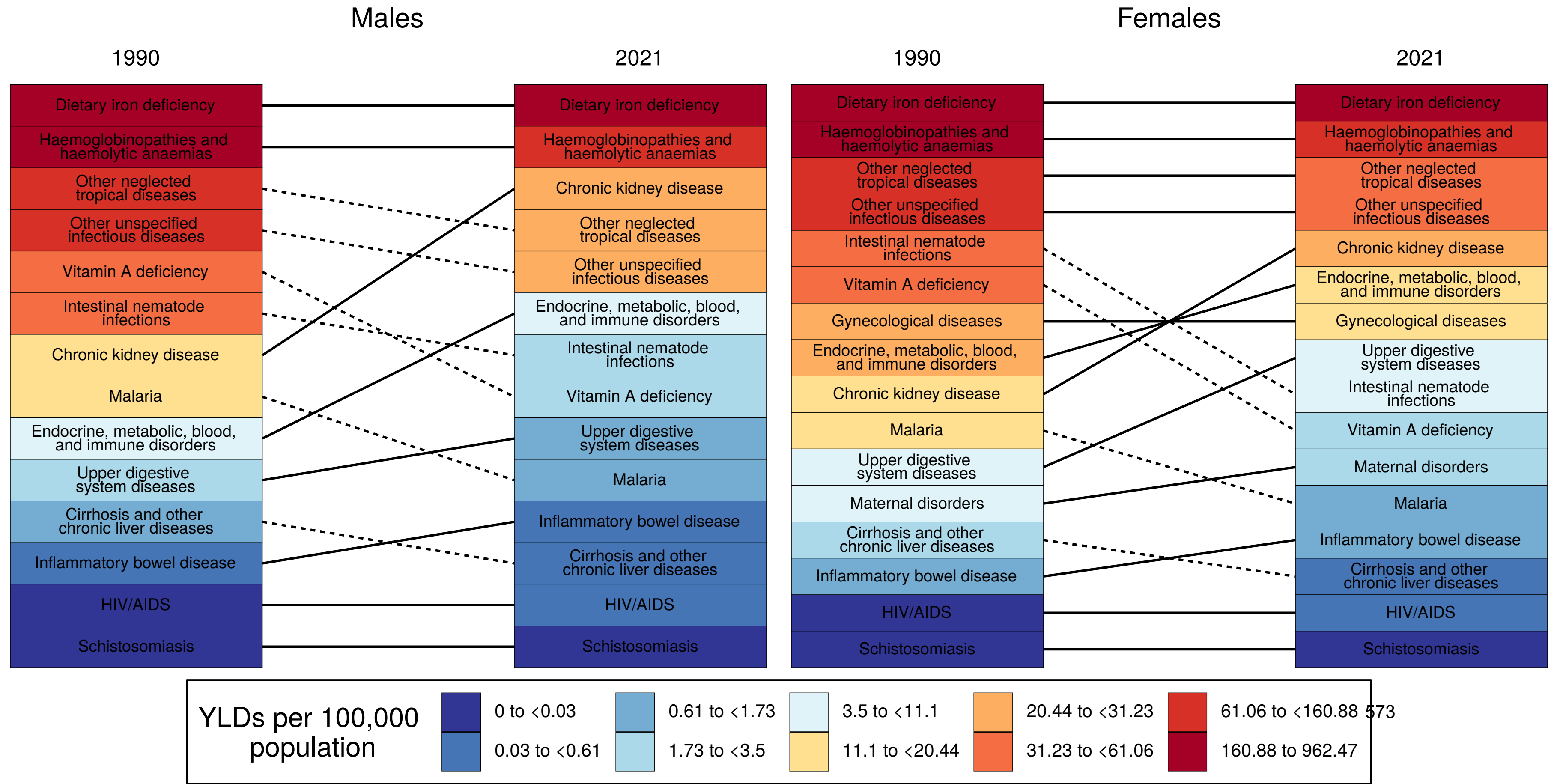
Males
Females
1990
2021
Dietary iron deficiency
Haemoglobinopathies and haemolytic anaemias
Other neglected tropical diseases
Other unspecified infectious diseases
Vitamin A deficiency
Intestinal nematode infections
Chronic kidney disease
Malaria
Endocrine, metabolic, blood, and immune disorders
Upper digestive system diseases
Cirrhosis and other chronic liver diseases
Inflammatory bowel disease
HIV/AIDS
Schistosomiasis
Gynecological diseases
Maternal disorders
YLDs per 100,000 population
0 to <0.03
0.03 to <0.61
0.61 to <1.73
1.73 to <3.5
3.5 to <11.1
11.1 to <20.44
20.44 to <31.23
31.23 to <61.06
61.06 to <160.88
160.88 to 962.47

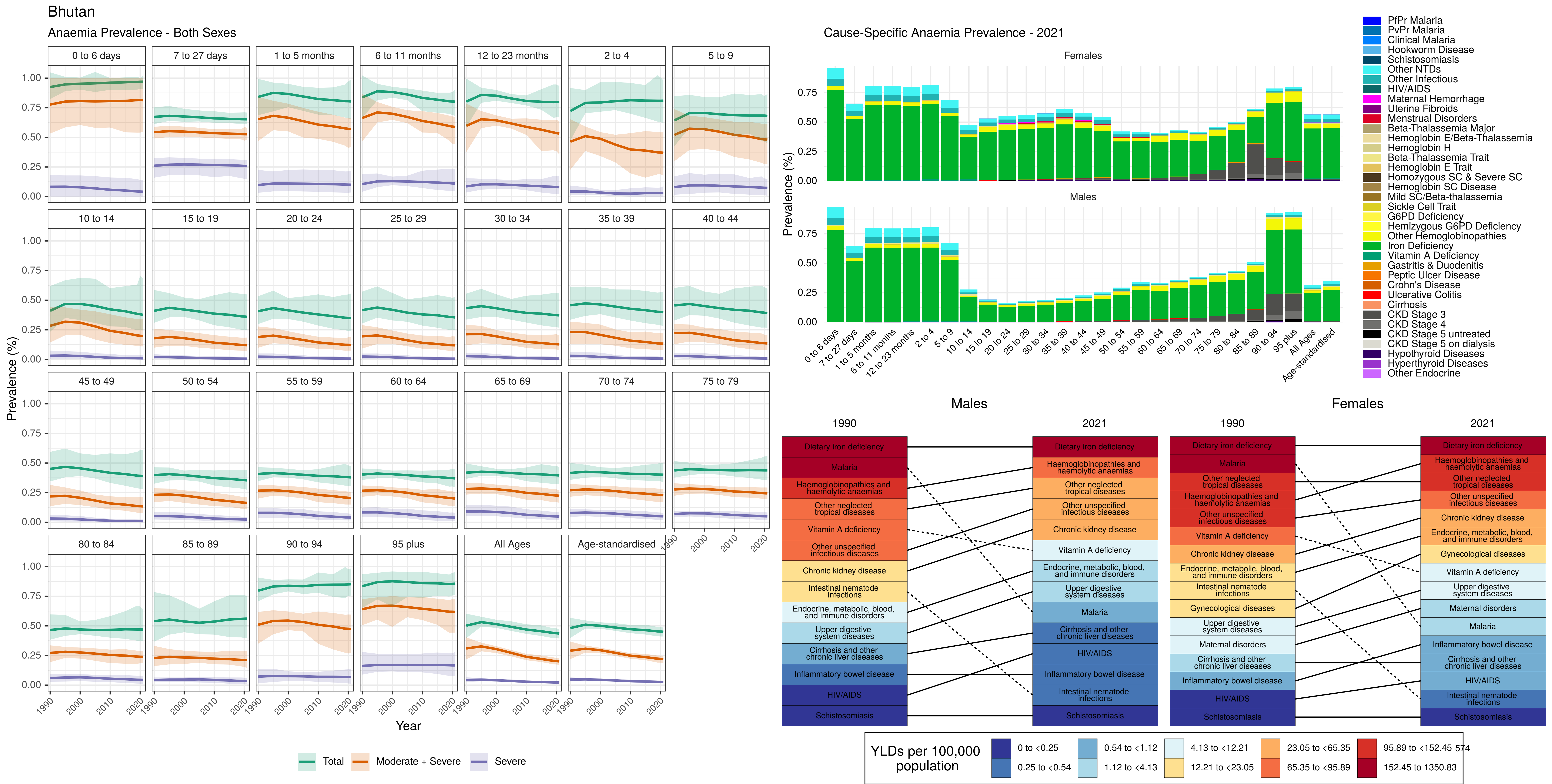

Bhutan
Anaemia Prevalence - Both Sexes
0 to 6 days
7 to 27 days
1 to 5 months
6 to 11 months
12 to 23 months
2 to 4
5 to 9
10 to 14
15 to 19
20 to 24
25 to 29
30 to 34
35 to 39
40 to 44
45 to 49
50 to 54
55 to 59
60 to 64
65 to 69
70 to 74
75 to 79
80 to 84
85 to 89
90 to 94
95 plus
All Ages
Age-standardised
Prevalence (%)
Year
Total
Moderate + Severe
Severe
Cause-Specific Anaemia Prevalence - 2021
Females
Males
PfPr Malaria
PvPr Malaria
Clinical Malaria
Hookworm Disease
Schistosomiasis
Other NTDs
Other Infectious
HIV/AIDS
Maternal Hemorrhage
Uterine Fibroids
Menstrual Disorders
Beta-Thalassemia Major
Hemoglobin E/Beta-Thalassemia
Hemoglobin H
Beta-Thalassemia Trait
Hemoglobin E Trait
Homozygous SC & Severe SC
Hemoglobin SC Disease
Mild SC/Beta-thalassemia
Sickle Cell Trait
G6PD Deficiency
Hemizygous G6PD Deficiency
Other Hemoglobinopathies
Iron Deficiency
Vitamin A Deficiency
Gastritis & Duodenitis
Peptic Ulcer Disease
Crohn's Disease
Ulcerative Colitis
Cirrhosis
CKD Stage 3
CKD Stage 4
CKD Stage 5 untreated
CKD Stage 5 on dialysis
Hypothyroid Diseases
Hyperthyroid Diseases
Other Endocrine
Males
Females
1990
2021
Dietary iron deficiency
Malaria
Haemoglobinopathies and haemolytic anaemias
Other neglected tropical diseases
Vitamin A deficiency
Other unspecified infectious diseases
Chronic kidney disease
Intestinal nematode infections
Endocrine, metabolic, blood, and immune disorders
Upper digestive system diseases
Cirrhosis and other chronic liver diseases
Inflammatory bowel disease
HIV/AIDS
Schistosomiasis
Gynecological diseases
Maternal disorders
YLDs per 100,000 population
0 to <0.25
0.25 to <0.54
0.54 to <1.12
1.12 to <4.13
4.13 to <12.21
12.21 to <23.05
23.05 to <65.35
65.35 to <95.89
95.89 to <152.45
152.45 to 1350.83

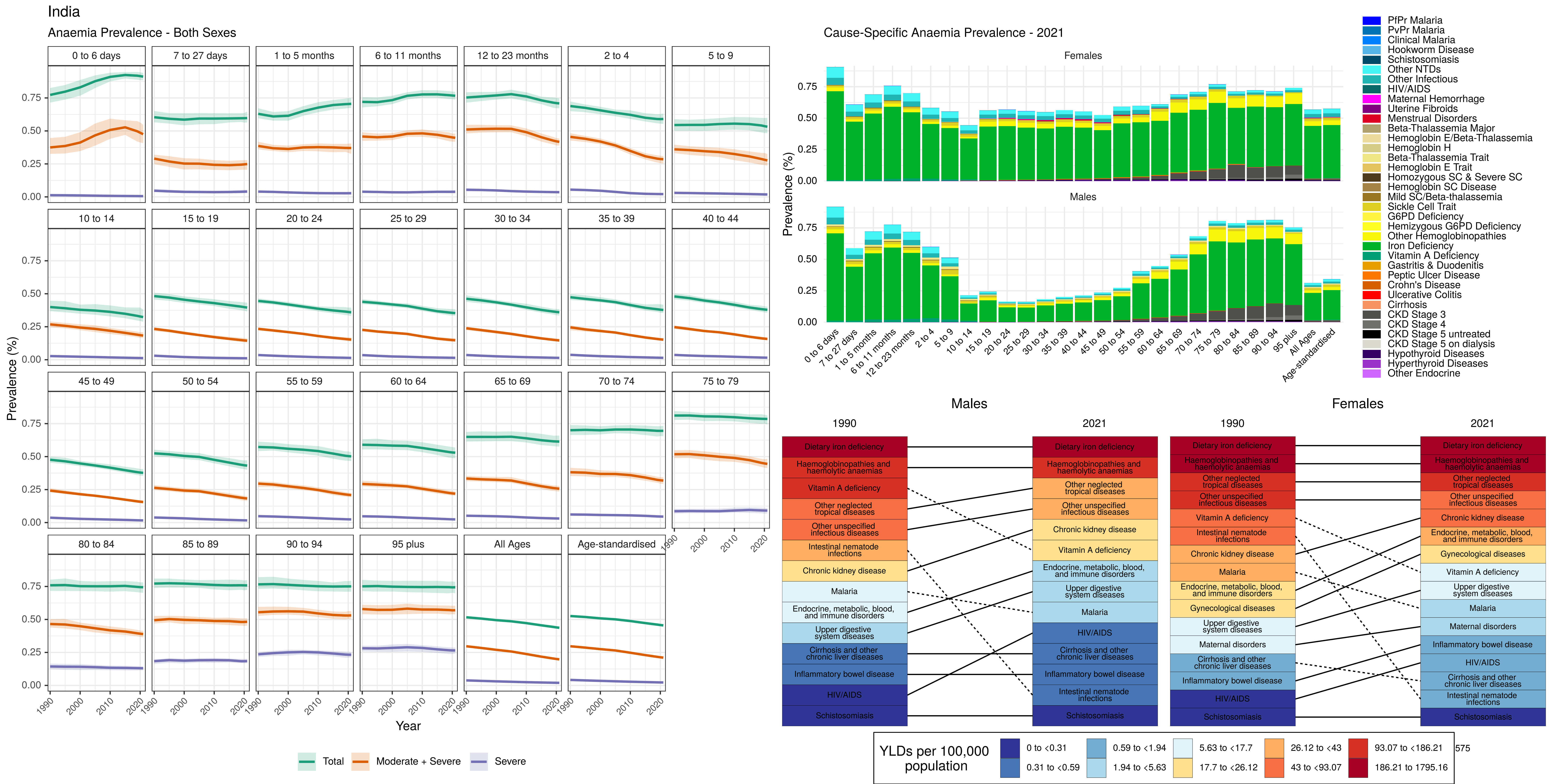
India
Anaemia Prevalence - Both Sexes
0 to 6 days
7 to 27 days
1 to 5 months
6 to 11 months
12 to 23 months
2 to 4
5 to 9
10 to 14
15 to 19
20 to 24
25 to 29
30 to 34
35 to 39
40 to 44
45 to 49
50 to 54
55 to 59
60 to 64
65 to 69
70 to 74
75 to 79
80 to 84
85 to 89
90 to 94
95 plus
All Ages
Age-standardised
Prevalence (%)
0.00
0.25
0.50
0.75
1990
2000
2010
2020
Year
Total
Moderate + Severe
Severe
Cause-Specific Anaemia Prevalence - 2021
Females
Males
Prevalence (%)
PfPr Malaria
PvPr Malaria
Clinical Malaria
Hookworm Disease
Schistosomiasis
Other NTDs
Other Infectious
HIV/AIDS
Maternal Hemorrhage
Uterine Fibroids
Menstrual Disorders
Beta-Thalassemia Major
Hemoglobin E/Beta-Thalassemia
Hemoglobin H
Beta-Thalassemia Trait
Hemoglobin E Trait
Homozygous SC & Severe SC
Hemoglobin SC Disease
Mild SC/Beta-thalassemia
Sickle Cell Trait
G6PD Deficiency
Hemizygous G6PD Deficiency
Other Hemoglobinopathies
Iron Deficiency
Vitamin A Deficiency
Gastritis & Duodenitis
Peptic Ulcer Disease
Crohn's Disease
Ulcerative Colitis
Cirrhosis
CKD Stage 3
CKD Stage 4
CKD Stage 5 untreated
CKD Stage 5 on dialysis
Hypothyroid Diseases
Hyperthyroid Diseases
Other Endocrine
Males
Females
1990
2021
Dietary iron deficiency
Haemoglobinopathies and haemolytic anaemias
Vitamin A deficiency
Other neglected tropical diseases
Other unspecified infectious diseases
Intestinal nematode infections
Chronic kidney disease
Malaria
Endocrine, metabolic, blood, and immune disorders
Upper digestive system diseases
Cirrhosis and other chronic liver diseases
Inflammatory bowel disease
HIV/AIDS
Schistosomiasis
Gynecological diseases
Maternal disorders
YLDs per 100,000 population
0 to <0.31
0.31 to <0.59
0.59 to <1.94
1.94 to <5.63
5.63 to <17.7
17.7 to <26.12
26.12 to <43
43 to <93.07
93.07 to <186.21
186.21 to 1795.16
575

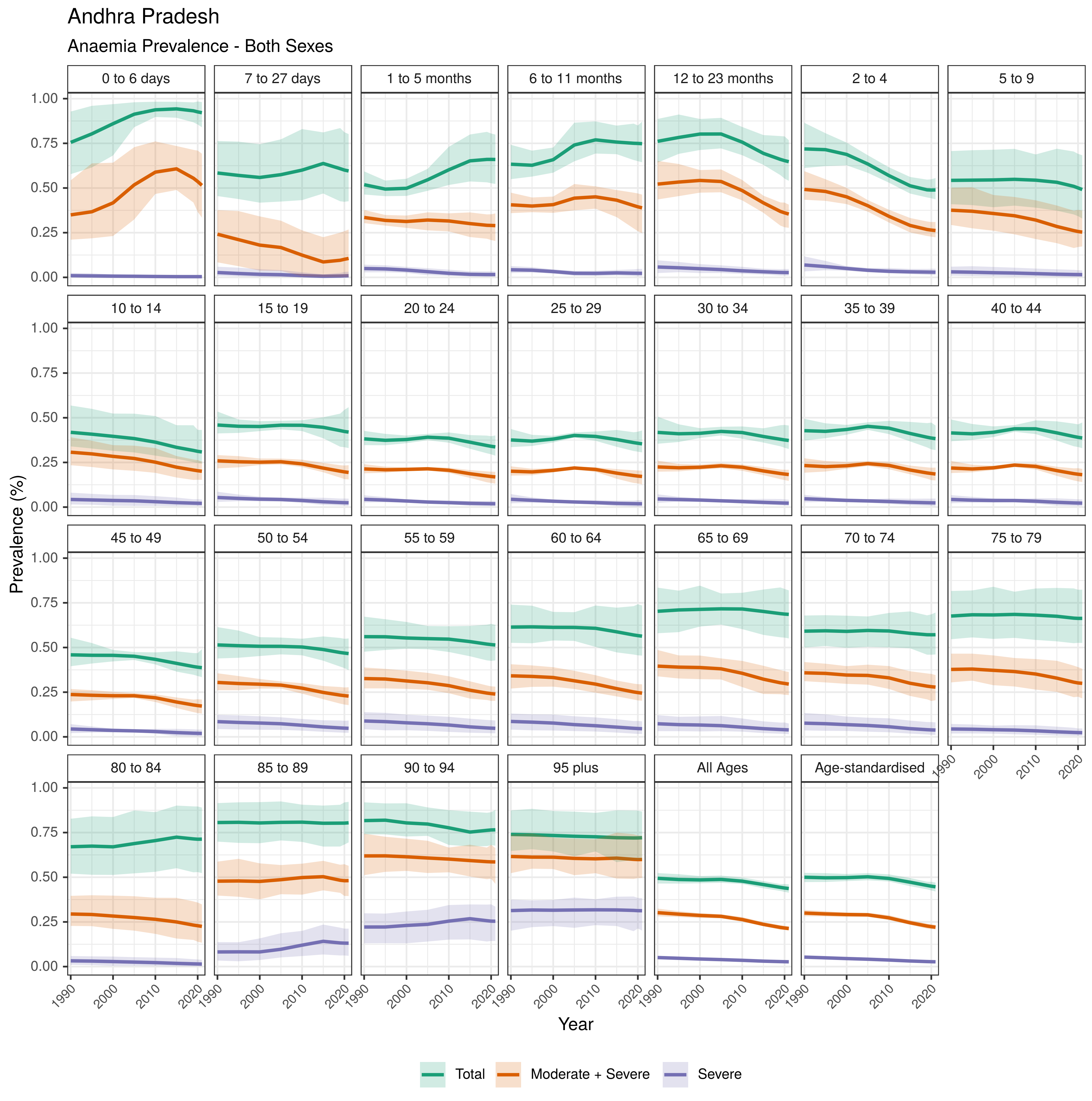
Andhra Pradesh
Anaemia Prevalence - Both Sexes
0 to 6 days
7 to 27 days
1 to 5 months
6 to 11 months
12 to 23 months
2 to 4
5 to 9
10 to 14
15 to 19
20 to 24
25 to 29
30 to 34
35 to 39
40 to 44
45 to 49
50 to 54
55 to 59
60 to 64
65 to 69
70 to 74
75 to 79
80 to 84
85 to 89
90 to 94
95 plus
All Ages
Age-standardised
Prevalence (%)
Year
Total
Moderate + Severe
Severe

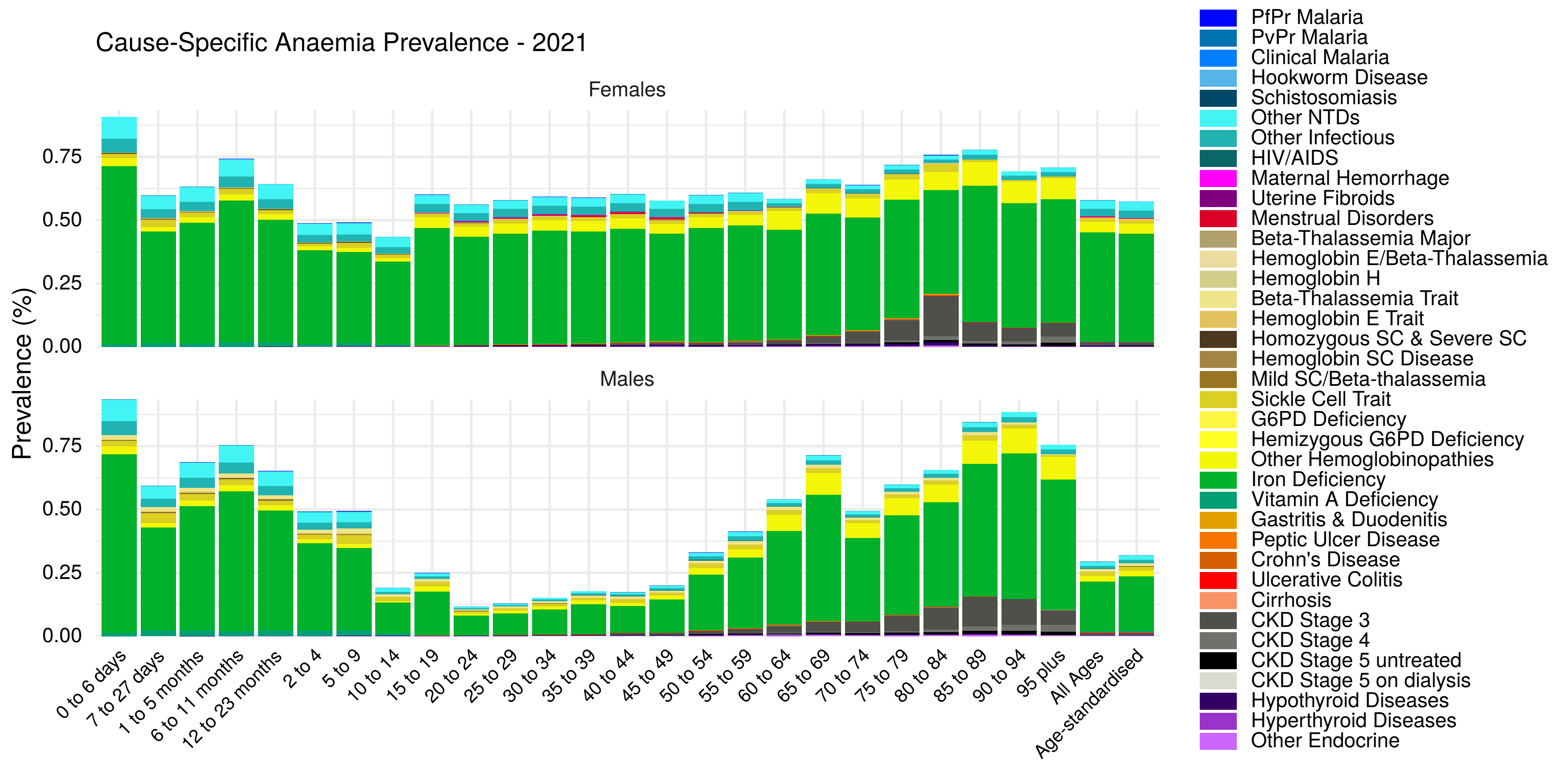
Cause-Specific Anaemia Prevalence - 2021
Females
Males
Prevalence (%)
PfPr Malaria
PvPr Malaria
Clinical Malaria
Hookworm Disease
Schistosomiasis
Other NTDs
Other Infectious
HIV/AIDS
Maternal Hemorrhage
Uterine Fibroids
Menstrual Disorders
Beta-Thalassemia Major
Hemoglobin E/Beta-Thalassemia
Hemoglobin H
Beta-Thalassemia Trait
Hemoglobin E Trait
Homozygous SC & Severe SC
Hemoglobin SC Disease
Mild SC/Beta-thalassemia
Sickle Cell Trait
G6PD Deficiency
Hemizygous G6PD Deficiency
Other Hemoglobinopathies
Iron Deficiency
Vitamin A Deficiency
Gastritis & Duodenitis
Peptic Ulcer Disease
Crohn's Disease
Ulcerative Colitis
Cirrhosis
CKD Stage 3
CKD Stage 4
CKD Stage 5 untreated
CKD Stage 5 on dialysis
Hypothyroid Diseases
Hyperthyroid Diseases
Other Endocrine

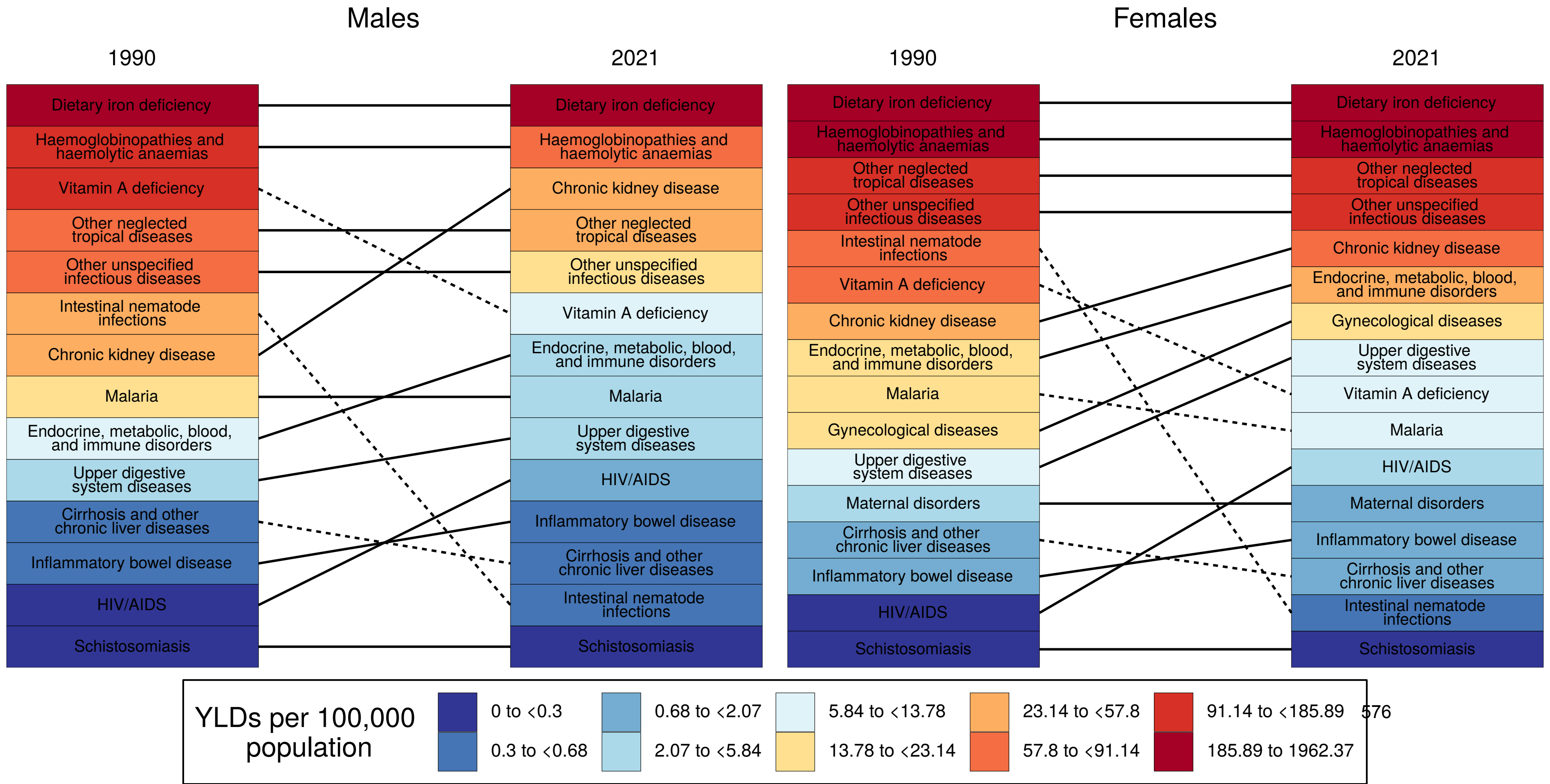
Males
Females
1990
2021
Dietary iron deficiency
Haemoglobinopathies and haemolytic anaemias
Vitamin A deficiency
Other neglected tropical diseases
Other unspecified infectious diseases
Intestinal nematode infections
Chronic kidney disease
Malaria
Endocrine, metabolic, blood, and immune disorders
Upper digestive system diseases
Cirrhosis and other chronic liver diseases
Inflammatory bowel disease
HIV/AIDS
Schistosomiasis
Gynecological diseases
Maternal disorders
YLDs per 100,000 population
0 to <0.3
0.3 to <0.68
0.68 to <2.07
2.07 to <5.84
5.84 to <13.78
13.78 to <23.14
23.14 to <57.8
57.8 to <91.14
91.14 to <185.89
185.89 to 1962.37

Arunachal Pradesh

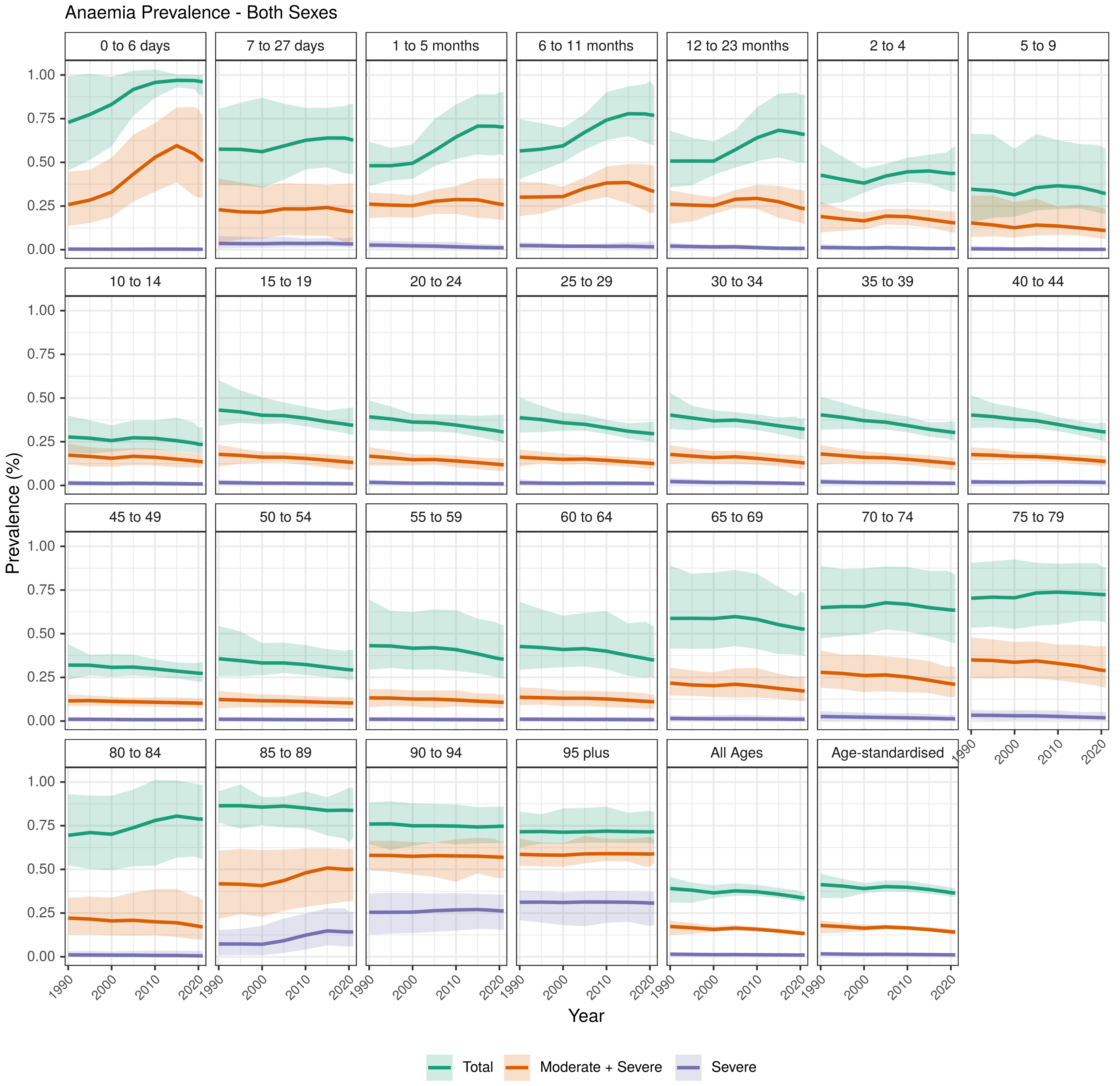

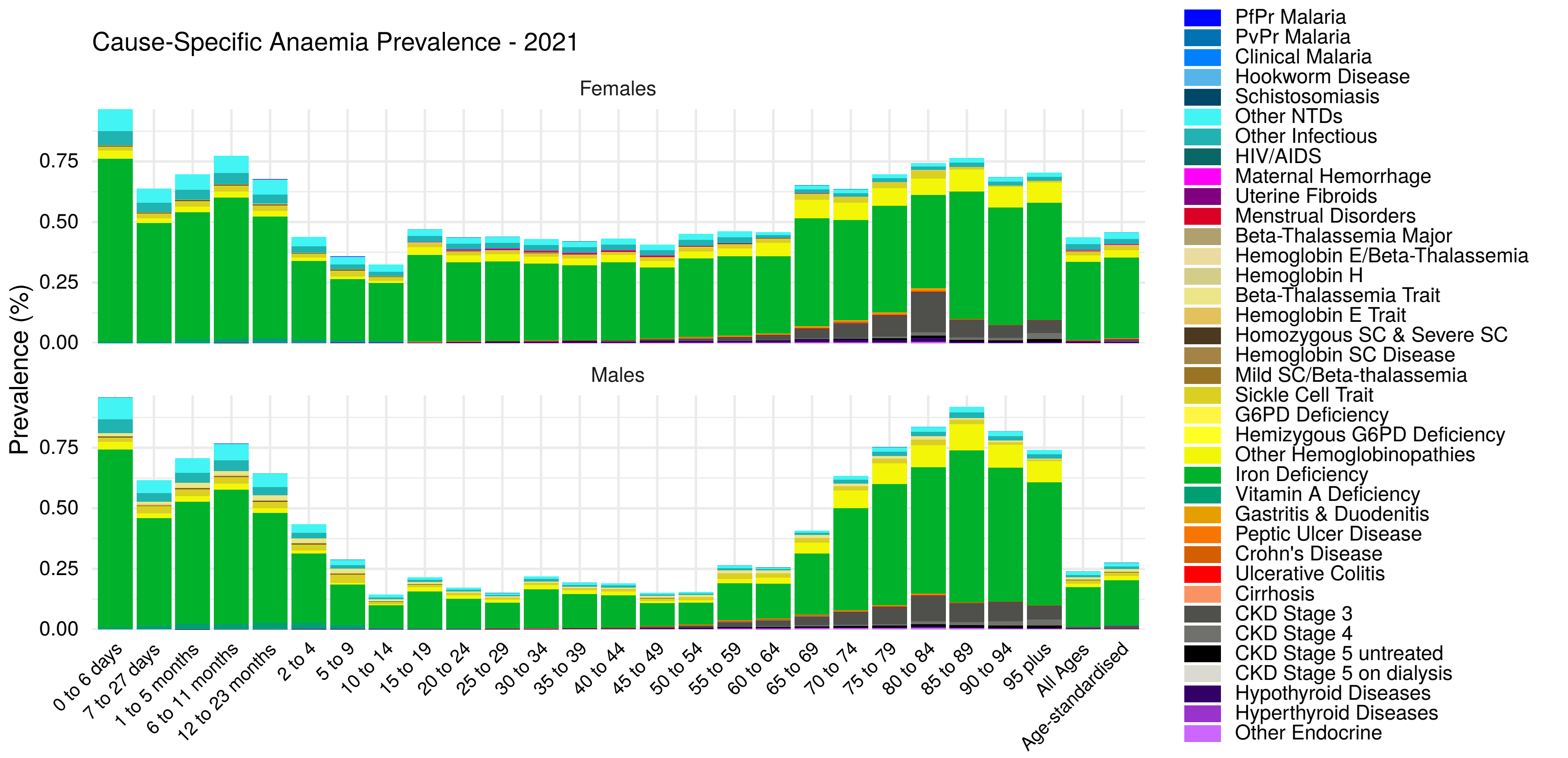

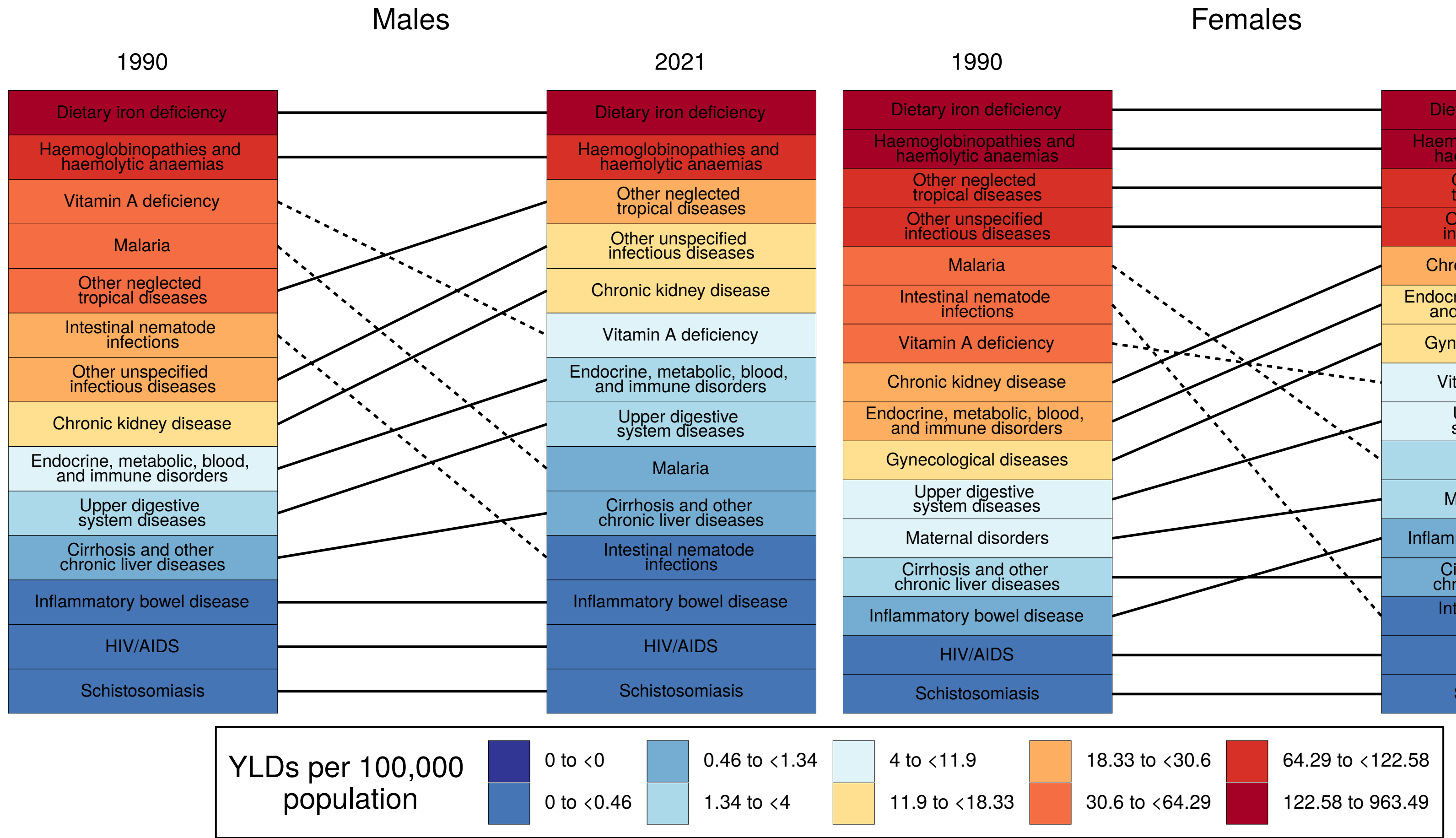

577

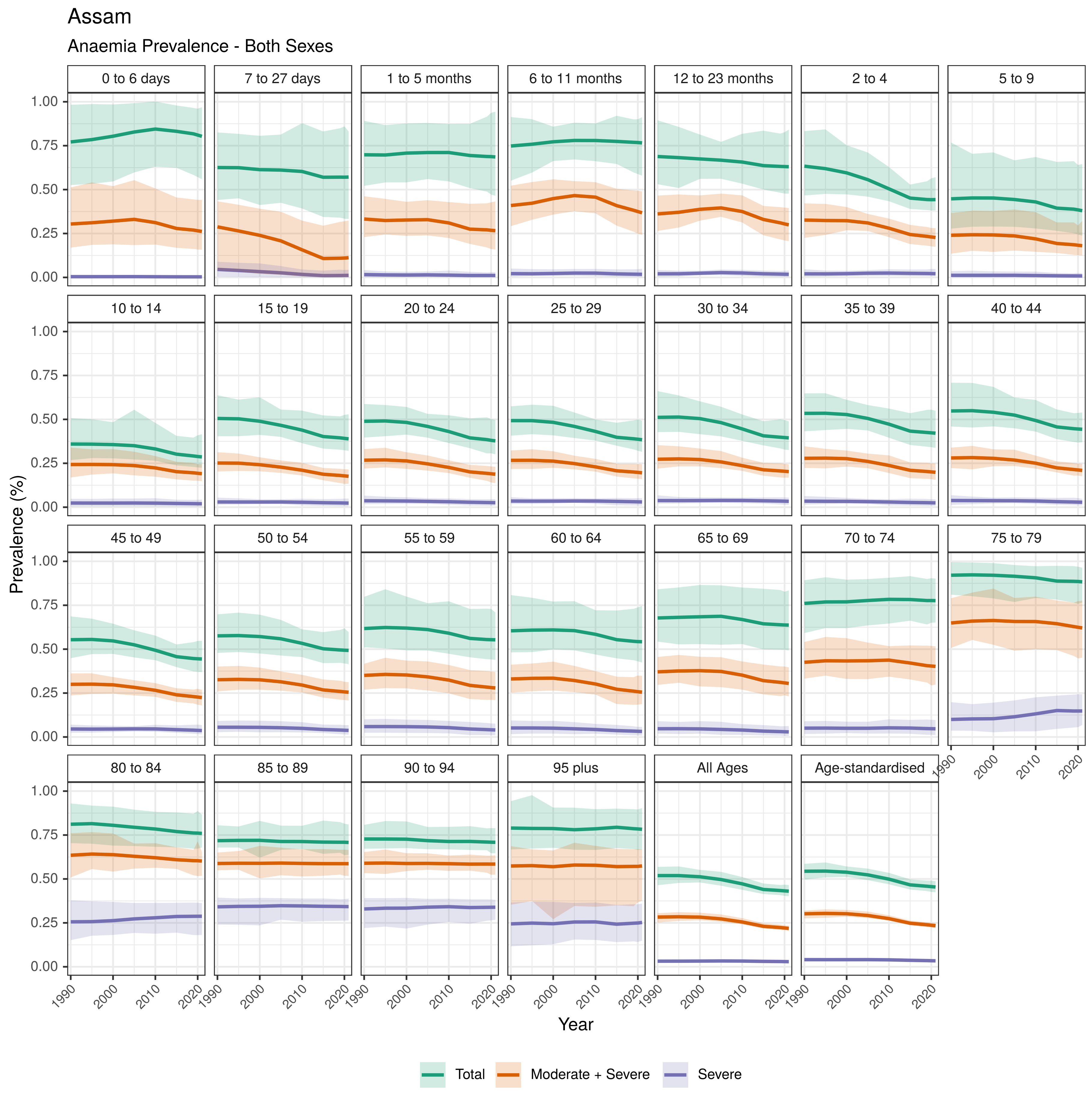
Assam
Anaemia Prevalence - Both Sexes
0 to 6 days
7 to 27 days
1 to 5 months
6 to 11 months
12 to 23 months
2 to 4
5 to 9
10 to 14
15 to 19
20 to 24
25 to 29
30 to 34
35 to 39
40 to 44
45 to 49
50 to 54
55 to 59
60 to 64
65 to 69
70 to 74
75 to 79
80 to 84
85 to 89
90 to 94
95 plus
All Ages
Age-standardised
Prevalence (%)
Year
Total
Moderate + Severe
Severe

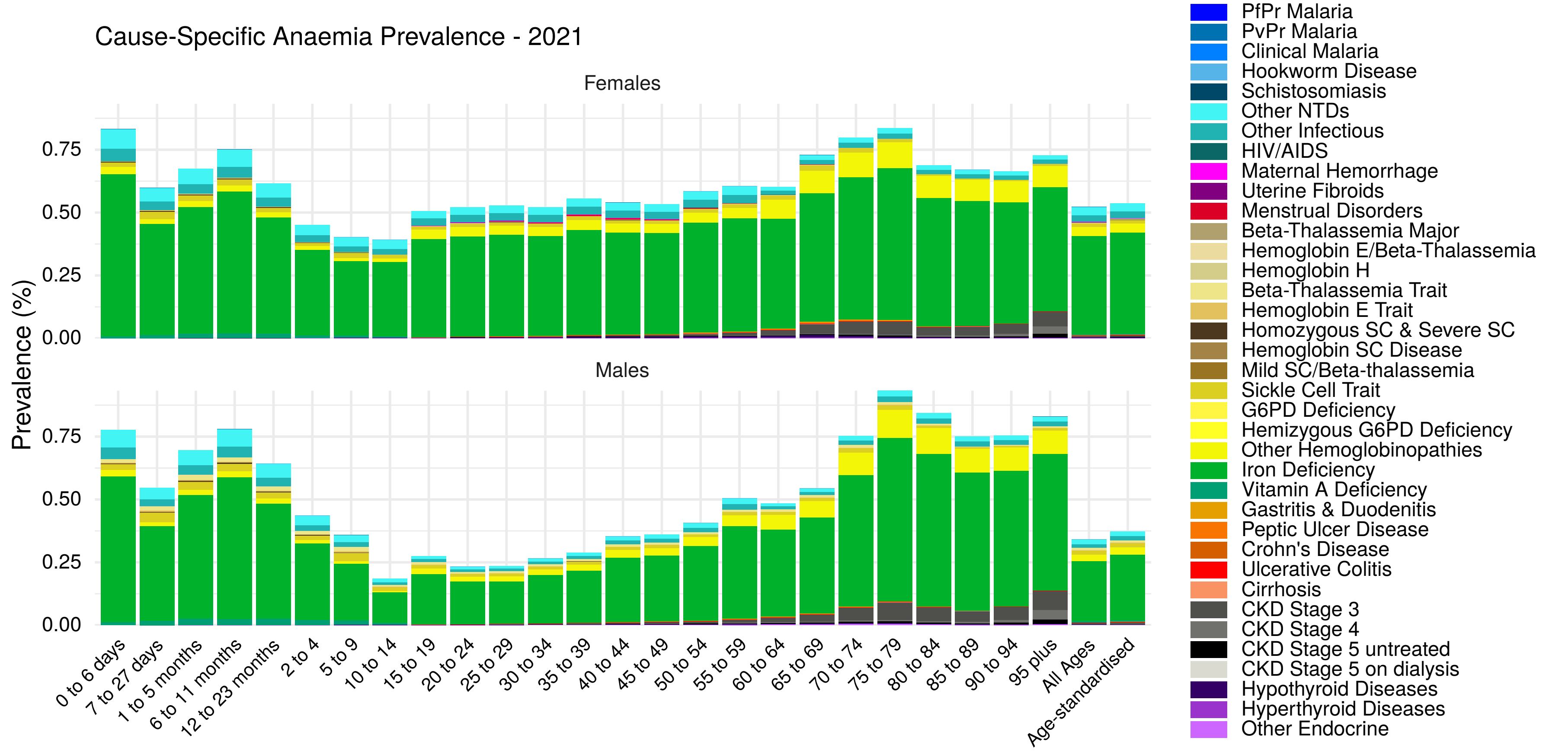
Cause-Specific Anaemia Prevalence - 2021
Females
Males
Prevalence (%)
PfPr Malaria
PvPr Malaria
Clinical Malaria
Hookworm Disease
Schistosomiasis
Other NTDs
Other Infectious
HIV/AIDS
Maternal Hemorrhage
Uterine Fibroids
Menstrual Disorders
Beta-Thalassemia Major
Hemoglobin E/Beta-Thalassemia
Hemoglobin H
Beta-Thalassemia Trait
Hemoglobin E Trait
Homozygous SC & Severe SC
Hemoglobin SC Disease
Mild SC/Beta-thalassemia
Sickle Cell Trait
G6PD Deficiency
Hemizygous G6PD Deficiency
Other Hemoglobinopathies
Iron Deficiency
Vitamin A Deficiency
Gastritis & Duodenitis
Peptic Ulcer Disease
Crohn's Disease
Ulcerative Colitis
Cirrhosis
CKD Stage 3
CKD Stage 4
CKD Stage 5 untreated
CKD Stage 5 on dialysis
Hypothyroid Diseases
Hyperthyroid Diseases
Other Endocrine

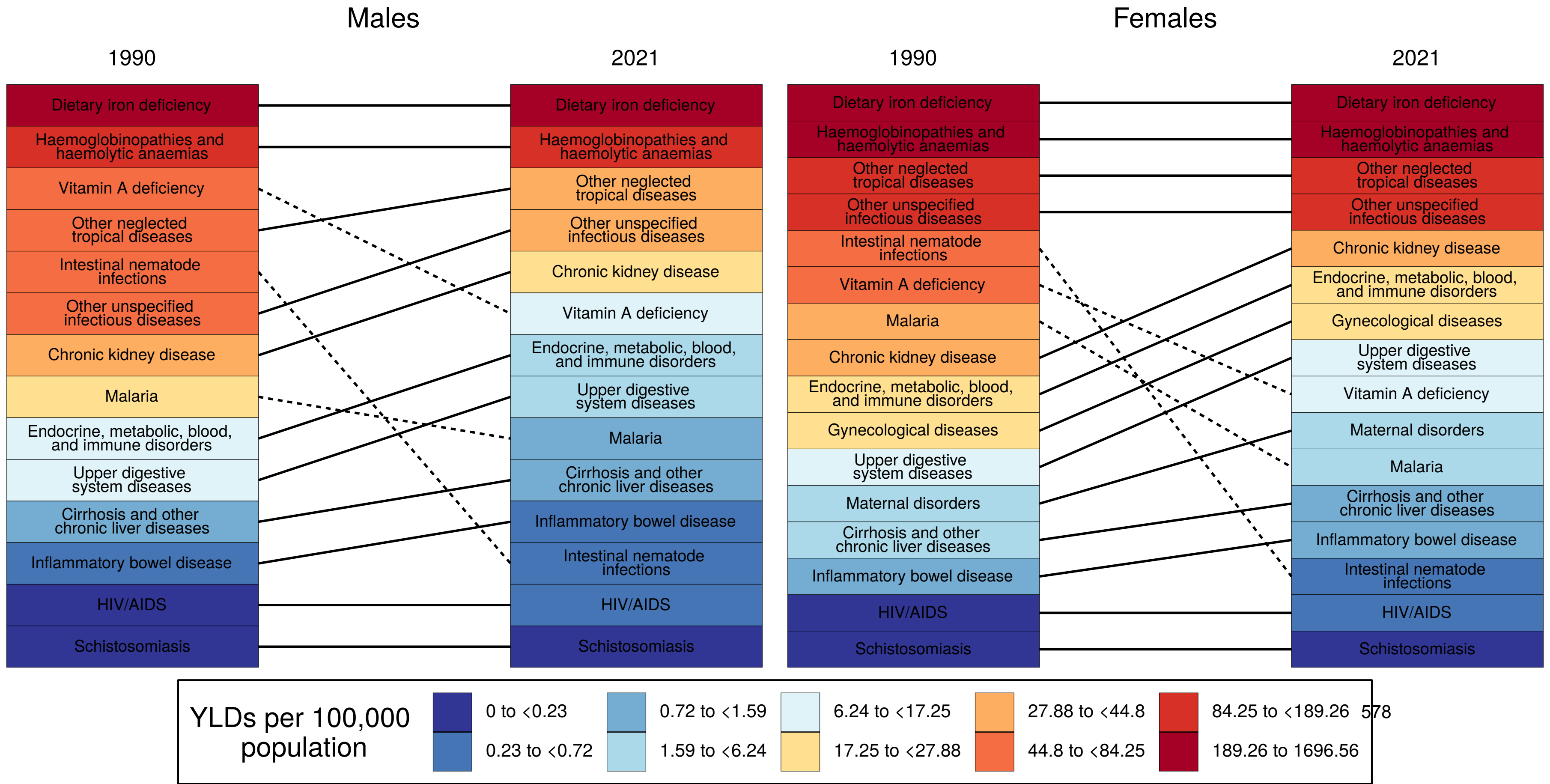
Males
Females
1990
2021
Dietary iron deficiency
Haemoglobinopathies and haemolytic anaemias
Vitamin A deficiency
Other neglected tropical diseases
Intestinal nematode infections
Other unspecified infectious diseases
Chronic kidney disease
Malaria
Endocrine, metabolic, blood, and immune disorders
Upper digestive system diseases
Cirrhosis and other chronic liver diseases
Inflammatory bowel disease
HIV/AIDS
Schistosomiasis
Gynecological diseases
Maternal disorders
YLDs per 100,000 population
0 to <0.23
0.23 to <0.72
0.72 to <1.59
1.59 to <6.24
6.24 to <17.25
17.25 to <27.88
27.88 to <44.8
44.8 to <84.25
84.25 to <189.26
189.26 to 1696.56

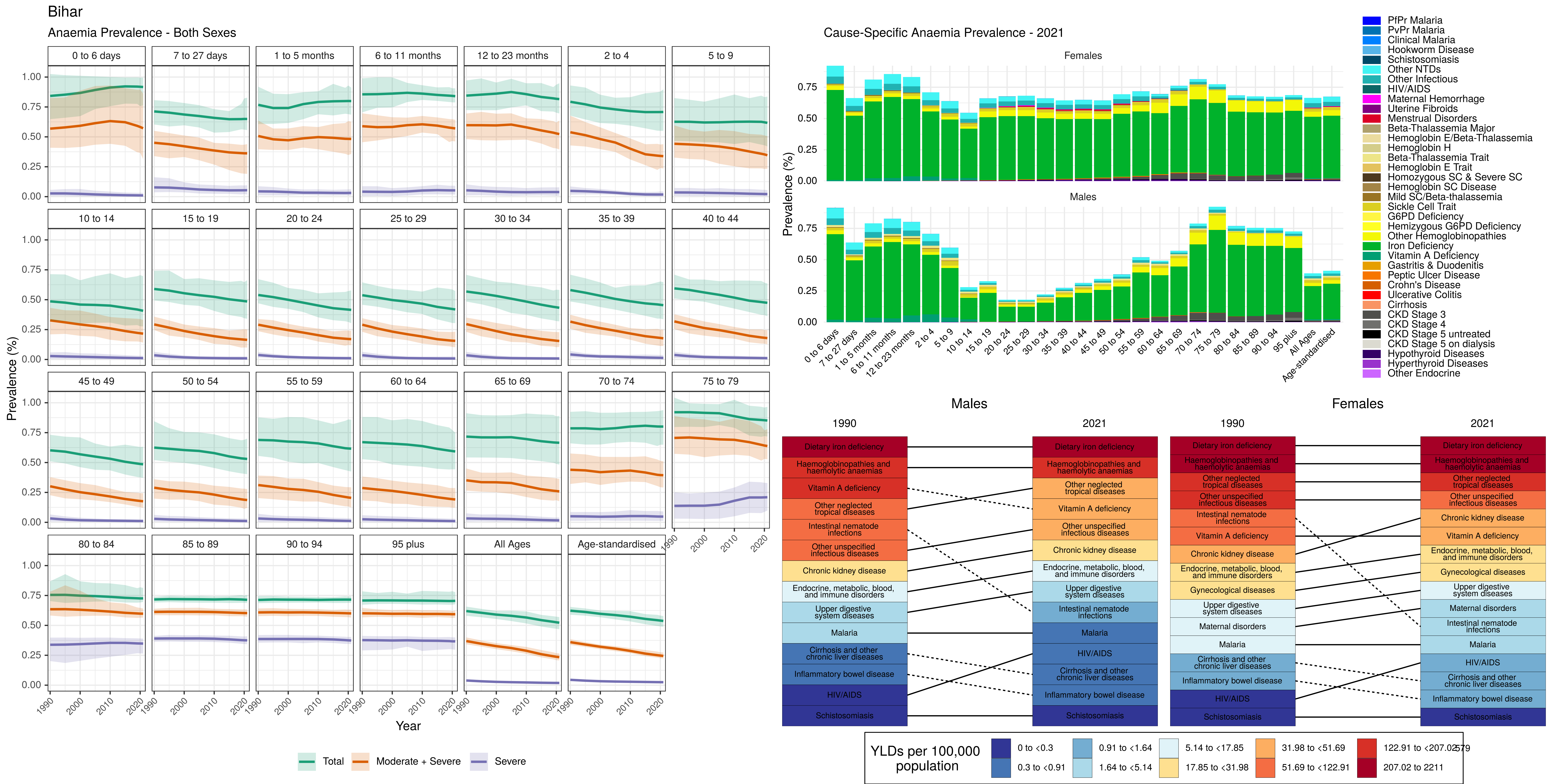

Bihar
Anaemia Prevalence - Both Sexes
0 to 6 days
7 to 27 days
1 to 5 months
6 to 11 months
12 to 23 months
2 to 4
5 to 9
10 to 14
15 to 19
20 to 24
25 to 29
30 to 34
35 to 39
40 to 44
45 to 49
50 to 54
55 to 59
60 to 64
65 to 69
70 to 74
75 to 79
80 to 84
85 to 89
90 to 94
95 plus
All Ages
Age-standardised
Prevalence (%)
Year
Total
Moderate + Severe
Severe
Cause-Specific Anaemia Prevalence - 2021
Females
Males
PfPr Malaria
PvPr Malaria
Clinical Malaria
Hookworm Disease
Schistosomiasis
Other NTDs
Other Infectious
HIV/AIDS
Maternal Hemorrhage
Uterine Fibroids
Menstrual Disorders
Beta-Thalassemia Major
Hemoglobin E/Beta-Thalassemia
Hemoglobin H
Beta-Thalassemia Trait
Hemoglobin E Trait
Homozygous SC & Severe SC
Hemoglobin SC Disease
Mild SC/Beta-thalassemia
Sickle Cell Trait
G6PD Deficiency
Hemizygous G6PD Deficiency
Other Hemoglobinopathies
Iron Deficiency
Vitamin A Deficiency
Gastritis & Duodenitis
Peptic Ulcer Disease
Crohn's Disease
Ulcerative Colitis
Cirrhosis
CKD Stage 3
CKD Stage 4
CKD Stage 5 untreated
CKD Stage 5 on dialysis
Hypothyroid Diseases
Hyperthyroid Diseases
Other Endocrine
Males
Females
1990
2021
Dietary iron deficiency
Haemoglobinopathies and haemolytic anaemias
Vitamin A deficiency
Other neglected tropical diseases
Intestinal nematode infections
Other unspecified infectious diseases
Chronic kidney disease
Endocrine, metabolic, blood, and immune disorders
Upper digestive system diseases
Malaria
Cirrhosis and other chronic liver diseases
Inflammatory bowel disease
HIV/AIDS
Schistosomiasis
Gynecological diseases
Maternal disorders
YLDs per 100,000 population
0 to <0.3
0.3 to <0.91
0.91 to <1.64
1.64 to <5.14
5.14 to <17.85
17.85 to <31.98
31.98 to <51.69
51.69 to <122.91
122.91 to <207.02
207.02 to 2211

Chhattisgarh

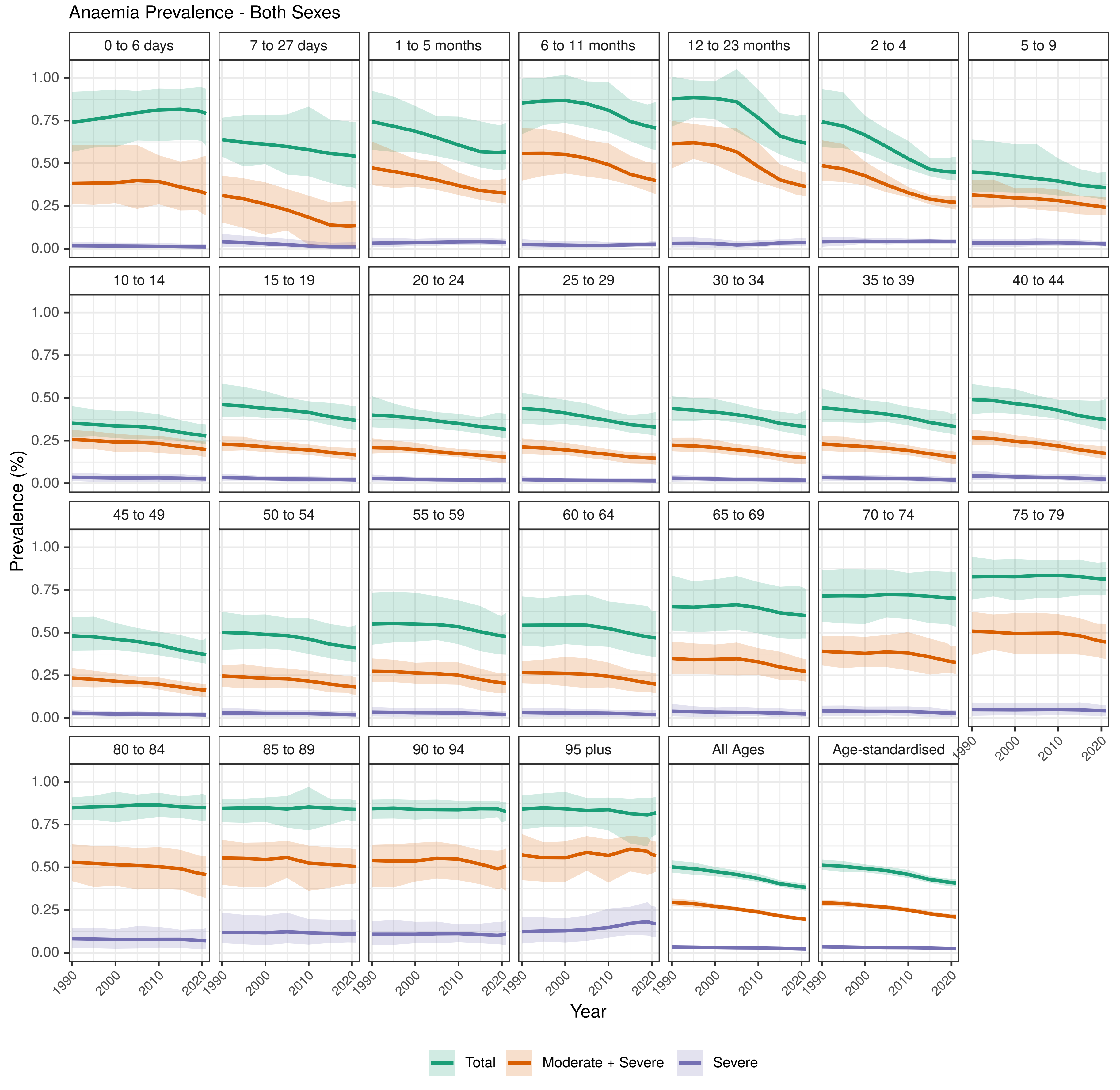

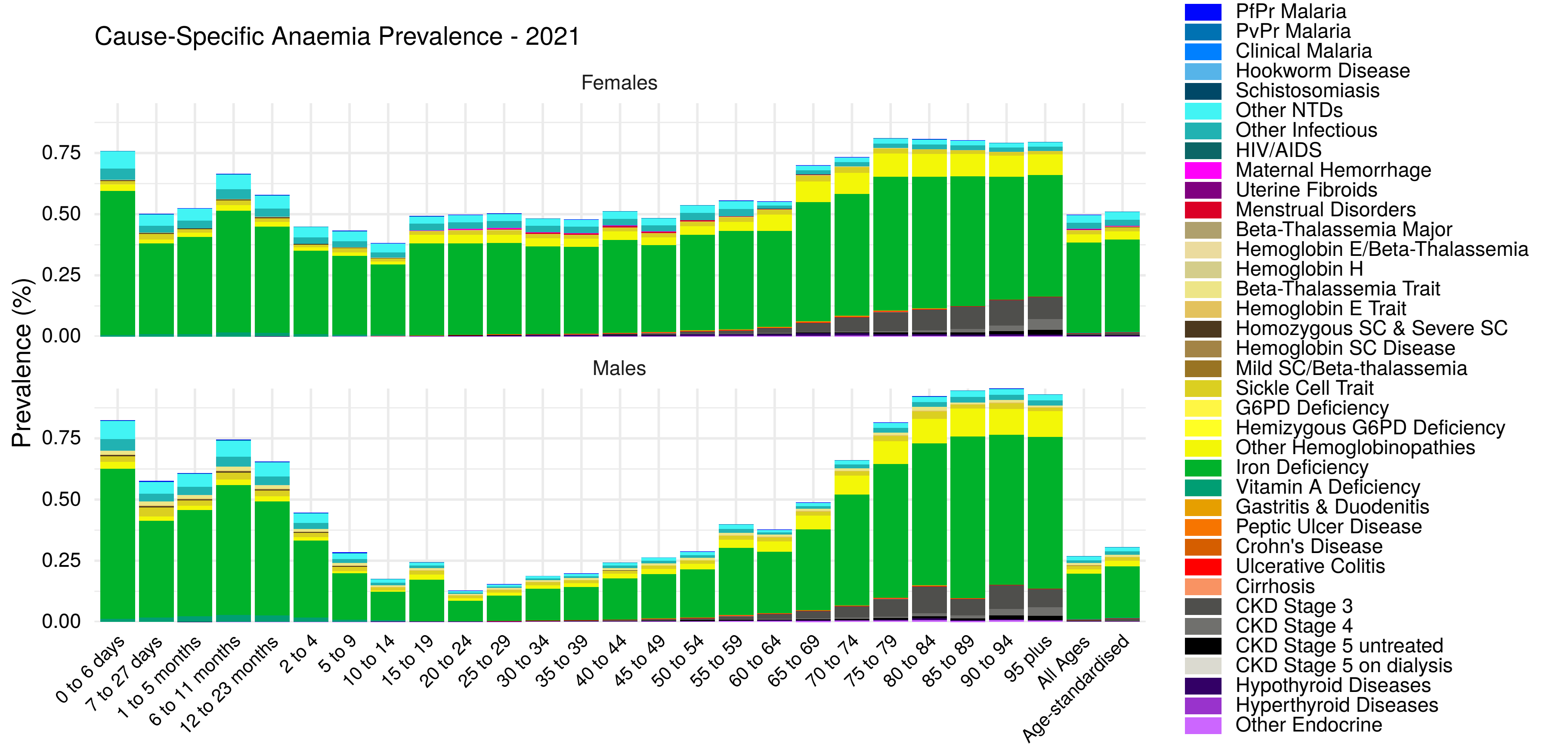

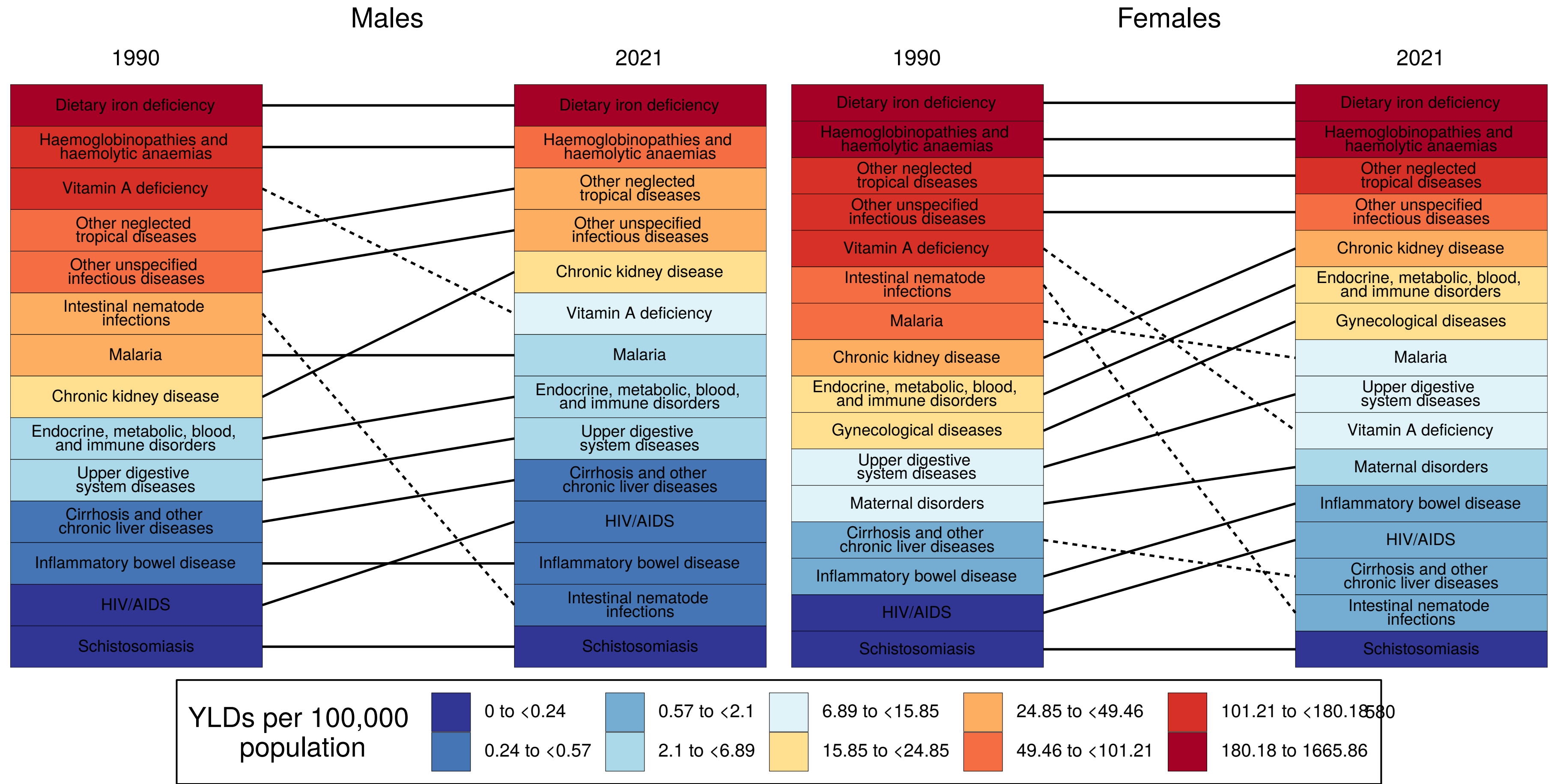

Delhi

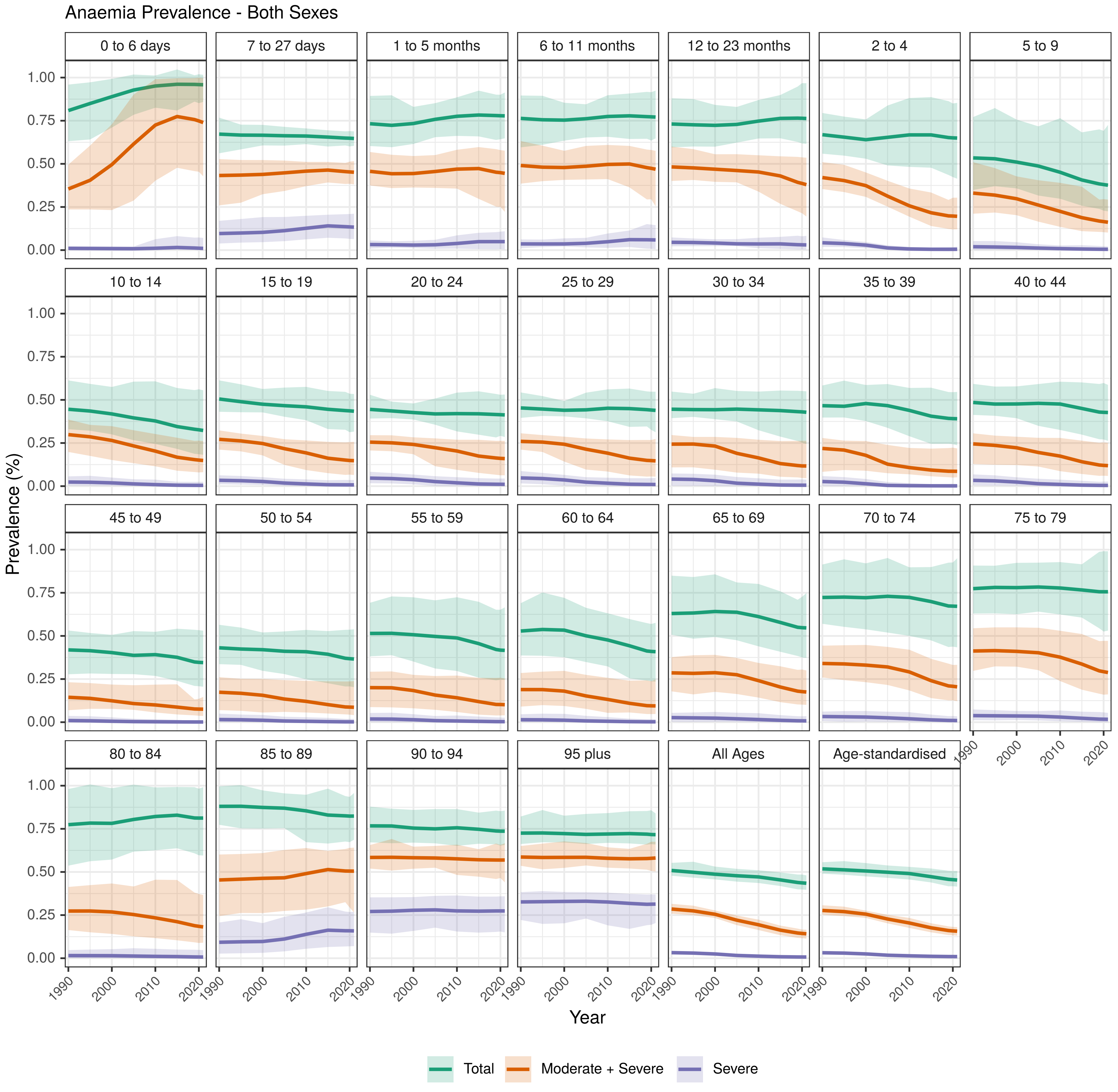
Anaemia Prevalence - Both Sexes
0 to 6 days
7 to 27 days
1 to 5 months
6 to 11 months
12 to 23 months
2 to 4
5 to 9
10 to 14
15 to 19
20 to 24
25 to 29
30 to 34
35 to 39
40 to 44
45 to 49
50 to 54
55 to 59
60 to 64
65 to 69
70 to 74
75 to 79
80 to 84
85 to 89
90 to 94
95 plus
All Ages
Age-standardised
Prevalence (%)
Year
Total
Moderate + Severe
Severe

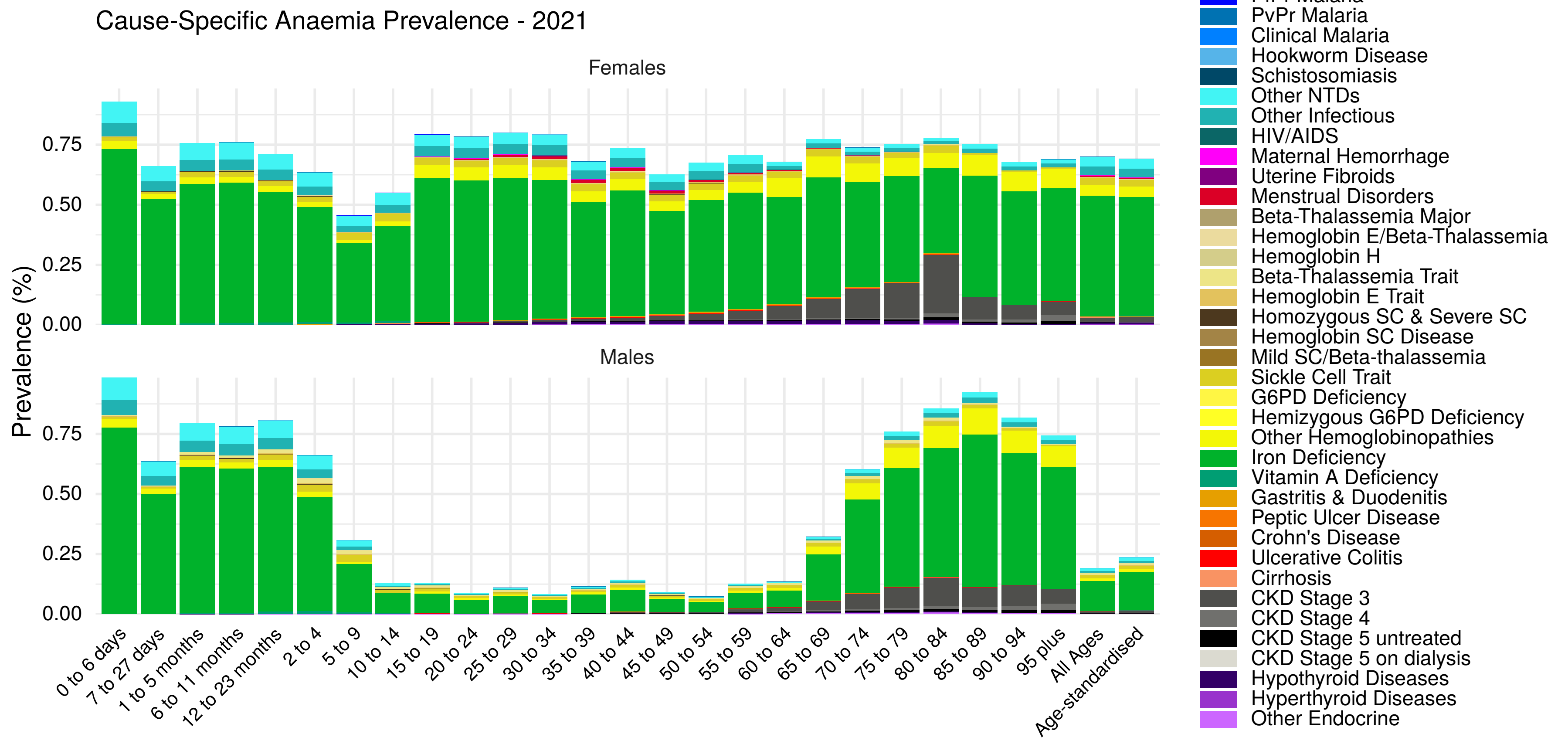
Cause-Specific Anaemia Prevalence - 2021
Females
Males
Prevalence (%)
PfPr Malaria
PvPr Malaria
Clinical Malaria
Hookworm Disease
Schistosomiasis
Other NTDs
Other Infectious
HIV/AIDS
Maternal Hemorrhage
Uterine Fibroids
Menstrual Disorders
Beta-Thalassemia Major
Hemoglobin E/Beta-Thalassemia
Hemoglobin H
Beta-Thalassemia Trait
Hemoglobin E Trait
Homozygous SC & Severe SC
Hemoglobin SC Disease
Mild SC/Beta-thalassemia
Sickle Cell Trait
G6PD Deficiency
Hemizygous G6PD Deficiency
Other Hemoglobinopathies
Iron Deficiency
Vitamin A Deficiency
Gastritis & Duodenitis
Peptic Ulcer Disease
Crohn's Disease
Ulcerative Colitis
Cirrhosis
CKD Stage 3
CKD Stage 4
CKD Stage 5 untreated
CKD Stage 5 on dialysis
Hypothyroid Diseases
Hyperthyroid Diseases
Other Endocrine

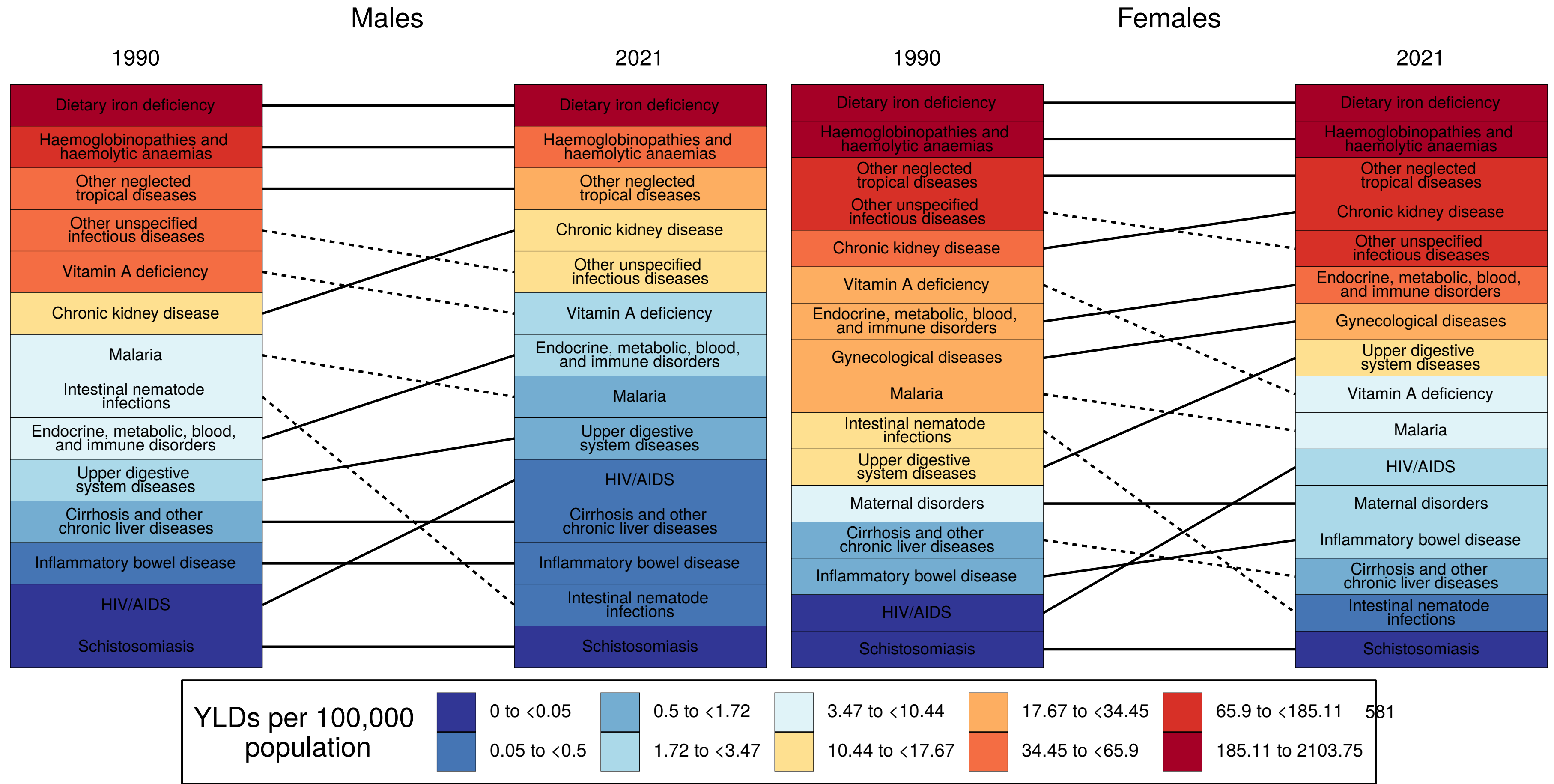
Males
Females
1990
2021
Dietary iron deficiency
Haemoglobinopathies and haemolytic anaemias
Other neglected tropical diseases
Other unspecified infectious diseases
Vitamin A deficiency
Chronic kidney disease
Malaria
Intestinal nematode infections
Endocrine, metabolic, blood, and immune disorders
Upper digestive system diseases
Cirrhosis and other chronic liver diseases
Inflammatory bowel disease
HIV/AIDS
Schistosomiasis
Gynecological diseases
Maternal disorders
YLDs per 100,000 population
0 to <0.05
0.05 to <0.5
0.5 to <1.72
1.72 to <3.47
3.47 to <10.44
10.44 to <17.67
17.67 to <34.45
34.45 to <65.9
65.9 to <185.11
185.11 to 2103.75

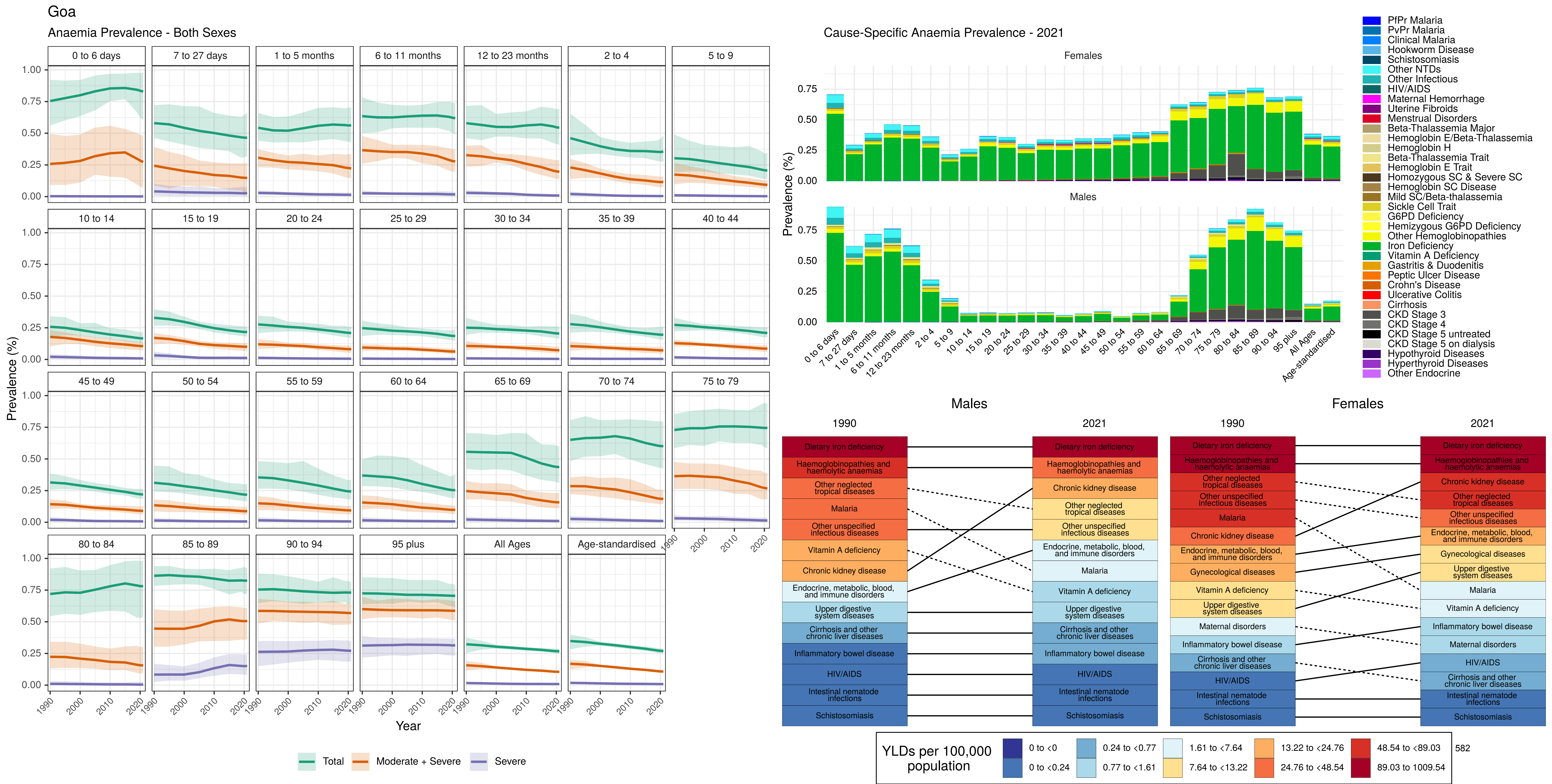

Goa
Anaemia Prevalence - Both Sexes
0 to 6 days
7 to 27 days
1 to 5 months
6 to 11 months
12 to 23 months
2 to 4
5 to 9
10 to 14
15 to 19
20 to 24
25 to 29
30 to 34
35 to 39
40 to 44
45 to 49
50 to 54
55 to 59
60 to 64
65 to 69
70 to 74
75 to 79
80 to 84
85 to 89
90 to 94
95 plus
All Ages
Age-standardised
Prevalence (%)
Year
Total
Moderate + Severe
Severe
Cause-Specific Anaemia Prevalence - 2021
Females
Males
PfPr Malaria
PvPr Malaria
Clinical Malaria
Hookworm Disease
Schistosomiasis
Other NTDs
Other Infectious
HIV/AIDS
Maternal Hemorrhage
Uterine Fibroids
Menstrual Disorders
Beta-Thalassemia Major
Hemoglobin E/Beta-Thalassemia
Hemoglobin H
Beta-Thalassemia Trait
Hemoglobin E Trait
Homozygous SC & Severe SC
Hemoglobin SC Disease
Mild SC/Beta-thalassemia
Sickle Cell Trait
G6PD Deficiency
Hemizygous G6PD Deficiency
Other Hemoglobinopathies
Iron Deficiency
Vitamin A Deficiency
Gastritis & Duodenitis
Peptic Ulcer Disease
Crohn's Disease
Ulcerative Colitis
Cirrhosis
CKD Stage 3
CKD Stage 4
CKD Stage 5 untreated
CKD Stage 5 on dialysis
Hypothyroid Diseases
Hyperthyroid Diseases
Other Endocrine
Males
Females
1990
2021
Dietary iron deficiency
Haemoglobinopathies and haemolytic anaemias
Other neglected tropical diseases
Malaria
Other unspecified infectious diseases
Vitamin A deficiency
Chronic kidney disease
Endocrine, metabolic, blood, and immune disorders
Upper digestive system diseases
Cirrhosis and other chronic liver diseases
Inflammatory bowel disease
HIV/AIDS
Intestinal nematode infections
Schistosomiasis
Gynecological diseases
Maternal disorders
YLDs per 100,000 population
0 to <0
0 to <0.24
0.24 to <0.77
0.77 to <1.61
1.61 to <7.64
7.64 to <13.22
13.22 to <24.76
24.76 to <48.54
48.54 to <89.03
89.03 to 1009.54

Gujarat

Anaemia Prevalence - Both Sexes

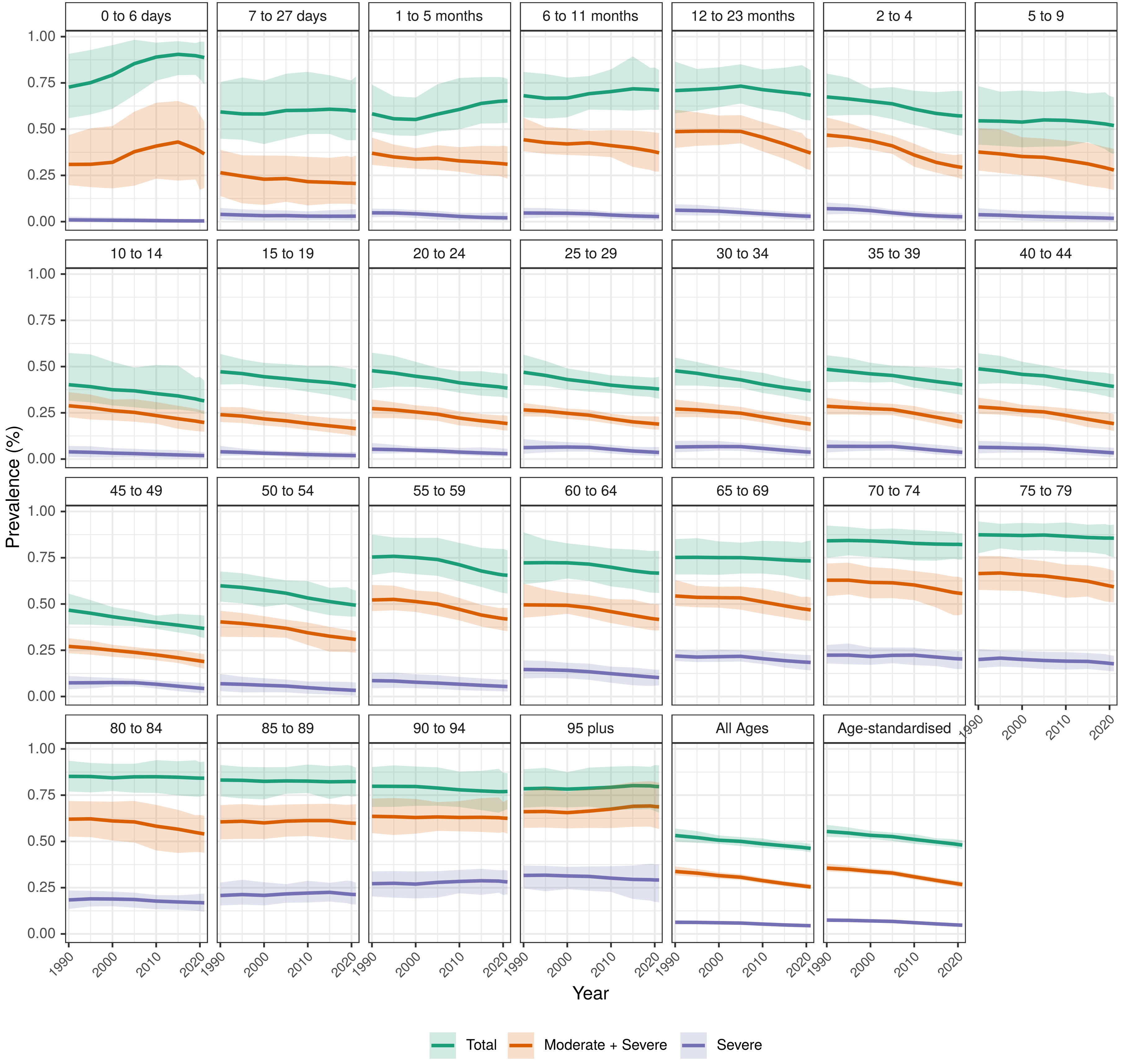

Cause-Specific Anaemia Prevalence - 2021

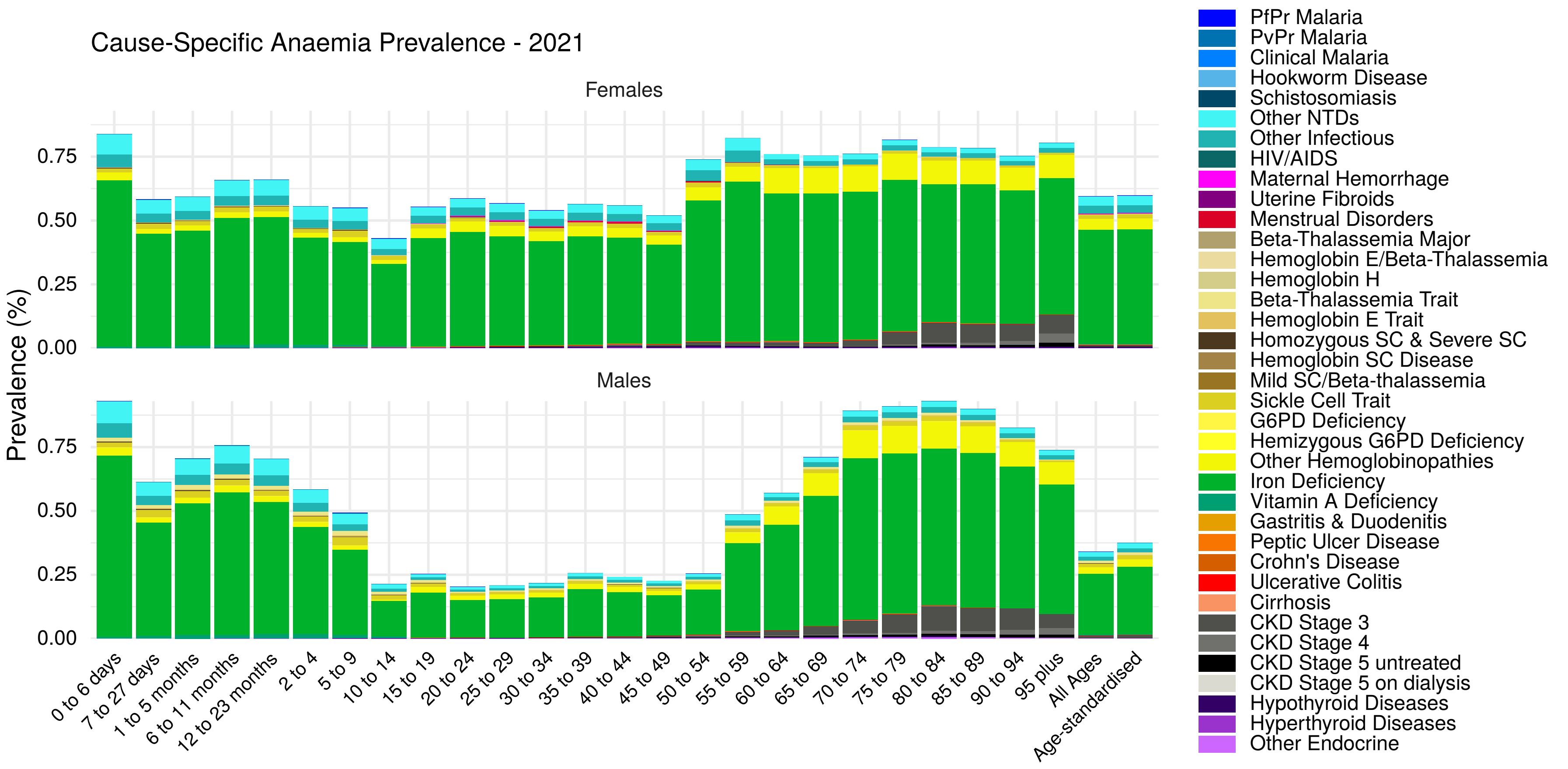

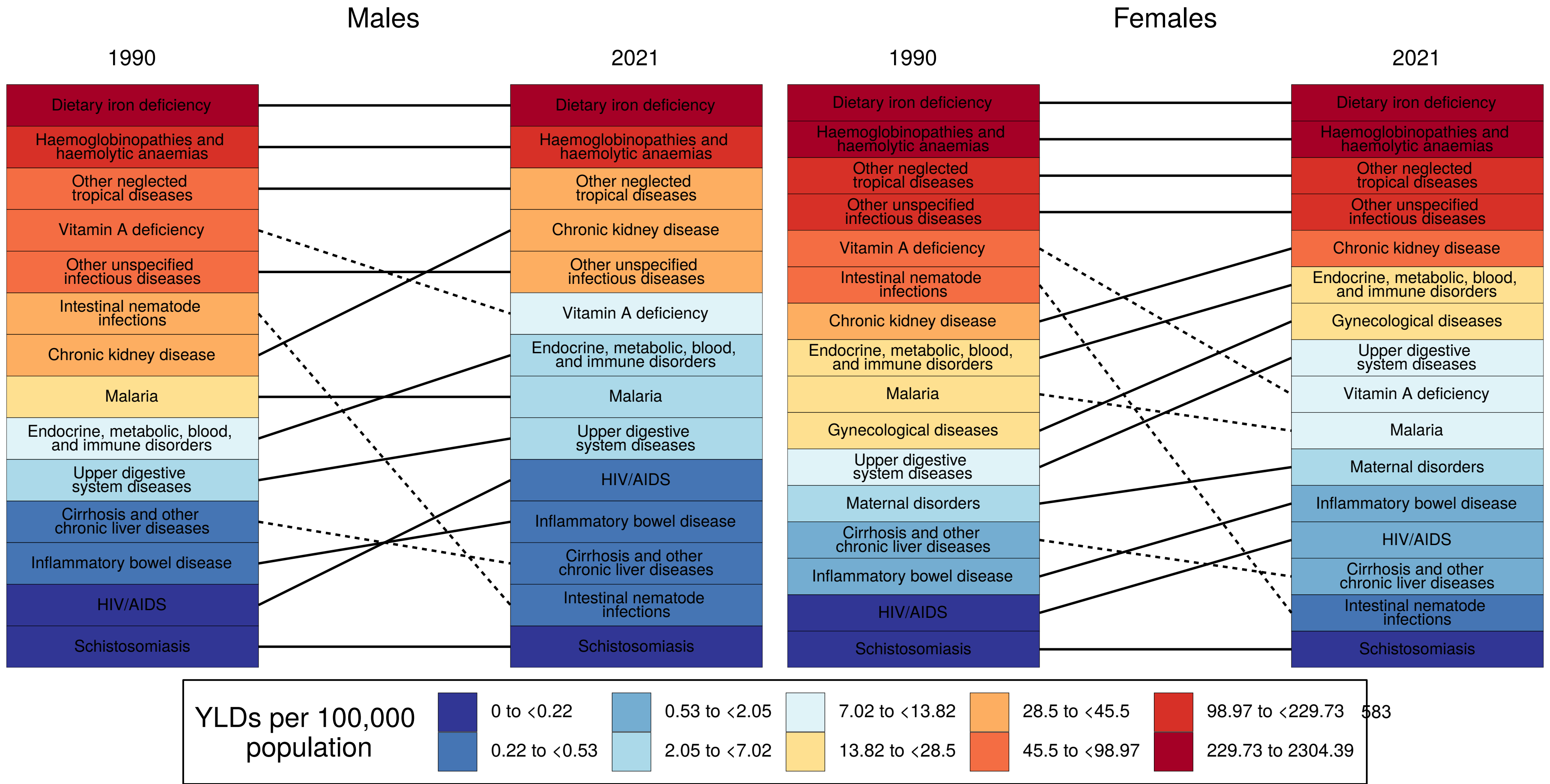

Haryana

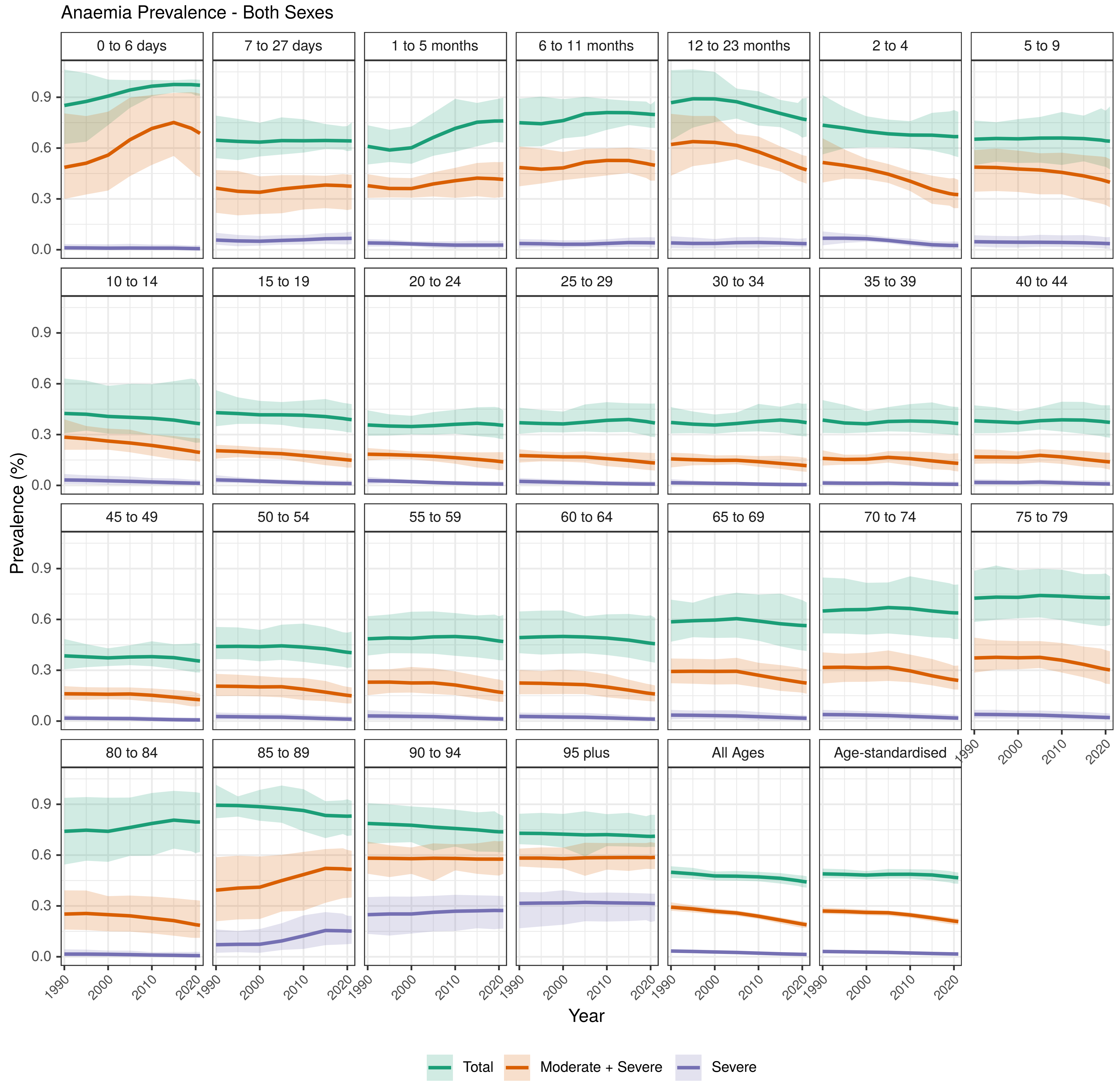

Anaemia Prevalence - Both Sexes
0 to 6 days
7 to 27 days
1 to 5 months
6 to 11 months
12 to 23 months
2 to 4
5 to 9
10 to 14
15 to 19
20 to 24
25 to 29
30 to 34
35 to 39
40 to 44
45 to 49
50 to 54
55 to 59
60 to 64
65 to 69
70 to 74
75 to 79
80 to 84
85 to 89
90 to 94
95 plus
All Ages
Age-standardised
Prevalence (%)
Year
Total
Moderate + Severe
Severe

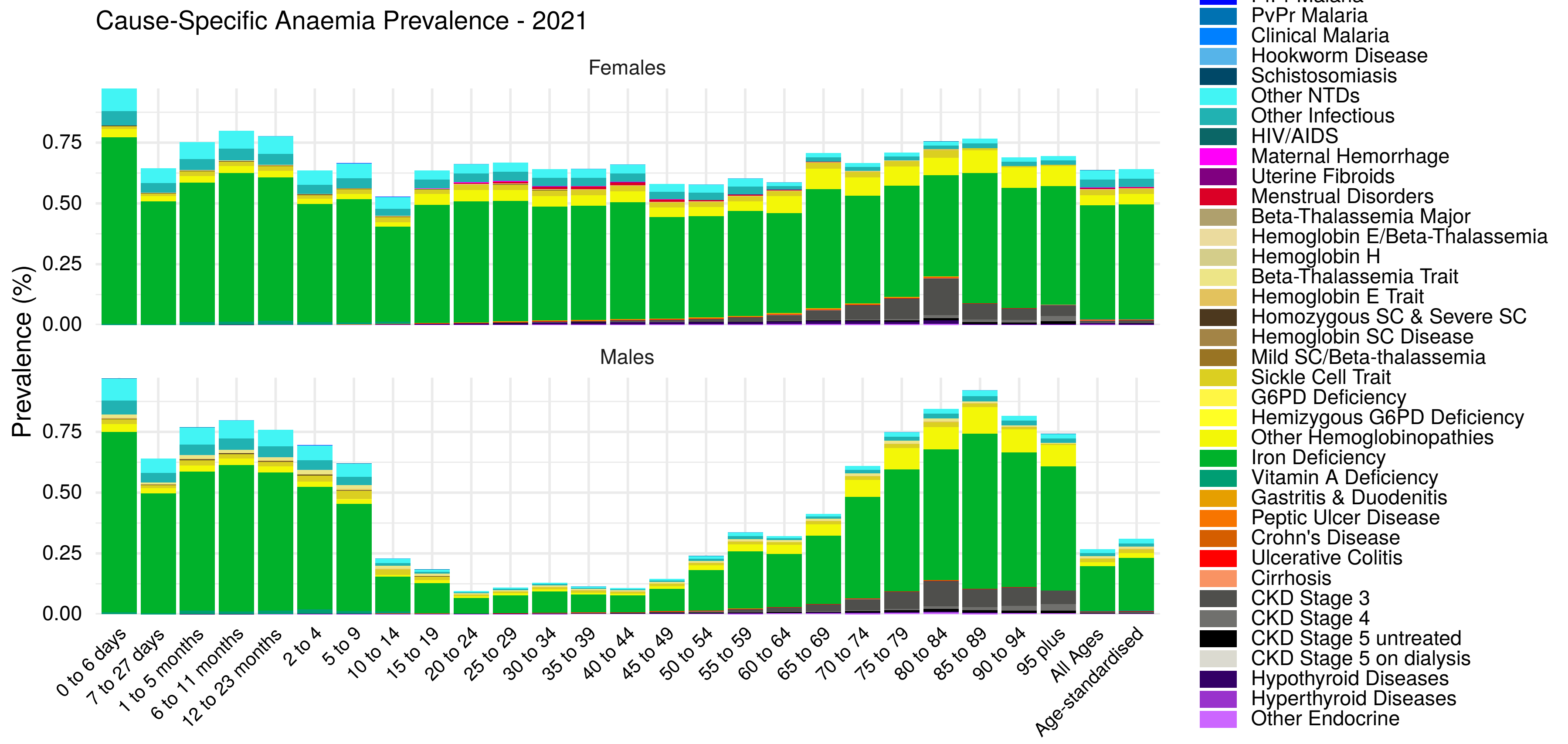

Cause-Specific Anaemia Prevalence - 2021
Females
Males
Prevalence (%)
PfPr Malaria
PvPr Malaria
Clinical Malaria
Hookworm Disease
Schistosomiasis
Other NTDs
Other Infectious
HIV/AIDS
Maternal Hemorrhage
Uterine Fibroids
Menstrual Disorders
Beta-Thalassemia Major
Hemoglobin E/Beta-Thalassemia
Hemoglobin H
Beta-Thalassemia Trait
Hemoglobin E Trait
Homozygous SC & Severe SC
Hemoglobin SC Disease
Mild SC/Beta-thalassemia
Sickle Cell Trait
G6PD Deficiency
Hemizygous G6PD Deficiency
Other Hemoglobinopathies
Iron Deficiency
Vitamin A Deficiency
Gastritis & Duodenitis
Peptic Ulcer Disease
Crohn's Disease
Ulcerative Colitis
Cirrhosis
CKD Stage 3
CKD Stage 4
CKD Stage 5 untreated
CKD Stage 5 on dialysis
Hypothyroid Diseases
Hyperthyroid Diseases
Other Endocrine

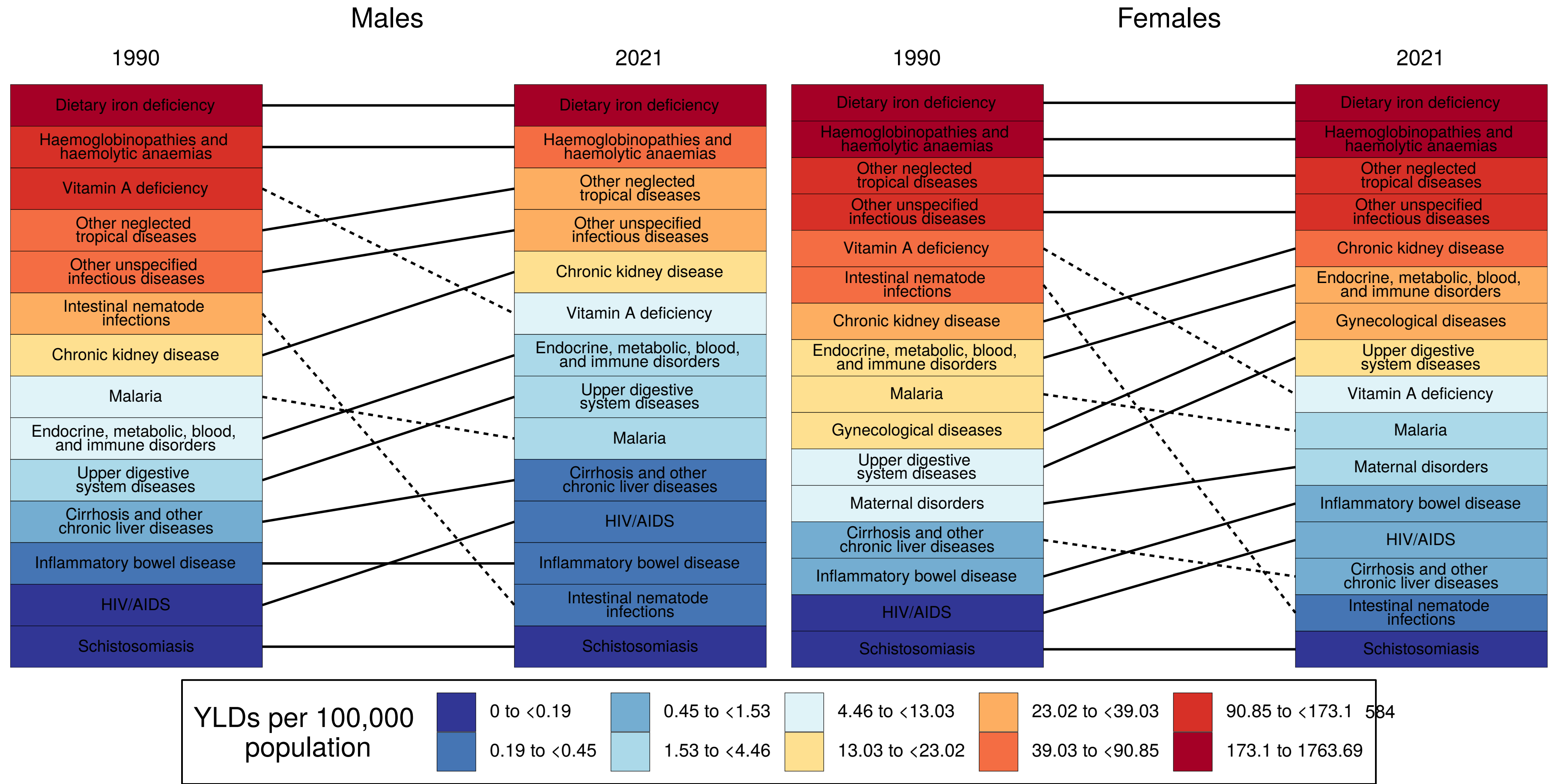

Males
Females
1990
2021
Dietary iron deficiency
Haemoglobinopathies and haemolytic anaemias
Vitamin A deficiency
Other neglected tropical diseases
Other unspecified infectious diseases
Intestinal nematode infections
Chronic kidney disease
Malaria
Endocrine, metabolic, blood, and immune disorders
Upper digestive system diseases
Cirrhosis and other chronic liver diseases
Inflammatory bowel disease
HIV/AIDS
Schistosomiasis
Gynecological diseases
Maternal disorders
YLDs per 100,000 population
0 to <0.19
0.19 to <0.45
0.45 to <1.53
1.53 to <4.46
4.46 to <13.03
13.03 to <23.02
23.02 to <39.03
39.03 to <90.85
90.85 to <173.1
173.1 to 1763.69

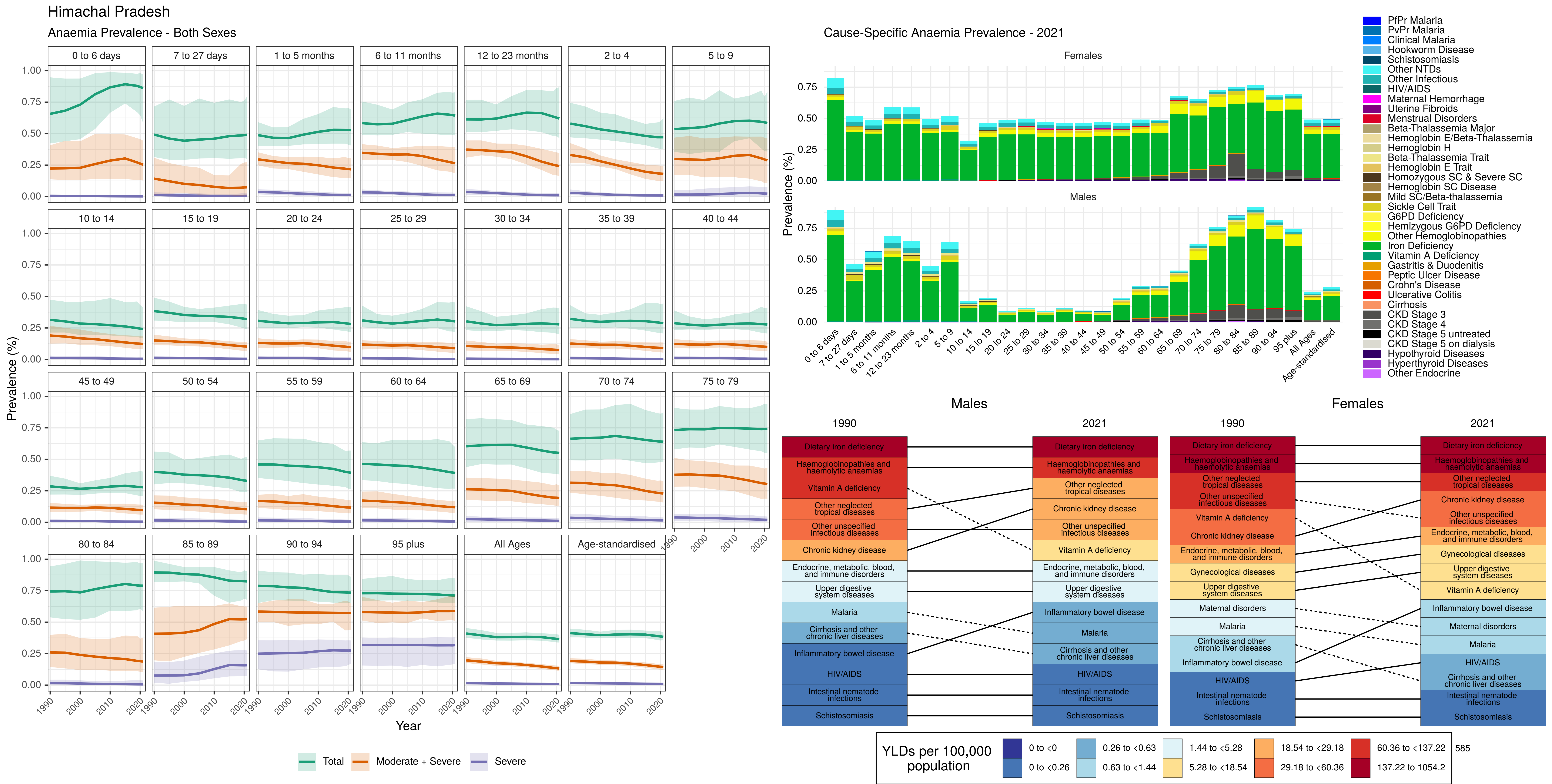

Himachal Pradesh
Anaemia Prevalence - Both Sexes
0 to 6 days
7 to 27 days
1 to 5 months
6 to 11 months
12 to 23 months
2 to 4
5 to 9
10 to 14
15 to 19
20 to 24
25 to 29
30 to 34
35 to 39
40 to 44
45 to 49
50 to 54
55 to 59
60 to 64
65 to 69
70 to 74
75 to 79
80 to 84
85 to 89
90 to 94
95 plus
All Ages
Age-standardised
Prevalence (%)
Year
Total
Moderate + Severe
Severe
Cause-Specific Anaemia Prevalence - 2021
Females
Males
PfPr Malaria
PvPr Malaria
Clinical Malaria
Hookworm Disease
Schistosomiasis
Other NTDs
Other Infectious
HIV/AIDS
Maternal Hemorrhage
Uterine Fibroids
Menstrual Disorders
Beta-Thalassemia Major
Hemoglobin E/Beta-Thalassemia
Hemoglobin H
Beta-Thalassemia Trait
Hemoglobin E Trait
Homozygous SC & Severe SC
Hemoglobin SC Disease
Mild SC/Beta-thalassemia
Sickle Cell Trait
G6PD Deficiency
Hemizygous G6PD Deficiency
Other Hemoglobinopathies
Iron Deficiency
Vitamin A Deficiency
Gastritis & Duodenitis
Peptic Ulcer Disease
Crohn's Disease
Ulcerative Colitis
Cirrhosis
CKD Stage 3
CKD Stage 4
CKD Stage 5 untreated
CKD Stage 5 on dialysis
Hypothyroid Diseases
Hyperthyroid Diseases
Other Endocrine
Males
1990
Dietary iron deficiency
Haemoglobinopathies and haemolytic anaemias
Vitamin A deficiency
Other neglected tropical diseases
Other unspecified infectious diseases
Chronic kidney disease
Endocrine, metabolic, blood, and immune disorders
Upper digestive system diseases
Malaria
Cirrhosis and other chronic liver diseases
Inflammatory bowel disease
HIV/AIDS
Intestinal nematode infections
Schistosomiasis
2021
Dietary iron deficiency
Haemoglobinopathies and haemolytic anaemias
Other neglected tropical diseases
Chronic kidney disease
Other unspecified infectious diseases
Vitamin A deficiency
Endocrine, metabolic, blood, and immune disorders
Upper digestive system diseases
Inflammatory bowel disease
Malaria
Cirrhosis and other chronic liver diseases
HIV/AIDS
Intestinal nematode infections
Schistosomiasis
Females
1990
Dietary iron deficiency
Haemoglobinopathies and haemolytic anaemias
Other neglected tropical diseases
Other unspecified infectious diseases
Vitamin A deficiency
Chronic kidney disease
Endocrine, metabolic, blood, and immune disorders
Gynecological diseases
Upper digestive system diseases
Maternal disorders
Malaria
Cirrhosis and other chronic liver diseases
Inflammatory bowel disease
HIV/AIDS
Intestinal nematode infections
Schistosomiasis
2021
Dietary iron deficiency
Haemoglobinopathies and haemolytic anaemias
Other neglected tropical diseases
Chronic kidney disease
Other unspecified infectious diseases
Endocrine, metabolic, blood, and immune disorders
Gynecological diseases
Upper digestive system diseases
Vitamin A deficiency
Inflammatory bowel disease
Maternal disorders
Malaria
HIV/AIDS
Cirrhosis and other chronic liver diseases
Intestinal nematode infections
Schistosomiasis
YLDs per 100,000 population
0 to <0
0 to <0.26
0.26 to <0.63
0.63 to <1.44
1.44 to <5.28
5.28 to <18.54
18.54 to <29.18
29.18 to <60.36
60.36 to <137.22
137.22 to 1054.2

Jammu & Kashmir and Ladakh

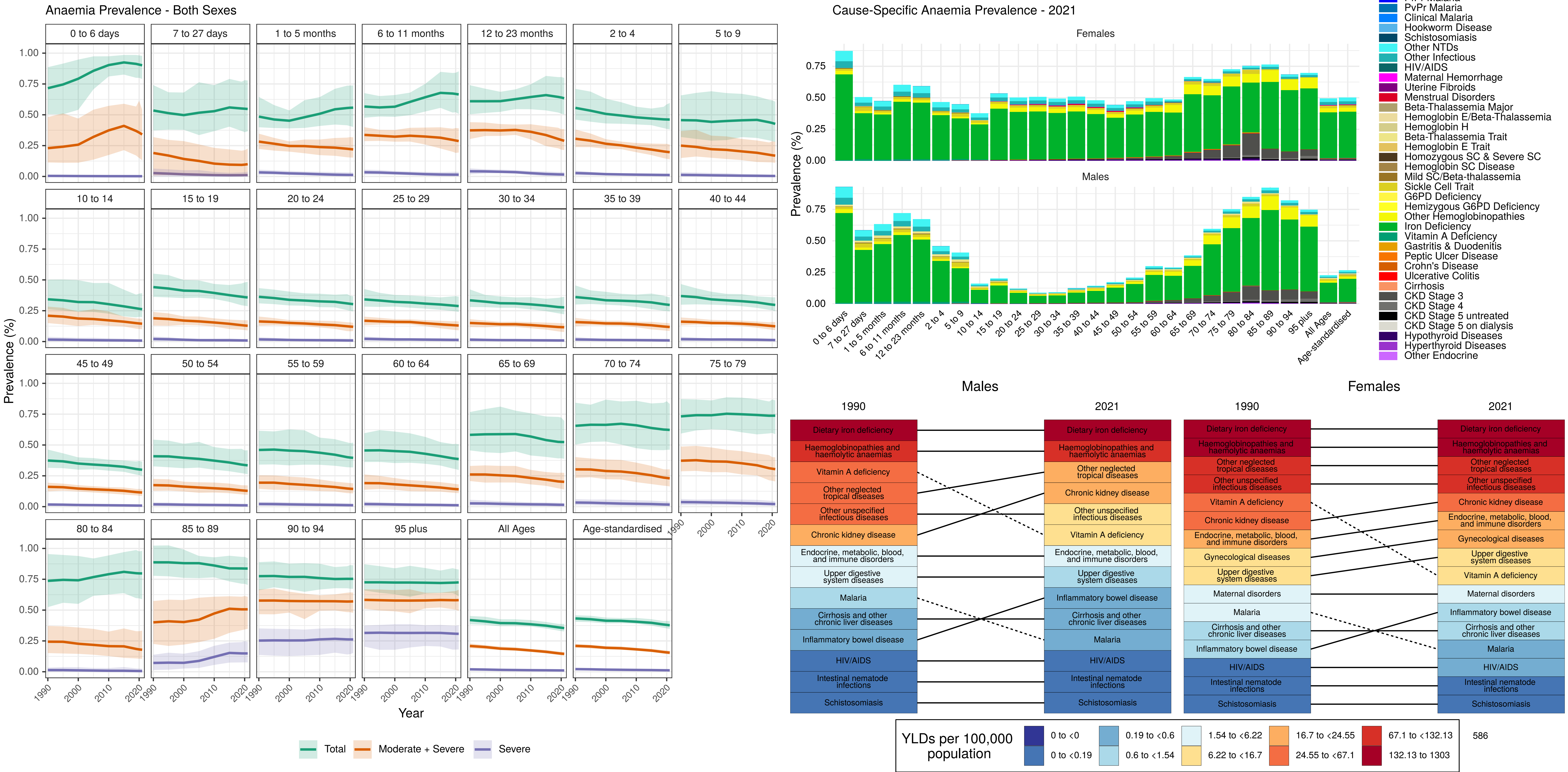

Jharkhand

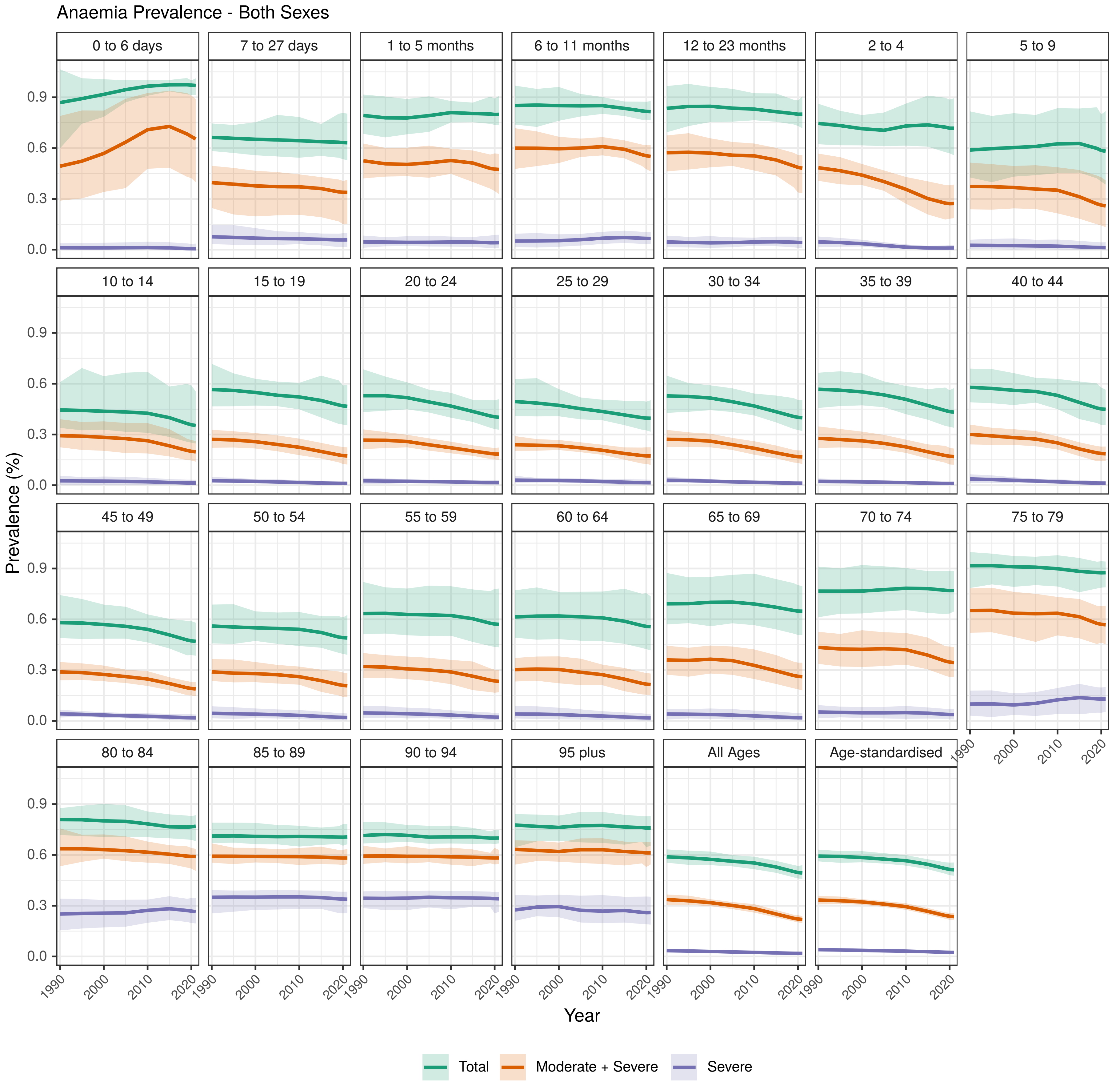

Anaemia Prevalence - Both Sexes
0 to 6 days
7 to 27 days
1 to 5 months
6 to 11 months
12 to 23 months
2 to 4
5 to 9
10 to 14
15 to 19
20 to 24
25 to 29
30 to 34
35 to 39
40 to 44
45 to 49
50 to 54
55 to 59
60 to 64
65 to 69
70 to 74
75 to 79
80 to 84
85 to 89
90 to 94
95 plus
All Ages
Age-standardised
Prevalence (%)
Year
Total
Moderate + Severe
Severe

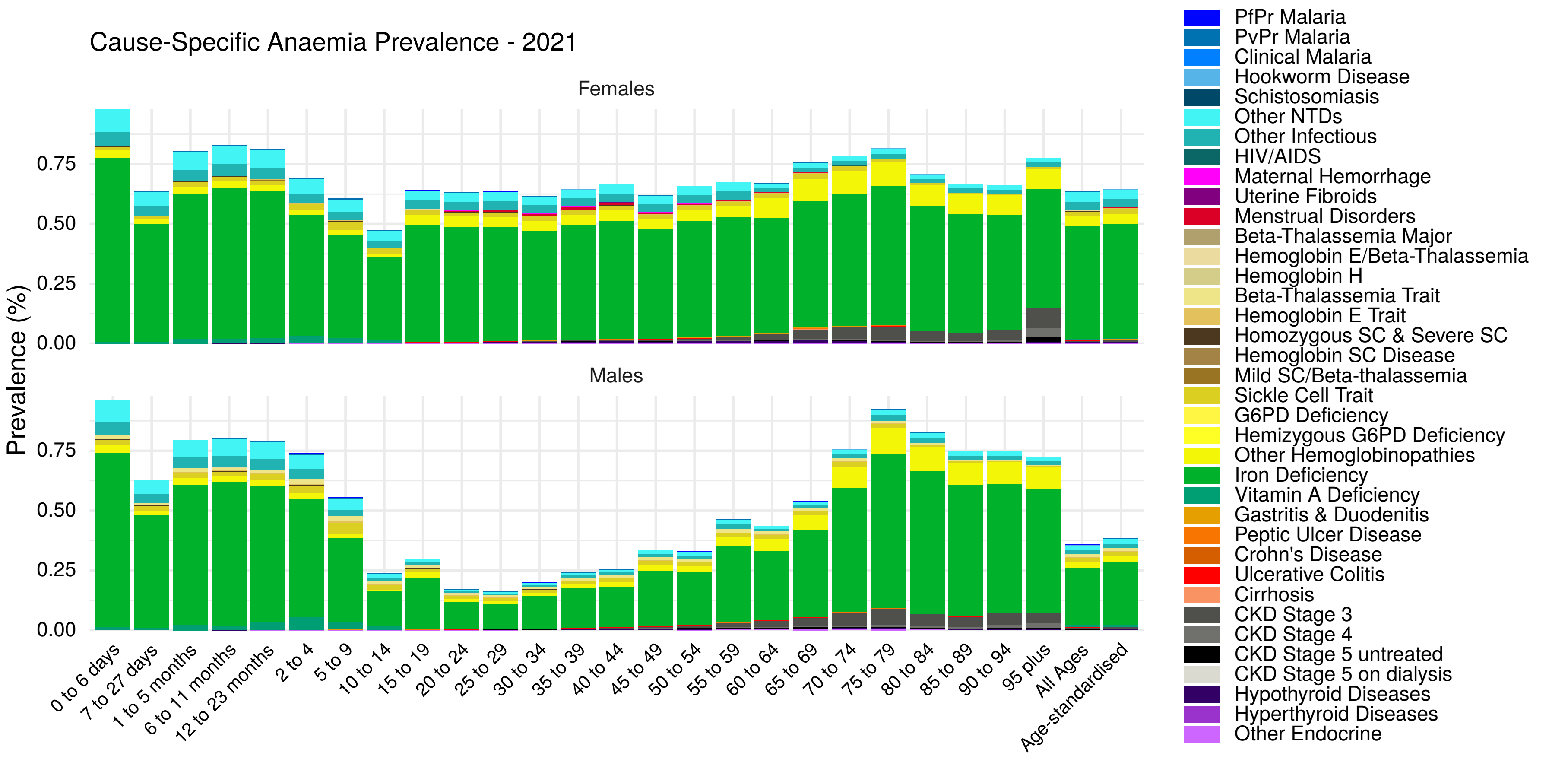

Cause-Specific Anaemia Prevalence - 2021
Females
Males
Prevalence (%)
PfPr Malaria
PvPr Malaria
Clinical Malaria
Hookworm Disease
Schistosomiasis
Other NTDs
Other Infectious
HIV/AIDS
Maternal Hemorrhage
Uterine Fibroids
Menstrual Disorders
Beta-Thalassemia Major
Hemoglobin E/Beta-Thalassemia
Hemoglobin H
Beta-Thalassemia Trait
Hemoglobin E Trait
Homozygous SC & Severe SC
Hemoglobin SC Disease
Mild SC/Beta-thalassemia
Sickle Cell Trait
G6PD Deficiency
Hemizygous G6PD Deficiency
Other Hemoglobinopathies
Iron Deficiency
Vitamin A Deficiency
Gastritis & Duodenitis
Peptic Ulcer Disease
Crohn's Disease
Ulcerative Colitis
Cirrhosis
CKD Stage 3
CKD Stage 4
CKD Stage 5 untreated
CKD Stage 5 on dialysis
Hypothyroid Diseases
Hyperthyroid Diseases
Other Endocrine

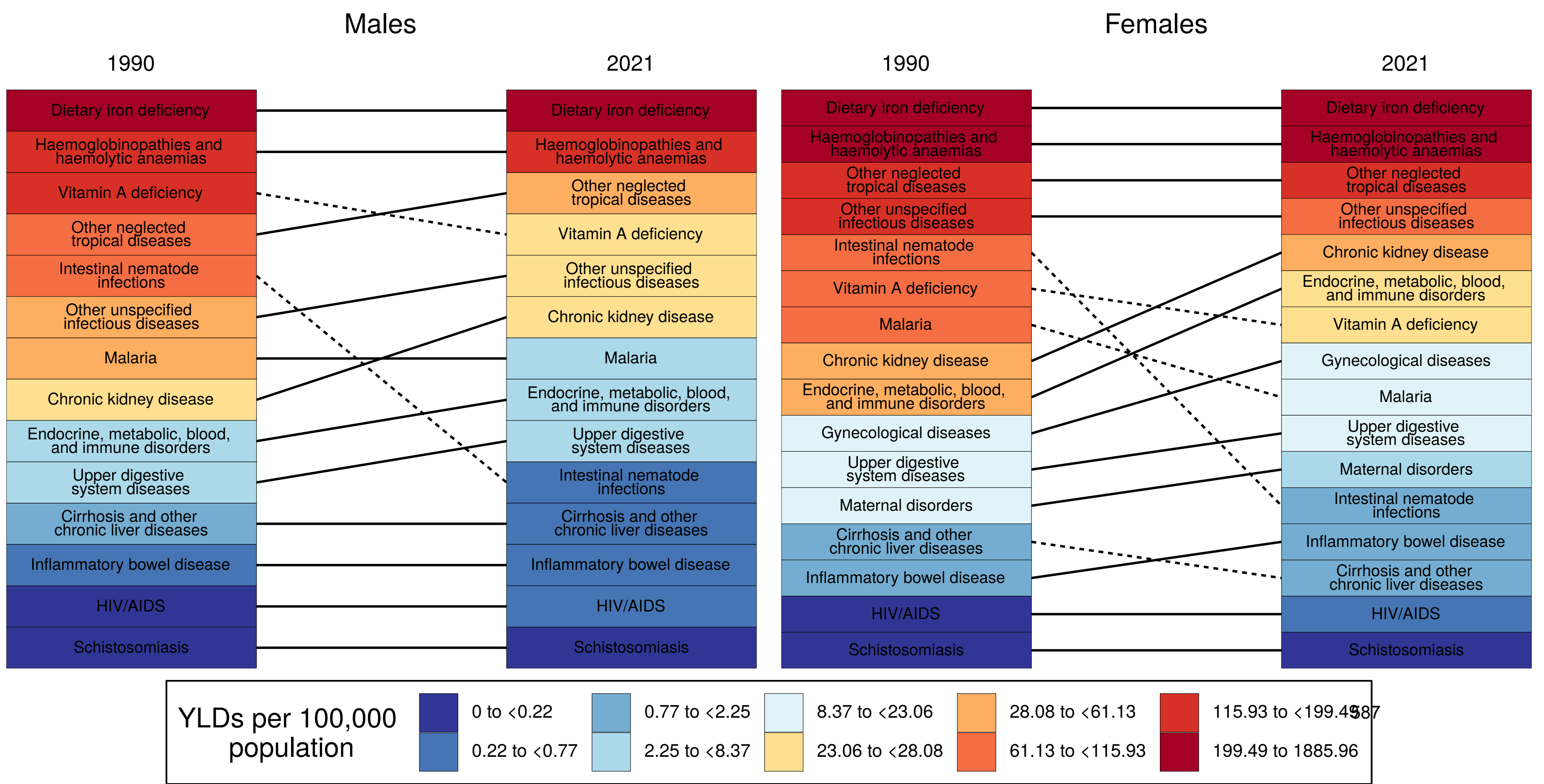

Males
Females
1990
2021
Dietary iron deficiency
Haemoglobinopathies and haemolytic anaemias
Vitamin A deficiency
Other neglected tropical diseases
Intestinal nematode infections
Other unspecified infectious diseases
Malaria
Chronic kidney disease
Endocrine, metabolic, blood, and immune disorders
Upper digestive system diseases
Cirrhosis and other chronic liver diseases
Inflammatory bowel disease
HIV/AIDS
Schistosomiasis
Gynecological diseases
Maternal disorders
YLDs per 100,000 population
0 to <0.22
0.22 to <0.77
0.77 to <2.25
2.25 to <8.37
8.37 to <23.06
23.06 to <28.08
28.08 to <61.13
61.13 to <115.93
115.93 to <199.4987
199.49 to 1885.96

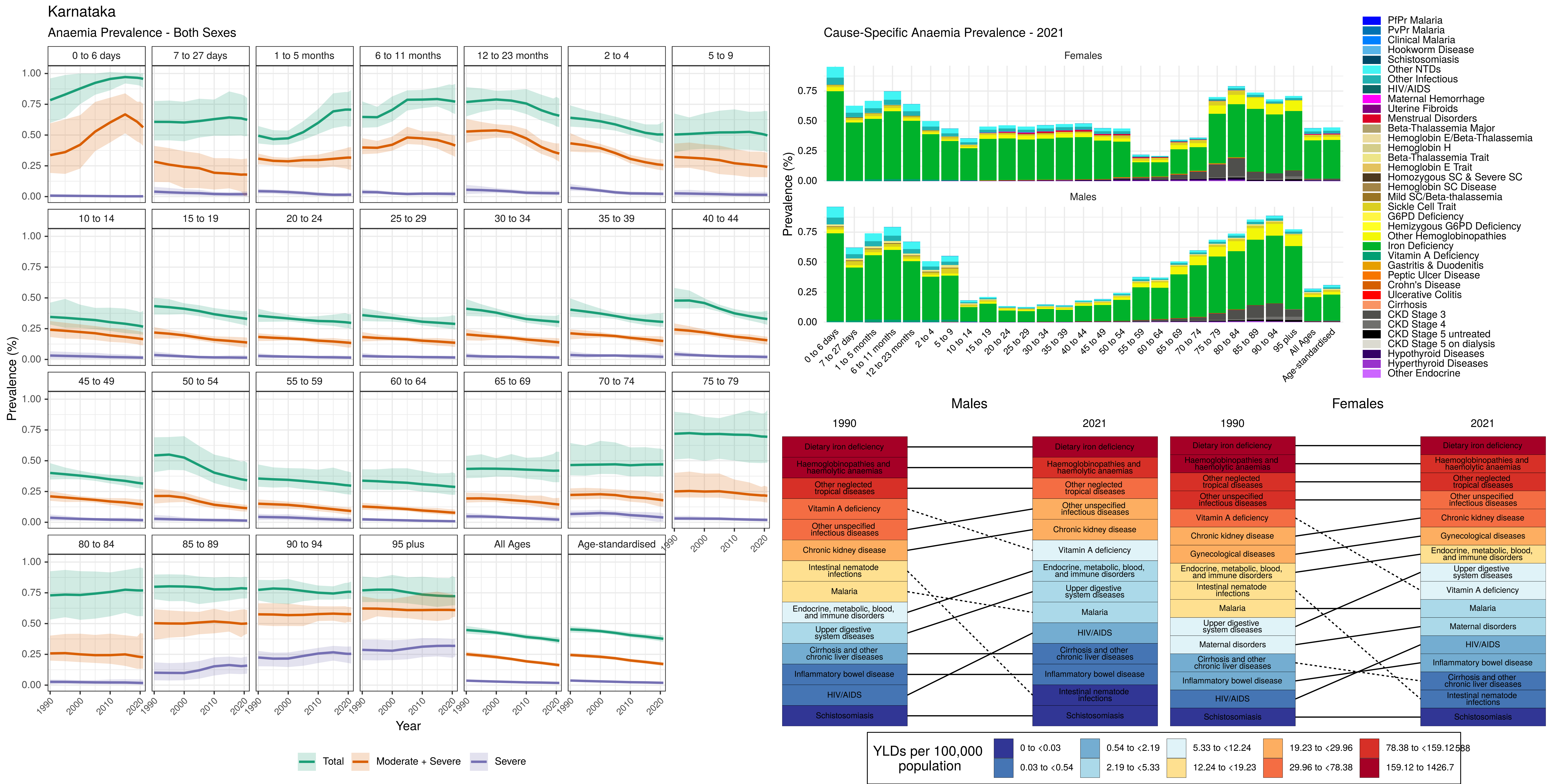
Karnataka
Anaemia Prevalence - Both Sexes
0 to 6 days
7 to 27 days
1 to 5 months
6 to 11 months
12 to 23 months
2 to 4
5 to 9
10 to 14
15 to 19
20 to 24
25 to 29
30 to 34
35 to 39
40 to 44
45 to 49
50 to 54
55 to 59
60 to 64
65 to 69
70 to 74
75 to 79
80 to 84
85 to 89
90 to 94
95 plus
All Ages
Age-standardised
Prevalence (%)
Year
Total
Moderate + Severe
Severe
Cause-Specific Anaemia Prevalence - 2021
Females
Males
PfPr Malaria
PvPr Malaria
Clinical Malaria
Hookworm Disease
Schistosomiasis
Other NTDs
Other Infectious
HIV/AIDS
Maternal Hemorrhage
Uterine Fibroids
Menstrual Disorders
Beta-Thalassemia Major
Hemoglobin E/Beta-Thalassemia
Hemoglobin H
Beta-Thalassemia Trait
Hemoglobin E Trait
Homozygous SC & Severe SC
Hemoglobin SC Disease
Mild SC/Beta-thalassemia
Sickle Cell Trait
G6PD Deficiency
Hemizygous G6PD Deficiency
Other Hemoglobinopathies
Iron Deficiency
Vitamin A Deficiency
Gastritis & Duodenitis
Peptic Ulcer Disease
Crohn's Disease
Ulcerative Colitis
Cirrhosis
CKD Stage 3
CKD Stage 4
CKD Stage 5 untreated
CKD Stage 5 on dialysis
Hypothyroid Diseases
Hyperthyroid Diseases
Other Endocrine
Males
Females
1990
2021
Dietary iron deficiency
Haemoglobinopathies and haemolytic anaemias
Other neglected tropical diseases
Vitamin A deficiency
Other unspecified infectious diseases
Chronic kidney disease
Intestinal nematode infections
Malaria
Endocrine, metabolic, blood, and immune disorders
Upper digestive system diseases
Cirrhosis and other chronic liver diseases
Inflammatory bowel disease
HIV/AIDS
Schistosomiasis
Gynecological diseases
Maternal disorders
YLDs per 100,000 population
0 to <0.03
0.03 to <0.54
0.54 to <2.19
2.19 to <5.33
5.33 to <12.24
12.24 to <19.23
19.23 to <29.96
29.96 to <78.38
78.38 to <159.12588
159.12 to 1426.7

Kerala

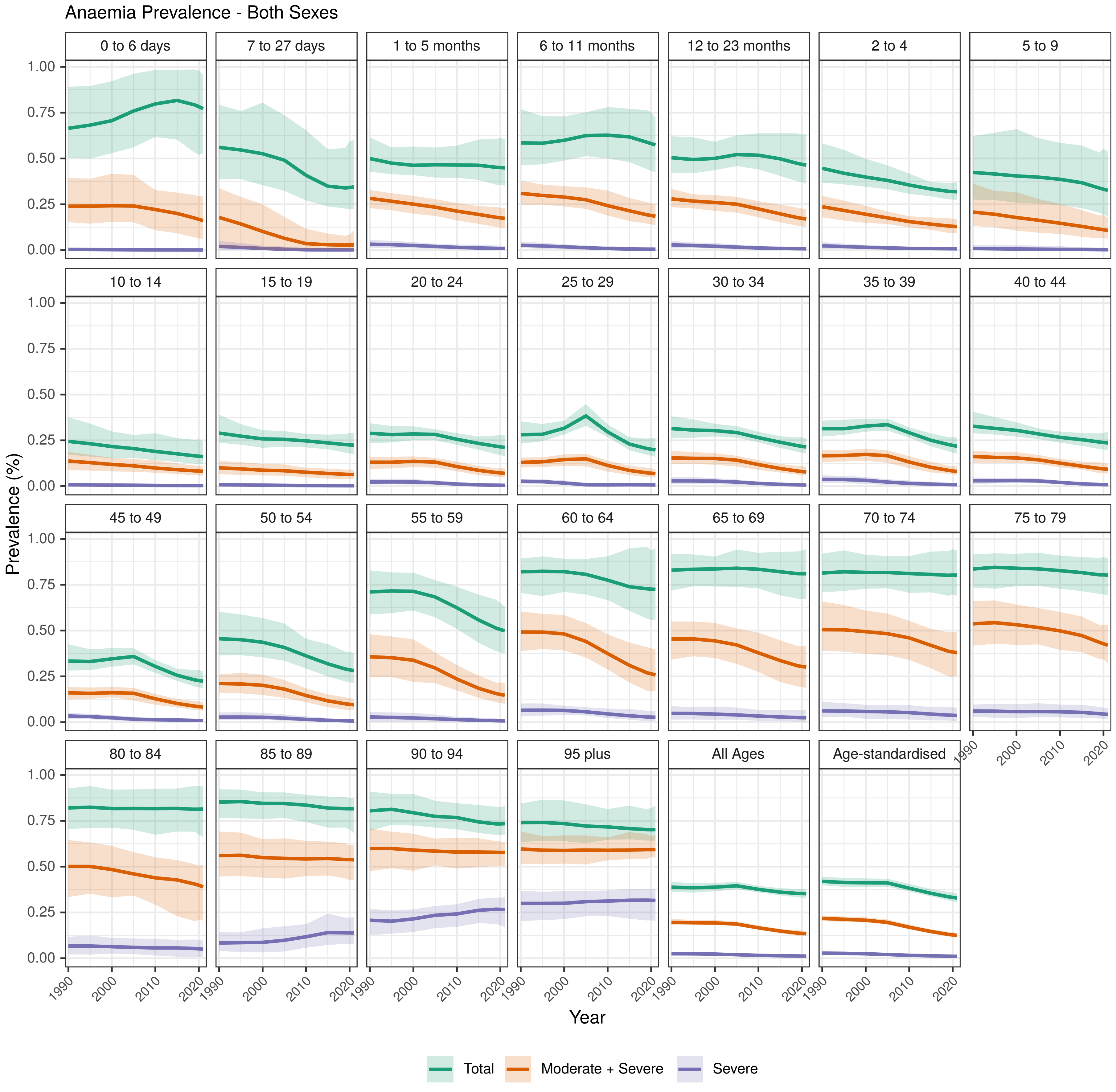

Anaemia Prevalence - Both Sexes
0 to 6 days
7 to 27 days
1 to 5 months
6 to 11 months
12 to 23 months
2 to 4
5 to 9
10 to 14
15 to 19
20 to 24
25 to 29
30 to 34
35 to 39
40 to 44
45 to 49
50 to 54
55 to 59
60 to 64
65 to 69
70 to 74
75 to 79
80 to 84
85 to 89
90 to 94
95 plus
All Ages
Age-standardised
Prevalence (%)
Year
Total
Moderate + Severe
Severe

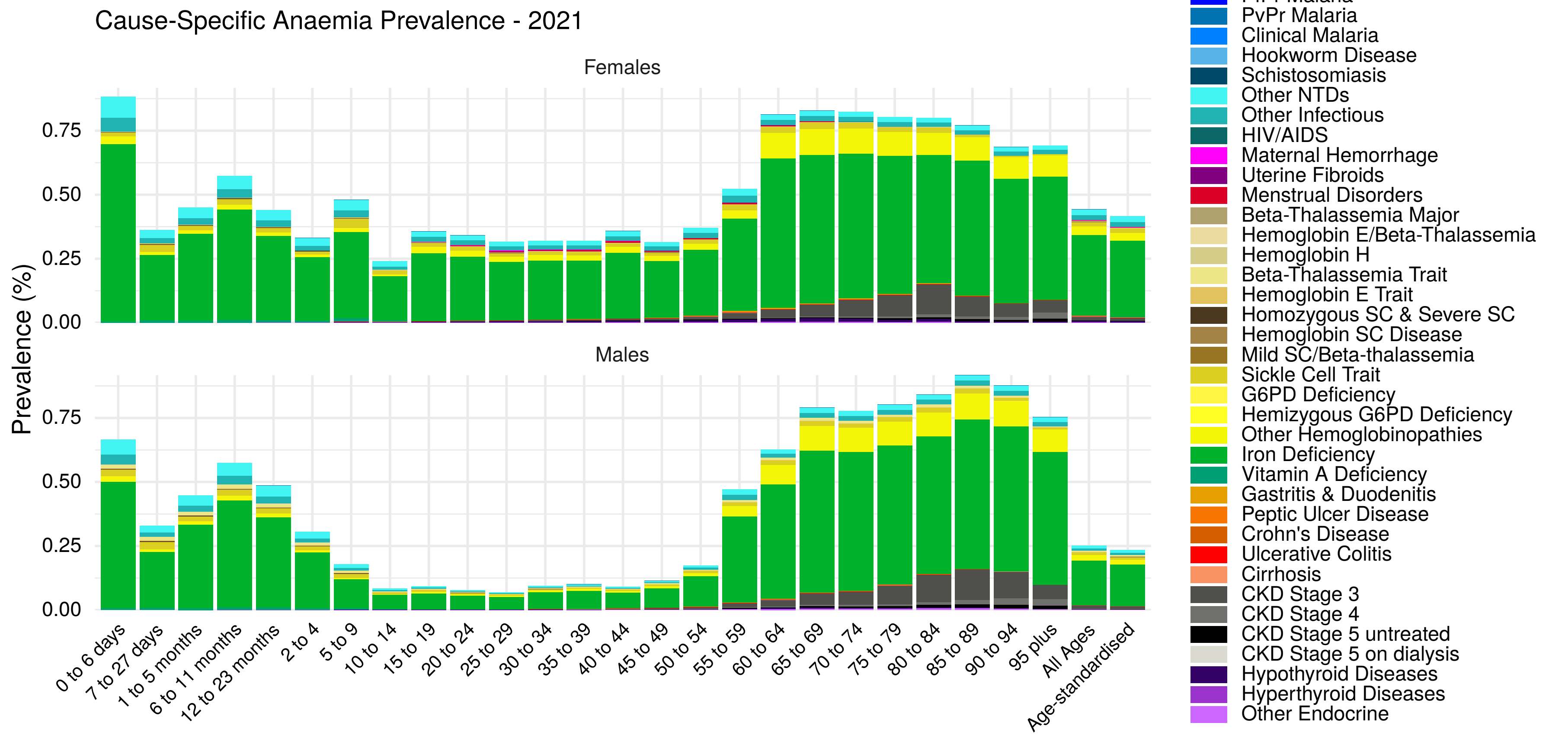

Cause-Specific Anaemia Prevalence - 2021
Females
Males
Prevalence (%)
0 to 6 days
7 to 27 days
1 to 5 months
6 to 11 months
12 to 23 months
2 to 4
5 to 9
10 to 14
15 to 19
20 to 24
25 to 29
30 to 34
35 to 39
40 to 44
45 to 49
50 to 54
55 to 59
60 to 64
65 to 69
70 to 74
75 to 79
80 to 84
85 to 89
90 to 94
95 plus
All Ages
Age-standardised
PfPr Malaria
PvPr Malaria
Clinical Malaria
Hookworm Disease
Schistosomiasis
Other NTDs
Other Infectious
HIV/AIDS
Maternal Hemorrhage
Uterine Fibroids
Menstrual Disorders
Beta-Thalassemia Major
Hemoglobin E/Beta-Thalassemia
Hemoglobin H
Beta-Thalassemia Trait
Hemoglobin E Trait
Homozygous SC & Severe SC
Hemoglobin SC Disease
Mild SC/Beta-thalassemia
Sickle Cell Trait
G6PD Deficiency
Hemizygous G6PD Deficiency
Other Hemoglobinopathies
Iron Deficiency
Vitamin A Deficiency
Gastritis & Duodenitis
Peptic Ulcer Disease
Crohn's Disease
Ulcerative Colitis
Cirrhosis
CKD Stage 3
CKD Stage 4
CKD Stage 5 untreated
CKD Stage 5 on dialysis
Hypothyroid Diseases
Hyperthyroid Diseases
Other Endocrine

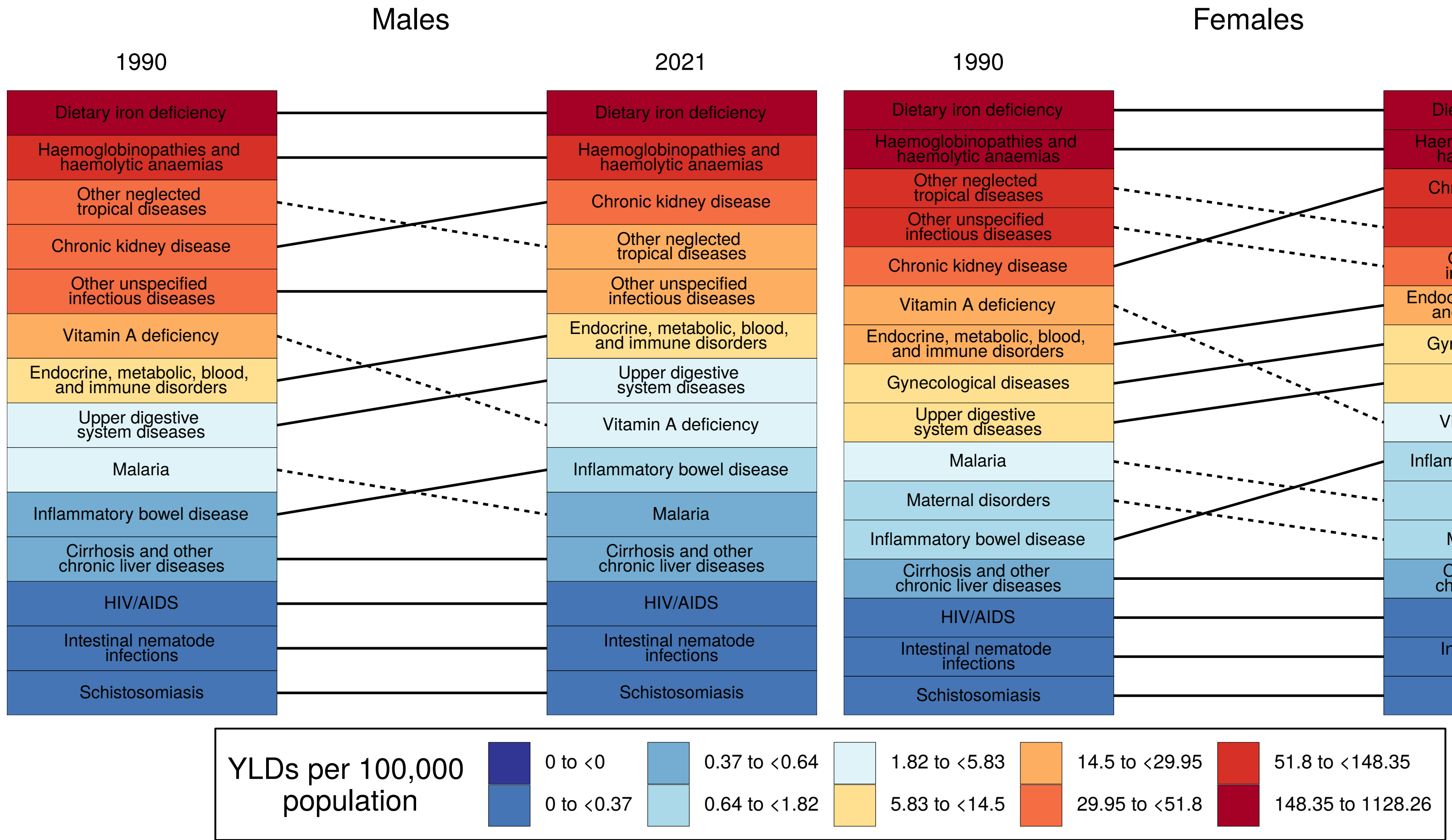

Males
Females
1990
2021
Dietary iron deficiency
Haemoglobinopathies and haemolytic anaemias
Other neglected tropical diseases
Chronic kidney disease
Other unspecified infectious diseases
Vitamin A deficiency
Endocrine, metabolic, blood, and immune disorders
Upper digestive system diseases
Malaria
Inflammatory bowel disease
Cirrhosis and other chronic liver diseases
HIV/AIDS
Intestinal nematode infections
Schistosomiasis
Gynecological diseases
Maternal disorders
YLDs per 100,000 population
0 to <0
0 to <0.37
0.37 to <0.64
0.64 to <1.82
1.82 to <5.83
5.83 to <14.5
14.5 to <29.95
29.95 to <51.8
51.8 to <148.35
148.35 to 1128.26

Madhya Pradesh

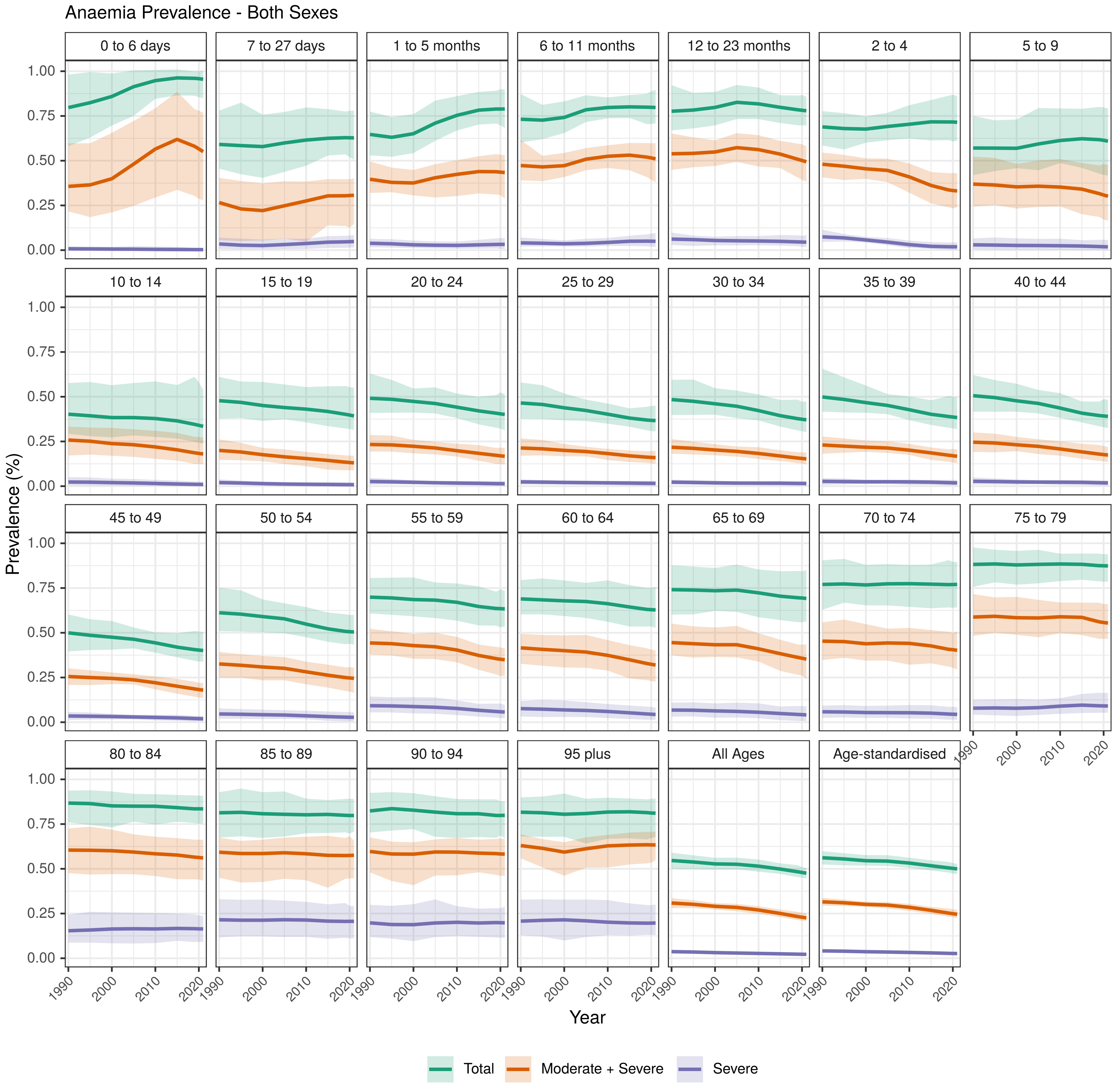

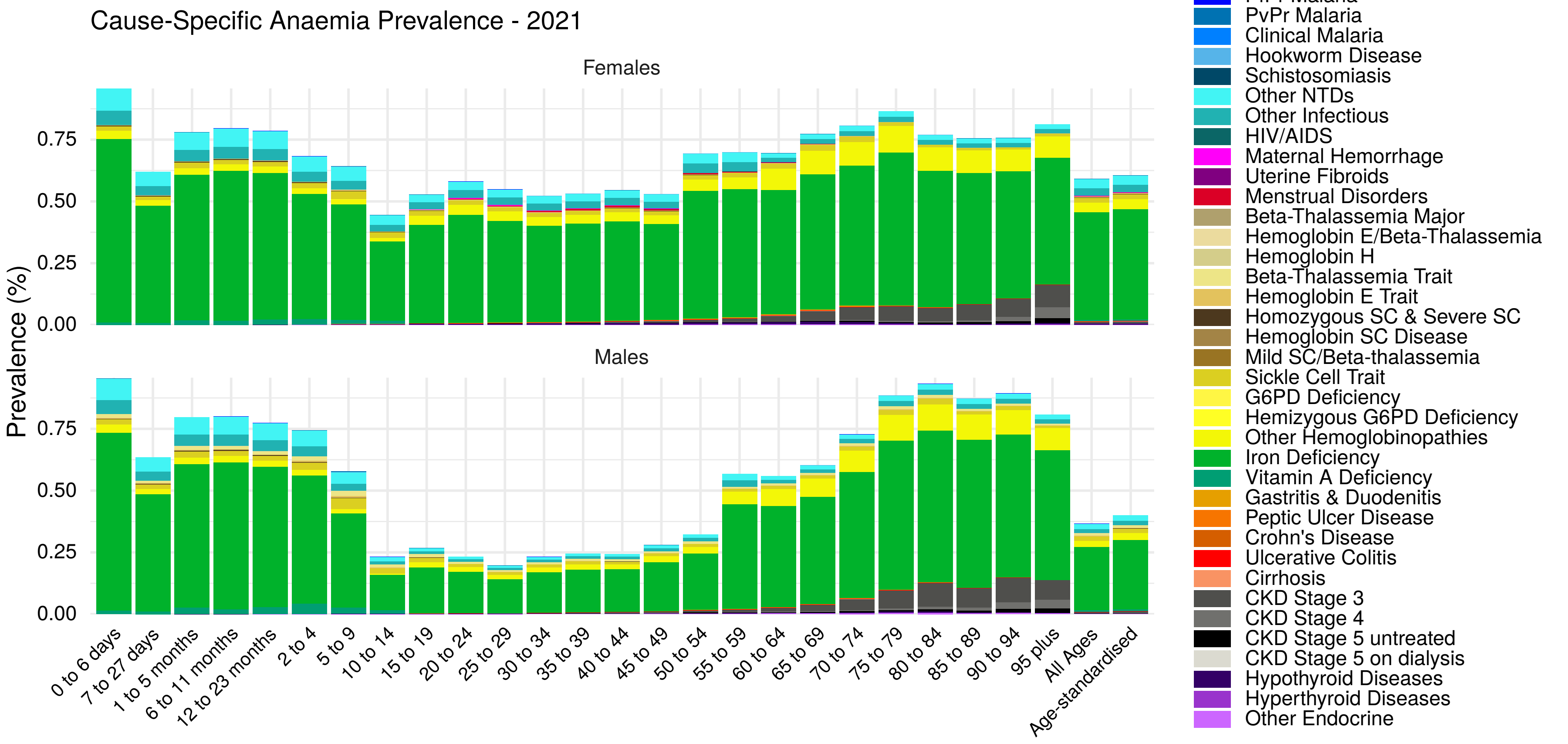

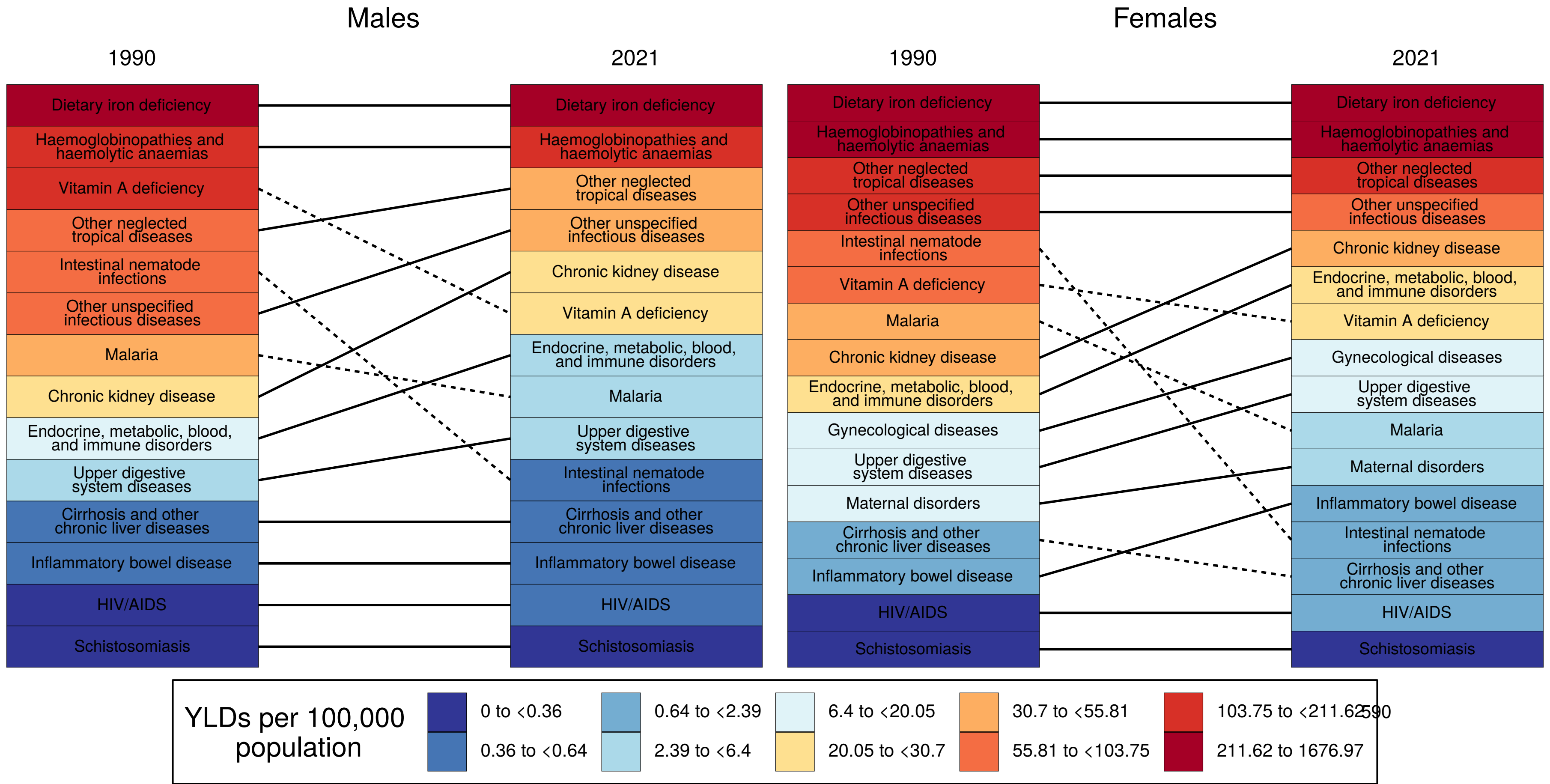

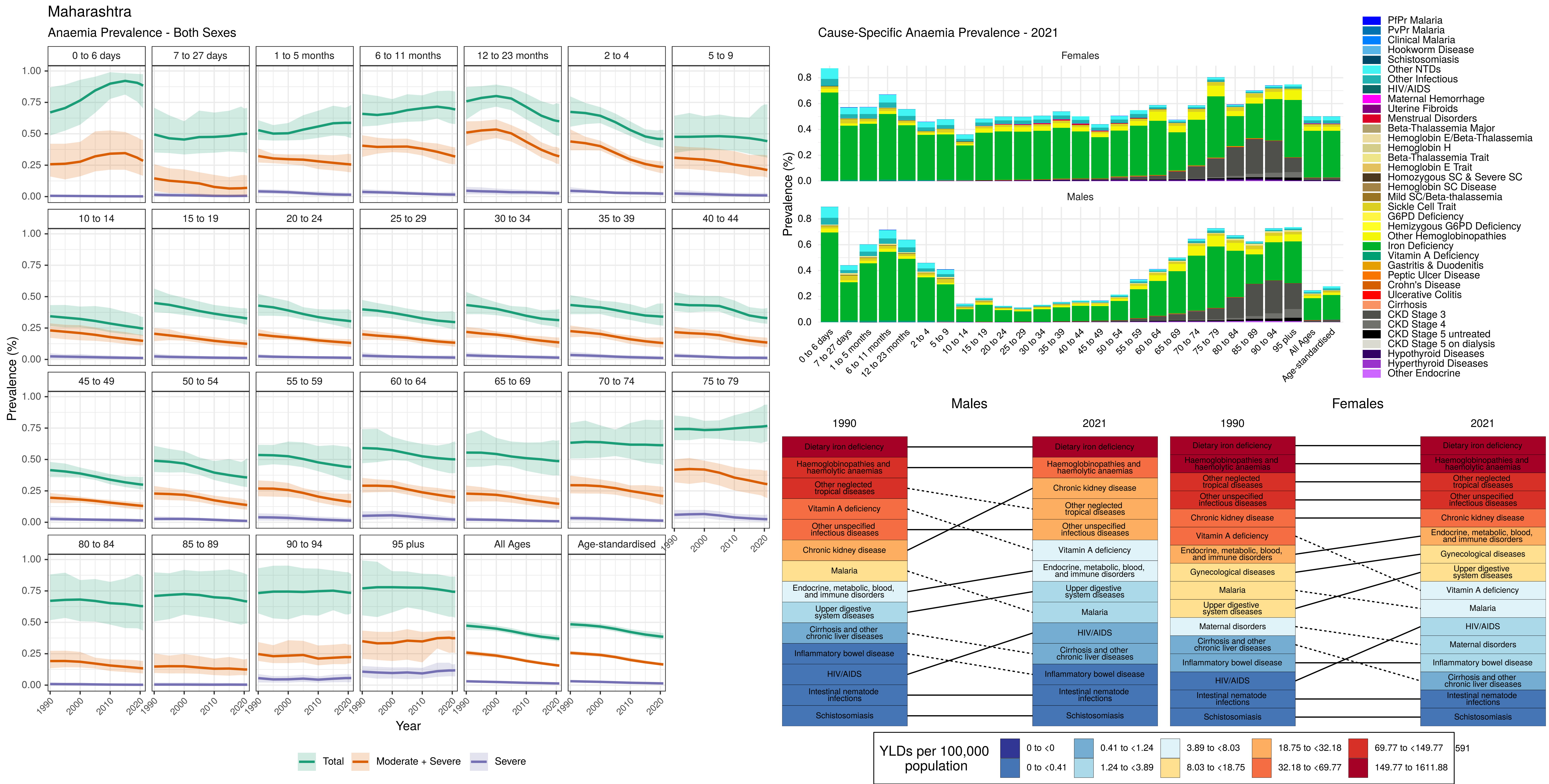
Maharashtra
Anaemia Prevalence - Both Sexes
0 to 6 days
7 to 27 days
1 to 5 months
6 to 11 months
12 to 23 months
2 to 4
5 to 9
10 to 14
15 to 19
20 to 24
25 to 29
30 to 34
35 to 39
40 to 44
45 to 49
50 to 54
55 to 59
60 to 64
65 to 69
70 to 74
75 to 79
80 to 84
85 to 89
90 to 94
95 plus
All Ages
Age-standardised
Prevalence (%)
Year
Total
Moderate + Severe
Severe
Cause-Specific Anaemia Prevalence - 2021
Females
Males
PfPr Malaria
PvPr Malaria
Clinical Malaria
Hookworm Disease
Schistosomiasis
Other NTDs
Other Infectious
HIV/AIDS
Maternal Hemorrhage
Uterine Fibroids
Menstrual Disorders
Beta-Thalassemia Major
Hemoglobin E/Beta-Thalassemia
Hemoglobin H
Beta-Thalassemia Trait
Hemoglobin E Trait
Homozygous SC & Severe SC
Hemoglobin SC Disease
Mild SC/Beta-thalassemia
Sickle Cell Trait
G6PD Deficiency
Hemizygous G6PD Deficiency
Other Hemoglobinopathies
Iron Deficiency
Vitamin A Deficiency
Gastritis & Duodenitis
Peptic Ulcer Disease
Crohn's Disease
Ulcerative Colitis
Cirrhosis
CKD Stage 3
CKD Stage 4
CKD Stage 5 untreated
CKD Stage 5 on dialysis
Hypothyroid Diseases
Hyperthyroid Diseases
Other Endocrine
Males
Females
1990
2021
Dietary iron deficiency
Haemoglobinopathies and haemolytic anaemias
Other neglected tropical diseases
Vitamin A deficiency
Other unspecified infectious diseases
Chronic kidney disease
Malaria
Endocrine, metabolic, blood, and immune disorders
Upper digestive system diseases
Cirrhosis and other chronic liver diseases
Inflammatory bowel disease
HIV/AIDS
Intestinal nematode infections
Schistosomiasis
Gynecological diseases
Maternal disorders
YLDs per 100,000 population
0 to <0
0 to <0.41
0.41 to <1.24
1.24 to <3.89
3.89 to <8.03
8.03 to <18.75
18.75 to <32.18
32.18 to <69.77
69.77 to <149.77
149.77 to 1611.88

Manipur

Anaemia Prevalence - Both Sexes

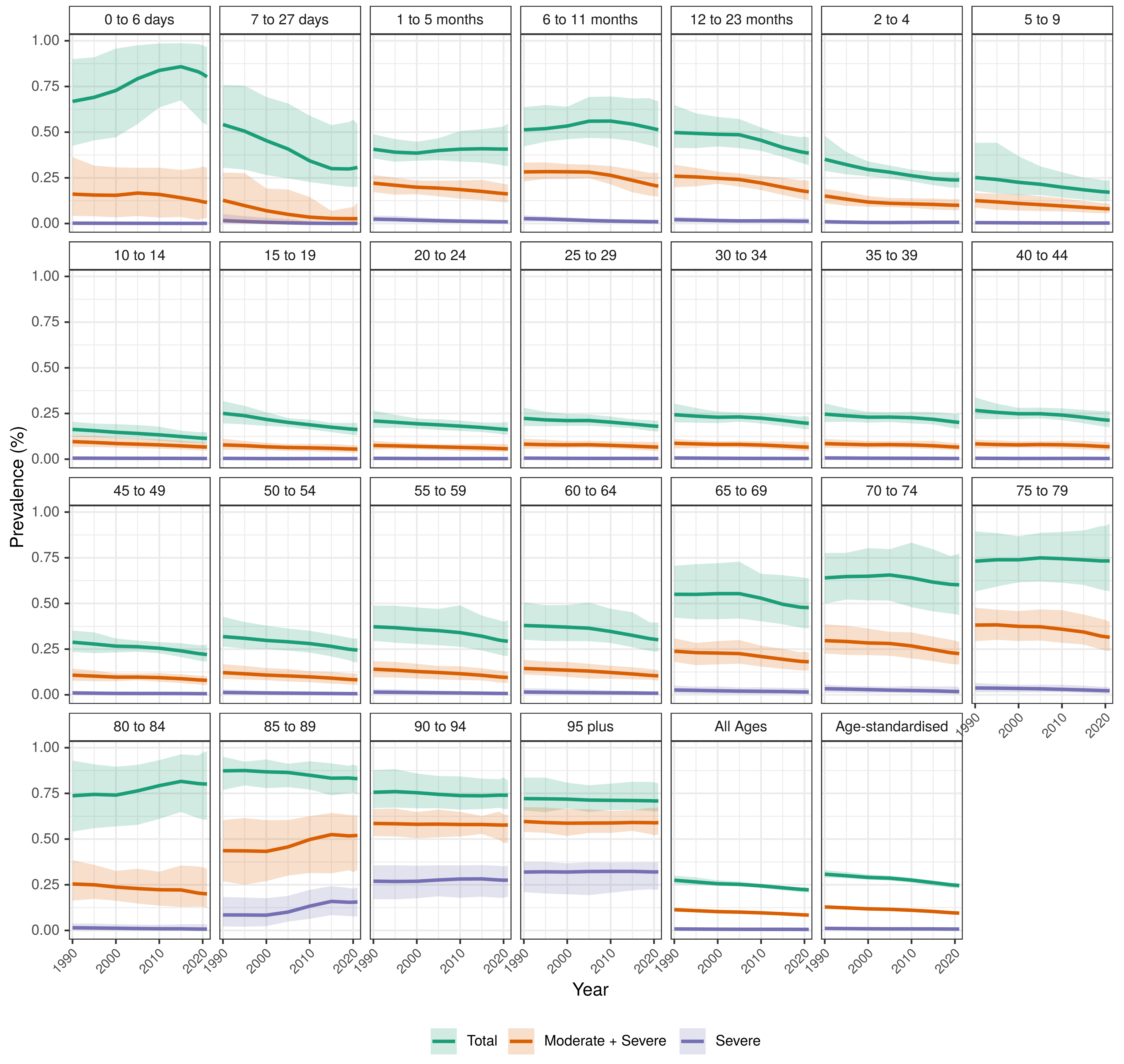

Cause-Specific Anaemia Prevalence - 2021

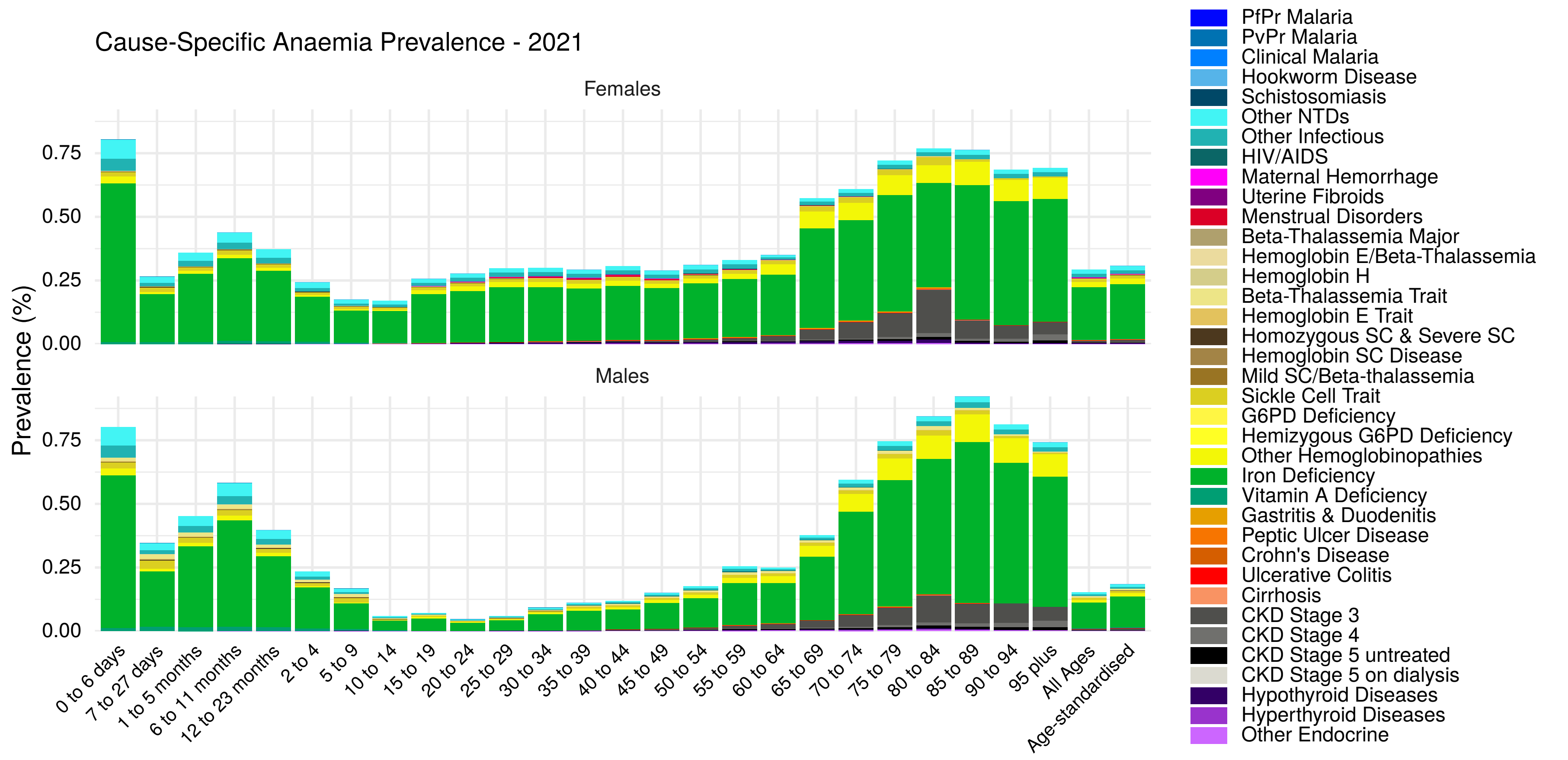

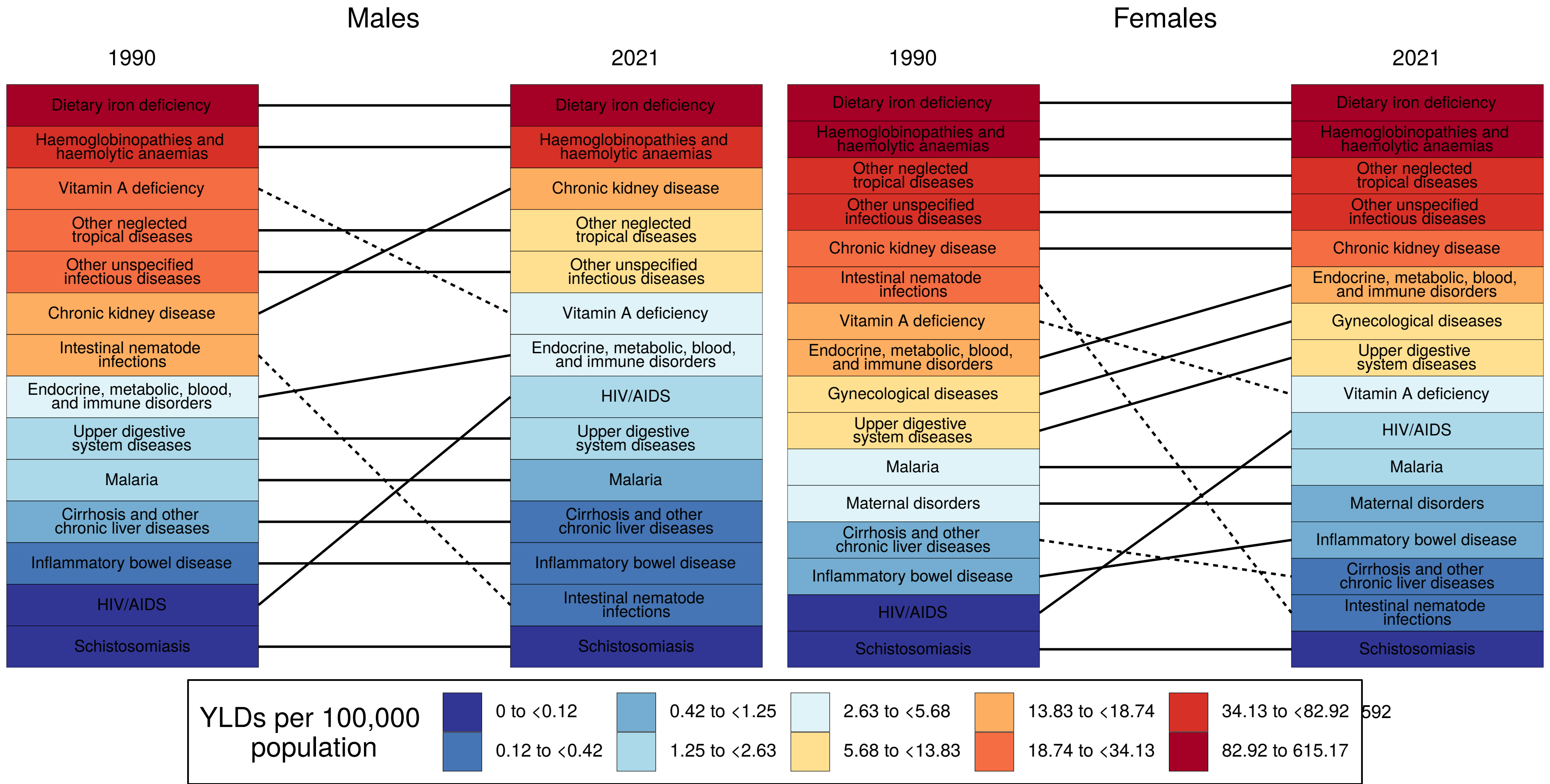

592

Meghalaya

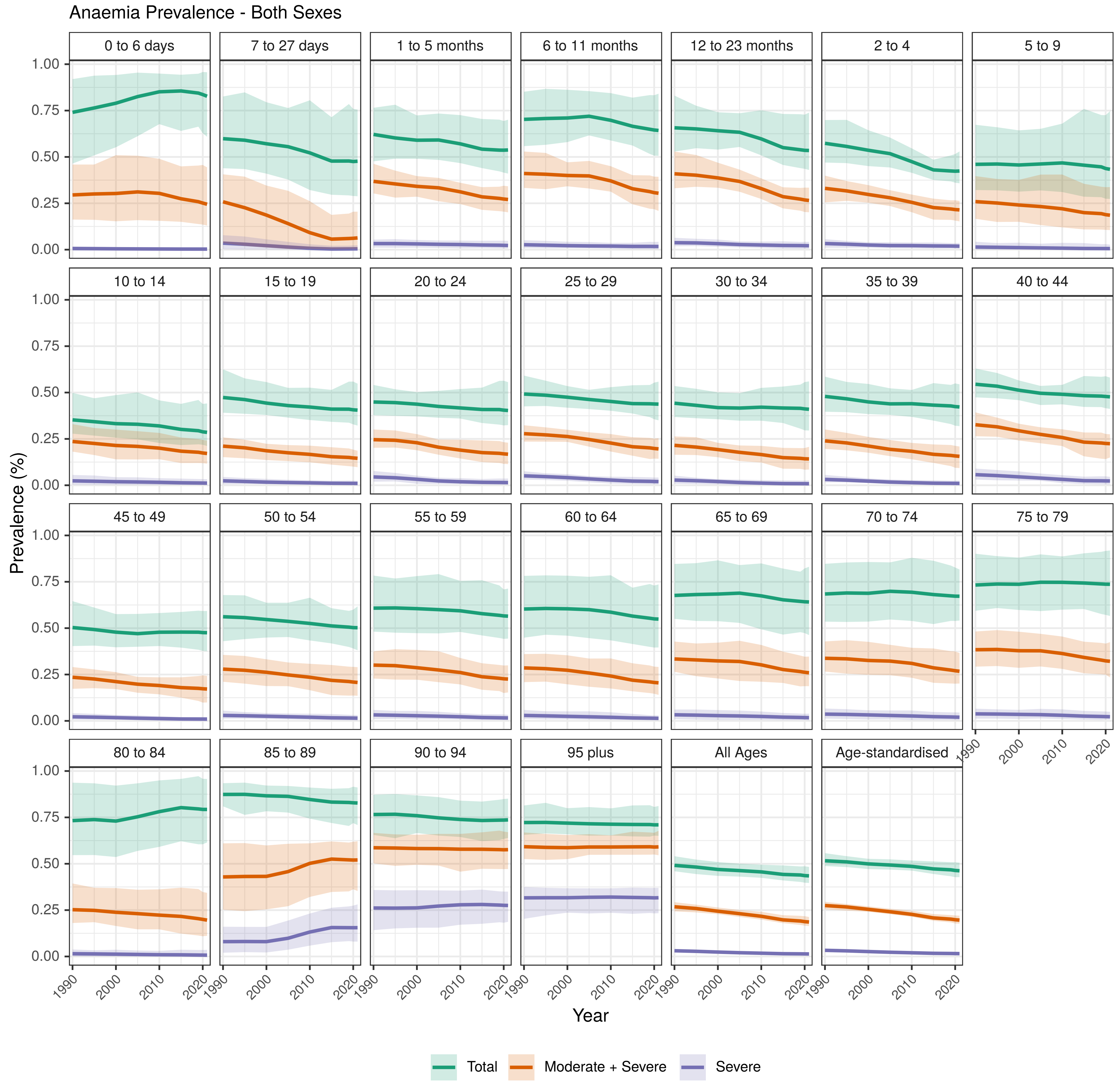

Anaemia Prevalence - Both Sexes
0 to 6 days
7 to 27 days
1 to 5 months
6 to 11 months
12 to 23 months
2 to 4
5 to 9
10 to 14
15 to 19
20 to 24
25 to 29
30 to 34
35 to 39
40 to 44
45 to 49
50 to 54
55 to 59
60 to 64
65 to 69
70 to 74
75 to 79
80 to 84
85 to 89
90 to 94
95 plus
All Ages
Age-standardised
1.00
0.75
0.50
0.25
0.00
Prevalence (%)
1990
2000
2010
2020
Year
Total
Moderate + Severe
Severe

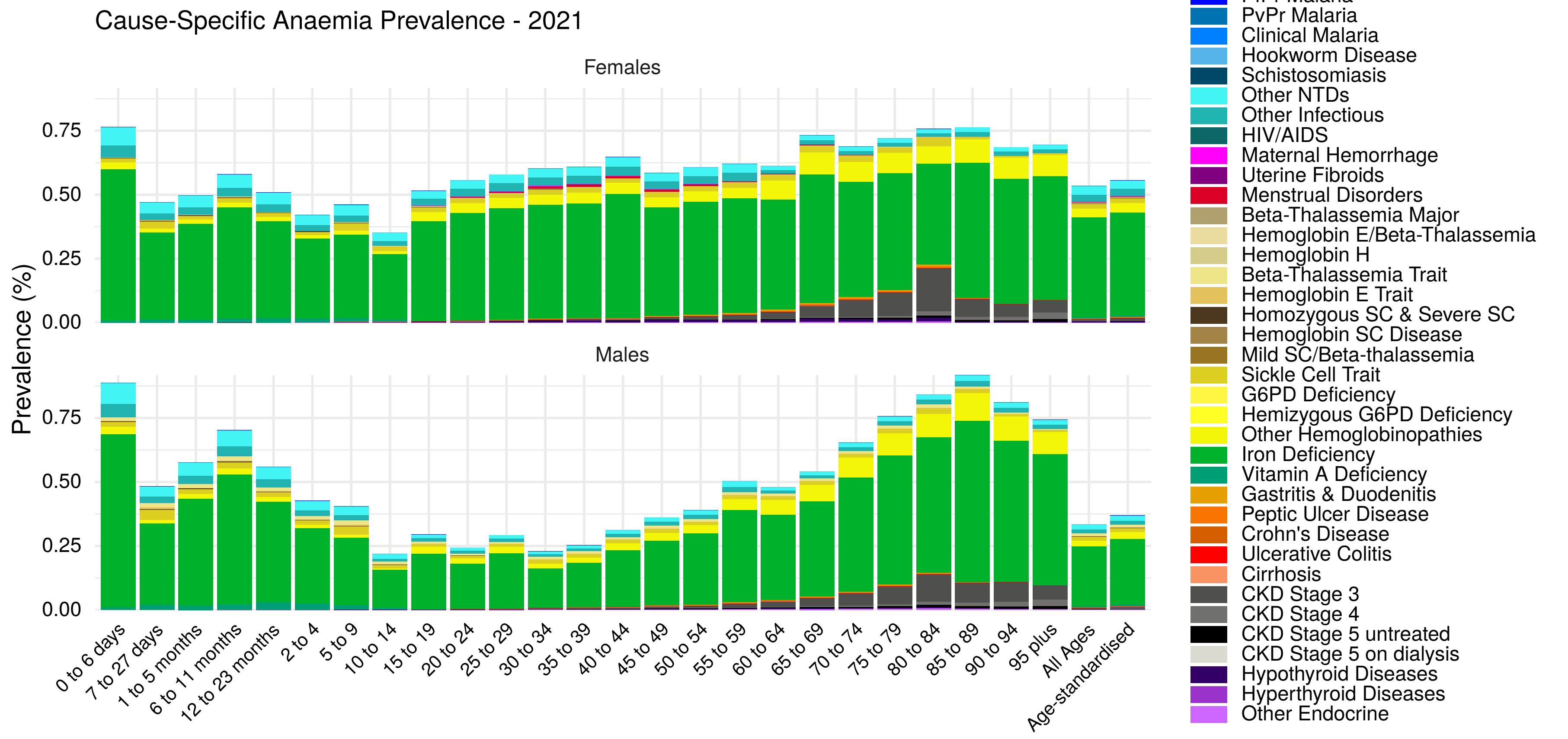

Cause-Specific Anaemia Prevalence - 2021
Females
Males
Prevalence (%)
0.75
0.50
0.25
0.00
0 to 6 days
7 to 27 days
1 to 5 months
6 to 11 months
12 to 23 months
2 to 4
5 to 9
10 to 14
15 to 19
20 to 24
25 to 29
30 to 34
35 to 39
40 to 44
45 to 49
50 to 54
55 to 59
60 to 64
65 to 69
70 to 74
75 to 79
80 to 84
85 to 89
90 to 94
95 plus
All Ages
Age-standardised
PfPr Malaria
PvPr Malaria
Clinical Malaria
Hookworm Disease
Schistosomiasis
Other NTDs
Other Infectious
HIV/AIDS
Maternal Hemorrhage
Uterine Fibroids
Menstrual Disorders
Beta-Thalassemia Major
Hemoglobin E/Beta-Thalassemia
Hemoglobin H
Beta-Thalassemia Trait
Hemoglobin E Trait
Homozygous SC & Severe SC
Hemoglobin SC Disease
Mild SC/Beta-thalassemia
Sickle Cell Trait
G6PD Deficiency
Hemizygous G6PD Deficiency
Other Hemoglobinopathies
Iron Deficiency
Vitamin A Deficiency
Gastritis & Duodenitis
Peptic Ulcer Disease
Crohn's Disease
Ulcerative Colitis
Cirrhosis
CKD Stage 3
CKD Stage 4
CKD Stage 5 untreated
CKD Stage 5 on dialysis
Hypothyroid Diseases
Hyperthyroid Diseases
Other Endocrine

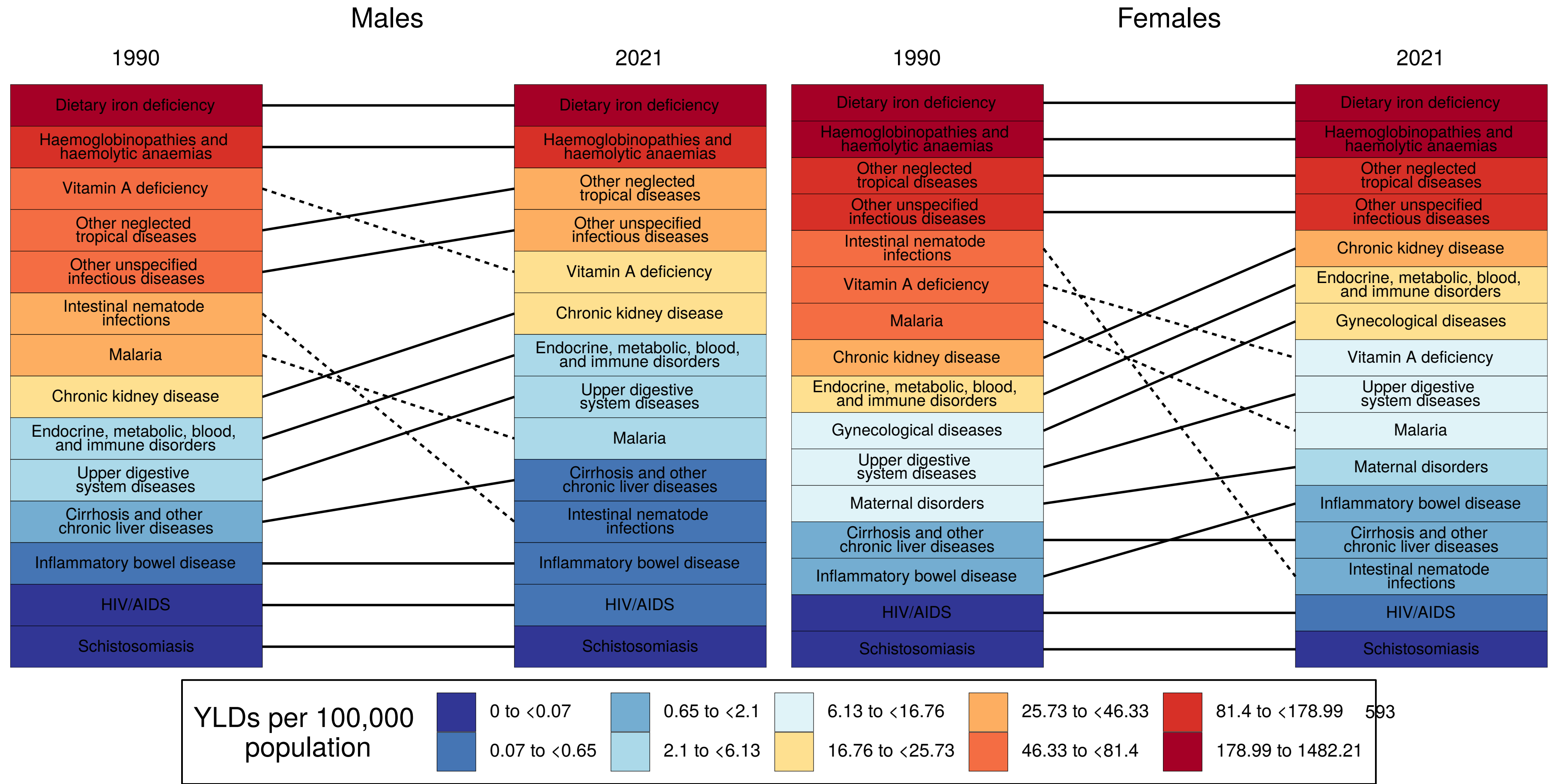

Males
Females
1990
2021
Dietary iron deficiency
Haemoglobinopathies and haemolytic anaemias
Vitamin A deficiency
Other neglected tropical diseases
Other unspecified infectious diseases
Intestinal nematode infections
Malaria
Chronic kidney disease
Endocrine, metabolic, blood, and immune disorders
Upper digestive system diseases
Cirrhosis and other chronic liver diseases
Inflammatory bowel disease
HIV/AIDS
Schistosomiasis
Gynecological diseases
Maternal disorders
YLDs per 100,000 population
0 to <0.07
0.07 to <0.65
0.65 to <2.1
2.1 to <6.13
6.13 to <16.76
16.76 to <25.73
25.73 to <46.33
46.33 to <81.4
81.4 to <178.99
178.99 to 1482.21

Mizoram

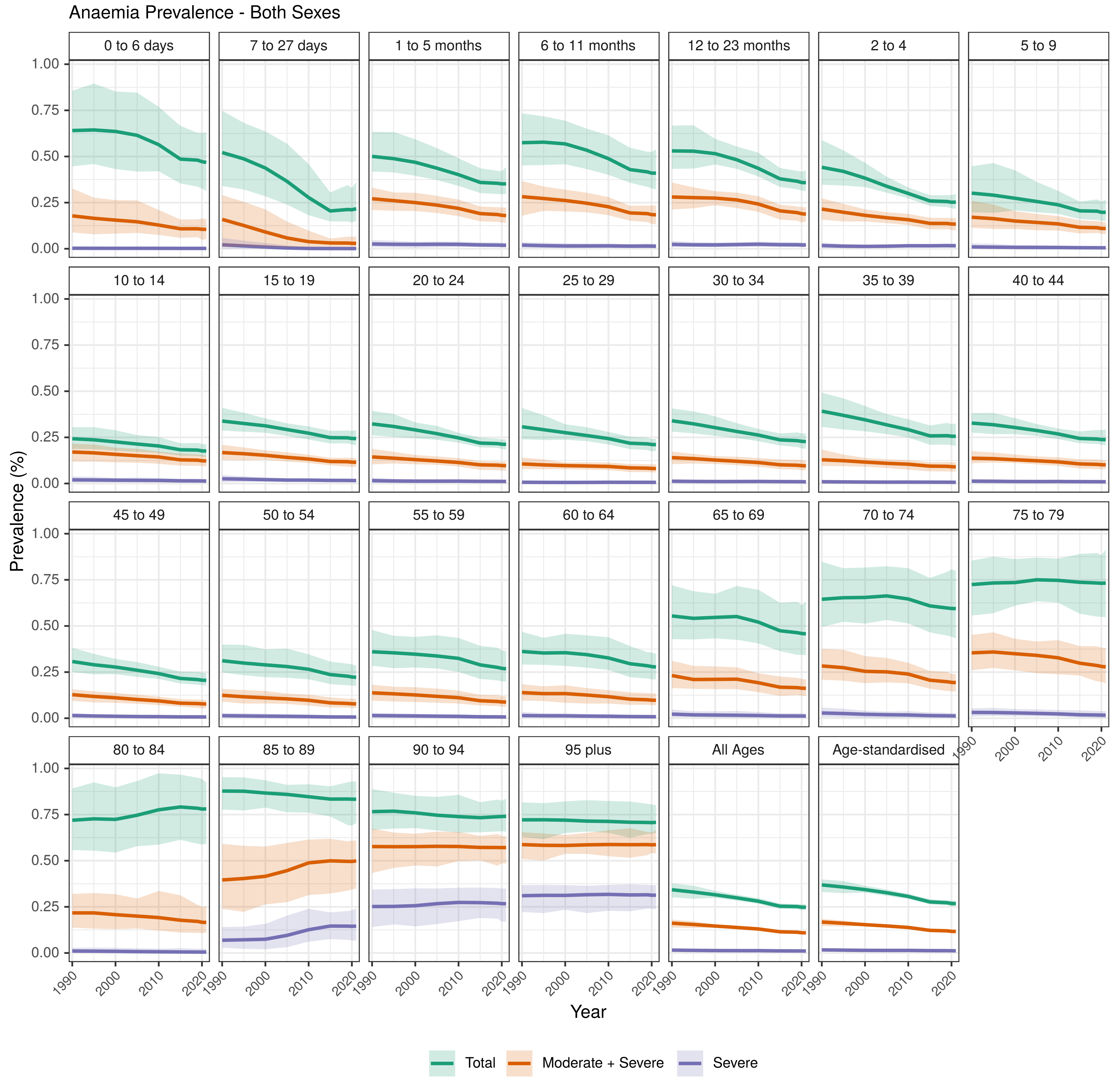
Anaemia Prevalence - Both Sexes
0 to 6 days
7 to 27 days
1 to 5 months
6 to 11 months
12 to 23 months
2 to 4
5 to 9
10 to 14
15 to 19
20 to 24
25 to 29
30 to 34
35 to 39
40 to 44
45 to 49
50 to 54
55 to 59
60 to 64
65 to 69
70 to 74
75 to 79
80 to 84
85 to 89
90 to 94
95 plus
All Ages
Age-standardised
Prevalence (%)
Year
1.00
0.75
0.50
0.25
0.00
1990
2000
2010
2020
Total
Moderate + Severe
Severe

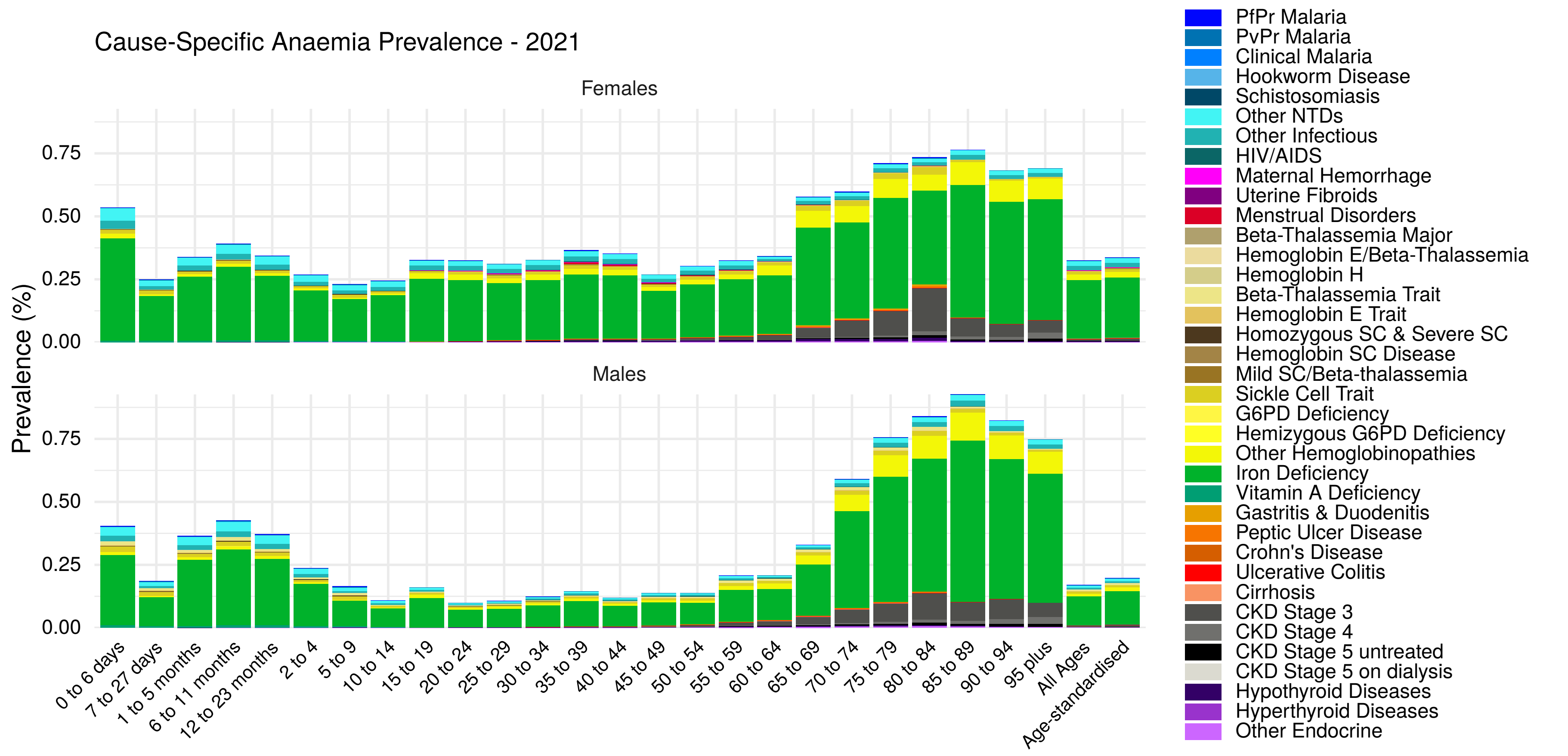
Cause-Specific Anaemia Prevalence - 2021
Females
Males
Prevalence (%)
0.75
0.50
0.25
0.00
0 to 6 days
7 to 27 days
1 to 5 months
6 to 11 months
12 to 23 months
2 to 4
5 to 9
10 to 14
15 to 19
20 to 24
25 to 29
30 to 34
35 to 39
40 to 44
45 to 49
50 to 54
55 to 59
60 to 64
65 to 69
70 to 74
75 to 79
80 to 84
85 to 89
90 to 94
95 plus
All Ages
Age-standardised
PfPr Malaria
PvPr Malaria
Clinical Malaria
Hookworm Disease
Schistosomiasis
Other NTDs
Other Infectious
HIV/AIDS
Maternal Hemorrhage
Uterine Fibroids
Menstrual Disorders
Beta-Thalassemia Major
Hemoglobin E/Beta-Thalassemia
Hemoglobin H
Beta-Thalassemia Trait
Hemoglobin E Trait
Homozygous SC & Severe SC
Hemoglobin SC Disease
Mild SC/Beta-thalassemia
Sickle Cell Trait
G6PD Deficiency
Hemizygous G6PD Deficiency
Other Hemoglobinopathies
Iron Deficiency
Vitamin A Deficiency
Gastritis & Duodenitis
Peptic Ulcer Disease
Crohn's Disease
Ulcerative Colitis
Cirrhosis
CKD Stage 3
CKD Stage 4
CKD Stage 5 untreated
CKD Stage 5 on dialysis
Hypothyroid Diseases
Hyperthyroid Diseases
Other Endocrine

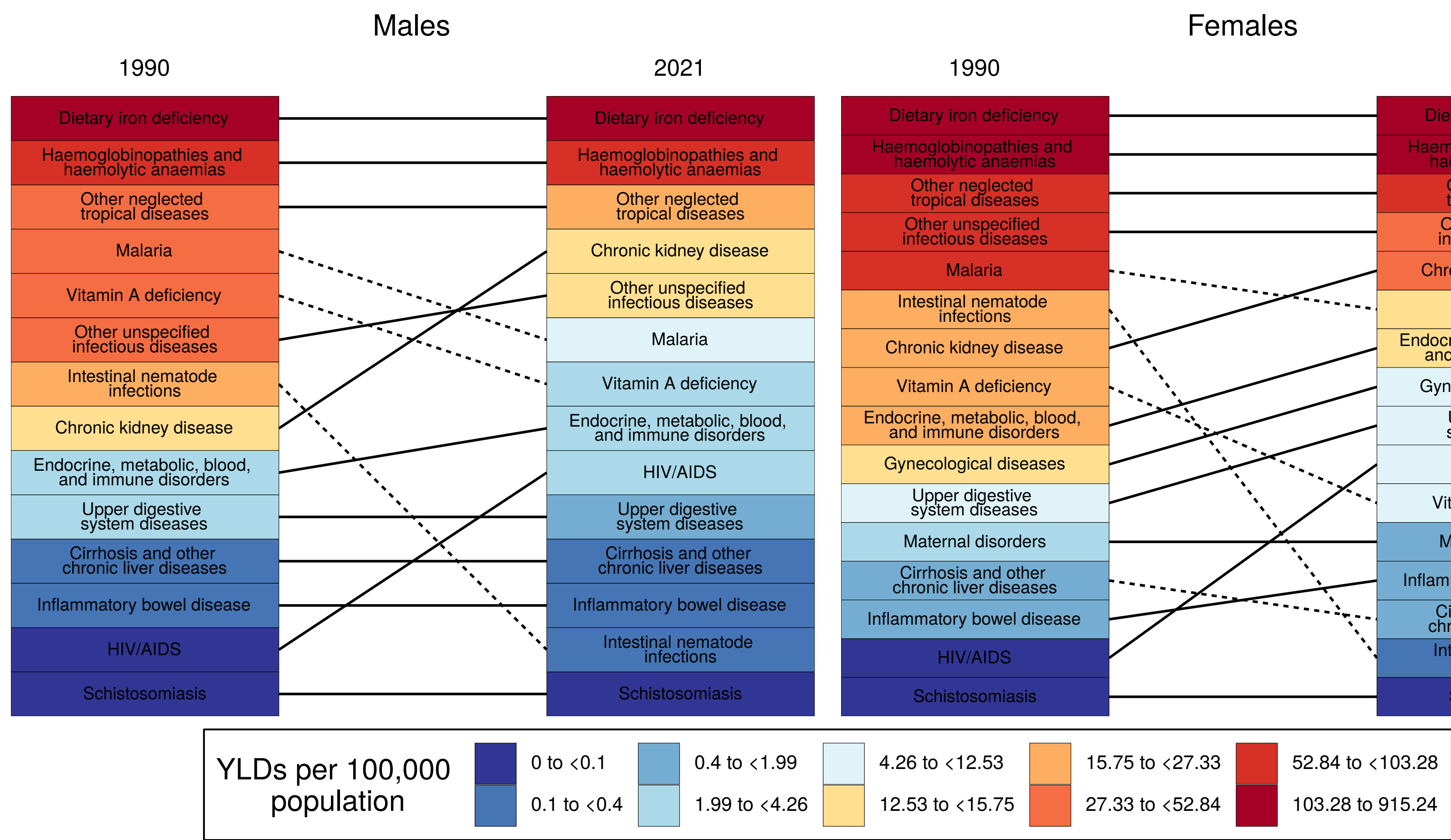
Males
Females
1990
2021
Dietary iron deficiency
Haemoglobinopathies and haemolytic anaemias
Other neglected tropical diseases
Malaria
Vitamin A deficiency
Other unspecified infectious diseases
Intestinal nematode infections
Chronic kidney disease
Endocrine, metabolic, blood, and immune disorders
Upper digestive system diseases
Cirrhosis and other chronic liver diseases
Inflammatory bowel disease
HIV/AIDS
Schistosomiasis
Gynecological diseases
Maternal disorders
YLDs per 100,000 population
0 to <0.1
0.1 to <0.4
0.4 to <1.99
1.99 to <4.26
4.26 to <12.53
12.53 to <15.75
15.75 to <27.33
27.33 to <52.84
52.84 to <103.28
103.28 to 915.24

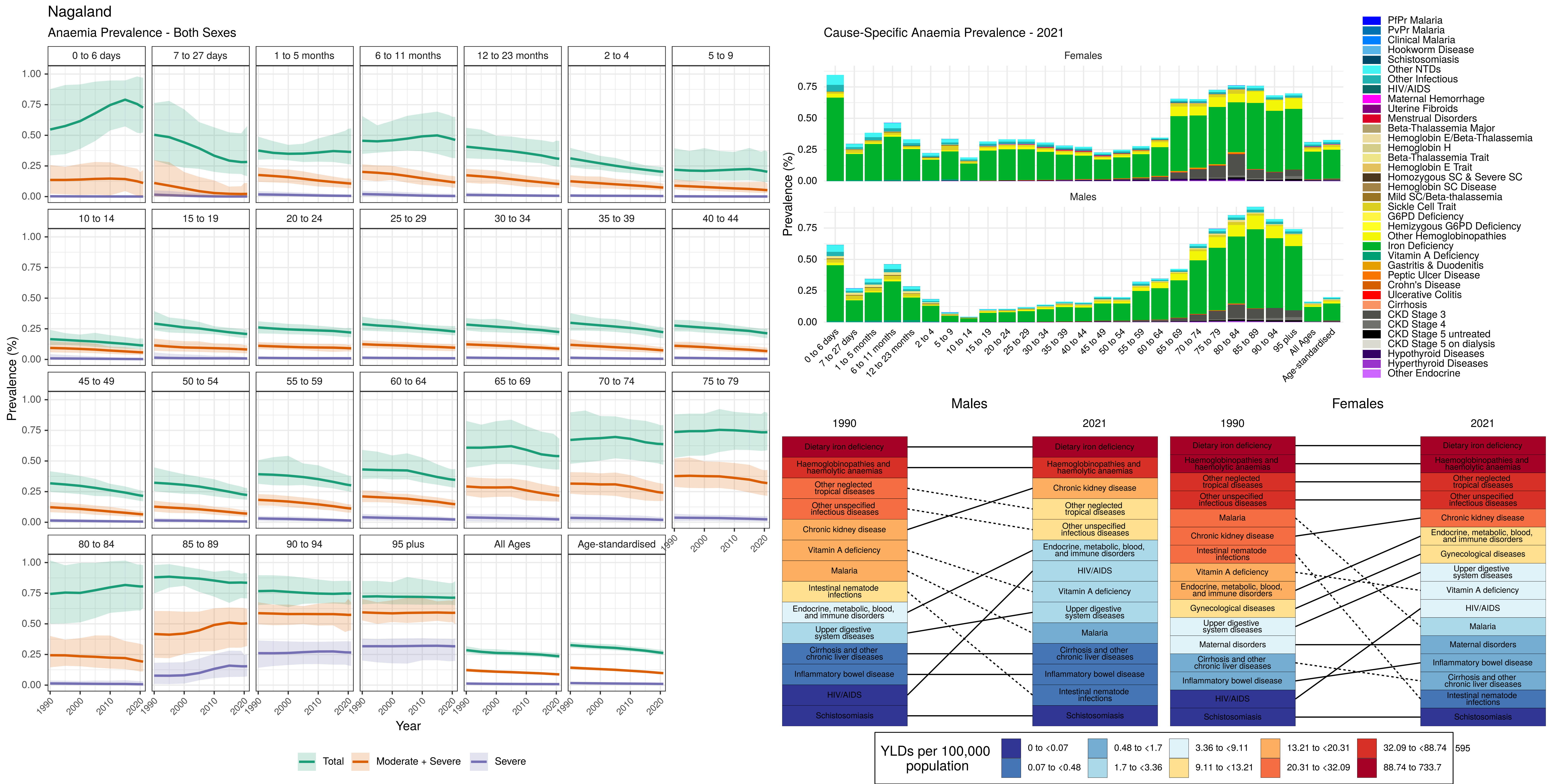
Nagaland
Anaemia Prevalence - Both Sexes
0 to 6 days
7 to 27 days
1 to 5 months
6 to 11 months
12 to 23 months
2 to 4
5 to 9
10 to 14
15 to 19
20 to 24
25 to 29
30 to 34
35 to 39
40 to 44
45 to 49
50 to 54
55 to 59
60 to 64
65 to 69
70 to 74
75 to 79
80 to 84
85 to 89
90 to 94
95 plus
All Ages
Age-standardised
Prevalence (%)
Year
Total
Moderate + Severe
Severe
Cause-Specific Anaemia Prevalence - 2021
Females
Males
PfPr Malaria
PvPr Malaria
Clinical Malaria
Hookworm Disease
Schistosomiasis
Other NTDs
Other Infectious
HIV/AIDS
Maternal Hemorrhage
Uterine Fibroids
Menstrual Disorders
Beta-Thalassemia Major
Hemoglobin E/Beta-Thalassemia
Hemoglobin H
Beta-Thalassemia Trait
Hemoglobin E Trait
Homozygous SC & Severe SC
Hemoglobin SC Disease
Mild SC/Beta-thalassemia
Sickle Cell Trait
G6PD Deficiency
Hemizygous G6PD Deficiency
Other Hemoglobinopathies
Iron Deficiency
Vitamin A Deficiency
Gastritis & Duodenitis
Peptic Ulcer Disease
Crohn's Disease
Ulcerative Colitis
Cirrhosis
CKD Stage 3
CKD Stage 4
CKD Stage 5 untreated
CKD Stage 5 on dialysis
Hypothyroid Diseases
Hyperthyroid Diseases
Other Endocrine
Males
Females
1990
2021
Dietary iron deficiency
Haemoglobinopathies and haemolytic anaemias
Other neglected tropical diseases
Other unspecified infectious diseases
Chronic kidney disease
Vitamin A deficiency
Malaria
Intestinal nematode infections
Endocrine, metabolic, blood, and immune disorders
Upper digestive system diseases
Cirrhosis and other chronic liver diseases
Inflammatory bowel disease
HIV/AIDS
Schistosomiasis
Gynecological diseases
Maternal disorders
YLDs per 100,000 population
0 to <0.07
0.07 to <0.48
0.48 to <1.7
1.7 to <3.36
3.36 to <9.11
9.11 to <13.21
13.21 to <20.31
20.31 to <32.09
32.09 to <88.74
88.74 to 733.7

Odisha

Anaemia Prevalence - Both Sexes

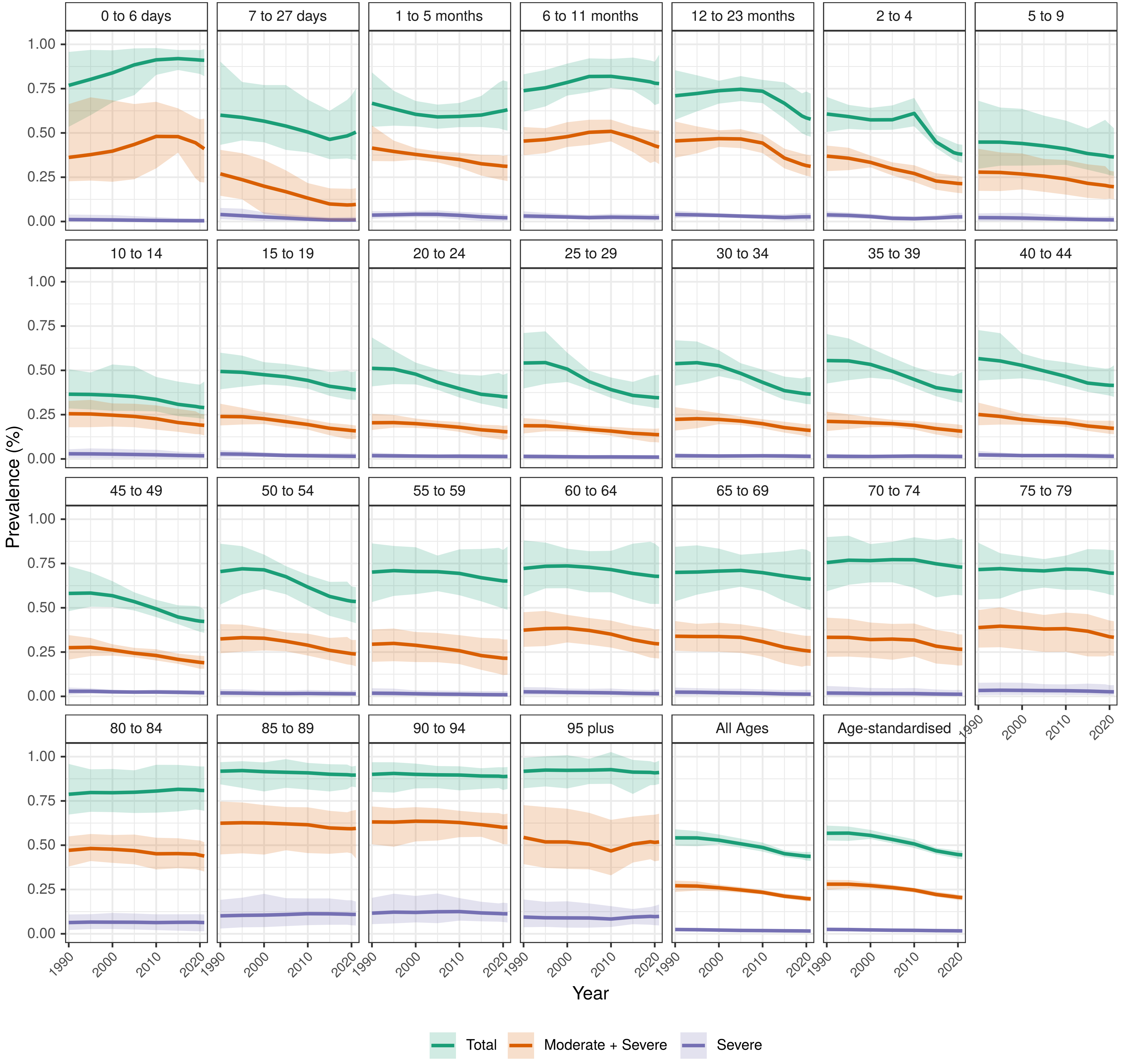

Cause-Specific Anaemia Prevalence - 2021

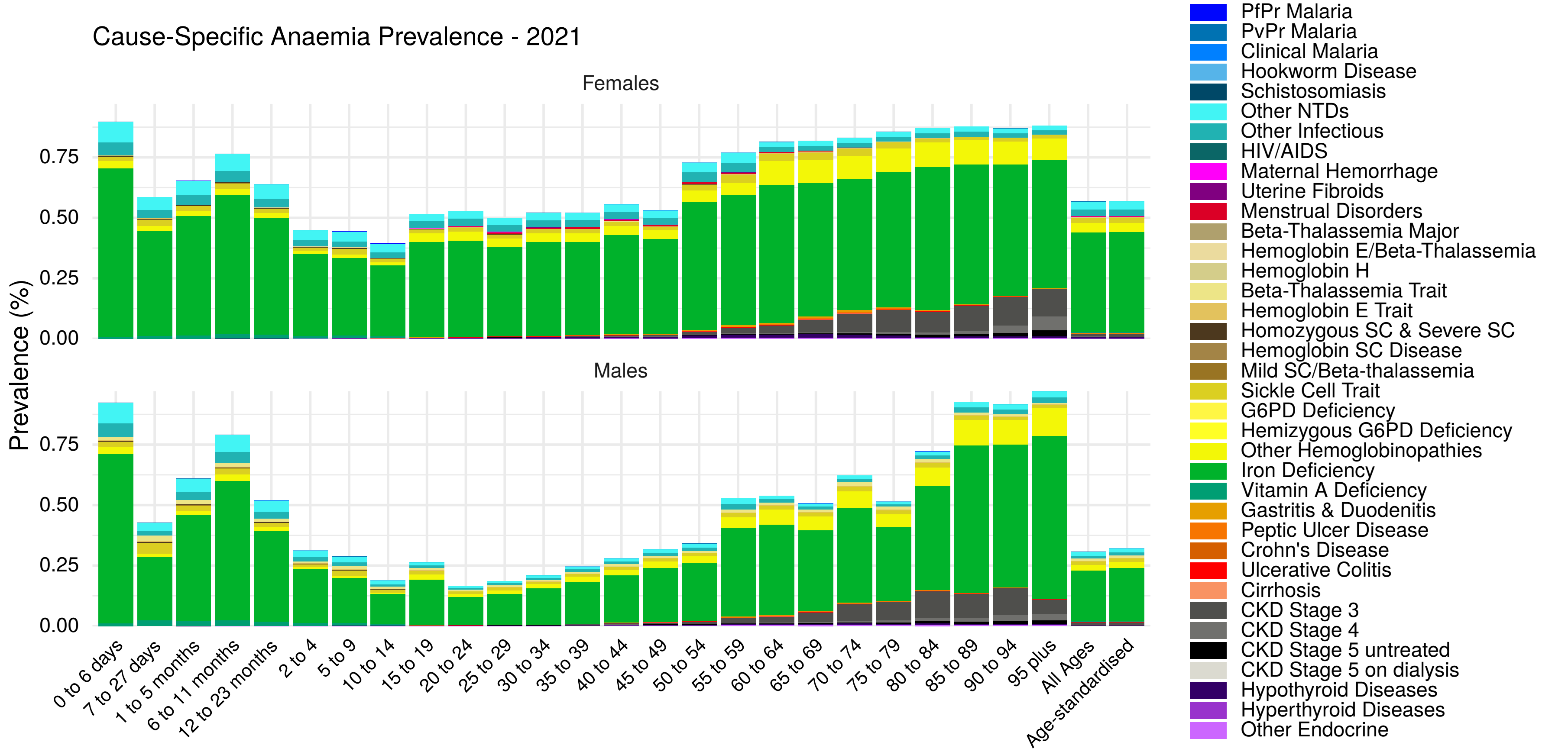

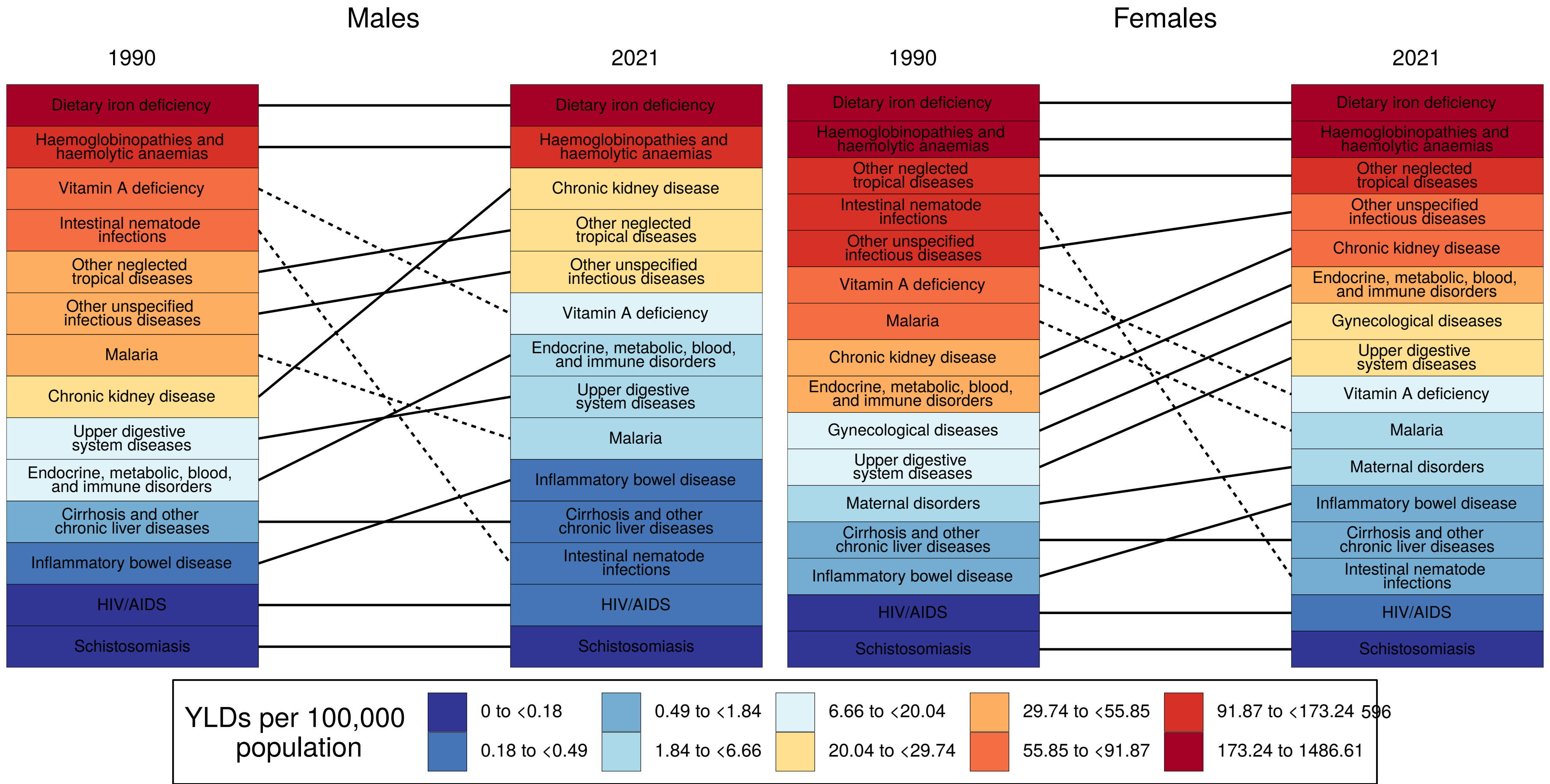

Other Union Territories

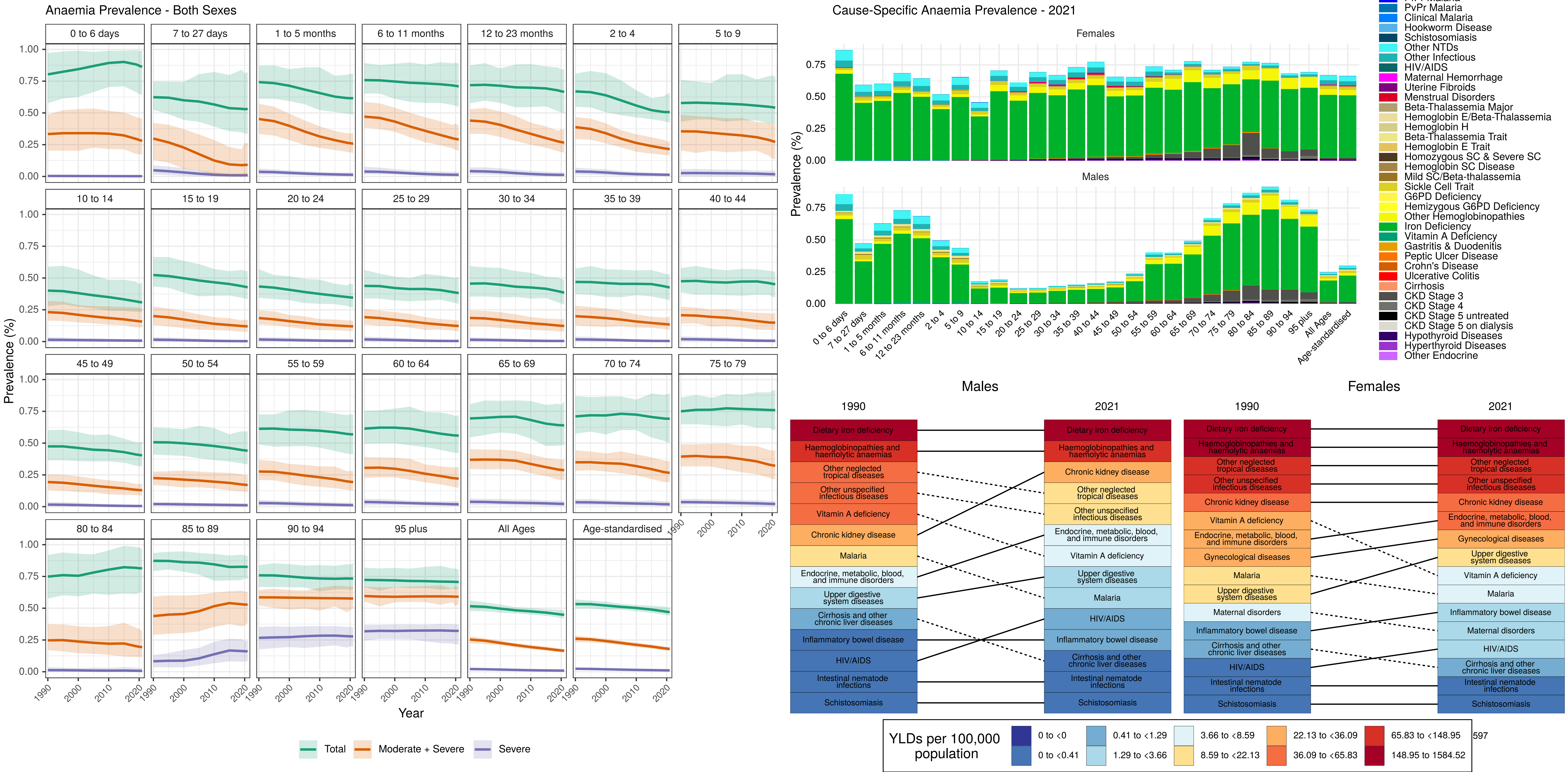

Punjab

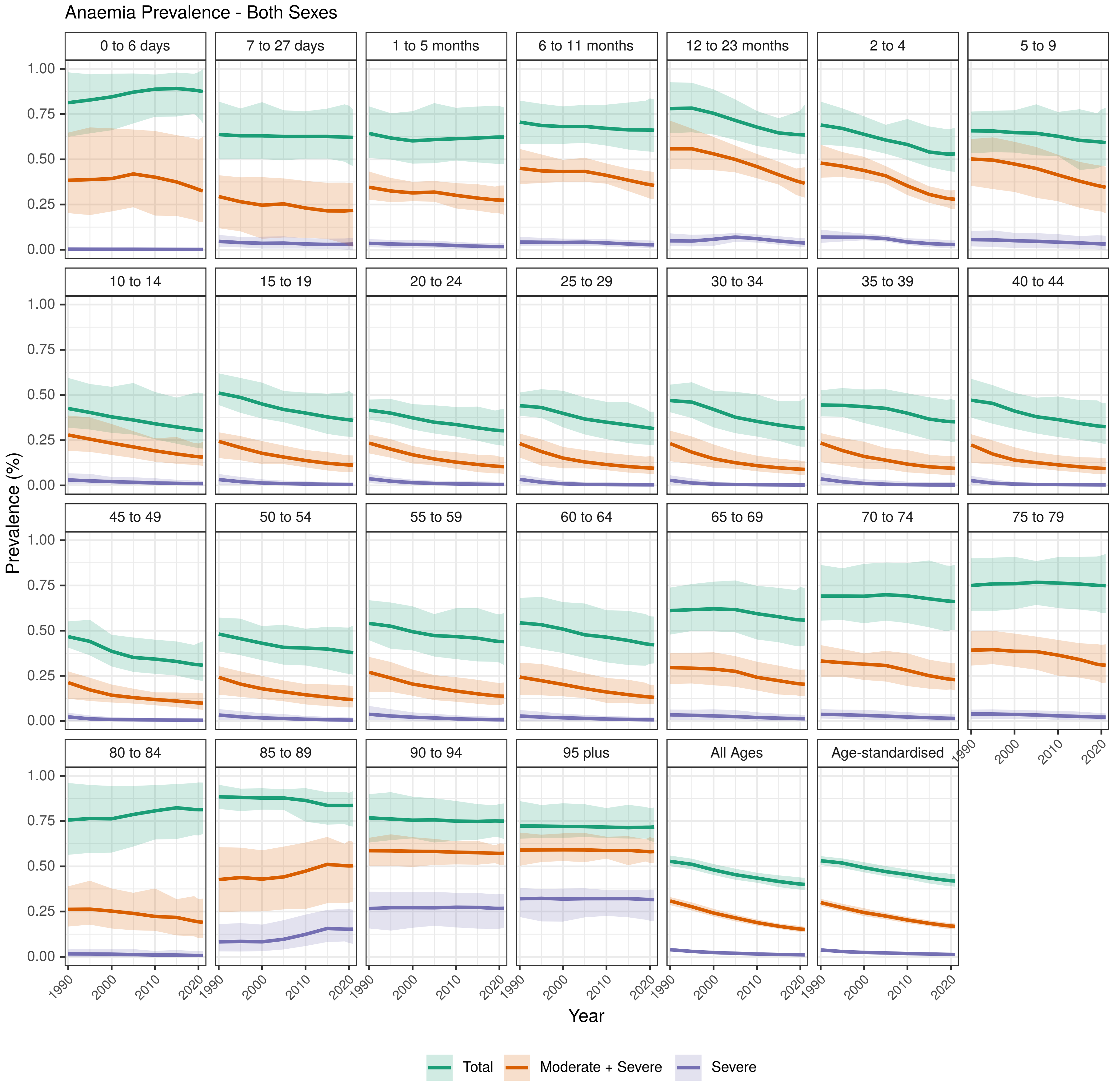

Anaemia Prevalence - Both Sexes
0 to 6 days
7 to 27 days
1 to 5 months
6 to 11 months
12 to 23 months
2 to 4
5 to 9
10 to 14
15 to 19
20 to 24
25 to 29
30 to 34
35 to 39
40 to 44
45 to 49
50 to 54
55 to 59
60 to 64
65 to 69
70 to 74
75 to 79
80 to 84
85 to 89
90 to 94
95 plus
All Ages
Age-standardised
Prevalence (%)
Year
Total
Moderate + Severe
Severe

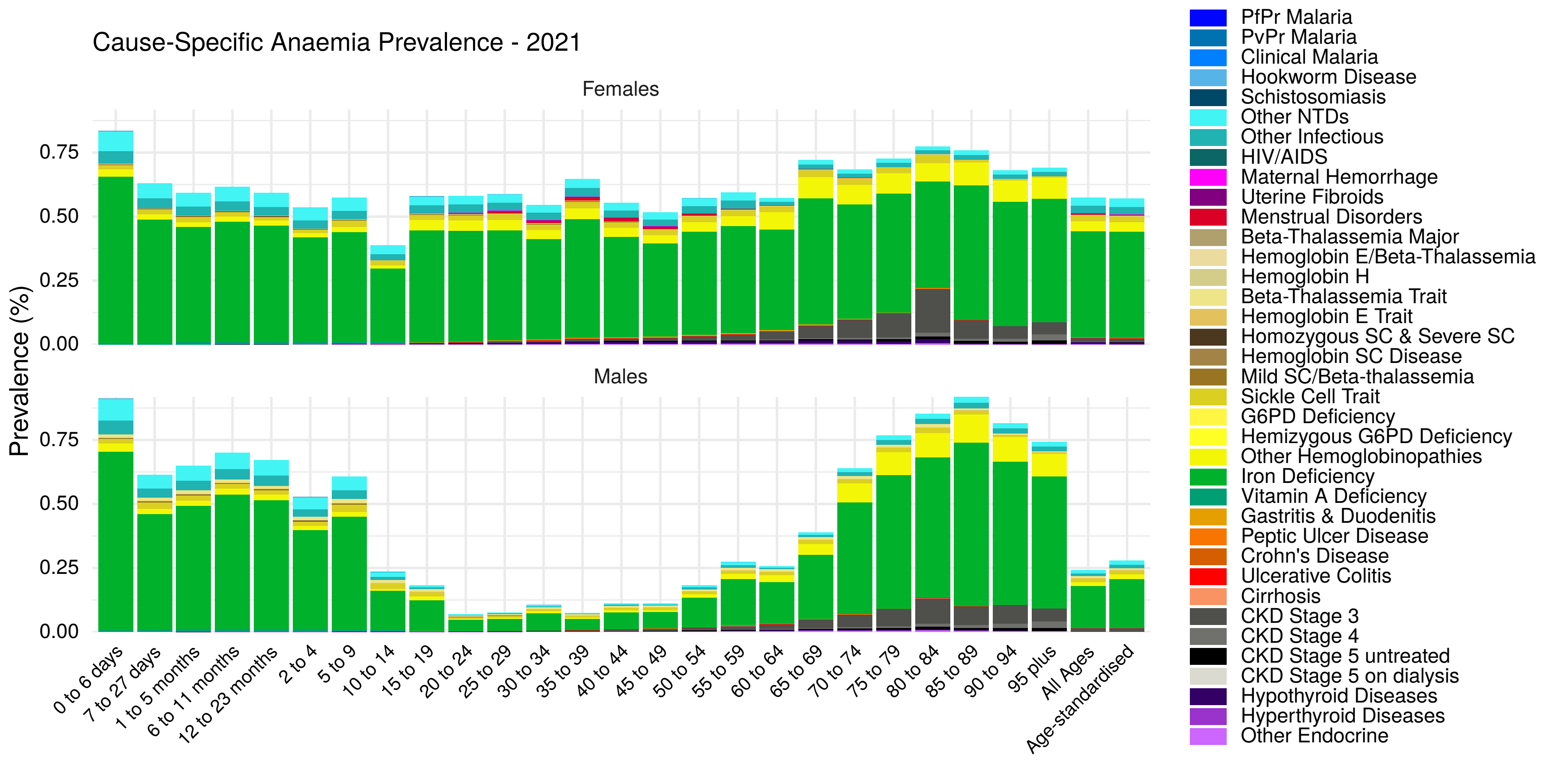

Cause-Specific Anaemia Prevalence - 2021
Females
Males
Prevalence (%)
PfPr Malaria
PvPr Malaria
Clinical Malaria
Hookworm Disease
Schistosomiasis
Other NTDs
Other Infectious
HIV/AIDS
Maternal Hemorrhage
Uterine Fibroids
Menstrual Disorders
Beta-Thalassemia Major
Hemoglobin E/Beta-Thalassemia
Hemoglobin H
Beta-Thalassemia Trait
Hemoglobin E Trait
Homozygous SC & Severe SC
Hemoglobin SC Disease
Mild SC/Beta-thalassemia
Sickle Cell Trait
G6PD Deficiency
Hemizygous G6PD Deficiency
Other Hemoglobinopathies
Iron Deficiency
Vitamin A Deficiency
Gastritis & Duodenitis
Peptic Ulcer Disease
Crohn's Disease
Ulcerative Colitis
Cirrhosis
CKD Stage 3
CKD Stage 4
CKD Stage 5 untreated
CKD Stage 5 on dialysis
Hypothyroid Diseases
Hyperthyroid Diseases
Other Endocrine

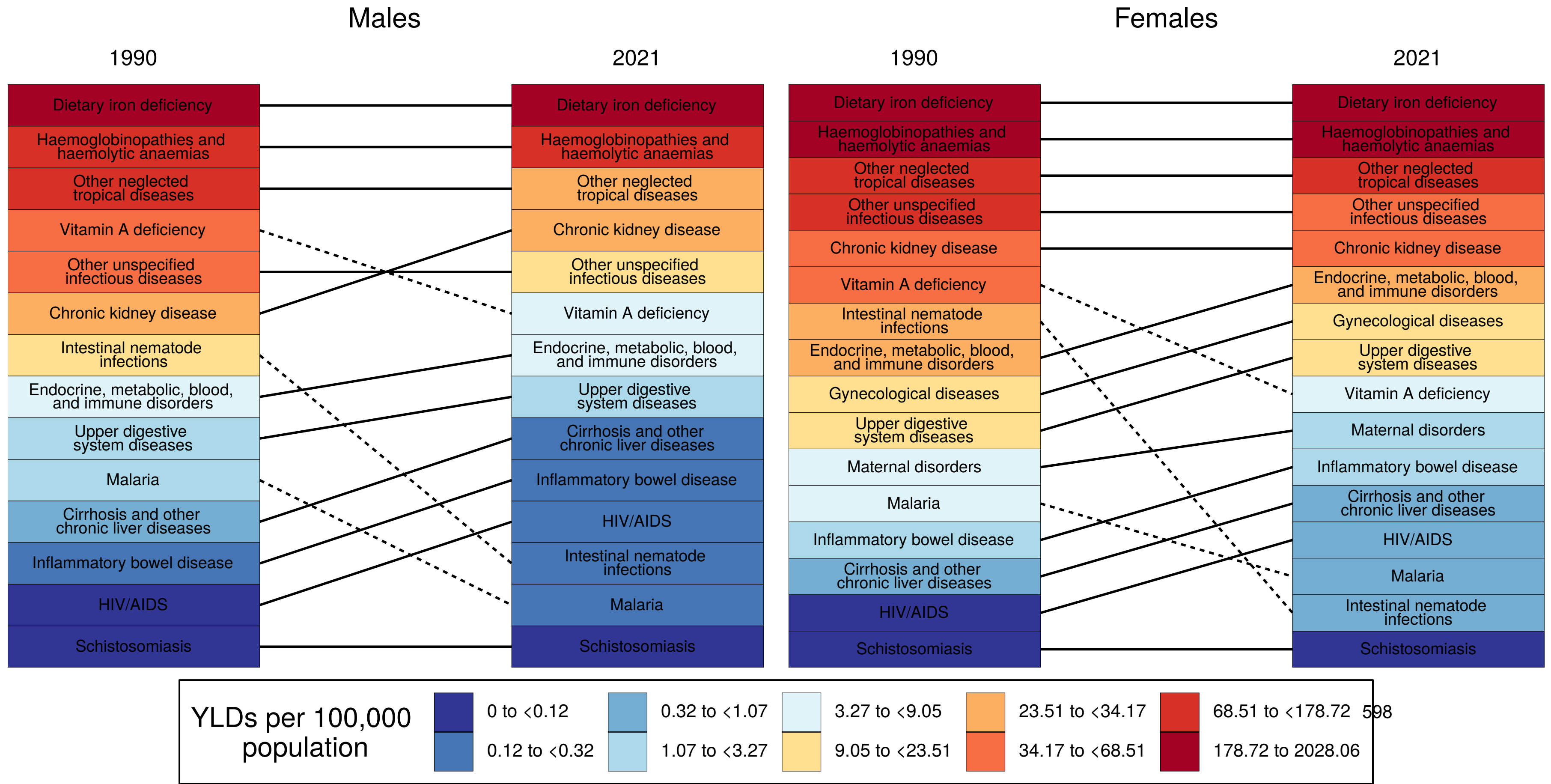

Males
Females
1990
2021
Dietary iron deficiency
Haemoglobinopathies and haemolytic anaemias
Other neglected tropical diseases
Vitamin A deficiency
Other unspecified infectious diseases
Chronic kidney disease
Intestinal nematode infections
Endocrine, metabolic, blood, and immune disorders
Upper digestive system diseases
Malaria
Cirrhosis and other chronic liver diseases
Inflammatory bowel disease
HIV/AIDS
Schistosomiasis
Gynecological diseases
Maternal disorders
YLDs per 100,000 population
0 to <0.12
0.12 to <0.32
0.32 to <1.07
1.07 to <3.27
3.27 to <9.05
9.05 to <23.51
23.51 to <34.17
34.17 to <68.51
68.51 to <178.72
178.72 to 2028.06

Rajasthan

Anaemia Prevalence - Both Sexes

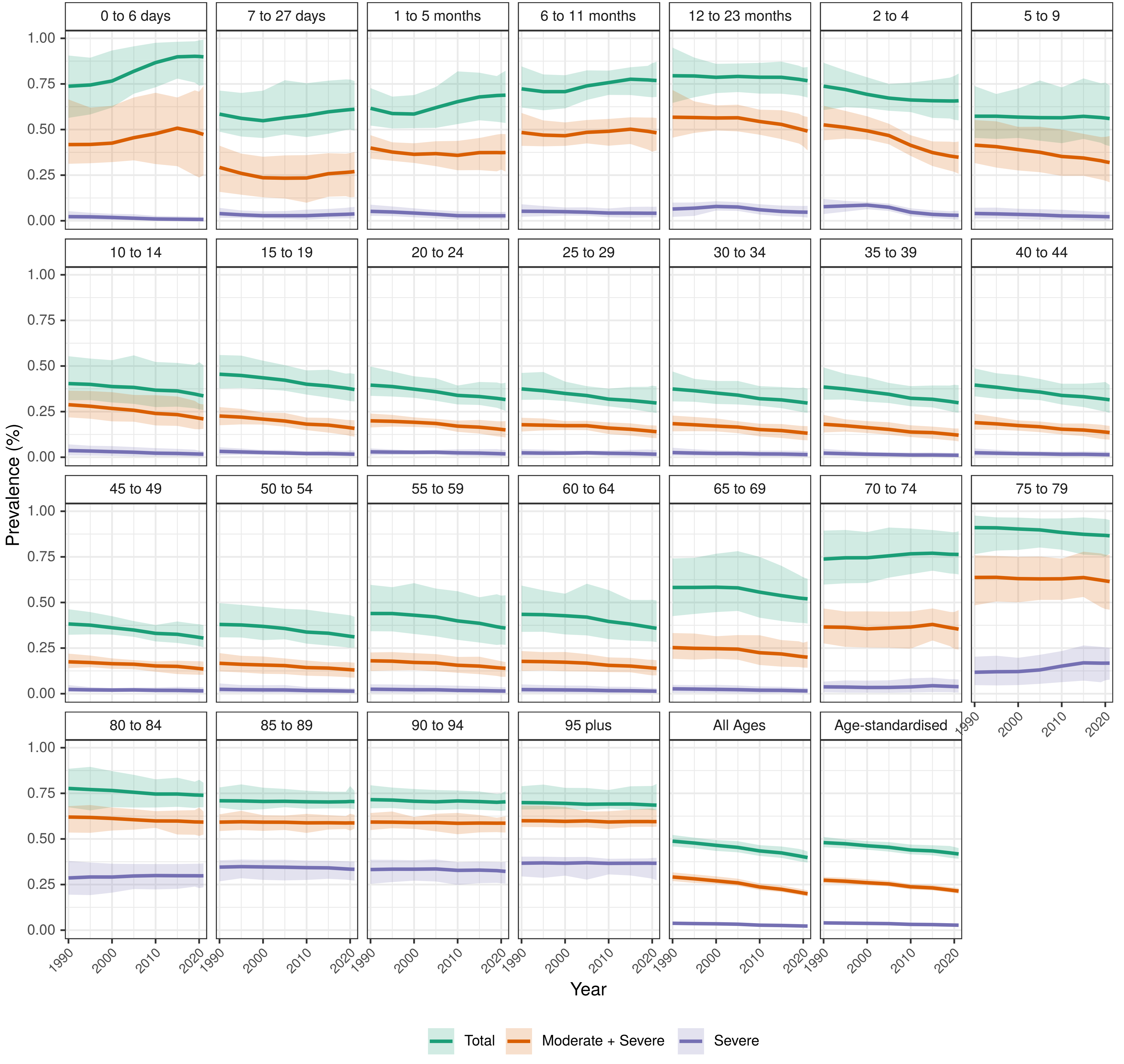

Cause-Specific Anaemia Prevalence - 2021

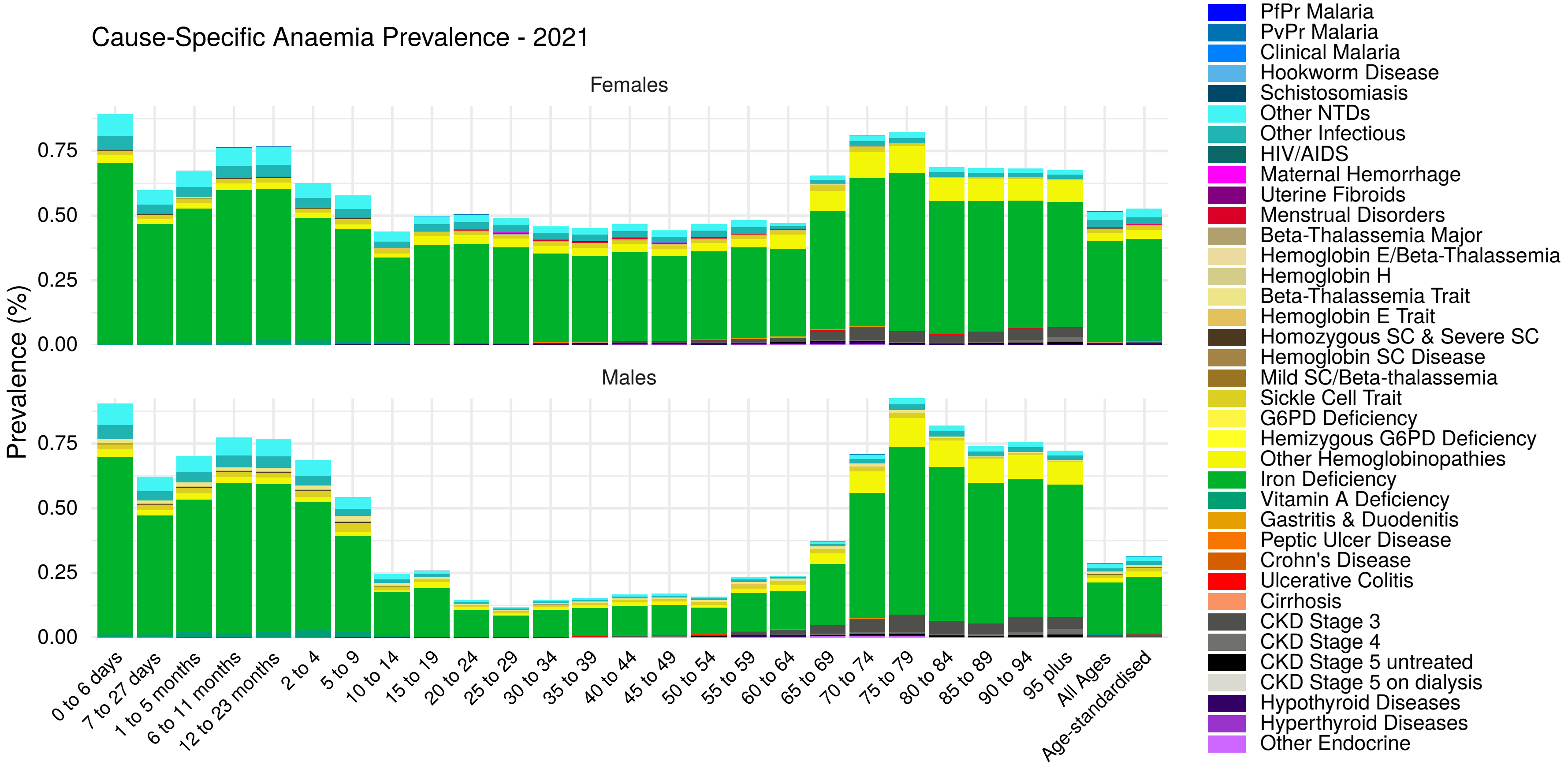

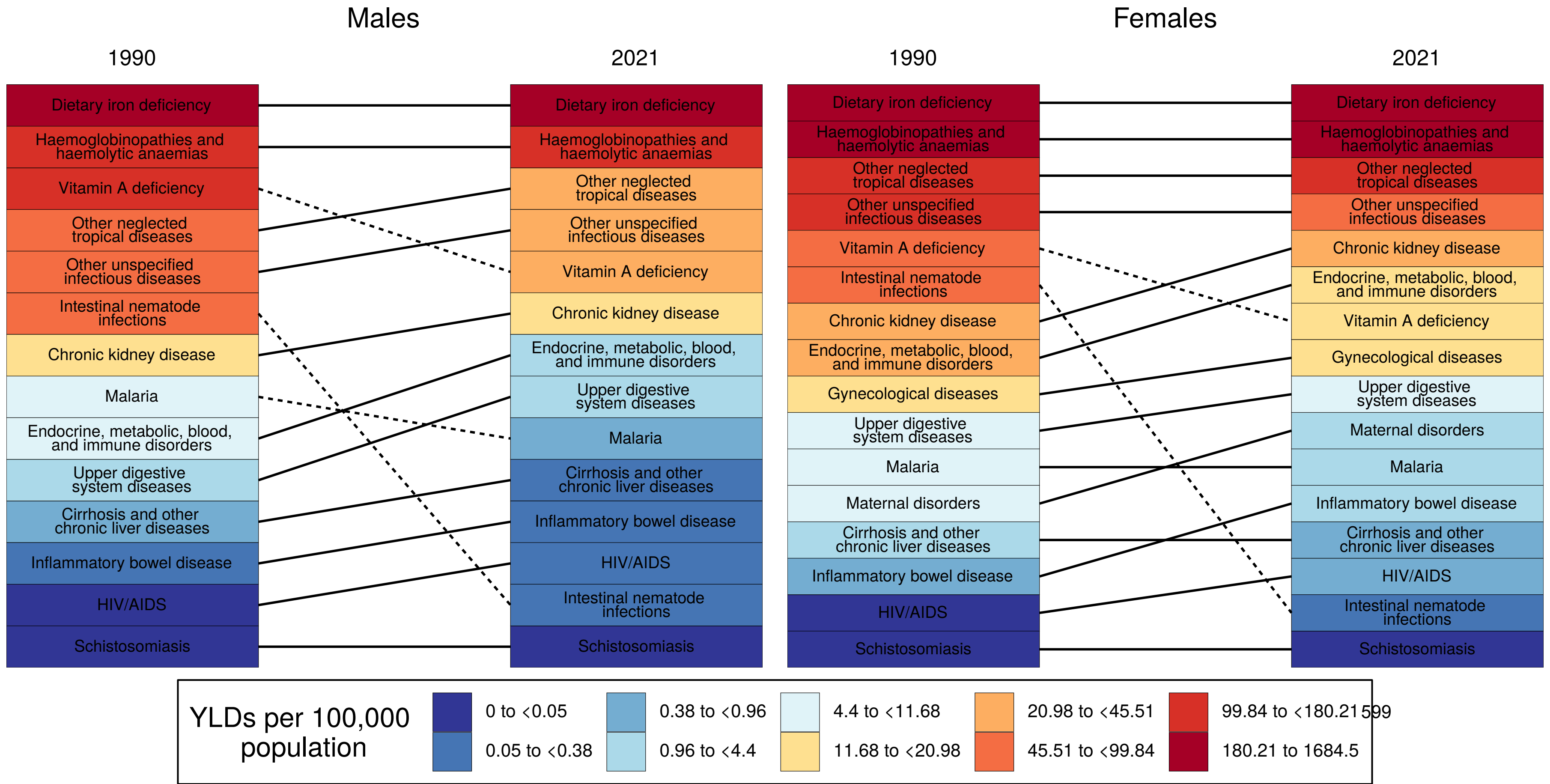

Sikkim

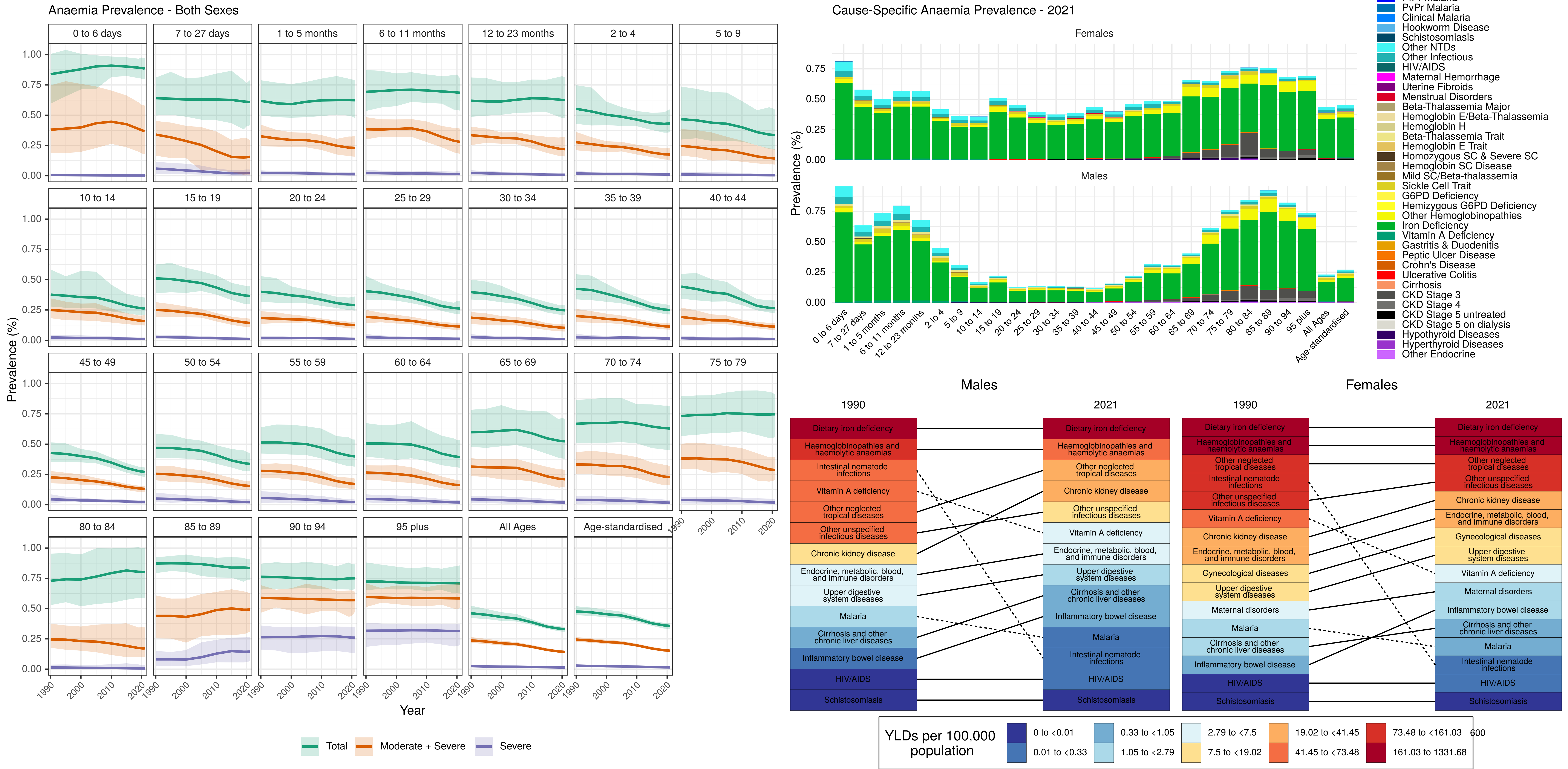
Anaemia Prevalence - Both Sexes
0 to 6 days
7 to 27 days
1 to 5 months
6 to 11 months
12 to 23 months
2 to 4
5 to 9
10 to 14
15 to 19
20 to 24
25 to 29
30 to 34
35 to 39
40 to 44
45 to 49
50 to 54
55 to 59
60 to 64
65 to 69
70 to 74
75 to 79
80 to 84
85 to 89
90 to 94
95 plus
All Ages
Age-standardised
Prevalence (%)
Year
Total
Moderate + Severe
Severe
Cause-Specific Anaemia Prevalence - 2021
Females
Males
Prevalence (%)
PfPr Malaria
PvPr Malaria
Clinical Malaria
Hookworm Disease
Schistosomiasis
Other NTDs
Other Infectious
HIV/AIDS
Maternal Hemorrhage
Uterine Fibroids
Menstrual Disorders
Beta-Thalassemia Major
Hemoglobin E/Beta-Thalassemia
Hemoglobin H
Beta-Thalassemia Trait
Hemoglobin E Trait
Homozygous SC & Severe SC
Hemoglobin SC Disease
Mild SC/Beta-thalassemia
Sickle Cell Trait
G6PD Deficiency
Hemizygous G6PD Deficiency
Other Hemoglobinopathies
Iron Deficiency
Vitamin A Deficiency
Gastritis & Duodenitis
Peptic Ulcer Disease
Crohn's Disease
Ulcerative Colitis
Cirrhosis
CKD Stage 3
CKD Stage 4
CKD Stage 5 untreated
CKD Stage 5 on dialysis
Hypothyroid Diseases
Hyperthyroid Diseases
Other Endocrine
Males
Females
1990
2021
Dietary iron deficiency
Haemoglobinopathies and haemolytic anaemias
Intestinal nematode infections
Vitamin A deficiency
Other neglected tropical diseases
Other unspecified infectious diseases
Chronic kidney disease
Endocrine, metabolic, blood, and immune disorders
Upper digestive system diseases
Malaria
Cirrhosis and other chronic liver diseases
Inflammatory bowel disease
HIV/AIDS
Schistosomiasis
Gynecological diseases
Maternal disorders
YLDs per 100,000 population
0 to <0.01
0.01 to <0.33
0.33 to <1.05
1.05 to <2.79
2.79 to <7.5
7.5 to <19.02
19.02 to <41.45
41.45 to <73.48
73.48 to <161.03
161.03 to 1331.68

Tamil Nadu

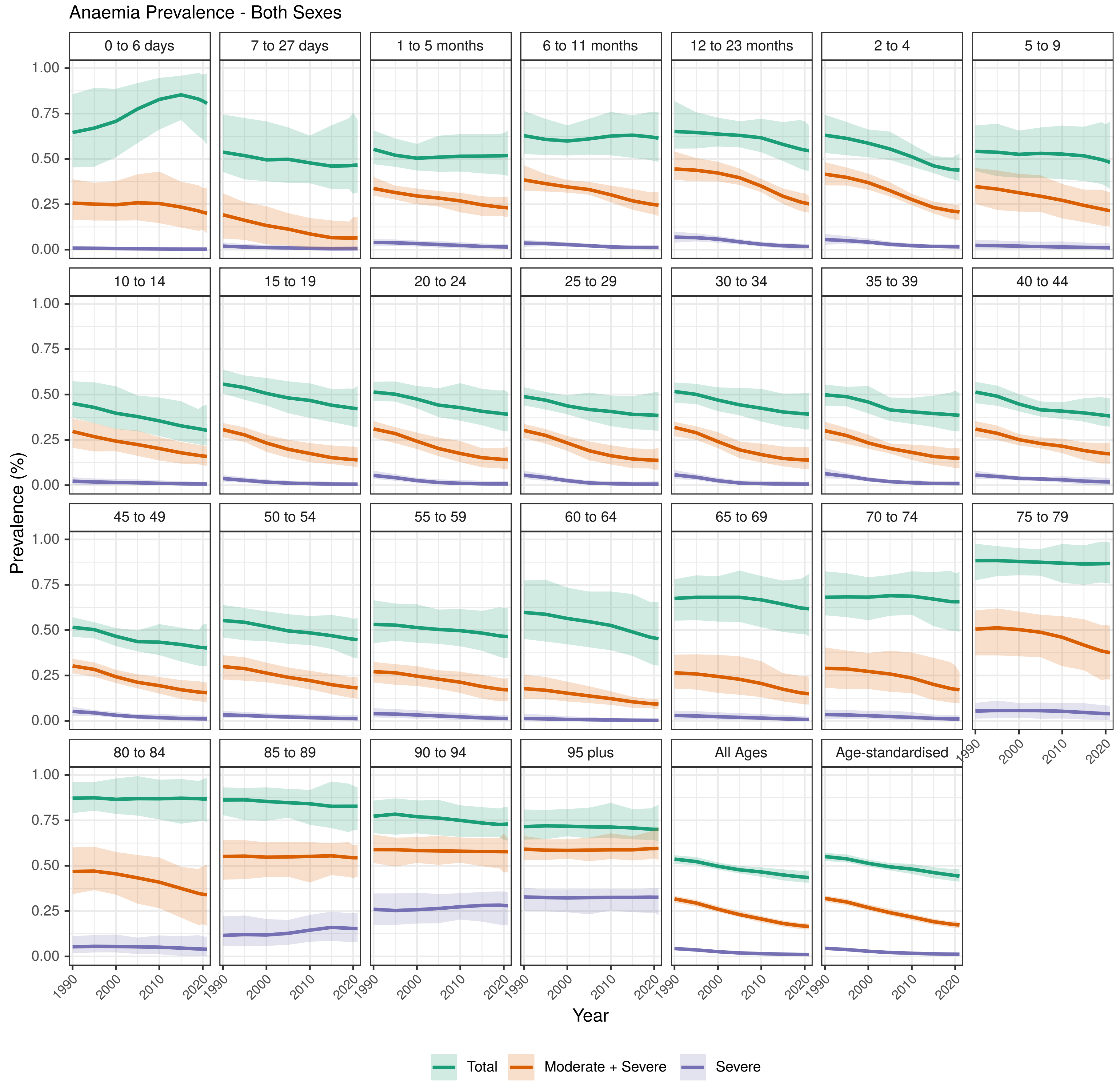

Anaemia Prevalence - Both Sexes
0 to 6 days
7 to 27 days
1 to 5 months
6 to 11 months
12 to 23 months
2 to 4
5 to 9
10 to 14
15 to 19
20 to 24
25 to 29
30 to 34
35 to 39
40 to 44
45 to 49
50 to 54
55 to 59
60 to 64
65 to 69
70 to 74
75 to 79
80 to 84
85 to 89
90 to 94
95 plus
All Ages
Age-standardised
Prevalence (%)
0.00
0.25
0.50
0.75
1.00
1990
2000
2010
2020
Year
Total
Moderate + Severe
Severe

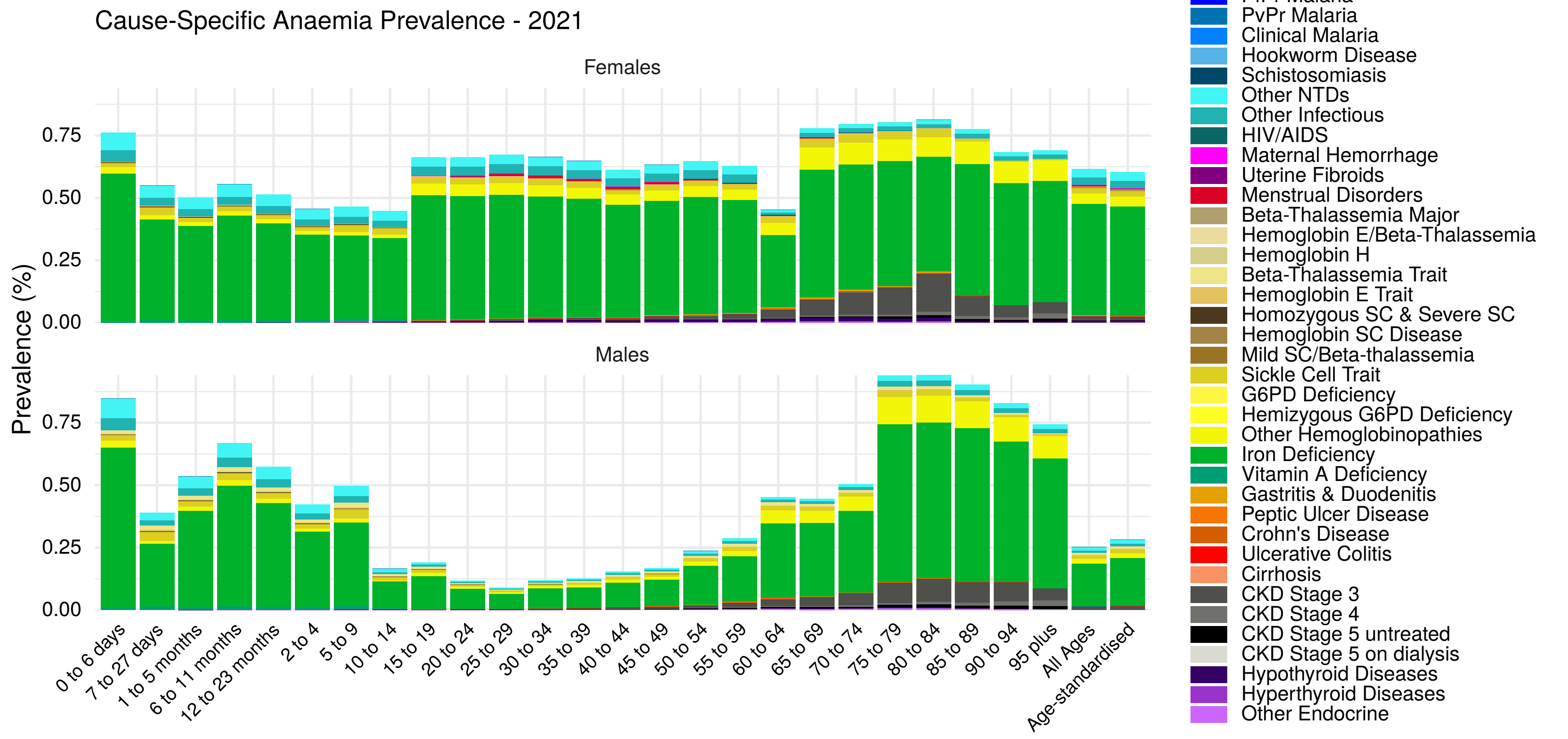

Cause-Specific Anaemia Prevalence - 2021
Females
Males
Prevalence (%)
0.00
0.25
0.50
0.75
0 to 6 days
7 to 27 days
1 to 5 months
6 to 11 months
12 to 23 months
2 to 4
5 to 9
10 to 14
15 to 19
20 to 24
25 to 29
30 to 34
35 to 39
40 to 44
45 to 49
50 to 54
55 to 59
60 to 64
65 to 69
70 to 74
75 to 79
80 to 84
85 to 89
90 to 94
95 plus
All Ages
Age-standardised
PfPr Malaria
PvPr Malaria
Clinical Malaria
Hookworm Disease
Schistosomiasis
Other NTDs
Other Infectious
HIV/AIDS
Maternal Hemorrhage
Uterine Fibroids
Menstrual Disorders
Beta-Thalassemia Major
Hemoglobin E/Beta-Thalassemia
Hemoglobin H
Beta-Thalassemia Trait
Hemoglobin E Trait
Homozygous SC & Severe SC
Hemoglobin SC Disease
Mild SC/Beta-thalassemia
Sickle Cell Trait
G6PD Deficiency
Hemizygous G6PD Deficiency
Other Hemoglobinopathies
Iron Deficiency
Vitamin A Deficiency
Gastritis & Duodenitis
Peptic Ulcer Disease
Crohn's Disease
Ulcerative Colitis
Cirrhosis
CKD Stage 3
CKD Stage 4
CKD Stage 5 untreated
CKD Stage 5 on dialysis
Hypothyroid Diseases
Hyperthyroid Diseases
Other Endocrine

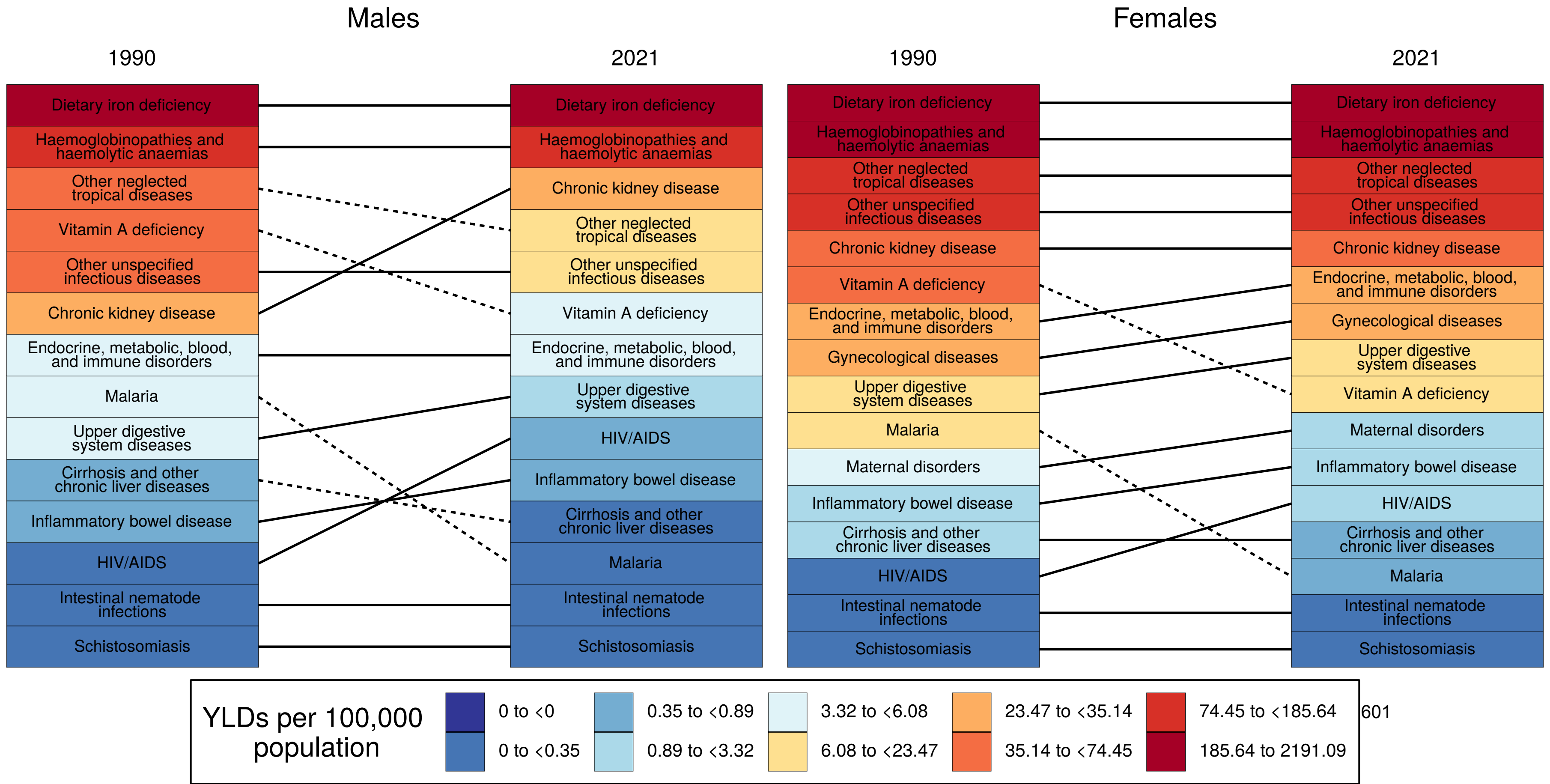

Males
Females
1990
2021
Dietary iron deficiency
Haemoglobinopathies and haemolytic anaemias
Other neglected tropical diseases
Vitamin A deficiency
Other unspecified infectious diseases
Chronic kidney disease
Endocrine, metabolic, blood, and immune disorders
Malaria
Upper digestive system diseases
Cirrhosis and other chronic liver diseases
Inflammatory bowel disease
HIV/AIDS
Intestinal nematode infections
Schistosomiasis
Gynecological diseases
Maternal disorders
YLDs per 100,000 population
0 to <0
0 to <0.35
0.35 to <0.89
0.89 to <3.32
3.32 to <6.08
6.08 to <23.47
23.47 to <35.14
35.14 to <74.45
74.45 to <185.64
185.64 to 2191.09

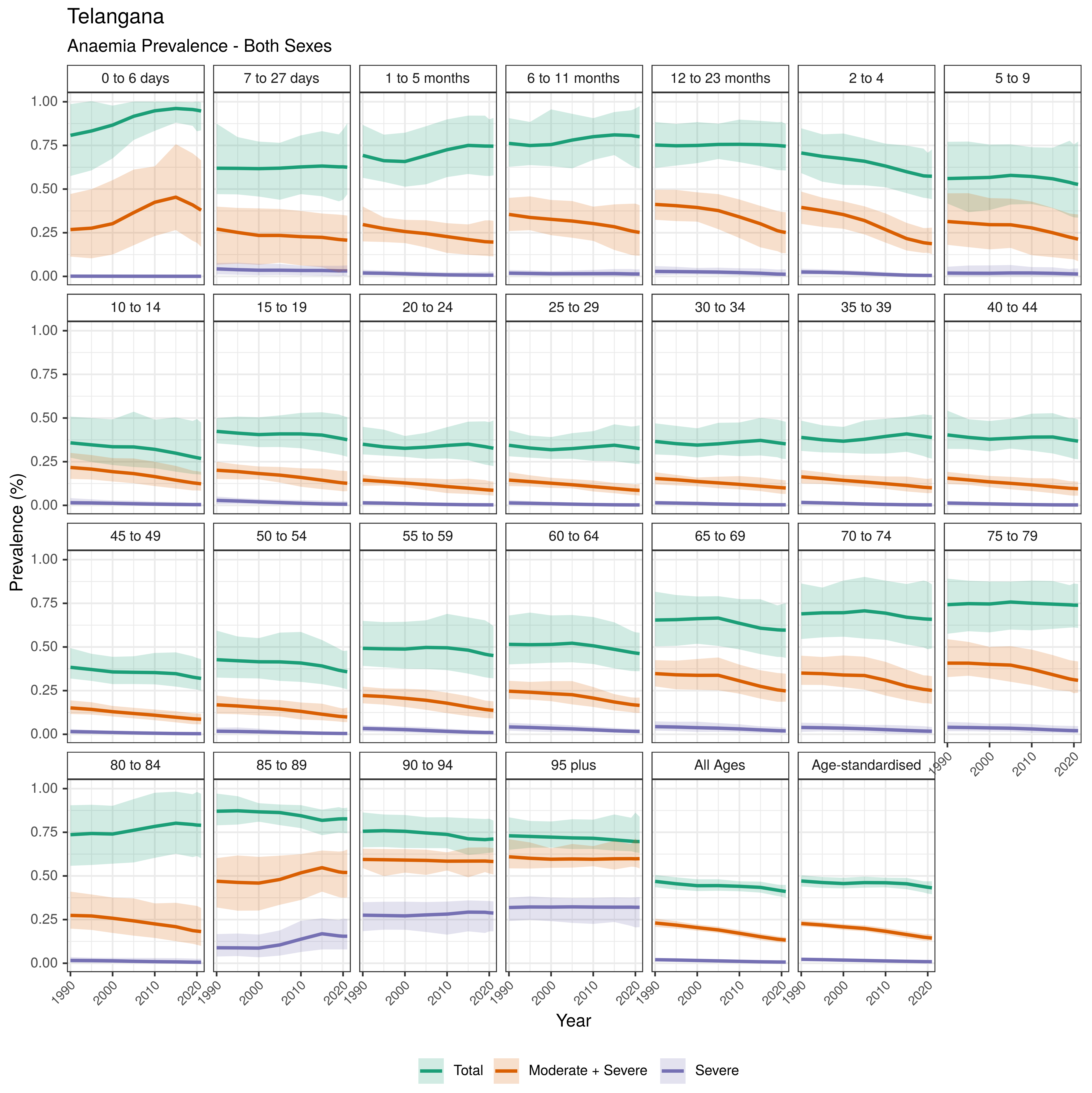

Telangana
Anaemia Prevalence - Both Sexes
0 to 6 days
7 to 27 days
1 to 5 months
6 to 11 months
12 to 23 months
2 to 4
5 to 9
10 to 14
15 to 19
20 to 24
25 to 29
30 to 34
35 to 39
40 to 44
45 to 49
50 to 54
55 to 59
60 to 64
65 to 69
70 to 74
75 to 79
80 to 84
85 to 89
90 to 94
95 plus
All Ages
Age-standardised
Prevalence (%)
1.00
0.75
0.50
0.25
0.00
1990
2000
2010
2020
Year
Total
Moderate + Severe
Severe

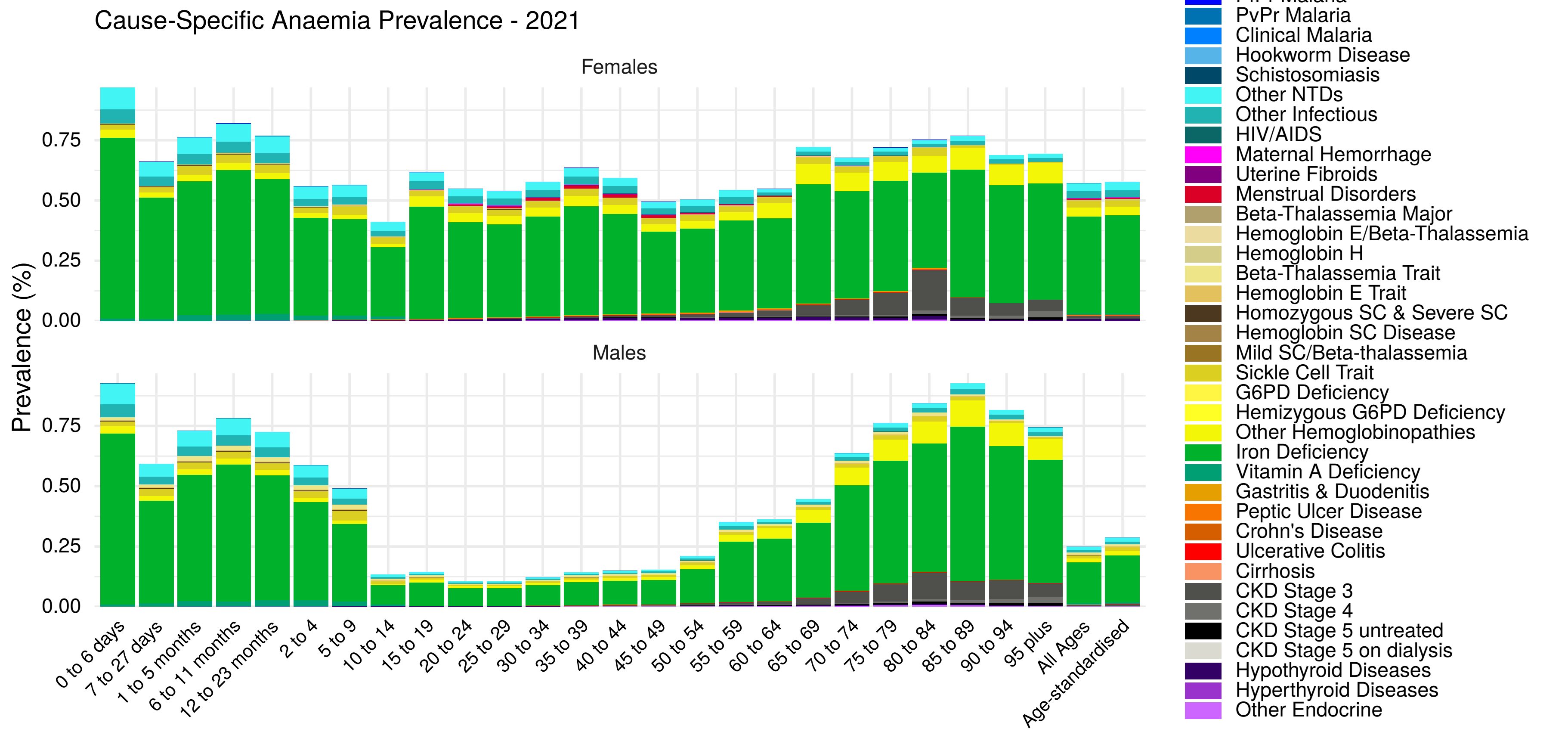

Cause-Specific Anaemia Prevalence - 2021
Females
Males
Prevalence (%)
0.75
0.50
0.25
0.00
0 to 6 days
7 to 27 days
1 to 5 months
6 to 11 months
12 to 23 months
2 to 4
5 to 9
10 to 14
15 to 19
20 to 24
25 to 29
30 to 34
35 to 39
40 to 44
45 to 49
50 to 54
55 to 59
60 to 64
65 to 69
70 to 74
75 to 79
80 to 84
85 to 89
90 to 94
95 plus
All Ages
Age-standardised
PfPr Malaria
PvPr Malaria
Clinical Malaria
Hookworm Disease
Schistosomiasis
Other NTDs
Other Infectious
HIV/AIDS
Maternal Hemorrhage
Uterine Fibroids
Menstrual Disorders
Beta-Thalassemia Major
Hemoglobin E/Beta-Thalassemia
Hemoglobin H
Beta-Thalassemia Trait
Hemoglobin E Trait
Homozygous SC & Severe SC
Hemoglobin SC Disease
Mild SC/Beta-thalassemia
Sickle Cell Trait
G6PD Deficiency
Hemizygous G6PD Deficiency
Other Hemoglobinopathies
Iron Deficiency
Vitamin A Deficiency
Gastritis & Duodenitis
Peptic Ulcer Disease
Crohn's Disease
Ulcerative Colitis
Cirrhosis
CKD Stage 3
CKD Stage 4
CKD Stage 5 untreated
CKD Stage 5 on dialysis
Hypothyroid Diseases
Hyperthyroid Diseases
Other Endocrine

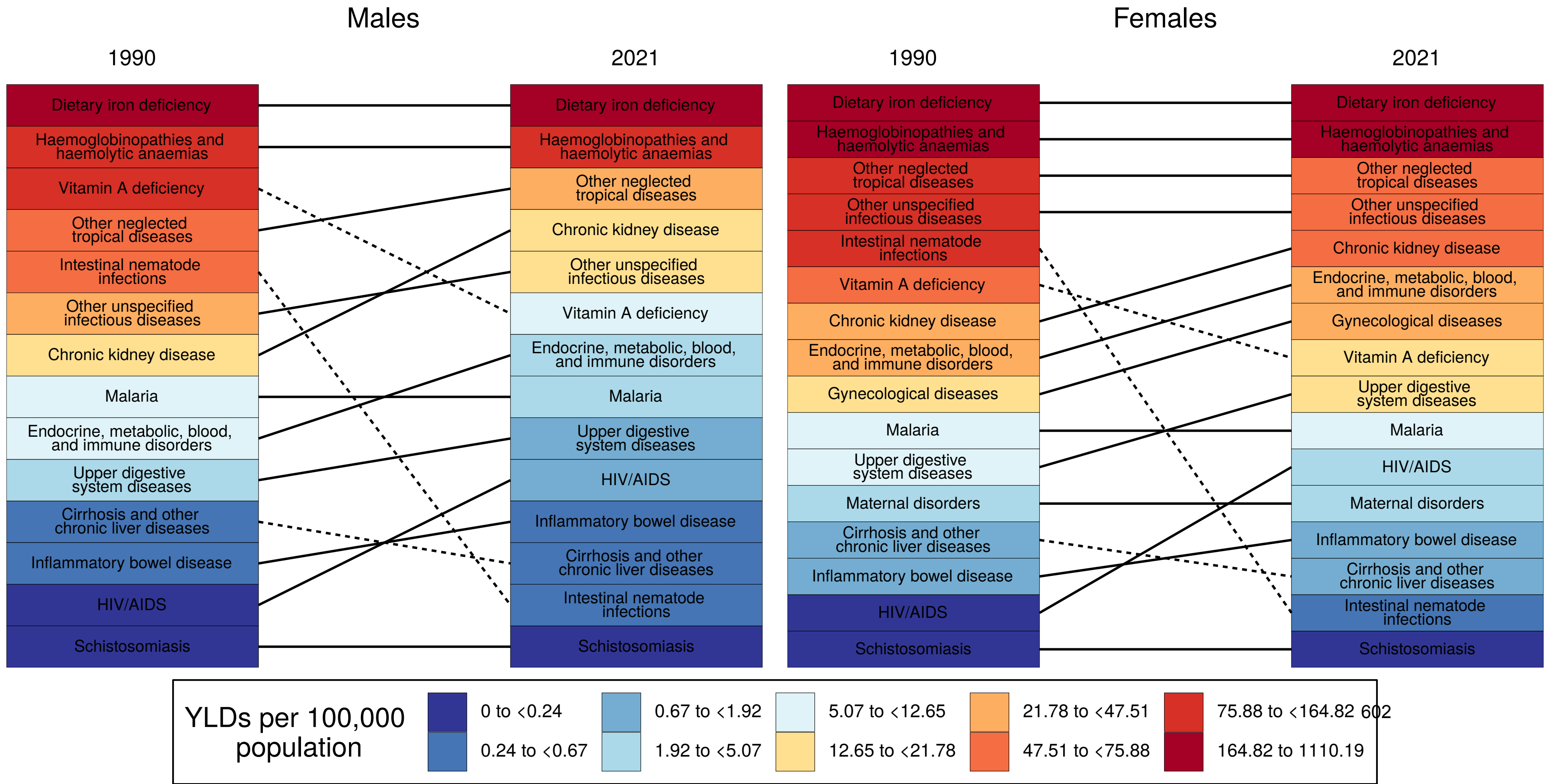

Males
Females
1990
2021
Dietary iron deficiency
Haemoglobinopathies and haemolytic anaemias
Vitamin A deficiency
Other neglected tropical diseases
Intestinal nematode infections
Other unspecified infectious diseases
Chronic kidney disease
Malaria
Endocrine, metabolic, blood, and immune disorders
Upper digestive system diseases
Cirrhosis and other chronic liver diseases
Inflammatory bowel disease
HIV/AIDS
Schistosomiasis
Gynecological diseases
Maternal disorders
YLDs per 100,000 population
0 to <0.24
0.24 to <0.67
0.67 to <1.92
1.92 to <5.07
5.07 to <12.65
12.65 to <21.78
21.78 to <47.51
47.51 to <75.88
75.88 to <164.82
164.82 to 1110.19

Tripura

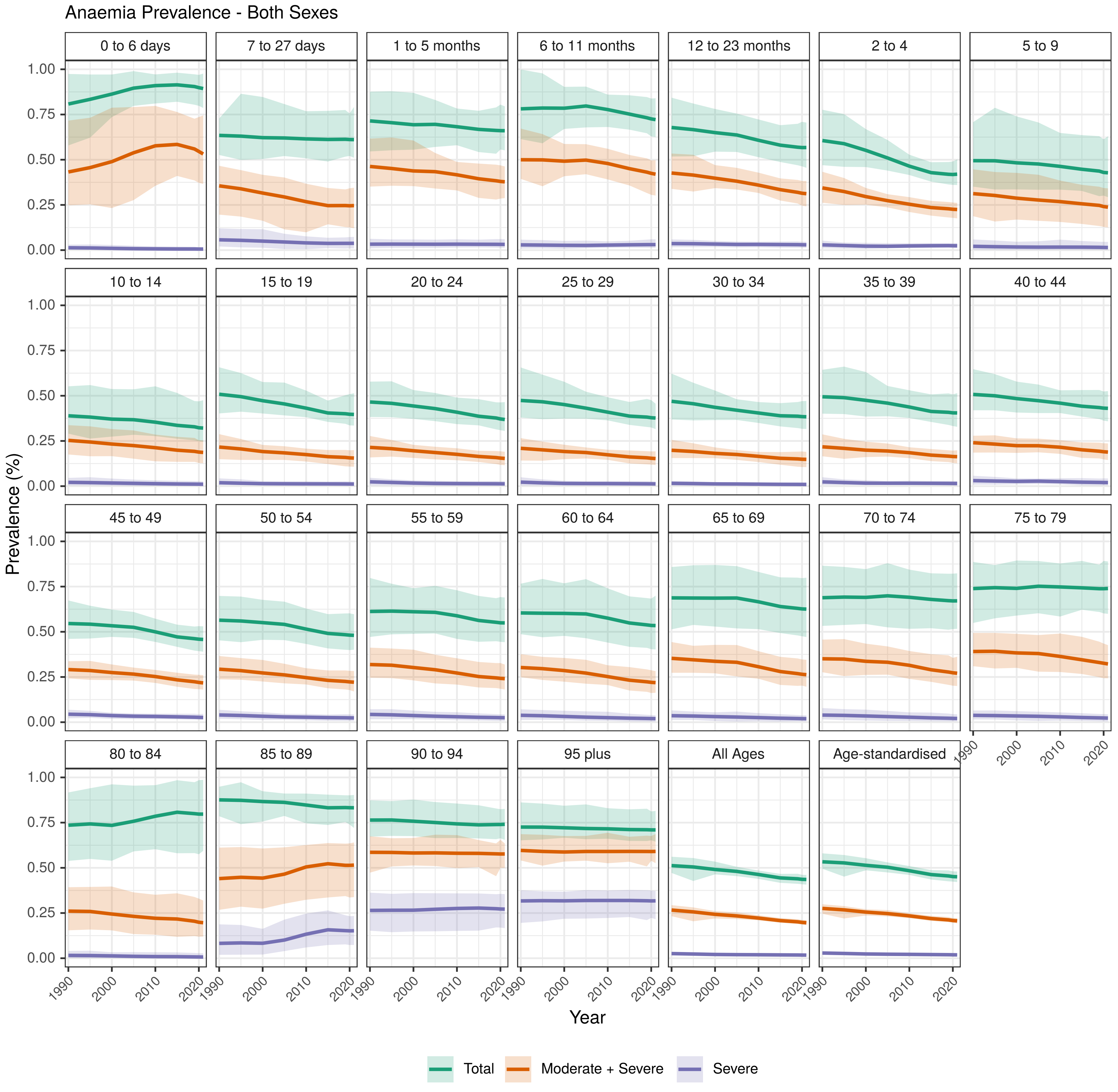
Anaemia Prevalence - Both Sexes
0 to 6 days
7 to 27 days
1 to 5 months
6 to 11 months
12 to 23 months
2 to 4
5 to 9
10 to 14
15 to 19
20 to 24
25 to 29
30 to 34
35 to 39
40 to 44
45 to 49
50 to 54
55 to 59
60 to 64
65 to 69
70 to 74
75 to 79
80 to 84
85 to 89
90 to 94
95 plus
All Ages
Age-standardised
Prevalence (%)
0.00
0.25
0.50
0.75
1.00
1990
2000
2010
2020
Year
Total
Moderate + Severe
Severe

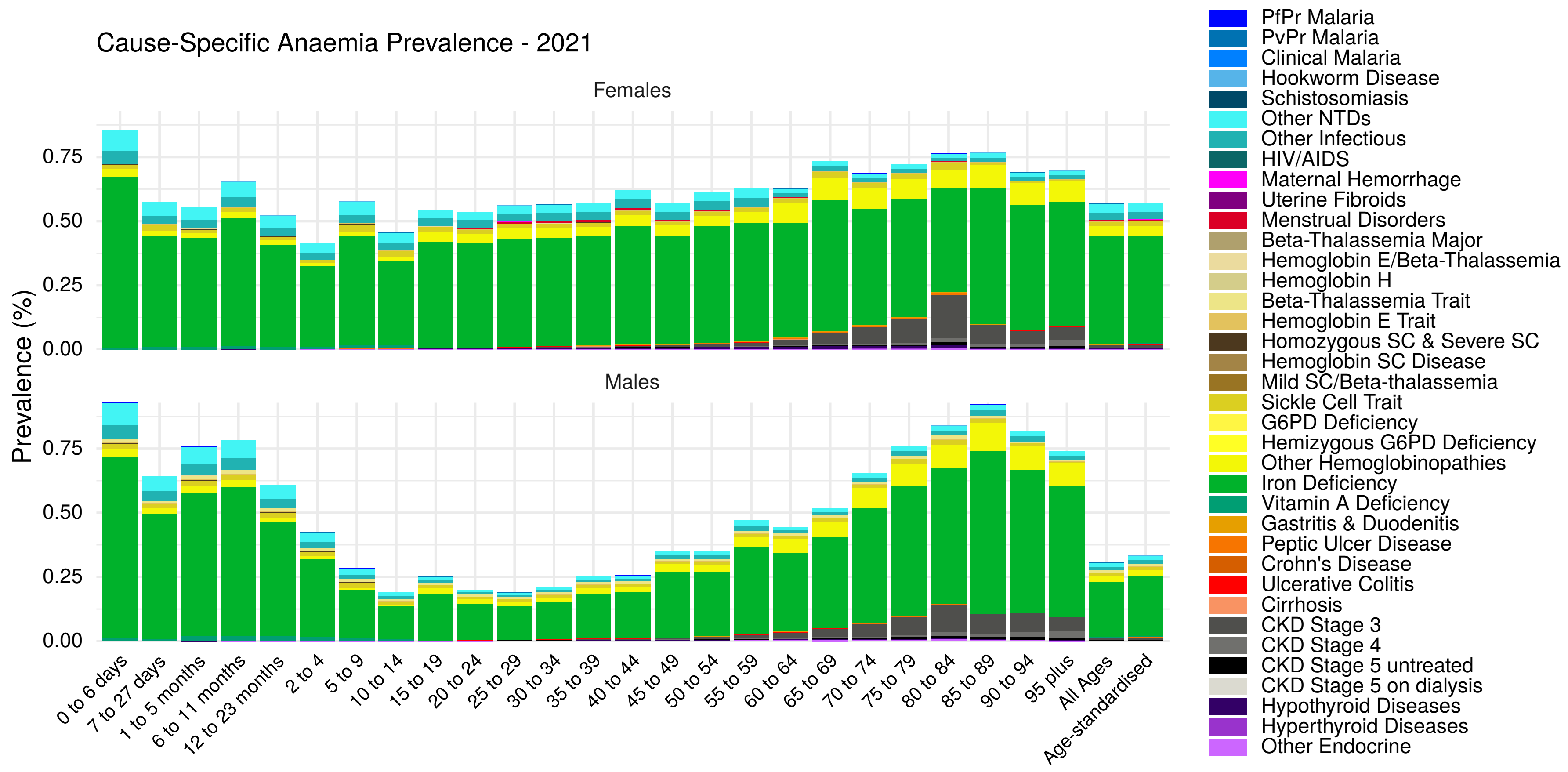
Cause-Specific Anaemia Prevalence - 2021
Females
Males
Prevalence (%)
0.00
0.25
0.50
0.75
0 to 6 days
7 to 27 days
1 to 5 months
6 to 11 months
12 to 23 months
2 to 4
5 to 9
10 to 14
15 to 19
20 to 24
25 to 29
30 to 34
35 to 39
40 to 44
45 to 49
50 to 54
55 to 59
60 to 64
65 to 69
70 to 74
75 to 79
80 to 84
85 to 89
90 to 94
95 plus
All Ages
Age-standardised
PfPr Malaria
PvPr Malaria
Clinical Malaria
Hookworm Disease
Schistosomiasis
Other NTDs
Other Infectious
HIV/AIDS
Maternal Hemorrhage
Uterine Fibroids
Menstrual Disorders
Beta-Thalassemia Major
Hemoglobin E/Beta-Thalassemia
Hemoglobin H
Beta-Thalassemia Trait
Hemoglobin E Trait
Homozygous SC & Severe SC
Hemoglobin SC Disease
Mild SC/Beta-thalassemia
Sickle Cell Trait
G6PD Deficiency
Hemizygous G6PD Deficiency
Other Hemoglobinopathies
Iron Deficiency
Vitamin A Deficiency
Gastritis & Duodenitis
Peptic Ulcer Disease
Crohn's Disease
Ulcerative Colitis
Cirrhosis
CKD Stage 3
CKD Stage 4
CKD Stage 5 untreated
CKD Stage 5 on dialysis
Hypothyroid Diseases
Hyperthyroid Diseases
Other Endocrine

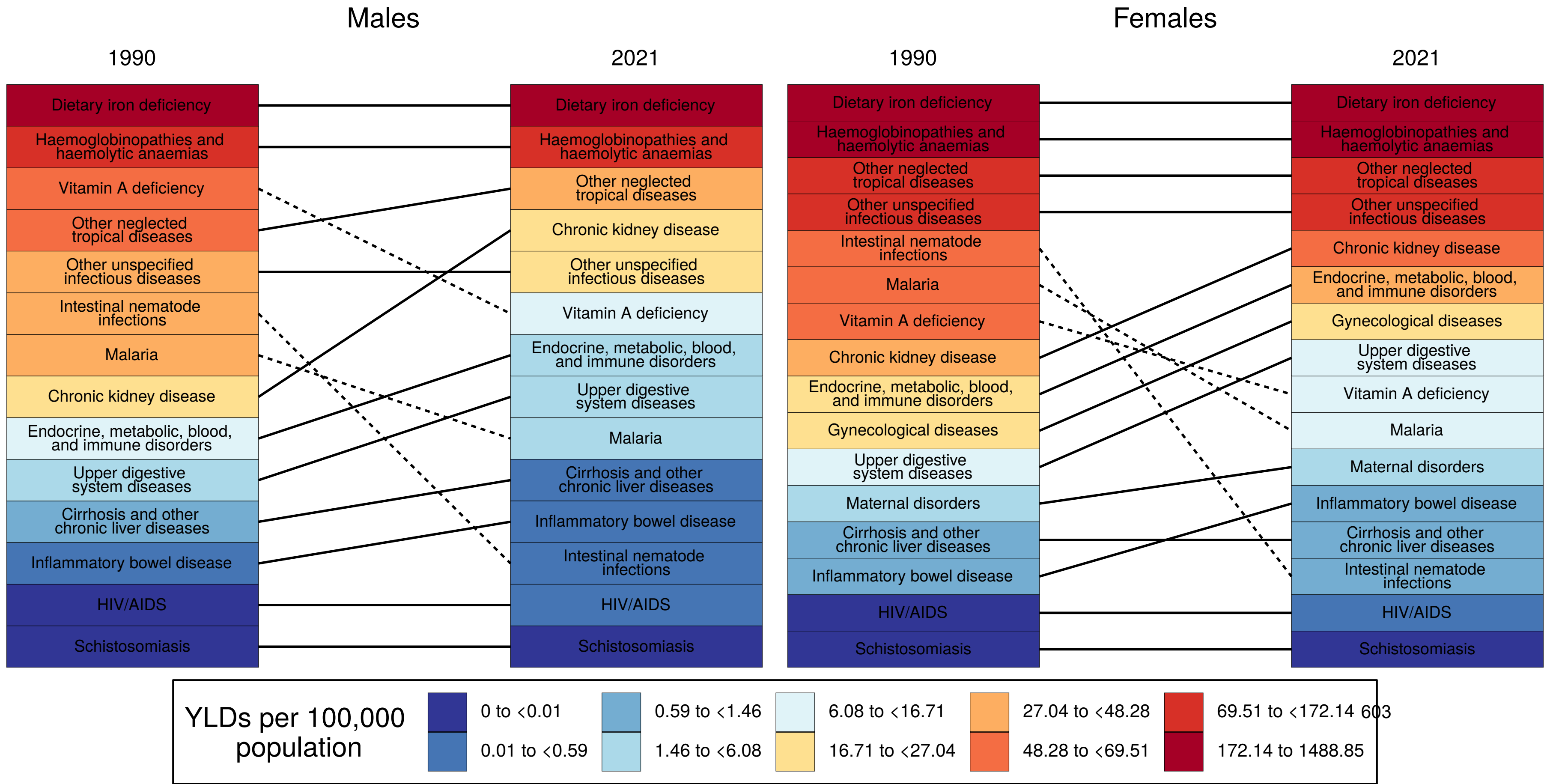
Males
1990
Dietary iron deficiency
Haemoglobinopathies and haemolytic anaemias
Vitamin A deficiency
Other neglected tropical diseases
Other unspecified infectious diseases
Intestinal nematode infections
Malaria
Chronic kidney disease
Endocrine, metabolic, blood, and immune disorders
Upper digestive system diseases
Cirrhosis and other chronic liver diseases
Inflammatory bowel disease
HIV/AIDS
Schistosomiasis
2021
Dietary iron deficiency
Haemoglobinopathies and haemolytic anaemias
Other neglected tropical diseases
Chronic kidney disease
Other unspecified infectious diseases
Vitamin A deficiency
Endocrine, metabolic, blood, and immune disorders
Upper digestive system diseases
Malaria
Cirrhosis and other chronic liver diseases
Inflammatory bowel disease
Intestinal nematode infections
HIV/AIDS
Schistosomiasis
Females
1990
Dietary iron deficiency
Haemoglobinopathies and haemolytic anaemias
Other neglected tropical diseases
Other unspecified infectious diseases
Intestinal nematode infections
Malaria
Vitamin A deficiency
Chronic kidney disease
Endocrine, metabolic, blood, and immune disorders
Gynecological diseases
Upper digestive system diseases
Maternal disorders
Cirrhosis and other chronic liver diseases
Inflammatory bowel disease
HIV/AIDS
Schistosomiasis
2021
Dietary iron deficiency
Haemoglobinopathies and haemolytic anaemias
Other neglected tropical diseases
Other unspecified infectious diseases
Chronic kidney disease
Endocrine, metabolic, blood, and immune disorders
Gynecological diseases
Upper digestive system diseases
Vitamin A deficiency
Malaria
Maternal disorders
Inflammatory bowel disease
Cirrhosis and other chronic liver diseases
Intestinal nematode infections
HIV/AIDS
Schistosomiasis
YLDs per 100,000 population
0 to <0.01
0.01 to <0.59
0.59 to <1.46
1.46 to <6.08
6.08 to <16.71
16.71 to <27.04
27.04 to <48.28
48.28 to <69.51
69.51 to <172.14
172.14 to 1488.85

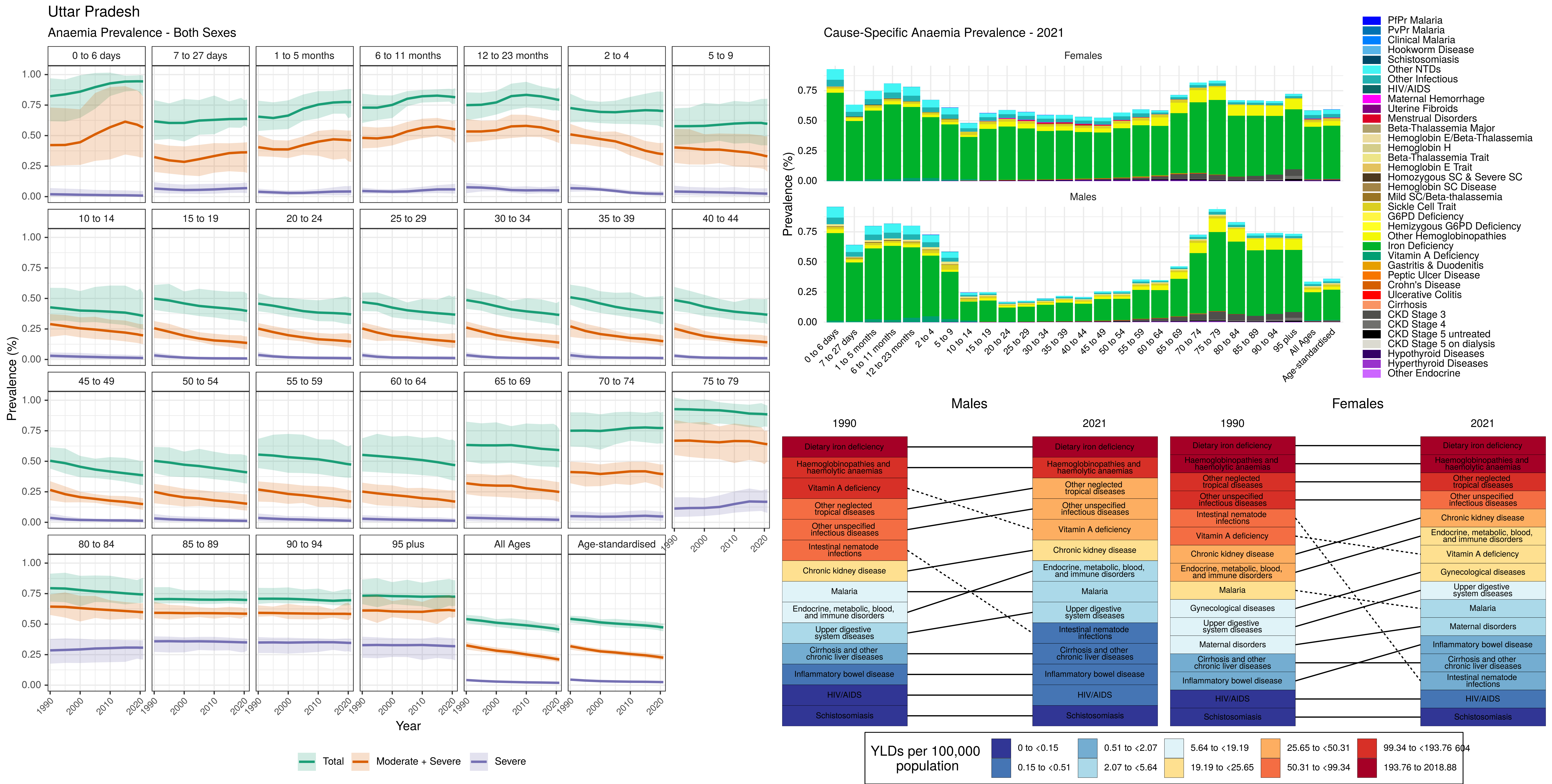

Uttar Pradesh
Anaemia Prevalence - Both Sexes
0 to 6 days
7 to 27 days
1 to 5 months
6 to 11 months
12 to 23 months
2 to 4
5 to 9
10 to 14
15 to 19
20 to 24
25 to 29
30 to 34
35 to 39
40 to 44
45 to 49
50 to 54
55 to 59
60 to 64
65 to 69
70 to 74
75 to 79
80 to 84
85 to 89
90 to 94
95 plus
All Ages
Age-standardised
Prevalence (%)
Year
Total
Moderate + Severe
Severe
Cause-Specific Anaemia Prevalence - 2021
Females
Males
PfPr Malaria
PvPr Malaria
Clinical Malaria
Hookworm Disease
Schistosomiasis
Other NTDs
Other Infectious
HIV/AIDS
Maternal Hemorrhage
Uterine Fibroids
Menstrual Disorders
Beta-Thalassemia Major
Hemoglobin E/Beta-Thalassemia
Hemoglobin H
Beta-Thalassemia Trait
Hemoglobin E Trait
Homozygous SC & Severe SC
Hemoglobin SC Disease
Mild SC/Beta-thalassemia
Sickle Cell Trait
G6PD Deficiency
Hemizygous G6PD Deficiency
Other Hemoglobinopathies
Iron Deficiency
Vitamin A Deficiency
Gastritis & Duodenitis
Peptic Ulcer Disease
Crohn's Disease
Ulcerative Colitis
Cirrhosis
CKD Stage 3
CKD Stage 4
CKD Stage 5 untreated
CKD Stage 5 on dialysis
Hypothyroid Diseases
Hyperthyroid Diseases
Other Endocrine
Males
Females
1990
2021
Dietary iron deficiency
Haemoglobinopathies and haemolytic anaemias
Vitamin A deficiency
Other neglected tropical diseases
Other unspecified infectious diseases
Intestinal nematode infections
Chronic kidney disease
Malaria
Endocrine, metabolic, blood, and immune disorders
Upper digestive system diseases
Cirrhosis and other chronic liver diseases
Inflammatory bowel disease
HIV/AIDS
Schistosomiasis
Gynecological diseases
Maternal disorders
YLDs per 100,000 population
0 to <0.15
0.15 to <0.51
0.51 to <2.07
2.07 to <5.64
5.64 to <19.19
19.19 to <25.65
25.65 to <50.31
50.31 to <99.34
99.34 to <193.76
193.76 to 2018.88

Uttarakhand

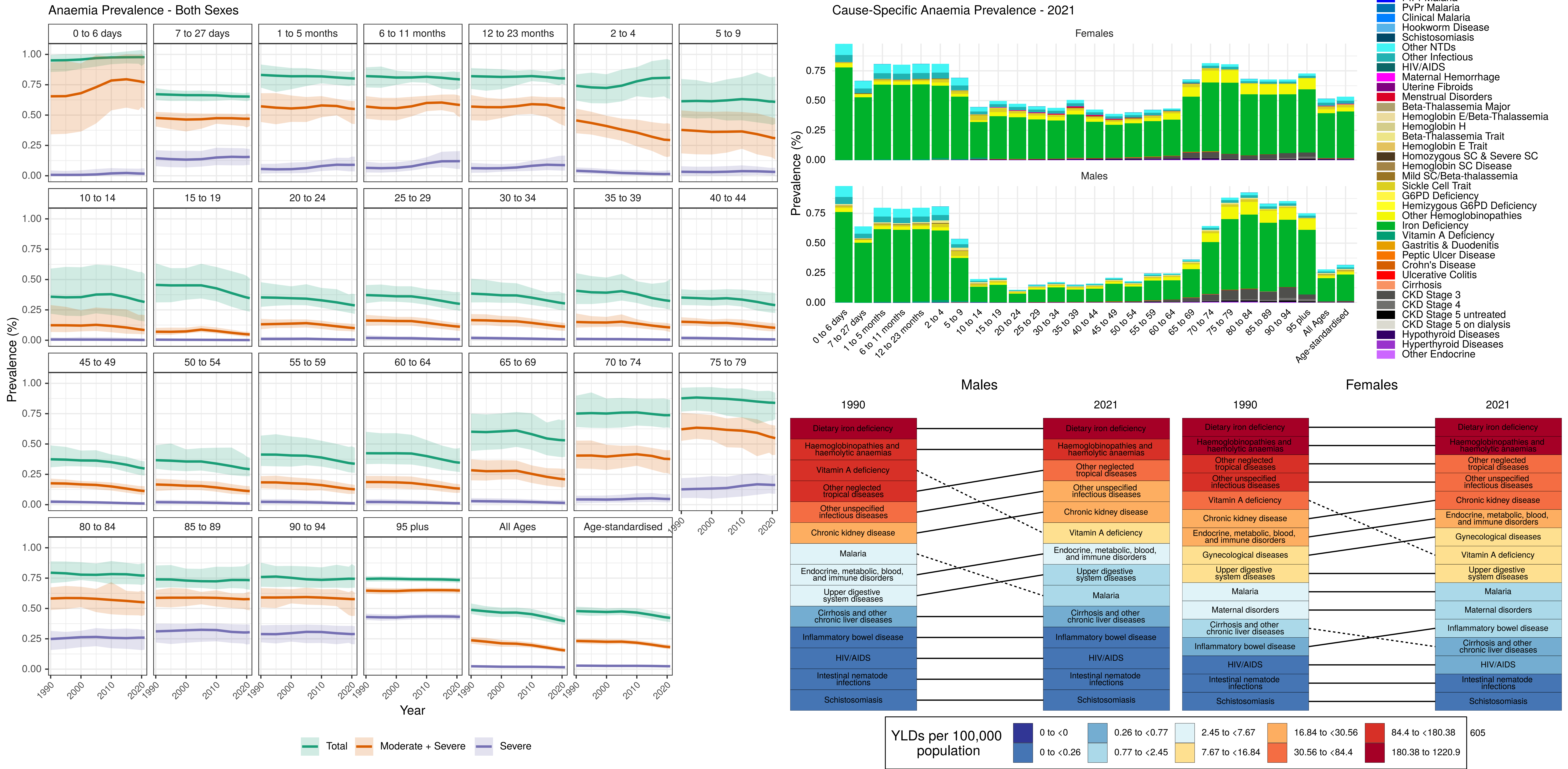

West Bengal

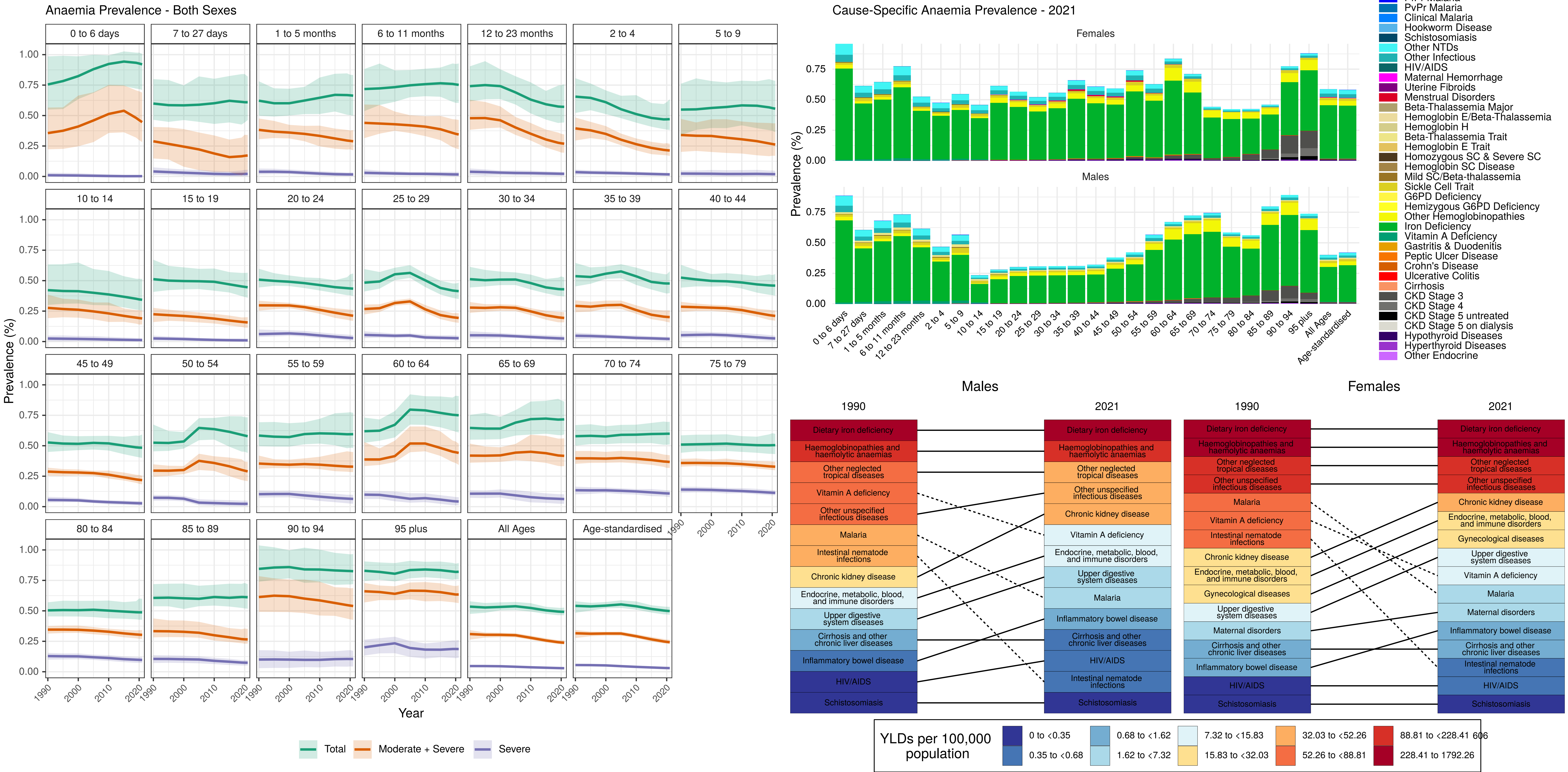
Anaemia Prevalence - Both Sexes
0 to 6 days
7 to 27 days
1 to 5 months
6 to 11 months
12 to 23 months
2 to 4
5 to 9
10 to 14
15 to 19
20 to 24
25 to 29
30 to 34
35 to 39
40 to 44
45 to 49
50 to 54
55 to 59
60 to 64
65 to 69
70 to 74
75 to 79
80 to 84
85 to 89
90 to 94
95 plus
All Ages
Age-standardised
Prevalence (%)
Year
Total
Moderate + Severe
Severe
Cause-Specific Anaemia Prevalence - 2021
Females
Males
Prevalence (%)
PfPr Malaria
PvPr Malaria
Clinical Malaria
Hookworm Disease
Schistosomiasis
Other NTDs
Other Infectious
HIV/AIDS
Maternal Hemorrhage
Uterine Fibroids
Menstrual Disorders
Beta-Thalassemia Major
Hemoglobin E/Beta-Thalassemia
Hemoglobin H
Beta-Thalassemia Trait
Hemoglobin E Trait
Homozygous SC & Severe SC
Hemoglobin SC Disease
Mild SC/Beta-thalassemia
Sickle Cell Trait
G6PD Deficiency
Hemizygous G6PD Deficiency
Other Hemoglobinopathies
Iron Deficiency
Vitamin A Deficiency
Gastritis & Duodenitis
Peptic Ulcer Disease
Crohn's Disease
Ulcerative Colitis
Cirrhosis
CKD Stage 3
CKD Stage 4
CKD Stage 5 untreated
CKD Stage 5 on dialysis
Hypothyroid Diseases
Hyperthyroid Diseases
Other Endocrine
Males
Females
1990
2021
Dietary iron deficiency
Haemoglobinopathies and haemolytic anaemias
Other neglected tropical diseases
Vitamin A deficiency
Other unspecified infectious diseases
Malaria
Intestinal nematode infections
Chronic kidney disease
Endocrine, metabolic, blood, and immune disorders
Upper digestive system diseases
Cirrhosis and other chronic liver diseases
Inflammatory bowel disease
HIV/AIDS
Schistosomiasis
Gynecological diseases
Maternal disorders
YLDs per 100,000 population
0 to <0.35
0.35 to <0.68
0.68 to <1.62
1.62 to <7.32
7.32 to <15.83
15.83 to <32.03
32.03 to <52.26
52.26 to <88.81
88.81 to <228.41
228.41 to 1792.26

Nepal

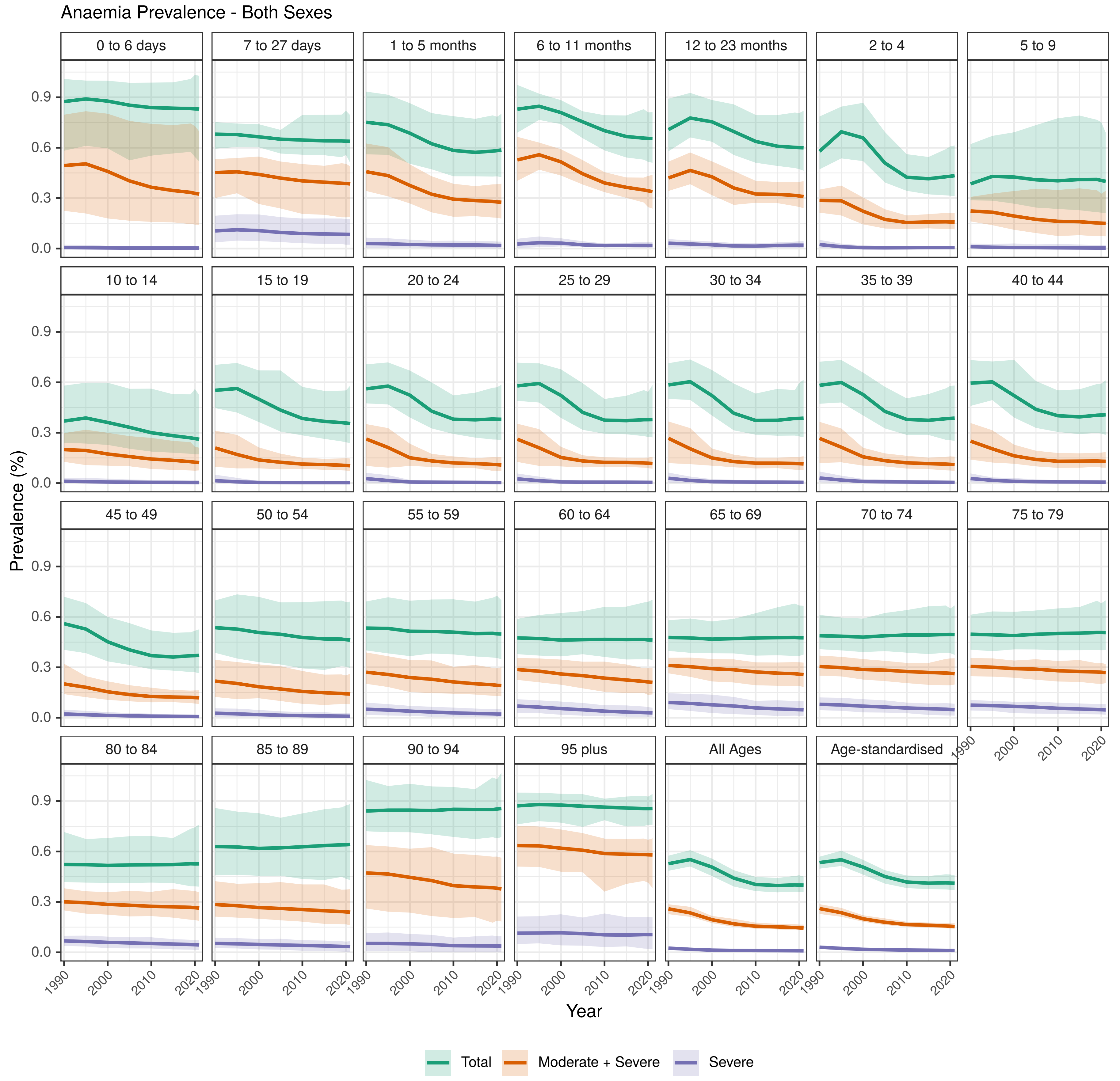
Anaemia Prevalence - Both Sexes
0 to 6 days
7 to 27 days
1 to 5 months
6 to 11 months
12 to 23 months
2 to 4
5 to 9
10 to 14
15 to 19
20 to 24
25 to 29
30 to 34
35 to 39
40 to 44
45 to 49
50 to 54
55 to 59
60 to 64
65 to 69
70 to 74
75 to 79
80 to 84
85 to 89
90 to 94
95 plus
All Ages
Age-standardised
Prevalence (%)
0.0
0.3
0.6
0.9
1990
2000
2010
2020
Year
Total
Moderate + Severe
Severe

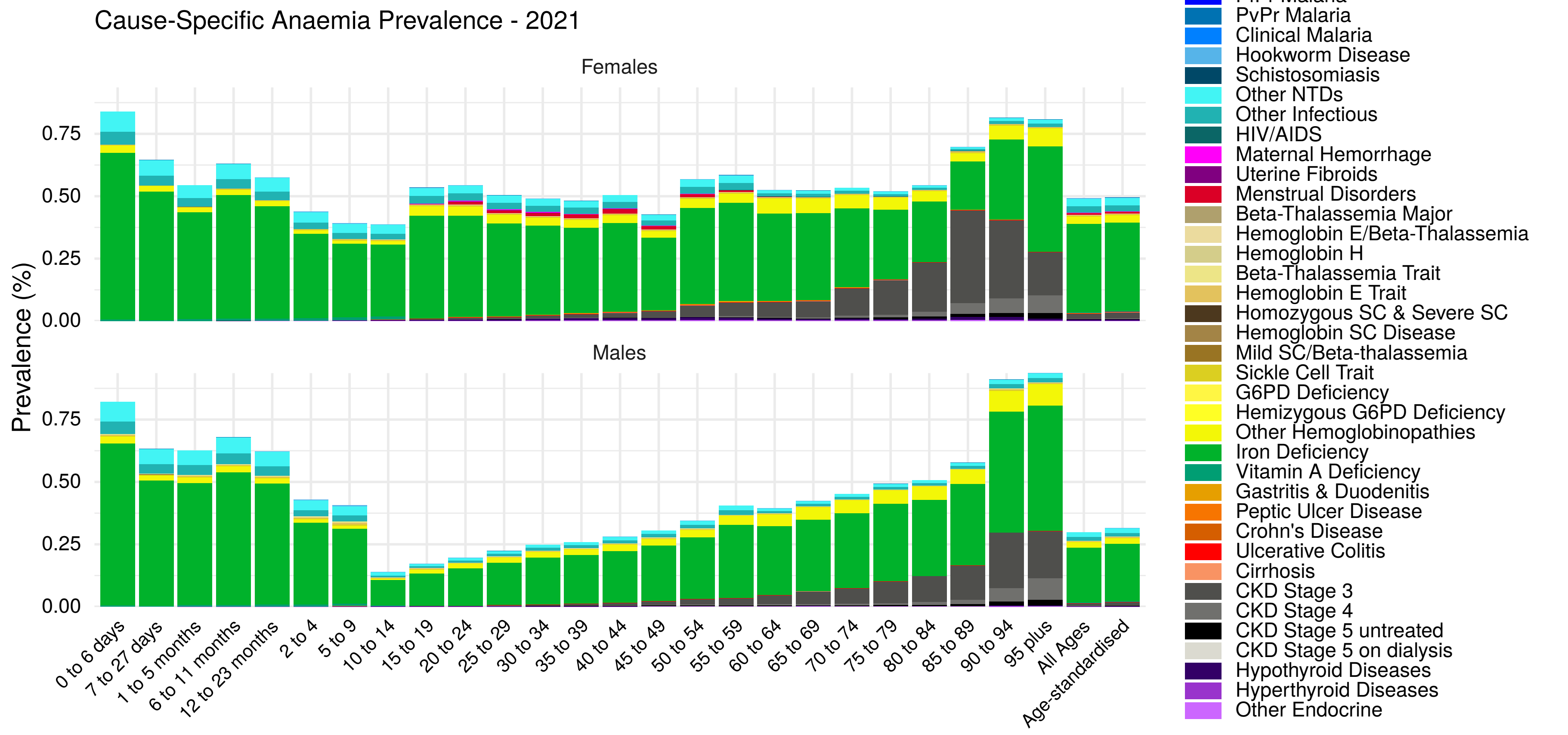
Cause-Specific Anaemia Prevalence - 2021
Females
Males
Prevalence (%)
0.00
0.25
0.50
0.75
0 to 6 days
7 to 27 days
1 to 5 months
6 to 11 months
12 to 23 months
2 to 4
5 to 9
10 to 14
15 to 19
20 to 24
25 to 29
30 to 34
35 to 39
40 to 44
45 to 49
50 to 54
55 to 59
60 to 64
65 to 69
70 to 74
75 to 79
80 to 84
85 to 89
90 to 94
95 plus
All Ages
Age-standardised
PfPr Malaria
PvPr Malaria
Clinical Malaria
Hookworm Disease
Schistosomiasis
Other NTDs
Other Infectious
HIV/AIDS
Maternal Hemorrhage
Uterine Fibroids
Menstrual Disorders
Beta-Thalassemia Major
Hemoglobin E/Beta-Thalassemia
Hemoglobin H
Beta-Thalassemia Trait
Hemoglobin E Trait
Homozygous SC & Severe SC
Hemoglobin SC Disease
Mild SC/Beta-thalassemia
Sickle Cell Trait
G6PD Deficiency
Hemizygous G6PD Deficiency
Other Hemoglobinopathies
Iron Deficiency
Vitamin A Deficiency
Gastritis & Duodenitis
Peptic Ulcer Disease
Crohn's Disease
Ulcerative Colitis
Cirrhosis
CKD Stage 3
CKD Stage 4
CKD Stage 5 untreated
CKD Stage 5 on dialysis
Hypothyroid Diseases
Hyperthyroid Diseases
Other Endocrine

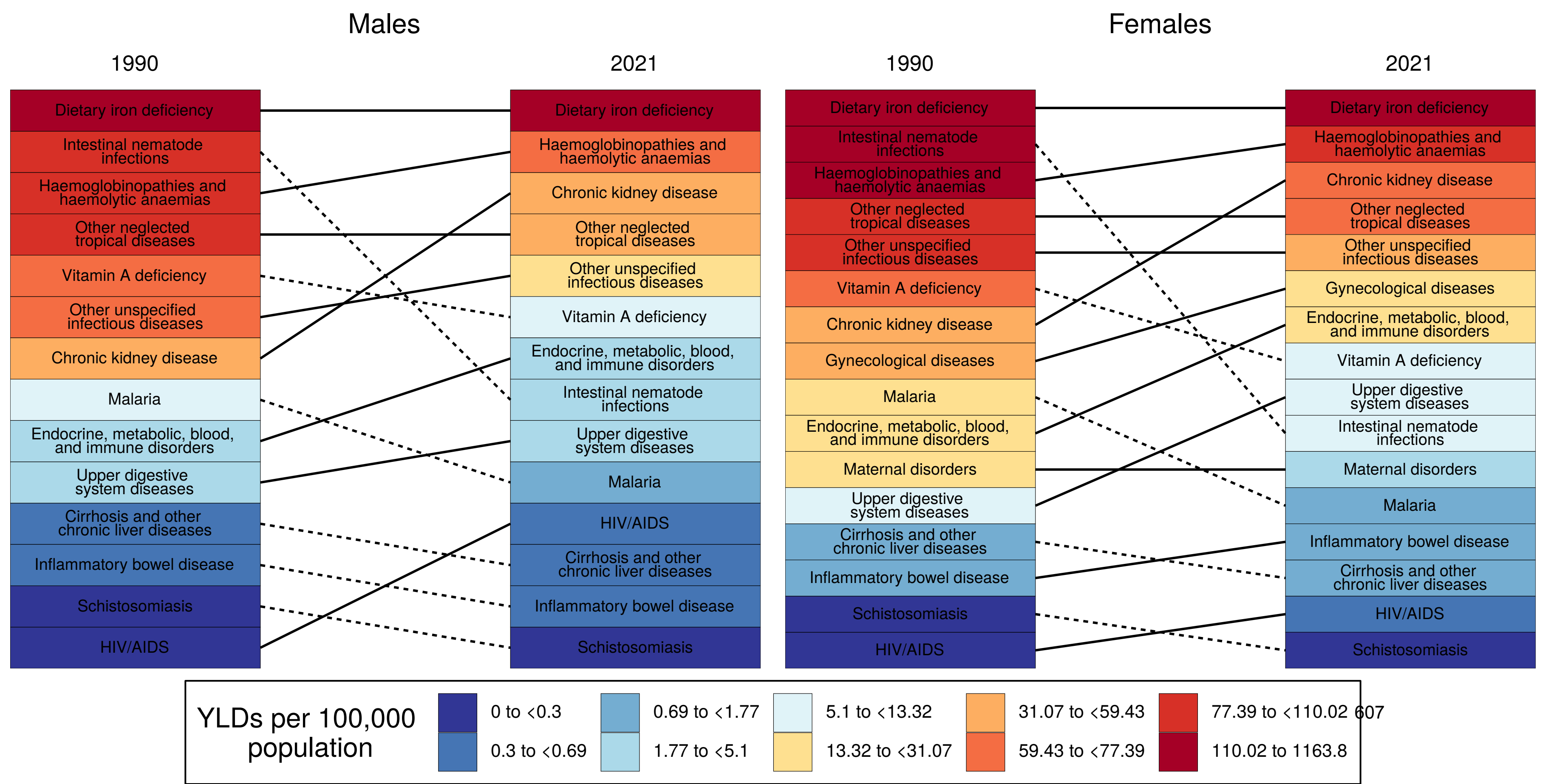
Males
1990
2021
Dietary iron deficiency
Intestinal nematode infections
Haemoglobinopathies and haemolytic anaemias
Other neglected tropical diseases
Vitamin A deficiency
Other unspecified infectious diseases
Chronic kidney disease
Malaria
Endocrine, metabolic, blood, and immune disorders
Upper digestive system diseases
Cirrhosis and other chronic liver diseases
Inflammatory bowel disease
Schistosomiasis
HIV/AIDS
Dietary iron deficiency
Haemoglobinopathies and haemolytic anaemias
Chronic kidney disease
Other neglected tropical diseases
Other unspecified infectious diseases
Vitamin A deficiency
Endocrine, metabolic, blood, and immune disorders
Intestinal nematode infections
Upper digestive system diseases
Malaria
HIV/AIDS
Cirrhosis and other chronic liver diseases
Inflammatory bowel disease
Schistosomiasis
Females
1990
2021
Dietary iron deficiency
Intestinal nematode infections
Haemoglobinopathies and haemolytic anaemias
Other neglected tropical diseases
Other unspecified infectious diseases
Vitamin A deficiency
Chronic kidney disease
Gynecological diseases
Malaria
Endocrine, metabolic, blood, and immune disorders
Maternal disorders
Upper digestive system diseases
Cirrhosis and other chronic liver diseases
Inflammatory bowel disease
Schistosomiasis
HIV/AIDS
Dietary iron deficiency
Haemoglobinopathies and haemolytic anaemias
Chronic kidney disease
Other neglected tropical diseases
Other unspecified infectious diseases
Gynecological diseases
Endocrine, metabolic, blood, and immune disorders
Vitamin A deficiency
Upper digestive system diseases
Intestinal nematode infections
Maternal disorders
Malaria
Inflammatory bowel disease
Cirrhosis and other chronic liver diseases
HIV/AIDS
Schistosomiasis
YLDs per 100,000 population
0 to <0.3
0.3 to <0.69
0.69 to <1.77
1.77 to <5.1
5.1 to <13.32
13.32 to <31.07
31.07 to <59.43
59.43 to <77.39
77.39 to <110.02
110.02 to 1163.8

607

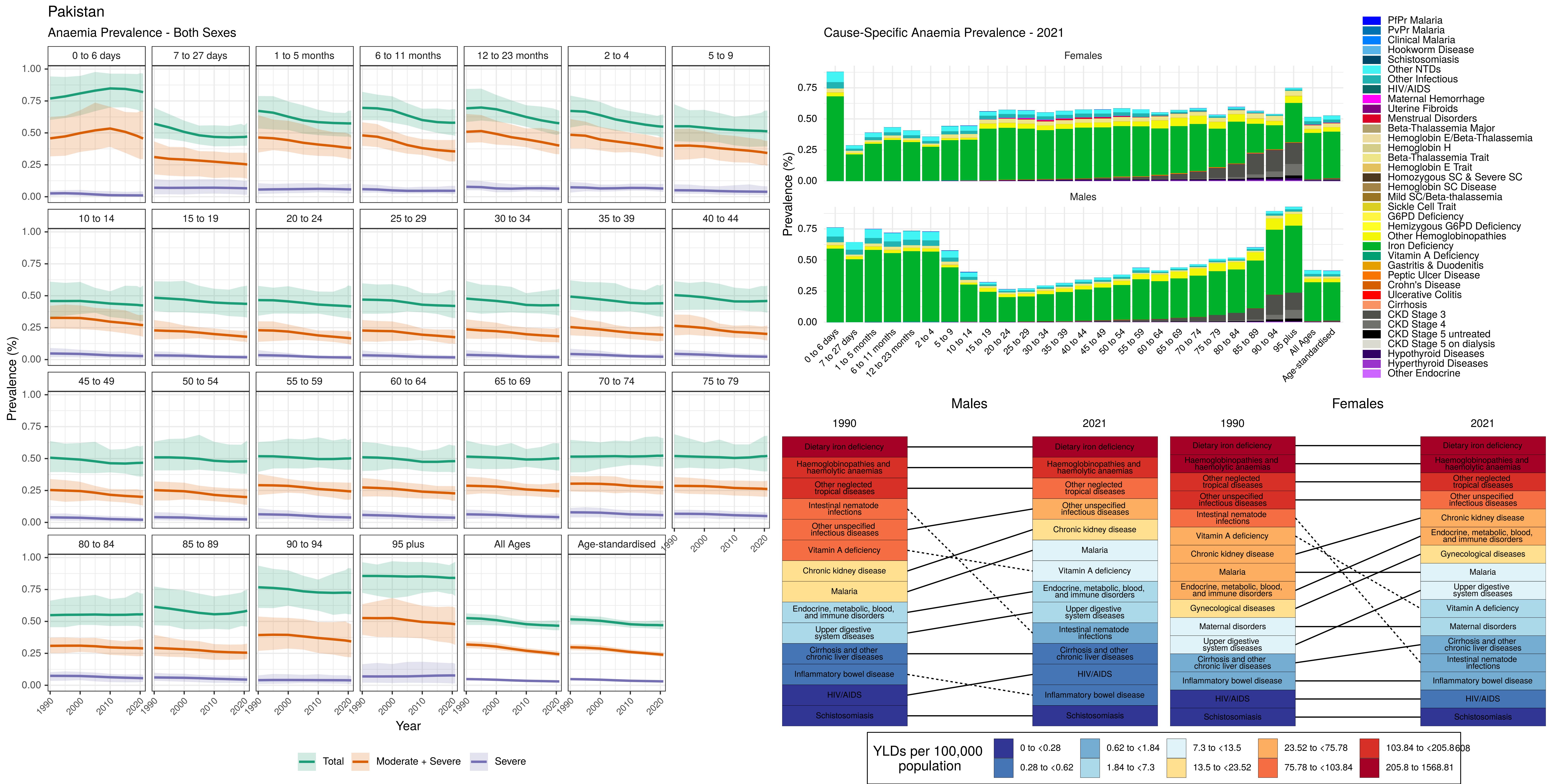

Pakistan
Anaemia Prevalence - Both Sexes
0 to 6 days
7 to 27 days
1 to 5 months
6 to 11 months
12 to 23 months
2 to 4
5 to 9
10 to 14
15 to 19
20 to 24
25 to 29
30 to 34
35 to 39
40 to 44
45 to 49
50 to 54
55 to 59
60 to 64
65 to 69
70 to 74
75 to 79
80 to 84
85 to 89
90 to 94
95 plus
All Ages
Age-standardised
Prevalence (%)
Year
Total
Moderate + Severe
Severe
Cause-Specific Anaemia Prevalence - 2021
Females
Males
PfPr Malaria
PvPr Malaria
Clinical Malaria
Hookworm Disease
Schistosomiasis
Other NTDs
Other Infectious
HIV/AIDS
Maternal Hemorrhage
Uterine Fibroids
Menstrual Disorders
Beta-Thalassemia Major
Hemoglobin E/Beta-Thalassemia
Hemoglobin H
Beta-Thalassemia Trait
Hemoglobin E Trait
Homozygous SC & Severe SC
Hemoglobin SC Disease
Mild SC/Beta-thalassemia
Sickle Cell Trait
G6PD Deficiency
Hemizygous G6PD Deficiency
Other Hemoglobinopathies
Iron Deficiency
Vitamin A Deficiency
Gastritis & Duodenitis
Peptic Ulcer Disease
Crohn's Disease
Ulcerative Colitis
Cirrhosis
CKD Stage 3
CKD Stage 4
CKD Stage 5 untreated
CKD Stage 5 on dialysis
Hypothyroid Diseases
Hyperthyroid Diseases
Other Endocrine
Males
Females
1990
2021
Dietary iron deficiency
Haemoglobinopathies and haemolytic anaemias
Other neglected tropical diseases
Intestinal nematode infections
Other unspecified infectious diseases
Vitamin A deficiency
Chronic kidney disease
Malaria
Endocrine, metabolic, blood, and immune disorders
Upper digestive system diseases
Cirrhosis and other chronic liver diseases
Inflammatory bowel disease
HIV/AIDS
Schistosomiasis
Gynecological diseases
Maternal disorders
YLDs per 100,000 population
0 to <0.28
0.28 to <0.62
0.62 to <1.84
1.84 to <7.3
7.3 to <13.5
13.5 to <23.52
23.52 to <75.78
75.78 to <103.84
103.84 to <205.8608
205.8 to 1568.81

Azad Jammu & Kashmir

Anaemia Prevalence - Both Sexes

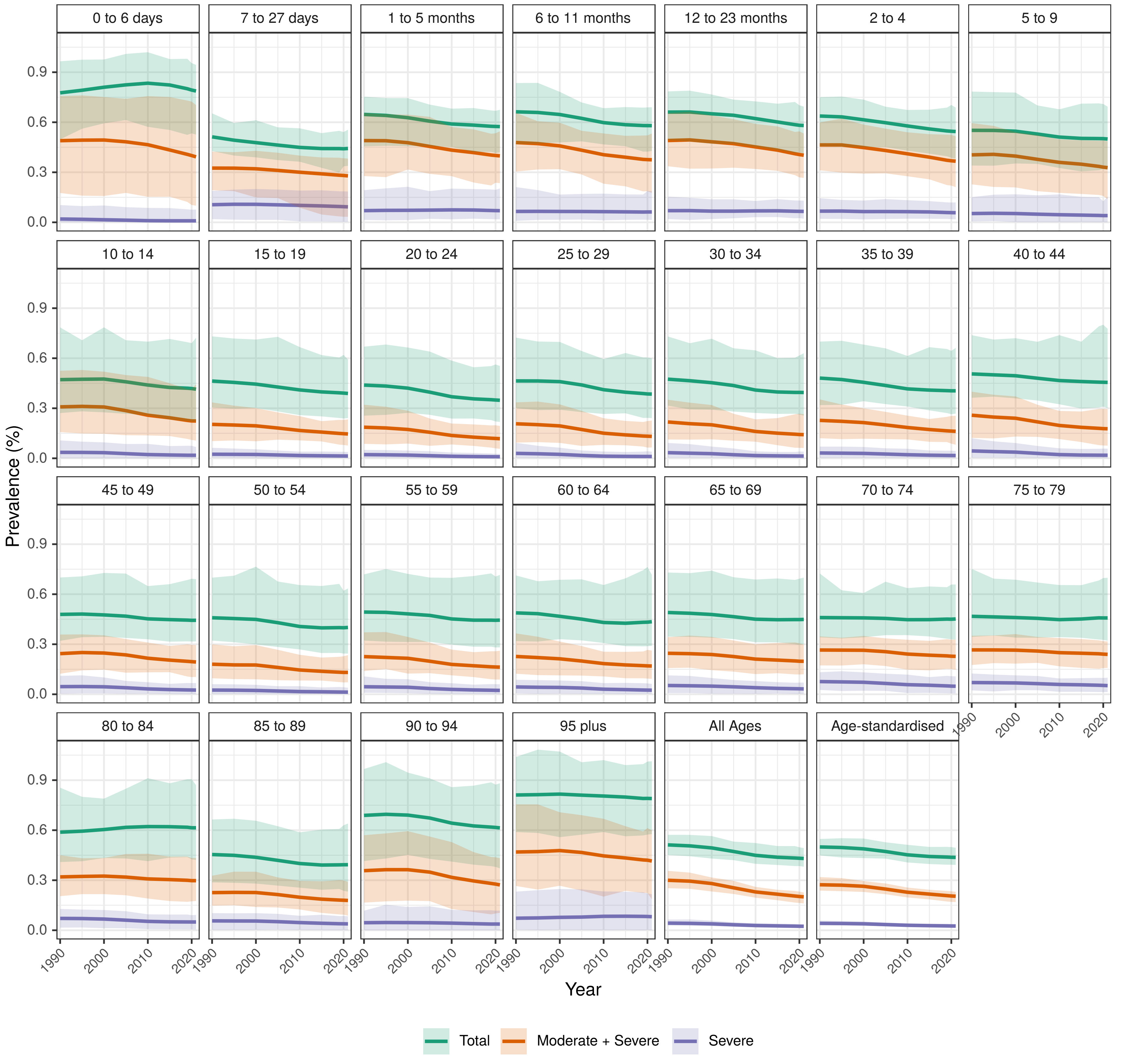

Cause-Specific Anaemia Prevalence - 2021

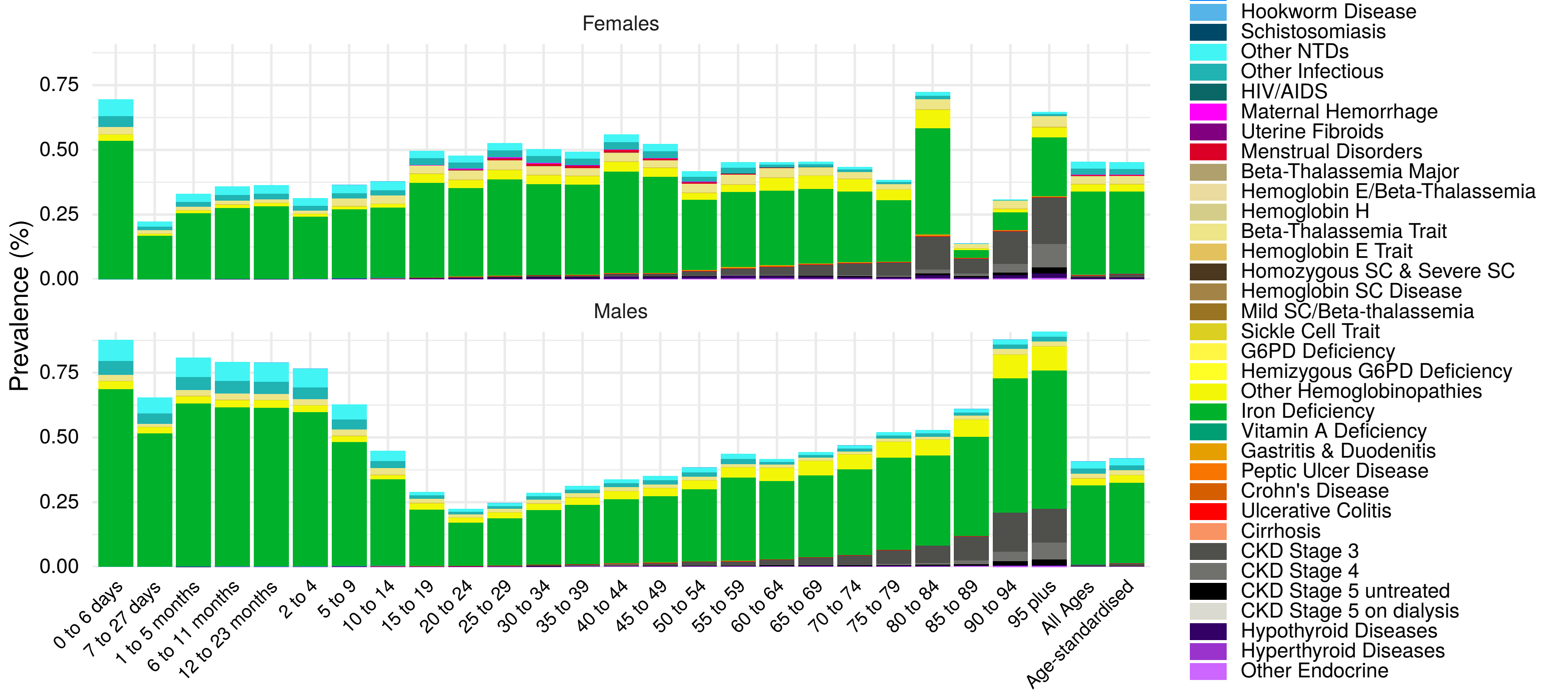

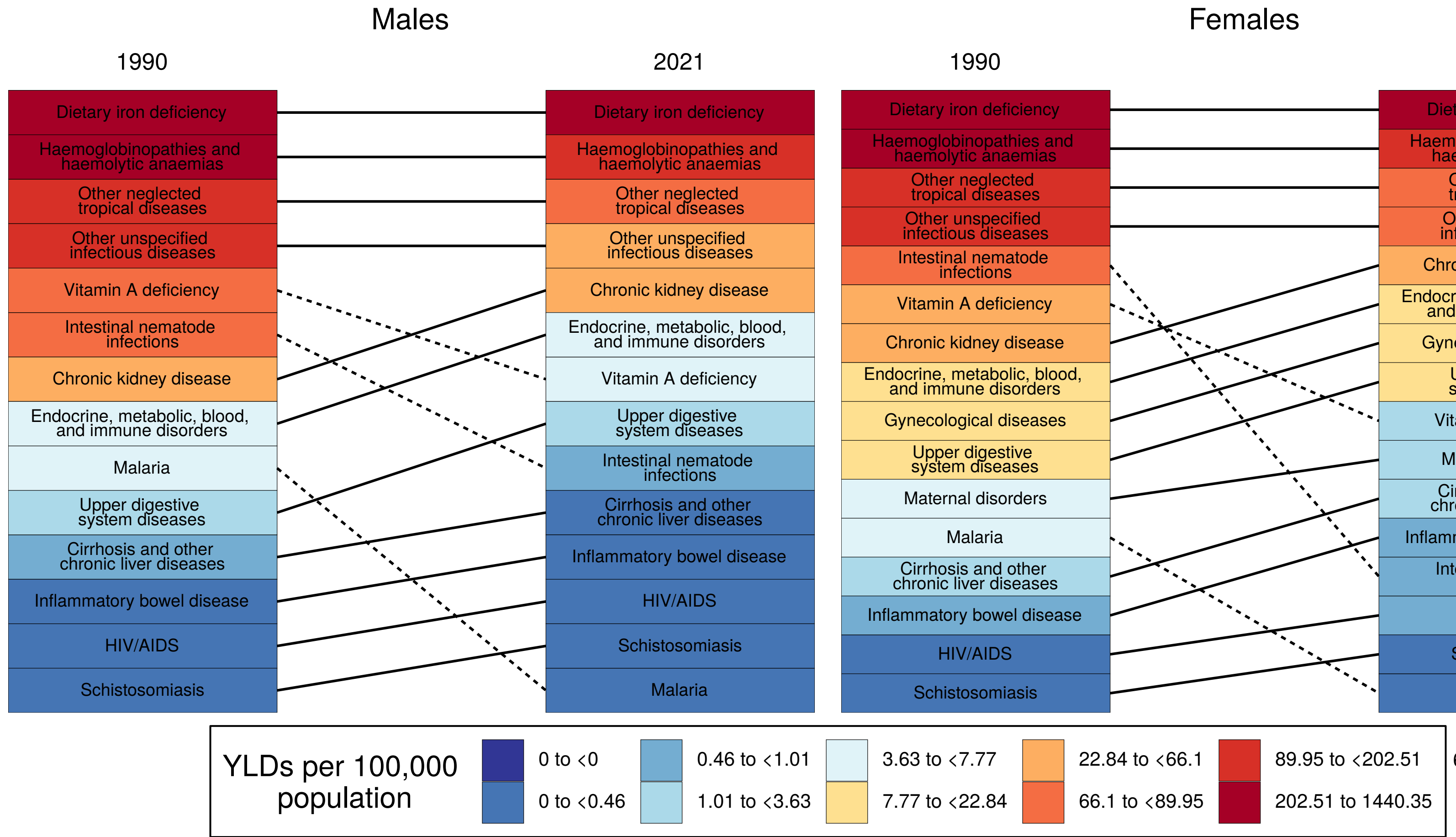

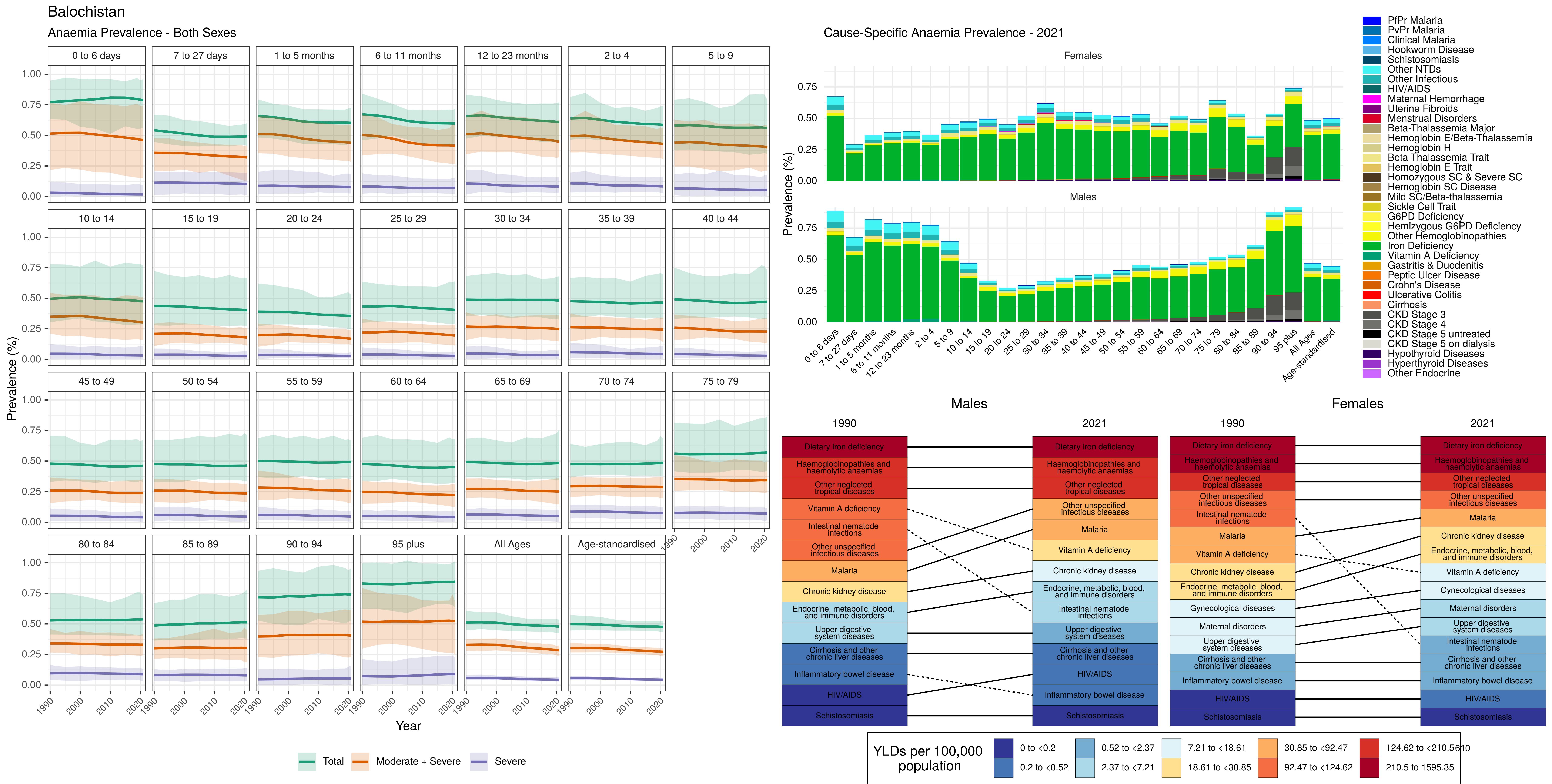

Balochistan
Anaemia Prevalence - Both Sexes
0 to 6 days
7 to 27 days
1 to 5 months
6 to 11 months
12 to 23 months
2 to 4
5 to 9
10 to 14
15 to 19
20 to 24
25 to 29
30 to 34
35 to 39
40 to 44
45 to 49
50 to 54
55 to 59
60 to 64
65 to 69
70 to 74
75 to 79
80 to 84
85 to 89
90 to 94
95 plus
All Ages
Age-standardised
Prevalence (%)
Year
Total
Moderate + Severe
Severe
Cause-Specific Anaemia Prevalence - 2021
Females
Males
Prevalence (%)
PfPr Malaria
PvPr Malaria
Clinical Malaria
Hookworm Disease
Schistosomiasis
Other NTDs
Other Infectious
HIV/AIDS
Maternal Hemorrhage
Uterine Fibroids
Menstrual Disorders
Beta-Thalassemia Major
Hemoglobin E/Beta-Thalassemia
Hemoglobin H
Beta-Thalassemia Trait
Hemoglobin E Trait
Homozygous SC & Severe SC
Hemoglobin SC Disease
Mild SC/Beta-thalassemia
Sickle Cell Trait
G6PD Deficiency
Hemizygous G6PD Deficiency
Other Hemoglobinopathies
Iron Deficiency
Vitamin A Deficiency
Gastritis & Duodenitis
Peptic Ulcer Disease
Crohn's Disease
Ulcerative Colitis
Cirrhosis
CKD Stage 3
CKD Stage 4
CKD Stage 5 untreated
CKD Stage 5 on dialysis
Hypothyroid Diseases
Hyperthyroid Diseases
Other Endocrine
Males
Females
1990
2021
Dietary iron deficiency
Haemoglobinopathies and haemolytic anaemias
Other neglected tropical diseases
Vitamin A deficiency
Intestinal nematode infections
Other unspecified infectious diseases
Malaria
Chronic kidney disease
Endocrine, metabolic, blood, and immune disorders
Upper digestive system diseases
Cirrhosis and other chronic liver diseases
Inflammatory bowel disease
HIV/AIDS
Schistosomiasis
Gynecological diseases
Maternal disorders
YLDs per 100,000 population
0 to <0.2
0.2 to <0.52
0.52 to <2.37
2.37 to <7.21
7.21 to <18.61
18.61 to <30.85
30.85 to <92.47
92.47 to <124.62
124.62 to <210.5610
210.5 to 1595.35

Gilgit-Baltistan

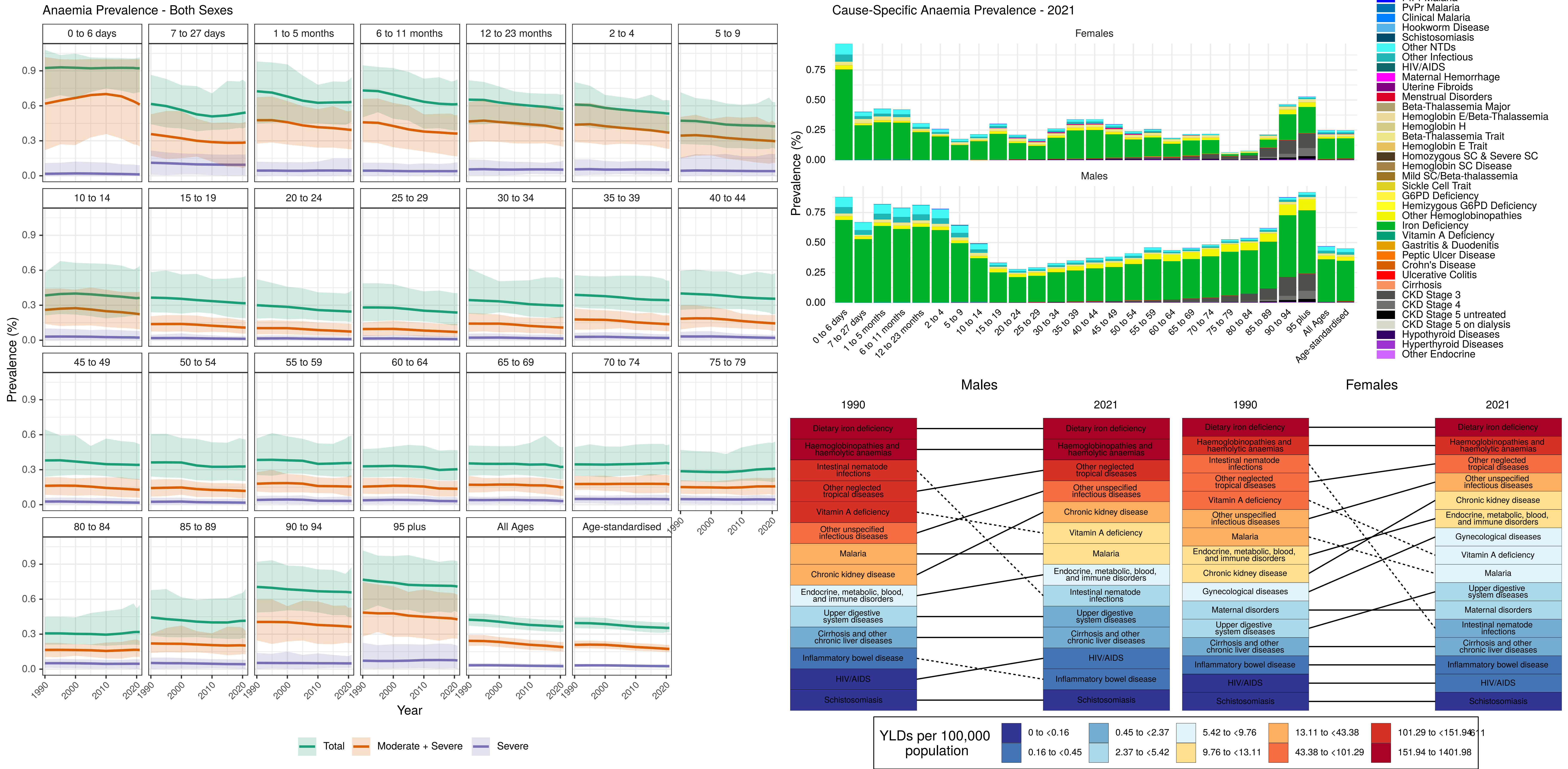

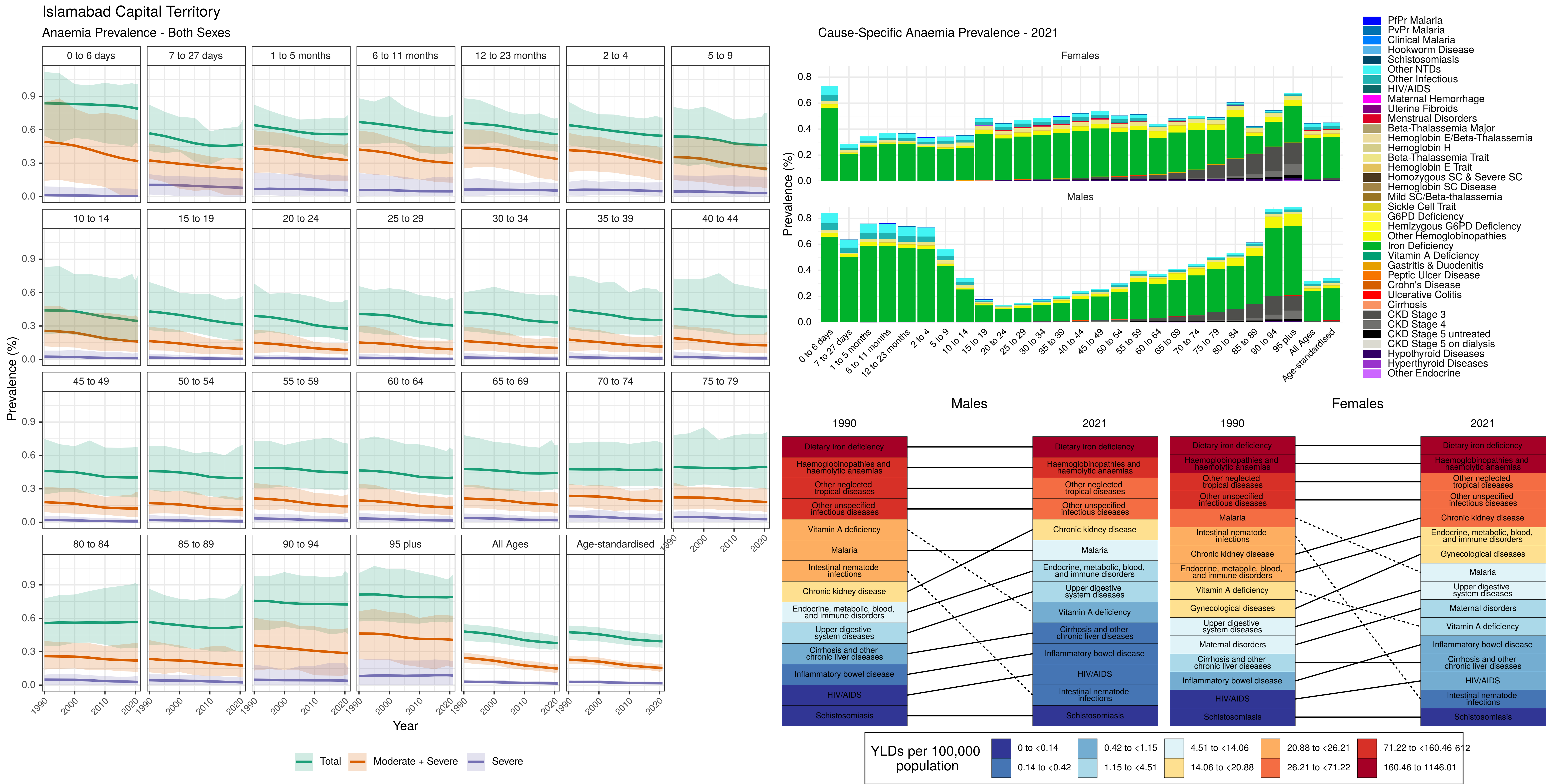
Islamabad Capital Territory
Anaemia Prevalence - Both Sexes
0 to 6 days
7 to 27 days
1 to 5 months
6 to 11 months
12 to 23 months
2 to 4
5 to 9
10 to 14
15 to 19
20 to 24
25 to 29
30 to 34
35 to 39
40 to 44
45 to 49
50 to 54
55 to 59
60 to 64
65 to 69
70 to 74
75 to 79
80 to 84
85 to 89
90 to 94
95 plus
All Ages
Age-standardised
Prevalence (%)
Year
Total
Moderate + Severe
Severe
Cause-Specific Anaemia Prevalence - 2021
Females
Males
Prevalence (%)
PfPr Malaria
PvPr Malaria
Clinical Malaria
Hookworm Disease
Schistosomiasis
Other NTDs
Other Infectious
HIV/AIDS
Maternal Hemorrhage
Uterine Fibroids
Menstrual Disorders
Beta-Thalassemia Major
Hemoglobin E/Beta-Thalassemia
Hemoglobin H
Beta-Thalassemia Trait
Hemoglobin E Trait
Homozygous SC & Severe SC
Hemoglobin SC Disease
Mild SC/Beta-thalassemia
Sickle Cell Trait
G6PD Deficiency
Hemizygous G6PD Deficiency
Other Hemoglobinopathies
Iron Deficiency
Vitamin A Deficiency
Gastritis & Duodenitis
Peptic Ulcer Disease
Crohn's Disease
Ulcerative Colitis
Cirrhosis
CKD Stage 3
CKD Stage 4
CKD Stage 5 untreated
CKD Stage 5 on dialysis
Hypothyroid Diseases
Hyperthyroid Diseases
Other Endocrine
Males
1990
Dietary iron deficiency
Haemoglobinopathies and haemolytic anaemias
Other neglected tropical diseases
Other unspecified infectious diseases
Vitamin A deficiency
Malaria
Intestinal nematode infections
Chronic kidney disease
Endocrine, metabolic, blood, and immune disorders
Upper digestive system diseases
Cirrhosis and other chronic liver diseases
Inflammatory bowel disease
HIV/AIDS
Schistosomiasis
2021
Dietary iron deficiency
Haemoglobinopathies and haemolytic anaemias
Other neglected tropical diseases
Other unspecified infectious diseases
Chronic kidney disease
Malaria
Endocrine, metabolic, blood, and immune disorders
Upper digestive system diseases
Vitamin A deficiency
Cirrhosis and other chronic liver diseases
Inflammatory bowel disease
HIV/AIDS
Intestinal nematode infections
Schistosomiasis
Females
1990
Dietary iron deficiency
Haemoglobinopathies and haemolytic anaemias
Other neglected tropical diseases
Other unspecified infectious diseases
Malaria
Intestinal nematode infections
Chronic kidney disease
Endocrine, metabolic, blood, and immune disorders
Vitamin A deficiency
Gynecological diseases
Upper digestive system diseases
Maternal disorders
Cirrhosis and other chronic liver diseases
Inflammatory bowel disease
HIV/AIDS
Schistosomiasis
2021
Dietary iron deficiency
Haemoglobinopathies and haemolytic anaemias
Other neglected tropical diseases
Other unspecified infectious diseases
Chronic kidney disease
Endocrine, metabolic, blood, and immune disorders
Gynecological diseases
Malaria
Upper digestive system diseases
Maternal disorders
Vitamin A deficiency
Inflammatory bowel disease
Cirrhosis and other chronic liver diseases
HIV/AIDS
Intestinal nematode infections
Schistosomiasis
YLDs per 100,000 population
0 to <0.14
0.14 to <0.42
0.42 to <1.15
1.15 to <4.51
4.51 to <14.06
14.06 to <20.88
20.88 to <26.21
26.21 to <71.22
71.22 to <160.46
160.46 to 1146.01

Khyber Pakhtunkhwa

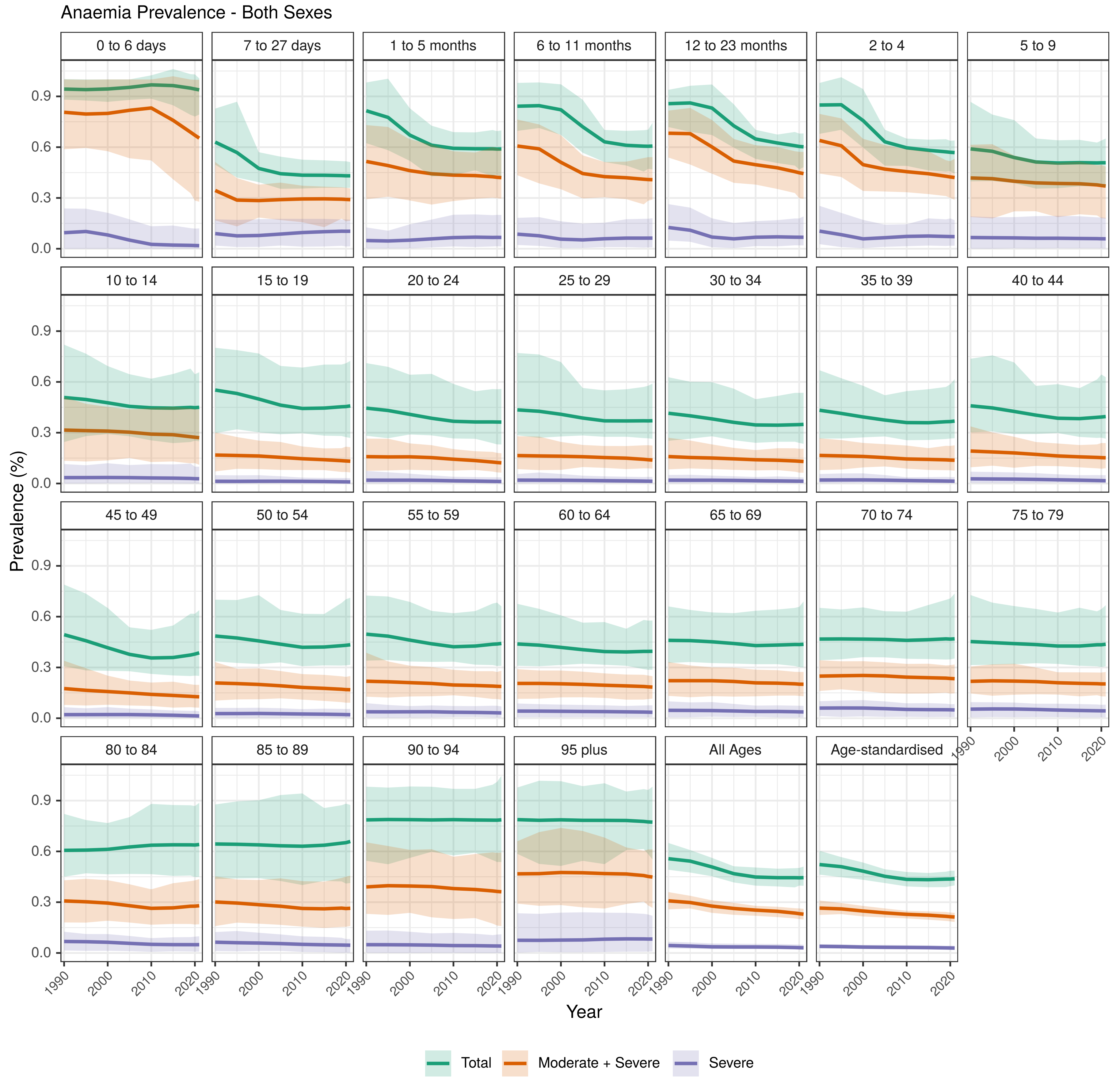

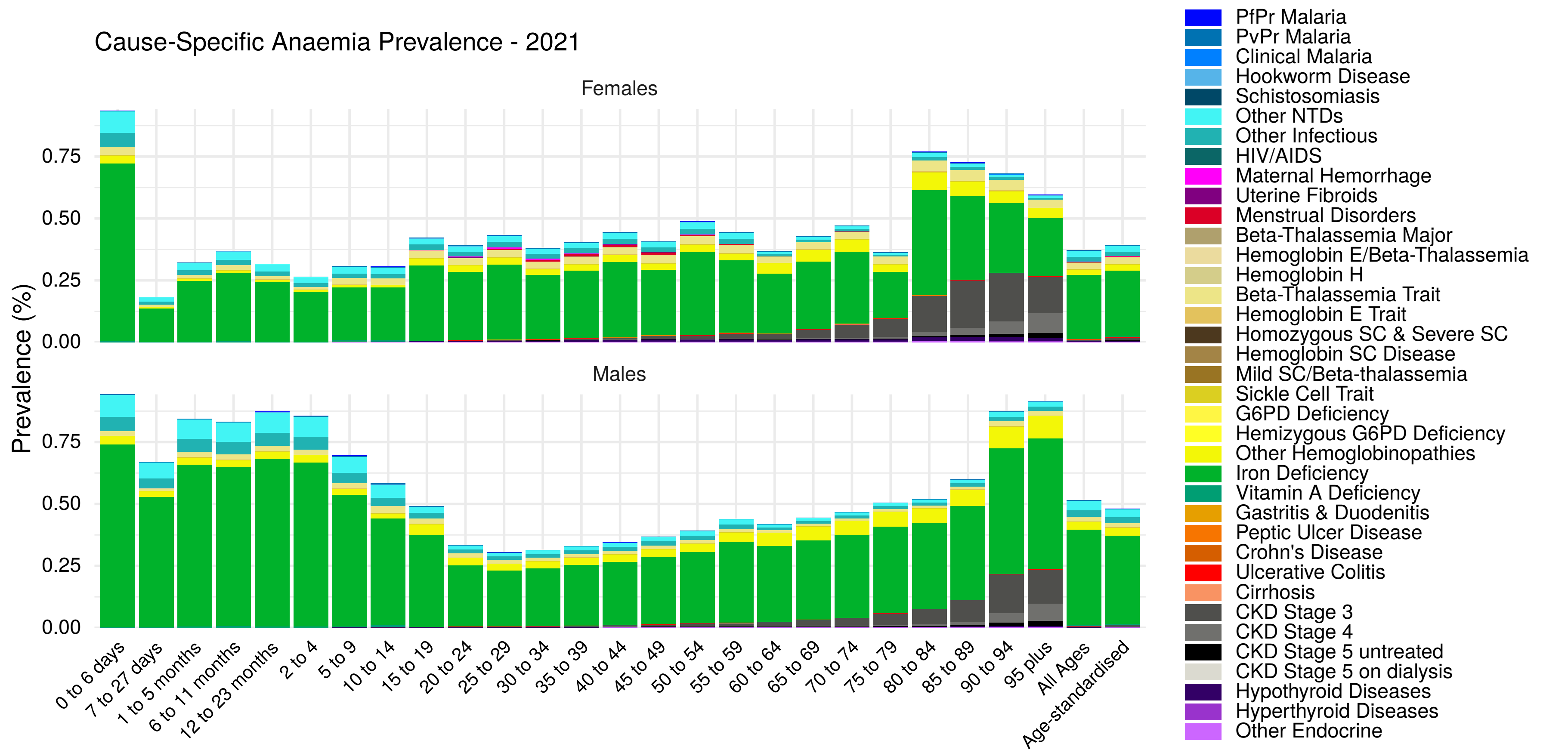

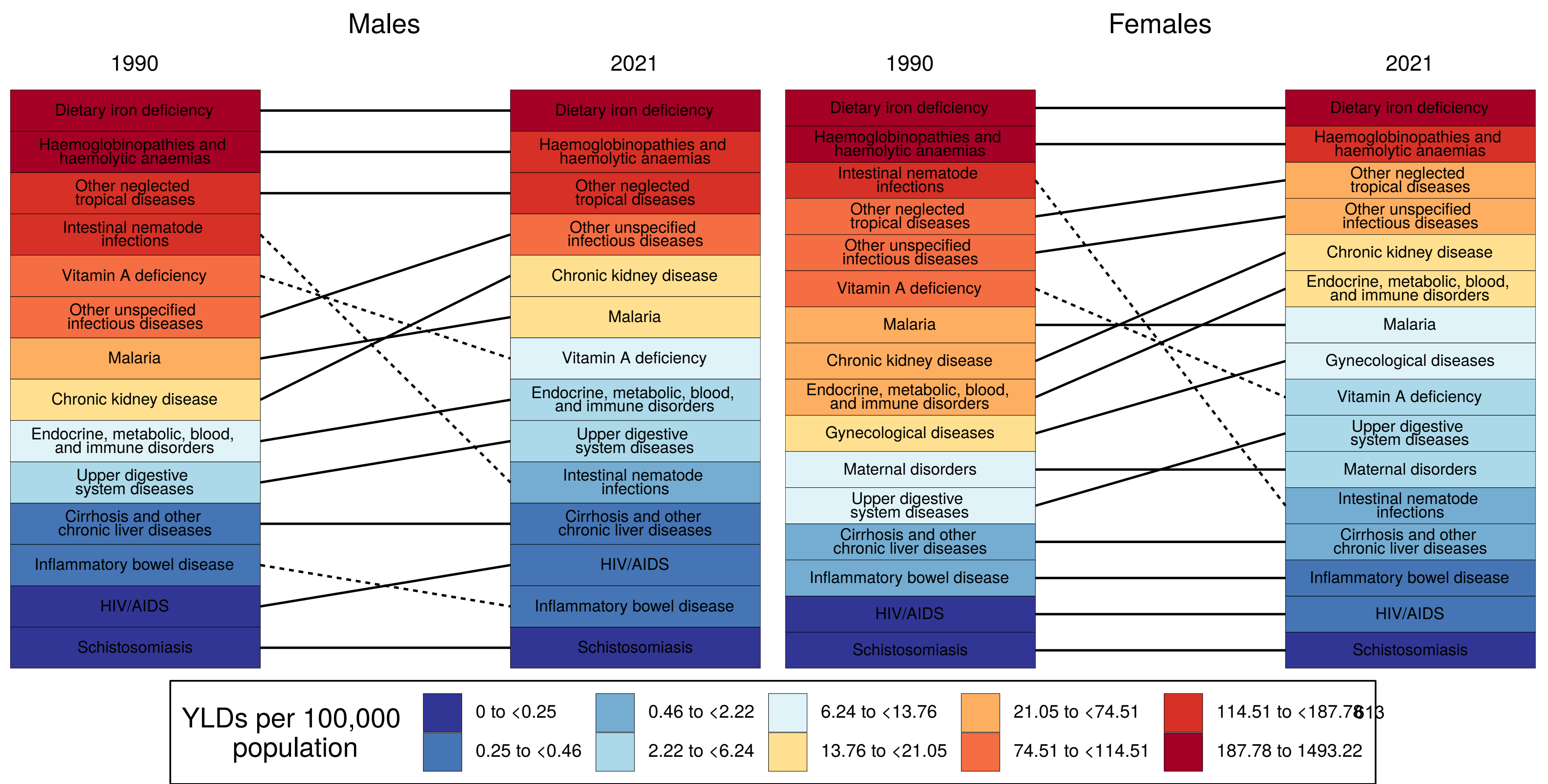

Punjab

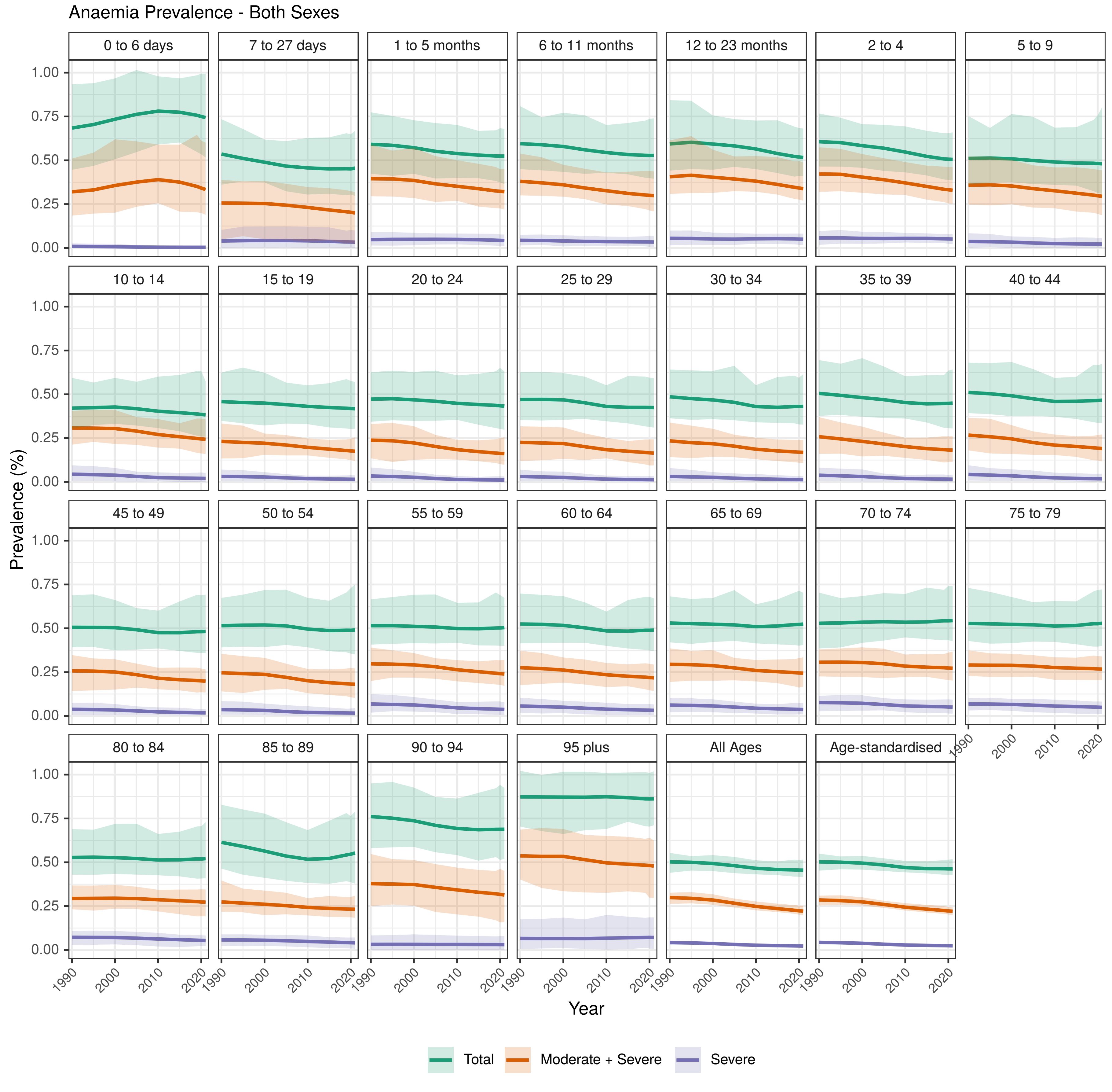

Anaemia Prevalence - Both Sexes
0 to 6 days
7 to 27 days
1 to 5 months
6 to 11 months
12 to 23 months
2 to 4
5 to 9
10 to 14
15 to 19
20 to 24
25 to 29
30 to 34
35 to 39
40 to 44
45 to 49
50 to 54
55 to 59
60 to 64
65 to 69
70 to 74
75 to 79
80 to 84
85 to 89
90 to 94
95 plus
All Ages
Age-standardised
Prevalence (%)
Year
Total
Moderate + Severe
Severe

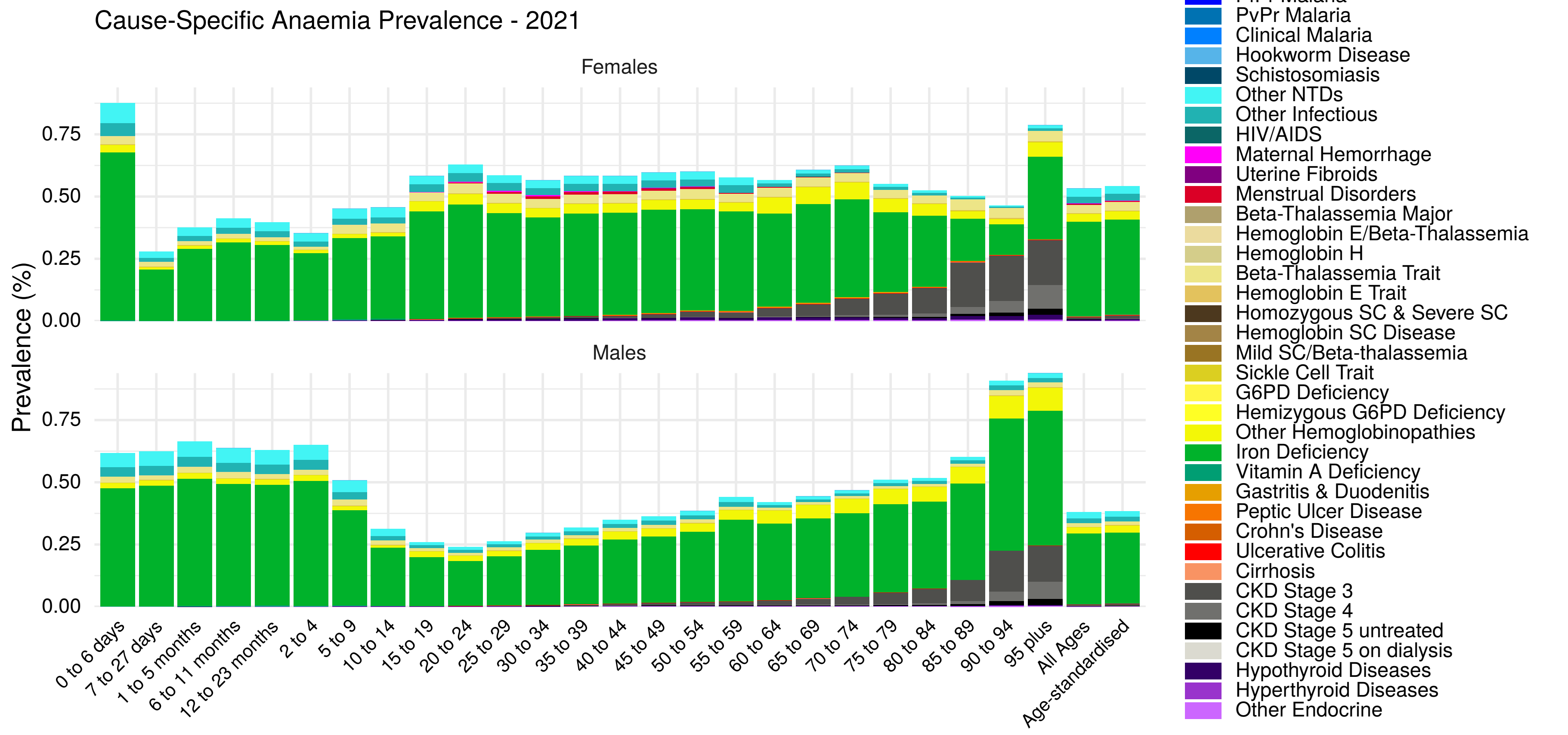

Cause-Specific Anaemia Prevalence - 2021
Females
Males
Prevalence (%)
PfPr Malaria
PvPr Malaria
Clinical Malaria
Hookworm Disease
Schistosomiasis
Other NTDs
Other Infectious
HIV/AIDS
Maternal Hemorrhage
Uterine Fibroids
Menstrual Disorders
Beta-Thalassemia Major
Hemoglobin E/Beta-Thalassemia
Hemoglobin H
Beta-Thalassemia Trait
Hemoglobin E Trait
Homozygous SC & Severe SC
Hemoglobin SC Disease
Mild SC/Beta-thalassemia
Sickle Cell Trait
G6PD Deficiency
Hemizygous G6PD Deficiency
Other Hemoglobinopathies
Iron Deficiency
Vitamin A Deficiency
Gastritis & Duodenitis
Peptic Ulcer Disease
Crohn's Disease
Ulcerative Colitis
Cirrhosis
CKD Stage 3
CKD Stage 4
CKD Stage 5 untreated
CKD Stage 5 on dialysis
Hypothyroid Diseases
Hyperthyroid Diseases
Other Endocrine

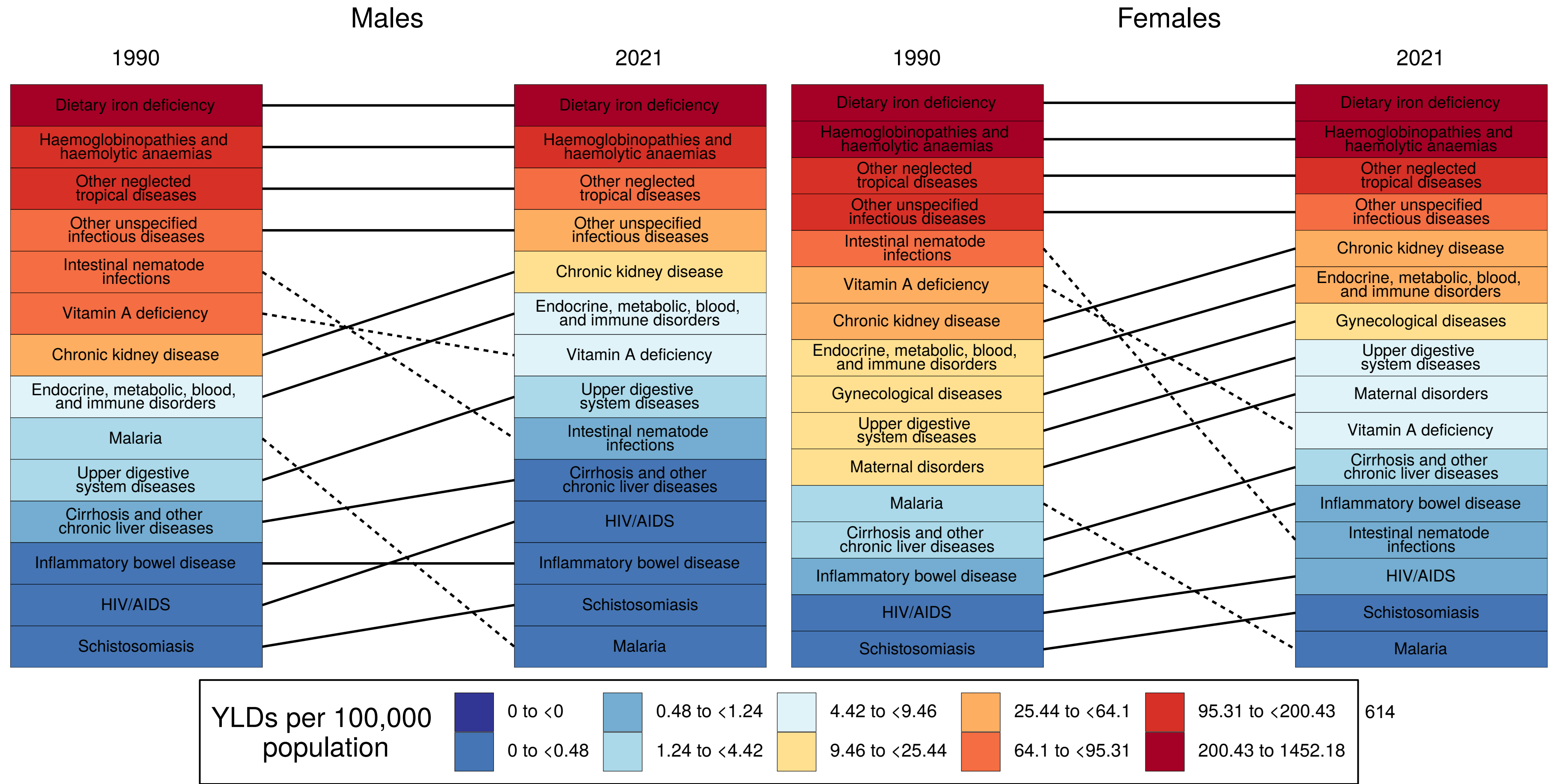

Males
Females
1990
2021
Dietary iron deficiency
Haemoglobinopathies and haemolytic anaemias
Other neglected tropical diseases
Other unspecified infectious diseases
Intestinal nematode infections
Vitamin A deficiency
Chronic kidney disease
Endocrine, metabolic, blood, and immune disorders
Malaria
Upper digestive system diseases
Cirrhosis and other chronic liver diseases
Inflammatory bowel disease
HIV/AIDS
Schistosomiasis
Gynecological diseases
Maternal disorders
YLDs per 100,000 population
0 to <0
0 to <0.48
0.48 to <1.24
1.24 to <4.42
4.42 to <9.46
9.46 to <25.44
25.44 to <64.1
64.1 to <95.31
95.31 to <200.43
200.43 to 1452.18

Sindh

Anaemia Prevalence - Both Sexes

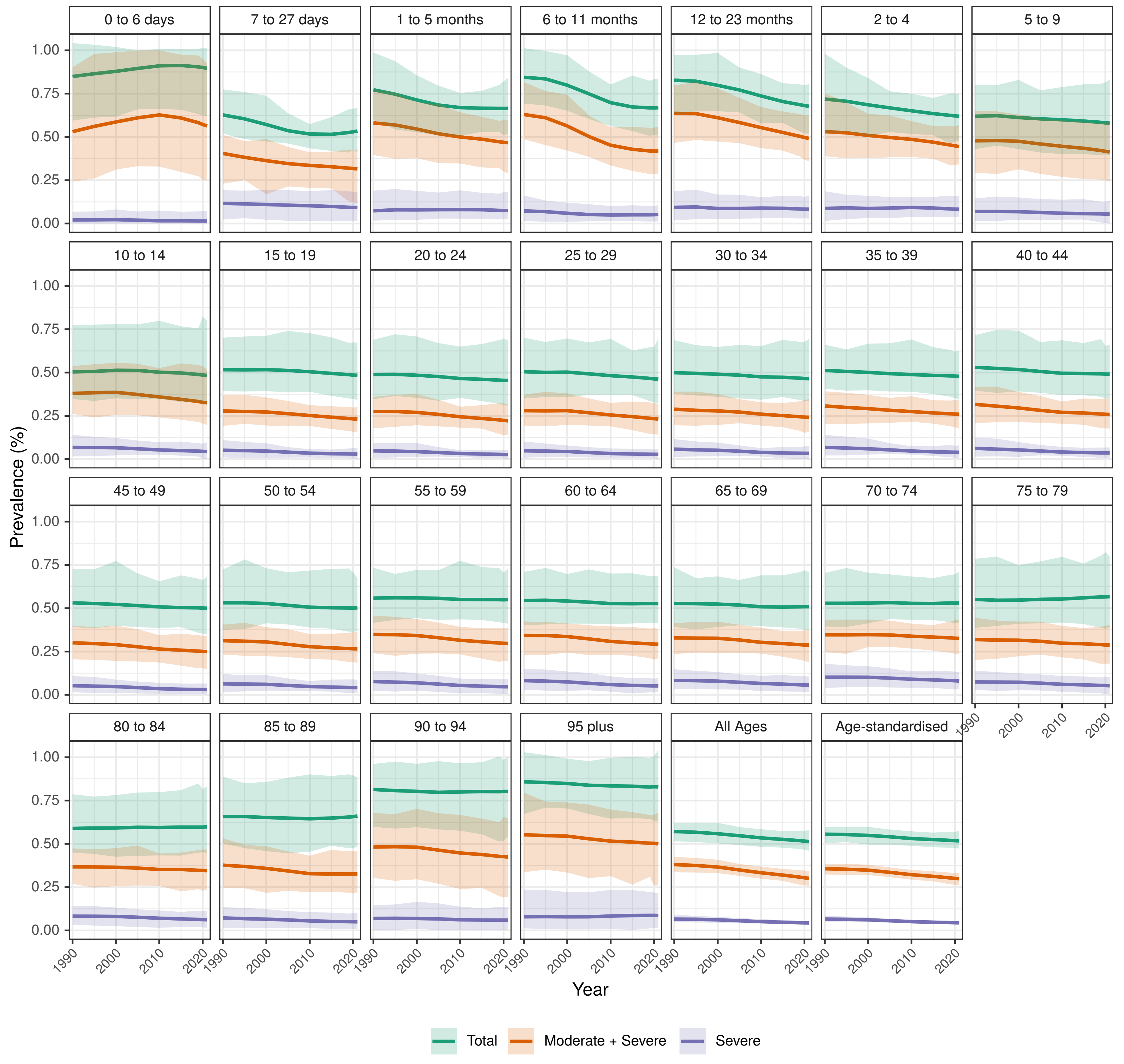

Cause-Specific Anaemia Prevalence - 2021

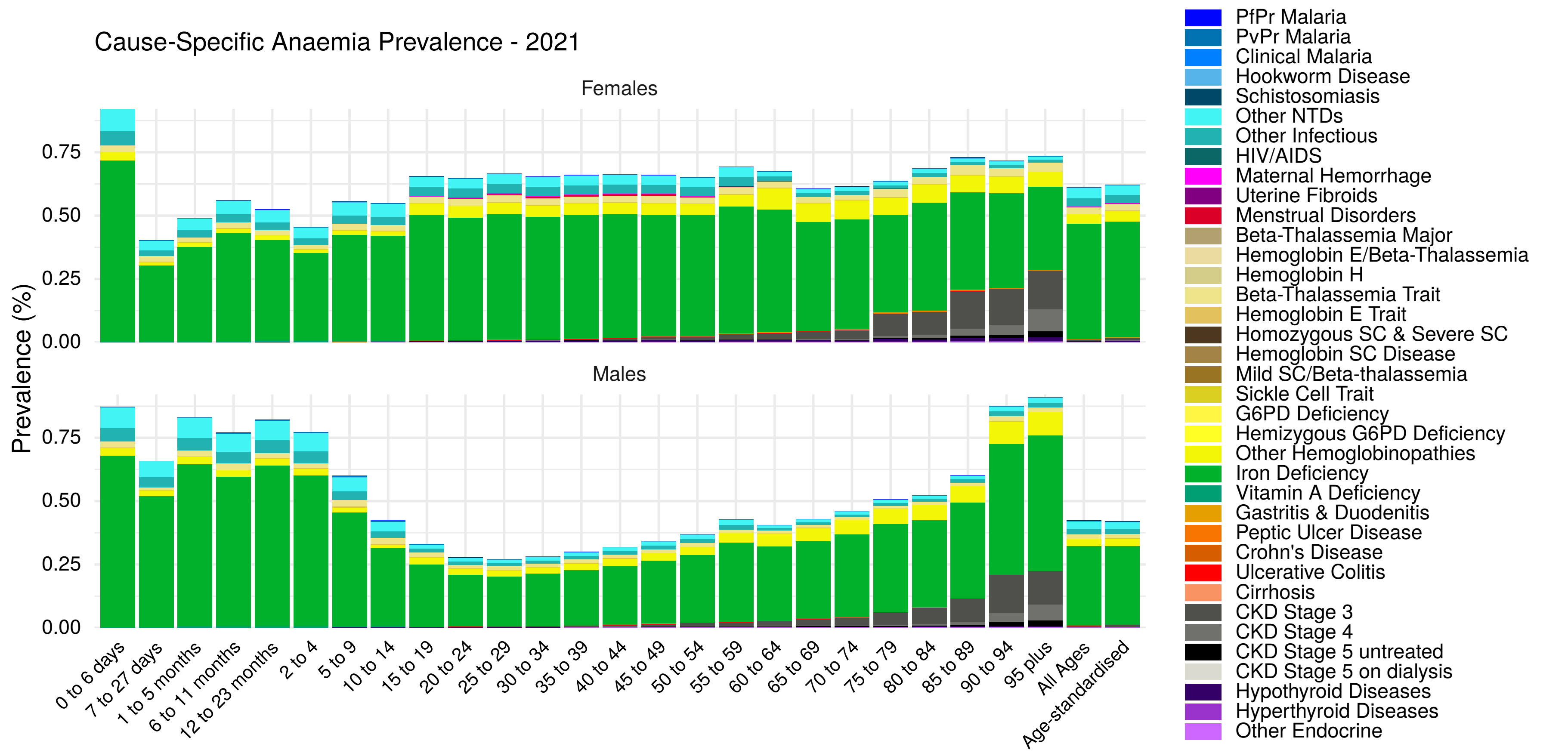

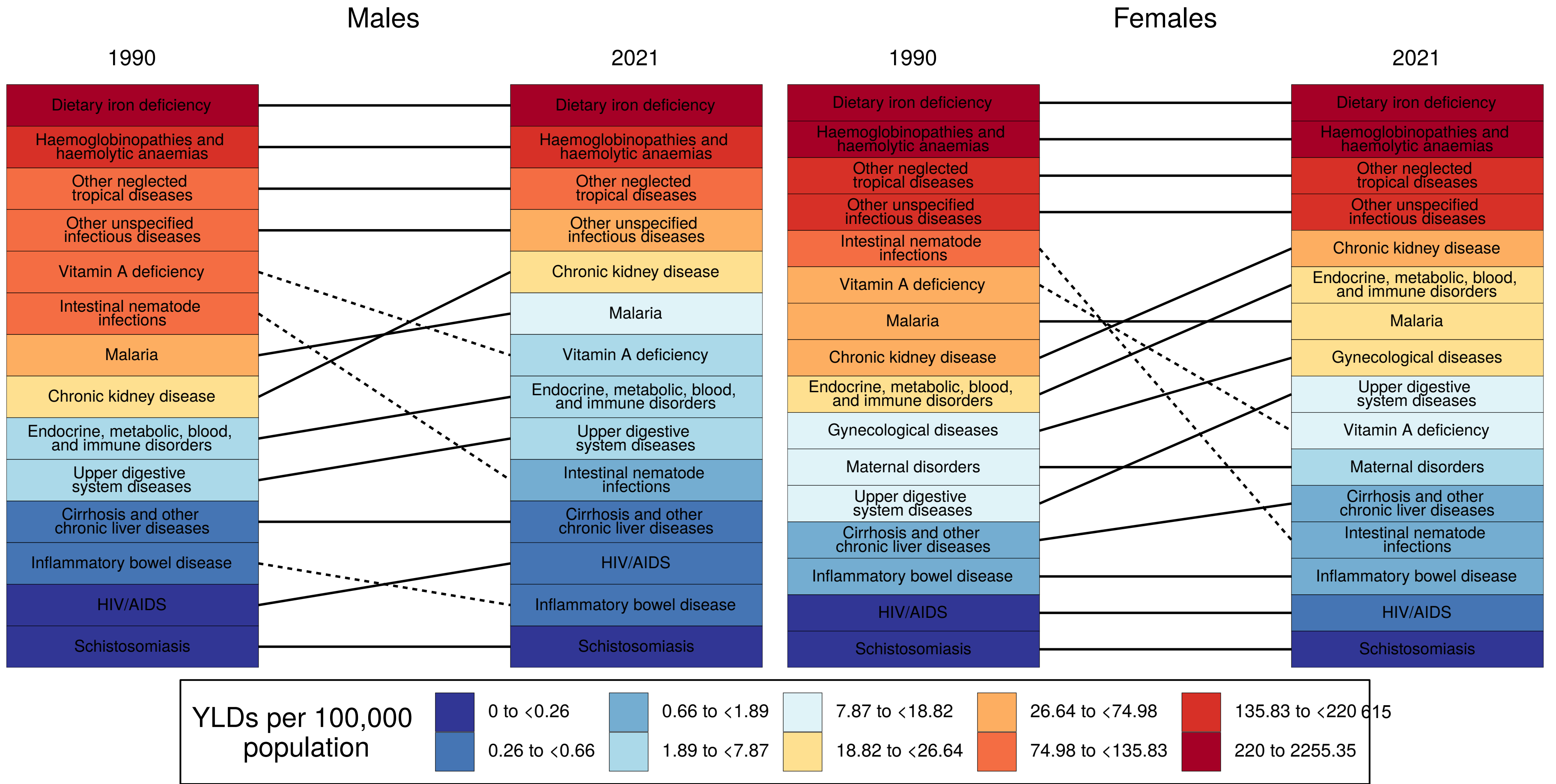

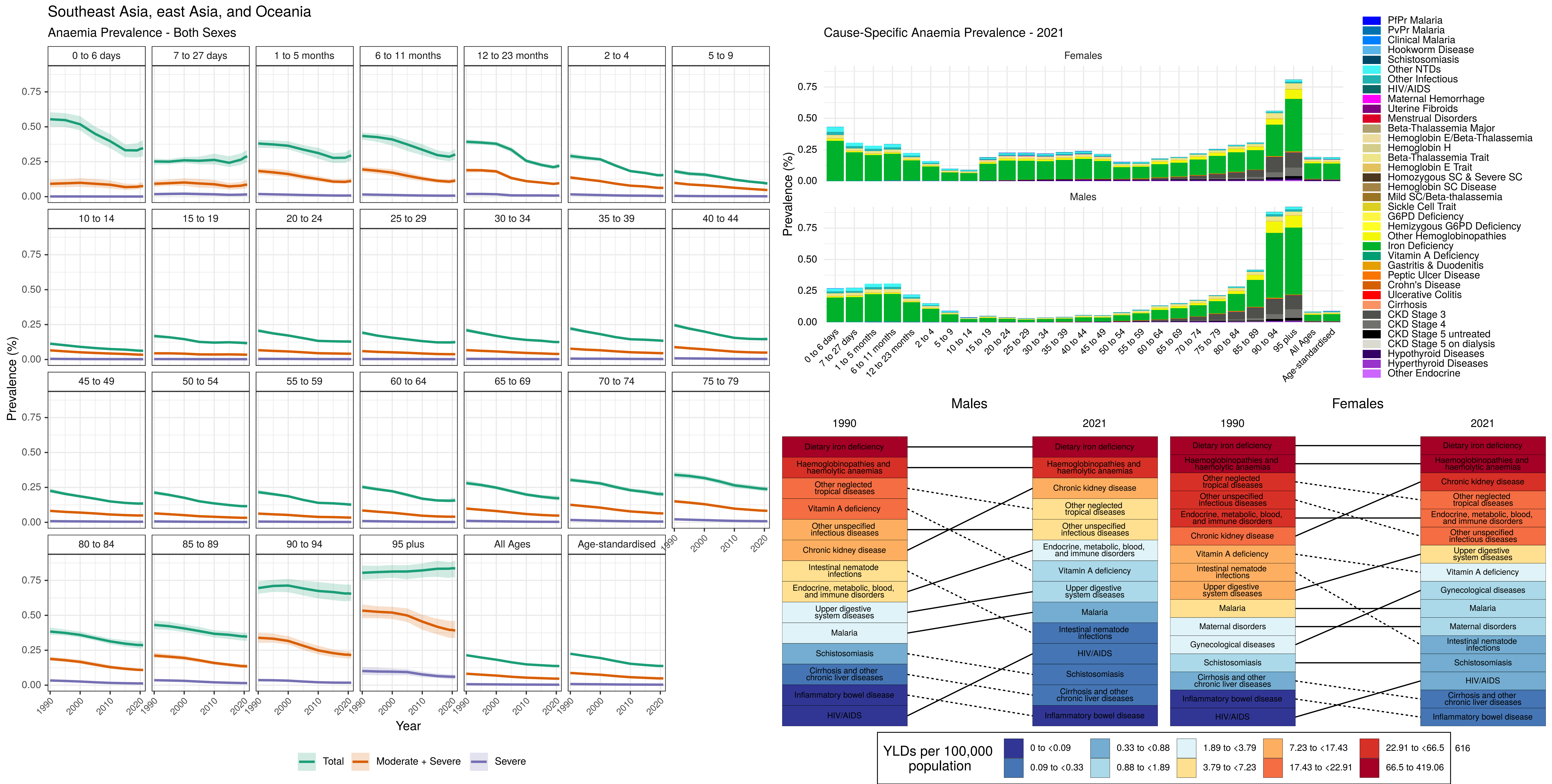

Southeast Asia, east Asia, and Oceania
Anaemia Prevalence - Both Sexes
0 to 6 days
7 to 27 days
1 to 5 months
6 to 11 months
12 to 23 months
2 to 4
5 to 9
10 to 14
15 to 19
20 to 24
25 to 29
30 to 34
35 to 39
40 to 44
45 to 49
50 to 54
55 to 59
60 to 64
65 to 69
70 to 74
75 to 79
80 to 84
85 to 89
90 to 94
95 plus
All Ages
Age-standardised
0.00
0.25
0.50
0.75
Prevalence (%)
1990
2000
2010
2020
Year
Total
Moderate + Severe
Severe
Cause-Specific Anaemia Prevalence - 2021
Females
Males
Prevalence (%)
PfPr Malaria
PvPr Malaria
Clinical Malaria
Hookworm Disease
Schistosomiasis
Other NTDs
Other Infectious
HIV/AIDS
Maternal Hemorrhage
Uterine Fibroids
Menstrual Disorders
Beta-Thalassemia Major
Hemoglobin E/Beta-Thalassemia
Hemoglobin H
Beta-Thalassemia Trait
Hemoglobin E Trait
Homozygous SC & Severe SC
Hemoglobin SC Disease
Mild SC/Beta-thalassemia
Sickle Cell Trait
G6PD Deficiency
Hemizygous G6PD Deficiency
Other Hemoglobinopathies
Iron Deficiency
Vitamin A Deficiency
Gastritis & Duodenitis
Peptic Ulcer Disease
Crohn's Disease
Ulcerative Colitis
Cirrhosis
CKD Stage 3
CKD Stage 4
CKD Stage 5 untreated
CKD Stage 5 on dialysis
Hypothyroid Diseases
Hyperthyroid Diseases
Other Endocrine
Males
Females
1990
2021
Dietary iron deficiency
Haemoglobinopathies and haemolytic anaemias
Other neglected tropical diseases
Vitamin A deficiency
Other unspecified infectious diseases
Chronic kidney disease
Intestinal nematode infections
Endocrine, metabolic, blood, and immune disorders
Upper digestive system diseases
Malaria
Schistosomiasis
Cirrhosis and other chronic liver diseases
Inflammatory bowel disease
HIV/AIDS
Maternal disorders
Gynecological diseases
YLDs per 100,000 population
0 to <0.09
0.09 to <0.33
0.33 to <0.88
0.88 to <1.89
1.89 to <3.79
3.79 to <7.23
7.23 to <17.43
17.43 to <22.91
22.91 to <66.5
66.5 to 419.06

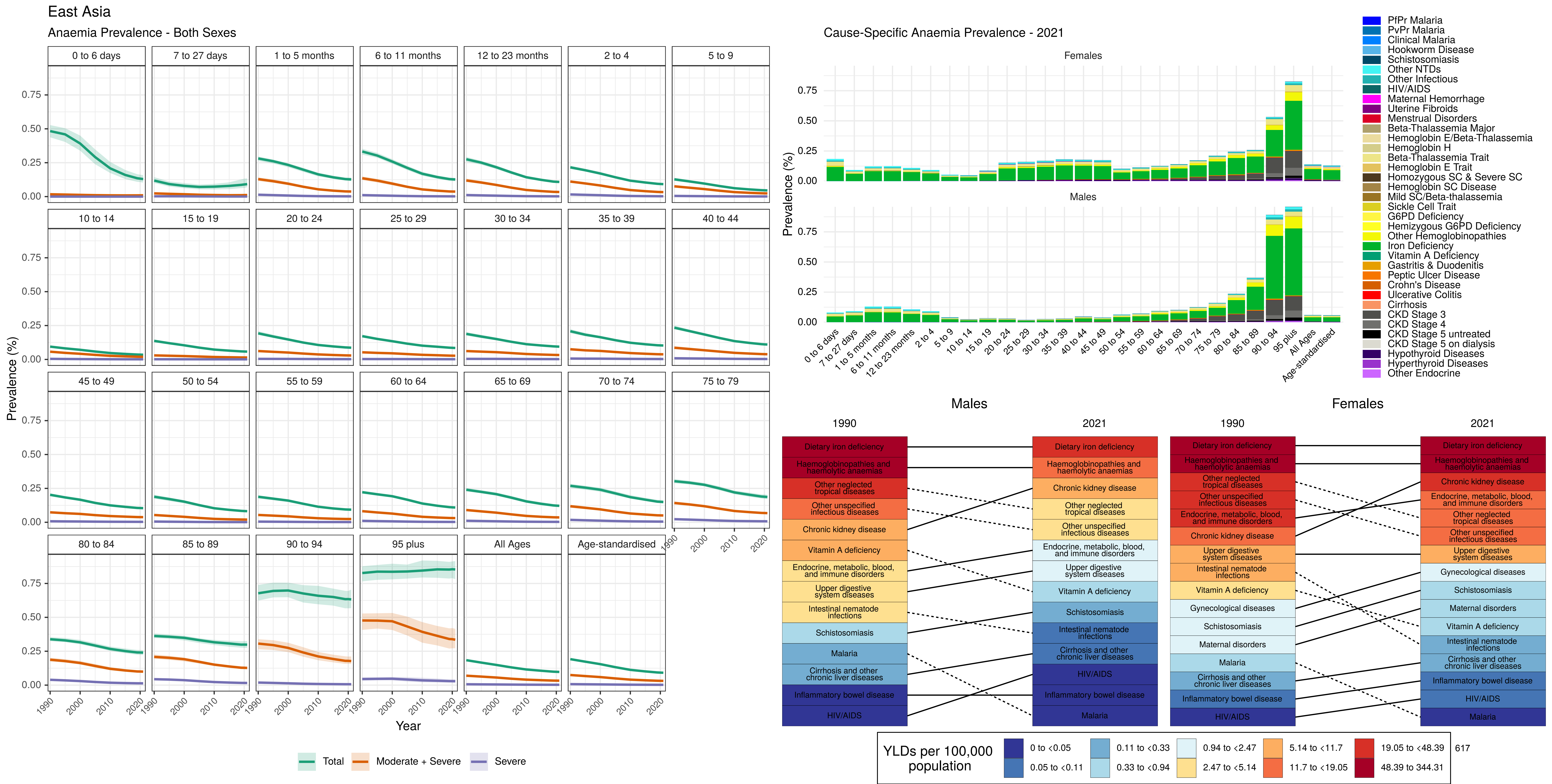
East Asia
Anaemia Prevalence - Both Sexes
0 to 6 days
7 to 27 days
1 to 5 months
6 to 11 months
12 to 23 months
2 to 4
5 to 9
10 to 14
15 to 19
20 to 24
25 to 29
30 to 34
35 to 39
40 to 44
45 to 49
50 to 54
55 to 59
60 to 64
65 to 69
70 to 74
75 to 79
80 to 84
85 to 89
90 to 94
95 plus
All Ages
Age-standardised
Prevalence (%)
Year
Total
Moderate + Severe
Severe
Cause-Specific Anaemia Prevalence - 2021
Females
Males
PfPr Malaria
PvPr Malaria
Clinical Malaria
Hookworm Disease
Schistosomiasis
Other NTDs
Other Infectious
HIV/AIDS
Maternal Hemorrhage
Uterine Fibroids
Menstrual Disorders
Beta-Thalassemia Major
Hemoglobin E/Beta-Thalassemia
Hemoglobin H
Beta-Thalassemia Trait
Hemoglobin E Trait
Homozygous SC & Severe SC
Hemoglobin SC Disease
Mild SC/Beta-thalassemia
Sickle Cell Trait
G6PD Deficiency
Hemizygous G6PD Deficiency
Other Hemoglobinopathies
Iron Deficiency
Vitamin A Deficiency
Gastritis & Duodenitis
Peptic Ulcer Disease
Crohn's Disease
Ulcerative Colitis
Cirrhosis
CKD Stage 3
CKD Stage 4
CKD Stage 5 untreated
CKD Stage 5 on dialysis
Hypothyroid Diseases
Hyperthyroid Diseases
Other Endocrine
Males
Females
1990
2021
Dietary iron deficiency
Haemoglobinopathies and haemolytic anaemias
Other neglected tropical diseases
Other unspecified infectious diseases
Chronic kidney disease
Vitamin A deficiency
Endocrine, metabolic, blood, and immune disorders
Upper digestive system diseases
Intestinal nematode infections
Schistosomiasis
Malaria
Cirrhosis and other chronic liver diseases
Inflammatory bowel disease
HIV/AIDS
Gynecological diseases
Maternal disorders
YLDs per 100,000 population
0 to <0.05
0.05 to <0.11
0.11 to <0.33
0.33 to <0.94
0.94 to <2.47
2.47 to <5.14
5.14 to <11.7
11.7 to <19.05
19.05 to <48.39
48.39 to 344.31

China

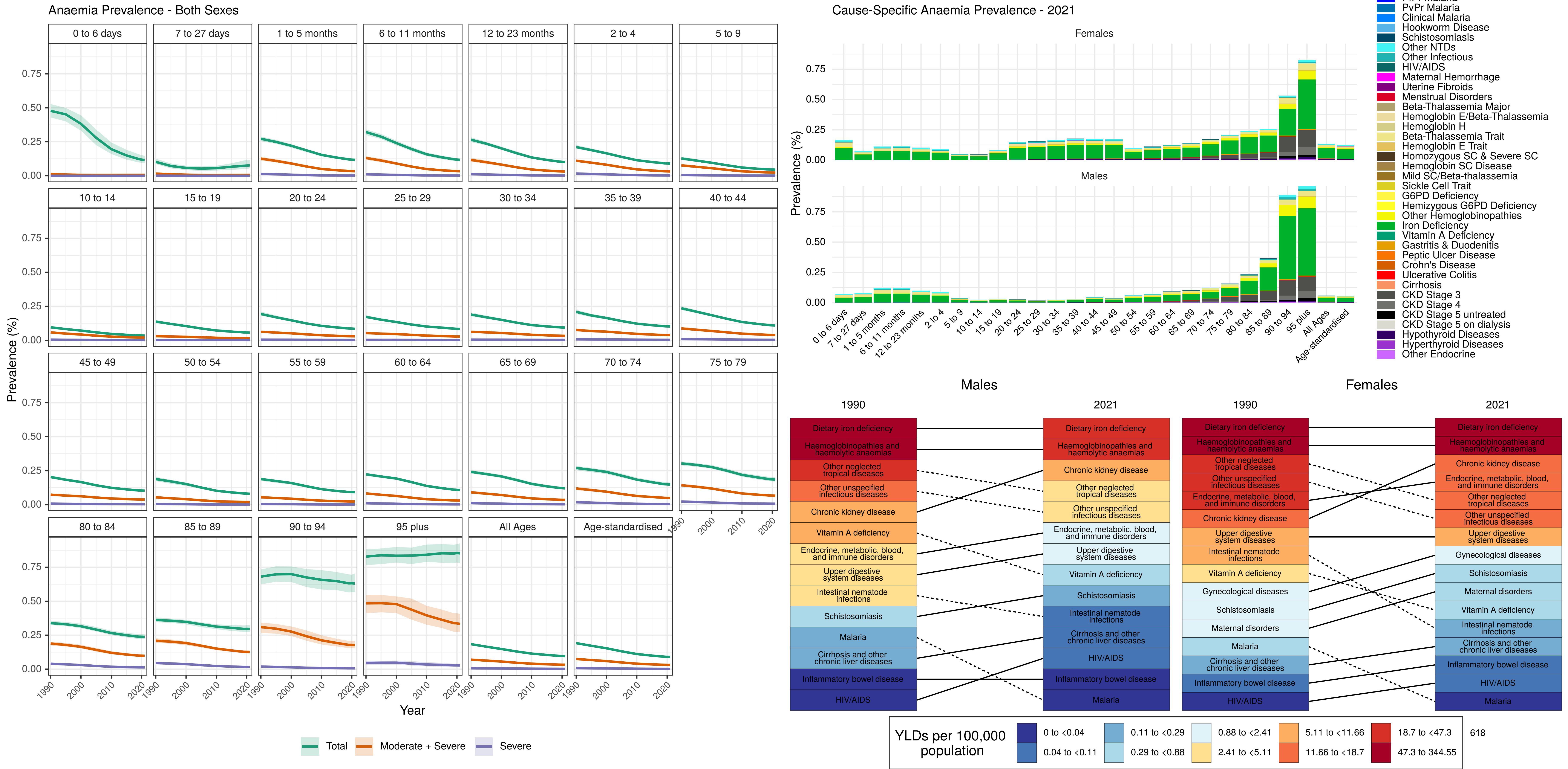

Anaemia Prevalence - Both Sexes
0 to 6 days
7 to 27 days
1 to 5 months
6 to 11 months
12 to 23 months
2 to 4
5 to 9
10 to 14
15 to 19
20 to 24
25 to 29
30 to 34
35 to 39
40 to 44
45 to 49
50 to 54
55 to 59
60 to 64
65 to 69
70 to 74
75 to 79
80 to 84
85 to 89
90 to 94
95 plus
All Ages
Age-standardised
Prevalence (%)
0.00
0.25
0.50
0.75
1990
2000
2010
2020
Year
Total
Moderate + Severe
Severe
Cause-Specific Anaemia Prevalence - 2021
Females
Males
PfPr Malaria
PvPr Malaria
Clinical Malaria
Hookworm Disease
Schistosomiasis
Other NTDs
Other Infectious
HIV/AIDS
Maternal Hemorrhage
Uterine Fibroids
Menstrual Disorders
Beta-Thalassemia Major
Hemoglobin E/Beta-Thalassemia
Hemoglobin H
Beta-Thalassemia Trait
Hemoglobin E Trait
Homozygous SC & Severe SC
Hemoglobin SC Disease
Mild SC/Beta-thalassemia
Sickle Cell Trait
G6PD Deficiency
Hemizygous G6PD Deficiency
Other Hemoglobinopathies
Iron Deficiency
Vitamin A Deficiency
Gastritis & Duodenitis
Peptic Ulcer Disease
Crohn's Disease
Ulcerative Colitis
Cirrhosis
CKD Stage 3
CKD Stage 4
CKD Stage 5 untreated
CKD Stage 5 on dialysis
Hypothyroid Diseases
Hyperthyroid Diseases
Other Endocrine
Males
Females
1990
2021
Dietary iron deficiency
Haemoglobinopathies and haemolytic anaemias
Other neglected tropical diseases
Other unspecified infectious diseases
Chronic kidney disease
Vitamin A deficiency
Endocrine, metabolic, blood, and immune disorders
Upper digestive system diseases
Intestinal nematode infections
Schistosomiasis
Malaria
Cirrhosis and other chronic liver diseases
Inflammatory bowel disease
HIV/AIDS
Gynecological diseases
Maternal disorders
YLDs per 100,000 population
0 to <0.04
0.04 to <0.11
0.11 to <0.29
0.29 to <0.88
0.88 to <2.41
2.41 to <5.11
5.11 to <11.66
11.66 to <18.7
18.7 to <47.3
47.3 to 344.55

618

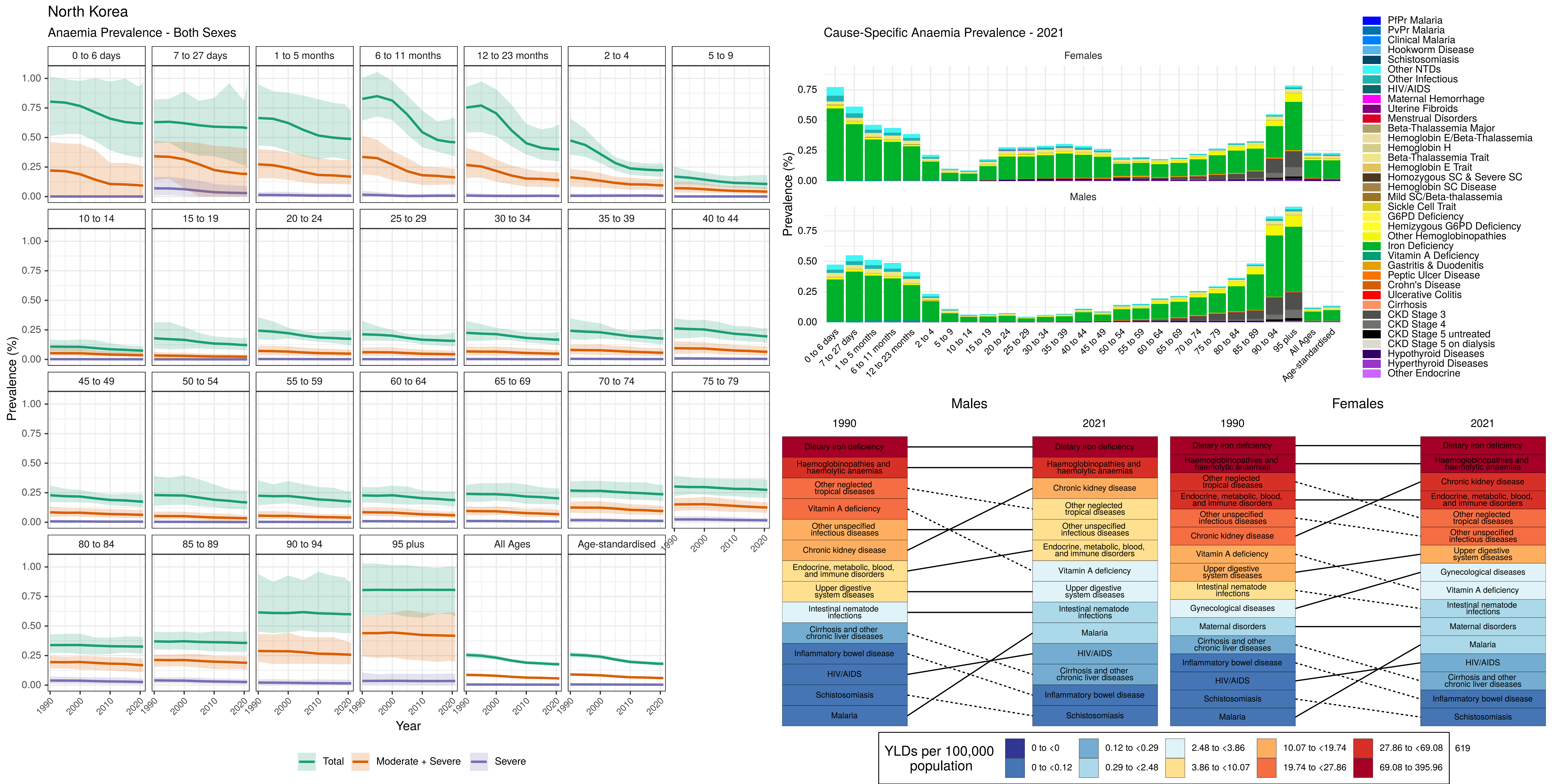

North Korea
Anaemia Prevalence - Both Sexes
0 to 6 days
7 to 27 days
1 to 5 months
6 to 11 months
12 to 23 months
2 to 4
5 to 9
10 to 14
15 to 19
20 to 24
25 to 29
30 to 34
35 to 39
40 to 44
45 to 49
50 to 54
55 to 59
60 to 64
65 to 69
70 to 74
75 to 79
80 to 84
85 to 89
90 to 94
95 plus
All Ages
Age-standardised
Prevalence (%)
Year
Total
Moderate + Severe
Severe
Cause-Specific Anaemia Prevalence - 2021
Females
Males
Prevalence (%)
PfPr Malaria
PvPr Malaria
Clinical Malaria
Hookworm Disease
Schistosomiasis
Other NTDs
Other Infectious
HIV/AIDS
Maternal Hemorrhage
Uterine Fibroids
Menstrual Disorders
Beta-Thalassemia Major
Hemoglobin E/Beta-Thalassemia
Hemoglobin H
Beta-Thalassemia Trait
Hemoglobin E Trait
Homozygous SC & Severe SC
Hemoglobin SC Disease
Mild SC/Beta-thalassemia
Sickle Cell Trait
G6PD Deficiency
Hemizygous G6PD Deficiency
Other Hemoglobinopathies
Iron Deficiency
Vitamin A Deficiency
Gastritis & Duodenitis
Peptic Ulcer Disease
Crohn's Disease
Ulcerative Colitis
Cirrhosis
CKD Stage 3
CKD Stage 4
CKD Stage 5 untreated
CKD Stage 5 on dialysis
Hypothyroid Diseases
Hyperthyroid Diseases
Other Endocrine
Males
1990
Dietary iron deficiency
Haemoglobinopathies and haemolytic anaemias
Other neglected tropical diseases
Vitamin A deficiency
Other unspecified infectious diseases
Chronic kidney disease
Endocrine, metabolic, blood, and immune disorders
Upper digestive system diseases
Intestinal nematode infections
Cirrhosis and other chronic liver diseases
Inflammatory bowel disease
HIV/AIDS
Schistosomiasis
Malaria
2021
Dietary iron deficiency
Haemoglobinopathies and haemolytic anaemias
Chronic kidney disease
Other neglected tropical diseases
Other unspecified infectious diseases
Endocrine, metabolic, blood, and immune disorders
Vitamin A deficiency
Upper digestive system diseases
Intestinal nematode infections
Malaria
HIV/AIDS
Cirrhosis and other chronic liver diseases
Inflammatory bowel disease
Schistosomiasis
Females
1990
Dietary iron deficiency
Haemoglobinopathies and haemolytic anaemias
Other neglected tropical diseases
Endocrine, metabolic, blood, and immune disorders
Other unspecified infectious diseases
Chronic kidney disease
Vitamin A deficiency
Upper digestive system diseases
Intestinal nematode infections
Gynecological diseases
Maternal disorders
Cirrhosis and other chronic liver diseases
Inflammatory bowel disease
HIV/AIDS
Schistosomiasis
Malaria
2021
Dietary iron deficiency
Haemoglobinopathies and haemolytic anaemias
Chronic kidney disease
Endocrine, metabolic, blood, and immune disorders
Other neglected tropical diseases
Other unspecified infectious diseases
Upper digestive system diseases
Gynecological diseases
Vitamin A deficiency
Intestinal nematode infections
Maternal disorders
Malaria
HIV/AIDS
Cirrhosis and other chronic liver diseases
Inflammatory bowel disease
Schistosomiasis
YLDs per 100,000 population
0 to <0
0 to <0.12
0.12 to <0.29
0.29 to <2.48
2.48 to <3.86
3.86 to <10.07
10.07 to <19.74
19.74 to <27.86
27.86 to <69.08
69.08 to 395.96
619

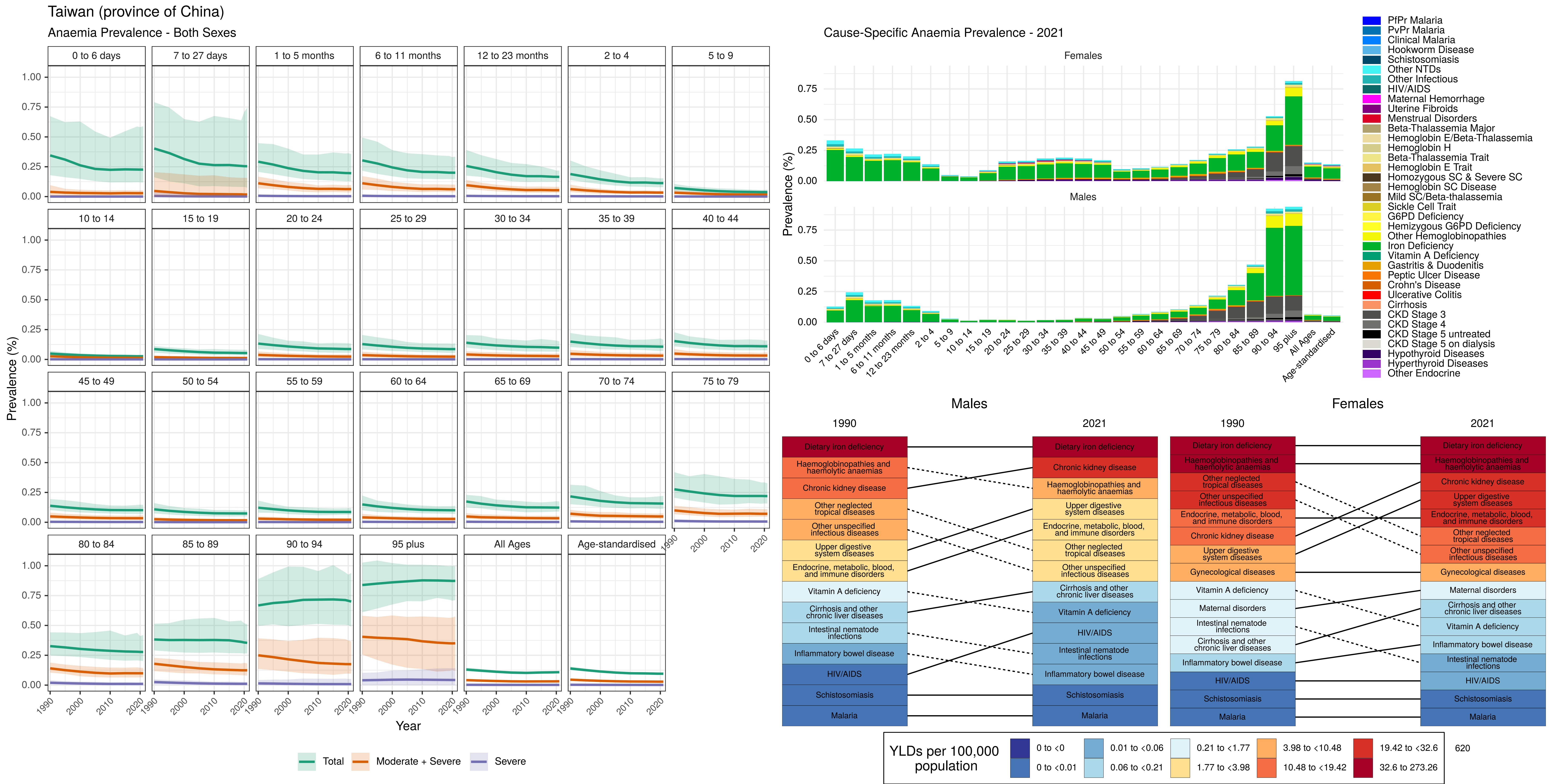
Taiwan (province of China)
Anaemia Prevalence - Both Sexes
0 to 6 days
7 to 27 days
1 to 5 months
6 to 11 months
12 to 23 months
2 to 4
5 to 9
10 to 14
15 to 19
20 to 24
25 to 29
30 to 34
35 to 39
40 to 44
45 to 49
50 to 54
55 to 59
60 to 64
65 to 69
70 to 74
75 to 79
80 to 84
85 to 89
90 to 94
95 plus
All Ages
Age-standardised
Prevalence (%)
Year
Total
Moderate + Severe
Severe
Cause-Specific Anaemia Prevalence - 2021
Females
Males
PfPr Malaria
PvPr Malaria
Clinical Malaria
Hookworm Disease
Schistosomiasis
Other NTDs
Other Infectious
HIV/AIDS
Maternal Hemorrhage
Uterine Fibroids
Menstrual Disorders
Beta-Thalassemia Major
Hemoglobin E/Beta-Thalassemia
Hemoglobin H
Beta-Thalassemia Trait
Hemoglobin E Trait
Homozygous SC & Severe SC
Hemoglobin SC Disease
Mild SC/Beta-thalassemia
Sickle Cell Trait
G6PD Deficiency
Hemizygous G6PD Deficiency
Other Hemoglobinopathies
Iron Deficiency
Vitamin A Deficiency
Gastritis & Duodenitis
Peptic Ulcer Disease
Crohn's Disease
Ulcerative Colitis
Cirrhosis
CKD Stage 3
CKD Stage 4
CKD Stage 5 untreated
CKD Stage 5 on dialysis
Hypothyroid Diseases
Hyperthyroid Diseases
Other Endocrine
Males
Females
1990
2021
Dietary iron deficiency
Haemoglobinopathies and haemolytic anaemias
Chronic kidney disease
Other neglected tropical diseases
Other unspecified infectious diseases
Upper digestive system diseases
Endocrine, metabolic, blood, and immune disorders
Vitamin A deficiency
Cirrhosis and other chronic liver diseases
Intestinal nematode infections
Inflammatory bowel disease
HIV/AIDS
Schistosomiasis
Malaria
Gynecological diseases
Maternal disorders
YLDs per 100,000 population
0 to <0
0 to <0.01
0.01 to <0.06
0.06 to <0.21
0.21 to <1.77
1.77 to <3.98
3.98 to <10.48
10.48 to <19.42
19.42 to <32.6
32.6 to 273.26
620

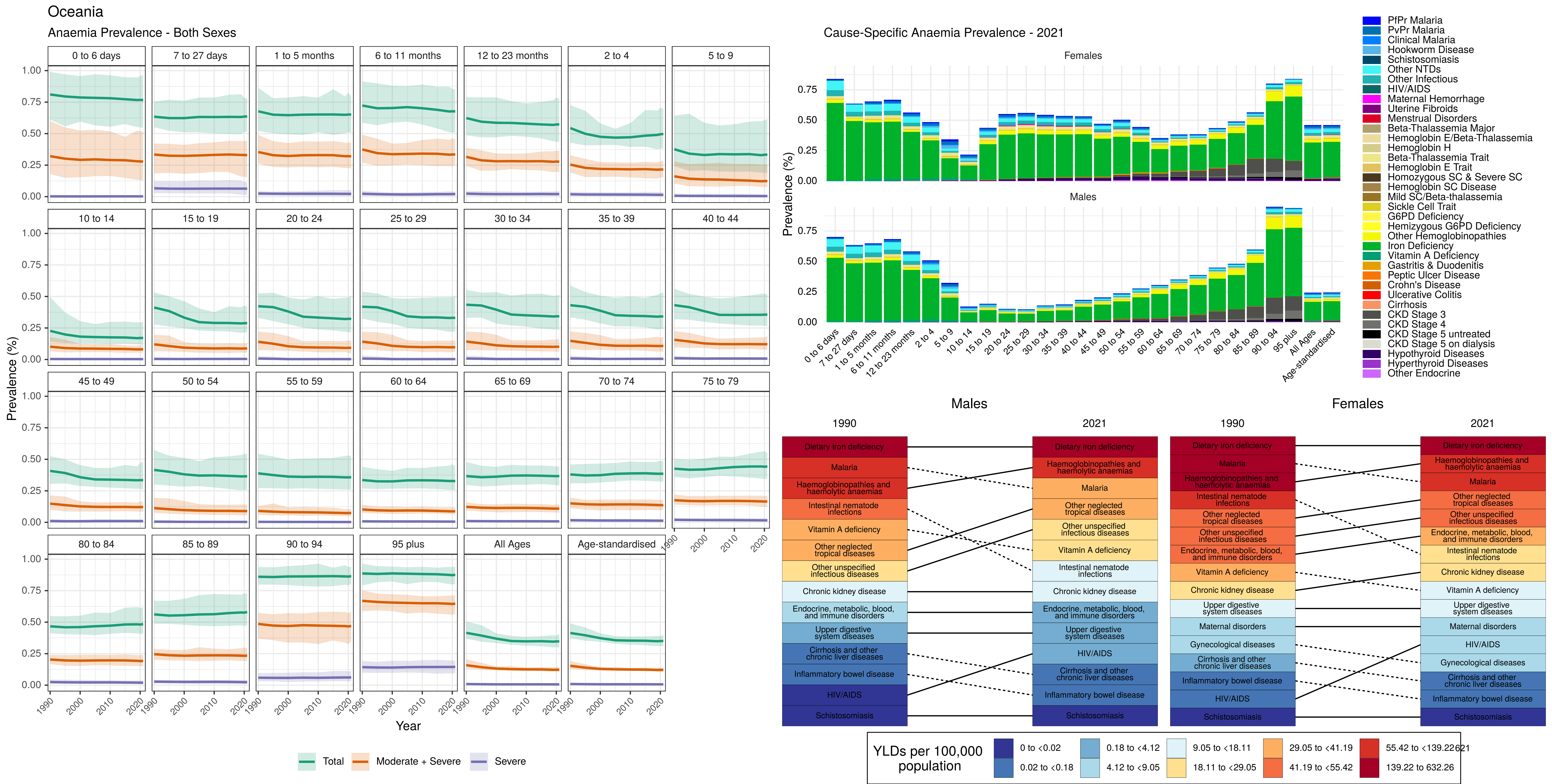

Oceania
Anaemia Prevalence - Both Sexes
0 to 6 days
7 to 27 days
1 to 5 months
6 to 11 months
12 to 23 months
2 to 4
5 to 9
10 to 14
15 to 19
20 to 24
25 to 29
30 to 34
35 to 39
40 to 44
45 to 49
50 to 54
55 to 59
60 to 64
65 to 69
70 to 74
75 to 79
80 to 84
85 to 89
90 to 94
95 plus
All Ages
Age-standardised
Prevalence (%)
Year
Total
Moderate + Severe
Severe
Cause-Specific Anaemia Prevalence - 2021
Females
Males
Prevalence (%)
PfPr Malaria
PvPr Malaria
Clinical Malaria
Hookworm Disease
Schistosomiasis
Other NTDs
Other Infectious
HIV/AIDS
Maternal Hemorrhage
Uterine Fibroids
Menstrual Disorders
Beta-Thalassemia Major
Hemoglobin E/Beta-Thalassemia
Hemoglobin H
Beta-Thalassemia Trait
Hemoglobin E Trait
Homozygous SC & Severe SC
Hemoglobin SC Disease
Mild SC/Beta-thalassemia
Sickle Cell Trait
G6PD Deficiency
Hemizygous G6PD Deficiency
Other Hemoglobinopathies
Iron Deficiency
Vitamin A Deficiency
Gastritis & Duodenitis
Peptic Ulcer Disease
Crohn's Disease
Ulcerative Colitis
Cirrhosis
CKD Stage 3
CKD Stage 4
CKD Stage 5 untreated
CKD Stage 5 on dialysis
Hypothyroid Diseases
Hyperthyroid Diseases
Other Endocrine
Males
Females
1990
2021
Dietary iron deficiency
Malaria
Haemoglobinopathies and haemolytic anaemias
Intestinal nematode infections
Vitamin A deficiency
Other neglected tropical diseases
Other unspecified infectious diseases
Chronic kidney disease
Endocrine, metabolic, blood, and immune disorders
Upper digestive system diseases
Cirrhosis and other chronic liver diseases
Inflammatory bowel disease
HIV/AIDS
Schistosomiasis
Maternal disorders
Gynecological diseases
YLDs per 100,000 population
0 to <0.02
0.02 to <0.18
0.18 to <4.12
4.12 to <9.05
9.05 to <18.11
18.11 to <29.05
29.05 to <41.19
41.19 to <55.42
55.42 to <139.22621
139.22 to 632.26

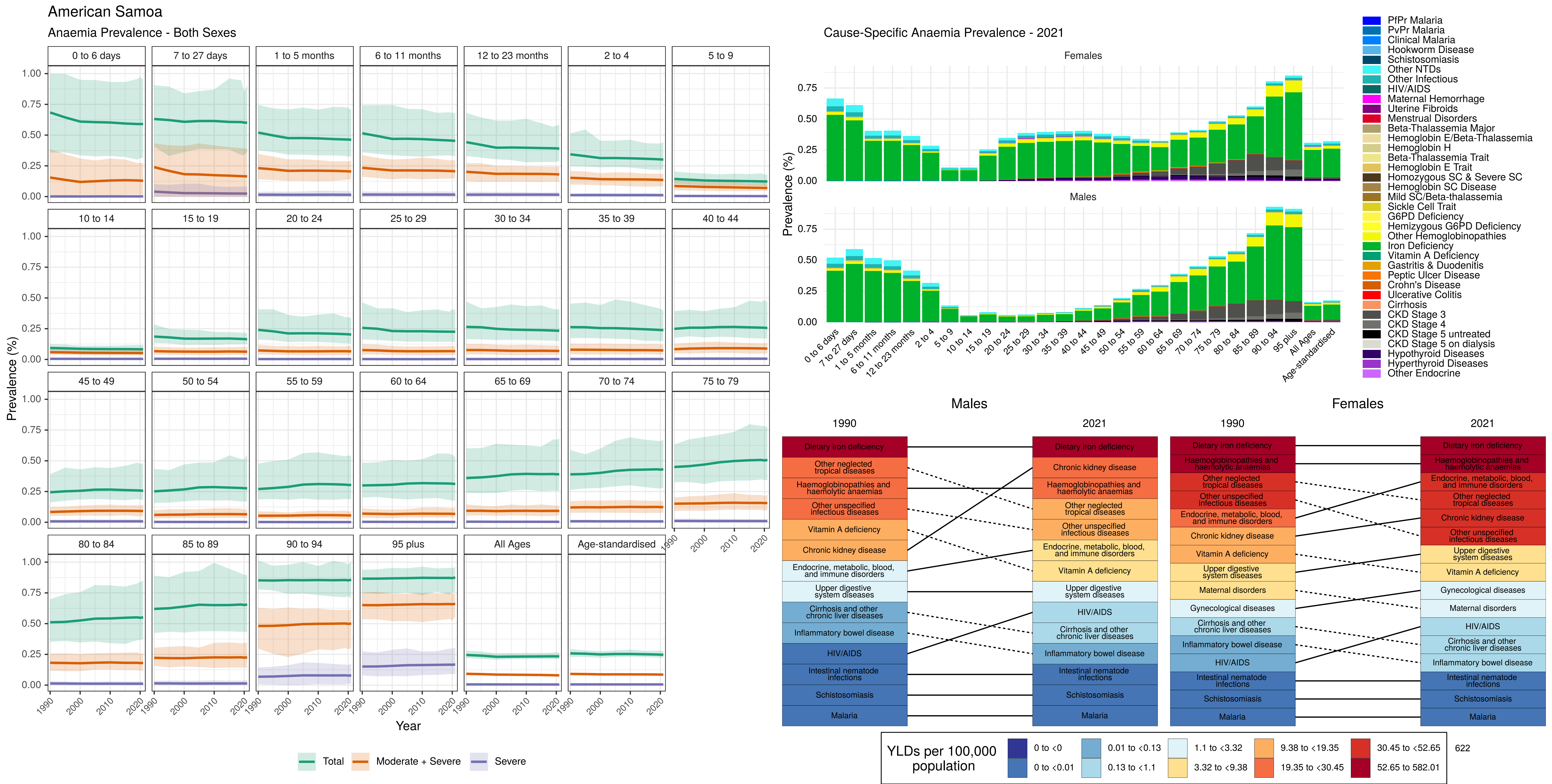
American Samoa
Anaemia Prevalence - Both Sexes
0 to 6 days
7 to 27 days
1 to 5 months
6 to 11 months
12 to 23 months
2 to 4
5 to 9
10 to 14
15 to 19
20 to 24
25 to 29
30 to 34
35 to 39
40 to 44
45 to 49
50 to 54
55 to 59
60 to 64
65 to 69
70 to 74
75 to 79
80 to 84
85 to 89
90 to 94
95 plus
All Ages
Age-standardised
Prevalence (%)
Year
Total
Moderate + Severe
Severe
Cause-Specific Anaemia Prevalence - 2021
Females
Males
PfPr Malaria
PvPr Malaria
Clinical Malaria
Hookworm Disease
Schistosomiasis
Other NTDs
Other Infectious
HIV/AIDS
Maternal Hemorrhage
Uterine Fibroids
Menstrual Disorders
Beta-Thalassemia Major
Hemoglobin E/Beta-Thalassemia
Hemoglobin H
Beta-Thalassemia Trait
Hemoglobin E Trait
Homozygous SC & Severe SC
Hemoglobin SC Disease
Mild SC/Beta-thalassemia
Sickle Cell Trait
G6PD Deficiency
Hemizygous G6PD Deficiency
Other Hemoglobinopathies
Iron Deficiency
Vitamin A Deficiency
Gastritis & Duodenitis
Peptic Ulcer Disease
Crohn's Disease
Ulcerative Colitis
Cirrhosis
CKD Stage 3
CKD Stage 4
CKD Stage 5 untreated
CKD Stage 5 on dialysis
Hypothyroid Diseases
Hyperthyroid Diseases
Other Endocrine
Males
Females
1990
2021
Dietary iron deficiency
Other neglected tropical diseases
Haemoglobinopathies and haemolytic anaemias
Other unspecified infectious diseases
Vitamin A deficiency
Chronic kidney disease
Endocrine, metabolic, blood, and immune disorders
Upper digestive system diseases
Cirrhosis and other chronic liver diseases
Inflammatory bowel disease
HIV/AIDS
Intestinal nematode infections
Schistosomiasis
Malaria
Maternal disorders
Gynecological diseases
YLDs per 100,000 population
0 to <0
0 to <0.01
0.01 to <0.13
0.13 to <1.1
1.1 to <3.32
3.32 to <9.38
9.38 to <19.35
19.35 to <30.45
30.45 to <52.65
52.65 to 582.01
622

Cook Islands

Anaemia Prevalence - Both Sexes

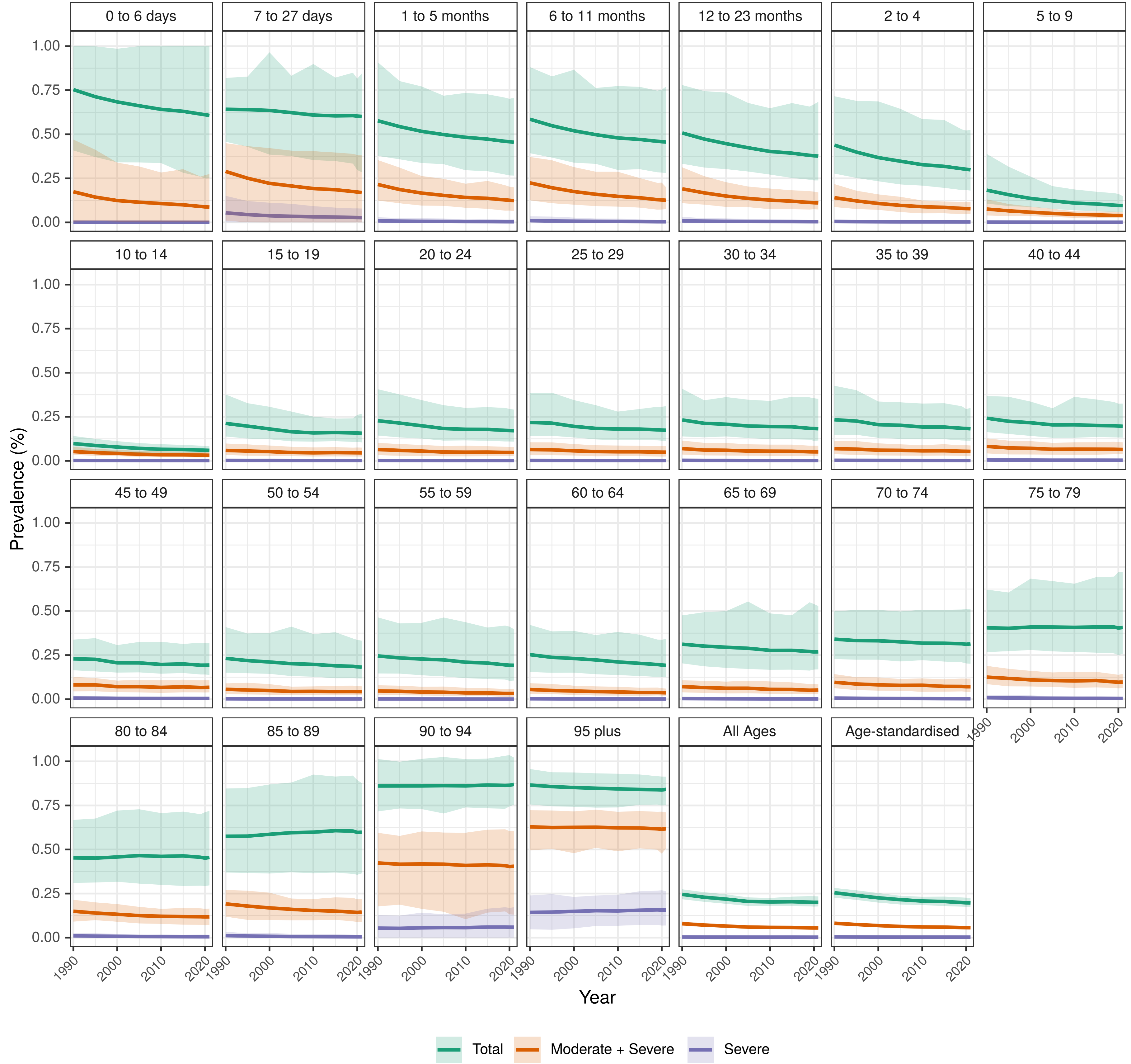

Cause-Specific Anaemia Prevalence - 2021

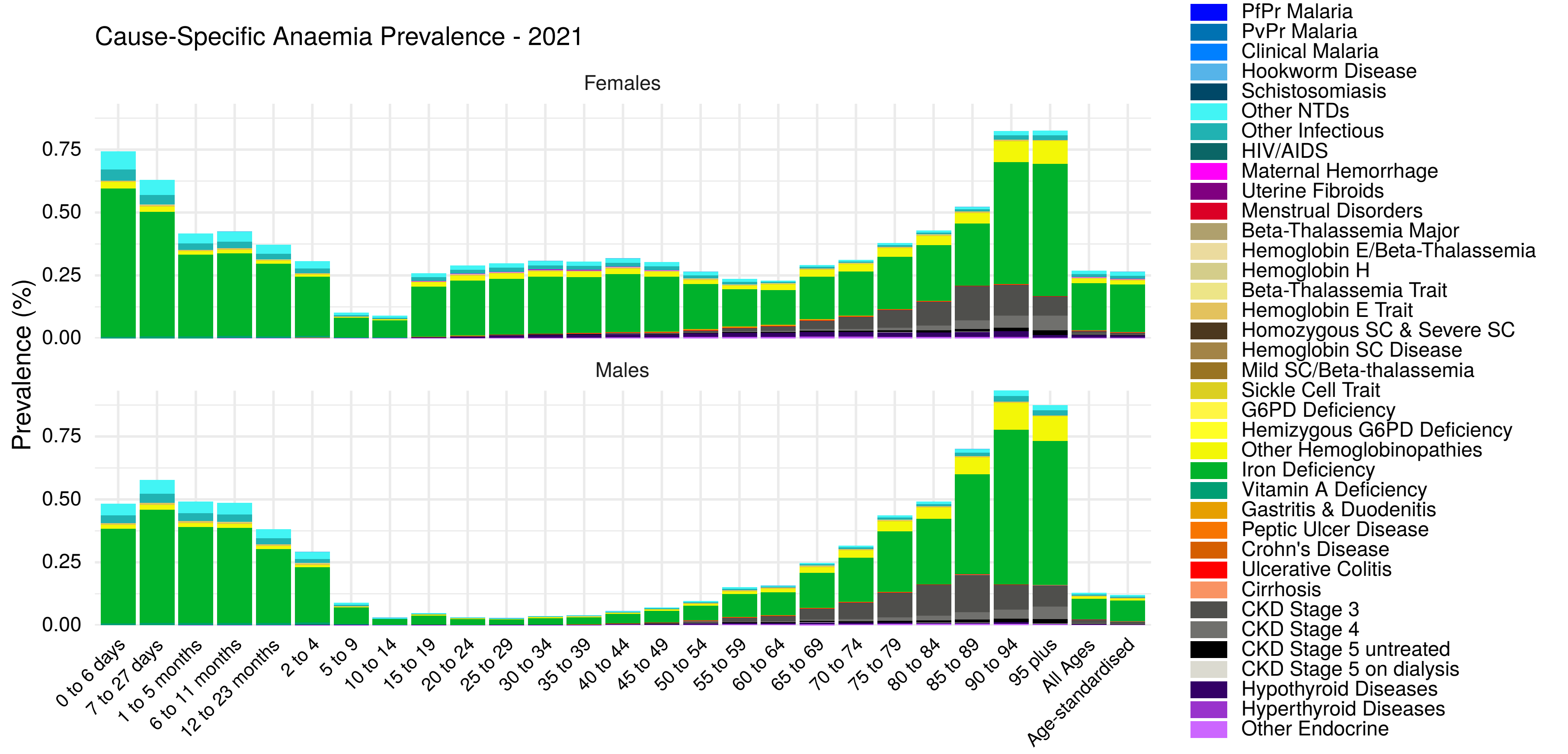

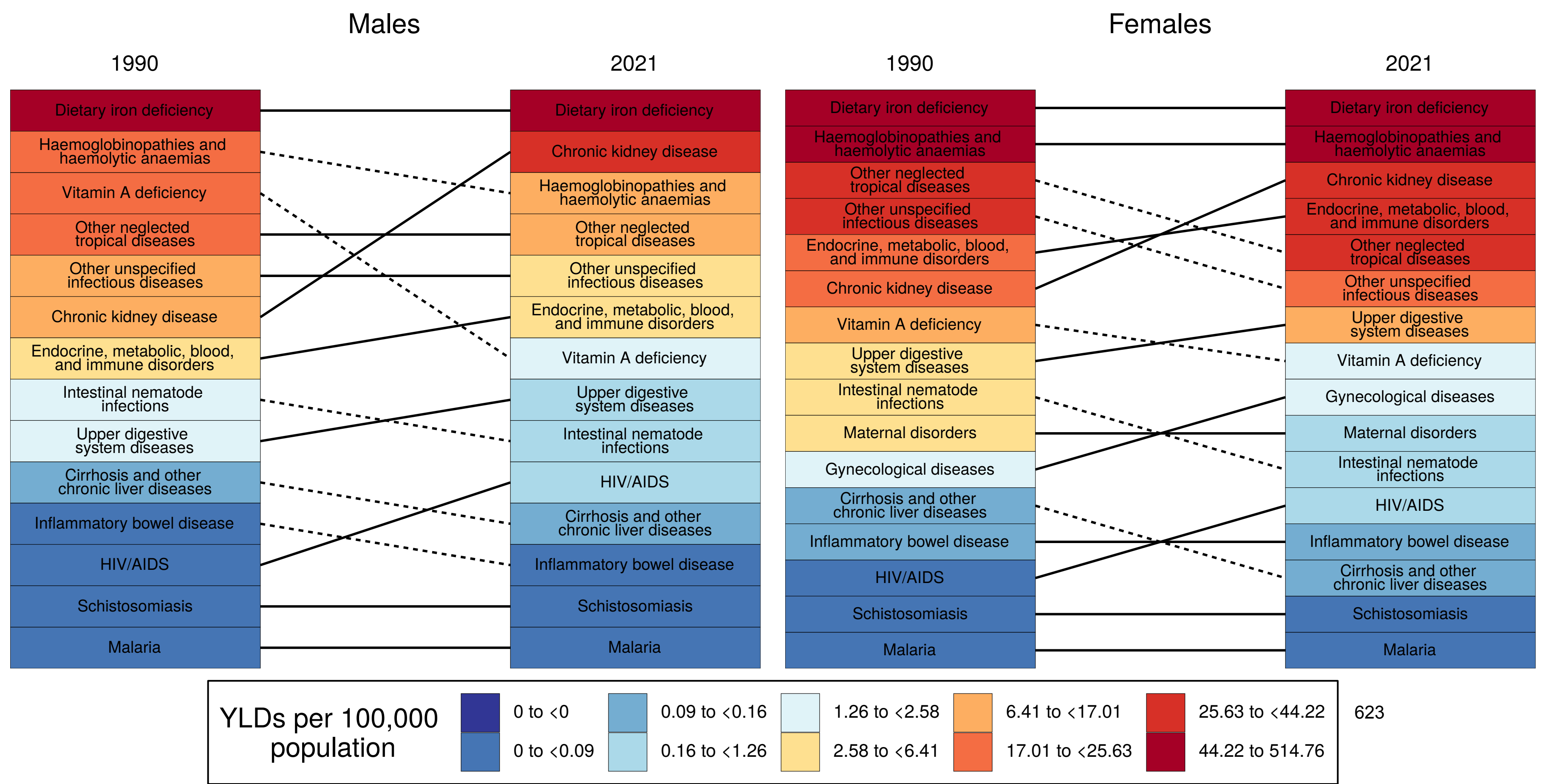

Fiji

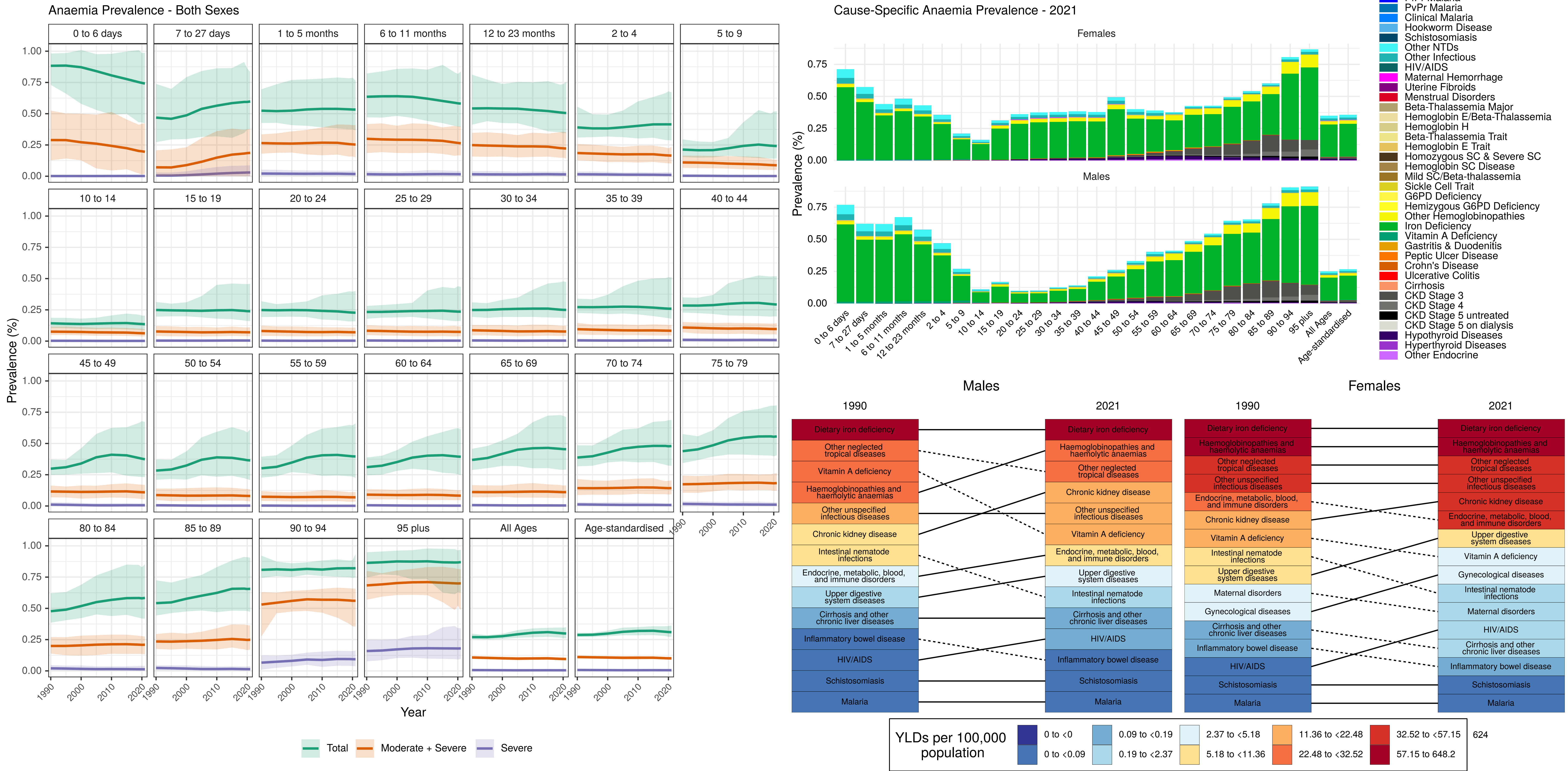

Anaemia Prevalence - Both Sexes
0 to 6 days
7 to 27 days
1 to 5 months
6 to 11 months
12 to 23 months
2 to 4
5 to 9
10 to 14
15 to 19
20 to 24
25 to 29
30 to 34
35 to 39
40 to 44
45 to 49
50 to 54
55 to 59
60 to 64
65 to 69
70 to 74
75 to 79
80 to 84
85 to 89
90 to 94
95 plus
All Ages
Age-standardised
Prevalence (%)
Year
Total
Moderate + Severe
Severe
Cause-Specific Anaemia Prevalence - 2021
Females
Males
PfPr Malaria
PvPr Malaria
Clinical Malaria
Hookworm Disease
Schistosomiasis
Other NTDs
Other Infectious
HIV/AIDS
Maternal Hemorrhage
Uterine Fibroids
Menstrual Disorders
Beta-Thalassemia Major
Hemoglobin E/Beta-Thalassemia
Hemoglobin H
Beta-Thalassemia Trait
Hemoglobin E Trait
Homozygous SC & Severe SC
Hemoglobin SC Disease
Mild SC/Beta-thalassemia
Sickle Cell Trait
G6PD Deficiency
Hemizygous G6PD Deficiency
Other Hemoglobinopathies
Iron Deficiency
Vitamin A Deficiency
Gastritis & Duodenitis
Peptic Ulcer Disease
Crohn's Disease
Ulcerative Colitis
Cirrhosis
CKD Stage 3
CKD Stage 4
CKD Stage 5 untreated
CKD Stage 5 on dialysis
Hypothyroid Diseases
Hyperthyroid Diseases
Other Endocrine
Males
Females
1990
2021
Dietary iron deficiency
Other neglected tropical diseases
Vitamin A deficiency
Haemoglobinopathies and haemolytic anaemias
Other unspecified infectious diseases
Chronic kidney disease
Intestinal nematode infections
Endocrine, metabolic, blood, and immune disorders
Upper digestive system diseases
Cirrhosis and other chronic liver diseases
Inflammatory bowel disease
HIV/AIDS
Schistosomiasis
Malaria
Maternal disorders
Gynecological diseases
YLDs per 100,000 population
0 to <0
0 to <0.09
0.09 to <0.19
0.19 to <2.37
2.37 to <5.18
5.18 to <11.36
11.36 to <22.48
22.48 to <32.52
32.52 to <57.15
57.15 to 648.2

Guam

Anaemia Prevalence - Both Sexes

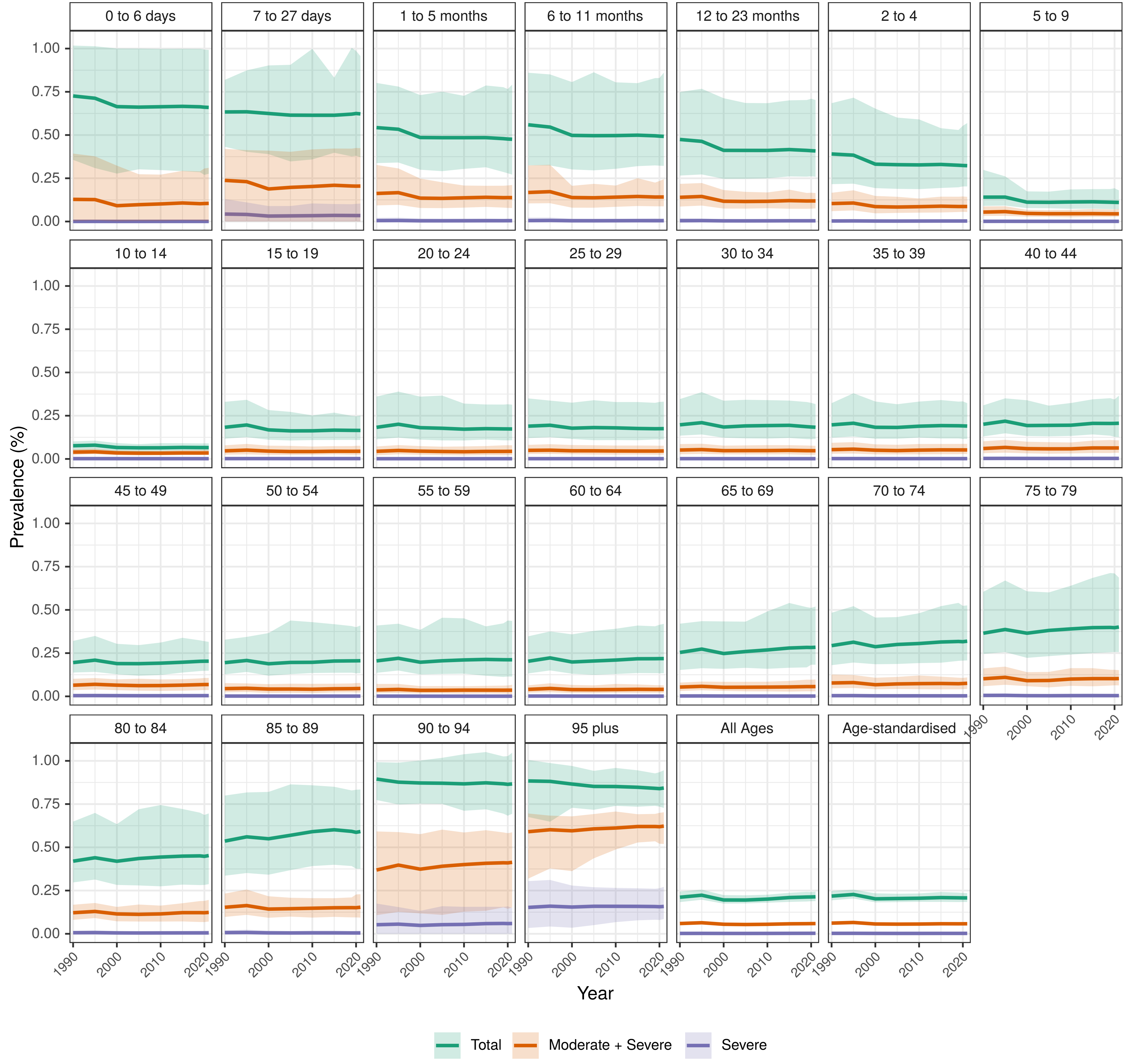

Cause-Specific Anaemia Prevalence - 2021

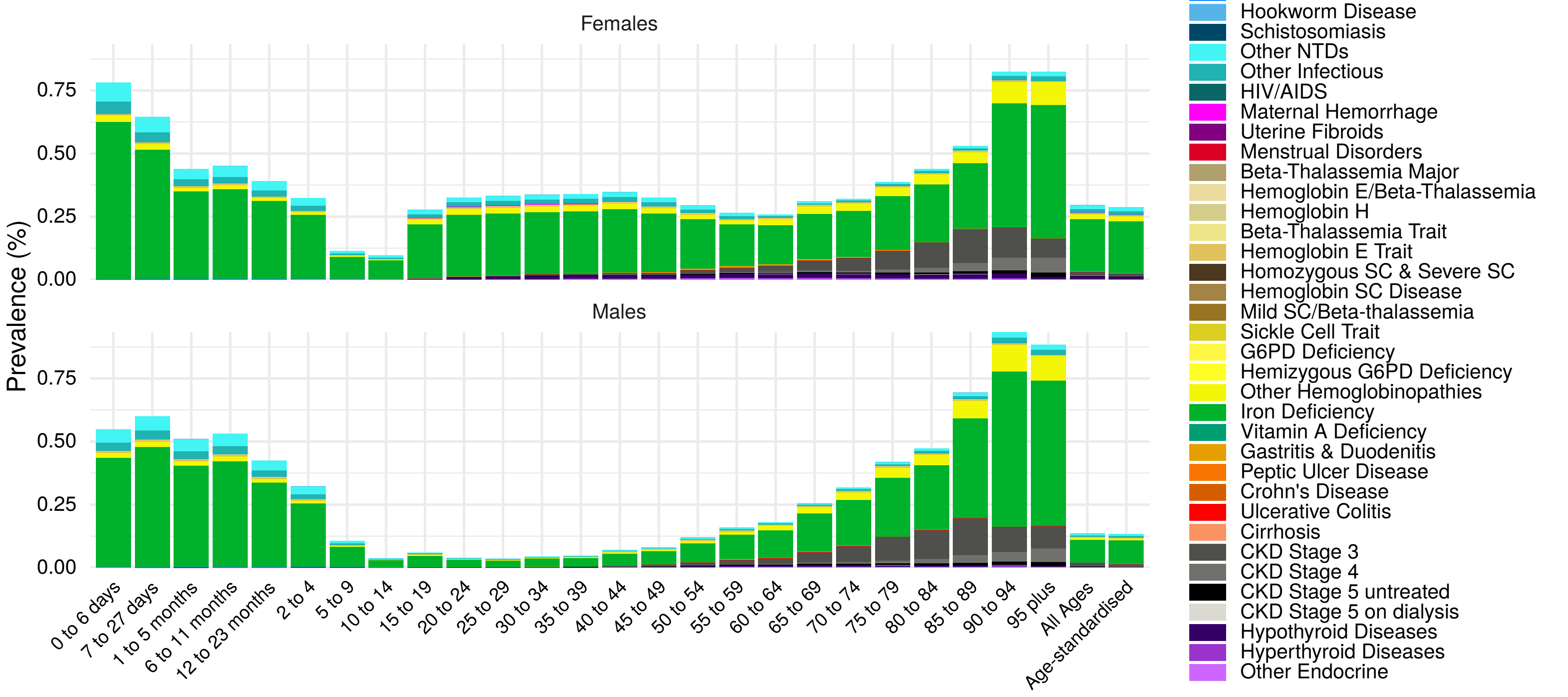

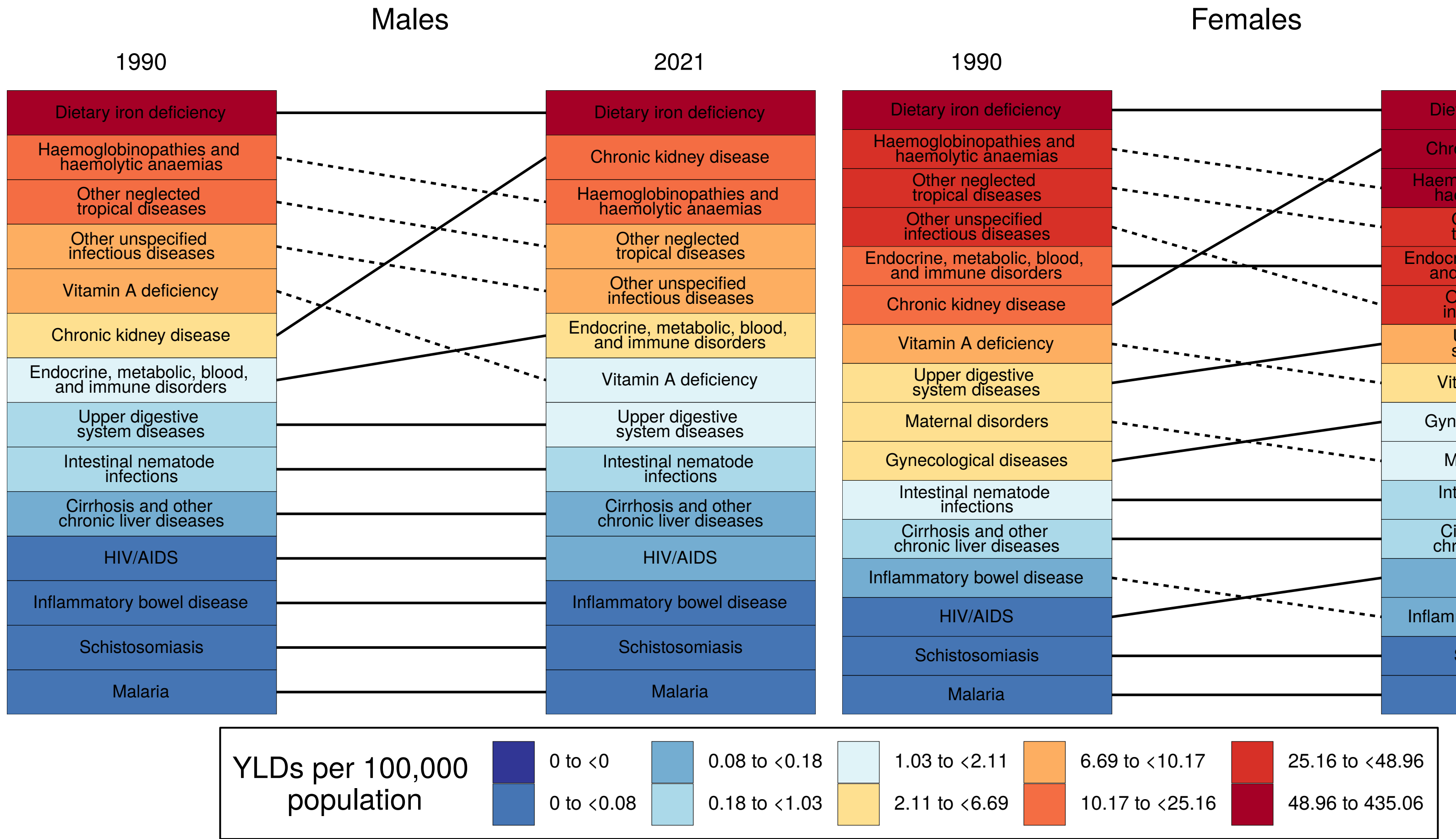

Kiribati

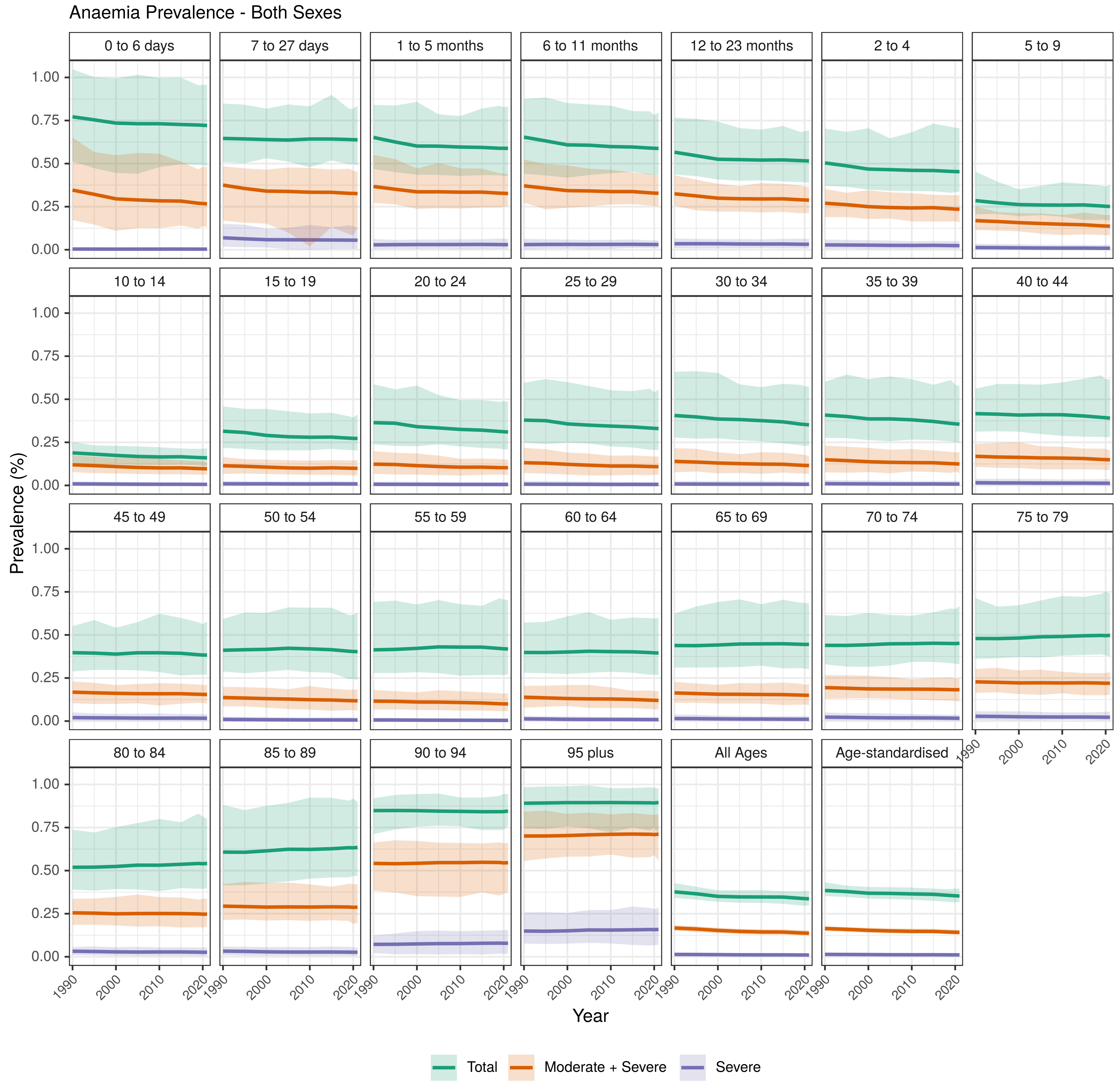

Anaemia Prevalence - Both Sexes
0 to 6 days
7 to 27 days
1 to 5 months
6 to 11 months
12 to 23 months
2 to 4
5 to 9
10 to 14
15 to 19
20 to 24
25 to 29
30 to 34
35 to 39
40 to 44
45 to 49
50 to 54
55 to 59
60 to 64
65 to 69
70 to 74
75 to 79
80 to 84
85 to 89
90 to 94
95 plus
All Ages
Age-standardised
Prevalence (%)
Year
Total
Moderate + Severe
Severe

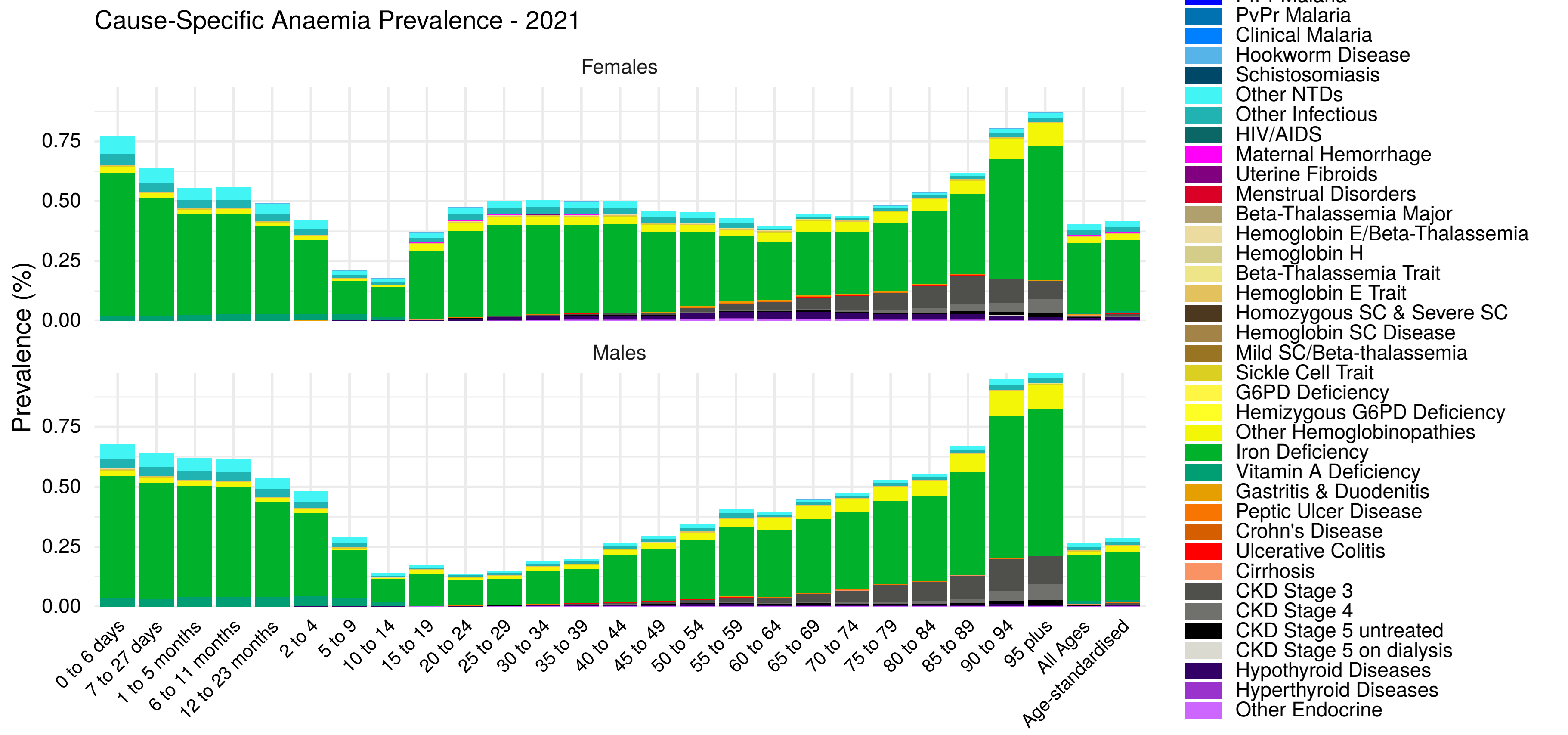

Cause-Specific Anaemia Prevalence - 2021
Females
Males
Prevalence (%)
PfPr Malaria
PvPr Malaria
Clinical Malaria
Hookworm Disease
Schistosomiasis
Other NTDs
Other Infectious
HIV/AIDS
Maternal Hemorrhage
Uterine Fibroids
Menstrual Disorders
Beta-Thalassemia Major
Hemoglobin E/Beta-Thalassemia
Hemoglobin H
Beta-Thalassemia Trait
Hemoglobin E Trait
Homozygous SC & Severe SC
Hemoglobin SC Disease
Mild SC/Beta-thalassemia
Sickle Cell Trait
G6PD Deficiency
Hemizygous G6PD Deficiency
Other Hemoglobinopathies
Iron Deficiency
Vitamin A Deficiency
Gastritis & Duodenitis
Peptic Ulcer Disease
Crohn's Disease
Ulcerative Colitis
Cirrhosis
CKD Stage 3
CKD Stage 4
CKD Stage 5 untreated
CKD Stage 5 on dialysis
Hypothyroid Diseases
Hyperthyroid Diseases
Other Endocrine

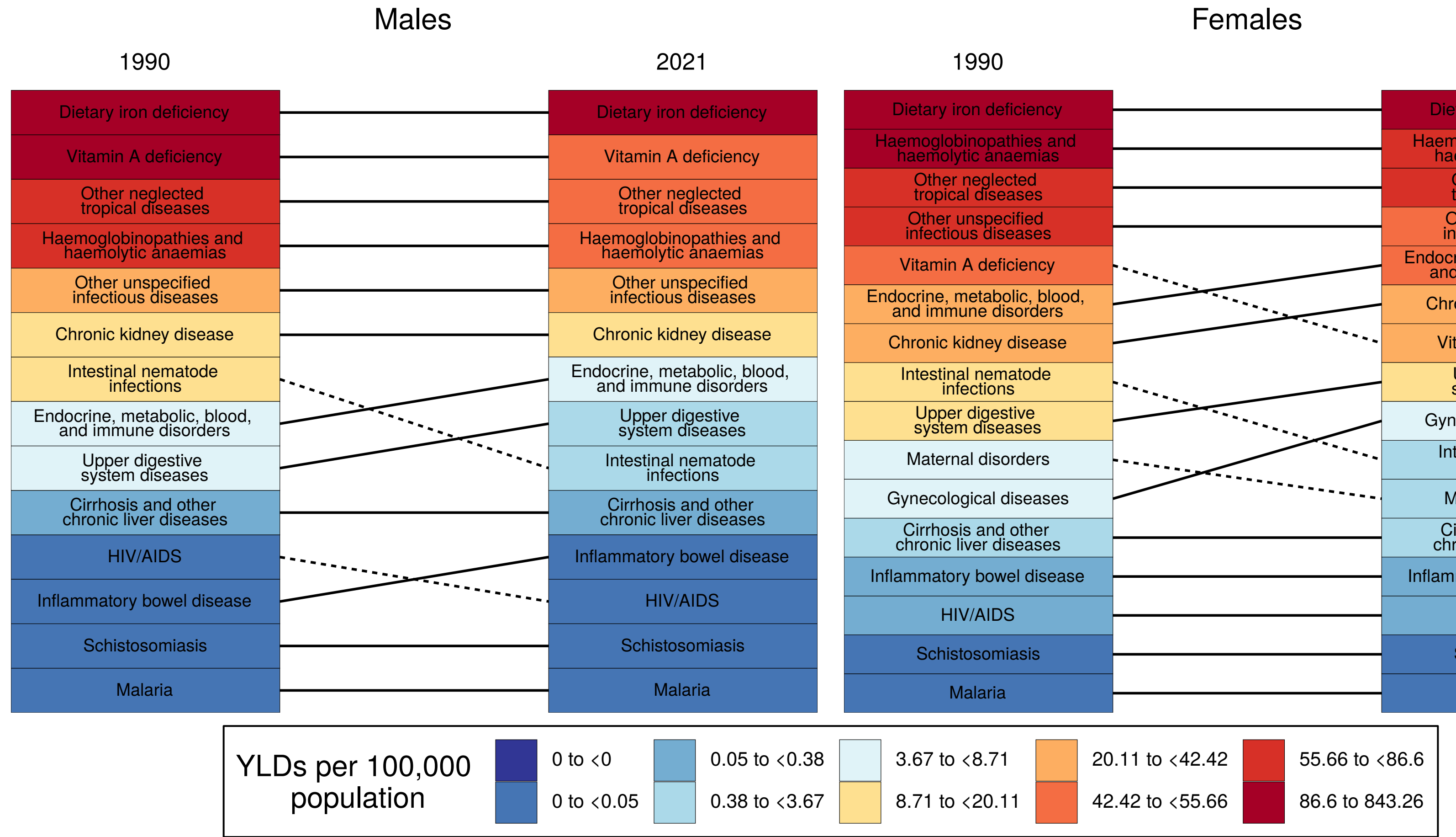

Males
Females
1990
2021
Dietary iron deficiency
Vitamin A deficiency
Other neglected tropical diseases
Haemoglobinopathies and haemolytic anaemias
Other unspecified infectious diseases
Chronic kidney disease
Intestinal nematode infections
Endocrine, metabolic, blood, and immune disorders
Upper digestive system diseases
Cirrhosis and other chronic liver diseases
HIV/AIDS
Inflammatory bowel disease
Schistosomiasis
Malaria
Maternal disorders
Gynecological diseases
YLDs per 100,000 population
0 to <0
0 to <0.05
0.05 to <0.38
0.38 to <3.67
3.67 to <8.71
8.71 to <20.11
20.11 to <42.42
42.42 to <55.66
55.66 to <86.6
86.6 to 843.26

Marshall Islands

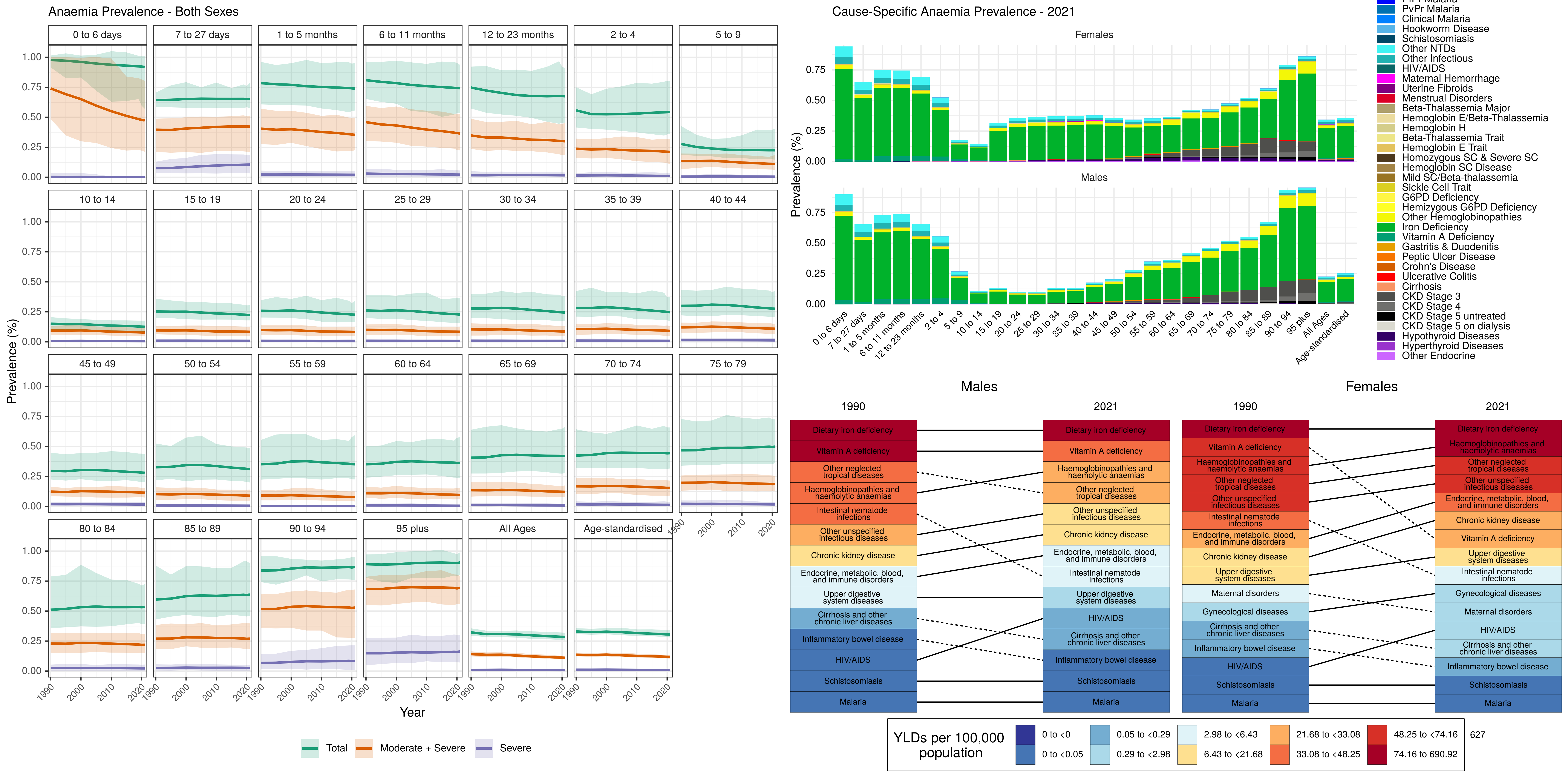

627

Federated States of Micronesia

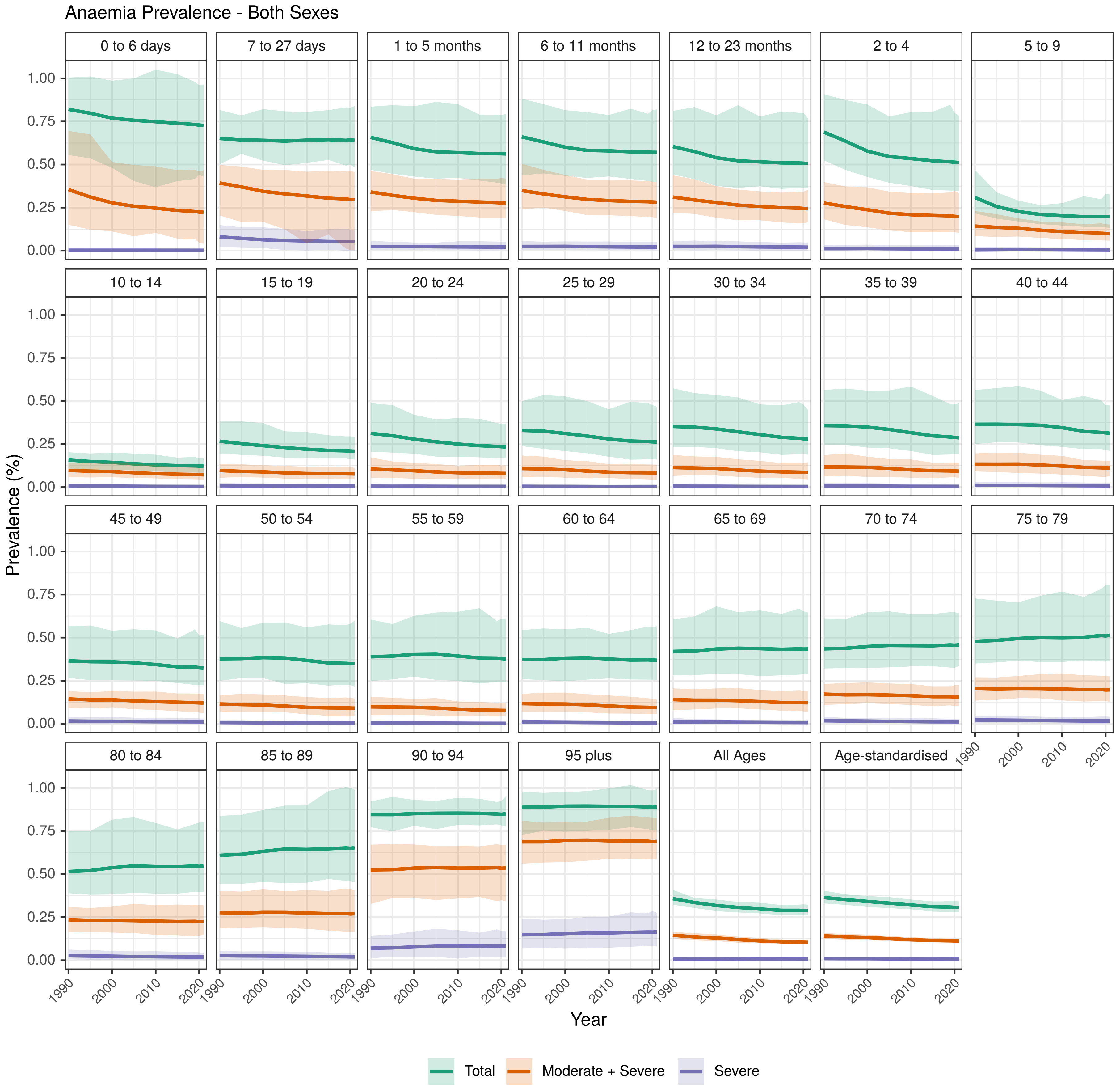

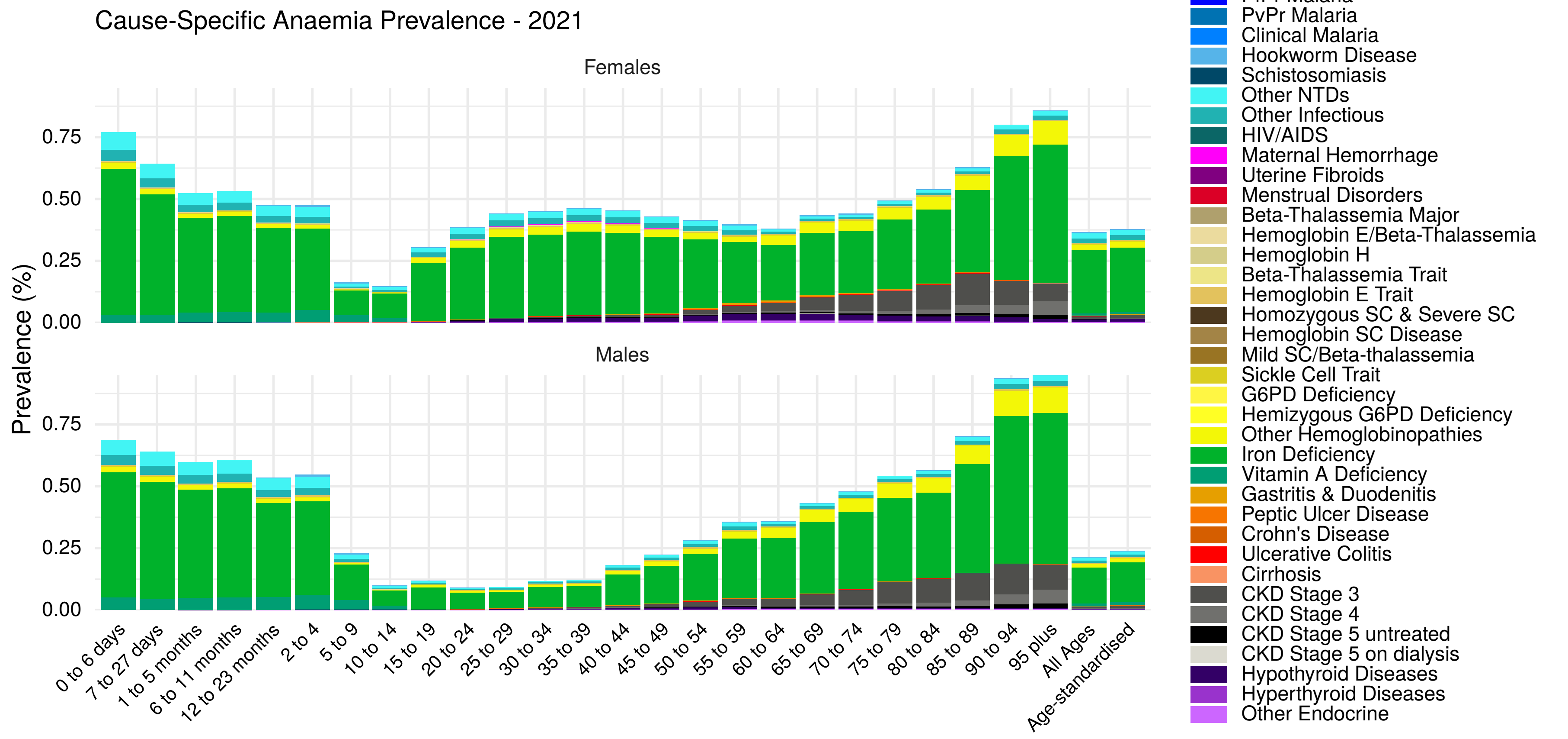

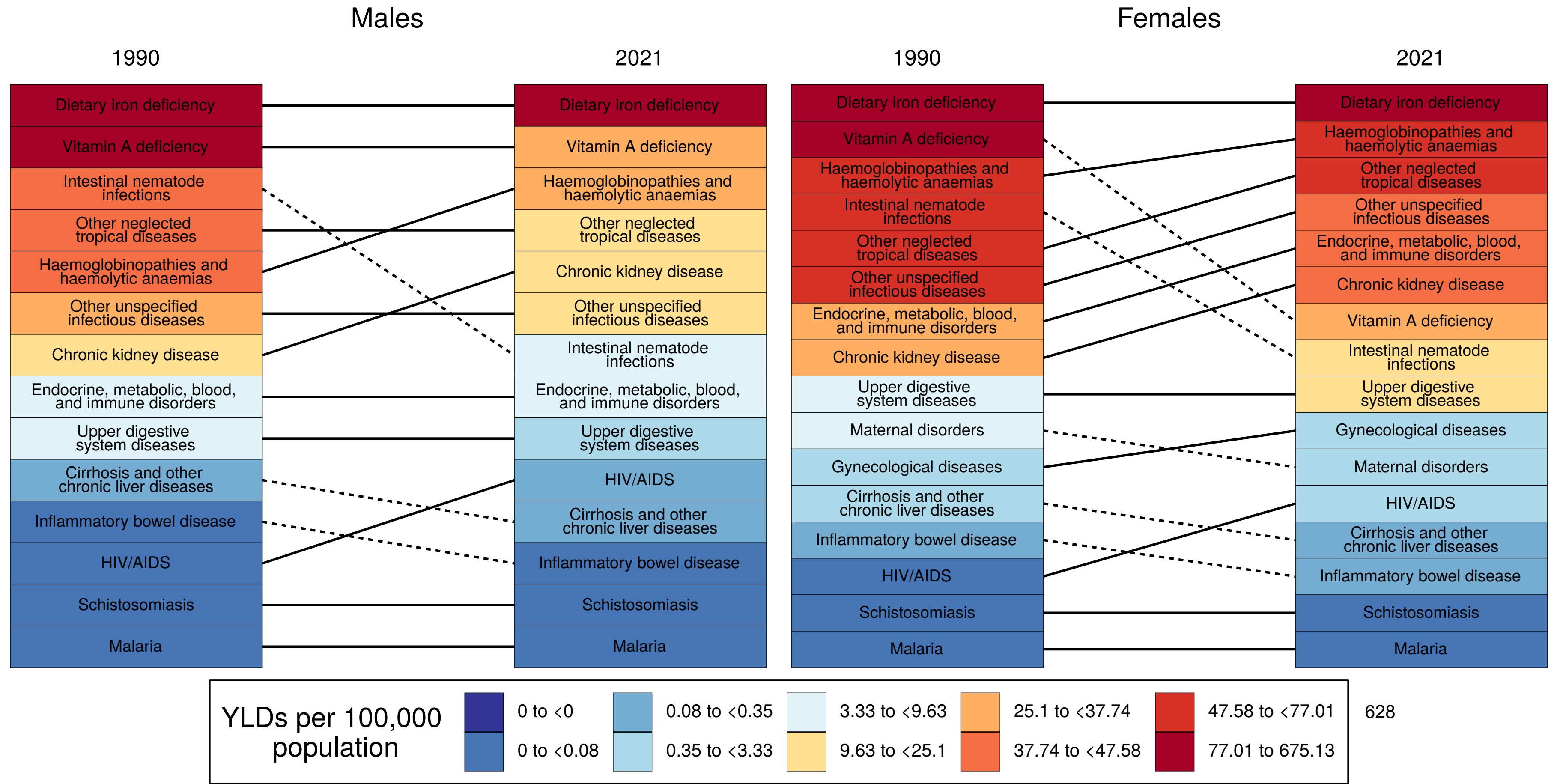

Nauru

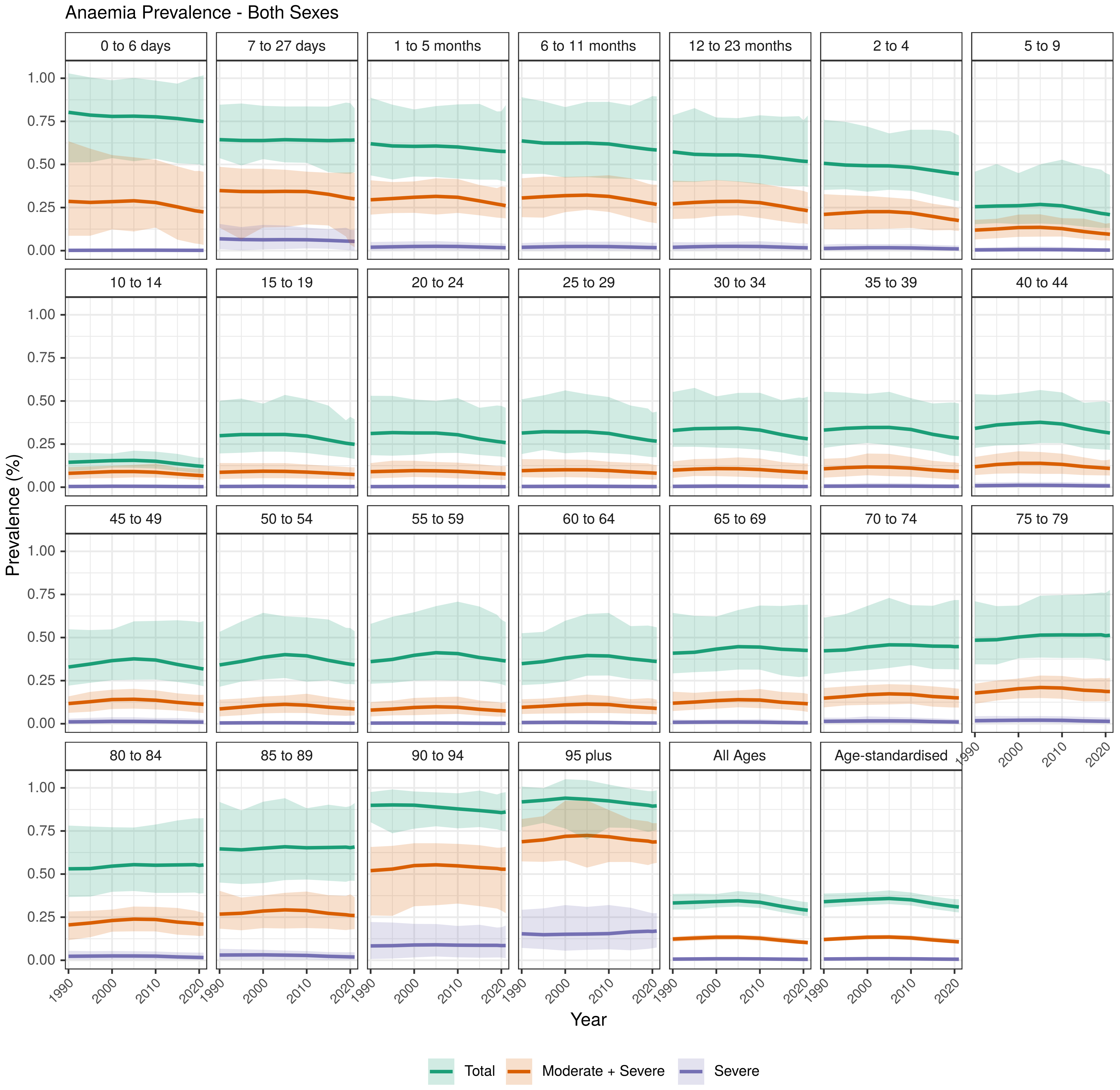

Anaemia Prevalence - Both Sexes
0 to 6 days
7 to 27 days
1 to 5 months
6 to 11 months
12 to 23 months
2 to 4
5 to 9
10 to 14
15 to 19
20 to 24
25 to 29
30 to 34
35 to 39
40 to 44
45 to 49
50 to 54
55 to 59
60 to 64
65 to 69
70 to 74
75 to 79
80 to 84
85 to 89
90 to 94
95 plus
All Ages
Age-standardised
Prevalence (%)
0.00
0.25
0.50
0.75
1.00
1990
2000
2010
2020
Year
Total
Moderate + Severe
Severe

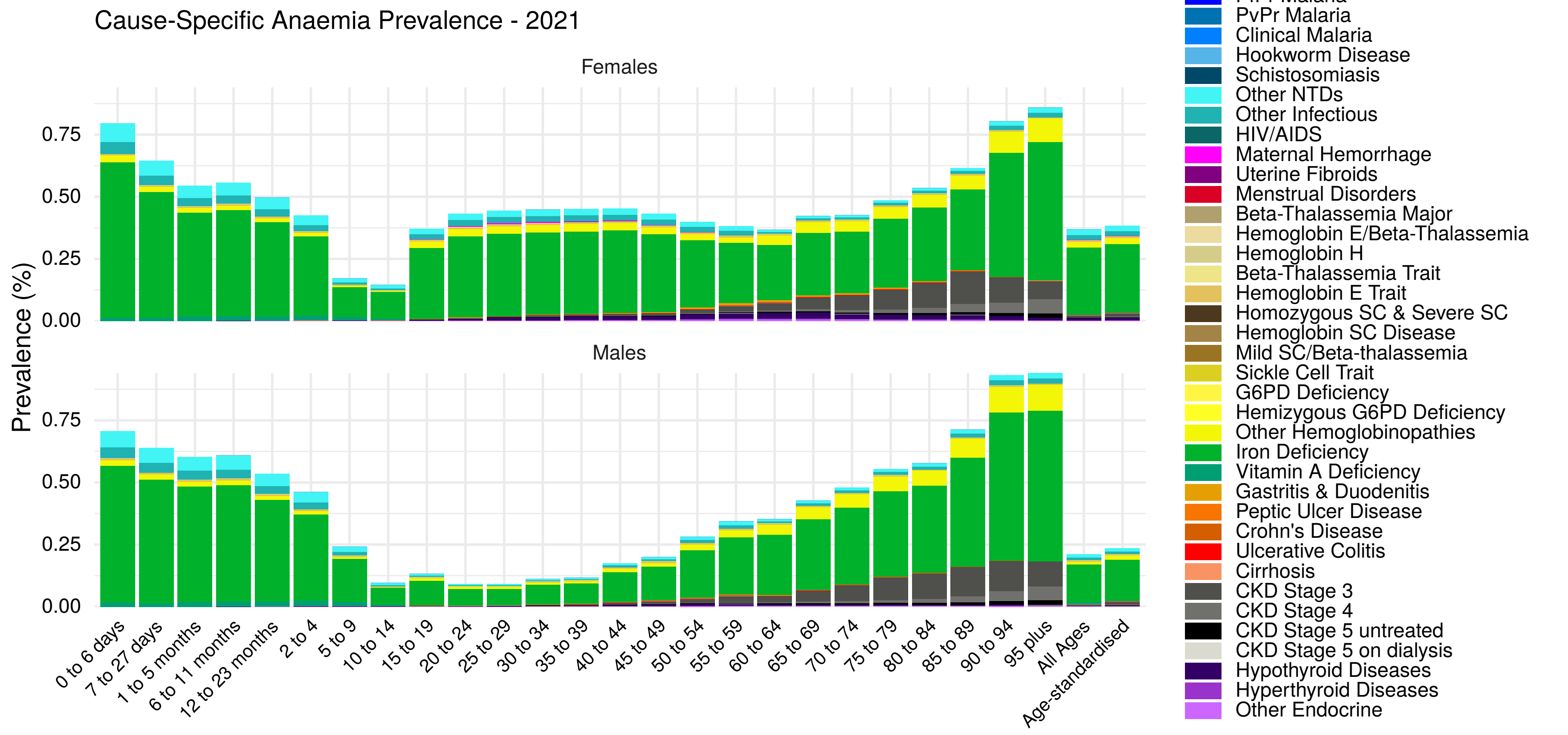

Cause-Specific Anaemia Prevalence - 2021
Females
Males
Prevalence (%)
0.00
0.25
0.50
0.75
0 to 6 days
7 to 27 days
1 to 5 months
6 to 11 months
12 to 23 months
2 to 4
5 to 9
10 to 14
15 to 19
20 to 24
25 to 29
30 to 34
35 to 39
40 to 44
45 to 49
50 to 54
55 to 59
60 to 64
65 to 69
70 to 74
75 to 79
80 to 84
85 to 89
90 to 94
95 plus
All Ages
Age-standardised
PfPr Malaria
PvPr Malaria
Clinical Malaria
Hookworm Disease
Schistosomiasis
Other NTDs
Other Infectious
HIV/AIDS
Maternal Hemorrhage
Uterine Fibroids
Menstrual Disorders
Beta-Thalassemia Major
Hemoglobin E/Beta-Thalassemia
Hemoglobin H
Beta-Thalassemia Trait
Hemoglobin E Trait
Homozygous SC & Severe SC
Hemoglobin SC Disease
Mild SC/Beta-thalassemia
Sickle Cell Trait
G6PD Deficiency
Hemizygous G6PD Deficiency
Other Hemoglobinopathies
Iron Deficiency
Vitamin A Deficiency
Gastritis & Duodenitis
Peptic Ulcer Disease
Crohn's Disease
Ulcerative Colitis
Cirrhosis
CKD Stage 3
CKD Stage 4
CKD Stage 5 untreated
CKD Stage 5 on dialysis
Hypothyroid Diseases
Hyperthyroid Diseases
Other Endocrine

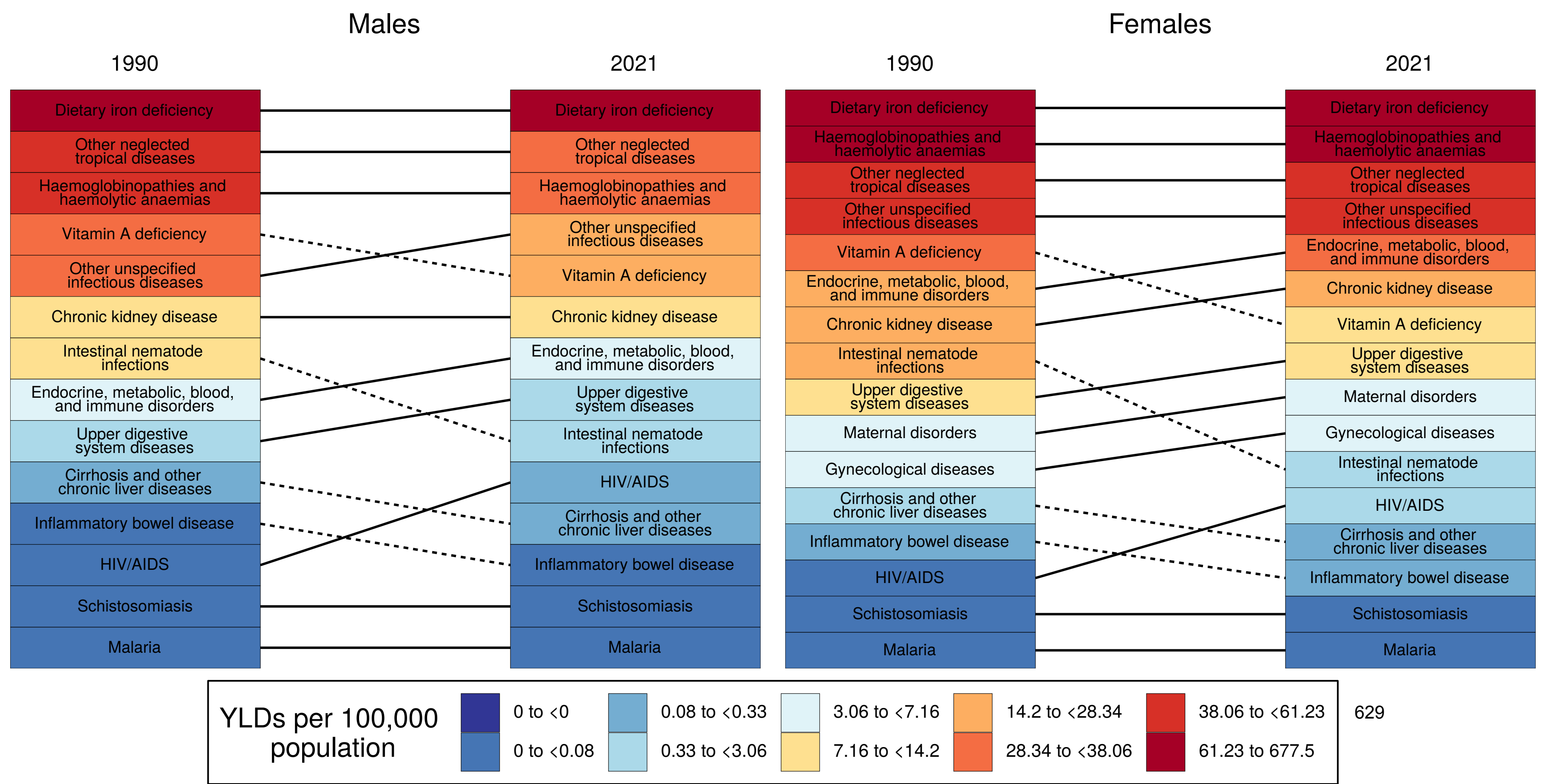

Males
Females
1990
2021
Dietary iron deficiency
Other neglected tropical diseases
Haemoglobinopathies and haemolytic anaemias
Vitamin A deficiency
Other unspecified infectious diseases
Chronic kidney disease
Intestinal nematode infections
Endocrine, metabolic, blood, and immune disorders
Upper digestive system diseases
Cirrhosis and other chronic liver diseases
Inflammatory bowel disease
HIV/AIDS
Schistosomiasis
Malaria
Maternal disorders
Gynecological diseases
YLDs per 100,000 population
0 to <0
0 to <0.08
0.08 to <0.33
0.33 to <3.06
3.06 to <7.16
7.16 to <14.2
14.2 to <28.34
28.34 to <38.06
38.06 to <61.23
61.23 to 677.5
629

Niue

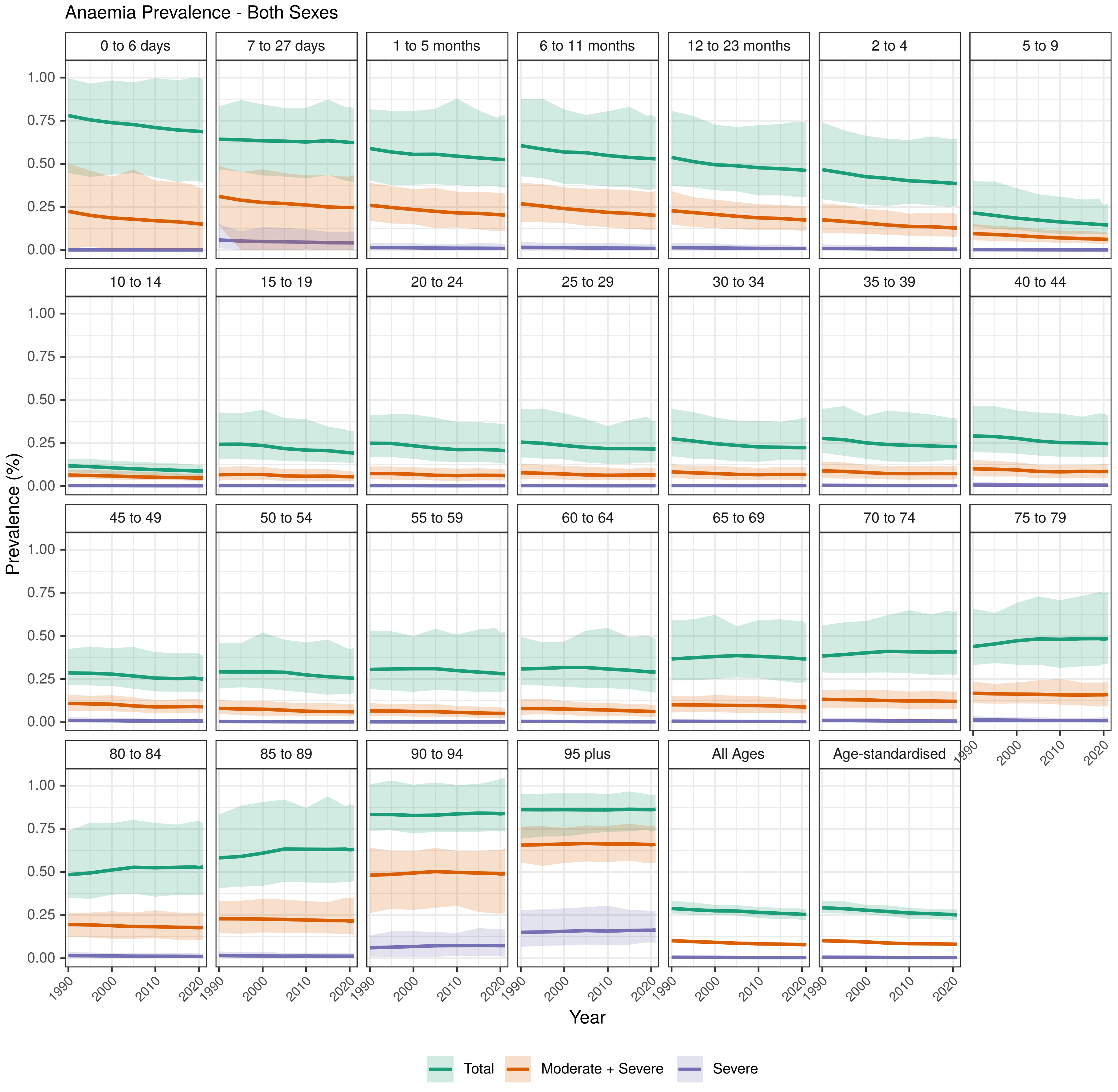
Anaemia Prevalence - Both Sexes
0 to 6 days
7 to 27 days
1 to 5 months
6 to 11 months
12 to 23 months
2 to 4
5 to 9
10 to 14
15 to 19
20 to 24
25 to 29
30 to 34
35 to 39
40 to 44
45 to 49
50 to 54
55 to 59
60 to 64
65 to 69
70 to 74
75 to 79
80 to 84
85 to 89
90 to 94
95 plus
All Ages
Age-standardised
Prevalence (%)
Year
Total
Moderate + Severe
Severe

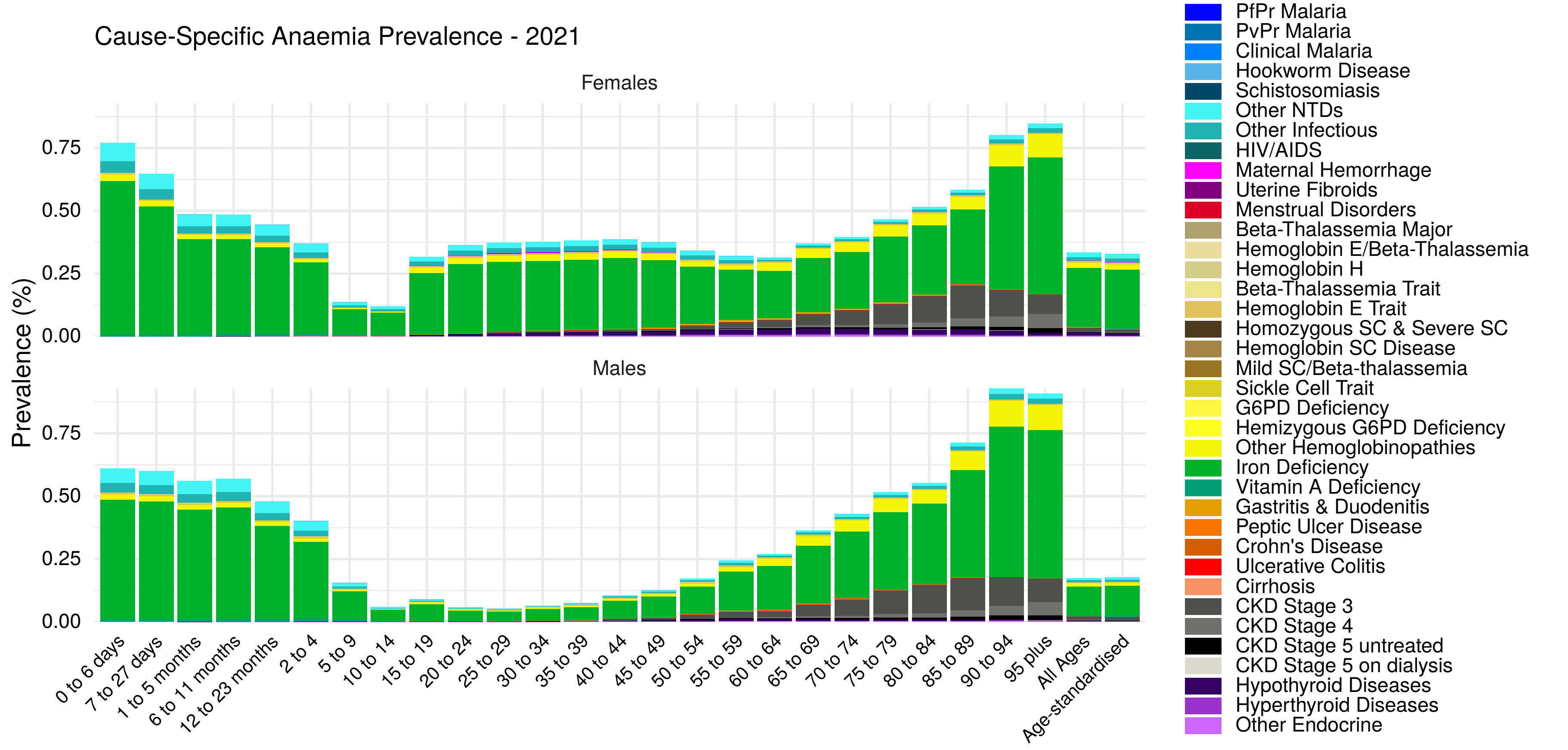
Cause-Specific Anaemia Prevalence - 2021
Females
Males
Prevalence (%)
PfPr Malaria
PvPr Malaria
Clinical Malaria
Hookworm Disease
Schistosomiasis
Other NTDs
Other Infectious
HIV/AIDS
Maternal Hemorrhage
Uterine Fibroids
Menstrual Disorders
Beta-Thalassemia Major
Hemoglobin E/Beta-Thalassemia
Hemoglobin H
Beta-Thalassemia Trait
Hemoglobin E Trait
Homozygous SC & Severe SC
Hemoglobin SC Disease
Mild SC/Beta-thalassemia
Sickle Cell Trait
G6PD Deficiency
Hemizygous G6PD Deficiency
Other Hemoglobinopathies
Iron Deficiency
Vitamin A Deficiency
Gastritis & Duodenitis
Peptic Ulcer Disease
Crohn's Disease
Ulcerative Colitis
Cirrhosis
CKD Stage 3
CKD Stage 4
CKD Stage 5 untreated
CKD Stage 5 on dialysis
Hypothyroid Diseases
Hyperthyroid Diseases
Other Endocrine

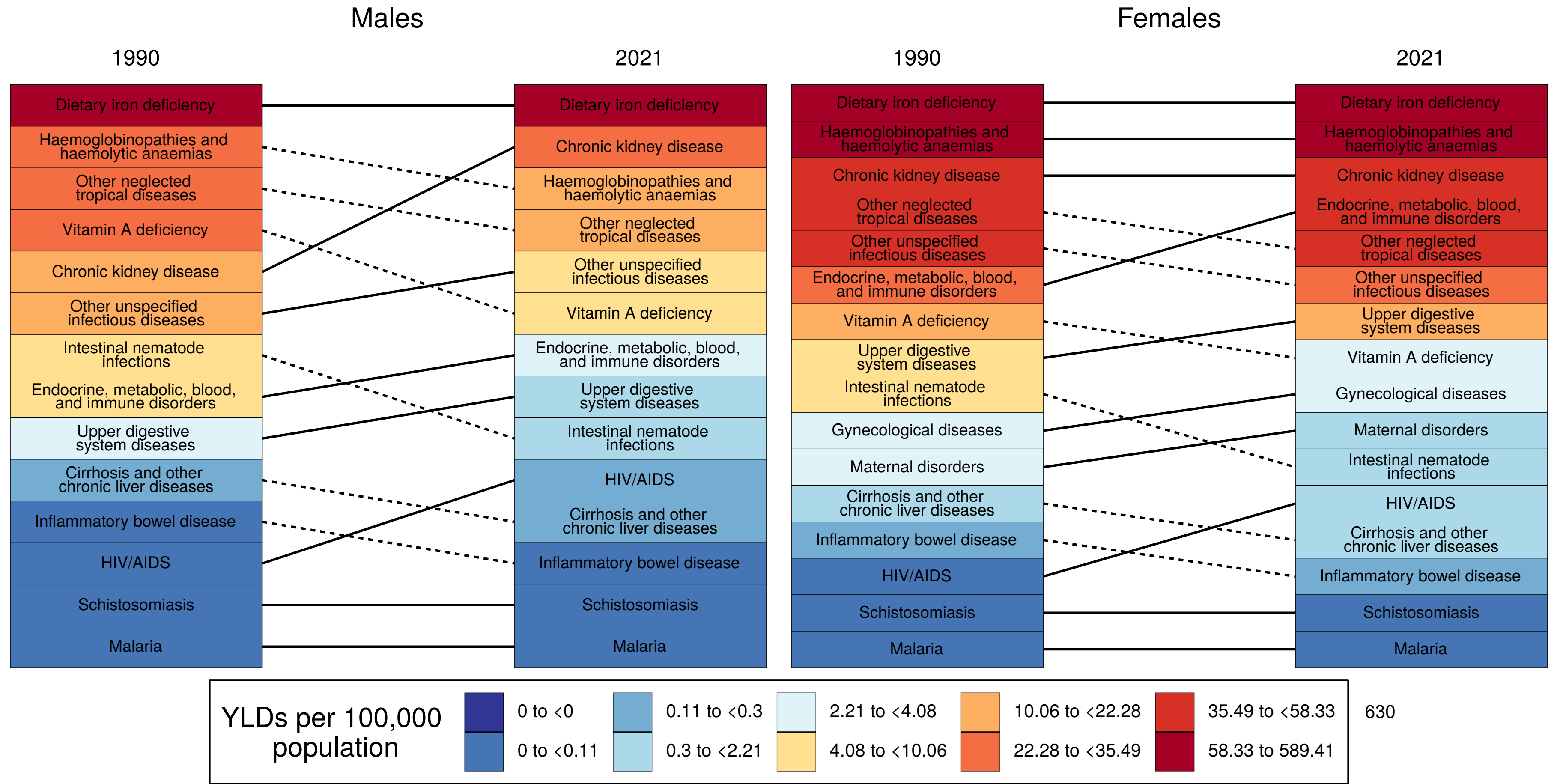
Males
Females
1990
2021
Dietary iron deficiency
Haemoglobinopathies and haemolytic anaemias
Other neglected tropical diseases
Vitamin A deficiency
Chronic kidney disease
Other unspecified infectious diseases
Intestinal nematode infections
Endocrine, metabolic, blood, and immune disorders
Upper digestive system diseases
Cirrhosis and other chronic liver diseases
Inflammatory bowel disease
HIV/AIDS
Schistosomiasis
Malaria
Gynecological diseases
Maternal disorders
YLDs per 100,000 population
0 to <0
0 to <0.11
0.11 to <0.3
0.3 to <2.21
2.21 to <4.08
4.08 to <10.06
10.06 to <22.28
22.28 to <35.49
35.49 to <58.33
58.33 to 589.41

630

Northern Mariana Islands

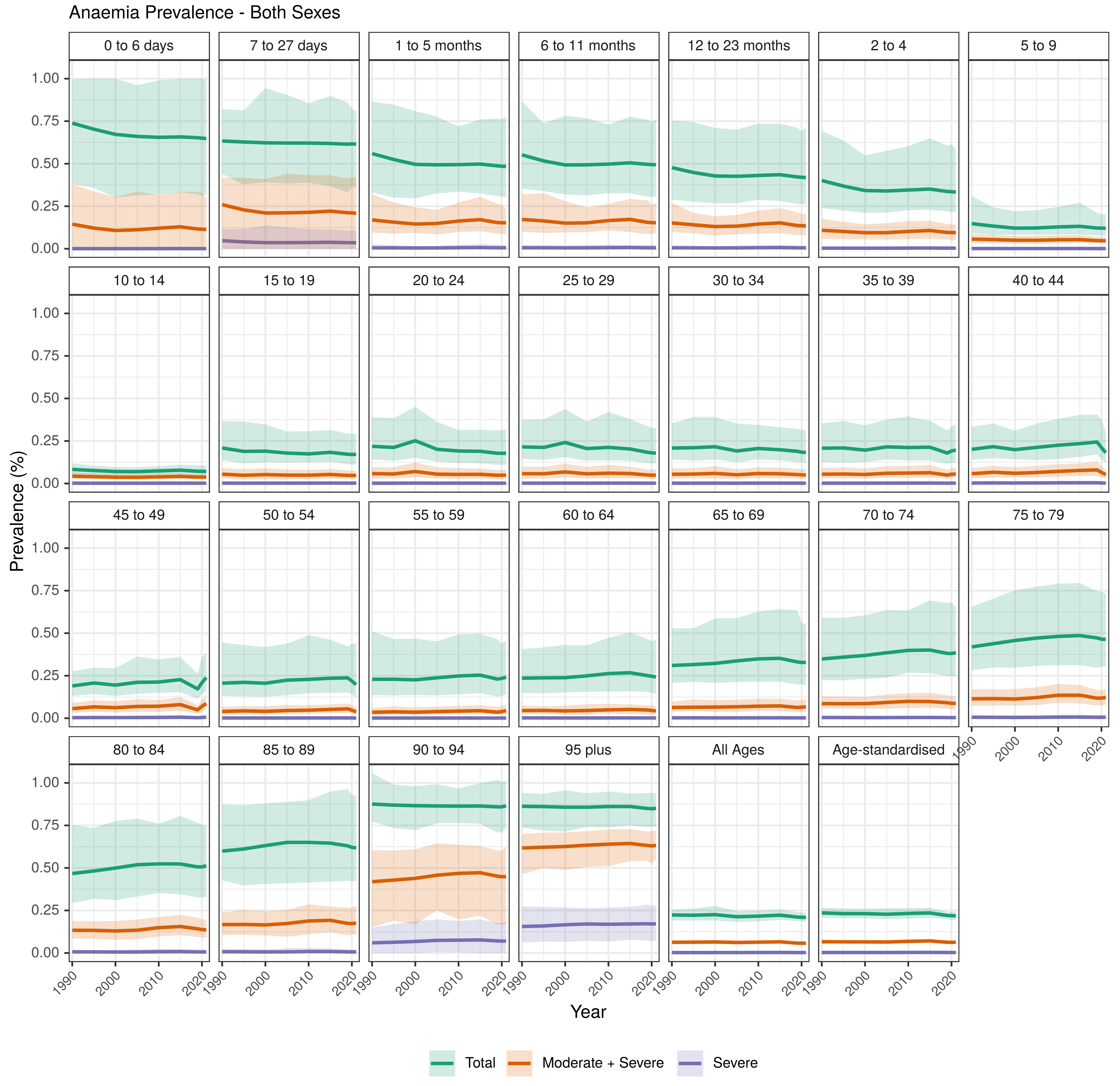

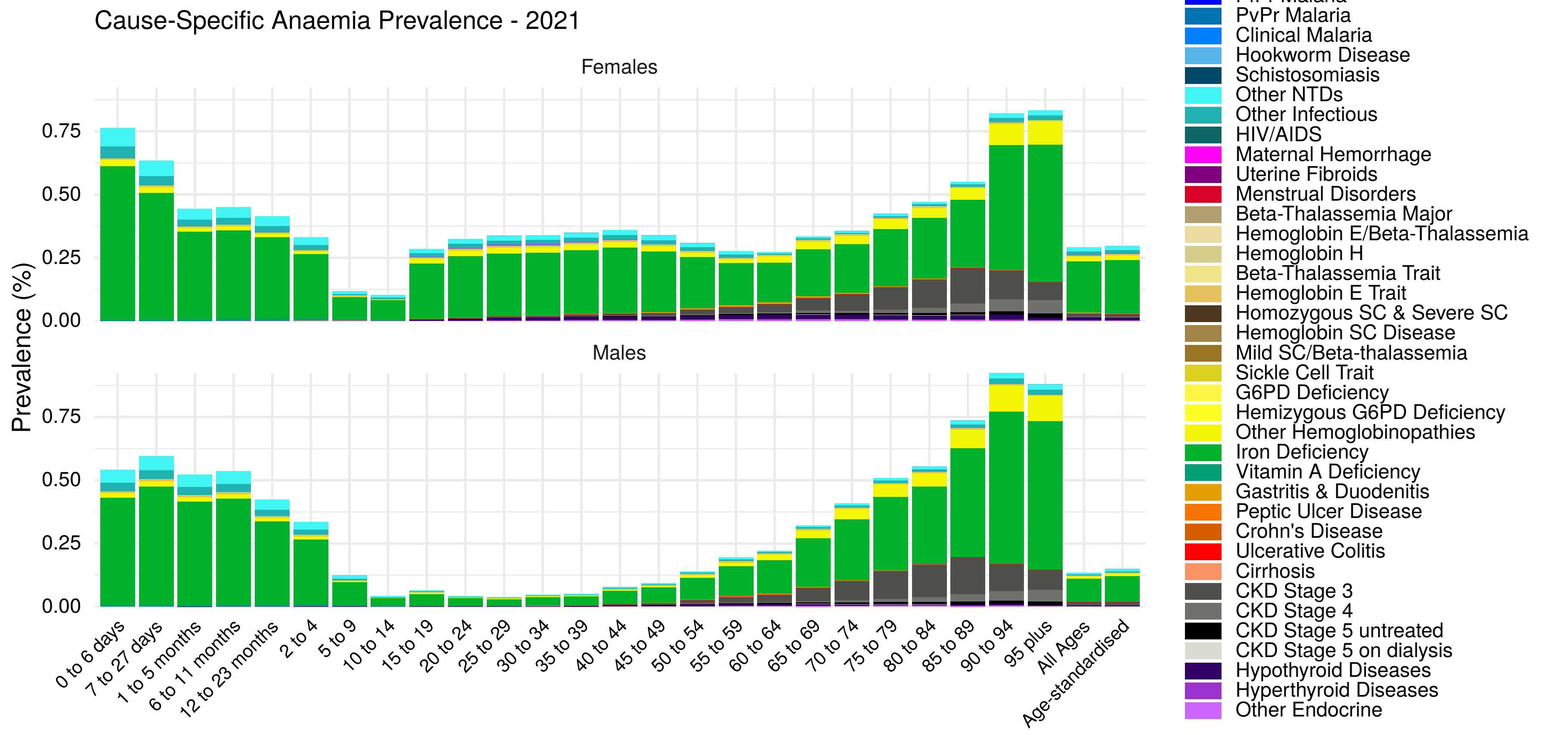

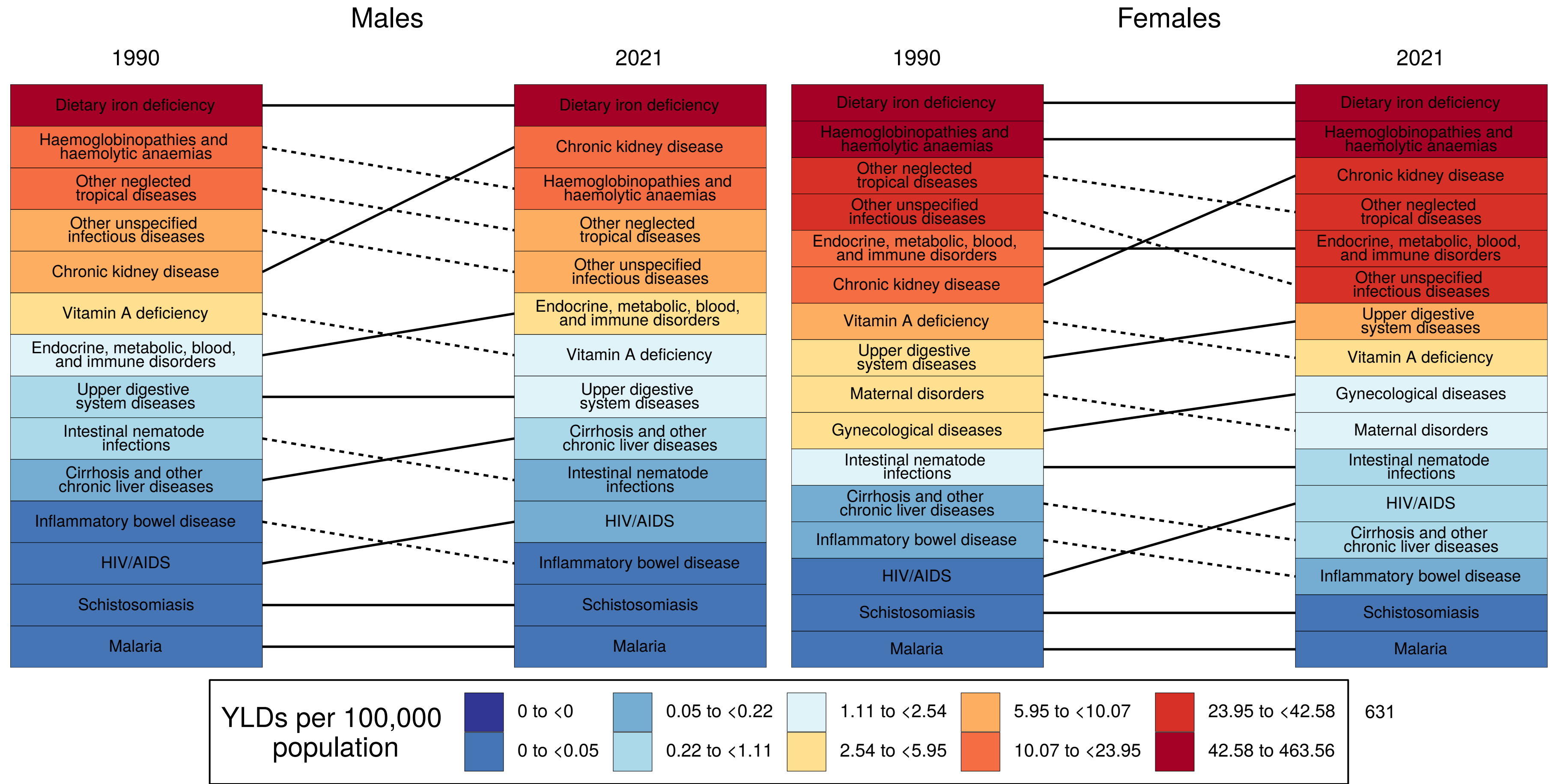

Palau

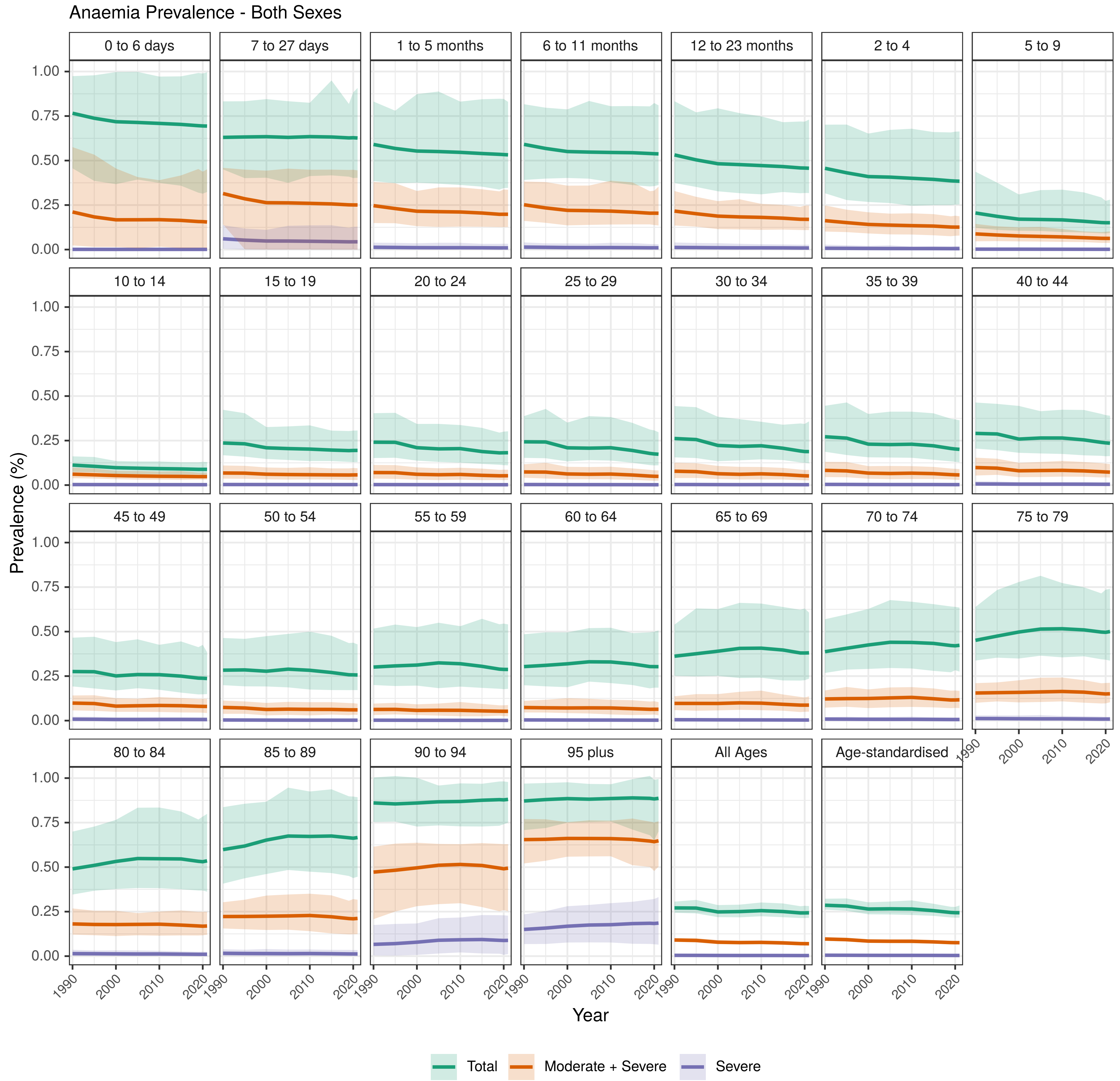

Anaemia Prevalence - Both Sexes
0 to 6 days
7 to 27 days
1 to 5 months
6 to 11 months
12 to 23 months
2 to 4
5 to 9
10 to 14
15 to 19
20 to 24
25 to 29
30 to 34
35 to 39
40 to 44
45 to 49
50 to 54
55 to 59
60 to 64
65 to 69
70 to 74
75 to 79
80 to 84
85 to 89
90 to 94
95 plus
All Ages
Age-standardised
Prevalence (%)
Year
Total
Moderate + Severe
Severe

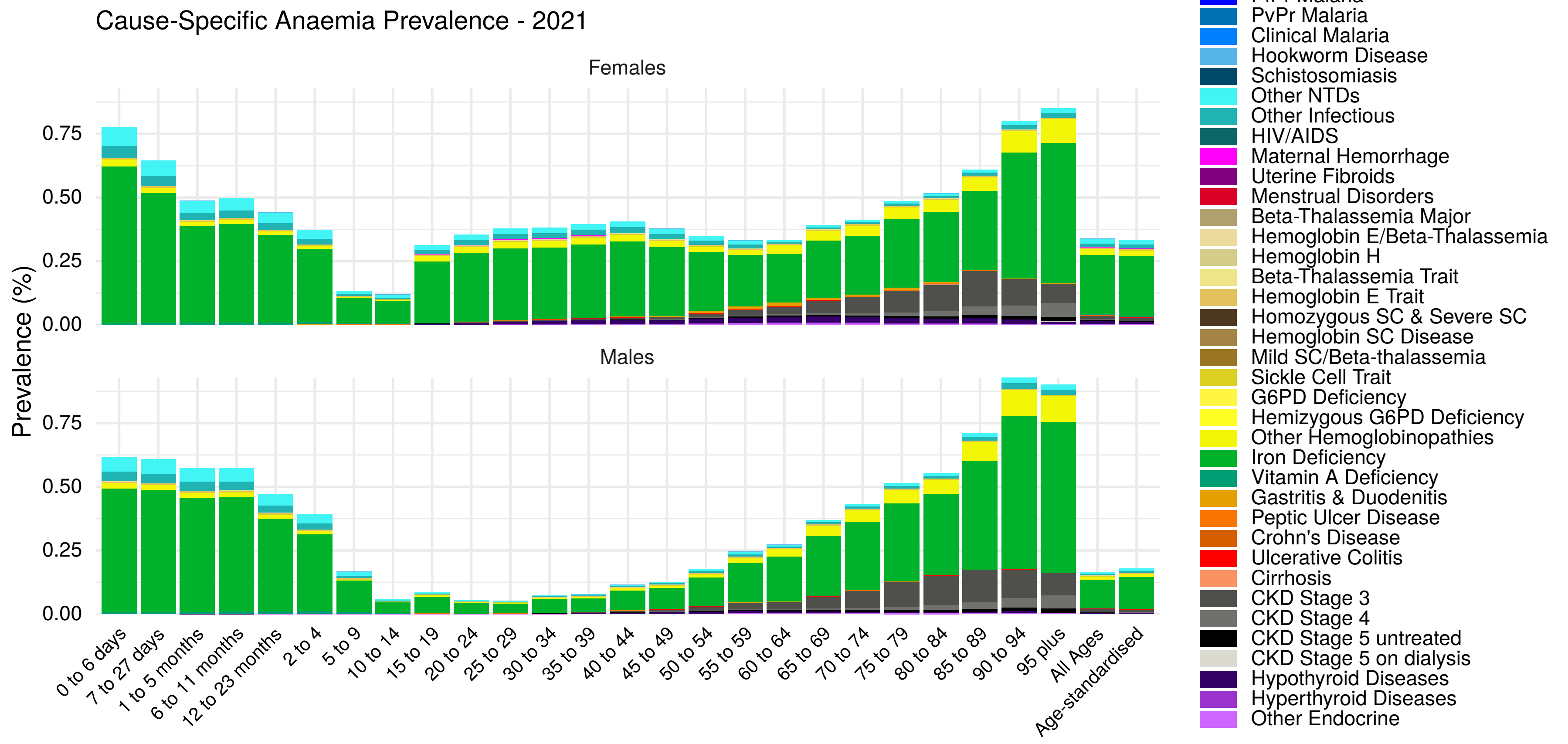

Cause-Specific Anaemia Prevalence - 2021
Females
Males
Prevalence (%)
PfPr Malaria
PvPr Malaria
Clinical Malaria
Hookworm Disease
Schistosomiasis
Other NTDs
Other Infectious
HIV/AIDS
Maternal Hemorrhage
Uterine Fibroids
Menstrual Disorders
Beta-Thalassemia Major
Hemoglobin E/Beta-Thalassemia
Hemoglobin H
Beta-Thalassemia Trait
Hemoglobin E Trait
Homozygous SC & Severe SC
Hemoglobin SC Disease
Mild SC/Beta-thalassemia
Sickle Cell Trait
G6PD Deficiency
Hemizygous G6PD Deficiency
Other Hemoglobinopathies
Iron Deficiency
Vitamin A Deficiency
Gastritis & Duodenitis
Peptic Ulcer Disease
Crohn's Disease
Ulcerative Colitis
Cirrhosis
CKD Stage 3
CKD Stage 4
CKD Stage 5 untreated
CKD Stage 5 on dialysis
Hypothyroid Diseases
Hyperthyroid Diseases
Other Endocrine

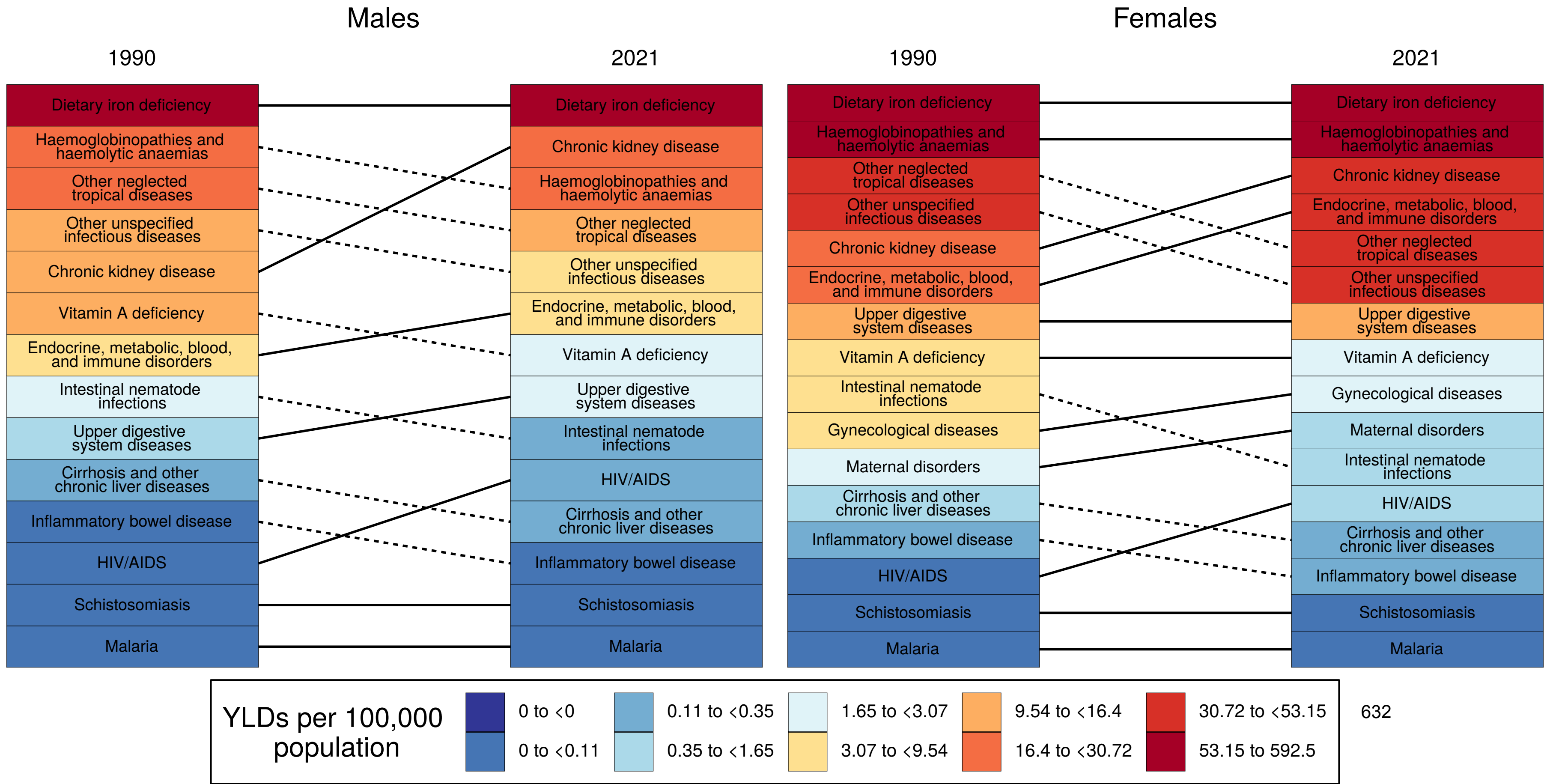

Males
Females
1990
2021
Dietary iron deficiency
Haemoglobinopathies and haemolytic anaemias
Other neglected tropical diseases
Other unspecified infectious diseases
Chronic kidney disease
Vitamin A deficiency
Endocrine, metabolic, blood, and immune disorders
Intestinal nematode infections
Upper digestive system diseases
Cirrhosis and other chronic liver diseases
Inflammatory bowel disease
HIV/AIDS
Schistosomiasis
Malaria
Gynecological diseases
Maternal disorders
YLDs per 100,000 population
0 to <0
0 to <0.11
0.11 to <0.35
0.35 to <1.65
1.65 to <3.07
3.07 to <9.54
9.54 to <16.4
16.4 to <30.72
30.72 to <53.15
53.15 to 592.5

632

Papua New Guinea

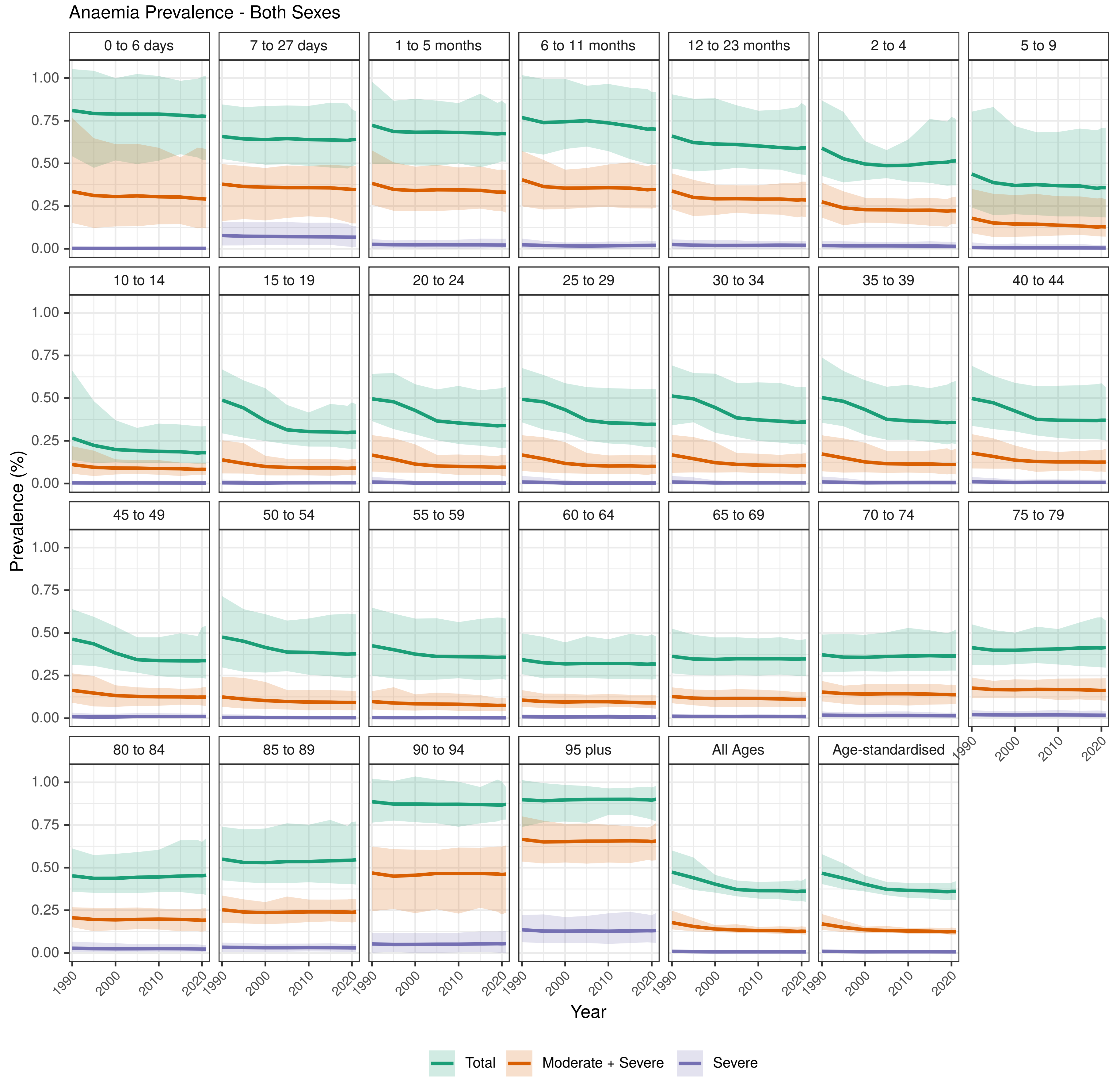

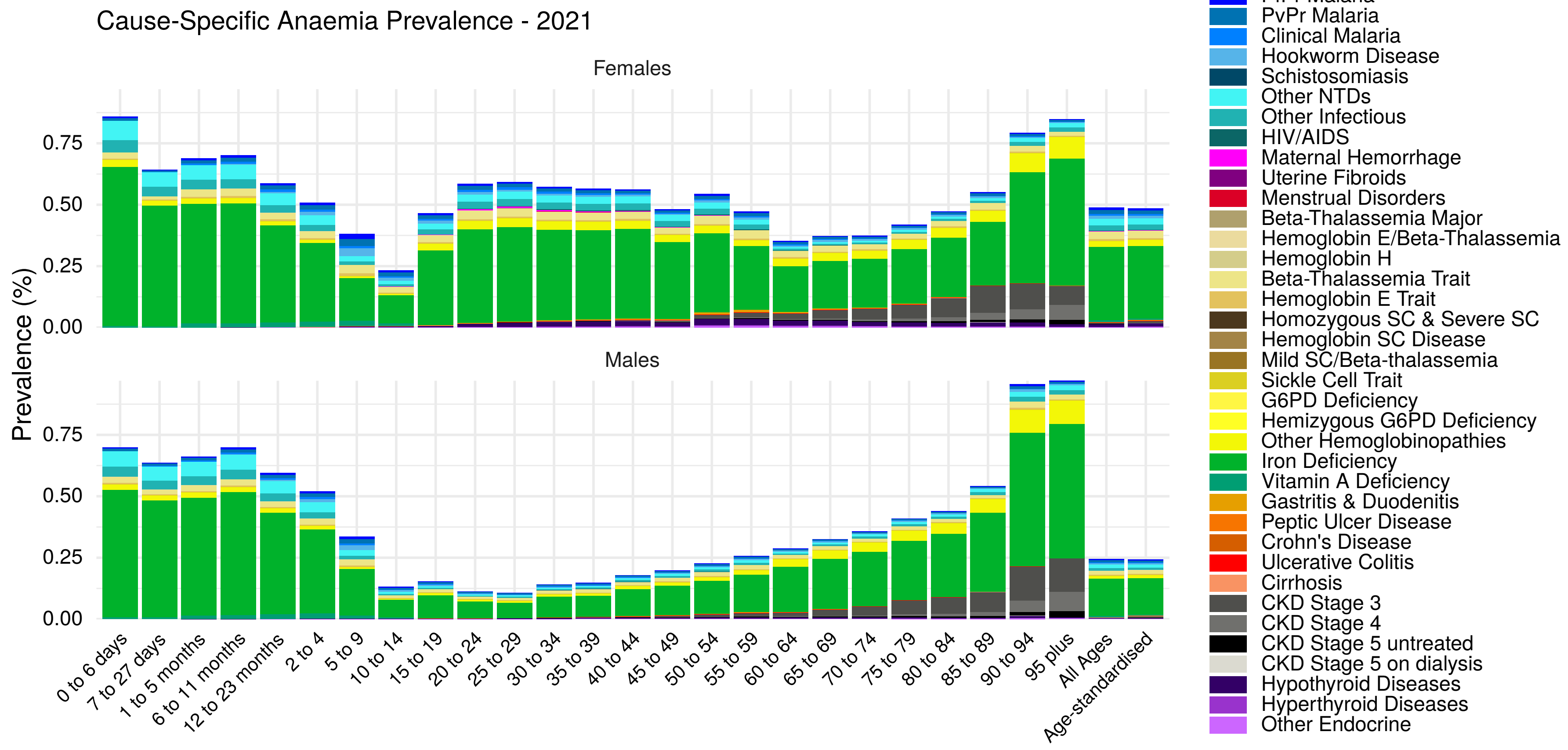

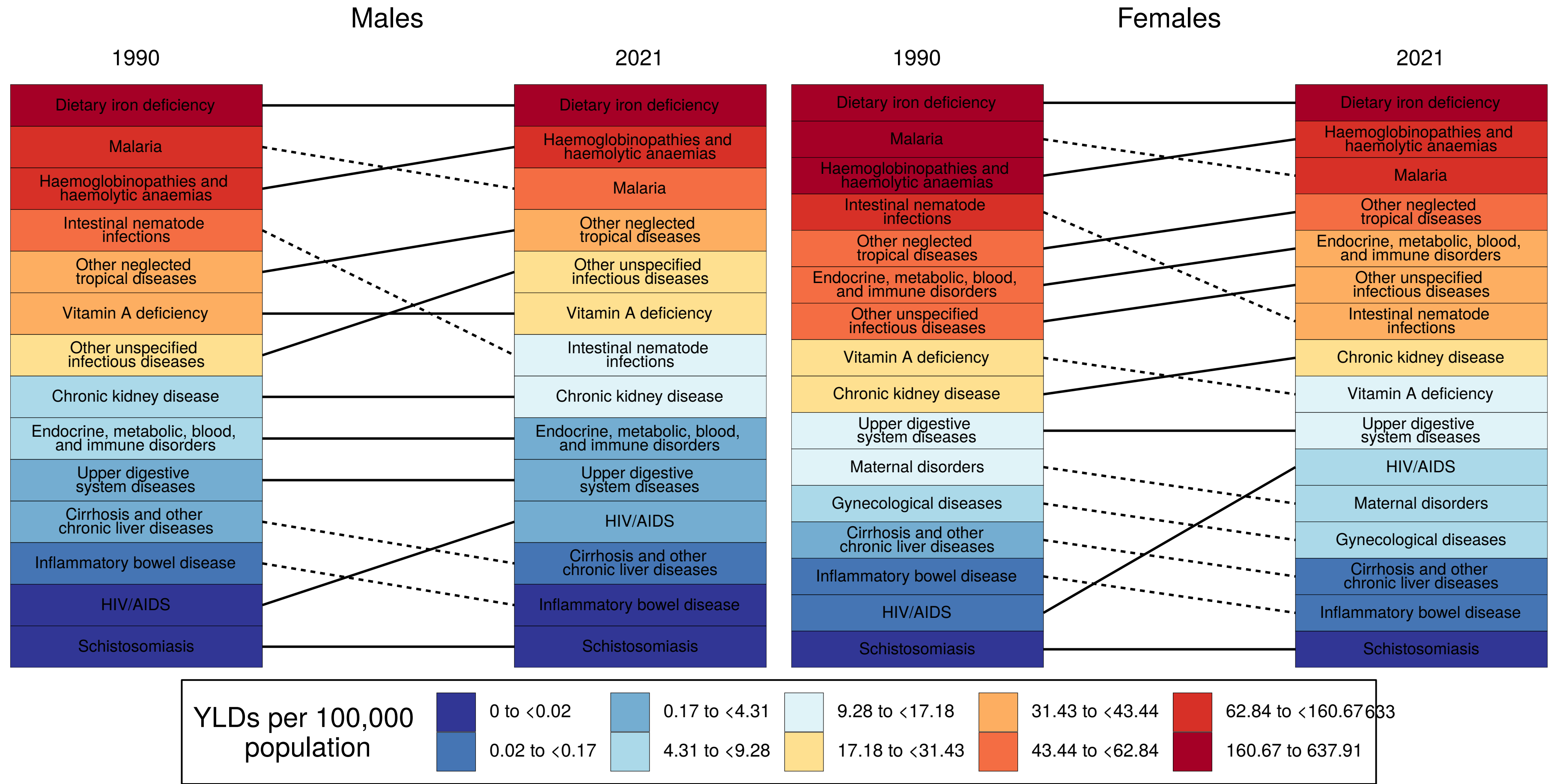

Samoa

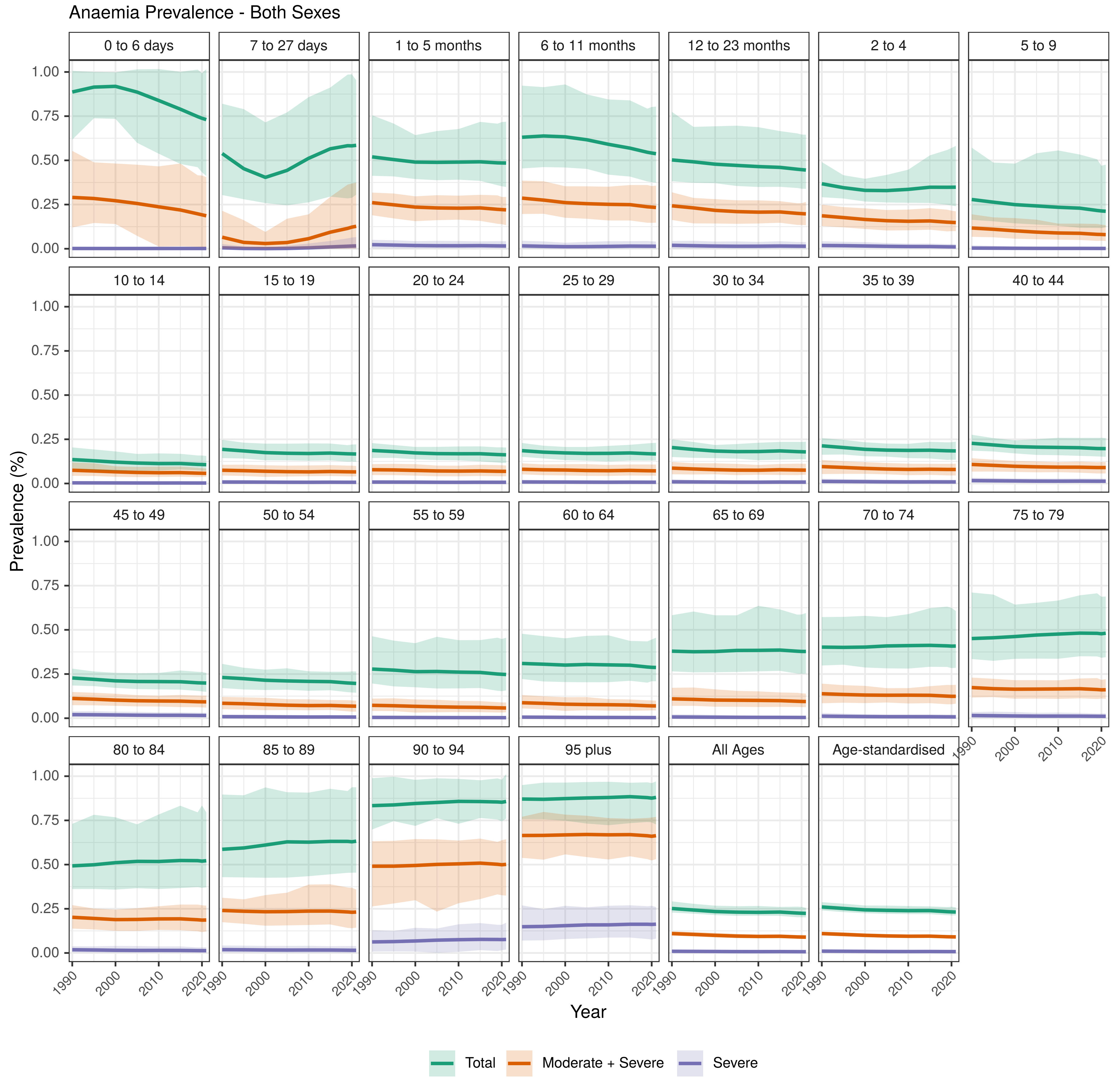

Anaemia Prevalence - Both Sexes
0 to 6 days
7 to 27 days
1 to 5 months
6 to 11 months
12 to 23 months
2 to 4
5 to 9
10 to 14
15 to 19
20 to 24
25 to 29
30 to 34
35 to 39
40 to 44
45 to 49
50 to 54
55 to 59
60 to 64
65 to 69
70 to 74
75 to 79
80 to 84
85 to 89
90 to 94
95 plus
All Ages
Age-standardised
1.00
0.75
0.50
0.25
0.00
Prevalence (%)
1990
2000
2010
2020
Year
Total
Moderate + Severe
Severe

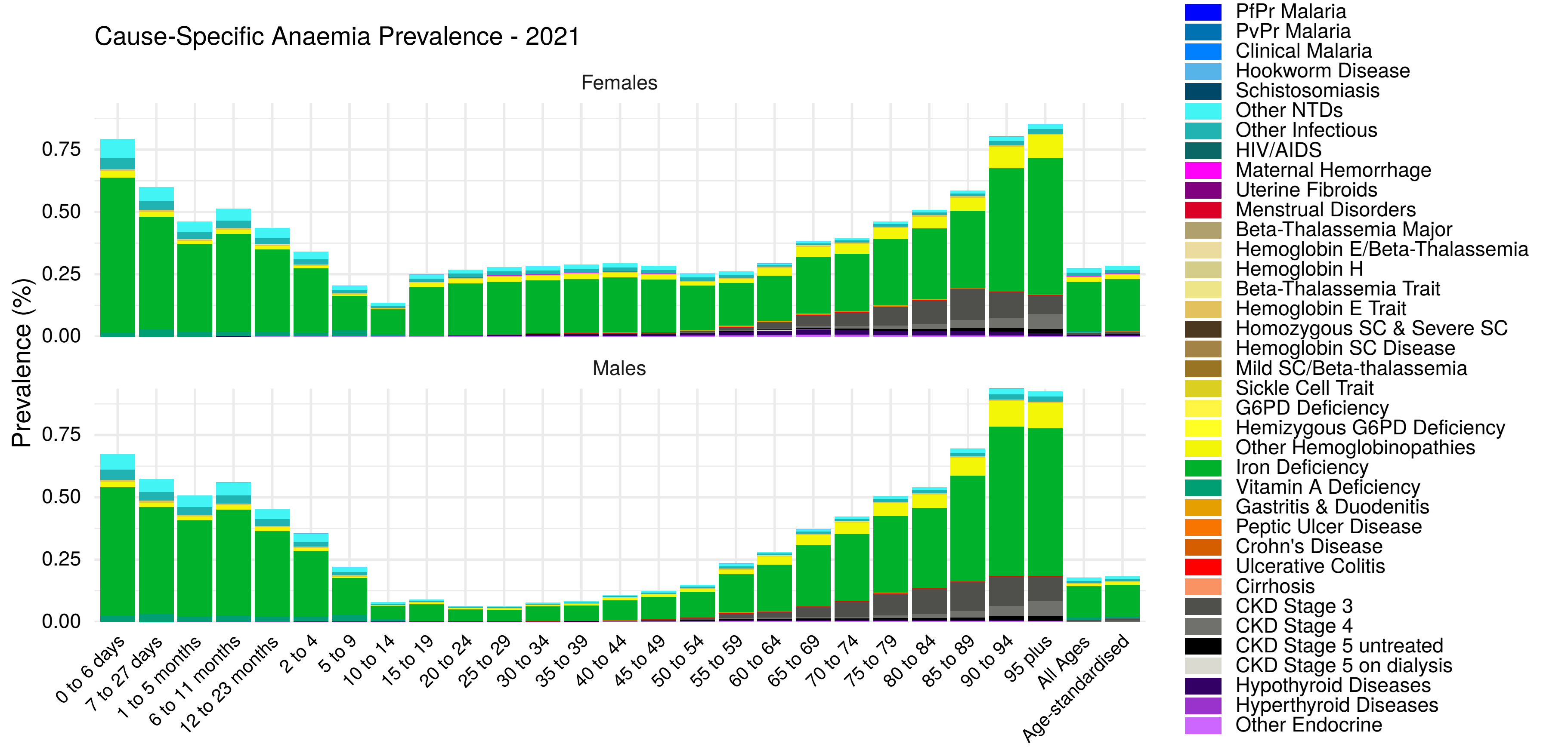

Cause-Specific Anaemia Prevalence - 2021
Females
Males
Prevalence (%)
0.75
0.50
0.25
0.00
0 to 6 days
7 to 27 days
1 to 5 months
6 to 11 months
12 to 23 months
2 to 4
5 to 9
10 to 14
15 to 19
20 to 24
25 to 29
30 to 34
35 to 39
40 to 44
45 to 49
50 to 54
55 to 59
60 to 64
65 to 69
70 to 74
75 to 79
80 to 84
85 to 89
90 to 94
95 plus
All Ages
Age-standardised
PfPr Malaria
PvPr Malaria
Clinical Malaria
Hookworm Disease
Schistosomiasis
Other NTDs
Other Infectious
HIV/AIDS
Maternal Hemorrhage
Uterine Fibroids
Menstrual Disorders
Beta-Thalassemia Major
Hemoglobin E/Beta-Thalassemia
Hemoglobin H
Beta-Thalassemia Trait
Hemoglobin E Trait
Homozygous SC & Severe SC
Hemoglobin SC Disease
Mild SC/Beta-thalassemia
Sickle Cell Trait
G6PD Deficiency
Hemizygous G6PD Deficiency
Other Hemoglobinopathies
Iron Deficiency
Vitamin A Deficiency
Gastritis & Duodenitis
Peptic Ulcer Disease
Crohn's Disease
Ulcerative Colitis
Cirrhosis
CKD Stage 3
CKD Stage 4
CKD Stage 5 untreated
CKD Stage 5 on dialysis
Hypothyroid Diseases
Hyperthyroid Diseases
Other Endocrine

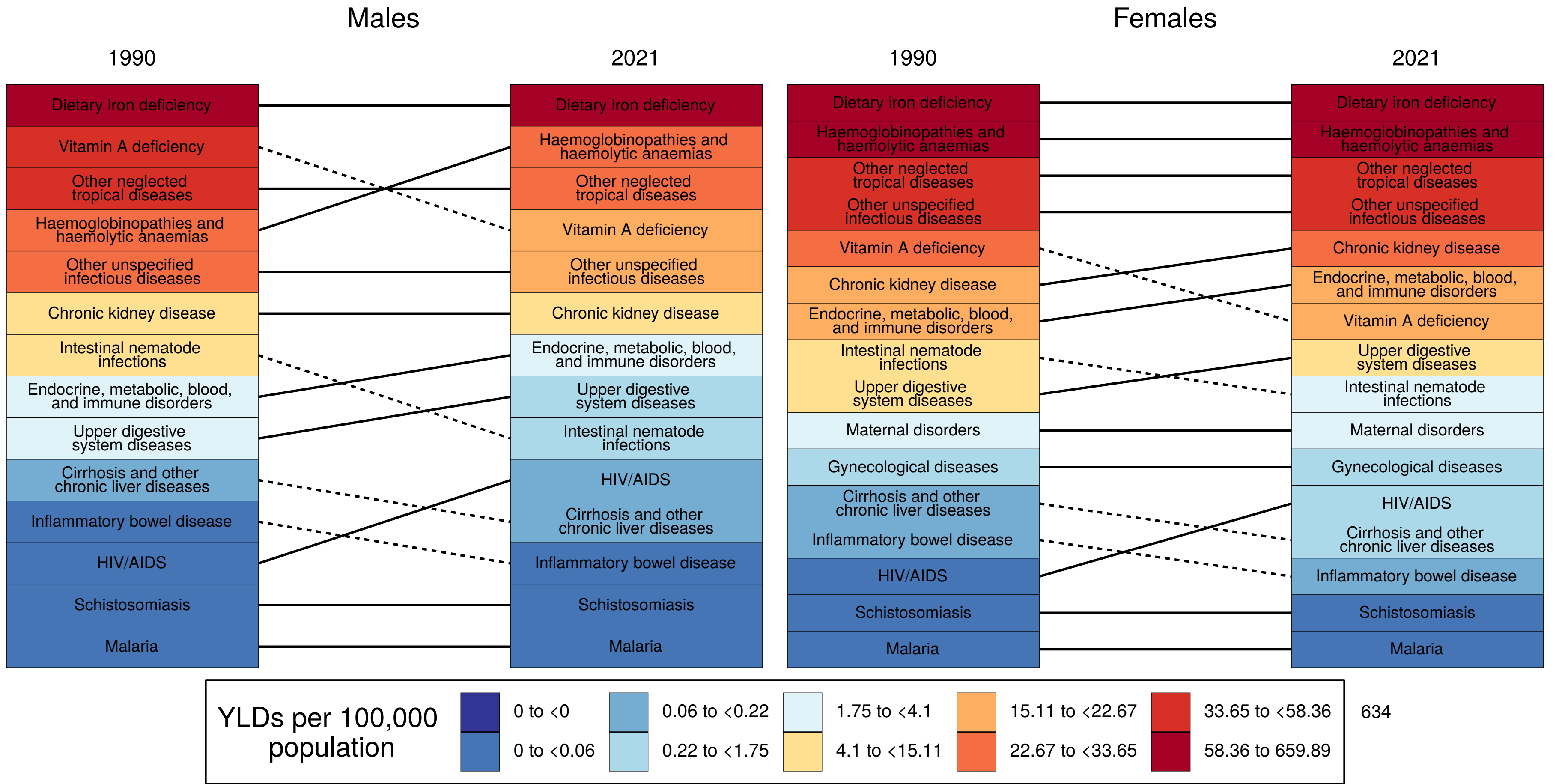

Males
Females
1990
2021
Dietary iron deficiency
Vitamin A deficiency
Other neglected tropical diseases
Haemoglobinopathies and haemolytic anaemias
Other unspecified infectious diseases
Chronic kidney disease
Intestinal nematode infections
Endocrine, metabolic, blood, and immune disorders
Upper digestive system diseases
Cirrhosis and other chronic liver diseases
Inflammatory bowel disease
HIV/AIDS
Schistosomiasis
Malaria
Maternal disorders
Gynecological diseases
YLDs per 100,000 population
0 to <0
0 to <0.06
0.06 to <0.22
0.22 to <1.75
1.75 to <4.1
4.1 to <15.11
15.11 to <22.67
22.67 to <33.65
33.65 to <58.36
58.36 to 659.89

Solomon Islands

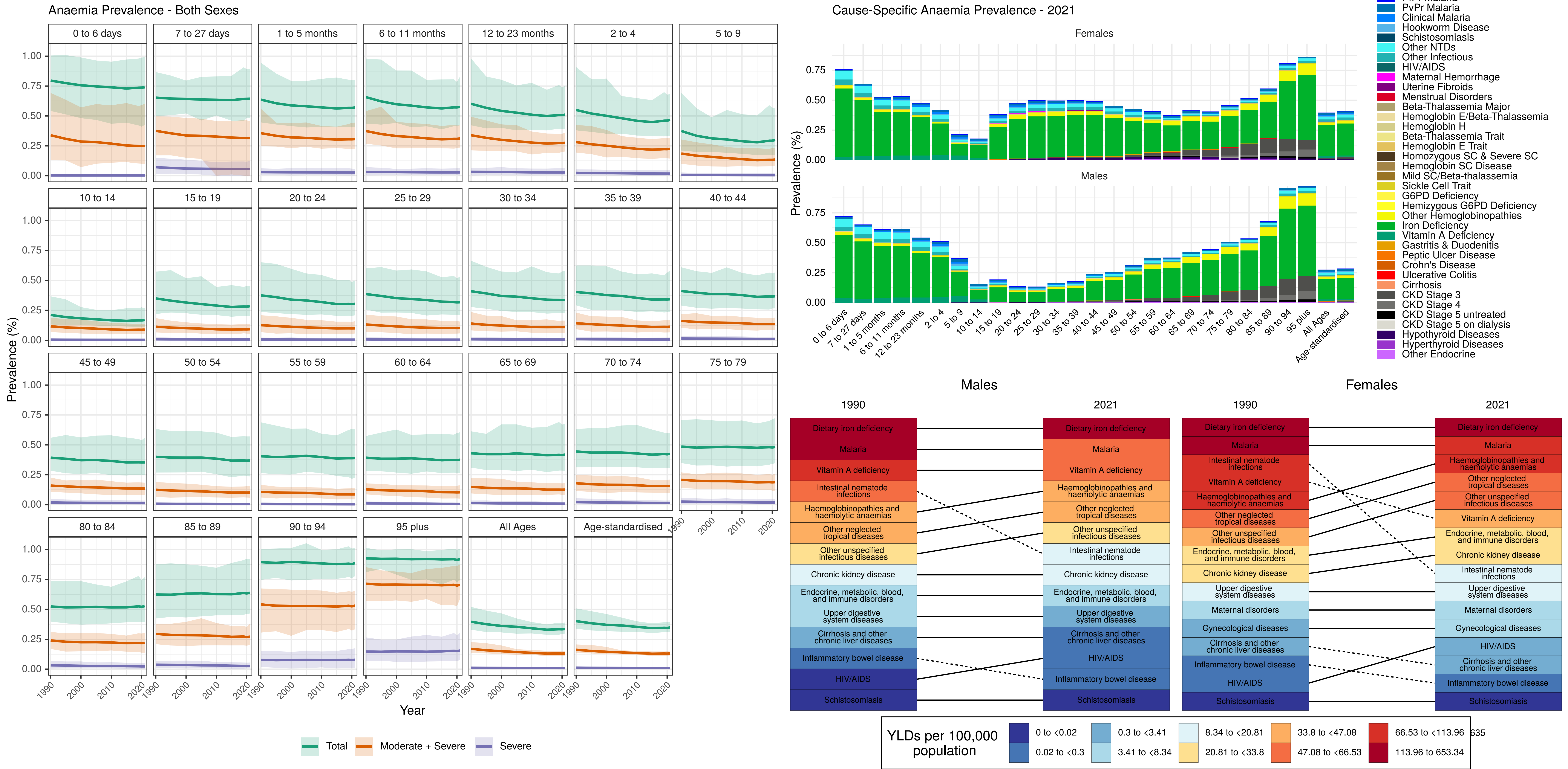

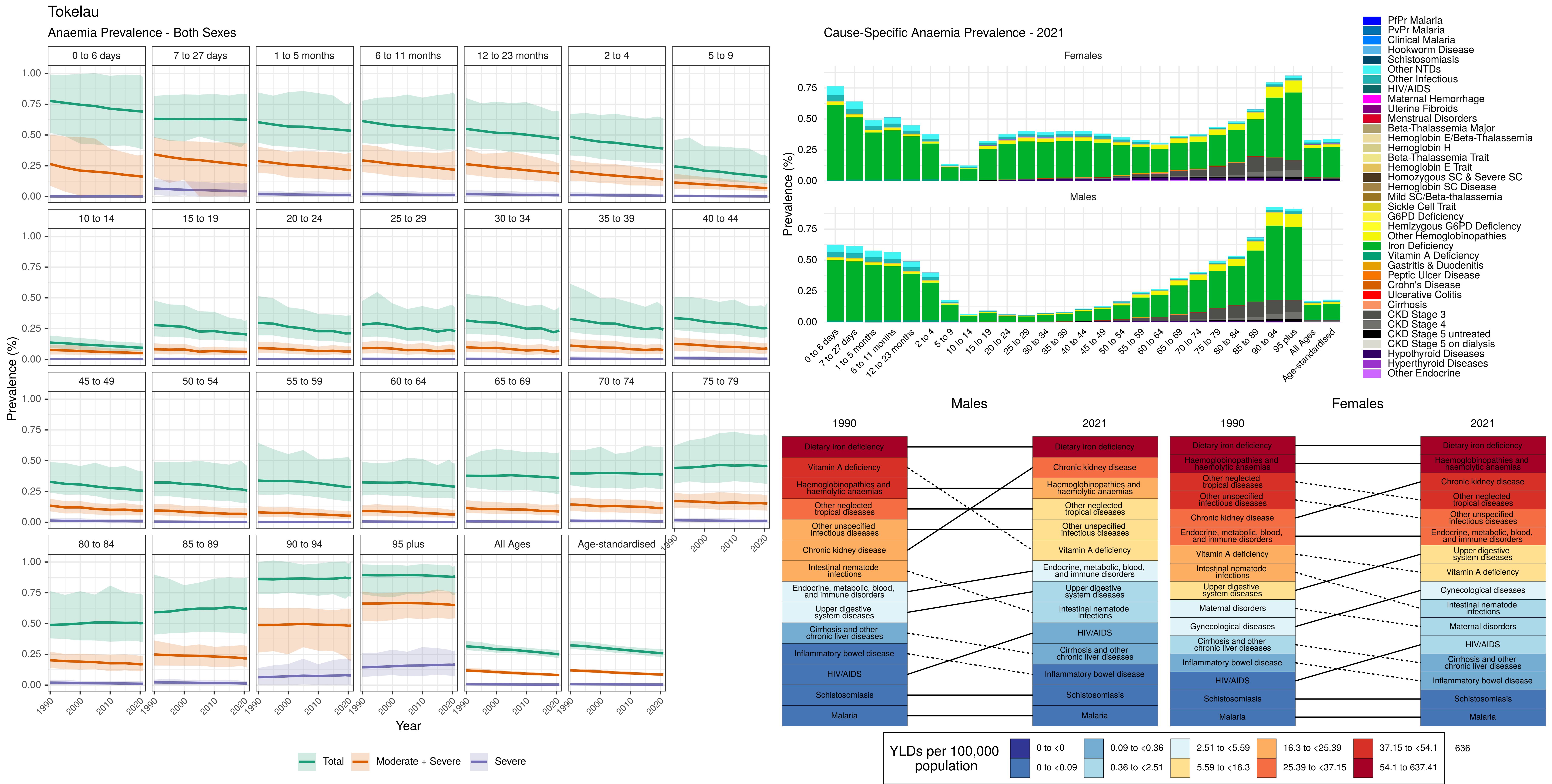
Tokelau
Anaemia Prevalence - Both Sexes
0 to 6 days
7 to 27 days
1 to 5 months
6 to 11 months
12 to 23 months
2 to 4
5 to 9
10 to 14
15 to 19
20 to 24
25 to 29
30 to 34
35 to 39
40 to 44
45 to 49
50 to 54
55 to 59
60 to 64
65 to 69
70 to 74
75 to 79
80 to 84
85 to 89
90 to 94
95 plus
All Ages
Age-standardised
Prevalence (%)
Year
Total
Moderate + Severe
Severe
Cause-Specific Anaemia Prevalence - 2021
Females
Males
PfPr Malaria
PvPr Malaria
Clinical Malaria
Hookworm Disease
Schistosomiasis
Other NTDs
Other Infectious
HIV/AIDS
Maternal Hemorrhage
Uterine Fibroids
Menstrual Disorders
Beta-Thalassemia Major
Hemoglobin E/Beta-Thalassemia
Hemoglobin H
Beta-Thalassemia Trait
Hemoglobin E Trait
Homozygous SC & Severe SC
Hemoglobin SC Disease
Mild SC/Beta-thalassemia
Sickle Cell Trait
G6PD Deficiency
Hemizygous G6PD Deficiency
Other Hemoglobinopathies
Iron Deficiency
Vitamin A Deficiency
Gastritis & Duodenitis
Peptic Ulcer Disease
Crohn's Disease
Ulcerative Colitis
Cirrhosis
CKD Stage 3
CKD Stage 4
CKD Stage 5 untreated
CKD Stage 5 on dialysis
Hypothyroid Diseases
Hyperthyroid Diseases
Other Endocrine
Males
Females
1990
2021
Dietary iron deficiency
Vitamin A deficiency
Haemoglobinopathies and haemolytic anaemias
Other neglected tropical diseases
Other unspecified infectious diseases
Chronic kidney disease
Intestinal nematode infections
Endocrine, metabolic, blood, and immune disorders
Upper digestive system diseases
Cirrhosis and other chronic liver diseases
Inflammatory bowel disease
HIV/AIDS
Schistosomiasis
Malaria
Maternal disorders
Gynecological diseases
YLDs per 100,000 population
0 to <0
0 to <0.09
0.09 to <0.36
0.36 to <2.51
2.51 to <5.59
5.59 to <16.3
16.3 to <25.39
25.39 to <37.15
37.15 to <54.1
54.1 to 637.41
636

Tonga

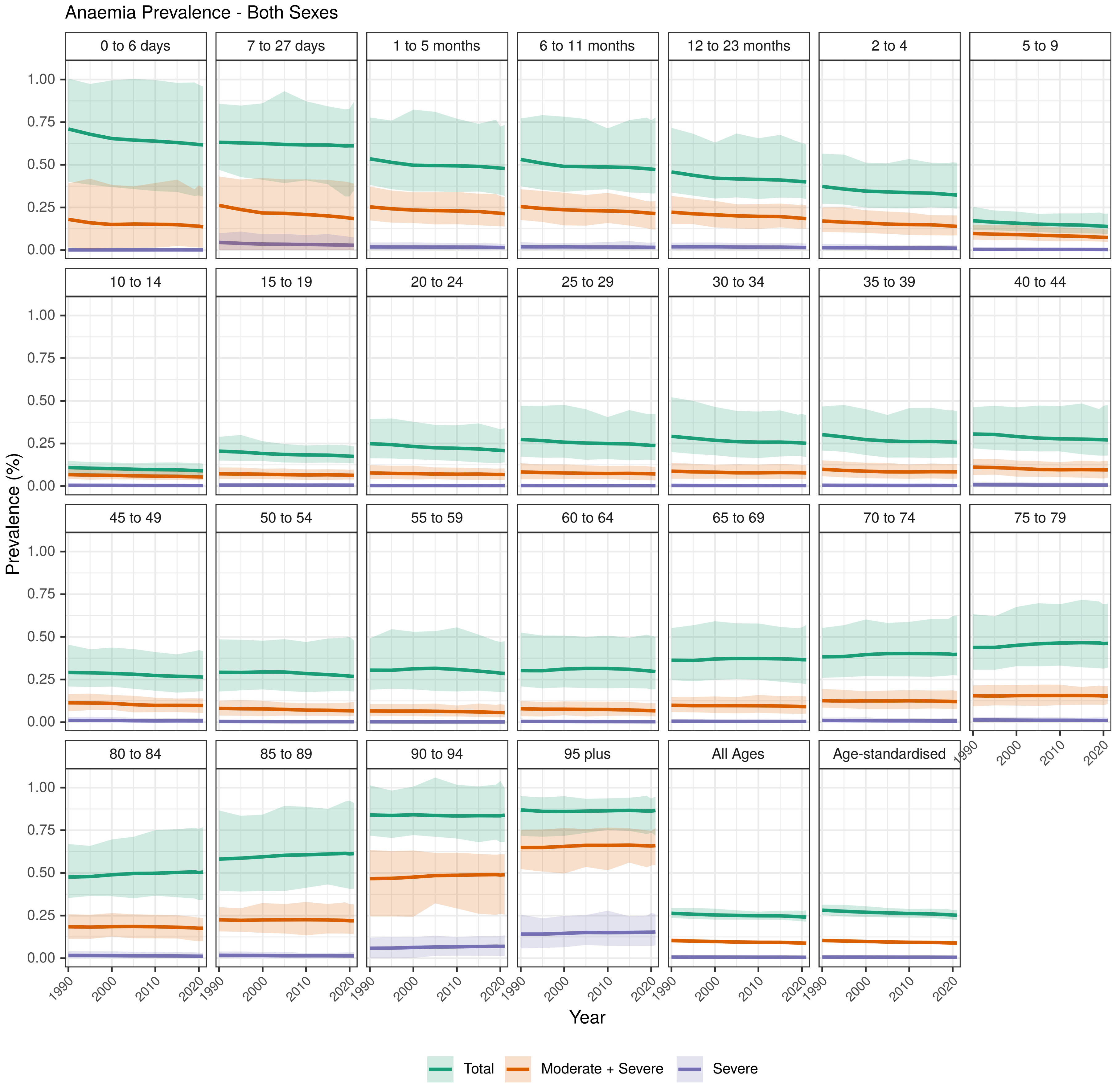

Anaemia Prevalence - Both Sexes
0 to 6 days
7 to 27 days
1 to 5 months
6 to 11 months
12 to 23 months
2 to 4
5 to 9
10 to 14
15 to 19
20 to 24
25 to 29
30 to 34
35 to 39
40 to 44
45 to 49
50 to 54
55 to 59
60 to 64
65 to 69
70 to 74
75 to 79
80 to 84
85 to 89
90 to 94
95 plus
All Ages
Age-standardised
Prevalence (%)
Year
1.00
0.75
0.50
0.25
0.00
1990
2000
2010
2020
Total
Moderate + Severe
Severe

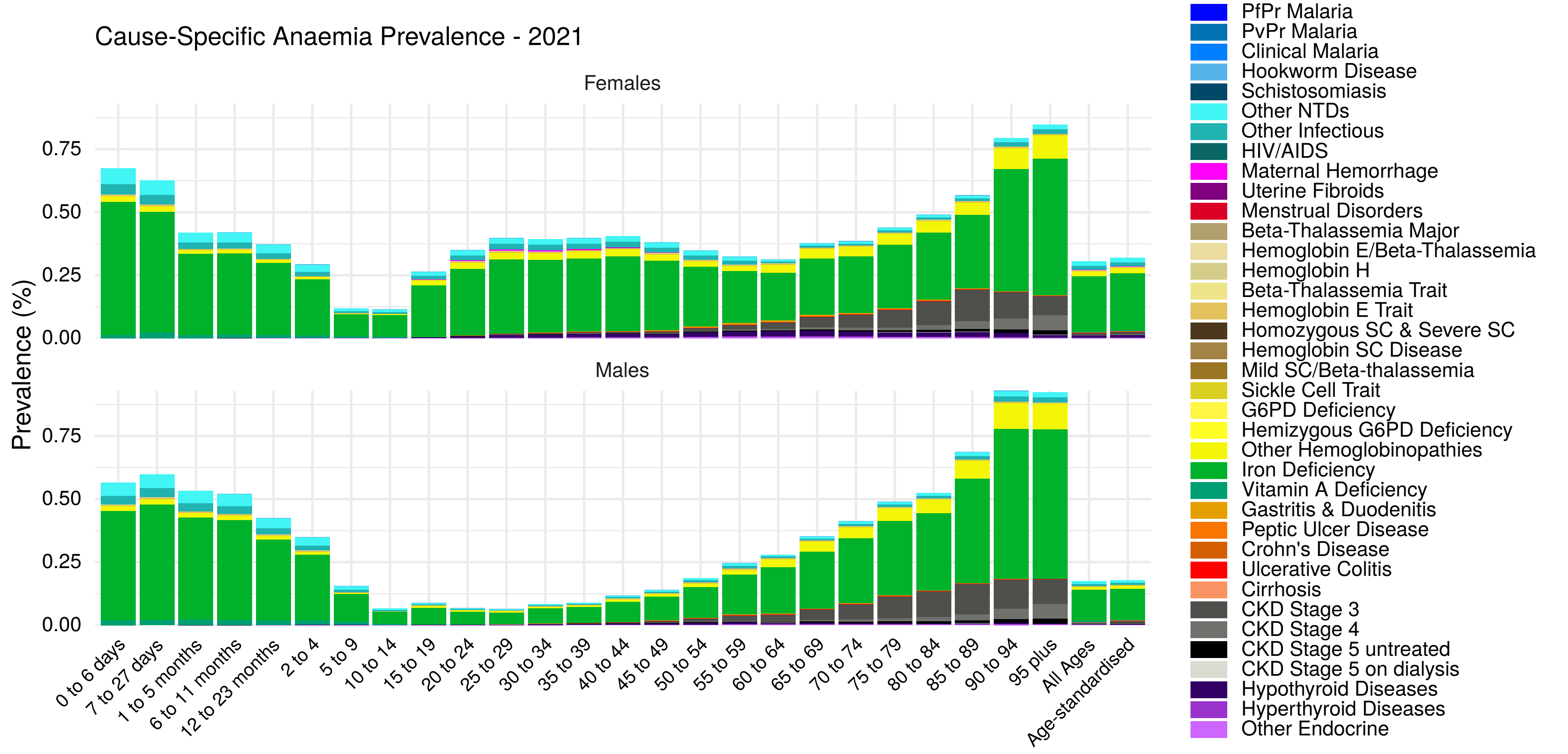

Cause-Specific Anaemia Prevalence - 2021
Females
Males
Prevalence (%)
0.75
0.50
0.25
0.00
0 to 6 days
7 to 27 days
1 to 5 months
6 to 11 months
12 to 23 months
2 to 4
5 to 9
10 to 14
15 to 19
20 to 24
25 to 29
30 to 34
35 to 39
40 to 44
45 to 49
50 to 54
55 to 59
60 to 64
65 to 69
70 to 74
75 to 79
80 to 84
85 to 89
90 to 94
95 plus
All Ages
Age-standardised
PfPr Malaria
PvPr Malaria
Clinical Malaria
Hookworm Disease
Schistosomiasis
Other NTDs
Other Infectious
HIV/AIDS
Maternal Hemorrhage
Uterine Fibroids
Menstrual Disorders
Beta-Thalassemia Major
Hemoglobin E/Beta-Thalassemia
Hemoglobin H
Beta-Thalassemia Trait
Hemoglobin E Trait
Homozygous SC & Severe SC
Hemoglobin SC Disease
Mild SC/Beta-thalassemia
Sickle Cell Trait
G6PD Deficiency
Hemizygous G6PD Deficiency
Other Hemoglobinopathies
Iron Deficiency
Vitamin A Deficiency
Gastritis & Duodenitis
Peptic Ulcer Disease
Crohn's Disease
Ulcerative Colitis
Cirrhosis
CKD Stage 3
CKD Stage 4
CKD Stage 5 untreated
CKD Stage 5 on dialysis
Hypothyroid Diseases
Hyperthyroid Diseases
Other Endocrine

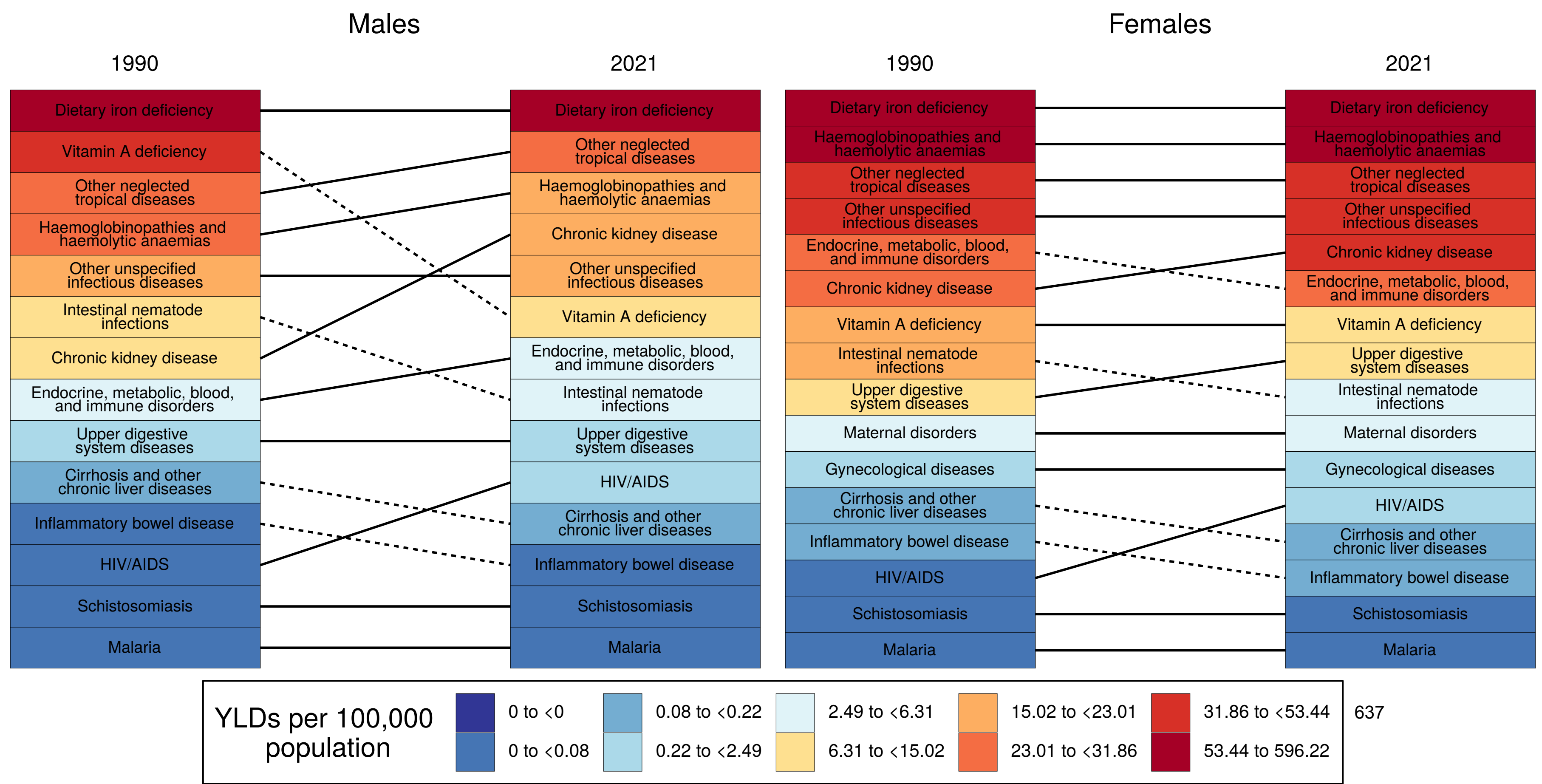

Males
Females
1990
2021
Dietary iron deficiency
Vitamin A deficiency
Other neglected tropical diseases
Haemoglobinopathies and haemolytic anaemias
Other unspecified infectious diseases
Intestinal nematode infections
Chronic kidney disease
Endocrine, metabolic, blood, and immune disorders
Upper digestive system diseases
Cirrhosis and other chronic liver diseases
Inflammatory bowel disease
HIV/AIDS
Schistosomiasis
Malaria
Maternal disorders
Gynecological diseases
YLDs per 100,000 population
0 to <0
0 to <0.08
0.08 to <0.22
0.22 to <2.49
2.49 to <6.31
6.31 to <15.02
15.02 to <23.01
23.01 to <31.86
31.86 to <53.44
53.44 to 596.22

637

Tuvalu

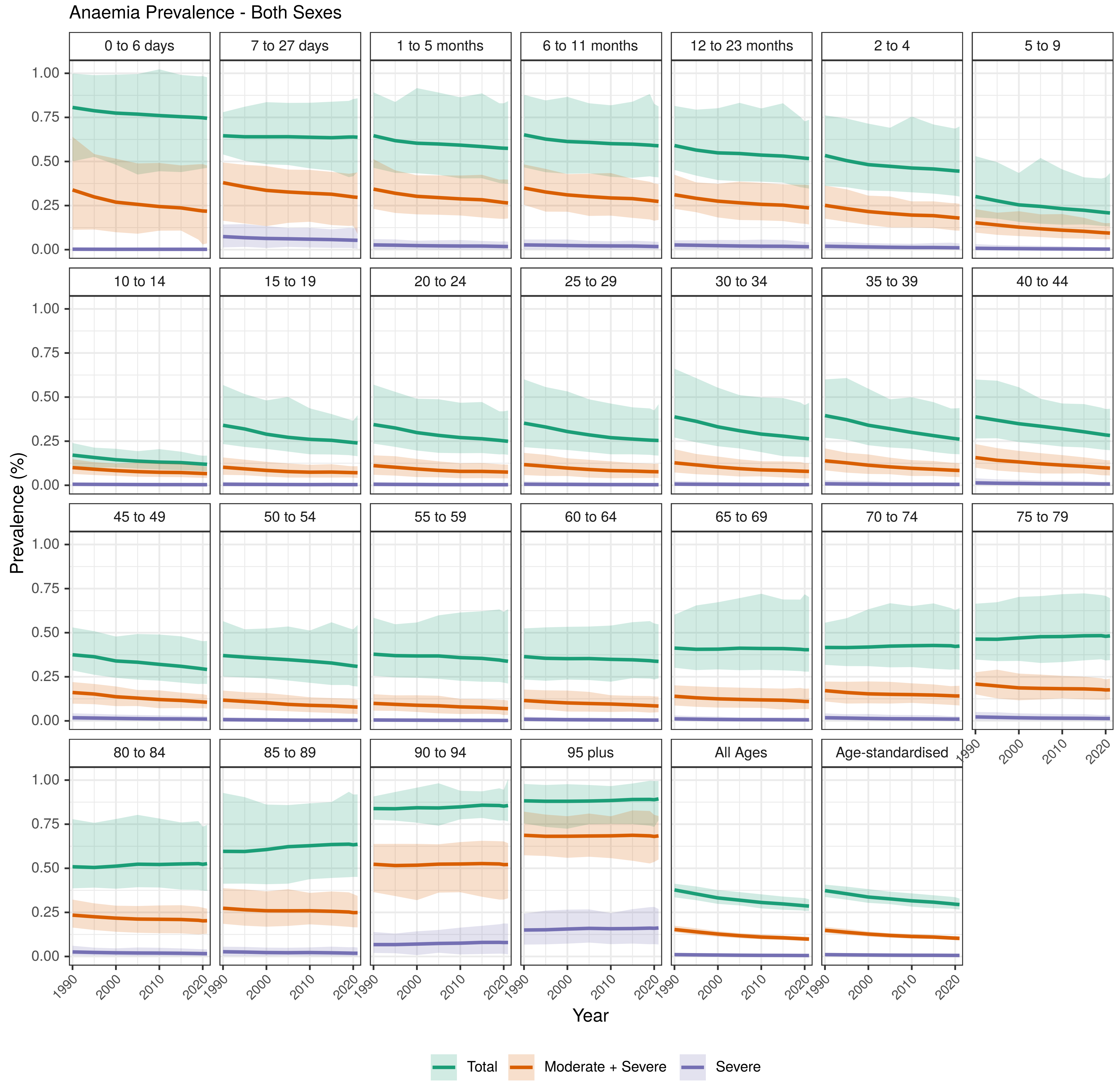

Anaemia Prevalence - Both Sexes
0 to 6 days
7 to 27 days
1 to 5 months
6 to 11 months
12 to 23 months
2 to 4
5 to 9
10 to 14
15 to 19
20 to 24
25 to 29
30 to 34
35 to 39
40 to 44
45 to 49
50 to 54
55 to 59
60 to 64
65 to 69
70 to 74
75 to 79
80 to 84
85 to 89
90 to 94
95 plus
All Ages
Age-standardised
Prevalence (%)
1.00
0.75
0.50
0.25
0.00
1990
2000
2010
2020
Year
Total
Moderate + Severe
Severe

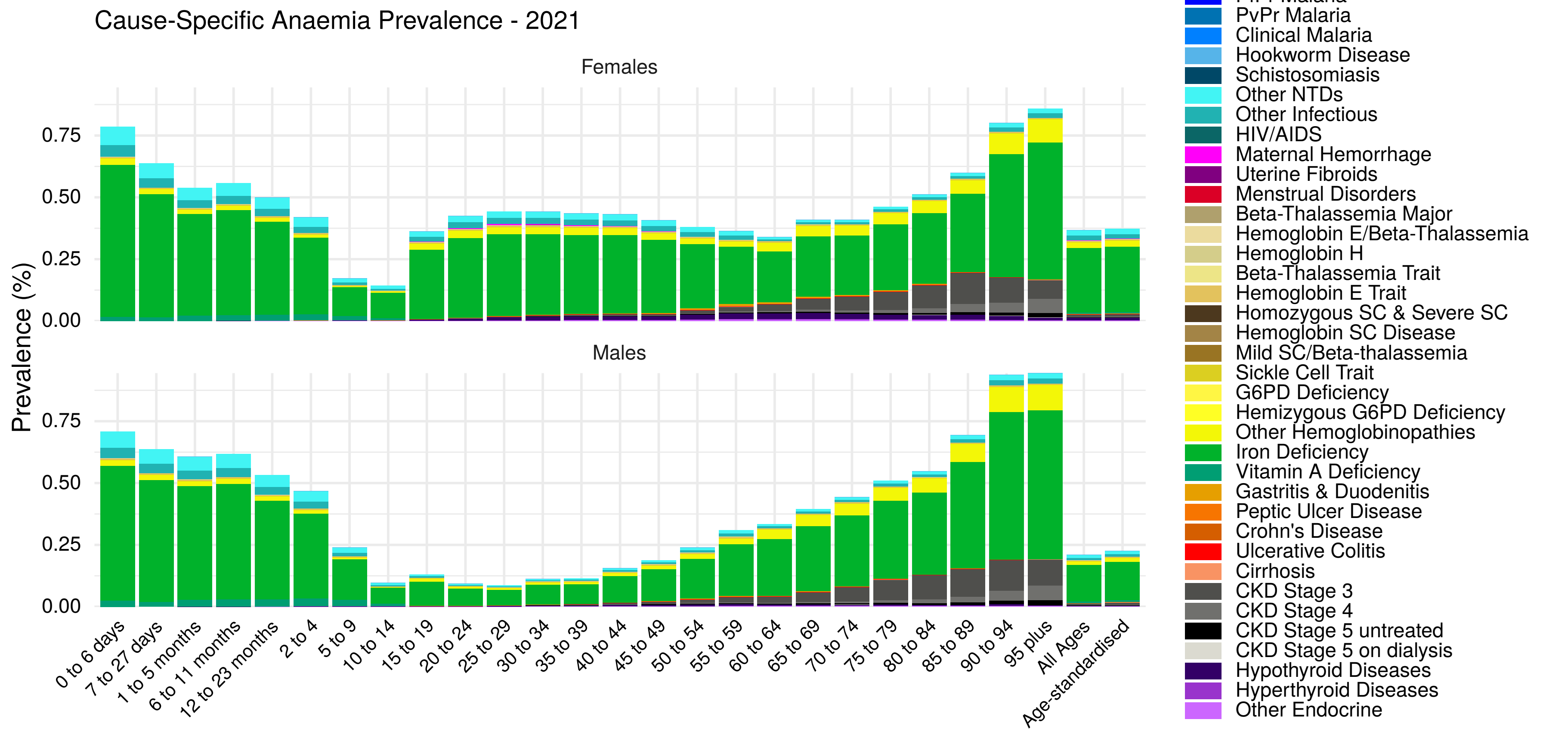

Cause-Specific Anaemia Prevalence - 2021
Females
Males
Prevalence (%)
0.75
0.50
0.25
0.00
0 to 6 days
7 to 27 days
1 to 5 months
6 to 11 months
12 to 23 months
2 to 4
5 to 9
10 to 14
15 to 19
20 to 24
25 to 29
30 to 34
35 to 39
40 to 44
45 to 49
50 to 54
55 to 59
60 to 64
65 to 69
70 to 74
75 to 79
80 to 84
85 to 89
90 to 94
95 plus
All Ages
Age-standardised
PfPr Malaria
PvPr Malaria
Clinical Malaria
Hookworm Disease
Schistosomiasis
Other NTDs
Other Infectious
HIV/AIDS
Maternal Hemorrhage
Uterine Fibroids
Menstrual Disorders
Beta-Thalassemia Major
Hemoglobin E/Beta-Thalassemia
Hemoglobin H
Beta-Thalassemia Trait
Hemoglobin E Trait
Homozygous SC & Severe SC
Hemoglobin SC Disease
Mild SC/Beta-thalassemia
Sickle Cell Trait
G6PD Deficiency
Hemizygous G6PD Deficiency
Other Hemoglobinopathies
Iron Deficiency
Vitamin A Deficiency
Gastritis & Duodenitis
Peptic Ulcer Disease
Crohn's Disease
Ulcerative Colitis
Cirrhosis
CKD Stage 3
CKD Stage 4
CKD Stage 5 untreated
CKD Stage 5 on dialysis
Hypothyroid Diseases
Hyperthyroid Diseases
Other Endocrine

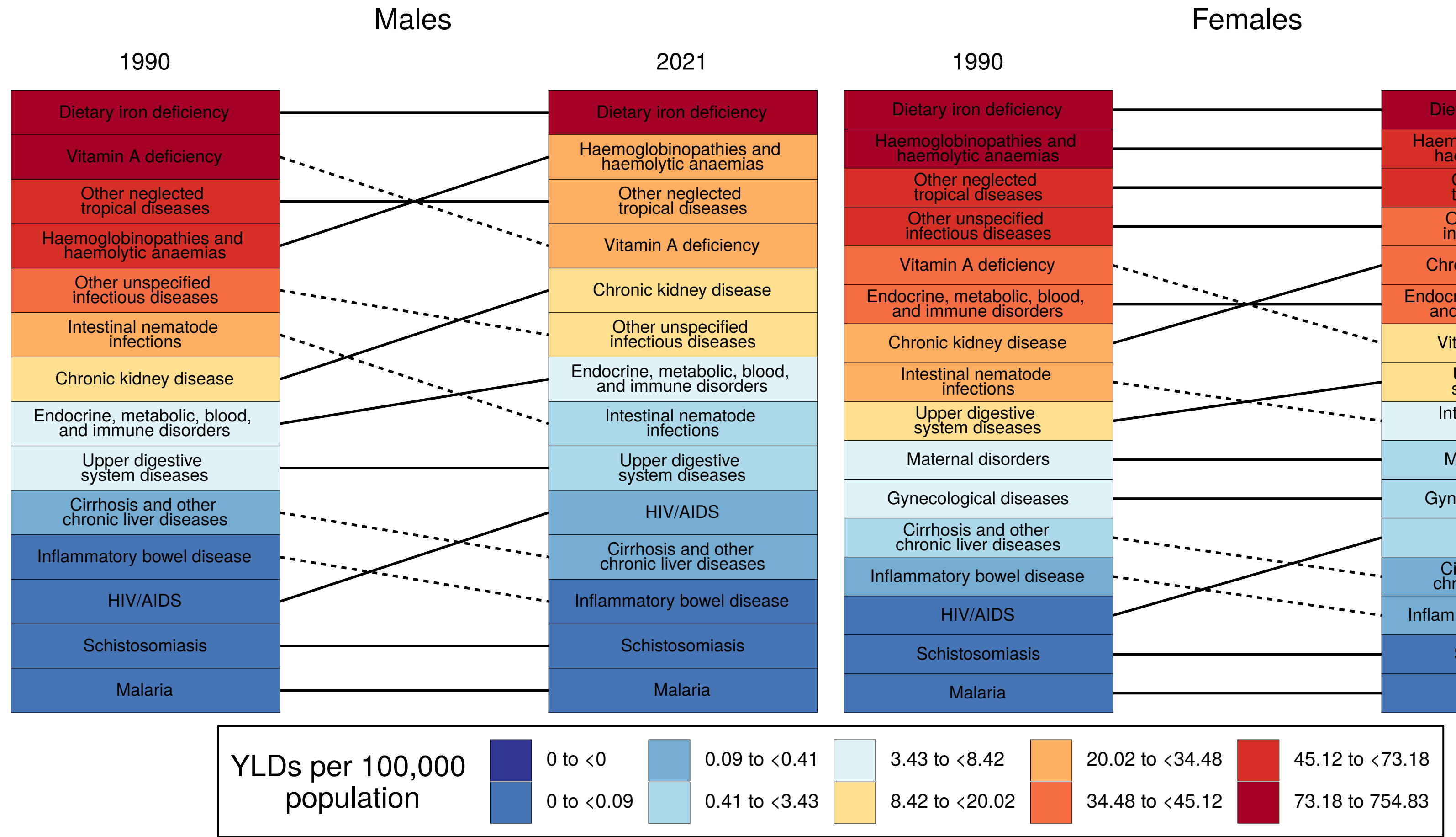

Males
1990
Dietary iron deficiency
Vitamin A deficiency
Other neglected tropical diseases
Haemoglobinopathies and haemolytic anaemias
Other unspecified infectious diseases
Intestinal nematode infections
Chronic kidney disease
Endocrine, metabolic, blood, and immune disorders
Upper digestive system diseases
Cirrhosis and other chronic liver diseases
Inflammatory bowel disease
HIV/AIDS
Schistosomiasis
Malaria
2021
Dietary iron deficiency
Haemoglobinopathies and haemolytic anaemias
Other neglected tropical diseases
Vitamin A deficiency
Chronic kidney disease
Other unspecified infectious diseases
Endocrine, metabolic, blood, and immune disorders
Intestinal nematode infections
Upper digestive system diseases
HIV/AIDS
Cirrhosis and other chronic liver diseases
Inflammatory bowel disease
Schistosomiasis
Malaria
Females
1990
Dietary iron deficiency
Haemoglobinopathies and haemolytic anaemias
Other neglected tropical diseases
Other unspecified infectious diseases
Vitamin A deficiency
Endocrine, metabolic, blood, and immune disorders
Chronic kidney disease
Intestinal nematode infections
Upper digestive system diseases
Maternal disorders
Gynecological diseases
Cirrhosis and other chronic liver diseases
Inflammatory bowel disease
HIV/AIDS
Schistosomiasis
Malaria
2021
Dietary iron deficiency
Haemoglobinopathies and haemolytic anaemias
Other neglected tropical diseases
Other unspecified infectious diseases
Chronic kidney disease
Endocrine, metabolic, blood, and immune disorders
Vitamin A deficiency
Upper digestive system diseases
Intestinal nematode infections
Maternal disorders
Gynecological diseases
HIV/AIDS
Cirrhosis and other chronic liver diseases
Inflammatory bowel disease
Schistosomiasis
Malaria
YLDs per 100,000 population
0 to <0
0 to <0.09
0.09 to <0.41
0.41 to <3.43
3.43 to <8.42
8.42 to <20.02
20.02 to <34.48
34.48 to <45.12
45.12 to <73.18
73.18 to 754.83

Vanuatu

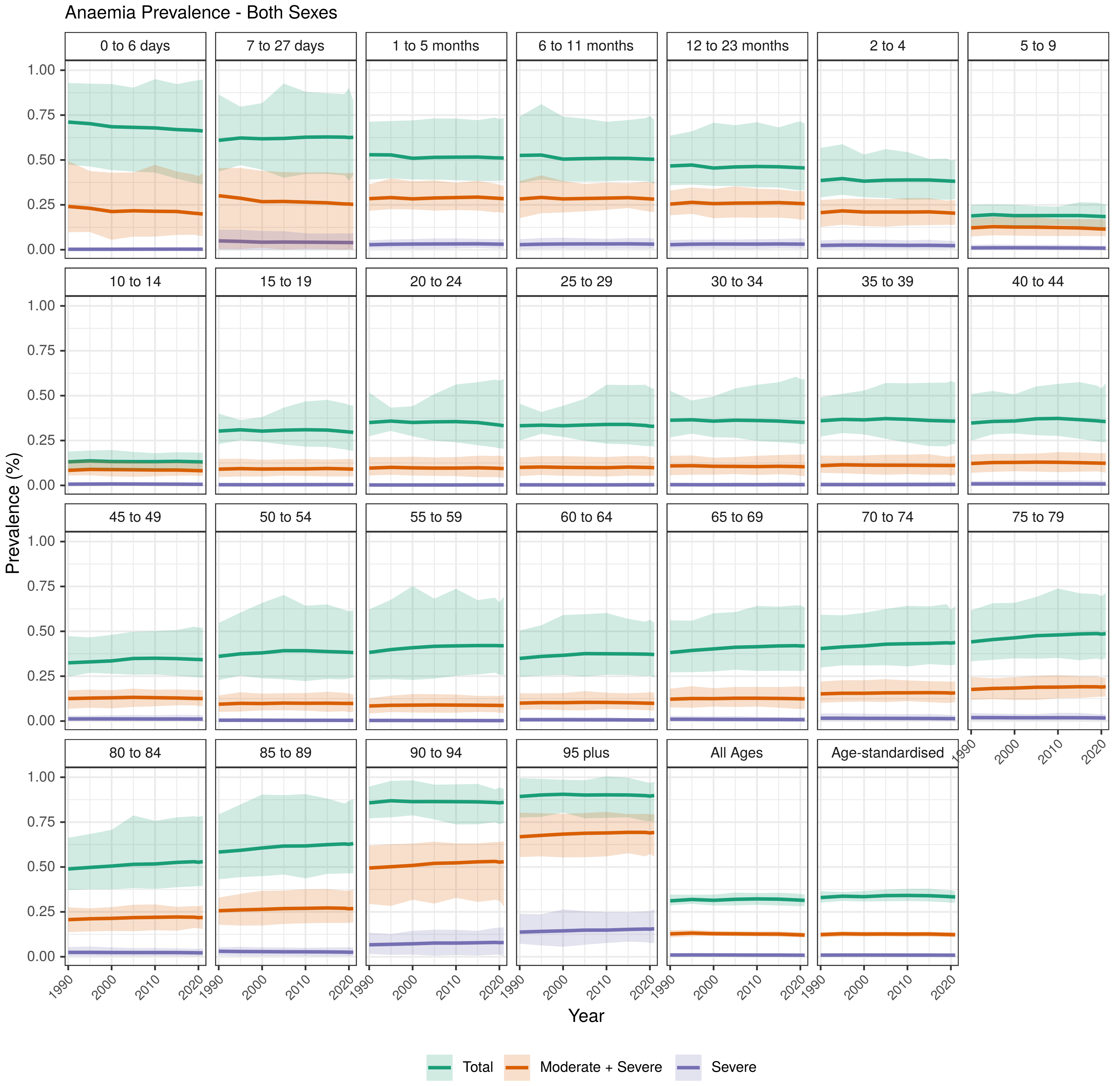
Anaemia Prevalence - Both Sexes
0 to 6 days
7 to 27 days
1 to 5 months
6 to 11 months
12 to 23 months
2 to 4
5 to 9
10 to 14
15 to 19
20 to 24
25 to 29
30 to 34
35 to 39
40 to 44
45 to 49
50 to 54
55 to 59
60 to 64
65 to 69
70 to 74
75 to 79
80 to 84
85 to 89
90 to 94
95 plus
All Ages
Age-standardised
1.00
0.75
0.50
0.25
0.00
Prevalence (%)
1990
2000
2010
2020
Year
Total
Moderate + Severe
Severe

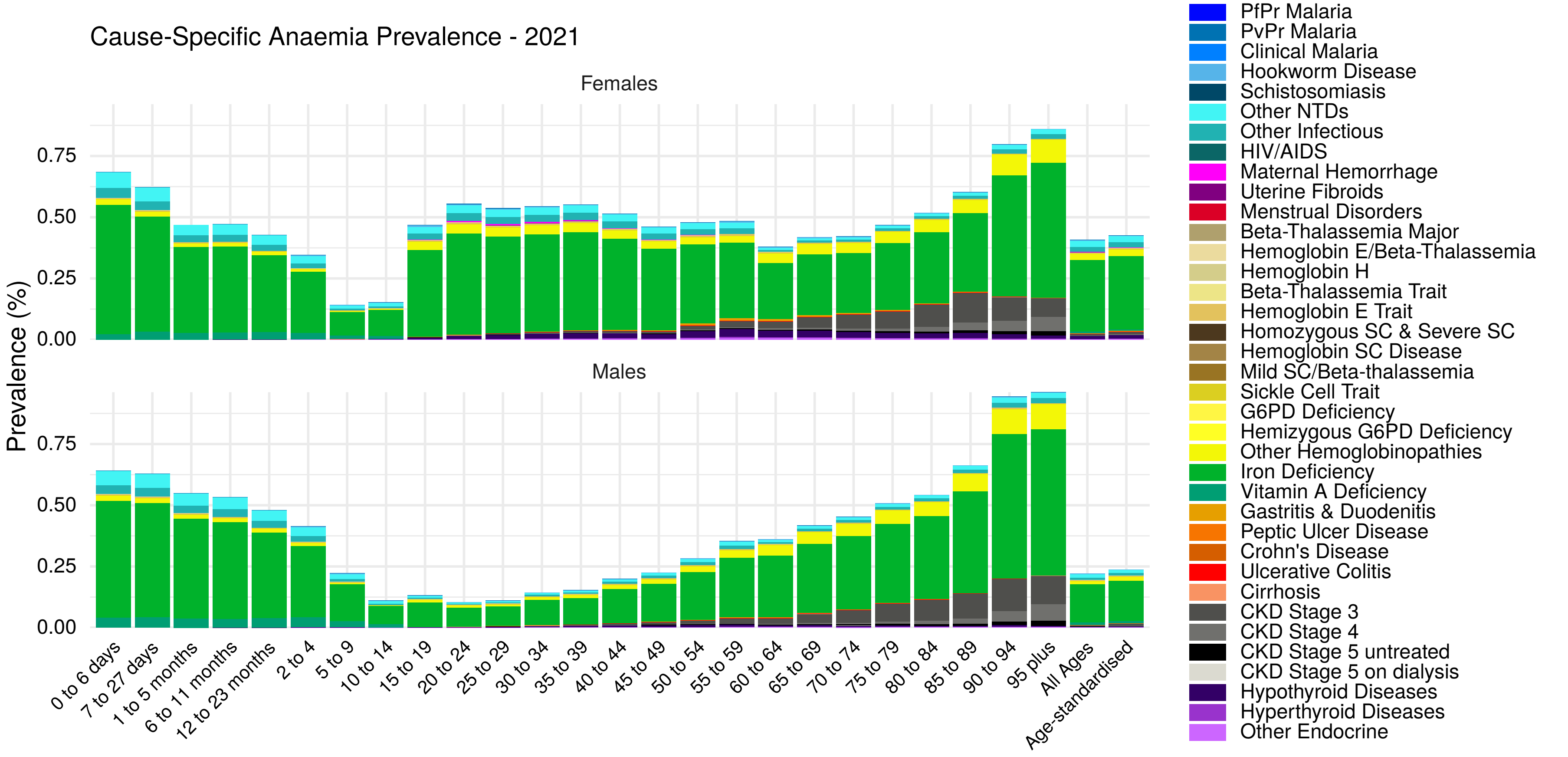
Cause-Specific Anaemia Prevalence - 2021
Females
Males
Prevalence (%)
0.75
0.50
0.25
0.00
0 to 6 days
7 to 27 days
1 to 5 months
6 to 11 months
12 to 23 months
2 to 4
5 to 9
10 to 14
15 to 19
20 to 24
25 to 29
30 to 34
35 to 39
40 to 44
45 to 49
50 to 54
55 to 59
60 to 64
65 to 69
70 to 74
75 to 79
80 to 84
85 to 89
90 to 94
95 plus
All Ages
Age-standardised
PfPr Malaria
PvPr Malaria
Clinical Malaria
Hookworm Disease
Schistosomiasis
Other NTDs
Other Infectious
HIV/AIDS
Maternal Hemorrhage
Uterine Fibroids
Menstrual Disorders
Beta-Thalassemia Major
Hemoglobin E/Beta-Thalassemia
Hemoglobin H
Beta-Thalassemia Trait
Hemoglobin E Trait
Homozygous SC & Severe SC
Hemoglobin SC Disease
Mild SC/Beta-thalassemia
Sickle Cell Trait
G6PD Deficiency
Hemizygous G6PD Deficiency
Other Hemoglobinopathies
Iron Deficiency
Vitamin A Deficiency
Gastritis & Duodenitis
Peptic Ulcer Disease
Crohn's Disease
Ulcerative Colitis
Cirrhosis
CKD Stage 3
CKD Stage 4
CKD Stage 5 untreated
CKD Stage 5 on dialysis
Hypothyroid Diseases
Hyperthyroid Diseases
Other Endocrine

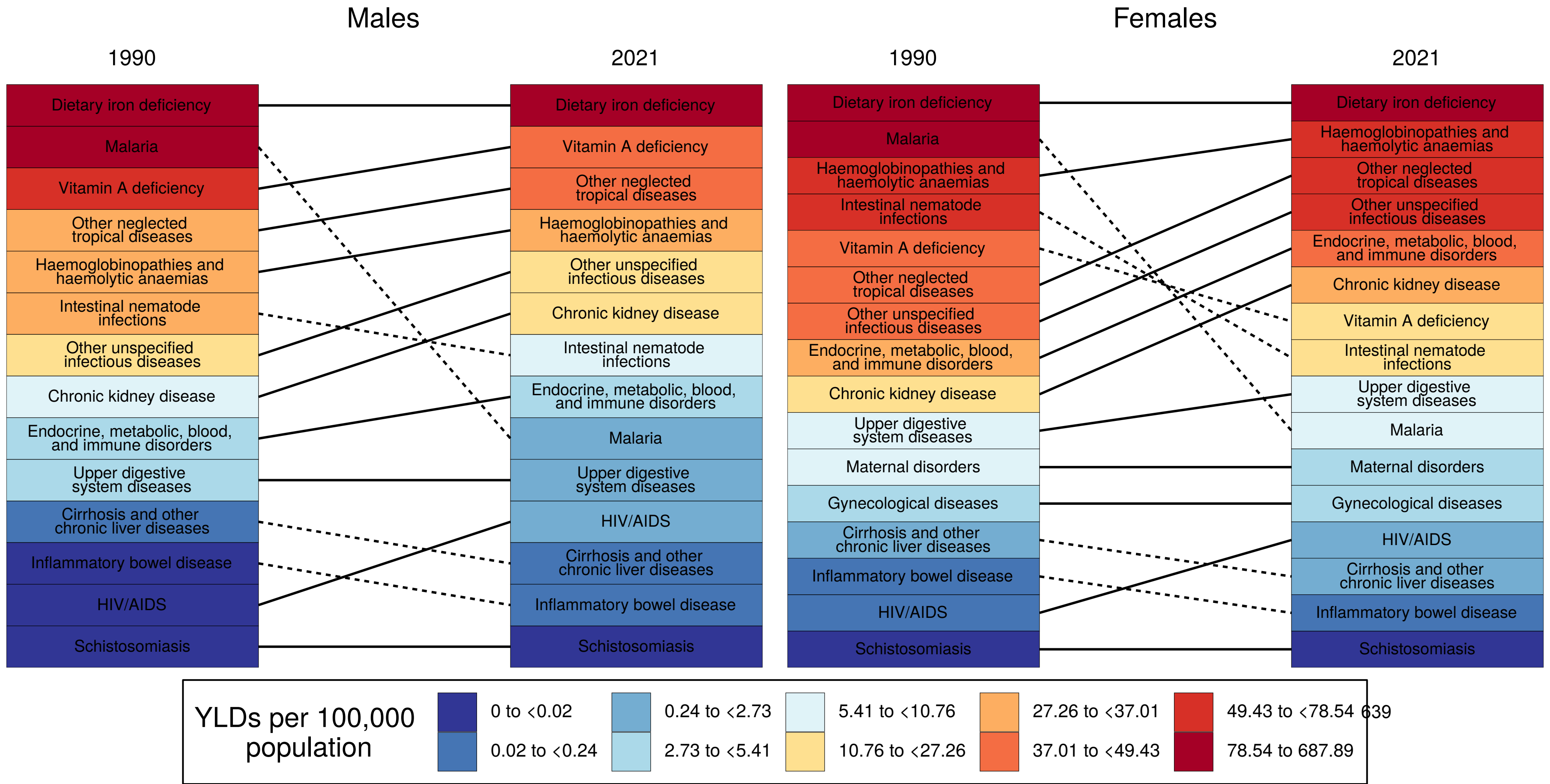
Males
Females
1990
2021
Dietary iron deficiency
Malaria
Vitamin A deficiency
Other neglected tropical diseases
Haemoglobinopathies and haemolytic anaemias
Intestinal nematode infections
Other unspecified infectious diseases
Chronic kidney disease
Endocrine, metabolic, blood, and immune disorders
Upper digestive system diseases
Cirrhosis and other chronic liver diseases
Inflammatory bowel disease
HIV/AIDS
Schistosomiasis
Maternal disorders
Gynecological diseases
YLDs per 100,000 population
0 to <0.02
0.02 to <0.24
0.24 to <2.73
2.73 to <5.41
5.41 to <10.76
10.76 to <27.26
27.26 to <37.01
37.01 to <49.43
49.43 to <78.54 639
78.54 to 687.89

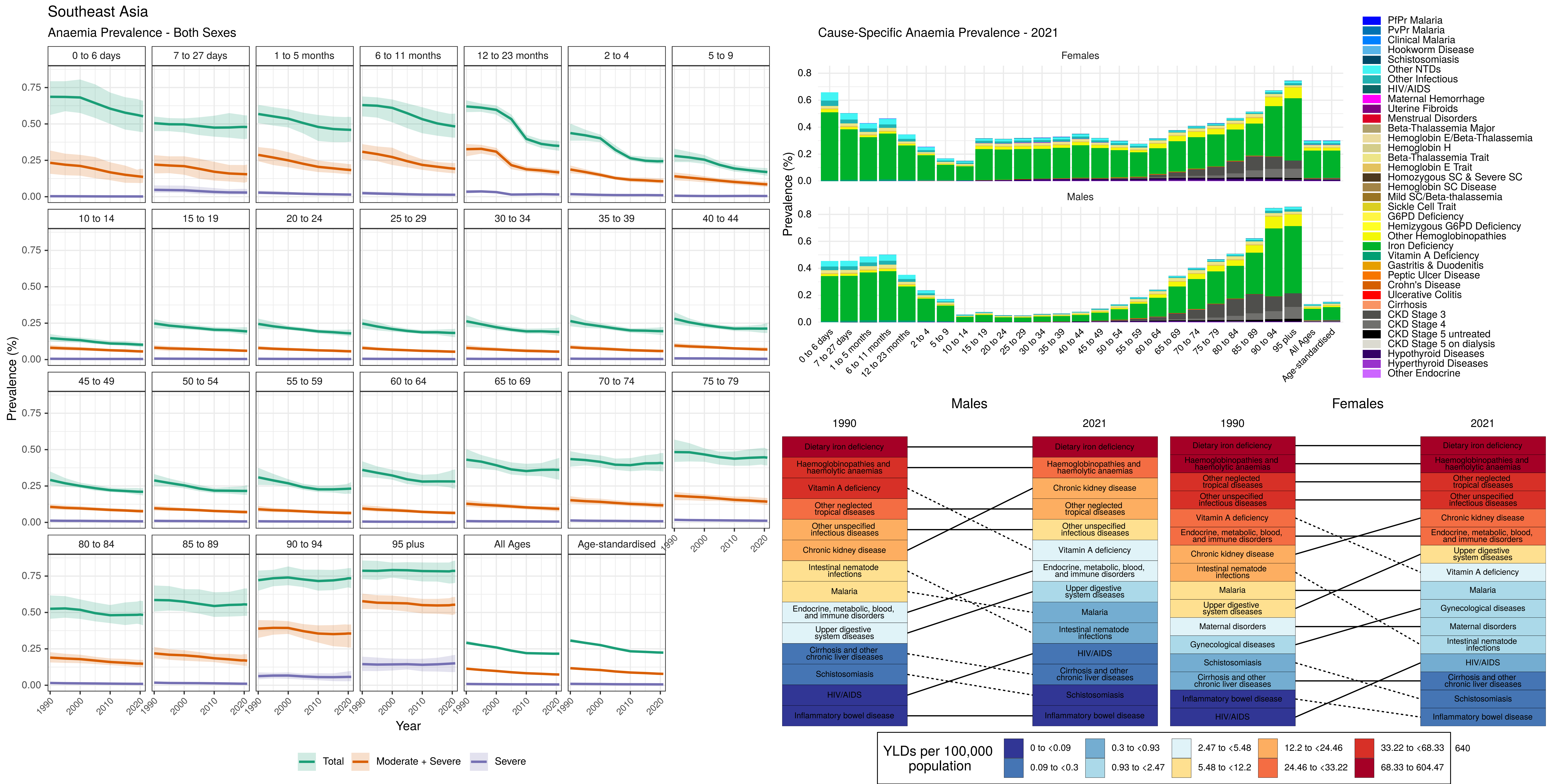
Southeast Asia
Anaemia Prevalence - Both Sexes
0 to 6 days
7 to 27 days
1 to 5 months
6 to 11 months
12 to 23 months
2 to 4
5 to 9
10 to 14
15 to 19
20 to 24
25 to 29
30 to 34
35 to 39
40 to 44
45 to 49
50 to 54
55 to 59
60 to 64
65 to 69
70 to 74
75 to 79
80 to 84
85 to 89
90 to 94
95 plus
All Ages
Age-standardised
Prevalence (%)
Year
Total
Moderate + Severe
Severe
Cause-Specific Anaemia Prevalence - 2021
Females
Males
PfPr Malaria
PvPr Malaria
Clinical Malaria
Hookworm Disease
Schistosomiasis
Other NTDs
Other Infectious
HIV/AIDS
Maternal Hemorrhage
Uterine Fibroids
Menstrual Disorders
Beta-Thalassemia Major
Hemoglobin E/Beta-Thalassemia
Hemoglobin H
Beta-Thalassemia Trait
Hemoglobin E Trait
Homozygous SC & Severe SC
Hemoglobin SC Disease
Mild SC/Beta-thalassemia
Sickle Cell Trait
G6PD Deficiency
Hemizygous G6PD Deficiency
Other Hemoglobinopathies
Iron Deficiency
Vitamin A Deficiency
Gastritis & Duodenitis
Peptic Ulcer Disease
Crohn's Disease
Ulcerative Colitis
Cirrhosis
CKD Stage 3
CKD Stage 4
CKD Stage 5 untreated
CKD Stage 5 on dialysis
Hypothyroid Diseases
Hyperthyroid Diseases
Other Endocrine
Males
Females
1990
2021
Dietary iron deficiency
Haemoglobinopathies and haemolytic anaemias
Vitamin A deficiency
Other neglected tropical diseases
Other unspecified infectious diseases
Chronic kidney disease
Intestinal nematode infections
Malaria
Endocrine, metabolic, blood, and immune disorders
Upper digestive system diseases
Cirrhosis and other chronic liver diseases
Schistosomiasis
HIV/AIDS
Inflammatory bowel disease
Maternal disorders
Gynecological diseases
YLDs per 100,000 population
0 to <0.09
0.09 to <0.3
0.3 to <0.93
0.93 to <2.47
2.47 to <5.48
5.48 to <12.2
12.2 to <24.46
24.46 to <33.22
33.22 to <68.33
68.33 to 604.47
640

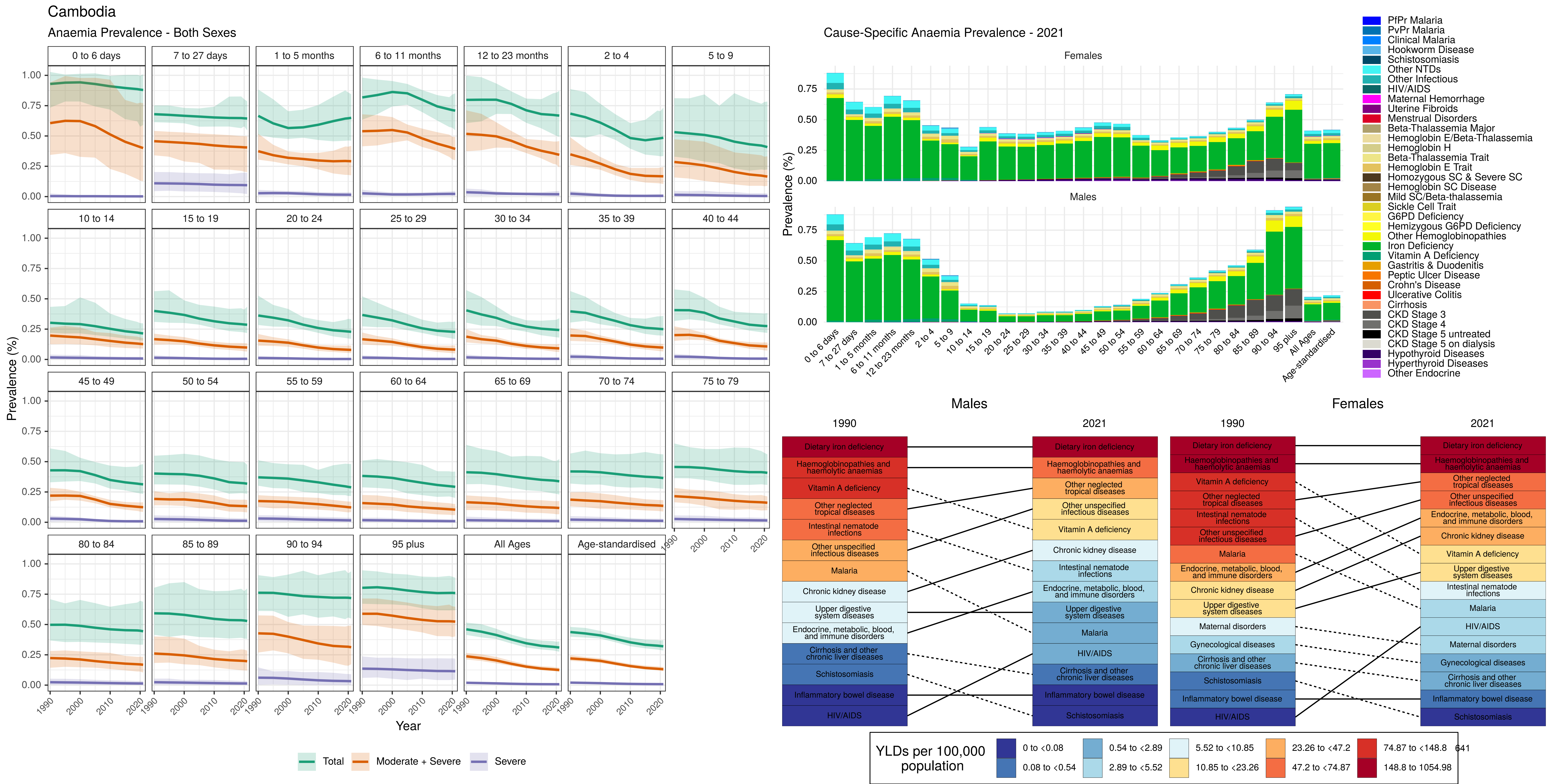
Cambodia
Anaemia Prevalence - Both Sexes
0 to 6 days
7 to 27 days
1 to 5 months
6 to 11 months
12 to 23 months
2 to 4
5 to 9
10 to 14
15 to 19
20 to 24
25 to 29
30 to 34
35 to 39
40 to 44
45 to 49
50 to 54
55 to 59
60 to 64
65 to 69
70 to 74
75 to 79
80 to 84
85 to 89
90 to 94
95 plus
All Ages
Age-standardised
Prevalence (%)
Year
Total
Moderate + Severe
Severe
Cause-Specific Anaemia Prevalence - 2021
Females
Males
Prevalence (%)
PfPr Malaria
PvPr Malaria
Clinical Malaria
Hookworm Disease
Schistosomiasis
Other NTDs
Other Infectious
HIV/AIDS
Maternal Hemorrhage
Uterine Fibroids
Menstrual Disorders
Beta-Thalassemia Major
Hemoglobin E/Beta-Thalassemia
Hemoglobin H
Beta-Thalassemia Trait
Hemoglobin E Trait
Homozygous SC & Severe SC
Hemoglobin SC Disease
Mild SC/Beta-thalassemia
Sickle Cell Trait
G6PD Deficiency
Hemizygous G6PD Deficiency
Other Hemoglobinopathies
Iron Deficiency
Vitamin A Deficiency
Gastritis & Duodenitis
Peptic Ulcer Disease
Crohn's Disease
Ulcerative Colitis
Cirrhosis
CKD Stage 3
CKD Stage 4
CKD Stage 5 untreated
CKD Stage 5 on dialysis
Hypothyroid Diseases
Hyperthyroid Diseases
Other Endocrine
Males
1990
Dietary iron deficiency
Haemoglobinopathies and haemolytic anaemias
Vitamin A deficiency
Other neglected tropical diseases
Intestinal nematode infections
Other unspecified infectious diseases
Malaria
Chronic kidney disease
Upper digestive system diseases
Endocrine, metabolic, blood, and immune disorders
Cirrhosis and other chronic liver diseases
Schistosomiasis
Inflammatory bowel disease
HIV/AIDS
2021
Dietary iron deficiency
Haemoglobinopathies and haemolytic anaemias
Other neglected tropical diseases
Other unspecified infectious diseases
Vitamin A deficiency
Chronic kidney disease
Intestinal nematode infections
Endocrine, metabolic, blood, and immune disorders
Upper digestive system diseases
Malaria
HIV/AIDS
Cirrhosis and other chronic liver diseases
Inflammatory bowel disease
Schistosomiasis
Females
1990
Dietary iron deficiency
Haemoglobinopathies and haemolytic anaemias
Vitamin A deficiency
Other neglected tropical diseases
Intestinal nematode infections
Other unspecified infectious diseases
Malaria
Endocrine, metabolic, blood, and immune disorders
Chronic kidney disease
Upper digestive system diseases
Maternal disorders
Gynecological diseases
Cirrhosis and other chronic liver diseases
Schistosomiasis
Inflammatory bowel disease
HIV/AIDS
2021
Dietary iron deficiency
Haemoglobinopathies and haemolytic anaemias
Other neglected tropical diseases
Other unspecified infectious diseases
Endocrine, metabolic, blood, and immune disorders
Chronic kidney disease
Vitamin A deficiency
Upper digestive system diseases
Intestinal nematode infections
Malaria
HIV/AIDS
Maternal disorders
Gynecological diseases
Cirrhosis and other chronic liver diseases
Inflammatory bowel disease
Schistosomiasis
YLDs per 100,000 population
0 to <0.08
0.08 to <0.54
0.54 to <2.89
2.89 to <5.52
5.52 to <10.85
10.85 to <23.26
23.26 to <47.2
47.2 to <74.87
74.87 to <148.8
148.8 to 1054.98

Indonesia

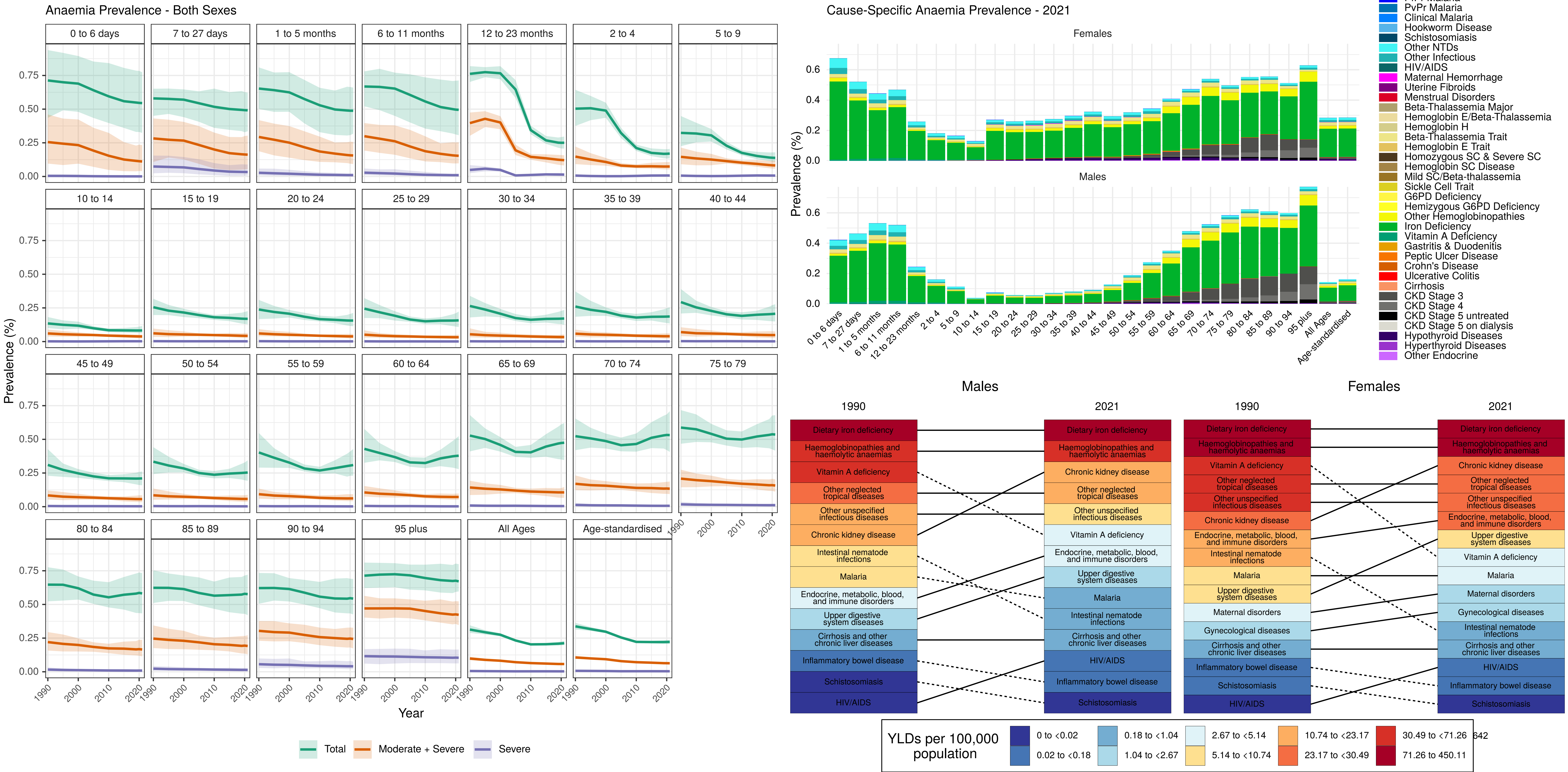
Anaemia Prevalence - Both Sexes
0 to 6 days
7 to 27 days
1 to 5 months
6 to 11 months
12 to 23 months
2 to 4
5 to 9
10 to 14
15 to 19
20 to 24
25 to 29
30 to 34
35 to 39
40 to 44
45 to 49
50 to 54
55 to 59
60 to 64
65 to 69
70 to 74
75 to 79
80 to 84
85 to 89
90 to 94
95 plus
All Ages
Age-standardised
Prevalence (%)
Year
Total
Moderate + Severe
Severe
Cause-Specific Anaemia Prevalence - 2021
Females
Males
PfPr Malaria
PvPr Malaria
Clinical Malaria
Hookworm Disease
Schistosomiasis
Other NTDs
Other Infectious
HIV/AIDS
Maternal Hemorrhage
Uterine Fibroids
Menstrual Disorders
Beta-Thalassemia Major
Hemoglobin E/Beta-Thalassemia
Hemoglobin H
Beta-Thalassemia Trait
Hemoglobin E Trait
Homozygous SC & Severe SC
Hemoglobin SC Disease
Mild SC/Beta-thalassemia
Sickle Cell Trait
G6PD Deficiency
Hemizygous G6PD Deficiency
Other Hemoglobinopathies
Iron Deficiency
Vitamin A Deficiency
Gastritis & Duodenitis
Peptic Ulcer Disease
Crohn's Disease
Ulcerative Colitis
Cirrhosis
CKD Stage 3
CKD Stage 4
CKD Stage 5 untreated
CKD Stage 5 on dialysis
Hypothyroid Diseases
Hyperthyroid Diseases
Other Endocrine
Males
Females
1990
2021
Dietary iron deficiency
Haemoglobinopathies and haemolytic anaemias
Vitamin A deficiency
Other neglected tropical diseases
Other unspecified infectious diseases
Chronic kidney disease
Intestinal nematode infections
Malaria
Endocrine, metabolic, blood, and immune disorders
Upper digestive system diseases
Cirrhosis and other chronic liver diseases
Inflammatory bowel disease
Schistosomiasis
HIV/AIDS
Maternal disorders
Gynecological diseases
YLDs per 100,000 population
0 to <0.02
0.02 to <0.18
0.18 to <1.04
1.04 to <2.67
2.67 to <5.14
5.14 to <10.74
10.74 to <23.17
23.17 to <30.49
30.49 to <71.26
71.26 to 450.11

Aceh

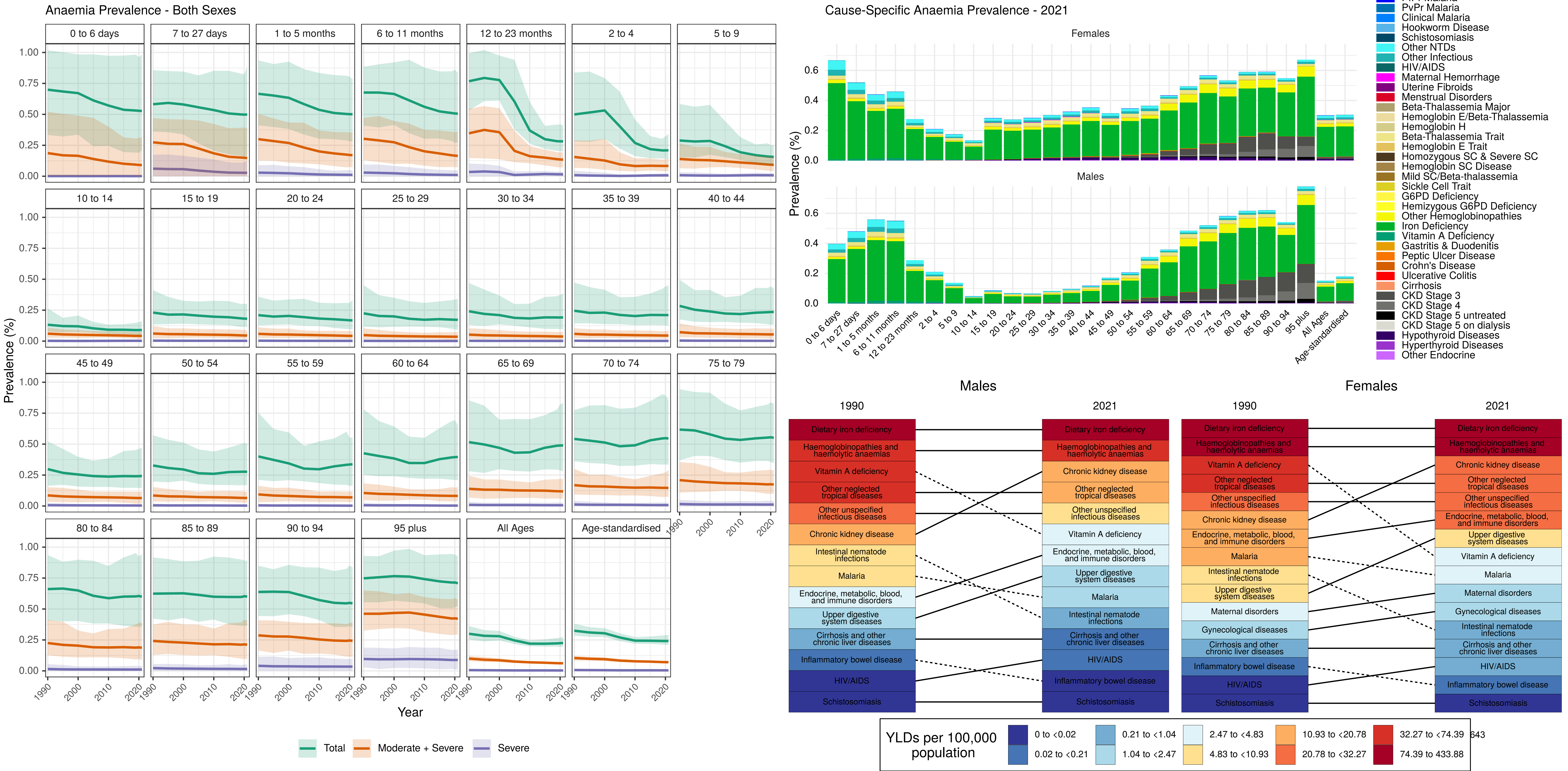

Anaemia Prevalence - Both Sexes
0 to 6 days
7 to 27 days
1 to 5 months
6 to 11 months
12 to 23 months
2 to 4
5 to 9
10 to 14
15 to 19
20 to 24
25 to 29
30 to 34
35 to 39
40 to 44
45 to 49
50 to 54
55 to 59
60 to 64
65 to 69
70 to 74
75 to 79
80 to 84
85 to 89
90 to 94
95 plus
All Ages
Age-standardised
Prevalence (%)
Year
Total
Moderate + Severe
Severe
Cause-Specific Anaemia Prevalence - 2021
Females
Males
PfPr Malaria
PvPr Malaria
Clinical Malaria
Hookworm Disease
Schistosomiasis
Other NTDs
Other Infectious
HIV/AIDS
Maternal Hemorrhage
Uterine Fibroids
Menstrual Disorders
Beta-Thalassemia Major
Hemoglobin E/Beta-Thalassemia
Hemoglobin H
Beta-Thalassemia Trait
Hemoglobin E Trait
Homozygous SC & Severe SC
Hemoglobin SC Disease
Mild SC/Beta-thalassemia
Sickle Cell Trait
G6PD Deficiency
Hemizygous G6PD Deficiency
Other Hemoglobinopathies
Iron Deficiency
Vitamin A Deficiency
Gastritis & Duodenitis
Peptic Ulcer Disease
Crohn's Disease
Ulcerative Colitis
Cirrhosis
CKD Stage 3
CKD Stage 4
CKD Stage 5 untreated
CKD Stage 5 on dialysis
Hypothyroid Diseases
Hyperthyroid Diseases
Other Endocrine
Males
Females
1990
2021
Dietary iron deficiency
Haemoglobinopathies and haemolytic anaemias
Vitamin A deficiency
Other neglected tropical diseases
Other unspecified infectious diseases
Chronic kidney disease
Intestinal nematode infections
Malaria
Endocrine, metabolic, blood, and immune disorders
Upper digestive system diseases
Cirrhosis and other chronic liver diseases
Inflammatory bowel disease
HIV/AIDS
Schistosomiasis
Maternal disorders
Gynecological diseases
YLDs per 100,000 population
0 to <0.02
0.02 to <0.21
0.21 to <1.04
1.04 to <2.47
2.47 to <4.83
4.83 to <10.93
10.93 to <20.78
20.78 to <32.27
32.27 to <74.39
74.39 to 433.88

643

Bali

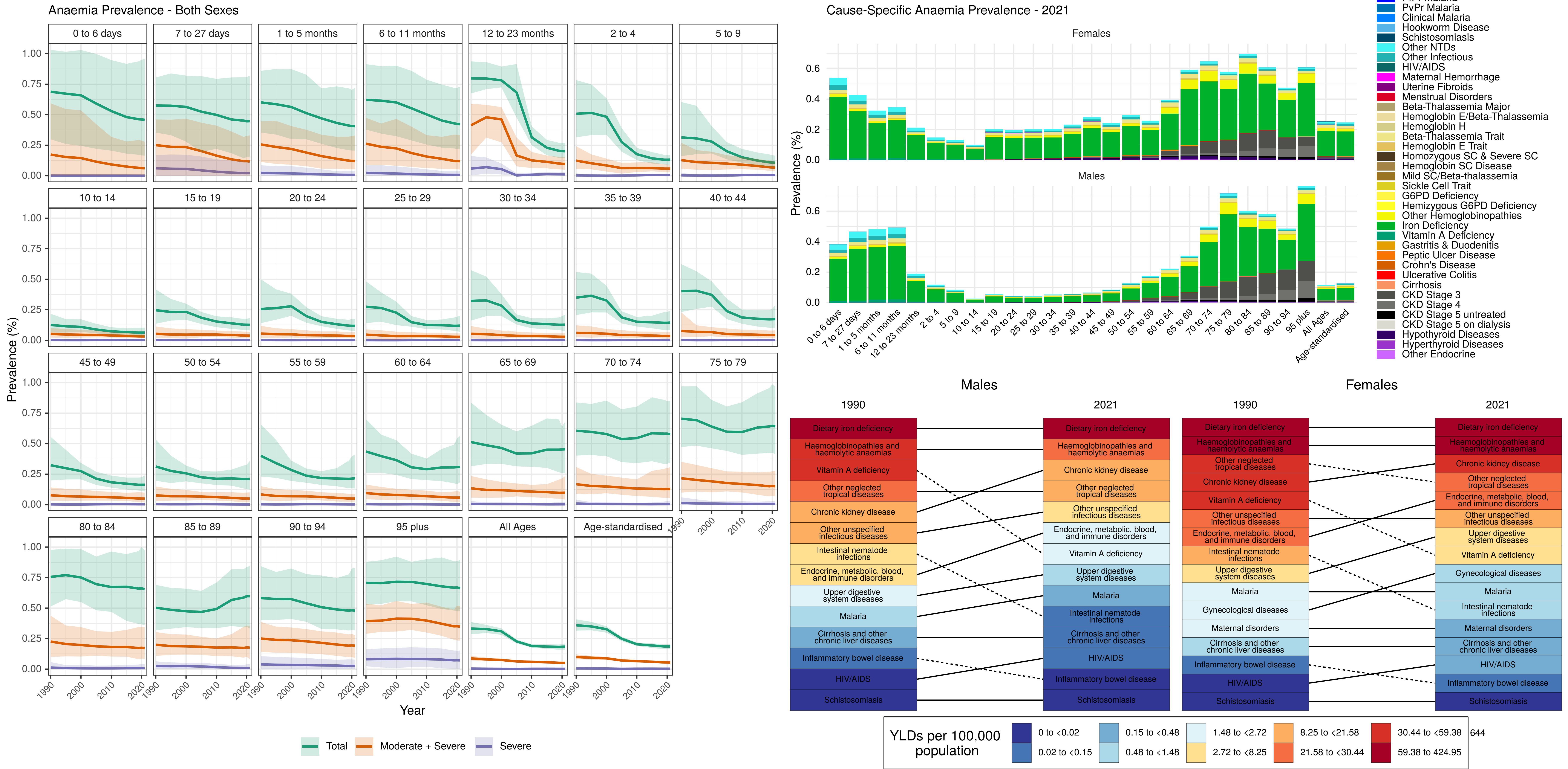

Anaemia Prevalence - Both Sexes
0 to 6 days
7 to 27 days
1 to 5 months
6 to 11 months
12 to 23 months
2 to 4
5 to 9
10 to 14
15 to 19
20 to 24
25 to 29
30 to 34
35 to 39
40 to 44
45 to 49
50 to 54
55 to 59
60 to 64
65 to 69
70 to 74
75 to 79
80 to 84
85 to 89
90 to 94
95 plus
All Ages
Age-standardised
Prevalence (%)
Year
Total
Moderate + Severe
Severe
Cause-Specific Anaemia Prevalence - 2021
Females
Males
Prevalence (%)
PfPr Malaria
PvPr Malaria
Clinical Malaria
Hookworm Disease
Schistosomiasis
Other NTDs
Other Infectious
HIV/AIDS
Maternal Hemorrhage
Uterine Fibroids
Menstrual Disorders
Beta-Thalassemia Major
Hemoglobin E/Beta-Thalassemia
Hemoglobin H
Beta-Thalassemia Trait
Hemoglobin E Trait
Homozygous SC & Severe SC
Hemoglobin SC Disease
Mild SC/Beta-thalassemia
Sickle Cell Trait
G6PD Deficiency
Hemizygous G6PD Deficiency
Other Hemoglobinopathies
Iron Deficiency
Vitamin A Deficiency
Gastritis & Duodenitis
Peptic Ulcer Disease
Crohn's Disease
Ulcerative Colitis
Cirrhosis
CKD Stage 3
CKD Stage 4
CKD Stage 5 untreated
CKD Stage 5 on dialysis
Hypothyroid Diseases
Hyperthyroid Diseases
Other Endocrine
Males
Females
1990
2021
Dietary iron deficiency
Haemoglobinopathies and haemolytic anaemias
Vitamin A deficiency
Other neglected tropical diseases
Chronic kidney disease
Other unspecified infectious diseases
Intestinal nematode infections
Endocrine, metabolic, blood, and immune disorders
Upper digestive system diseases
Malaria
Cirrhosis and other chronic liver diseases
Inflammatory bowel disease
HIV/AIDS
Schistosomiasis
Gynecological diseases
Maternal disorders
YLDs per 100,000 population
0 to <0.02
0.02 to <0.15
0.15 to <0.48
0.48 to <1.48
1.48 to <2.72
2.72 to <8.25
8.25 to <21.58
21.58 to <30.44
30.44 to <59.38
59.38 to 424.95
644

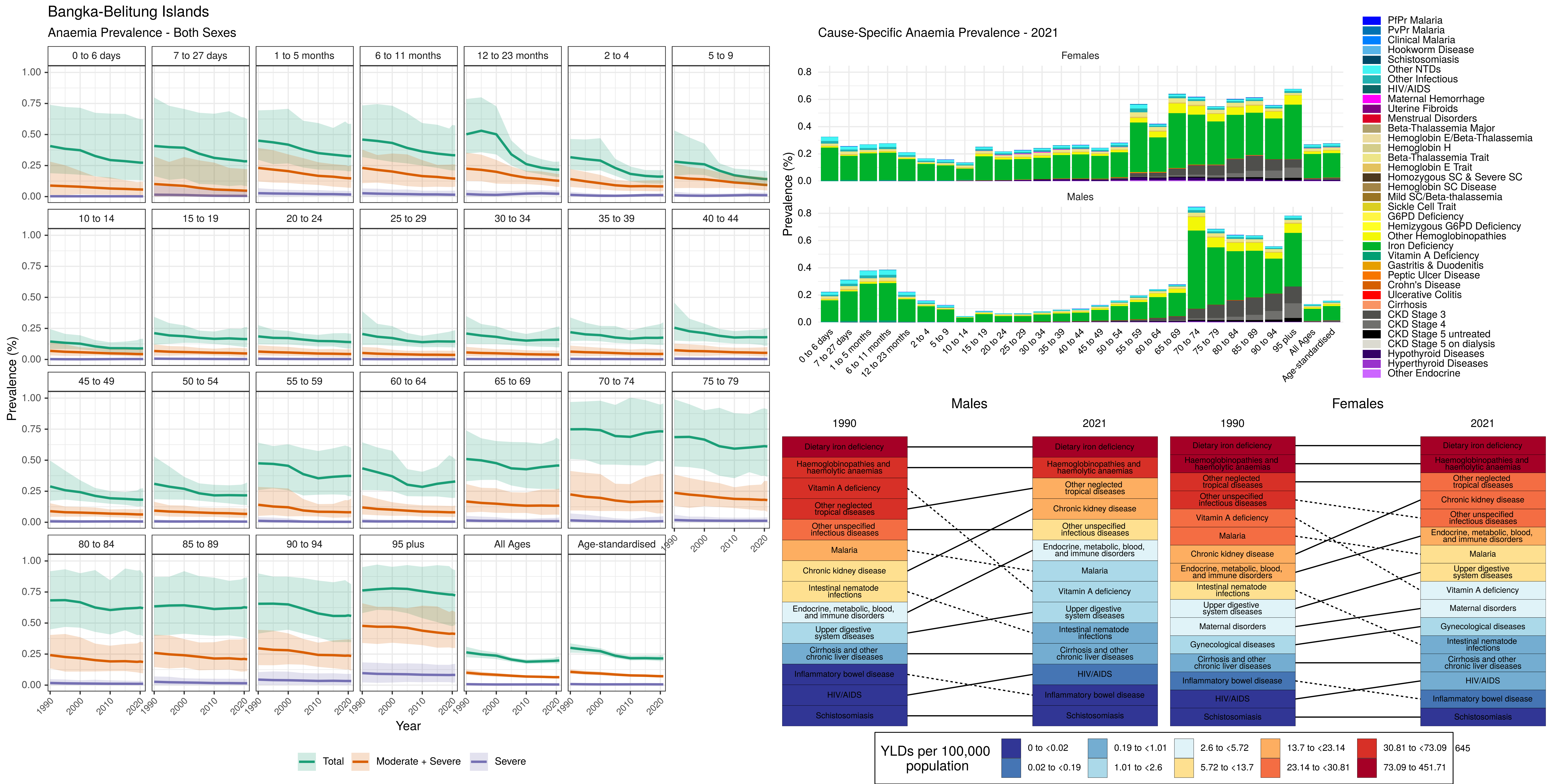
Bangka-Belitung Islands
Anaemia Prevalence - Both Sexes
0 to 6 days
7 to 27 days
1 to 5 months
6 to 11 months
12 to 23 months
2 to 4
5 to 9
10 to 14
15 to 19
20 to 24
25 to 29
30 to 34
35 to 39
40 to 44
45 to 49
50 to 54
55 to 59
60 to 64
65 to 69
70 to 74
75 to 79
80 to 84
85 to 89
90 to 94
95 plus
All Ages
Age-standardised
Prevalence (%)
Year
Total
Moderate + Severe
Severe
Cause-Specific Anaemia Prevalence - 2021
Females
Males
Prevalence (%)
PfPr Malaria
PvPr Malaria
Clinical Malaria
Hookworm Disease
Schistosomiasis
Other NTDs
Other Infectious
HIV/AIDS
Maternal Hemorrhage
Uterine Fibroids
Menstrual Disorders
Beta-Thalassemia Major
Hemoglobin E/Beta-Thalassemia
Hemoglobin H
Beta-Thalassemia Trait
Hemoglobin E Trait
Homozygous SC & Severe SC
Hemoglobin SC Disease
Mild SC/Beta-thalassemia
Sickle Cell Trait
G6PD Deficiency
Hemizygous G6PD Deficiency
Other Hemoglobinopathies
Iron Deficiency
Vitamin A Deficiency
Gastritis & Duodenitis
Peptic Ulcer Disease
Crohn's Disease
Ulcerative Colitis
Cirrhosis
CKD Stage 3
CKD Stage 4
CKD Stage 5 untreated
CKD Stage 5 on dialysis
Hypothyroid Diseases
Hyperthyroid Diseases
Other Endocrine
Males
Females
1990
2021
Males 1990: Dietary iron deficiency; Haemoglobinopathies and haemolytic anaemias; Vitamin A deficiency; Other neglected tropical diseases; Other unspecified infectious diseases; Malaria; Chronic kidney disease; Intestinal nematode infections; Endocrine, metabolic, blood, and immune disorders; Upper digestive system diseases; Cirrhosis and other chronic liver diseases; Inflammatory bowel disease; HIV/AIDS; Schistosomiasis
Males 2021: Dietary iron deficiency; Haemoglobinopathies and haemolytic anaemias; Other neglected tropical diseases; Chronic kidney disease; Other unspecified infectious diseases; Endocrine, metabolic, blood, and immune disorders; Malaria; Vitamin A deficiency; Upper digestive system diseases; Intestinal nematode infections; Cirrhosis and other chronic liver diseases; HIV/AIDS; Inflammatory bowel disease; Schistosomiasis
Females 1990: Dietary iron deficiency; Haemoglobinopathies and haemolytic anaemias; Other neglected tropical diseases; Other unspecified infectious diseases; Vitamin A deficiency; Malaria; Chronic kidney disease; Endocrine, metabolic, blood, and immune disorders; Intestinal nematode infections; Upper digestive system diseases; Maternal disorders; Gynecological diseases; Cirrhosis and other chronic liver diseases; Inflammatory bowel disease; HIV/AIDS; Schistosomiasis
Females 2021: Dietary iron deficiency; Haemoglobinopathies and haemolytic anaemias; Other neglected tropical diseases; Chronic kidney disease; Other unspecified infectious diseases; Endocrine, metabolic, blood, and immune disorders; Malaria; Upper digestive system diseases; Vitamin A deficiency; Maternal disorders; Gynecological diseases; Intestinal nematode infections; Cirrhosis and other chronic liver diseases; HIV/AIDS; Inflammatory bowel disease; Schistosomiasis
YLDs per 100,000 population
0 to <0.02
0.02 to <0.19
0.19 to <1.01
1.01 to <2.6
2.6 to <5.72
5.72 to <13.7
13.7 to <23.14
23.14 to <30.81
30.81 to <73.09
73.09 to 451.71

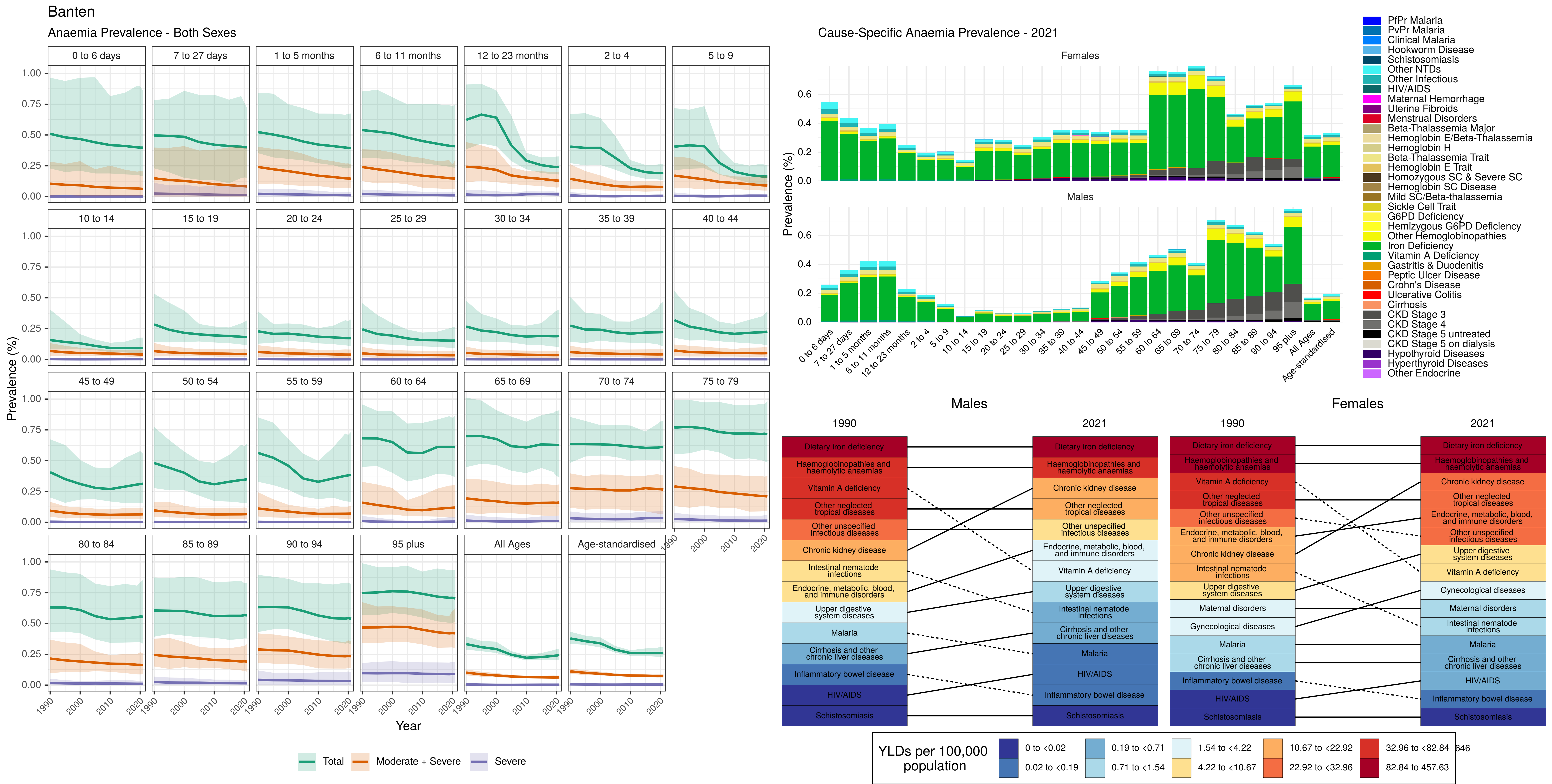

Banten
Anaemia Prevalence - Both Sexes
0 to 6 days
7 to 27 days
1 to 5 months
6 to 11 months
12 to 23 months
2 to 4
5 to 9
10 to 14
15 to 19
20 to 24
25 to 29
30 to 34
35 to 39
40 to 44
45 to 49
50 to 54
55 to 59
60 to 64
65 to 69
70 to 74
75 to 79
80 to 84
85 to 89
90 to 94
95 plus
All Ages
Age-standardised
Prevalence (%)
Year
Total
Moderate + Severe
Severe
Cause-Specific Anaemia Prevalence - 2021
Females
Males
PfPr Malaria
PvPr Malaria
Clinical Malaria
Hookworm Disease
Schistosomiasis
Other NTDs
Other Infectious
HIV/AIDS
Maternal Hemorrhage
Uterine Fibroids
Menstrual Disorders
Beta-Thalassemia Major
Hemoglobin E/Beta-Thalassemia
Hemoglobin H
Beta-Thalassemia Trait
Hemoglobin E Trait
Homozygous SC & Severe SC
Hemoglobin SC Disease
Mild SC/Beta-thalassemia
Sickle Cell Trait
G6PD Deficiency
Hemizygous G6PD Deficiency
Other Hemoglobinopathies
Iron Deficiency
Vitamin A Deficiency
Gastritis & Duodenitis
Peptic Ulcer Disease
Crohn's Disease
Ulcerative Colitis
Cirrhosis
CKD Stage 3
CKD Stage 4
CKD Stage 5 untreated
CKD Stage 5 on dialysis
Hypothyroid Diseases
Hyperthyroid Diseases
Other Endocrine
Males
Females
1990
2021
Dietary iron deficiency
Haemoglobinopathies and haemolytic anaemias
Vitamin A deficiency
Other neglected tropical diseases
Other unspecified infectious diseases
Chronic kidney disease
Intestinal nematode infections
Endocrine, metabolic, blood, and immune disorders
Upper digestive system diseases
Malaria
Cirrhosis and other chronic liver diseases
Inflammatory bowel disease
HIV/AIDS
Schistosomiasis
Maternal disorders
Gynecological diseases
YLDs per 100,000 population
0 to <0.02
0.02 to <0.19
0.19 to <0.71
0.71 to <1.54
1.54 to <4.22
4.22 to <10.67
10.67 to <22.92
22.92 to <32.96
32.96 to <82.84
82.84 to 457.63
646

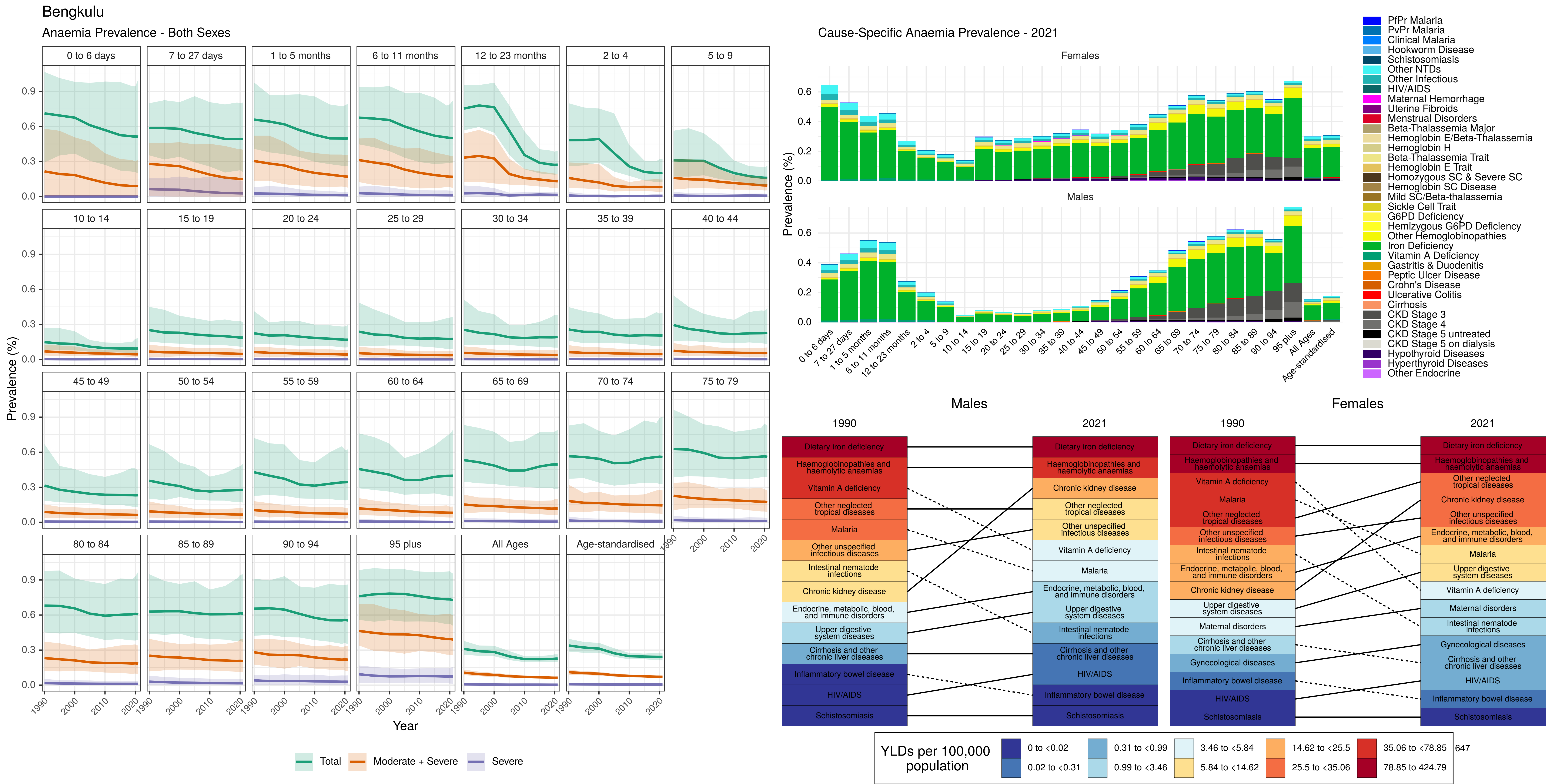

Bengkulu
Anaemia Prevalence - Both Sexes
0 to 6 days
7 to 27 days
1 to 5 months
6 to 11 months
12 to 23 months
2 to 4
5 to 9
10 to 14
15 to 19
20 to 24
25 to 29
30 to 34
35 to 39
40 to 44
45 to 49
50 to 54
55 to 59
60 to 64
65 to 69
70 to 74
75 to 79
80 to 84
85 to 89
90 to 94
95 plus
All Ages
Age-standardised
Prevalence (%)
Year
Total
Moderate + Severe
Severe
Cause-Specific Anaemia Prevalence - 2021
Females
Males
PfPr Malaria
PvPr Malaria
Clinical Malaria
Hookworm Disease
Schistosomiasis
Other NTDs
Other Infectious
HIV/AIDS
Maternal Hemorrhage
Uterine Fibroids
Menstrual Disorders
Beta-Thalassemia Major
Hemoglobin E/Beta-Thalassemia
Hemoglobin H
Beta-Thalassemia Trait
Hemoglobin E Trait
Homozygous SC & Severe SC
Hemoglobin SC Disease
Mild SC/Beta-thalassemia
Sickle Cell Trait
G6PD Deficiency
Hemizygous G6PD Deficiency
Other Hemoglobinopathies
Iron Deficiency
Vitamin A Deficiency
Gastritis & Duodenitis
Peptic Ulcer Disease
Crohn's Disease
Ulcerative Colitis
Cirrhosis
CKD Stage 3
CKD Stage 4
CKD Stage 5 untreated
CKD Stage 5 on dialysis
Hypothyroid Diseases
Hyperthyroid Diseases
Other Endocrine
Males
Females
1990
2021
Dietary iron deficiency
Haemoglobinopathies and haemolytic anaemias
Vitamin A deficiency
Other neglected tropical diseases
Malaria
Other unspecified infectious diseases
Intestinal nematode infections
Chronic kidney disease
Endocrine, metabolic, blood, and immune disorders
Upper digestive system diseases
Cirrhosis and other chronic liver diseases
Inflammatory bowel disease
HIV/AIDS
Schistosomiasis
Maternal disorders
Gynecological diseases
YLDs per 100,000 population
0 to <0.02
0.02 to <0.31
0.31 to <0.99
0.99 to <3.46
3.46 to <5.84
5.84 to <14.62
14.62 to <25.5
25.5 to <35.06
35.06 to <78.85
78.85 to 424.79

Gorontalo

Anaemia Prevalence - Both Sexes

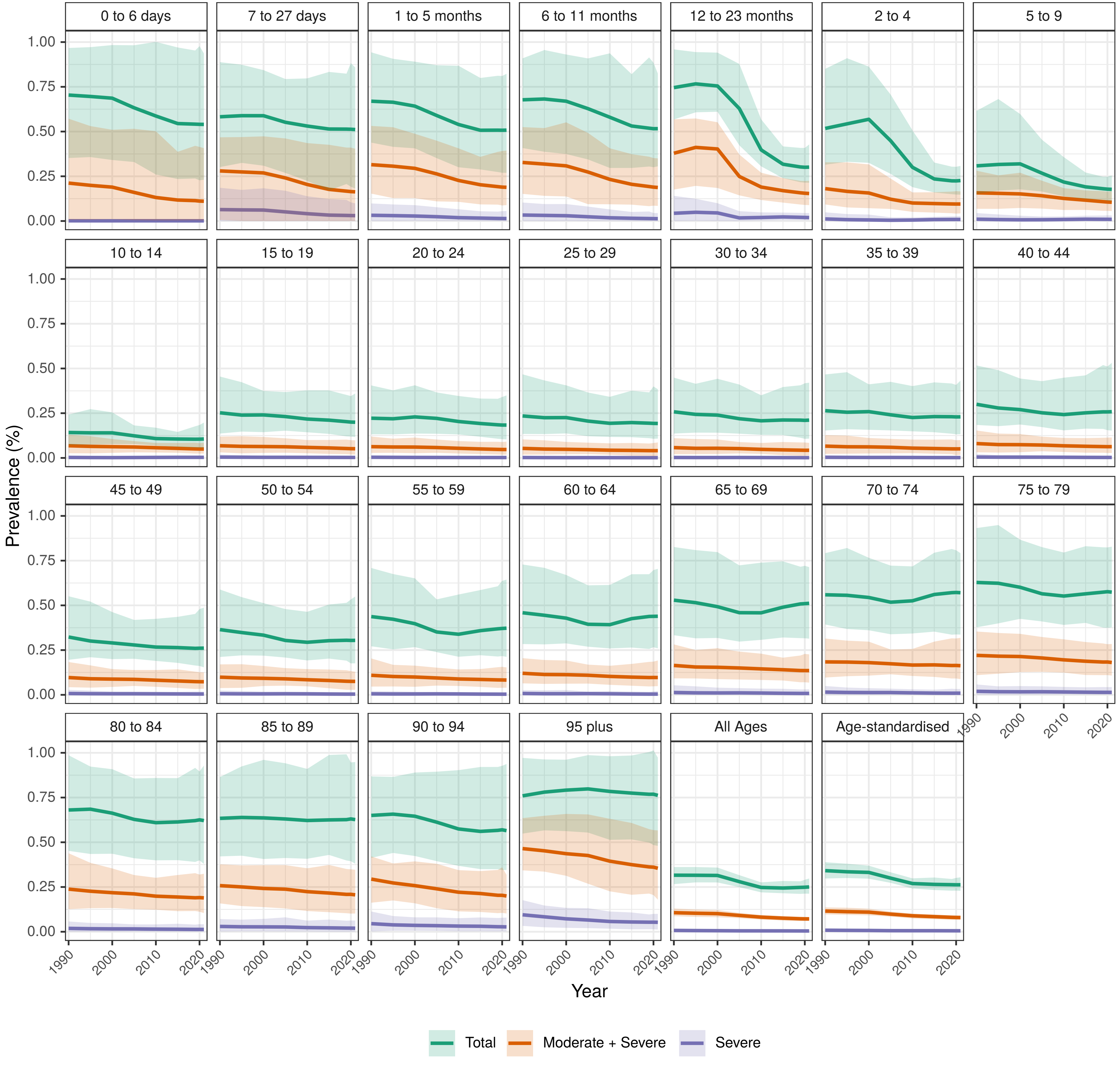

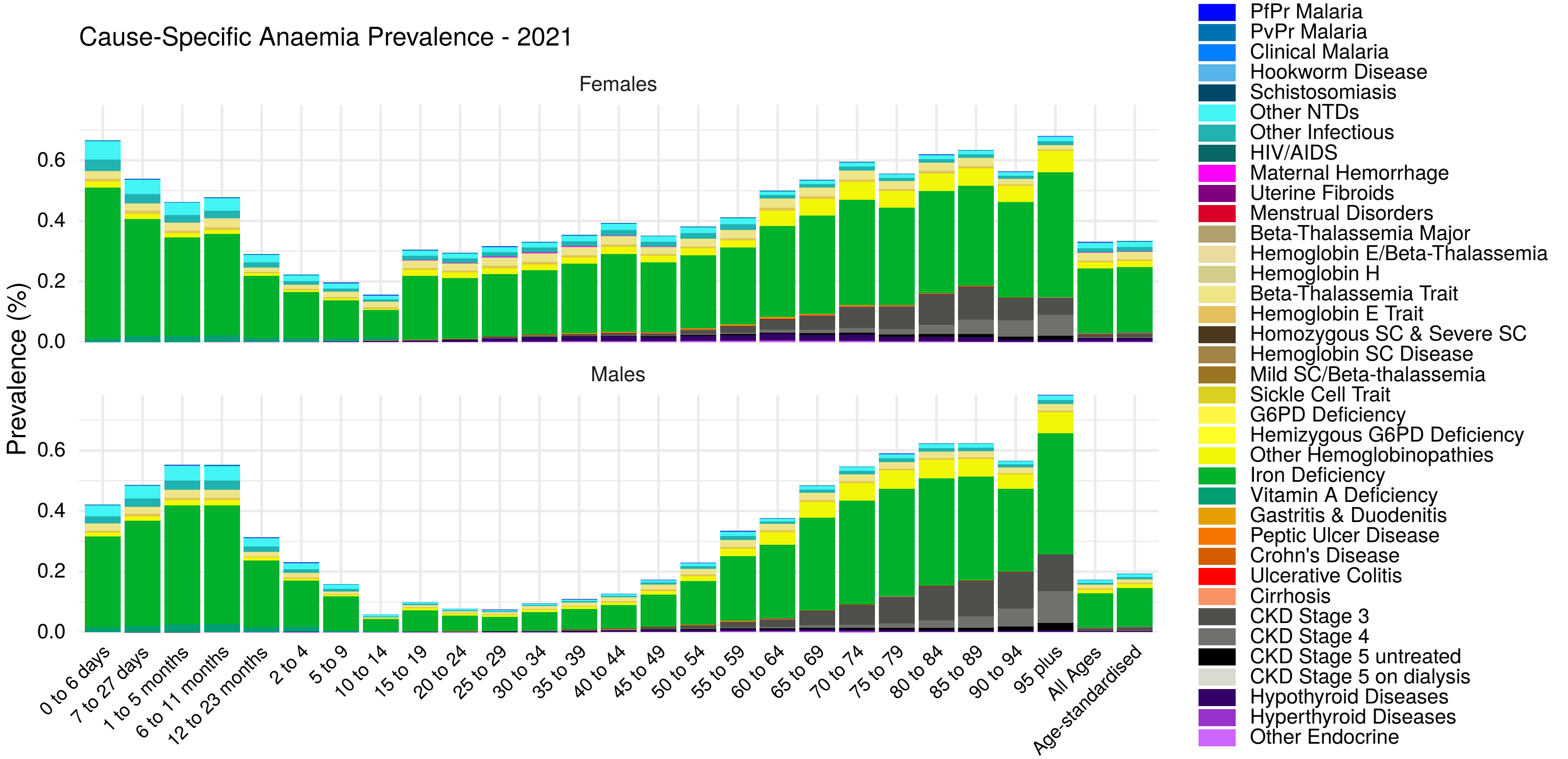

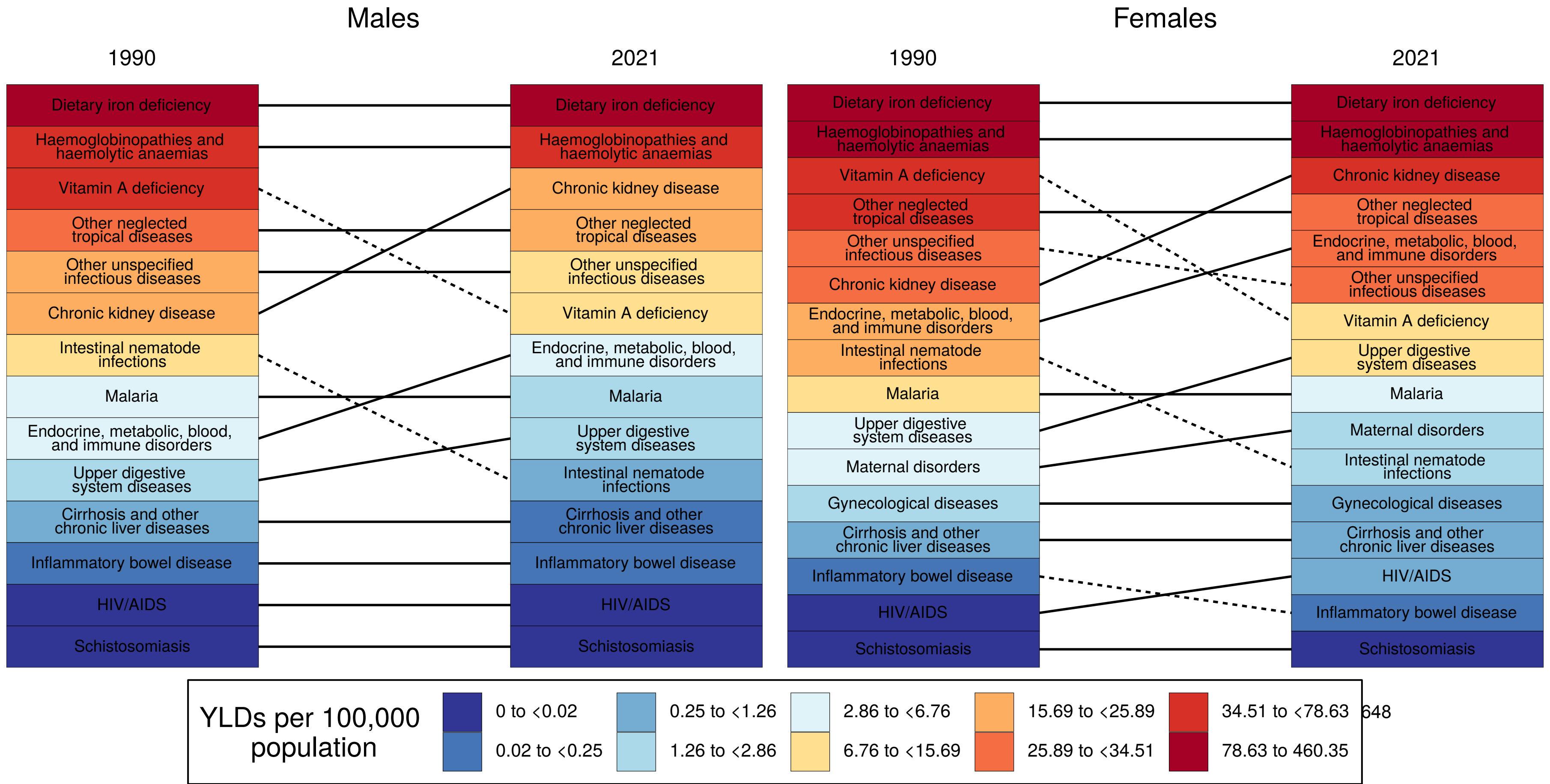

648

Jakarta

Anaemia Prevalence - Both Sexes

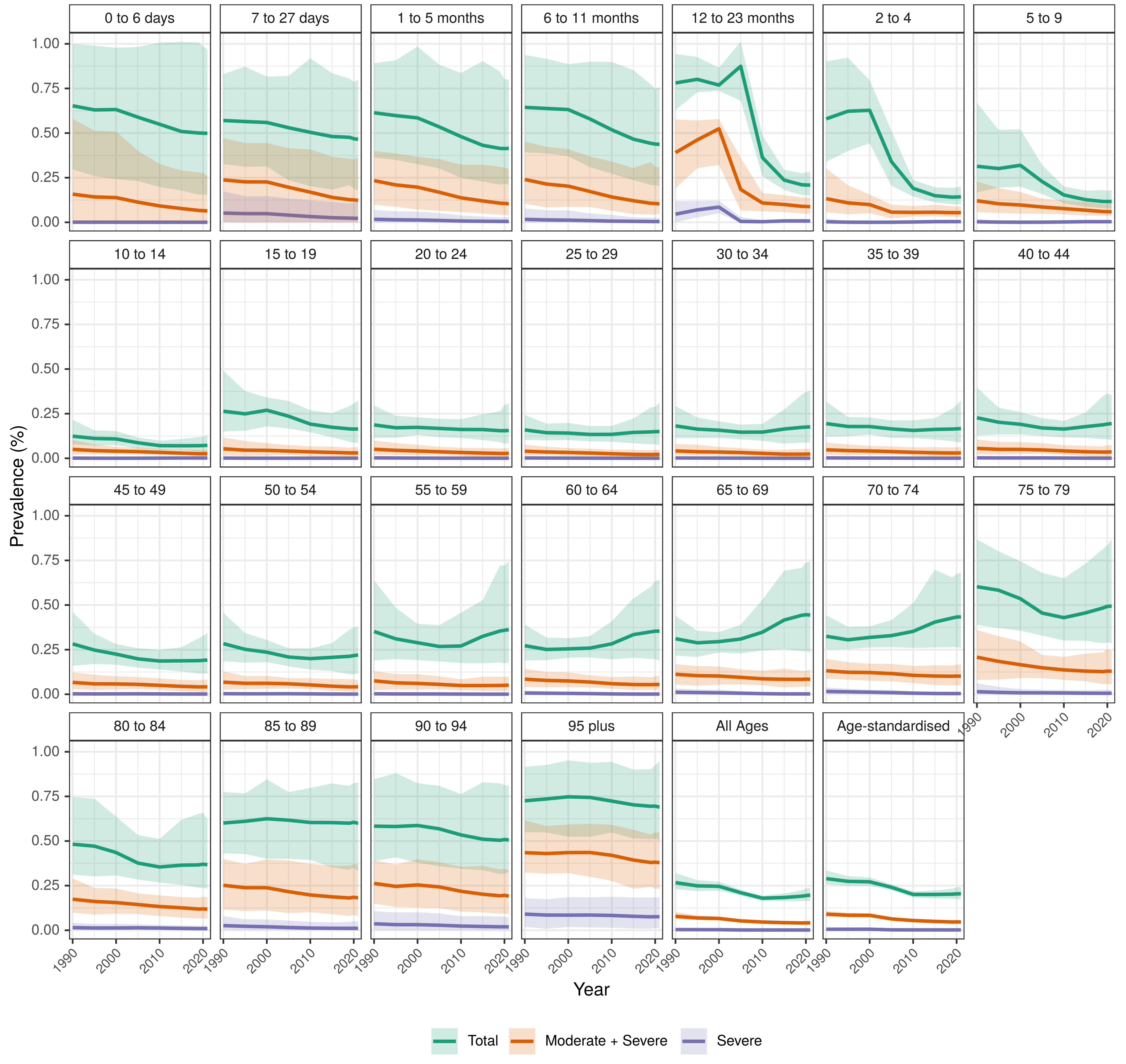

Cause-Specific Anaemia Prevalence - 2021

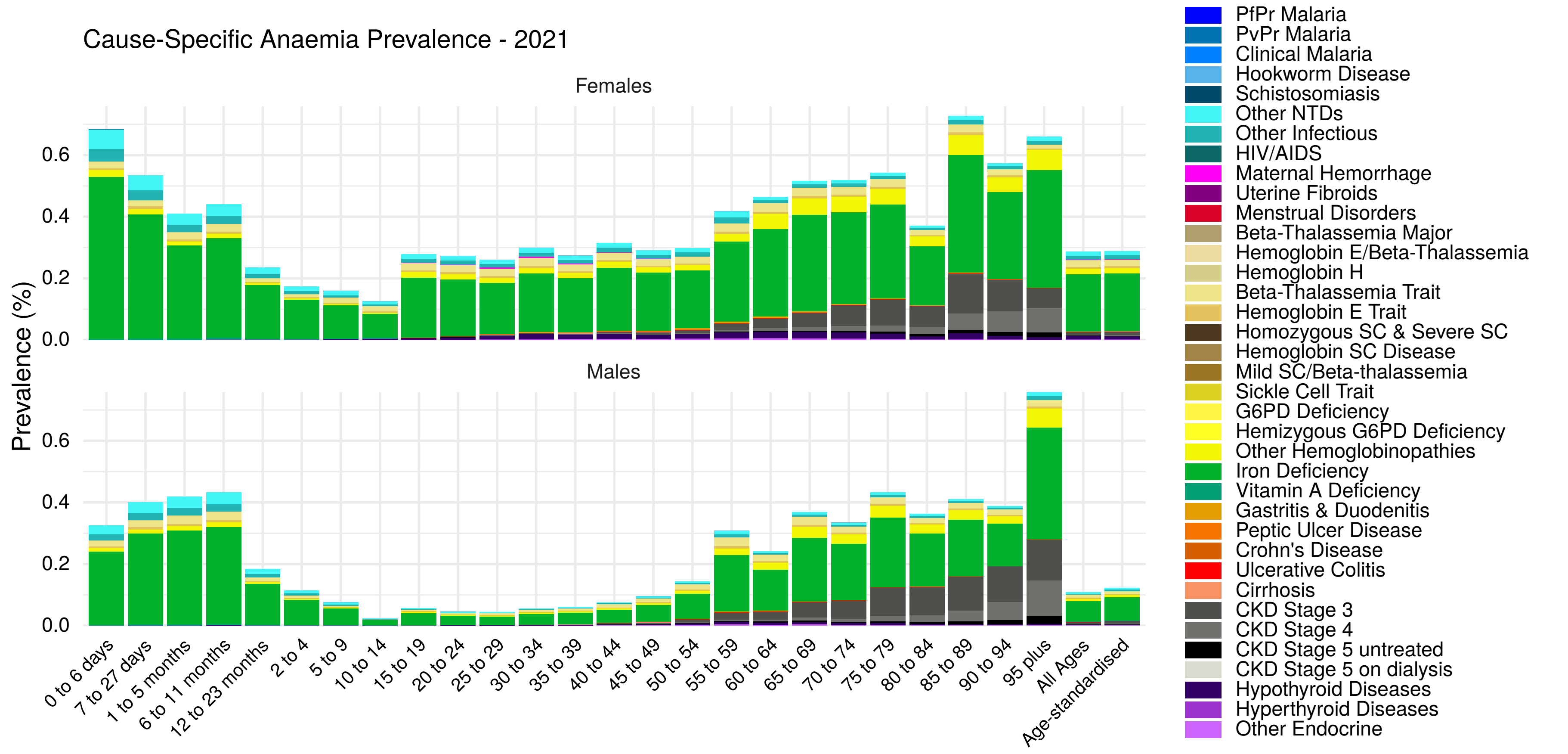

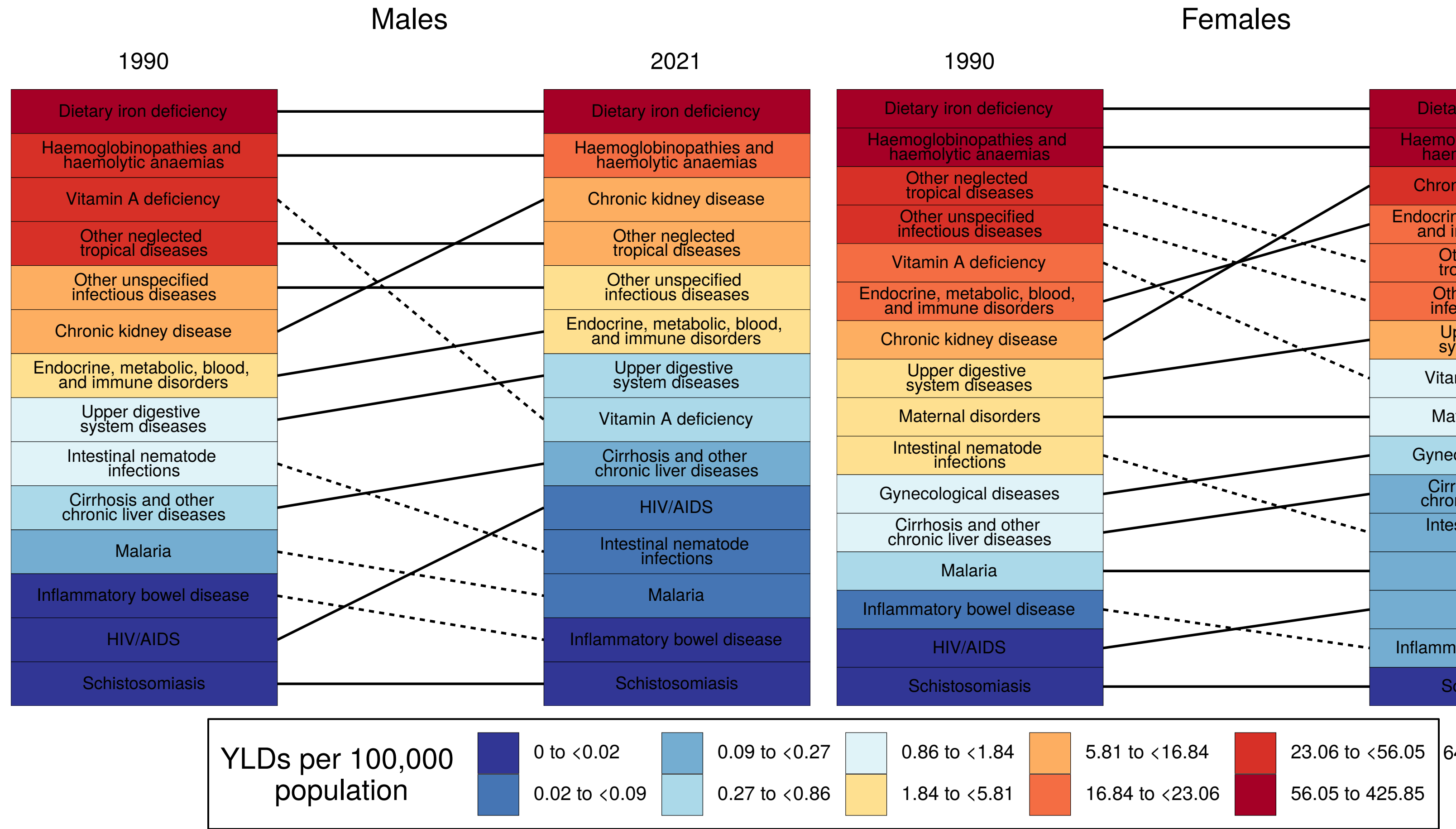

649

Jambi

Anaemia Prevalence - Both Sexes

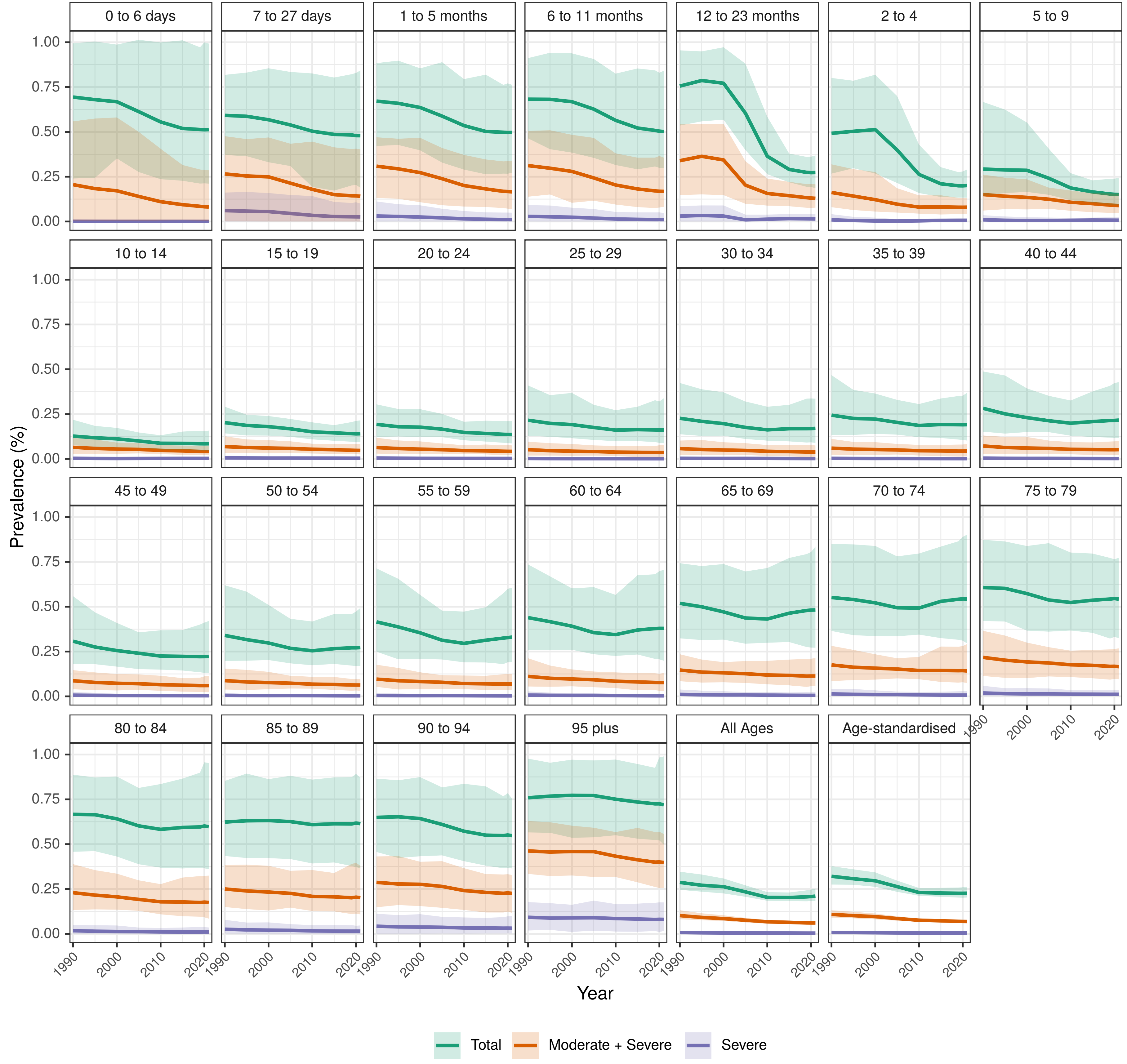

Cause-Specific Anaemia Prevalence - 2021

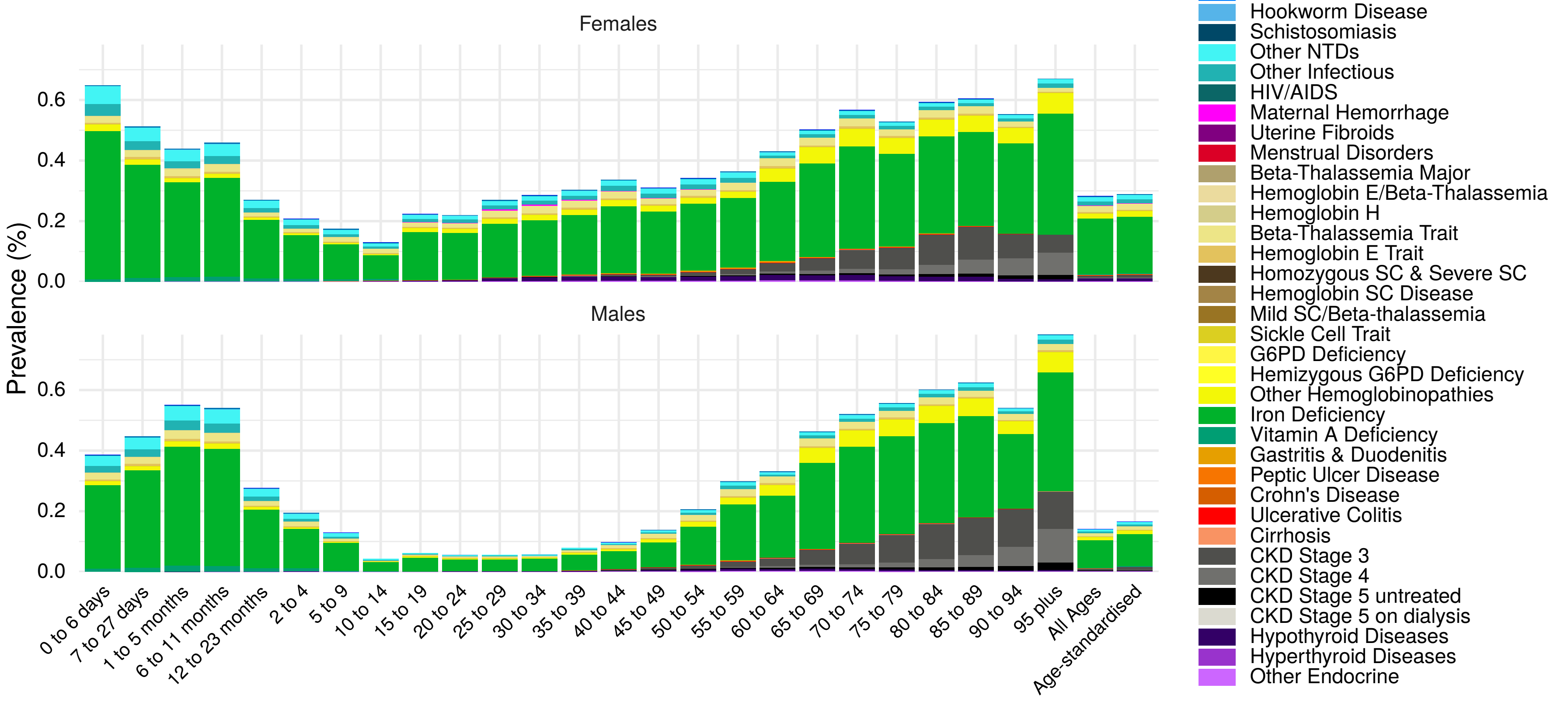

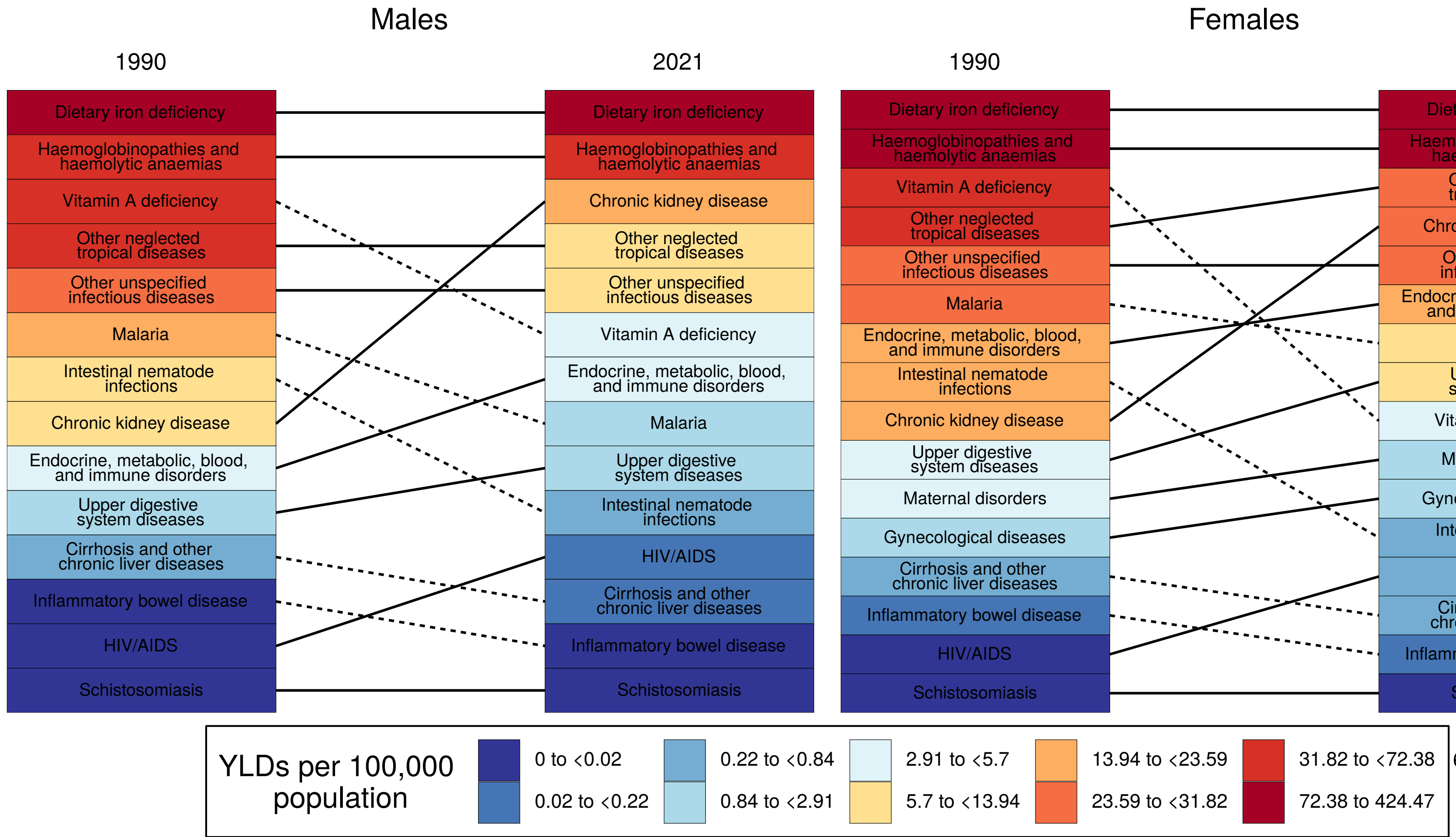

West Java

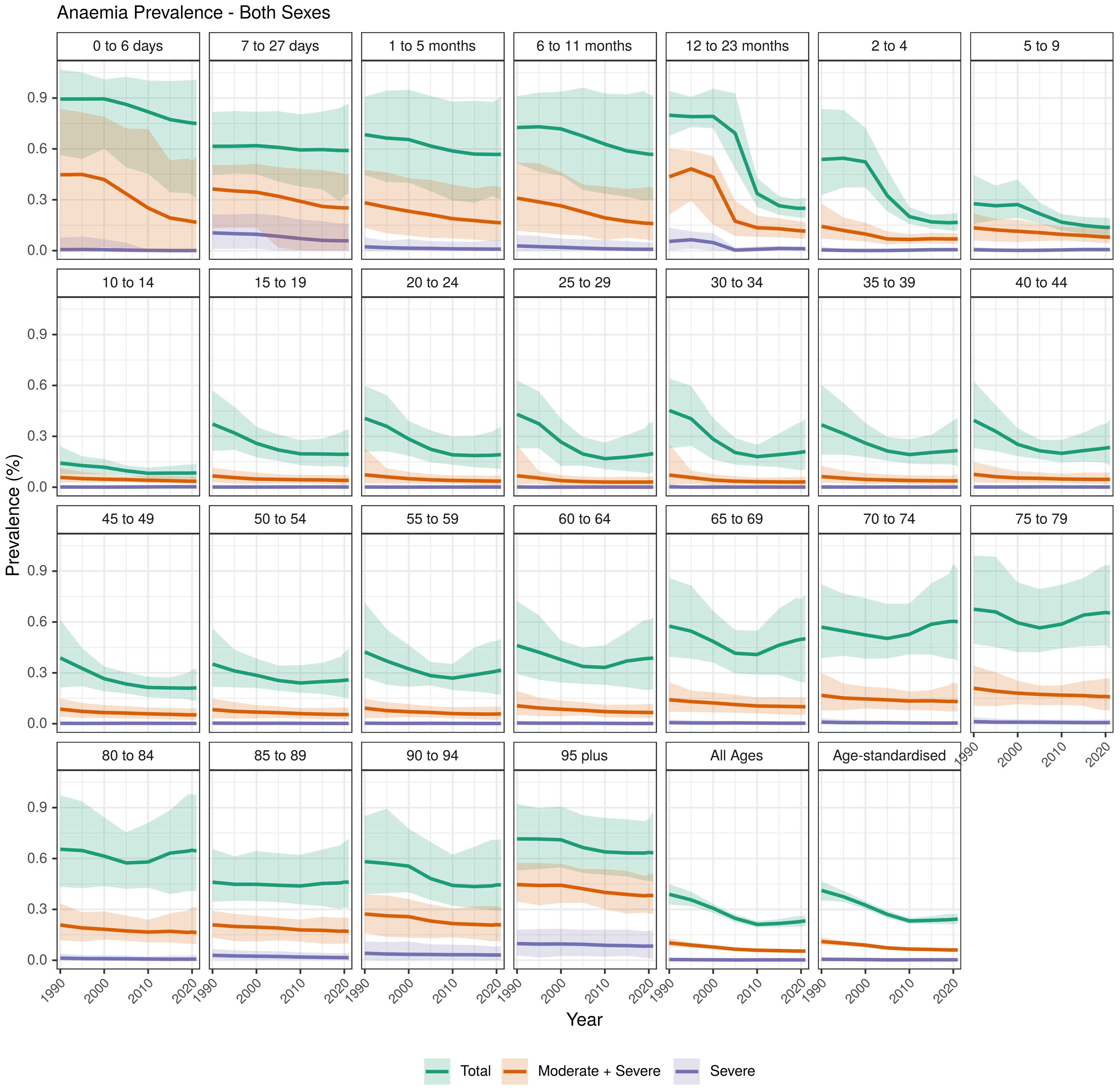

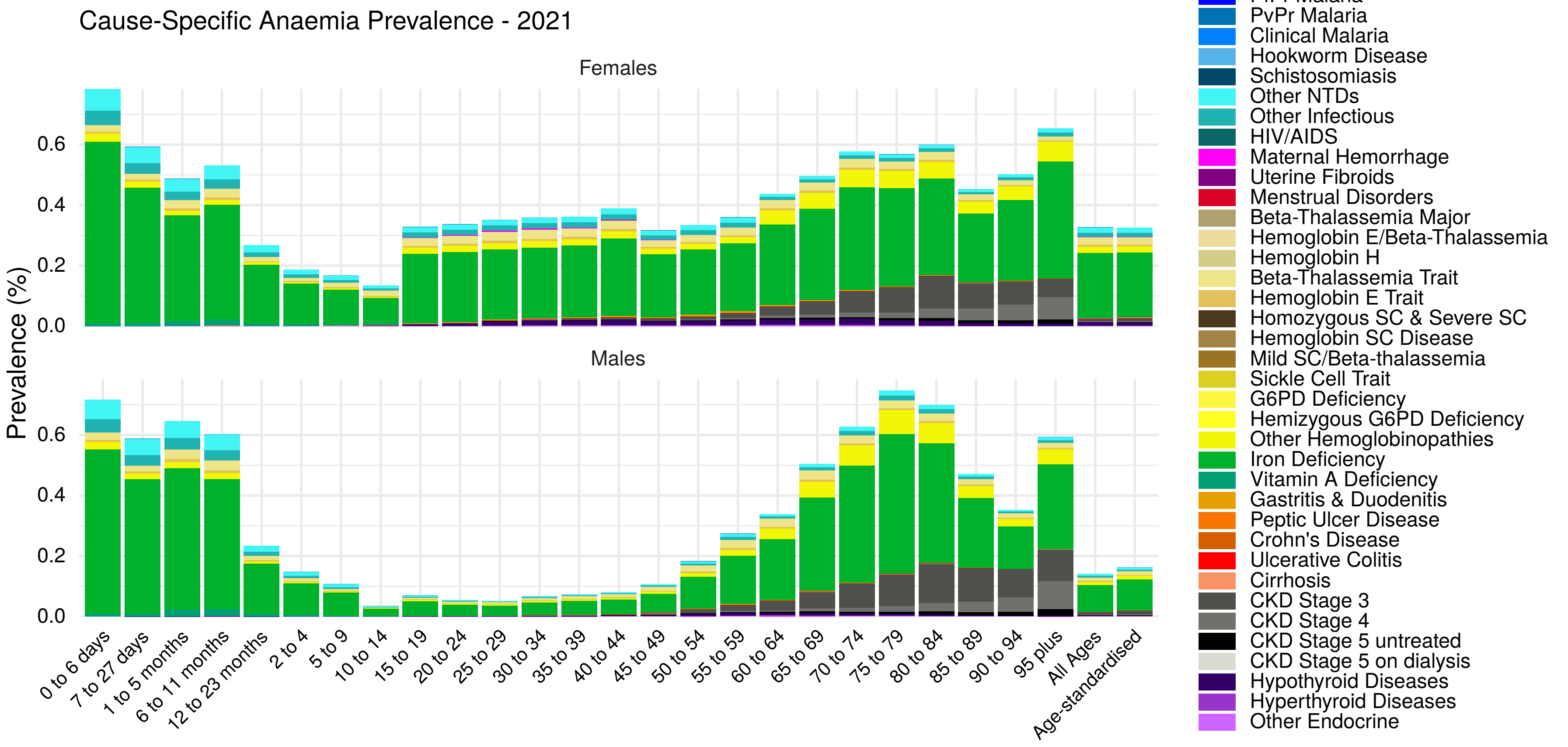

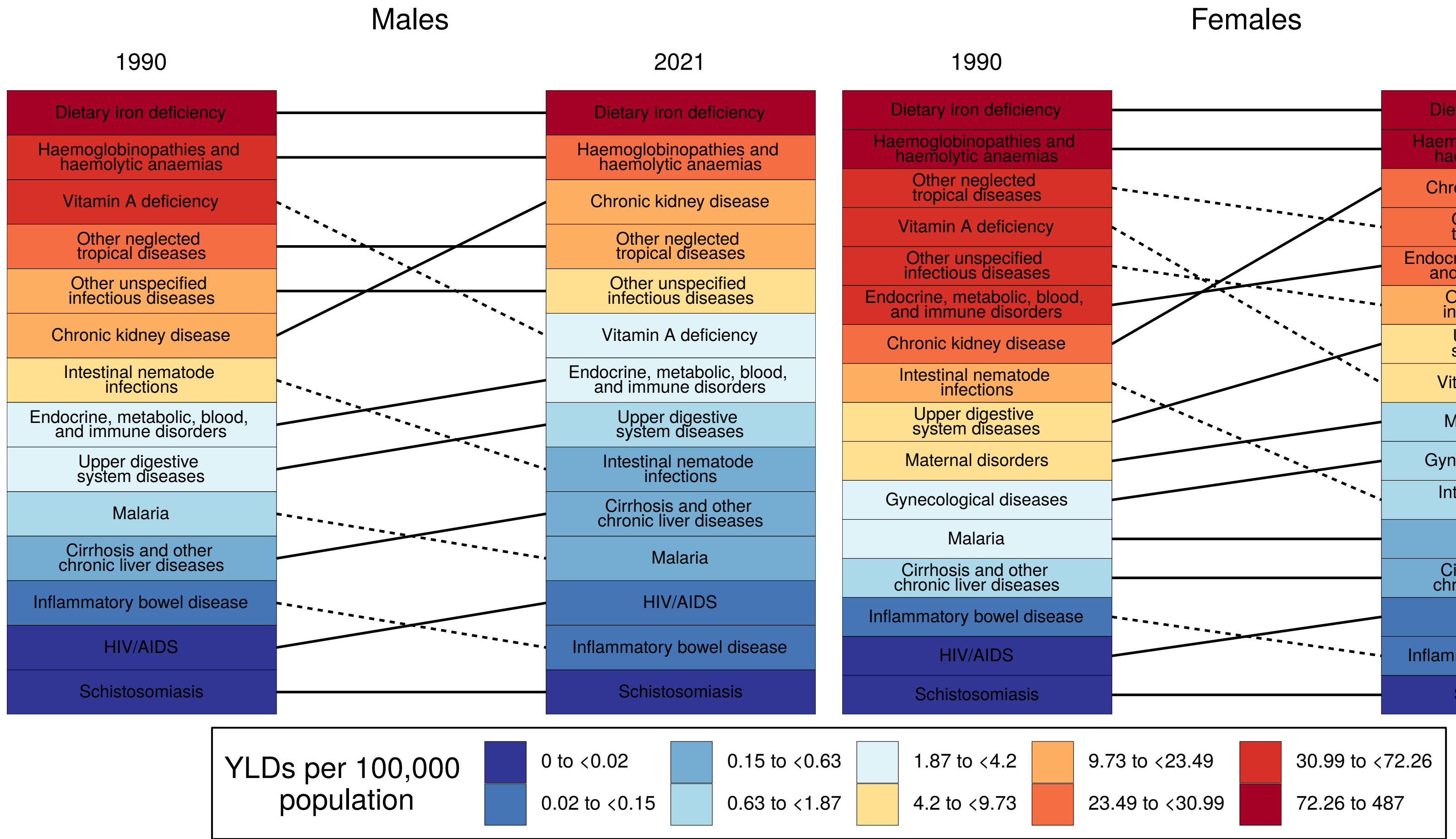

651

Central Java

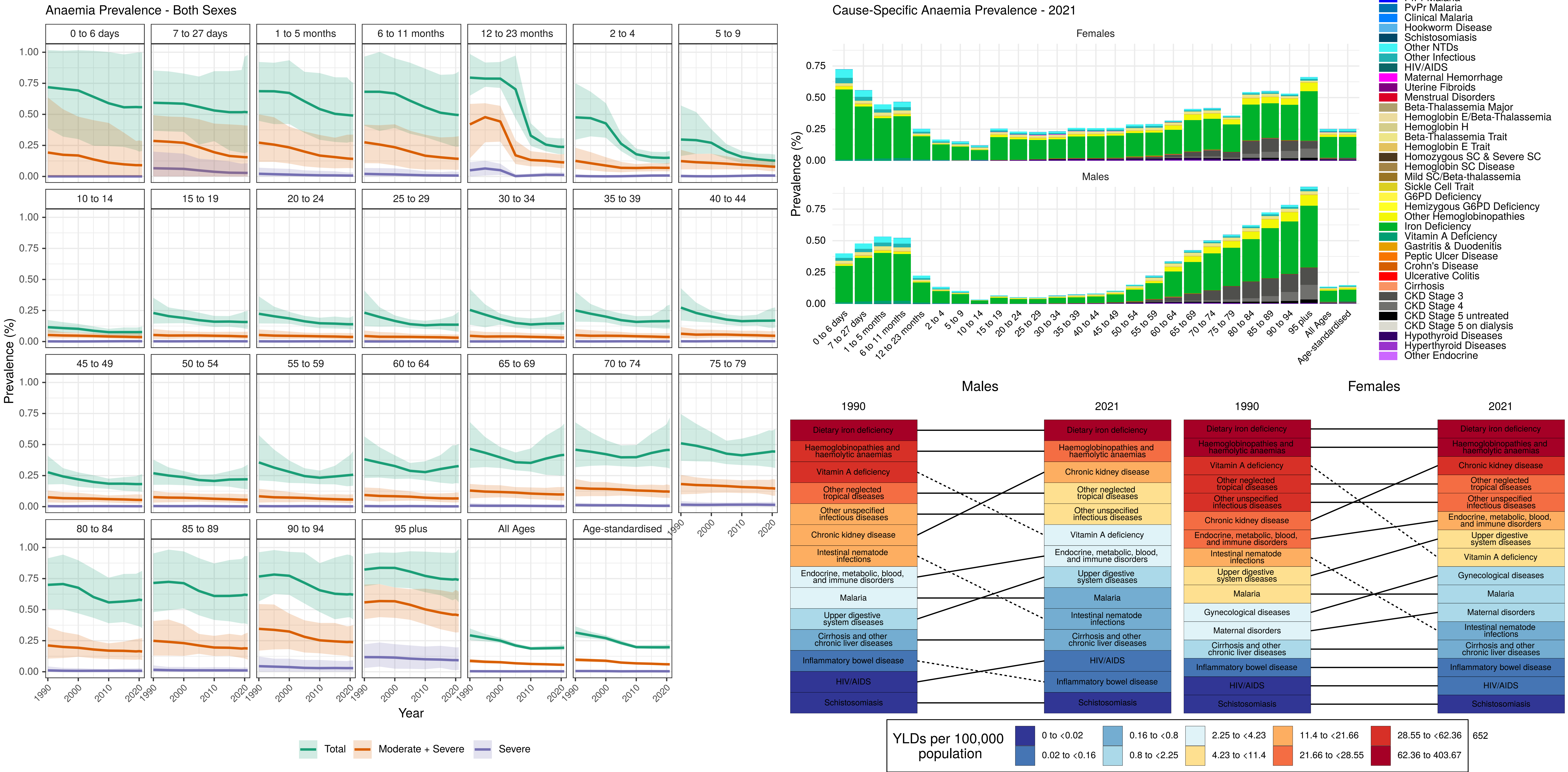

East Java

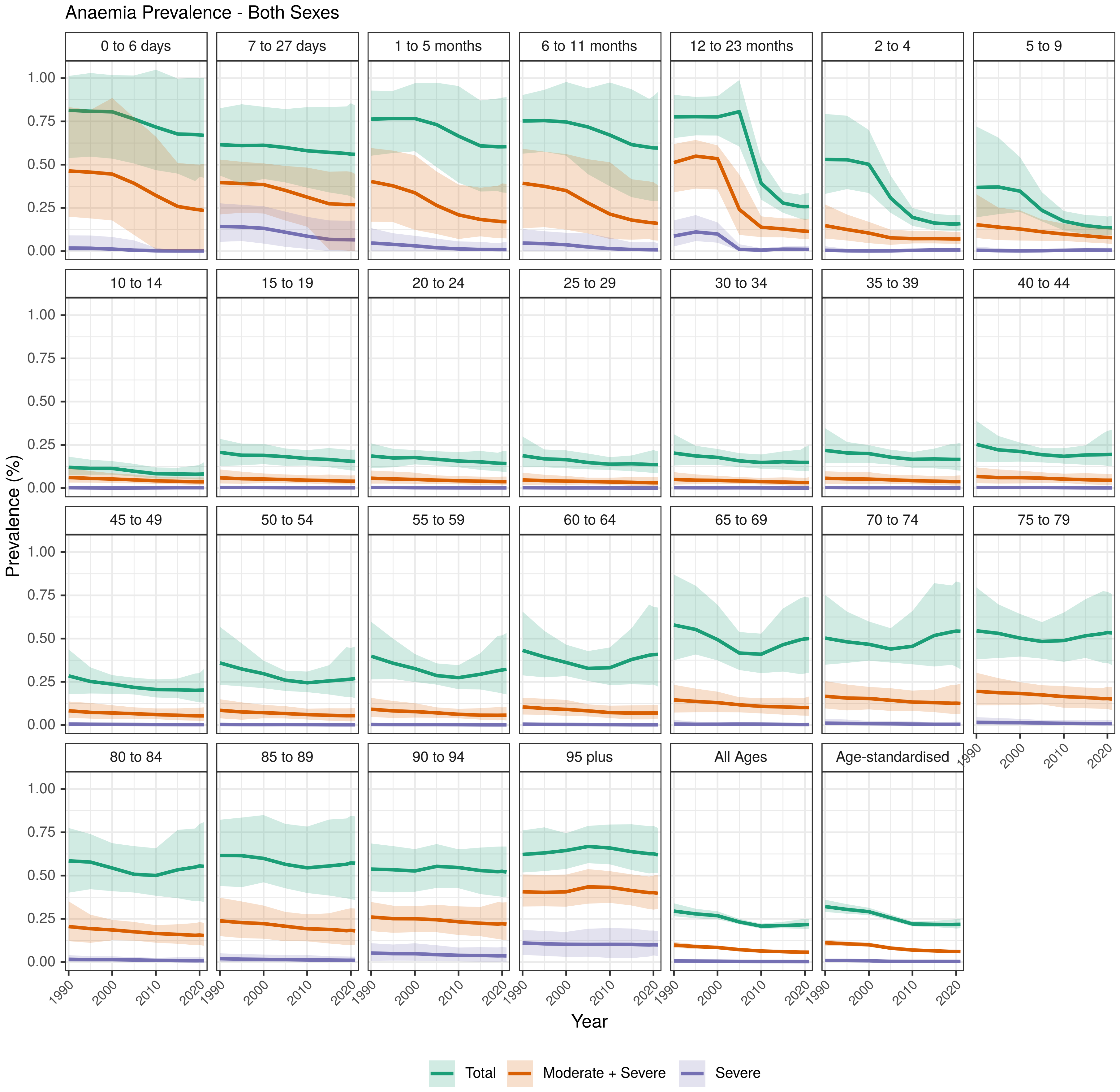

Anaemia Prevalence - Both Sexes
0 to 6 days
7 to 27 days
1 to 5 months
6 to 11 months
12 to 23 months
2 to 4
5 to 9
10 to 14
15 to 19
20 to 24
25 to 29
30 to 34
35 to 39
40 to 44
45 to 49
50 to 54
55 to 59
60 to 64
65 to 69
70 to 74
75 to 79
80 to 84
85 to 89
90 to 94
95 plus
All Ages
Age-standardised
Prevalence (%)
Year
Total
Moderate + Severe
Severe

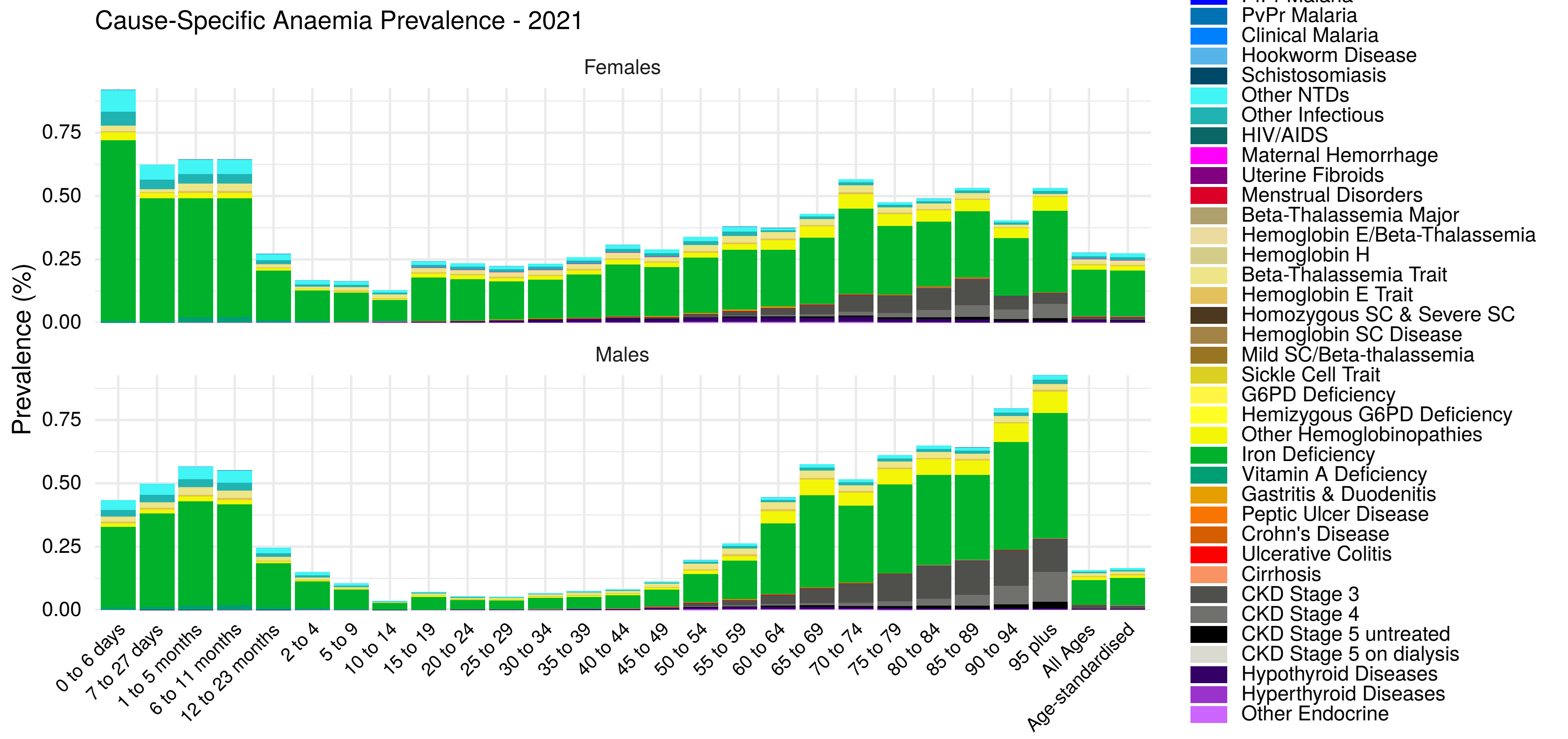

Cause-Specific Anaemia Prevalence - 2021
Females
Males
Prevalence (%)
PfPr Malaria
PvPr Malaria
Clinical Malaria
Hookworm Disease
Schistosomiasis
Other NTDs
Other Infectious
HIV/AIDS
Maternal Hemorrhage
Uterine Fibroids
Menstrual Disorders
Beta-Thalassemia Major
Hemoglobin E/Beta-Thalassemia
Hemoglobin H
Beta-Thalassemia Trait
Hemoglobin E Trait
Homozygous SC & Severe SC
Hemoglobin SC Disease
Mild SC/Beta-thalassemia
Sickle Cell Trait
G6PD Deficiency
Hemizygous G6PD Deficiency
Other Hemoglobinopathies
Iron Deficiency
Vitamin A Deficiency
Gastritis & Duodenitis
Peptic Ulcer Disease
Crohn's Disease
Ulcerative Colitis
Cirrhosis
CKD Stage 3
CKD Stage 4
CKD Stage 5 untreated
CKD Stage 5 on dialysis
Hypothyroid Diseases
Hyperthyroid Diseases
Other Endocrine

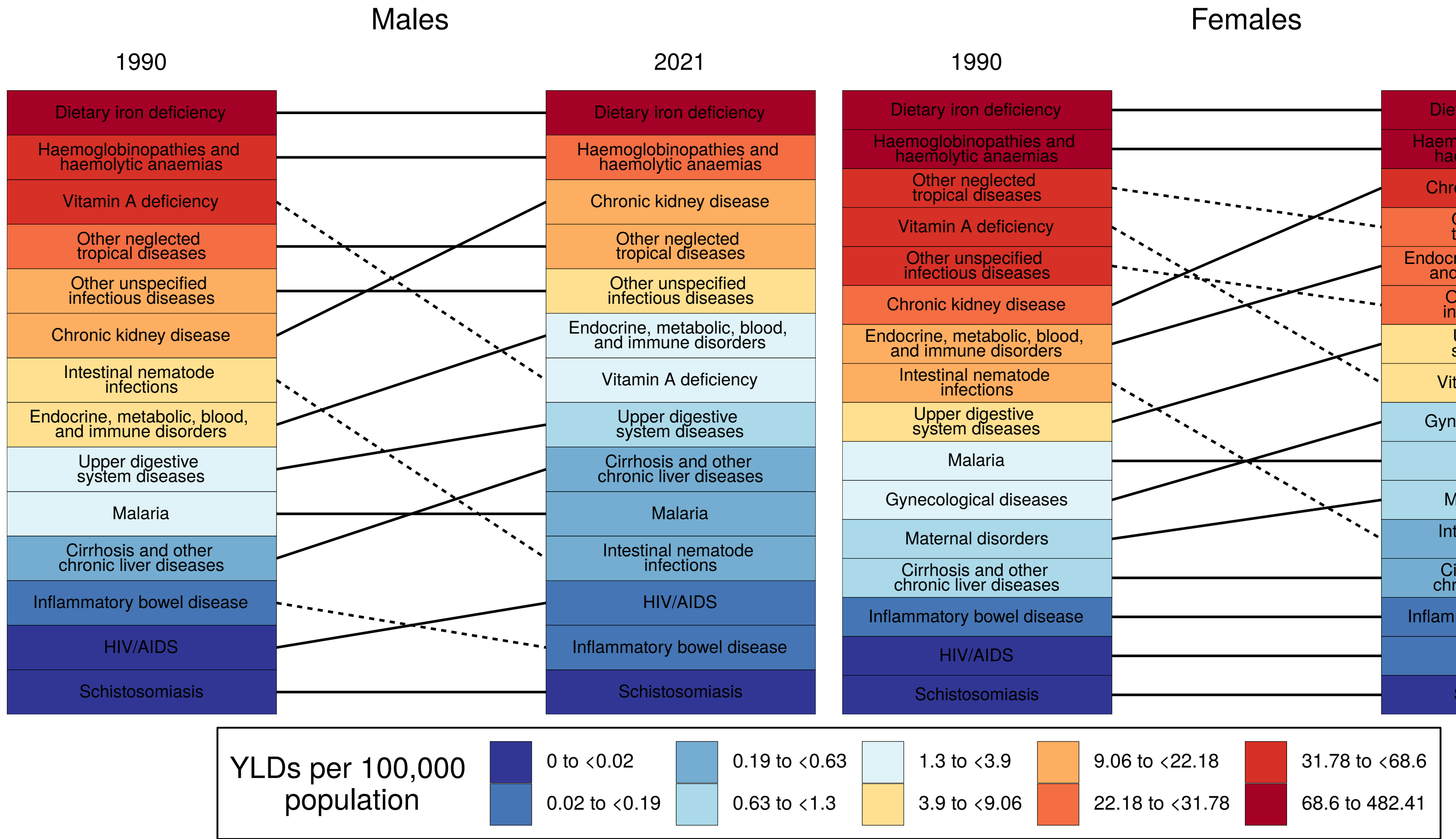

Males
Females
1990
2021
Dietary iron deficiency
Haemoglobinopathies and haemolytic anaemias
Vitamin A deficiency
Other neglected tropical diseases
Other unspecified infectious diseases
Chronic kidney disease
Intestinal nematode infections
Endocrine, metabolic, blood, and immune disorders
Upper digestive system diseases
Malaria
Cirrhosis and other chronic liver diseases
Inflammatory bowel disease
HIV/AIDS
Schistosomiasis
Gynecological diseases
Maternal disorders
YLDs per 100,000 population
0 to <0.02
0.02 to <0.19
0.19 to <0.63
0.63 to <1.3
1.3 to <3.9
3.9 to <9.06
9.06 to <22.18
22.18 to <31.78
31.78 to <68.6
68.6 to 482.41

West Kalimantan

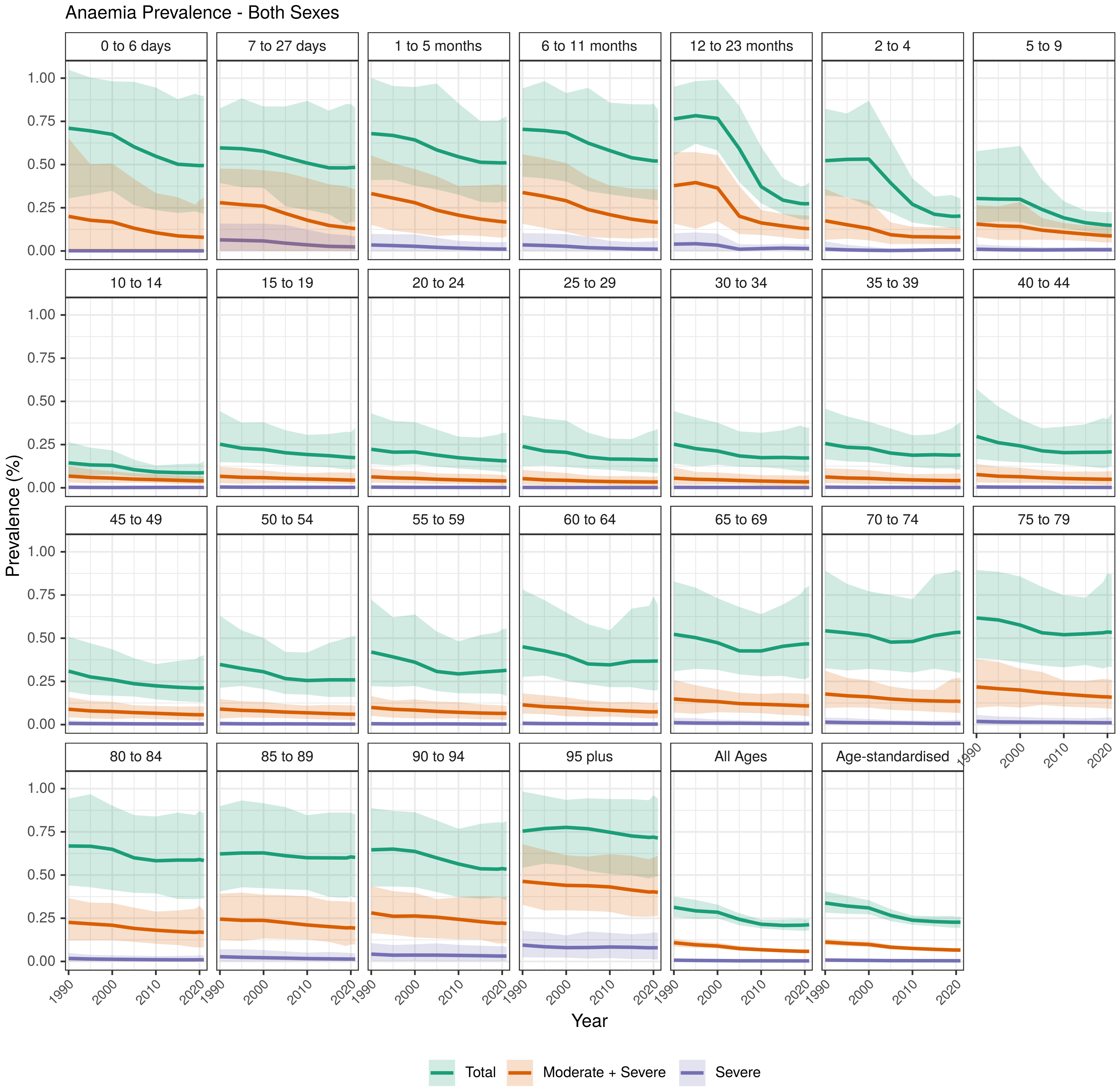

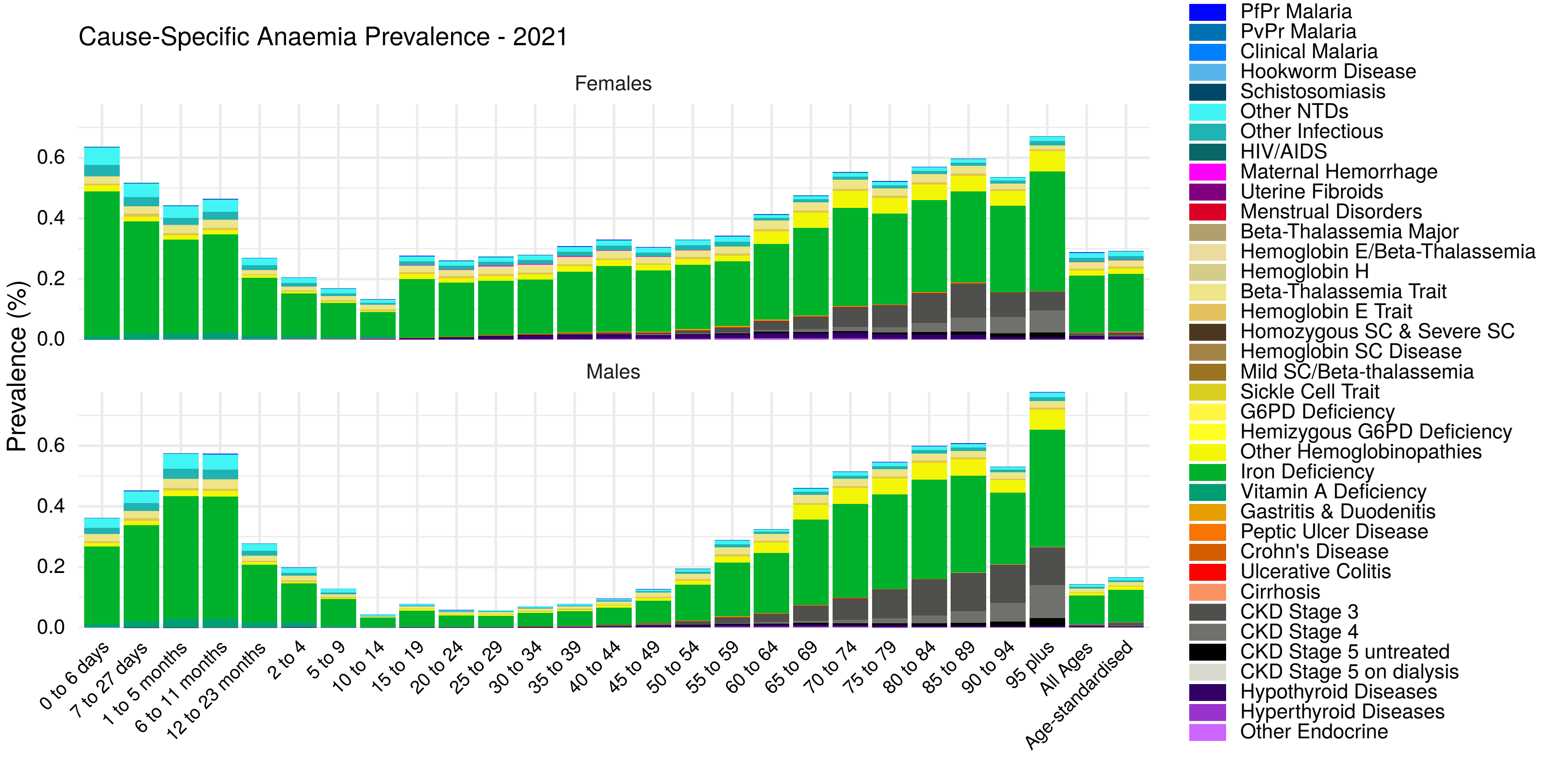

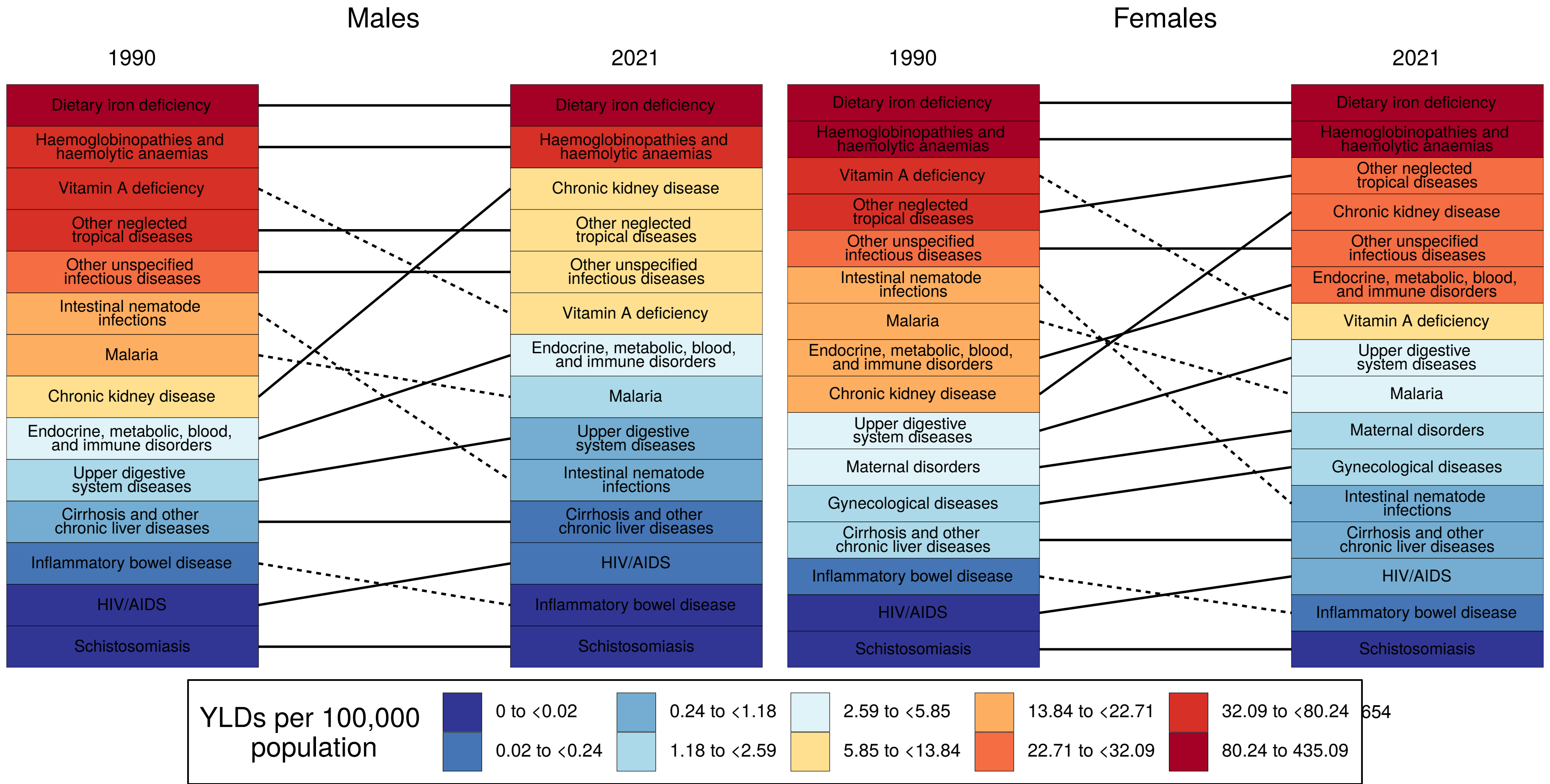

South Kalimantan

Anaemia Prevalence - Both Sexes

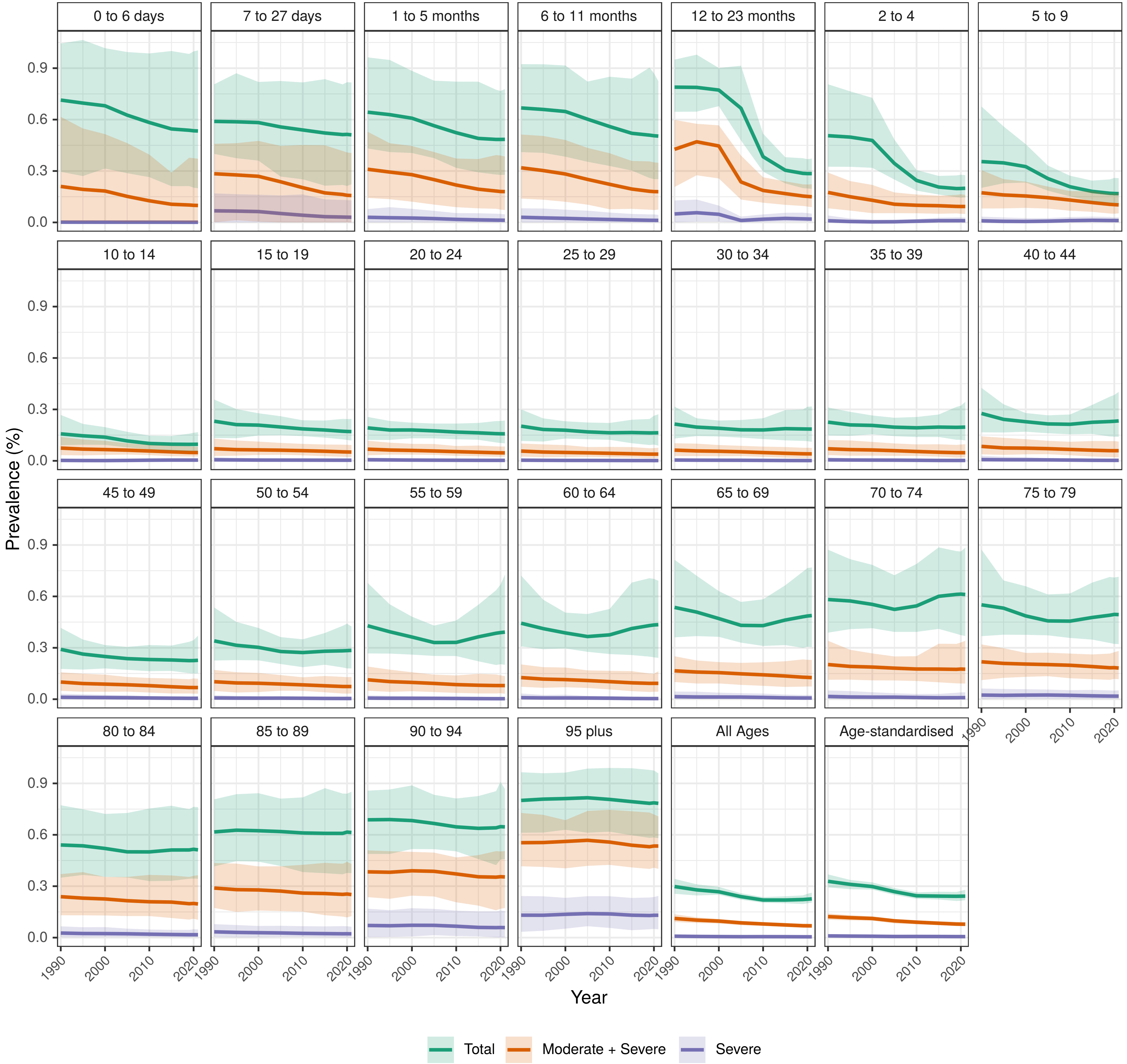

Cause-Specific Anaemia Prevalence - 2021

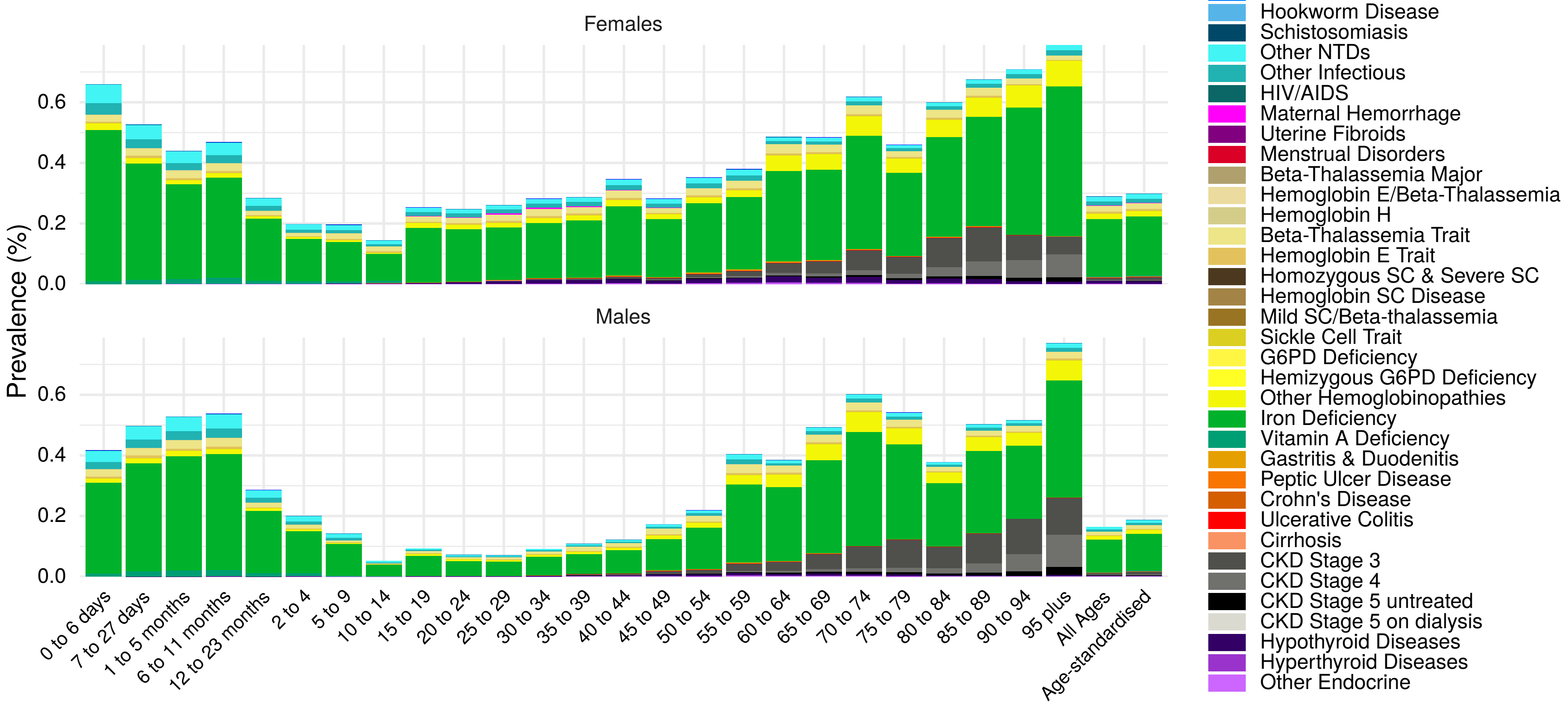

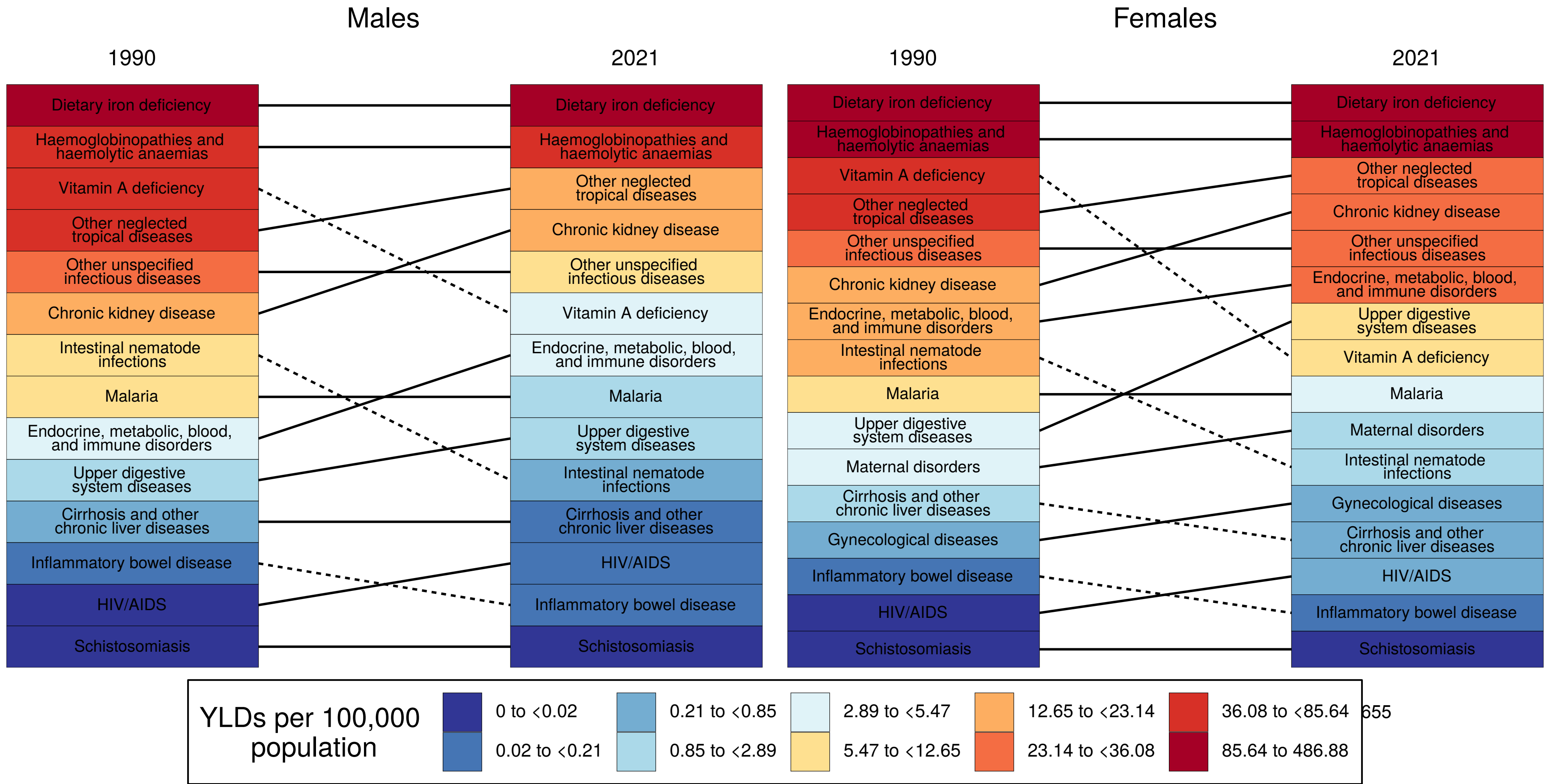

655

Central Kalimantan

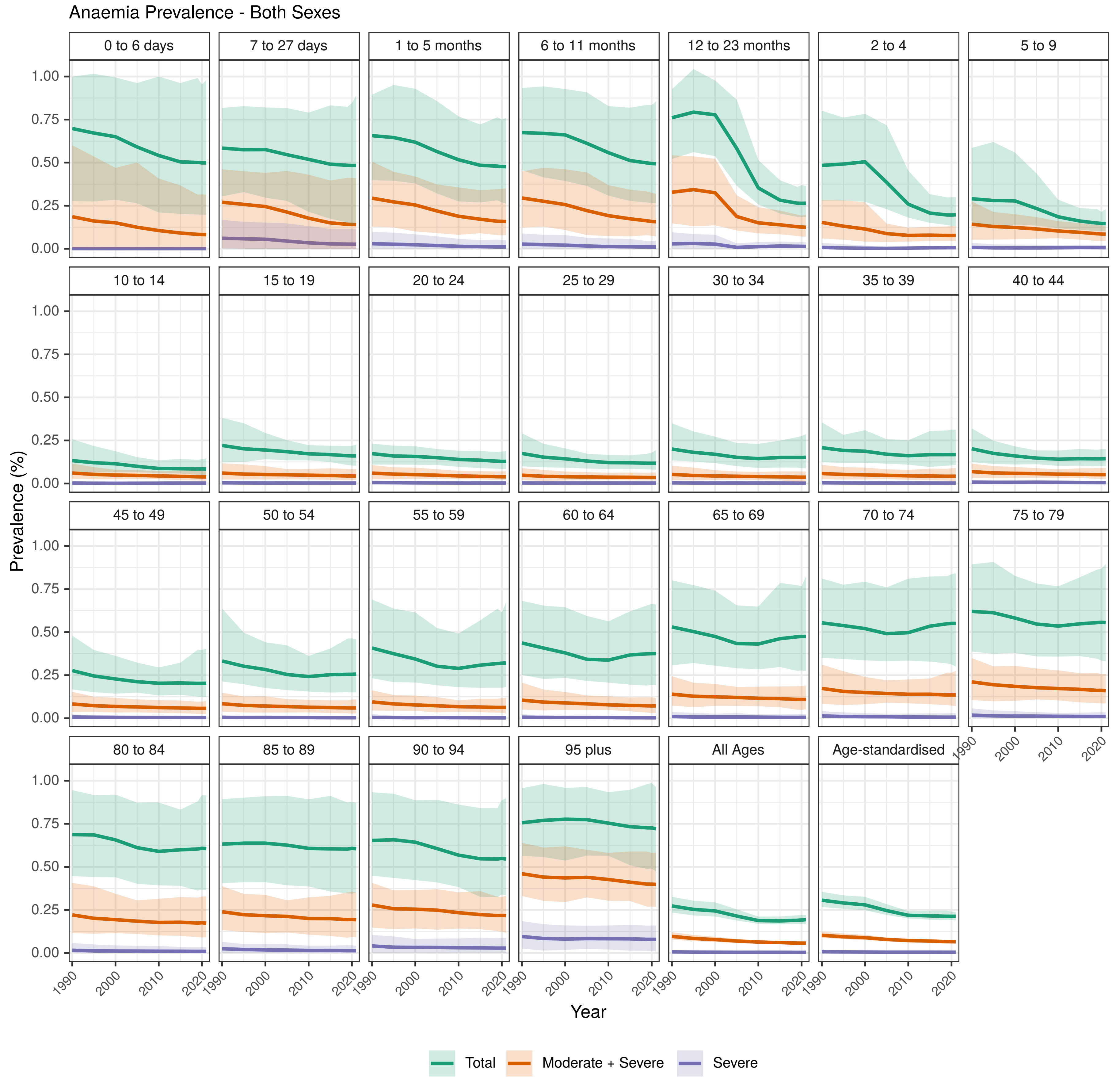

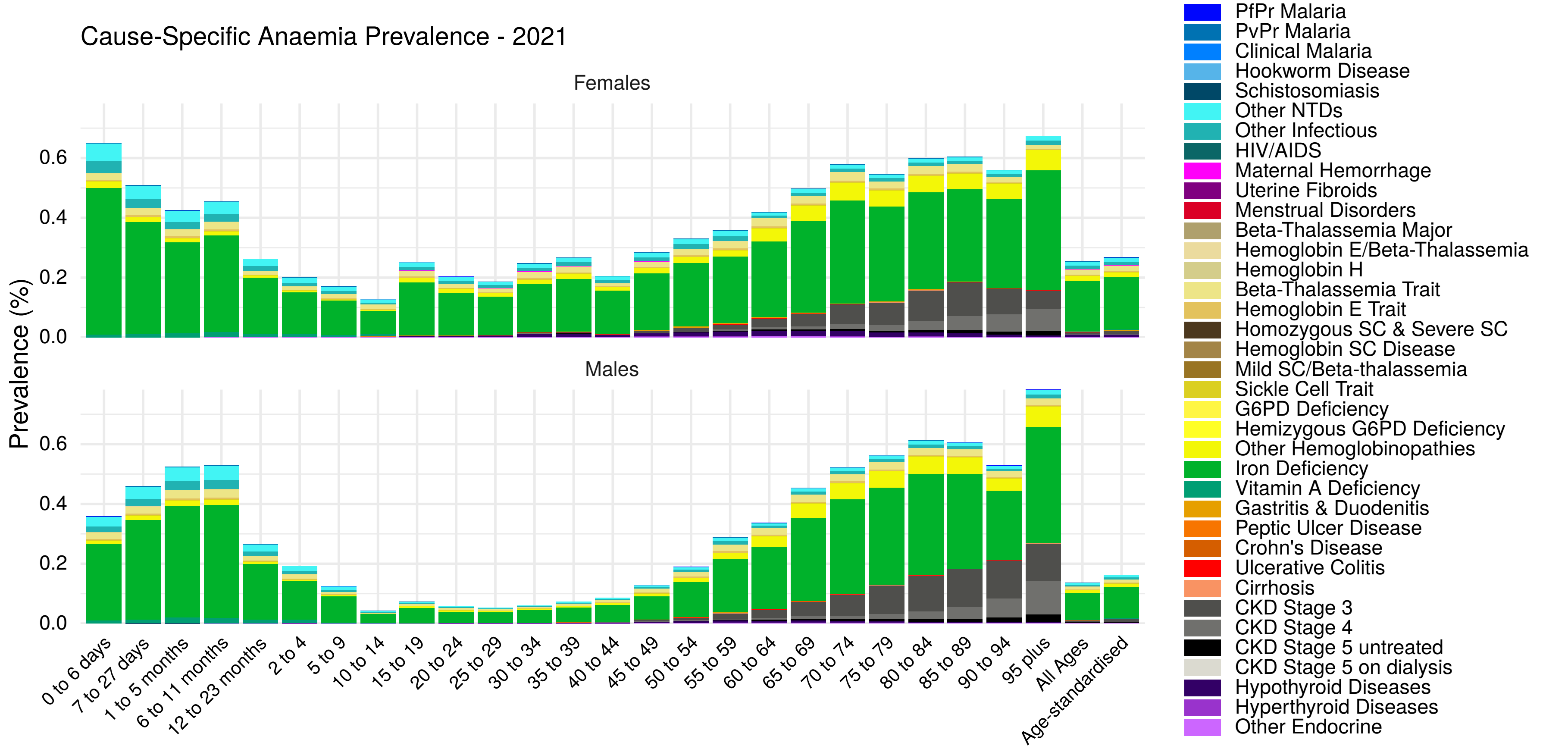

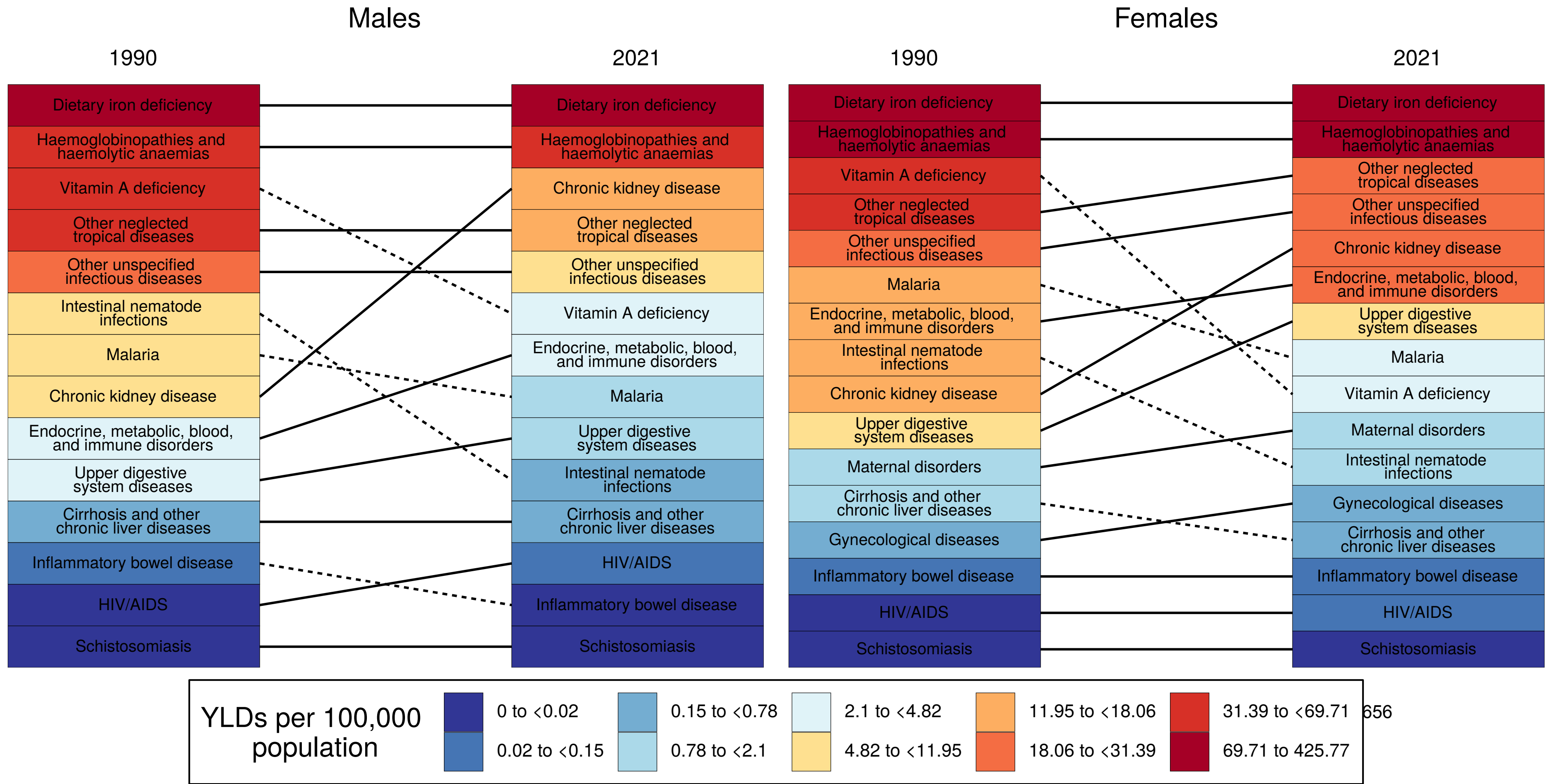

656

East Kalimantan

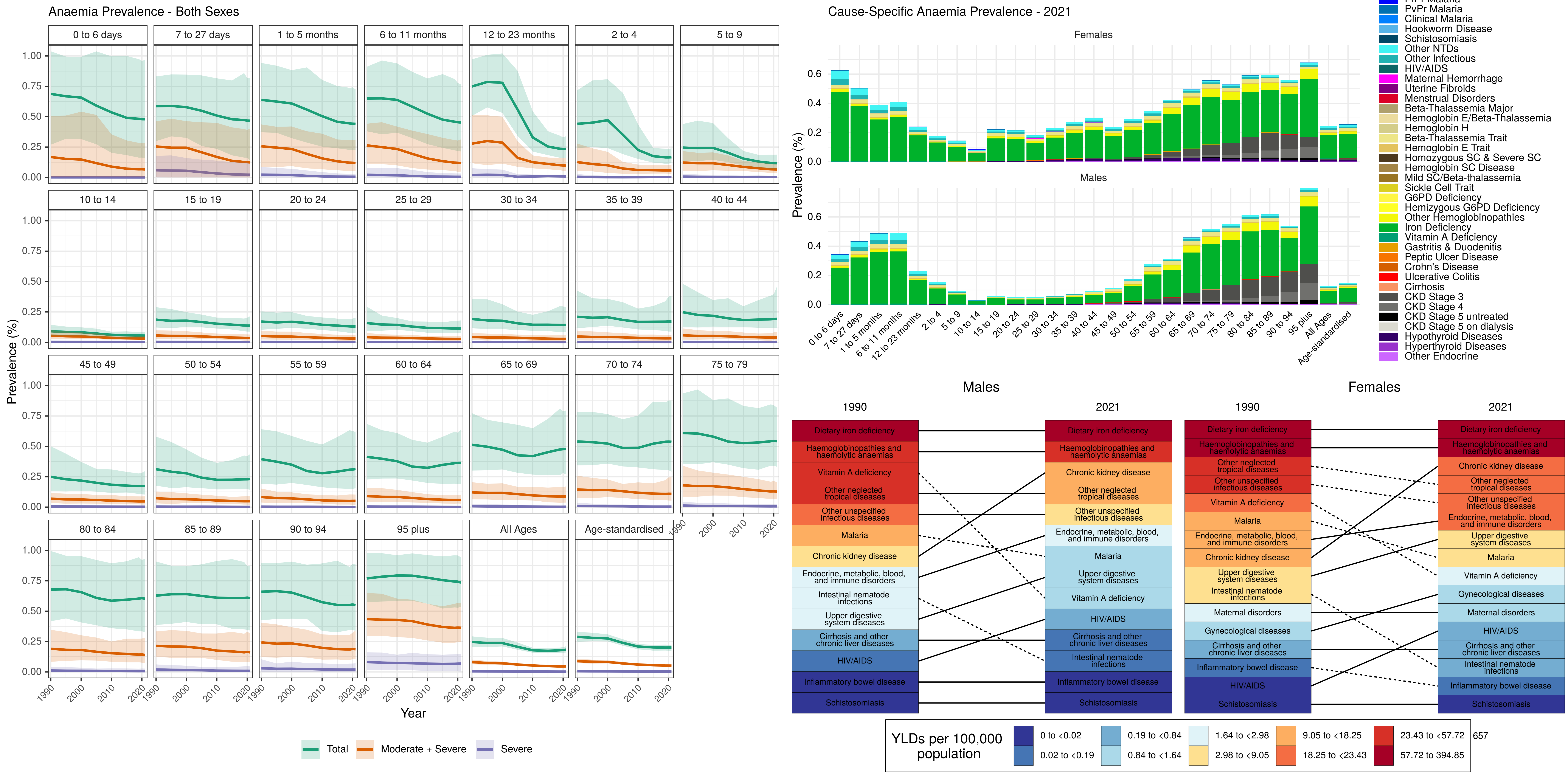

North Kalimantan

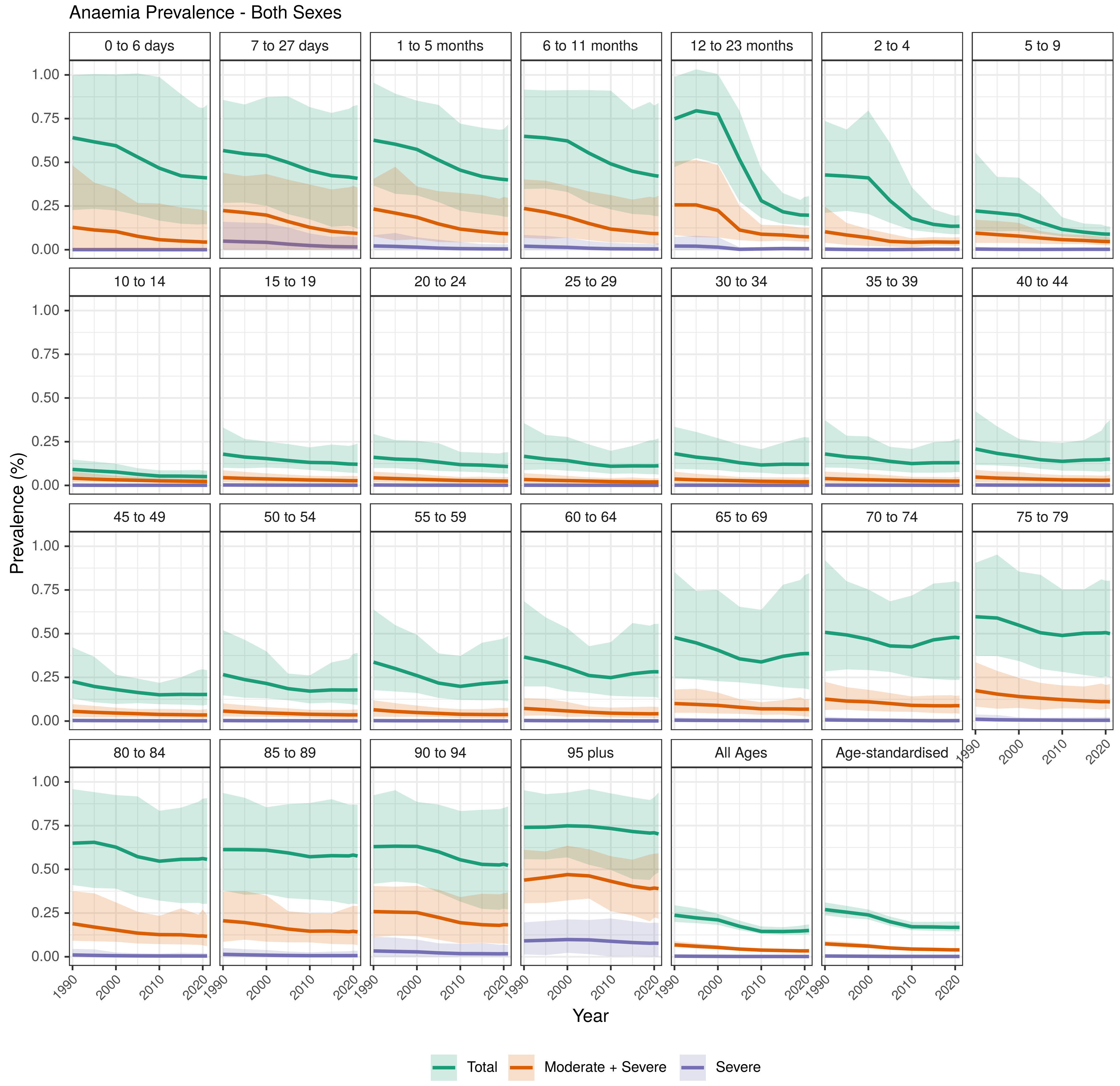

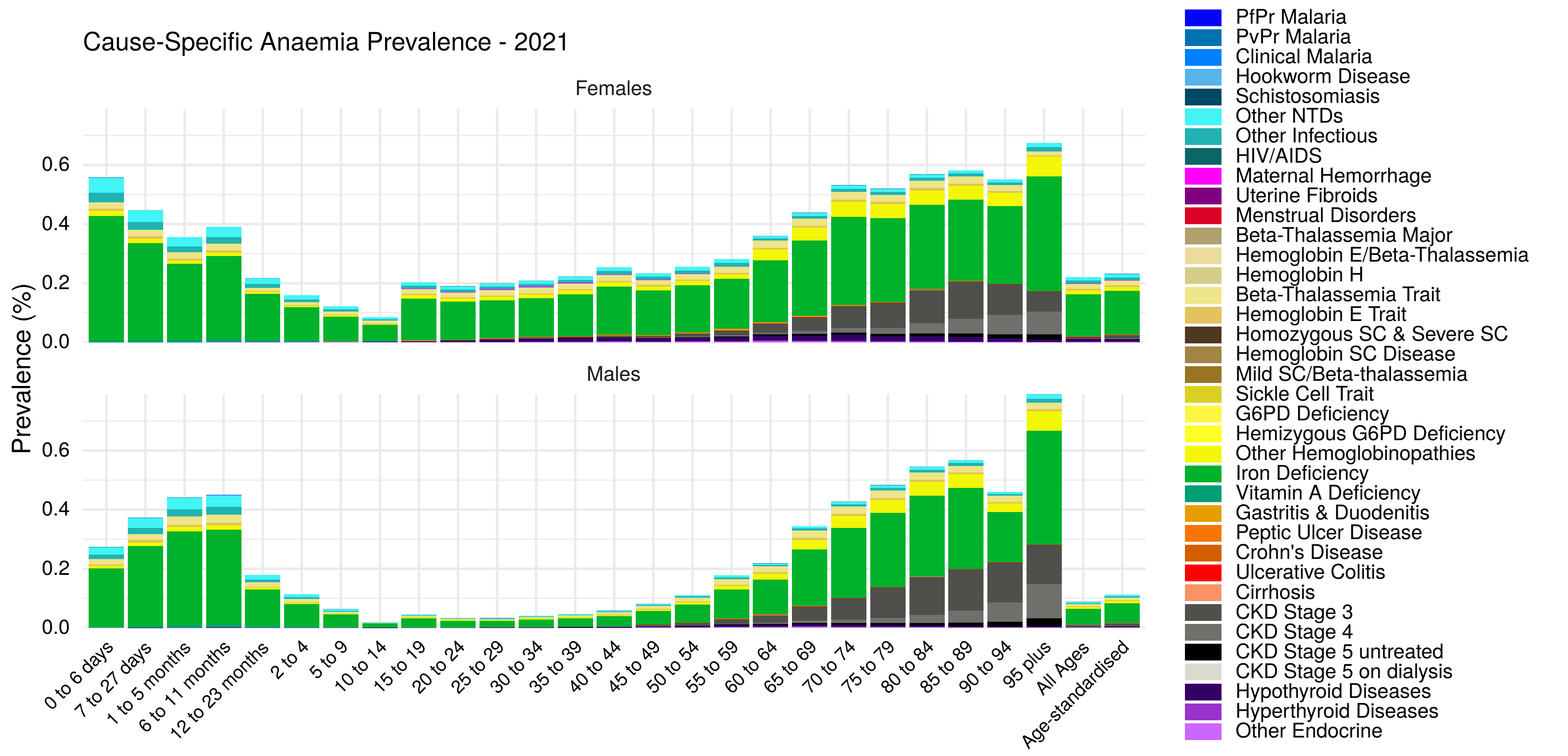

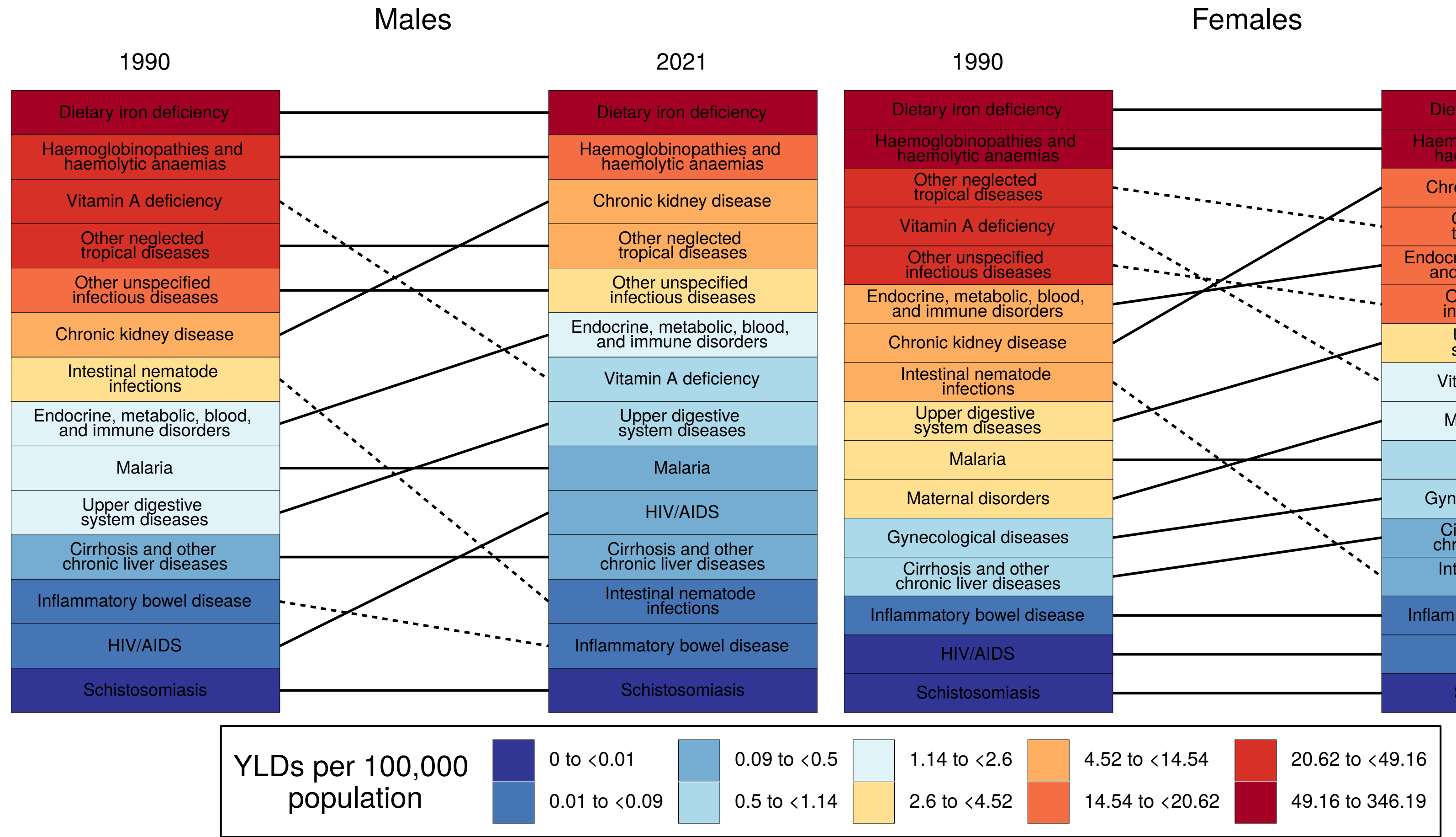

Riau Islands

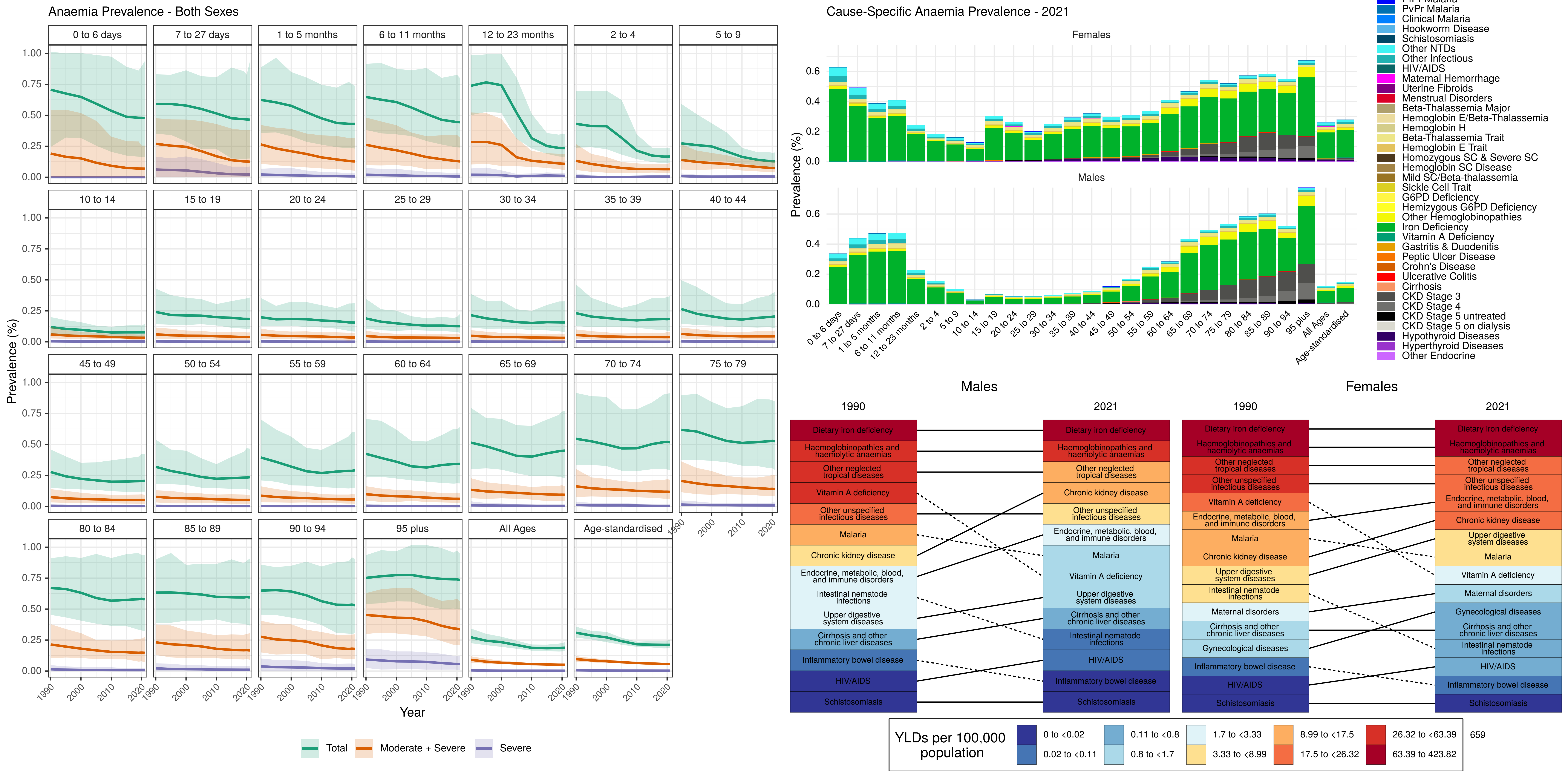

659

Lampung

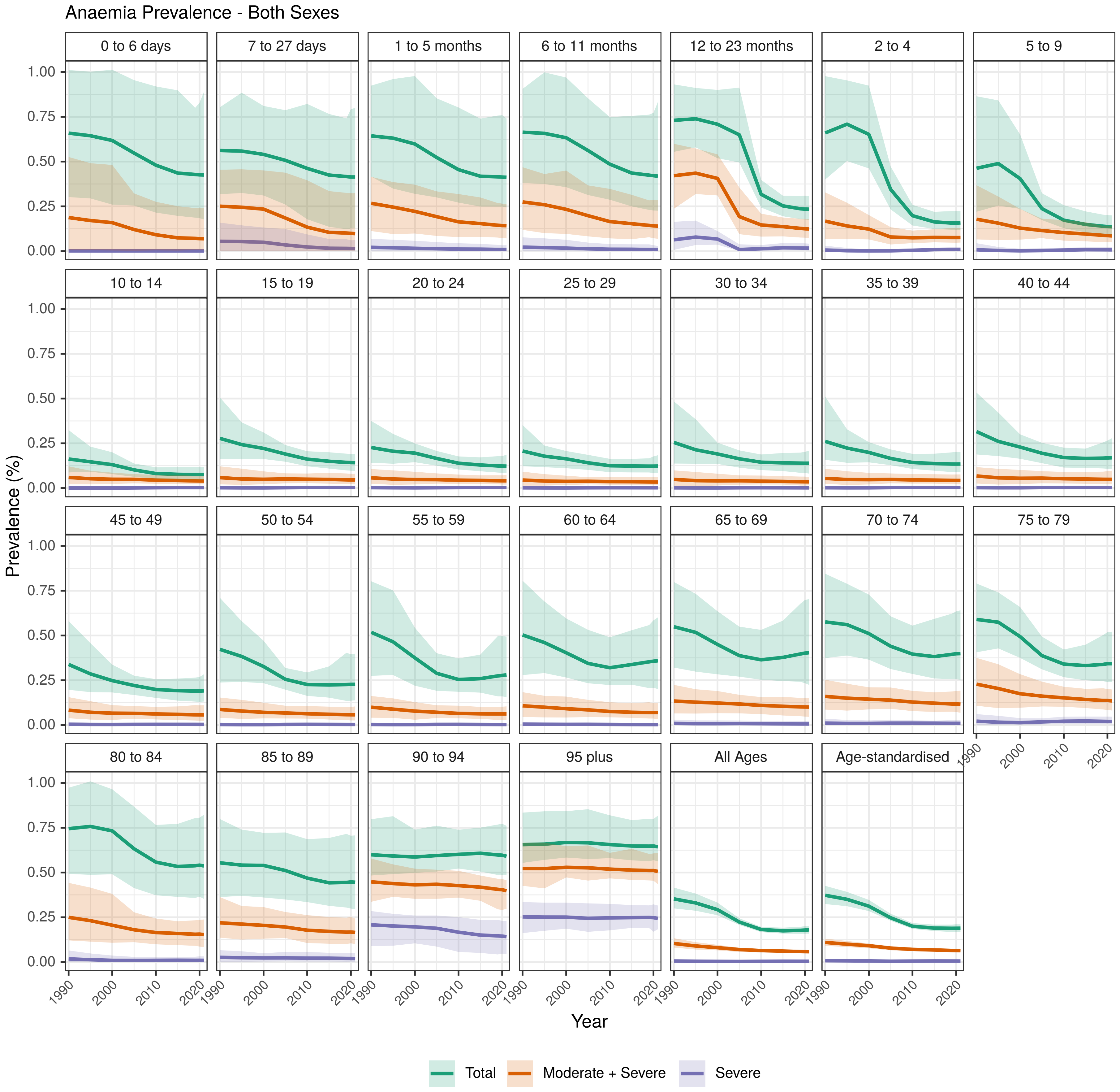
Anaemia Prevalence - Both Sexes
0 to 6 days
7 to 27 days
1 to 5 months
6 to 11 months
12 to 23 months
2 to 4
5 to 9
10 to 14
15 to 19
20 to 24
25 to 29
30 to 34
35 to 39
40 to 44
45 to 49
50 to 54
55 to 59
60 to 64
65 to 69
70 to 74
75 to 79
80 to 84
85 to 89
90 to 94
95 plus
All Ages
Age-standardised
Prevalence (%)
0.00
0.25
0.50
0.75
1.00
1990
2000
2010
2020
Year
Total
Moderate + Severe
Severe

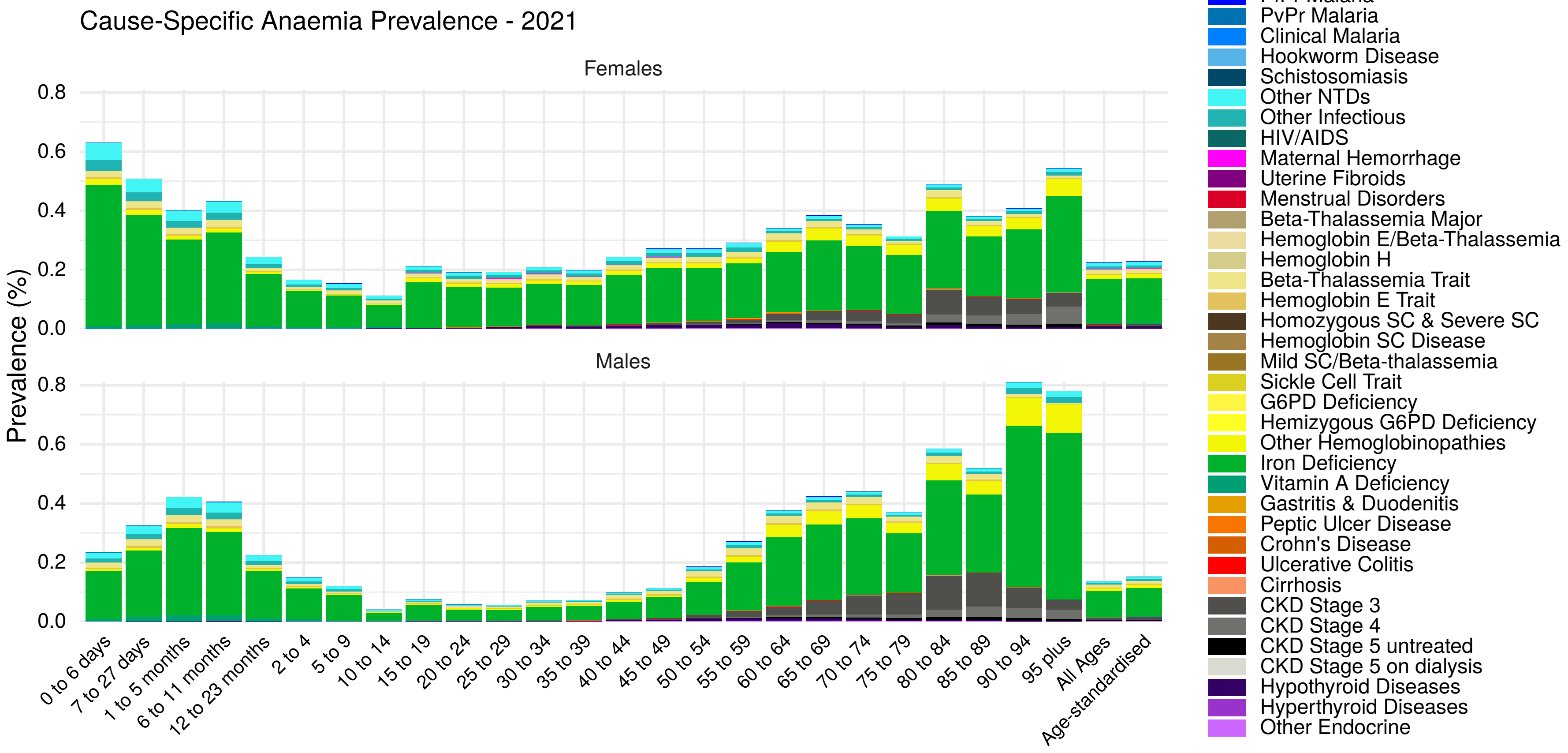
Cause-Specific Anaemia Prevalence - 2021
Females
Males
Prevalence (%)
0.0
0.2
0.4
0.6
0.8
0 to 6 days
7 to 27 days
1 to 5 months
6 to 11 months
12 to 23 months
2 to 4
5 to 9
10 to 14
15 to 19
20 to 24
25 to 29
30 to 34
35 to 39
40 to 44
45 to 49
50 to 54
55 to 59
60 to 64
65 to 69
70 to 74
75 to 79
80 to 84
85 to 89
90 to 94
95 plus
All Ages
Age-standardised
PfPr Malaria
PvPr Malaria
Clinical Malaria
Hookworm Disease
Schistosomiasis
Other NTDs
Other Infectious
HIV/AIDS
Maternal Hemorrhage
Uterine Fibroids
Menstrual Disorders
Beta-Thalassemia Major
Hemoglobin E/Beta-Thalassemia
Hemoglobin H
Beta-Thalassemia Trait
Hemoglobin E Trait
Homozygous SC & Severe SC
Hemoglobin SC Disease
Mild SC/Beta-thalassemia
Sickle Cell Trait
G6PD Deficiency
Hemizygous G6PD Deficiency
Other Hemoglobinopathies
Iron Deficiency
Vitamin A Deficiency
Gastritis & Duodenitis
Peptic Ulcer Disease
Crohn's Disease
Ulcerative Colitis
Cirrhosis
CKD Stage 3
CKD Stage 4
CKD Stage 5 untreated
CKD Stage 5 on dialysis
Hypothyroid Diseases
Hyperthyroid Diseases
Other Endocrine

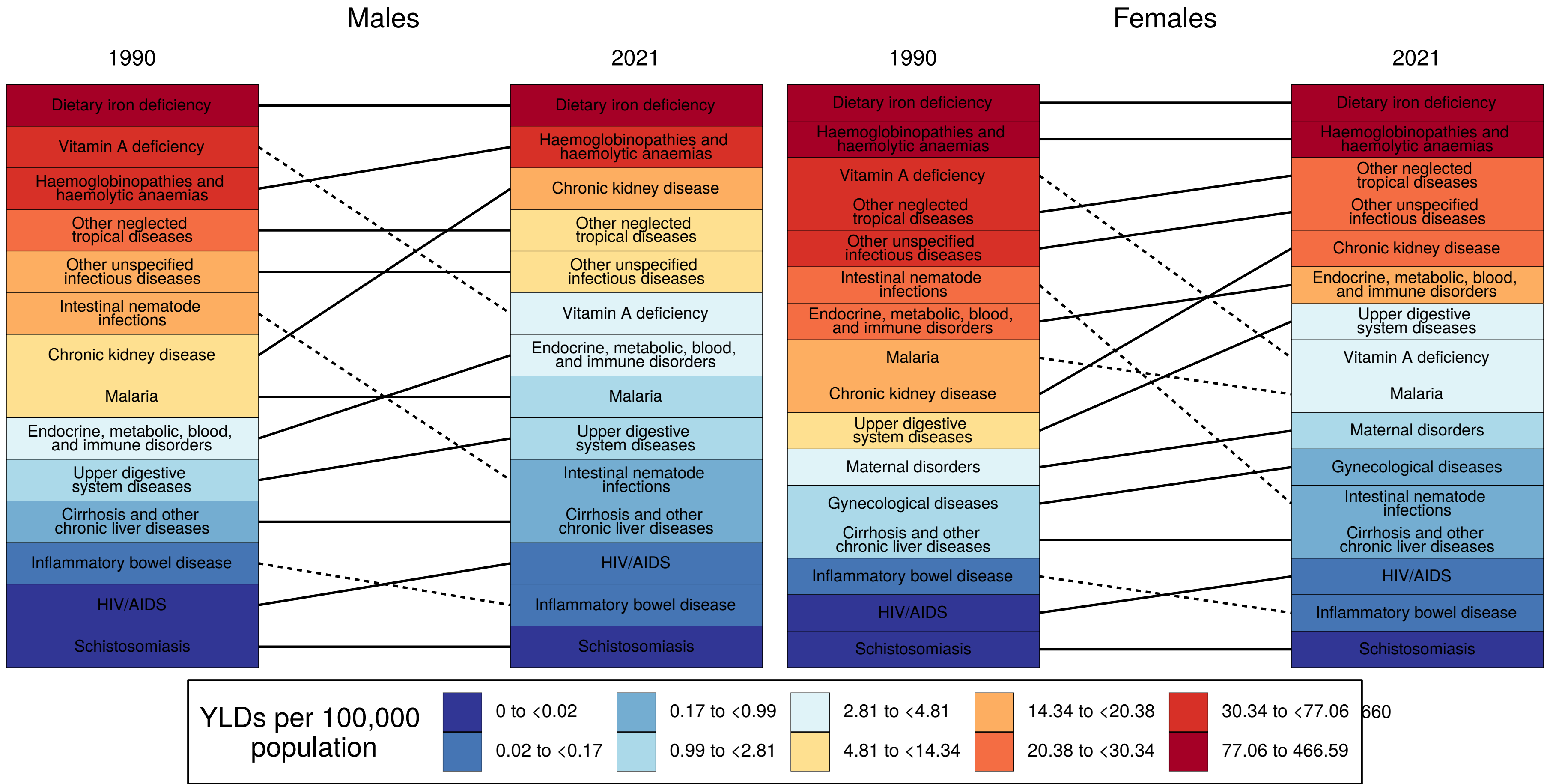
Males
Females
1990
2021
Dietary iron deficiency
Vitamin A deficiency
Haemoglobinopathies and haemolytic anaemias
Other neglected tropical diseases
Other unspecified infectious diseases
Intestinal nematode infections
Chronic kidney disease
Malaria
Endocrine, metabolic, blood, and immune disorders
Upper digestive system diseases
Cirrhosis and other chronic liver diseases
Inflammatory bowel disease
HIV/AIDS
Schistosomiasis
Maternal disorders
Gynecological diseases
YLDs per 100,000 population
0 to <0.02
0.02 to <0.17
0.17 to <0.99
0.99 to <2.81
2.81 to <4.81
4.81 to <14.34
14.34 to <20.38
20.38 to <30.34
30.34 to <77.06
77.06 to 466.59

660

Maluku

Anaemia Prevalence - Both Sexes

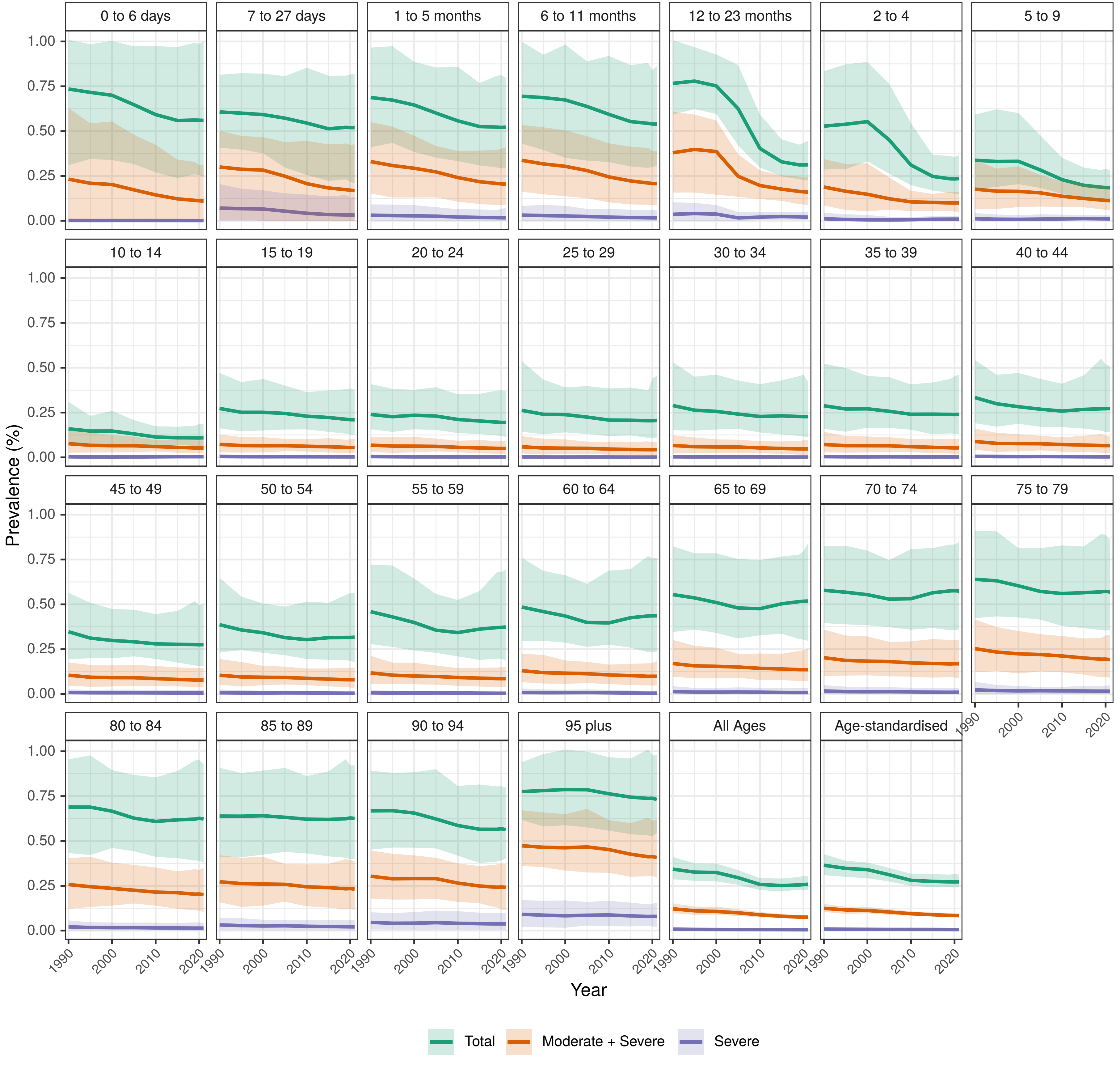

Cause-Specific Anaemia Prevalence - 2021

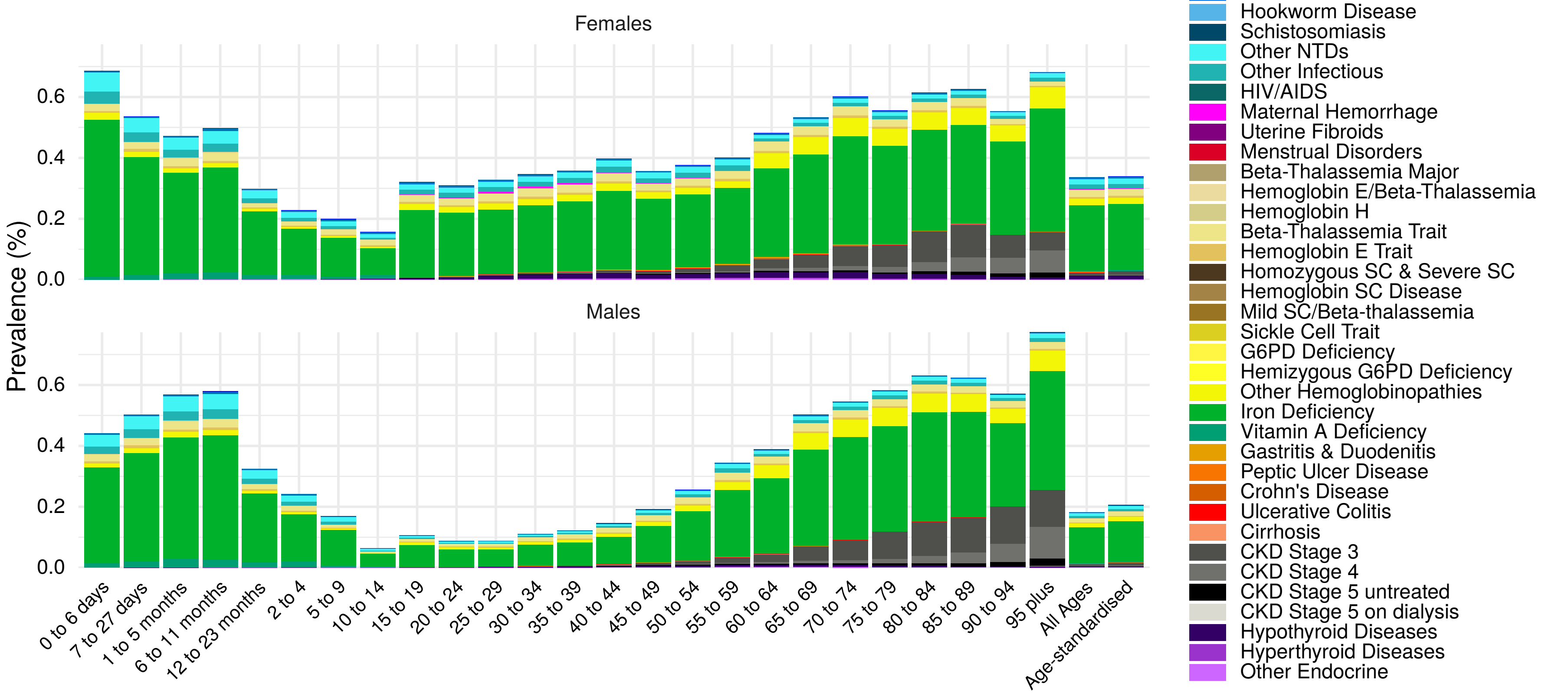

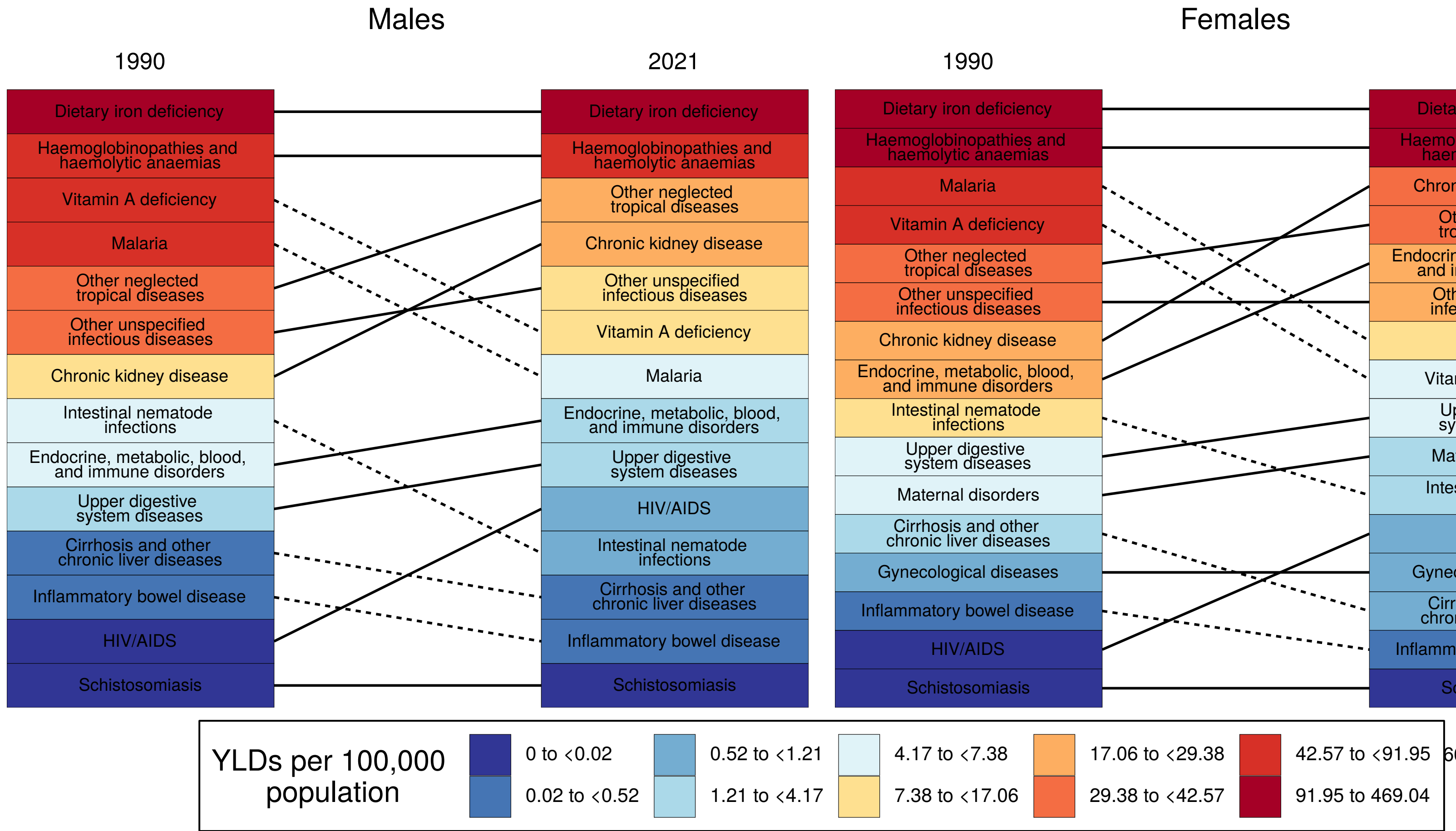

661

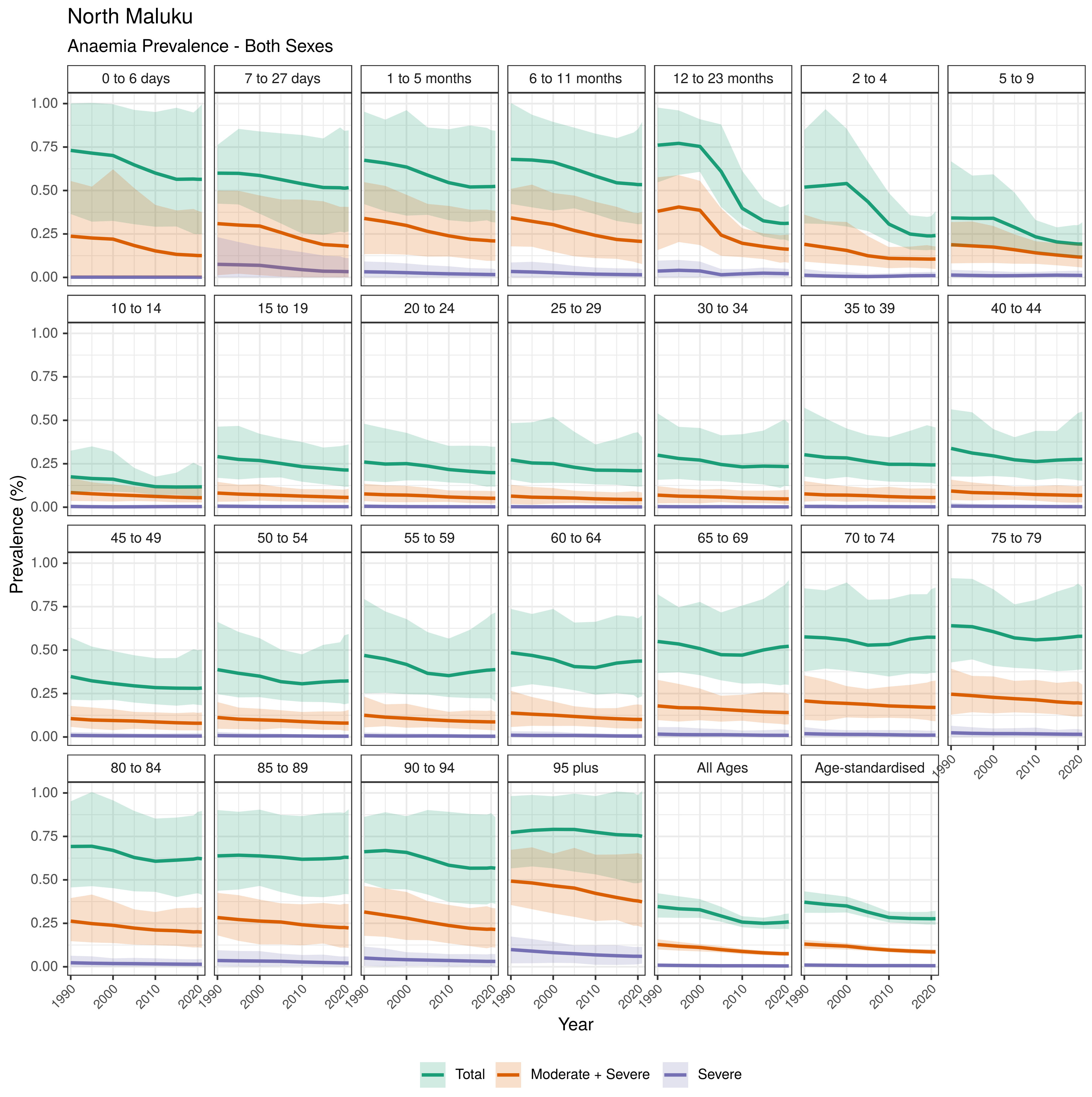
North Maluku
Anaemia Prevalence - Both Sexes
0 to 6 days
7 to 27 days
1 to 5 months
6 to 11 months
12 to 23 months
2 to 4
5 to 9
10 to 14
15 to 19
20 to 24
25 to 29
30 to 34
35 to 39
40 to 44
45 to 49
50 to 54
55 to 59
60 to 64
65 to 69
70 to 74
75 to 79
80 to 84
85 to 89
90 to 94
95 plus
All Ages
Age-standardised
Prevalence (%)
Year
Total
Moderate + Severe
Severe

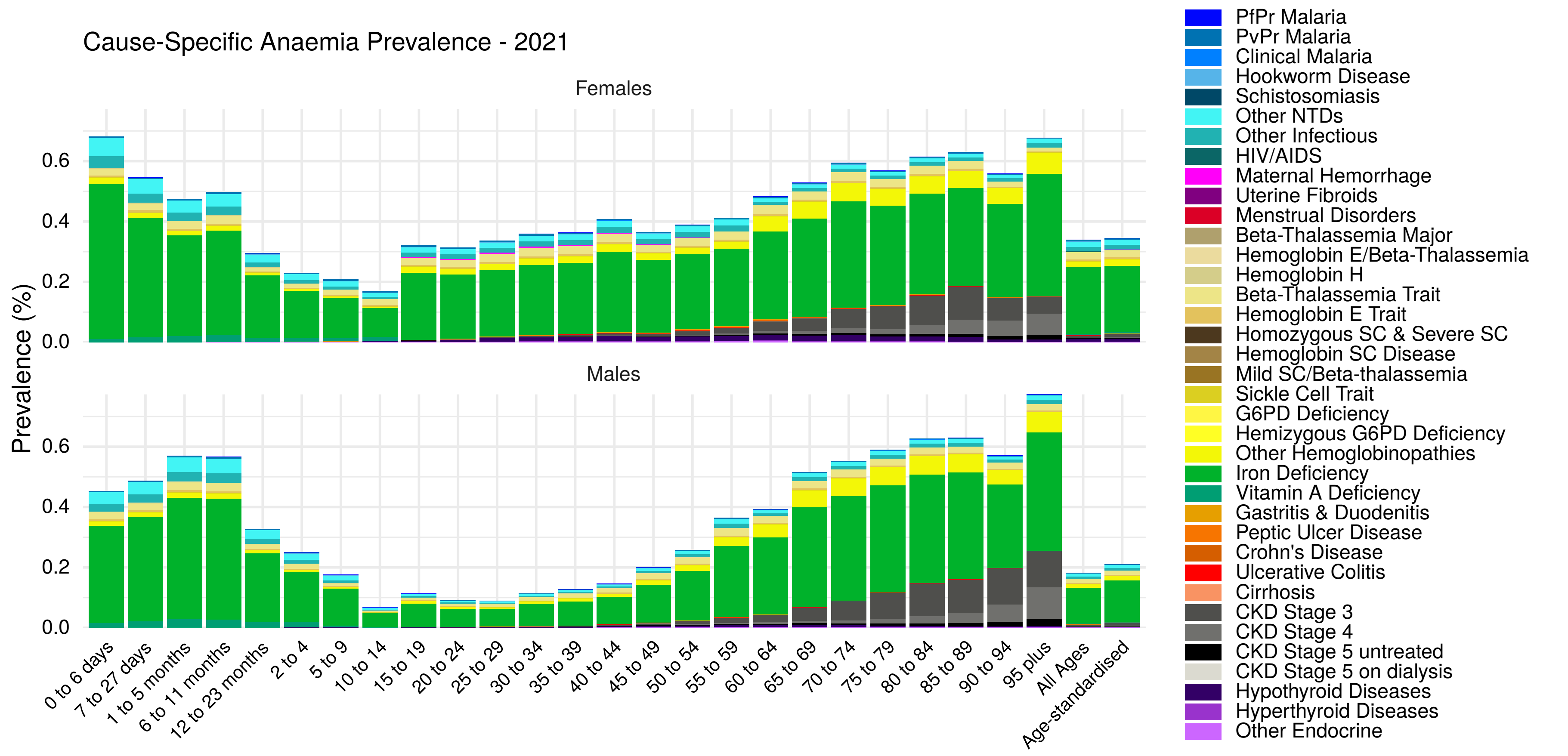
Cause-Specific Anaemia Prevalence - 2021
Females
Males
Prevalence (%)
PfPr Malaria
PvPr Malaria
Clinical Malaria
Hookworm Disease
Schistosomiasis
Other NTDs
Other Infectious
HIV/AIDS
Maternal Hemorrhage
Uterine Fibroids
Menstrual Disorders
Beta-Thalassemia Major
Hemoglobin E/Beta-Thalassemia
Hemoglobin H
Beta-Thalassemia Trait
Hemoglobin E Trait
Homozygous SC & Severe SC
Hemoglobin SC Disease
Mild SC/Beta-thalassemia
Sickle Cell Trait
G6PD Deficiency
Hemizygous G6PD Deficiency
Other Hemoglobinopathies
Iron Deficiency
Vitamin A Deficiency
Gastritis & Duodenitis
Peptic Ulcer Disease
Crohn's Disease
Ulcerative Colitis
Cirrhosis
CKD Stage 3
CKD Stage 4
CKD Stage 5 untreated
CKD Stage 5 on dialysis
Hypothyroid Diseases
Hyperthyroid Diseases
Other Endocrine

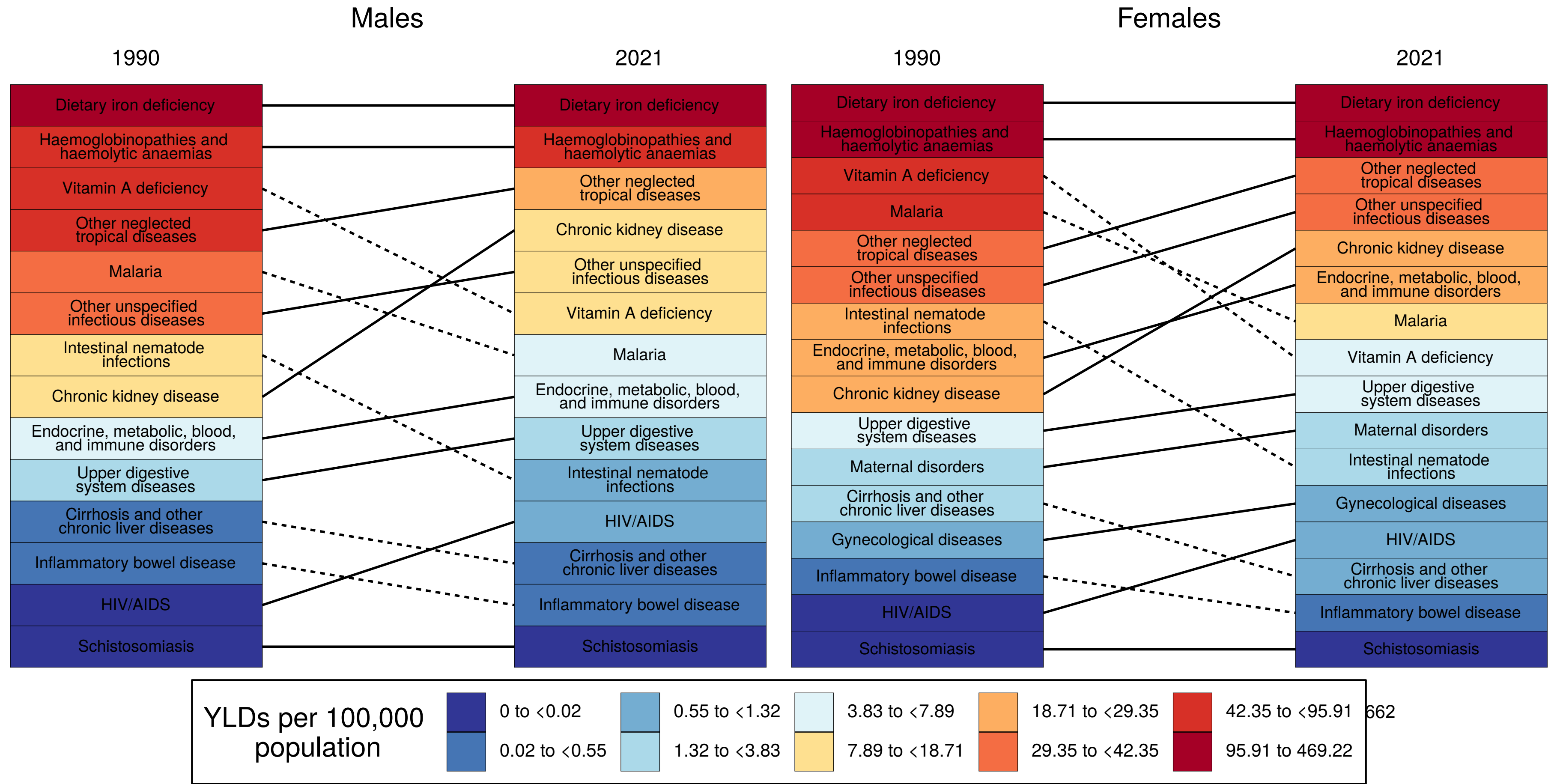
Males
Females
1990
2021
Dietary iron deficiency
Haemoglobinopathies and haemolytic anaemias
Vitamin A deficiency
Other neglected tropical diseases
Malaria
Other unspecified infectious diseases
Intestinal nematode infections
Chronic kidney disease
Endocrine, metabolic, blood, and immune disorders
Upper digestive system diseases
Cirrhosis and other chronic liver diseases
Inflammatory bowel disease
HIV/AIDS
Schistosomiasis
Maternal disorders
Gynecological diseases
YLDs per 100,000 population
0 to <0.02
0.02 to <0.55
0.55 to <1.32
1.32 to <3.83
3.83 to <7.89
7.89 to <18.71
18.71 to <29.35
29.35 to <42.35
42.35 to <95.91
95.91 to 469.22

West Nusa Tenggara

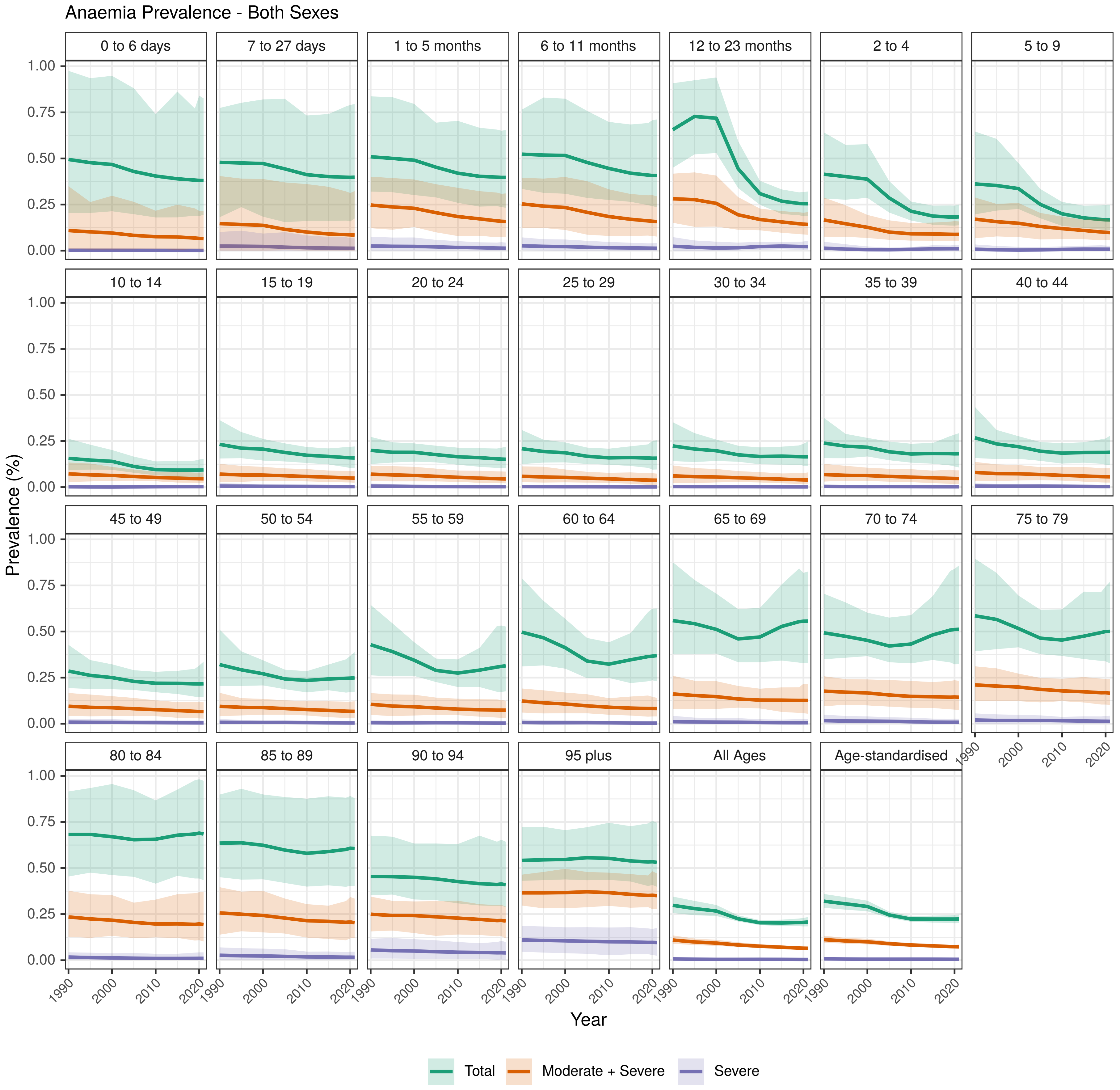

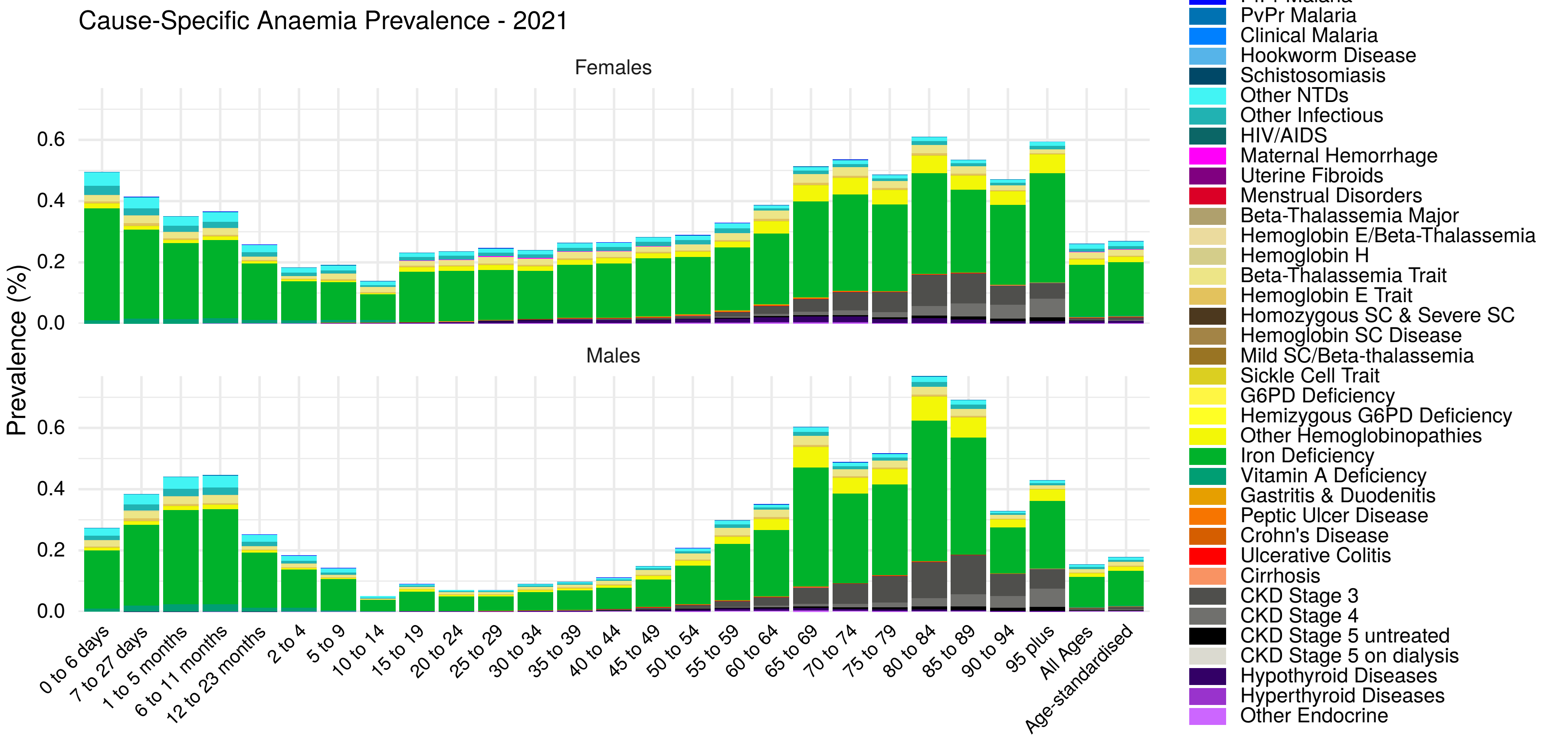

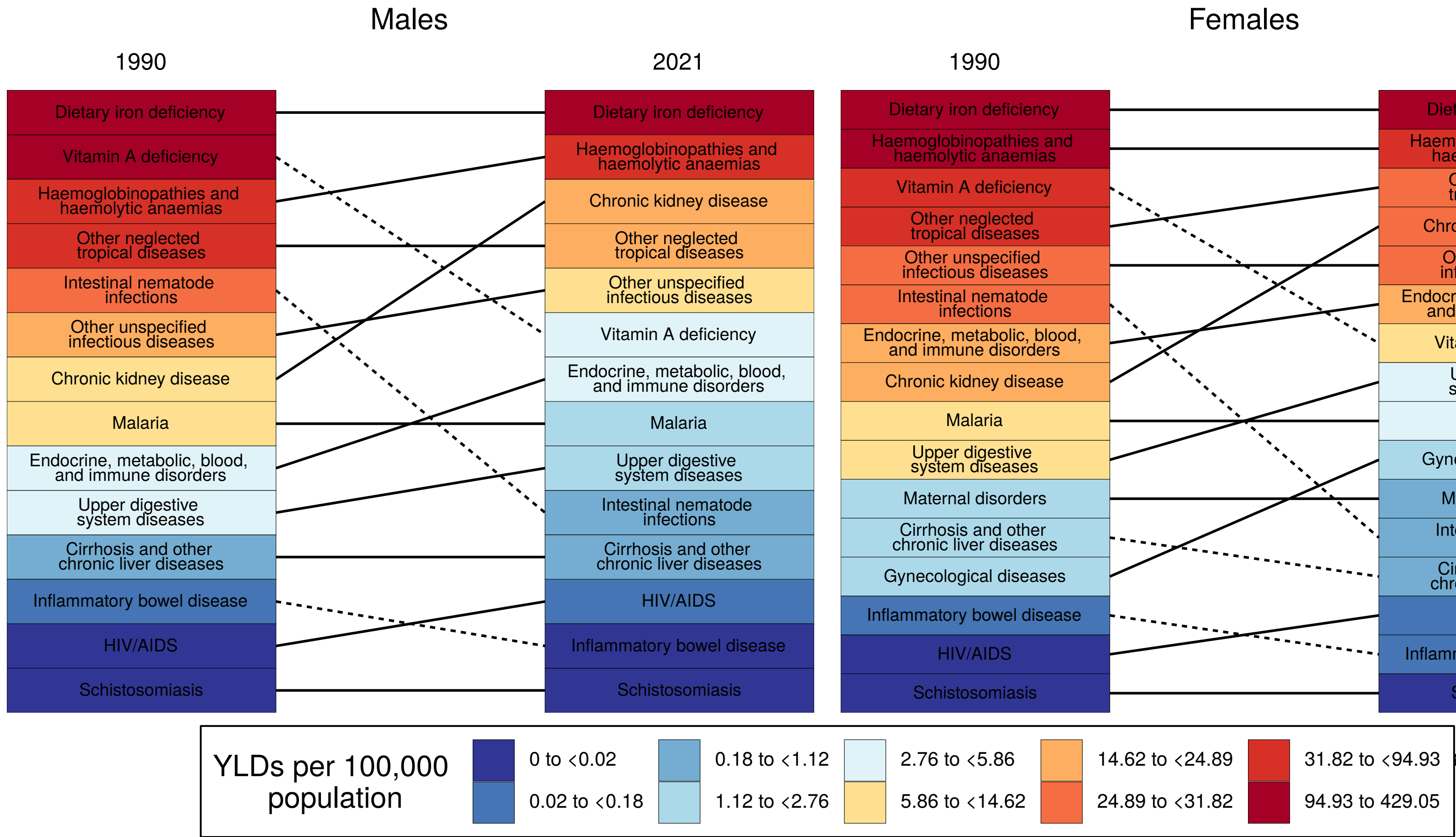

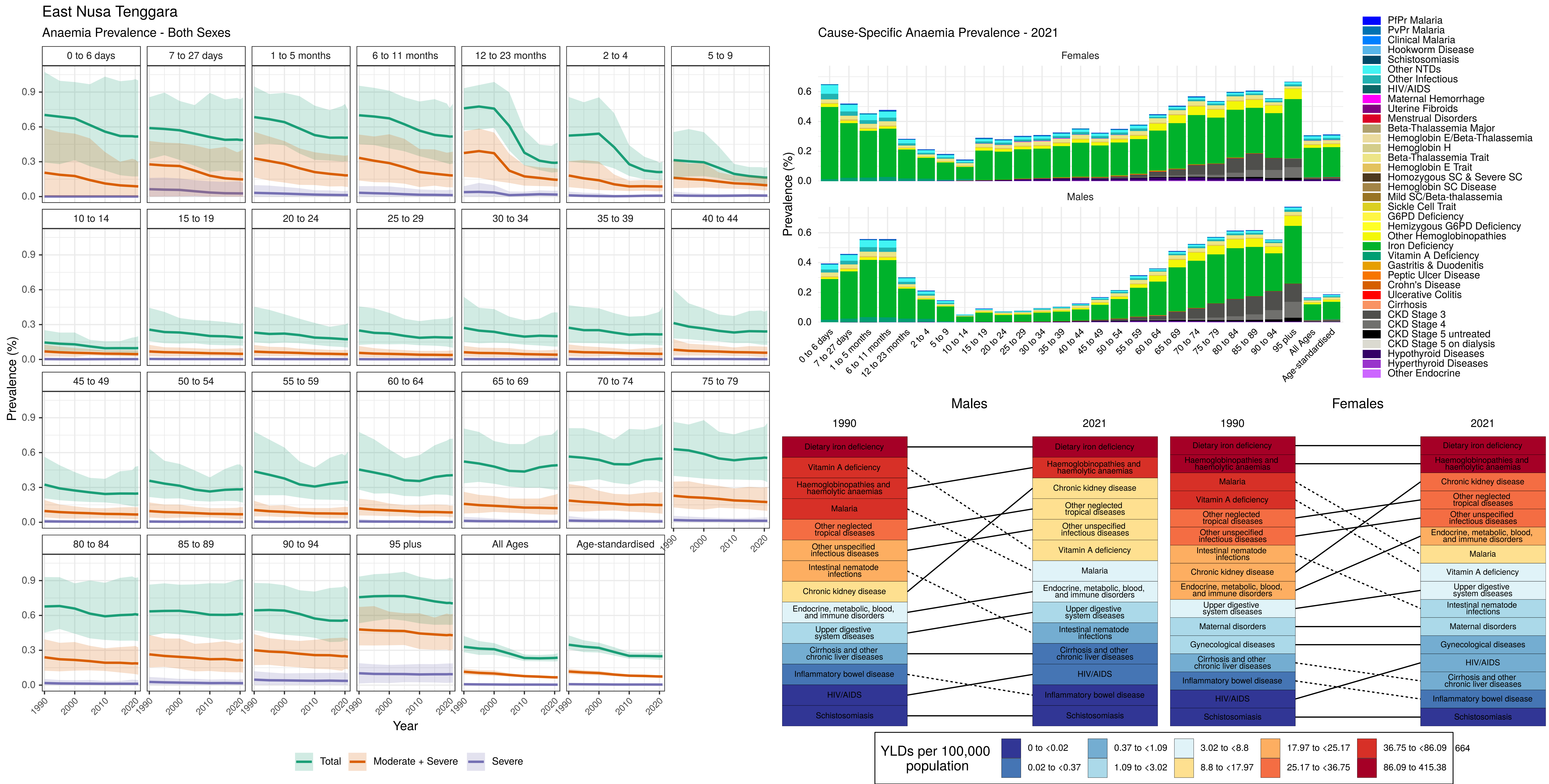

East Nusa Tenggara
Anaemia Prevalence - Both Sexes
0 to 6 days
7 to 27 days
1 to 5 months
6 to 11 months
12 to 23 months
2 to 4
5 to 9
10 to 14
15 to 19
20 to 24
25 to 29
30 to 34
35 to 39
40 to 44
45 to 49
50 to 54
55 to 59
60 to 64
65 to 69
70 to 74
75 to 79
80 to 84
85 to 89
90 to 94
95 plus
All Ages
Age-standardised
Prevalence (%)
Year
Total
Moderate + Severe
Severe
Cause-Specific Anaemia Prevalence - 2021
Females
Males
PfPr Malaria
PvPr Malaria
Clinical Malaria
Hookworm Disease
Schistosomiasis
Other NTDs
Other Infectious
HIV/AIDS
Maternal Hemorrhage
Uterine Fibroids
Menstrual Disorders
Beta-Thalassemia Major
Hemoglobin E/Beta-Thalassemia
Hemoglobin H
Beta-Thalassemia Trait
Hemoglobin E Trait
Homozygous SC & Severe SC
Hemoglobin SC Disease
Mild SC/Beta-thalassemia
Sickle Cell Trait
G6PD Deficiency
Hemizygous G6PD Deficiency
Other Hemoglobinopathies
Iron Deficiency
Vitamin A Deficiency
Gastritis & Duodenitis
Peptic Ulcer Disease
Crohn's Disease
Ulcerative Colitis
Cirrhosis
CKD Stage 3
CKD Stage 4
CKD Stage 5 untreated
CKD Stage 5 on dialysis
Hypothyroid Diseases
Hyperthyroid Diseases
Other Endocrine
Males
Females
1990
2021
Dietary iron deficiency
Vitamin A deficiency
Haemoglobinopathies and haemolytic anaemias
Malaria
Other neglected tropical diseases
Other unspecified infectious diseases
Intestinal nematode infections
Chronic kidney disease
Endocrine, metabolic, blood, and immune disorders
Upper digestive system diseases
Cirrhosis and other chronic liver diseases
Inflammatory bowel disease
HIV/AIDS
Schistosomiasis
Maternal disorders
Gynecological diseases
YLDs per 100,000 population
0 to <0.02
0.02 to <0.37
0.37 to <1.09
1.09 to <3.02
3.02 to <8.8
8.8 to <17.97
17.97 to <25.17
25.17 to <36.75
36.75 to <86.09
86.09 to 415.38

Papua

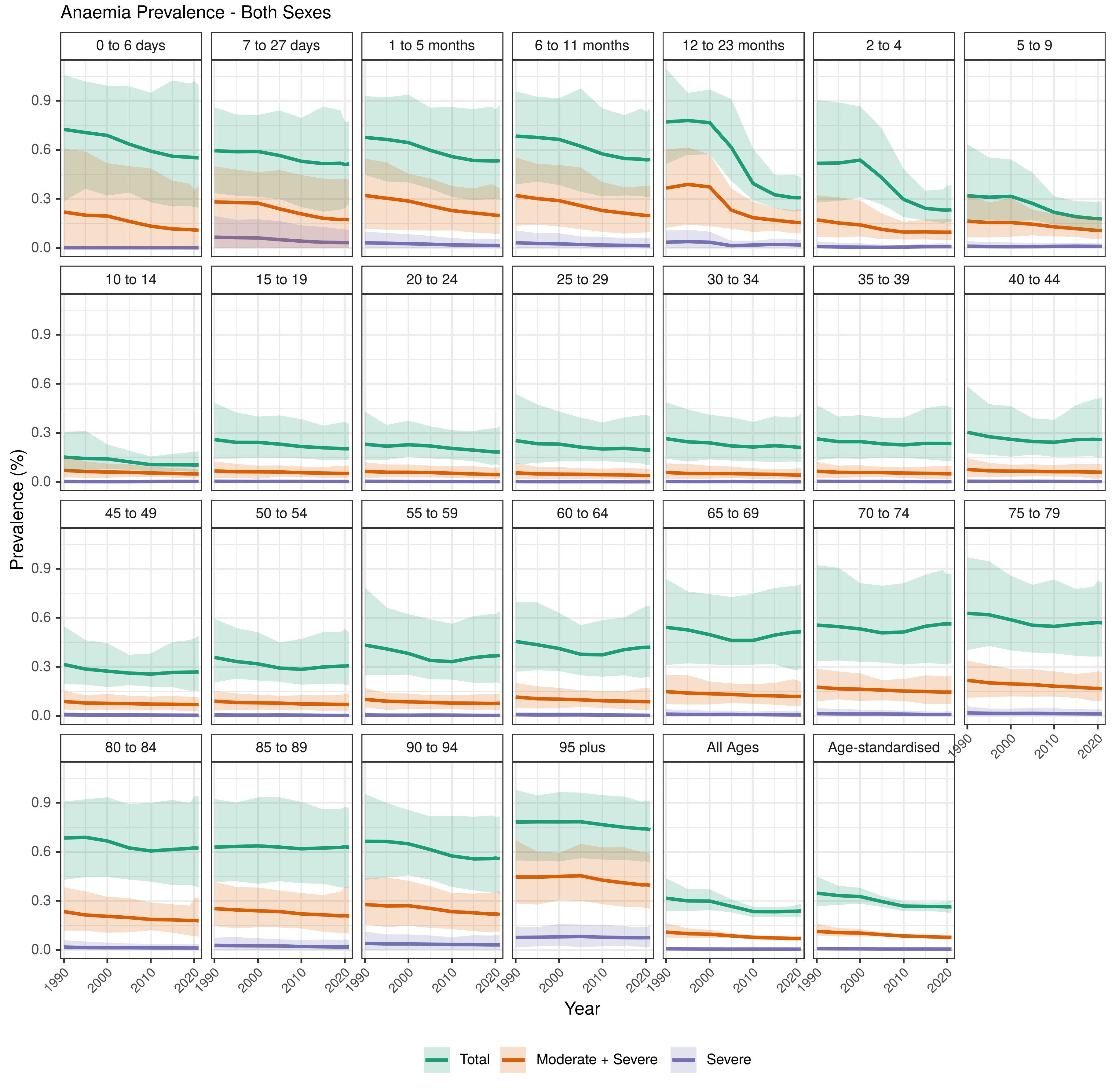
Anaemia Prevalence - Both Sexes
0 to 6 days
7 to 27 days
1 to 5 months
6 to 11 months
12 to 23 months
2 to 4
5 to 9
10 to 14
15 to 19
20 to 24
25 to 29
30 to 34
35 to 39
40 to 44
45 to 49
50 to 54
55 to 59
60 to 64
65 to 69
70 to 74
75 to 79
80 to 84
85 to 89
90 to 94
95 plus
All Ages
Age-standardised
Prevalence (%)
0.0
0.3
0.6
0.9
1990
2000
2010
2020
Year
Total
Moderate + Severe
Severe

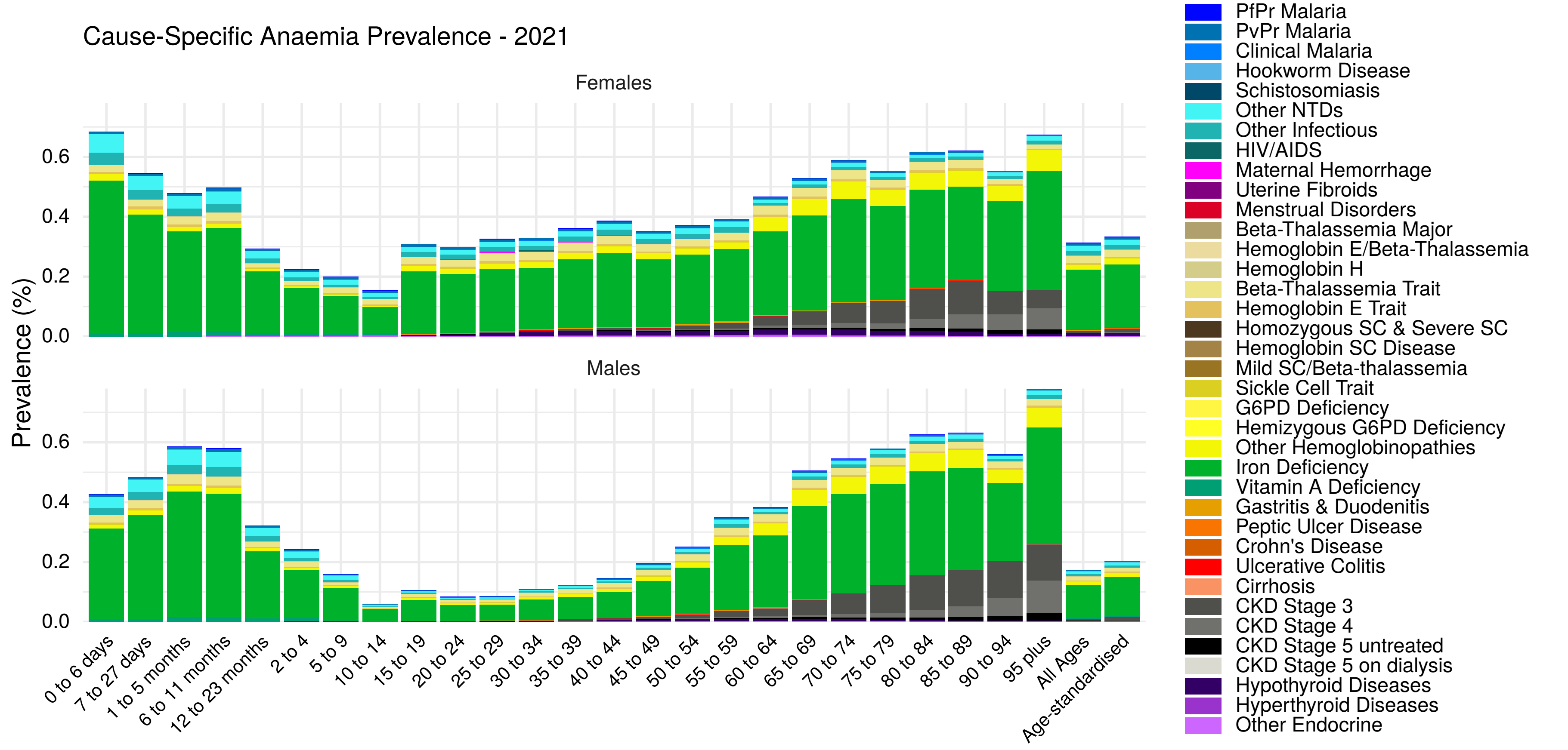
Cause-Specific Anaemia Prevalence - 2021
Females
Males
Prevalence (%)
0.0
0.2
0.4
0.6
0 to 6 days
7 to 27 days
1 to 5 months
6 to 11 months
12 to 23 months
2 to 4
5 to 9
10 to 14
15 to 19
20 to 24
25 to 29
30 to 34
35 to 39
40 to 44
45 to 49
50 to 54
55 to 59
60 to 64
65 to 69
70 to 74
75 to 79
80 to 84
85 to 89
90 to 94
95 plus
All Ages
Age-standardised
PfPr Malaria
PvPr Malaria
Clinical Malaria
Hookworm Disease
Schistosomiasis
Other NTDs
Other Infectious
HIV/AIDS
Maternal Hemorrhage
Uterine Fibroids
Menstrual Disorders
Beta-Thalassemia Major
Hemoglobin E/Beta-Thalassemia
Hemoglobin H
Beta-Thalassemia Trait
Hemoglobin E Trait
Homozygous SC & Severe SC
Hemoglobin SC Disease
Mild SC/Beta-thalassemia
Sickle Cell Trait
G6PD Deficiency
Hemizygous G6PD Deficiency
Other Hemoglobinopathies
Iron Deficiency
Vitamin A Deficiency
Gastritis & Duodenitis
Peptic Ulcer Disease
Crohn's Disease
Ulcerative Colitis
Cirrhosis
CKD Stage 3
CKD Stage 4
CKD Stage 5 untreated
CKD Stage 5 on dialysis
Hypothyroid Diseases
Hyperthyroid Diseases
Other Endocrine

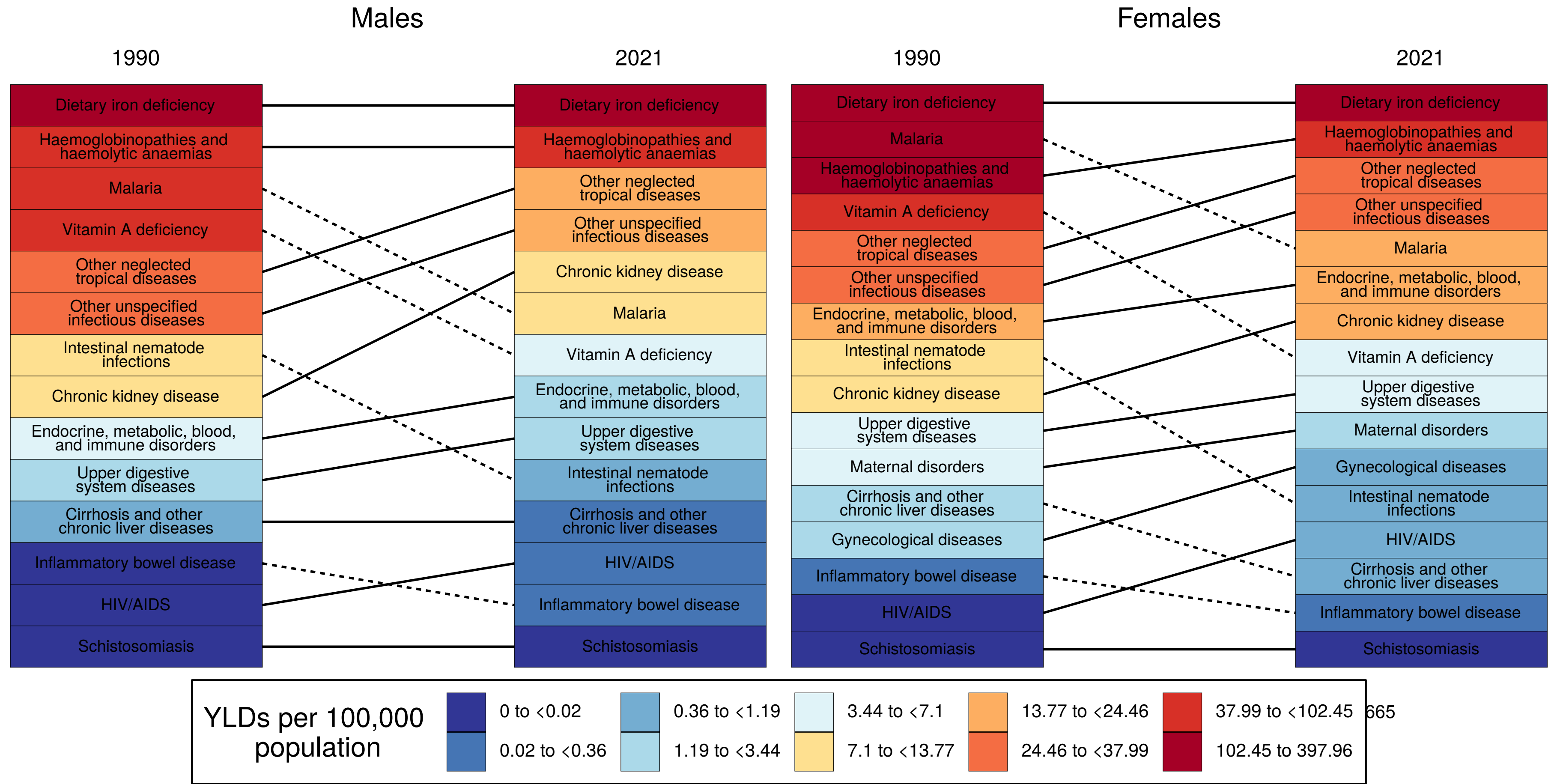
Males
Females
1990
2021
Males 1990:
Dietary iron deficiency
Haemoglobinopathies and haemolytic anaemias
Malaria
Vitamin A deficiency
Other neglected tropical diseases
Other unspecified infectious diseases
Intestinal nematode infections
Chronic kidney disease
Endocrine, metabolic, blood, and immune disorders
Upper digestive system diseases
Cirrhosis and other chronic liver diseases
Inflammatory bowel disease
HIV/AIDS
Schistosomiasis
Males 2021:
Dietary iron deficiency
Haemoglobinopathies and haemolytic anaemias
Other neglected tropical diseases
Other unspecified infectious diseases
Chronic kidney disease
Malaria
Vitamin A deficiency
Endocrine, metabolic, blood, and immune disorders
Upper digestive system diseases
Intestinal nematode infections
Cirrhosis and other chronic liver diseases
HIV/AIDS
Inflammatory bowel disease
Schistosomiasis
Females 1990:
Dietary iron deficiency
Malaria
Haemoglobinopathies and haemolytic anaemias
Vitamin A deficiency
Other neglected tropical diseases
Other unspecified infectious diseases
Endocrine, metabolic, blood, and immune disorders
Intestinal nematode infections
Chronic kidney disease
Upper digestive system diseases
Maternal disorders
Cirrhosis and other chronic liver diseases
Gynecological diseases
Inflammatory bowel disease
HIV/AIDS
Schistosomiasis
Females 2021:
Dietary iron deficiency
Haemoglobinopathies and haemolytic anaemias
Other neglected tropical diseases
Other unspecified infectious diseases
Malaria
Endocrine, metabolic, blood, and immune disorders
Chronic kidney disease
Vitamin A deficiency
Upper digestive system diseases
Maternal disorders
Gynecological diseases
Intestinal nematode infections
HIV/AIDS
Cirrhosis and other chronic liver diseases
Inflammatory bowel disease
Schistosomiasis
YLDs per 100,000 population
0 to <0.02
0.02 to <0.36
0.36 to <1.19
1.19 to <3.44
3.44 to <7.1
7.1 to <13.77
13.77 to <24.46
24.46 to <37.99
37.99 to <102.45
102.45 to 397.96

West Papua

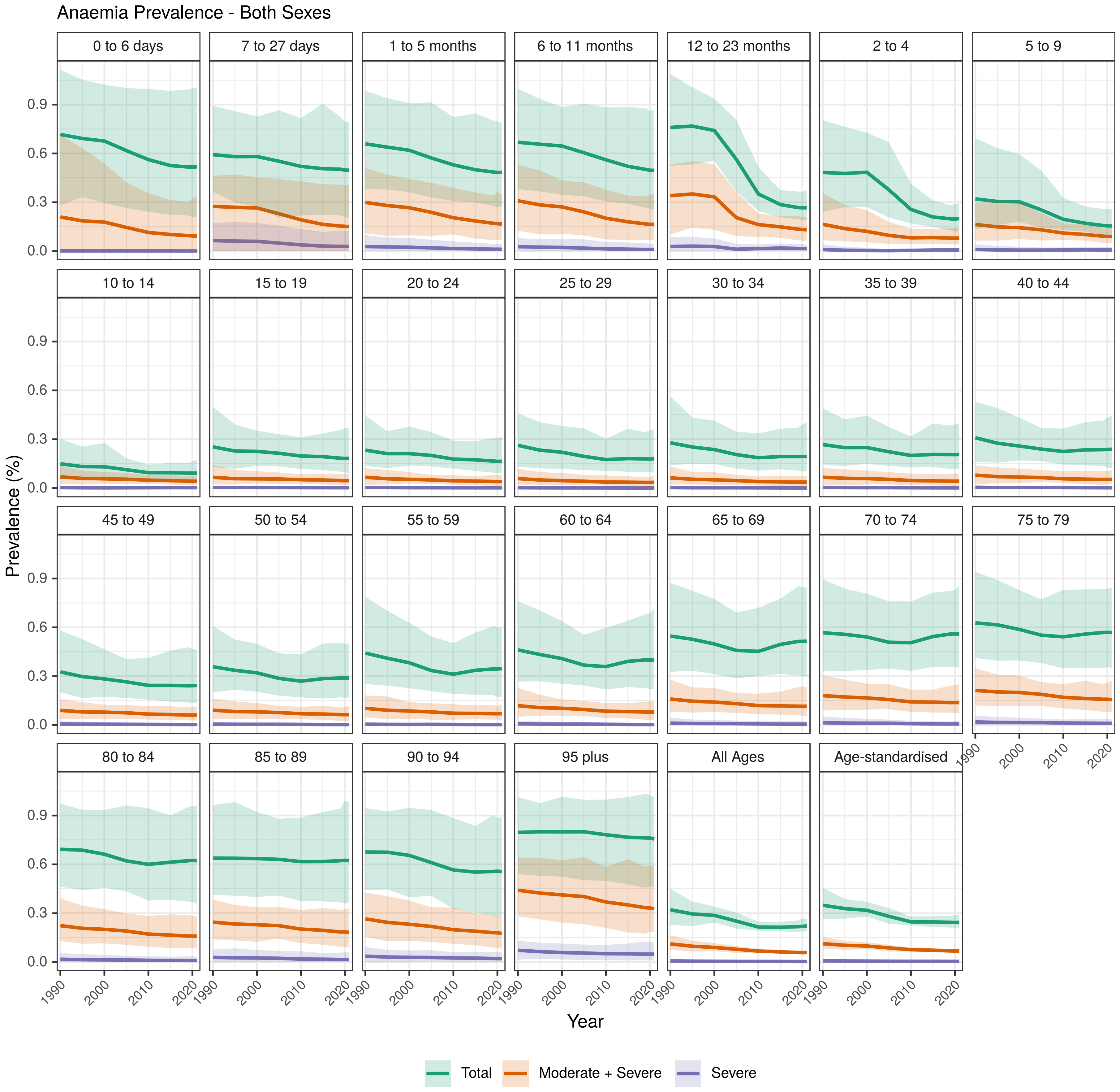

Anaemia Prevalence - Both Sexes
0 to 6 days
7 to 27 days
1 to 5 months
6 to 11 months
12 to 23 months
2 to 4
5 to 9
10 to 14
15 to 19
20 to 24
25 to 29
30 to 34
35 to 39
40 to 44
45 to 49
50 to 54
55 to 59
60 to 64
65 to 69
70 to 74
75 to 79
80 to 84
85 to 89
90 to 94
95 plus
All Ages
Age-standardised
Prevalence (%)
Year
1990
2000
2010
2020
0.0
0.3
0.6
0.9
Total
Moderate + Severe
Severe

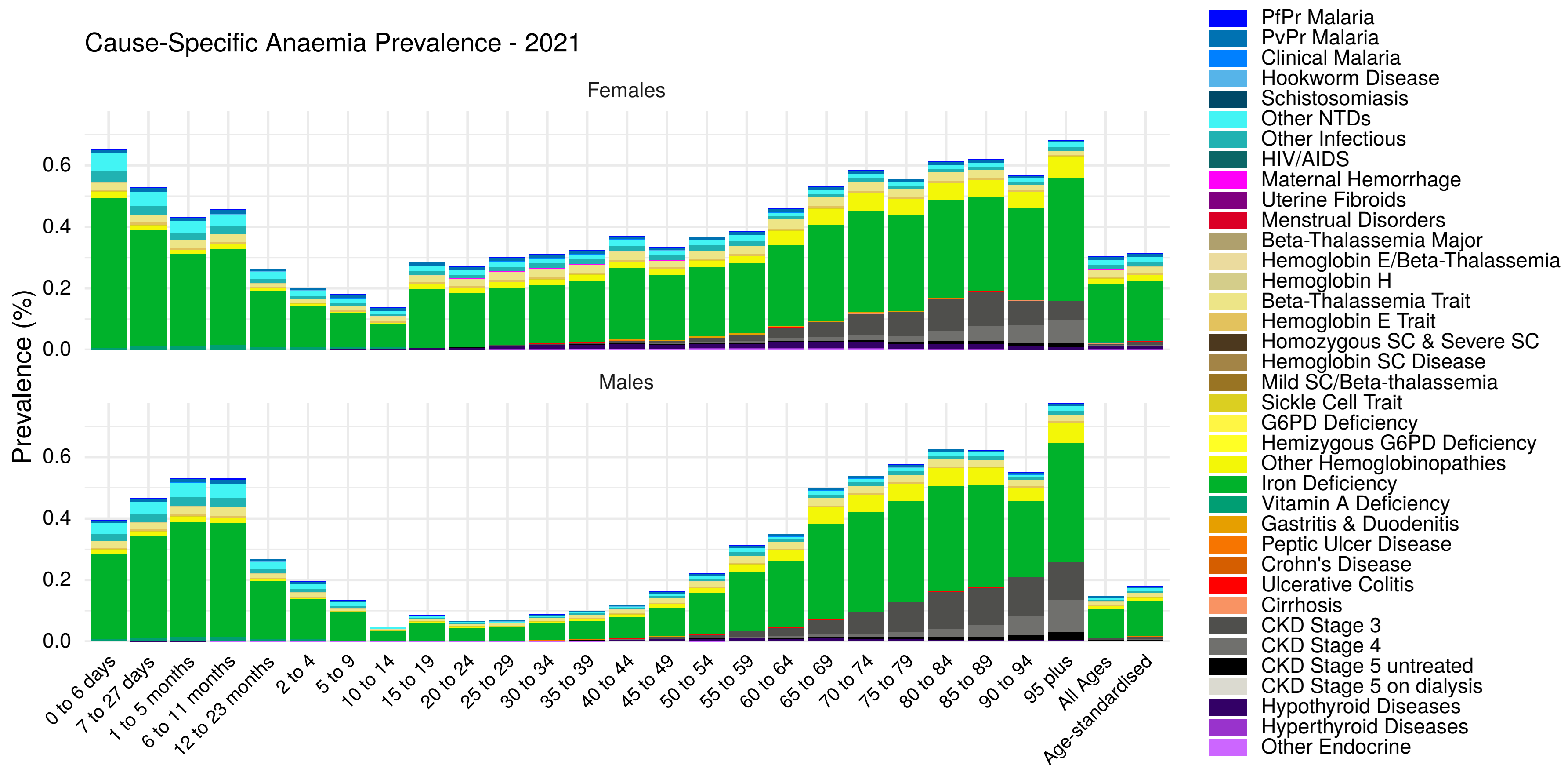

Cause-Specific Anaemia Prevalence - 2021
Females
Males
Prevalence (%)
0.0
0.2
0.4
0.6
0 to 6 days
7 to 27 days
1 to 5 months
6 to 11 months
12 to 23 months
2 to 4
5 to 9
10 to 14
15 to 19
20 to 24
25 to 29
30 to 34
35 to 39
40 to 44
45 to 49
50 to 54
55 to 59
60 to 64
65 to 69
70 to 74
75 to 79
80 to 84
85 to 89
90 to 94
95 plus
All Ages
Age-standardised
PfPr Malaria
PvPr Malaria
Clinical Malaria
Hookworm Disease
Schistosomiasis
Other NTDs
Other Infectious
HIV/AIDS
Maternal Hemorrhage
Uterine Fibroids
Menstrual Disorders
Beta-Thalassemia Major
Hemoglobin E/Beta-Thalassemia
Hemoglobin H
Beta-Thalassemia Trait
Hemoglobin E Trait
Homozygous SC & Severe SC
Hemoglobin SC Disease
Mild SC/Beta-thalassemia
Sickle Cell Trait
G6PD Deficiency
Hemizygous G6PD Deficiency
Other Hemoglobinopathies
Iron Deficiency
Vitamin A Deficiency
Gastritis & Duodenitis
Peptic Ulcer Disease
Crohn's Disease
Ulcerative Colitis
Cirrhosis
CKD Stage 3
CKD Stage 4
CKD Stage 5 untreated
CKD Stage 5 on dialysis
Hypothyroid Diseases
Hyperthyroid Diseases
Other Endocrine

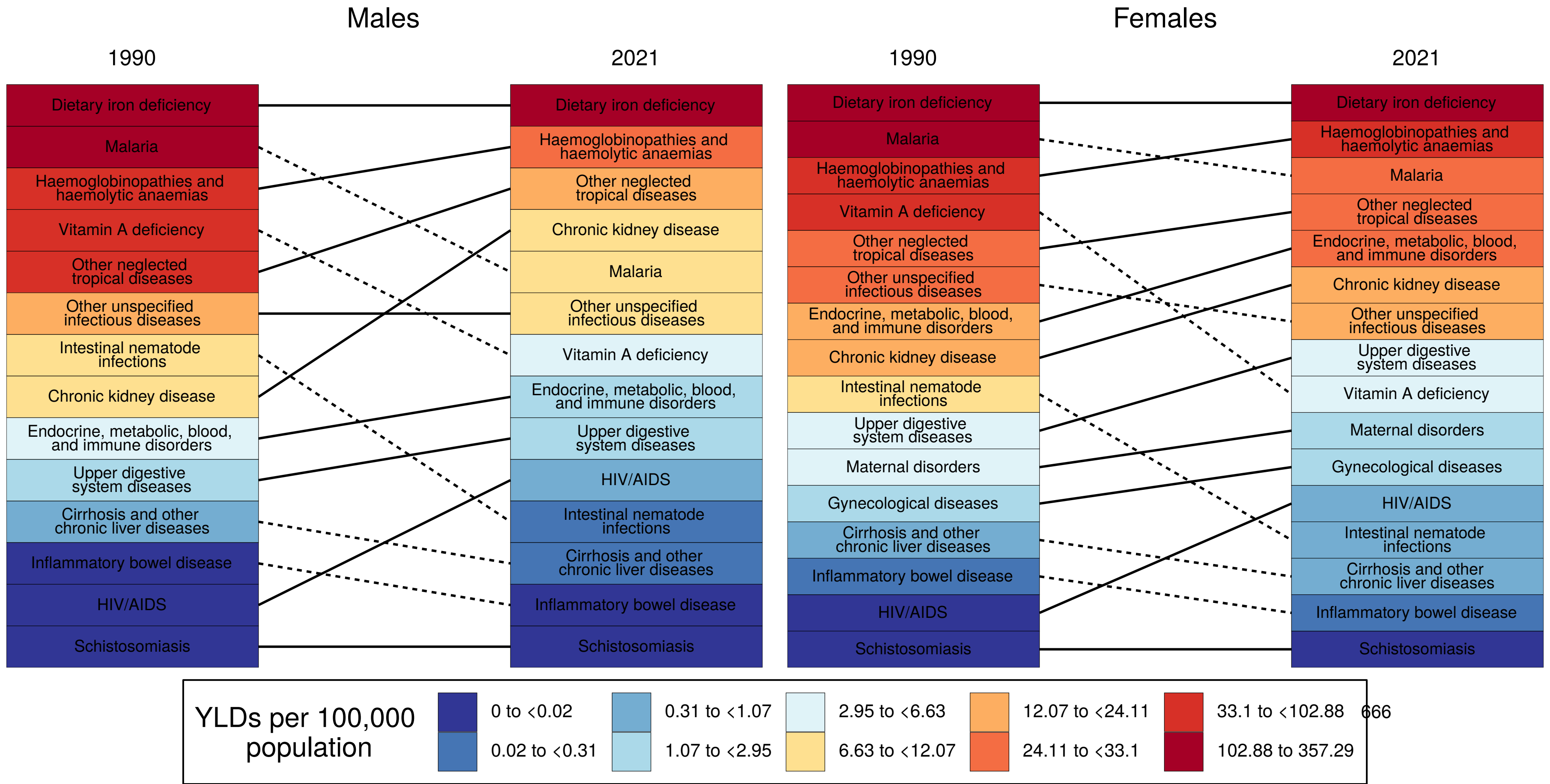

Males
Females
1990
2021
Dietary iron deficiency
Malaria
Haemoglobinopathies and haemolytic anaemias
Vitamin A deficiency
Other neglected tropical diseases
Other unspecified infectious diseases
Intestinal nematode infections
Chronic kidney disease
Endocrine, metabolic, blood, and immune disorders
Upper digestive system diseases
Cirrhosis and other chronic liver diseases
Inflammatory bowel disease
HIV/AIDS
Schistosomiasis
Maternal disorders
Gynecological diseases
YLDs per 100,000 population
0 to <0.02
0.02 to <0.31
0.31 to <1.07
1.07 to <2.95
2.95 to <6.63
6.63 to <12.07
12.07 to <24.11
24.11 to <33.1
33.1 to <102.88
102.88 to 357.29

Riau

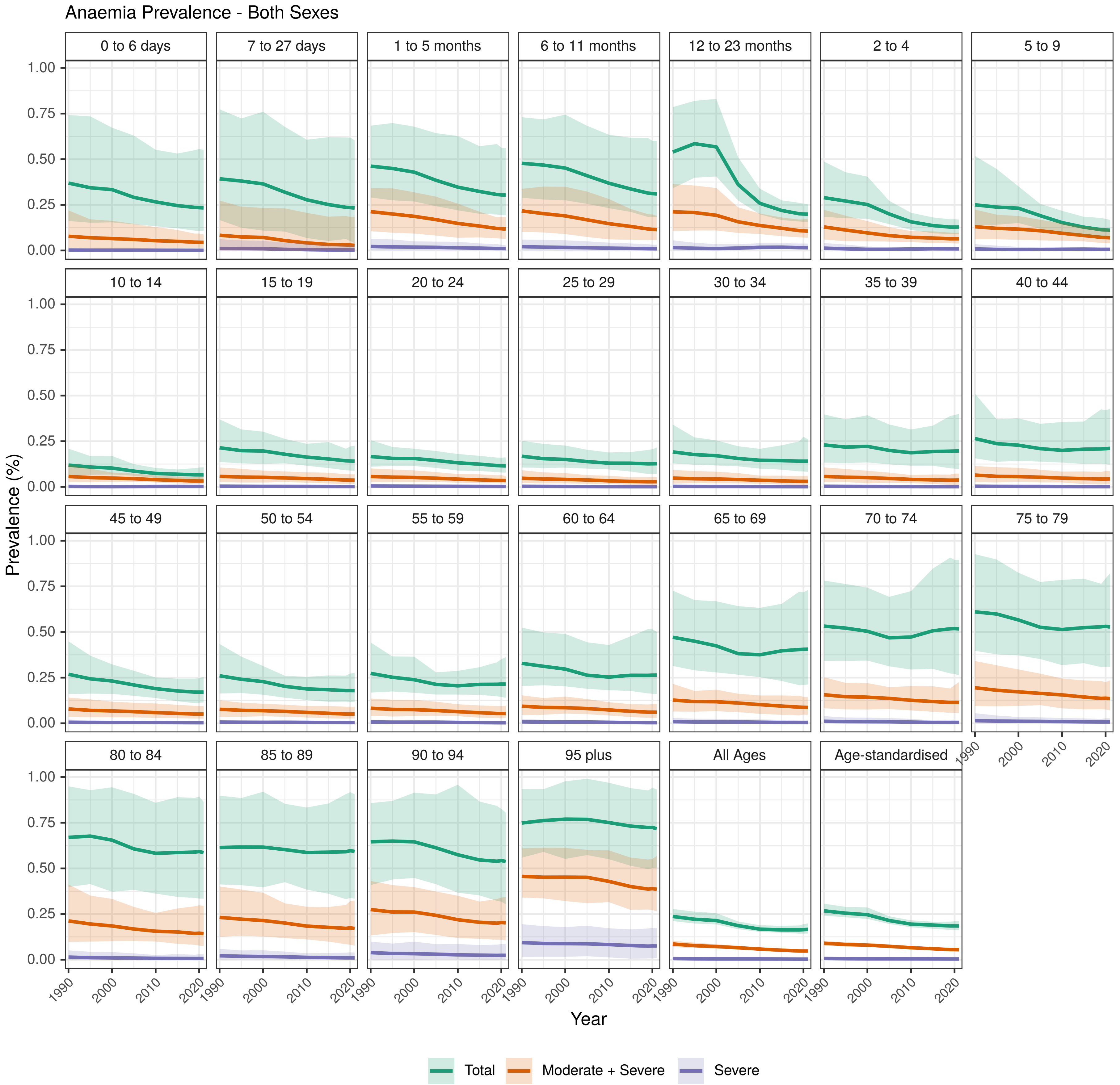
Anaemia Prevalence - Both Sexes
0 to 6 days
7 to 27 days
1 to 5 months
6 to 11 months
12 to 23 months
2 to 4
5 to 9
10 to 14
15 to 19
20 to 24
25 to 29
30 to 34
35 to 39
40 to 44
45 to 49
50 to 54
55 to 59
60 to 64
65 to 69
70 to 74
75 to 79
80 to 84
85 to 89
90 to 94
95 plus
All Ages
Age-standardised
Prevalence (%)
Year
Total
Moderate + Severe
Severe

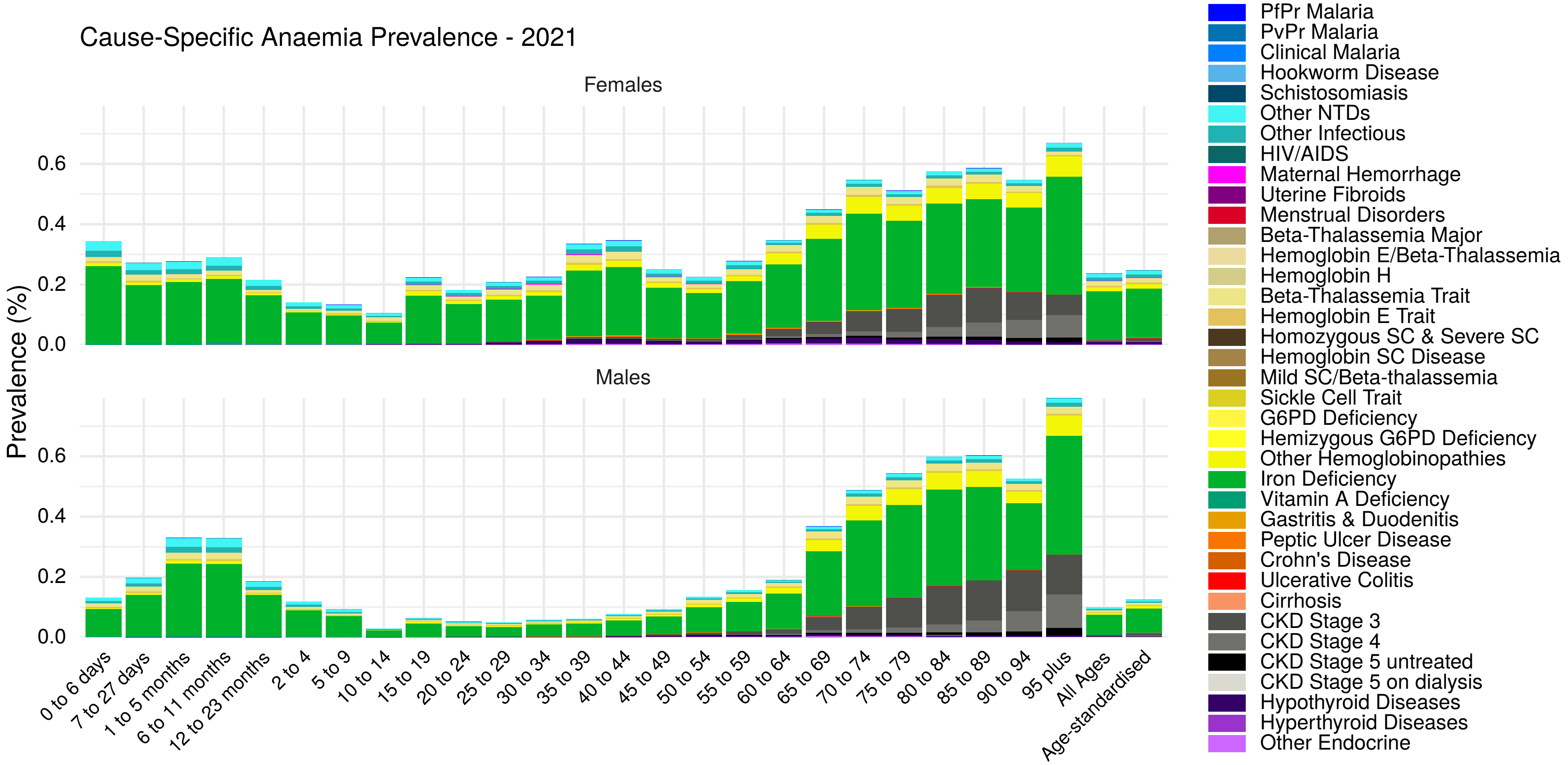
Cause-Specific Anaemia Prevalence - 2021
Females
Males
Prevalence (%)
PfPr Malaria
PvPr Malaria
Clinical Malaria
Hookworm Disease
Schistosomiasis
Other NTDs
Other Infectious
HIV/AIDS
Maternal Hemorrhage
Uterine Fibroids
Menstrual Disorders
Beta-Thalassemia Major
Hemoglobin E/Beta-Thalassemia
Hemoglobin H
Beta-Thalassemia Trait
Hemoglobin E Trait
Homozygous SC & Severe SC
Hemoglobin SC Disease
Mild SC/Beta-thalassemia
Sickle Cell Trait
G6PD Deficiency
Hemizygous G6PD Deficiency
Other Hemoglobinopathies
Iron Deficiency
Vitamin A Deficiency
Gastritis & Duodenitis
Peptic Ulcer Disease
Crohn's Disease
Ulcerative Colitis
Cirrhosis
CKD Stage 3
CKD Stage 4
CKD Stage 5 untreated
CKD Stage 5 on dialysis
Hypothyroid Diseases
Hyperthyroid Diseases
Other Endocrine

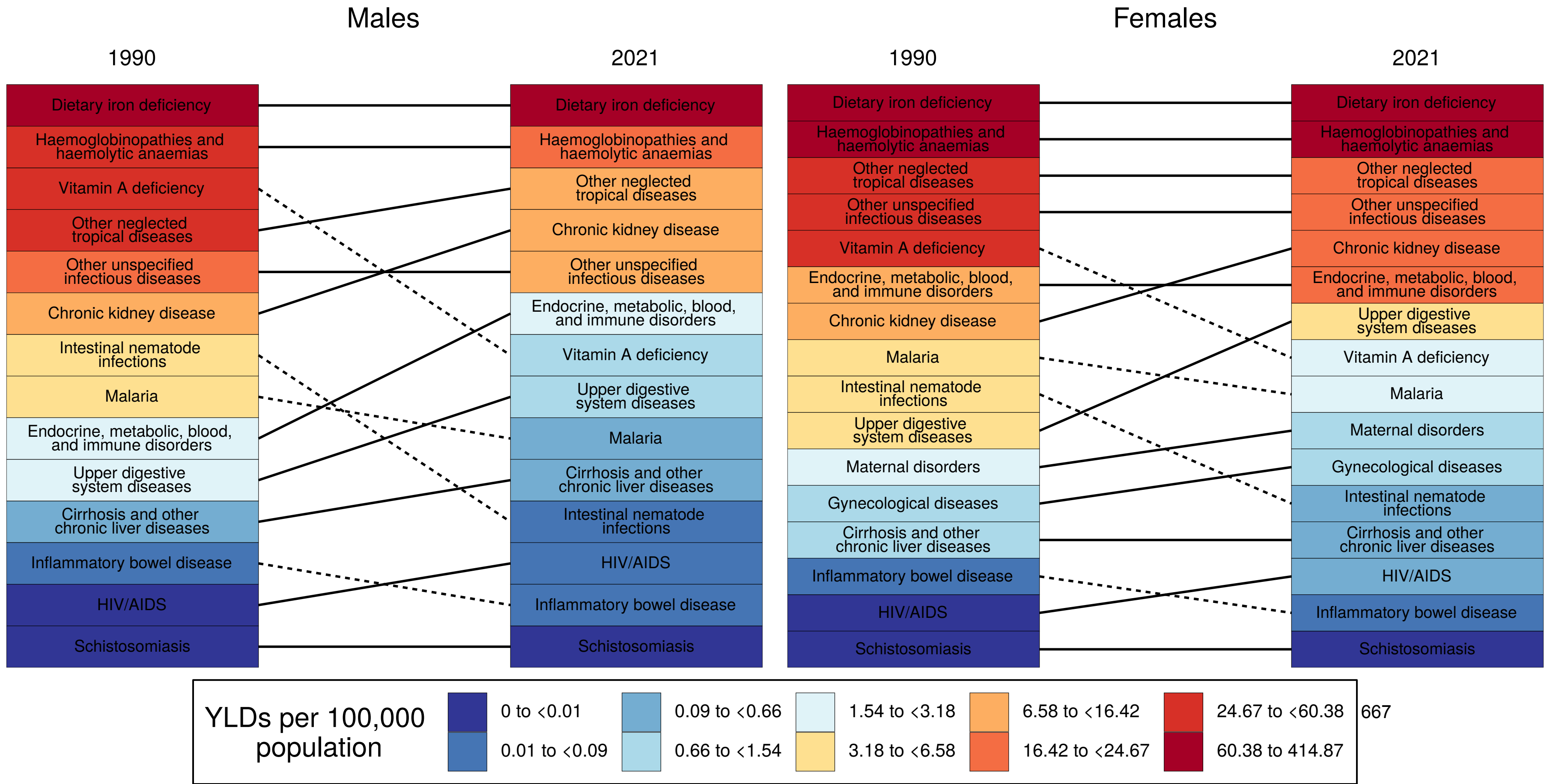
Males
Females
1990
2021
Dietary iron deficiency
Haemoglobinopathies and haemolytic anaemias
Vitamin A deficiency
Other neglected tropical diseases
Other unspecified infectious diseases
Chronic kidney disease
Intestinal nematode infections
Malaria
Endocrine, metabolic, blood, and immune disorders
Upper digestive system diseases
Cirrhosis and other chronic liver diseases
Inflammatory bowel disease
HIV/AIDS
Schistosomiasis
Maternal disorders
Gynecological diseases
YLDs per 100,000 population
0 to <0.01
0.01 to <0.09
0.09 to <0.66
0.66 to <1.54
1.54 to <3.18
3.18 to <6.58
6.58 to <16.42
16.42 to <24.67
24.67 to <60.38
60.38 to 414.87

667

West Sulawesi

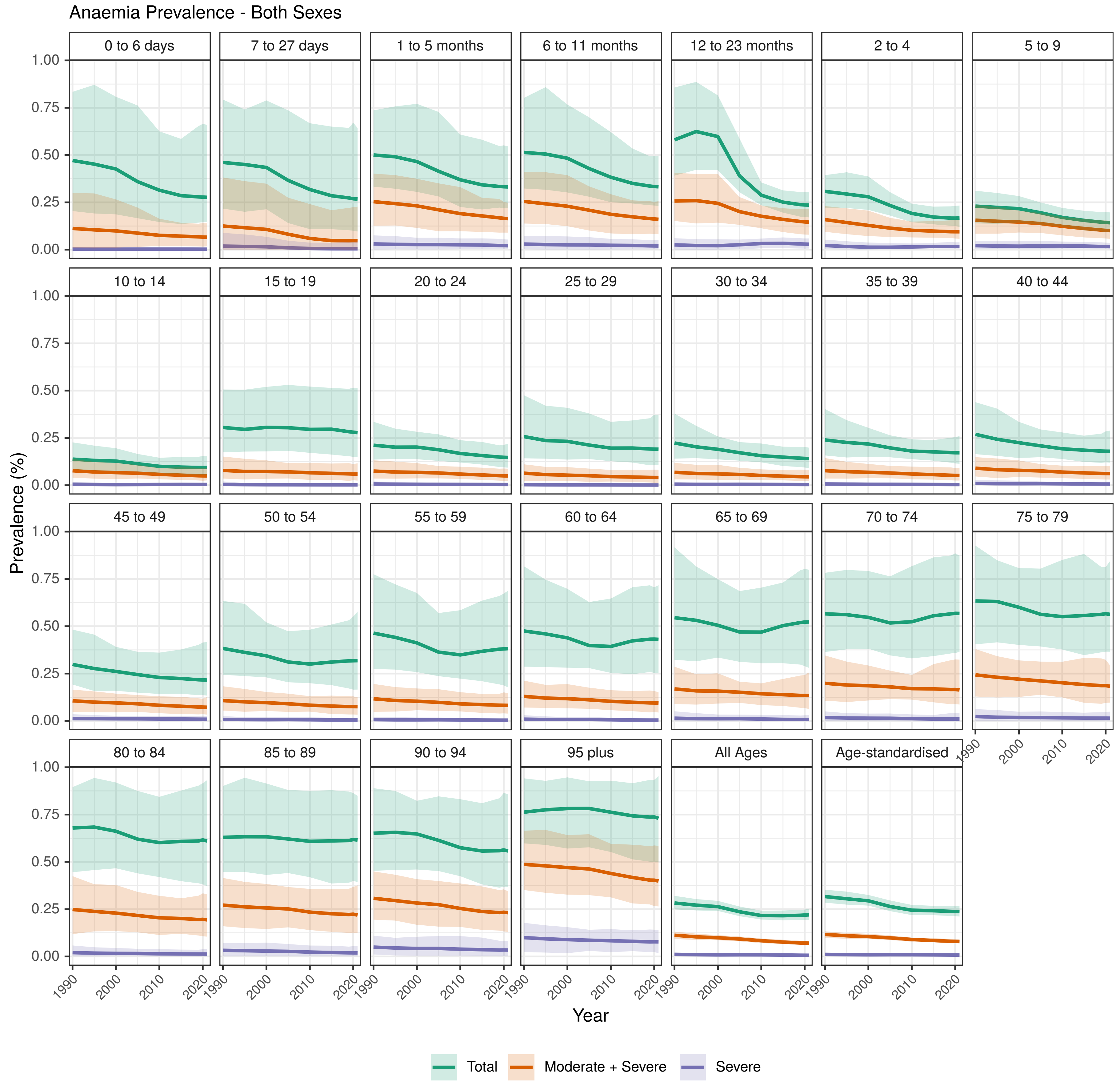

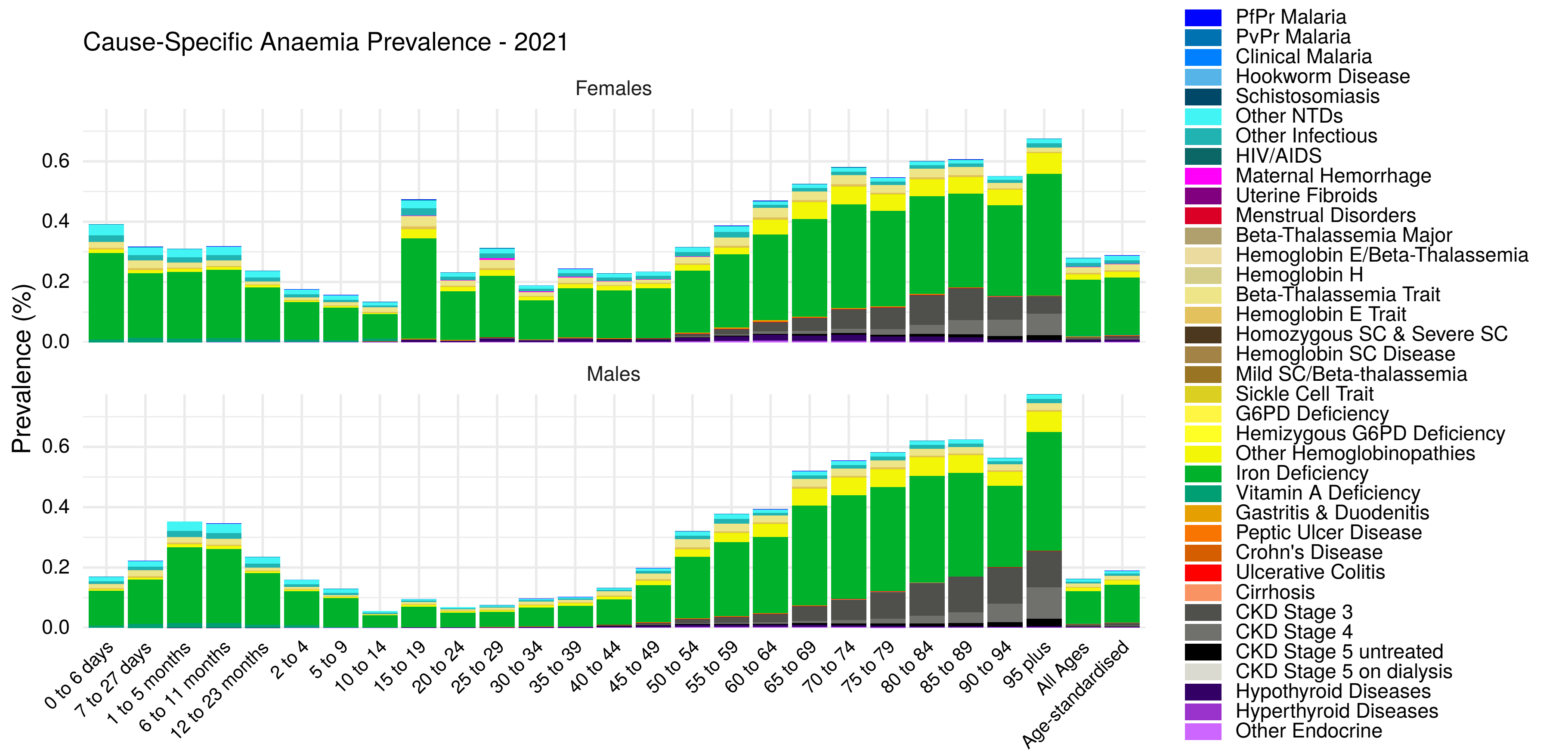

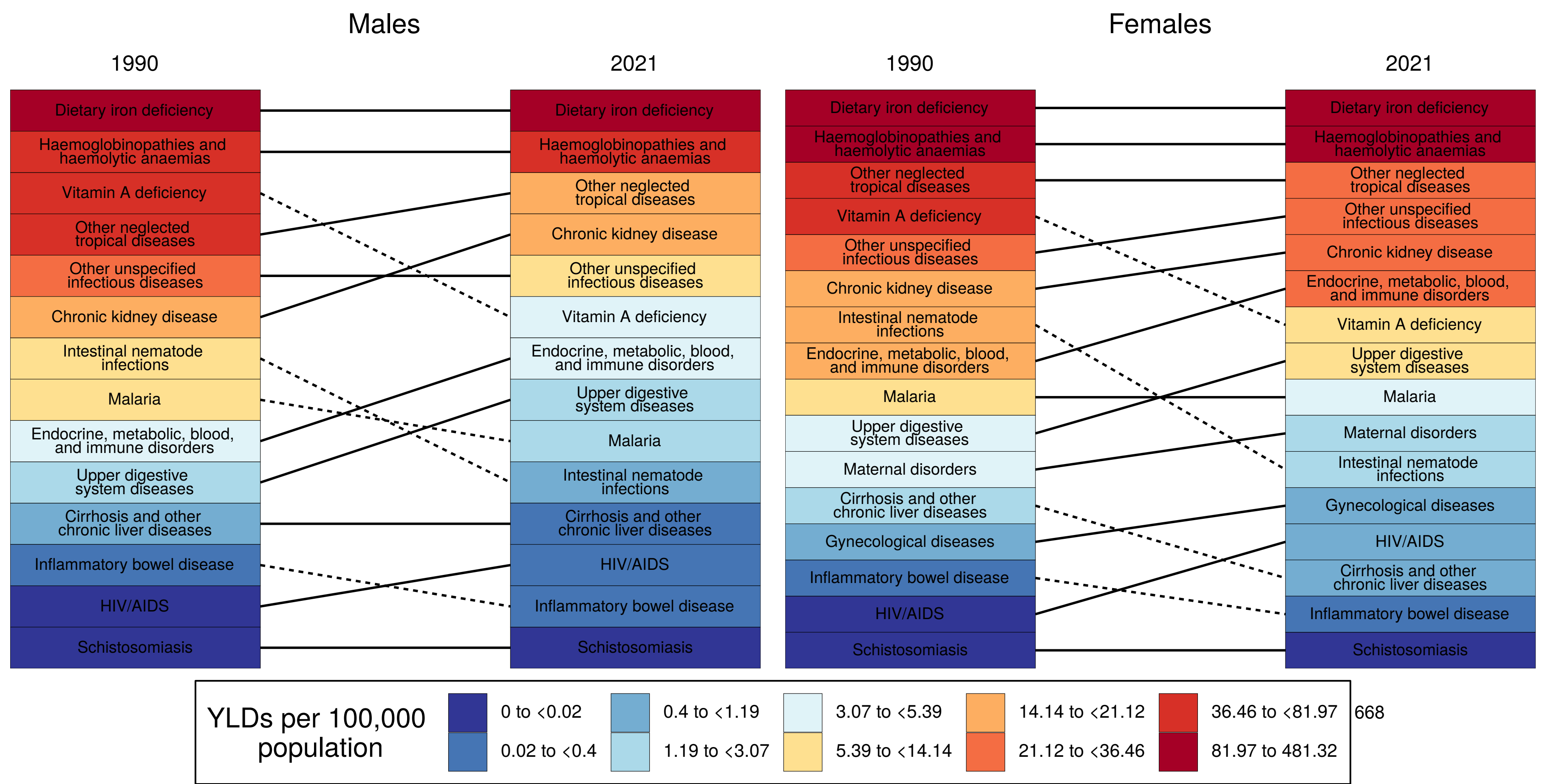

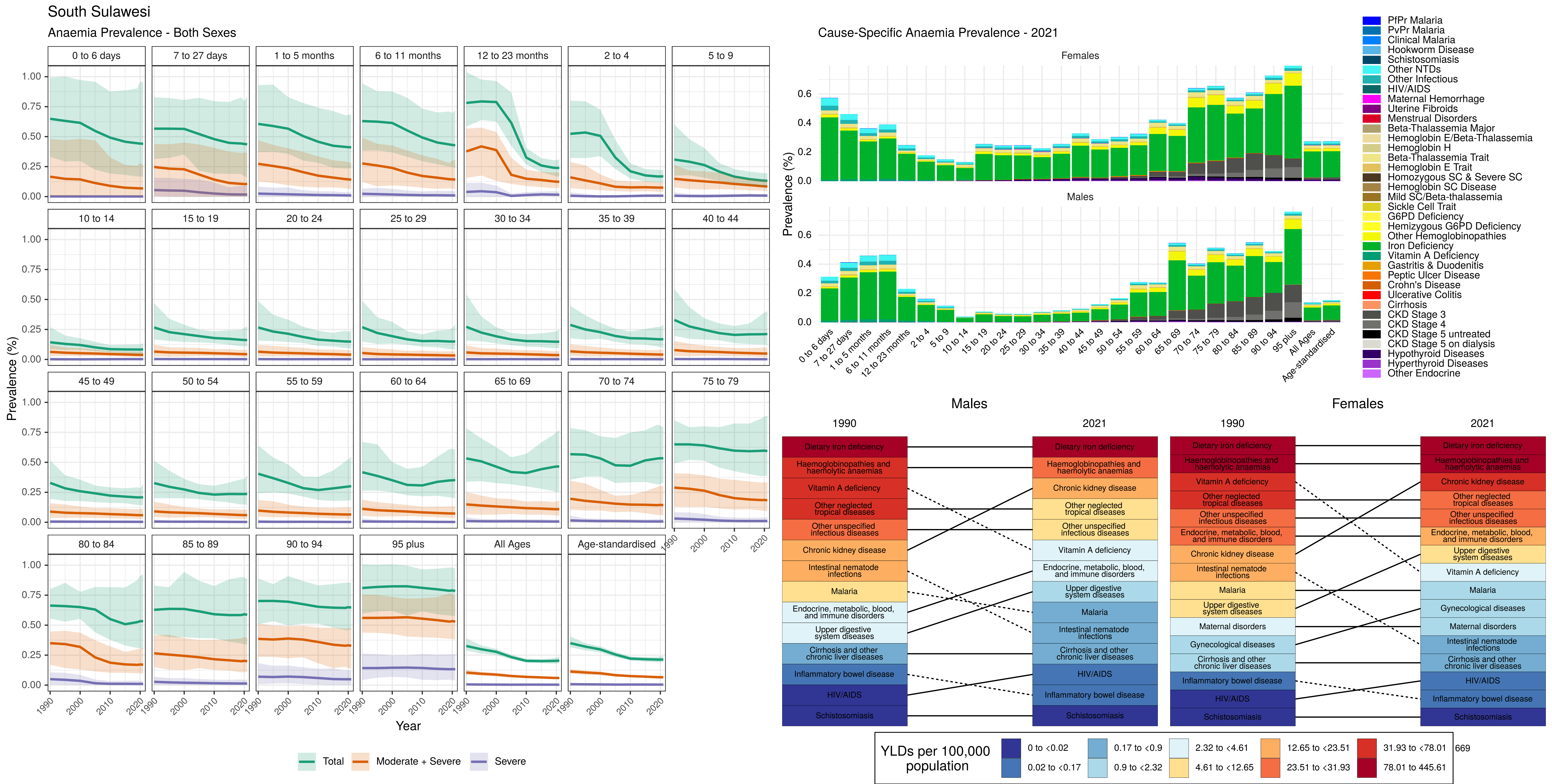
South Sulawesi
Anaemia Prevalence - Both Sexes
0 to 6 days
7 to 27 days
1 to 5 months
6 to 11 months
12 to 23 months
2 to 4
5 to 9
10 to 14
15 to 19
20 to 24
25 to 29
30 to 34
35 to 39
40 to 44
45 to 49
50 to 54
55 to 59
60 to 64
65 to 69
70 to 74
75 to 79
80 to 84
85 to 89
90 to 94
95 plus
All Ages
Age-standardised
Prevalence (%)
Year
Total
Moderate + Severe
Severe
Cause-Specific Anaemia Prevalence - 2021
Females
Males
PfPr Malaria
PvPr Malaria
Clinical Malaria
Hookworm Disease
Schistosomiasis
Other NTDs
Other Infectious
HIV/AIDS
Maternal Hemorrhage
Uterine Fibroids
Menstrual Disorders
Beta-Thalassemia Major
Hemoglobin E/Beta-Thalassemia
Hemoglobin H
Beta-Thalassemia Trait
Hemoglobin E Trait
Homozygous SC & Severe SC
Hemoglobin SC Disease
Mild SC/Beta-thalassemia
Sickle Cell Trait
G6PD Deficiency
Hemizygous G6PD Deficiency
Other Hemoglobinopathies
Iron Deficiency
Vitamin A Deficiency
Gastritis & Duodenitis
Peptic Ulcer Disease
Crohn's Disease
Ulcerative Colitis
Cirrhosis
CKD Stage 3
CKD Stage 4
CKD Stage 5 untreated
CKD Stage 5 on dialysis
Hypothyroid Diseases
Hyperthyroid Diseases
Other Endocrine
Males
Females
1990
2021
Dietary iron deficiency
Haemoglobinopathies and haemolytic anaemias
Vitamin A deficiency
Other neglected tropical diseases
Other unspecified infectious diseases
Chronic kidney disease
Intestinal nematode infections
Malaria
Endocrine, metabolic, blood, and immune disorders
Upper digestive system diseases
Cirrhosis and other chronic liver diseases
Inflammatory bowel disease
HIV/AIDS
Schistosomiasis
Maternal disorders
Gynecological diseases
YLDs per 100,000 population
0 to <0.02
0.02 to <0.17
0.17 to <0.9
0.9 to <2.32
2.32 to <4.61
4.61 to <12.65
12.65 to <23.51
23.51 to <31.93
31.93 to <78.01
78.01 to 445.61

669

Central Sulawesi

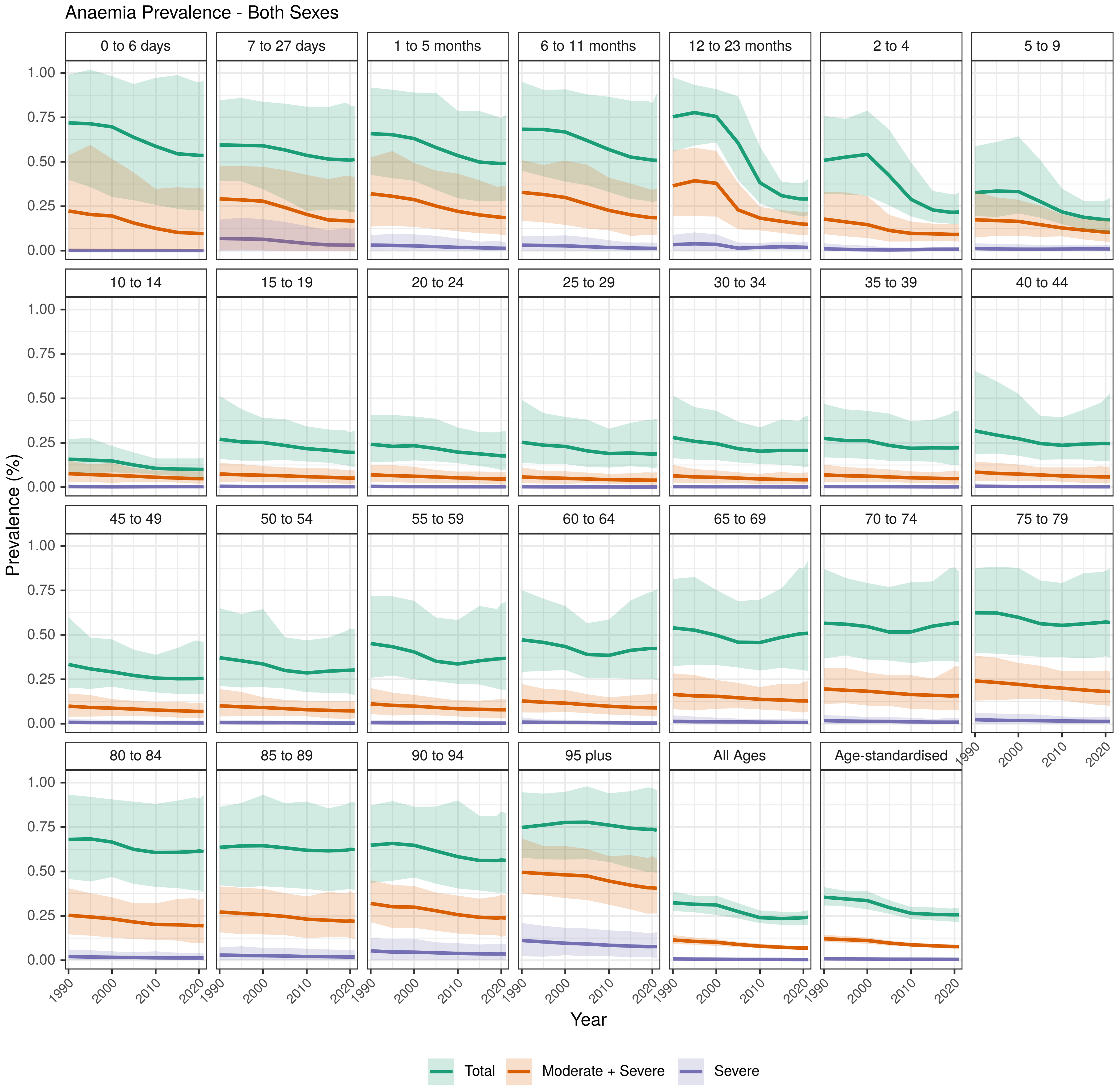

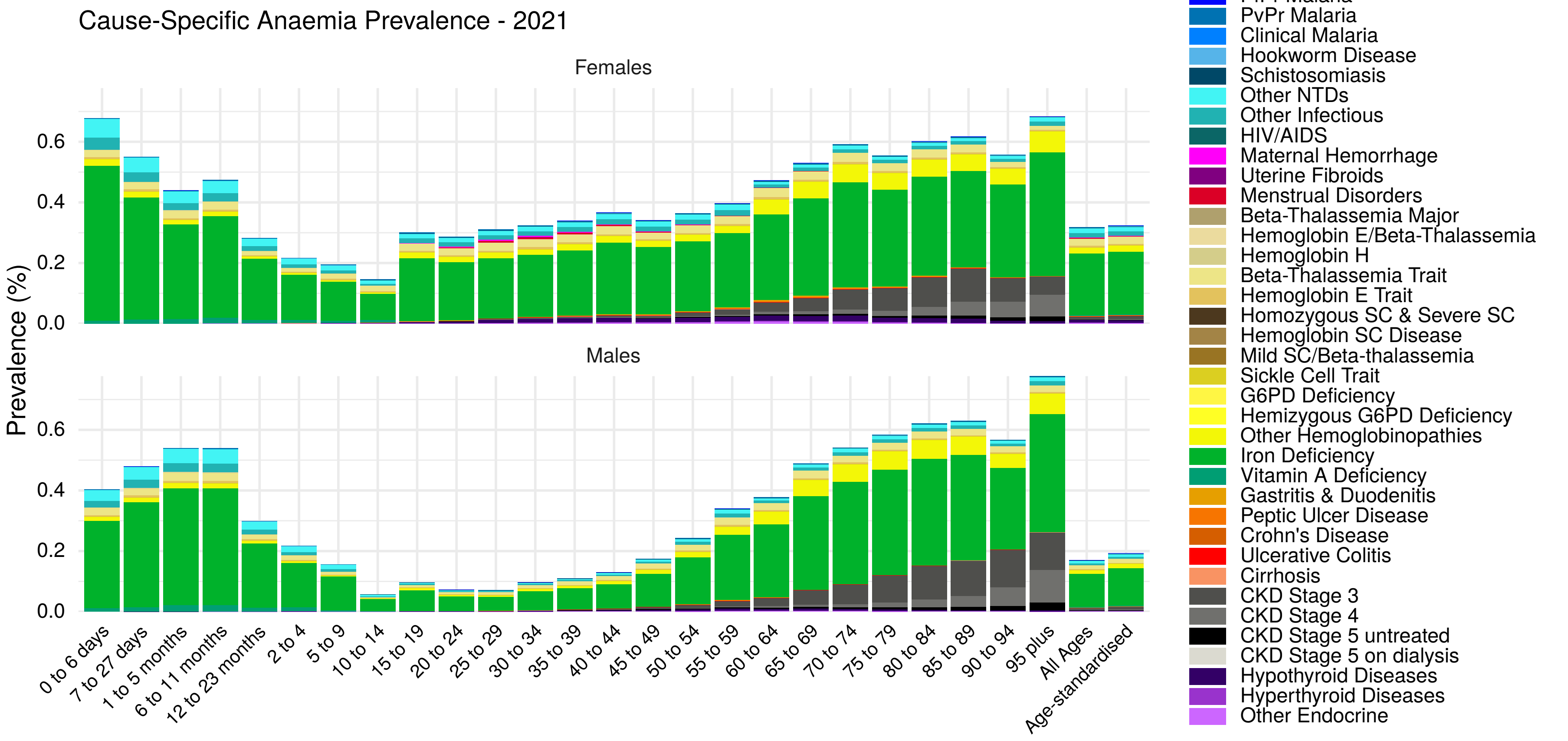

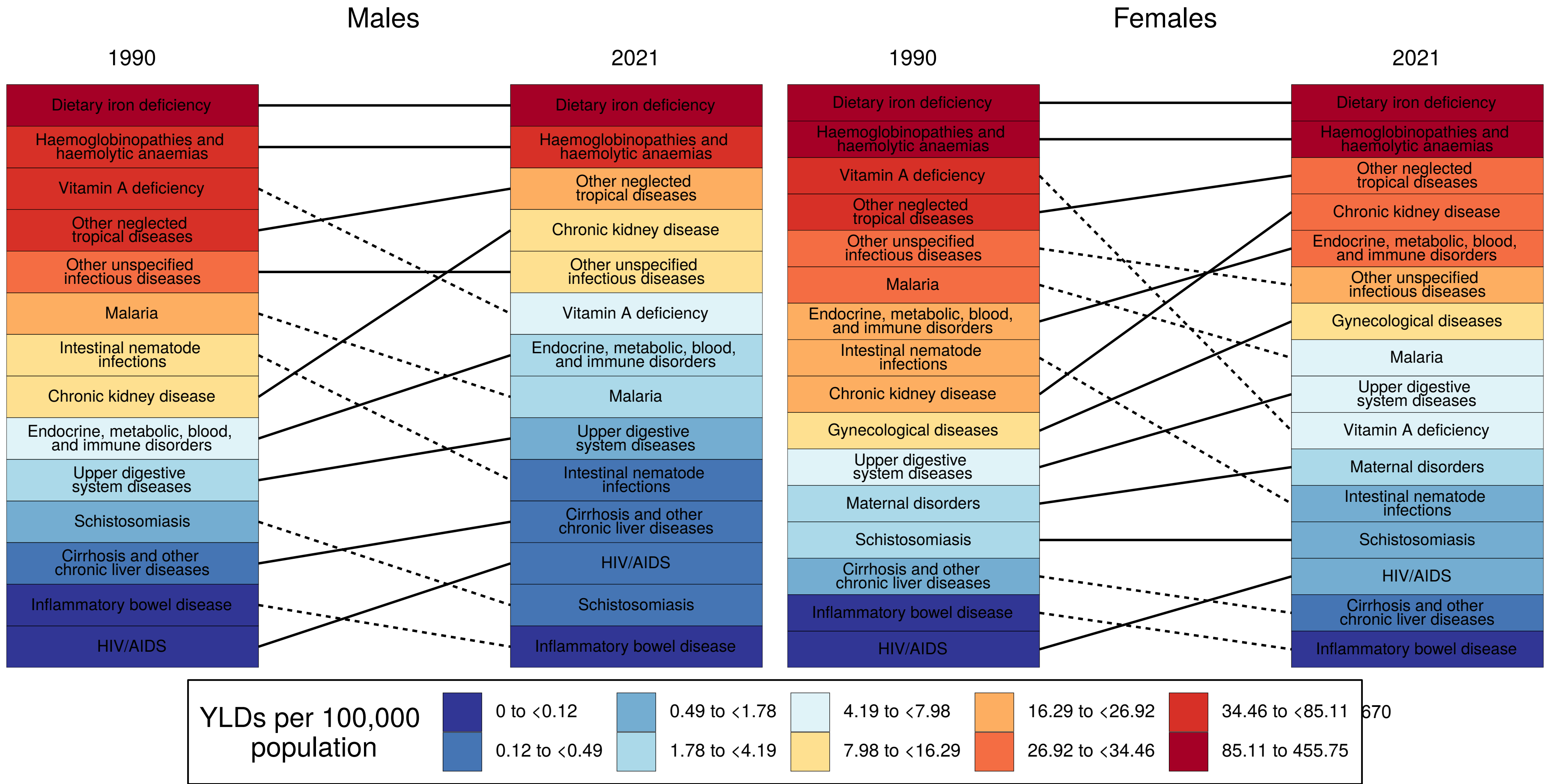

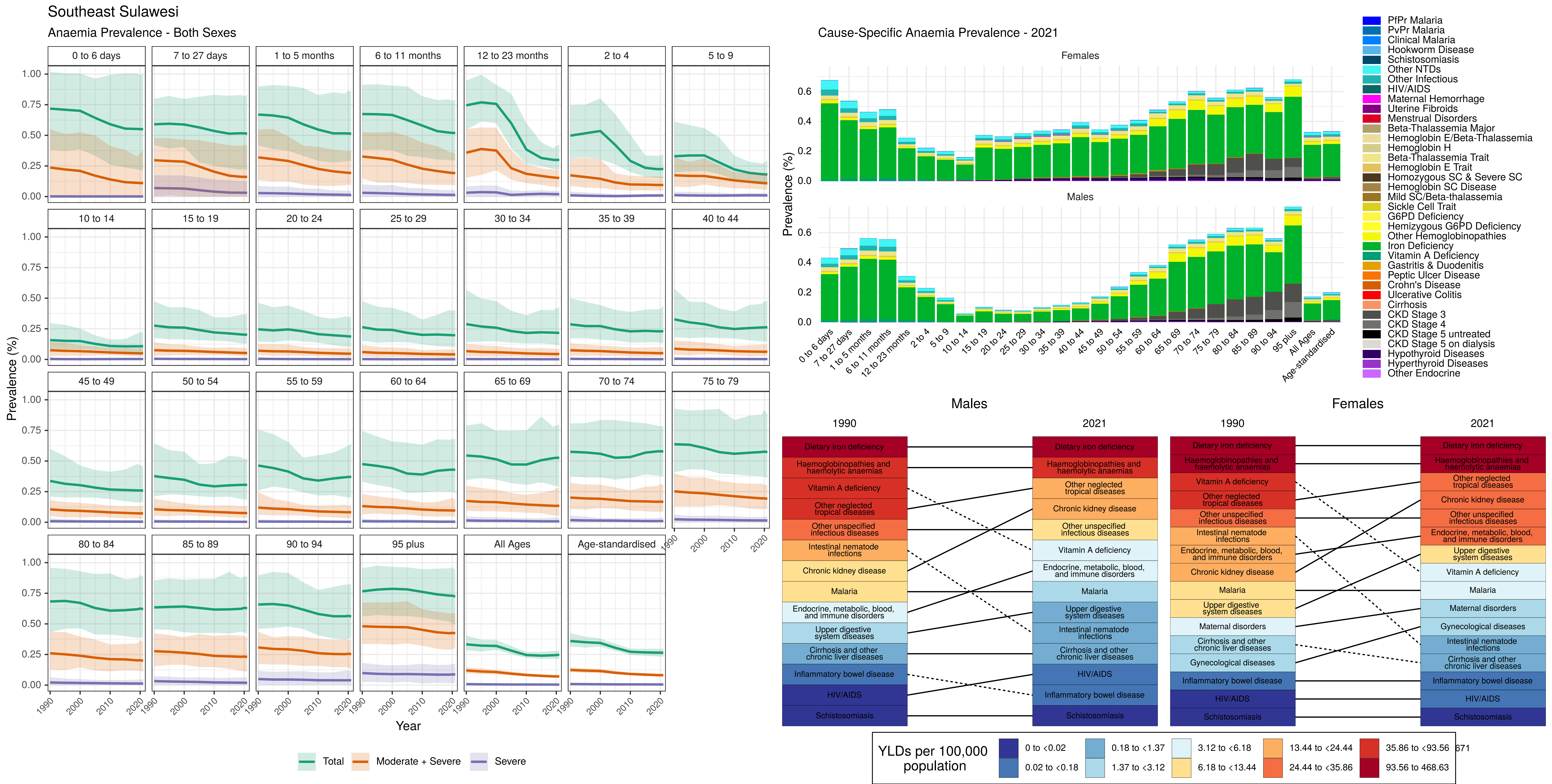

Southeast Sulawesi
Anaemia Prevalence - Both Sexes
0 to 6 days
7 to 27 days
1 to 5 months
6 to 11 months
12 to 23 months
2 to 4
5 to 9
10 to 14
15 to 19
20 to 24
25 to 29
30 to 34
35 to 39
40 to 44
45 to 49
50 to 54
55 to 59
60 to 64
65 to 69
70 to 74
75 to 79
80 to 84
85 to 89
90 to 94
95 plus
All Ages
Age-standardised
Prevalence (%)
Year
Total
Moderate + Severe
Severe
Cause-Specific Anaemia Prevalence - 2021
Females
Males
Prevalence (%)
PfPr Malaria
PvPr Malaria
Clinical Malaria
Hookworm Disease
Schistosomiasis
Other NTDs
Other Infectious
HIV/AIDS
Maternal Hemorrhage
Uterine Fibroids
Menstrual Disorders
Beta-Thalassemia Major
Hemoglobin E/Beta-Thalassemia
Hemoglobin H
Beta-Thalassemia Trait
Hemoglobin E Trait
Homozygous SC & Severe SC
Hemoglobin SC Disease
Mild SC/Beta-thalassemia
Sickle Cell Trait
G6PD Deficiency
Hemizygous G6PD Deficiency
Other Hemoglobinopathies
Iron Deficiency
Vitamin A Deficiency
Gastritis & Duodenitis
Peptic Ulcer Disease
Crohn's Disease
Ulcerative Colitis
Cirrhosis
CKD Stage 3
CKD Stage 4
CKD Stage 5 untreated
CKD Stage 5 on dialysis
Hypothyroid Diseases
Hyperthyroid Diseases
Other Endocrine
Males
Females
1990
2021
Dietary iron deficiency
Haemoglobinopathies and haemolytic anaemias
Vitamin A deficiency
Other neglected tropical diseases
Other unspecified infectious diseases
Intestinal nematode infections
Chronic kidney disease
Malaria
Endocrine, metabolic, blood, and immune disorders
Upper digestive system diseases
Cirrhosis and other chronic liver diseases
Inflammatory bowel disease
HIV/AIDS
Schistosomiasis
Maternal disorders
Gynecological diseases
YLDs per 100,000 population
0 to <0.02
0.02 to <0.18
0.18 to <1.37
1.37 to <3.12
3.12 to <6.18
6.18 to <13.44
13.44 to <24.44
24.44 to <35.86
35.86 to <93.56
93.56 to 468.63
671

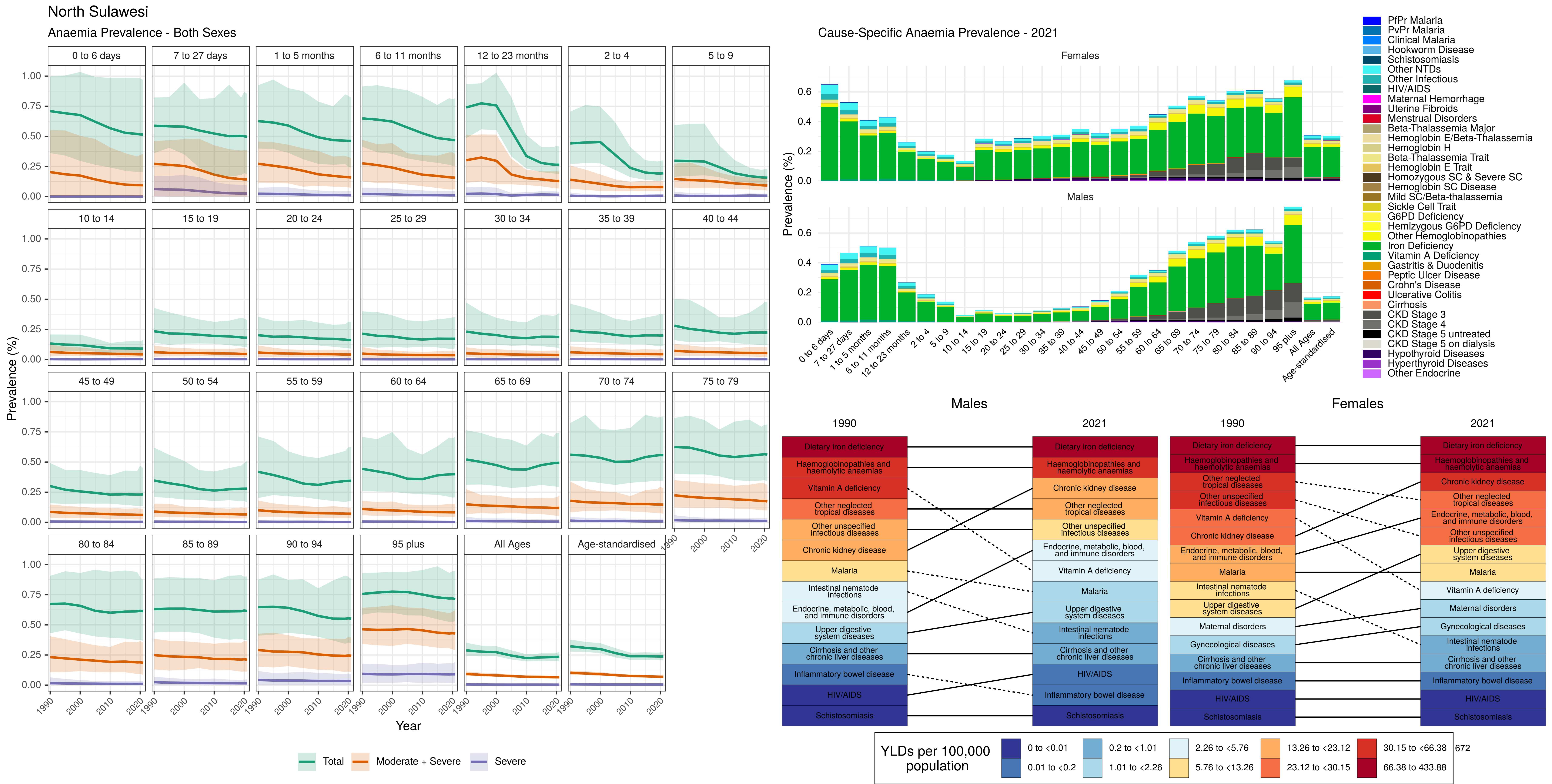

North Sulawesi
Anaemia Prevalence - Both Sexes
0 to 6 days
7 to 27 days
1 to 5 months
6 to 11 months
12 to 23 months
2 to 4
5 to 9
10 to 14
15 to 19
20 to 24
25 to 29
30 to 34
35 to 39
40 to 44
45 to 49
50 to 54
55 to 59
60 to 64
65 to 69
70 to 74
75 to 79
80 to 84
85 to 89
90 to 94
95 plus
All Ages
Age-standardised
Prevalence (%)
Year
Total
Moderate + Severe
Severe
Cause-Specific Anaemia Prevalence - 2021
Females
Males
PfPr Malaria
PvPr Malaria
Clinical Malaria
Hookworm Disease
Schistosomiasis
Other NTDs
Other Infectious
HIV/AIDS
Maternal Hemorrhage
Uterine Fibroids
Menstrual Disorders
Beta-Thalassemia Major
Hemoglobin E/Beta-Thalassemia
Hemoglobin H
Beta-Thalassemia Trait
Hemoglobin E Trait
Homozygous SC & Severe SC
Hemoglobin SC Disease
Mild SC/Beta-thalassemia
Sickle Cell Trait
G6PD Deficiency
Hemizygous G6PD Deficiency
Other Hemoglobinopathies
Iron Deficiency
Vitamin A Deficiency
Gastritis & Duodenitis
Peptic Ulcer Disease
Crohn's Disease
Cirrhosis
Ulcerative Colitis
CKD Stage 3
CKD Stage 4
CKD Stage 5 untreated
CKD Stage 5 on dialysis
Hypothyroid Diseases
Hyperthyroid Diseases
Other Endocrine
Males
Females
1990
2021
Dietary iron deficiency
Haemoglobinopathies and haemolytic anaemias
Vitamin A deficiency
Other neglected tropical diseases
Other unspecified infectious diseases
Chronic kidney disease
Malaria
Intestinal nematode infections
Endocrine, metabolic, blood, and immune disorders
Upper digestive system diseases
Cirrhosis and other chronic liver diseases
Inflammatory bowel disease
HIV/AIDS
Schistosomiasis
Maternal disorders
Gynecological diseases
YLDs per 100,000 population
0 to <0.01
0.01 to <0.2
0.2 to <1.01
1.01 to <2.26
2.26 to <5.76
5.76 to <13.26
13.26 to <23.12
23.12 to <30.15
30.15 to <66.38
66.38 to 433.88
672

West Sumatra

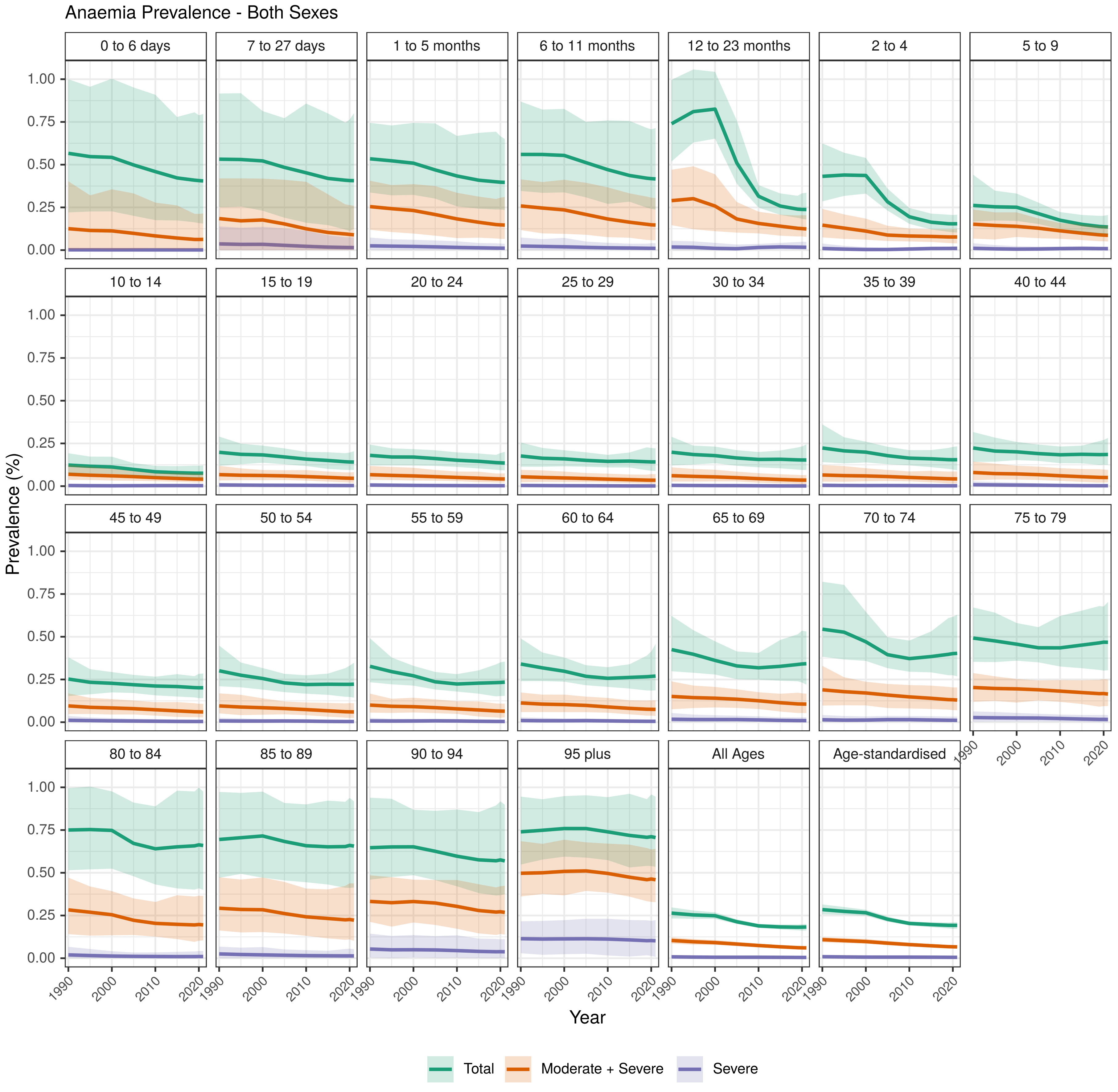

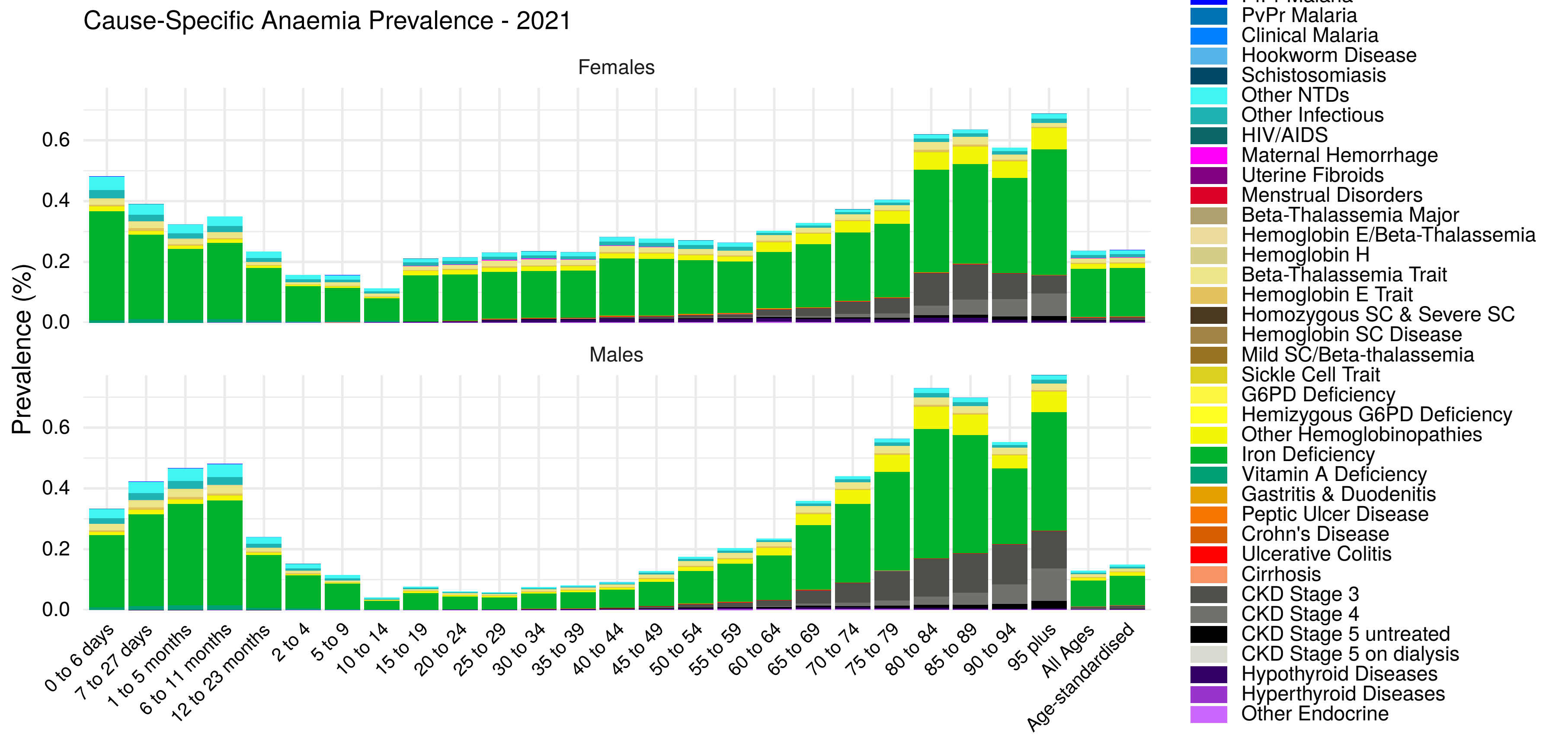

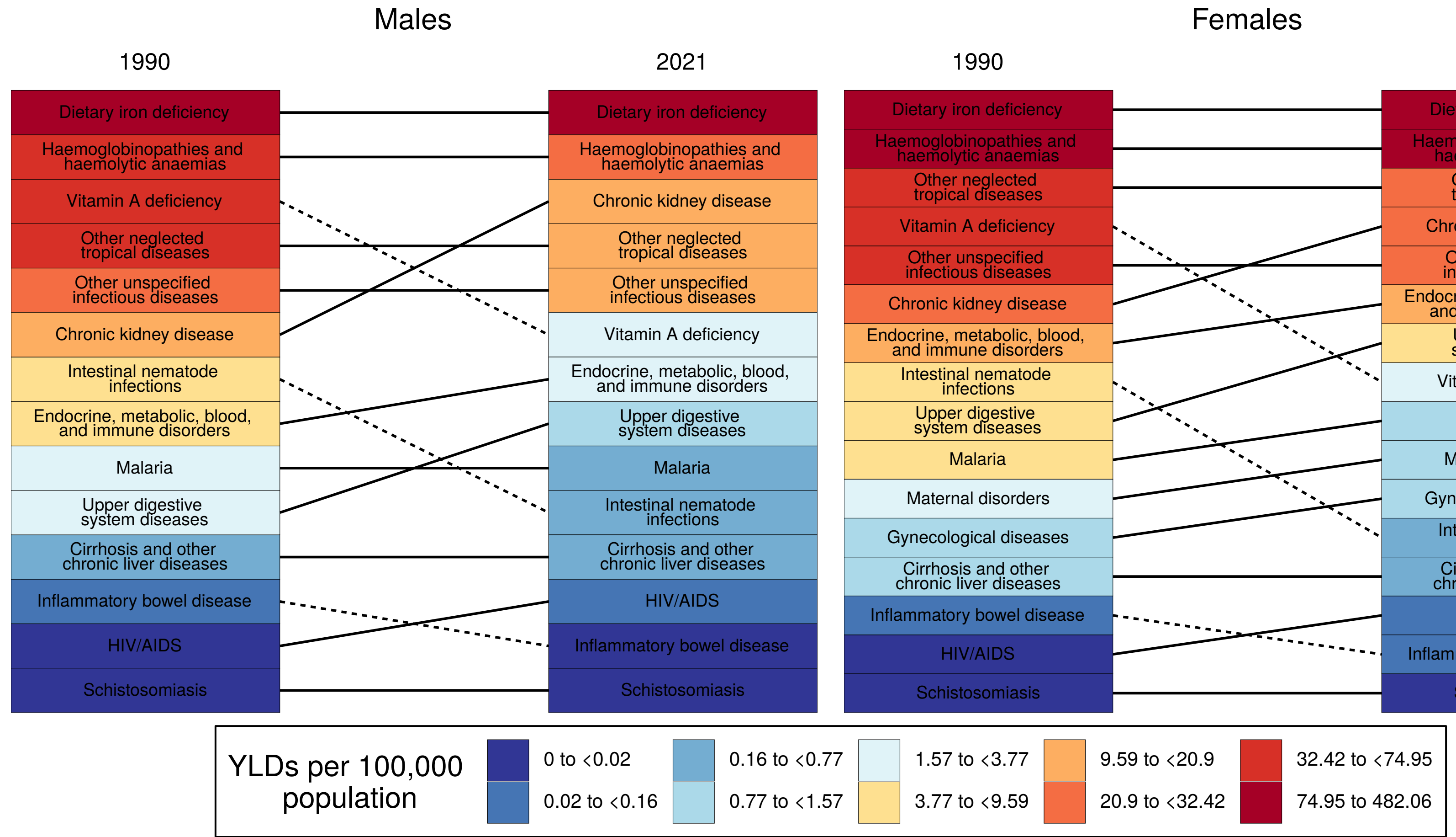

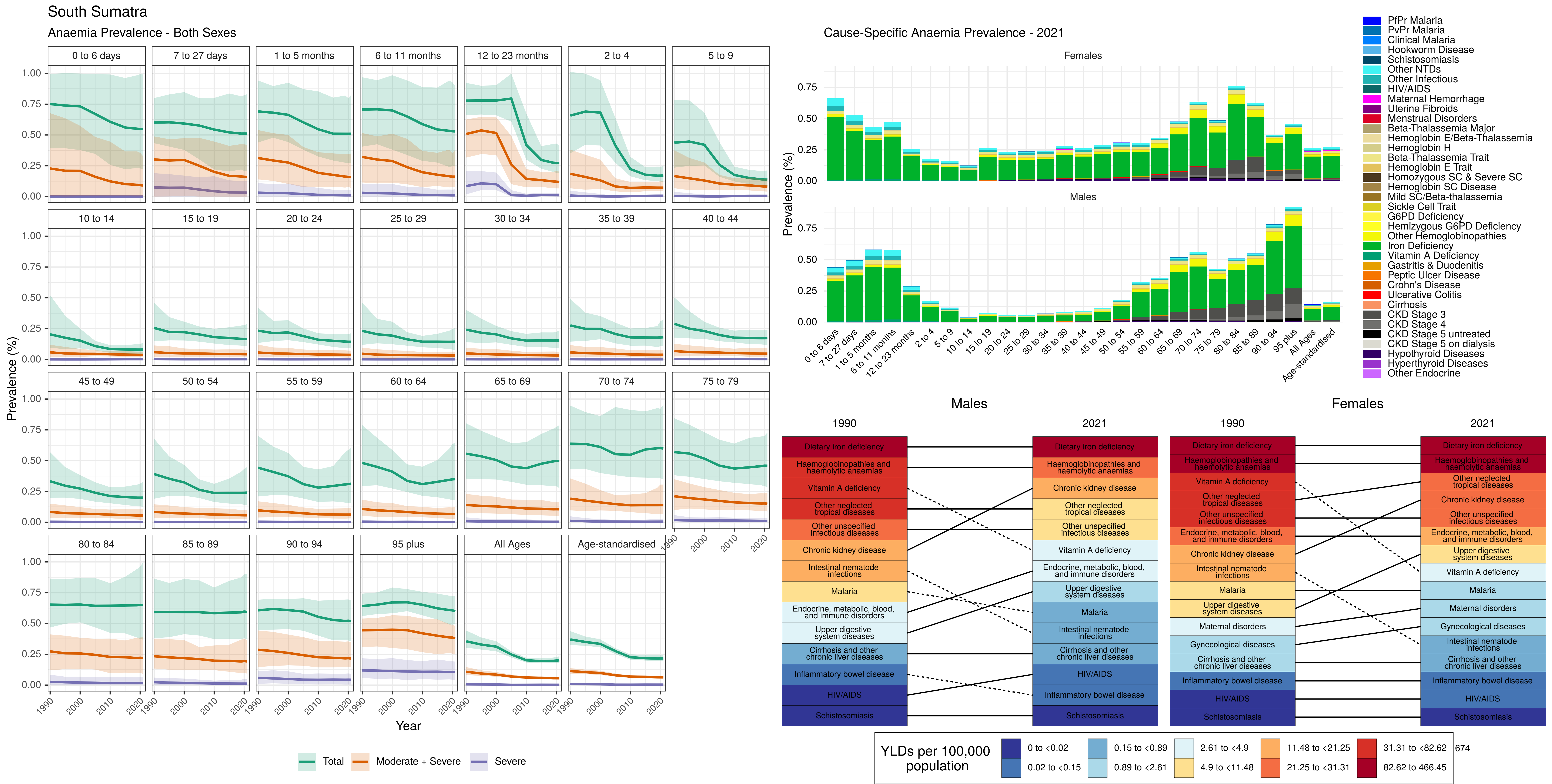
South Sumatra
Anaemia Prevalence - Both Sexes
0 to 6 days
7 to 27 days
1 to 5 months
6 to 11 months
12 to 23 months
2 to 4
5 to 9
10 to 14
15 to 19
20 to 24
25 to 29
30 to 34
35 to 39
40 to 44
45 to 49
50 to 54
55 to 59
60 to 64
65 to 69
70 to 74
75 to 79
80 to 84
85 to 89
90 to 94
95 plus
All Ages
Age-standardised
Prevalence (%)
Year
Total
Moderate + Severe
Severe
Cause-Specific Anaemia Prevalence - 2021
Females
Males
Prevalence (%)
PfPr Malaria
PvPr Malaria
Clinical Malaria
Hookworm Disease
Schistosomiasis
Other NTDs
Other Infectious
HIV/AIDS
Maternal Hemorrhage
Uterine Fibroids
Menstrual Disorders
Beta-Thalassemia Major
Hemoglobin E/Beta-Thalassemia
Hemoglobin H
Beta-Thalassemia Trait
Hemoglobin E Trait
Homozygous SC & Severe SC
Hemoglobin SC Disease
Mild SC/Beta-thalassemia
Sickle Cell Trait
G6PD Deficiency
Hemizygous G6PD Deficiency
Other Hemoglobinopathies
Iron Deficiency
Vitamin A Deficiency
Gastritis & Duodenitis
Peptic Ulcer Disease
Crohn's Disease
Ulcerative Colitis
Cirrhosis
CKD Stage 3
CKD Stage 4
CKD Stage 5 untreated
CKD Stage 5 on dialysis
Hypothyroid Diseases
Hyperthyroid Diseases
Other Endocrine
Males
Females
1990
2021
Dietary iron deficiency
Haemoglobinopathies and haemolytic anaemias
Vitamin A deficiency
Other neglected tropical diseases
Other unspecified infectious diseases
Chronic kidney disease
Intestinal nematode infections
Malaria
Endocrine, metabolic, blood, and immune disorders
Upper digestive system diseases
Cirrhosis and other chronic liver diseases
Inflammatory bowel disease
HIV/AIDS
Schistosomiasis
Maternal disorders
Gynecological diseases
YLDs per 100,000 population
0 to <0.02
0.02 to <0.15
0.15 to <0.89
0.89 to <2.61
2.61 to <4.9
4.9 to <11.48
11.48 to <21.25
21.25 to <31.31
31.31 to <82.62
82.62 to 466.45

North Sumatra

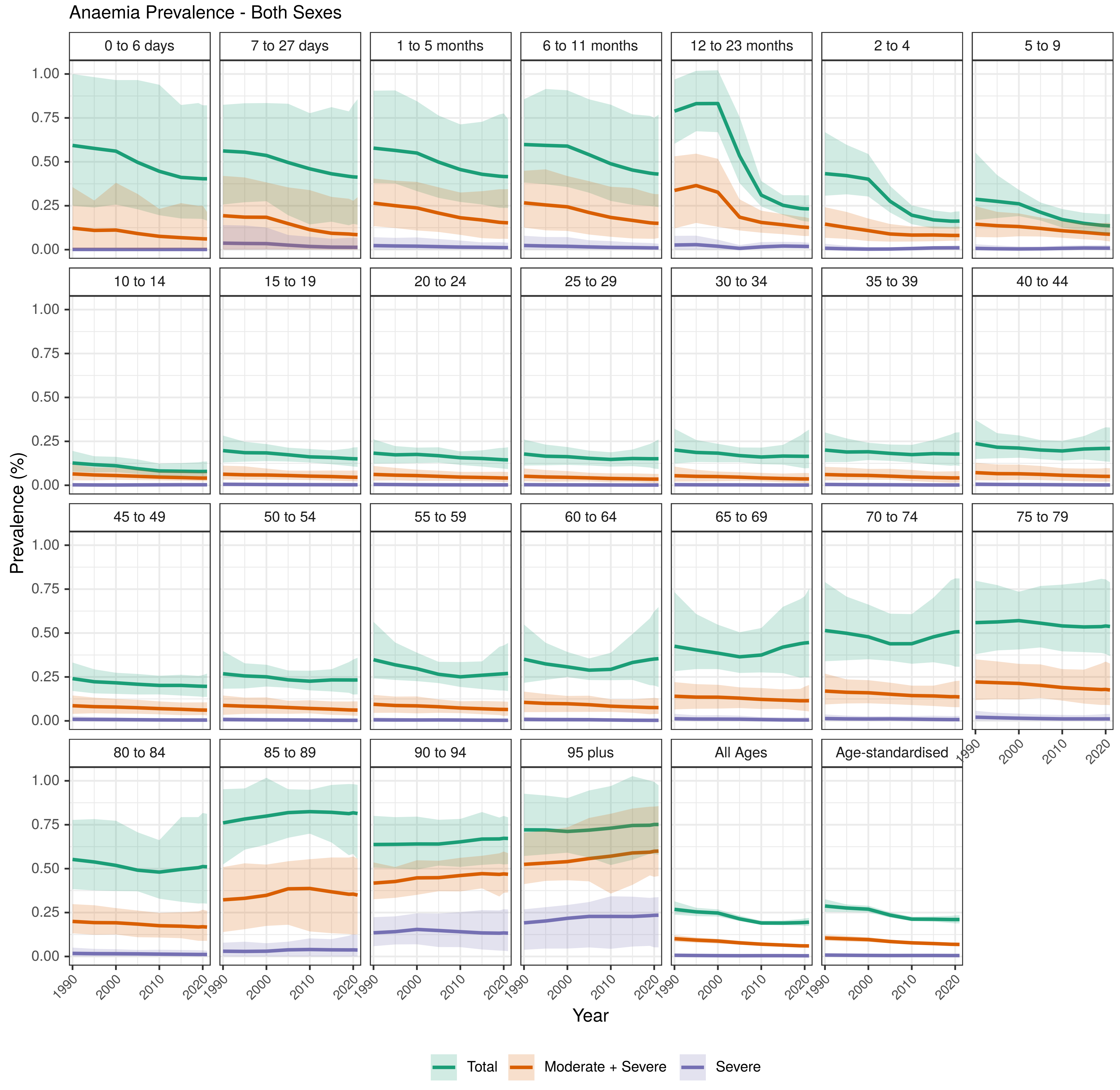

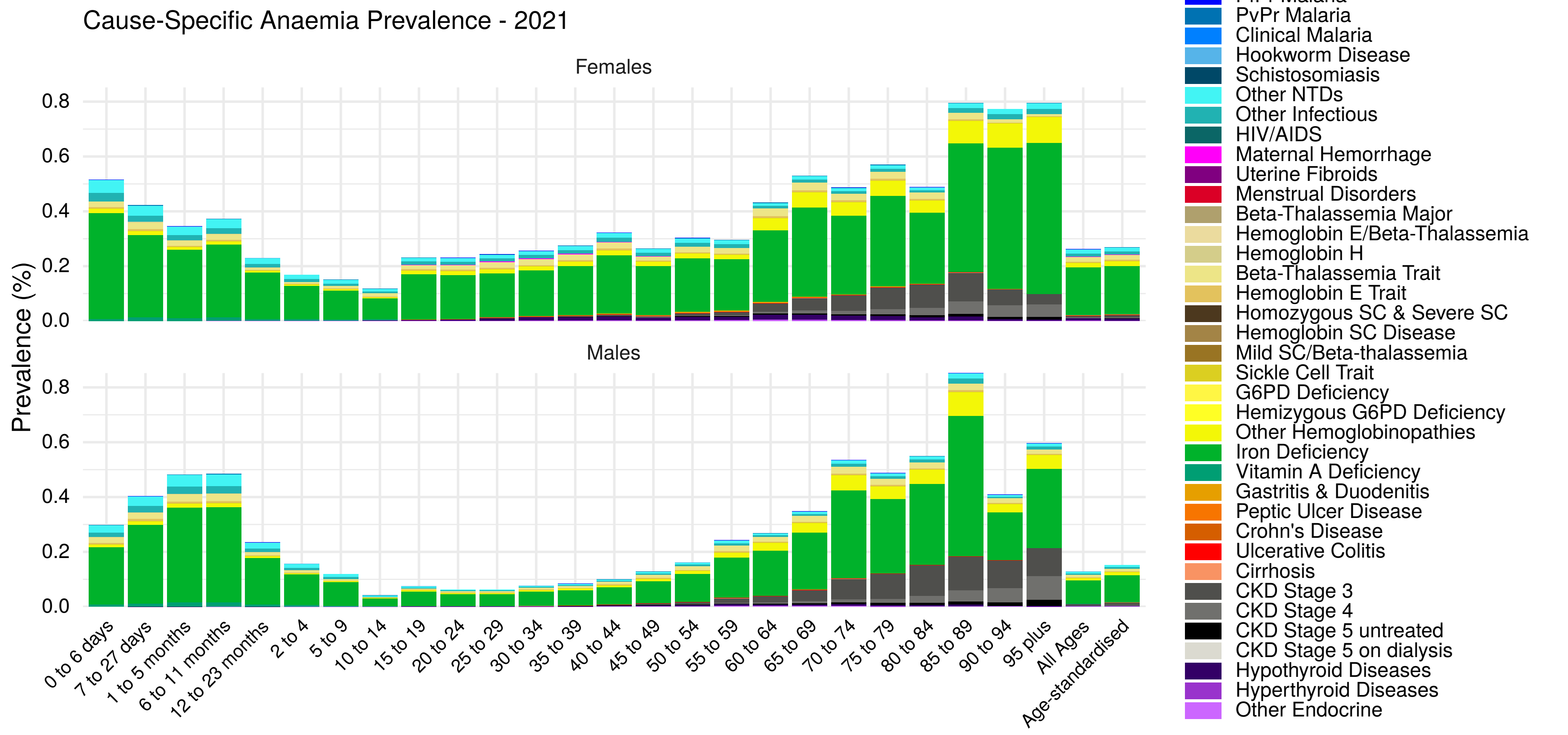

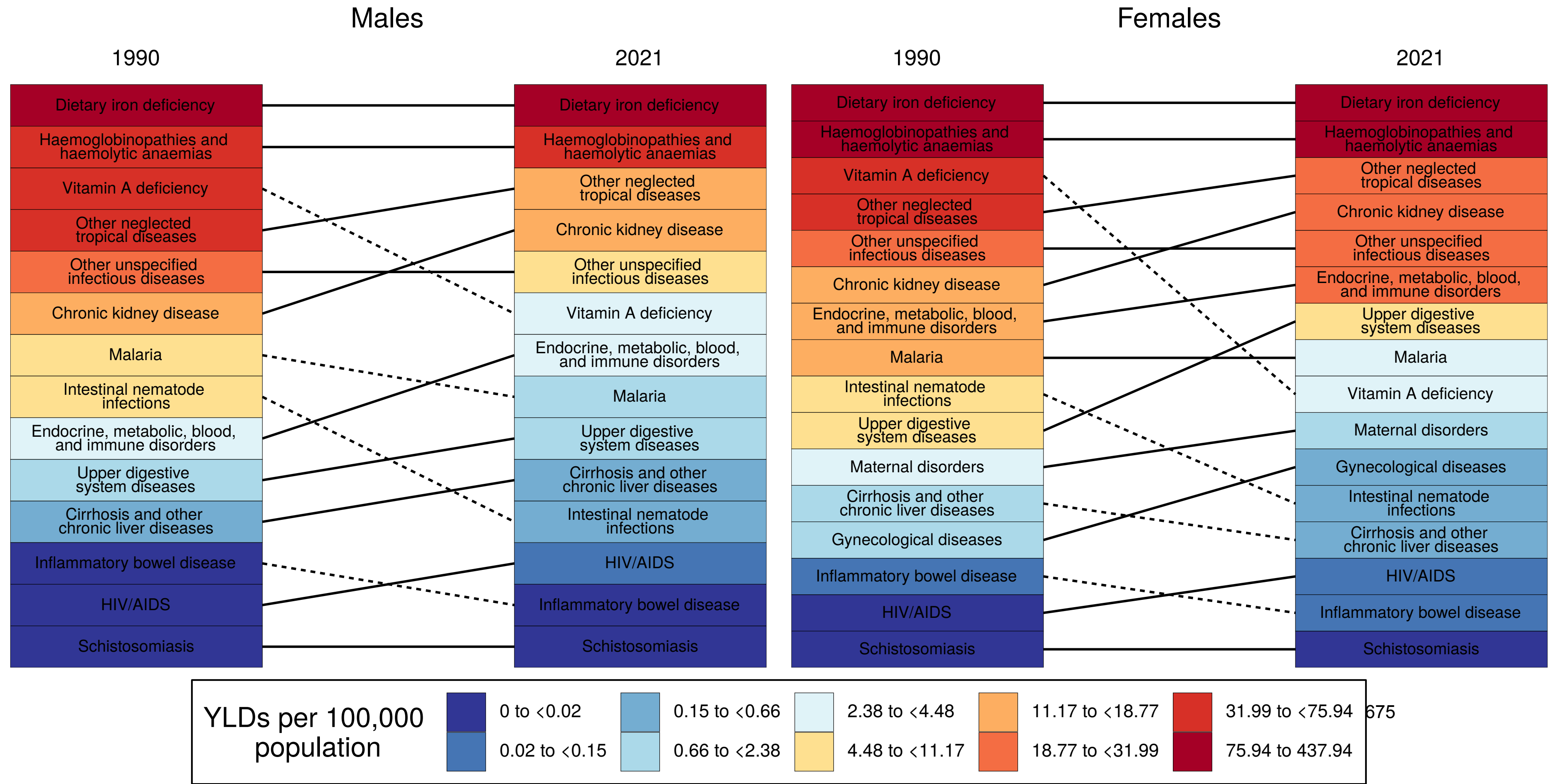

Yogyakarta

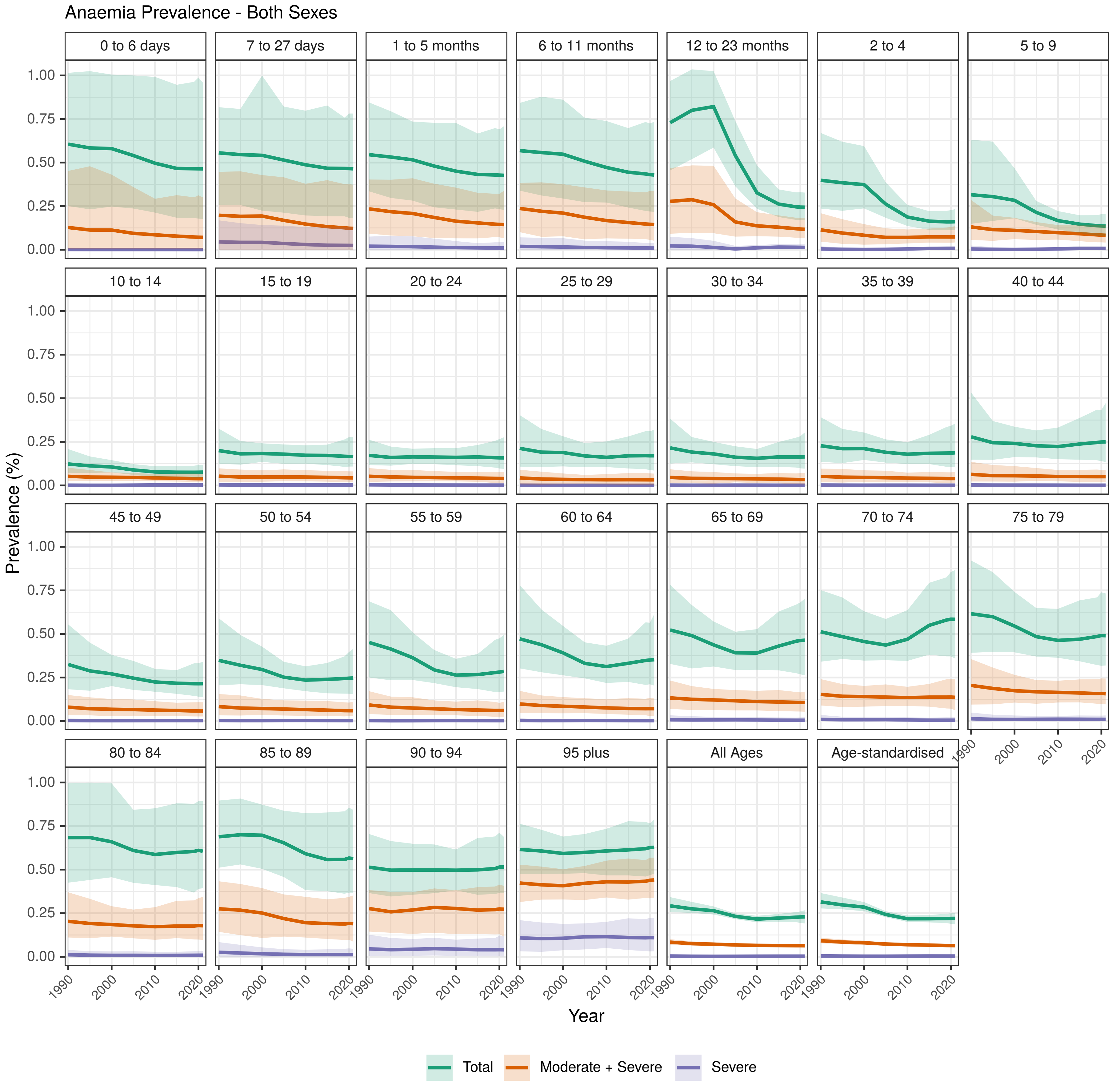

Anaemia Prevalence - Both Sexes
0 to 6 days
7 to 27 days
1 to 5 months
6 to 11 months
12 to 23 months
2 to 4
5 to 9
10 to 14
15 to 19
20 to 24
25 to 29
30 to 34
35 to 39
40 to 44
45 to 49
50 to 54
55 to 59
60 to 64
65 to 69
70 to 74
75 to 79
80 to 84
85 to 89
90 to 94
95 plus
All Ages
Age-standardised
Prevalence (%)
Year
Total
Moderate + Severe
Severe

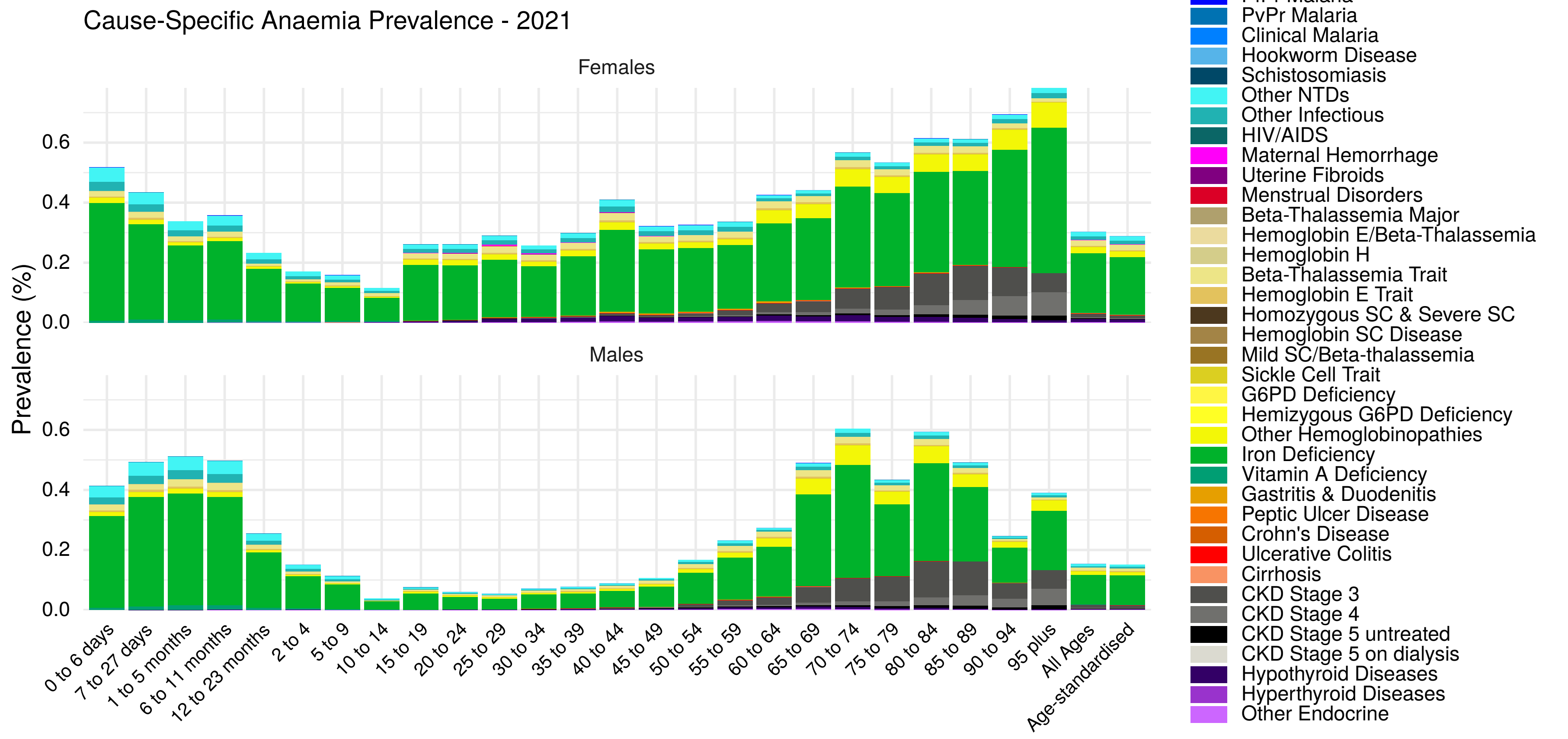

Cause-Specific Anaemia Prevalence - 2021
Females
Males
Prevalence (%)
PfPr Malaria
PvPr Malaria
Clinical Malaria
Hookworm Disease
Schistosomiasis
Other NTDs
Other Infectious
HIV/AIDS
Maternal Hemorrhage
Uterine Fibroids
Menstrual Disorders
Beta-Thalassemia Major
Hemoglobin E/Beta-Thalassemia
Hemoglobin H
Beta-Thalassemia Trait
Hemoglobin E Trait
Homozygous SC & Severe SC
Hemoglobin SC Disease
Mild SC/Beta-thalassemia
Sickle Cell Trait
G6PD Deficiency
Hemizygous G6PD Deficiency
Other Hemoglobinopathies
Iron Deficiency
Vitamin A Deficiency
Gastritis & Duodenitis
Peptic Ulcer Disease
Crohn's Disease
Ulcerative Colitis
Cirrhosis
CKD Stage 3
CKD Stage 4
CKD Stage 5 untreated
CKD Stage 5 on dialysis
Hypothyroid Diseases
Hyperthyroid Diseases
Other Endocrine

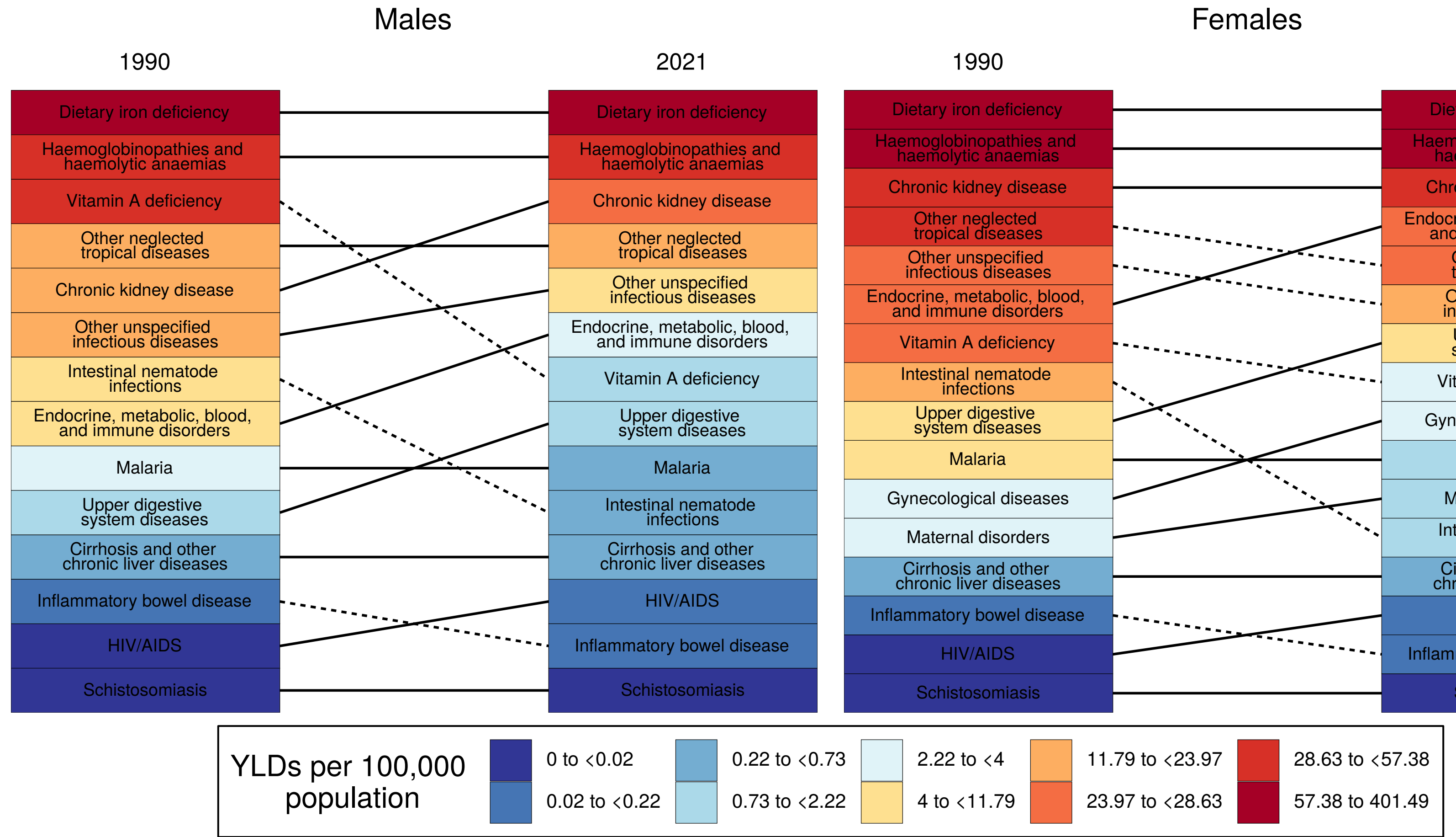

Males
Females
1990
2021
Dietary iron deficiency
Haemoglobinopathies and haemolytic anaemias
Vitamin A deficiency
Other neglected tropical diseases
Chronic kidney disease
Other unspecified infectious diseases
Intestinal nematode infections
Endocrine, metabolic, blood, and immune disorders
Malaria
Upper digestive system diseases
Cirrhosis and other chronic liver diseases
Inflammatory bowel disease
HIV/AIDS
Schistosomiasis
Gynecological diseases
Maternal disorders
YLDs per 100,000 population
0 to <0.02
0.02 to <0.22
0.22 to <0.73
0.73 to <2.22
2.22 to <4
4 to <11.79
11.79 to <23.97
23.97 to <28.63
28.63 to <57.38
57.38 to 401.49

Laos

Anaemia Prevalence - Both Sexes

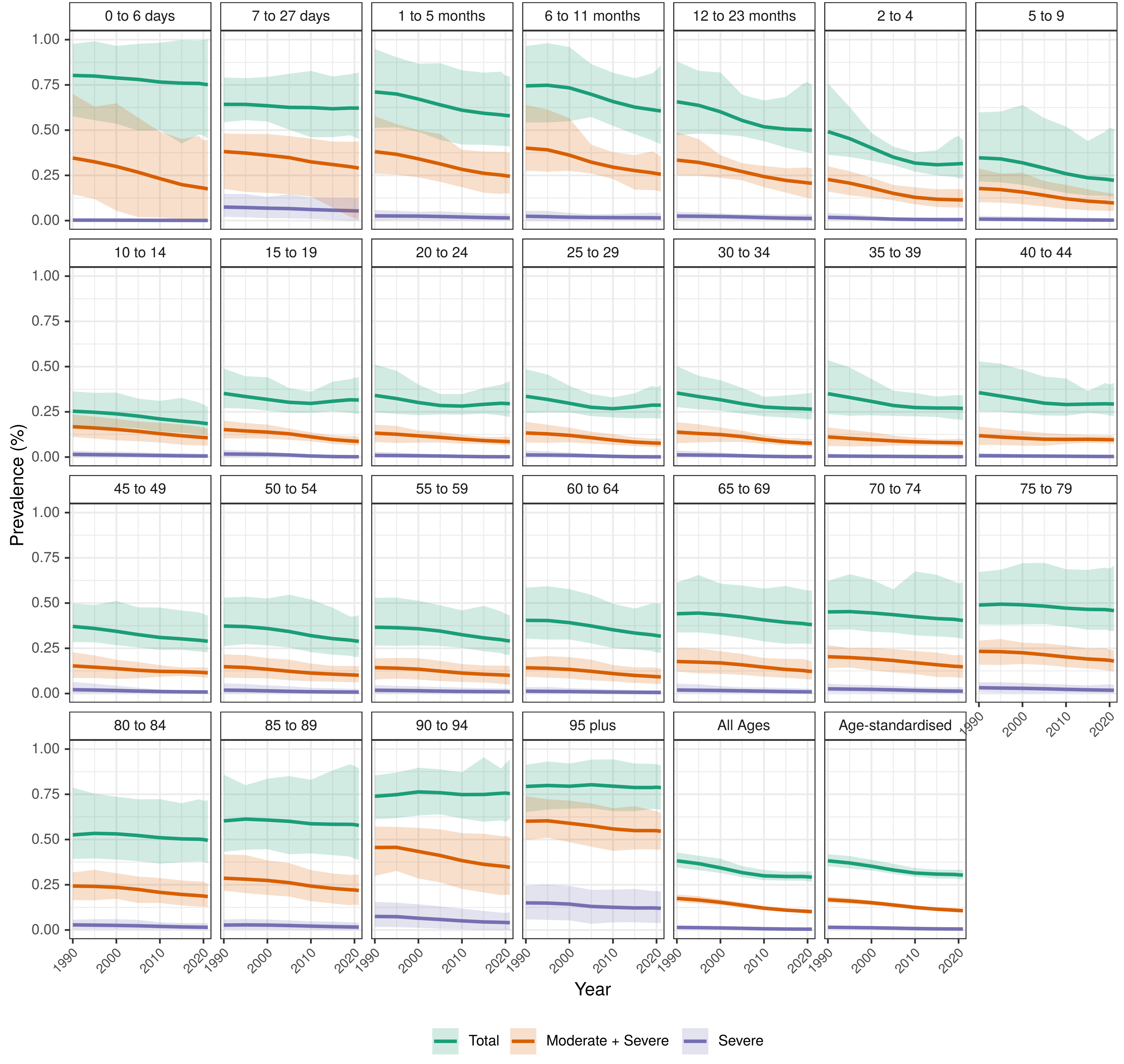

Cause-Specific Anaemia Prevalence - 2021

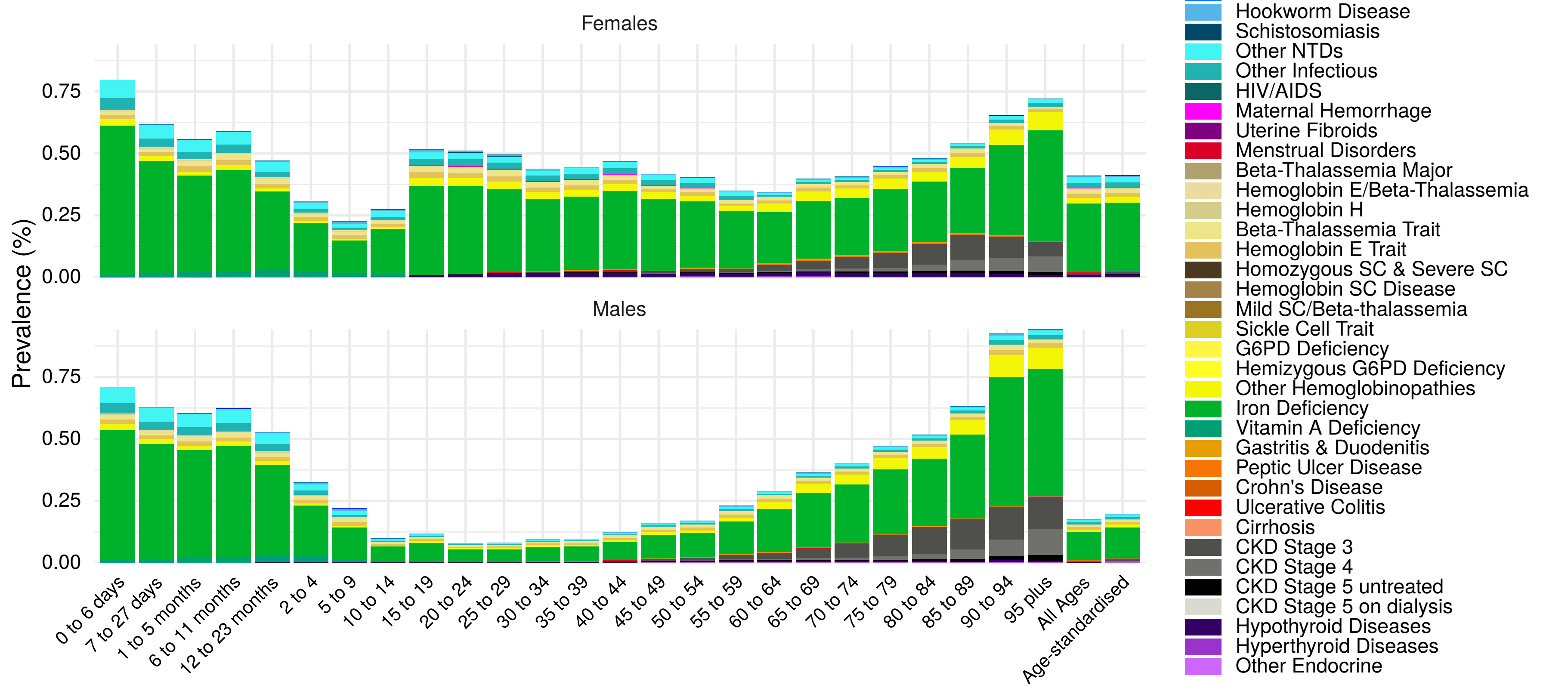

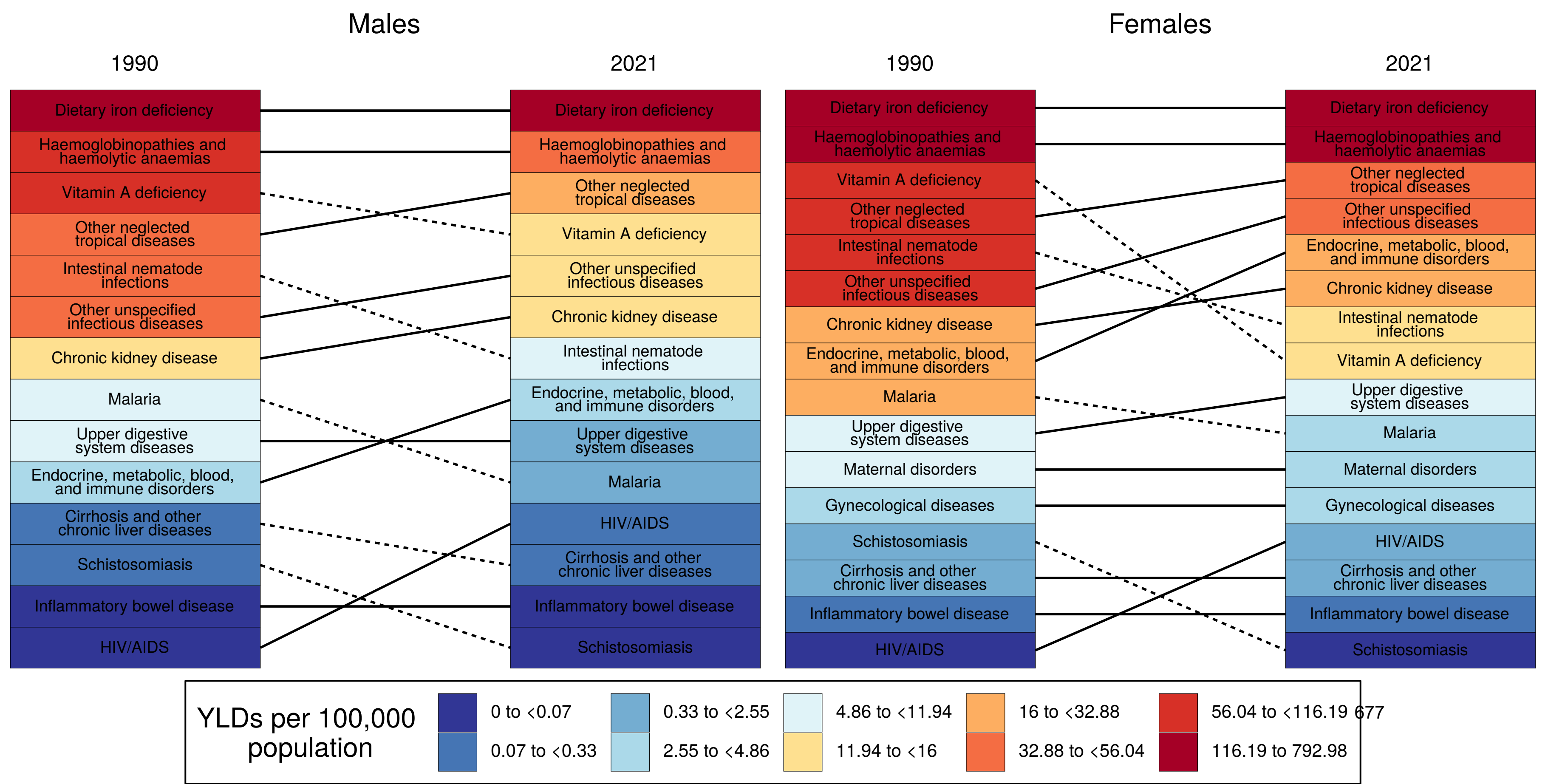

677

Malaysia

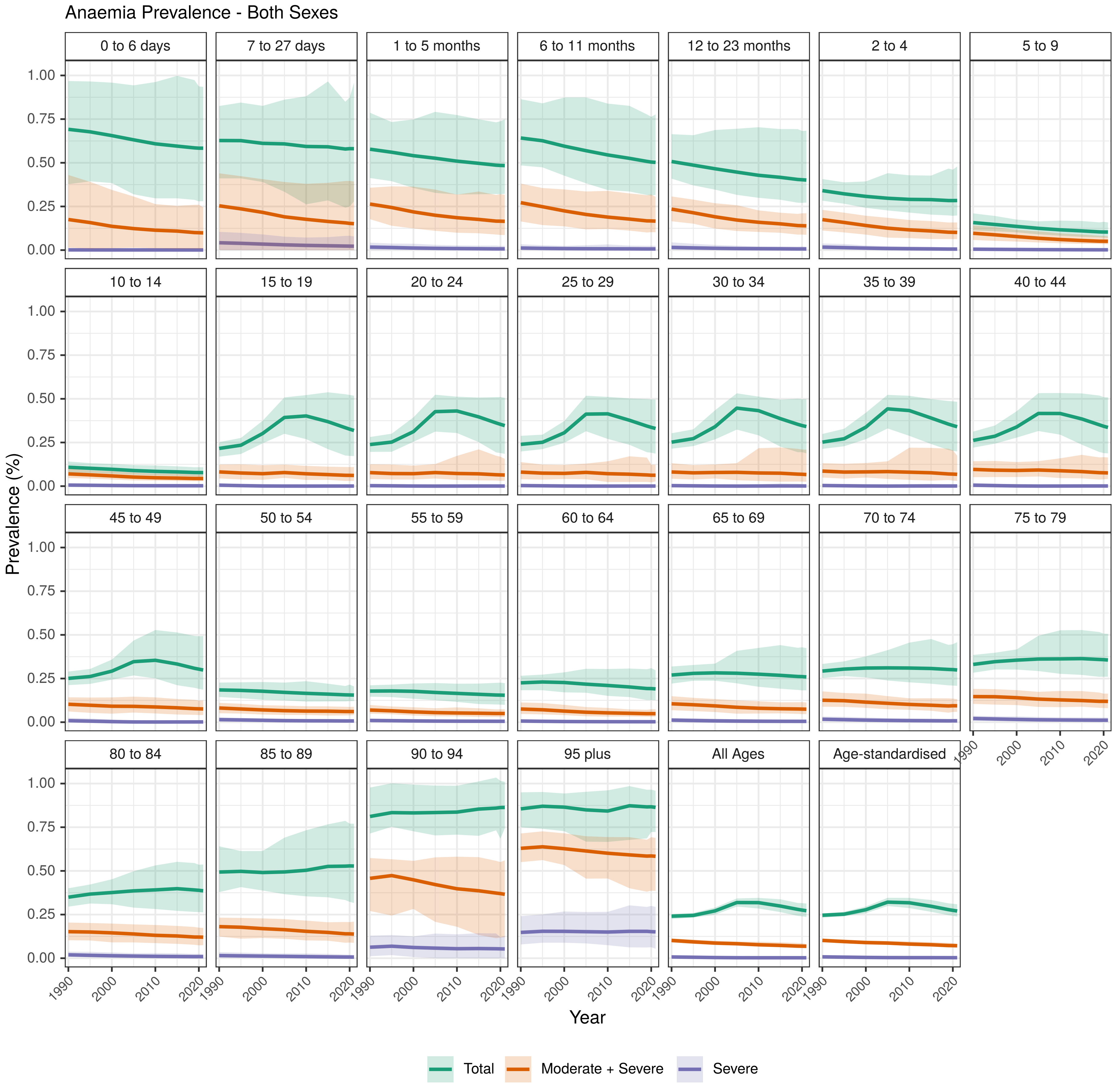

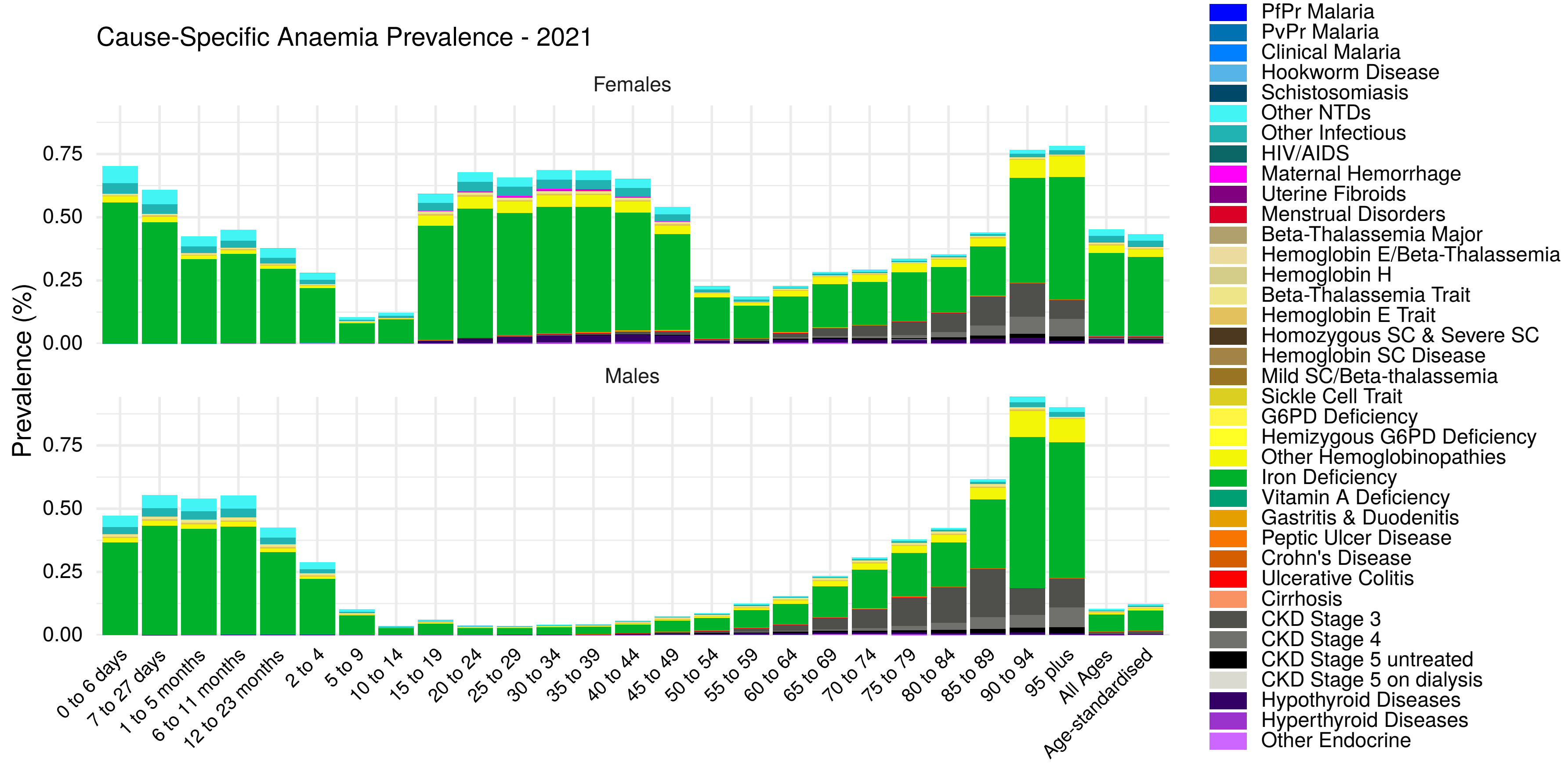

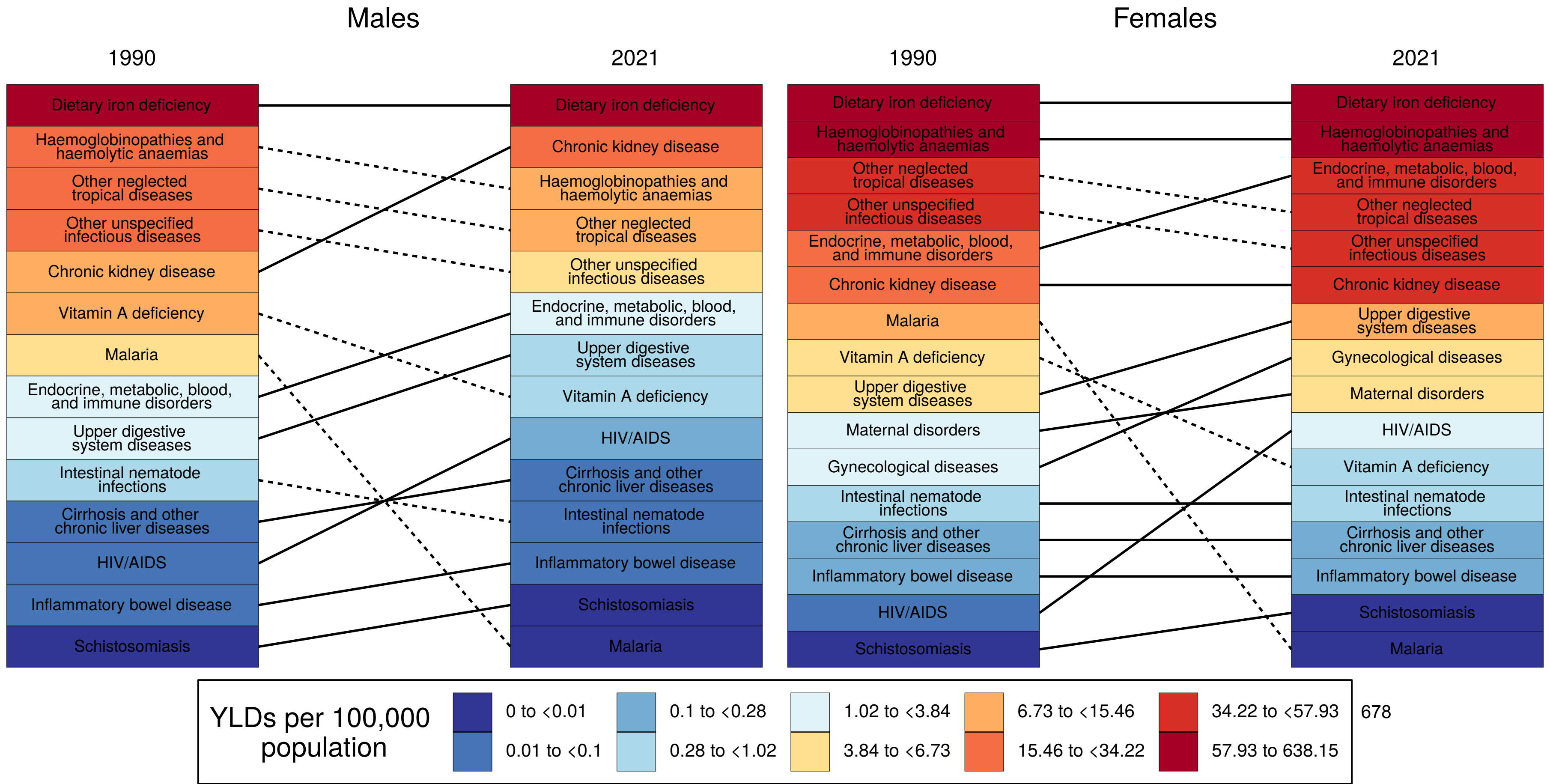

678

Maldives

Anaemia Prevalence - Both Sexes

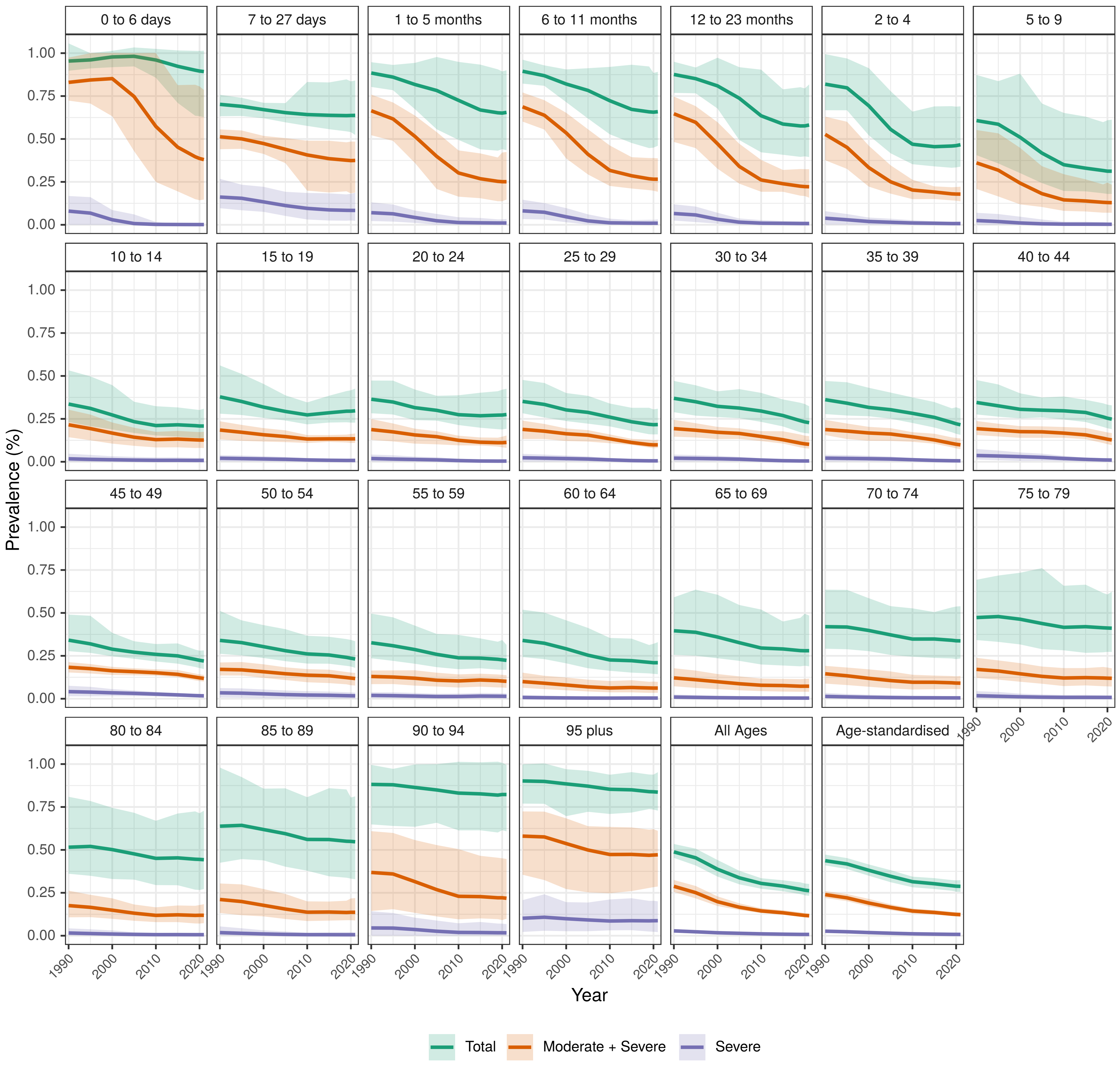

Cause-Specific Anaemia Prevalence - 2021

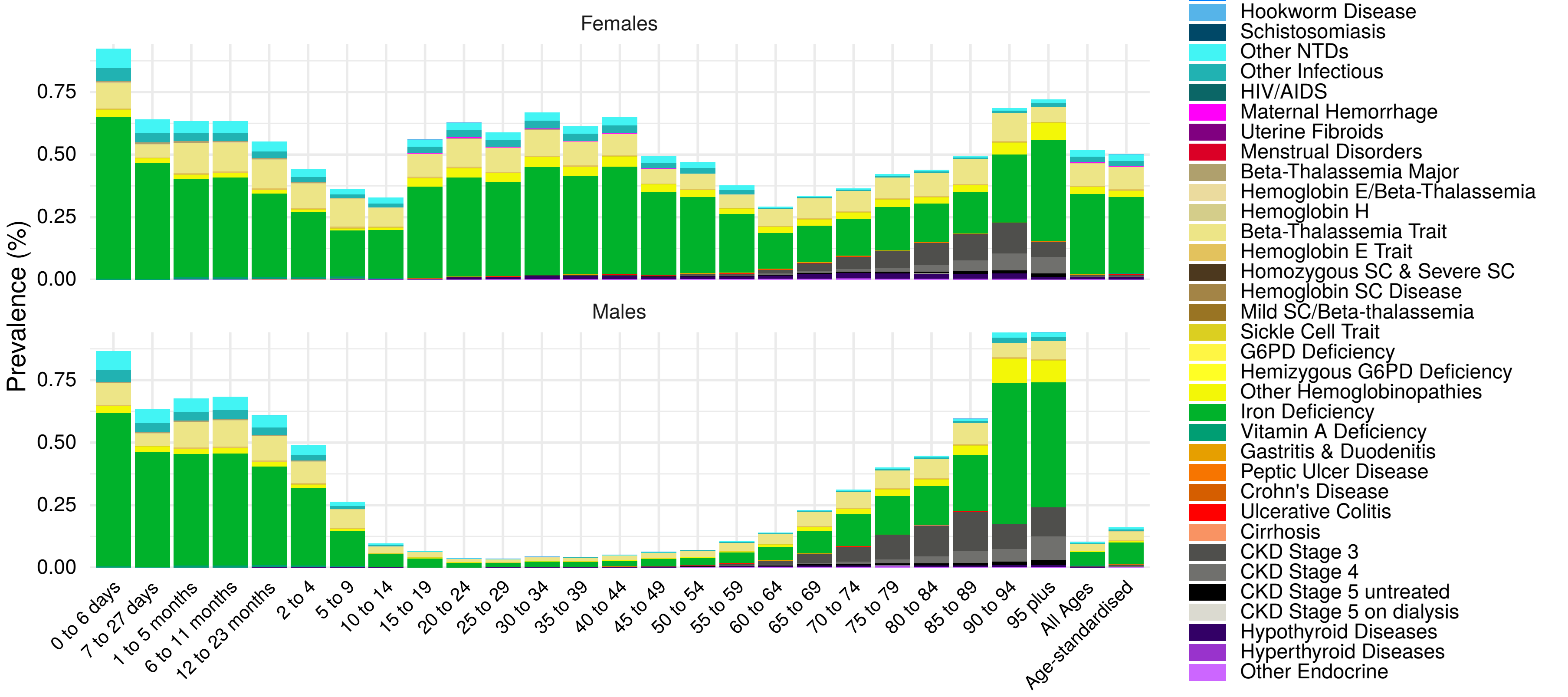

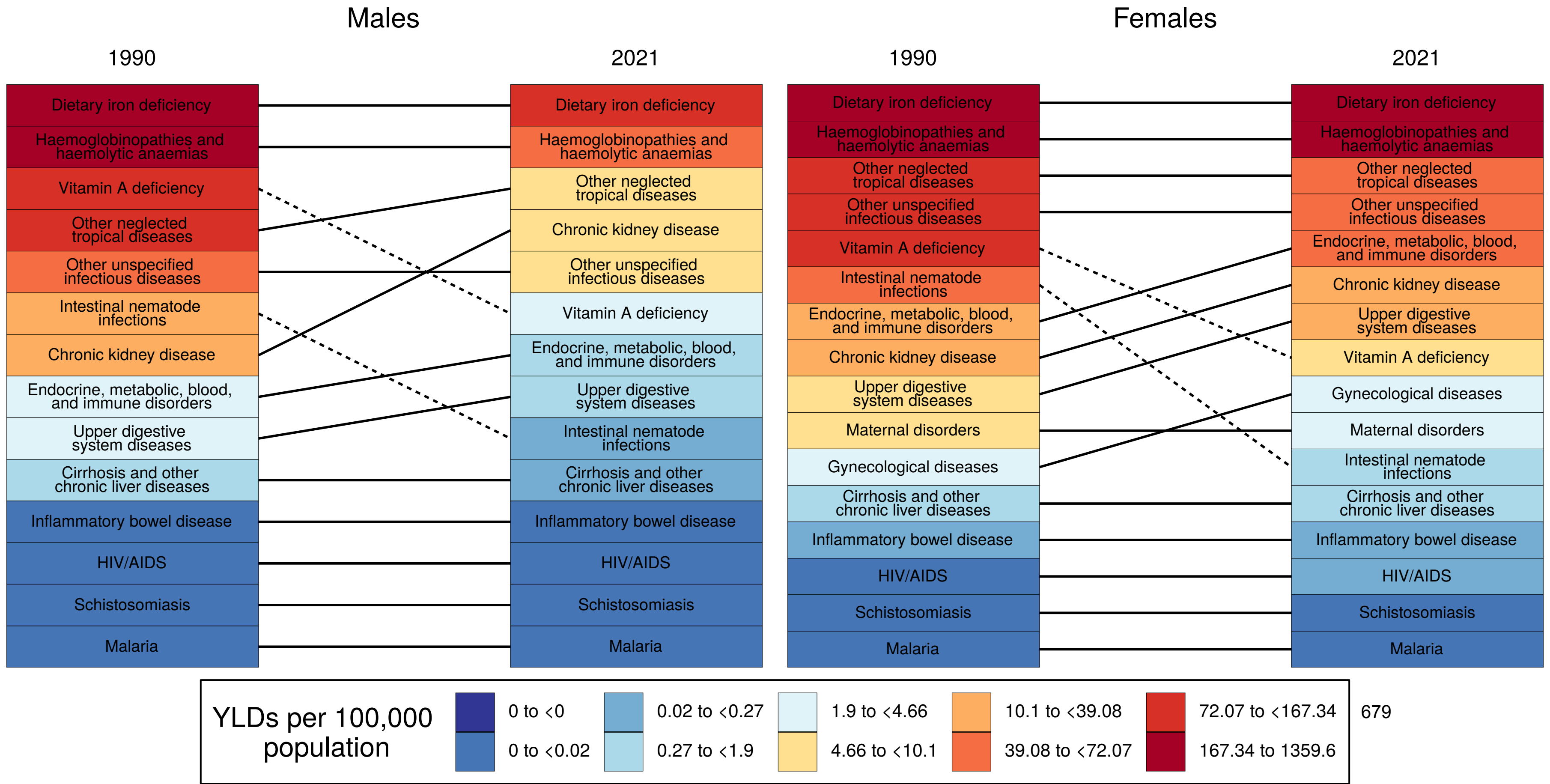

Mauritius

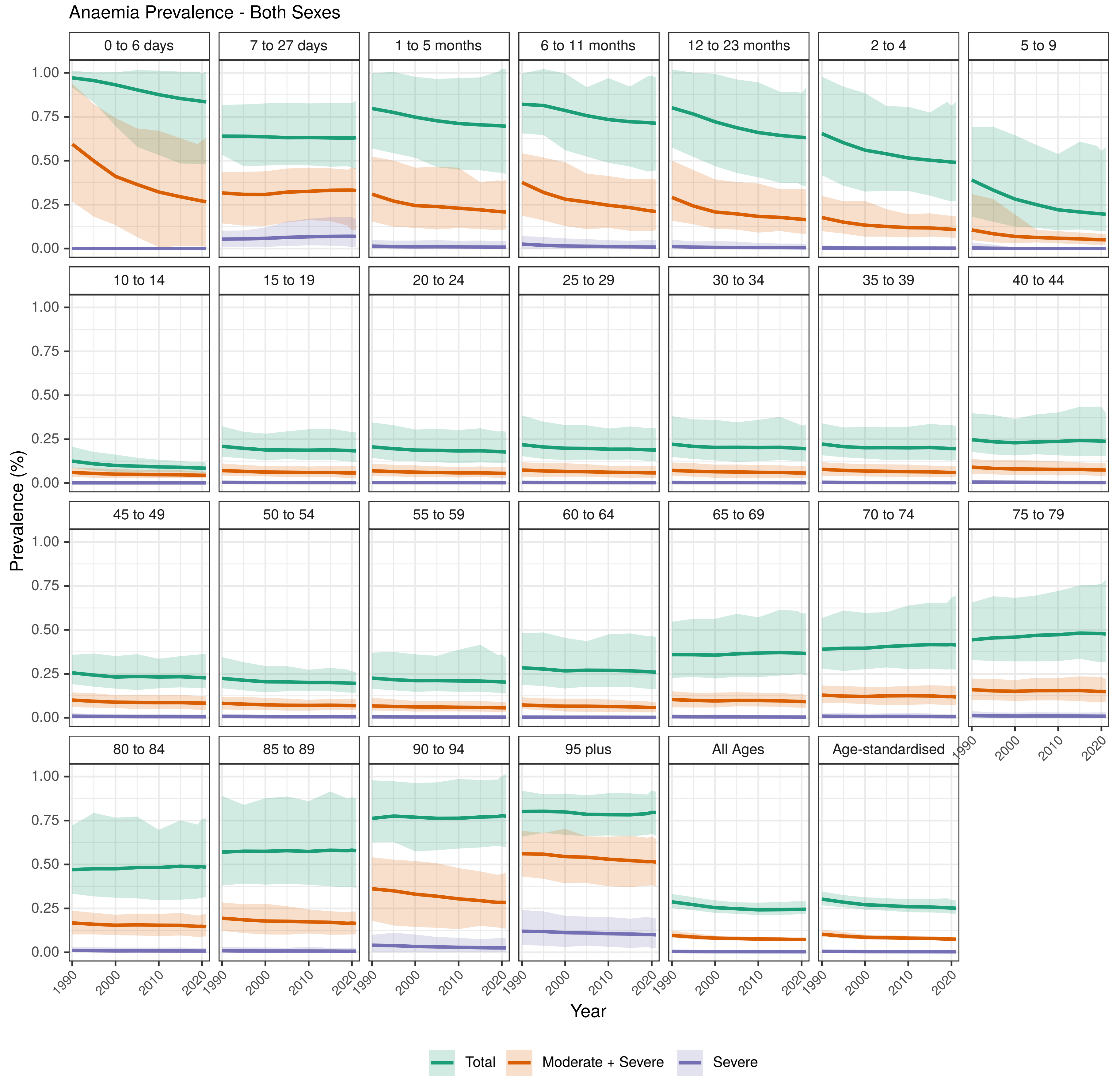

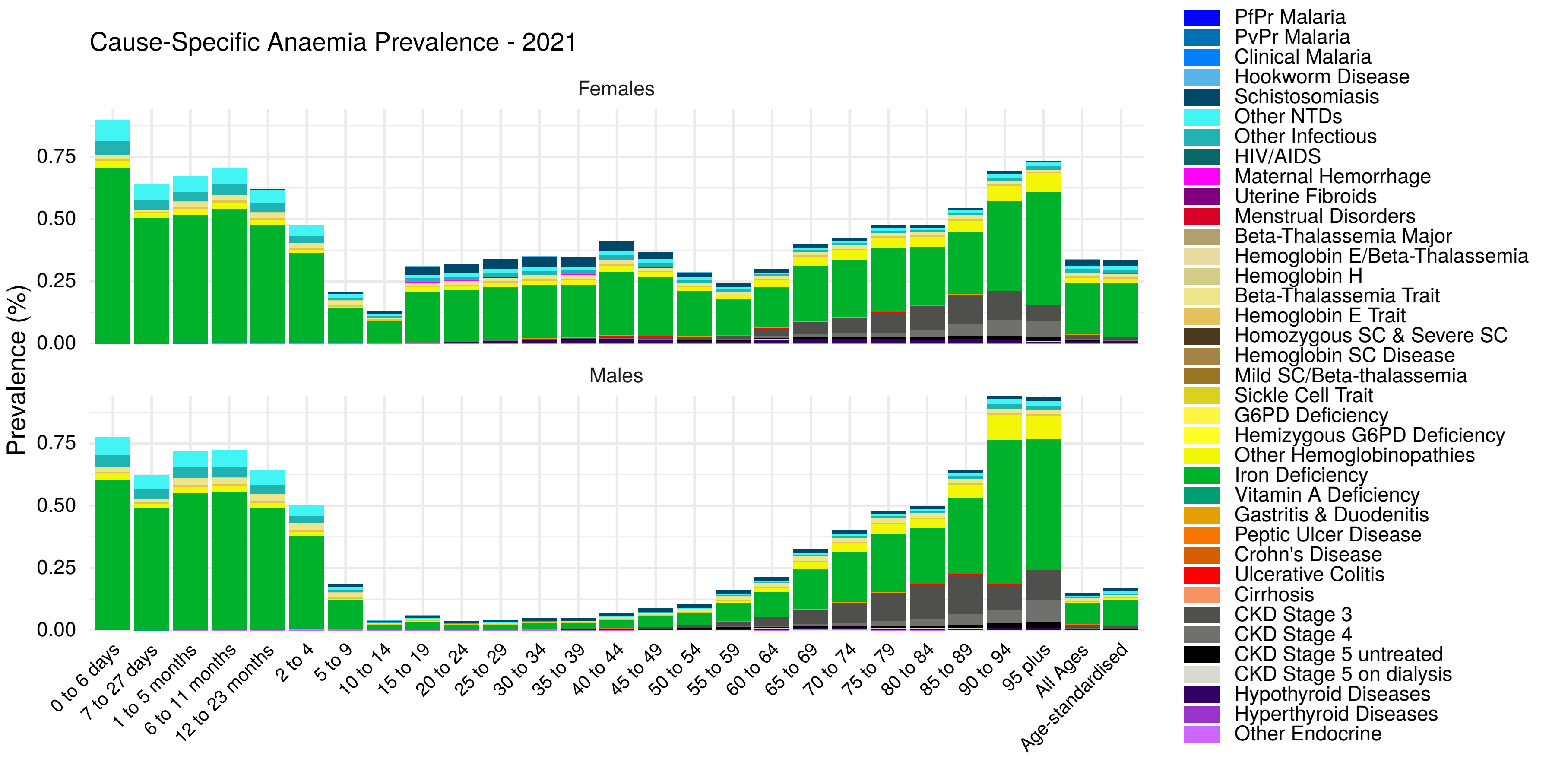

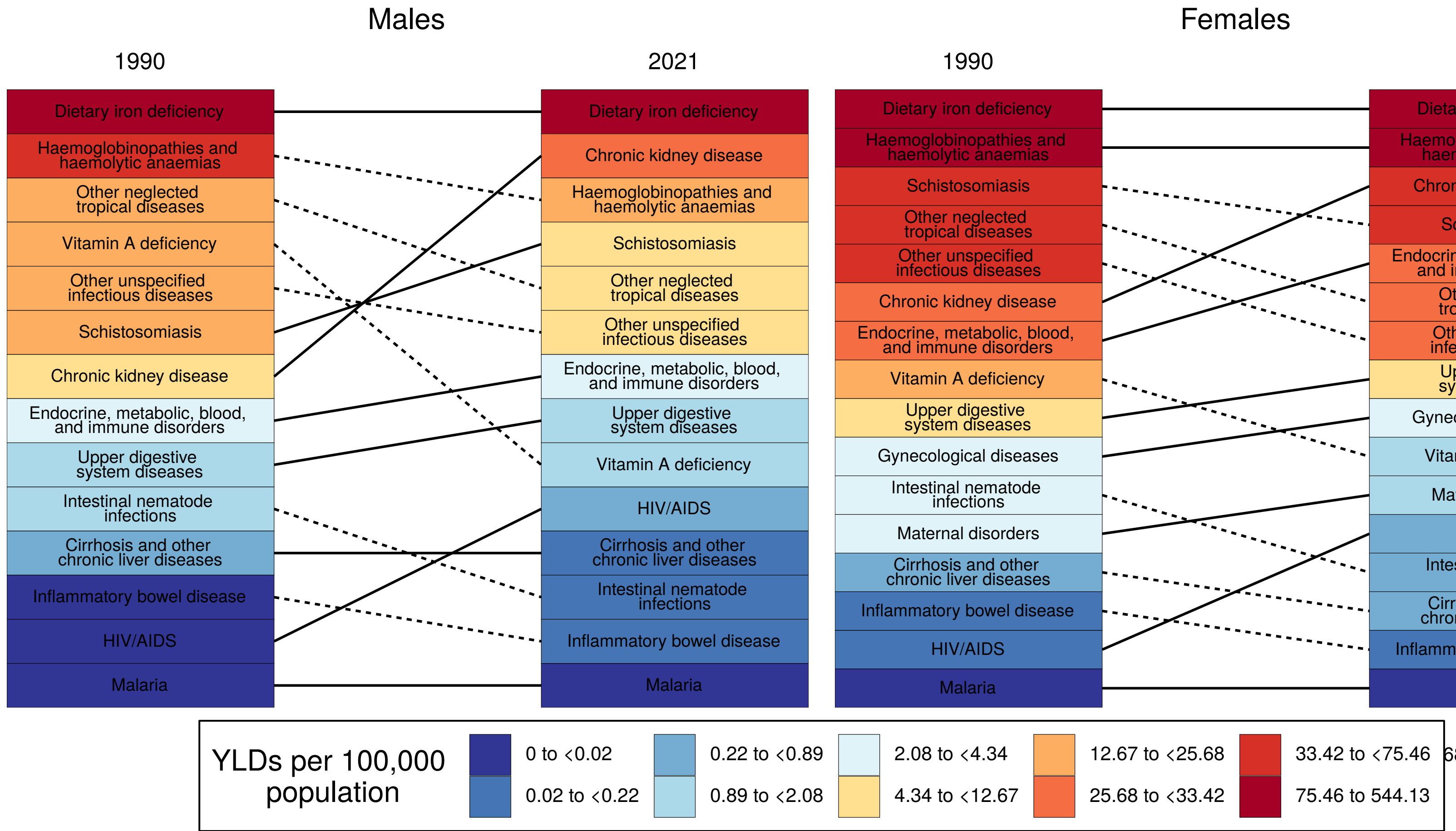

680

Myanmar

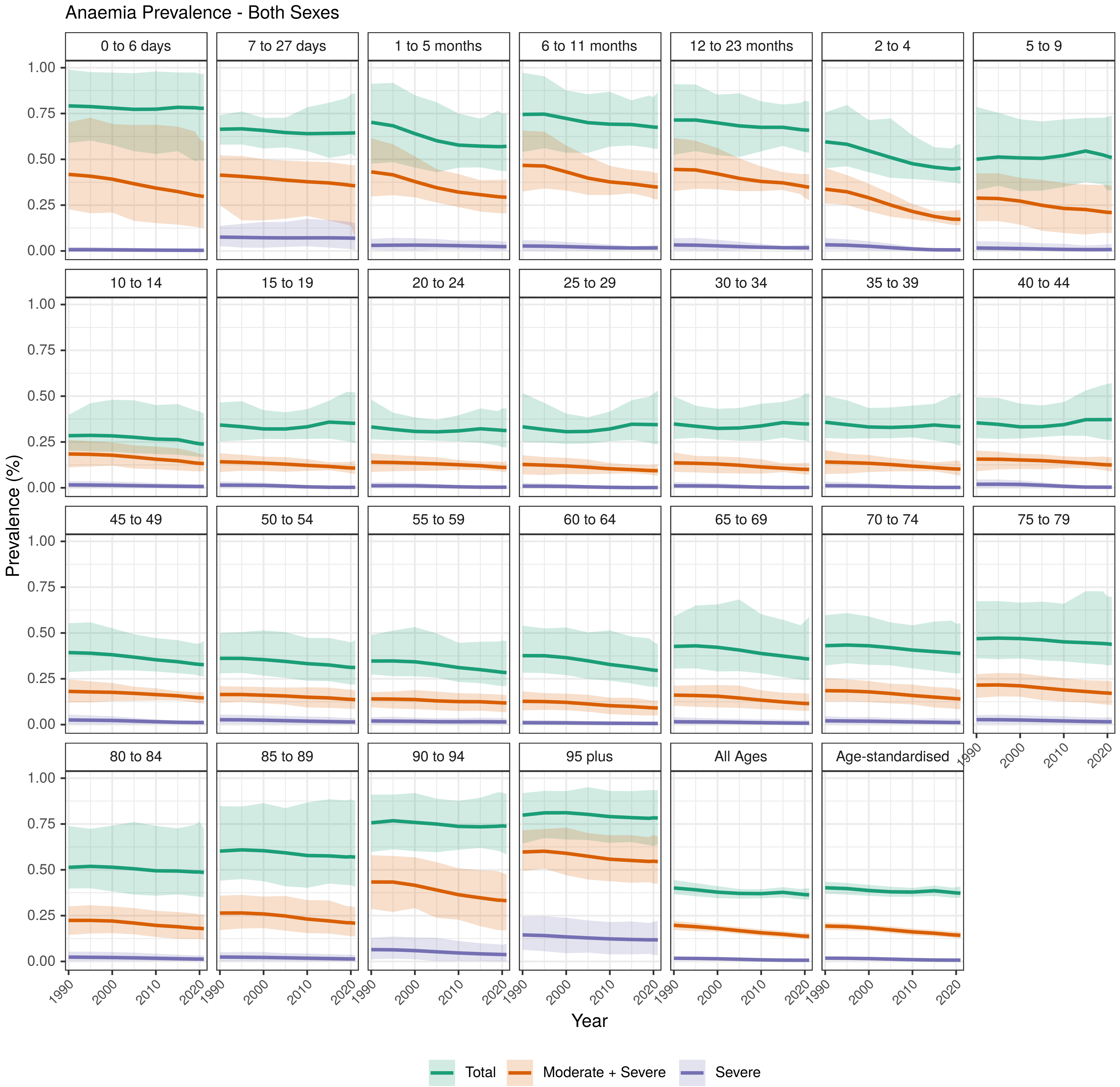

Anaemia Prevalence - Both Sexes
0 to 6 days
7 to 27 days
1 to 5 months
6 to 11 months
12 to 23 months
2 to 4
5 to 9
10 to 14
15 to 19
20 to 24
25 to 29
30 to 34
35 to 39
40 to 44
45 to 49
50 to 54
55 to 59
60 to 64
65 to 69
70 to 74
75 to 79
80 to 84
85 to 89
90 to 94
95 plus
All Ages
Age-standardised
Prevalence (%)
Year
1990
2000
2010
2020
0.00
0.25
0.50
0.75
1.00
Total
Moderate + Severe
Severe

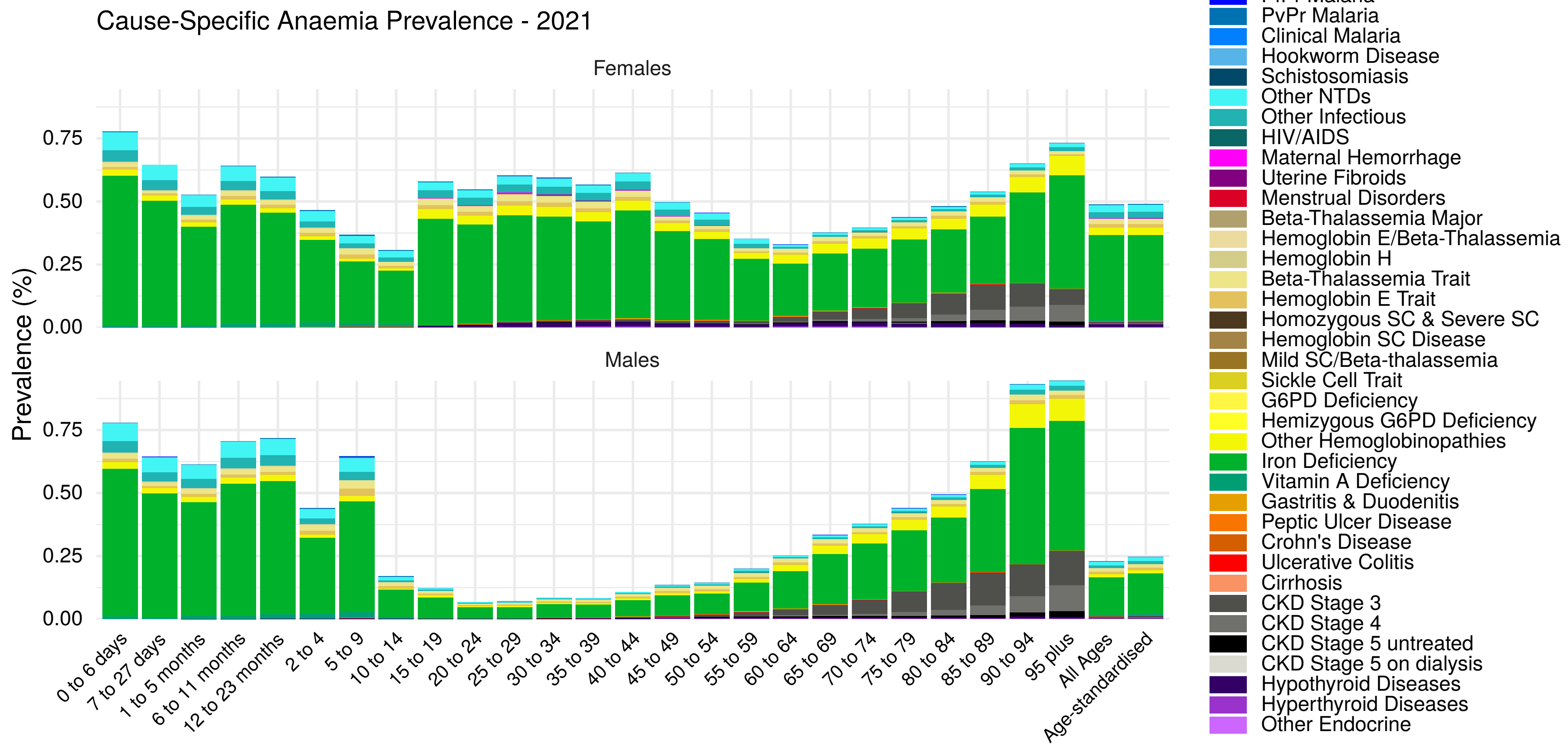

Cause-Specific Anaemia Prevalence - 2021
Females
Males
Prevalence (%)
0.00
0.25
0.50
0.75
0 to 6 days
7 to 27 days
1 to 5 months
6 to 11 months
12 to 23 months
2 to 4
5 to 9
10 to 14
15 to 19
20 to 24
25 to 29
30 to 34
35 to 39
40 to 44
45 to 49
50 to 54
55 to 59
60 to 64
65 to 69
70 to 74
75 to 79
80 to 84
85 to 89
90 to 94
95 plus
All Ages
Age-standardised
PfPr Malaria
PvPr Malaria
Clinical Malaria
Hookworm Disease
Schistosomiasis
Other NTDs
Other Infectious
HIV/AIDS
Maternal Hemorrhage
Uterine Fibroids
Menstrual Disorders
Beta-Thalassemia Major
Hemoglobin E/Beta-Thalassemia
Hemoglobin H
Beta-Thalassemia Trait
Hemoglobin E Trait
Homozygous SC & Severe SC
Hemoglobin SC Disease
Mild SC/Beta-thalassemia
Sickle Cell Trait
G6PD Deficiency
Hemizygous G6PD Deficiency
Other Hemoglobinopathies
Iron Deficiency
Vitamin A Deficiency
Gastritis & Duodenitis
Peptic Ulcer Disease
Crohn's Disease
Ulcerative Colitis
Cirrhosis
CKD Stage 3
CKD Stage 4
CKD Stage 5 untreated
CKD Stage 5 on dialysis
Hypothyroid Diseases
Hyperthyroid Diseases
Other Endocrine

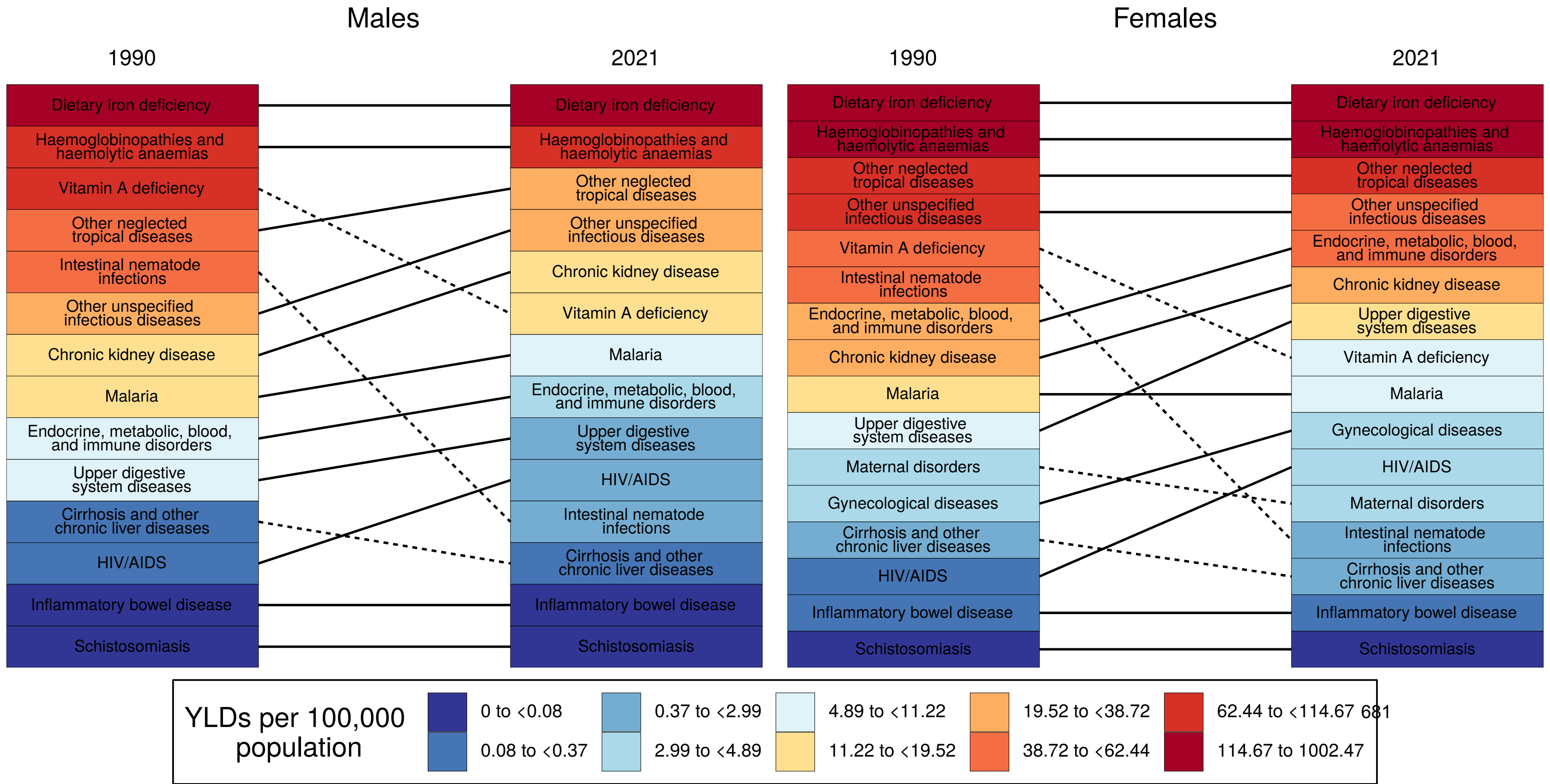

Males
1990
Dietary iron deficiency
Haemoglobinopathies and haemolytic anaemias
Vitamin A deficiency
Other neglected tropical diseases
Intestinal nematode infections
Other unspecified infectious diseases
Chronic kidney disease
Malaria
Endocrine, metabolic, blood, and immune disorders
Upper digestive system diseases
Cirrhosis and other chronic liver diseases
HIV/AIDS
Inflammatory bowel disease
Schistosomiasis
2021
Dietary iron deficiency
Haemoglobinopathies and haemolytic anaemias
Other neglected tropical diseases
Other unspecified infectious diseases
Chronic kidney disease
Vitamin A deficiency
Malaria
Endocrine, metabolic, blood, and immune disorders
Upper digestive system diseases
HIV/AIDS
Intestinal nematode infections
Cirrhosis and other chronic liver diseases
Inflammatory bowel disease
Schistosomiasis
Females
1990
Dietary iron deficiency
Haemoglobinopathies and haemolytic anaemias
Other neglected tropical diseases
Other unspecified infectious diseases
Vitamin A deficiency
Intestinal nematode infections
Endocrine, metabolic, blood, and immune disorders
Chronic kidney disease
Malaria
Upper digestive system diseases
Maternal disorders
Gynecological diseases
Cirrhosis and other chronic liver diseases
HIV/AIDS
Inflammatory bowel disease
Schistosomiasis
2021
Dietary iron deficiency
Haemoglobinopathies and haemolytic anaemias
Other neglected tropical diseases
Other unspecified infectious diseases
Endocrine, metabolic, blood, and immune disorders
Chronic kidney disease
Upper digestive system diseases
Vitamin A deficiency
Malaria
Gynecological diseases
HIV/AIDS
Maternal disorders
Intestinal nematode infections
Cirrhosis and other chronic liver diseases
Inflammatory bowel disease
Schistosomiasis
YLDs per 100,000 population
0 to <0.08
0.08 to <0.37
0.37 to <2.99
2.99 to <4.89
4.89 to <11.22
11.22 to <19.52
19.52 to <38.72
38.72 to <62.44
62.44 to <114.67
114.67 to 1002.47

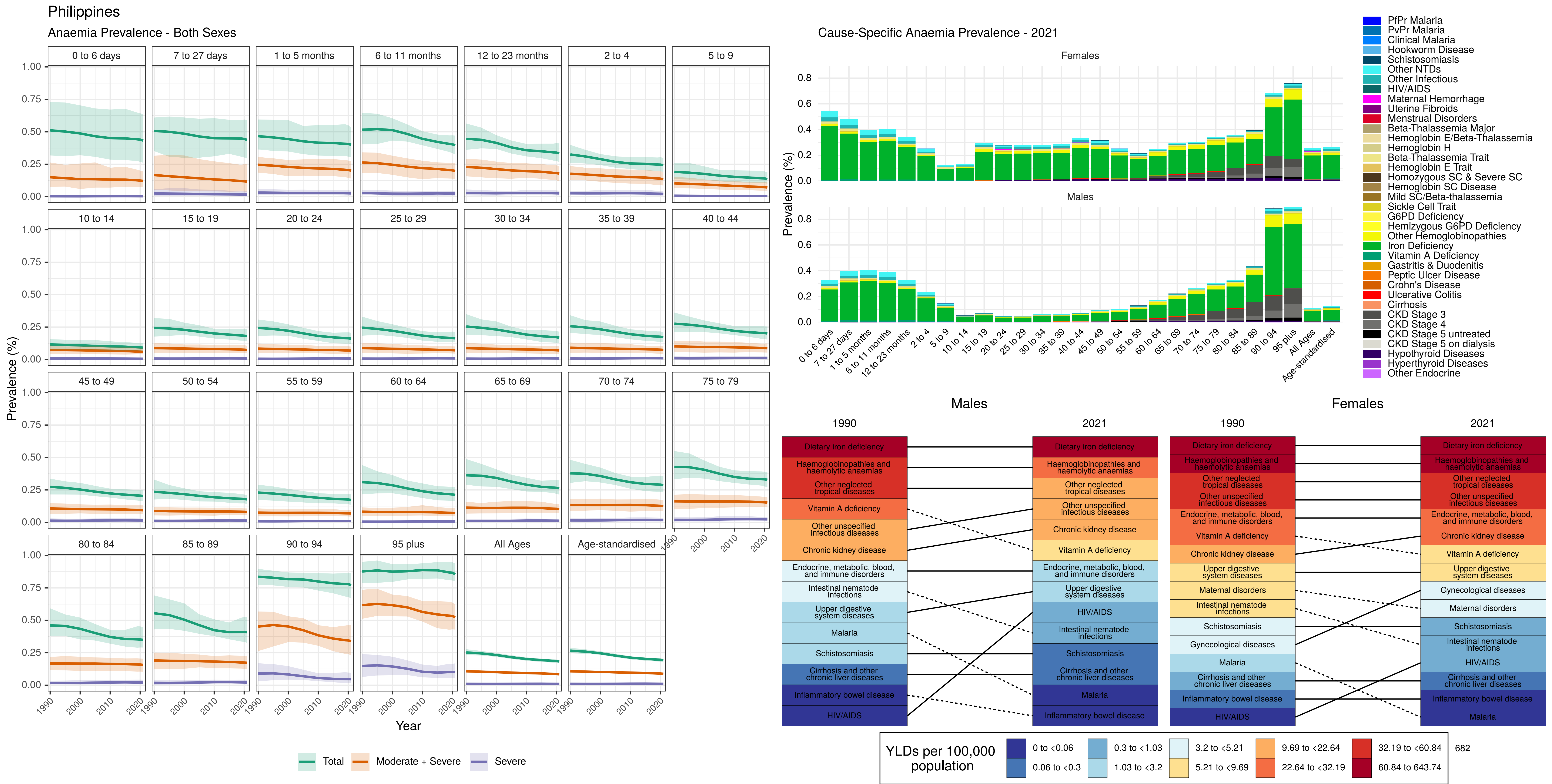

Philippines
Anaemia Prevalence - Both Sexes
0 to 6 days
7 to 27 days
1 to 5 months
6 to 11 months
12 to 23 months
2 to 4
5 to 9
10 to 14
15 to 19
20 to 24
25 to 29
30 to 34
35 to 39
40 to 44
45 to 49
50 to 54
55 to 59
60 to 64
65 to 69
70 to 74
75 to 79
80 to 84
85 to 89
90 to 94
95 plus
All Ages
Age-standardised
Prevalence (%)
Year
Total
Moderate + Severe
Severe
Cause-Specific Anaemia Prevalence - 2021
Females
Males
PfPr Malaria
PvPr Malaria
Clinical Malaria
Hookworm Disease
Schistosomiasis
Other NTDs
Other Infectious
HIV/AIDS
Maternal Hemorrhage
Uterine Fibroids
Menstrual Disorders
Beta-Thalassemia Major
Hemoglobin E/Beta-Thalassemia
Hemoglobin H
Beta-Thalassemia Trait
Hemoglobin E Trait
Homozygous SC & Severe SC
Hemoglobin SC Disease
Mild SC/Beta-thalassemia
Sickle Cell Trait
G6PD Deficiency
Hemizygous G6PD Deficiency
Other Hemoglobinopathies
Iron Deficiency
Vitamin A Deficiency
Gastritis & Duodenitis
Peptic Ulcer Disease
Crohn's Disease
Ulcerative Colitis
Cirrhosis
CKD Stage 3
CKD Stage 4
CKD Stage 5 untreated
CKD Stage 5 on dialysis
Hypothyroid Diseases
Hyperthyroid Diseases
Other Endocrine
Males
Females
1990
2021
Dietary iron deficiency
Haemoglobinopathies and haemolytic anaemias
Other neglected tropical diseases
Vitamin A deficiency
Other unspecified infectious diseases
Chronic kidney disease
Endocrine, metabolic, blood, and immune disorders
Intestinal nematode infections
Upper digestive system diseases
Malaria
Schistosomiasis
Cirrhosis and other chronic liver diseases
Inflammatory bowel disease
HIV/AIDS
Maternal disorders
Gynecological diseases
YLDs per 100,000 population
0 to <0.06
0.06 to <0.3
0.3 to <1.03
1.03 to <3.2
3.2 to <5.21
5.21 to <9.69
9.69 to <22.64
22.64 to <32.19
32.19 to <60.84
60.84 to 643.74
682

Abra

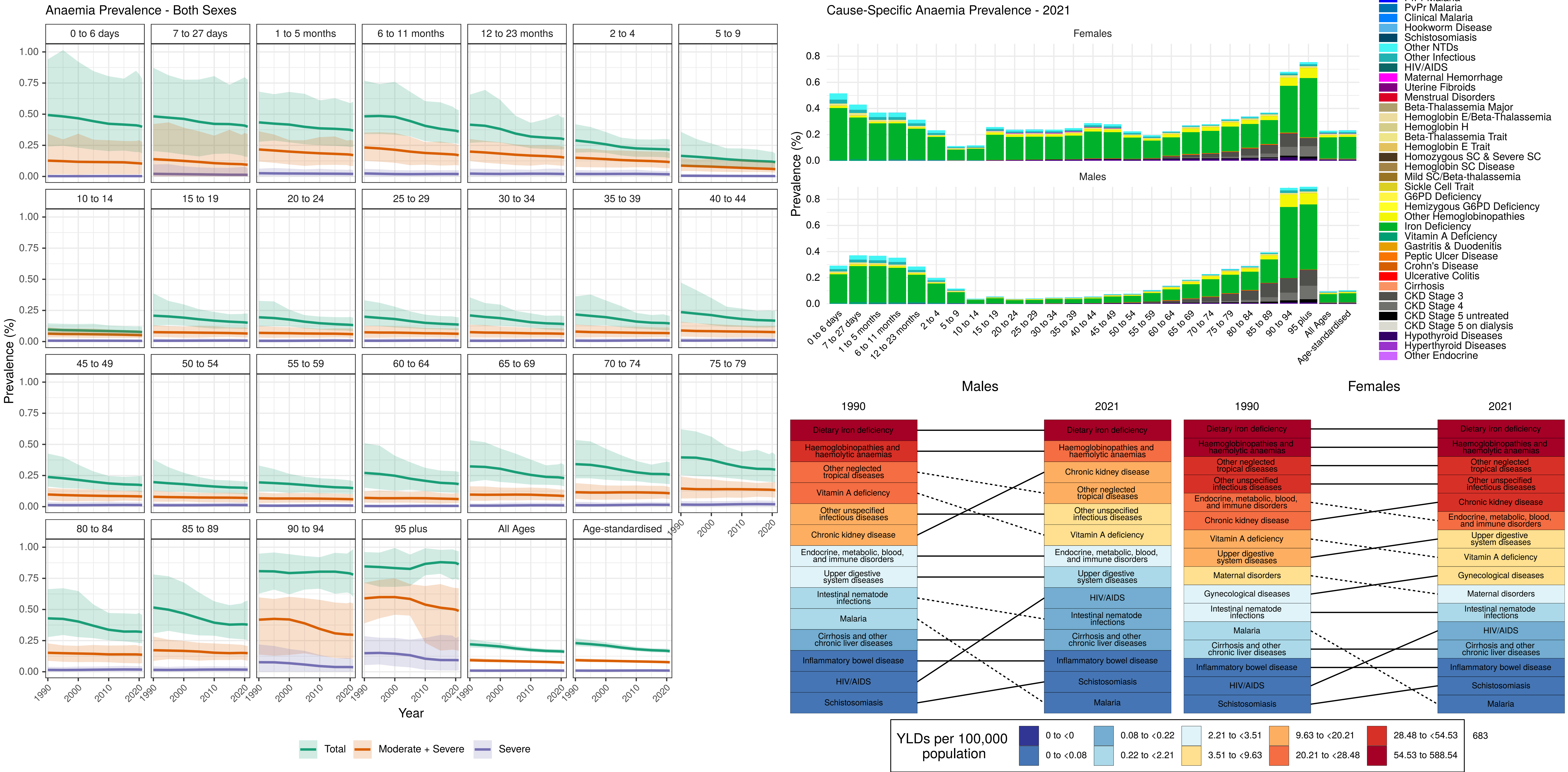
Anaemia Prevalence - Both Sexes
0 to 6 days
7 to 27 days
1 to 5 months
6 to 11 months
12 to 23 months
2 to 4
5 to 9
10 to 14
15 to 19
20 to 24
25 to 29
30 to 34
35 to 39
40 to 44
45 to 49
50 to 54
55 to 59
60 to 64
65 to 69
70 to 74
75 to 79
80 to 84
85 to 89
90 to 94
95 plus
All Ages
Age-standardised
Prevalence (%)
0.00
0.25
0.50
0.75
1.00
1990
2000
2010
2020
Year
Total
Moderate + Severe
Severe
Cause-Specific Anaemia Prevalence - 2021
Females
Males
Prevalence (%)
0.0
0.2
0.4
0.6
0.8
0 to 6 days
7 to 27 days
1 to 5 months
6 to 11 months
12 to 23 months
2 to 4
5 to 9
10 to 14
15 to 19
20 to 24
25 to 29
30 to 34
35 to 39
40 to 44
45 to 49
50 to 54
55 to 59
60 to 64
65 to 69
70 to 74
75 to 79
80 to 84
85 to 89
90 to 94
95 plus
All Ages
Age-standardised
PfPr Malaria
PvPr Malaria
Clinical Malaria
Hookworm Disease
Schistosomiasis
Other NTDs
Other Infectious
HIV/AIDS
Maternal Hemorrhage
Uterine Fibroids
Menstrual Disorders
Beta-Thalassemia Major
Hemoglobin E/Beta-Thalassemia
Hemoglobin H
Beta-Thalassemia Trait
Hemoglobin E Trait
Homozygous SC & Severe SC
Hemoglobin SC Disease
Mild SC/Beta-thalassemia
Sickle Cell Trait
G6PD Deficiency
Hemizygous G6PD Deficiency
Other Hemoglobinopathies
Iron Deficiency
Vitamin A Deficiency
Gastritis & Duodenitis
Peptic Ulcer Disease
Crohn's Disease
Ulcerative Colitis
Cirrhosis
CKD Stage 3
CKD Stage 4
CKD Stage 5 untreated
CKD Stage 5 on dialysis
Hypothyroid Diseases
Hyperthyroid Diseases
Other Endocrine
Males
1990
Dietary iron deficiency
Haemoglobinopathies and haemolytic anaemias
Other neglected tropical diseases
Vitamin A deficiency
Other unspecified infectious diseases
Chronic kidney disease
Endocrine, metabolic, blood, and immune disorders
Upper digestive system diseases
Intestinal nematode infections
Malaria
Cirrhosis and other chronic liver diseases
Inflammatory bowel disease
HIV/AIDS
Schistosomiasis
2021
Dietary iron deficiency
Haemoglobinopathies and haemolytic anaemias
Chronic kidney disease
Other neglected tropical diseases
Other unspecified infectious diseases
Vitamin A deficiency
Endocrine, metabolic, blood, and immune disorders
Upper digestive system diseases
HIV/AIDS
Intestinal nematode infections
Cirrhosis and other chronic liver diseases
Inflammatory bowel disease
Schistosomiasis
Malaria
Females
1990
Dietary iron deficiency
Haemoglobinopathies and haemolytic anaemias
Other neglected tropical diseases
Other unspecified infectious diseases
Endocrine, metabolic, blood, and immune disorders
Chronic kidney disease
Vitamin A deficiency
Upper digestive system diseases
Maternal disorders
Gynecological diseases
Intestinal nematode infections
Malaria
Cirrhosis and other chronic liver diseases
Inflammatory bowel disease
HIV/AIDS
Schistosomiasis
2021
Dietary iron deficiency
Haemoglobinopathies and haemolytic anaemias
Other neglected tropical diseases
Other unspecified infectious diseases
Chronic kidney disease
Endocrine, metabolic, blood, and immune disorders
Upper digestive system diseases
Vitamin A deficiency
Gynecological diseases
Maternal disorders
Intestinal nematode infections
HIV/AIDS
Cirrhosis and other chronic liver diseases
Inflammatory bowel disease
Schistosomiasis
Malaria
YLDs per 100,000 population
0 to <0
0 to <0.08
0.08 to <0.22
0.22 to <2.21
2.21 to <3.51
3.51 to <9.63
9.63 to <20.21
20.21 to <28.48
28.48 to <54.53
54.53 to 588.54

Agusan Del Norte

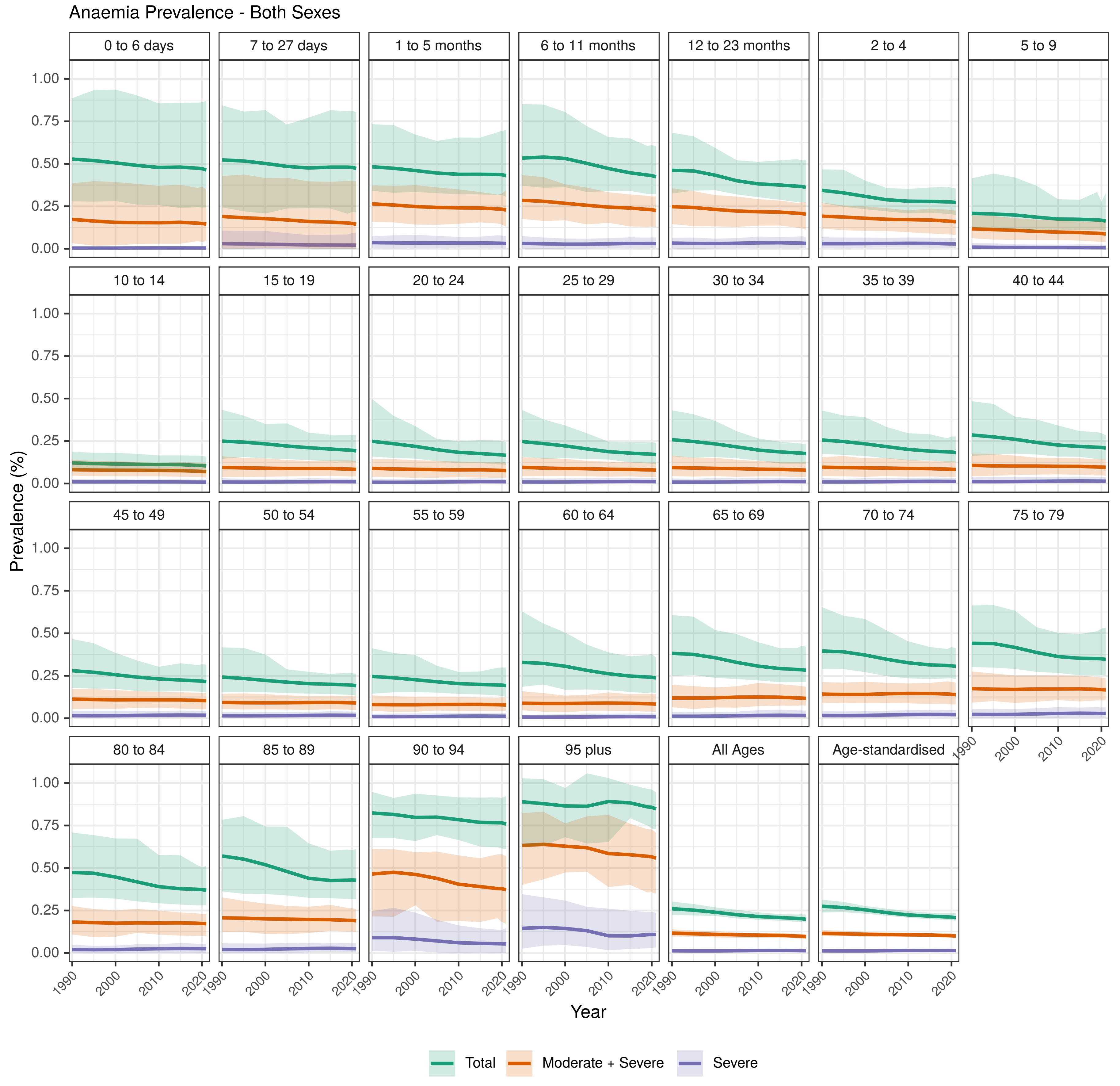

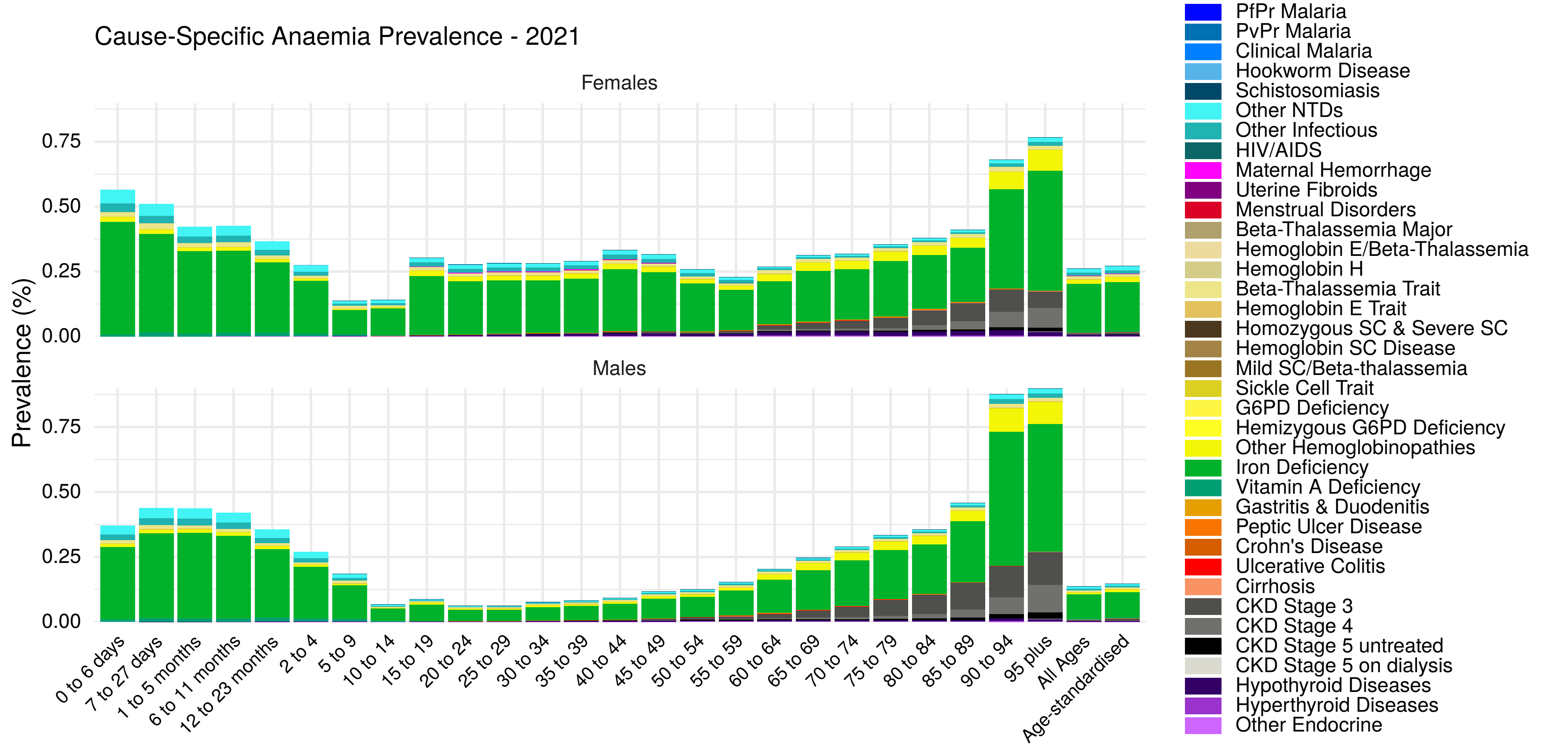

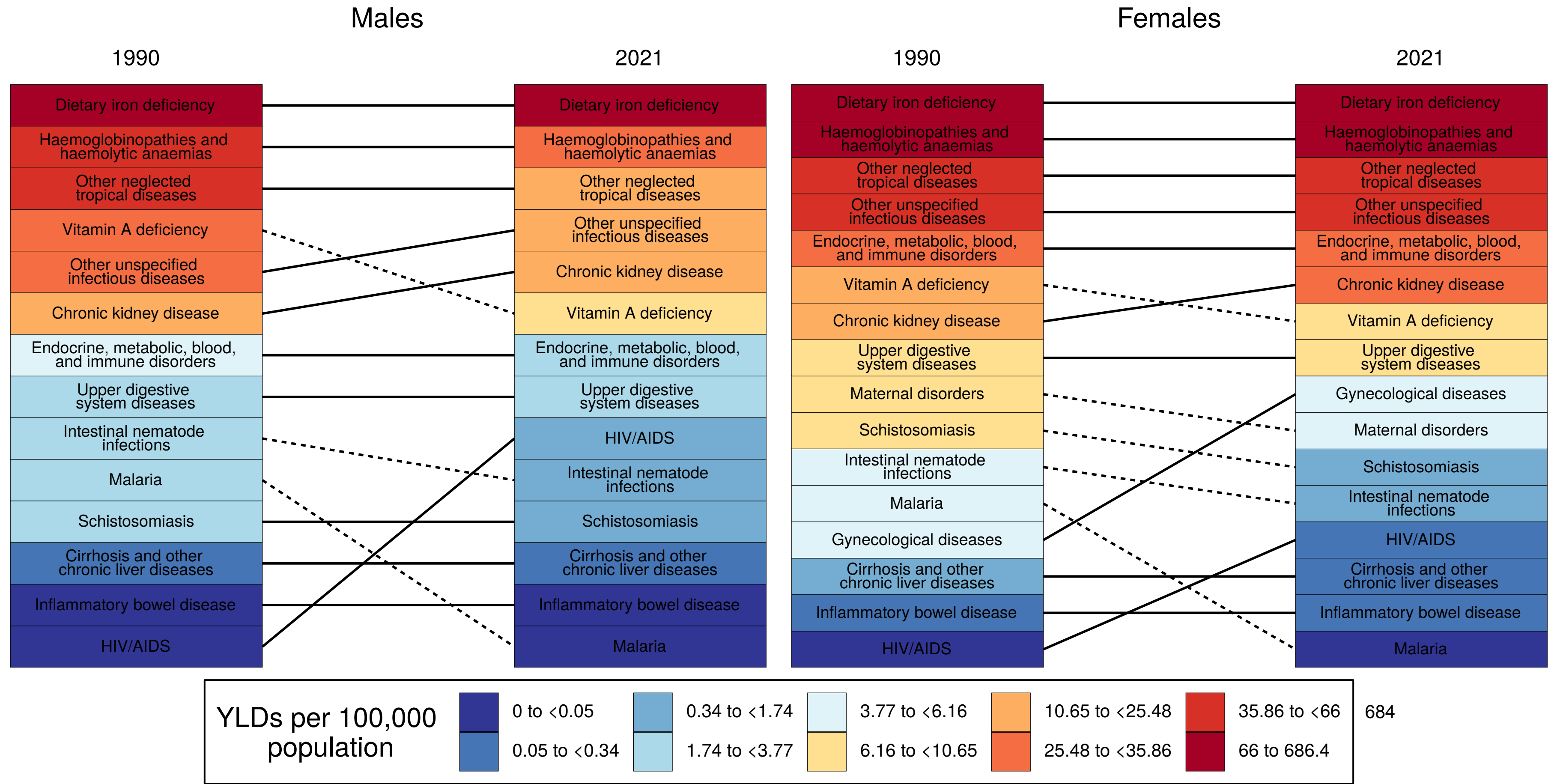

Agusan Del Sur

Anaemia Prevalence - Both Sexes

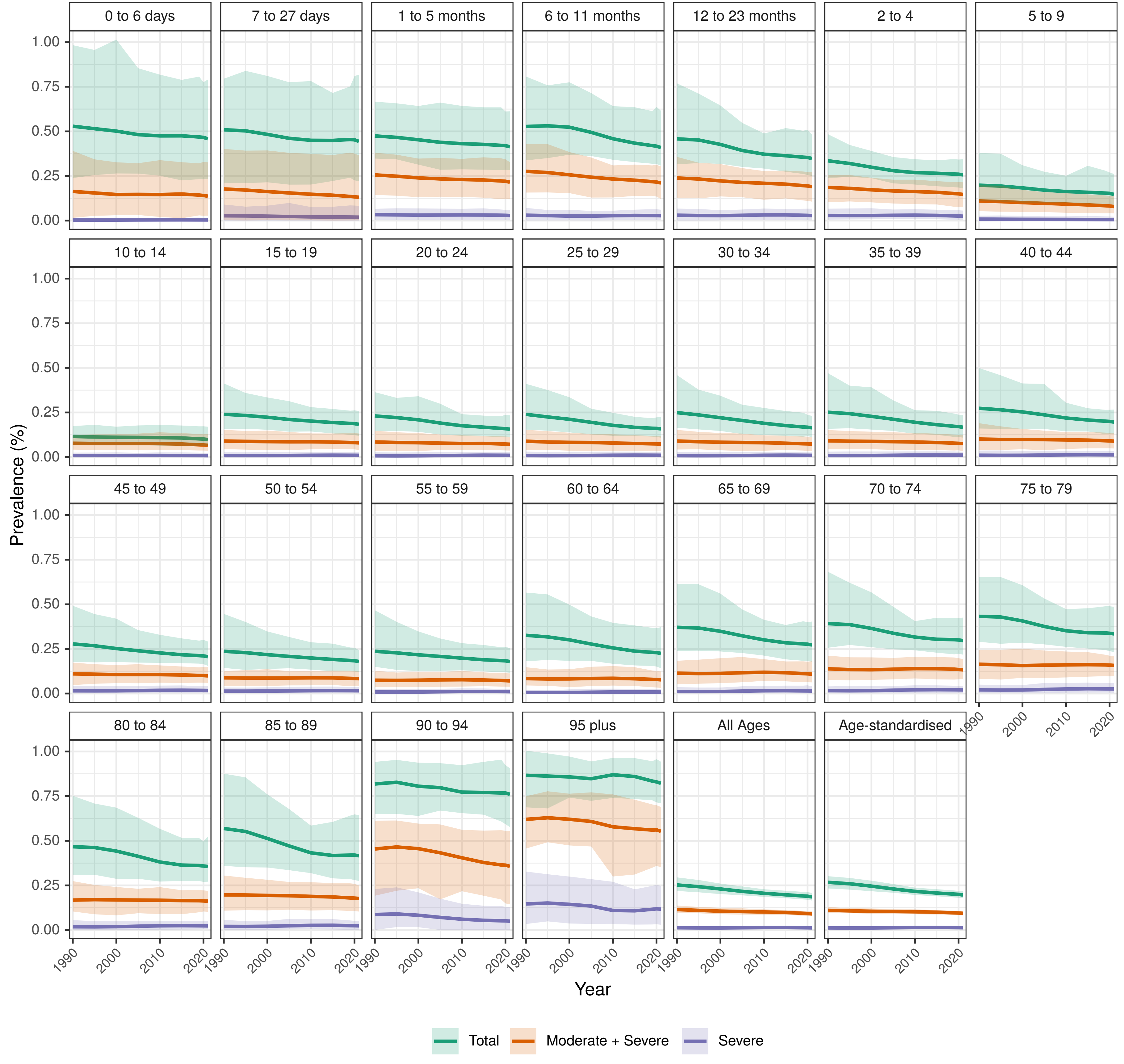

Cause-Specific Anaemia Prevalence - 2021

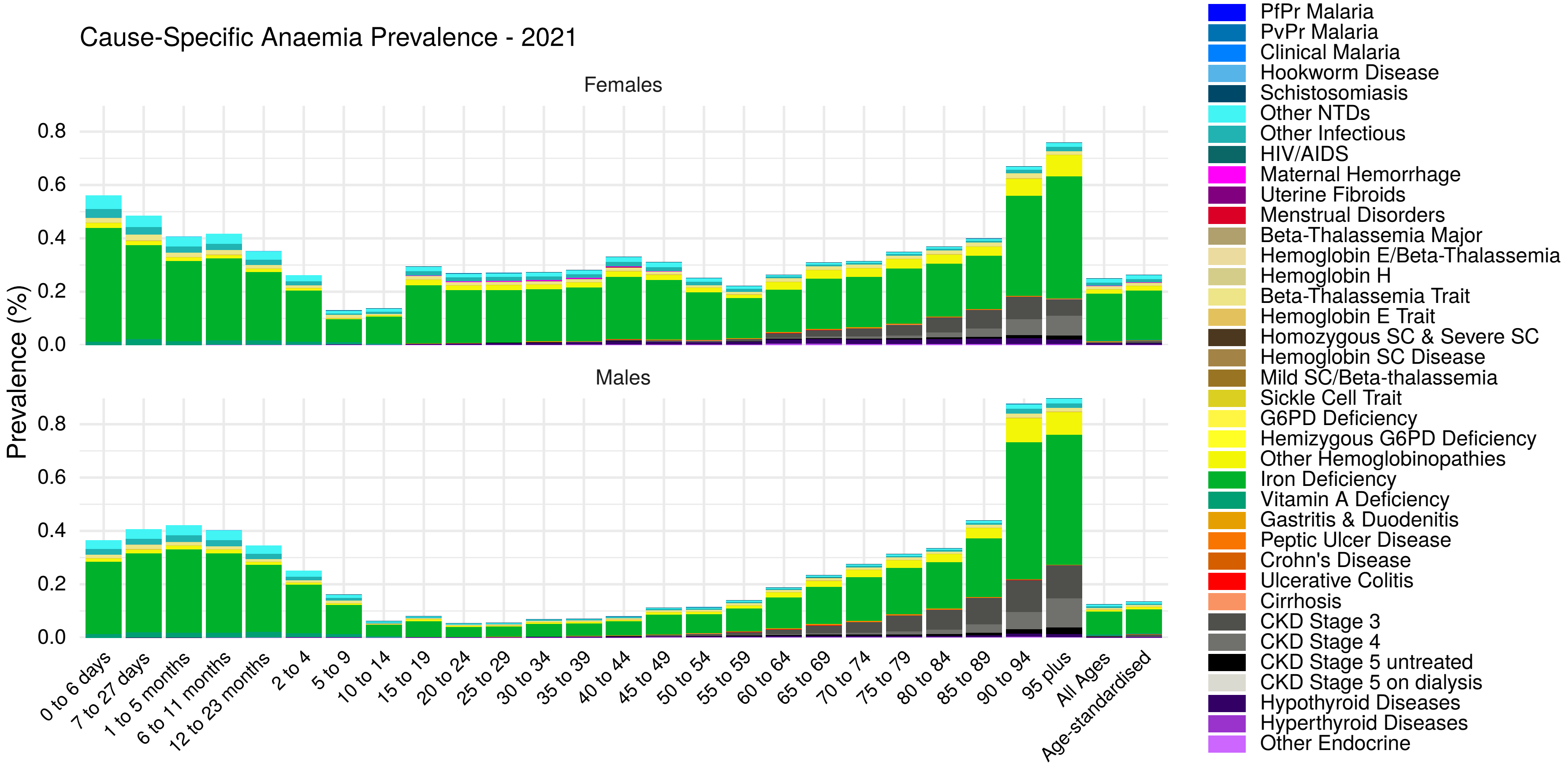

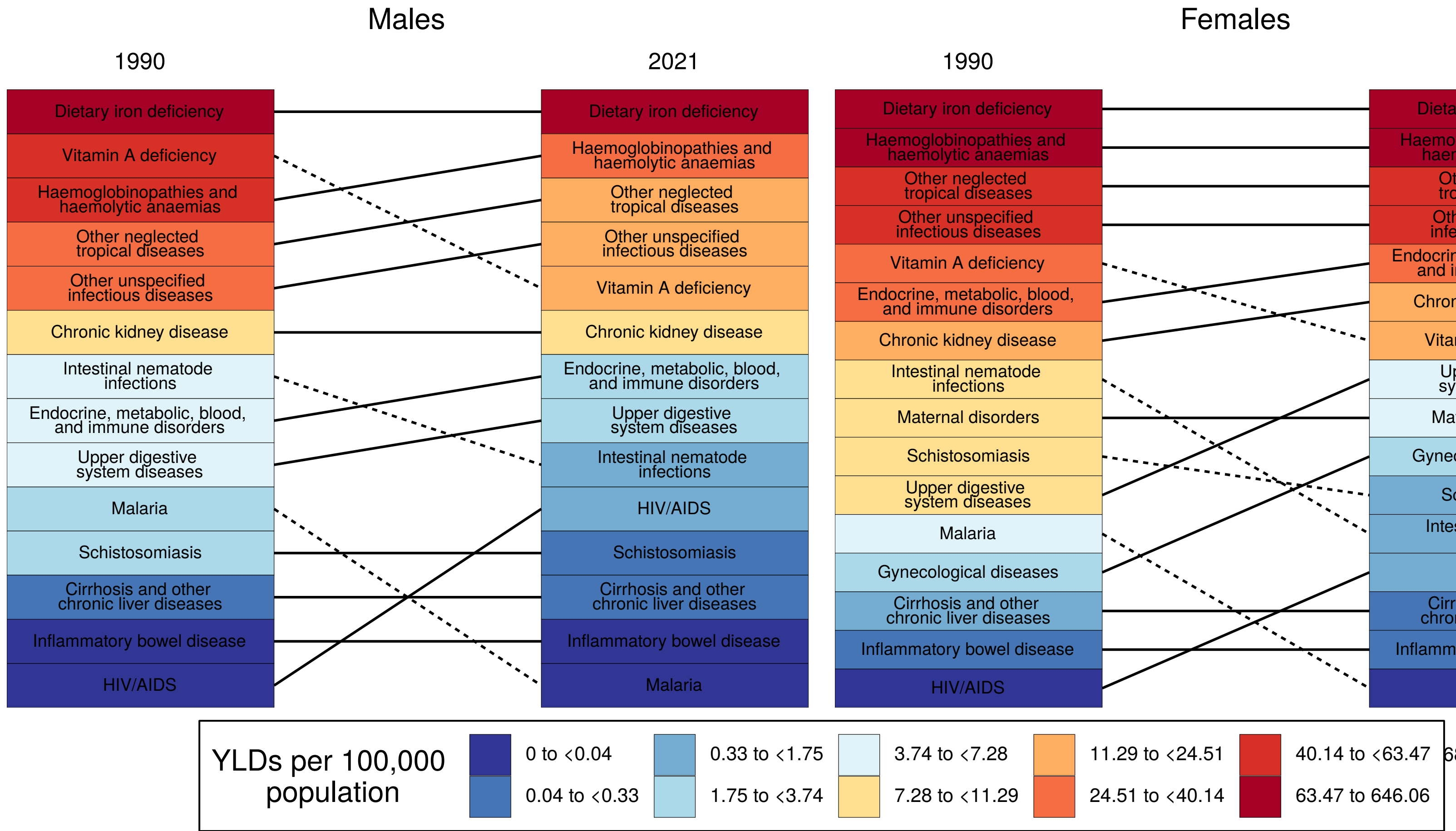

Aklan

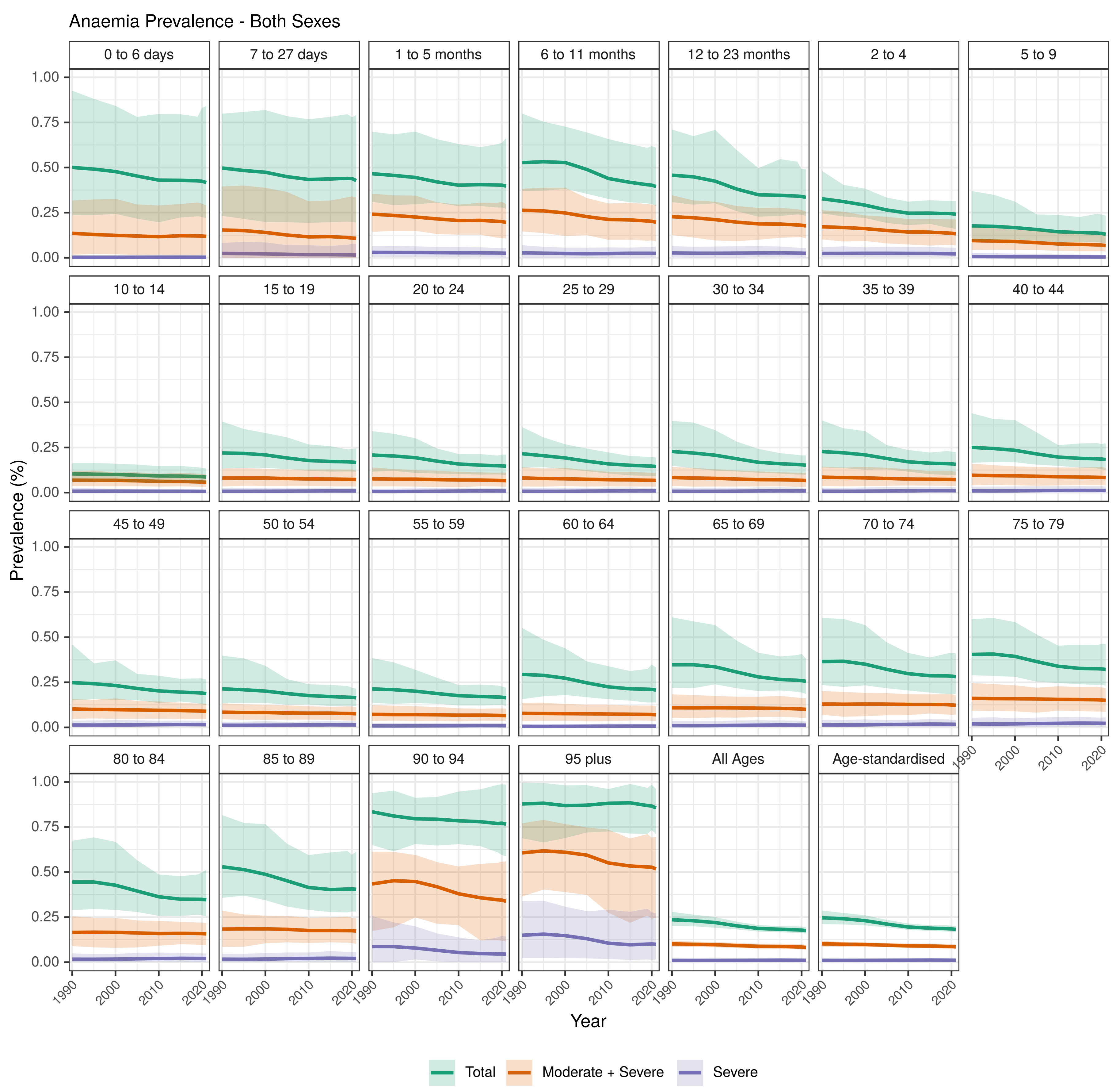
Anaemia Prevalence - Both Sexes
0 to 6 days
7 to 27 days
1 to 5 months
6 to 11 months
12 to 23 months
2 to 4
5 to 9
10 to 14
15 to 19
20 to 24
25 to 29
30 to 34
35 to 39
40 to 44
45 to 49
50 to 54
55 to 59
60 to 64
65 to 69
70 to 74
75 to 79
80 to 84
85 to 89
90 to 94
95 plus
All Ages
Age-standardised
Prevalence (%)
0.00
0.25
0.50
0.75
1.00
1990
2000
2010
2020
Year
Total
Moderate + Severe
Severe

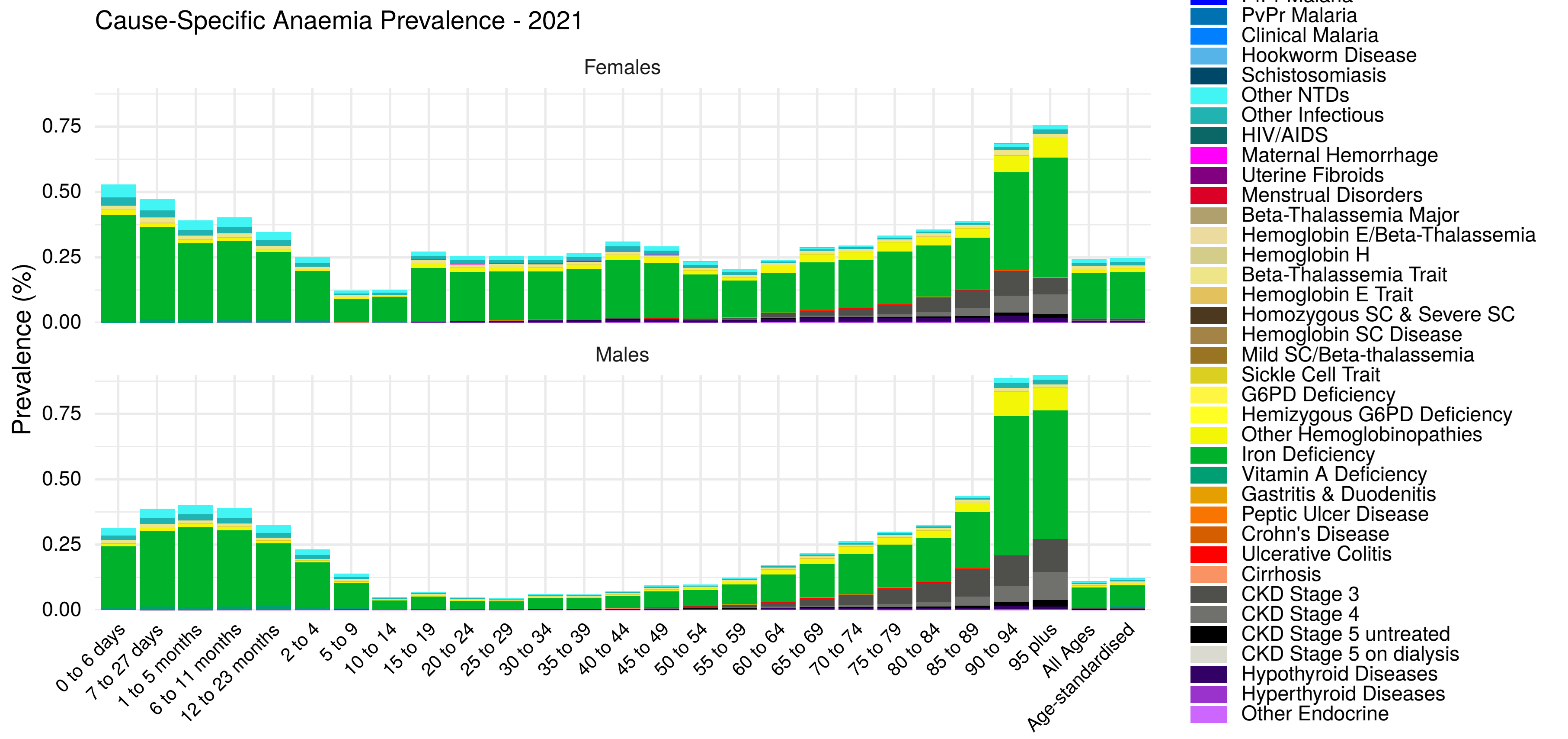
Cause-Specific Anaemia Prevalence - 2021
Females
Males
Prevalence (%)
0.00
0.25
0.50
0.75
0 to 6 days
7 to 27 days
1 to 5 months
6 to 11 months
12 to 23 months
2 to 4
5 to 9
10 to 14
15 to 19
20 to 24
25 to 29
30 to 34
35 to 39
40 to 44
45 to 49
50 to 54
55 to 59
60 to 64
65 to 69
70 to 74
75 to 79
80 to 84
85 to 89
90 to 94
95 plus
All Ages
Age-standardised
PfPr Malaria
PvPr Malaria
Clinical Malaria
Hookworm Disease
Schistosomiasis
Other NTDs
Other Infectious
HIV/AIDS
Maternal Hemorrhage
Uterine Fibroids
Menstrual Disorders
Beta-Thalassemia Major
Hemoglobin E/Beta-Thalassemia
Hemoglobin H
Beta-Thalassemia Trait
Hemoglobin E Trait
Homozygous SC & Severe SC
Hemoglobin SC Disease
Mild SC/Beta-thalassemia
Sickle Cell Trait
G6PD Deficiency
Hemizygous G6PD Deficiency
Other Hemoglobinopathies
Iron Deficiency
Vitamin A Deficiency
Gastritis & Duodenitis
Peptic Ulcer Disease
Crohn's Disease
Ulcerative Colitis
Cirrhosis
CKD Stage 3
CKD Stage 4
CKD Stage 5 untreated
CKD Stage 5 on dialysis
Hypothyroid Diseases
Hyperthyroid Diseases
Other Endocrine

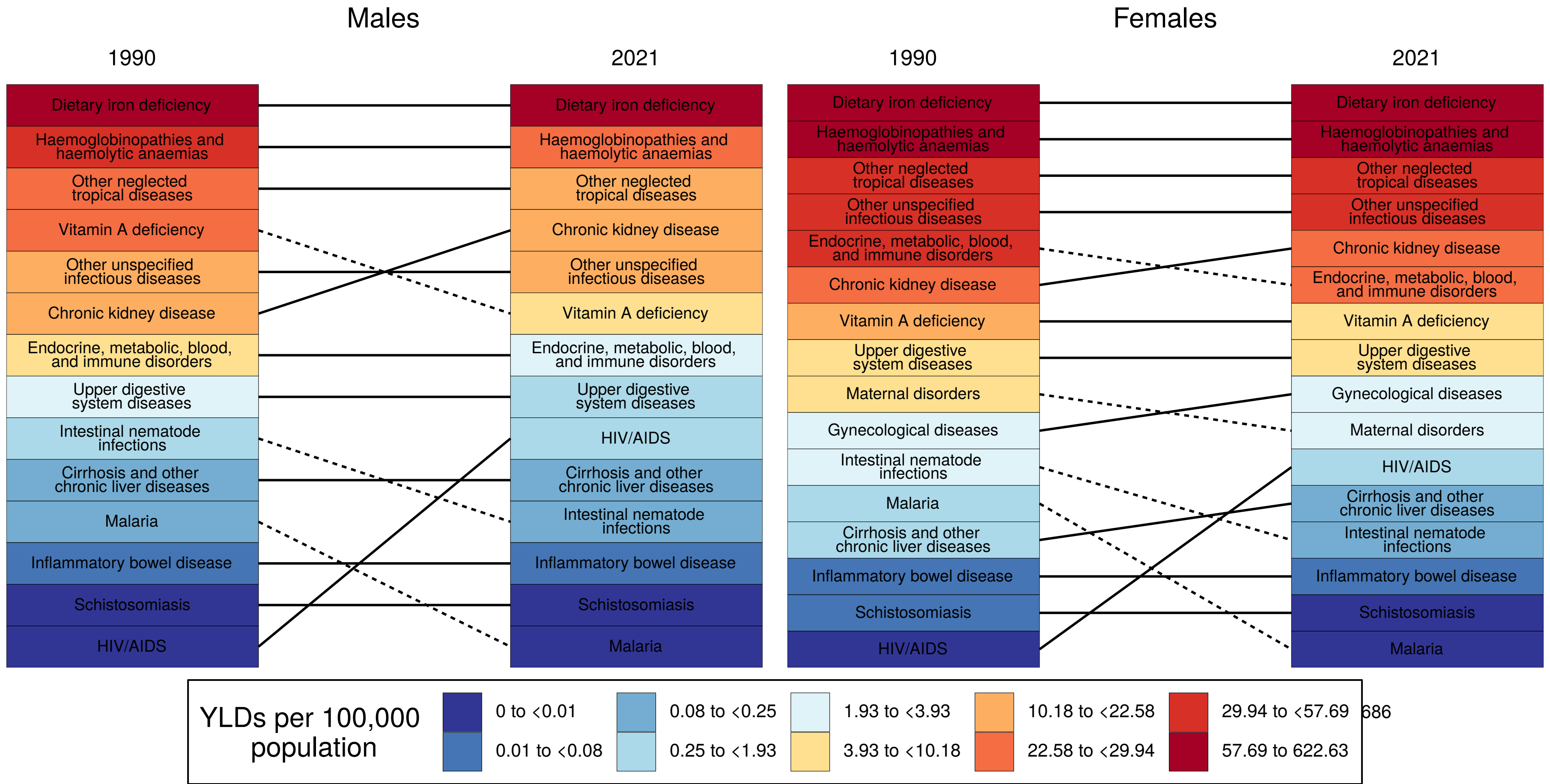
Males
1990
2021
Dietary iron deficiency
Haemoglobinopathies and haemolytic anaemias
Other neglected tropical diseases
Vitamin A deficiency
Other unspecified infectious diseases
Chronic kidney disease
Endocrine, metabolic, blood, and immune disorders
Upper digestive system diseases
Intestinal nematode infections
Cirrhosis and other chronic liver diseases
Malaria
Inflammatory bowel disease
Schistosomiasis
HIV/AIDS
Dietary iron deficiency
Haemoglobinopathies and haemolytic anaemias
Other neglected tropical diseases
Chronic kidney disease
Other unspecified infectious diseases
Vitamin A deficiency
Endocrine, metabolic, blood, and immune disorders
Upper digestive system diseases
HIV/AIDS
Cirrhosis and other chronic liver diseases
Intestinal nematode infections
Inflammatory bowel disease
Schistosomiasis
Malaria
Females
1990
2021
Dietary iron deficiency
Haemoglobinopathies and haemolytic anaemias
Other neglected tropical diseases
Other unspecified infectious diseases
Endocrine, metabolic, blood, and immune disorders
Chronic kidney disease
Vitamin A deficiency
Upper digestive system diseases
Maternal disorders
Gynecological diseases
Intestinal nematode infections
Malaria
Cirrhosis and other chronic liver diseases
Inflammatory bowel disease
Schistosomiasis
HIV/AIDS
Dietary iron deficiency
Haemoglobinopathies and haemolytic anaemias
Other neglected tropical diseases
Other unspecified infectious diseases
Chronic kidney disease
Endocrine, metabolic, blood, and immune disorders
Vitamin A deficiency
Upper digestive system diseases
Gynecological diseases
Maternal disorders
HIV/AIDS
Cirrhosis and other chronic liver diseases
Intestinal nematode infections
Inflammatory bowel disease
Schistosomiasis
Malaria
YLDs per 100,000 population
0 to <0.01
0.01 to <0.08
0.08 to <0.25
0.25 to <1.93
1.93 to <3.93
3.93 to <10.18
10.18 to <22.58
22.58 to <29.94
29.94 to <57.69
57.69 to 622.63
686

Albay

Anaemia Prevalence - Both Sexes

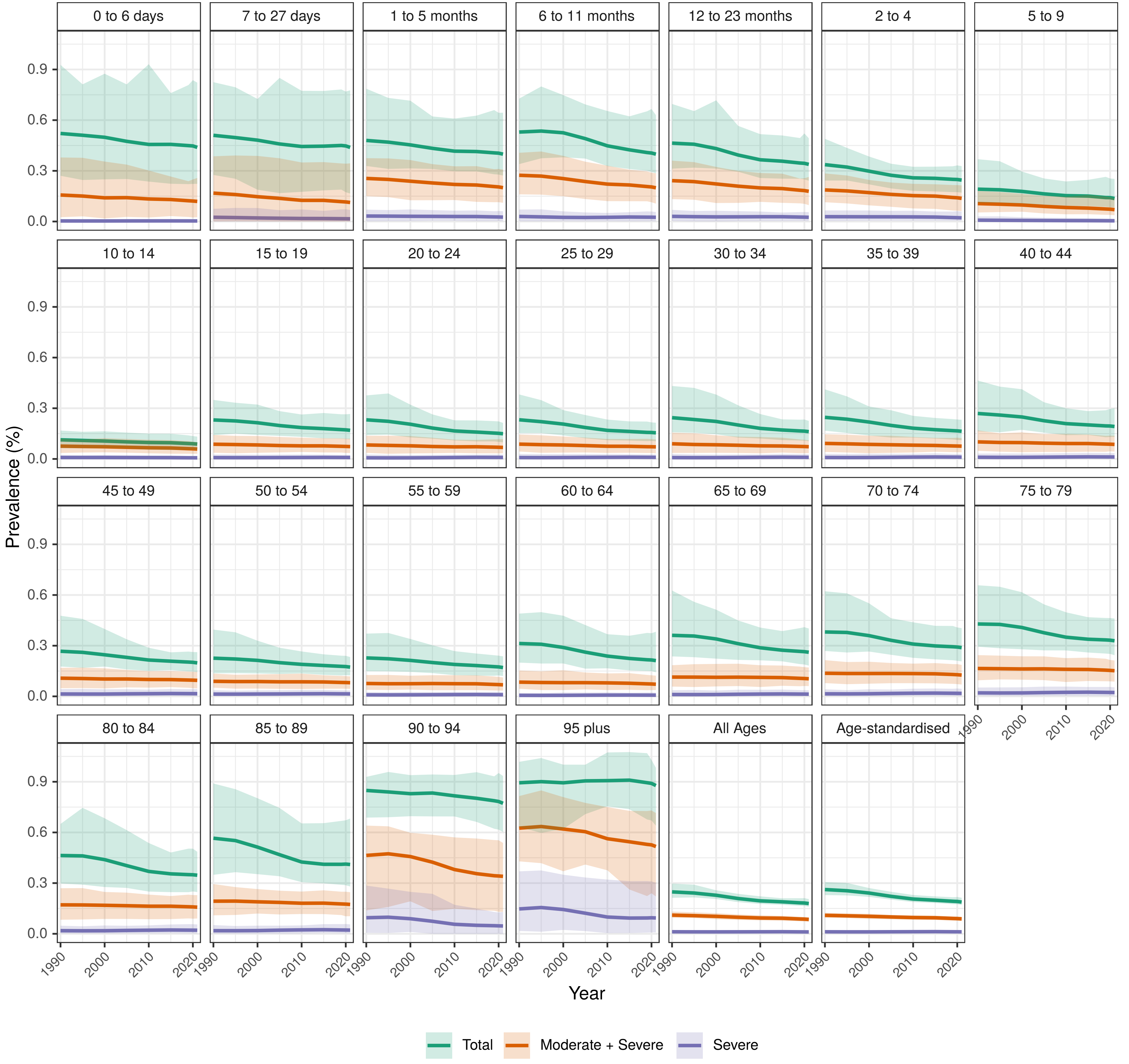

Cause-Specific Anaemia Prevalence - 2021

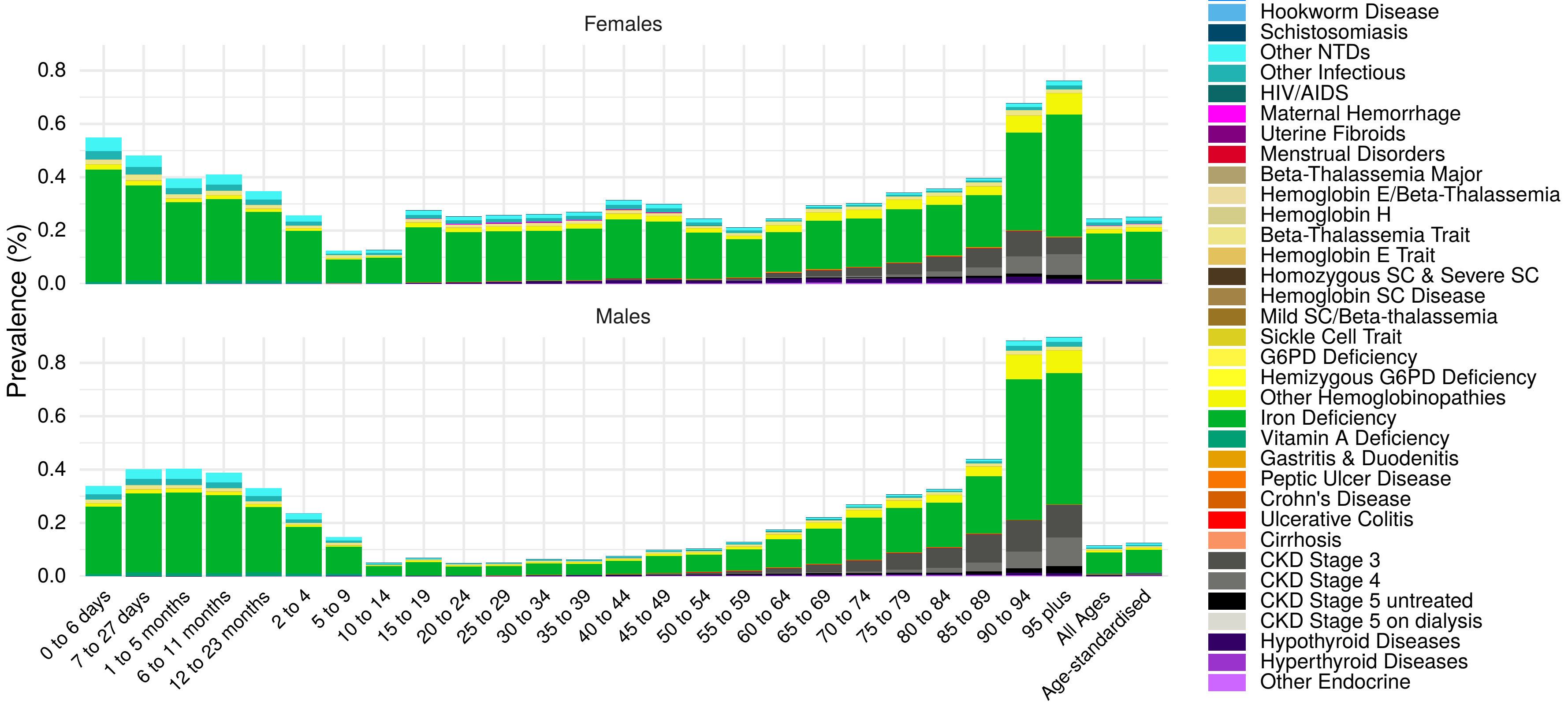

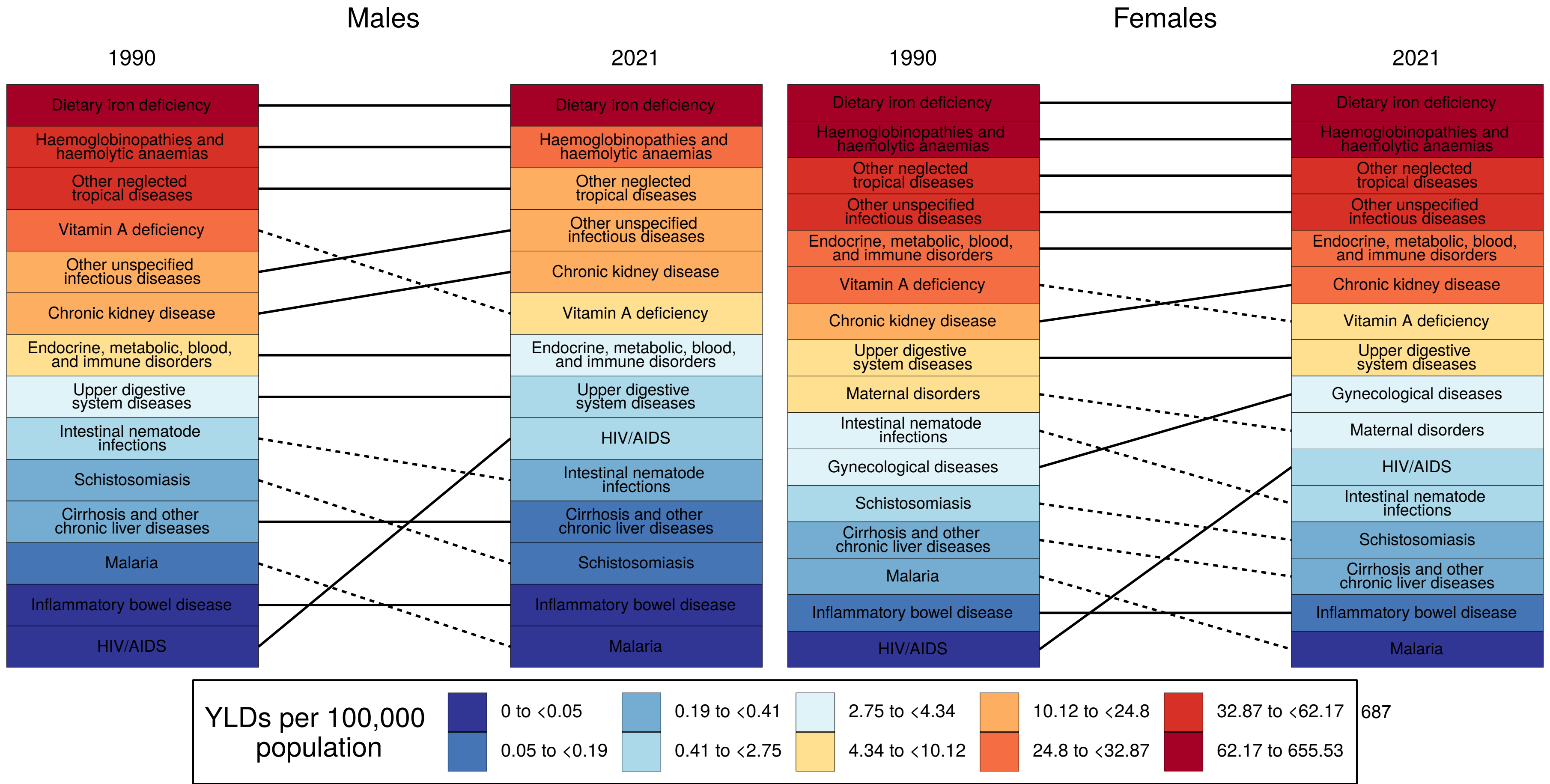

Antique

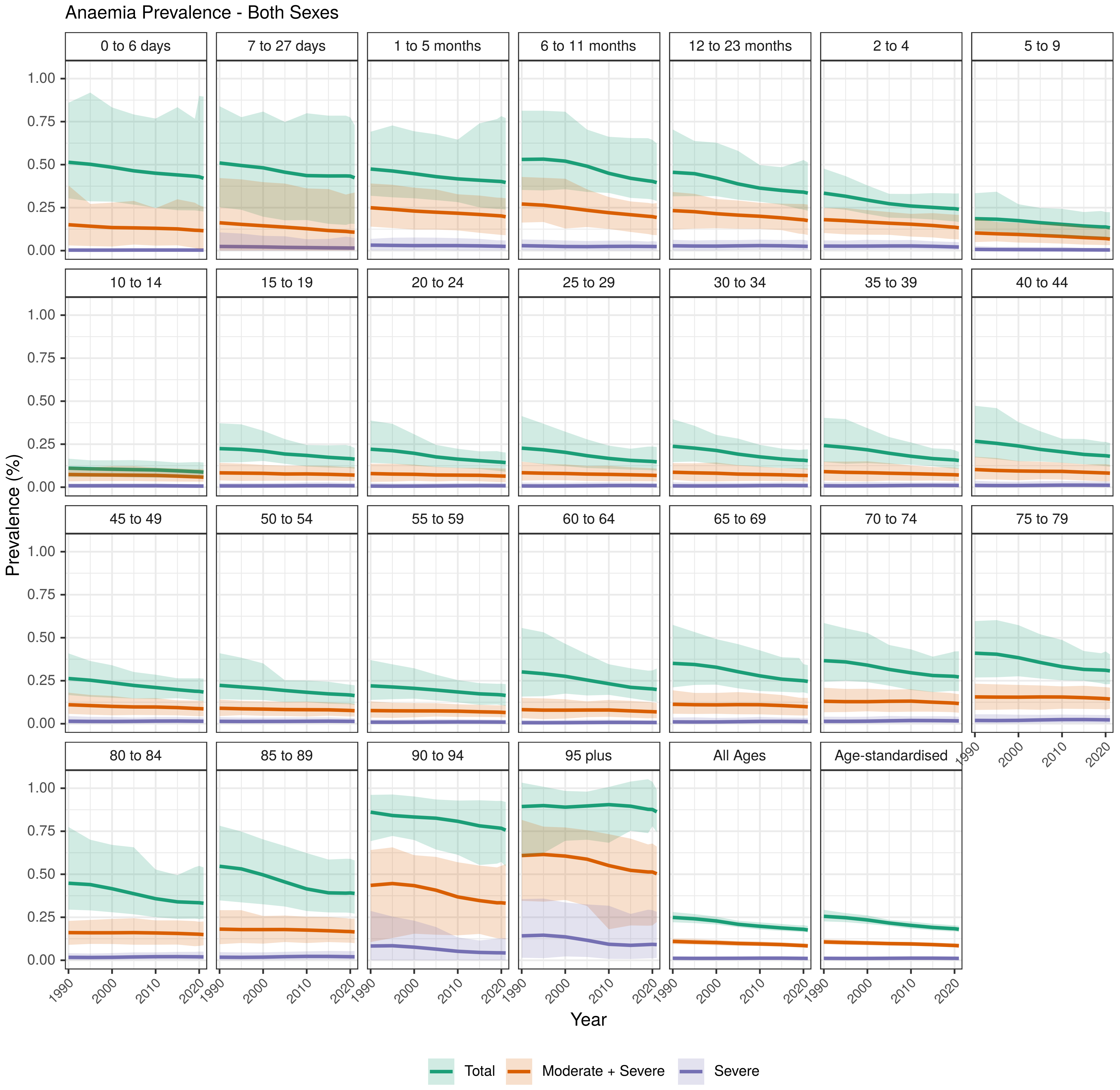
Anaemia Prevalence - Both Sexes
0 to 6 days
7 to 27 days
1 to 5 months
6 to 11 months
12 to 23 months
2 to 4
5 to 9
10 to 14
15 to 19
20 to 24
25 to 29
30 to 34
35 to 39
40 to 44
45 to 49
50 to 54
55 to 59
60 to 64
65 to 69
70 to 74
75 to 79
80 to 84
85 to 89
90 to 94
95 plus
All Ages
Age-standardised
Prevalence (%)
Year
Total
Moderate + Severe
Severe

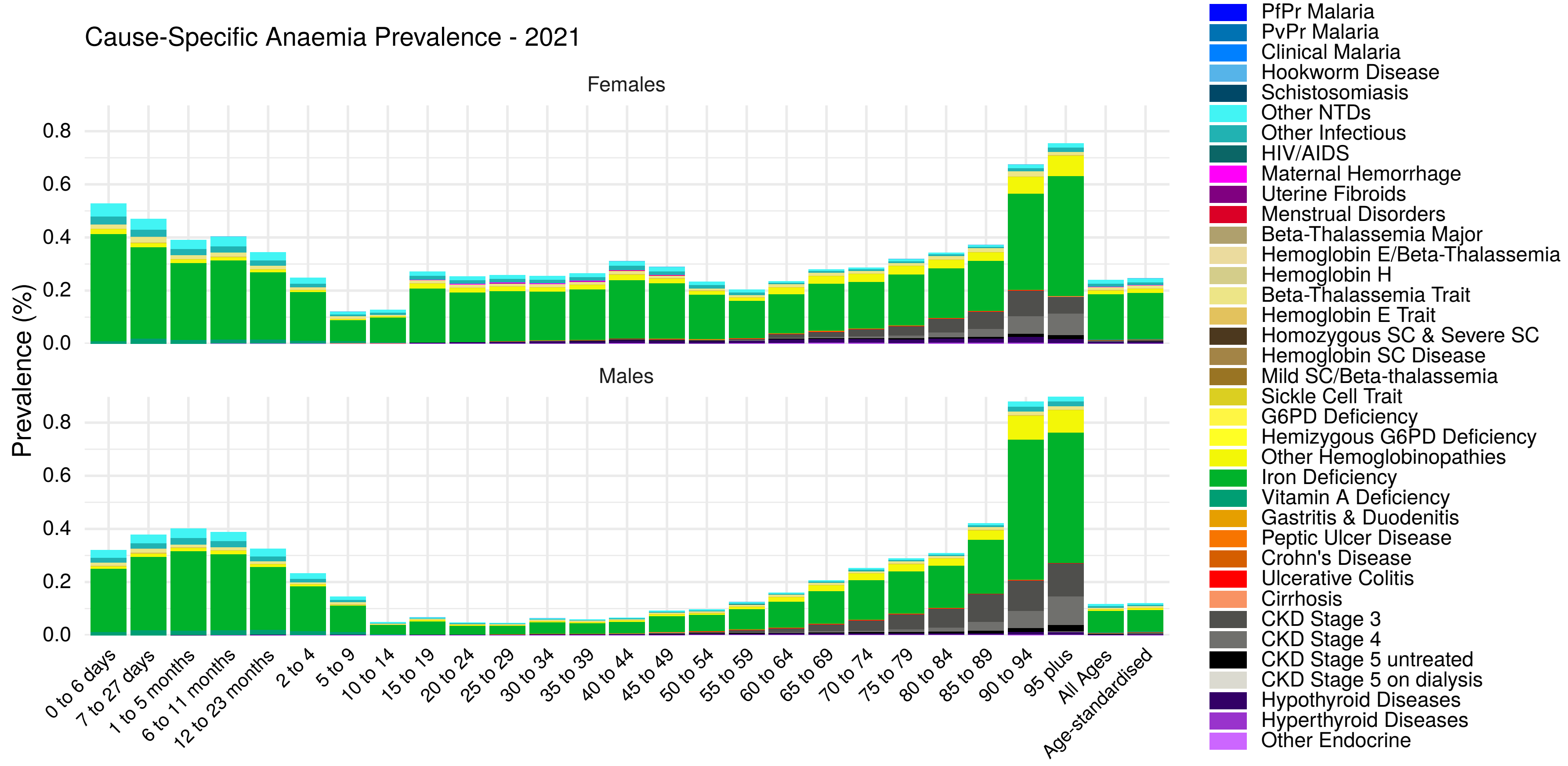
Cause-Specific Anaemia Prevalence - 2021
Females
Males
Prevalence (%)
PfPr Malaria
PvPr Malaria
Clinical Malaria
Hookworm Disease
Schistosomiasis
Other NTDs
Other Infectious
HIV/AIDS
Maternal Hemorrhage
Uterine Fibroids
Menstrual Disorders
Beta-Thalassemia Major
Hemoglobin E/Beta-Thalassemia
Hemoglobin H
Beta-Thalassemia Trait
Hemoglobin E Trait
Homozygous SC & Severe SC
Hemoglobin SC Disease
Mild SC/Beta-thalassemia
Sickle Cell Trait
G6PD Deficiency
Hemizygous G6PD Deficiency
Other Hemoglobinopathies
Iron Deficiency
Vitamin A Deficiency
Gastritis & Duodenitis
Peptic Ulcer Disease
Crohn's Disease
Ulcerative Colitis
Cirrhosis
CKD Stage 3
CKD Stage 4
CKD Stage 5 untreated
CKD Stage 5 on dialysis
Hypothyroid Diseases
Hyperthyroid Diseases
Other Endocrine

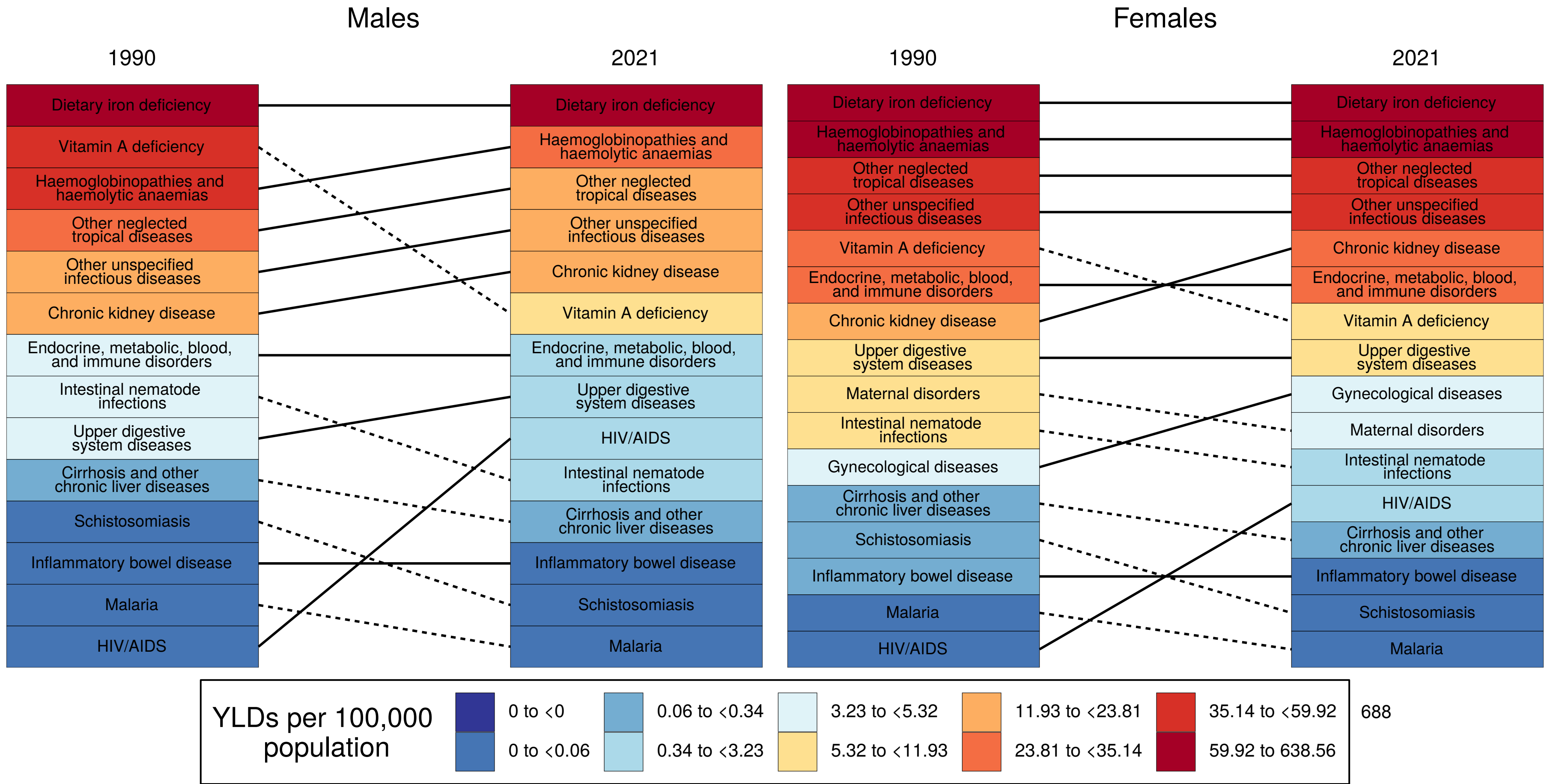
Males
Females
1990
2021
Dietary iron deficiency
Vitamin A deficiency
Haemoglobinopathies and haemolytic anaemias
Other neglected tropical diseases
Other unspecified infectious diseases
Chronic kidney disease
Endocrine, metabolic, blood, and immune disorders
Intestinal nematode infections
Upper digestive system diseases
Cirrhosis and other chronic liver diseases
Schistosomiasis
Inflammatory bowel disease
Malaria
HIV/AIDS
Maternal disorders
Gynecological diseases
YLDs per 100,000 population
0 to <0
0 to <0.06
0.06 to <0.34
0.34 to <3.23
3.23 to <5.32
5.32 to <11.93
11.93 to <23.81
23.81 to <35.14
35.14 to <59.92
59.92 to 638.56

Apayao

Anaemia Prevalence - Both Sexes

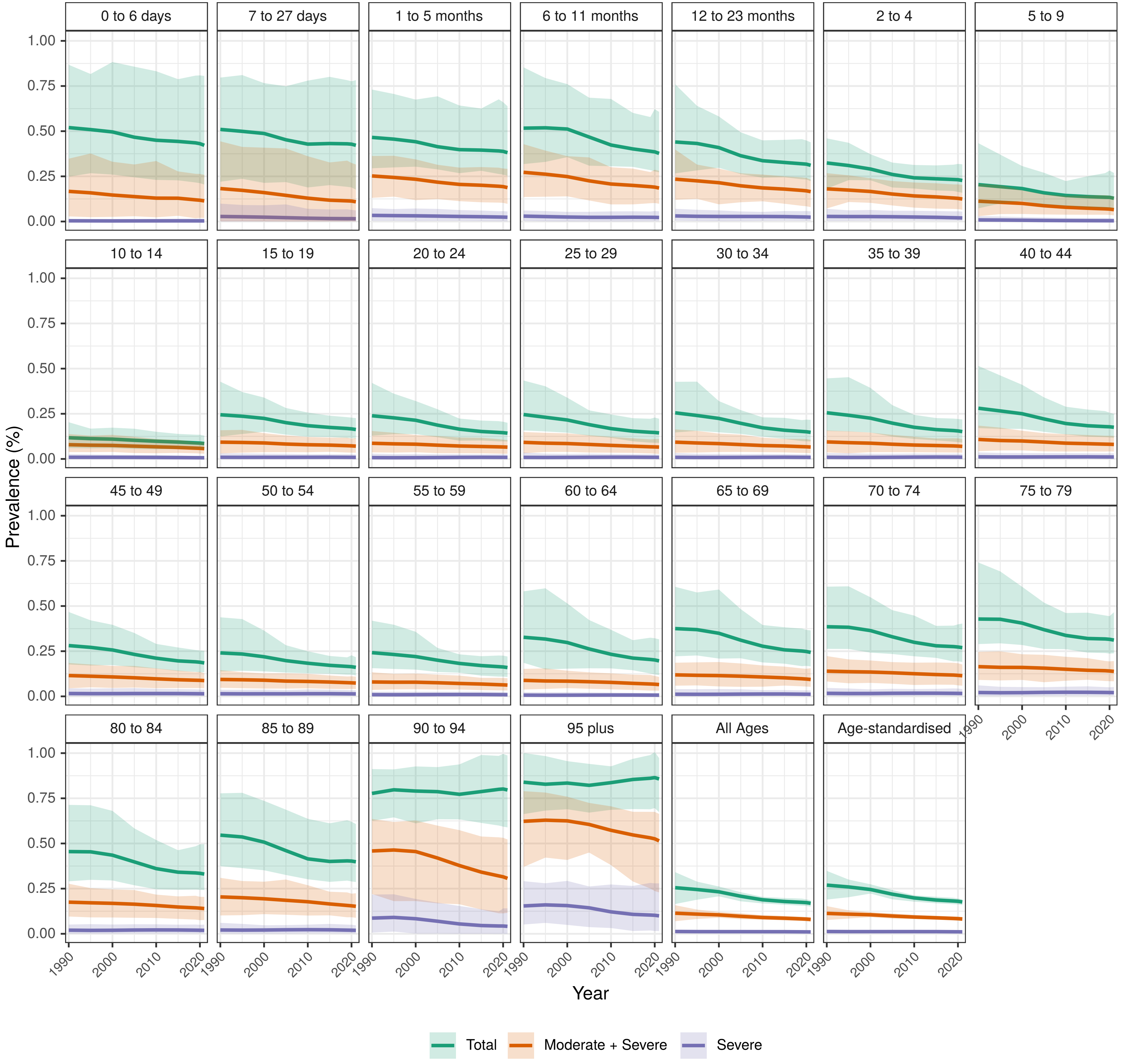

Cause-Specific Anaemia Prevalence - 2021

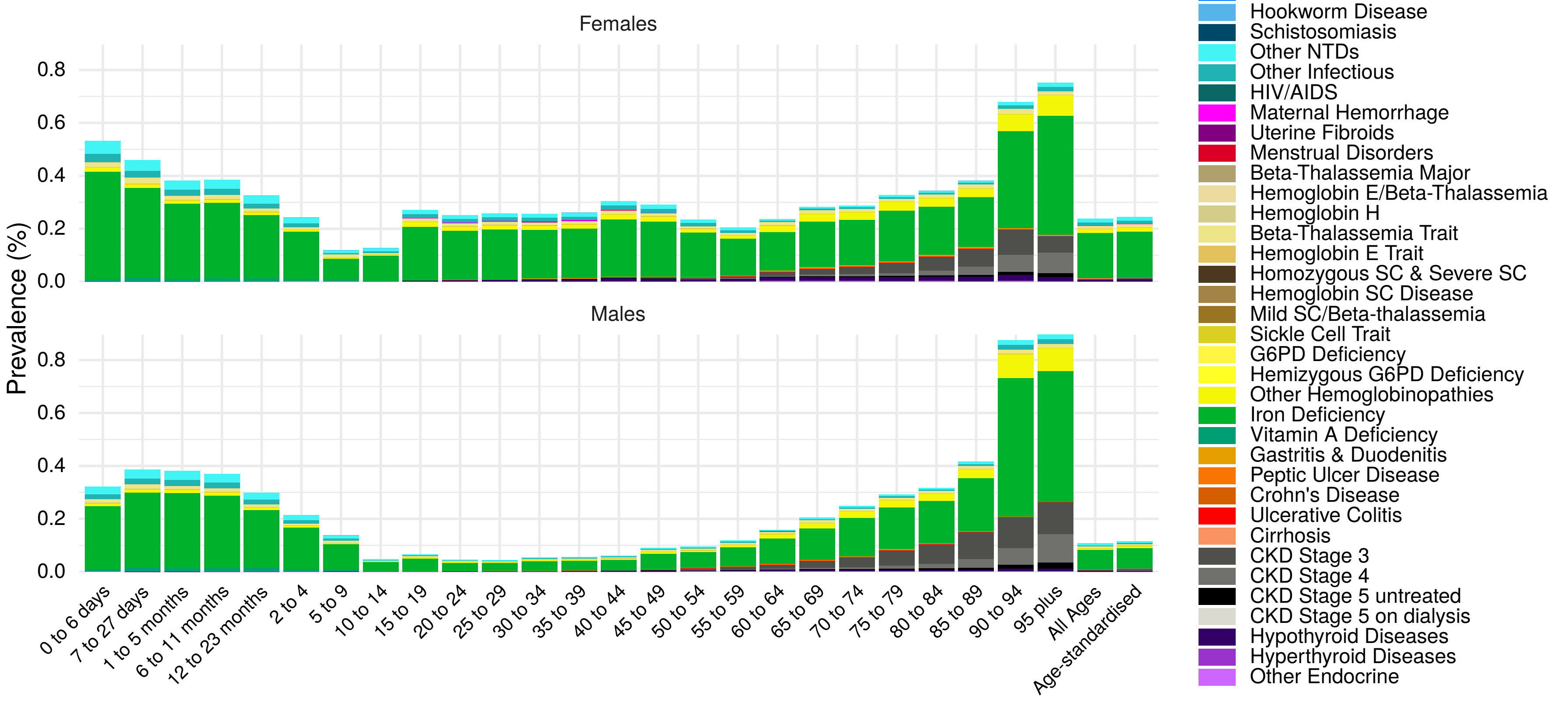

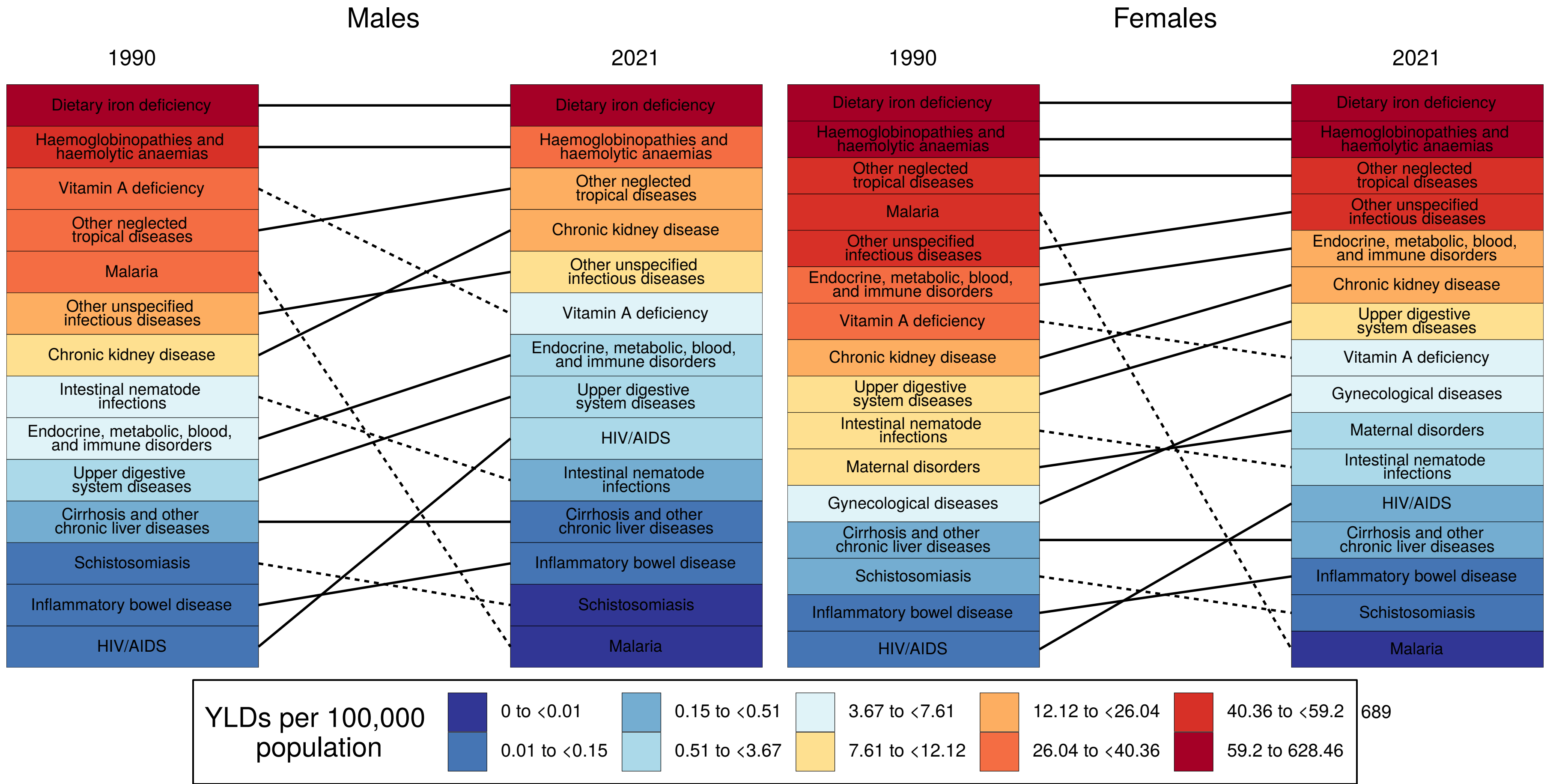

Aurora

Anaemia Prevalence - Both Sexes

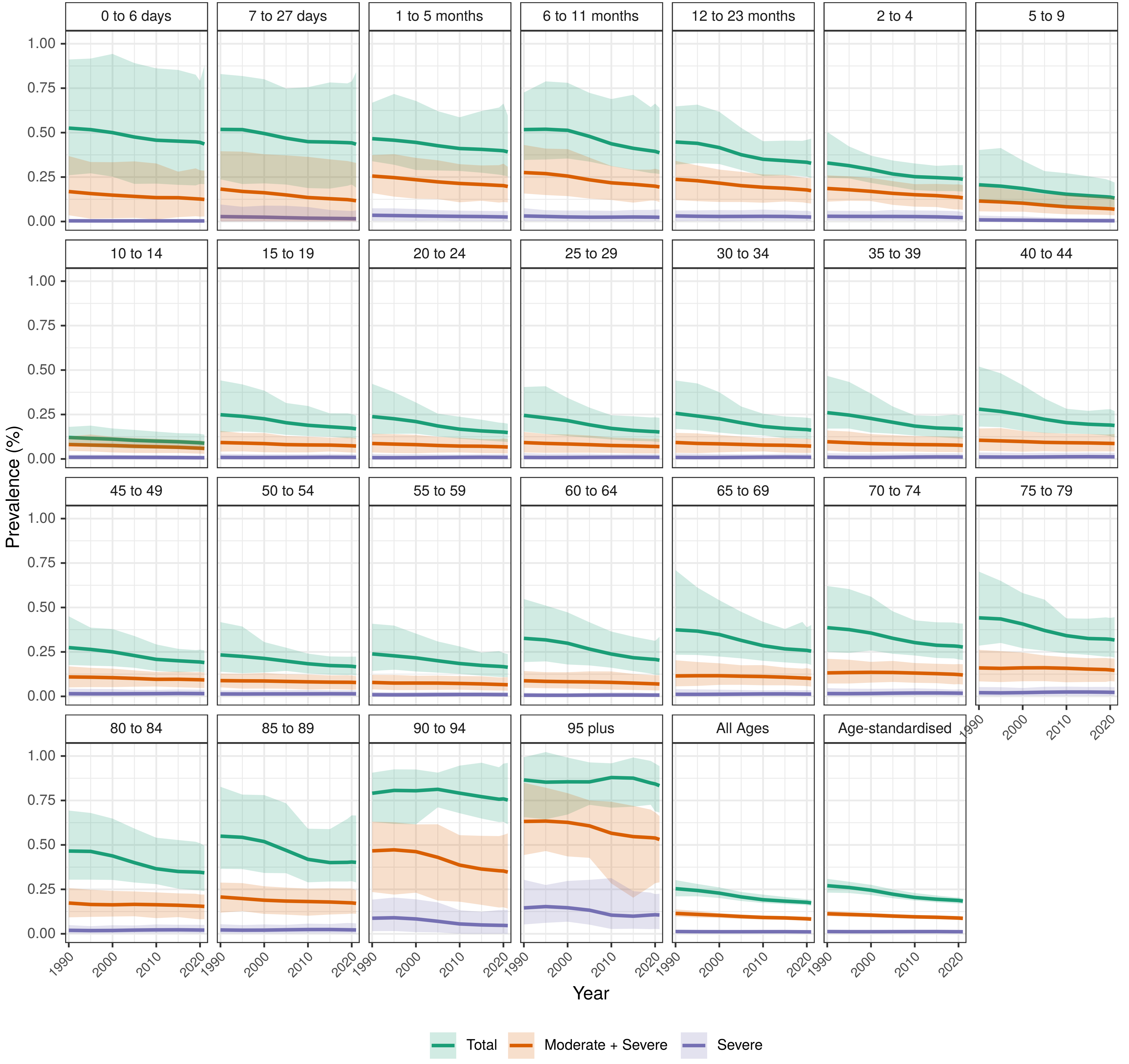

Cause-Specific Anaemia Prevalence - 2021

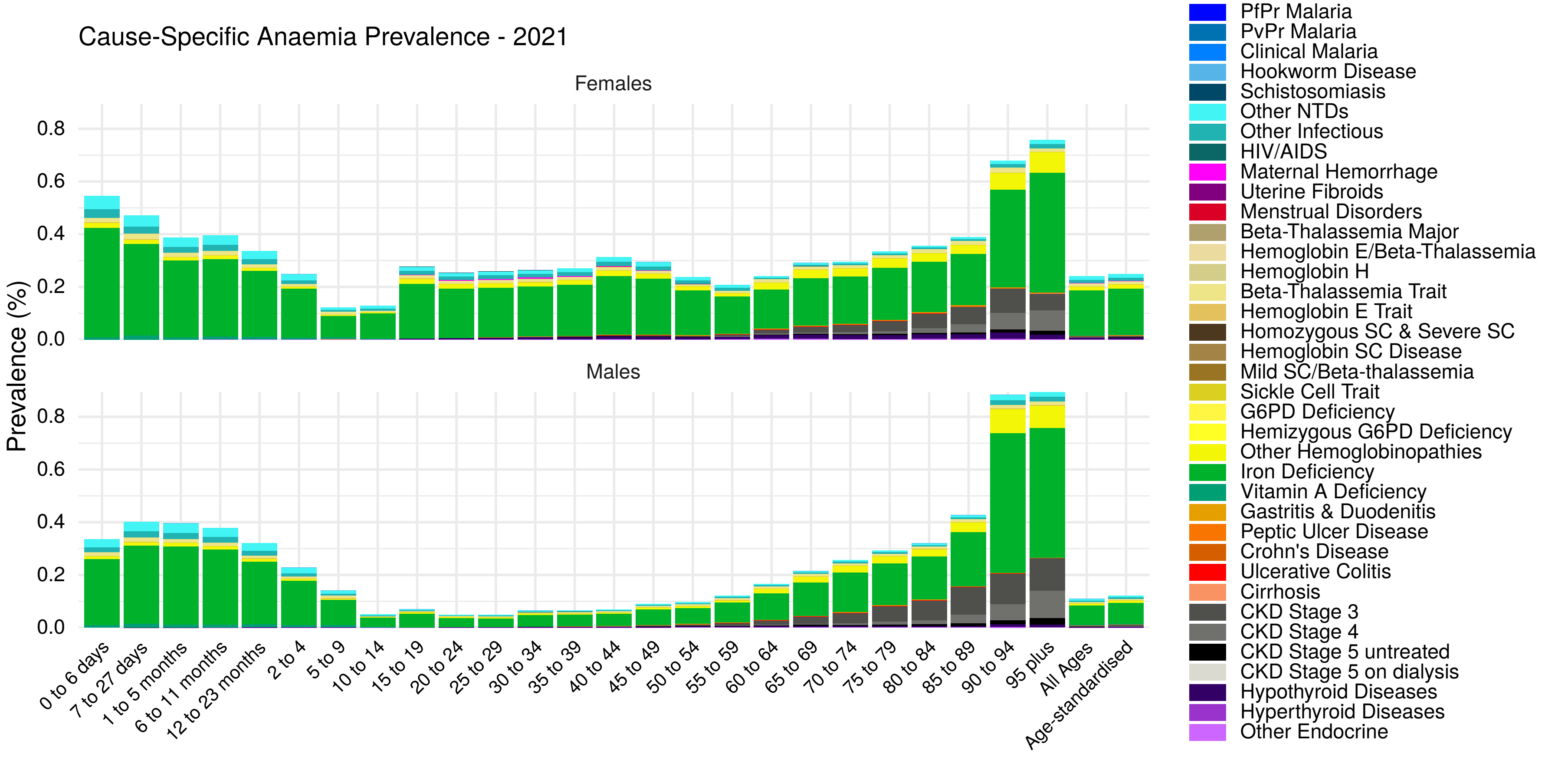

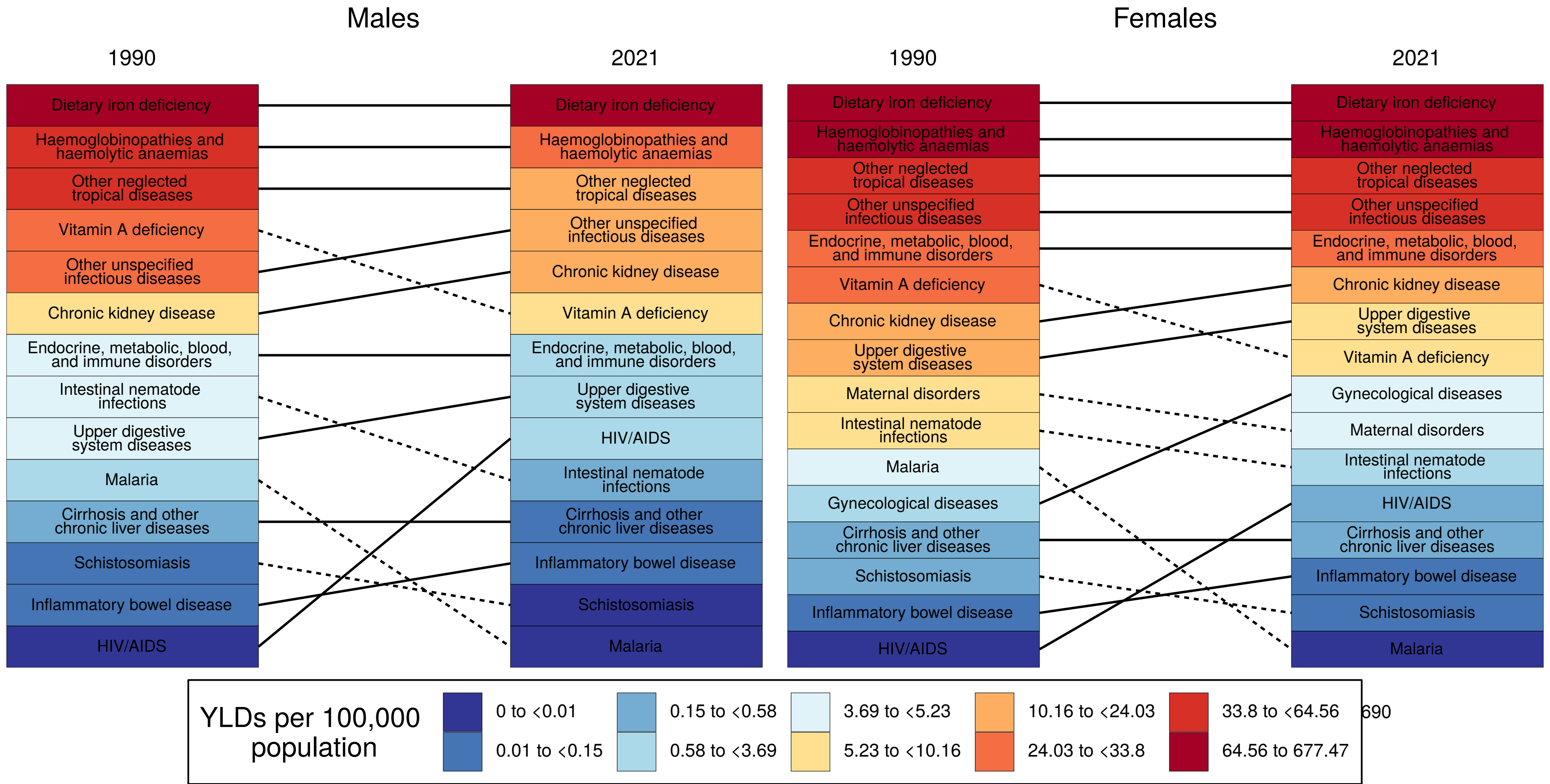

690

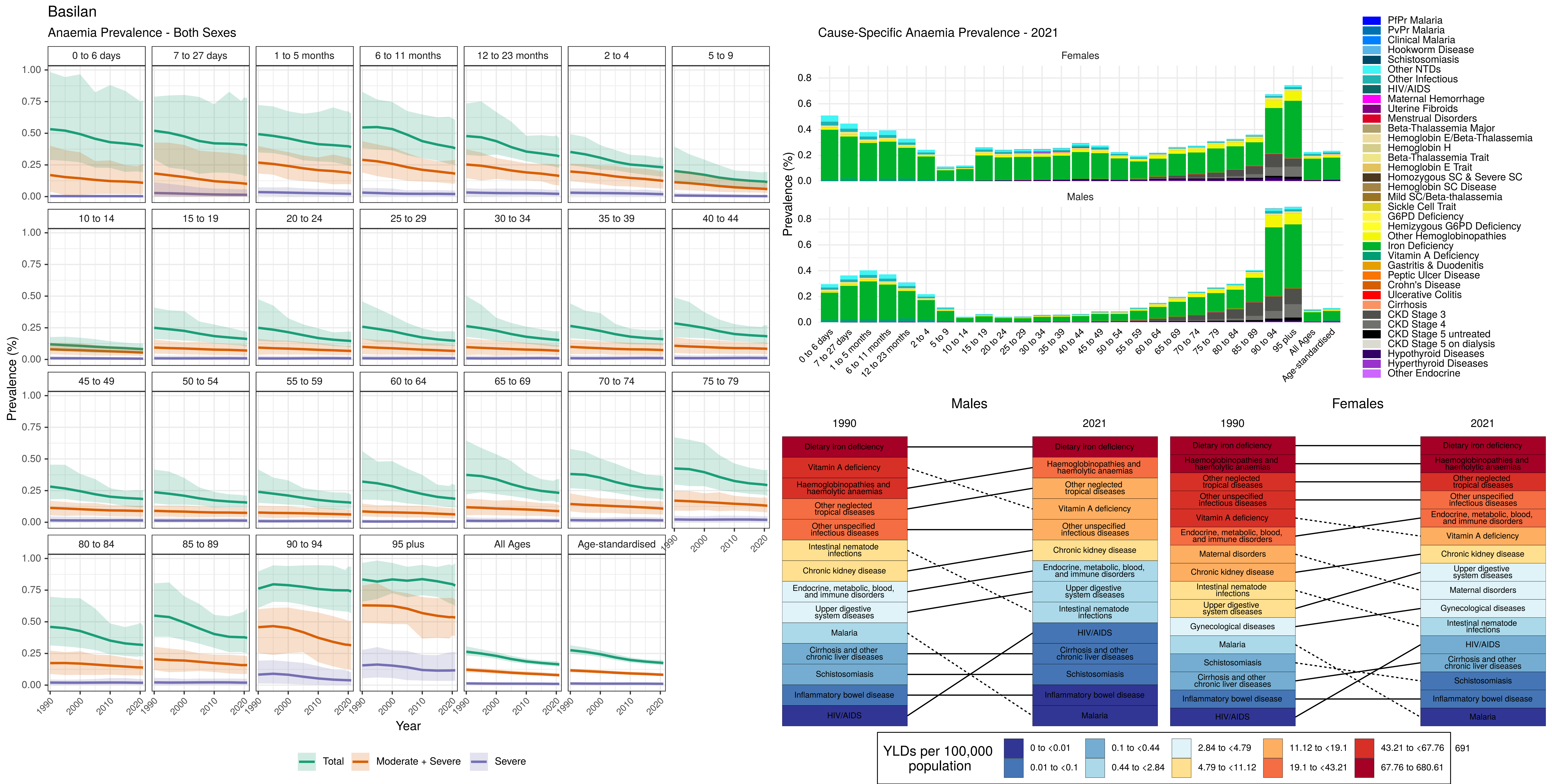
Basilan
Anaemia Prevalence - Both Sexes
0 to 6 days
7 to 27 days
1 to 5 months
6 to 11 months
12 to 23 months
2 to 4
5 to 9
10 to 14
15 to 19
20 to 24
25 to 29
30 to 34
35 to 39
40 to 44
45 to 49
50 to 54
55 to 59
60 to 64
65 to 69
70 to 74
75 to 79
80 to 84
85 to 89
90 to 94
95 plus
All Ages
Age-standardised
Prevalence (%)
Year
Total
Moderate + Severe
Severe
Cause-Specific Anaemia Prevalence - 2021
Females
Males
PfPr Malaria
PvPr Malaria
Clinical Malaria
Hookworm Disease
Schistosomiasis
Other NTDs
Other Infectious
HIV/AIDS
Maternal Hemorrhage
Uterine Fibroids
Menstrual Disorders
Beta-Thalassemia Major
Hemoglobin E/Beta-Thalassemia
Hemoglobin H
Beta-Thalassemia Trait
Hemoglobin E Trait
Homozygous SC & Severe SC
Hemoglobin SC Disease
Mild SC/Beta-thalassemia
Sickle Cell Trait
G6PD Deficiency
Hemizygous G6PD Deficiency
Other Hemoglobinopathies
Iron Deficiency
Vitamin A Deficiency
Gastritis & Duodenitis
Peptic Ulcer Disease
Crohn's Disease
Ulcerative Colitis
Cirrhosis
CKD Stage 3
CKD Stage 4
CKD Stage 5 untreated
CKD Stage 5 on dialysis
Hypothyroid Diseases
Hyperthyroid Diseases
Other Endocrine
Males
Females
1990
2021
Dietary iron deficiency
Vitamin A deficiency
Haemoglobinopathies and haemolytic anaemias
Other neglected tropical diseases
Other unspecified infectious diseases
Intestinal nematode infections
Chronic kidney disease
Endocrine, metabolic, blood, and immune disorders
Upper digestive system diseases
Malaria
Cirrhosis and other chronic liver diseases
Schistosomiasis
Inflammatory bowel disease
HIV/AIDS
Maternal disorders
Gynecological diseases
YLDs per 100,000 population
0 to <0.01
0.01 to <0.1
0.1 to <0.44
0.44 to <2.84
2.84 to <4.79
4.79 to <11.12
11.12 to <19.1
19.1 to <43.21
43.21 to <67.76
67.76 to 680.61

Bataan

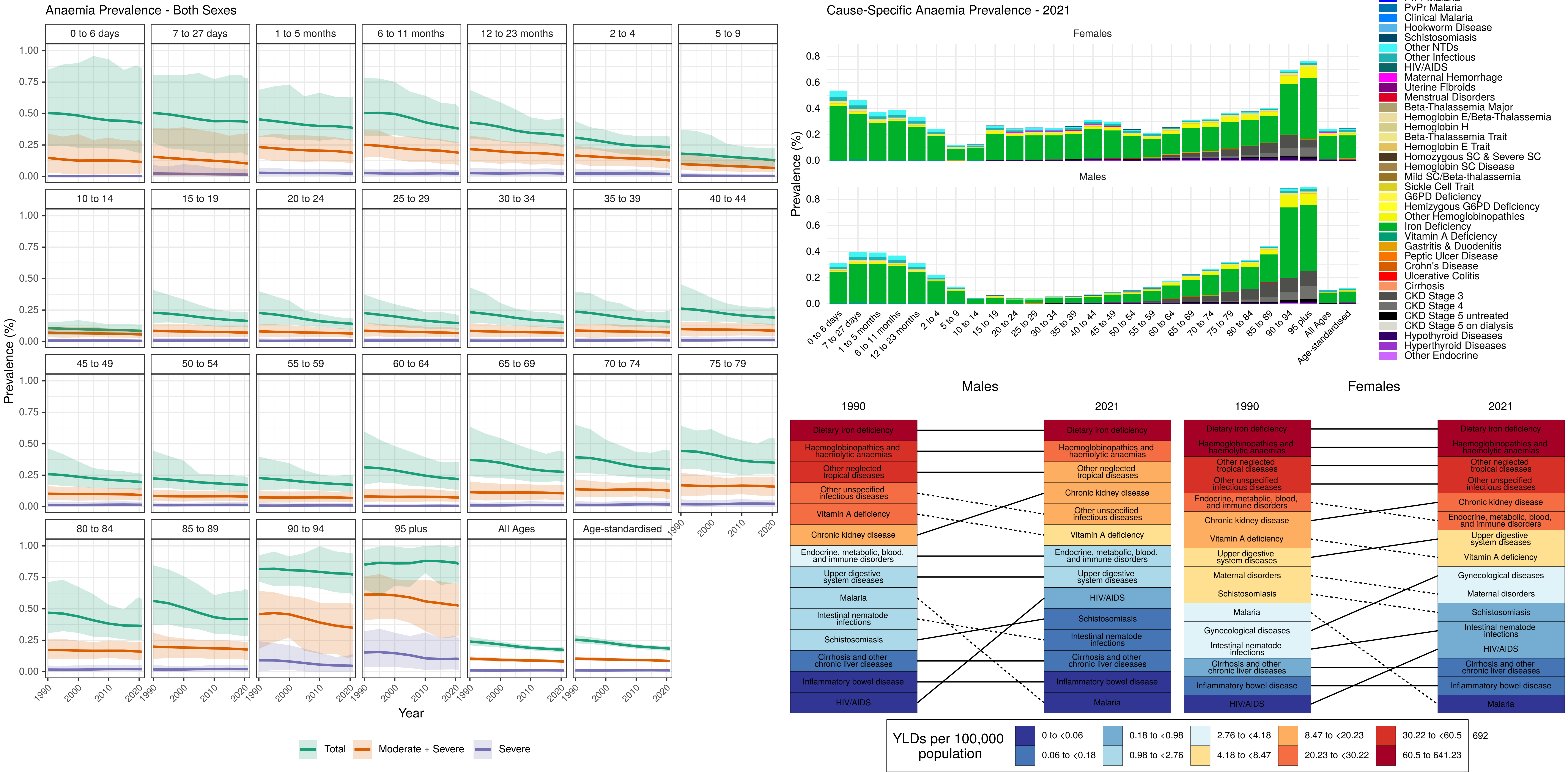
Anaemia Prevalence - Both Sexes
0 to 6 days
7 to 27 days
1 to 5 months
6 to 11 months
12 to 23 months
2 to 4
5 to 9
10 to 14
15 to 19
20 to 24
25 to 29
30 to 34
35 to 39
40 to 44
45 to 49
50 to 54
55 to 59
60 to 64
65 to 69
70 to 74
75 to 79
80 to 84
85 to 89
90 to 94
95 plus
All Ages
Age-standardised
Prevalence (%)
Year
Total
Moderate + Severe
Severe
Cause-Specific Anaemia Prevalence - 2021
Females
Males
Prevalence (%)
PfPr Malaria
PvPr Malaria
Clinical Malaria
Hookworm Disease
Schistosomiasis
Other NTDs
Other Infectious
HIV/AIDS
Maternal Hemorrhage
Uterine Fibroids
Menstrual Disorders
Beta-Thalassemia Major
Hemoglobin E/Beta-Thalassemia
Hemoglobin H
Beta-Thalassemia Trait
Hemoglobin E Trait
Homozygous SC & Severe SC
Hemoglobin SC Disease
Mild SC/Beta-thalassemia
Sickle Cell Trait
G6PD Deficiency
Hemizygous G6PD Deficiency
Other Hemoglobinopathies
Iron Deficiency
Vitamin A Deficiency
Gastritis & Duodenitis
Peptic Ulcer Disease
Crohn's Disease
Ulcerative Colitis
Cirrhosis
CKD Stage 3
CKD Stage 4
CKD Stage 5 untreated
CKD Stage 5 on dialysis
Hypothyroid Diseases
Hyperthyroid Diseases
Other Endocrine
Males
Females
1990
2021
Dietary iron deficiency
Haemoglobinopathies and haemolytic anaemias
Other neglected tropical diseases
Other unspecified infectious diseases
Vitamin A deficiency
Chronic kidney disease
Endocrine, metabolic, blood, and immune disorders
Upper digestive system diseases
Malaria
Intestinal nematode infections
Schistosomiasis
Cirrhosis and other chronic liver diseases
Inflammatory bowel disease
HIV/AIDS
Maternal disorders
Gynecological diseases
YLDs per 100,000 population
0 to <0.06
0.06 to <0.18
0.18 to <0.98
0.98 to <2.76
2.76 to <4.18
4.18 to <8.47
8.47 to <20.23
20.23 to <30.22
30.22 to <60.5
60.5 to 641.23
692

Batanes

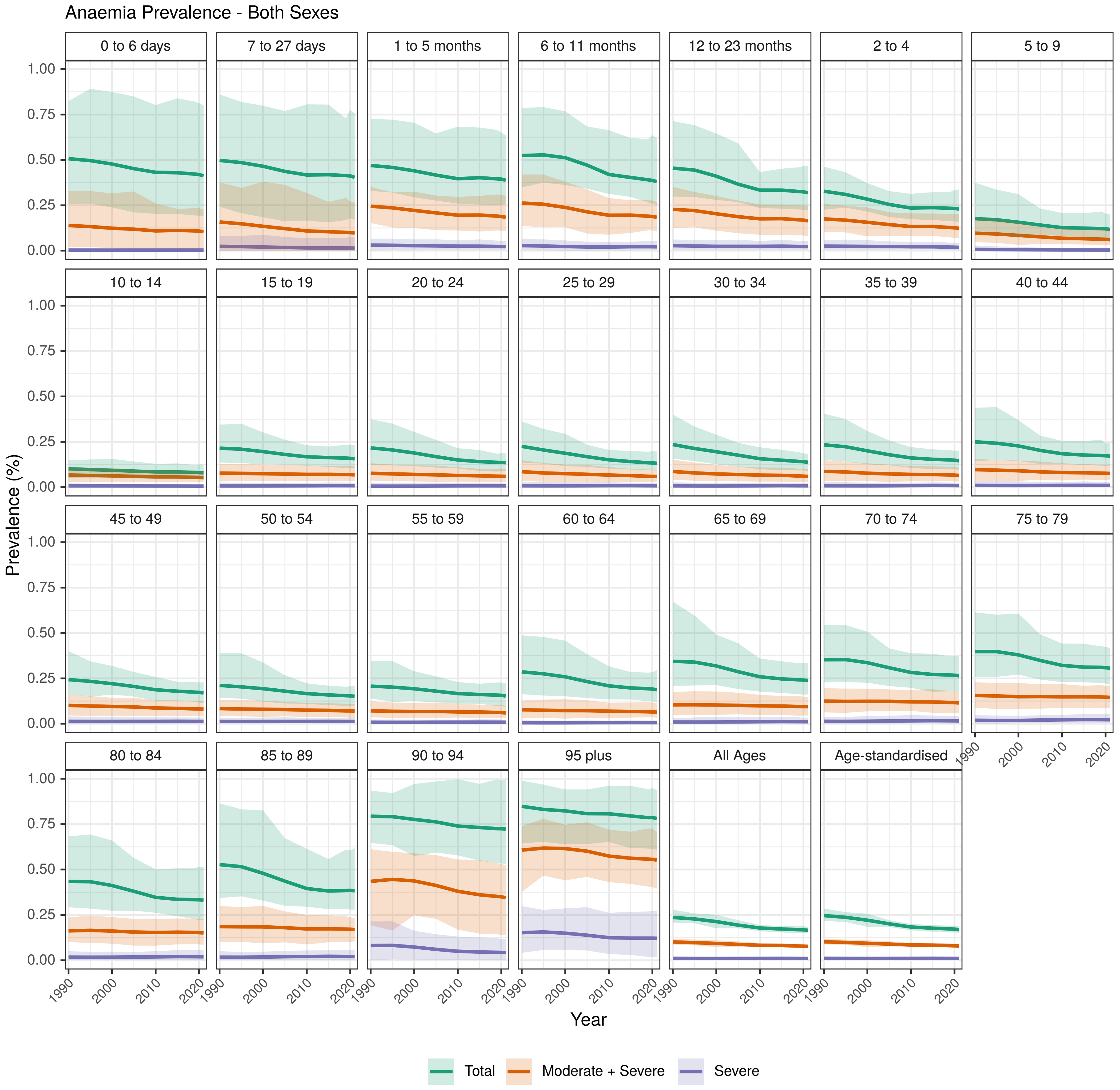

Anaemia Prevalence - Both Sexes
0 to 6 days
7 to 27 days
1 to 5 months
6 to 11 months
12 to 23 months
2 to 4
5 to 9
10 to 14
15 to 19
20 to 24
25 to 29
30 to 34
35 to 39
40 to 44
45 to 49
50 to 54
55 to 59
60 to 64
65 to 69
70 to 74
75 to 79
80 to 84
85 to 89
90 to 94
95 plus
All Ages
Age-standardised
Prevalence (%)
Year
Total
Moderate + Severe
Severe

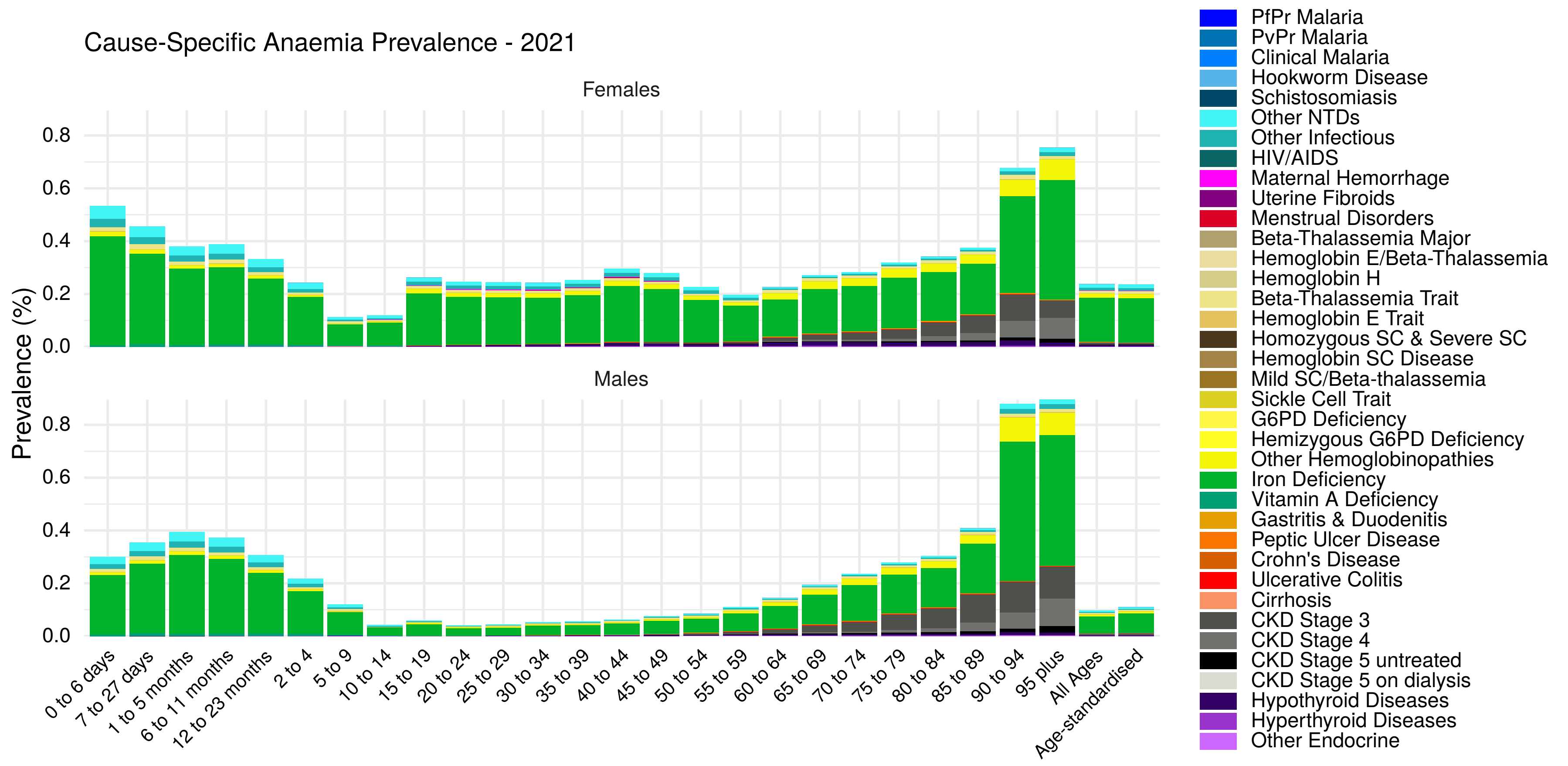

Cause-Specific Anaemia Prevalence - 2021
Females
Males
Prevalence (%)
PfPr Malaria
PvPr Malaria
Clinical Malaria
Hookworm Disease
Schistosomiasis
Other NTDs
Other Infectious
HIV/AIDS
Maternal Hemorrhage
Uterine Fibroids
Menstrual Disorders
Beta-Thalassemia Major
Hemoglobin E/Beta-Thalassemia
Hemoglobin H
Beta-Thalassemia Trait
Hemoglobin E Trait
Homozygous SC & Severe SC
Hemoglobin SC Disease
Mild SC/Beta-thalassemia
Sickle Cell Trait
G6PD Deficiency
Hemizygous G6PD Deficiency
Other Hemoglobinopathies
Iron Deficiency
Vitamin A Deficiency
Gastritis & Duodenitis
Peptic Ulcer Disease
Crohn's Disease
Ulcerative Colitis
Cirrhosis
CKD Stage 3
CKD Stage 4
CKD Stage 5 untreated
CKD Stage 5 on dialysis
Hypothyroid Diseases
Hyperthyroid Diseases
Other Endocrine

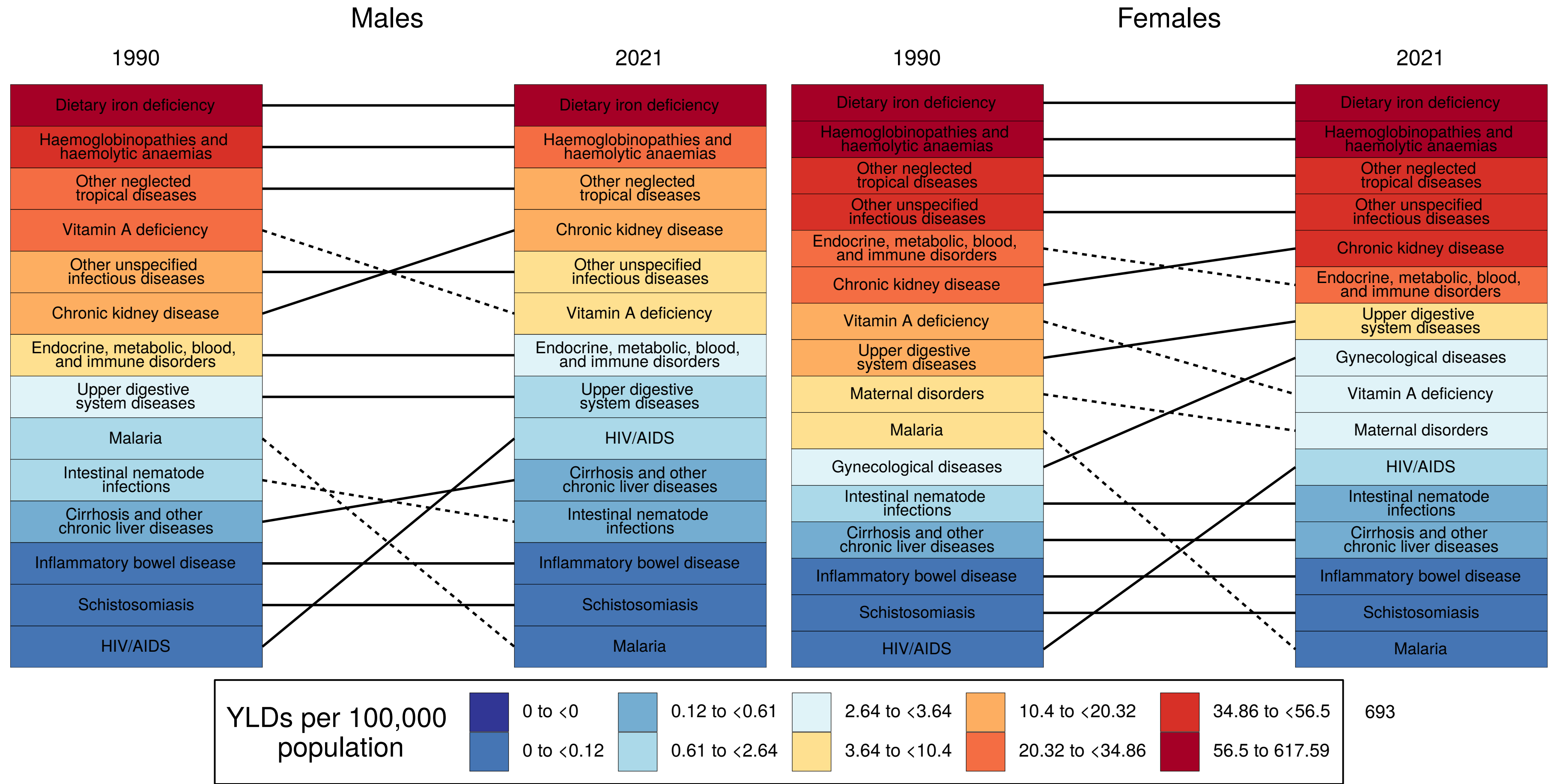

Males
Females
1990
2021
Dietary iron deficiency
Haemoglobinopathies and haemolytic anaemias
Other neglected tropical diseases
Vitamin A deficiency
Other unspecified infectious diseases
Chronic kidney disease
Endocrine, metabolic, blood, and immune disorders
Upper digestive system diseases
Malaria
Intestinal nematode infections
Cirrhosis and other chronic liver diseases
Inflammatory bowel disease
Schistosomiasis
HIV/AIDS
Maternal disorders
Gynecological diseases
YLDs per 100,000 population
0 to <0
0 to <0.12
0.12 to <0.61
0.61 to <2.64
2.64 to <3.64
3.64 to <10.4
10.4 to <20.32
20.32 to <34.86
34.86 to <56.5
56.5 to 617.59

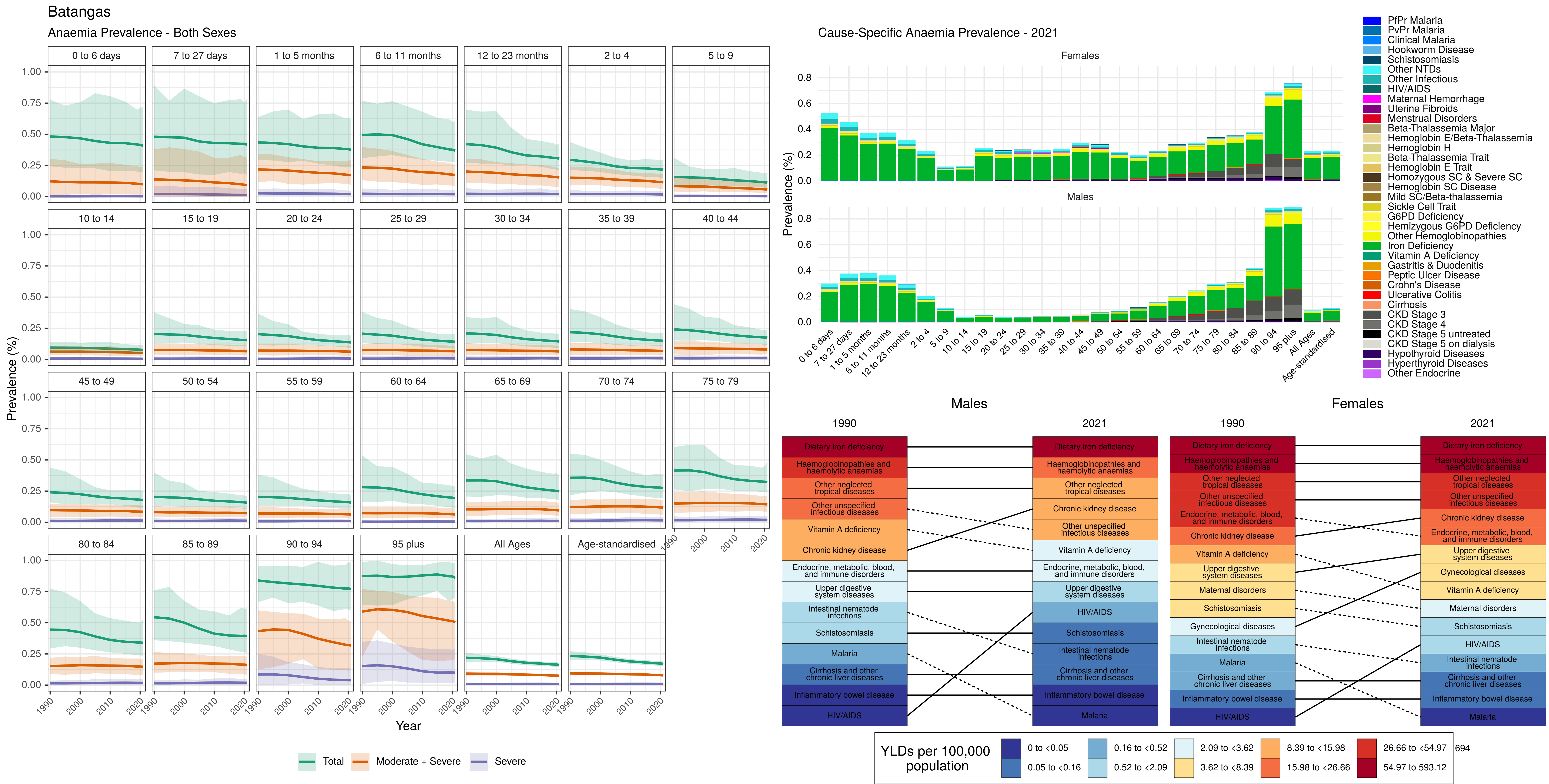
Batangas
Anaemia Prevalence - Both Sexes
0 to 6 days
7 to 27 days
1 to 5 months
6 to 11 months
12 to 23 months
2 to 4
5 to 9
10 to 14
15 to 19
20 to 24
25 to 29
30 to 34
35 to 39
40 to 44
45 to 49
50 to 54
55 to 59
60 to 64
65 to 69
70 to 74
75 to 79
80 to 84
85 to 89
90 to 94
95 plus
All Ages
Age-standardised
Prevalence (%)
Year
Total
Moderate + Severe
Severe
Cause-Specific Anaemia Prevalence - 2021
Females
Males
PfPr Malaria
PvPr Malaria
Clinical Malaria
Hookworm Disease
Schistosomiasis
Other NTDs
Other Infectious
HIV/AIDS
Maternal Hemorrhage
Uterine Fibroids
Menstrual Disorders
Beta-Thalassemia Major
Hemoglobin E/Beta-Thalassemia
Hemoglobin H
Beta-Thalassemia Trait
Hemoglobin E Trait
Homozygous SC & Severe SC
Hemoglobin SC Disease
Mild SC/Beta-thalassemia
Sickle Cell Trait
G6PD Deficiency
Hemizygous G6PD Deficiency
Other Hemoglobinopathies
Iron Deficiency
Vitamin A Deficiency
Gastritis & Duodenitis
Peptic Ulcer Disease
Crohn's Disease
Ulcerative Colitis
Cirrhosis
CKD Stage 3
CKD Stage 4
CKD Stage 5 untreated
CKD Stage 5 on dialysis
Hypothyroid Diseases
Hyperthyroid Diseases
Other Endocrine
Males
Females
1990
2021
Dietary iron deficiency
Haemoglobinopathies and haemolytic anaemias
Other neglected tropical diseases
Other unspecified infectious diseases
Vitamin A deficiency
Chronic kidney disease
Endocrine, metabolic, blood, and immune disorders
Upper digestive system diseases
Intestinal nematode infections
Schistosomiasis
Malaria
Cirrhosis and other chronic liver diseases
Inflammatory bowel disease
HIV/AIDS
Maternal disorders
Gynecological diseases
YLDs per 100,000 population
0 to <0.05
0.05 to <0.16
0.16 to <0.52
0.52 to <2.09
2.09 to <3.62
3.62 to <8.39
8.39 to <15.98
15.98 to <26.66
26.66 to <54.97
54.97 to 593.12

Benguet

Anaemia Prevalence - Both Sexes

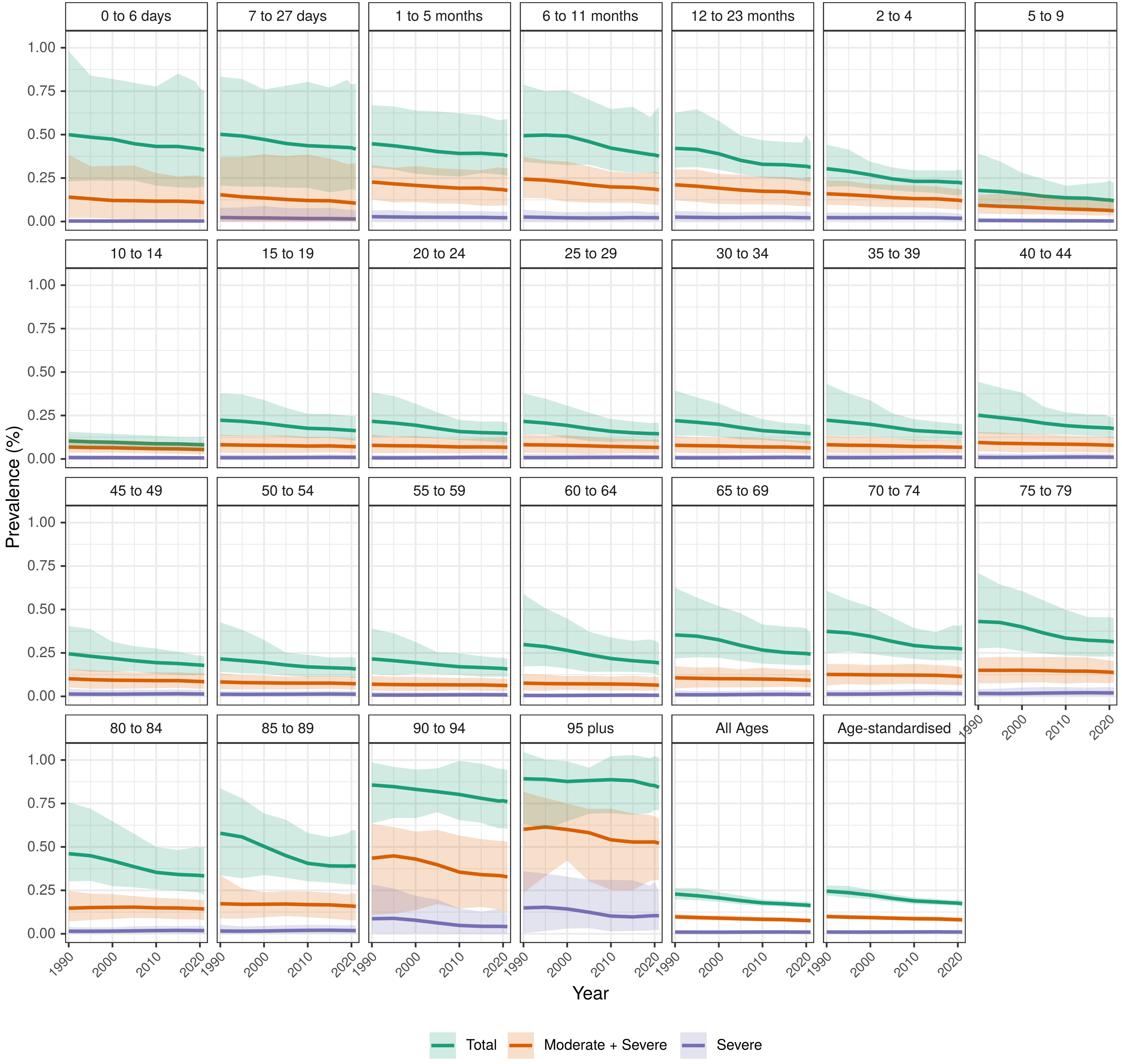

Cause-Specific Anaemia Prevalence - 2021

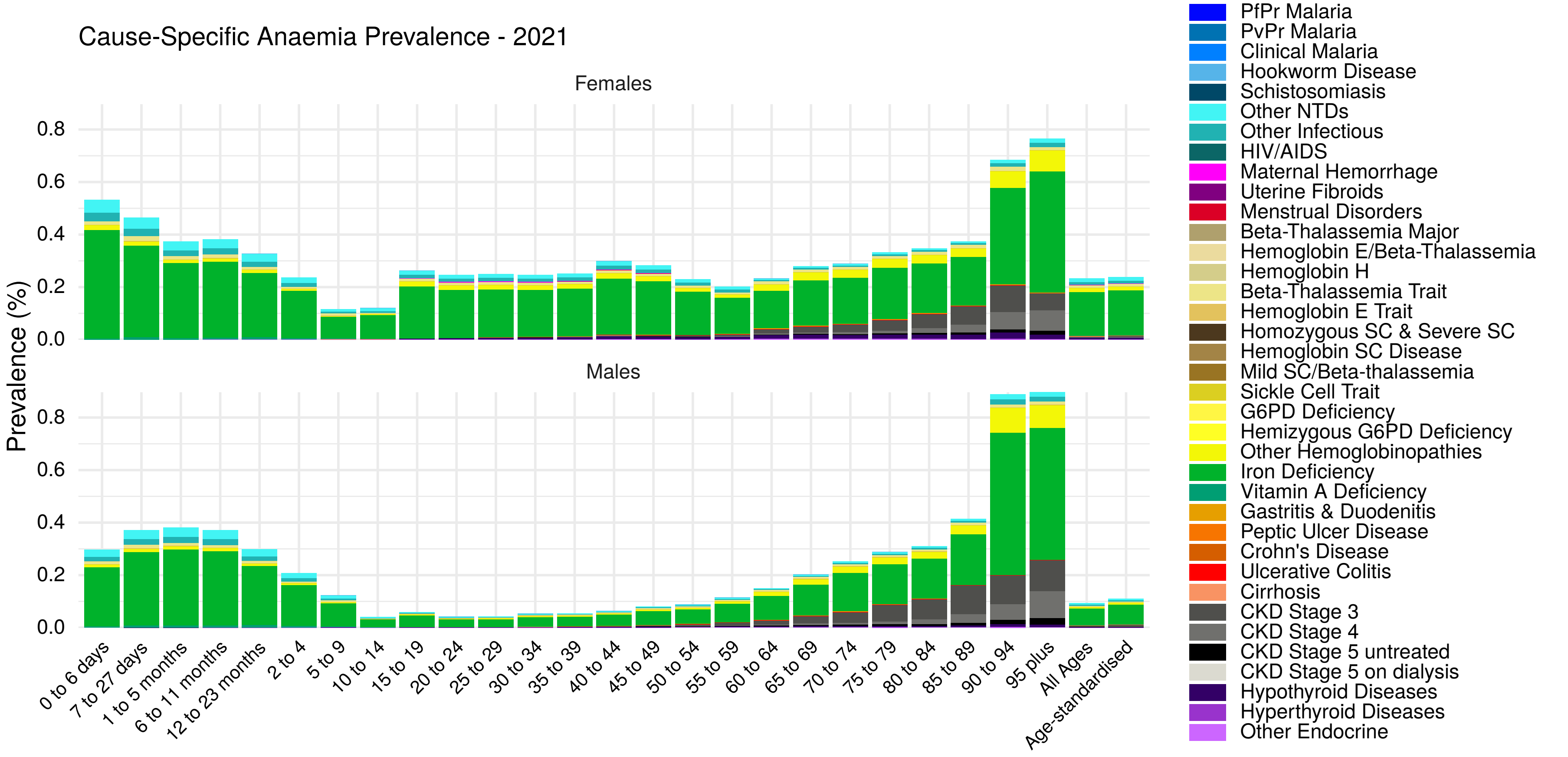

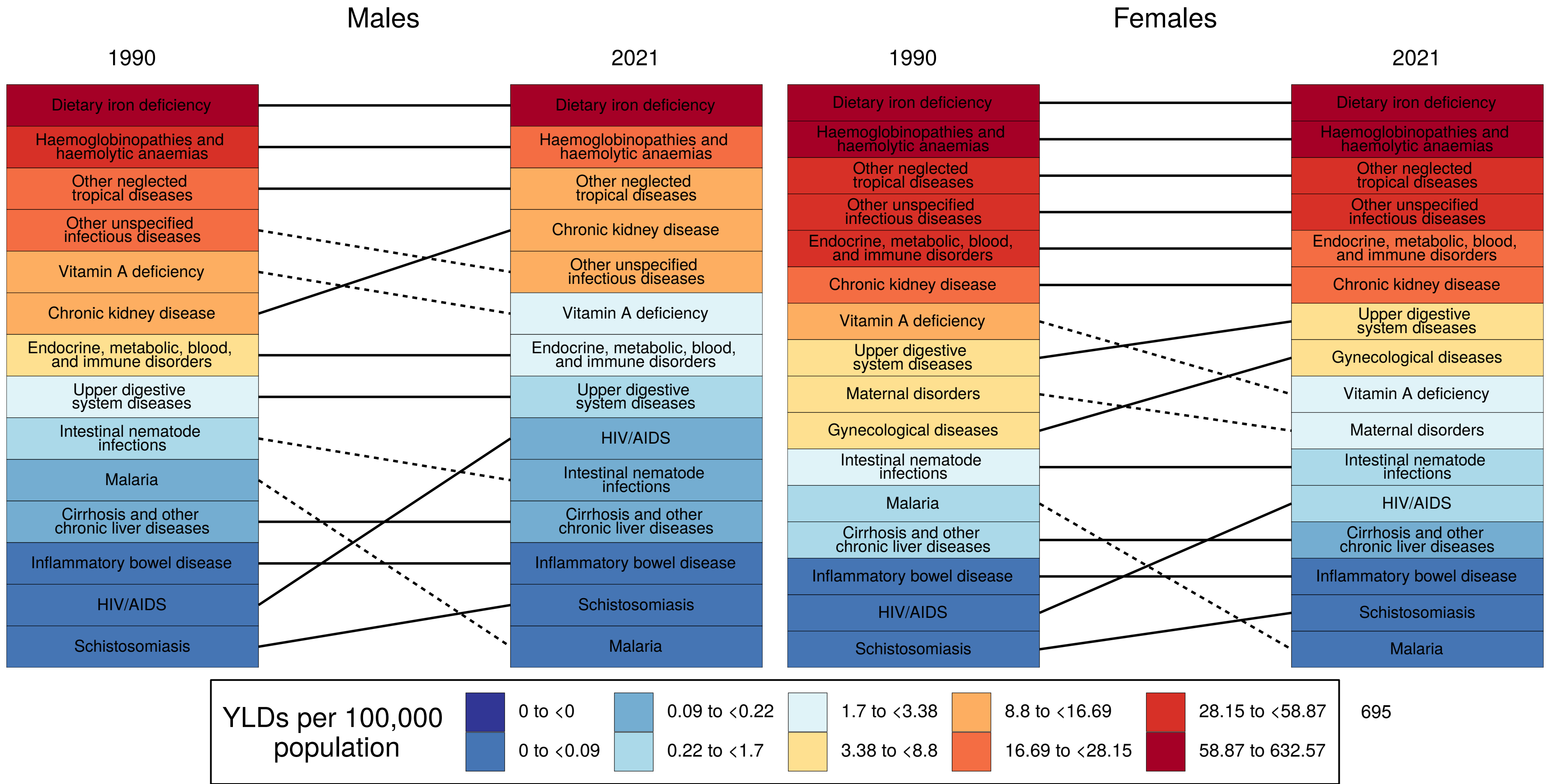

Biliran

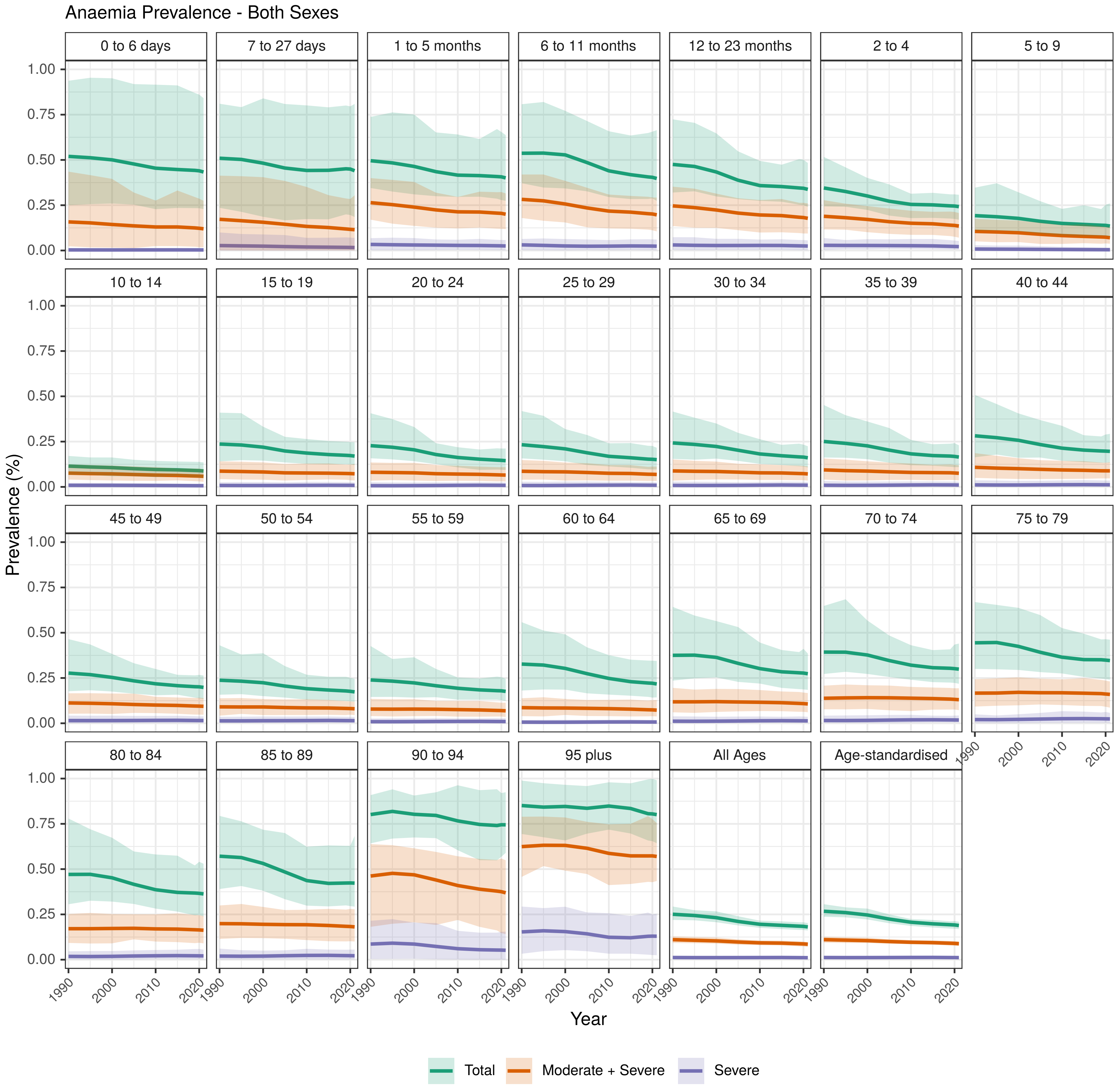
Anaemia Prevalence - Both Sexes
0 to 6 days
7 to 27 days
1 to 5 months
6 to 11 months
12 to 23 months
2 to 4
5 to 9
10 to 14
15 to 19
20 to 24
25 to 29
30 to 34
35 to 39
40 to 44
45 to 49
50 to 54
55 to 59
60 to 64
65 to 69
70 to 74
75 to 79
80 to 84
85 to 89
90 to 94
95 plus
All Ages
Age-standardised
Prevalence (%)
Year
Total
Moderate + Severe
Severe

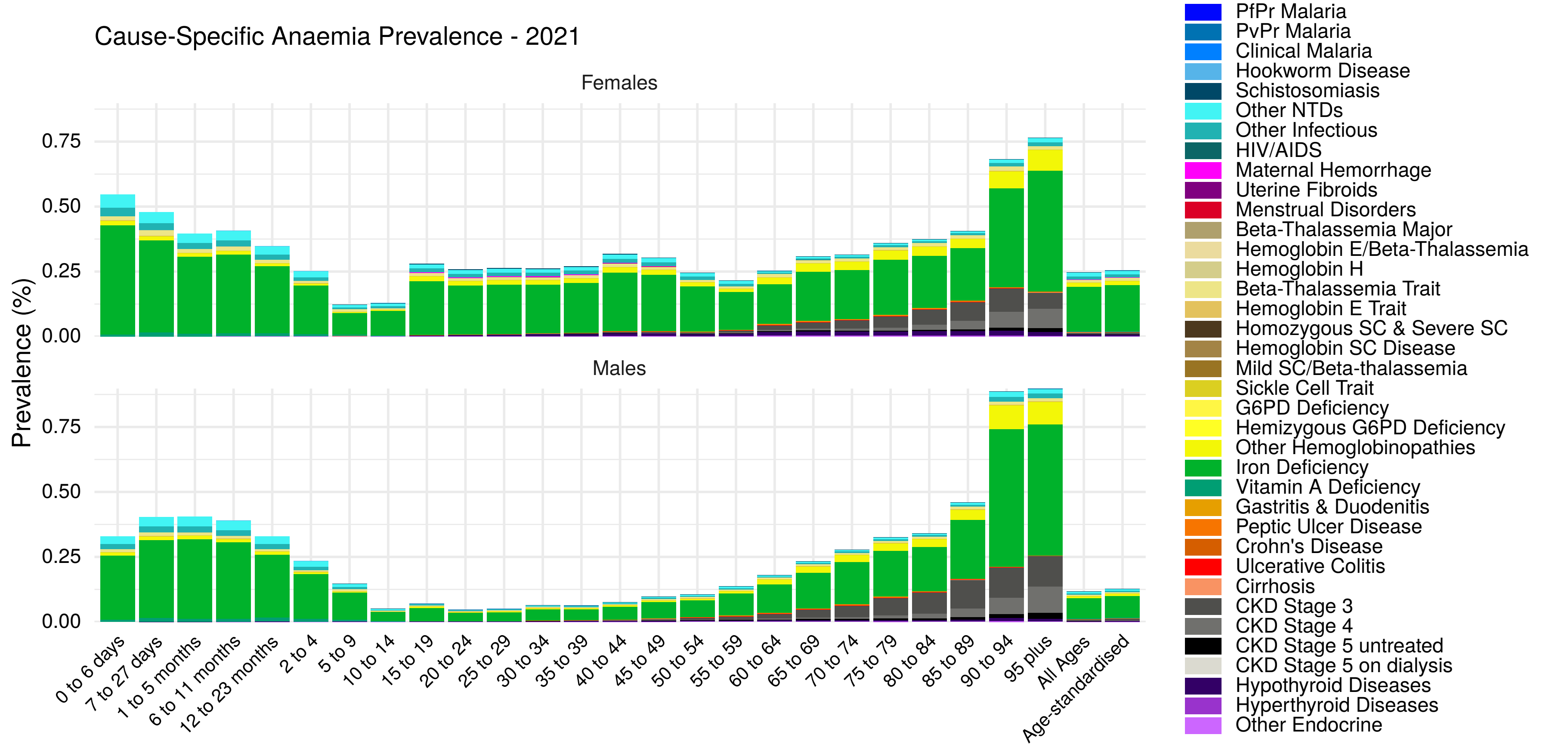
Cause-Specific Anaemia Prevalence - 2021
Females
Males
Prevalence (%)
PfPr Malaria
PvPr Malaria
Clinical Malaria
Hookworm Disease
Schistosomiasis
Other NTDs
Other Infectious
HIV/AIDS
Maternal Hemorrhage
Uterine Fibroids
Menstrual Disorders
Beta-Thalassemia Major
Hemoglobin E/Beta-Thalassemia
Hemoglobin H
Beta-Thalassemia Trait
Hemoglobin E Trait
Homozygous SC & Severe SC
Hemoglobin SC Disease
Mild SC/Beta-thalassemia
Sickle Cell Trait
G6PD Deficiency
Hemizygous G6PD Deficiency
Other Hemoglobinopathies
Iron Deficiency
Vitamin A Deficiency
Gastritis & Duodenitis
Peptic Ulcer Disease
Crohn's Disease
Ulcerative Colitis
Cirrhosis
CKD Stage 3
CKD Stage 4
CKD Stage 5 untreated
CKD Stage 5 on dialysis
Hypothyroid Diseases
Hyperthyroid Diseases
Other Endocrine

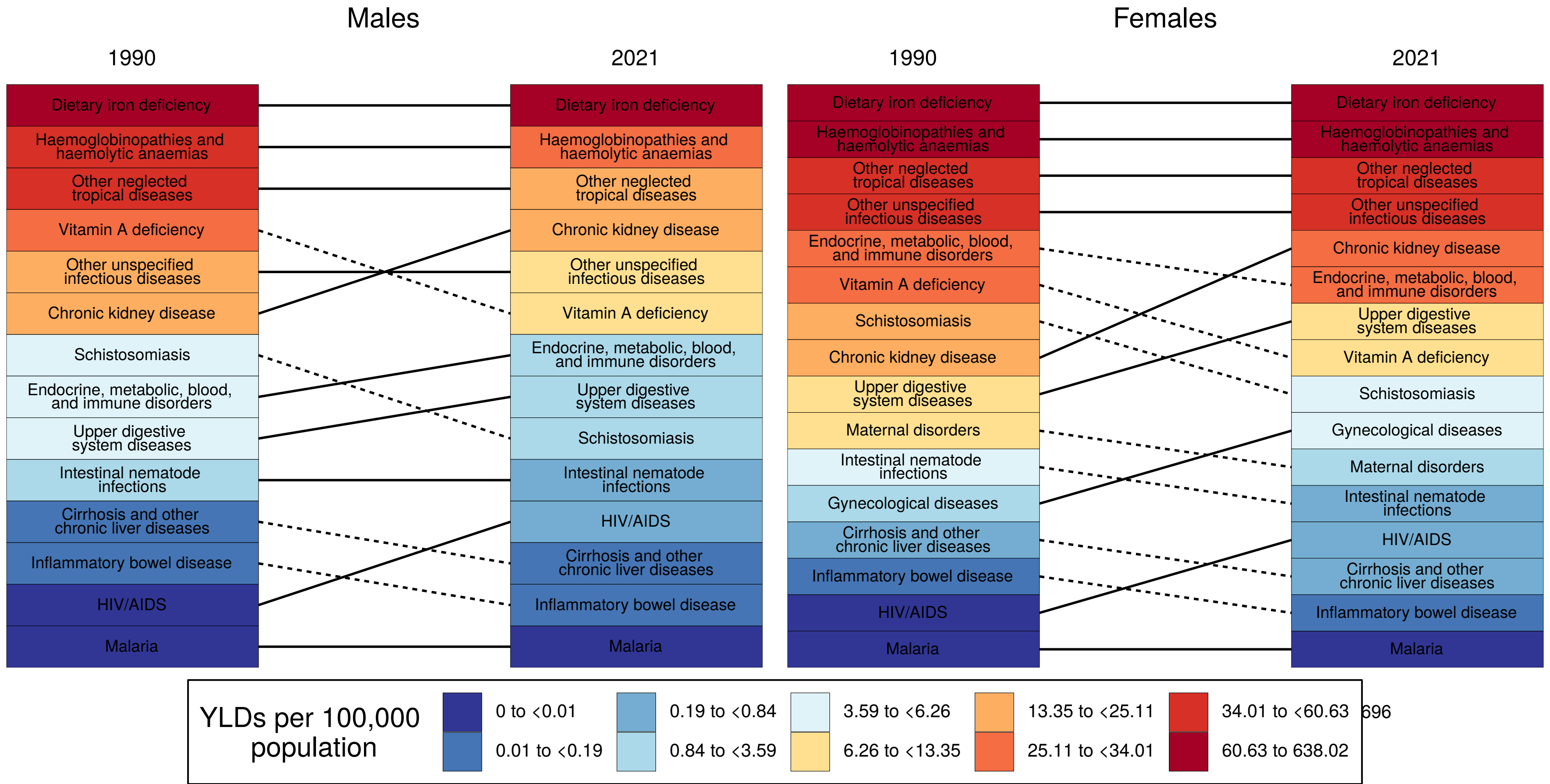
Males
Females
1990
2021
Dietary iron deficiency
Haemoglobinopathies and haemolytic anaemias
Other neglected tropical diseases
Vitamin A deficiency
Other unspecified infectious diseases
Chronic kidney disease
Schistosomiasis
Endocrine, metabolic, blood, and immune disorders
Upper digestive system diseases
Intestinal nematode infections
Cirrhosis and other chronic liver diseases
Inflammatory bowel disease
HIV/AIDS
Malaria
Maternal disorders
Gynecological diseases
YLDs per 100,000 population
0 to <0.01
0.01 to <0.19
0.19 to <0.84
0.84 to <3.59
3.59 to <6.26
6.26 to <13.35
13.35 to <25.11
25.11 to <34.01
34.01 to <60.63
60.63 to 638.02

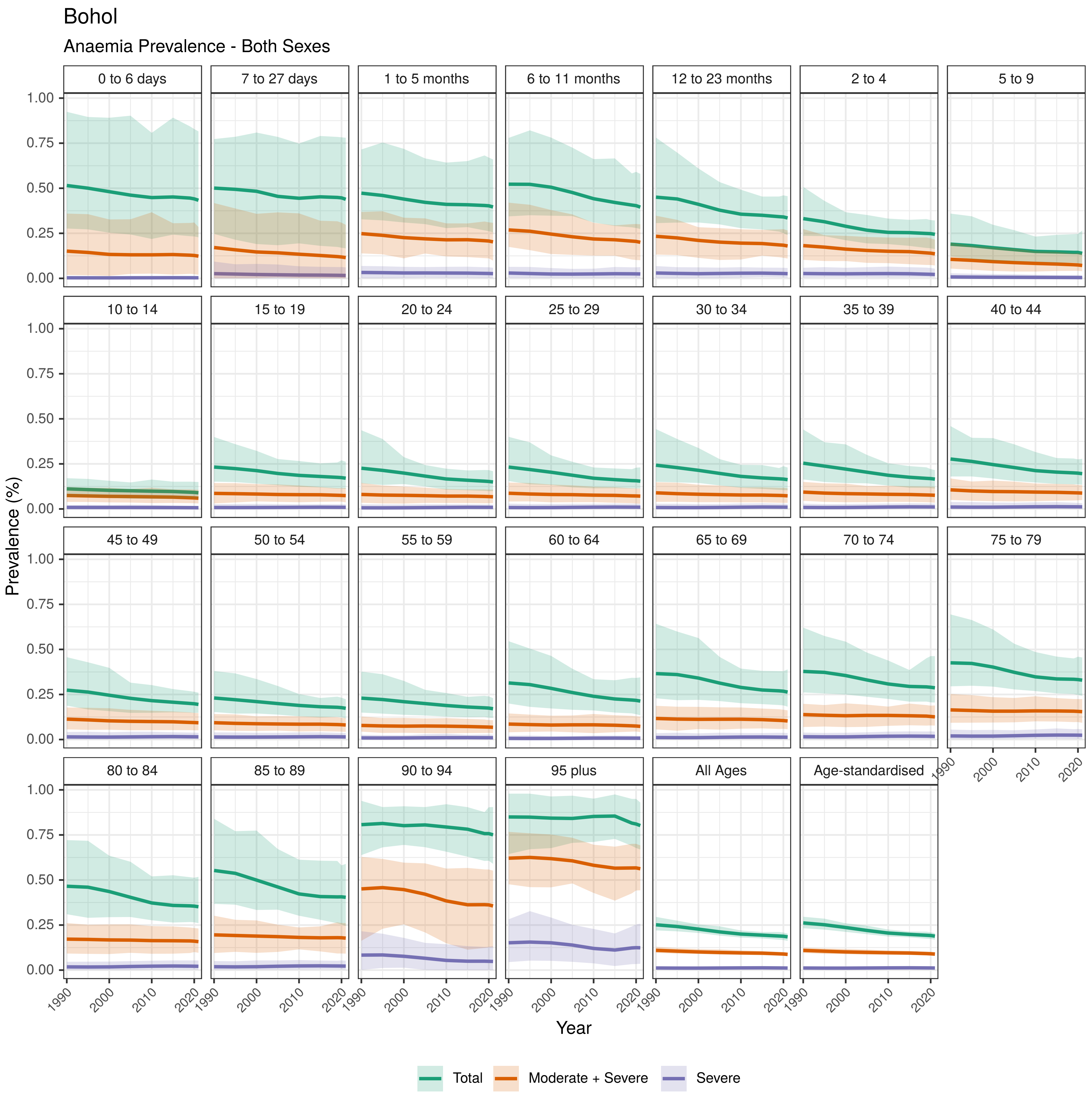
Bohol
Anaemia Prevalence - Both Sexes
0 to 6 days
7 to 27 days
1 to 5 months
6 to 11 months
12 to 23 months
2 to 4
5 to 9
10 to 14
15 to 19
20 to 24
25 to 29
30 to 34
35 to 39
40 to 44
45 to 49
50 to 54
55 to 59
60 to 64
65 to 69
70 to 74
75 to 79
80 to 84
85 to 89
90 to 94
95 plus
All Ages
Age-standardised
0.00
0.25
0.50
0.75
1.00
1990
2000
2010
2020
Prevalence (%)
Year
Total
Moderate + Severe
Severe

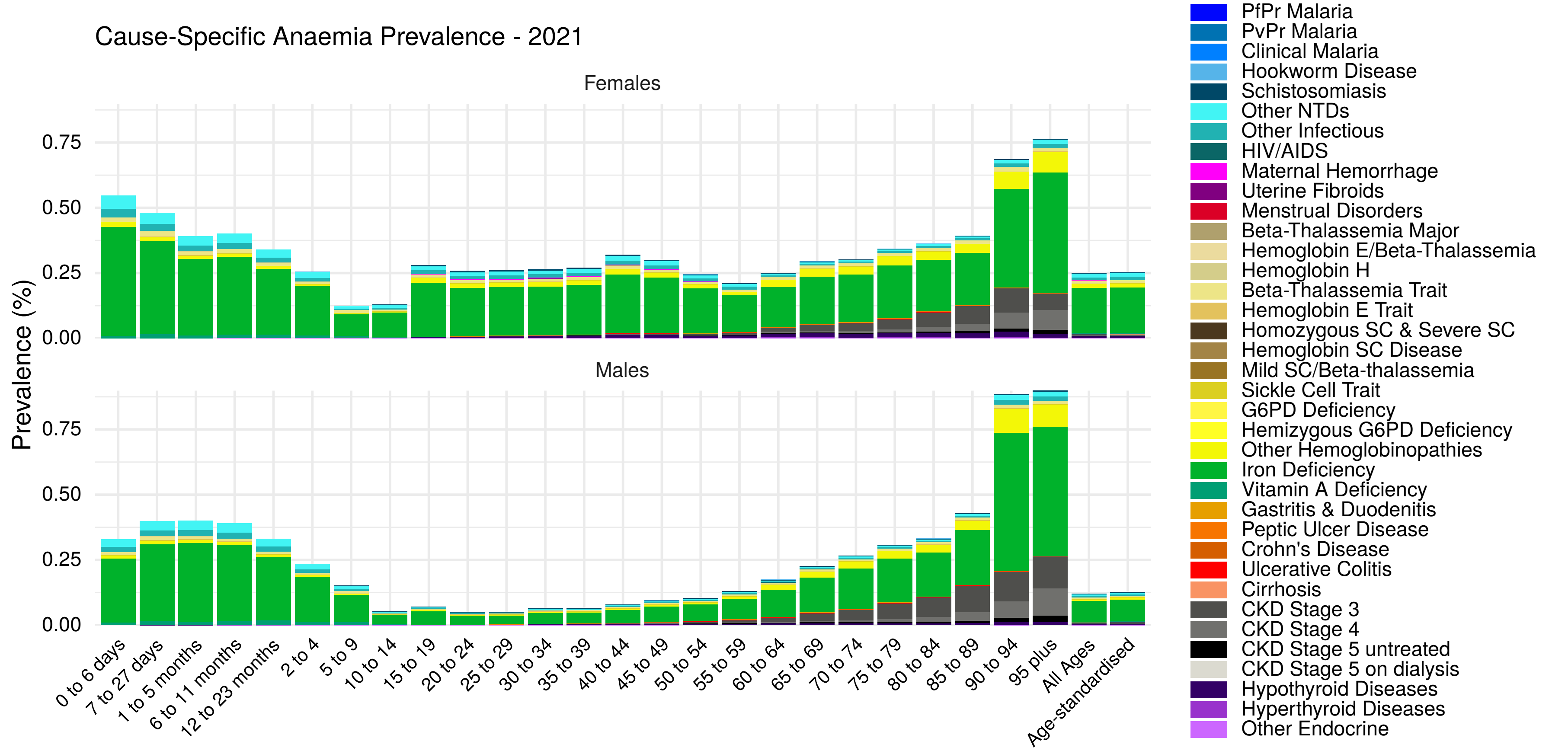
Cause-Specific Anaemia Prevalence - 2021
Females
Males
Prevalence (%)
0.00
0.25
0.50
0.75
0 to 6 days
7 to 27 days
1 to 5 months
6 to 11 months
12 to 23 months
2 to 4
5 to 9
10 to 14
15 to 19
20 to 24
25 to 29
30 to 34
35 to 39
40 to 44
45 to 49
50 to 54
55 to 59
60 to 64
65 to 69
70 to 74
75 to 79
80 to 84
85 to 89
90 to 94
95 plus
All Ages
Age-standardised
PfPr Malaria
PvPr Malaria
Clinical Malaria
Hookworm Disease
Schistosomiasis
Other NTDs
Other Infectious
HIV/AIDS
Maternal Hemorrhage
Uterine Fibroids
Menstrual Disorders
Beta-Thalassemia Major
Hemoglobin E/Beta-Thalassemia
Hemoglobin H
Beta-Thalassemia Trait
Hemoglobin E Trait
Homozygous SC & Severe SC
Hemoglobin SC Disease
Mild SC/Beta-thalassemia
Sickle Cell Trait
G6PD Deficiency
Hemizygous G6PD Deficiency
Other Hemoglobinopathies
Iron Deficiency
Vitamin A Deficiency
Gastritis & Duodenitis
Peptic Ulcer Disease
Crohn's Disease
Ulcerative Colitis
Cirrhosis
CKD Stage 3
CKD Stage 4
CKD Stage 5 untreated
CKD Stage 5 on dialysis
Hypothyroid Diseases
Hyperthyroid Diseases
Other Endocrine

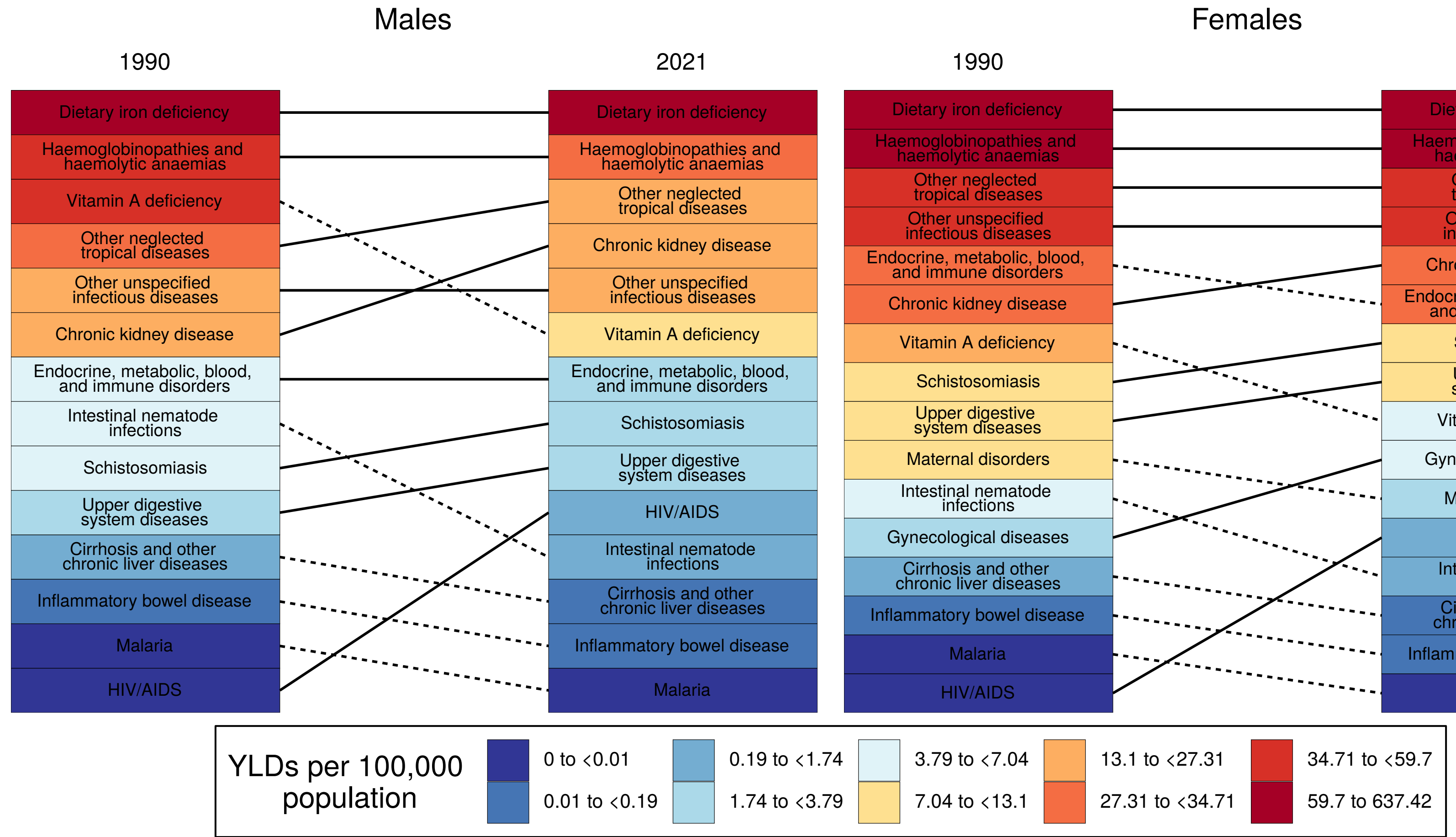
Males
Females
1990
2021
Dietary iron deficiency
Haemoglobinopathies and haemolytic anaemias
Vitamin A deficiency
Other neglected tropical diseases
Other unspecified infectious diseases
Chronic kidney disease
Endocrine, metabolic, blood, and immune disorders
Intestinal nematode infections
Schistosomiasis
Upper digestive system diseases
Cirrhosis and other chronic liver diseases
Inflammatory bowel disease
Malaria
HIV/AIDS
Maternal disorders
Gynecological diseases
YLDs per 100,000 population
0 to <0.01
0.01 to <0.19
0.19 to <1.74
1.74 to <3.79
3.79 to <7.04
7.04 to <13.1
13.1 to <27.31
27.31 to <34.71
34.71 to <59.7
59.7 to 637.42

Bukidnon

Anaemia Prevalence - Both Sexes

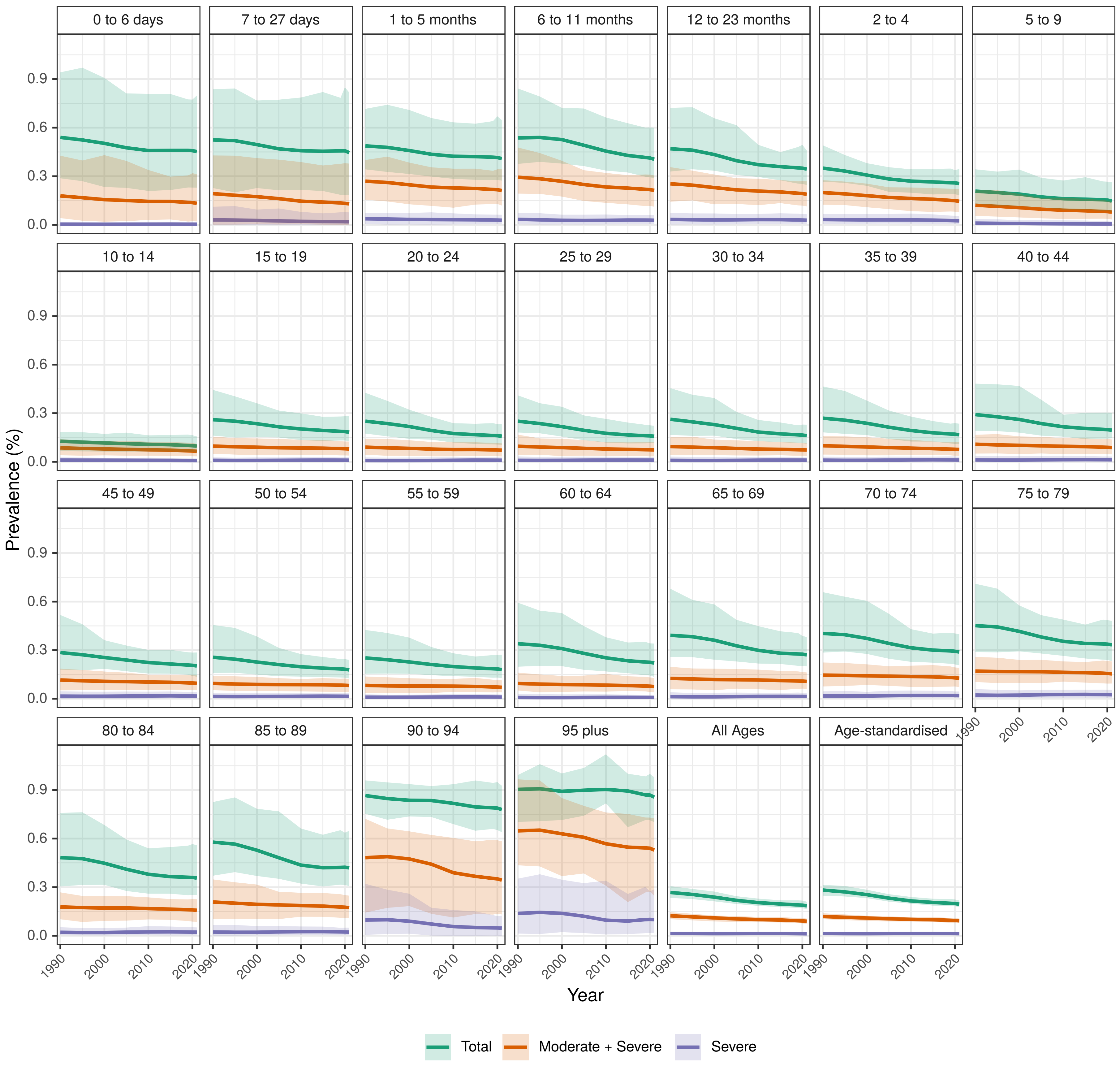

Cause-Specific Anaemia Prevalence - 2021

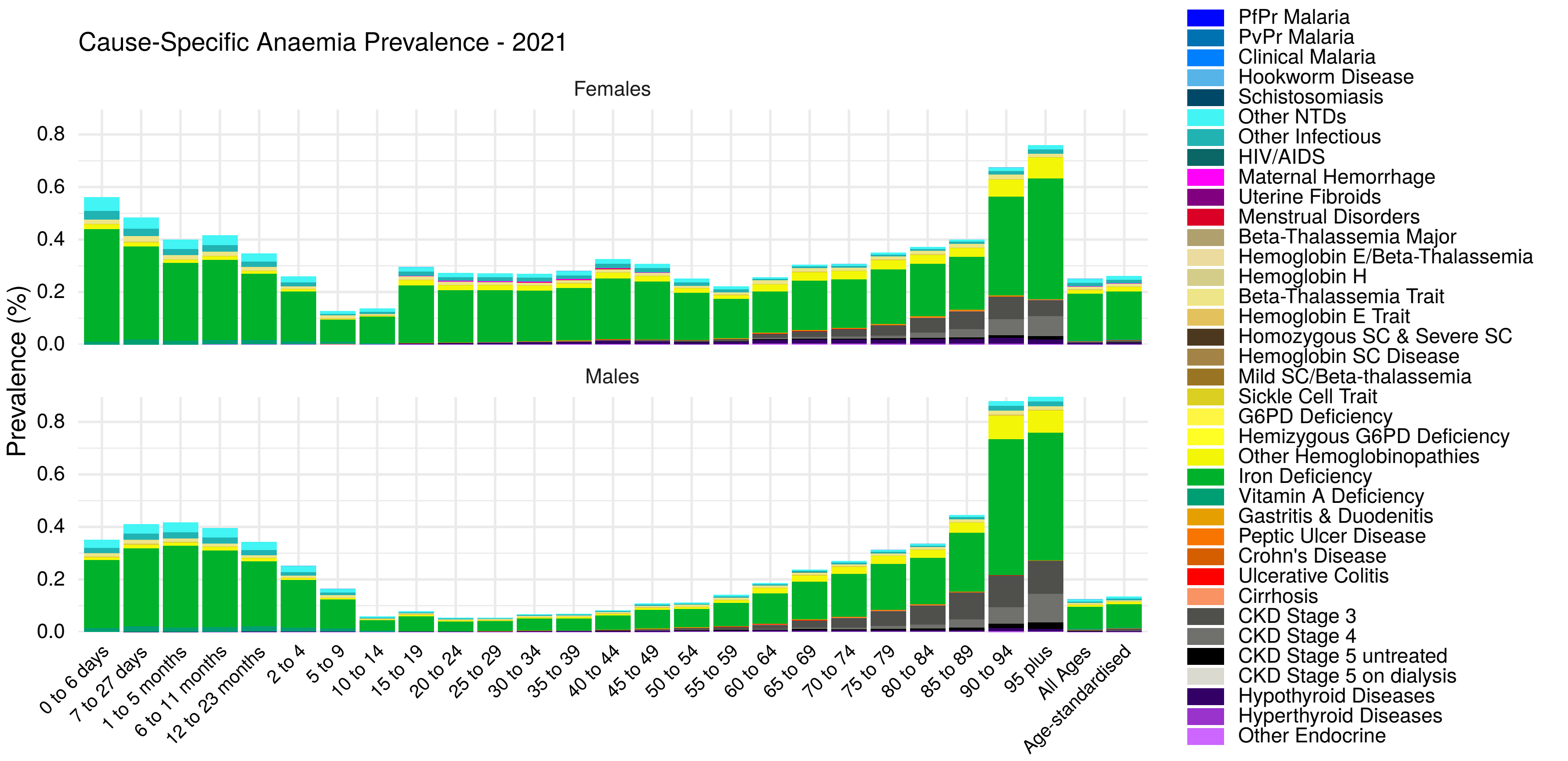

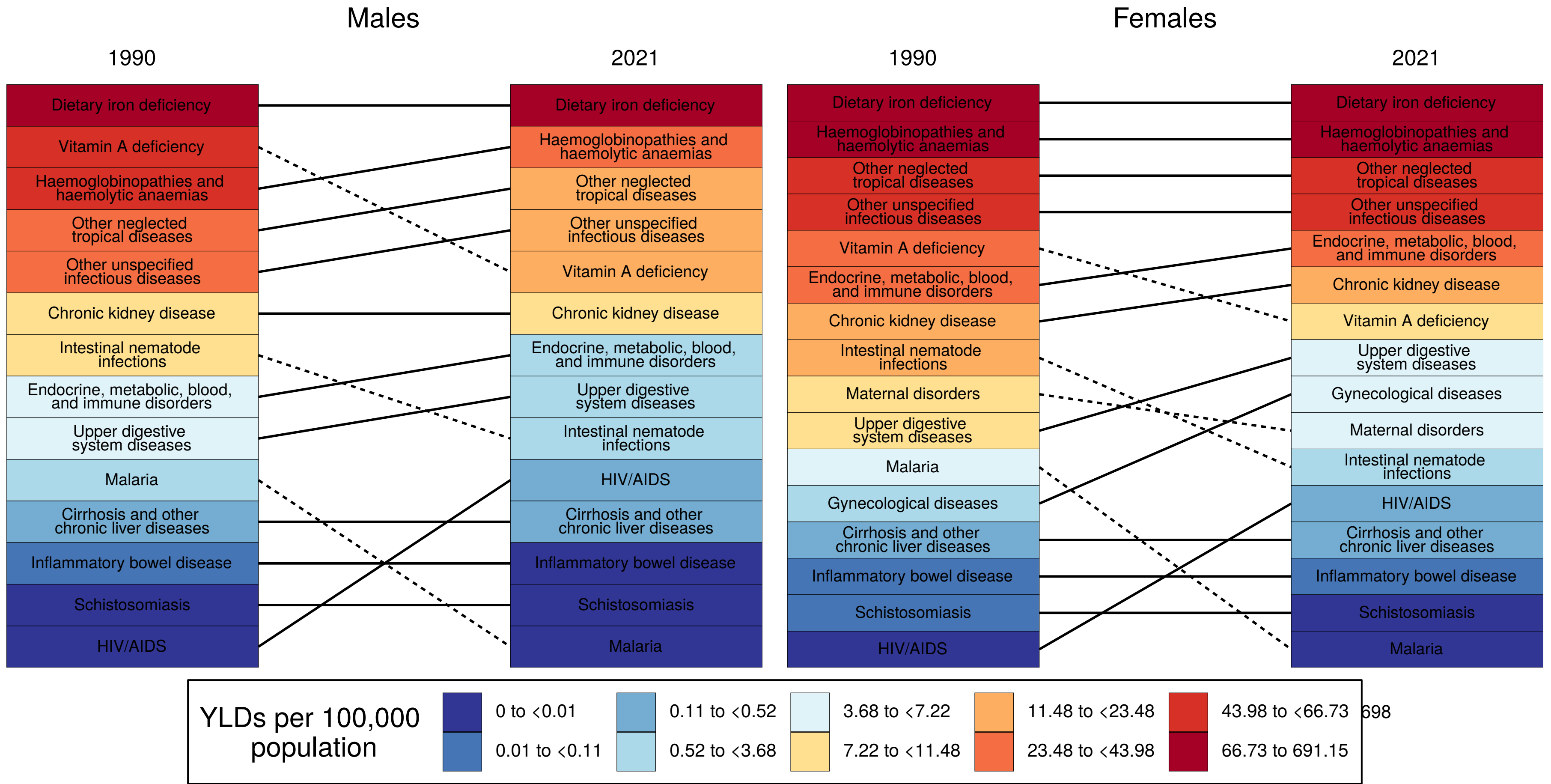

Bulacan

Anaemia Prevalence - Both Sexes

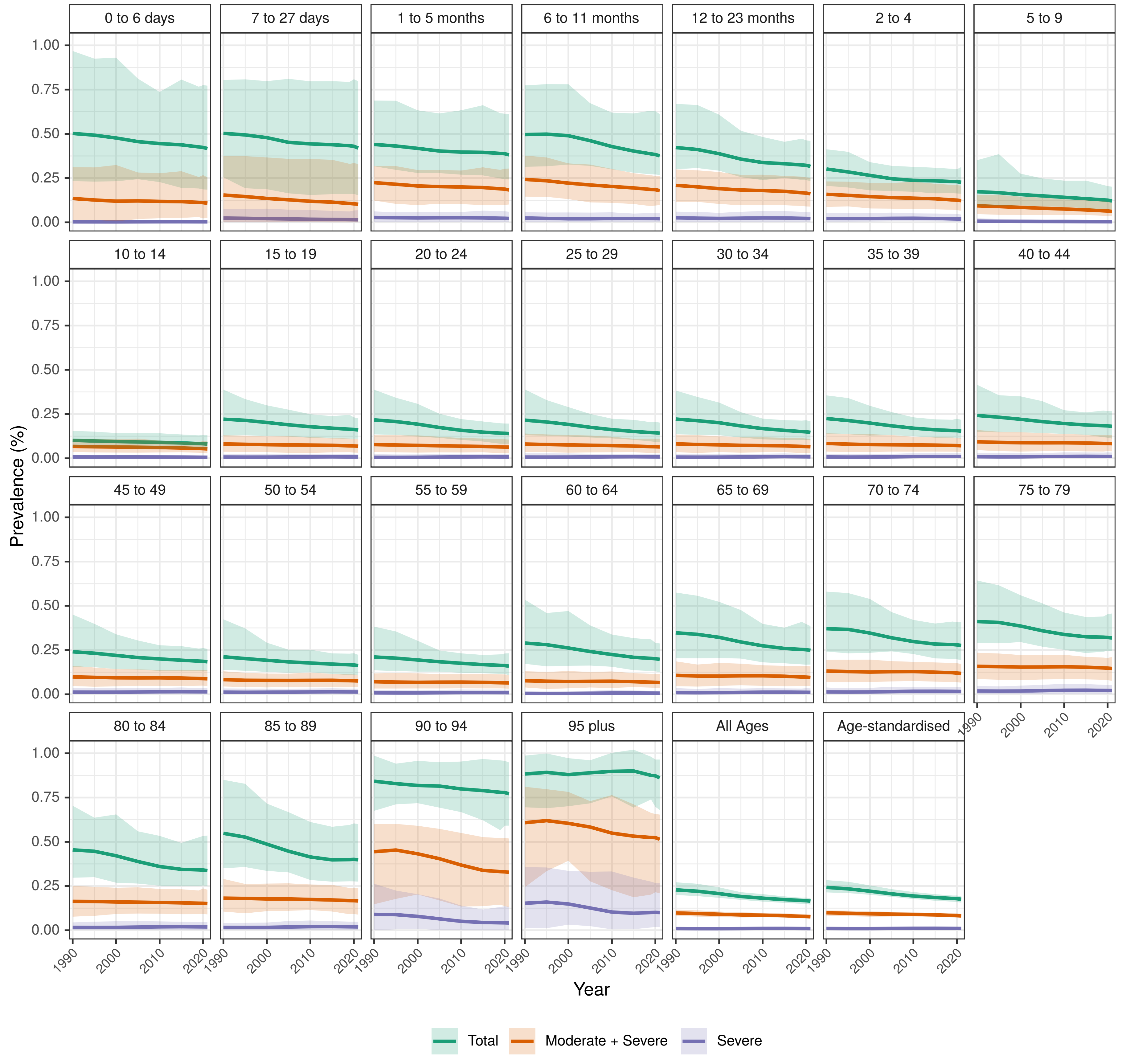

Cause-Specific Anaemia Prevalence - 2021

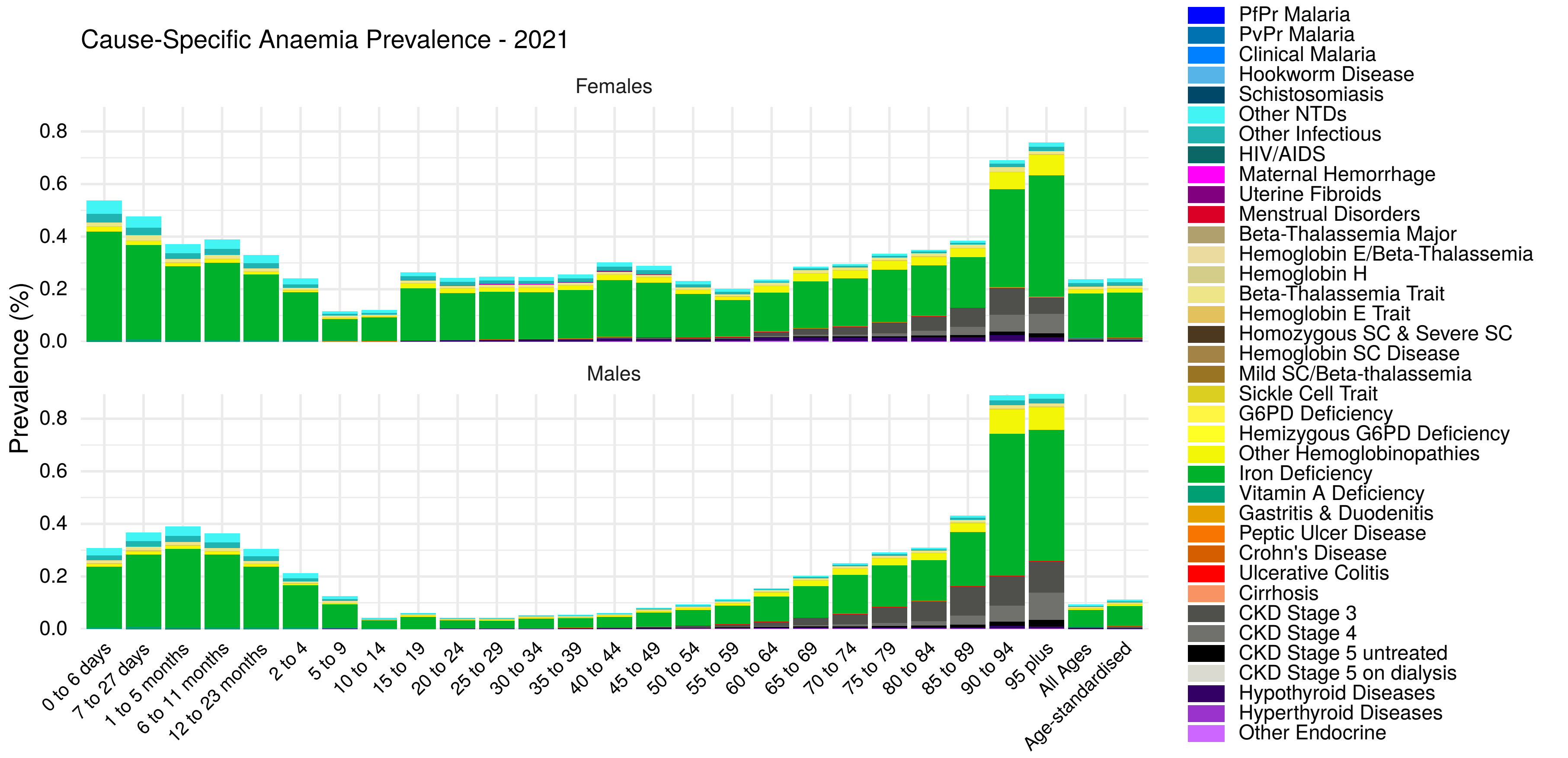

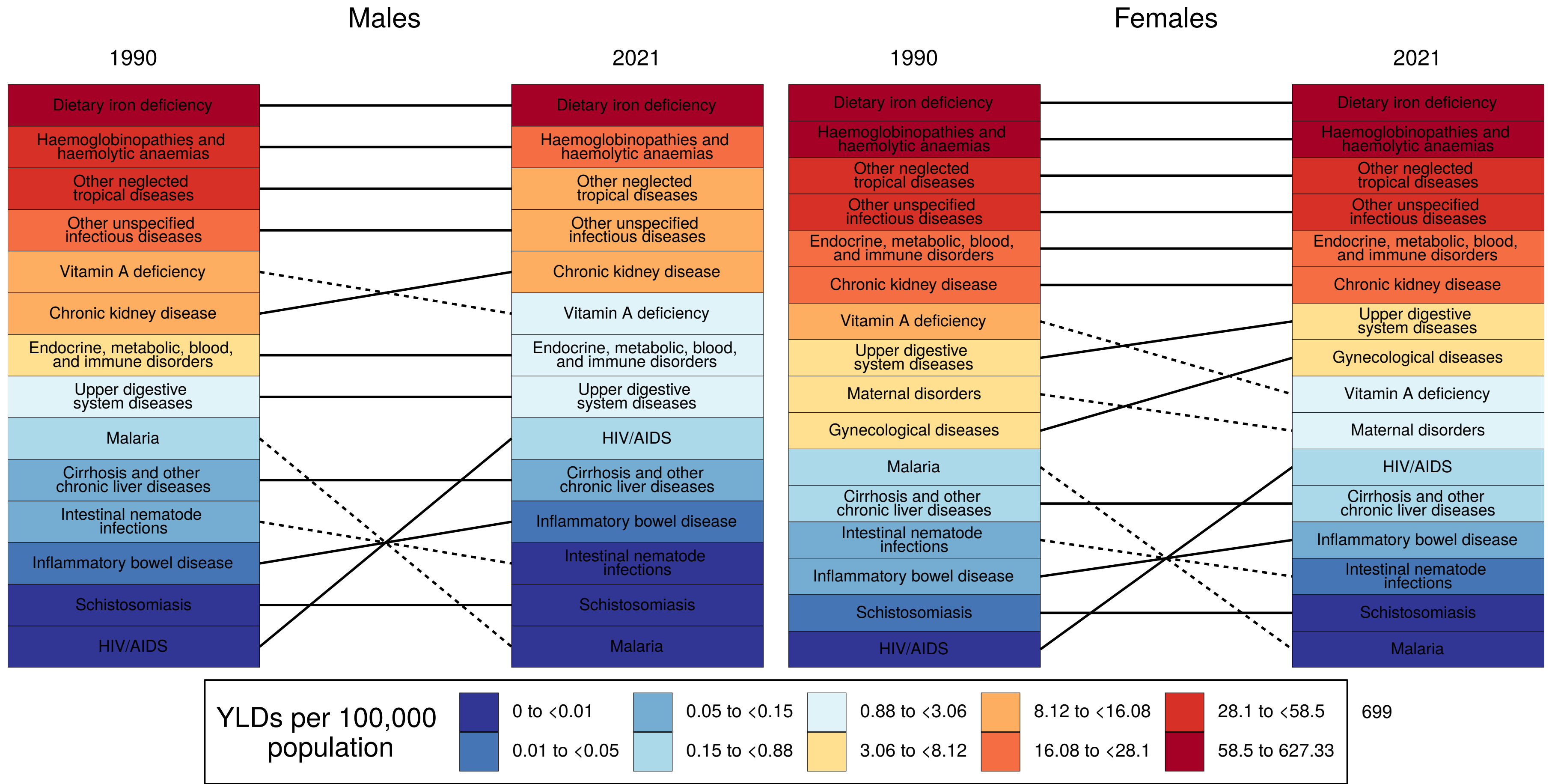

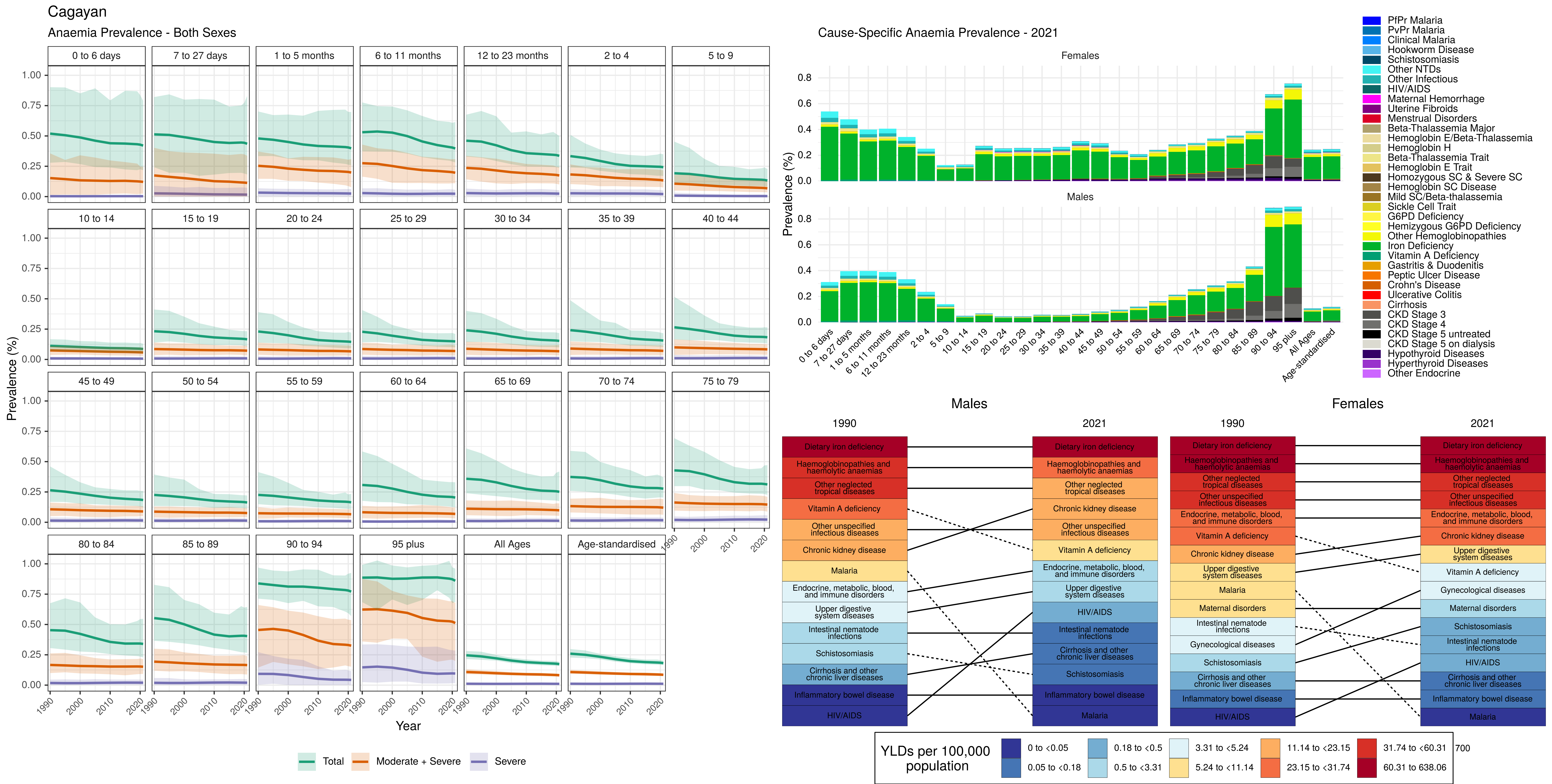

Cagayan
Anaemia Prevalence - Both Sexes
0 to 6 days
7 to 27 days
1 to 5 months
6 to 11 months
12 to 23 months
2 to 4
5 to 9
10 to 14
15 to 19
20 to 24
25 to 29
30 to 34
35 to 39
40 to 44
45 to 49
50 to 54
55 to 59
60 to 64
65 to 69
70 to 74
75 to 79
80 to 84
85 to 89
90 to 94
95 plus
All Ages
Age-standardised
Prevalence (%)
Year
Total
Moderate + Severe
Severe
Cause-Specific Anaemia Prevalence - 2021
Females
Males
PfPr Malaria
PvPr Malaria
Clinical Malaria
Hookworm Disease
Schistosomiasis
Other NTDs
Other Infectious
HIV/AIDS
Maternal Hemorrhage
Uterine Fibroids
Menstrual Disorders
Beta-Thalassemia Major
Hemoglobin E/Beta-Thalassemia
Hemoglobin H
Beta-Thalassemia Trait
Hemoglobin E Trait
Homozygous SC & Severe SC
Hemoglobin SC Disease
Mild SC/Beta-thalassemia
Sickle Cell Trait
G6PD Deficiency
Hemizygous G6PD Deficiency
Other Hemoglobinopathies
Iron Deficiency
Vitamin A Deficiency
Gastritis & Duodenitis
Peptic Ulcer Disease
Crohn's Disease
Ulcerative Colitis
Cirrhosis
CKD Stage 3
CKD Stage 4
CKD Stage 5 untreated
CKD Stage 5 on dialysis
Hypothyroid Diseases
Hyperthyroid Diseases
Other Endocrine
Males
Females
1990
2021
Dietary iron deficiency
Haemoglobinopathies and haemolytic anaemias
Other neglected tropical diseases
Vitamin A deficiency
Other unspecified infectious diseases
Chronic kidney disease
Malaria
Endocrine, metabolic, blood, and immune disorders
Upper digestive system diseases
Intestinal nematode infections
Schistosomiasis
Cirrhosis and other chronic liver diseases
Inflammatory bowel disease
HIV/AIDS
Maternal disorders
Gynecological diseases
YLDs per 100,000 population
0 to <0.05
0.05 to <0.18
0.18 to <0.5
0.5 to <3.31
3.31 to <5.24
5.24 to <11.14
11.14 to <23.15
23.15 to <31.74
31.74 to <60.31
60.31 to 638.06

Camarines Norte

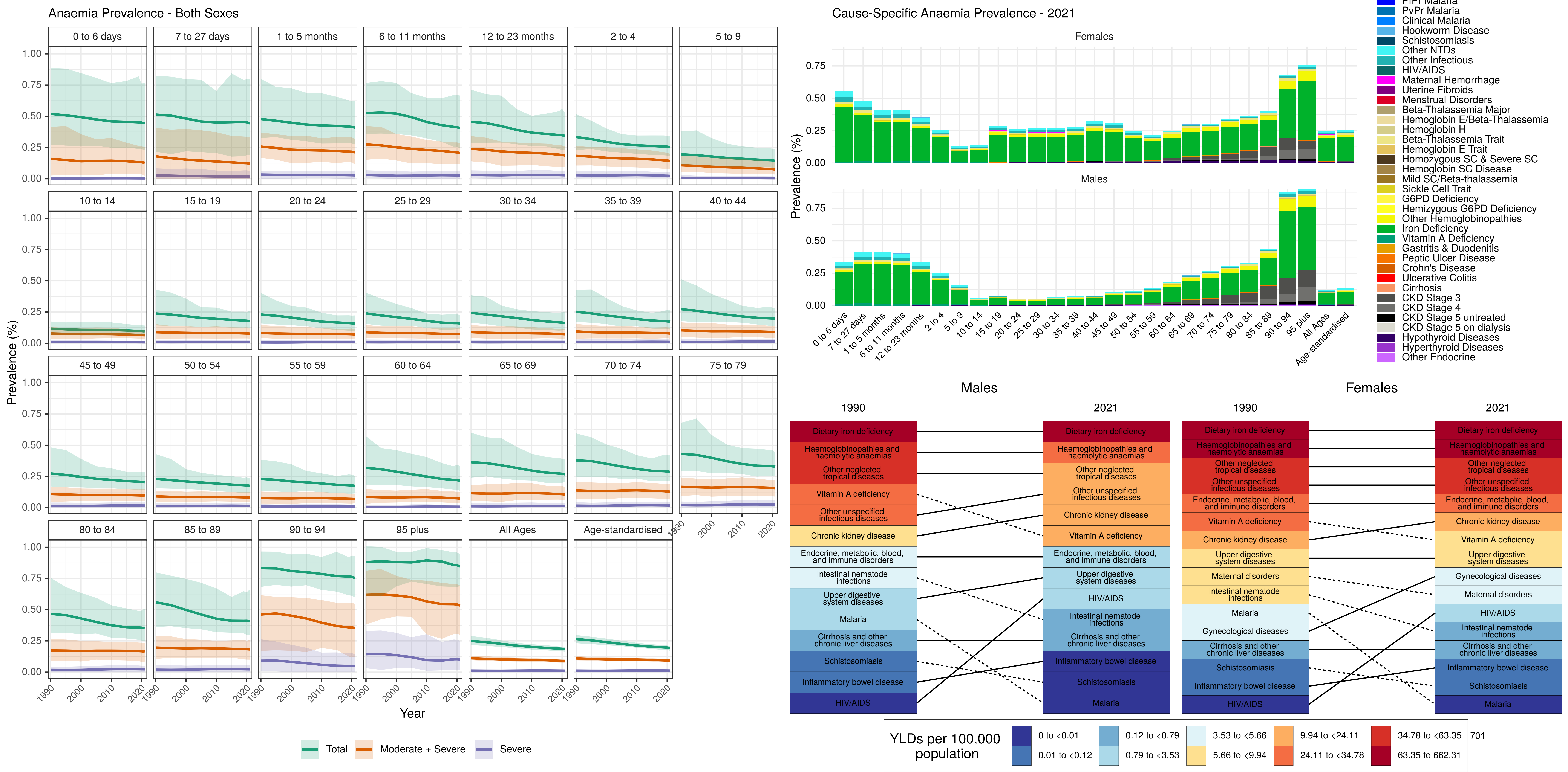

Camarines Sur

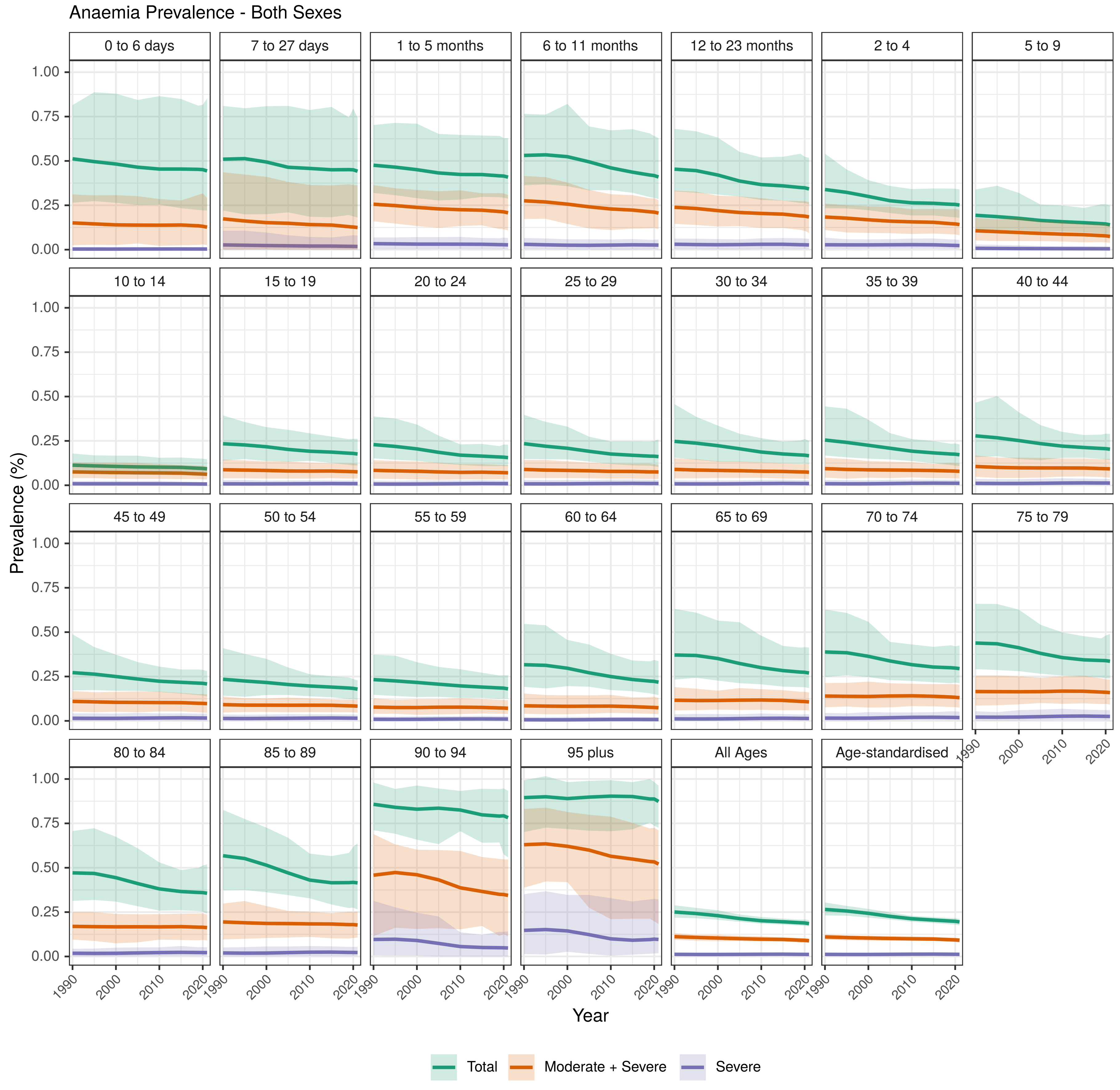

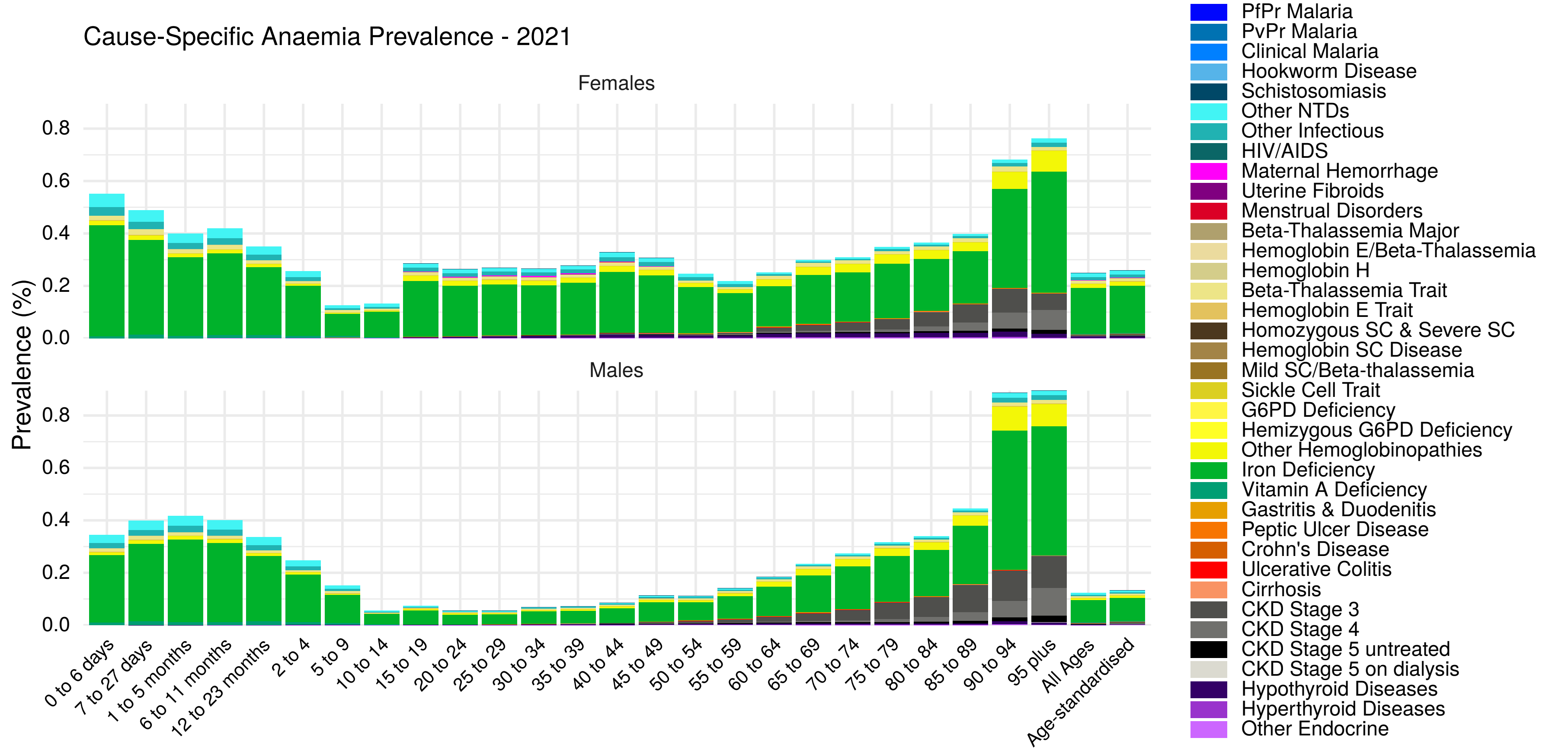

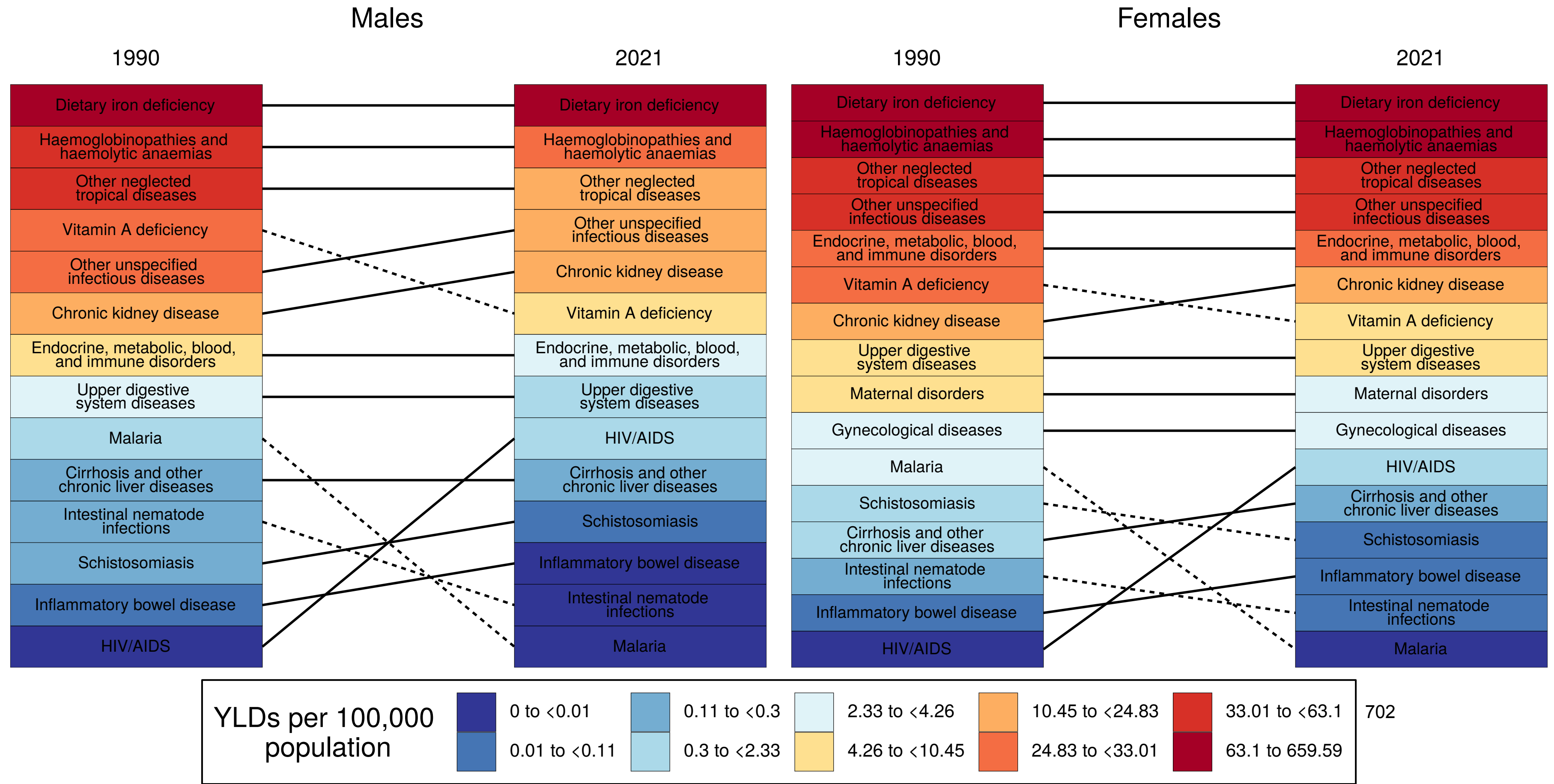

Camiguin

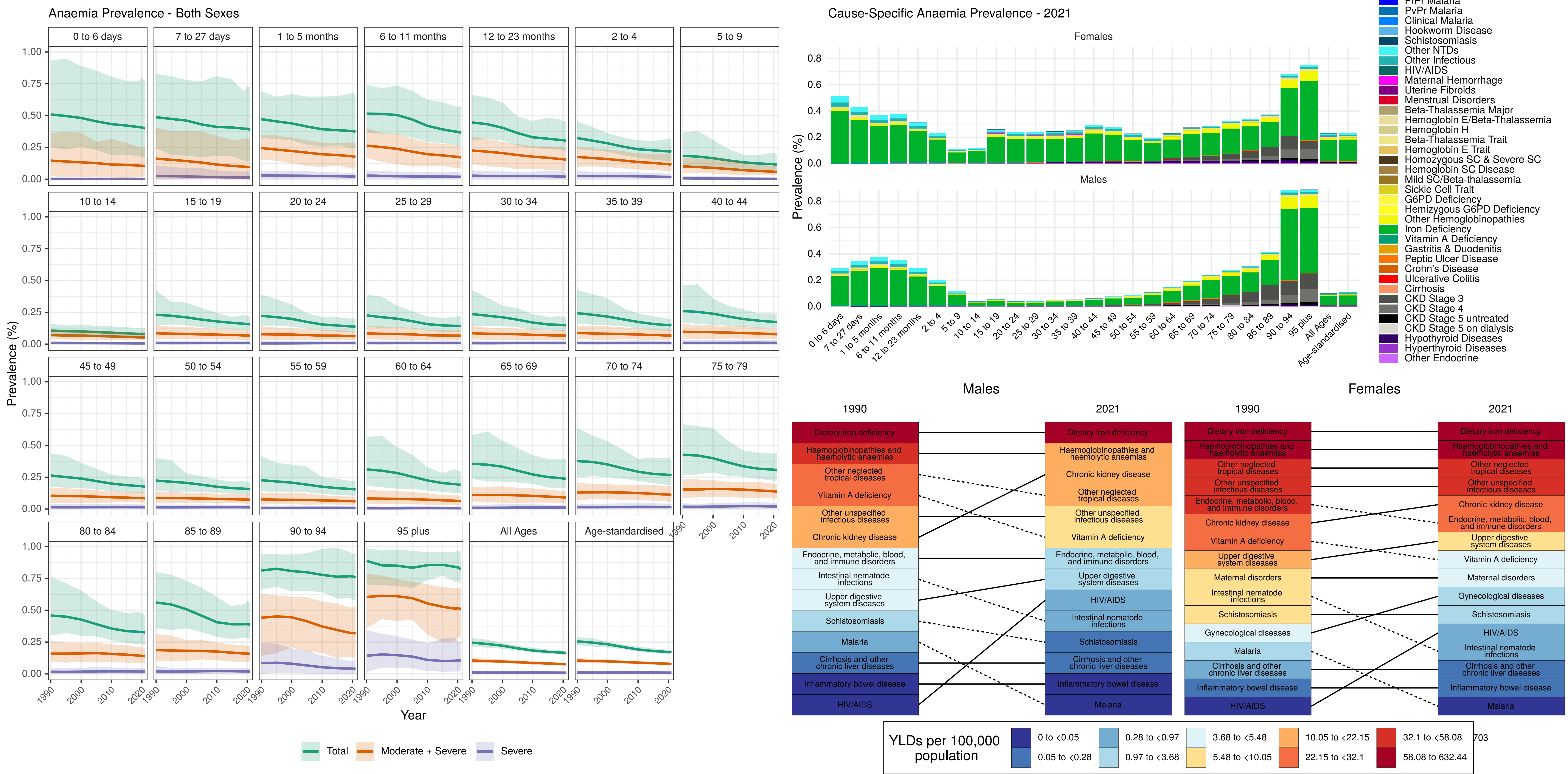

Anaemia Prevalence - Both Sexes
0 to 6 days
7 to 27 days
1 to 5 months
6 to 11 months
12 to 23 months
2 to 4
5 to 9
10 to 14
15 to 19
20 to 24
25 to 29
30 to 34
35 to 39
40 to 44
45 to 49
50 to 54
55 to 59
60 to 64
65 to 69
70 to 74
75 to 79
80 to 84
85 to 89
90 to 94
95 plus
All Ages
Age-standardised
Prevalence (%)
Year
Total
Moderate + Severe
Severe
Cause-Specific Anaemia Prevalence - 2021
Females
Males
PfPr Malaria
PvPr Malaria
Clinical Malaria
Hookworm Disease
Schistosomiasis
Other NTDs
Other Infectious
HIV/AIDS
Maternal Hemorrhage
Uterine Fibroids
Menstrual Disorders
Beta-Thalassemia Major
Hemoglobin E/Beta-Thalassemia
Hemoglobin H
Beta-Thalassemia Trait
Hemoglobin E Trait
Homozygous SC & Severe SC
Hemoglobin SC Disease
Mild SC/Beta-thalassemia
Sickle Cell Trait
G6PD Deficiency
Hemizygous G6PD Deficiency
Other Hemoglobinopathies
Iron Deficiency
Vitamin A Deficiency
Gastritis & Duodenitis
Peptic Ulcer Disease
Crohn's Disease
Ulcerative Colitis
Cirrhosis
CKD Stage 3
CKD Stage 4
CKD Stage 5 untreated
CKD Stage 5 on dialysis
Hypothyroid Diseases
Hyperthyroid Diseases
Other Endocrine
Males
1990
Dietary iron deficiency
Haemoglobinopathies and haemolytic anaemias
Other neglected tropical diseases
Vitamin A deficiency
Other unspecified infectious diseases
Chronic kidney disease
Endocrine, metabolic, blood, and immune disorders
Intestinal nematode infections
Upper digestive system diseases
Schistosomiasis
Malaria
Cirrhosis and other chronic liver diseases
Inflammatory bowel disease
HIV/AIDS
2021
Dietary iron deficiency
Haemoglobinopathies and haemolytic anaemias
Chronic kidney disease
Other neglected tropical diseases
Other unspecified infectious diseases
Vitamin A deficiency
Endocrine, metabolic, blood, and immune disorders
Upper digestive system diseases
HIV/AIDS
Intestinal nematode infections
Schistosomiasis
Cirrhosis and other chronic liver diseases
Inflammatory bowel disease
Malaria
Females
1990
Dietary iron deficiency
Haemoglobinopathies and haemolytic anaemias
Other neglected tropical diseases
Other unspecified infectious diseases
Endocrine, metabolic, blood, and immune disorders
Chronic kidney disease
Vitamin A deficiency
Upper digestive system diseases
Maternal disorders
Intestinal nematode infections
Schistosomiasis
Gynecological diseases
Malaria
Cirrhosis and other chronic liver diseases
Inflammatory bowel disease
HIV/AIDS
2021
Dietary iron deficiency
Haemoglobinopathies and haemolytic anaemias
Other neglected tropical diseases
Other unspecified infectious diseases
Chronic kidney disease
Endocrine, metabolic, blood, and immune disorders
Upper digestive system diseases
Vitamin A deficiency
Maternal disorders
Gynecological diseases
Schistosomiasis
HIV/AIDS
Intestinal nematode infections
Cirrhosis and other chronic liver diseases
Inflammatory bowel disease
Malaria
YLDs per 100,000 population
0 to <0.05
0.05 to <0.28
0.28 to <0.97
0.97 to <3.68
3.68 to <5.48
5.48 to <10.05
10.05 to <22.15
22.15 to <32.1
32.1 to <58.08
58.08 to 632.44

Capiz

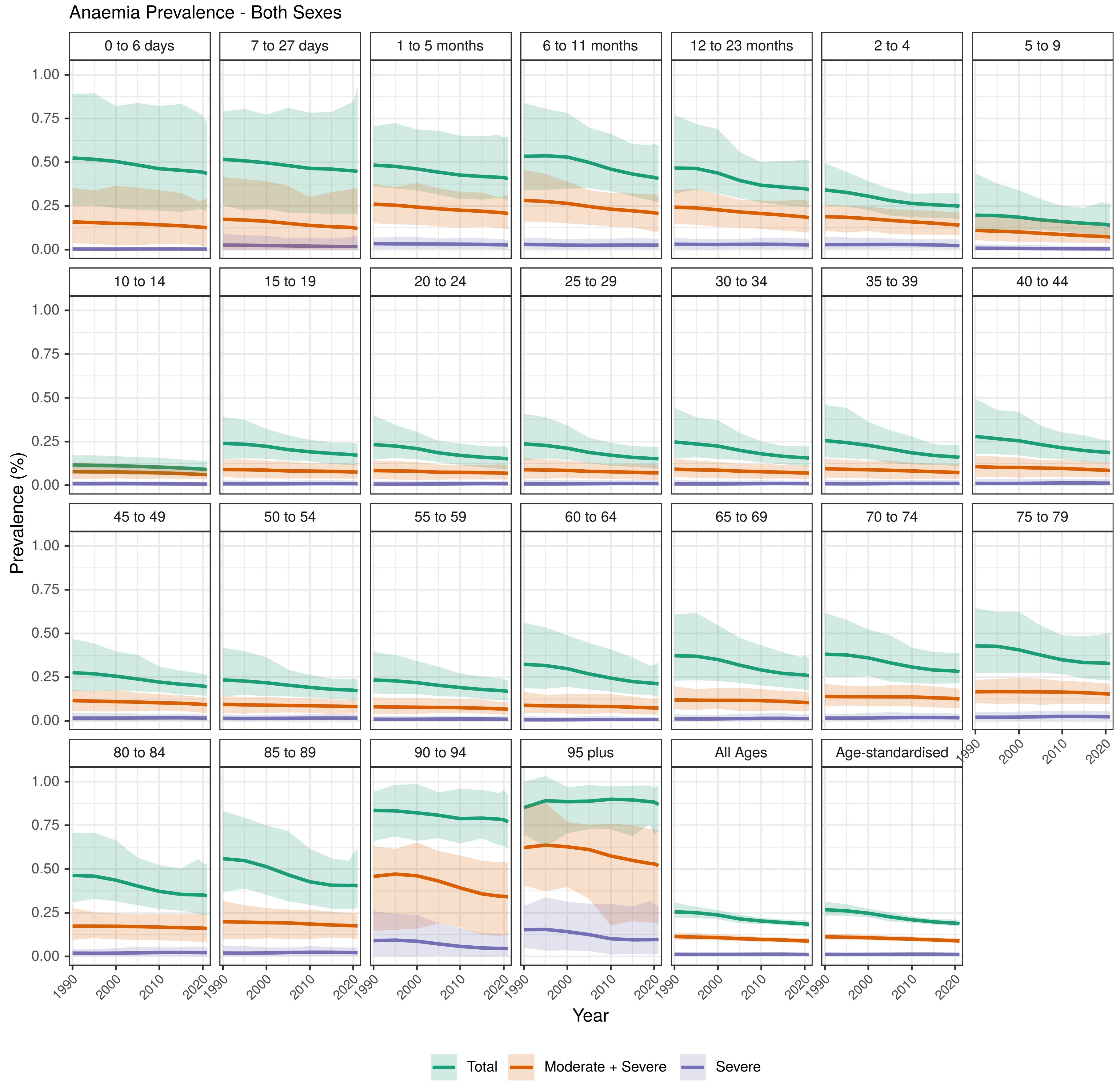

Anaemia Prevalence - Both Sexes
0 to 6 days
7 to 27 days
1 to 5 months
6 to 11 months
12 to 23 months
2 to 4
5 to 9
10 to 14
15 to 19
20 to 24
25 to 29
30 to 34
35 to 39
40 to 44
45 to 49
50 to 54
55 to 59
60 to 64
65 to 69
70 to 74
75 to 79
80 to 84
85 to 89
90 to 94
95 plus
All Ages
Age-standardised
Prevalence (%)
Year
Total
Moderate + Severe
Severe

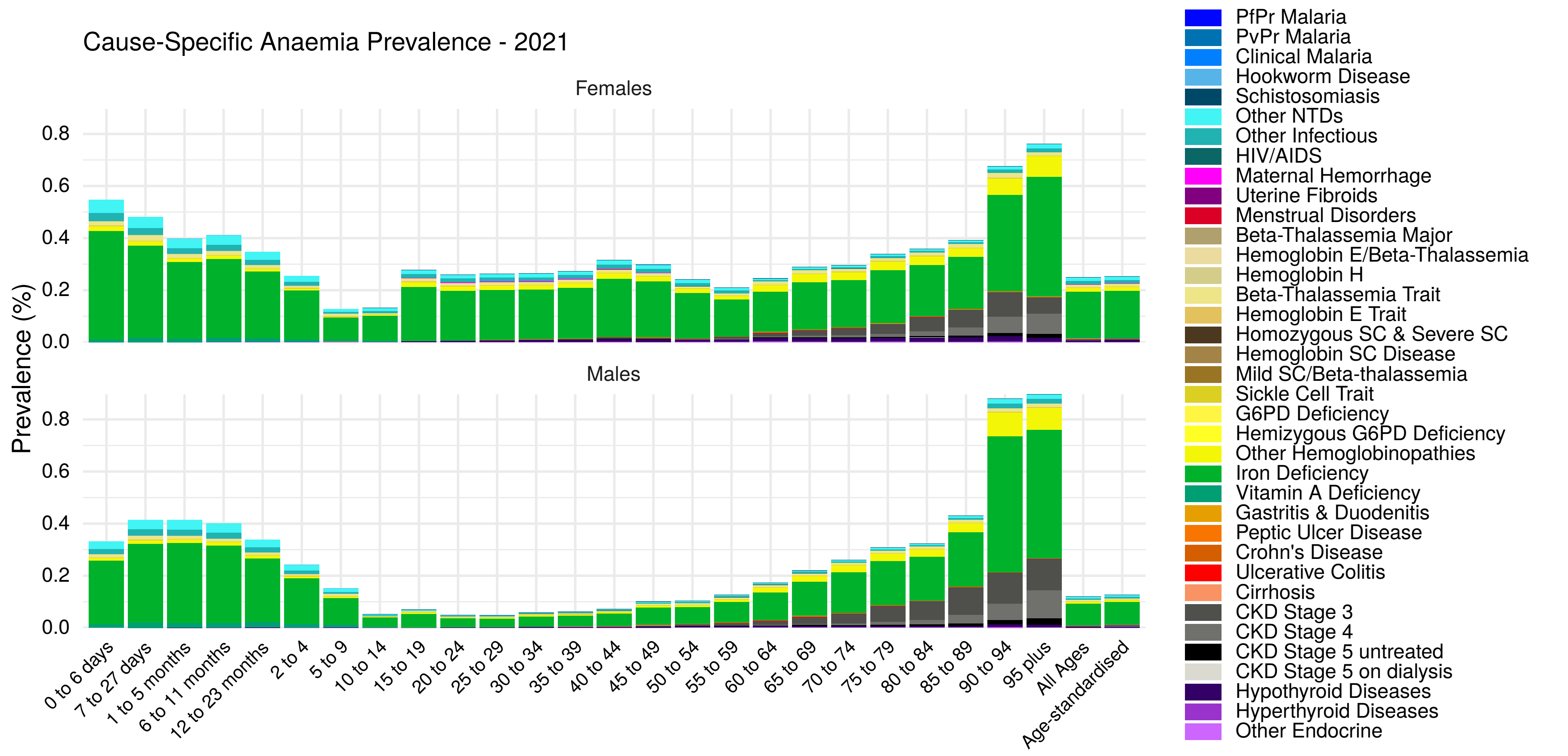

Cause-Specific Anaemia Prevalence - 2021
Females
Males
Prevalence (%)
PfPr Malaria
PvPr Malaria
Clinical Malaria
Hookworm Disease
Schistosomiasis
Other NTDs
Other Infectious
HIV/AIDS
Maternal Hemorrhage
Uterine Fibroids
Menstrual Disorders
Beta-Thalassemia Major
Hemoglobin E/Beta-Thalassemia
Hemoglobin H
Beta-Thalassemia Trait
Hemoglobin E Trait
Homozygous SC & Severe SC
Hemoglobin SC Disease
Mild SC/Beta-thalassemia
Sickle Cell Trait
G6PD Deficiency
Hemizygous G6PD Deficiency
Other Hemoglobinopathies
Iron Deficiency
Vitamin A Deficiency
Gastritis & Duodenitis
Peptic Ulcer Disease
Crohn's Disease
Ulcerative Colitis
Cirrhosis
CKD Stage 3
CKD Stage 4
CKD Stage 5 untreated
CKD Stage 5 on dialysis
Hypothyroid Diseases
Hyperthyroid Diseases
Other Endocrine

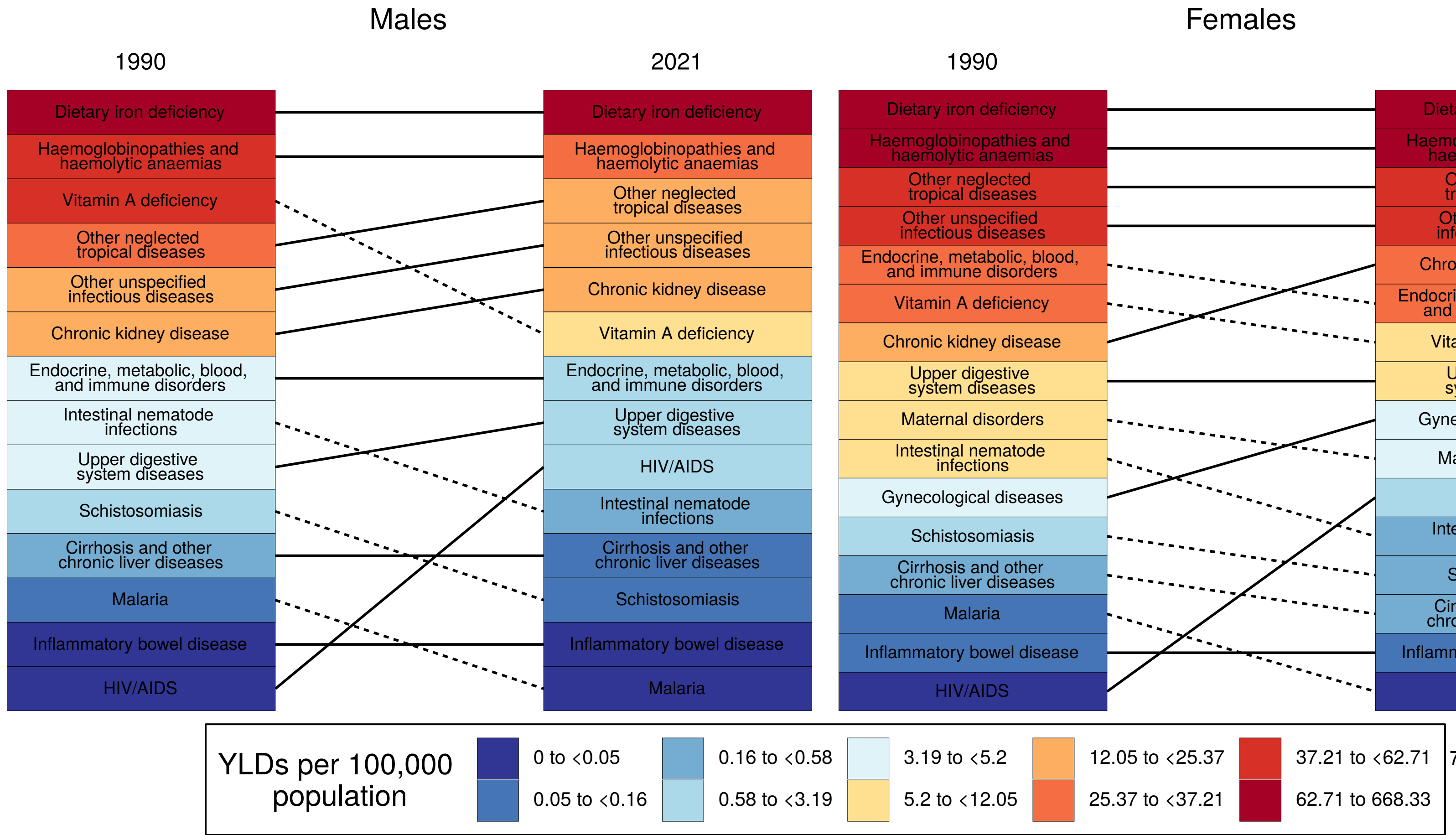

Males
Females
1990
2021
Dietary iron deficiency
Haemoglobinopathies and haemolytic anaemias
Vitamin A deficiency
Other neglected tropical diseases
Other unspecified infectious diseases
Chronic kidney disease
Endocrine, metabolic, blood, and immune disorders
Intestinal nematode infections
Upper digestive system diseases
Schistosomiasis
Cirrhosis and other chronic liver diseases
Malaria
Inflammatory bowel disease
HIV/AIDS
Maternal disorders
Gynecological diseases
YLDs per 100,000 population
0 to <0.05
0.05 to <0.16
0.16 to <0.58
0.58 to <3.19
3.19 to <5.2
5.2 to <12.05
12.05 to <25.37
25.37 to <37.21
37.21 to <62.71
62.71 to 668.33

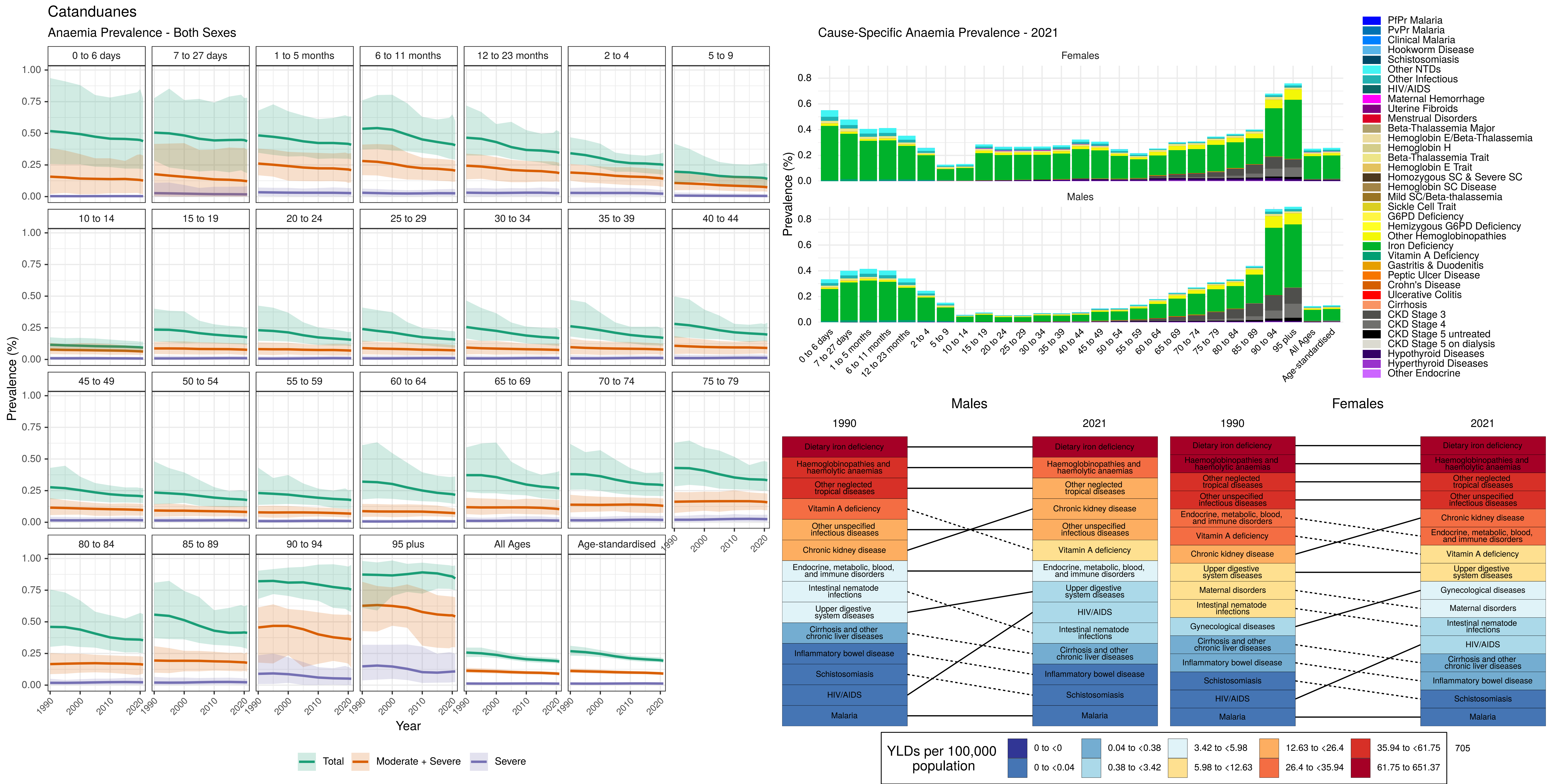

Catanduanes
Anaemia Prevalence - Both Sexes
0 to 6 days
7 to 27 days
1 to 5 months
6 to 11 months
12 to 23 months
2 to 4
5 to 9
10 to 14
15 to 19
20 to 24
25 to 29
30 to 34
35 to 39
40 to 44
45 to 49
50 to 54
55 to 59
60 to 64
65 to 69
70 to 74
75 to 79
80 to 84
85 to 89
90 to 94
95 plus
All Ages
Age-standardised
Prevalence (%)
Year
Total
Moderate + Severe
Severe
Cause-Specific Anaemia Prevalence - 2021
Females
Males
Prevalence (%)
PfPr Malaria
PvPr Malaria
Clinical Malaria
Hookworm Disease
Schistosomiasis
Other NTDs
Other Infectious
HIV/AIDS
Maternal Hemorrhage
Uterine Fibroids
Menstrual Disorders
Beta-Thalassemia Major
Hemoglobin E/Beta-Thalassemia
Hemoglobin H
Beta-Thalassemia Trait
Hemoglobin E Trait
Homozygous SC & Severe SC
Hemoglobin SC Disease
Mild SC/Beta-thalassemia
Sickle Cell Trait
G6PD Deficiency
Hemizygous G6PD Deficiency
Other Hemoglobinopathies
Iron Deficiency
Vitamin A Deficiency
Gastritis & Duodenitis
Peptic Ulcer Disease
Crohn's Disease
Ulcerative Colitis
Cirrhosis
CKD Stage 3
CKD Stage 4
CKD Stage 5 untreated
CKD Stage 5 on dialysis
Hypothyroid Diseases
Hyperthyroid Diseases
Other Endocrine
Males
Females
1990
2021
Dietary iron deficiency
Haemoglobinopathies and haemolytic anaemias
Other neglected tropical diseases
Vitamin A deficiency
Other unspecified infectious diseases
Chronic kidney disease
Endocrine, metabolic, blood, and immune disorders
Intestinal nematode infections
Upper digestive system diseases
Cirrhosis and other chronic liver diseases
Inflammatory bowel disease
Schistosomiasis
HIV/AIDS
Malaria
Maternal disorders
Gynecological diseases
YLDs per 100,000 population
0 to <0
0 to <0.04
0.04 to <0.38
0.38 to <3.42
3.42 to <5.98
5.98 to <12.63
12.63 to <26.4
26.4 to <35.94
35.94 to <61.75
61.75 to 651.37

Cavite

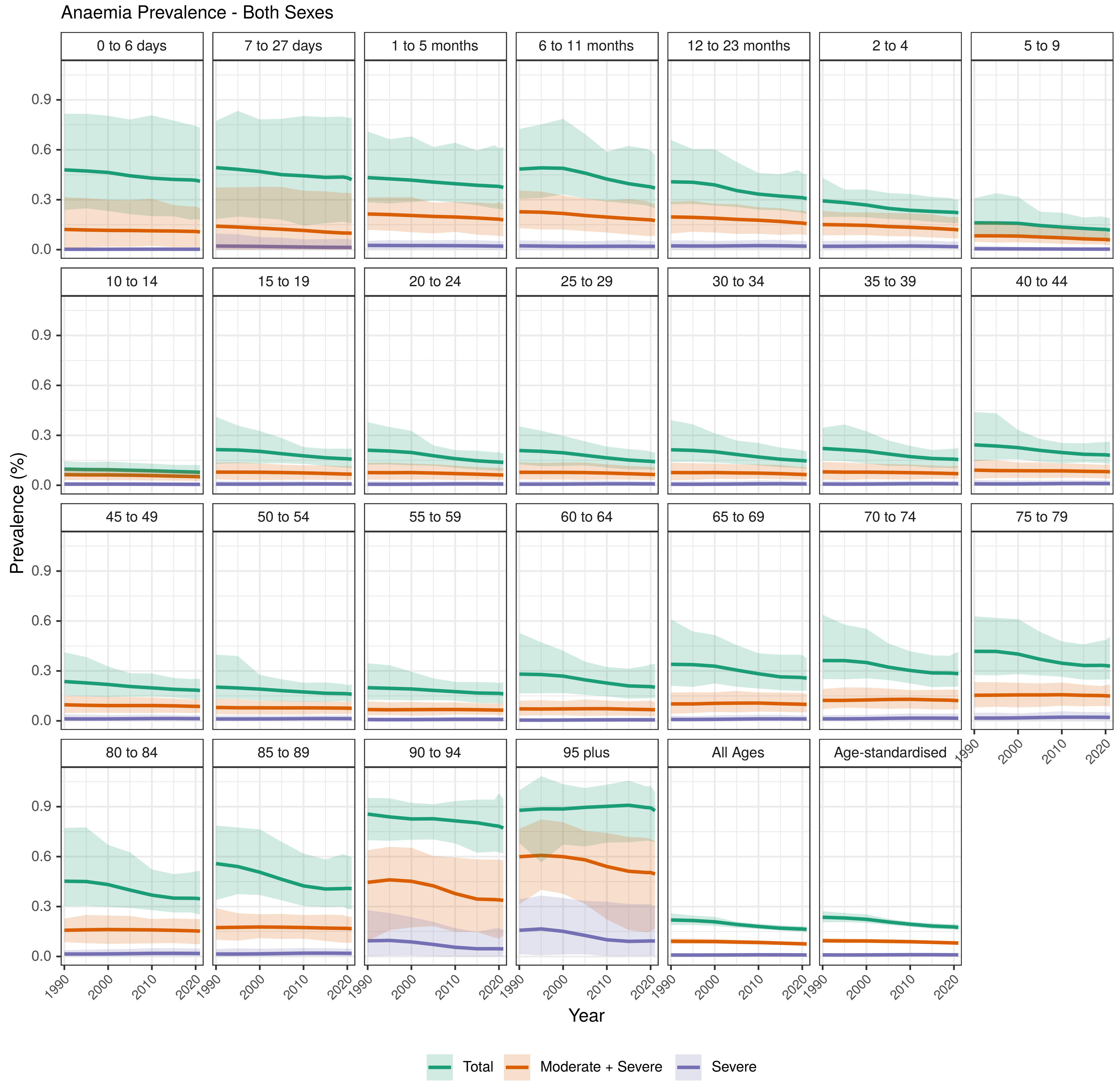

Anaemia Prevalence - Both Sexes
0 to 6 days
7 to 27 days
1 to 5 months
6 to 11 months
12 to 23 months
2 to 4
5 to 9
10 to 14
15 to 19
20 to 24
25 to 29
30 to 34
35 to 39
40 to 44
45 to 49
50 to 54
55 to 59
60 to 64
65 to 69
70 to 74
75 to 79
80 to 84
85 to 89
90 to 94
95 plus
All Ages
Age-standardised
Prevalence (%)
0.0
0.3
0.6
0.9
1990
2000
2010
2020
Year
Total
Moderate + Severe
Severe

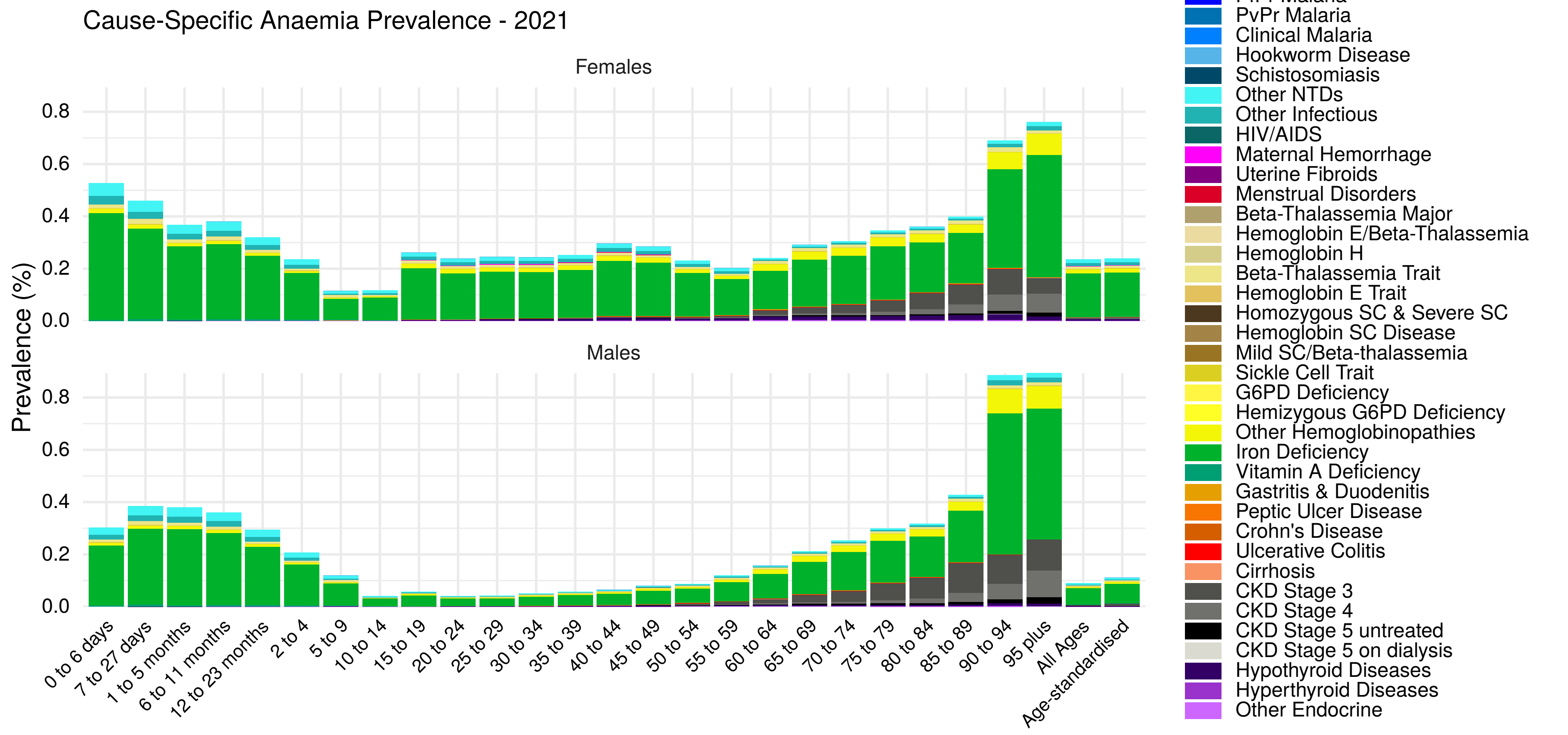

Cause-Specific Anaemia Prevalence - 2021
Females
Males
Prevalence (%)
0.0
0.2
0.4
0.6
0.8
0 to 6 days
7 to 27 days
1 to 5 months
6 to 11 months
12 to 23 months
2 to 4
5 to 9
10 to 14
15 to 19
20 to 24
25 to 29
30 to 34
35 to 39
40 to 44
45 to 49
50 to 54
55 to 59
60 to 64
65 to 69
70 to 74
75 to 79
80 to 84
85 to 89
90 to 94
95 plus
All Ages
Age-standardised
PfPr Malaria
PvPr Malaria
Clinical Malaria
Hookworm Disease
Schistosomiasis
Other NTDs
Other Infectious
HIV/AIDS
Maternal Hemorrhage
Uterine Fibroids
Menstrual Disorders
Beta-Thalassemia Major
Hemoglobin E/Beta-Thalassemia
Hemoglobin H
Beta-Thalassemia Trait
Hemoglobin E Trait
Homozygous SC & Severe SC
Hemoglobin SC Disease
Mild SC/Beta-thalassemia
Sickle Cell Trait
G6PD Deficiency
Hemizygous G6PD Deficiency
Other Hemoglobinopathies
Iron Deficiency
Vitamin A Deficiency
Gastritis & Duodenitis
Peptic Ulcer Disease
Crohn's Disease
Ulcerative Colitis
Cirrhosis
CKD Stage 3
CKD Stage 4
CKD Stage 5 untreated
CKD Stage 5 on dialysis
Hypothyroid Diseases
Hyperthyroid Diseases
Other Endocrine

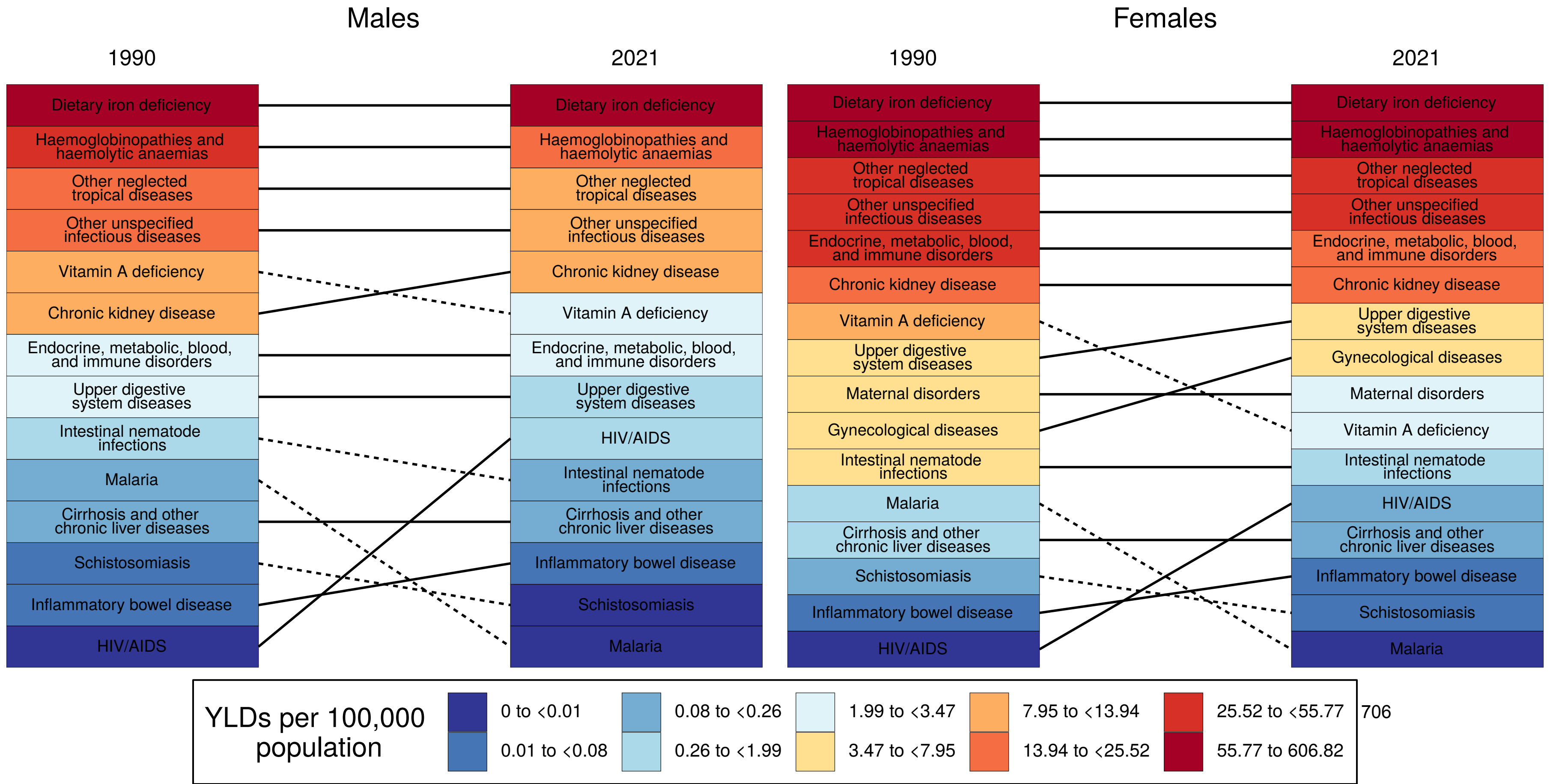

Males
Females
1990
2021
Males 1990:
Dietary iron deficiency
Haemoglobinopathies and haemolytic anaemias
Other neglected tropical diseases
Other unspecified infectious diseases
Vitamin A deficiency
Chronic kidney disease
Endocrine, metabolic, blood, and immune disorders
Upper digestive system diseases
Intestinal nematode infections
Malaria
Cirrhosis and other chronic liver diseases
Schistosomiasis
Inflammatory bowel disease
HIV/AIDS
Males 2021:
Dietary iron deficiency
Haemoglobinopathies and haemolytic anaemias
Other neglected tropical diseases
Other unspecified infectious diseases
Chronic kidney disease
Vitamin A deficiency
Endocrine, metabolic, blood, and immune disorders
Upper digestive system diseases
HIV/AIDS
Intestinal nematode infections
Cirrhosis and other chronic liver diseases
Inflammatory bowel disease
Schistosomiasis
Malaria
Females 1990:
Dietary iron deficiency
Haemoglobinopathies and haemolytic anaemias
Other neglected tropical diseases
Other unspecified infectious diseases
Endocrine, metabolic, blood, and immune disorders
Chronic kidney disease
Vitamin A deficiency
Upper digestive system diseases
Maternal disorders
Gynecological diseases
Intestinal nematode infections
Malaria
Cirrhosis and other chronic liver diseases
Schistosomiasis
Inflammatory bowel disease
HIV/AIDS
Females 2021:
Dietary iron deficiency
Haemoglobinopathies and haemolytic anaemias
Other neglected tropical diseases
Other unspecified infectious diseases
Endocrine, metabolic, blood, and immune disorders
Chronic kidney disease
Upper digestive system diseases
Gynecological diseases
Maternal disorders
Vitamin A deficiency
Intestinal nematode infections
HIV/AIDS
Cirrhosis and other chronic liver diseases
Inflammatory bowel disease
Schistosomiasis
Malaria
YLDs per 100,000 population
0 to <0.01
0.01 to <0.08
0.08 to <0.26
0.26 to <1.99
1.99 to <3.47
3.47 to <7.95
7.95 to <13.94
13.94 to <25.52
25.52 to <55.77
55.77 to 606.82

Cebu

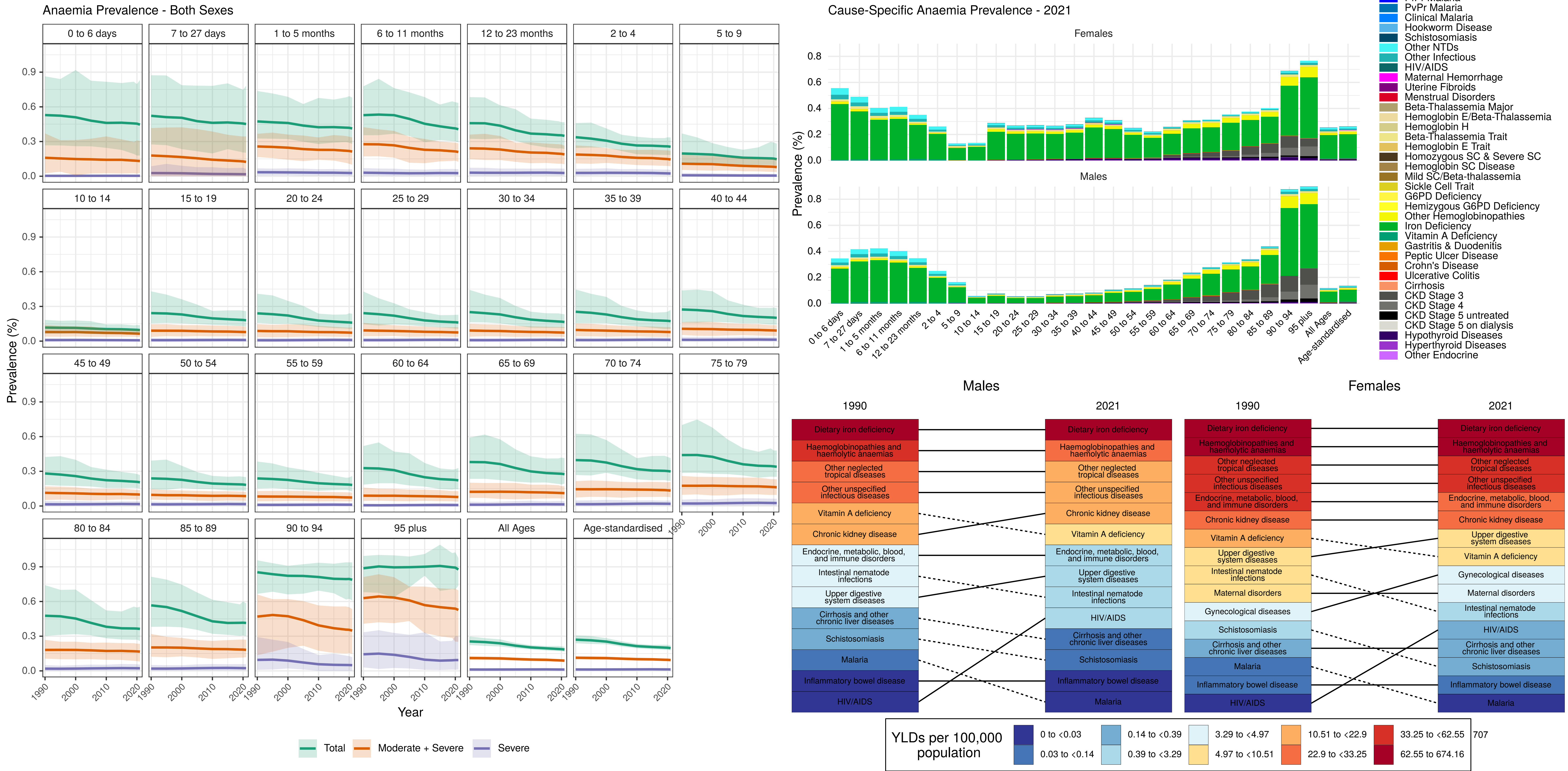

Anaemia Prevalence - Both Sexes
0 to 6 days
7 to 27 days
1 to 5 months
6 to 11 months
12 to 23 months
2 to 4
5 to 9
10 to 14
15 to 19
20 to 24
25 to 29
30 to 34
35 to 39
40 to 44
45 to 49
50 to 54
55 to 59
60 to 64
65 to 69
70 to 74
75 to 79
80 to 84
85 to 89
90 to 94
95 plus
All Ages
Age-standardised
Prevalence (%)
Year
Total
Moderate + Severe
Severe
Cause-Specific Anaemia Prevalence - 2021
Females
Males
Prevalence (%)
PfPr Malaria
PvPr Malaria
Clinical Malaria
Hookworm Disease
Schistosomiasis
Other NTDs
Other Infectious
HIV/AIDS
Maternal Hemorrhage
Uterine Fibroids
Menstrual Disorders
Beta-Thalassemia Major
Hemoglobin E/Beta-Thalassemia
Hemoglobin H
Beta-Thalassemia Trait
Hemoglobin E Trait
Homozygous SC & Severe SC
Hemoglobin SC Disease
Mild SC/Beta-thalassemia
Sickle Cell Trait
G6PD Deficiency
Hemizygous G6PD Deficiency
Other Hemoglobinopathies
Iron Deficiency
Vitamin A Deficiency
Gastritis & Duodenitis
Peptic Ulcer Disease
Crohn's Disease
Ulcerative Colitis
Cirrhosis
CKD Stage 3
CKD Stage 4
CKD Stage 5 untreated
CKD Stage 5 on dialysis
Hypothyroid Diseases
Hyperthyroid Diseases
Other Endocrine
Males
Females
1990
2021
Dietary iron deficiency
Haemoglobinopathies and haemolytic anaemias
Other neglected tropical diseases
Other unspecified infectious diseases
Vitamin A deficiency
Chronic kidney disease
Endocrine, metabolic, blood, and immune disorders
Intestinal nematode infections
Upper digestive system diseases
Cirrhosis and other chronic liver diseases
Schistosomiasis
Malaria
Inflammatory bowel disease
HIV/AIDS
Maternal disorders
Gynecological diseases
YLDs per 100,000 population
0 to <0.03
0.03 to <0.14
0.14 to <0.39
0.39 to <3.29
3.29 to <4.97
4.97 to <10.51
10.51 to <22.9
22.9 to <33.25
33.25 to <62.55
62.55 to 674.16

Cotabato (North Cotabato)

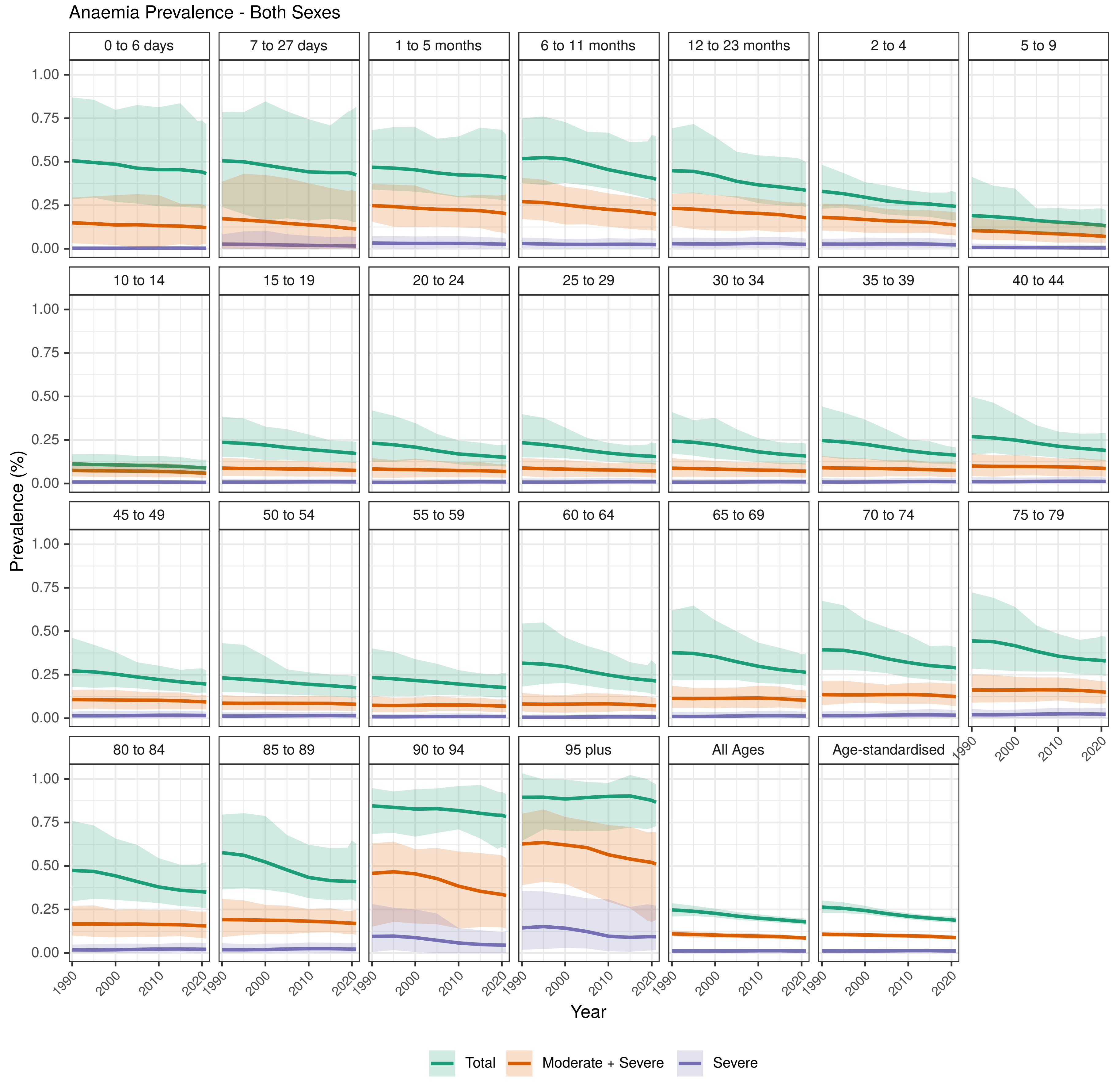

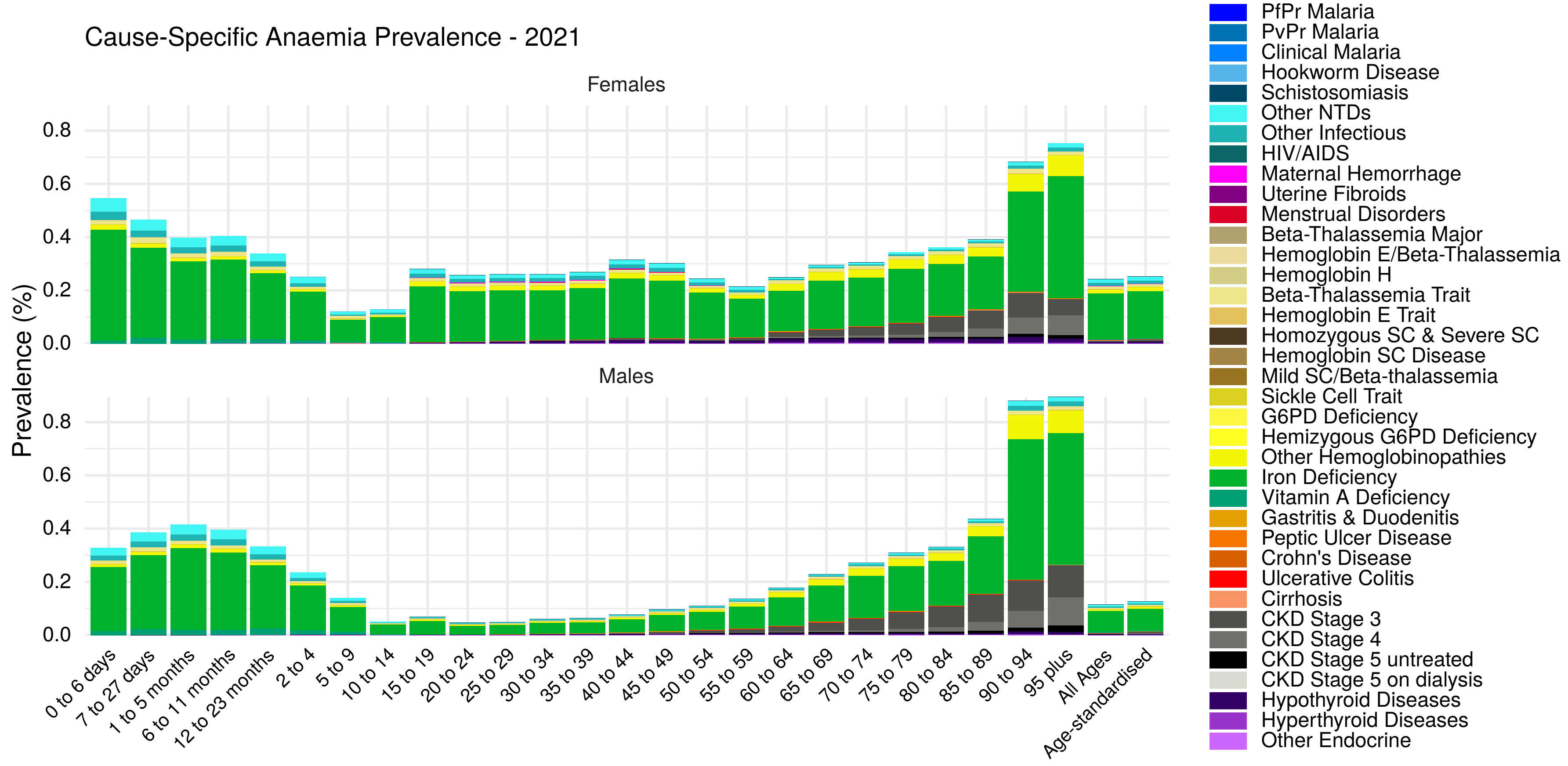

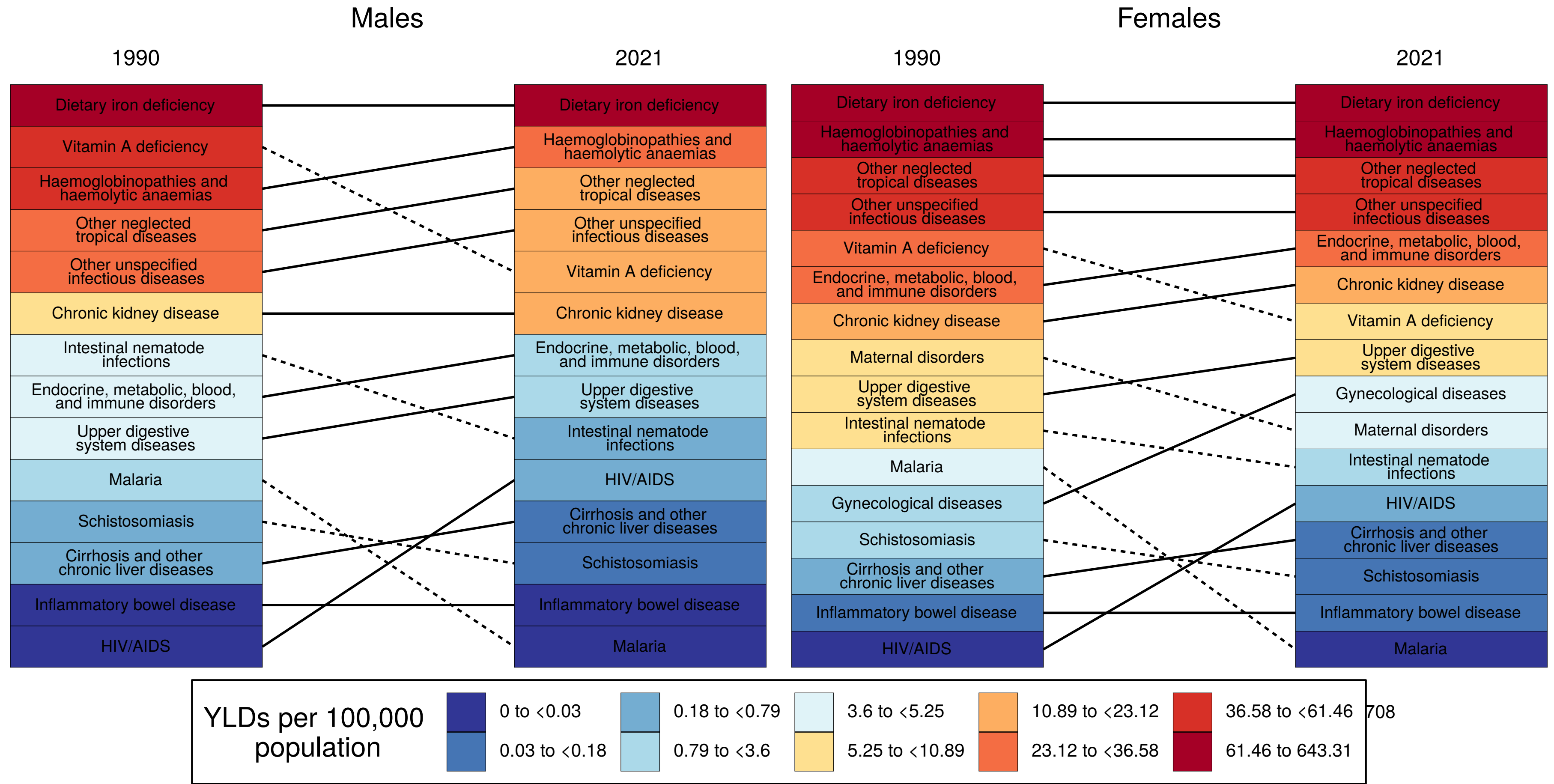

Davao de Oro

Anaemia Prevalence - Both Sexes

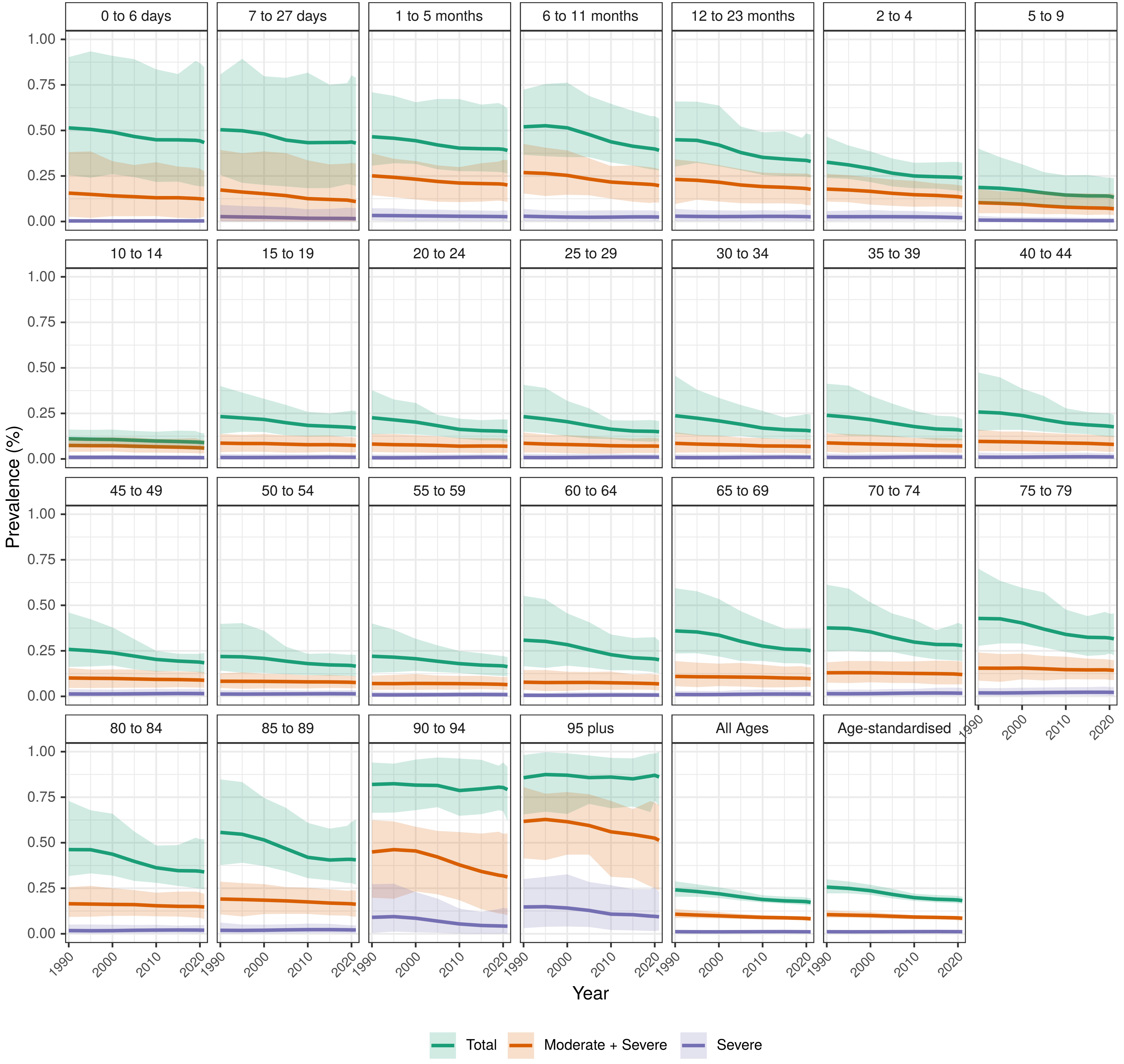

Cause-Specific Anaemia Prevalence - 2021

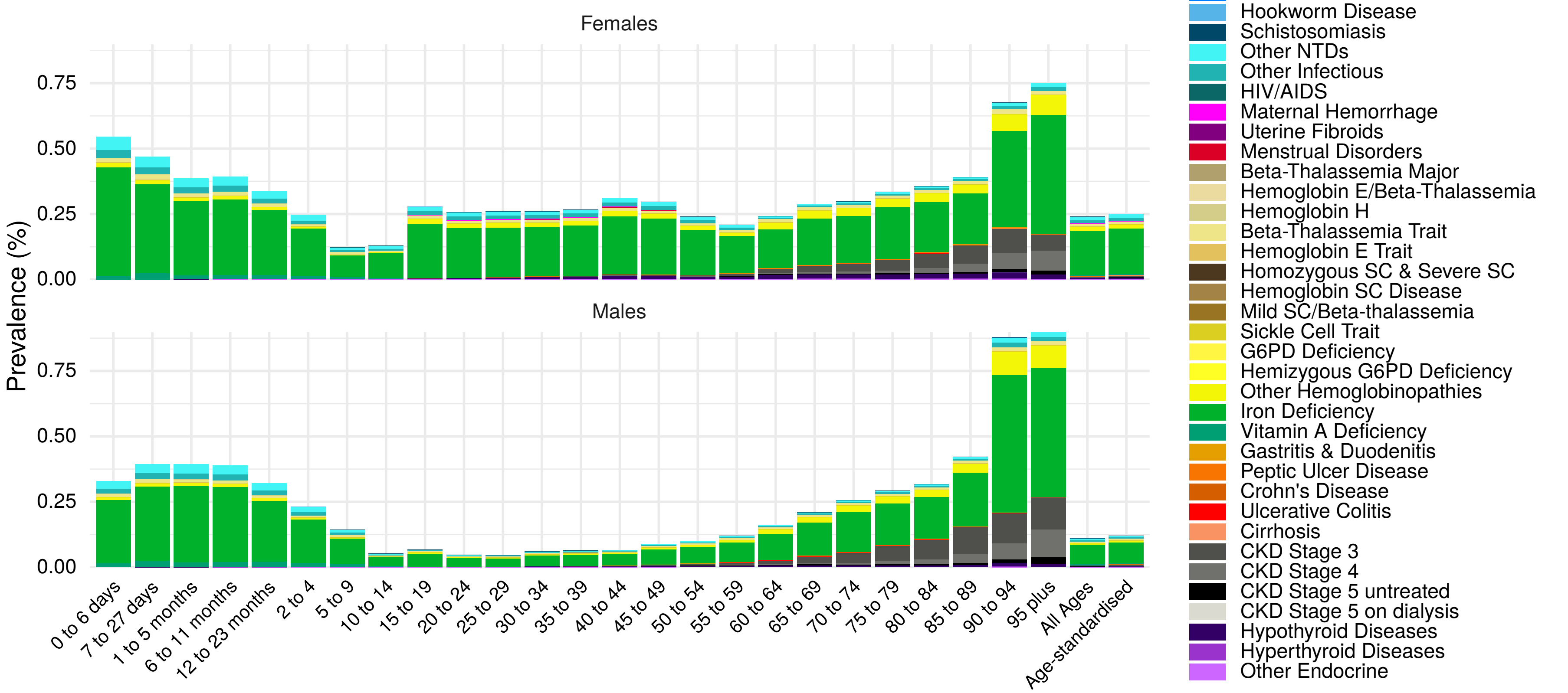

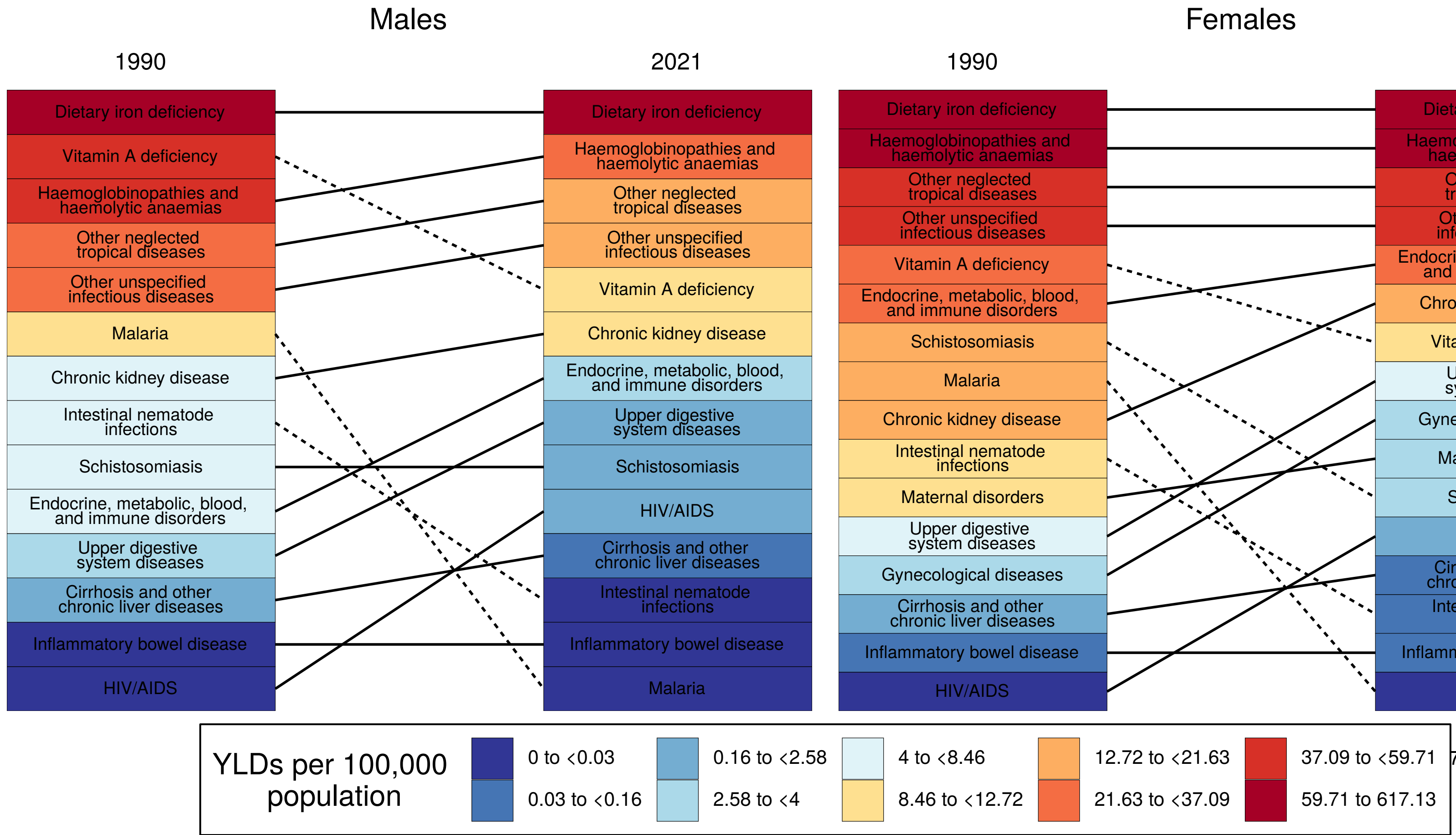

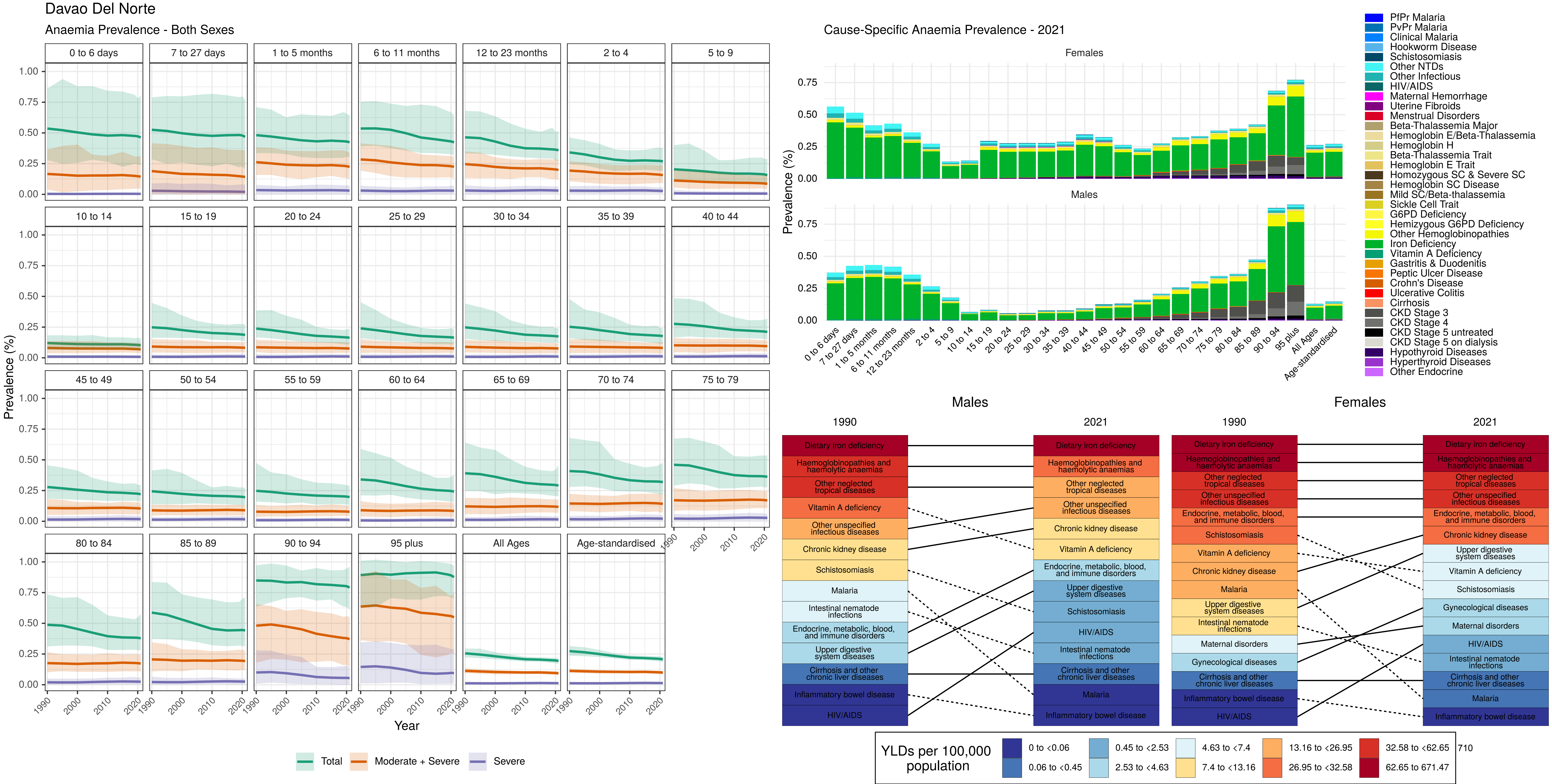
Davao Del Norte
Anaemia Prevalence - Both Sexes
0 to 6 days
7 to 27 days
1 to 5 months
6 to 11 months
12 to 23 months
2 to 4
5 to 9
10 to 14
15 to 19
20 to 24
25 to 29
30 to 34
35 to 39
40 to 44
45 to 49
50 to 54
55 to 59
60 to 64
65 to 69
70 to 74
75 to 79
80 to 84
85 to 89
90 to 94
95 plus
All Ages
Age-standardised
Prevalence (%)
Year
Total
Moderate + Severe
Severe
Cause-Specific Anaemia Prevalence - 2021
Females
Males
PfPr Malaria
PvPr Malaria
Clinical Malaria
Hookworm Disease
Schistosomiasis
Other NTDs
Other Infectious
HIV/AIDS
Maternal Hemorrhage
Uterine Fibroids
Menstrual Disorders
Beta-Thalassemia Major
Hemoglobin E/Beta-Thalassemia
Hemoglobin H
Beta-Thalassemia Trait
Hemoglobin E Trait
Homozygous SC & Severe SC
Hemoglobin SC Disease
Mild SC/Beta-thalassemia
Sickle Cell Trait
G6PD Deficiency
Hemizygous G6PD Deficiency
Other Hemoglobinopathies
Iron Deficiency
Vitamin A Deficiency
Gastritis & Duodenitis
Peptic Ulcer Disease
Crohn's Disease
Ulcerative Colitis
Cirrhosis
CKD Stage 3
CKD Stage 4
CKD Stage 5 untreated
CKD Stage 5 on dialysis
Hypothyroid Diseases
Hyperthyroid Diseases
Other Endocrine
Males
Females
1990
2021
Dietary iron deficiency
Haemoglobinopathies and haemolytic anaemias
Other neglected tropical diseases
Vitamin A deficiency
Other unspecified infectious diseases
Chronic kidney disease
Schistosomiasis
Malaria
Intestinal nematode infections
Endocrine, metabolic, blood, and immune disorders
Upper digestive system diseases
Cirrhosis and other chronic liver diseases
Inflammatory bowel disease
HIV/AIDS
Maternal disorders
Gynecological diseases
YLDs per 100,000 population
0 to <0.06
0.06 to <0.45
0.45 to <2.53
2.53 to <4.63
4.63 to <7.4
7.4 to <13.16
13.16 to <26.95
26.95 to <32.58
32.58 to <62.65
62.65 to 671.47

Davao Del Sur

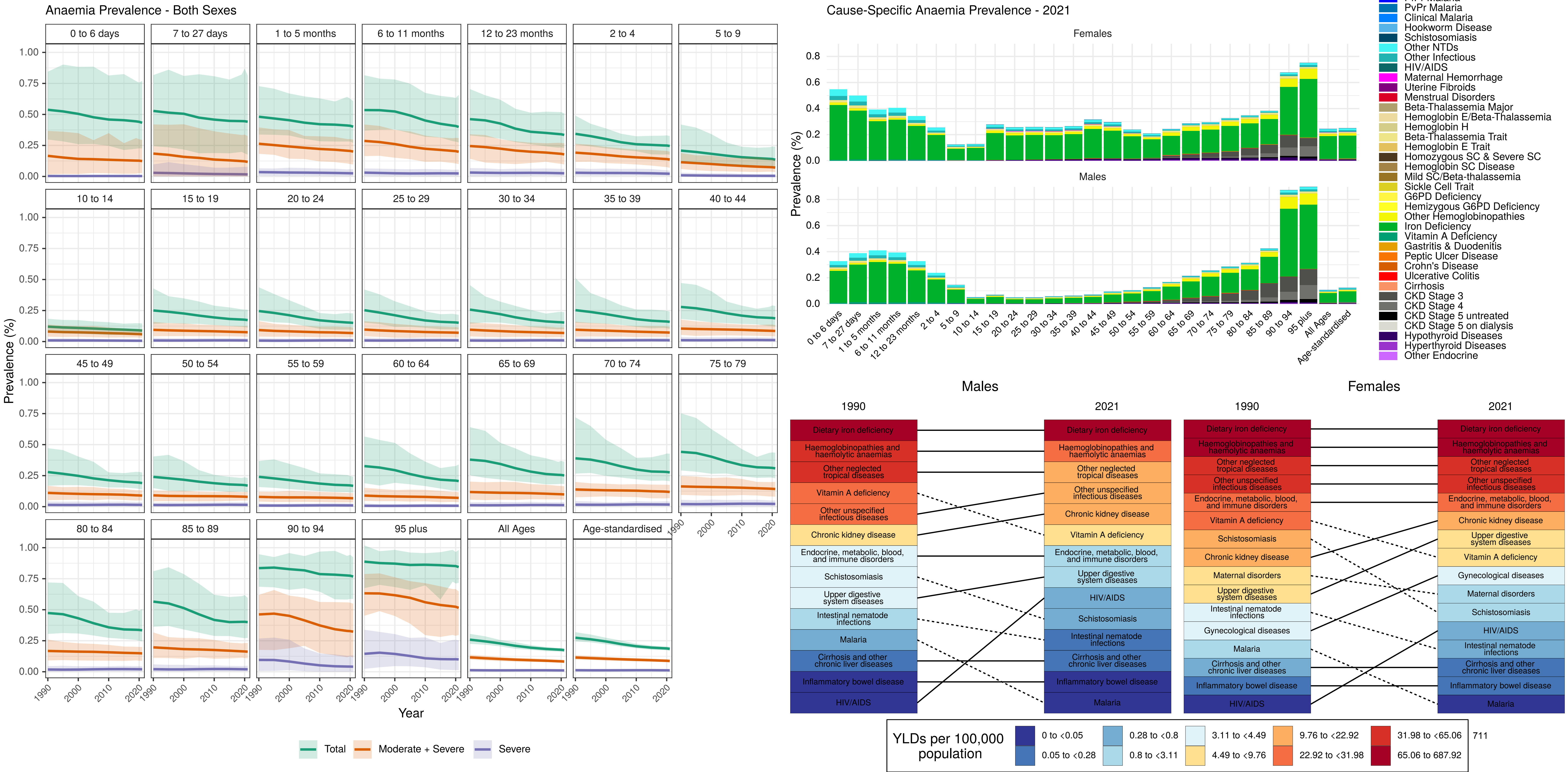

Davao Occidental

Anaemia Prevalence - Both Sexes

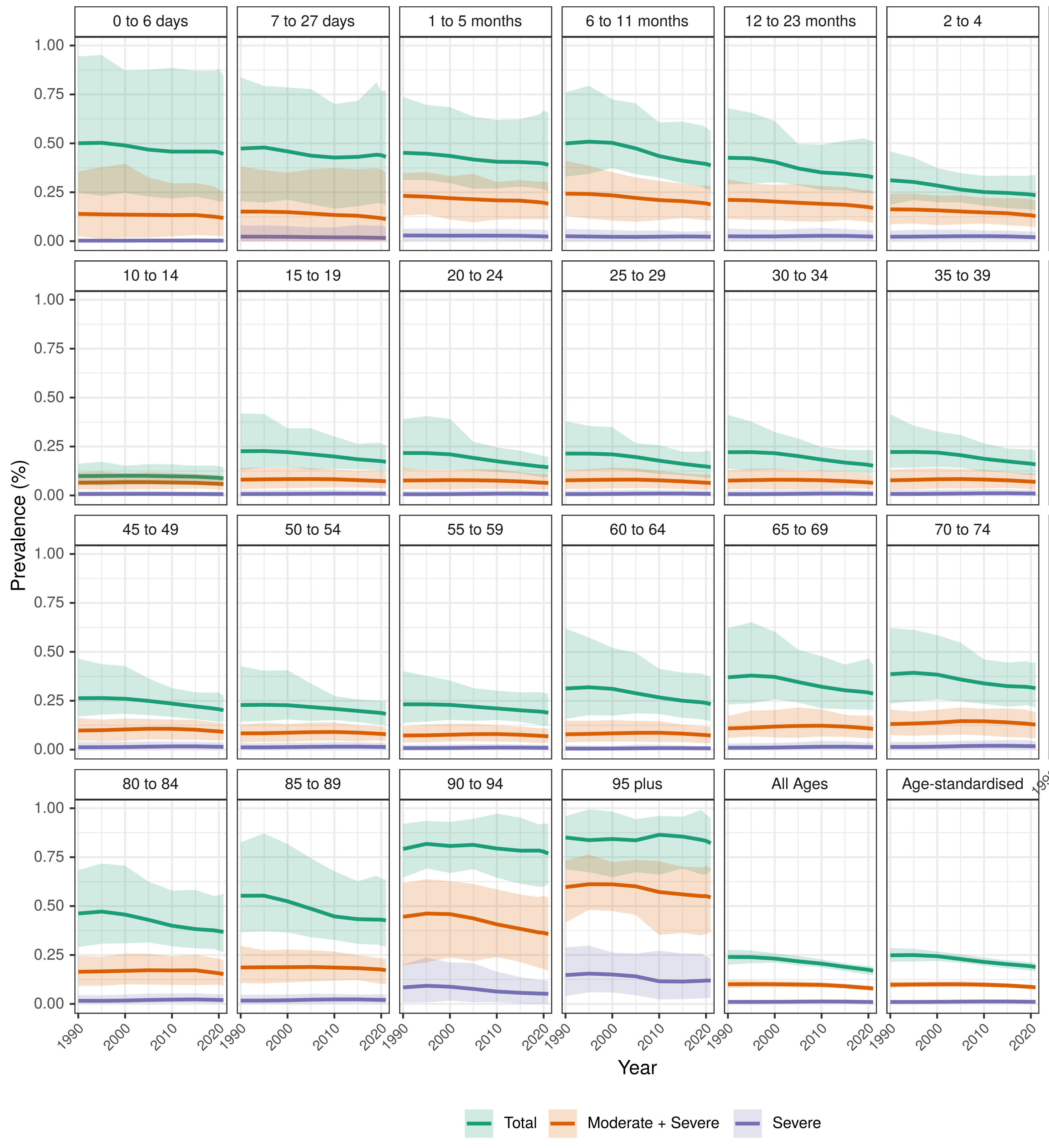

Cause-Specific Anaemia Prevalence - 2021

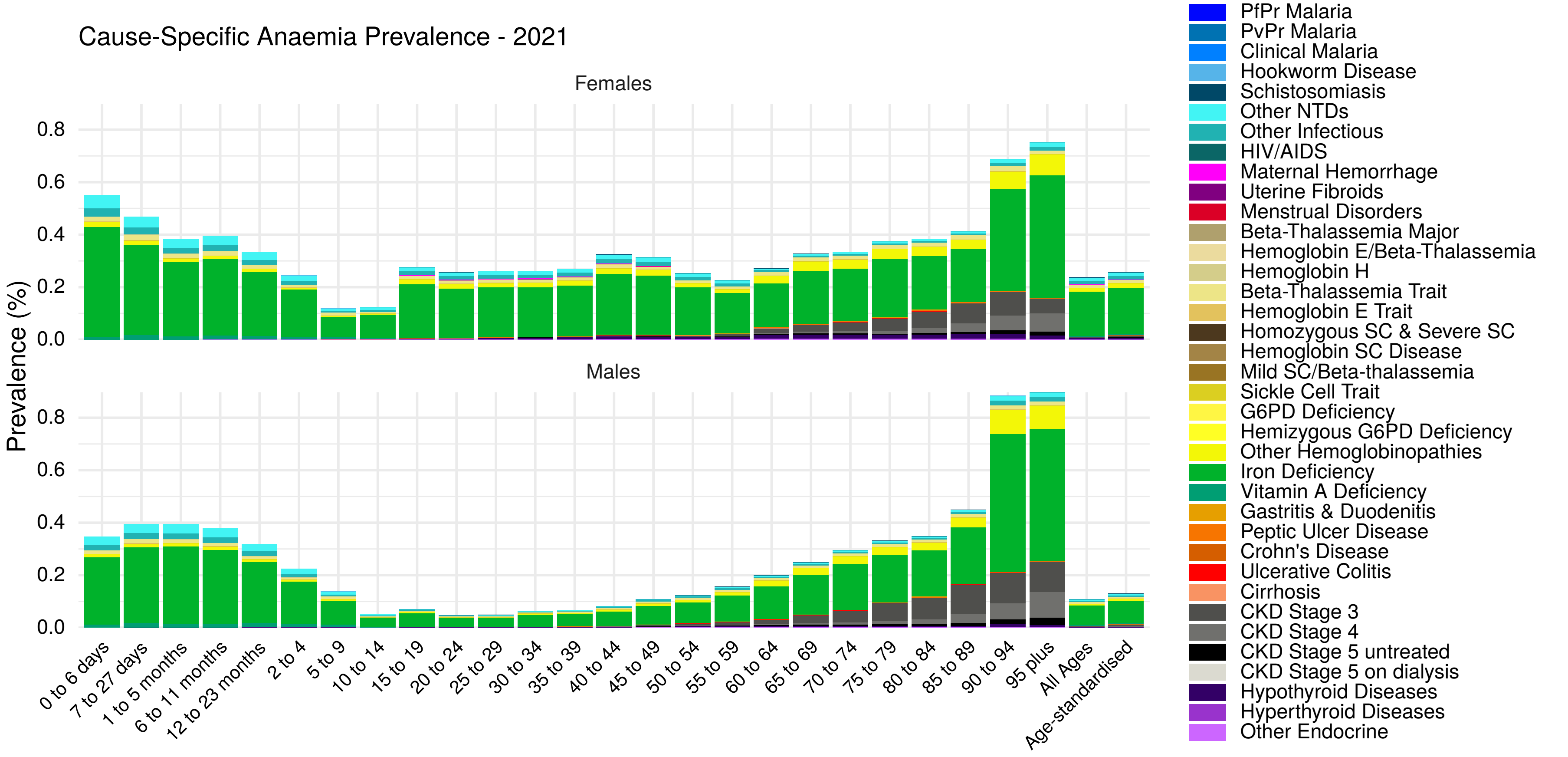

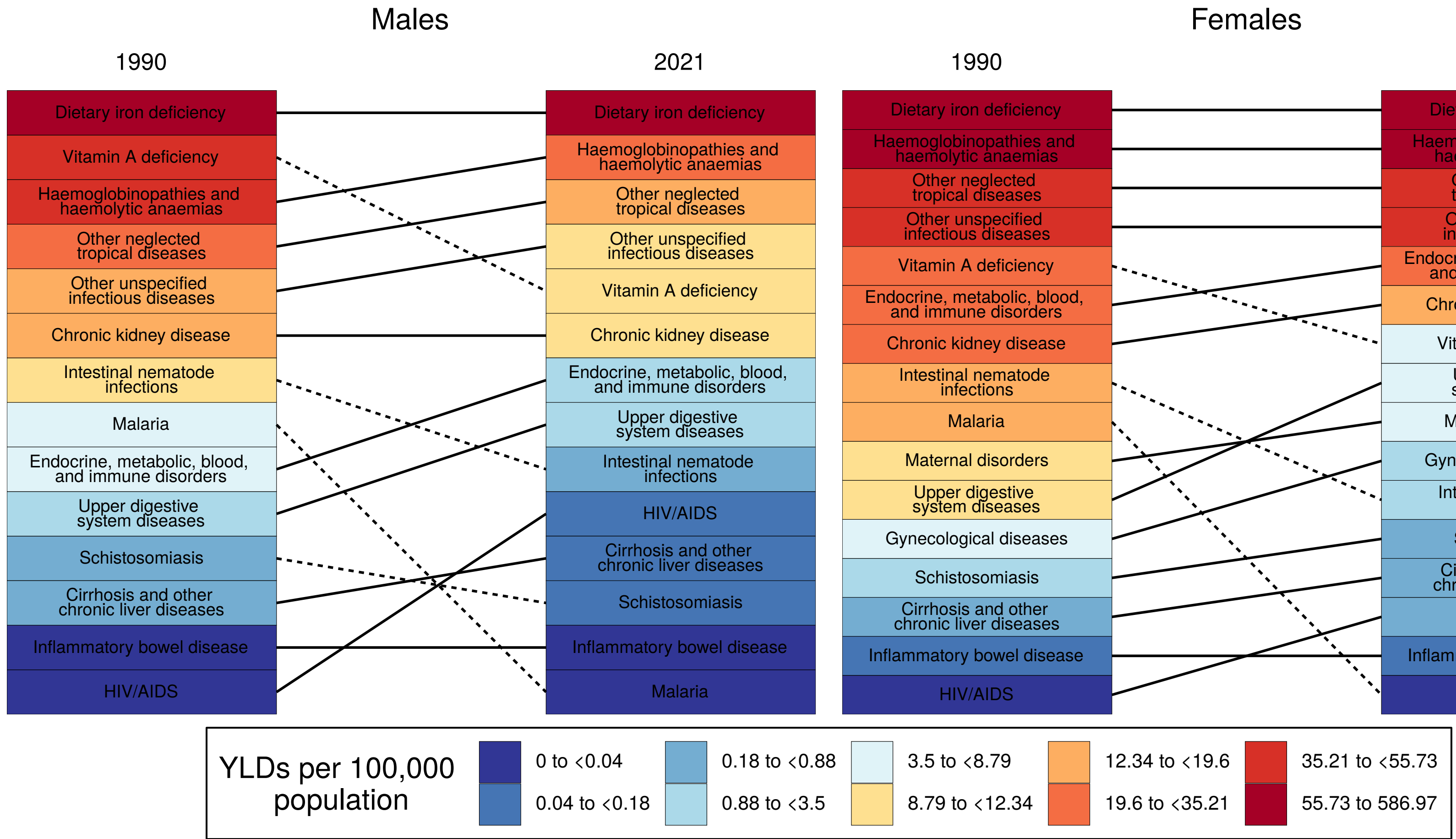

Davao Oriental

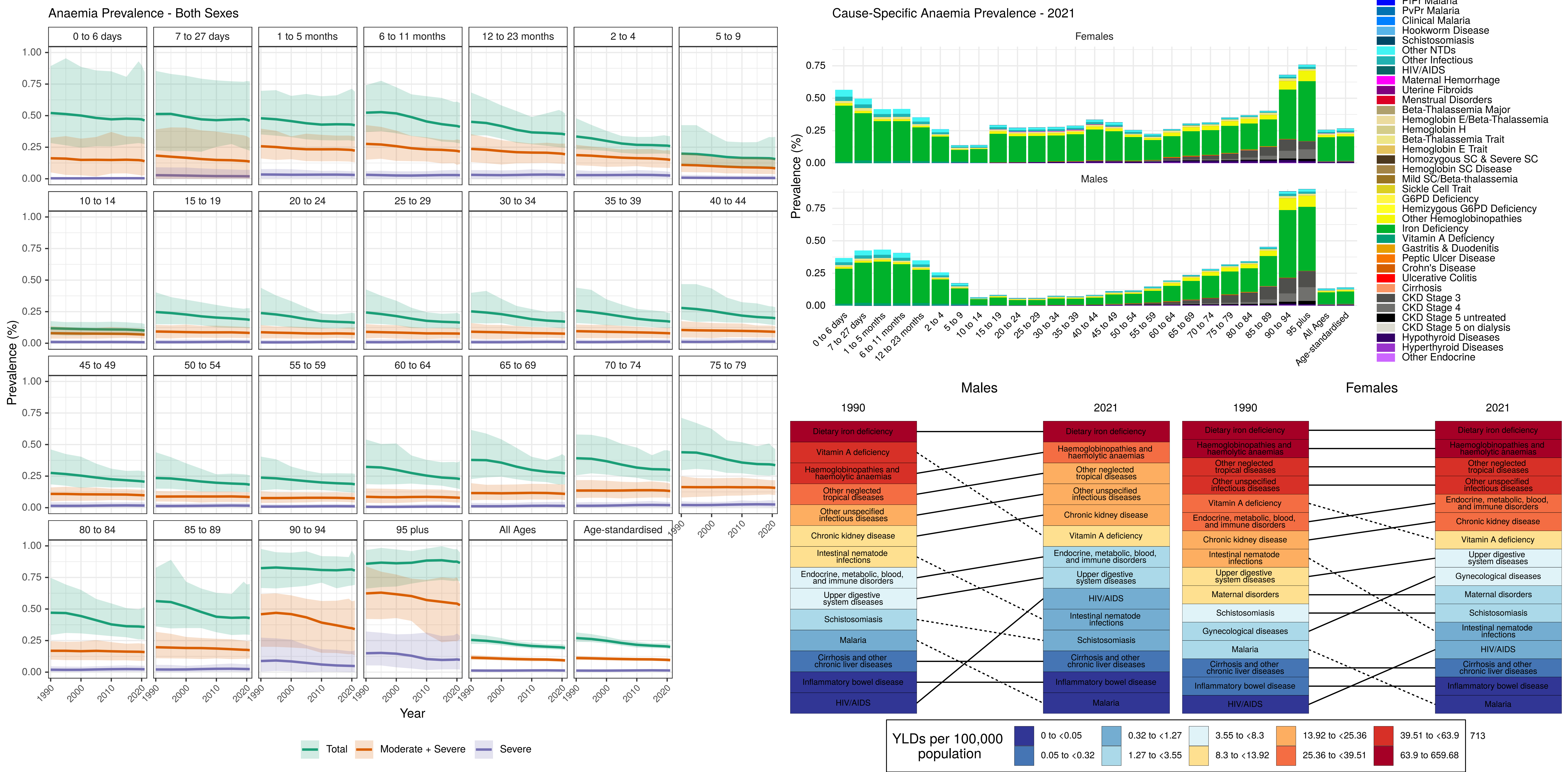

Dinagat Islands

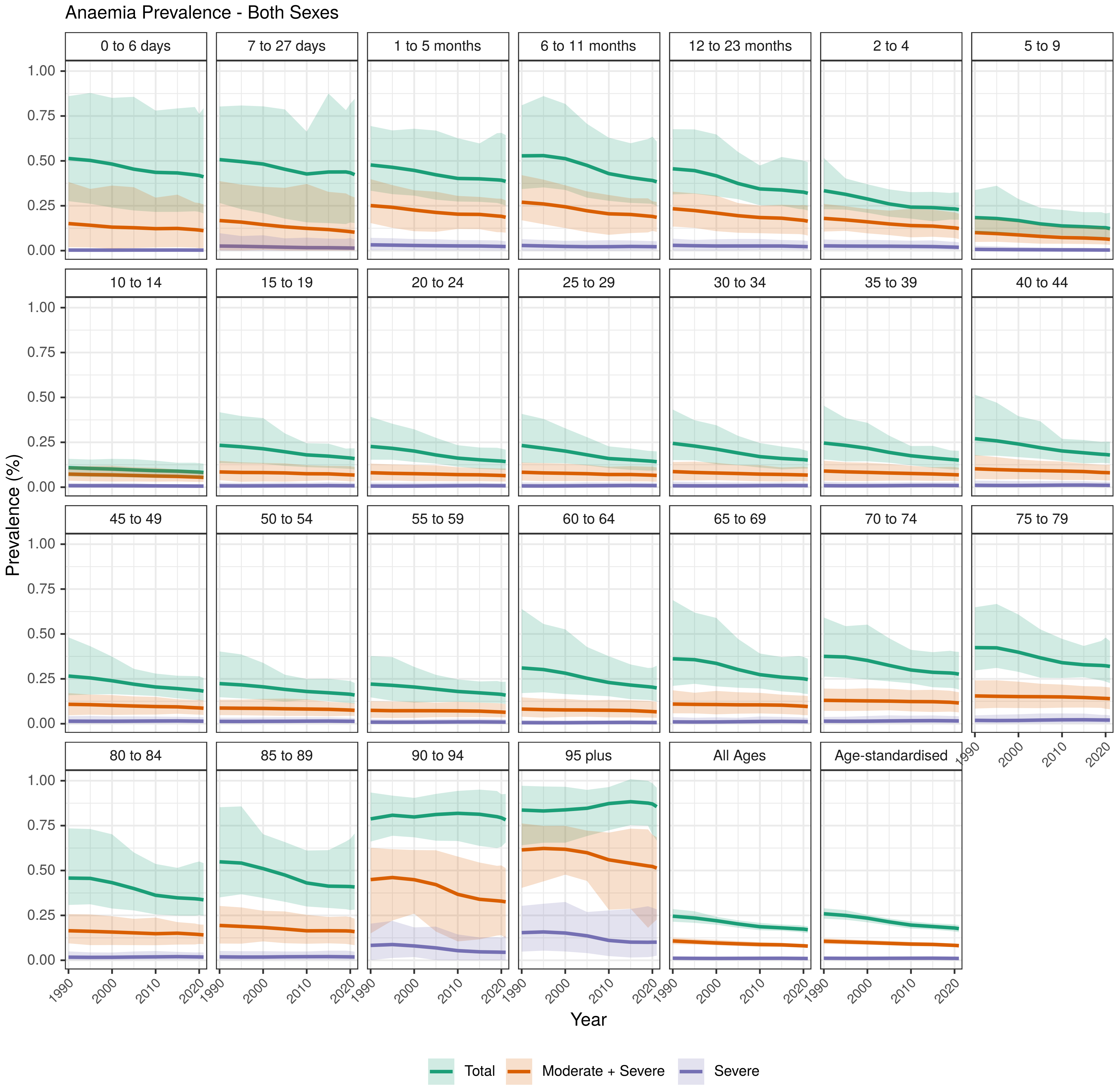

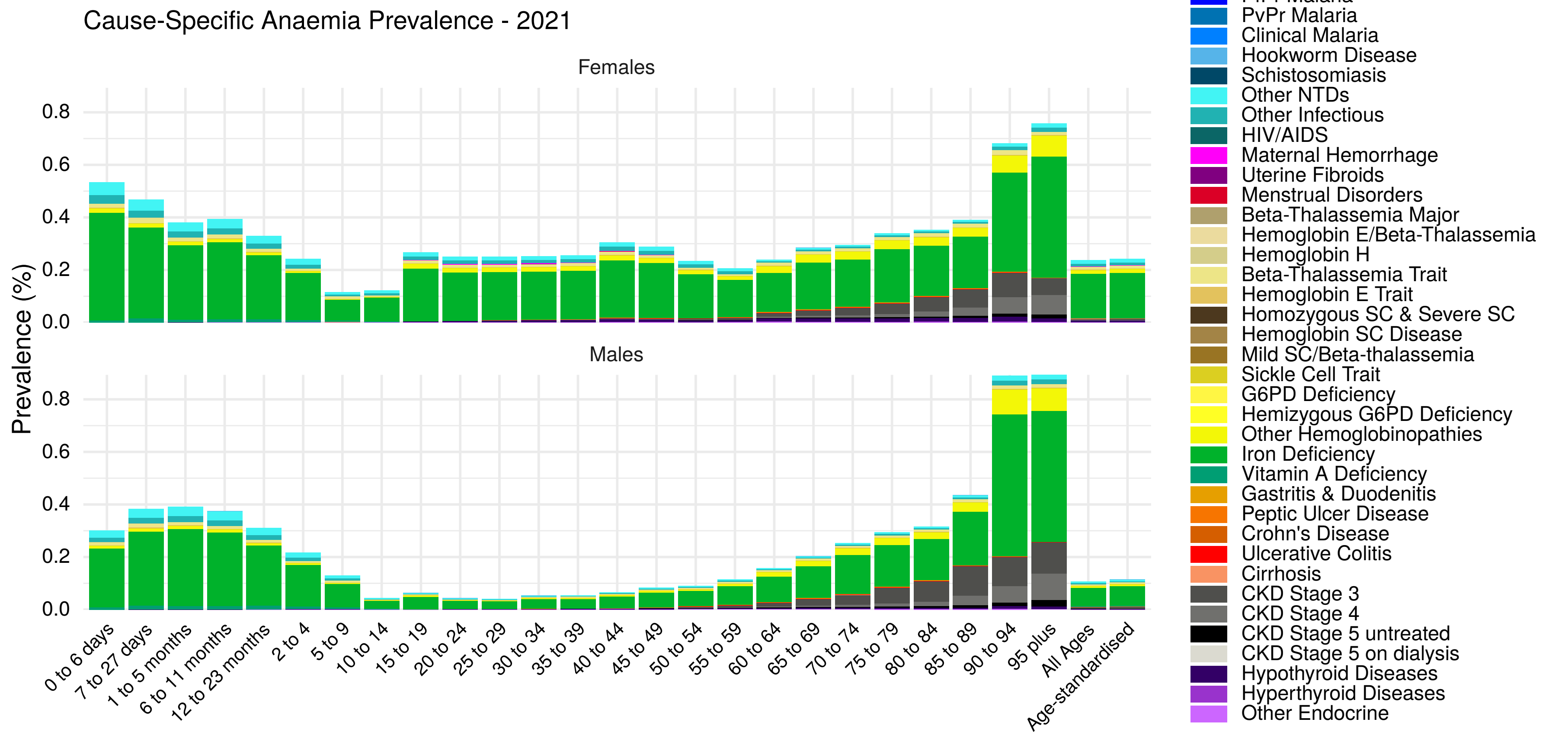

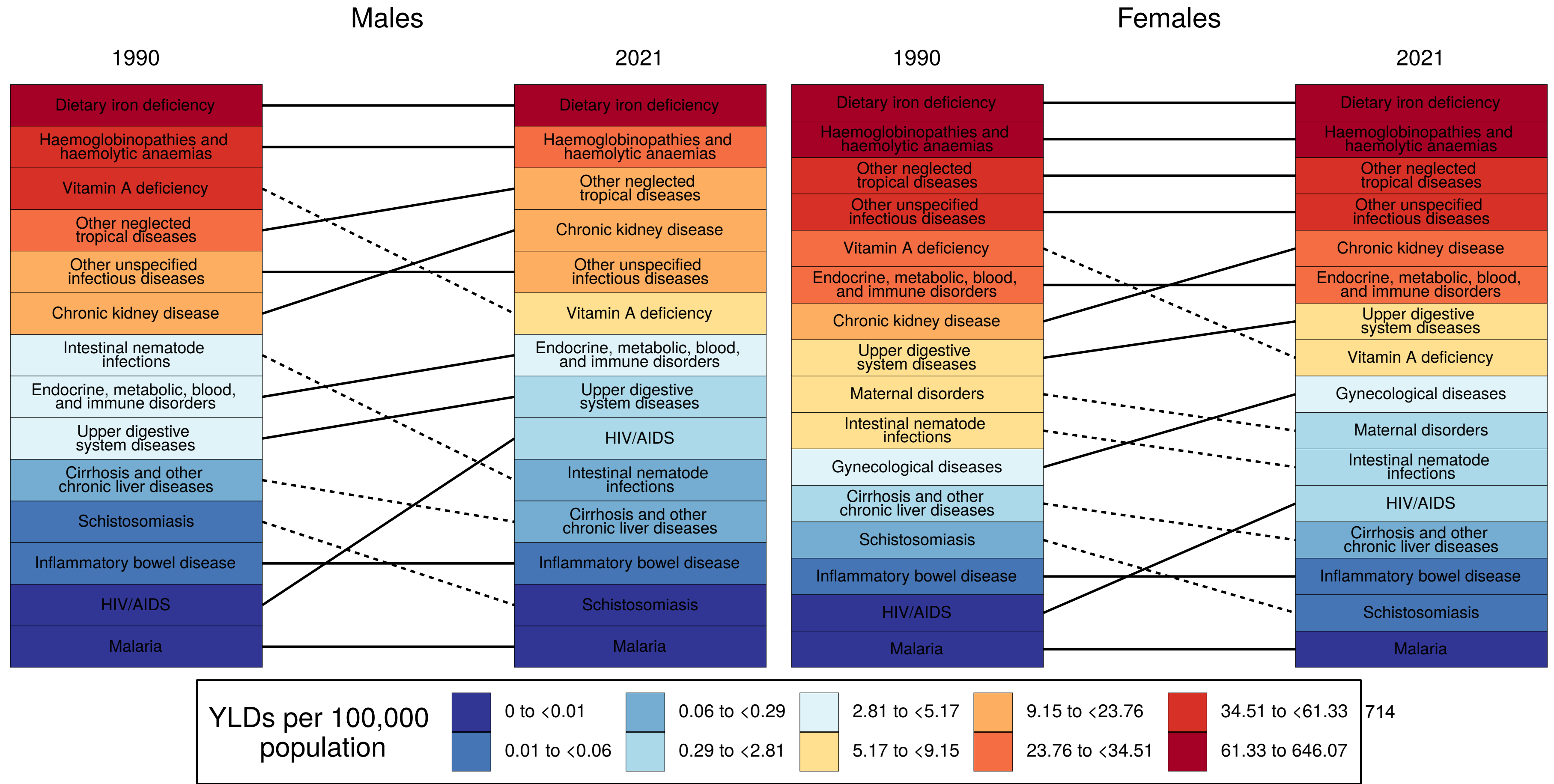

Eastern Samar

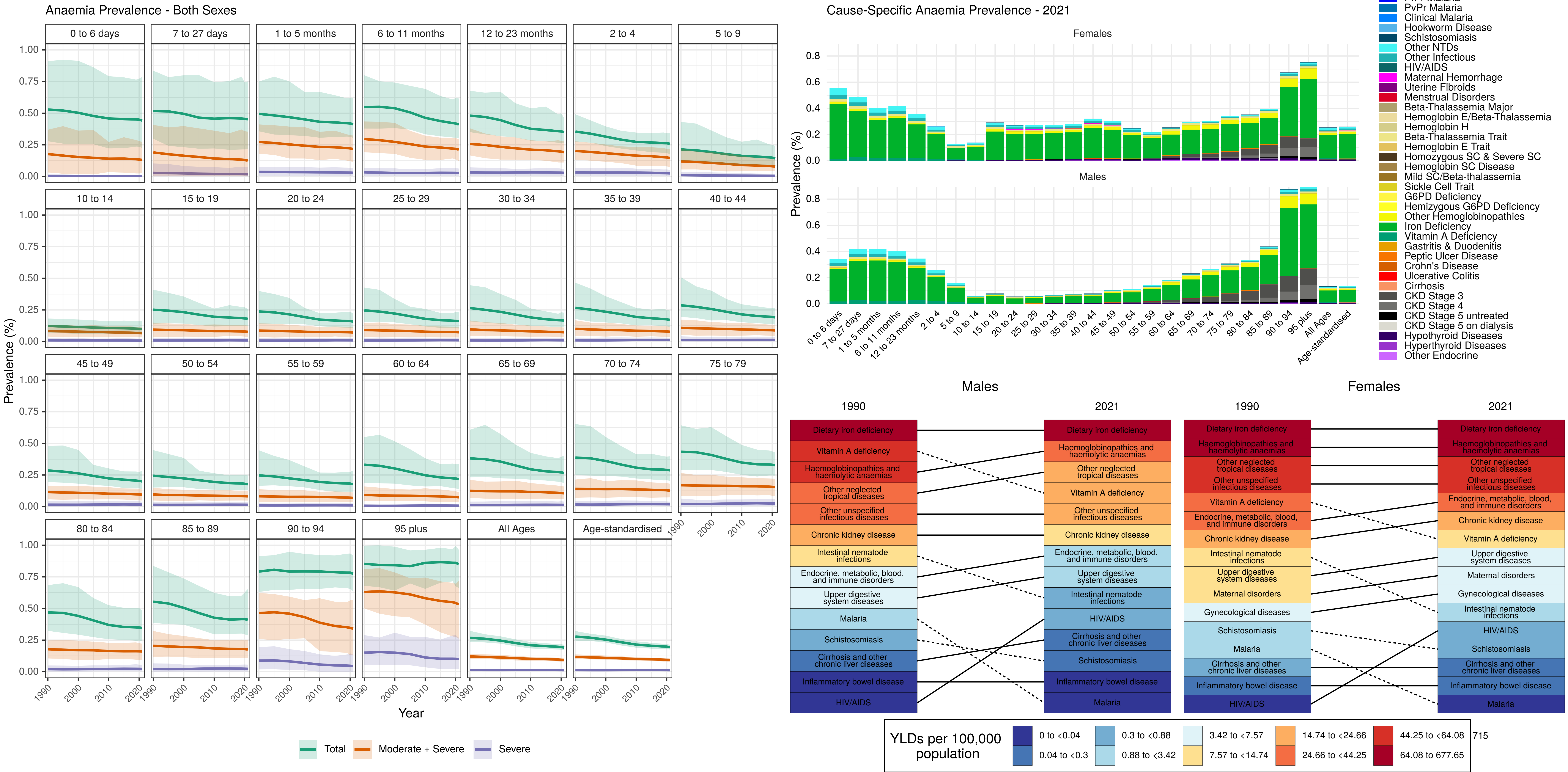

Guimaras

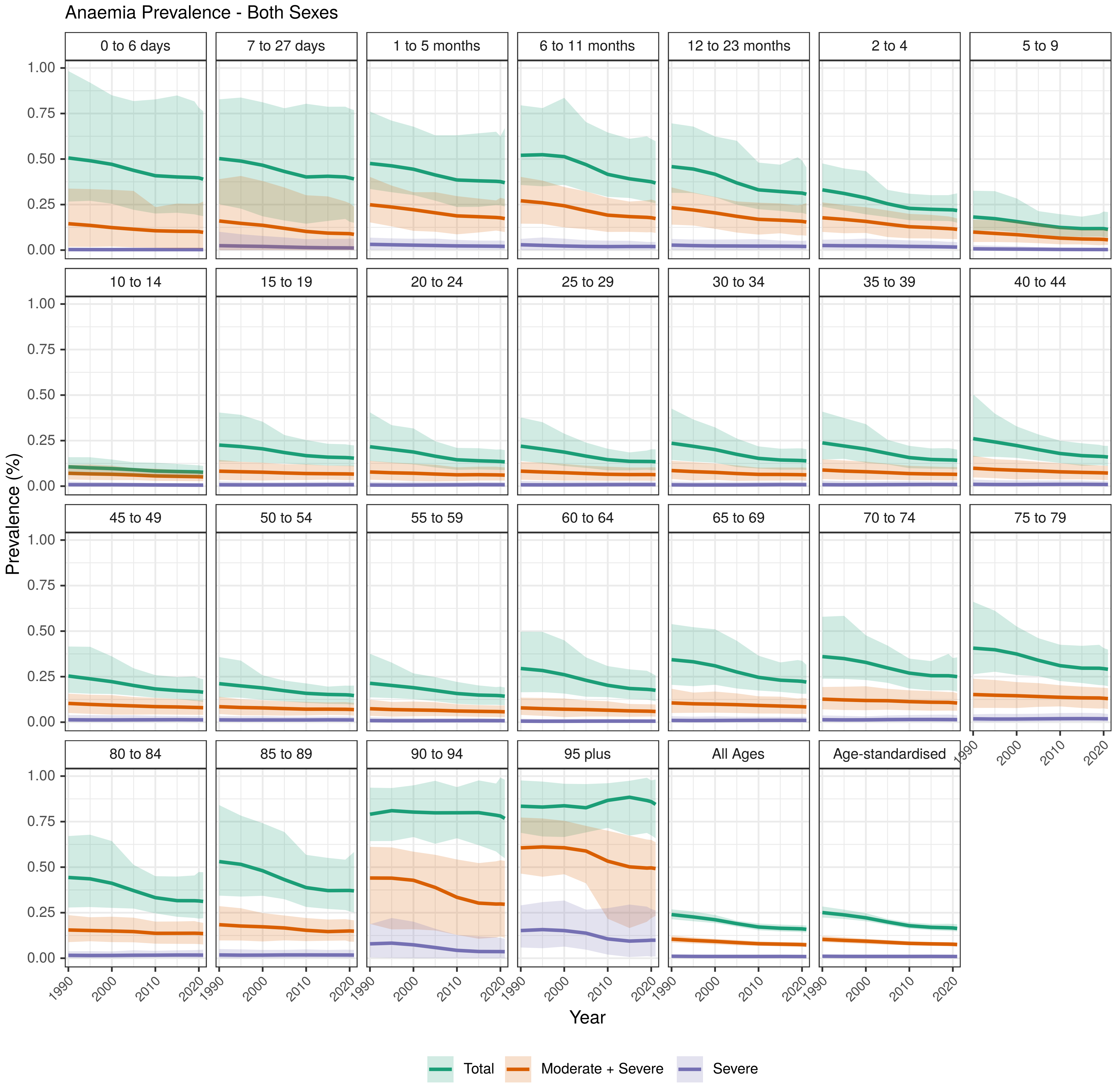
Anaemia Prevalence - Both Sexes
0 to 6 days
7 to 27 days
1 to 5 months
6 to 11 months
12 to 23 months
2 to 4
5 to 9
10 to 14
15 to 19
20 to 24
25 to 29
30 to 34
35 to 39
40 to 44
45 to 49
50 to 54
55 to 59
60 to 64
65 to 69
70 to 74
75 to 79
80 to 84
85 to 89
90 to 94
95 plus
All Ages
Age-standardised
Prevalence (%)
Year
Total
Moderate + Severe
Severe

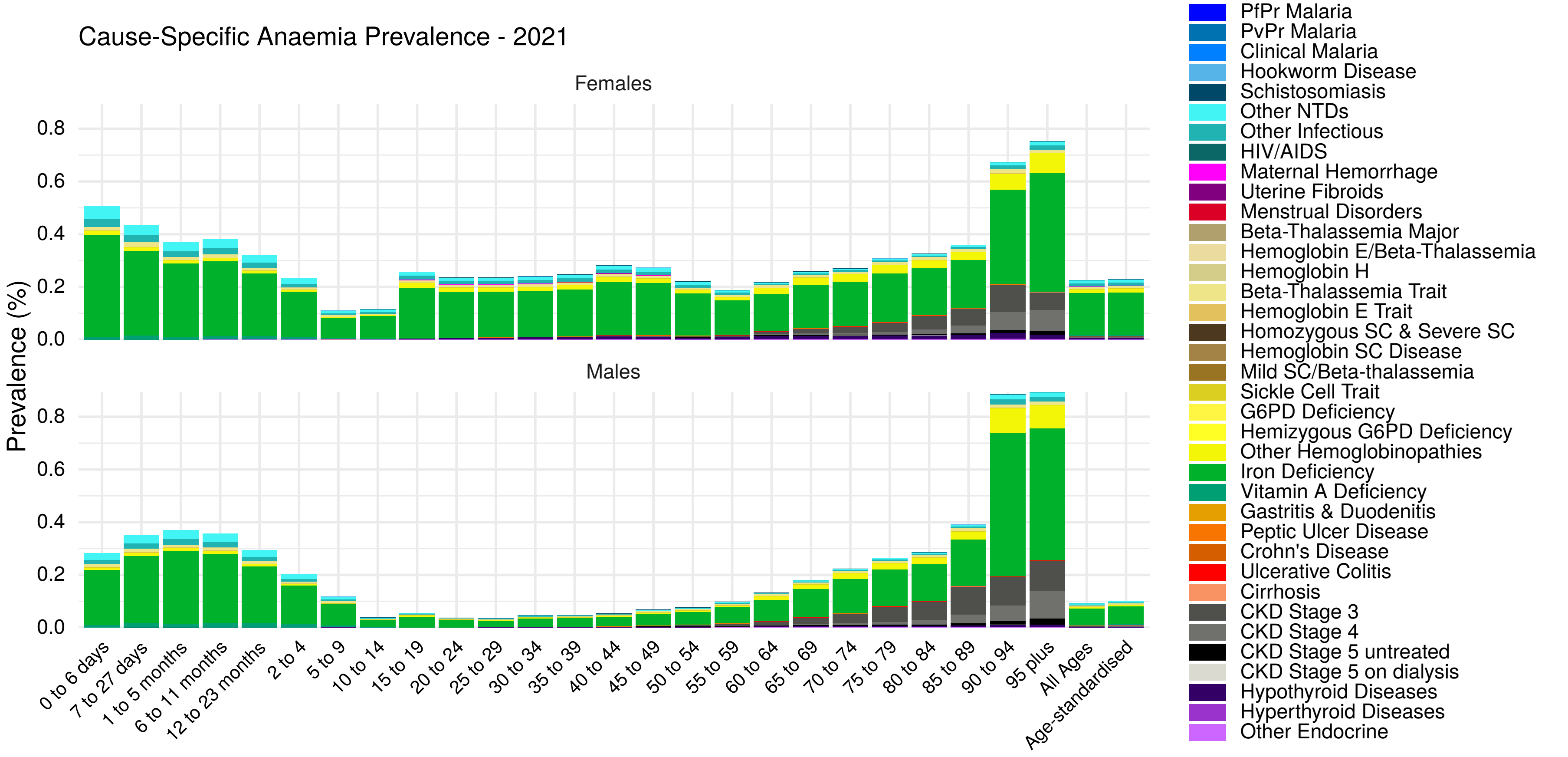
Cause-Specific Anaemia Prevalence - 2021
Females
Males
Prevalence (%)
PfPr Malaria
PvPr Malaria
Clinical Malaria
Hookworm Disease
Schistosomiasis
Other NTDs
Other Infectious
HIV/AIDS
Maternal Hemorrhage
Uterine Fibroids
Menstrual Disorders
Beta-Thalassemia Major
Hemoglobin E/Beta-Thalassemia
Hemoglobin H
Beta-Thalassemia Trait
Hemoglobin E Trait
Homozygous SC & Severe SC
Hemoglobin SC Disease
Mild SC/Beta-thalassemia
Sickle Cell Trait
G6PD Deficiency
Hemizygous G6PD Deficiency
Other Hemoglobinopathies
Iron Deficiency
Vitamin A Deficiency
Gastritis & Duodenitis
Peptic Ulcer Disease
Crohn's Disease
Ulcerative Colitis
Cirrhosis
CKD Stage 3
CKD Stage 4
CKD Stage 5 untreated
CKD Stage 5 on dialysis
Hypothyroid Diseases
Hyperthyroid Diseases
Other Endocrine

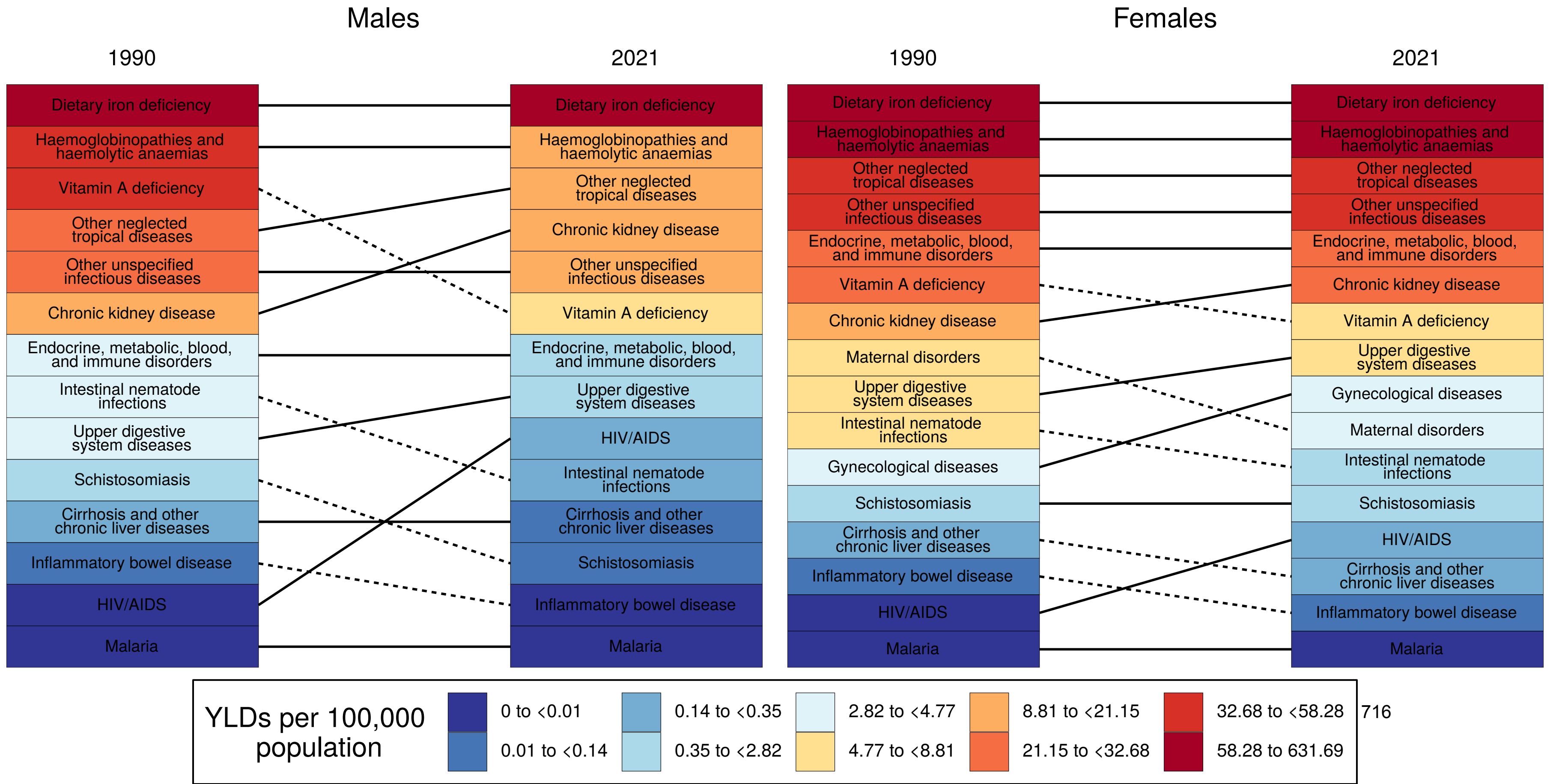
Males
Females
1990
2021
Dietary iron deficiency
Haemoglobinopathies and haemolytic anaemias
Vitamin A deficiency
Other neglected tropical diseases
Other unspecified infectious diseases
Chronic kidney disease
Endocrine, metabolic, blood, and immune disorders
Intestinal nematode infections
Upper digestive system diseases
Schistosomiasis
Cirrhosis and other chronic liver diseases
Inflammatory bowel disease
HIV/AIDS
Malaria
Maternal disorders
Gynecological diseases
YLDs per 100,000 population
0 to <0.01
0.01 to <0.14
0.14 to <0.35
0.35 to <2.82
2.82 to <4.77
4.77 to <8.81
8.81 to <21.15
21.15 to <32.68
32.68 to <58.28
58.28 to 631.69

Ifugao

Anaemia Prevalence - Both Sexes

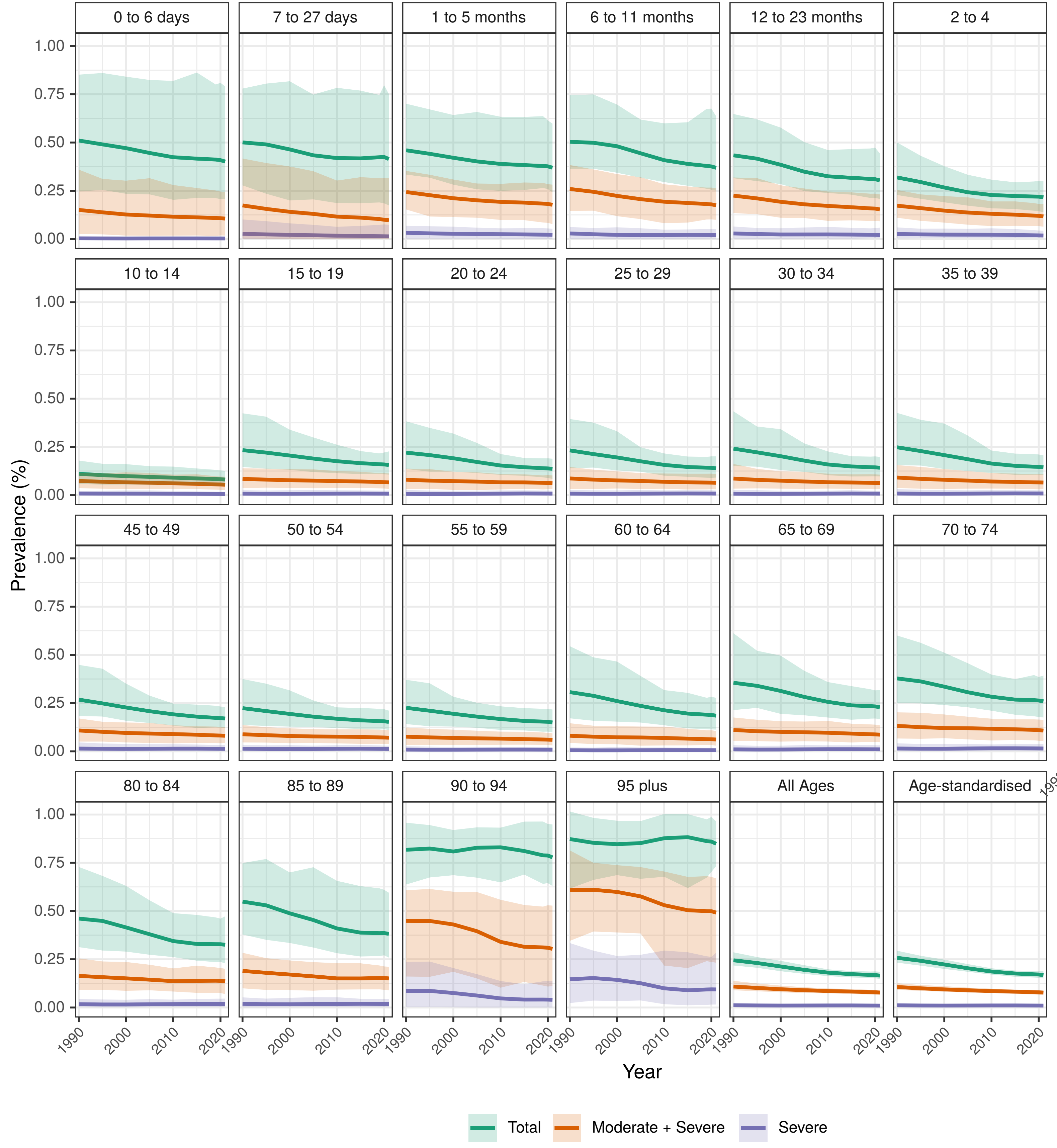

Cause-Specific Anaemia Prevalence - 2021

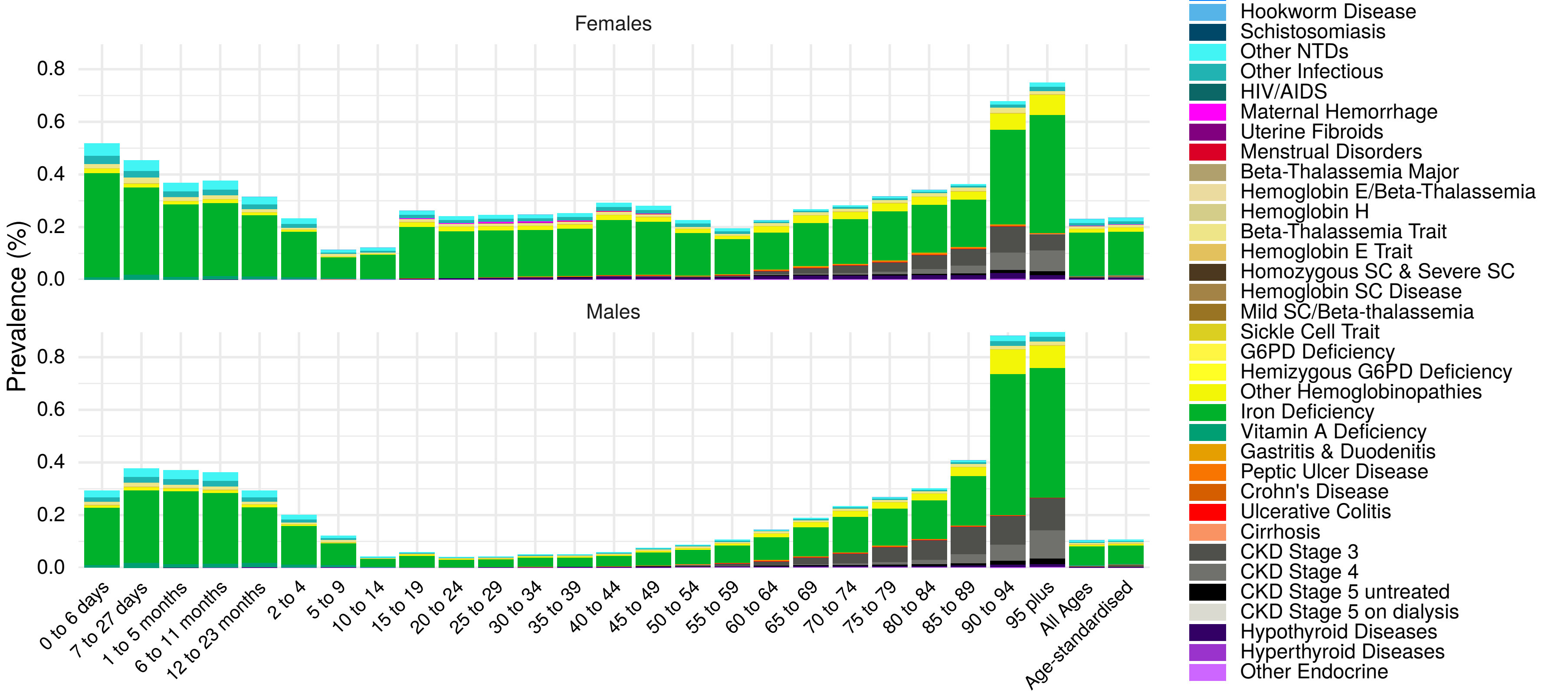

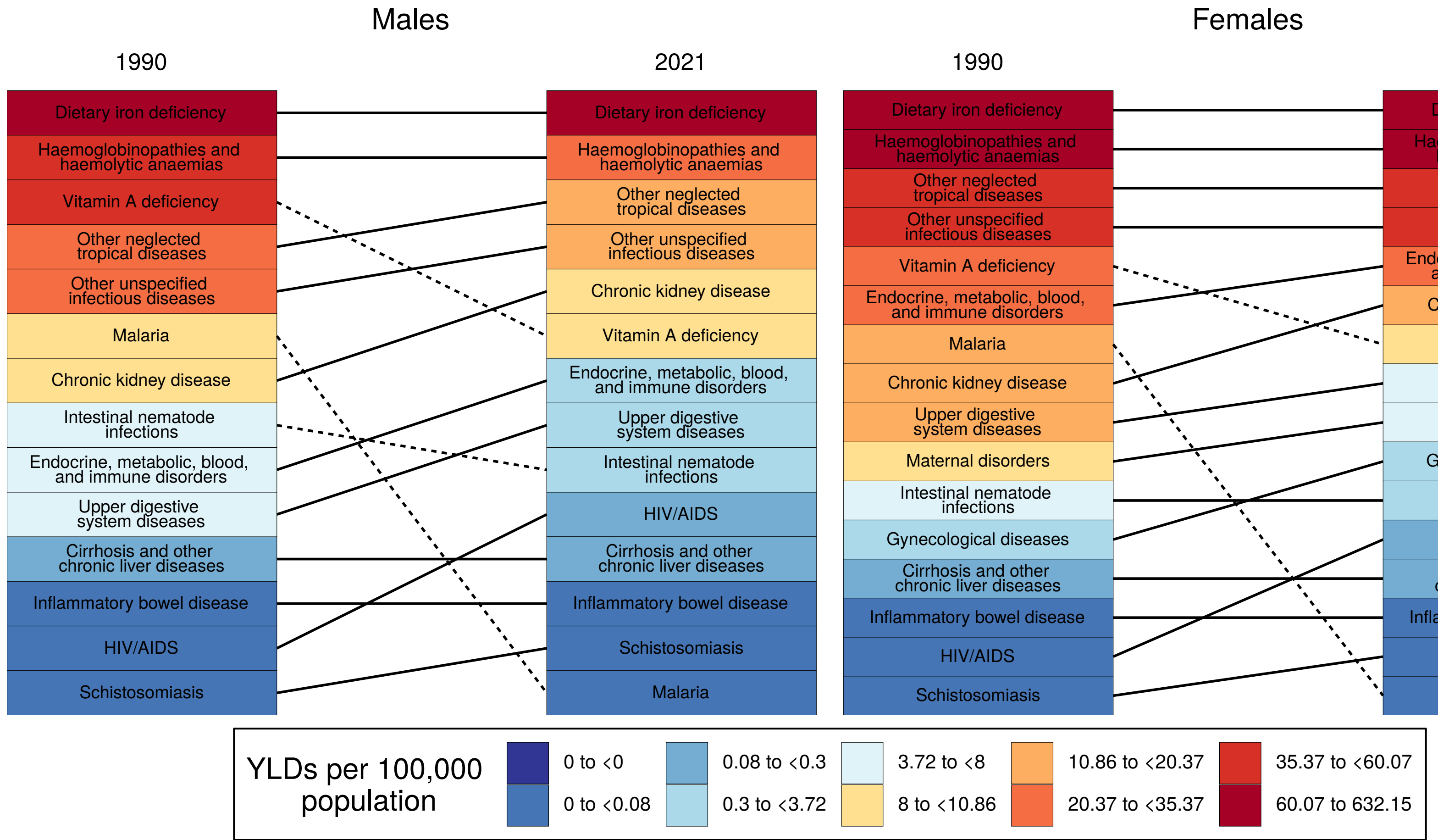

Ilocos Norte

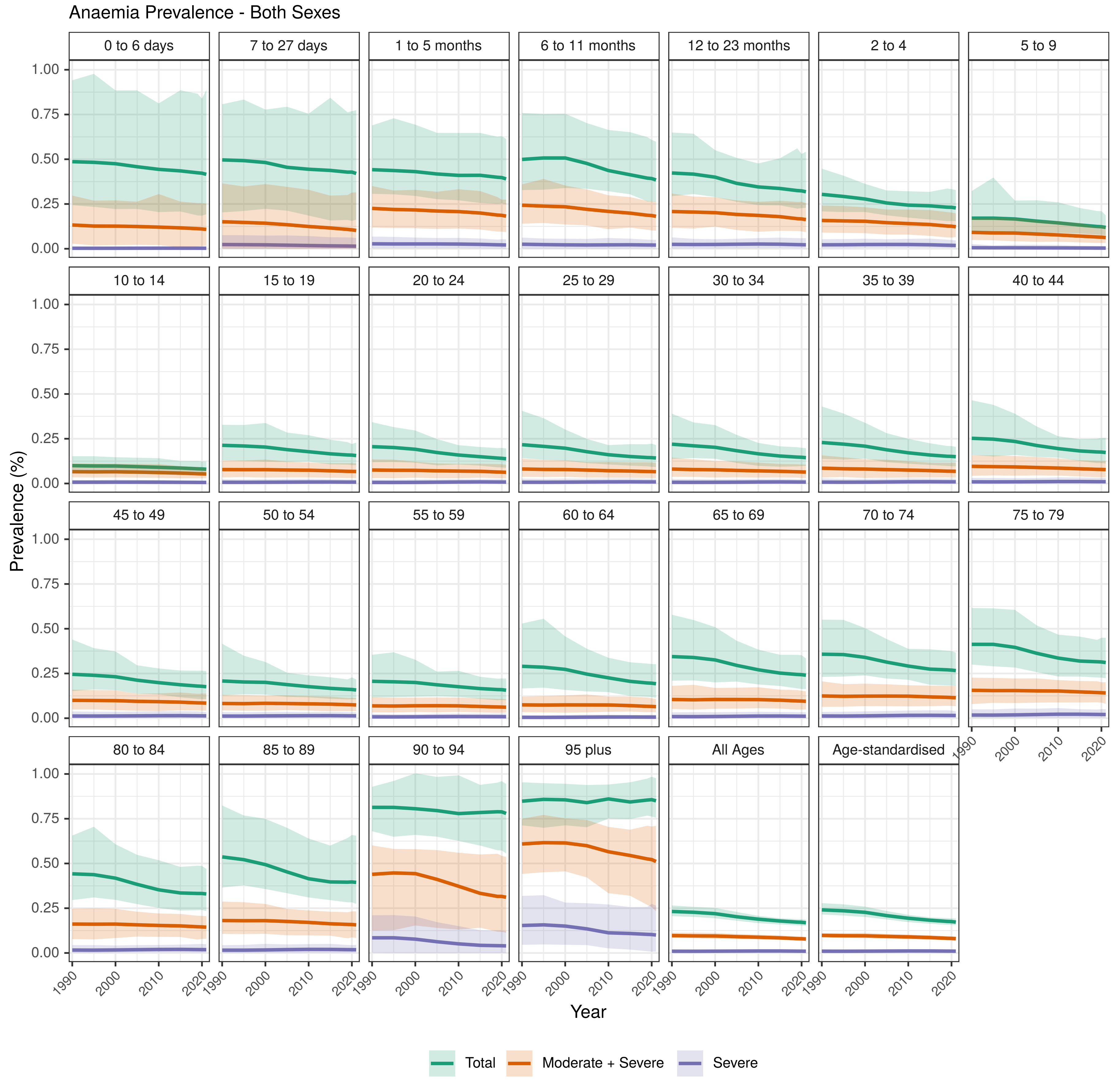

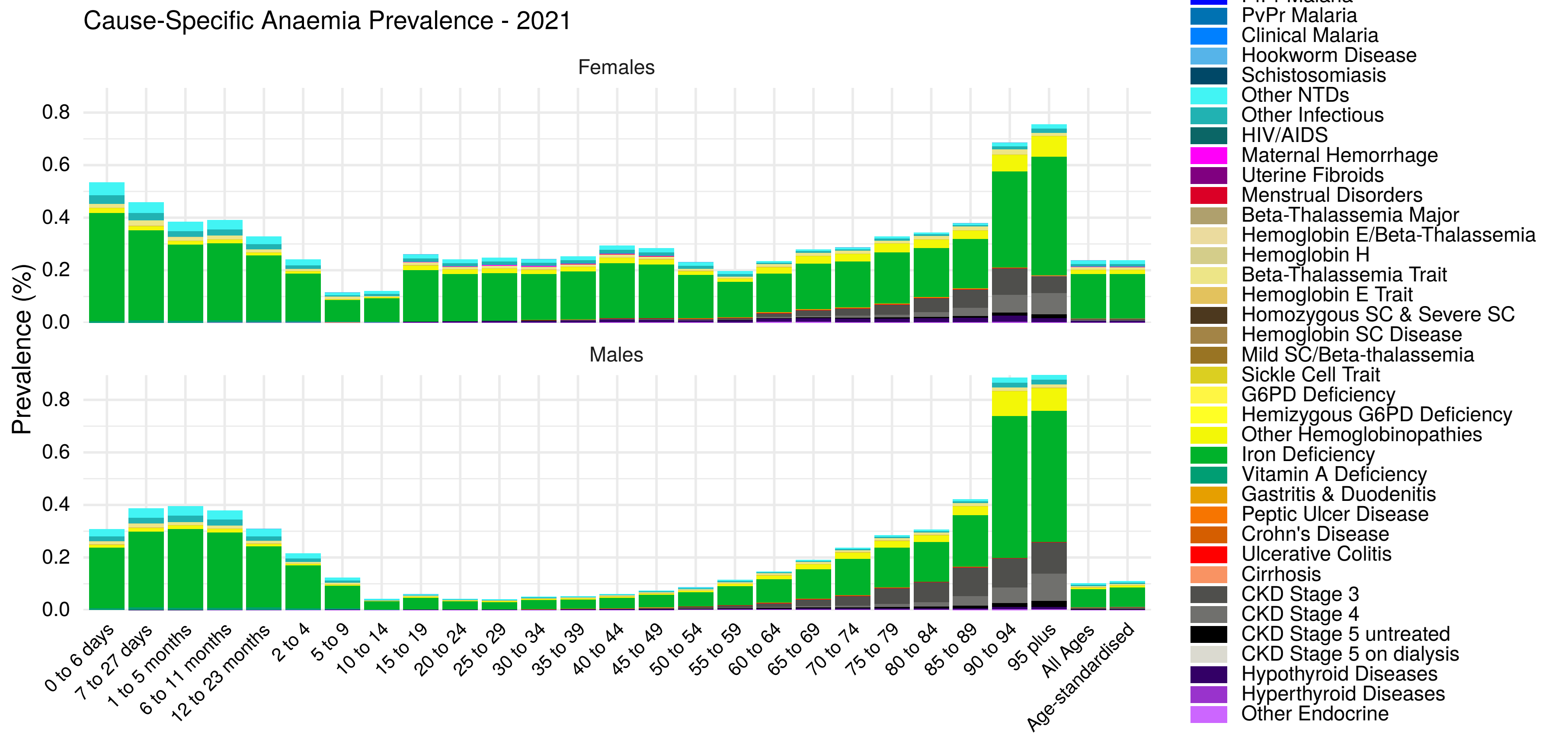

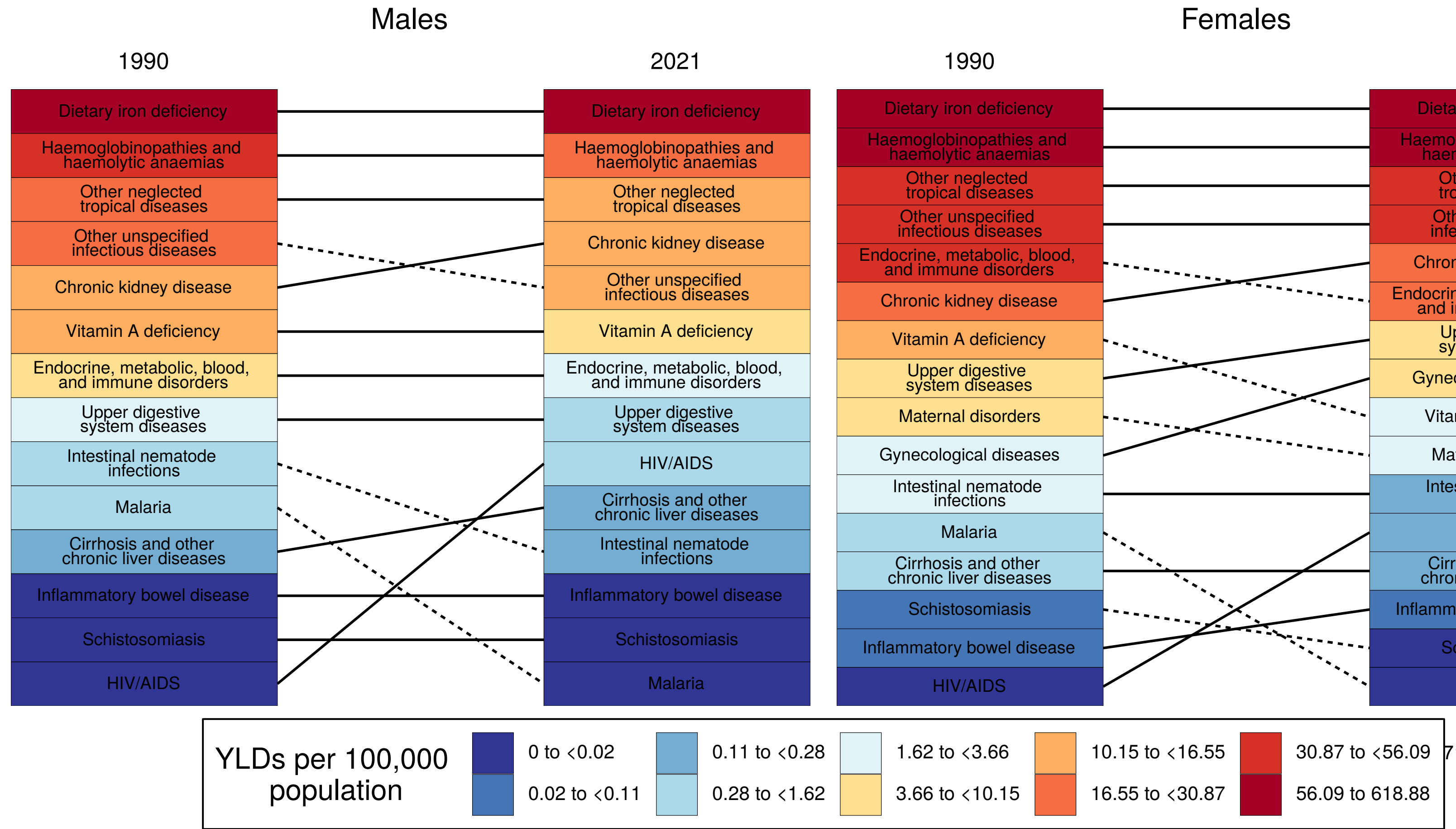

Ilocos Sur

Anaemia Prevalence - Both Sexes

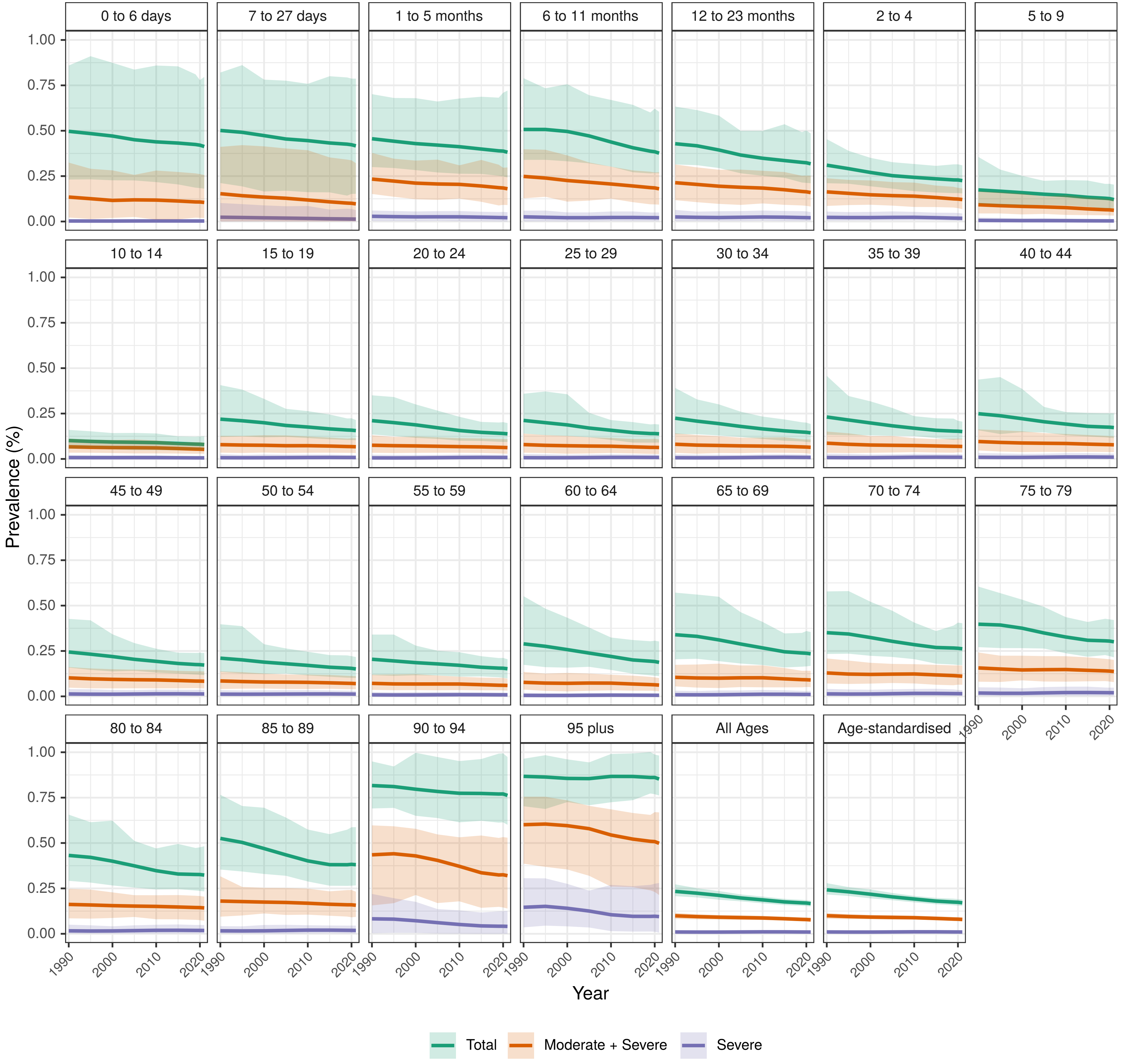

Cause-Specific Anaemia Prevalence - 2021

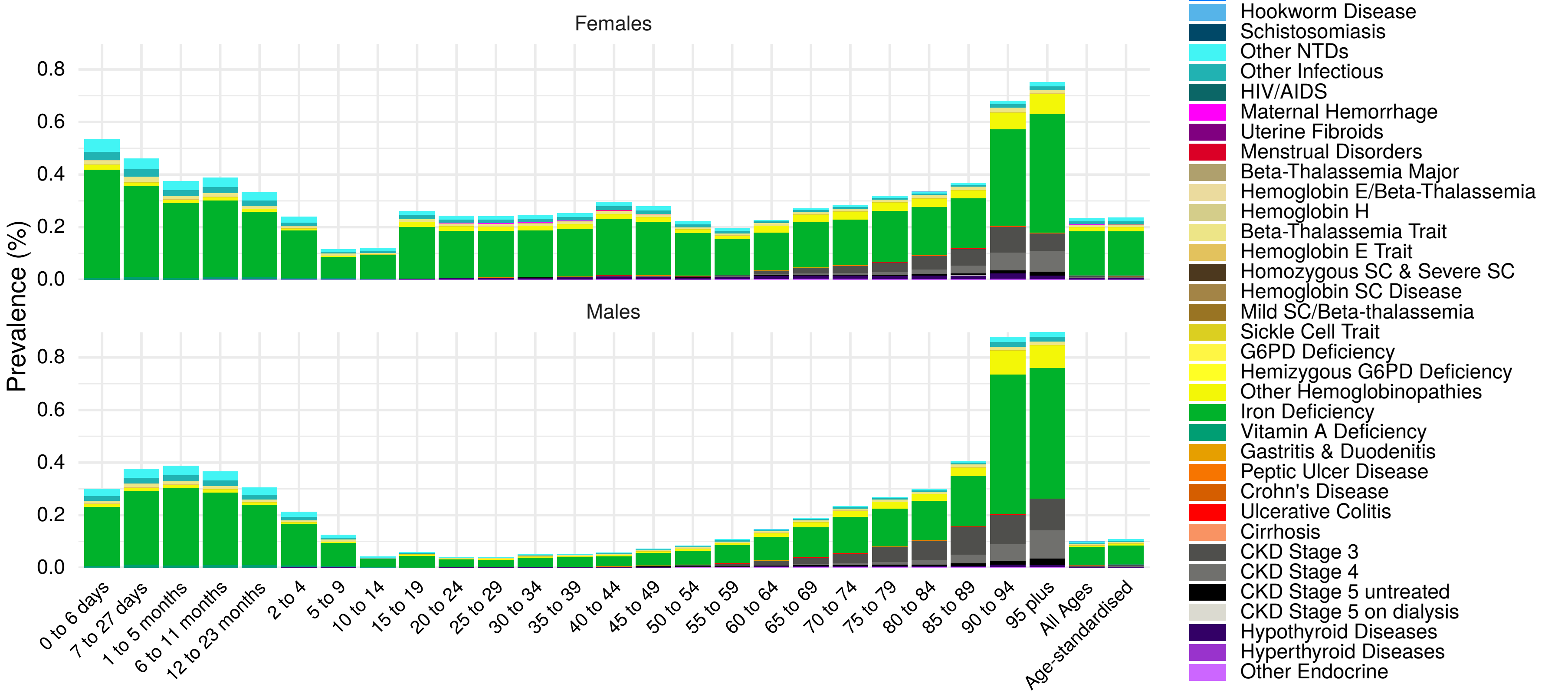

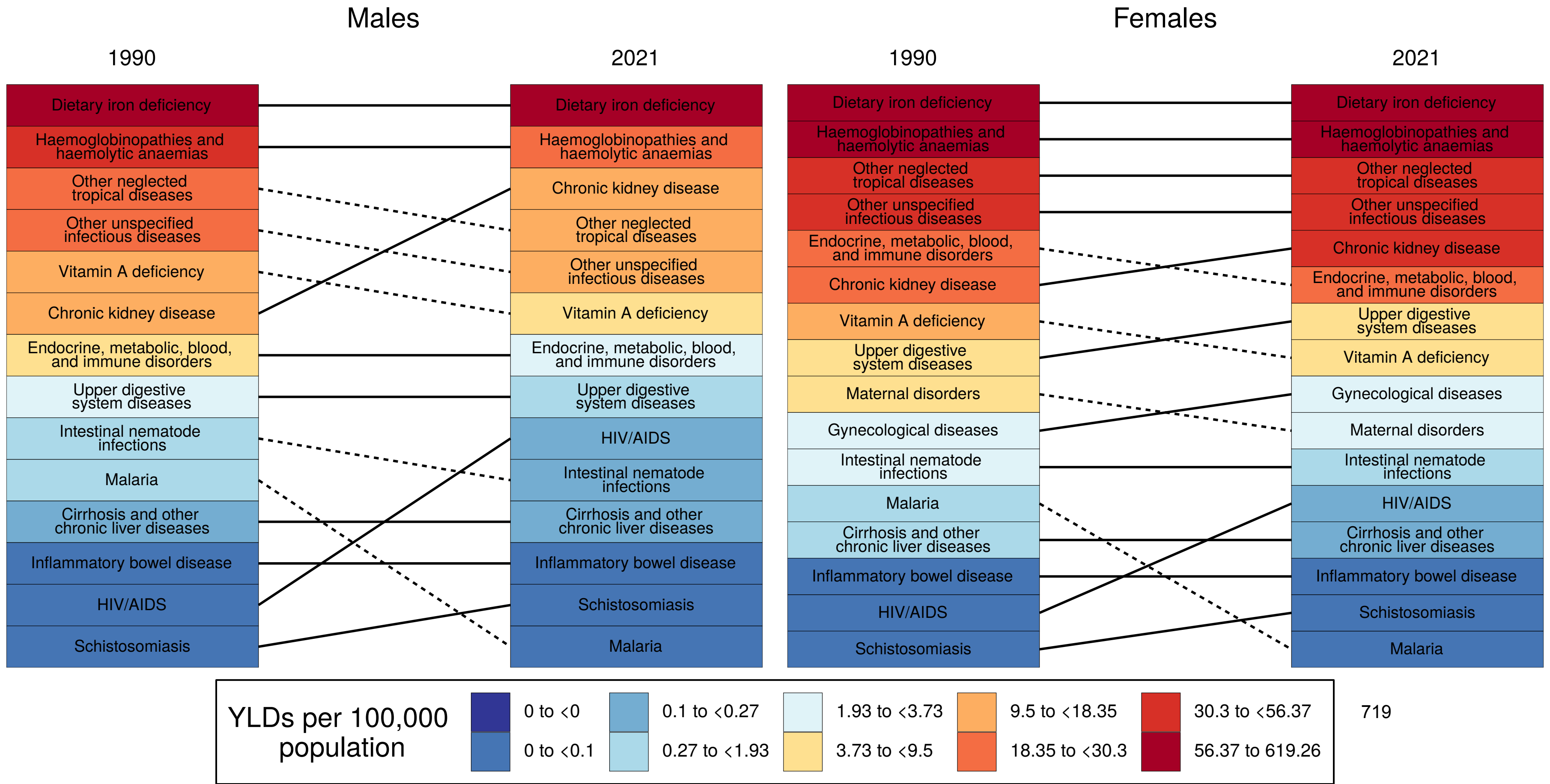

Iloilo

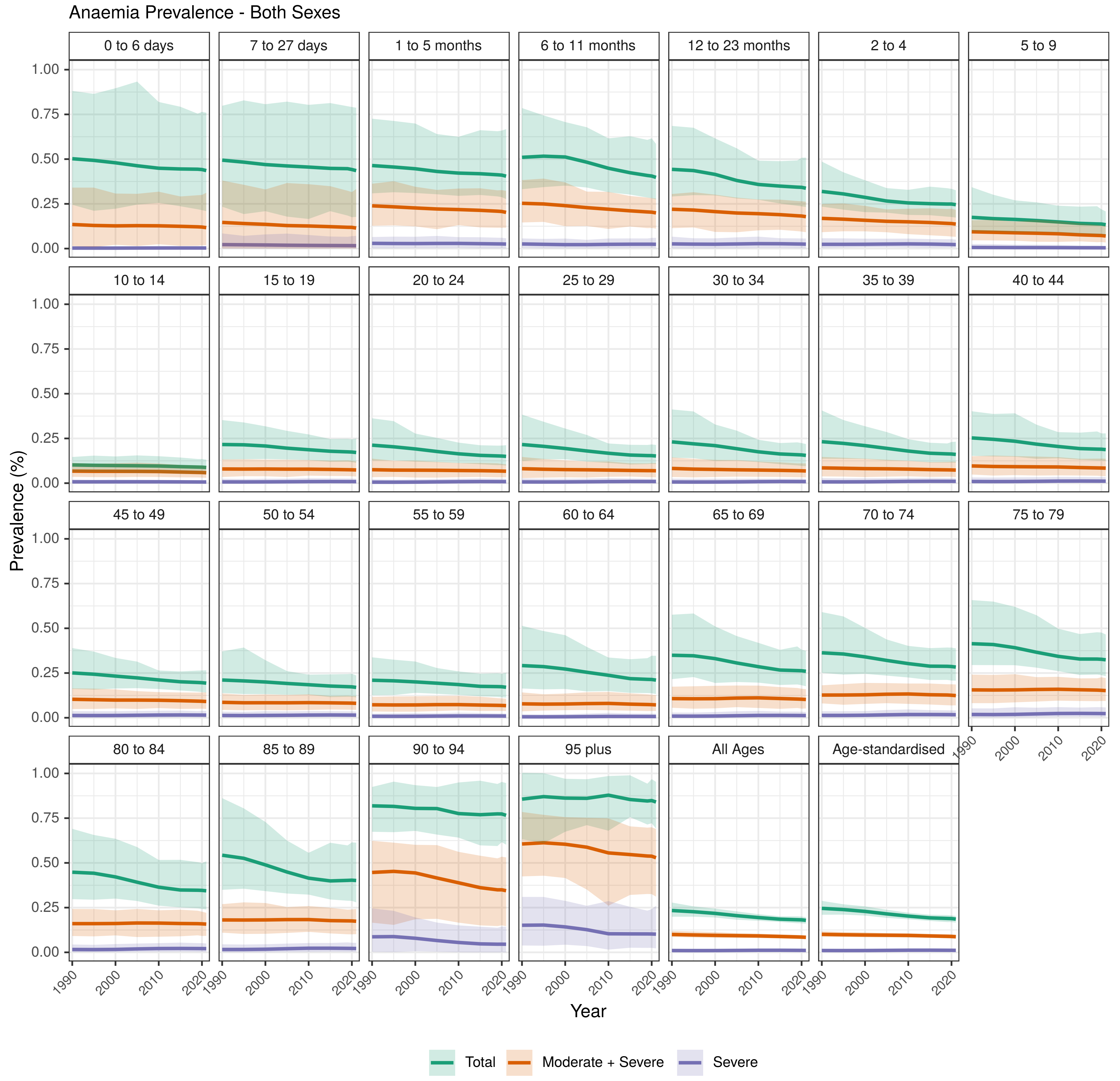

Anaemia Prevalence - Both Sexes
0 to 6 days
7 to 27 days
1 to 5 months
6 to 11 months
12 to 23 months
2 to 4
5 to 9
10 to 14
15 to 19
20 to 24
25 to 29
30 to 34
35 to 39
40 to 44
45 to 49
50 to 54
55 to 59
60 to 64
65 to 69
70 to 74
75 to 79
80 to 84
85 to 89
90 to 94
95 plus
All Ages
Age-standardised
Prevalence (%)
0.00
0.25
0.50
0.75
1.00
1990
2000
2010
2020
Year
Total
Moderate + Severe
Severe

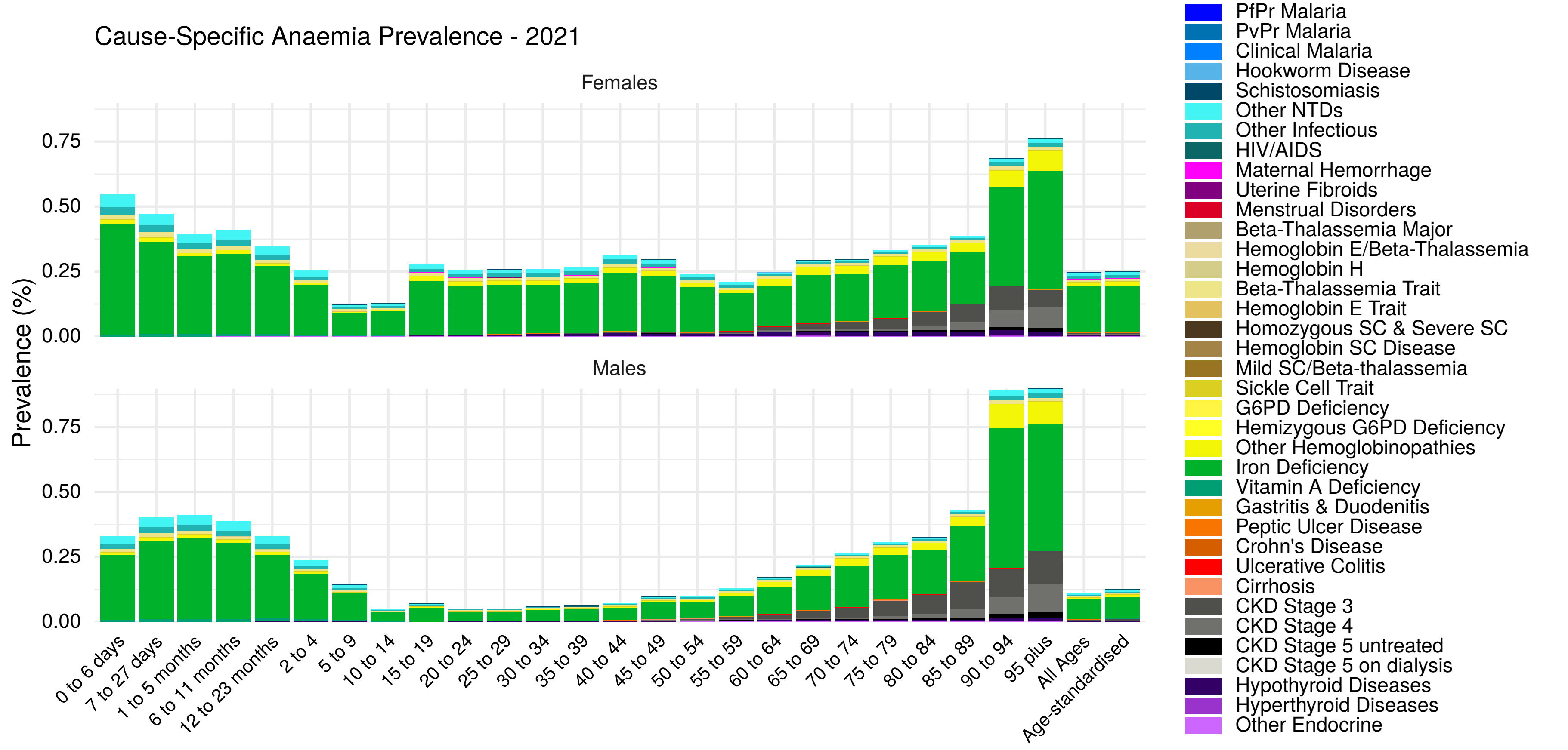

Cause-Specific Anaemia Prevalence - 2021
Females
Males
Prevalence (%)
0.00
0.25
0.50
0.75
0 to 6 days
7 to 27 days
1 to 5 months
6 to 11 months
12 to 23 months
2 to 4
5 to 9
10 to 14
15 to 19
20 to 24
25 to 29
30 to 34
35 to 39
40 to 44
45 to 49
50 to 54
55 to 59
60 to 64
65 to 69
70 to 74
75 to 79
80 to 84
85 to 89
90 to 94
95 plus
All Ages
Age-standardised
PfPr Malaria
PvPr Malaria
Clinical Malaria
Hookworm Disease
Schistosomiasis
Other NTDs
Other Infectious
HIV/AIDS
Maternal Hemorrhage
Uterine Fibroids
Menstrual Disorders
Beta-Thalassemia Major
Hemoglobin E/Beta-Thalassemia
Hemoglobin H
Beta-Thalassemia Trait
Hemoglobin E Trait
Homozygous SC & Severe SC
Hemoglobin SC Disease
Mild SC/Beta-thalassemia
Sickle Cell Trait
G6PD Deficiency
Hemizygous G6PD Deficiency
Other Hemoglobinopathies
Iron Deficiency
Vitamin A Deficiency
Gastritis & Duodenitis
Peptic Ulcer Disease
Crohn's Disease
Ulcerative Colitis
Cirrhosis
CKD Stage 3
CKD Stage 4
CKD Stage 5 untreated
CKD Stage 5 on dialysis
Hypothyroid Diseases
Hyperthyroid Diseases
Other Endocrine

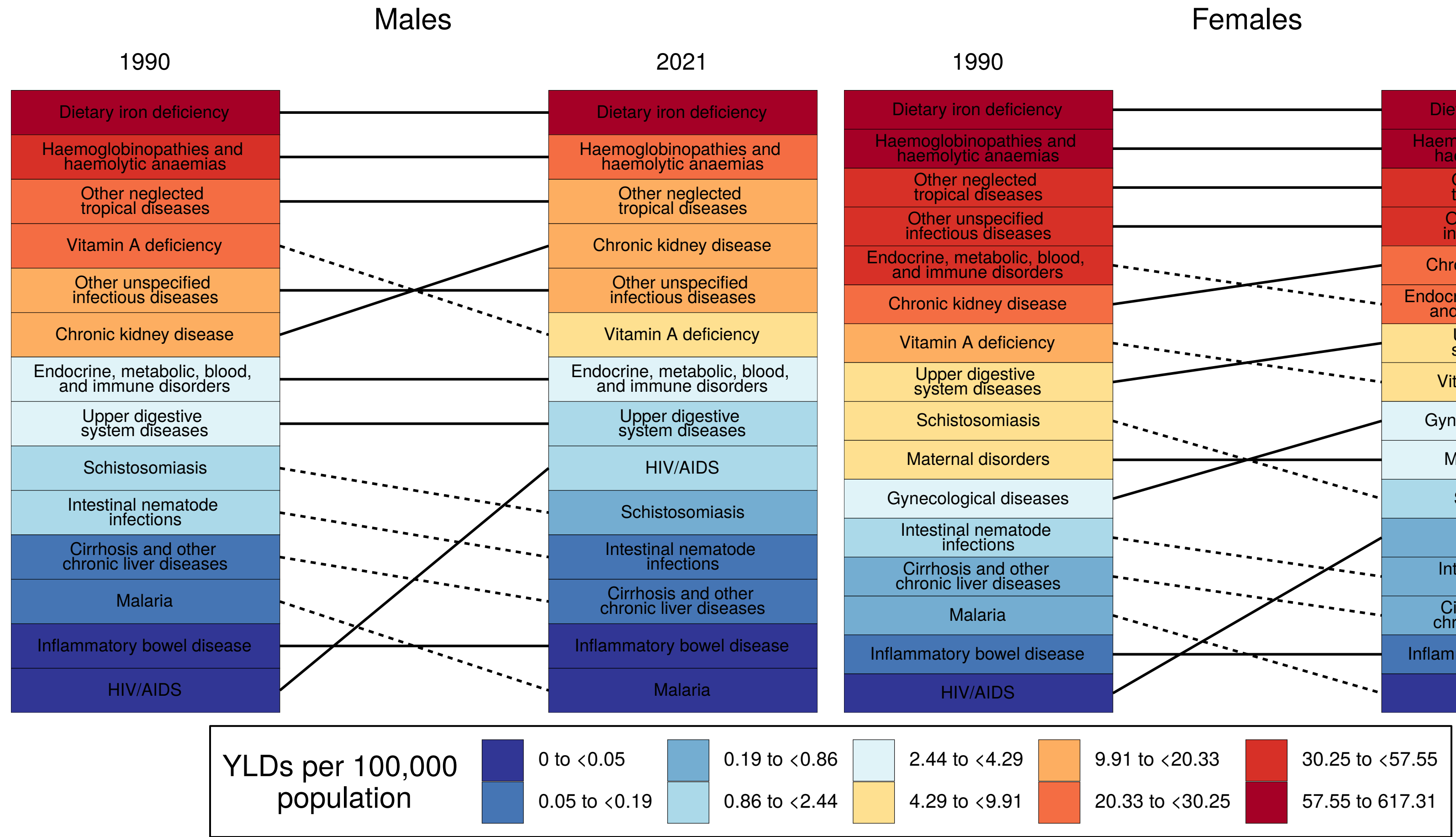

Males
Females
1990
2021
Dietary iron deficiency
Haemoglobinopathies and haemolytic anaemias
Other neglected tropical diseases
Vitamin A deficiency
Other unspecified infectious diseases
Chronic kidney disease
Endocrine, metabolic, blood, and immune disorders
Upper digestive system diseases
Schistosomiasis
Intestinal nematode infections
Cirrhosis and other chronic liver diseases
Malaria
Inflammatory bowel disease
HIV/AIDS
Gynecological diseases
Maternal disorders
YLDs per 100,000 population
0 to <0.05
0.05 to <0.19
0.19 to <0.86
0.86 to <2.44
2.44 to <4.29
4.29 to <9.91
9.91 to <20.33
20.33 to <30.25
30.25 to <57.55
57.55 to 617.31

720

Isabela

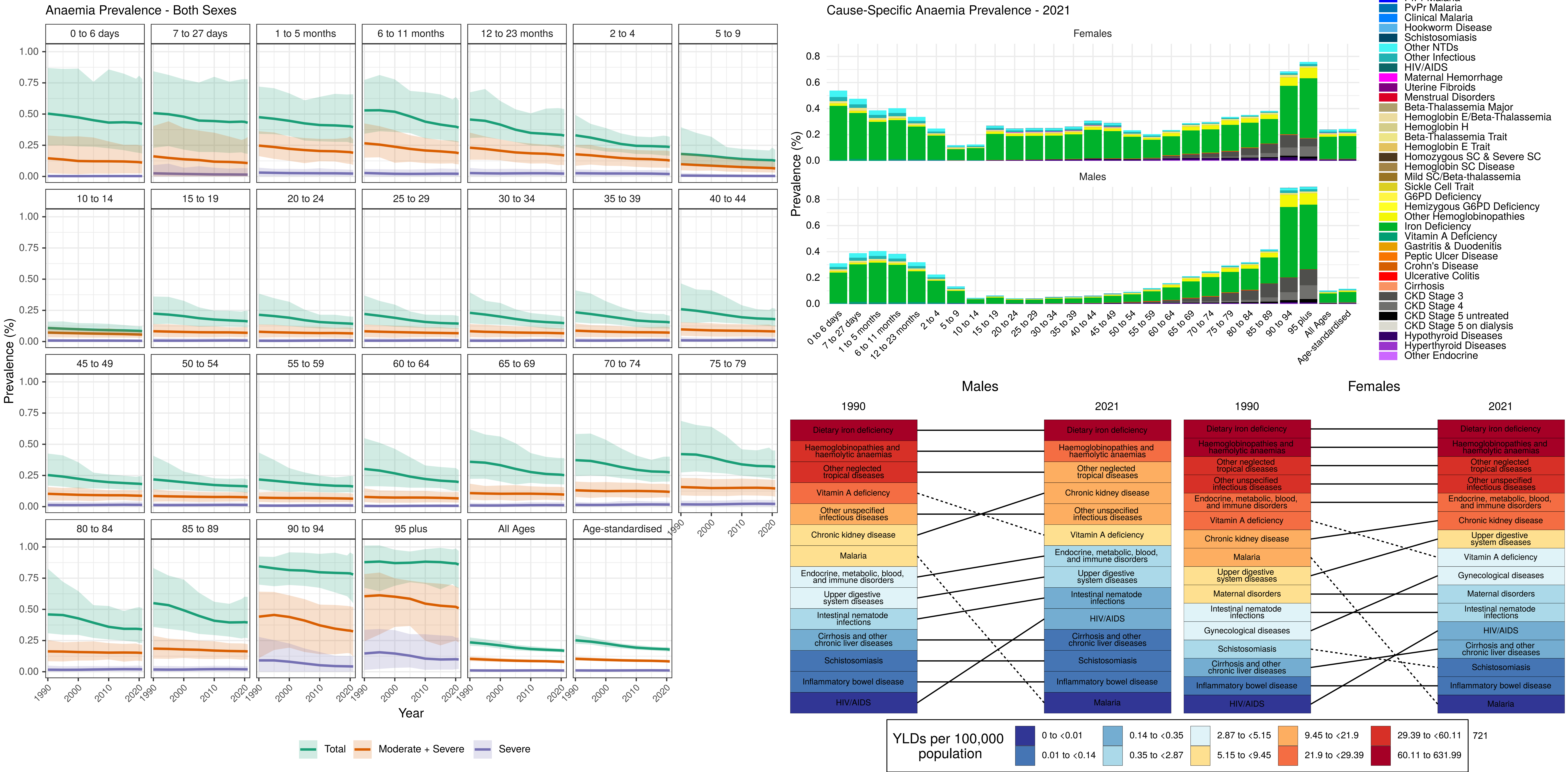

Anaemia Prevalence - Both Sexes
0 to 6 days
7 to 27 days
1 to 5 months
6 to 11 months
12 to 23 months
2 to 4
5 to 9
10 to 14
15 to 19
20 to 24
25 to 29
30 to 34
35 to 39
40 to 44
45 to 49
50 to 54
55 to 59
60 to 64
65 to 69
70 to 74
75 to 79
80 to 84
85 to 89
90 to 94
95 plus
All Ages
Age-standardised
Prevalence (%)
Year
Total
Moderate + Severe
Severe
Cause-Specific Anaemia Prevalence - 2021
Females
Males
Prevalence (%)
PfPr Malaria
PvPr Malaria
Clinical Malaria
Hookworm Disease
Schistosomiasis
Other NTDs
Other Infectious
HIV/AIDS
Maternal Hemorrhage
Uterine Fibroids
Menstrual Disorders
Beta-Thalassemia Major
Hemoglobin E/Beta-Thalassemia
Hemoglobin H
Beta-Thalassemia Trait
Hemoglobin E Trait
Homozygous SC & Severe SC
Hemoglobin SC Disease
Mild SC/Beta-thalassemia
Sickle Cell Trait
G6PD Deficiency
Hemizygous G6PD Deficiency
Other Hemoglobinopathies
Iron Deficiency
Vitamin A Deficiency
Gastritis & Duodenitis
Peptic Ulcer Disease
Crohn's Disease
Ulcerative Colitis
Cirrhosis
CKD Stage 3
CKD Stage 4
CKD Stage 5 untreated
CKD Stage 5 on dialysis
Hypothyroid Diseases
Hyperthyroid Diseases
Other Endocrine
Males
Females
1990
2021
Dietary iron deficiency
Haemoglobinopathies and haemolytic anaemias
Other neglected tropical diseases
Vitamin A deficiency
Other unspecified infectious diseases
Chronic kidney disease
Malaria
Endocrine, metabolic, blood, and immune disorders
Upper digestive system diseases
Intestinal nematode infections
Cirrhosis and other chronic liver diseases
Schistosomiasis
Inflammatory bowel disease
HIV/AIDS
Maternal disorders
Gynecological diseases
YLDs per 100,000 population
0 to <0.01
0.01 to <0.14
0.14 to <0.35
0.35 to <2.87
2.87 to <5.15
5.15 to <9.45
9.45 to <21.9
21.9 to <29.39
29.39 to <60.11
60.11 to 631.99

Kalinga

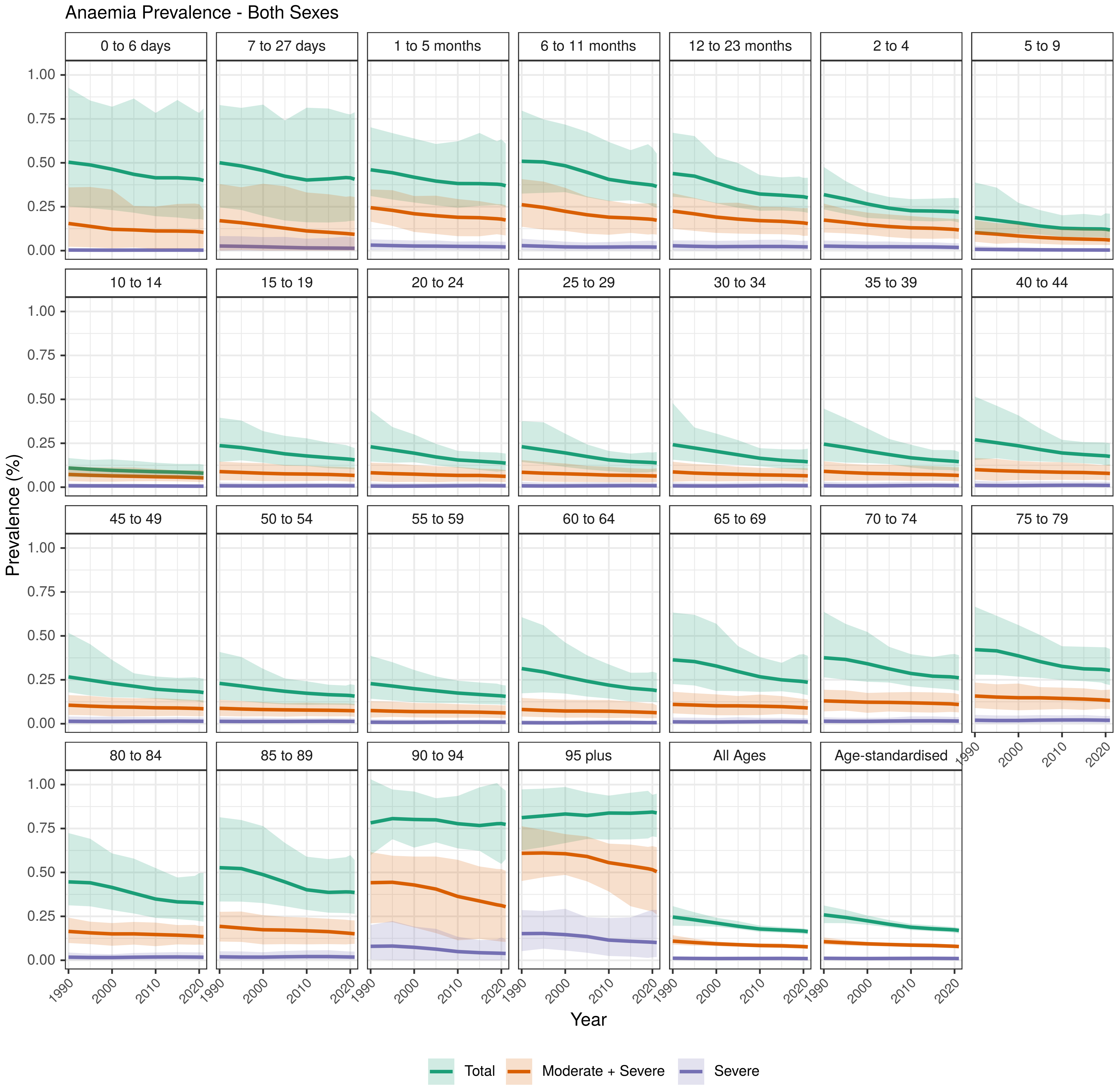

Anaemia Prevalence - Both Sexes
0 to 6 days
7 to 27 days
1 to 5 months
6 to 11 months
12 to 23 months
2 to 4
5 to 9
10 to 14
15 to 19
20 to 24
25 to 29
30 to 34
35 to 39
40 to 44
45 to 49
50 to 54
55 to 59
60 to 64
65 to 69
70 to 74
75 to 79
80 to 84
85 to 89
90 to 94
95 plus
All Ages
Age-standardised
Prevalence (%)
Year
Total
Moderate + Severe
Severe

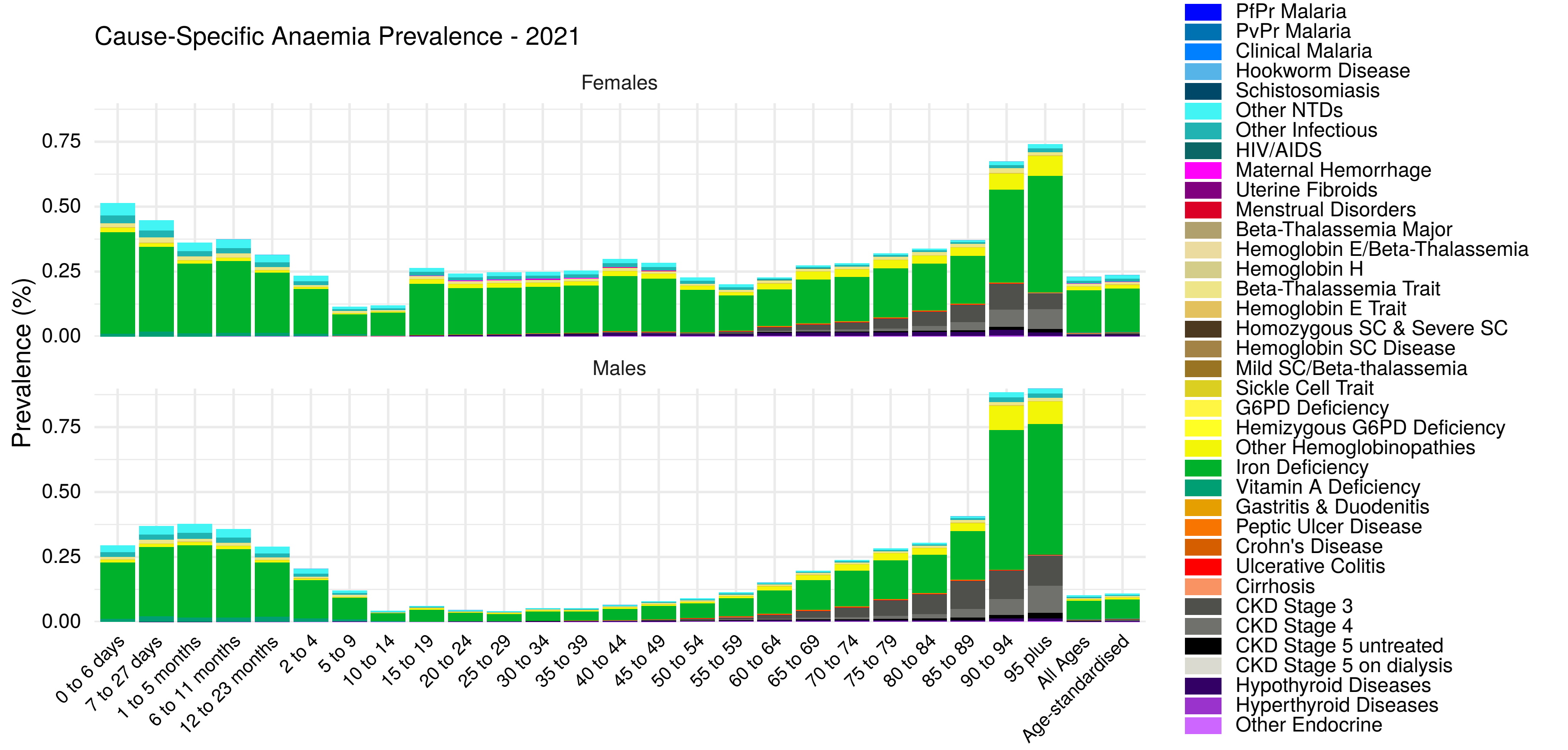

Cause-Specific Anaemia Prevalence - 2021
Females
Males
Prevalence (%)
PfPr Malaria
PvPr Malaria
Clinical Malaria
Hookworm Disease
Schistosomiasis
Other NTDs
Other Infectious
HIV/AIDS
Maternal Hemorrhage
Uterine Fibroids
Menstrual Disorders
Beta-Thalassemia Major
Hemoglobin E/Beta-Thalassemia
Hemoglobin H
Beta-Thalassemia Trait
Hemoglobin E Trait
Homozygous SC & Severe SC
Hemoglobin SC Disease
Mild SC/Beta-thalassemia
Sickle Cell Trait
G6PD Deficiency
Hemizygous G6PD Deficiency
Other Hemoglobinopathies
Iron Deficiency
Vitamin A Deficiency
Gastritis & Duodenitis
Peptic Ulcer Disease
Crohn's Disease
Ulcerative Colitis
Cirrhosis
CKD Stage 3
CKD Stage 4
CKD Stage 5 untreated
CKD Stage 5 on dialysis
Hypothyroid Diseases
Hyperthyroid Diseases
Other Endocrine

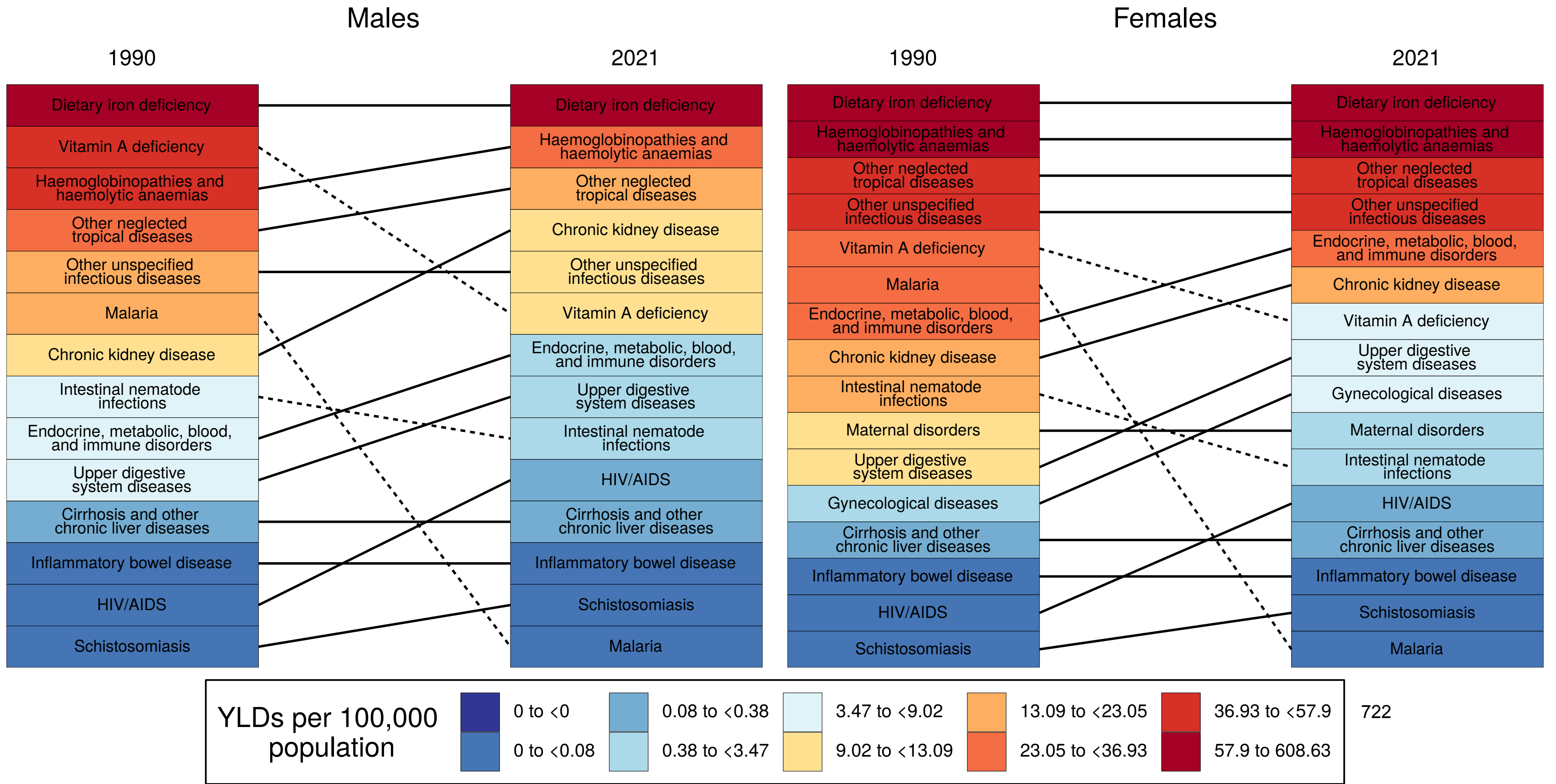

Males
Females
1990
2021
Dietary iron deficiency
Vitamin A deficiency
Haemoglobinopathies and haemolytic anaemias
Other neglected tropical diseases
Other unspecified infectious diseases
Malaria
Chronic kidney disease
Intestinal nematode infections
Endocrine, metabolic, blood, and immune disorders
Upper digestive system diseases
Cirrhosis and other chronic liver diseases
Inflammatory bowel disease
HIV/AIDS
Schistosomiasis
Maternal disorders
Gynecological diseases
YLDs per 100,000 population
0 to <0
0 to <0.08
0.08 to <0.38
0.38 to <3.47
3.47 to <9.02
9.02 to <13.09
13.09 to <23.05
23.05 to <36.93
36.93 to <57.9
57.9 to 608.63

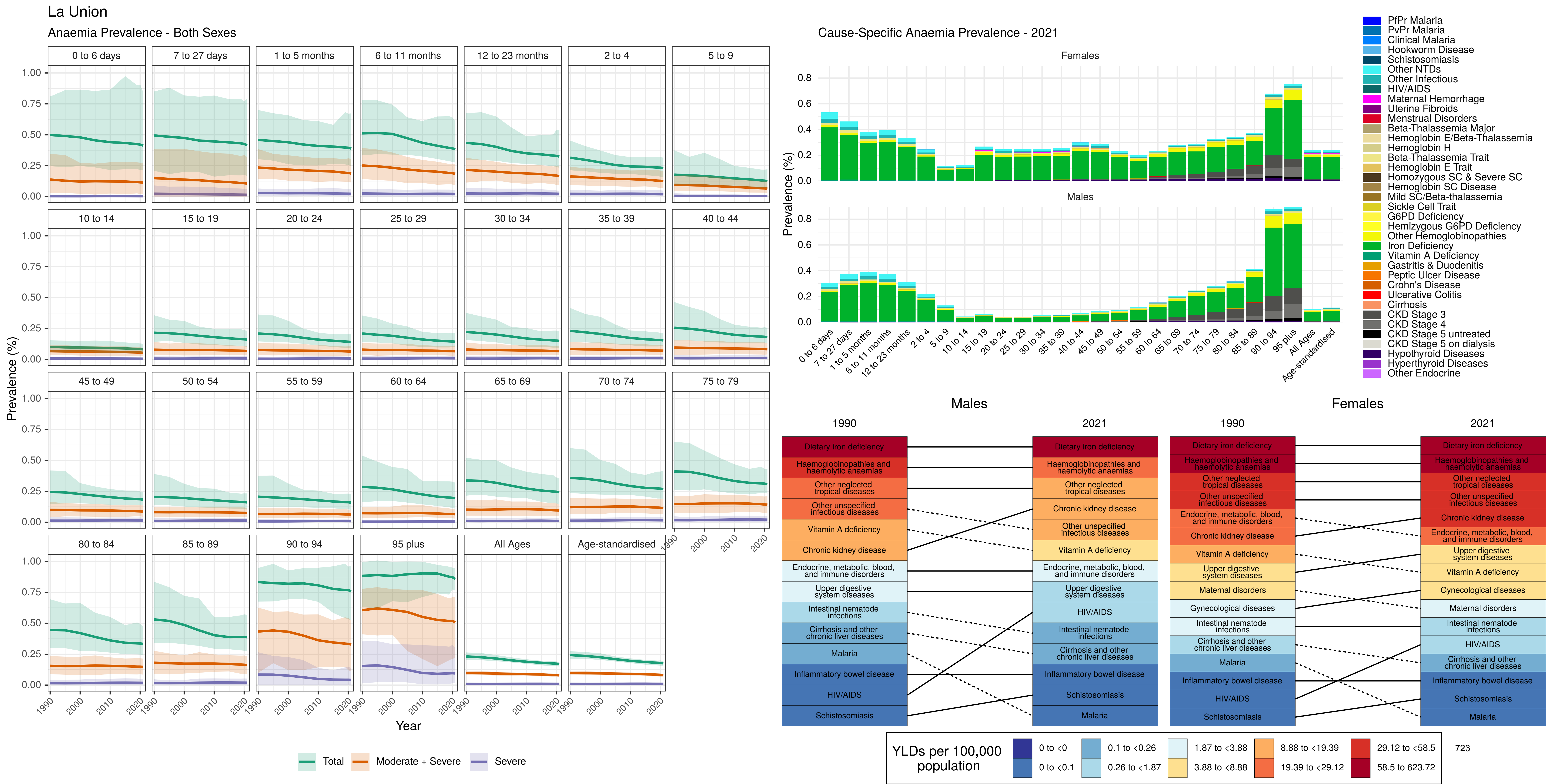

La Union
Anaemia Prevalence - Both Sexes
0 to 6 days
7 to 27 days
1 to 5 months
6 to 11 months
12 to 23 months
2 to 4
5 to 9
10 to 14
15 to 19
20 to 24
25 to 29
30 to 34
35 to 39
40 to 44
45 to 49
50 to 54
55 to 59
60 to 64
65 to 69
70 to 74
75 to 79
80 to 84
85 to 89
90 to 94
95 plus
All Ages
Age-standardised
Prevalence (%)
Year
Total
Moderate + Severe
Severe
Cause-Specific Anaemia Prevalence - 2021
Females
Males
PfPr Malaria
PvPr Malaria
Clinical Malaria
Hookworm Disease
Schistosomiasis
Other NTDs
Other Infectious
HIV/AIDS
Maternal Hemorrhage
Uterine Fibroids
Menstrual Disorders
Beta-Thalassemia Major
Hemoglobin E/Beta-Thalassemia
Hemoglobin H
Beta-Thalassemia Trait
Hemoglobin E Trait
Homozygous SC & Severe SC
Hemoglobin SC Disease
Mild SC/Beta-thalassemia
Sickle Cell Trait
G6PD Deficiency
Hemizygous G6PD Deficiency
Other Hemoglobinopathies
Iron Deficiency
Vitamin A Deficiency
Gastritis & Duodenitis
Peptic Ulcer Disease
Crohn's Disease
Ulcerative Colitis
Cirrhosis
CKD Stage 3
CKD Stage 4
CKD Stage 5 untreated
CKD Stage 5 on dialysis
Hypothyroid Diseases
Hyperthyroid Diseases
Other Endocrine
Males
1990
2021
Dietary iron deficiency
Haemoglobinopathies and haemolytic anaemias
Other neglected tropical diseases
Other unspecified infectious diseases
Vitamin A deficiency
Chronic kidney disease
Endocrine, metabolic, blood, and immune disorders
Upper digestive system diseases
Intestinal nematode infections
Cirrhosis and other chronic liver diseases
Malaria
Inflammatory bowel disease
HIV/AIDS
Schistosomiasis
Females
Maternal disorders
Gynecological diseases
YLDs per 100,000 population
0 to <0
0 to <0.1
0.1 to <0.26
0.26 to <1.87
1.87 to <3.88
3.88 to <8.88
8.88 to <19.39
19.39 to <29.12
29.12 to <58.5
58.5 to 623.72

Laguna

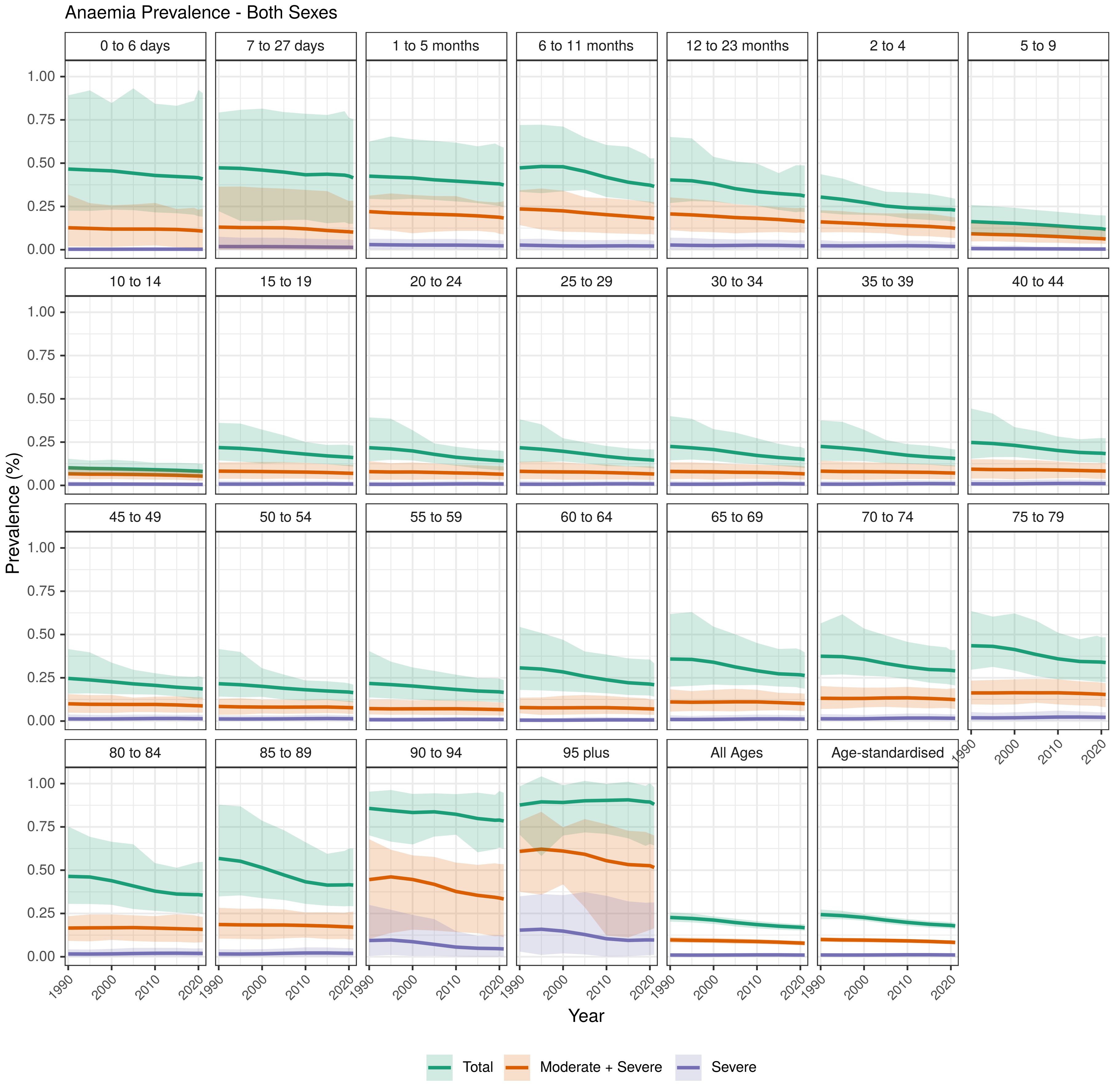

Anaemia Prevalence - Both Sexes
0 to 6 days
7 to 27 days
1 to 5 months
6 to 11 months
12 to 23 months
2 to 4
5 to 9
10 to 14
15 to 19
20 to 24
25 to 29
30 to 34
35 to 39
40 to 44
45 to 49
50 to 54
55 to 59
60 to 64
65 to 69
70 to 74
75 to 79
80 to 84
85 to 89
90 to 94
95 plus
All Ages
Age-standardised
Prevalence (%)
1.00
0.75
0.50
0.25
0.00
1990
2000
2010
2020
Year
Total
Moderate + Severe
Severe

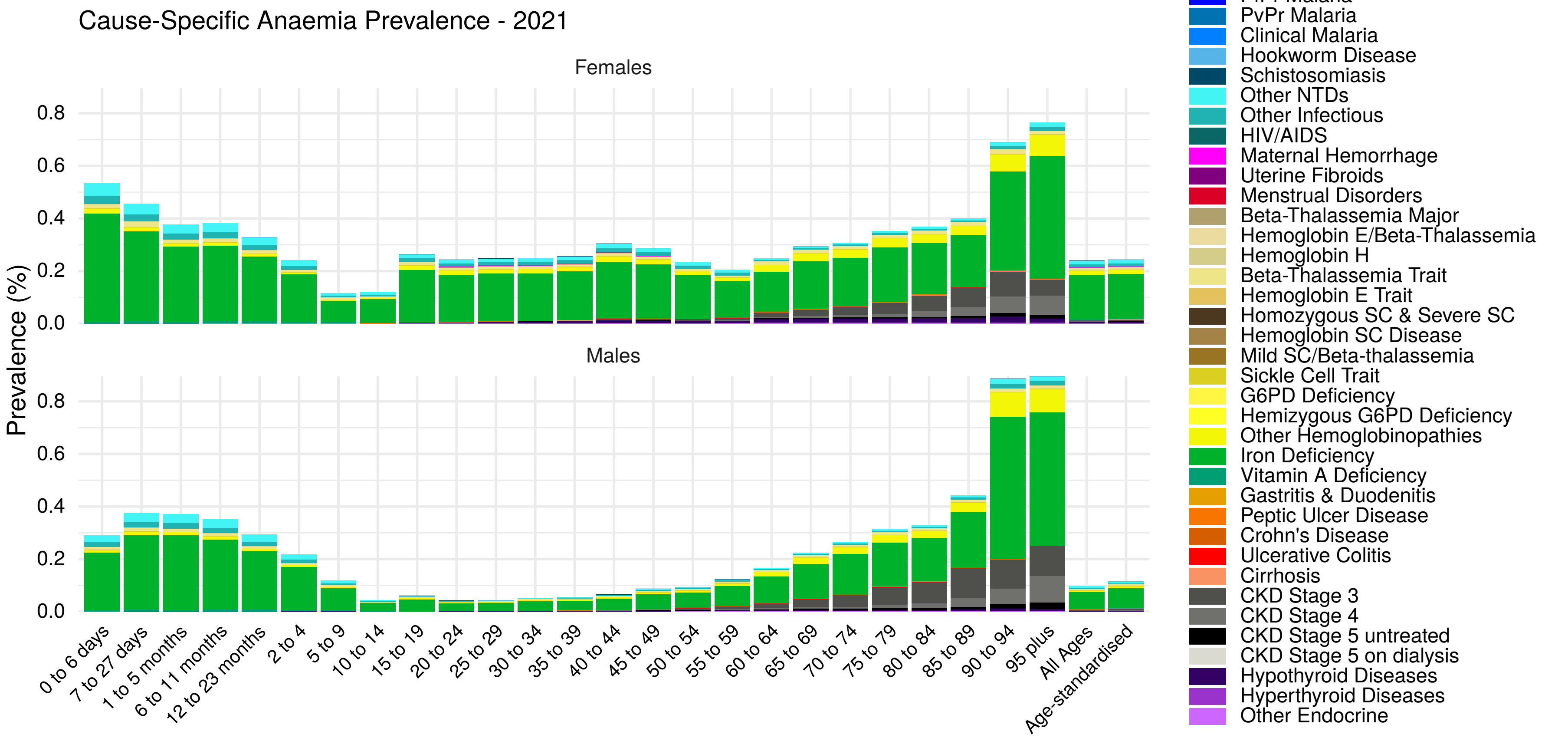

Cause-Specific Anaemia Prevalence - 2021
Females
Males
Prevalence (%)
0.8
0.6
0.4
0.2
0.0
0 to 6 days
7 to 27 days
1 to 5 months
6 to 11 months
12 to 23 months
2 to 4
5 to 9
10 to 14
15 to 19
20 to 24
25 to 29
30 to 34
35 to 39
40 to 44
45 to 49
50 to 54
55 to 59
60 to 64
65 to 69
70 to 74
75 to 79
80 to 84
85 to 89
90 to 94
95 plus
All Ages
Age-standardised
PfPr Malaria
PvPr Malaria
Clinical Malaria
Hookworm Disease
Schistosomiasis
Other NTDs
Other Infectious
HIV/AIDS
Maternal Hemorrhage
Uterine Fibroids
Menstrual Disorders
Beta-Thalassemia Major
Hemoglobin E/Beta-Thalassemia
Hemoglobin H
Beta-Thalassemia Trait
Hemoglobin E Trait
Homozygous SC & Severe SC
Hemoglobin SC Disease
Mild SC/Beta-thalassemia
Sickle Cell Trait
G6PD Deficiency
Hemizygous G6PD Deficiency
Other Hemoglobinopathies
Iron Deficiency
Vitamin A Deficiency
Gastritis & Duodenitis
Peptic Ulcer Disease
Crohn's Disease
Ulcerative Colitis
Cirrhosis
CKD Stage 3
CKD Stage 4
CKD Stage 5 untreated
CKD Stage 5 on dialysis
Hypothyroid Diseases
Hyperthyroid Diseases
Other Endocrine

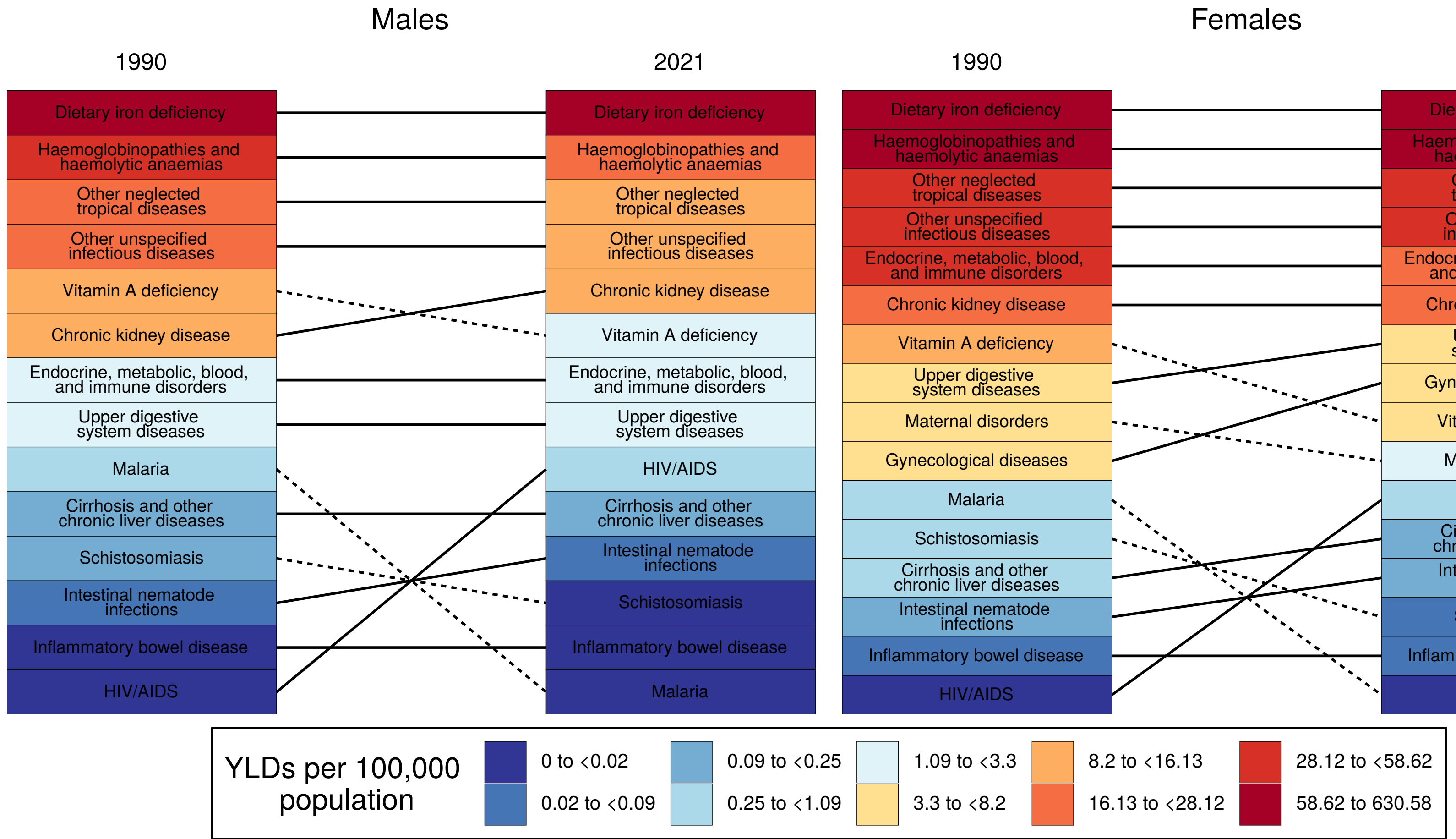

Males
Females
1990
2021
Dietary iron deficiency
Haemoglobinopathies and haemolytic anaemias
Other neglected tropical diseases
Other unspecified infectious diseases
Vitamin A deficiency
Chronic kidney disease
Endocrine, metabolic, blood, and immune disorders
Upper digestive system diseases
Malaria
Cirrhosis and other chronic liver diseases
Schistosomiasis
Intestinal nematode infections
Inflammatory bowel disease
HIV/AIDS
Maternal disorders
Gynecological diseases
YLDs per 100,000 population
0 to <0.02
0.02 to <0.09
0.09 to <0.25
0.25 to <1.09
1.09 to <3.3
3.3 to <8.2
8.2 to <16.13
16.13 to <28.12
28.12 to <58.62
58.62 to 630.58

Lanao Del Norte

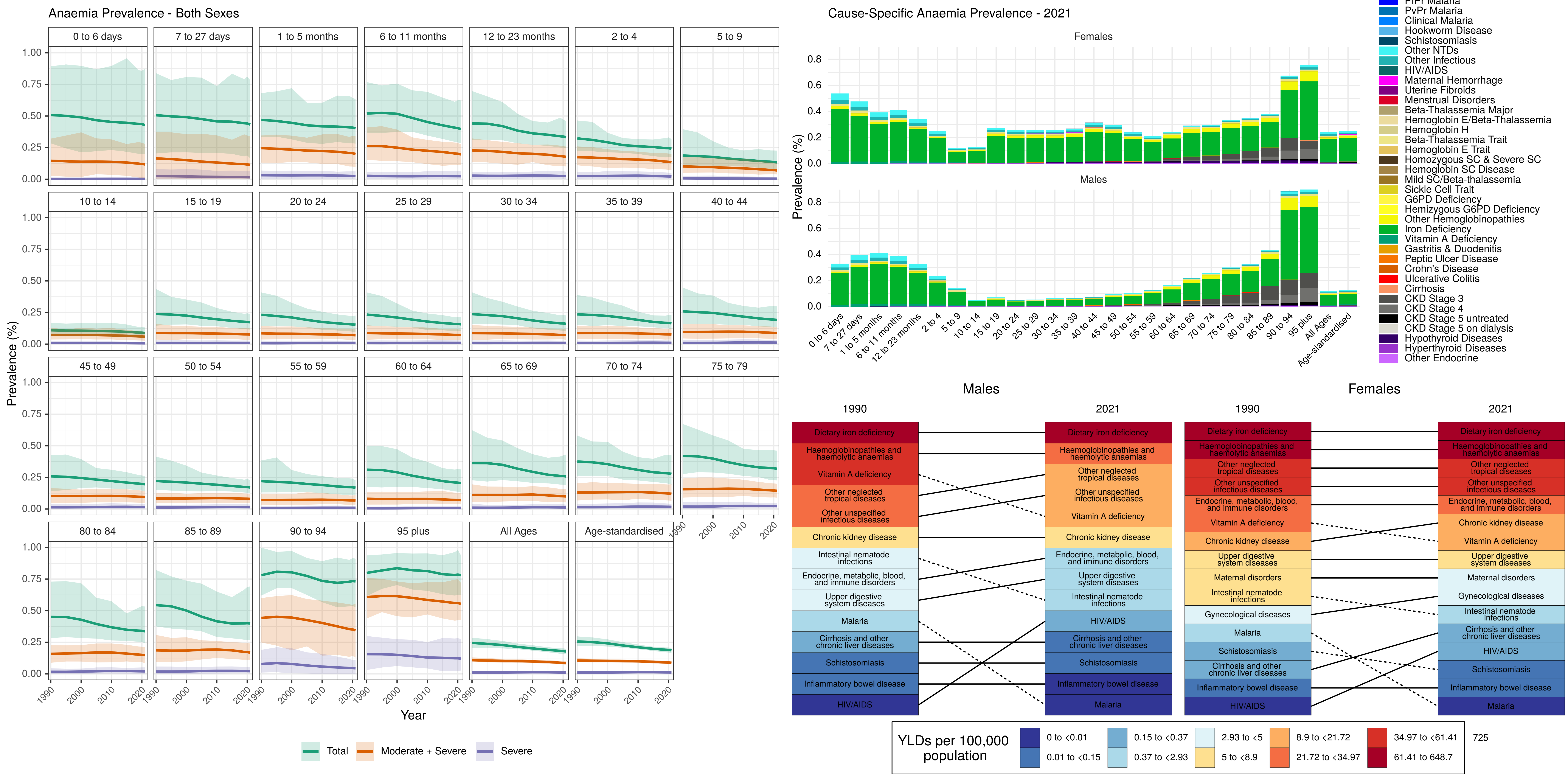

Lanao Del Sur

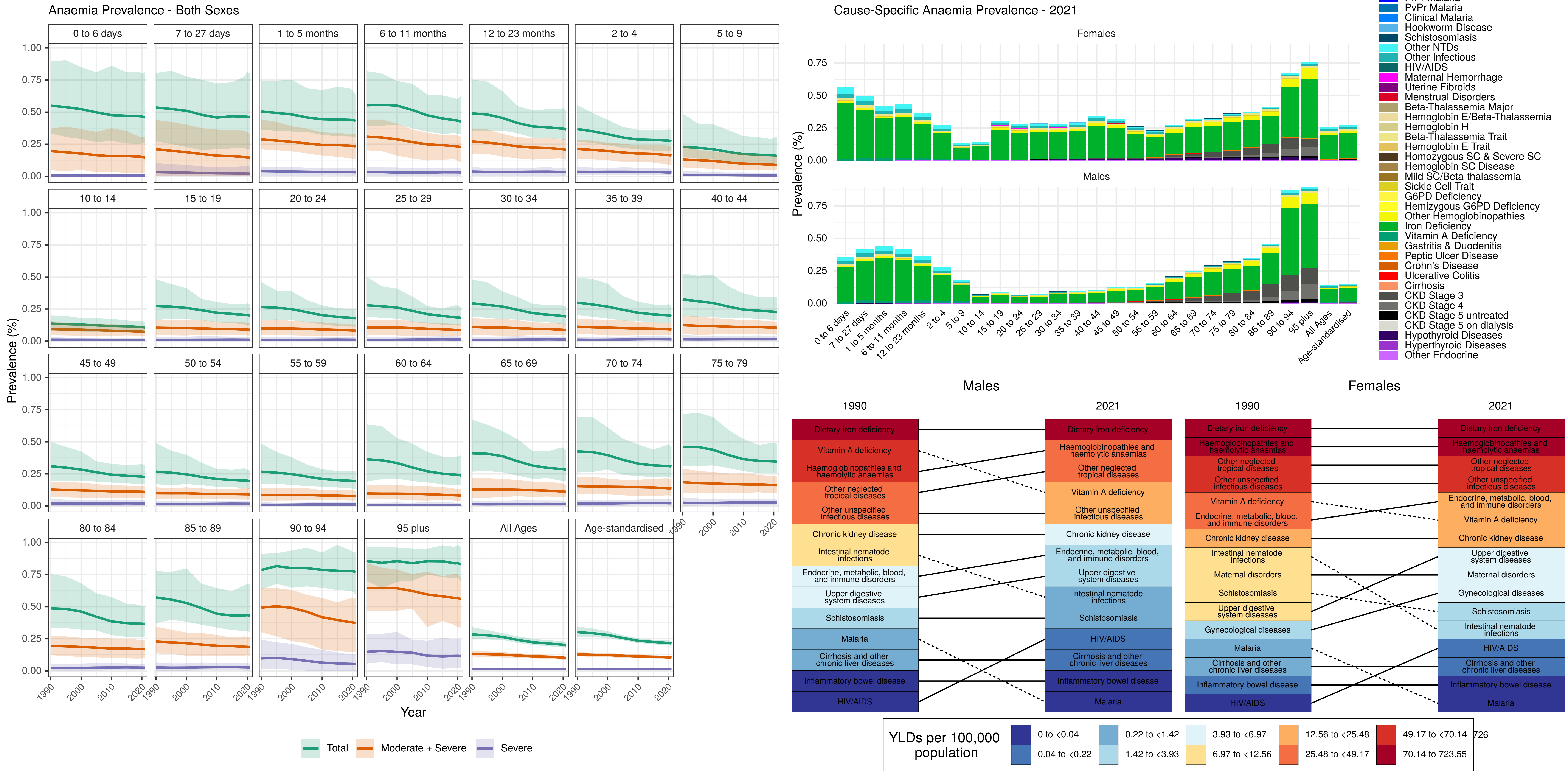

Leyte

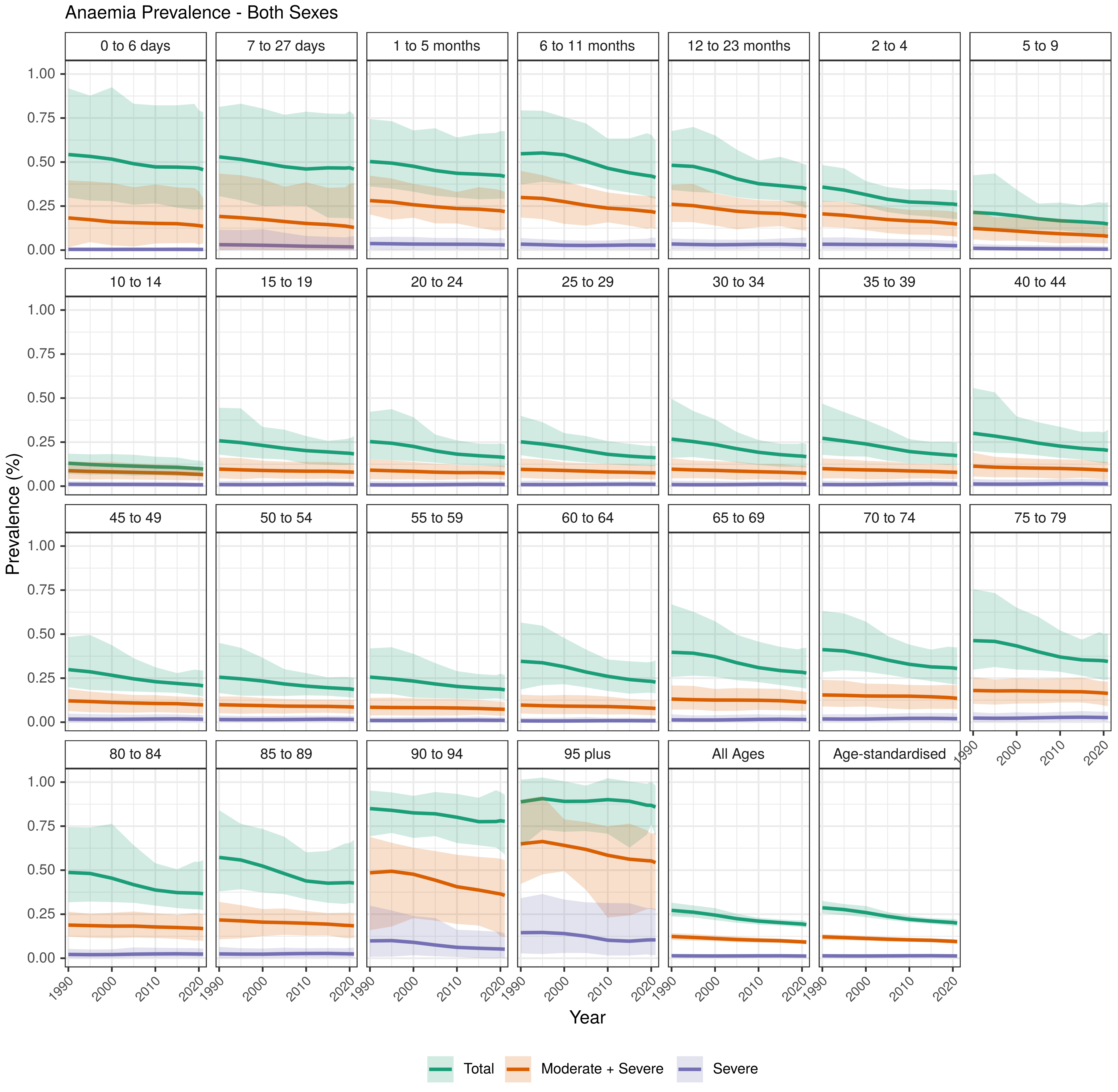

Anaemia Prevalence - Both Sexes
0 to 6 days
7 to 27 days
1 to 5 months
6 to 11 months
12 to 23 months
2 to 4
5 to 9
10 to 14
15 to 19
20 to 24
25 to 29
30 to 34
35 to 39
40 to 44
45 to 49
50 to 54
55 to 59
60 to 64
65 to 69
70 to 74
75 to 79
80 to 84
85 to 89
90 to 94
95 plus
All Ages
Age-standardised
Prevalence (%)
0.00
0.25
0.50
0.75
1.00
1990
2000
2010
2020
Year
Total
Moderate + Severe
Severe

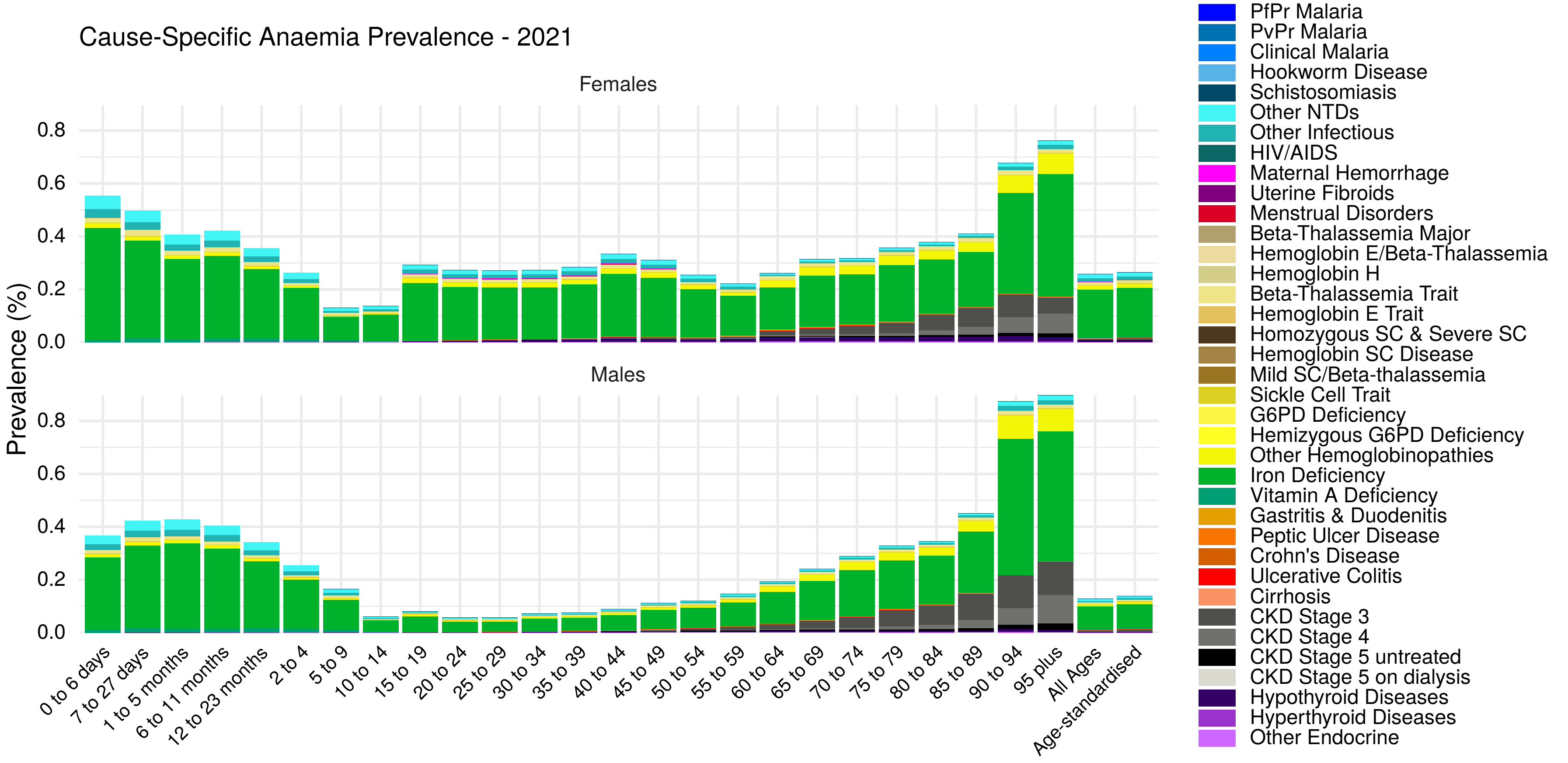

Cause-Specific Anaemia Prevalence - 2021
Females
Males
Prevalence (%)
0.0
0.2
0.4
0.6
0.8
0 to 6 days
7 to 27 days
1 to 5 months
6 to 11 months
12 to 23 months
2 to 4
5 to 9
10 to 14
15 to 19
20 to 24
25 to 29
30 to 34
35 to 39
40 to 44
45 to 49
50 to 54
55 to 59
60 to 64
65 to 69
70 to 74
75 to 79
80 to 84
85 to 89
90 to 94
95 plus
All Ages
Age-standardised
PfPr Malaria
PvPr Malaria
Clinical Malaria
Hookworm Disease
Schistosomiasis
Other NTDs
Other Infectious
HIV/AIDS
Maternal Hemorrhage
Uterine Fibroids
Menstrual Disorders
Beta-Thalassemia Major
Hemoglobin E/Beta-Thalassemia
Hemoglobin H
Beta-Thalassemia Trait
Hemoglobin E Trait
Homozygous SC & Severe SC
Hemoglobin SC Disease
Mild SC/Beta-thalassemia
Sickle Cell Trait
G6PD Deficiency
Hemizygous G6PD Deficiency
Other Hemoglobinopathies
Iron Deficiency
Vitamin A Deficiency
Gastritis & Duodenitis
Peptic Ulcer Disease
Crohn's Disease
Ulcerative Colitis
Cirrhosis
CKD Stage 3
CKD Stage 4
CKD Stage 5 untreated
CKD Stage 5 on dialysis
Hypothyroid Diseases
Hyperthyroid Diseases
Other Endocrine

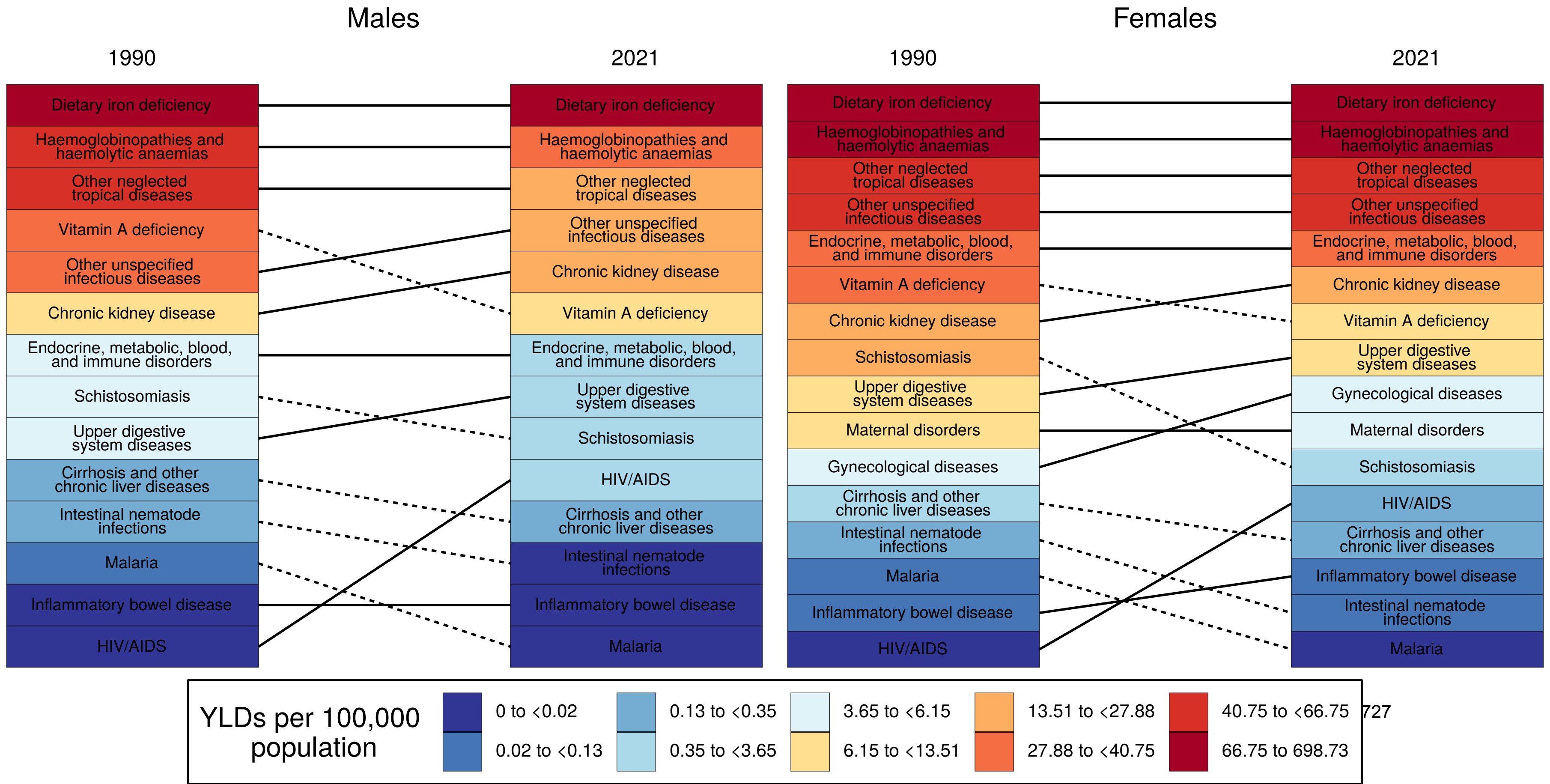

Males
Females
1990
2021
Dietary iron deficiency
Haemoglobinopathies and haemolytic anaemias
Other neglected tropical diseases
Vitamin A deficiency
Other unspecified infectious diseases
Chronic kidney disease
Endocrine, metabolic, blood, and immune disorders
Schistosomiasis
Upper digestive system diseases
Cirrhosis and other chronic liver diseases
Intestinal nematode infections
Malaria
Inflammatory bowel disease
HIV/AIDS
Maternal disorders
Gynecological diseases
YLDs per 100,000 population
0 to <0.02
0.02 to <0.13
0.13 to <0.35
0.35 to <3.65
3.65 to <6.15
6.15 to <13.51
13.51 to <27.88
27.88 to <40.75
40.75 to <66.75
66.75 to 698.73

727

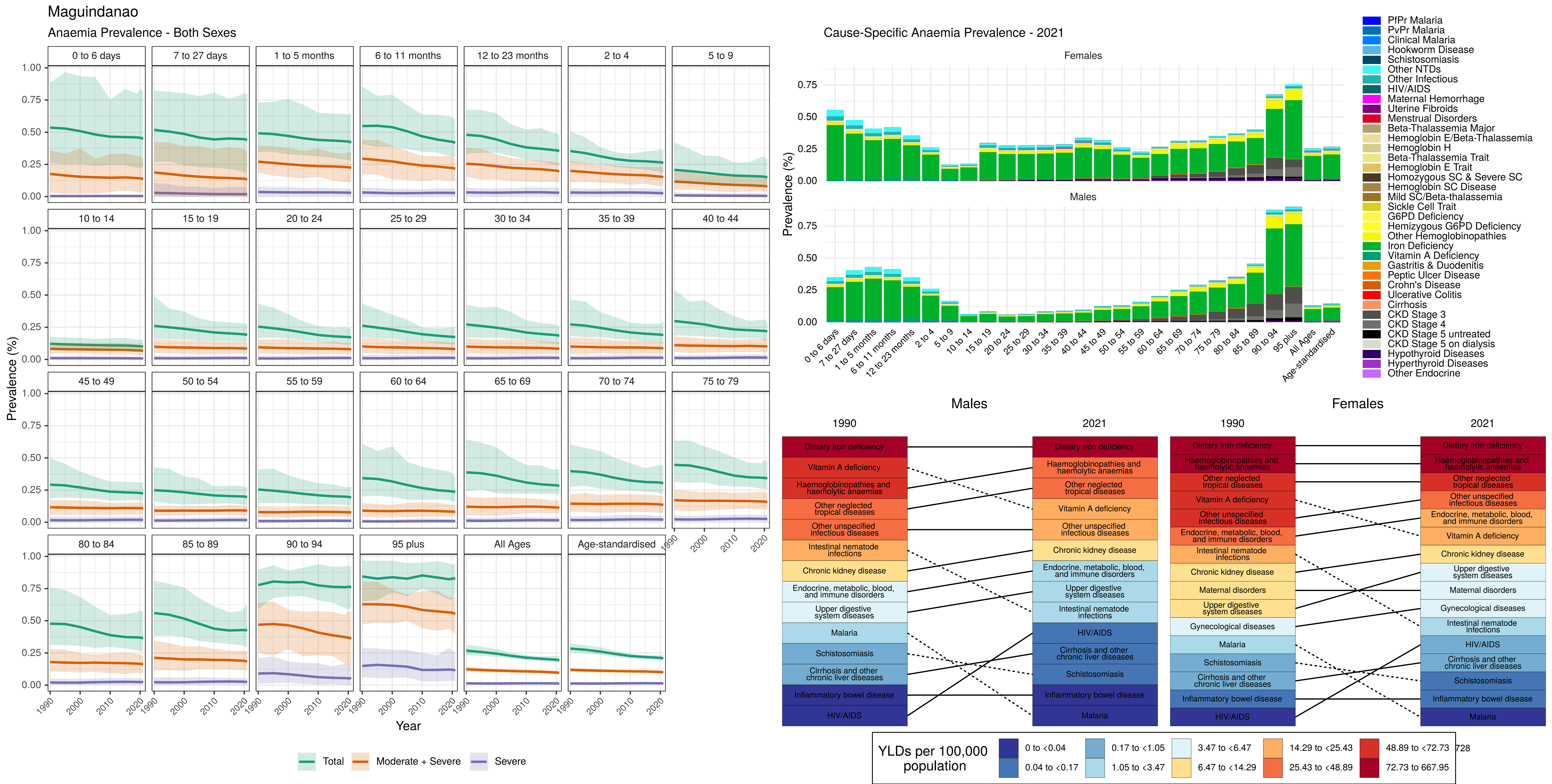
Maguindanao
Anaemia Prevalence - Both Sexes
0 to 6 days
7 to 27 days
1 to 5 months
6 to 11 months
12 to 23 months
2 to 4
5 to 9
10 to 14
15 to 19
20 to 24
25 to 29
30 to 34
35 to 39
40 to 44
45 to 49
50 to 54
55 to 59
60 to 64
65 to 69
70 to 74
75 to 79
80 to 84
85 to 89
90 to 94
95 plus
All Ages
Age-standardised
Prevalence (%)
Year
Total
Moderate + Severe
Severe
Cause-Specific Anaemia Prevalence - 2021
Females
Males
PfPr Malaria
PvPr Malaria
Clinical Malaria
Hookworm Disease
Schistosomiasis
Other NTDs
Other Infectious
HIV/AIDS
Maternal Hemorrhage
Uterine Fibroids
Menstrual Disorders
Beta-Thalassemia Major
Hemoglobin E/Beta-Thalassemia
Hemoglobin H
Beta-Thalassemia Trait
Hemoglobin E Trait
Homozygous SC & Severe SC
Hemoglobin SC Disease
Mild SC/Beta-thalassemia
Sickle Cell Trait
G6PD Deficiency
Hemizygous G6PD Deficiency
Other Hemoglobinopathies
Iron Deficiency
Vitamin A Deficiency
Gastritis & Duodenitis
Peptic Ulcer Disease
Crohn's Disease
Ulcerative Colitis
Cirrhosis
CKD Stage 3
CKD Stage 4
CKD Stage 5 untreated
CKD Stage 5 on dialysis
Hypothyroid Diseases
Hyperthyroid Diseases
Other Endocrine
Males
Females
1990
2021
Dietary iron deficiency
Vitamin A deficiency
Haemoglobinopathies and haemolytic anaemias
Other neglected tropical diseases
Other unspecified infectious diseases
Intestinal nematode infections
Chronic kidney disease
Endocrine, metabolic, blood, and immune disorders
Upper digestive system diseases
Malaria
Schistosomiasis
Cirrhosis and other chronic liver diseases
Inflammatory bowel disease
HIV/AIDS
Maternal disorders
Gynecological diseases
YLDs per 100,000 population
0 to <0.04
0.04 to <0.17
0.17 to <1.05
1.05 to <3.47
3.47 to <6.47
6.47 to <14.29
14.29 to <25.43
25.43 to <48.89
48.89 to <72.73
72.73 to 667.95

728

Marinduque

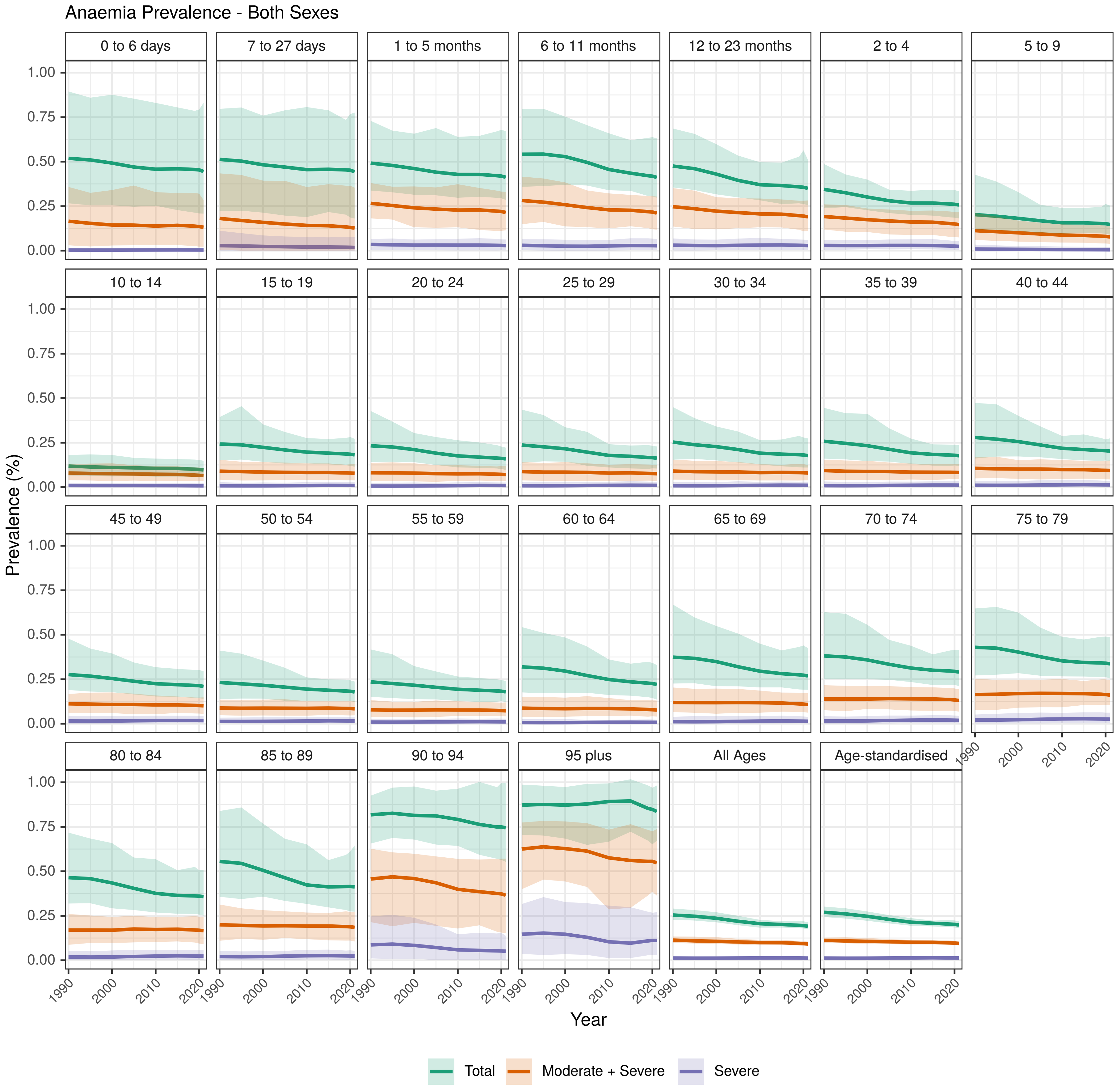

Anaemia Prevalence - Both Sexes
0 to 6 days
7 to 27 days
1 to 5 months
6 to 11 months
12 to 23 months
2 to 4
5 to 9
10 to 14
15 to 19
20 to 24
25 to 29
30 to 34
35 to 39
40 to 44
45 to 49
50 to 54
55 to 59
60 to 64
65 to 69
70 to 74
75 to 79
80 to 84
85 to 89
90 to 94
95 plus
All Ages
Age-standardised
Prevalence (%)
Year
1990
2000
2010
2020
0.00
0.25
0.50
0.75
1.00
Total
Moderate + Severe
Severe

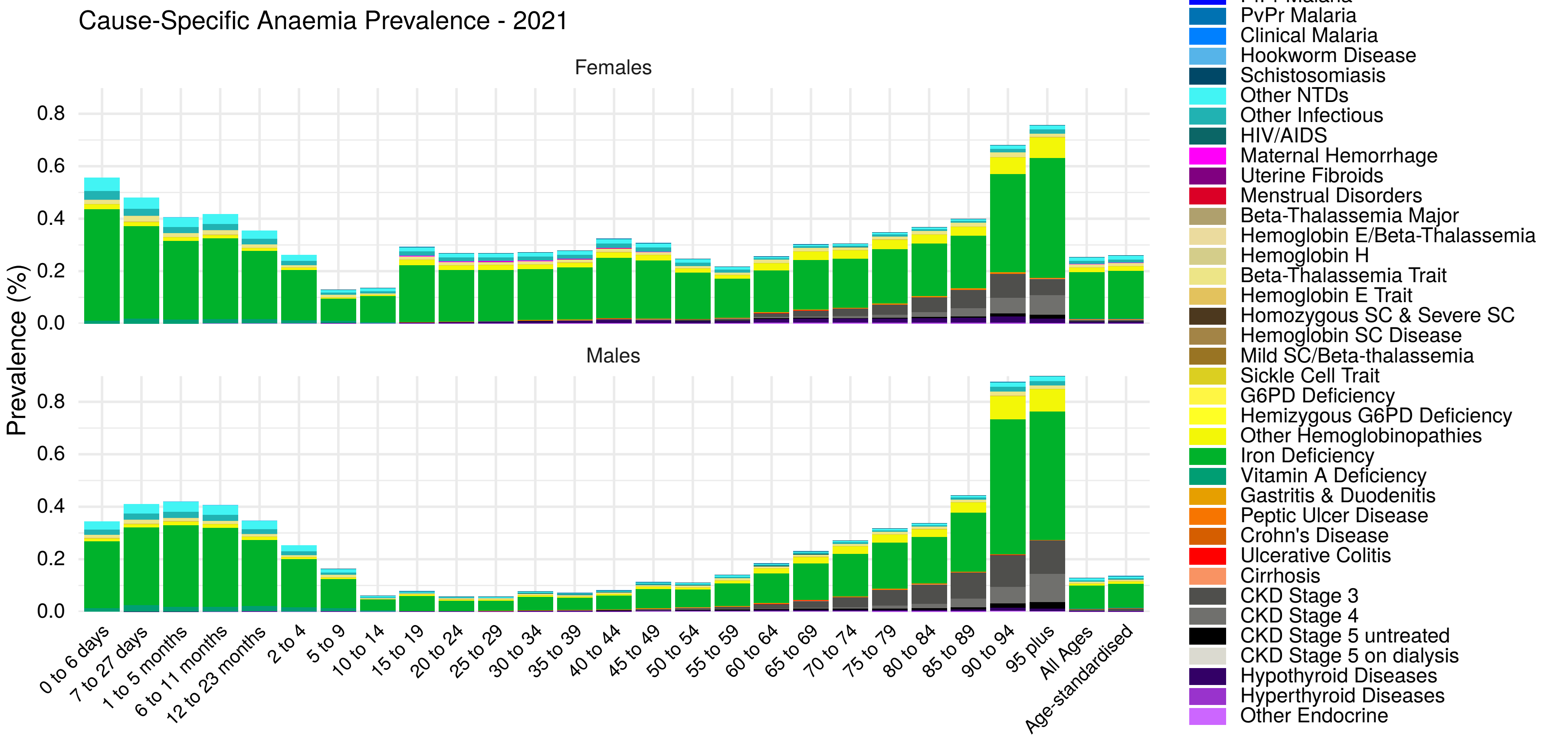

Cause-Specific Anaemia Prevalence - 2021
Females
Males
Prevalence (%)
0.0
0.2
0.4
0.6
0.8
0 to 6 days
7 to 27 days
1 to 5 months
6 to 11 months
12 to 23 months
2 to 4
5 to 9
10 to 14
15 to 19
20 to 24
25 to 29
30 to 34
35 to 39
40 to 44
45 to 49
50 to 54
55 to 59
60 to 64
65 to 69
70 to 74
75 to 79
80 to 84
85 to 89
90 to 94
95 plus
All Ages
Age-standardised
PfPr Malaria
PvPr Malaria
Clinical Malaria
Hookworm Disease
Schistosomiasis
Other NTDs
Other Infectious
HIV/AIDS
Maternal Hemorrhage
Uterine Fibroids
Menstrual Disorders
Beta-Thalassemia Major
Hemoglobin E/Beta-Thalassemia
Hemoglobin H
Beta-Thalassemia Trait
Hemoglobin E Trait
Homozygous SC & Severe SC
Hemoglobin SC Disease
Mild SC/Beta-thalassemia
Sickle Cell Trait
G6PD Deficiency
Hemizygous G6PD Deficiency
Other Hemoglobinopathies
Iron Deficiency
Vitamin A Deficiency
Gastritis & Duodenitis
Peptic Ulcer Disease
Crohn's Disease
Ulcerative Colitis
Cirrhosis
CKD Stage 3
CKD Stage 4
CKD Stage 5 untreated
CKD Stage 5 on dialysis
Hypothyroid Diseases
Hyperthyroid Diseases
Other Endocrine

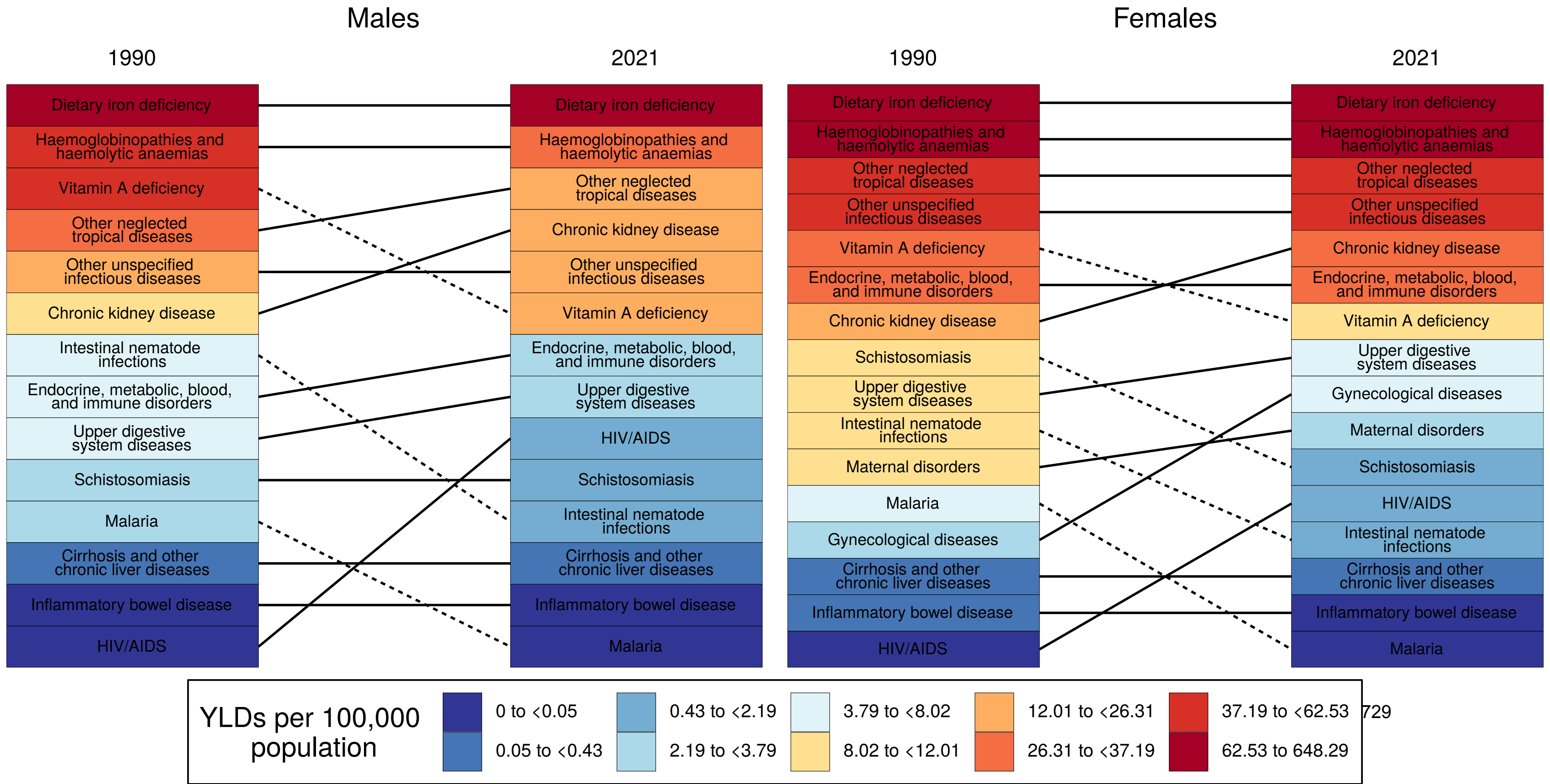

Males
1990
Dietary iron deficiency
Haemoglobinopathies and haemolytic anaemias
Vitamin A deficiency
Other neglected tropical diseases
Other unspecified infectious diseases
Chronic kidney disease
Intestinal nematode infections
Endocrine, metabolic, blood, and immune disorders
Upper digestive system diseases
Schistosomiasis
Malaria
Cirrhosis and other chronic liver diseases
Inflammatory bowel disease
HIV/AIDS
2021
Dietary iron deficiency
Haemoglobinopathies and haemolytic anaemias
Other neglected tropical diseases
Chronic kidney disease
Other unspecified infectious diseases
Vitamin A deficiency
Endocrine, metabolic, blood, and immune disorders
Upper digestive system diseases
HIV/AIDS
Schistosomiasis
Intestinal nematode infections
Cirrhosis and other chronic liver diseases
Inflammatory bowel disease
Malaria
Females
1990
Dietary iron deficiency
Haemoglobinopathies and haemolytic anaemias
Other neglected tropical diseases
Other unspecified infectious diseases
Vitamin A deficiency
Endocrine, metabolic, blood, and immune disorders
Chronic kidney disease
Schistosomiasis
Upper digestive system diseases
Intestinal nematode infections
Maternal disorders
Malaria
Gynecological diseases
Cirrhosis and other chronic liver diseases
Inflammatory bowel disease
HIV/AIDS
2021
Dietary iron deficiency
Haemoglobinopathies and haemolytic anaemias
Other neglected tropical diseases
Other unspecified infectious diseases
Chronic kidney disease
Endocrine, metabolic, blood, and immune disorders
Vitamin A deficiency
Upper digestive system diseases
Gynecological diseases
Maternal disorders
Schistosomiasis
HIV/AIDS
Intestinal nematode infections
Cirrhosis and other chronic liver diseases
Inflammatory bowel disease
Malaria
YLDs per 100,000 population
0 to <0.05
0.05 to <0.43
0.43 to <2.19
2.19 to <3.79
3.79 to <8.02
8.02 to <12.01
12.01 to <26.31
26.31 to <37.19
37.19 to <62.53
62.53 to 648.29

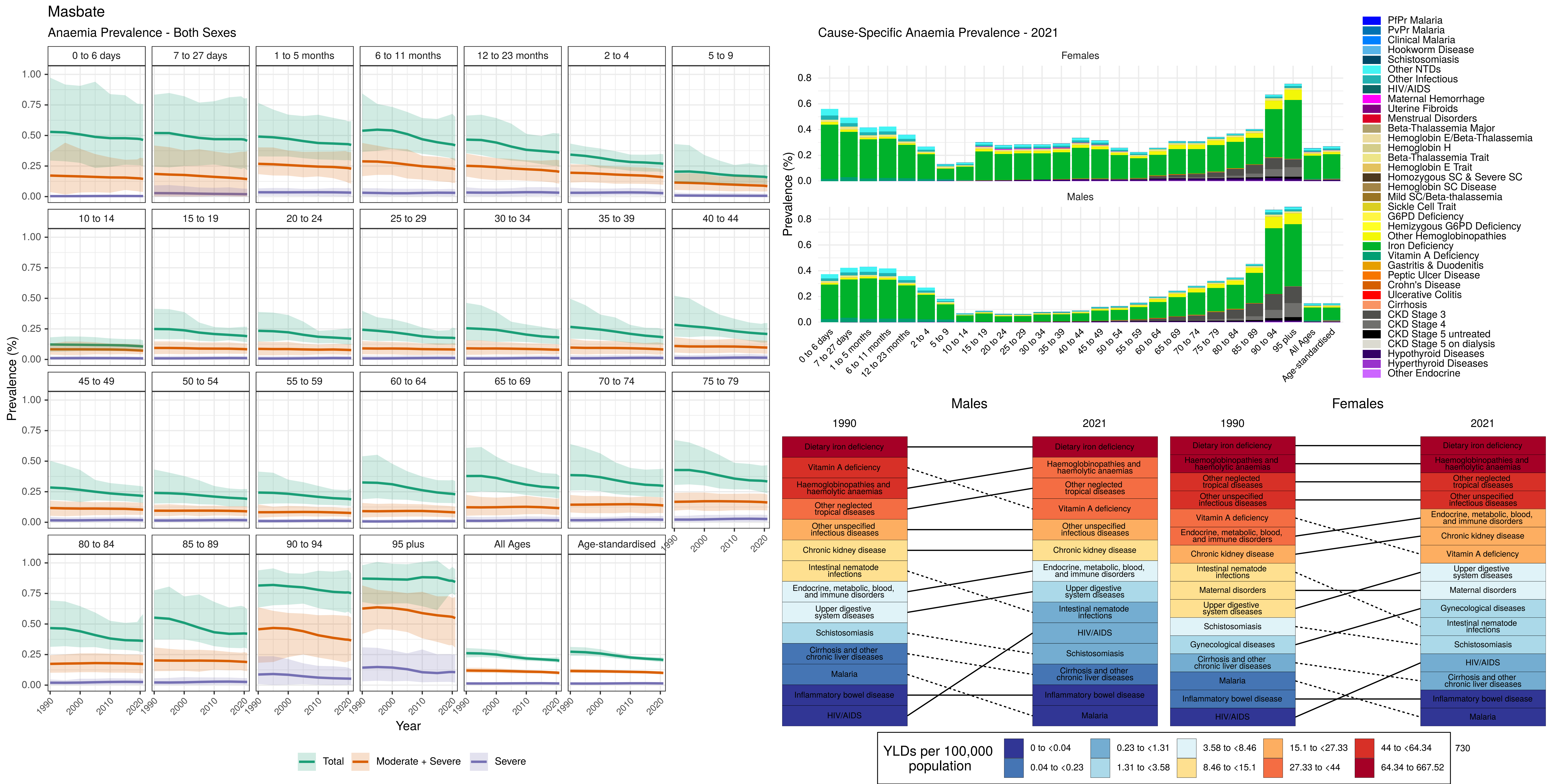
Masbate
Anaemia Prevalence - Both Sexes
0 to 6 days
7 to 27 days
1 to 5 months
6 to 11 months
12 to 23 months
2 to 4
5 to 9
10 to 14
15 to 19
20 to 24
25 to 29
30 to 34
35 to 39
40 to 44
45 to 49
50 to 54
55 to 59
60 to 64
65 to 69
70 to 74
75 to 79
80 to 84
85 to 89
90 to 94
95 plus
All Ages
Age-standardised
Prevalence (%)
Year
Total
Moderate + Severe
Severe
Cause-Specific Anaemia Prevalence - 2021
Females
Males
PfPr Malaria
PvPr Malaria
Clinical Malaria
Hookworm Disease
Schistosomiasis
Other NTDs
Other Infectious
HIV/AIDS
Maternal Hemorrhage
Uterine Fibroids
Menstrual Disorders
Beta-Thalassemia Major
Hemoglobin E/Beta-Thalassemia
Hemoglobin H
Beta-Thalassemia Trait
Hemoglobin E Trait
Homozygous SC & Severe SC
Hemoglobin SC Disease
Mild SC/Beta-thalassemia
Sickle Cell Trait
G6PD Deficiency
Hemizygous G6PD Deficiency
Other Hemoglobinopathies
Iron Deficiency
Vitamin A Deficiency
Gastritis & Duodenitis
Peptic Ulcer Disease
Crohn's Disease
Ulcerative Colitis
Cirrhosis
CKD Stage 3
CKD Stage 4
CKD Stage 5 untreated
CKD Stage 5 on dialysis
Hypothyroid Diseases
Hyperthyroid Diseases
Other Endocrine
Males
Females
1990
2021
Dietary iron deficiency
Vitamin A deficiency
Haemoglobinopathies and haemolytic anaemias
Other neglected tropical diseases
Other unspecified infectious diseases
Chronic kidney disease
Intestinal nematode infections
Endocrine, metabolic, blood, and immune disorders
Upper digestive system diseases
Schistosomiasis
Cirrhosis and other chronic liver diseases
Malaria
Inflammatory bowel disease
HIV/AIDS
Maternal disorders
Gynecological diseases
YLDs per 100,000 population
0 to <0.04
0.04 to <0.23
0.23 to <1.31
1.31 to <3.58
3.58 to <8.46
8.46 to <15.1
15.1 to <27.33
27.33 to <44
44 to <64.34
64.34 to 667.52
730

Misamis Occidental

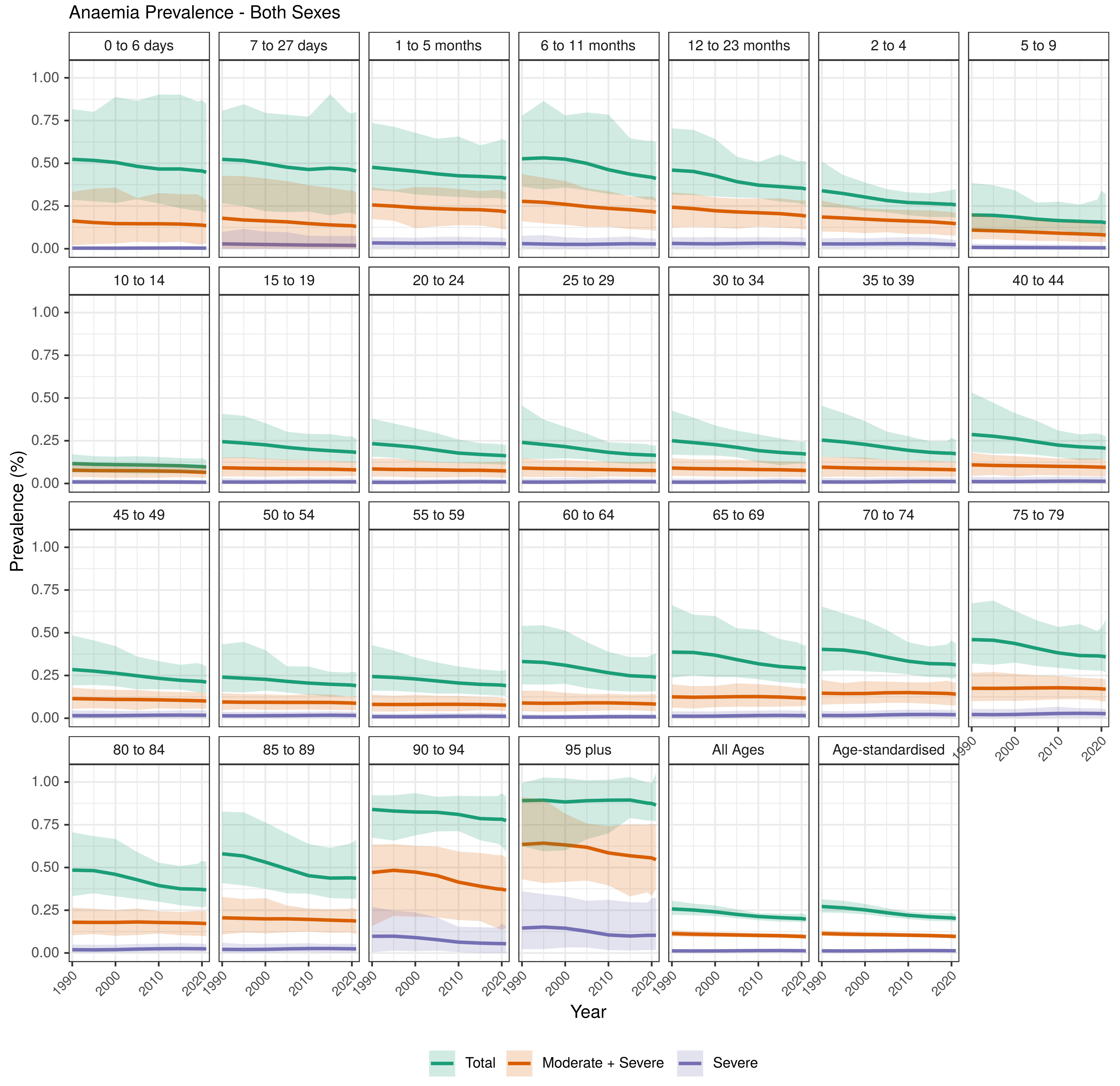

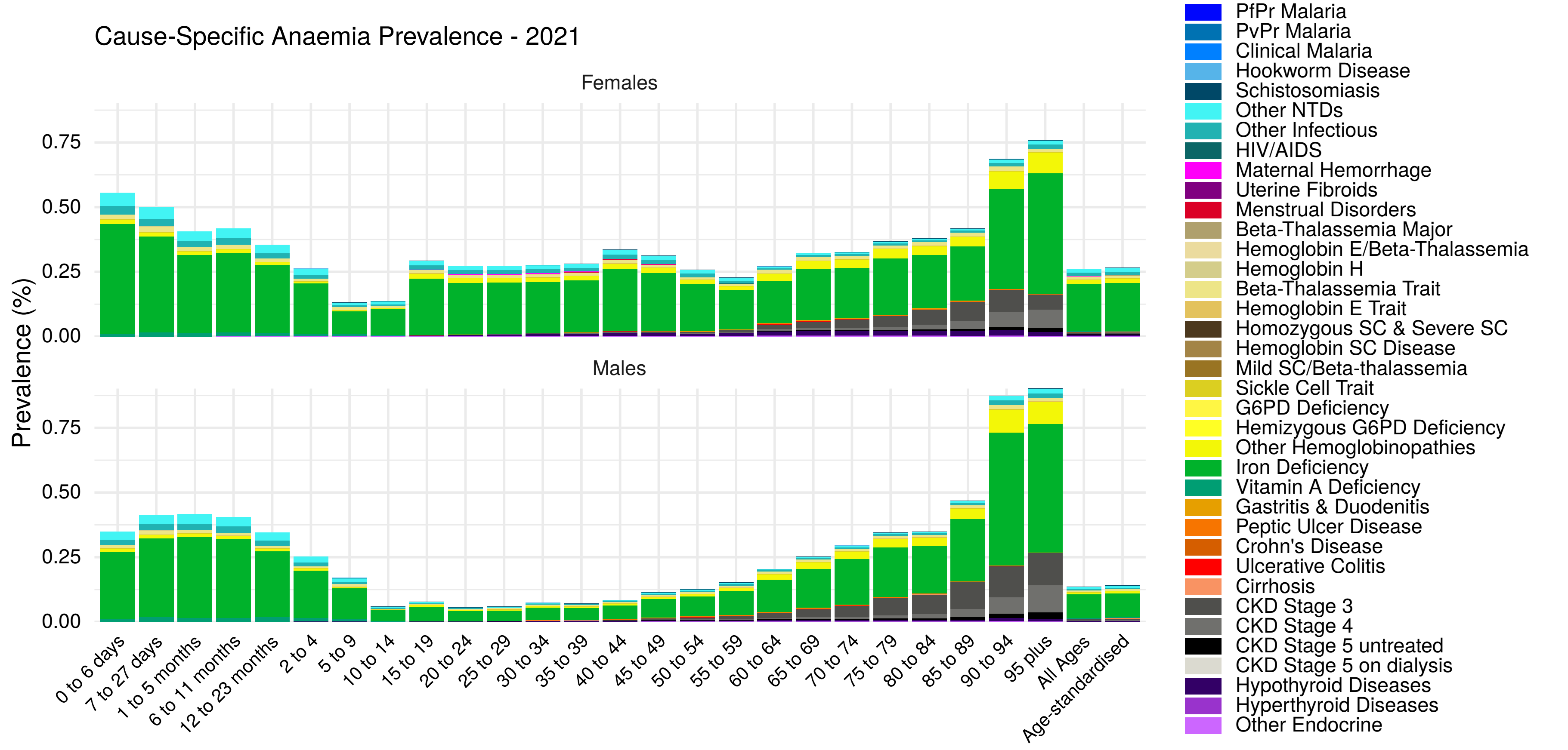

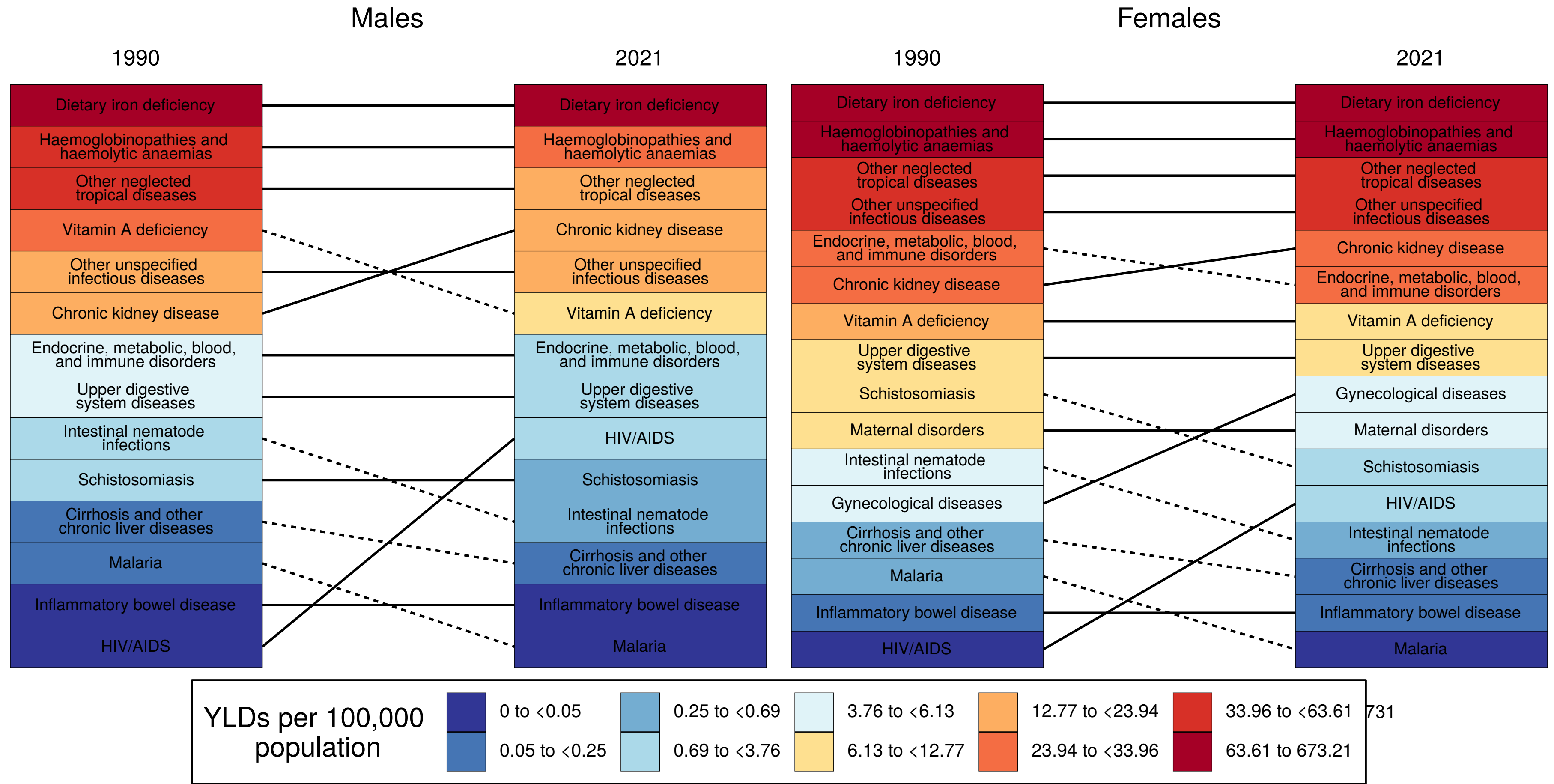

Misamis Oriental

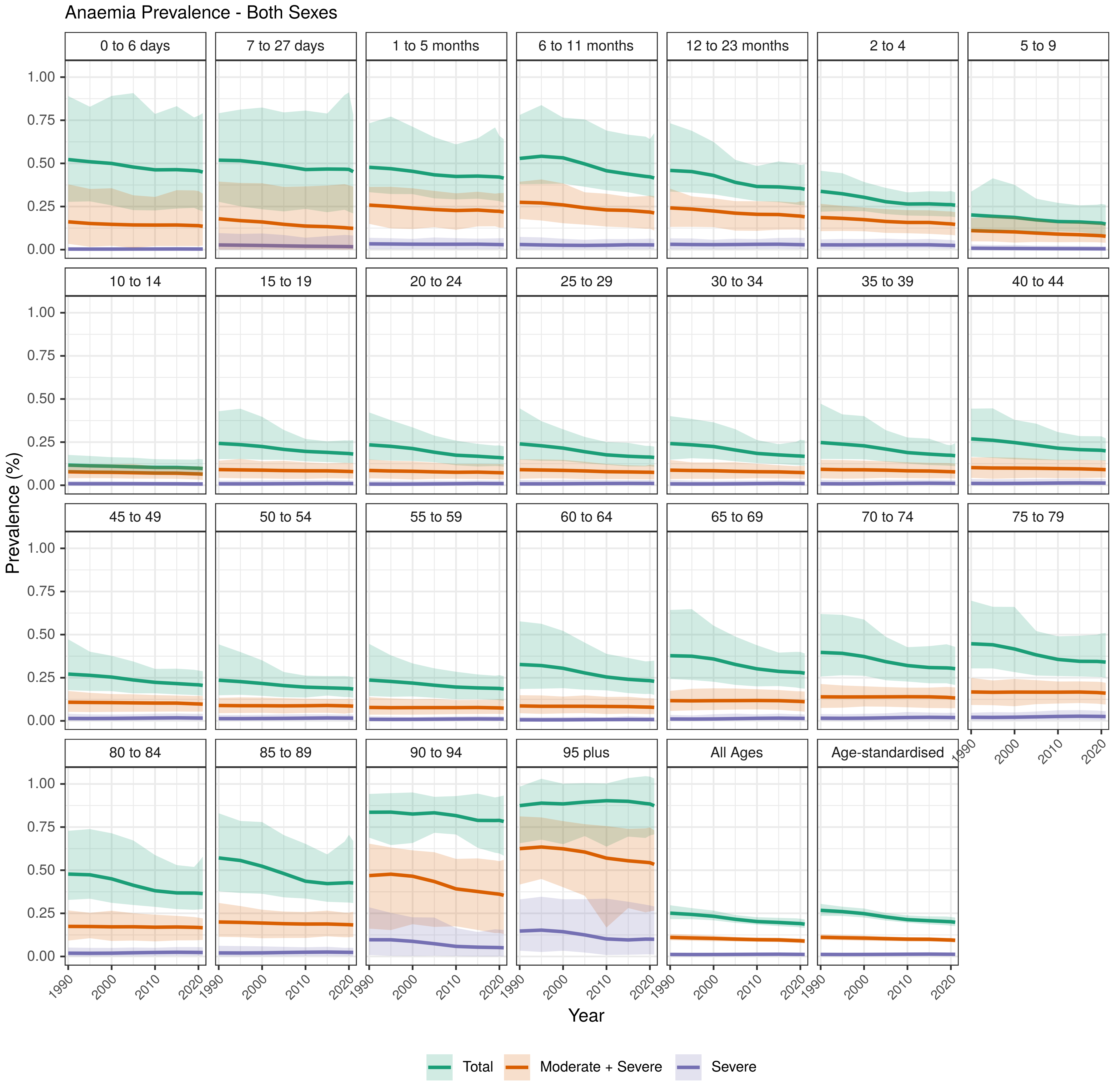

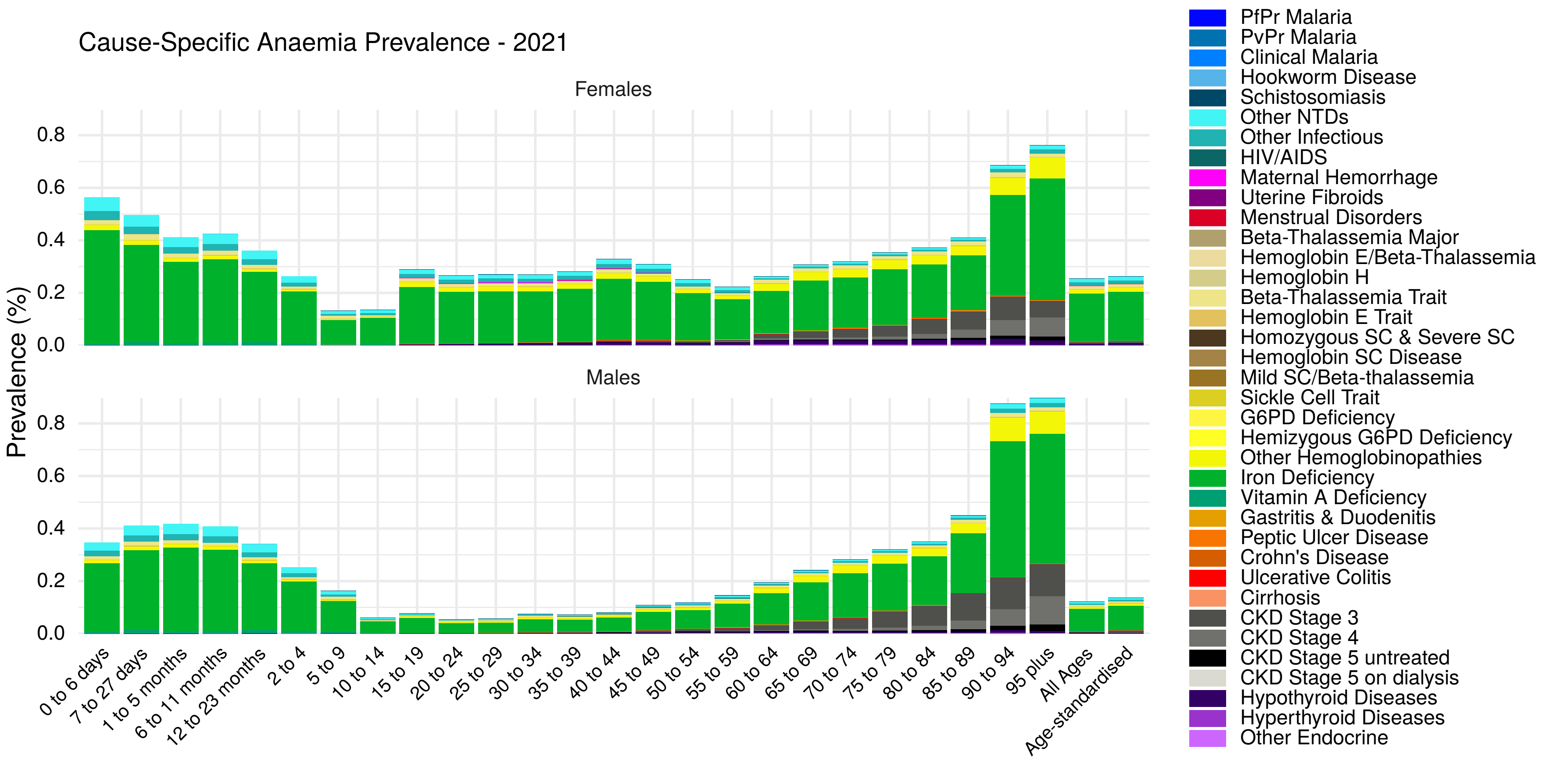

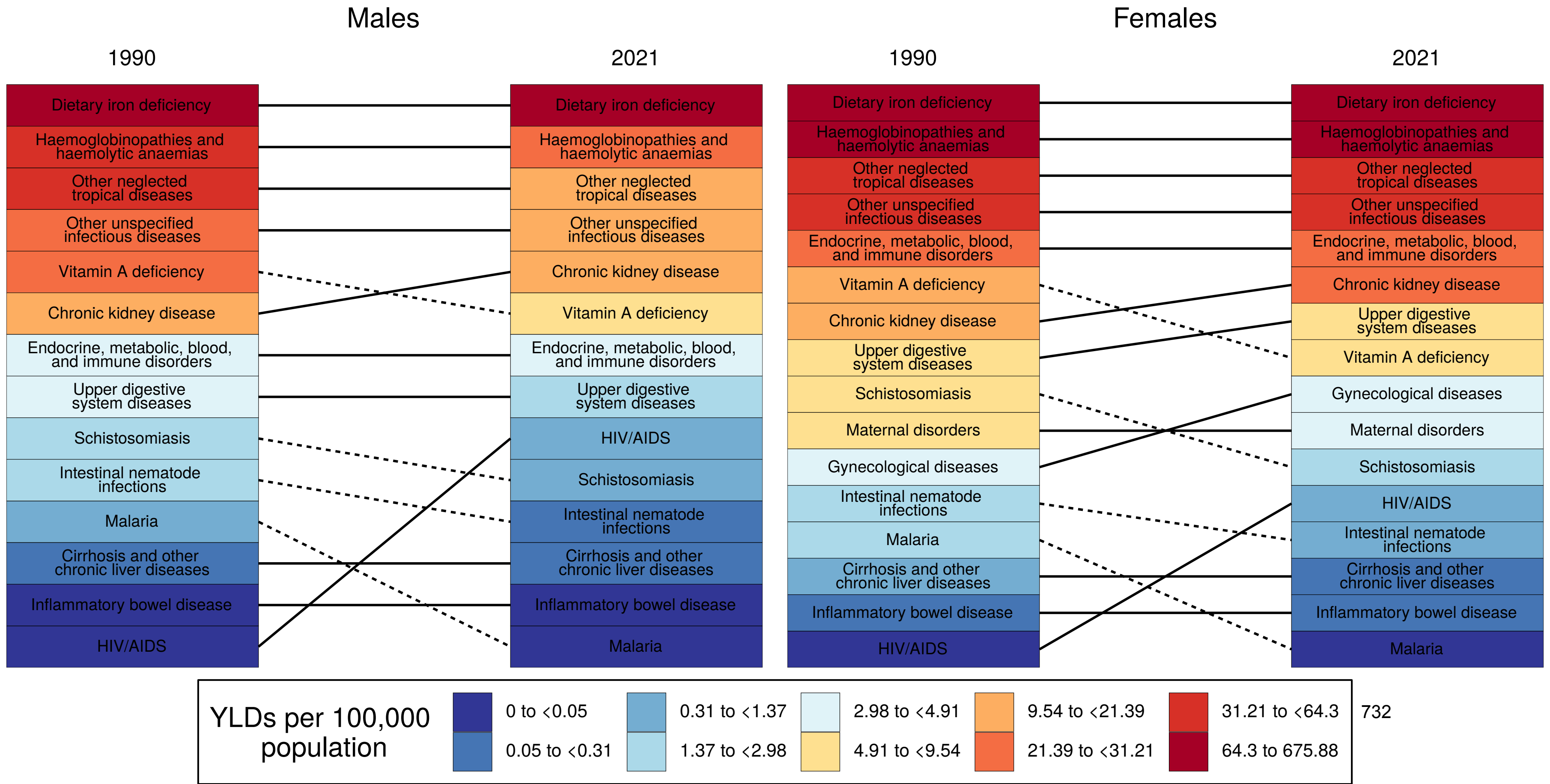

Mountain Province

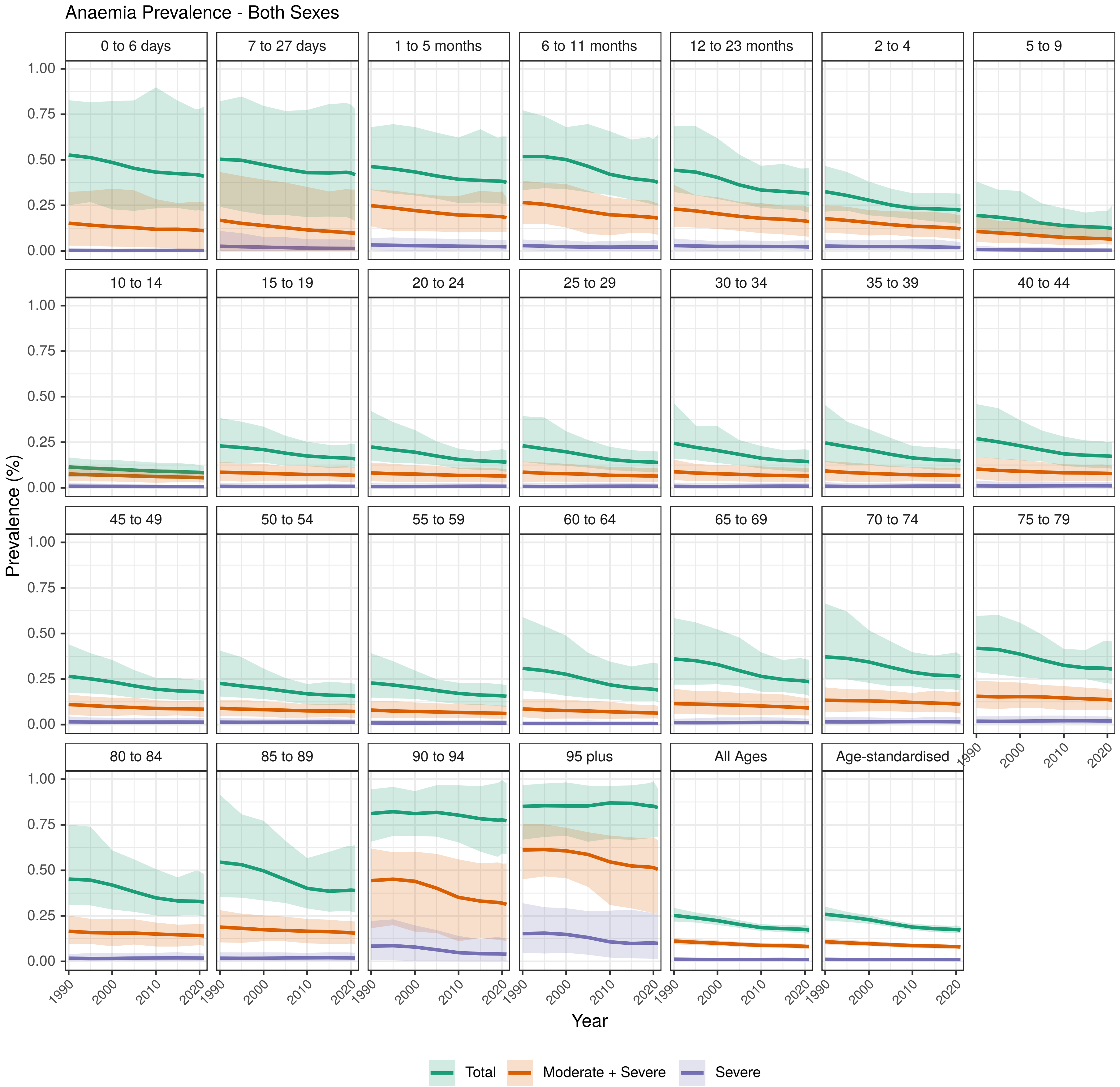

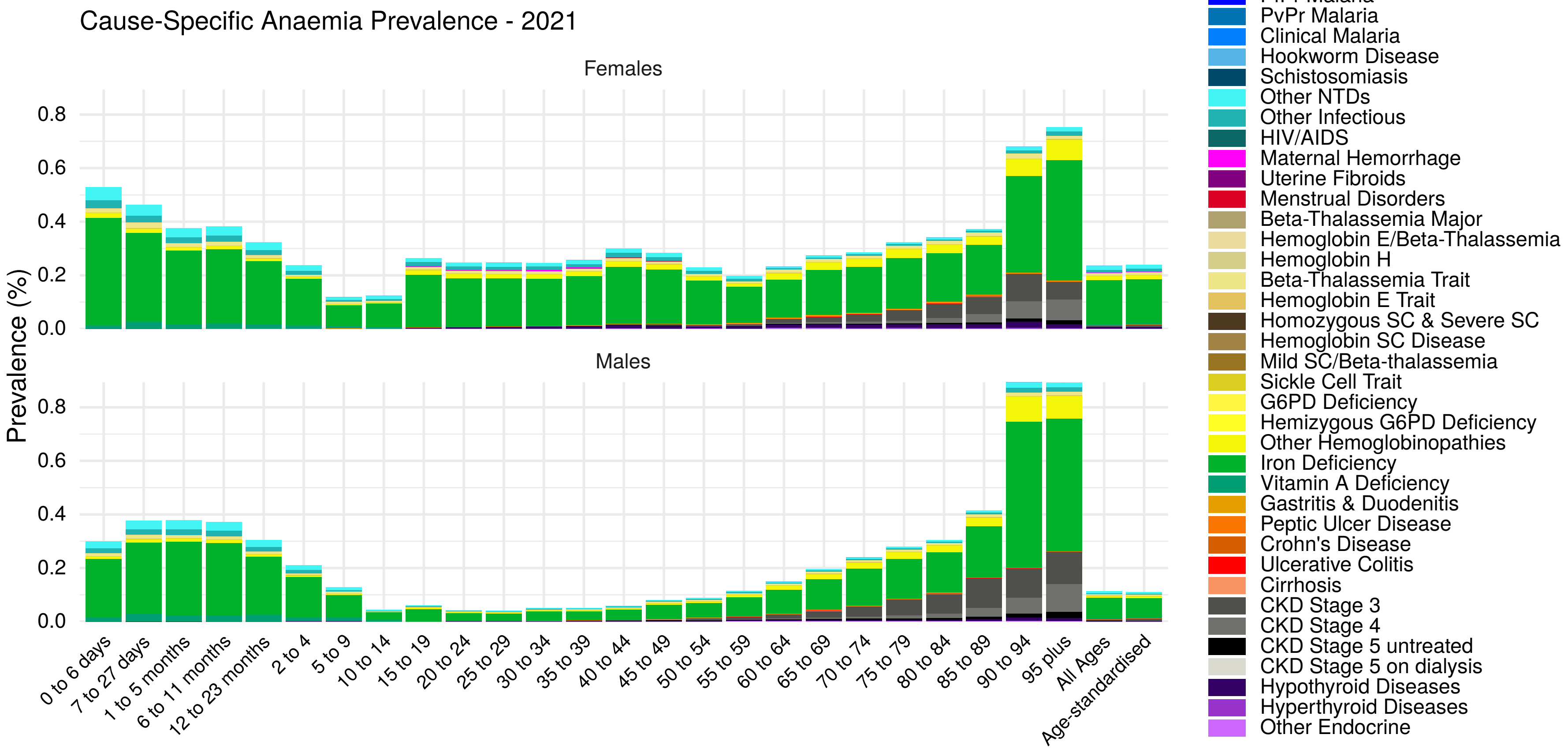

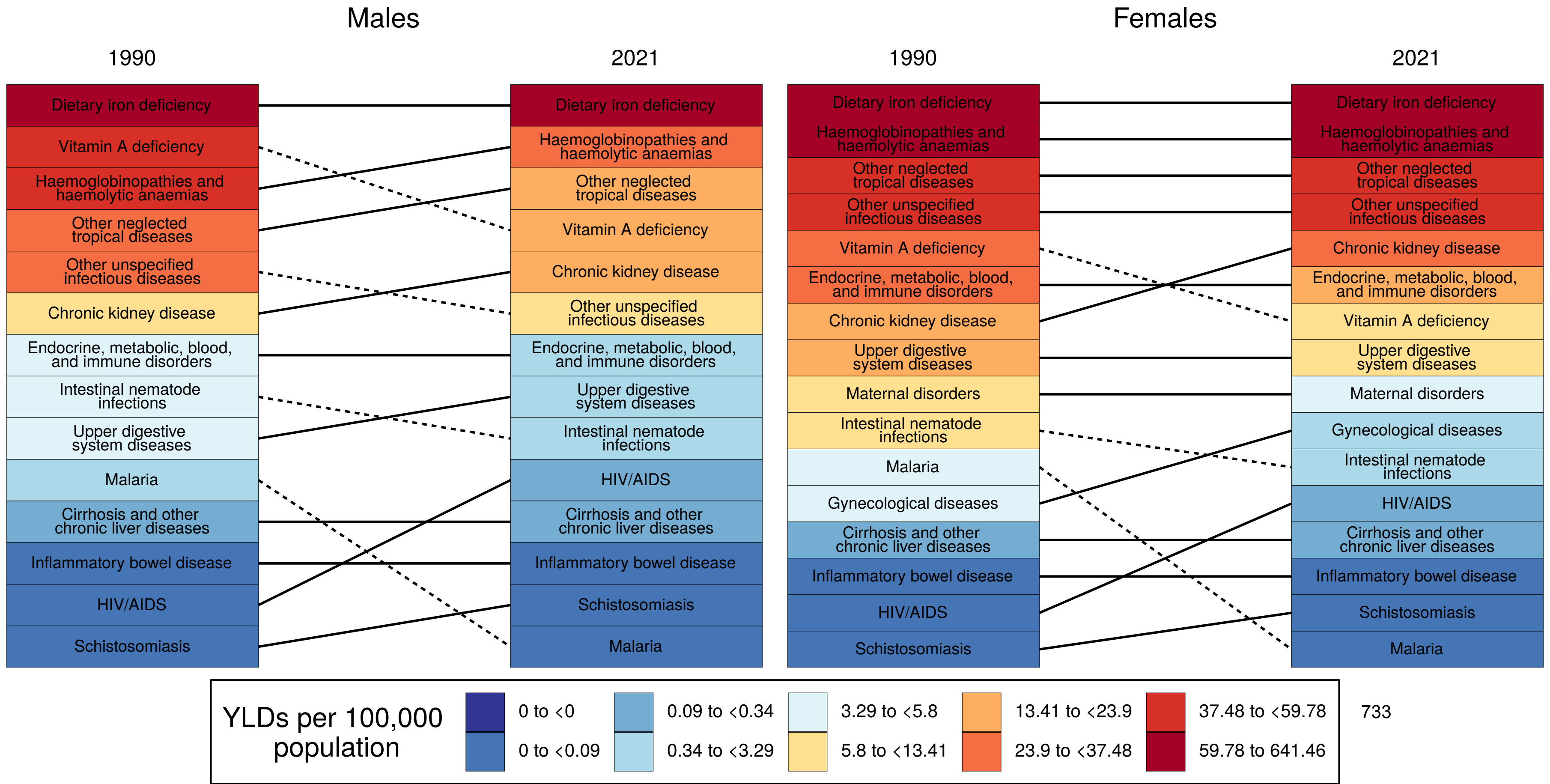

National Capital Region

Anaemia Prevalence - Both Sexes

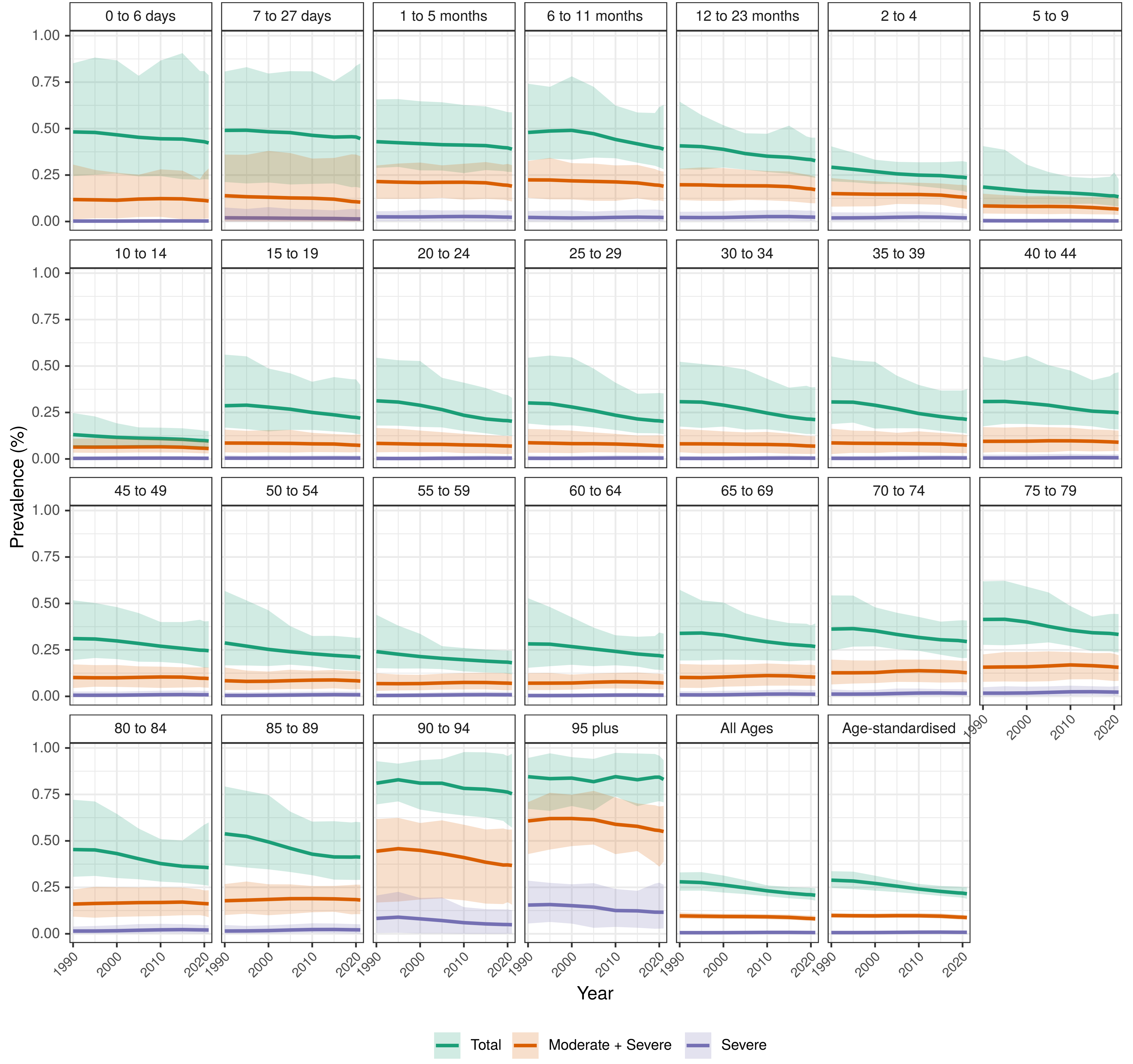

Cause-Specific Anaemia Prevalence - 2021

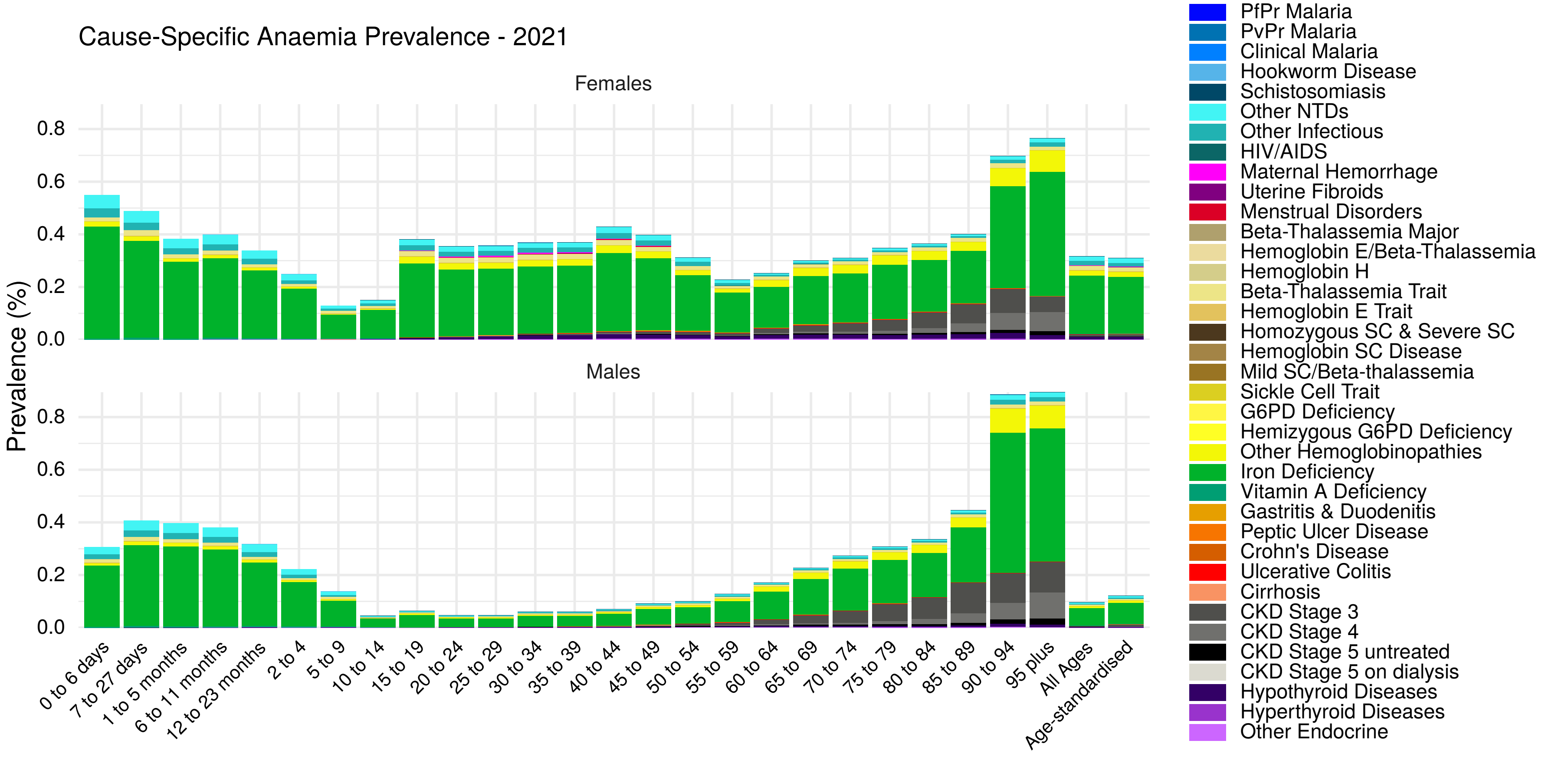

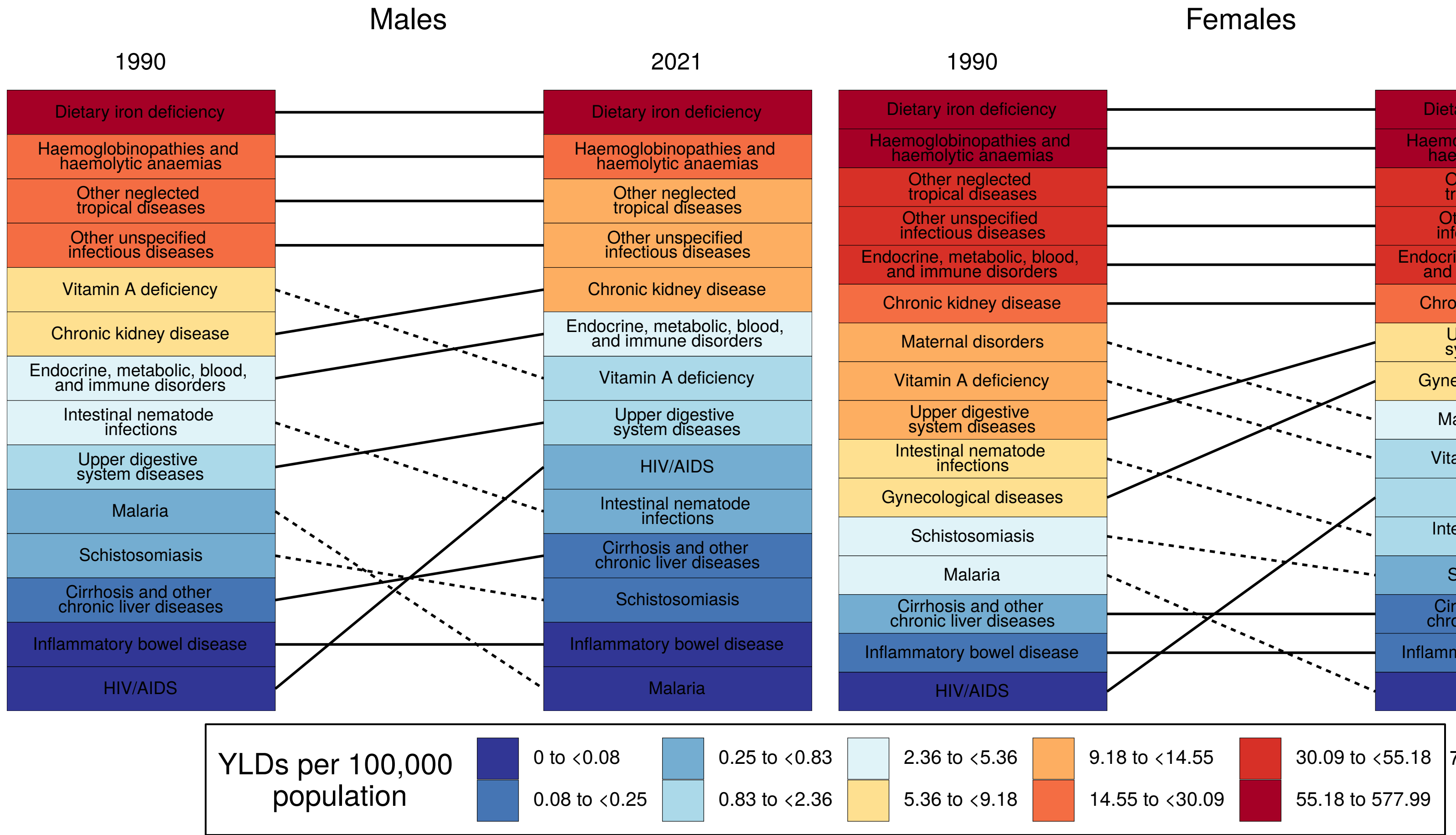

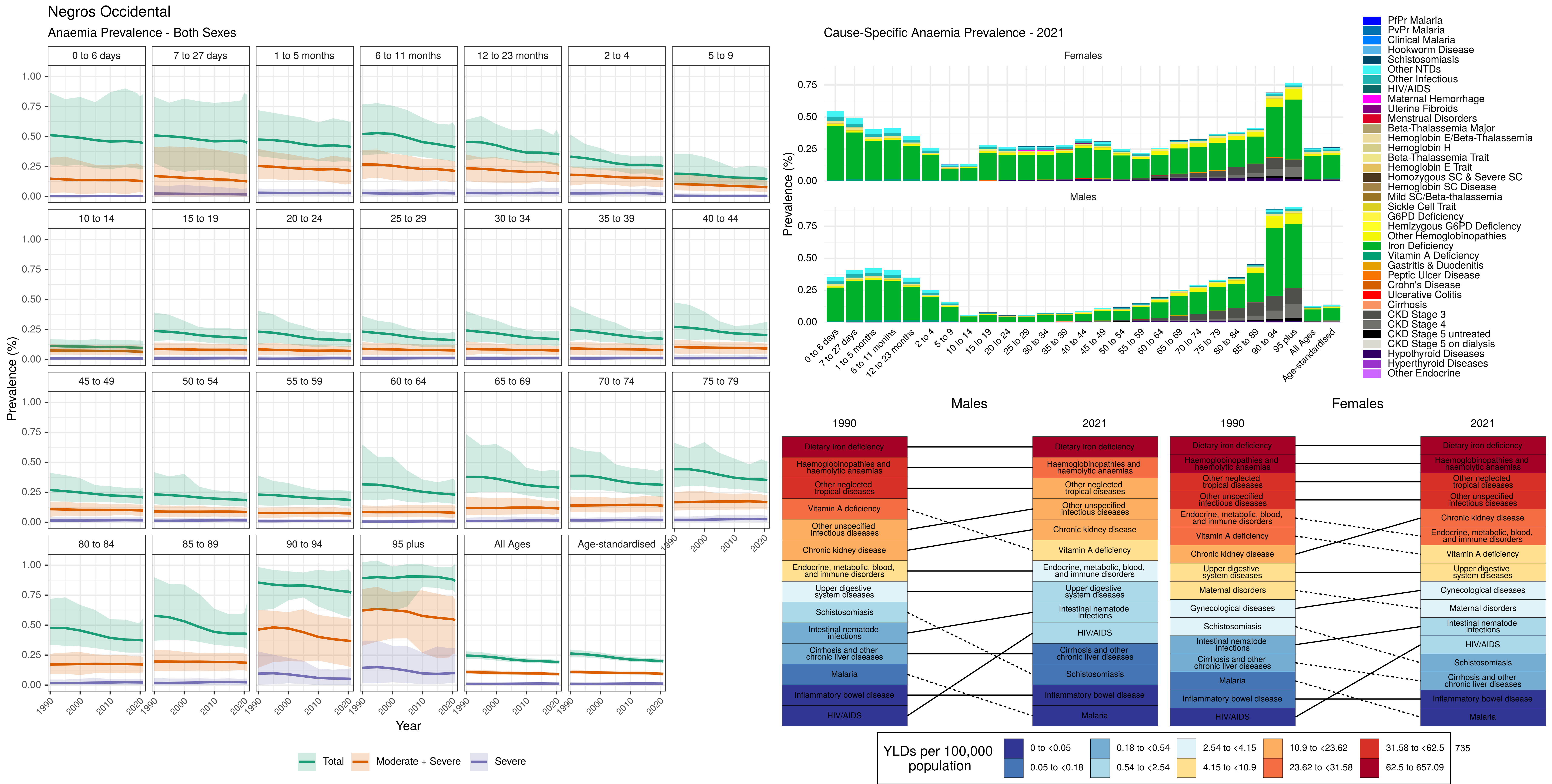
Negros Occidental
Anaemia Prevalence - Both Sexes
0 to 6 days
7 to 27 days
1 to 5 months
6 to 11 months
12 to 23 months
2 to 4
5 to 9
10 to 14
15 to 19
20 to 24
25 to 29
30 to 34
35 to 39
40 to 44
45 to 49
50 to 54
55 to 59
60 to 64
65 to 69
70 to 74
75 to 79
80 to 84
85 to 89
90 to 94
95 plus
All Ages
Age-standardised
Prevalence (%)
Year
Total
Moderate + Severe
Severe
Cause-Specific Anaemia Prevalence - 2021
Females
Males
PfPr Malaria
PvPr Malaria
Clinical Malaria
Hookworm Disease
Schistosomiasis
Other NTDs
Other Infectious
HIV/AIDS
Maternal Hemorrhage
Uterine Fibroids
Menstrual Disorders
Beta-Thalassemia Major
Hemoglobin E/Beta-Thalassemia
Hemoglobin H
Beta-Thalassemia Trait
Hemoglobin E Trait
Homozygous SC & Severe SC
Hemoglobin SC Disease
Mild SC/Beta-thalassemia
Sickle Cell Trait
G6PD Deficiency
Hemizygous G6PD Deficiency
Other Hemoglobinopathies
Iron Deficiency
Vitamin A Deficiency
Gastritis & Duodenitis
Peptic Ulcer Disease
Crohn's Disease
Ulcerative Colitis
Cirrhosis
CKD Stage 3
CKD Stage 4
CKD Stage 5 untreated
CKD Stage 5 on dialysis
Hypothyroid Diseases
Hyperthyroid Diseases
Other Endocrine
Males
Females
1990
2021
Dietary iron deficiency
Haemoglobinopathies and haemolytic anaemias
Other neglected tropical diseases
Vitamin A deficiency
Other unspecified infectious diseases
Chronic kidney disease
Endocrine, metabolic, blood, and immune disorders
Upper digestive system diseases
Schistosomiasis
Intestinal nematode infections
Cirrhosis and other chronic liver diseases
Malaria
Inflammatory bowel disease
HIV/AIDS
Maternal disorders
Gynecological diseases
YLDs per 100,000 population
0 to <0.05
0.05 to <0.18
0.18 to <0.54
0.54 to <2.54
2.54 to <4.15
4.15 to <10.9
10.9 to <23.62
23.62 to <31.58
31.58 to <62.5
62.5 to 657.09
735

Negros Oriental

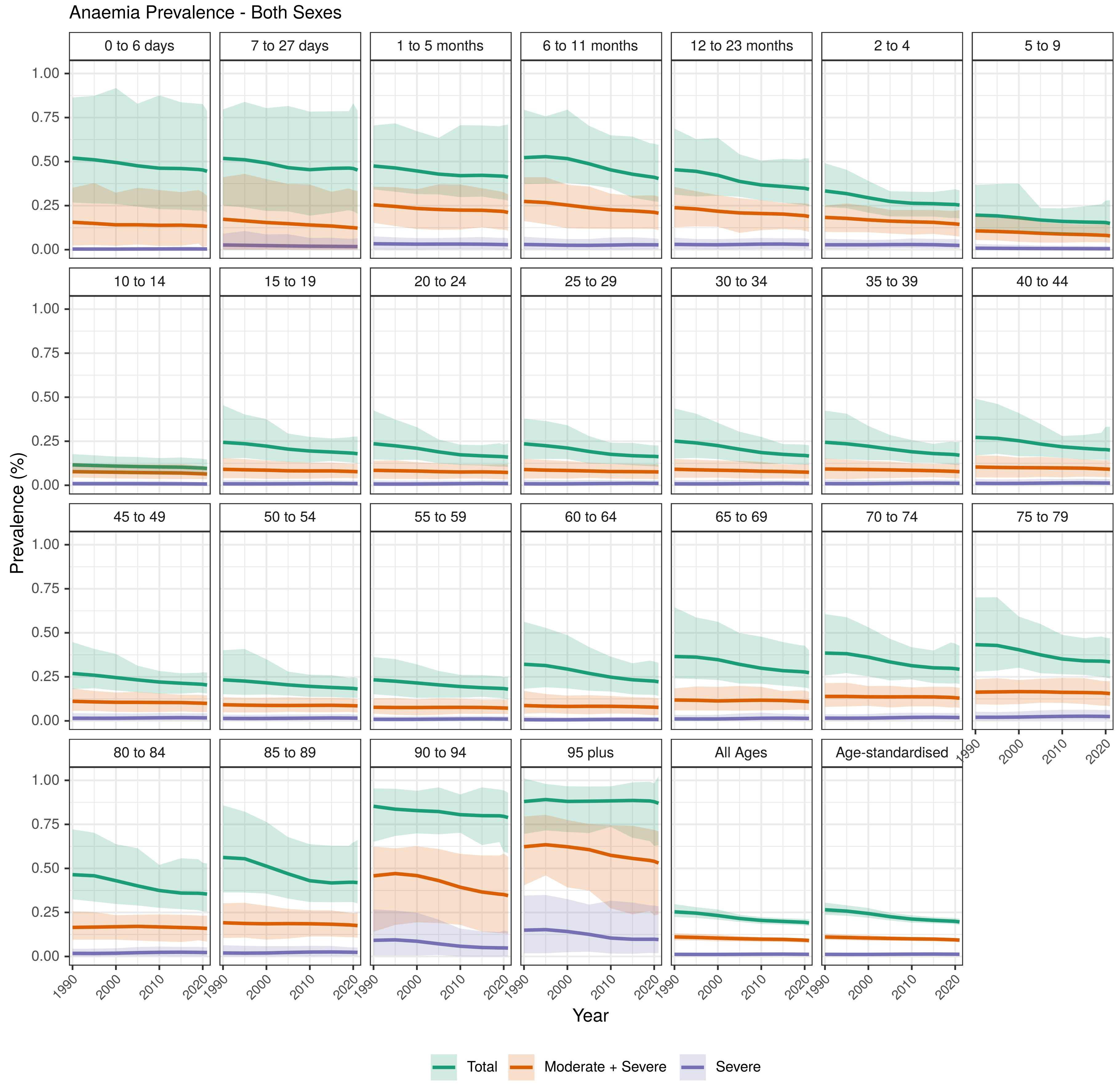

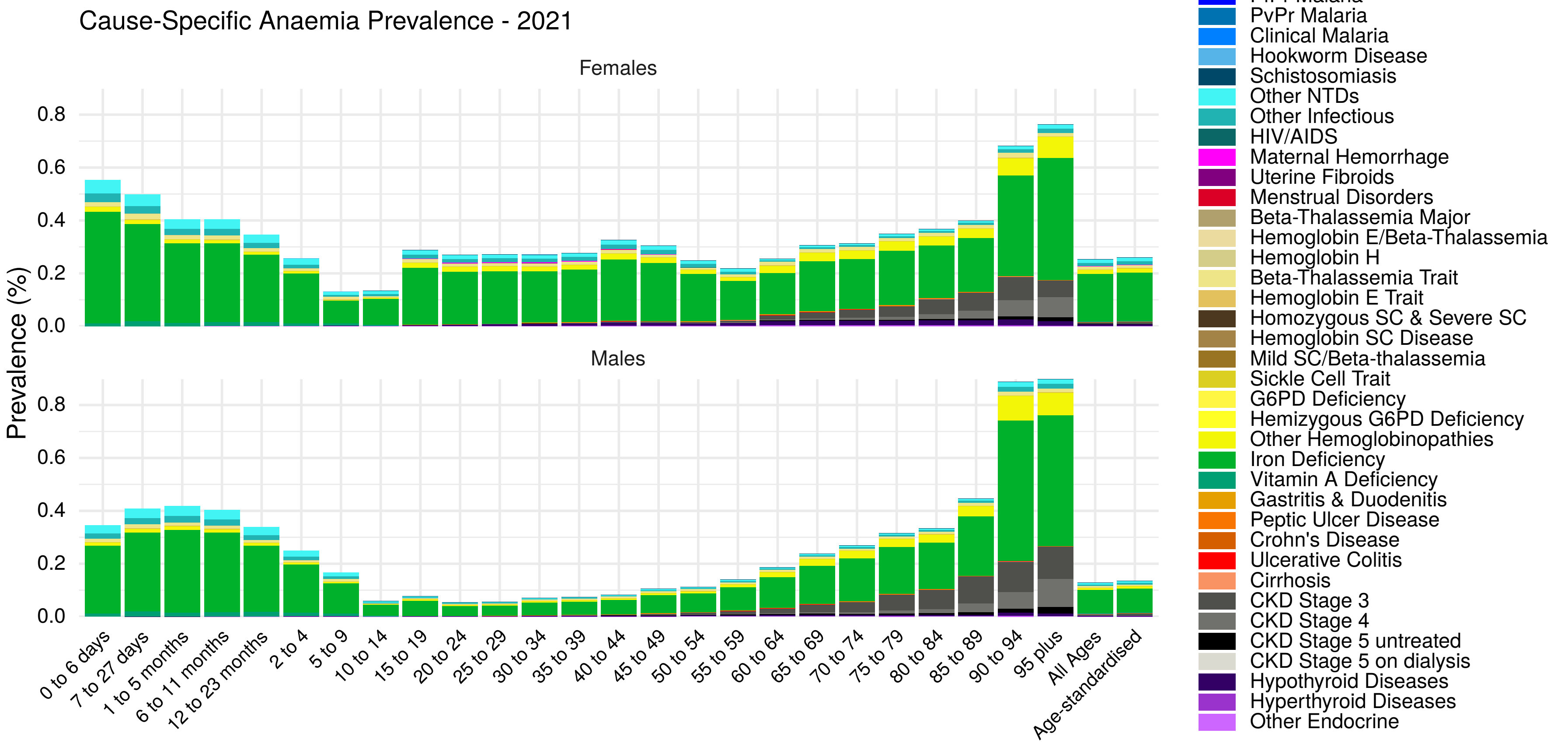

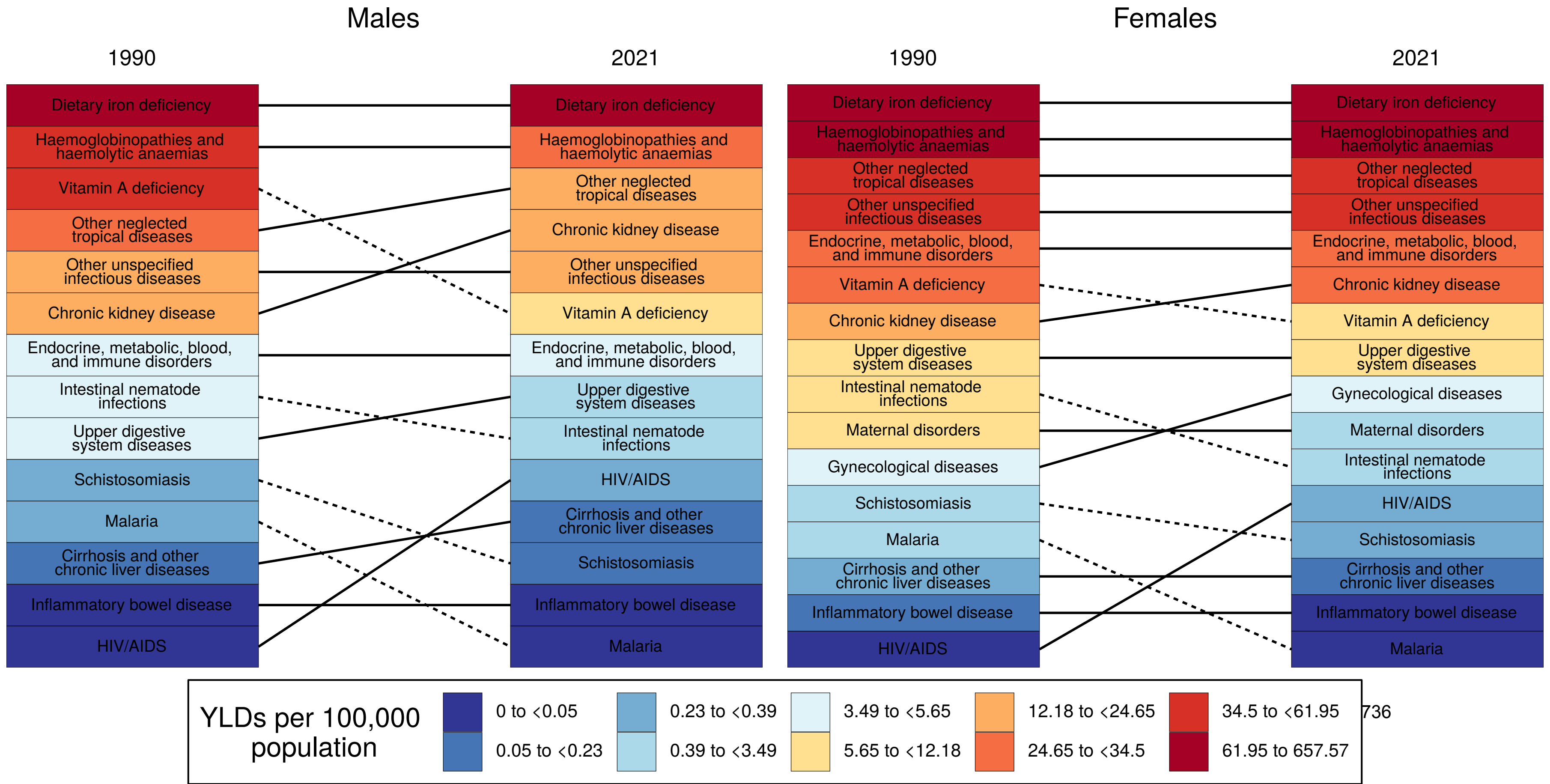

Northern Samar

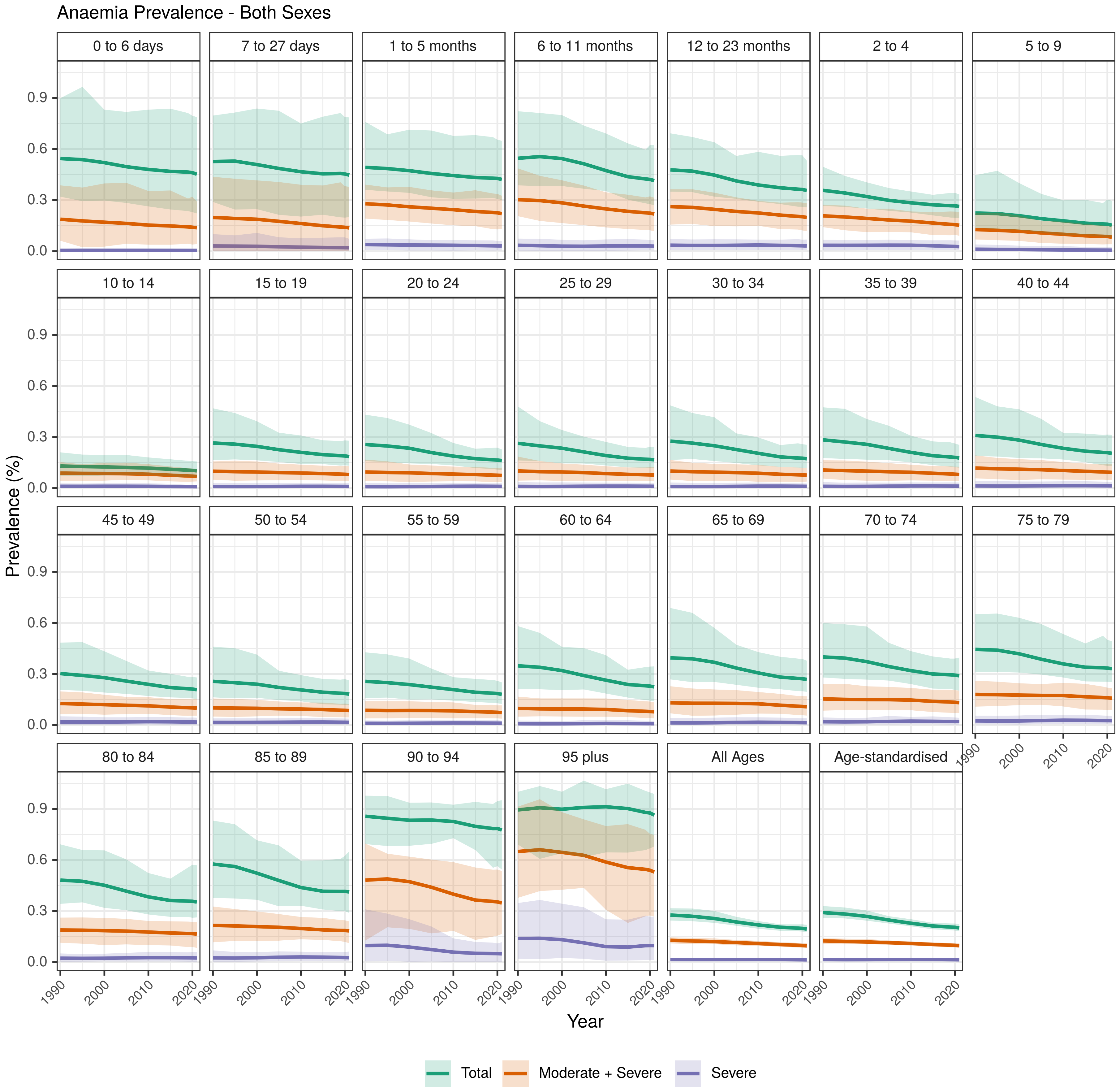

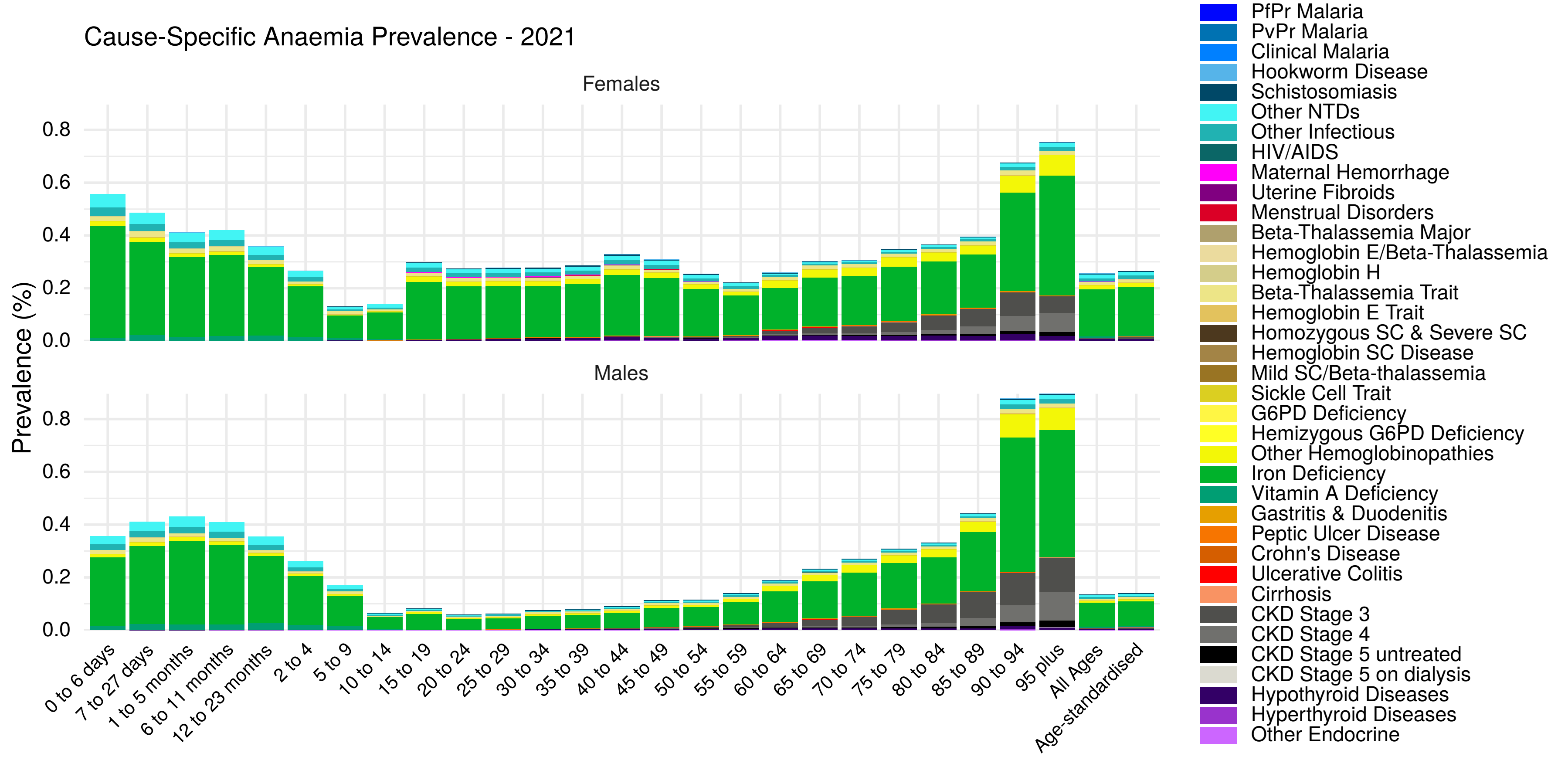

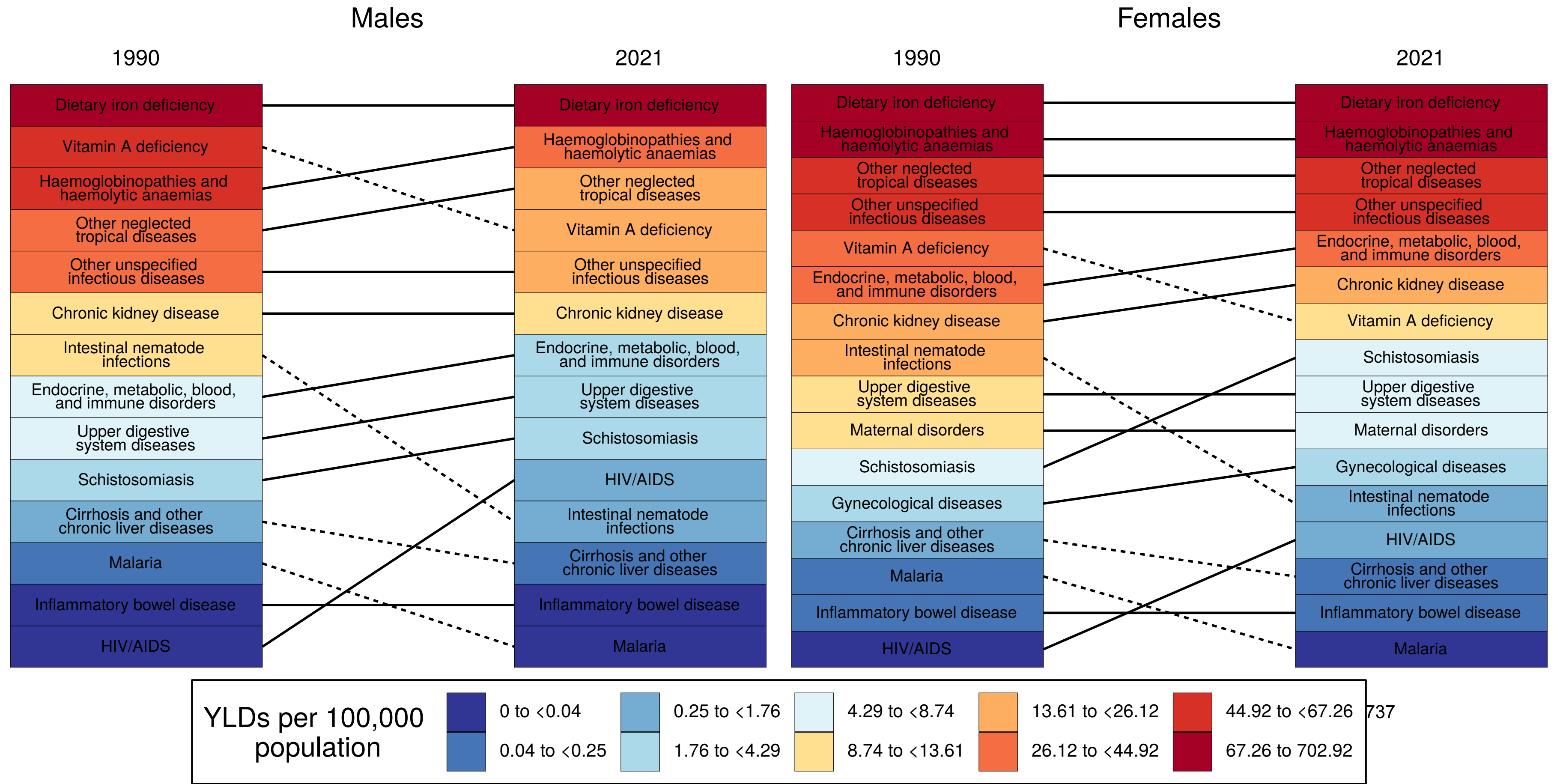

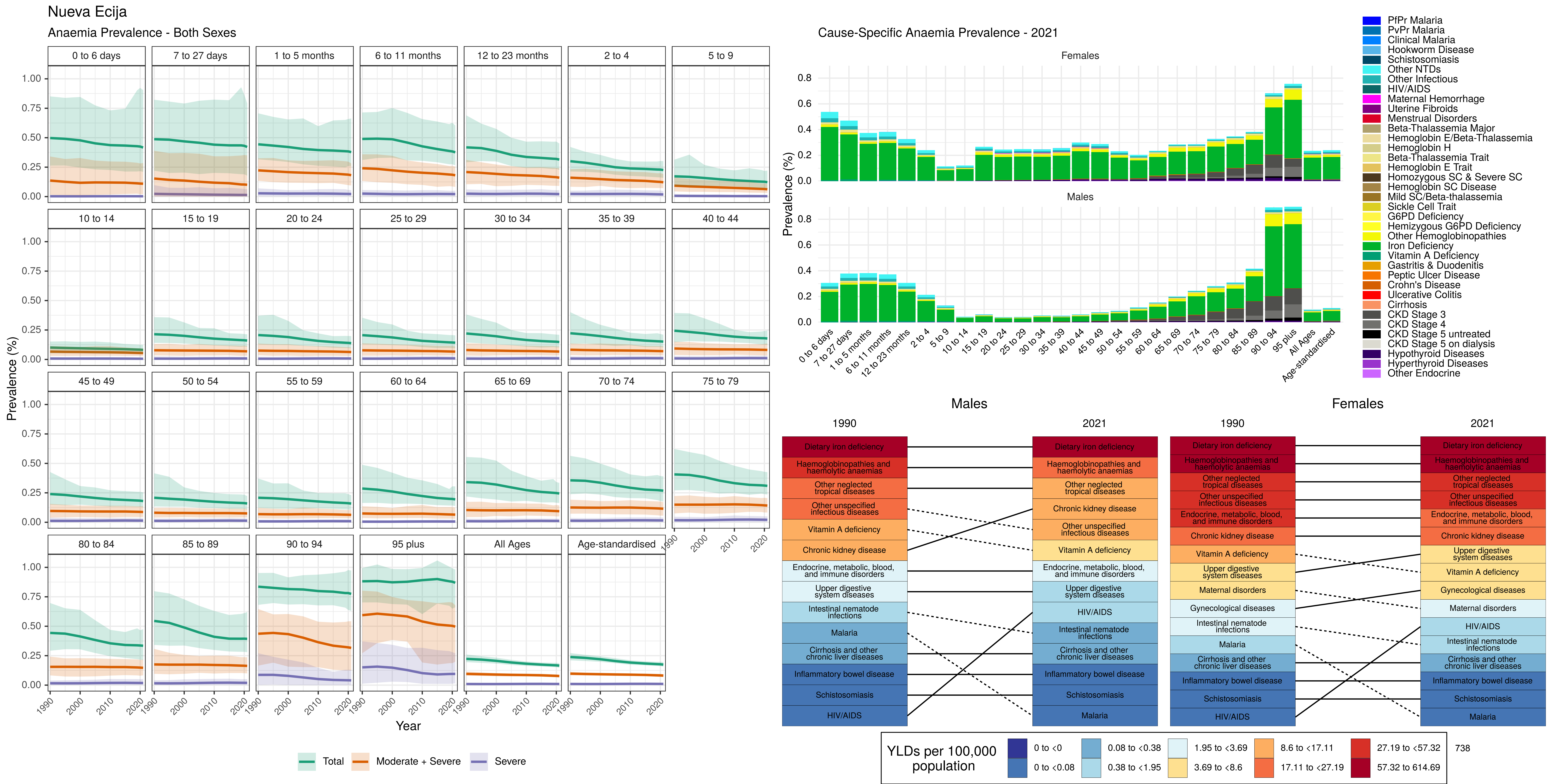

Nueva Ecija
Anaemia Prevalence - Both Sexes
0 to 6 days
7 to 27 days
1 to 5 months
6 to 11 months
12 to 23 months
2 to 4
5 to 9
10 to 14
15 to 19
20 to 24
25 to 29
30 to 34
35 to 39
40 to 44
45 to 49
50 to 54
55 to 59
60 to 64
65 to 69
70 to 74
75 to 79
80 to 84
85 to 89
90 to 94
95 plus
All Ages
Age-standardised
Prevalence (%)
Year
Total
Moderate + Severe
Severe
Cause-Specific Anaemia Prevalence - 2021
Females
Males
PfPr Malaria
PvPr Malaria
Clinical Malaria
Hookworm Disease
Schistosomiasis
Other NTDs
Other Infectious
HIV/AIDS
Maternal Hemorrhage
Uterine Fibroids
Menstrual Disorders
Beta-Thalassemia Major
Hemoglobin E/Beta-Thalassemia
Hemoglobin H
Beta-Thalassemia Trait
Hemoglobin E Trait
Homozygous SC & Severe SC
Hemoglobin SC Disease
Mild SC/Beta-thalassemia
Sickle Cell Trait
G6PD Deficiency
Hemizygous G6PD Deficiency
Other Hemoglobinopathies
Iron Deficiency
Vitamin A Deficiency
Gastritis & Duodenitis
Peptic Ulcer Disease
Crohn's Disease
Ulcerative Colitis
Cirrhosis
CKD Stage 3
CKD Stage 4
CKD Stage 5 untreated
CKD Stage 5 on dialysis
Hypothyroid Diseases
Hyperthyroid Diseases
Other Endocrine
Males
Females
1990
2021
Dietary iron deficiency
Haemoglobinopathies and haemolytic anaemias
Other neglected tropical diseases
Other unspecified infectious diseases
Vitamin A deficiency
Chronic kidney disease
Endocrine, metabolic, blood, and immune disorders
Upper digestive system diseases
Intestinal nematode infections
Malaria
Cirrhosis and other chronic liver diseases
Inflammatory bowel disease
Schistosomiasis
HIV/AIDS
Maternal disorders
Gynecological diseases
YLDs per 100,000 population
0 to <0
0 to <0.08
0.08 to <0.38
0.38 to <1.95
1.95 to <3.69
3.69 to <8.6
8.6 to <17.11
17.11 to <27.19
27.19 to <57.32
57.32 to 614.69

Nueva Vizcaya

Anaemia Prevalence - Both Sexes

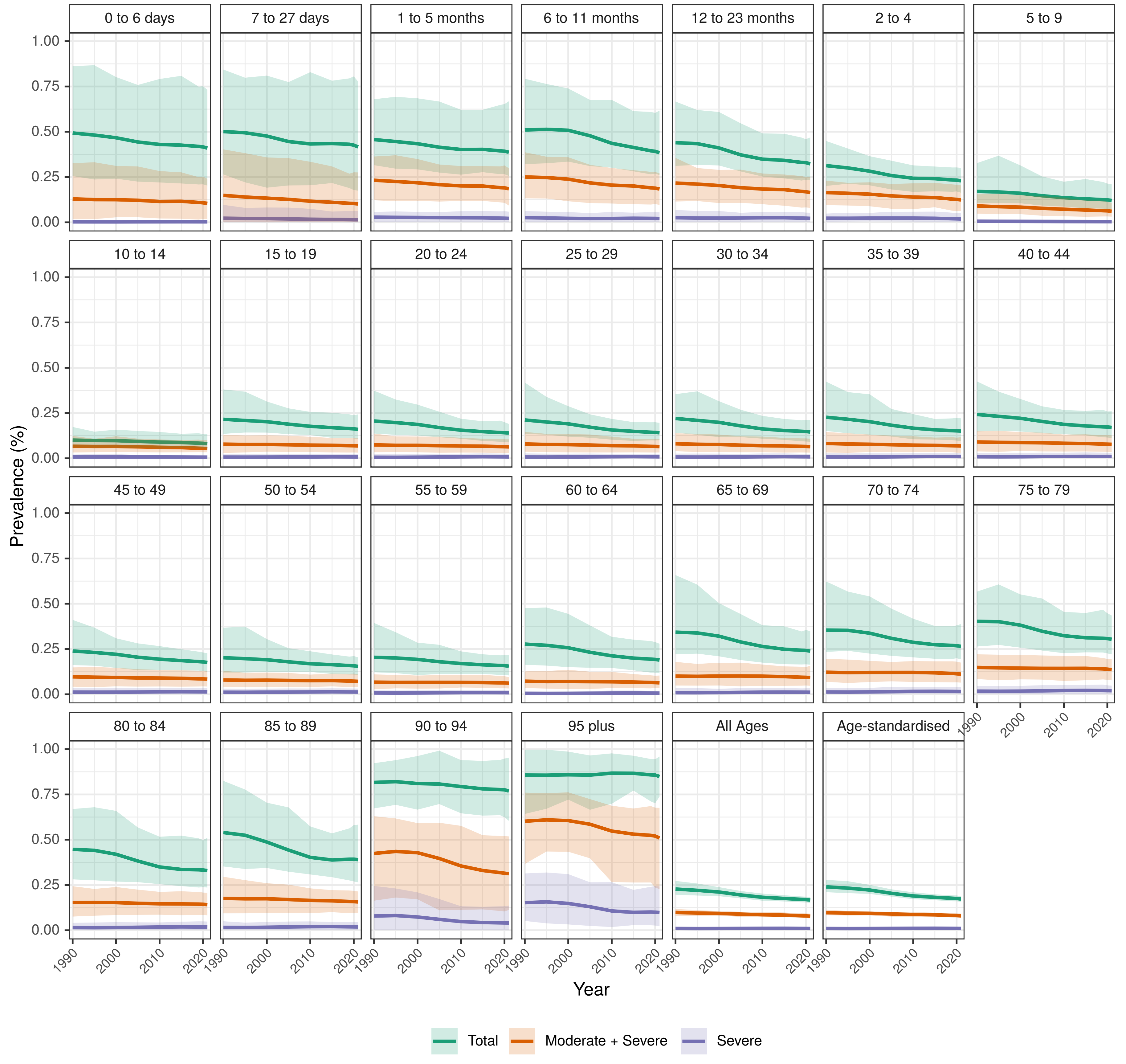

Cause-Specific Anaemia Prevalence - 2021

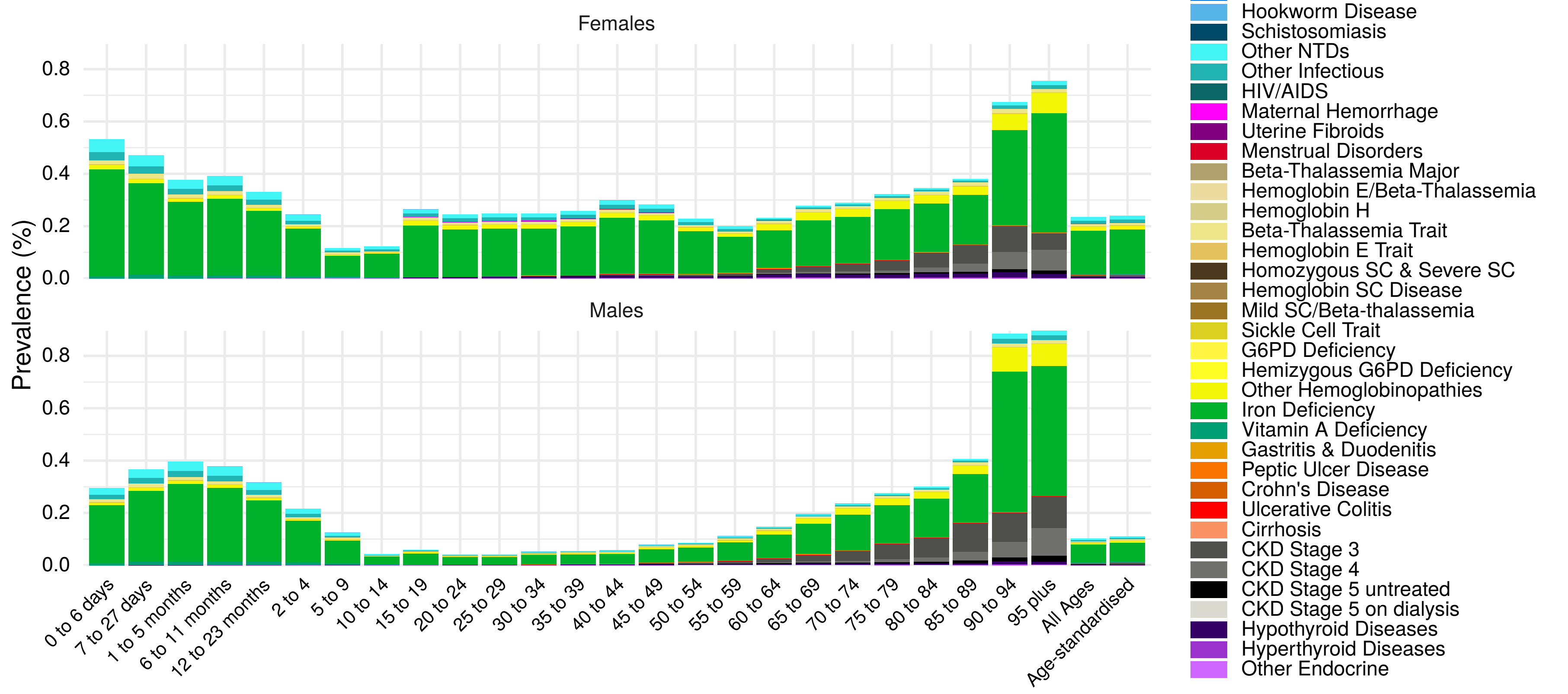

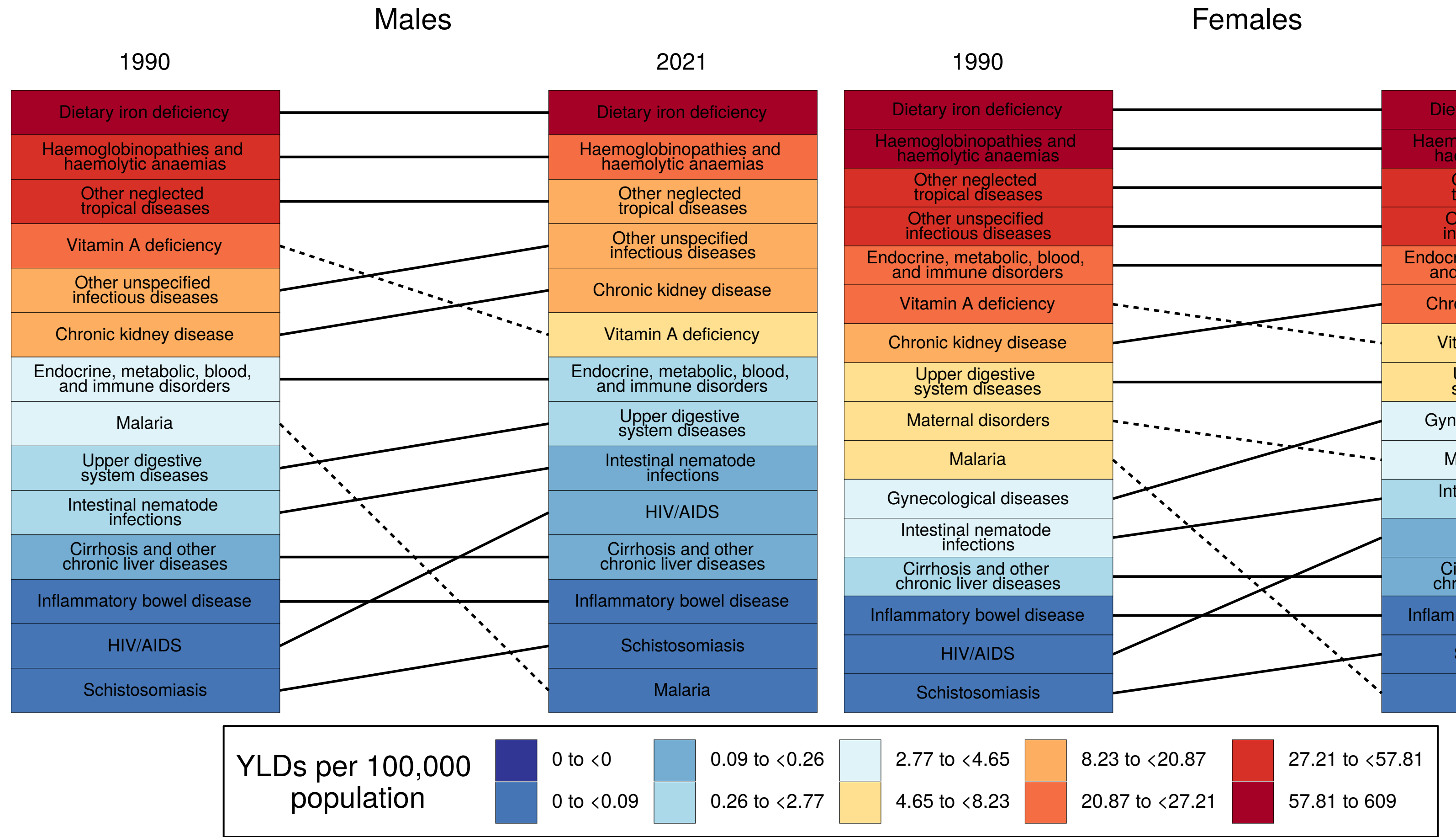

Occidental Mindoro

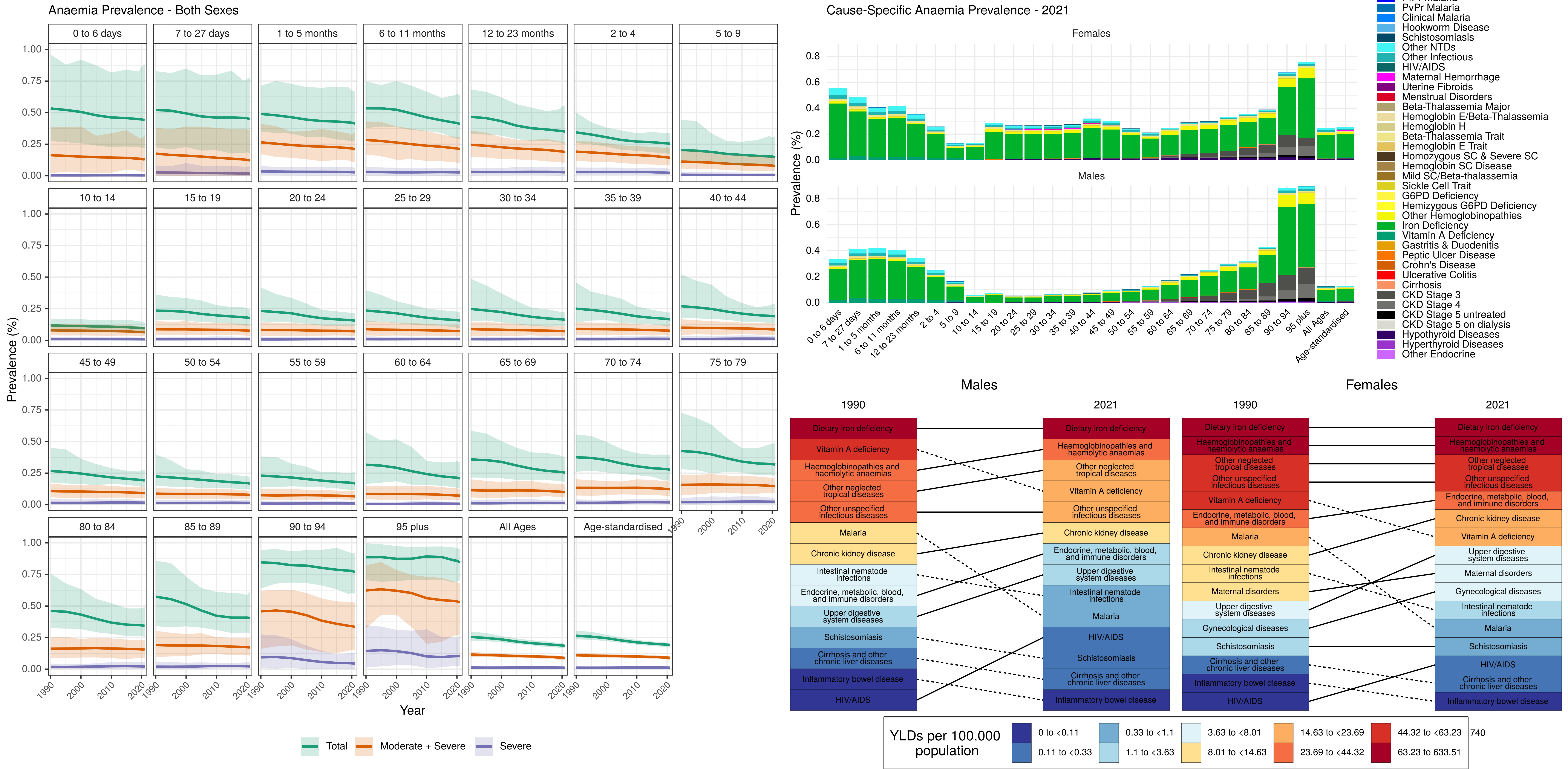

Oriental Mindoro

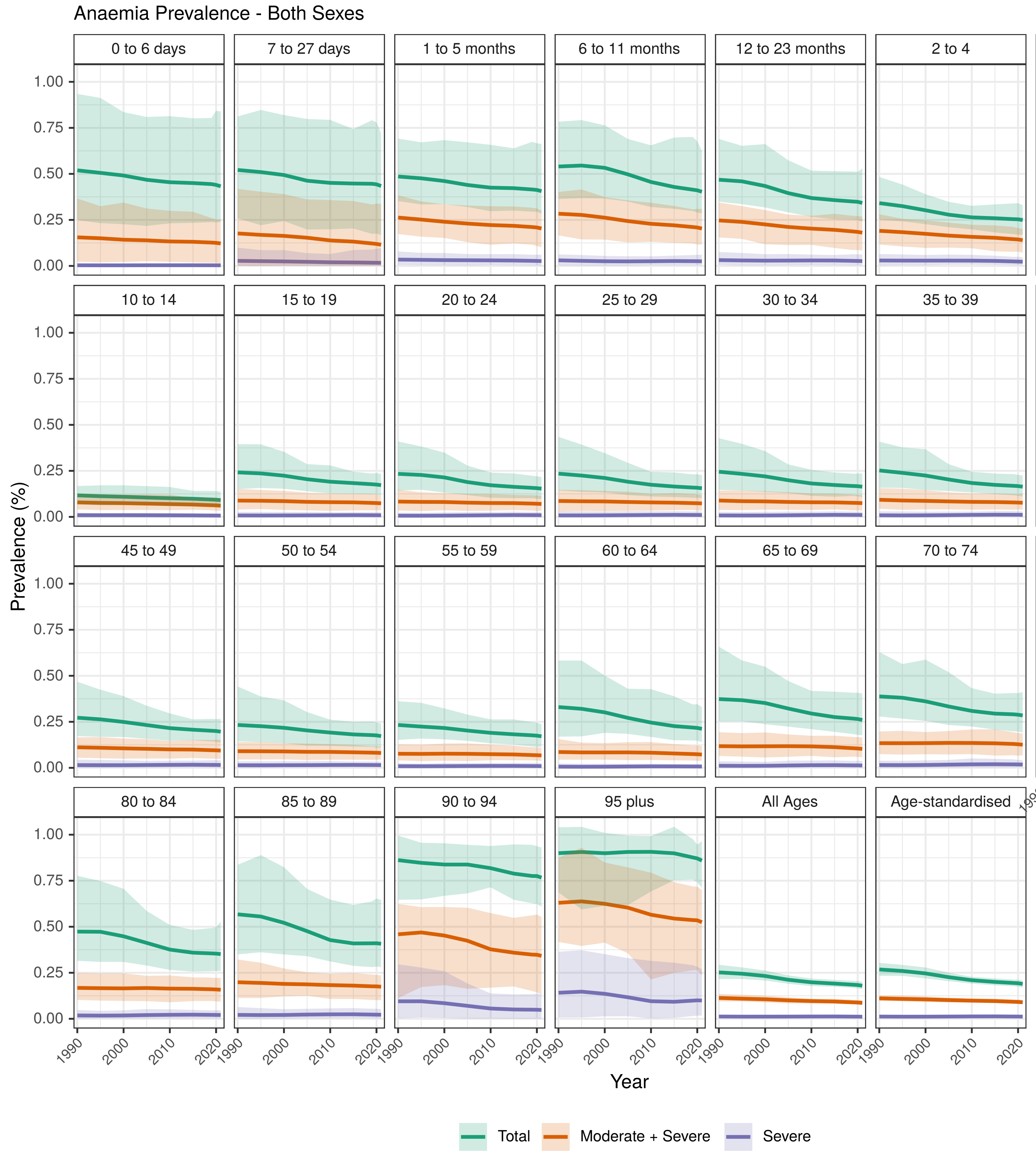

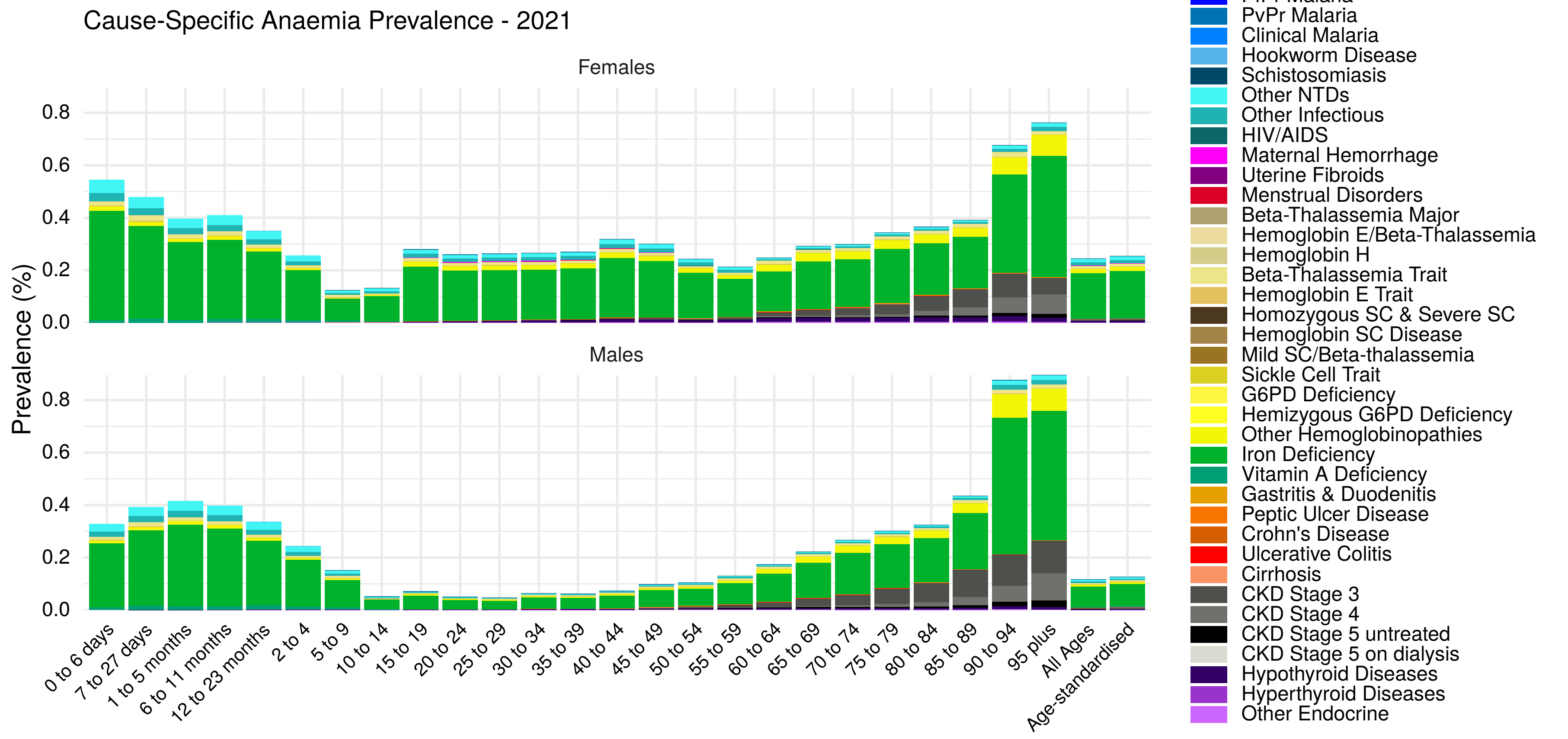

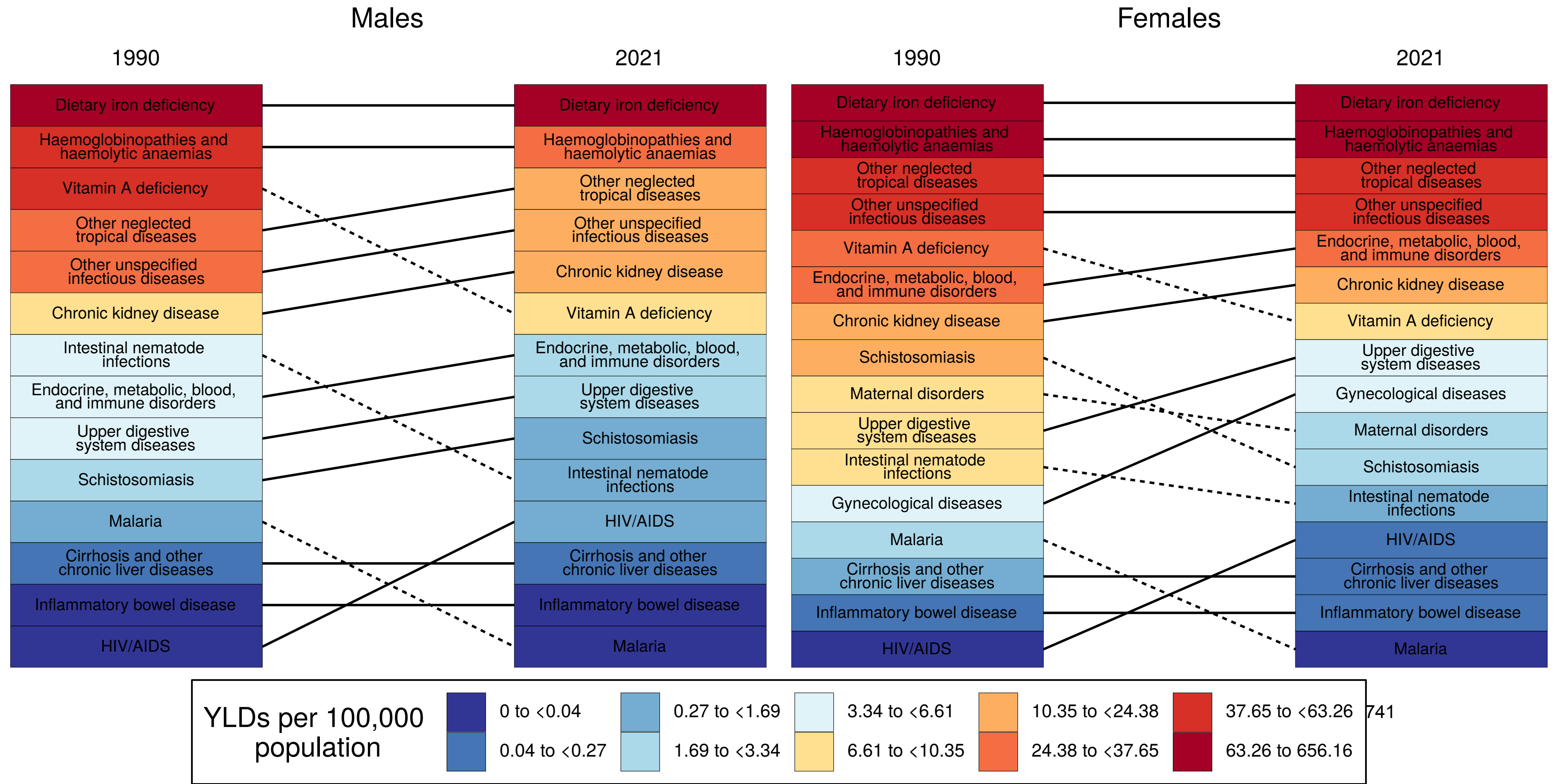

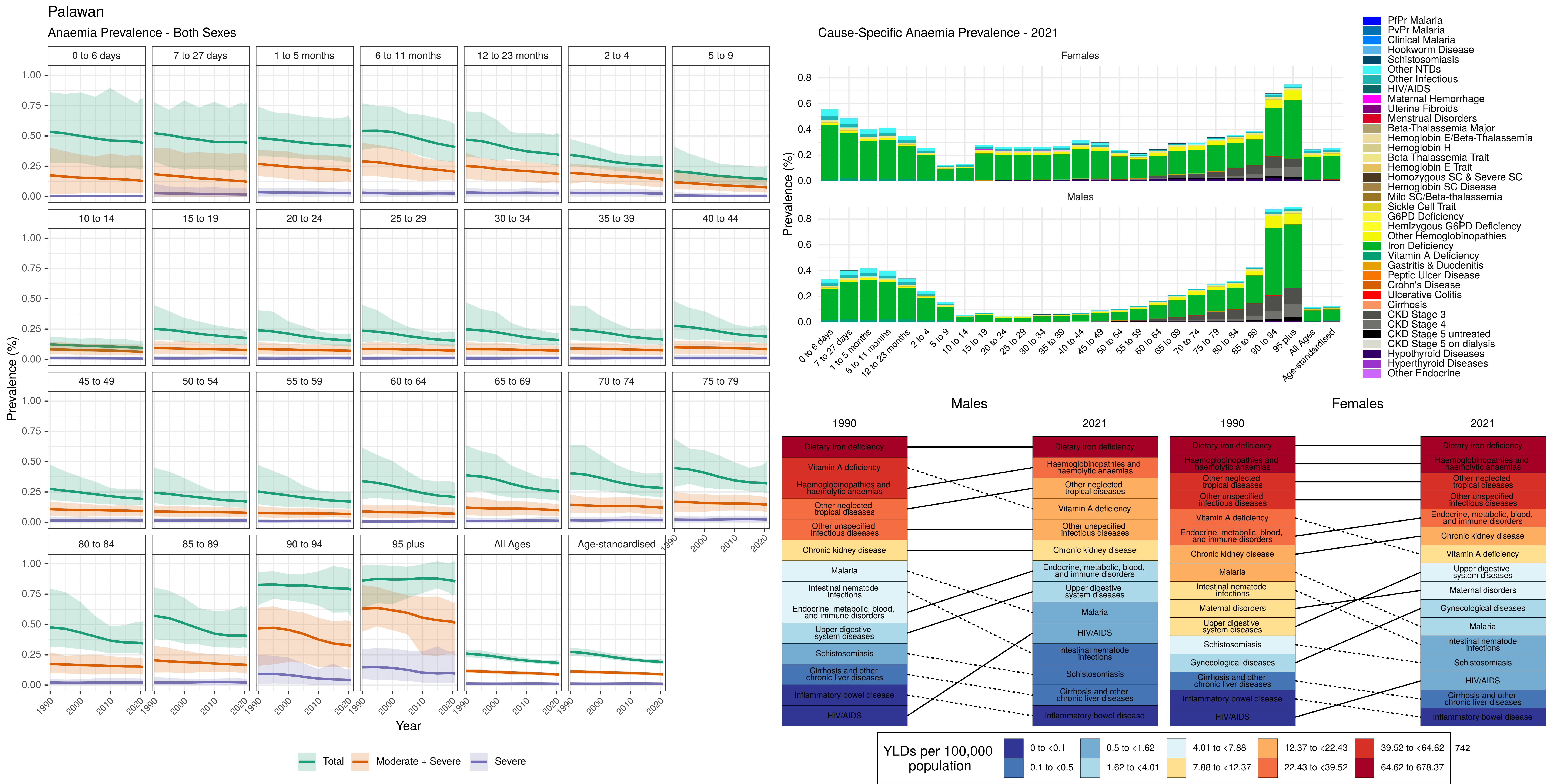
Palawan
Anaemia Prevalence - Both Sexes
0 to 6 days
7 to 27 days
1 to 5 months
6 to 11 months
12 to 23 months
2 to 4
5 to 9
10 to 14
15 to 19
20 to 24
25 to 29
30 to 34
35 to 39
40 to 44
45 to 49
50 to 54
55 to 59
60 to 64
65 to 69
70 to 74
75 to 79
80 to 84
85 to 89
90 to 94
95 plus
All Ages
Age-standardised
Prevalence (%)
Year
Total
Moderate + Severe
Severe
Cause-Specific Anaemia Prevalence - 2021
Females
Males
PfPr Malaria
PvPr Malaria
Clinical Malaria
Hookworm Disease
Schistosomiasis
Other NTDs
Other Infectious
HIV/AIDS
Maternal Hemorrhage
Uterine Fibroids
Menstrual Disorders
Beta-Thalassemia Major
Hemoglobin E/Beta-Thalassemia
Hemoglobin H
Beta-Thalassemia Trait
Hemoglobin E Trait
Homozygous SC & Severe SC
Hemoglobin SC Disease
Mild SC/Beta-thalassemia
Sickle Cell Trait
G6PD Deficiency
Hemizygous G6PD Deficiency
Other Hemoglobinopathies
Iron Deficiency
Vitamin A Deficiency
Gastritis & Duodenitis
Peptic Ulcer Disease
Crohn's Disease
Ulcerative Colitis
Cirrhosis
CKD Stage 3
CKD Stage 4
CKD Stage 5 untreated
CKD Stage 5 on dialysis
Hypothyroid Diseases
Hyperthyroid Diseases
Other Endocrine
Males
Females
1990
2021
Dietary iron deficiency
Vitamin A deficiency
Haemoglobinopathies and haemolytic anaemias
Other neglected tropical diseases
Other unspecified infectious diseases
Chronic kidney disease
Malaria
Intestinal nematode infections
Endocrine, metabolic, blood, and immune disorders
Upper digestive system diseases
Schistosomiasis
Cirrhosis and other chronic liver diseases
Inflammatory bowel disease
HIV/AIDS
Maternal disorders
Gynecological diseases
YLDs per 100,000 population
0 to <0.1
0.1 to <0.5
0.5 to <1.62
1.62 to <4.01
4.01 to <7.88
7.88 to <12.37
12.37 to <22.43
22.43 to <39.52
39.52 to <64.62
64.62 to 678.37

742

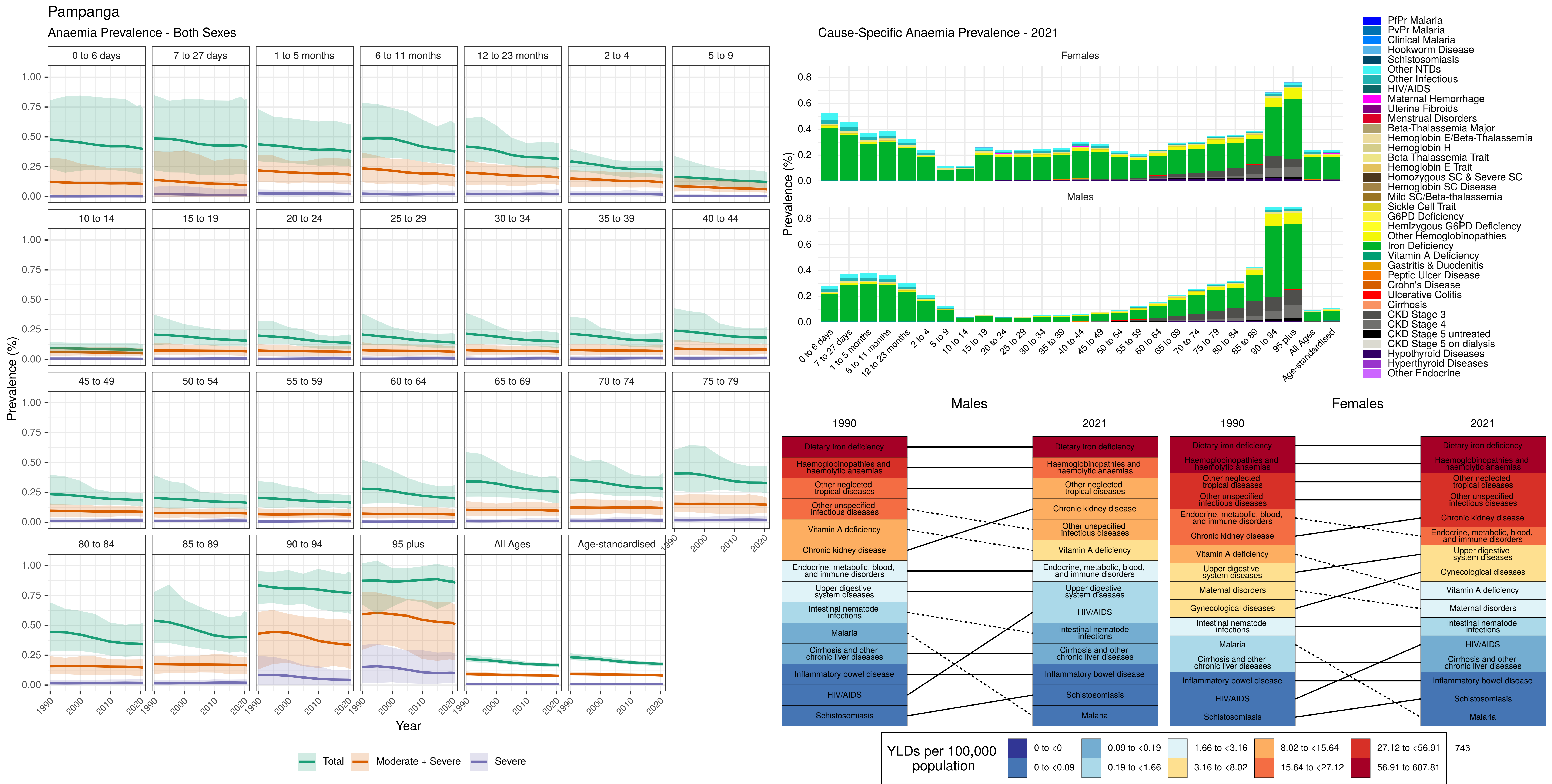

Pampanga
Anaemia Prevalence - Both Sexes
0 to 6 days
7 to 27 days
1 to 5 months
6 to 11 months
12 to 23 months
2 to 4
5 to 9
10 to 14
15 to 19
20 to 24
25 to 29
30 to 34
35 to 39
40 to 44
45 to 49
50 to 54
55 to 59
60 to 64
65 to 69
70 to 74
75 to 79
80 to 84
85 to 89
90 to 94
95 plus
All Ages
Age-standardised
Prevalence (%)
Year
Total
Moderate + Severe
Severe
Cause-Specific Anaemia Prevalence - 2021
Females
Males
Prevalence (%)
PfPr Malaria
PvPr Malaria
Clinical Malaria
Hookworm Disease
Schistosomiasis
Other NTDs
Other Infectious
HIV/AIDS
Maternal Hemorrhage
Uterine Fibroids
Menstrual Disorders
Beta-Thalassemia Major
Hemoglobin E/Beta-Thalassemia
Hemoglobin H
Beta-Thalassemia Trait
Hemoglobin E Trait
Homozygous SC & Severe SC
Hemoglobin SC Disease
Mild SC/Beta-thalassemia
Sickle Cell Trait
G6PD Deficiency
Hemizygous G6PD Deficiency
Other Hemoglobinopathies
Iron Deficiency
Vitamin A Deficiency
Gastritis & Duodenitis
Peptic Ulcer Disease
Crohn's Disease
Ulcerative Colitis
Cirrhosis
CKD Stage 3
CKD Stage 4
CKD Stage 5 untreated
CKD Stage 5 on dialysis
Hypothyroid Diseases
Hyperthyroid Diseases
Other Endocrine
Males
Females
1990
2021
Males 1990: Dietary iron deficiency; Haemoglobinopathies and haemolytic anaemias; Other neglected tropical diseases; Other unspecified infectious diseases; Vitamin A deficiency; Chronic kidney disease; Endocrine, metabolic, blood, and immune disorders; Upper digestive system diseases; Intestinal nematode infections; Malaria; Cirrhosis and other chronic liver diseases; Inflammatory bowel disease; HIV/AIDS; Schistosomiasis
Males 2021: Dietary iron deficiency; Haemoglobinopathies and haemolytic anaemias; Other neglected tropical diseases; Chronic kidney disease; Other unspecified infectious diseases; Vitamin A deficiency; Endocrine, metabolic, blood, and immune disorders; Upper digestive system diseases; HIV/AIDS; Intestinal nematode infections; Cirrhosis and other chronic liver diseases; Inflammatory bowel disease; Schistosomiasis; Malaria
Females 1990: Dietary iron deficiency; Haemoglobinopathies and haemolytic anaemias; Other neglected tropical diseases; Other unspecified infectious diseases; Endocrine, metabolic, blood, and immune disorders; Chronic kidney disease; Vitamin A deficiency; Upper digestive system diseases; Maternal disorders; Gynecological diseases; Intestinal nematode infections; Malaria; Cirrhosis and other chronic liver diseases; Inflammatory bowel disease; HIV/AIDS; Schistosomiasis
Females 2021: Dietary iron deficiency; Haemoglobinopathies and haemolytic anaemias; Other neglected tropical diseases; Other unspecified infectious diseases; Chronic kidney disease; Endocrine, metabolic, blood, and immune disorders; Upper digestive system diseases; Gynecological diseases; Vitamin A deficiency; Maternal disorders; Intestinal nematode infections; HIV/AIDS; Cirrhosis and other chronic liver diseases; Inflammatory bowel disease; Schistosomiasis; Malaria
YLDs per 100,000 population
0 to <0
0 to <0.09
0.09 to <0.19
0.19 to <1.66
1.66 to <3.16
3.16 to <8.02
8.02 to <15.64
15.64 to <27.12
27.12 to <56.91
56.91 to 607.81

Pangasinan

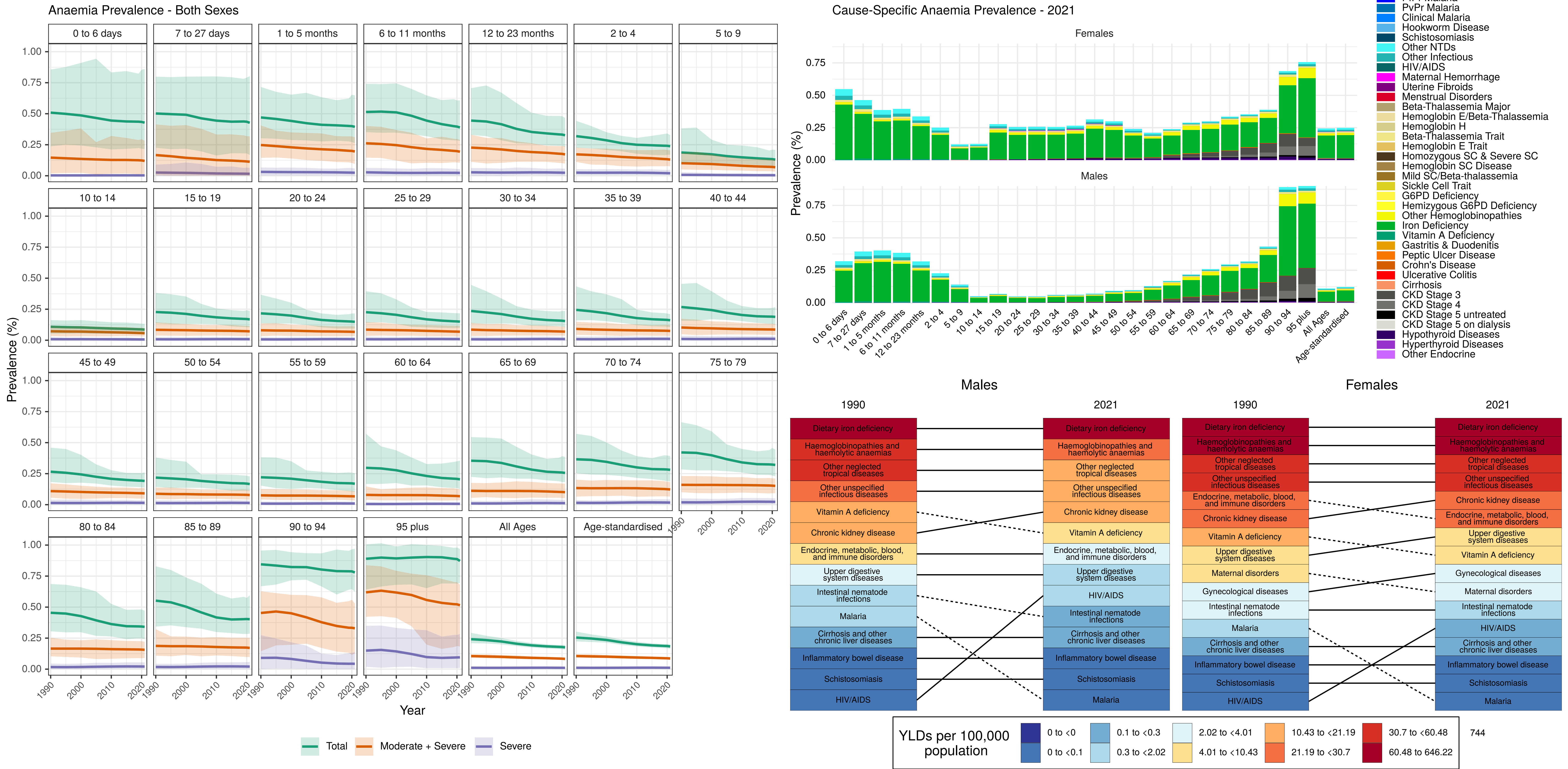

Anaemia Prevalence - Both Sexes
0 to 6 days
7 to 27 days
1 to 5 months
6 to 11 months
12 to 23 months
2 to 4
5 to 9
10 to 14
15 to 19
20 to 24
25 to 29
30 to 34
35 to 39
40 to 44
45 to 49
50 to 54
55 to 59
60 to 64
65 to 69
70 to 74
75 to 79
80 to 84
85 to 89
90 to 94
95 plus
All Ages
Age-standardised
Prevalence (%)
Year
Total
Moderate + Severe
Severe
Cause-Specific Anaemia Prevalence - 2021
Females
Males
PfPr Malaria
PvPr Malaria
Clinical Malaria
Hookworm Disease
Schistosomiasis
Other NTDs
Other Infectious
HIV/AIDS
Maternal Hemorrhage
Uterine Fibroids
Menstrual Disorders
Beta-Thalassemia Major
Hemoglobin E/Beta-Thalassemia
Hemoglobin H
Beta-Thalassemia Trait
Hemoglobin E Trait
Homozygous SC & Severe SC
Hemoglobin SC Disease
Mild SC/Beta-thalassemia
Sickle Cell Trait
G6PD Deficiency
Hemizygous G6PD Deficiency
Other Hemoglobinopathies
Iron Deficiency
Vitamin A Deficiency
Gastritis & Duodenitis
Peptic Ulcer Disease
Crohn's Disease
Ulcerative Colitis
Cirrhosis
CKD Stage 3
CKD Stage 4
CKD Stage 5 untreated
CKD Stage 5 on dialysis
Hypothyroid Diseases
Hyperthyroid Diseases
Other Endocrine
Males
Females
1990
2021
Dietary iron deficiency
Haemoglobinopathies and haemolytic anaemias
Other neglected tropical diseases
Other unspecified infectious diseases
Vitamin A deficiency
Chronic kidney disease
Endocrine, metabolic, blood, and immune disorders
Upper digestive system diseases
Intestinal nematode infections
Malaria
Cirrhosis and other chronic liver diseases
Inflammatory bowel disease
Schistosomiasis
HIV/AIDS
Maternal disorders
Gynecological diseases
YLDs per 100,000 population
0 to <0
0 to <0.1
0.1 to <0.3
0.3 to <2.02
2.02 to <4.01
4.01 to <10.43
10.43 to <21.19
21.19 to <30.7
30.7 to <60.48
60.48 to 646.22

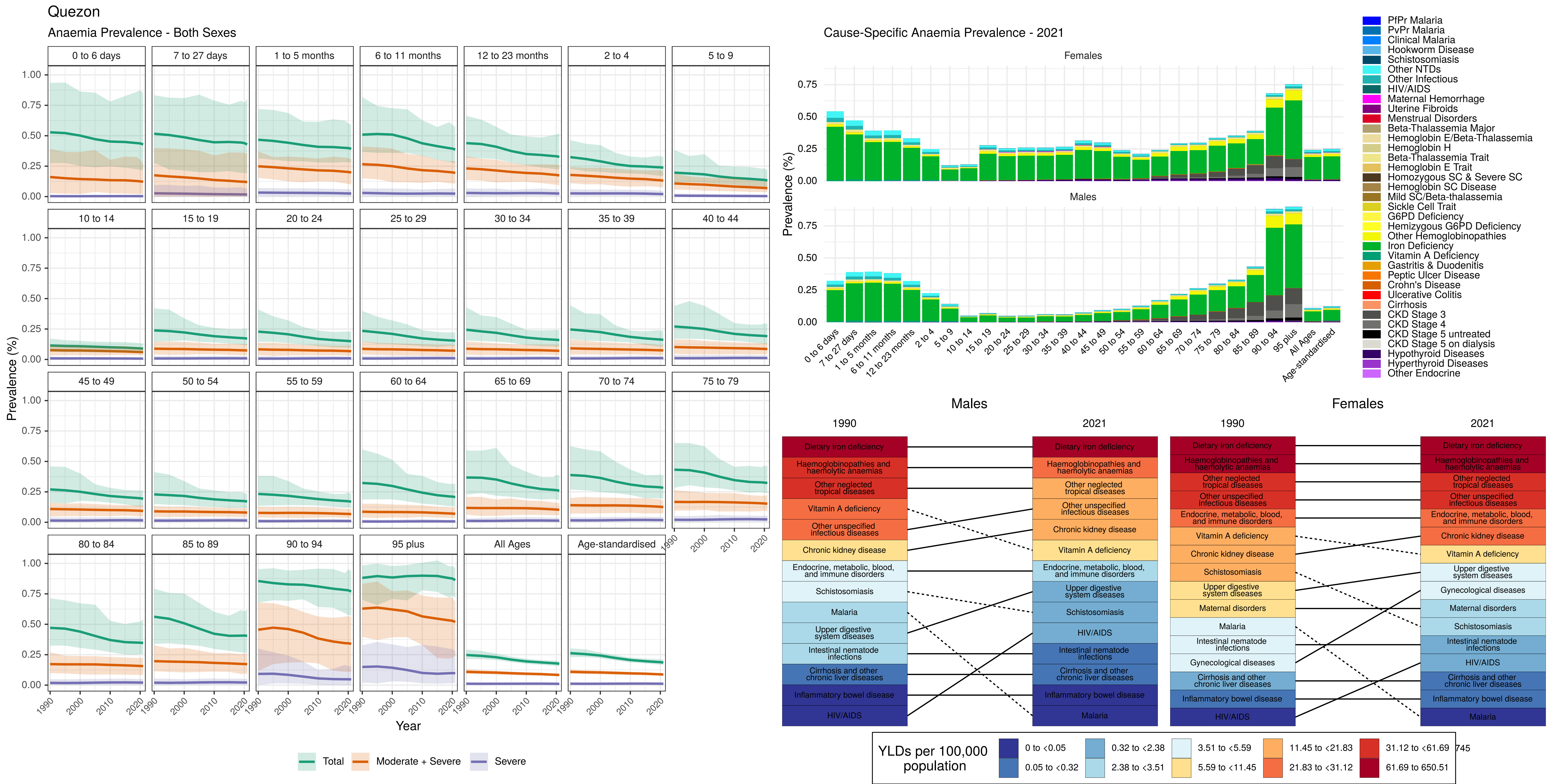
Quezon
Anaemia Prevalence - Both Sexes
0 to 6 days
7 to 27 days
1 to 5 months
6 to 11 months
12 to 23 months
2 to 4
5 to 9
10 to 14
15 to 19
20 to 24
25 to 29
30 to 34
35 to 39
40 to 44
45 to 49
50 to 54
55 to 59
60 to 64
65 to 69
70 to 74
75 to 79
80 to 84
85 to 89
90 to 94
95 plus
All Ages
Age-standardised
Prevalence (%)
Year
Total
Moderate + Severe
Severe
Cause-Specific Anaemia Prevalence - 2021
Females
Males
PfPr Malaria
PvPr Malaria
Clinical Malaria
Hookworm Disease
Schistosomiasis
Other NTDs
Other Infectious
HIV/AIDS
Maternal Hemorrhage
Uterine Fibroids
Menstrual Disorders
Beta-Thalassemia Major
Hemoglobin E/Beta-Thalassemia
Hemoglobin H
Beta-Thalassemia Trait
Hemoglobin E Trait
Homozygous SC & Severe SC
Hemoglobin SC Disease
Mild SC/Beta-thalassemia
Sickle Cell Trait
G6PD Deficiency
Hemizygous G6PD Deficiency
Other Hemoglobinopathies
Iron Deficiency
Vitamin A Deficiency
Gastritis & Duodenitis
Peptic Ulcer Disease
Crohn's Disease
Ulcerative Colitis
Cirrhosis
CKD Stage 3
CKD Stage 4
CKD Stage 5 untreated
CKD Stage 5 on dialysis
Hypothyroid Diseases
Hyperthyroid Diseases
Other Endocrine
Males
Females
1990
2021
Dietary iron deficiency
Haemoglobinopathies and haemolytic anaemias
Other neglected tropical diseases
Vitamin A deficiency
Other unspecified infectious diseases
Chronic kidney disease
Endocrine, metabolic, blood, and immune disorders
Schistosomiasis
Malaria
Upper digestive system diseases
Intestinal nematode infections
Cirrhosis and other chronic liver diseases
Inflammatory bowel disease
HIV/AIDS
Maternal disorders
Gynecological diseases
YLDs per 100,000 population
0 to <0.05
0.05 to <0.32
0.32 to <2.38
2.38 to <3.51
3.51 to <5.59
5.59 to <11.45
11.45 to <21.83
21.83 to <31.12
31.12 to <61.69
61.69 to 650.51

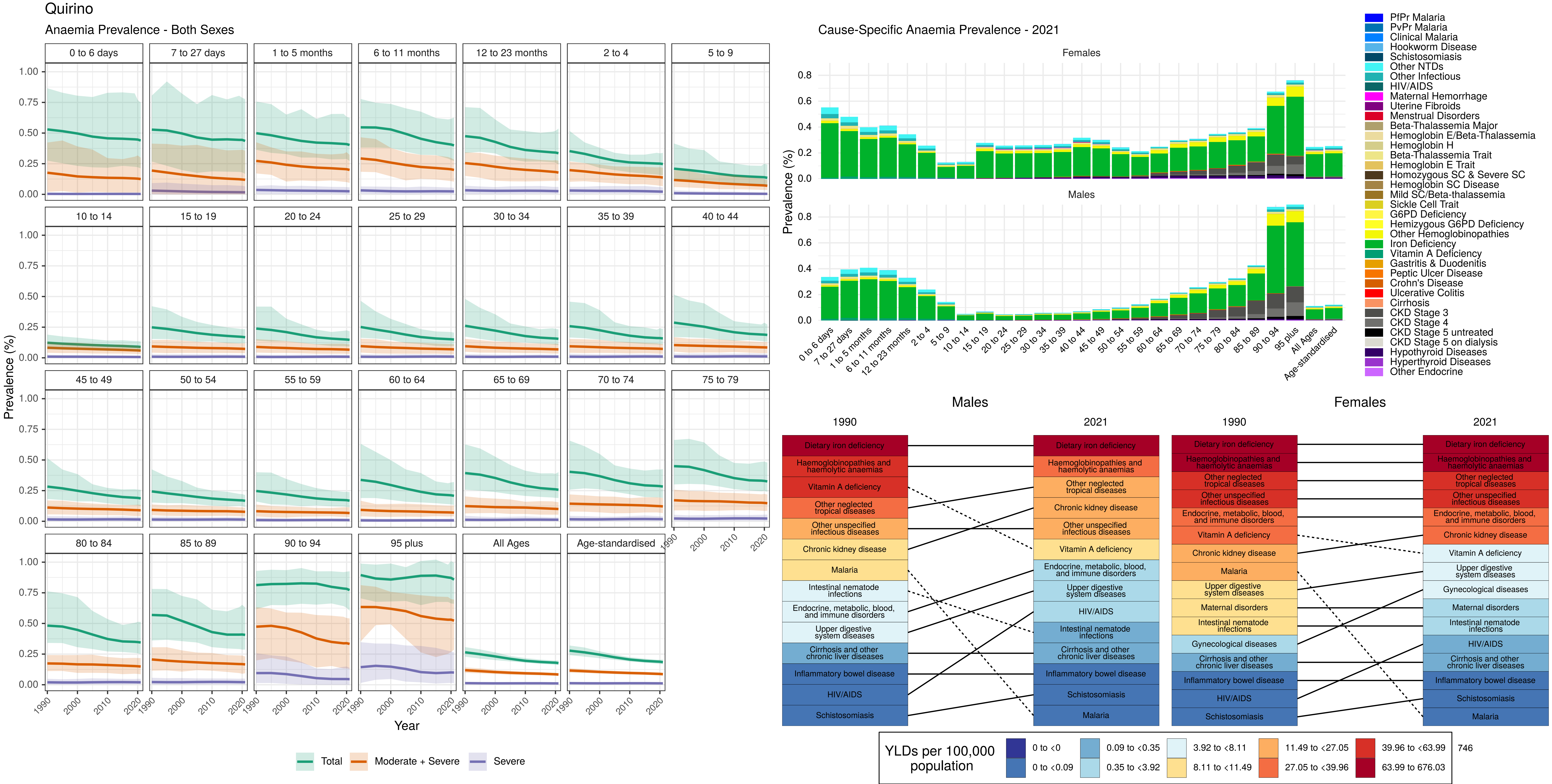

Quirino
Anaemia Prevalence - Both Sexes
0 to 6 days
7 to 27 days
1 to 5 months
6 to 11 months
12 to 23 months
2 to 4
5 to 9
10 to 14
15 to 19
20 to 24
25 to 29
30 to 34
35 to 39
40 to 44
45 to 49
50 to 54
55 to 59
60 to 64
65 to 69
70 to 74
75 to 79
80 to 84
85 to 89
90 to 94
95 plus
All Ages
Age-standardised
0.00 0.25 0.50 0.75 1.00
1990 2000 2010 2020
Prevalence (%)
Year
Total
Moderate + Severe
Severe
Cause-Specific Anaemia Prevalence - 2021
Females
Males
Prevalence (%)
0.0 0.2 0.4 0.6 0.8
PfPr Malaria
PvPr Malaria
Clinical Malaria
Hookworm Disease
Schistosomiasis
Other NTDs
Other Infectious
HIV/AIDS
Maternal Hemorrhage
Uterine Fibroids
Menstrual Disorders
Beta-Thalassemia Major
Hemoglobin E/Beta-Thalassemia
Hemoglobin H
Beta-Thalassemia Trait
Hemoglobin E Trait
Homozygous SC & Severe SC
Hemoglobin SC Disease
Mild SC/Beta-thalassemia
Sickle Cell Trait
G6PD Deficiency
Hemizygous G6PD Deficiency
Other Hemoglobinopathies
Iron Deficiency
Vitamin A Deficiency
Gastritis & Duodenitis
Peptic Ulcer Disease
Crohn's Disease
Ulcerative Colitis
Cirrhosis
CKD Stage 3
CKD Stage 4
CKD Stage 5 untreated
CKD Stage 5 on dialysis
Hypothyroid Diseases
Hyperthyroid Diseases
Other Endocrine
Males
Females
1990
2021
Males 1990: Dietary iron deficiency; Haemoglobinopathies and haemolytic anaemias; Vitamin A deficiency; Other neglected tropical diseases; Other unspecified infectious diseases; Chronic kidney disease; Malaria; Intestinal nematode infections; Endocrine, metabolic, blood, and immune disorders; Upper digestive system diseases; Cirrhosis and other chronic liver diseases; Inflammatory bowel disease; HIV/AIDS; Schistosomiasis
Males 2021: Dietary iron deficiency; Haemoglobinopathies and haemolytic anaemias; Other neglected tropical diseases; Chronic kidney disease; Other unspecified infectious diseases; Vitamin A deficiency; Endocrine, metabolic, blood, and immune disorders; Upper digestive system diseases; HIV/AIDS; Intestinal nematode infections; Cirrhosis and other chronic liver diseases; Inflammatory bowel disease; Schistosomiasis; Malaria
Females 1990: Dietary iron deficiency; Haemoglobinopathies and haemolytic anaemias; Other neglected tropical diseases; Other unspecified infectious diseases; Endocrine, metabolic, blood, and immune disorders; Vitamin A deficiency; Chronic kidney disease; Malaria; Upper digestive system diseases; Maternal disorders; Intestinal nematode infections; Gynecological diseases; Cirrhosis and other chronic liver diseases; Inflammatory bowel disease; HIV/AIDS; Schistosomiasis
Females 2021: Dietary iron deficiency; Haemoglobinopathies and haemolytic anaemias; Other neglected tropical diseases; Other unspecified infectious diseases; Endocrine, metabolic, blood, and immune disorders; Chronic kidney disease; Vitamin A deficiency; Upper digestive system diseases; Gynecological diseases; Maternal disorders; Intestinal nematode infections; HIV/AIDS; Cirrhosis and other chronic liver diseases; Inflammatory bowel disease; Schistosomiasis; Malaria
YLDs per 100,000 population
0 to <0
0 to <0.09
0.09 to <0.35
0.35 to <3.92
3.92 to <8.11
8.11 to <11.49
11.49 to <27.05
27.05 to <39.96
39.96 to <63.99
63.99 to 676.03
746

Rizal

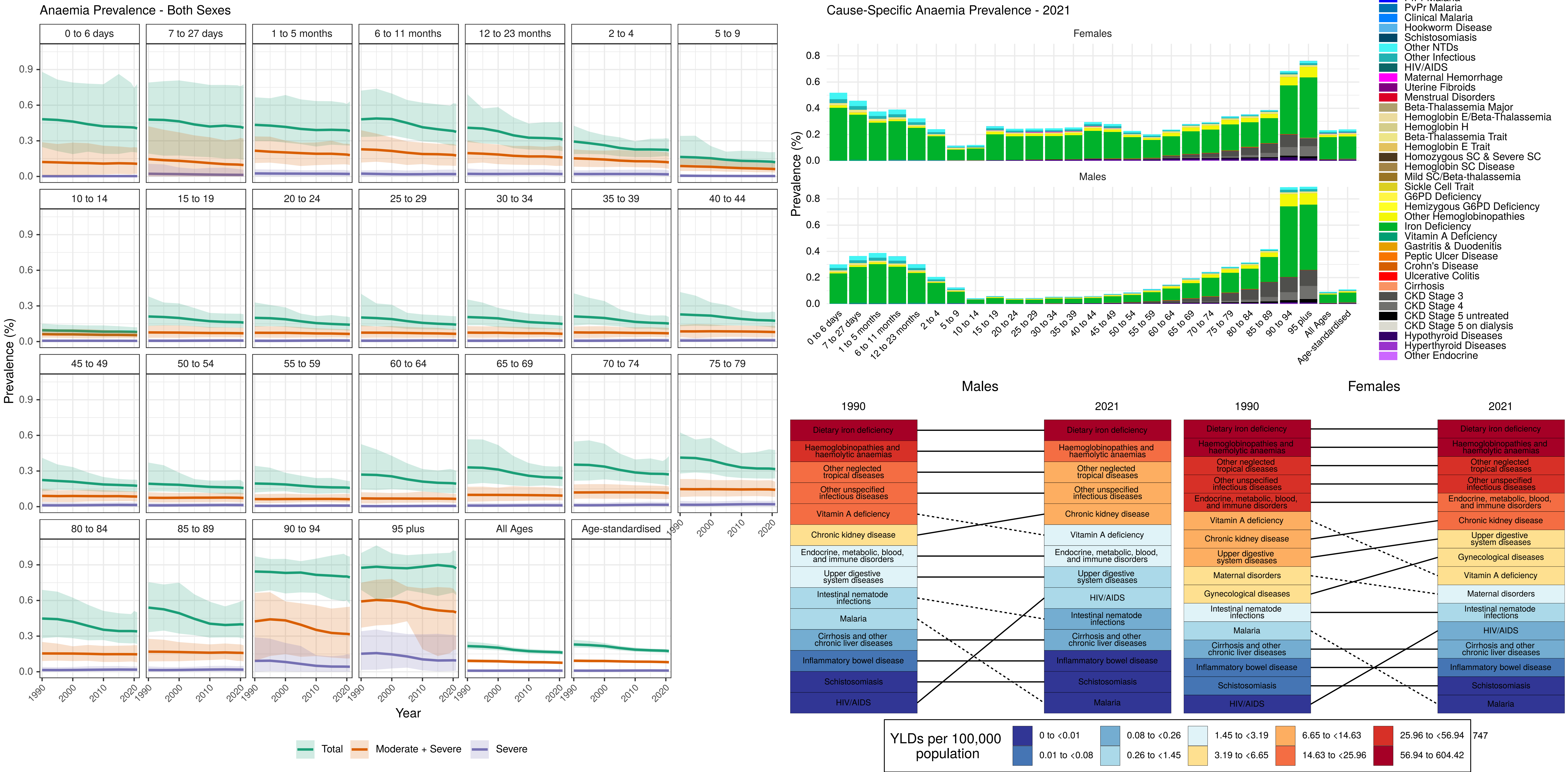
Anaemia Prevalence - Both Sexes
0 to 6 days
7 to 27 days
1 to 5 months
6 to 11 months
12 to 23 months
2 to 4
5 to 9
10 to 14
15 to 19
20 to 24
25 to 29
30 to 34
35 to 39
40 to 44
45 to 49
50 to 54
55 to 59
60 to 64
65 to 69
70 to 74
75 to 79
80 to 84
85 to 89
90 to 94
95 plus
All Ages
Age-standardised
Prevalence (%)
Year
Total
Moderate + Severe
Severe
Cause-Specific Anaemia Prevalence - 2021
Females
Males
PfPr Malaria
PvPr Malaria
Clinical Malaria
Hookworm Disease
Schistosomiasis
Other NTDs
Other Infectious
HIV/AIDS
Maternal Hemorrhage
Uterine Fibroids
Menstrual Disorders
Beta-Thalassemia Major
Hemoglobin E/Beta-Thalassemia
Hemoglobin H
Beta-Thalassemia Trait
Hemoglobin E Trait
Homozygous SC & Severe SC
Hemoglobin SC Disease
Mild SC/Beta-thalassemia
Sickle Cell Trait
G6PD Deficiency
Hemizygous G6PD Deficiency
Other Hemoglobinopathies
Iron Deficiency
Vitamin A Deficiency
Gastritis & Duodenitis
Peptic Ulcer Disease
Crohn's Disease
Ulcerative Colitis
Cirrhosis
CKD Stage 3
CKD Stage 4
CKD Stage 5 untreated
CKD Stage 5 on dialysis
Hypothyroid Diseases
Hyperthyroid Diseases
Other Endocrine
Males
Females
1990
2021
Dietary iron deficiency
Haemoglobinopathies and haemolytic anaemias
Other neglected tropical diseases
Other unspecified infectious diseases
Vitamin A deficiency
Chronic kidney disease
Endocrine, metabolic, blood, and immune disorders
Upper digestive system diseases
Intestinal nematode infections
Malaria
Cirrhosis and other chronic liver diseases
Inflammatory bowel disease
Schistosomiasis
HIV/AIDS
Maternal disorders
Gynecological diseases
YLDs per 100,000 population
0 to <0.01
0.01 to <0.08
0.08 to <0.26
0.26 to <1.45
1.45 to <3.19
3.19 to <6.65
6.65 to <14.63
14.63 to <25.96
25.96 to <56.94
56.94 to 604.42

Romblon

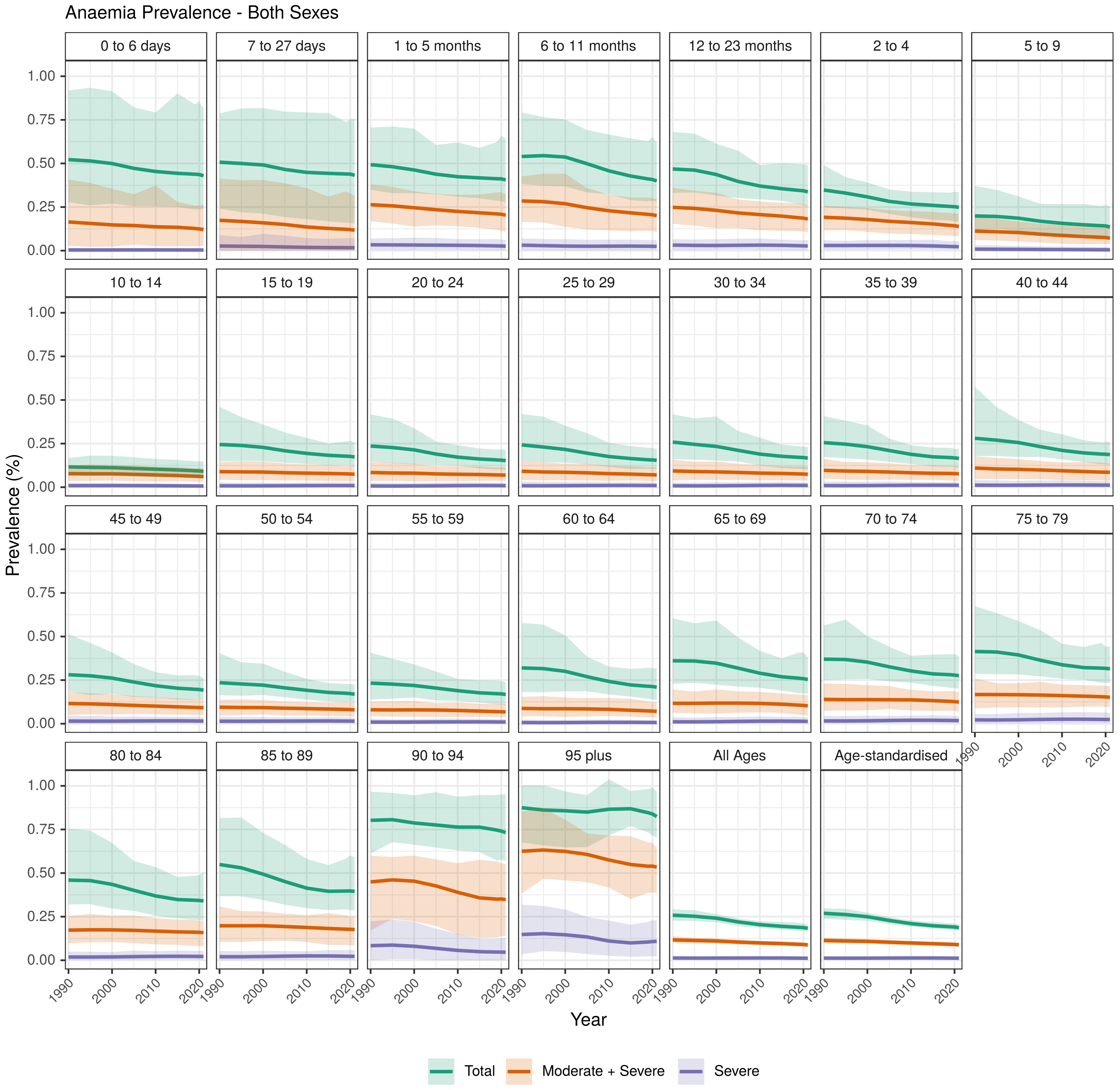

Anaemia Prevalence - Both Sexes
0 to 6 days
7 to 27 days
1 to 5 months
6 to 11 months
12 to 23 months
2 to 4
5 to 9
10 to 14
15 to 19
20 to 24
25 to 29
30 to 34
35 to 39
40 to 44
45 to 49
50 to 54
55 to 59
60 to 64
65 to 69
70 to 74
75 to 79
80 to 84
85 to 89
90 to 94
95 plus
All Ages
Age-standardised
Prevalence (%)
1.00
0.75
0.50
0.25
0.00
1990
2000
2010
2020
Year
Total
Moderate + Severe
Severe

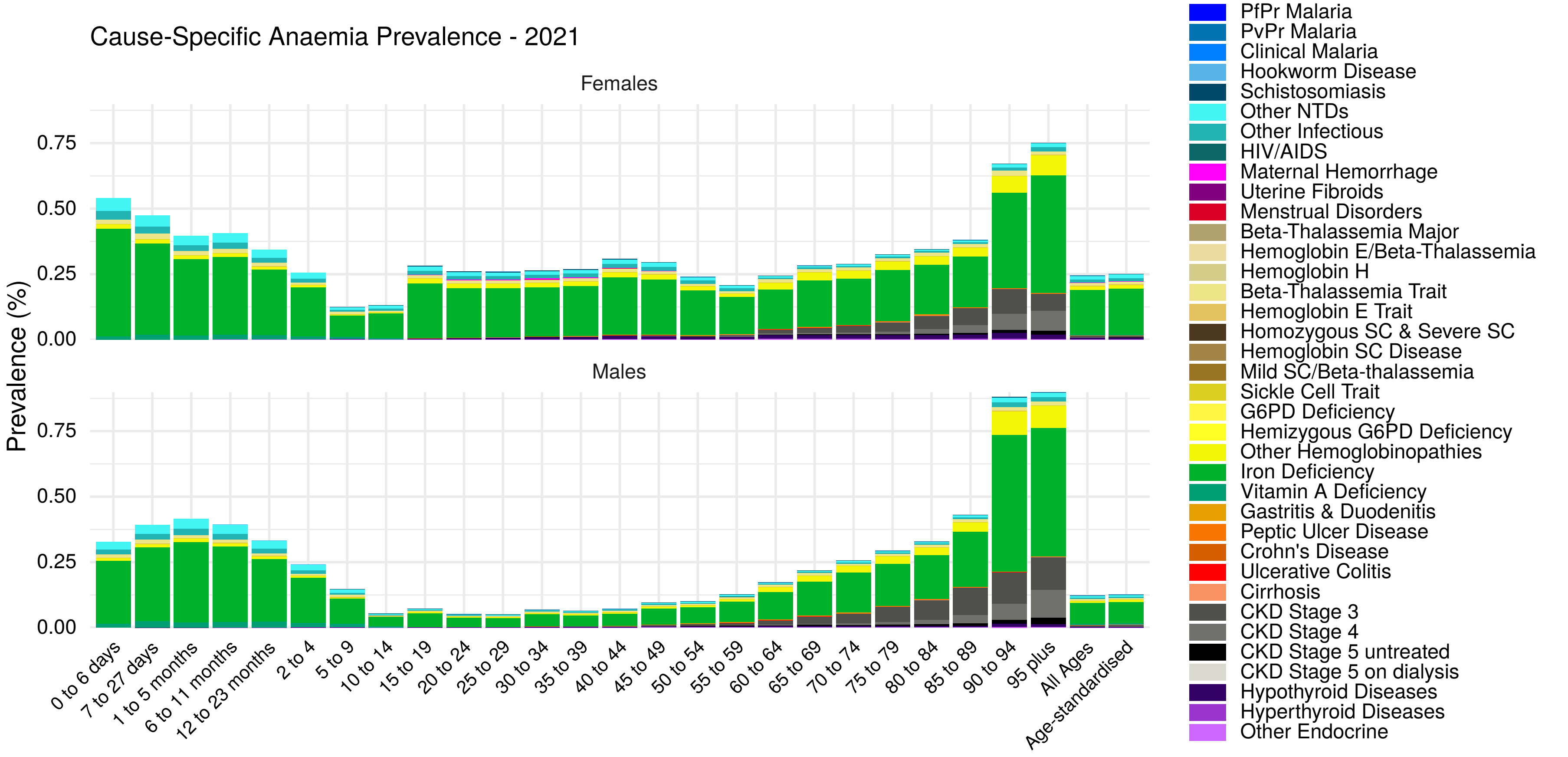

Cause-Specific Anaemia Prevalence - 2021
Females
Males
Prevalence (%)
0.75
0.50
0.25
0.00
0 to 6 days
7 to 27 days
1 to 5 months
6 to 11 months
12 to 23 months
2 to 4
5 to 9
10 to 14
15 to 19
20 to 24
25 to 29
30 to 34
35 to 39
40 to 44
45 to 49
50 to 54
55 to 59
60 to 64
65 to 69
70 to 74
75 to 79
80 to 84
85 to 89
90 to 94
95 plus
All Ages
Age-standardised
PfPr Malaria
PvPr Malaria
Clinical Malaria
Hookworm Disease
Schistosomiasis
Other NTDs
Other Infectious
HIV/AIDS
Maternal Hemorrhage
Uterine Fibroids
Menstrual Disorders
Beta-Thalassemia Major
Hemoglobin E/Beta-Thalassemia
Hemoglobin H
Beta-Thalassemia Trait
Hemoglobin E Trait
Homozygous SC & Severe SC
Hemoglobin SC Disease
Mild SC/Beta-thalassemia
Sickle Cell Trait
G6PD Deficiency
Hemizygous G6PD Deficiency
Other Hemoglobinopathies
Iron Deficiency
Vitamin A Deficiency
Gastritis & Duodenitis
Peptic Ulcer Disease
Crohn's Disease
Ulcerative Colitis
Cirrhosis
CKD Stage 3
CKD Stage 4
CKD Stage 5 untreated
CKD Stage 5 on dialysis
Hypothyroid Diseases
Hyperthyroid Diseases
Other Endocrine

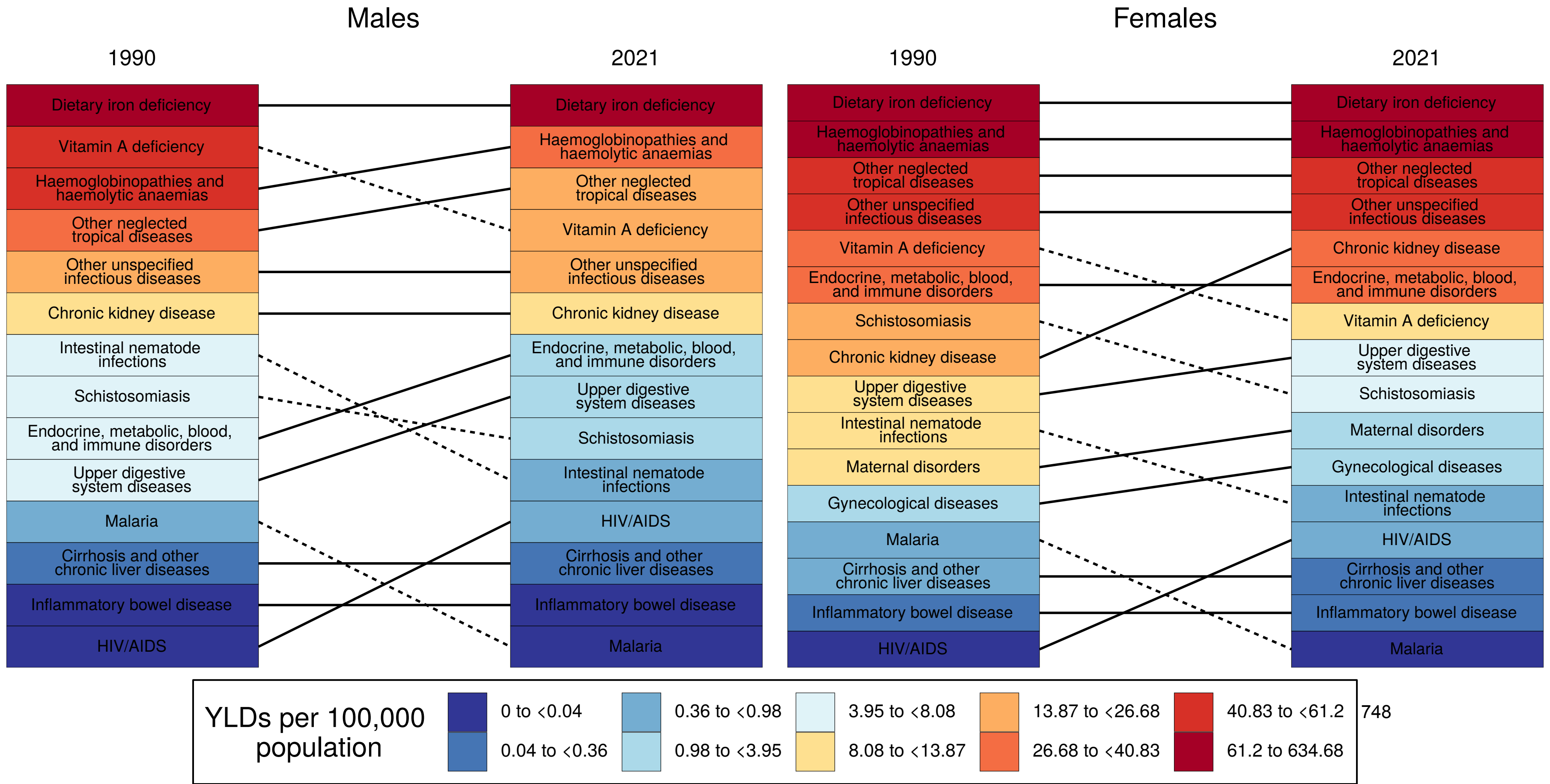

Males
Females
1990
2021
Dietary iron deficiency
Vitamin A deficiency
Haemoglobinopathies and haemolytic anaemias
Other neglected tropical diseases
Other unspecified infectious diseases
Chronic kidney disease
Intestinal nematode infections
Schistosomiasis
Endocrine, metabolic, blood, and immune disorders
Upper digestive system diseases
Malaria
Cirrhosis and other chronic liver diseases
Inflammatory bowel disease
HIV/AIDS
Maternal disorders
Gynecological diseases
YLDs per 100,000 population
0 to <0.04
0.04 to <0.36
0.36 to <0.98
0.98 to <3.95
3.95 to <8.08
8.08 to <13.87
13.87 to <26.68
26.68 to <40.83
40.83 to <61.2
61.2 to 634.68

748

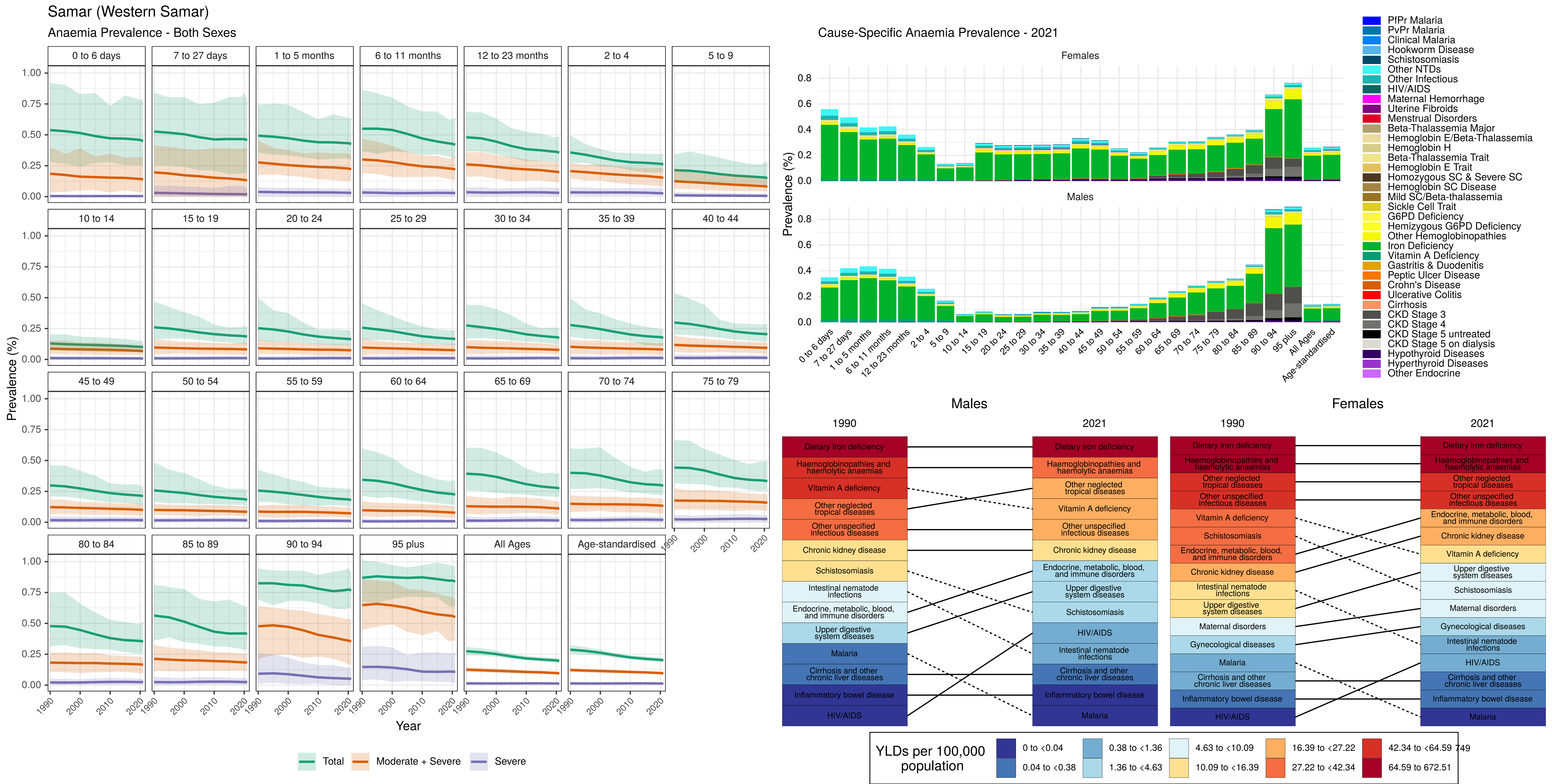
Samar (Western Samar)
Anaemia Prevalence - Both Sexes
0 to 6 days
7 to 27 days
1 to 5 months
6 to 11 months
12 to 23 months
2 to 4
5 to 9
10 to 14
15 to 19
20 to 24
25 to 29
30 to 34
35 to 39
40 to 44
45 to 49
50 to 54
55 to 59
60 to 64
65 to 69
70 to 74
75 to 79
80 to 84
85 to 89
90 to 94
95 plus
All Ages
Age-standardised
Prevalence (%)
Year
Total
Moderate + Severe
Severe
Cause-Specific Anaemia Prevalence - 2021
Females
Males
PfPr Malaria
PvPr Malaria
Clinical Malaria
Hookworm Disease
Schistosomiasis
Other NTDs
Other Infectious
HIV/AIDS
Maternal Hemorrhage
Uterine Fibroids
Menstrual Disorders
Beta-Thalassemia Major
Hemoglobin E/Beta-Thalassemia
Hemoglobin H
Beta-Thalassemia Trait
Hemoglobin E Trait
Homozygous SC & Severe SC
Hemoglobin SC Disease
Mild SC/Beta-thalassemia
Sickle Cell Trait
G6PD Deficiency
Hemizygous G6PD Deficiency
Other Hemoglobinopathies
Iron Deficiency
Vitamin A Deficiency
Gastritis & Duodenitis
Peptic Ulcer Disease
Crohn's Disease
Ulcerative Colitis
Cirrhosis
CKD Stage 3
CKD Stage 4
CKD Stage 5 untreated
CKD Stage 5 on dialysis
Hypothyroid Diseases
Hyperthyroid Diseases
Other Endocrine
Males
Females
1990
2021
Males 1990: Dietary iron deficiency; Haemoglobinopathies and haemolytic anaemias; Vitamin A deficiency; Other neglected tropical diseases; Other unspecified infectious diseases; Chronic kidney disease; Schistosomiasis; Intestinal nematode infections; Endocrine, metabolic, blood, and immune disorders; Upper digestive system diseases; Malaria; Cirrhosis and other chronic liver diseases; Inflammatory bowel disease; HIV/AIDS
Males 2021: Dietary iron deficiency; Haemoglobinopathies and haemolytic anaemias; Other neglected tropical diseases; Vitamin A deficiency; Other unspecified infectious diseases; Chronic kidney disease; Endocrine, metabolic, blood, and immune disorders; Upper digestive system diseases; Schistosomiasis; HIV/AIDS; Intestinal nematode infections; Cirrhosis and other chronic liver diseases; Inflammatory bowel disease; Malaria
Females 1990: Dietary iron deficiency; Haemoglobinopathies and haemolytic anaemias; Other neglected tropical diseases; Other unspecified infectious diseases; Vitamin A deficiency; Schistosomiasis; Endocrine, metabolic, blood, and immune disorders; Chronic kidney disease; Intestinal nematode infections; Upper digestive system diseases; Maternal disorders; Gynecological diseases; Malaria; Cirrhosis and other chronic liver diseases; Inflammatory bowel disease; HIV/AIDS
Females 2021: Dietary iron deficiency; Haemoglobinopathies and haemolytic anaemias; Other neglected tropical diseases; Other unspecified infectious diseases; Endocrine, metabolic, blood, and immune disorders; Chronic kidney disease; Vitamin A deficiency; Upper digestive system diseases; Schistosomiasis; Maternal disorders; Gynecological diseases; Intestinal nematode infections; HIV/AIDS; Cirrhosis and other chronic liver diseases; Inflammatory bowel disease; Malaria
YLDs per 100,000 population
0 to <0.04
0.04 to <0.38
0.38 to <1.36
1.36 to <4.63
4.63 to <10.09
10.09 to <16.39
16.39 to <27.22
27.22 to <42.34
42.34 to <64.59
64.59 to 672.51

Sarangani

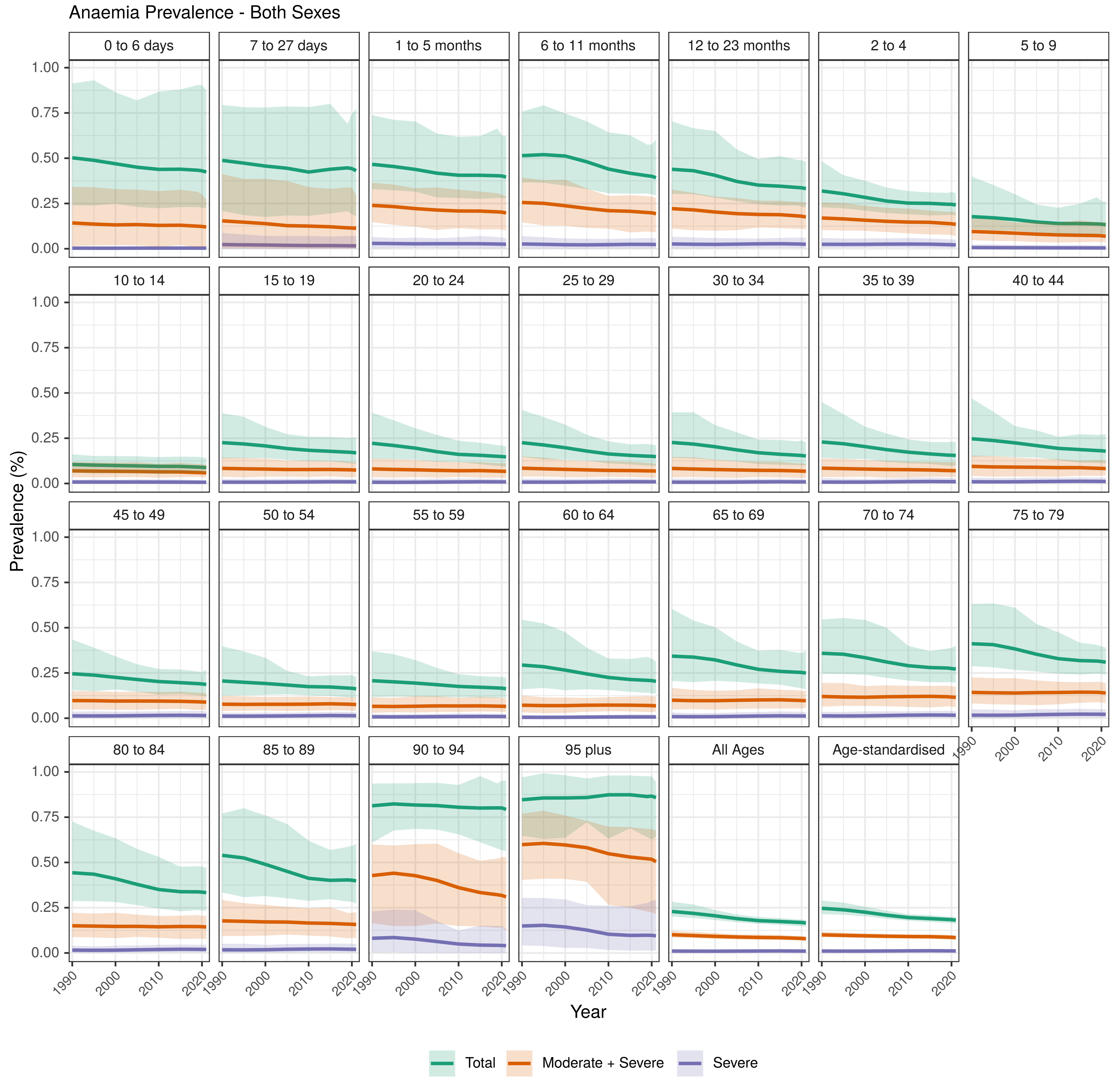

Anaemia Prevalence - Both Sexes
0 to 6 days
7 to 27 days
1 to 5 months
6 to 11 months
12 to 23 months
2 to 4
5 to 9
10 to 14
15 to 19
20 to 24
25 to 29
30 to 34
35 to 39
40 to 44
45 to 49
50 to 54
55 to 59
60 to 64
65 to 69
70 to 74
75 to 79
80 to 84
85 to 89
90 to 94
95 plus
All Ages
Age-standardised
Prevalence (%)
Year
Total
Moderate + Severe
Severe

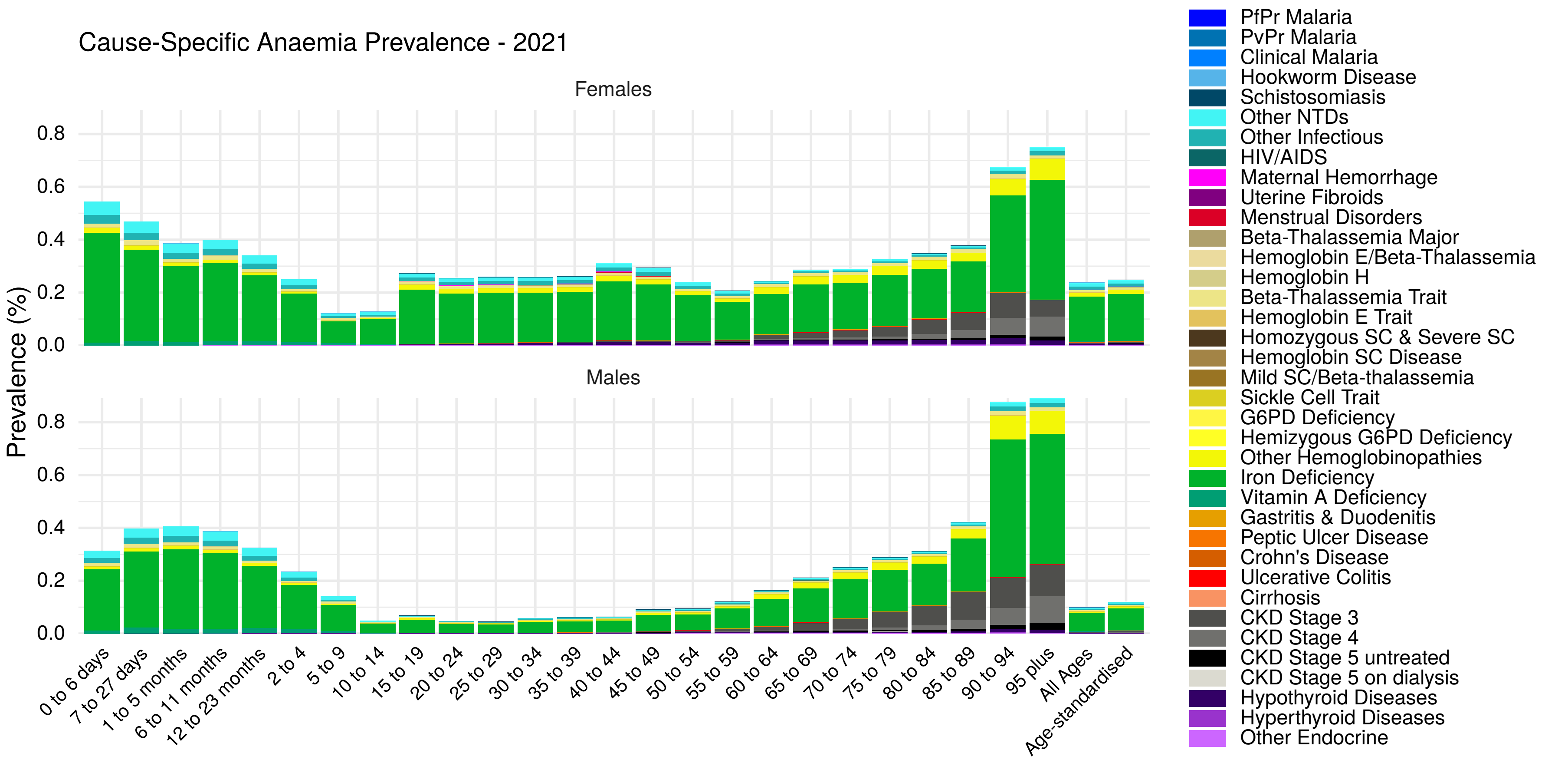

Cause-Specific Anaemia Prevalence - 2021
Females
Males
Prevalence (%)
PfPr Malaria
PvPr Malaria
Clinical Malaria
Hookworm Disease
Schistosomiasis
Other NTDs
Other Infectious
HIV/AIDS
Maternal Hemorrhage
Uterine Fibroids
Menstrual Disorders
Beta-Thalassemia Major
Hemoglobin E/Beta-Thalassemia
Hemoglobin H
Beta-Thalassemia Trait
Hemoglobin E Trait
Homozygous SC & Severe SC
Hemoglobin SC Disease
Mild SC/Beta-thalassemia
Sickle Cell Trait
G6PD Deficiency
Hemizygous G6PD Deficiency
Other Hemoglobinopathies
Iron Deficiency
Vitamin A Deficiency
Gastritis & Duodenitis
Peptic Ulcer Disease
Crohn's Disease
Ulcerative Colitis
Cirrhosis
CKD Stage 3
CKD Stage 4
CKD Stage 5 untreated
CKD Stage 5 on dialysis
Hypothyroid Diseases
Hyperthyroid Diseases
Other Endocrine

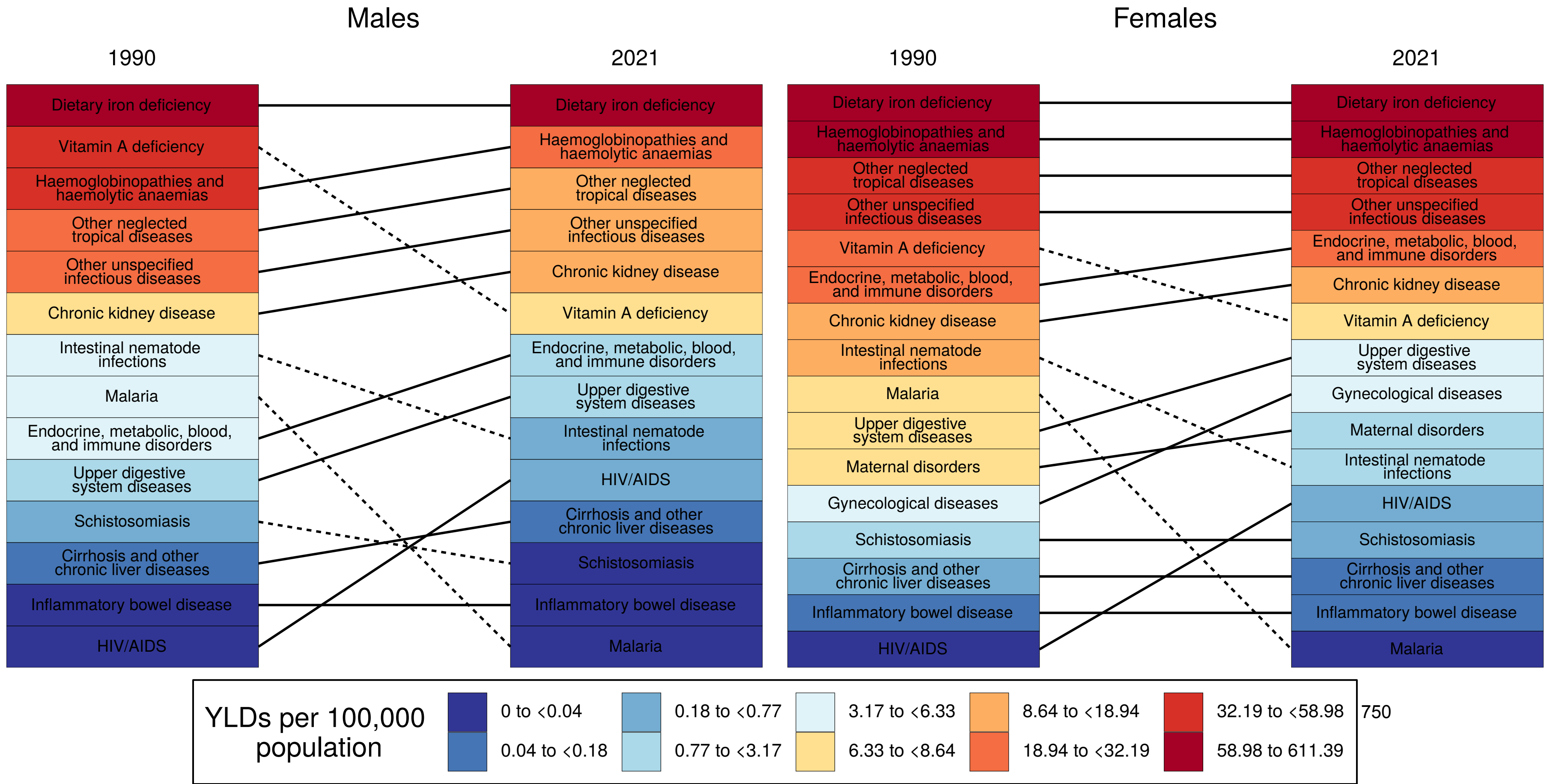

Males
Females
1990
2021
Dietary iron deficiency
Vitamin A deficiency
Haemoglobinopathies and haemolytic anaemias
Other neglected tropical diseases
Other unspecified infectious diseases
Chronic kidney disease
Intestinal nematode infections
Malaria
Endocrine, metabolic, blood, and immune disorders
Upper digestive system diseases
Schistosomiasis
Cirrhosis and other chronic liver diseases
Inflammatory bowel disease
HIV/AIDS
Maternal disorders
Gynecological diseases
YLDs per 100,000 population
0 to <0.04
0.04 to <0.18
0.18 to <0.77
0.77 to <3.17
3.17 to <6.33
6.33 to <8.64
8.64 to <18.94
18.94 to <32.19
32.19 to <58.98
58.98 to 611.39
750

Siquijor

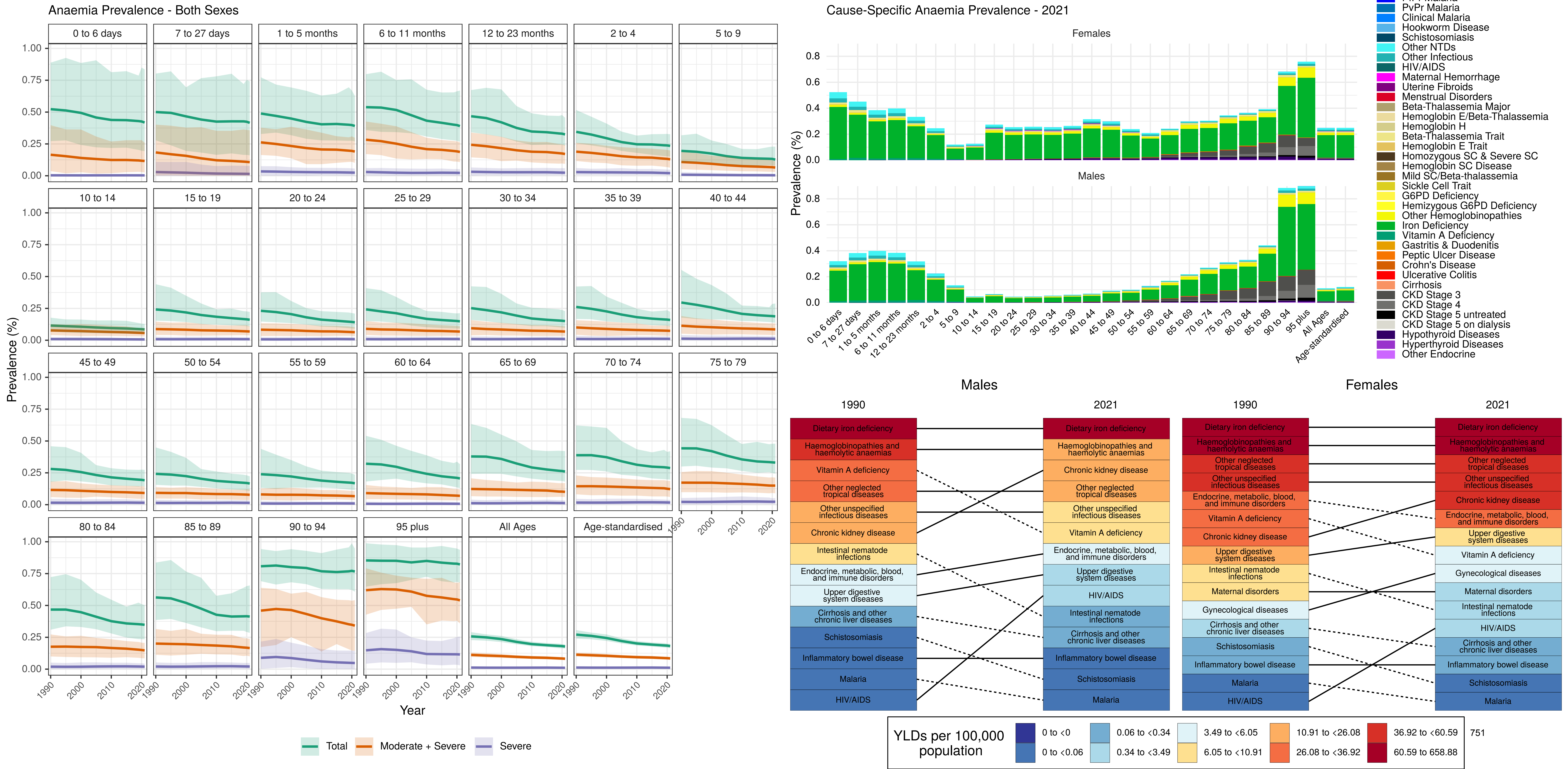
Anaemia Prevalence - Both Sexes
0 to 6 days
7 to 27 days
1 to 5 months
6 to 11 months
12 to 23 months
2 to 4
5 to 9
10 to 14
15 to 19
20 to 24
25 to 29
30 to 34
35 to 39
40 to 44
45 to 49
50 to 54
55 to 59
60 to 64
65 to 69
70 to 74
75 to 79
80 to 84
85 to 89
90 to 94
95 plus
All Ages
Age-standardised
Prevalence (%)
Year
Total
Moderate + Severe
Severe
Cause-Specific Anaemia Prevalence - 2021
Females
Males
Prevalence (%)
PfPr Malaria
PvPr Malaria
Clinical Malaria
Hookworm Disease
Schistosomiasis
Other NTDs
Other Infectious
HIV/AIDS
Maternal Hemorrhage
Uterine Fibroids
Menstrual Disorders
Beta-Thalassemia Major
Hemoglobin E/Beta-Thalassemia
Hemoglobin H
Beta-Thalassemia Trait
Hemoglobin E Trait
Homozygous SC & Severe SC
Hemoglobin SC Disease
Mild SC/Beta-thalassemia
Sickle Cell Trait
G6PD Deficiency
Hemizygous G6PD Deficiency
Other Hemoglobinopathies
Iron Deficiency
Vitamin A Deficiency
Gastritis & Duodenitis
Peptic Ulcer Disease
Crohn's Disease
Ulcerative Colitis
Cirrhosis
CKD Stage 3
CKD Stage 4
CKD Stage 5 untreated
CKD Stage 5 on dialysis
Hypothyroid Diseases
Hyperthyroid Diseases
Other Endocrine
Males
Females
1990
2021
Dietary iron deficiency
Haemoglobinopathies and haemolytic anaemias
Vitamin A deficiency
Other neglected tropical diseases
Other unspecified infectious diseases
Chronic kidney disease
Intestinal nematode infections
Endocrine, metabolic, blood, and immune disorders
Upper digestive system diseases
Cirrhosis and other chronic liver diseases
Schistosomiasis
Inflammatory bowel disease
Malaria
HIV/AIDS
Maternal disorders
Gynecological diseases
YLDs per 100,000 population
0 to <0
0 to <0.06
0.06 to <0.34
0.34 to <3.49
3.49 to <6.05
6.05 to <10.91
10.91 to <26.08
26.08 to <36.92
36.92 to <60.59
60.59 to 658.88

Sorsogon

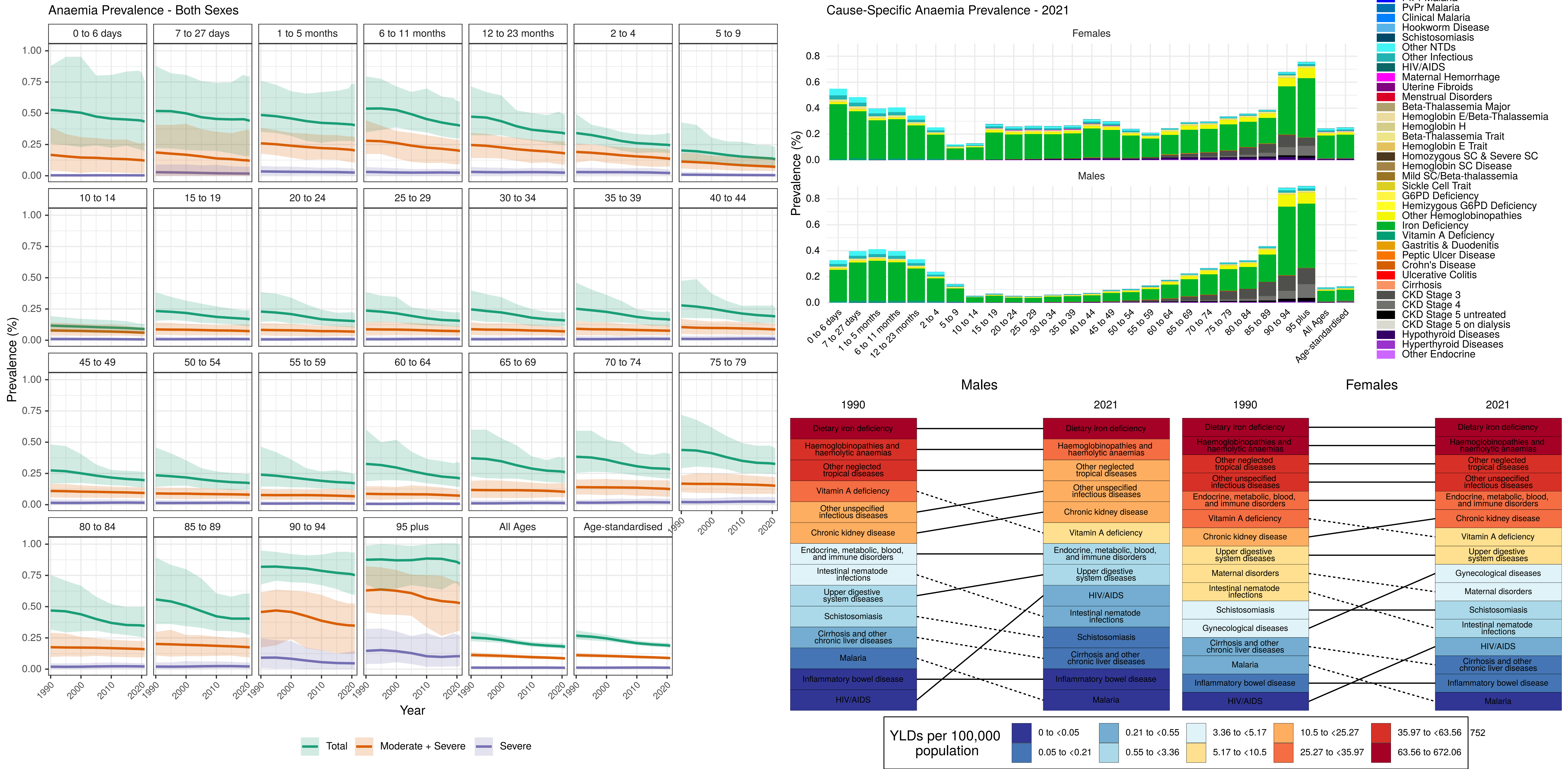

Anaemia Prevalence - Both Sexes
0 to 6 days
7 to 27 days
1 to 5 months
6 to 11 months
12 to 23 months
2 to 4
5 to 9
10 to 14
15 to 19
20 to 24
25 to 29
30 to 34
35 to 39
40 to 44
45 to 49
50 to 54
55 to 59
60 to 64
65 to 69
70 to 74
75 to 79
80 to 84
85 to 89
90 to 94
95 plus
All Ages
Age-standardised
Prevalence (%)
Year
Total
Moderate + Severe
Severe
Cause-Specific Anaemia Prevalence - 2021
Females
Males
PfPr Malaria
PvPr Malaria
Clinical Malaria
Hookworm Disease
Schistosomiasis
Other NTDs
Other Infectious
HIV/AIDS
Maternal Hemorrhage
Uterine Fibroids
Menstrual Disorders
Beta-Thalassemia Major
Hemoglobin E/Beta-Thalassemia
Hemoglobin H
Beta-Thalassemia Trait
Hemoglobin E Trait
Homozygous SC & Severe SC
Hemoglobin SC Disease
Mild SC/Beta-thalassemia
Sickle Cell Trait
G6PD Deficiency
Hemizygous G6PD Deficiency
Other Hemoglobinopathies
Iron Deficiency
Vitamin A Deficiency
Gastritis & Duodenitis
Peptic Ulcer Disease
Crohn's Disease
Ulcerative Colitis
Cirrhosis
CKD Stage 3
CKD Stage 4
CKD Stage 5 untreated
CKD Stage 5 on dialysis
Hypothyroid Diseases
Hyperthyroid Diseases
Other Endocrine
Males
Females
1990
2021
Dietary iron deficiency
Haemoglobinopathies and haemolytic anaemias
Other neglected tropical diseases
Vitamin A deficiency
Other unspecified infectious diseases
Chronic kidney disease
Endocrine, metabolic, blood, and immune disorders
Intestinal nematode infections
Upper digestive system diseases
Schistosomiasis
Cirrhosis and other chronic liver diseases
Malaria
Inflammatory bowel disease
HIV/AIDS
Maternal disorders
Gynecological diseases
YLDs per 100,000 population
0 to <0.05
0.05 to <0.21
0.21 to <0.55
0.55 to <3.36
3.36 to <5.17
5.17 to <10.5
10.5 to <25.27
25.27 to <35.97
35.97 to <63.56
63.56 to 672.06

South Cotabato

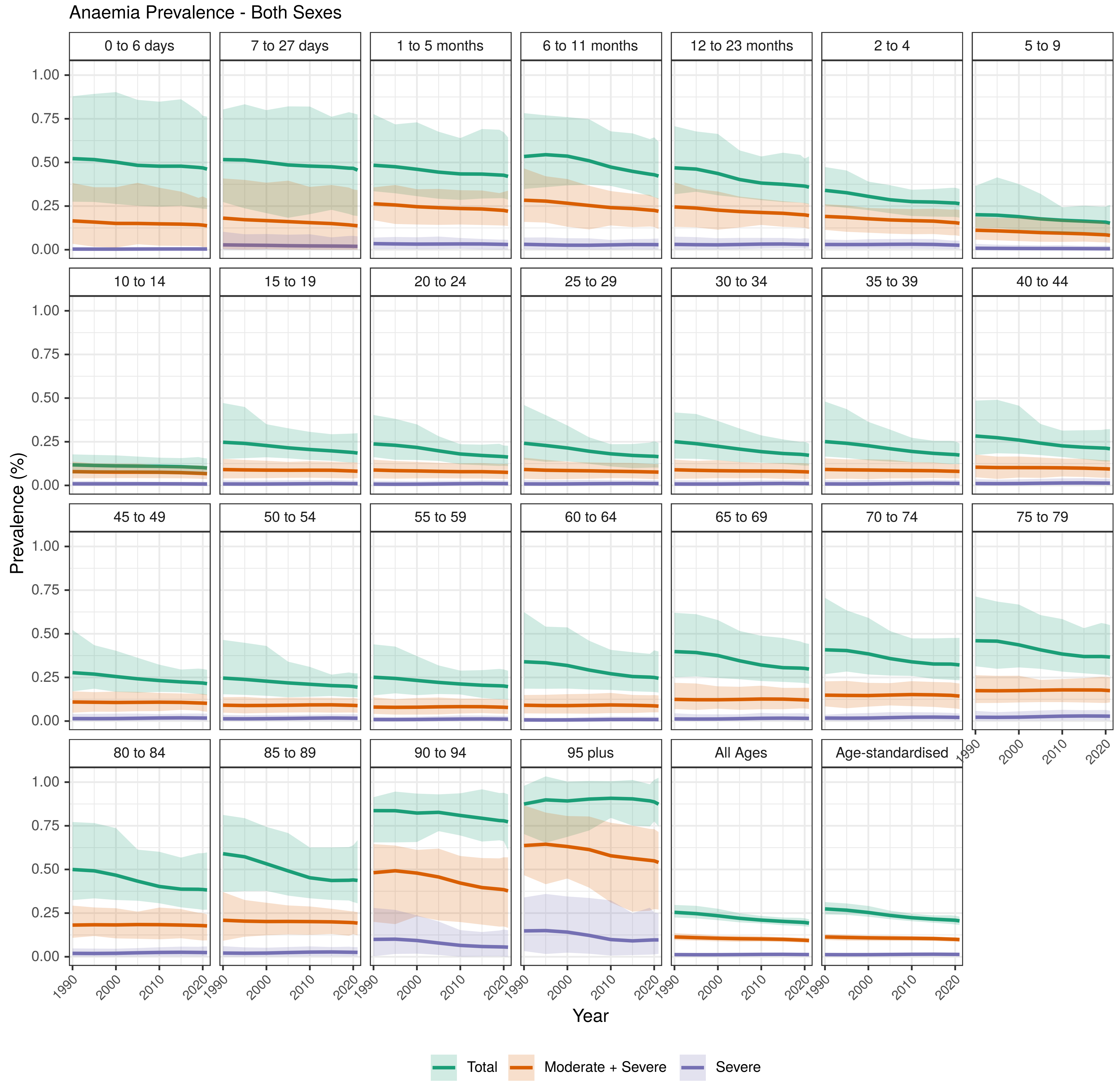

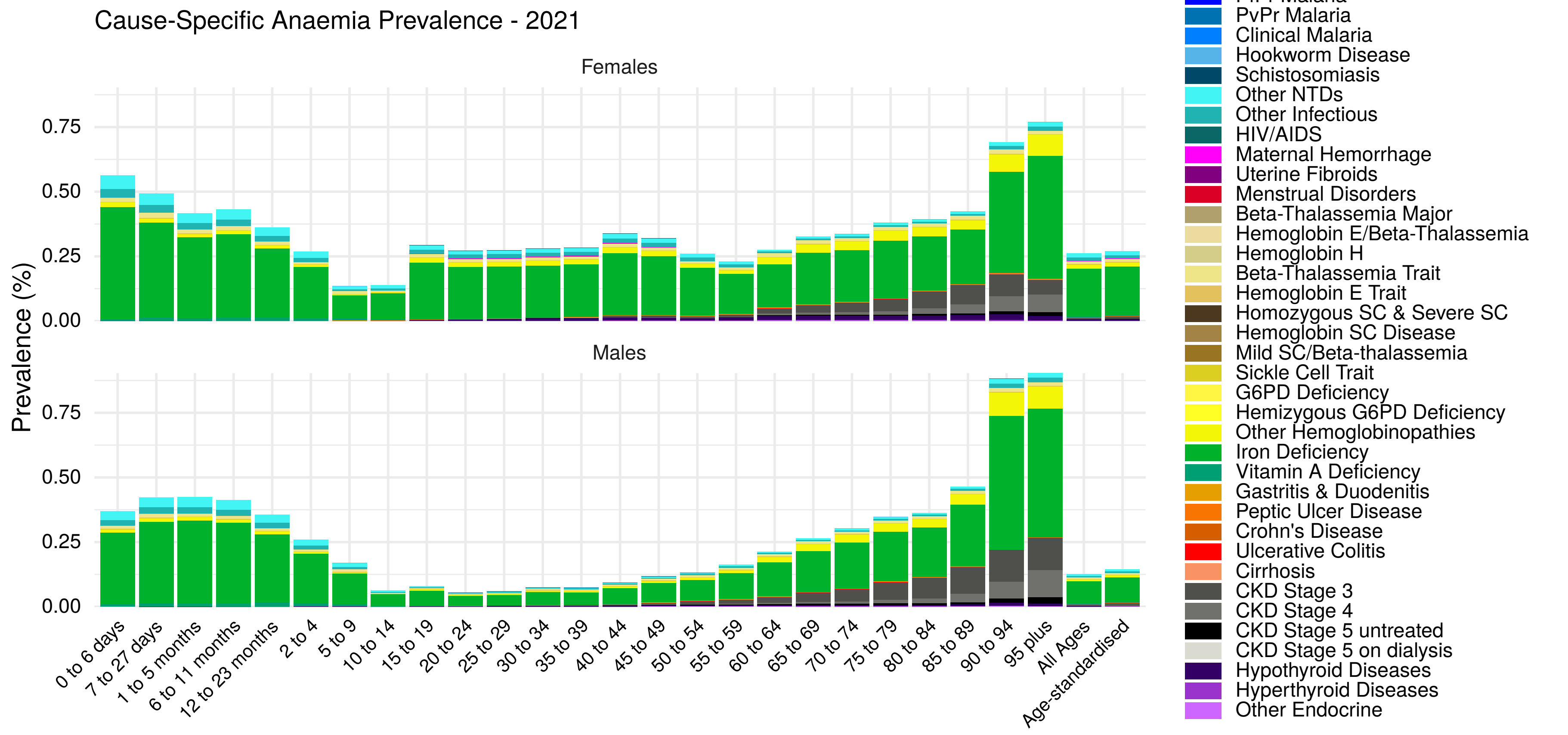

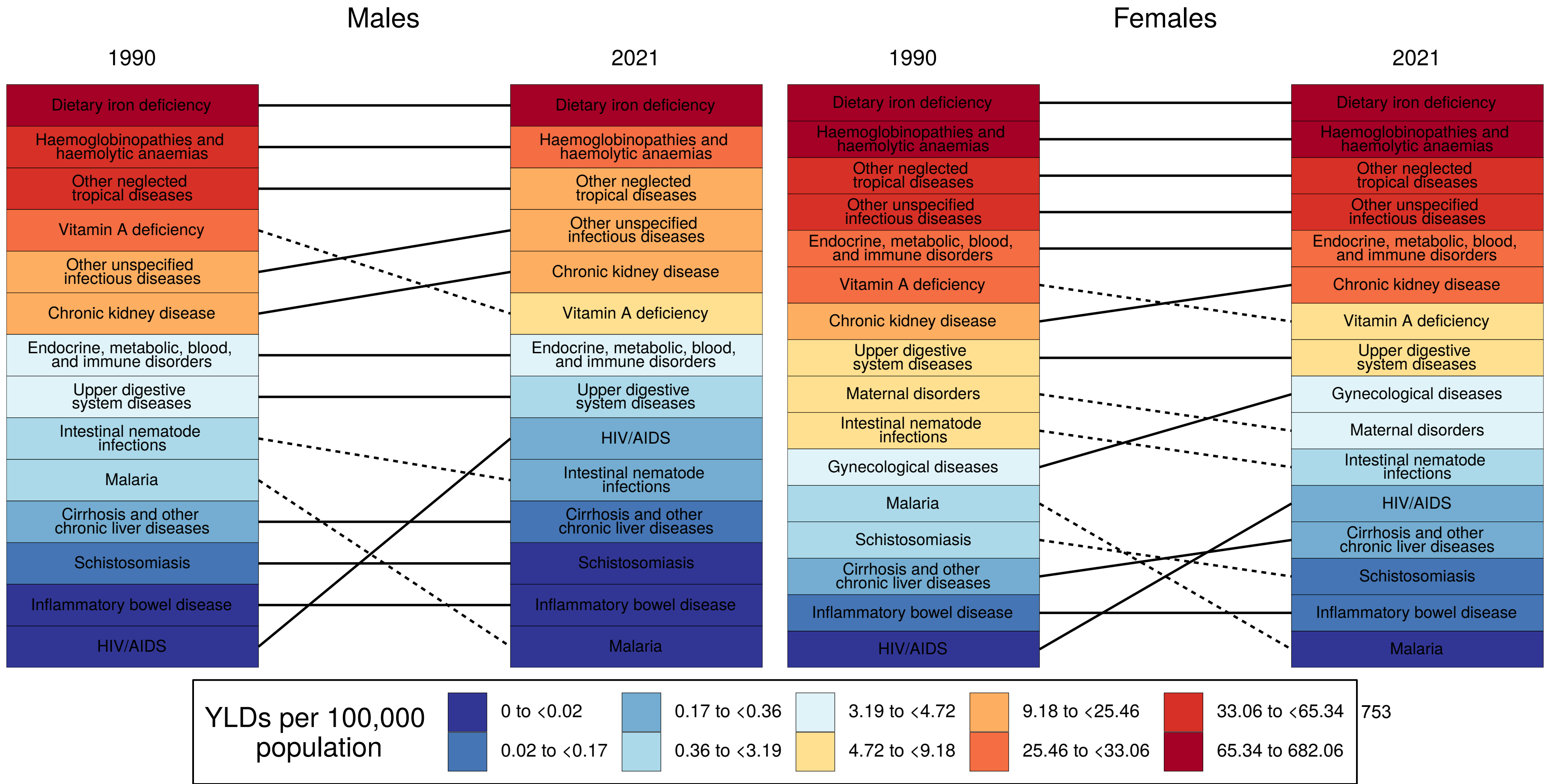

Southern Leyte

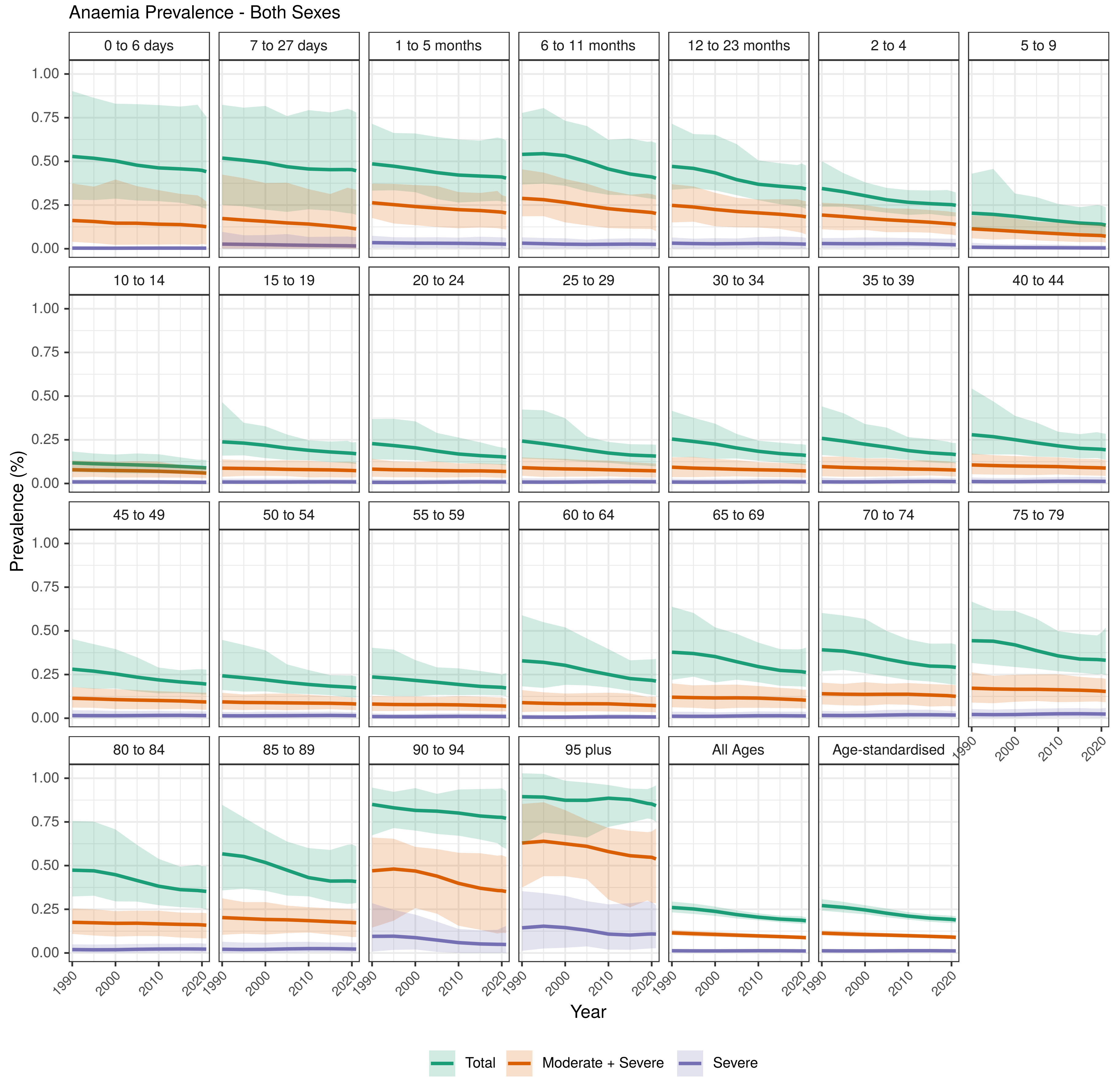

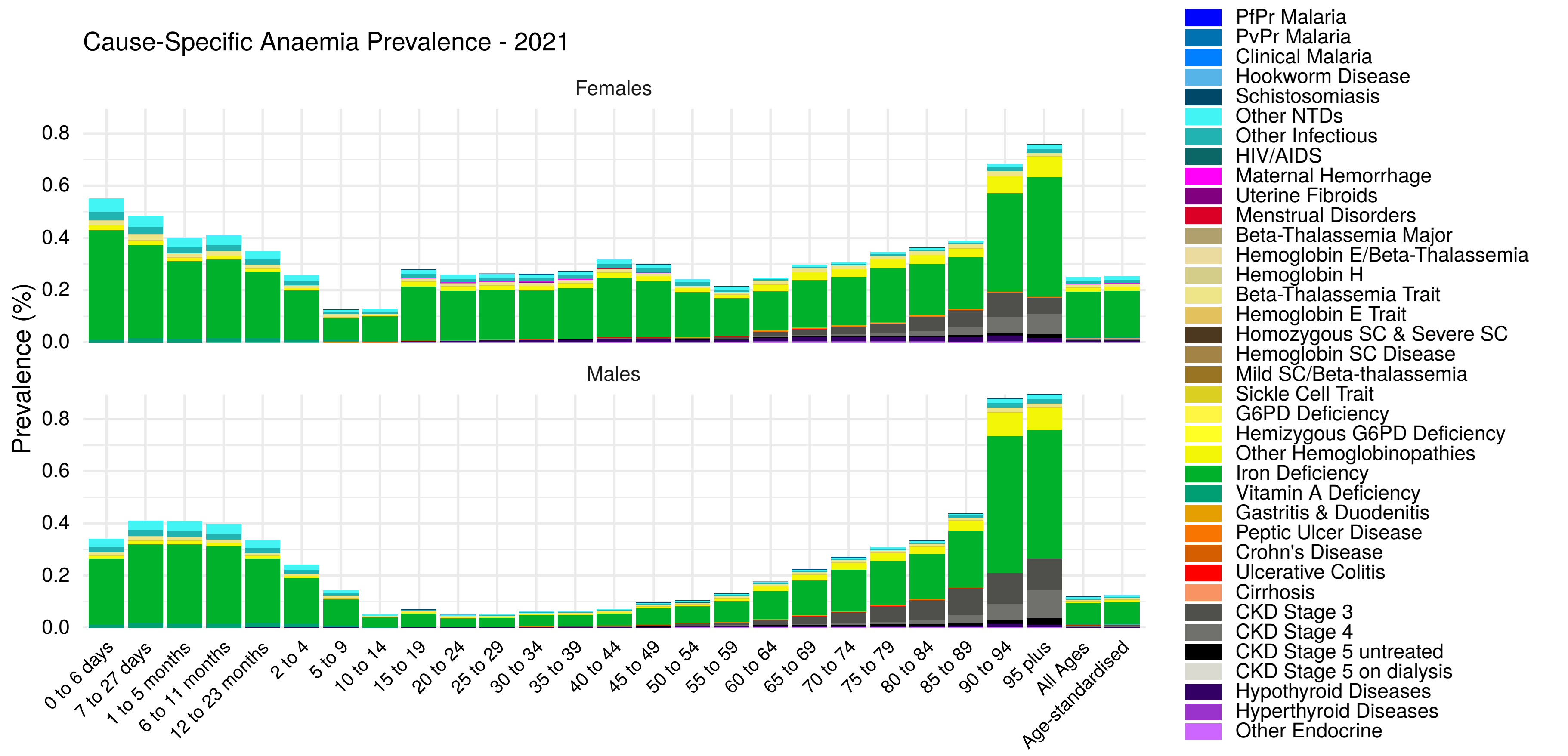

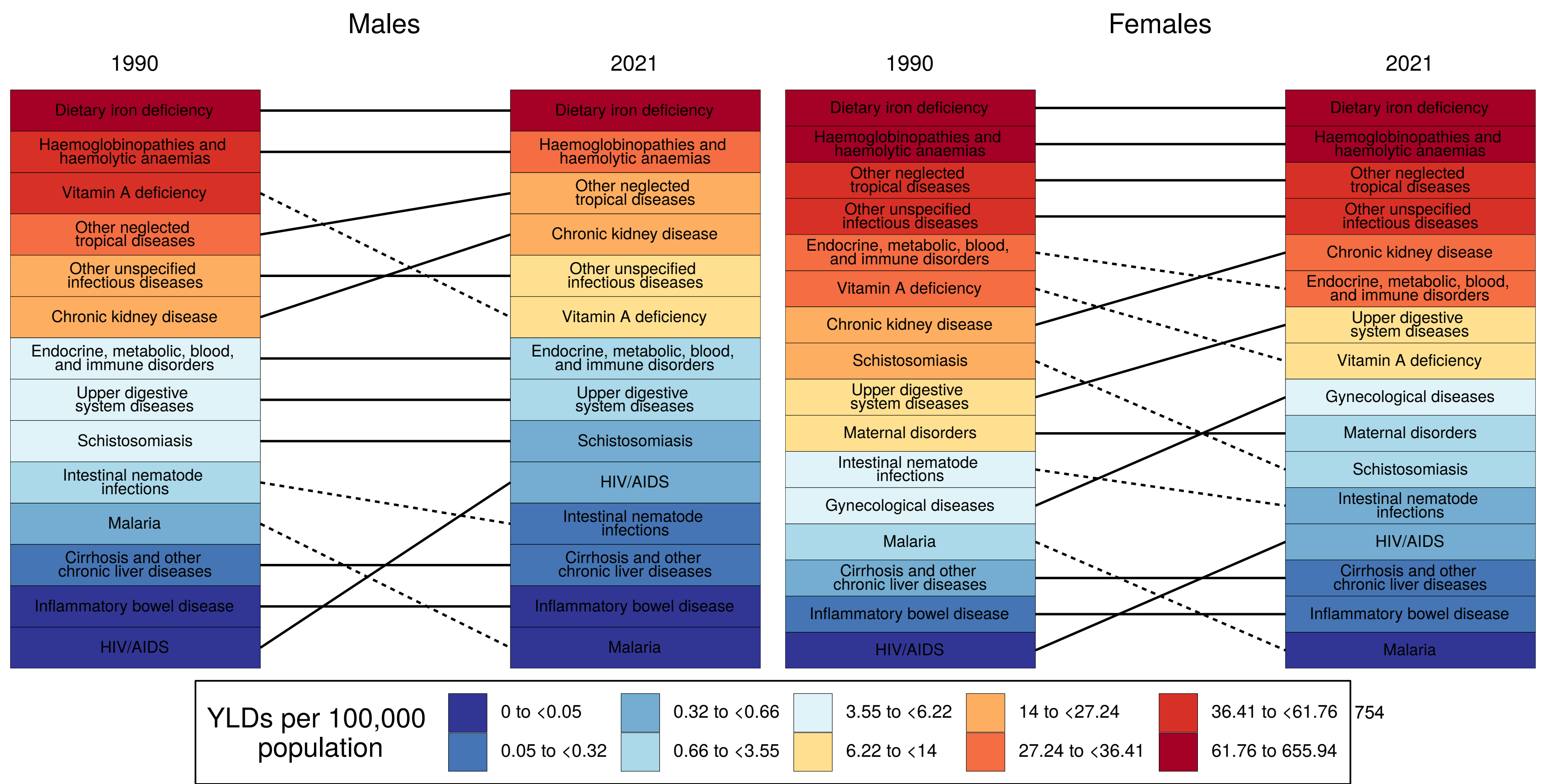

Sultan Kudarat

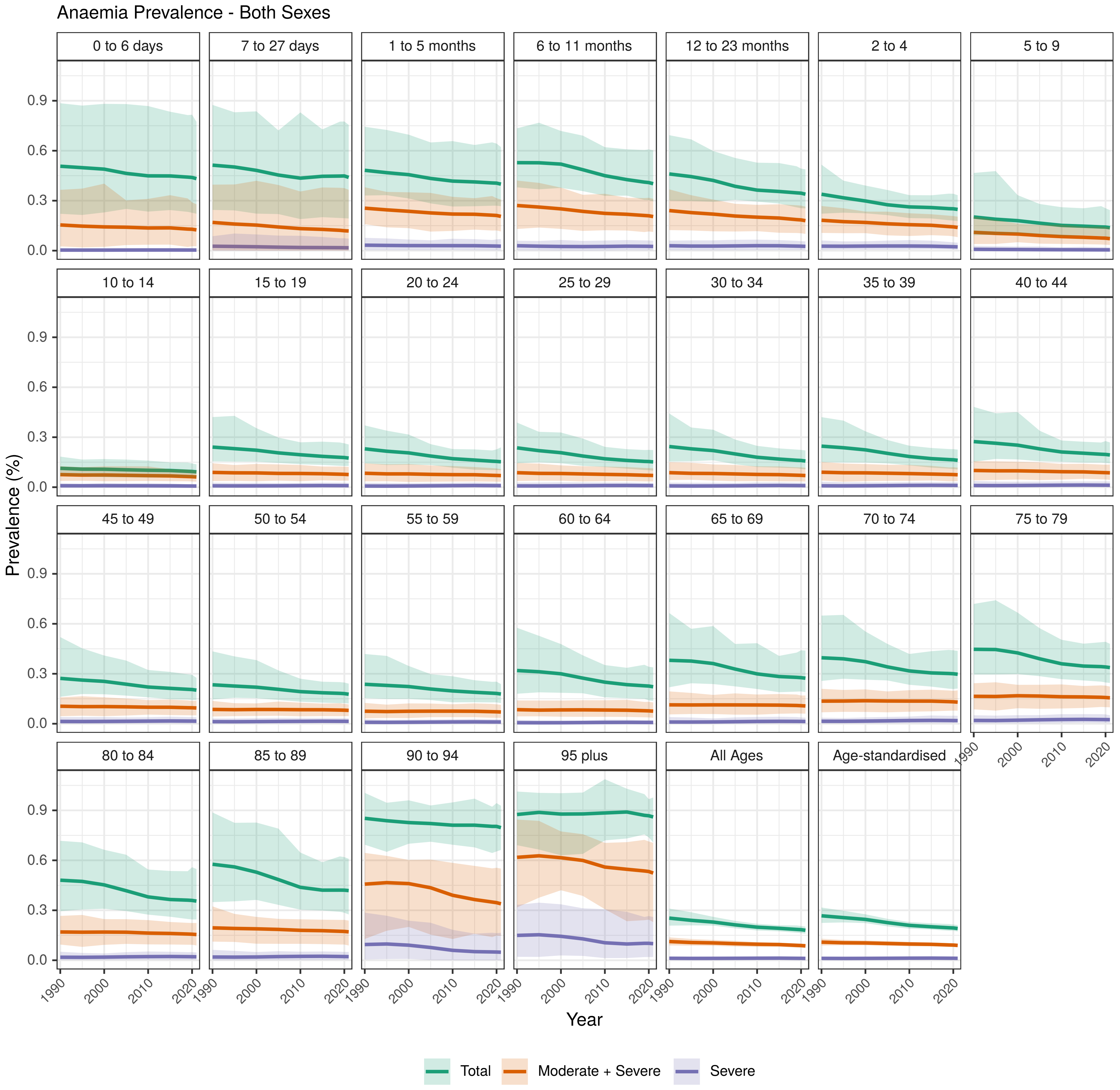

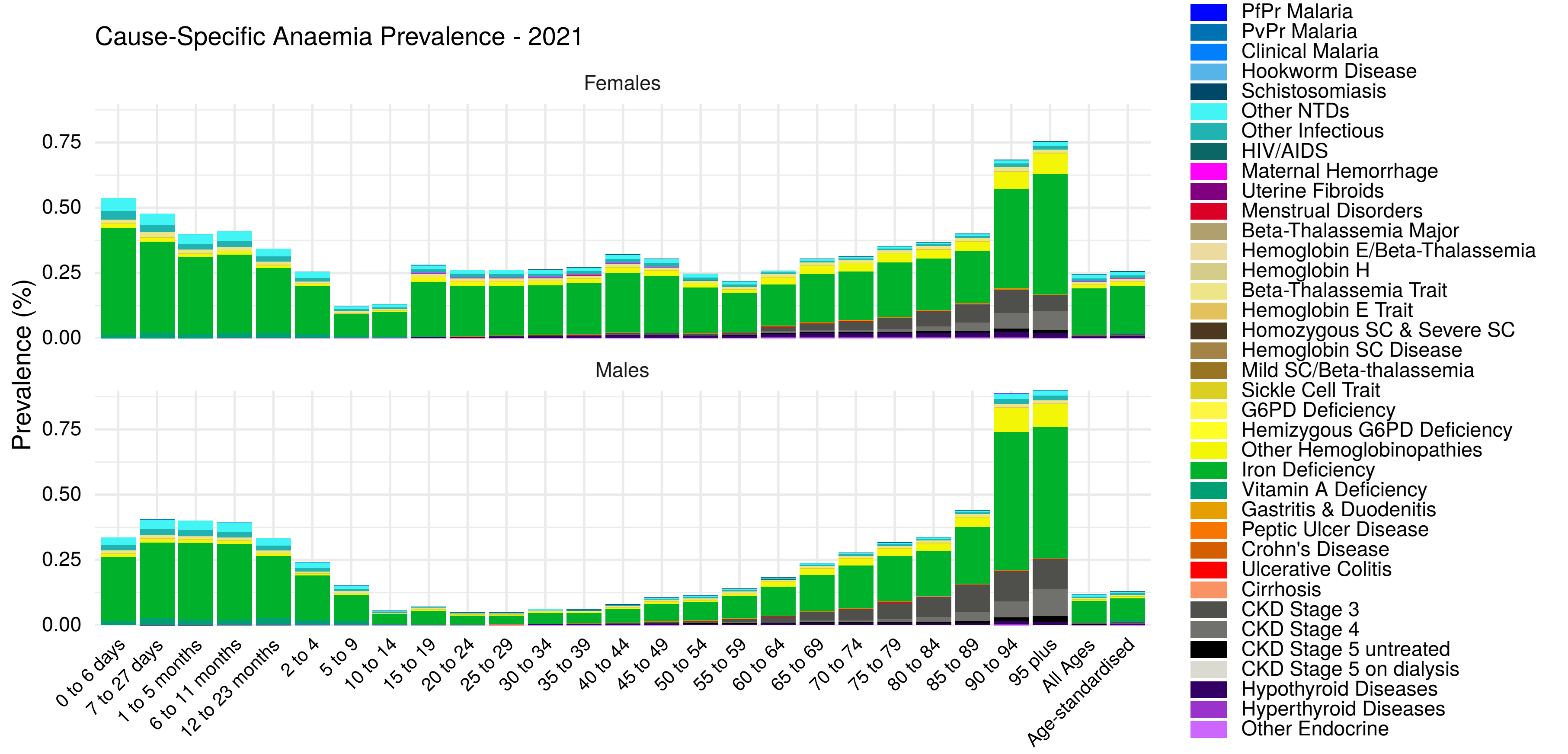

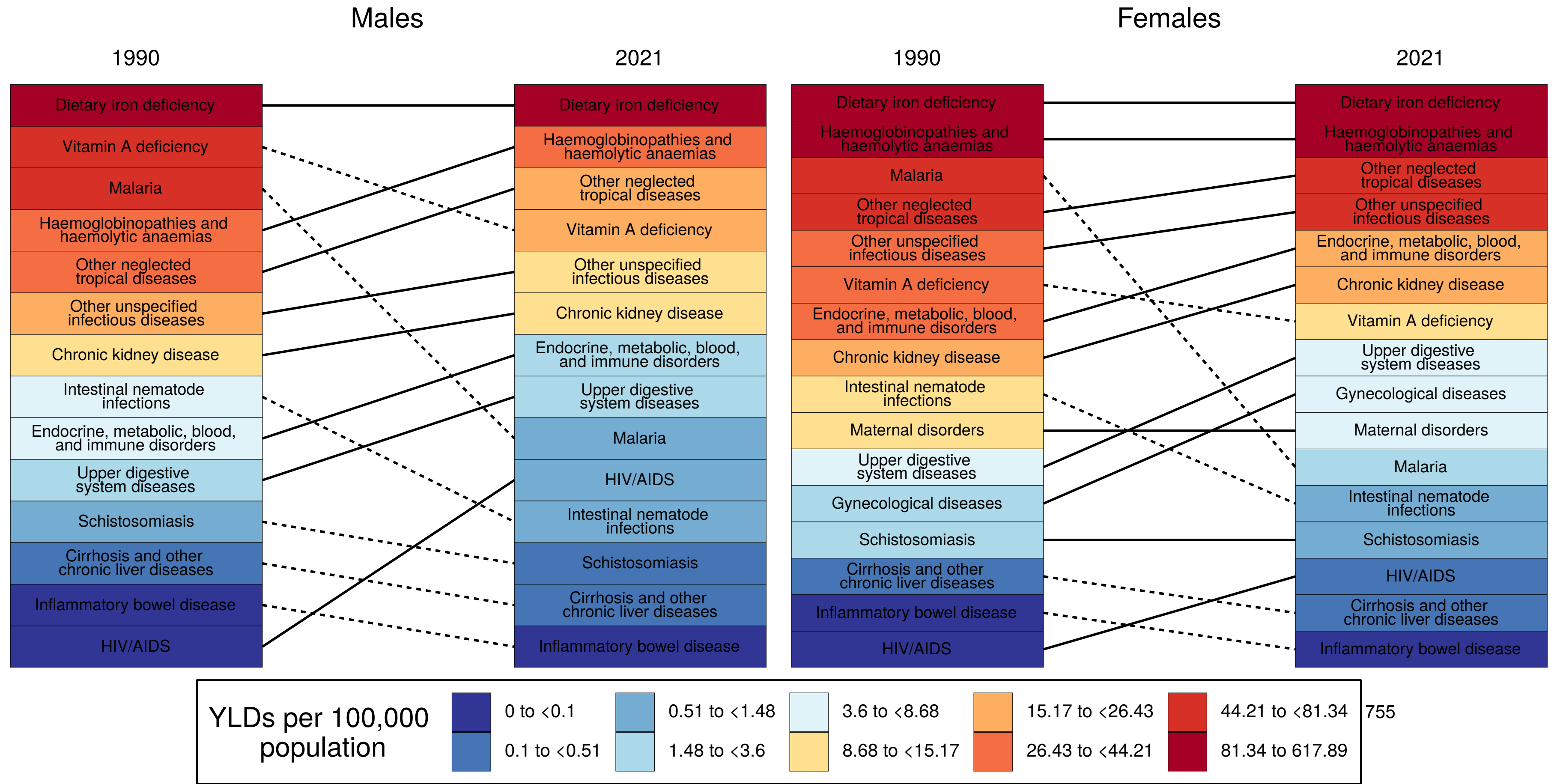

Sulu

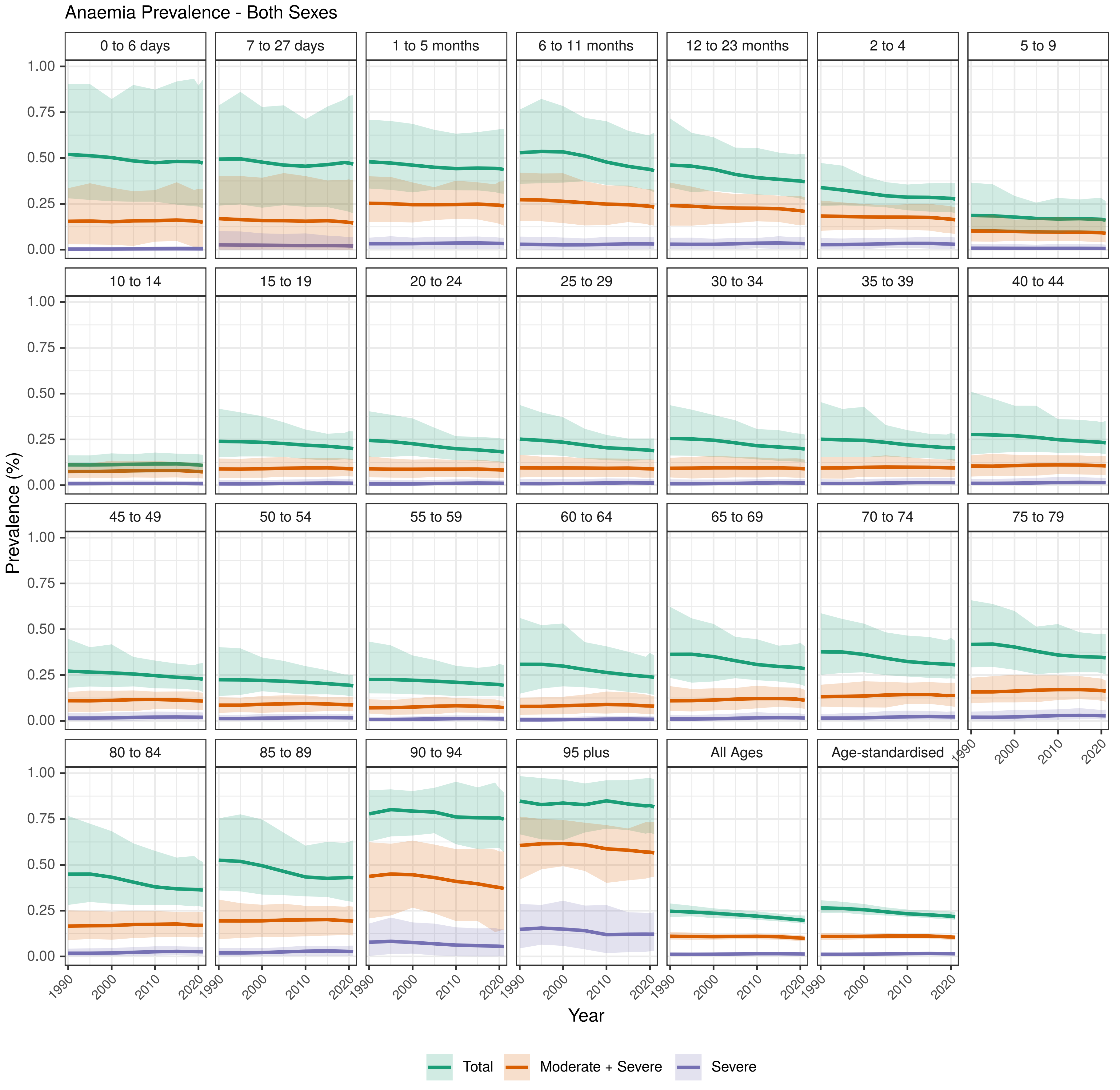
Anaemia Prevalence - Both Sexes
0 to 6 days
7 to 27 days
1 to 5 months
6 to 11 months
12 to 23 months
2 to 4
5 to 9
10 to 14
15 to 19
20 to 24
25 to 29
30 to 34
35 to 39
40 to 44
45 to 49
50 to 54
55 to 59
60 to 64
65 to 69
70 to 74
75 to 79
80 to 84
85 to 89
90 to 94
95 plus
All Ages
Age-standardised
Prevalence (%)
Year
Total
Moderate + Severe
Severe

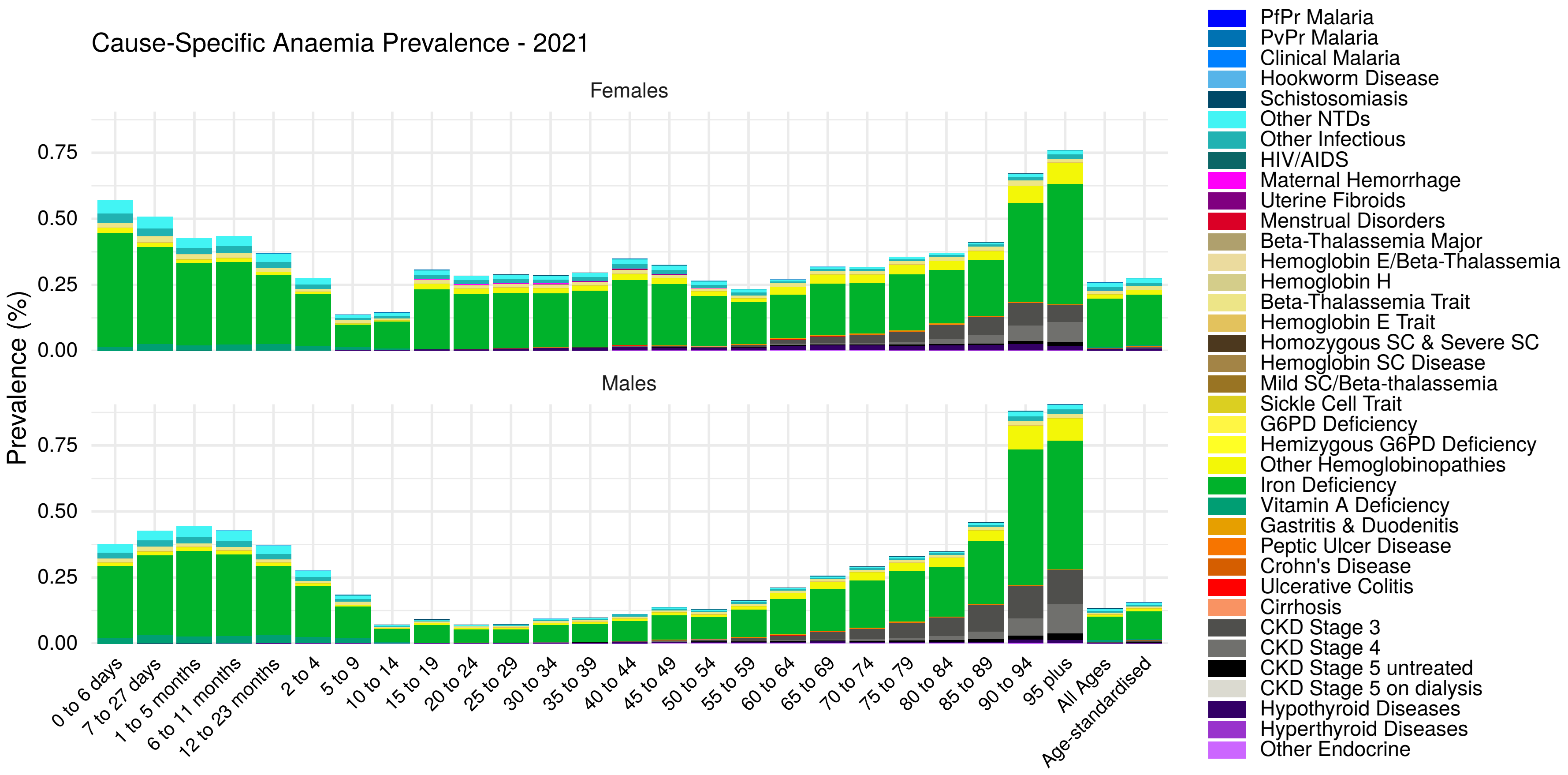
Cause-Specific Anaemia Prevalence - 2021
Females
Males
Prevalence (%)
PfPr Malaria
PvPr Malaria
Clinical Malaria
Hookworm Disease
Schistosomiasis
Other NTDs
Other Infectious
HIV/AIDS
Maternal Hemorrhage
Uterine Fibroids
Menstrual Disorders
Beta-Thalassemia Major
Hemoglobin E/Beta-Thalassemia
Hemoglobin H
Beta-Thalassemia Trait
Hemoglobin E Trait
Homozygous SC & Severe SC
Hemoglobin SC Disease
Mild SC/Beta-thalassemia
Sickle Cell Trait
G6PD Deficiency
Hemizygous G6PD Deficiency
Other Hemoglobinopathies
Iron Deficiency
Vitamin A Deficiency
Gastritis & Duodenitis
Peptic Ulcer Disease
Crohn's Disease
Ulcerative Colitis
Cirrhosis
CKD Stage 3
CKD Stage 4
CKD Stage 5 untreated
CKD Stage 5 on dialysis
Hypothyroid Diseases
Hyperthyroid Diseases
Other Endocrine

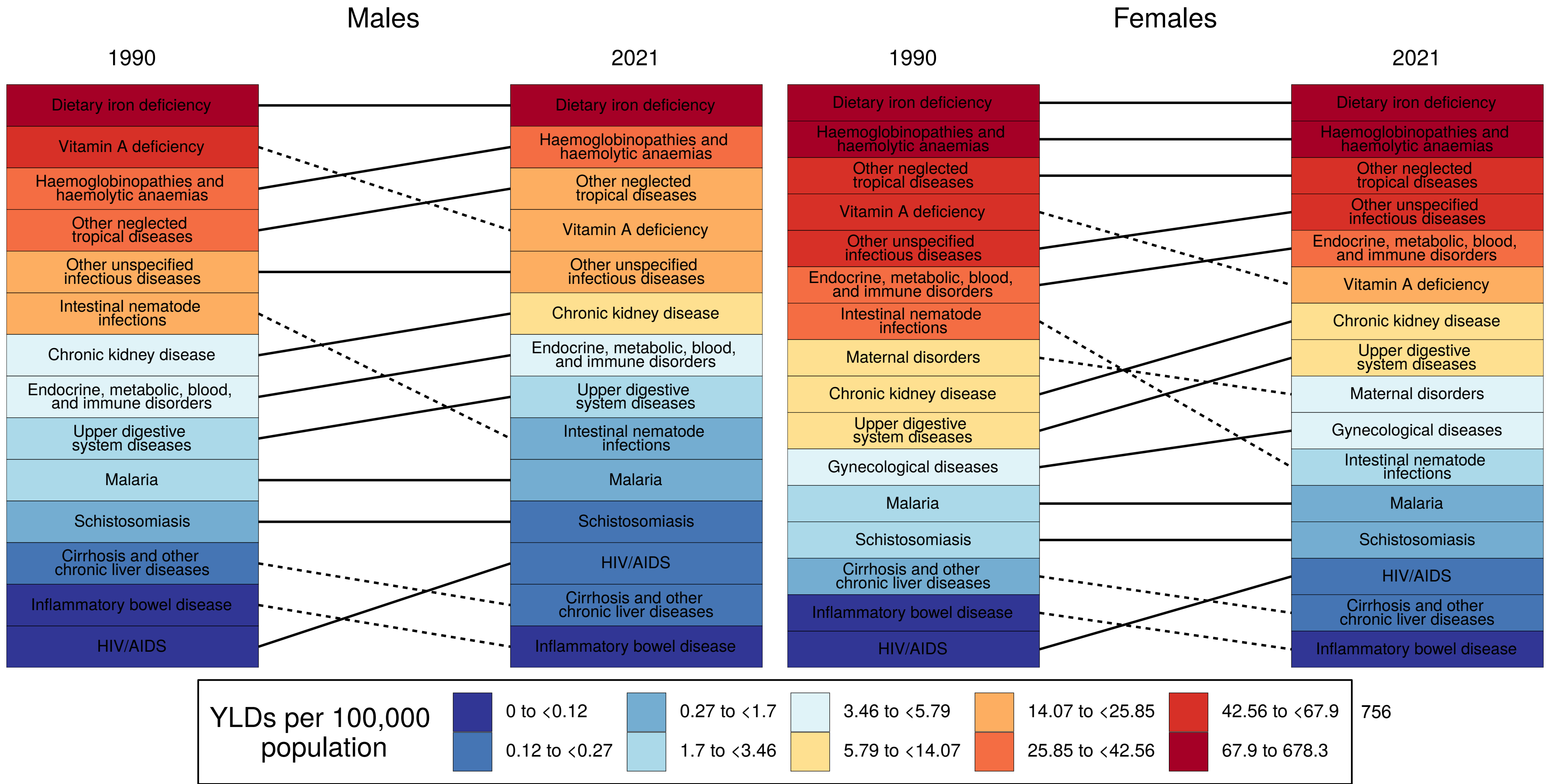
Males
Females
1990
2021
Dietary iron deficiency
Vitamin A deficiency
Haemoglobinopathies and haemolytic anaemias
Other neglected tropical diseases
Other unspecified infectious diseases
Intestinal nematode infections
Chronic kidney disease
Endocrine, metabolic, blood, and immune disorders
Upper digestive system diseases
Malaria
Schistosomiasis
Cirrhosis and other chronic liver diseases
Inflammatory bowel disease
HIV/AIDS
Maternal disorders
Gynecological diseases
YLDs per 100,000 population
0 to <0.12
0.12 to <0.27
0.27 to <1.7
1.7 to <3.46
3.46 to <5.79
5.79 to <14.07
14.07 to <25.85
25.85 to <42.56
42.56 to <67.9
67.9 to 678.3

Surigao Del Norte

Anaemia Prevalence - Both Sexes

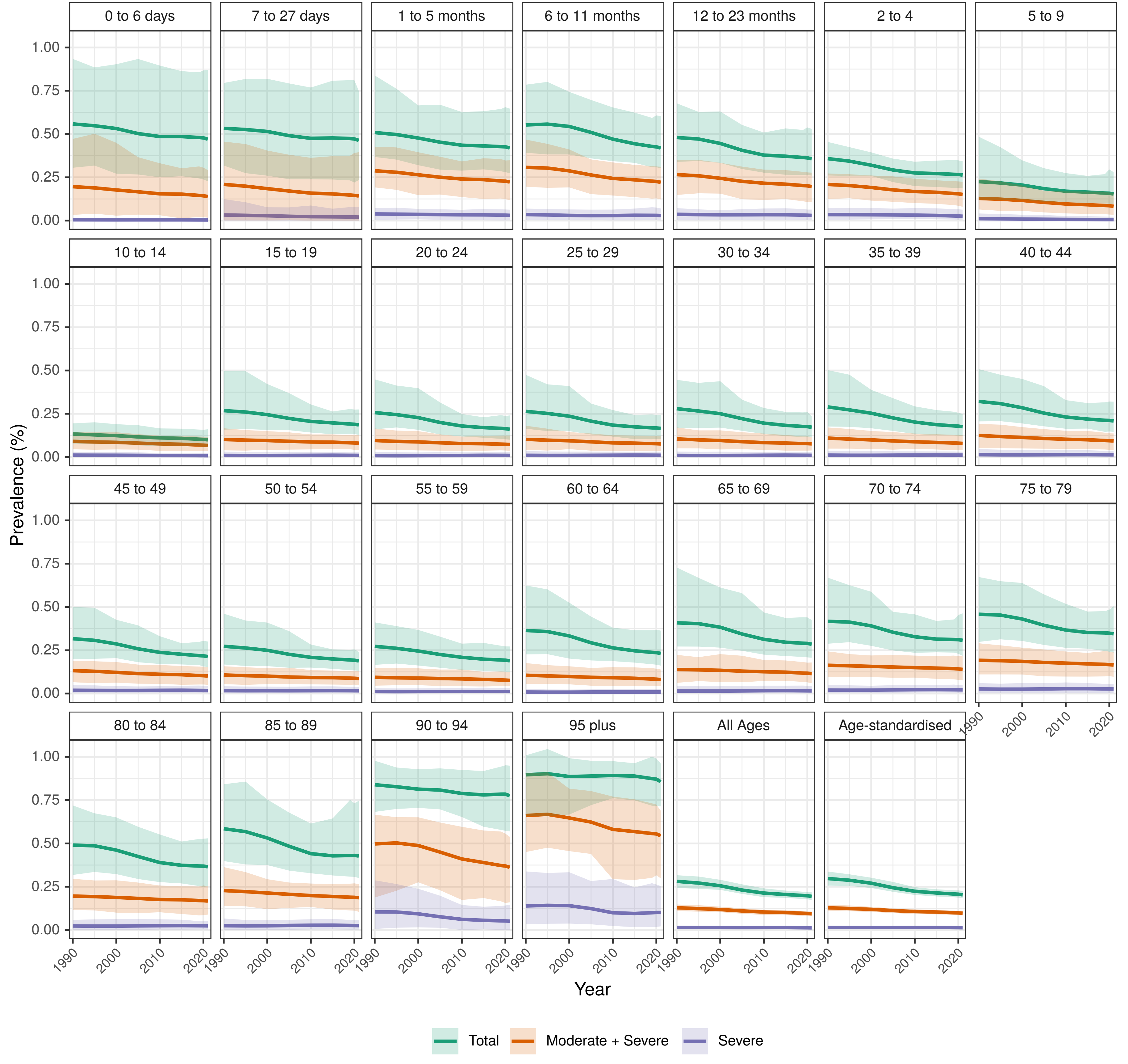

Cause-Specific Anaemia Prevalence - 2021

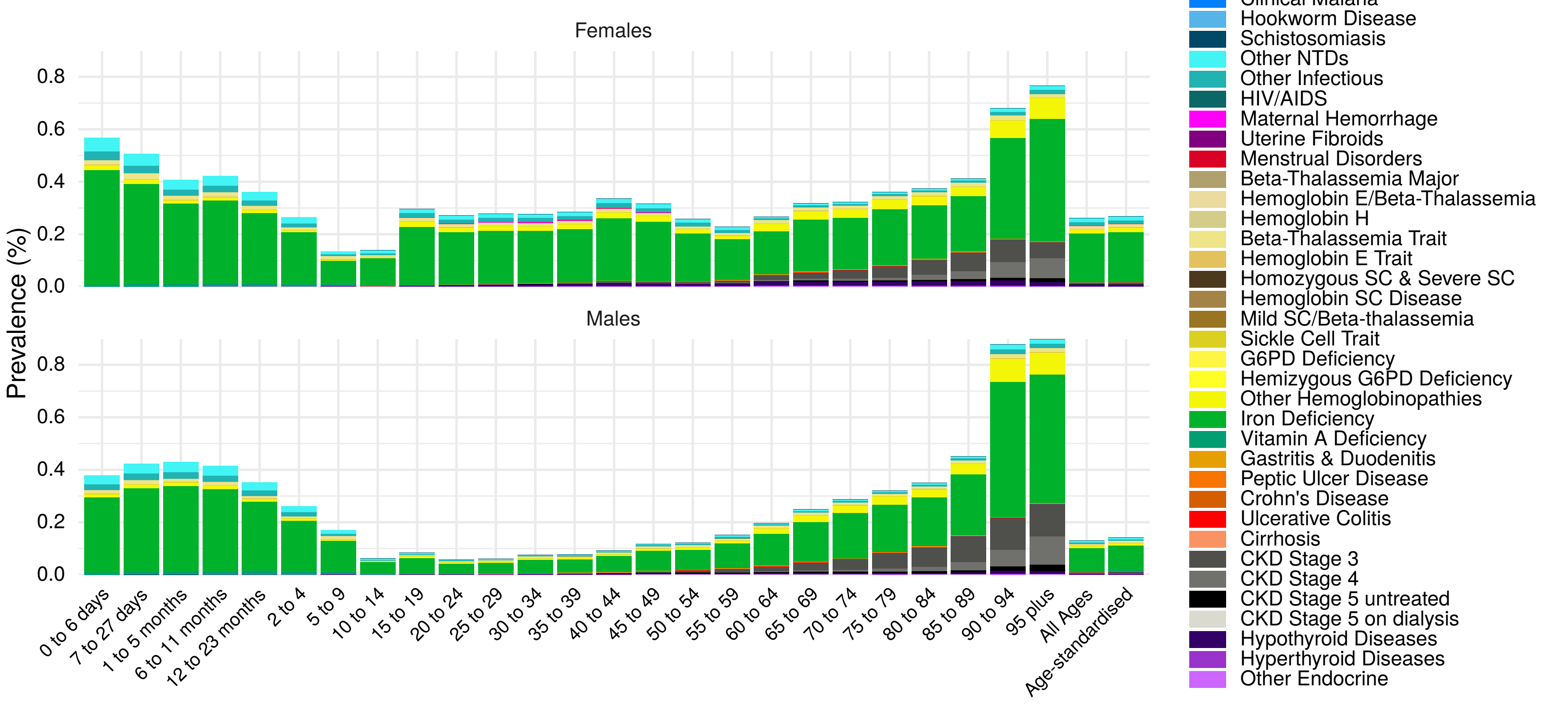

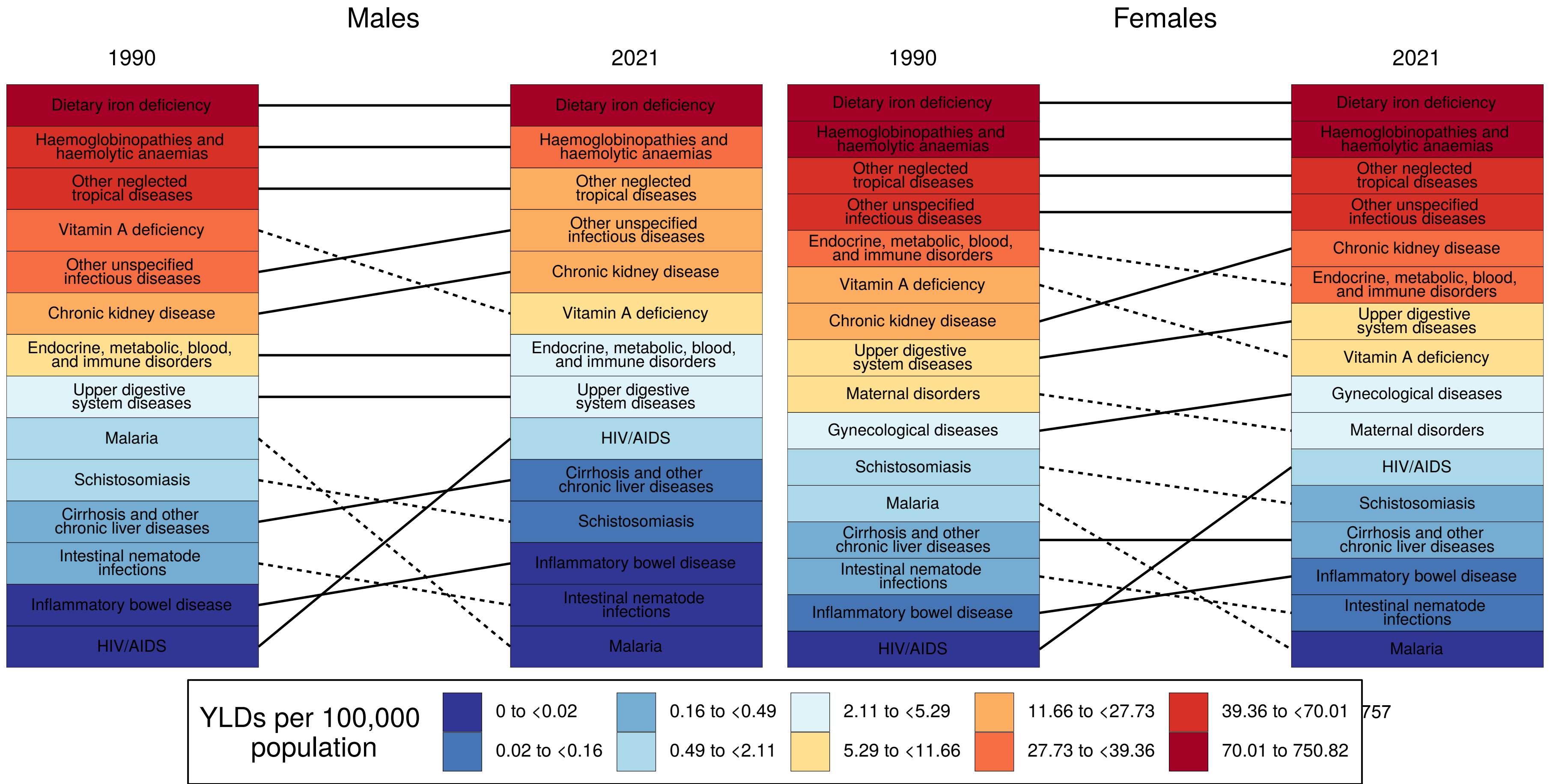

Surigao Del Sur

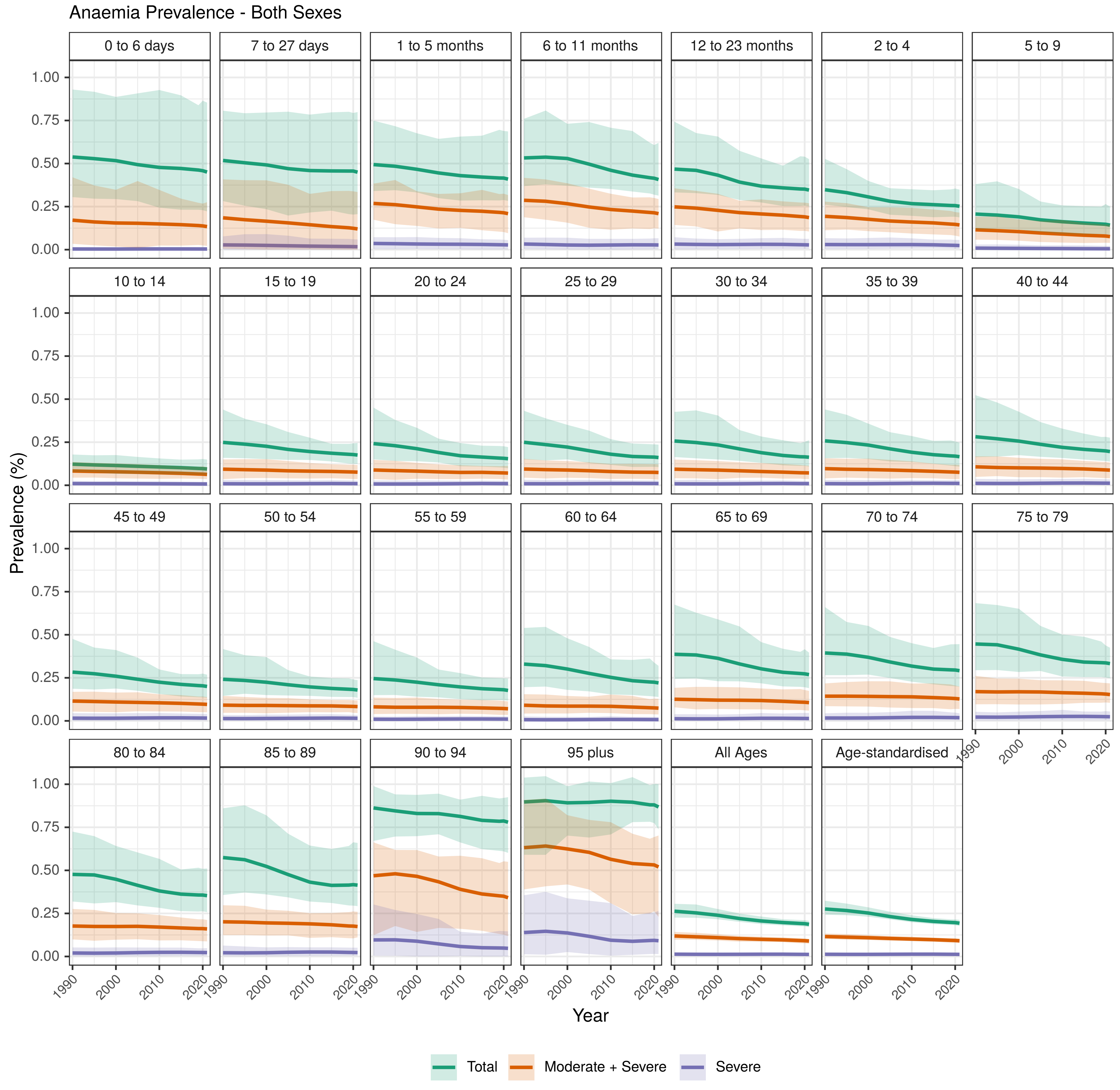

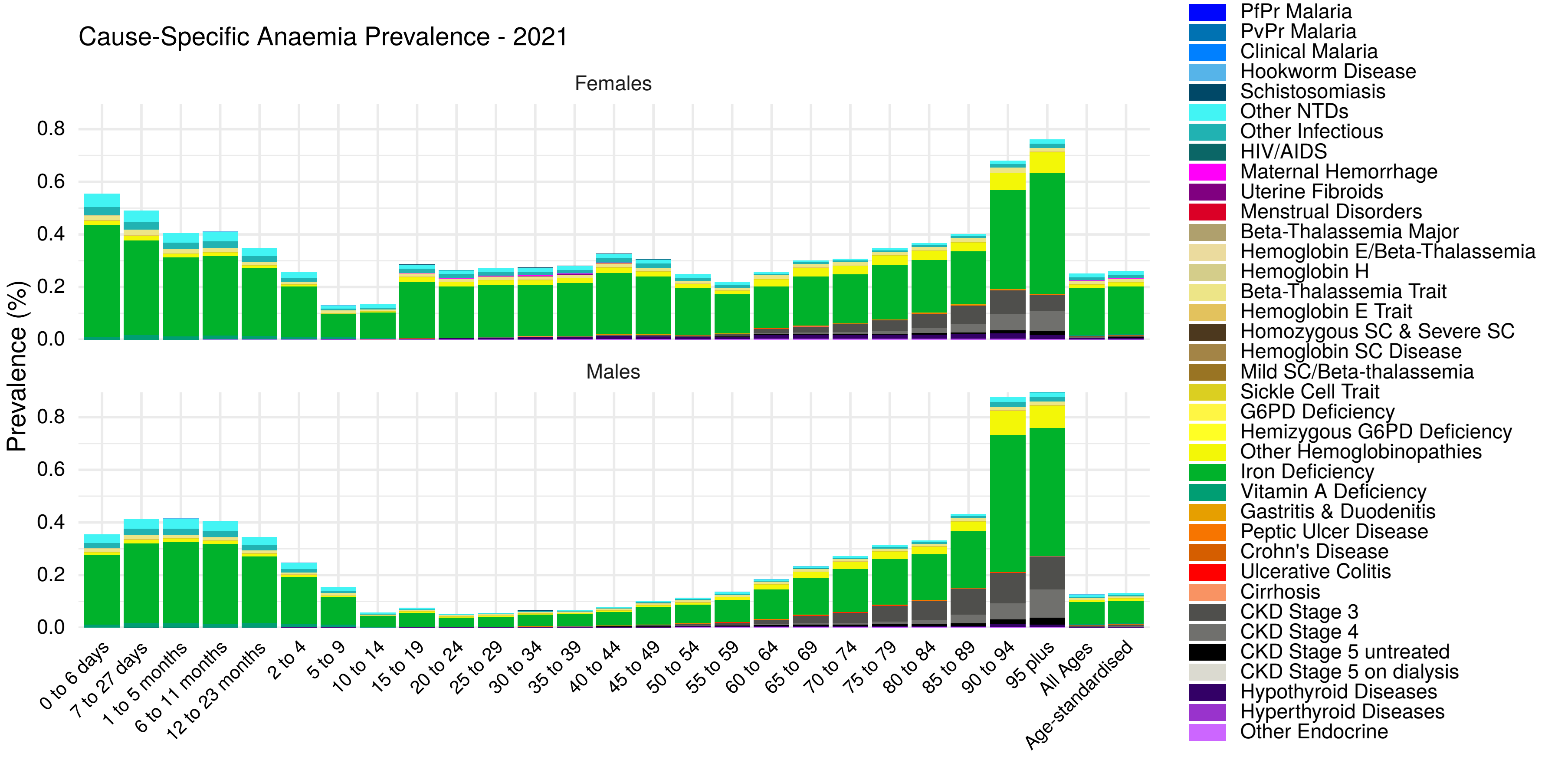

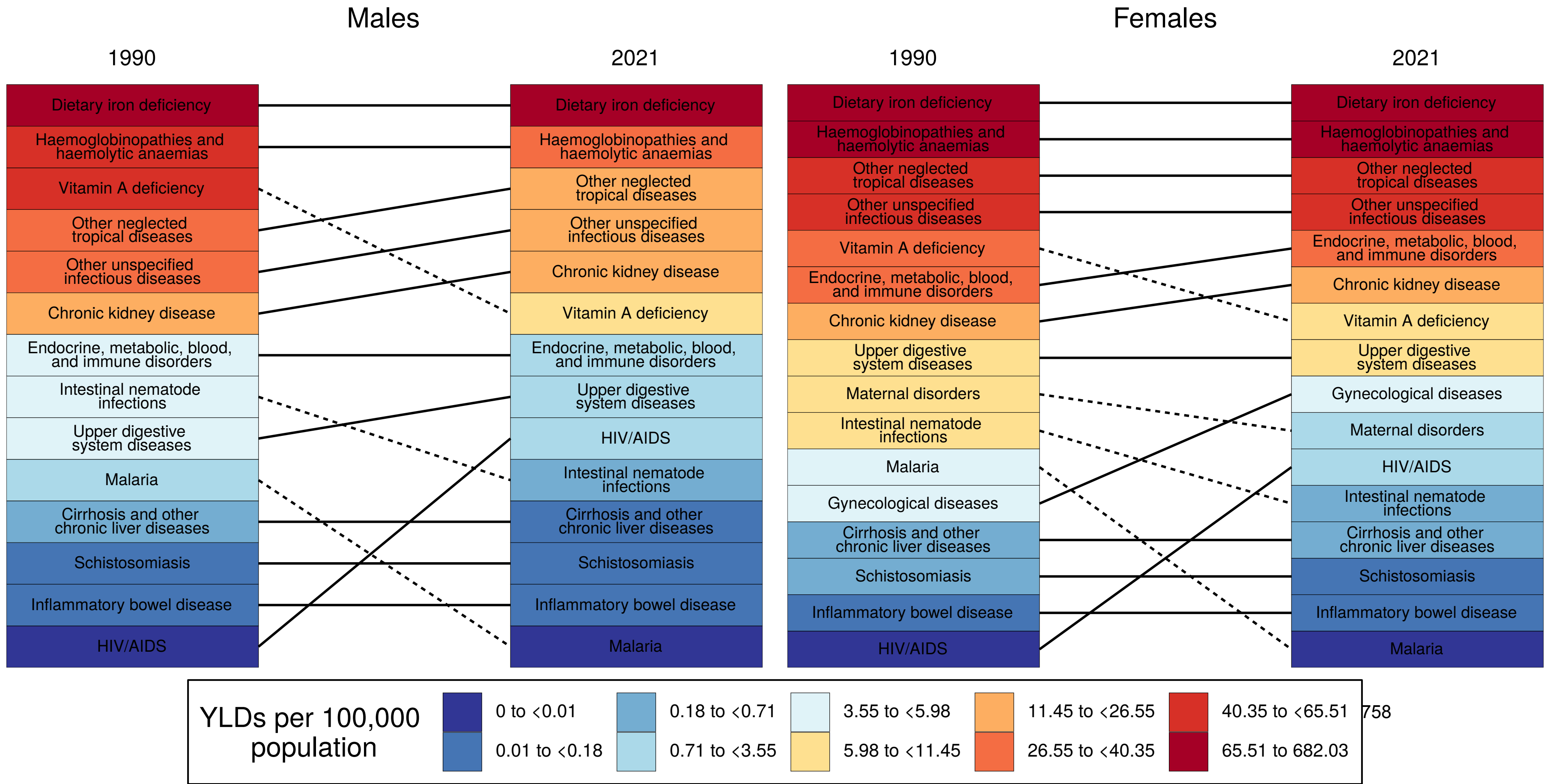

758

Tarlac

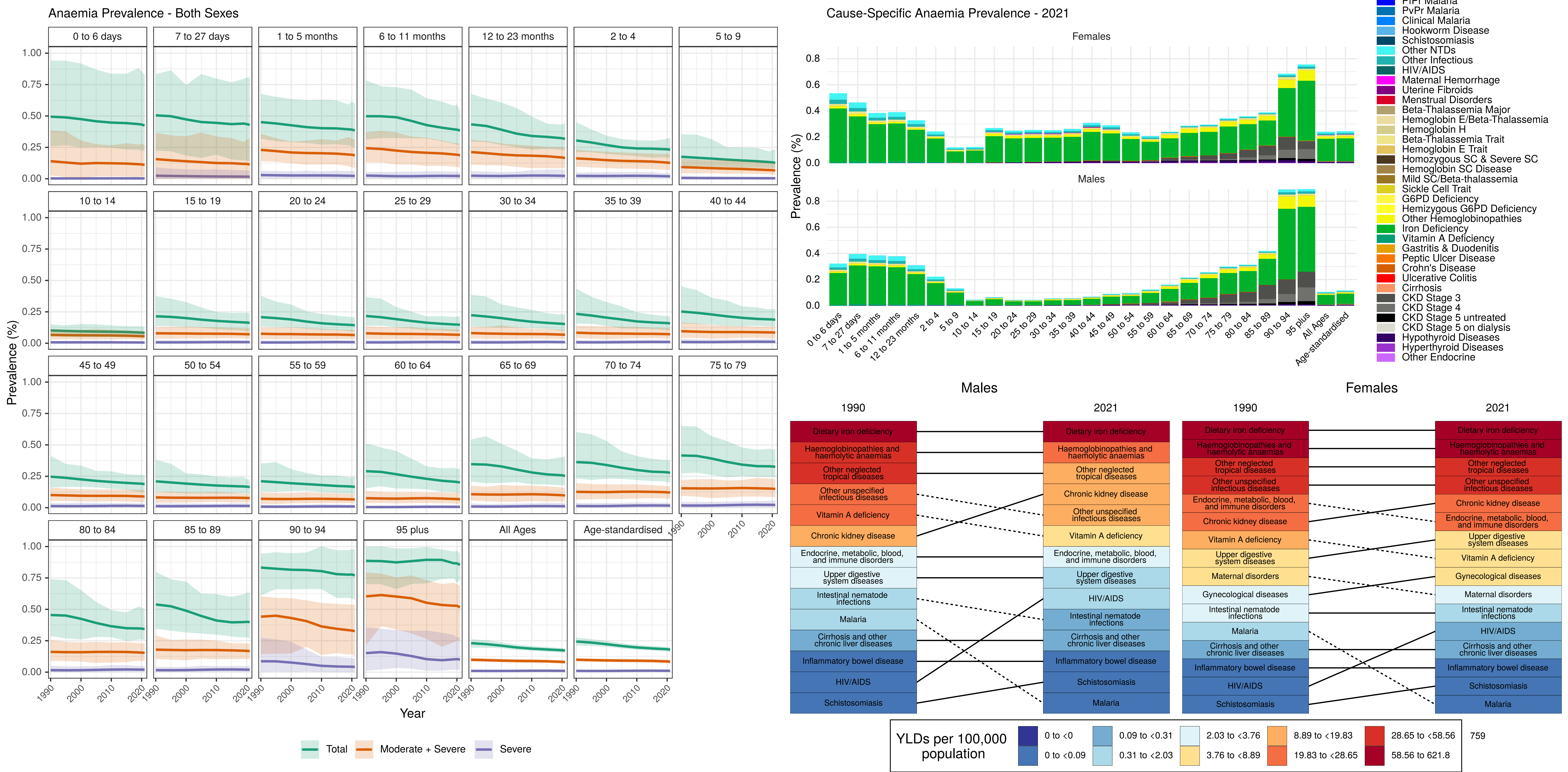

Anaemia Prevalence - Both Sexes
0 to 6 days
7 to 27 days
1 to 5 months
6 to 11 months
12 to 23 months
2 to 4
5 to 9
10 to 14
15 to 19
20 to 24
25 to 29
30 to 34
35 to 39
40 to 44
45 to 49
50 to 54
55 to 59
60 to 64
65 to 69
70 to 74
75 to 79
80 to 84
85 to 89
90 to 94
95 plus
All Ages
Age-standardised
Prevalence (%)
Year
Total
Moderate + Severe
Severe
Cause-Specific Anaemia Prevalence - 2021
Females
Males
Prevalence (%)
PfPr Malaria
PvPr Malaria
Clinical Malaria
Hookworm Disease
Schistosomiasis
Other NTDs
Other Infectious
HIV/AIDS
Maternal Hemorrhage
Uterine Fibroids
Menstrual Disorders
Beta-Thalassemia Major
Hemoglobin E/Beta-Thalassemia
Hemoglobin H
Beta-Thalassemia Trait
Hemoglobin E Trait
Homozygous SC & Severe SC
Hemoglobin SC Disease
Mild SC/Beta-thalassemia
Sickle Cell Trait
G6PD Deficiency
Hemizygous G6PD Deficiency
Other Hemoglobinopathies
Iron Deficiency
Vitamin A Deficiency
Gastritis & Duodenitis
Peptic Ulcer Disease
Crohn's Disease
Ulcerative Colitis
Cirrhosis
CKD Stage 3
CKD Stage 4
CKD Stage 5 untreated
CKD Stage 5 on dialysis
Hypothyroid Diseases
Hyperthyroid Diseases
Other Endocrine
Males
Females
1990
2021
Males 1990: Dietary iron deficiency; Haemoglobinopathies and haemolytic anaemias; Other neglected tropical diseases; Other unspecified infectious diseases; Vitamin A deficiency; Chronic kidney disease; Endocrine, metabolic, blood, and immune disorders; Upper digestive system diseases; Intestinal nematode infections; Malaria; Cirrhosis and other chronic liver diseases; Inflammatory bowel disease; HIV/AIDS; Schistosomiasis
Males 2021: Dietary iron deficiency; Haemoglobinopathies and haemolytic anaemias; Other neglected tropical diseases; Chronic kidney disease; Other unspecified infectious diseases; Vitamin A deficiency; Endocrine, metabolic, blood, and immune disorders; Upper digestive system diseases; HIV/AIDS; Intestinal nematode infections; Cirrhosis and other chronic liver diseases; Inflammatory bowel disease; Schistosomiasis; Malaria
Females 1990: Dietary iron deficiency; Haemoglobinopathies and haemolytic anaemias; Other neglected tropical diseases; Other unspecified infectious diseases; Endocrine, metabolic, blood, and immune disorders; Chronic kidney disease; Vitamin A deficiency; Upper digestive system diseases; Maternal disorders; Gynecological diseases; Intestinal nematode infections; Malaria; Cirrhosis and other chronic liver diseases; Inflammatory bowel disease; HIV/AIDS; Schistosomiasis
Females 2021: Dietary iron deficiency; Haemoglobinopathies and haemolytic anaemias; Other neglected tropical diseases; Other unspecified infectious diseases; Chronic kidney disease; Endocrine, metabolic, blood, and immune disorders; Upper digestive system diseases; Vitamin A deficiency; Gynecological diseases; Maternal disorders; Intestinal nematode infections; HIV/AIDS; Cirrhosis and other chronic liver diseases; Inflammatory bowel disease; Schistosomiasis; Malaria
YLDs per 100,000 population
0 to <0
0 to <0.09
0.09 to <0.31
0.31 to <2.03
2.03 to <3.76
3.76 to <8.89
8.89 to <19.83
19.83 to <28.65
28.65 to <58.56
58.56 to 621.8

759

Tawi-Tawi

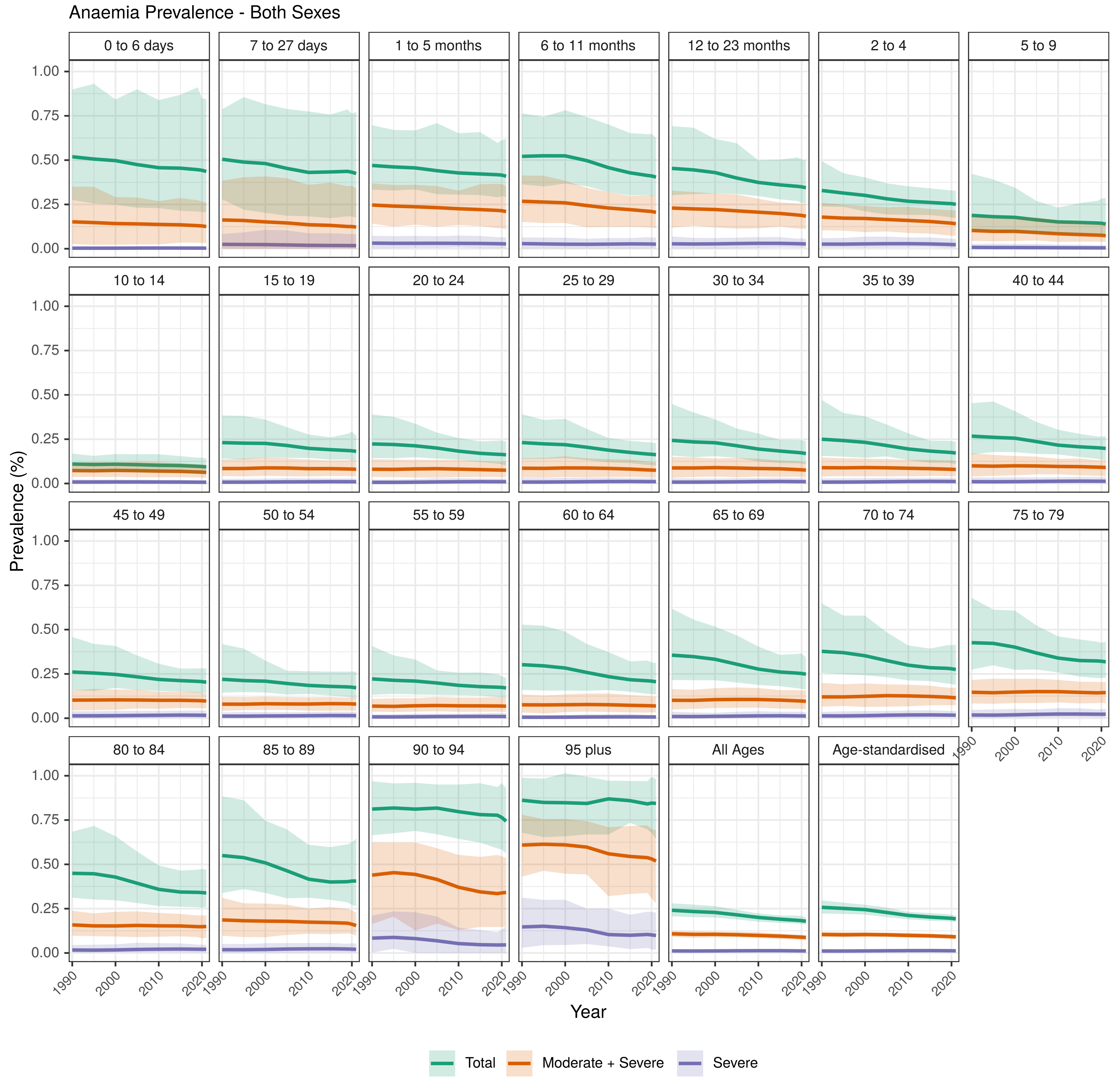

Anaemia Prevalence - Both Sexes
0 to 6 days
7 to 27 days
1 to 5 months
6 to 11 months
12 to 23 months
2 to 4
5 to 9
10 to 14
15 to 19
20 to 24
25 to 29
30 to 34
35 to 39
40 to 44
45 to 49
50 to 54
55 to 59
60 to 64
65 to 69
70 to 74
75 to 79
80 to 84
85 to 89
90 to 94
95 plus
All Ages
Age-standardised
Prevalence (%)
0.00
0.25
0.50
0.75
1.00
1990
2000
2010
2020
Year
Total
Moderate + Severe
Severe

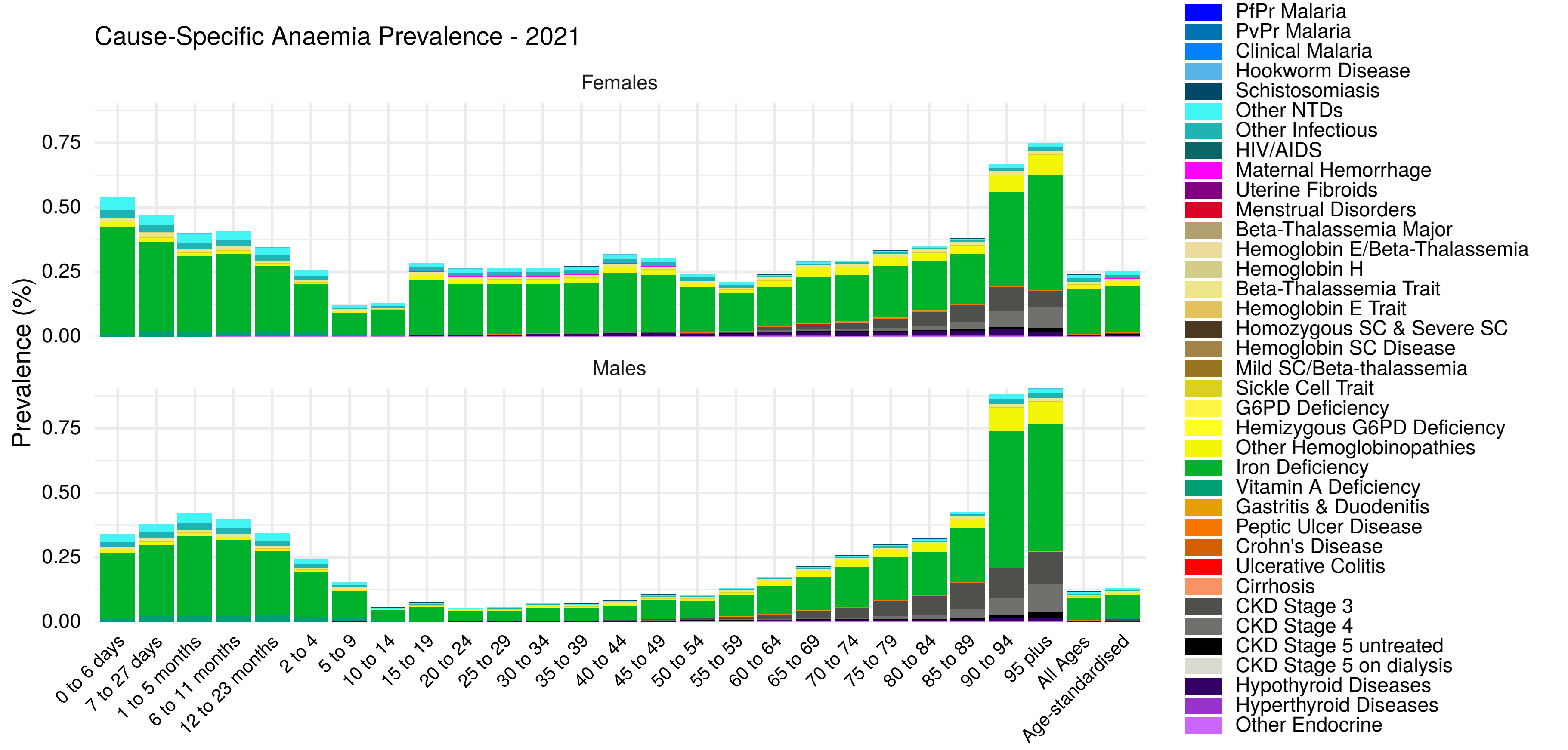

Cause-Specific Anaemia Prevalence - 2021
Females
Males
Prevalence (%)
0.00
0.25
0.50
0.75
0 to 6 days
7 to 27 days
1 to 5 months
6 to 11 months
12 to 23 months
2 to 4
5 to 9
10 to 14
15 to 19
20 to 24
25 to 29
30 to 34
35 to 39
40 to 44
45 to 49
50 to 54
55 to 59
60 to 64
65 to 69
70 to 74
75 to 79
80 to 84
85 to 89
90 to 94
95 plus
All Ages
Age-standardised
PfPr Malaria
PvPr Malaria
Clinical Malaria
Hookworm Disease
Schistosomiasis
Other NTDs
Other Infectious
HIV/AIDS
Maternal Hemorrhage
Uterine Fibroids
Menstrual Disorders
Beta-Thalassemia Major
Hemoglobin E/Beta-Thalassemia
Hemoglobin H
Beta-Thalassemia Trait
Hemoglobin E Trait
Homozygous SC & Severe SC
Hemoglobin SC Disease
Mild SC/Beta-thalassemia
Sickle Cell Trait
G6PD Deficiency
Hemizygous G6PD Deficiency
Other Hemoglobinopathies
Iron Deficiency
Vitamin A Deficiency
Gastritis & Duodenitis
Peptic Ulcer Disease
Crohn's Disease
Ulcerative Colitis
Cirrhosis
CKD Stage 3
CKD Stage 4
CKD Stage 5 untreated
CKD Stage 5 on dialysis
Hypothyroid Diseases
Hyperthyroid Diseases
Other Endocrine

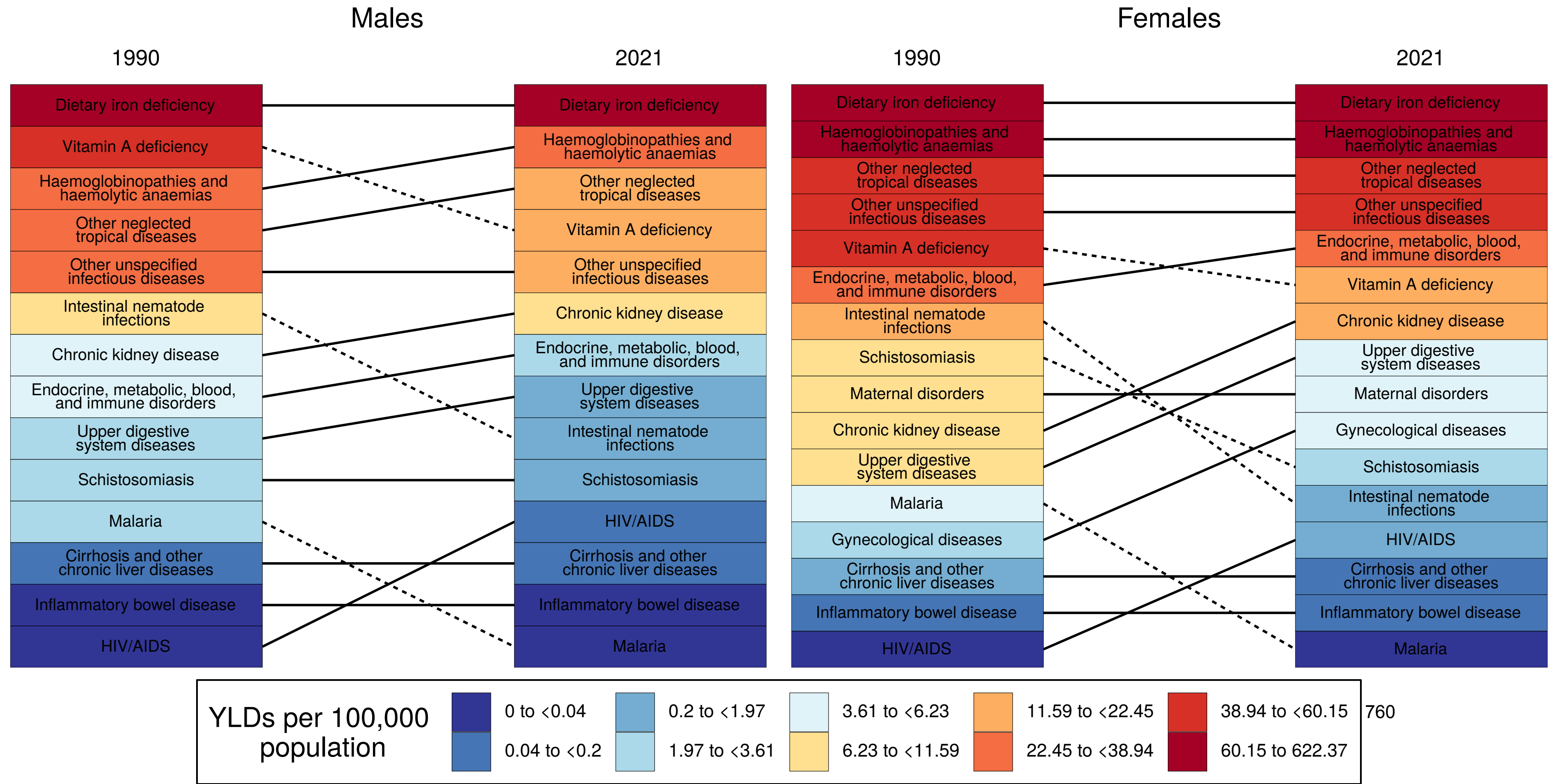

Males
Females
1990
2021
Dietary iron deficiency
Vitamin A deficiency
Haemoglobinopathies and haemolytic anaemias
Other neglected tropical diseases
Other unspecified infectious diseases
Intestinal nematode infections
Chronic kidney disease
Endocrine, metabolic, blood, and immune disorders
Upper digestive system diseases
Schistosomiasis
Malaria
Cirrhosis and other chronic liver diseases
Inflammatory bowel disease
HIV/AIDS
Maternal disorders
Gynecological diseases
YLDs per 100,000 population
0 to <0.04
0.04 to <0.2
0.2 to <1.97
1.97 to <3.61
3.61 to <6.23
6.23 to <11.59
11.59 to <22.45
22.45 to <38.94
38.94 to <60.15
60.15 to 622.37
760

Zambales

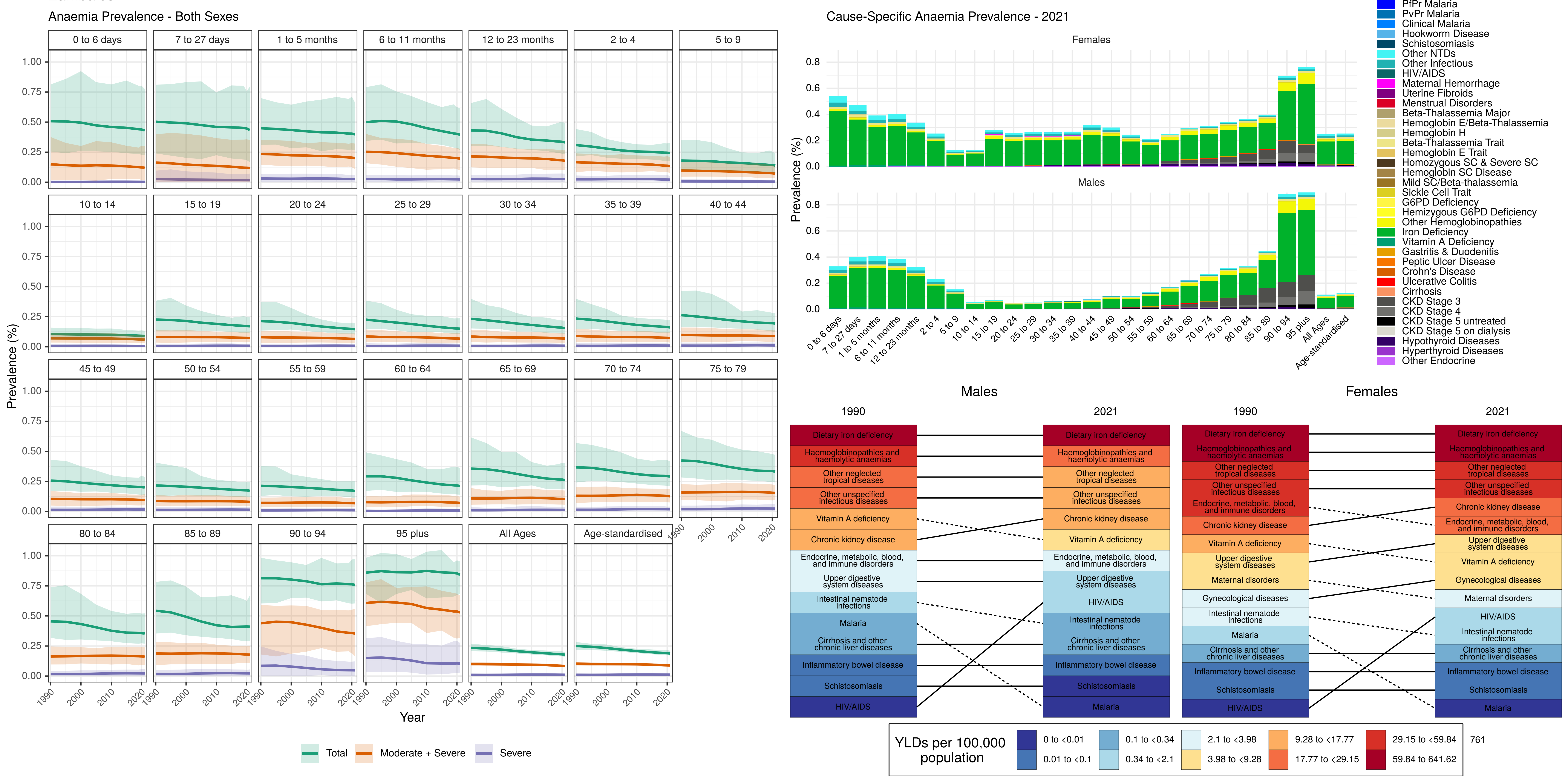

Anaemia Prevalence - Both Sexes
0 to 6 days
7 to 27 days
1 to 5 months
6 to 11 months
12 to 23 months
2 to 4
5 to 9
10 to 14
15 to 19
20 to 24
25 to 29
30 to 34
35 to 39
40 to 44
45 to 49
50 to 54
55 to 59
60 to 64
65 to 69
70 to 74
75 to 79
80 to 84
85 to 89
90 to 94
95 plus
All Ages
Age-standardised
Prevalence (%)
Year
Total
Moderate + Severe
Severe
Cause-Specific Anaemia Prevalence - 2021
Females
Males
PfPr Malaria
PvPr Malaria
Clinical Malaria
Hookworm Disease
Schistosomiasis
Other NTDs
Other Infectious
HIV/AIDS
Maternal Hemorrhage
Uterine Fibroids
Menstrual Disorders
Beta-Thalassemia Major
Hemoglobin E/Beta-Thalassemia
Hemoglobin H
Beta-Thalassemia Trait
Hemoglobin E Trait
Homozygous SC & Severe SC
Hemoglobin SC Disease
Mild SC/Beta-thalassemia
Sickle Cell Trait
G6PD Deficiency
Hemizygous G6PD Deficiency
Other Hemoglobinopathies
Iron Deficiency
Vitamin A Deficiency
Gastritis & Duodenitis
Peptic Ulcer Disease
Crohn's Disease
Ulcerative Colitis
Cirrhosis
CKD Stage 3
CKD Stage 4
CKD Stage 5 untreated
CKD Stage 5 on dialysis
Hypothyroid Diseases
Hyperthyroid Diseases
Other Endocrine
Males
Females
1990
2021
Dietary iron deficiency
Haemoglobinopathies and haemolytic anaemias
Other neglected tropical diseases
Other unspecified infectious diseases
Vitamin A deficiency
Chronic kidney disease
Endocrine, metabolic, blood, and immune disorders
Upper digestive system diseases
Intestinal nematode infections
Malaria
Cirrhosis and other chronic liver diseases
Inflammatory bowel disease
Schistosomiasis
HIV/AIDS
Maternal disorders
Gynecological diseases
YLDs per 100,000 population
0 to <0.01
0.01 to <0.1
0.1 to <0.34
0.34 to <2.1
2.1 to <3.98
3.98 to <9.28
9.28 to <17.77
17.77 to <29.15
29.15 to <59.84
59.84 to 641.62

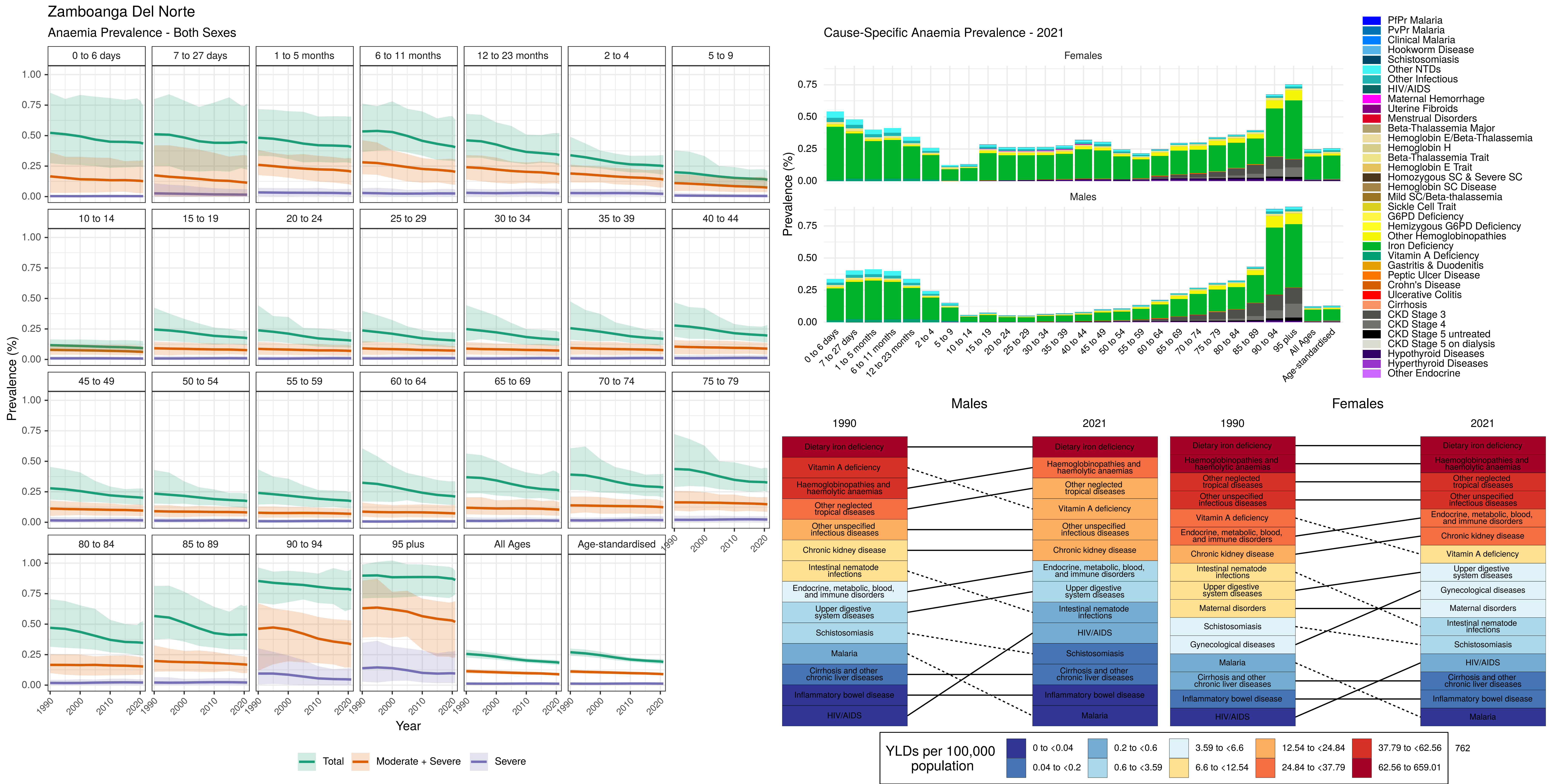

Zamboanga Del Norte
Anaemia Prevalence - Both Sexes
0 to 6 days
7 to 27 days
1 to 5 months
6 to 11 months
12 to 23 months
2 to 4
5 to 9
10 to 14
15 to 19
20 to 24
25 to 29
30 to 34
35 to 39
40 to 44
45 to 49
50 to 54
55 to 59
60 to 64
65 to 69
70 to 74
75 to 79
80 to 84
85 to 89
90 to 94
95 plus
All Ages
Age-standardised
Prevalence (%)
Year
Total
Moderate + Severe
Severe
Cause-Specific Anaemia Prevalence - 2021
Females
Males
PfPr Malaria
PvPr Malaria
Clinical Malaria
Hookworm Disease
Schistosomiasis
Other NTDs
Other Infectious
HIV/AIDS
Maternal Hemorrhage
Uterine Fibroids
Menstrual Disorders
Beta-Thalassemia Major
Hemoglobin E/Beta-Thalassemia
Hemoglobin H
Beta-Thalassemia Trait
Hemoglobin E Trait
Homozygous SC & Severe SC
Hemoglobin SC Disease
Mild SC/Beta-thalassemia
Sickle Cell Trait
G6PD Deficiency
Hemizygous G6PD Deficiency
Other Hemoglobinopathies
Iron Deficiency
Vitamin A Deficiency
Gastritis & Duodenitis
Peptic Ulcer Disease
Crohn's Disease
Ulcerative Colitis
Cirrhosis
CKD Stage 3
CKD Stage 4
CKD Stage 5 untreated
CKD Stage 5 on dialysis
Hypothyroid Diseases
Hyperthyroid Diseases
Other Endocrine
Males
Females
1990
2021
Dietary iron deficiency
Vitamin A deficiency
Haemoglobinopathies and haemolytic anaemias
Other neglected tropical diseases
Other unspecified infectious diseases
Chronic kidney disease
Intestinal nematode infections
Endocrine, metabolic, blood, and immune disorders
Upper digestive system diseases
Schistosomiasis
Malaria
Cirrhosis and other chronic liver diseases
Inflammatory bowel disease
HIV/AIDS
Maternal disorders
Gynecological diseases
YLDs per 100,000 population
0 to <0.04
0.04 to <0.2
0.2 to <0.6
0.6 to <3.59
3.59 to <6.6
6.6 to <12.54
12.54 to <24.84
24.84 to <37.79
37.79 to <62.56
62.56 to 659.01

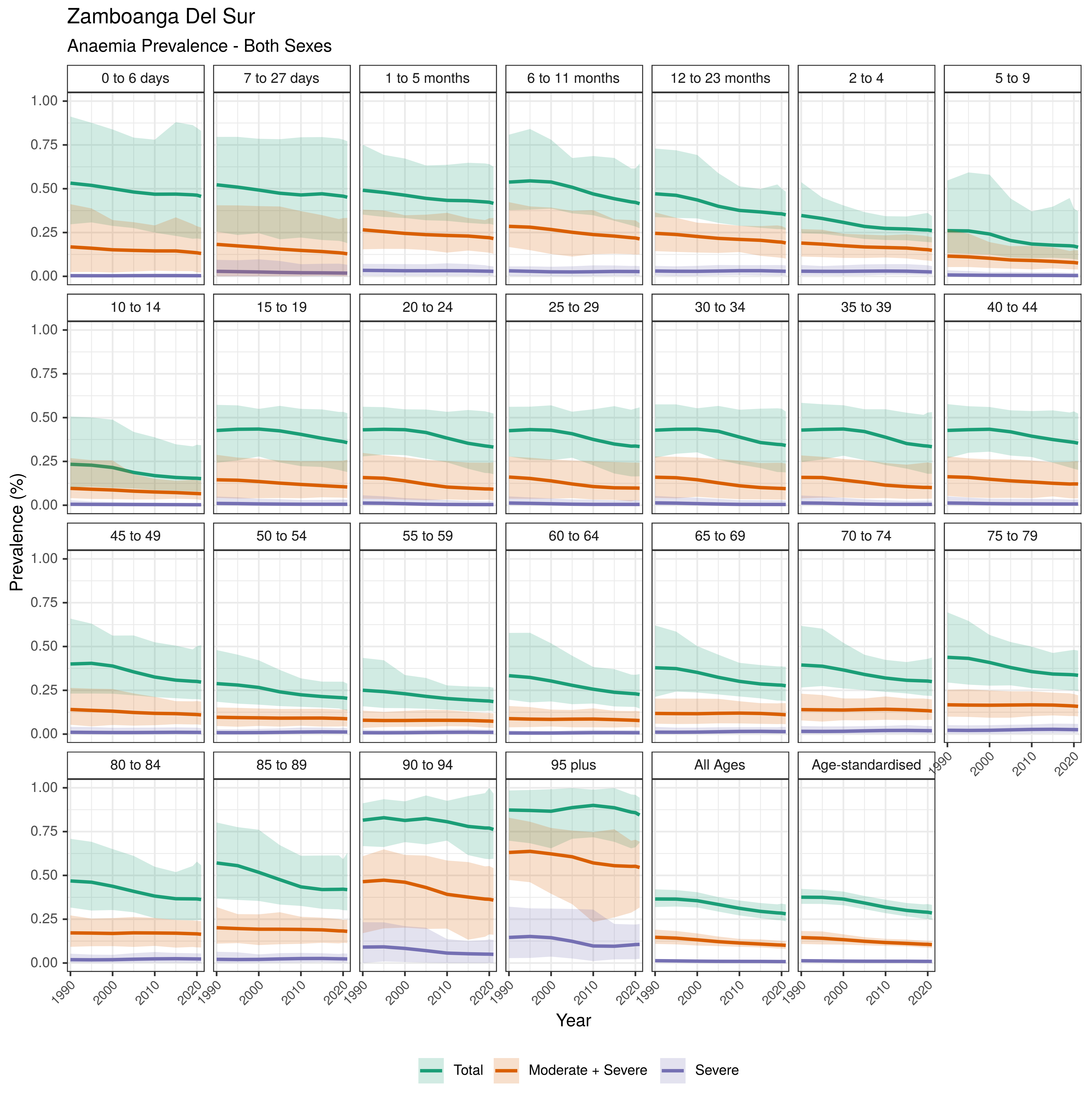

Zamboanga Del Sur
Anaemia Prevalence - Both Sexes
0 to 6 days
7 to 27 days
1 to 5 months
6 to 11 months
12 to 23 months
2 to 4
5 to 9
10 to 14
15 to 19
20 to 24
25 to 29
30 to 34
35 to 39
40 to 44
45 to 49
50 to 54
55 to 59
60 to 64
65 to 69
70 to 74
75 to 79
80 to 84
85 to 89
90 to 94
95 plus
All Ages
Age-standardised
1.00
0.75
0.50
0.25
0.00
1990
2000
2010
2020
Prevalence (%)
Year
Total
Moderate + Severe
Severe

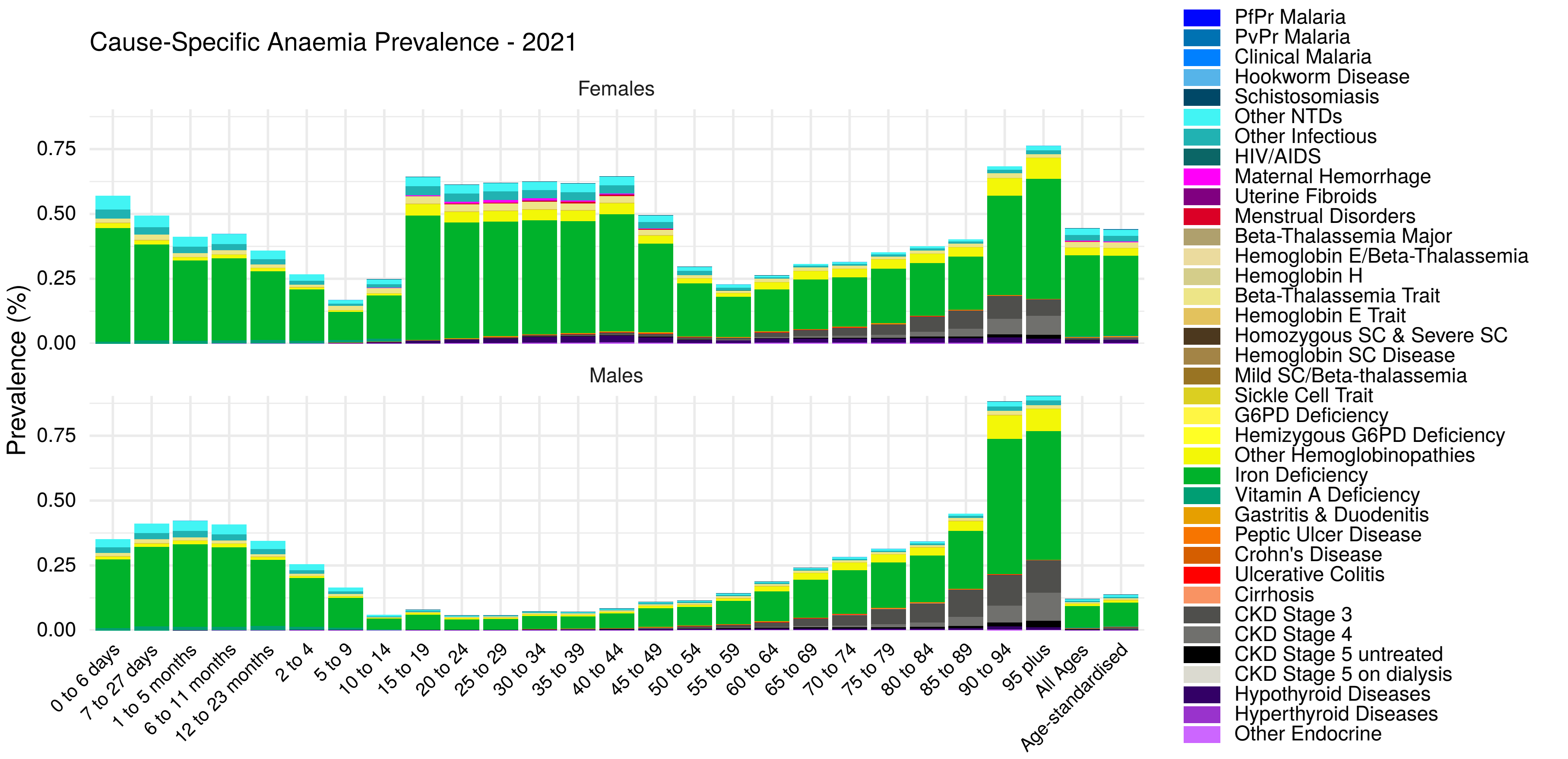

Cause-Specific Anaemia Prevalence - 2021
Females
Males
Prevalence (%)
0.75
0.50
0.25
0.00
0 to 6 days
7 to 27 days
1 to 5 months
6 to 11 months
12 to 23 months
2 to 4
5 to 9
10 to 14
15 to 19
20 to 24
25 to 29
30 to 34
35 to 39
40 to 44
45 to 49
50 to 54
55 to 59
60 to 64
65 to 69
70 to 74
75 to 79
80 to 84
85 to 89
90 to 94
95 plus
All Ages
Age-standardised
PfPr Malaria
PvPr Malaria
Clinical Malaria
Hookworm Disease
Schistosomiasis
Other NTDs
Other Infectious
HIV/AIDS
Maternal Hemorrhage
Uterine Fibroids
Menstrual Disorders
Beta-Thalassemia Major
Hemoglobin E/Beta-Thalassemia
Hemoglobin H
Beta-Thalassemia Trait
Hemoglobin E Trait
Homozygous SC & Severe SC
Hemoglobin SC Disease
Mild SC/Beta-thalassemia
Sickle Cell Trait
G6PD Deficiency
Hemizygous G6PD Deficiency
Other Hemoglobinopathies
Iron Deficiency
Vitamin A Deficiency
Gastritis & Duodenitis
Peptic Ulcer Disease
Crohn's Disease
Ulcerative Colitis
Cirrhosis
CKD Stage 3
CKD Stage 4
CKD Stage 5 untreated
CKD Stage 5 on dialysis
Hypothyroid Diseases
Hyperthyroid Diseases
Other Endocrine

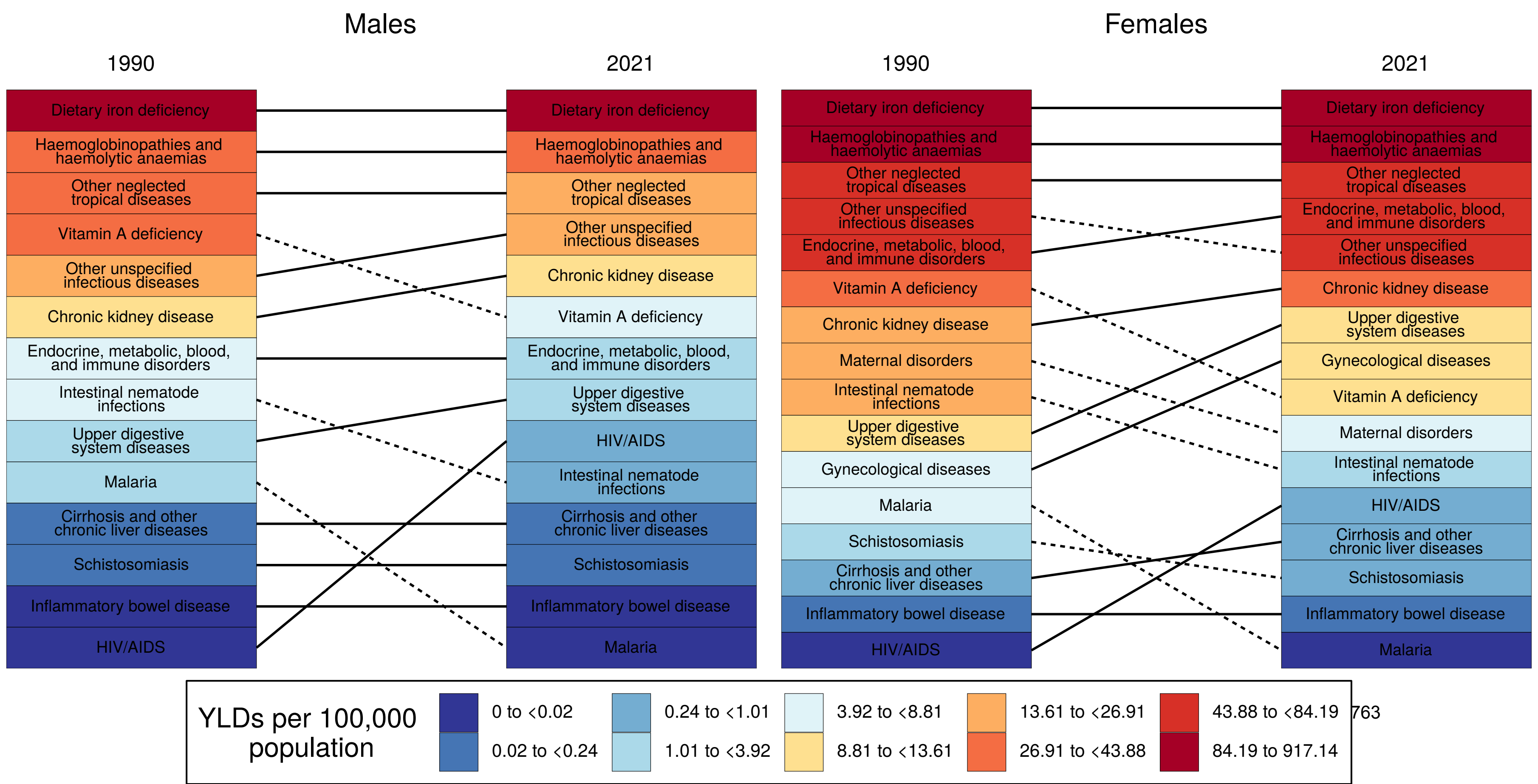

Males
Females
1990
2021
Dietary iron deficiency
Haemoglobinopathies and haemolytic anaemias
Other neglected tropical diseases
Vitamin A deficiency
Other unspecified infectious diseases
Chronic kidney disease
Endocrine, metabolic, blood, and immune disorders
Intestinal nematode infections
Upper digestive system diseases
Malaria
Cirrhosis and other chronic liver diseases
Schistosomiasis
Inflammatory bowel disease
HIV/AIDS
Maternal disorders
Gynecological diseases
YLDs per 100,000 population
0 to <0.02
0.02 to <0.24
0.24 to <1.01
1.01 to <3.92
3.92 to <8.81
8.81 to <13.61
13.61 to <26.91
26.91 to <43.88
43.88 to <84.19
84.19 to 917.14

763

Zamboanga Sibugay

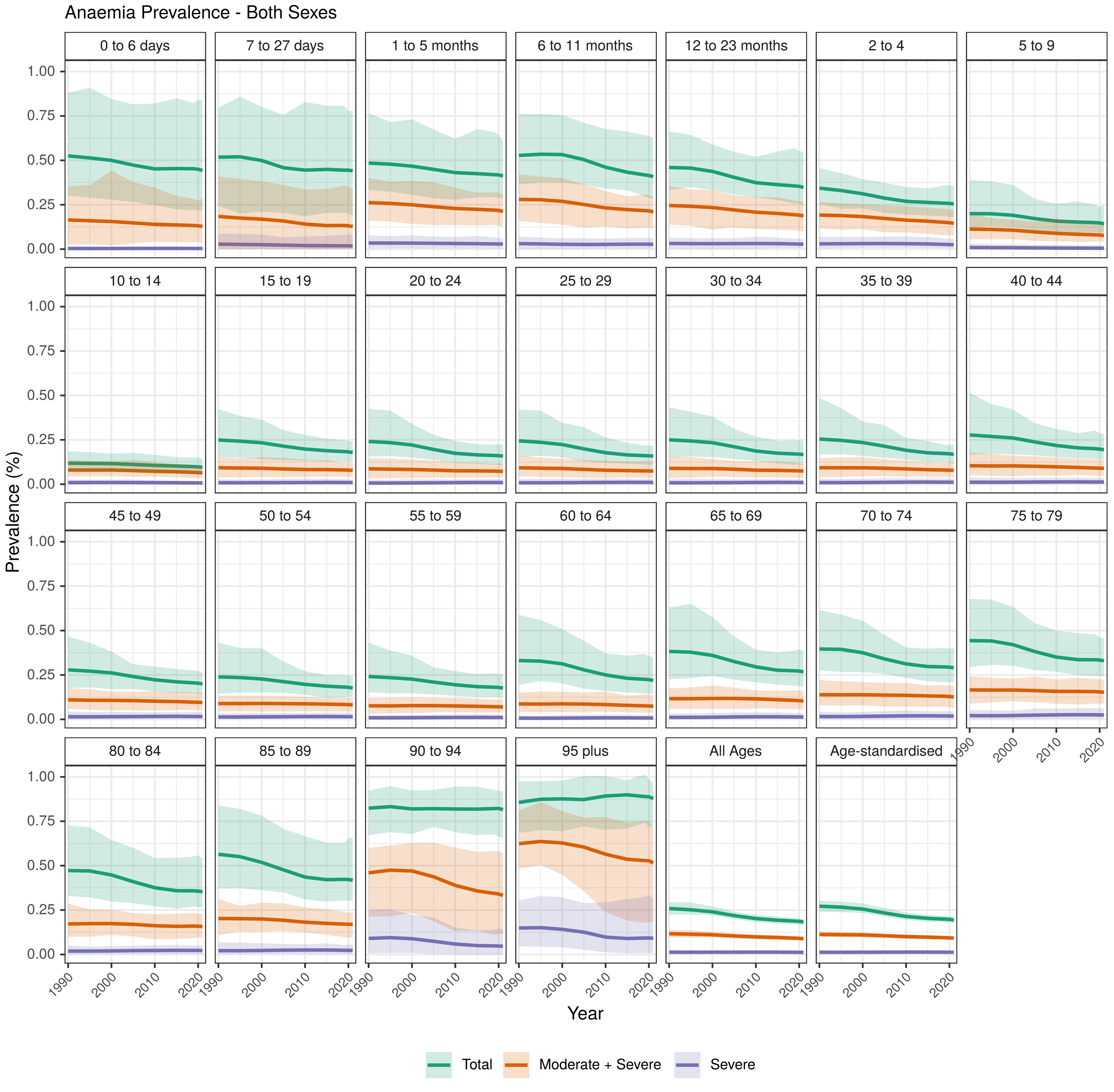

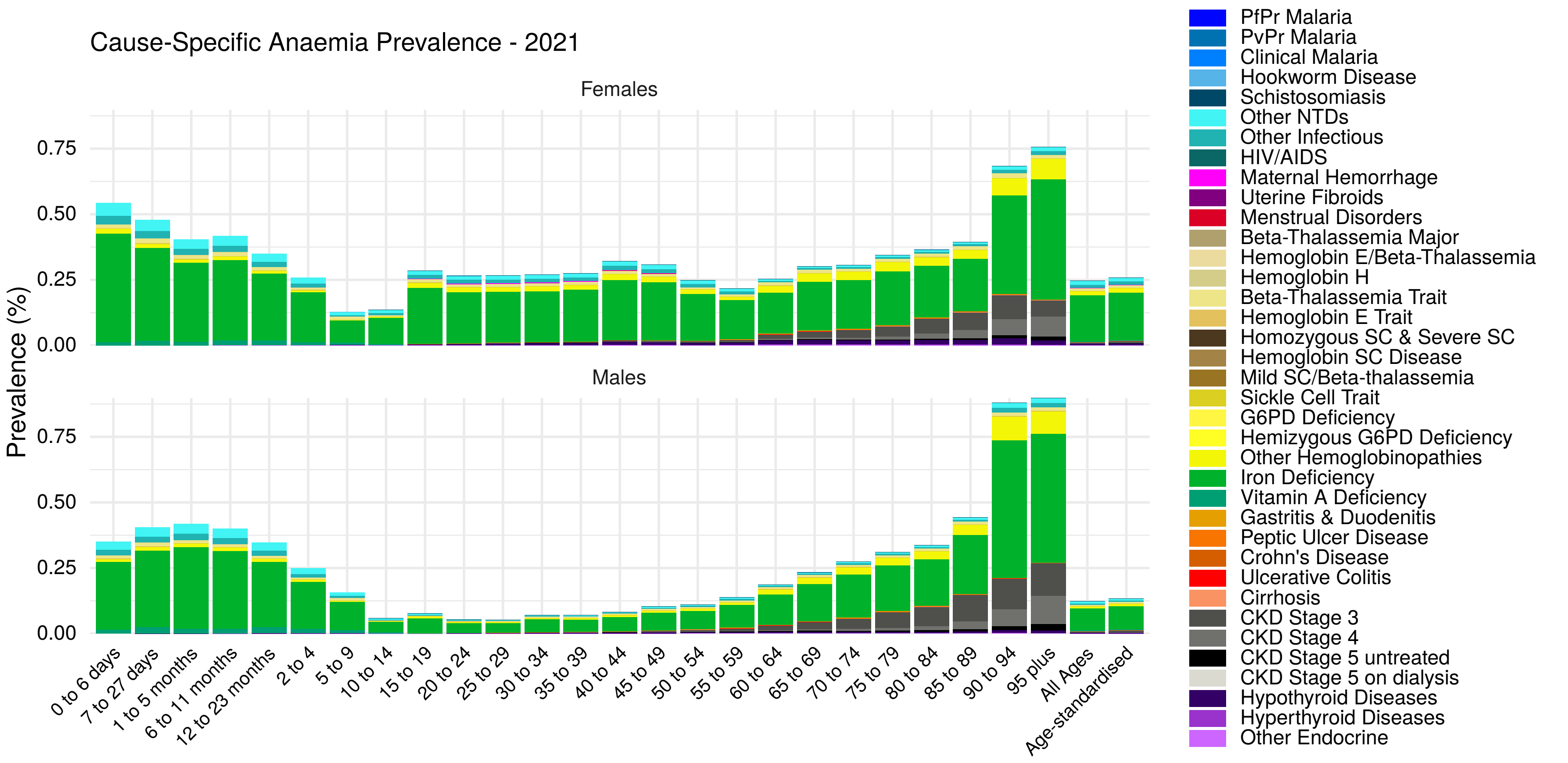

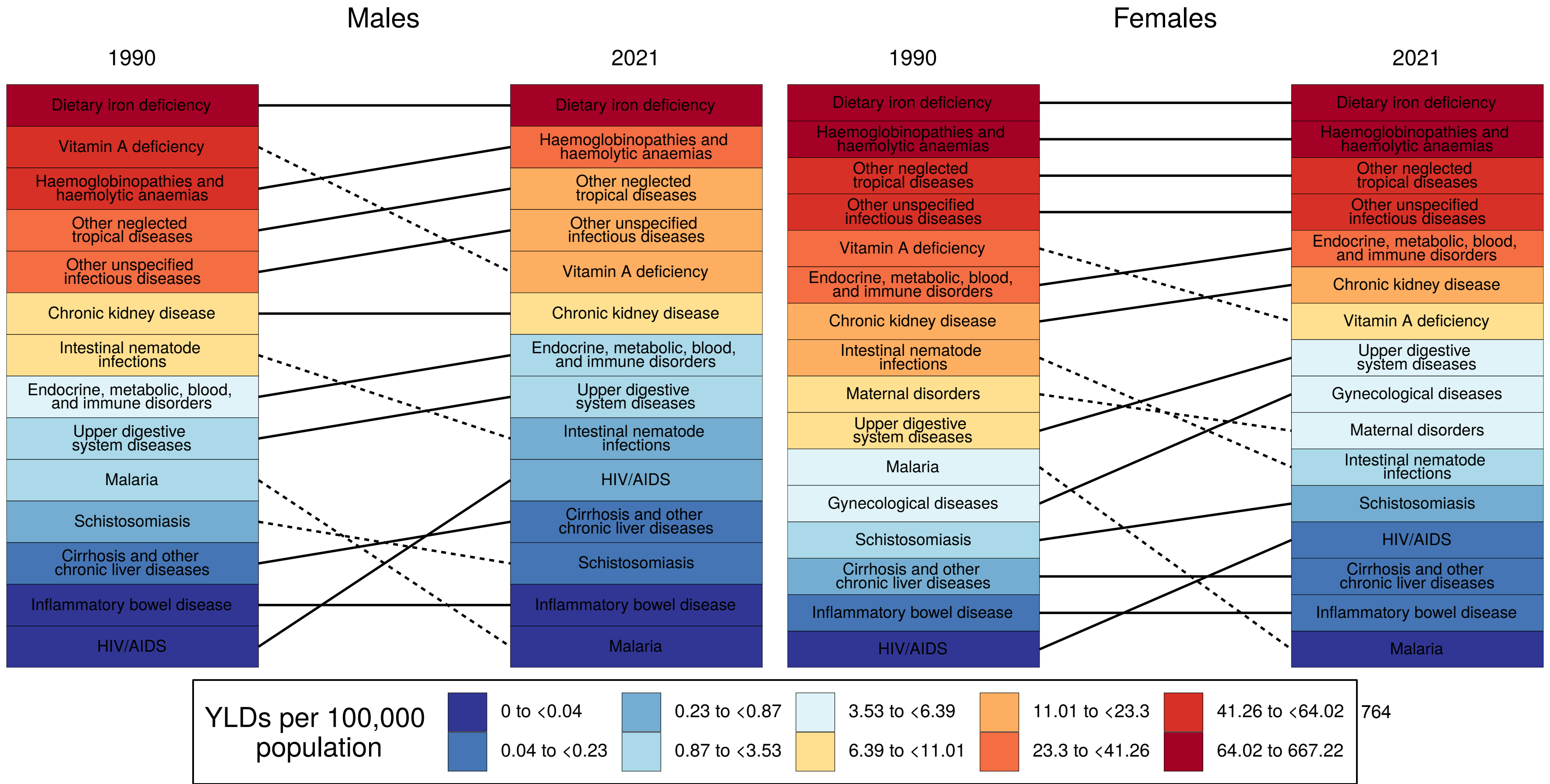

Seychelles

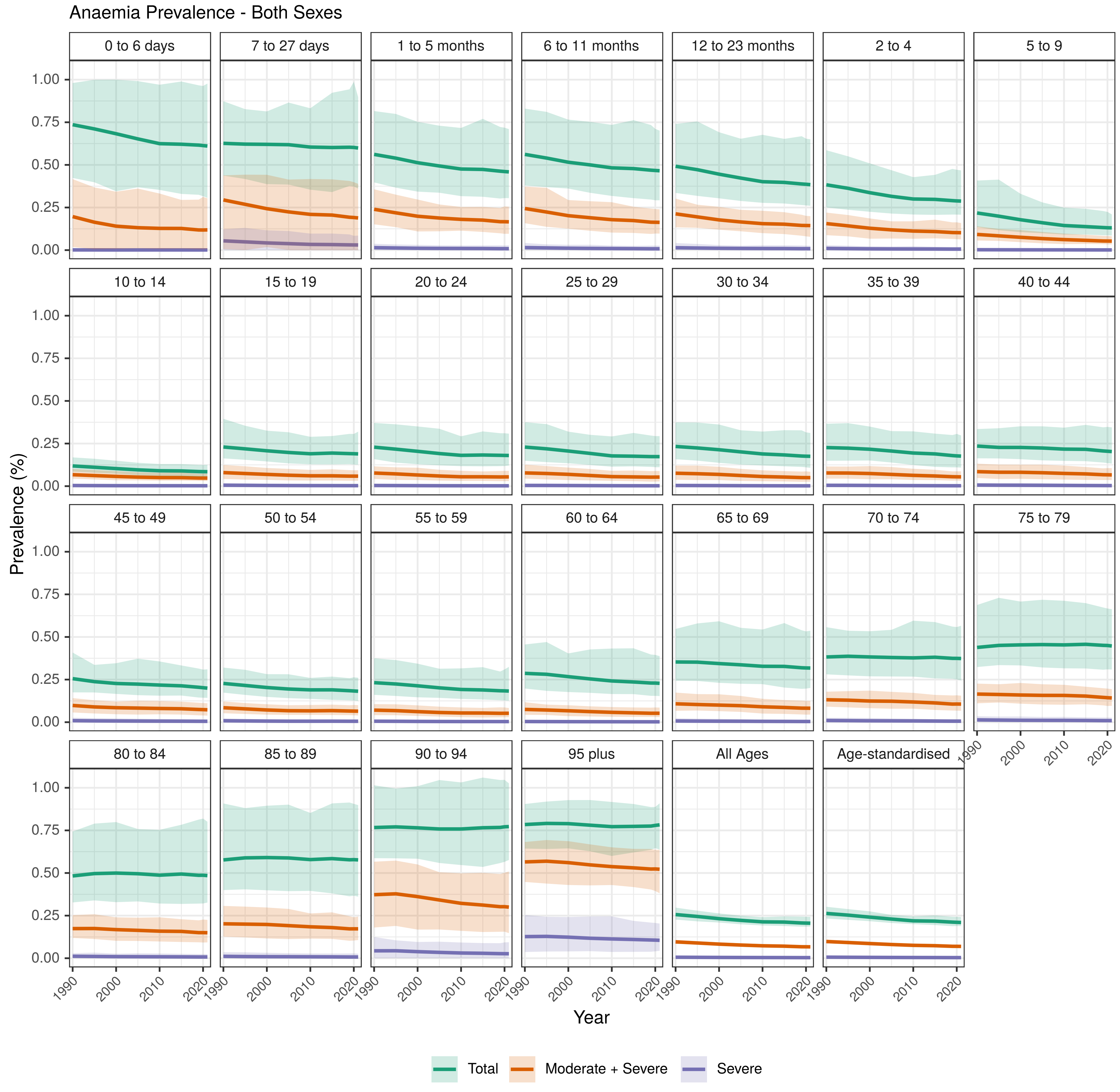

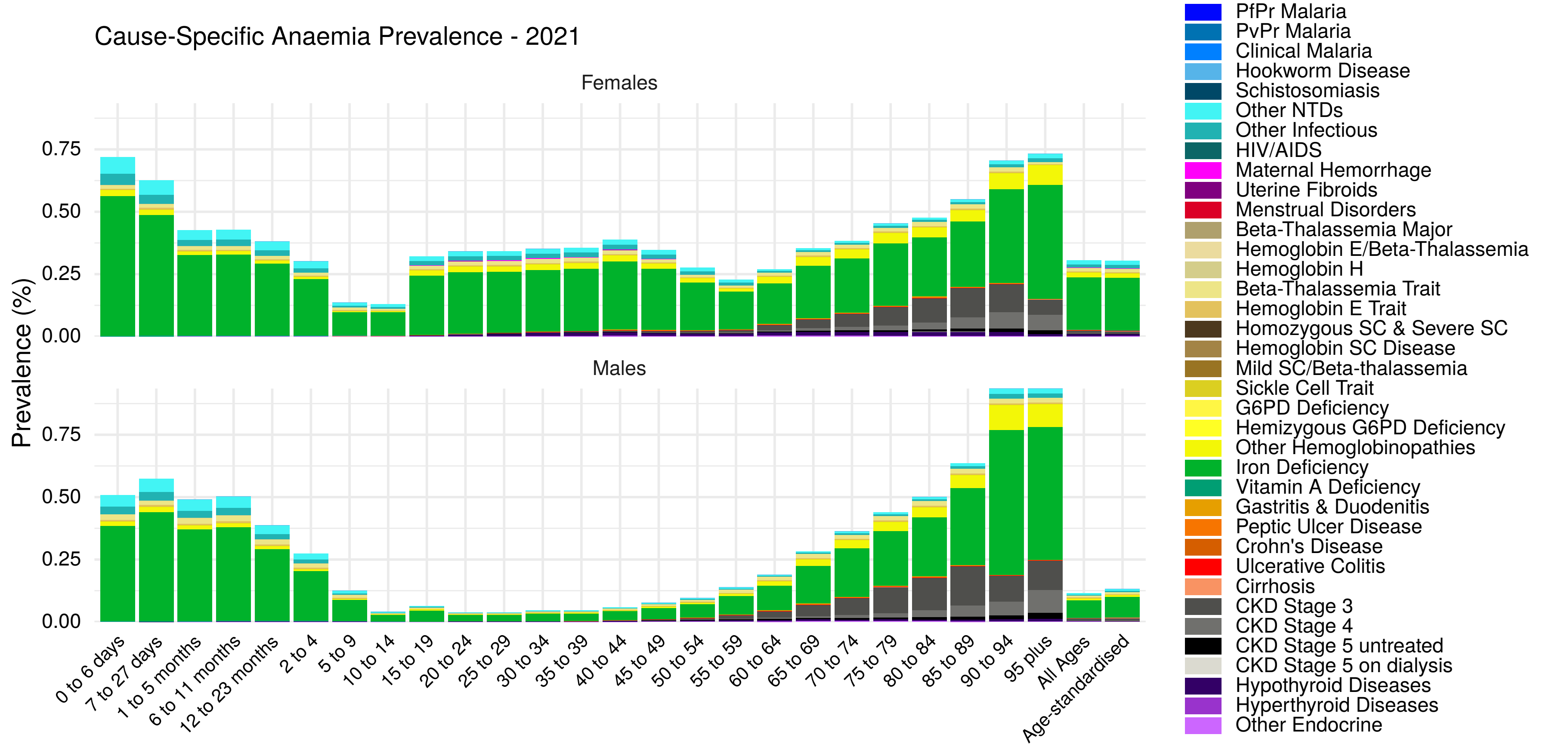

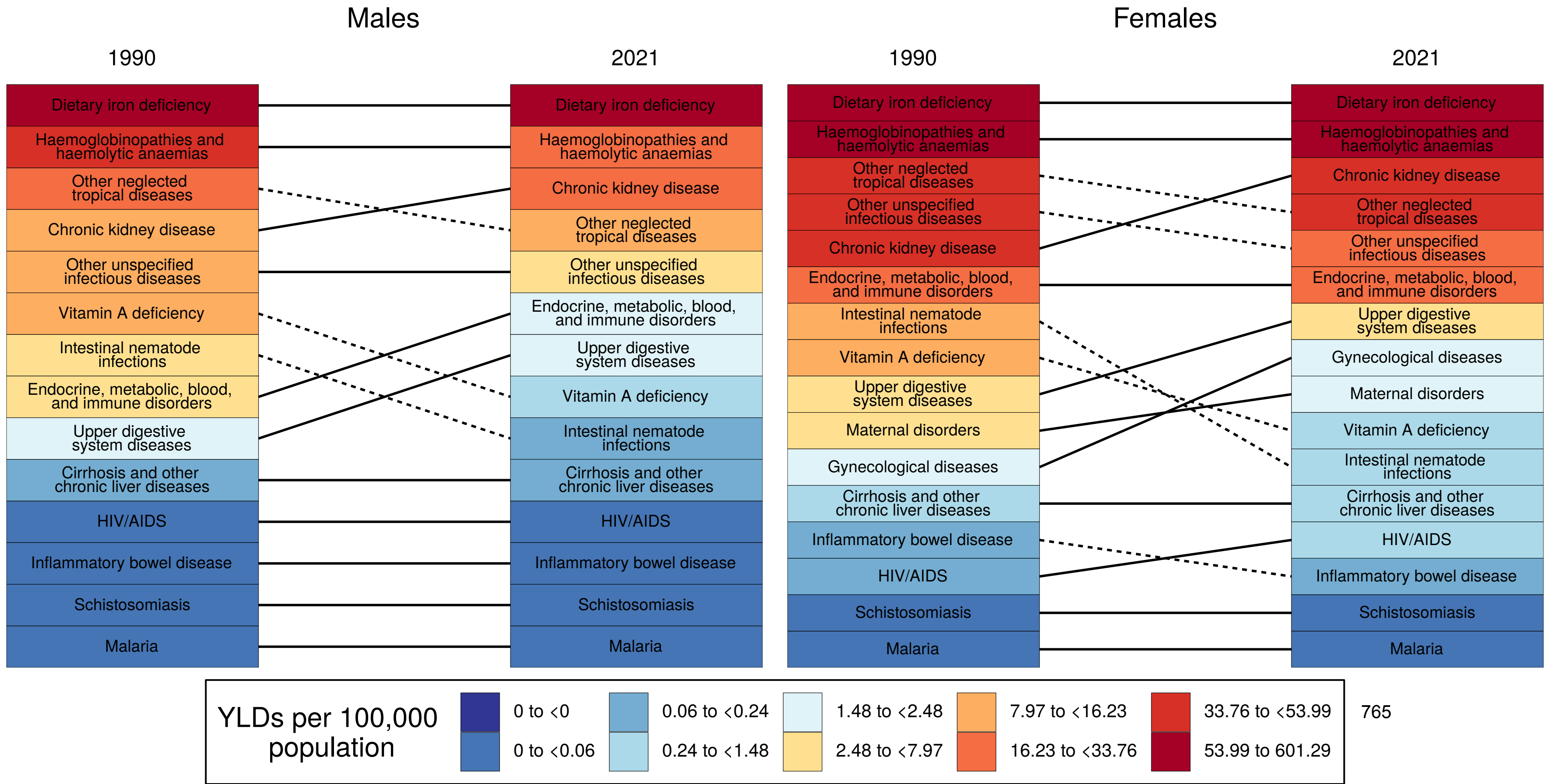

765

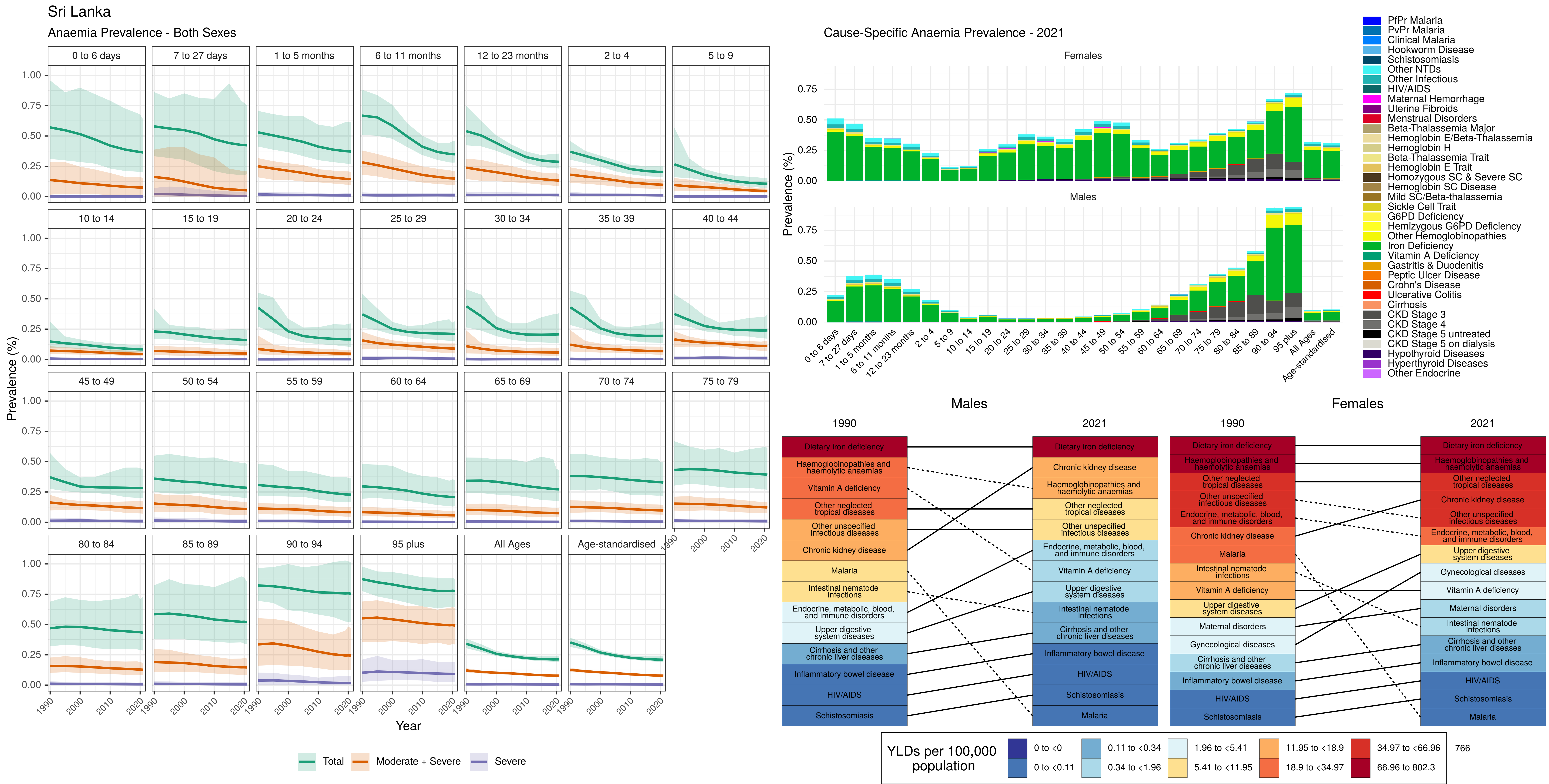

Sri Lanka
Anaemia Prevalence - Both Sexes
0 to 6 days
7 to 27 days
1 to 5 months
6 to 11 months
12 to 23 months
2 to 4
5 to 9
10 to 14
15 to 19
20 to 24
25 to 29
30 to 34
35 to 39
40 to 44
45 to 49
50 to 54
55 to 59
60 to 64
65 to 69
70 to 74
75 to 79
80 to 84
85 to 89
90 to 94
95 plus
All Ages
Age-standardised
Prevalence (%)
Year
Total
Moderate + Severe
Severe
Cause-Specific Anaemia Prevalence - 2021
Females
Males
PfPr Malaria
PvPr Malaria
Clinical Malaria
Hookworm Disease
Schistosomiasis
Other NTDs
Other Infectious
HIV/AIDS
Maternal Hemorrhage
Uterine Fibroids
Menstrual Disorders
Beta-Thalassemia Major
Hemoglobin E/Beta-Thalassemia
Hemoglobin H
Beta-Thalassemia Trait
Hemoglobin E Trait
Homozygous SC & Severe SC
Hemoglobin SC Disease
Mild SC/Beta-thalassemia
Sickle Cell Trait
G6PD Deficiency
Hemizygous G6PD Deficiency
Other Hemoglobinopathies
Iron Deficiency
Vitamin A Deficiency
Gastritis & Duodenitis
Peptic Ulcer Disease
Crohn's Disease
Ulcerative Colitis
Cirrhosis
CKD Stage 3
CKD Stage 4
CKD Stage 5 untreated
CKD Stage 5 on dialysis
Hypothyroid Diseases
Hyperthyroid Diseases
Other Endocrine
Males
Females
1990
2021
Dietary iron deficiency
Haemoglobinopathies and haemolytic anaemias
Vitamin A deficiency
Other neglected tropical diseases
Other unspecified infectious diseases
Chronic kidney disease
Malaria
Intestinal nematode infections
Endocrine, metabolic, blood, and immune disorders
Upper digestive system diseases
Cirrhosis and other chronic liver diseases
Inflammatory bowel disease
HIV/AIDS
Schistosomiasis
Maternal disorders
Gynecological diseases
YLDs per 100,000 population
0 to <0
0 to <0.11
0.11 to <0.34
0.34 to <1.96
1.96 to <5.41
5.41 to <11.95
11.95 to <18.9
18.9 to <34.97
34.97 to <66.96
66.96 to 802.3

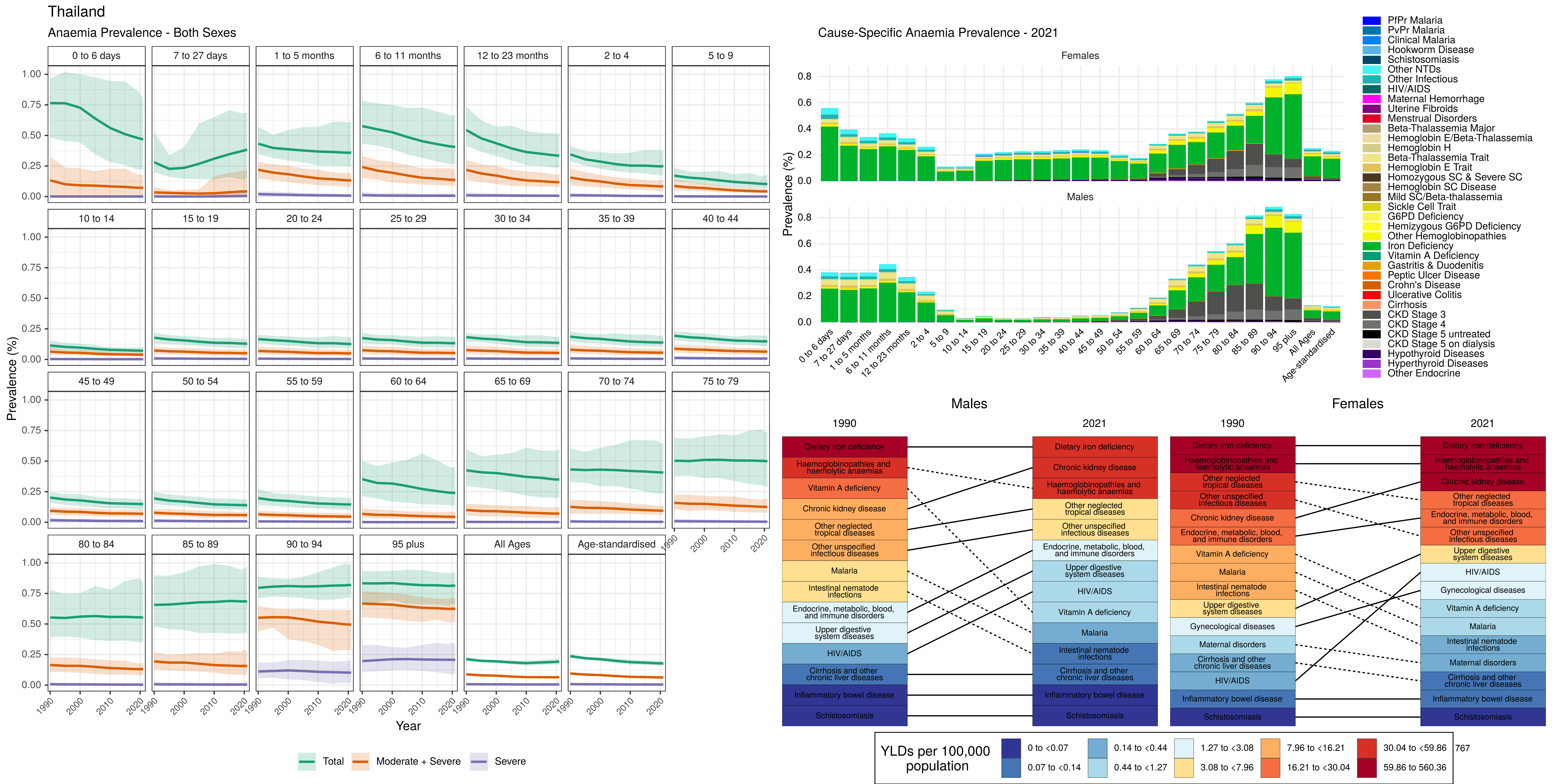
Thailand
Anaemia Prevalence - Both Sexes
0 to 6 days
7 to 27 days
1 to 5 months
6 to 11 months
12 to 23 months
2 to 4
5 to 9
10 to 14
15 to 19
20 to 24
25 to 29
30 to 34
35 to 39
40 to 44
45 to 49
50 to 54
55 to 59
60 to 64
65 to 69
70 to 74
75 to 79
80 to 84
85 to 89
90 to 94
95 plus
All Ages
Age-standardised
Prevalence (%)
Year
1990
2000
2010
2020
0.00
0.25
0.50
0.75
1.00
Total
Moderate + Severe
Severe
Cause-Specific Anaemia Prevalence - 2021
Females
Males
Prevalence (%)
0.0
0.2
0.4
0.6
0.8
PfPr Malaria
PvPr Malaria
Clinical Malaria
Hookworm Disease
Schistosomiasis
Other NTDs
Other Infectious
HIV/AIDS
Maternal Hemorrhage
Uterine Fibroids
Menstrual Disorders
Beta-Thalassemia Major
Hemoglobin E/Beta-Thalassemia
Hemoglobin H
Beta-Thalassemia Trait
Hemoglobin E Trait
Homozygous SC & Severe SC
Hemoglobin SC Disease
Mild SC/Beta-thalassemia
Sickle Cell Trait
G6PD Deficiency
Hemizygous G6PD Deficiency
Other Hemoglobinopathies
Iron Deficiency
Vitamin A Deficiency
Gastritis & Duodenitis
Peptic Ulcer Disease
Crohn's Disease
Ulcerative Colitis
Cirrhosis
CKD Stage 3
CKD Stage 4
CKD Stage 5 untreated
CKD Stage 5 on dialysis
Hypothyroid Diseases
Hyperthyroid Diseases
Other Endocrine
Males
Females
1990
2021
Males 1990:
Dietary iron deficiency
Haemoglobinopathies and haemolytic anaemias
Vitamin A deficiency
Chronic kidney disease
Other neglected tropical diseases
Other unspecified infectious diseases
Malaria
Intestinal nematode infections
Endocrine, metabolic, blood, and immune disorders
Upper digestive system diseases
HIV/AIDS
Cirrhosis and other chronic liver diseases
Inflammatory bowel disease
Schistosomiasis
Males 2021:
Dietary iron deficiency
Chronic kidney disease
Haemoglobinopathies and haemolytic anaemias
Other neglected tropical diseases
Other unspecified infectious diseases
Endocrine, metabolic, blood, and immune disorders
Upper digestive system diseases
HIV/AIDS
Vitamin A deficiency
Malaria
Intestinal nematode infections
Cirrhosis and other chronic liver diseases
Inflammatory bowel disease
Schistosomiasis
Females 1990:
Dietary iron deficiency
Haemoglobinopathies and haemolytic anaemias
Other neglected tropical diseases
Other unspecified infectious diseases
Chronic kidney disease
Endocrine, metabolic, blood, and immune disorders
Vitamin A deficiency
Malaria
Intestinal nematode infections
Upper digestive system diseases
Gynecological diseases
Maternal disorders
Cirrhosis and other chronic liver diseases
HIV/AIDS
Inflammatory bowel disease
Schistosomiasis
Females 2021:
Dietary iron deficiency
Haemoglobinopathies and haemolytic anaemias
Chronic kidney disease
Other neglected tropical diseases
Endocrine, metabolic, blood, and immune disorders
Other unspecified infectious diseases
Upper digestive system diseases
HIV/AIDS
Gynecological diseases
Vitamin A deficiency
Malaria
Intestinal nematode infections
Maternal disorders
Cirrhosis and other chronic liver diseases
Inflammatory bowel disease
Schistosomiasis
YLDs per 100,000 population
0 to <0.07
0.07 to <0.14
0.14 to <0.44
0.44 to <1.27
1.27 to <3.08
3.08 to <7.96
7.96 to <16.21
16.21 to <30.04
30.04 to <59.86
59.86 to 560.36
767

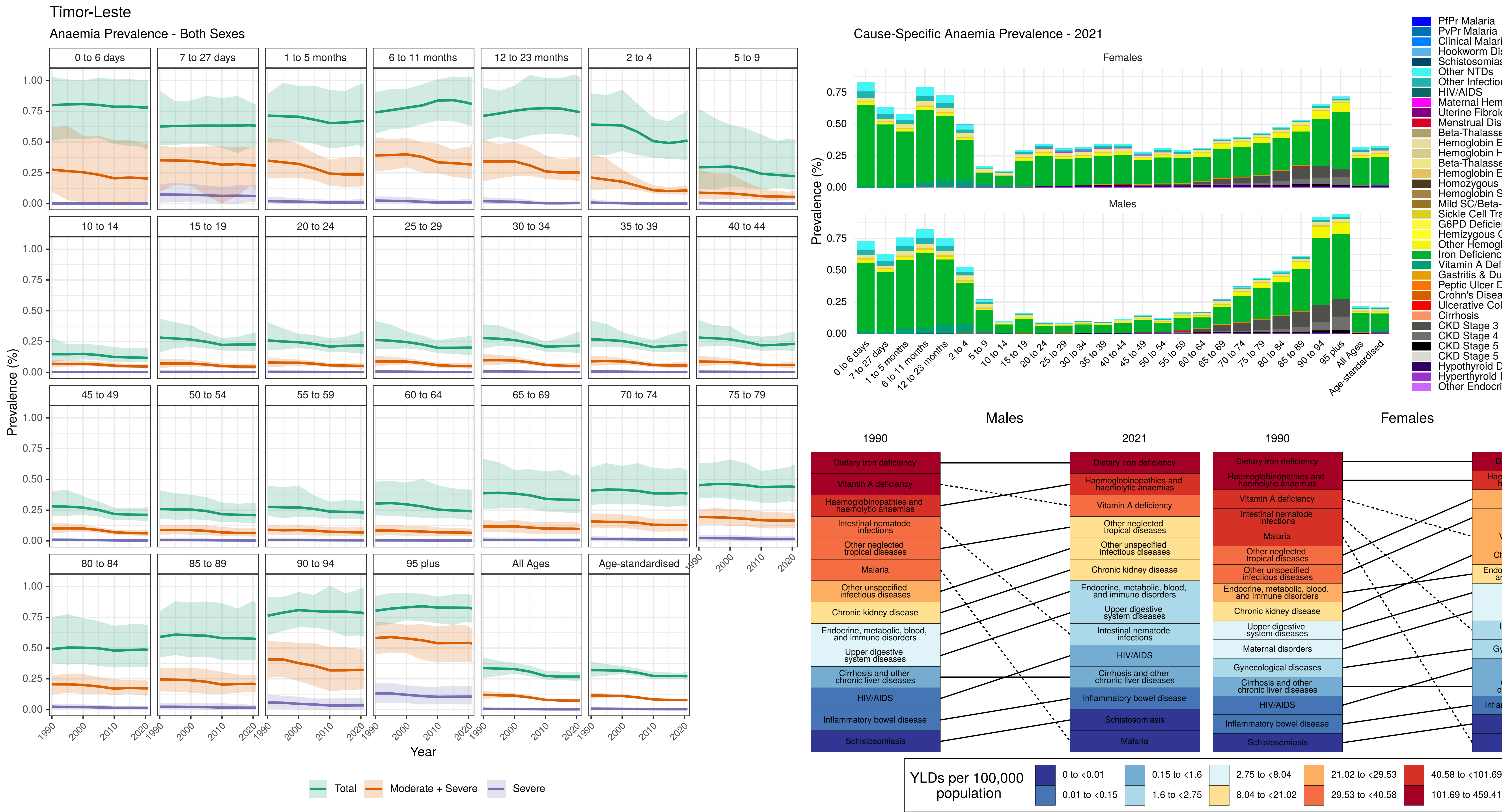

Timor-Leste
Anaemia Prevalence - Both Sexes
0 to 6 days
7 to 27 days
1 to 5 months
6 to 11 months
12 to 23 months
2 to 4
5 to 9
10 to 14
15 to 19
20 to 24
25 to 29
30 to 34
35 to 39
40 to 44
45 to 49
50 to 54
55 to 59
60 to 64
65 to 69
70 to 74
75 to 79
80 to 84
85 to 89
90 to 94
95 plus
All Ages
Age-standardised
Prevalence (%)
Year
Total
Moderate + Severe
Severe
Cause-Specific Anaemia Prevalence - 2021
Females
Males
PfPr Malaria
PvPr Malaria
Clinical Malaria
Hookworm Disease
Schistosomiasis
Other NTDs
Other Infectious
HIV/AIDS
Maternal Hemorrhage
Uterine Fibroids
Menstrual Disorders
Beta-Thalassemia Major
Hemoglobin E/Beta-Thalassemia
Hemoglobin H
Beta-Thalassemia Trait
Hemoglobin E Trait
Homozygous SC & Severe SC
Hemoglobin SC Disease
Mild SC/Beta-thalassemia
Sickle Cell Trait
G6PD Deficiency
Hemizygous G6PD Deficiency
Other Hemoglobinopathies
Iron Deficiency
Vitamin A Deficiency
Gastritis & Duodenitis
Peptic Ulcer Disease
Crohn's Disease
Ulcerative Colitis
Cirrhosis
CKD Stage 3
CKD Stage 4
CKD Stage 5 untreated
CKD Stage 5 on dialysis
Hypothyroid Diseases
Hyperthyroid Diseases
Other Endocrine
Males
Females
1990
2021
Dietary iron deficiency
Vitamin A deficiency
Haemoglobinopathies and haemolytic anaemias
Intestinal nematode infections
Other neglected tropical diseases
Malaria
Other unspecified infectious diseases
Chronic kidney disease
Endocrine, metabolic, blood, and immune disorders
Upper digestive system diseases
Cirrhosis and other chronic liver diseases
HIV/AIDS
Inflammatory bowel disease
Schistosomiasis
Maternal disorders
Gynecological diseases
YLDs per 100,000 population
0 to <0.01
0.01 to <0.15
0.15 to <1.6
1.6 to <2.75
2.75 to <8.04
8.04 to <21.02
21.02 to <29.53
29.53 to <40.58
40.58 to <101.69
101.69 to 459.41

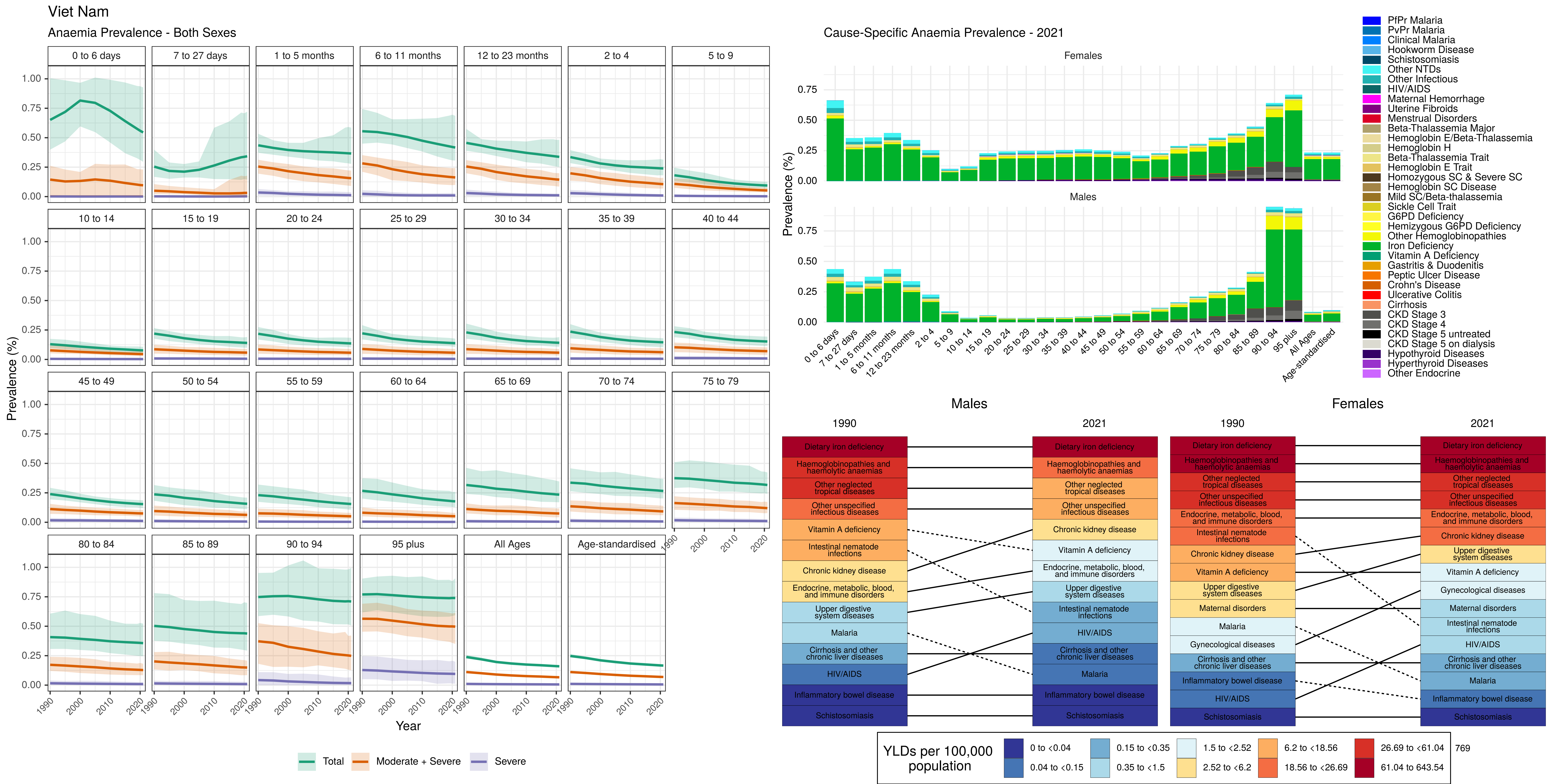
Viet Nam
Anaemia Prevalence - Both Sexes
0 to 6 days
7 to 27 days
1 to 5 months
6 to 11 months
12 to 23 months
2 to 4
5 to 9
10 to 14
15 to 19
20 to 24
25 to 29
30 to 34
35 to 39
40 to 44
45 to 49
50 to 54
55 to 59
60 to 64
65 to 69
70 to 74
75 to 79
80 to 84
85 to 89
90 to 94
95 plus
All Ages
Age-standardised
Prevalence (%)
Year
Total
Moderate + Severe
Severe
Cause-Specific Anaemia Prevalence - 2021
Females
Males
PfPr Malaria
PvPr Malaria
Clinical Malaria
Hookworm Disease
Schistosomiasis
Other NTDs
Other Infectious
HIV/AIDS
Maternal Hemorrhage
Uterine Fibroids
Menstrual Disorders
Beta-Thalassemia Major
Hemoglobin E/Beta-Thalassemia
Hemoglobin H
Beta-Thalassemia Trait
Hemoglobin E Trait
Homozygous SC & Severe SC
Hemoglobin SC Disease
Mild SC/Beta-thalassemia
Sickle Cell Trait
G6PD Deficiency
Hemizygous G6PD Deficiency
Other Hemoglobinopathies
Iron Deficiency
Vitamin A Deficiency
Gastritis & Duodenitis
Peptic Ulcer Disease
Crohn's Disease
Ulcerative Colitis
Cirrhosis
CKD Stage 3
CKD Stage 4
CKD Stage 5 untreated
CKD Stage 5 on dialysis
Hypothyroid Diseases
Hyperthyroid Diseases
Other Endocrine
Males
Females
1990
2021
Dietary iron deficiency
Haemoglobinopathies and haemolytic anaemias
Other neglected tropical diseases
Other unspecified infectious diseases
Vitamin A deficiency
Intestinal nematode infections
Chronic kidney disease
Endocrine, metabolic, blood, and immune disorders
Upper digestive system diseases
Malaria
Cirrhosis and other chronic liver diseases
HIV/AIDS
Inflammatory bowel disease
Schistosomiasis
Maternal disorders
Gynecological diseases
YLDs per 100,000 population
0 to <0.04
0.04 to <0.15
0.15 to <0.35
0.35 to <1.5
1.5 to <2.52
2.52 to <6.2
6.2 to <18.56
18.56 to <26.69
26.69 to <61.04
61.04 to 643.54
769

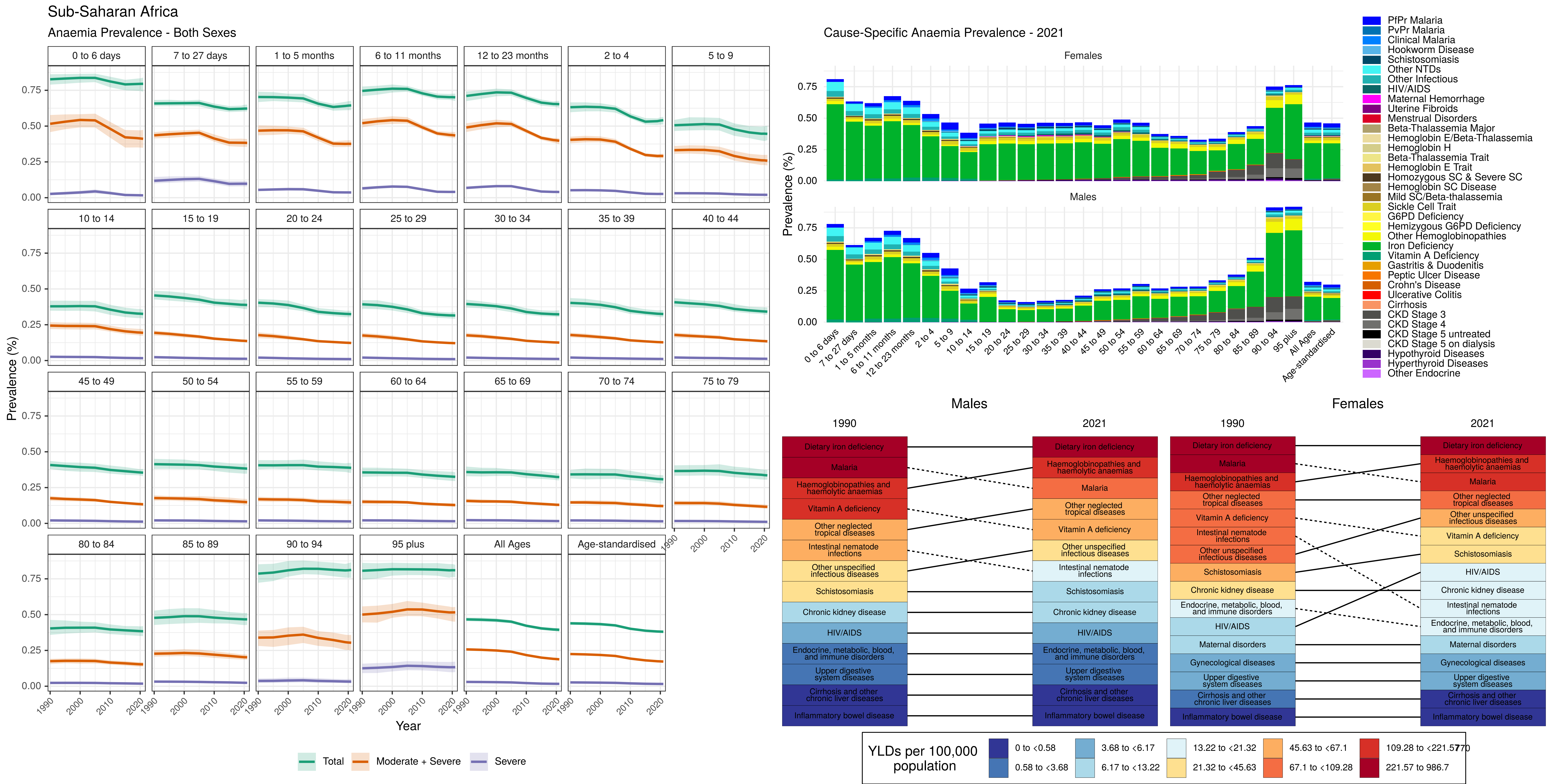

Sub-Saharan Africa
Anaemia Prevalence - Both Sexes
0 to 6 days
7 to 27 days
1 to 5 months
6 to 11 months
12 to 23 months
2 to 4
5 to 9
10 to 14
15 to 19
20 to 24
25 to 29
30 to 34
35 to 39
40 to 44
45 to 49
50 to 54
55 to 59
60 to 64
65 to 69
70 to 74
75 to 79
80 to 84
85 to 89
90 to 94
95 plus
All Ages
Age-standardised
Prevalence (%)
0.00
0.25
0.50
0.75
1990
2000
2010
2020
Year
Total
Moderate + Severe
Severe
Cause-Specific Anaemia Prevalence - 2021
Females
Males
PfPr Malaria
PvPr Malaria
Clinical Malaria
Hookworm Disease
Schistosomiasis
Other NTDs
Other Infectious
HIV/AIDS
Maternal Hemorrhage
Uterine Fibroids
Menstrual Disorders
Beta-Thalassemia Major
Hemoglobin E/Beta-Thalassemia
Hemoglobin H
Beta-Thalassemia Trait
Hemoglobin E Trait
Homozygous SC & Severe SC
Hemoglobin SC Disease
Mild SC/Beta-thalassemia
Sickle Cell Trait
G6PD Deficiency
Hemizygous G6PD Deficiency
Other Hemoglobinopathies
Iron Deficiency
Vitamin A Deficiency
Gastritis & Duodenitis
Peptic Ulcer Disease
Crohn's Disease
Ulcerative Colitis
Cirrhosis
CKD Stage 3
CKD Stage 4
CKD Stage 5 untreated
CKD Stage 5 on dialysis
Hypothyroid Diseases
Hyperthyroid Diseases
Other Endocrine
Males
Females
1990
2021
Dietary iron deficiency
Malaria
Haemoglobinopathies and haemolytic anaemias
Vitamin A deficiency
Other neglected tropical diseases
Intestinal nematode infections
Other unspecified infectious diseases
Schistosomiasis
Chronic kidney disease
HIV/AIDS
Endocrine, metabolic, blood, and immune disorders
Upper digestive system diseases
Cirrhosis and other chronic liver diseases
Inflammatory bowel disease
Maternal disorders
Gynecological diseases
YLDs per 100,000 population
0 to <0.58
0.58 to <3.68
3.68 to <6.17
6.17 to <13.22
13.22 to <21.32
21.32 to <45.63
45.63 to <67.1
67.1 to <109.28
109.28 to <221.5770
221.57 to 986.7

Central sub-Saharan Africa

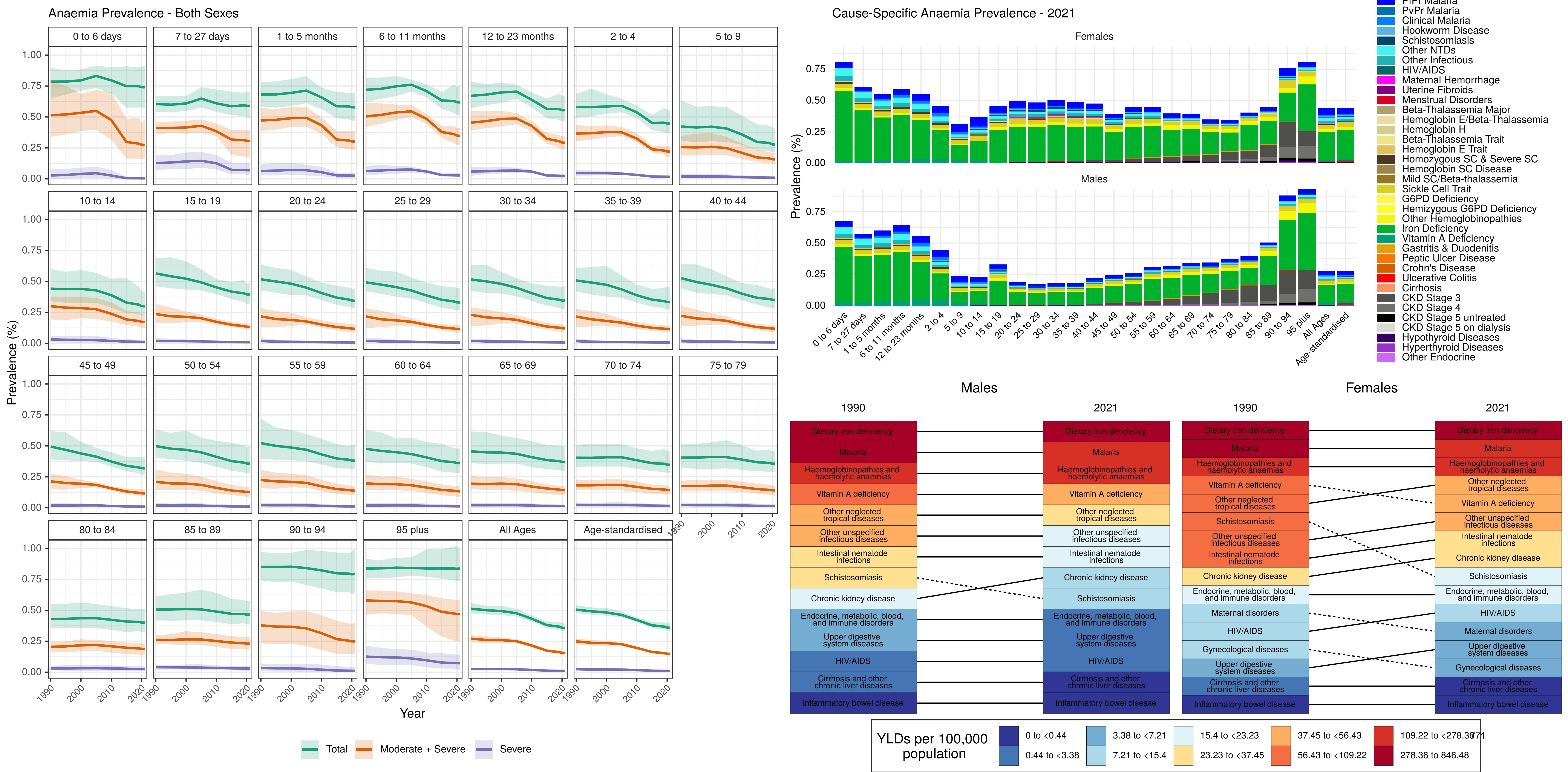

Angola

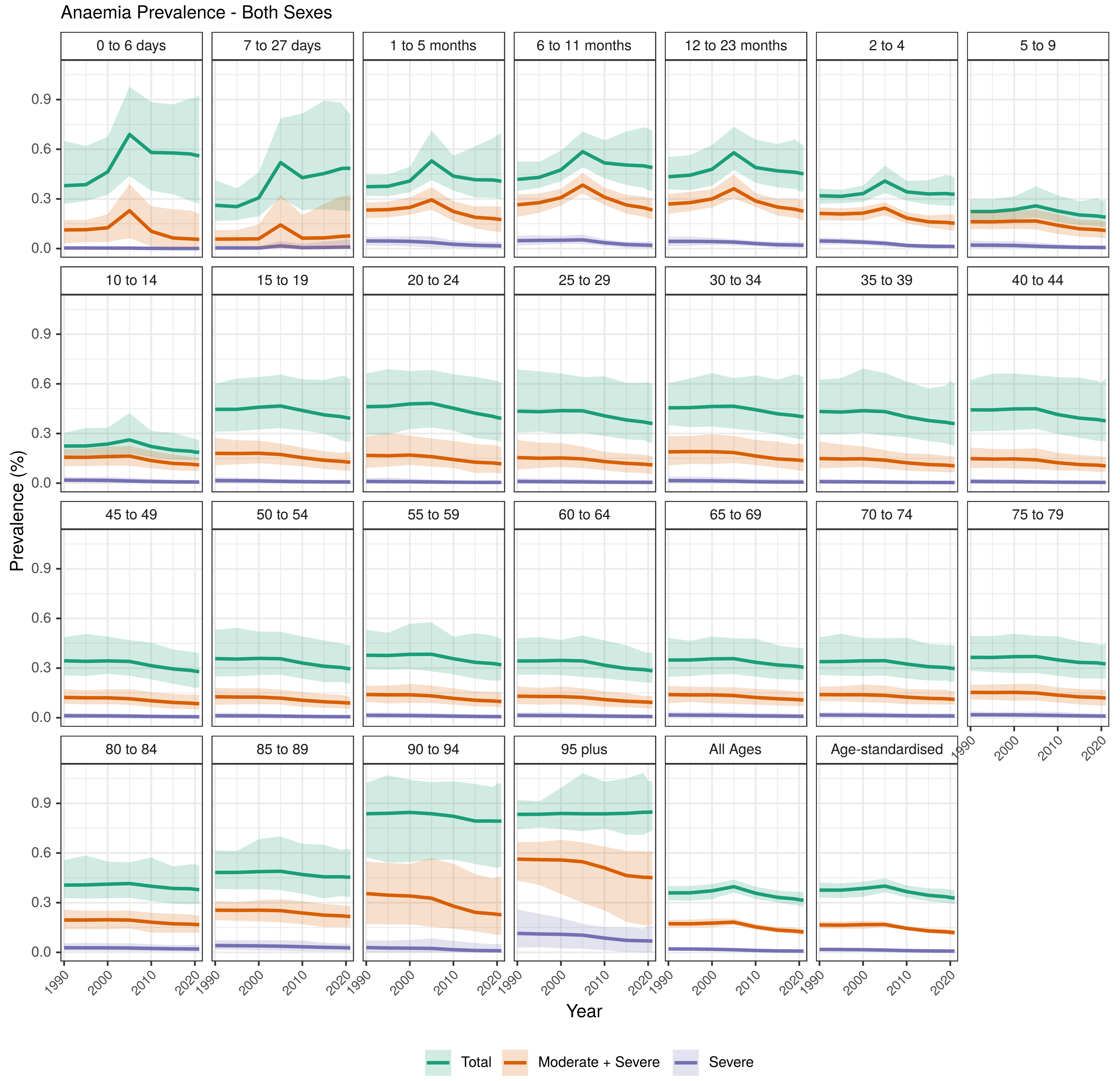
Anaemia Prevalence - Both Sexes
0 to 6 days
7 to 27 days
1 to 5 months
6 to 11 months
12 to 23 months
2 to 4
5 to 9
10 to 14
15 to 19
20 to 24
25 to 29
30 to 34
35 to 39
40 to 44
45 to 49
50 to 54
55 to 59
60 to 64
65 to 69
70 to 74
75 to 79
80 to 84
85 to 89
90 to 94
95 plus
All Ages
Age-standardised
Prevalence (%)
Year
Total
Moderate + Severe
Severe

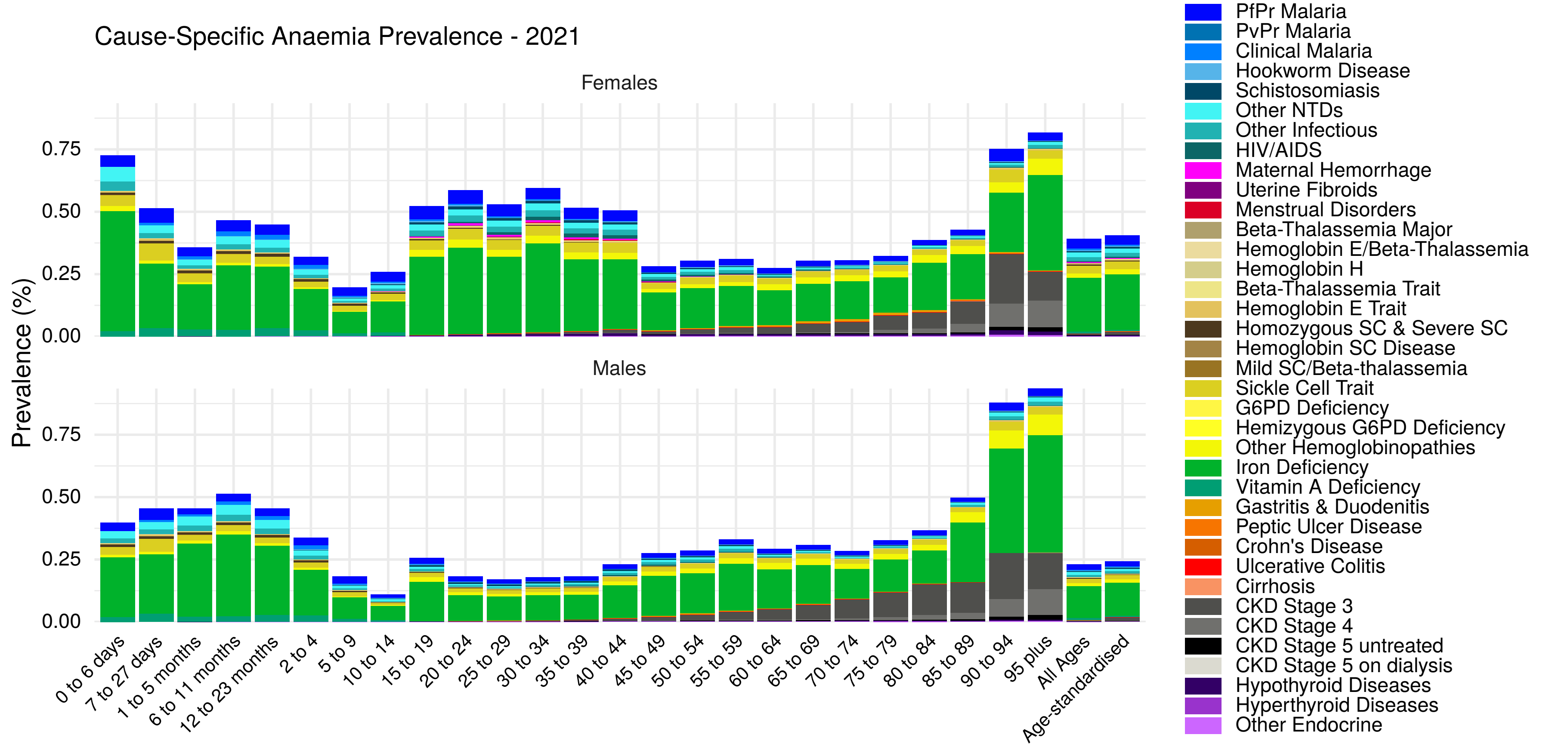
Cause-Specific Anaemia Prevalence - 2021
Females
Males
Prevalence (%)
PfPr Malaria
PvPr Malaria
Clinical Malaria
Hookworm Disease
Schistosomiasis
Other NTDs
Other Infectious
HIV/AIDS
Maternal Hemorrhage
Uterine Fibroids
Menstrual Disorders
Beta-Thalassemia Major
Hemoglobin E/Beta-Thalassemia
Hemoglobin H
Beta-Thalassemia Trait
Hemoglobin E Trait
Homozygous SC & Severe SC
Hemoglobin SC Disease
Mild SC/Beta-thalassemia
Sickle Cell Trait
G6PD Deficiency
Hemizygous G6PD Deficiency
Other Hemoglobinopathies
Iron Deficiency
Vitamin A Deficiency
Gastritis & Duodenitis
Peptic Ulcer Disease
Crohn's Disease
Ulcerative Colitis
Cirrhosis
CKD Stage 3
CKD Stage 4
CKD Stage 5 untreated
CKD Stage 5 on dialysis
Hypothyroid Diseases
Hyperthyroid Diseases
Other Endocrine

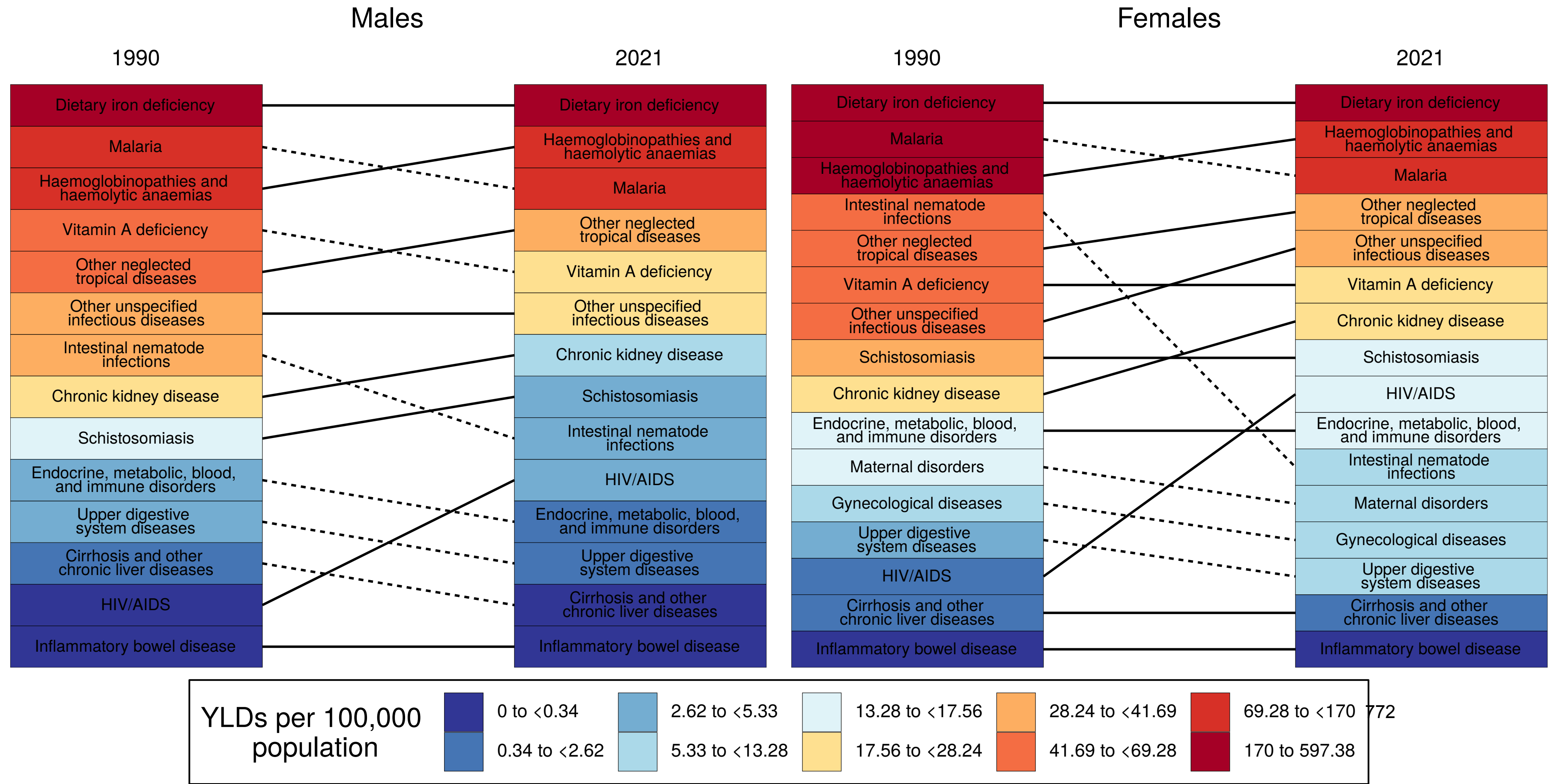
Males
Females
1990
2021
Dietary iron deficiency
Malaria
Haemoglobinopathies and haemolytic anaemias
Vitamin A deficiency
Other neglected tropical diseases
Other unspecified infectious diseases
Intestinal nematode infections
Chronic kidney disease
Schistosomiasis
Endocrine, metabolic, blood, and immune disorders
Upper digestive system diseases
Cirrhosis and other chronic liver diseases
HIV/AIDS
Inflammatory bowel disease
Maternal disorders
Gynecological diseases
YLDs per 100,000 population
0 to <0.34
0.34 to <2.62
2.62 to <5.33
5.33 to <13.28
13.28 to <17.56
17.56 to <28.24
28.24 to <41.69
41.69 to <69.28
69.28 to <170 772
170 to 597.38

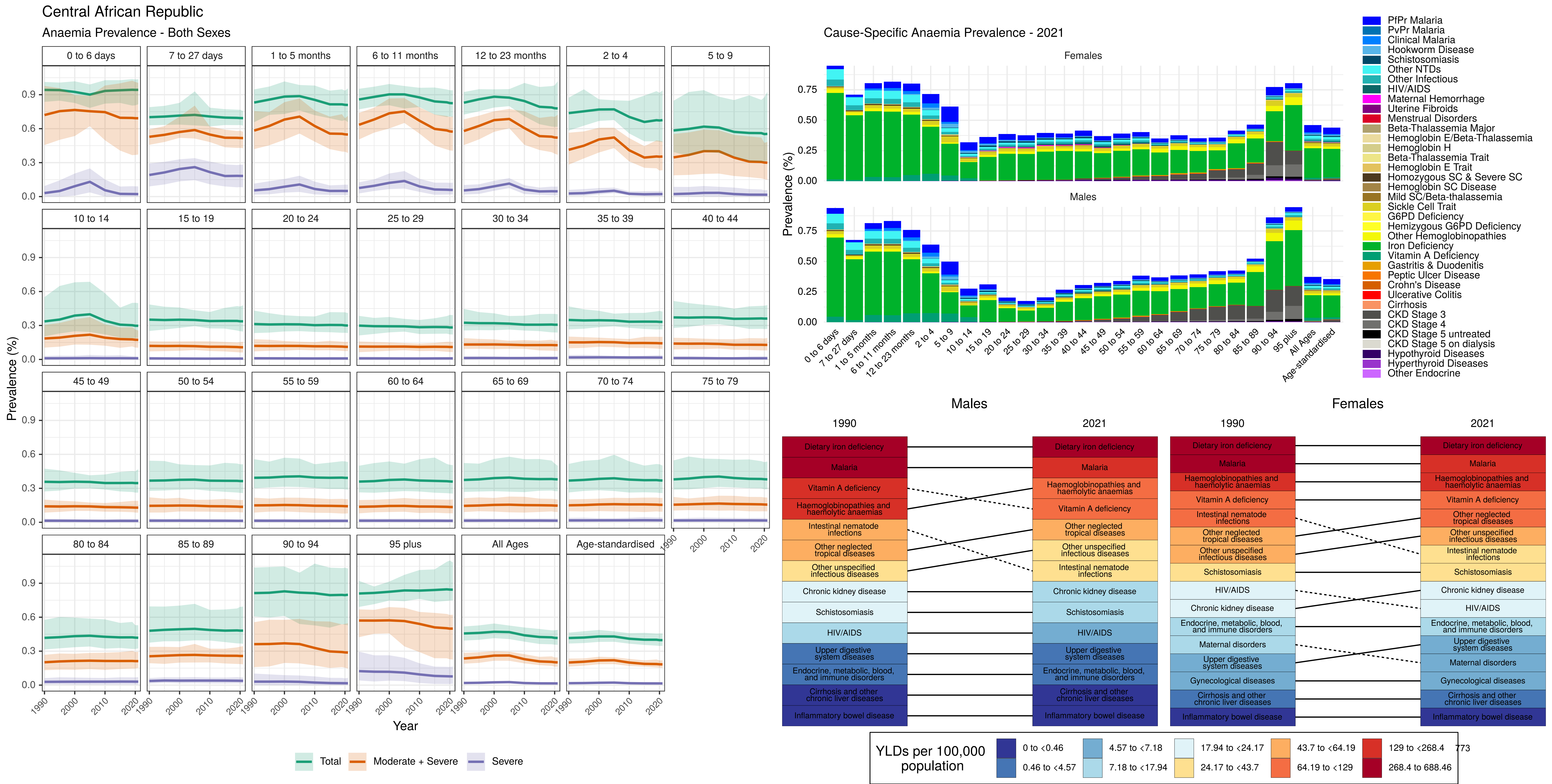
Central African Republic
Anaemia Prevalence - Both Sexes
0 to 6 days
7 to 27 days
1 to 5 months
6 to 11 months
12 to 23 months
2 to 4
5 to 9
10 to 14
15 to 19
20 to 24
25 to 29
30 to 34
35 to 39
40 to 44
45 to 49
50 to 54
55 to 59
60 to 64
65 to 69
70 to 74
75 to 79
80 to 84
85 to 89
90 to 94
95 plus
All Ages
Age-standardised
Prevalence (%)
Year
Total
Moderate + Severe
Severe
Cause-Specific Anaemia Prevalence - 2021
Females
Males
PfPr Malaria
PvPr Malaria
Clinical Malaria
Hookworm Disease
Schistosomiasis
Other NTDs
Other Infectious
HIV/AIDS
Maternal Hemorrhage
Uterine Fibroids
Menstrual Disorders
Beta-Thalassemia Major
Hemoglobin E/Beta-Thalassemia
Hemoglobin H
Beta-Thalassemia Trait
Hemoglobin E Trait
Homozygous SC & Severe SC
Hemoglobin SC Disease
Mild SC/Beta-thalassemia
Sickle Cell Trait
G6PD Deficiency
Hemizygous G6PD Deficiency
Other Hemoglobinopathies
Iron Deficiency
Vitamin A Deficiency
Gastritis & Duodenitis
Peptic Ulcer Disease
Crohn's Disease
Cirrhosis
Ulcerative Colitis
CKD Stage 3
CKD Stage 4
CKD Stage 5 untreated
CKD Stage 5 on dialysis
Hypothyroid Diseases
Hyperthyroid Diseases
Other Endocrine
Males
Females
1990
2021
Dietary iron deficiency
Malaria
Vitamin A deficiency
Haemoglobinopathies and haemolytic anaemias
Intestinal nematode infections
Other neglected tropical diseases
Other unspecified infectious diseases
Chronic kidney disease
Schistosomiasis
HIV/AIDS
Upper digestive system diseases
Endocrine, metabolic, blood, and immune disorders
Cirrhosis and other chronic liver diseases
Inflammatory bowel disease
Maternal disorders
Gynecological diseases
YLDs per 100,000 population
0 to <0.46
0.46 to <4.57
4.57 to <7.18
7.18 to <17.94
17.94 to <24.17
24.17 to <43.7
43.7 to <64.19
64.19 to <129
129 to <268.4
268.4 to 688.46

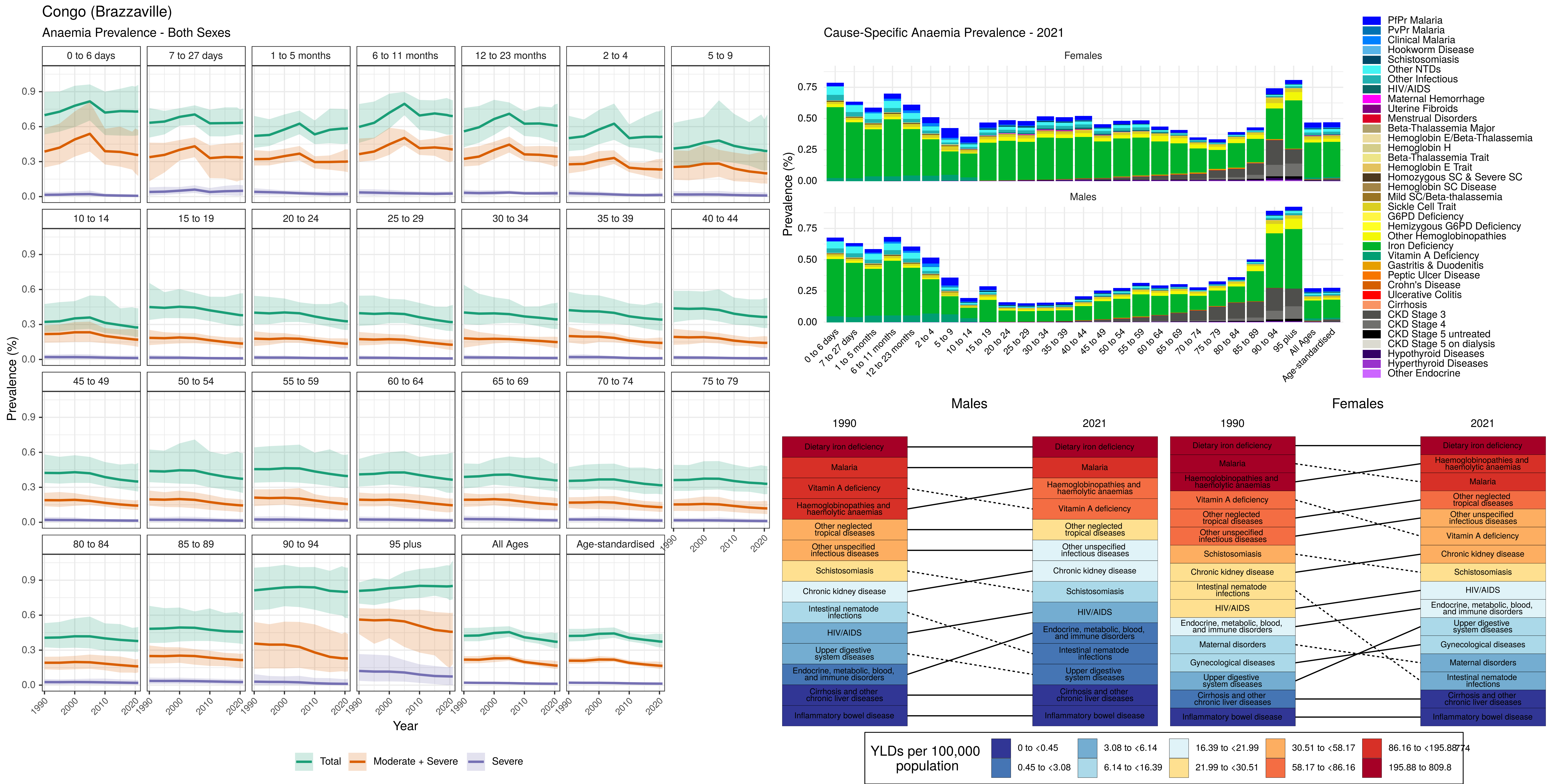

Congo (Brazzaville)
Anaemia Prevalence - Both Sexes
0 to 6 days
7 to 27 days
1 to 5 months
6 to 11 months
12 to 23 months
2 to 4
5 to 9
10 to 14
15 to 19
20 to 24
25 to 29
30 to 34
35 to 39
40 to 44
45 to 49
50 to 54
55 to 59
60 to 64
65 to 69
70 to 74
75 to 79
80 to 84
85 to 89
90 to 94
95 plus
All Ages
Age-standardised
Prevalence (%)
Year
Total
Moderate + Severe
Severe
Cause-Specific Anaemia Prevalence - 2021
Females
Males
PfPr Malaria
PvPr Malaria
Clinical Malaria
Hookworm Disease
Schistosomiasis
Other NTDs
Other Infectious
HIV/AIDS
Maternal Hemorrhage
Uterine Fibroids
Menstrual Disorders
Beta-Thalassemia Major
Hemoglobin E/Beta-Thalassemia
Hemoglobin H
Beta-Thalassemia Trait
Hemoglobin E Trait
Homozygous SC & Severe SC
Hemoglobin SC Disease
Mild SC/Beta-thalassemia
Sickle Cell Trait
G6PD Deficiency
Hemizygous G6PD Deficiency
Other Hemoglobinopathies
Iron Deficiency
Vitamin A Deficiency
Gastritis & Duodenitis
Peptic Ulcer Disease
Crohn's Disease
Ulcerative Colitis
Cirrhosis
CKD Stage 3
CKD Stage 4
CKD Stage 5 untreated
CKD Stage 5 on dialysis
Hypothyroid Diseases
Hyperthyroid Diseases
Other Endocrine
Males
Females
1990
2021
Dietary iron deficiency
Malaria
Vitamin A deficiency
Haemoglobinopathies and haemolytic anaemias
Other neglected tropical diseases
Other unspecified infectious diseases
Schistosomiasis
Chronic kidney disease
Intestinal nematode infections
HIV/AIDS
Upper digestive system diseases
Endocrine, metabolic, blood, and immune disorders
Cirrhosis and other chronic liver diseases
Inflammatory bowel disease
Maternal disorders
Gynecological diseases
YLDs per 100,000 population
0 to <0.45
0.45 to <3.08
3.08 to <6.14
6.14 to <16.39
16.39 to <21.99
21.99 to <30.51
30.51 to <58.17
58.17 to <86.16
86.16 to <195.88774
195.88 to 809.8

DR Congo

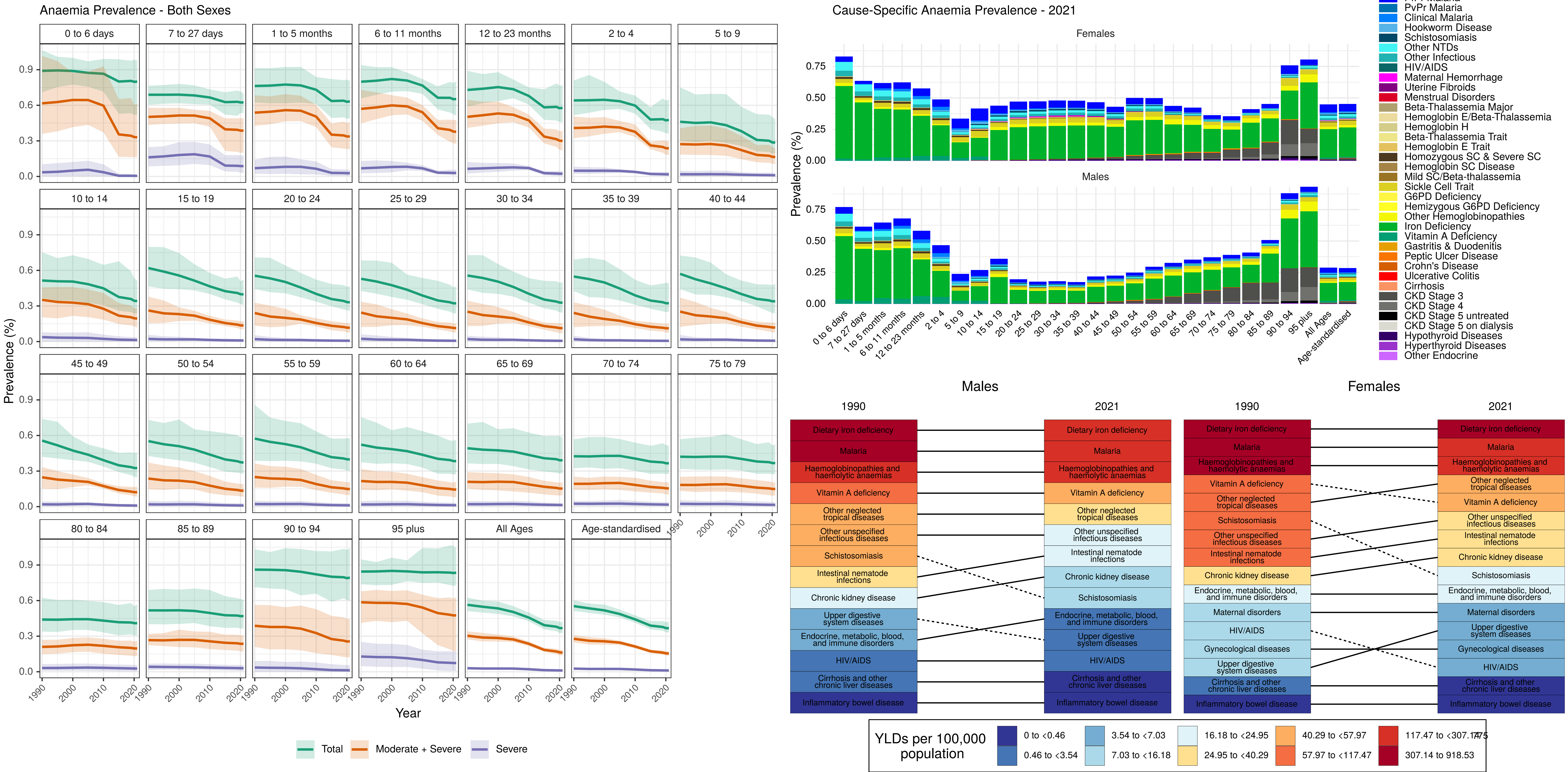

Anaemia Prevalence - Both Sexes
0 to 6 days
7 to 27 days
1 to 5 months
6 to 11 months
12 to 23 months
2 to 4
5 to 9
10 to 14
15 to 19
20 to 24
25 to 29
30 to 34
35 to 39
40 to 44
45 to 49
50 to 54
55 to 59
60 to 64
65 to 69
70 to 74
75 to 79
80 to 84
85 to 89
90 to 94
95 plus
All Ages
Age-standardised
Prevalence (%)
Year
Total
Moderate + Severe
Severe
Cause-Specific Anaemia Prevalence - 2021
Females
Males
Prevalence (%)
PfPr Malaria
PvPr Malaria
Clinical Malaria
Hookworm Disease
Schistosomiasis
Other NTDs
Other Infectious
HIV/AIDS
Maternal Hemorrhage
Uterine Fibroids
Menstrual Disorders
Beta-Thalassemia Major
Hemoglobin E/Beta-Thalassemia
Hemoglobin H
Beta-Thalassemia Trait
Hemoglobin E Trait
Homozygous SC & Severe SC
Hemoglobin SC Disease
Mild SC/Beta-thalassemia
Sickle Cell Trait
G6PD Deficiency
Hemizygous G6PD Deficiency
Other Hemoglobinopathies
Iron Deficiency
Vitamin A Deficiency
Gastritis & Duodenitis
Peptic Ulcer Disease
Crohn's Disease
Ulcerative Colitis
Cirrhosis
CKD Stage 3
CKD Stage 4
CKD Stage 5 untreated
CKD Stage 5 on dialysis
Hypothyroid Diseases
Hyperthyroid Diseases
Other Endocrine
Males
Females
1990
2021
Dietary iron deficiency
Malaria
Haemoglobinopathies and haemolytic anaemias
Vitamin A deficiency
Other neglected tropical diseases
Other unspecified infectious diseases
Schistosomiasis
Intestinal nematode infections
Chronic kidney disease
Upper digestive system diseases
Endocrine, metabolic, blood, and immune disorders
HIV/AIDS
Cirrhosis and other chronic liver diseases
Inflammatory bowel disease
Maternal disorders
Gynecological diseases
YLDs per 100,000 population
0 to <0.46
0.46 to <3.54
3.54 to <7.03
7.03 to <16.18
16.18 to <24.95
24.95 to <40.29
40.29 to <57.97
57.97 to <117.47
117.47 to <307.14
307.14 to 918.53

Equatorial Guinea

Anaemia Prevalence - Both Sexes

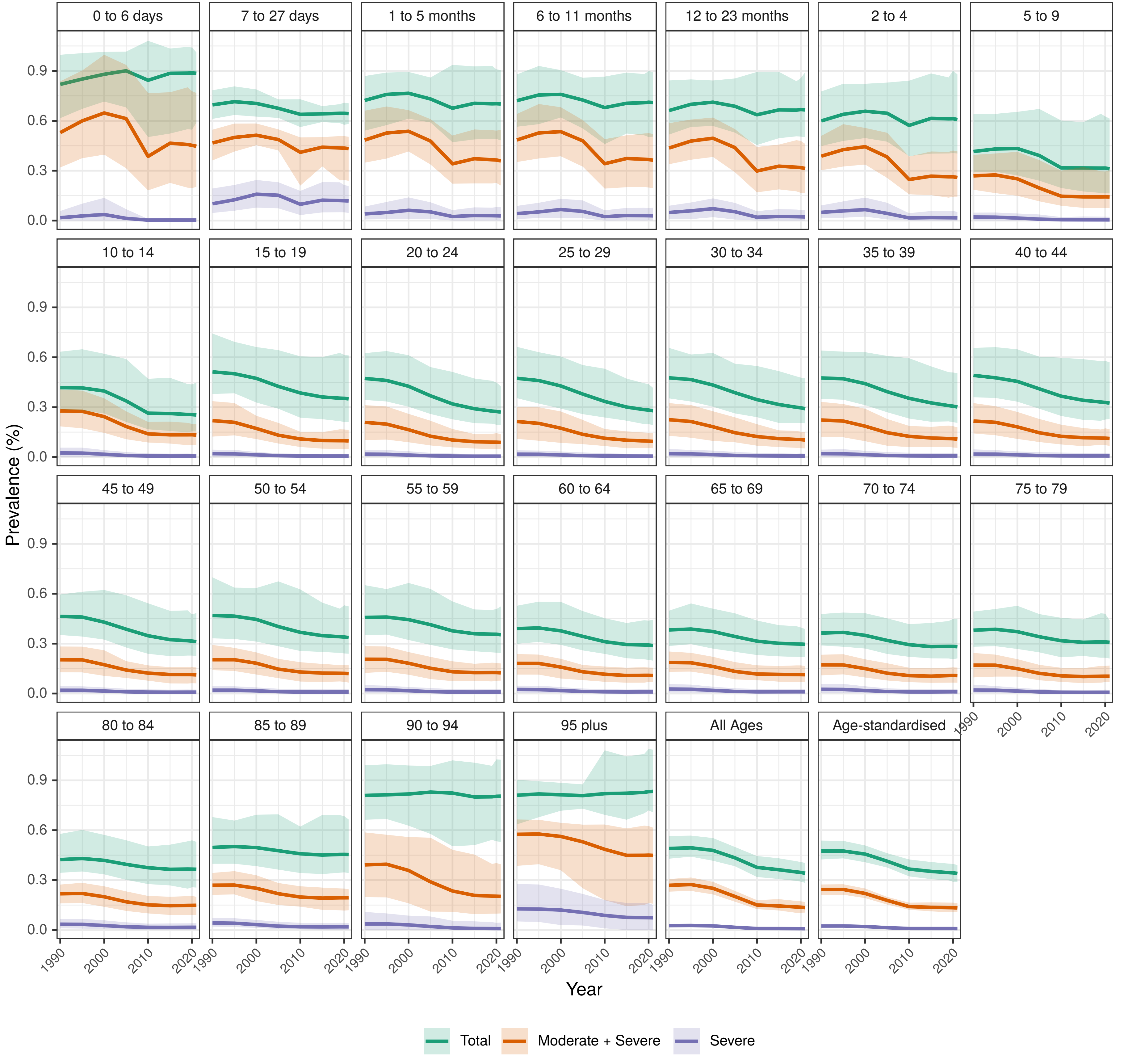

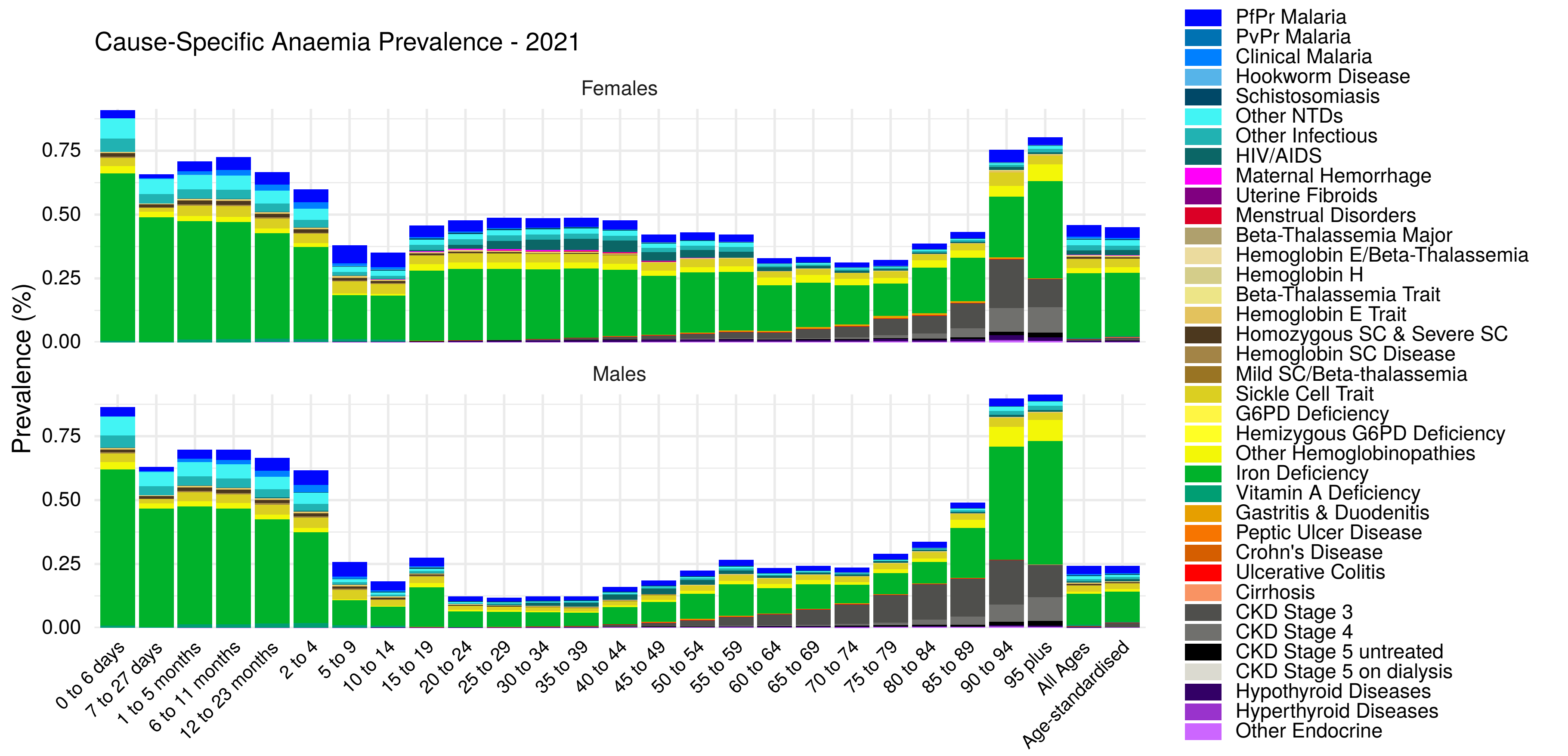

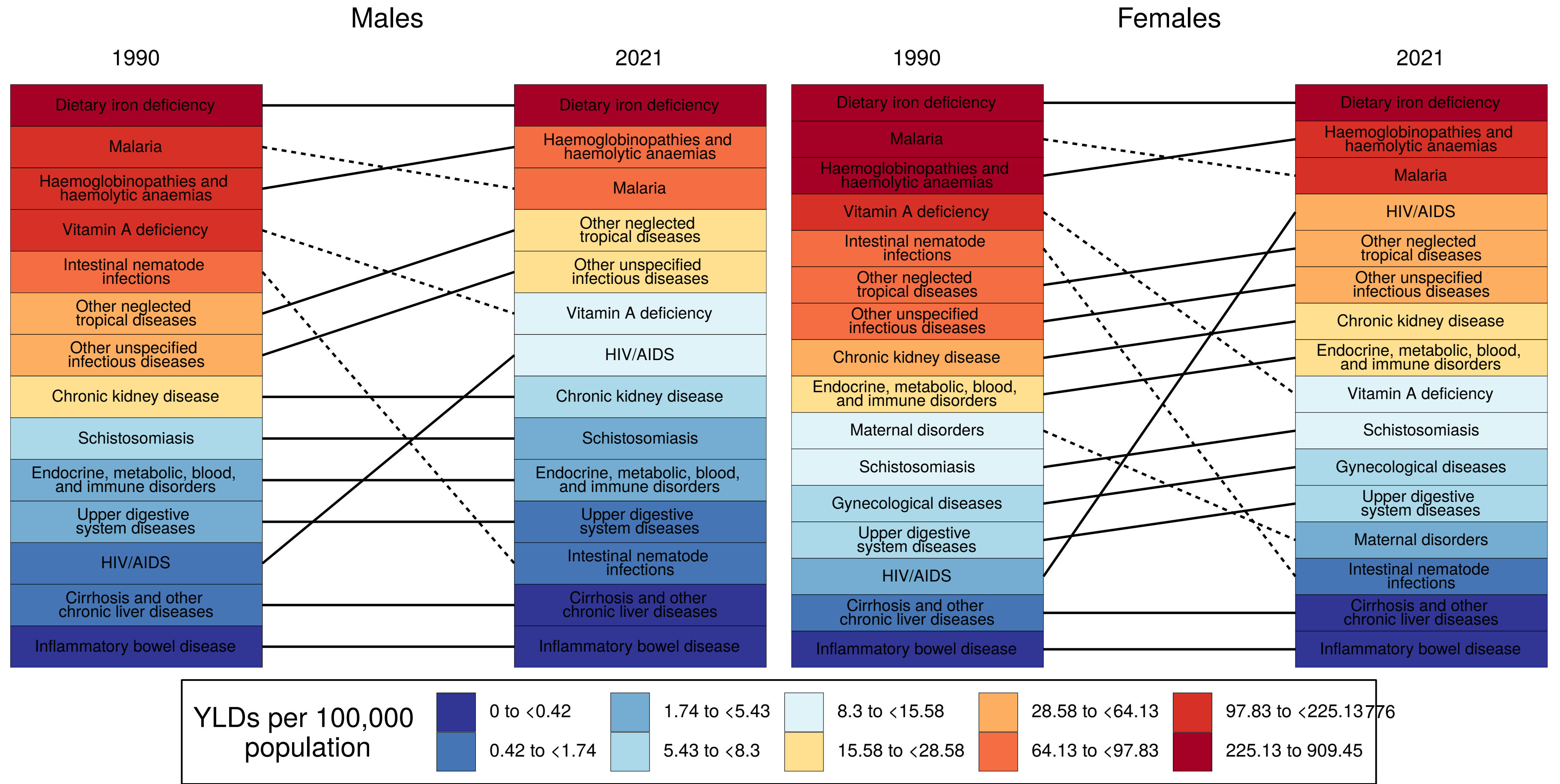

Gabon

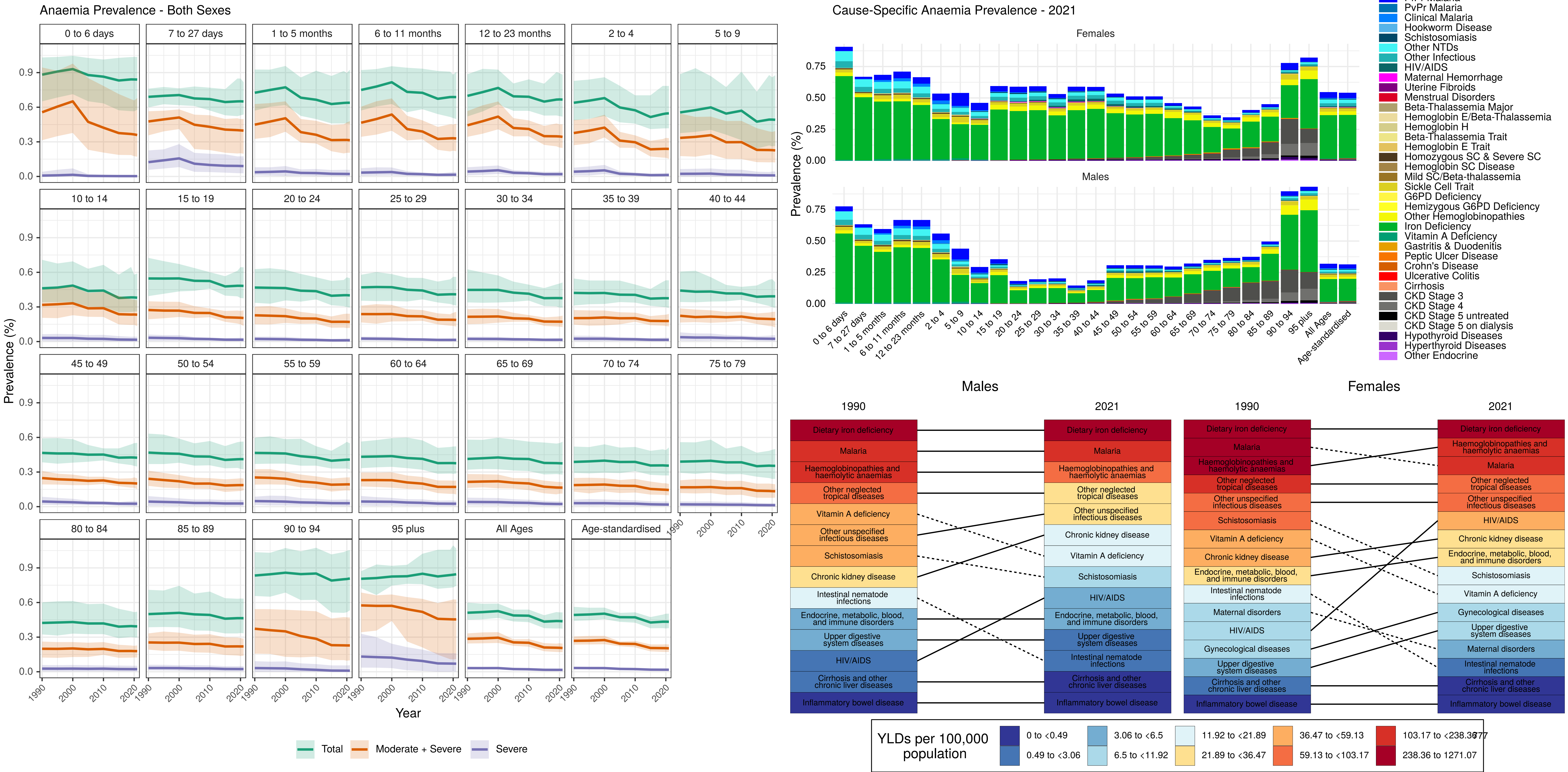
Anaemia Prevalence - Both Sexes
0 to 6 days
7 to 27 days
1 to 5 months
6 to 11 months
12 to 23 months
2 to 4
5 to 9
10 to 14
15 to 19
20 to 24
25 to 29
30 to 34
35 to 39
40 to 44
45 to 49
50 to 54
55 to 59
60 to 64
65 to 69
70 to 74
75 to 79
80 to 84
85 to 89
90 to 94
95 plus
All Ages
Age-standardised
Prevalence (%)
Year
Total
Moderate + Severe
Severe
Cause-Specific Anaemia Prevalence - 2021
Females
Males
PfPr Malaria
PvPr Malaria
Clinical Malaria
Hookworm Disease
Schistosomiasis
Other NTDs
Other Infectious
HIV/AIDS
Maternal Hemorrhage
Uterine Fibroids
Menstrual Disorders
Beta-Thalassemia Major
Hemoglobin E/Beta-Thalassemia
Hemoglobin H
Beta-Thalassemia Trait
Hemoglobin E Trait
Homozygous SC & Severe SC
Hemoglobin SC Disease
Mild SC/Beta-thalassemia
Sickle Cell Trait
G6PD Deficiency
Hemizygous G6PD Deficiency
Other Hemoglobinopathies
Iron Deficiency
Vitamin A Deficiency
Gastritis & Duodenitis
Peptic Ulcer Disease
Crohn's Disease
Ulcerative Colitis
Cirrhosis
CKD Stage 3
CKD Stage 4
CKD Stage 5 untreated
CKD Stage 5 on dialysis
Hypothyroid Diseases
Hyperthyroid Diseases
Other Endocrine
Males
Females
1990
2021
Dietary iron deficiency
Malaria
Haemoglobinopathies and haemolytic anaemias
Other neglected tropical diseases
Vitamin A deficiency
Other unspecified infectious diseases
Schistosomiasis
Chronic kidney disease
Intestinal nematode infections
Endocrine, metabolic, blood, and immune disorders
Upper digestive system diseases
HIV/AIDS
Cirrhosis and other chronic liver diseases
Inflammatory bowel disease
Maternal disorders
Gynecological diseases
YLDs per 100,000 population
0 to <0.49
0.49 to <3.06
3.06 to <6.5
6.5 to <11.92
11.92 to <21.89
21.89 to <36.47
36.47 to <59.13
59.13 to <103.17
103.17 to <238.3677
238.36 to 1271.07

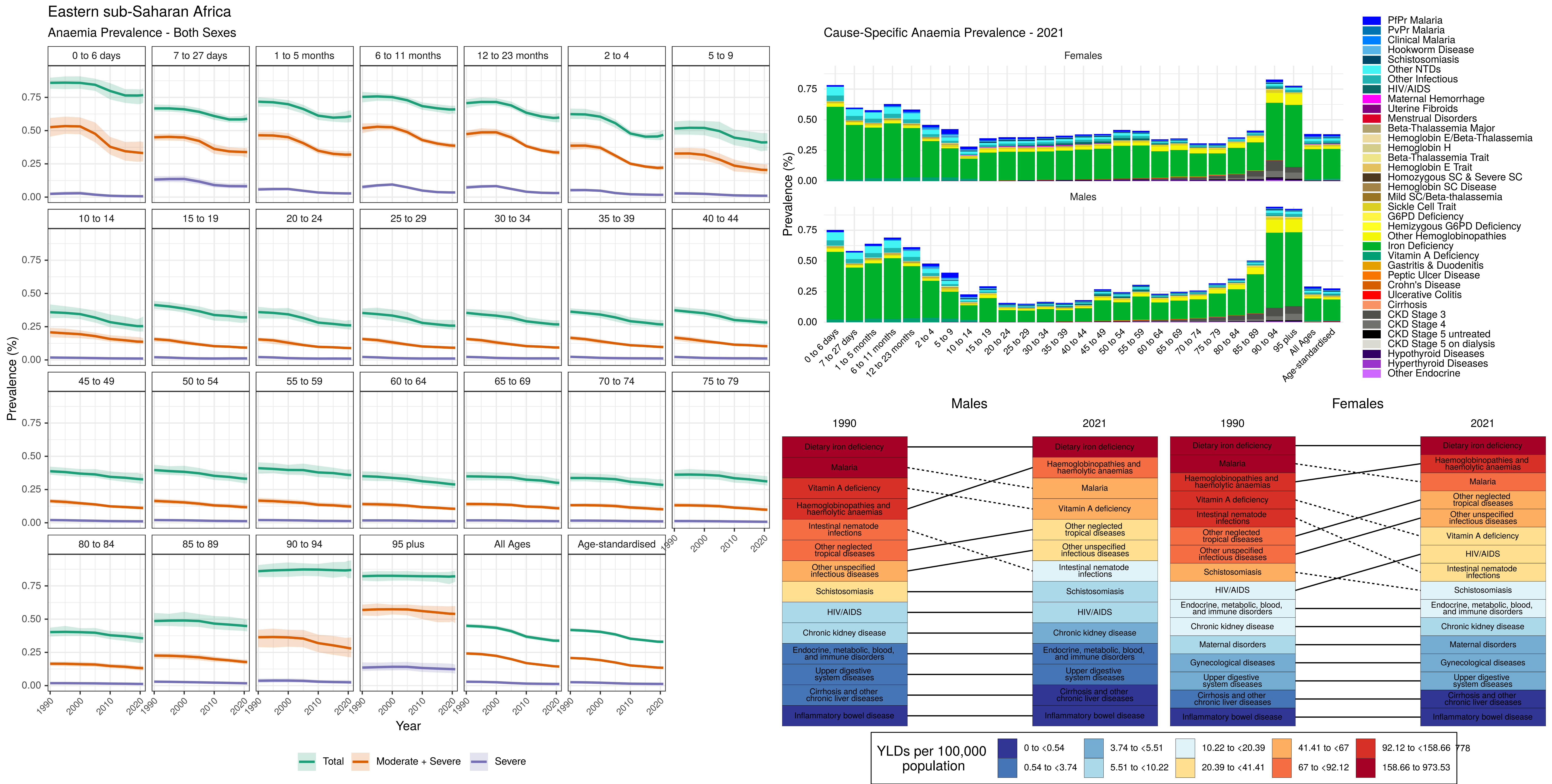

Eastern sub-Saharan Africa
Anaemia Prevalence - Both Sexes
0 to 6 days
7 to 27 days
1 to 5 months
6 to 11 months
12 to 23 months
2 to 4
5 to 9
10 to 14
15 to 19
20 to 24
25 to 29
30 to 34
35 to 39
40 to 44
45 to 49
50 to 54
55 to 59
60 to 64
65 to 69
70 to 74
75 to 79
80 to 84
85 to 89
90 to 94
95 plus
All Ages
Age-standardised
Prevalence (%)
Year
Total
Moderate + Severe
Severe
Cause-Specific Anaemia Prevalence - 2021
Females
Males
PfPr Malaria
PvPr Malaria
Clinical Malaria
Hookworm Disease
Schistosomiasis
Other NTDs
Other Infectious
HIV/AIDS
Maternal Hemorrhage
Uterine Fibroids
Menstrual Disorders
Beta-Thalassemia Major
Hemoglobin E/Beta-Thalassemia
Hemoglobin H
Beta-Thalassemia Trait
Hemoglobin E Trait
Homozygous SC & Severe SC
Hemoglobin SC Disease
Mild SC/Beta-thalassemia
Sickle Cell Trait
G6PD Deficiency
Hemizygous G6PD Deficiency
Other Hemoglobinopathies
Iron Deficiency
Vitamin A Deficiency
Gastritis & Duodenitis
Peptic Ulcer Disease
Crohn's Disease
Ulcerative Colitis
Cirrhosis
CKD Stage 3
CKD Stage 4
CKD Stage 5 untreated
CKD Stage 5 on dialysis
Hypothyroid Diseases
Hyperthyroid Diseases
Other Endocrine
Males
Females
1990
2021
Dietary iron deficiency
Malaria
Vitamin A deficiency
Haemoglobinopathies and haemolytic anaemias
Intestinal nematode infections
Other neglected tropical diseases
Other unspecified infectious diseases
Schistosomiasis
HIV/AIDS
Chronic kidney disease
Endocrine, metabolic, blood, and immune disorders
Upper digestive system diseases
Cirrhosis and other chronic liver diseases
Inflammatory bowel disease
Maternal disorders
Gynecological diseases
YLDs per 100,000 population
0 to <0.54
0.54 to <3.74
3.74 to <5.51
5.51 to <10.22
10.22 to <20.39
20.39 to <41.41
41.41 to <67
67 to <92.12
92.12 to <158.66
158.66 to 973.53

Burundi

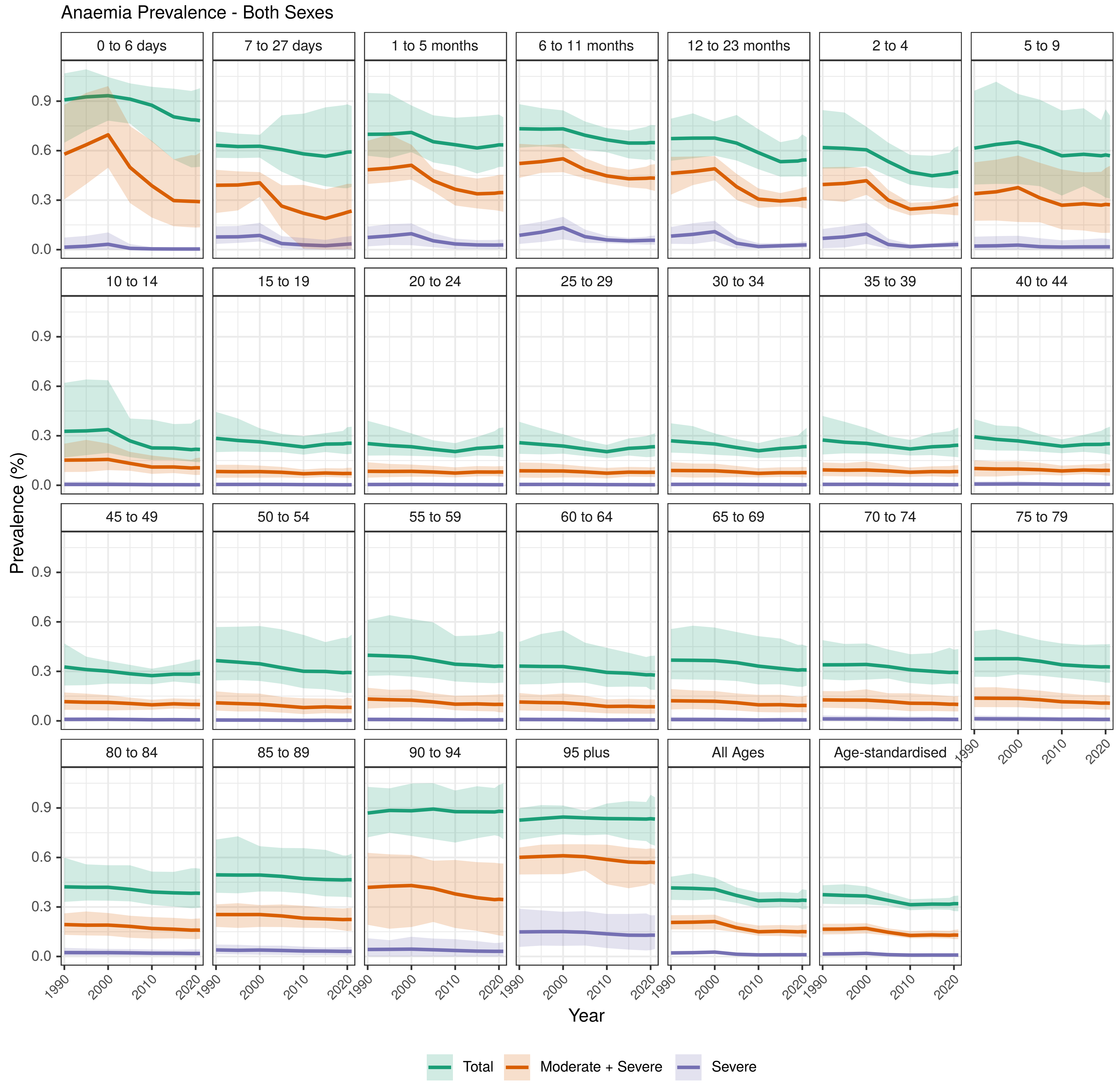

Anaemia Prevalence - Both Sexes
0 to 6 days
7 to 27 days
1 to 5 months
6 to 11 months
12 to 23 months
2 to 4
5 to 9
10 to 14
15 to 19
20 to 24
25 to 29
30 to 34
35 to 39
40 to 44
45 to 49
50 to 54
55 to 59
60 to 64
65 to 69
70 to 74
75 to 79
80 to 84
85 to 89
90 to 94
95 plus
All Ages
Age-standardised
Prevalence (%)
Year
Total
Moderate + Severe
Severe

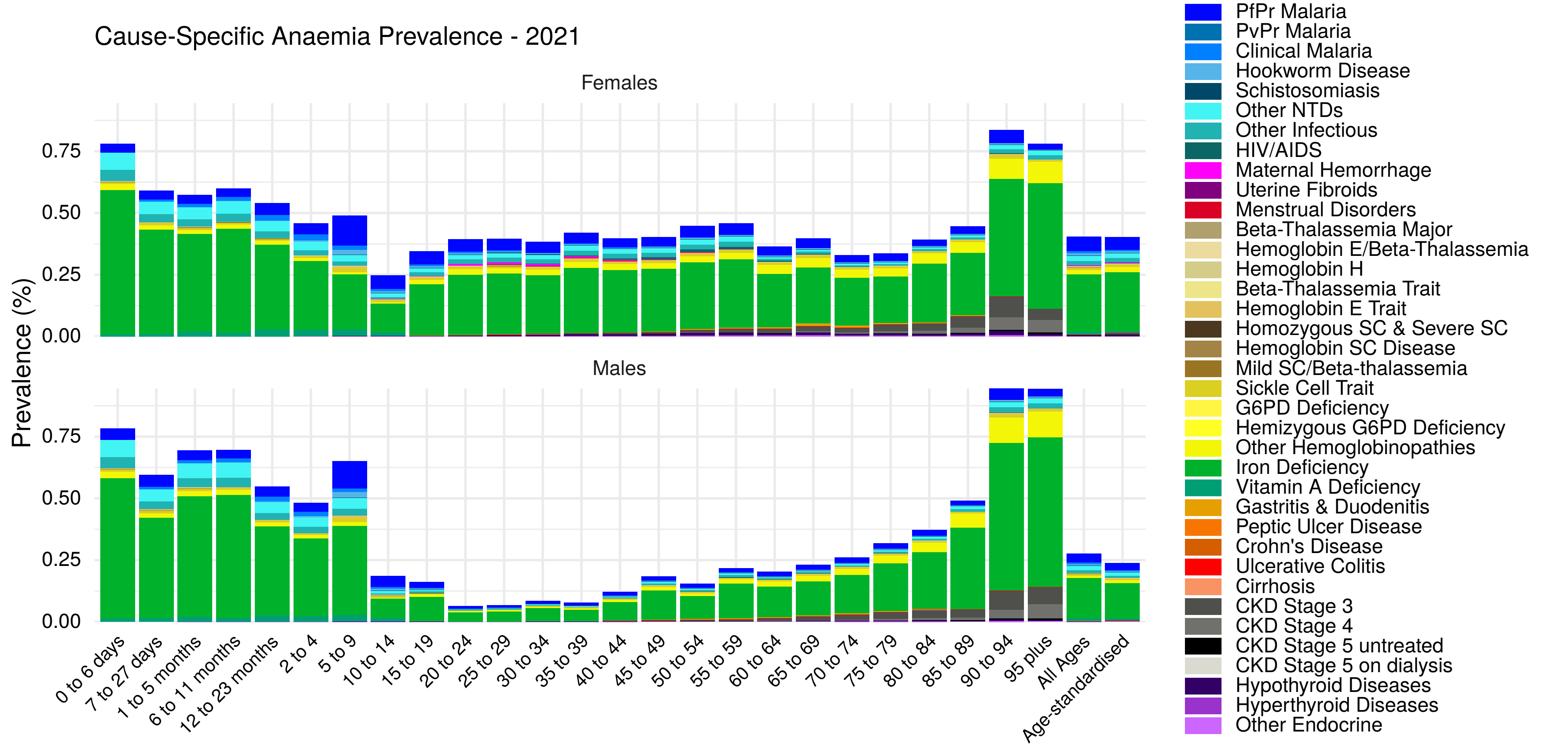

Cause-Specific Anaemia Prevalence - 2021
Females
Males
Prevalence (%)
PfPr Malaria
PvPr Malaria
Clinical Malaria
Hookworm Disease
Schistosomiasis
Other NTDs
Other Infectious
HIV/AIDS
Maternal Hemorrhage
Uterine Fibroids
Menstrual Disorders
Beta-Thalassemia Major
Hemoglobin E/Beta-Thalassemia
Hemoglobin H
Beta-Thalassemia Trait
Hemoglobin E Trait
Homozygous SC & Severe SC
Hemoglobin SC Disease
Mild SC/Beta-thalassemia
Sickle Cell Trait
G6PD Deficiency
Hemizygous G6PD Deficiency
Other Hemoglobinopathies
Iron Deficiency
Vitamin A Deficiency
Gastritis & Duodenitis
Peptic Ulcer Disease
Crohn's Disease
Ulcerative Colitis
Cirrhosis
CKD Stage 3
CKD Stage 4
CKD Stage 5 untreated
CKD Stage 5 on dialysis
Hypothyroid Diseases
Hyperthyroid Diseases
Other Endocrine

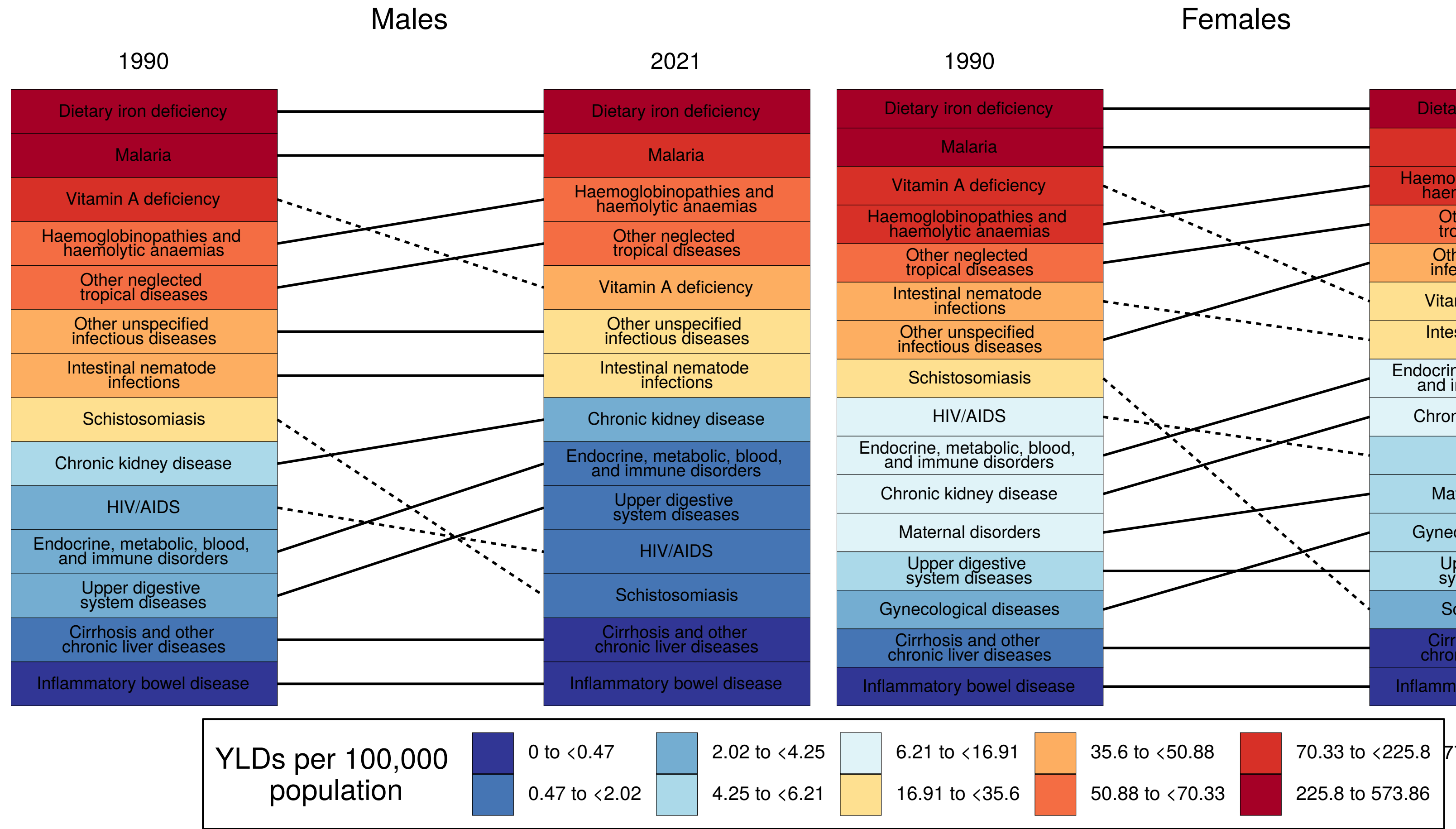

Males
Females
1990
2021
Dietary iron deficiency
Malaria
Vitamin A deficiency
Haemoglobinopathies and haemolytic anaemias
Other neglected tropical diseases
Other unspecified infectious diseases
Intestinal nematode infections
Schistosomiasis
Chronic kidney disease
HIV/AIDS
Endocrine, metabolic, blood, and immune disorders
Upper digestive system diseases
Cirrhosis and other chronic liver diseases
Inflammatory bowel disease
Maternal disorders
Gynecological diseases
YLDs per 100,000 population
0 to <0.47
0.47 to <2.02
2.02 to <4.25
4.25 to <6.21
6.21 to <16.91
16.91 to <35.6
35.6 to <50.88
50.88 to <70.33
70.33 to <225.8
225.8 to 573.86

Comoros

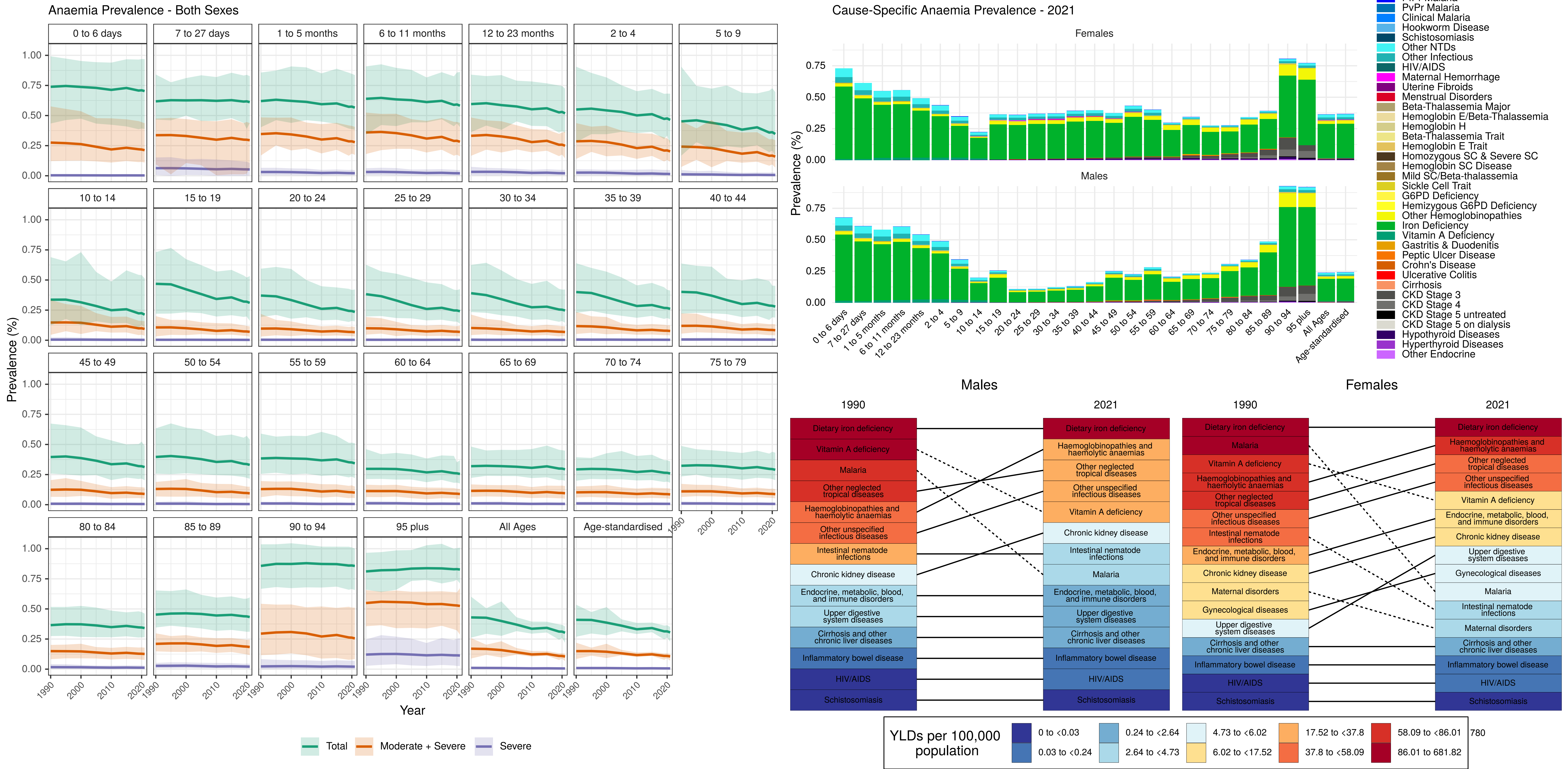

Anaemia Prevalence - Both Sexes
0 to 6 days
7 to 27 days
1 to 5 months
6 to 11 months
12 to 23 months
2 to 4
5 to 9
10 to 14
15 to 19
20 to 24
25 to 29
30 to 34
35 to 39
40 to 44
45 to 49
50 to 54
55 to 59
60 to 64
65 to 69
70 to 74
75 to 79
80 to 84
85 to 89
90 to 94
95 plus
All Ages
Age-standardised
Prevalence (%)
Year
Total
Moderate + Severe
Severe
Cause-Specific Anaemia Prevalence - 2021
Females
Males
PfPr Malaria
PvPr Malaria
Clinical Malaria
Hookworm Disease
Schistosomiasis
Other NTDs
Other Infectious
HIV/AIDS
Maternal Hemorrhage
Uterine Fibroids
Menstrual Disorders
Beta-Thalassemia Major
Hemoglobin E/Beta-Thalassemia
Hemoglobin H
Beta-Thalassemia Trait
Hemoglobin E Trait
Homozygous SC & Severe SC
Hemoglobin SC Disease
Mild SC/Beta-thalassemia
Sickle Cell Trait
G6PD Deficiency
Hemizygous G6PD Deficiency
Other Hemoglobinopathies
Iron Deficiency
Vitamin A Deficiency
Gastritis & Duodenitis
Peptic Ulcer Disease
Crohn's Disease
Ulcerative Colitis
Cirrhosis
CKD Stage 3
CKD Stage 4
CKD Stage 5 untreated
CKD Stage 5 on dialysis
Hypothyroid Diseases
Hyperthyroid Diseases
Other Endocrine
Males
Females
1990
2021
Dietary iron deficiency
Vitamin A deficiency
Malaria
Other neglected tropical diseases
Haemoglobinopathies and haemolytic anaemias
Other unspecified infectious diseases
Intestinal nematode infections
Chronic kidney disease
Endocrine, metabolic, blood, and immune disorders
Upper digestive system diseases
Cirrhosis and other chronic liver diseases
Inflammatory bowel disease
HIV/AIDS
Schistosomiasis
Maternal disorders
Gynecological diseases
YLDs per 100,000 population
0 to <0.03
0.03 to <0.24
0.24 to <2.64
2.64 to <4.73
4.73 to <6.02
6.02 to <17.52
17.52 to <37.8
37.8 to <58.09
58.09 to <86.01
86.01 to 681.82

Djibouti

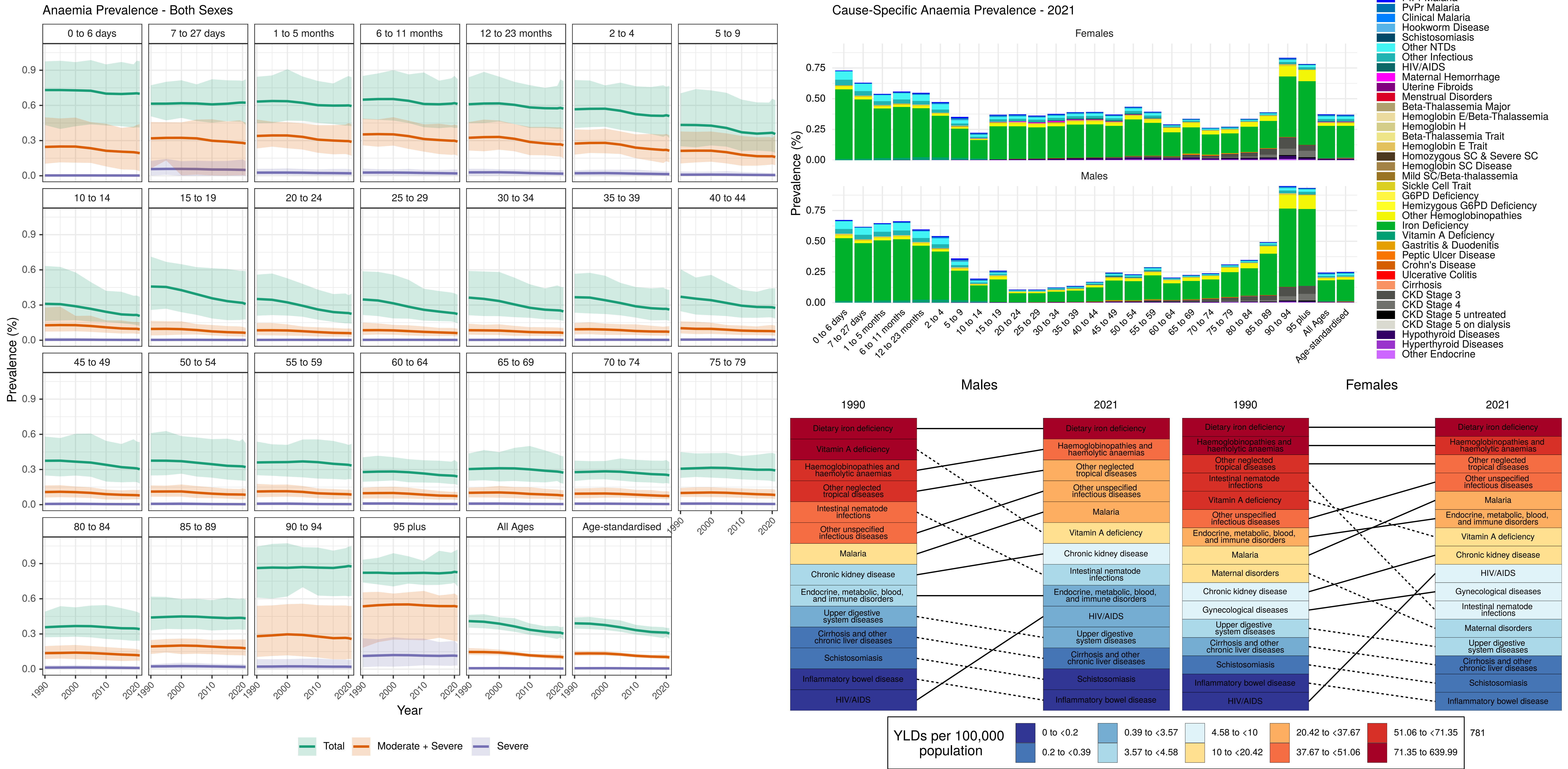

Anaemia Prevalence - Both Sexes
0 to 6 days
7 to 27 days
1 to 5 months
6 to 11 months
12 to 23 months
2 to 4
5 to 9
10 to 14
15 to 19
20 to 24
25 to 29
30 to 34
35 to 39
40 to 44
45 to 49
50 to 54
55 to 59
60 to 64
65 to 69
70 to 74
75 to 79
80 to 84
85 to 89
90 to 94
95 plus
All Ages
Age-standardised
Prevalence (%)
0.0
0.3
0.6
0.9
1990
2000
2010
2020
Year
Total
Moderate + Severe
Severe
Cause-Specific Anaemia Prevalence - 2021
Females
Males
Prevalence (%)
0.00
0.25
0.50
0.75
PfPr Malaria
PvPr Malaria
Clinical Malaria
Hookworm Disease
Schistosomiasis
Other NTDs
Other Infectious
HIV/AIDS
Maternal Hemorrhage
Uterine Fibroids
Menstrual Disorders
Beta-Thalassemia Major
Hemoglobin E/Beta-Thalassemia
Hemoglobin H
Beta-Thalassemia Trait
Hemoglobin E Trait
Homozygous SC & Severe SC
Hemoglobin SC Disease
Mild SC/Beta-thalassemia
Sickle Cell Trait
G6PD Deficiency
Hemizygous G6PD Deficiency
Other Hemoglobinopathies
Iron Deficiency
Vitamin A Deficiency
Gastritis & Duodenitis
Peptic Ulcer Disease
Crohn's Disease
Ulcerative Colitis
Cirrhosis
CKD Stage 3
CKD Stage 4
CKD Stage 5 untreated
CKD Stage 5 on dialysis
Hypothyroid Diseases
Hyperthyroid Diseases
Other Endocrine
Males
Females
1990
2021
Dietary iron deficiency
Vitamin A deficiency
Haemoglobinopathies and haemolytic anaemias
Other neglected tropical diseases
Intestinal nematode infections
Other unspecified infectious diseases
Malaria
Chronic kidney disease
Endocrine, metabolic, blood, and immune disorders
Upper digestive system diseases
Cirrhosis and other chronic liver diseases
Schistosomiasis
Inflammatory bowel disease
HIV/AIDS
Maternal disorders
Gynecological diseases
YLDs per 100,000 population
0 to <0.2
0.2 to <0.39
0.39 to <3.57
3.57 to <4.58
4.58 to <10
10 to <20.42
20.42 to <37.67
37.67 to <51.06
51.06 to <71.35
71.35 to 639.99
781

Eritrea

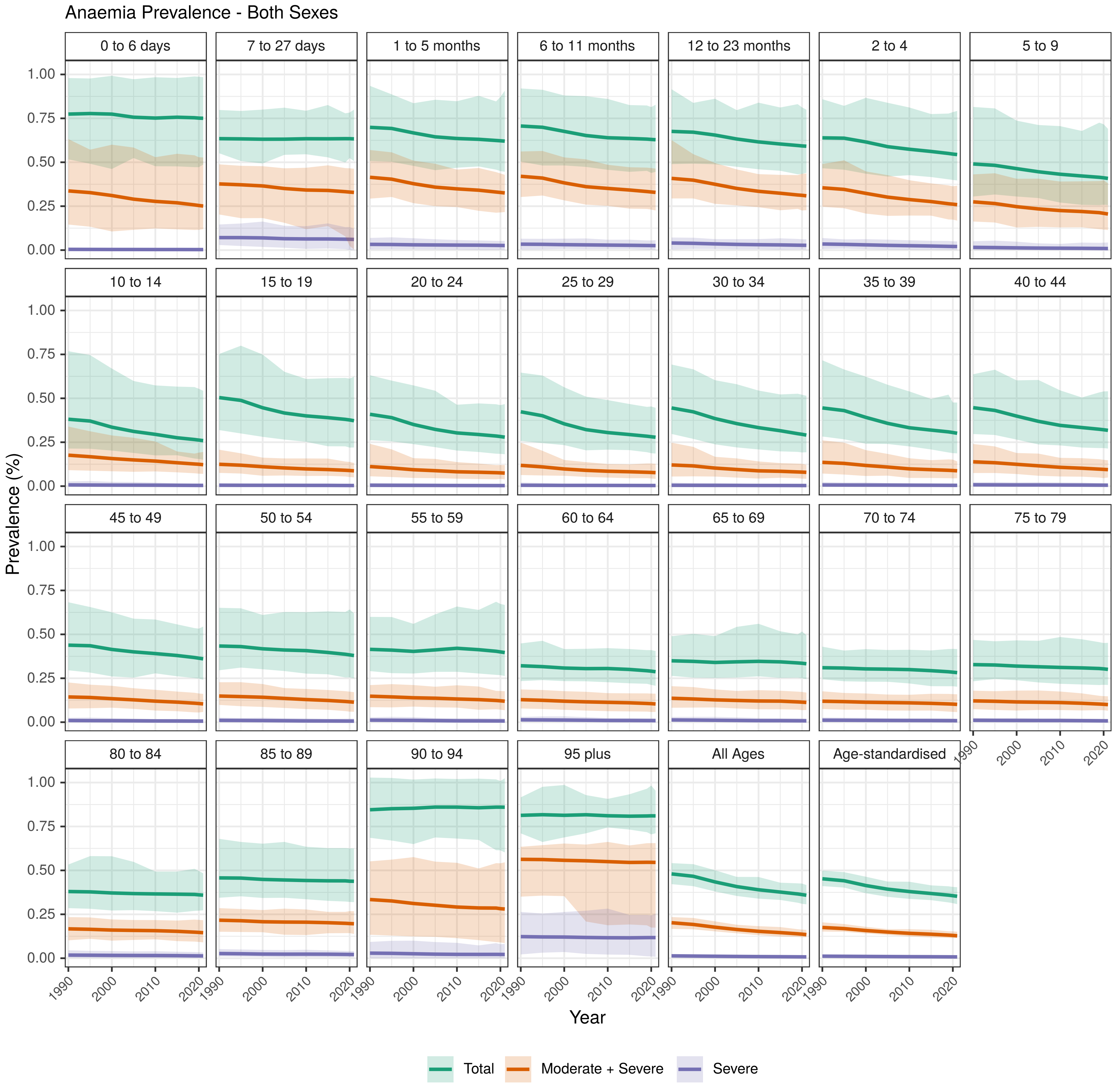

Anaemia Prevalence - Both Sexes
0 to 6 days
7 to 27 days
1 to 5 months
6 to 11 months
12 to 23 months
2 to 4
5 to 9
10 to 14
15 to 19
20 to 24
25 to 29
30 to 34
35 to 39
40 to 44
45 to 49
50 to 54
55 to 59
60 to 64
65 to 69
70 to 74
75 to 79
80 to 84
85 to 89
90 to 94
95 plus
All Ages
Age-standardised
Prevalence (%)
0.00
0.25
0.50
0.75
1.00
1990
2000
2010
2020
Year
Total
Moderate + Severe
Severe

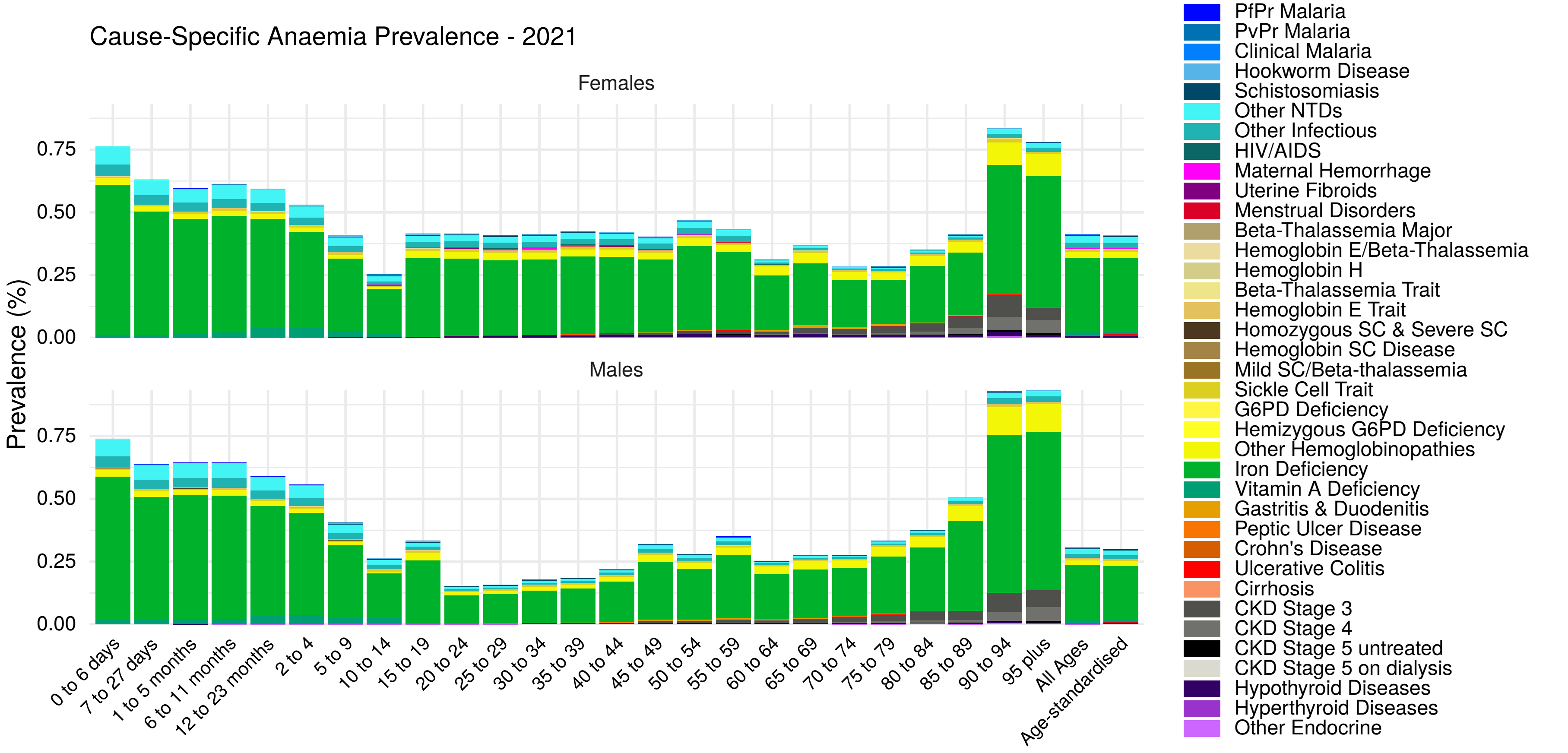

Cause-Specific Anaemia Prevalence - 2021
Females
Males
Prevalence (%)
0.00
0.25
0.50
0.75
0 to 6 days
7 to 27 days
1 to 5 months
6 to 11 months
12 to 23 months
2 to 4
5 to 9
10 to 14
15 to 19
20 to 24
25 to 29
30 to 34
35 to 39
40 to 44
45 to 49
50 to 54
55 to 59
60 to 64
65 to 69
70 to 74
75 to 79
80 to 84
85 to 89
90 to 94
95 plus
All Ages
Age-standardised
PfPr Malaria
PvPr Malaria
Clinical Malaria
Hookworm Disease
Schistosomiasis
Other NTDs
Other Infectious
HIV/AIDS
Maternal Hemorrhage
Uterine Fibroids
Menstrual Disorders
Beta-Thalassemia Major
Hemoglobin E/Beta-Thalassemia
Hemoglobin H
Beta-Thalassemia Trait
Hemoglobin E Trait
Homozygous SC & Severe SC
Hemoglobin SC Disease
Mild SC/Beta-thalassemia
Sickle Cell Trait
G6PD Deficiency
Hemizygous G6PD Deficiency
Other Hemoglobinopathies
Iron Deficiency
Vitamin A Deficiency
Gastritis & Duodenitis
Peptic Ulcer Disease
Crohn's Disease
Ulcerative Colitis
Cirrhosis
CKD Stage 3
CKD Stage 4
CKD Stage 5 untreated
CKD Stage 5 on dialysis
Hypothyroid Diseases
Hyperthyroid Diseases
Other Endocrine

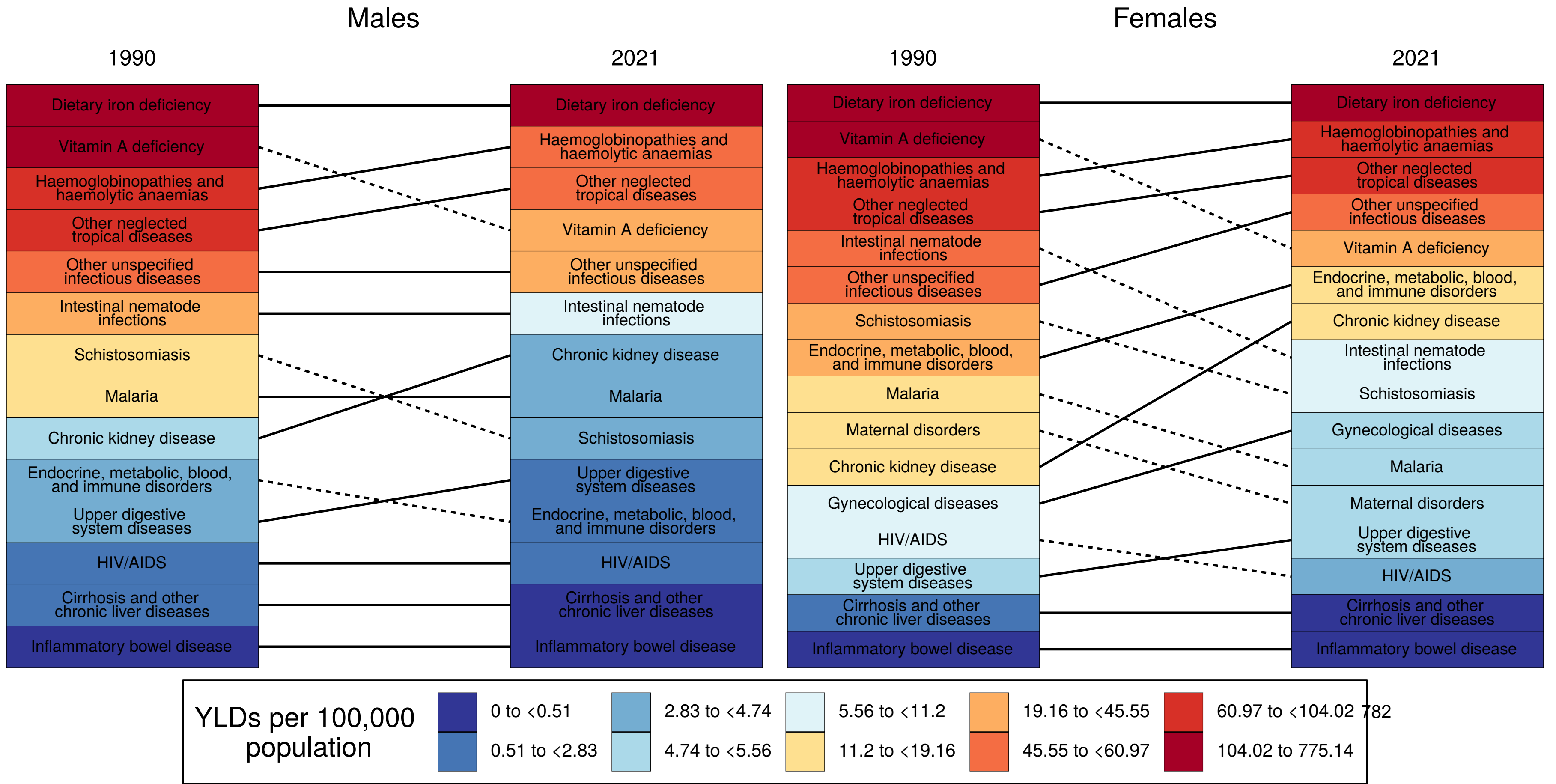

Males
Females
1990
2021
Dietary iron deficiency
Vitamin A deficiency
Haemoglobinopathies and haemolytic anaemias
Other neglected tropical diseases
Other unspecified infectious diseases
Intestinal nematode infections
Schistosomiasis
Malaria
Chronic kidney disease
Endocrine, metabolic, blood, and immune disorders
Upper digestive system diseases
HIV/AIDS
Cirrhosis and other chronic liver diseases
Inflammatory bowel disease
Maternal disorders
Gynecological diseases
YLDs per 100,000 population
0 to <0.51
0.51 to <2.83
2.83 to <4.74
4.74 to <5.56
5.56 to <11.2
11.2 to <19.16
19.16 to <45.55
45.55 to <60.97
60.97 to <104.02
104.02 to 775.14

782

Ethiopia

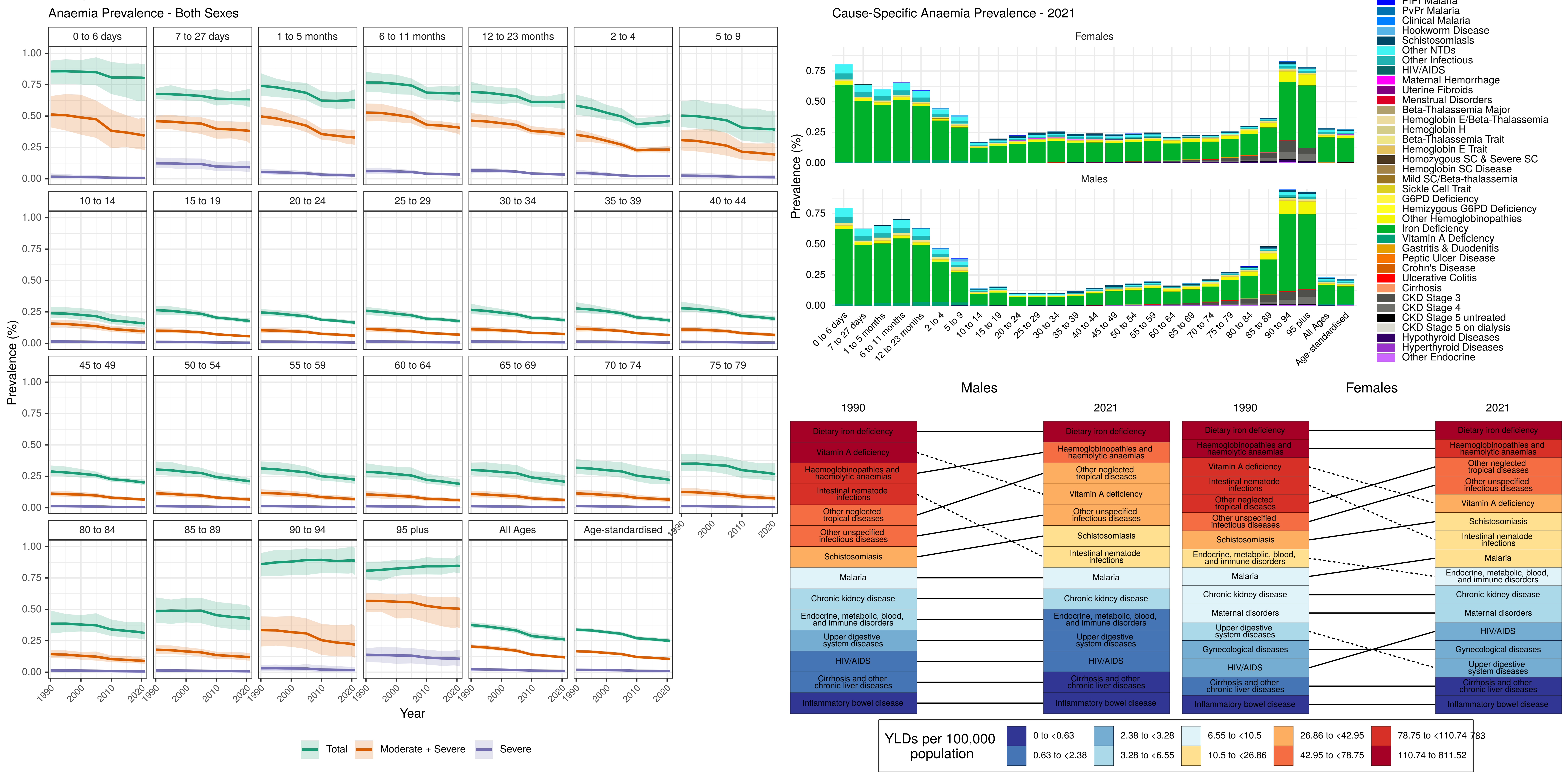

Anaemia Prevalence - Both Sexes
0 to 6 days
7 to 27 days
1 to 5 months
6 to 11 months
12 to 23 months
2 to 4
5 to 9
10 to 14
15 to 19
20 to 24
25 to 29
30 to 34
35 to 39
40 to 44
45 to 49
50 to 54
55 to 59
60 to 64
65 to 69
70 to 74
75 to 79
80 to 84
85 to 89
90 to 94
95 plus
All Ages
Age-standardised
Prevalence (%)
Year
Total
Moderate + Severe
Severe
Cause-Specific Anaemia Prevalence - 2021
Females
Males
Prevalence (%)
PfPr Malaria
PvPr Malaria
Clinical Malaria
Hookworm Disease
Schistosomiasis
Other NTDs
Other Infectious
HIV/AIDS
Maternal Hemorrhage
Uterine Fibroids
Menstrual Disorders
Beta-Thalassemia Major
Hemoglobin E/Beta-Thalassemia
Hemoglobin H
Beta-Thalassemia Trait
Hemoglobin E Trait
Homozygous SC & Severe SC
Hemoglobin SC Disease
Mild SC/Beta-thalassemia
Sickle Cell Trait
G6PD Deficiency
Hemizygous G6PD Deficiency
Other Hemoglobinopathies
Iron Deficiency
Vitamin A Deficiency
Gastritis & Duodenitis
Peptic Ulcer Disease
Crohn's Disease
Ulcerative Colitis
Cirrhosis
CKD Stage 3
CKD Stage 4
CKD Stage 5 untreated
CKD Stage 5 on dialysis
Hypothyroid Diseases
Hyperthyroid Diseases
Other Endocrine
Males
Females
1990
2021
Dietary iron deficiency
Vitamin A deficiency
Haemoglobinopathies and haemolytic anaemias
Intestinal nematode infections
Other neglected tropical diseases
Other unspecified infectious diseases
Schistosomiasis
Malaria
Chronic kidney disease
Endocrine, metabolic, blood, and immune disorders
Upper digestive system diseases
HIV/AIDS
Cirrhosis and other chronic liver diseases
Inflammatory bowel disease
Maternal disorders
Gynecological diseases
YLDs per 100,000 population
0 to <0.63
0.63 to <2.38
2.38 to <3.28
3.28 to <6.55
6.55 to <10.5
10.5 to <26.86
26.86 to <42.95
42.95 to <78.75
78.75 to <110.74
110.74 to 811.52

Addis Ababa

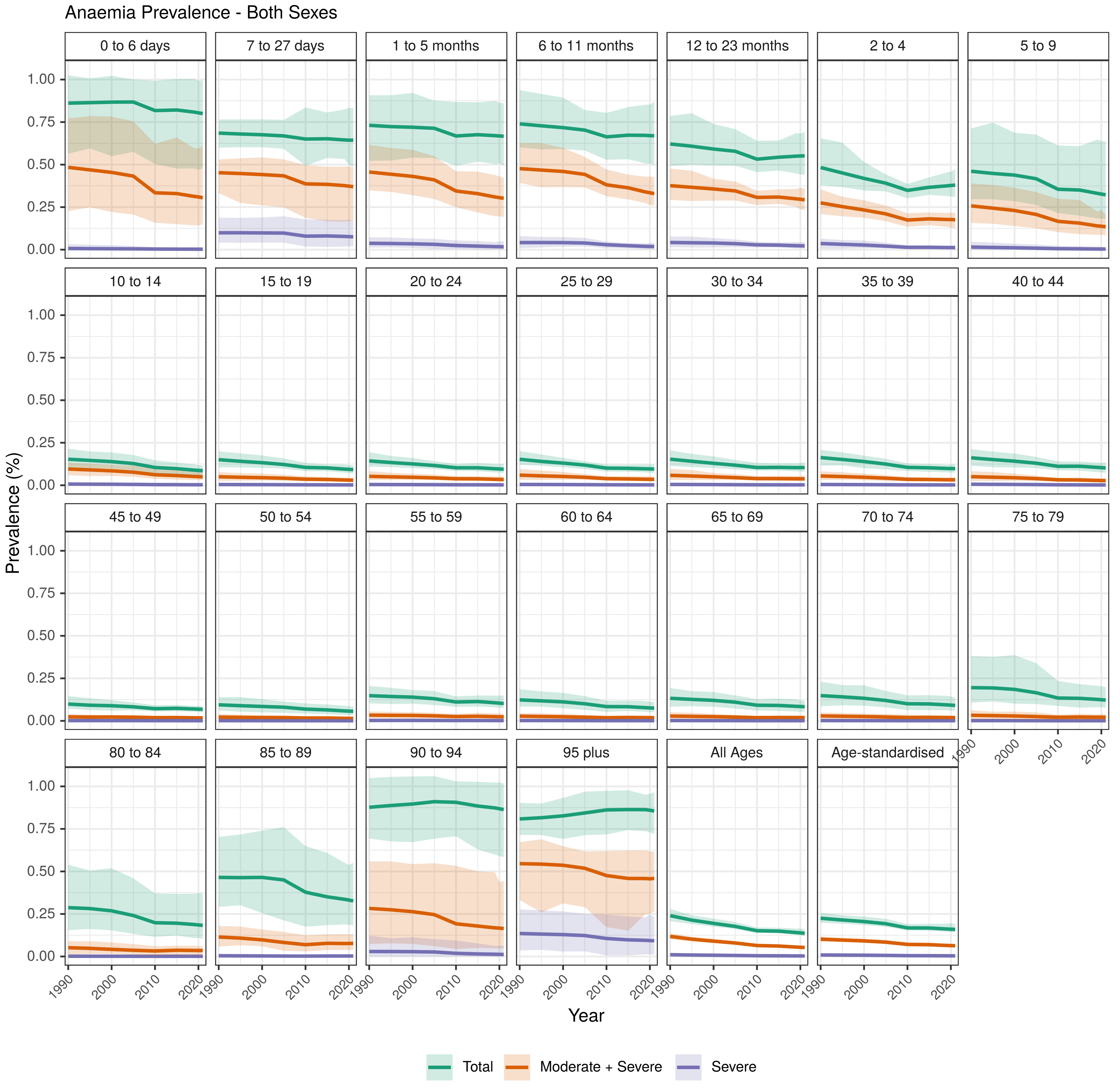
Anaemia Prevalence - Both Sexes
0 to 6 days
7 to 27 days
1 to 5 months
6 to 11 months
12 to 23 months
2 to 4
5 to 9
10 to 14
15 to 19
20 to 24
25 to 29
30 to 34
35 to 39
40 to 44
45 to 49
50 to 54
55 to 59
60 to 64
65 to 69
70 to 74
75 to 79
80 to 84
85 to 89
90 to 94
95 plus
All Ages
Age-standardised
Prevalence (%)
Year
Total
Moderate + Severe
Severe

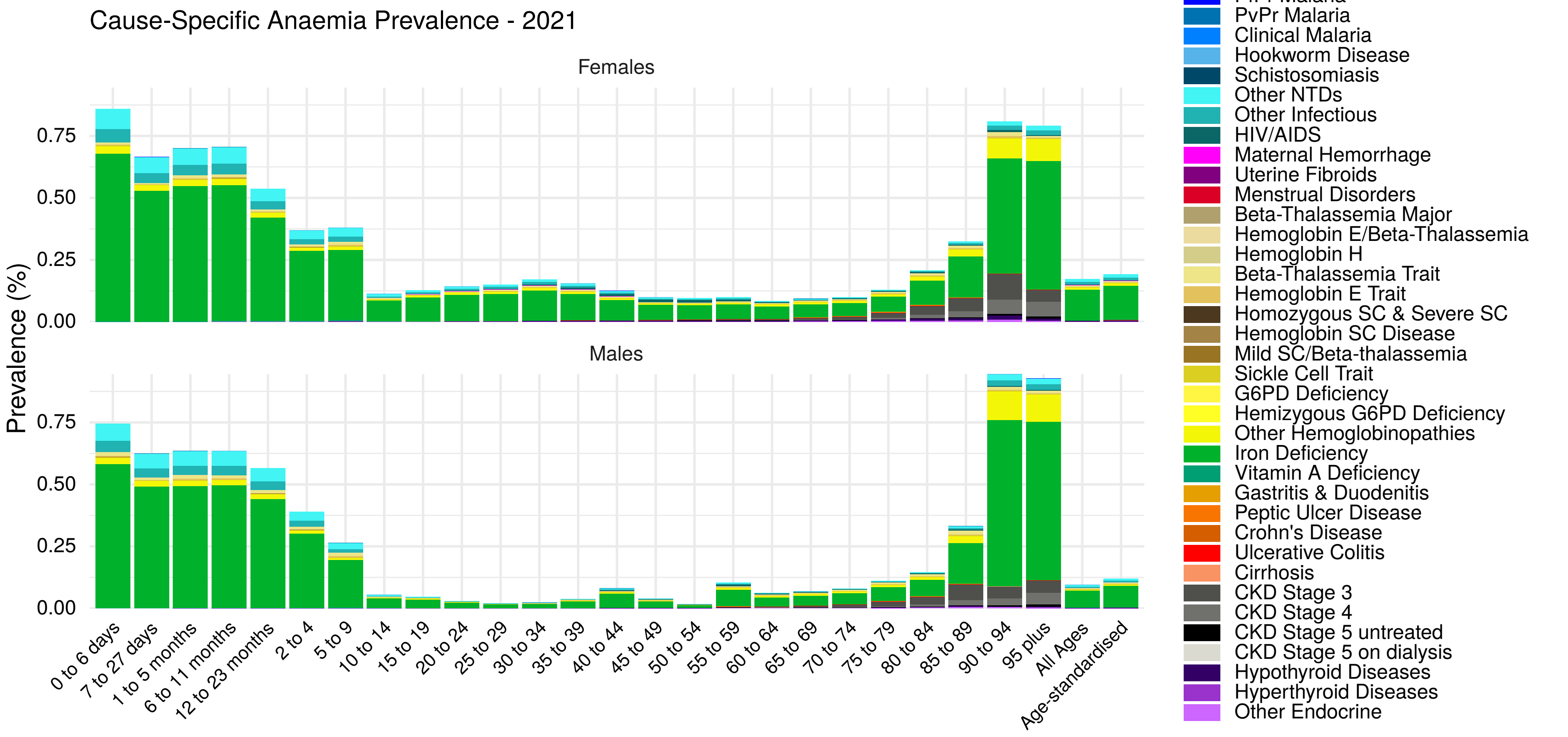
Cause-Specific Anaemia Prevalence - 2021
Females
Males
Prevalence (%)
PfPr Malaria
PvPr Malaria
Clinical Malaria
Hookworm Disease
Schistosomiasis
Other NTDs
Other Infectious
HIV/AIDS
Maternal Hemorrhage
Uterine Fibroids
Menstrual Disorders
Beta-Thalassemia Major
Hemoglobin E/Beta-Thalassemia
Hemoglobin H
Beta-Thalassemia Trait
Hemoglobin E Trait
Homozygous SC & Severe SC
Hemoglobin SC Disease
Mild SC/Beta-thalassemia
Sickle Cell Trait
G6PD Deficiency
Hemizygous G6PD Deficiency
Other Hemoglobinopathies
Iron Deficiency
Vitamin A Deficiency
Gastritis & Duodenitis
Peptic Ulcer Disease
Crohn's Disease
Ulcerative Colitis
Cirrhosis
CKD Stage 3
CKD Stage 4
CKD Stage 5 untreated
CKD Stage 5 on dialysis
Hypothyroid Diseases
Hyperthyroid Diseases
Other Endocrine

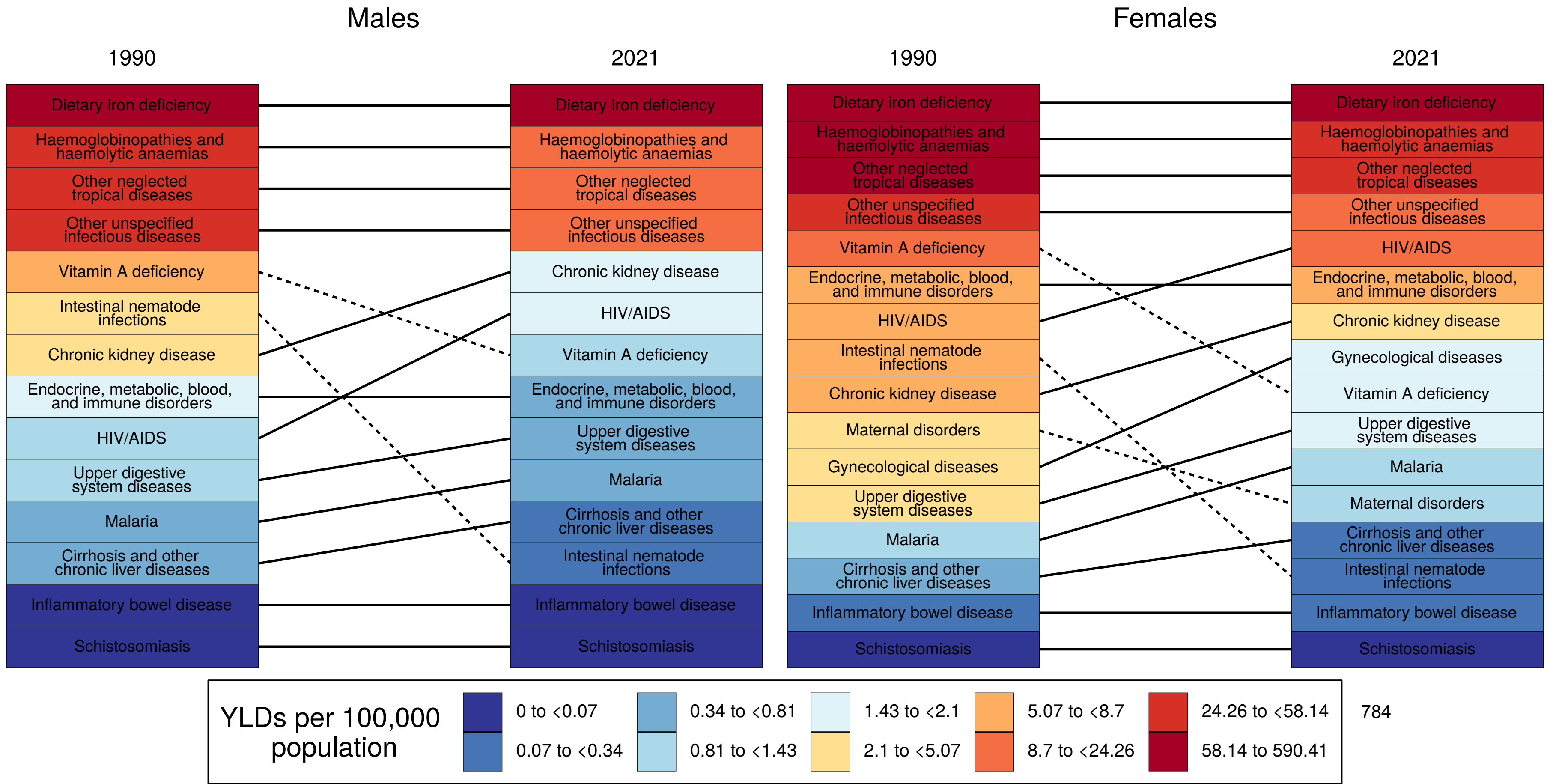
Males
Females
1990
2021
Dietary iron deficiency
Haemoglobinopathies and haemolytic anaemias
Other neglected tropical diseases
Other unspecified infectious diseases
Vitamin A deficiency
Intestinal nematode infections
Chronic kidney disease
Endocrine, metabolic, blood, and immune disorders
HIV/AIDS
Upper digestive system diseases
Malaria
Cirrhosis and other chronic liver diseases
Inflammatory bowel disease
Schistosomiasis
Maternal disorders
Gynecological diseases
YLDs per 100,000 population
0 to <0.07
0.07 to <0.34
0.34 to <0.81
0.81 to <1.43
1.43 to <2.1
2.1 to <5.07
5.07 to <8.7
8.7 to <24.26
24.26 to <58.14
58.14 to 590.41

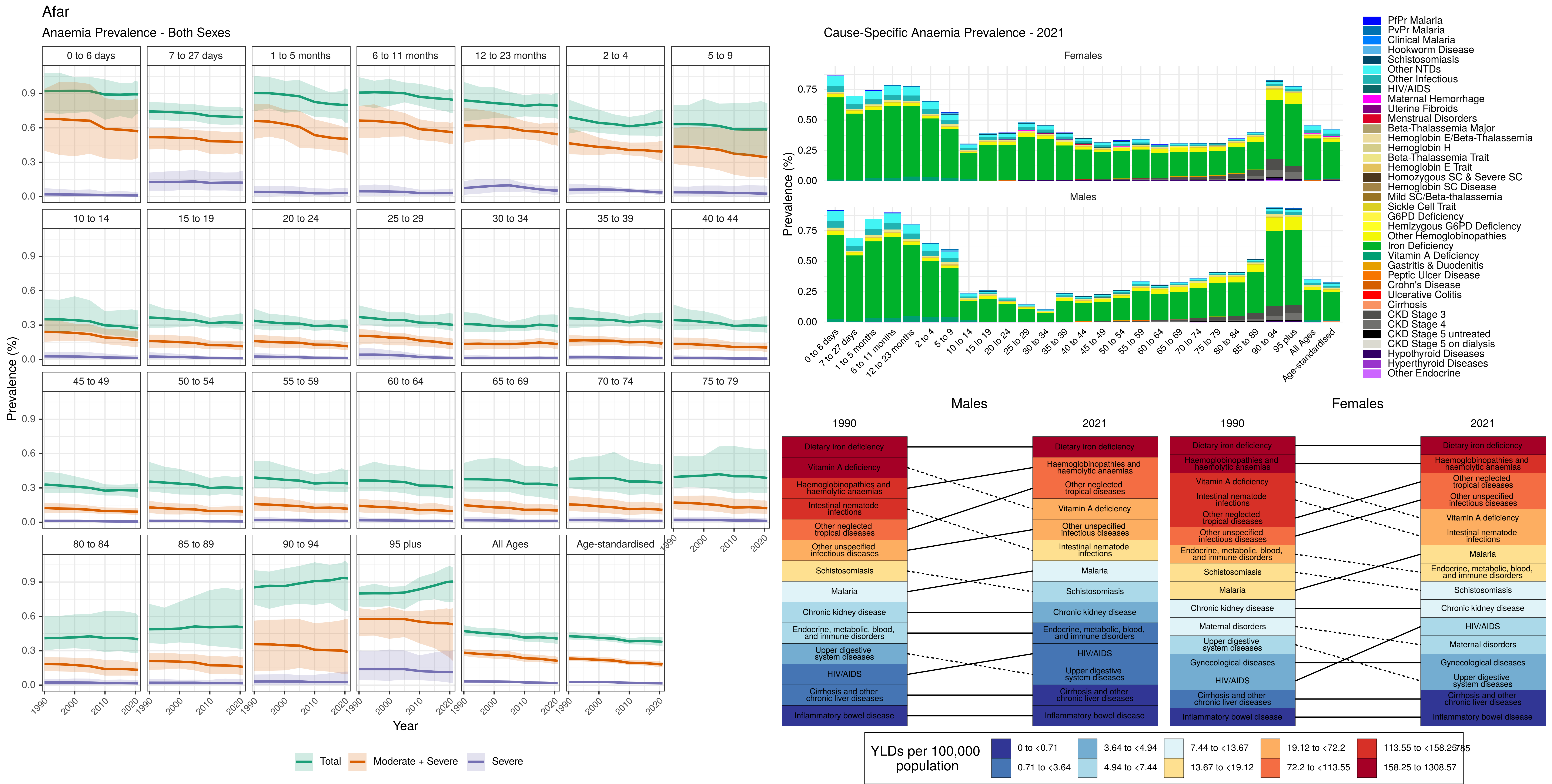

Afar
Anaemia Prevalence - Both Sexes
0 to 6 days
7 to 27 days
1 to 5 months
6 to 11 months
12 to 23 months
2 to 4
5 to 9
10 to 14
15 to 19
20 to 24
25 to 29
30 to 34
35 to 39
40 to 44
45 to 49
50 to 54
55 to 59
60 to 64
65 to 69
70 to 74
75 to 79
80 to 84
85 to 89
90 to 94
95 plus
All Ages
Age-standardised
Prevalence (%)
Year
Total
Moderate + Severe
Severe
Cause-Specific Anaemia Prevalence - 2021
Females
Males
PfPr Malaria
PvPr Malaria
Clinical Malaria
Hookworm Disease
Schistosomiasis
Other NTDs
Other Infectious
HIV/AIDS
Maternal Hemorrhage
Uterine Fibroids
Menstrual Disorders
Beta-Thalassemia Major
Hemoglobin E/Beta-Thalassemia
Hemoglobin H
Beta-Thalassemia Trait
Hemoglobin E Trait
Homozygous SC & Severe SC
Hemoglobin SC Disease
Mild SC/Beta-thalassemia
Sickle Cell Trait
G6PD Deficiency
Hemizygous G6PD Deficiency
Other Hemoglobinopathies
Iron Deficiency
Vitamin A Deficiency
Gastritis & Duodenitis
Peptic Ulcer Disease
Crohn's Disease
Ulcerative Colitis
Cirrhosis
CKD Stage 3
CKD Stage 4
CKD Stage 5 untreated
CKD Stage 5 on dialysis
Hypothyroid Diseases
Hyperthyroid Diseases
Other Endocrine
Males
Females
1990
2021
Dietary iron deficiency
Vitamin A deficiency
Haemoglobinopathies and haemolytic anaemias
Intestinal nematode infections
Other neglected tropical diseases
Other unspecified infectious diseases
Schistosomiasis
Malaria
Chronic kidney disease
Endocrine, metabolic, blood, and immune disorders
Upper digestive system diseases
HIV/AIDS
Cirrhosis and other chronic liver diseases
Inflammatory bowel disease
Maternal disorders
Gynecological diseases
YLDs per 100,000 population
0 to <0.71
0.71 to <3.64
3.64 to <4.94
4.94 to <7.44
7.44 to <13.67
13.67 to <19.12
19.12 to <72.2
72.2 to <113.55
113.55 to <158.25
158.25 to 1308.57

Amhara

Anaemia Prevalence - Both Sexes

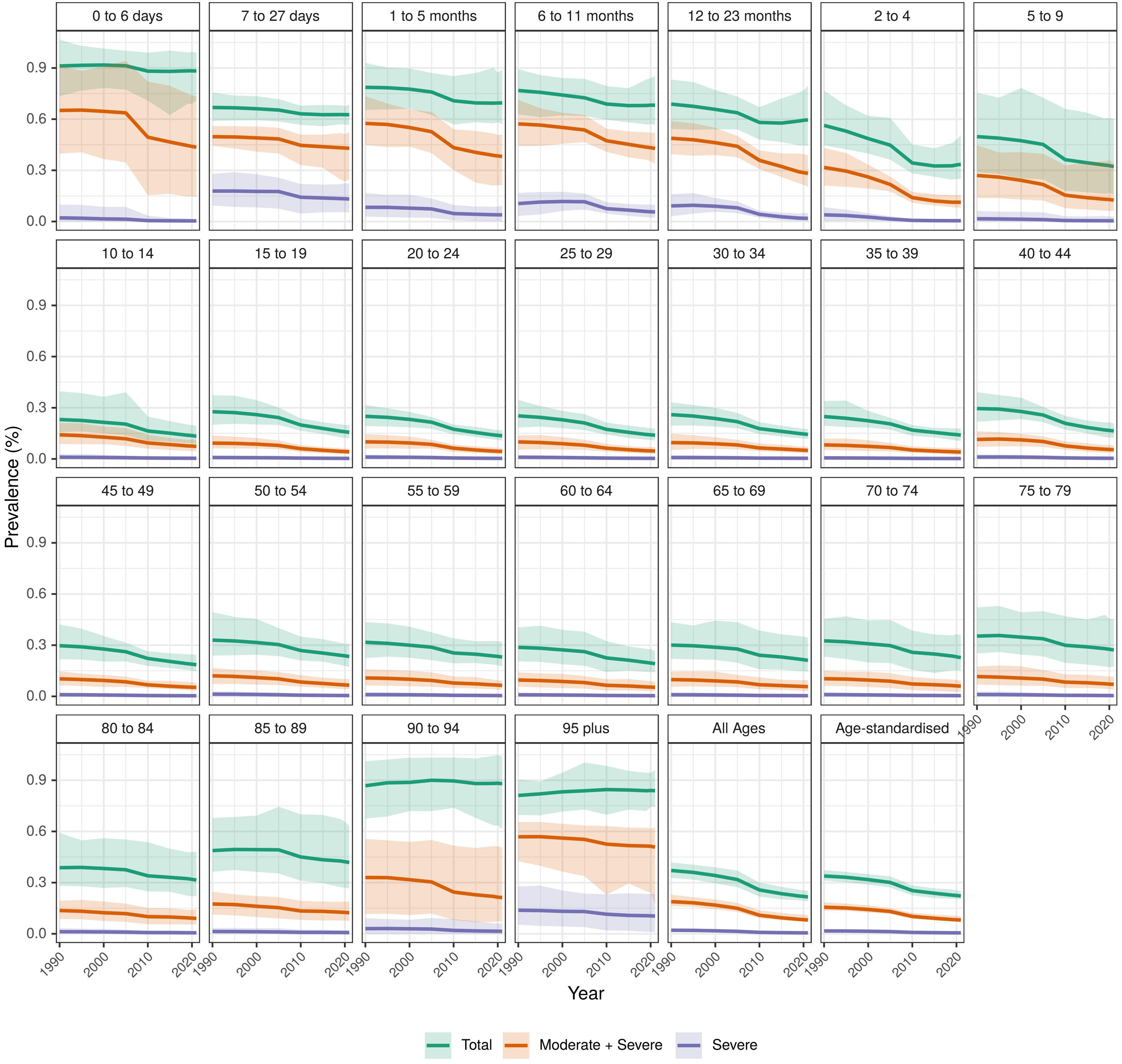

Cause-Specific Anaemia Prevalence - 2021

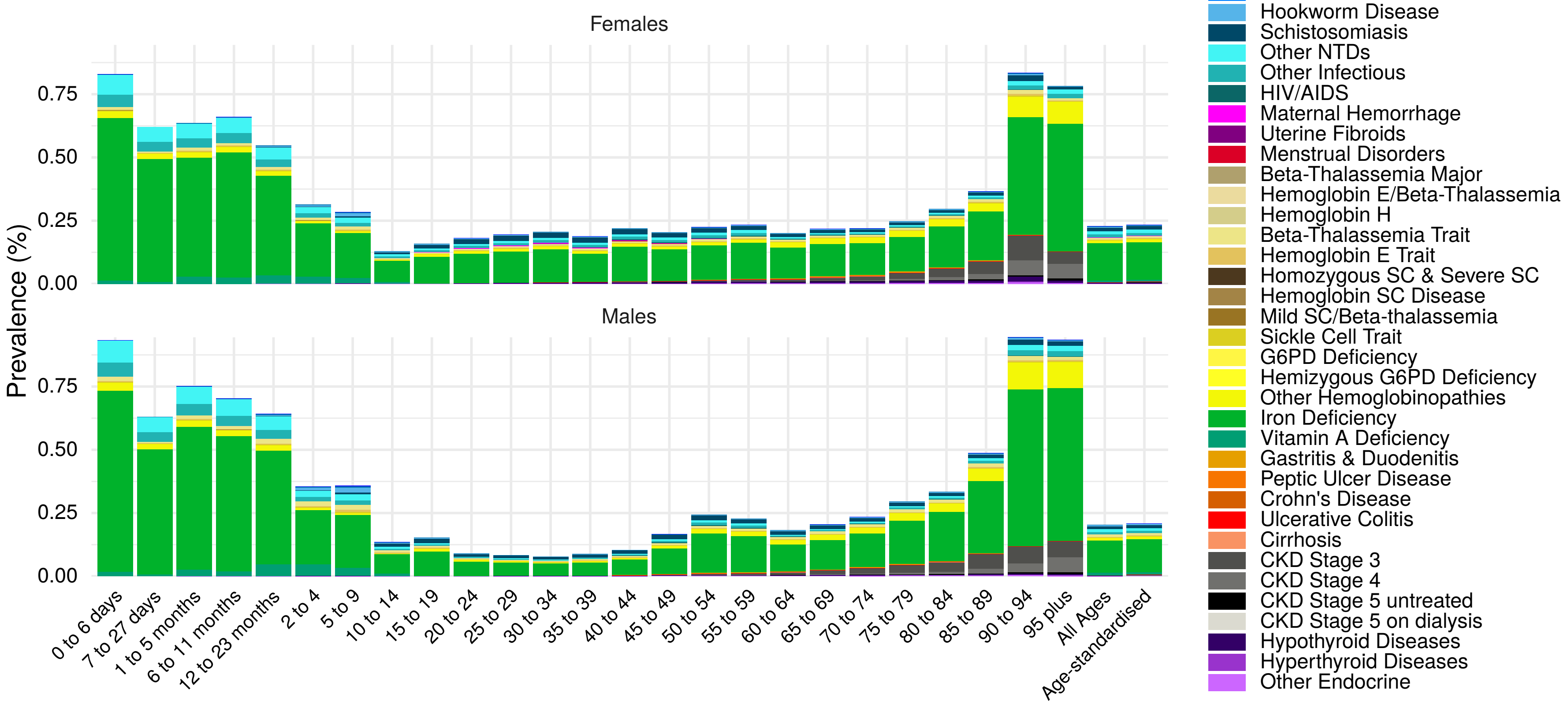

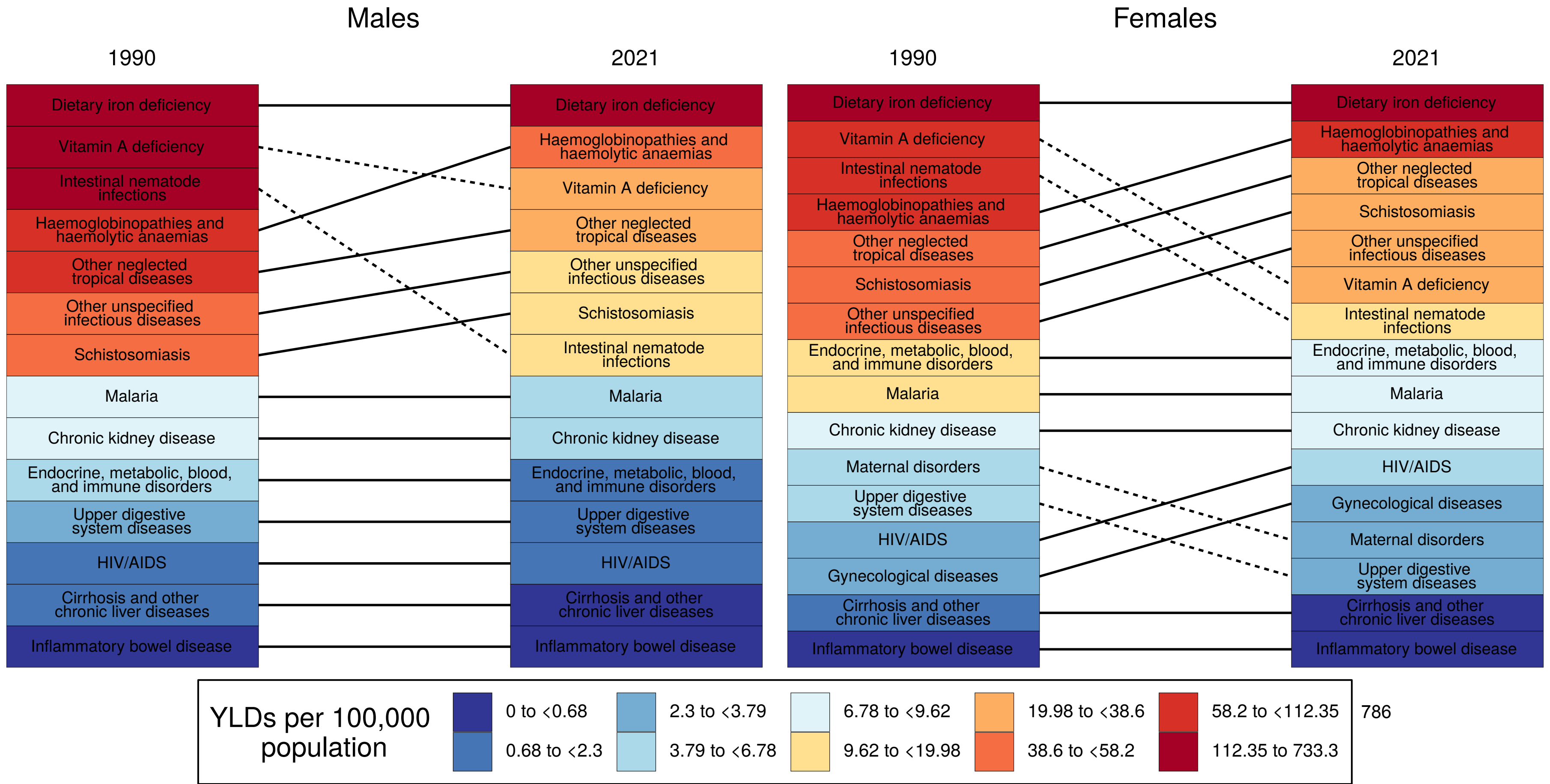

Benishangul-Gumuz

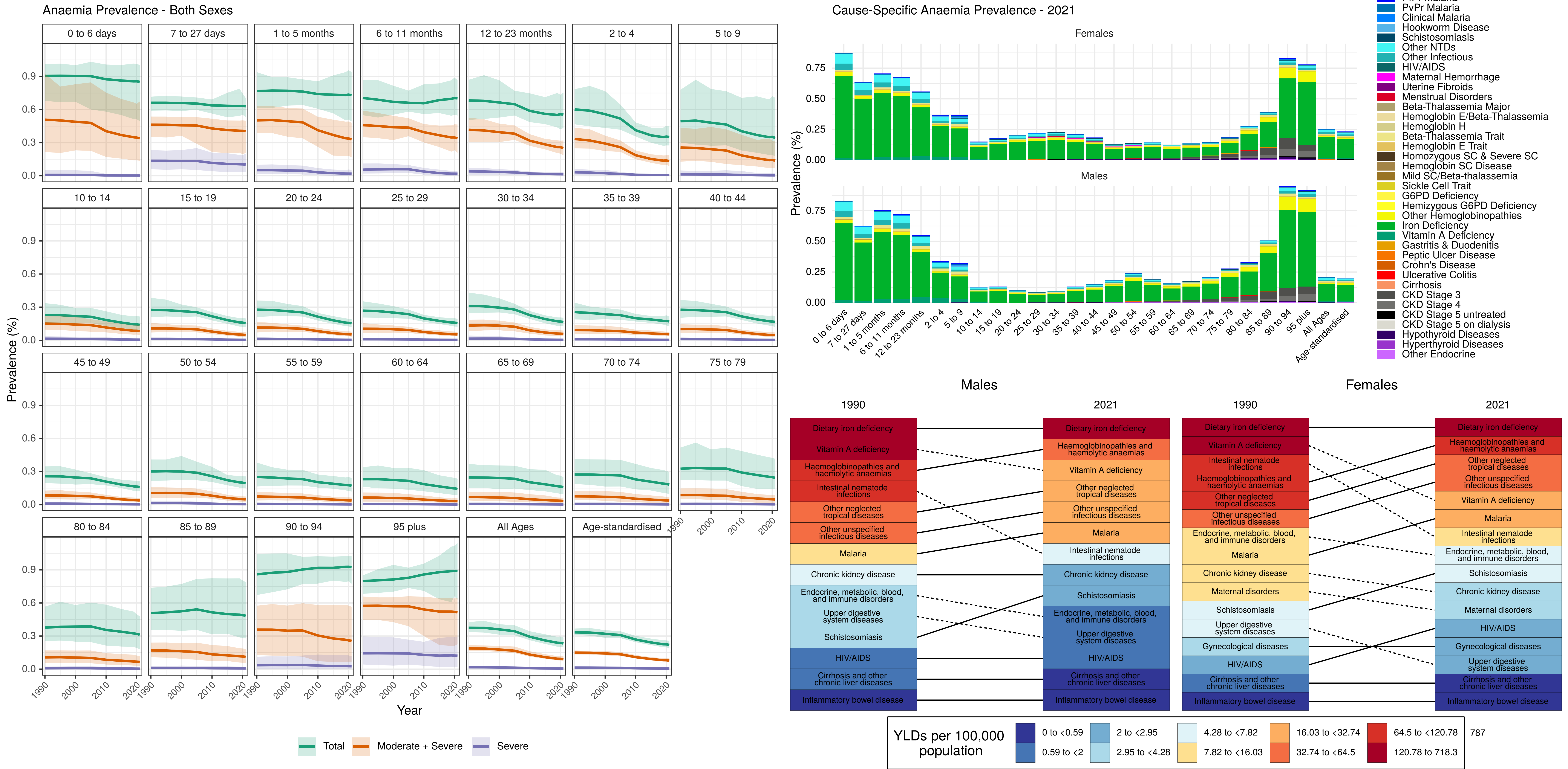

Dire Dawa

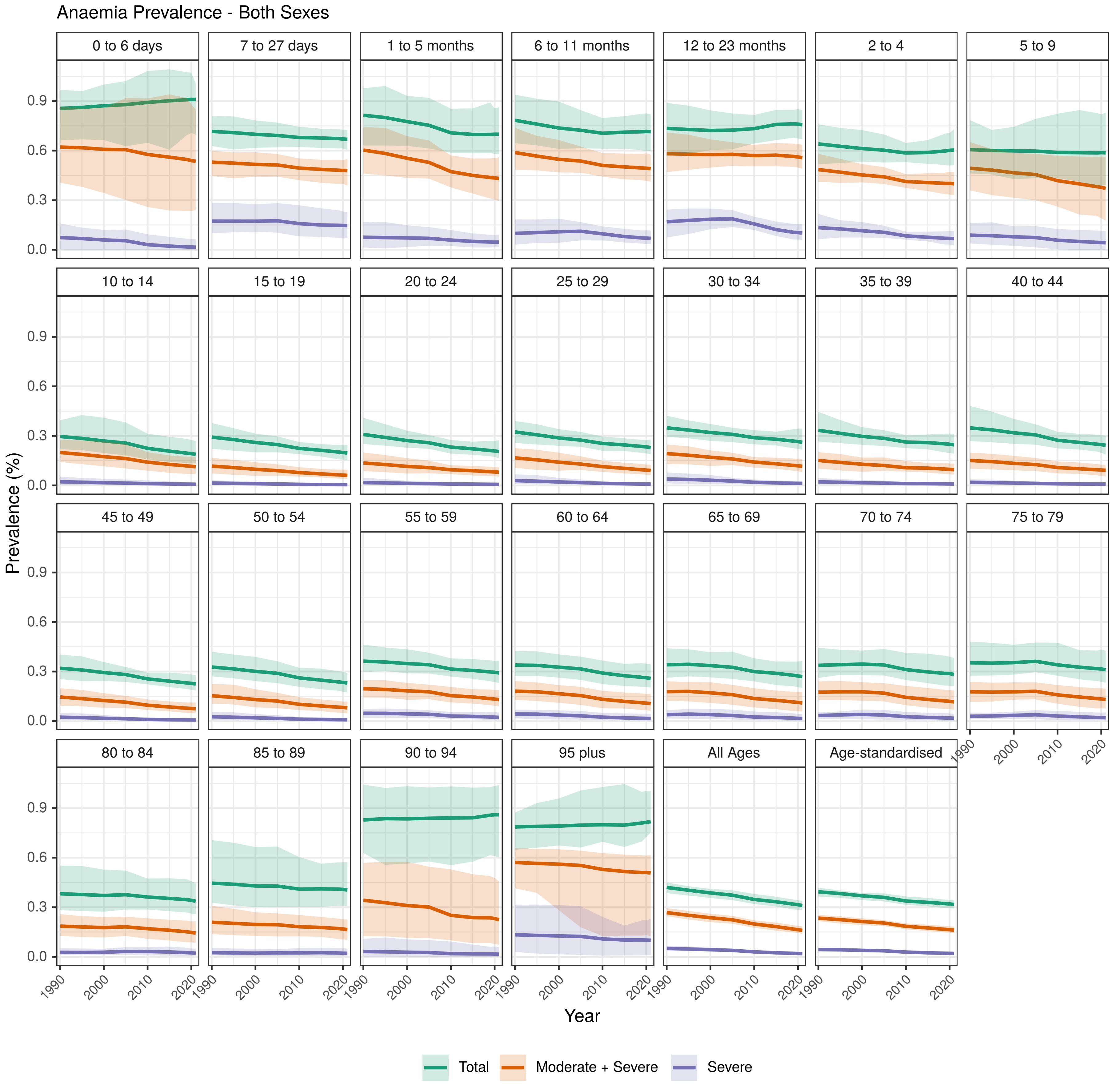

Anaemia Prevalence - Both Sexes
0 to 6 days
7 to 27 days
1 to 5 months
6 to 11 months
12 to 23 months
2 to 4
5 to 9
10 to 14
15 to 19
20 to 24
25 to 29
30 to 34
35 to 39
40 to 44
45 to 49
50 to 54
55 to 59
60 to 64
65 to 69
70 to 74
75 to 79
80 to 84
85 to 89
90 to 94
95 plus
All Ages
Age-standardised
Prevalence (%)
Year
Total
Moderate + Severe
Severe

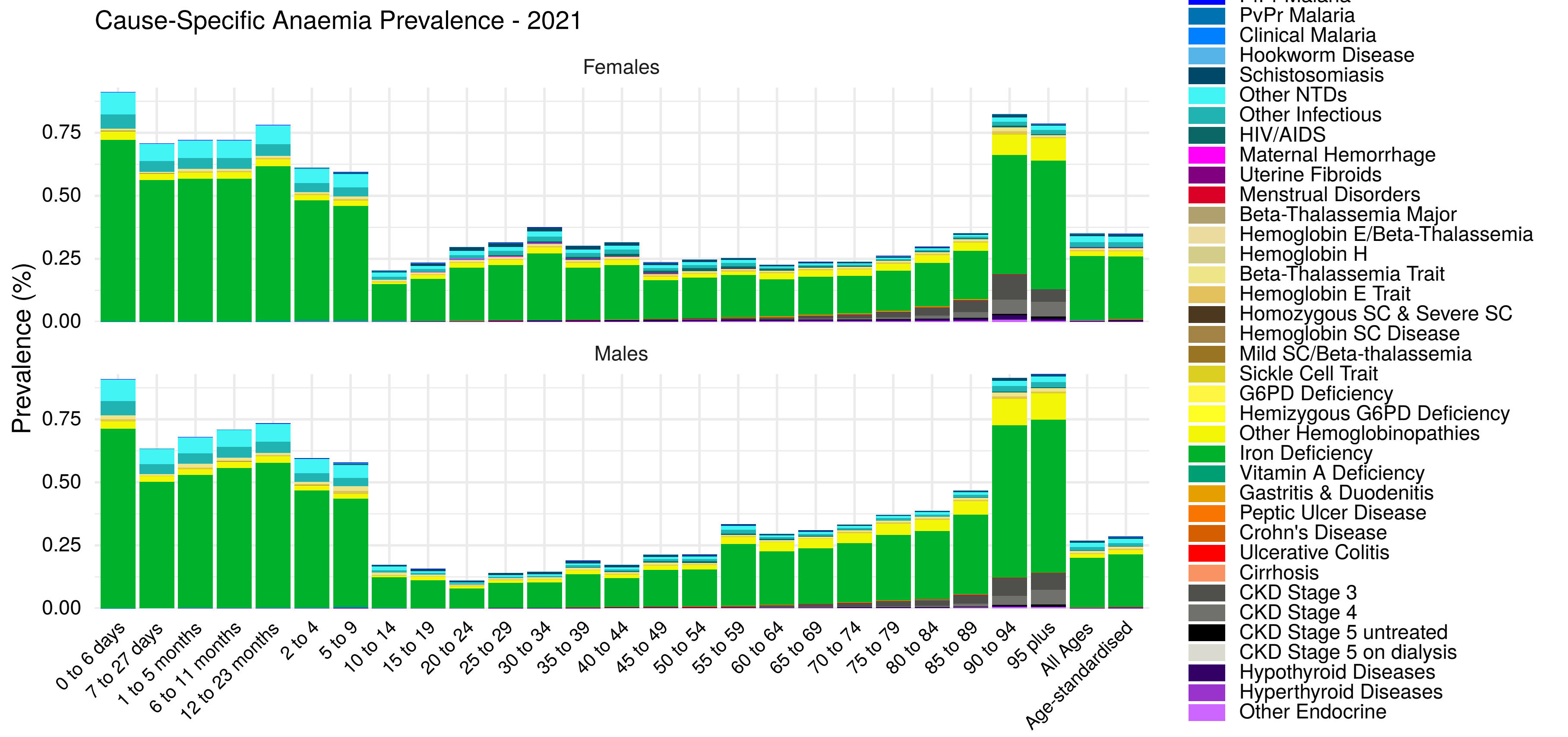

Cause-Specific Anaemia Prevalence - 2021
Females
Males
Prevalence (%)
PfPr Malaria
PvPr Malaria
Clinical Malaria
Hookworm Disease
Schistosomiasis
Other NTDs
Other Infectious
HIV/AIDS
Maternal Hemorrhage
Uterine Fibroids
Menstrual Disorders
Beta-Thalassemia Major
Hemoglobin E/Beta-Thalassemia
Hemoglobin H
Beta-Thalassemia Trait
Hemoglobin E Trait
Homozygous SC & Severe SC
Hemoglobin SC Disease
Mild SC/Beta-thalassemia
Sickle Cell Trait
G6PD Deficiency
Hemizygous G6PD Deficiency
Other Hemoglobinopathies
Iron Deficiency
Vitamin A Deficiency
Gastritis & Duodenitis
Peptic Ulcer Disease
Crohn's Disease
Ulcerative Colitis
Cirrhosis
CKD Stage 3
CKD Stage 4
CKD Stage 5 untreated
CKD Stage 5 on dialysis
Hypothyroid Diseases
Hyperthyroid Diseases
Other Endocrine

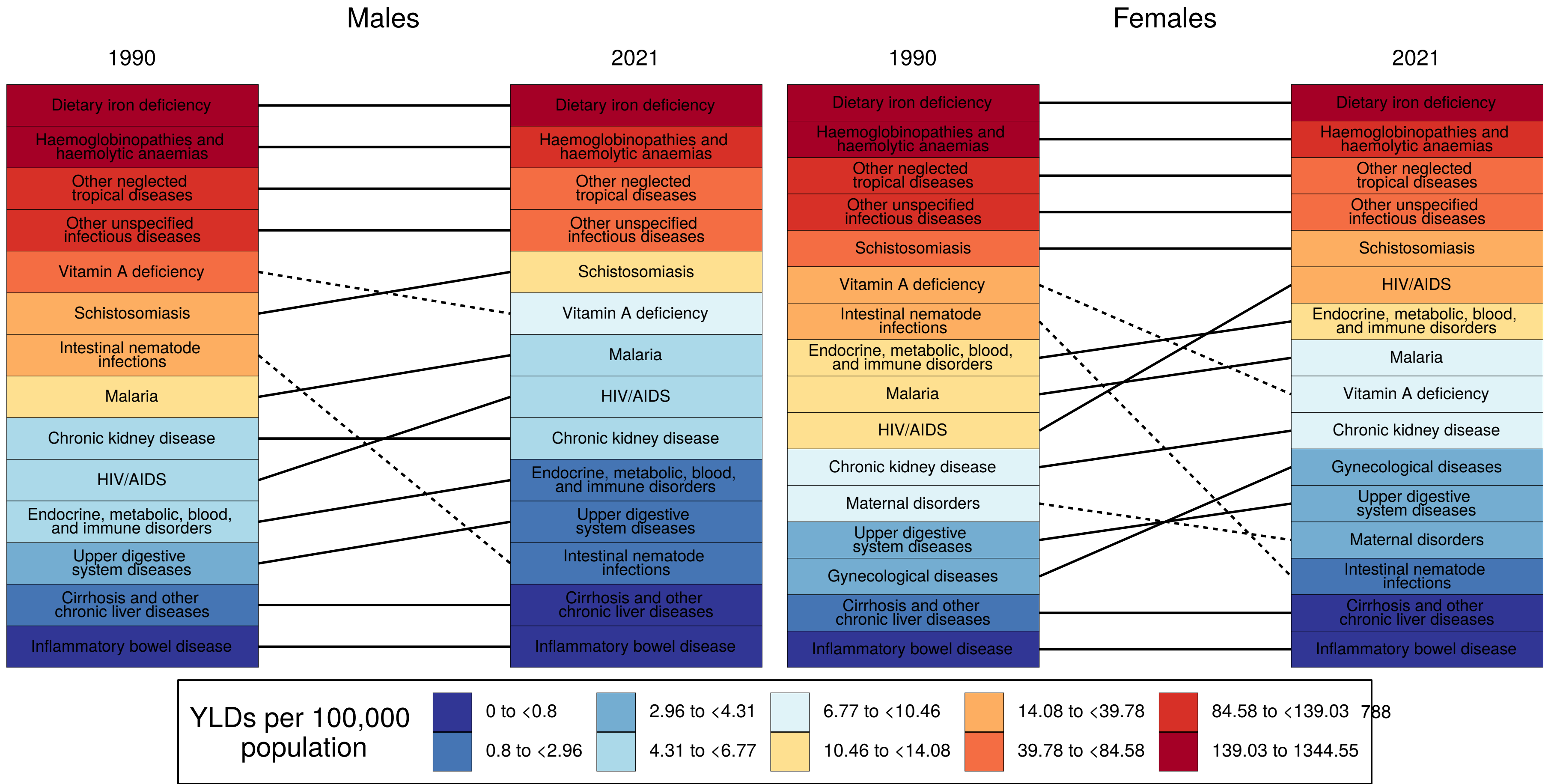

Males
Females
1990
2021
Dietary iron deficiency
Haemoglobinopathies and haemolytic anaemias
Other neglected tropical diseases
Other unspecified infectious diseases
Vitamin A deficiency
Schistosomiasis
Intestinal nematode infections
Malaria
Chronic kidney disease
HIV/AIDS
Endocrine, metabolic, blood, and immune disorders
Upper digestive system diseases
Cirrhosis and other chronic liver diseases
Inflammatory bowel disease
Maternal disorders
Gynecological diseases
YLDs per 100,000 population
0 to <0.8
0.8 to <2.96
2.96 to <4.31
4.31 to <6.77
6.77 to <10.46
10.46 to <14.08
14.08 to <39.78
39.78 to <84.58
84.58 to <139.03
139.03 to 1344.55

Gambella

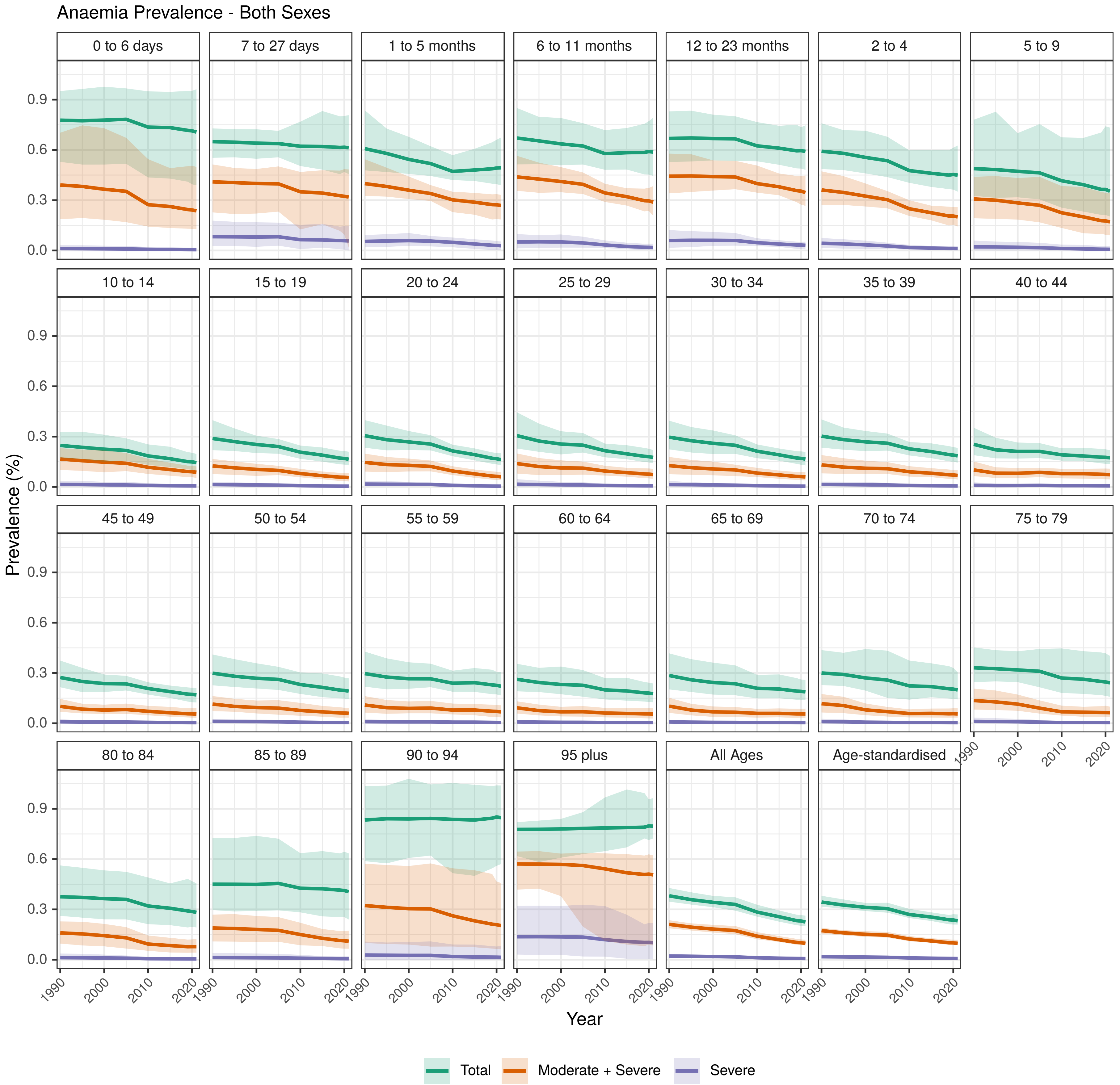

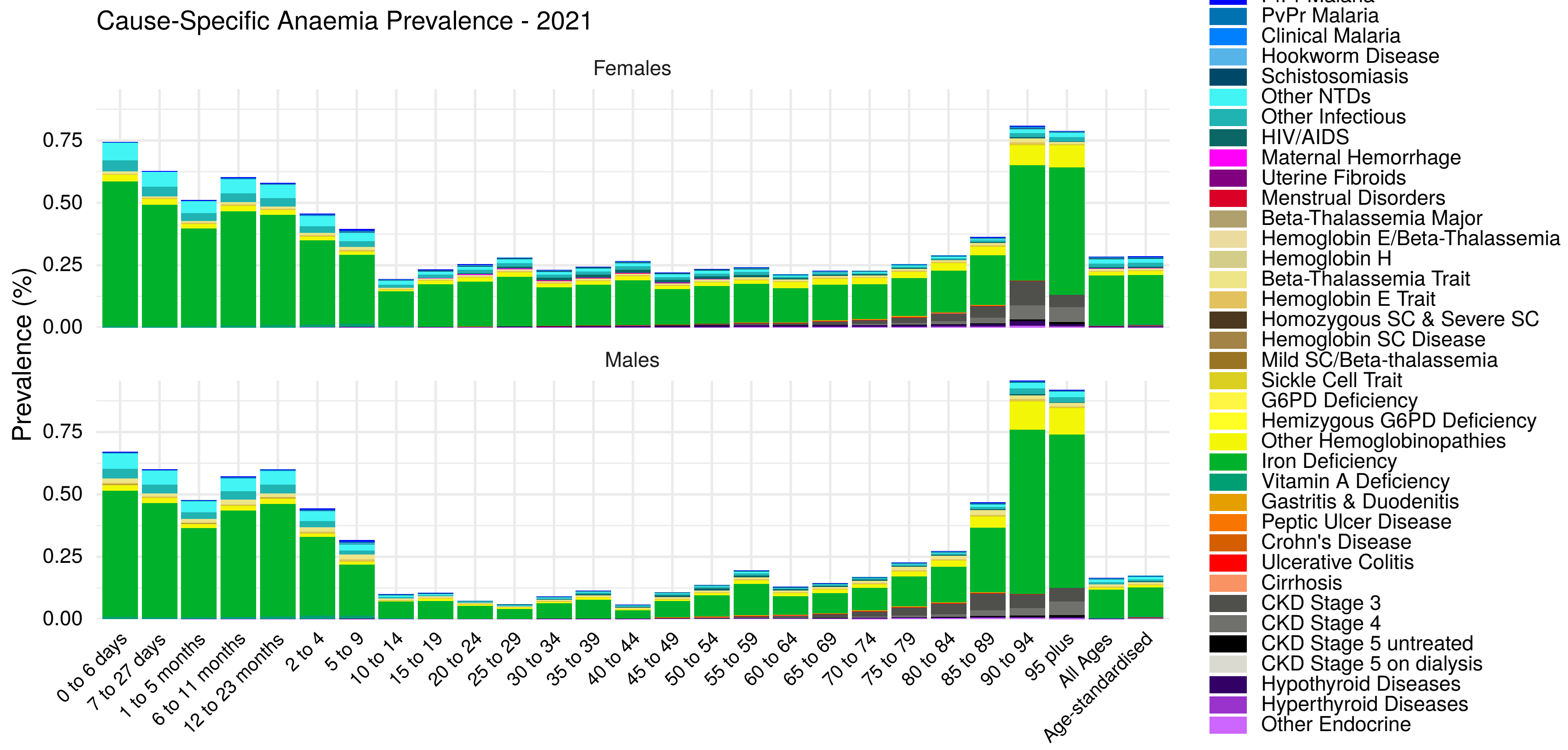

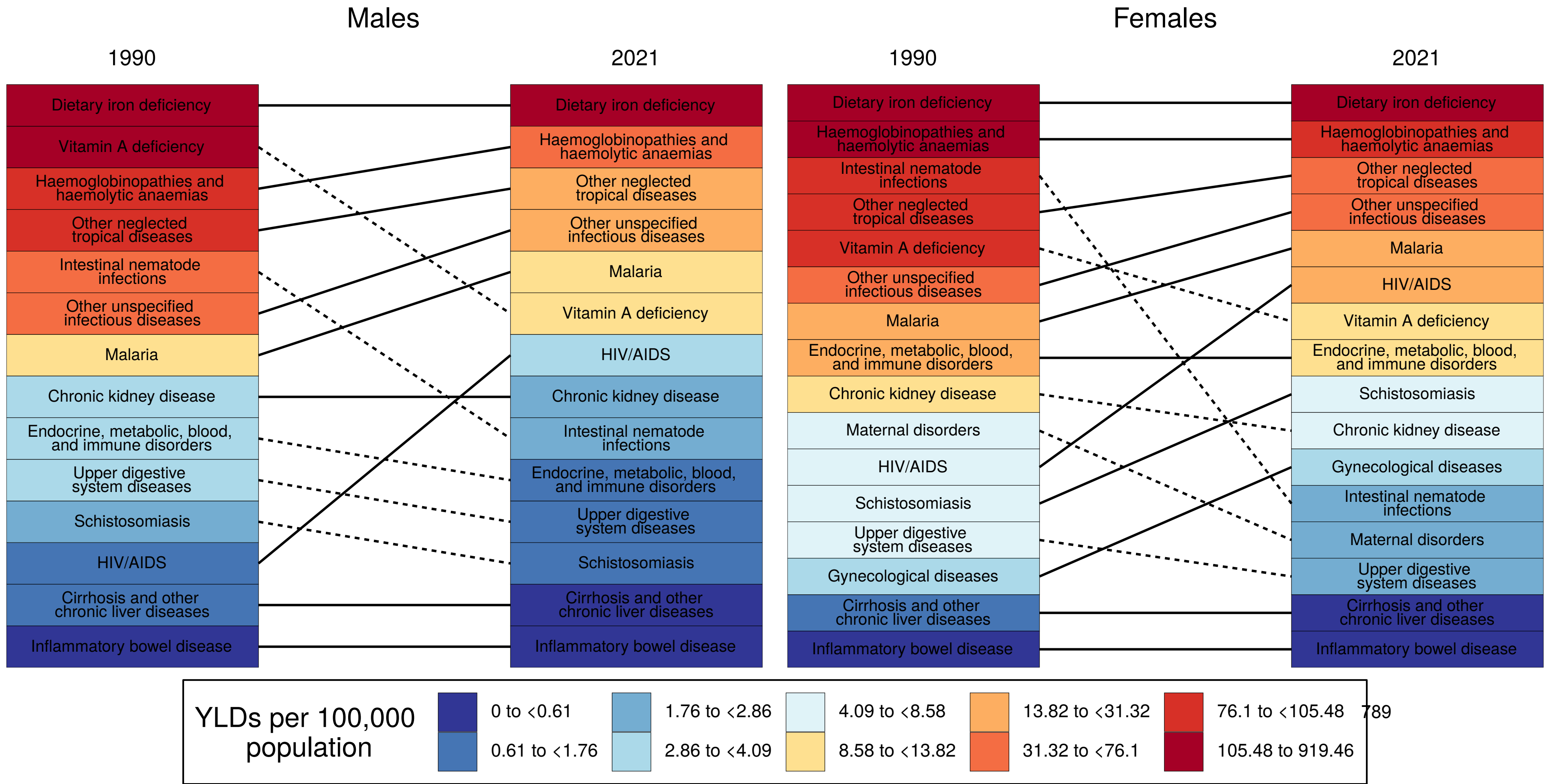

789

Harari

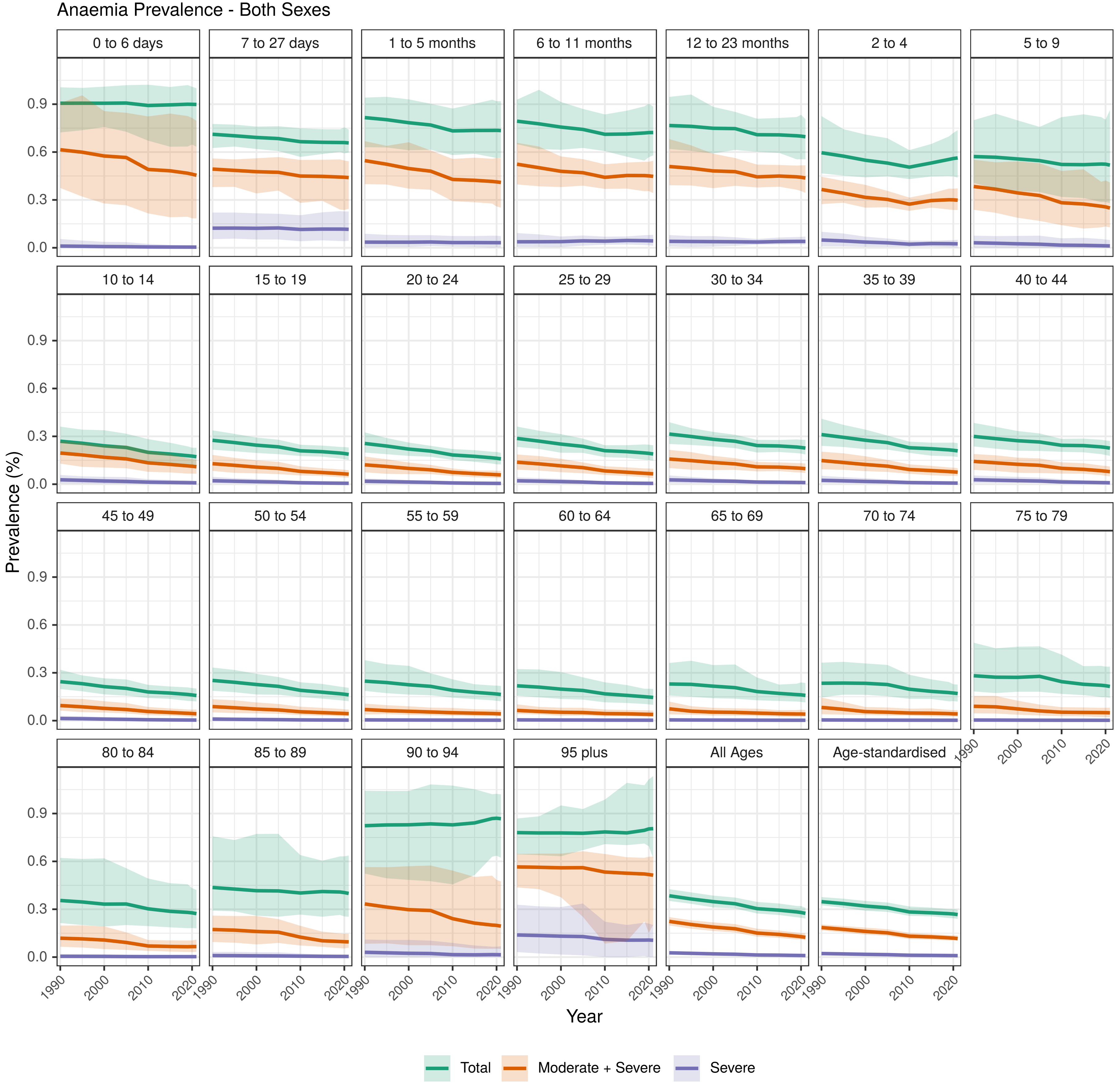
Anaemia Prevalence - Both Sexes
0 to 6 days
7 to 27 days
1 to 5 months
6 to 11 months
12 to 23 months
2 to 4
5 to 9
10 to 14
15 to 19
20 to 24
25 to 29
30 to 34
35 to 39
40 to 44
45 to 49
50 to 54
55 to 59
60 to 64
65 to 69
70 to 74
75 to 79
80 to 84
85 to 89
90 to 94
95 plus
All Ages
Age-standardised
Prevalence (%)
0.0
0.3
0.6
0.9
1990
2000
2010
2020
Year
Total
Moderate + Severe
Severe

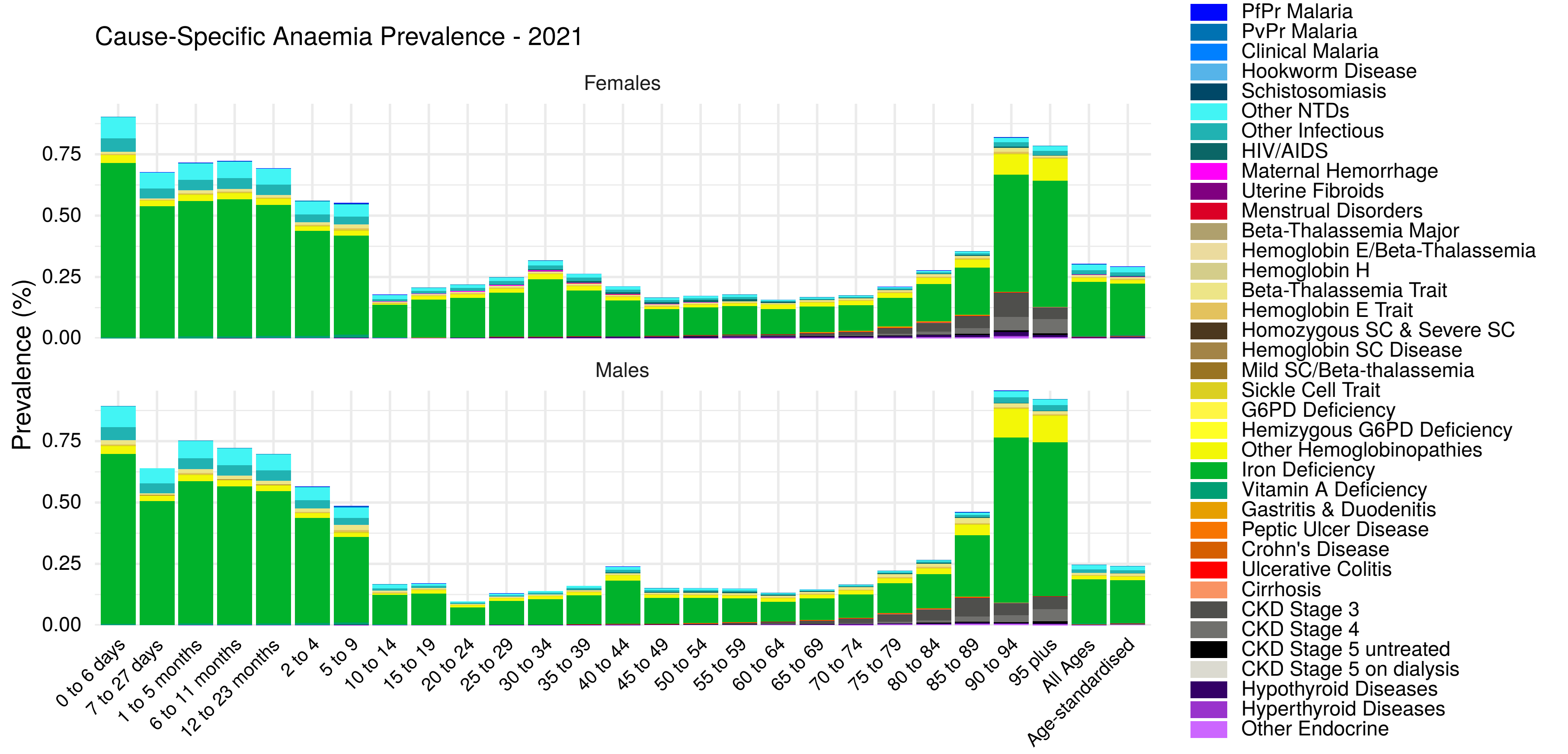
Cause-Specific Anaemia Prevalence - 2021
Females
Males
Prevalence (%)
0.00
0.25
0.50
0.75
0 to 6 days
7 to 27 days
1 to 5 months
6 to 11 months
12 to 23 months
2 to 4
5 to 9
10 to 14
15 to 19
20 to 24
25 to 29
30 to 34
35 to 39
40 to 44
45 to 49
50 to 54
55 to 59
60 to 64
65 to 69
70 to 74
75 to 79
80 to 84
85 to 89
90 to 94
95 plus
All Ages
Age-standardised
PfPr Malaria
PvPr Malaria
Clinical Malaria
Hookworm Disease
Schistosomiasis
Other NTDs
Other Infectious
HIV/AIDS
Maternal Hemorrhage
Uterine Fibroids
Menstrual Disorders
Beta-Thalassemia Major
Hemoglobin E/Beta-Thalassemia
Hemoglobin H
Beta-Thalassemia Trait
Hemoglobin E Trait
Homozygous SC & Severe SC
Hemoglobin SC Disease
Mild SC/Beta-thalassemia
Sickle Cell Trait
G6PD Deficiency
Hemizygous G6PD Deficiency
Other Hemoglobinopathies
Iron Deficiency
Vitamin A Deficiency
Gastritis & Duodenitis
Peptic Ulcer Disease
Crohn's Disease
Ulcerative Colitis
Cirrhosis
CKD Stage 3
CKD Stage 4
CKD Stage 5 untreated
CKD Stage 5 on dialysis
Hypothyroid Diseases
Hyperthyroid Diseases
Other Endocrine

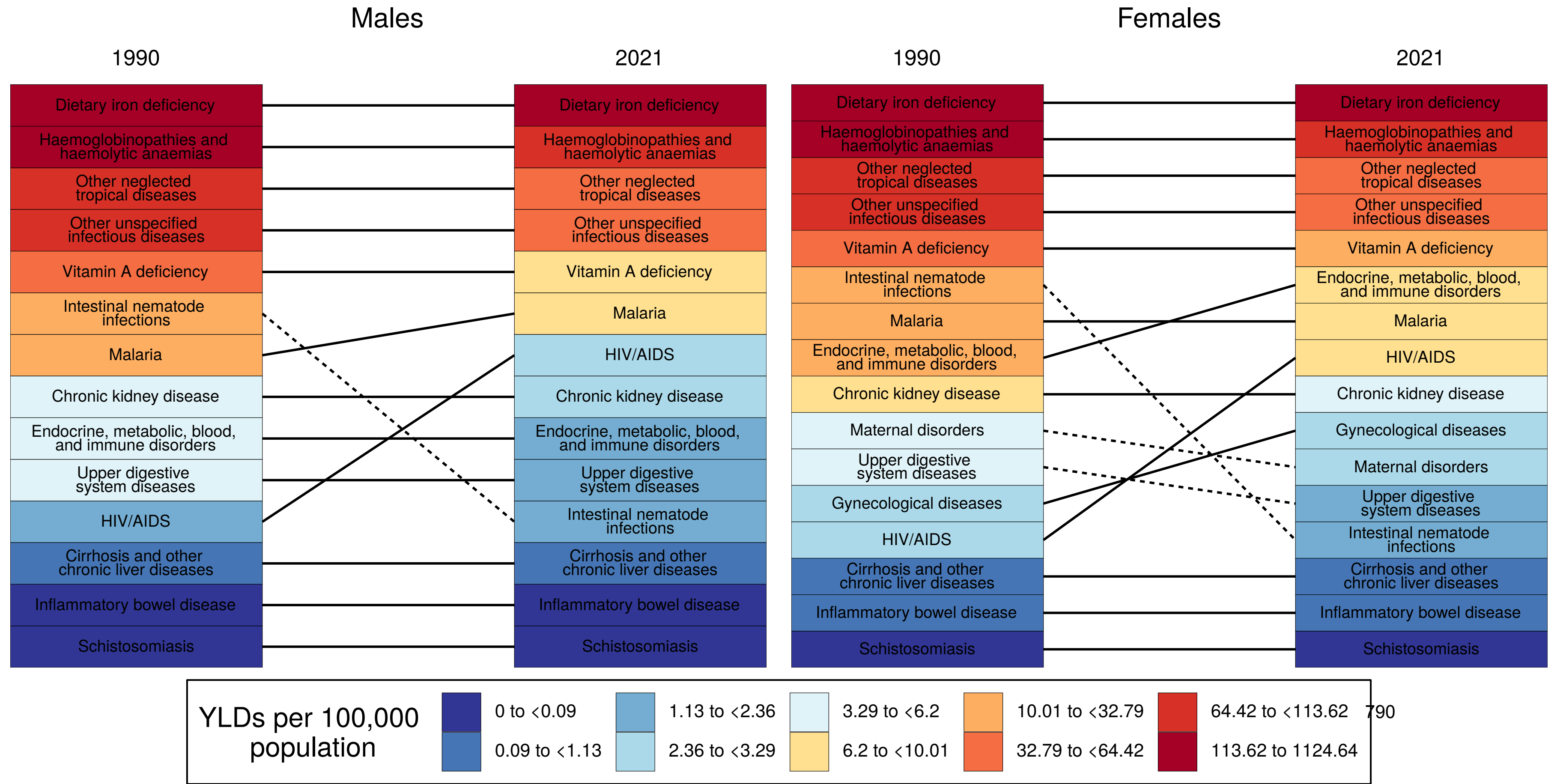
Males
Females
1990
2021
Dietary iron deficiency
Haemoglobinopathies and haemolytic anaemias
Other neglected tropical diseases
Other unspecified infectious diseases
Vitamin A deficiency
Intestinal nematode infections
Malaria
Chronic kidney disease
Endocrine, metabolic, blood, and immune disorders
Upper digestive system diseases
HIV/AIDS
Cirrhosis and other chronic liver diseases
Inflammatory bowel disease
Schistosomiasis
Maternal disorders
Gynecological diseases
YLDs per 100,000 population
0 to <0.09
0.09 to <1.13
1.13 to <2.36
2.36 to <3.29
3.29 to <6.2
6.2 to <10.01
10.01 to <32.79
32.79 to <64.42
64.42 to <113.62
113.62 to 1124.64

Oromia

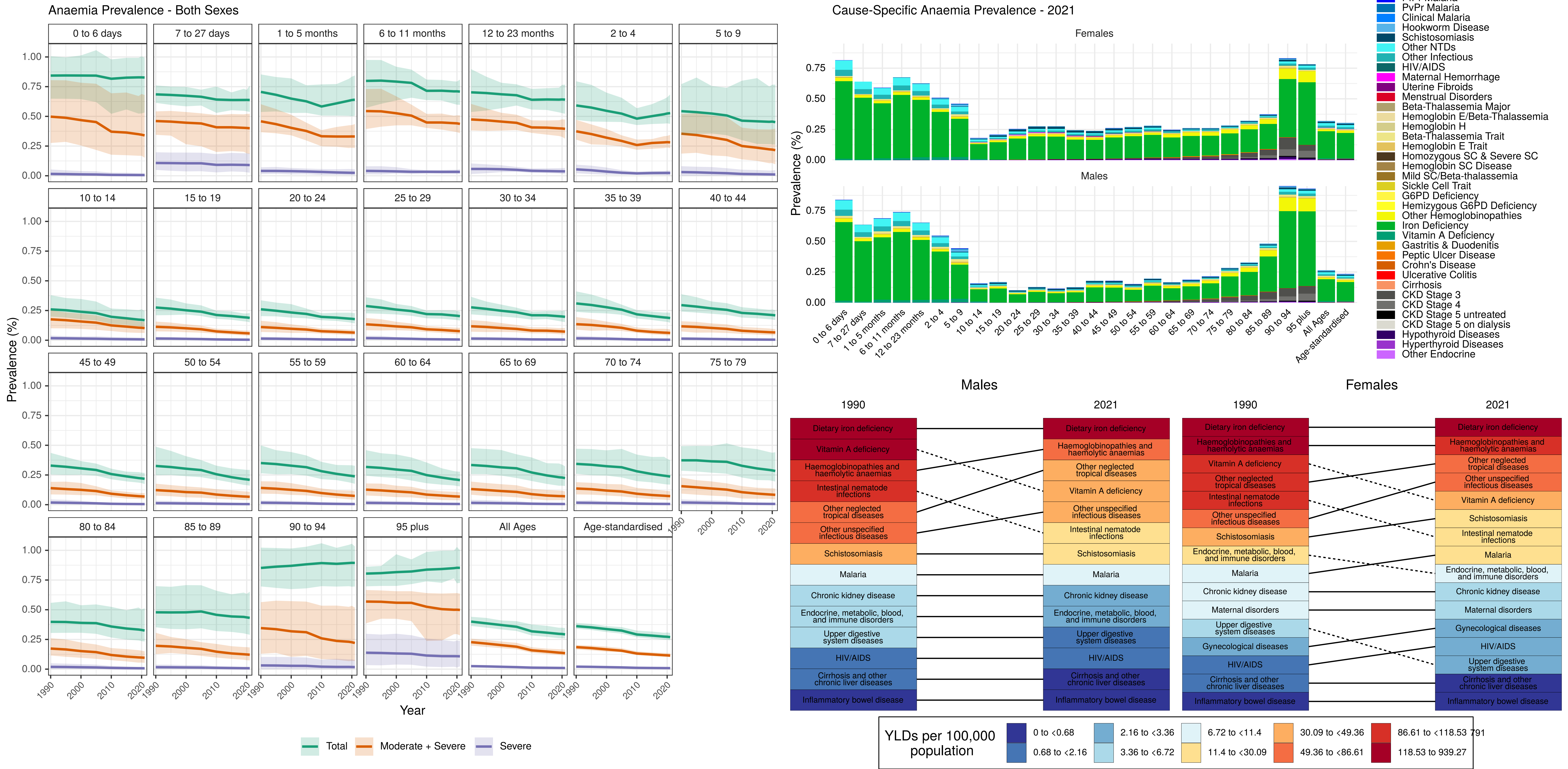

Anaemia Prevalence - Both Sexes
0 to 6 days
7 to 27 days
1 to 5 months
6 to 11 months
12 to 23 months
2 to 4
5 to 9
10 to 14
15 to 19
20 to 24
25 to 29
30 to 34
35 to 39
40 to 44
45 to 49
50 to 54
55 to 59
60 to 64
65 to 69
70 to 74
75 to 79
80 to 84
85 to 89
90 to 94
95 plus
All Ages
Age-standardised
Prevalence (%)
Year
Total
Moderate + Severe
Severe
Cause-Specific Anaemia Prevalence - 2021
Females
Males
PfPr Malaria
PvPr Malaria
Clinical Malaria
Hookworm Disease
Schistosomiasis
Other NTDs
Other Infectious
HIV/AIDS
Maternal Hemorrhage
Uterine Fibroids
Menstrual Disorders
Beta-Thalassemia Major
Hemoglobin E/Beta-Thalassemia
Hemoglobin H
Beta-Thalassemia Trait
Hemoglobin E Trait
Homozygous SC & Severe SC
Hemoglobin SC Disease
Mild SC/Beta-thalassemia
Sickle Cell Trait
G6PD Deficiency
Hemizygous G6PD Deficiency
Other Hemoglobinopathies
Iron Deficiency
Vitamin A Deficiency
Gastritis & Duodenitis
Peptic Ulcer Disease
Crohn's Disease
Ulcerative Colitis
Cirrhosis
CKD Stage 3
CKD Stage 4
CKD Stage 5 untreated
CKD Stage 5 on dialysis
Hypothyroid Diseases
Hyperthyroid Diseases
Other Endocrine
Males
Females
1990
2021
Dietary iron deficiency
Vitamin A deficiency
Haemoglobinopathies and haemolytic anaemias
Intestinal nematode infections
Other neglected tropical diseases
Other unspecified infectious diseases
Schistosomiasis
Malaria
Chronic kidney disease
Endocrine, metabolic, blood, and immune disorders
Upper digestive system diseases
HIV/AIDS
Cirrhosis and other chronic liver diseases
Inflammatory bowel disease
Maternal disorders
Gynecological diseases
YLDs per 100,000 population
0 to <0.68
0.68 to <2.16
2.16 to <3.36
3.36 to <6.72
6.72 to <11.4
11.4 to <30.09
30.09 to <49.36
49.36 to <86.61
86.61 to <118.53
118.53 to 939.27

Somali

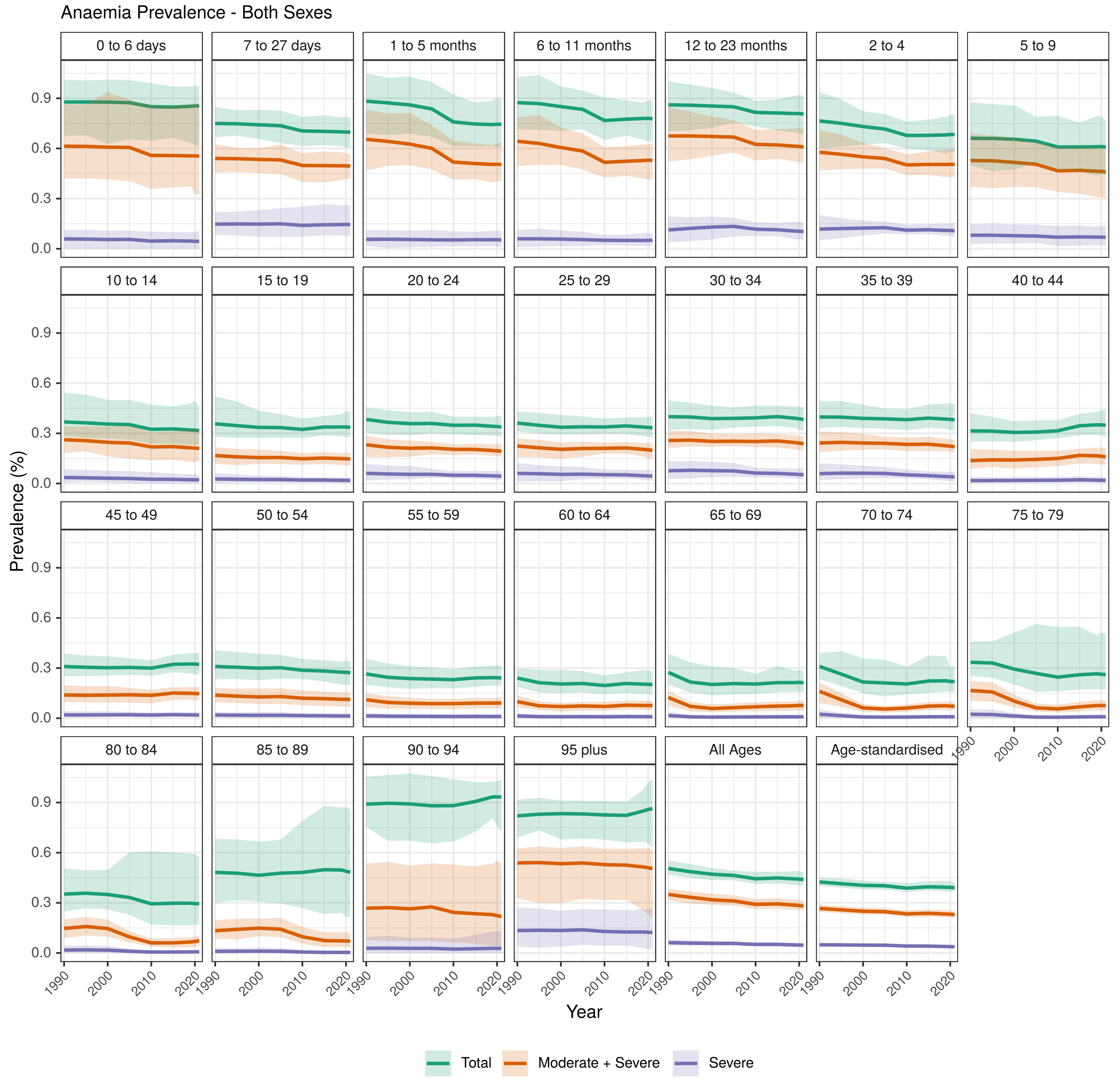
Anaemia Prevalence - Both Sexes
0 to 6 days
7 to 27 days
1 to 5 months
6 to 11 months
12 to 23 months
2 to 4
5 to 9
10 to 14
15 to 19
20 to 24
25 to 29
30 to 34
35 to 39
40 to 44
45 to 49
50 to 54
55 to 59
60 to 64
65 to 69
70 to 74
75 to 79
80 to 84
85 to 89
90 to 94
95 plus
All Ages
Age-standardised
Prevalence (%)
Year
Total
Moderate + Severe
Severe

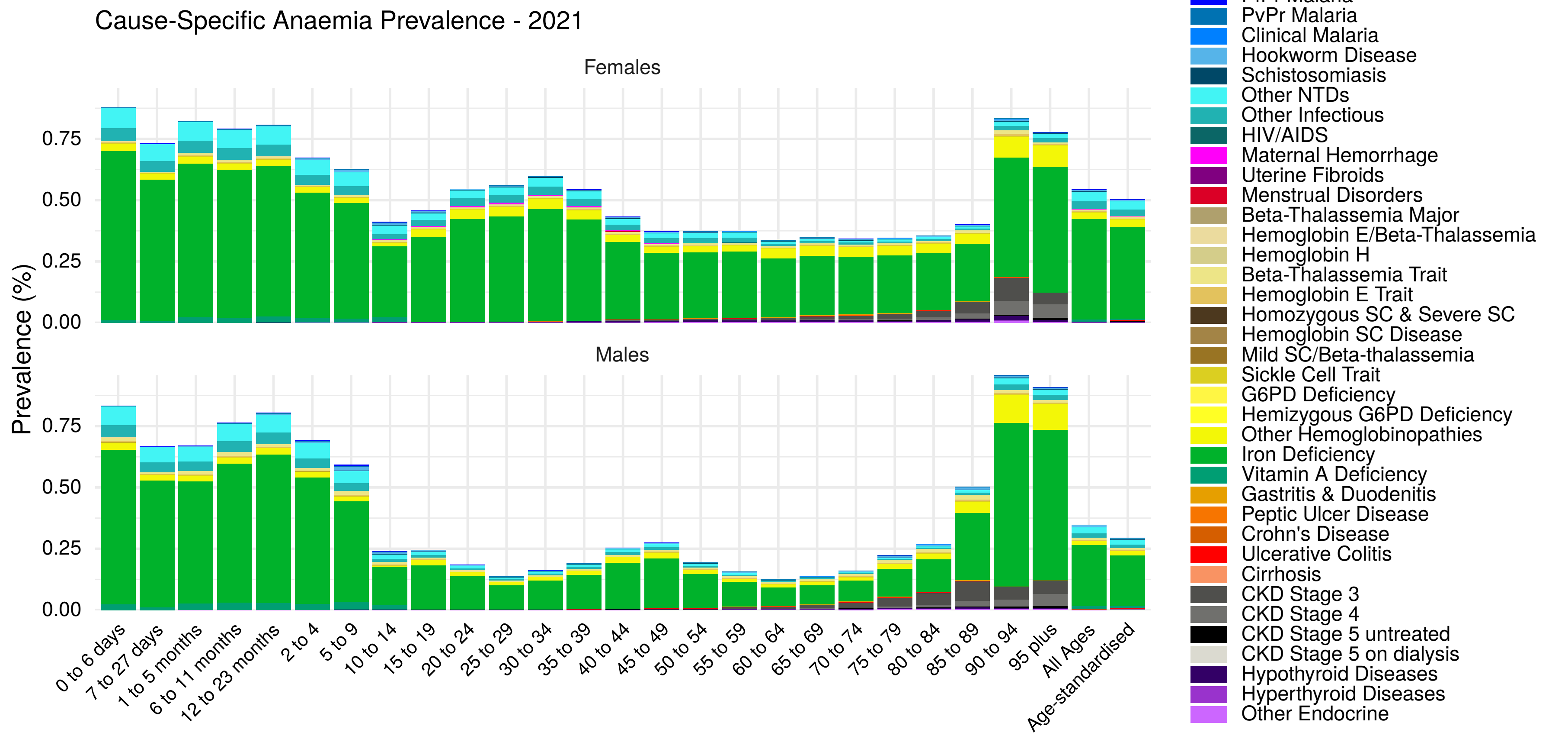
Cause-Specific Anaemia Prevalence - 2021
Females
Males
Prevalence (%)
PfPr Malaria
PvPr Malaria
Clinical Malaria
Hookworm Disease
Schistosomiasis
Other NTDs
Other Infectious
HIV/AIDS
Maternal Hemorrhage
Uterine Fibroids
Menstrual Disorders
Beta-Thalassemia Major
Hemoglobin E/Beta-Thalassemia
Hemoglobin H
Beta-Thalassemia Trait
Hemoglobin E Trait
Homozygous SC & Severe SC
Hemoglobin SC Disease
Mild SC/Beta-thalassemia
Sickle Cell Trait
G6PD Deficiency
Hemizygous G6PD Deficiency
Other Hemoglobinopathies
Iron Deficiency
Vitamin A Deficiency
Gastritis & Duodenitis
Peptic Ulcer Disease
Crohn's Disease
Ulcerative Colitis
Cirrhosis
CKD Stage 3
CKD Stage 4
CKD Stage 5 untreated
CKD Stage 5 on dialysis
Hypothyroid Diseases
Hyperthyroid Diseases
Other Endocrine

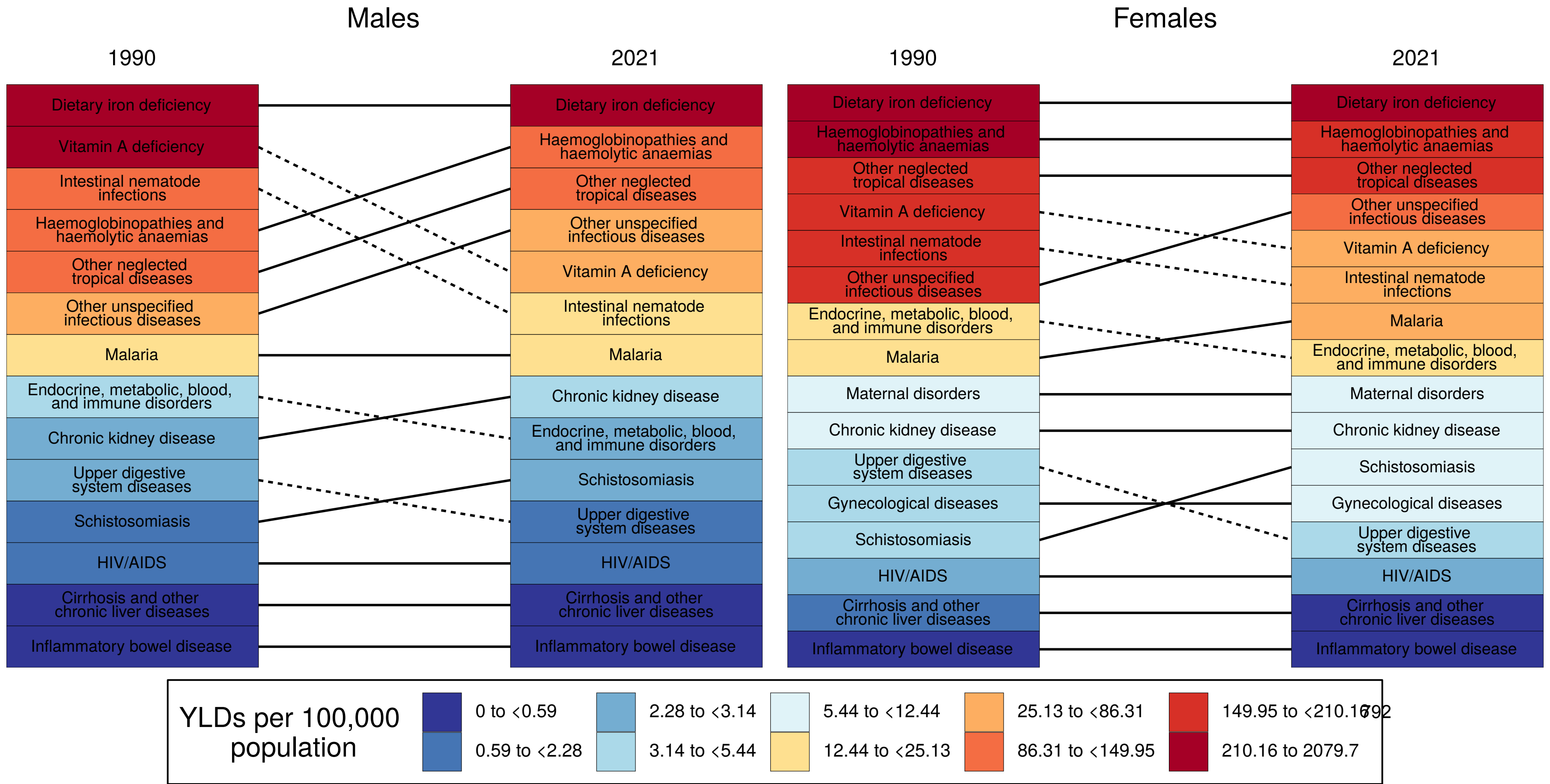
Males
Females
1990
2021
Dietary iron deficiency
Vitamin A deficiency
Intestinal nematode infections
Haemoglobinopathies and haemolytic anaemias
Other neglected tropical diseases
Other unspecified infectious diseases
Malaria
Endocrine, metabolic, blood, and immune disorders
Chronic kidney disease
Upper digestive system diseases
Schistosomiasis
HIV/AIDS
Cirrhosis and other chronic liver diseases
Inflammatory bowel disease
Maternal disorders
Gynecological diseases
YLDs per 100,000 population
0 to <0.59
0.59 to <2.28
2.28 to <3.14
3.14 to <5.44
5.44 to <12.44
12.44 to <25.13
25.13 to <86.31
86.31 to <149.95
149.95 to <210.16
210.16 to 2079.7

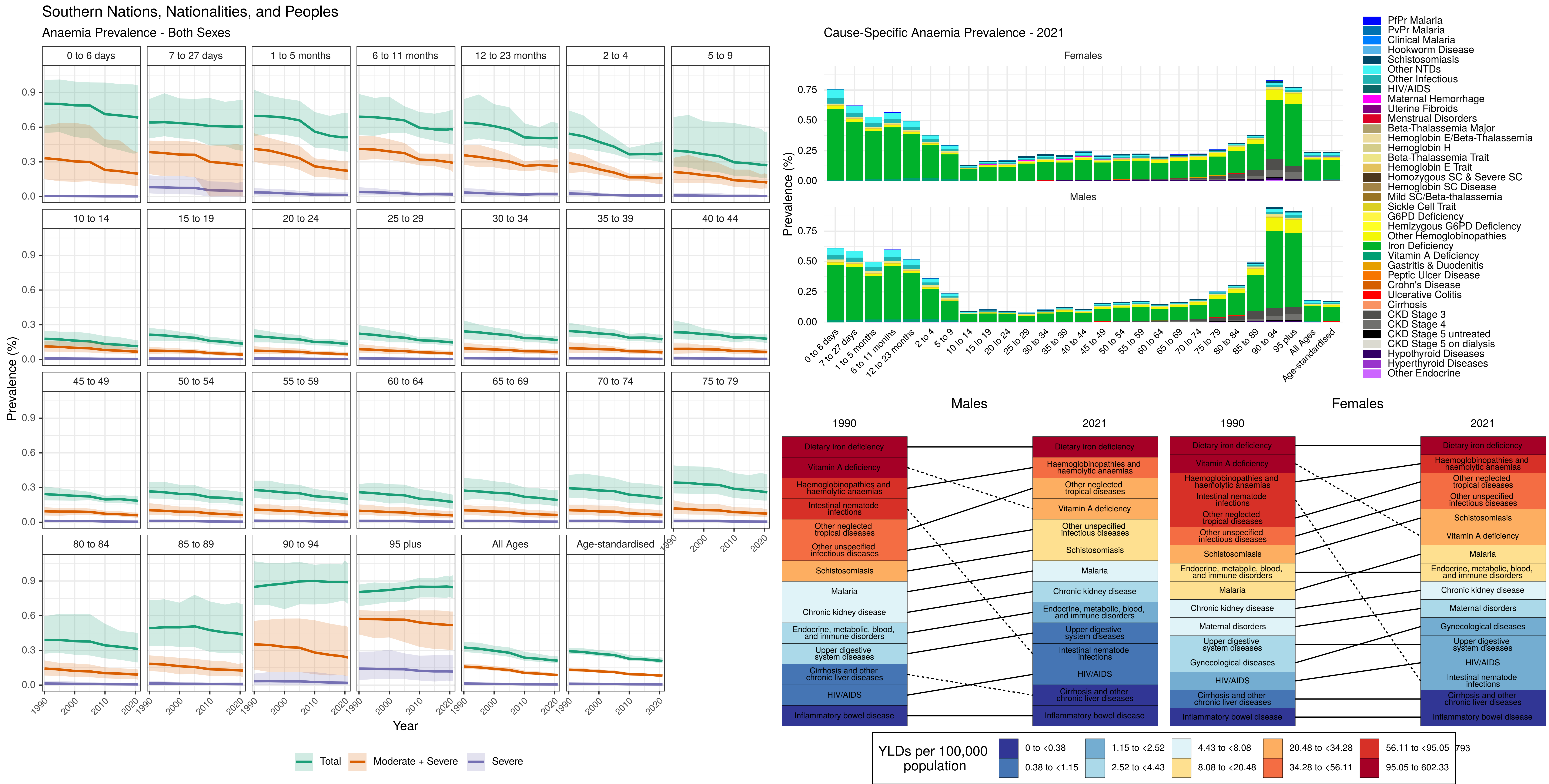
Southern Nations, Nationalities, and Peoples
Anaemia Prevalence - Both Sexes
0 to 6 days
7 to 27 days
1 to 5 months
6 to 11 months
12 to 23 months
2 to 4
5 to 9
10 to 14
15 to 19
20 to 24
25 to 29
30 to 34
35 to 39
40 to 44
45 to 49
50 to 54
55 to 59
60 to 64
65 to 69
70 to 74
75 to 79
80 to 84
85 to 89
90 to 94
95 plus
All Ages
Age-standardised
Prevalence (%)
Year
Total
Moderate + Severe
Severe
Cause-Specific Anaemia Prevalence - 2021
Females
Males
PfPr Malaria
PvPr Malaria
Clinical Malaria
Hookworm Disease
Schistosomiasis
Other NTDs
Other Infectious
HIV/AIDS
Maternal Hemorrhage
Uterine Fibroids
Menstrual Disorders
Beta-Thalassemia Major
Hemoglobin E/Beta-Thalassemia
Hemoglobin H
Beta-Thalassemia Trait
Hemoglobin E Trait
Homozygous SC & Severe SC
Hemoglobin SC Disease
Mild SC/Beta-thalassemia
Sickle Cell Trait
G6PD Deficiency
Hemizygous G6PD Deficiency
Other Hemoglobinopathies
Iron Deficiency
Vitamin A Deficiency
Gastritis & Duodenitis
Peptic Ulcer Disease
Crohn's Disease
Ulcerative Colitis
Cirrhosis
CKD Stage 3
CKD Stage 4
CKD Stage 5 untreated
CKD Stage 5 on dialysis
Hypothyroid Diseases
Hyperthyroid Diseases
Other Endocrine
Males
Females
1990
2021
Dietary iron deficiency
Vitamin A deficiency
Haemoglobinopathies and haemolytic anaemias
Intestinal nematode infections
Other neglected tropical diseases
Other unspecified infectious diseases
Schistosomiasis
Malaria
Chronic kidney disease
Endocrine, metabolic, blood, and immune disorders
Upper digestive system diseases
Cirrhosis and other chronic liver diseases
HIV/AIDS
Inflammatory bowel disease
Maternal disorders
Gynecological diseases
YLDs per 100,000 population
0 to <0.38
0.38 to <1.15
1.15 to <2.52
2.52 to <4.43
4.43 to <8.08
8.08 to <20.48
20.48 to <34.28
34.28 to <56.11
56.11 to <95.05
95.05 to 602.33

Tigray

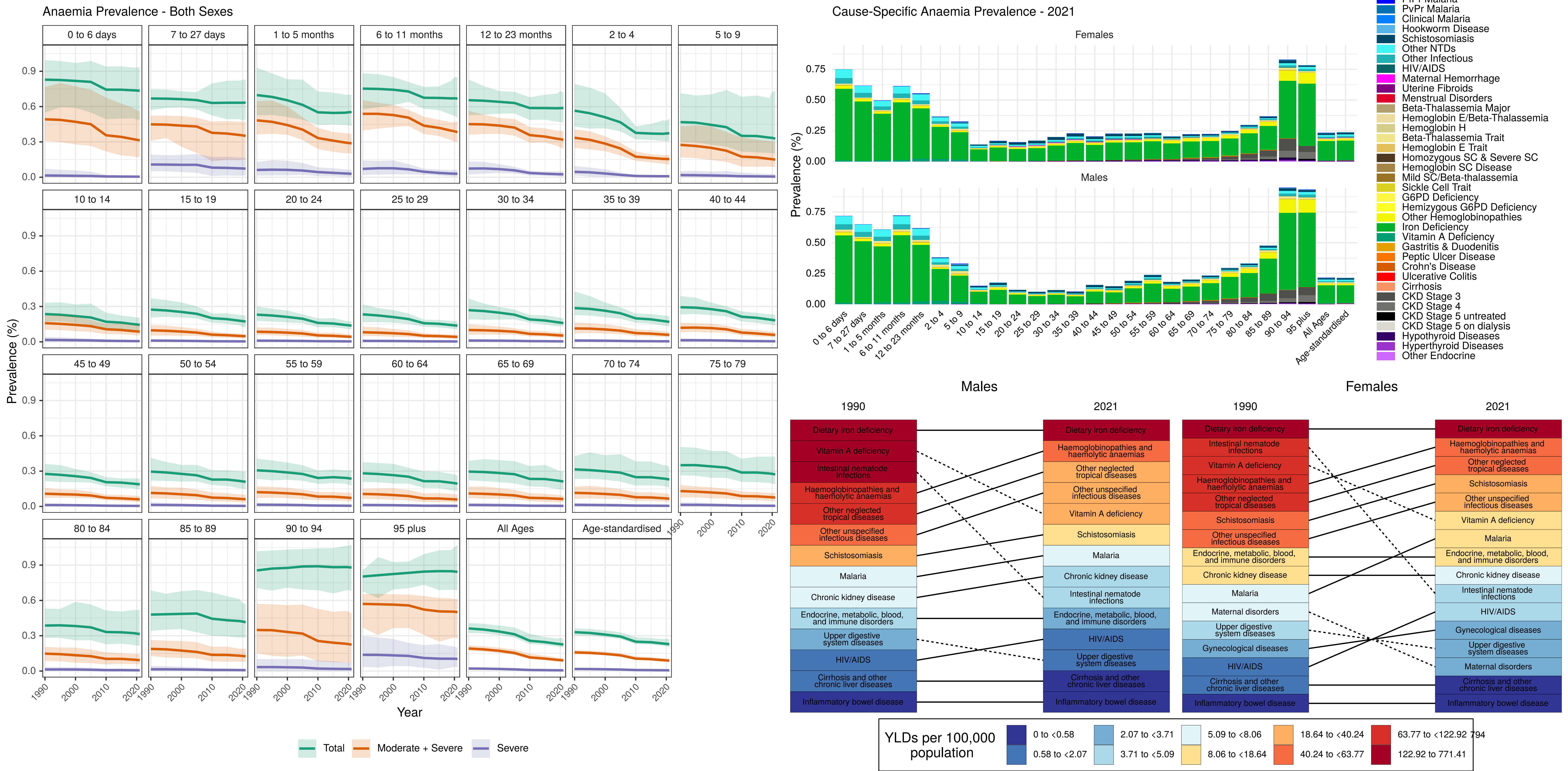
Anaemia Prevalence - Both Sexes
0 to 6 days
7 to 27 days
1 to 5 months
6 to 11 months
12 to 23 months
2 to 4
5 to 9
10 to 14
15 to 19
20 to 24
25 to 29
30 to 34
35 to 39
40 to 44
45 to 49
50 to 54
55 to 59
60 to 64
65 to 69
70 to 74
75 to 79
80 to 84
85 to 89
90 to 94
95 plus
All Ages
Age-standardised
Prevalence (%)
Year
Total
Moderate + Severe
Severe
Cause-Specific Anaemia Prevalence - 2021
Females
Males
PfPr Malaria
PvPr Malaria
Clinical Malaria
Hookworm Disease
Schistosomiasis
Other NTDs
Other Infectious
HIV/AIDS
Maternal Hemorrhage
Uterine Fibroids
Menstrual Disorders
Beta-Thalassemia Major
Hemoglobin E/Beta-Thalassemia
Hemoglobin H
Beta-Thalassemia Trait
Hemoglobin E Trait
Homozygous SC & Severe SC
Hemoglobin SC Disease
Mild SC/Beta-thalassemia
Sickle Cell Trait
G6PD Deficiency
Hemizygous G6PD Deficiency
Other Hemoglobinopathies
Iron Deficiency
Vitamin A Deficiency
Gastritis & Duodenitis
Peptic Ulcer Disease
Crohn's Disease
Ulcerative Colitis
Cirrhosis
CKD Stage 3
CKD Stage 4
CKD Stage 5 untreated
CKD Stage 5 on dialysis
Hypothyroid Diseases
Hyperthyroid Diseases
Other Endocrine
Males
Females
1990
2021
Dietary iron deficiency
Vitamin A deficiency
Intestinal nematode infections
Haemoglobinopathies and haemolytic anaemias
Other neglected tropical diseases
Other unspecified infectious diseases
Schistosomiasis
Malaria
Chronic kidney disease
Endocrine, metabolic, blood, and immune disorders
Upper digestive system diseases
HIV/AIDS
Cirrhosis and other chronic liver diseases
Inflammatory bowel disease
Maternal disorders
Gynecological diseases
YLDs per 100,000 population
0 to <0.58
0.58 to <2.07
2.07 to <3.71
3.71 to <5.09
5.09 to <8.06
8.06 to <18.64
18.64 to <40.24
40.24 to <63.77
63.77 to <122.92
122.92 to 771.41

Kenya

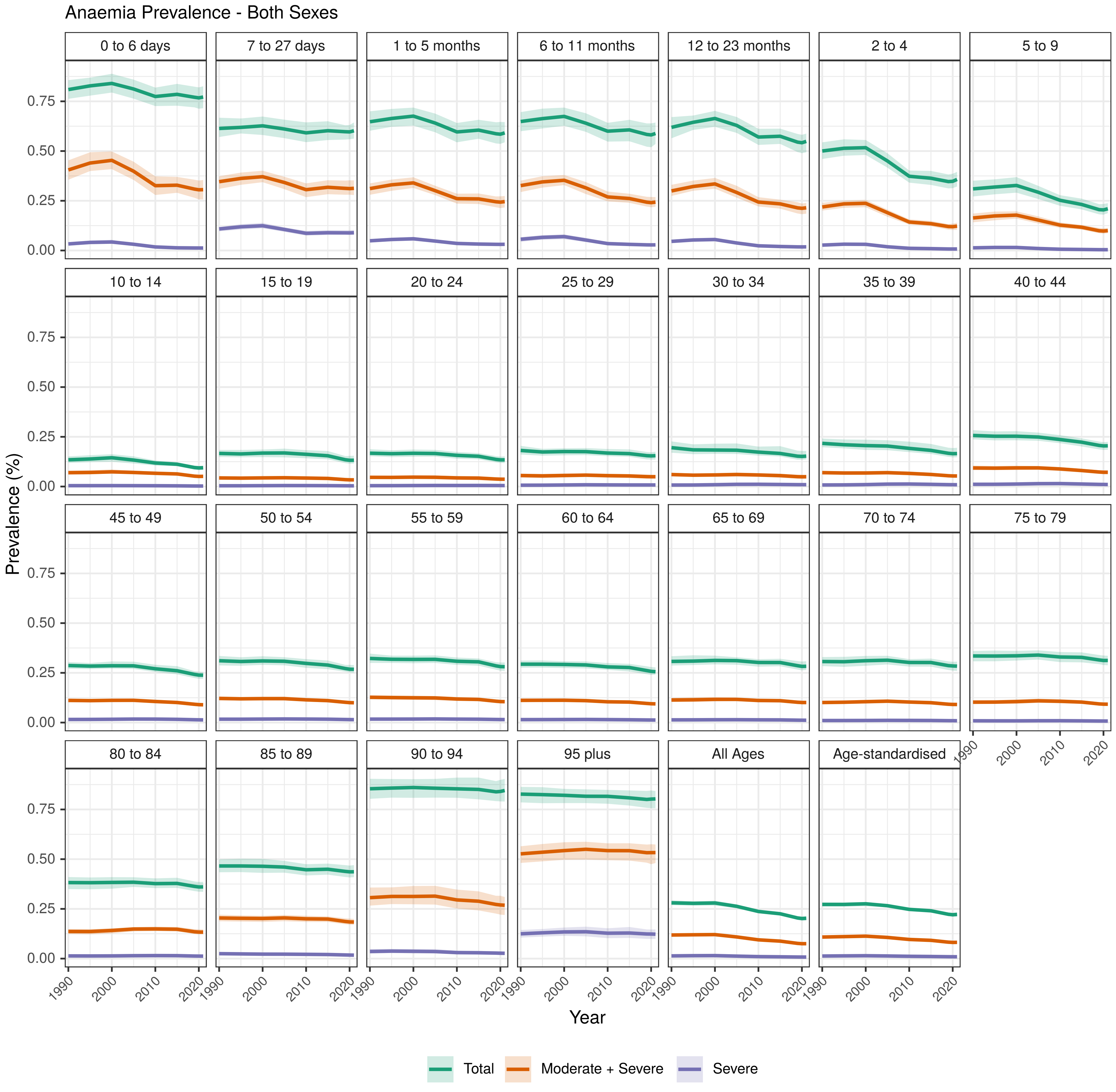

Anaemia Prevalence - Both Sexes
0 to 6 days
7 to 27 days
1 to 5 months
6 to 11 months
12 to 23 months
2 to 4
5 to 9
10 to 14
15 to 19
20 to 24
25 to 29
30 to 34
35 to 39
40 to 44
45 to 49
50 to 54
55 to 59
60 to 64
65 to 69
70 to 74
75 to 79
80 to 84
85 to 89
90 to 94
95 plus
All Ages
Age-standardised
Prevalence (%)
Year
Total
Moderate + Severe
Severe

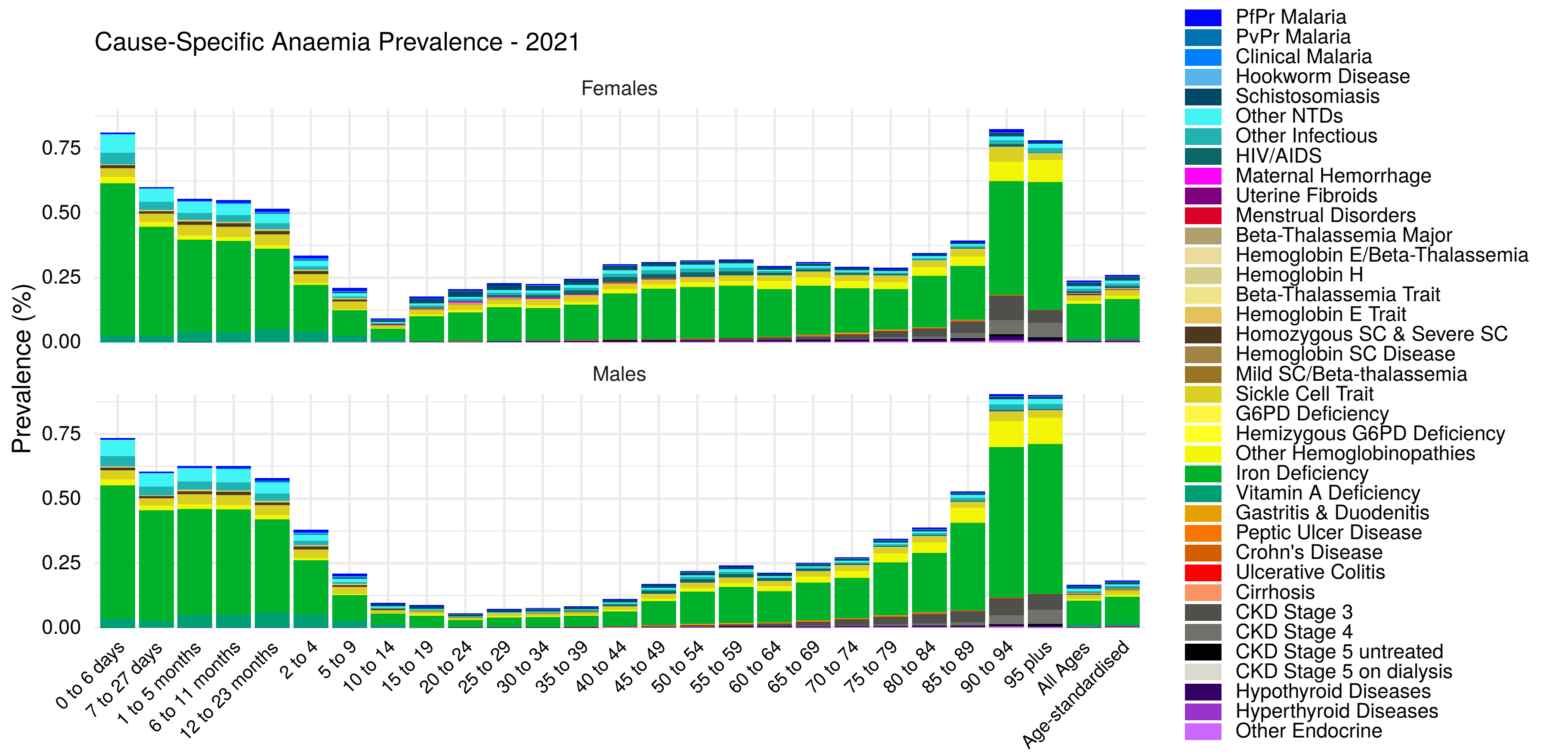

Cause-Specific Anaemia Prevalence - 2021
Females
Males
Prevalence (%)
PfPr Malaria
PvPr Malaria
Clinical Malaria
Hookworm Disease
Schistosomiasis
Other NTDs
Other Infectious
HIV/AIDS
Maternal Hemorrhage
Uterine Fibroids
Menstrual Disorders
Beta-Thalassemia Major
Hemoglobin E/Beta-Thalassemia
Hemoglobin H
Beta-Thalassemia Trait
Hemoglobin E Trait
Homozygous SC & Severe SC
Hemoglobin SC Disease
Mild SC/Beta-thalassemia
Sickle Cell Trait
G6PD Deficiency
Hemizygous G6PD Deficiency
Other Hemoglobinopathies
Iron Deficiency
Vitamin A Deficiency
Gastritis & Duodenitis
Peptic Ulcer Disease
Crohn's Disease
Ulcerative Colitis
Cirrhosis
CKD Stage 3
CKD Stage 4
CKD Stage 5 untreated
CKD Stage 5 on dialysis
Hypothyroid Diseases
Hyperthyroid Diseases
Other Endocrine

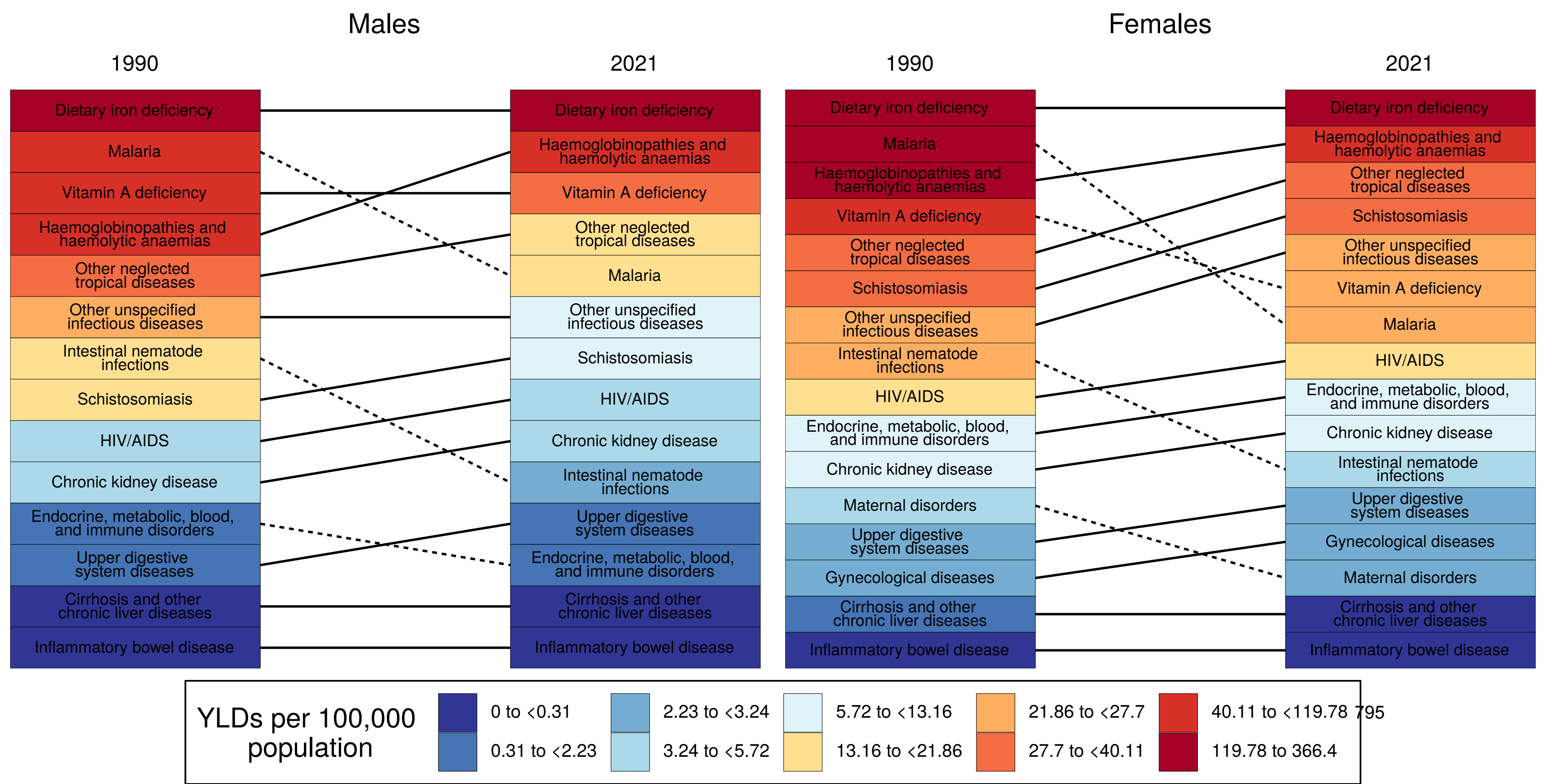

Males
Females
1990
2021
Dietary iron deficiency
Malaria
Vitamin A deficiency
Haemoglobinopathies and haemolytic anaemias
Other neglected tropical diseases
Other unspecified infectious diseases
Intestinal nematode infections
Schistosomiasis
HIV/AIDS
Chronic kidney disease
Endocrine, metabolic, blood, and immune disorders
Upper digestive system diseases
Cirrhosis and other chronic liver diseases
Inflammatory bowel disease
Maternal disorders
Gynecological diseases
YLDs per 100,000 population
0 to <0.31
0.31 to <2.23
2.23 to <3.24
3.24 to <5.72
5.72 to <13.16
13.16 to <21.86
21.86 to <27.7
27.7 to <40.11
40.11 to <119.78
119.78 to 366.4

Baringo

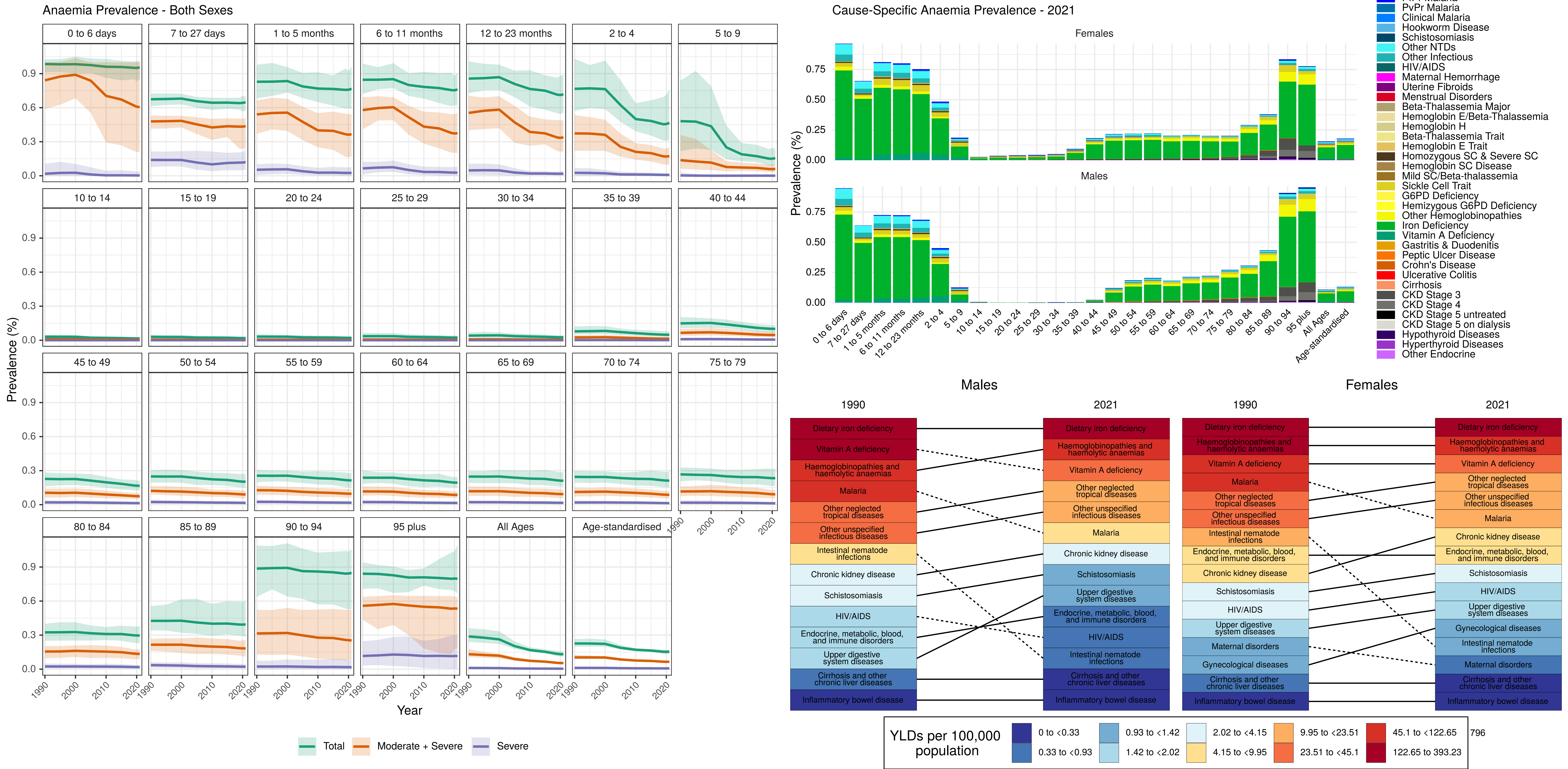
Anaemia Prevalence - Both Sexes
0 to 6 days
7 to 27 days
1 to 5 months
6 to 11 months
12 to 23 months
2 to 4
5 to 9
10 to 14
15 to 19
20 to 24
25 to 29
30 to 34
35 to 39
40 to 44
45 to 49
50 to 54
55 to 59
60 to 64
65 to 69
70 to 74
75 to 79
80 to 84
85 to 89
90 to 94
95 plus
All Ages
Age-standardised
Prevalence (%)
Year
Total
Moderate + Severe
Severe
Cause-Specific Anaemia Prevalence - 2021
Females
Males
PfPr Malaria
PvPr Malaria
Clinical Malaria
Hookworm Disease
Schistosomiasis
Other NTDs
Other Infectious
HIV/AIDS
Maternal Hemorrhage
Uterine Fibroids
Menstrual Disorders
Beta-Thalassemia Major
Hemoglobin E/Beta-Thalassemia
Hemoglobin H
Beta-Thalassemia Trait
Hemoglobin E Trait
Homozygous SC & Severe SC
Hemoglobin SC Disease
Mild SC/Beta-thalassemia
Sickle Cell Trait
G6PD Deficiency
Hemizygous G6PD Deficiency
Other Hemoglobinopathies
Iron Deficiency
Vitamin A Deficiency
Gastritis & Duodenitis
Peptic Ulcer Disease
Crohn's Disease
Ulcerative Colitis
Cirrhosis
CKD Stage 3
CKD Stage 4
CKD Stage 5 untreated
CKD Stage 5 on dialysis
Hypothyroid Diseases
Hyperthyroid Diseases
Other Endocrine
Males
Females
1990
2021
Dietary iron deficiency
Vitamin A deficiency
Haemoglobinopathies and haemolytic anaemias
Malaria
Other neglected tropical diseases
Other unspecified infectious diseases
Intestinal nematode infections
Chronic kidney disease
Schistosomiasis
HIV/AIDS
Endocrine, metabolic, blood, and immune disorders
Upper digestive system diseases
Cirrhosis and other chronic liver diseases
Inflammatory bowel disease
Maternal disorders
Gynecological diseases
YLDs per 100,000 population
0 to <0.33
0.33 to <0.93
0.93 to <1.42
1.42 to <2.02
2.02 to <4.15
4.15 to <9.95
9.95 to <23.51
23.51 to <45.1
45.1 to <122.65
122.65 to 393.23

Bomet

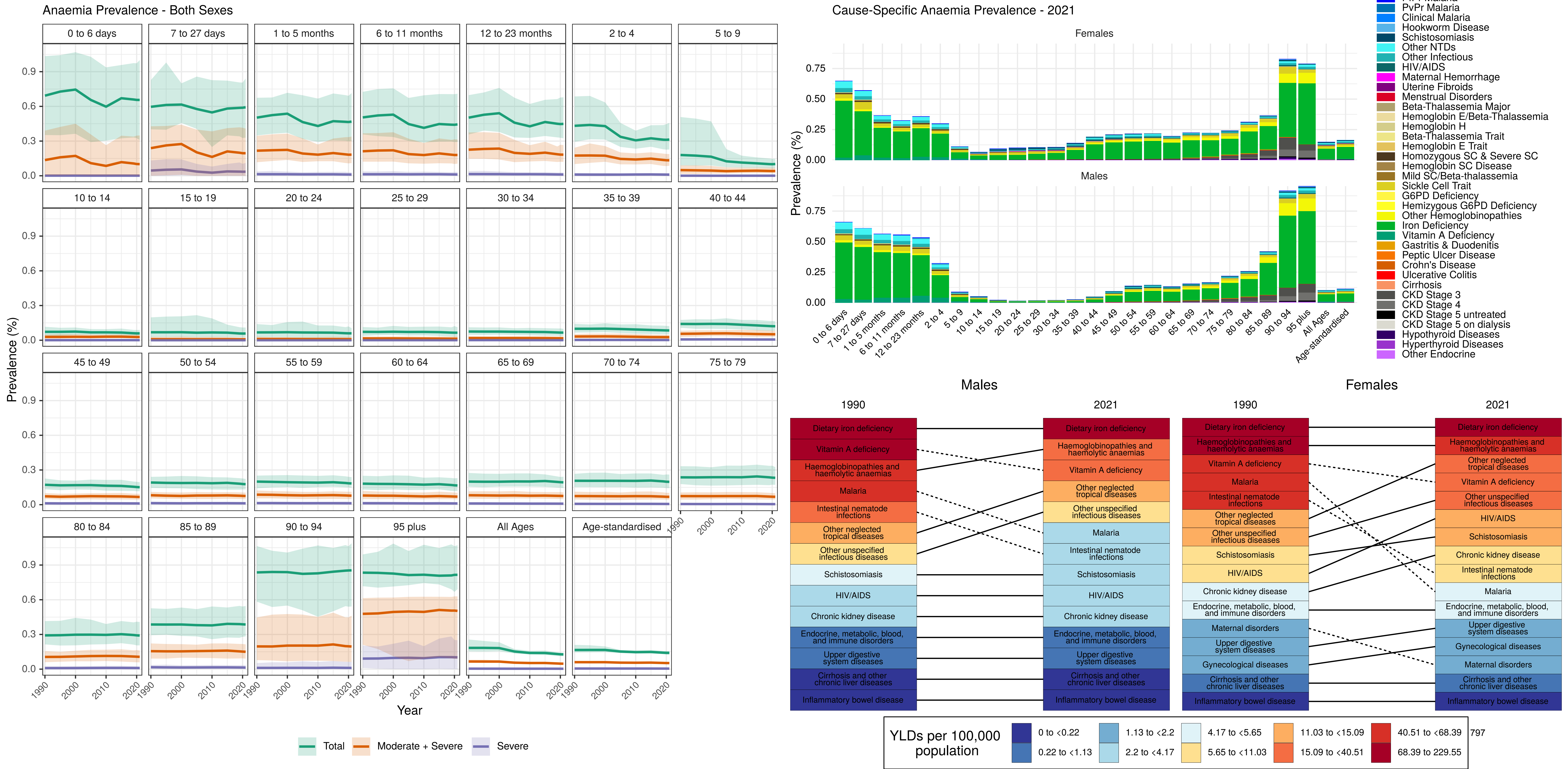

Anaemia Prevalence - Both Sexes
0 to 6 days
7 to 27 days
1 to 5 months
6 to 11 months
12 to 23 months
2 to 4
5 to 9
10 to 14
15 to 19
20 to 24
25 to 29
30 to 34
35 to 39
40 to 44
45 to 49
50 to 54
55 to 59
60 to 64
65 to 69
70 to 74
75 to 79
80 to 84
85 to 89
90 to 94
95 plus
All Ages
Age-standardised
Prevalence (%)
Year
Total
Moderate + Severe
Severe
Cause-Specific Anaemia Prevalence - 2021
Females
Males
PfPr Malaria
PvPr Malaria
Clinical Malaria
Hookworm Disease
Schistosomiasis
Other NTDs
Other Infectious
HIV/AIDS
Maternal Hemorrhage
Uterine Fibroids
Menstrual Disorders
Beta-Thalassemia Major
Hemoglobin E/Beta-Thalassemia
Hemoglobin H
Beta-Thalassemia Trait
Hemoglobin E Trait
Homozygous SC & Severe SC
Hemoglobin SC Disease
Mild SC/Beta-thalassemia
Sickle Cell Trait
G6PD Deficiency
Hemizygous G6PD Deficiency
Other Hemoglobinopathies
Iron Deficiency
Vitamin A Deficiency
Gastritis & Duodenitis
Peptic Ulcer Disease
Crohn's Disease
Ulcerative Colitis
Cirrhosis
CKD Stage 3
CKD Stage 4
CKD Stage 5 untreated
CKD Stage 5 on dialysis
Hypothyroid Diseases
Hyperthyroid Diseases
Other Endocrine
Males
Females
1990
2021
Dietary iron deficiency
Vitamin A deficiency
Haemoglobinopathies and haemolytic anaemias
Malaria
Intestinal nematode infections
Other neglected tropical diseases
Other unspecified infectious diseases
Schistosomiasis
HIV/AIDS
Chronic kidney disease
Endocrine, metabolic, blood, and immune disorders
Upper digestive system diseases
Cirrhosis and other chronic liver diseases
Inflammatory bowel disease
Maternal disorders
Gynecological diseases
YLDs per 100,000 population
0 to <0.22
0.22 to <1.13
1.13 to <2.2
2.2 to <4.17
4.17 to <5.65
5.65 to <11.03
11.03 to <15.09
15.09 to <40.51
40.51 to <68.39
68.39 to 229.55

Bungoma

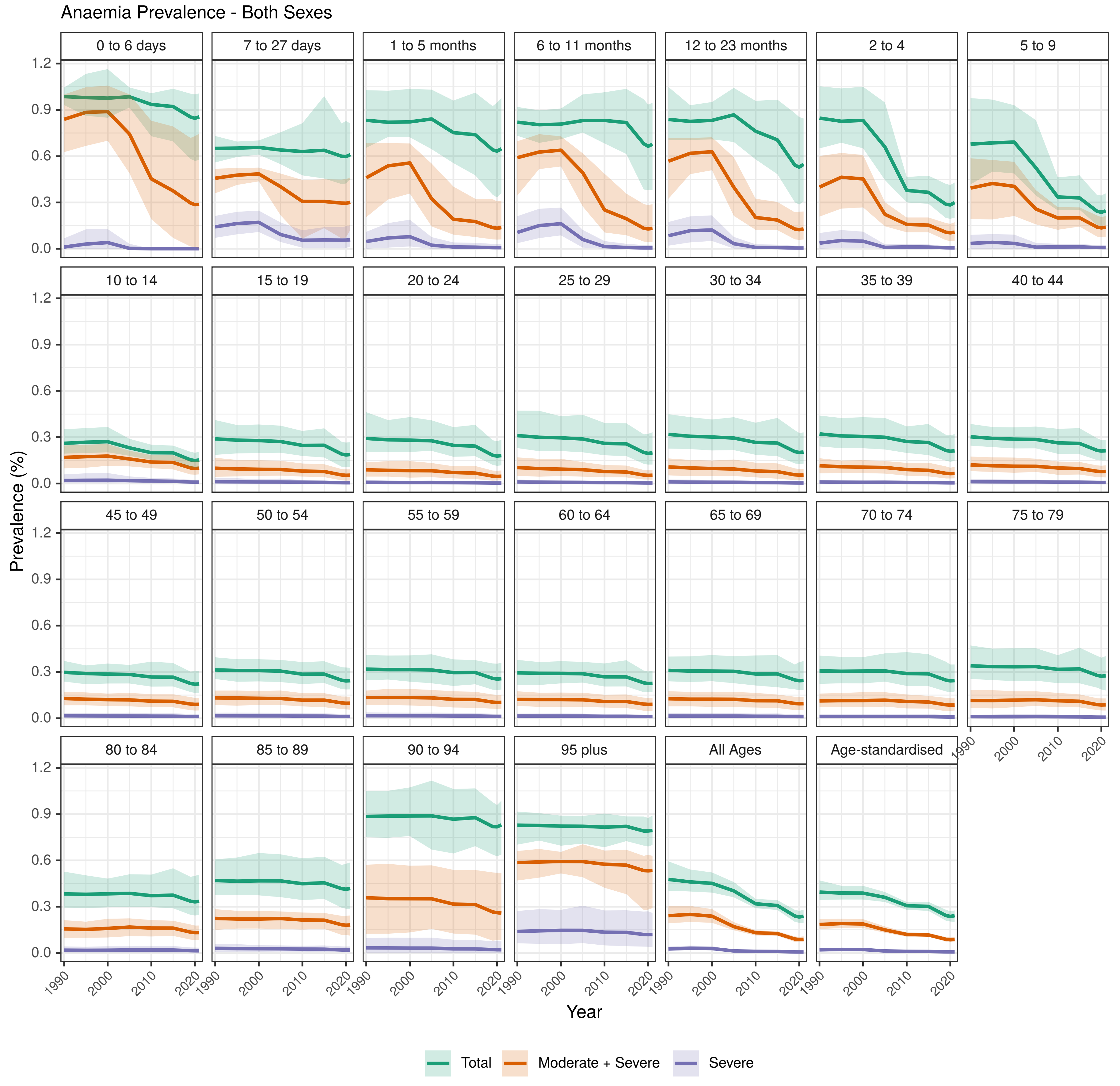

Anaemia Prevalence - Both Sexes
0 to 6 days
7 to 27 days
1 to 5 months
6 to 11 months
12 to 23 months
2 to 4
5 to 9
10 to 14
15 to 19
20 to 24
25 to 29
30 to 34
35 to 39
40 to 44
45 to 49
50 to 54
55 to 59
60 to 64
65 to 69
70 to 74
75 to 79
80 to 84
85 to 89
90 to 94
95 plus
All Ages
Age-standardised
Prevalence (%)
Year
Total
Moderate + Severe
Severe

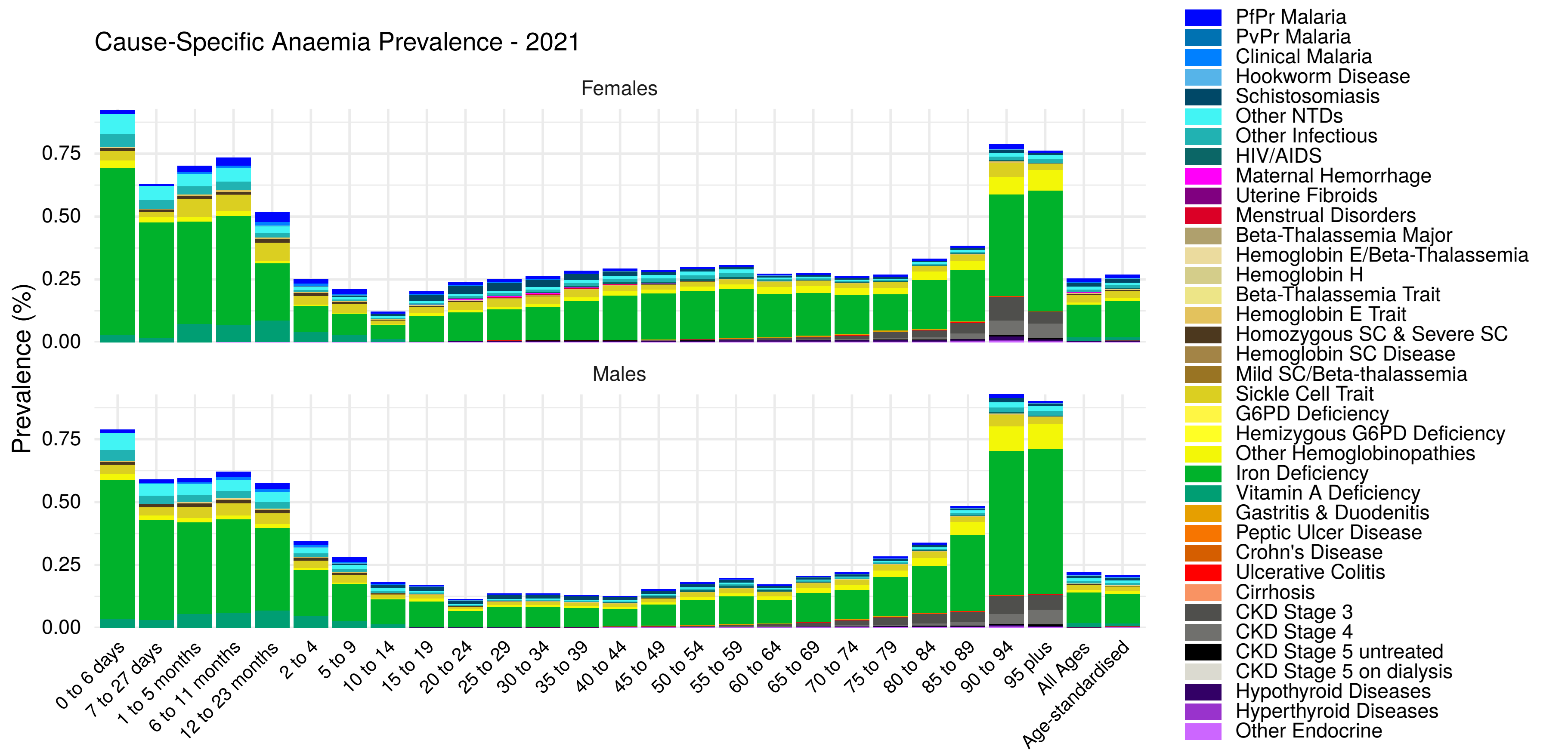

Cause-Specific Anaemia Prevalence - 2021
Females
Males
Prevalence (%)
PfPr Malaria
PvPr Malaria
Clinical Malaria
Hookworm Disease
Schistosomiasis
Other NTDs
Other Infectious
HIV/AIDS
Maternal Hemorrhage
Uterine Fibroids
Menstrual Disorders
Beta-Thalassemia Major
Hemoglobin E/Beta-Thalassemia
Hemoglobin H
Beta-Thalassemia Trait
Hemoglobin E Trait
Homozygous SC & Severe SC
Hemoglobin SC Disease
Mild SC/Beta-thalassemia
Sickle Cell Trait
G6PD Deficiency
Hemizygous G6PD Deficiency
Other Hemoglobinopathies
Iron Deficiency
Vitamin A Deficiency
Gastritis & Duodenitis
Peptic Ulcer Disease
Crohn's Disease
Ulcerative Colitis
Cirrhosis
CKD Stage 3
CKD Stage 4
CKD Stage 5 untreated
CKD Stage 5 on dialysis
Hypothyroid Diseases
Hyperthyroid Diseases
Other Endocrine

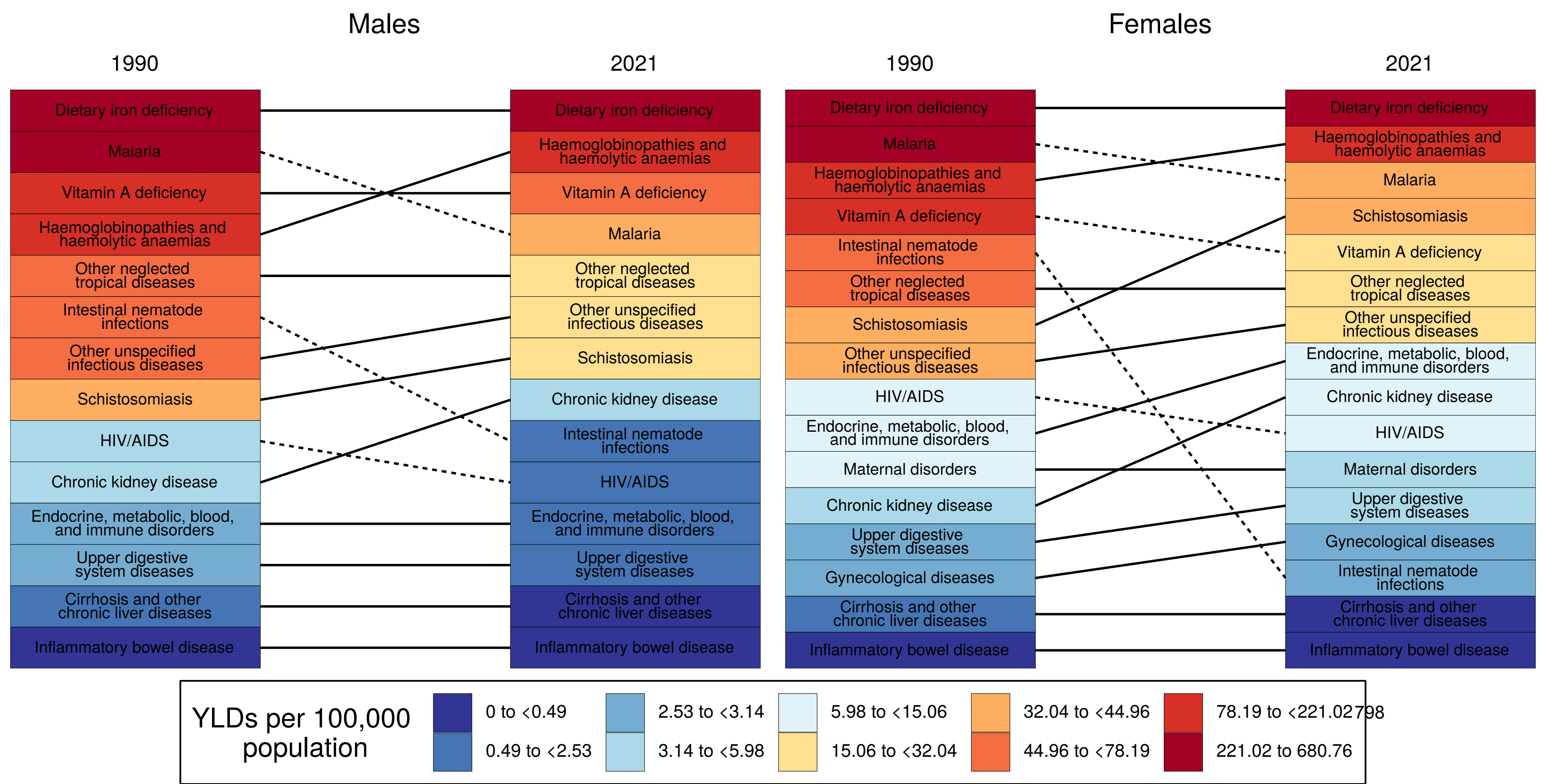

Males
Females
1990
2021
Dietary iron deficiency
Malaria
Vitamin A deficiency
Haemoglobinopathies and haemolytic anaemias
Other neglected tropical diseases
Intestinal nematode infections
Other unspecified infectious diseases
Schistosomiasis
HIV/AIDS
Chronic kidney disease
Endocrine, metabolic, blood, and immune disorders
Upper digestive system diseases
Cirrhosis and other chronic liver diseases
Inflammatory bowel disease
Maternal disorders
Gynecological diseases
YLDs per 100,000 population
0 to <0.49
0.49 to <2.53
2.53 to <3.14
3.14 to <5.98
5.98 to <15.06
15.06 to <32.04
32.04 to <44.96
44.96 to <78.19
78.19 to <221.02798
221.02 to 680.76

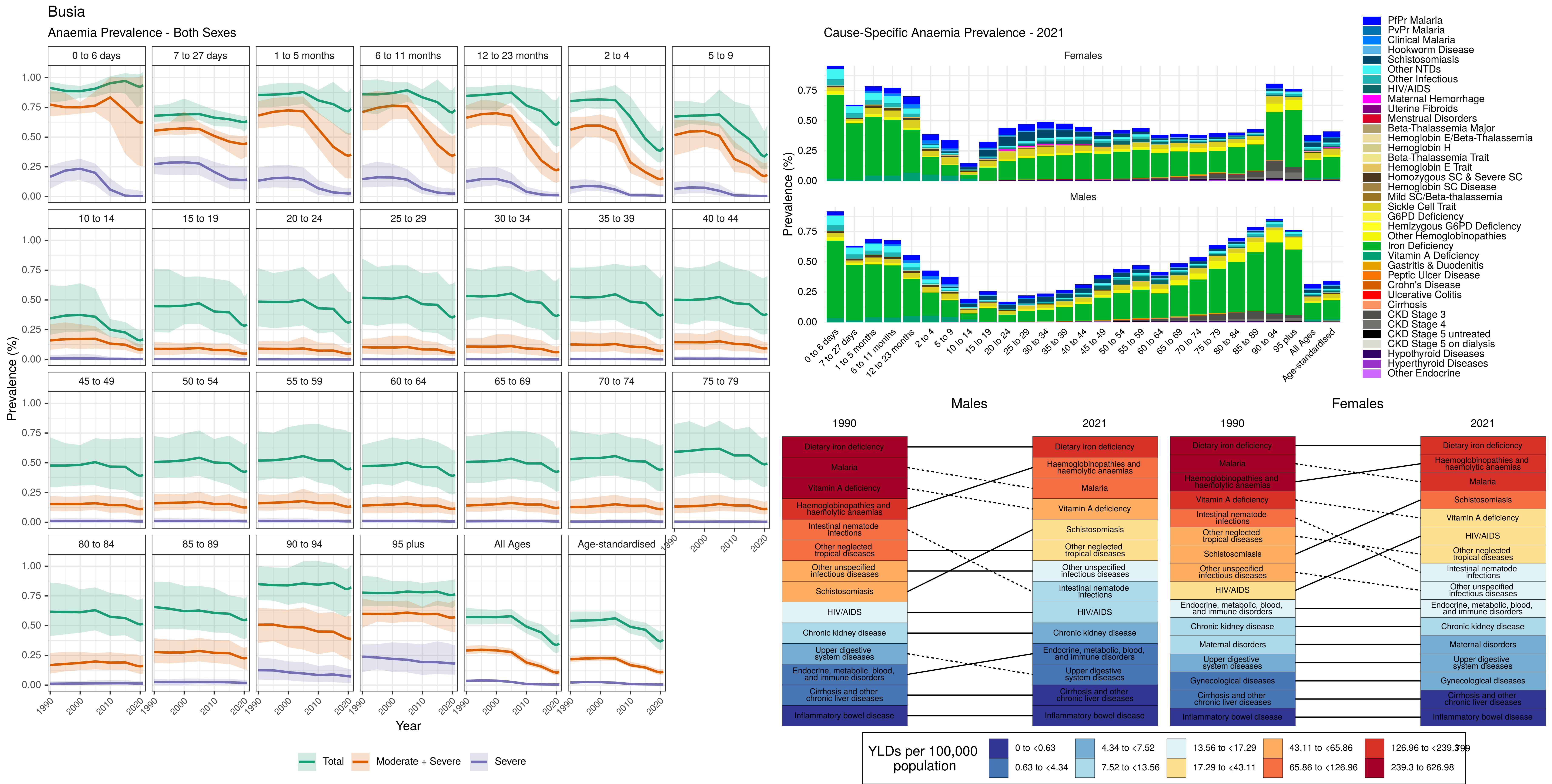

Busia
Anaemia Prevalence - Both Sexes
0 to 6 days
7 to 27 days
1 to 5 months
6 to 11 months
12 to 23 months
2 to 4
5 to 9
10 to 14
15 to 19
20 to 24
25 to 29
30 to 34
35 to 39
40 to 44
45 to 49
50 to 54
55 to 59
60 to 64
65 to 69
70 to 74
75 to 79
80 to 84
85 to 89
90 to 94
95 plus
All Ages
Age-standardised
Prevalence (%)
Year
Total
Moderate + Severe
Severe
Cause-Specific Anaemia Prevalence - 2021
Females
Males
PfPr Malaria
PvPr Malaria
Clinical Malaria
Hookworm Disease
Schistosomiasis
Other NTDs
Other Infectious
HIV/AIDS
Maternal Hemorrhage
Uterine Fibroids
Menstrual Disorders
Beta-Thalassemia Major
Hemoglobin E/Beta-Thalassemia
Hemoglobin H
Beta-Thalassemia Trait
Hemoglobin E Trait
Homozygous SC & Severe SC
Hemoglobin SC Disease
Mild SC/Beta-thalassemia
Sickle Cell Trait
G6PD Deficiency
Hemizygous G6PD Deficiency
Other Hemoglobinopathies
Iron Deficiency
Vitamin A Deficiency
Gastritis & Duodenitis
Peptic Ulcer Disease
Crohn's Disease
Ulcerative Colitis
Cirrhosis
CKD Stage 3
CKD Stage 4
CKD Stage 5 untreated
CKD Stage 5 on dialysis
Hypothyroid Diseases
Hyperthyroid Diseases
Other Endocrine
Males
Females
1990
2021
Dietary iron deficiency
Malaria
Vitamin A deficiency
Haemoglobinopathies and haemolytic anaemias
Intestinal nematode infections
Other neglected tropical diseases
Other unspecified infectious diseases
Schistosomiasis
HIV/AIDS
Chronic kidney disease
Upper digestive system diseases
Endocrine, metabolic, blood, and immune disorders
Cirrhosis and other chronic liver diseases
Inflammatory bowel disease
Maternal disorders
Gynecological diseases
YLDs per 100,000 population
0 to <0.63
0.63 to <4.34
4.34 to <7.52
7.52 to <13.56
13.56 to <17.29
17.29 to <43.11
43.11 to <65.86
65.86 to <126.96
126.96 to <239.399
239.3 to 626.98

Elgeyo Marakwet

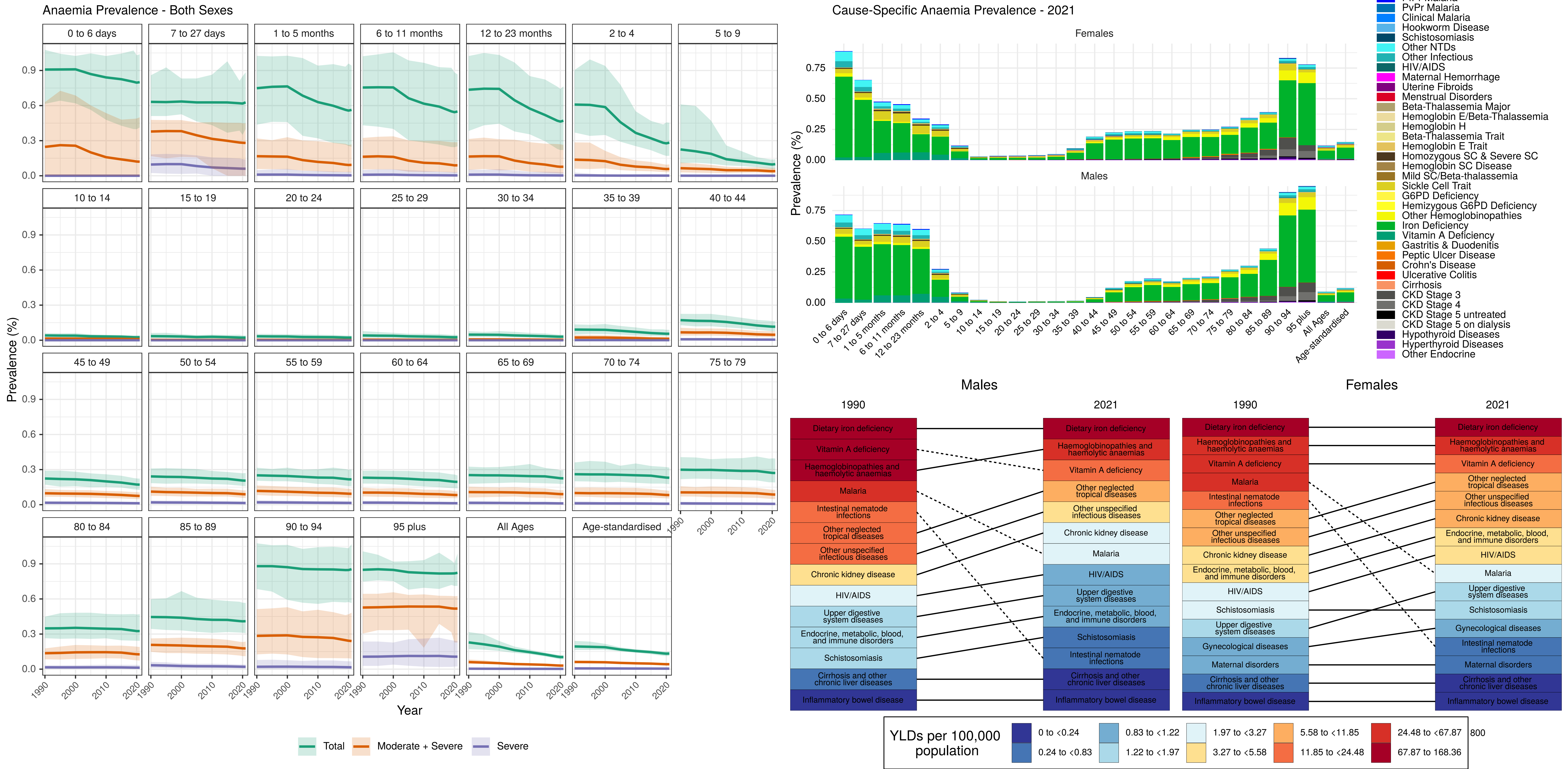

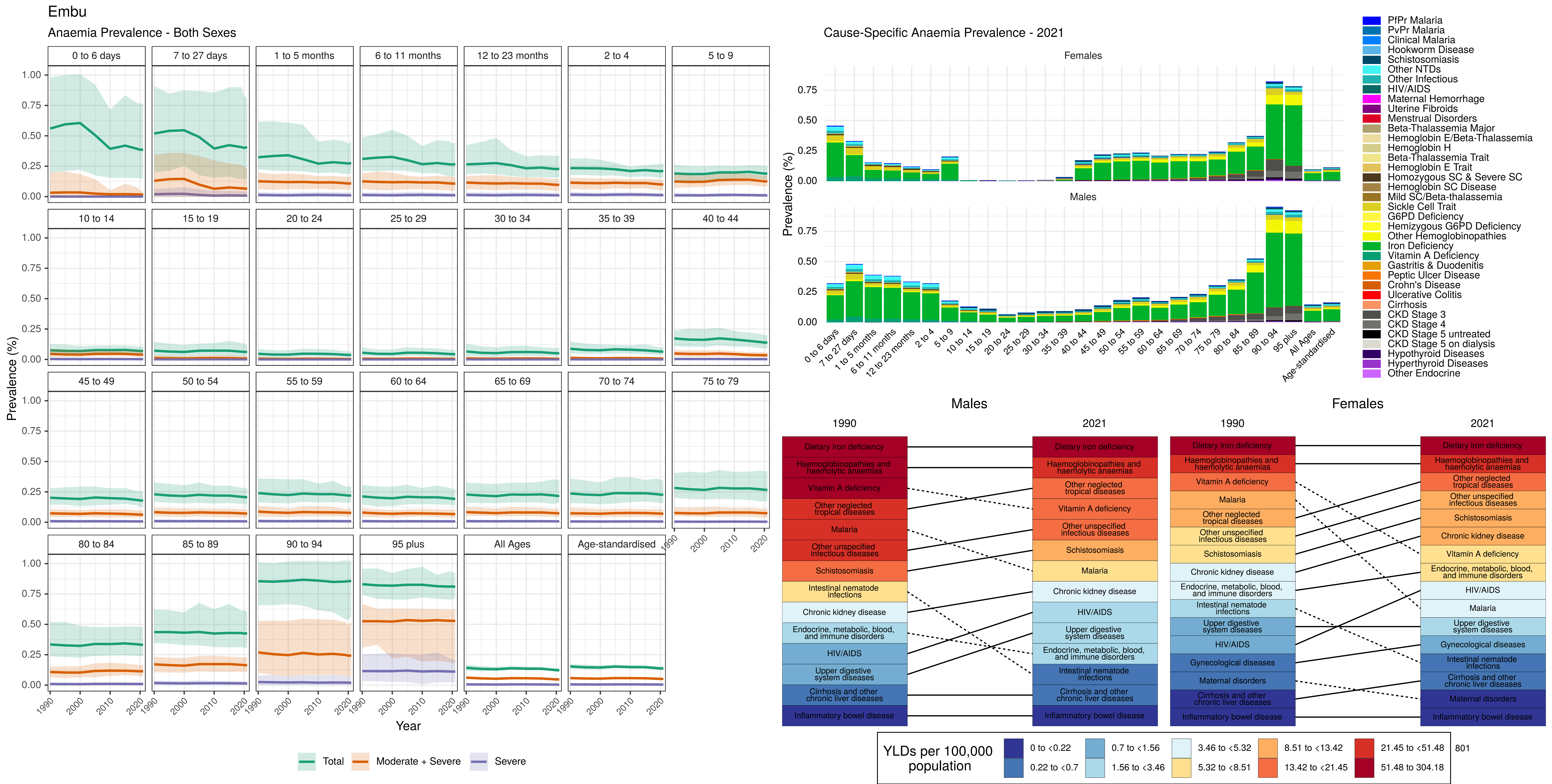
Embu
Anaemia Prevalence - Both Sexes
0 to 6 days
7 to 27 days
1 to 5 months
6 to 11 months
12 to 23 months
2 to 4
5 to 9
10 to 14
15 to 19
20 to 24
25 to 29
30 to 34
35 to 39
40 to 44
45 to 49
50 to 54
55 to 59
60 to 64
65 to 69
70 to 74
75 to 79
80 to 84
85 to 89
90 to 94
95 plus
All Ages
Age-standardised
Prevalence (%)
Year
Total
Moderate + Severe
Severe
Cause-Specific Anaemia Prevalence - 2021
Females
Males
PfPr Malaria
PvPr Malaria
Clinical Malaria
Hookworm Disease
Schistosomiasis
Other NTDs
Other Infectious
HIV/AIDS
Maternal Hemorrhage
Uterine Fibroids
Menstrual Disorders
Beta-Thalassemia Major
Hemoglobin E/Beta-Thalassemia
Hemoglobin H
Beta-Thalassemia Trait
Hemoglobin E Trait
Homozygous SC & Severe SC
Hemoglobin SC Disease
Mild SC/Beta-thalassemia
Sickle Cell Trait
G6PD Deficiency
Hemizygous G6PD Deficiency
Other Hemoglobinopathies
Iron Deficiency
Vitamin A Deficiency
Gastritis & Duodenitis
Peptic Ulcer Disease
Crohn's Disease
Ulcerative Colitis
Cirrhosis
CKD Stage 3
CKD Stage 4
CKD Stage 5 untreated
CKD Stage 5 on dialysis
Hypothyroid Diseases
Hyperthyroid Diseases
Other Endocrine
Males
Females
1990
2021
Dietary iron deficiency
Haemoglobinopathies and haemolytic anaemias
Vitamin A deficiency
Other neglected tropical diseases
Malaria
Other unspecified infectious diseases
Schistosomiasis
Intestinal nematode infections
Chronic kidney disease
Endocrine, metabolic, blood, and immune disorders
HIV/AIDS
Upper digestive system diseases
Cirrhosis and other chronic liver diseases
Inflammatory bowel disease
Gynecological diseases
Maternal disorders
YLDs per 100,000 population
0 to <0.22
0.22 to <0.7
0.7 to <1.56
1.56 to <3.46
3.46 to <5.32
5.32 to <8.51
8.51 to <13.42
13.42 to <21.45
21.45 to <51.48
51.48 to 304.18
801

Garissa

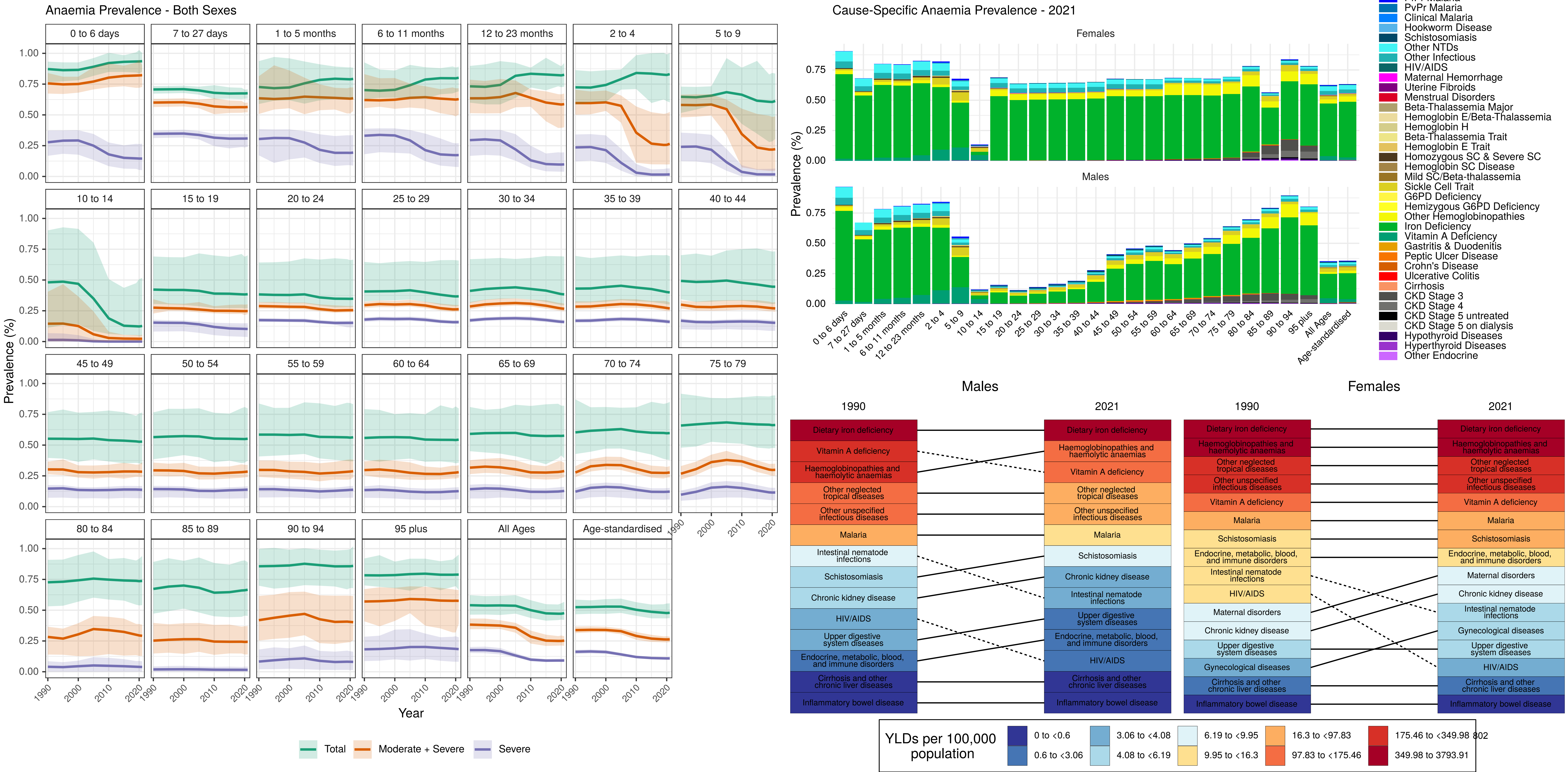

Anaemia Prevalence - Both Sexes
0 to 6 days
7 to 27 days
1 to 5 months
6 to 11 months
12 to 23 months
2 to 4
5 to 9
10 to 14
15 to 19
20 to 24
25 to 29
30 to 34
35 to 39
40 to 44
45 to 49
50 to 54
55 to 59
60 to 64
65 to 69
70 to 74
75 to 79
80 to 84
85 to 89
90 to 94
95 plus
All Ages
Age-standardised
Prevalence (%)
Year
Total
Moderate + Severe
Severe
Cause-Specific Anaemia Prevalence - 2021
Females
Males
Prevalence (%)
PfPr Malaria
PvPr Malaria
Clinical Malaria
Hookworm Disease
Schistosomiasis
Other NTDs
Other Infectious
HIV/AIDS
Maternal Hemorrhage
Uterine Fibroids
Menstrual Disorders
Beta-Thalassemia Major
Hemoglobin E/Beta-Thalassemia
Hemoglobin H
Beta-Thalassemia Trait
Hemoglobin E Trait
Homozygous SC & Severe SC
Hemoglobin SC Disease
Mild SC/Beta-thalassemia
Sickle Cell Trait
G6PD Deficiency
Hemizygous G6PD Deficiency
Other Hemoglobinopathies
Iron Deficiency
Vitamin A Deficiency
Gastritis & Duodenitis
Peptic Ulcer Disease
Crohn's Disease
Ulcerative Colitis
Cirrhosis
CKD Stage 3
CKD Stage 4
CKD Stage 5 untreated
CKD Stage 5 on dialysis
Hypothyroid Diseases
Hyperthyroid Diseases
Other Endocrine
Males
Females
1990
2021
Dietary iron deficiency
Vitamin A deficiency
Haemoglobinopathies and haemolytic anaemias
Other neglected tropical diseases
Other unspecified infectious diseases
Malaria
Intestinal nematode infections
Schistosomiasis
Chronic kidney disease
HIV/AIDS
Upper digestive system diseases
Endocrine, metabolic, blood, and immune disorders
Cirrhosis and other chronic liver diseases
Inflammatory bowel disease
Maternal disorders
Gynecological diseases
YLDs per 100,000 population
0 to <0.6
0.6 to <3.06
3.06 to <4.08
4.08 to <6.19
6.19 to <9.95
9.95 to <16.3
16.3 to <97.83
97.83 to <175.46
175.46 to <349.98 802
349.98 to 3793.91

Homa Bay

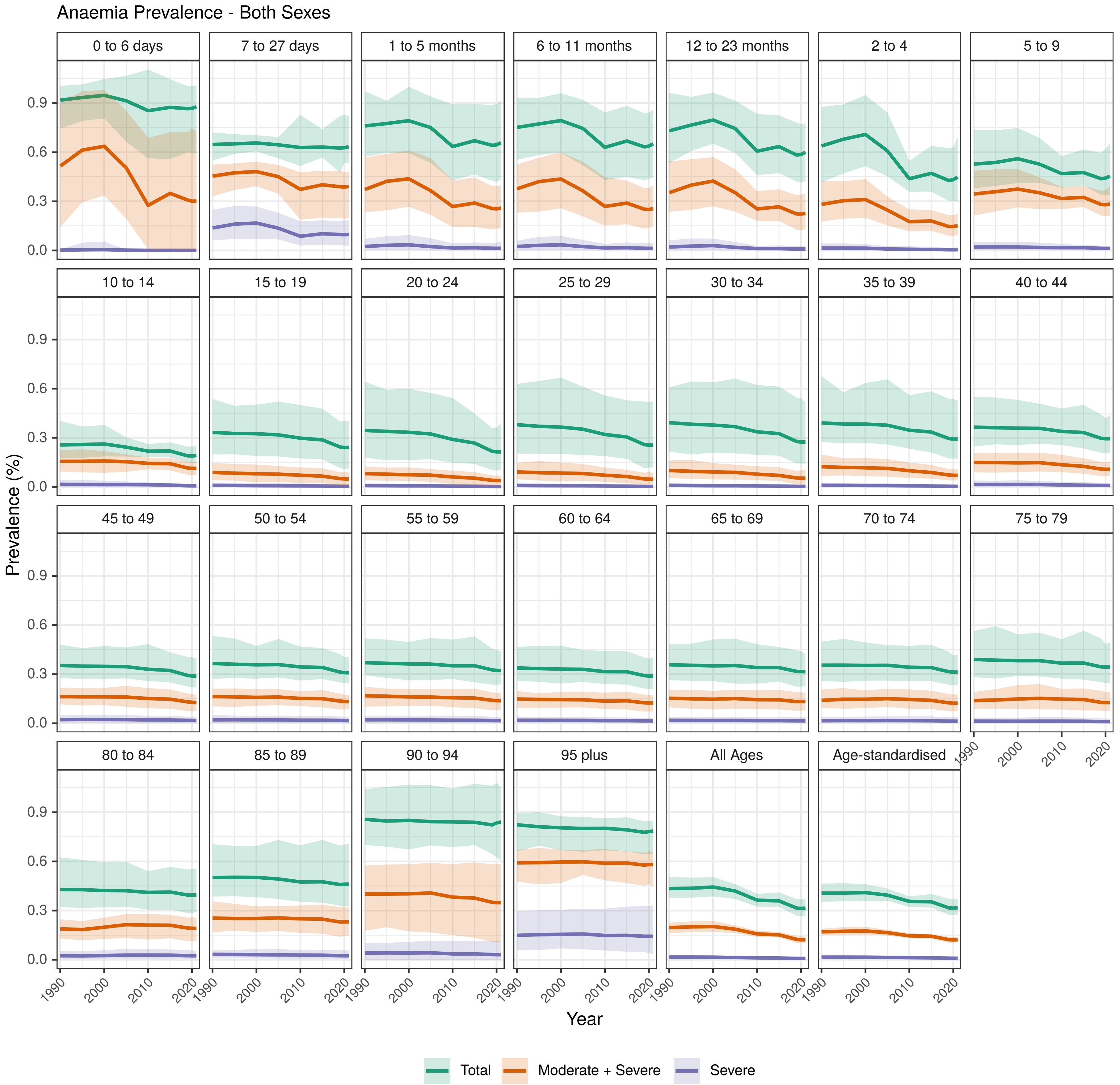
Anaemia Prevalence - Both Sexes
0 to 6 days
7 to 27 days
1 to 5 months
6 to 11 months
12 to 23 months
2 to 4
5 to 9
10 to 14
15 to 19
20 to 24
25 to 29
30 to 34
35 to 39
40 to 44
45 to 49
50 to 54
55 to 59
60 to 64
65 to 69
70 to 74
75 to 79
80 to 84
85 to 89
90 to 94
95 plus
All Ages
Age-standardised
Prevalence (%)
Year
Total
Moderate + Severe
Severe

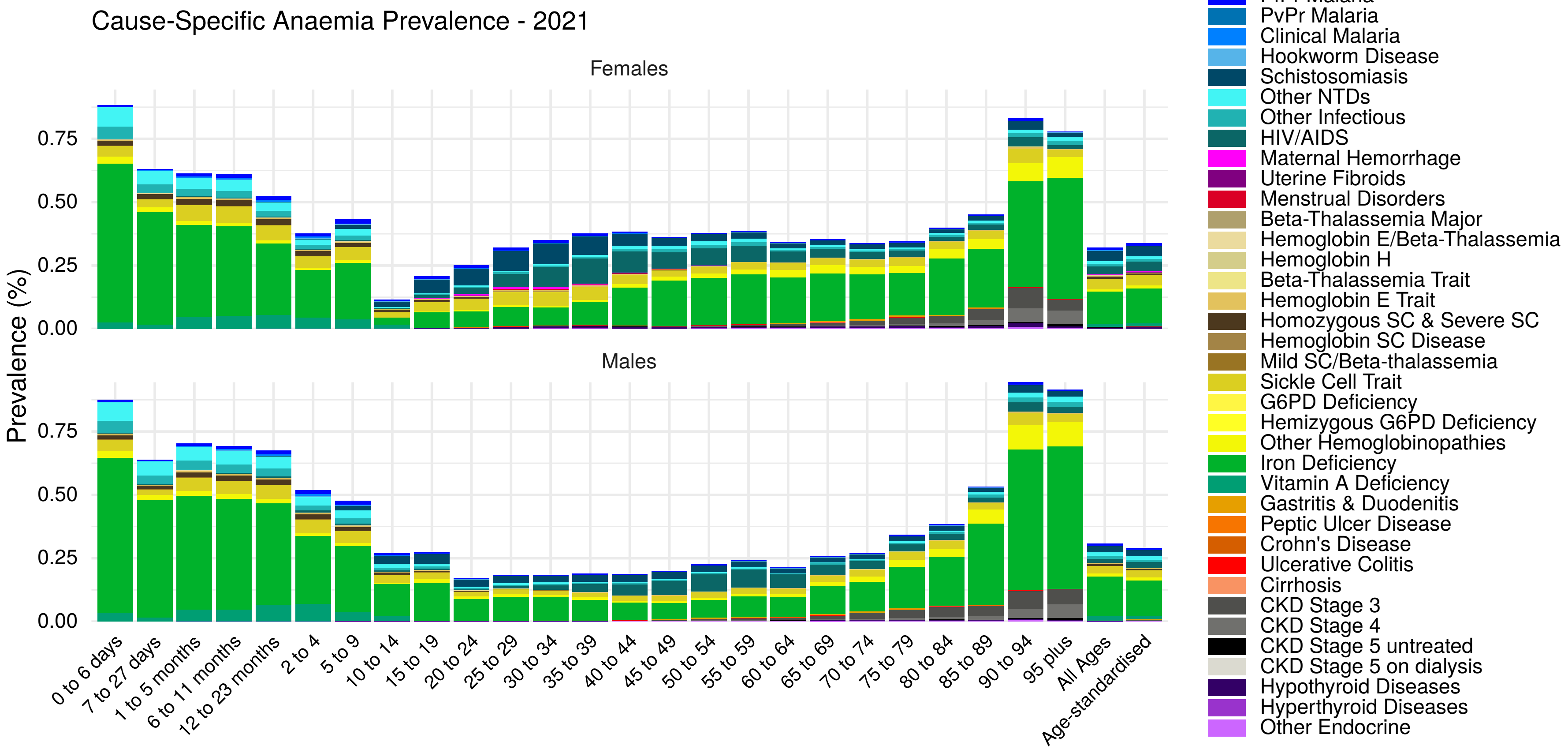
Cause-Specific Anaemia Prevalence - 2021
Females
Males
Prevalence (%)
PfPr Malaria
PvPr Malaria
Clinical Malaria
Hookworm Disease
Schistosomiasis
Other NTDs
Other Infectious
HIV/AIDS
Maternal Hemorrhage
Uterine Fibroids
Menstrual Disorders
Beta-Thalassemia Major
Hemoglobin E/Beta-Thalassemia
Hemoglobin H
Beta-Thalassemia Trait
Hemoglobin E Trait
Homozygous SC & Severe SC
Hemoglobin SC Disease
Mild SC/Beta-thalassemia
Sickle Cell Trait
G6PD Deficiency
Hemizygous G6PD Deficiency
Other Hemoglobinopathies
Iron Deficiency
Vitamin A Deficiency
Gastritis & Duodenitis
Peptic Ulcer Disease
Crohn's Disease
Ulcerative Colitis
Cirrhosis
CKD Stage 3
CKD Stage 4
CKD Stage 5 untreated
CKD Stage 5 on dialysis
Hypothyroid Diseases
Hyperthyroid Diseases
Other Endocrine

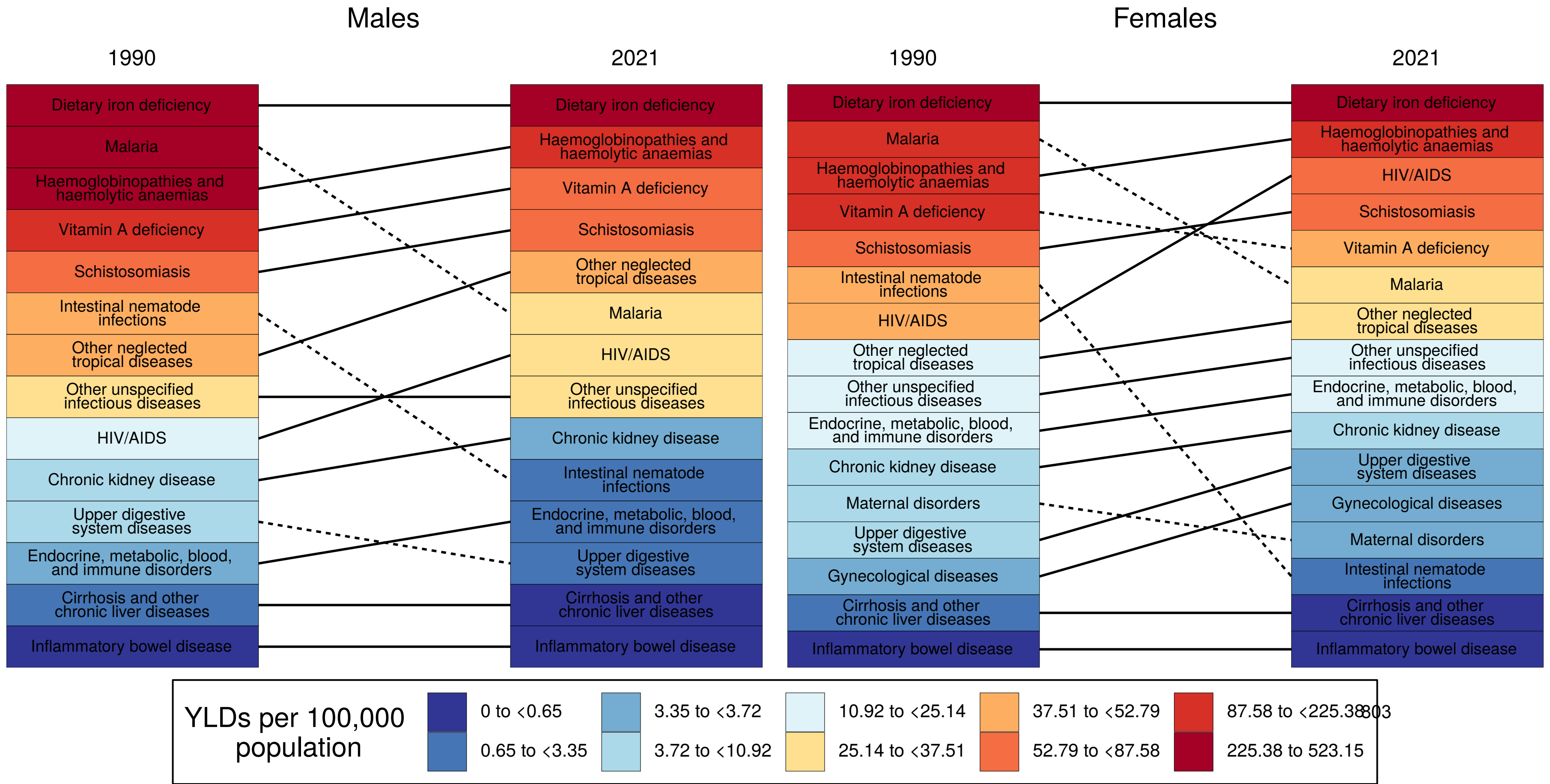
Males
Females
1990
2021
Dietary iron deficiency
Malaria
Haemoglobinopathies and haemolytic anaemias
Vitamin A deficiency
Schistosomiasis
Intestinal nematode infections
Other neglected tropical diseases
Other unspecified infectious diseases
HIV/AIDS
Chronic kidney disease
Upper digestive system diseases
Endocrine, metabolic, blood, and immune disorders
Cirrhosis and other chronic liver diseases
Inflammatory bowel disease
Maternal disorders
Gynecological diseases
YLDs per 100,000 population
0 to <0.65
0.65 to <3.35
3.35 to <3.72
3.72 to <10.92
10.92 to <25.14
25.14 to <37.51
37.51 to <52.79
52.79 to <87.58
87.58 to <225.38
225.38 to 523.15

803

Isiolo

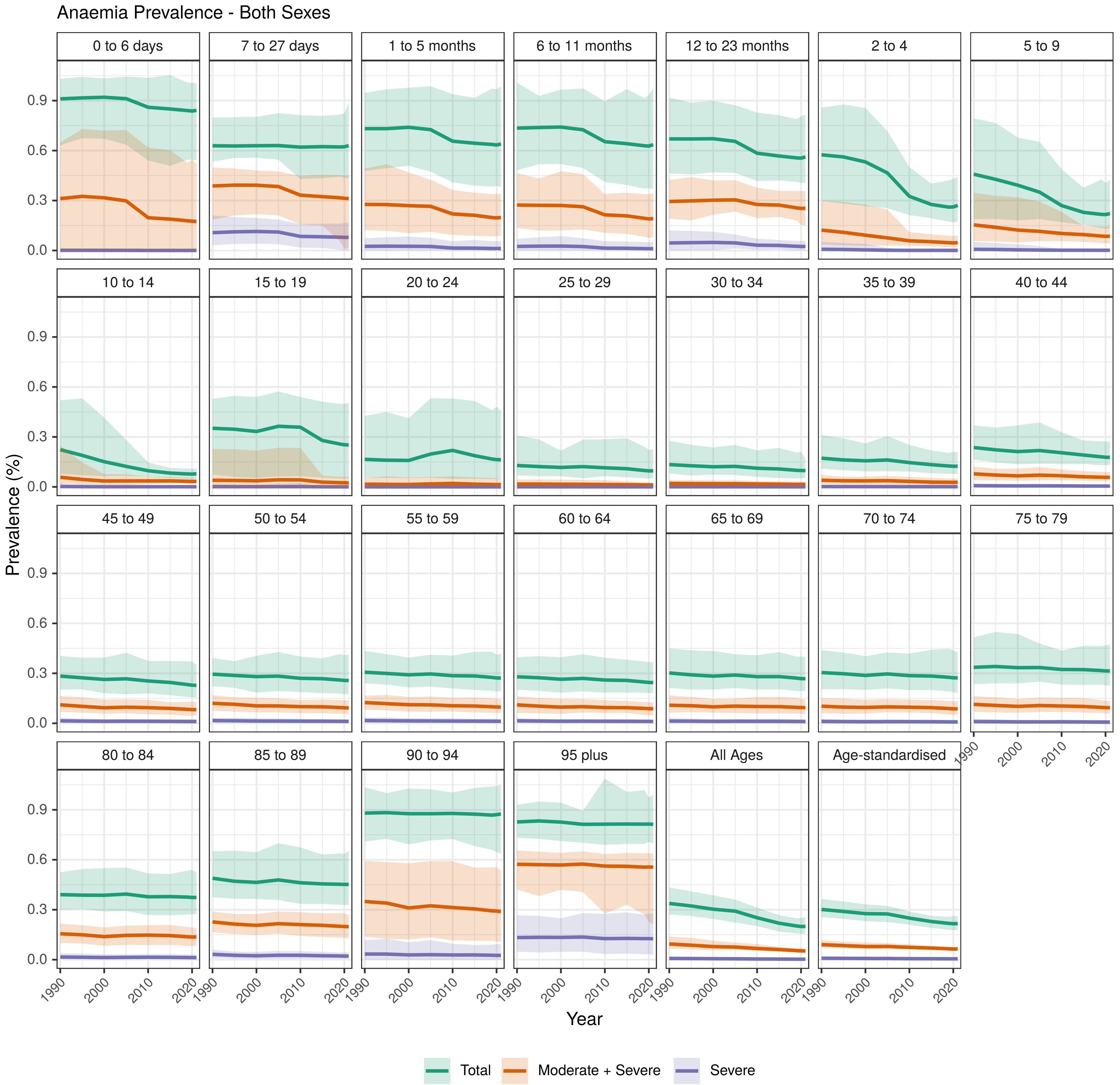

Anaemia Prevalence - Both Sexes
0 to 6 days
7 to 27 days
1 to 5 months
6 to 11 months
12 to 23 months
2 to 4
5 to 9
10 to 14
15 to 19
20 to 24
25 to 29
30 to 34
35 to 39
40 to 44
45 to 49
50 to 54
55 to 59
60 to 64
65 to 69
70 to 74
75 to 79
80 to 84
85 to 89
90 to 94
95 plus
All Ages
Age-standardised
Prevalence (%)
0.0
0.3
0.6
0.9
1990
2000
2010
2020
Year
Total
Moderate + Severe
Severe

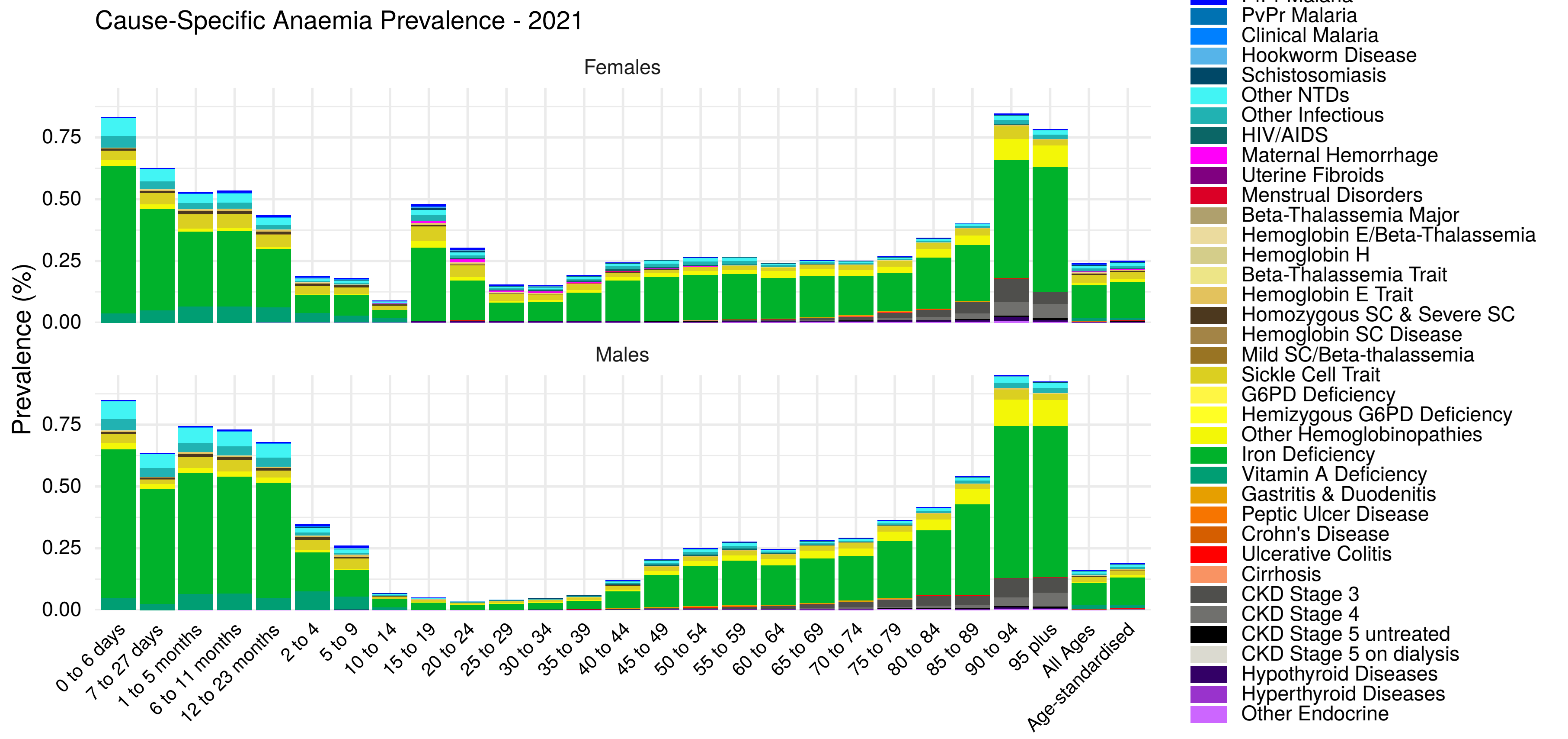

Cause-Specific Anaemia Prevalence - 2021
Females
Males
Prevalence (%)
0.00
0.25
0.50
0.75
0 to 6 days
7 to 27 days
1 to 5 months
6 to 11 months
12 to 23 months
2 to 4
5 to 9
10 to 14
15 to 19
20 to 24
25 to 29
30 to 34
35 to 39
40 to 44
45 to 49
50 to 54
55 to 59
60 to 64
65 to 69
70 to 74
75 to 79
80 to 84
85 to 89
90 to 94
95 plus
All Ages
Age-standardised
PfPr Malaria
PvPr Malaria
Clinical Malaria
Hookworm Disease
Schistosomiasis
Other NTDs
Other Infectious
HIV/AIDS
Maternal Hemorrhage
Uterine Fibroids
Menstrual Disorders
Beta-Thalassemia Major
Hemoglobin E/Beta-Thalassemia
Hemoglobin H
Beta-Thalassemia Trait
Hemoglobin E Trait
Homozygous SC & Severe SC
Hemoglobin SC Disease
Mild SC/Beta-thalassemia
Sickle Cell Trait
G6PD Deficiency
Hemizygous G6PD Deficiency
Other Hemoglobinopathies
Iron Deficiency
Vitamin A Deficiency
Gastritis & Duodenitis
Peptic Ulcer Disease
Crohn's Disease
Ulcerative Colitis
Cirrhosis
CKD Stage 3
CKD Stage 4
CKD Stage 5 untreated
CKD Stage 5 on dialysis
Hypothyroid Diseases
Hyperthyroid Diseases
Other Endocrine

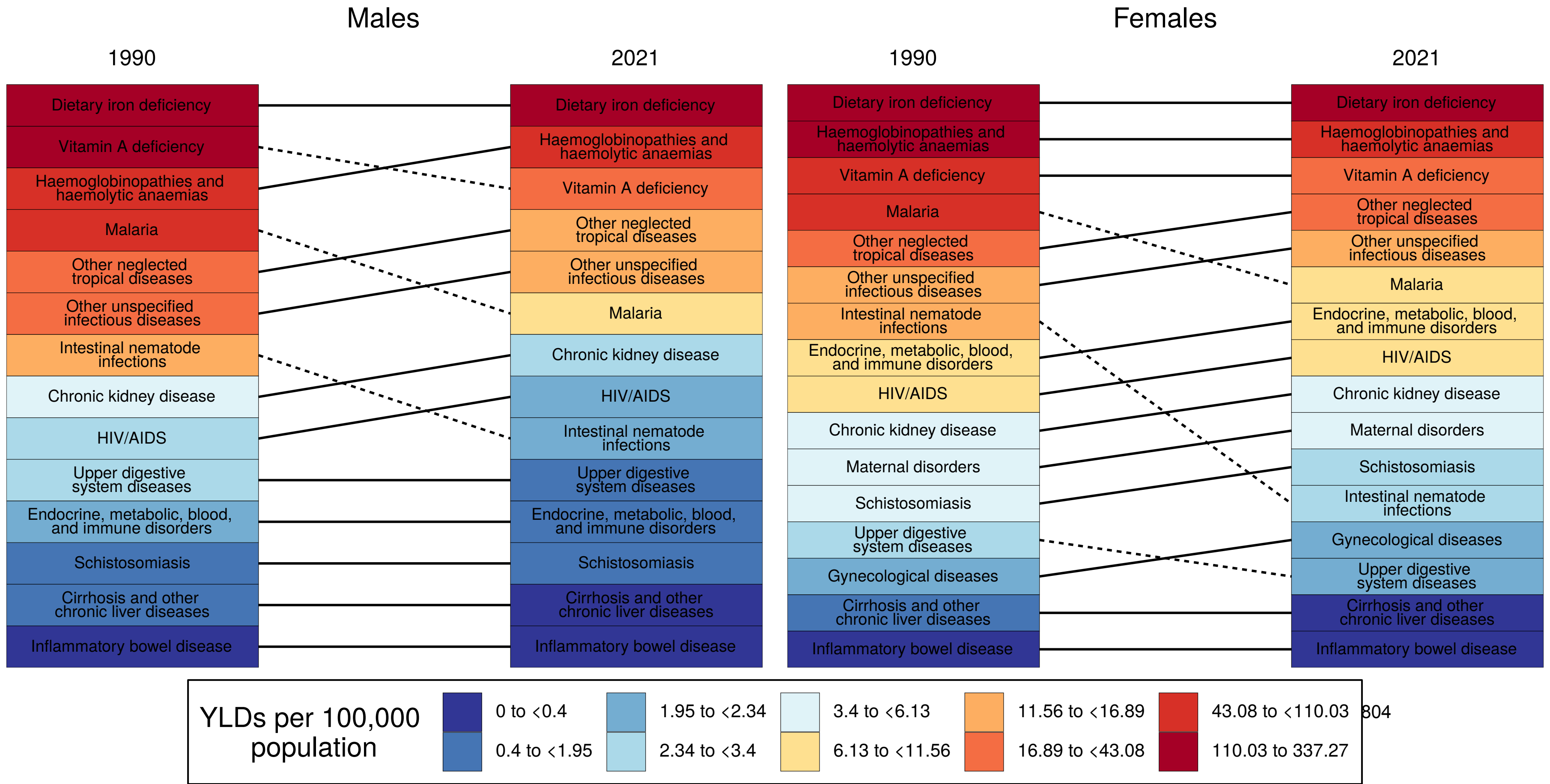

Males
Females
1990
2021
Dietary iron deficiency
Vitamin A deficiency
Haemoglobinopathies and haemolytic anaemias
Malaria
Other neglected tropical diseases
Other unspecified infectious diseases
Intestinal nematode infections
Chronic kidney disease
HIV/AIDS
Upper digestive system diseases
Endocrine, metabolic, blood, and immune disorders
Schistosomiasis
Cirrhosis and other chronic liver diseases
Inflammatory bowel disease
Maternal disorders
Gynecological diseases
YLDs per 100,000 population
0 to <0.4
0.4 to <1.95
1.95 to <2.34
2.34 to <3.4
3.4 to <6.13
6.13 to <11.56
11.56 to <16.89
16.89 to <43.08
43.08 to <110.03
110.03 to 337.27

Kajiado

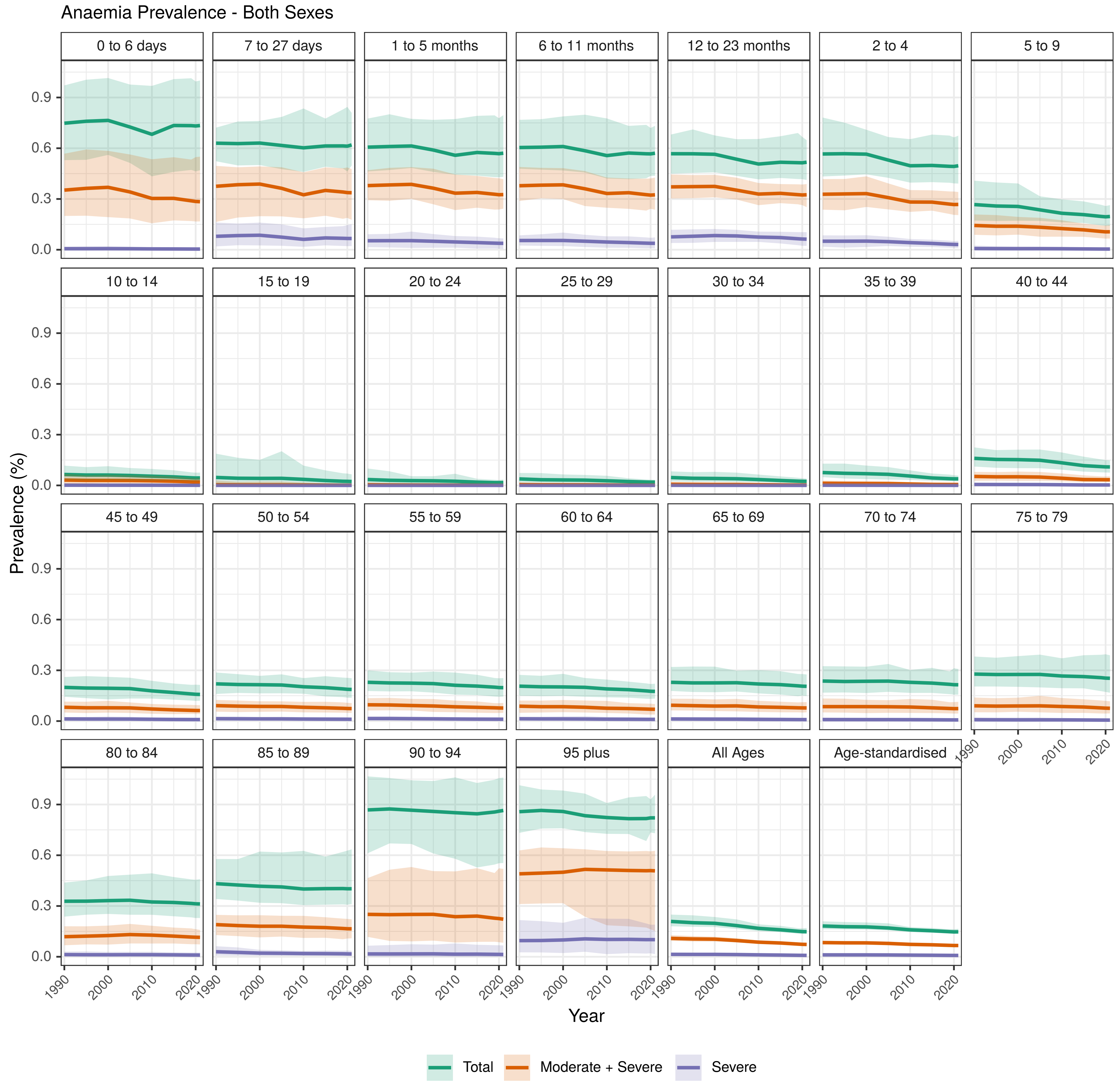
Anaemia Prevalence - Both Sexes
0 to 6 days
7 to 27 days
1 to 5 months
6 to 11 months
12 to 23 months
2 to 4
5 to 9
10 to 14
15 to 19
20 to 24
25 to 29
30 to 34
35 to 39
40 to 44
45 to 49
50 to 54
55 to 59
60 to 64
65 to 69
70 to 74
75 to 79
80 to 84
85 to 89
90 to 94
95 plus
All Ages
Age-standardised
Prevalence (%)
0.0
0.3
0.6
0.9
1990
2000
2010
2020
Year
Total
Moderate + Severe
Severe

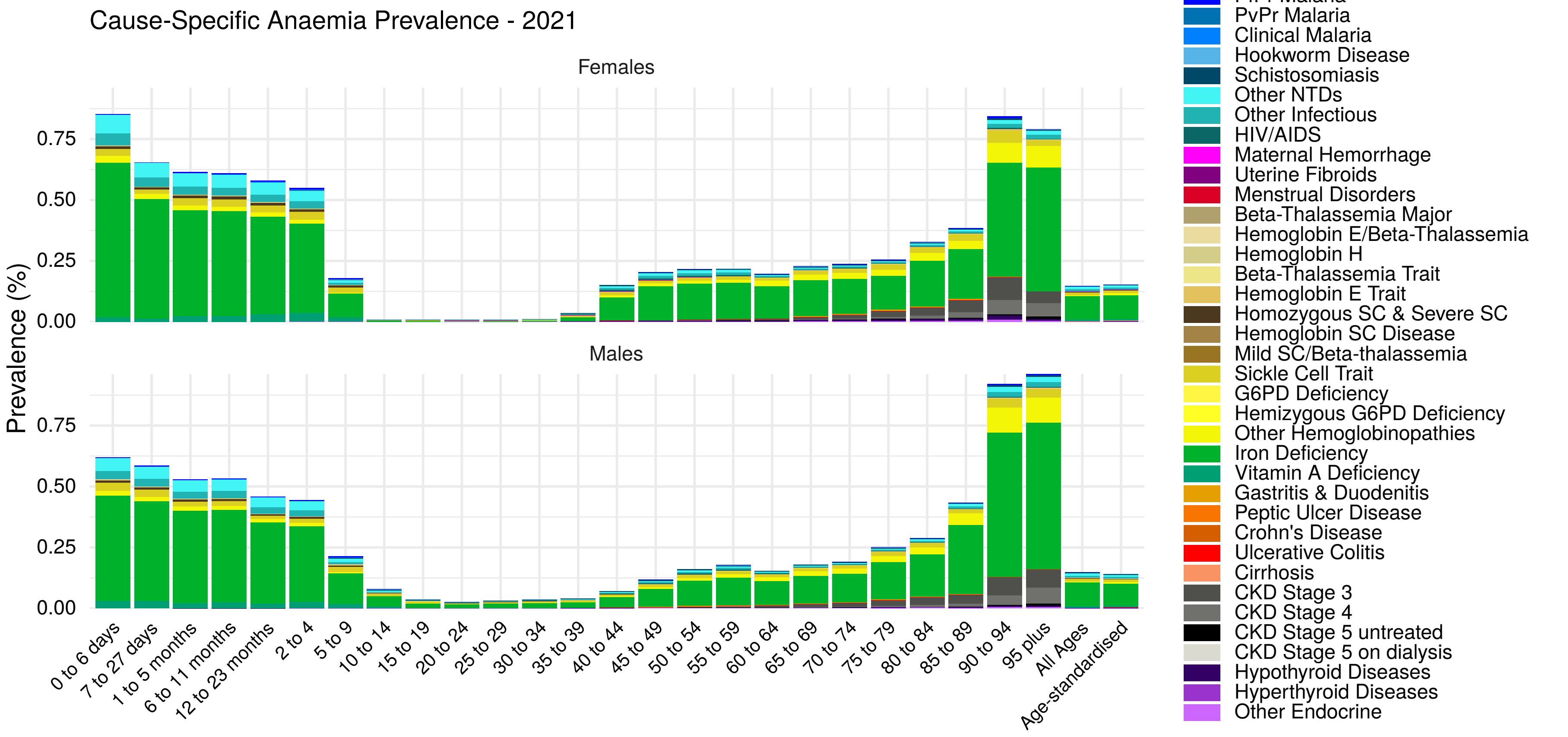
Cause-Specific Anaemia Prevalence - 2021
Females
Males
Prevalence (%)
0.00
0.25
0.50
0.75
0 to 6 days
7 to 27 days
1 to 5 months
6 to 11 months
12 to 23 months
2 to 4
5 to 9
10 to 14
15 to 19
20 to 24
25 to 29
30 to 34
35 to 39
40 to 44
45 to 49
50 to 54
55 to 59
60 to 64
65 to 69
70 to 74
75 to 79
80 to 84
85 to 89
90 to 94
95 plus
All Ages
Age-standardised
PfPr Malaria
PvPr Malaria
Clinical Malaria
Hookworm Disease
Schistosomiasis
Other NTDs
Other Infectious
HIV/AIDS
Maternal Hemorrhage
Uterine Fibroids
Menstrual Disorders
Beta-Thalassemia Major
Hemoglobin E/Beta-Thalassemia
Hemoglobin H
Beta-Thalassemia Trait
Hemoglobin E Trait
Homozygous SC & Severe SC
Hemoglobin SC Disease
Mild SC/Beta-thalassemia
Sickle Cell Trait
G6PD Deficiency
Hemizygous G6PD Deficiency
Other Hemoglobinopathies
Iron Deficiency
Vitamin A Deficiency
Gastritis & Duodenitis
Peptic Ulcer Disease
Crohn's Disease
Ulcerative Colitis
Cirrhosis
CKD Stage 3
CKD Stage 4
CKD Stage 5 untreated
CKD Stage 5 on dialysis
Hypothyroid Diseases
Hyperthyroid Diseases
Other Endocrine

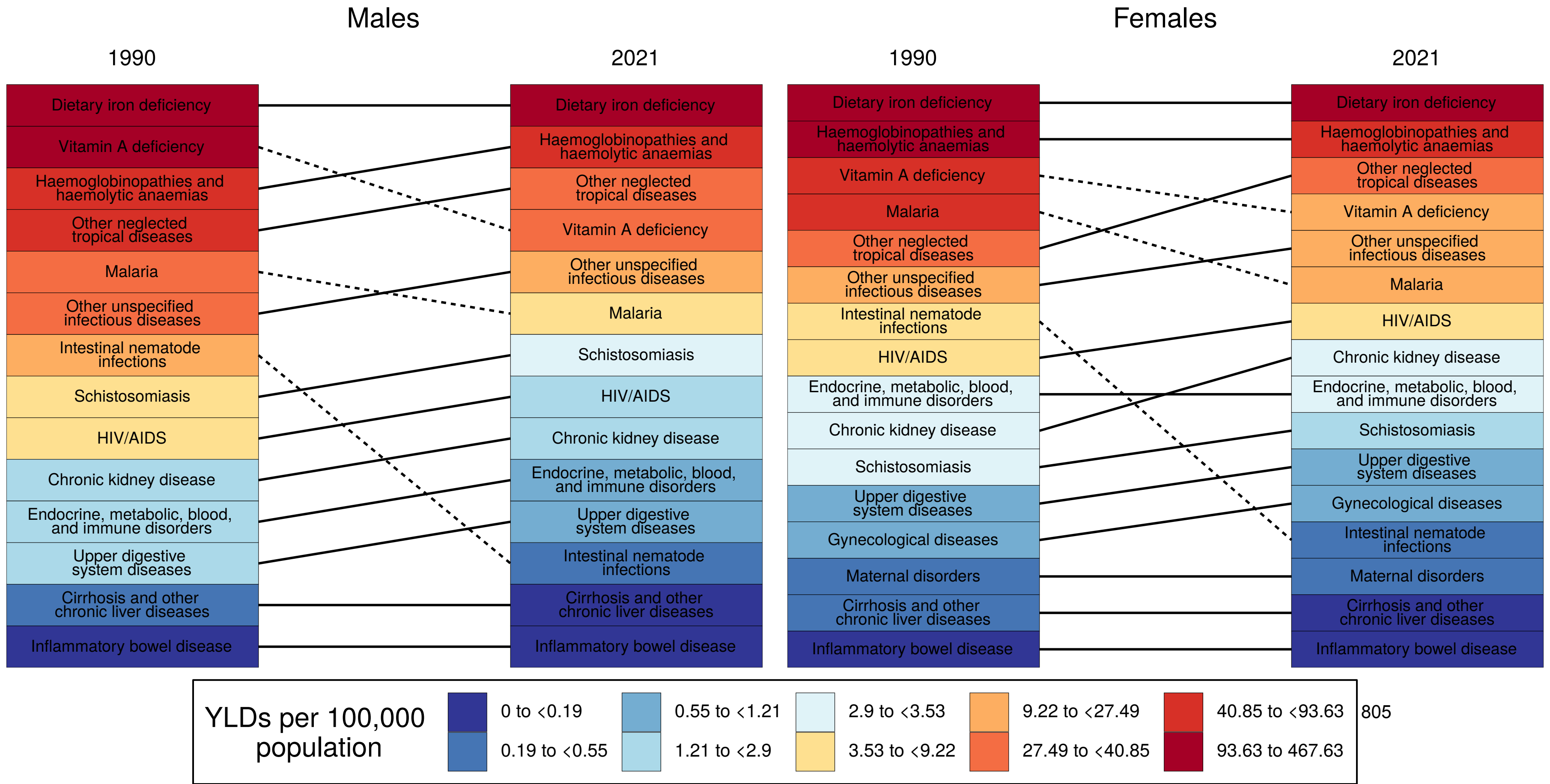
Males
Females
1990
2021
Dietary iron deficiency
Vitamin A deficiency
Haemoglobinopathies and haemolytic anaemias
Other neglected tropical diseases
Malaria
Other unspecified infectious diseases
Intestinal nematode infections
Schistosomiasis
HIV/AIDS
Chronic kidney disease
Endocrine, metabolic, blood, and immune disorders
Upper digestive system diseases
Cirrhosis and other chronic liver diseases
Inflammatory bowel disease
Gynecological diseases
Maternal disorders
YLDs per 100,000 population
0 to <0.19
0.19 to <0.55
0.55 to <1.21
1.21 to <2.9
2.9 to <3.53
3.53 to <9.22
9.22 to <27.49
27.49 to <40.85
40.85 to <93.63
93.63 to 467.63

Kakamega

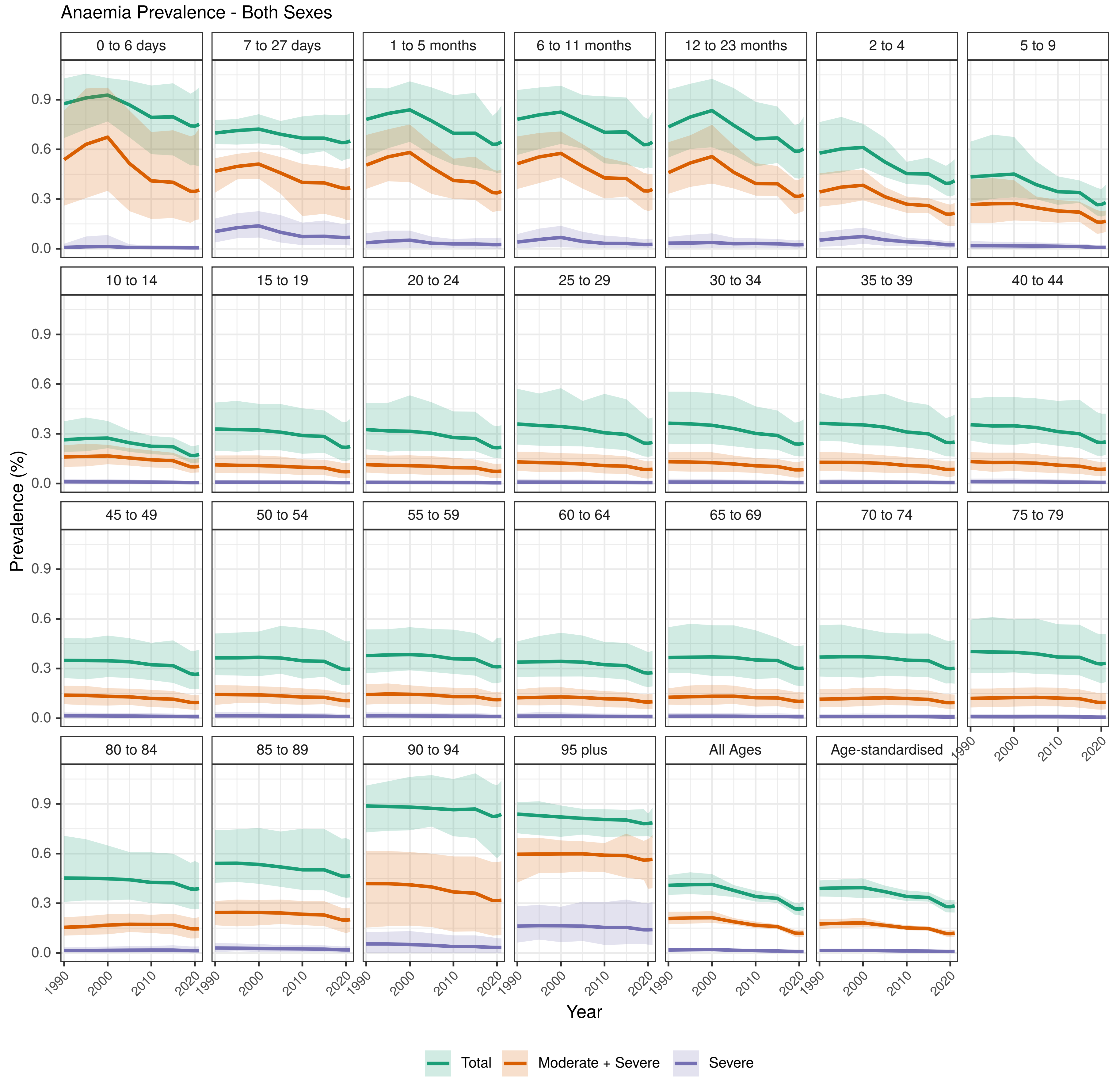
Anaemia Prevalence - Both Sexes
0 to 6 days
7 to 27 days
1 to 5 months
6 to 11 months
12 to 23 months
2 to 4
5 to 9
10 to 14
15 to 19
20 to 24
25 to 29
30 to 34
35 to 39
40 to 44
45 to 49
50 to 54
55 to 59
60 to 64
65 to 69
70 to 74
75 to 79
80 to 84
85 to 89
90 to 94
95 plus
All Ages
Age-standardised
Prevalence (%)
Year
Total
Moderate + Severe
Severe

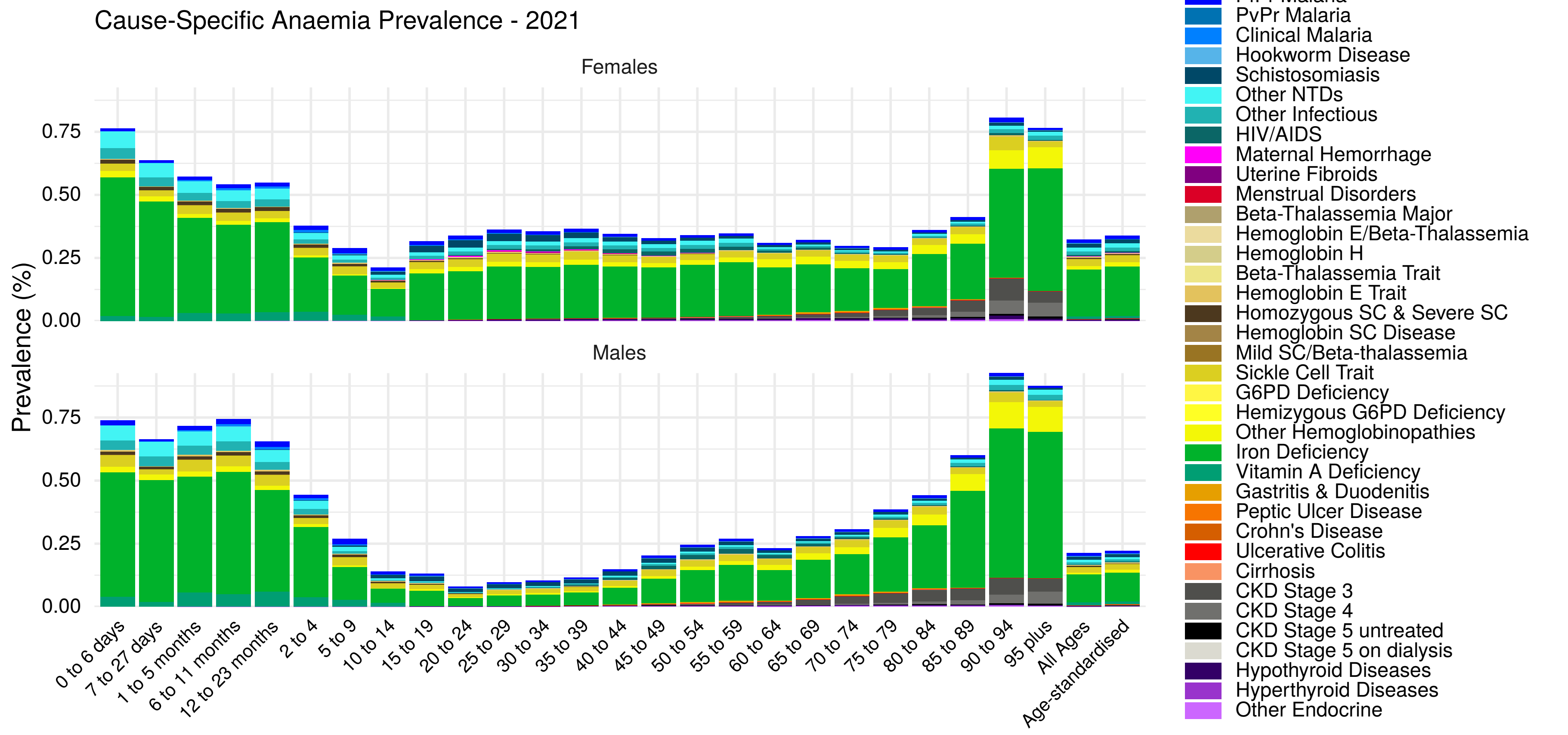
Cause-Specific Anaemia Prevalence - 2021
Females
Males
Prevalence (%)
PfPr Malaria
PvPr Malaria
Clinical Malaria
Hookworm Disease
Schistosomiasis
Other NTDs
Other Infectious
HIV/AIDS
Maternal Hemorrhage
Uterine Fibroids
Menstrual Disorders
Beta-Thalassemia Major
Hemoglobin E/Beta-Thalassemia
Hemoglobin H
Beta-Thalassemia Trait
Hemoglobin E Trait
Homozygous SC & Severe SC
Hemoglobin SC Disease
Mild SC/Beta-thalassemia
Sickle Cell Trait
G6PD Deficiency
Hemizygous G6PD Deficiency
Other Hemoglobinopathies
Iron Deficiency
Vitamin A Deficiency
Gastritis & Duodenitis
Peptic Ulcer Disease
Crohn's Disease
Ulcerative Colitis
Cirrhosis
CKD Stage 3
CKD Stage 4
CKD Stage 5 untreated
CKD Stage 5 on dialysis
Hypothyroid Diseases
Hyperthyroid Diseases
Other Endocrine

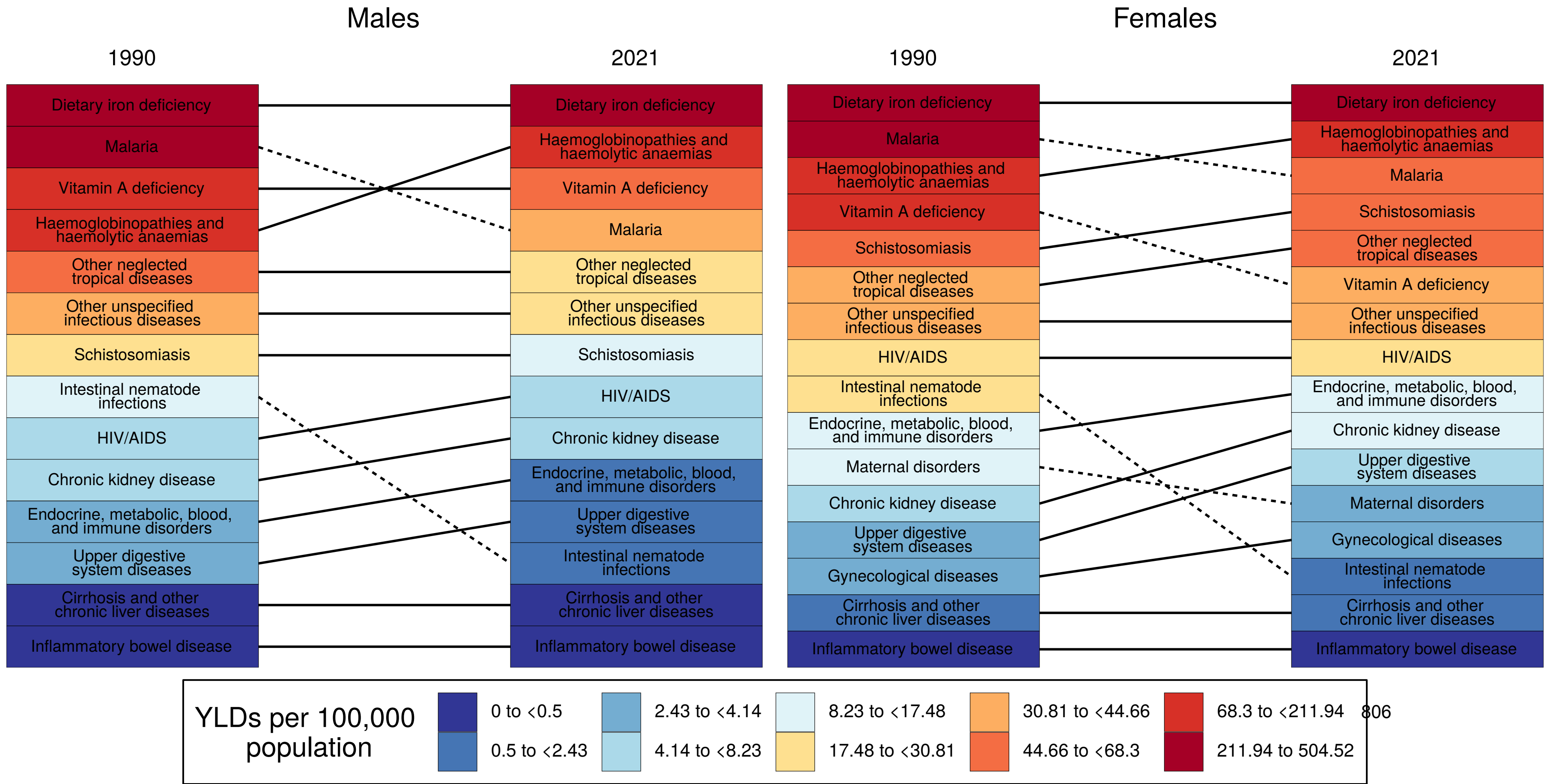
Males
Females
1990
2021
Dietary iron deficiency
Malaria
Vitamin A deficiency
Haemoglobinopathies and haemolytic anaemias
Other neglected tropical diseases
Other unspecified infectious diseases
Schistosomiasis
Intestinal nematode infections
HIV/AIDS
Chronic kidney disease
Endocrine, metabolic, blood, and immune disorders
Upper digestive system diseases
Cirrhosis and other chronic liver diseases
Inflammatory bowel disease
Maternal disorders
Gynecological diseases
YLDs per 100,000 population
0 to <0.5
0.5 to <2.43
2.43 to <4.14
4.14 to <8.23
8.23 to <17.48
17.48 to <30.81
30.81 to <44.66
44.66 to <68.3
68.3 to <211.94
211.94 to 504.52

Kericho

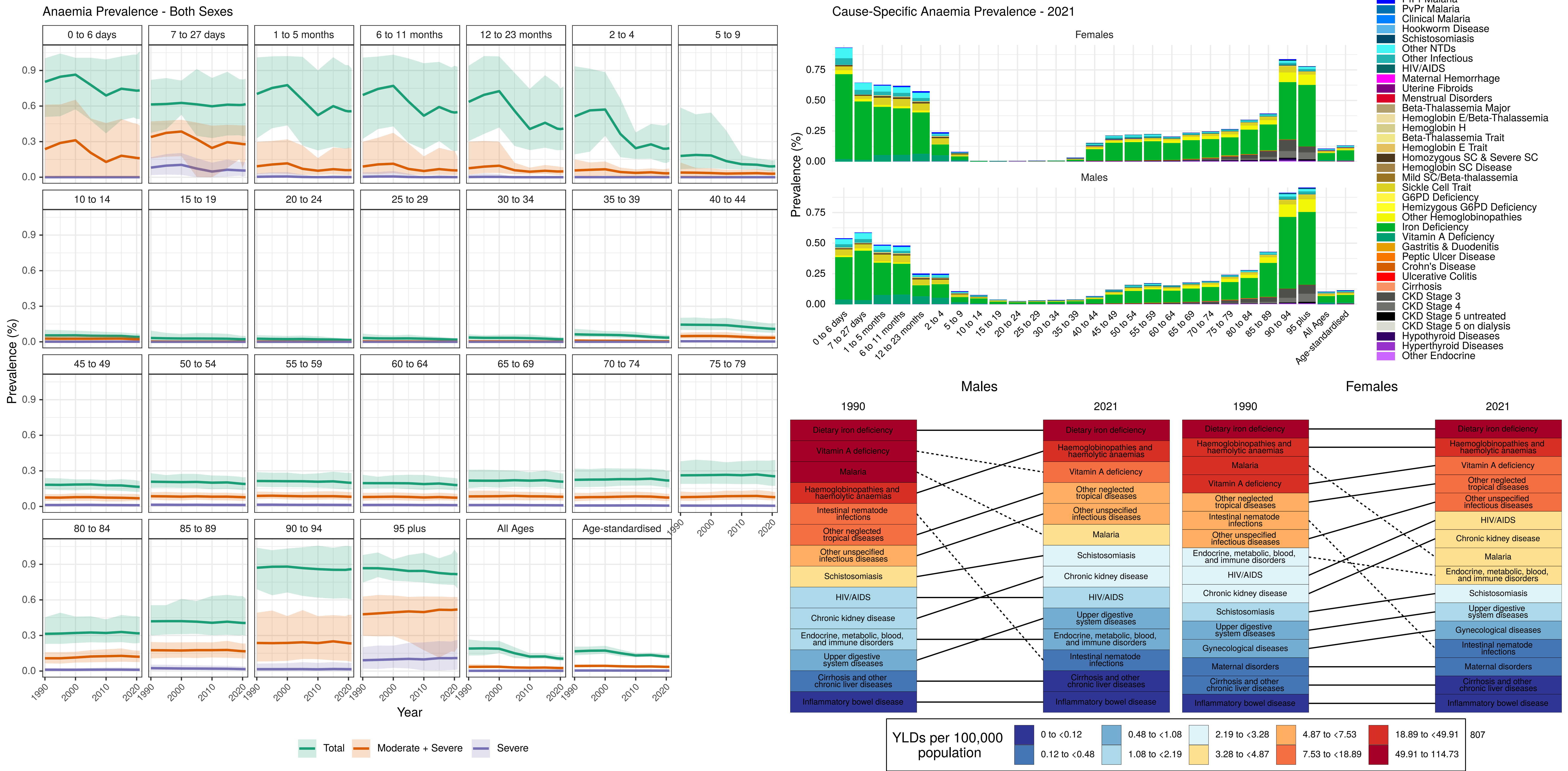

Anaemia Prevalence - Both Sexes
0 to 6 days
7 to 27 days
1 to 5 months
6 to 11 months
12 to 23 months
2 to 4
5 to 9
10 to 14
15 to 19
20 to 24
25 to 29
30 to 34
35 to 39
40 to 44
45 to 49
50 to 54
55 to 59
60 to 64
65 to 69
70 to 74
75 to 79
80 to 84
85 to 89
90 to 94
95 plus
All Ages
Age-standardised
Prevalence (%)
Year
Total
Moderate + Severe
Severe
Cause-Specific Anaemia Prevalence - 2021
Females
Males
Prevalence (%)
PfPr Malaria
PvPr Malaria
Clinical Malaria
Hookworm Disease
Schistosomiasis
Other NTDs
Other Infectious
HIV/AIDS
Maternal Hemorrhage
Uterine Fibroids
Menstrual Disorders
Beta-Thalassemia Major
Hemoglobin E/Beta-Thalassemia
Hemoglobin H
Beta-Thalassemia Trait
Hemoglobin E Trait
Homozygous SC & Severe SC
Hemoglobin SC Disease
Mild SC/Beta-thalassemia
Sickle Cell Trait
G6PD Deficiency
Hemizygous G6PD Deficiency
Other Hemoglobinopathies
Iron Deficiency
Vitamin A Deficiency
Gastritis & Duodenitis
Peptic Ulcer Disease
Crohn's Disease
Ulcerative Colitis
Cirrhosis
CKD Stage 3
CKD Stage 4
CKD Stage 5 untreated
CKD Stage 5 on dialysis
Hypothyroid Diseases
Hyperthyroid Diseases
Other Endocrine
Males
Females
1990
2021
Dietary iron deficiency
Vitamin A deficiency
Malaria
Haemoglobinopathies and haemolytic anaemias
Intestinal nematode infections
Other neglected tropical diseases
Other unspecified infectious diseases
Schistosomiasis
HIV/AIDS
Chronic kidney disease
Endocrine, metabolic, blood, and immune disorders
Upper digestive system diseases
Cirrhosis and other chronic liver diseases
Inflammatory bowel disease
Gynecological diseases
Maternal disorders
YLDs per 100,000 population
0 to <0.12
0.12 to <0.48
0.48 to <1.08
1.08 to <2.19
2.19 to <3.28
3.28 to <4.87
4.87 to <7.53
7.53 to <18.89
18.89 to <49.91
49.91 to 114.73

Kiambu

Anaemia Prevalence - Both Sexes

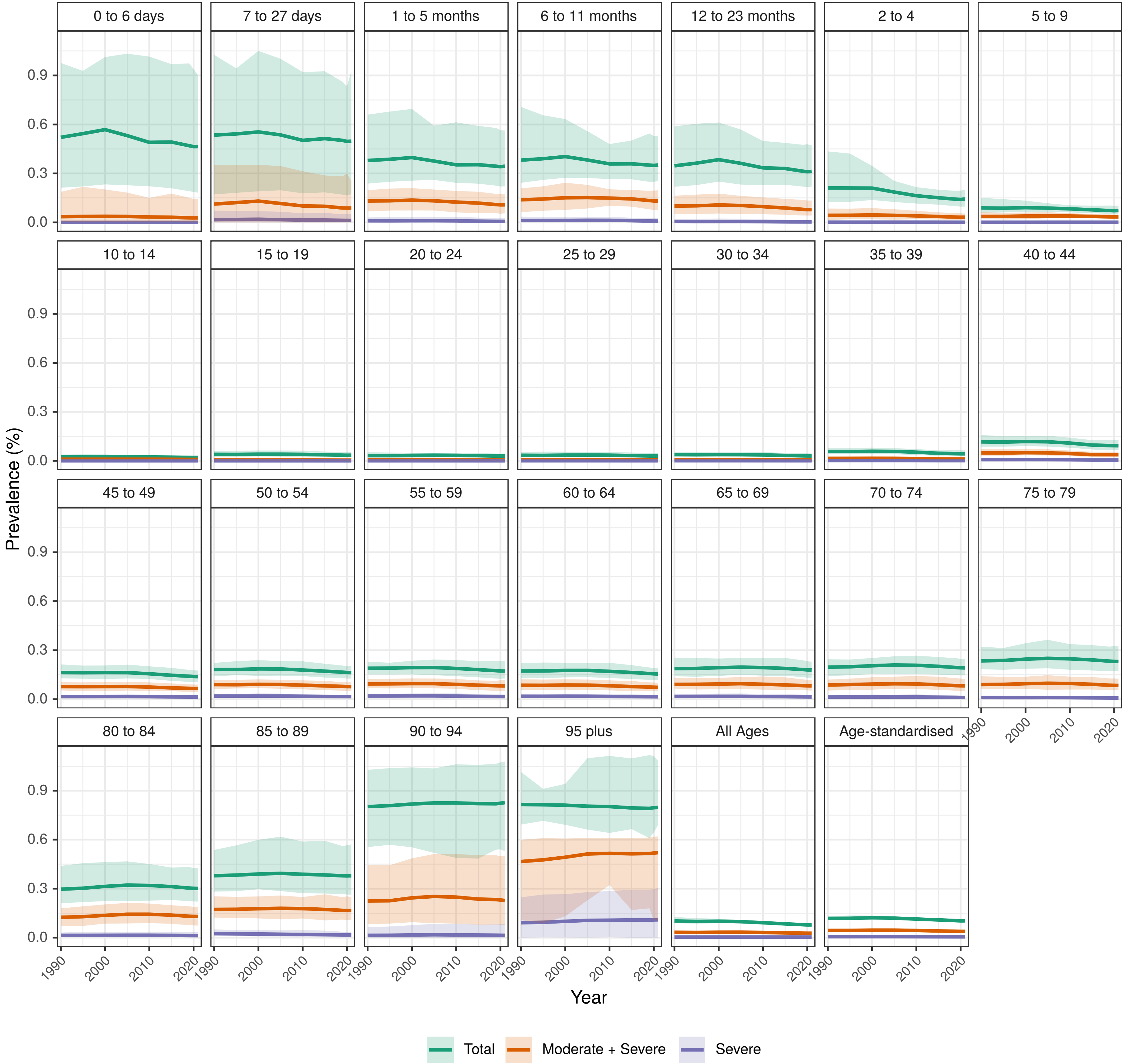

Cause-Specific Anaemia Prevalence - 2021

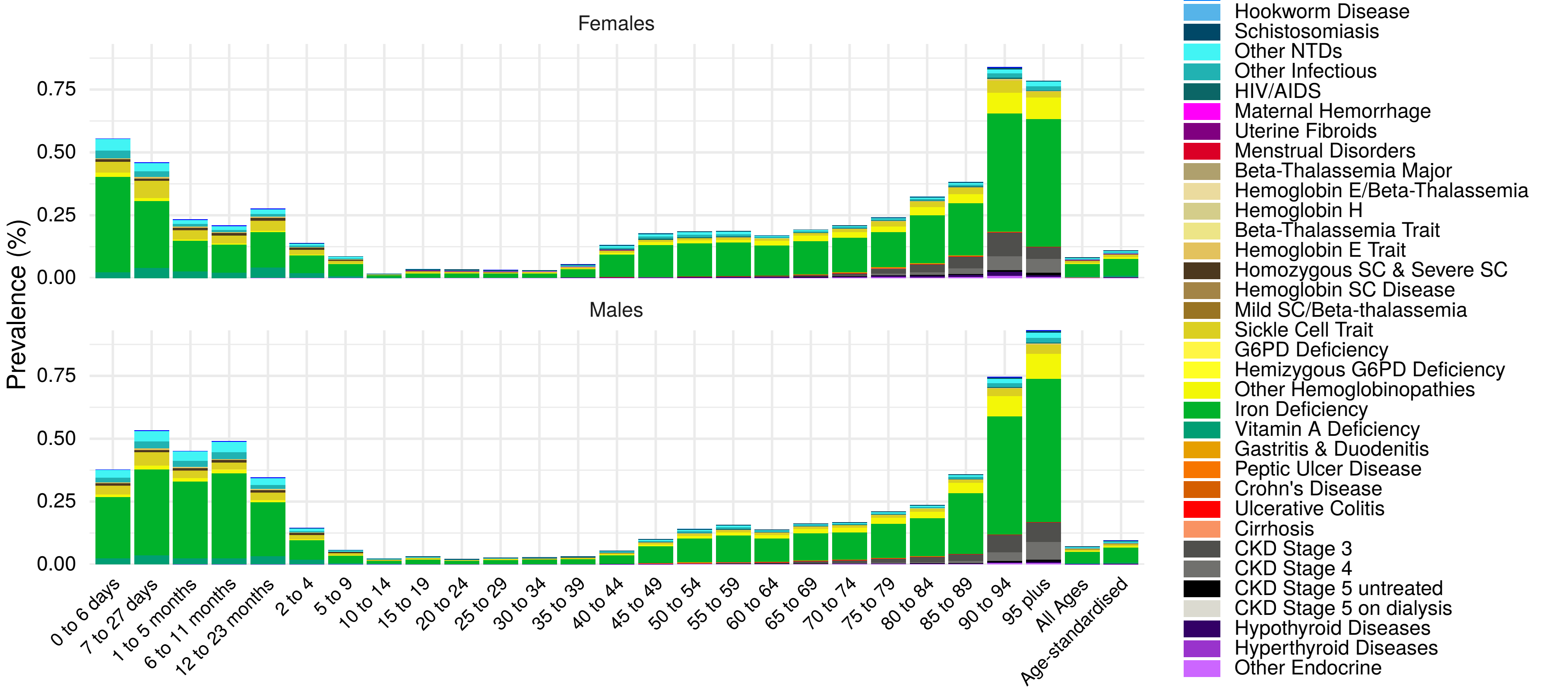

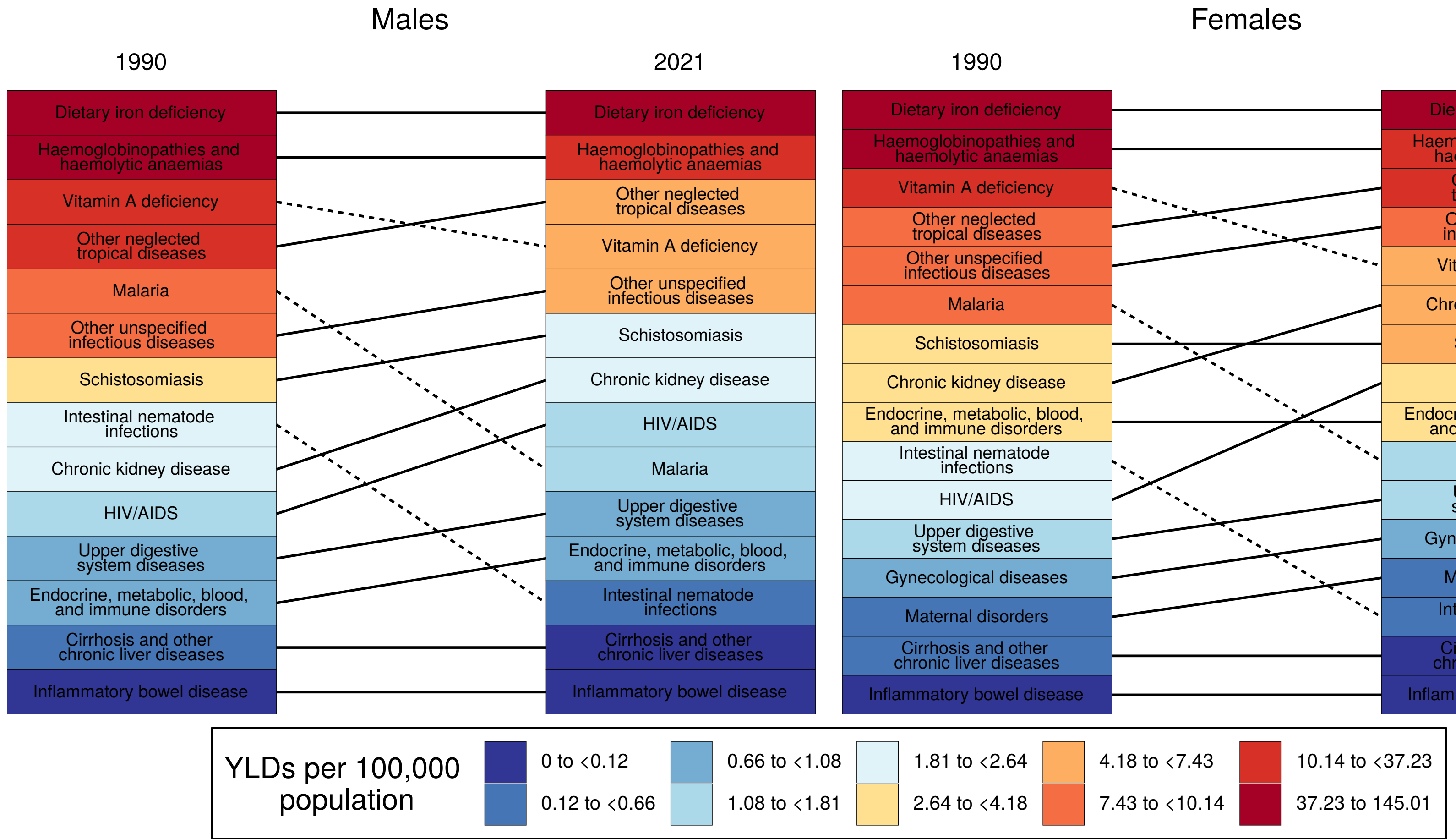

Kilifi

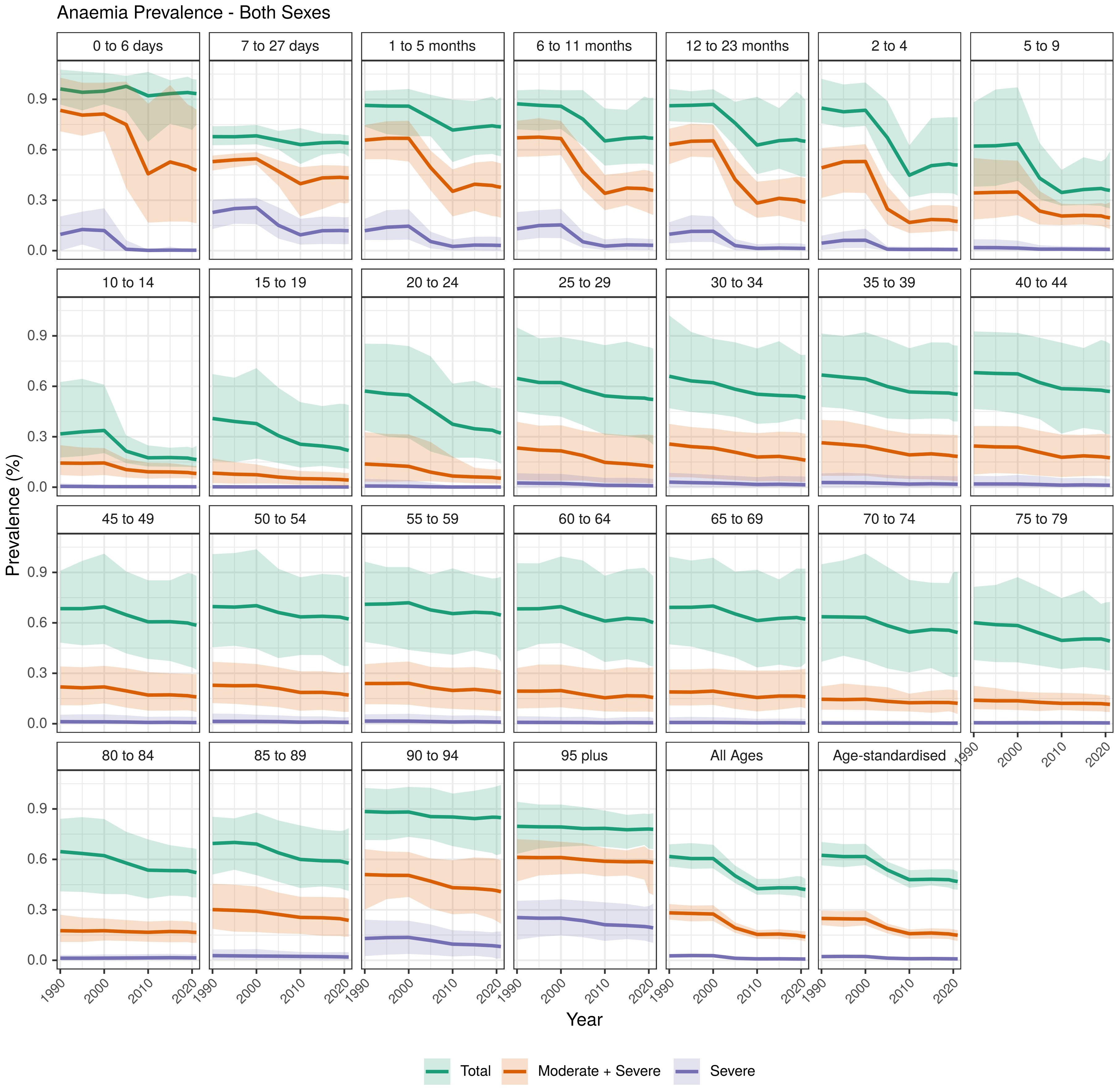
Anaemia Prevalence - Both Sexes
0 to 6 days
7 to 27 days
1 to 5 months
6 to 11 months
12 to 23 months
2 to 4
5 to 9
10 to 14
15 to 19
20 to 24
25 to 29
30 to 34
35 to 39
40 to 44
45 to 49
50 to 54
55 to 59
60 to 64
65 to 69
70 to 74
75 to 79
80 to 84
85 to 89
90 to 94
95 plus
All Ages
Age-standardised
Prevalence (%)
Year
Total
Moderate + Severe
Severe

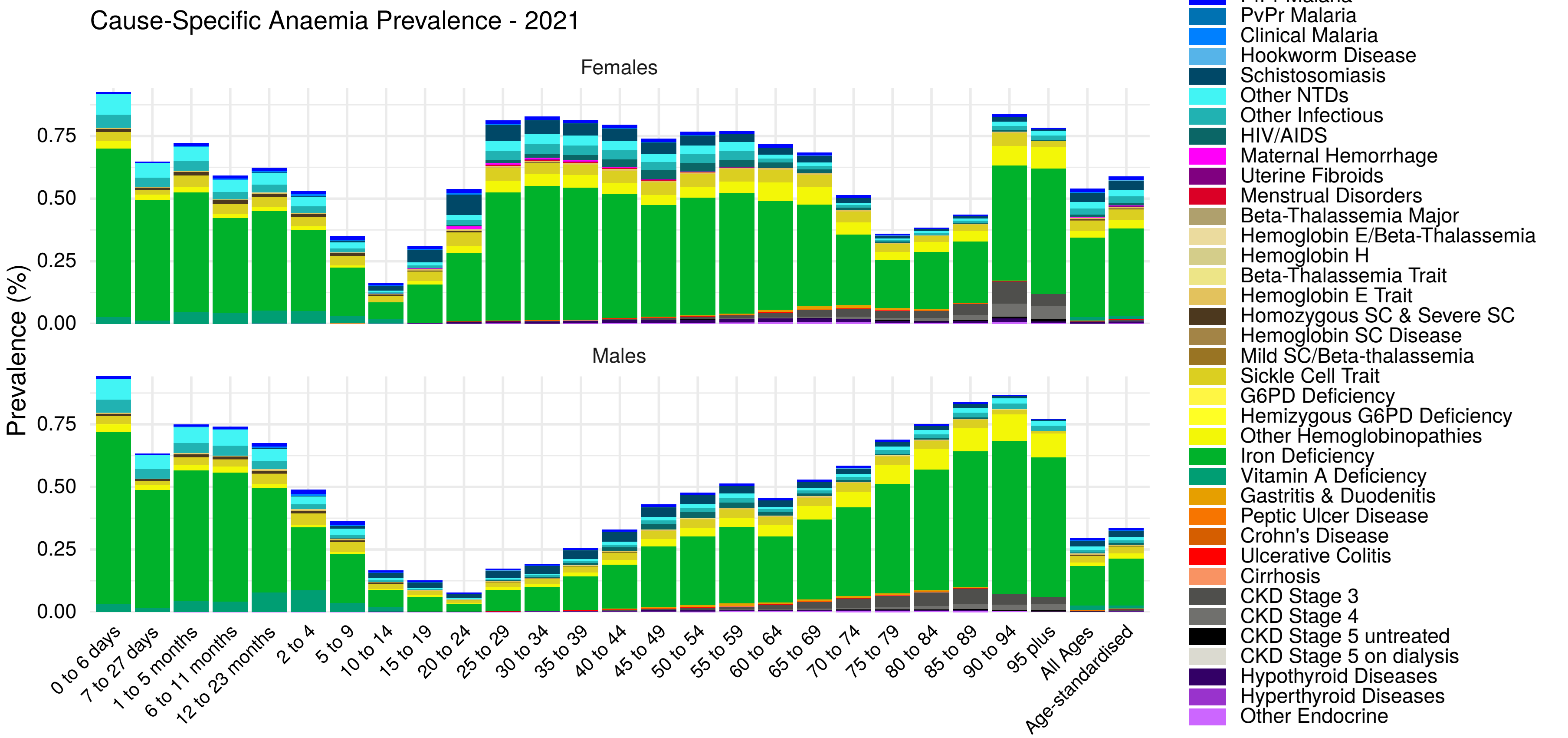
Cause-Specific Anaemia Prevalence - 2021
Females
Males
Prevalence (%)
PfPr Malaria
PvPr Malaria
Clinical Malaria
Hookworm Disease
Schistosomiasis
Other NTDs
Other Infectious
HIV/AIDS
Maternal Hemorrhage
Uterine Fibroids
Menstrual Disorders
Beta-Thalassemia Major
Hemoglobin E/Beta-Thalassemia
Hemoglobin H
Beta-Thalassemia Trait
Hemoglobin E Trait
Homozygous SC & Severe SC
Hemoglobin SC Disease
Mild SC/Beta-thalassemia
Sickle Cell Trait
G6PD Deficiency
Hemizygous G6PD Deficiency
Other Hemoglobinopathies
Iron Deficiency
Vitamin A Deficiency
Gastritis & Duodenitis
Peptic Ulcer Disease
Crohn's Disease
Ulcerative Colitis
Cirrhosis
CKD Stage 3
CKD Stage 4
CKD Stage 5 untreated
CKD Stage 5 on dialysis
Hypothyroid Diseases
Hyperthyroid Diseases
Other Endocrine

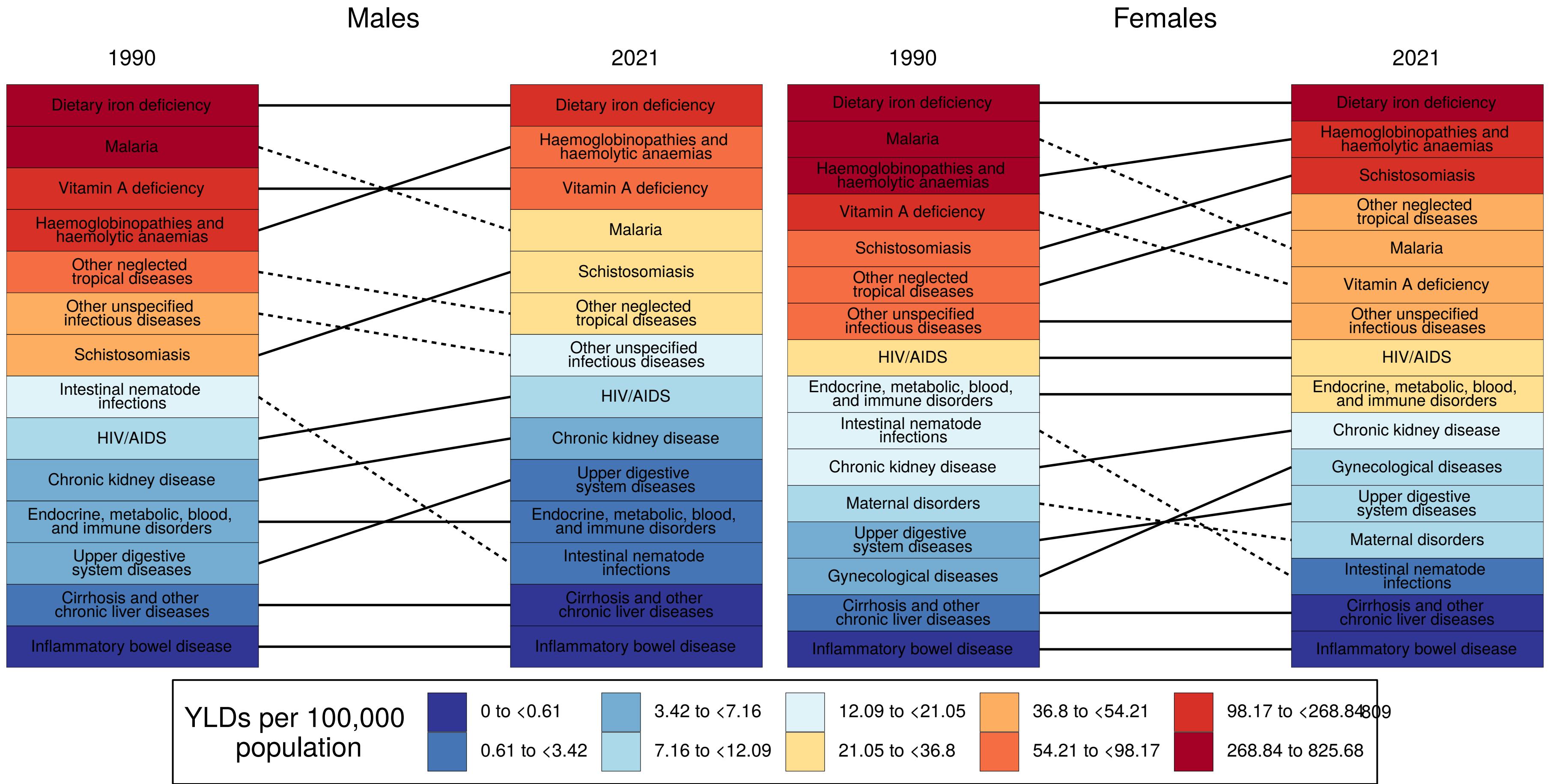
Males
Females
1990
2021
Dietary iron deficiency
Malaria
Vitamin A deficiency
Haemoglobinopathies and haemolytic anaemias
Other neglected tropical diseases
Other unspecified infectious diseases
Schistosomiasis
Intestinal nematode infections
HIV/AIDS
Chronic kidney disease
Endocrine, metabolic, blood, and immune disorders
Upper digestive system diseases
Cirrhosis and other chronic liver diseases
Inflammatory bowel disease
Maternal disorders
Gynecological diseases
YLDs per 100,000 population
0 to <0.61
0.61 to <3.42
3.42 to <7.16
7.16 to <12.09
12.09 to <21.05
21.05 to <36.8
36.8 to <54.21
54.21 to <98.17
98.17 to <268.84
268.84 to 825.68

Kirinyaga

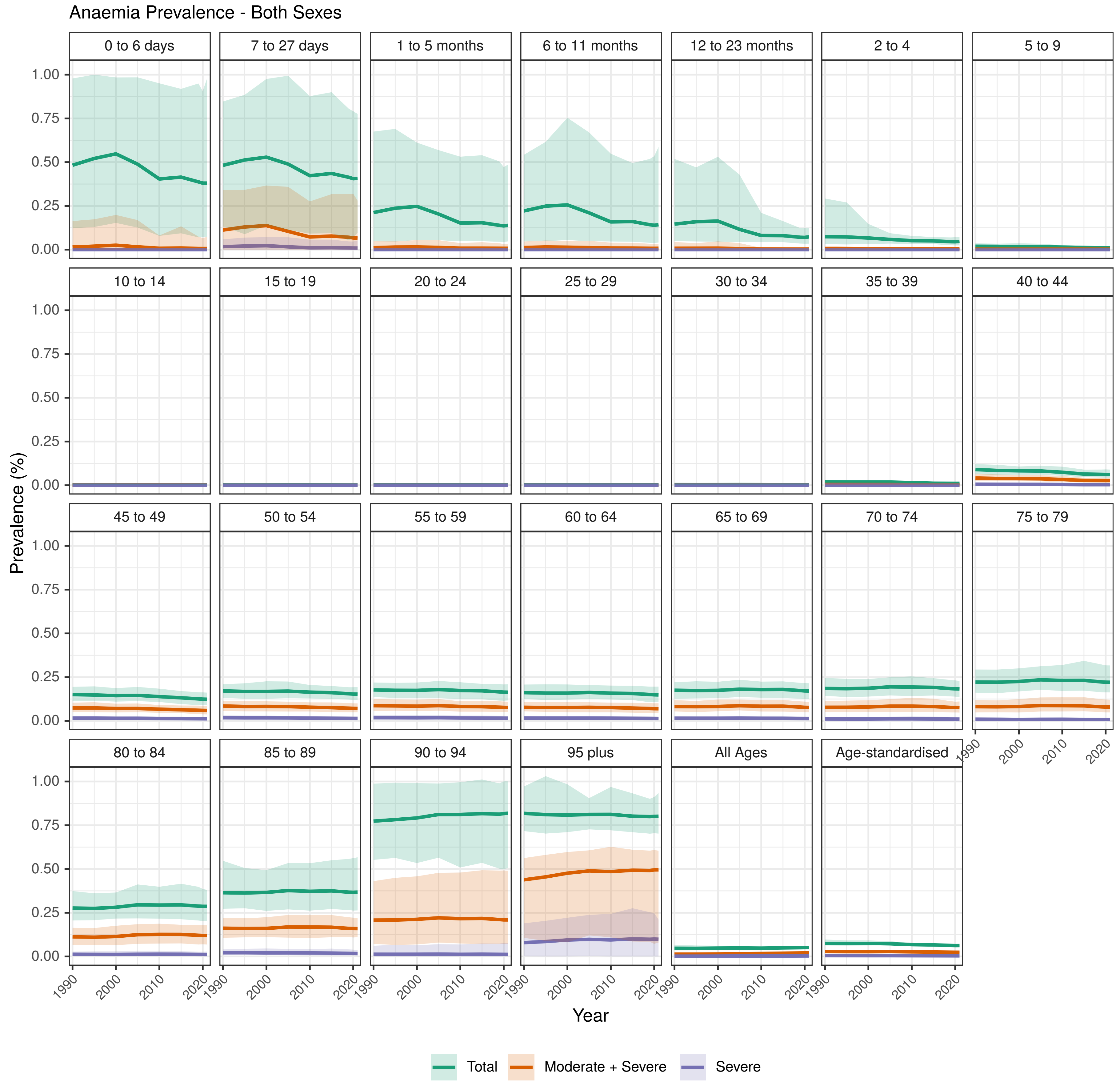

Anaemia Prevalence - Both Sexes
0 to 6 days
7 to 27 days
1 to 5 months
6 to 11 months
12 to 23 months
2 to 4
5 to 9
10 to 14
15 to 19
20 to 24
25 to 29
30 to 34
35 to 39
40 to 44
45 to 49
50 to 54
55 to 59
60 to 64
65 to 69
70 to 74
75 to 79
80 to 84
85 to 89
90 to 94
95 plus
All Ages
Age-standardised
Prevalence (%)
0.00
0.25
0.50
0.75
1.00
1990
2000
2010
2020
Year
Total
Moderate + Severe
Severe

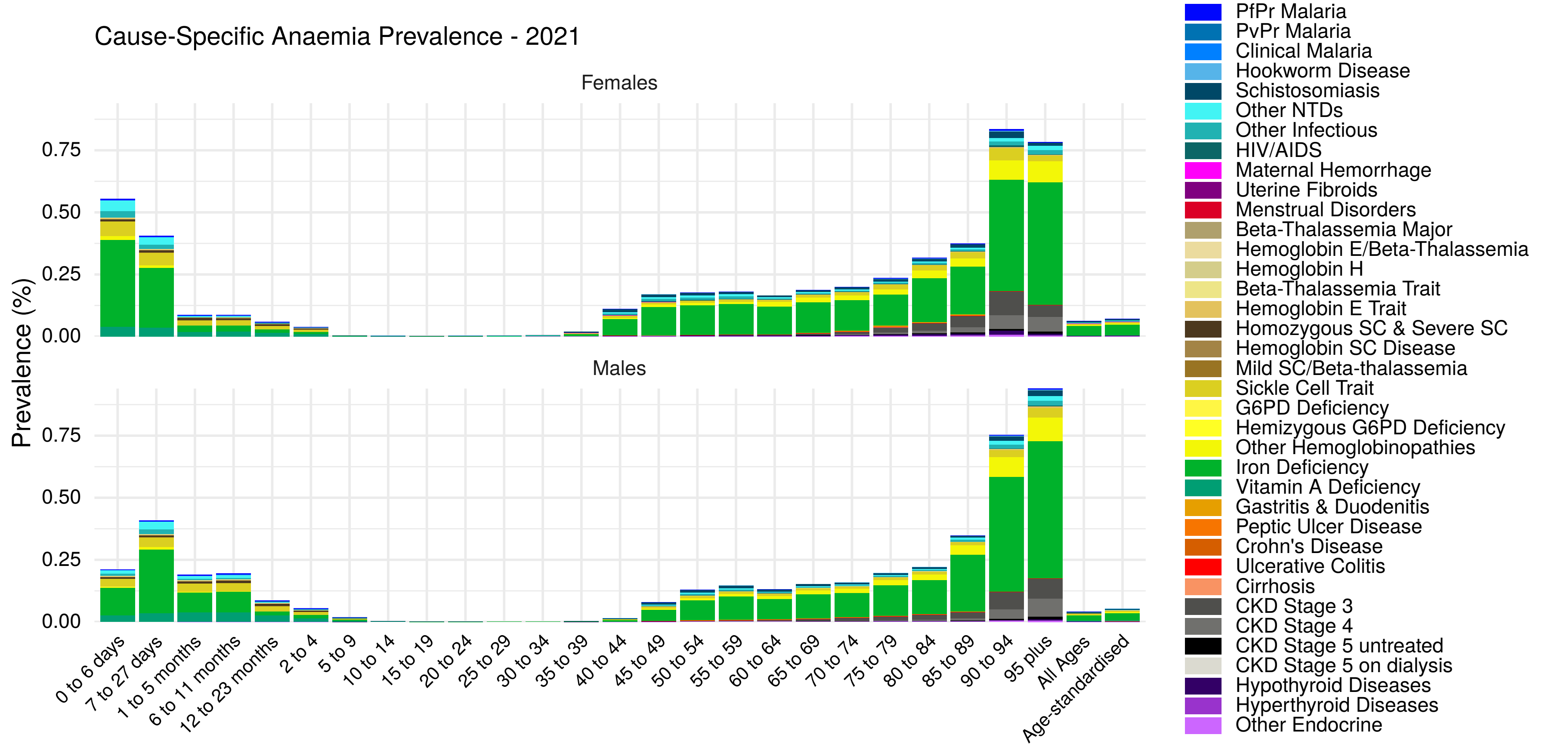

Cause-Specific Anaemia Prevalence - 2021
Females
Males
Prevalence (%)
0.00
0.25
0.50
0.75
0 to 6 days
7 to 27 days
1 to 5 months
6 to 11 months
12 to 23 months
2 to 4
5 to 9
10 to 14
15 to 19
20 to 24
25 to 29
30 to 34
35 to 39
40 to 44
45 to 49
50 to 54
55 to 59
60 to 64
65 to 69
70 to 74
75 to 79
80 to 84
85 to 89
90 to 94
95 plus
All Ages
Age-standardised
PfPr Malaria
PvPr Malaria
Clinical Malaria
Hookworm Disease
Schistosomiasis
Other NTDs
Other Infectious
HIV/AIDS
Maternal Hemorrhage
Uterine Fibroids
Menstrual Disorders
Beta-Thalassemia Major
Hemoglobin E/Beta-Thalassemia
Hemoglobin H
Beta-Thalassemia Trait
Hemoglobin E Trait
Homozygous SC & Severe SC
Hemoglobin SC Disease
Mild SC/Beta-thalassemia
Sickle Cell Trait
G6PD Deficiency
Hemizygous G6PD Deficiency
Other Hemoglobinopathies
Iron Deficiency
Vitamin A Deficiency
Gastritis & Duodenitis
Peptic Ulcer Disease
Crohn's Disease
Ulcerative Colitis
Cirrhosis
CKD Stage 3
CKD Stage 4
CKD Stage 5 untreated
CKD Stage 5 on dialysis
Hypothyroid Diseases
Hyperthyroid Diseases
Other Endocrine

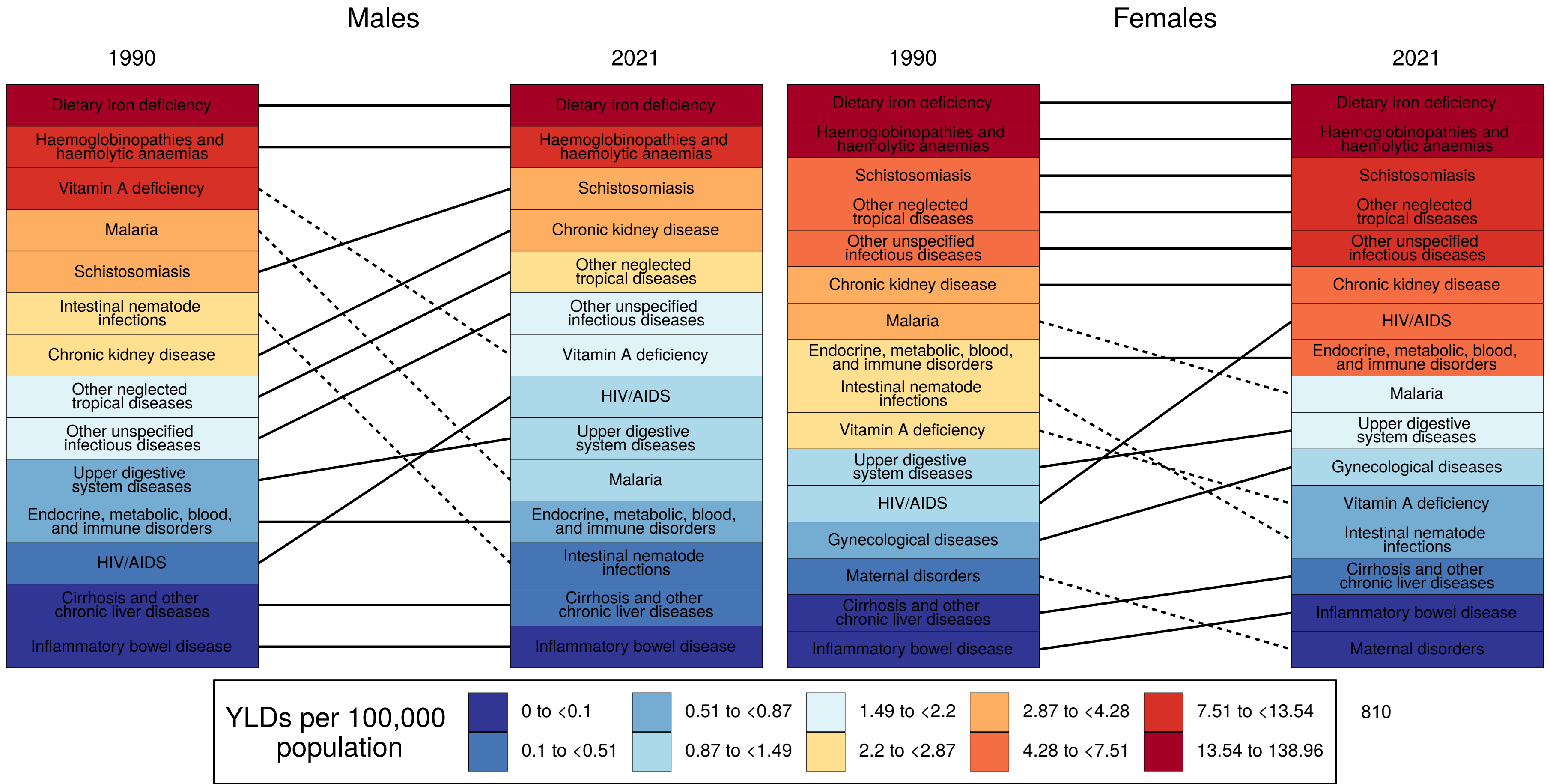

Males
Females
1990
2021
Dietary iron deficiency
Haemoglobinopathies and haemolytic anaemias
Vitamin A deficiency
Malaria
Schistosomiasis
Intestinal nematode infections
Chronic kidney disease
Other neglected tropical diseases
Other unspecified infectious diseases
Upper digestive system diseases
Endocrine, metabolic, blood, and immune disorders
HIV/AIDS
Cirrhosis and other chronic liver diseases
Inflammatory bowel disease
Gynecological diseases
Maternal disorders
YLDs per 100,000 population
0 to <0.1
0.1 to <0.51
0.51 to <0.87
0.87 to <1.49
1.49 to <2.2
2.2 to <2.87
2.87 to <4.28
4.28 to <7.51
7.51 to <13.54
13.54 to 138.96

Kisii

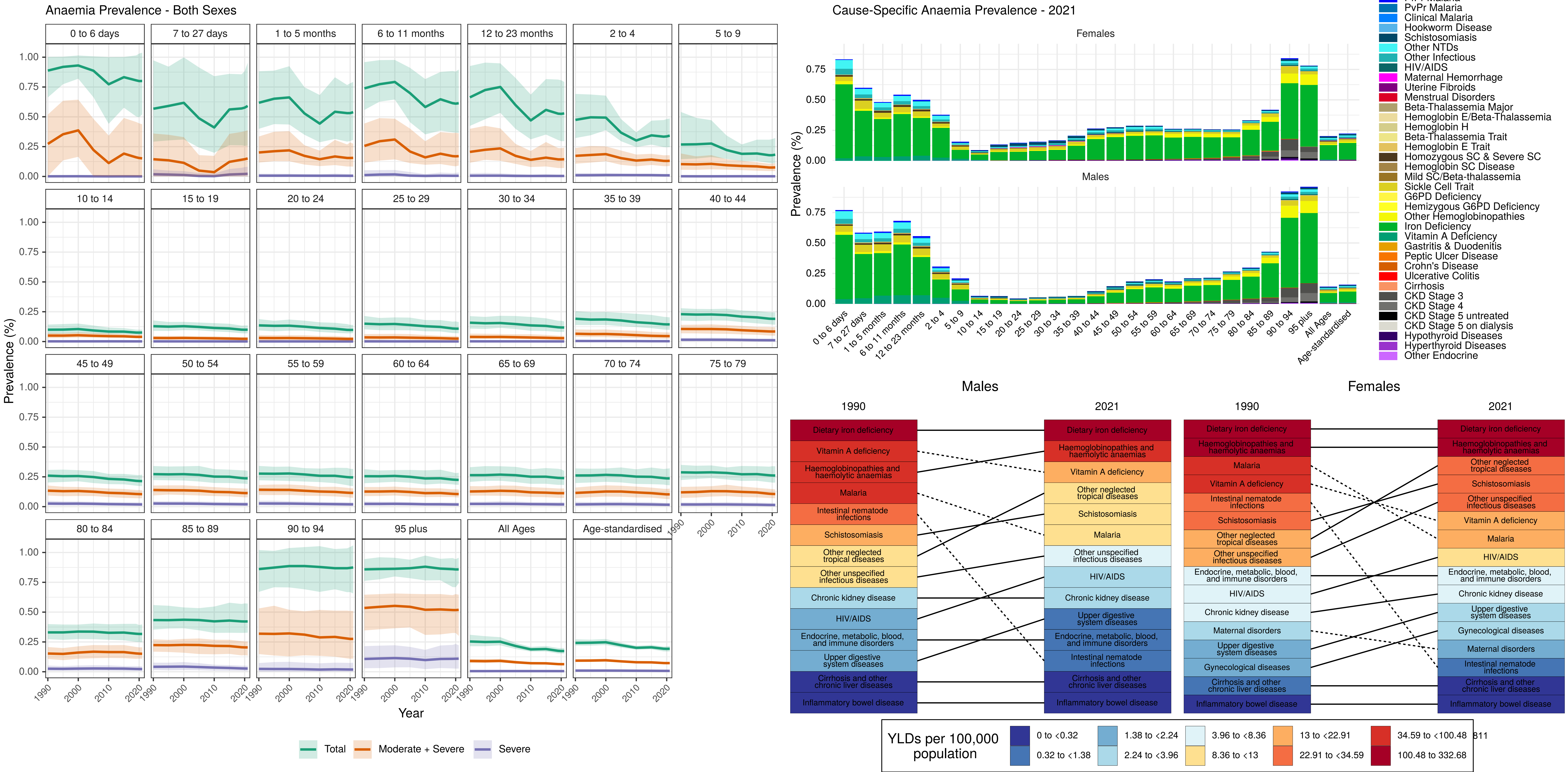

Anaemia Prevalence - Both Sexes
0 to 6 days
7 to 27 days
1 to 5 months
6 to 11 months
12 to 23 months
2 to 4
5 to 9
10 to 14
15 to 19
20 to 24
25 to 29
30 to 34
35 to 39
40 to 44
45 to 49
50 to 54
55 to 59
60 to 64
65 to 69
70 to 74
75 to 79
80 to 84
85 to 89
90 to 94
95 plus
All Ages
Age-standardised
Prevalence (%)
Year
Total
Moderate + Severe
Severe
Cause-Specific Anaemia Prevalence - 2021
Females
Males
PfPr Malaria
PvPr Malaria
Clinical Malaria
Hookworm Disease
Schistosomiasis
Other NTDs
Other Infectious
HIV/AIDS
Maternal Hemorrhage
Uterine Fibroids
Menstrual Disorders
Beta-Thalassemia Major
Hemoglobin E/Beta-Thalassemia
Hemoglobin H
Beta-Thalassemia Trait
Hemoglobin E Trait
Homozygous SC & Severe SC
Hemoglobin SC Disease
Mild SC/Beta-thalassemia
Sickle Cell Trait
G6PD Deficiency
Hemizygous G6PD Deficiency
Other Hemoglobinopathies
Iron Deficiency
Vitamin A Deficiency
Gastritis & Duodenitis
Peptic Ulcer Disease
Crohn's Disease
Ulcerative Colitis
Cirrhosis
CKD Stage 3
CKD Stage 4
CKD Stage 5 untreated
CKD Stage 5 on dialysis
Hypothyroid Diseases
Hyperthyroid Diseases
Other Endocrine
Males
Females
1990
2021
Dietary iron deficiency
Vitamin A deficiency
Haemoglobinopathies and haemolytic anaemias
Malaria
Intestinal nematode infections
Schistosomiasis
Other neglected tropical diseases
Other unspecified infectious diseases
Chronic kidney disease
HIV/AIDS
Endocrine, metabolic, blood, and immune disorders
Upper digestive system diseases
Cirrhosis and other chronic liver diseases
Inflammatory bowel disease
Maternal disorders
Gynecological diseases
YLDs per 100,000 population
0 to <0.32
0.32 to <1.38
1.38 to <2.24
2.24 to <3.96
3.96 to <8.36
8.36 to <13
13 to <22.91
22.91 to <34.59
34.59 to <100.48
100.48 to 332.68

Kisumu

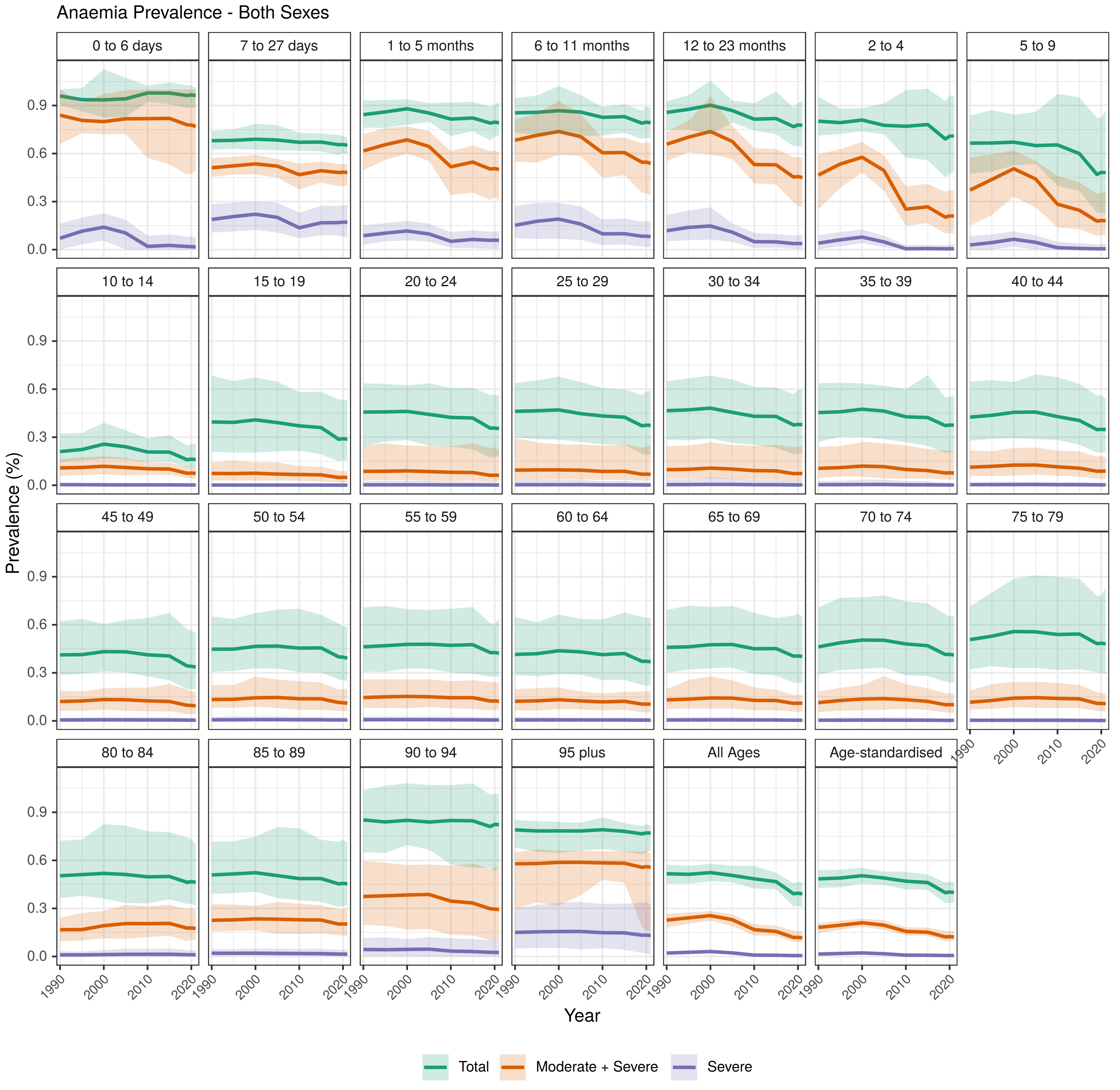

Anaemia Prevalence - Both Sexes
0 to 6 days
7 to 27 days
1 to 5 months
6 to 11 months
12 to 23 months
2 to 4
5 to 9
10 to 14
15 to 19
20 to 24
25 to 29
30 to 34
35 to 39
40 to 44
45 to 49
50 to 54
55 to 59
60 to 64
65 to 69
70 to 74
75 to 79
80 to 84
85 to 89
90 to 94
95 plus
All Ages
Age-standardised
Prevalence (%)
Year
Total
Moderate + Severe
Severe

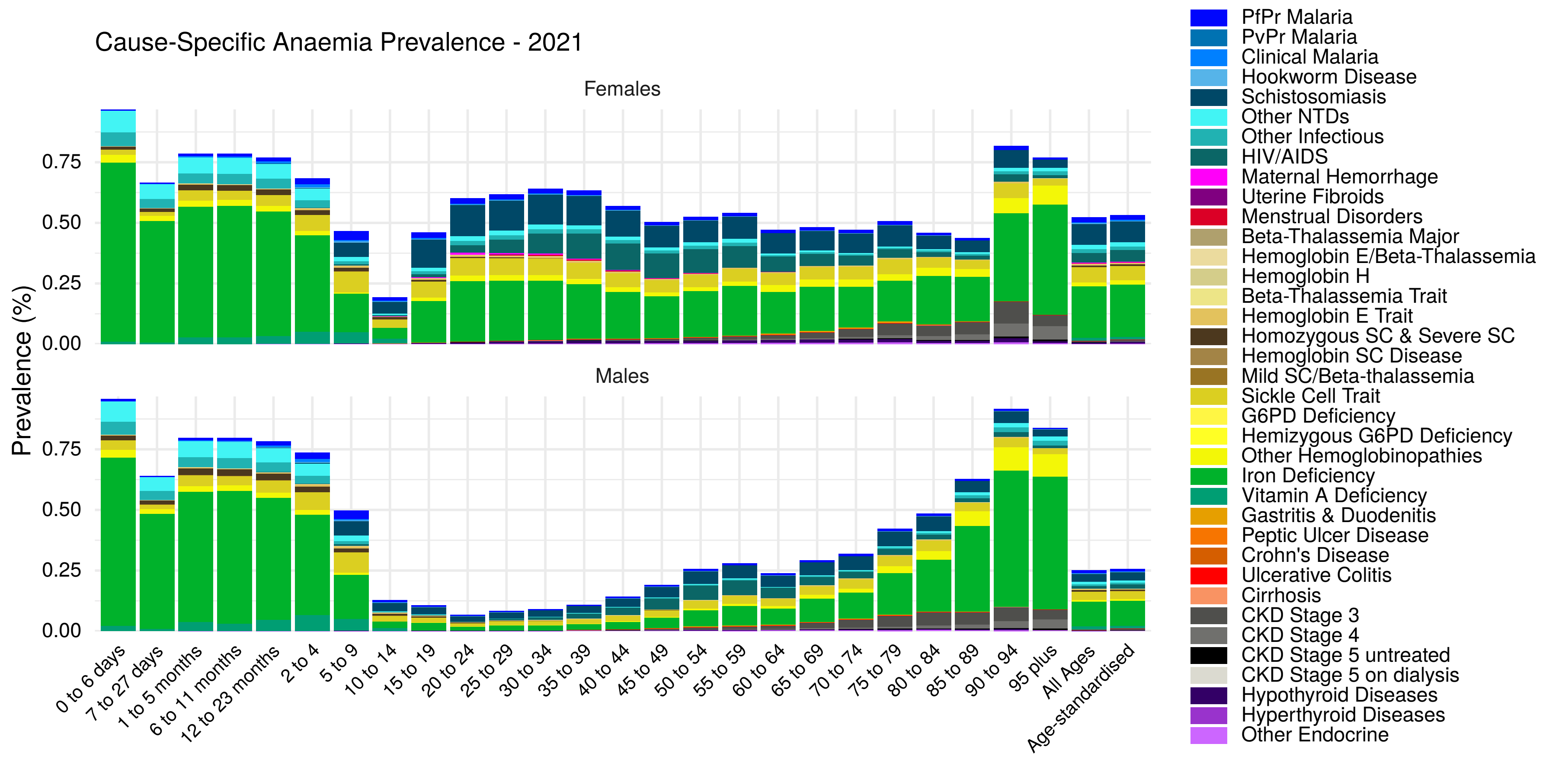

Cause-Specific Anaemia Prevalence - 2021
Females
Males
Prevalence (%)
PfPr Malaria
PvPr Malaria
Clinical Malaria
Hookworm Disease
Schistosomiasis
Other NTDs
Other Infectious
HIV/AIDS
Maternal Hemorrhage
Uterine Fibroids
Menstrual Disorders
Beta-Thalassemia Major
Hemoglobin E/Beta-Thalassemia
Hemoglobin H
Beta-Thalassemia Trait
Hemoglobin E Trait
Homozygous SC & Severe SC
Hemoglobin SC Disease
Mild SC/Beta-thalassemia
Sickle Cell Trait
G6PD Deficiency
Hemizygous G6PD Deficiency
Other Hemoglobinopathies
Iron Deficiency
Vitamin A Deficiency
Gastritis & Duodenitis
Peptic Ulcer Disease
Crohn's Disease
Ulcerative Colitis
Cirrhosis
CKD Stage 3
CKD Stage 4
CKD Stage 5 untreated
CKD Stage 5 on dialysis
Hypothyroid Diseases
Hyperthyroid Diseases
Other Endocrine

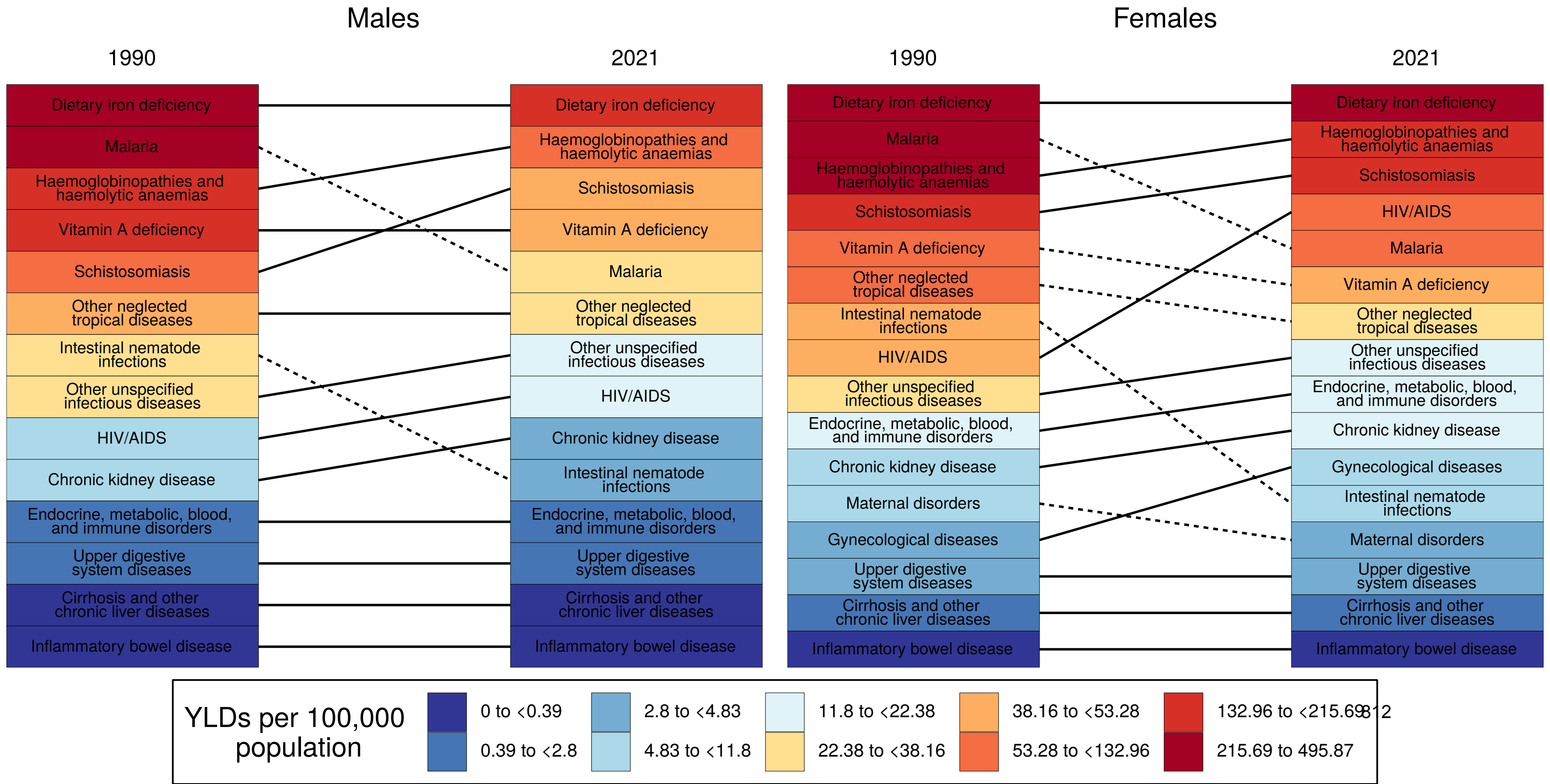

Males
Females
1990
2021
Dietary iron deficiency
Malaria
Haemoglobinopathies and haemolytic anaemias
Vitamin A deficiency
Schistosomiasis
Other neglected tropical diseases
Intestinal nematode infections
Other unspecified infectious diseases
HIV/AIDS
Chronic kidney disease
Endocrine, metabolic, blood, and immune disorders
Upper digestive system diseases
Cirrhosis and other chronic liver diseases
Inflammatory bowel disease
Maternal disorders
Gynecological diseases
YLDs per 100,000 population
0 to <0.39
0.39 to <2.8
2.8 to <4.83
4.83 to <11.8
11.8 to <22.38
22.38 to <38.16
38.16 to <53.28
53.28 to <132.96
132.96 to <215.69
215.69 to 495.87

Kitui

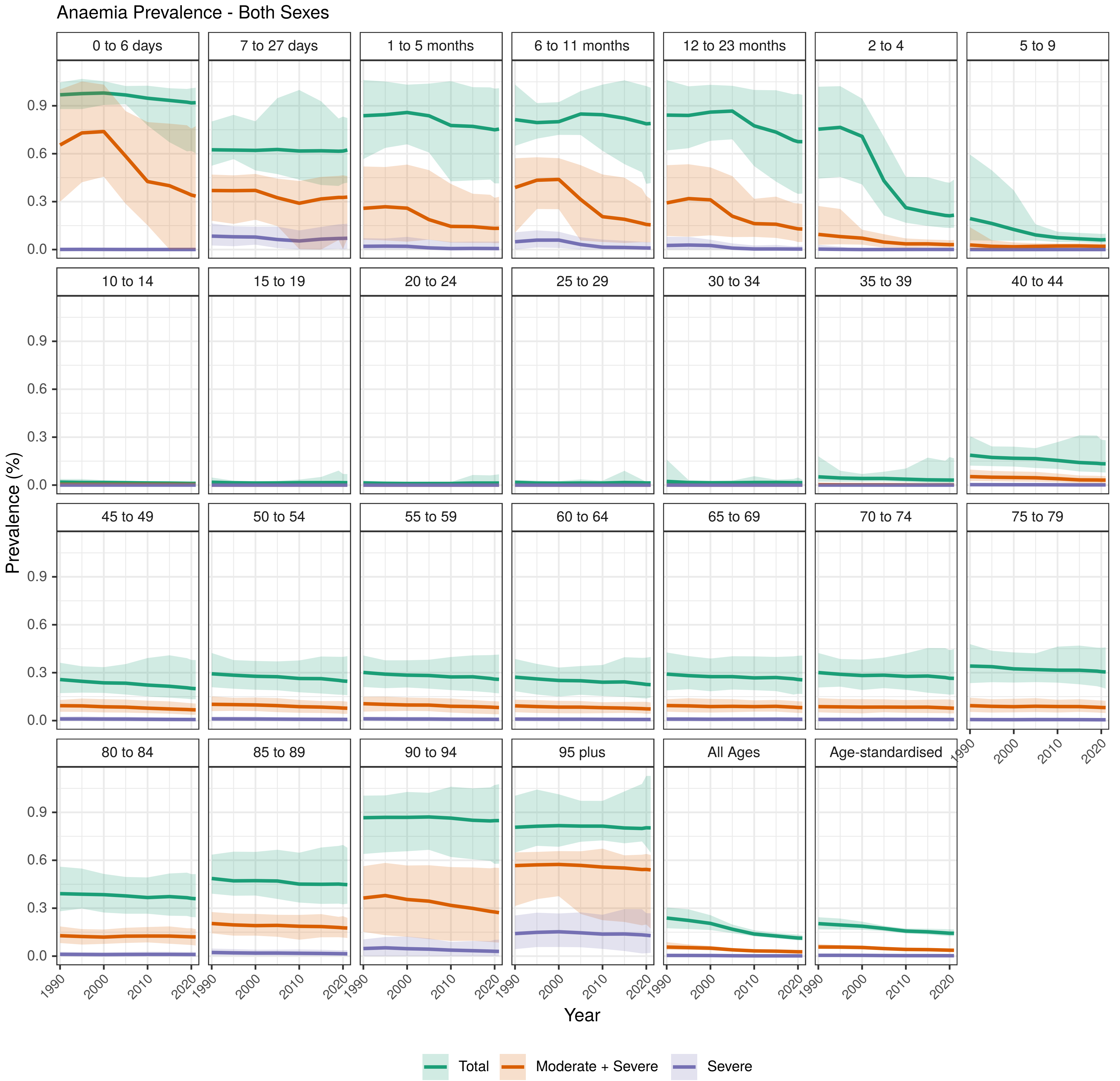
Anaemia Prevalence - Both Sexes
0 to 6 days
7 to 27 days
1 to 5 months
6 to 11 months
12 to 23 months
2 to 4
5 to 9
10 to 14
15 to 19
20 to 24
25 to 29
30 to 34
35 to 39
40 to 44
45 to 49
50 to 54
55 to 59
60 to 64
65 to 69
70 to 74
75 to 79
80 to 84
85 to 89
90 to 94
95 plus
All Ages
Age-standardised
Prevalence (%)
Year
Total
Moderate + Severe
Severe

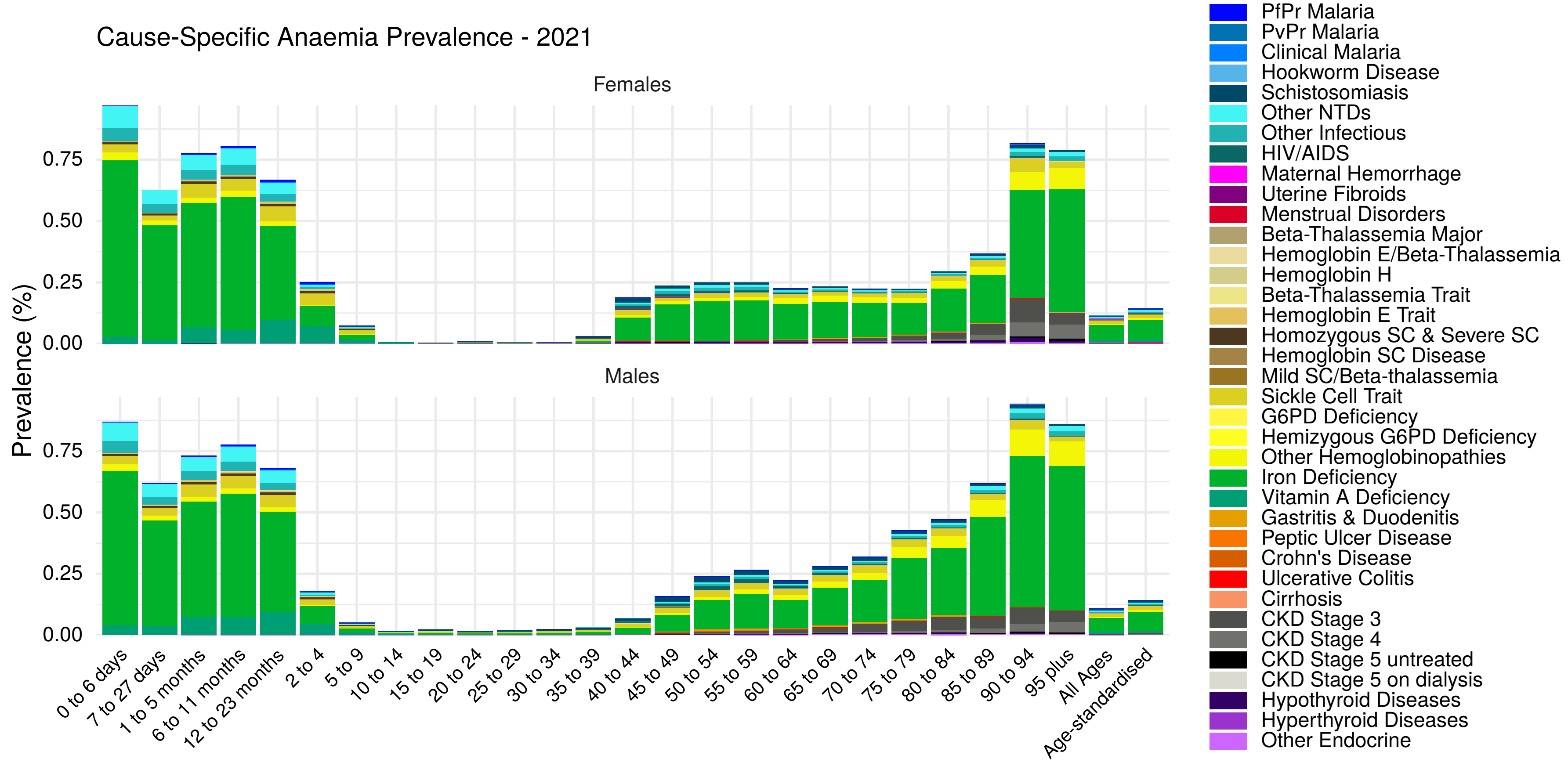
Cause-Specific Anaemia Prevalence - 2021
Females
Males
Prevalence (%)
PfPr Malaria
PvPr Malaria
Clinical Malaria
Hookworm Disease
Schistosomiasis
Other NTDs
Other Infectious
HIV/AIDS
Maternal Hemorrhage
Uterine Fibroids
Menstrual Disorders
Beta-Thalassemia Major
Hemoglobin E/Beta-Thalassemia
Hemoglobin H
Beta-Thalassemia Trait
Hemoglobin E Trait
Homozygous SC & Severe SC
Hemoglobin SC Disease
Mild SC/Beta-thalassemia
Sickle Cell Trait
G6PD Deficiency
Hemizygous G6PD Deficiency
Other Hemoglobinopathies
Iron Deficiency
Vitamin A Deficiency
Gastritis & Duodenitis
Peptic Ulcer Disease
Crohn's Disease
Ulcerative Colitis
Cirrhosis
CKD Stage 3
CKD Stage 4
CKD Stage 5 untreated
CKD Stage 5 on dialysis
Hypothyroid Diseases
Hyperthyroid Diseases
Other Endocrine

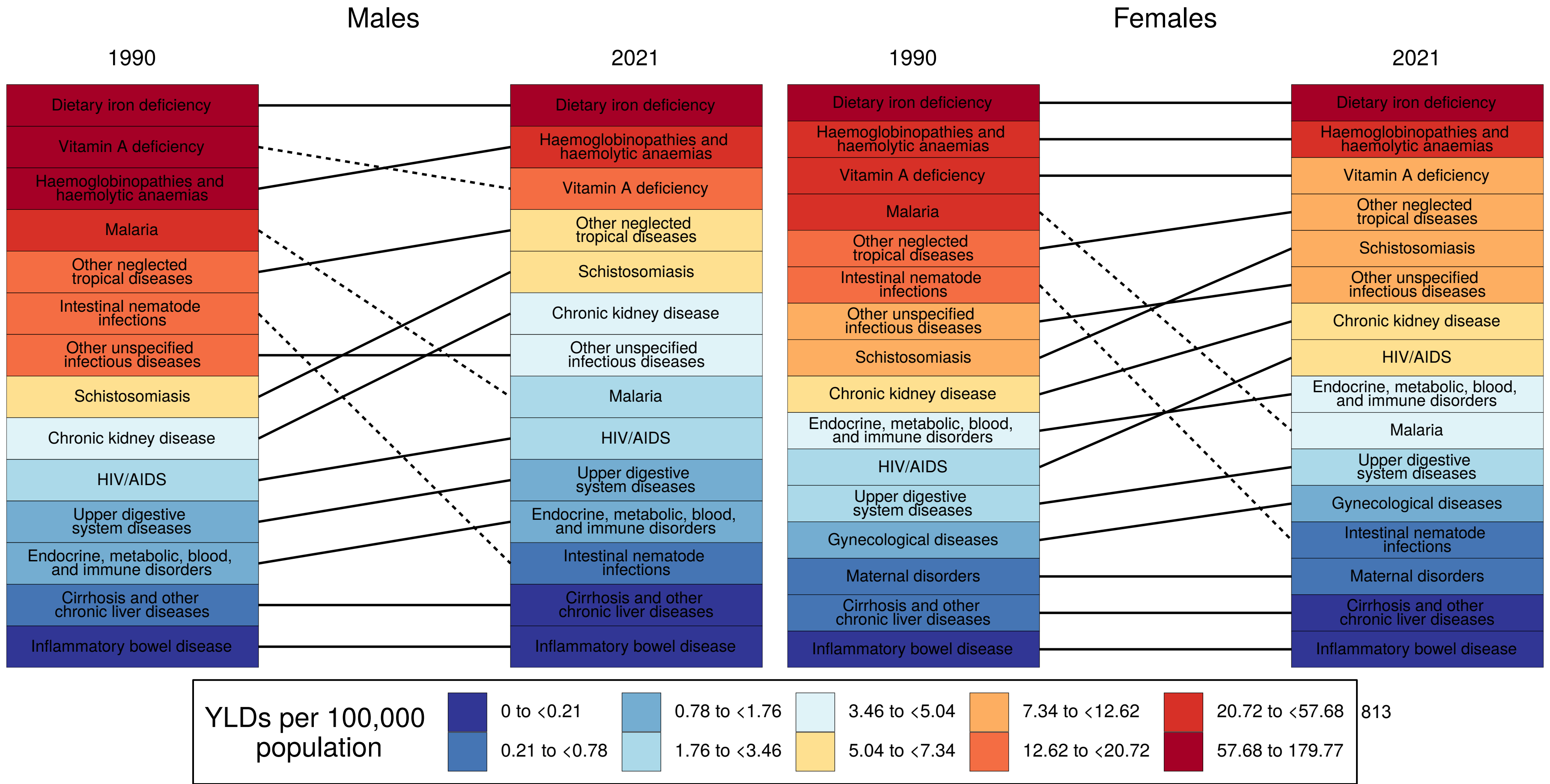
Males
Females
1990
2021
Dietary iron deficiency
Vitamin A deficiency
Haemoglobinopathies and haemolytic anaemias
Malaria
Other neglected tropical diseases
Intestinal nematode infections
Other unspecified infectious diseases
Schistosomiasis
Chronic kidney disease
HIV/AIDS
Upper digestive system diseases
Endocrine, metabolic, blood, and immune disorders
Cirrhosis and other chronic liver diseases
Inflammatory bowel disease
Gynecological diseases
Maternal disorders
YLDs per 100,000 population
0 to <0.21
0.21 to <0.78
0.78 to <1.76
1.76 to <3.46
3.46 to <5.04
5.04 to <7.34
7.34 to <12.62
12.62 to <20.72
20.72 to <57.68
57.68 to 179.77

Kwale

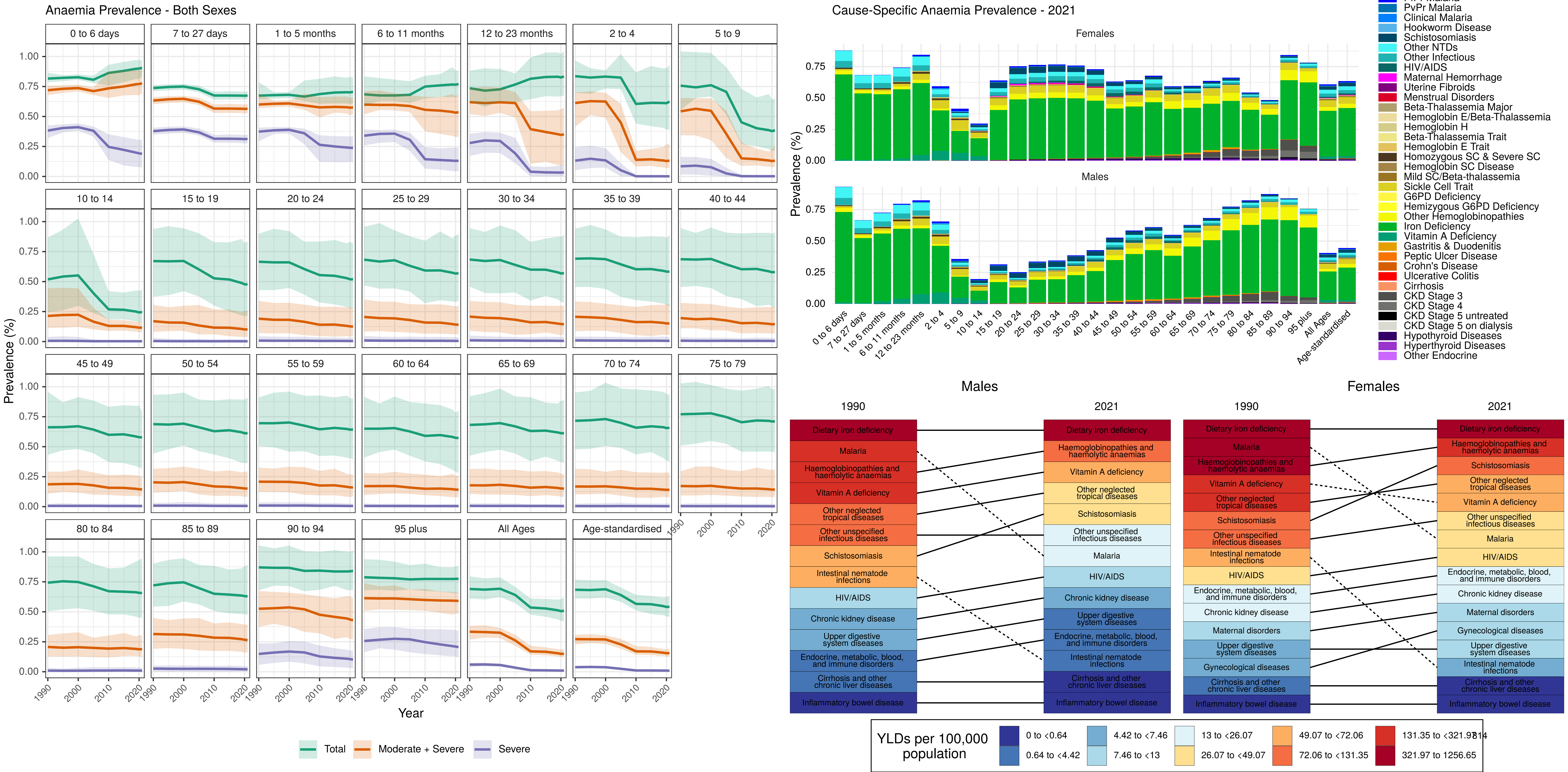

Anaemia Prevalence - Both Sexes
0 to 6 days
7 to 27 days
1 to 5 months
6 to 11 months
12 to 23 months
2 to 4
5 to 9
10 to 14
15 to 19
20 to 24
25 to 29
30 to 34
35 to 39
40 to 44
45 to 49
50 to 54
55 to 59
60 to 64
65 to 69
70 to 74
75 to 79
80 to 84
85 to 89
90 to 94
95 plus
All Ages
Age-standardised
Prevalence (%)
0.00
0.25
0.50
0.75
1.00
1990
2000
2010
2020
Year
Total
Moderate + Severe
Severe
Cause-Specific Anaemia Prevalence - 2021
Females
Males
Prevalence (%)
PfPr Malaria
PvPr Malaria
Clinical Malaria
Hookworm Disease
Schistosomiasis
Other NTDs
Other Infectious
HIV/AIDS
Maternal Hemorrhage
Uterine Fibroids
Menstrual Disorders
Beta-Thalassemia Major
Hemoglobin E/Beta-Thalassemia
Hemoglobin H
Beta-Thalassemia Trait
Hemoglobin E Trait
Homozygous SC & Severe SC
Hemoglobin SC Disease
Mild SC/Beta-thalassemia
Sickle Cell Trait
G6PD Deficiency
Hemizygous G6PD Deficiency
Other Hemoglobinopathies
Iron Deficiency
Vitamin A Deficiency
Gastritis & Duodenitis
Peptic Ulcer Disease
Crohn's Disease
Ulcerative Colitis
Cirrhosis
CKD Stage 3
CKD Stage 4
CKD Stage 5 untreated
CKD Stage 5 on dialysis
Hypothyroid Diseases
Hyperthyroid Diseases
Other Endocrine
Males
Females
1990
2021
Dietary iron deficiency
Malaria
Haemoglobinopathies and haemolytic anaemias
Vitamin A deficiency
Other neglected tropical diseases
Other unspecified infectious diseases
Schistosomiasis
Intestinal nematode infections
HIV/AIDS
Chronic kidney disease
Upper digestive system diseases
Endocrine, metabolic, blood, and immune disorders
Cirrhosis and other chronic liver diseases
Inflammatory bowel disease
Maternal disorders
Gynecological diseases
YLDs per 100,000 population
0 to <0.64
0.64 to <4.42
4.42 to <7.46
7.46 to <13
13 to <26.07
26.07 to <49.07
49.07 to <72.06
72.06 to <131.35
131.35 to <321.97
321.97 to 1256.65

Laikipia

Anaemia Prevalence - Both Sexes

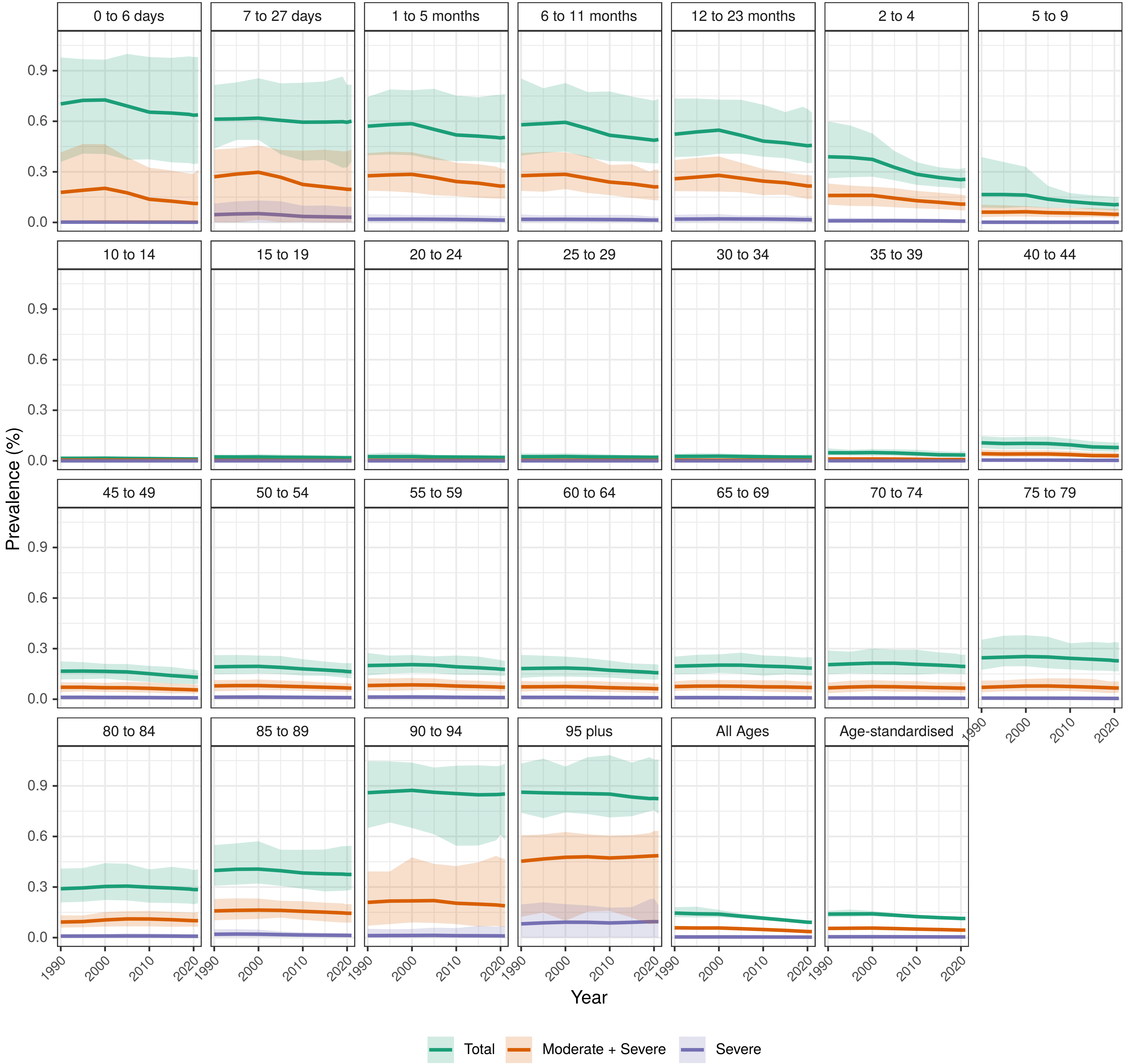

Cause-Specific Anaemia Prevalence - 2021

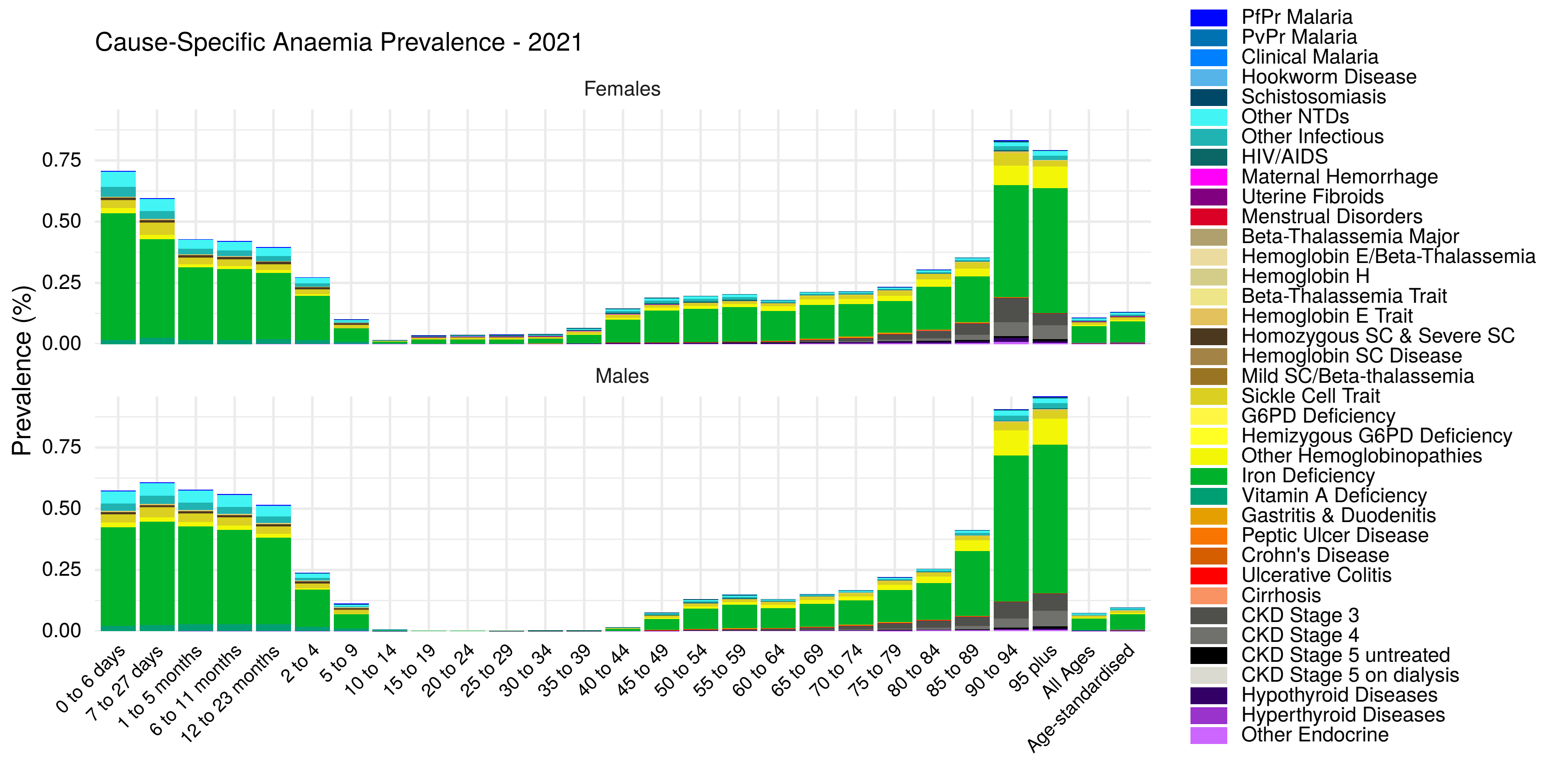

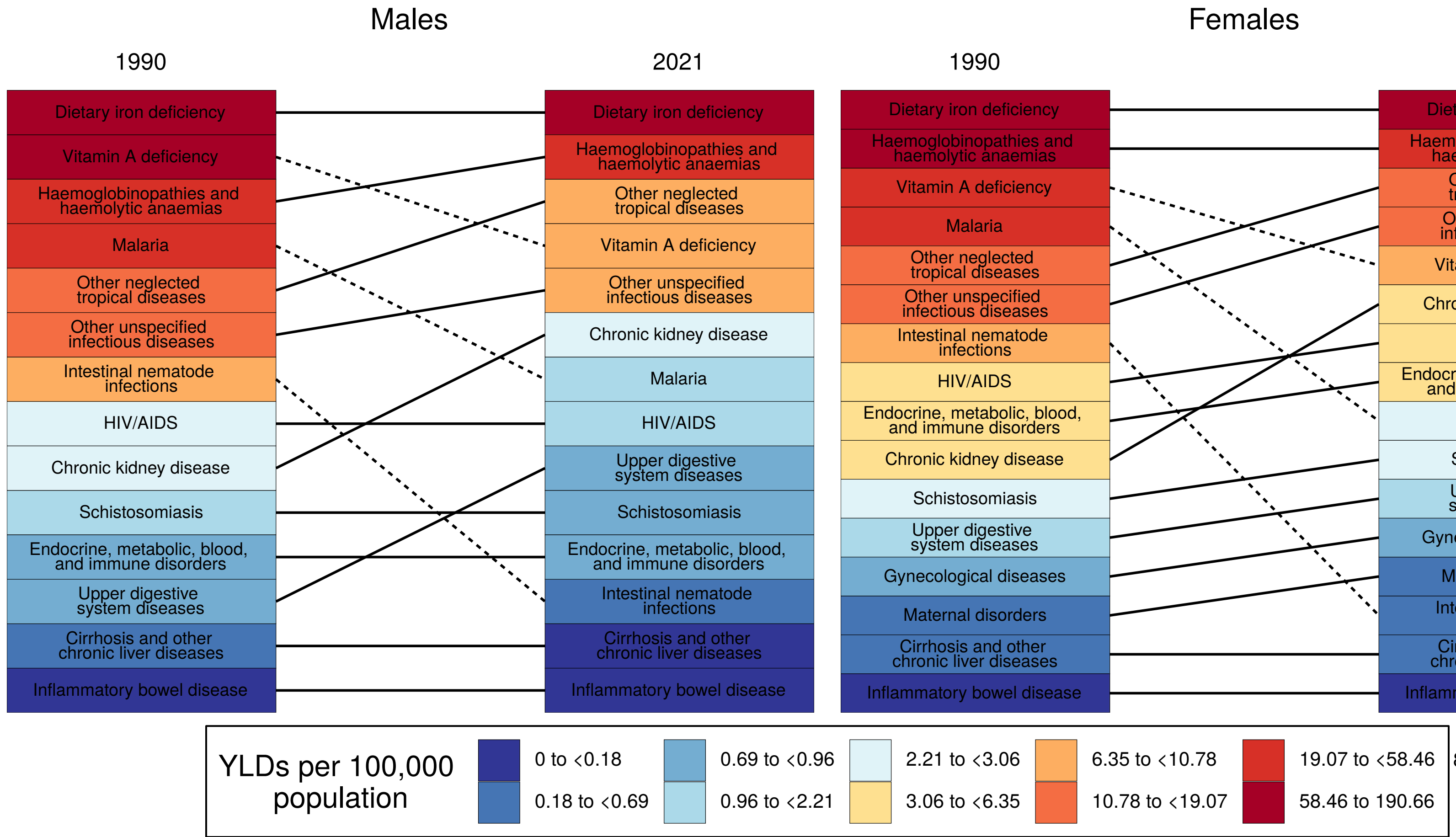

Lamu

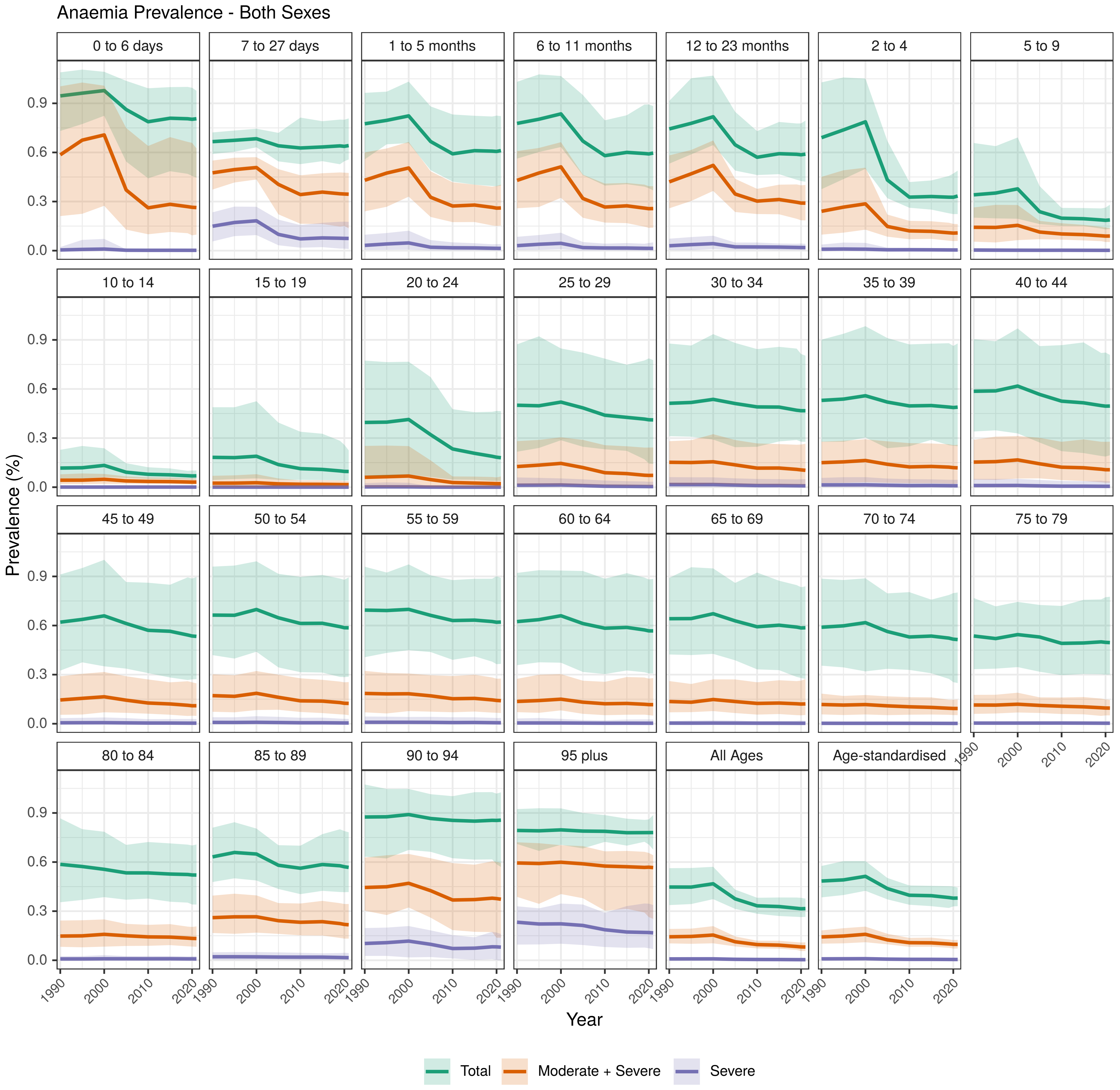

Anaemia Prevalence - Both Sexes
0 to 6 days
7 to 27 days
1 to 5 months
6 to 11 months
12 to 23 months
2 to 4
5 to 9
10 to 14
15 to 19
20 to 24
25 to 29
30 to 34
35 to 39
40 to 44
45 to 49
50 to 54
55 to 59
60 to 64
65 to 69
70 to 74
75 to 79
80 to 84
85 to 89
90 to 94
95 plus
All Ages
Age-standardised
Prevalence (%)
0.0
0.3
0.6
0.9
1990
2000
2010
2020
Year
Total
Moderate + Severe
Severe

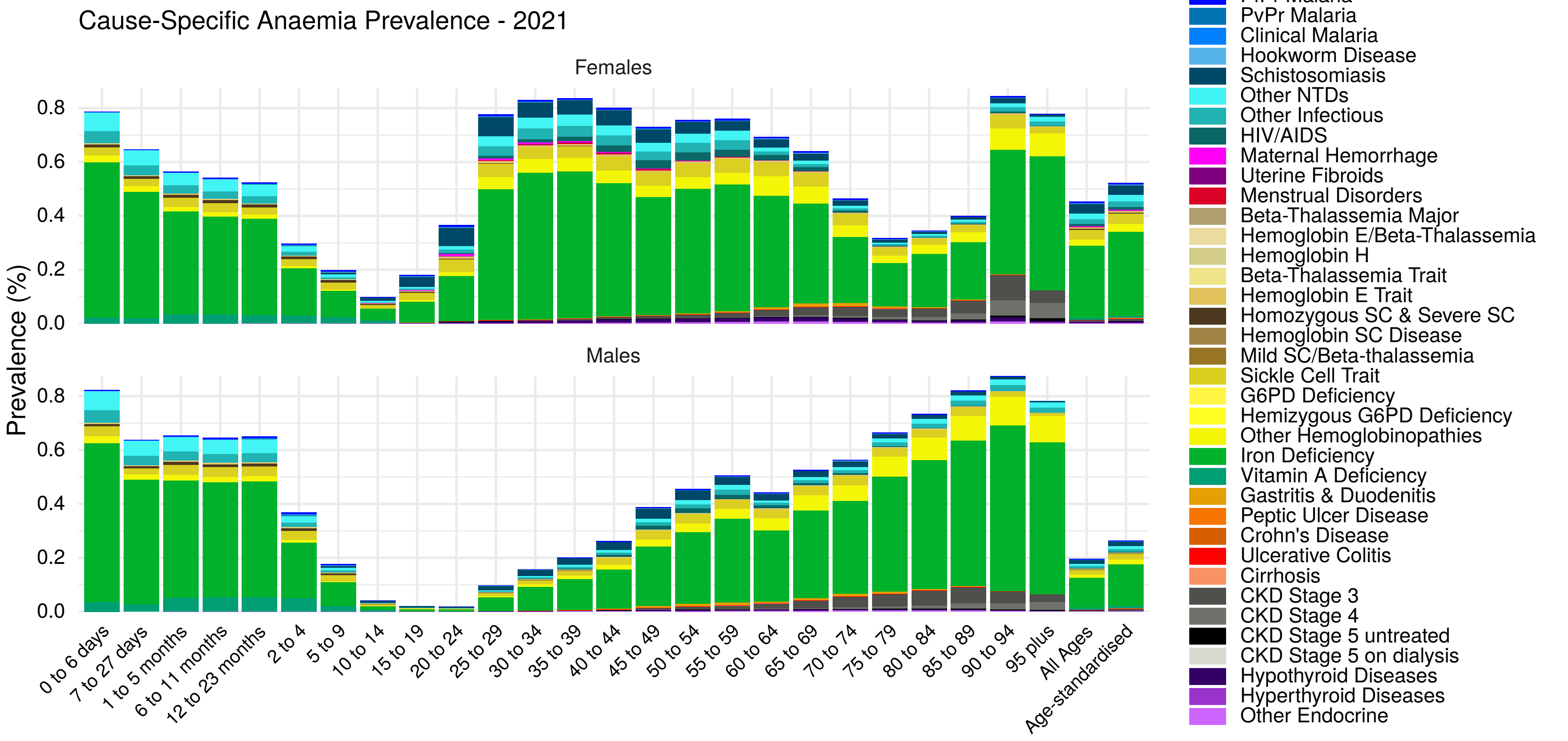

Cause-Specific Anaemia Prevalence - 2021
Females
Males
Prevalence (%)
0.0
0.2
0.4
0.6
0.8
0 to 6 days
7 to 27 days
1 to 5 months
6 to 11 months
12 to 23 months
2 to 4
5 to 9
10 to 14
15 to 19
20 to 24
25 to 29
30 to 34
35 to 39
40 to 44
45 to 49
50 to 54
55 to 59
60 to 64
65 to 69
70 to 74
75 to 79
80 to 84
85 to 89
90 to 94
95 plus
All Ages
Age-standardised
PfPr Malaria
PvPr Malaria
Clinical Malaria
Hookworm Disease
Schistosomiasis
Other NTDs
Other Infectious
HIV/AIDS
Maternal Hemorrhage
Uterine Fibroids
Menstrual Disorders
Beta-Thalassemia Major
Hemoglobin E/Beta-Thalassemia
Hemoglobin H
Beta-Thalassemia Trait
Hemoglobin E Trait
Homozygous SC & Severe SC
Hemoglobin SC Disease
Mild SC/Beta-thalassemia
Sickle Cell Trait
G6PD Deficiency
Hemizygous G6PD Deficiency
Other Hemoglobinopathies
Iron Deficiency
Vitamin A Deficiency
Gastritis & Duodenitis
Peptic Ulcer Disease
Crohn's Disease
Ulcerative Colitis
Cirrhosis
CKD Stage 3
CKD Stage 4
CKD Stage 5 untreated
CKD Stage 5 on dialysis
Hypothyroid Diseases
Hyperthyroid Diseases
Other Endocrine

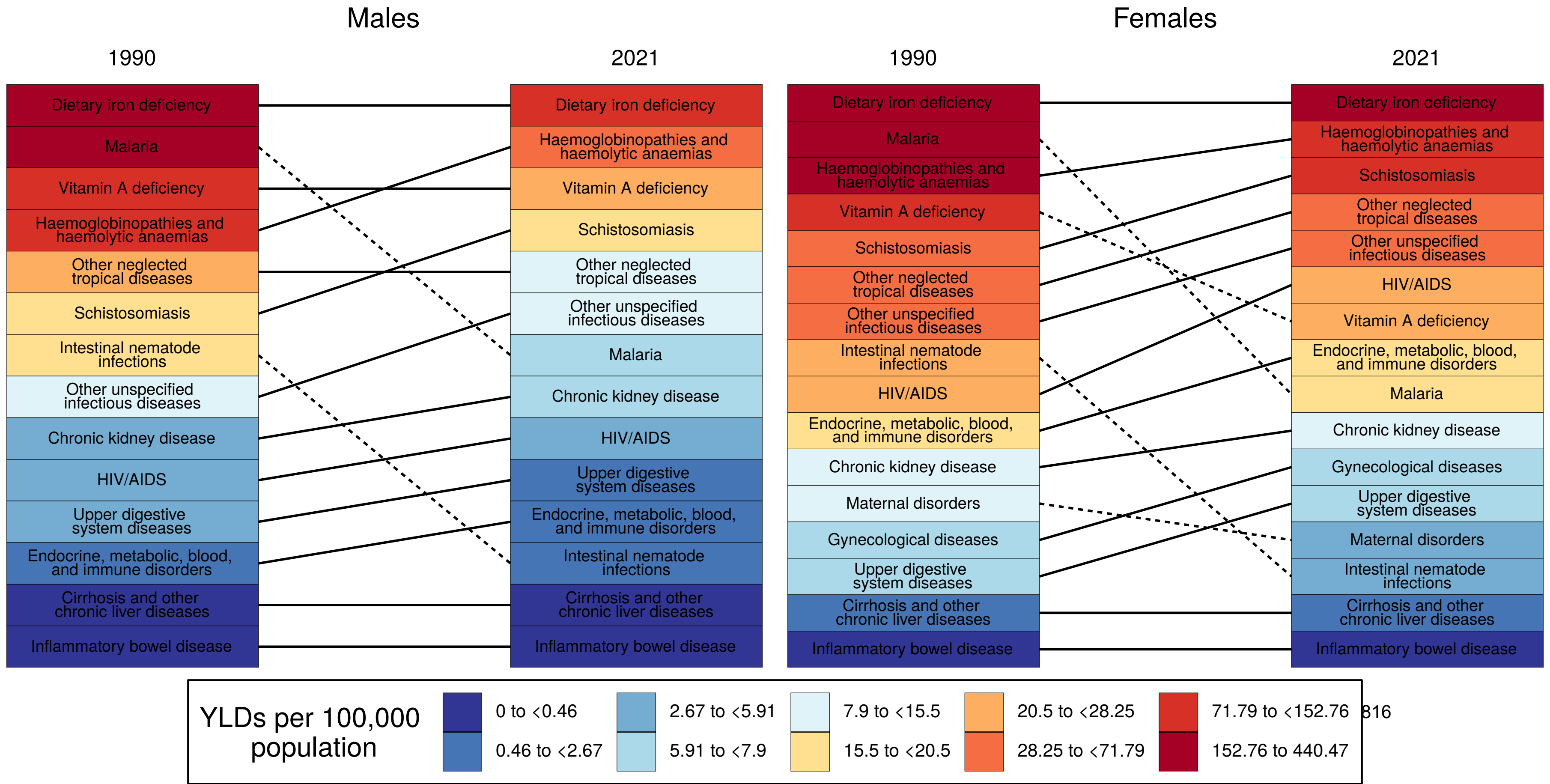

Males
Females
1990
2021
Dietary iron deficiency
Malaria
Vitamin A deficiency
Haemoglobinopathies and haemolytic anaemias
Other neglected tropical diseases
Schistosomiasis
Intestinal nematode infections
Other unspecified infectious diseases
Chronic kidney disease
HIV/AIDS
Upper digestive system diseases
Endocrine, metabolic, blood, and immune disorders
Cirrhosis and other chronic liver diseases
Inflammatory bowel disease
Maternal disorders
Gynecological diseases
YLDs per 100,000 population
0 to <0.46
0.46 to <2.67
2.67 to <5.91
5.91 to <7.9
7.9 to <15.5
15.5 to <20.5
20.5 to <28.25
28.25 to <71.79
71.79 to <152.76
152.76 to 440.47

Machakos

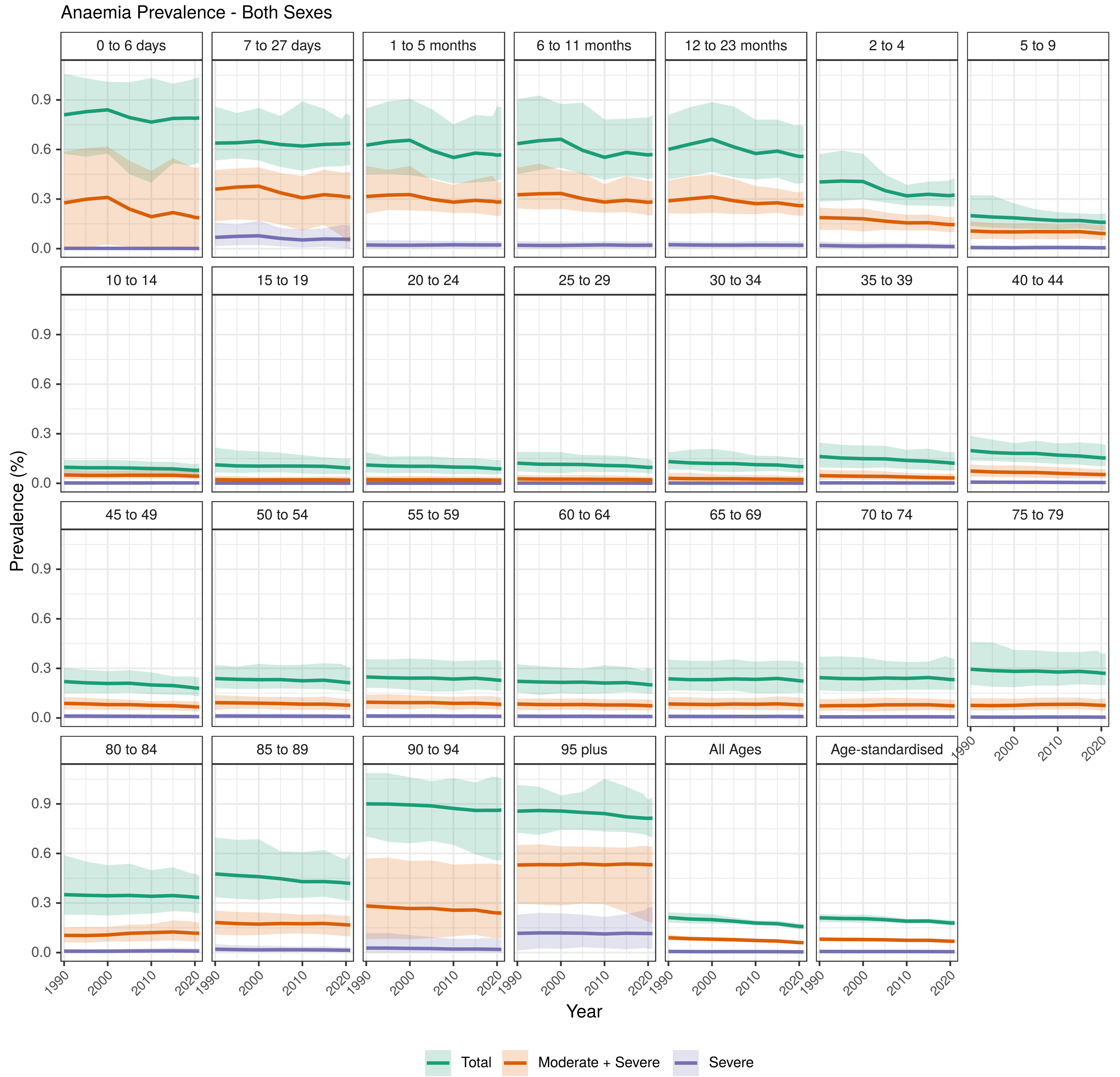

Anaemia Prevalence - Both Sexes
0 to 6 days
7 to 27 days
1 to 5 months
6 to 11 months
12 to 23 months
2 to 4
5 to 9
10 to 14
15 to 19
20 to 24
25 to 29
30 to 34
35 to 39
40 to 44
45 to 49
50 to 54
55 to 59
60 to 64
65 to 69
70 to 74
75 to 79
80 to 84
85 to 89
90 to 94
95 plus
All Ages
Age-standardised
Prevalence (%)
Year
Total
Moderate + Severe
Severe

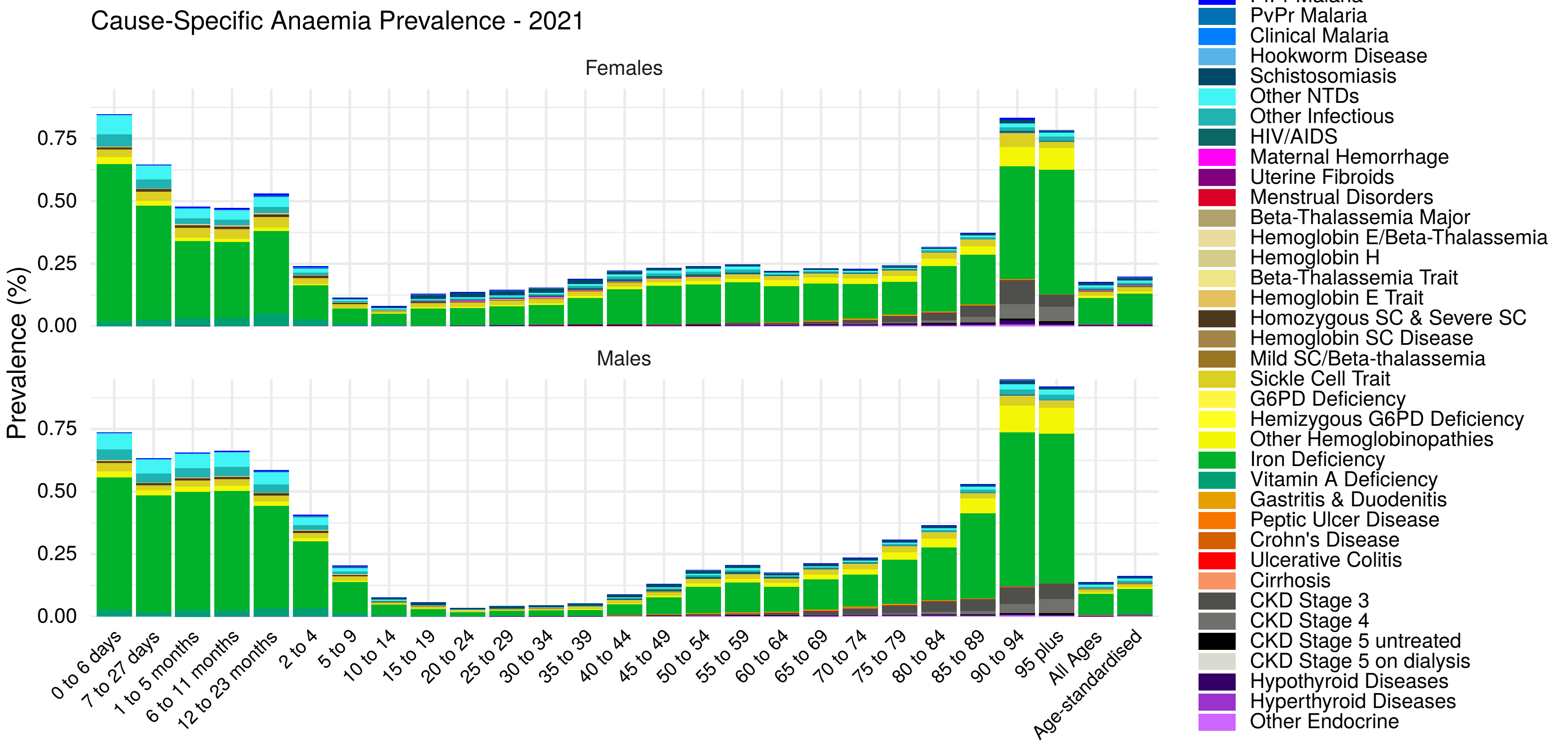

Cause-Specific Anaemia Prevalence - 2021
Females
Males
Prevalence (%)
PfPr Malaria
PvPr Malaria
Clinical Malaria
Hookworm Disease
Schistosomiasis
Other NTDs
Other Infectious
HIV/AIDS
Maternal Hemorrhage
Uterine Fibroids
Menstrual Disorders
Beta-Thalassemia Major
Hemoglobin E/Beta-Thalassemia
Hemoglobin H
Beta-Thalassemia Trait
Hemoglobin E Trait
Homozygous SC & Severe SC
Hemoglobin SC Disease
Mild SC/Beta-thalassemia
Sickle Cell Trait
G6PD Deficiency
Hemizygous G6PD Deficiency
Other Hemoglobinopathies
Iron Deficiency
Vitamin A Deficiency
Gastritis & Duodenitis
Peptic Ulcer Disease
Crohn's Disease
Ulcerative Colitis
Cirrhosis
CKD Stage 3
CKD Stage 4
CKD Stage 5 untreated
CKD Stage 5 on dialysis
Hypothyroid Diseases
Hyperthyroid Diseases
Other Endocrine

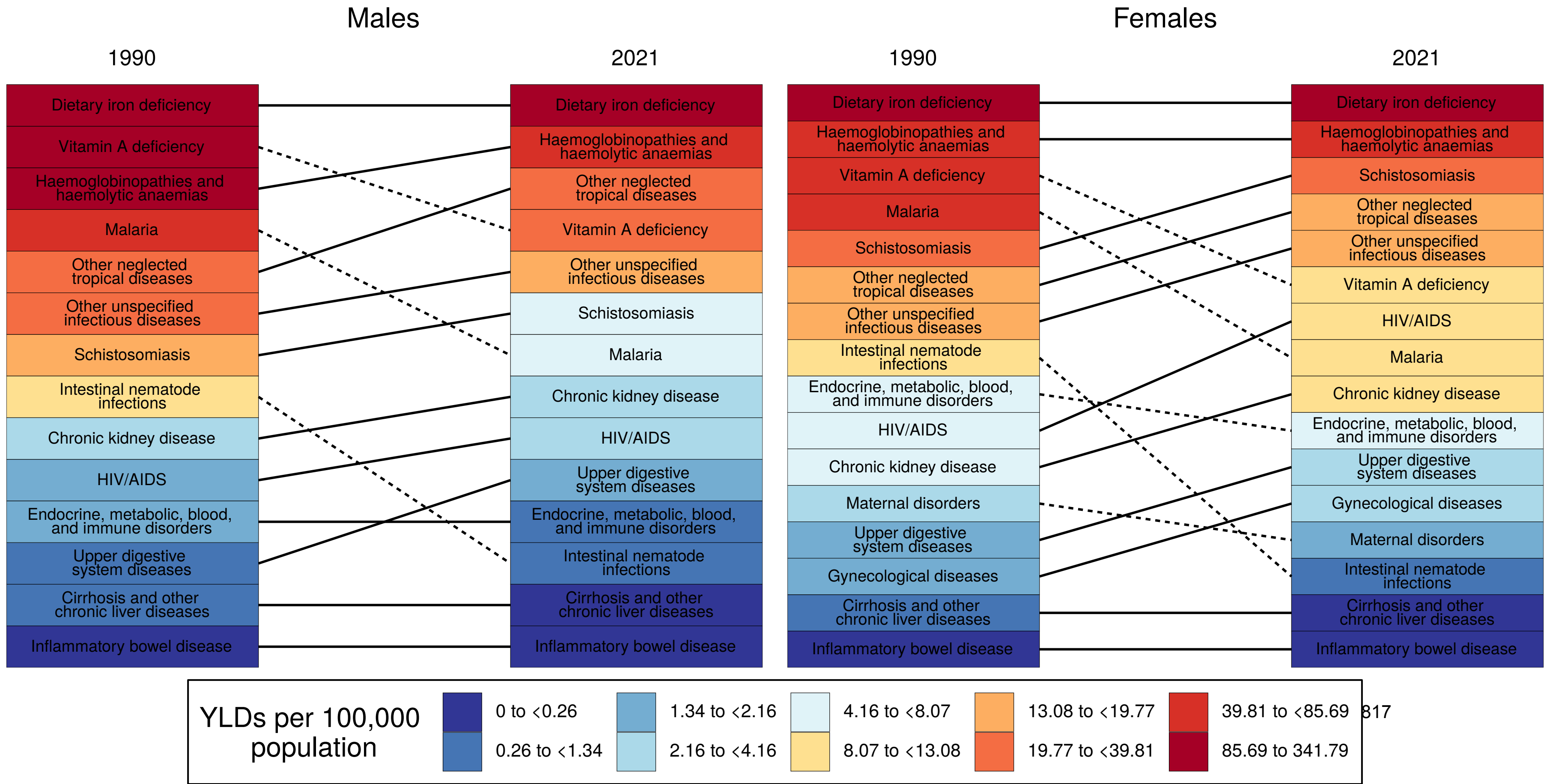

Males
Females
1990
2021
Dietary iron deficiency
Vitamin A deficiency
Haemoglobinopathies and haemolytic anaemias
Malaria
Other neglected tropical diseases
Other unspecified infectious diseases
Schistosomiasis
Intestinal nematode infections
Chronic kidney disease
HIV/AIDS
Endocrine, metabolic, blood, and immune disorders
Upper digestive system diseases
Cirrhosis and other chronic liver diseases
Inflammatory bowel disease
Maternal disorders
Gynecological diseases
YLDs per 100,000 population
0 to <0.26
0.26 to <1.34
1.34 to <2.16
2.16 to <4.16
4.16 to <8.07
8.07 to <13.08
13.08 to <19.77
19.77 to <39.81
39.81 to <85.69
85.69 to 341.79

Makueni

Anaemia Prevalence - Both Sexes

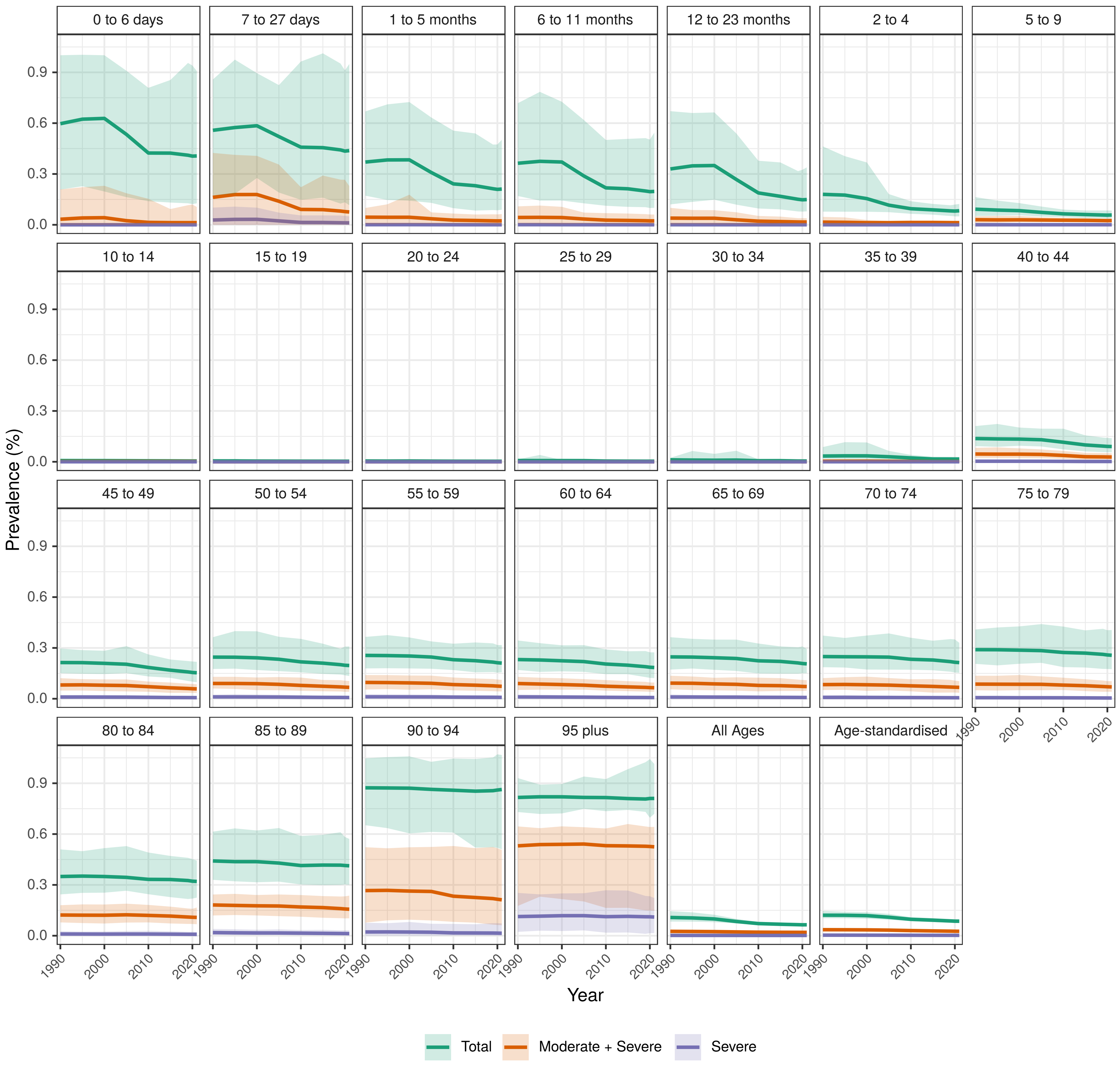

Cause-Specific Anaemia Prevalence - 2021

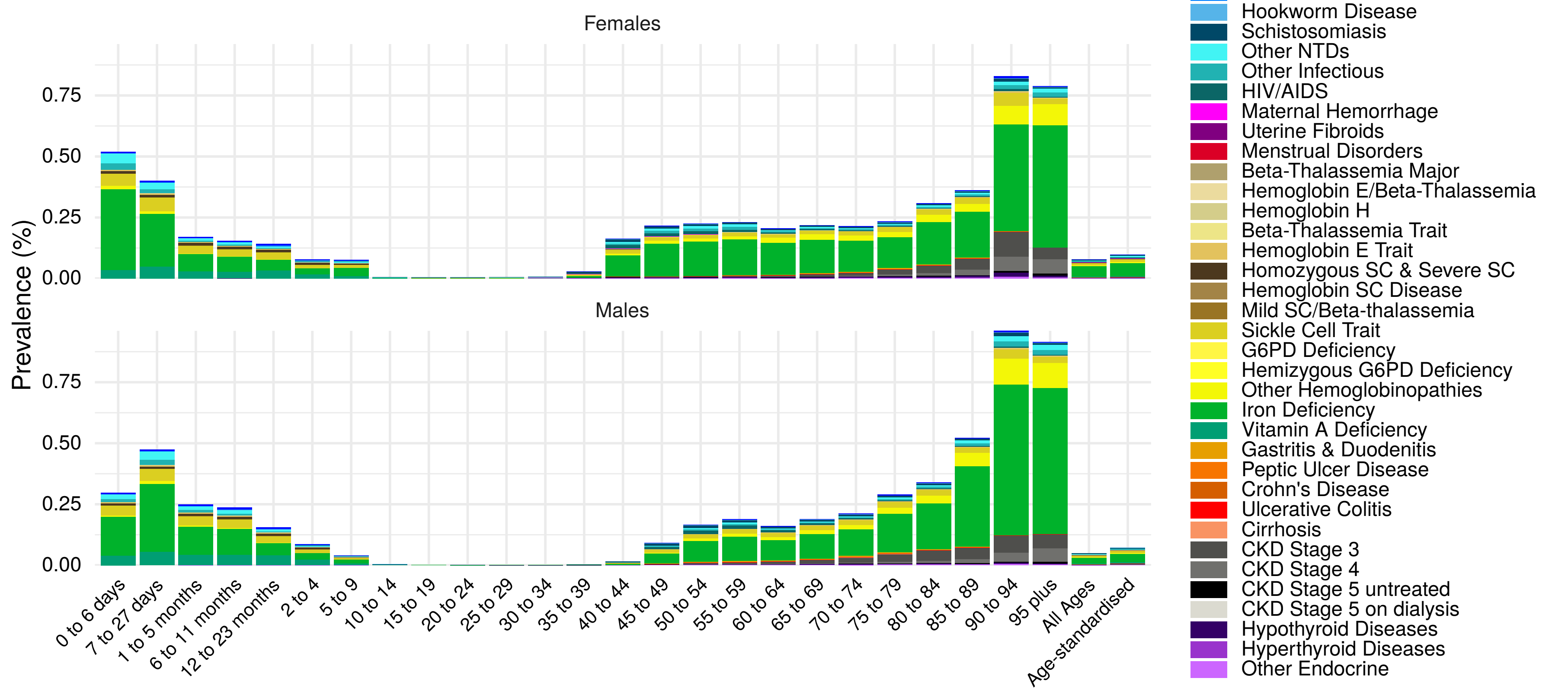

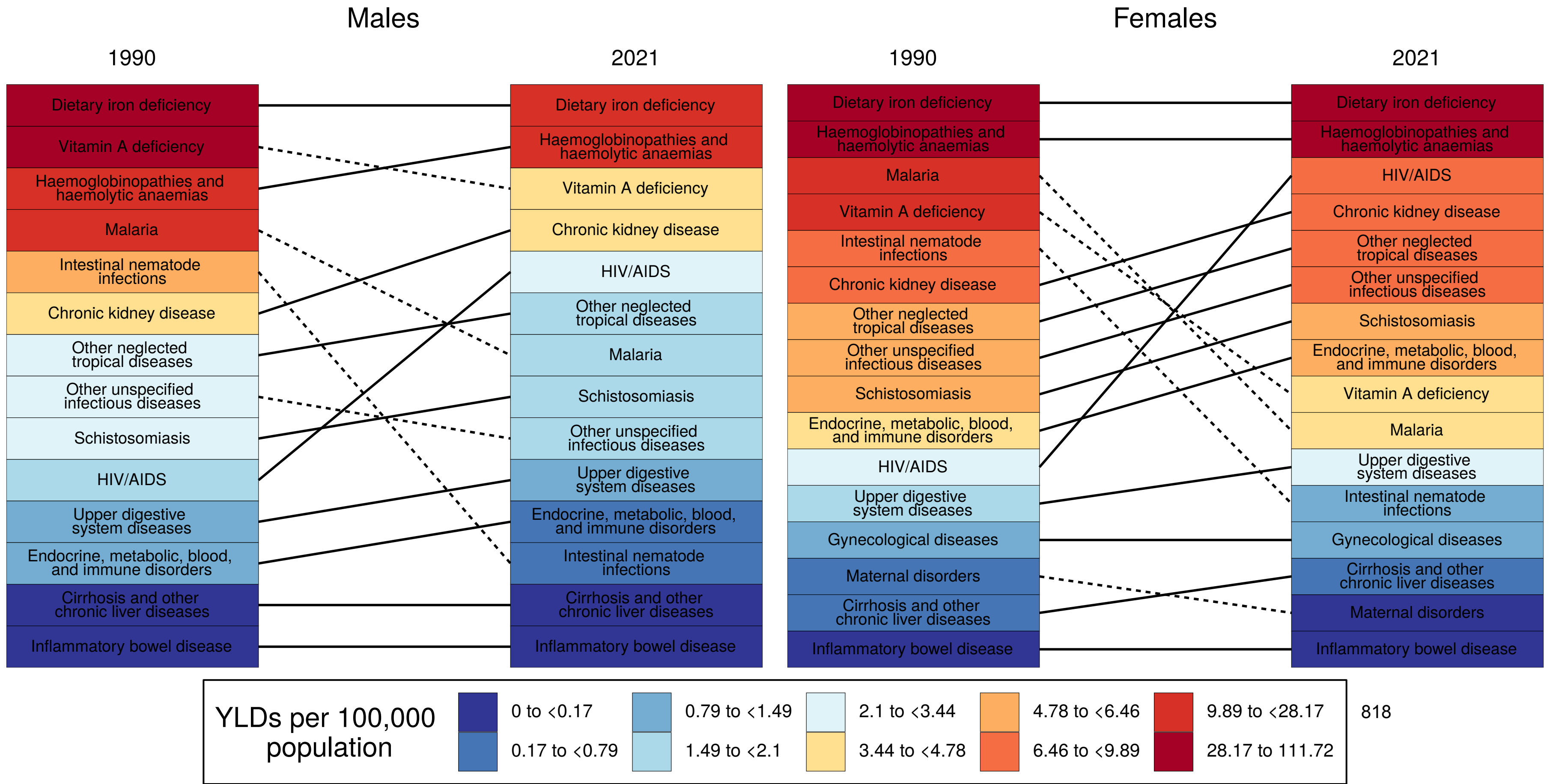

818

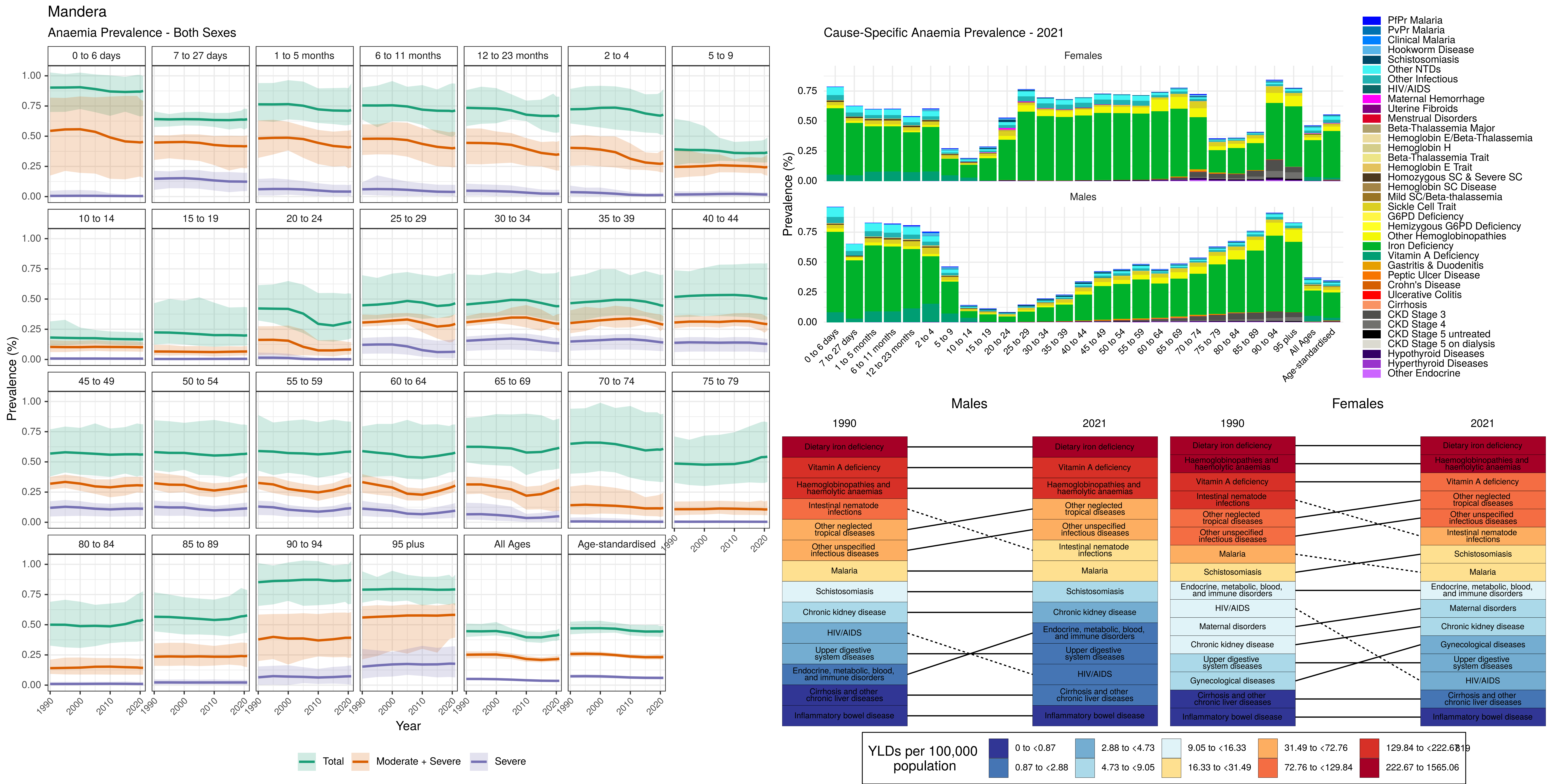
Mandera
Anaemia Prevalence - Both Sexes
0 to 6 days
7 to 27 days
1 to 5 months
6 to 11 months
12 to 23 months
2 to 4
5 to 9
10 to 14
15 to 19
20 to 24
25 to 29
30 to 34
35 to 39
40 to 44
45 to 49
50 to 54
55 to 59
60 to 64
65 to 69
70 to 74
75 to 79
80 to 84
85 to 89
90 to 94
95 plus
All Ages
Age-standardised
Prevalence (%)
Year
Total
Moderate + Severe
Severe
Cause-Specific Anaemia Prevalence - 2021
Females
Males
PfPr Malaria
PvPr Malaria
Clinical Malaria
Hookworm Disease
Schistosomiasis
Other NTDs
Other Infectious
HIV/AIDS
Maternal Hemorrhage
Uterine Fibroids
Menstrual Disorders
Beta-Thalassemia Major
Hemoglobin E/Beta-Thalassemia
Hemoglobin H
Beta-Thalassemia Trait
Hemoglobin E Trait
Homozygous SC & Severe SC
Hemoglobin SC Disease
Mild SC/Beta-thalassemia
Sickle Cell Trait
G6PD Deficiency
Hemizygous G6PD Deficiency
Other Hemoglobinopathies
Iron Deficiency
Vitamin A Deficiency
Gastritis & Duodenitis
Peptic Ulcer Disease
Crohn's Disease
Ulcerative Colitis
Cirrhosis
CKD Stage 3
CKD Stage 4
CKD Stage 5 untreated
CKD Stage 5 on dialysis
Hypothyroid Diseases
Hyperthyroid Diseases
Other Endocrine
Males
Females
1990
2021
Dietary iron deficiency
Vitamin A deficiency
Haemoglobinopathies and haemolytic anaemias
Intestinal nematode infections
Other neglected tropical diseases
Other unspecified infectious diseases
Malaria
Schistosomiasis
Chronic kidney disease
HIV/AIDS
Upper digestive system diseases
Endocrine, metabolic, blood, and immune disorders
Cirrhosis and other chronic liver diseases
Inflammatory bowel disease
Maternal disorders
Gynecological diseases
YLDs per 100,000 population
0 to <0.87
0.87 to <2.88
2.88 to <4.73
4.73 to <9.05
9.05 to <16.33
16.33 to <31.49
31.49 to <72.76
72.76 to <129.84
129.84 to <222.67
222.67 to 1565.06

Marsabit

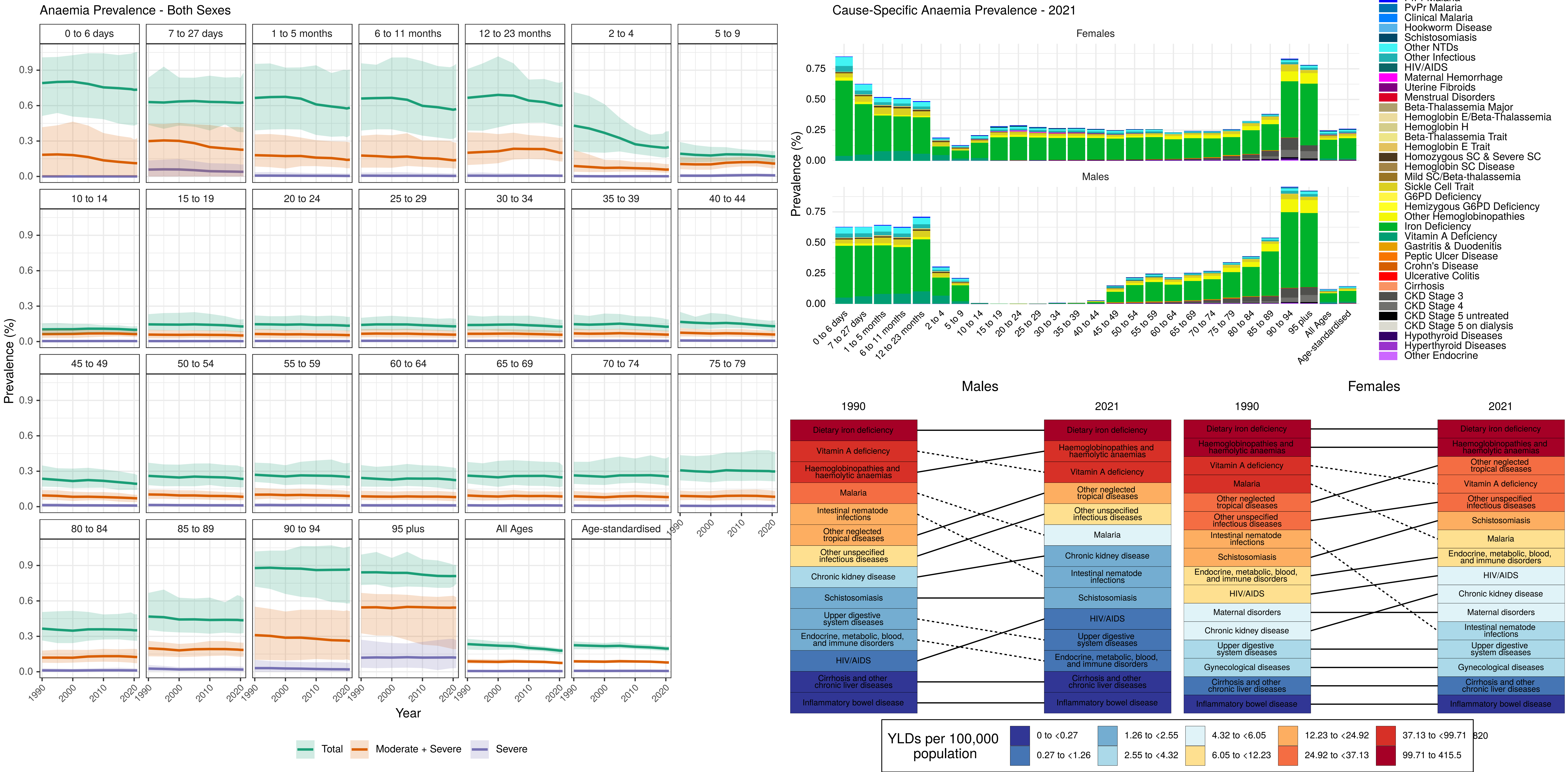

Anaemia Prevalence - Both Sexes
0 to 6 days
7 to 27 days
1 to 5 months
6 to 11 months
12 to 23 months
2 to 4
5 to 9
10 to 14
15 to 19
20 to 24
25 to 29
30 to 34
35 to 39
40 to 44
45 to 49
50 to 54
55 to 59
60 to 64
65 to 69
70 to 74
75 to 79
80 to 84
85 to 89
90 to 94
95 plus
All Ages
Age-standardised
Prevalence (%)
Year
Total
Moderate + Severe
Severe
Cause-Specific Anaemia Prevalence - 2021
Females
Males
Prevalence (%)
PfPr Malaria
PvPr Malaria
Clinical Malaria
Hookworm Disease
Schistosomiasis
Other NTDs
Other Infectious
HIV/AIDS
Maternal Hemorrhage
Uterine Fibroids
Menstrual Disorders
Beta-Thalassemia Major
Hemoglobin E/Beta-Thalassemia
Hemoglobin H
Beta-Thalassemia Trait
Hemoglobin E Trait
Homozygous SC & Severe SC
Hemoglobin SC Disease
Mild SC/Beta-thalassemia
Sickle Cell Trait
G6PD Deficiency
Hemizygous G6PD Deficiency
Other Hemoglobinopathies
Iron Deficiency
Vitamin A Deficiency
Gastritis & Duodenitis
Peptic Ulcer Disease
Crohn's Disease
Ulcerative Colitis
Cirrhosis
CKD Stage 3
CKD Stage 4
CKD Stage 5 untreated
CKD Stage 5 on dialysis
Hypothyroid Diseases
Hyperthyroid Diseases
Other Endocrine
Males
Females
1990
2021
Dietary iron deficiency
Vitamin A deficiency
Haemoglobinopathies and haemolytic anaemias
Malaria
Intestinal nematode infections
Other neglected tropical diseases
Other unspecified infectious diseases
Chronic kidney disease
Schistosomiasis
Upper digestive system diseases
Endocrine, metabolic, blood, and immune disorders
HIV/AIDS
Cirrhosis and other chronic liver diseases
Inflammatory bowel disease
Maternal disorders
Gynecological diseases
YLDs per 100,000 population
0 to <0.27
0.27 to <1.26
1.26 to <2.55
2.55 to <4.32
4.32 to <6.05
6.05 to <12.23
12.23 to <24.92
24.92 to <37.13
37.13 to <99.71
99.71 to 415.5

Meru

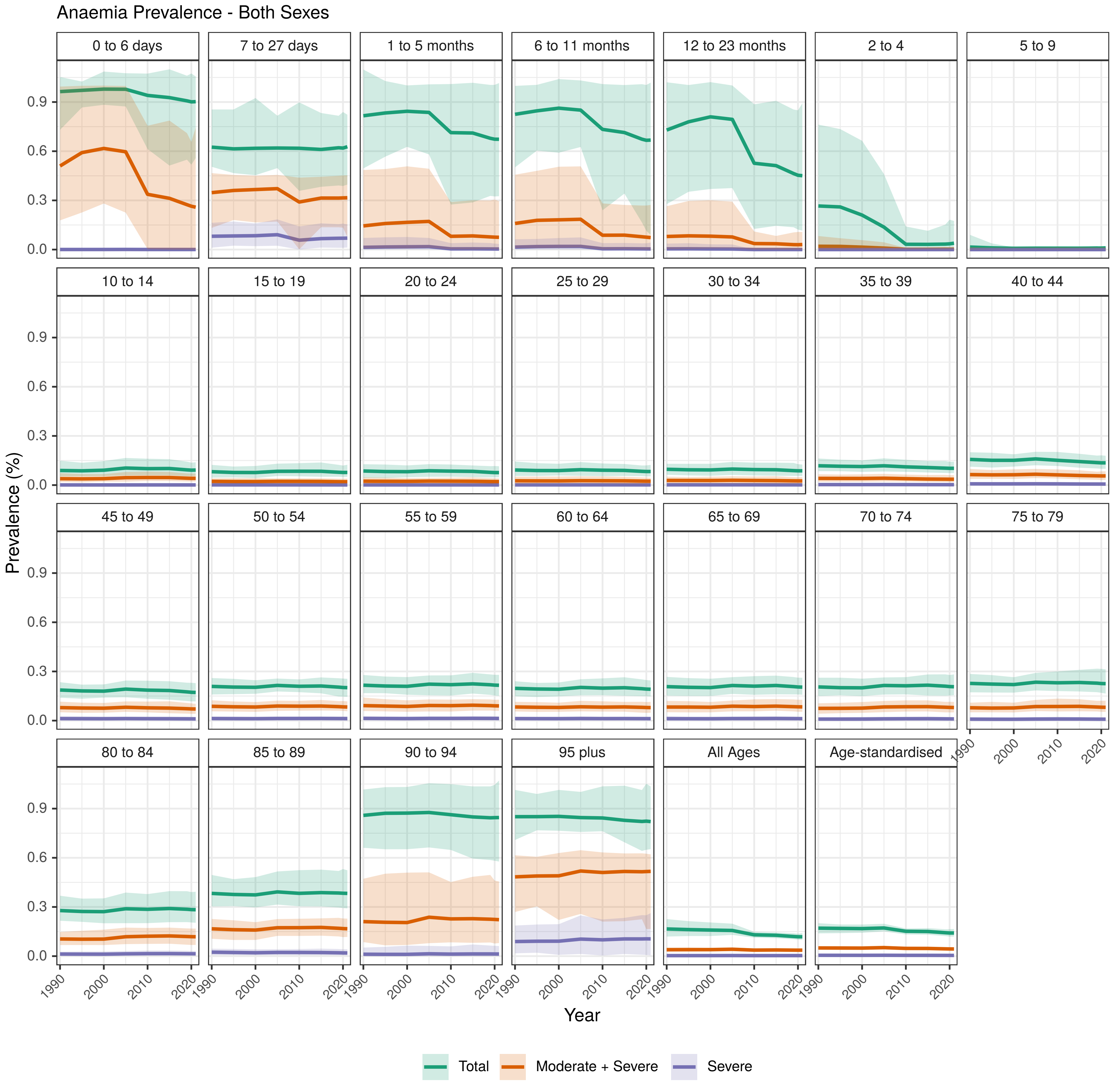
Anaemia Prevalence - Both Sexes
0 to 6 days
7 to 27 days
1 to 5 months
6 to 11 months
12 to 23 months
2 to 4
5 to 9
10 to 14
15 to 19
20 to 24
25 to 29
30 to 34
35 to 39
40 to 44
45 to 49
50 to 54
55 to 59
60 to 64
65 to 69
70 to 74
75 to 79
80 to 84
85 to 89
90 to 94
95 plus
All Ages
Age-standardised
Prevalence (%)
0.0
0.3
0.6
0.9
1990
2000
2010
2020
Year
Total
Moderate + Severe
Severe

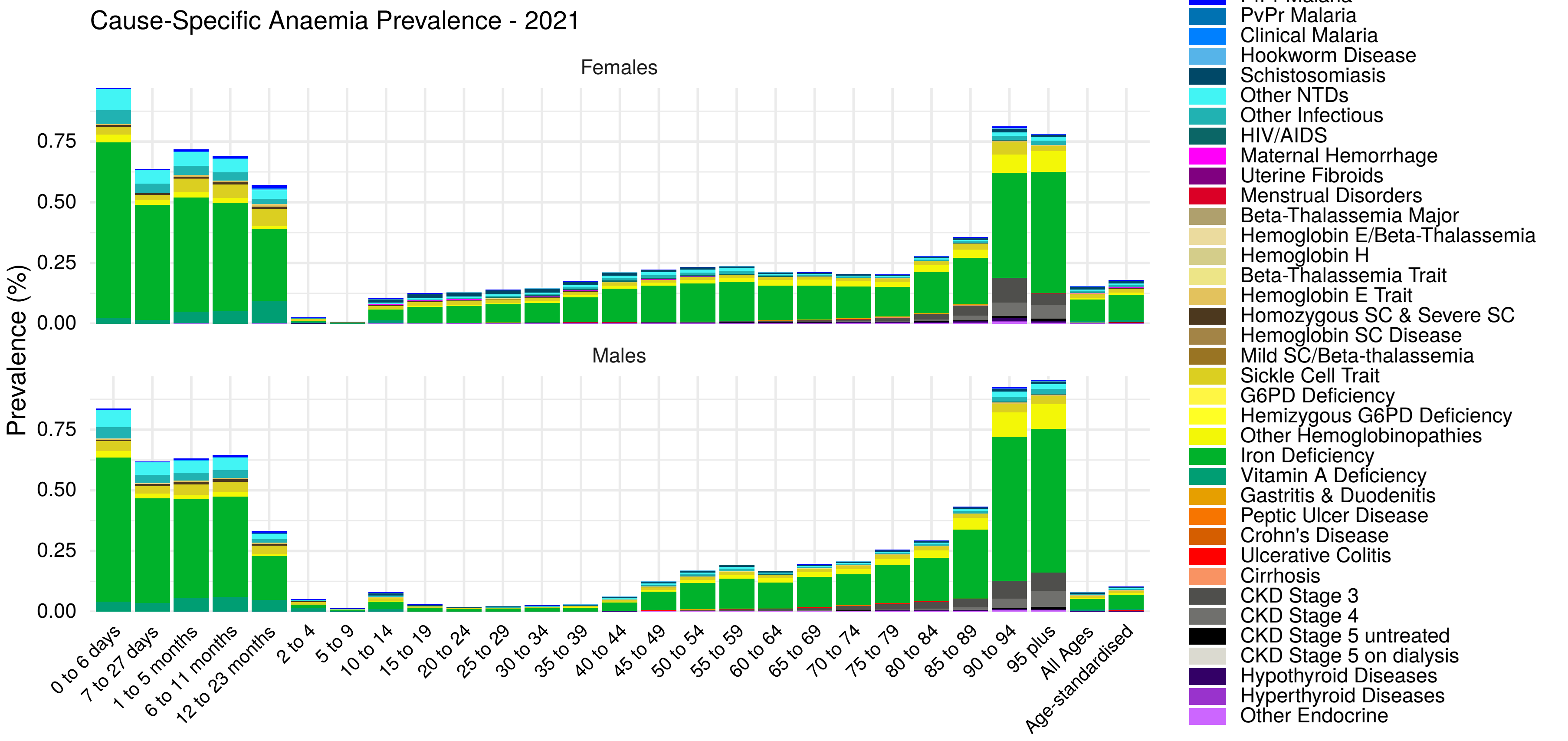
Cause-Specific Anaemia Prevalence - 2021
Females
Males
Prevalence (%)
0.00
0.25
0.50
0.75
0 to 6 days
7 to 27 days
1 to 5 months
6 to 11 months
12 to 23 months
2 to 4
5 to 9
10 to 14
15 to 19
20 to 24
25 to 29
30 to 34
35 to 39
40 to 44
45 to 49
50 to 54
55 to 59
60 to 64
65 to 69
70 to 74
75 to 79
80 to 84
85 to 89
90 to 94
95 plus
All Ages
Age-standardised
PfPr Malaria
PvPr Malaria
Clinical Malaria
Hookworm Disease
Schistosomiasis
Other NTDs
Other Infectious
HIV/AIDS
Maternal Hemorrhage
Uterine Fibroids
Menstrual Disorders
Beta-Thalassemia Major
Hemoglobin E/Beta-Thalassemia
Hemoglobin H
Beta-Thalassemia Trait
Hemoglobin E Trait
Homozygous SC & Severe SC
Hemoglobin SC Disease
Mild SC/Beta-thalassemia
Sickle Cell Trait
G6PD Deficiency
Hemizygous G6PD Deficiency
Other Hemoglobinopathies
Iron Deficiency
Vitamin A Deficiency
Gastritis & Duodenitis
Peptic Ulcer Disease
Crohn's Disease
Ulcerative Colitis
Cirrhosis
CKD Stage 3
CKD Stage 4
CKD Stage 5 untreated
CKD Stage 5 on dialysis
Hypothyroid Diseases
Hyperthyroid Diseases
Other Endocrine

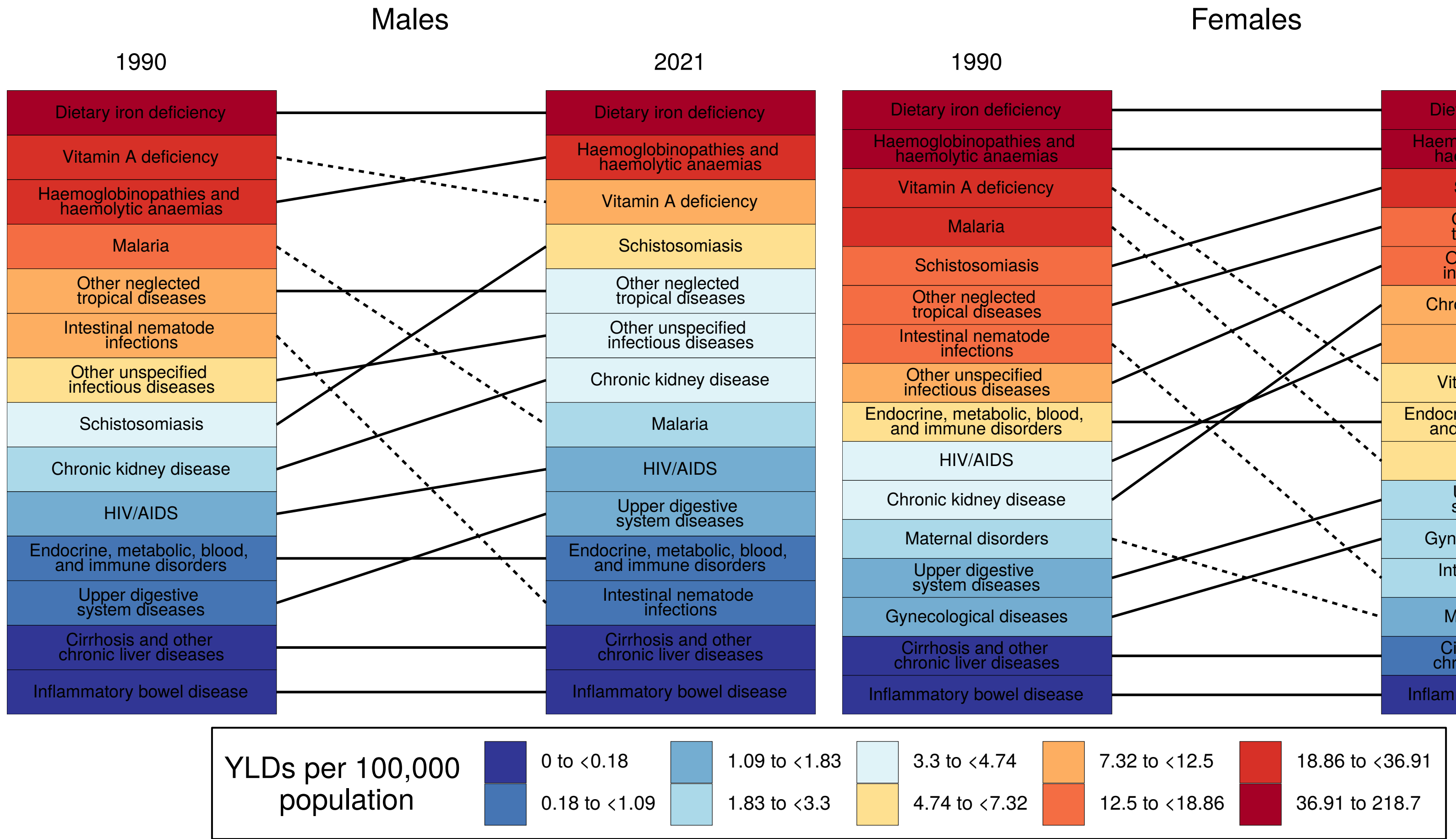
Males
Females
1990
2021
Dietary iron deficiency
Vitamin A deficiency
Haemoglobinopathies and haemolytic anaemias
Malaria
Other neglected tropical diseases
Intestinal nematode infections
Other unspecified infectious diseases
Schistosomiasis
Chronic kidney disease
HIV/AIDS
Endocrine, metabolic, blood, and immune disorders
Upper digestive system diseases
Cirrhosis and other chronic liver diseases
Inflammatory bowel disease
Maternal disorders
Gynecological diseases
YLDs per 100,000 population
0 to <0.18
0.18 to <1.09
1.09 to <1.83
1.83 to <3.3
3.3 to <4.74
4.74 to <7.32
7.32 to <12.5
12.5 to <18.86
18.86 to <36.91
36.91 to 218.7

821

Migori

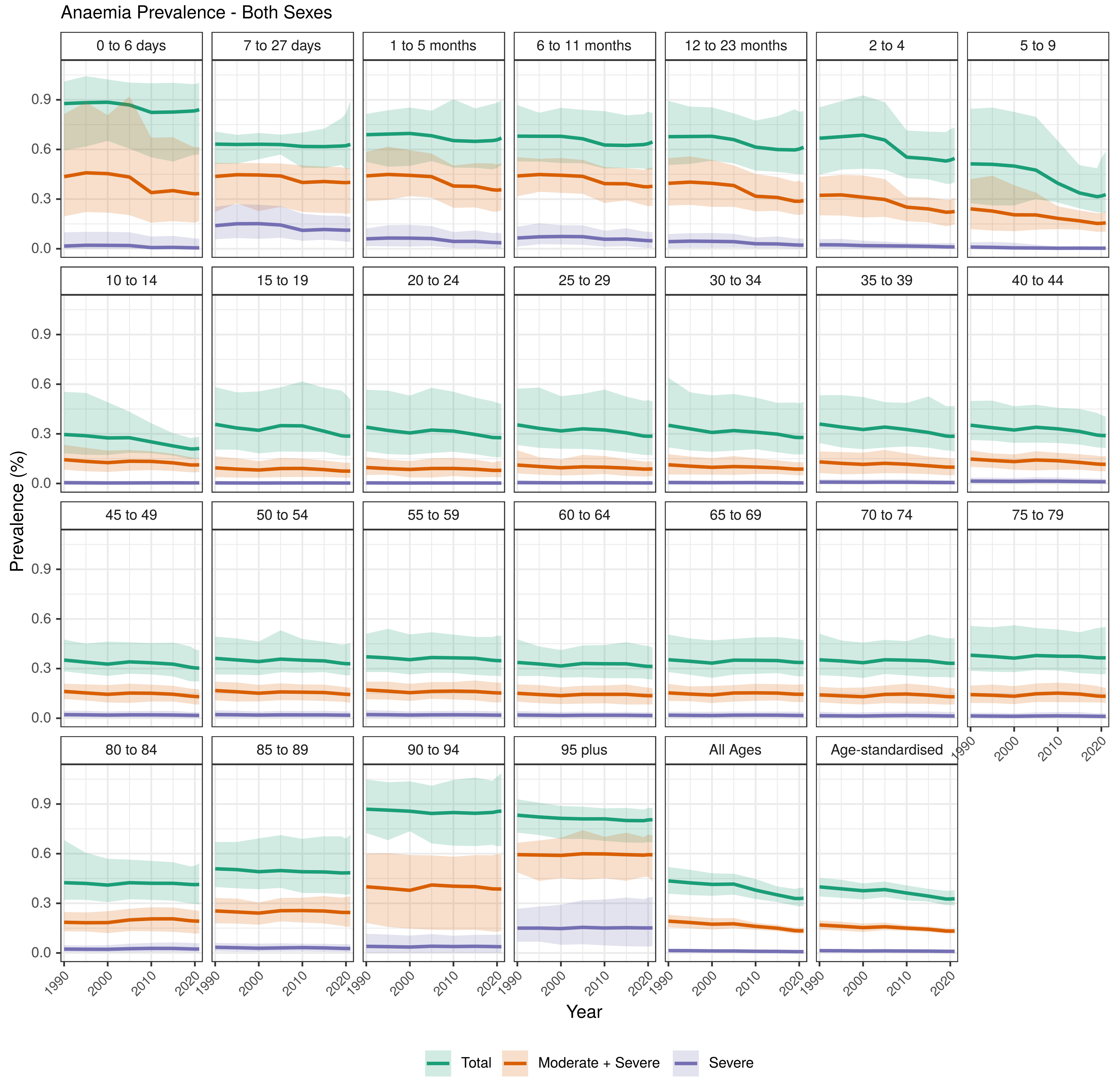

Anaemia Prevalence - Both Sexes
0 to 6 days
7 to 27 days
1 to 5 months
6 to 11 months
12 to 23 months
2 to 4
5 to 9
10 to 14
15 to 19
20 to 24
25 to 29
30 to 34
35 to 39
40 to 44
45 to 49
50 to 54
55 to 59
60 to 64
65 to 69
70 to 74
75 to 79
80 to 84
85 to 89
90 to 94
95 plus
All Ages
Age-standardised
Prevalence (%)
Year
Total
Moderate + Severe
Severe

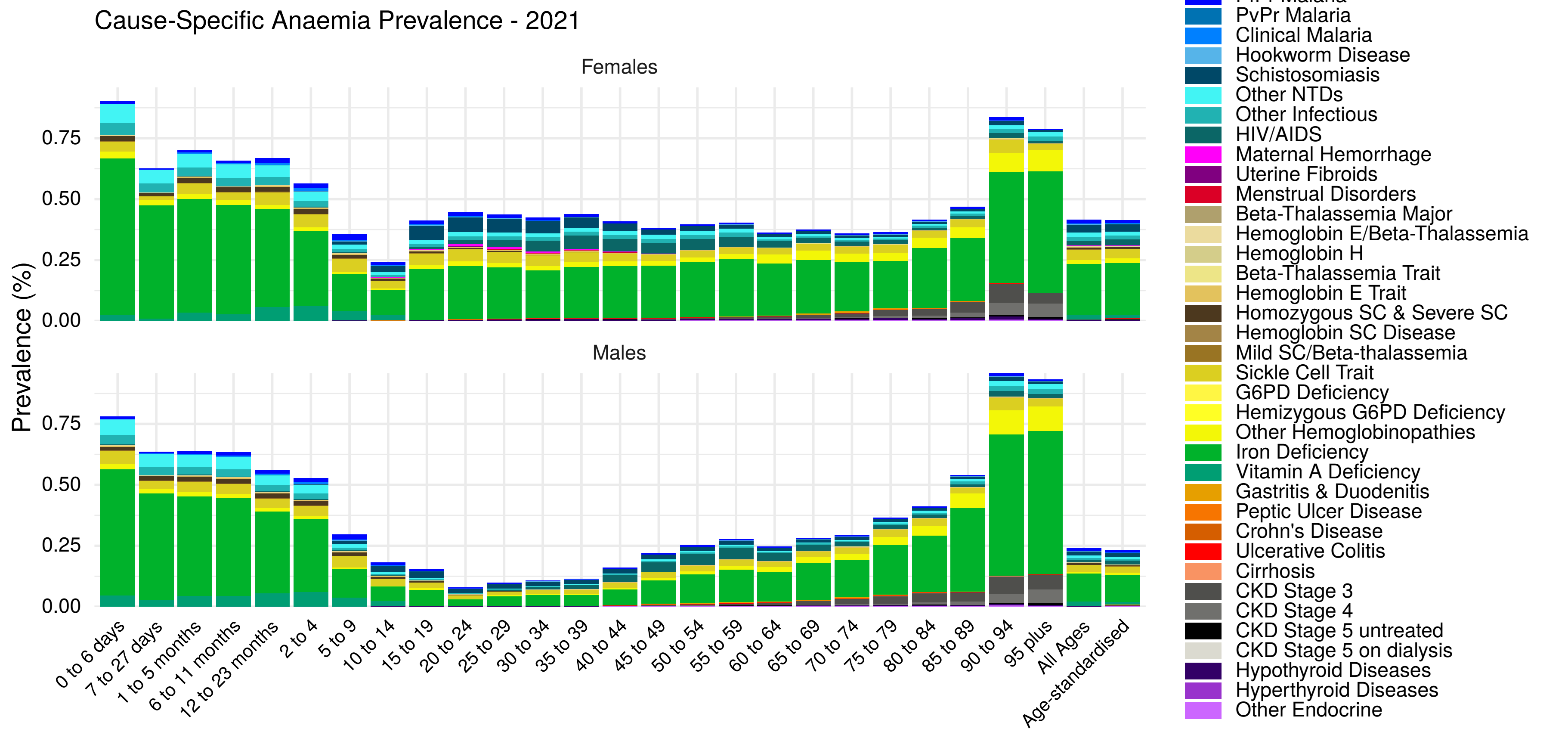

Cause-Specific Anaemia Prevalence - 2021
Females
Males
Prevalence (%)
0 to 6 days
7 to 27 days
1 to 5 months
6 to 11 months
12 to 23 months
2 to 4
5 to 9
10 to 14
15 to 19
20 to 24
25 to 29
30 to 34
35 to 39
40 to 44
45 to 49
50 to 54
55 to 59
60 to 64
65 to 69
70 to 74
75 to 79
80 to 84
85 to 89
90 to 94
95 plus
All Ages
Age-standardised
PfPr Malaria
PvPr Malaria
Clinical Malaria
Hookworm Disease
Schistosomiasis
Other NTDs
Other Infectious
HIV/AIDS
Maternal Hemorrhage
Uterine Fibroids
Menstrual Disorders
Beta-Thalassemia Major
Hemoglobin E/Beta-Thalassemia
Hemoglobin H
Beta-Thalassemia Trait
Hemoglobin E Trait
Homozygous SC & Severe SC
Hemoglobin SC Disease
Mild SC/Beta-thalassemia
Sickle Cell Trait
G6PD Deficiency
Hemizygous G6PD Deficiency
Other Hemoglobinopathies
Iron Deficiency
Vitamin A Deficiency
Gastritis & Duodenitis
Peptic Ulcer Disease
Crohn's Disease
Ulcerative Colitis
Cirrhosis
CKD Stage 3
CKD Stage 4
CKD Stage 5 untreated
CKD Stage 5 on dialysis
Hypothyroid Diseases
Hyperthyroid Diseases
Other Endocrine

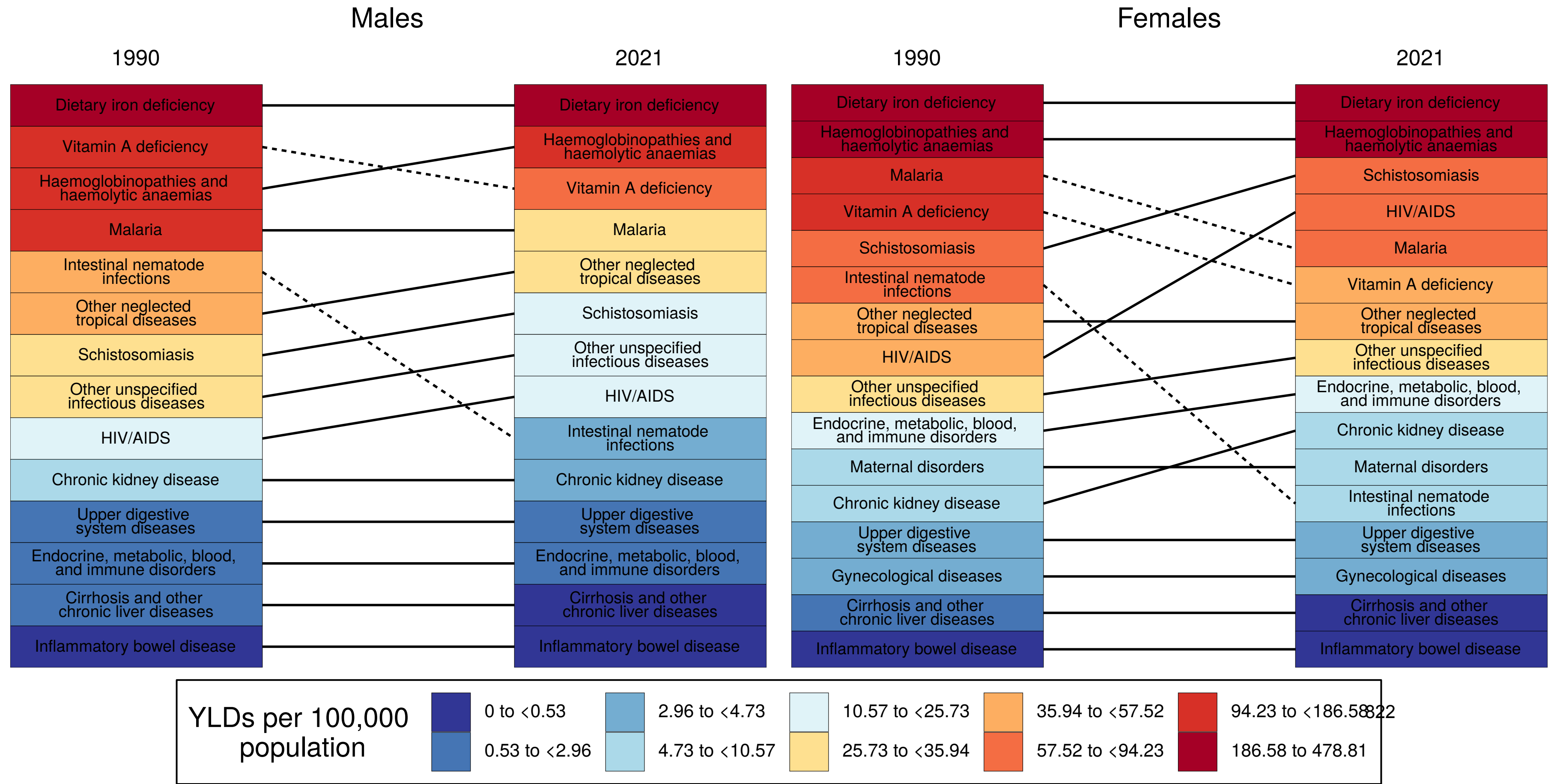

Males
Females
1990
2021
Dietary iron deficiency
Vitamin A deficiency
Haemoglobinopathies and haemolytic anaemias
Malaria
Intestinal nematode infections
Other neglected tropical diseases
Schistosomiasis
Other unspecified infectious diseases
HIV/AIDS
Chronic kidney disease
Upper digestive system diseases
Endocrine, metabolic, blood, and immune disorders
Cirrhosis and other chronic liver diseases
Inflammatory bowel disease
Maternal disorders
Gynecological diseases
YLDs per 100,000 population
0 to <0.53
0.53 to <2.96
2.96 to <4.73
4.73 to <10.57
10.57 to <25.73
25.73 to <35.94
35.94 to <57.52
57.52 to <94.23
94.23 to <186.58
186.58 to 478.81

Mombasa

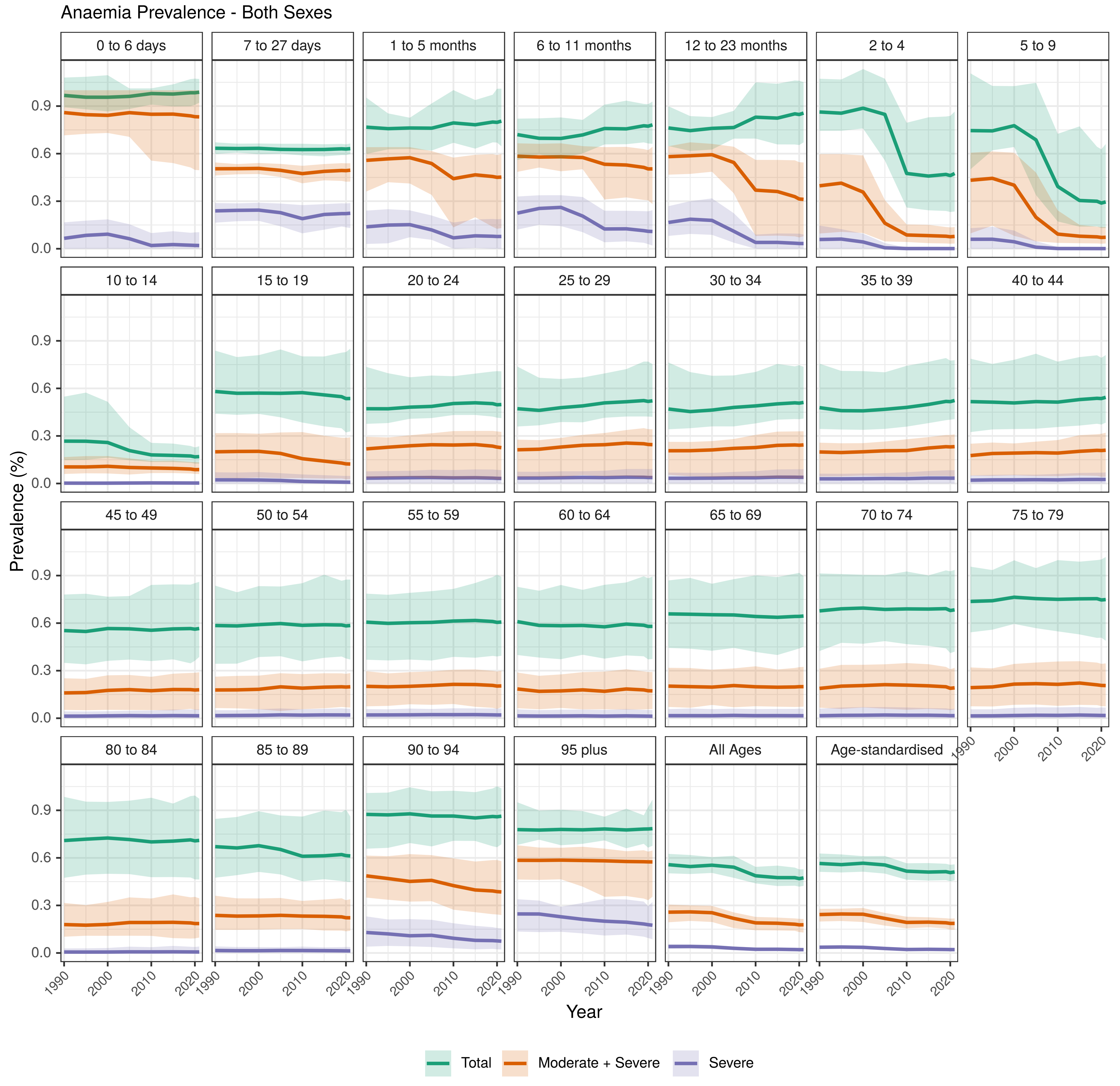
Anaemia Prevalence - Both Sexes
0 to 6 days
7 to 27 days
1 to 5 months
6 to 11 months
12 to 23 months
2 to 4
5 to 9
10 to 14
15 to 19
20 to 24
25 to 29
30 to 34
35 to 39
40 to 44
45 to 49
50 to 54
55 to 59
60 to 64
65 to 69
70 to 74
75 to 79
80 to 84
85 to 89
90 to 94
95 plus
All Ages
Age-standardised
Prevalence (%)
Year
Total
Moderate + Severe
Severe

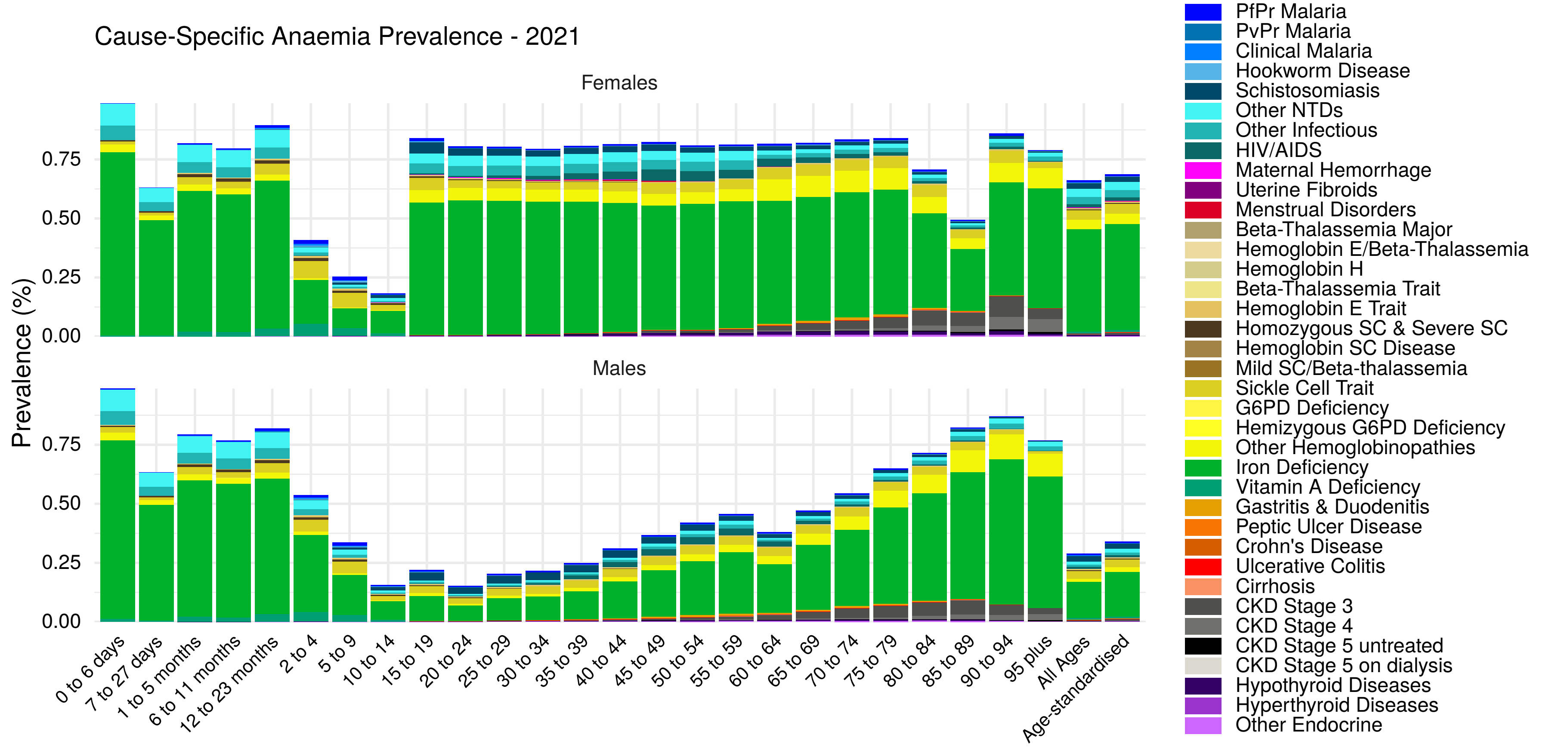
Cause-Specific Anaemia Prevalence - 2021
Females
Males
Prevalence (%)
PfPr Malaria
PvPr Malaria
Clinical Malaria
Hookworm Disease
Schistosomiasis
Other NTDs
Other Infectious
HIV/AIDS
Maternal Hemorrhage
Uterine Fibroids
Menstrual Disorders
Beta-Thalassemia Major
Hemoglobin E/Beta-Thalassemia
Hemoglobin H
Beta-Thalassemia Trait
Hemoglobin E Trait
Homozygous SC & Severe SC
Hemoglobin SC Disease
Mild SC/Beta-thalassemia
Sickle Cell Trait
G6PD Deficiency
Hemizygous G6PD Deficiency
Other Hemoglobinopathies
Iron Deficiency
Vitamin A Deficiency
Gastritis & Duodenitis
Peptic Ulcer Disease
Crohn's Disease
Ulcerative Colitis
Cirrhosis
CKD Stage 3
CKD Stage 4
CKD Stage 5 untreated
CKD Stage 5 on dialysis
Hypothyroid Diseases
Hyperthyroid Diseases
Other Endocrine

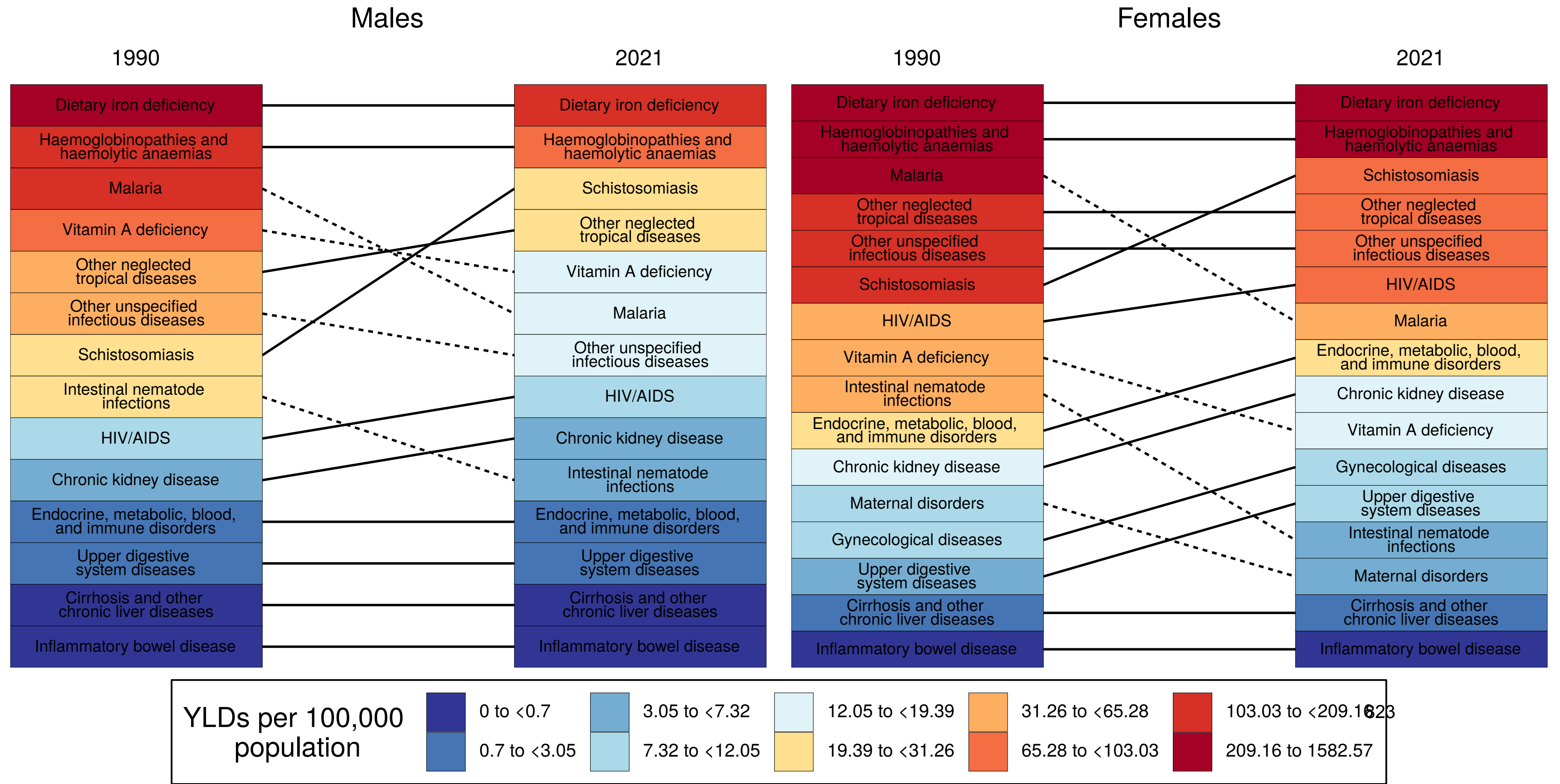
Males
Females
1990
2021
Dietary iron deficiency
Haemoglobinopathies and haemolytic anaemias
Malaria
Vitamin A deficiency
Other neglected tropical diseases
Other unspecified infectious diseases
Schistosomiasis
Intestinal nematode infections
HIV/AIDS
Chronic kidney disease
Endocrine, metabolic, blood, and immune disorders
Upper digestive system diseases
Cirrhosis and other chronic liver diseases
Inflammatory bowel disease
Maternal disorders
Gynecological diseases
YLDs per 100,000 population
0 to <0.7
0.7 to <3.05
3.05 to <7.32
7.32 to <12.05
12.05 to <19.39
19.39 to <31.26
31.26 to <65.28
65.28 to <103.03
103.03 to <209.16
209.16 to 1582.57

Murang'a

Anaemia Prevalence - Both Sexes

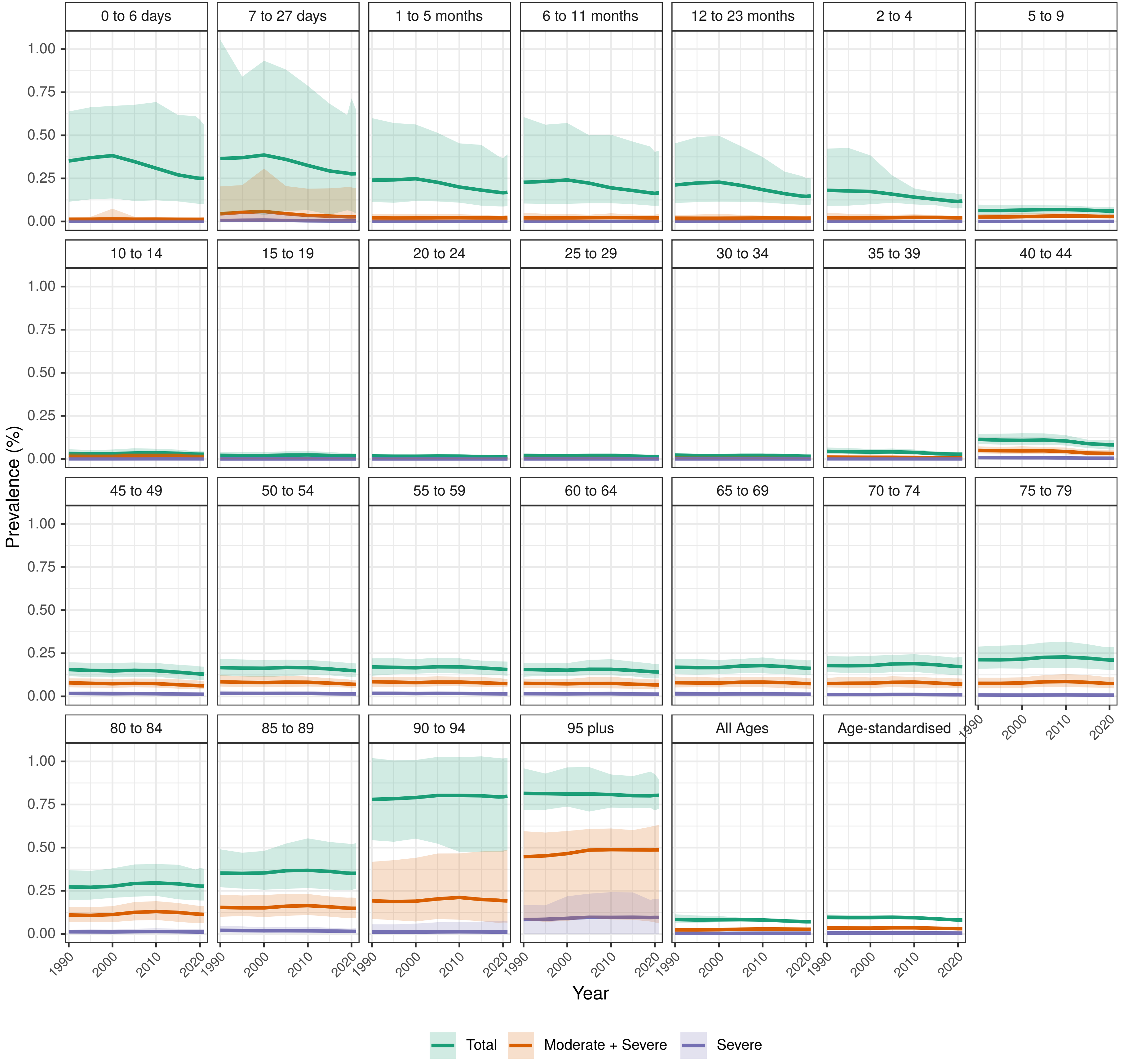

Cause-Specific Anaemia Prevalence - 2021

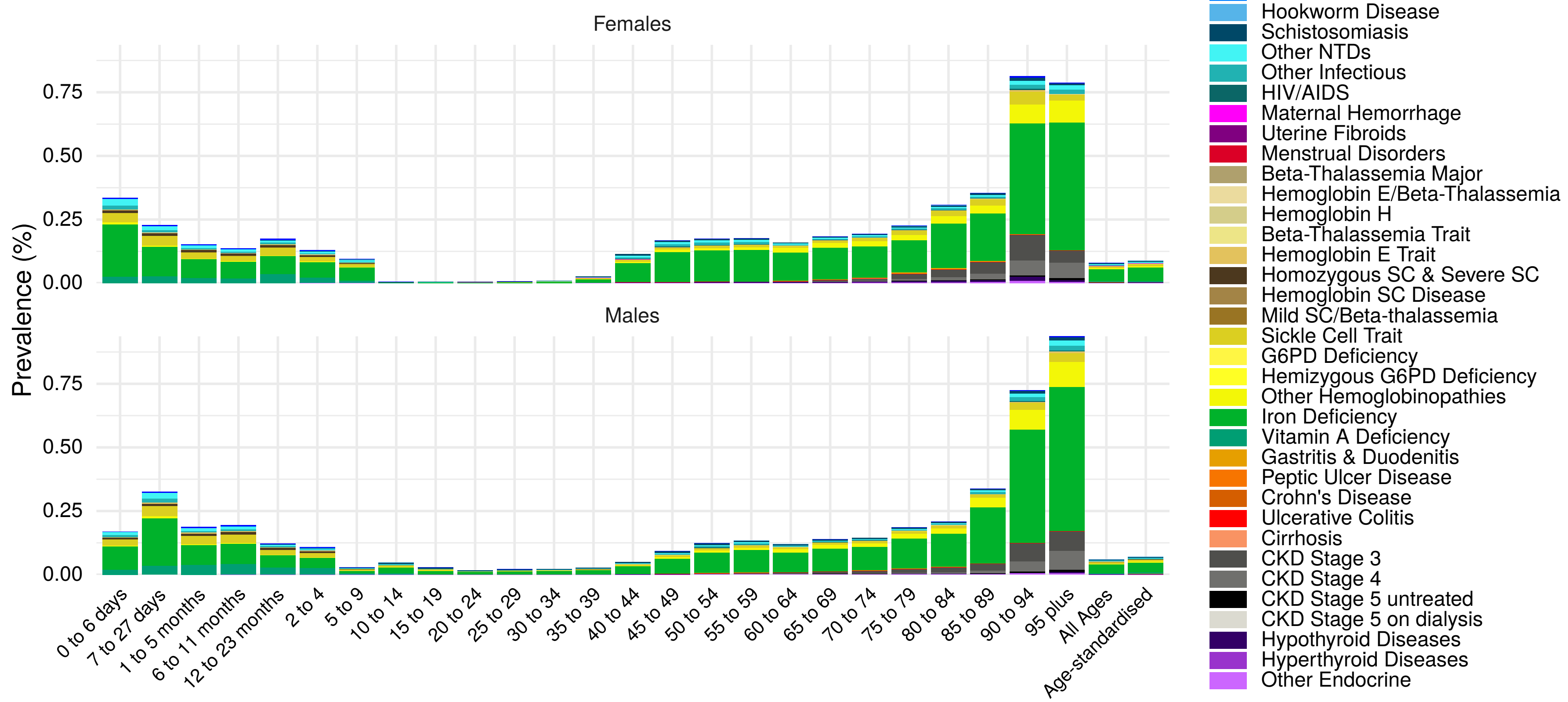

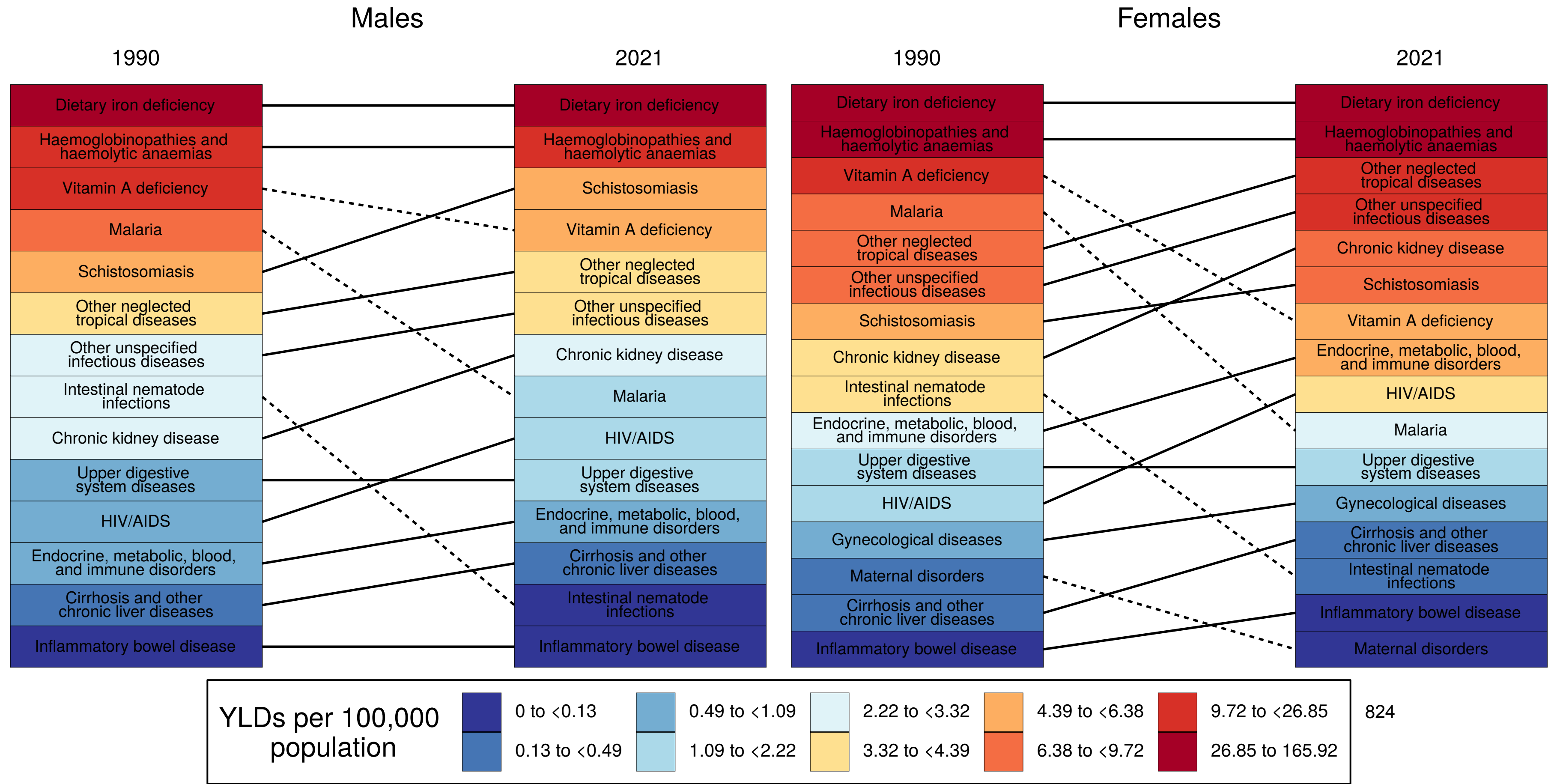

Nairobi

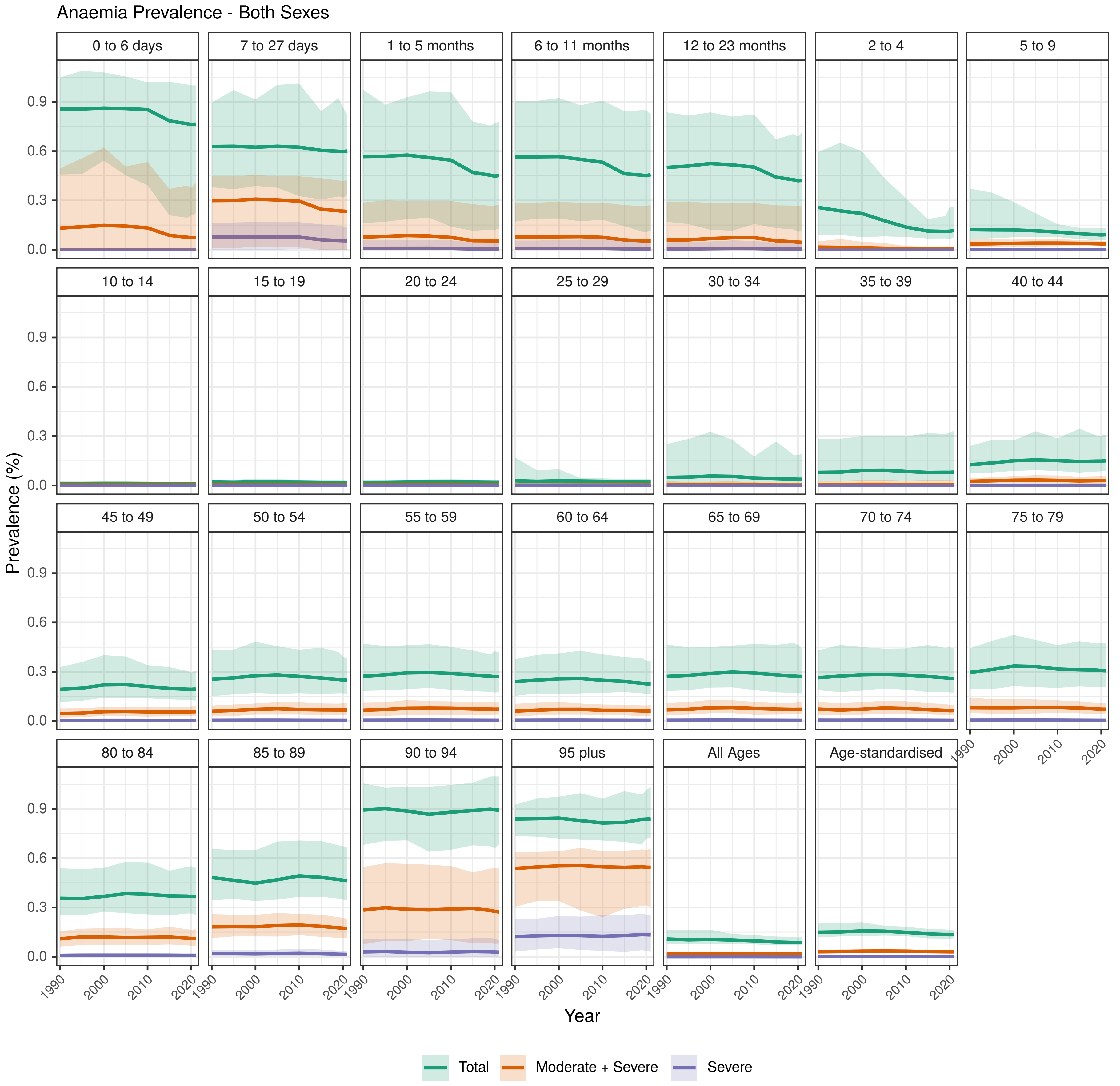
Anaemia Prevalence - Both Sexes
0 to 6 days
7 to 27 days
1 to 5 months
6 to 11 months
12 to 23 months
2 to 4
5 to 9
10 to 14
15 to 19
20 to 24
25 to 29
30 to 34
35 to 39
40 to 44
45 to 49
50 to 54
55 to 59
60 to 64
65 to 69
70 to 74
75 to 79
80 to 84
85 to 89
90 to 94
95 plus
All Ages
Age-standardised
Prevalence (%)
Year
Total
Moderate + Severe
Severe

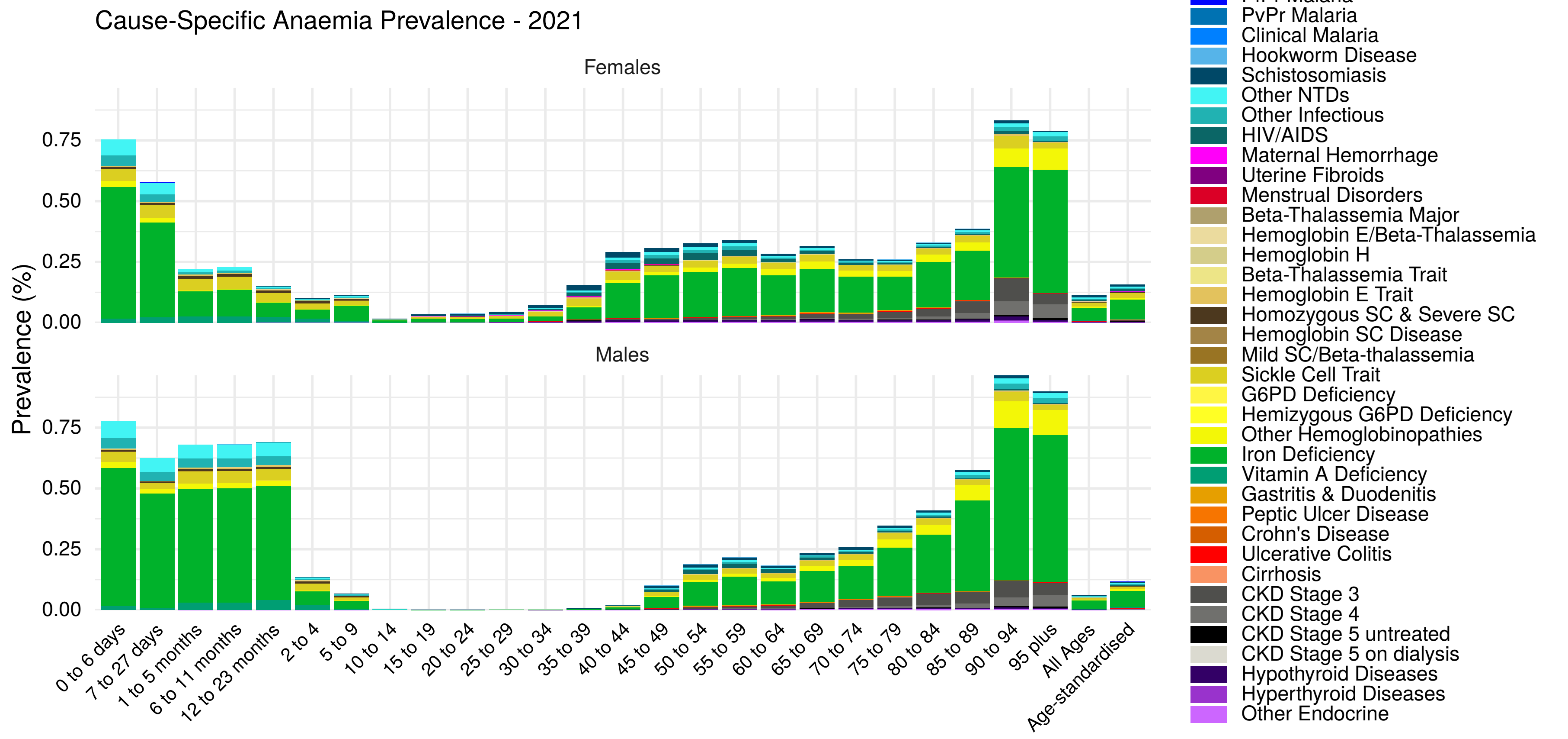
Cause-Specific Anaemia Prevalence - 2021
Females
Males
Prevalence (%)
PfPr Malaria
PvPr Malaria
Clinical Malaria
Hookworm Disease
Schistosomiasis
Other NTDs
Other Infectious
HIV/AIDS
Maternal Hemorrhage
Uterine Fibroids
Menstrual Disorders
Beta-Thalassemia Major
Hemoglobin E/Beta-Thalassemia
Hemoglobin H
Beta-Thalassemia Trait
Hemoglobin E Trait
Homozygous SC & Severe SC
Hemoglobin SC Disease
Mild SC/Beta-thalassemia
Sickle Cell Trait
G6PD Deficiency
Hemizygous G6PD Deficiency
Other Hemoglobinopathies
Iron Deficiency
Vitamin A Deficiency
Gastritis & Duodenitis
Peptic Ulcer Disease
Crohn's Disease
Ulcerative Colitis
Cirrhosis
CKD Stage 3
CKD Stage 4
CKD Stage 5 untreated
CKD Stage 5 on dialysis
Hypothyroid Diseases
Hyperthyroid Diseases
Other Endocrine

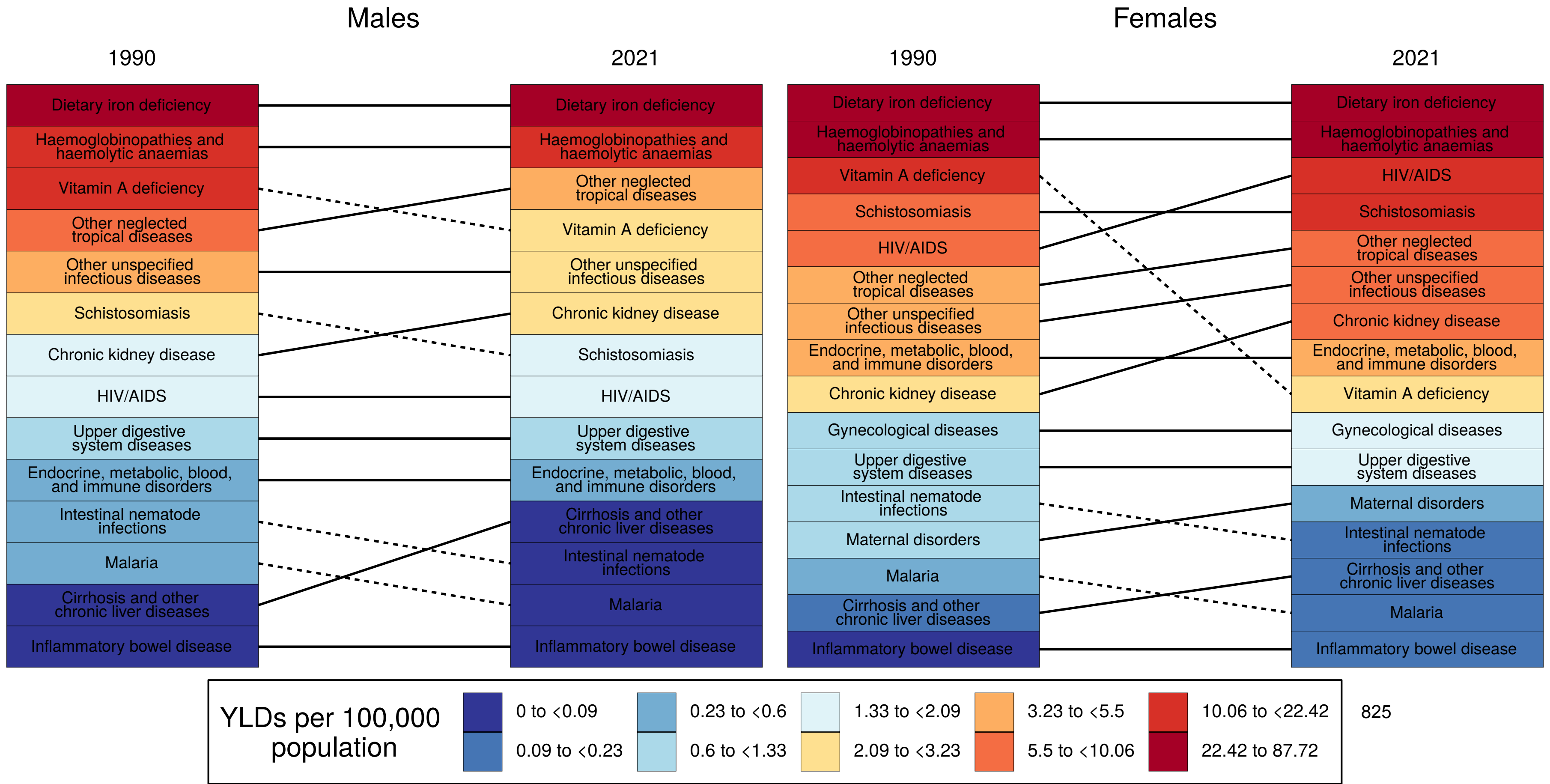
Males
Females
1990
2021
Dietary iron deficiency
Haemoglobinopathies and haemolytic anaemias
Vitamin A deficiency
Other neglected tropical diseases
Other unspecified infectious diseases
Schistosomiasis
Chronic kidney disease
HIV/AIDS
Upper digestive system diseases
Endocrine, metabolic, blood, and immune disorders
Intestinal nematode infections
Malaria
Cirrhosis and other chronic liver diseases
Inflammatory bowel disease
Gynecological diseases
Maternal disorders
YLDs per 100,000 population
0 to <0.09
0.09 to <0.23
0.23 to <0.6
0.6 to <1.33
1.33 to <2.09
2.09 to <3.23
3.23 to <5.5
5.5 to <10.06
10.06 to <22.42
22.42 to 87.72

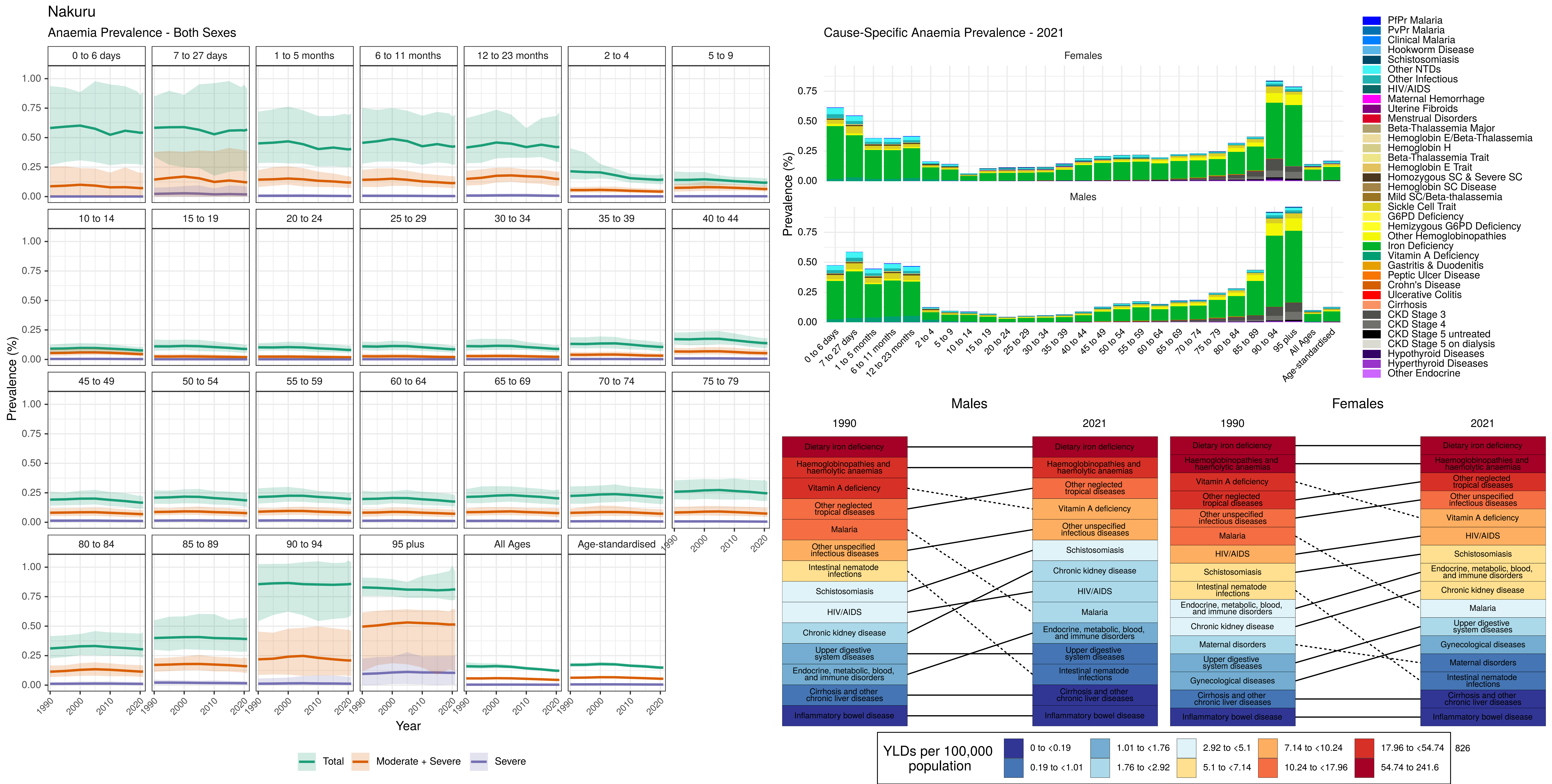
Nakuru
Anaemia Prevalence - Both Sexes
0 to 6 days
7 to 27 days
1 to 5 months
6 to 11 months
12 to 23 months
2 to 4
5 to 9
10 to 14
15 to 19
20 to 24
25 to 29
30 to 34
35 to 39
40 to 44
45 to 49
50 to 54
55 to 59
60 to 64
65 to 69
70 to 74
75 to 79
80 to 84
85 to 89
90 to 94
95 plus
All Ages
Age-standardised
Prevalence (%)
Year
Total
Moderate + Severe
Severe
Cause-Specific Anaemia Prevalence - 2021
Females
Males
PfPr Malaria
PvPr Malaria
Clinical Malaria
Hookworm Disease
Schistosomiasis
Other NTDs
Other Infectious
HIV/AIDS
Maternal Hemorrhage
Uterine Fibroids
Menstrual Disorders
Beta-Thalassemia Major
Hemoglobin E/Beta-Thalassemia
Hemoglobin H
Beta-Thalassemia Trait
Hemoglobin E Trait
Homozygous SC & Severe SC
Hemoglobin SC Disease
Mild SC/Beta-thalassemia
Sickle Cell Trait
G6PD Deficiency
Hemizygous G6PD Deficiency
Other Hemoglobinopathies
Iron Deficiency
Vitamin A Deficiency
Gastritis & Duodenitis
Peptic Ulcer Disease
Crohn's Disease
Ulcerative Colitis
Cirrhosis
CKD Stage 3
CKD Stage 4
CKD Stage 5 untreated
CKD Stage 5 on dialysis
Hypothyroid Diseases
Hyperthyroid Diseases
Other Endocrine
Males
1990
Dietary iron deficiency
Haemoglobinopathies and haemolytic anaemias
Vitamin A deficiency
Other neglected tropical diseases
Malaria
Other unspecified infectious diseases
Intestinal nematode infections
Schistosomiasis
HIV/AIDS
Chronic kidney disease
Upper digestive system diseases
Endocrine, metabolic, blood, and immune disorders
Cirrhosis and other chronic liver diseases
Inflammatory bowel disease
2021
Dietary iron deficiency
Haemoglobinopathies and haemolytic anaemias
Other neglected tropical diseases
Vitamin A deficiency
Other unspecified infectious diseases
Schistosomiasis
Chronic kidney disease
HIV/AIDS
Malaria
Endocrine, metabolic, blood, and immune disorders
Upper digestive system diseases
Intestinal nematode infections
Cirrhosis and other chronic liver diseases
Inflammatory bowel disease
Females
1990
Dietary iron deficiency
Haemoglobinopathies and haemolytic anaemias
Vitamin A deficiency
Other neglected tropical diseases
Other unspecified infectious diseases
Malaria
HIV/AIDS
Schistosomiasis
Intestinal nematode infections
Endocrine, metabolic, blood, and immune disorders
Chronic kidney disease
Maternal disorders
Upper digestive system diseases
Gynecological diseases
Cirrhosis and other chronic liver diseases
Inflammatory bowel disease
2021
Dietary iron deficiency
Haemoglobinopathies and haemolytic anaemias
Other neglected tropical diseases
Other unspecified infectious diseases
Vitamin A deficiency
HIV/AIDS
Schistosomiasis
Endocrine, metabolic, blood, and immune disorders
Chronic kidney disease
Malaria
Upper digestive system diseases
Gynecological diseases
Maternal disorders
Intestinal nematode infections
Cirrhosis and other chronic liver diseases
Inflammatory bowel disease
YLDs per 100,000 population
0 to <0.19
0.19 to <1.01
1.01 to <1.76
1.76 to <2.92
2.92 to <5.1
5.1 to <7.14
7.14 to <10.24
10.24 to <17.96
17.96 to <54.74
54.74 to 241.6

Nandi

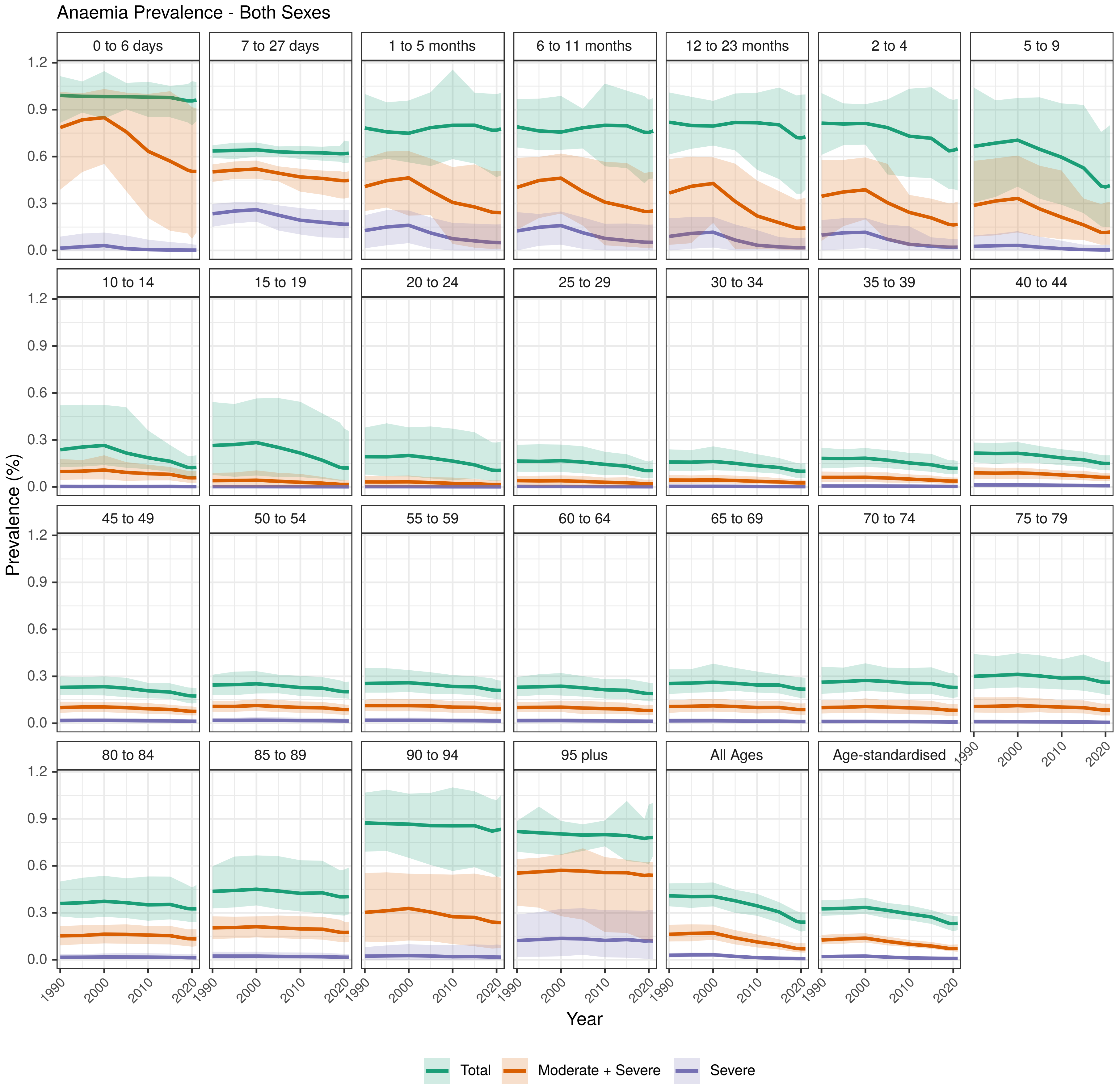

Anaemia Prevalence - Both Sexes
0 to 6 days
7 to 27 days
1 to 5 months
6 to 11 months
12 to 23 months
2 to 4
5 to 9
10 to 14
15 to 19
20 to 24
25 to 29
30 to 34
35 to 39
40 to 44
45 to 49
50 to 54
55 to 59
60 to 64
65 to 69
70 to 74
75 to 79
80 to 84
85 to 89
90 to 94
95 plus
All Ages
Age-standardised
0.0
0.3
0.6
0.9
1.2
1990
2000
2010
2020
Prevalence (%)
Year
Total
Moderate + Severe
Severe

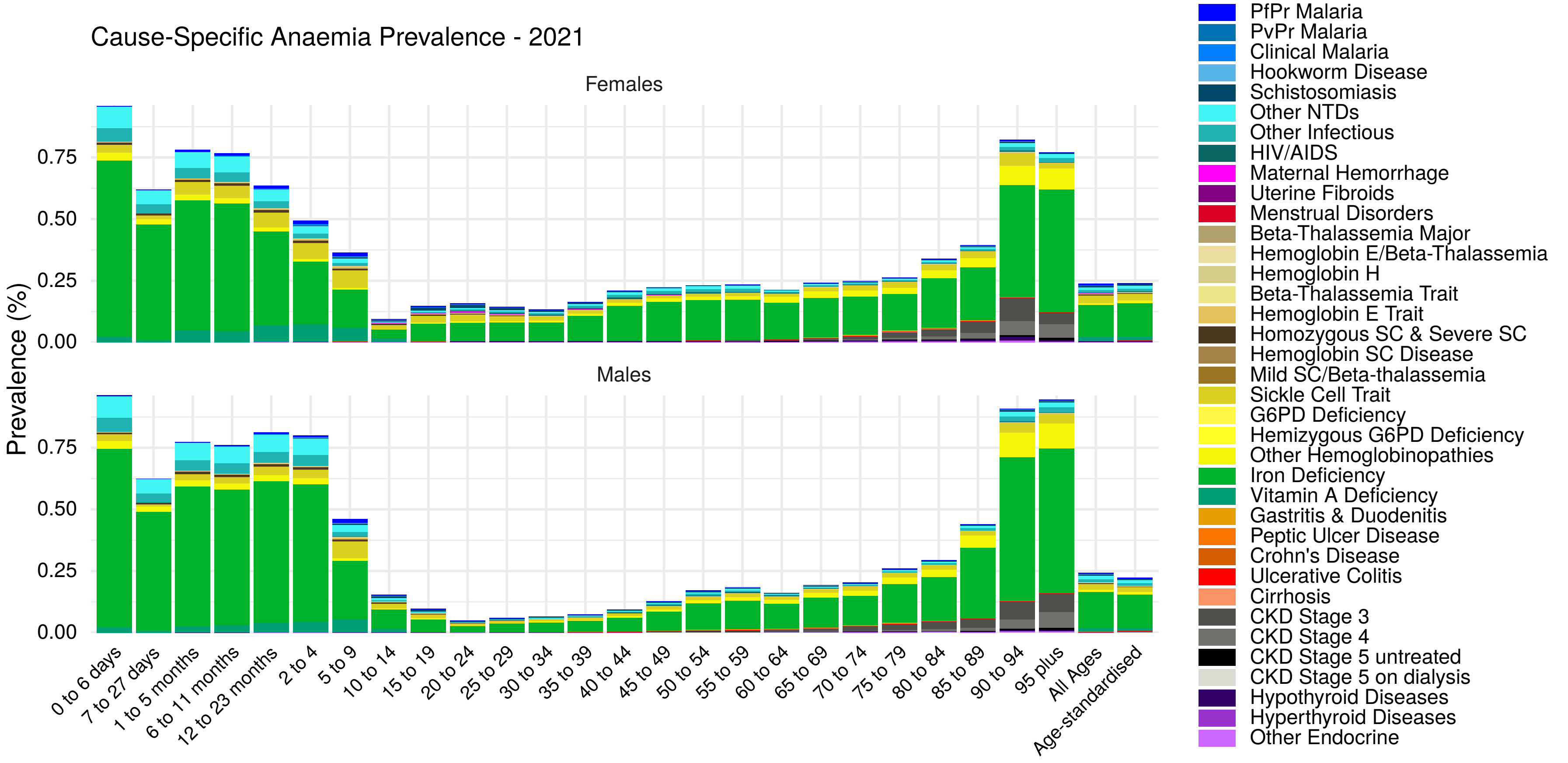

Cause-Specific Anaemia Prevalence - 2021
Females
Males
Prevalence (%)
0.00
0.25
0.50
0.75
0 to 6 days
7 to 27 days
1 to 5 months
6 to 11 months
12 to 23 months
2 to 4
5 to 9
10 to 14
15 to 19
20 to 24
25 to 29
30 to 34
35 to 39
40 to 44
45 to 49
50 to 54
55 to 59
60 to 64
65 to 69
70 to 74
75 to 79
80 to 84
85 to 89
90 to 94
95 plus
All Ages
Age-standardised
PfPr Malaria
PvPr Malaria
Clinical Malaria
Hookworm Disease
Schistosomiasis
Other NTDs
Other Infectious
HIV/AIDS
Maternal Hemorrhage
Uterine Fibroids
Menstrual Disorders
Beta-Thalassemia Major
Hemoglobin E/Beta-Thalassemia
Hemoglobin H
Beta-Thalassemia Trait
Hemoglobin E Trait
Homozygous SC & Severe SC
Hemoglobin SC Disease
Mild SC/Beta-thalassemia
Sickle Cell Trait
G6PD Deficiency
Hemizygous G6PD Deficiency
Other Hemoglobinopathies
Iron Deficiency
Vitamin A Deficiency
Gastritis & Duodenitis
Peptic Ulcer Disease
Crohn's Disease
Ulcerative Colitis
Cirrhosis
CKD Stage 3
CKD Stage 4
CKD Stage 5 untreated
CKD Stage 5 on dialysis
Hypothyroid Diseases
Hyperthyroid Diseases
Other Endocrine

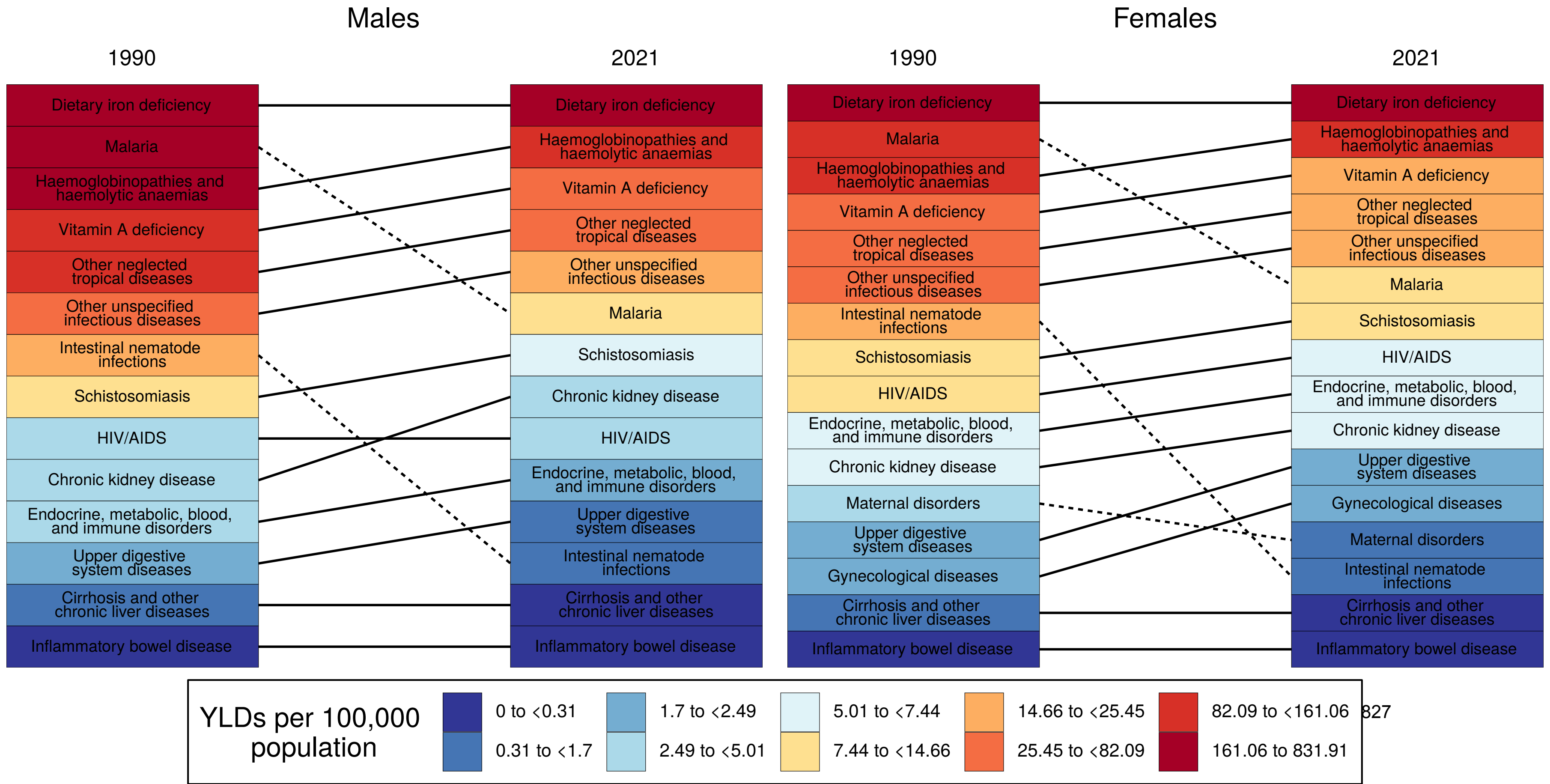

Males
Females
1990
2021
Dietary iron deficiency
Malaria
Haemoglobinopathies and haemolytic anaemias
Vitamin A deficiency
Other neglected tropical diseases
Other unspecified infectious diseases
Intestinal nematode infections
Schistosomiasis
HIV/AIDS
Chronic kidney disease
Endocrine, metabolic, blood, and immune disorders
Upper digestive system diseases
Cirrhosis and other chronic liver diseases
Inflammatory bowel disease
Maternal disorders
Gynecological diseases
YLDs per 100,000 population
0 to <0.31
0.31 to <1.7
1.7 to <2.49
2.49 to <5.01
5.01 to <7.44
7.44 to <14.66
14.66 to <25.45
25.45 to <82.09
82.09 to <161.06
161.06 to 831.91

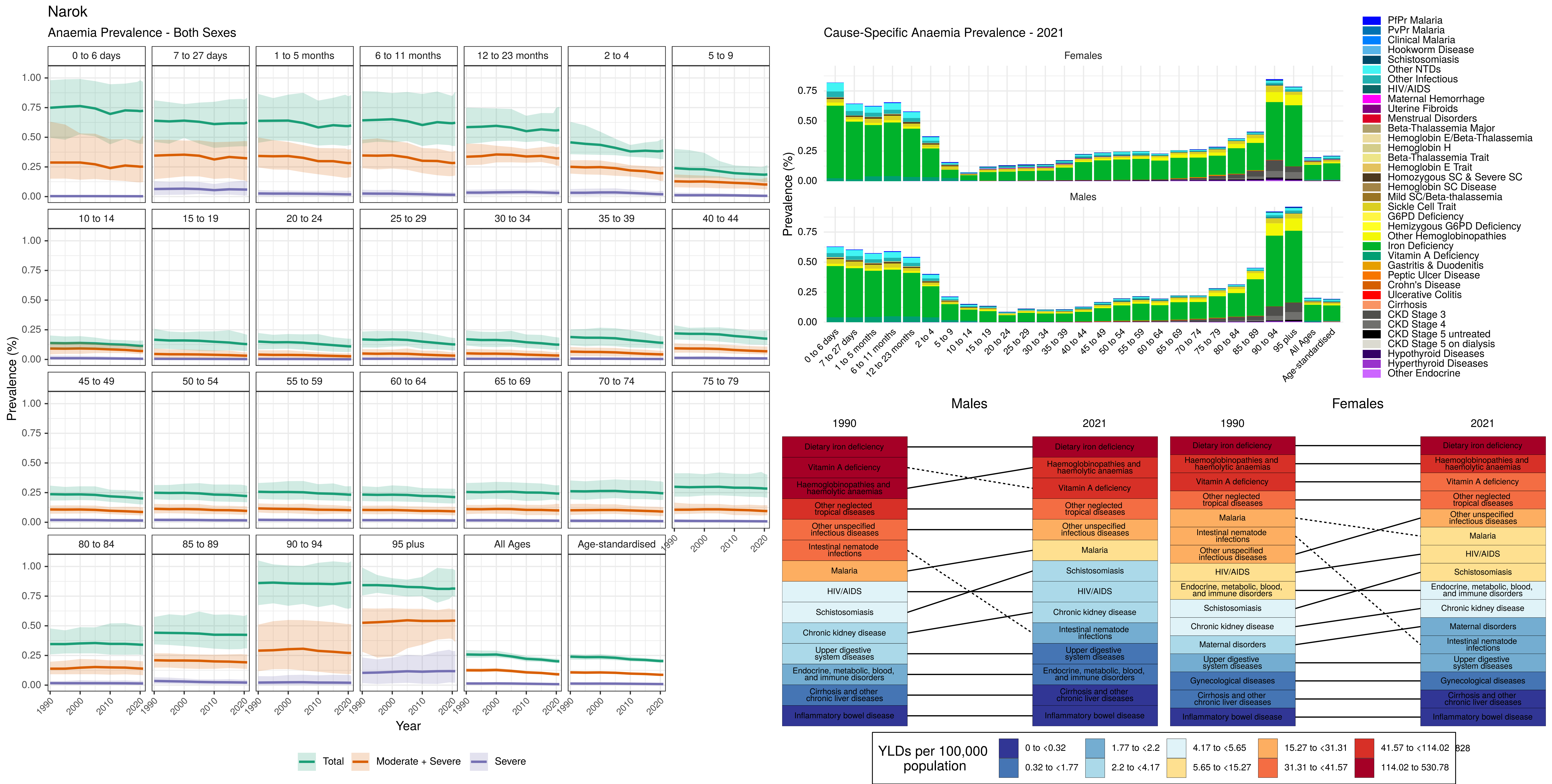

Narok
Anaemia Prevalence - Both Sexes
0 to 6 days
7 to 27 days
1 to 5 months
6 to 11 months
12 to 23 months
2 to 4
5 to 9
10 to 14
15 to 19
20 to 24
25 to 29
30 to 34
35 to 39
40 to 44
45 to 49
50 to 54
55 to 59
60 to 64
65 to 69
70 to 74
75 to 79
80 to 84
85 to 89
90 to 94
95 plus
All Ages
Age-standardised
Prevalence (%)
Year
Total
Moderate + Severe
Severe
Cause-Specific Anaemia Prevalence - 2021
Females
Males
PfPr Malaria
PvPr Malaria
Clinical Malaria
Hookworm Disease
Schistosomiasis
Other NTDs
Other Infectious
HIV/AIDS
Maternal Hemorrhage
Uterine Fibroids
Menstrual Disorders
Beta-Thalassemia Major
Hemoglobin E/Beta-Thalassemia
Hemoglobin H
Beta-Thalassemia Trait
Hemoglobin E Trait
Homozygous SC & Severe SC
Hemoglobin SC Disease
Mild SC/Beta-thalassemia
Sickle Cell Trait
G6PD Deficiency
Hemizygous G6PD Deficiency
Other Hemoglobinopathies
Iron Deficiency
Vitamin A Deficiency
Gastritis & Duodenitis
Peptic Ulcer Disease
Crohn's Disease
Ulcerative Colitis
Cirrhosis
CKD Stage 3
CKD Stage 4
CKD Stage 5 untreated
CKD Stage 5 on dialysis
Hypothyroid Diseases
Hyperthyroid Diseases
Other Endocrine
Males
Females
1990
2021
Dietary iron deficiency
Vitamin A deficiency
Haemoglobinopathies and haemolytic anaemias
Other neglected tropical diseases
Other unspecified infectious diseases
Intestinal nematode infections
Malaria
HIV/AIDS
Schistosomiasis
Chronic kidney disease
Upper digestive system diseases
Endocrine, metabolic, blood, and immune disorders
Cirrhosis and other chronic liver diseases
Inflammatory bowel disease
Maternal disorders
Gynecological diseases
YLDs per 100,000 population
0 to <0.32
0.32 to <1.77
1.77 to <2.2
2.2 to <4.17
4.17 to <5.65
5.65 to <15.27
15.27 to <31.31
31.31 to <41.57
41.57 to <114.02
114.02 to 530.78

Nyamira

Anaemia Prevalence - Both Sexes

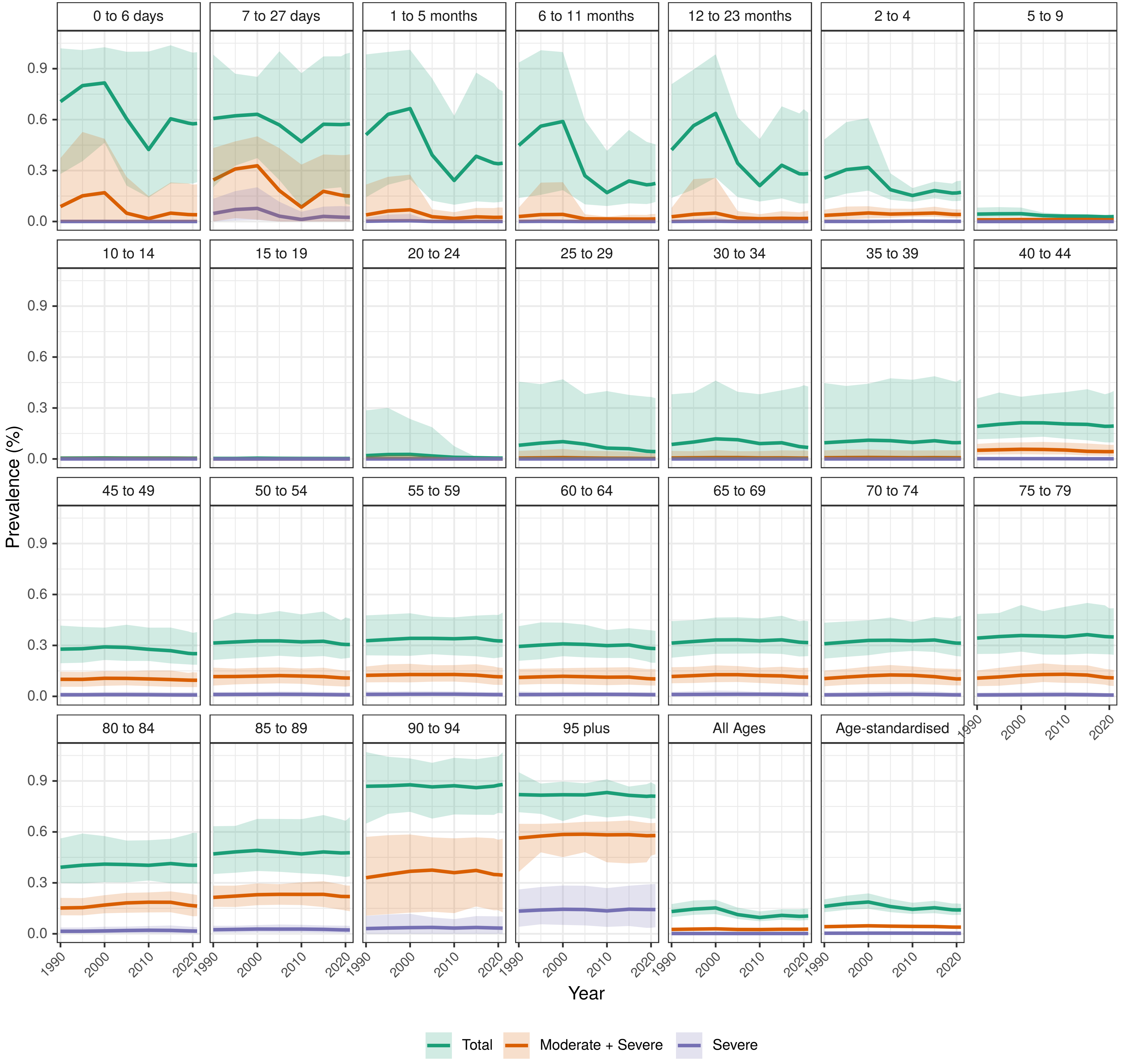

Cause-Specific Anaemia Prevalence - 2021

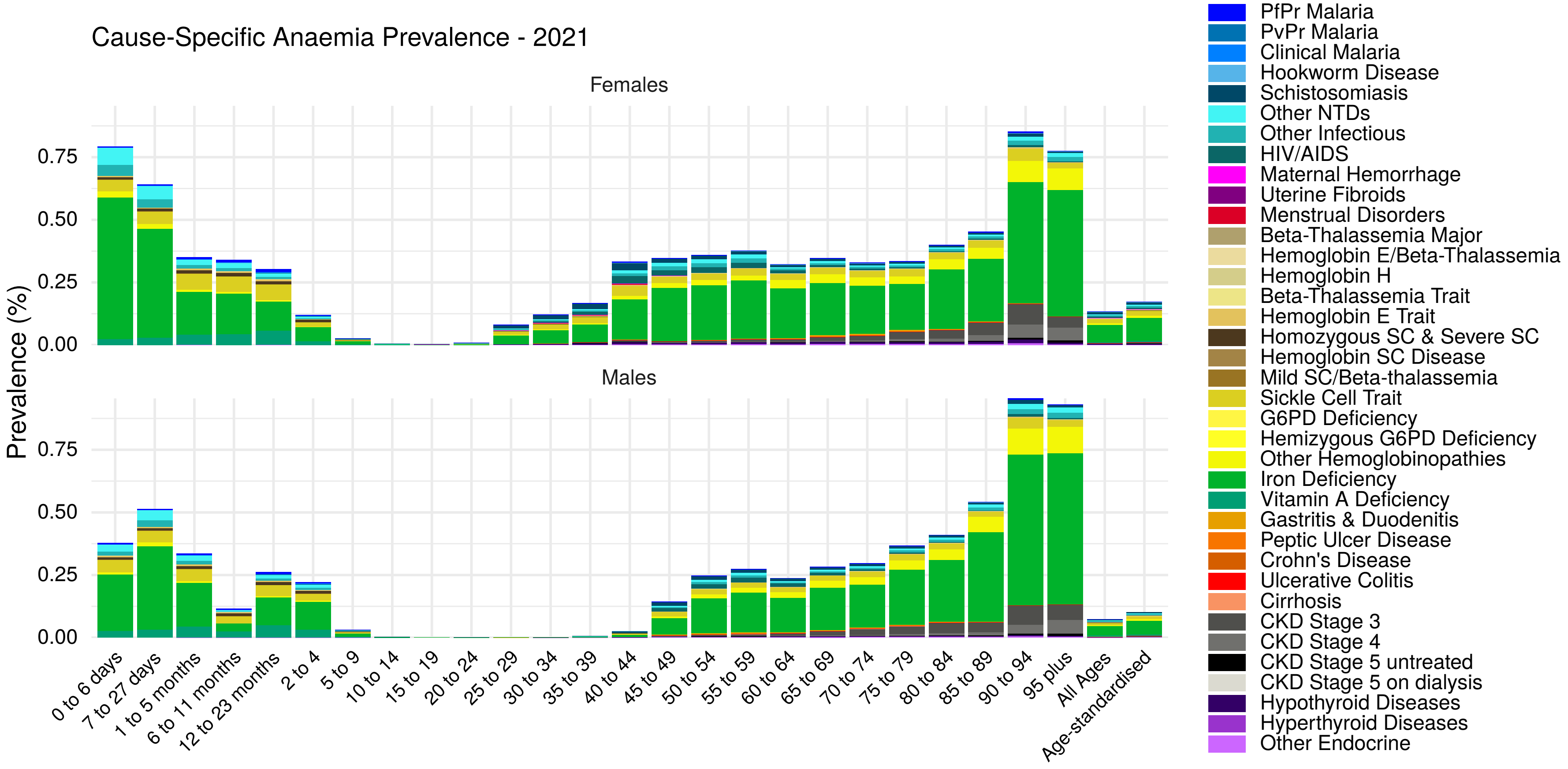

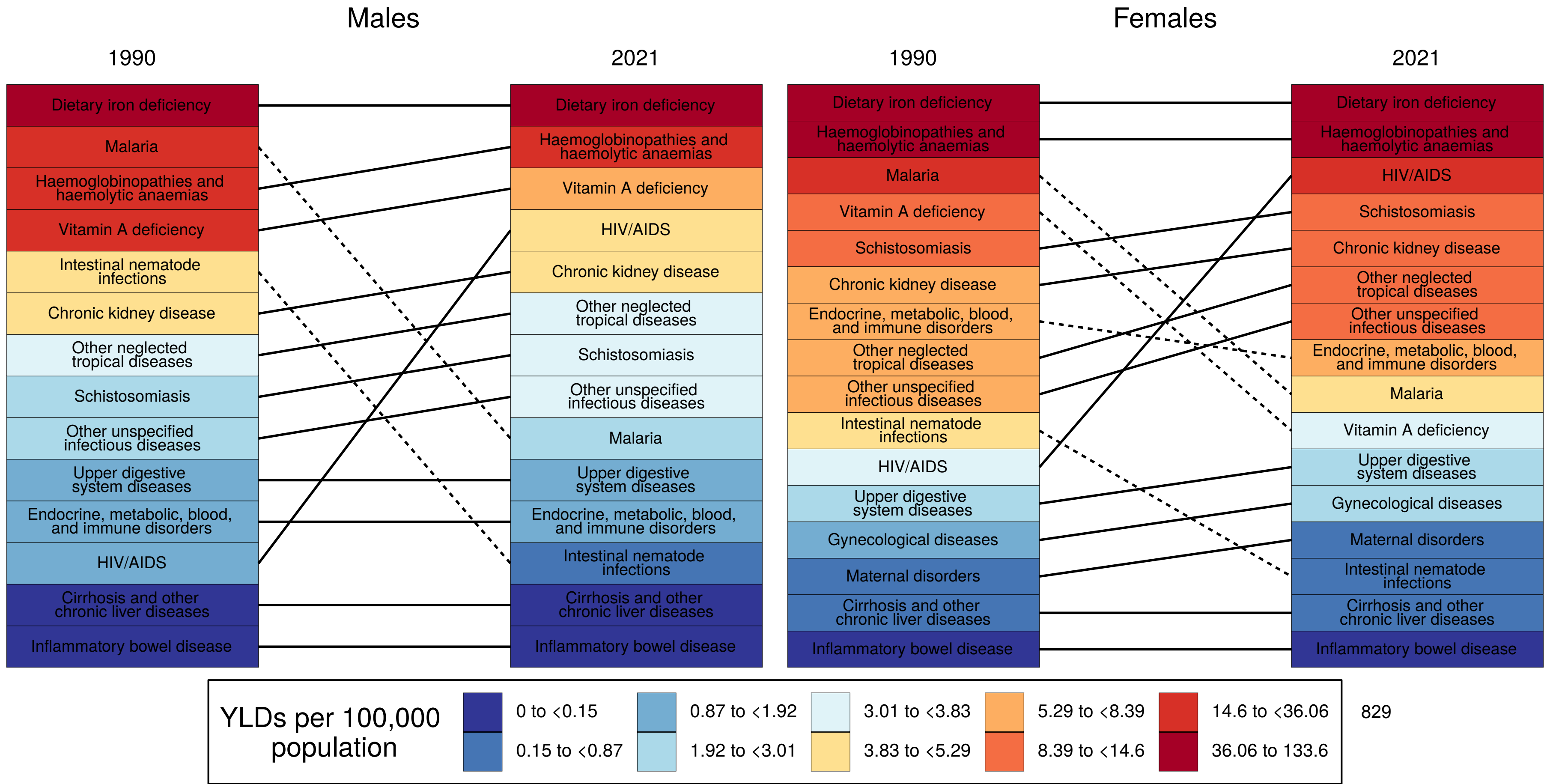

Nyandarua

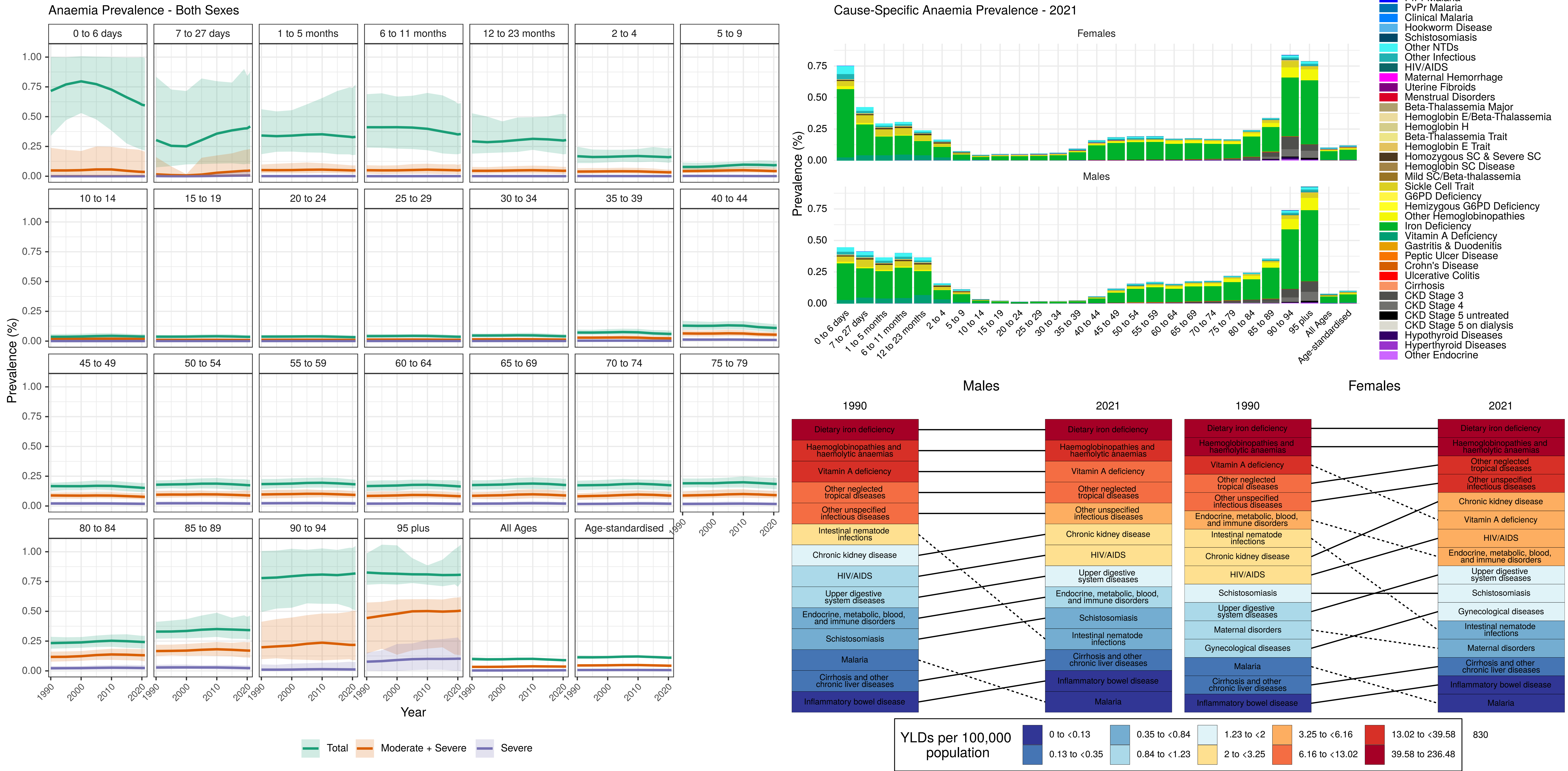

Anaemia Prevalence - Both Sexes
0 to 6 days
7 to 27 days
1 to 5 months
6 to 11 months
12 to 23 months
2 to 4
5 to 9
10 to 14
15 to 19
20 to 24
25 to 29
30 to 34
35 to 39
40 to 44
45 to 49
50 to 54
55 to 59
60 to 64
65 to 69
70 to 74
75 to 79
80 to 84
85 to 89
90 to 94
95 plus
All Ages
Age-standardised
Prevalence (%)
Year
Total
Moderate + Severe
Severe
Cause-Specific Anaemia Prevalence - 2021
Females
Males
PfPr Malaria
PvPr Malaria
Clinical Malaria
Hookworm Disease
Schistosomiasis
Other NTDs
Other Infectious
HIV/AIDS
Maternal Hemorrhage
Uterine Fibroids
Menstrual Disorders
Beta-Thalassemia Major
Hemoglobin E/Beta-Thalassemia
Hemoglobin H
Beta-Thalassemia Trait
Hemoglobin E Trait
Homozygous SC & Severe SC
Hemoglobin SC Disease
Mild SC/Beta-thalassemia
Sickle Cell Trait
G6PD Deficiency
Hemizygous G6PD Deficiency
Other Hemoglobinopathies
Iron Deficiency
Vitamin A Deficiency
Gastritis & Duodenitis
Peptic Ulcer Disease
Crohn's Disease
Ulcerative Colitis
Cirrhosis
CKD Stage 3
CKD Stage 4
CKD Stage 5 untreated
CKD Stage 5 on dialysis
Hypothyroid Diseases
Hyperthyroid Diseases
Other Endocrine
Males
Females
1990
2021
Males 1990: Dietary iron deficiency; Haemoglobinopathies and haemolytic anaemias; Vitamin A deficiency; Other neglected tropical diseases; Other unspecified infectious diseases; Intestinal nematode infections; Chronic kidney disease; HIV/AIDS; Upper digestive system diseases; Endocrine, metabolic, blood, and immune disorders; Schistosomiasis; Malaria; Cirrhosis and other chronic liver diseases; Inflammatory bowel disease
Males 2021: Dietary iron deficiency; Haemoglobinopathies and haemolytic anaemias; Vitamin A deficiency; Other neglected tropical diseases; Other unspecified infectious diseases; Chronic kidney disease; HIV/AIDS; Upper digestive system diseases; Endocrine, metabolic, blood, and immune disorders; Schistosomiasis; Intestinal nematode infections; Cirrhosis and other chronic liver diseases; Inflammatory bowel disease; Malaria
Females 1990: Dietary iron deficiency; Haemoglobinopathies and haemolytic anaemias; Vitamin A deficiency; Other neglected tropical diseases; Other unspecified infectious diseases; Endocrine, metabolic, blood, and immune disorders; Intestinal nematode infections; Chronic kidney disease; HIV/AIDS; Schistosomiasis; Upper digestive system diseases; Maternal disorders; Gynecological diseases; Malaria; Cirrhosis and other chronic liver diseases; Inflammatory bowel disease
Females 2021: Dietary iron deficiency; Haemoglobinopathies and haemolytic anaemias; Other neglected tropical diseases; Other unspecified infectious diseases; Chronic kidney disease; Vitamin A deficiency; HIV/AIDS; Endocrine, metabolic, blood, and immune disorders; Upper digestive system diseases; Schistosomiasis; Gynecological diseases; Intestinal nematode infections; Maternal disorders; Cirrhosis and other chronic liver diseases; Inflammatory bowel disease; Malaria
YLDs per 100,000 population
0 to <0.13
0.13 to <0.35
0.35 to <0.84
0.84 to <1.23
1.23 to <2
2 to <3.25
3.25 to <6.16
6.16 to <13.02
13.02 to <39.58
39.58 to 236.48

Nyeri

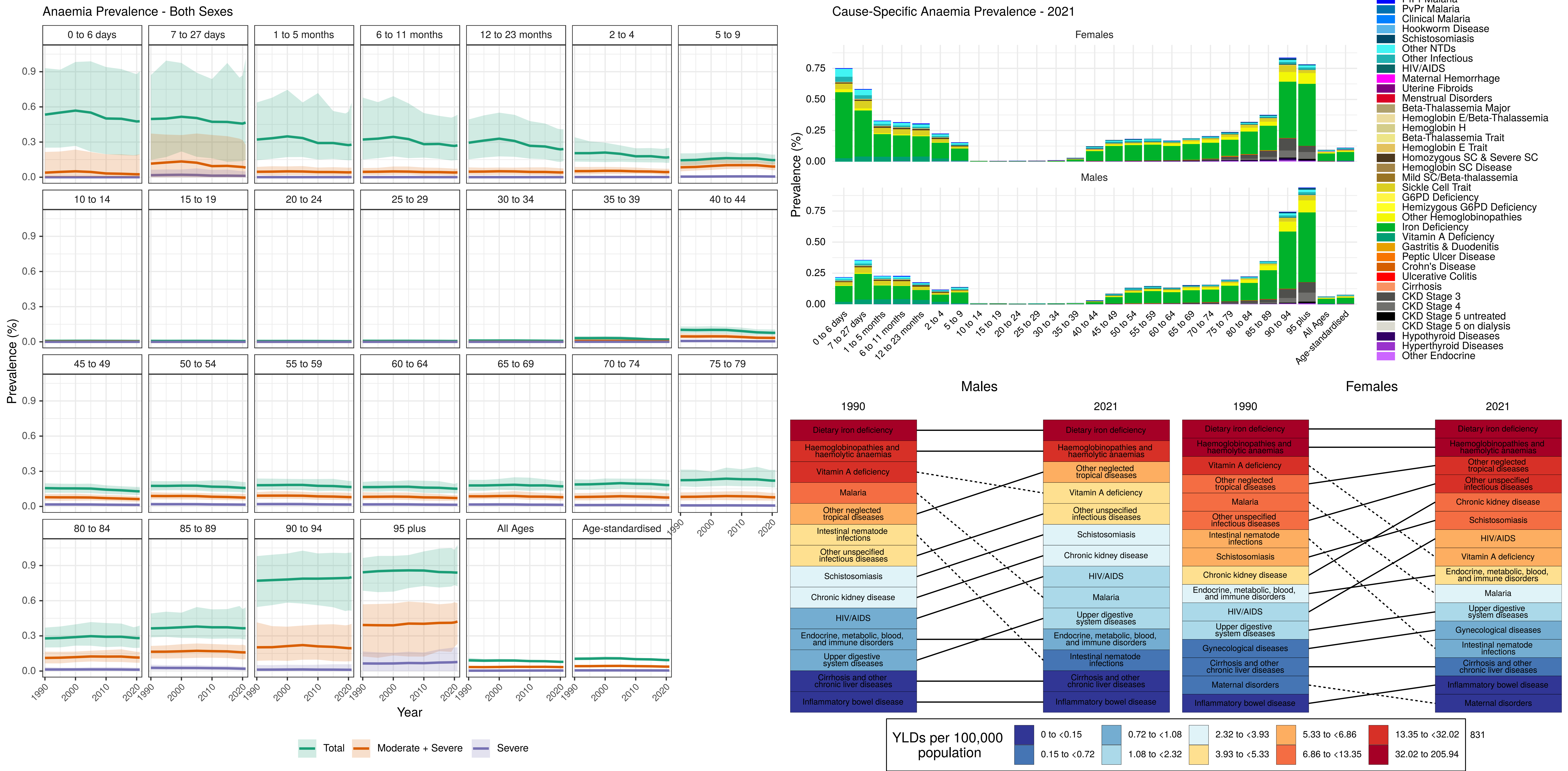

Anaemia Prevalence - Both Sexes
0 to 6 days
7 to 27 days
1 to 5 months
6 to 11 months
12 to 23 months
2 to 4
5 to 9
10 to 14
15 to 19
20 to 24
25 to 29
30 to 34
35 to 39
40 to 44
45 to 49
50 to 54
55 to 59
60 to 64
65 to 69
70 to 74
75 to 79
80 to 84
85 to 89
90 to 94
95 plus
All Ages
Age-standardised
Prevalence (%)
Year
Total
Moderate + Severe
Severe
Cause-Specific Anaemia Prevalence - 2021
Females
Males
PfPr Malaria
PvPr Malaria
Clinical Malaria
Hookworm Disease
Schistosomiasis
Other NTDs
Other Infectious
HIV/AIDS
Maternal Hemorrhage
Uterine Fibroids
Menstrual Disorders
Beta-Thalassemia Major
Hemoglobin E/Beta-Thalassemia
Hemoglobin H
Beta-Thalassemia Trait
Hemoglobin E Trait
Homozygous SC & Severe SC
Hemoglobin SC Disease
Mild SC/Beta-thalassemia
Sickle Cell Trait
G6PD Deficiency
Hemizygous G6PD Deficiency
Other Hemoglobinopathies
Iron Deficiency
Vitamin A Deficiency
Gastritis & Duodenitis
Peptic Ulcer Disease
Crohn's Disease
Ulcerative Colitis
Cirrhosis
CKD Stage 3
CKD Stage 4
CKD Stage 5 untreated
CKD Stage 5 on dialysis
Hypothyroid Diseases
Hyperthyroid Diseases
Other Endocrine
Males
Females
1990
2021
Dietary iron deficiency
Haemoglobinopathies and haemolytic anaemias
Vitamin A deficiency
Malaria
Other neglected tropical diseases
Intestinal nematode infections
Other unspecified infectious diseases
Schistosomiasis
Chronic kidney disease
HIV/AIDS
Endocrine, metabolic, blood, and immune disorders
Upper digestive system diseases
Cirrhosis and other chronic liver diseases
Inflammatory bowel disease
Gynecological diseases
Maternal disorders
YLDs per 100,000 population
0 to <0.15
0.15 to <0.72
0.72 to <1.08
1.08 to <2.32
2.32 to <3.93
3.93 to <5.33
5.33 to <6.86
6.86 to <13.35
13.35 to <32.02
32.02 to 205.94

Samburu

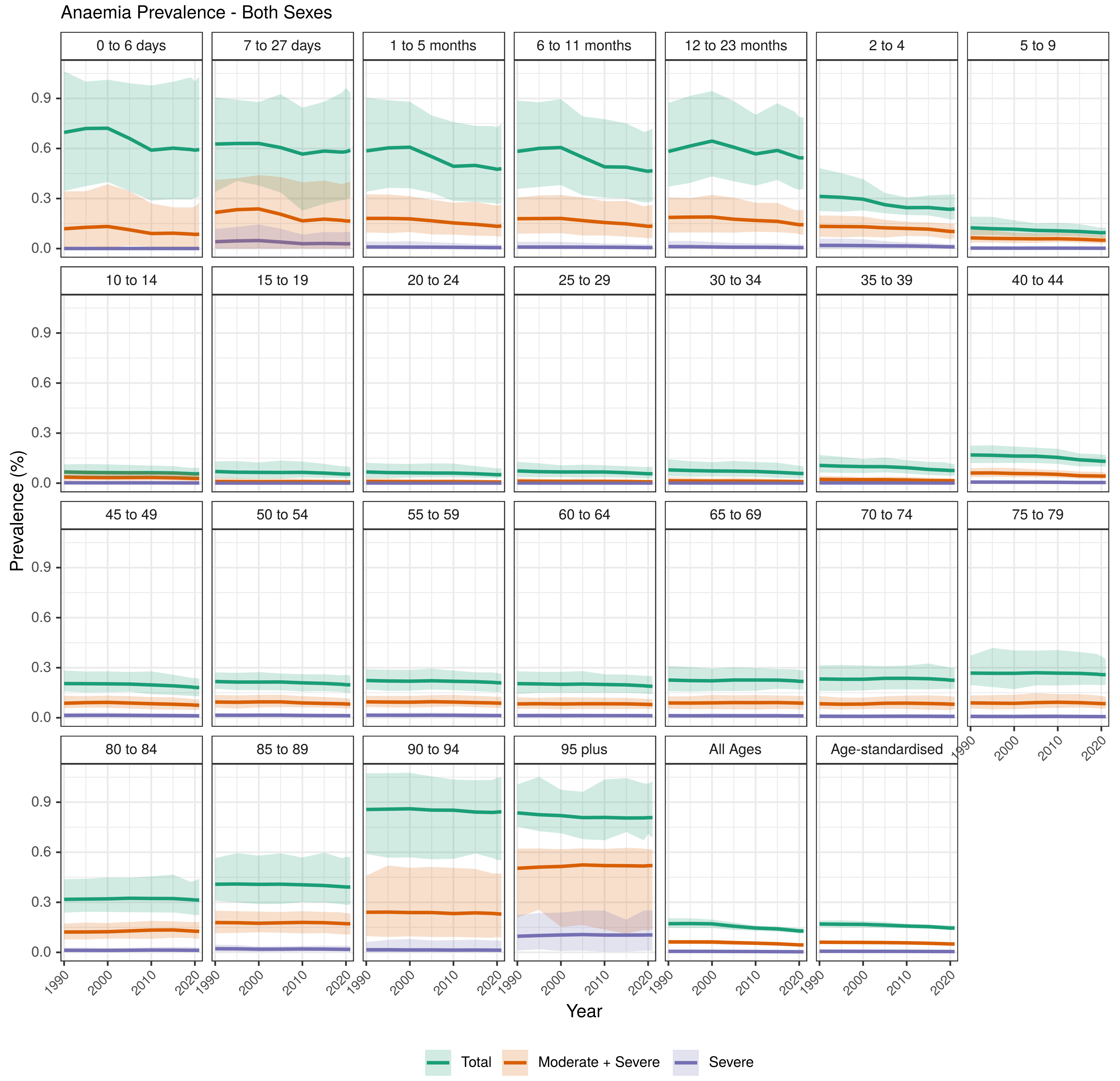

Anaemia Prevalence - Both Sexes
0 to 6 days
7 to 27 days
1 to 5 months
6 to 11 months
12 to 23 months
2 to 4
5 to 9
10 to 14
15 to 19
20 to 24
25 to 29
30 to 34
35 to 39
40 to 44
45 to 49
50 to 54
55 to 59
60 to 64
65 to 69
70 to 74
75 to 79
80 to 84
85 to 89
90 to 94
95 plus
All Ages
Age-standardised
Prevalence (%)
Year
Total
Moderate + Severe
Severe

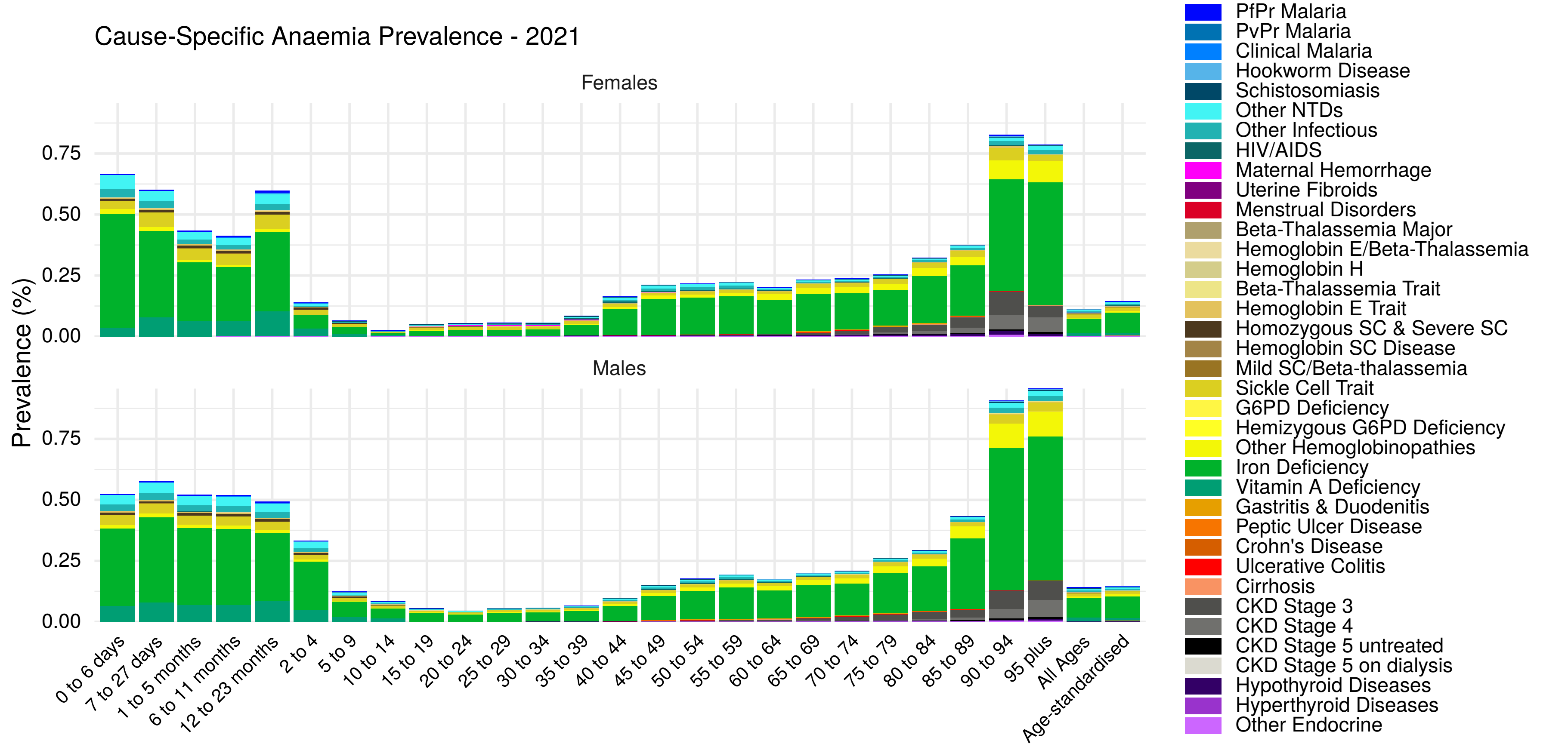

Cause-Specific Anaemia Prevalence - 2021
Females
Males
Prevalence (%)
PfPr Malaria
PvPr Malaria
Clinical Malaria
Hookworm Disease
Schistosomiasis
Other NTDs
Other Infectious
HIV/AIDS
Maternal Hemorrhage
Uterine Fibroids
Menstrual Disorders
Beta-Thalassemia Major
Hemoglobin E/Beta-Thalassemia
Hemoglobin H
Beta-Thalassemia Trait
Hemoglobin E Trait
Homozygous SC & Severe SC
Hemoglobin SC Disease
Mild SC/Beta-thalassemia
Sickle Cell Trait
G6PD Deficiency
Hemizygous G6PD Deficiency
Other Hemoglobinopathies
Iron Deficiency
Vitamin A Deficiency
Gastritis & Duodenitis
Peptic Ulcer Disease
Crohn's Disease
Ulcerative Colitis
Cirrhosis
CKD Stage 3
CKD Stage 4
CKD Stage 5 untreated
CKD Stage 5 on dialysis
Hypothyroid Diseases
Hyperthyroid Diseases
Other Endocrine

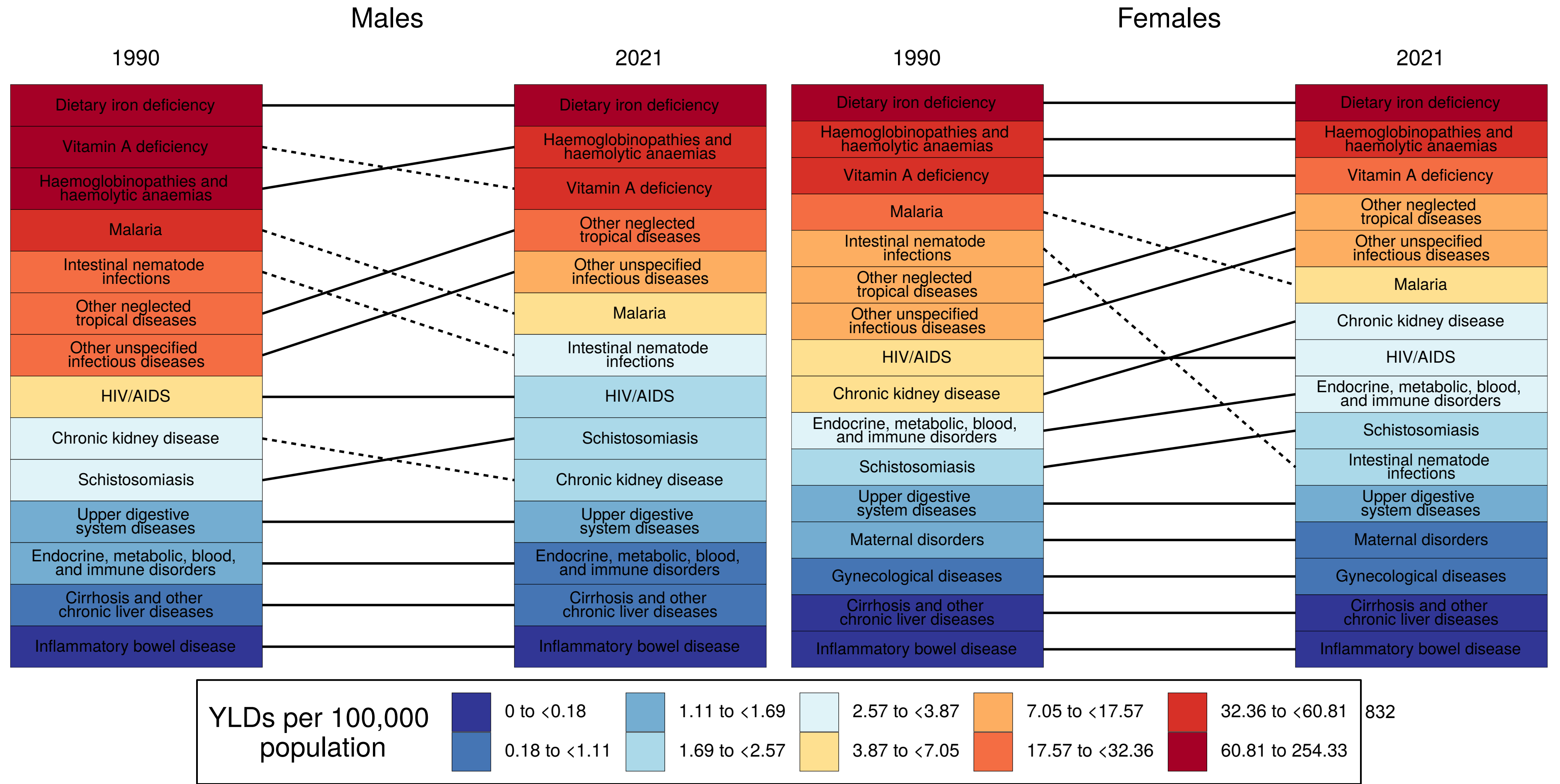

Males
Females
1990
2021
Dietary iron deficiency
Vitamin A deficiency
Haemoglobinopathies and haemolytic anaemias
Malaria
Intestinal nematode infections
Other neglected tropical diseases
Other unspecified infectious diseases
HIV/AIDS
Chronic kidney disease
Schistosomiasis
Upper digestive system diseases
Endocrine, metabolic, blood, and immune disorders
Cirrhosis and other chronic liver diseases
Inflammatory bowel disease
Maternal disorders
Gynecological diseases
YLDs per 100,000 population
0 to <0.18
0.18 to <1.11
1.11 to <1.69
1.69 to <2.57
2.57 to <3.87
3.87 to <7.05
7.05 to <17.57
17.57 to <32.36
32.36 to <60.81
60.81 to 254.33

832

Siaya

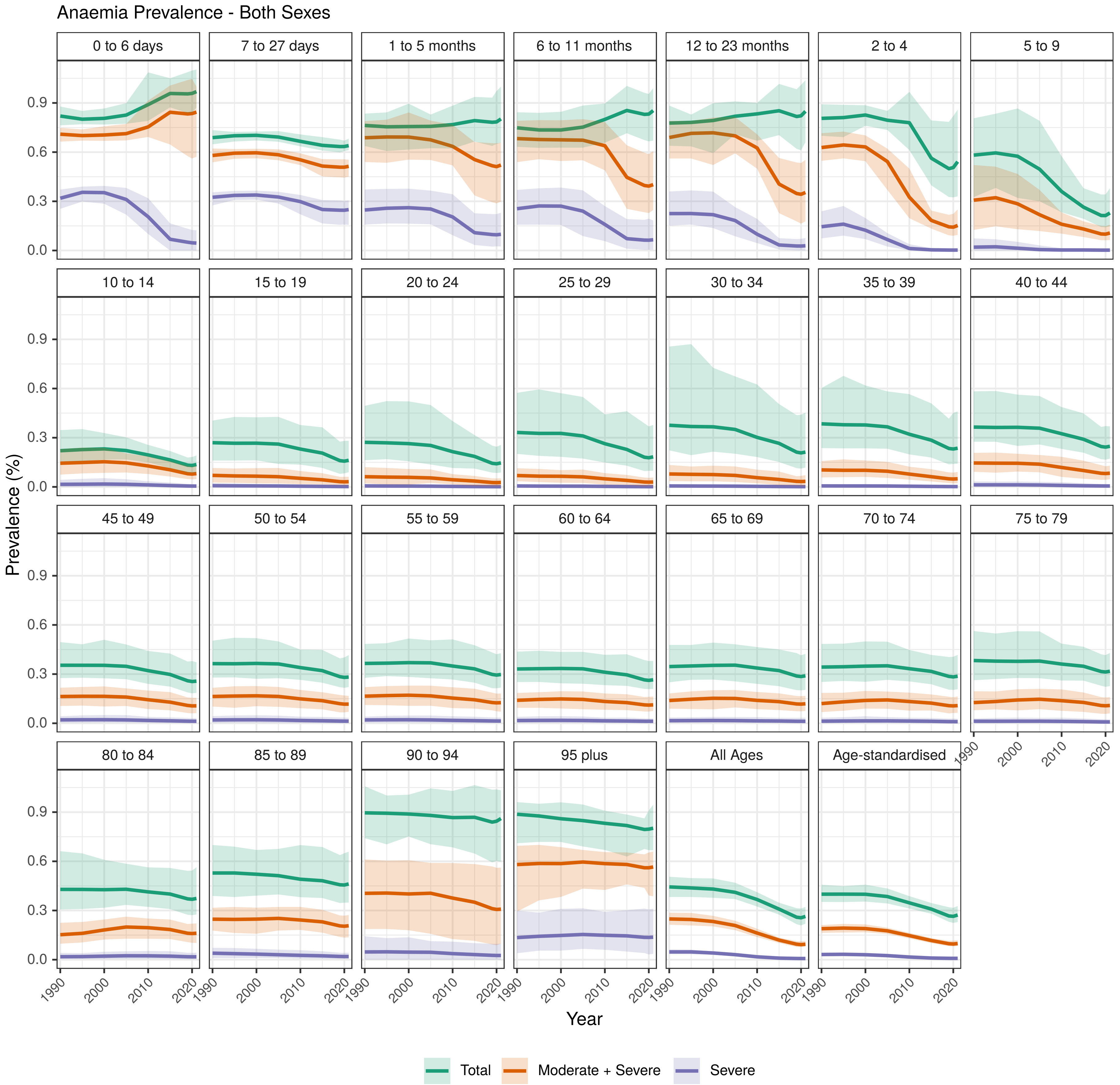

Anaemia Prevalence - Both Sexes
0 to 6 days
7 to 27 days
1 to 5 months
6 to 11 months
12 to 23 months
2 to 4
5 to 9
10 to 14
15 to 19
20 to 24
25 to 29
30 to 34
35 to 39
40 to 44
45 to 49
50 to 54
55 to 59
60 to 64
65 to 69
70 to 74
75 to 79
80 to 84
85 to 89
90 to 94
95 plus
All Ages
Age-standardised
Prevalence (%)
Year
Total
Moderate + Severe
Severe

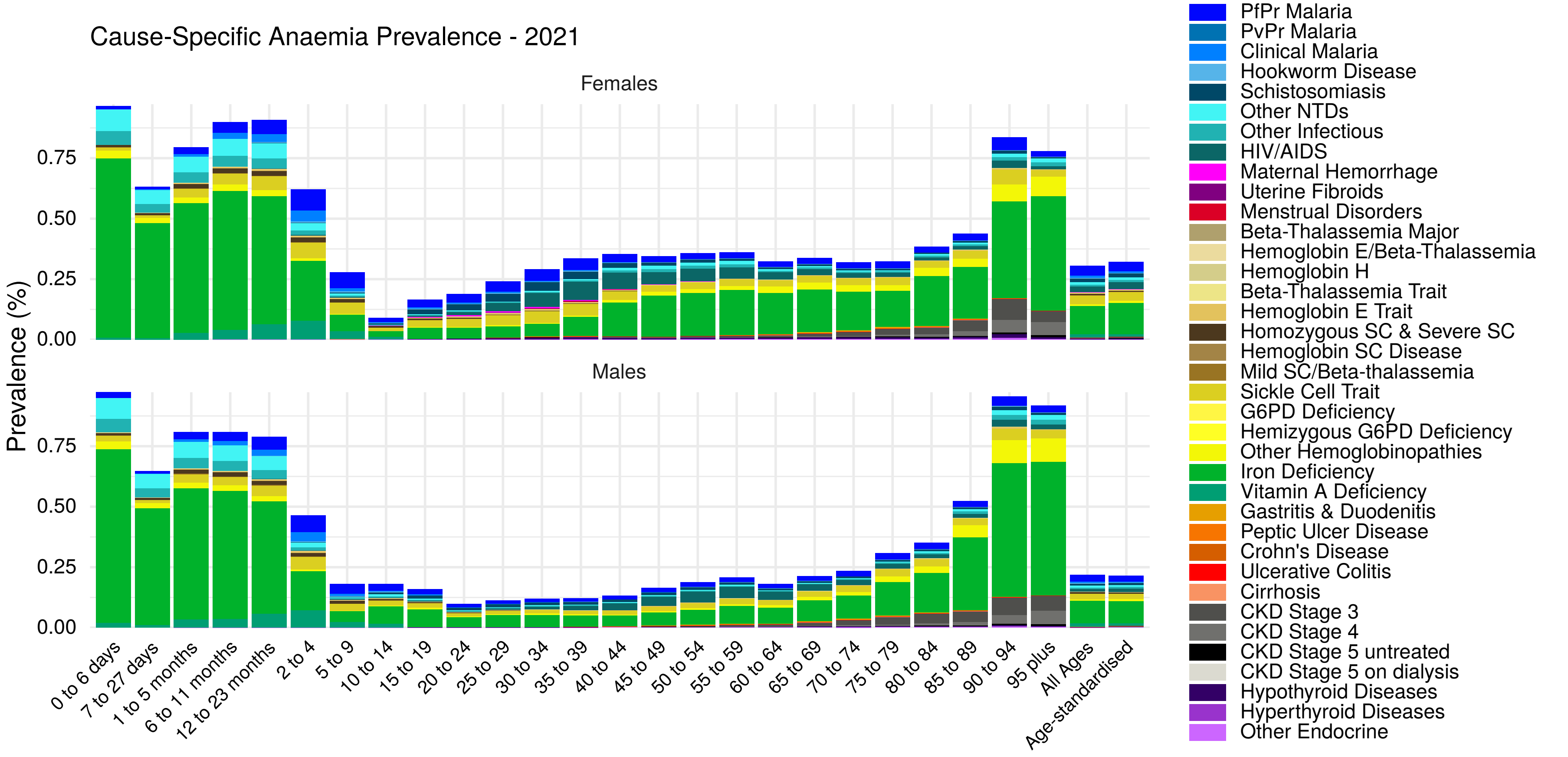

Cause-Specific Anaemia Prevalence - 2021
Females
Males
Prevalence (%)
PfPr Malaria
PvPr Malaria
Clinical Malaria
Hookworm Disease
Schistosomiasis
Other NTDs
Other Infectious
HIV/AIDS
Maternal Hemorrhage
Uterine Fibroids
Menstrual Disorders
Beta-Thalassemia Major
Hemoglobin E/Beta-Thalassemia
Hemoglobin H
Beta-Thalassemia Trait
Hemoglobin E Trait
Homozygous SC & Severe SC
Hemoglobin SC Disease
Mild SC/Beta-thalassemia
Sickle Cell Trait
G6PD Deficiency
Hemizygous G6PD Deficiency
Other Hemoglobinopathies
Iron Deficiency
Vitamin A Deficiency
Gastritis & Duodenitis
Peptic Ulcer Disease
Crohn's Disease
Ulcerative Colitis
Cirrhosis
CKD Stage 3
CKD Stage 4
CKD Stage 5 untreated
CKD Stage 5 on dialysis
Hypothyroid Diseases
Hyperthyroid Diseases
Other Endocrine

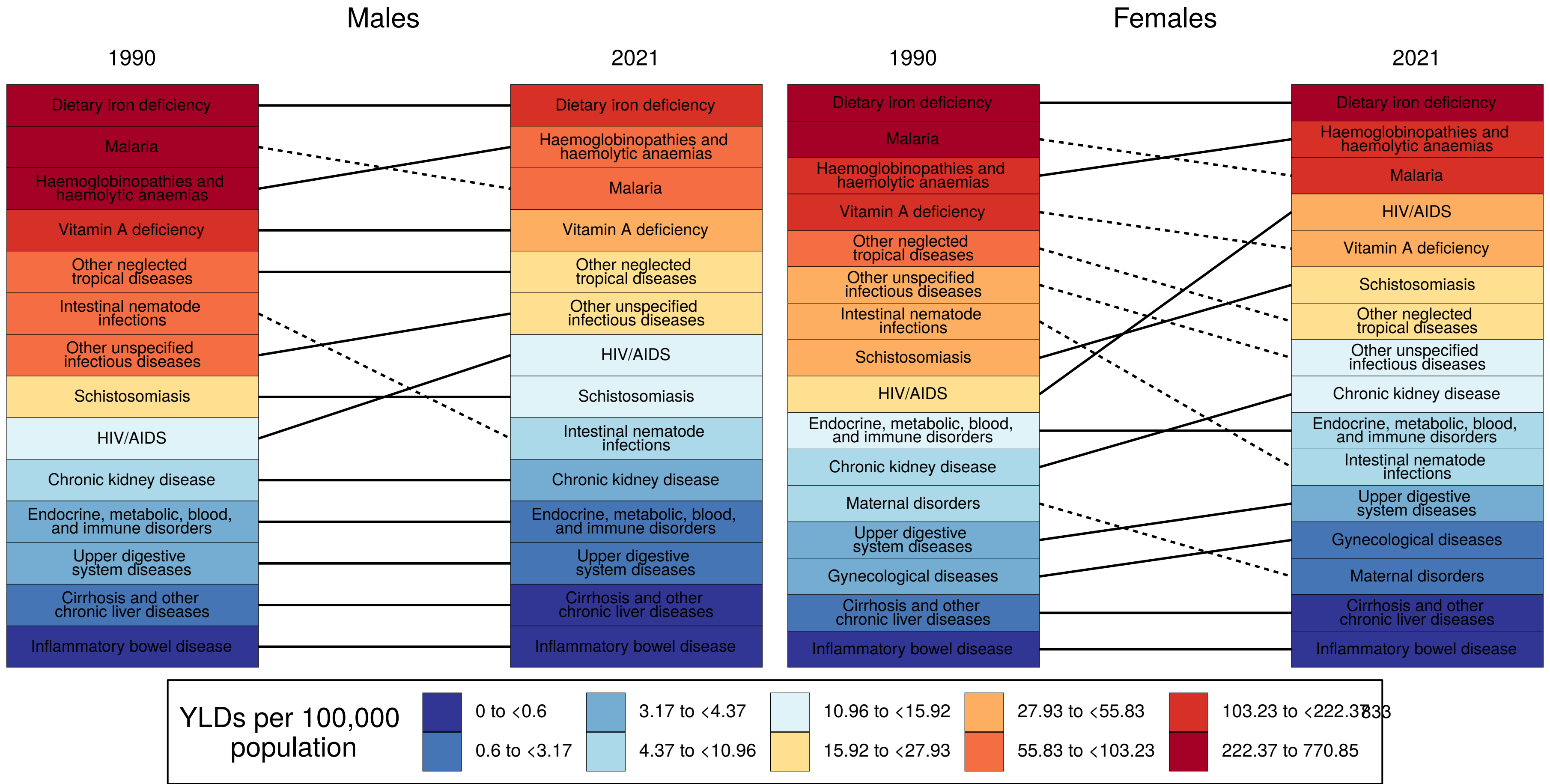

Males
Females
1990
2021
Dietary iron deficiency
Malaria
Haemoglobinopathies and haemolytic anaemias
Vitamin A deficiency
Other neglected tropical diseases
Intestinal nematode infections
Other unspecified infectious diseases
Schistosomiasis
HIV/AIDS
Chronic kidney disease
Endocrine, metabolic, blood, and immune disorders
Upper digestive system diseases
Cirrhosis and other chronic liver diseases
Inflammatory bowel disease
Maternal disorders
Gynecological diseases
YLDs per 100,000 population
0 to <0.6
0.6 to <3.17
3.17 to <4.37
4.37 to <10.96
10.96 to <15.92
15.92 to <27.93
27.93 to <55.83
55.83 to <103.23
103.23 to <222.37
222.37 to 770.85

Taita Taveta

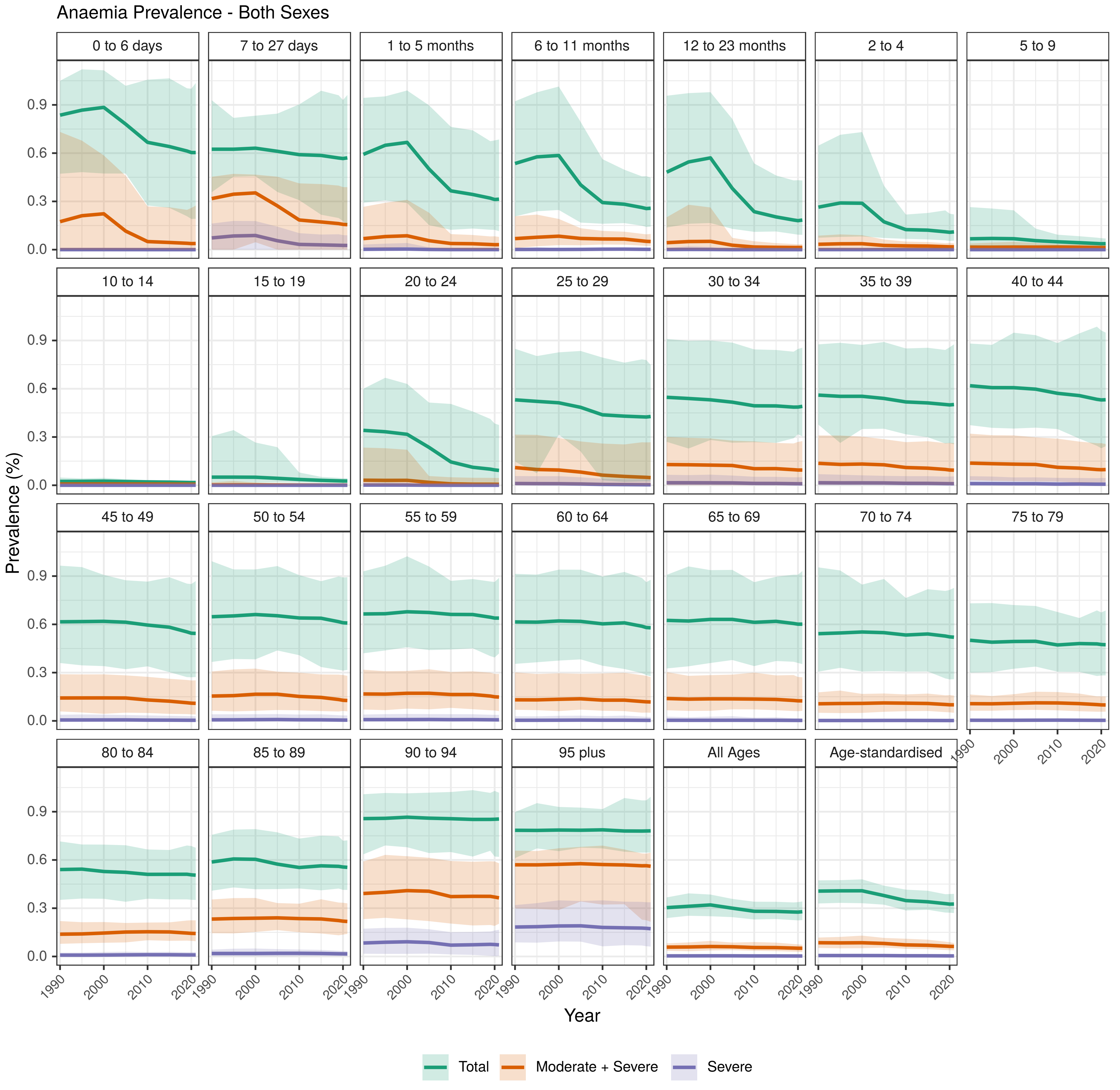

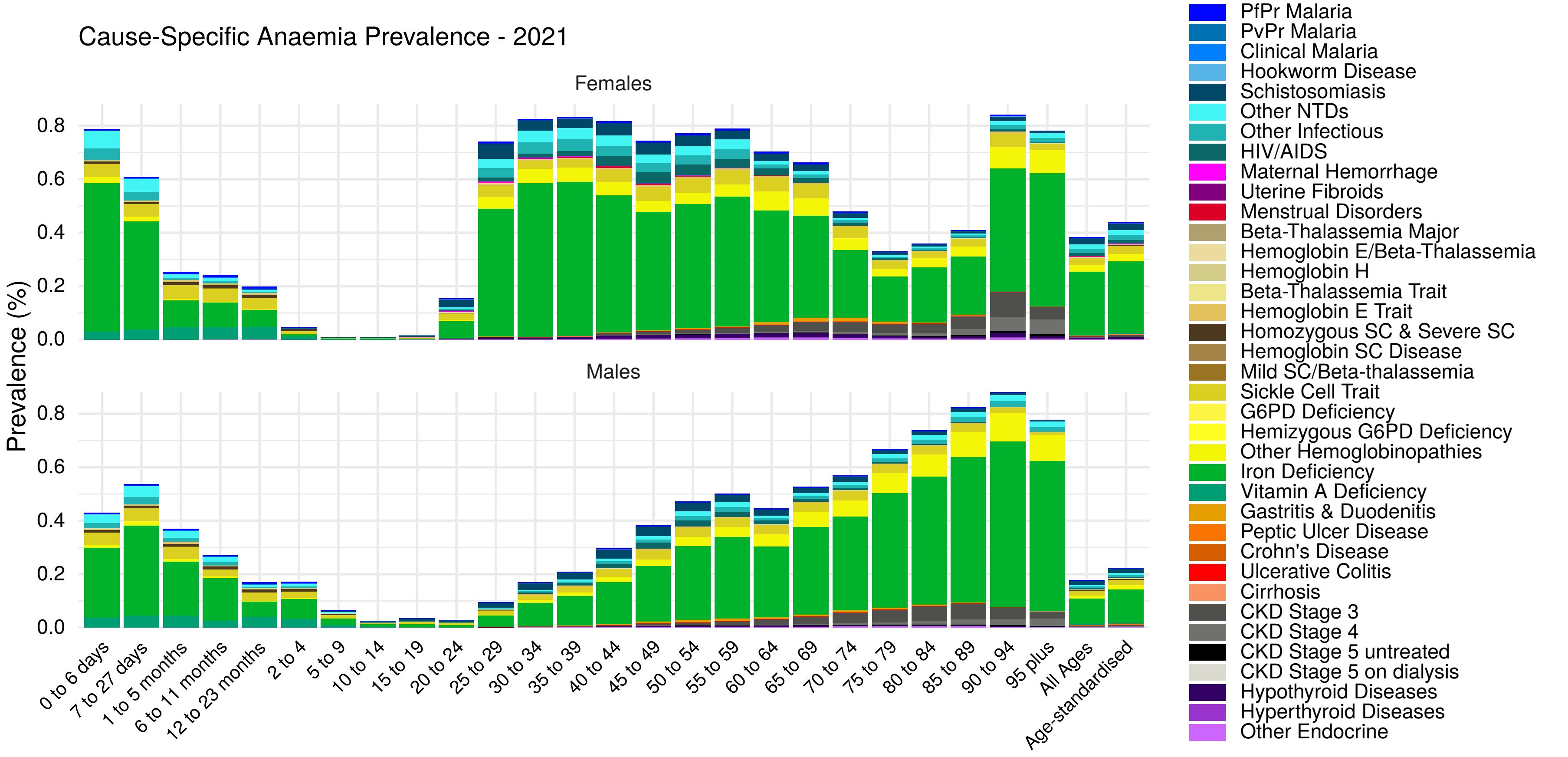

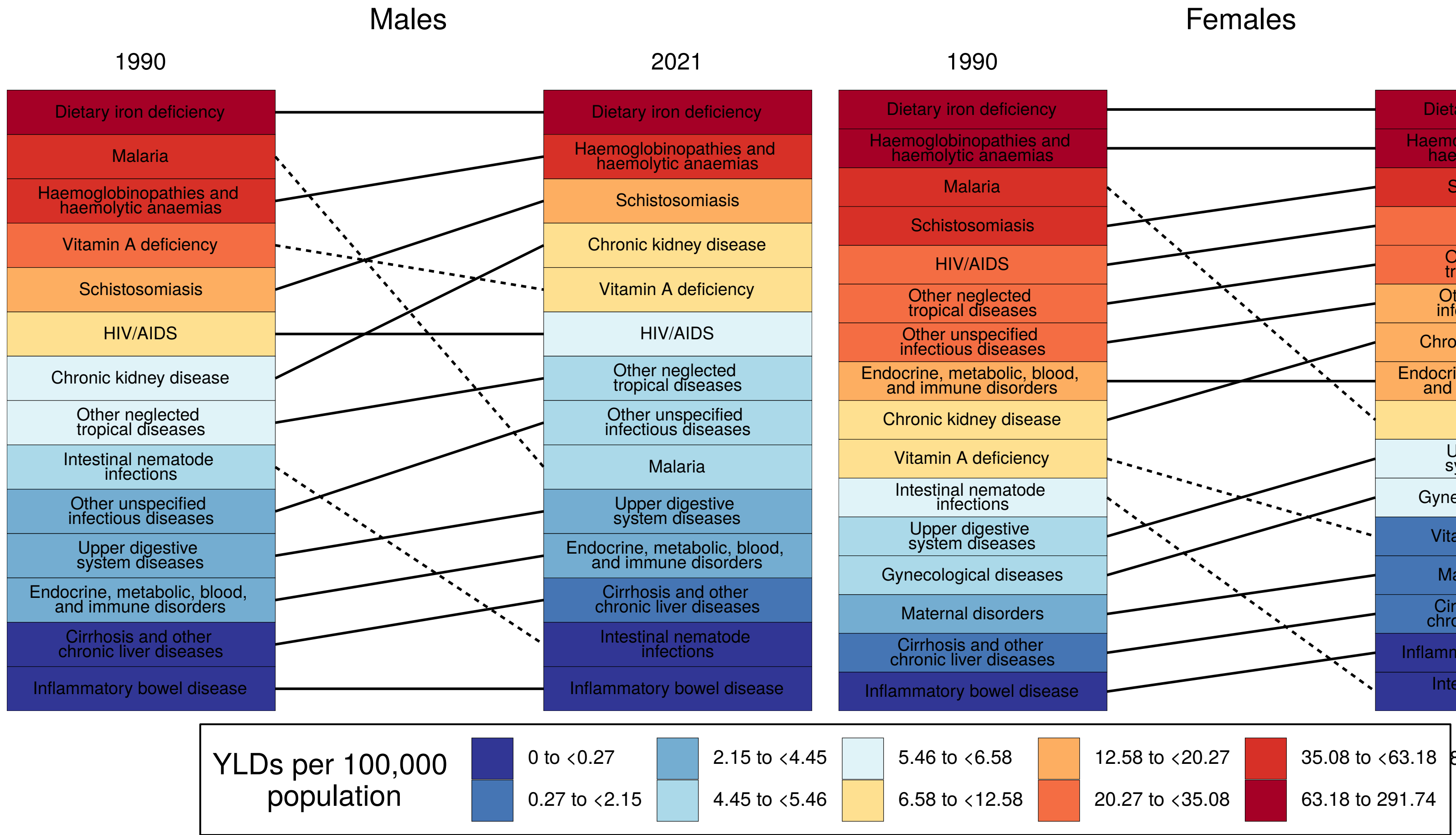

Tana River

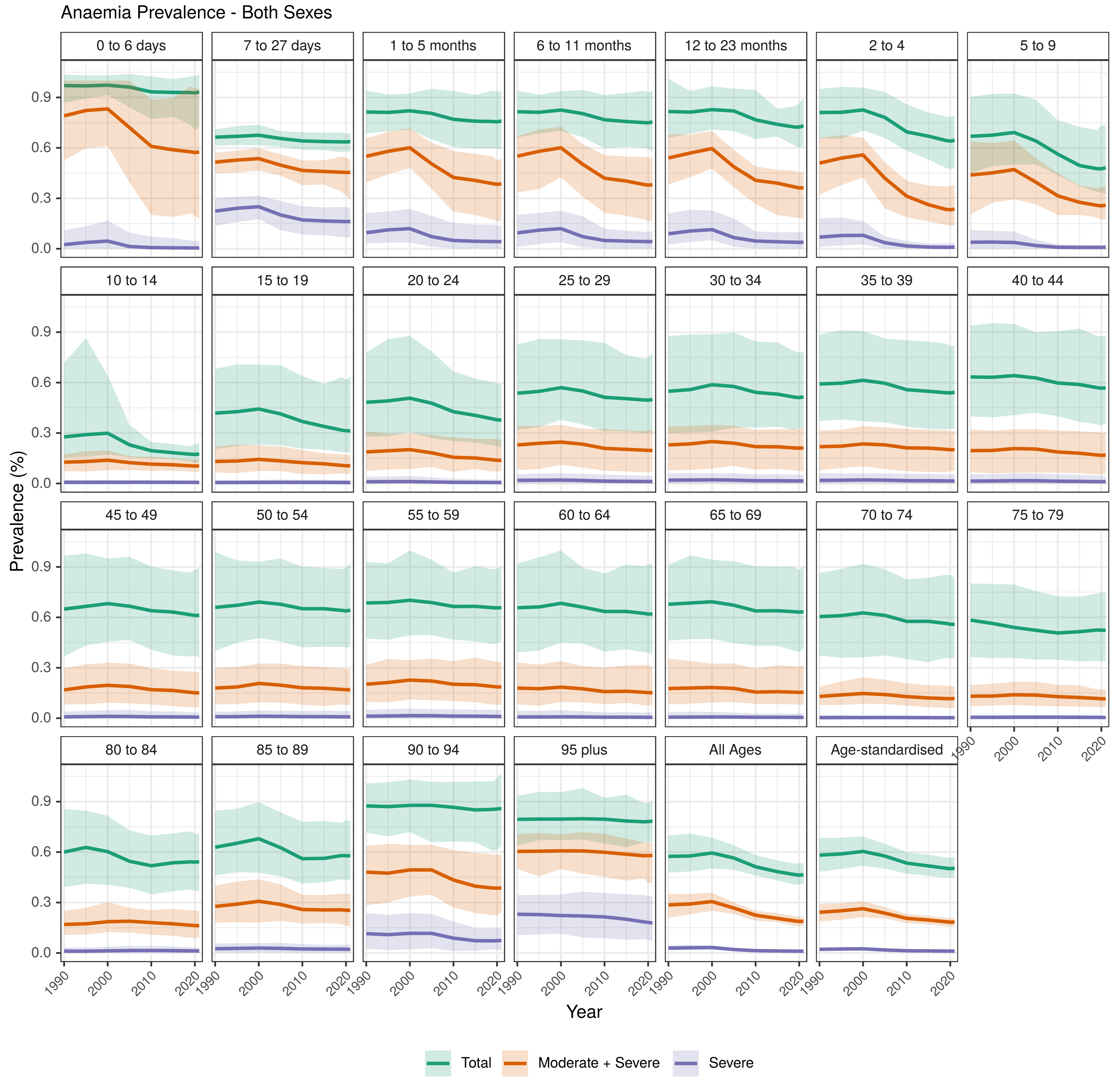

Anaemia Prevalence - Both Sexes
0 to 6 days
7 to 27 days
1 to 5 months
6 to 11 months
12 to 23 months
2 to 4
5 to 9
10 to 14
15 to 19
20 to 24
25 to 29
30 to 34
35 to 39
40 to 44
45 to 49
50 to 54
55 to 59
60 to 64
65 to 69
70 to 74
75 to 79
80 to 84
85 to 89
90 to 94
95 plus
All Ages
Age-standardised
Prevalence (%)
Year
Total
Moderate + Severe
Severe

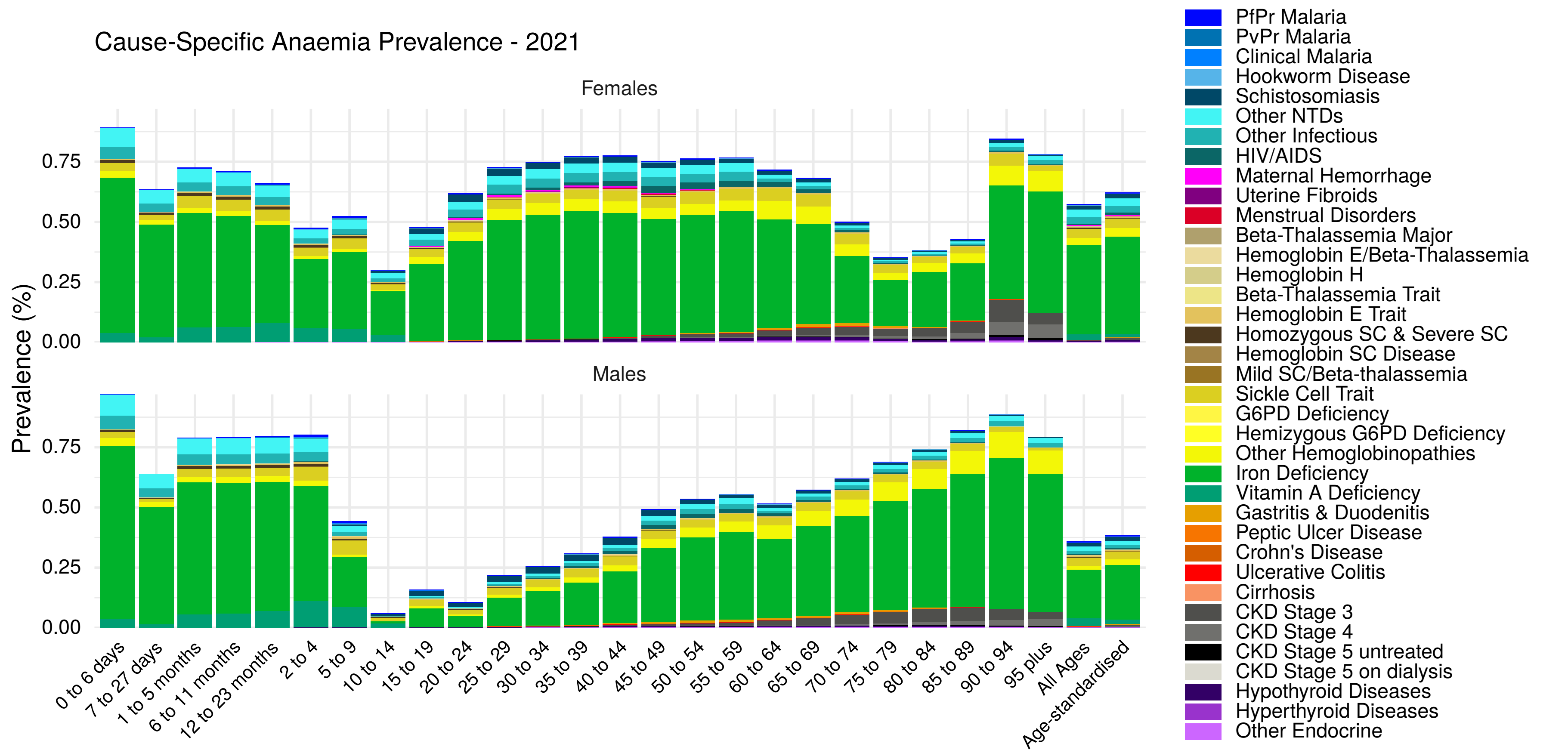

Cause-Specific Anaemia Prevalence - 2021
Females
Males
Prevalence (%)
PfPr Malaria
PvPr Malaria
Clinical Malaria
Hookworm Disease
Schistosomiasis
Other NTDs
Other Infectious
HIV/AIDS
Maternal Hemorrhage
Uterine Fibroids
Menstrual Disorders
Beta-Thalassemia Major
Hemoglobin E/Beta-Thalassemia
Hemoglobin H
Beta-Thalassemia Trait
Hemoglobin E Trait
Homozygous SC & Severe SC
Hemoglobin SC Disease
Mild SC/Beta-thalassemia
Sickle Cell Trait
G6PD Deficiency
Hemizygous G6PD Deficiency
Other Hemoglobinopathies
Iron Deficiency
Vitamin A Deficiency
Gastritis & Duodenitis
Peptic Ulcer Disease
Crohn's Disease
Ulcerative Colitis
Cirrhosis
CKD Stage 3
CKD Stage 4
CKD Stage 5 untreated
CKD Stage 5 on dialysis
Hypothyroid Diseases
Hyperthyroid Diseases
Other Endocrine

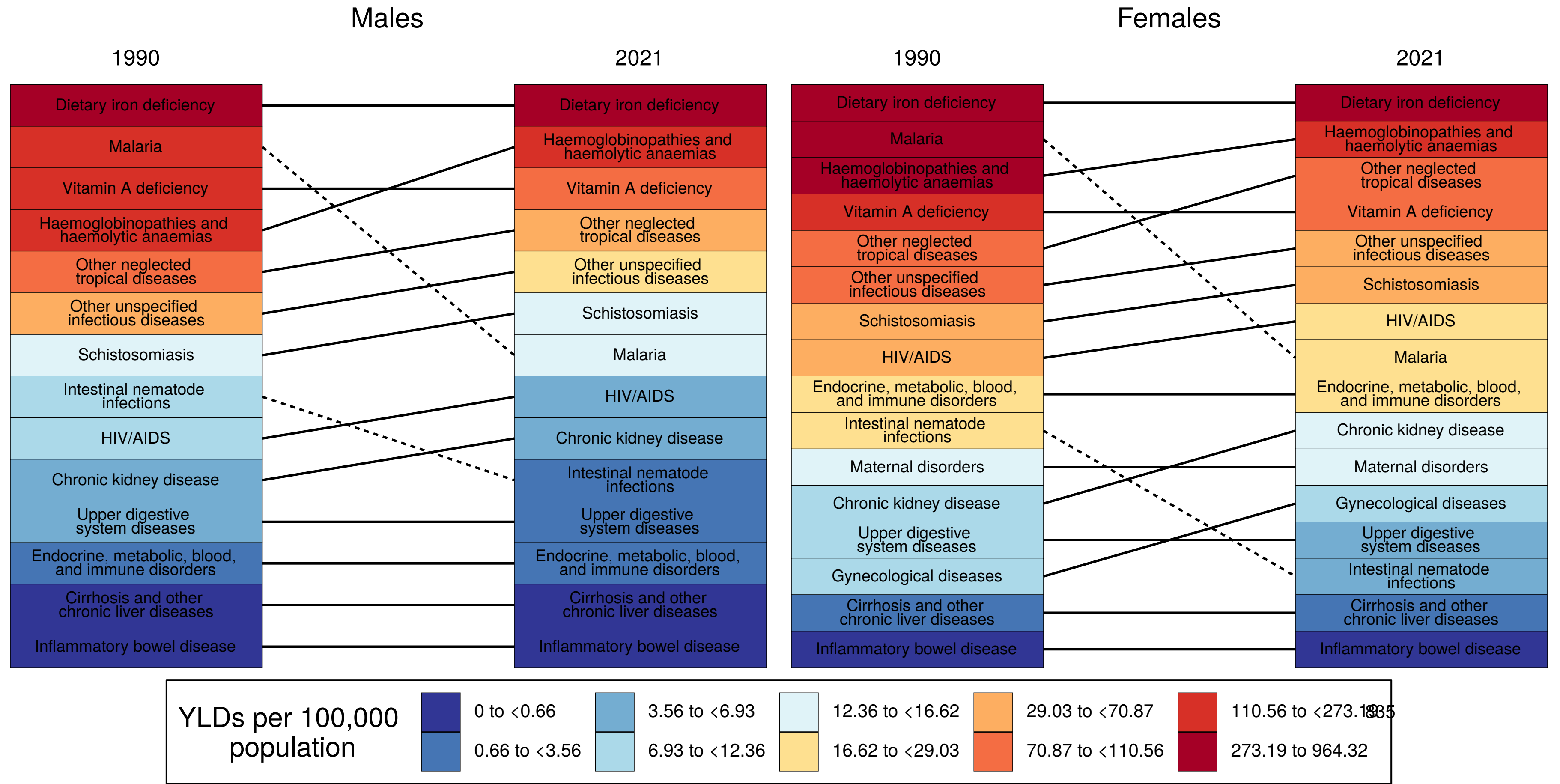

Males
Females
1990
2021
Dietary iron deficiency
Malaria
Vitamin A deficiency
Haemoglobinopathies and haemolytic anaemias
Other neglected tropical diseases
Other unspecified infectious diseases
Schistosomiasis
Intestinal nematode infections
HIV/AIDS
Chronic kidney disease
Upper digestive system diseases
Endocrine, metabolic, blood, and immune disorders
Cirrhosis and other chronic liver diseases
Inflammatory bowel disease
Maternal disorders
Gynecological diseases
YLDs per 100,000 population
0 to <0.66
0.66 to <3.56
3.56 to <6.93
6.93 to <12.36
12.36 to <16.62
16.62 to <29.03
29.03 to <70.87
70.87 to <110.56
110.56 to <273.19
273.19 to 964.32

Tharaka Nithi

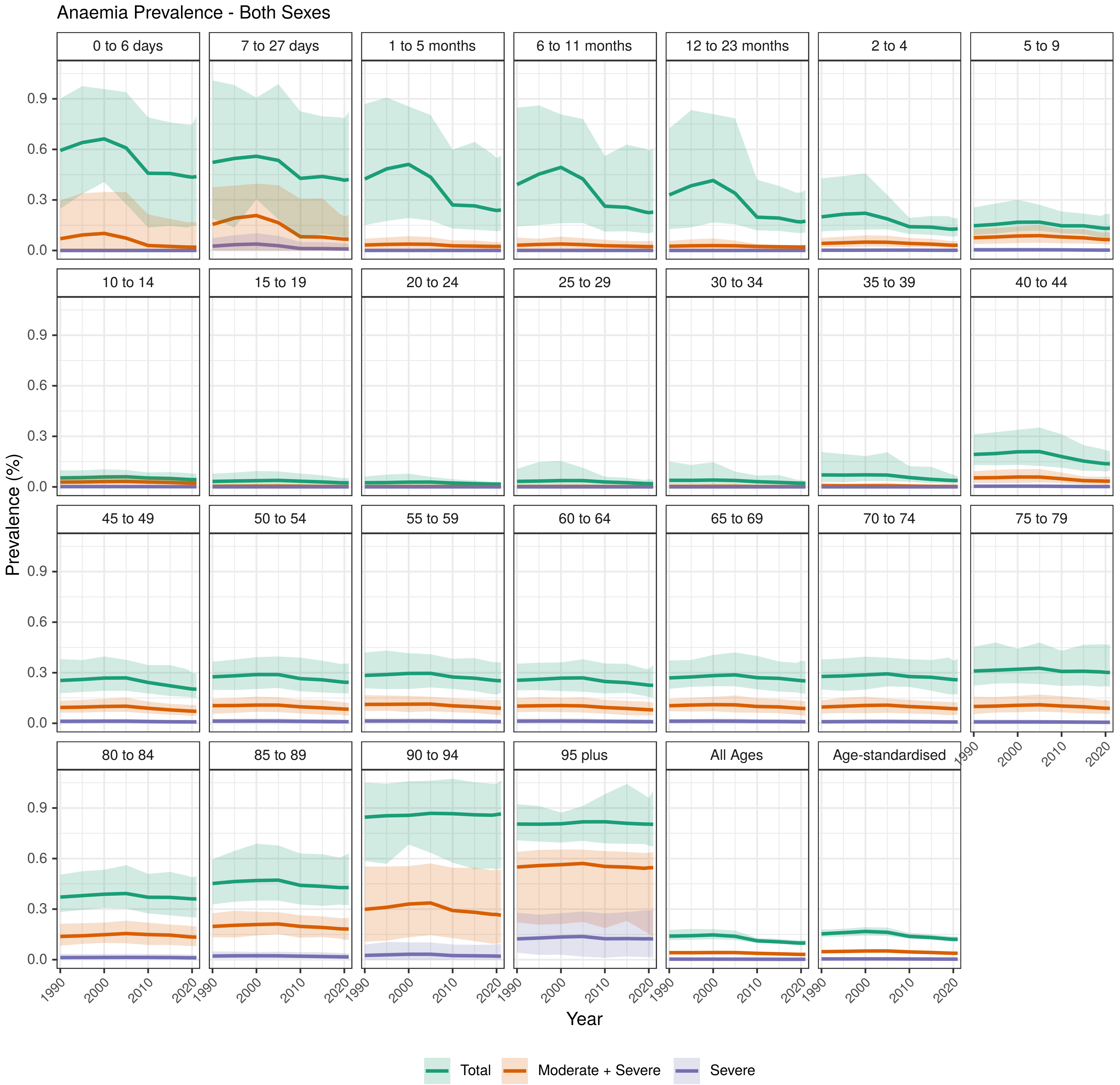

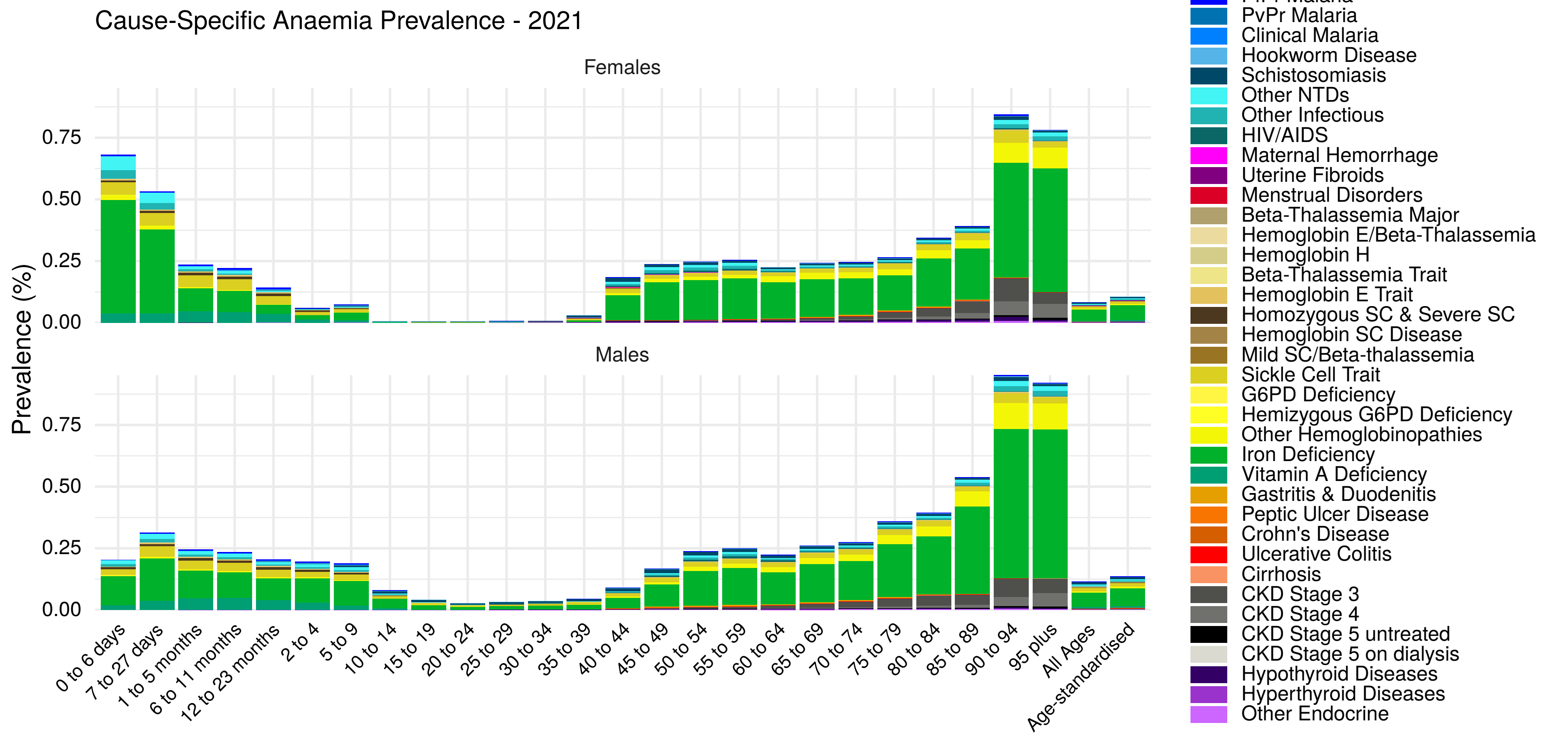

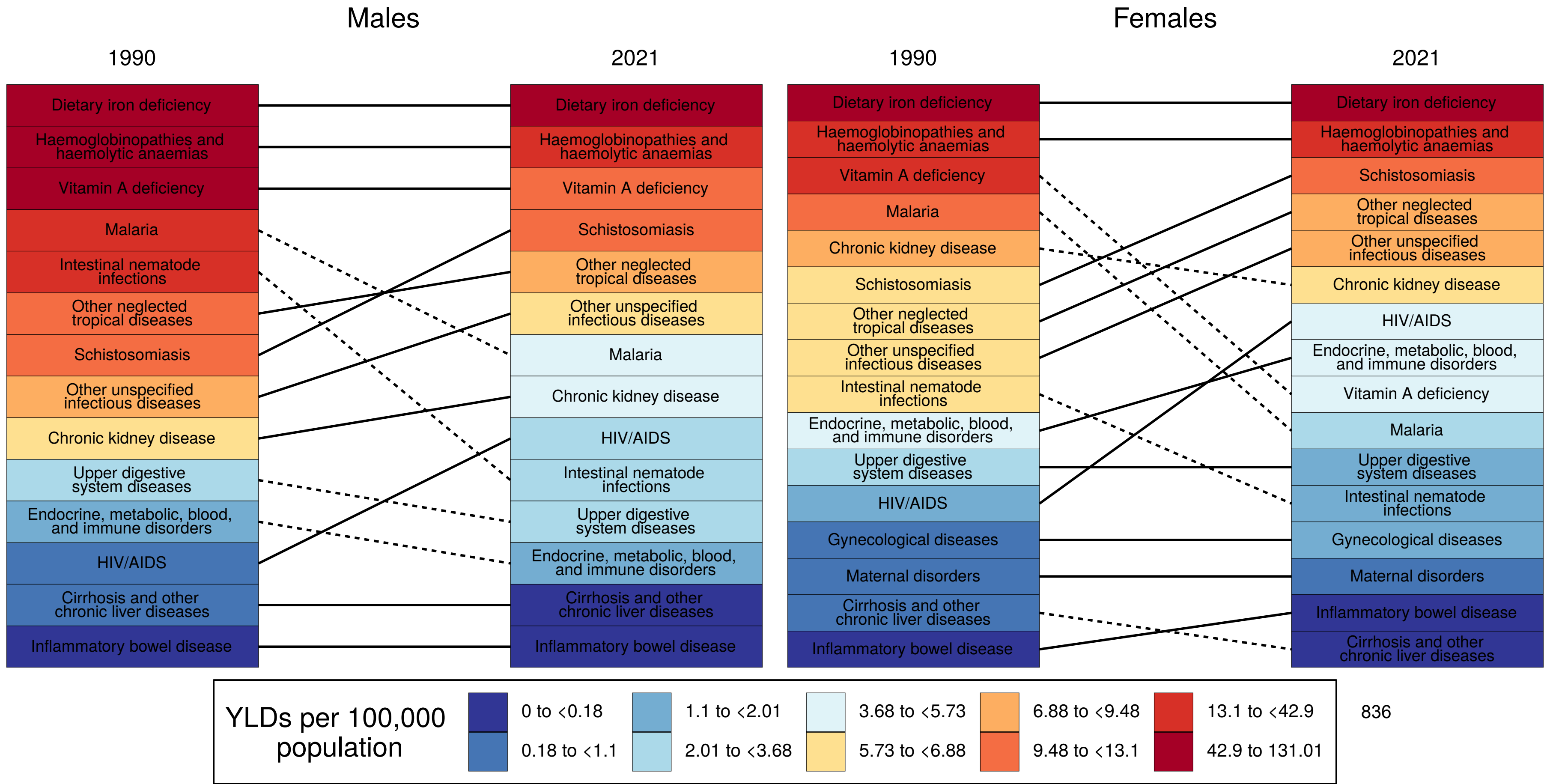

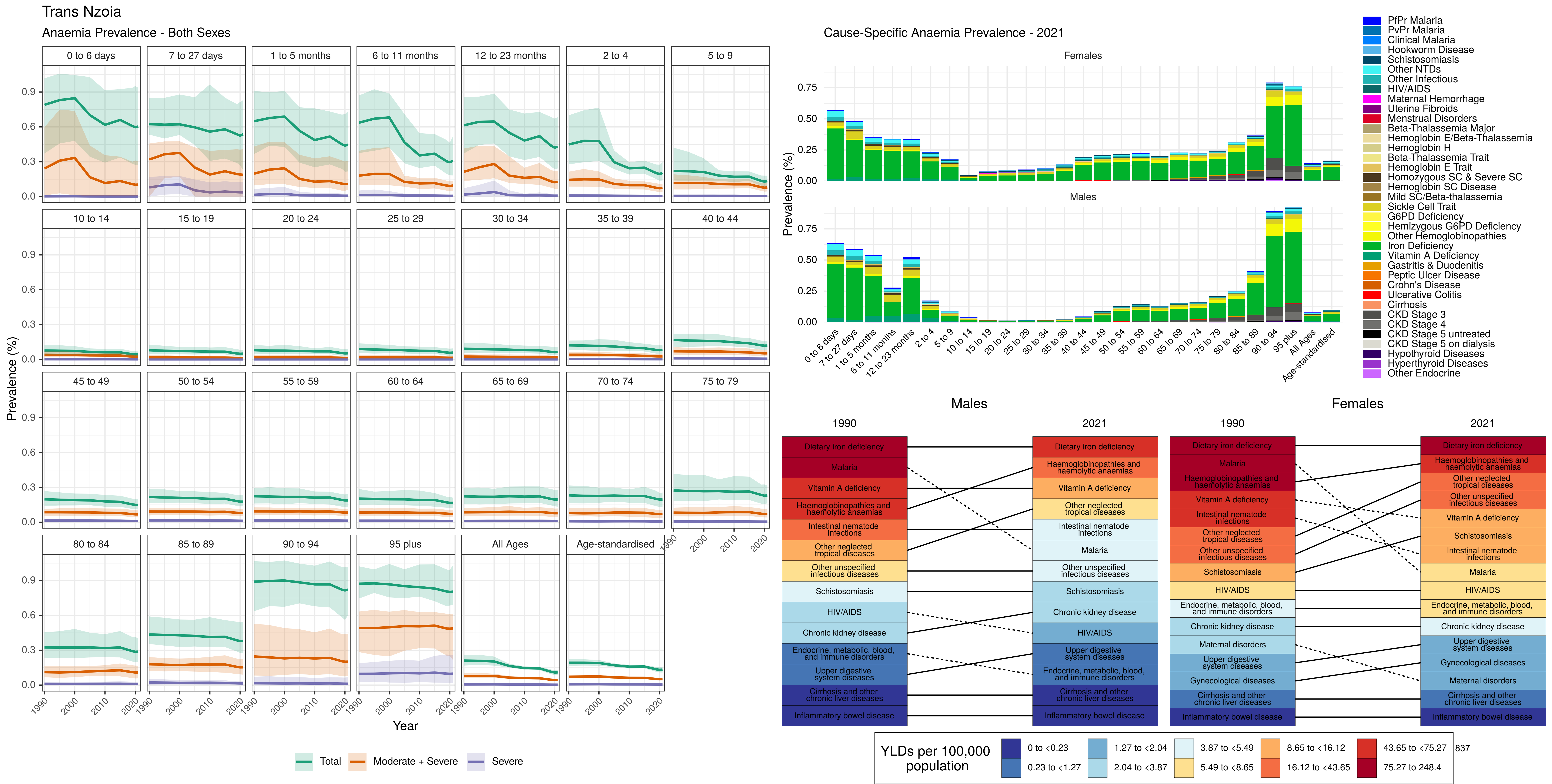
Trans Nzoia
Anaemia Prevalence - Both Sexes
0 to 6 days
7 to 27 days
1 to 5 months
6 to 11 months
12 to 23 months
2 to 4
5 to 9
10 to 14
15 to 19
20 to 24
25 to 29
30 to 34
35 to 39
40 to 44
45 to 49
50 to 54
55 to 59
60 to 64
65 to 69
70 to 74
75 to 79
80 to 84
85 to 89
90 to 94
95 plus
All Ages
Age-standardised
Prevalence (%)
Year
Total
Moderate + Severe
Severe
Cause-Specific Anaemia Prevalence - 2021
Females
Males
Prevalence (%)
PfPr Malaria
PvPr Malaria
Clinical Malaria
Hookworm Disease
Schistosomiasis
Other NTDs
Other Infectious
HIV/AIDS
Maternal Hemorrhage
Uterine Fibroids
Menstrual Disorders
Beta-Thalassemia Major
Hemoglobin E/Beta-Thalassemia
Hemoglobin H
Beta-Thalassemia Trait
Hemoglobin E Trait
Homozygous SC & Severe SC
Hemoglobin SC Disease
Mild SC/Beta-thalassemia
Sickle Cell Trait
G6PD Deficiency
Hemizygous G6PD Deficiency
Other Hemoglobinopathies
Iron Deficiency
Vitamin A Deficiency
Gastritis & Duodenitis
Peptic Ulcer Disease
Crohn's Disease
Ulcerative Colitis
Cirrhosis
CKD Stage 3
CKD Stage 4
CKD Stage 5 untreated
CKD Stage 5 on dialysis
Hypothyroid Diseases
Hyperthyroid Diseases
Other Endocrine
Males
Females
1990
2021
Dietary iron deficiency
Malaria
Vitamin A deficiency
Haemoglobinopathies and haemolytic anaemias
Intestinal nematode infections
Other neglected tropical diseases
Other unspecified infectious diseases
Schistosomiasis
HIV/AIDS
Chronic kidney disease
Endocrine, metabolic, blood, and immune disorders
Upper digestive system diseases
Cirrhosis and other chronic liver diseases
Inflammatory bowel disease
Maternal disorders
Gynecological diseases
YLDs per 100,000 population
0 to <0.23
0.23 to <1.27
1.27 to <2.04
2.04 to <3.87
3.87 to <5.49
5.49 to <8.65
8.65 to <16.12
16.12 to <43.65
43.65 to <75.27
75.27 to 248.4

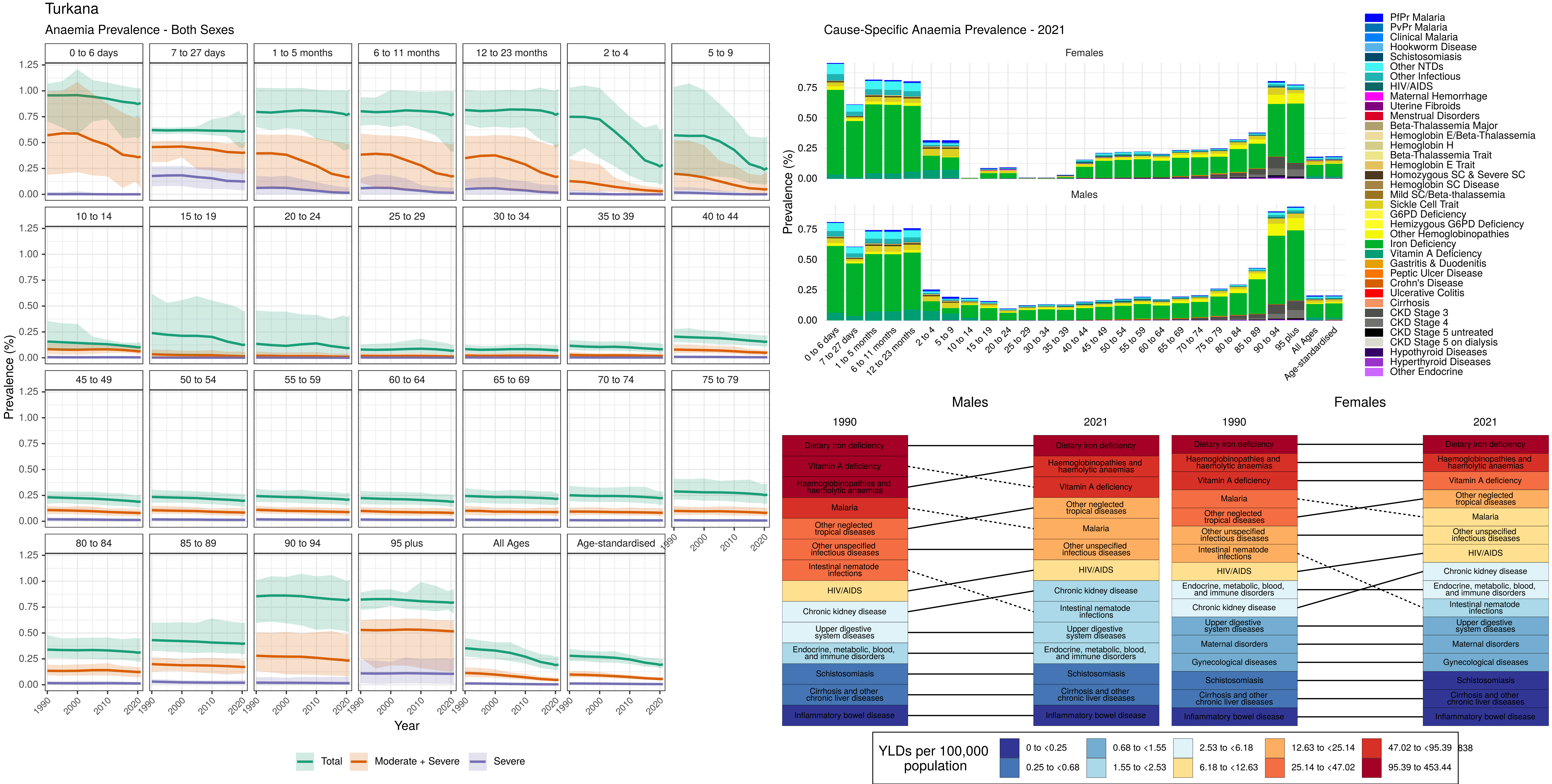
Turkana
Anaemia Prevalence - Both Sexes
0 to 6 days
7 to 27 days
1 to 5 months
6 to 11 months
12 to 23 months
2 to 4
5 to 9
10 to 14
15 to 19
20 to 24
25 to 29
30 to 34
35 to 39
40 to 44
45 to 49
50 to 54
55 to 59
60 to 64
65 to 69
70 to 74
75 to 79
80 to 84
85 to 89
90 to 94
95 plus
All Ages
Age-standardised
Prevalence (%)
Year
Total
Moderate + Severe
Severe
Cause-Specific Anaemia Prevalence - 2021
Females
Males
Prevalence (%)
PfPr Malaria
PvPr Malaria
Clinical Malaria
Hookworm Disease
Schistosomiasis
Other NTDs
Other Infectious
HIV/AIDS
Maternal Hemorrhage
Uterine Fibroids
Menstrual Disorders
Beta-Thalassemia Major
Hemoglobin E/Beta-Thalassemia
Hemoglobin H
Beta-Thalassemia Trait
Hemoglobin E Trait
Homozygous SC & Severe SC
Hemoglobin SC Disease
Mild SC/Beta-thalassemia
Sickle Cell Trait
G6PD Deficiency
Hemizygous G6PD Deficiency
Other Hemoglobinopathies
Iron Deficiency
Vitamin A Deficiency
Gastritis & Duodenitis
Peptic Ulcer Disease
Crohn's Disease
Ulcerative Colitis
Cirrhosis
CKD Stage 3
CKD Stage 4
CKD Stage 5 untreated
CKD Stage 5 on dialysis
Hypothyroid Diseases
Hyperthyroid Diseases
Other Endocrine
Males
Females
1990
2021
Dietary iron deficiency
Vitamin A deficiency
Haemoglobinopathies and haemolytic anaemias
Malaria
Other neglected tropical diseases
Other unspecified infectious diseases
Intestinal nematode infections
HIV/AIDS
Chronic kidney disease
Upper digestive system diseases
Endocrine, metabolic, blood, and immune disorders
Schistosomiasis
Cirrhosis and other chronic liver diseases
Inflammatory bowel disease
Maternal disorders
Gynecological diseases
YLDs per 100,000 population
0 to <0.25
0.25 to <0.68
0.68 to <1.55
1.55 to <2.53
2.53 to <6.18
6.18 to <12.63
12.63 to <25.14
25.14 to <47.02
47.02 to <95.39
95.39 to 453.44

Uasin Gishu

Anaemia Prevalence - Both Sexes

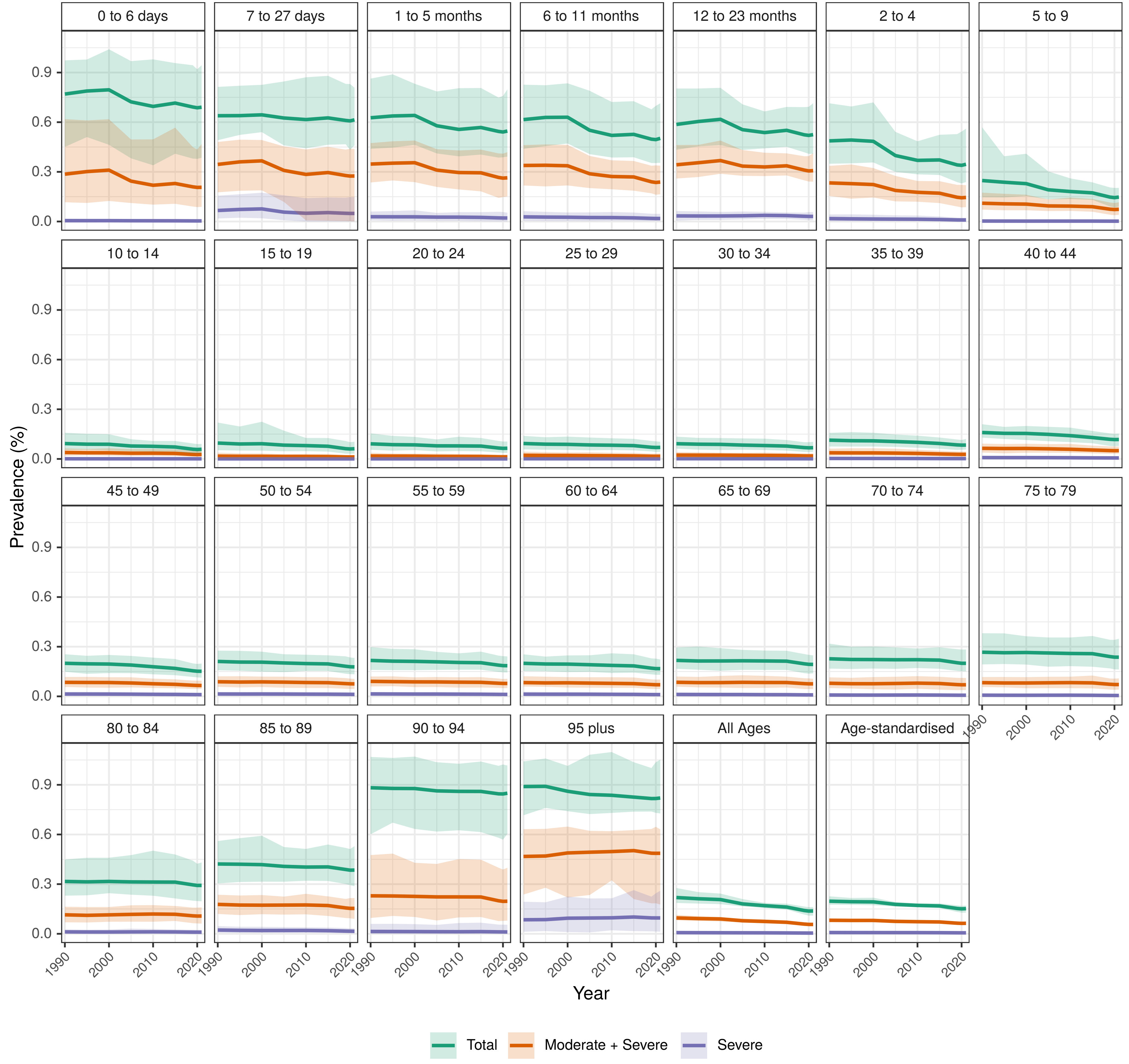

Cause-Specific Anaemia Prevalence - 2021

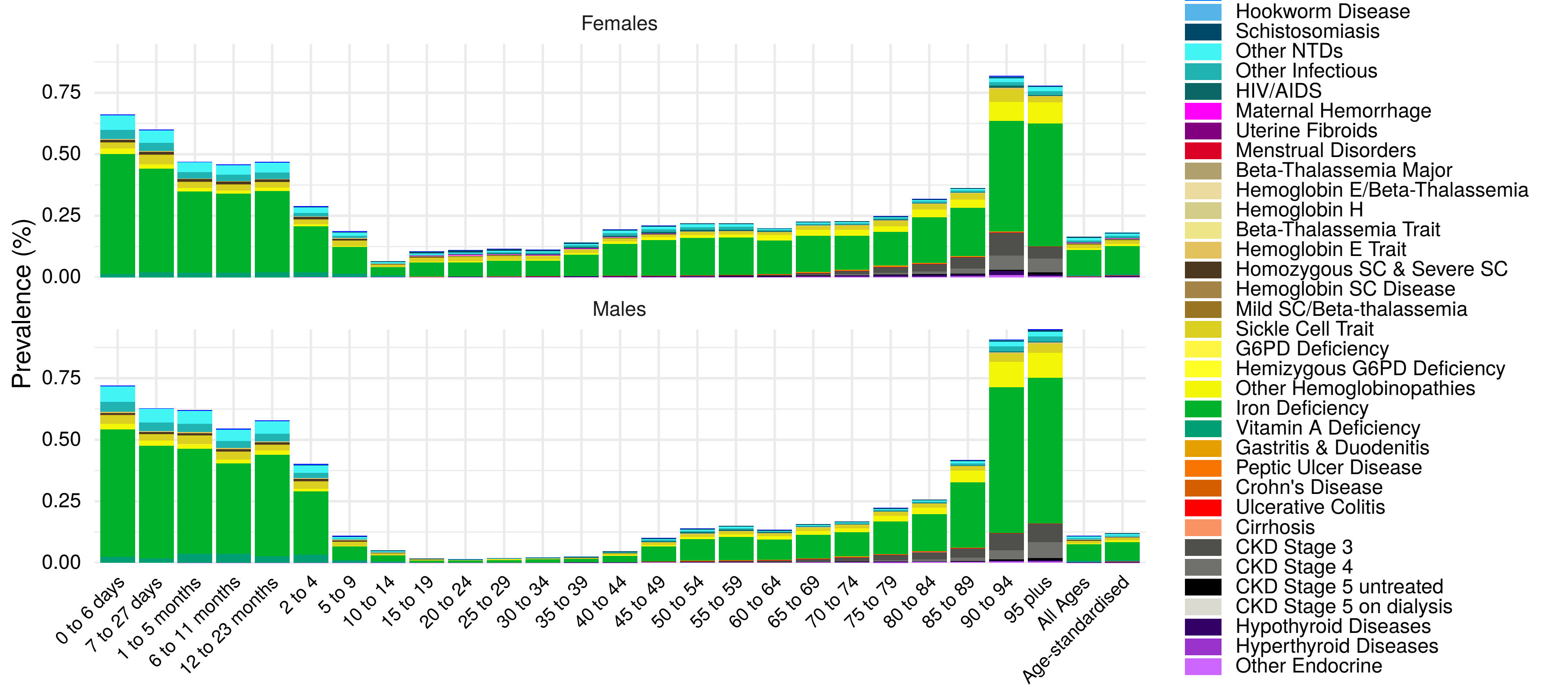

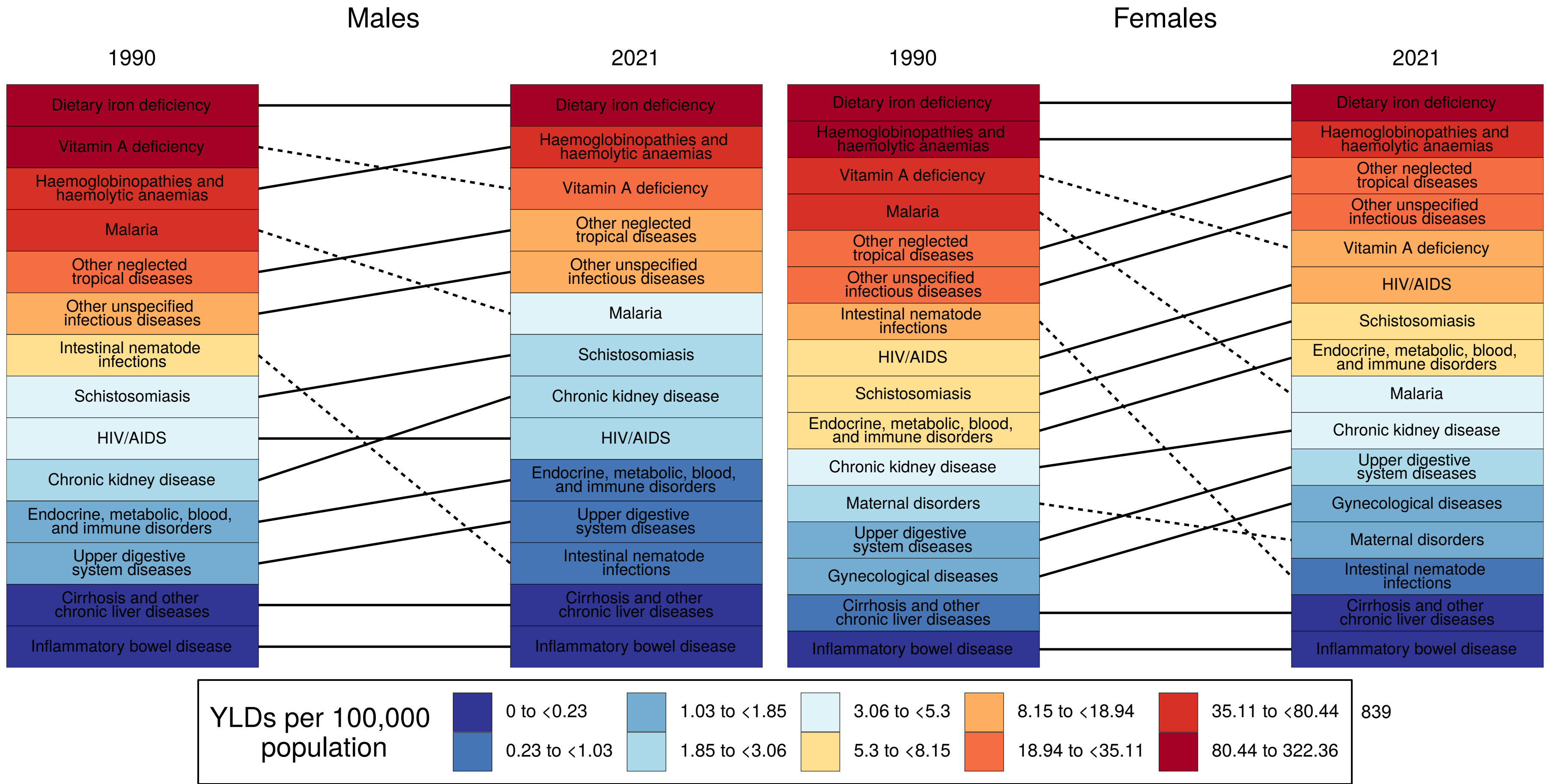

Vihiga

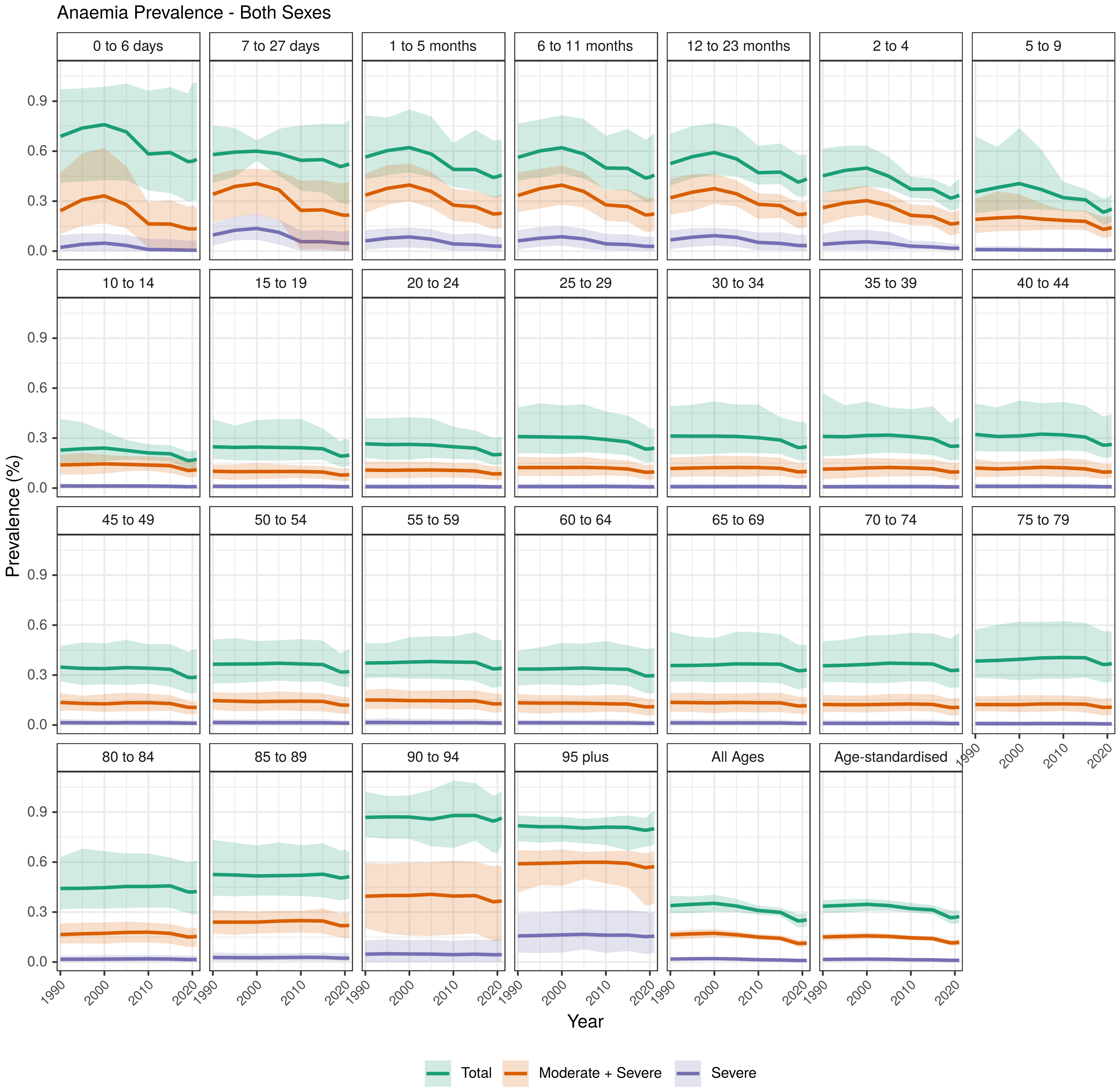

Anaemia Prevalence - Both Sexes
0 to 6 days
7 to 27 days
1 to 5 months
6 to 11 months
12 to 23 months
2 to 4
5 to 9
10 to 14
15 to 19
20 to 24
25 to 29
30 to 34
35 to 39
40 to 44
45 to 49
50 to 54
55 to 59
60 to 64
65 to 69
70 to 74
75 to 79
80 to 84
85 to 89
90 to 94
95 plus
All Ages
Age-standardised
Prevalence (%)
0.0
0.3
0.6
0.9
1990
2000
2010
2020
Year
Total
Moderate + Severe
Severe

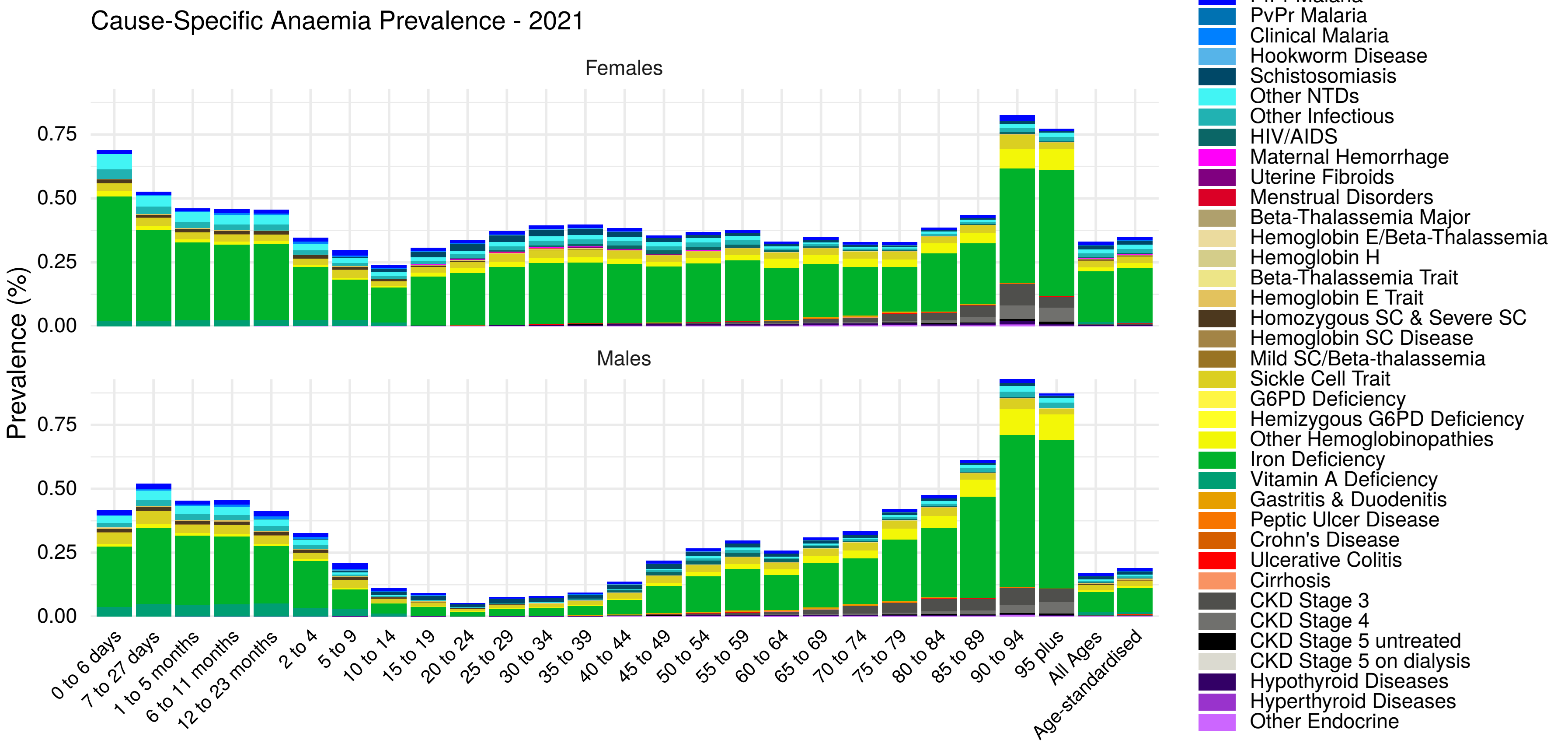

Cause-Specific Anaemia Prevalence - 2021
Females
Males
Prevalence (%)
0.00
0.25
0.50
0.75
0 to 6 days
7 to 27 days
1 to 5 months
6 to 11 months
12 to 23 months
2 to 4
5 to 9
10 to 14
15 to 19
20 to 24
25 to 29
30 to 34
35 to 39
40 to 44
45 to 49
50 to 54
55 to 59
60 to 64
65 to 69
70 to 74
75 to 79
80 to 84
85 to 89
90 to 94
95 plus
All Ages
Age-standardised
PfPr Malaria
PvPr Malaria
Clinical Malaria
Hookworm Disease
Schistosomiasis
Other NTDs
Other Infectious
HIV/AIDS
Maternal Hemorrhage
Uterine Fibroids
Menstrual Disorders
Beta-Thalassemia Major
Hemoglobin E/Beta-Thalassemia
Hemoglobin H
Beta-Thalassemia Trait
Hemoglobin E Trait
Homozygous SC & Severe SC
Hemoglobin SC Disease
Mild SC/Beta-thalassemia
Sickle Cell Trait
G6PD Deficiency
Hemizygous G6PD Deficiency
Other Hemoglobinopathies
Iron Deficiency
Vitamin A Deficiency
Gastritis & Duodenitis
Peptic Ulcer Disease
Crohn's Disease
Ulcerative Colitis
Cirrhosis
CKD Stage 3
CKD Stage 4
CKD Stage 5 untreated
CKD Stage 5 on dialysis
Hypothyroid Diseases
Hyperthyroid Diseases
Other Endocrine

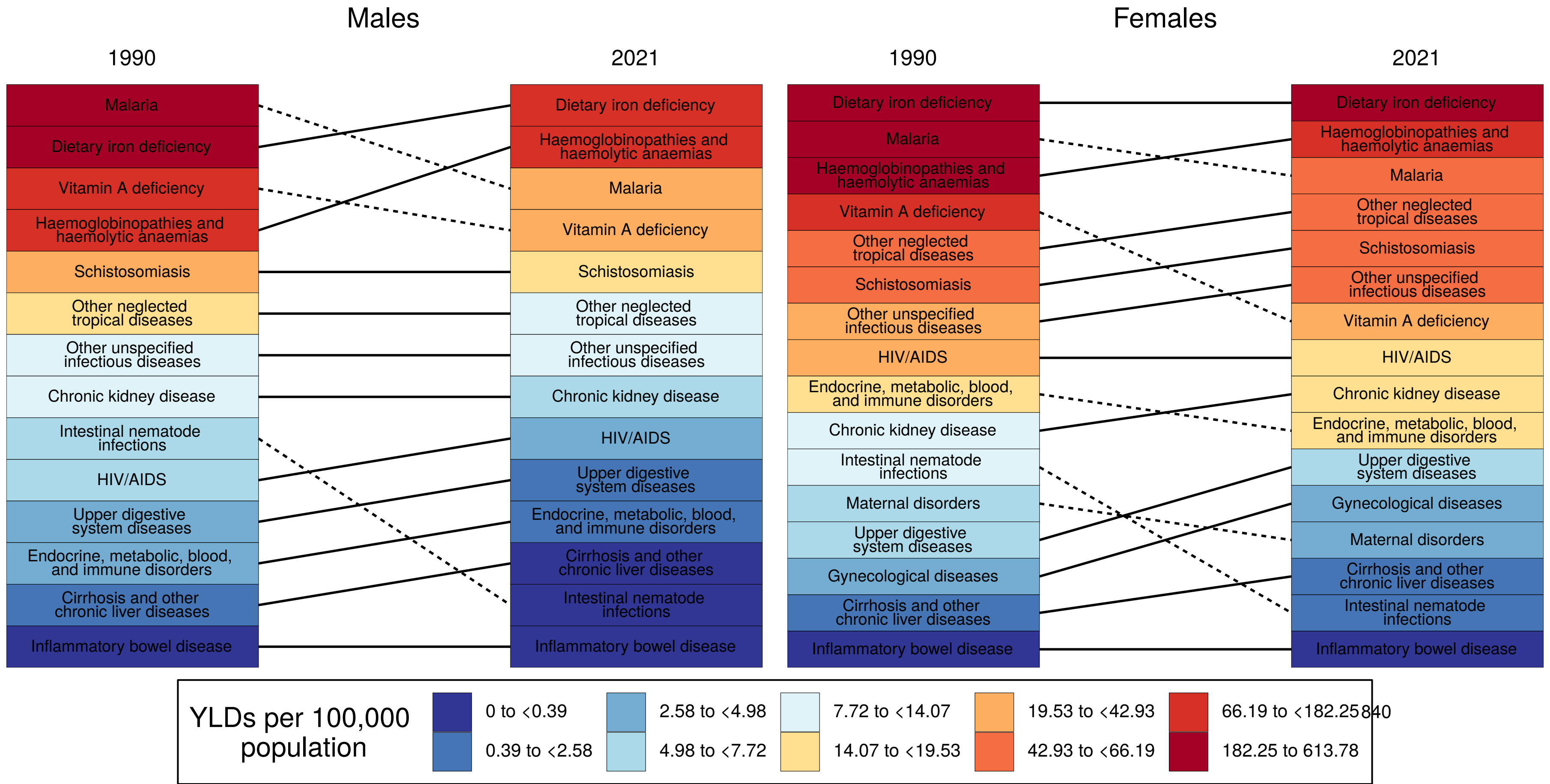

Males
Females
1990
2021
Malaria
Dietary iron deficiency
Vitamin A deficiency
Haemoglobinopathies and haemolytic anaemias
Schistosomiasis
Other neglected tropical diseases
Other unspecified infectious diseases
Chronic kidney disease
Intestinal nematode infections
HIV/AIDS
Upper digestive system diseases
Endocrine, metabolic, blood, and immune disorders
Cirrhosis and other chronic liver diseases
Inflammatory bowel disease
Maternal disorders
Gynecological diseases
YLDs per 100,000 population
0 to <0.39
0.39 to <2.58
2.58 to <4.98
4.98 to <7.72
7.72 to <14.07
14.07 to <19.53
19.53 to <42.93
42.93 to <66.19
66.19 to <182.25
182.25 to 613.78

Wajir

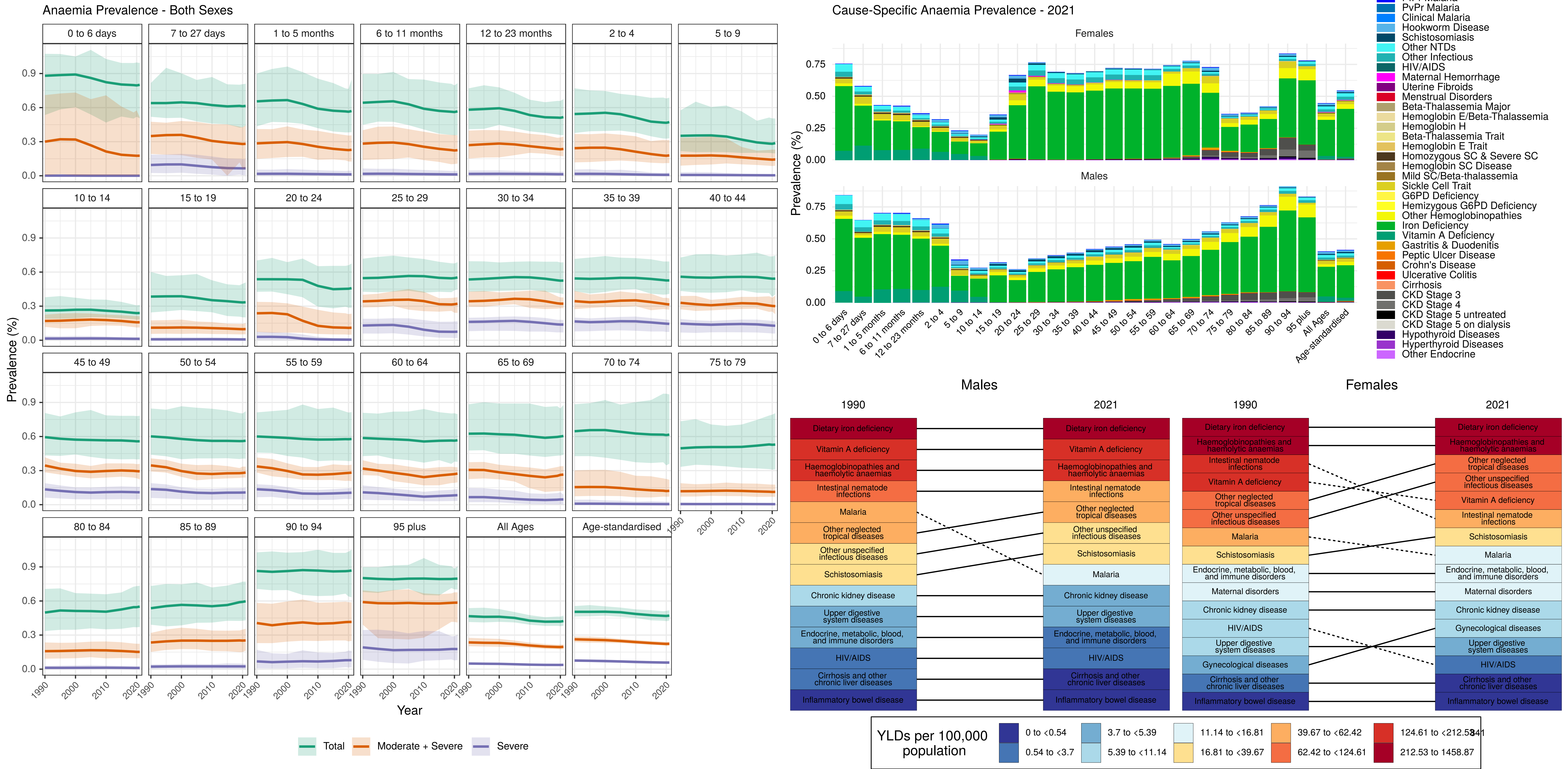

Anaemia Prevalence - Both Sexes
0 to 6 days
7 to 27 days
1 to 5 months
6 to 11 months
12 to 23 months
2 to 4
5 to 9
10 to 14
15 to 19
20 to 24
25 to 29
30 to 34
35 to 39
40 to 44
45 to 49
50 to 54
55 to 59
60 to 64
65 to 69
70 to 74
75 to 79
80 to 84
85 to 89
90 to 94
95 plus
All Ages
Age-standardised
Prevalence (%)
Year
Total
Moderate + Severe
Severe
Cause-Specific Anaemia Prevalence - 2021
Females
Males
Prevalence (%)
PfPr Malaria
PvPr Malaria
Clinical Malaria
Hookworm Disease
Schistosomiasis
Other NTDs
Other Infectious
HIV/AIDS
Maternal Hemorrhage
Uterine Fibroids
Menstrual Disorders
Beta-Thalassemia Major
Hemoglobin E/Beta-Thalassemia
Hemoglobin H
Beta-Thalassemia Trait
Hemoglobin E Trait
Homozygous SC & Severe SC
Hemoglobin SC Disease
Mild SC/Beta-thalassemia
Sickle Cell Trait
G6PD Deficiency
Hemizygous G6PD Deficiency
Other Hemoglobinopathies
Iron Deficiency
Vitamin A Deficiency
Gastritis & Duodenitis
Peptic Ulcer Disease
Crohn's Disease
Ulcerative Colitis
Cirrhosis
CKD Stage 3
CKD Stage 4
CKD Stage 5 untreated
CKD Stage 5 on dialysis
Hypothyroid Diseases
Hyperthyroid Diseases
Other Endocrine
Males
Females
1990
2021
Dietary iron deficiency
Vitamin A deficiency
Haemoglobinopathies and haemolytic anaemias
Intestinal nematode infections
Malaria
Other neglected tropical diseases
Other unspecified infectious diseases
Schistosomiasis
Chronic kidney disease
Upper digestive system diseases
Endocrine, metabolic, blood, and immune disorders
HIV/AIDS
Cirrhosis and other chronic liver diseases
Inflammatory bowel disease
Maternal disorders
Gynecological diseases
YLDs per 100,000 population
0 to <0.54
0.54 to <3.7
3.7 to <5.39
5.39 to <11.14
11.14 to <16.81
16.81 to <39.67
39.67 to <62.42
62.42 to <124.61
124.61 to <212.53
212.53 to 1458.87

West Pokot

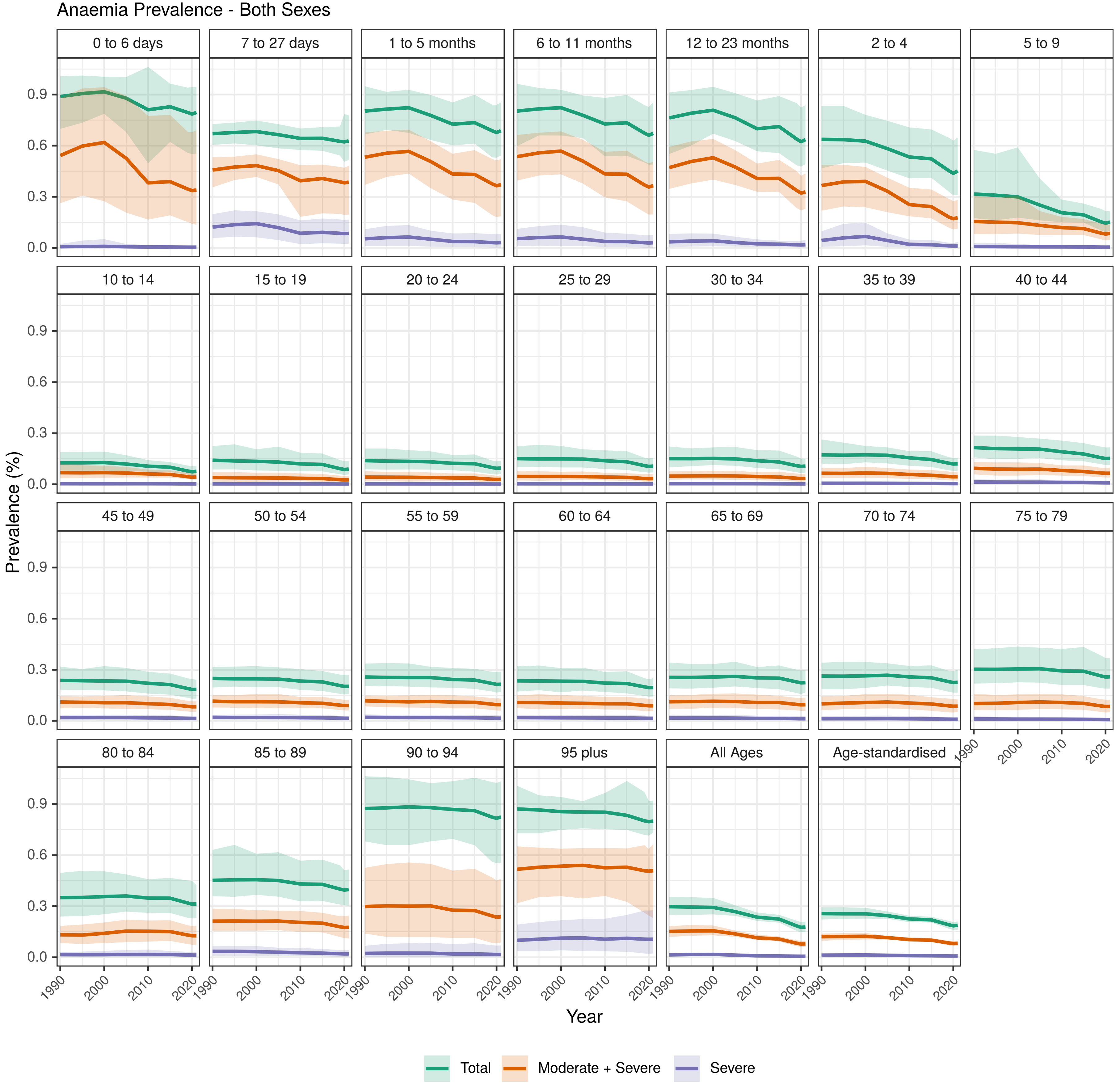

Anaemia Prevalence - Both Sexes
0 to 6 days
7 to 27 days
1 to 5 months
6 to 11 months
12 to 23 months
2 to 4
5 to 9
10 to 14
15 to 19
20 to 24
25 to 29
30 to 34
35 to 39
40 to 44
45 to 49
50 to 54
55 to 59
60 to 64
65 to 69
70 to 74
75 to 79
80 to 84
85 to 89
90 to 94
95 plus
All Ages
Age-standardised
Prevalence (%)
Year
Total
Moderate + Severe
Severe

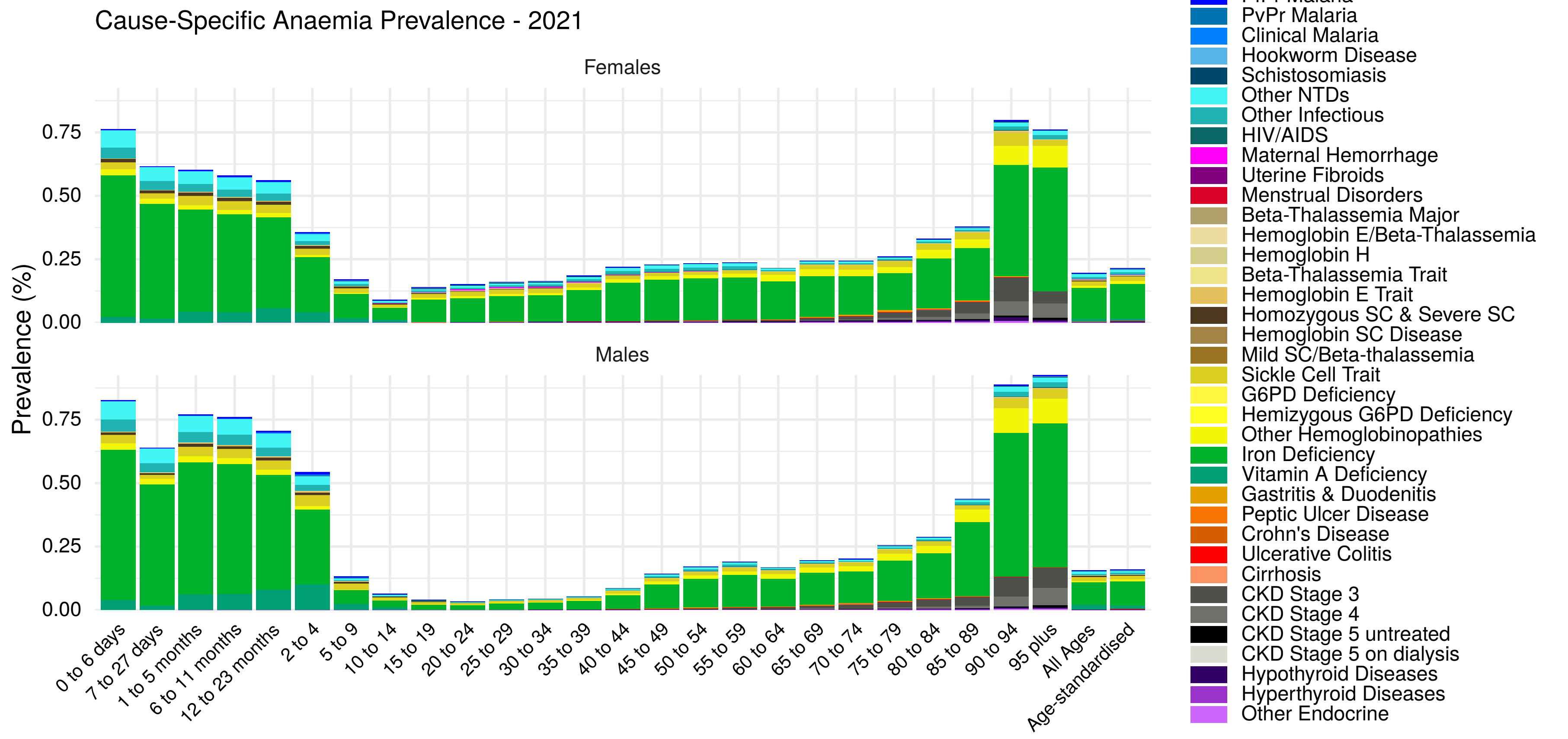

Cause-Specific Anaemia Prevalence - 2021
Females
Males
Prevalence (%)
PfPr Malaria
PvPr Malaria
Clinical Malaria
Hookworm Disease
Schistosomiasis
Other NTDs
Other Infectious
HIV/AIDS
Maternal Hemorrhage
Uterine Fibroids
Menstrual Disorders
Beta-Thalassemia Major
Hemoglobin E/Beta-Thalassemia
Hemoglobin H
Beta-Thalassemia Trait
Hemoglobin E Trait
Homozygous SC & Severe SC
Hemoglobin SC Disease
Mild SC/Beta-thalassemia
Sickle Cell Trait
G6PD Deficiency
Hemizygous G6PD Deficiency
Other Hemoglobinopathies
Iron Deficiency
Vitamin A Deficiency
Gastritis & Duodenitis
Peptic Ulcer Disease
Crohn's Disease
Ulcerative Colitis
Cirrhosis
CKD Stage 3
CKD Stage 4
CKD Stage 5 untreated
CKD Stage 5 on dialysis
Hypothyroid Diseases
Hyperthyroid Diseases
Other Endocrine

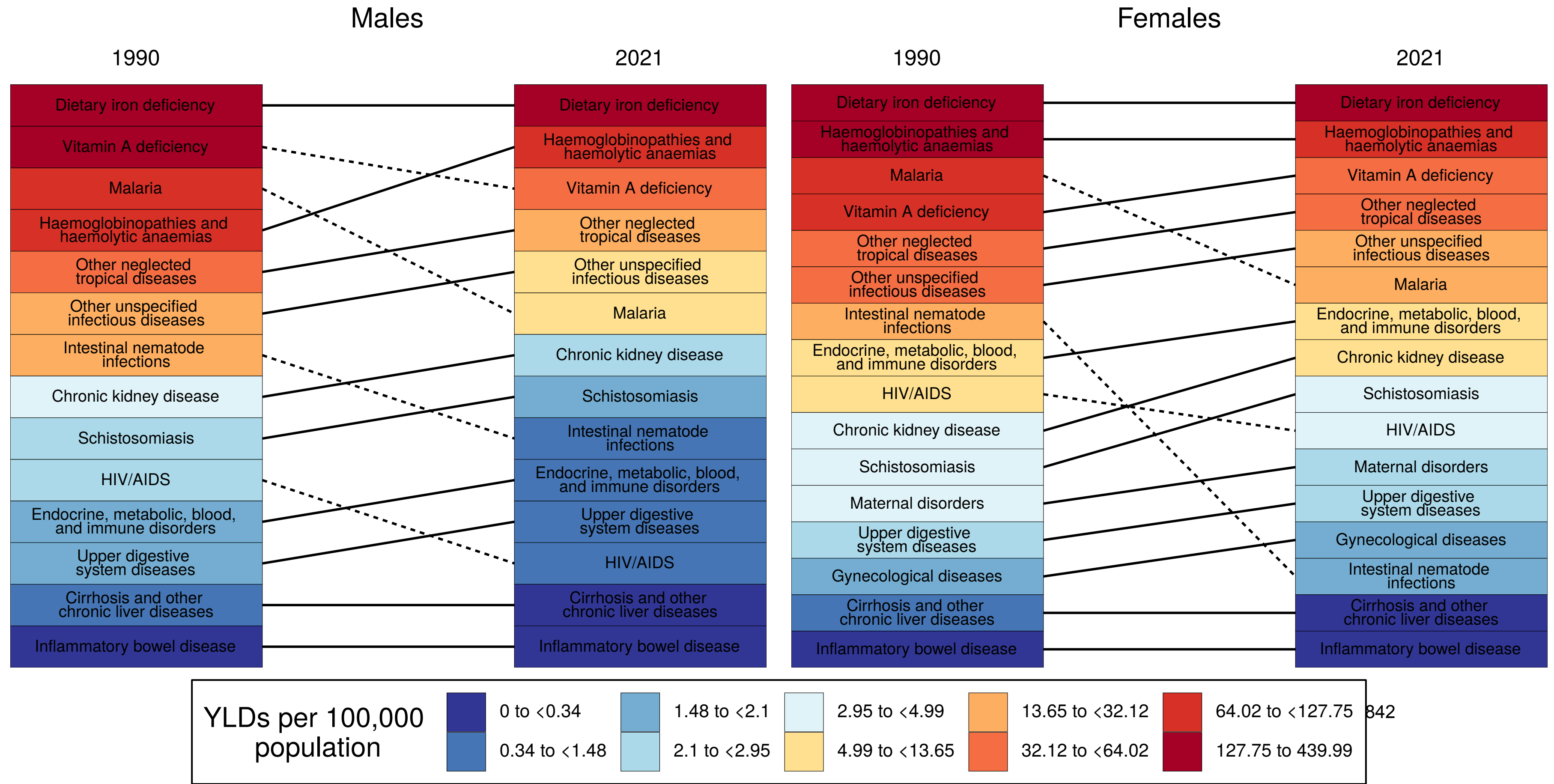

Males
Females
1990
2021
Males 1990: Dietary iron deficiency; Vitamin A deficiency; Malaria; Haemoglobinopathies and haemolytic anaemias; Other neglected tropical diseases; Other unspecified infectious diseases; Intestinal nematode infections; Chronic kidney disease; Schistosomiasis; HIV/AIDS; Endocrine, metabolic, blood, and immune disorders; Upper digestive system diseases; Cirrhosis and other chronic liver diseases; Inflammatory bowel disease
Males 2021: Dietary iron deficiency; Haemoglobinopathies and haemolytic anaemias; Vitamin A deficiency; Other neglected tropical diseases; Other unspecified infectious diseases; Malaria; Chronic kidney disease; Schistosomiasis; Intestinal nematode infections; Endocrine, metabolic, blood, and immune disorders; Upper digestive system diseases; HIV/AIDS; Cirrhosis and other chronic liver diseases; Inflammatory bowel disease
Females 1990: Dietary iron deficiency; Haemoglobinopathies and haemolytic anaemias; Malaria; Vitamin A deficiency; Other neglected tropical diseases; Other unspecified infectious diseases; Intestinal nematode infections; Endocrine, metabolic, blood, and immune disorders; HIV/AIDS; Chronic kidney disease; Schistosomiasis; Maternal disorders; Upper digestive system diseases; Gynecological diseases; Cirrhosis and other chronic liver diseases; Inflammatory bowel disease
Females 2021: Dietary iron deficiency; Haemoglobinopathies and haemolytic anaemias; Vitamin A deficiency; Other neglected tropical diseases; Other unspecified infectious diseases; Malaria; Endocrine, metabolic, blood, and immune disorders; Chronic kidney disease; Schistosomiasis; HIV/AIDS; Maternal disorders; Upper digestive system diseases; Gynecological diseases; Intestinal nematode infections; Cirrhosis and other chronic liver diseases; Inflammatory bowel disease
YLDs per 100,000 population
0 to <0.34
0.34 to <1.48
1.48 to <2.1
2.1 to <2.95
2.95 to <4.99
4.99 to <13.65
13.65 to <32.12
32.12 to <64.02
64.02 to <127.75
127.75 to 439.99

Madagascar

Anaemia Prevalence - Both Sexes

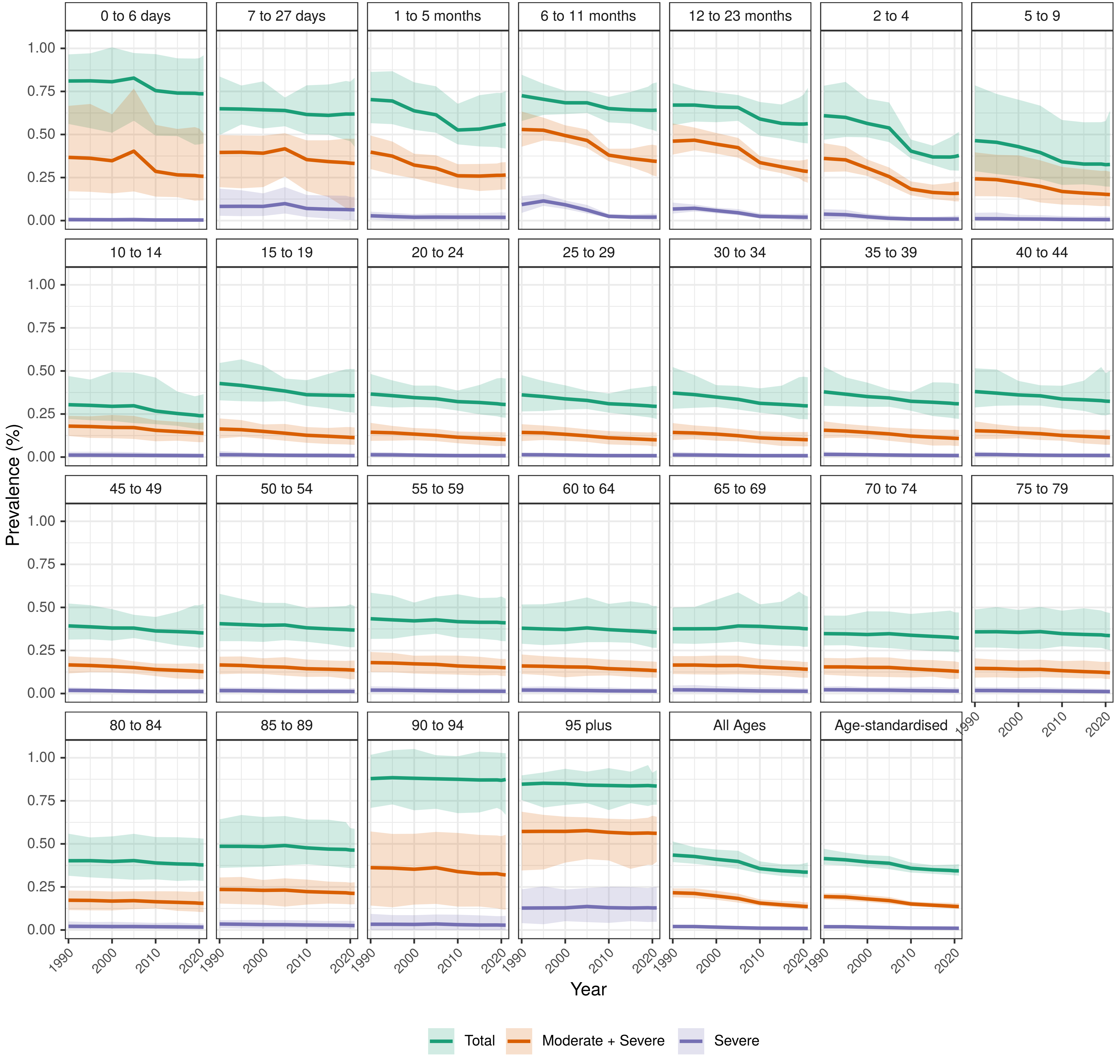

Cause-Specific Anaemia Prevalence - 2021

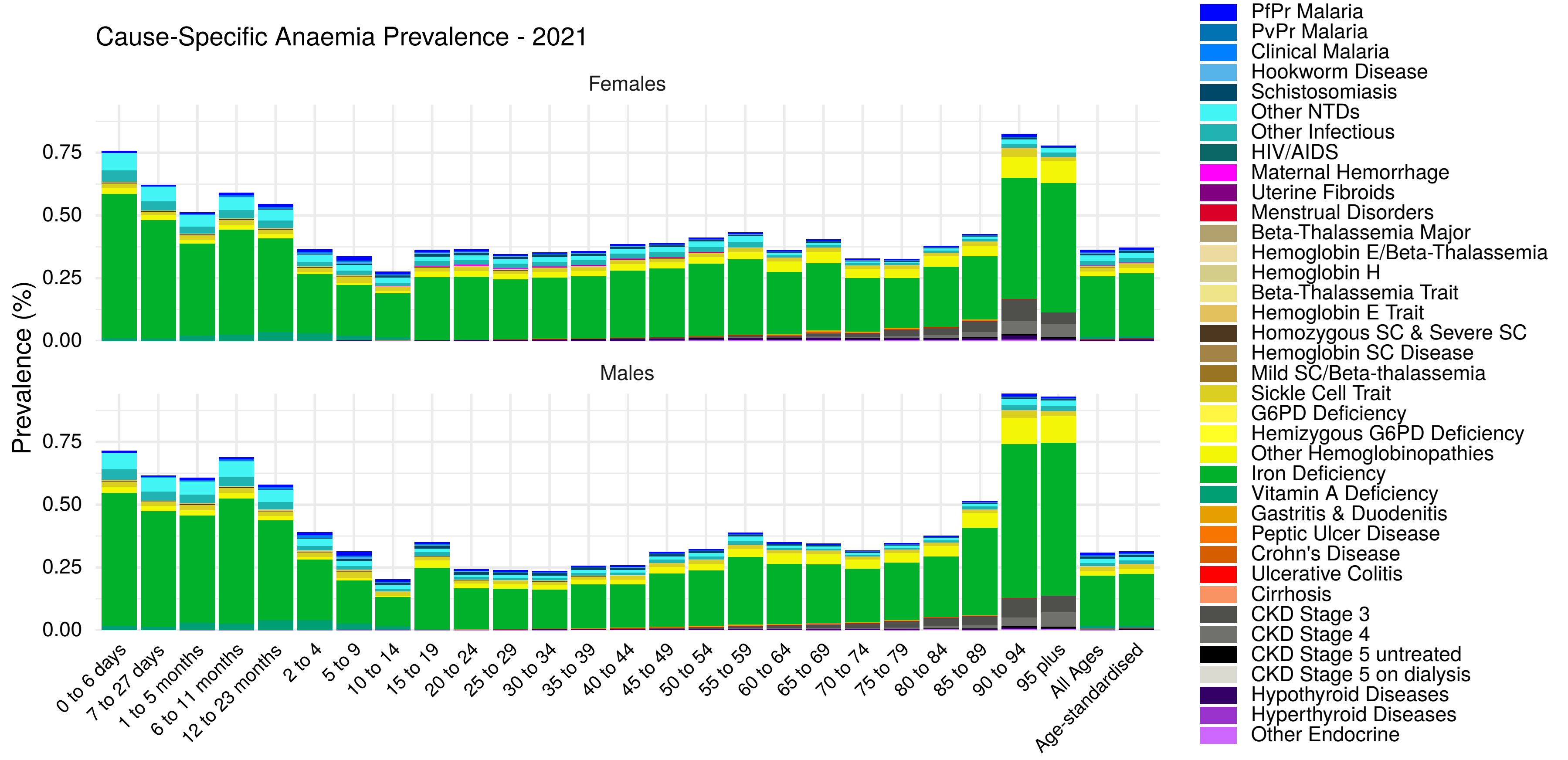

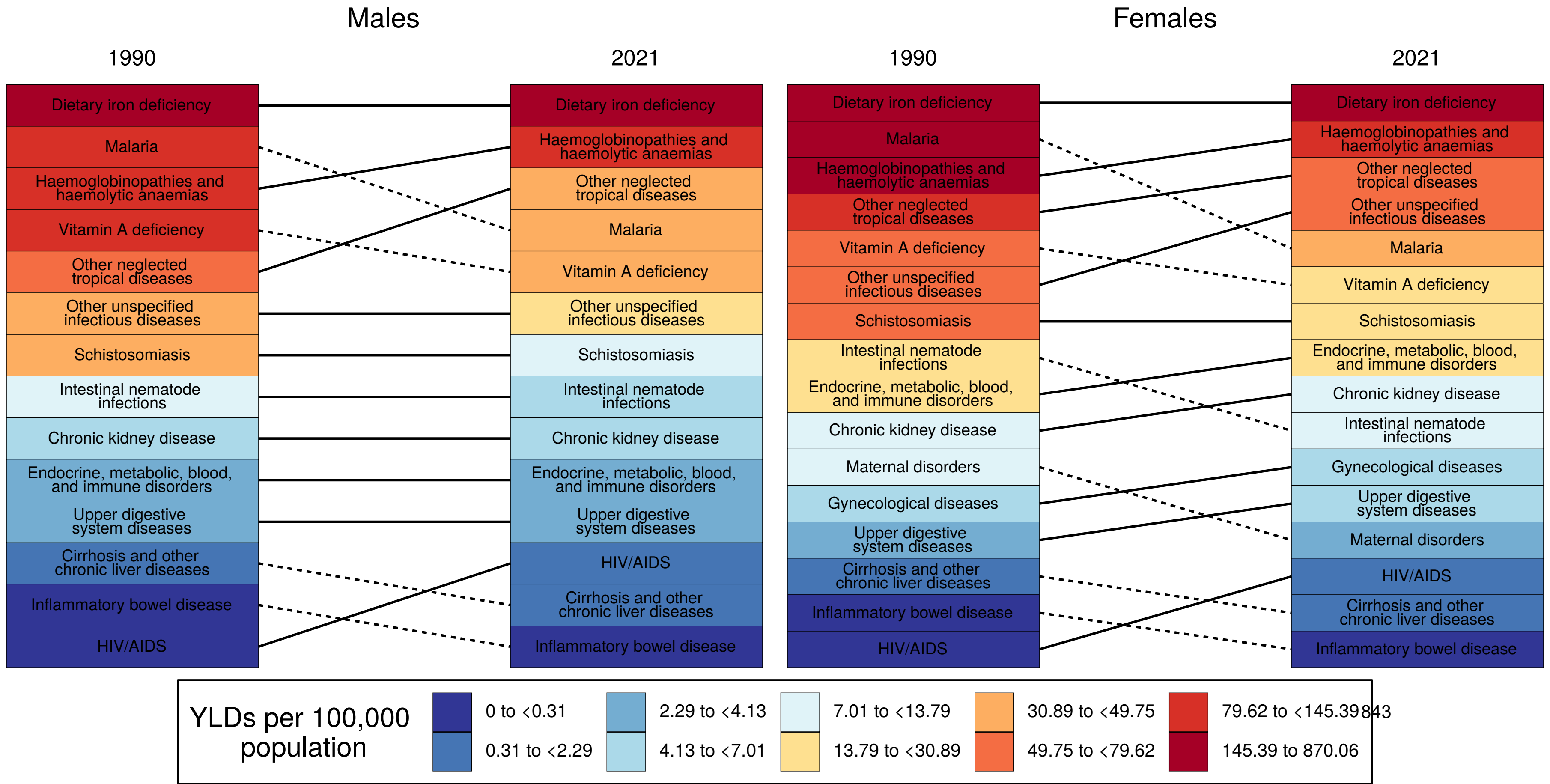

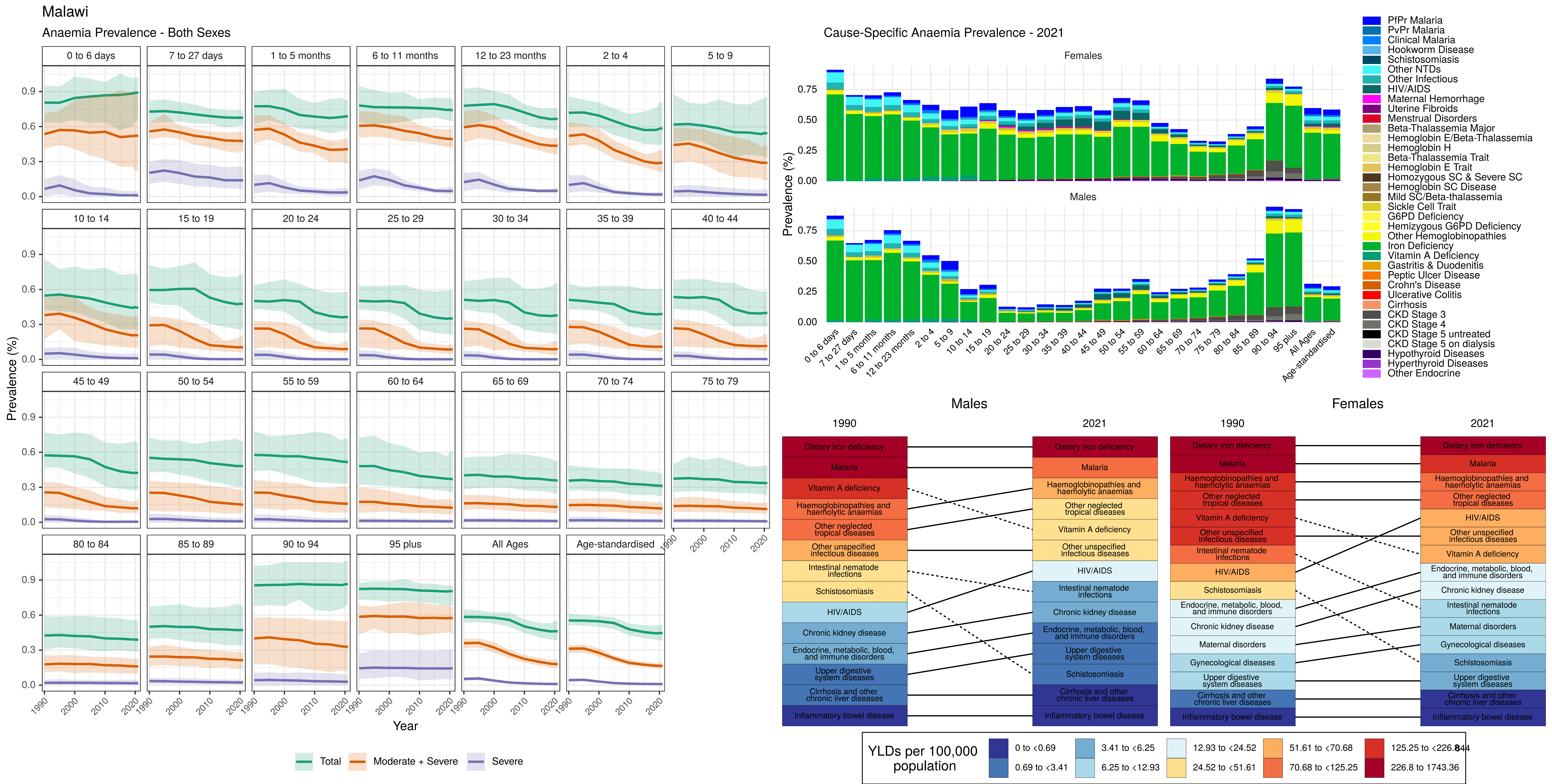
Malawi
Anaemia Prevalence - Both Sexes
0 to 6 days
7 to 27 days
1 to 5 months
6 to 11 months
12 to 23 months
2 to 4
5 to 9
10 to 14
15 to 19
20 to 24
25 to 29
30 to 34
35 to 39
40 to 44
45 to 49
50 to 54
55 to 59
60 to 64
65 to 69
70 to 74
75 to 79
80 to 84
85 to 89
90 to 94
95 plus
All Ages
Age-standardised
Prevalence (%)
0.0
0.3
0.6
0.9
1990
2000
2010
2020
Year
Total
Moderate + Severe
Severe
Cause-Specific Anaemia Prevalence - 2021
Females
Males
Prevalence (%)
0.00
0.25
0.50
0.75
PfPr Malaria
PvPr Malaria
Clinical Malaria
Hookworm Disease
Schistosomiasis
Other NTDs
Other Infectious
HIV/AIDS
Maternal Hemorrhage
Uterine Fibroids
Menstrual Disorders
Beta-Thalassemia Major
Hemoglobin E/Beta-Thalassemia
Hemoglobin H
Beta-Thalassemia Trait
Hemoglobin E Trait
Homozygous SC & Severe SC
Hemoglobin SC Disease
Mild SC/Beta-thalassemia
Sickle Cell Trait
G6PD Deficiency
Hemizygous G6PD Deficiency
Other Hemoglobinopathies
Iron Deficiency
Vitamin A Deficiency
Gastritis & Duodenitis
Peptic Ulcer Disease
Crohn's Disease
Ulcerative Colitis
Cirrhosis
CKD Stage 3
CKD Stage 4
CKD Stage 5 untreated
CKD Stage 5 on dialysis
Hypothyroid Diseases
Hyperthyroid Diseases
Other Endocrine
Males
Females
1990
2021
Dietary iron deficiency
Malaria
Vitamin A deficiency
Haemoglobinopathies and haemolytic anaemias
Other neglected tropical diseases
Other unspecified infectious diseases
Intestinal nematode infections
Schistosomiasis
HIV/AIDS
Chronic kidney disease
Endocrine, metabolic, blood, and immune disorders
Upper digestive system diseases
Cirrhosis and other chronic liver diseases
Inflammatory bowel disease
Maternal disorders
Gynecological diseases
YLDs per 100,000 population
0 to <0.69
0.69 to <3.41
3.41 to <6.25
6.25 to <12.93
12.93 to <24.52
24.52 to <51.61
51.61 to <70.68
70.68 to <125.25
125.25 to <226.8
226.8 to 1743.36
844

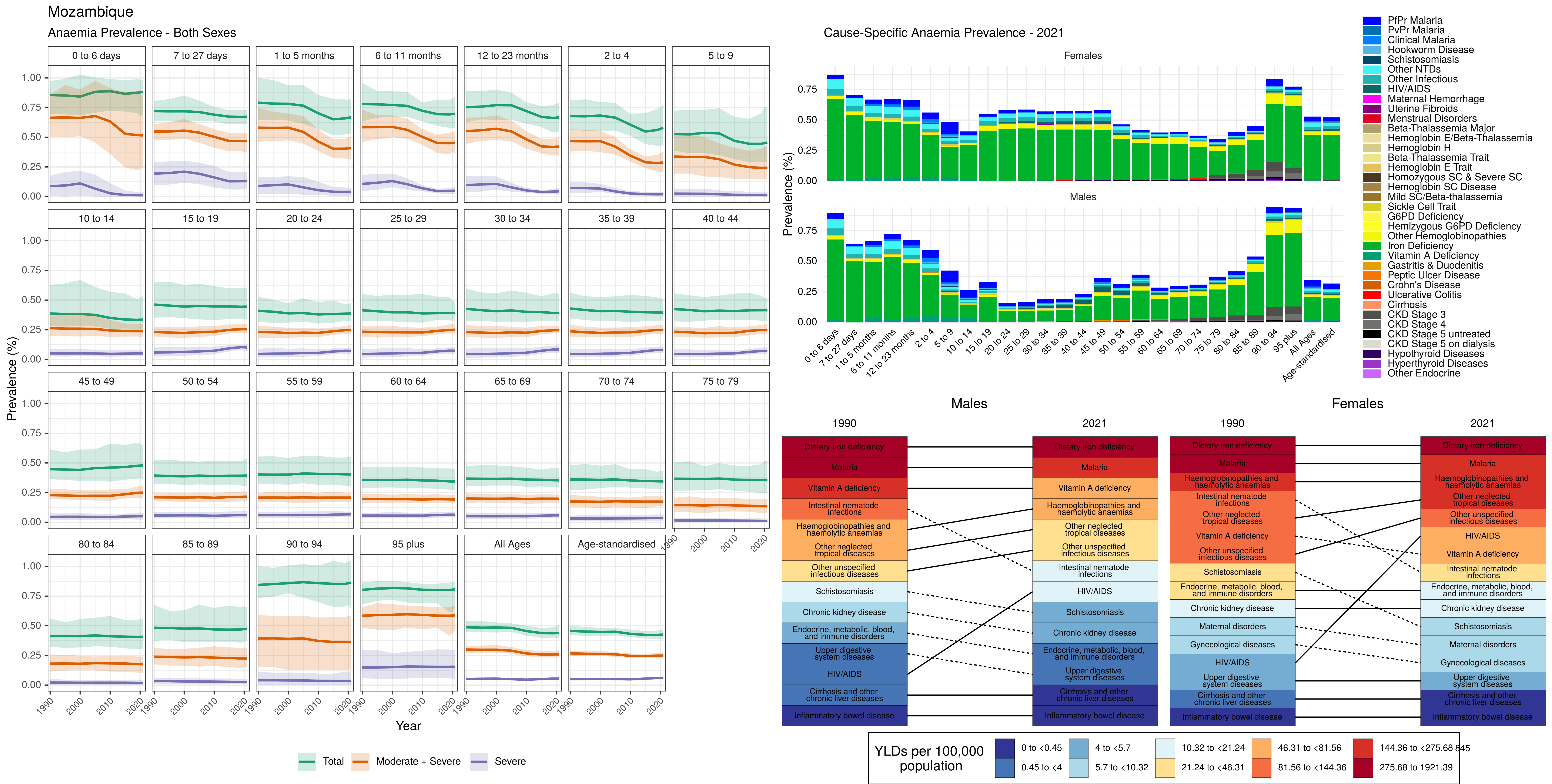

Mozambique
Anaemia Prevalence - Both Sexes
0 to 6 days
7 to 27 days
1 to 5 months
6 to 11 months
12 to 23 months
2 to 4
5 to 9
10 to 14
15 to 19
20 to 24
25 to 29
30 to 34
35 to 39
40 to 44
45 to 49
50 to 54
55 to 59
60 to 64
65 to 69
70 to 74
75 to 79
80 to 84
85 to 89
90 to 94
95 plus
All Ages
Age-standardised
Prevalence (%)
Year
Total
Moderate + Severe
Severe
Cause-Specific Anaemia Prevalence - 2021
Females
Males
PfPr Malaria
PvPr Malaria
Clinical Malaria
Hookworm Disease
Schistosomiasis
Other NTDs
Other Infectious
HIV/AIDS
Maternal Hemorrhage
Uterine Fibroids
Menstrual Disorders
Beta-Thalassemia Major
Hemoglobin E/Beta-Thalassemia
Hemoglobin H
Beta-Thalassemia Trait
Hemoglobin E Trait
Homozygous SC & Severe SC
Hemoglobin SC Disease
Mild SC/Beta-thalassemia
Sickle Cell Trait
G6PD Deficiency
Hemizygous G6PD Deficiency
Other Hemoglobinopathies
Iron Deficiency
Vitamin A Deficiency
Gastritis & Duodenitis
Peptic Ulcer Disease
Crohn's Disease
Ulcerative Colitis
Cirrhosis
CKD Stage 3
CKD Stage 4
CKD Stage 5 untreated
CKD Stage 5 on dialysis
Hypothyroid Diseases
Hyperthyroid Diseases
Other Endocrine
Males
Females
1990
2021
Dietary iron deficiency
Malaria
Vitamin A deficiency
Intestinal nematode infections
Haemoglobinopathies and haemolytic anaemias
Other neglected tropical diseases
Other unspecified infectious diseases
Schistosomiasis
Chronic kidney disease
Endocrine, metabolic, blood, and immune disorders
Upper digestive system diseases
HIV/AIDS
Cirrhosis and other chronic liver diseases
Inflammatory bowel disease
Maternal disorders
Gynecological diseases
YLDs per 100,000 population
0 to <0.45
0.45 to <4
4 to <5.7
5.7 to <10.32
10.32 to <21.24
21.24 to <46.31
46.31 to <81.56
81.56 to <144.36
144.36 to <275.68
275.68 to 1921.39

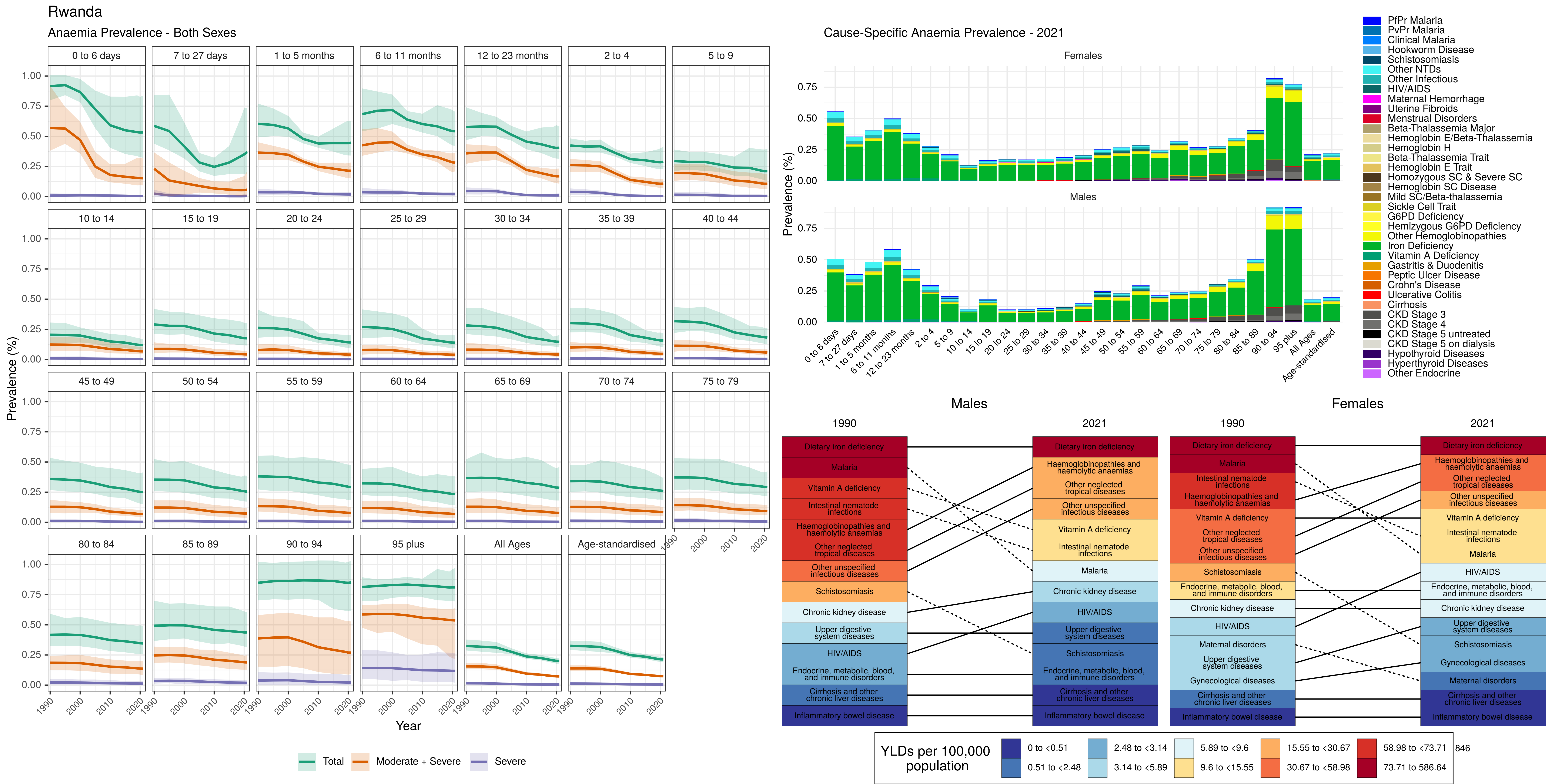

Rwanda
Anaemia Prevalence - Both Sexes
0 to 6 days
7 to 27 days
1 to 5 months
6 to 11 months
12 to 23 months
2 to 4
5 to 9
10 to 14
15 to 19
20 to 24
25 to 29
30 to 34
35 to 39
40 to 44
45 to 49
50 to 54
55 to 59
60 to 64
65 to 69
70 to 74
75 to 79
80 to 84
85 to 89
90 to 94
95 plus
All Ages
Age-standardised
Prevalence (%)
Year
Total
Moderate + Severe
Severe
Cause-Specific Anaemia Prevalence - 2021
Females
Males
PfPr Malaria
PvPr Malaria
Clinical Malaria
Hookworm Disease
Schistosomiasis
Other NTDs
Other Infectious
HIV/AIDS
Maternal Hemorrhage
Uterine Fibroids
Menstrual Disorders
Beta-Thalassemia Major
Hemoglobin E/Beta-Thalassemia
Hemoglobin H
Beta-Thalassemia Trait
Hemoglobin E Trait
Homozygous SC & Severe SC
Hemoglobin SC Disease
Mild SC/Beta-thalassemia
Sickle Cell Trait
G6PD Deficiency
Hemizygous G6PD Deficiency
Other Hemoglobinopathies
Iron Deficiency
Vitamin A Deficiency
Gastritis & Duodenitis
Peptic Ulcer Disease
Crohn's Disease
Ulcerative Colitis
Cirrhosis
CKD Stage 3
CKD Stage 4
CKD Stage 5 untreated
CKD Stage 5 on dialysis
Hypothyroid Diseases
Hyperthyroid Diseases
Other Endocrine
Males
Females
1990
2021
Dietary iron deficiency
Malaria
Vitamin A deficiency
Intestinal nematode infections
Haemoglobinopathies and haemolytic anaemias
Other neglected tropical diseases
Other unspecified infectious diseases
Schistosomiasis
Chronic kidney disease
Upper digestive system diseases
HIV/AIDS
Endocrine, metabolic, blood, and immune disorders
Cirrhosis and other chronic liver diseases
Inflammatory bowel disease
Maternal disorders
Gynecological diseases
YLDs per 100,000 population
0 to <0.51
0.51 to <2.48
2.48 to <3.14
3.14 to <5.89
5.89 to <9.6
9.6 to <15.55
15.55 to <30.67
30.67 to <58.98
58.98 to <73.71
73.71 to 586.64

Somalia

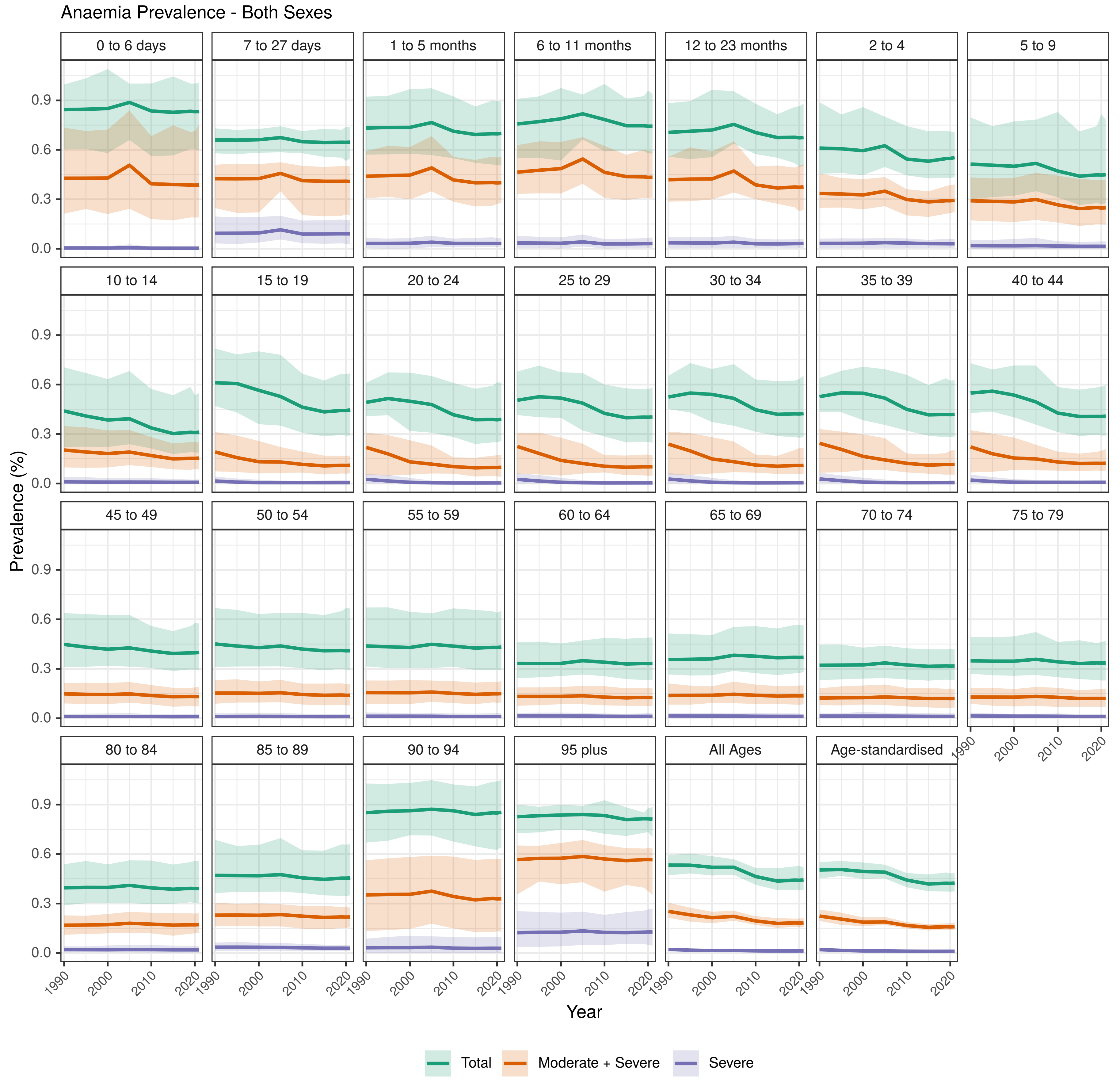

Anaemia Prevalence - Both Sexes
0 to 6 days
7 to 27 days
1 to 5 months
6 to 11 months
12 to 23 months
2 to 4
5 to 9
10 to 14
15 to 19
20 to 24
25 to 29
30 to 34
35 to 39
40 to 44
45 to 49
50 to 54
55 to 59
60 to 64
65 to 69
70 to 74
75 to 79
80 to 84
85 to 89
90 to 94
95 plus
All Ages
Age-standardised
0.0
0.3
0.6
0.9
Prevalence (%)
1990
2000
2010
2020
Year
Total
Moderate + Severe
Severe

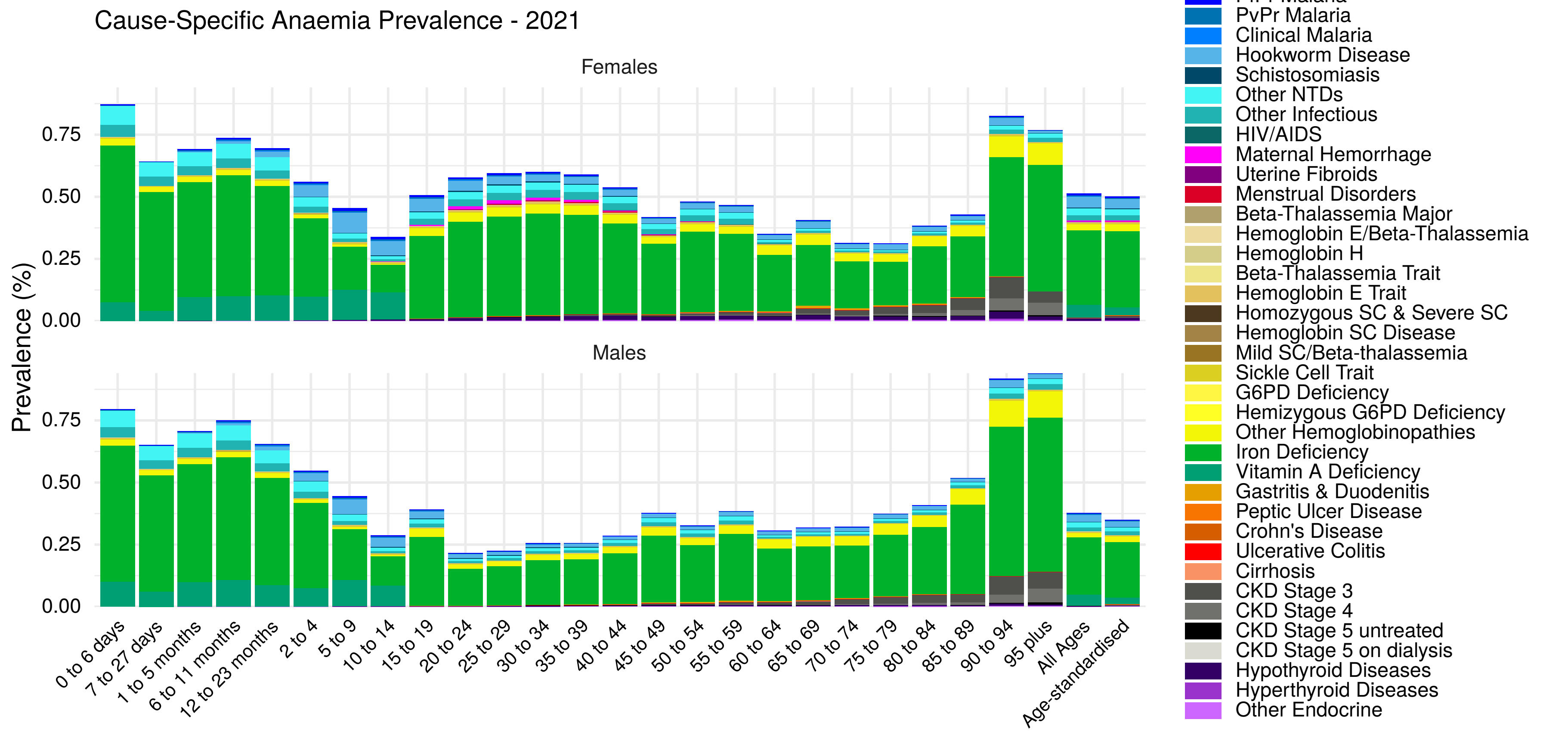

Cause-Specific Anaemia Prevalence - 2021
Females
Males
Prevalence (%)
0.00
0.25
0.50
0.75
0 to 6 days
7 to 27 days
1 to 5 months
6 to 11 months
12 to 23 months
2 to 4
5 to 9
10 to 14
15 to 19
20 to 24
25 to 29
30 to 34
35 to 39
40 to 44
45 to 49
50 to 54
55 to 59
60 to 64
65 to 69
70 to 74
75 to 79
80 to 84
85 to 89
90 to 94
95 plus
All Ages
Age-standardised
PfPr Malaria
PvPr Malaria
Clinical Malaria
Hookworm Disease
Schistosomiasis
Other NTDs
Other Infectious
HIV/AIDS
Maternal Hemorrhage
Uterine Fibroids
Menstrual Disorders
Beta-Thalassemia Major
Hemoglobin E/Beta-Thalassemia
Hemoglobin H
Beta-Thalassemia Trait
Hemoglobin E Trait
Homozygous SC & Severe SC
Hemoglobin SC Disease
Mild SC/Beta-thalassemia
Sickle Cell Trait
G6PD Deficiency
Hemizygous G6PD Deficiency
Other Hemoglobinopathies
Iron Deficiency
Vitamin A Deficiency
Gastritis & Duodenitis
Peptic Ulcer Disease
Crohn's Disease
Ulcerative Colitis
Cirrhosis
CKD Stage 3
CKD Stage 4
CKD Stage 5 untreated
CKD Stage 5 on dialysis
Hypothyroid Diseases
Hyperthyroid Diseases
Other Endocrine

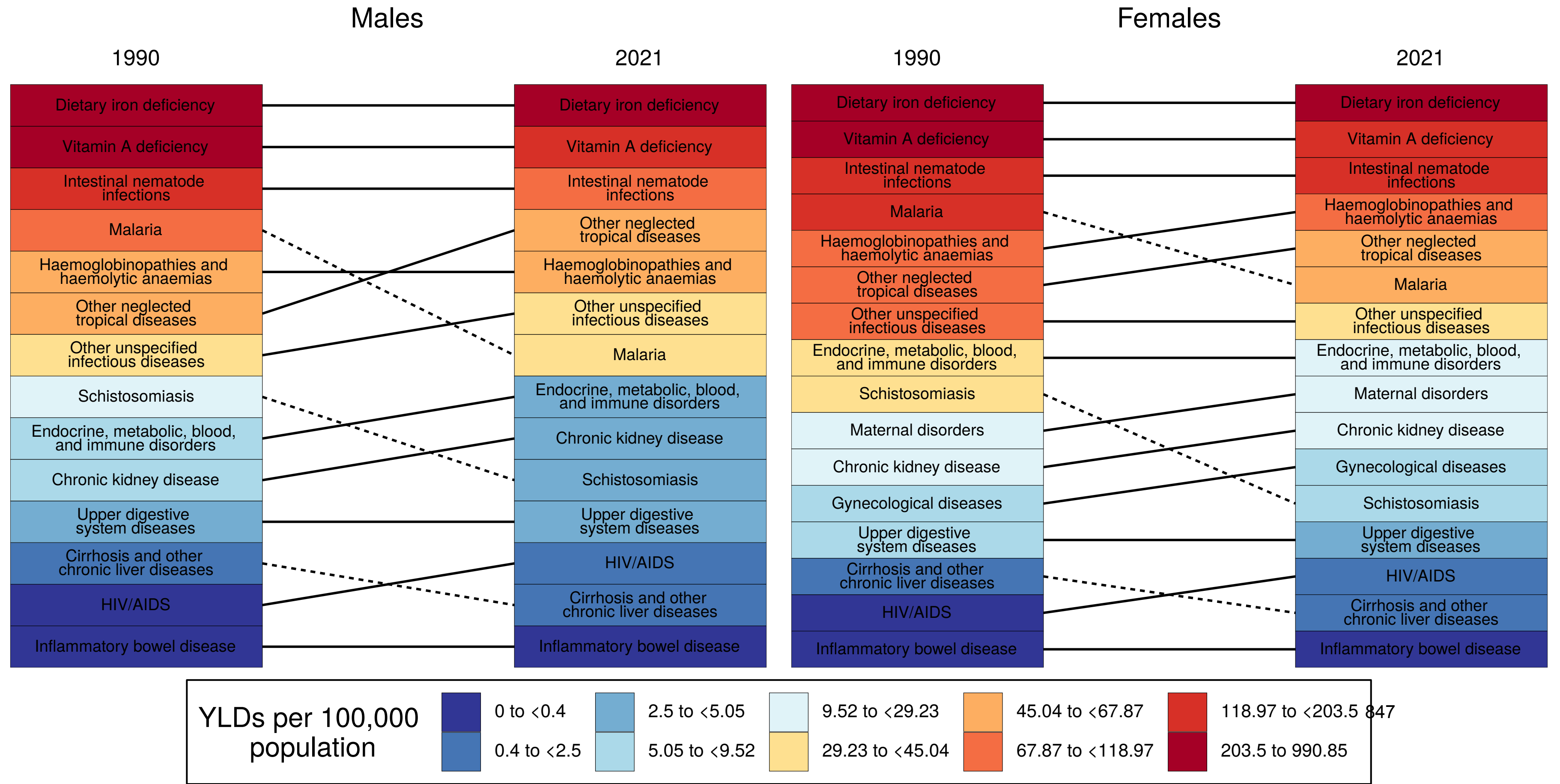

Males
Females
1990
2021
Dietary iron deficiency
Vitamin A deficiency
Intestinal nematode infections
Malaria
Haemoglobinopathies and haemolytic anaemias
Other neglected tropical diseases
Other unspecified infectious diseases
Schistosomiasis
Endocrine, metabolic, blood, and immune disorders
Chronic kidney disease
Upper digestive system diseases
Cirrhosis and other chronic liver diseases
HIV/AIDS
Inflammatory bowel disease
Maternal disorders
Gynecological diseases
YLDs per 100,000 population
0 to <0.4
0.4 to <2.5
2.5 to <5.05
5.05 to <9.52
9.52 to <29.23
29.23 to <45.04
45.04 to <67.87
67.87 to <118.97
118.97 to <203.5
203.5 to 990.85

South Sudan

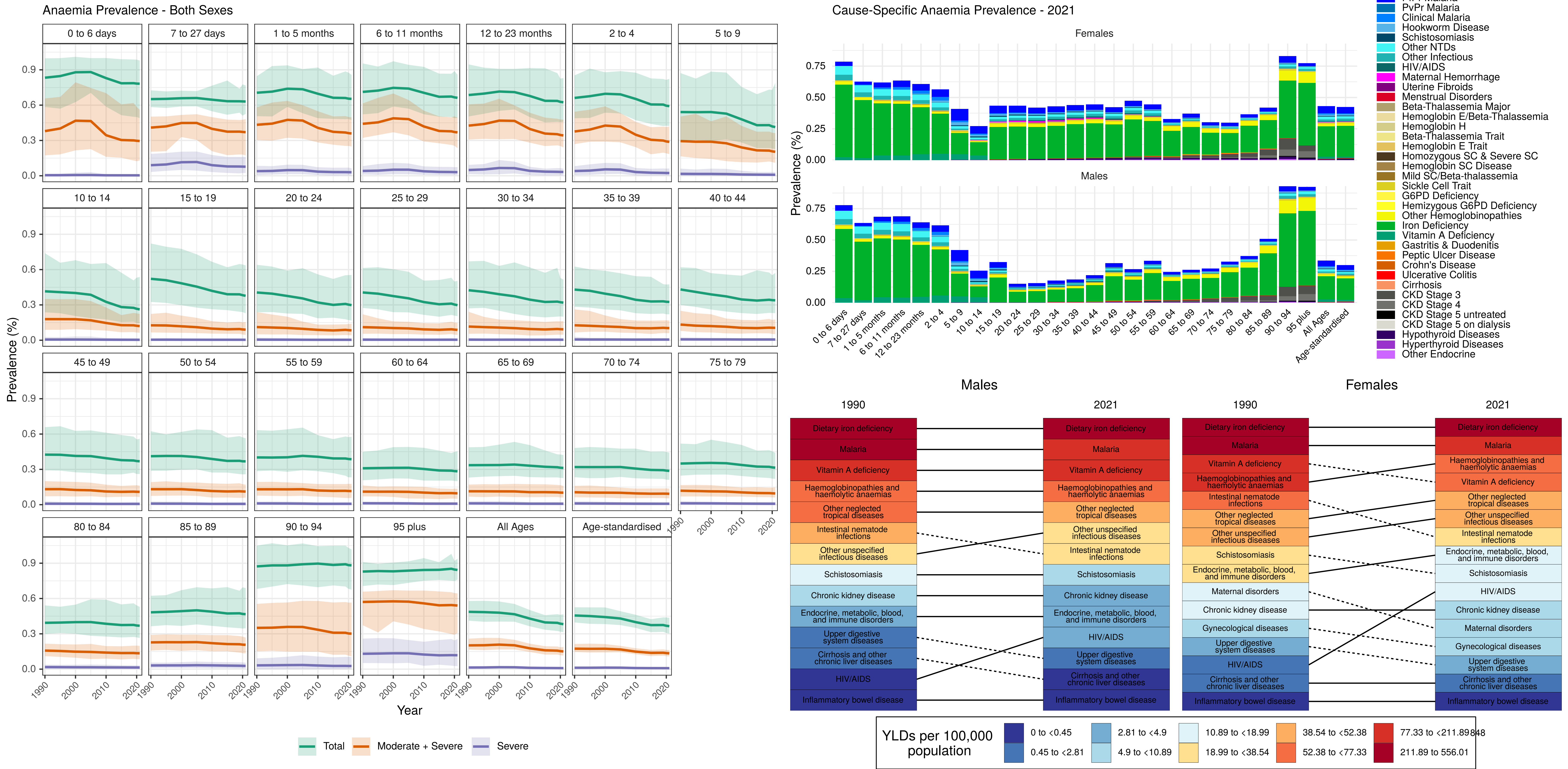

Anaemia Prevalence - Both Sexes
0 to 6 days
7 to 27 days
1 to 5 months
6 to 11 months
12 to 23 months
2 to 4
5 to 9
10 to 14
15 to 19
20 to 24
25 to 29
30 to 34
35 to 39
40 to 44
45 to 49
50 to 54
55 to 59
60 to 64
65 to 69
70 to 74
75 to 79
80 to 84
85 to 89
90 to 94
95 plus
All Ages
Age-standardised
Prevalence (%)
Year
Total
Moderate + Severe
Severe
Cause-Specific Anaemia Prevalence - 2021
Females
Males
Prevalence (%)
PfPr Malaria
PvPr Malaria
Clinical Malaria
Hookworm Disease
Schistosomiasis
Other NTDs
Other Infectious
HIV/AIDS
Maternal Hemorrhage
Uterine Fibroids
Menstrual Disorders
Beta-Thalassemia Major
Hemoglobin E/Beta-Thalassemia
Hemoglobin H
Beta-Thalassemia Trait
Hemoglobin E Trait
Homozygous SC & Severe SC
Hemoglobin SC Disease
Mild SC/Beta-thalassemia
Sickle Cell Trait
G6PD Deficiency
Hemizygous G6PD Deficiency
Other Hemoglobinopathies
Iron Deficiency
Vitamin A Deficiency
Gastritis & Duodenitis
Peptic Ulcer Disease
Crohn's Disease
Ulcerative Colitis
Cirrhosis
CKD Stage 3
CKD Stage 4
CKD Stage 5 untreated
CKD Stage 5 on dialysis
Hypothyroid Diseases
Hyperthyroid Diseases
Other Endocrine
Males
1990
Dietary iron deficiency
Malaria
Vitamin A deficiency
Haemoglobinopathies and haemolytic anaemias
Other neglected tropical diseases
Intestinal nematode infections
Other unspecified infectious diseases
Schistosomiasis
Chronic kidney disease
Endocrine, metabolic, blood, and immune disorders
Upper digestive system diseases
Cirrhosis and other chronic liver diseases
HIV/AIDS
Inflammatory bowel disease
2021
Dietary iron deficiency
Malaria
Vitamin A deficiency
Haemoglobinopathies and haemolytic anaemias
Other neglected tropical diseases
Other unspecified infectious diseases
Intestinal nematode infections
Schistosomiasis
Chronic kidney disease
Endocrine, metabolic, blood, and immune disorders
HIV/AIDS
Upper digestive system diseases
Cirrhosis and other chronic liver diseases
Inflammatory bowel disease
Females
1990
Dietary iron deficiency
Malaria
Vitamin A deficiency
Haemoglobinopathies and haemolytic anaemias
Intestinal nematode infections
Other neglected tropical diseases
Other unspecified infectious diseases
Schistosomiasis
Endocrine, metabolic, blood, and immune disorders
Maternal disorders
Chronic kidney disease
Gynecological diseases
Upper digestive system diseases
HIV/AIDS
Cirrhosis and other chronic liver diseases
Inflammatory bowel disease
2021
Dietary iron deficiency
Malaria
Haemoglobinopathies and haemolytic anaemias
Vitamin A deficiency
Other neglected tropical diseases
Other unspecified infectious diseases
Intestinal nematode infections
Endocrine, metabolic, blood, and immune disorders
Schistosomiasis
HIV/AIDS
Chronic kidney disease
Maternal disorders
Gynecological diseases
Upper digestive system diseases
Cirrhosis and other chronic liver diseases
Inflammatory bowel disease
YLDs per 100,000 population
0 to <0.45
0.45 to <2.81
2.81 to <4.9
4.9 to <10.89
10.89 to <18.99
18.99 to <38.54
38.54 to <52.38
52.38 to <77.33
77.33 to <211.89
211.89 to 556.01

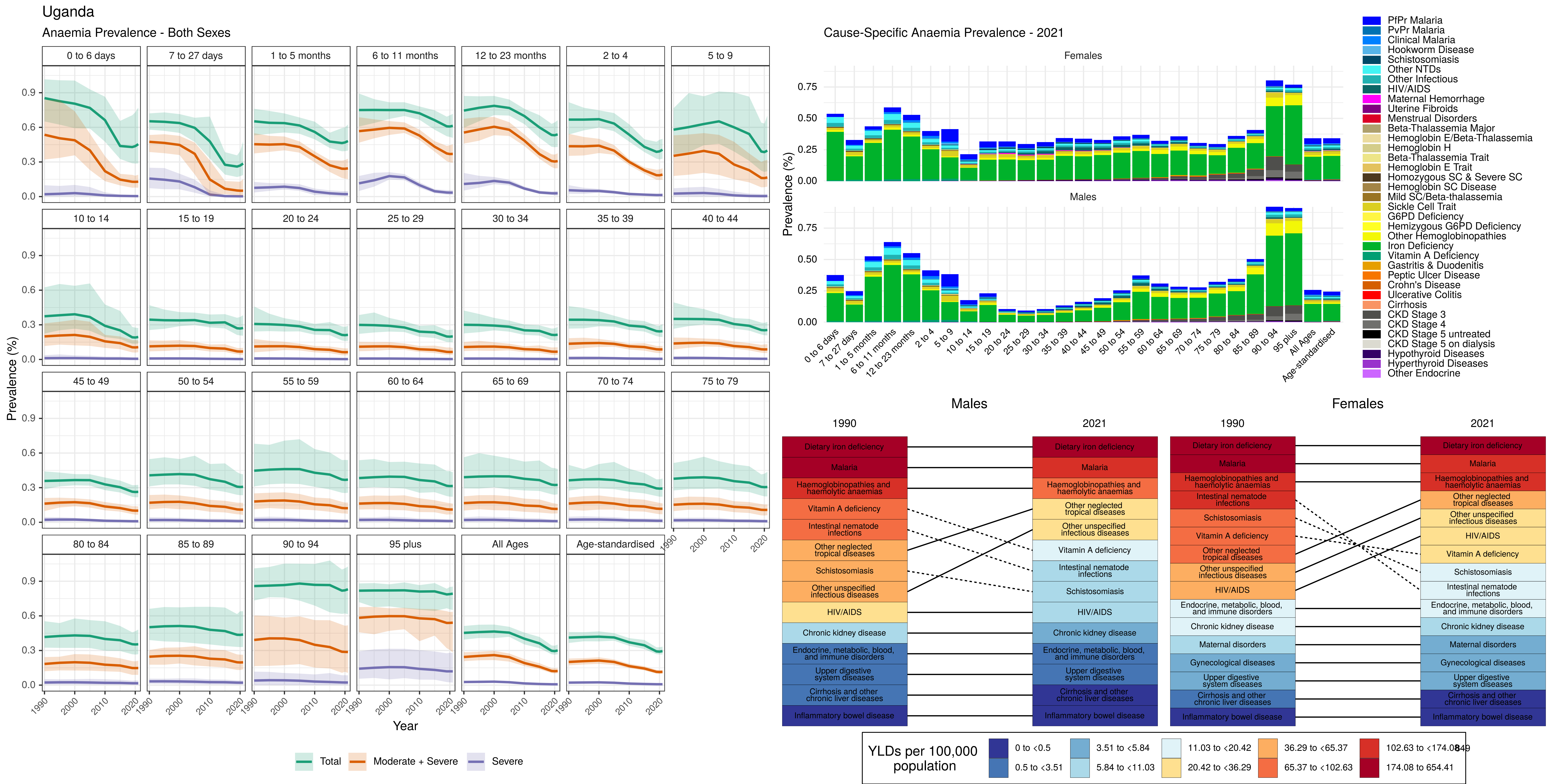
Uganda
Anaemia Prevalence - Both Sexes
0 to 6 days
7 to 27 days
1 to 5 months
6 to 11 months
12 to 23 months
2 to 4
5 to 9
10 to 14
15 to 19
20 to 24
25 to 29
30 to 34
35 to 39
40 to 44
45 to 49
50 to 54
55 to 59
60 to 64
65 to 69
70 to 74
75 to 79
80 to 84
85 to 89
90 to 94
95 plus
All Ages
Age-standardised
Prevalence (%)
Year
Total
Moderate + Severe
Severe
Cause-Specific Anaemia Prevalence - 2021
Females
Males
PfPr Malaria
PvPr Malaria
Clinical Malaria
Hookworm Disease
Schistosomiasis
Other NTDs
Other Infectious
HIV/AIDS
Maternal Hemorrhage
Uterine Fibroids
Menstrual Disorders
Beta-Thalassemia Major
Hemoglobin E/Beta-Thalassemia
Hemoglobin H
Beta-Thalassemia Trait
Hemoglobin E Trait
Homozygous SC & Severe SC
Hemoglobin SC Disease
Mild SC/Beta-thalassemia
Sickle Cell Trait
G6PD Deficiency
Hemizygous G6PD Deficiency
Other Hemoglobinopathies
Iron Deficiency
Vitamin A Deficiency
Gastritis & Duodenitis
Peptic Ulcer Disease
Crohn's Disease
Ulcerative Colitis
Cirrhosis
CKD Stage 3
CKD Stage 4
CKD Stage 5 untreated
CKD Stage 5 on dialysis
Hypothyroid Diseases
Hyperthyroid Diseases
Other Endocrine
Males
Females
1990
2021
Dietary iron deficiency
Malaria
Haemoglobinopathies and haemolytic anaemias
Vitamin A deficiency
Intestinal nematode infections
Other neglected tropical diseases
Schistosomiasis
Other unspecified infectious diseases
HIV/AIDS
Chronic kidney disease
Endocrine, metabolic, blood, and immune disorders
Upper digestive system diseases
Cirrhosis and other chronic liver diseases
Inflammatory bowel disease
Maternal disorders
Gynecological diseases
YLDs per 100,000 population
0 to <0.5
0.5 to <3.51
3.51 to <5.84
5.84 to <11.03
11.03 to <20.42
20.42 to <36.29
36.29 to <65.37
65.37 to <102.63
102.63 to <174.08
174.08 to 654.41

Tanzania

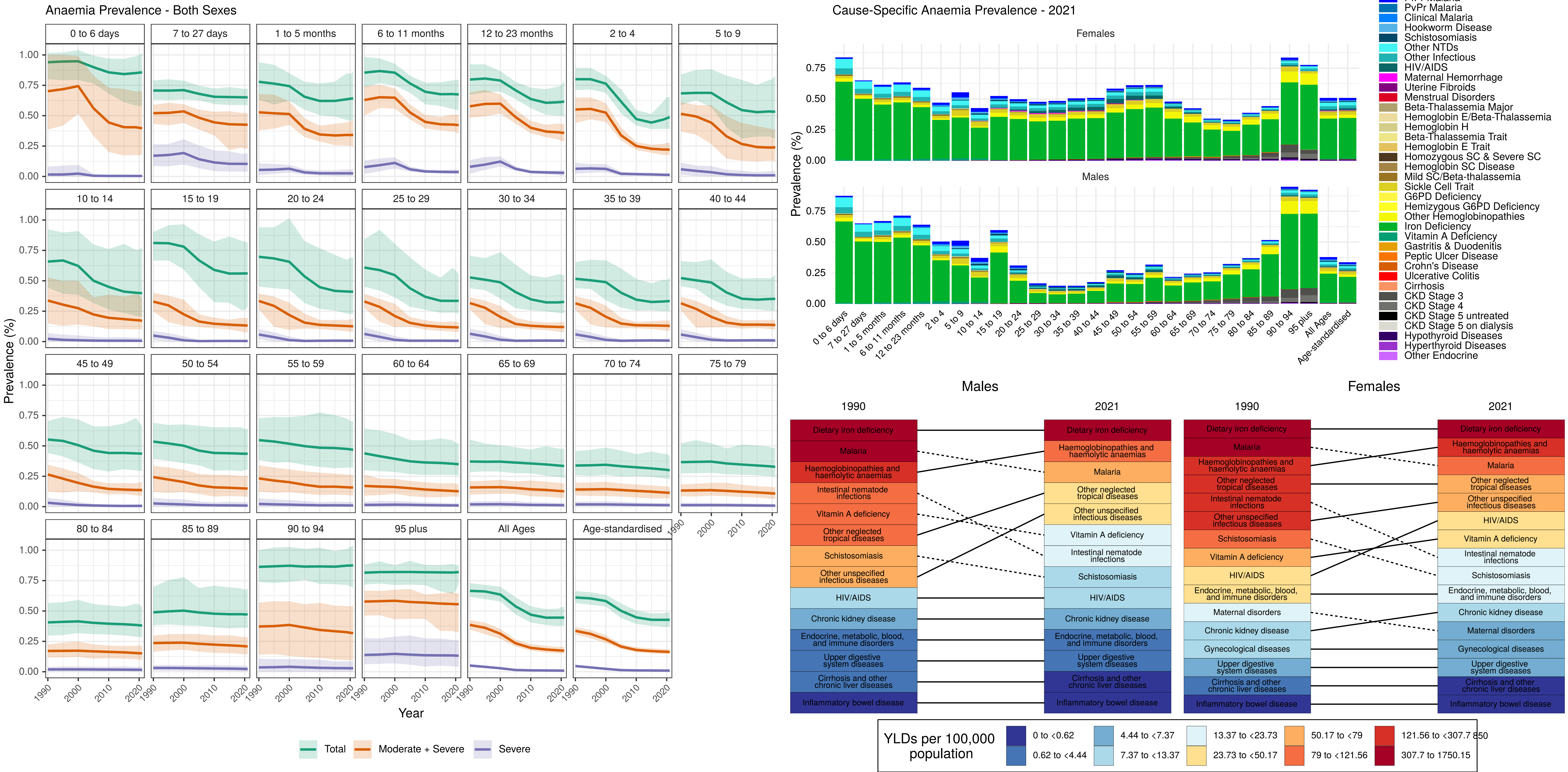

Anaemia Prevalence - Both Sexes
0 to 6 days
7 to 27 days
1 to 5 months
6 to 11 months
12 to 23 months
2 to 4
5 to 9
10 to 14
15 to 19
20 to 24
25 to 29
30 to 34
35 to 39
40 to 44
45 to 49
50 to 54
55 to 59
60 to 64
65 to 69
70 to 74
75 to 79
80 to 84
85 to 89
90 to 94
95 plus
All Ages
Age-standardised
Prevalence (%)
Year
Total
Moderate + Severe
Severe
Cause-Specific Anaemia Prevalence - 2021
Females
Males
Prevalence (%)
PfPr Malaria
PvPr Malaria
Clinical Malaria
Hookworm Disease
Schistosomiasis
Other NTDs
Other Infectious
HIV/AIDS
Maternal Hemorrhage
Uterine Fibroids
Menstrual Disorders
Beta-Thalassemia Major
Hemoglobin E/Beta-Thalassemia
Hemoglobin H
Beta-Thalassemia Trait
Hemoglobin E Trait
Homozygous SC & Severe SC
Hemoglobin SC Disease
Mild SC/Beta-thalassemia
Sickle Cell Trait
G6PD Deficiency
Hemizygous G6PD Deficiency
Other Hemoglobinopathies
Iron Deficiency
Vitamin A Deficiency
Gastritis & Duodenitis
Peptic Ulcer Disease
Crohn's Disease
Ulcerative Colitis
Cirrhosis
CKD Stage 3
CKD Stage 4
CKD Stage 5 untreated
CKD Stage 5 on dialysis
Hypothyroid Diseases
Hyperthyroid Diseases
Other Endocrine
Males
Females
1990
2021
Dietary iron deficiency
Malaria
Haemoglobinopathies and haemolytic anaemias
Intestinal nematode infections
Vitamin A deficiency
Other neglected tropical diseases
Schistosomiasis
Other unspecified infectious diseases
HIV/AIDS
Chronic kidney disease
Endocrine, metabolic, blood, and immune disorders
Upper digestive system diseases
Cirrhosis and other chronic liver diseases
Inflammatory bowel disease
Maternal disorders
Gynecological diseases
YLDs per 100,000 population
0 to <0.62
0.62 to <4.44
4.44 to <7.37
7.37 to <13.37
13.37 to <23.73
23.73 to <50.17
50.17 to <79
79 to <121.56
121.56 to <307.7 850
307.7 to 1750.15

Zambia

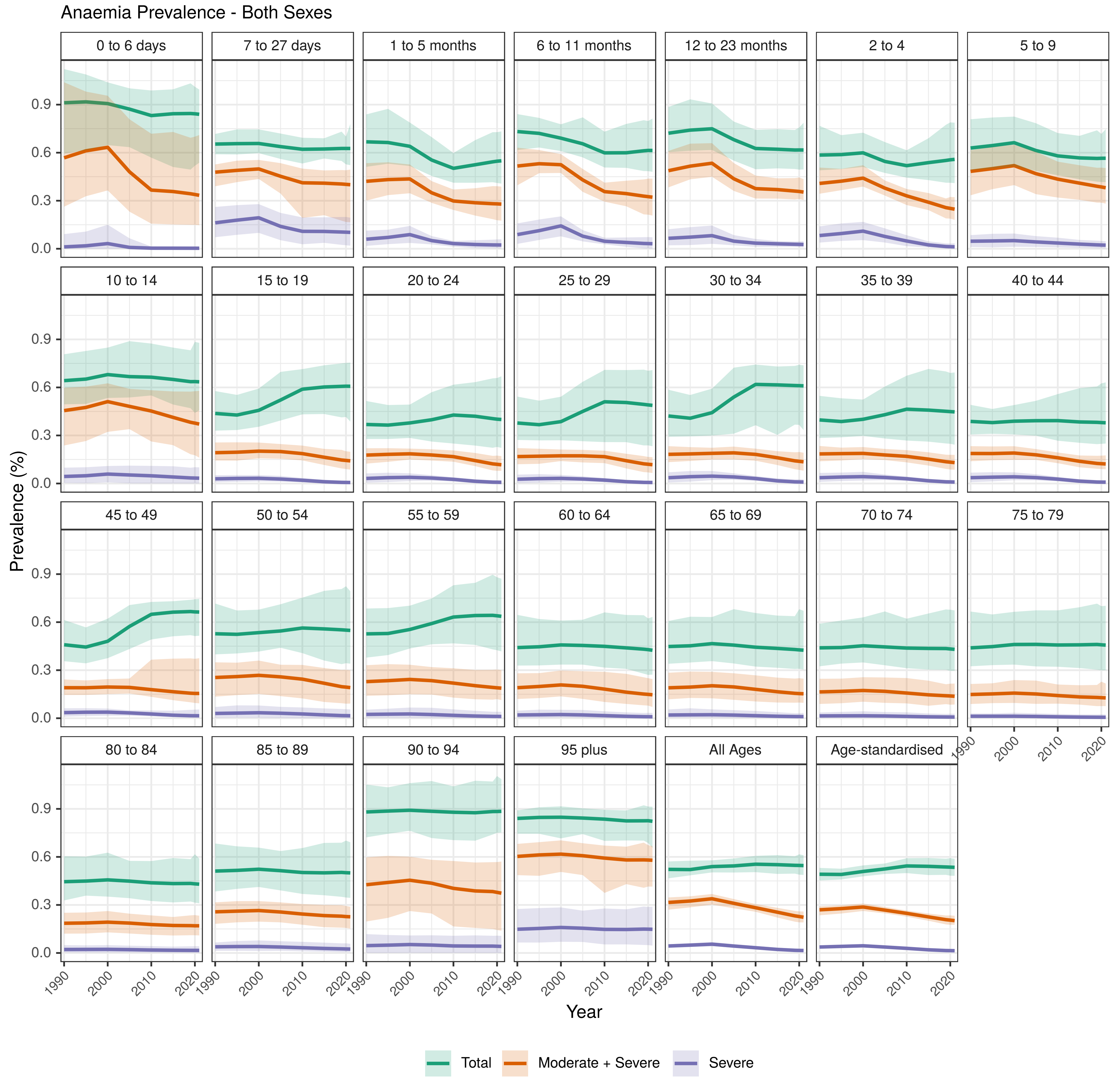

Anaemia Prevalence - Both Sexes
0 to 6 days
7 to 27 days
1 to 5 months
6 to 11 months
12 to 23 months
2 to 4
5 to 9
10 to 14
15 to 19
20 to 24
25 to 29
30 to 34
35 to 39
40 to 44
45 to 49
50 to 54
55 to 59
60 to 64
65 to 69
70 to 74
75 to 79
80 to 84
85 to 89
90 to 94
95 plus
All Ages
Age-standardised
0.0
0.3
0.6
0.9
Prevalence (%)
1990
2000
2010
2020
Year
Total
Moderate + Severe
Severe

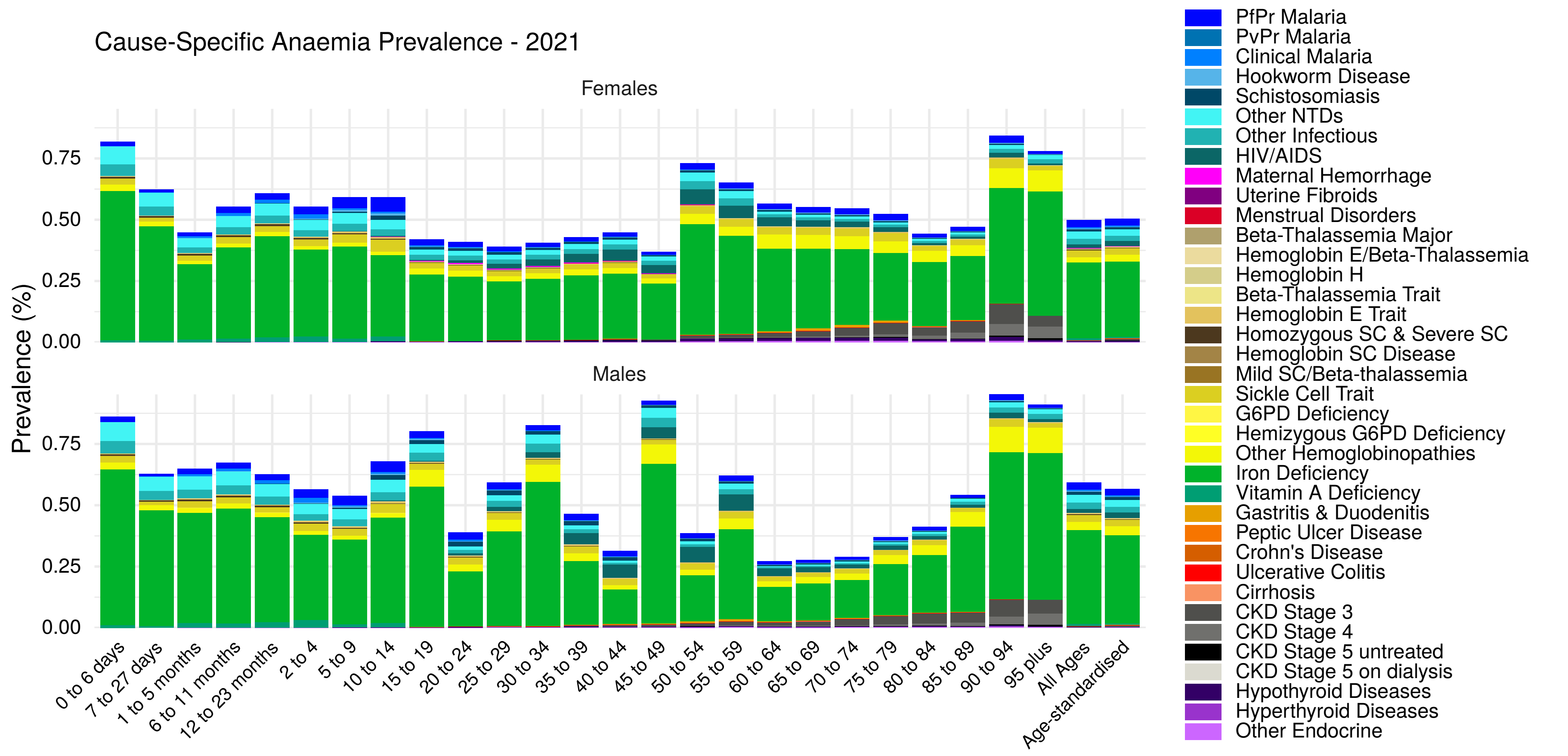

Cause-Specific Anaemia Prevalence - 2021
Females
Males
0.00
0.25
0.50
0.75
Prevalence (%)
0 to 6 days
7 to 27 days
1 to 5 months
6 to 11 months
12 to 23 months
2 to 4
5 to 9
10 to 14
15 to 19
20 to 24
25 to 29
30 to 34
35 to 39
40 to 44
45 to 49
50 to 54
55 to 59
60 to 64
65 to 69
70 to 74
75 to 79
80 to 84
85 to 89
90 to 94
95 plus
All Ages
Age-standardised
PfPr Malaria
PvPr Malaria
Clinical Malaria
Hookworm Disease
Schistosomiasis
Other NTDs
Other Infectious
HIV/AIDS
Maternal Hemorrhage
Uterine Fibroids
Menstrual Disorders
Beta-Thalassemia Major
Hemoglobin E/Beta-Thalassemia
Hemoglobin H
Beta-Thalassemia Trait
Hemoglobin E Trait
Homozygous SC & Severe SC
Hemoglobin SC Disease
Mild SC/Beta-thalassemia
Sickle Cell Trait
G6PD Deficiency
Hemizygous G6PD Deficiency
Other Hemoglobinopathies
Iron Deficiency
Vitamin A Deficiency
Gastritis & Duodenitis
Peptic Ulcer Disease
Crohn's Disease
Ulcerative Colitis
Cirrhosis
CKD Stage 3
CKD Stage 4
CKD Stage 5 untreated
CKD Stage 5 on dialysis
Hypothyroid Diseases
Hyperthyroid Diseases
Other Endocrine

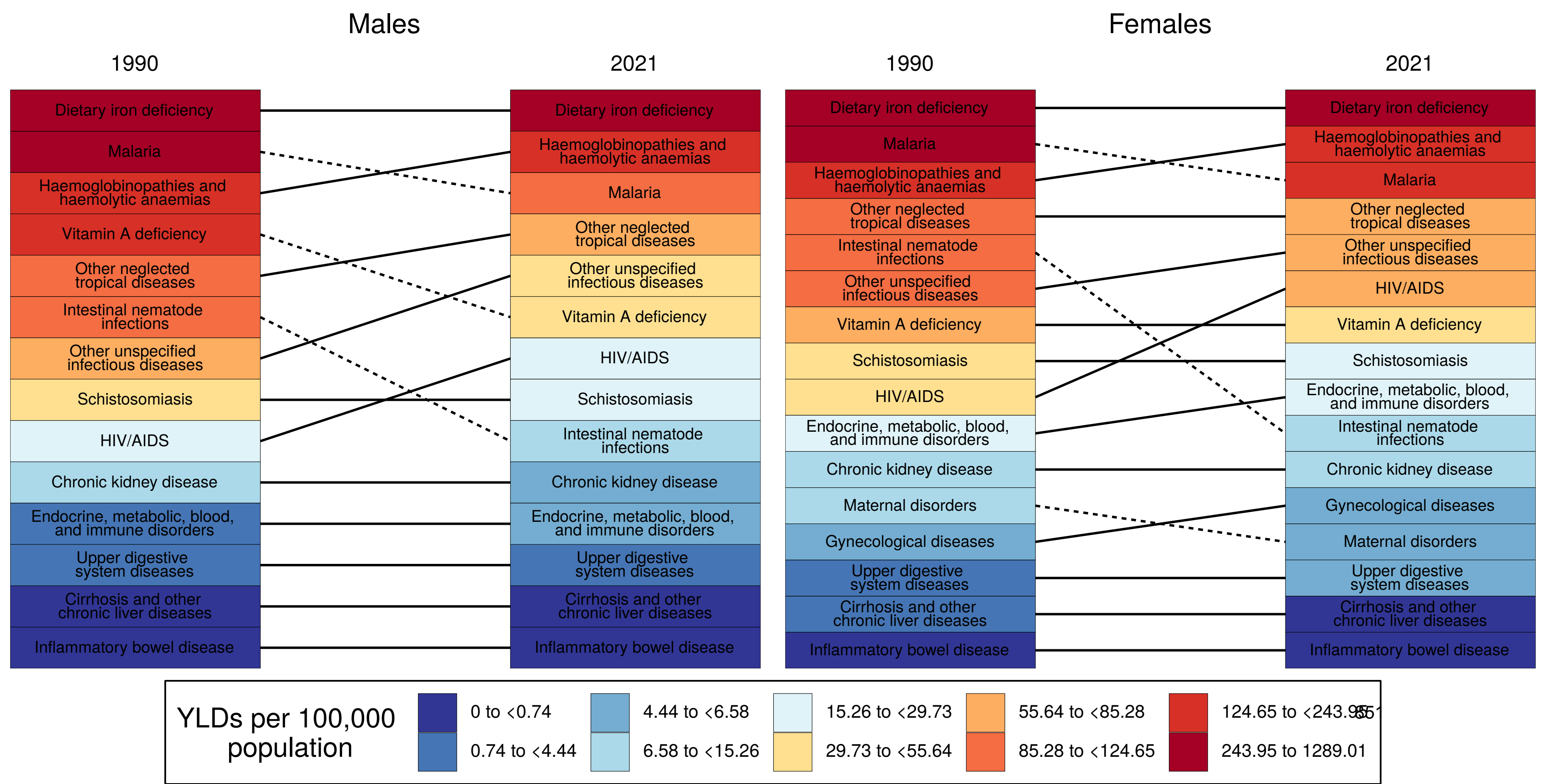

Males
Females
1990
2021
Dietary iron deficiency
Malaria
Haemoglobinopathies and haemolytic anaemias
Vitamin A deficiency
Other neglected tropical diseases
Intestinal nematode infections
Other unspecified infectious diseases
Schistosomiasis
HIV/AIDS
Chronic kidney disease
Endocrine, metabolic, blood, and immune disorders
Upper digestive system diseases
Cirrhosis and other chronic liver diseases
Inflammatory bowel disease
Maternal disorders
Gynecological diseases
YLDs per 100,000 population
0 to <0.74
0.74 to <4.44
4.44 to <6.58
6.58 to <15.26
15.26 to <29.73
29.73 to <55.64
55.64 to <85.28
85.28 to <124.65
124.65 to <243.95
243.95 to 1289.01

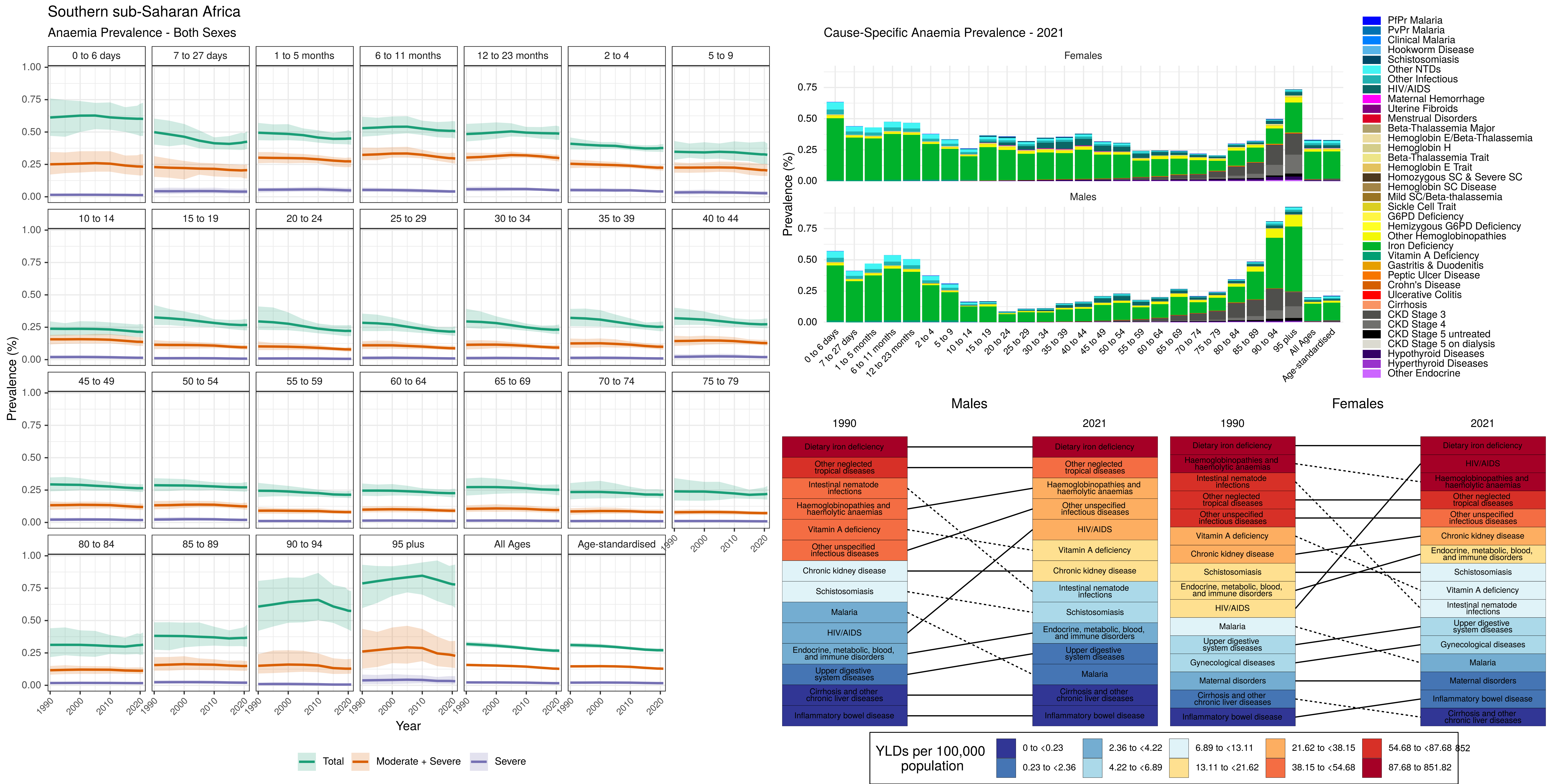
Southern sub-Saharan Africa
Anaemia Prevalence - Both Sexes
0 to 6 days
7 to 27 days
1 to 5 months
6 to 11 months
12 to 23 months
2 to 4
5 to 9
10 to 14
15 to 19
20 to 24
25 to 29
30 to 34
35 to 39
40 to 44
45 to 49
50 to 54
55 to 59
60 to 64
65 to 69
70 to 74
75 to 79
80 to 84
85 to 89
90 to 94
95 plus
All Ages
Age-standardised
Prevalence (%)
Year
Total
Moderate + Severe
Severe
Cause-Specific Anaemia Prevalence - 2021
Females
Males
PfPr Malaria
PvPr Malaria
Clinical Malaria
Hookworm Disease
Schistosomiasis
Other NTDs
Other Infectious
HIV/AIDS
Maternal Hemorrhage
Uterine Fibroids
Menstrual Disorders
Beta-Thalassemia Major
Hemoglobin E/Beta-Thalassemia
Hemoglobin H
Beta-Thalassemia Trait
Hemoglobin E Trait
Homozygous SC & Severe SC
Hemoglobin SC Disease
Mild SC/Beta-thalassemia
Sickle Cell Trait
G6PD Deficiency
Hemizygous G6PD Deficiency
Other Hemoglobinopathies
Iron Deficiency
Vitamin A Deficiency
Gastritis & Duodenitis
Peptic Ulcer Disease
Crohn's Disease
Ulcerative Colitis
Cirrhosis
CKD Stage 3
CKD Stage 4
CKD Stage 5 untreated
CKD Stage 5 on dialysis
Hypothyroid Diseases
Hyperthyroid Diseases
Other Endocrine
Males
Females
1990
2021
Dietary iron deficiency
Other neglected tropical diseases
Intestinal nematode infections
Haemoglobinopathies and haemolytic anaemias
Vitamin A deficiency
Other unspecified infectious diseases
Chronic kidney disease
Schistosomiasis
Malaria
HIV/AIDS
Endocrine, metabolic, blood, and immune disorders
Upper digestive system diseases
Cirrhosis and other chronic liver diseases
Inflammatory bowel disease
Gynecological diseases
Maternal disorders
YLDs per 100,000 population
0 to <0.23
0.23 to <2.36
2.36 to <4.22
4.22 to <6.89
6.89 to <13.11
13.11 to <21.62
21.62 to <38.15
38.15 to <54.68
54.68 to <87.68
87.68 to 851.82

Botswana

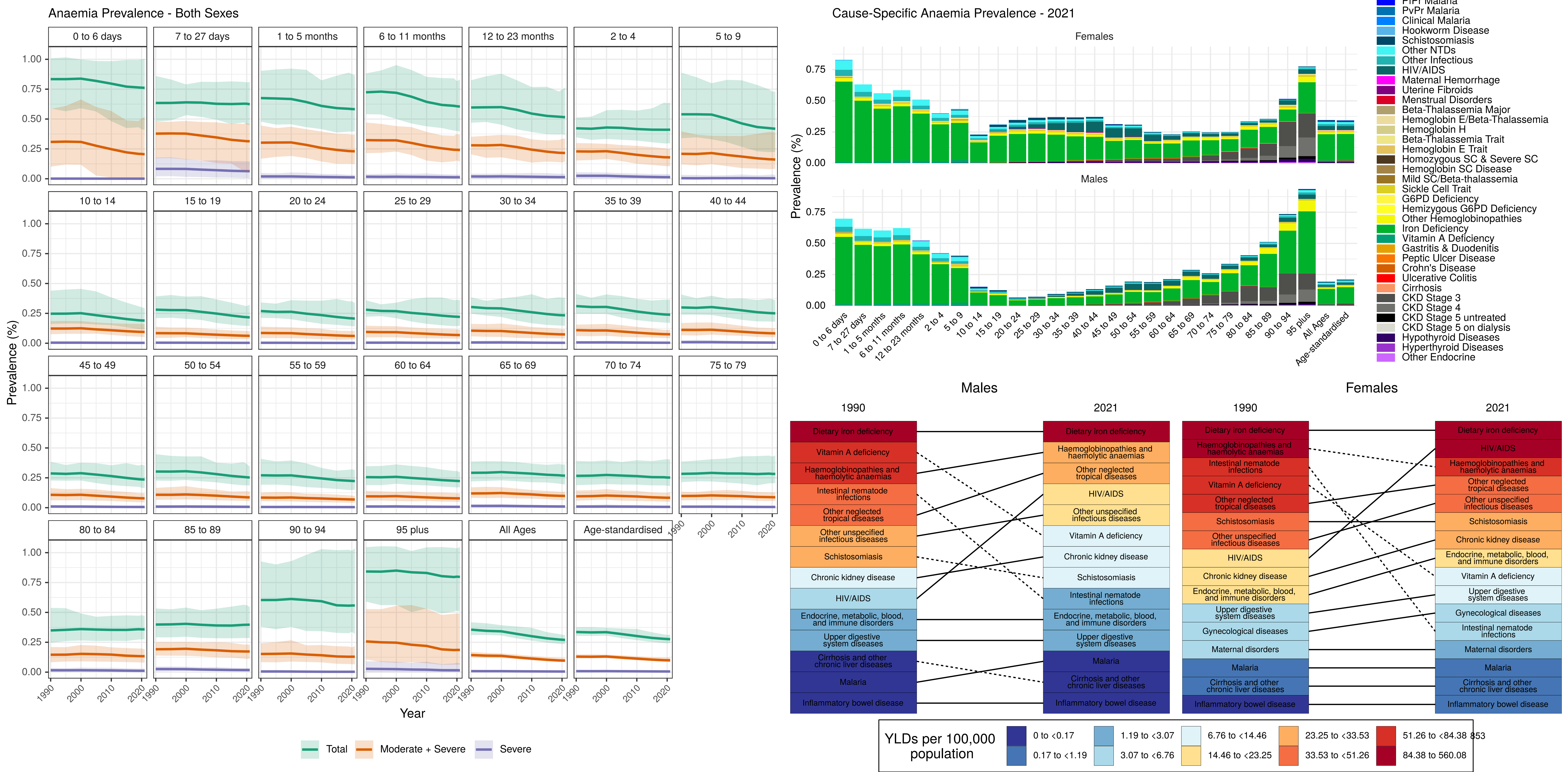

Anaemia Prevalence - Both Sexes
0 to 6 days
7 to 27 days
1 to 5 months
6 to 11 months
12 to 23 months
2 to 4
5 to 9
10 to 14
15 to 19
20 to 24
25 to 29
30 to 34
35 to 39
40 to 44
45 to 49
50 to 54
55 to 59
60 to 64
65 to 69
70 to 74
75 to 79
80 to 84
85 to 89
90 to 94
95 plus
All Ages
Age-standardised
Prevalence (%)
Year
Total
Moderate + Severe
Severe
Cause-Specific Anaemia Prevalence - 2021
Females
Males
PfPr Malaria
PvPr Malaria
Clinical Malaria
Hookworm Disease
Schistosomiasis
Other NTDs
Other Infectious
HIV/AIDS
Maternal Hemorrhage
Uterine Fibroids
Menstrual Disorders
Beta-Thalassemia Major
Hemoglobin E/Beta-Thalassemia
Hemoglobin H
Beta-Thalassemia Trait
Hemoglobin E Trait
Homozygous SC & Severe SC
Hemoglobin SC Disease
Mild SC/Beta-thalassemia
Sickle Cell Trait
G6PD Deficiency
Hemizygous G6PD Deficiency
Other Hemoglobinopathies
Iron Deficiency
Vitamin A Deficiency
Gastritis & Duodenitis
Peptic Ulcer Disease
Crohn's Disease
Ulcerative Colitis
Cirrhosis
CKD Stage 3
CKD Stage 4
CKD Stage 5 untreated
CKD Stage 5 on dialysis
Hypothyroid Diseases
Hyperthyroid Diseases
Other Endocrine
Males
Females
1990
2021
Dietary iron deficiency
Vitamin A deficiency
Haemoglobinopathies and haemolytic anaemias
Intestinal nematode infections
Other neglected tropical diseases
Other unspecified infectious diseases
Schistosomiasis
Chronic kidney disease
HIV/AIDS
Endocrine, metabolic, blood, and immune disorders
Upper digestive system diseases
Cirrhosis and other chronic liver diseases
Malaria
Inflammatory bowel disease
Gynecological diseases
Maternal disorders
YLDs per 100,000 population
0 to <0.17
0.17 to <1.19
1.19 to <3.07
3.07 to <6.76
6.76 to <14.46
14.46 to <23.25
23.25 to <33.53
33.53 to <51.26
51.26 to <84.38
84.38 to 560.08

Eswatini

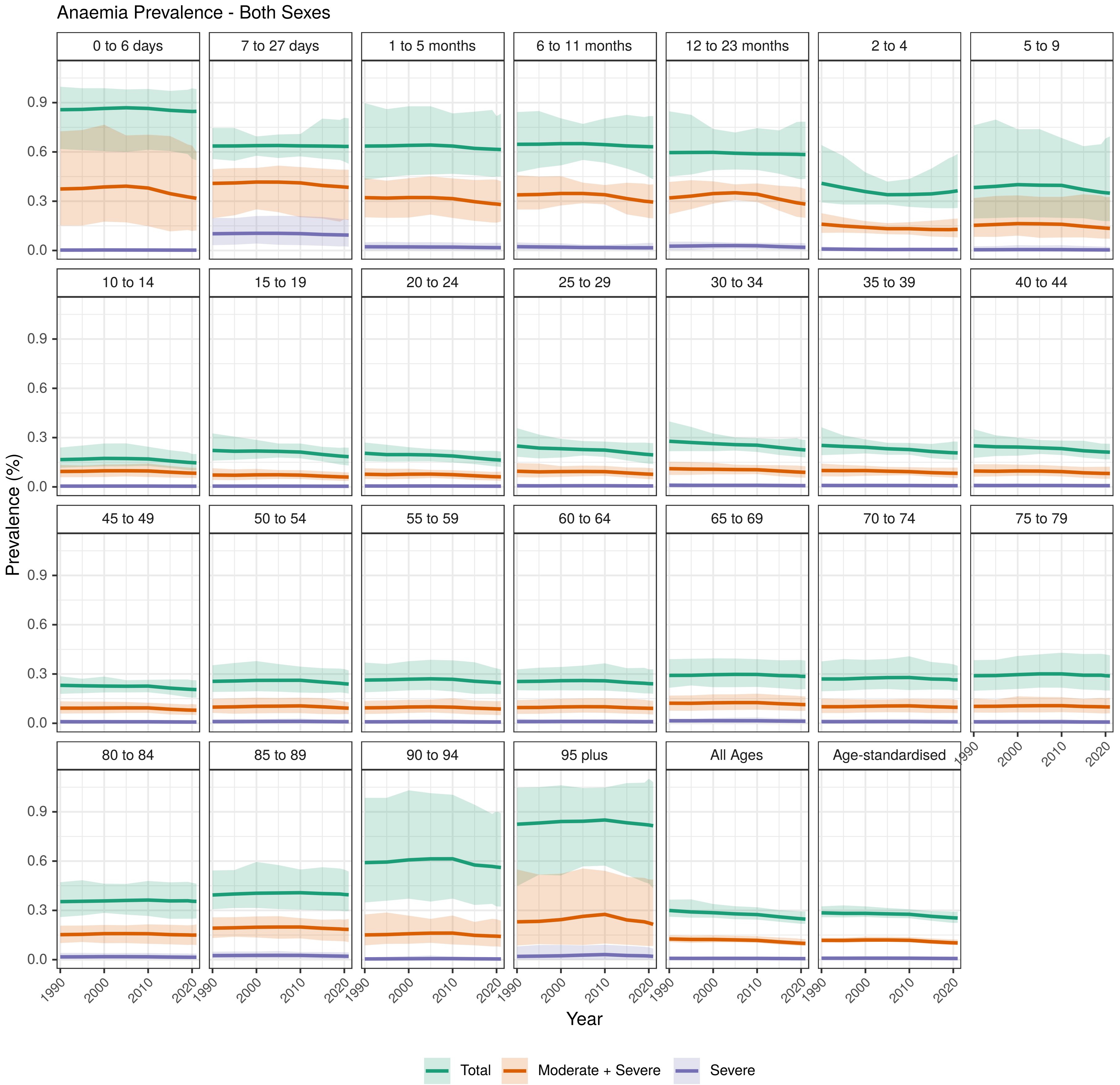
Anaemia Prevalence - Both Sexes
0 to 6 days
7 to 27 days
1 to 5 months
6 to 11 months
12 to 23 months
2 to 4
5 to 9
10 to 14
15 to 19
20 to 24
25 to 29
30 to 34
35 to 39
40 to 44
45 to 49
50 to 54
55 to 59
60 to 64
65 to 69
70 to 74
75 to 79
80 to 84
85 to 89
90 to 94
95 plus
All Ages
Age-standardised
Prevalence (%)
Year
Total
Moderate + Severe
Severe

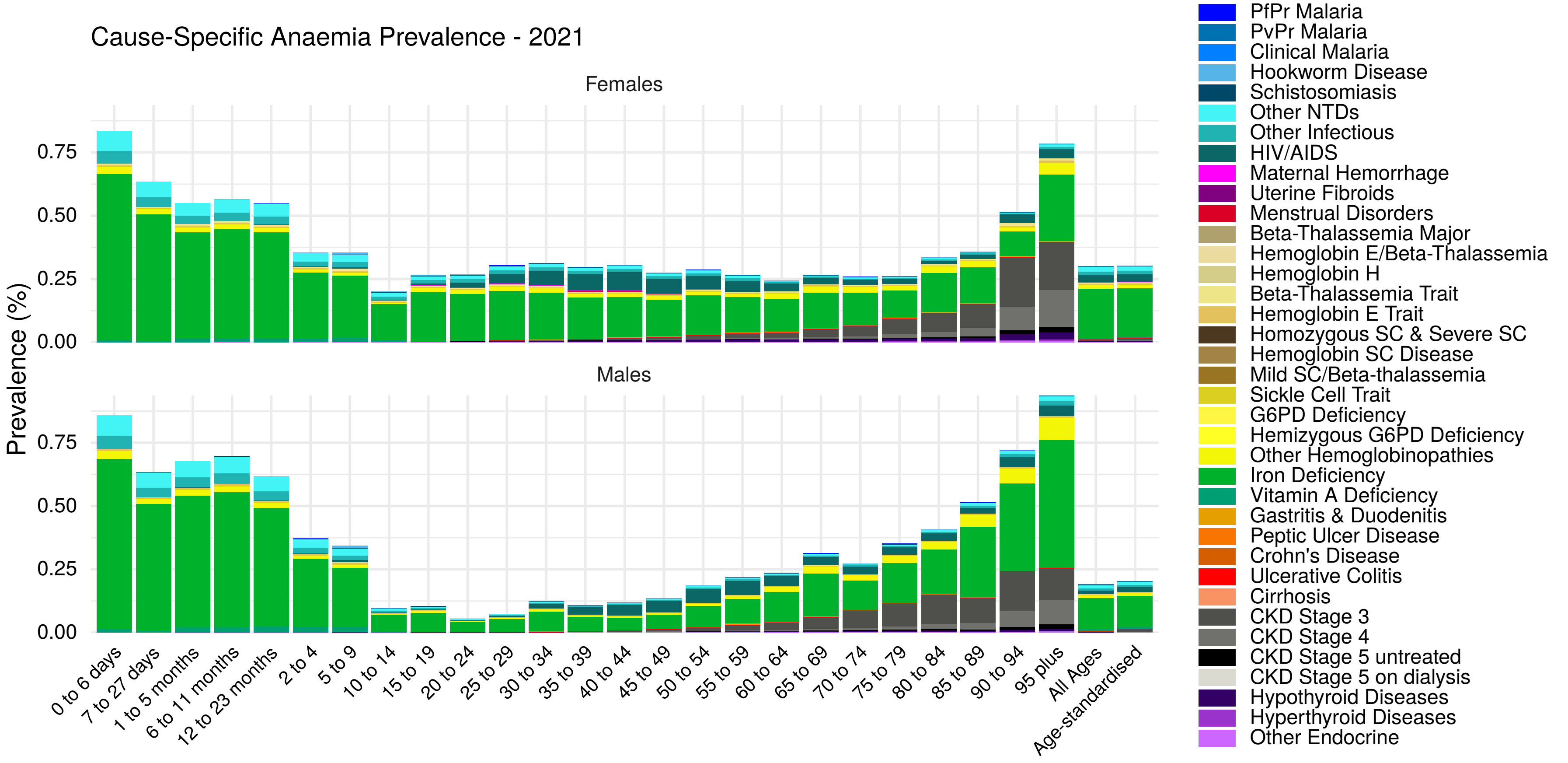
Cause-Specific Anaemia Prevalence - 2021
Females
Males
Prevalence (%)
PfPr Malaria
PvPr Malaria
Clinical Malaria
Hookworm Disease
Schistosomiasis
Other NTDs
Other Infectious
HIV/AIDS
Maternal Hemorrhage
Uterine Fibroids
Menstrual Disorders
Beta-Thalassemia Major
Hemoglobin E/Beta-Thalassemia
Hemoglobin H
Beta-Thalassemia Trait
Hemoglobin E Trait
Homozygous SC & Severe SC
Hemoglobin SC Disease
Mild SC/Beta-thalassemia
Sickle Cell Trait
G6PD Deficiency
Hemizygous G6PD Deficiency
Other Hemoglobinopathies
Iron Deficiency
Vitamin A Deficiency
Gastritis & Duodenitis
Peptic Ulcer Disease
Crohn's Disease
Ulcerative Colitis
Cirrhosis
CKD Stage 3
CKD Stage 4
CKD Stage 5 untreated
CKD Stage 5 on dialysis
Hypothyroid Diseases
Hyperthyroid Diseases
Other Endocrine

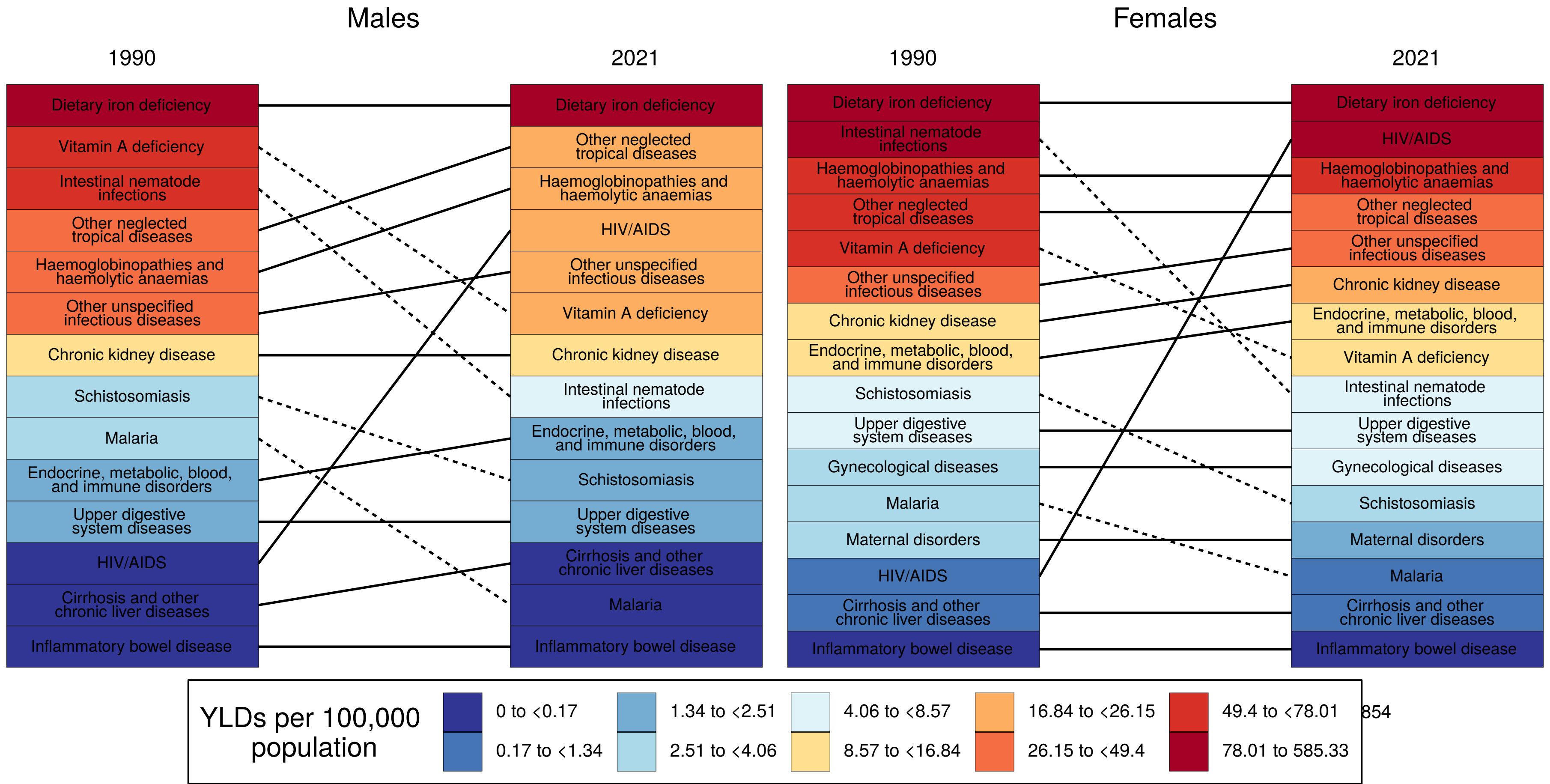
Males
Females
1990
2021
Males 1990: Dietary iron deficiency; Vitamin A deficiency; Intestinal nematode infections; Other neglected tropical diseases; Haemoglobinopathies and haemolytic anaemias; Other unspecified infectious diseases; Chronic kidney disease; Schistosomiasis; Malaria; Endocrine, metabolic, blood, and immune disorders; Upper digestive system diseases; HIV/AIDS; Cirrhosis and other chronic liver diseases; Inflammatory bowel disease
Males 2021: Dietary iron deficiency; Other neglected tropical diseases; Haemoglobinopathies and haemolytic anaemias; HIV/AIDS; Other unspecified infectious diseases; Vitamin A deficiency; Chronic kidney disease; Intestinal nematode infections; Endocrine, metabolic, blood, and immune disorders; Schistosomiasis; Upper digestive system diseases; Cirrhosis and other chronic liver diseases; Malaria; Inflammatory bowel disease
Females 1990: Dietary iron deficiency; Intestinal nematode infections; Haemoglobinopathies and haemolytic anaemias; Other neglected tropical diseases; Vitamin A deficiency; Other unspecified infectious diseases; Chronic kidney disease; Endocrine, metabolic, blood, and immune disorders; Schistosomiasis; Upper digestive system diseases; Gynecological diseases; Malaria; Maternal disorders; HIV/AIDS; Cirrhosis and other chronic liver diseases; Inflammatory bowel disease
Females 2021: Dietary iron deficiency; HIV/AIDS; Haemoglobinopathies and haemolytic anaemias; Other neglected tropical diseases; Other unspecified infectious diseases; Chronic kidney disease; Endocrine, metabolic, blood, and immune disorders; Vitamin A deficiency; Intestinal nematode infections; Upper digestive system diseases; Gynecological diseases; Schistosomiasis; Maternal disorders; Malaria; Cirrhosis and other chronic liver diseases; Inflammatory bowel disease
YLDs per 100,000 population
0 to <0.17
0.17 to <1.34
1.34 to <2.51
2.51 to <4.06
4.06 to <8.57
8.57 to <16.84
16.84 to <26.15
26.15 to <49.4
49.4 to <78.01
78.01 to 585.33

Lesotho

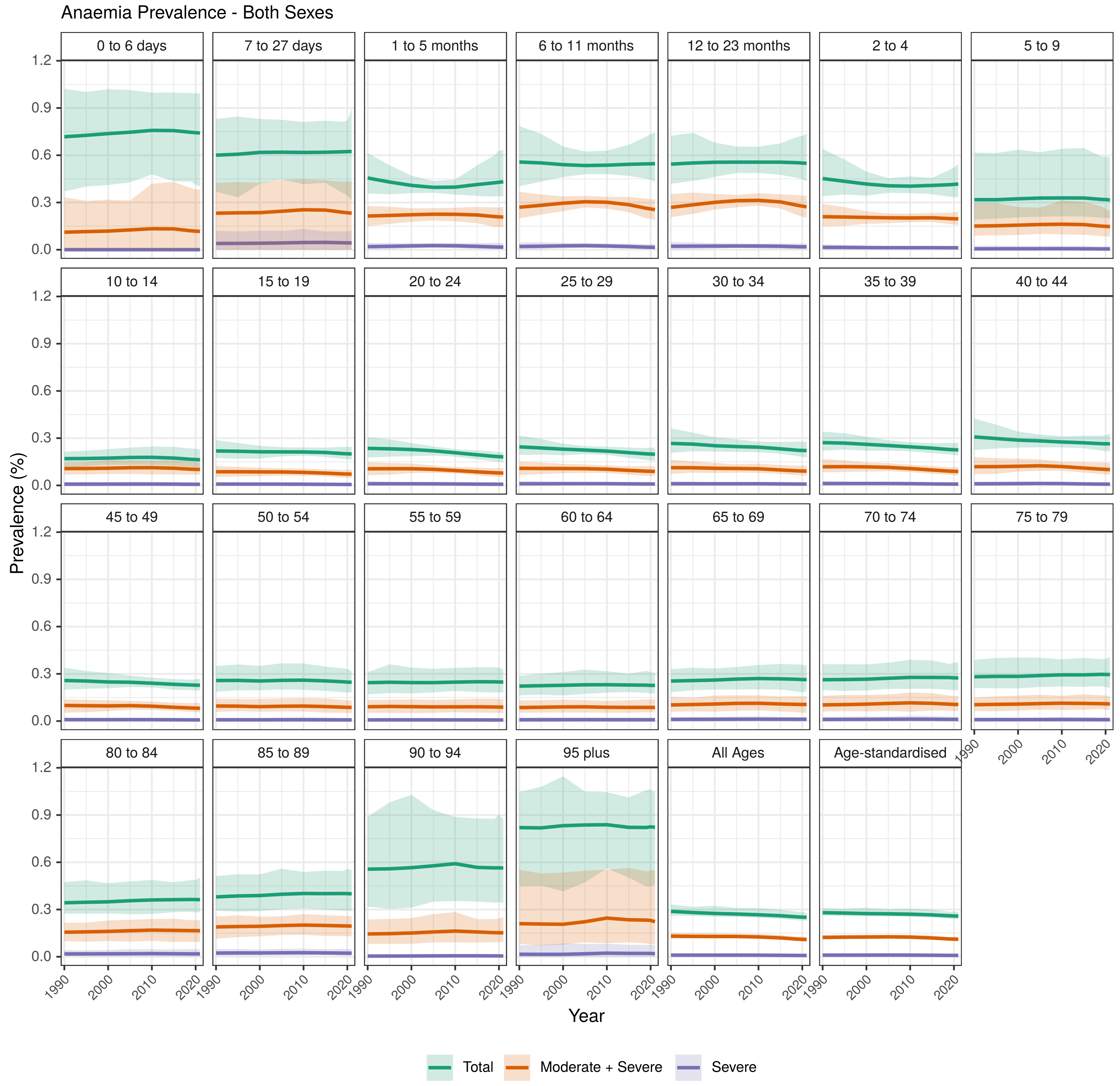
Anaemia Prevalence - Both Sexes
0 to 6 days
7 to 27 days
1 to 5 months
6 to 11 months
12 to 23 months
2 to 4
5 to 9
10 to 14
15 to 19
20 to 24
25 to 29
30 to 34
35 to 39
40 to 44
45 to 49
50 to 54
55 to 59
60 to 64
65 to 69
70 to 74
75 to 79
80 to 84
85 to 89
90 to 94
95 plus
All Ages
Age-standardised
Prevalence (%)
Year
Total
Moderate + Severe
Severe

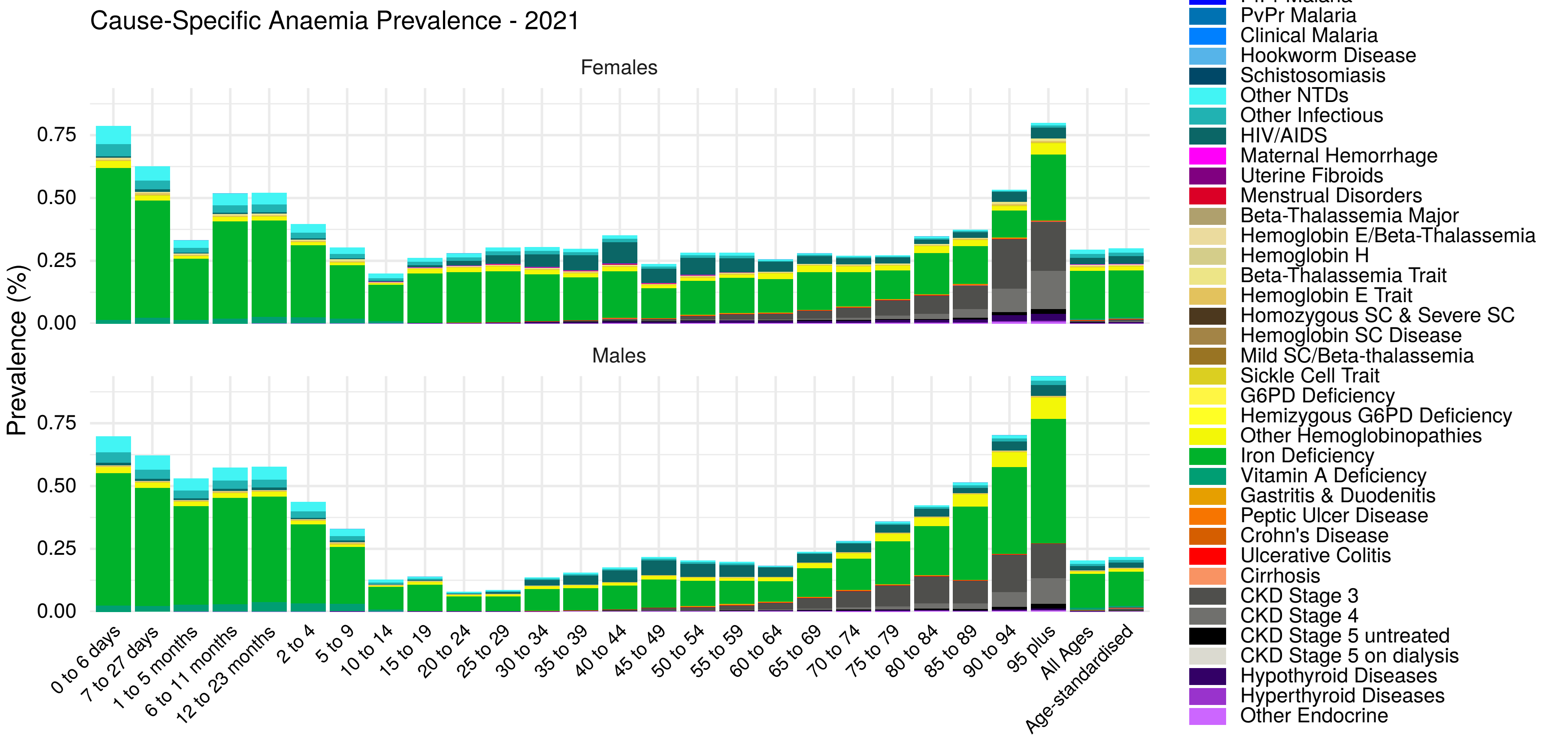
Cause-Specific Anaemia Prevalence - 2021
Females
Males
Prevalence (%)
PfPr Malaria
PvPr Malaria
Clinical Malaria
Hookworm Disease
Schistosomiasis
Other NTDs
Other Infectious
HIV/AIDS
Maternal Hemorrhage
Uterine Fibroids
Menstrual Disorders
Beta-Thalassemia Major
Hemoglobin E/Beta-Thalassemia
Hemoglobin H
Beta-Thalassemia Trait
Hemoglobin E Trait
Homozygous SC & Severe SC
Hemoglobin SC Disease
Mild SC/Beta-thalassemia
Sickle Cell Trait
G6PD Deficiency
Hemizygous G6PD Deficiency
Other Hemoglobinopathies
Iron Deficiency
Vitamin A Deficiency
Gastritis & Duodenitis
Peptic Ulcer Disease
Crohn's Disease
Ulcerative Colitis
Cirrhosis
CKD Stage 3
CKD Stage 4
CKD Stage 5 untreated
CKD Stage 5 on dialysis
Hypothyroid Diseases
Hyperthyroid Diseases
Other Endocrine

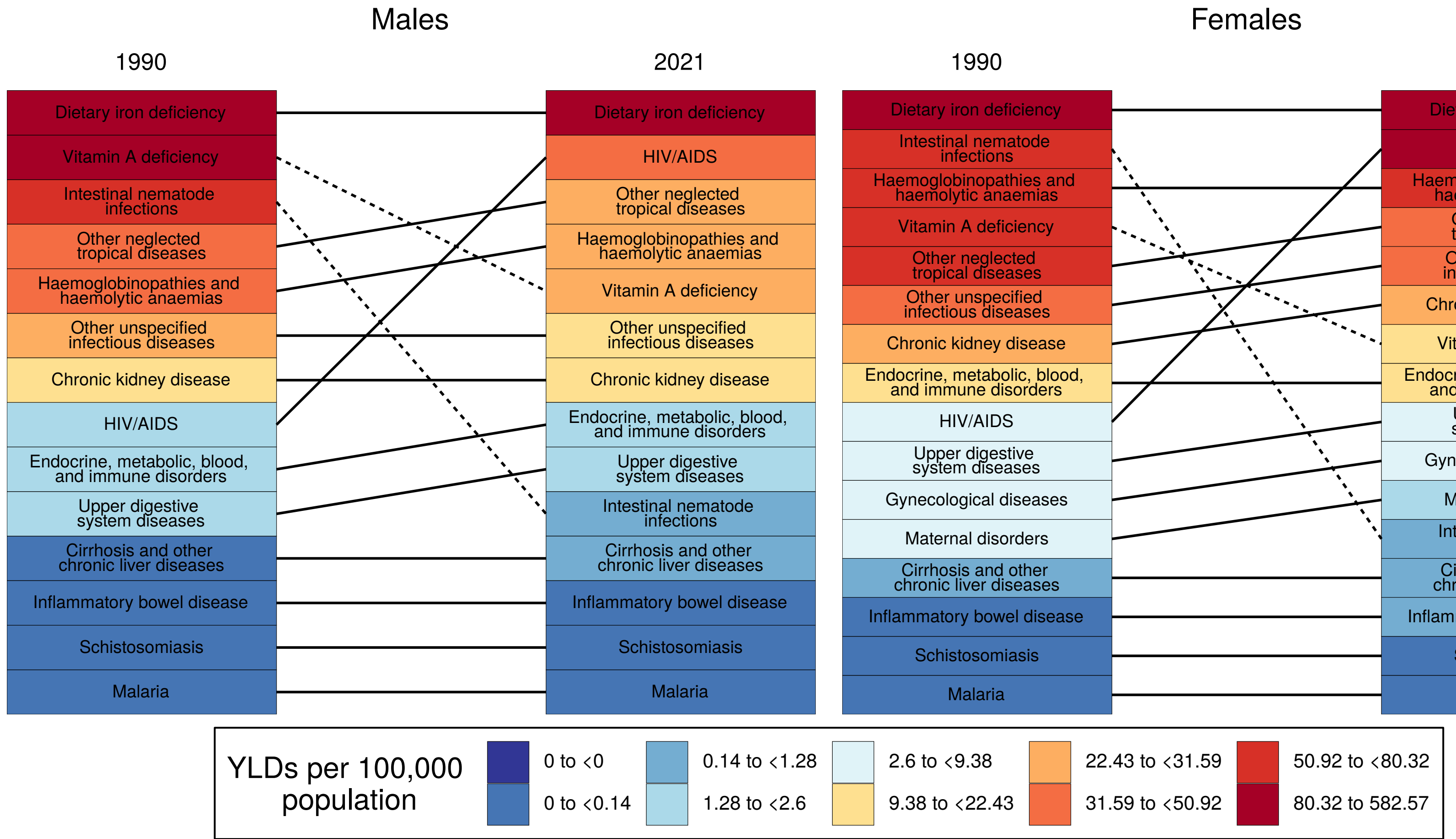
Males
Females
1990
2021
Dietary iron deficiency
Vitamin A deficiency
Intestinal nematode infections
Other neglected tropical diseases
Haemoglobinopathies and haemolytic anaemias
Other unspecified infectious diseases
Chronic kidney disease
HIV/AIDS
Endocrine, metabolic, blood, and immune disorders
Upper digestive system diseases
Cirrhosis and other chronic liver diseases
Inflammatory bowel disease
Schistosomiasis
Malaria
Gynecological diseases
Maternal disorders
YLDs per 100,000 population
0 to <0
0 to <0.14
0.14 to <1.28
1.28 to <2.6
2.6 to <9.38
9.38 to <22.43
22.43 to <31.59
31.59 to <50.92
50.92 to <80.32
80.32 to 582.57

Namibia

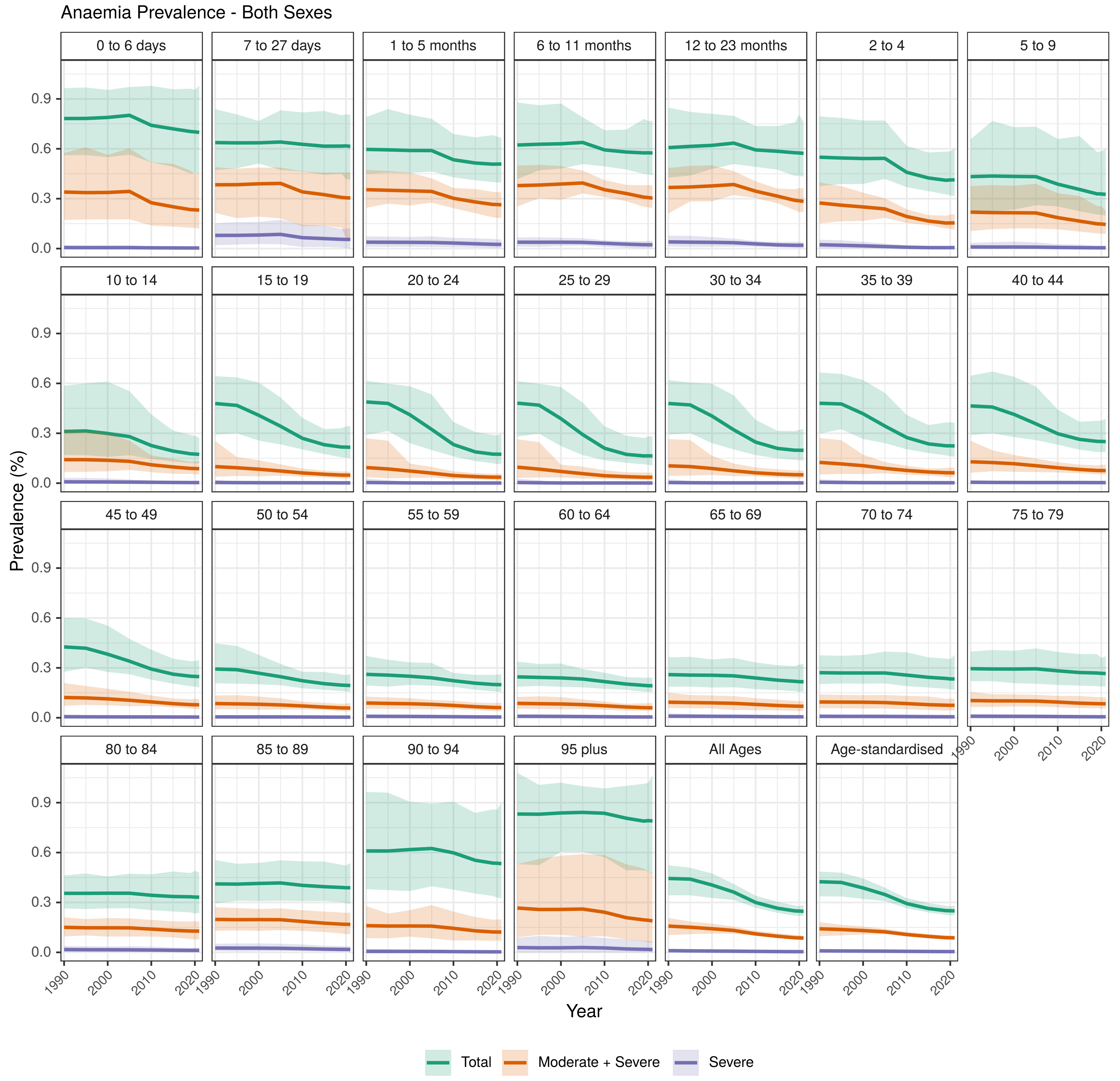

Anaemia Prevalence - Both Sexes
0 to 6 days
7 to 27 days
1 to 5 months
6 to 11 months
12 to 23 months
2 to 4
5 to 9
10 to 14
15 to 19
20 to 24
25 to 29
30 to 34
35 to 39
40 to 44
45 to 49
50 to 54
55 to 59
60 to 64
65 to 69
70 to 74
75 to 79
80 to 84
85 to 89
90 to 94
95 plus
All Ages
Age-standardised
Prevalence (%)
0.0
0.3
0.6
0.9
1990
2000
2010
2020
Year
Total
Moderate + Severe
Severe

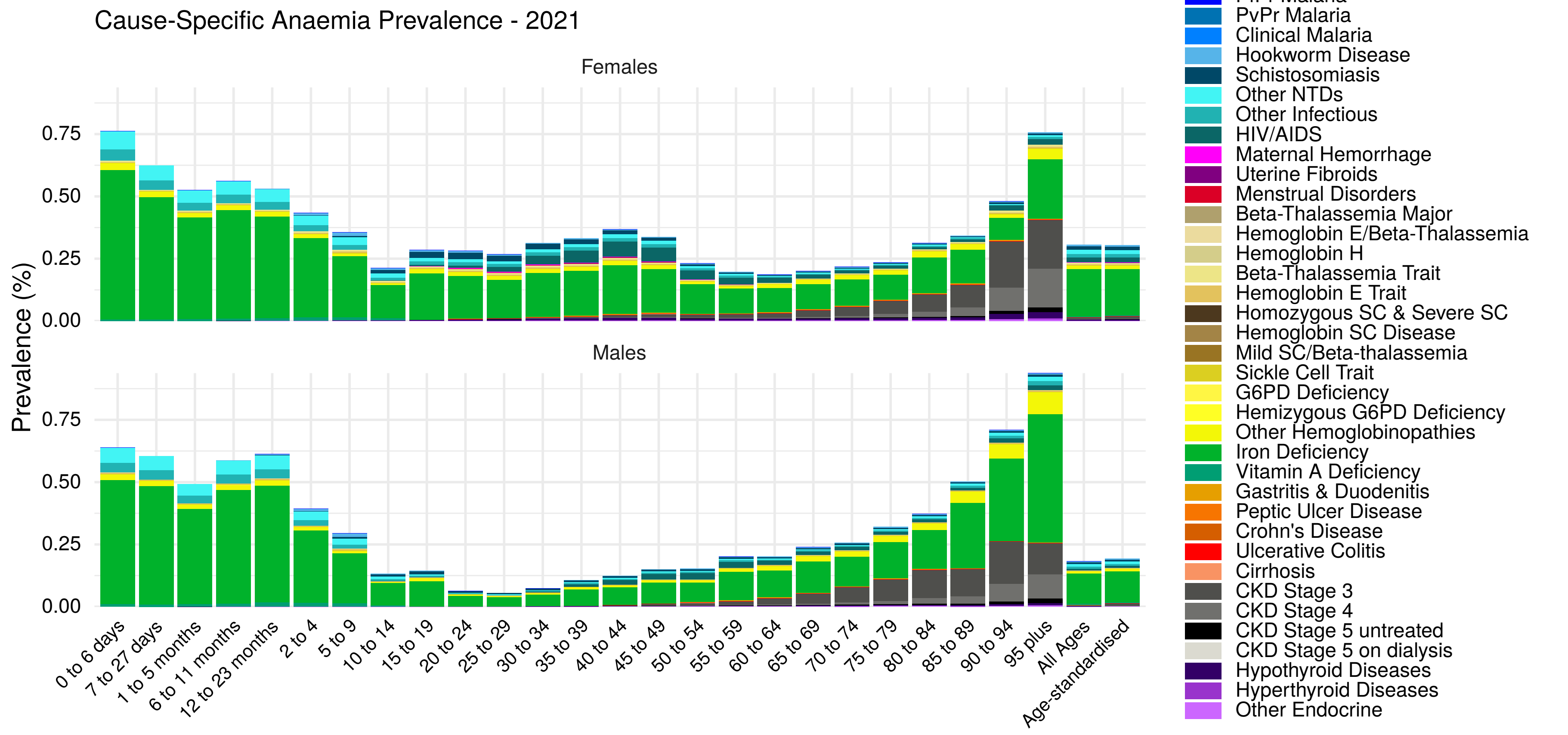

Cause-Specific Anaemia Prevalence - 2021
Females
Males
Prevalence (%)
0.00
0.25
0.50
0.75
0 to 6 days
7 to 27 days
1 to 5 months
6 to 11 months
12 to 23 months
2 to 4
5 to 9
10 to 14
15 to 19
20 to 24
25 to 29
30 to 34
35 to 39
40 to 44
45 to 49
50 to 54
55 to 59
60 to 64
65 to 69
70 to 74
75 to 79
80 to 84
85 to 89
90 to 94
95 plus
All Ages
Age-standardised
PfPr Malaria
PvPr Malaria
Clinical Malaria
Hookworm Disease
Schistosomiasis
Other NTDs
Other Infectious
HIV/AIDS
Maternal Hemorrhage
Uterine Fibroids
Menstrual Disorders
Beta-Thalassemia Major
Hemoglobin E/Beta-Thalassemia
Hemoglobin H
Beta-Thalassemia Trait
Hemoglobin E Trait
Homozygous SC & Severe SC
Hemoglobin SC Disease
Mild SC/Beta-thalassemia
Sickle Cell Trait
G6PD Deficiency
Hemizygous G6PD Deficiency
Other Hemoglobinopathies
Iron Deficiency
Vitamin A Deficiency
Gastritis & Duodenitis
Peptic Ulcer Disease
Crohn's Disease
Ulcerative Colitis
Cirrhosis
CKD Stage 3
CKD Stage 4
CKD Stage 5 untreated
CKD Stage 5 on dialysis
Hypothyroid Diseases
Hyperthyroid Diseases
Other Endocrine

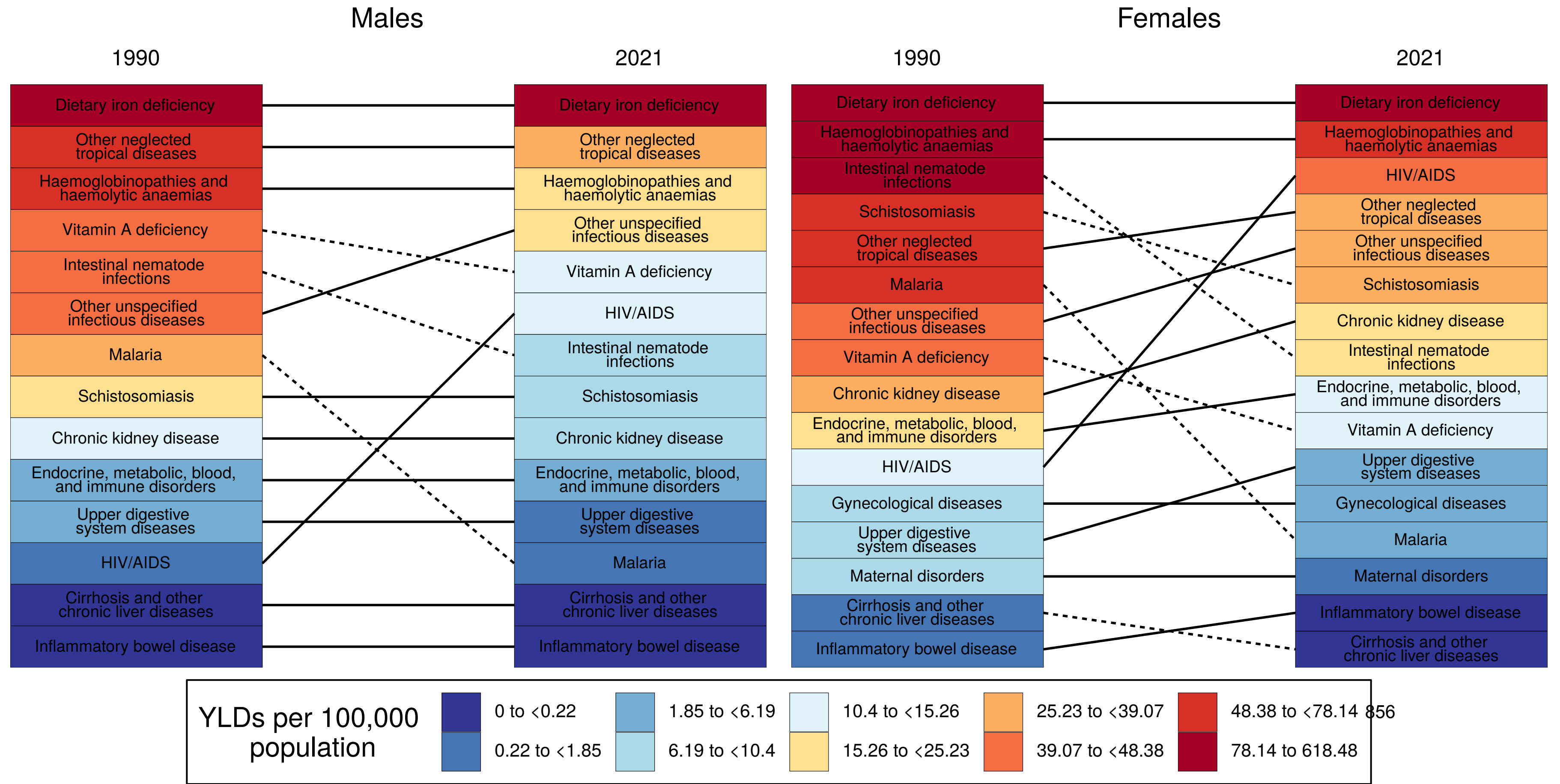

Males
1990
Dietary iron deficiency
Other neglected tropical diseases
Haemoglobinopathies and haemolytic anaemias
Vitamin A deficiency
Intestinal nematode infections
Other unspecified infectious diseases
Malaria
Schistosomiasis
Chronic kidney disease
Endocrine, metabolic, blood, and immune disorders
Upper digestive system diseases
HIV/AIDS
Cirrhosis and other chronic liver diseases
Inflammatory bowel disease
2021
Dietary iron deficiency
Other neglected tropical diseases
Haemoglobinopathies and haemolytic anaemias
Other unspecified infectious diseases
Vitamin A deficiency
HIV/AIDS
Intestinal nematode infections
Schistosomiasis
Chronic kidney disease
Endocrine, metabolic, blood, and immune disorders
Upper digestive system diseases
Malaria
Cirrhosis and other chronic liver diseases
Inflammatory bowel disease
Females
1990
Dietary iron deficiency
Haemoglobinopathies and haemolytic anaemias
Intestinal nematode infections
Schistosomiasis
Other neglected tropical diseases
Malaria
Other unspecified infectious diseases
Vitamin A deficiency
Chronic kidney disease
Endocrine, metabolic, blood, and immune disorders
HIV/AIDS
Gynecological diseases
Upper digestive system diseases
Maternal disorders
Cirrhosis and other chronic liver diseases
Inflammatory bowel disease
2021
Dietary iron deficiency
Haemoglobinopathies and haemolytic anaemias
HIV/AIDS
Other neglected tropical diseases
Other unspecified infectious diseases
Schistosomiasis
Chronic kidney disease
Intestinal nematode infections
Endocrine, metabolic, blood, and immune disorders
Vitamin A deficiency
Upper digestive system diseases
Gynecological diseases
Malaria
Maternal disorders
Inflammatory bowel disease
Cirrhosis and other chronic liver diseases
YLDs per 100,000 population
0 to <0.22
0.22 to <1.85
1.85 to <6.19
6.19 to <10.4
10.4 to <15.26
15.26 to <25.23
25.23 to <39.07
39.07 to <48.38
48.38 to <78.14 856
78.14 to 618.48

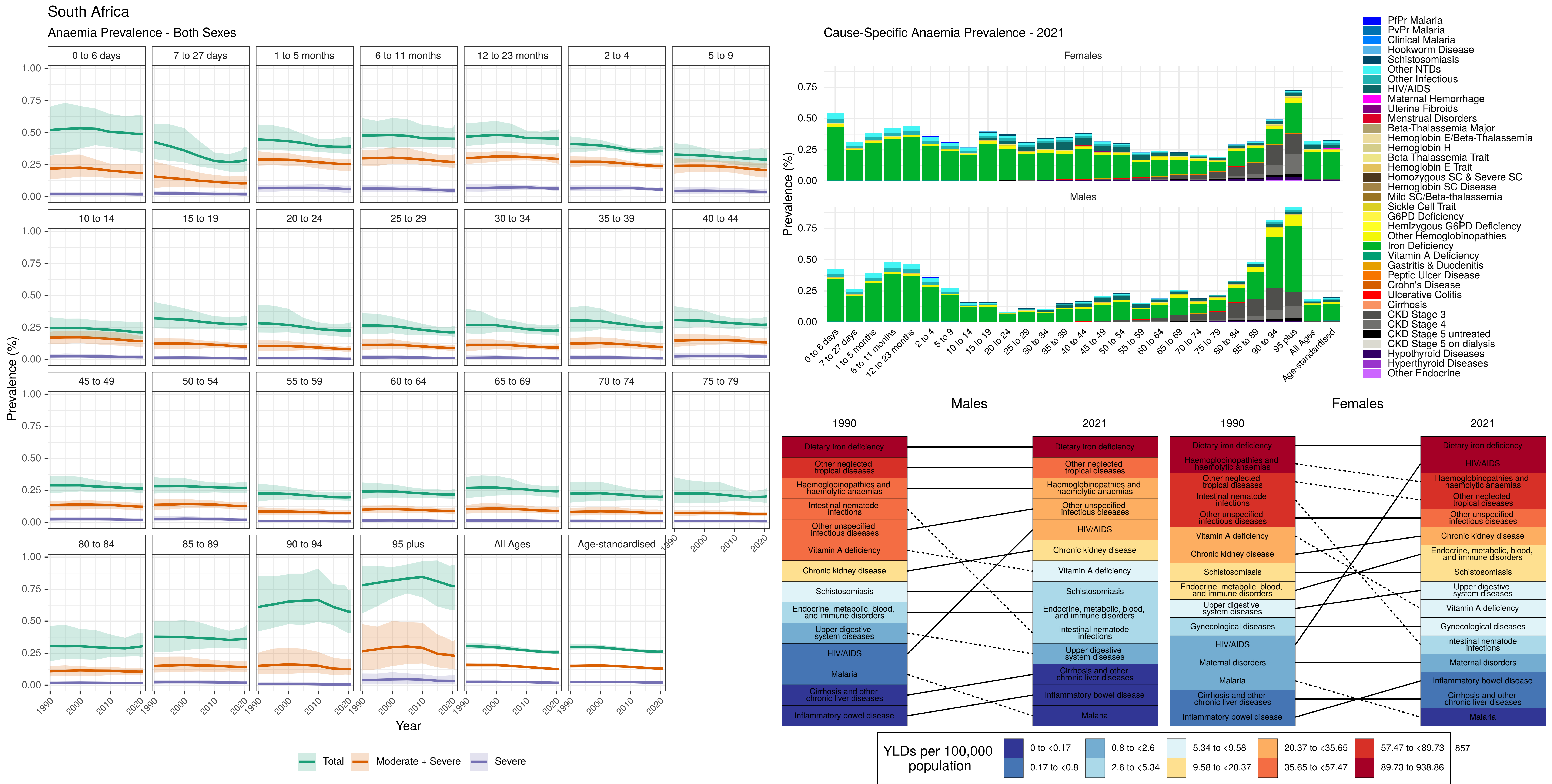
South Africa
Anaemia Prevalence - Both Sexes
0 to 6 days
7 to 27 days
1 to 5 months
6 to 11 months
12 to 23 months
2 to 4
5 to 9
10 to 14
15 to 19
20 to 24
25 to 29
30 to 34
35 to 39
40 to 44
45 to 49
50 to 54
55 to 59
60 to 64
65 to 69
70 to 74
75 to 79
80 to 84
85 to 89
90 to 94
95 plus
All Ages
Age-standardised
Prevalence (%)
Year
Total
Moderate + Severe
Severe
Cause-Specific Anaemia Prevalence - 2021
Females
Males
PfPr Malaria
PvPr Malaria
Clinical Malaria
Hookworm Disease
Schistosomiasis
Other NTDs
Other Infectious
HIV/AIDS
Maternal Hemorrhage
Uterine Fibroids
Menstrual Disorders
Beta-Thalassemia Major
Hemoglobin E/Beta-Thalassemia
Hemoglobin H
Beta-Thalassemia Trait
Hemoglobin E Trait
Homozygous SC & Severe SC
Hemoglobin SC Disease
Mild SC/Beta-thalassemia
Sickle Cell Trait
G6PD Deficiency
Hemizygous G6PD Deficiency
Other Hemoglobinopathies
Iron Deficiency
Vitamin A Deficiency
Gastritis & Duodenitis
Peptic Ulcer Disease
Crohn's Disease
Ulcerative Colitis
Cirrhosis
CKD Stage 3
CKD Stage 4
CKD Stage 5 untreated
CKD Stage 5 on dialysis
Hypothyroid Diseases
Hyperthyroid Diseases
Other Endocrine
Males
Females
1990
2021
Dietary iron deficiency
Other neglected tropical diseases
Haemoglobinopathies and haemolytic anaemias
Intestinal nematode infections
Other unspecified infectious diseases
Vitamin A deficiency
Chronic kidney disease
Schistosomiasis
Endocrine, metabolic, blood, and immune disorders
Upper digestive system diseases
HIV/AIDS
Malaria
Cirrhosis and other chronic liver diseases
Inflammatory bowel disease
Gynecological diseases
Maternal disorders
YLDs per 100,000 population
0 to <0.17
0.17 to <0.8
0.8 to <2.6
2.6 to <5.34
5.34 to <9.58
9.58 to <20.37
20.37 to <35.65
35.65 to <57.47
57.47 to <89.73
89.73 to 938.86

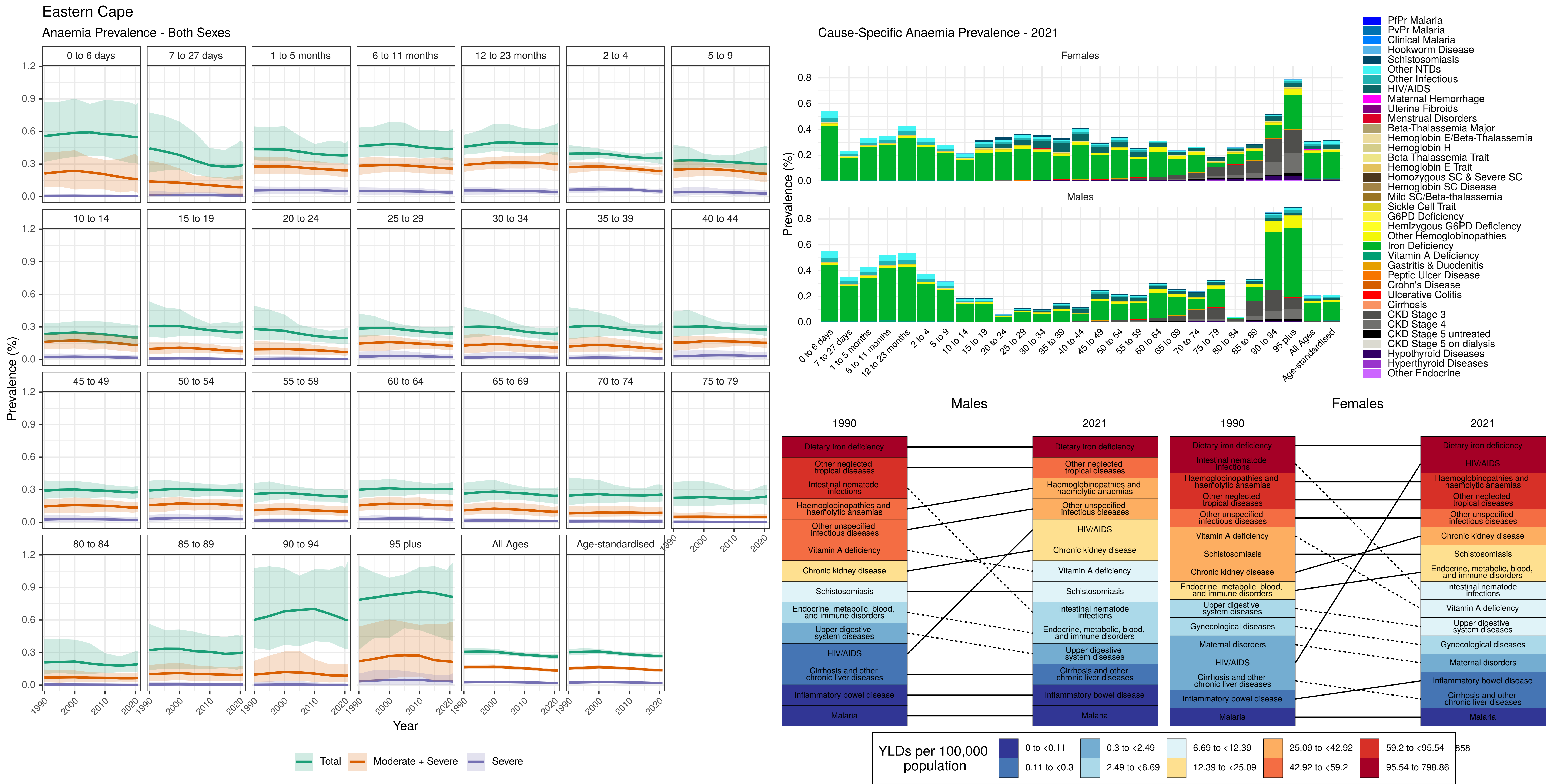
Eastern Cape
Anaemia Prevalence - Both Sexes
0 to 6 days
7 to 27 days
1 to 5 months
6 to 11 months
12 to 23 months
2 to 4
5 to 9
10 to 14
15 to 19
20 to 24
25 to 29
30 to 34
35 to 39
40 to 44
45 to 49
50 to 54
55 to 59
60 to 64
65 to 69
70 to 74
75 to 79
80 to 84
85 to 89
90 to 94
95 plus
All Ages
Age-standardised
Prevalence (%)
Year
Total
Moderate + Severe
Severe
Cause-Specific Anaemia Prevalence - 2021
Females
Males
PfPr Malaria
PvPr Malaria
Clinical Malaria
Hookworm Disease
Schistosomiasis
Other NTDs
Other Infectious
HIV/AIDS
Maternal Hemorrhage
Uterine Fibroids
Menstrual Disorders
Beta-Thalassemia Major
Hemoglobin E/Beta-Thalassemia
Hemoglobin H
Beta-Thalassemia Trait
Hemoglobin E Trait
Homozygous SC & Severe SC
Hemoglobin SC Disease
Mild SC/Beta-thalassemia
Sickle Cell Trait
G6PD Deficiency
Hemizygous G6PD Deficiency
Other Hemoglobinopathies
Iron Deficiency
Vitamin A Deficiency
Gastritis & Duodenitis
Peptic Ulcer Disease
Crohn's Disease
Ulcerative Colitis
Cirrhosis
CKD Stage 3
CKD Stage 4
CKD Stage 5 untreated
CKD Stage 5 on dialysis
Hypothyroid Diseases
Hyperthyroid Diseases
Other Endocrine
Males
1990
Dietary iron deficiency
Other neglected tropical diseases
Intestinal nematode infections
Haemoglobinopathies and haemolytic anaemias
Other unspecified infectious diseases
Vitamin A deficiency
Chronic kidney disease
Schistosomiasis
Endocrine, metabolic, blood, and immune disorders
Upper digestive system diseases
HIV/AIDS
Cirrhosis and other chronic liver diseases
Inflammatory bowel disease
Malaria
2021
Dietary iron deficiency
Other neglected tropical diseases
Haemoglobinopathies and haemolytic anaemias
Other unspecified infectious diseases
HIV/AIDS
Chronic kidney disease
Vitamin A deficiency
Schistosomiasis
Intestinal nematode infections
Endocrine, metabolic, blood, and immune disorders
Upper digestive system diseases
Cirrhosis and other chronic liver diseases
Inflammatory bowel disease
Malaria
Females
1990
Dietary iron deficiency
Intestinal nematode infections
Haemoglobinopathies and haemolytic anaemias
Other neglected tropical diseases
Other unspecified infectious diseases
Vitamin A deficiency
Schistosomiasis
Chronic kidney disease
Endocrine, metabolic, blood, and immune disorders
Upper digestive system diseases
Gynecological diseases
Maternal disorders
HIV/AIDS
Cirrhosis and other chronic liver diseases
Inflammatory bowel disease
Malaria
2021
Dietary iron deficiency
HIV/AIDS
Haemoglobinopathies and haemolytic anaemias
Other neglected tropical diseases
Other unspecified infectious diseases
Chronic kidney disease
Schistosomiasis
Endocrine, metabolic, blood, and immune disorders
Intestinal nematode infections
Vitamin A deficiency
Upper digestive system diseases
Gynecological diseases
Maternal disorders
Inflammatory bowel disease
Cirrhosis and other chronic liver diseases
Malaria
YLDs per 100,000 population
0 to <0.11
0.11 to <0.3
0.3 to <2.49
2.49 to <6.69
6.69 to <12.39
12.39 to <25.09
25.09 to <42.92
42.92 to <59.2
59.2 to <95.54
95.54 to 798.86

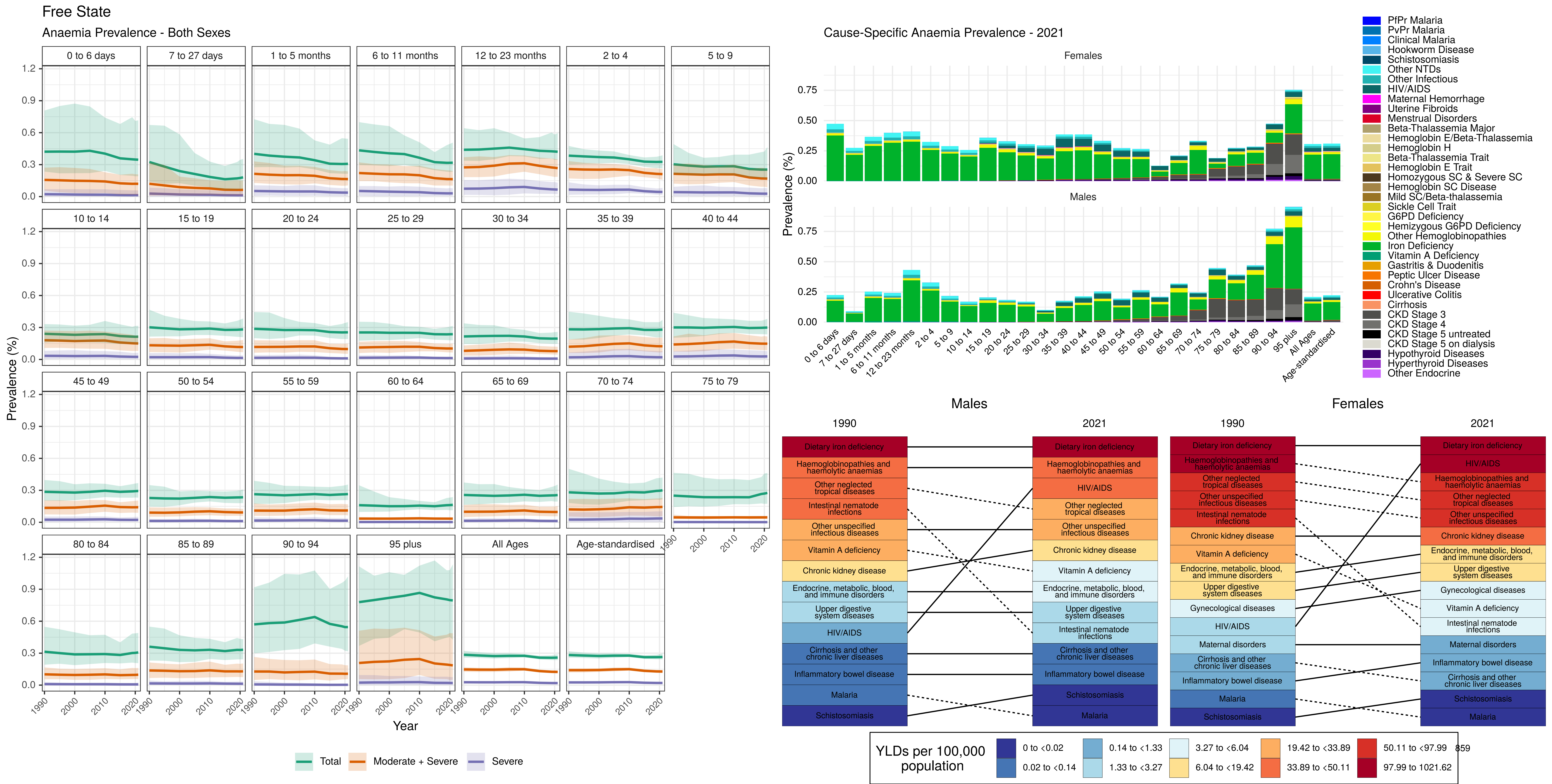
Free State
Anaemia Prevalence - Both Sexes
0 to 6 days
7 to 27 days
1 to 5 months
6 to 11 months
12 to 23 months
2 to 4
5 to 9
10 to 14
15 to 19
20 to 24
25 to 29
30 to 34
35 to 39
40 to 44
45 to 49
50 to 54
55 to 59
60 to 64
65 to 69
70 to 74
75 to 79
80 to 84
85 to 89
90 to 94
95 plus
All Ages
Age-standardised
Prevalence (%)
Year
Total
Moderate + Severe
Severe
Cause-Specific Anaemia Prevalence - 2021
Females
Males
PfPr Malaria
PvPr Malaria
Clinical Malaria
Hookworm Disease
Schistosomiasis
Other NTDs
Other Infectious
HIV/AIDS
Maternal Hemorrhage
Uterine Fibroids
Menstrual Disorders
Beta-Thalassemia Major
Hemoglobin E/Beta-Thalassemia
Hemoglobin H
Beta-Thalassemia Trait
Hemoglobin E Trait
Homozygous SC & Severe SC
Hemoglobin SC Disease
Mild SC/Beta-thalassemia
Sickle Cell Trait
G6PD Deficiency
Hemizygous G6PD Deficiency
Other Hemoglobinopathies
Iron Deficiency
Vitamin A Deficiency
Gastritis & Duodenitis
Peptic Ulcer Disease
Crohn's Disease
Ulcerative Colitis
Cirrhosis
CKD Stage 3
CKD Stage 4
CKD Stage 5 untreated
CKD Stage 5 on dialysis
Hypothyroid Diseases
Hyperthyroid Diseases
Other Endocrine
Males
Females
1990
2021
Dietary iron deficiency
Haemoglobinopathies and haemolytic anaemias
Other neglected tropical diseases
Intestinal nematode infections
Other unspecified infectious diseases
Vitamin A deficiency
Chronic kidney disease
Endocrine, metabolic, blood, and immune disorders
Upper digestive system diseases
HIV/AIDS
Cirrhosis and other chronic liver diseases
Inflammatory bowel disease
Malaria
Schistosomiasis
Gynecological diseases
Maternal disorders
YLDs per 100,000 population
0 to <0.02
0.02 to <0.14
0.14 to <1.33
1.33 to <3.27
3.27 to <6.04
6.04 to <19.42
19.42 to <33.89
33.89 to <50.11
50.11 to <97.99
97.99 to 1021.62

Gauteng

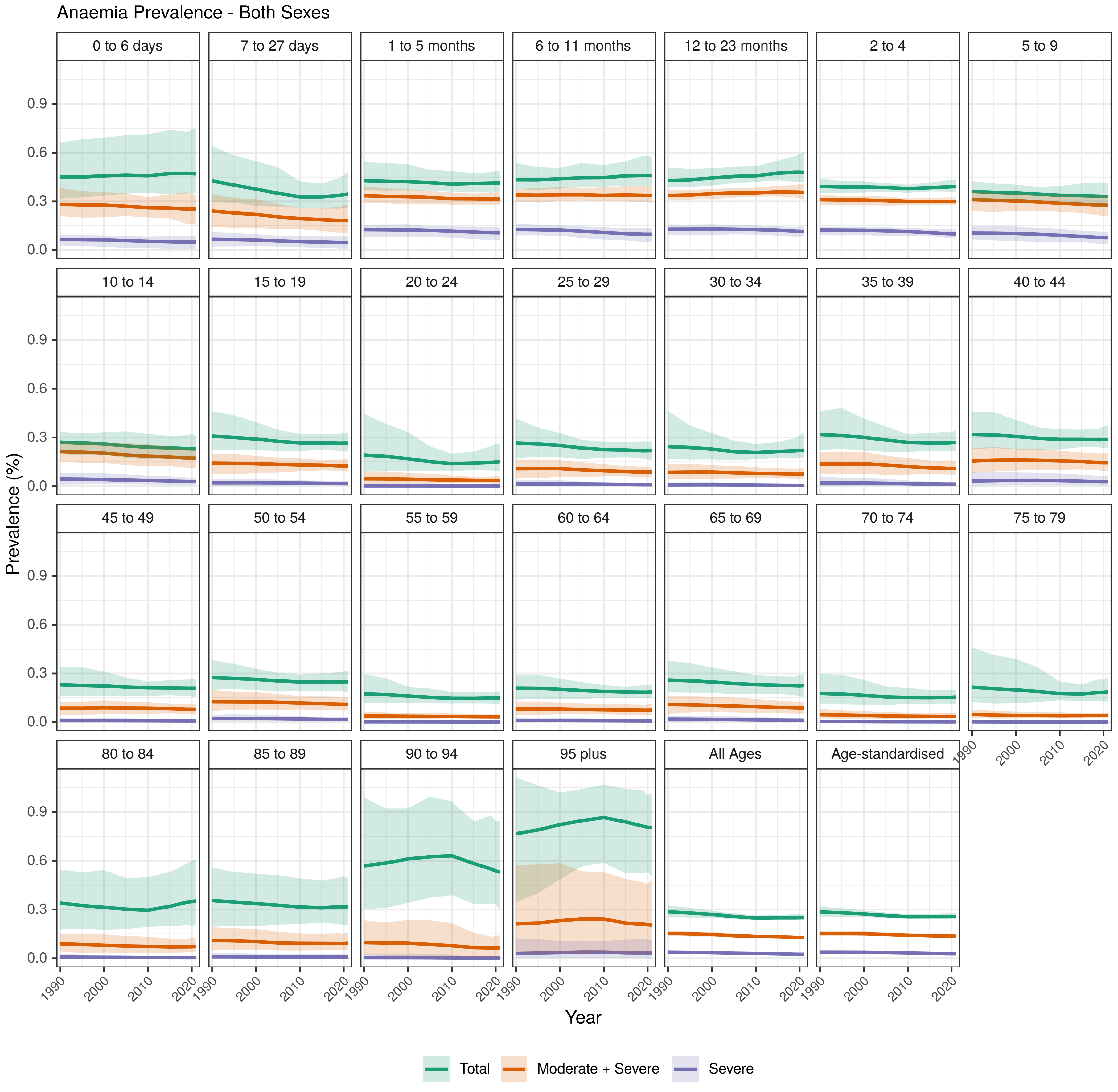

Anaemia Prevalence - Both Sexes
0 to 6 days
7 to 27 days
1 to 5 months
6 to 11 months
12 to 23 months
2 to 4
5 to 9
10 to 14
15 to 19
20 to 24
25 to 29
30 to 34
35 to 39
40 to 44
45 to 49
50 to 54
55 to 59
60 to 64
65 to 69
70 to 74
75 to 79
80 to 84
85 to 89
90 to 94
95 plus
All Ages
Age-standardised
Prevalence (%)
0.0
0.3
0.6
0.9
1990
2000
2010
2020
Year
Total
Moderate + Severe
Severe

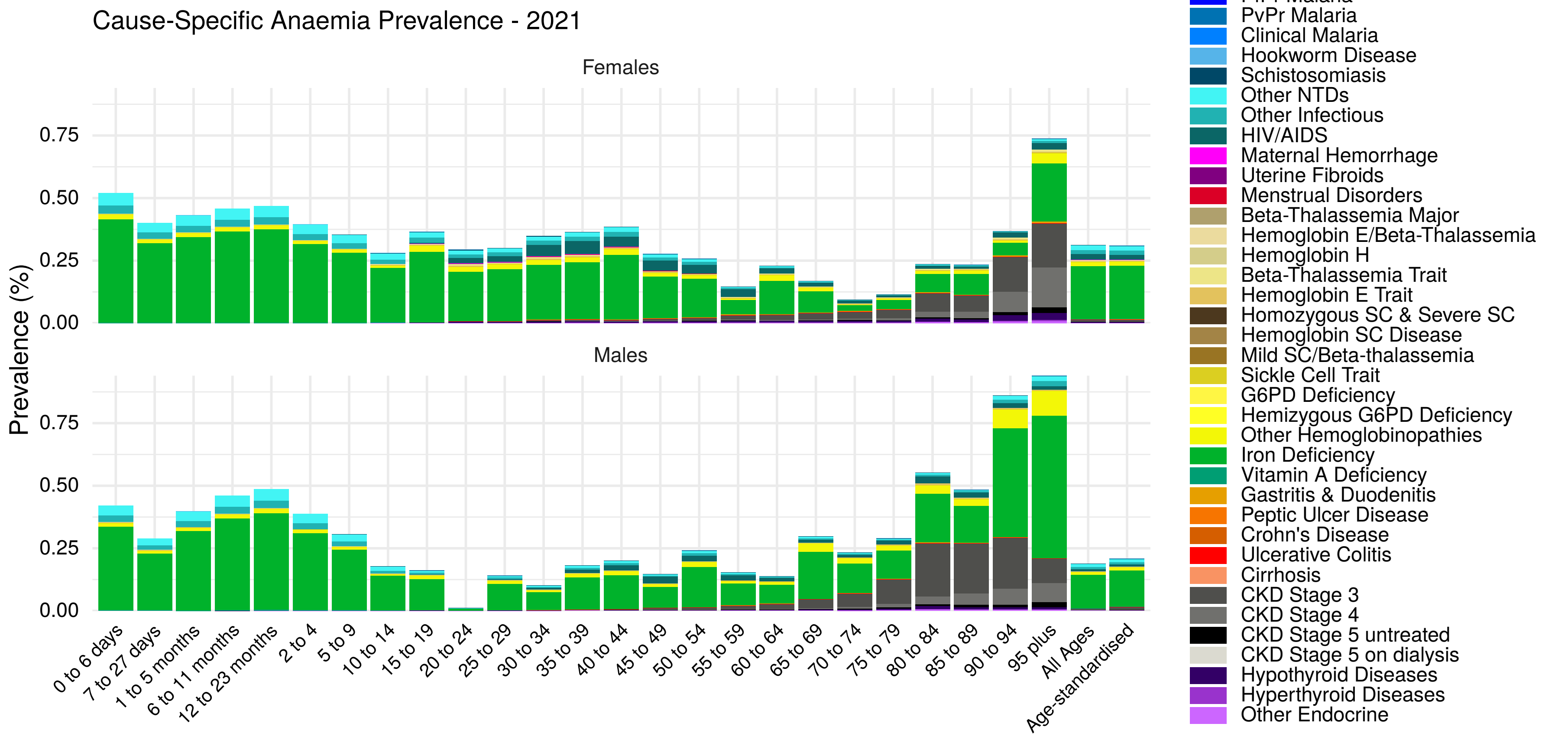

Cause-Specific Anaemia Prevalence - 2021
Females
Males
Prevalence (%)
0.00
0.25
0.50
0.75
0 to 6 days
7 to 27 days
1 to 5 months
6 to 11 months
12 to 23 months
2 to 4
5 to 9
10 to 14
15 to 19
20 to 24
25 to 29
30 to 34
35 to 39
40 to 44
45 to 49
50 to 54
55 to 59
60 to 64
65 to 69
70 to 74
75 to 79
80 to 84
85 to 89
90 to 94
95 plus
All Ages
Age-standardised
PfPr Malaria
PvPr Malaria
Clinical Malaria
Hookworm Disease
Schistosomiasis
Other NTDs
Other Infectious
HIV/AIDS
Maternal Hemorrhage
Uterine Fibroids
Menstrual Disorders
Beta-Thalassemia Major
Hemoglobin E/Beta-Thalassemia
Hemoglobin H
Beta-Thalassemia Trait
Hemoglobin E Trait
Homozygous SC & Severe SC
Hemoglobin SC Disease
Mild SC/Beta-thalassemia
Sickle Cell Trait
G6PD Deficiency
Hemizygous G6PD Deficiency
Other Hemoglobinopathies
Iron Deficiency
Vitamin A Deficiency
Gastritis & Duodenitis
Peptic Ulcer Disease
Crohn's Disease
Ulcerative Colitis
Cirrhosis
CKD Stage 3
CKD Stage 4
CKD Stage 5 untreated
CKD Stage 5 on dialysis
Hypothyroid Diseases
Hyperthyroid Diseases
Other Endocrine

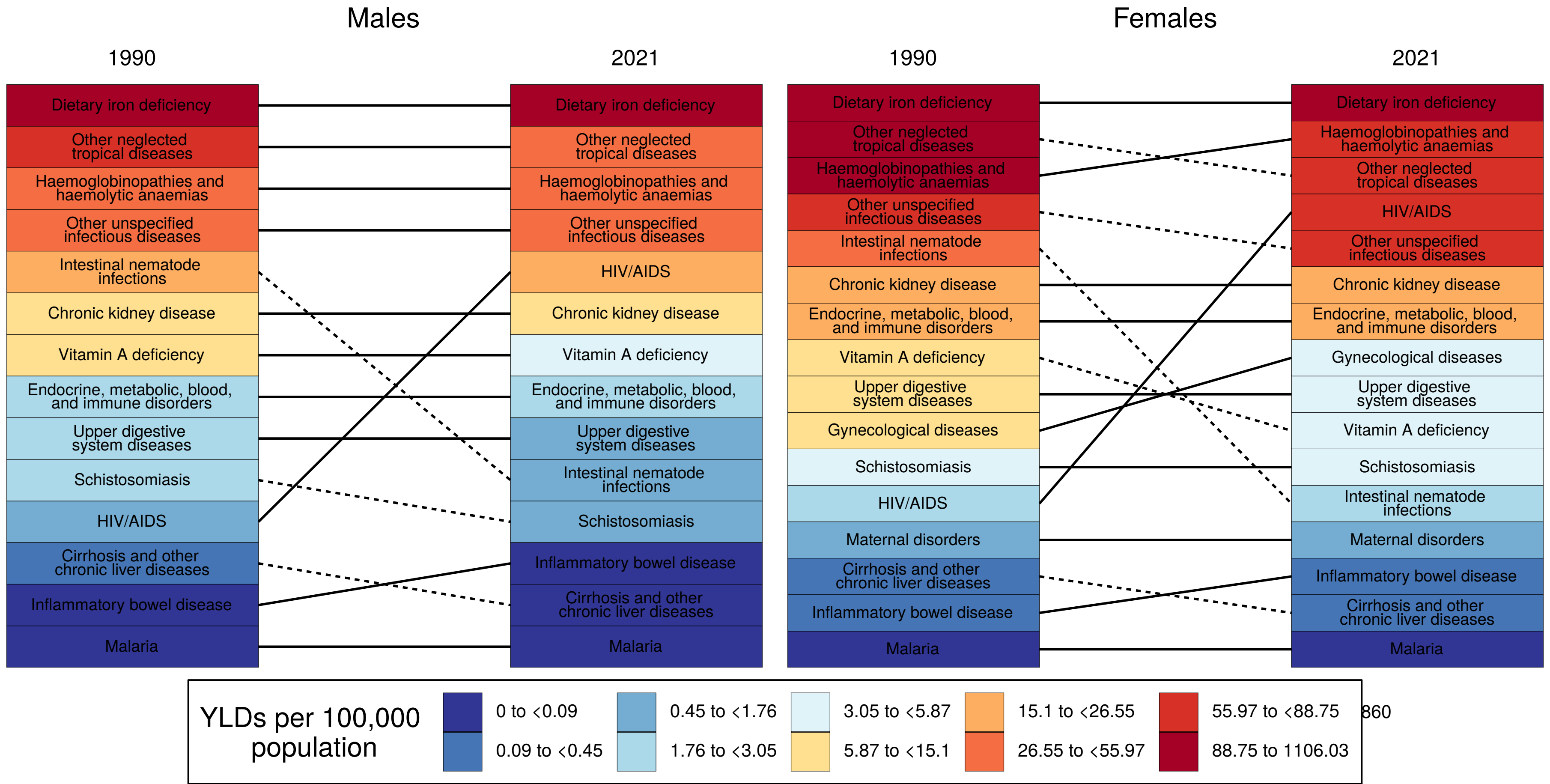

Males
Females
1990
2021
Dietary iron deficiency
Other neglected tropical diseases
Haemoglobinopathies and haemolytic anaemias
Other unspecified infectious diseases
Intestinal nematode infections
Chronic kidney disease
Vitamin A deficiency
Endocrine, metabolic, blood, and immune disorders
Upper digestive system diseases
Schistosomiasis
HIV/AIDS
Cirrhosis and other chronic liver diseases
Inflammatory bowel disease
Malaria
Gynecological diseases
Maternal disorders
YLDs per 100,000 population
0 to <0.09
0.09 to <0.45
0.45 to <1.76
1.76 to <3.05
3.05 to <5.87
5.87 to <15.1
15.1 to <26.55
26.55 to <55.97
55.97 to <88.75
88.75 to 1106.03

KwaZulu-Natal

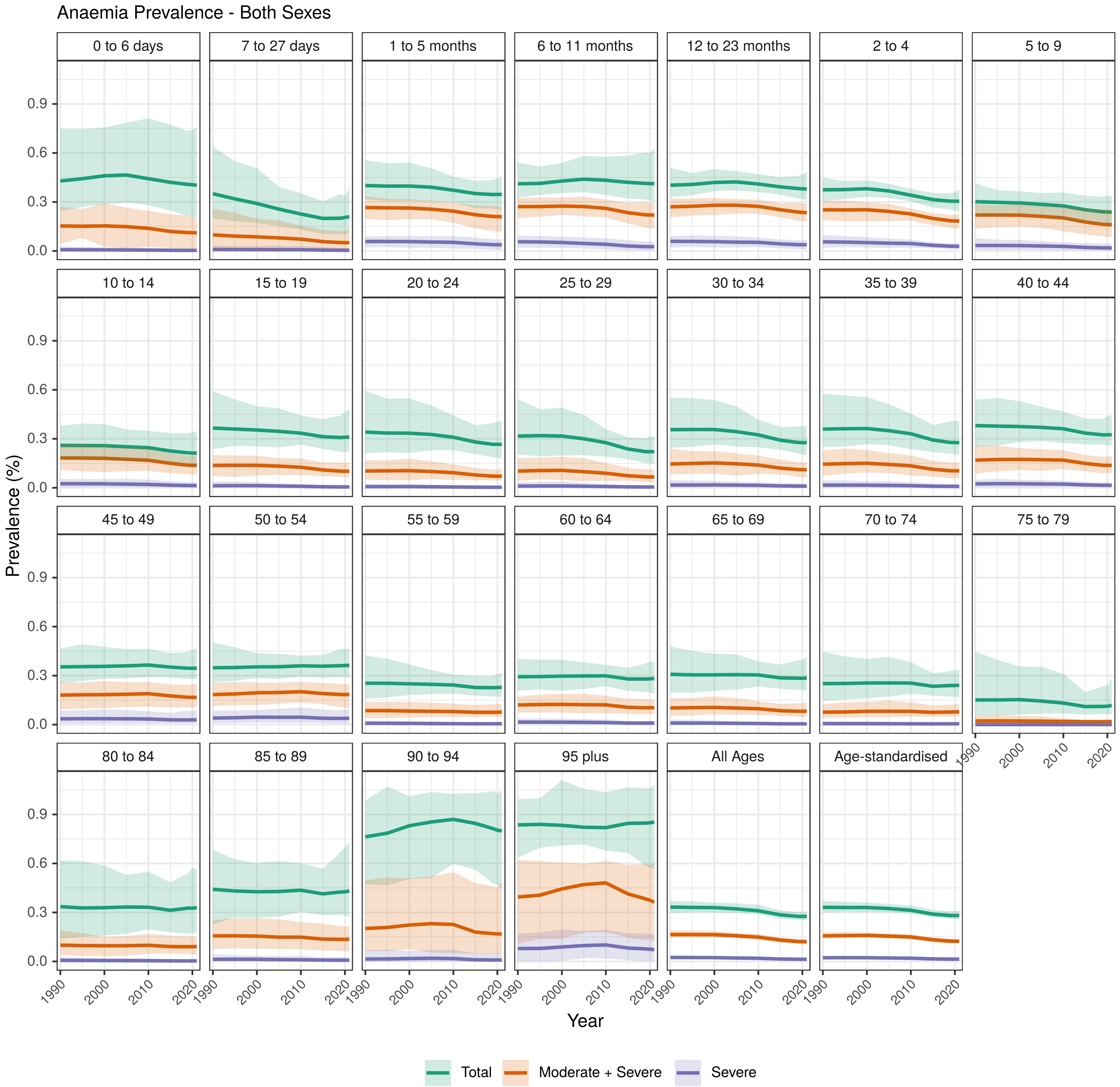

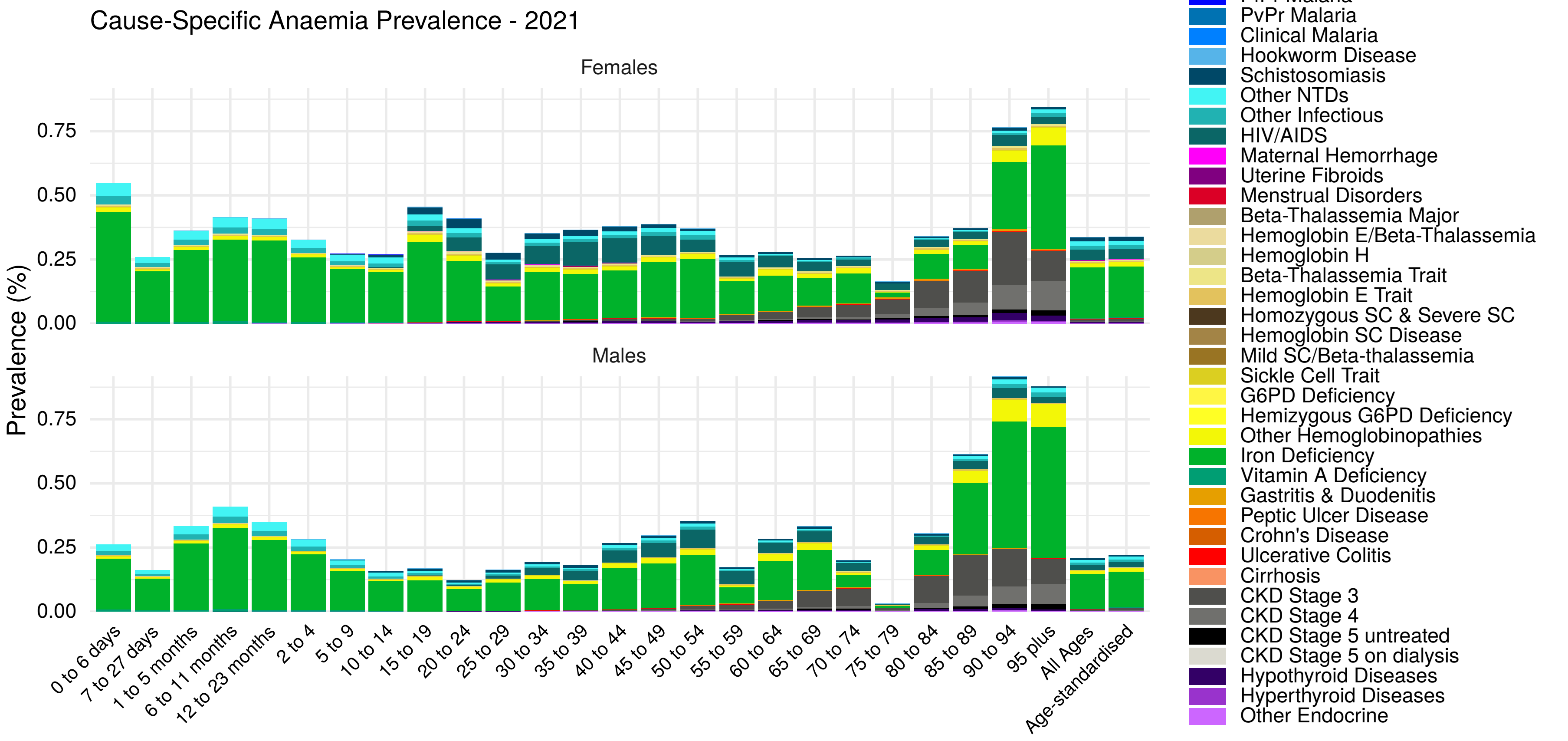

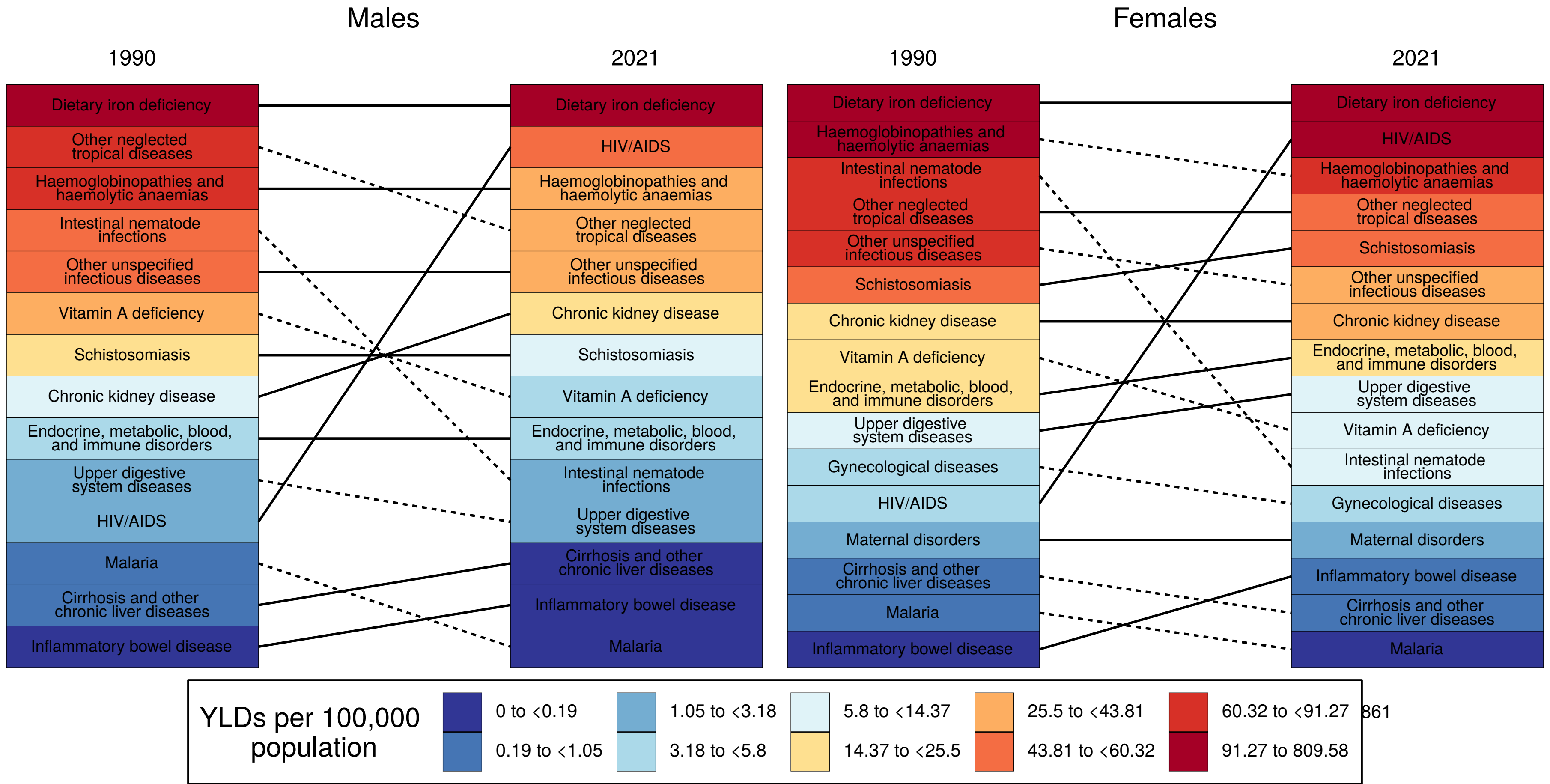

Limpopo

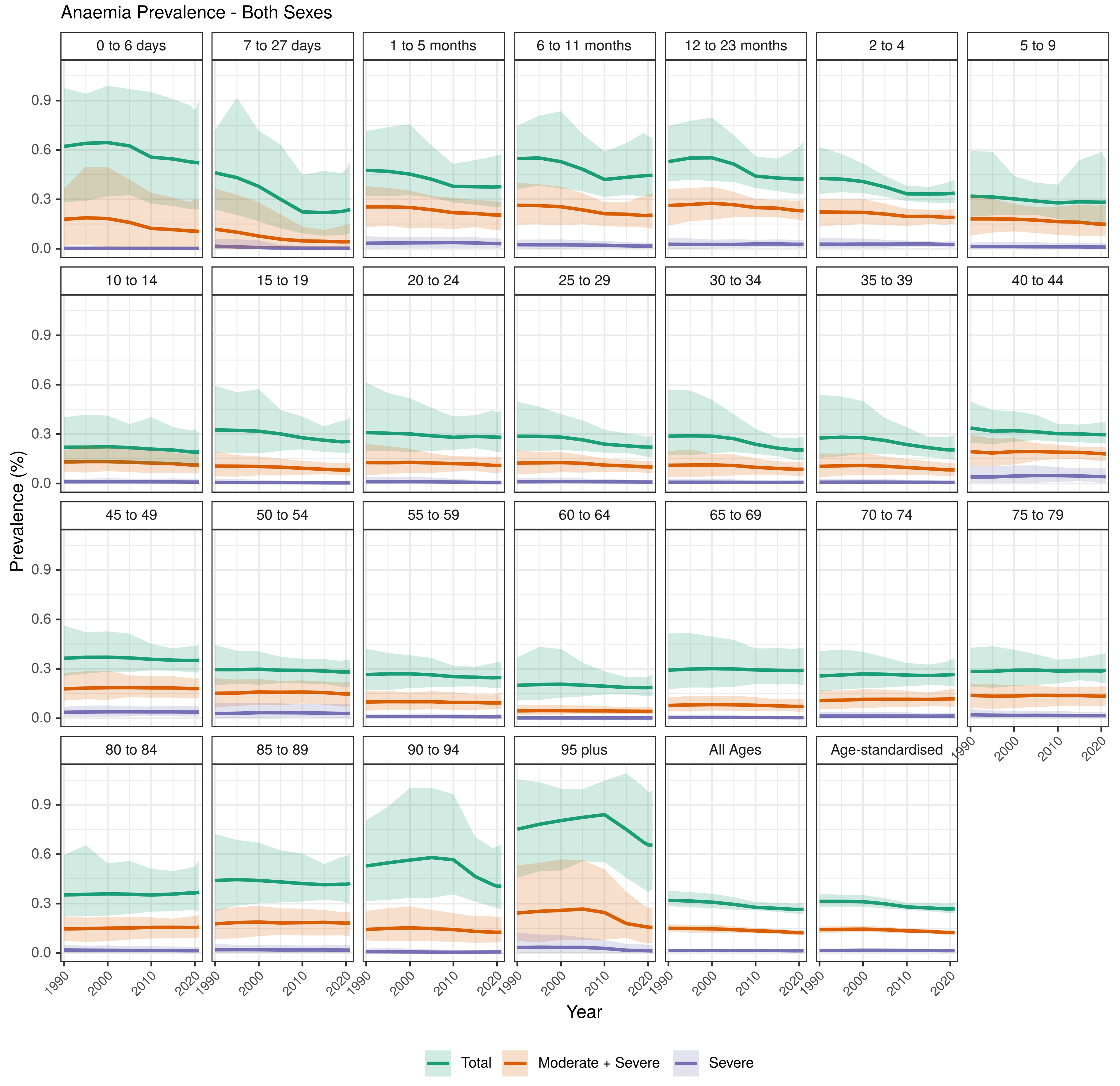
Anaemia Prevalence - Both Sexes
0 to 6 days
7 to 27 days
1 to 5 months
6 to 11 months
12 to 23 months
2 to 4
5 to 9
10 to 14
15 to 19
20 to 24
25 to 29
30 to 34
35 to 39
40 to 44
45 to 49
50 to 54
55 to 59
60 to 64
65 to 69
70 to 74
75 to 79
80 to 84
85 to 89
90 to 94
95 plus
All Ages
Age-standardised
Prevalence (%)
0.0
0.3
0.6
0.9
1990
2000
2010
2020
Year
Total
Moderate + Severe
Severe

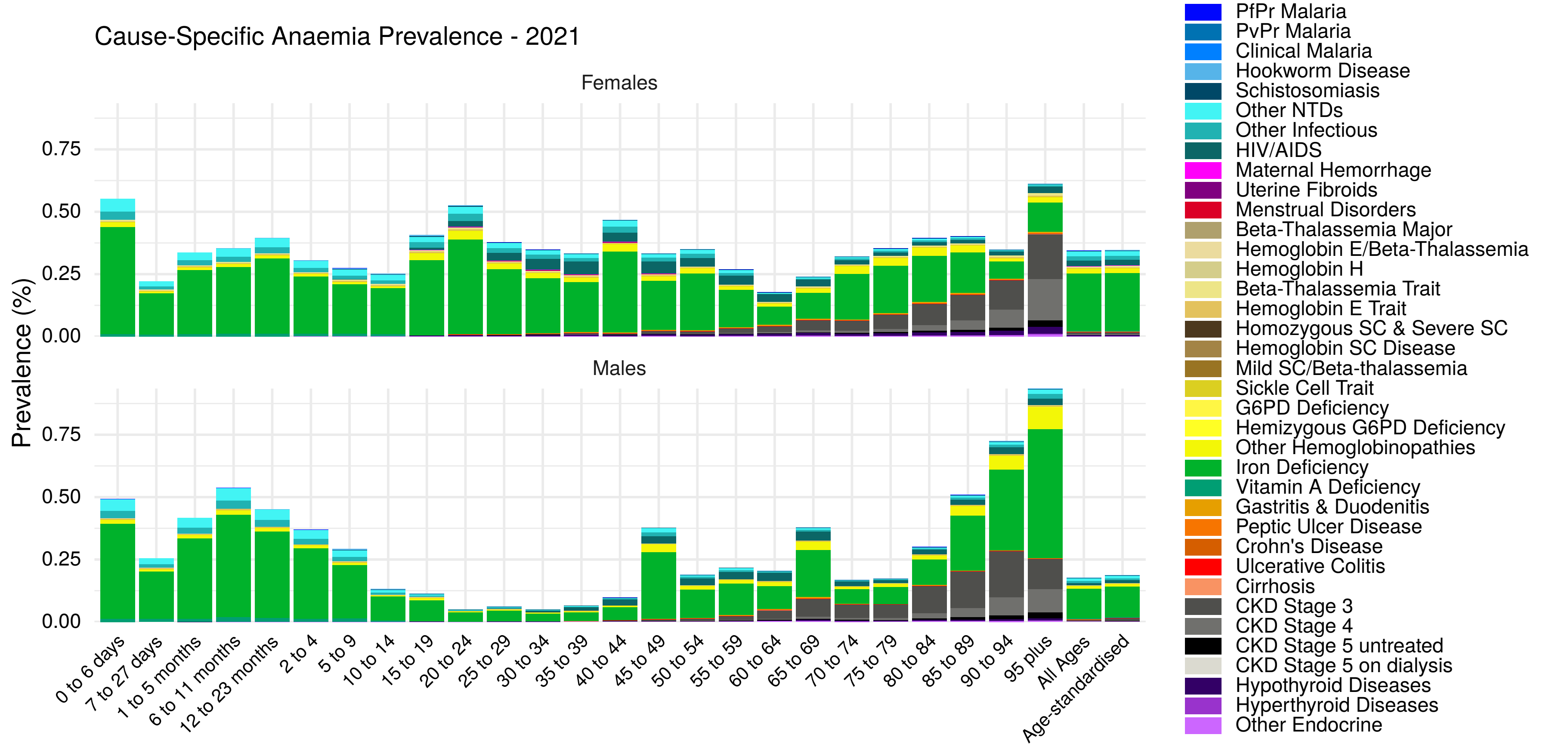
Cause-Specific Anaemia Prevalence - 2021
Females
Males
Prevalence (%)
0.00
0.25
0.50
0.75
0 to 6 days
7 to 27 days
1 to 5 months
6 to 11 months
12 to 23 months
2 to 4
5 to 9
10 to 14
15 to 19
20 to 24
25 to 29
30 to 34
35 to 39
40 to 44
45 to 49
50 to 54
55 to 59
60 to 64
65 to 69
70 to 74
75 to 79
80 to 84
85 to 89
90 to 94
95 plus
All Ages
Age-standardised
PfPr Malaria
PvPr Malaria
Clinical Malaria
Hookworm Disease
Schistosomiasis
Other NTDs
Other Infectious
HIV/AIDS
Maternal Hemorrhage
Uterine Fibroids
Menstrual Disorders
Beta-Thalassemia Major
Hemoglobin E/Beta-Thalassemia
Hemoglobin H
Beta-Thalassemia Trait
Hemoglobin E Trait
Homozygous SC & Severe SC
Hemoglobin SC Disease
Mild SC/Beta-thalassemia
Sickle Cell Trait
G6PD Deficiency
Hemizygous G6PD Deficiency
Other Hemoglobinopathies
Iron Deficiency
Vitamin A Deficiency
Gastritis & Duodenitis
Peptic Ulcer Disease
Crohn's Disease
Ulcerative Colitis
Cirrhosis
CKD Stage 3
CKD Stage 4
CKD Stage 5 untreated
CKD Stage 5 on dialysis
Hypothyroid Diseases
Hyperthyroid Diseases
Other Endocrine

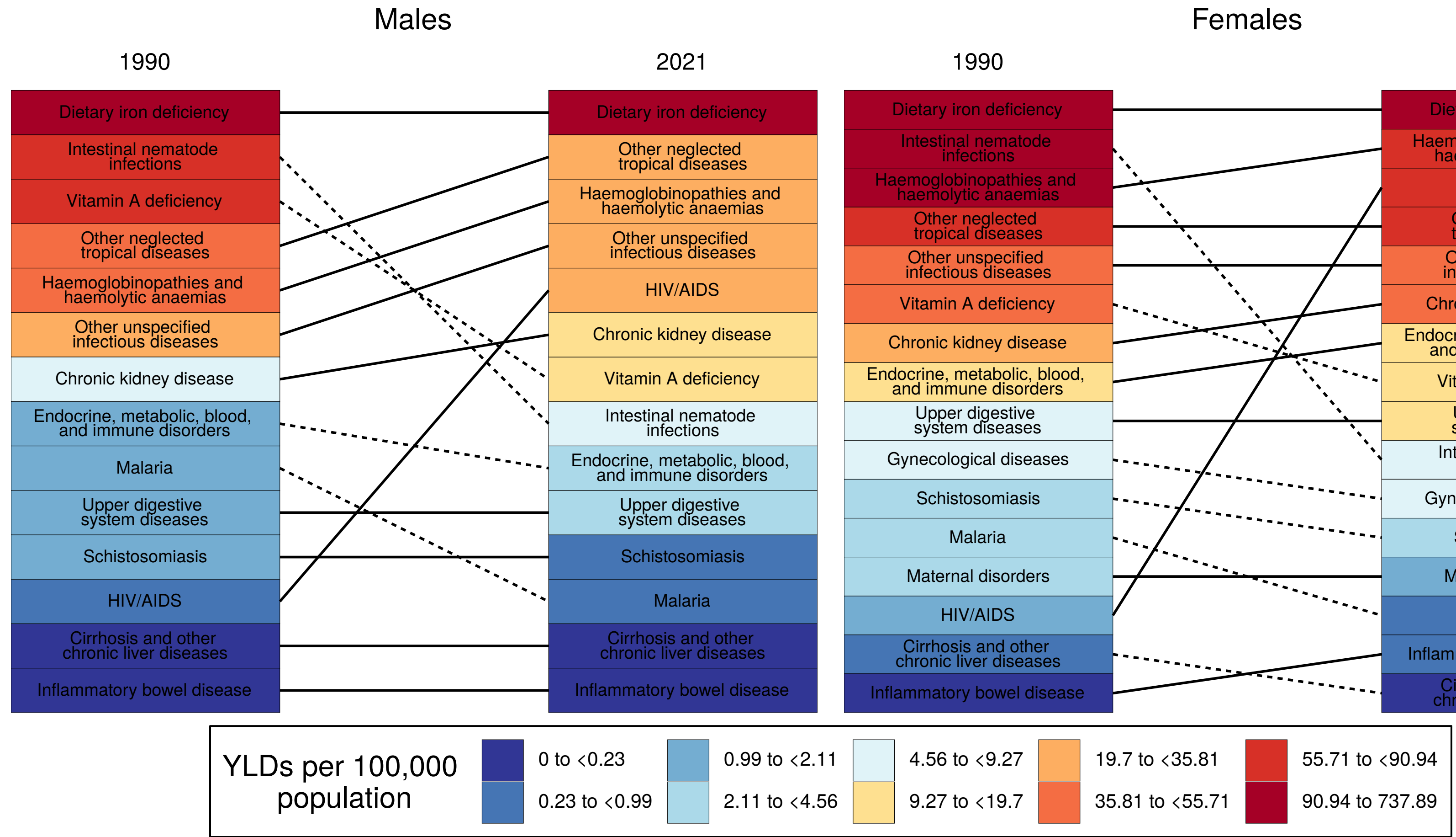
Males
Females
1990
2021
Dietary iron deficiency
Intestinal nematode infections
Vitamin A deficiency
Other neglected tropical diseases
Haemoglobinopathies and haemolytic anaemias
Other unspecified infectious diseases
Chronic kidney disease
Endocrine, metabolic, blood, and immune disorders
Malaria
Upper digestive system diseases
Schistosomiasis
HIV/AIDS
Cirrhosis and other chronic liver diseases
Inflammatory bowel disease
Gynecological diseases
Maternal disorders
YLDs per 100,000 population
0 to <0.23
0.23 to <0.99
0.99 to <2.11
2.11 to <4.56
4.56 to <9.27
9.27 to <19.7
19.7 to <35.81
35.81 to <55.71
55.71 to <90.94
90.94 to 737.89

Mpumalanga

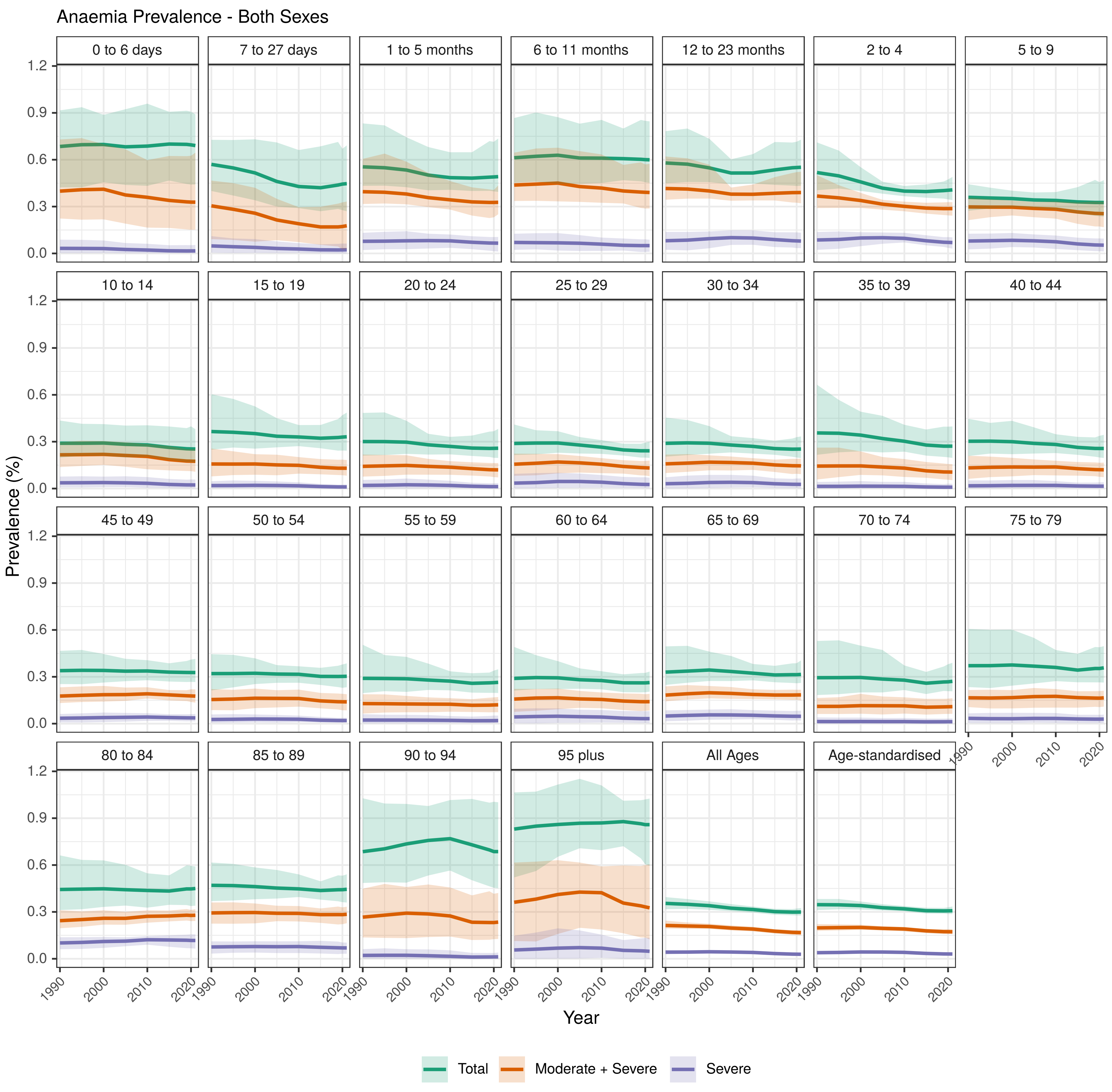
Anaemia Prevalence - Both Sexes
0 to 6 days
7 to 27 days
1 to 5 months
6 to 11 months
12 to 23 months
2 to 4
5 to 9
10 to 14
15 to 19
20 to 24
25 to 29
30 to 34
35 to 39
40 to 44
45 to 49
50 to 54
55 to 59
60 to 64
65 to 69
70 to 74
75 to 79
80 to 84
85 to 89
90 to 94
95 plus
All Ages
Age-standardised
Prevalence (%)
Year
Total
Moderate + Severe
Severe

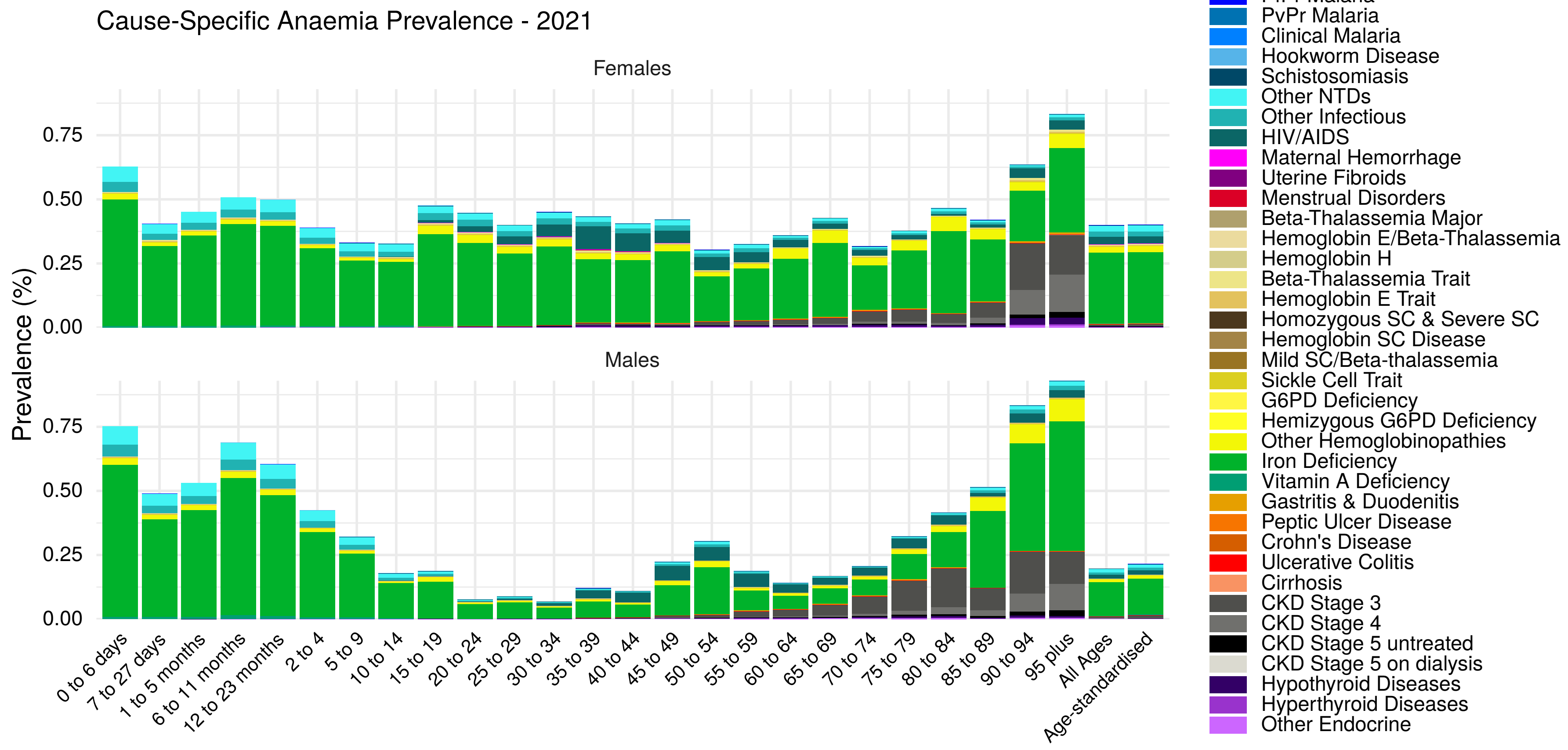
Cause-Specific Anaemia Prevalence - 2021
Females
Males
Prevalence (%)
PfPr Malaria
PvPr Malaria
Clinical Malaria
Hookworm Disease
Schistosomiasis
Other NTDs
Other Infectious
HIV/AIDS
Maternal Hemorrhage
Uterine Fibroids
Menstrual Disorders
Beta-Thalassemia Major
Hemoglobin E/Beta-Thalassemia
Hemoglobin H
Beta-Thalassemia Trait
Hemoglobin E Trait
Homozygous SC & Severe SC
Hemoglobin SC Disease
Mild SC/Beta-thalassemia
Sickle Cell Trait
G6PD Deficiency
Hemizygous G6PD Deficiency
Other Hemoglobinopathies
Iron Deficiency
Vitamin A Deficiency
Gastritis & Duodenitis
Peptic Ulcer Disease
Crohn's Disease
Ulcerative Colitis
Cirrhosis
CKD Stage 3
CKD Stage 4
CKD Stage 5 untreated
CKD Stage 5 on dialysis
Hypothyroid Diseases
Hyperthyroid Diseases
Other Endocrine

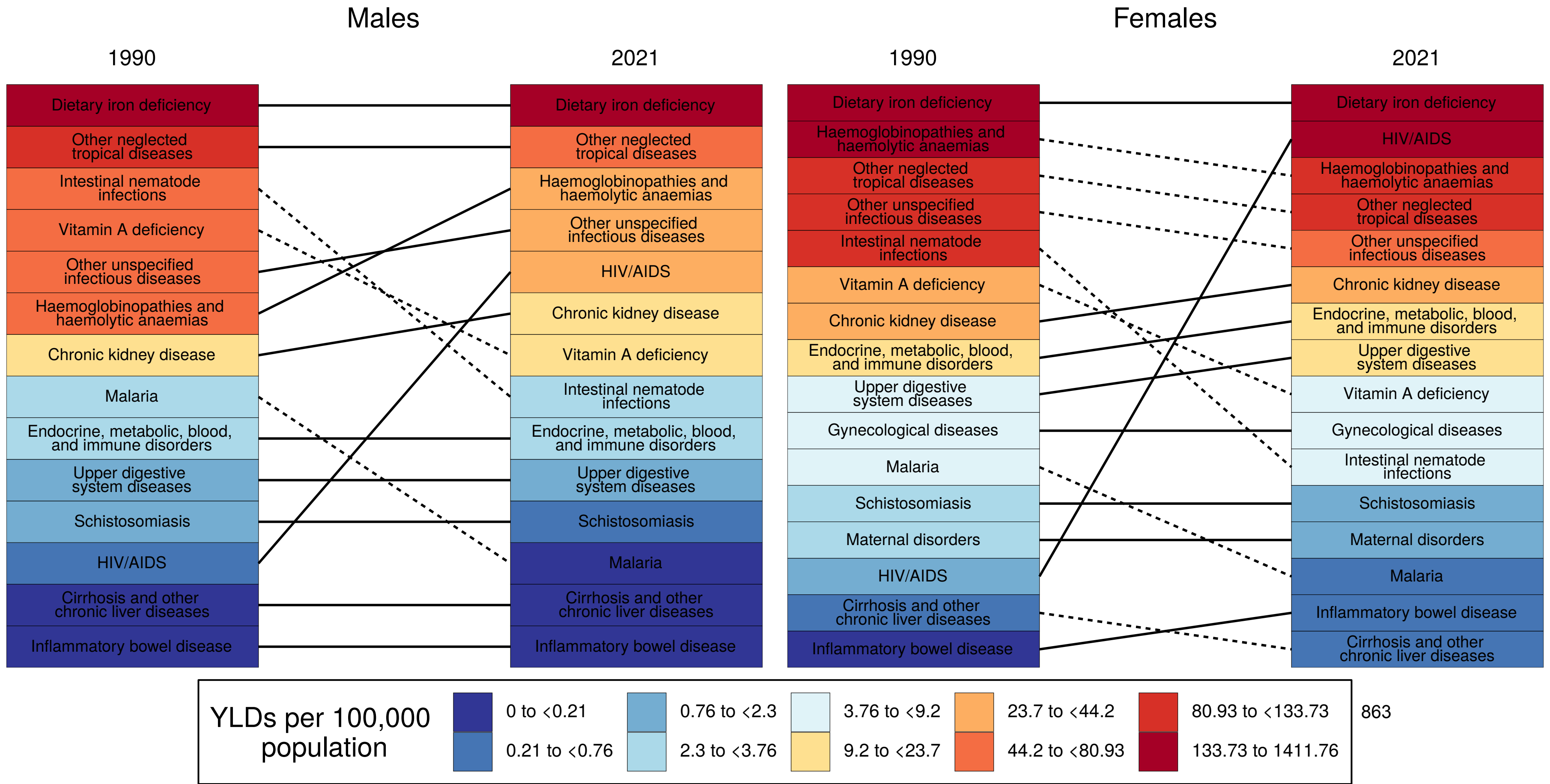
Males
Females
1990
2021
Dietary iron deficiency
Other neglected tropical diseases
Intestinal nematode infections
Vitamin A deficiency
Other unspecified infectious diseases
Haemoglobinopathies and haemolytic anaemias
Chronic kidney disease
Malaria
Endocrine, metabolic, blood, and immune disorders
Upper digestive system diseases
Schistosomiasis
HIV/AIDS
Cirrhosis and other chronic liver diseases
Inflammatory bowel disease
Gynecological diseases
Maternal disorders
YLDs per 100,000 population
0 to <0.21
0.21 to <0.76
0.76 to <2.3
2.3 to <3.76
3.76 to <9.2
9.2 to <23.7
23.7 to <44.2
44.2 to <80.93
80.93 to <133.73
133.73 to 1411.76

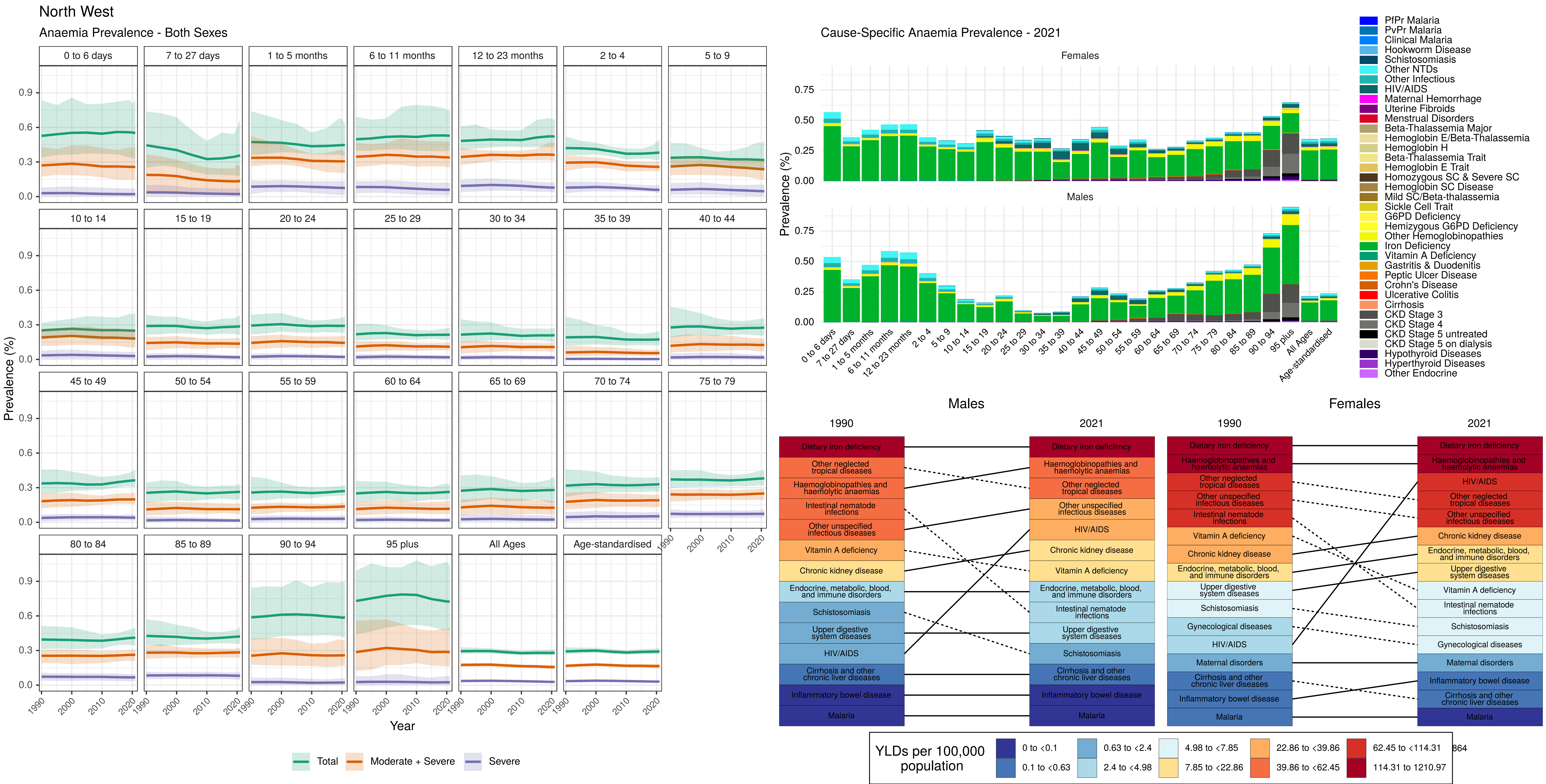
North West
Anaemia Prevalence - Both Sexes
0 to 6 days
7 to 27 days
1 to 5 months
6 to 11 months
12 to 23 months
2 to 4
5 to 9
10 to 14
15 to 19
20 to 24
25 to 29
30 to 34
35 to 39
40 to 44
45 to 49
50 to 54
55 to 59
60 to 64
65 to 69
70 to 74
75 to 79
80 to 84
85 to 89
90 to 94
95 plus
All Ages
Age-standardised
Prevalence (%)
Year
Total
Moderate + Severe
Severe
Cause-Specific Anaemia Prevalence - 2021
Females
Males
PfPr Malaria
PvPr Malaria
Clinical Malaria
Hookworm Disease
Schistosomiasis
Other NTDs
Other Infectious
HIV/AIDS
Maternal Hemorrhage
Uterine Fibroids
Menstrual Disorders
Beta-Thalassemia Major
Hemoglobin E/Beta-Thalassemia
Hemoglobin H
Beta-Thalassemia Trait
Hemoglobin E Trait
Homozygous SC & Severe SC
Hemoglobin SC Disease
Mild SC/Beta-thalassemia
Sickle Cell Trait
G6PD Deficiency
Hemizygous G6PD Deficiency
Other Hemoglobinopathies
Iron Deficiency
Vitamin A Deficiency
Gastritis & Duodenitis
Peptic Ulcer Disease
Crohn's Disease
Ulcerative Colitis
Cirrhosis
CKD Stage 3
CKD Stage 4
CKD Stage 5 untreated
CKD Stage 5 on dialysis
Hypothyroid Diseases
Hyperthyroid Diseases
Other Endocrine
Males
Females
1990
2021
Dietary iron deficiency
Other neglected tropical diseases
Haemoglobinopathies and haemolytic anaemias
Intestinal nematode infections
Other unspecified infectious diseases
Vitamin A deficiency
Chronic kidney disease
Endocrine, metabolic, blood, and immune disorders
Schistosomiasis
Upper digestive system diseases
HIV/AIDS
Cirrhosis and other chronic liver diseases
Inflammatory bowel disease
Malaria
Gynecological diseases
Maternal disorders
YLDs per 100,000 population
0 to <0.1
0.1 to <0.63
0.63 to <2.4
2.4 to <4.98
4.98 to <7.85
7.85 to <22.86
22.86 to <39.86
39.86 to <62.45
62.45 to <114.31
114.31 to 1210.97

Northern Cape

Anaemia Prevalence - Both Sexes

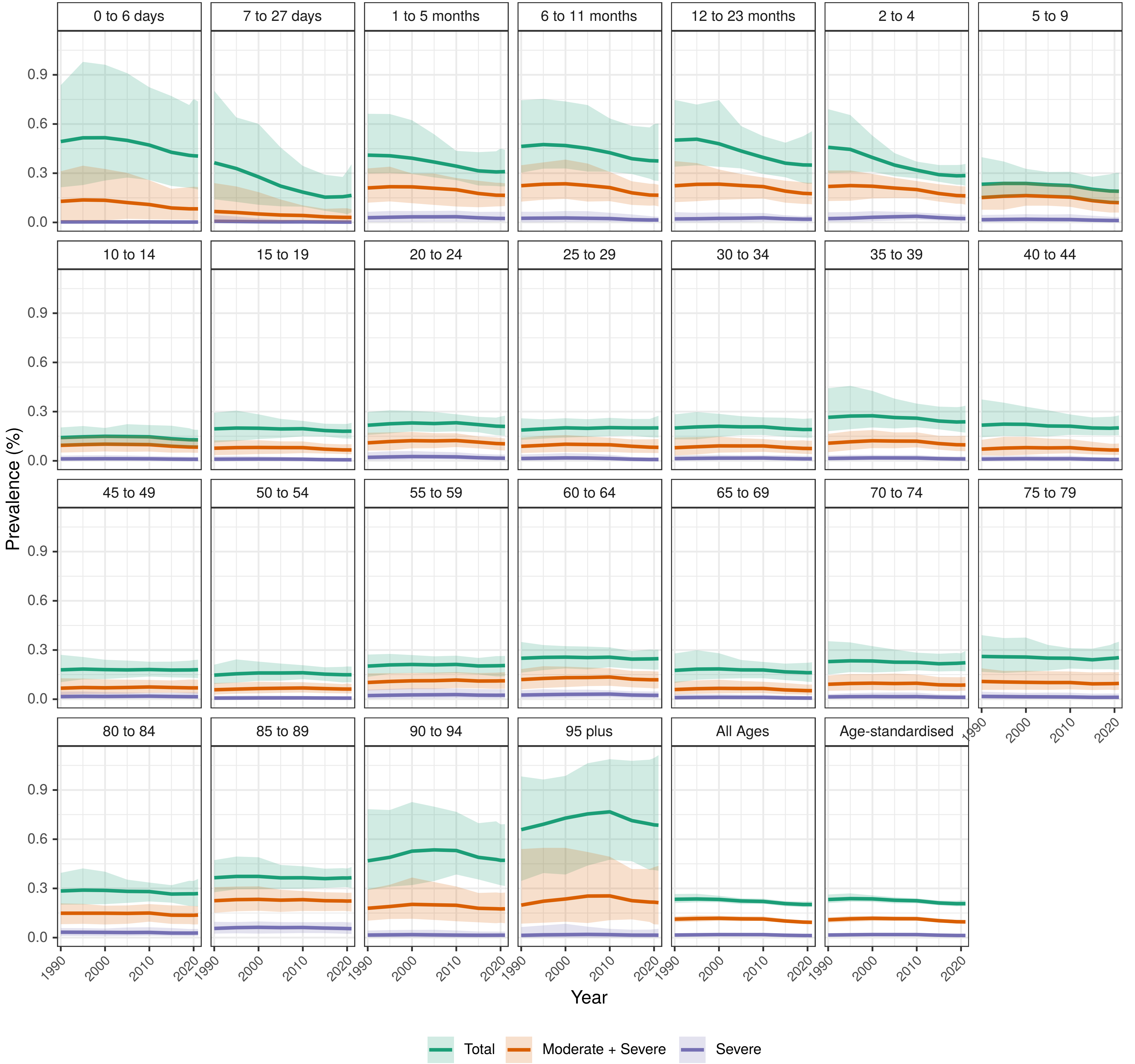

Cause-Specific Anaemia Prevalence - 2021

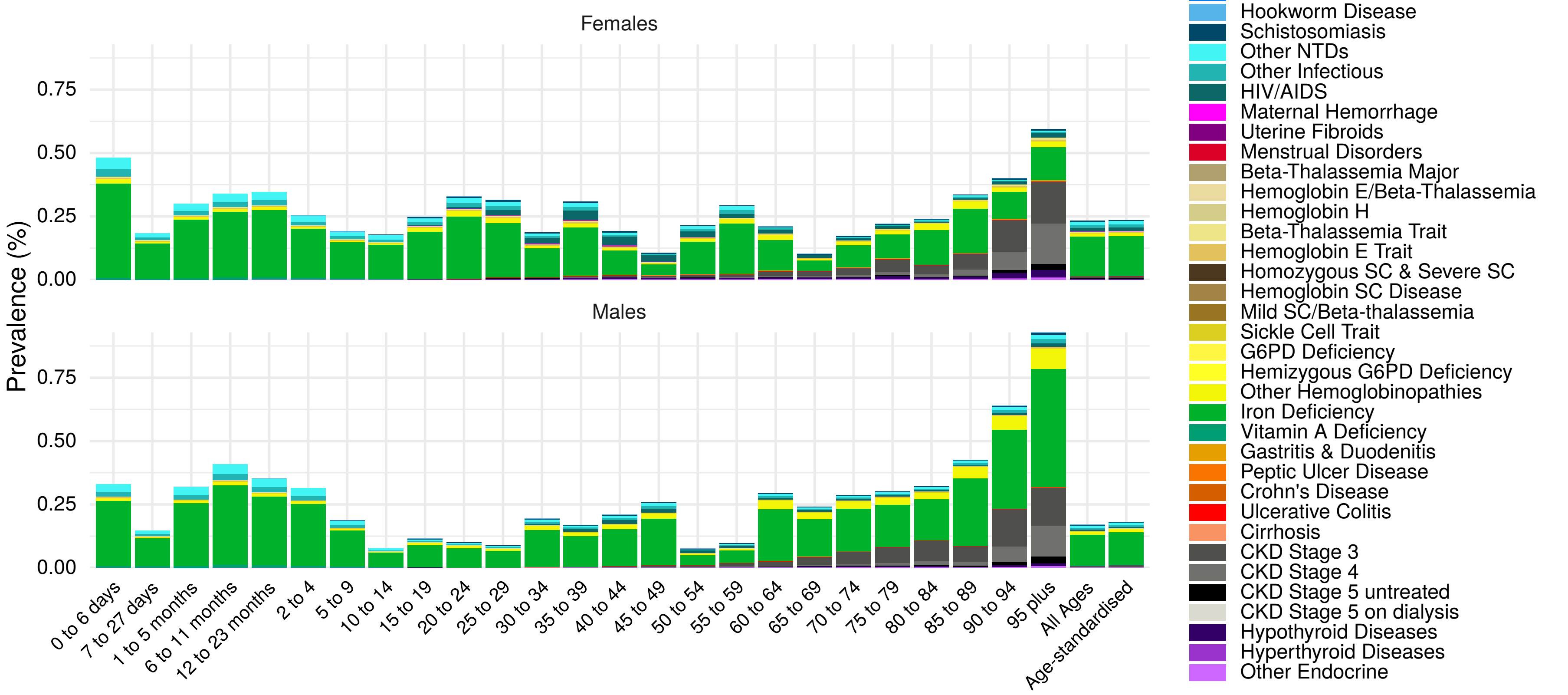

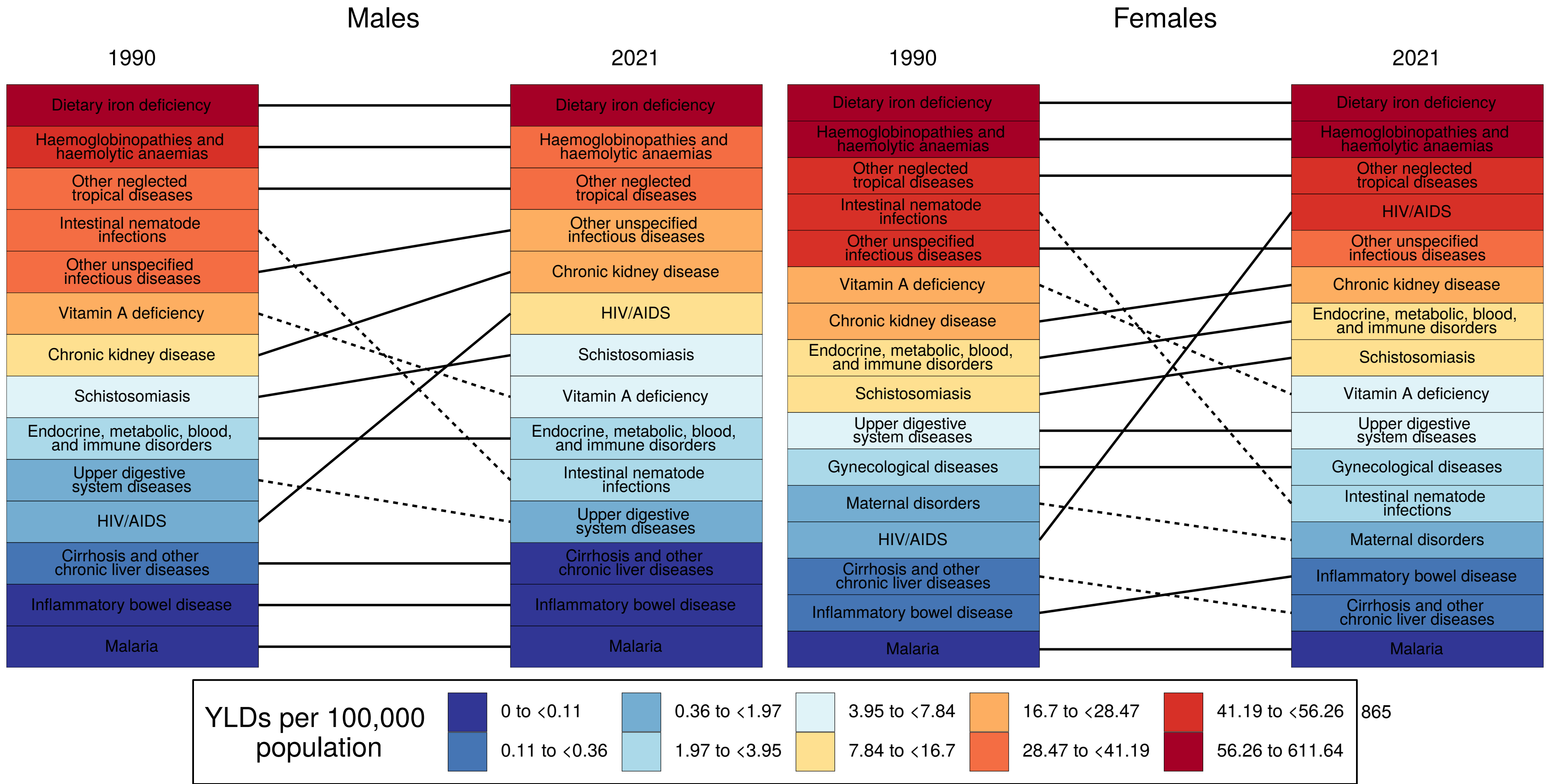

Western Cape

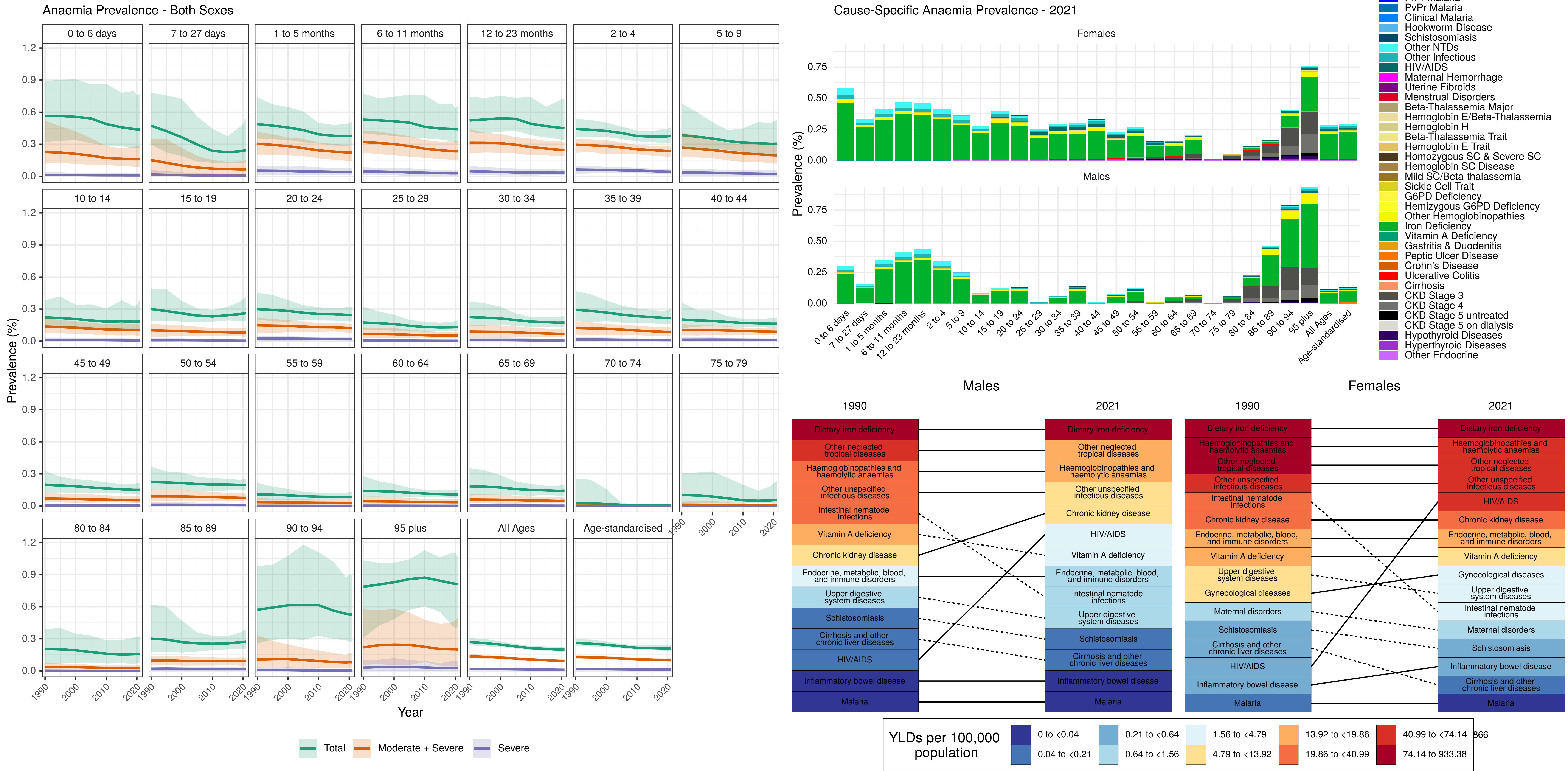

Zimbabwe

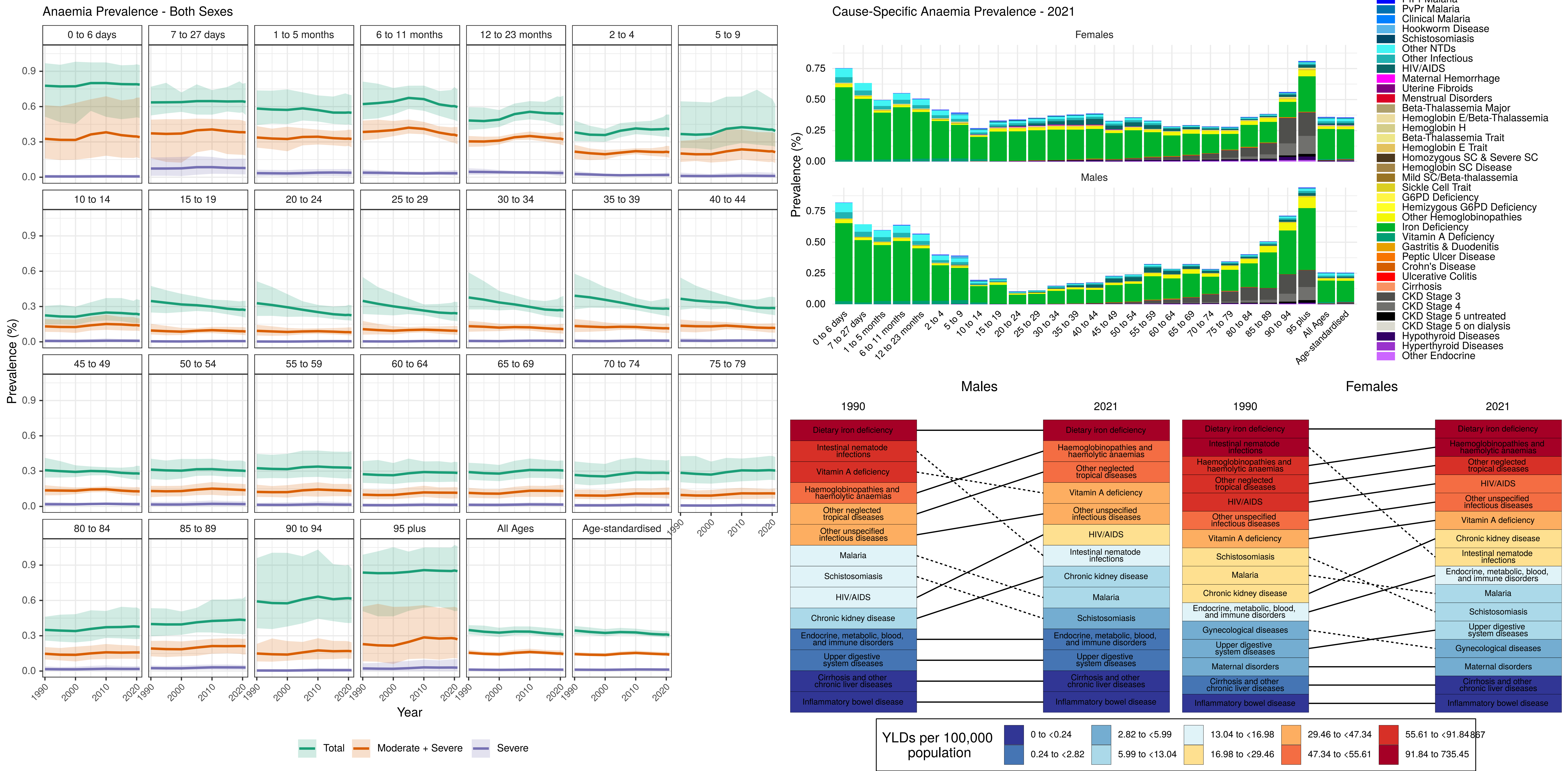
Anaemia Prevalence - Both Sexes
0 to 6 days
7 to 27 days
1 to 5 months
6 to 11 months
12 to 23 months
2 to 4
5 to 9
10 to 14
15 to 19
20 to 24
25 to 29
30 to 34
35 to 39
40 to 44
45 to 49
50 to 54
55 to 59
60 to 64
65 to 69
70 to 74
75 to 79
80 to 84
85 to 89
90 to 94
95 plus
All Ages
Age-standardised
Prevalence (%)
Year
Total
Moderate + Severe
Severe
Cause-Specific Anaemia Prevalence - 2021
Females
Males
PfPr Malaria
PvPr Malaria
Clinical Malaria
Hookworm Disease
Schistosomiasis
Other NTDs
Other Infectious
HIV/AIDS
Maternal Hemorrhage
Uterine Fibroids
Menstrual Disorders
Beta-Thalassemia Major
Hemoglobin E/Beta-Thalassemia
Hemoglobin H
Beta-Thalassemia Trait
Hemoglobin E Trait
Homozygous SC & Severe SC
Hemoglobin SC Disease
Mild SC/Beta-thalassemia
Sickle Cell Trait
G6PD Deficiency
Hemizygous G6PD Deficiency
Other Hemoglobinopathies
Iron Deficiency
Vitamin A Deficiency
Gastritis & Duodenitis
Peptic Ulcer Disease
Crohn's Disease
Ulcerative Colitis
Cirrhosis
CKD Stage 3
CKD Stage 4
CKD Stage 5 untreated
CKD Stage 5 on dialysis
Hypothyroid Diseases
Hyperthyroid Diseases
Other Endocrine
Males
Females
1990
2021
Dietary iron deficiency
Intestinal nematode infections
Vitamin A deficiency
Haemoglobinopathies and haemolytic anaemias
Other neglected tropical diseases
Other unspecified infectious diseases
Malaria
Schistosomiasis
HIV/AIDS
Chronic kidney disease
Endocrine, metabolic, blood, and immune disorders
Upper digestive system diseases
Cirrhosis and other chronic liver diseases
Inflammatory bowel disease
Gynecological diseases
Maternal disorders
YLDs per 100,000 population
0 to <0.24
0.24 to <2.82
2.82 to <5.99
5.99 to <13.04
13.04 to <16.98
16.98 to <29.46
29.46 to <47.34
47.34 to <55.61
55.61 to <91.84867
91.84 to 735.45

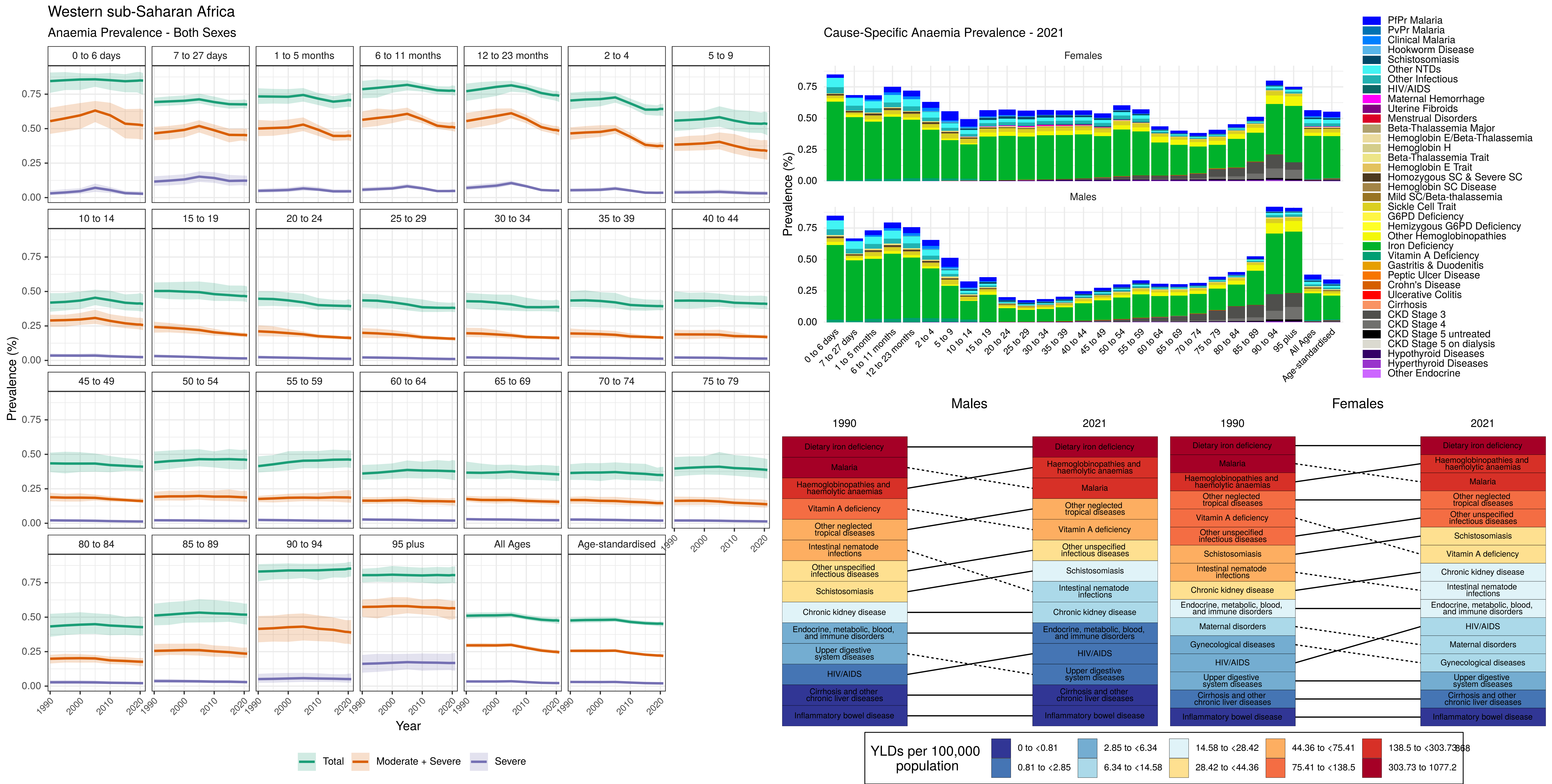

Western sub-Saharan Africa
Anaemia Prevalence - Both Sexes
0 to 6 days
7 to 27 days
1 to 5 months
6 to 11 months
12 to 23 months
2 to 4
5 to 9
10 to 14
15 to 19
20 to 24
25 to 29
30 to 34
35 to 39
40 to 44
45 to 49
50 to 54
55 to 59
60 to 64
65 to 69
70 to 74
75 to 79
80 to 84
85 to 89
90 to 94
95 plus
All Ages
Age-standardised
Prevalence (%)
Year
Total
Moderate + Severe
Severe
Cause-Specific Anaemia Prevalence - 2021
Females
Males
PfPr Malaria
PvPr Malaria
Clinical Malaria
Hookworm Disease
Schistosomiasis
Other NTDs
Other Infectious
HIV/AIDS
Maternal Hemorrhage
Uterine Fibroids
Menstrual Disorders
Beta-Thalassemia Major
Hemoglobin E/Beta-Thalassemia
Hemoglobin H
Beta-Thalassemia Trait
Hemoglobin E Trait
Homozygous SC & Severe SC
Hemoglobin SC Disease
Mild SC/Beta-thalassemia
Sickle Cell Trait
G6PD Deficiency
Hemizygous G6PD Deficiency
Other Hemoglobinopathies
Iron Deficiency
Vitamin A Deficiency
Gastritis & Duodenitis
Peptic Ulcer Disease
Crohn's Disease
Ulcerative Colitis
Cirrhosis
CKD Stage 3
CKD Stage 4
CKD Stage 5 untreated
CKD Stage 5 on dialysis
Hypothyroid Diseases
Hyperthyroid Diseases
Other Endocrine
Males
Females
1990
2021
Dietary iron deficiency
Malaria
Haemoglobinopathies and haemolytic anaemias
Vitamin A deficiency
Other neglected tropical diseases
Intestinal nematode infections
Other unspecified infectious diseases
Schistosomiasis
Chronic kidney disease
Endocrine, metabolic, blood, and immune disorders
Upper digestive system diseases
HIV/AIDS
Cirrhosis and other chronic liver diseases
Inflammatory bowel disease
Maternal disorders
Gynecological diseases
YLDs per 100,000 population
0 to <0.81
0.81 to <2.85
2.85 to <6.34
6.34 to <14.58
14.58 to <28.42
28.42 to <44.36
44.36 to <75.41
75.41 to <138.5
138.5 to <303.73
303.73 to 1077.2

Benin

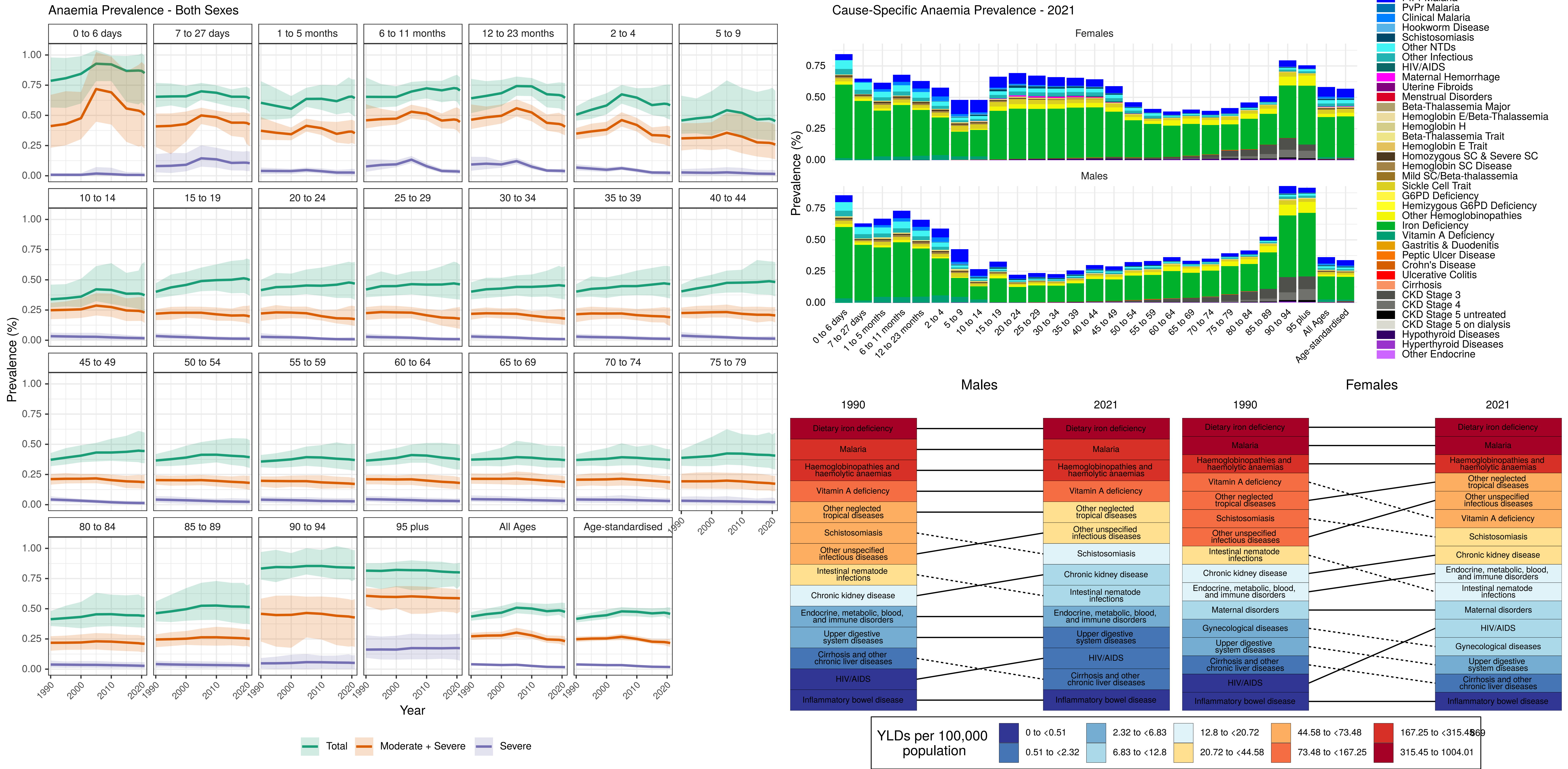

Anaemia Prevalence - Both Sexes
0 to 6 days
7 to 27 days
1 to 5 months
6 to 11 months
12 to 23 months
2 to 4
5 to 9
10 to 14
15 to 19
20 to 24
25 to 29
30 to 34
35 to 39
40 to 44
45 to 49
50 to 54
55 to 59
60 to 64
65 to 69
70 to 74
75 to 79
80 to 84
85 to 89
90 to 94
95 plus
All Ages
Age-standardised
Prevalence (%)
Year
Total
Moderate + Severe
Severe
Cause-Specific Anaemia Prevalence - 2021
Females
Males
PfPr Malaria
PvPr Malaria
Clinical Malaria
Hookworm Disease
Schistosomiasis
Other NTDs
Other Infectious
HIV/AIDS
Maternal Hemorrhage
Uterine Fibroids
Menstrual Disorders
Beta-Thalassemia Major
Hemoglobin E/Beta-Thalassemia
Hemoglobin H
Beta-Thalassemia Trait
Hemoglobin E Trait
Homozygous SC & Severe SC
Hemoglobin SC Disease
Mild SC/Beta-thalassemia
Sickle Cell Trait
G6PD Deficiency
Hemizygous G6PD Deficiency
Other Hemoglobinopathies
Iron Deficiency
Vitamin A Deficiency
Gastritis & Duodenitis
Peptic Ulcer Disease
Crohn's Disease
Ulcerative Colitis
Cirrhosis
CKD Stage 3
CKD Stage 4
CKD Stage 5 untreated
CKD Stage 5 on dialysis
Hypothyroid Diseases
Hyperthyroid Diseases
Other Endocrine
Males
Females
1990
2021
Dietary iron deficiency
Malaria
Haemoglobinopathies and haemolytic anaemias
Vitamin A deficiency
Other neglected tropical diseases
Schistosomiasis
Other unspecified infectious diseases
Intestinal nematode infections
Chronic kidney disease
Endocrine, metabolic, blood, and immune disorders
Upper digestive system diseases
Cirrhosis and other chronic liver diseases
HIV/AIDS
Inflammatory bowel disease
Maternal disorders
Gynecological diseases
YLDs per 100,000 population
0 to <0.51
0.51 to <2.32
2.32 to <6.83
6.83 to <12.8
12.8 to <20.72
20.72 to <44.58
44.58 to <73.48
73.48 to <167.25
167.25 to <315.45
315.45 to 1004.01

Burkina Faso

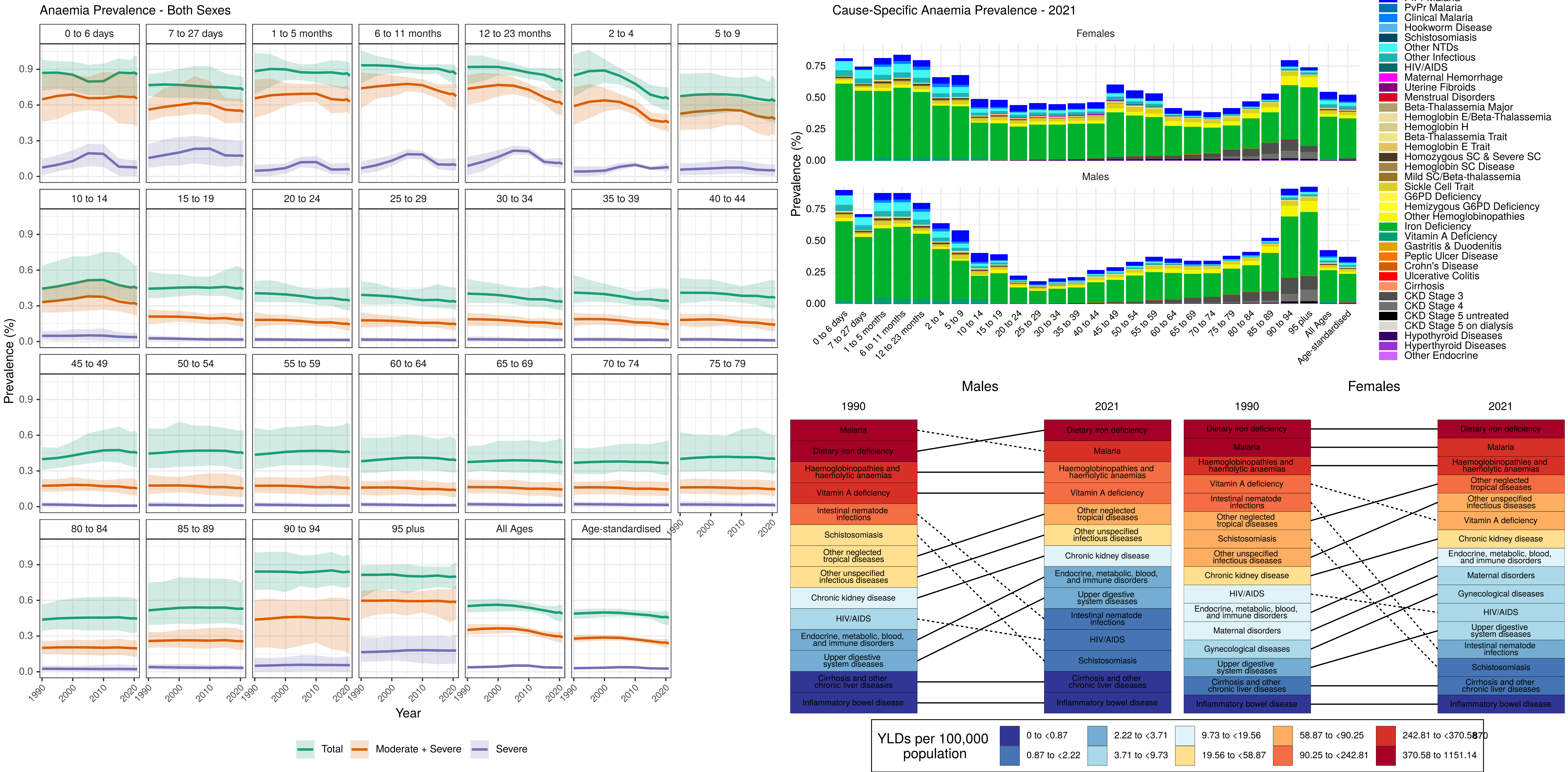

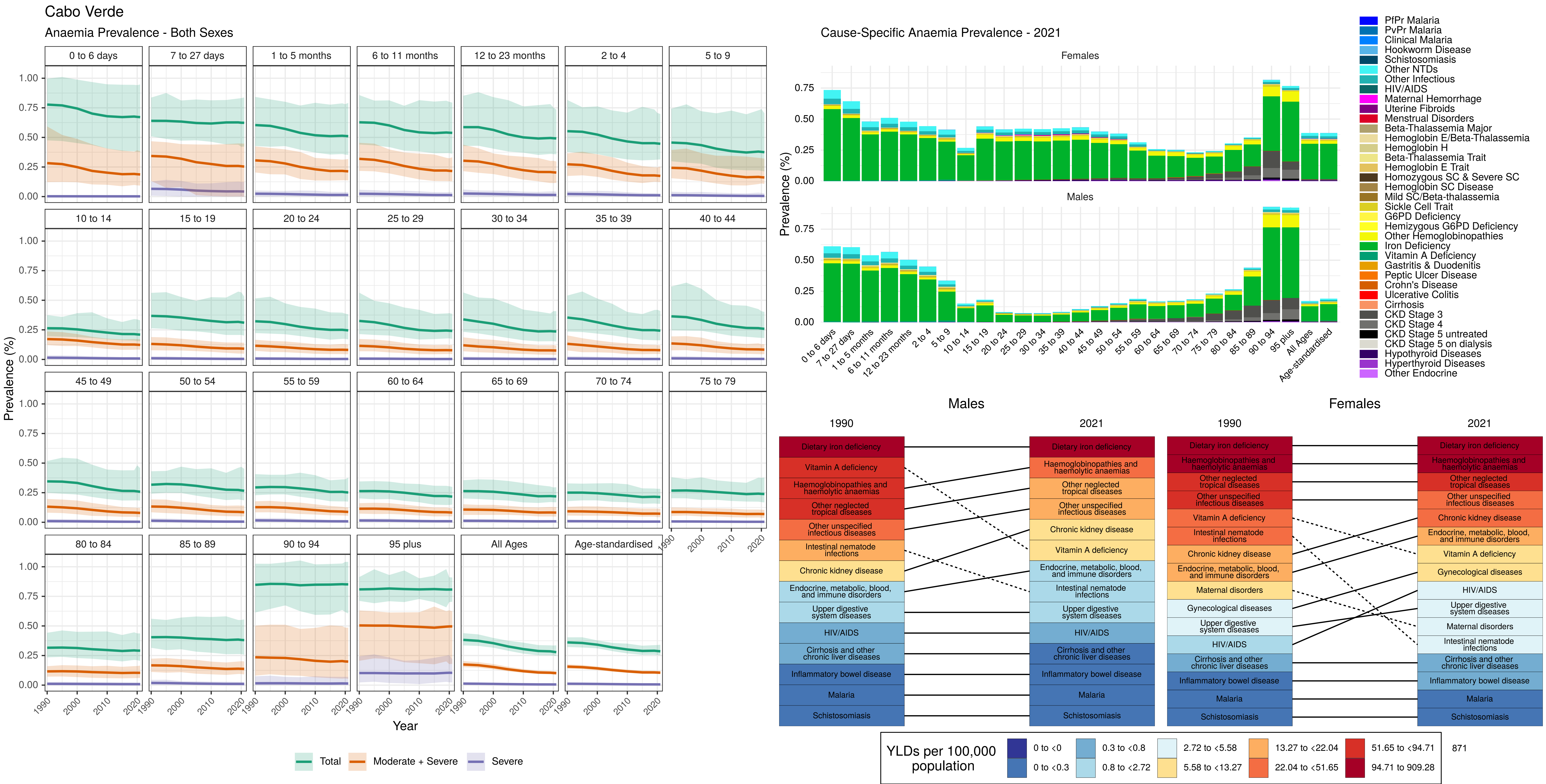
Cabo Verde
Anaemia Prevalence - Both Sexes
0 to 6 days
7 to 27 days
1 to 5 months
6 to 11 months
12 to 23 months
2 to 4
5 to 9
10 to 14
15 to 19
20 to 24
25 to 29
30 to 34
35 to 39
40 to 44
45 to 49
50 to 54
55 to 59
60 to 64
65 to 69
70 to 74
75 to 79
80 to 84
85 to 89
90 to 94
95 plus
All Ages
Age-standardised
Prevalence (%)
Year
Total
Moderate + Severe
Severe
Cause-Specific Anaemia Prevalence - 2021
Females
Males
PfPr Malaria
PvPr Malaria
Clinical Malaria
Hookworm Disease
Schistosomiasis
Other NTDs
Other Infectious
HIV/AIDS
Maternal Hemorrhage
Uterine Fibroids
Menstrual Disorders
Beta-Thalassemia Major
Hemoglobin E/Beta-Thalassemia
Hemoglobin H
Beta-Thalassemia Trait
Hemoglobin E Trait
Homozygous SC & Severe SC
Hemoglobin SC Disease
Mild SC/Beta-thalassemia
Sickle Cell Trait
G6PD Deficiency
Hemizygous G6PD Deficiency
Other Hemoglobinopathies
Iron Deficiency
Vitamin A Deficiency
Gastritis & Duodenitis
Peptic Ulcer Disease
Crohn's Disease
Ulcerative Colitis
Cirrhosis
CKD Stage 3
CKD Stage 4
CKD Stage 5 untreated
CKD Stage 5 on dialysis
Hypothyroid Diseases
Hyperthyroid Diseases
Other Endocrine
Males
Females
1990
2021
Dietary iron deficiency
Vitamin A deficiency
Haemoglobinopathies and haemolytic anaemias
Other neglected tropical diseases
Other unspecified infectious diseases
Intestinal nematode infections
Chronic kidney disease
Endocrine, metabolic, blood, and immune disorders
Upper digestive system diseases
HIV/AIDS
Cirrhosis and other chronic liver diseases
Inflammatory bowel disease
Malaria
Schistosomiasis
Maternal disorders
Gynecological diseases
YLDs per 100,000 population
0 to <0
0 to <0.3
0.3 to <0.8
0.8 to <2.72
2.72 to <5.58
5.58 to <13.27
13.27 to <22.04
22.04 to <51.65
51.65 to <94.71
94.71 to 909.28

871

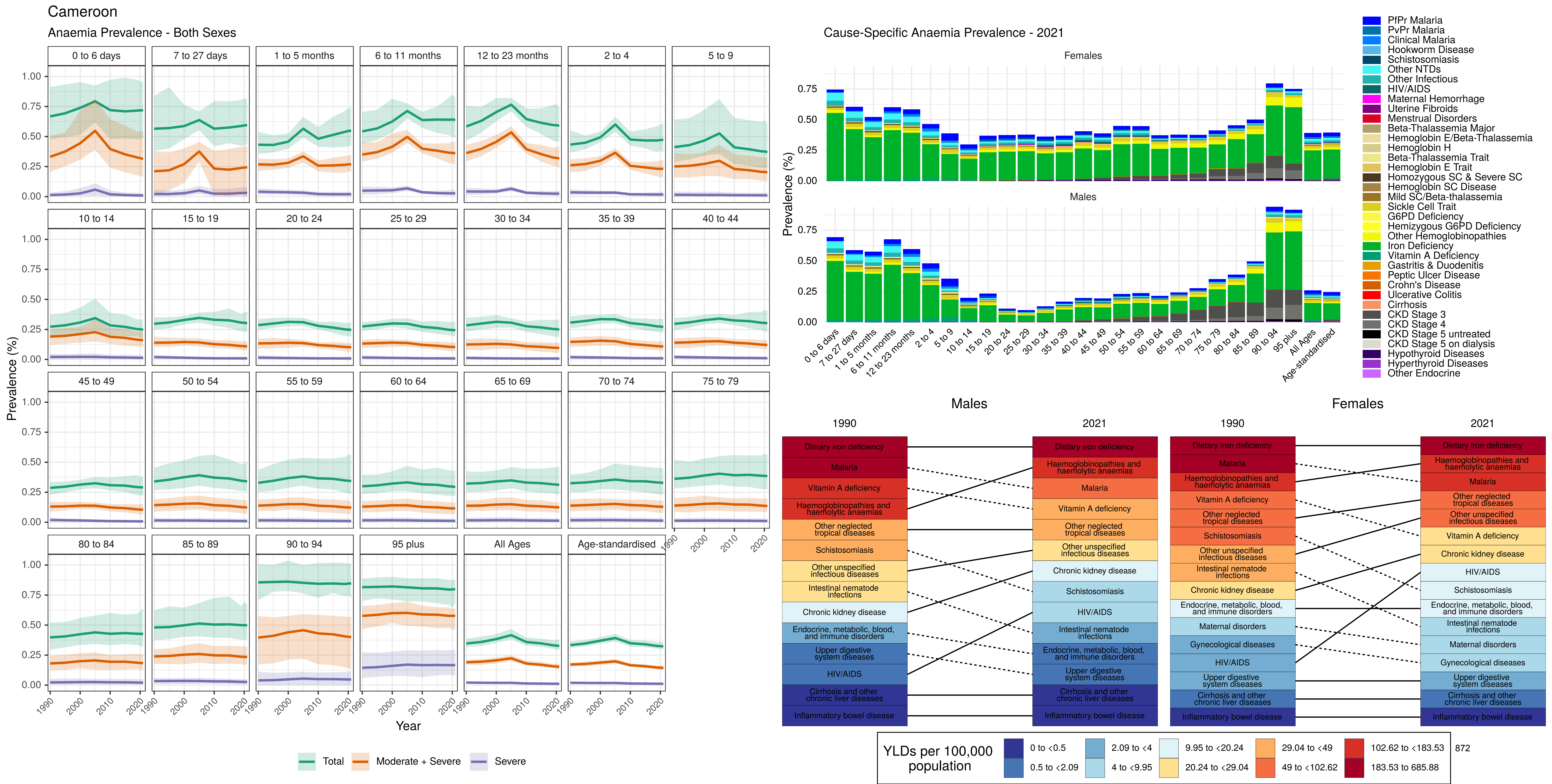
Cameroon
Anaemia Prevalence - Both Sexes
0 to 6 days
7 to 27 days
1 to 5 months
6 to 11 months
12 to 23 months
2 to 4
5 to 9
10 to 14
15 to 19
20 to 24
25 to 29
30 to 34
35 to 39
40 to 44
45 to 49
50 to 54
55 to 59
60 to 64
65 to 69
70 to 74
75 to 79
80 to 84
85 to 89
90 to 94
95 plus
All Ages
Age-standardised
Prevalence (%)
Year
Total
Moderate + Severe
Severe
Cause-Specific Anaemia Prevalence - 2021
Females
Males
PfPr Malaria
PvPr Malaria
Clinical Malaria
Hookworm Disease
Schistosomiasis
Other NTDs
Other Infectious
HIV/AIDS
Maternal Hemorrhage
Uterine Fibroids
Menstrual Disorders
Beta-Thalassemia Major
Hemoglobin E/Beta-Thalassemia
Hemoglobin H
Beta-Thalassemia Trait
Hemoglobin E Trait
Homozygous SC & Severe SC
Hemoglobin SC Disease
Mild SC/Beta-thalassemia
Sickle Cell Trait
G6PD Deficiency
Hemizygous G6PD Deficiency
Other Hemoglobinopathies
Iron Deficiency
Vitamin A Deficiency
Gastritis & Duodenitis
Peptic Ulcer Disease
Crohn's Disease
Ulcerative Colitis
Cirrhosis
CKD Stage 3
CKD Stage 4
CKD Stage 5 untreated
CKD Stage 5 on dialysis
Hypothyroid Diseases
Hyperthyroid Diseases
Other Endocrine
Males
Females
1990
2021
Dietary iron deficiency
Malaria
Vitamin A deficiency
Haemoglobinopathies and haemolytic anaemias
Other neglected tropical diseases
Schistosomiasis
Other unspecified infectious diseases
Intestinal nematode infections
Chronic kidney disease
Endocrine, metabolic, blood, and immune disorders
Upper digestive system diseases
HIV/AIDS
Cirrhosis and other chronic liver diseases
Inflammatory bowel disease
Maternal disorders
Gynecological diseases
YLDs per 100,000 population
0 to <0.5
0.5 to <2.09
2.09 to <4
4 to <9.95
9.95 to <20.24
20.24 to <29.04
29.04 to <49
49 to <102.62
102.62 to <183.53
183.53 to 685.88

Chad

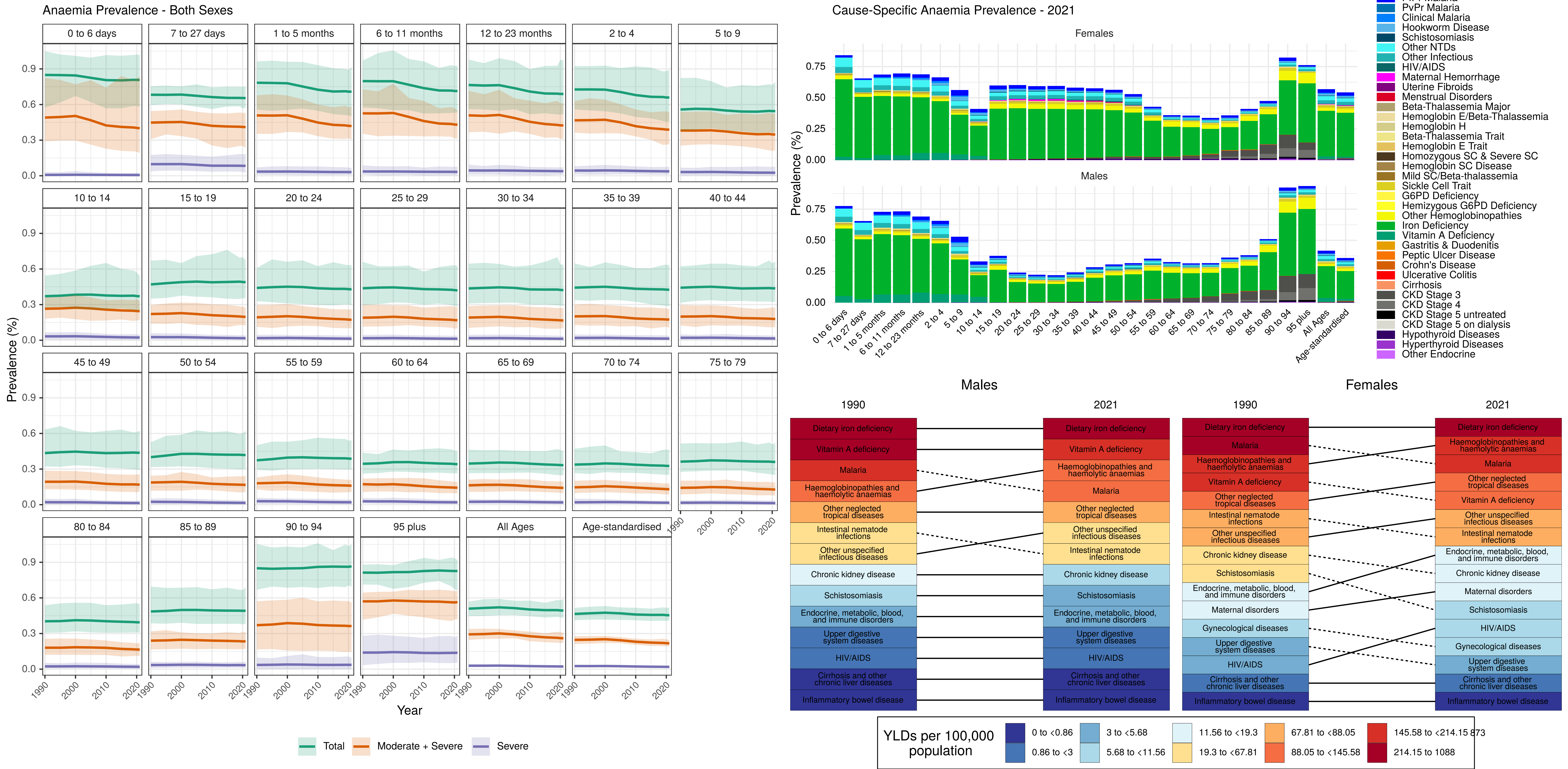

Anaemia Prevalence - Both Sexes
0 to 6 days
7 to 27 days
1 to 5 months
6 to 11 months
12 to 23 months
2 to 4
5 to 9
10 to 14
15 to 19
20 to 24
25 to 29
30 to 34
35 to 39
40 to 44
45 to 49
50 to 54
55 to 59
60 to 64
65 to 69
70 to 74
75 to 79
80 to 84
85 to 89
90 to 94
95 plus
All Ages
Age-standardised
Prevalence (%)
Year
Total
Moderate + Severe
Severe
Cause-Specific Anaemia Prevalence - 2021
Females
Males
PfPr Malaria
PvPr Malaria
Clinical Malaria
Hookworm Disease
Schistosomiasis
Other NTDs
Other Infectious
HIV/AIDS
Maternal Hemorrhage
Uterine Fibroids
Menstrual Disorders
Beta-Thalassemia Major
Hemoglobin E/Beta-Thalassemia
Hemoglobin H
Beta-Thalassemia Trait
Hemoglobin E Trait
Homozygous SC & Severe SC
Hemoglobin SC Disease
Mild SC/Beta-thalassemia
Sickle Cell Trait
G6PD Deficiency
Hemizygous G6PD Deficiency
Other Hemoglobinopathies
Iron Deficiency
Vitamin A Deficiency
Gastritis & Duodenitis
Peptic Ulcer Disease
Crohn's Disease
Ulcerative Colitis
Cirrhosis
CKD Stage 3
CKD Stage 4
CKD Stage 5 untreated
CKD Stage 5 on dialysis
Hypothyroid Diseases
Hyperthyroid Diseases
Other Endocrine
Males
Females
1990
2021
Dietary iron deficiency
Vitamin A deficiency
Malaria
Haemoglobinopathies and haemolytic anaemias
Other neglected tropical diseases
Intestinal nematode infections
Other unspecified infectious diseases
Chronic kidney disease
Schistosomiasis
Endocrine, metabolic, blood, and immune disorders
Upper digestive system diseases
HIV/AIDS
Cirrhosis and other chronic liver diseases
Inflammatory bowel disease
Maternal disorders
Gynecological diseases
YLDs per 100,000 population
0 to <0.86
0.86 to <3
3 to <5.68
5.68 to <11.56
11.56 to <19.3
19.3 to <67.81
67.81 to <88.05
88.05 to <145.58
145.58 to <214.15
214.15 to 1088

Côte d'Ivoire

Anaemia Prevalence - Both Sexes

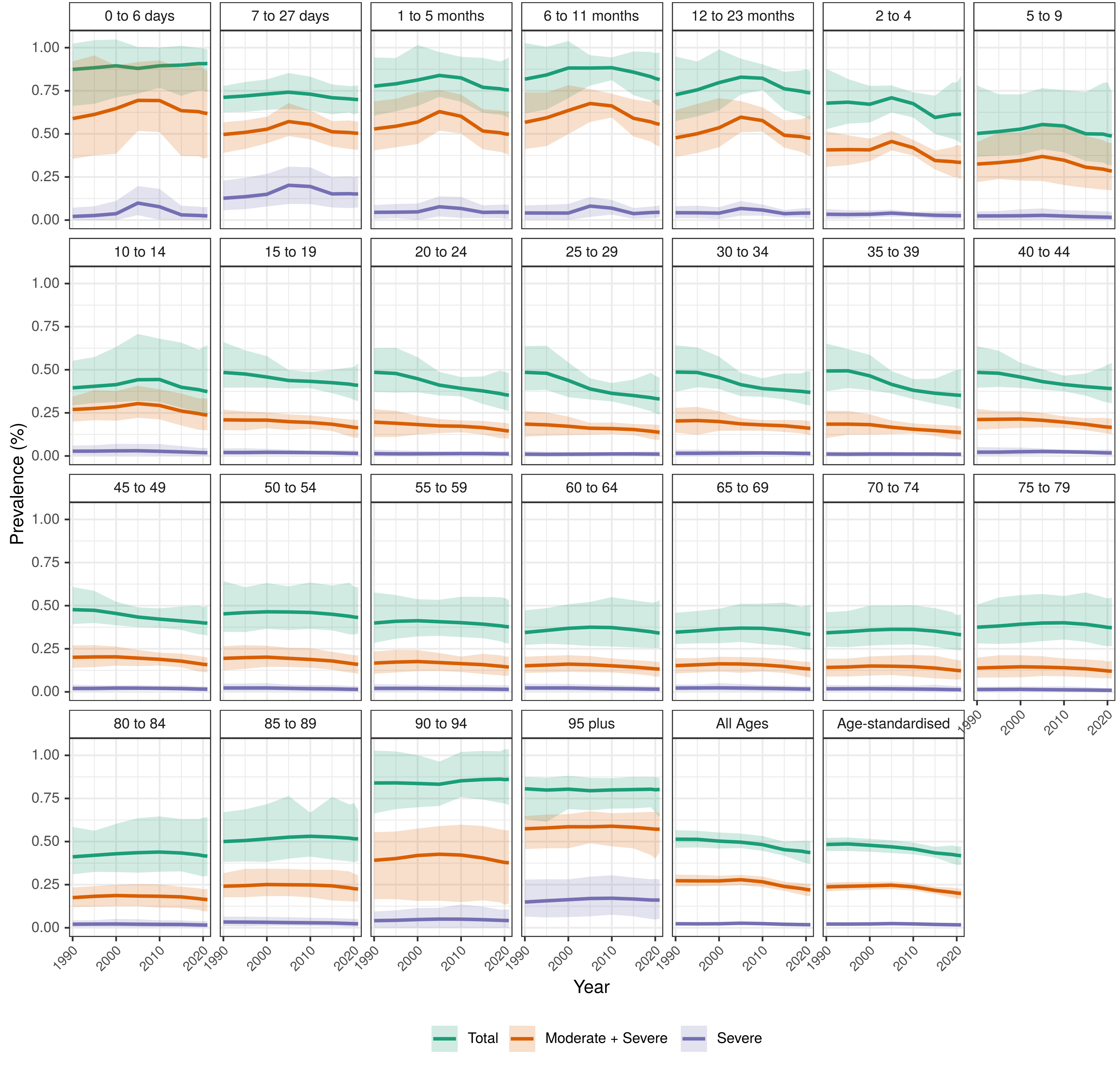

Cause-Specific Anaemia Prevalence - 2021

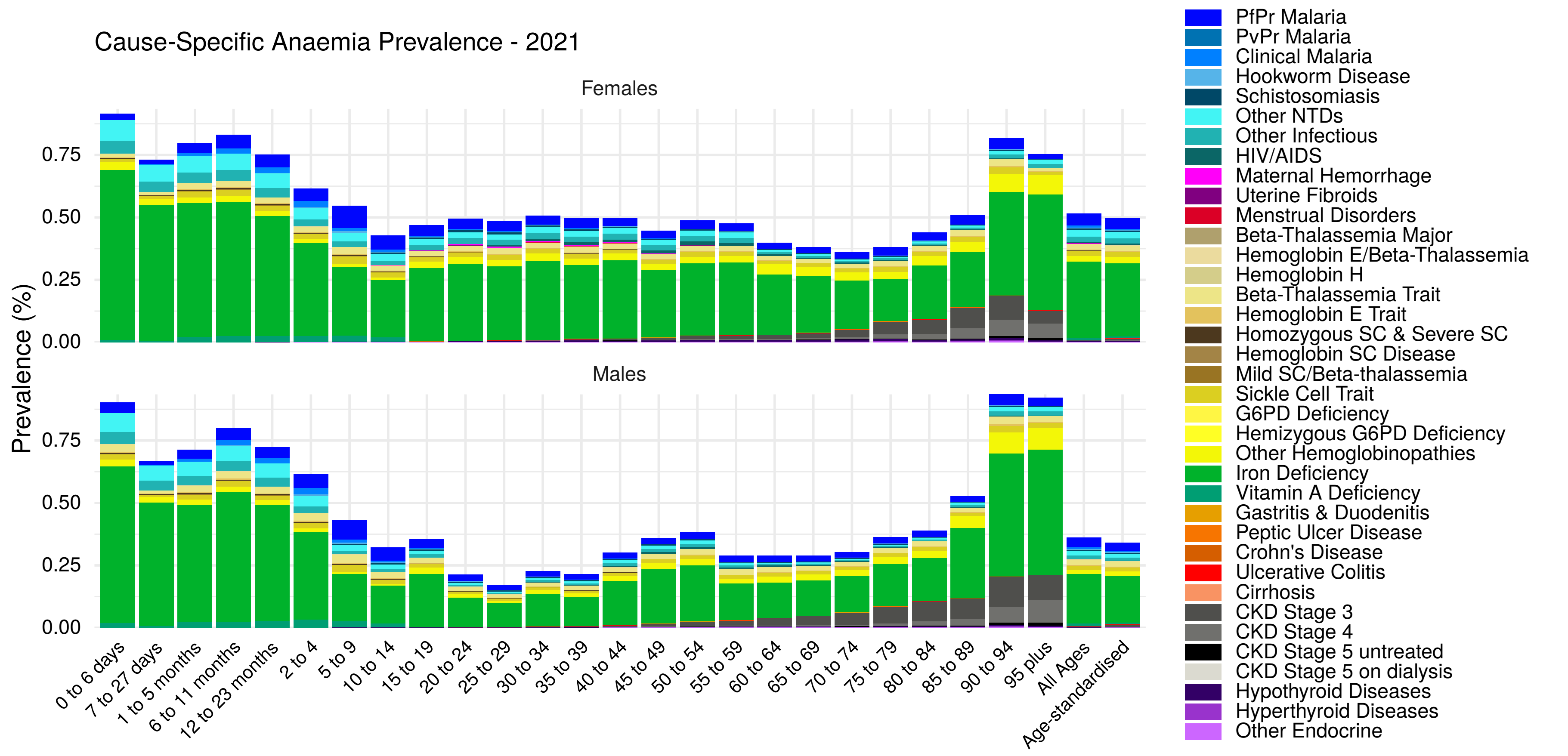

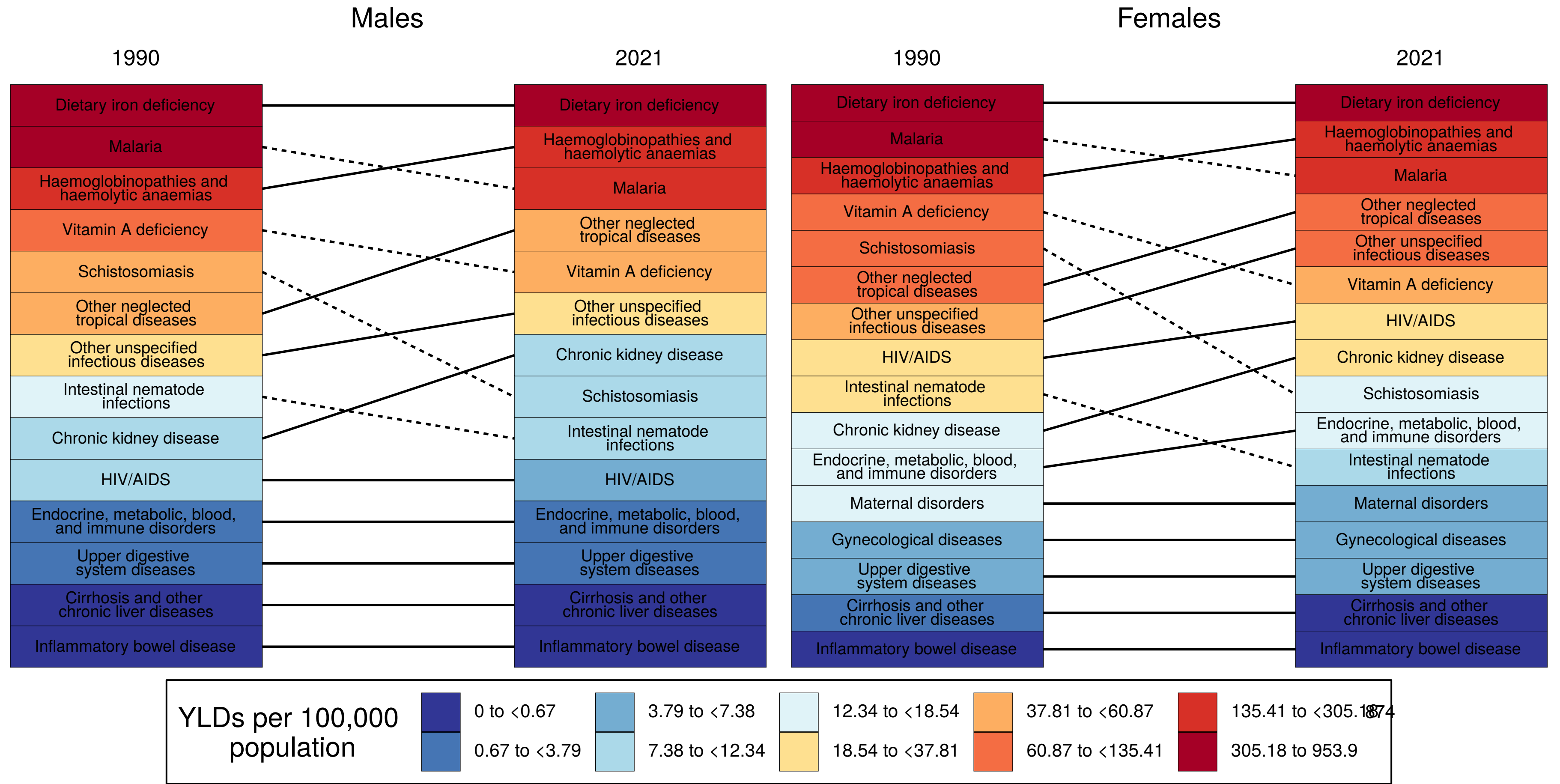

The Gambia

Anaemia Prevalence - Both Sexes

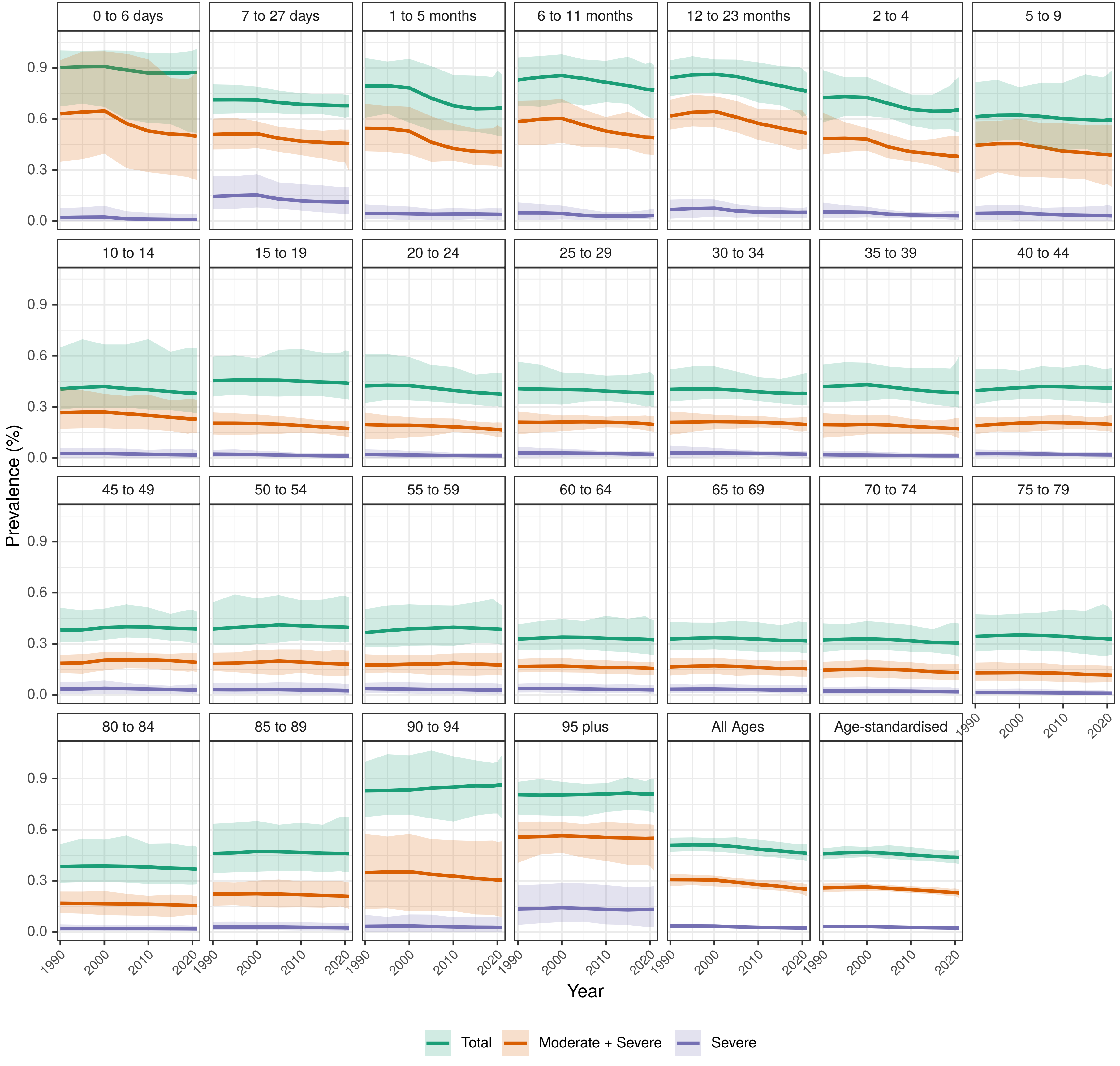

Cause-Specific Anaemia Prevalence - 2021

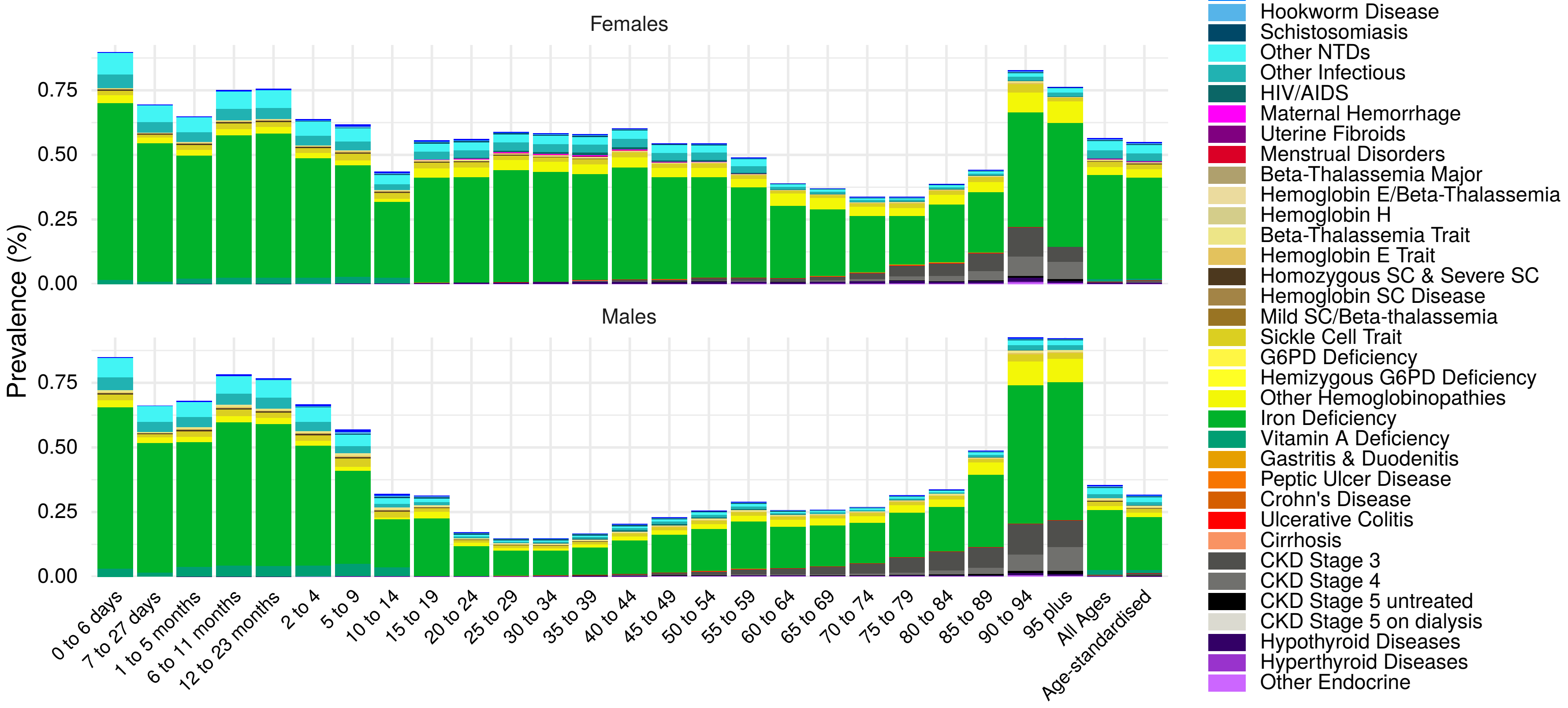

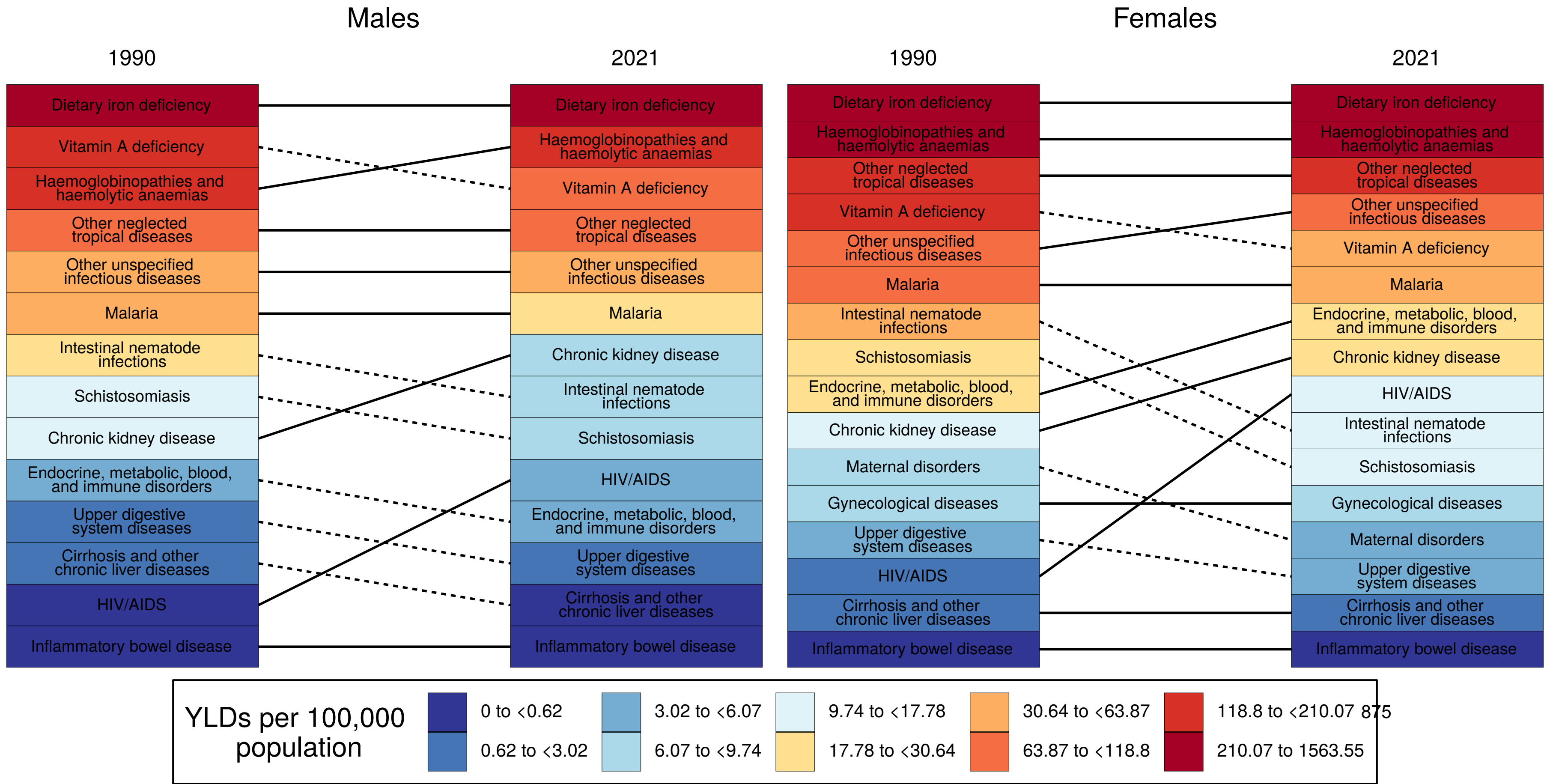

Ghana

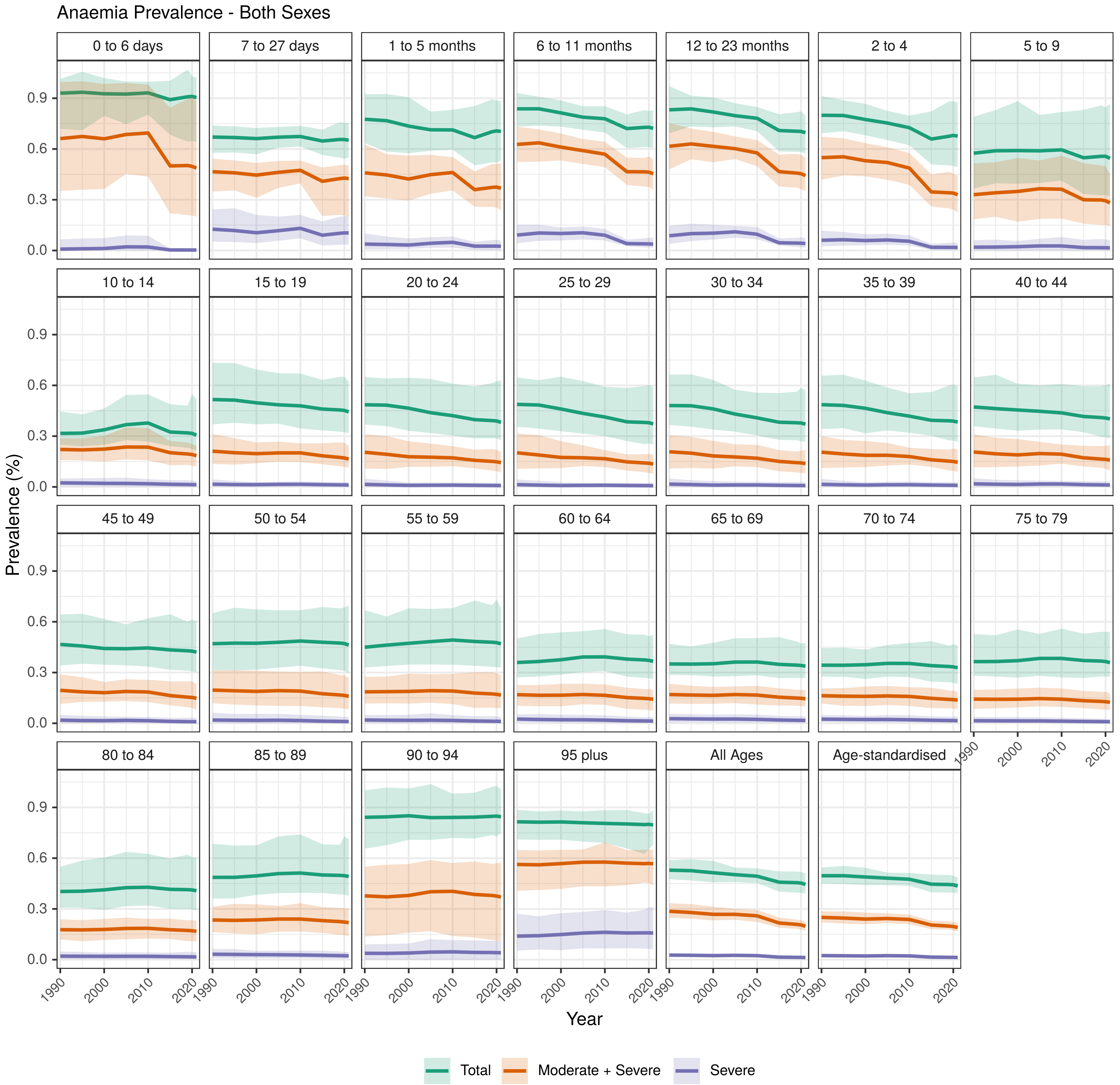

Anaemia Prevalence - Both Sexes
0 to 6 days
7 to 27 days
1 to 5 months
6 to 11 months
12 to 23 months
2 to 4
5 to 9
10 to 14
15 to 19
20 to 24
25 to 29
30 to 34
35 to 39
40 to 44
45 to 49
50 to 54
55 to 59
60 to 64
65 to 69
70 to 74
75 to 79
80 to 84
85 to 89
90 to 94
95 plus
All Ages
Age-standardised
Prevalence (%)
Year
Total
Moderate + Severe
Severe

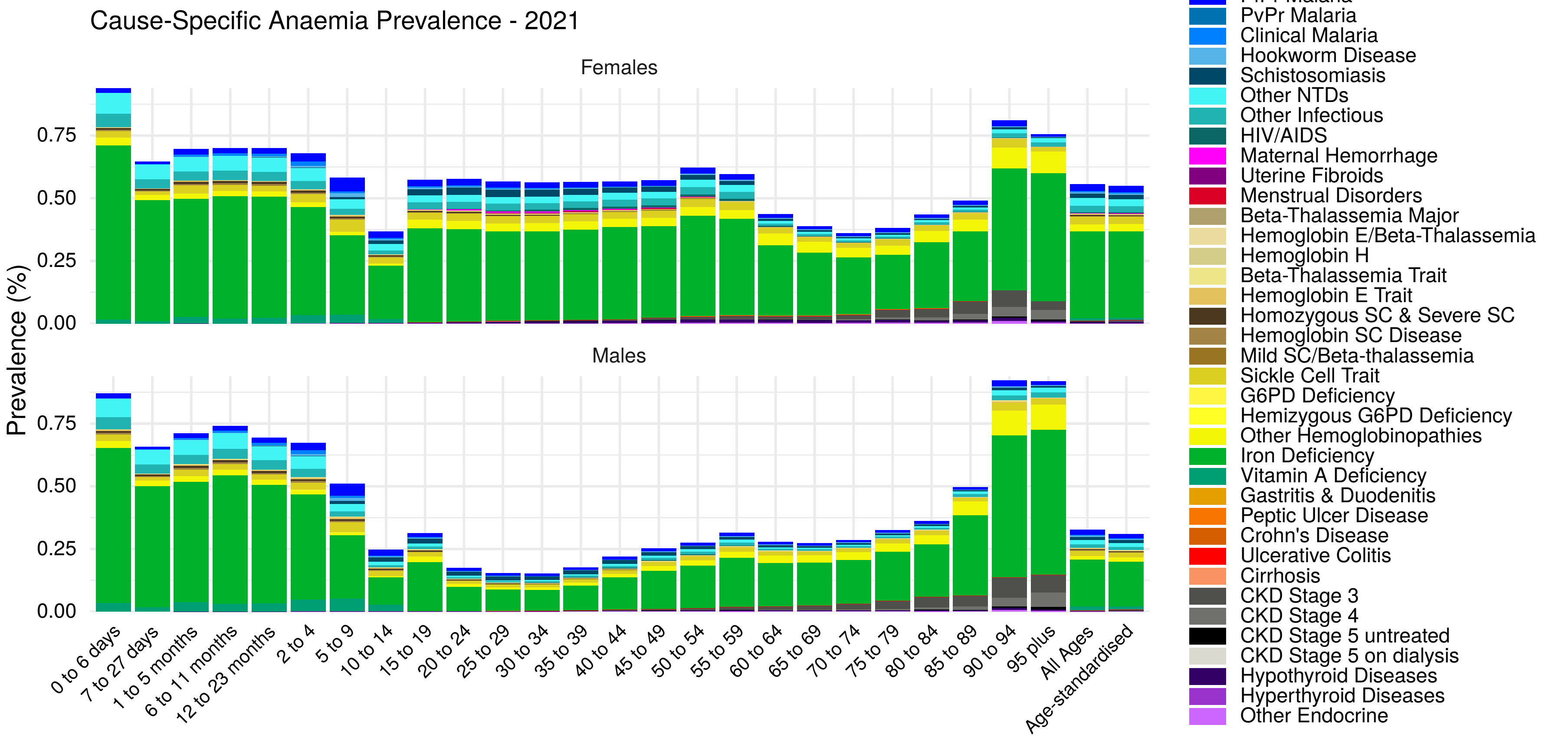

Cause-Specific Anaemia Prevalence - 2021
Females
Males
Prevalence (%)
PfPr Malaria
PvPr Malaria
Clinical Malaria
Hookworm Disease
Schistosomiasis
Other NTDs
Other Infectious
HIV/AIDS
Maternal Hemorrhage
Uterine Fibroids
Menstrual Disorders
Beta-Thalassemia Major
Hemoglobin E/Beta-Thalassemia
Hemoglobin H
Beta-Thalassemia Trait
Hemoglobin E Trait
Homozygous SC & Severe SC
Hemoglobin SC Disease
Mild SC/Beta-thalassemia
Sickle Cell Trait
G6PD Deficiency
Hemizygous G6PD Deficiency
Other Hemoglobinopathies
Iron Deficiency
Vitamin A Deficiency
Gastritis & Duodenitis
Peptic Ulcer Disease
Crohn's Disease
Ulcerative Colitis
Cirrhosis
CKD Stage 3
CKD Stage 4
CKD Stage 5 untreated
CKD Stage 5 on dialysis
Hypothyroid Diseases
Hyperthyroid Diseases
Other Endocrine

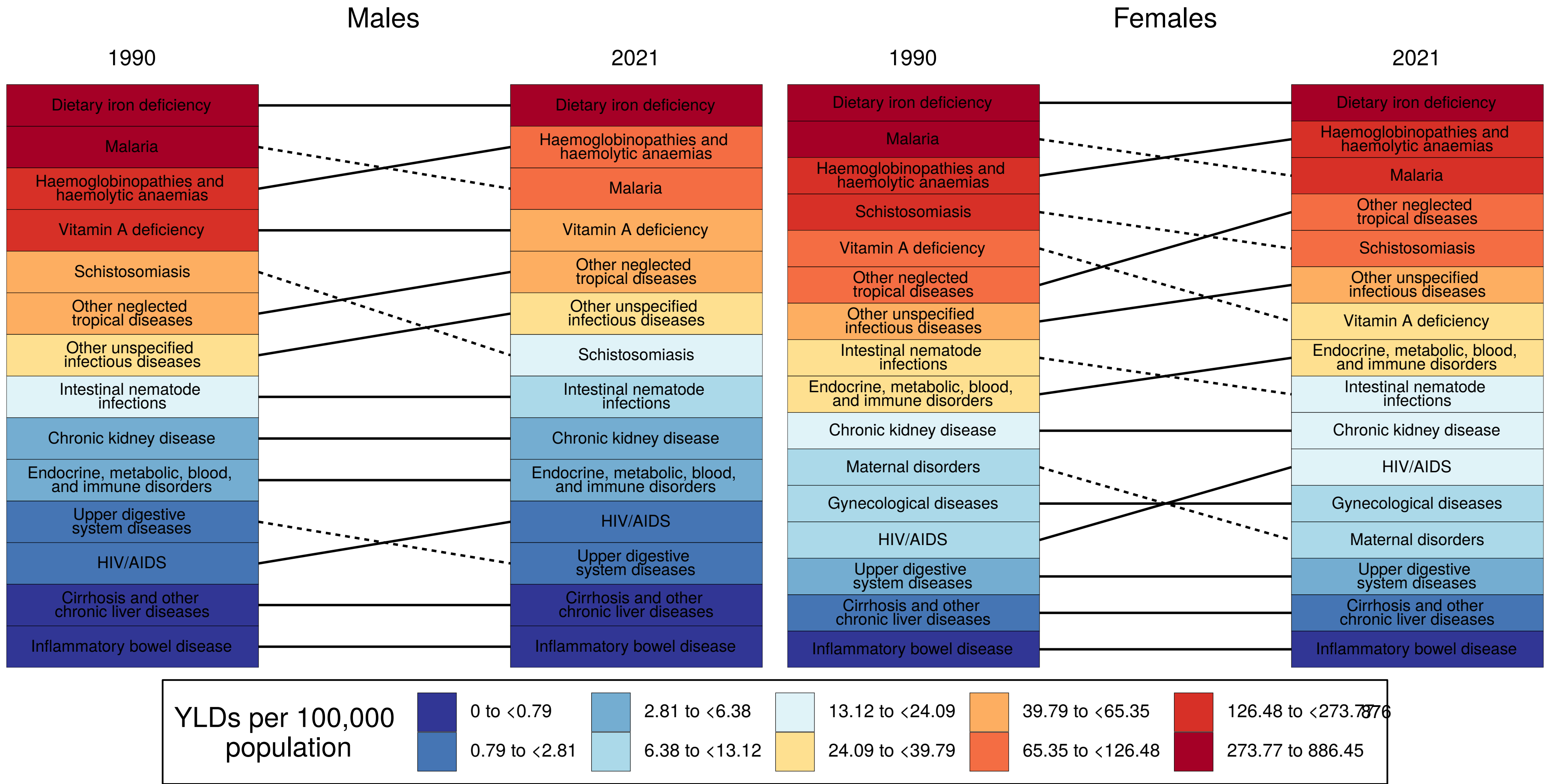

Males
Females
1990
2021
Dietary iron deficiency
Malaria
Haemoglobinopathies and haemolytic anaemias
Vitamin A deficiency
Schistosomiasis
Other neglected tropical diseases
Other unspecified infectious diseases
Intestinal nematode infections
Chronic kidney disease
Endocrine, metabolic, blood, and immune disorders
Upper digestive system diseases
HIV/AIDS
Cirrhosis and other chronic liver diseases
Inflammatory bowel disease
Maternal disorders
Gynecological diseases
YLDs per 100,000 population
0 to <0.79
0.79 to <2.81
2.81 to <6.38
6.38 to <13.12
13.12 to <24.09
24.09 to <39.79
39.79 to <65.35
65.35 to <126.48
126.48 to <273.77
273.77 to 886.45

Guinea

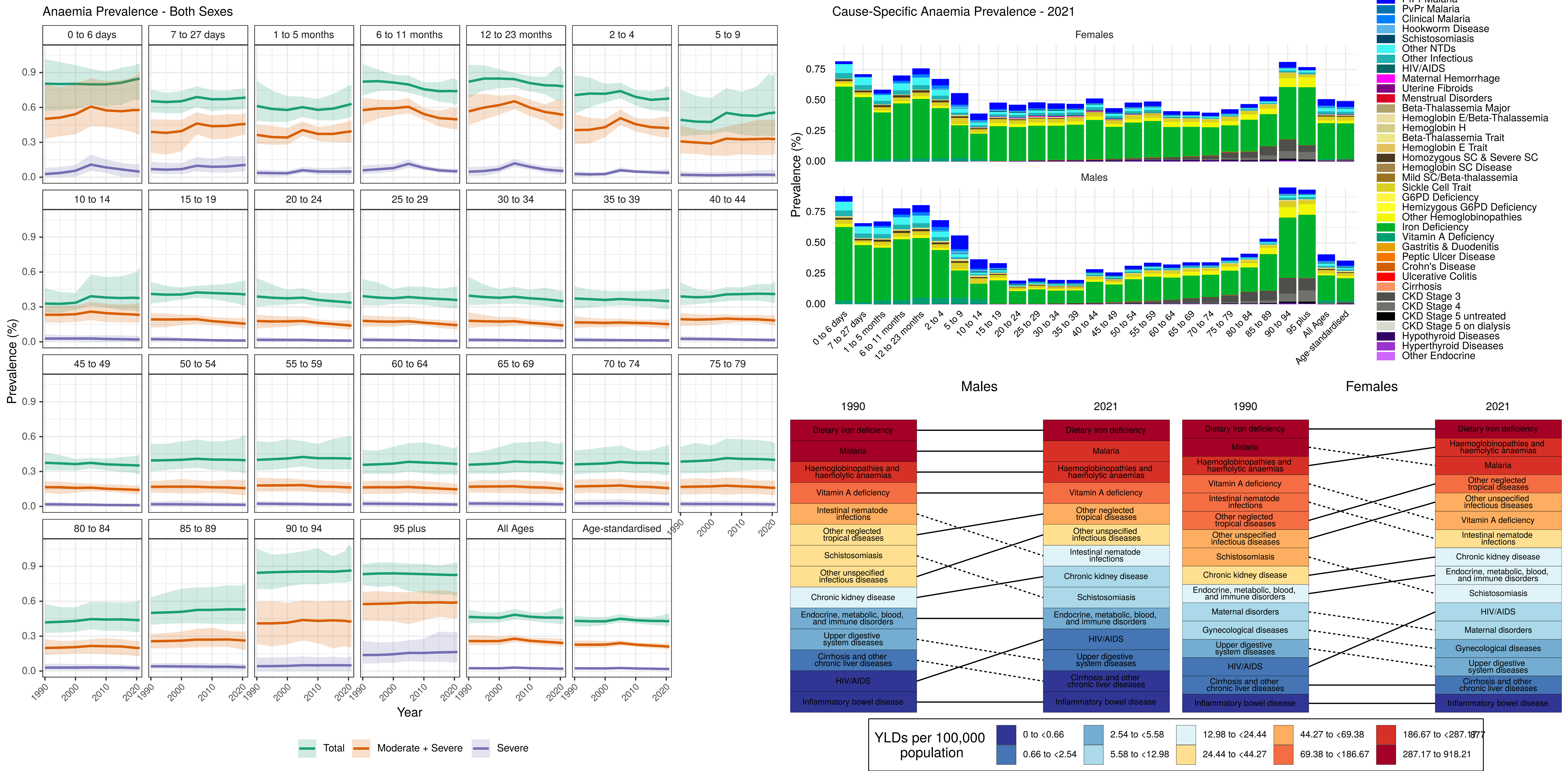
Anaemia Prevalence - Both Sexes
0 to 6 days
7 to 27 days
1 to 5 months
6 to 11 months
12 to 23 months
2 to 4
5 to 9
10 to 14
15 to 19
20 to 24
25 to 29
30 to 34
35 to 39
40 to 44
45 to 49
50 to 54
55 to 59
60 to 64
65 to 69
70 to 74
75 to 79
80 to 84
85 to 89
90 to 94
95 plus
All Ages
Age-standardised
Prevalence (%)
Year
Total
Moderate + Severe
Severe
Cause-Specific Anaemia Prevalence - 2021
Females
Males
PfPr Malaria
PvPr Malaria
Clinical Malaria
Hookworm Disease
Schistosomiasis
Other NTDs
Other Infectious
HIV/AIDS
Maternal Hemorrhage
Uterine Fibroids
Menstrual Disorders
Beta-Thalassemia Major
Hemoglobin E/Beta-Thalassemia
Hemoglobin H
Beta-Thalassemia Trait
Hemoglobin E Trait
Homozygous SC & Severe SC
Hemoglobin SC Disease
Mild SC/Beta-thalassemia
Sickle Cell Trait
G6PD Deficiency
Hemizygous G6PD Deficiency
Other Hemoglobinopathies
Iron Deficiency
Vitamin A Deficiency
Gastritis & Duodenitis
Peptic Ulcer Disease
Crohn's Disease
Ulcerative Colitis
Cirrhosis
CKD Stage 3
CKD Stage 4
CKD Stage 5 untreated
CKD Stage 5 on dialysis
Hypothyroid Diseases
Hyperthyroid Diseases
Other Endocrine
Males
Females
1990
2021
Dietary iron deficiency
Malaria
Haemoglobinopathies and haemolytic anaemias
Vitamin A deficiency
Intestinal nematode infections
Other neglected tropical diseases
Schistosomiasis
Other unspecified infectious diseases
Chronic kidney disease
Endocrine, metabolic, blood, and immune disorders
Upper digestive system diseases
Cirrhosis and other chronic liver diseases
HIV/AIDS
Inflammatory bowel disease
Maternal disorders
Gynecological diseases
YLDs per 100,000 population
0 to <0.66
0.66 to <2.54
2.54 to <5.58
5.58 to <12.98
12.98 to <24.44
24.44 to <44.27
44.27 to <69.38
69.38 to <186.67
186.67 to <287.1877
287.17 to 918.21

Guinea-Bissau

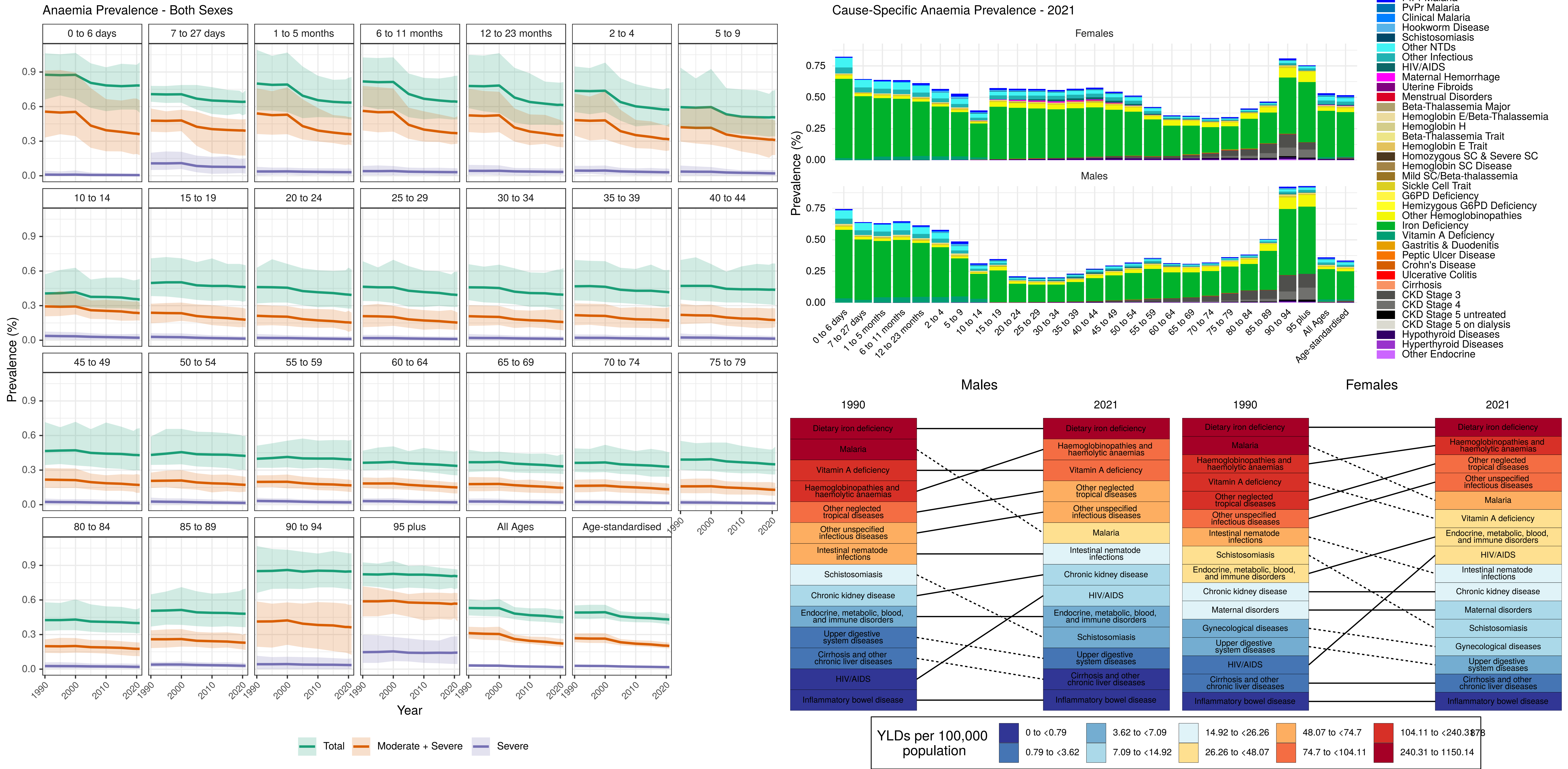

Liberia

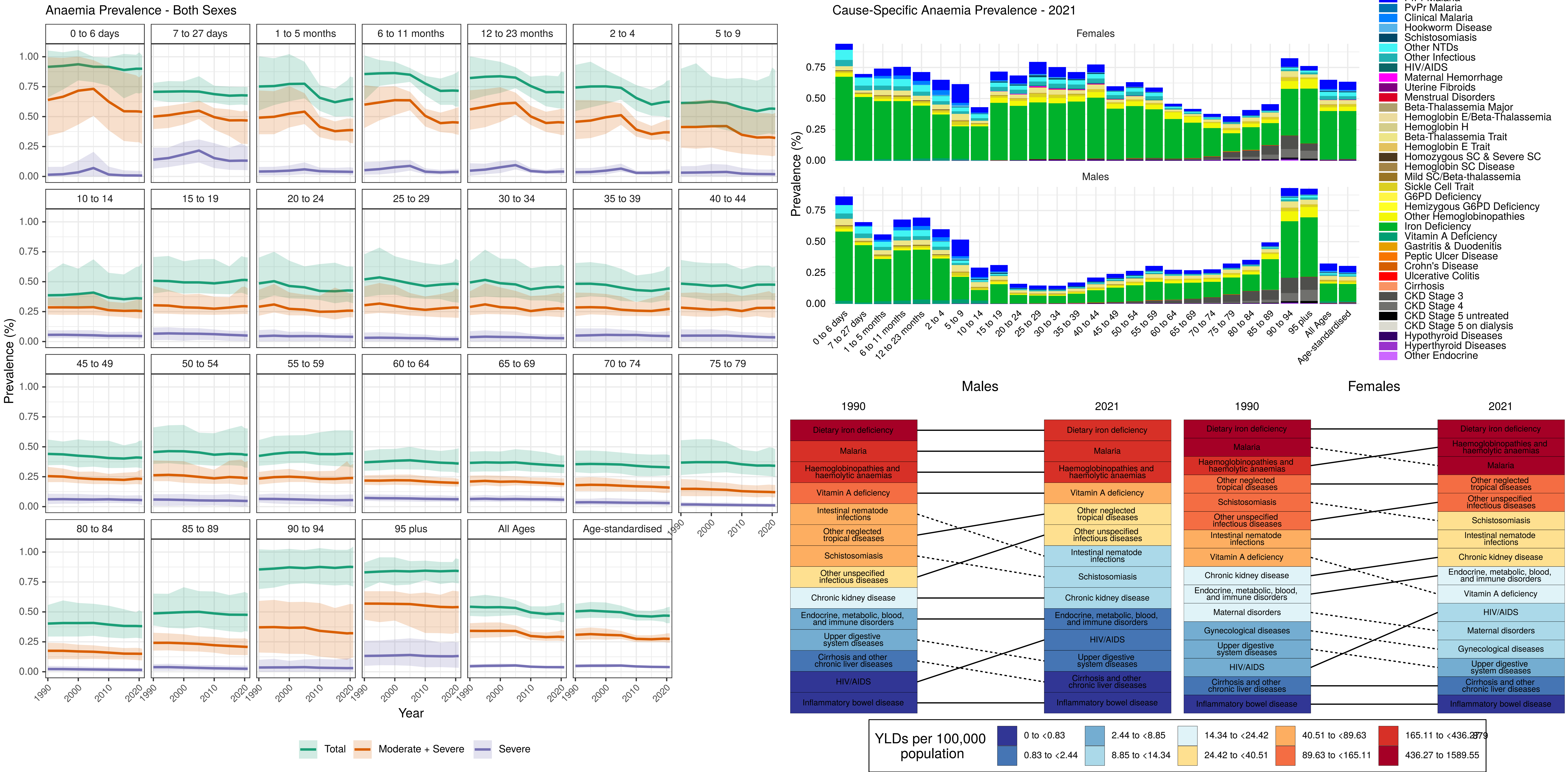

Anaemia Prevalence - Both Sexes
0 to 6 days
7 to 27 days
1 to 5 months
6 to 11 months
12 to 23 months
2 to 4
5 to 9
10 to 14
15 to 19
20 to 24
25 to 29
30 to 34
35 to 39
40 to 44
45 to 49
50 to 54
55 to 59
60 to 64
65 to 69
70 to 74
75 to 79
80 to 84
85 to 89
90 to 94
95 plus
All Ages
Age-standardised
Prevalence (%)
Year
Total
Moderate + Severe
Severe
Cause-Specific Anaemia Prevalence - 2021
Females
Males
PfPr Malaria
PvPr Malaria
Clinical Malaria
Hookworm Disease
Schistosomiasis
Other NTDs
Other Infectious
HIV/AIDS
Maternal Hemorrhage
Uterine Fibroids
Menstrual Disorders
Beta-Thalassemia Major
Hemoglobin E/Beta-Thalassemia
Hemoglobin H
Beta-Thalassemia Trait
Hemoglobin E Trait
Homozygous SC & Severe SC
Hemoglobin SC Disease
Mild SC/Beta-thalassemia
Sickle Cell Trait
G6PD Deficiency
Hemizygous G6PD Deficiency
Other Hemoglobinopathies
Iron Deficiency
Vitamin A Deficiency
Gastritis & Duodenitis
Peptic Ulcer Disease
Crohn's Disease
Ulcerative Colitis
Cirrhosis
CKD Stage 3
CKD Stage 4
CKD Stage 5 untreated
CKD Stage 5 on dialysis
Hypothyroid Diseases
Hyperthyroid Diseases
Other Endocrine
Males
Females
1990
2021
Dietary iron deficiency
Malaria
Haemoglobinopathies and haemolytic anaemias
Vitamin A deficiency
Intestinal nematode infections
Other neglected tropical diseases
Schistosomiasis
Other unspecified infectious diseases
Chronic kidney disease
Endocrine, metabolic, blood, and immune disorders
Upper digestive system diseases
Cirrhosis and other chronic liver diseases
HIV/AIDS
Inflammatory bowel disease
Maternal disorders
Gynecological diseases
YLDs per 100,000 population
0 to <0.83
0.83 to <2.44
2.44 to <8.85
8.85 to <14.34
14.34 to <24.42
24.42 to <40.51
40.51 to <89.63
89.63 to <165.11
165.11 to <436.27
436.27 to 1589.55

Mali

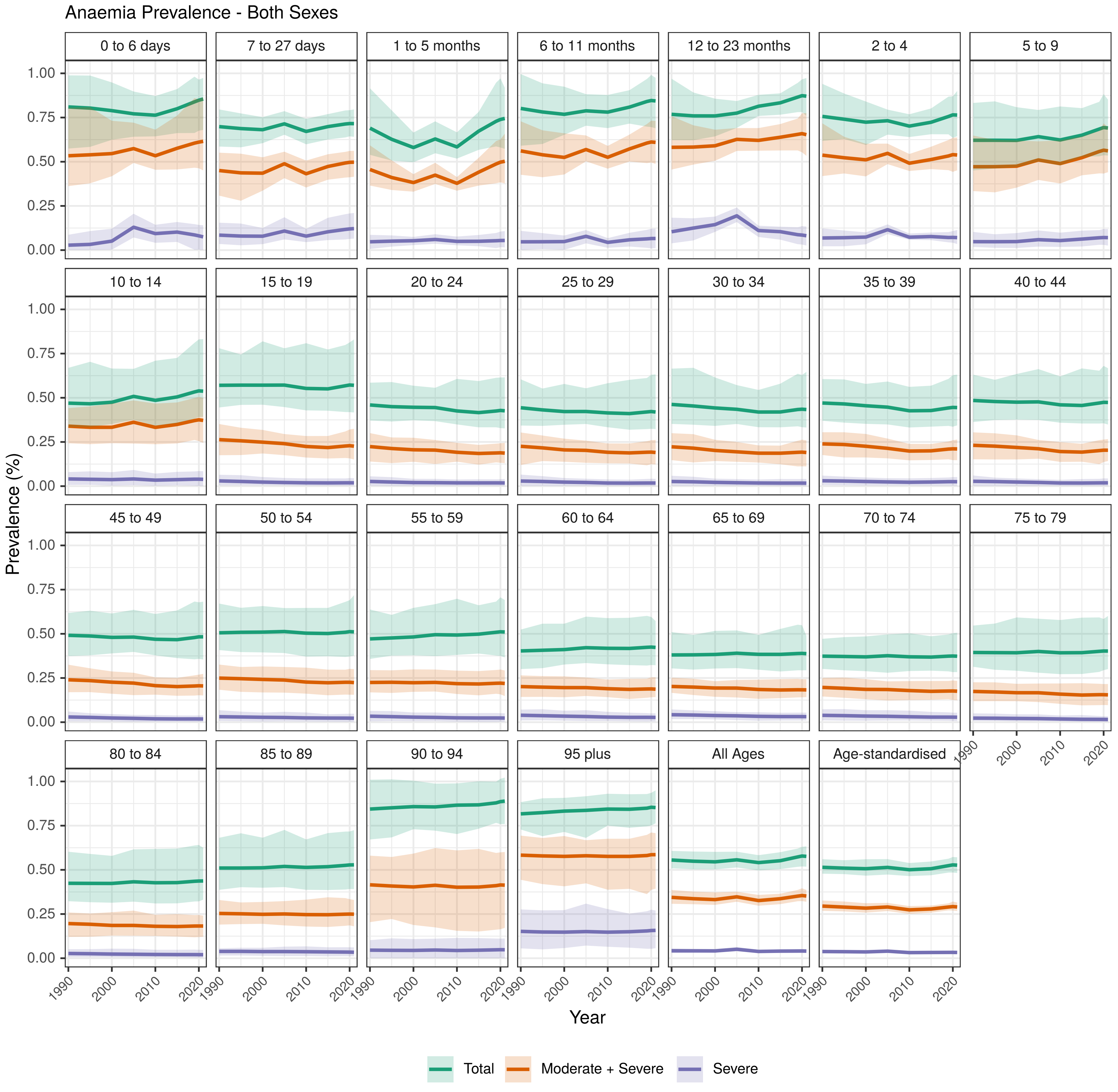
Anaemia Prevalence - Both Sexes
0 to 6 days
7 to 27 days
1 to 5 months
6 to 11 months
12 to 23 months
2 to 4
5 to 9
10 to 14
15 to 19
20 to 24
25 to 29
30 to 34
35 to 39
40 to 44
45 to 49
50 to 54
55 to 59
60 to 64
65 to 69
70 to 74
75 to 79
80 to 84
85 to 89
90 to 94
95 plus
All Ages
Age-standardised
1.00
0.75
0.50
0.25
0.00
Prevalence (%)
1990
2000
2010
2020
Year
Total
Moderate + Severe
Severe

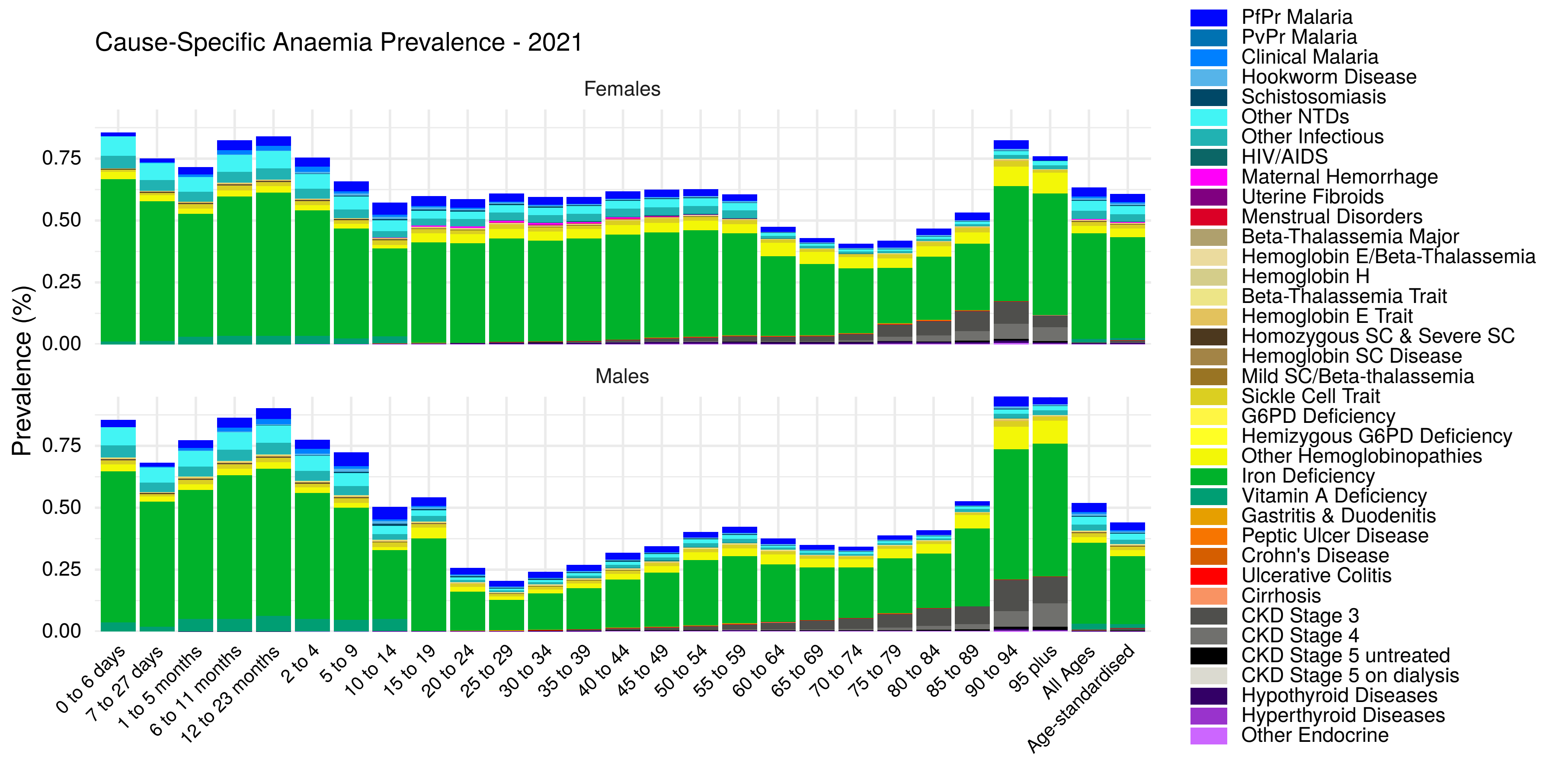
Cause-Specific Anaemia Prevalence - 2021
Females
Males
Prevalence (%)
0.75
0.50
0.25
0.00
0 to 6 days
7 to 27 days
1 to 5 months
6 to 11 months
12 to 23 months
2 to 4
5 to 9
10 to 14
15 to 19
20 to 24
25 to 29
30 to 34
35 to 39
40 to 44
45 to 49
50 to 54
55 to 59
60 to 64
65 to 69
70 to 74
75 to 79
80 to 84
85 to 89
90 to 94
95 plus
All Ages
Age-standardised
PfPr Malaria
PvPr Malaria
Clinical Malaria
Hookworm Disease
Schistosomiasis
Other NTDs
Other Infectious
HIV/AIDS
Maternal Hemorrhage
Uterine Fibroids
Menstrual Disorders
Beta-Thalassemia Major
Hemoglobin E/Beta-Thalassemia
Hemoglobin H
Beta-Thalassemia Trait
Hemoglobin E Trait
Homozygous SC & Severe SC
Hemoglobin SC Disease
Mild SC/Beta-thalassemia
Sickle Cell Trait
G6PD Deficiency
Hemizygous G6PD Deficiency
Other Hemoglobinopathies
Iron Deficiency
Vitamin A Deficiency
Gastritis & Duodenitis
Peptic Ulcer Disease
Crohn's Disease
Ulcerative Colitis
Cirrhosis
CKD Stage 3
CKD Stage 4
CKD Stage 5 untreated
CKD Stage 5 on dialysis
Hypothyroid Diseases
Hyperthyroid Diseases
Other Endocrine

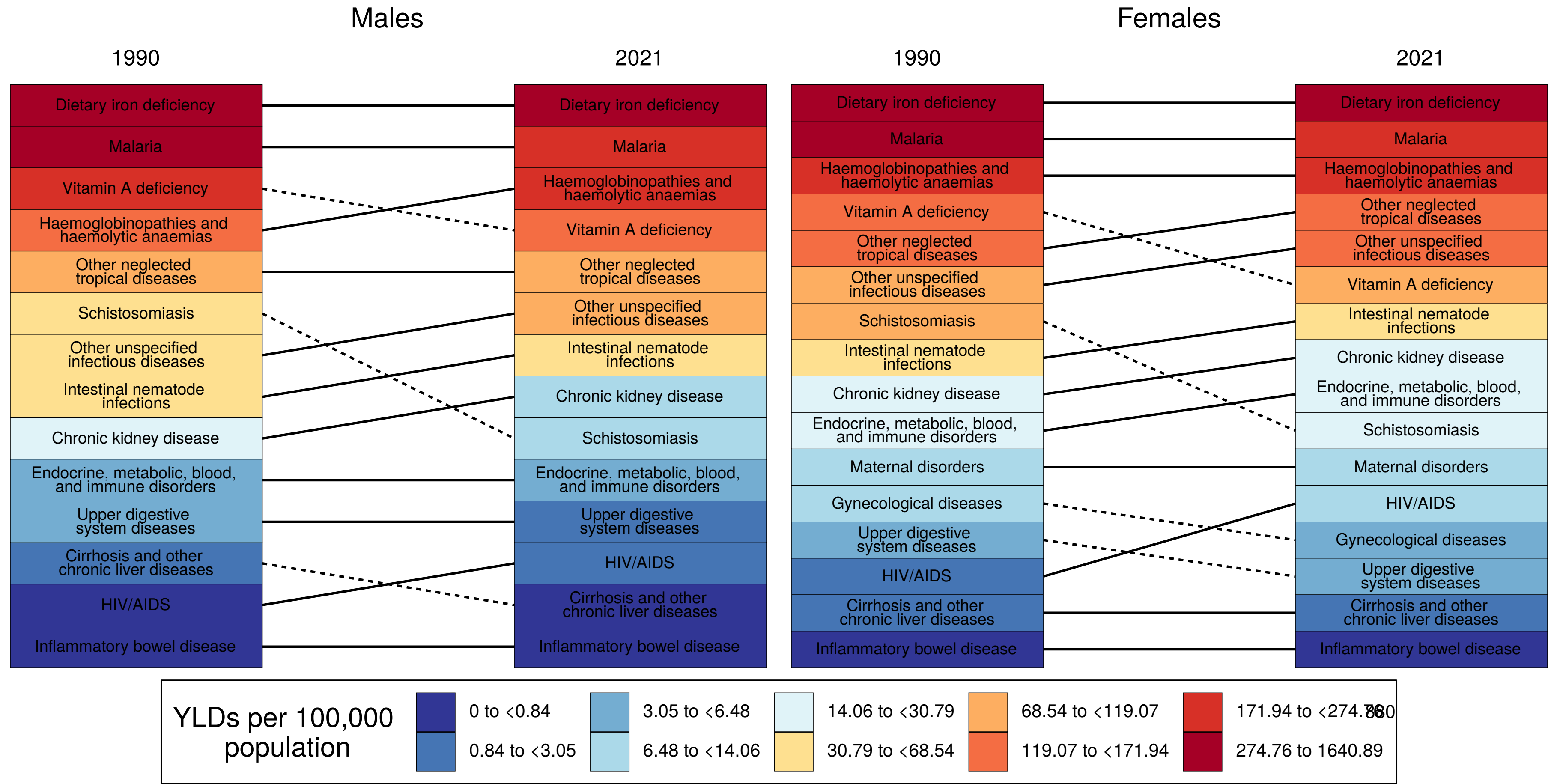
Males
Females
1990
2021
Dietary iron deficiency
Malaria
Vitamin A deficiency
Haemoglobinopathies and haemolytic anaemias
Other neglected tropical diseases
Schistosomiasis
Other unspecified infectious diseases
Intestinal nematode infections
Chronic kidney disease
Endocrine, metabolic, blood, and immune disorders
Upper digestive system diseases
Cirrhosis and other chronic liver diseases
HIV/AIDS
Inflammatory bowel disease
Maternal disorders
Gynecological diseases
YLDs per 100,000 population
0 to <0.84
0.84 to <3.05
3.05 to <6.48
6.48 to <14.06
14.06 to <30.79
30.79 to <68.54
68.54 to <119.07
119.07 to <171.94
171.94 to <274.76
274.76 to 1640.89

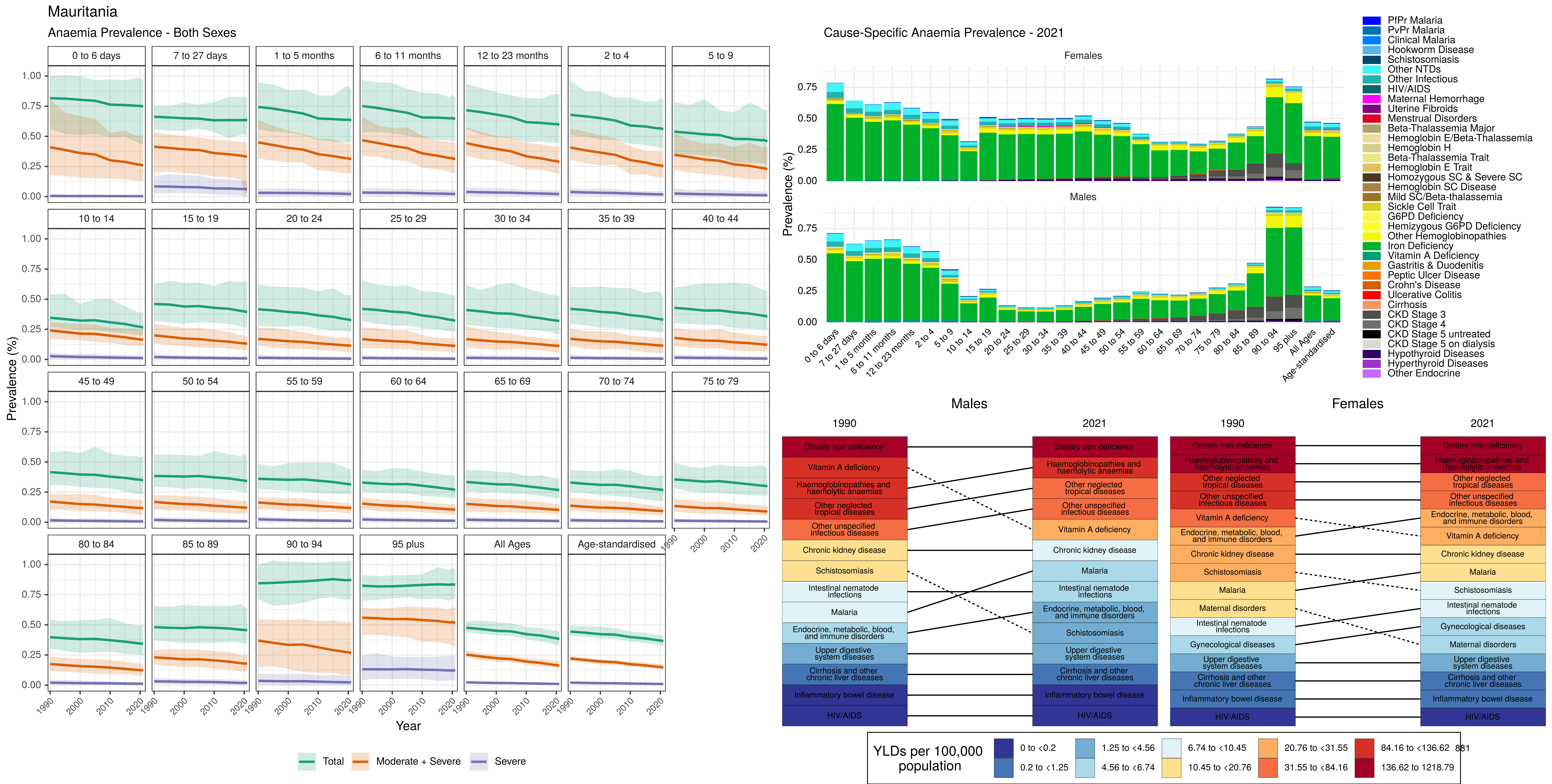
Mauritania
Anaemia Prevalence - Both Sexes
0 to 6 days
7 to 27 days
1 to 5 months
6 to 11 months
12 to 23 months
2 to 4
5 to 9
10 to 14
15 to 19
20 to 24
25 to 29
30 to 34
35 to 39
40 to 44
45 to 49
50 to 54
55 to 59
60 to 64
65 to 69
70 to 74
75 to 79
80 to 84
85 to 89
90 to 94
95 plus
All Ages
Age-standardised
Prevalence (%)
Year
Total
Moderate + Severe
Severe
Cause-Specific Anaemia Prevalence - 2021
Females
Males
PfPr Malaria
PvPr Malaria
Clinical Malaria
Hookworm Disease
Schistosomiasis
Other NTDs
Other Infectious
HIV/AIDS
Maternal Hemorrhage
Uterine Fibroids
Menstrual Disorders
Beta-Thalassemia Major
Hemoglobin E/Beta-Thalassemia
Hemoglobin H
Beta-Thalassemia Trait
Hemoglobin E Trait
Homozygous SC & Severe SC
Hemoglobin SC Disease
Mild SC/Beta-thalassemia
Sickle Cell Trait
G6PD Deficiency
Hemizygous G6PD Deficiency
Other Hemoglobinopathies
Iron Deficiency
Vitamin A Deficiency
Gastritis & Duodenitis
Peptic Ulcer Disease
Crohn's Disease
Ulcerative Colitis
Cirrhosis
CKD Stage 3
CKD Stage 4
CKD Stage 5 untreated
CKD Stage 5 on dialysis
Hypothyroid Diseases
Hyperthyroid Diseases
Other Endocrine
Males
Females
1990
2021
Dietary iron deficiency
Vitamin A deficiency
Haemoglobinopathies and haemolytic anaemias
Other neglected tropical diseases
Other unspecified infectious diseases
Chronic kidney disease
Schistosomiasis
Intestinal nematode infections
Malaria
Endocrine, metabolic, blood, and immune disorders
Upper digestive system diseases
Cirrhosis and other chronic liver diseases
Inflammatory bowel disease
HIV/AIDS
Maternal disorders
Gynecological diseases
YLDs per 100,000 population
0 to <0.2
0.2 to <1.25
1.25 to <4.56
4.56 to <6.74
6.74 to <10.45
10.45 to <20.76
20.76 to <31.55
31.55 to <84.16
84.16 to <136.62
136.62 to 1218.79

Niger

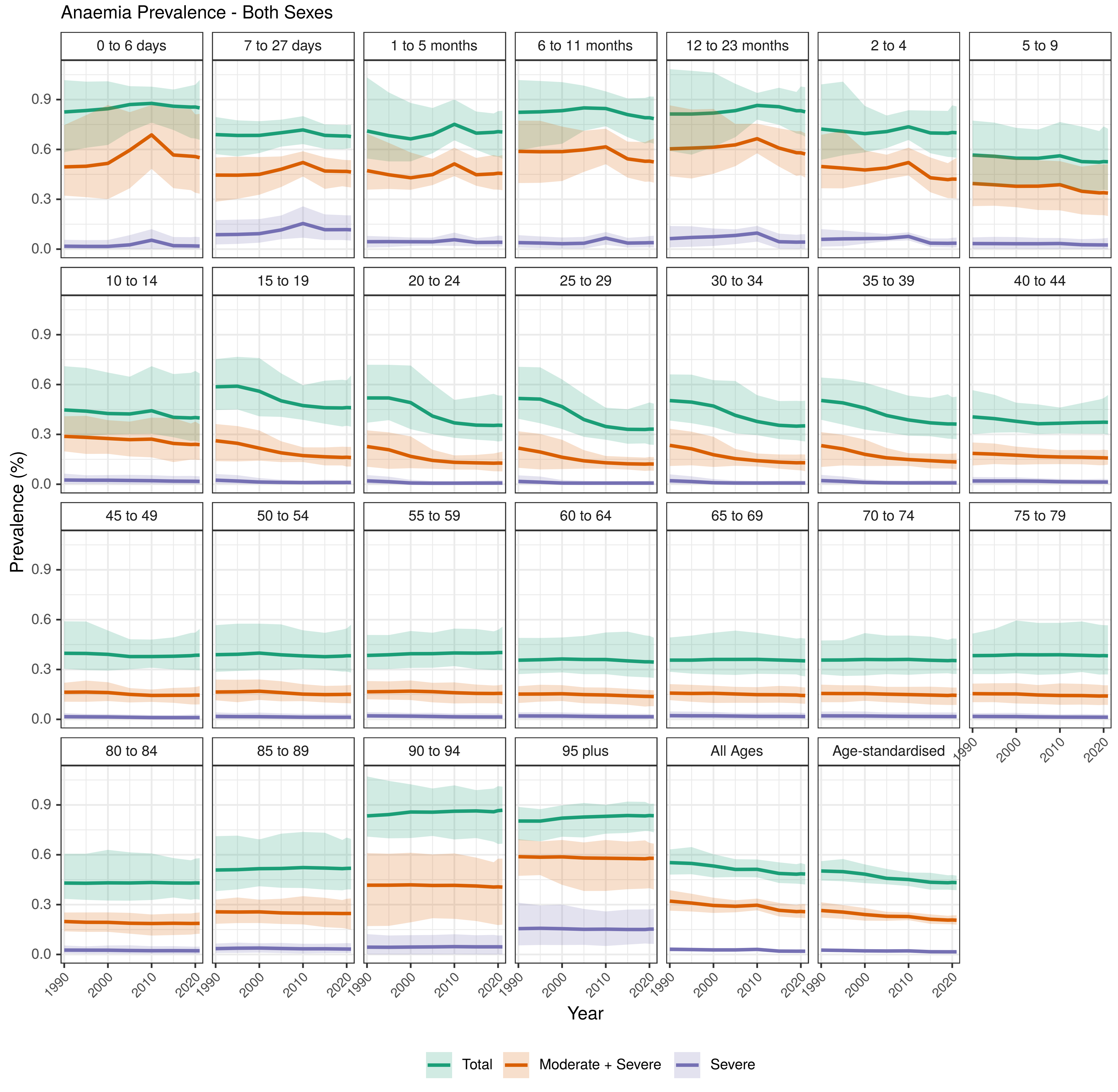
Anaemia Prevalence - Both Sexes
0 to 6 days
7 to 27 days
1 to 5 months
6 to 11 months
12 to 23 months
2 to 4
5 to 9
10 to 14
15 to 19
20 to 24
25 to 29
30 to 34
35 to 39
40 to 44
45 to 49
50 to 54
55 to 59
60 to 64
65 to 69
70 to 74
75 to 79
80 to 84
85 to 89
90 to 94
95 plus
All Ages
Age-standardised
Prevalence (%)
Year
Total
Moderate + Severe
Severe

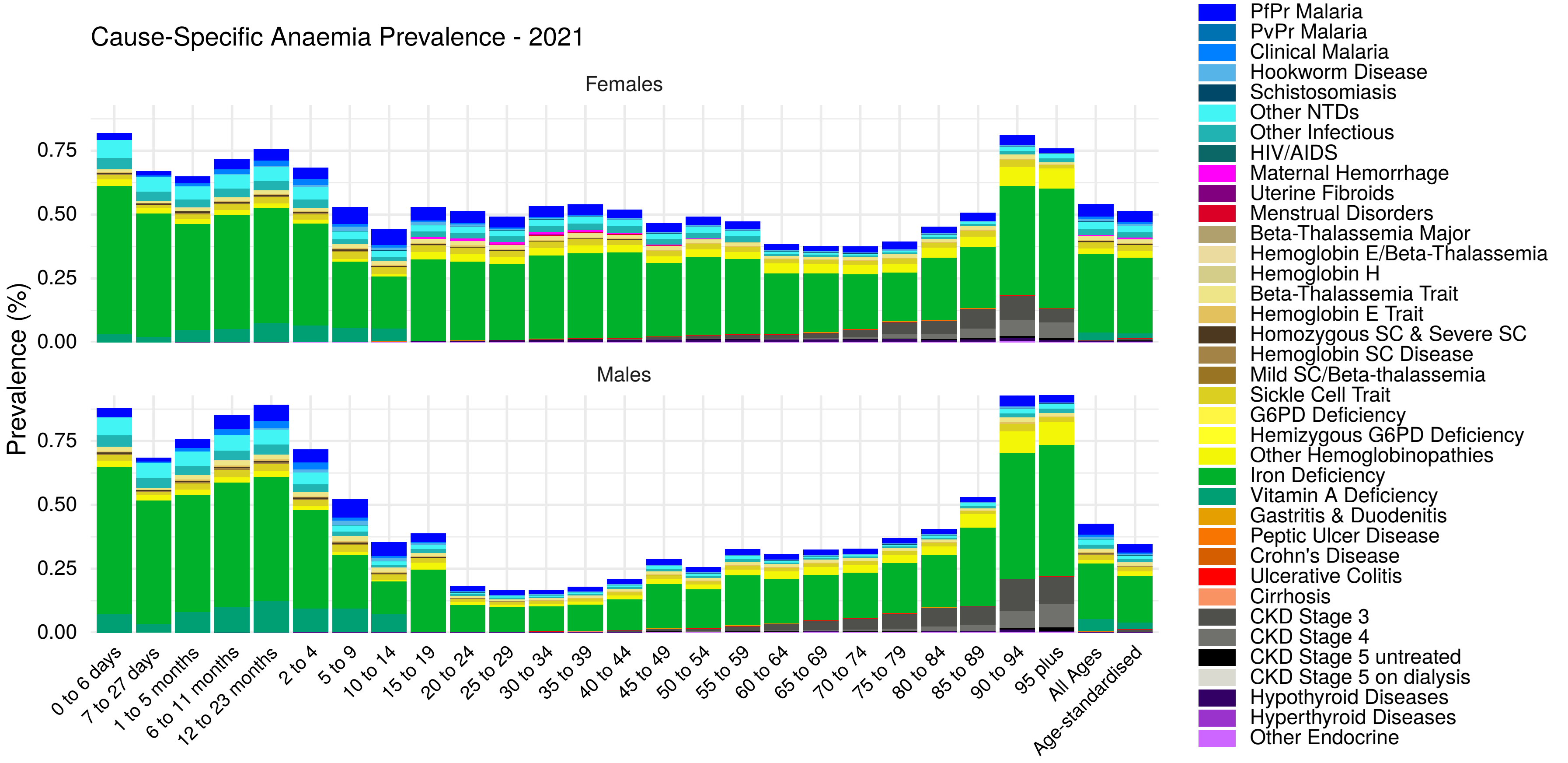
Cause-Specific Anaemia Prevalence - 2021
Females
Males
Prevalence (%)
PfPr Malaria
PvPr Malaria
Clinical Malaria
Hookworm Disease
Schistosomiasis
Other NTDs
Other Infectious
HIV/AIDS
Maternal Hemorrhage
Uterine Fibroids
Menstrual Disorders
Beta-Thalassemia Major
Hemoglobin E/Beta-Thalassemia
Hemoglobin H
Beta-Thalassemia Trait
Hemoglobin E Trait
Homozygous SC & Severe SC
Hemoglobin SC Disease
Mild SC/Beta-thalassemia
Sickle Cell Trait
G6PD Deficiency
Hemizygous G6PD Deficiency
Other Hemoglobinopathies
Iron Deficiency
Vitamin A Deficiency
Gastritis & Duodenitis
Peptic Ulcer Disease
Crohn's Disease
Ulcerative Colitis
Cirrhosis
CKD Stage 3
CKD Stage 4
CKD Stage 5 untreated
CKD Stage 5 on dialysis
Hypothyroid Diseases
Hyperthyroid Diseases
Other Endocrine

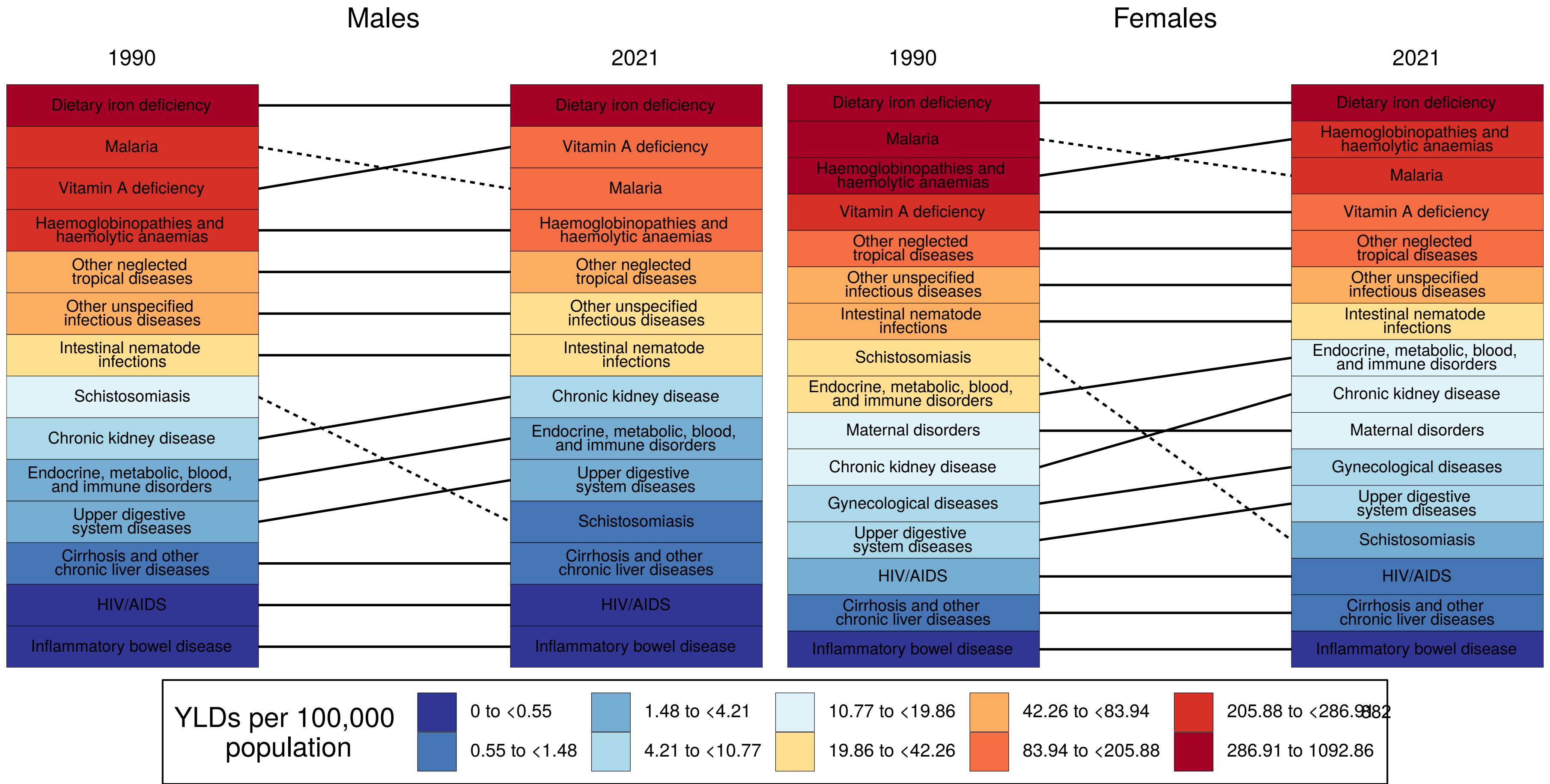
Males
Females
1990
2021
Dietary iron deficiency
Malaria
Vitamin A deficiency
Haemoglobinopathies and haemolytic anaemias
Other neglected tropical diseases
Other unspecified infectious diseases
Intestinal nematode infections
Schistosomiasis
Chronic kidney disease
Endocrine, metabolic, blood, and immune disorders
Upper digestive system diseases
Cirrhosis and other chronic liver diseases
HIV/AIDS
Inflammatory bowel disease
Maternal disorders
Gynecological diseases
YLDs per 100,000 population
0 to <0.55
0.55 to <1.48
1.48 to <4.21
4.21 to <10.77
10.77 to <19.86
19.86 to <42.26
42.26 to <83.94
83.94 to <205.88
205.88 to <286.91
286.91 to 1092.86

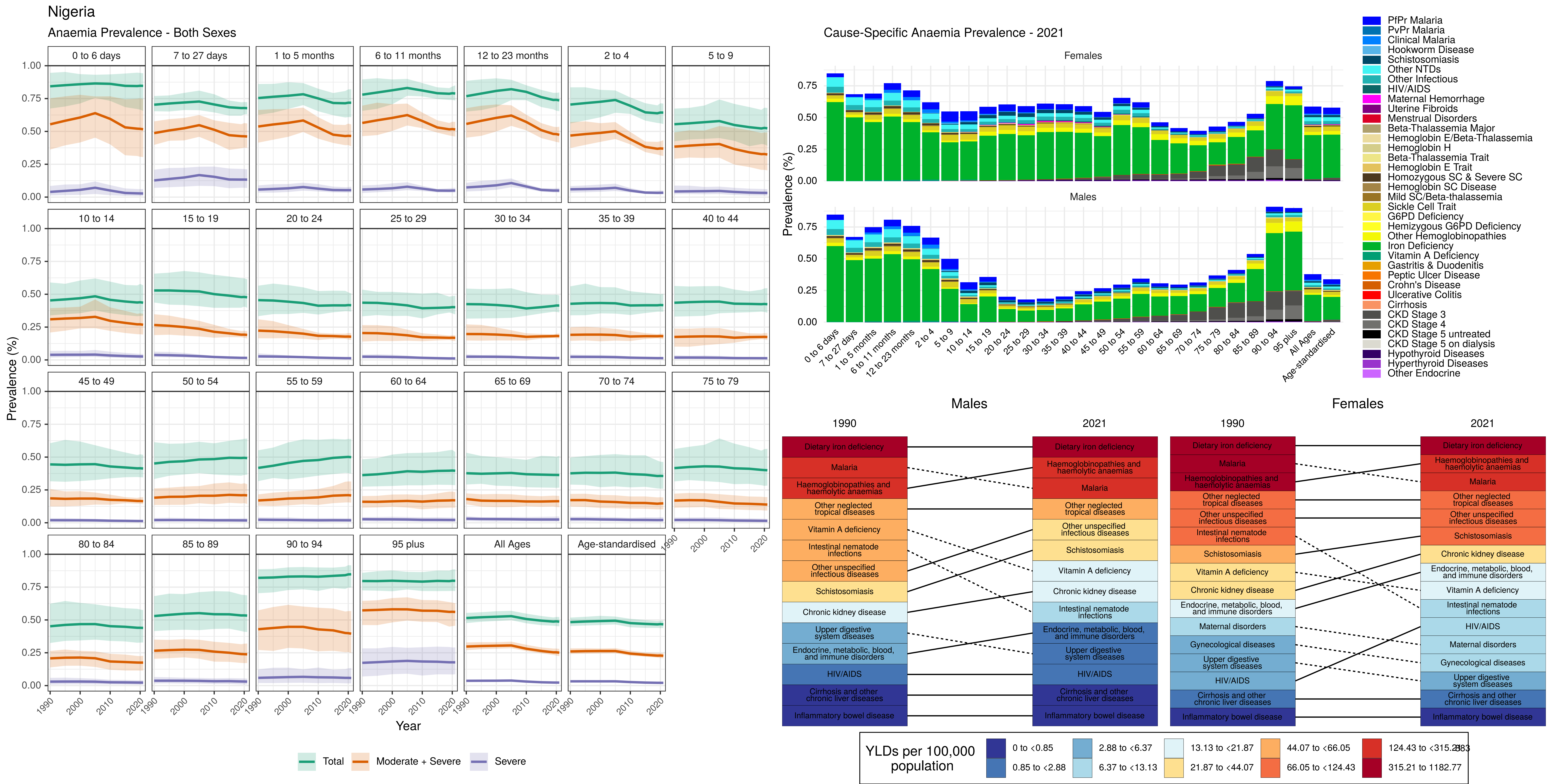
Nigeria
Anaemia Prevalence - Both Sexes
0 to 6 days
7 to 27 days
1 to 5 months
6 to 11 months
12 to 23 months
2 to 4
5 to 9
10 to 14
15 to 19
20 to 24
25 to 29
30 to 34
35 to 39
40 to 44
45 to 49
50 to 54
55 to 59
60 to 64
65 to 69
70 to 74
75 to 79
80 to 84
85 to 89
90 to 94
95 plus
All Ages
Age-standardised
Prevalence (%)
Year
Total
Moderate + Severe
Severe
Cause-Specific Anaemia Prevalence - 2021
Females
Males
PfPr Malaria
PvPr Malaria
Clinical Malaria
Hookworm Disease
Schistosomiasis
Other NTDs
Other Infectious
HIV/AIDS
Maternal Hemorrhage
Uterine Fibroids
Menstrual Disorders
Beta-Thalassemia Major
Hemoglobin E/Beta-Thalassemia
Hemoglobin H
Beta-Thalassemia Trait
Hemoglobin E Trait
Homozygous SC & Severe SC
Hemoglobin SC Disease
Mild SC/Beta-thalassemia
Sickle Cell Trait
G6PD Deficiency
Hemizygous G6PD Deficiency
Other Hemoglobinopathies
Iron Deficiency
Vitamin A Deficiency
Gastritis & Duodenitis
Peptic Ulcer Disease
Crohn's Disease
Ulcerative Colitis
Cirrhosis
CKD Stage 3
CKD Stage 4
CKD Stage 5 untreated
CKD Stage 5 on dialysis
Hypothyroid Diseases
Hyperthyroid Diseases
Other Endocrine
Males
Females
1990
2021
Dietary iron deficiency
Malaria
Haemoglobinopathies and haemolytic anaemias
Other neglected tropical diseases
Vitamin A deficiency
Intestinal nematode infections
Other unspecified infectious diseases
Schistosomiasis
Chronic kidney disease
Upper digestive system diseases
Endocrine, metabolic, blood, and immune disorders
HIV/AIDS
Cirrhosis and other chronic liver diseases
Inflammatory bowel disease
Maternal disorders
Gynecological diseases
YLDs per 100,000 population
0 to <0.85
0.85 to <2.88
2.88 to <6.37
6.37 to <13.13
13.13 to <21.87
21.87 to <44.07
44.07 to <66.05
66.05 to <124.43
315.21 to 1182.77

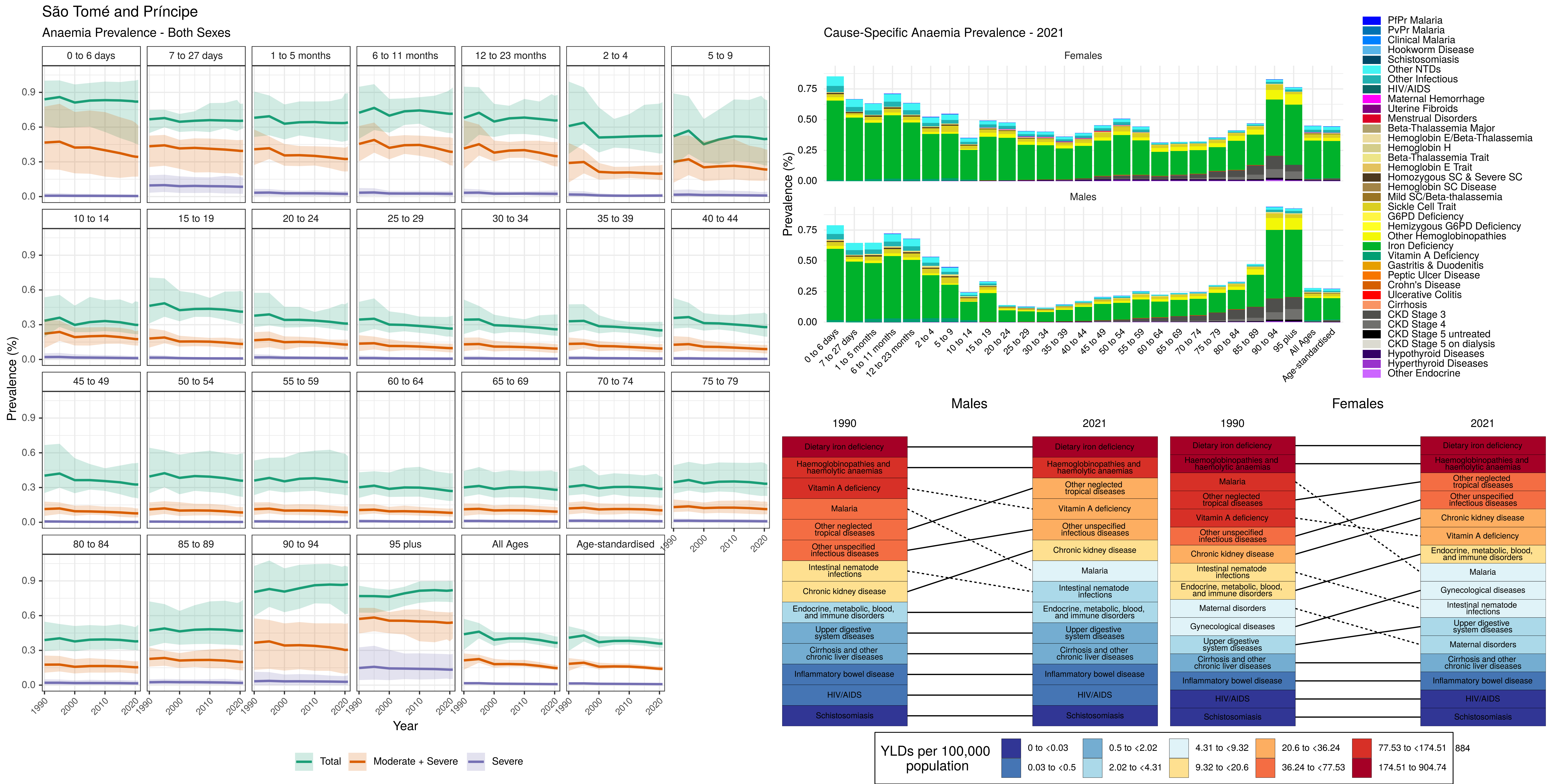
São Tomé and Príncipe
Anaemia Prevalence - Both Sexes
0 to 6 days
7 to 27 days
1 to 5 months
6 to 11 months
12 to 23 months
2 to 4
5 to 9
10 to 14
15 to 19
20 to 24
25 to 29
30 to 34
35 to 39
40 to 44
45 to 49
50 to 54
55 to 59
60 to 64
65 to 69
70 to 74
75 to 79
80 to 84
85 to 89
90 to 94
95 plus
All Ages
Age-standardised
Prevalence (%)
Year
Total
Moderate + Severe
Severe
Cause-Specific Anaemia Prevalence - 2021
Females
Males
PfPr Malaria
PvPr Malaria
Clinical Malaria
Hookworm Disease
Schistosomiasis
Other NTDs
Other Infectious
HIV/AIDS
Maternal Hemorrhage
Uterine Fibroids
Menstrual Disorders
Beta-Thalassemia Major
Hemoglobin E/Beta-Thalassemia
Hemoglobin H
Beta-Thalassemia Trait
Hemoglobin E Trait
Homozygous SC & Severe SC
Hemoglobin SC Disease
Mild SC/Beta-thalassemia
Sickle Cell Trait
G6PD Deficiency
Hemizygous G6PD Deficiency
Other Hemoglobinopathies
Iron Deficiency
Vitamin A Deficiency
Gastritis & Duodenitis
Peptic Ulcer Disease
Crohn's Disease
Ulcerative Colitis
Cirrhosis
CKD Stage 3
CKD Stage 4
CKD Stage 5 untreated
CKD Stage 5 on dialysis
Hypothyroid Diseases
Hyperthyroid Diseases
Other Endocrine
Males
Females
1990
2021
Dietary iron deficiency
Haemoglobinopathies and haemolytic anaemias
Vitamin A deficiency
Malaria
Other neglected tropical diseases
Other unspecified infectious diseases
Intestinal nematode infections
Chronic kidney disease
Endocrine, metabolic, blood, and immune disorders
Upper digestive system diseases
Cirrhosis and other chronic liver diseases
Inflammatory bowel disease
HIV/AIDS
Schistosomiasis
Maternal disorders
Gynecological diseases
YLDs per 100,000 population
0 to <0.03
0.03 to <0.5
0.5 to <2.02
2.02 to <4.31
4.31 to <9.32
9.32 to <20.6
20.6 to <36.24
36.24 to <77.53
77.53 to <174.51
174.51 to 904.74

Senegal

Anaemia Prevalence - Both Sexes

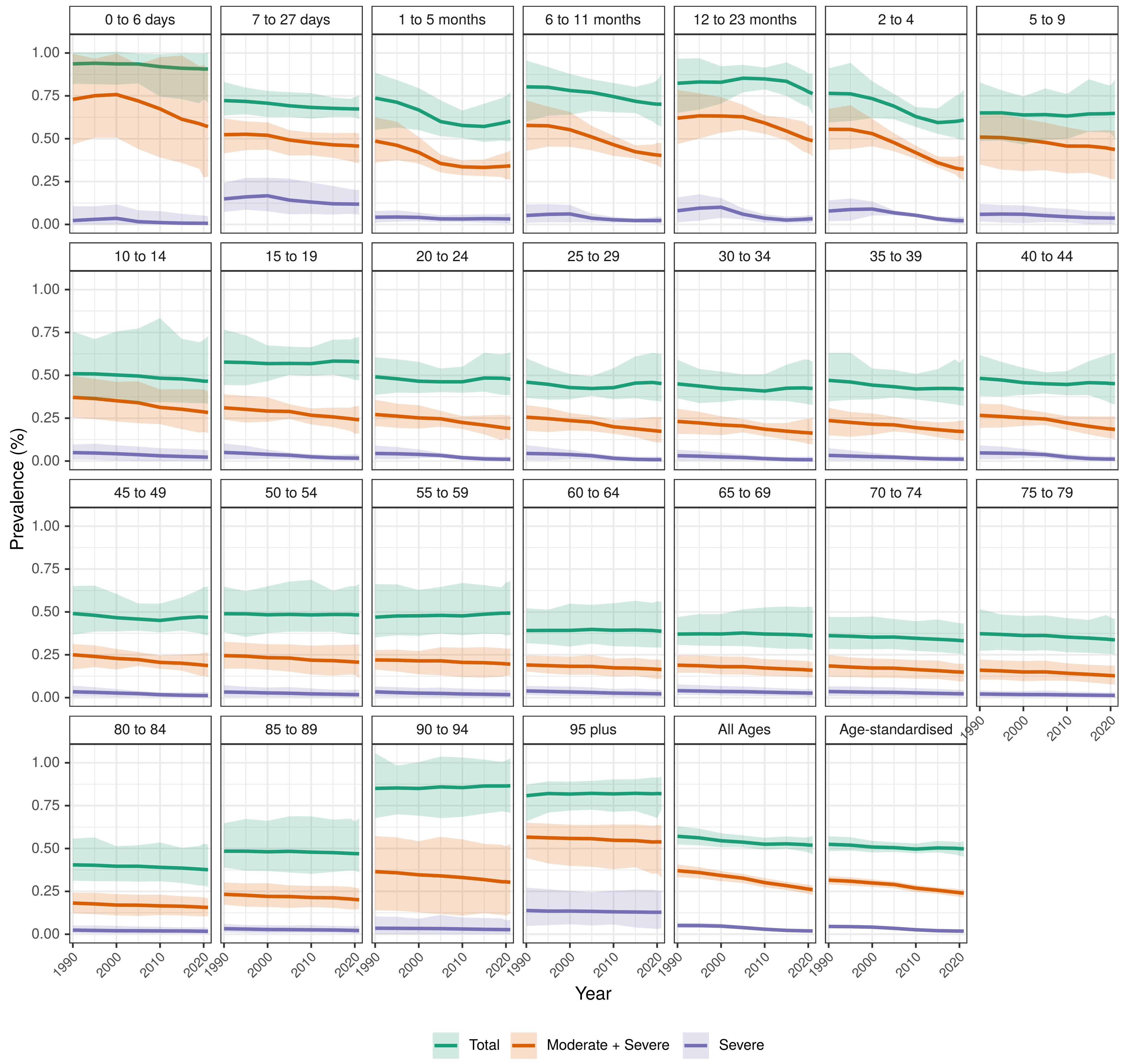

Cause-Specific Anaemia Prevalence - 2021

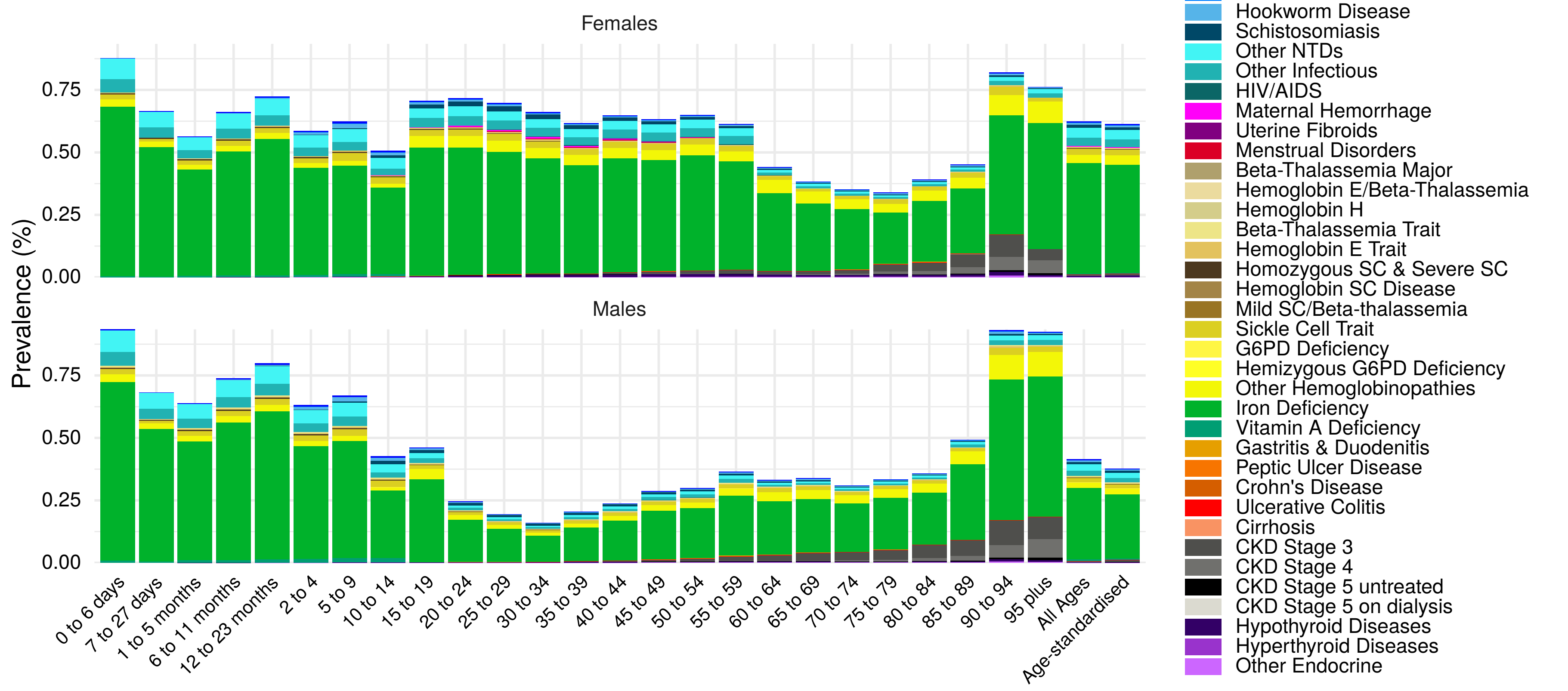

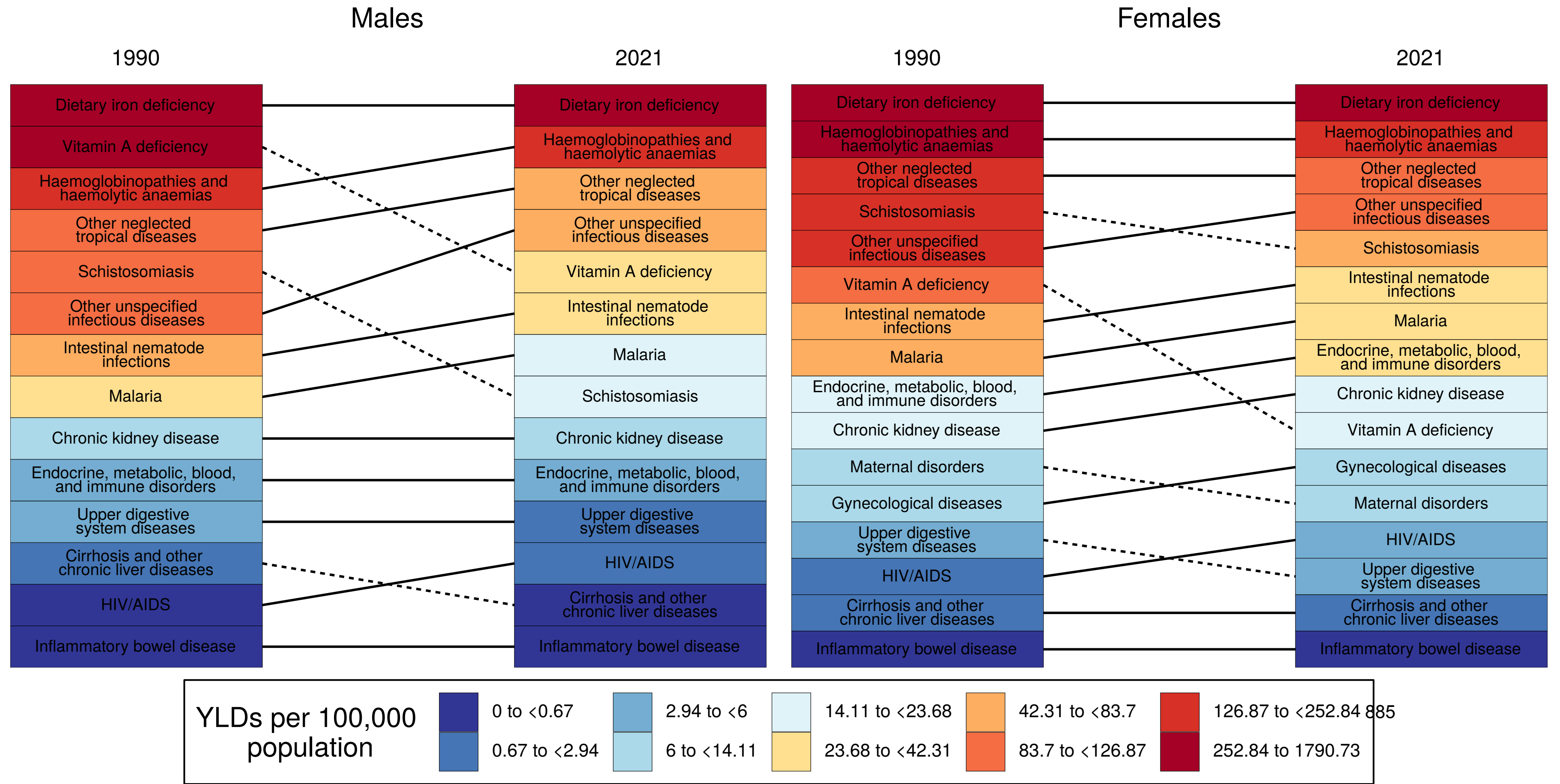

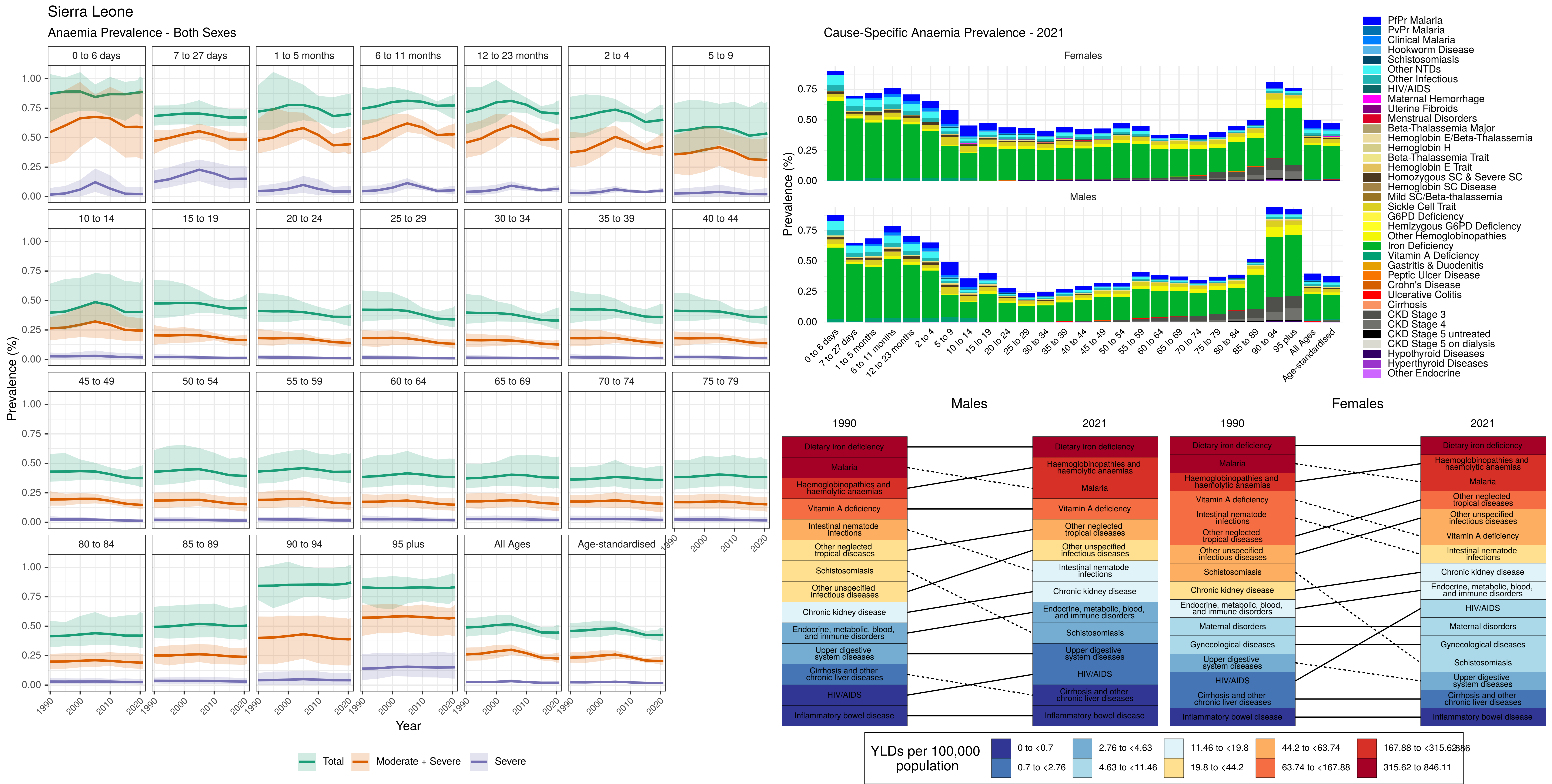
Sierra Leone
Anaemia Prevalence - Both Sexes
0 to 6 days
7 to 27 days
1 to 5 months
6 to 11 months
12 to 23 months
2 to 4
5 to 9
10 to 14
15 to 19
20 to 24
25 to 29
30 to 34
35 to 39
40 to 44
45 to 49
50 to 54
55 to 59
60 to 64
65 to 69
70 to 74
75 to 79
80 to 84
85 to 89
90 to 94
95 plus
All Ages
Age-standardised
Prevalence (%)
Year
Total
Moderate + Severe
Severe
Cause-Specific Anaemia Prevalence - 2021
Females
Males
PfPr Malaria
PvPr Malaria
Clinical Malaria
Hookworm Disease
Schistosomiasis
Other NTDs
Other Infectious
HIV/AIDS
Maternal Hemorrhage
Uterine Fibroids
Menstrual Disorders
Beta-Thalassemia Major
Hemoglobin E/Beta-Thalassemia
Hemoglobin H
Beta-Thalassemia Trait
Hemoglobin E Trait
Homozygous SC & Severe SC
Hemoglobin SC Disease
Mild SC/Beta-thalassemia
Sickle Cell Trait
G6PD Deficiency
Hemizygous G6PD Deficiency
Other Hemoglobinopathies
Iron Deficiency
Vitamin A Deficiency
Gastritis & Duodenitis
Peptic Ulcer Disease
Crohn's Disease
Ulcerative Colitis
Cirrhosis
CKD Stage 3
CKD Stage 4
CKD Stage 5 untreated
CKD Stage 5 on dialysis
Hypothyroid Diseases
Hyperthyroid Diseases
Other Endocrine
Males
Females
1990
2021
Dietary iron deficiency
Malaria
Haemoglobinopathies and haemolytic anaemias
Vitamin A deficiency
Intestinal nematode infections
Other neglected tropical diseases
Schistosomiasis
Other unspecified infectious diseases
Chronic kidney disease
Endocrine, metabolic, blood, and immune disorders
Upper digestive system diseases
Cirrhosis and other chronic liver diseases
HIV/AIDS
Inflammatory bowel disease
Maternal disorders
Gynecological diseases
YLDs per 100,000 population
0 to <0.7
0.7 to <2.76
2.76 to <4.63
4.63 to <11.46
11.46 to <19.8
19.8 to <44.2
44.2 to <63.74
63.74 to <167.88
167.88 to <315.62886
315.62 to 846.11

Togo

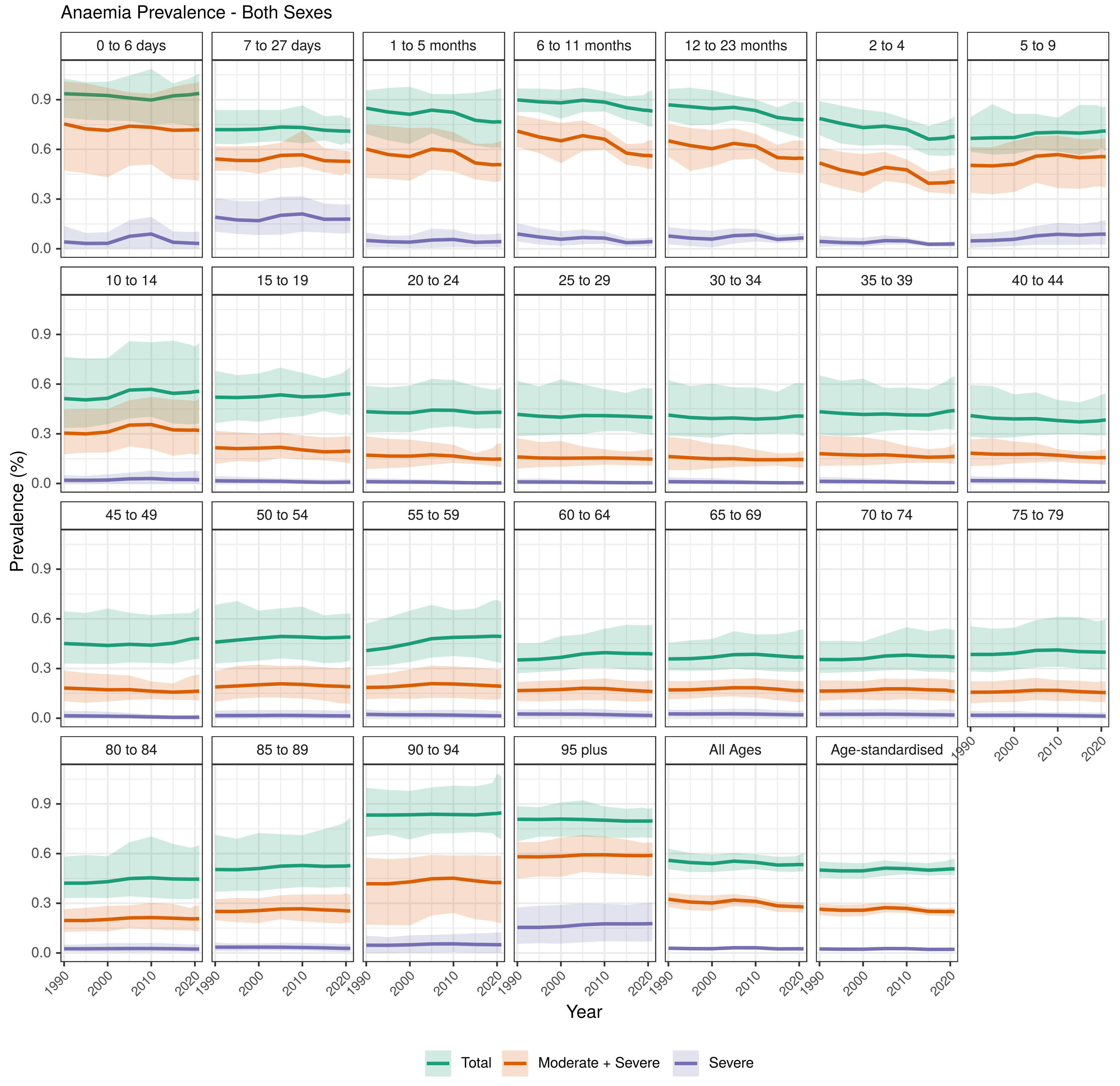

Anaemia Prevalence - Both Sexes
0 to 6 days
7 to 27 days
1 to 5 months
6 to 11 months
12 to 23 months
2 to 4
5 to 9
10 to 14
15 to 19
20 to 24
25 to 29
30 to 34
35 to 39
40 to 44
45 to 49
50 to 54
55 to 59
60 to 64
65 to 69
70 to 74
75 to 79
80 to 84
85 to 89
90 to 94
95 plus
All Ages
Age-standardised
Prevalence (%)
Year
Total
Moderate + Severe
Severe

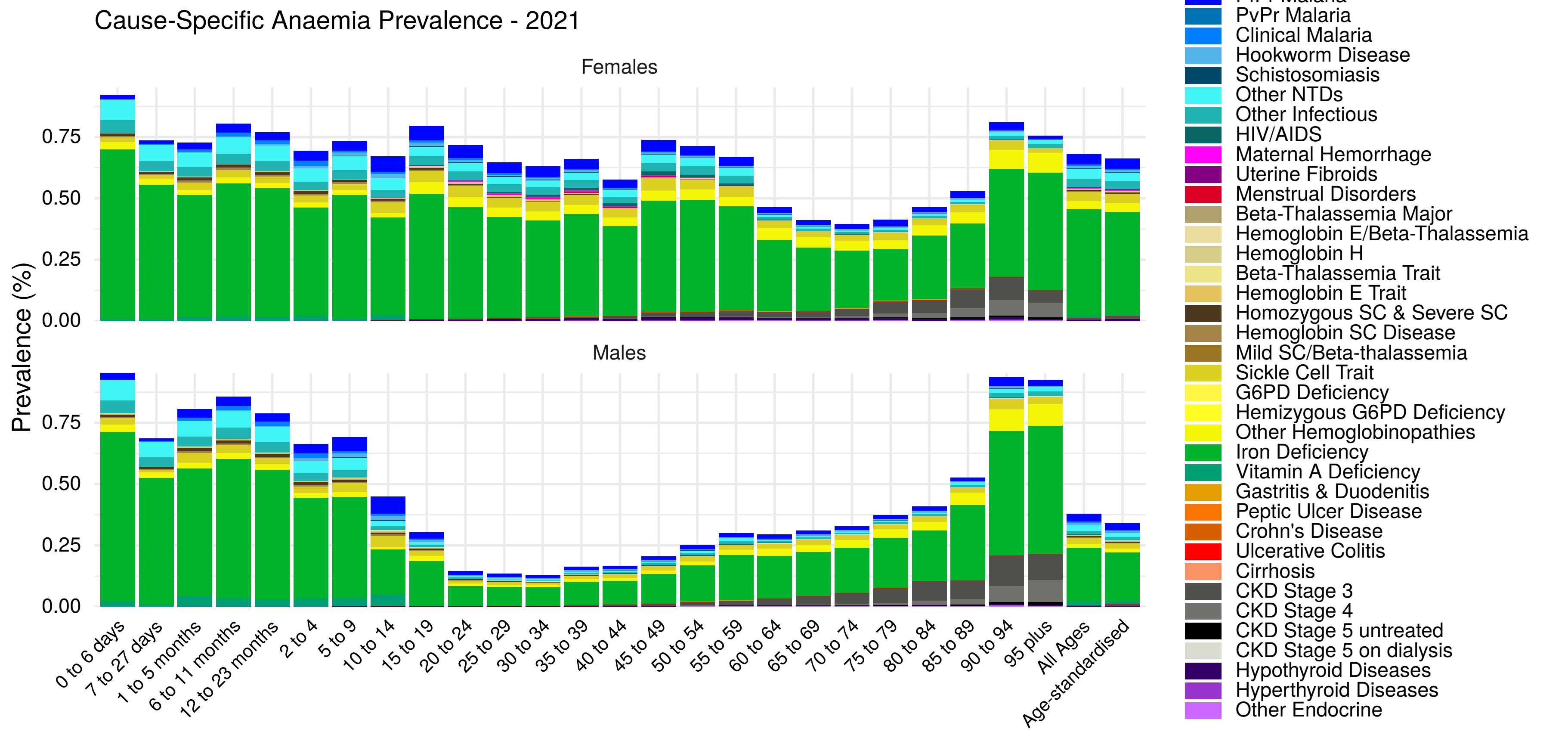

Cause-Specific Anaemia Prevalence - 2021
Females
Males
Prevalence (%)
PfPr Malaria
PvPr Malaria
Clinical Malaria
Hookworm Disease
Schistosomiasis
Other NTDs
Other Infectious
HIV/AIDS
Maternal Hemorrhage
Uterine Fibroids
Menstrual Disorders
Beta-Thalassemia Major
Hemoglobin E/Beta-Thalassemia
Hemoglobin H
Beta-Thalassemia Trait
Hemoglobin E Trait
Homozygous SC & Severe SC
Hemoglobin SC Disease
Mild SC/Beta-thalassemia
Sickle Cell Trait
G6PD Deficiency
Hemizygous G6PD Deficiency
Other Hemoglobinopathies
Iron Deficiency
Vitamin A Deficiency
Gastritis & Duodenitis
Peptic Ulcer Disease
Crohn's Disease
Ulcerative Colitis
Cirrhosis
CKD Stage 3
CKD Stage 4
CKD Stage 5 untreated
CKD Stage 5 on dialysis
Hypothyroid Diseases
Hyperthyroid Diseases
Other Endocrine

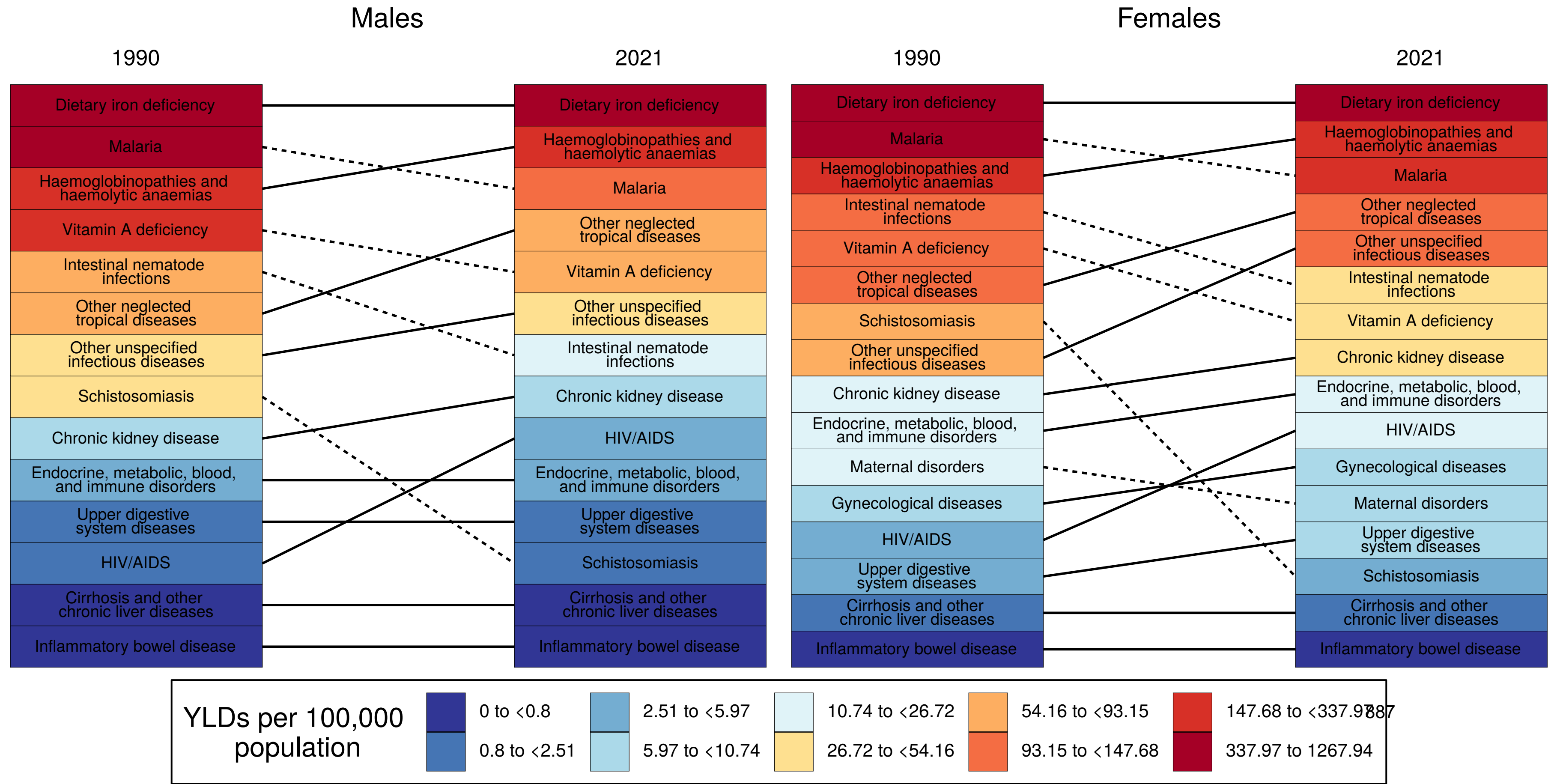

Males
Females
1990
2021
Dietary iron deficiency
Malaria
Haemoglobinopathies and haemolytic anaemias
Vitamin A deficiency
Intestinal nematode infections
Other neglected tropical diseases
Other unspecified infectious diseases
Schistosomiasis
Chronic kidney disease
Endocrine, metabolic, blood, and immune disorders
Upper digestive system diseases
HIV/AIDS
Cirrhosis and other chronic liver diseases
Inflammatory bowel disease
Maternal disorders
Gynecological diseases
YLDs per 100,000 population
0 to <0.8
0.8 to <2.51
2.51 to <5.97
5.97 to <10.74
10.74 to <26.72
26.72 to <54.16
54.16 to <93.15
93.15 to <147.68
147.68 to <337.97
337.97 to 1267.94

Section 7: Anaemia prevalence input data sources

Table S9. Anaemia Envelope Input Data Sources

Source name	Location	NID (unique GHDx identifier)
Central Statistics Organization (Afghanistan), United Nations Children's Fund (UNICEF). Afghanistan Multiple Indicator Cluster Survey 2010-2011. New York, United States of America: United Nations Children's Fund (UNICEF), 2013.	Afghanistan	56830
Centers for Disease Control and Prevention (CDC), Ministry of Public Health (Afghanistan), National Institute for Research on Food and Nutrition (INRAN) (Italy), Tufts University, United Nations Children's Fund (UNICEF). Afghanistan National Nutrition Survey 2004.	Afghanistan	135163
HOPE worldwide, Ministry of Public Health (Afghanistan), World Health Organization (WHO). Afghanistan National Malaria Prevalence Survey 2002.	Afghanistan	139286
Petry N, Olofin I, Hurrell RF, Boy E, Wirth JP, Moursi M, Angel MD, Rohner F. The Proportion of Anemia Associated with Iron Deficiency in Low, Medium, and High Human Development Index Countries: A Systematic Analysis of National Surveys. Nutrients. 2016; 8(11).	Afghanistan, Azerbaijan, Bangladesh, Cambodia, Cameroon, Cote d'Ivoire, Dominican Republic, Ecuador, Georgia, Iraq, Kenya, Lao People's Democratic Republic, Liberia, Mexico, Mongolia, Mozambique, Nicaragua, Oman, Philippines, Sierra Leone, South Africa, Tajikistan, Viet Nam	294288
ICF Macro, Institute of Public Health (Albania), Institute of Statistics (Albania). Albania Demographic and Health Survey 2008-2009. Fairfax, United States of America: ICF International, 2009.	Albania	18834
Buonomo E, Cenko F, Altan AMD, Godo A, Marazzi MC, Palombi L. Iron deficiency anemia and feeding practices in Albanian children. Ann Ig. 2005; 17(1): 27-33.	Albania	143687
Albania Institute of Public Health (IPH), Albania Institute of Statistics (INSTAT), ICF International. Albania Demographic and Health Survey 2017-2018. Fairfax, United States of America: ICF International, 2019.	Albania	393876
COSEP-Consulting Ltd., Consaúde Ltd., Macro International, Inc, Ministry of Health (Angola). Angola Malaria Indicator Survey 2006-2007. Fairfax, United States of America: ICF International.	Angola	672
COSEP-Consulting Ltd., Consaúde Ltd., ICF International, National Malaria Control (Angola), President's Malaria Initiative (PMI). Angola Malaria Indicator Survey 2011. Fairfax, United States of America: ICF International.	Angola	56169
ICF International, Ministry of Health (Angola), National Institute of Statistics (Angola), United Nations Children's Fund (UNICEF). Angola Demographic and Health Survey 2015-2016. Fairfax, United States of America: ICF International, 2017.	Angola	218555
Micronutrient Working Group. Iron and vitamin A status in five Caribbean countries. Cajanus. 2002; 4-34.	Antigua and Barbuda, Dominica	143689
Morasso M del C, Molero J, Vinocur P, Acosta L, Paccussi N, Raselli S, Falivene G, Viteri FE. [Iron deficiency and anemia in pregnant women from Chaco, Argentina]. Arch Latinoam Nutr. 2002; 52(4): 336-43.	Argentina	143692
Calvo EB, Sosa EM. Iron status in non-pregnant women of child-bearing age living at Greater Buenos Aires. Eur J Clin Nutr. 1991; 45(4): 215-20.	Argentina	143693

Marín GH, Fazio P, Rubbo S, Baistrocchi A, Sager G, Gelemur A. [Prevalence of anaemia in pregnancy and analysis of the underlying factors]. Aten Primaria. 2002; 29(3): 158-63.	Argentina	143694
Ministry of Health and Environment (Argentina), United Nations Development Programme (UNDP). Argentina National Survey of Nutrition and Health 2004-2005. Argentina: Ministry of Health and Environment (Argentina).	Argentina	56715
Morasso M del C, Molero J, Vinocur P, Acosta L, Paccussi N, Raselli S, Falivene G, Viteri FE. [Iron and vitamin A deficiencies and prevalence of anemia in boys and girls between 6 to 24 months of age in Chaco, Argentina]. Arch Latinoam Nutr. 2003; 53(1): 21-7.	Argentina	140949
Calvo EB, Gnazzo N. Prevalence of iron deficiency in children aged 9-24 mo from a large urban area of Argentina. Am J Clin Nutr. 1990; 52(3): 534-40.	Argentina	143690
Calvo E, Islam J, Gnazzo N, Ibáñez M, de Martínez C, de Vacaliuc R, Quintana E, Carmuega E. Encuesta nutricional en niños de 2 años de la provincia de misiones, II: indicadores dietéticos y hematológicos. Arch Argent Pediatr. 1987; 260-9.	Argentina	143691
Center for Studies on Infant Nutrition (CESNI) (Argentina), Jorge Macri Foundation (Argentina). Argentina - Tierra del Fuego Project Baseline Health and Nutrition Survey 1995. Buenos Aires, Argentina: Jorge Macri Foundation (Argentina), 1995.	Argentina	137334
Ministry of Health (Armenia), National Statistical Service (NSS), ORC Macro. Armenia Demographic and Health Survey 2000. Fairfax, United States of America: ICF International.	Armenia	18843
MOH Center for Health Information and Statistics, Macro International, Inc, National Statistical Service (NSS). Armenia Demographic and Health Survey 2005. Fairfax, United States of America: ICF International.	Armenia	18854
Rangan AM, Aitkin I, Blight GD, Binns CW. Factors affecting iron status in 15-30 year old female students. Asia Pac J Clin Nutr. 1997; 6(4): 291-5.	Australia	143697
Fleming AF, Martin JD, Stenhouse NS. Pregnancy anaemia, iron and folate deficiency in Western Australia. Med J Aust. 1974; 2(13): 479-84.	Australia	143699
Macro International, Inc, State Statistical Committee of Azerbaijan. Azerbaijan Demographic and Health Survey 2006. Fairfax, United States of America: ICF International.	Azerbaijan	18865
Adventist Development and Relief Agency (ADRA), Azerbaijan Ministry of Health, State Statistical Committee of Azerbaijan, and Centers for Disease Control and Prevention (CDC). (2003) Azerbaijan Reproductive Health Survey 2001. Atlanta, United States: Centers for Disease Control and Prevention (CDC).	Azerbaijan	27338
Azerbaijan Ministry of Health, GroundWork LLC, State Statistical Committee of Azerbaijan, United Nations Children's Fund (UNICEF). Azerbaijan Nutrition Survey 2013.	Azerbaijan	230882
Ministry of Health (Bahrain). Bahrain National Nutrition Survey 1998-1999.	Bahrain	111628
ICF Macro, Mitra and Associates, National Institute of Population Research and Training (NIPORT). Bangladesh Demographic and Health Survey 2011-2012. Calverton, United States of America: ICF Macro.	Bangladesh	55956
Helen Keller International. Bangladesh Nutritional Surveillance Project Bulletin No. 10, March 2002. New York City, United States of America: Helen Keller International, 2002.	Bangladesh	139421
Institute of Public Health Nutrition (Bangladesh), International Centre for Diarrhoeal Disease Research, Bangladesh (ICDDR,B), National Institute of Population Research and Training (NIPORT). Bangladesh National Nutrition Programme Baseline Survey 2004-2005.	Bangladesh	139424
Helen Keller International. Bangladesh Nutritional Surveillance Project Bulletin No. 16, April 2006. New York City, United States of America: Helen Keller International, 2006.	Bangladesh	139426
Stoltzfus R, Chakraborty J, Rice A, Briere B, Francisco A. Plausible evidence of effectiveness of an iron supplementation pr ogramme for pregnant and post-partum women in rural Bangladesh. Food Nutr Bull. 1998; 197-204.	Bangladesh	143701

Ahmed F, Khan MR, Karim R, Taj S, Hyderi T, Faruque MO, Margetts BM, Jackson AA. Serum retinol and biochemical measures of iron status in adolescent schoolgirls in urban Bangladesh. Eur J Clin Nutr. 1996; 50(6): 346-51.	Bangladesh	143702
Ahmed F, Mahmuda I, Sattar A, Akhtaruzzaman M. Anaemia and vitamin A deficiency in poor urban pregnant women of Bangladesh. Asia Pac J Clin Nutr. 2003; 12(4): 460-6.	Bangladesh	143703
Bhargava A, Bouis HE, Scrimshaw NS. Dietary intakes and socioeconomic factors are associated with the hemoglobin concentration of Bangladeshi women. J Nutr. 2001; 131(3): 758-64.	Bangladesh	143704
Kolsteren P, Rahman SR, Hilderbrand K, Diniz A. Treatment for iron deficiency anaemia with a combined supplementation of iron, vitamin A and zinc in women of Dinajpur, Bangladesh. Eur J Clin Nutr. 1999; 53(2): 102-6.	Bangladesh	143706
Hasin A, Begum R, Khan MR, Ahmed F. Relationship between birth weight and biochemical measures of maternal nutritional status at delivery in Bangladeshi urban poors. Int J Food Sci Nutr. 1996; 47(3): 273-9.	Bangladesh	143707
Rahman S, Hilderbrand K, Kolsteren P, Diniz A. A nutritional profile of non-pregnant women from the slums of Dinajpur, Bangladesh. Trop Doct. 1999; 29(4): 221-4.	Bangladesh	143708
Ziauddin Hyder S, Persson Lk, Chowdhury A, Ekström EC. Anaemia among non-pregnant women in rural Bangladesh. Public Health Nutr. 2001; 4(1): 79-83.	Bangladesh	143710
Monawarul Islam AI, Siddiqua R, Rahman A, Rahman A, Ara I. Iron-deficiency anaemia in pregnancy: (biochemical investigations of iron-deficiency and nutritional status). Bangladesh Med Res Counc Bull. 1977; 3(1): 1-8.	Bangladesh	143711
Hussain MA, Khan AK, Abedin Z, Ferdous Z, Ahmad K. Studies on the nutritional status of expectant mothers and newborn babies. Bangladesh Med Res Counc Bull. 1976; 2(2): 120-6.	Bangladesh	143712
Ahmed F, Khan MR, Islam M, Kabir I, Fuchs GJ. Anaemia and iron deficiency among adolescent schoolgirls in peri-urban Bangladesh. Eur J Clin Nutr. 2000; 54(9): 678-83.	Bangladesh	143713
Helen Keller International. High Anemia Prevalence Among Bangladeshi Children in Urban Slums: An Ethical and Economic Rationale for Multi-micronutrient Supplementation. New York City, United States: Helen Keller International, 2000. (HKI-Nutrition Surveillance Project Bulletin, no. 1).	Bangladesh	139422
Global Alliance for Improved Nutrition (GAIN), Institute of Public Health Nutrition (Bangladesh), International Centre for Diarrhoeal Disease Research, Bangladesh (ICDDR,B), United Nations Children's Fund (UNICEF). Bangladesh National Micronutrients Status Survey 2011.	Bangladesh	262810
Fogarty International Center, National Institutes of Health (NIH), Foundation for the National Institutes of Health (FNIH), International Centre for Diarrhoeal Disease Research, Bangladesh (ICDDR,B). Bangladesh - Dhaka Malnutrition and Enteric Disease Study 2009-2014.	Bangladesh	261683
Caribbean Food and Nutrition Institute, Pan American Health Organization (PAHO), Ministry of Health (Barbados). Barbados National Health and Nutrition Survey 1981.	Barbados	139427
Massot C, Vanderpas J. A survey of iron deficiency anaemia during pregnancy in Belgium: analysis of routine hospital laboratory data in Mons. Acta Clin Belg. 2003; 58(3): 169-77.	Belgium	143716
Elwood PC, Hughes J, Abernethy M, Davies R, Gough R, Johnson AP, Dubourg AY. An international haematological survey. Bull World Health Organ. 1976; 54(1): 87-95.	Belgium, Czechia, France, Hungary, Ireland, Italy, Norway, Spain, United Kingdom	143717
Dirren H, Decarli B, Lesourd B, Schlienger JL, Deslypere JP, Kiepurski A. Nutritional status: haematology and albumin. Eur J Clin Nutr. 1991; 45 (Suppl 3): 43-52.	Belgium, Denmark, France, Netherlands, Portugal, Spain, Switzerland	143715
National Institute of Statistics and Economic Analysis (INSAE) (Benin), ORC Macro. Benin Demographic and Health Survey 2001. Fairfax, United States of America: ICF International.	Benin	18950

Macro International, Inc, National Institute of Statistics and Economic Analysis (INSAE) (Benin), National Program Against AIDS (PNLS) (Benin). Benin Demographic and Health Survey 2006. Fairfax, United States of America: ICF International.	Benin	18959
ICF International, National Institute of Statistics and Economic Analysis (INSAE) (Benin), National Program Against AIDS (PNLS) (Benin). Benin Demographic and Health Survey 2011-2012. Fairfax, United States of America: ICF International, 2014.	Benin	79839
Hercberg S, Chauliac M, Galán P, Devanlay M, Zohoun I, Agboton Y, Soustre Y, Bories C, Christides JP, Potier de Courcy G. Relationship between anaemia, iron and folacin deficiency, haemoglobinopathies and parasitic infection. Hum Nutr Clin Nutr. 1986; 40(5): 371-9.	Benin	143719
Hercberg S, Chauliac M, Galan P, Devanlay M, Zohoun I, Agboton Y, Soustre Y, Auvert B, Masse-Raimbault AM, Dupin H. Prevalence of iron deficiency and iron-deficiency anaemia in Benin. Public Health. 1988; 102(1): 73-83.	Benin	143720
Hercberg S, Chauliac M, Devanlay M, Galán P, Pureur J, Soustre Y, Houdegbe A, Agboton Y, Zohoun I, Masse-Raimbault AM, Dupin H. Evaluation of the iron status of a rural population in south Benin. Nutr Res. 1986; 627-34.	Benin	143721
Ogbeide O, Wagbatsoma V, Orhue A. Anaemia in pregnancy. East Afr Med J. 1994; 672-3.	Benin	143722
Hubert Koutoukou Maga National University Hospital Center (CNHU-HKM)(Benin), ICF International, National Institute of Statistics and Economic Analysis (INSAE) (Benin), National Malaria Control Program, Ministry of Health (Benin), Permanent Secretariat of the Food Council and Nutrition (SP-CAN)(Benin). Benin Demographic and Health Survey 2017-2018. Fairfax, United States of America: ICF International, 2018.	Benin	218565
Macro International, Inc, National Institute of Statistics (Bolivia). Bolivia Demographic and Health Survey 1998. Fairfax, United States of America: ICF International.	Bolivia	18971
Macro International, Inc, Ministry of Health and Sports (Bolivia), National Institute of Statistics (Bolivia). Bolivia Demographic and Health Survey 2003-2004. Fairfax, United States of America: ICF International.	Bolivia	19001
Macro International, Inc, Ministry of Health and Sports (Bolivia), National Institute of Statistics (Bolivia). Bolivia Demographic and Health Survey 2008. Fairfax, United States of America: ICF International.	Bolivia	19016
Ministry of Health (Bolivia), Unit for Analysis of Social and Economic Policies (UDAPE) (Bolivia). Bolivia Health and Nutrition Assessment Survey 2012. La Paz, Bolivia: Unit for Analysis of Social and Economic Policies (UDAPE) (Bolivia).	Bolivia	285880
Ministry of Health (Bolivia), National Institute of Statistics (Bolivia). Bolivia Demographic and Health Survey 2016. La Paz, Bolivia: National Institute of Statistics (Bolivia), 2017.	Bolivia	323944
Stefanini MLR. Merenda escolar: história, evolução e contribuição no atendimento das necessidades nutricionais da criança [School Lunch: History, Evolution and Contribution in Addressing the Nutritional Needs of the Child] [dissertation]. Presented at: University of São Paulo; 1998; São Paulo, Brazil.	Brazil	139467
Brazilian Center for Analysis and Planning (CEBRAP), Brazilian Institute of Public Opinion and Statistics (IBOPE), Ministry of Health (Brazil). Brazil National Demographic and Health Survey of Children and Women 2006-2007. Rio de Janeiro, Brazil: Ministry of Health (Brazil).	Brazil	141948
Rondo PH, Abbott R, Rodrigues LC, Tomkins AM. Vitamin A, folate, and iron concentrations in cord and maternal blood of intra-uterine growth retarded and appropriate birth weight babies. Eur J Clin Nutr. 1995; 49(6): 391-9.	Brazil	143742
Muniz-Junqueira MI, Queiroz EFO. Relationship between protein-energy malnutrition, vitamin A, and parasitoses in living in Brasília. Rev Soc Bras Med Trop. 2002; 35(2): 133-41.	Brazil	143730
Osório MM, Lira PI, Batista-Filho M, Ashworth A. Prevalence of anemia in children 6-59 months old in the state of Pernambuco, Brazil. Rev Panam Salud Publica. 2001; 10(2): 101-7.	Brazil	143731

Assis AMO, Gaudenzi EN, Gomes G, Ribeiro R de C, Szarfarc SC, Souza SB de. [Hemoglobin concentration, breastfeeding and complementary feeding in the first year of life]. Rev Saude Publica. 2004; 38(4): 543-51.	Brazil	143753
Fogarty International Center, National Institutes of Health (NIH), Foundation for the National Institutes of Health (FNIH), National Institute of Science, Technology, and Biomedicine of Semi-Arid Brazil (INCT-IBISAB). Brazil - Fortaleza Malnutrition and Enteric Disease Study 2009-2014.	Brazil	261873
Almeida APC, Zandonade E, Arantes MM, Lamounier JA. Deficiência de ferro e anemia ferropriva na população de 6 meses a 6 anos em Vitória, Espírito Santo, Sudeste do Brasil [Iron Deficiency and Iron Deficiency Anemia in the Population of 6 Months to 6 Years in Vitória, Espírito Santo, Southeastern Brazil] [master's thesis]. Presented at: UFMG, Belo Horizonte, Brazil; 2000.	Brazil	139466
Piaui State Government, United Nations Children's Fund (UNICEF). Brazil - Piauí Children and Adolescents: Health, Education, and Work 1991. Teresina, Brazil: Piaui State Government, 1992.	Brazil	139457
Macro International, Inc, National Institute of Statistics and Demography (Burkina Faso). Burkina Faso Demographic and Health Survey 2003. Fairfax, United States of America: ICF International.	Burkina Faso	19088
ICF Macro, Ministry of Health (Burkina Faso), National Institute of Statistics and Demography (Burkina Faso). Burkina Faso Demographic and Health Survey 2010-2011. Fairfax, United States of America: ICF International.	Burkina Faso	19133
Meda N, Cousens S, Kanki B. Anaemia among women of reproductive age in Burkina Faso. World Health Forum. 1996; 17(4): 369-72.	Burkina Faso	143771
ICF International, National Center for Research and Training on Malaria (CNRFP) (Burkina Faso), National Institute of Statistics and Demography (Burkina Faso), National Program for the Fight Against Malaria (PNLP) (Burkina Faso). Burkina Faso Malaria Indicator Survey 2014. Fairfax, United States of America: ICF International, 2015.	Burkina Faso	188785
ICF International, National Center for Research and Training on Malaria (CNRFP) (Burkina Faso), National Institute of Statistics and Demography (Burkina Faso), National Program for the Fight Against Malaria (PNLP) (Burkina Faso). Burkina Faso Malaria Indicator Survey 2017-2018. Fairfax, United States of America: ICF International, 2019.	Burkina Faso	413556
Institut de Recherche pour le Développement (IRD); Institut de Recherche en Sciences de la Santé (IRSS); and HarvestPlus, International Food Policy Research Institute (IFPRI). 2016. Food consumption and iron status survey in two provinces of rural Burkina Faso. Washington, DC: International Food Policy Research Institute (IFPRI). http://dx.doi.org/10.7910/DVN/5CXCLX	Burkina Faso	283273
Burundi Institute of Statistics and Economic Studies, ICF International, Ministry of Public Health and the Fight Against AIDS (Burundi). Burundi Demographic and Health Survey 2010-2011. Fairfax, United States of America: ICF International, 2012.	Burundi	30431
Burundi Institute of Statistics and Economic Studies, ICF International, Ministry of Public Health and the Fight Against AIDS (Burundi). Burundi Demographic and Health Survey 2016-2017. Fairfax, United States of America: ICF International, 2018.	Burundi	286766
ICF International, Ministry of the Fight Against AIDS (Côte d'Ivoire), National Institute of Statistics (Côte d'Ivoire). Côte d'Ivoire Demographic and Health Survey 2011-2012. Fairfax, United States of America: ICF International.	Cote d'Ivoire	18533
Asobayire FS, Adou P, Davidsson L, Cook JD, Hurrell RF. Prevalence of iron deficiency with and without concurrent anemia in population groups with high prevalences of malaria and other infections: a study in Côte d'Ivoire. Am J Clin Nutr. 2001; 74(6): 776-82.	Cote d'Ivoire	143826
Reinhardt MC. Maternal anaemia in Abidjan--Its influence on placenta and newborns. Helv Paediatr Acta Suppl. 1978; 43-63.	Cote d'Ivoire	143828
Reinhardt MC, Marti HR. Haematological data of African newborns and their mothers in Abidjan. Helv Paediatr Acta Suppl. 1978; 85-99.	Cote d'Ivoire	143829

Rohner F, Northrop-Clewes C, Tschannen AB, Bosso PE, Kouassi-Gohou V, Erhardt JG, Bui M, Zimmermann MB, Mascie-Taylor CG. Prevalence and public health relevance of micronutrient deficiencies and undernutrition in pre-school children and women of reproductive age in Côte d'Ivoire, west Africa. Public Health Nutr. 2013; 17(9): 2016-28.	Cote d'Ivoire	294293
Macro International, Inc, Ministry of Health (Cambodia), National Institute of Statistics (Cambodia). Cambodia Demographic and Health Survey 2000. Fairfax, United States of America: ICF International.	Cambodia	19156
Macro International, Inc, National Institute of Public Health (Cambodia), National Institute of Statistics (Cambodia). Cambodia Demographic and Health Survey 2005-2006. Fairfax, United States of America: ICF International.	Cambodia	19167
ICF Macro, Ministry of Health (Cambodia), National Institute of Statistics (Cambodia). Cambodia Demographic and Health Survey 2010-2011. Fairfax, United States of America: ICF International.	Cambodia	30379
United Nations Children's Fund (UNICEF). Cambodia Seth Koma Follow-up Survey 2003. 2003.	Cambodia	139614
ICF International, Ministry of Health (Cambodia), National Institute of Statistics (Cambodia). Cambodia Demographic and Health Survey 2014. Fairfax, United States of America: ICF International, 2017.	Cambodia	157024
Helen Keller International. Iron deficiency in Cambodia: The Need for Iron Supplementation Among Preschool-aged Children. HKI Cambodia Nutrition Bulletin. 2001; 2: 1-4.	Cambodia	139612
Macro International, Inc, National Institute of Statistics (Cameroon). Cameroon Demographic and Health Survey 2004. Fairfax, United States of America: ICF International.	Cameroon	19211
ICF International, Ministry of Economy, Planning and Regional Development (Cameroon), Ministry of Public Health (Cameroon), National Institute of Statistics (Cameroon), Pasteur Center of Cameroon. Cameroon Demographic and Health Survey 2011. Fairfax, United States of America: ICF International.	Cameroon	19274
Coulibaly M, Costagliola D, Zittoun J, Mary JY. Modifications of hemato-biological parameters in pregnant women in a migrating population in northern Cameroon: prevalence of anemia, iron and folates deficiencies. Int J Vitam Nutr Res. 1987; 57(2): 173-8.	Cameroon	143774
Engle-Stone R, Nankap M, Ndjebayi AO, Erhardt JG, Brown KH. Plasma ferritin and soluble transferrin receptor concentrations and body iron stores identify similar risk factors for iron deficiency but result in different estimates of the national prevalence of iron deficiency and iron-deficiency anemia. J Nutr. 2013; 143(3): 369-77.	Cameroon	294308
Valberg LS, Sorbie J, Ludwig J, Pelletier O. Serum ferritin and the iron status of Canadians. CMAJ. 1976; 114(5): 417-21.	Canada	143775
Hodgins S, Dewailly E, Chatwood S, Bruneau S, Bernier F. Iron-deficiency anemia in Nunavik: pregnancy and infancy. Int J Circumpolar Health. 1998; 57 (Suppl 1): 135-40.	Canada	143776
Brault-Dubuc M, Nadeau M, Dickie J. Iron status of French-Canadian children: a three year follow-up study. Hum Nutr Appl Nutr. 1983; 37 A(3): 210-21.	Canada	143777
Yeung GS, Zlotkin SH. Efficacy of meat and iron-fortified commercial cereal to prevent iron depletion in cow milk-fed infants 6 to 12 months of age: a randomized controlled trial. Can J Public Health. 2000; 91(4): 263-7.	Canada	143780
Lehmann F, Gray-Donald K, Mongeon M, Di Tommaso S. Iron deficiency anemia in 1-year-old children of disadvantaged families in Montreal. CMAJ. 1992; 146(9): 1571-7.	Canada	143781
United Nations Children's Fund (UNICEF). Central African Republic Multiple Indicator Cluster Survey 2006. New York, United States: United Nations Children's Fund (UNICEF).	Central African Republic	2223
Department of Public Health, Pontifical Catholic University of Chile, Institute of Nutrition and Food Technology (INTA), University of Chile, Ministry of Health (Chile), Pan American Health Organization (PAHO). Chile National Health Survey 2003.	Chile	111907

Mardones F, Rioseco A, Ocqueteau M, Urrutia MT, Javet L, Rojas I, Villarroel del L. [Anemia in pregnant women from the community of Puente Alto, Chile]. Rev Med Chil. 2003; 131(5): 520-5.	Chile	143789
Hertrampf E, Olivares M, Letelier A, Castillo C. [Iron nutritional status in pregnant adolescents at the beginning of gestation]. Rev Med Chil. 1994; 122(12): 1372-7.	Chile	143790
Hertrampf E, Dinamarca M, Llaguno TM, Stekel A. [Iron nutrition and breast feeding in Chilean infants]. Rev Chil Pediatr. 1987; 58(3): 193-7.	Chile	143794
Díaz A, Guerra H, Campos S, Letelier C, Olivares G. Prevalencia de deficiencia de hierro en preescolares de la comuna la Pintana [Prevalence of iron deficiency in preschool children from la Pintana county]. Rev Chilena Nutr. 2002; 10-3.	Chile	143797
Ministry of Public Health (China). China National Nutrition Survey 1992 - China CDC.	China	119643
Cai MQ, Yan WY. Study on iron nutritional status in adolescence. Biomed Environ Sci. 1990; 3(1): 113-9.	China	143801
Gao J, Zeng S, Sun BL, Fan HM, Han LH. Menstrual blood loss and hematologic indices in healthy Chinese women. J Reprod Med. 1987; 32(11): 822-6.	China	143814
Zhou LM, Yang WW, Hua JZ, Deng CQ, Tao X, Stoltzfus RJ. Relation of hemoglobin measured at different times in pregnancy to preterm birth and low birth weight in Shanghai, China. Am J Epidemiol. 1998; 148(10): 998-1006.	China	143816
Ministry of Health (China), National Bureau of Statistics of China. China National Nutrition Survey 2002 - China CDC.	China	124479
Wang W, Chen X, Liu D. Hematologic response to iron and ascorbic acid administration in preschool children with anemia. Nutr Res. 1986; 241-8.	China	143808
Carolina Population Center, University of North Carolina at Chapel Hill, Chinese Center for Disease Control and Prevention (CCDC). China Health and Nutrition Survey 1989-2015. Chapel Hill, United States of America: Carolina Population Center, University of North Carolina at Chapel Hill.	China	414197
Dang S, Yan H, Yamamoto S, Wang X, Zeng L. Poor nutritional status of younger Tibetan children living at high altitudes. Eur J Clin Nutr. 2004; 58(6): 938-46.	China	143045
Agudelo GM, Cardona OL, Posada M, Montoya MN, Ocampo NE, Marín CM, Correa MC, López C. [Prevalence of iron-deficiency anemia in schoolchildren and adolescents, Medellín, Colombia, 1999]. Rev Panam Salud Publica. 2003; 13(6): 376-86.	Colombia	143818
Colombian Family Welfare Institute, National Institute of Health (Colombia), Pan American Health Organization (PAHO), Profamilia (Colombia), University of Antioquia. Colombia National Survey of the Nutritional Situation 2004-2005. Bogotá, Colombia: Colombian Family Welfare Institute.	Colombia	124467
Colombian Family Welfare Institute, Ministry of Social Protection (Colombia), National Institute of Health (Colombia), Profamilia (Colombia). Colombia National Survey of the Nutritional Situation 2010. Bogotá, Colombia: Colombian Family Welfare Institute.	Colombia	126331
Macro International, Inc, National Center for Statistics and Economic Studies (Congo, Rep.). Congo Demographic and Health Survey 2005. Fairfax, United States of America: ICF International.	Congo	19391
ICF International, Ministry of Health (Congo, Rep.), National Center for Statistics and Economic Studies (Congo, Rep.). Congo Demographic and Health Survey 2011-2012. Fairfax, United States of America: ICF International.	Congo	56151
Rodríguez S, Blanco A, Cunningham L, Ascencio M, Chávez M, Muñoz L. [Prevalence of nutritional anemia in women of reproductive age. Costa Rica. National nutrition survey, 1996]. Arch Latinoam Nutr. 2001; 51(1): 19-24.	Costa Rica	143819
Villarejos VM, Bickers J, Alvaro Riveraaa S, Peña Chavarria A, Hunter GW 3rd, Kotcher E. Pathogenesis of anemia in Costa Rica. Epidemiologic study of hemoglobin and serum protein levels and hookworm infection in children. Am J Trop Med Hyg. 1970; 19(4): 603-9.	Costa Rica	143820
Jiménez E, Brittenham GM, Jiménez R, Lozoff B, Mora L, Gómez I, Carrillo JM. [Iron deficiency anemia in unweaned infants: a prospective study]. Bol Med Hosp Infant Mex. 1987; 44(6): 309-15.	Costa Rica	143821

Cunningham L, Blanco A, Rodríguez S, Ascencio M. [Prevalence of anemia, iron and folate deficiency in children 7 years smaller. Costa Rica, 1996]. Arch Latinoam Nutr. 2001; 51(1): 37-43.	Costa Rica	143822
Prendes Labrada M, Baños Rodríguez A, Toledo Dieppa O, Lescay Megret O. Prevalencia de anemia en gestantes en un área de sa lud [Prevalence of anemia in pregnant women in a health area]. Rev Cubana Med General Integral. 2000; 25-30.	Cuba	143834
Rodriguez J, Perez J, Hernández A, Triana M, Chong A, Sá chez M. Anemia nutricional en un grupo de niños aparentemente sanos de 2 a 4 años de edad. Rev Cubana Aliment Nutr. 2002; 31-4.	Cuba	143832
Reboso Pérez J, Jiménez Acosta S, Gay Rodríguez J, Cabrera A, Sánchez M. [Anemia in a group of children of 14 to 57 months of age, apparently health]. Rev Cub Salud Publica. 2003; 128-31.	Cuba	143833
Osancová K, Hejda S. Nutrition and its reflection on the health status of the population. Rev Czech Med. 1968; 14(2): 101-17.	Czechia	143837
European Union (EU), Government of North Korea, United Nations Children's Fund (UNICEF), World Food Programme (WFP). North Korea Multiple Indicator Cluster Survey 1998.	Democratic People's Republic of Korea	7465
Central Bureau of Statistics (North Korea), United Nations Children's Fund (UNICEF), World Food Programme (WFP). North Korea Nutrition Assessment 2004.	Democratic People's Republic of Korea	58954
Central Bureau of Statistics (North Korea), United Nations Children's Fund (UNICEF), World Food Programme (WFP). North Korea Nutrition Assessment 2012.	Democratic People's Republic of Korea	68833
Macro International, Inc, Ministry of Planning (Congo, DR). Democratic Republic of the Congo Demographic and Health Survey 2007. Fairfax, United States of America: ICF International.	Democratic Republic of the Congo	19381
ICF International, Ministry of Planning and Monitoring Implementation of the Revolution of Modernity (Congo, DR), Ministry of Public Health (Congo, DR), National Institute of Statistics (Congo, DR). Democratic Republic of the Congo Demographic and Health Survey 2013-2014. Fairfax, United States of America: ICF International, 2014.	Democratic Republic of the Congo	76878
Jackson DJ, Klee EB, Green SD, Mokili JL, Elton RA, Cutting WA. Severe anaemia in pregnancy: a problem of primigravidae in rural Zaire. Trans R Soc Trop Med Hyg. 1991; 85(6): 829-32.	Democratic Republic of the Congo	143839
Milman N, Cohn J, Pedersen NS. Serum ferritin and iron status of children in the Faroe Islands. Eur J Pediatr. 1984; 142(2): 89-92.	Denmark	143840
Hagerup L, Eriksen M, Schroll M, Hollnagel H, Agner E, Larsen S. The Glostrup population studies. Collection of epidemiologic tables. Reference values for use in cardiovascular population studies. Scand J Soc Med Suppl. 1981; 1-112.	Denmark	143841
Milman N, Heitman B, Lyhne N, Rosdahl N, Jensen K, Graudal N. Iron status in 1113 Danish men and women aged 35-65 years: relation to dietary and supplemental iron intake. Scand J Nutr. 1993; 98-103.	Denmark	143842
Milman N, Agger AO, Nielsen OJ. Iron supplementation during pregnancy. Effect on iron status markers, serum erythropoietin and human placental lactogen. A placebo controlled study in 207 Danish women. Dan Med Bull. 1991; 38(6): 471-6.	Denmark	143843
Milman N, Clausen JO, Jordal R. Iron status in young Danish men and women: a population survey comprising 548 individuals. Ann Hematol. 1995; 70(4): 215-21.	Denmark	143848
Milman N, Thomsen H, Mathiassen B. Serum ferritin, iron status and plasma ascorbic acid in 40- to 49-year-old males in the Faroe Islands. Scand J Clin Lab Invest. 1990; 50(5): 559-64.	Denmark	143849
Marner T. Haemoglobin, erythrocyres and serum iron values in normal children 3-6 years of age. Acta Paediatr Scand. 1969; 58(4): 363-8.	Denmark	143850
Center for Social and Demographic Studies (Dominican Republic) (CESDEM), Centers for Disease Control and Prevention (CDC), Institute of Nutrition of Central America and Panama, Ministry of Public Health and Social Assistance (Dominican Republic), UNICEF International Child Development Center (ICDC), World Bank. Dominican Republic National Micronutrient Survey 2009.	Dominican Republic	262907
Quizhpe E, San Sebastián M, Hurtig AK, Llamas A. [Prevalence of anaemia in schoolchildren in the Amazon area of Ecuador]. Rev Panam Salud Publica. 2003; 13(6): 355-61.	Ecuador	143852

Ministry of Public Health (Ecuador), National Institute of Statistics and Censuses (Ecuador). Ecuador National Health and Nutrition Survey 2012.	Ecuador	153674
Macro International, Inc, Population Council (Egypt). Egypt Demographic and Health Survey 2000. Fairfax, United States of America: ICF International.	Egypt	19511
El-Zanaty and Associates, Macro International, Inc, Ministry of Health and Population (Egypt), Population Council (Egypt). Egypt Demographic and Health Survey 2005. Fairfax, United States of America: ICF International.	Egypt	19521
Centers for Disease Control and Prevention (CDC), Ministry of Health and Population (Egypt), National Nutrition Institute (Egypt), United States Agency for International Development (USAID). Egypt National Nutrition Survey 1978.	Egypt	139723
el-Sahn F, Sallam S, Mandil A, Galal O. Anaemia among Egyptian adolescents: prevalence and determinants. East Mediterr Health J. 2000; 6(5-6): 1017-25.	Egypt	143854
Abdou IA, Ali HE, Said AK, Mousa WA, Demian HG, Soliman AM, el-Hawary LH. Incidence of nutritional deficiencies, goitre and dental caries among school children in Cairo. J Egypt Public Health Assoc. 1967; 42(4): 175-84.	Egypt	143858
El-Zanaty and Associates, ICF International, Ministry of Health and Population (Egypt). Egypt Demographic and Health Survey 2014. Fairfax, United States of America: ICF International.	Egypt	154897
Shukry AS, Labib FM, Kamel LM. Assessment of the health and nutritional standards of infants and preschool children in high density Cairo community. Gaz Egypt Paediatr Assoc. 1973; 21(3): 47-56.	Egypt	143859
El Salvador Demographic Association (ADS), Division of Reproductive Health-Centers for Disease Control and Prevention (CDC). El Salvador Reproductive Health Survey 1998. Atlanta, United States: Centers for Disease Control and Prevention (CDC).	El Salvador	27590
Asociación Demográfica Salvadoreña (ADS), Division of Reproductive Health-Centers for Disease Control and Prevention (CDC). (2004) El Salvador Reproductive Health Survey 2002-2003. San Salvador, El Salvador: ADS.	El Salvador	27599
Asociación Demográfica Salvadoreña (ADS), Division of Reproductive Health-Centers for Disease Control and Prevention (CDC). (2009) El Salvador Reproductive Health Survey 2008. San Salvador, El Salvador: ADS.	El Salvador	27606
El Salvador Demographic Association (ADS), Institute of Nutrition of Central America and Panama, Ministry of Public Health and Social Assistance (El Salvador). El Salvador Assessment of Nutritional Food Situation 1988. Guatemala City, Guatemala: Institute of Nutrition of Central America and Panama, 1990.	El Salvador	139894
General Administration of Statistics and Censuses (El Salvador), Ministry of Health (El Salvador), United Nations Children's Fund (UNICEF). El Salvador Multiple Indicator Cluster Survey 2014. New York, United States of America: United Nations Children's Fund (UNICEF), 2017.	El Salvador	200636
Ross SM. Haemoglobin and haematocrit values in pregnant women on a high iron intake and living at a high altitude. J Obstet Gynaecol Br Commonw. 1972; 79(12): 1103-7.	Ethiopia	143869
Haidar J, Nekatibeb H, Urga K. Iron deficiency anemia in pregnant and lactating mothers in rural Ethiopia. East Afr Med J. 1999; 76(11): 618-22.	Ethiopia	143870
Desalegn S. Prevalence of anaemia in pregnancy in Jima town, southwestern Ethiopia. Ethiop Med J. 1993; 31(4): 251-8.	Ethiopia	143871
Gies S, Brabin BJ, Yassin MA, Cuevas LE. Comparison of screening methods for anaemia in pregnant women in Awassa, Ethiopia. Trop Med Int Health. 2003; 8(4): 301-9.	Ethiopia	143872
Macro International, Inc, Population and Housing Census Commissions Office (PHCCO). Ethiopia Demographic and Health Survey 2005. Fairfax, United States of America: ICF International.	Ethiopia	19557
Central Statistics Agency (Ethiopia), ICF Macro, Ministry of Health (Ethiopia). Ethiopia Demographic and Health Survey 2010-2011. Fairfax, United States of America: ICF International.	Ethiopia	21301
Central Statistics Agency (Ethiopia), ICF International. Ethiopia Demographic and Health Survey 2016. Fairfax, United States of America: ICF International, 2017.	Ethiopia	218568

Ethiopian Public Health Institute (EPHI), Ministry of Health (Ethiopia). Ethiopia National Micronutrient Survey 2015.	Ethiopia	408685
National Food and Nutrition Centre (Fiji). Fiji National Nutrition Survey 2004.	Fiji	124447
National Food and Nutrition Committee (Fiji). Fiji National Nutrition Survey 1993.	Fiji	124448
Hill Laboratories (New Zealand), National Food and Nutrition Centre (Fiji), TropicHealth Laboratories, World Health Organization (WHO). Fiji Impact Study of Iron Fortified Flour 2010.	Fiji	135526
Lahti-Koski M, Valsta LM, Alfthan G, Tapanainen H, Aro A. Iron status of adults in the capital area of Finland. Eur J Nutr. 2003; 42(5): 287-92.	Finland	143875
Takkunen H. Iron deficiency in the Finnish adult population: an epidemiological survey from 1967 to 1972 inclusive. Scand J Haematol Suppl. 1976; 1-91.	Finland	143876
Widholm O, Vartiainen E, Tenhunen T. On iron requirement in menstruating teen-age girls. Iron study among teen-agers. I. Acta Obstet Gynecol Scand. 1967; 46(Suppl 1): 29-46.	Finland	143880
Siimes MA, Saarinen UM, Dallman PR. Relationship between hemoglobin concentration and transferrin saturation in iron-sufficient infants. Am J Clin Nutr. 1979; 32(11): 2295-300.	Finland	143877
Knuiman JT, Westenbrink S, van der Heyden L, West CE, Burema J, de Boer J, Hautvast JG, Räsänen L, Virkkunen L, Viikari J. Determinants of total and high density lipoprotein cholesterol in boys from Finland, The Netherlands, Italy, the Philippines and Ghana with special reference to diet. Hum Nutr Clin Nutr. 1983; 37(4): 237-54.	Finland, Ghana, Italy, Netherlands, Philippines	143879
Galan P, Yoon HC, Preziosi P, Viteri F, Valeix P, Fieux B, Briançon S, Malvy D, Roussel AM, Favier A, Hercberg S. Determining factors in the iron status of adult women in the SU.VI.MAX study. SUpplementation en VItamines et Minéraux AntioXydants. Eur J Clin Nutr. 1998; 52(6): 383-8.	France	143883
Galán P, Hercberg S, Soustre Y, Dop MC, Dupin H. Factors affecting iron stores in French female students. Hum Nutr Clin Nutr. 1985; 39(4): 279-87.	France	143887
Blot I, Papiernik E, Kaltwasser JP, Werner E, Tchernia G. Influence of routine administration of folic acid and iron during pregnancy. Gynecol Obstet Invest. 1981; 12(6): 294-304.	France	143888
Blot I, Rey A, Kaltwasser JP, Francoual J, Papiernik E, Tchernia G. Folate and iron deficiencies in mothers and their newborn children. Blut. 1982; 44(5): 297-303.	France	143892
Hercberg S, Galán P, Soustre Y, Dop MC, Devanlay M, Dupin H. Effects of iron supplementation on serum ferritin and other hematological indices of iron status in menstruating women. Ann Nutr Metab. 1985; 29(4): 232-8.	France	143893
Zittoun J, Blot I, Hill C, Zittoun R, Papiernik E, Tchernia G. Iron supplements versus placebo during pregnancy: its effects on iron and folate status on mothers and newborns. Ann Nutr Metab. 1983; 27(4): 320-7.	France	143894
General Directorate of Statistics (Gabon), ICF International, Ministry of Economy, Employment and Sustainable Development (Gabon), Ministry of Health (Gabon). Gabon Demographic and Health Survey 2012. Fairfax, United States of America: ICF International, 2013.	Gabon	76706
Gambia Bureau of Statistics (GBOS), ICF International, Ministry of Health and Social Welfare (The Gambia). Gambia Demographic and Health Survey 2013. Fairfax, United States of America: ICF International, 2015.	Gambia	77384
National Center for Disease Control (Georgia), State Department of Statistics of Georgia, United Nations Children's Fund (UNICEF). Georgia Multiple Indicator Cluster Survey 2005. New York, United States of America: United Nations Children's Fund (UNICEF).	Georgia	3970
National Center for Disease Control and Public Health (Georgia), United Nations Children's Fund (UNICEF). Georgia National Nutrition Survey 2009.	Georgia	141238
Robert Koch Institute. Germany Health Interview and Examination Survey for Children and Adolescents 2003-2006. Berlin, Germany: Robert Koch Institute, 2008.	Germany	236076

Bergmann RL, Gravens-Müller L, Hertwig K, Hinkel J, Andres B, Bergmann KE, Dudenhausen JW. Iron deficiency is prevalent in a sample of pregnant women at delivery in Germany. Eur J Obstet Gynecol Reprod Biol. 2002; 102(2): 155-60.	Germany	143895
Ghana Statistical Service, Macro International, Inc. Ghana Demographic and Health Survey 2003. Fairfax, United States of America: ICF International.	Ghana	19627
Ghana Statistical Service, Macro International, Inc, Ministry of Health (Ghana). Ghana Demographic and Health Survey 2008. Fairfax, United States of America: ICF International.	Ghana	21188
Mockenhaupt FP, Rong B, Günther M, Beck S, Till H, Kohne E, Thompson WN, Bienzle U. Anaemia in pregnant Ghanaian women: importance of malaria, iron deficiency, and haemoglobinopathies. Trans R Soc Trop Med Hyg. 2000; 94(5): 477-83.	Ghana	142049
Agyepong IA, Wellington EK, Abbey MA. A comparative study of clinical and sociocultural aspects of anaemia among adolescent girls in rural Ghana. Addict Behav Rep. 1997; 65(3): 123-38.	Ghana	143897
Lassey AT, Klufio CA, Annan BD, Wilson JB. Antenatal haemoglobin profile at Korle-Bu Teaching Hospital. East Afr Med J. 1999; 76(4): 228-32.	Ghana	143898
Ghana Health Service, Ghana Statistical Service, ICF International. Ghana Demographic and Health Survey 2014. Fairfax, United States of America: ICF International, 2016.	Ghana	157027
Centers for Disease Control and Prevention (CDC), Ghana Statistical Service, Government of Japan, ICF Macro, Ministry of Health (Ghana), Navrongo Health Research Centre, United Nations Children's Fund (UNICEF), United Nations Population Fund (UNFPA), United States Agency for International Development (USAID). Ghana Multiple Indicator Cluster Survey 2011. New York, United States of America: United Nations Children's Fund (UNICEF), 2013.	Ghana	63993
Koram KA, Owusu-Agyei S, Utz G, Binka FN, Baird JK, Hoffman SL, Nkrumah FK. Severe anemia in young children after high and low malaria transmission seasons in the Kassena-Nankana district of northern Ghana. Am J Trop Med Hyg. 2000; 62(6): 670-4.	Ghana	255115
Ghana Health Service, Ghana Statistical Service, ICF International, National Malaria Control Programme (Ghana), National Public Health and Reference Laboratory (NHPRL) (Ghana). Ghana Malaria Indicator Survey 2016. Fairfax, United States of America: ICF International.	Ghana	286788
The Partnership for Child Development. The Health and Nutritional Status of Schoolchildren in Africa: Evidence from School-based Health Programmes in Ghana and Tanzania. Trans R Soc Trop Med Hyg. 1998; 92(3): 254-61.	Ghana, United Republic of Tanzania	139599
World Health Organization (WHO). WHO Global Database on Anemia, Nutrition Landscape Information System. Geneva, Switzerland: World Health Organization (WHO).	Global	144314
Kafatos A, Diacatou A, Labadarios D, Kounali D, Apostolaki J, Vlachonikolis J, Mamalakis G, Megremis S. Nutrition status of the elderly in Anogia, Crete, Greece. J Am Coll Nutr. 1993; 12(6): 685-92.	Greece	143899
Kattamis C, Metaxotou-Mavromnati A, Konidaris C, Touliatos N, Constantsas N, Matsaniotis N. Iron deficiency in Greece. Epidemiologic and hematologic studies. J Pediatr. 1974; 84(5): 666-71.	Greece	143900
Guatemala Ministry of Health and Social Assistance, University of Valle and Division of Reproductive Health-Centers for Disease Control and Prevention (CDC). Guatemala Reproductive Health Survey 2008-2009. Atlanta, United States: Centers for Disease Control and Prevention (CDC).	Guatemala	4779
Guatemala Ministry of Health and Social Assistance, University of Valle, Division of Reproductive Health-Centers for Disease Control and Prevention (CDC). (2003) Guatemala Reproductive Health Survey 2002. Atlanta, United States: Centers for Disease Control and Prevention (CDC).	Guatemala	27563
Guatemala Ministry of Health and Social Assistance, Macro International, Inc, National Statistics Institute (Guatemala). Guatemala Interim Demographic and Health Survey 1998-1999. Fairfax, United States of America: ICF International.	Guatemala	19656

ICF International, Institute of Nutrition of Central America and Panama, Ministry of Public Health and Social Assistance (Guatemala), National Statistics Institute (Guatemala), Secretary of Planning and Programming of the Presidency (Segeplán) (Guatemala). Guatemala Demographic and Health Survey 2014-2015. Fairfax, United States of America: ICF International, 2017.	Guatemala	157031
Macro International, Inc, National Statistics Directorate (Guinea). Guinea Demographic and Health Survey 2005. Fairfax, United States of America: ICF International.	Guinea	19683
ICF Macro, Ministry of Health and Public Hygiene (Guinea), National Institute of Statistics (Guinea). Guinea Demographic and Health Survey 2012. Fairfax, United States of America: ICF International.	Guinea	69761
Ministry of Health (Guinea), Ministry of Planning and Economic Development (Guinea), National Institute of Statistics (Guinea). Guinea Demographic and Health Survey 2018. Fairfax, United States of America: ICF International, 2019.	Guinea	396957
Bureau of Statistics (Guyana), ICF Macro, Ministry of Health (Guyana). Guyana Demographic and Health Survey 2009. Fairfax, United States of America: ICF International, 2011.	Guyana	21348
Johnson AA, Latham MC, Roe DA. The prevalence and the etiology of the nutritional anemias in Guyana. Am J Clin Nutr. 1982; 35(2): 309-18.	Guyana	143903
Haitian Institute of Childhood (IHE), Macro International, Inc. Haiti Demographic and Health Survey 2000. Fairfax, United States of America: ICF International.	Haiti	19708
Haitian Institute of Childhood (IHE), Haitian Institute of Statistics and Informatics, Macro International, Inc. Haiti Demographic and Health Survey 2005-2006. Fairfax, United States of America: ICF International.	Haiti	19720
Centers for Disease Control and Prevention (CDC), Haitian Institute of Childhood (IHE), Haitian Institute of Statistics and Informatics, Macro International, Inc. Haiti Demographic and Health Survey 2012. Fairfax, United States of America: ICF International.	Haiti	65118
Nicklas TA, Kuvibidila S, Gatewood LC, Metzinger AB, Frempong KO. Prevalence of anaemia and iron deficiency in urban Haïtian children two to five years of age. J Trop Pediatr. 1998; 44(3): 133-8.	Haiti	143904
Haitian Institute of Childhood (IHE), Haitian Institute of Statistics and Informatics, ICF International, Ministry of Public Health and Population (Haiti). Haiti Demographic and Health Survey 2016-2017. Fairfax, United States of America: ICF International.	Haiti	218574
Macro International, Inc, National Institute of Statistics (Honduras), Secretary of Health (Honduras). Honduras Demographic and Health Survey 2005-2006. Fairfax, United States of America: ICF International.	Honduras	19728
ICF Macro, National Institute of Statistics (Honduras). Honduras Demographic and Health Survey 2011-2012. Fairfax, United States of America: ICF International.	Honduras	95440
Honduras Family Planning Association (ASHONPLAFA), Ministry of Health (Honduras), and Division of Reproductive Health-Centers for Disease Control and Prevention (CDC). Honduras Reproductive Health Survey 2001. Tegucigalpa, Honduras: Honduras Family Planning Association (ASHONPLAFA).	Honduras	27551
Domellöf M, Cohen RJ, Dewey KG, Hernell O, Rivera LL, Lönnerdal B. Iron supplementation of breast-fed Honduran and Swedish infants from 4 to 9 months of age. J Pediatr. 2001; 138(5): 679-87.	Honduras, Sweden	143905
Antal M, Regöly-Mérei A, Biró L, Greiner E, Sági K, Agfalvy R, Nagy K, Biró G. Iron status in a healthy population of Hungarian secondary school boys and girls. Ann Nutr Metab. 1998; 42(1): 33-9.	Hungary	143906
Jonsson JJ, Johannesson GM, Sigfusson N, Magnusson B, Thjodleifsson B, Magnusson S. Prevalence of iron deficiency and iron overload in the adult Icelandic population. J Clin Epidemiol. 1991; 44(12): 1289-97.	Iceland	143907
International Institute for Population Sciences (India), Macro International, Inc. India Demographic and Health Survey 1998-1999. Calverton, United States of America: Macro International, Inc.	India	19950

International Institute for Population Sciences (India), Macro International, Inc. India Demographic and Health Survey 2005-2006. Fairfax, United States of America: ICF International.	India	19963
National Nutrition Monitoring Bureau (India). India Micronutrient Deficiency Survey 2003-2006.	India	133801
Kapil U, Pathak P, Tandon M, Singh C, Pradhan R, Dwivedi SN. Micronutrient deficiency disorders amongst pregnant women in three urban slum communities of Delhi. Indian Pediatr. 1999; 36(10): 983-9.	India	139573
Pathak P, Singh P, Kapil U, Raghuvanshi RS. Prevalence of iron, vitamin A, and iodine deficiencies amongst adolescent pregnant mothers. Indian J Pediatr. 2003; 70(4): 299-301.	India	139585
Pathak P, Tandon M, Kapil U, Singh C. Prevalence of iron deficiency anemia amongst pregnant women in urban slum communities of Delhi. Indian Pediatr. 1999; 36(3): 322-3.	India	143912
Brabin L, Nicholas S, Gogate A, Gogate S, Karande A. High prevalence of anaemia among women in Mumbai, India. Food Nutr Bull. 1998; 205-9.	India	143916
Verma M, Chhatwal J, Kaur G. Prevalence of anemia among urban school children of Punjab. Indian Pediatr. 1998; 35(12): 1181-6.	India	143917
Abel R, Rajaratnam J, Kalaimani A, Kirubakaran S. Can iron status be improved in each of the three trimesters? A community-based study. Eur J Clin Nutr. 2000; 54(6): 490-3.	India	143921
Agarwal KN, Gomber S, Bisht H, Som M. Anemia prophylaxis in adolescent school girls by weekly or daily iron-folate supplementation. Indian Pediatr. 2003; 40(4): 296-301.	India	143923
Sethi V, Goindi G, Kapil U. Prevalence of anemia amongst primary school age children (6-11 years) in National Capital Territory of Delhi. Indian J Pediatr. 2003; 70(6): 519-20.	India	143925
Agarwal DK, Bhardwaj B, Singla PN, Tripathi AM, Agarwal KN. Etiology of maternal and early childhood deficiency anemia. Indian J Pediatr. 1986; 53(3): 389-95.	India	143926
Agarwal DK, Agarwal KN, Tripathi AM. Nutritional status in rural pregnant women of Bihar and Uttar Pradesh. Indian Pediatr. 1987; 24(2): 119-25.	India	143927
Sharma JB, Soni D, Murthy NS, Malhotra M. Effect of dietary habits on prevalence of anemia in pregnant women of Delhi. J Obstet Gynaecol Res. 2003; 29(2): 73-8.	India	143931
Agarwal AK, Sen AK, Kalra NK, Gupta N. Prevalence of anaemia during pregnancy in district Burdwan, West Bengal. Indian J Public Health. 1999; 43(1): 26-31.	India	143932
Rajaratnam J, Abel R, Asokan JS, Jonathan P. Prevalence of anemia among adolescent girls of rural Tamilnadu. Indian Pediatr. 2000; 37(5): 532-6.	India	143933
Rajaratnam J, Abel R, Ganesan C, Jayaseelan SA. Maternal anaemia: a persistent problem in rural Tamil Nadu. Natl Med J India. 2000; 13(5): 242-5.	India	143934
Chiplonkar S, Agte V, Mengale S. Relative importance of micronutrient deficiencies in iron deficiency anemia. Nutr Res. 2003; 1355-67.	India	143937
Reddaiah VP, Raj PP, Ramachandran K, Nath LM, Sood SK, Madan N, Rusia U. Supplementary iron dose in pregnancy anemia prophylaxis. Indian J Pediatr. 1989; 56(1): 109-14.	India	143939
Brahmam G, Madhavan NK, Laxmaiah A, Reddy C, Ranganathan S, Vishnuvardhana Rao M, Nadamuni Naidu A, Vijayaraghavan K, Sivakumar B, Kamala Krishnaswamy, Gowarinth Sastry J, Mohan Ram M, Praldad Rao N, Vinodini R. Community Trials with Iron and Iodine Fortified Salt (Double Fortified Salt). In: Geertman RM, ed. Proceedings of the 8th World Salt Symposium. Amsterdam, The Netherlands: Elsevier, 2000. 955-60.	India	143940
Abel R, Rajaratnam J, Gnanasekaran VJ, Jayaraman P. Prevalence of anaemia and iron deficiency in three trimesters in Rural Vellore district, South India. Trop Doct. 2001; 31(2): 86-9.	India	143941
Thilothammal N, Sujaritha R, Banu K, Ratnam S, Ezhlarasi. The nutritional status of south Indian women in child bearing age and its influence on birth weight and maturity of offspring. J Obstet Gynaecol India. 1993; 871-7.	India	143942

Bhargava M, Iyer PU, Kumar R, Ramji S, Kapani V, Bhargava SK. Relationship of maternal serum ferritin with foetal serum ferritin, birth weight and gestation. J Trop Pediatr. 1991; 37(4): 149-52.	India	143946
Sarin A. Severe anemia of pregnancy, recent experience. Int J Gynaecol Obstet. 1995; 50(Suppl 2): 45-49.	India	143947
Kumar CSV, Anand Kumar H, Sunita V, Kapur I. Prevalence of anemia and worm infestation in school going girls at Gulbargha, Karnataka. Indian Pediatr. 2003; 40(1): 70-2.	India	143948
Bains K, Mann S. Physical fitness in relation to energy and iron status of female college students. Food Nutr Bull. 2000; 305-10.	India	143955
Thangaleela T, Vijayalakshmi P. Prevalence of anaemia in pregnancy. Indian J Nutr Diet. 1994; 26-9.	India	143957
Koen M, Lemson M, Sampathkumar V, Abel R. Prevalence of anaemia among pregnant mothers in a rural South Indian population. J Obstet Gynaecol India. 1992; 283-7.	India	143963
Bharati P, Ghosh R, Gupta R. Socioeconomic Condition and Anaemia among the Mahishya Population of Southern West Bengal, India. Malays J Nutr. 2004; 10(1): 23-30.	India	143967
Swain S, Singh S, Bhatia BD, Pandey S, Krishna M. Maternal hemoglobin and serum albumin and fetal growth. Indian Pediatr. 1994; 31(7): 777-82.	India	143969
Ray SK, Mallick S, Kumar S, Biswas B. A rapid assessment of anaemia in pregnancy in West Bengal with special reference to care seeking behaviour of mothers. Indian J Public Health. 2000; 44(2): 58-64.	India	143970
International Institute for Population Sciences (India). India District Level Household Survey 2012-2014. New Delhi, India: Ministry of Health and Family Welfare (India).	India	165390
International Institute for Population Sciences (India). India District Level Household Survey 2002-2005. Mumbai, India: International Institute for Population Sciences (India).	India	23219
Jain S, Chopra H, Garg SK, Bhatnagar M, Singh JV. Anemia in children: early iron supplementation. Indian J Pediatr. 2000; 67(1): 19-21.	India	143919
ICF International, International Institute for Population Sciences (India), Ministry of Health and Family Welfare (India). India Demographic and Health Survey 2015-2016. Fairfax, United States of America: ICF International, 2018.	India	157050
National Nutrition Monitoring Bureau (India). India Rural Survey of Diet and Nutritional Status 2004-2006.	India, Andhra Pradesh, Gujarat, Karnataka, Kerala, Madhya Pradesh, Maharashtra, Odisha, Tamil Nadu, West Bengal	129905
Indian Council of Medical Research (ICMR), National Institute of Health and Family Welfare (India), Nutrition Foundation of India, Office of the Registrar General and Census Commissioner (India). India Clinical, Anthropometric and Bio-chemical Survey 2014.	India, Assam, Bihar, Chhattisgarh, Jharkhand, Madhya Pradesh, Odisha, Rajasthan, Uttar Pradesh, Uttarakhand	233917
Nangia V, Jonas JB, Sinha A, Matin A, Kulkarni M, Panda-Jonas S. Ocular axial length and its associations in an adult population of Central Rural India. The Central India Eye and Medical Study [Unpublished data]. Ophthalmology 2010;117(7):1360-6.	India, Maharashtra	133397
Government of Orissa, Micronutritent Initiative, United Nations Children's Fund (UNICEF), World Health Organization (WHO). India - Orissa Impact of Vitamin A Supplementation Delivered with Oral Polio Vaccine as Part of Immunization Campaign [draft final report]. Bhubheshwar, Government of Orissa, 2001.	India, Odisha	140228
Fogarty International Center, National Institutes of Health (NIH), Foundation for the National Institutes of Health (FNIH). India - Vellore Malnutrition and Enteric Disease Study 2009-2014.	India, Tamil Nadu	261875
Macro International, Inc, RAND Corporation, University of California, Los Angeles (UCLA), University of Indonesia. Indonesia Family Life Survey 1997.	Indonesia	5827

Center for Population and Policy Studies, Gadjah Mada University (Indonesia), RAND Corporation. Indonesia Family Life Survey 2000. Santa Monica, United States of America: RAND Corporation.	Indonesia	6111
Center for Population and Policy Studies, Gadjah Mada University (Indonesia), RAND Corporation, SurveyMETER. Indonesia Family Life Survey 2007-2008. Santa Monica, United States of America: RAND Corporation.	Indonesia	6464
Dillon, DHS, Verhoef H, West CE, Hautvast JGAJ, Burema J, Meester HJ, Bos MB. Concentration of Hemoglobin and Plasma Ferritin are not Related to Riboflavin Status but to Vitamin A Status in Indonesian Adolescent Schoolgirls. Wageningen, In: Dillon DHS, ed., Nutrition Health of Indonesian Adolescent Girls: The Role of Riboflavin and Vitamin A on Iron Status. Wageningen, The Netherlands: Wageningen University, 2005. 61-75.	Indonesia	140221
Sari M, de Pee S, Martini E, Herman S, Sugiatmi, Bloem MW, Yip R. Estimating the prevalence of anaemia: a comparison of three methods. Bull World Health Organ. 2001; 79(6): 506-11.	Indonesia	143972
Pegelow K, Gross R, Pietrzik K, Lukito W, Richards AL, Fryauff DJ. Parasitological and nutritional situation of school children in the Sukaraja district, West Java, Indonesia. Southeast Asian J Trop Med Public Health. 1997; 28(1): 173-90.	Indonesia	143974
De Pee S, Bloem MW, Sari M, Kiess L, Yip R, Kosen S. The high prevalence of low hemoglobin concentration among Indonesian infants aged 3-5 months is related to maternal anemia. J Nutr. 2002; 132(8): 2215-21.	Indonesia	143975
Soekarjo DD, de Pee S, Bloem MW, Tjiong R, Yip R, Schreurs WH, Muhilal. Socio-economic status and puberty are the main factors determining anaemia in adolescent girls and boys in East Java, Indonesia. Eur J Clin Nutr. 2001; 55(11): 932-9.	Indonesia	143977
Muslimatun S, Schmidt MK, Schultink W, West CE, Hautvast JA, Gross R, Muhilal. Weekly supplementation with iron and vitamin A during pregnancy increases hemoglobin concentration but decreases serum ferritin concentration in Indonesian pregnant women. J Nutr. 2001; 131(1): 85-90.	Indonesia	143978
Suega K, Dharmayuda TG, Sutarga IM, Bakta IM. Iron-deficiency anemia in pregnant women in Bali, Indonesia: a profile of risk factors and epidemiology. Southeast Asian J Trop Med Public Health. 2002; 33(3): 604-7.	Indonesia	143979
Thorand B, Schultink W, Gross R, Sastroamidjojo S, Wentzel S. Efficiency of the iron supplementation programme for pregnant women in Jeneponto, Sulawesi, Indonesia. Asia Pac J Clin Nutr. 1994; 3(4): 211-5.	Indonesia	143982
Andrade Juguan J, Lukito W, Schultink W. Thiamine deficiency is prevalent in a selected group of urban Indonesian elderly people. J Nutr. 1999; 129(2): 366-71.	Indonesia	143983
Angeles-Agdeppa I, Schultink W, Sastroamidjojo S, Gross R, Karyadi D. Weekly micronutrient supplementation to build iron stores in female Indonesian adolescents. Am J Clin Nutr. 1997; 66(1): 177-83.	Indonesia	143984
Nurdia DS, Sumarni S, Suyoko, Hakim M, Winkvist A. Impact of intestinal helminth infection on anemia and iron status during pregnancy: a community based study in Indonesia. Southeast Asian J Trop Med Public Health. 2001; 32(1): 14-22.	Indonesia	143986
Dijkhuizen MA, Wieringa FT, West CE, Muherdiyantiningsih, Muhilal. Concurrent micronutrient deficiencies in lactating mothers and their infants in Indonesia. Am J Clin Nutr. 2001; 73(4): 786-91.	Indonesia	143987
Ridwan E, Schultink W, Dillon D, Gross R. Effects of weekly iron supplementation on pregnant Indonesian women are similar to those of daily supplementation. Am J Clin Nutr. 1996; 63(6): 884-90.	Indonesia	143988
Suharno D, West CE, Muhilal, Logman MH, de Waart FG, Karyadi D, Hautvast JG. Cross-sectional study on the iron and vitamin A status of pregnant women in West Java, Indonesia. Am J Clin Nutr. 1992; 56(6): 988-93.	Indonesia	143989
RAND Corporation, SurveyMETER. Indonesia Family Life Survey 2014-2015. Santa Monica, United States of America: RAND Corporation, 2016.	Indonesia	264956
Lind T, Lönnerdal B, Stenlund H, Ismail D, Seswandhana R, Ekström E-C, Persson L-A. A community-based randomized controlled trial of iron and zinc supplementation in Indonesian infants: interactions between iron and zinc. Am J Clin Nutr. 2003; 77(4): 883-90.	Indonesia	143980

Palupi L, Schultink W, Achadi E, Gross R. Effective community intervention to improve hemoglobin status in preschoolers receiving once-weekly iron supplementation. Am J Clin Nutr. 1997; 65(4): 1057-61.	Indonesia	143985
Institute of Nutrition and Food Technology, Shaheed Beheshti Medical University (Iran), Pasteur Institute of Iran, United Nations Children's Fund (UNICEF). Iran National Integrated Micronutrient Survey 2001. Tehran, Iran (Islamic Republic of): Ministry of Health and Medical Education (Iran).	Iran (Islamic Republic of)	140090
Karimi M, Kadivar R, Yarmohammadi H. Assessment of the prevalence of iron deficiency anemia, by serum ferritin, in pregnant women of Southern Iran. Med Sci Monit. 2002; 8(7): 488-492.	Iran (Islamic Republic of)	143993
Ministry of Health and Medical Education (Iran), Tehran University of Medical Sciences, United Nations Children's Fund (UNICEF). Iran National Integrated Micronutrient Survey 2012.	Iran (Islamic Republic of)	262117
Moshirfatemi M, Faghihi M, Salimi M, Banan A, Khatibi A. Hemoglobin levels in an obstetric population in Isfahan, Iran. Iran J Public Health. 1976; 113-20.	Iran (Islamic Republic of)	143990
Digestive Diseases Research Institute (DDRI) (Iran), Shiraz University of Medical Sciences. Iran - Pars Cohort Study.	Iran (Islamic Republic of)	305739
Froozani MD, Vahdani F, Montazami K, Maiekafzali H. Maternal and newborn iron status in a public and a private maternity hospital at delivery in Tehran. J Trop Pediatr Environ Child Health. 1978; 24(4): 182-6.	Iran (Islamic Republic of)	143992
Al-Sharbatti SS, Al-Ward NJ, Al-Timimi DJ. Anemia among adolescents. Saudi Med J. 2003; 24(2): 189-94.	Iraq	143996
Armstrong PL. Iron deficiency in adolescents. BMJ. 1989; 298(6672): 499.	Ireland	143997
Shehab S, Nutenker K, Ron M, Salahov E, Tulchinsky TH. Hemoglobin levels among Arab and Druze children aged 1-2 years in Akko subdistrict. Public Health Rev. 2000; 28(1-4): 75-81.	Israel	143998
Inelmen EM, D'Alessio M, Gatto MR, Baggio MB, Jimenez G, Bizzotto MG, Enzi G. Descriptive analysis of the prevalence of anemia in a randomly selected sample of elderly people living at home: some results of an Italian multicentric study. Aging (Milano). 1994; 6(2): 81-9.	Italy	143999
Fidanza F, Losito G. Nutritional status of the elderly. Anthropometry, diet and clinical data from old pensioners in Perugia, Italy. Bibl Nutr Dieta. 1981; 70-80.	Italy	144000
Miall WE, Milner PF, Lovell HG, Standard KL. Haematological investigations of population samples in Jamaica. Br J Prev Soc Med. 1967; 21(2): 45-55.	Jamaica	96462
Simmons WK, Jutsum PJ, Fox K, Spence M, Gueri M, Paradis R, Gurney JM. A survey of the anemia status of preschool age children and pregnant and lactating women in Jamaica. Am J Clin Nutr. 1982; 35(2): 319-26.	Jamaica	144003
Grantham-McGregor SM, Desai P, Milner PF. Haematological levels in Jamaican infants. Arch Dis Child. 1974; 49(7): 525-30.	Jamaica	144002
Fujii M, Miyoshi T, Tada T, Goto H, Ishimoto H. Incidence of anemia in women in fishing villages. Tokushima J Exp Med. 1993; 40(3-4): 183-9.	Japan	144008
Watanabe T, Ishihara N, Miyasaka M, Koizumi A, Fujita H, Ikeda M. Hemoglobin levels among Japanese farmers: with special reference to climate and work intensity. Hum Biol. 1986; 58(2): 197-208.	Japan	144009
Hokama T. A study of the hemoglobin levels in breast-fed infants in one village of Okinawa prefecture. Acta Paediatr Jpn. 1993; 35(2): 138-40.	Japan	144010
Department of Statistics (Jordan), Macro International, Inc. Jordan Demographic and Health Survey 2002. Fairfax, United States of America: ICF International.	Jordan	20073
Department of Statistics (Jordan), Macro International, Inc. Jordan Demographic and Health Survey 2007. Fairfax, United States of America: ICF International.	Jordan	20083
Department of Statistics (Jordan), ICF Macro. Jordan Interim Demographic and Health Survey 2009. Fairfax, United States of America: ICF International, 2010.	Jordan	21206
Department of Statistics (Jordan), ICF International. Jordan Demographic and Health Survey 2012. Fairfax, United States of America: ICF International.	Jordan	77517
Albsoul-Younes AM, Al-Ramahi RJ, Al-Safi SA. Frequency of anemia in pregnancy in Northern Jordan. Saudi Med J. 2004; 25(10): 1525-7.	Jordan	144011

Mawajdeh SM, Al-Qutob R, Schmidt A. Measuring reproductive morbidity: a community-based approach, Jordan. Health Care Women Int. 2003; 24(7): 635-49.	Jordan	144012
Centers for Disease Control and Prevention (CDC), Global Alliance for Improved Nutrition (GAIN), Ministry of Health (Jordan), United Nations Children's Fund (UNICEF). Jordan National Micronutrient Survey 2010.	Jordan	149068
Department of Statistics (Jordan), ICF International. Jordan Demographic and Health Survey 2017-2018. Fairfax, United States of America: ICF International, 2019.	Jordan	356955
Macro International, Inc, National Institute of Nutrition (Kazakhstan). Kazakhstan Demographic and Health Survey 1995. Fairfax, United States of America: ICF International.	Kazakhstan	20092
Academy of Preventive Medicine (Kazakhstan), Macro International, Inc. Kazakhstan Demographic and Health Survey 1999. Fairfax, United States of America: ICF International.	Kazakhstan	20103
Dangour AD, Hill HL, Ismail SJ. Haemoglobin status of adult non-pregnant Kazakh women living in Kzyl-Orda region, Kazakhstan. Eur J Clin Nutr. 2001; 55(12): 1068-75.	Kazakhstan	144013
Dangour AD, Hill HL, Ismail SJ. Height, weight and haemoglobin status of 6 to 59-month-old Kazakh children living in Kzyl-Orda region, Kazakhstan. Eur J Clin Nutr. 2002; 56(10): 1030-8.	Kazakhstan	143098
Centers for Disease Control and Prevention (CDC), ICF Macro, Kenya Medical Research Institute (KEMRI), Kenya National Bureau of Statistics, Measure DHS, Ministry of Public Health and Sanitation (Kenya), Population Services International (PSI), President's Malaria Initiative (PMI), United Nations Children's Fund (UNICEF), Walter Reed Project, World Health Organization (WHO). Kenya Malaria Indicator Survey 2010. Nairobi, Kenya: Kenya National Bureau of Statistics.	Kenya	58006
Kenya Medical Research Institute (KEMRI), Ministry of Health (Kenya), Social Sciences and Medicine Africa Network (SOMA-NET), United Nations Children's Fund (UNICEF), University of Nairobi. Kenya National Micronutrient Survey 1999.	Kenya	140093
Olsen A, Magnussen P, Ouma JH, Andreassen J, Friis H. The contribution of hookworm and other parasitic infections to haemoglobin and iron status among children and adults in western Kenya. Trans R Soc Trop Med Hyg. 1998; 92(6): 643-9.	Kenya	144017
Leenstra T, Kariuki SK, Kurtis JD, Oloo AJ, Kager PA, ter Kuile FO. Prevalence and severity of anemia and iron deficiency: cross-sectional studies in adolescent schoolgirls in western Kenya. Eur J Clin Nutr. 2004; 58(4): 681-91.	Kenya	144020
Friis H, Mwaniki D, Omondi B, Muniu E, Thiong'o F, Ouma J, Magnussen P, Geissler PW, Michaelsen KF. Effects on haemoglobin of multi-micronutrient supplementation and multi-helminth chemotherapy: a randomized, controlled trial in Kenyan school children. Eur J Clin Nutr. 2003; 57(4): 573-9.	Kenya	144021
Shulman CE, Levene M, Morison L, Dorman E, Peshu N, Marsh K. Screening for severe anaemia in pregnancy in Kenya, using pallor examination and self-reported morbidity. Trans R Soc Trop Med Hyg. 2001; 95(3): 250-5.	Kenya	144022
Zucker JR, Lackritz EM, Ruebush TK, Hightower AW, Adungosi JE, Were JB, Campbell CC. Anaemia, blood transfusion practices, HIV and mortality among women of reproductive age in western Kenya. Trans R Soc Trop Med Hyg. 1994; 88(2): 173-6.	Kenya	144025
Shulman CE, Graham WJ, Jilo H, Lowe BS, New L, Obiero J, Snow RW, Marsh K. Malaria is an important cause of anaemia in primigravidae: evidence from a district hospital in coastal Kenya. Trans R Soc Trop Med Hyg. 1996; 90(5): 535-9.	Kenya	144026
ICF International, Kenya National Bureau of Statistics, National Malaria Control Program (NMCP) (Kenya). Kenya Malaria Indicator Survey 2015. Fairfax, United States of America: ICF International, 2015.	Kenya	218579
Newton CR, Warn PA, Winstanley PA, Peshu N, Snow RW, Pasvol G, Marsh K. Severe anaemia in children living in a malaria endemic area of Kenya. Trop Med Int Health. 1997; 2(2): 165-78.	Kenya	144015

Akhwale WS, Lum JK, Kaneko A, Eto H, Obonyo C, Björkman A, Kobayakawa T. Anemia and malaria at different altitudes in the western highlands of Kenya. Addict Behav Rep. 2004; 91(2): 167-75.	Kenya	144019
Ter Kuile FO, Terlouw DJ, Phillips-Howard PA, Hawley WA, Friedman JF, Kolczak MS, Kariuki SK, Shi YP, Kwena AM, Vulule JM, Nahlen BL. Impact of permethrin-treated bed nets on malaria and all-cause morbidity in young children in an area of intense perennial malaria transmission in western Kenya: cross-sectional survey. Am J Trop Med Hyg. 2003; 68(4 Suppl): 100-7.	Kenya	144024
Al-Mousa Z, Jackson RT, Al-Raqua M, Prakash P. The Epidemiology of Anaemia in Kuwait. In: Musaiger AO, ed. Micronutrient Deficiencies and Diet-related Chronic Diseases in the Arab Gulf Countries. Manama, Bahrain: Bahrain Centre for Studies and Research, 2000. 13-24.	Kuwait	140203
Jackson R, Al Hamad N, Al-Somaie M, Al Guoad N, Prakash P. Gender and age differences in anemia prevalence during the lifecycle in Kuwait. Ecol Food Nutr. 2004; 61-75.	Kuwait	144029
Al-Haifi A, Huffman F. Feeding practices influence weight and hemoglobin status in Kuwaiti infants. Ecol Food Nutr. 2004; 41-59.	Kuwait	144030
Jackson RT, Al-Mousa Z. Iron deficiency is a more important cause of anemia than hemoglobinopathies in Kuwaiti adolescent girls. J Nutr. 2000; 130(5): 1212-6.	Kuwait	144031
Al Mousa Z, Prakash P, Jackson R, Al Raqua M. A comparison of selected nutrient intakes in anemic and nonanemic adolescent girls in Kuwait. Nutr Res. 2003; 425-433.	Kuwait	144034
Amine EK, Al-Awadi F. Nutritional status survey of preschool children in Kuwait. East Mediterr Health J. 1996; 2(3): 386-95.	Kuwait	143103
Macro International, Inc, Ministry of Health (Kyrgyzstan), Research Institute of Obstetrics and Pediatrics (Kyrgyzstan). Kyrgyzstan Demographic and Health Survey 1997. Fairfax, United States of America: ICF International.	Kyrgyzstan	20154
ICF International, Ministry of Health (Kyrgyzstan), National Statistical Committee of the Kyrgyz Republic. Kyrgyzstan Demographic and Health Survey 2012. Fairfax, United States of America: ICF International.	Kyrgyzstan	77518
United Nations Children's Fund (UNICEF), Department of Statistics (Laos), Ministry of Health (Laos). Laos Multiple Indicator Cluster Survey 2006. New York, United States: United Nations Children's Fund (UNICEF).	Lao People's Democratic Republic	7629
Knowles J, Thurnham DI, Phengdy B, Houamboun K, Philavong K, Keomoungkhone I, Keovilay K. Impact of inflammation on the biomarkers of iron status in a cross-sectional survey of Lao women and children. Br J Nutr. 2013; 110(12): 2285-97.	Lao People's Democratic Republic	294314
Lao Statistics Bureau, Ministry of Education and Sports (Laos), Ministry of Health (Laos), United Nations Children's Fund (UNICEF). Laos Multiple Indicator Cluster Survey 2017. New York, United States of America: United Nations Children's Fund (UNICEF), 2018.	Lao People's Democratic Republic	375362
Al Khatib L, Obeid O, Sibai A-M, Batal M, Adra N, Hwalla N. Folate deficiency is associated with nutritional anaemia in Lebanese women of childbearing age. Public Health Nutr. 2006; 9(7): 921-7.	Lebanon	144036
Hwalla N, Adra N, Jackson RT. Iron Deficiency Is an Important Contributor to Anemia Among Reproductive Age Women in Lebanon. Ecol Food Nutr. 2004; 43(1–2): 77–92.	Lebanon	295863
Bureau of Statistics (Lesotho), Macro International, Inc, Ministry of Health and Social Welfare (Lesotho). Lesotho Demographic and Health Survey 2004-2005. Fairfax, United States of America: ICF International.	Lesotho	20167
ICF Macro, Ministry of Health and Social Welfare (Lesotho). Lesotho Demographic and Health Survey 2009-2010. Fairfax, United States of America: ICF International.	Lesotho	21382
ICF International, Ministry of Health and Social Welfare (Lesotho). Lesotho Demographic and Health Survey 2014. Fairfax, United States of America: ICF International.	Lesotho	157058
Jackson RT. Hemoglobin levels and anemia-associated symptoms in pregnant Liberian women. Nutrition. 1992; 8(6): 430-3.	Liberia	144037

ICF Macro, Liberia Institute for Statistics and Geo-information Services (LISGIS), National Malaria Control Program (Liberia). Liberia Malaria Indicator Survey 2008-2009. Fairfax, United States of America: ICF International.	Liberia	34279
ICF International, Liberia Institute for Statistics and Geo-information Services (LISGIS), National Malaria Control Program (Liberia). Liberia Malaria Indicator Survey 2011. Fairfax, United States of America: ICF International, 2012.	Liberia	56828
ICF International, Liberia Institute for Statistics and Geo-information Services (LISGIS), National Malaria Control Program (Liberia). Liberia Malaria Indicator Survey 2016. Fairfax, United States of America: ICF International, 2017.	Liberia	286768
Institute of Public Health (Macedonia), UNICEF Macedonia. Macedonia National Nutrition Survey 2011.	Macedonia	261352
Macro International, Inc, National Institute of Statistics (Madagascar). Madagascar Demographic and Health Survey 1997. Fairfax, United States of America: ICF International.	Madagascar	20212
Macro International, Inc, National Institute of Statistics (Madagascar). Madagascar Demographic and Health Survey 2003-2004. Fairfax, United States of America: ICF International.	Madagascar	20223
ICF Macro, National Institute of Statistics (Madagascar). Madagascar Demographic and Health Survey 2008-2009. Fairfax, United States of America: ICF International.	Madagascar	21409
ICF International, National Institute of Statistics (Madagascar), National Program for the Fight Against Malaria (PNLP) (Madagascar), Pasteur Institute of Madagascar (IPM). Madagascar Malaria Indicator Survey 2011. Fairfax, United States of America: ICF International.	Madagascar	69806
ICF International, National Institute of Statistics (Madagascar), National Program for the Fight Against Malaria (PNLP) (Madagascar), Pasteur Institute of Madagascar (IPM). Madagascar Malaria Indicator Survey 2013. Fairfax, United States of America: ICF International, 2013.	Madagascar	111438
ICF International, Ministry of Public Health (Madagascar), National Institute of Statistics (Madagascar), National Program for the Fight Against Malaria (PNLP) (Madagascar), Pasteur Institute of Madagascar (IPM). Madagascar Malaria Indicator Survey 2016. Fairfax, United States of America: ICF International, 2017.	Madagascar	218580
Macro International, Inc, National Statistical Office of Malawi. Malawi Demographic and Health Survey 2004-2005. Fairfax, United States of America: ICF International.	Malawi	20263
ICF Macro, National Statistical Office of Malawi. Malawi Demographic and Health Survey 2010. Fairfax, United States of America: ICF International.	Malawi	21393
Verhoeff FH, Brabin BJ, Chimsuku L, Kazembe P, Broadhead RL. Malaria in pregnancy and its consequences for the infant in rural Malawi. Ann Trop Med Parasitol. 1999; 25-33.	Malawi	144040
Van den Broek NR, Rogerson SJ, Mhango CG, Kambala B, White SA, Molyneux ME. Anaemia in pregnancy in southern Malawi: prevalence and risk factors. BJOG. 2000; 107(4): 445-51.	Malawi	144041
Semba RD, Kumwenda N, Taha TE, Mtimavalye L, Broadhead R, Garrett E, Miotti PG, Chiphangwi JD. Impact of vitamin A supplementation on anaemia and plasma erythropoietin concentrations in pregnant women: a controlled clinical trial. Eur J Haematol. 2001; 66(6): 389-95.	Malawi	144043
Fazio-Tirrozzo G, Brabin L, Brabin B, Agbaje O, Harper G, Broadhead R. A community based study of vitamin A and vitamin E status of adolescent girls living in the Shire Valley, Southern Malawi. Eur J Clin Nutr. 1998; 52(9): 637-42.	Malawi	144045
Van den Broek NR, Ntonya C, Mhango E, White SA. Diagnosing anaemia in pregnancy in rural clinics: assessing the potential of the Haemoglobin Colour Scale. Bull World Health Organ. 1999; 77(1): 15-21.	Malawi	144046
Emory University and Centers for Disease Control & Prevention Collaboration, ICF International, Ministry of Health (Malawi), National Statistical Office of Malawi. Malawi Demographic and Health Survey 2015-2016. Fairfax, United States of America: ICF International, 2017.	Malawi	218581
ICF International, National Malaria Control Program (Malawi). Malawi Malaria Indicator Survey 2012. Fairfax, United States of America: ICF International.	Malawi	77387

Mukiibi JM, Mtimavalye LA, Broadhead R, Mzula E, Dzinyemba WE, Merrick R, Khoromana CO, Ching'ani GW. Some haematological parameters in Malawian neonates. East Afr Med J. 1995; 72(1): 10-4.	Malawi	144042
ICF International, Ministry of Health (Malawi), National Malaria Control Program (Malawi), National Statistical Office of Malawi. Malawi Malaria Indicator Survey 2014. Fairfax, United States of America: ICF International, 2015.	Malawi	157059
ICF International, National Malaria Control Program (Malawi). Malawi Malaria Indicator Survey 2017. Fairfax, United States of America: ICF International, 2018.	Malawi	286769
Sagin DD, Ismail G, Mohamad M, Pang EKH, Sya OT. Anemia in remote interior communities in Sarawak, Malaysia. Southeast Asian J Trop Med Public Health. 2002; 33(2): 373-7.	Malaysia	144047
Tee ES Jr, Khor G, Tony N, Yassin Z, Chee H, Mdyusof S. Nutritional assessment of rural villages and estates in Peninsular Malaysia*. III. Prevalence of anaemia. Malays J Nutr. 1998; 4(1): 1-29.	Malaysia	144048
Ahmad Z Jr, Jaafar R, Mohd Hassan M, Othman M, Hashim A. Anaemia during pregnancy in rural Kelantan. Malays J Nutr. 1997; 3(1): 83-90.	Malaysia	144049
Foo LH, Khor GL, Tee E-S, Prabakaran D. Iron status and dietary iron intake of adolescents from a rural community in Sabah, Malaysia. Asia Pac J Clin Nutr. 2004; 13(1): 48-55.	Malaysia	144050
Shahar S, Earland J, Powers HJ, Rahman SA. Nutritional status of rural elderly Malays: dietary and biochemical findings. Int J Vitam Nutr Res. 1999; 69(4): 277-84.	Malaysia	144053
Ministry of Health (Maldives), Republic of Maldives, United Nations Children's Fund (UNICEF). Maldives Multiple Indicator Cluster Survey 2001.	Maldives	8033
ICF International, Ministry of Health (Maldives). Maldives Demographic and Health Survey 2016-2017. Fairfax, United States of America: ICF International, 2019.	Maldives	218582
Aga Khan University, Ministry of Health (Maldives), United Nations Children's Fund (UNICEF). Maldives National Micronutrient Survey 2007-2008.	Maldives	236130
Macro International, Inc, Ministry of Health (Mali), National Directorate of Statistics and Informatics (DNSI) (Mali). Mali Demographic and Health Survey 2006. Fairfax, United States of America: ICF International.	Mali	20274
Macro International, Inc, National Directorate of Statistics and Informatics (DNSI) (Mali), Planning and Statistics Unit, Ministry of Health (Mali). Mali Demographic and Health Survey 2001. Fairfax, United States of America: ICF International.	Mali	20315
ICF International, INFO-STAT (Mali), Ministry of Health (Mali), National Institute of Statistics (INSTAT) (Mali), Planning and Statistics Unit, Ministry of Health (Mali). Mali Demographic and Health Survey 2012-2013. Fairfax, United States of America: ICF International, 2014.	Mali	77388
ICF Macro, INFO-STAT (Mali), National Program for the Fight Against Malaria (Mali). Mali Special Demographic and Health Survey 2010. Fairfax, United States of America: ICF International, 2011.	Mali	30777
Ministry of Health (Mali), National Institute of Statistics (INSTAT) (Mali), United Nations Children's Fund (UNICEF). Mali Multiple Indicator Cluster Survey 2009-2010. New York, United States of America: United Nations Children's Fund (UNICEF), 2017.	Mali	270627
ICF International, National Institute of Statistics (INSTAT) (Mali). Mali Demographic and Health Survey 2018. Fairfax, United States of America: ICF International, 2019.	Mali	398033
ICF International, INFO-STAT (Mali), Ministry of Health and Public Hygiene (Mali), National Institute of Public Health Research (INRSP) (Mali), National Institute of Statistics (INSTAT) (Mali), National Program for the Fight Against Malaria (Mali). Mali Malaria Indicator Survey 2015. Fairfax, United States of America: ICF International, 2016.	Mali	218587
Ministry of Health (Marshall Islands), United Nations Children's Fund (UNICEF). Marshall Islands National Nutrition Survey 1990-1991.	Marshall Islands	140118
Palafox NA, Gamble MV, Dancheck B, Ricks MO, Briand K, Semba RD. Vitamin A deficiency, iron deficiency, and anemia among preschool children in the Republic of the Marshall Islands. Nutrition. 2003; 19(5): 405-8.	Marshall Islands	144057

Ministry of Health and Quality of Life (Mauritius), United Nations Children's Fund (UNICEF). Mauritius Survey on the Nutritional Status of Preschool Children 1985.	Mauritius	140116
National Institute of Public Health (Mexico). Mexico National Survey of Health and Nutrition 2005-2006. Cuernavaca, Mexico: National Institute of Public Health (Mexico).	Mexico	8618
National Institute of Public Health (Mexico). Mexico National Survey of Health and Nutrition 2011-2012. Cuernavaca, Mexico: National Institute of Public Health (Mexico).	Mexico	81748
National Institute of Public Health (Mexico). Mexico National Nutrition Survey 1988. Cuernavaca, Mexico: National Institute of Public Health (Mexico).	Mexico	105806
Secretariat of Health (Mexico), Secretary of Public Education (Mexico). Mexico National School Health Survey 2008. Cuernavaca, Mexico: National Institute of Public Health (Mexico), 2010.	Mexico	135527
Martínez H, González-Cossío T, Flores M, Rivera-Dommarco J, Lezana MA, Sepúlveda-Amor J. [Anemia in women of reproductive age. The results of a national probability survey]. Salud Publica Mex. 1995; 37(2): 108-19.	Mexico	144063
Ramakrishnan U, González-Cossio T, Neufeld L, Rivera J, Martorell R. Multiple micronutrient supplementation during pregnancy does not lead to greater infant birth size than does iron-only supplementation: a randomized controlled trial in a semirural community in Mexico. Am J Clin Nutr. 2003; 720-5.	Mexico	144065
Vásquez-Molina ME, Corral-Terrazas M, Apezteguia MA, Carmona-Sawasky J, Levario-Carrillo M. [Relationship between maternal and neonatal iron stores]. Salud Publica Mex. 2001; 43(5): 402-7.	Mexico	144070
Black AK, Allen LH, Pelto GH, de Mata MP, Chávez A. Iron, vitamin B-12 and folate status in Mexico: associated factors in men and women and during pregnancy and lactation. J Nutr. 1994; 124(8): 1179-88.	Mexico	144071
Ramírez-Mateos C, Loría A, Nieto-Gómez M, Malacara JM, Piedras J. [Anemia and iron deficiency in 490 Mexican pregnant women]. Rev Invest Clin. 1998; 50(2): 119-26.	Mexico	144072
Jaime-Pérez JC, Gómez-Almaguer D. Iron stores in low-income pregnant Mexican women at term. Arch Med Res. 2002; 33(1): 81-4.	Mexico	144074
Vásquez-Garibay EM, Romero-Velarde E, Nápoles-Rodríguez F, Nuño-Cosío ME, Trujillo-Contreras F, Sánchez-Mercado O. [Prevalence of iron and iodine deficiency, and parasitosis among children from Arandas, Jalisco, Mexico]. Salud Publica Mex. 2002; 44(3): 195-200.	Mexico	144061
Kordas K, Lopez P, Rosado JL, García Vargas G, Alatorre Rico J, Ronquillo D, Cebrián ME, Stoltzfus RJ. Blood lead, anemia, and short stature are independently associated with cognitive performance in Mexican school children. J Nutr. 2004; 134(2): 363-71.	Mexico	144062
Tejas AR, Wyatt CJ, Ramírez MJ. Prevalence of undernutrition and iron deficiency in pre-school children from different socioeconomic regions in the city of Oaxaca, Oaxaca, Mexico. J Nutr Sci Vitaminol. 2001; 47(1): 47-51.	Mexico	144064
Maulen-Radovan I, Villagómez S, Soler E, Villicaña R, Hernández-Ronquillo L, Rosado JL. [Nutritional impact of whole milk supplemented with vitamins and minerals in children]]. Salud Publica Mex. 1999; 41(5): 389-96.	Mexico	144069
Allen LH, Rosado JL, Casterline JE, López P, Muñoz E, Garcia OP, Martinez H. Lack of hemoglobin response to iron supplementation in anemic mexican preschoolers with multiple micronutrient deficiencies. Am J Clin Nutr. 2000; 71(6): 1485-94.	Mexico	144076
Lloyd-Puryear M, Mahoney J, Humphrey J, Mahoney F, Siren N, Moorman C, West KJ. Vitamin A deficiency in Micronesia: a statewide survey in Chuuk. Nutr Res. 1991; 1101-1110.	Micronesia (Federated States of)	143873
Republic of Kiribati. Pacific Helminths Initiative 2001-2002.	Micronesia (Federated States of), Fiji, French Polynesia, Kiribati, Marshall Islands, Nauru, New Zealand, Cook Islands, Niue, Samoa, Solomon Islands, Tonga,	142784

	Tuvalu, United States of America, American Samoa, Vanuatu	
Public Health Institute, Ministry of Health (Mongolia), United Nations Children's Fund (UNICEF). Mongolia National Nutrition Survey 2004.	Mongolia	137528
Centers for Disease Control and Prevention (CDC), Ministry of Health (Mongolia), United Nations Children's Fund (UNICEF), World Health Organization (WHO). Mongolia Survey Assessing the Nutritional Consequences of the Dzud 2001.	Mongolia	139872
ICF Macro, Manhica Health Research Center (CISM), Ministry of Health (Mozambique), National Institute of Statistics (INE) (Mozambique). Mozambique Demographic and Health Survey 2011. Fairfax, United States of America: ICF International.	Mozambique	55975
Liljestrand J, Bergström S, Birgegård G. Anaemia of pregnancy in Mozambique. Trans R Soc Trop Med Hyg. 1986; 80(2): 249-55.	Mozambique	144078
Centers for Disease Control and Prevention (CDC), ICF International, Ministry of Health (Mozambique), National Institute of Health (Mozambique), National Institute of Statistics (INE) (Mozambique). Mozambique AIDS Indicator Survey 2015. Fairfax, United States of America: ICF International, 2018.	Mozambique	157060
ICF International, National Institute of Health (Mozambique), National Institute of Statistics (INE) (Mozambique), National Malaria Control Program (Mozambique). Mozambique Malaria Indicator Survey 2018. Fairfax, United States of America: ICF International, 2019.	Mozambique	413934
Helen Keller International, Micronutrient Initiative. Mozambique - Manica School-based Iron/Folate Supplementation for Girls Pilot Project Baseline Survey 2001.	Mozambique	140214
ICF International, Ministry of Health and Sports (Myanmar). Myanmar Demographic and Health Survey 2015-2016. Fairfax, United States of America: ICF International, 2017.	Myanmar	157061
Thomson J. Anaemia in pregnant women in eastern Caprivi, Namibia. S Afr Med J. 1997; 87(11): 1544-7.	Namibia	144082
ICF International, Ministry of Health and Social Services (Namibia), Namibia Institute of Pathology, Namibia Statistics Agency. Namibia Demographic and Health Survey 2013. Fairfax, United States of America: ICF International.	Namibia	150382
Macro International, Inc, Ministry of Health and Population (Nepal), New ERA. Nepal Demographic and Health Survey 2006. Fairfax, United States of America: ICF International.	Nepal	20462
ICF Macro, Ministry of Health and Population (Nepal), New ERA. Nepal Demographic and Health Survey 2011. Fairfax, United States of America: ICF International.	Nepal	21240
Micronutrient Initiative, Ministry of Health and Population (Nepal), National Planning Commission (Nepal), New ERA, United Nations Children's Fund (UNICEF), World Health Organization (WHO). Nepal Micronutrient Status Survey 1997-1998.	Nepal	137462
Christian P, Shrestha J, LeClerq SC, Khatry SK, Jiang T, Wagner T, Katz J, West KP Jr. Supplementation with micronutrients in addition to iron and folic acid does not further improve the hematologic status of pregnant women in rural Nepal. J Nutr. 2003; 133(11): 3492-8.	Nepal	144083
Shrestha PN. Anemia in children of rural Kathmandu. Indian J Pediatr. 1986; 53(5): 647-50.	Nepal	144084
Jiang T, Christian P, Khatry SK, Wu L, West KP Jr. Micronutrient deficiencies in early pregnancy are common, concurrent, and vary by season among rural Nepali pregnant women. J Nutr. 2005; 135(5): 1106-12.	Nepal	144089
Dreyfuss ML, Stoltzfus RJ, Shrestha JB, Pradhan EK, LeClerq SC, Khatry SK, Shrestha SR, Katz J, Albonico M, West KP Jr. Hookworms, malaria and vitamin A deficiency contribute to anemia and iron deficiency among pregnant women in the plains of Nepal. J Nutr. 2000; 130(10): 2527-36.	Nepal	144090
Curtale F, Tilden R, Muhilal, Vaidya Y, Pokhrel RP, Guerra R. Intestinal helminths and risk of anaemia among Nepalese children. Panminerva Med. 1993; 35(3): 159-66.	Nepal	144088

ICF International, Ministry of Health (Nepal), New ERA. Nepal Demographic and Health Survey 2016-2017. Fairfax, United States of America: ICF International, 2017.	Nepal	286782
Fogarty International Center, National Institutes of Health (NIH), Foundation for the National Institutes of Health (FNIH), Institute of Medicine, Tribhuvan University, University of Bergen, Walter Reed/AFRIMS Research Unit Nepal (WARUN). Nepal - Bhaktapur Malnutrition and Enteric Disease Study 2009-2014.	Nepal	261880
Ministry of Health (New Zealand), University of Otago (New Zealand). New Zealand National Nutrition Survey 1996-1997.	New Zealand	124477
Fawcett JP, Brooke M, Beresford CH. Iron deficiency and anaemia in a longitudinal study of New Zealanders at ages 11 and 21 years. N Z Med J. 1998; 111(1076): 400-2.	New Zealand	144091
Schaaf D, Scragg R, Metcalf P, Grant C, Buchanan J. Prevalence of iron deficiency in Auckland high school students. N Z Med J. 2000; 113(1116): 347-50.	New Zealand	144095
Heath AL, Skeaff CM, Williams S, Gibson RS. The role of blood loss and diet in the aetiology of mild iron deficiency in premenopausal adult New Zealand women. Public Health Nutr. 2001; 4(2): 197-206.	New Zealand	144098
Soh P, Ferguson EL, McKenzie JE, Homs MYV, Gibson RS. Iron deficiency and risk factors for lower iron stores in 6-24-month-old New Zealanders. Eur J Clin Nutr. 2004; 58(1): 71-9.	New Zealand	144093
CBG Health Research Ltd., Ministry of Health (New Zealand). New Zealand Health Survey 2014-2015. Wellington, New Zealand: Ministry of Health (New Zealand).	New Zealand	264325
Ministry of Health (Nicaragua). Nicaragua National Micronutrient Survey 2000.	Nicaragua	139860
Sullivan KM, Venugopalan B, Jefferds ME, Boy E, Bonilla J, Sandino I, Halleslevens P. Association of elevated α_1-acid glycoprotein (AGP) and the prevalence of anemia in Nicaraguan preschool children. Food Nutr Bull. 2012; 33(2): 137-41.	Nicaragua	294378
Department of Statistics and National Accounts (Niger), Macro International, Inc. Niger Demographic and Health Survey 2006. Fairfax, United States of America: ICF International.	Niger	20499
ICF International, Ministry of Public Health (Niger), National Institute of Statistics (Niger). Niger Demographic and Health Survey 2012. Fairfax, United States of America: ICF International, 2019.	Niger	74393
Preziosi P, Prual A, Galan P, Daouda H, Boureima H, Hercberg S. Effect of iron supplementation on the iron status of pregnant women: consequences for newborns. Am J Clin Nutr. 1997; 66(5): 1178-82.	Niger	144101
Lyko Z, Lyko J, Gaertner H. Anemia in pregnancy in Nigeria: hemoglobin level in pregnant patients of the Ife region hospital. Hamdard Medicus. 1993; 116-24.	Nigeria	144103
Reid H. Physiological changes in packed-cell volume and haemoglobin levels in Nigerian Igbo women during pregnancy. East Afr Med J. 1996; 201-7.	Nigeria	144104
Brabin L, Ikimalo J, Dollimore N, Kemp J, Ikokwu-Wonodi C, Babatunde S, Obunge O, Briggs N. How do they grow? A study of south-eastern Nigerian adolescent girls. Acta Paediatr. 1997; 86(10): 1114-20.	Nigeria	144106
Onadeko MO, Avokey F, Lawoyin TO. Observations of stillbirths, birthweight and maternal haemoglobin in teenage pregnancy in Ibadan, Nigeria. Afr J Med Sci. 1996; 25(1): 81-6.	Nigeria	144107
Barr F, Brabin L, Agbaje S, Buseri F, Ikimalo J, Briggs N. Reducing iron deficiency anaemia due to heavy menstrual blood loss in Nigerian rural adolescents. Public Health Nutr. 1998; 1(4): 249-57.	Nigeria	144108
Gwarzo MY, Sen KK, Atiku MK. Diet and serum iron status in pregnant and lactating Hausa women in Kano State, Nigeria. Ann Trop Med Parasitol. 1994; 88(6): 673-6.	Nigeria	144109
ICF Macro, National Malaria Control Programme (Nigeria), National Population Commission of Nigeria. Nigeria Malaria Indicator Survey 2010. Fairfax, United States of America: ICF International.	Nigeria	30991
Federal Ministry of Health (Nigeria), ICF International, National Population Commission (NPC). Nigeria Demographic and Health Survey 2018. Fairfax, United States of America: ICF International, 2020.	Nigeria	408484

International Institute of Tropical Agriculture (IITA), and HarvestPlus of International Food Policy Research Institute (IFPRI). 2015. Dietary intakes, vitamin A, and iron status of women of childbearing age and children 6-59 months of age from Akwa Ibom state in Nigeria. Washington, DC: International Food Policy Research Institute (IFPRI) [datasets]. http://dx.doi.org/10.7910/DVN/29604	Nigeria	283272
Borch-Iohnsen B, Sandstad B, Asberg A. Iron status among 3005 women aged 20-55 years in Central Norway: the Nord-Trøndelag Health Study (the HUNT study). Scand J Clin Lab Invest. 2005; 65(1): 45-54.	Norway	144111
Eskeland B, Hunskaar S. Anaemia and iron deficiency screening in adolescence: a pilot study of iron stores and haemoglobin response to iron treatment in a population of 14-15-year-olds in Norway. Acta Paediatr. 1999; 88(8): 815-21.	Norway	144112
Centers for Disease Control and Prevention (CDC), Ministry of Health (Oman), United Nations Children's Fund (UNICEF), World Health Organization Regional Office for the Eastern Mediterranean (EMRO-WHO). Oman Food Fortification Study 2004. Muscat, Oman: Ministry of Health (Oman).	Oman	30030
Ministry of Health (Oman). Oman National Health Survey 2000. Muscat, Oman: Ministry of Health (Oman).	Oman	30033
Alasfoor DH. Nutritional Anemia and Vitamin A Deficiency in Oman: A Multivariate Analysis [thesis]. Ithaca (NY), United States: Cornell University; 1996.	Oman	139852
Al-Riyami A, Ebrahim GJ. Genetic Blood Disorders Survey in the Sultanate of Oman. J Trop Pediatr. 2003; 49(Suppl 1): 1-20.	Oman	110030
Agha F, Hasan TJ, Khan RA, Jafarey S. Iron stores in maternal and cord blood. Asia Oceania J Obstet Gynaecol. 1988; 14(4): 405-9.	Pakistan	144119
Aga Khan University, Ministry of National Health Services, Regulations & Coordination (Pakistan), Ministry of Planning and Development (Pakistan), Pakistan Medical Research Council, United Nations Children's Fund (UNICEF). Pakistan National Nutrition Survey 2011.	Pakistan	141521
Paracha PI, Hameed A, Simon J, Jamil A, Nawab G. Prevalence of anaemia in semi-urban areas of Peshawar, Pakistan: a challenge for health professionals and policy makers. J Pak Med Assoc. 1997; 47(2): 49-53.	Pakistan	144115
Molla A, Khurshid M, Molla AM. Prevalence of iron deficiency anaemia in children of the urban slums of Karachi. J Pak Med Assoc. 1992; 42(5): 118-21.	Pakistan	144116
Hassan K, Hayat A, Khan U, Ikram N, Dodhy M, Afghan R, Tahir M. Reference values of hematological parameters in full term normal neonates in Rawalpindi and Islamabad. Pak J Obstet Gynecol. 1994; 29-31.	Pakistan	144120
Baig L, Thaver IH, Iqbal R. Risk factors associated with anaemia in children less than 5 years of age residing in a squatter settlement of Karachi. Pak J Med Res. 1996; 35(4): 176-178.	Pakistan	144121
Aga Khan University, Fogarty International Center, National Institutes of Health (NIH), Foundation for the National Institutes of Health (FNIH). Pakistan - Naushahro Feroze Malnutrition and Enteric Disease Study 2009-2014.	Pakistan	261883
Birzeit University, Palestinian Central Bureau of Statistics. Palestine Nutrition Survey 2002. Ramallah, Palestine: Palestinian Central Bureau of Statistics.	Palestine	9989
Ministry of Health (Palestine), Palestinian Central Bureau of Statistics, United Nations Children's Fund (UNICEF), United Nations Population Fund (UNFPA). Palestine Multiple Indicator Cluster Survey 2010. New York, United States of America: United Nations Children's Fund (UNICEF), 2014.	Palestine	125591
Ministry of Health (Palestine), United Nations Children's Fund (UNICEF), University of Vienna. Palestine Micronutrient Survey 2013.	Palestine	285322
Ministry of Health (Panama). Panama National Survey on Vitamin A 1992.	Panama	139825
Centers for Disease Control and Prevention (CDC), National Department of Health (Papua New Guinea), United Nations Children's Fund (UNICEF), University of Papua New Guinea. Papua New Guinea National Nutrition Survey 2005.	Papua New Guinea	135757
Sill PR, White JC, Cheetham JM. A survey of haemoglobin concentration in pregnancy in Port Moresby, Papua New Guinea. P N G Med J. 1986; 29(3): 221-4.	Papua New Guinea	144122
Brabin B, Piper C. Anaemia- and malaria-attributable low birthweight in two populations in Papua New Guinea. Ann Hum Biol. 1997; 24(6): 547-55.	Papua New Guinea	144124

Mola G, Permezel M, Amoa AB, Klufio CA. Anaemia and perinatal outcome in Port Moresby. Aust N Z J Obstet Gynaecol. 1999; 39(1): 31-4.	Papua New Guinea	144125
Brabin BJ, Ginny M, Sapau J, Galme K, Paino J. Consequences of maternal anaemia on outcome of pregnancy in a malaria endemic area in Papua New Guinea. Ann Trop Med Parasitol. 1990; 84(1): 11-24.	Papua New Guinea	144128
Amoa AB, Klufio CA, Kariwiga G, Heywood S. Antenatal haemoglobin profile at the Port Moresby General Hospital. P N G Med J. 1998; 41(3-4): 119-25.	Papua New Guinea	144129
Macro International, Inc, National Institute of Statistics (Peru). Peru Demographic and Health Survey 1996. Fairfax, United States of America: ICF International.	Peru	20638
Macro International, Inc, National Institute of Statistics (Peru). Peru Demographic and Health Survey 2000. Fairfax, United States of America: ICF International.	Peru	20649
National Institute of Health (Peru). Behavior of Nutrition Indicators in Peru: Monitoring National Nutritional Indicators 2002-2004. Lima, Peru: National Institute of Health (Peru), 2007.	Peru	139818
Zavaleta N, Caulfield LE, Garcia T. Changes in iron status during pregnancy in Peruvian women receiving prenatal iron and folic acid supplements with or without zinc. Am J Clin Nutr. 2000; 71(4): 956-61.	Peru	144130
Becerra C, Gonzales GF, Villena A, de la Cruz D, Florián A. [Prevalence of anemia in pregnancy, Pucallpa Regional Hospital, Perú]. Rev Panam Salud Publica. 1998; 3(5): 285-92.	Peru	144133
National Institute of Statistics and Informatics (Peru), ORC Macro. Peru Continuous Demographic and Health Survey 2009. Fairfax, United States of America: ICF International.	Peru	270404
National Institute of Statistics and Informatics (Peru). Peru Continuous Demographic and Health Survey 2010. Fairfax, United States of America: ICF International.	Peru	270469
Macro International, Inc, National Institute of Statistics and Informatics (Peru). Peru Continuous Demographic and Health Survey 2011. Fairfax, United States of America: ICF International.	Peru	270470
Macro International, Inc, National Institute of Statistics and Informatics (Peru). Peru Continuous Demographic and Health Survey 2012. Fairfax, United States of America: ICF International.	Peru	270471
Ministry of Economy and Finance (Peru), National Institute of Statistics and Informatics (Peru), ORC Macro. Peru Continuous Demographic and Health Survey 2003-2008. Fairfax, United States of America: ICF International.	Peru	275090
Ministry of Health (Peru), National Center for Food and Nutrition, National Institute of Health (Peru), National Institute of Health (Peru). Peru National Survey on Nutritional, Biochemical, Socioeconomic Indicators Related to Chronic Degenerative Diseases 2004-2005.	Peru	139823
López de Romaña G, Cusirramos S, López de Romaña D, Gross R. Efficacy of multiple micronutrient supplementation for improving anemia, micronutrient status, growth, and morbidity of Peruvian infants. J Nutr. 2005; 135(3): 646-652.	Peru	144131
Alarcon K, Kolsteren PW, Prada AM, Chian AM, Velarde RE, Pecho IL, Hoeree TF. Effects of separate delivery of zinc or zinc and vitamin A on hemoglobin response, growth, and diarrhea in young Peruvian children receiving iron therapy for anemia. Am J Clin Nutr. 2004; 80(5): 1276-82.	Peru	144136
ICF International, Ministry of Health (Peru), National Institute of Statistics and Informatics (Peru), National Police of Peru (PNP). Peru Continuous Demographic and Health Survey 2014 - INEI. Lima, Peru: National Institute of Statistics and Informatics (Peru), 2015.	Peru	210182
ICF International, National Institute of Statistics and Informatics (Peru). Peru Continuous Demographic and Health Survey 2013 - INEI. Lima, Peru: National Institute of Statistics and Informatics (Peru), 2014.	Peru	210231
National Institute of Statistics and Informatics (Peru). Peru Demographic and Family Health Survey 2015. Lima, Peru: National Institute of Statistics and Informatics (Peru), 2017.	Peru	303663

National Institute of Statistics and Informatics (Peru). Peru Demographic and Family Health Survey 2016. Lima, Peru: National Institute of Statistics and Informatics (Peru), 2017.	Peru	303664
National Center for Food and Nutrition, National Institute of Health (Peru), National Institute of Statistics and Informatics (Peru), National Police of Peru (PNP). Peru Demographic and Family Health Survey 2017. Lima, Peru: National Institute of Statistics and Informatics (Peru).	Peru	358824
Executive Directorate of Food and Nutritional Surveillance (DEVAN) (Peru), Ministry of Health (Peru), National Center for Food and Nutrition, National Institute of Health (Peru), National Institute of Health (Peru), National Institute of Statistics and Informatics (Peru). Peru Monitoring of Nutritional Indicators in the National Household Survey 2009-2011.	Peru	359146
Executive Directorate of Food and Nutritional Surveillance (DEVAN) (Peru), Ministry of Health (Peru), National Center for Food and Nutrition, National Institute of Health (Peru), National Institute of Health (Peru), National Institute of Statistics and Informatics (Peru). Peru Monitoring of Nutritional Indicators in the National Household Survey 2007-2008.	Peru	359163
Executive Directorate of Food and Nutritional Surveillance (DEVAN) (Peru), Ministry of Health (Peru), National Center for Food and Nutrition, National Institute of Health (Peru), National Institute of Health (Peru), National Institute of Statistics and Informatics (Peru). Peru Monitoring of Nutritional Indicators in the National Household Survey 2011-2012.	Peru	359165
National Institute of Statistics and Informatics (Peru). Peru Demographic and Family Health Survey 2018. Lima, Peru: National Institute of Statistics and Informatics (Peru), 2019.	Peru	407869
Fogarty International Center, National Institutes of Health (NIH), Foundation for the National Institutes of Health (FNIH), Johns Hopkins Bloomberg School of Public Health. Peru - Loreto Malnutrition and Enteric Disease Study 2009-2014.	Peru	261879
Food and Nutrition Research Institute, Department of Science and Technology (Philippines). Philippines National Nutrition Survey 1998.	Philippines	10950
Food and Nutrition Research Institute, Department of Science and Technology (Philippines). Philippines National Nutrition Survey 2003.	Philippines	124455
Food and Nutrition Research Institute, Department of Science and Technology (Philippines). Philippines National Nutrition Survey 2008-2009.	Philippines	126395
De Jong N, Romano ABA, Gibson RS. Zinc and iron status during pregnancy of Filipino women. Asia Pac J Clin Nutr. 2002; 11(3): 186-93.	Philippines	144140
Food and Nutrition Research Institute, Department of Science and Technology (Philippines). Philippines National Nutrition Survey 2013-2014.	Philippines	249262
Kuizon MD, Cheong RL, Ancheta LP, Desnacido JA, Macapinlac MP, Baens JS. Effect of anaemia and other maternal characteristics on birthweight. Hum Nutr Clin Nutr. 1985; 39(6): 419-26.	Philippines	254226
Rohner F, Woodruff BA, Aaron GJ, Yakes EA, Lebanan MA, Rayco-Solon P, Saniel OP. Infant and young child feeding practices in urban Philippines and their associations with stunting, anemia, and deficiencies of iron and vitamin A. Food Nutr Bull. 2013; 34(2 Suppl): S17-34.	Philippines	294316
Spodaryk K. Disparity between dietary iron intake and iron status of children aged 10-12 years. Arch Physiol Biochem. 1999; 107(5): 361-6.	Poland	144142
Choi CW, Lee J, Park KH, Yoon SY, Choi IK, Oh SC, Seo JH, Kim BS, Shin SW, Kim YH, Kim JS. Prevalence and characteristics of anemia in the elderly: cross-sectional study of three urban Korean population samples. Am J Hematol. 2004; 77(1): 26-30.	Republic of Korea	144177
Choi JW, Kim CS, Pai SH. Erythropoietic activity and soluble transferrin receptor level in neonates and maternal blood. Acta Paediatr. 2000; 89(6): 675-9.	Republic of Korea	144178
Macro International, Inc, National Scientific and Applied Center for Preventive Medicine (Moldova). Moldova Demographic and Health Survey 2005. Fairfax, United States of America: ICF International.	Republic of Moldova	20339
Ministry of Health (Moldova), National Bureau of Statistics (Moldova), United Nations Children's Fund (UNICEF). Moldova Multiple Indicator Cluster Survey 2012.	Republic of Moldova	140200

New York, United States of America: United Nations Children's Fund (UNICEF), 2015.		
Centers for Disease Control and Prevention (CDC), Ministry of Health (Romania), United Nations Children's Fund (UNICEF). Romania National Nutrition Survey 1991.	Romania	139132
Rubin CH, Esteban E, Reissman DB, Daley WR, Noonan GP, Karpati A, Gurvitch E, Kuzmin SV, Privalova LI, Zukov A, Zlepko A. Lead poisoning among young children in Russia: concurrent evaluation of childhood lead exposure in Ekaterinburg, Krasnouralsk, and Volgograd. Environ Health Perspect. 2002; 110(6): 559-62.	Russian Federation	122634
Ufa Eye Research Institute. Russia - Ural Eye and Medical Study 2015-2017.	Russian Federation	304148
Macro International, Inc, National Institute of Statistics of Rwanda. Rwanda Demographic and Health Survey 2005. Fairfax, United States of America: ICF International.	Rwanda	20740
ICF Macro, Ministry of Health (Rwanda), National Institute of Statistics of Rwanda. Rwanda Demographic and Health Survey 2010-2011. Fairfax, United States of America: ICF International.	Rwanda	56040
ICF International, Ministry of Health (Rwanda), National Institute of Statistics of Rwanda. Rwanda Demographic and Health Survey 2014-2015. Fairfax, United States of America: ICF International, 2016.	Rwanda	157063
ICF Macro, MEASURE Evaluation Project, Carolina Population Center, University of North Carolina, Ministry of Health (Rwanda), National Institute of Statistics of Rwanda, School of Public Health, University of Rwanda. Rwanda Child Verbal Autopsy Study 2008.	Rwanda	274703
Macro International, Inc, Ministry of Health (Rwanda), National Institute of Statistics of Rwanda. Rwanda Interim Demographic and Health Survey 2007-2008. Fairfax, United States of America: ICF International.	Rwanda	21222
ICF Macro, Ministry of Health (Sao Tome and Principe), National Institute of Statistics (Sao Tome and Principe). Sao Tome and Principe Demographic and Health Survey 2008-2009. Fairfax, United States of America: ICF International.	Sao Tome and Principe	26866
Ghafouri H, Al Fares A, Islam S, Ahmed A, Jan M. Haematological reference values assessed from birth to adolescence in Saudi subjects in the area of Jeddah. Saudi Med J. 1987; 575-82.	Saudi Arabia	144147
Hawa L, Rowland A, Ahmed G, Dery J. Haemoglobin and iron status in mothers and their babies at delivery. Saudi Med J. 1993; 110-5.	Saudi Arabia	144148
Mahfouz AA, el-Said MM, Alakija W, Badawi IA, al-Erian RA, Moneim MA. Anemia among pregnant women in the Asir region, Saudi Arabia: an epidemiologic study. Southeast Asian J Trop Med Public Health. 1994; 25(1): 84-7.	Saudi Arabia	144152
Al Fawaz I. Surveillance for iron deficiency anemia at a well baby clinic in Riyadh, Saudi Arabia. Saudi Med J. 1993; 27-31.	Saudi Arabia	144146
Ahmed MM. Haematological values and parasitic infections in school children in Riyadh, Saudi Arabia. J Egypt Soc Parasitol. 1991; 21(3): 831-8.	Saudi Arabia	144149
al-Momen AK, al-Meshari A, al-Nuaim L, Saddique A, Abotalib Z, Khashogji T, Abbas M. Intravenous iron sucrose complex in the treatment of iron deficiency anemia during pregnancy. Eur J Obstet Gynecol Reprod Biol. 1996; 69(2): 121-4.	Saudi Arabia	144158
Mahfouz AA, el-Said MM, al-Erian RA, Hamid AM. Teenage pregnancy: are teenagers a high risk group? Eur J Obstet Gynecol Reprod Biol. 1995; 59(1): 17-20.	Saudi Arabia	144308
Alquaiz AM, Gad Mohamed A, Khoja TAM, Alsharif A, Shaikh SA, Al Mane H, Aldiris A, Kazi A, Hammad D. Prevalence of anemia and associated factors in childbearing age women in Riyadh, Saudi Arabia. J Nutr Metab. 2013; 2013: 636585.	Saudi Arabia	295204
Ministry of Health and Prevention (Senegal), Research Center for Human Development (Senegal). Senegal Demographic and Health Survey 2005. Fairfax, United States of America: ICF International.	Senegal	26855
Center for Research in Human Development (CRDH), Cheikh Anta Diop University, Hospital Aristide Le Dantec, ICF Macro, National Agency of Statistics and Demography (Senegal). Senegal Demographic and Health Survey 2010-2011. Fairfax, United States of America: ICF International.	Senegal	56063

Cheikh Anta Diop University, ICF International, National Agency of Statistics and Demography (Senegal). Senegal Continuous Demographic and Health Survey 2015. Fairfax, United States of America: ICF International, 2016.	Senegal	218592
Macro International, Inc, Research Center for Human Development (Senegal). Senegal Malaria Indicator Survey 2008-2009. Fairfax, United States of America: ICF International.	Senegal	11540
ICF International, Ministry of Health and Social Action (Senegal), National Agency of Statistics and Demography (Senegal). Senegal Continuous Demographic and Health Survey 2012-2013. Fairfax, United States of America: ICF International.	Senegal	111432
Cheikh Anta Diop University, ICF International, National Agency of Statistics and Demography (Senegal). Senegal Continuous Demographic and Health Survey 2014. Fairfax, United States of America: ICF International.	Senegal	191270
ICF International, Ministry of Health and Social Action (Senegal), National Agency of Statistics and Demography (Senegal). Senegal Continuous Demographic and Health Survey 2016. Fairfax, United States of America: ICF International, 2017.	Senegal	286772
ICF International, Ministry of Health and Social Action (Senegal), National Agency of Statistics and Demography (Senegal), Unit for the Fight Against Malnutrition (Senegal). Senegal Continuous Demographic and Health Survey 2017. Fairfax, United States of America: ICF International, 2018.	Senegal	353526
Macro International, Inc, Statistics Sierra Leone. Sierra Leone Demographic and Health Survey 2008. Fairfax, United States of America: ICF International.	Sierra Leone	21258
ICF International, Ministry of Health and Sanitation (Sierra Leone), Statistics Sierra Leone. Sierra Leone Demographic and Health Survey 2013. Fairfax, United States of America: ICF International, 2014.	Sierra Leone	131467
Catholic Relief Services (CRS), College of Medicine and Allied Health Sciences, University of Sierra Leone (COMAHS), ICF International, National Malaria Control Programme (Sierra Leone), Roll Back Malaria Partnership, Statistics Sierra Leone. Sierra Leone Malaria Indicator Survey 2016. Fairfax, United States of America: ICF International, 2017.	Sierra Leone	286773
Wirth JP, Rohner F, Woodruff BA, Chiwile F, Yankson H, Koroma AS, Russel F, Sesay F, Dominguez E, Petry N, Shahab-Ferdows S, de Onis M, Hodges MH. Anemia, Micronutrient Deficiencies, and Malaria in Children and Women in Sierra Leone Prior to the Ebola Outbreak - Findings of a Cross-Sectional Study. PLoS One. 2016; 11(5): e0155031.	Sierra Leone	294311
Singh K, Fong YF, Arulkumaran S. Anaemia in pregnancy--a cross-sectional study in Singapore. Eur J Clin Nutr. 1998; 52(1): 65-70.	Singapore	144162
Centre for International Health and Development, Institute of Child Health (CIHD), Food and Nutrition Security Analysis Unit (Somalia), Ministry of Health (Somalia), Ministry of Health and Labor (Somaliland), Puntland Ministry of Health (Somalia), United Nations Children's Fund (UNICEF), World Food Programme (WFP), World Health Organization (WHO). Somalia National Micronutrient and Anthropometric Survey 2009.	Somalia	152968
Omar MM, Högberg U, Bergström B. Maternal health and child survival in relation to socioeconomic factors. Gynecol Obstet Invest. 1994; 38(2): 107-12.	Somalia	254295
South African Vitamin A Consultative Group (SAVACG). South Africa Anthropometric, Vitamin A, Iron and Immunisation Coverage Status in Children Aged 6 to 71 Months 1994.	South Africa	139810
Oelofse A, Faber M, Benadé JG, Benadé AJ, Kenoyer DG. The nutritional status of a rural community in KwaZulu-Natal, South Africa: the Ndunakazi project. Cent Afr J Med. 1999; 45(1): 14-9.	South Africa	144167
Faber M, Jogessar VB, Benadé AJ. Nutritional status and dietary intakes of children aged 2-5 years and their caregivers in a rural South African community. Int J Food Sci Nutr. 2001; 52(5): 401-11.	South Africa	144174
Human Sciences Research Council, South African Medical Research Council. South Africa National Health and Nutrition Examination Survey 2012.	South Africa	155628
Department of Health (South Africa), ICF International, South African Medical Research Council, Statistics South Africa. South Africa Demographic and Health Survey 2016. Fairfax, United States of America: ICF International, 2019.	South Africa	157064

Mayet FGH, Schutte CHJ, Reinach SG. Anaemia among the inhabitants of a rural area in northern Natal. S Afr Med J. 1985; 67(12): 458-62.	South Africa	255129
Van der Westhuyzen J, Van Tonder SV, Gilbertson I, Metz J. Iron, folate and vitamin B12 nutrition and anaemia in black preschool children in the northern Transvaal. S Afr Med J. 1986; 70(3): 143-6.	South Africa	255133
Department of Health (South Africa), North-West University, South African Medical Research Council, Stellenbosch University, University of Cape Town, University of Kwazulu-Natal, University of Limpopo, University of Pretoria, University of the Free State, University of the Western Cape. South Africa National Food Consumption Survey - Fortification Baseline 2005.	South Africa	261367
Fogarty International Center, National Institutes of Health (NIH), Foundation for the National Institutes of Health (FNIH), University of Venda. South Africa - Venda Malnutrition and Enteric Disease Study 2009-2014.	South Africa	261887
Henríquez Sánchez P, Díaz Romero C, Rodríguez E, López Blanco F, Alvárez León E, Díaz Cremádes J, Pastor Ferrer MC, Serra Majem L. [Biochemical assessment of nutritional status in the Canary Islands population (1998)]. Arch Latinoam Nutr. 2000; 50(1 Suppl 1): 43-54.	Spain	144179
Medical Research Institute (Sri Lanka), United Nations Children's Fund (UNICEF), World Food Programme (WFP). Sri Lanka Nutrition and Food Security Assessment 2009.	Sri Lanka	141592
Department of Census and Statistics (Sri Lanka), Macro International, Inc. Sri Lanka Demographic and Health Survey 2006-2007. 2009.	Sri Lanka	18815
Mudalige R, Nestel P. Prevalence of anaemia in Sri Lanka. Ceylon J Med Sci. 1996; 9-16.	Sri Lanka	144180
Fernando SD, Paranavitane SR, Rajakaruna J, Weerasinghe S, Silva D, Wickremasinghe AR. The health and nutritional status of school children in two rural communities in Sri Lanka. Trop Med Int Health. 2000; 5(6): 450-2.	Sri Lanka	144182
Goonewardene M, Seekkuge J, Liyanage C. Iron stores and its correlation to haemoglobin levels in pregnant women attending an antenatal clinic. Ceylon Med J. 1995; 40(2): 67-9.	Sri Lanka	144183
Atukorala TM, Lanerolle P. Soil-transmitted helminthic infection and its effect on nutritional status of adolescent schoolgirls of low socioeconomic status in Sri Lanka. J Trop Pediatr. 1999; 45(1): 18-22.	Sri Lanka	144184
Medical Research Institute (Sri Lanka), United Nations Children's Fund (UNICEF). Sri Lanka National Nutrition and Micronutrient Survey 2012.	Sri Lanka	153000
Atukorala TM, de Silva LDR. Iron status of adolescent females in three schools in an urban area of Sri Lanka. J Trop Pediatr. 1990; 36(6): 316-21.	Sri Lanka	255138
Elnour A, Hambraeus L, Eltom M, Dramaix M, Bourdoux P. Endemic goiter with iodine sufficiency: a possible role for the consumption of pearl millet in the etiology of endemic goiter. Am J Clin Nutr. 2000; 71(1): 59-66.	Sudan	137249
Central Bureau of Statistics (Sudan), Federal Ministry of Health (Sudan), United Nations Children's Fund (UNICEF). Sudan Multiple Indicator Cluster Survey 2014. New York, United States of America: United Nations Children's Fund (UNICEF), 2016.	Sudan	200617
Central Statistical Office (Swaziland), Macro International, Inc. Swaziland Demographic and Health Survey 2006-2007. Fairfax, United States of America: ICF International.	Swaziland	20829
Akesson A, Bjellerup P, Berglund M, Bremme K, Vahter M. Serum transferrin receptor: a specific marker of iron deficiency in pregnancy. Am J Clin Nutr. 1998; 68(6): 1241-6.	Sweden	144186
Hallberg L, Hultén L, Lindstedt G, Lundberg PA, Mark A, Purens J, Svanberg B, Swolin B. Prevalence of iron deficiency in Swedish adolescents. Pediatr Res. 1993; 34(5): 680-7.	Sweden	144187
Samuelson G, Bratteby LE, Berggren K, Elverby JE, Kempe B. Dietary iron intake and iron status in adolescents. Acta Paediatr. 1996; 85(9): 1033-8.	Sweden	144188
Hess SY, Zimmermann MB, Brogli S, Hurrell RF. A national survey of iron and folate status in pregnant women in Switzerland. Int J Vitam Nutr Res. 2001; 71(5): 268-73.	Switzerland	144189

Shaw NS. Iron deficiency and anemia in school children and adolescents. J Formos Med Assoc. 1996; 95(9): 692-8.	Taiwan (Province of China)	143810
Ministry of Health (Tajikistan), United Nations Children's Fund (UNICEF). Tajikistan Micronutrient Status Survey 2009.	Tajikistan	126399
ICF International, Statistical Agency under the President of the Republic of Tajikistan. Tajikistan Demographic and Health Survey 2017. Fairfax, United States of America: ICF International, 2018.	Tajikistan	341838
Ministry of Public Health (Thailand). Thailand National Nutrition Survey 1995.	Thailand	124416
Bloem MW, Wedel M, Egger RJ, Speek AJ, Schrijver J, Saowakontha S, Schreurs WH. Iron metabolism and vitamin A deficiency in children in northeast Thailand. Am J Clin Nutr. 1989; 50(2): 332-8.	Thailand	144199
Saowakontha S, Pongpaew P, Schelp FP, Rojsathaporn K, Sriboonlue P, Vudhivai N, Intarakhao C, Pipitgool V, Mahaweerawat U, Supawan V. Quetelet index, hemoglobin and parasitic infection of rural women in northeast Thailand. Southeast Asian J Trop Med Public Health. 1994; 25(3): 474-8.	Thailand	144202
Linpisarn S, Thanangkul O, Suwanraj C, Kaewvichit R, Kricka LJ, Whitehead TP. Iron deficiency in a Northern Thai population: the effects of iron supplements studied by means of plasma ferritin estimations. Ann Clin Biochem. 1984; 21 (Pt 4): 268-74.	Thailand	144207
Sanchaisuriya P, Pongpaew P, Saowakontha S, Supawan V, Migasena P, Schelp FP. Nutritional health and parasitic infection of rural Thai women of the child bearing age. J Med Assoc Thai. 1993; 76(3): 138-45.	Thailand	144209
Linpisarn S, Tienboon P, Promtet N, Putsyainunt P, Santawanpat S, Fuchs GJ. Iron deficiency and anaemia in children with a high prevalence of haemoglobinopathies: implications for screening. Int J Epidemiol. 1996; 25(6): 1262-6.	Thailand	144213
Tungtrongchitr R, Pongpaew P, Schelp FP, Phonrat B, Mahaweerawat U, Paksanont S, Sanchaisuriya P, Jotking P, Intarakhao C, Saowakhontha S. Vitamin B12, folic acid, ferritin and haemoglobin status of rural women in child-bearing age in northeast Thailand. J Med Assoc Thai. 1997; 80(12): 785-90.	Thailand	144214
ICF Macro, Ministry of Finance (Timor-Leste), National Statistics Directorate (Timor-Leste). Timor-Leste Demographic and Health Survey 2009-2010. Fairfax, United States of America: ICF International.	Timor-Leste	21274
ACIL Australia Pty Ltd., Australian National University, Ministry of Health (Timor-Leste), National Statistics Directorate (Timor-Leste), University of Newcastle (Australia). Timor-Leste Demographic and Health Survey 2003. Newcastle, Australia: University of Newcastle (Australia).	Timor-Leste	20888
Ministry of Health (Timor-Leste). Timor-Leste Food and Nutrition Survey 2013.	Timor-Leste	286211
ICF International, National Statistics Directorate (Timor-Leste). Timor-Leste Demographic and Health Survey 2016. Fairfax, United States of America: ICF International, 2018.	Timor-Leste	286785
Directorate General of Statistics and National Accounts (Togo), ICF International, Ministry of Health (Togo), Ministry of Planning, Development and Zoning (Togo). Togo Demographic and Health Survey 2013-2014. Fairfax, United States of America: ICF International, 2015.	Togo	77515
Berger J, Dyck JL, Galan P, Aplogan A, Schneider D, Traissac P, Hercberg S. Effect of daily iron supplementation on iron status, cell-mediated immunity, and incidence of infections in 6-36 month old Togolese children. Eur J Clin Nutr. 2000; 54(1): 29-35.	Togo	144216
ICF International, National Institute of Hygiene, Ministry of Health (Togo), National Institute of Statistics and Economic and Demographic Studies (INSEED) (Togo), National Program to Fight Malaria, Ministry of Health (Togo). Togo Malaria Indicator Survey 2017. Fairfax, United States of America: ICF International, 2018.	Togo	359318
Kilinç M, Yüregir GT, Ekerbiçer H. Anaemia and iron-deficiency anaemia in south-east Anatolia. Eur J Haematol. 2002; 69(5-6): 280-3.	Turkey	144220
Macro International, Inc, Uganda Bureau of Statistics. Uganda Demographic and Health Survey 2000-2001. Fairfax, United States of America: ICF International.	Uganda	20993

MEASURE Evaluation Project, Carolina Population Center, University of North Carolina, Macro International, Inc, Ministry of Health (Uganda), Uganda Bureau of Statistics. Uganda Child Verbal Autopsy Study 2007. Calverton, United States of America: Macro International, Inc.	Uganda	23289
ICF Macro, Uganda Bureau of Statistics. Uganda Demographic and Health Survey 2011. Fairfax, United States of America: ICF International.	Uganda	56021
ICF International, National Malaria Control Program, Ministry of Health (Uganda), Uganda Bureau of Statistics. Uganda Malaria Indicator Survey 2014-2015. Fairfax, United States of America: ICF International.	Uganda	157065
ICF Macro, Ministry of Health (Uganda), Mulago Hospital, Uganda Bureau of Statistics, United Nations Children's Fund (UNICEF), World Health Organization (WHO). Uganda Malaria Indicator Survey 2009-2010. Fairfax, United States of America: ICF International.	Uganda	13109
Macro International, Inc, Uganda Bureau of Statistics. Uganda Demographic and Health Survey 2006. Fairfax, United States of America: ICF International.	Uganda	21014
Kiwanuka GN, Isharaza WK, Mahmoud S. Iron status of pregnant women at first antenatal booking in Mbarara University Teaching Hospital. Trop Doct. 1999; 29(4): 228-30.	Uganda	144221
ICF International, Uganda Bureau of Statistics. Uganda Demographic and Health Survey 2016. Fairfax, United States of America: ICF International, 2018.	Uganda	286780
Hossain MM, Bakir M, Pugh RN, Sheekh-Hussen M, Bin Ishaq SA, Berg DB, Lindblad BS. The prevalence and correlates of anaemia among young children and women of childbearing age in Al Ain, United Arab Emirates. Ann Trop Paediatr. 1995; 15(3): 227-35.	United Arab Emirates	144223
Hughes PF, Morrison J. Pregnancy outcome data in a United Arab Emirates population: what can they tell us? Asia Oceania J Obstet Gynaecol. 1994; 20(2): 183-90.	United Arab Emirates	144307
Human Nutrition Research, Medical Research Council, National Centre for Social Research (NatCen), Northern Ireland Statistics and Research Agency (NISRA), University College London Medical School. United Kingdom National Diet and Nutrition Survey 2008-2011 - Public Health England.	United Kingdom	120995
NatCen Social Research et al. , Diet and Nutrition Survey of Infants and Young Children, 2011 [computer file]. 2nd Edition. Colchester, Essex: UK Data Archive [distributor], October 2013. SN: 7263 , http://dx.doi.org/10.5255/UKDA-SN-7263-2.	United Kingdom	129281
Fischbacher C, Bhopal R, Patel S, White M, Unwin N, Alberti KG. Anaemia in Chinese, South Asian, and European populations in Newcastle upon Tyne: cross sectional study. BMJ. 2001; 322(7292): 958-9.	United Kingdom	144224
Wright CM, Kelly J, Trail A, Parkinson KN, Summerfield G. The diagnosis of borderline iron deficiency: results of a therapeutic trial. Arch Dis Child. 2004; 89(11): 1028-31.	United Kingdom	144226
Nelson M, White J, Rhodes C. Haemoglobin, ferritin, and iron intakes in British children aged 12-14 years: a preliminary investigation. Br J Nutr. 1993; 70(1): 147-55.	United Kingdom	144229
Clarke R, Refsum H, Birks J, Evans JG, Johnston C, Sherliker P, Ueland PM, Schneede J, McPartlin J, Nexo E, Scott JM. Screening for vitamin B-12 and folate deficiency in older persons. Am J Clin Nutr. 2003; 77(5): 1241-7.	United Kingdom	144230
Sherriff A, Emond A, Hawkins N, Golding J. Haemoglobin and ferritin concentrations in children aged 12 and 18 months. ALSPAC Children in Focus Study Team. Arch Dis Child. 1999; 80(2): 153-7.	United Kingdom	144231
Emond AM, Hawkins N, Pennock C, Golding J. Haemoglobin and ferritin concentrations in infants at 8 months of age. Arch Dis Child. 1996; 74(1): 36-9.	United Kingdom	144232
Robinson S, Godfrey K, Denne J, Cox V. The determinants of iron status in early pregnancy. Br J Nutr. 1998; 79(3): 249-55.	United Kingdom	144234
Nelson M, Bakaliou F, Trivedi A. Iron-deficiency anaemia and physical performance in adolescent girls from different ethnic backgrounds. Br J Nutr. 1994; 72(3): 427-33.	United Kingdom	144237

Office of Population Censuses and Surveys. Social Survey Division, Health Survey for England, 1991-1992: Combined Data File. Colchester, Essex: UK Data Archive [distributor], October 1997. SN: 3238, http://dx.doi.org/10.5255/UKDA-SN-3238-1	United Kingdom, England	22286
Office of Population Censuses and Surveys. Social Survey Division, Health Survey for England, 1993 [Computer file]. Colchester, Essex: UK Data Archive [distributor], April 1995. SN: 3316, http://dx.doi.org/10.5255/UKDA-SN-3316-1	United Kingdom, England	22293
Joint Health Surveys Unit of Social and Community Planning Research and University College London, Health Survey for England, 1994 [computer file]. 4th ed. Colchester, Essex: UK Data Archive [distributor], 26 March 2001. SN: 3640.	United Kingdom, England	22306
Joint Health Surveys Unit of Social and Community Planning Research and University College London, Health Survey for England, 1995 [computer file]. 3rd ed. Colchester, Essex: UK Data Archive [distributor], 26 March 2001. SN: 3796.	United Kingdom, England	22317
Joint Health Surveys Unit of Social and Community Planning Research and University College London, Health Survey for England, 1996 [computer file]. 3rd ed. Colchester, Essex: UK Data Archive [distributor], March 2001. SN: 3886.	United Kingdom, England	22329
Joint Health Surveys Unit of Social and Community Planning Research and University College London, Health Survey for England, 1997 [computer file]. 2nd ed. Colchester, Essex: UK Data Archive [distributor], 4 December 2000. SN: 3979.	United Kingdom, England	22341
National Centre for Social Research, University College London Department of Epidemiology and Public Health, Health Survey for England, 1998 [computer file]. 4th ed. Colchester, Essex: UK Data Archive [distributor], 30 November 2002. SN: 4150.	United Kingdom, England	22352
National Centre for Social Research and University College London. Department of Epidemiology and Public Health, Health Survey for England, 1999 [computer file]. 3rd Edition. Colchester, Essex: UK Data Archive [distributor], February 2002. SN: 4365.	United Kingdom, England	22364
National Centre for Social Research, University College London Department of Epidemiology and Public Health, Health Survey for England, 2000 [computer file]. Colchester, Essex: UK Data Archive [distributor], 23 April 2002. SN: 4487.	United Kingdom, England	22374
National Centre for Social Research and University College London. Department of Epidemiology and Public Health, Health Survey for England, 2001 [computer file]. 2nd Edition. Colchester, Essex: UK Data Archive [distributor], June 2004. SN: 4628.	United Kingdom, England	22388
National Centre for Social Research and University College London. Department of Epidemiology and Public Health, Health Survey for England, 2002 [computer file]. Colchester, Essex: UK Data Archive [distributor], May 2004. SN: 4912.	United Kingdom, England	22403
National Centre for Social Research and University College London. Department of Epidemiology and Public Health, Health Survey for England, 2004 [computer file]. Colchester, Essex: UK Data Archive [distributor], July 2006. SN: 5439.	United Kingdom, England	22449
National Centre for Social Research and University College London. Department of Epidemiology and Public Health, Health Survey for England, 2005 [computer file]. Colchester, Essex: UK Data Archive [distributor], July 2007. SN: 5675.	United Kingdom, England	22463
National Centre for Social Research and University College London. Department of Epidemiology and Public Health, Health Survey for England, 2006 [computer file]. 4th Edition. Colchester, Essex: UK Data Archive [distributor], July 2011. SN: 5809, http://dx.doi.org/10.5255/UKDA-SN-5809-1	United Kingdom, England	22476
National Centre for Social Research and University College London. Department of Epidemiology and Public Health, Health Survey for England, 2009 [computer file]. 2nd Edition. Colchester, Essex: UK Data Archive [distributor], July 2011. SN: 6732, http://dx.doi.org/10.5255/UKDA-SN-6732-1	United Kingdom, England	95630
Marmot, M. et al. , English Longitudinal Study of Ageing: Waves 0-5, 1998-2011 [computer file]. 20th Edition. Colchester, Essex: UK Data Archive [distributor], October 2013. SN: 5050, http://dx.doi.org/10.5255/UKDA-SN-5050-6	United Kingdom, England	134368
Office for National Statistics. Social and Vital Statistics Division and Food Standards Agency, National Diet and Nutrition Survey : Adults Aged 19 to 64 Years, 2000-2001 [computer file]. Colchester, Essex: UK Data Archive [distributor], May 2005. SN: 5140 , http://dx.doi.org/10.5255/UKDA-SN-5140-1.	United Kingdom, England, Scotland, Wales	124433

Office for National Statistics. Social Survey Division et al. , National Diet and Nutrition Survey : Young People Aged 4 to 18 Years, 1997 [computer file]. Colchester, Essex: UK Data Archive [distributor], January 2001. SN: 4243, http://dx.doi.org/10.5255/UKDA-SN-4243-1.	United Kingdom, England, Scotland, Wales	129421
Strain JJ, Thompson KA, Barker ME, Carville DGM. Iron sufficiency in the population of Northern Ireland: estimates from blood measurements. Br J Nutr. 1990; 64(1): 219-24.	United Kingdom, Northern Ireland	255142
Joint Health Surveys Unit of Social and Community Planning Research and University College London, Scottish Health Survey, 1998 [computer file]. Colchester, Essex: UK Data Archive [distributor], July 2001. SN: 4379 , http://dx.doi.org/10.5255/UKDA-SN-4379-1.	United Kingdom, Scotland	13265
Joint Health Surveys Unit of Social and Community Planning Research and University College London, Scottish Health Survey, 1995 [computer file]. 3rd ed. Colchester, Essex: UK Data Archive [distributor], 11 February 1999. SN: 3807.	United Kingdom, Scotland	13290
Macro International, Inc, National Bureau of Statistics (Tanzania). Tanzania Demographic and Health Survey 2004-2005. Fairfax, United States of America: ICF International.	United Republic of Tanzania	20875
ICF Macro, National Bureau of Statistics (Tanzania). Tanzania Demographic and Health Survey 2009-2010. Fairfax, United States of America: ICF International.	United Republic of Tanzania	21331
Lwambo NJ, Brooker S, Siza JE, Bundy DA, Guyatt H. Age patterns in stunting and anaemia in African schoolchildren: a cross-sectional study in Tanzania. Eur J Clin Nutr. 2000; 54(1): 36-40.	United Republic of Tanzania	110765
Tatala S, Svanberg U, Mduma B. Low dietary iron availability is a major cause of anemia: a nutrition survey in the Lindi District of Tanzania. Am J Clin Nutr. 1998; 68(1): 171-8.	United Republic of Tanzania	110784
Macro International, Inc, National Bureau of Statistics (Tanzania), Office of the Chief Government Statistician (OCGS) (Zanzibar), Tanzania Commission for AIDS (TACAIDS), Zanzibar AIDS Commission (ZAC). Tanzania HIV/AIDS and Malaria Indicator Survey 2007-2008. Fairfax, United States of America: ICF International.	United Republic of Tanzania	12644
ICF International, National Bureau of Statistics (Tanzania), Office of the Chief Government Statistician (OCGS) (Zanzibar), Tanzania Commission for AIDS (TACAIDS), Zanzibar AIDS Commission (ZAC). Tanzania AIDS Indicator Survey 2011-2012. Fairfax, United States of America: ICF International, 2013.	United Republic of Tanzania	77395
Kitange H, Swai A, Kilima P, Masuki G, Alberti K, McLarty D. Anaemia is a major public health problem in Tanzania. Health Policy Plan. 1993; 413-24.	United Republic of Tanzania	144190
Massawe SN, Urassa EN, Nyström L, Lindmark G. Effectiveness of primary level antenatal care in decreasing anemia at term in Tanzania. Acta Obstet Gynecol Scand. 1999; 78(7): 573-9.	United Republic of Tanzania	144192
Stoltzfus RJ, Chwaya HM, Tielsch JM, Schulze KJ, Albonico M, Savioli L. Epidemiology of iron deficiency anemia in Zanzibari schoolchildren: the importance of hookworms. Am J Clin Nutr. 1997; 65(1): 153-9.	United Republic of Tanzania	144194
Bergsjo P, Seha AM, Ole-King'ori N. Hemoglobin concentration in pregnant women. Experience from Moshi, Tanzania. Acta Obstet Gynecol Scand. 1996; 75(3): 241-4.	United Republic of Tanzania	144195
Stoltzfus RJ, Edward-Raj A, Dreyfuss ML, Albonico M, Montresor A, Dhoj Thapa M, West KP Jr, Chwaya HM, Savioli L, Tielsch J. Clinical pallor is useful to detect severe anemia in populations where anemia is prevalent and severe. J Nutr. 1999; 129(9): 1675-81.	United Republic of Tanzania	144196
ICF International, Ministry of Health (Zanzibar), Ministry of Health, Community Development, Gender, Elderly and Children (MoHCDEC) (Tanzania), National Bureau of Statistics (Tanzania), Office of the Chief Government Statistician (OCGS) (Zanzibar). Tanzania Malaria Indicator Survey 2017. Fairfax, United States of America: ICF International, 2018.	United Republic of Tanzania	350798
Fogarty International Center, National Institutes of Health (NIH), Foundation for the National Institutes of Health (FNIH), Haydom Lutheran Hospital. Tanzania - Haydom Malnutrition and Enteric Disease Study 2009-2014.	United Republic of Tanzania	261889
ICF International, Ministry of Health (Zanzibar), Ministry of Health, Community Development, Gender, Elderly and Children (MoHCDEC) (Tanzania), National	United Republic of Tanzania	218593

Bureau of Statistics (Tanzania), Office of the Chief Government Statistician (OCGS) (Zanzibar). Tanzania Demographic and Health Survey 2015-2016. Fairfax, United States of America: ICF International, 2016.		
National Center for Health Statistics (NCHS), Centers for Disease Control and Prevention (CDC). United States National Health and Nutrition Examination Survey 2007-2008. Hyattsville, United States: National Center for Health Statistics (NCHS), Centers for Disease Control and Prevention (CDC), 2009.	United States of America	25914
National Center for Health Statistics (NCHS), Centers for Disease Control and Prevention (CDC). United States National Health and Nutrition Examination Survey 2005-2006. Hyattsville, United States: National Center for Health Statistics (NCHS), Centers for Disease Control and Prevention (CDC), 2007.	United States of America	47478
National Center for Health Statistics (NCHS), Centers for Disease Control and Prevention (CDC). United States National Health and Nutrition Examination Survey 2003-2004. Hyattsville, United States: National Center for Health Statistics (NCHS), Centers for Disease Control and Prevention (CDC).	United States of America	47962
National Center for Health Statistics (NCHS), Centers for Disease Control and Prevention (CDC). United States National Health and Nutrition Examination Survey 2009-2010. Hyattsville, United States: National Center for Health Statistics (NCHS), Centers for Disease Control and Prevention (CDC), 2011.	United States of America	48332
National Center for Health Statistics (NCHS), Centers for Disease Control and Prevention (CDC). United States National Health and Nutrition Examination Survey 1988-1994. Hyattsville, United States: National Center for Health Statistics (NCHS), Centers for Disease Control and Prevention (CDC).	United States of America	48604
National Center for Health Statistics (NCHS), Centers for Disease Control and Prevention (CDC). United States National Health and Nutrition Examination Survey 2001-2002. Hyattsville, United States: National Center for Health Statistics (NCHS), Centers for Disease Control and Prevention (CDC).	United States of America	49205
National Center for Health Statistics (NCHS), Centers for Disease Control and Prevention (CDC). United States National Health and Nutrition Examination Survey 1999-2000. Hyattsville, United States: National Center for Health Statistics (NCHS), Centers for Disease Control and Prevention (CDC).	United States of America	52110
National Center for Health Statistics, Centers for Disease Control and Prevention. United States National Health and Nutrition Examination Survey 2011-2012. Hyattsville, United States of America: National Center for Health Statistics, Centers for Disease Control and Prevention, 2013.	United States of America	110300
National Center for Health Statistics, Centers for Disease Control and Prevention. United States National Health and Nutrition Examination Survey 2013-2014. Hyattsville, United States of America: National Center for Health Statistics, Centers for Disease Control and Prevention.	United States of America	165892
Penrod JC, Anderson K, Acosta PB. Impact on iron status of introducing cow's milk in the second six months of life. J Pediatr Gastroenterol Nutr. 1990; 10(4): 462-7.	United States of America	144249
Bodnar LM, Scanlon KS, Freedman DS, Siega-Riz AM, Cogswell ME. High prevalence of postpartum anemia among low-income women in the United States. Am J Obstet Gynecol. 2001; 185(2): 438-43.	United States of America	144254
Boutry M, Needlman R. Use of diet history in the screening of iron deficiency. Pediatrics. 1996; 98(6 Pt 1): 1138-42.	United States of America	144256
Bogen DL, Duggan AK, Dover GJ, Wilson MH. Screening for iron deficiency anemia by dietary history in a high-risk population. Pediatrics. 2000; 105(6): 1254-9.	United States of America	144259
Carriaga MT, Skikne BS, Finley B, Cutler B, Cook JD. Serum transferrin receptor for the detection of iron deficiency in pregnancy. Am J Clin Nutr. 1991; 54(6): 1077-81.	United States of America	144261
Adams WG, Geva J, Coffman J, Palfrey S, Bauchner H. Anemia and elevated lead levels in underimmunized inner-city children. Pediatrics. 1998; 101(3): 6.	United States of America	144262
Wright RO, Tsaih S-W, Schwartz J, Wright RJ, Hu H. Association between iron deficiency and blood lead level in a longitudinal analysis of children followed in an urban primary care clinic. J Pediatr. 2003; 142(1): 9-14.	United States of America	144263
Salive ME, Cornoni-Huntley J, Guralnik JM, Phillips CL, Wallace RB, Ostfeld AM, Cohen HJ. Anemia and hemoglobin levels in older persons: relationship with age, gender, and health status. J Am Geriatr Soc. 1992; 40(5): 489-96.	United States of America	144264

Fleming DJ, Jacques PF, Tucker KL, Massaro JM, D'Agostino RB Sr, Wilson PW, Wood RJ. Iron status of the free-living, elderly Framingham Heart Study cohort: an iron-replete population with a high prevalence of elevated iron stores. Am J Clin Nutr. 2001; 73(3): 638-46.	United States of America	144265
Alper BS, Kimber R, Reddy AK. Using ferritin levels to determine iron-deficiency anemia in pregnancy. J Fam Pract. 2000; 49(9): 829-32.	United States of America	144267
Gupta S, Venkateswaran R, Gorenflo DW, Eyler AE. Childhood iron deficiency anemia, maternal nutritional knowledge, and maternal feeding practices in a high-risk population. Prev Med. 1999; 29(3): 152-6.	United States of America	144277
Irigoyen M, Davidson LL, Carriero D, Seaman C. Randomized, placebo-controlled trial of iron supplementation in infants with low hemoglobin levels fed iron-fortified formula. Pediatrics. 1991; 88(2): 320-6.	United States of America	144279
Mitchell MC, Lerner E. Maternal hematologic measures and pregnancy outcome. J Am Diet Assoc. 1992; 92(4): 484-6.	United States of America	144283
Sarnak MJ, Tighiouart H, Manjunath G, MacLeod B, Griffith J, Salem D, Levey AS. Anemia as a risk factor for cardiovascular disease in The Atherosclerosis Risk in Communities (ARIC) study. J Am Coll Cardiol. 2002; 40(1): 27-33.	United States of America	144284
Bogen DL, Krause JP, Serwint JR. Outcome of children identified as anemic by routine screening in an inner-city clinic. Arch Pediatr Adolesc Med. 2001; 155(3): 366-71.	United States of America	144285
Institute of Obstetrics and Gynecology, Ministry of Health (Uzbekistan), Macro International, Inc, Ministry of Health (Uzbekistan). Uzbekistan Demographic and Health Survey 1996. Fairfax, United States of America: ICF International.	Uzbekistan	21033
Jalaludin B, Taylor R, Levy S, Montaville B, Gee K. Prevalence of anaemia and iron deficiency at different levels of urbanization in Vanuatu. P N G Med J. 1992; 35(2): 128-36.	Vanuatu	144287
Layrisse M, Chaves JF, Mendez-Castellano, Bosch V, Tropper E, Bastardo B, González E. Early response to the effect of iron fortification in the Venezuelan population. Am J Clin Nutr. 1996; 64(6): 903-7.	Venezuela (Bolivarian Republic of)	144289
Castejon HV, Ortega P, Amaya D, Gomez G, Leal J, Castejon OJ. Co-existence of anemia, vitamin A deficiency and growth retardation among children 24-84 months old in Maracaibo, Venezuela. Nutr Neurosci. 2004; 7(2): 113-9.	Venezuela (Bolivarian Republic of)	144292
Diez-Ewald M, Torrés-Guerra E, Leets I, Layrisse M, Vizcaíno G, Arteaga Vizcaíno M. [Anemia in indigenous population of the West of Venezuela]. Invest Clin. 1999; 40(3): 191-202.	Venezuela (Bolivarian Republic of)	144294
Martí-Carvajal A, Peña-Martí G, Comunian G, Muñoz S. Prevalence of anemia during pregnancy: results of Valencia (Venezuela) anemia during pregnancy study. Arch Latinoam Nutr. 2002; 52(1): 5-11.	Venezuela (Bolivarian Republic of)	144295
Peña E, Sánchez A, Solano L. [Profile of nutritional risk in pregnant adolescents]. Arch Latinoam Nutr. 2003; 53(2): 141-9.	Venezuela (Bolivarian Republic of)	144296
Thu BD, Schultink W, Dillon D, Gross R, Leswara ND, Khoi HH. Effect of daily and weekly micronutrient supplementation on micronutrient deficiencies and growth in young Vietnamese children. Am J Clin Nutr. 1999; 69(1): 80-6.	Viet Nam	144297
Laillou A, Pham TV, Tran NT, Le HT, Wieringa F, Rohner F, Fortin S, Le MB, Tran do T, Moench-Pfanner R, Berger J. Micronutrient Deficits Are Still Public Health Issues among Women and Young Children in Vietnam. PLoS One. 2012; 7(4): e34906.	Viet Nam	261370
Central Statistical Organization (Yemen), ICF International, Ministry of Public Health and Population (Yemen). Yemen Demographic and Health Survey 2013. Fairfax, United States of America: ICF International.	Yemen	112500
Raja'a YA, Sulaiman SM, Elkarib SA, Mubarak JS. Nutritional status of Yemeni schoolchildren in Al-Mahweet Governorate. East Mediterr Health J. 2001; 7(1-2): 204-10.	Yemen	144299
Hautvast JL, Tolboom JJ, Kafwembe EM, Musonda RM, Mwanakasale V, van Staveren WA, van 't Hof MA, Sauerwein RW, Willems JL, Monnens LA. Severe linear growth retardation in rural Zambian children: the influence of biological variables. Am J Clin Nutr. 2000; 71(2): 550-9.	Zambia	255147
Ezeilo GC. Haemotological values in pregnant Zambian women. Trop Geogr Med. 1972; 24(2): 152-6.	Zambia	255159

Central Statistical Office (Zambia), ICF International, Ministry of Health (Zambia), University Teaching Hospital (Zambia), University of Zambia. Zambia Demographic and Health Survey 2018-2019. Fairfax, United States of America: ICF International, 2020.	Zambia	411301
Government of Zambia, National Malaria Control Centre (Zambia), World Bank. Zambia Access to ACT Initiative Survey 2009. Washington DC, United States of America: World Bank.	Zambia	162031
Mel and Enid Zuckerman College of Public Health, University of Arizona, National Food and Nutrition Commission (Zambia), Tropical Diseases Research Centre, UNICEF International Child Development Center (ICDC). Zambia Food Consumption and Micronutrient Survey 2008.	Zambia	262998
Central Statistical Office (Zimbabwe), Macro International, Inc. Zimbabwe Demographic and Health Survey 2005-2006. Fairfax, United States of America: ICF International.	Zimbabwe	21163
ICF Macro, Zimbabwe National Statistics Agency. Zimbabwe Demographic and Health Survey 2010-2011. Calverton, United States of America: ICF Macro, 2012.	Zimbabwe	55992
ICF International, National Microbiology Reference Laboratory, Harare Central Hospital (NMRL) (Zimbabwe), Zimbabwe National Statistics Agency. Zimbabwe Demographic and Health Survey 2015. Fairfax, United States of America: ICF International, 2016.	Zimbabwe	157066
Friis H, Gomo E, Koestel P, Ndhlovu P, Nyazema N, Krarup H, Michaelsen KF. HIV and other predictors of serum folate, serum ferritin, and hemoglobin in pregnancy: a cross-sectional study in Zimbabwe. Am J Clin Nutr. 2001; 73(6): 1066-73.	Zimbabwe	144301
Ncube TN, Malaba L, Greiner T, Gebre-Medhin M. Evidence of grave vitamin A deficiency among lactating women in the semi-arid rural area of Makhaza in Zimbabwe. A population-based study. Eur J Clin Nutr. 2001; 55(4): 229-34.	Zimbabwe	144303

Authors' Contributions

Managing the overall research enterprise

Nicholas J Kassebaum, Christopher J L Murray.

Writing the first draft of the manuscript

William M Gardner, Hailey Hagins, Conor Hennessy, Nicholas J Kassebaum, and Theresa A McHugh.

Primary responsibility for applying analytical methods to produce estimates

Kelly M Cercy, Catherine S Chen, William M Gardner, Hailey Hagins, Conor Hennessy, Nicholas J Kassebaum, Stephen S Lim, Christopher J L Murray, Christian Razo, Heather Jean Taylor, Victor M Vilchis-Tella, and Leo Zucker Zoeckler.

Primary responsibility for seeking, cataloguing, extracting, or cleaning data; designing or coding figures and tables

Kelly M Cercy, William M Gardner, Conor Hennessy, Nicholas J Kassebaum, Christian Razo, Heather Jean Taylor, Victor M Vilchis-Tella, and Leo Zucker Zoeckler.

Providing data or critical feedback on data sources

Kalkidan Hassen Abate, Sherief Abd-Elsalam, Ame Mehadi Abdurehman, Hassan Abidi, Richard Gyan Aboagye, Hassan Abolhassani, Yonas Derso Abtew, Denberu Eshetie Adane Adane, Qorinah Estiningtyas Sakilah Adnani, Muhammad Sohail Afzal, Saira Afzal, Bright Opoku Ahinkorah, Sajjad Ahmad, Sepideh Ahmadi, Ayman Ahmed, Tarik Ahmed Rashid, Fares Alahdab, Astawus Alemayehu, Robert Kaba Alhassan, Sami Almustanyir, Khalid A Altirkawi, Nelson Alvis-Guzman, Hubert Amu, Tadele Fentabil Anagaw, Maxwell Hubert Antwi, Davood Anvari, Jalal Arabloo, Hany Ariffin, Timur Aripov, Judie Arulappan, Tahira Ashraf, Melash Belachew Asresie, Seyyed Shamsadin Athari, Daniel Atlaw, Sara Bagherieh, Atif Amin Baig, Maciej Banach, Palash Chandra Banik, Amadou Barrow, Abdul-Monim Mohammad Batiha, Melaku Ashagrie Belete, Ajay Nagesh Bhat, Zulfiqar A Bhutta, Boris Bikbov, Sk Masum Billah, Jessica Devin Bishai, Archith Boloor, Francieli Cembranel, Gashaw Sisay Chanie, Vijay Kumar Chattu, Eunice Chung, Michael H Criqui, Natália Cruz-Martins, Xiaochen Dai, Lalit Dandona, Rakhi Dandona, Aso Mohammad Darwesh, Hardik Dineshbhai Desai, Markos Desalegn Desalegn, Fikadu Nugusu Dessalegn, Gashaw Dessie, Samath Dhamminda Dharmaratne, Meghnath Dhimal, Sameer Dhingra, M Ashworth Dirac, Saeid Doaei, Milad Dodangeh, Paul Narh Doku, Haneil Larson Dsouza, Michael Ekholuenetale, Samuel B Ewald, Adeniyi Francis Fagbamigbe, Ali Fatehizadeh, Ginenus Fekadu, Ryan Fitzgerald, Muktar A. Gadanya, Santosh Gaihre, Yaseen Galali, William M. Gardner, Tushar Garg, Lemma Getacher, Kazem Ghaffari, Mansour Ghafourifard, Ahmad Ghashghaee, Sherief Ghozy, Abraham Tamirat T Gizaw, James C Glasbey, Mahaveer Golechha, Pouya Goleij, Mohamad Golitaleb, Alessandra C Goulart, Habtamu Alganeh Guadie, Zewdie Gudisa, Rahul Gupta, Sapna Gupta, Vivek Kumar Gupta, Alemu Guta, Harapan Harapan, Ahmed I. Hasaballah, Hadi Hassankhani, Sheikh Jamal Hossain, Mehdi Hosseinzadeh, Segun Emmanuel Ibitoye, Sheikh Mohammed Shariful Islam, Nahlah Elkudssiah Ismail, Linda Merin J, Manthan Dilipkumar Janodia, Shubha Jayaram, Jost B. Jonas, Jacek Jerzy Jozwiak, Rohollah Kalhor, Himal Kandel, Tesfaye K Kanko, Nicholas J Kassebaum, Joonas H Kauppila, Harkiran Kaur, Gbenga A. Kayode, Yousef Saleh Khader, Himanshu Khajuria, Nauman Khalid, Mohammad Khammarnia, Moien AB Khan, Khaled Khatab, Zaher Khazaei, Jagdish Khubchandani, Yun Jin Kim, Sezer Kisa, Soewarta Kosen, Sindhura Lakshmi Koulmane Laxminarayana, Kewal Krishan, Barthelemy Kuate Defo, G Anil Kumar, Om P Kurmi, Dharmesh Kumar Lal, Kamaluddin Latief, Caterina Ledda, Sang-woong Lee, Samson Mideksa Legesse, Stephen S Lim, Xuefeng Liu, Preetam Bhalchandra Mahajan, Ata Mahmoodpoor, Deborah Carvalho Malta, Fabiola Mejia-Rodriguez, Walter Mendoza, Oliver Mendoza-Cano, Haftu Asmerom Meresa, Moonis Mirza, Awoke Misganaw, Shafiu Mohammed, Syam Mohan, Nagabhishek Moka, Ali H

Mokdad, Lorenzo Monasta, Mohammad Ali Moni, Maryam Moradi, Ebrahim Mostafavi, Temesgen Muche, Francesk Mulita, Getaneh Baye B Mulu, Christopher J L Murray, Ahamarshan Jayaraman Nagarajan, Sreenivas Narasimha Swamy, Zuhair S Natto, Biswa Prakash Nayak, Shumaila Naz, Ionut Negoi, Ruxandra Irina Negoi, Josephine W. Ngunjiri, Robina Khan Niazi, Dieta Nurrika, Khan M Nuruzzaman, Ogochukwu Janet Nzoputam, Bogdan Oancea, Mohammed Mohammed Suleiman Suleiman Obsa, Julius Nyerere Odhiambo, Osaretin Christabel Okonji, Andrew T Olagunju, Ahmed Omar Bali, Abidemi E Emmanuel Omonisi, Alberto Ortiz, Mayowa O Owolabi, Jagadish Rao Padubidri, Anamika Pandey, Shahina Pardhan, Aslam Ramjan Pathan, Uttam Paudel, Shrikant Pawar, Simone Perna, Navaraj Perumalsamy, Zahra Zahid Piracha, Zahiruddin Quazi Syed, Fakher Rahim, Mehran Rahimi, Amir Masoud Rahmani, Pradhum Ram, Juwel Rana, Chythra R Rao, Sowmya J Rao, Sina Rashedi, Salman Rawaf, Lal Rawal, Rabail Zehra Raza, Elrashdy Moustafa Mohamed Redwan, Maryam Rezaei, Jefferson Antonio Buendia Rodriguez, Leonardo Roever, Godfrey M Rwegerera, Aly M A Saad, Basema Saddik, Umar Saeed, Harihar Sahoo, Marwa Rashad Salem, Abdallah M Samy, Itamar S Santos, Maheswar Satpathy, Ganesh Kumar Saya, Subramanian Senthilkumaran, Allen Seylani, Pritik A Shah, Masood Ali Shaikh, Mohd Shanawaz, Mohammed Shannawaz, Mequannent Melaku Sharew Sharew, Purva Sharma, B Suresh Kumar Shetty, Pavanchand H Shetty, Jae Il Shin, Parnian Shobeiri, Wudneh Simegn, Jasvinder A. Singh, Anna Aleksandrovna Skryabina, Jeffrey D Stanaway, Parminder S Suchdev, Mindy D Szeto, Ker-Kan Tan, Elvis Enowbeyang Tarkang, Yibekal Manaye Tefera, Amir Tiyuri, Ruoyan Tobe-Gai, Marcos Roberto Tovani-Palone, Mai Thi Ngoc Tran, Irfan Ullah, Abdurezak Adem Umer, Bhaskaran Unnikrishnan, Sahel Valadan Tahbaz, Pascual R Valdez, Shoban Babu Varthya, Linh Gia Vu, Birhanu Wagaye, Melat Woldemariam, Tewodros Eshete Wonde, Gedif Ashebir Wubetie, Dong Keon Yon, Naohiro Yonemoto, Melissa F. Young, Chuanhua Yu, Burhan Abdullah Zaman, Iman Zare, Zahra Zareshahrabadi, Zhi-Jiang Zhang, and Mohammad Zoladl.

Developing methods or computational machinery

Yonas Derso Abtew, Qorinah Estiningtyas Sakilah Adnani, Saira Afzal, Tarik Ahmed Rashid, Hubert Amu, Davood Anvari, Aleksandr Y Aravkin, Judie Arulappan, Atif Amin Baig, Azadeh Bashiri, Abdul-Monim Mohammad Batiha, Akshaya Srikanth Bhagavathula, Xiaochen Dai, Aso Mohammad Darwesh, Hardik Dineshbhai Desai, Fikadu Nugusu Dessalegn, Saeid Doaei, Ahmed Elabbas Mustafa Elagali, Ali Fatehizadeh, Ryan Fitzgerald, William M. Gardner, Ketema Bizuwork Gebremedhin, Kazem Ghaffari, Mahaveer Golechha, Mohamad Golitaleb, Girma Garedew Goyomsa, Rajat Das Gupta, Simon I Hay, Mehdi Hosseinzadeh, Linda Merin J, Nicholas J Kassebaum, Zaher Khazaei, Sang-woong Lee, Seyedeh Zahra Masoumi, Moonis Mirza, Nagabhishek Moka, Ali H Mokdad, Mohammad Ali Moni, Francesk Mulita, Christopher J L Murray, Josephine W. Ngunjiri, Ahmed Omar Bali, Brandon V Pickering, Zahra Zahid Piracha, Zahiruddin Quazi Syed, Amir Masoud Rahmani, Maryam Rezaei, Siamak Sabour, Umar Saeed, Abdallah M Samy, Maheswar Satpathy, Ganesh Kumar Saya, Mohammed Shannawaz, Mustafa Kamal Sikder, Reed J D Sorensen, Jeffrey D Stanaway, Yibekal Manaye Tefera, Mai Thi Ngoc Tran, Irfan Ullah, Tewodros Eshete Wonde, and Peng Zheng.

Providing critical feedback on methods or results

Amirali Aali, Sherief Abd-Elsalam, Ame Mehadi Abdurehman, Hassan Abidi, Richard Gyan Aboagye, Hassan Abolhassani, Yonas Derso Abtew, Denberu Eshetie Adane Adane, Tigist Demssew Adane, Isaac Yeboah Addo, Miracle Ayomikun Adesina, Daniel Adedayo Adeyinka, Qorinah Estiningtyas Sakilah Adnani, Muhammad Sohail Afzal, Saira Afzal, Bright Opoku Ahinkorah, Aqeel Ahmad, Sajjad Ahmad, Ayman Ahmed, Tarik Ahmed Rashid, Wajeeha Aiman, Hossein Akbarialiabad, Fares Alahdab, Ziyad Al-Aly, Nazmul Alam, Astawus Alemayehu, Robert Kaba Alhassan, Muhammad Ashar Ali, Sami Almustanyir, Rajaa M Al-Raddadi, Rami H. Hani Al-Rifai, Khalid A Altirkawi, Saba Alvand, Nelson Alvis-Guzman, Edward Kwabena Ameyaw, Hubert Amu, Tadele Fentabil Anagaw, Ali Arash Anoushirvani, Maxwell Hubert

Antwi, Davood Anvari, Jalal Arabloo, Hany Ariffin, Timur Aripov, Asrat Arja, Michael Benjamin Arndt, Judie Arulappan, Raphael Taiwo Aruleba, Tahira Ashraf, Melash Belachew Asresie, Seyyed Shamsadin Athari, Daniel Atlaw, Avinash Aujayeb, Andargie Abate Awoke, Mamaru Ayenew Awoke, Sina Azadnajafabad, Mohammadreza Azangou-Khyavy, Darshan B B, Alaa Badawi, Ashish D Badiye, Nayereh Baghcheghi, Nasser Bagheri, Sara Bagherieh, Atif Amin Baig, Maciej Banach, Palash Chandra Banik, Abere Tilahun Bantie, Ronald D Barr, Amadou Barrow, Azadeh Bashiri, Saurav Basu, Abdul-Monim Mohammad Batiha, Tahmina Begum, Melaku Ashagrie Belete, Alemshet Yirga Berhie, Akshaya Srikanth Bhagavathula, Nikha Bhardwaj, Pankaj Bhardwaj, Ajay Nagesh Bhat, Zulfiqar A Bhutta, Sk Masum Billah, Setognal Birara, Jessica Devin Bishai, Archith Boloor, João Silva Botelho Botelho, Katrin Burkart, Francieli Cembranel, Promit Ananyo Chakraborty, Gashaw Sisay Chanie, Vijay Kumar Chattu, Isaac Sunday Chukwu, Michael H Criqui, Natália Cruz-Martins, Omid Dadras, Gizachew Worku Dagnew, Xiaochen Dai, Hadi A Danawi, Lalit Dandona, Rakhi Dandona, Aso Mohammad Darwesh, Jai K Das, Saswati Das, Vanessa De la Cruz-Góngora, Fitsum Wolde Demisse, Solomon Demissie, Desalegn Getnet Demsie, Hardik Dineshbhai Desai, Markos Desalegn Desalegn, Fikadu Nugusu Dessalegn, Gashaw Dessie, Samath Dhamminda Dharmaratne, Meghnath Dhimal, Sameer Dhingra, Daniel Diaz, Mojtaba Didehdar, M Ashworth Dirac, Mengistie Diress, Saeid Doaei, Milad Dodangeh, Paul Narh Doku, Deepa Dongarwar, Bezabih Terefe Dora, Haneil Larson Dsouza, Hisham Atan Edinur, Michael Ekholuenetale, Ahmed Elabbas Mustafa Elagali, Ghada Metwally Tawfik ElGohary, Muhammed Elhadi, Mohamed A Elmonem, Daniel Berhanie Enyew, Habitu Birhan Eshetu, Rana Ezzeddini, Adeniyi Francis Fagbamigbe, Abidemi Omolara Fasanmi, Ali Fatehizadeh, Ginenus Fekadu, Bikila Regassa Feyisa, Florian Fischer, Ryan Fitzgerald, Masoud Foroutan, Kayode Raphael Fowobaje, Muktar A. Gadanya, Abhay Motiramji Gaidhane, Santosh Gaihre, Abduzhappar Gaipov, Yaseen Galali, William M. Gardner, Priyanka Garg, Tushar Garg, Yosef Haile Gebremariam, Berhe Gebremichael, Kazem Ghaffari, Mansour Ghafourifard, Mohammad Ghasemi Nour, Ahmad Ghashghaee, Maryam Gholamalizadeh, Sherief Ghozy, Abraham Tamirat T Gizaw, James C Glasbey, Mahaveer Golechha, Mohamad Golitaleb, Alessandra C Goulart, Girma Garedew Goyomsa, Habtamu Alganeh Guadie, Mohammed Ibrahim Mohialdeen Gubari, Damitha Asanga Gunawardane, Rahul Gupta, Rajat Das Gupta, Sapna Gupta, Vivek Kumar Gupta, Alemu Guta, Parham Habibzadeh, Samer Hamidi, Alexis J Handal, Asif Hanif, Harapan Harapan, Mehdi Harorani, Ahmed I. Hasaballah, Md. Mehedi Hasan, Hamidreza Hasani, Hadi Hassankhani, Mohammed Bheser Hassen, Simon I Hay, Khezar Hayat, Golnaz Heidari, Demisu Zenbaba Heyi, Kamal Hezam, Yuta Hiraike, Ramesh Holla, Sheikh Jamal Hossain, Kaveh Hosseini, Mohammad-Salar Hosseini, Mehdi Hosseinzadeh, Mihaela Hostiuc, Salman Hussain, Foziya Mohammed Hussien, Segun Emmanuel Ibitoye, Olayinka Stephen Ilesanmi, Irena M. Ilic, Mustapha Immurana, Leeberk Raja Inbaraj, Sheikh Mohammed Shariful Islam, Nahlah Elkudssiah Ismail, Linda Merin J, Elham Jamshidi, Umesh Jayarajah, Shubha Jayaram, Rime Jebai, Bedru Jemal, Angeline Jeyakumar, Ravi Prakash Jha, Jost B. Jonas, Jacek Jerzy Jozwiak, Ali Kabir, Laleh R. Kalankesh, Rohollah Kalhor, Vineet Kumar Kamal, Himal Kandel, Tesfaye K Kanko, Ibraheem M Karaye, Faizan Zaffar Kashoo, Nicholas J Kassebaum, Patrick D.M.C. Katoto, Joonas H Kauppila, Harkiran Kaur, Gbenga A. Kayode, Adera Debella Kebede, Vikash Ranjan Keshri, Mohammad Keykhaei, Yousef Saleh Khader, Himanshu Khajuria, Nauman Khalid, Imteyaz A Khan, Moien AB Khan, Khaled Khatab, Zaher Khazaei, Jagdish Khubchandani, Yun Jin Kim, Ruth W Kimokoti, Sezer Kisa, Farzad Kompani, Sindhura Lakshmi Koulmane Laxminarayana, Kewal Krishan, Barthelemy Kuate Defo, Mohammed Kuddus, G Anil Kumar, Naveen Kumar, Nithin Kumar, Om P Kurmi, Oluwatosin Kuti, Dharmesh Kumar Lal, Anders O Larsson, Zohra S Lassi, Kamaluddin Latief, Avula Laxmaiah, Caterina Ledda, Sang-woong Lee, Samson Mideksa Legesse, Stephen S Lim, Xuefeng Liu, László Lorenzovici, Vanessa Sintra Machado Machado, Preetam Bhalchandra Mahajan, Ata Mahmoodpoor, Elham Mahmoudi, Elaheh Malakan Rad, Tauqeer Hussain Mallhi, Deborah Carvalho Malta, Sahar Masoudi, John Robert Carabeo Medina, José João João Mendes Mendes, Walter Mendoza, Oliver Mendoza-Cano, Alexios-Fotios A. Mentis, Haftu Asmerom Meresa, Tomislav Mestrovic, Tomasz Miazgowski, Andreea Mirica, Moonis Mirza, Awoke Misganaw, Sanjeev Misra, Dara K.

Mohammad, Shafiu Mohammed, Syam Mohan, Nagabhishek Moka, Ali H Mokdad, Sara Momtazmanesh, Mohammad Ali Moni, Delaram Moosavi, Maryam Moradi, Ebrahim Mostafavi, Temesgen Muche, Francesk Mulita, Getaneh Baye B Mulu, Christopher J L Murray, Ana-Maria Musina, Ghulam Mustafa, Ahamarshan Jayaraman Nagarajan, Tapas Sadasivan Nair, Sreenivas Narasimha Swamy, Hasan Nassereldine, Zuhair S Natto, Biswa Prakash Nayak, Shumaila Naz, Ionut Negoi, Ruxandra Irina Negoi, Georges Nguefack-Tsague, Josephine W. Ngunjiri, Robina Khan Niazi, Ali Nowroozi, Dieta Nurrika, Khan M Nuruzzaman, Ogochukwu Janet Nzoputam, Bogdan Oancea, Rahman Md Obaidur, Mohammed Mohammed Suleiman Suleiman Obsa, Julius Nyerere Odhiambo, Ropo Ebenezer Ogunsakin, Hassan Okati-Aliabad, Osaretin Christabel Okonji, Adeolu Olufunso Oladunjoye, Olubunmi Omotola Oladunjoye, Andrew T Olagunju, Isaac Iyinoluwa Olufadewa, Ahmed Omar Bali, Abidemi E Emmanuel Omonisi, Alberto Ortiz, Mayowa O Owolabi, Jagadish Rao Padubidri, Reza Pakzad, Tamás Palicz, Anamika Pandey, Apurva Kumar Pandya, Paraskevi Papadopoulou, Shahina Pardhan, Jay Patel, Ashish Pathak, Aslam Ramjan Pathan, Rajan Paudel, Uttam Paudel, Shrikant Pawar, Gavin Pereira, Simone Perna, Nandita Perumal, Navaraj Perumalsamy, Ionela-Roxana Petcu, Zahra Zahid Piracha, Richard Charles G Pollok, Pranil Man Singh Pradhan, Akila Prashant, Ibrahim Qattea, Zahiruddin Quazi Syed, Fakher Rahim, Mehran Rahimi, Azizur Rahman, Mosiur Rahman, Amir Masoud Rahmani, Shayan Rahmani, Rajesh Kumar Rai, Sathish Rajaa, Pradhum Ram, Juwel Rana, Muhammad Modassar Ali Nawaz Ranjha, Chythra R Rao, Sowmya J Rao, Sina Rashedi, Mohammad-Mahdi Rashidi, Salman Rawaf, Lal Rawal, Rabail Zehra Raza, Elrashdy Moustafa Mohamed Redwan, Maryam Rezaei, Nazila Rezaei, Negar Rezaei, Toby Richards, Jennifer Rickard, Jefferson Antonio Buendia Rodriguez, Leonardo Roever, Gholamreza Roshandel, Godfrey M Rwegerera, Aly M A Saad, Siamak Sabour, Basema Saddik, Malihe Sadeghi, Saeid Sadeghian, Umar Saeed, Harihar Sahoo, Marwa Rashad Salem, Abdallah M Samy, Senthilkumar Sankararaman, Rocco Santoro, Itamar S Santos, Maheswar Satpathy, Ganesh Kumar Saya, Binyam Tariku Seboka, Anbissa Muleta Senbeta, Melika Shafeghat, Pritik A Shah, Masood Ali Shaikh, Mohd Shanawaz, Mohammed Shannawaz, Mequannent Melaku Sharew Sharew, Purva Sharma, Rahim Ali Sheikhi, Suchitra M Shenoy, Adithi Shetty, B Suresh Kumar Shetty, Jeevan K Shetty, Jae Il Shin, Siddharudha Shivalli, Velizar Shivarov, Parnian Shobeiri, Seyed Afshin Shorofi, Wudneh Simegn, Jasvinder A. Singh, Narinder Pal Singh, Paramdeep Singh, Surjit Singh, Md Shahjahan Siraj, Yordanos Sisay, Anna Aleksandrovna Skryabina, Yonatan Solomon, Yi Song, Jeffrey D Stanaway, Parminder S Suchdev, Mu'awiyyah Babale Sufiyan, Saima Sultana, Mindy D Szeto, Seidamir Pasha Tabaeian, Alireza Tahamtan, Majid Taheri, Moslem Taheri Soodejani, Zemenu Tamir, Ker-Kan Tan, Md. Tariqujjaman, Elvis Enowbeyang Tarkang, Yibekal Manaye Tefera, Mohamad-Hani Temsah, Rekha Thapar, Arulmani Thiyagarajan, Jansje Henny Vera Ticoalu, Bereket M Tigabu, Amir Tiyuri, Ruoyan Tobe-Gai, Marcos Roberto Tovani-Palone, Mai Thi Ngoc Tran, Biruk Shalmeno Tusa, Irfan Ullah, Abdurezak Adem Umer, Bhaskaran Unnikrishnan, Sahel Valadan Tahbaz, Pascual R Valdez, Priya Vart, Shoban Babu Varthya, Siavash Vaziri, Madhur verma Verma, Massimiliano Veroux, Dominique Vervoort, Linh Gia Vu, Birhanu Wagaye, Fitsum Weldegebreal, Nuwan Darshana Wickramasinghe, Melat Woldemariam, Tewodros Eshete Wonde, Gedif Ashebir Wubetie, Xiaoyue Xu, Fereshteh Yazdanpanah, Sisay Shewasinad Yehualashet, Arzu Yigit, Vahit Yiğit, Eshetu Yisihak, Dong Keon Yon, Naohiro Yonemoto, Melissa F. Young, Chuanhua Yu, Ismaeel Yunusa, Mazyar Zahir, Burhan Abdullah Zaman, Nelson Zamora, Getachew Assefa Zenebe, and Mohammad Zoladl.

Drafting the work or revising it critically for important intellectual content

Amirali Aali, Kalkidan Hassen Abate, Sherief Abd-Elsalam, Ame Mehadi Abdurehman, Getachew Abebe, Hassan Abidi, Hassan Abolhassani, Denberu Eshetie Adane Adane, Isaac Yeboah Addo, Daniel Adedayo Adeyinka, Qorinah Estiningtyas Sakilah Adnani, Muhammad Sohail Afzal, Saira Afzal, Bright Opoku Ahinkorah, Sepideh Ahmadi, Ayman Ahmed, Wajeeha Aiman, Marjan Ajami, Hossein Akbarialiabad, Fares Alahdab, Robert Kaba Alhassan, Muhammad Ashar Ali, Sami Almustanyir, Rami H. Hani Al-Rifai,

Saba Alvand, Nelson Alvis-Guzman, Yasser Sami Abdel Dayem Amer, Hubert Amu, Tadele Fentabil Anagaw, Robert Ancuceanu, Ali Arash Anoushirvani, Jalal Arabloo, Hany Ariffin, Timur Aripov, Michael Benjamin Arndt, Judie Arulappan, Raphael Taiwo Aruleba, Melash Belachew Asresie, Seyyed Shamsadin Athari, Daniel Atlaw, Avinash Aujayeb, Andargie Abate Awoke, Mamaru Ayenew Awoke, Sina Azadnajafabad, Mohammadreza Azangou-Khyavy, Darshan B B, Alaa Badawi, Ashish D Badiye, Sara Bagherieh, Atif Amin Baig, Maciej Banach, Palash Chandra Banik, Abere Tilahun Bantie, Ronald D Barr, Amadou Barrow, Saurav Basu, Abdul-Monim Mohammad Batiha, Tahmina Begum, Melaku Ashagrie Belete, Luis Belo, Isabela M Bensenor, Akshaya Srikanth Bhagavathula, Boris Bikbov, Saeid Bitaraf, João Silva Botelho Botelho, Daniela Calina, Francieli Cembranel, Vijay Kumar Chattu, Ju-Huei Chien, Michael H Criqui, Natália Cruz-Martins, Fitsum Wolde Demisse, Hardik Dineshbhai Desai, Fikadu Nugusu Dessalegn, Samath Dhamminda Dharmaratne, Meghnath Dhimal, Sameer Dhingra, Daniel Diaz, Mojtaba Didehdar, M Ashworth Dirac, Saeid Doaei, Milad Dodangeh, Paul Narh Doku, Deepa Dongarwar, Bezabih Terefe Dora, Haneil Larson Dsouza, Mostafa Ahmed Elbahnasawy, Iffat Elbarazi, Ghada Metwally Tawfik ElGohary, Muhammed Elhadi, Waseem El-Huneidi, Mohamed A Elmonem, Rana Ezzeddini, Adeniyi Francis Fagbamigbe, Ali Fatehizadeh, Florian Fischer, Masoud Foroutan, Muktar A. Gadanya, Santosh Gaihre, Yaseen Galali, Nasrin Galehdar, William M. Gardner, Priyanka Garg, Tushar Garg, Yibeltal Yismaw Gela, Urge Gerema, Lemma Getacher, Kazem Ghaffari, Seyyed-Hadi Ghamari, Mohammad Ghasemi Nour, Sherief Ghozy, Abraham Tamirat T Gizaw, James C Glasbey, Alessandra C Goulart, Girma Garedew Goyomsa, Habtamu Alganeh Guadie, Rahul Gupta, Rajat Das Gupta, Sapna Gupta, Vivek Kumar Gupta, Alemu Guta, Parham Habibzadeh, Alexis J Handal, Md. Abdul Hannan, Harapan Harapan, Ahmed I. Hasaballah, Simon I Hay, Khezar Hayat, Golnaz Heidari, Demisu Zenbaba Heyi, Kamal Hezam, Yuta Hiraike, Ramesh Holla, Sheikh Jamal Hossain, Kaveh Hosseini, Mohammad-Salar Hosseini, Sorin Hostiuc, Junjie Huang, Salman Hussain, Foziya Mohammed Hussien, Segun Emmanuel Ibitoye, Olayinka Stephen Ilesanmi, Irena M. Ilic, Mustapha Immurana, Sheikh Mohammed Shariful Islam, Nahlah Elkudssiah Ismail, Linda Merin J, Manthan Dilipkumar Janodia, Shubha Jayaram, Rime Jebai, Bedru Jemal, Ravi Prakash Jha, Jost B. Jonas, Nitin Joseph, Jacek Jerzy Jozwiak, Ali Kabir, Laleh R. Kalankesh, Vineet Kumar Kamal, Himal Kandel, Tesfaye K Kanko, Faizan Zaffar Kashoo, Nicholas J Kassebaum, Patrick D.M.C. Katoto, Joonas H Kauppila, Gbenga A. Kayode, Vikash Ranjan Keshri, Yousef Saleh Khader, Moien AB Khan, Khaled Khatab, Zaher Khazaei, Jagdish Khubchandani, Yun Jin Kim, Sezer Kisa, Farzad Kompani, Sindhura Lakshmi Koulmane Laxminarayana, Kewal Krishan, Barthelemy Kuate Defo, Mohammed Kuddus, Naveen Kumar, Om P Kurmi, Oluwatosin Kuti, Iván Landires, Anders O Larsson, Kamaluddin Latief, Samson Mideksa Legesse, László Lorenzovici, Vanessa Sintra Machado Machado, Preetam Bhalchandra Mahajan, Soleiman Mahjoub, Ata Mahmoodpoor, Elham Mahmoudi, Elaheh Malakan Rad, Tauqeer Hussain Mallhi, Deborah Carvalho Malta, Theresa A McHugh, José João João Mendes Mendes, Walter Mendoza, Oliver Mendoza-Cano, Alexios-Fotios A. Mentis, Haftu Asmerom Meresa, Tomislav Mestrovic, Tomasz Miazgowski, Mojgan Mirghafourvand, Moonis Mirza, Awoke Misganaw, Dara K. Mohammad, Shadieh Mohammadi, Shafiu Mohammed, Nagabhishek Moka, Ali H Mokdad, Sara Momtazmanesh, Lorenzo Monasta, Mohammad Ali Moni, Delaram Moosavi, Maryam Moradi, Abbas Mosapour, Ebrahim Mostafavi, Christopher J L Murray, Ghulam Mustafa, Ahamarshan Jayaraman Nagarajan, Sreenivas Narasimha Swamy, Hasan Nassereldine, Zuhair S Natto, Biswa Prakash Nayak, Shumaila Naz, Ionut Negoi, Ruxandra Irina Negoi, Georges Nguefack-Tsague, Josephine W. Ngunjiri, Robina Khan Niazi, Ali Nowroozi, Dieta Nurrika, Khan M Nuruzzaman, Ogochukwu Janet Nzoputam, Bogdan Oancea, Osaretin Christabel Okonji, Andrew T Olagunju, Abidemi E Emmanuel Omonisi, Alberto Ortiz, Mayowa O Owolabi, Jagadish Rao Padubidri, Reza Pakzad, Tamás Palicz, Apurva Kumar Pandya, Paraskevi Papadopoulou, Shahina Pardhan, Shrikant Pawar, Gavin Pereira, Norberto Perico, Simone Perna, Ionela-Roxana Petcu, Zahra Zahid Piracha, Richard Charles G Pollok, Pranil Man Singh Pradhan, Akila Prashant, Ibrahim Qattea, Zahiruddin Quazi Syed, Fakher Rahim, Mehran Rahimi, Azizur Rahman, Mohammad Hifz Ur Rahman, Shayan Rahmani, ivano raimondo, Sathish Rajaa, Pradhum Ram, Muhammad Modassar Ali Nawaz

Ranjha, Chythra R Rao, Sowmya J Rao, Salman Rawaf, Lal Rawal, Rabail Zehra Raza, Christian Razo, Elrashdy Moustafa Mohamed Redwan, Giuseppe Remuzzi, Maryam Rezaei, Nazila Rezaei, Toby Richards, Jennifer Rickard, Jefferson Antonio Buendia Rodriguez, Leonardo Roever, Gholamreza Roshandel, Bedanta Roy, Godfrey M Rwegerera, Aly M A Saad, Siamak Sabour, Basema Saddik, Malihe Sadeghi, Umar Saeed, Amirhossein Sahebkar, Marwa Rashad Salem, Abdallah M Samy, Itamar S Santos, Maheswar Satpathy, Ganesh Kumar Saya, Allen Seylani, Melika Shafeghat, Mohd Shanawaz, Mohammed Shannawaz, Mequannent Melaku Sharew Sharew, Purva Sharma, Suchitra M Shenoy, Jeevan K Shetty, Pavanchand H Shetty, Siddharudha Shivalli, Parnian Shobeiri, Seyed Afshin Shorofi, Ali Reza Sima, Jasvinder A. Singh, Paramdeep Singh, Anna Aleksandrovna Skryabina, Yonatan Solomon, Yi Song, Jeffrey D Stanaway, Parminder S Suchdev, Mu'awiyyah Babale Sufiyan, Saima Sultana, Mindy D Szeto, Seidamir Pasha Tabaeian, Alireza Tahamtan, Majid Taheri, Ker-Kan Tan, Elvis Enowbeyang Tarkang, Nathan Y. Tat, Yibekal Manaye Tefera, Mohamad-Hani Temsah, Jansje Henny Vera Ticoalu, Bereket M Tigabu, Marcos Roberto Tovani-Palone, Mai Thi Ngoc Tran, Biruk Shalmeno Tusa, Irfan Ullah, Abdurezak Adem Umer, Bhaskaran Unnikrishnan, Marco Vacante, Sahel Valadan Tahbaz, Priya Vart, Shoban Babu Varthya, Madhur verma Verma, Massimiliano Veroux, Dominique Vervoort, Linh Gia Vu, Birhanu Wagaye, Fitsum Weldegebreal, Nuwan Darshana Wickramasinghe, Tewodros Eshete Wonde, Gedif Ashebir Wubetie, Kheirollah Yari, Sisay Shewasinad Yehualashet, Arzu Yigit, Vahit Yiğit, Naohiro Yonemoto, Mazyar Zahir, Leila Zaki, Burhan Abdullah Zaman, Nelson Zamora, Iman Zare, Getachew Assefa Zenebe, Zhi-Jiang Zhang, and Mohammad Zoladl.

Managing the estimation or publications process

Denberu Eshetie Adane Adane, Tadele Fentabil Anagaw, Francieli Cembranel, Fikadu Nugusu Dessalegn, Mojtaba Didehdar, Saeid Doaei, Ali Fatehizadeh, Kia Fuller, William M. Gardner, Kazem Ghaffari, Simon I Hay, Nicholas J Kassebaum, Zaher Khazaei, Samson Mideksa Legesse, Theresa A McHugh, Moonis Mirza, Ali H Mokdad, Christopher J L Murray, Zahiruddin Quazi Syed, Maryam Rezaei, Aly M A Saad, Abdallah M Samy, Jeffrey D Stanaway, Yibekal Manaye Tefera, Mai Thi Ngoc Tran, and Gedif Ashebir Wubetie.
